# A novel matrix of sequence descriptors for predicting protein-protein interactions from amino acid sequences

**DOI:** 10.1371/journal.pone.0217312

**Published:** 2019-06-07

**Authors:** Xue Wang, Yuejin Wu, Rujing Wang, Yuanyuan Wei, Yuanmiao Gui

**Affiliations:** 1 Institute of Technical Biology & Agriculture Engineering, Hefei Institutes of Physical Science, Chinese Academy of Sciences, HeFei City, AnHui Province, China; 2 University of Science and Technology of China, HeFei City, AnHui Province, China; 3 Institute of Intelligent Machine, Hefei Institutes of Physical Science, Chinese Academy of Sciences, HeFei City, AnHui Province, China; Newcastle University, UNITED KINGDOM

## Abstract

Protein-protein interactions (PPIs) play an important role in the life activities of organisms. With the availability of large amounts of protein sequence data, PPIs prediction methods have attracted increasing attention. A variety of protein sequence coding methods have emerged, but the training of these methods is particularly time consuming. To solve this issue, we have proposed a novel matrix sequence coding method. Based on deep neural network (DNN) and a novel matrix protein sequence descriptor, we constructed a protein interaction prediction model for predicting PPIs. When performed on human PPIs data, the method achieved an accuracy of 94.34%, a recall of 98.28%, an area under the curve (AUC) of 97.79% and a loss of 23.25%. A non-redundant dataset was used to evaluate this prediction model, and the prediction accuracy is 88.29%. These results indicate that the matrix of sequence (MOS) descriptor can enhance the predictive power of PPIs and reduce training time, which can be a useful complement for future proteomics research. The experimental code and experimental results can be found at https://github.com/smalltalkman/hppi-tensorflow.

## Introduction

Protein-protein interactions (PPIs) are useful for elucidating the changing mechanisms of organisms in physiological or pathological conditions and are important for disease prevention and drug development. In the last decade, numerous methods for studying protein-protein interactions, such as yeast two-hybrid screens [1], hybrid approaches [2] and protein chips [3], have emerged. However, all of these experimental methods have the disadvantage of being time-consuming and costly. Therefore, using computational approaches to predict unknown PPIs has become an important research topic in bioinformatics. In recent years, many computer prediction methods have been proposed to predict PPIs based on a phylogenetic profile method [4], amino acid index distribution [5] and gene fusion events [6, 7]. However, these methods are not universal because the reliability of these methods depends on a priori information about the protein pairs.

In recent years, a large amount of protein sequence information has been accumulated, and numerous computer calculation methods and sequence-based methods have become more universal and acceptable [8–18], such as support vector machines (SVM) [8–10], Naïve Bayes [11, 12], decision trees [13–14], random forests [15–16], and deep learning [17–18]. From the above methods, the accuracy of PPIs prediction is not only related to machine learning methods but also to protein coding methods. Protein coding methods and classification algorithms are the core steps of PPI prediction and have become primary tasks of current life science research. Until now, many efficient protein coding methods have been proposed for inferring PPIs based on protein sequence, such as the conjoint triad method (CT) [19], the auto covariance method (AC) [20] and local descriptor (LD) [21]. Among them, the conjoint triad method (CT) [19] considers considered the order relationship of three amino acids. In such a protein coding method, the 20 amino acids are clustered into seven classes according to the dipoles and volumes of the side chains. Auto covariance (AC) [20] considers the order relationship of 30 amino acids. Local descriptor (LD) is an alignment-free approach, and its effectiveness depends largely on the underlying amino acid groups, and only considers the neighbouring effect of two adjacent types of amino acids [21].

Though the various methods described above for protein coding methods are useful, one of the drawbacks is that the order relationship of the entire amino acid sequence is not considered. To overcome this problem, we propose a sequence-based method based on a novel representation of the matrix of sequence (MOS). The MOS descriptor is first classified into 7 classes according to the successful use of classification in Shen et al. [20]. Then, we combine this classification with a novel representation of protein sequence descriptors. Next, we constructed a (deep neural network- matrix of sequence) DNN-MOS model by combining the DNN and MOS. Finally, we evaluated the performance of the DNN-MOS protein prediction model. When performed on human data, our method had an accuracy of 94.34%, a recall of 98.28%, an area under the curve (AUC) of 97.79% and a loss of 23.25%. To prove the effectiveness of MOS, we compared MOS with existing protein coding methods. We found that the MOS can greatly reduce the loss and training time, and the prediction performance is improved. Additionally, we found that MOS achieves better performance in other classifiers such as decision tree, k-neighbors and random forest.

## Materials and methods

### Data set construction

(1) Benchmark dataset: The benchmark PPIs dataset was used in our experiment, which was provided by Pan et al. [22]. Among this benchmark dataset, the positive samples were taken from the Human Protein Reference Database (HPRD) 2007 version, and the negative samples were taken from the Swiss Swiss-Prot database 57.3 version. These positive samples are usually verified by reliable methods [23–24]. The negative samples (non-interacting pairs of proteins) were generated by pairing proteins found in different subcellular locations, according to the following requirements [19, 25]: (1) the non-interactive pairs cannot appear in interacting data sets; (2) sequences annotated with ambiguous or uncertain subcellular location terms were excluded to construct the negative samples; (3) sequences annotated by two or more locations were excluded due to lack of the uniqueness. After removing the self-interactions and duplicate interactions of the positive dataset, we finally obtained 36,630 positive pairs and 36,480 negative pairs. Protein pairs with unusual amino acids and <50 amino acids were excluded, such as B, J, O, U, X and Z to yield 36,591 positive samples and 36,324 negative samples to form the benchmark dataset. We mixed the positive and negative samples in the benchmark dataset and randomly selected 60,000 pairs (30,000 positive samples, 30,000 negative samples as training datasets for models, with the remainder constituting the training set as a hold-out test set to validate the model).

(2) Non-redundant dataset: This dataset was provided by Pan et al. [22]. The protein pairs of this dataset exclude proteins with ≥25% sequence identity from the benchmark dataset. This dataset contains 3,899 positive protein pairs and 4,262 negative protein pairs.

### Matrix of sequence (MOS)

#### Classification of amino acids

According to Shen et al. [19], 20 amino acids can be divided into seven different groups based on their dipole and side chain volumes. The seven different amino acid classifications are shown in Table 1. Then, a protein sequence is represented by these seven groups according to Table 1. For example, the protein sequence "AGCRQTSPLGVKSE" would be represented as “11754332211536”.

**Table 1:** Amino acid classification based on their dipole and side chain volumes.

Number	Amino Acids
1	A, G, V
2	I, L, F, P
3	Y, M, T, S
4	H, N, Q, W
5	R, K
6	D, E
7	C

#### Related definitions

Vector of protein sequence (VOS): Hypothetical non-empty finite set: Ω = {*w*_1_, …, *w*_7_}, where *w*_*i*_ is amino acid classification. Given sequence: S = *S*_1_, *S*_2_, …,*S*_*L*_, where L represents the length of sequence S, S_i_ ∈ Ω, 1≤i≤L. The sequence vector of a given sequence S can be expressed as: $VOS=(C_{W_{1}},\ldots ,C_{W_{N}})$, where $C_{W_{1}}$ is the number of occurrences of the *w*_*i*_ in the sequence S. Based on the definition of the sequence vector, the sum of all elements in the sequence matrix is equal to L.

Matrix of sequence (MOS): Hypothetical non-empty finite set: Ω = {*w*_1_, …, *W*_*N*_}, where N is the number of categories of the sequence. Given sequence: S = *S*_1_, *S*_2_, …, *S*_*L*_, where L represents the length of sequence S, S_i_ ∈ Ω, 1≤i≤L. The sequence matrix of a given sequence S can be expressed as: MOS = [*m*_*ij*_]_*N*×*N*_.

mij={…wi…or…wi…wi…Thenumberofoccurrences,i=j…wi…wj…Thenumberofoccurrences,i≠j (1)

Based on the definition of the sequence matrix, the sum of all elements in the sequence matrix is equal to $\frac{L(L+1)}{2}$, $m_{ij}=\frac{C_{w_{i}}(C_{w_{i}}+1)}{2}(1\leq i\leq N)$, $m_{ij}+m_{ji}=C_{w_{i}}C_{w_{j}}(i\neq j)$. Thus, for any two sequences, when the sequence lengths are different or the sequence lengths are the same but at least one element contains different numbers of elements, the corresponding sequence squares are different.

#### Algorithm of sequence matrix

Hypothetical non-empty finite set: Ω = {*w*_1_, …, *W*_*N*_}, where N is the number of categories of the sequence. Given sequence: S = *S*_1_, *S*_2_, …, *S*_*L*_, where L represents the length of sequence S, S_i_ ∈ Ω, 1≤i≤L. The sequence matrix of a given sequence S can be expressed as:

Input sequence: S = *S*_1_, *S*_2_, …, *S*_*L*_;

Output sequence matrix: MOS = [*m*_*ij*_]_*N*×*N*_.

The sequence matrix algorithm is calculated as follows:

Step 1. Initial value is set up: i ← L, VOS ← *VOS*_0_ = 0, MOS ← *MOS*_0_ = 0.

Step 2. VOS[*s*_*i*_] ← VOS[*s*_*i*_] + 1.

Step 3. MOS[*s*_*i*_] ← MOS[*s*_*i*_] + *VOS*.

Step 4. i ← i − 1.

Step 5. If i≥1, go to step 2.

#### Protein feature representation

In this article, we present a novel method of protein feature representation by combining sequence matrix descriptors with the amino acid classification method. To reduce the computational vector, we first classify 20 amino acids into 7 classes according to the amino acid classification method in Table 1. Thus, a protein sequence can be represented by a matrix of 7×7, as shown in Eq 2.

[mij]7×7={m11m12…m17m21m22…m27…m71m72…m77 (2)

The next step is to standardize *m*_*ij*_ of each matrix element ranging from 0 to 1. To solve this problem, we defined a new parameter *p*_*ij*_, by normalizing *m*_*ij*_ with Eq 3:

$$p_{ij}=\frac{m_{ij}}{\sum m_{ij}}$$ (3)

$$\sum m_{ij}=\frac{L(L+1)}{2}$$ (4)

where L is the length of the protein sequence. The numerical value of *p*_*ij*_ of each protein ranges from 0 to 1. The elements in the diagonal of the matrix and the elements above the diagonal are combined into a 28-dimensional vector. To distinguish the lengths of the sequences, a sequence tag is added, and the sequence tags are represented by the reciprocal of the length of the protein. Finally, a total 29-dimensional vector has been built to represent each protein sequence.

### Deep neural network (DNN)

**Fig 1:**
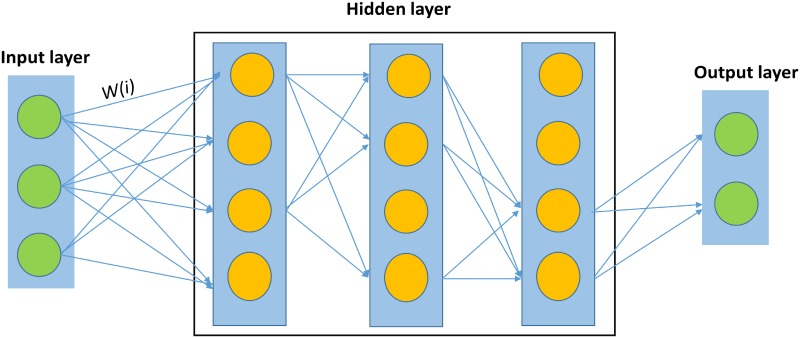
Neural network training procedure.

A deep neural network is a popular type of deep learning algorithm with three or more hidden layers. The basic structure of a deep neural network is similar to the basic structure of a shallow neural network and consists of an input layer, middle hidden layers, and an output layer. However, the parameters, calculation units and algorithms of deep neural networks are more abundant than traditional shallow neural networks. As shown in Fig 1, input data (x) are given to the input layer, processed layer by layer through the hidden layer, and then transmitted to the output layer. The weights *w*_*(i)*_ between neurons are free parameters that capture the model’s representation of the data and are learned from input/output samples. Each neuron computes a weighted sum of its inputs and applies a nonlinear activation function to calculate its outputs. The formulation of input data in forward propagation is calculated according to Eq 1:

$$a_{i}^{\left(l+1\right)}=\delta (Z_{i})$$ (5)

$$Z_{i}=w_{i}^{\left(l+1\right)}a^{l}+b_{i}^{\left(l+1\right)}$$ (6)

where *a*^*(l+1)*^ is the input data of the *(l+1)-th* layer, δ denotes the activation of the *(l+1)-th* layer, *w*^*(l+1)*^ is the connection weight matrix between the (*l)-th* layer and the *(l+1)-th* layer, *a*^*l*^ is the input data of the *(l)-th* layer, and *b*^*(l+1)*^ is the bias term in the *(l+1)-th* layer.

Back propagation is the propagation of the output through the hidden layer to the input layer, and the error is distributed to all of the cells of each layer, to obtain the error signal of each layer. In general, ReLU (rectified linear unit) is used as the activation function for neurons in DNN. The ReLU can change all negative values to zero while leaving the positive values unchanged. Compared to other activation functions, ReLU has a few advantages [26, 27]. For linear functions, ReLU is more expressive, especially in deep networks. For non-linear functions, ReLU does not have the disadvantage of gradient disappearance and can thereby maintain the convergence speed of the model at a stable level.

### Evaluation measure

The performance of the models was evaluated by a series of evaluation indicators, including the accuracy, recall, AUC and loss in this study. Their criterion functions are defined, respectively, by:

$$Accuracy=\frac{TP+TN}{TP+TN+FP+FN}$$ (7)

$$Recall=\frac{TP}{TP+FN}$$ (8)

where *TP*, *TN*, *FP*, and *FN* represent the true positive, true negative, false positive, and false negative, respectively. AUC was calculated using an open source code [28]. Loss was calculated according to the following cross-entropy function:

$$loss(y,y^{*})=-\frac{1}{n}\sum_{i=1}^{n}\left(y_{i}^{*}\text{ln}y_{i}+(1-y_{i}^{*})\text{ln}(1-y)\right)$$ (9)

where y = (y_1_,y_2_,y_3_……y_n_) represents the actual output and $\text{y}*=(y_{1}^{*},y_{2}^{*},y_{3}^{*},\ldots \ldots ,y_{n}^{*})$ represents the desired output.

## Results

### Selecting optimal parameters

Selecting an optimal parameter is an important step in the model training process and one of the key elements in training a robust model. In this experiment, ReLU was selected as the activation function, Adam as the optimizer, and cross entropy as the cost function. Compared with the Sigmoid and the Tanh activation functions, ReLU has a simple operation, has the sparse expression ability and learning ability of a neural network, and has a faster convergence speed during the gradient descent. Due to the above advantages, ReLU was used as the activation function for this model [27]. Adam combines the advantages of the RMSprop and Adagrad algorithms for the improved handling of noise, which led us to choose it as the optimizer [29, 30]. The cross-entropy cost function can measure the predicted and actual values in a deep neural network, and it can compensate for the defects caused by the easy saturation of the sigmoid function, thus causing the training set to converge faster. In this experiment, we chose to use the cross-entropy cost function.

In our method, the three parameters of learning rate, network width and network depth must be determined. To determine the learning rate, the number of hidden layer nodes is set to 64, the activation function is ReLU, the optimization algorithm is Adam, the batch size is 128, the dropout is 0, and the number of iterations is 300,000. The results of adjusting the learning rate are shown in Table 2. From Table 2, we found that when the learning rate is 0.01, it has the best predictive performance in the context of PPIs prediction. Therefore, here, 0.01 was chosen as the learning rate for our experiment.

**Table 2:** Adjusting the learning rate of our model.

Learning rate	Accuracy (%)	AUC (%)	Recall (%)	Loss (%)	Train time(s/100 steps)
0.01	0.7926±0.0256	0.8807±0.023	0.8923±0.0239	0.4373±0.0377	0.1296±0.001
0.001	0.7553±0.0377	0.8495±0.0367	0.8603±0.0393	0.4900±0.0489	0.1263±0.0012
0.0001	0.6784±0.0273	0.7584±0.0307	0.7616±0.0337	0.5871±0.0271	0.1307±0.0011
0.00001	0.6363±0.0076	0.6921±0.0096	0.687±0.01	0.6443±0.0193	0.1303±0.001

To determine the network width of the model, the learning rate is set to 0.01, the other parameters are unchanged, and the network width results of our model are shown in Table 3. According to Table 3, when the network width is 512, the performance of the model is better than that of several other network widths. Therefore, the network width of this model is set to 512.

**Table 3:** Adjusting of the network width of our model.

Width	Accuracy (%)	AUC (%)	Recall (%)	Loss (%)	Train time(s/100 steps)
128	0.7191±0.02	0.8139±0.0169	0.8232±0.0182	0.542±0.0236	0.1594±0.0034
256	0.7476±0.0199	0.8455±0.0161	0.8618±0.0182	0.4966±0.0276	0.1875±0.0014
512	0.8277±0.0352	0.9107±0.0296	0.9211±0.0288	0.3826±0.0527	0.2497±0.0026
1024	0.8234±0.0317	0.9081±0.0283	0.9192±0.0278	0.3882±0.0484	0.3483±0.0034

After the learning rate and network width are determined, the next step is to determine the depth of the model. The depth adjustment results are shown in Table 4. From Table 4, the model performance is best when the depth is 512 × 512 × 512. Therefore, 512 x 512 x 512 was chosen as our model depth.

**Table 4:** Adjusting the network width of our model.

Depth	Accuracy (%)	AUC (%)	Recall (%)	Loss (%)	Train time(s/100 steps)
512×512	0.8262±0.0376	0.913±0.0316	0.9241±0.0303	0.3904±0.0513	0.8478±0.0081
512×512×512	0.9159±0.0563	0.9621±0.0379	0.9689±0.035	0.2598±0.075	1.4374±0.0159
512×512×512×512	0.7988±0.0407	0.891±0.0335	0.9068±0.0333	0.428±0.0544	2.0333±0.0087
512×512×512×512×512	0.7104±0.0898	0.7976±0.1201	0.8447±0.0483	0.5276±0.0785	2.6208±0.0126

### Performance of MOS on PPIs

#### Results on benchmark dataset

The proposed DNN-MOS model (protein sequences coded by MOS descriptors) was applied to the human dataset. To investigate the contribution of the novel MOS descriptor, we separately trained DNN based on CT, AC, LD, and MOS. Among them, the parameter settings of DNN-MOS are shown in 3.1. The parameters of DNN-CT (deep neural network- conjoint triad), DNN-AC (deep neural network-auto covariance) and DNN-LD (deep neural network-local descriptor) are set as follows: the activation function was ReLU, the optimization algorithm was Adam, the batch size was 128, the dropout was 0, the number of hidden layer nodes was set to 256, the network depth was [256-256-256], the learning rate was 0.001, the number of times to repeat the hold-out-validation was 30 and the number of times was 10,000 per iteration.

The results of each prediction model are shown in Table 5. From Table 5, we can observe that the predictive performance using MOS is not superior to other descriptors for almost all evaluation metrics. The accuracy and AUC of DNN-MOS are 94.34% and 98.28%, lower than those of DNN-CT and DNN-AC. The AUC of DNN-MOS is slightly higher than that of DNN-LD and significantly lower than those of DNN-CT and DNN-AC. However, the loss of MOS is significantly better than the other three encoding methods.

**Table 5:** Results based on DNN with CT, AC, LD, and MOS on the benchmark dataset.

Method	Accuracy	Recall	AUC	Loss
DNN-CT	0.9711±0.0038	0.9891±0.0009	0.9835±0.0018	0.2747±0.0686
DNN-AC	0.9684±0.0013	0.9867±0.0013	0.9802±0.0022	0.6591±0.3178
DNN-LD	0.953±0.0087	0.9828±0.003	0.9757±0.0043	0.3623±0.0924
DNN-MOS	0.9434±0.0078	0.9828±0.0023	0.9779±0.0028	0.2325±0.0154

The training time is related to the parameters, such as the width and depth of the model. To compare the training time of each code, we set the parameters the same. The parameters of DNN-MOS, DNN-CT, DNN-AC and DNN-LD are set as follows: the number of hidden layer nodes was set to 64, the activation function was ReLU, the optimization algorithm was Adam, the batch size was 128, the dropout was 0, the learning rate was 0.001, the number of times to repeat the hold-out-validation was 30 and the number of times was 10,000 per iteration. The results of the training time are shown in Table 6. As shown in Table 6, the DNN-MOS has the lowest training time per 1000 steps, only 0.1261 seconds. The training time of DNN-MOS is nearly 2 times faster than DNN-AC’s training time, more than 2 times faster than DNN-CT’s and more than 3 times faster than DNN-LD’s. From Table 6, we found that the difference in test time was small, but the test time trend was the same as the training time. Therefore, we found that MOS can significantly save training time and test time. From Table 6, we can also see that the larger the vector dimension, the more training time was required.

**Table 6:** Results based on DNN with CT, AC, LD, and MOS on the benchmark dataset.

Method	Train time (s)	Test time (s)	The dimensions of vector space	Data set
DNN-CT	0.2852±0.0039	1.39E-05	686	HPRD (36591) + Swiss-Port (36324)
DNN-AC	0.2186±0.0014	1.32E-05	420	HPRD (36591) + Swiss-Port (36324)
DNN-LD	0.4045±0.0141	1.48E-05	1260	HPRD (36591) + Swiss-Port (36324)
DNN-MOS	0.1261±0.0039	1.28E-05	58	HPRD (36591) + Swiss-Port (36324)

#### Results on non-redundant dataset

To further assess the practical prediction ability of DNN-MOS, we trained the models of DNN-MOS, DNN-CT and DNN-AC on a non-redundant dataset (removing the samples that has ≥25% sequence identity to any sample in the pre-training set). The prediction results are shown in Table 7. From Table 7, we can observe that the accuracy of DNN-MOS, DNN-CT and DNN-AC on the non-redundant dataset are 88.29%, 89.88% and 93.35%, respectively. Shen et al. [17] studied the PPIs of the dataset using a deep learning algorithm, achieving an accuracy of 85.84%, which is lower than our results.

**Table 7:** Results of DNN with different feature extraction method on a non-redundant dataset.

Methods	Accuracy	Recall	AUC
**DNN-MOS**	88.29%	93.63%	92.23%
**DNN-CT**	89.88%	93.79%	91.78%
**DNN-AC**	93.35%	96.24%	94.99%
**Shen’s work [17]**	85.84%	N/A	N/A

### Comparison with different classifiers

In order to verify the effectiveness of the feature extraction method of MOS on PPIs, we combined the MOS with Decision Tree (DT), K-Neighbors (KN) and Random Forest (RF) on human data to construct three models of DT-MOS (decision tree—matrix of sequence), KN-MOS (K-Neighbors—matrix of sequence) and RF-MOS (random forest—matrix of sequence). The results are shown in Table 8. From Table 8, we can see that these methods present an accuracy of 83.01–97.29%, and the accuracies of DT-MOS, KN-MOS and RF-MOS are 94.36%, 83.01%, and 97.29%, respectively. These results show that the novel MOS of our proposed are also effective in other classifiers such as DT, KN and RF.

**Table 8:** Comparison of the performances of MOS based on different classifiers using the human dataset.

Methods	Accuracy	Recall	AUC
DT-MOS	0.9436	0.9365	0.9436
KN-MOS	0.8301	0.6973	0.8298
RF-MOS	0.9729	0.9611	0.9729

## Discussion

We have presented a novel protein sequence coding approach for PPIs prediction. Of note, we propose a strategy for projecting protein sequences into a vector space, which is used to represent the matrix space of PPI information. Specifically, we first classify 20 amino acids into 7 amino acids according to their physicochemical properties (Table 1). The dimensions of the matrix space can be significantly reduced, from 20×20 to 7×7. Next, we combine the elements on the 7×7 matrix diagonal and the elements above the diagonal into a 28-dimensional vector. To distinguish the length of a sequence, a sequence label is added. Finally, a 29-dimensional vector can represent a protein sequence. We combined MOS with DT, KN and RF and achieved good results. The experimental results show that the proposed MOS feature extraction method is effective. However, the disadvantage of the novel matrix sequence descriptor is that the sequence matrix cannot be in one-to-one correspondence with the protein sequence. For any given two sequences, the corresponding sequence matrices are different when the sequence lengths are different, or the sequence lengths are the same but at least one element contains different numbers of elements. Therefore, pre-processing data is required to remove protein pairs with the same protein sequence length and the same number of elements.

Recently, new feature extraction approaches for PPIs have been developed [30–33]. Among them, Li et al. [30] proposed a new method for predicting self-interacting proteins (SIPs) based on amino acid sequences, achieving high precisions of 86.86 and 91.30% on the *Saccharomyces cerevisiae* and human SIPs datasets, respectively. Wang et al. [31] reported a novel method of PPIs based on pseudo position specific scoring matrix (PSSM) feature descriptors and an ensemble rotation forest (RF) learning system from protein amino acid sequences. Their method achieved accuracies of 98.38%, 89.75%, and 96.25% on the yeast, *H*. *pylori*, and independent datasets, respectively. Li et al. [32] developed a new hybrid method of physical chemistry and evolution-based feature extraction methods, which can capture discriminant features from evolution-based information and physicochemical features. An et al. [33] explored a new feature representation method based on local binary pattern (LBP), which not only considers the amino acid sequence information but also the evolutionary information of multiple sequence alignments. The above studies show that effective feature extraction methods can mine useful information on protein pairs and improve the performance of PPIs prediction. In this study, although we found that the performance of DNN-MOS is not prominent in Table 5, DNN-MOS can greatly reduce loss and training time (Table 6). In addition, Table 8 show that the novel MOS of our proposed are also effective in other classifiers such as DT, KN and RF. Overall, although the performance of DNN-MOS is not prominent, it can be a useful supplement to PPIs predictions. The reason why the accuracy of DNN-MOS is lower than that of DNN-CT, DNN-AC and DNN-LD may be due to the loss of part of the information when converting the protein sequence into a matrix vector. In future research, we will try our best to solve this problem and improve the predictive performance of DNN-MOS.

## Conclusion

With the increasing number of PPI calculation methods, the coding methods of various amino acid feature vectors are also emerging. Although the various protein encoding methods such as AC, CT, and LD are useful, one of the disadvantages is that the order relationship of the entire amino acid sequence is not considered. The CT [19] considers considered the order relationship of three amino acids. AC [20] considers the order relationship of 30 amino acids. LD only considers the neighbouring effect of two adjacent types of amino acids [21]. To overcome this problem, we propose an efficient method for predicting PPIs from amino acid sequences by a novel matrix sequence descriptor feature representation with deep neural network. The novel protein feature extraction method we have proposed considers the order relationship of the entire amino acid sequence. When performed on human PPIs data, DNN-MOS, DT-MOS, KN-MOS and RF-MOS have achieved good results. Additionally, the model was used to evaluate this prediction model on a non-redundant dataset and the prediction accuracy is 88.29%. The experimental results show that the matrix sequence descriptor is promising for predicting PPIs and can be used as a complementary supplement to other methods.

## Supporting information

S1 File The positive protein-protein interaction. There are 36,630 protein-protein pairs from total 9476 proteins, and the first column is protein ID from HPRD, the second column is the other protein ID and the two proteins constitute the positive Protein-protein interaction.
(DOC)

S2 File The negative protein-protein interaction. There are 36,480 protein-protein pairs from total 2184 proteins, and the first column is protein ID, the second column is the other protein ID and the two proteins constitute the negative Protein-protein interaction.
(DOC)

S3 File The identity of positive protein-protein interaction is below 25%. There are 3899 protein-protein pairs from total 2502 proteins, and the first column is protein ID from HPRD, the second column is the other protein ID and the two proteins constitute the positive Protein-protein interaction and protein identity of all the proteins from S3 file is below 25%.
(DOC)

S4 File The identity of negative protein-protein interaction is below 25%. There are 4262 protein-protein pairs from total 661 proteins, and the first column is protein ID from HPRD, the second column is the other protein ID and the two proteins constitute the positive Protein-protein interaction and protein identity of all the proteins from S4 file is below 25%.
(DOC)
