# Supplementary material for: A novel matrix of sequence descriptors for predicting protein-protein interactions from amino acid sequences
Source: PLoS One. 2019 Jun 7;14(6):e0217312. doi: 10.1371/journal.pone.0217312 (PMC6555512; doi:10.1371/journal.pone.0217312)
Supplement: S3 File — There are 3899 protein-protein pairs from total 2502 proteins, and the first column is protein ID from HPRD, the second column is the other protein ID and the two proteins constitute the positive Protein-protein interaction and protein identity of all the proteins from S3 file is below 25%. (DOC) [file pone.0217312.s003.doc]

**Online support information C**

There are 3899 protein-protein pairs from total 2502 proteins, and the first column is protein ID from HPRD, the second column is the other protein ID and the two proteins constitute the positive Protein-protein interaction and protein identity of all the proteins from Supp-C and Supp-D is below 25%;

Index Protein_1_ID protein_2_ID

1 NP_612384.1 NP_055105.2

>NP_612384.1

MLSKRGCHARIYADFPIRRLISQRSSLETLEDIEENAPLRRCRTLSGSPRPKNFKKIHFIKNMRQHDTRNGRIVLISGRRSFCSIFSVLPYRDSTQVGDLKLDGGRQSTGAVSLKEIIGLEGVELGADGKTVSYTQFLLPTNAFGARRNTIDSTSSFSQFRNLSHRSLSIGRASGTQGSLDTGSDLGDFMDYDPNLLDDPQWPCGKHKRVLIFPSYMTTVIDYVKPSDLKKDMNETFKEKFPHIKLTLSKIRSLKREMRKLAQEDCGLEEPTVAMAFVYFEKLALKGKLNKQNRKLCAGACVLLAAKIGSDLKKHEVKHLIDKLEEKFRLNRRELIAFEFPVLVALEFALHLPEHEVMPHYRRLVQSS

>NP_055105.2

MLEGDLVSKMLRAVLQSHKNGVALPRLQGEYRSLTGDWIPFKQLGFPTLEAYLRSVPAVVRIETSRSGEITCYAMACTETARIAQLVARQRSSKRKTGRQVNCQMRVKKTMPFFLEGKPKATLRQPGFASNFSVGKKPNPAPLRDKGNSVGVKPDAEMSPYMLHTTLGNEAFKDIPVQRHVTMSTNNRFSPKASLQPPLQMHLSRTSTKEMSDNLNQTVEKPNVKPPASYTYKMDEVQNRIKEILNKHNNGIWISKLPHFYKELYKEDLNQGILQQFEHWPHICTVEKPCSGGQDLLLYPAKRKQLLRSELDTEKVPLSPLPGPKQTPPLKGCPTVMAGDFKEKVADLLVKYTSGLWASALPKAFEEMYKVKFPEDALKNLASLSDVCSIDYISGNPQKAILYAKLPLPTDKIQKDAGQAHGDNDIKAMVEQEYLQVEESIAESANTFMEDITVPPLMIPTEASPSVLVVELSNTNEVVIRYVGKDYSAAQELMEDEMKEYYSKNPKITPVQAVNVGQLLAVNAEEDAWLRAQVISTEENKIKVCYVDYGFSENVEKSKAYKLNPKFCSLSFQATKCKLAGLEVLSDDPDLVKVVESLTCGKIFAVEILDKADIPLVVLYDTSGEDDININATCLKAICDKSLEVHLQVDAMYTNVKVTNICSDGTLYCQVPCKGLNKLSDLLRKIEDYFHCKHMTSECFVSLPFCGKICLFHCKGKWLRVEITNVHSSRALDVQFLDSGTVTSVKVSELREIPPRFLQEMIAIPPQAIKCCLADLPQSIGMWTPDAVLWLRDSVLNCSDCSIKVTKVDETRGIAHVYLFTPKNFPDPHRSINRQITNADLWKHQKDVFLSAISSGADSPNSKNGNMPMSGNTGENFRKNLTDVIKKSMVDHTSAFSTEELPPPVHLSKPGEHMDVYVPVACHPGYFVIQPWQEIHKLEVLMEEMILYYSVSEERHIAVEKDQVYAAKVENKWHRVLLKGILTNGLVSVYELDYGKHELVNIRKVQPLVDMFRKLPFQAVTAQLAGVKCNQWSEEASMVFRNHVEKKPLVALVQTVIENANPWDRKVVVYLVDTSLPDTDTWIHDFMSEYLIELSKVN

2 NP_002855.1 AAA50404.1

>NP_002855.1

MRKDRLLHLCLVLLLILLSASDSNSTEPQYMVLVPSLLHTEAPKKGCVLLSHLNETVTVSASLESGRENRSLFTDLVAEKDLFHCVSFTLPRISASSEVAFLSIQIKGPTQDFRKRNTVLVLNTQSLVFVQTDKPMYKPGQTVRFRVVSVDENFRPRNELIPLIYLENPRRNRIAQWQSLKLEAGINQLSFPLSSEPIQGSYRVVVQTESGGRIQHPFTVEEFVLPKFEVKVQVPKIISIMDEKVNITVCGEYTYGKPVPGLATVSLCRKLSRVLNCDKQEVCEEFSQQLNSNGCITQQVHTKMLQITNTGFEMKLRVEARIREEGTDLEVTANRISEITNIVSKLKFVKVDSHFRQGIPFFAQVLLVDGKGVPIPNKLFFISVNDANYYSNATTNEQGLAQFSINTTSISVNKLFVRVFTVHPNLCFHYSWVAEDHQGAQHTANRVFSLSGSYIHLEPVAGTLPCGHTETITAHYTLNRQAMGELSELSFHYLIMAKGVIVRSGTHTLPVESGDMKGSFALSFPVESDVAPIARMFIFAILPDGEVVGDSEKFEIENCLANKVDLSFSPAQSPPASHAHLQVAAAPQSLCALRAVDQSVLLMKPEAELSVSSVYNLLTVKDLTNFPDNVDQQEEEQGHCPRPFFIHNGAIYVPLSSNEADIYSFLKGMGLKVFTNSKIRKPKSCSVIPSVSAGAVGQGYYGAGLGVVERPYVPQLGTYNVIPLNNEQSSGPVPETVRSYFPETWIWELVAVNSSGVAEVGVTVPDTITEWKAGAFCLSEDAGLGISSTASLRAFQPFFVELTMPYSVIRGEVFTLKATVLNYLPKCIRVSVQLKASPAFLASQNTKGEESYCICGSERQTLSWTVTPKTLGNVNFSVSAEAMQSLELCGNEVVEVPEIKRKDTVIKTLLVEAEGIEQEKTFSSMTCASGANVSEQLSLKLPSNVVKESARASFSVLGDILGSAMQNIQNLLQMPYGCGEQNMVLFAPNIYVLNYLNETQQLTQEIKAKAVGYLITGYQRQLNYKHQDGSYSTFGERYGRNQGNTWLTAFVLKTFAQARSYIFIDEAHITQSLTWLSQMQKDNGCFRSSGSLLNNAIKGGVEDEATLSAYVTIALLEIPLPVTNPIVRNALFCLESAWNVAKEGTHGSHVYTKALLAYAFSLLGKQNQNREILNSLDKEAVKEDNLVHWERPQRPKAPVGHLYQTQAPSAEVEMTSYVLLAYLTAQPAPTSGDLTSATNIVKWIMKQQNAQGGFSSTQDTVVALHALSRYGAATFTRTEKTAQVTVQDSQTFSTNFQVDNNNLLLLQQISLPELPGEYVITVTGERCVYLQTSMKYNILPEKEDSPFALKVQTVPQTCDGHKAHTSFQISLTISYTGNRPASNMVIVDVKMVSGFIPLKPTVKMLERSSSVSRTEVSNNHVLIYVEQVTNQTLSFSFMVLQDIPVGDLKPAIVKVYDYYETDESVVAEYIAPCSTDTEHGNV

>AAA50404.1

MHYCVLSAFLILHLVTVALSLSTCSTLDMDQFMRKRIEAIRGQILSKLKLTSPPEDYPEPEEVPPEVISIYNSTRDLLQEKASRRAAACERERSDEEYYAKEVYKIDMPPFFPSETVCPVVTTPSGSVGSLCSRQSQVLCGYLDAIPPTFYRPYFRIVRFDVSAMEKNASNLVKAEFRVFRLQNPKARVPEQRIELYQILKSKDLTSPTQRYIDSKVVKTRAEGEWLSFDVTDAVHEWLHHKDRNLGFKISLHCPCCTFVPSNNYIIPNKSEELEARFAGIDGTSTYTSGDQKTIKSTRKKNSGKTPHLLLMLLPSYRLESQQTNRRKKRALDAAYCFRNVQDNCCLRPLYIDFKRDLGWKWIHEPKGYNANFCAGACPYLWSSDTQHSRVLSLYNTINPEASASPCCVSQDLEPLTILYYIGKTPKIEQLSNMIVKSCKCS

3 NP_004636.1 NP_004851.1

>NP_004636.1

MAASETVRLRLQFDYPPPATPHCTAFWLLVDLNRCRVVTDLISLIRQRFGFSSGAFLGLYLEGGLLPPAESARLVRDNDCLRVKLEERGVAENSVVISNGDINLSLRKAKKRAFQLEEGEETEPDCKYSKKHWKSRENNNNNEKVLDLEPKAVTDQTVSKKNKRKNKATCGTVGDDNEEAKRKSPKKKEKCEYKKKAKNPKSPKVQAVKDWANQRCSSPKGSARNSLVKAKRKGSVSVCSKESPSSSSESESCDESISDGPSKVTLEARNSSEKLPTELSKEEPSTKNTTADKLAIKLGFSLTPSKGKTSGTTSSSSDSSAESDDQCLMSSSTPECAAGFLKTVGLFAGRGRPGPGLSSQTAGAAGWRRSGSNGGGQAPGASPSVSLPASLGRGWGREENLFSWKGAKGRGMRGRGRGRGHPVSCVVNRSTDNQRQQQLNDVVKNSSTIIQNPVETPKKDYSLLPLLAAAPQVGEKIAFKLLELTSSYSPDVSDYKEGRILSHNPETQQVDIEILSSLPALREPGKFDLVYHNENGAEVVEYAVTQESKITVFWKELIDPRLIIESPSNTSSTEPA

>NP_004851.1

MGGLASGGDVEPGLPVEVRGSNGAFYKGFVKDVHEDSVTIFFENNWQSERQIPFGDVRLPPPADYNKEITEGDEVEVYSRANEQEPCGWWLARVRMMKGDFYVIEYAACDATYNEIVTLERLRPVNPNPLATKGSFFKVTMAVPEDLREACSNENVHKEFKKALGANCIFLNITNSELFILSTTEAPVKRASLLGDMHFRSLRTKLLLMSRNEEATKHLETSKQLAAAFQEEFTVREDLMGLAIGTHGANIQQARKVPGVTAIELGEETCTFRIYGETPEACRQARSYLEFSEDSVQVPRNLVGKVIGKNGKVIQEIVDKSGVVRVRVEGDNDKKNPREEGMVPFIFVGTRENISNAQALLEYHLSYLQEVEQLRLERLQIDEQLRQIGLGFRPPGSGRGSGGSDKAGYSTDESSSSSLHATRTYGGSYGGRGRGRRTGGPAYGPSSDVSTASETESEKREEPNRAGPGDRDPPTRGEESRRRPTGGRGRGPPPAPRPTSRYNSSSISSVLKDPDSNPYSLLDTSEPEPPVDSEPGEPPPASARRRRSRRRRTDEDRTVMDGGLESDGPNMTENGLEDESRPQRRNRSRRRRNRGNRTDGSISGDRQPVTVADYISRAESQSRQSAPLERTKPSEDSLSGQKGDSVSKLPKGPSENGELSAPLELGSMVNGVS

4 NP_478126.1 NP_002784.1

>NP_478126.1

MPGDHRRIRGPEESQPPQLYAADEEEAPGTRDPTRLRPVYARAGLLSQAKGSAYLEAGGTKVLCAVSGPRQAEGGERGGGPAGAGGEAPAALRGRLLCDFRRAPFAGRRRRAPPGGCEERELALALQEALEPAVRLGRYPRAQLEVSALLLEDGGSALAAALTAAALALADAGVEMYDLVVGCGLSLAPGPAPTWLLDPTRLEEERAAAGLTVALMPVLNQVAGLLGSGEGGLTESWAEAVRLGLEGCQRLYPVLQQSLVRAARRRGAAAQP

>NP_002784.1

MLSSTAMYSAPGRDLGMEPHRAAGPLQLRFSPYVFNGGTILAIAGEDFAIVASDTRLSEGFSIHTRDSPKCYKLTDKTVIGCSGFHGDCLTLTKIIEARLKMYKHSNNKAMTTGAIAAMLSTILYSRRFFPYYVYNIIGGLDEEGKGAVYSFDPVGSYQRDSFKAGGSASAMLQPLLDNQVGFKNMQNVEHVPLSLDRAMRLVKDVFISAAERDVYTGDALRICIVTKEGIREETVSLRKD

5 NP_066921.2 NP_001014797.1

>NP_066921.2

MTEGARAADEVRVPLGAPPPGPAALVGASPESPGAPGREAERGSELGVSPSESPAAERGAELGADEEQRVPYPALAATVFFCLGQTTRPRSWCLRLVCNPWFEHVSMLVIMLNCVTLGMFRPCEDVECGSERCNILEAFDAFIFAFFAVEMVIKMVALGLFGQKCYLGDTWNRLDFFIVVAGMMEYSLDGHNVSLSAIRTVRVLRPLRAINRVPSMRILVTLLLDTLPMLGNVLLLCFFVFFIFGIVGVQLWAGLLRNRCFLDSAFVRNNNLTFLRPYYQTEEGEENPFICSSRRDNGMQKCSHIPGRRELRMPCTLGWEAYTQPQAEGVGAARNACINWNQYYNVCRSGDSNPHNGAINFDNIGYAWIAIFQVITLEGWVDIMYYVMDAHSFYNFIYFILLIIVGSFFMINLCLVVIATQFSETKQRESQLMREQRARHLSNDSTLASFSEPGSCYEELLKYVGHIFRKVKRRSLRLYARWQSRWRKKVDPSAVQGQGPGHRQRRAGRHTASVHHLVYHHHHHHHHHYHFSHGSPRRPGPEPGACDTRLVRAGAPPSPPSPGRGPPDAESVHSIYHADCHIEGPQERARVAHAAATAAASLRLATGLGTMNYPTILPSGVGSGKGSTSPGPKGKWAGGPPGTGGHGPLSLNSPDPYEKIPHVVGEHGLGQAPGHLSGLSVPCPLPSPPAGTLTCELKSCPYCTRALEDPEGELSGSESGDSDGRGVYEFTQDVRHGDRWDPTRPPRATDTPGPGPGSPQRRAQQRAAPGEPGWMGRLWVTFSGKLRRIVDSKYFSRGIMMAILVNTLSMGVEYHEQPEELTNALEISNIVFTSMFALEMLLKLLACGPLGYIRNPYNIFDGIIVVISVWEIVGQADGGLSVLRTFRLLRVLKLVRFLPALRRQLVVLVKTMDNVATFCTLLMLFIFIFSILGMHLFGCKFSLKTDTGDTVPDRKNFDSLLWAIVTVFQILTQEDWNVVLYNGMASTSSWAALYFVALMTFGNYVLFNLLVAILVEGFQAEGDANRSDTDEDKTSVHFEEDFHKLRELQTTELKMCSLAVTPNGHLEGRGSLSPPLIMCTAATPMPTPKSSPFLDAAPSLPDSRRGSSSSGDPPLGDQKPPASLRSSPCAPWGPSGAWSSRRSSWSSLGRAPSLKRRGQCGERESLLSGEGKGSTDDEAEDGRAAPGPRATPLRRAESLDPRPLRPAALPPTKCRDRDGQVVALPSDFFLRIDSHREDAAELDDDSEDSCCLRLHKVLEPYKPQWCRSREAWALYLFSPQNRFRVSCQKVITHKMFDHVVLVFIFLNCVTIALERPDIDPGSTERVFLSVSNYIFTAIFVAEMMVKVVALGLLSGEHAYLQSSWNLLDGLLVLVSLVDIVVAMASAGGAKILGVLRVLRLLRTLRPLRVISRAPGLKLVVETLISSLRPIGNIVLICCAFFIIFGILGVQLFKGKFYYCEGPDTRNISTKAQCRAAHYRWVRRKYNFDNLGQALMSLFVLSSKDGWVNIMYDGLDAVGVDQQPVQNHNPWMLLYFISFLLIVSFFVLNMFVGVVVENFHKCRQHQEAEEARRREEKRLRRLERRRRSTFPSPEAQRRPYYADYSPTRRSIHSLCTSHYLDLFITFIICVNVITMSMEHYNQPKSLDEALKYCNYVFTIVFVFEAALKLVAFGFRRFFKDRWNQLDLAIVLLSLMGITLEEIEMSAALPINPTIIRIMRVLRIARVLKLLKMATGMRALLDTVVQALPQVGNLGLLFMLLFFIYAALGVELFGRLECSEDNPCEGLSRHATFSNFGMAFLTLFRVSTGDNWNGIMKDTLRECSREDKHCLSYLPALSPVYFVTFVLVAQFVLVNVVVAVLMKHLEESNKEAREDAELDAEIELEMAQGPGSARRVDADRPPLPQESPGARDAPNLVARKVSVSRMLSLPNDSYMFRPVVPASAPHPRPLQEVEMETYGAGTPLGSVASVHSPPAESCASLQIPLAVSSPARSGEPLHALSPRGTARSPSLSRLLCRQEAVHTDSLEGKIDSPRDTLDPAEPGEKTPVRPVTQGGSLQSPPRSPRPASVRTRKHTFGQRCVSSRPAAPGGEEAEASDPADEEVSHITSSACPWQPTAEPHGPEASPVAGGERDLRRLYSVDAQGFLDKPGRADEQWRPSAELGSGEPGEAKAWGPEAEPALGARRKKKMSPPCISVEPPAEDEGSARPSAAEGGSTTLRRRTPSCEATPHRDSLEPTEGSGAGGDPAAKGERWGQASCRAEHLTVPSFAFEPLDLGVPSGDPFLDGSHSVTPESRASSSGAIVPLEPPESEPPMPVGDPPEKRRGLYLTVPQCPLEKPGSPSATPAPGGGADDPV

>NP_001014797.1

MANGGGGGGGSSGGGGGGGGSSLRMSSNIHANHLSLDASSSSSSSSSSSSSSSSSSSSSSVHEPKMDALIIPVTMEVPCDSRGQRMWWAFLASSMVTFFGGLFIILLWRTLKYLWTVCCHCGGKTKEAQKINNGSSQADGTLKPVDEKEEAVAAEVGWMTSVKDWAGVMISAQTLTGRVLVVLVFALSIGALVIYFIDSSNPIESCQNFYKDFTLQIDMAFNVFFLLYFGLRFIAANDKLWFWLEVNSVVDFFTVPPVFVSVYLNRSWLGLRFLRALRLIQFSEILQFLNILKTSNSIKLVNLLSIFISTWLTAAGFIHLVENSGDPWENFQNNQALTYWECVYLLMVTMSTVGYGDVYAKTTLGRLFMVFFILGGLAMFASYVPEIIELIGNRKKYGGSYSAVSGRKHIVVCGHITLESVSNFLKDFLHKDRDDVNVEIVFLHNISPNLELEALFKRHFTQVEFYQGSVLNPHDLARVKIESADACLILANKYCADPDAEDASNIMRVISIKNYHPKIRIITQMLQYHNKAHLLNIPSWNWKEGDDAICLAELKLGFIAQSCLAQGLSTMLANLFSMRSFIKIEEDTWQKYYLEGVSNEMYTEYLSSAFVGLSFPTVCELCFVKLKLLMIAIEYKSANRESRSRKRILINPGNHLKIQEGTLGFFIASDAKEVKRAFFYCKACHDDITDPKRIKKCGCKRLEDEQPSTLSPKKKQRNGGMRNSPNTSPKLMRHDPLLIPGNDQIDNMDSNVKKYDSTGMFHWCAPKEIEKVILTRSEAAMTVLSGHVVVCIFGDVSSALIGLRNLVMPLRASNFHYHELKHIVFVGSIEYLKREWETLHNFPKVSILPGTPLSRADLRAVNINLCDMCVILSANQNNIDDTSLQDKECILASLNIKSMQFDDSIGVLQANSQGFTPPGMDRSSPDNSPVHGMLRQPSITTGVNIPIITELVNDTNVQFLDQDDDDDPDTELYLTQPFACGTAFAVSVLDSLMSATYFNDNILTLIRTLVTGGATPELEALIAEENALRGGYSTPQTLANRDRCRVAQLALLDGPFADLGDGGCYGDLFCKALKTYNMLCFGIYRLRDAHLSTPSQCTKRYVITNPPYEFELVPTDLIFCLMQFDHNAGQSRASLSHSSHSSQSSSKKSSSVHSIPSTANRQNRPKSRESRDKQNRKEMVYR

6 NP_001886.1 NP_932070.1

>NP_001886.1

MSGPVPSRARVYTDVNTHRPREYWDYESHVVEWGNQDDYQLVRKLGRGKYSEVFEAINITNNEKVVVKILKPVKKKKIKREIKILENLRGGPNIITLADIVKDPVSRTPALVFEHVNNTDFKQLYQTLTDYDIRFYMYEILKALDYCHSMGIMHRDVKPHNVMIDHEHRKLRLIDWGLAEFYHPGQEYNVRVASRYFKGPELLVDYQMYDYSLDMWSLGCMLASMIFRKEPFFHGHDNYDQLVRIAKVLGTEDLYDYIDKYNIELDPRFNDILGRHSRKRWERFVHSENQHLVSPEALDFLDKLLRYDHQSRLTAREAMEHPYFYTVVKDQARMGSSSMPGGSTPVSSANMMSGISSVPTPSPLGPLAGSPVIAAANPLGMPVPAAAGAQQ

>NP_932070.1

MCSGAGVMMARWAARGRAGWRSTVRILSPLGHCEPGVSRSCRAAQAMDCEVNNGSSLRDECITNLLVFGFLQSCSDNSFRRELDALGHELPVLAPQWEGYDELQTDGNRSSHSRLGRIEADSESQEDIIRNIARHLAQVGDSMDRSIPPGLVNGLALQLRNTSRSEEDRNRDLATALEQLLQAYPRDMEKEKTMLVLALLLAKKVASHTPSLLRDVFHTTVNFINQNLRTYVRSLARNGMD

7 NP_892117.1 NP_006618.1

>NP_892117.1

MAAAPQAPGRGSLRKTRPLVVKTSLNNPYIIRWSALESEDMHFILQTLEDRLKAIGLQKIEDKKKKNKTPFLKKESREKCSIAVDISENLKEKKTDAKQQVSGWTPAHVRKQLAIGVNEVTRALERRELLLVLVCKSVKPAMITSHLIQLSLSRSVPACQVPRLSERIAPVIGLKCVLALAFKKNTTDFVDEVRAIIPRVPSLSVPWLQDRIEDSGENLETEPLESQDRELLDTSFEDLSKPKRKLADGRQASVTLQPLKIKKLIPNPNKIRKPPKSKKATPK

>NP_006618.1

MKSVIYHALSQKEANDSDVQPSGAQRAEAFVRAFLKRSTPRMSPQAREDQLQRKAVVLEYFTRHKRKEKKKKAKGLSARQRRELRLFDIKPEQQRYSLFLPLHELWKQYIRDLCSGLKPDTQPQMIQAKLLKADLHGAIISVTKSKCPSYVGITGILLQETKHIFKIITKEDRLKVIPKLNCVFTVETDGFISYIYGSKFQLRSSERSAKKFKAKGTIDL

8 NP_006752.1 NP_068660.1

>NP_006752.1

MDDREDLVYQAKLAEQAERYDEMVESMKKVAGMDVELTVEERNLLSVAYKNVIGARRASWRIISSIEQKEENKGGEDKLKMIREYRQMVETELKLICCDILDVLDKHLIPAANTGESKVFYYKMKGDYHRYLAEFATGNDRKEAAENSLVAYKAASDIAMTELPPTHPIRLGLALNFSVFYYEILNSPDRACRLAKAAFDDAIAELDTLSEESYKDSTLIMQLLRDNLTLWTSDMQGDGEEQNKEALQDVEDENQ

>NP_068660.1

MEVPQPEPAPGSALSPAGVCGGAQRPGHLPGLLLGSHGLLGSPVRAAASSPVTTLTQTMHDLAGLGSRSRLTHLSLSRRASESSLSSESSESSDAGLCMDSPSPMDPHMAEQTFEQAIQAASRIIRNEQFAIRRFQSMPVRLLGHSPVLRNITNSQAPDGRRKSEAGSGAASSSGEDKENVRFWKAGVGALREEEGACWGGSLACEDPPLPSWLQDGFVFKMPWKPTHPSSTHALAEWASRREAFAQRPSSAPDLMCLSPDRKMEVEELSPLALGRFSLTPAEGDTEEDDGFVDILESDLKDDDAVPPGMESLISAPLVKTLEKEEEKDLVMYSKCQRLFRSPSMPCSVIRPILKRLERPQDRDTPVQNKRRRSVTPPEEQQEAEEPKARVLRSKSLCHDEIENLLDSDHRELIGDYSKAFLLQTVDGKHQDLKYISPETMVALLTGKFSNIVDKFVIVDCRYPYEYEGGHIKTAVNLPLERDAESFLLKSPIAPCSLDKRVILIFHCEFSSERGPRMCRFIRERDRAVNDYPSLYYPEMYILKGGYKEFFPQHPNFCEPQDYRPMNHEAFKDELKTFRLKTRSWAGERSRRELCSRLQDQ

9 NP_005569.1 NP_542159.2

>NP_005569.1

MSHPSWLPPKSTGEPLGHVPARMETTHSFGNPSISVSTQQPPKKFAPVVAPKPKYNPYKQPGGEGDFLPPPPPPLDDSSALPSISGNFPPPPPLDEEAFKVQGNPGGKTLEERRSSLDAEIDSLTSILADLECSSPYKPRPPQSSTGSTASPPVSTPVTGHKRMVIPNQPPLTATKKSTLKPQPAPQAGPIPVAPIGTLKPQPQPVPASYTTASTSSRPTFNVQVKSAQPSPHYMAAPSSGQIYGSGPQGYNTQPVPVSGQCPPPSTRGGMDYAYIPPPGLQPEPGYGYAPNQGRYYEGYYAAGPGYGGRNDSDPTYGQQGHPNTWKREPGYTPPGAGNQNPPGMYPVTGPKKTYITDPVSAPCAPPLQPKGGHSGQLGPSSVAPSFRPEDELEHLTKKMLYDMENPPADEYFGRCARCGENVVGEGTGCTAMDQVFHVDCFTCIICNNKLRGQPFYAVEKKAYCEPCYINTLEQCNVCSKPIMERILRATGKAYHPHCFTCVMCHRSLDGIPFTVDAGGLIHCIEDFHKKFAPRCSVCKEPIMPAPGQEETVRIVALDRDFHVHCYRCEDCGGLLSEGDNQGCYPLDGHILCKTCNSARIRVLTAKASTDL

>NP_542159.2

MKTSAELHEQEKPPSSPRATGPGRLGHARGRGPDALRGGAAGPGRASSGAPRERKMAPHGPGSLTTLVPWAAALLLALGVERALALPEICTQCPGSVQNLSKVAFYCKTTRELMLHARCCLNQKGTILGLDLQNCSLEDPGPNFHQAHTTVIIDLQANPLKGDLANTFRGFTQLQTLILPQHVNCPGGINAWNTITSYIDNQICQGQKNLCNNTGDPEMCPENGSCVPDGPGLLQCVCADGFHGYKCMRQGSFSLLMFFGILGATTLSVSILLWATQRRKAKTS

10 NP_001005.1 NP_008998.1

>NP_001005.1

MLMPKKNRIAIYELLFKEGVMVAKKDVHMPKHPELADKNVPNLHVMKAMQSLKSRGYVKEQFAWRHFYWYLTNEGIQYLRDYLHLPPEIVPATLRRSRPETGRPRPKGLEGERPARLTRGEADRDTYRRSAVPPGADKKAEAGAGSATEFQFRGGFGRGRGQPPQ

>NP_008998.1

MPRRKRNAGSSSDGTEDSDFSTDLEHTDSSESDGTSRRSARVTRSSARLSQSSQDSSPVRNLQSFGTEEPAYSTRRVTRSQQQPTPVTPKKYPLRQTRSSGSETEQVVDFSDRETKNTADHDESPPRTPTGNAPSSESDIDISSPNVSHDESIAKDMSLKDSGSDLSHRPKRRRFHESYNFNMKCPTPGCNSLGHLTGKHERHFSISGCPLYHNLSADECKVRAQSRDKQIEERMLSHRQDDNNRHATRHQAPTERQLRYKEKVAELRKKRNSGLSKEQKEKYMEHRQTYGNTREPLLENLTSEYDLDLFRRAQARASEDLEKLRLQGQITEGSNMIKTIAFGRYELDTWYHSPYPEEYARLGRLYMCEFCLKYMKSQTILRRHMAKCVWKHPPGDEIYRKGSISVFEVDGKKNKIYCQNLCLLAKLFLDHKTLYYDVEPFLFYVMTEADNTGCHLIGYFSKEKNSFLNYNVSCILTMPQYMRQGYGKMLIDFSYLLSKVEEKVGSPERPLSDLGLISYRSYWKEVLLRYLHNFQGKEISIKEISQETAVNPVDIVSTLQALQMLKYWKGKHLVLKRQDLIDEWIAKEAKRSNSNKTMDPSCLKWTPPKGT

11 AAH32474.1 NP_055109.1

>AAH32474.1

MASSTSLPAPGSRPKKPLGKMADWFRQTLLKKPKKRPNSPESTSSDASQPTSQDSPLPPSLSSVTSPSLPPTHASDSGSSRWSKDYDVCVCHSEEDLVAAQDLVSYLEGSTASLRCFLQLRDATPGGAIVSELCQALSSSHCRVLLITPGFLQDPWCKYQMLQALTEAPGAEGCTIPLLSGLSRAAYPPELRFMYYVDGRGPDGGFRQVKEAVMRYLQTLSWHLLYHGTPEIGVKLETENPCRASDSHKCDKRYRE

>NP_055109.1

MAIRKKSTKSPPVLSHEFVLQNHADIVSCVAMVFLLGLMFEITAKASIIFVTLQYNVTLPATEEQATESVSLYYYGIKDLATVFFYMLVAIIIHAVIQEYMLDKINRRMHFSKTKHSKFNESGQLSAFYLFACVWGTFILISENYISDPTILWRAYPHNLMTFQMKFFYISQLAYWLHAFPELYFQKTKKEDIPRQLVYIGLYLFHIAGAYLLNLNHLGLVLLVLHYFVEFLFHISRLFYFSNEKYQKGFSLWAVLFVLGRLLTLILSVLTVGFGLARAENQKLDFSTGNFNVLAVRIAVLASICVTQAFMMWKFINFQLRRWREHSAFQAPAVKKKPTVTKGRSSKKGTENGVNGTLTSNVADSPRNKKEKSS

12 NP_003173.1 NP_060541.3

>NP_003173.1

MKILVALAVFFLVSTQLFAEEIGANDDLNYWSDWYDSDQIKEELPEPFEHLLQRIARRPKPQQFFGLMGKRDADSSIEKQVALLKALYGHGQISHKRHKTDSFVGLMGKRALNSVAYERSAMQNYERRR

>NP_060541.3

MEPEPVEDCVQSTLAALYPPFEATAPTLLGQVFQVVERTYREDALRYTLDFLVPAKHLLAKVQQEACAQYSGFLFFHEGWPLCLHEQVVVQLAALPWQLLRPGDFYLQVVPSAAQAPRLALKCLAPGGGRVQEVPVPNEACAYLFTPEWLQGINKDRPTGRLSTCLLSAPSGIQRLPWAELICPRFVHKEGLMVGHQPSTLPPELPSGPPGLPSPPLPEEALGTRSPGDGHNAPVEGPEGEYVELLEVTLPVRGSPTDAEGSPGLSRVRTVPTRKGAGGKGRHRRHRAWMHQKGLGPRGQDGARPPGEGSSTGASPESPPGAEAVPEAAVLEVSEPPAEAVGEASGSCPLRPGELRGGGGGGQGAEGPPGTPRRTGKGNRRKKRAAGRGALSRGGDSAPLSPGDKEDASHQEALGNLPSPSEHKLPECHLVKEEYEGSGKPESEPKELKTAGEKEPQLSEACGPTEEGAGERELEGPGLLCMAGHTGPEGPLSDTPTPPLETVQEGKGDNIPEEALAVSVSDHPDVAWDLMASGFLILTGGVDQSGRALLTITPPCPPEEPPPSRDTLNTTLHYLHSLLRPDLQTLGLSVLLDLRQAPPLPPALIPALSQLQDSGDPPLVQRLLILIHDDLPTELCGFQGAEVLSENDLKRVAKPEELQWELGGHRDPSPSHWVEIHQEVVRLCRLCQGVLGSVRQAIEELEGAAEPEEEEAVGMPKPLQKVLADPRLTALQRDGGAILMRLRSTPSSKLEGQGPATLYQEVDEAIHQLVRLSNLHVQQQEQRQCLRRLQQVLQWLSGPGEEQLASFAMPGDTLSALQETELRFRAFSAEVQERLAQAREALALEENATSQKVLDIFEQRLEQVESGLHRALRLQRFFQQAHEWVDEGFARLAGAGPGREAVLAALALRRAPEPSAGTFQEMRALALDLGSPAALREWGRCQARCQELERRIQQHVGEEASPRGYRRRRADGASSGGAQWGPRSPSPSLSSLLLPSSPGPRPAPSHCSLAPCGEDYEEEGPELAPEAEGRPPRAVLIRGLEVTSTEVVDRTCSPREHVLLGRARGPDGPWGVGTPRMERKRSISAQQRLVSELIACEQDYVATLSEPVPPPGPELTPELRGTWAAALSARERLRSFHRTHFLRELQGCATHPLRIGACFLRHGDQFSLYAQYVKHRHKLENGLAALSPLSKGSMEAGPYLPRALQQPLEQLTRYGRLLEELLREAGPELSSECRALGAAVQLLREQEARGRDLLAVEAVRGCEIDLKEQGQLLHRDPFTVICGRKKCLRHVFLFEHLLLFSKLKGPEGGSEMFVYKQAFKTADMGLTENIGDSGLCFELWFRRRRAREAYTLQATSPEIKLKWTSSIAQLLWRQAAHNKELRVQQMVSMGIGNKPFLDIKALGERTLSALLTGRAARTRASVAVSSFEHAGPSLPGLSPGACSLPARVEEEAWDLDVKQISLAPETLDSSGDVSPGPRNSPSLQPPHPGSSTPTLASRGILGLSRQSHARALSDPTTPL

13 NP_006592.3 NP_001017963.1

>NP_006592.3

MQPASAKWYDRRDYVFIEFCVEDSKDVNVNFEKSKLTFSCLGGSDNFKHLNEIDLFHCIDPNDSKHKRTDRSILCCLRKGESGQSWPRLTKERAKLNWLSVDFNNWKDWEDDSDEDMSNFDRFSEMMNNMGGDEDVDLPEVDGADDDSQDSDDEKMPDLE

>NP_001017963.1

MPPCSGGDGSTPPGPSLRDRDCPAQSAEYPRDRLDPRPGSPSEASSPPFLRSRAPVNWYQEKAQVFLWHLLVSGSTTLLCLWKQPFHVSAFPVTASLAFRQSQGAGQHLYKDLQPFILLRLLMPEETQTQDQPMEEEEVETFAFQAEIAQLMSLIINTFYSNKEIFLRELISNSSDALDKIRYESLTDPSKLDSGRELHINLIPNKQGRTLTIVDTGIGMTKADLINNLGTIAKSGTKAFMEALQAGADISMIGQFGVGFYSAYLVAEKVTVITKHNDDEQYAWESSAGGSFTVRTDTGEPMGRGTKVILHLKEDQTEYLEERRIKEIVKKHSQFIGYPITLFVEKERDKEVSDDEAEEKEDKEEEKEKEEKESEDKPEIEDVGSDEEEEKKDGDKKKKKKIKEKYIDQEELNKTKPIWTRNPDDITNEEYGEFYKSLTNDWEDHLAVKHFSVEGQLEFRALLFVPRRAPFDLFENRKKKNNIKLYVRRVFIMDNCEELIPEYLNFIRGVVDSEDLPLNISREMLQQSKILKVIRKNLVKKCLELFTELAEDKENYKKFYEQFSKNIKLGIHEDSQNRKKLSELLRYYTSASGDEMVSLKDYCTRMKENQKHIYYITGETKDQVANSAFVERLRKHGLEVIYMIEPIDEYCVQQLKEFEGKTLVSVTKEGLELPEDEEEKKKQEEKKTKFENLCKIMKDILEKKVEKVVVSNRLVTSPCCIVTSTYGWTANMERIMKAQALRDNSTMGYMAAKKHLEINPDHSIIETLRQKAEADKNDKSVKDLVILLYETALLSSGFSLEDPQTHANRIYRMIKLGLGIDEDDPTADDTSAAVTEEMPPLEGDDDTSRMEEVD

14 NP_000466.2 NP_004227.1

>NP_000466.2

MAGENHQWQGSILYNMLMSAKQTRAAPEAPETRLVDQCWGCSCGDEPGVGREGLLGGRNVALLYRCCFCGKDHPRQGSILYSMLTSAKQTYAAPKAPEATLGPCWGCSCGSDPGVGRAGLPGGRPVALLYRCCFCGEDHPRQGSILYSLLTSSKQTHVAPAAPEARPGGAWWDRSYFAQRPGGKEALPGGRATALLYRCCFCGEDHPQQGSTLYCVPTSTNQAQAAPEERPRAPWWDTSSGALRPVALKSPQVVCEAASAGLLKTLRFVKYLPCFQVLPLDQQLVLVRNCWASLLMLELAQDRLQFETVEVSEPSMLQKILTTRRRETGGNEPLPVPTLQHHLAPPAEARKVPSASQVQAIKCFLSKCWSLNISTKEYAYLKGTVLFNPDVPGLQCVKYIQGLQWGTQQILSEHTRMTHQGPHDRFIELNSTLFLLRFINANVIAELFFRPIIGTVSMDDMMLEMLCTKI

>NP_004227.1

MSDMEDDFMCDDEEDYDLEYSEDSNSEPNVDLENQYYNSKALKEDDPKAALSSFQKVLELEGEKGEWGFKALKQMIKINFKLTNFPEMMNRYKQLLTYIRSAVTRNYSEKSINSILDYISTSKQMDLLQEFYETTLEALKDAKNDRLWFKTNTKLGKLYLEREEYGKLQKILRQLHQSCQTDDGEDDLKKGTQLLEIYALEIQMYTAQKNNKKLKALYEQSLHIKSAIPHPLIMGVIRECGGKMHLREGEFEKAHTDFFEAFKNYDESGSPRRTTCLKYLVLANMLMKSGINPFDSQEAKPYKNDPEILAMTNLVSAYQNNDITEFEKILKTNHSNIMDDPFIREHIEELLRNIRTQVLIKLIKPYTRIHIPFISKELNIDVADVESLLVQCILDNTIHGRIDQVNQLLELDHQKRGGARYTALDKWTNQLNSLNQAVVSKLA

15 NP_060848.2 NP_733779.1

>NP_060848.2

MAEPGEGLPEEVLALIFRHLSLRDRAAAARVCRAWAAAATCSAVWHDTKISCECELEGMLPPYLSACLDHIHNLRLEFEPSRKPSRRAAIELLMVLAGRAPGLRGLRLECRGEKPLFDAGRDVLEAVHAVCGAASQLRHLDLRRLSFTLDDALVLQAARSCPELHSLFLDNSTLVGSVGPGSVLELLEACPRLRALGLHLASLSHAILEALAAPDRAPFALLALRCACPEDARASPLPNEAWVALRRRHPGLAVELELEPALPAESVTRVLQPAVPVAALRLNLSGDTVGPVRFAAHHYAATLCALEVRAAASAELNAALEELAARCAALREVHCFCVVSHSVLDAFRAHCPRLRTYTLKLTREPHPWRPTLVA

>NP_733779.1

MPSIKLQSSDGEIFEVDVEIAKQSVTIKTMLEDLGMDDEGDDDPVPLPNVNAAILKKVIQWCTHHKDDPPPPEDDENKEKRTDDIPVWDQEFLKVDQGTLFELILAANYLDIKGLLDVTCKTVANMIKGKTPEEIRKTFNIKNDFTEEEEAQVRKENQWCEEK

16 NP_006218.1 NP_000032.1

>NP_006218.1

MALFGALFLALLAGAHAEFPGCKIRVTSKALELVKQEGLRFLEQELETITIPDLRGKEGHFYYNISEVKVTELQLTSSELDFQPQQELMLQITNASLGLRFRRQLLYWFFYDGGYINASAEGVSIRTGLELSRDPAGRMKVSNVSCQASVSRMHAAFGGTFKKVYDFLSTFITSGMRFLLNQQICPVLYHAGTVLLNSLLDTVPVRSSVDELVGIDYSLMKDPVASTSNLDMDFRGAFFPLTERNWSLPNRAVEPQLQEEERMVYVAFSEFFFDSAMESYFRAGALQLLLVGDKVPHDLDMLLRATYFGSIVLLSPAVIDSPLKLELRVLAPPRCTIKPSGTTISVTASVTIALVPPDQPEVQLSSMTMDARLSAKMALRGKALRTQLDLRRFRIYSNHSALESLALIPLQAPLKTMLQIGVMPMLNERTWRGVQIPLPEGINFVHEVVTNHAGFLTIGADLHFAKGLREVIEKNRPADVRASTAPTPSTAAV

>NP_000032.1

MKVLWAALLVTFLAGCQAKVEQAVETEPEPELRQQTEWQSGQRWELALGRFWDYLRWVQTLSEQVQEELLSSQVTQELRALMDETMKELKAYKSELEEQLTPVAEETRARLSKELQAAQARLGADMEDVCGRLVQYRGEVQAMLGQSTEELRVRLASHLRKLRKRLLRDADDLQKRLAVYQAGAREGAERGLSAIRERLGPLVEQGRVRAATVGSLAGQPLQERAQAWGERLRARMEEMGSRTRDRLDEVKEQVAEVRAKLEEQAQQIRLQAEAFQARLKSWFEPLVEDMQRQWAGLVEKVQAAVGTSAAPVPSDNH

17 NP_001020272.1 NP_000323.2

>NP_001020272.1

MGNHAGKRELNAEKASTNSETNRGESEKKRNLGELSRTTSEDNEVFGEADANQNNGTSSQDTAVTDSKRTADPKNAWQDAHPADPGSRPHLIRLFSRDAPGREDNTFKDRPSESDELQTIQEDSAATSESLDVMASQKRPSQRHGSKYLATASTMDHARHGFLPRHRDTGILDSIGRFFGGDRGAPKRGSGKDSHHPARTAHYGSLPQKSHGRTQDENPVVHFFKNIVTPRTPPPSQGKGRGLSLSRFSWGAEGQRPGFGYGGRASDYKSAHKGFKGVDAQGTLSKIFKLGGRDSRSGSPMARR

>NP_000323.2

MKSNQERSNECLPPKKREIPATSRSSEEKAPTLPSDNHRVEGTAWLPGNPGGRGHGGGRHGPAGTSVELGLQQGIGLHKALSTGLDYSPPSAPRSVPVATTLPAAYATPQPGTPVSPVQYAHLPHTFQFIGSSQYSGTYASFIPSQLIPPTANPVTSAVASAAGATTPSQRSQLEAYSTLLANMGSLSQTPGHKAEQQQQQQQQQQQQHQHQQQQQQQQQQQQQQHLSRAPGLITPGSPPPAQQNQYVHISSSPQNTGRTASPPAIPVHLHPHQTMIPHTLTLGPPSQVVMQYADSGSHFVPREATKKAESSRLQQAIQAKEVLNGEMEKSRRYGAPSSADLGLGKAGGKSVPHPYESRHVVVHPSPSDYSSRDPSGVRASVMVLPNSNTPAADLEVQQATHREASPSTLNDKSGLHLGKPGHRSYALSPHTVIQTTHSASEPLPVGLPATAFYAGTQPPVIGYLSGQQQAITYAGSLPQHLVIPGTQPLLIPVGSTDMEASGAAPAIVTSSPQFAAVPHTFVTTALPKSENFNPEALVTQAAYPAMVQAQIHLPVVQSVASPAAAPPTLPPYFMKGSIIQLANGELKKVEDLKTEDFIQSAEISNDLKIDSSTVERIEDSHSPGVAVIQFAVGEHRAQVSVEVLVEYPFFVFGQGWSSCCPERTSQLFDLPCSKLSVGDVCISLTLKNLKNGSVKKGQPVDPASVLLKHSKADGLAGSRHRYAEQENGINQGSAQMLSENGELKFPEKMGLPAAPFLTKIEPSKPAATRKRRWSAPESRKLEKSEDEPPLTLPKPSLIPQEVKICIEGRSNVGK

18 NP_057368.3 NP_055330.1

>NP_057368.3

MNLDSLSLALSQISYLVDNLTKKNYRASQQEIQHIVNRHGPEADRHLLRCLFSHVDFSGDGKSSGKDFHQTQFLIQECALLITKPNFISTLSYAIDNPLHYQKSLKPAPHLFAQLSKVLKLSKVQEVIFGLALLNSSSSDLRGFAAQFIKQKLPDLLRSYIDADVSGNQEGGFQDIAIEVLHLLLSHLLFGQKGAFGVGQEQIDAFLKTLRRDFPQERCPVVLAPLLYPEKRDILMDRILPDSGGVAKTMMESSLADFMQEVGYGFCASIEECRNIIVQFGVREVTAAQVARVLGMMARTHSGLTDGIPLQSISAPGSGIWSDGKDKSDGAQAHTWNVEVLIDVLKELNPSLNFKEVTYELDHPGFQIRDSKGLHNVVYGIQRGLGMEVFPVDLIYRPWKHAEGQLSFIQHSLINPEIFCFADYPCHTVATDILKAPPEDDNREIATWKSLDLIESLLRLAEVGQYEQVKQLFSFPIKHCPDMLVLALLQINTSWHTLRHELISTLMPIFLGNHPNSAIILHYAWHGQGQSPSIRQLIMHAMAEWYMRGEQYDQAKLSRILDVAQDLKALSMLLNGTPFAFVIDLAALASRREYLKLDKWLTDKIREHGEPFIQACMTFLKRRCPSILGGLAPEKDQPKSAQLPPETLATMLACLQACAGSVSQELSETILTMVANCSNVMNKARQPPPGVMPKGRPPSASSLDAISPVQIDPLAGMTSLSIGGSAAPHTQSMQGFPPNLGSAFSTPQSPAKAFPPLSTPNQTTAFSGIGGLSSQLPVGGLGTGSLTGIGTGALGLPAVNNDPFVQRKLGTSGLNQPTFQQSKMKPSDLSQVWPEANQHFSKEIDDEANSYFQRIYNHPPHPTMSVDEVLEMLQRFKDSTIKREREVFNCMLRNLFEEYRFFPQYPDKELHITACLFGGIIEKGLVTYMALGLALRYVLEALRKPFGSKMYYFGIAALDRFKNRLKDYPQYCQHLASISHFMQFPHHLQEYIEYGQQSRDPPVKMQGSITTPGSIALAQAQAQAQVPAKAPLAGQVSTMVTTSTTTTVAKTVTVTRPTGVSFKKDVPPSINTTNIDTLLVATDQTERIVEPPENIQEKIAFIFNNLSQSNMTQKVEELKETVKEEFMPWVSQYLVMKRVSIEPNFHSLYSNFLDTLKNPEFNKMVLNETYRNIKVLLTSDKAAANFSDRSLLKNLGHWLGMITLAKNKPILHTDLDVKSLLLEAYVKGQQELLYVVPFVAKVLESSIRSVVFRPPNPWTMAIMNVLAELHQEHDLKLNLKFEIEVLCKNLALDINELKPGNLLKDKDRLKNLDEQLSAPKKDVKQPEELPPITTTTTSTTPATNTTCTATVPPQPQYSYHDINVYSLAGLAPHITLNPTIPLFQAHPQLKQCVRQAIERAVQELVHPVVDRSIKIAMTTCEQIVRKDFALDSEESRMRIAAHHMMRNLTAGMAMITCREPLLMSISTNLKNSFASALRTASPQQREMMDQAAAQLAQDNCELACCFIQKTAVEKAGPEMDKRLATEFELRKHARQEGRRYCDPVVLTYQAERMPEQIRLKVGGVDPKQLAVYEEFARNVPGFLPTNDLSQPTGFLAQPMKQAWATDDVAQIYDKCITELEQHLHAIPPTLAMNPQAQALRSLLEVVVLSRNSRDAIAALGLLQKAVEGLLDATSGADADLLLRYRECHLLVLKALQDGRAYGSPWCNKQITRCLIECRDEYKYNVEAVELLIRNHLVNMQQYDLHLAQSMENGLNYMAVAFAMQLVKILLVDERSVAHVTEADLFHTIETLMRINAHSRGNAPEGLPQLMEVVRSNYEAMIDRAHGGPNFMMHSGISQASEYDDPPGLREKAEYLLREWVNLYHSAAAGRDSTKAFSAFVGQMHQQGILKTDDLITRFFRLCTEMCVEISYRAQAEQQHNPAANPTMIRAKCYHNLDAFVRLIALLVKHSGEATNTVTKINLLNKVLGIVVGVLLQDHDVRQSEFQQLPYHRIFIMLLLELNAPEHVLETINFQTLTAFCNTFHILRPTKAPGFVYAWLELISHRIFIARMLAHTPQQKGWPMYAQLLIDLFKYLAPFLRNVELTKPMQILYKGTLRVLLVLLHDFPEFLCDYHYGFCDVIPPNCIQLRNLILSAFPRNMRLPDPFTPNLKVDMLSEINIAPRILTNFTGVMPPQFKKDLDSYLKTRSPVTFLSDLRSNLQVSNEPGNRYNLQLINALVLYVGTQAIAHIHNKGSTPSMSTITHSAHMDIFQNLAVDLDTEGRYLFLNAIANQLRYPNSHTHYFSCTMLYLFAEANTEAIQEQITRVLLERLIVNRPHPWGLLITFIELIKNPAFKFWNHEFVHCAPEIEKLFQSVAQCCMGQKQAQQVMEGTGAS

>NP_055330.1

MVRTDGHTLSEKRNYQVTNSMFGASRKKFVEGVDSDYHDENMYYSQSSMFPHRSEKDMLASPSTSGQLSQFGASLYGQQSALGLPMRGMSNNTPQLNRSLSQGTQLPSHVTPTTGVPTMSLHTPPSPSRGILPMNPRNMMNHSQVGQGIGIPSRTNSMSSSGLGSPNRSSPSIICMPKQQPSRQPFTVNSMSGFGMNRNQAFGMNNSLSSNIFNGTDGSENVTGLDLSDFPALADRNRREGSGNPTPLINPLAGRAPYVGMVTKPANEQSQDFSIHNEDFPALPGSSYKDPTSSNDDSKSNLNTSGKTTSSTDGPKFPGDKSSTTQNNNQQKKGIQVLPDGRVTNIPQGMVTDQFGMIGLLTFIRAAETDPGMVHLALGSDLTTLGLNLNSPENLYPKFASPWASSPCRPQDIDFHVPSEYLTNIHIRDKLAAIKLGRYGEDLLFYLYYMNGGDVLQLLAAVELFNRDWRYHKEERVWITRAPGMEPTMKTNTYERGTYYFFDCLNWRKVAKEFHLEYDKLEERPHLPSTFNYNPAQQAF

19 NP_079115.1 NP_006404.1

>NP_079115.1

MAGPVKDREAFQRLNFLYQAAHCVLAQDPENQALARFYCYTERTIAKRLVLRRDPSVKRTLCRGCSSLLVPGLTCTQRQRRCRGQRWTVQTCLTCQRSQRFLNDPGHLLWGDRPEAQLGSQADSKPLQPLPNTAHSISDRLPEEKMQTQGSSNQ

>NP_006404.1

MAVFADLDLRAGSDLKALRGLVETAAHLGYSVVAINHIVDFKEKKQEIEKPVAVSELFTTLPIVQGKSRPIKILTRLTIIVSDPSHCNVLRATSSRARLYDVVAVFPKTEKLFHIACTHLDVDLVCITVTEKLPFYFKRPPINVAIDRGLAFELVYSPAIKDSTMRRYTISSALNLMQICKGKNVIISSAAERPLEIRGPYDVANLGLLFGLSESDAKAAVSTNCRAALLHGETRKTAFGIISTVKKPRPSEGDEDCLPASKKAKCEG

20 NP_004171.2 NP_079355.1

>NP_004171.2

MDKNIGEQLNKAYEAFRQACMDRDSAVKELQQKTENYEQRIREQQEQLSLQQTIIDKLKSQLLLVNSTQDNNYGCVPLLEDSETRKNNLTLDQPQDKVISGIAREKLPKVRRQEVSSPRKETSARSLGSPLLHERGNIEKTFWDLKEEFHKICMLAKAQKDHLSKLNIPDTATETQCSVPIQCTDKTDKQEALFKPQAKDDINRGAPSITSVTPRGLCRDEEDTSFESLSKFNVKFPPMDNDSTFLHSTPERPGILSPATSEAVCQEKFNMEFRDNPGNFVKTEETLFEIQGIDPIASAIQNLKTTDKTKPSNLVNTCIRTTLDRAACLPPGDHNALYVNSFPLLDPSDAPFPSLDSPGKAIRGPQQPIWKPFPNQDSDSVVLSGTDSELHIPRVCEFCQAVFPPSITSRGDFLRHLNSHFNGET

>NP_079355.1

MSGPCGEKPVLEASPTMSLWEFEDSHSRQGTPRPGQELAAEEASALELQMKVDFFRKLGYSSTEIHSVLQKLGVQADTNTVLGELVKHGTATERERQTSPDPCPQLPLVPRGGGTPKAPNLEPPLPEEEKEGSDLRPVVIDGSNVAMSHGNKEVFSCRGILLAVNWFLERGHTDITVFVPSWRKEQPRPDVPITDQHILRELEKKKILVFTPSRRVGGKRVVCYDDRFIVKLAYESDGIVVSNDTYRDLQGERQEWKRFIEERLLMYSFVNDKFMPPDDPLGRHGPSLDNFLRKKPLTLEHRKQPCPYGRKCTYGIKCRFFHPERPSCPQRSVADELRANALLSPPRAPSKDKNGRRPSPSSQSSSLLTESEQCSLDGKKLGAQASPGSRQEGLTQTYAPSGRSLAPSGGSGSSFGPTDWLPQTLDSLPYVSQDCLDSGIGSLESQMSELWGVRGGGPGEPGPPRAPYTGYSPYGSELPATAAFSAFGRAMGAGHFSVPADYPPAPPAFPPREYWSEPYPLPPPTSVLQEPPVQSPGAGRSPWGRADSLAKEQASVYTKLCGVFPPHLVEAVMGRFPQLLDPQQLAAEILSYKSQHPSE

21 NP_001424.2 NP_001017963.1

>NP_001424.2

MPARRLLLLLTLLLPGLGIFGSTSTVTLPETLLFVSTLDGSLHAVSKRTGSIKWTLKEDPVLQVPTHVEEPAFLPDPNDGSLYTLGSKNNEGLTKLPFTIPELVQASPCRSSDGILYMGKKQDIWYVIDLLTGEKQQTLSSAFADSLCPSTSLLYLGRTEYTITMYDTKTRELRWNATYFDYAASLPEDDVDYKMSHFVSNGDGLVVTVDSESGDVLWIQNYASPVVAFYVWQREGLRKVMHINVAVETLRYLTFMSGEVGRITKWKYPFPKETEAKSKLTPTLYVGKYSTSLYASPSMVHEGVAVVPRGSTLPLLEGPQTDGVTIGDKGECVITPSTDVKFDPGLKSKNKLNYLRNYWLLIGHHETPLSASTKMLERFPNNLPKHRENVIPADSEKKSFEEVINLVDQTSENAPTTVSRDVEEKPAHAPARPEAPVDSMLKDMATIILSTFLLIGWVAFIITYPLSMHQQQQLQHQQFQKELEKIQLLQQQQQQLPFHPPGDTAQDGELLDTSGPYSESSGTSSPSTSPRASNHSLCSGSSASKAGSSPSLEQDDGDEETSVVIVGKISFCPKDVLGHGAEGTIVYRGMFDNRDVAVKRILPECFSFADREVQLLRESDEHPNVIRYFCTEKDRQFQYIAIELCAATLQEYVEQKDFAHLGLEPITLLQQTTSGLAHLHSLNIVHRDLKPHNILISMPNAHGKIKAMISDFGLCKKLAVGRHSFSRRSGVPGTEGWIAPEMLSEDCKENPTYTVDIFSAGCVFYYVISEGSHPFGKSLQRQANILLGACSLDCLHPEKHEDVIARELIEKMIAMDPQKRPSAKHVLKHPFFWSLEKQLQFFQDVSDRIEKESLDGPIVKQLERGGRAVVKMDWRENITDPLQTDLRKFRTYKGGSVRDLLRAMRNKKHHYRELPAEVRETLGTLPDDFVCYFTSRFPHLLAHTYRAMELCSHERLFQPYYFHEPPEPQPPVTPDAL

>NP_001017963.1

MPPCSGGDGSTPPGPSLRDRDCPAQSAEYPRDRLDPRPGSPSEASSPPFLRSRAPVNWYQEKAQVFLWHLLVSGSTTLLCLWKQPFHVSAFPVTASLAFRQSQGAGQHLYKDLQPFILLRLLMPEETQTQDQPMEEEEVETFAFQAEIAQLMSLIINTFYSNKEIFLRELISNSSDALDKIRYESLTDPSKLDSGRELHINLIPNKQGRTLTIVDTGIGMTKADLINNLGTIAKSGTKAFMEALQAGADISMIGQFGVGFYSAYLVAEKVTVITKHNDDEQYAWESSAGGSFTVRTDTGEPMGRGTKVILHLKEDQTEYLEERRIKEIVKKHSQFIGYPITLFVEKERDKEVSDDEAEEKEDKEEEKEKEEKESEDKPEIEDVGSDEEEEKKDGDKKKKKKIKEKYIDQEELNKTKPIWTRNPDDITNEEYGEFYKSLTNDWEDHLAVKHFSVEGQLEFRALLFVPRRAPFDLFENRKKKNNIKLYVRRVFIMDNCEELIPEYLNFIRGVVDSEDLPLNISREMLQQSKILKVIRKNLVKKCLELFTELAEDKENYKKFYEQFSKNIKLGIHEDSQNRKKLSELLRYYTSASGDEMVSLKDYCTRMKENQKHIYYITGETKDQVANSAFVERLRKHGLEVIYMIEPIDEYCVQQLKEFEGKTLVSVTKEGLELPEDEEEKKKQEEKKTKFENLCKIMKDILEKKVEKVVVSNRLVTSPCCIVTSTYGWTANMERIMKAQALRDNSTMGYMAAKKHLEINPDHSIIETLRQKAEADKNDKSVKDLVILLYETALLSSGFSLEDPQTHANRIYRMIKLGLGIDEDDPTADDTSAAVTEEMPPLEGDDDTSRMEEVD

22 NP_008973.1 NP_116269.2

>NP_008973.1

MPAPAATYERVVYKNPSEYHYMKVCLEFQDCGVGLNAAQFKQLLISAVKDLFGEVDAALPLDILTYEEKTLSAILRICSSGLVKLWSSLTLLGSYKGKKCAFRVIQVSPFLLALSGNSRELVLD

>NP_116269.2

MRTAPSLRRCVCLLLAAILDLARGYLTVNIEPLPPVVAGDAVTLKCNFKTDGRMREIVWYRVTDGGTIKQKIFTFDAMFSTNYSHMENYRKREDLVYQSTVRLPEVRISDNGPYECHVGIYDRATREKVVLASGNIFLNVMAPPTSIEVVAADTPAPFSRYQAQNFTLVCIVSGGKPAPMVYFKRDGEPIDAVPLSEPPAASSGPLQDSRPFRSLLHRDLDDTKMQKSLSLLDAENRGGRPYTERPSRGLTPDPNILLQPTTENIPETVVSREFPRWVHSAEPTYFLRHSRTPSSDGTVEVRALLTWTLNPQIDNEALFSCEVKHPALSMPMQAEVTLVAPKGPKIVMTPSRARVGDTVRILVHGFQNGVFPEPMFTWTRVGSRLLDGSAEFDGKELVLERVPAELNGSMYRCTAQNPLGSTDTHTRLIVFENPNIPRGTEDSNGSIGPTGARLTLVLALTVILELT

23 NP_066964.1 NP_003160.2

>NP_066964.1

MVRSGNKAAVVLCMDVGFTMSNSIPGIESPFEQAKKVITMFVQRQVFAENKDEIALVLFGTDGTDNPLSGGDQYQNITVHRHLMLPDFDLLEDIESKIQPGSQQADFLDALIVSMDVIQHETIGKKFEKRHIEIFTDLSSRFSKSQLDIIIHSLKKCDISLQFFLPFSLGKEDGSGDRGDGPFRLGGHGPSFPLKGITEQQKEGLEIVKMVMISLEGEDGLDEIYSFSESLRKLCVFKKIERHSIHWPCRLTIGSNLSIRIAAYKSILQERVKKTWTVVDAKTLKKEDIQKETVYCLNDDDETEVLKEDIIQGFRYGSDIVPFSKVDEEQMKYKSEGKCFSVLGFCKSSQVQRRFFMGNQVLKVFAARDDEAAAVALSSLIHALDDLDMVAIVRYAYDKRANPQVGVAFPHIKHNYECLVYVQLPFMEDLRQYMFSSLKNSKKYAPTEAQLNAVDALIDSMSLAKKDEKTDTLEDLFPTTKIPNPRFQRLFQCLLHRALHPREPLPPIQQHIWNMLNPPAEVTTKSQIPLSKIKTLFPLIEAKKKDQVTAQEIFQDNHEDGPTAKKLKTEQGGAHFSVSSLAEGSVTSVGSVNPAENFRVLVKQKKASFEEASNQLINHIEQFLDTNETPYFMKSIDCIRAFREEAIKFSEEQRFNNFLKALQEKVEIKQLNHFWEIVVQDGITLITKEEASGSSVTAEEAKKFLAPKDKPSGDTAAVFEEGGDVDDLLDMI

>NP_003160.2

MSDSEDSNFSEEEDSERSSDGEEAEVDEERRSAAGSEKEEEPEDEEEEEEEEEYDEEEEEEDDDRPPKKPRHGGFILDEADVDDEYEDEDQWEDGAEDILEKEEIEASNIDNVVLDEDRSGARRLQNLWRDQREEELGEYYMKKYAKSSVGETVYGGSDELSDDITQQQLLPGVKDPNLWTVKCKIGEERATAISLMRKFIAYQFTDTPLQIKSVVAPEHVKGYIYVEAYKQTHVKQAIEGVGNLRLGYWNQQMVPIKEMTDVLKVVKEVANLKPKSWVRLKRGIYKDDIAQVDYVEPSQNTISLKMIPRIDYDRIKARMSLKDWFAKRKKFKRPPQRLFDAEKIRSLGGDVASDGDFLIFEGNRYSRKGFLFKSFAMSAVITEGVKPTLSELEKFEDQPEGIDLEVVTESTGKEREHNFQPGDNVEVCEGELINLQGKILSVDGNKITIMPKHEDLKDMLEFPAQELRKYFKMGDHVKVIAGRFEGDTGLIVRVEENFVILFSDLTMHELKVLPRDLQLCSETASGVDVGGQHEWGELVQLDPQTVGVIVRLERETFQVLNMYGKVVTVRHQAVTRKKDNRFAVALDSEQNNIHVKDIVKVIDGPHSGREGEIRHLFRSFAFLHCKKLVENGGMFVCKTRHLVLAGGSKPRDVTNFTVGGFAPMSPRISSPMHPSAGGQRGGFGSPGGGSGGMSRGRGRRDNELIGQTVRISQGPYKGYIGVVKDATESTARVELHSTCQTISVDRQRLTTVGSRRPGGMTSTYGRTPMYGSQTPMYGSGSRTPMYGSQTPLQDGSRTPHYGSQTPLHDGSRTPAQSGAWDPNNPNTPSRAEEEYEYAFDDEPTPSPQAYGGTPNPQTPGYPDPSSPQVNPQYNPQTPGTPAMYNTDQFSPYAAPSPQGSYQPSPSPQSYHQVAPSPAGYQNTHSPASYHPTPSPMAYQASPSPSPVGYSPMTPGAPSPGGYNPHTPGSGIEQNSSDWVTTDIQVKVRDTYLDTQVVGQTGVIRSVTGGMCSVYLKDSEKVVSISSEHLEPITPTKNNKVKVILGEDREATGVLLSIDGEDGIVRMDLDEQLKILNLRFLGKLLEA

24 NP_004718.1 NP_000078.2

>NP_004718.1

MGKLIRMGPQERWLLRTKRLHWSRLLFLLGMLIIGSTYQHLRRPRGLSSLWAAVSSHQPIKLASRDLSSEEMMMMSSSPSKPSSEMGGKMLVPQASVGSDEATLSMTVENIPSMPKRTAKMIPTTTKNNYSPTAAGTERRKEDTPTSSRTLTYYTSTSSRQIVKKYTPTPRGEMKSYSPTQVREKVKYTPSPRGRRVGTYVPSTFMTMETSHAITPRTTVKDSDITATYKILETNSLKRIMEETTPTTLKGMFDSTPTFLTHEVEANVLTSPRSVMEKNNLFPPRRVESNSSAHPWGLVGKSNPKTPQGTVLLHTPATSEGQVTISTMTGSSPAETKAFTAAWSLRNPSPRTSVSAIKTAPAIVWRLAKKPSTAPSTSTTPTVRAKLTMQVHHCVVVKPTPAMLTTPSPSLTTALLPEELSPSPSVLPPSLPDLHPKGEYPPDLFSVEERRQGWVVLHVFGMMYVFVALAIVCDEYFVPALGVITDKLQISEDVAGATFMAAGGSAPELFTSLIGVFISHSNVGIGTIVGSAVFNILFVIGTCSLFSREILNLTWWPLFRDVSFYILDLIMLILFFLDSLIAWWESLLLLLAYAFYVFTMKWNKHIEVWVKEQLSRRPVAKVMALEDLSKPGDGAIAVDELQDNKKLKLPSLLTRGSSSTSLHNSTIRSTIYQLMLHSLDPLREVRLAKEKEEESLNQGARAQPQAKAESKPEEEEPAKLPAVTVTPAPVPDIKGDQKENPGGQEDVAEAESTGEMPGEEGETAGEGETEEKSGGETQPEGEGETETQGKGEECEDENEAEGKGDNEGEDEGEIHAEDGEMKGNEGETESQELSAENHGEAKNDEKGVEDGGGSDGGDSEEEEEEEEEQEEEEEEEEQEEEEEEEEEEEEKGNEEPLSLDWPETRQKQAIYLFLLPIVFPLWLTVPDVRRQESRKFFVFTFLGSIMWIAMFSYLMVWWAHQVGETIGISEEIMGLTILAAGTSIPDLITSVIVARKGLGDMAVSSSVGSNIFDITVGLPVPWLLFSLINGLQPVPVSSNGLFCAIVLLFLMLLFVISSIASCKWRMNKILGFTMFLLYFVFLIISVMLEDRIISCPVSV

>NP_000078.2

MKLSMKNNIINTQQSFVTMPNVIVPDIEKEIRRMENGACSSFSEDDDSASTSEESENENPHARGSFSYKSLRKGGPSQREQYLPGAIALFNVNNSSNKDQEPEEKKKKKKEKKSKSDDKNENKNDPEKKKKKKDKEKKKKEEKSKDKKEEEKKEVVVIDPSGNTYYNWLFCITLPVMYNWTMVIARACFDELQSDYLEYWLILDYVSDIVYLIDMFVRTRTGYLEQGLLVKEELKLINKYKSNLQFKLDVLSLIPTDLLYFKLGWNYPEIRLNRLLRFSRMFEFFQRTETRTNYPNIFRISNLVMYIVIIIHWNACVFYSISKAIGFGNDTWVYPDINDPEFGRLARKYVYSLYWSTLTLTTIGETPPPVRDSEYVFVVVDFLIGVLIFATIVGNIGSMISNMNAARAEFQARIDAIKQYMHFRNVSKDMEKRVIKWFDYLWTNKKTVDEKEVLKYLPDKLRAEIAINVHLDTLKKVRIFADCEAGLLVELVLKLQPQVYSPGDYICKKGDIGREMYIIKEGKLAVVADDGVTQFVVLSDGSYFGEISILNIKGSKAGNRRTANIKSIGYSDLFCLSKDDLMEALTEYPDAKTMLEEKGKQILMKDGLLDLNIANAGSDPKDLEEKVTRMEGSVDLLQTRFARILAEYESMQQKLKQRLTKVEKFLKPLIDTEFSSIEGPGAESGPIDST

25 NP_620305.1 NP_060362.3

>NP_620305.1

MATNIEQIFRSFVVSKFREIQQELSSGRNEGQLNGETNTPIEGNQAGDAAASARSLPNEEIVQKIEEVLSGVLDTELRYKPDLKEGSRKSRCVSVQTDPTDEIPTKKSKKHKKHKNKKKKKKKEKEKKYKRQPEESESKTKSHDDGNIDLESDSFLKFDSEPSAVALELPTRAFGPSETNESPAVVLEPPVVSMEVSEPHILETLKPATKTAELSVVSTSVISEQSEQSVAVMPEPSMTKILDSFAAAPVPTTTLVLKSSEPVVTMSVEYQMKSVLKSVESTSPEPSKIMLVEPPVAKVLEPSETLVVSSETPTEVYPEPSTSTTMDFPESSAIEALRLPEQPVDVPSEIADSSMTRPQELPELPKTTALELQESSVASAMELPGPPATSMPELQGPPVTPVLELPGPSATPVPELPGPLSTPVPELPGPPATAVPELPGPSVTPVPQLSQELPGLPAPSMGLEPPQEVPEPPVMAQELPGLPLVTAAVELPEQPAVTVAMELTEQPVTTTELEQPVGMTTVEHPGHPEVTTATGLLGQPEATMVLELPGQPVATTALELPGQPSVTGVPELPGLPSATRALELSGQPVATGALELPGPLMAAGALEFSGQSGAAGALELLGQPLATGVLELPGQPGAPELPGQPVATVALEISVQSVVTTSELSTMTVSQSLEVPSTTALESYNTVAQELPTTLVGETSVTVGVDPLMAPESHILASNTMETHILASNTMDSQMLASNTMDSQMLASNTMDSQMLASSTMDSQMLATSSMDSQMLATSSMDSQMLATSTMDSQMLATSSMDSQMLATSSMDSQMLATSSMDSQMLATSSMDSQMLATSTMDSQMLATSTMDSQMLATSSMDSQMLASGTMDSQMLASGTMDAQMLASGTMDAQMLASSTQDSAMLGSKSPDPYRLAQDPYRLAQDPYRLGHDPYRLGHDAYRLGQDPYRLGHDPYRLTPDPYRMSPRPYRIAPRSYRIAPRPYRLAPRPLMLASRRSMMMSYAAERSMMSSYERSMMSYERSMMSPMAERSMMSAYERSMMSAYERSMMSPMAERSMMSAYERSMMSAYERSMMSPMADRSMMSMGADRSMMSSYSAADRSMMSSYSAADRSMMSSYTADRSMMSMAADSYTDSYTDTYTEAYMVPPLPPEEPPTMPPLPPEEPPMTPPLPPEEPPEGPALPTEQSALTAENTWPTEVPSSPSEESVSQPEPPVSQSEISEPSAVPTDYSVSASDPSVLVSEAAVTVPEPPPEPESSITLTPVESAVVAEEHEVVPERPVTCMVSETPAMSAEPTVLASEPPVMSETAETFDSMRASGHVASEVSTSLLVPAVTTPVLAESILEPPAMAAPESSAMAVLESSAVTVLESSTVTVLESSTVTVLEPSVVTVPEPPVVAEPDYVTIPVPVVSALEPSVPVLEPAVSVLQPSMIVSEPSVSVQESTVTVSEPAVTVSEQTQVIPTEVAIESTPMILESSIMSSHVMKGINLSSGDQNLAPEIGMQEIALHSGEEPHAEEHLKGDFYESEHGINIDLNINNHLIAKEMEHNTVCAAGTSPVGEIGEEKILPTSETKQRTVLDTYPGVSEADAGETLSSTGPFALEPDATGTSKGIEFTTASTLSLVNKYDVDLSLTTQDTEHDMVISTSPSGGSEADIEGPLPAKDIHLDLPSNNNLVSKDTEEPLPVKESDQTLAALLSPKESSGGEKEVPPPPKETLPDSGFSANIEDINEADLVRPLLPKDMERLTSLRAGIEGPLLASDVGRDRSAASPVVSSMPERASESSSEEKDDYEIFVKVKDTHEKSKKNKNRDKGEKEKKRDSSLRSRSKRSKSSEHKSRKRTSESRSRARKRSSKSKSHRSQTRSRSRSRRRRRSSRSRSKSRGRRSVSKEKRKRSPKHRSKSRERKRKRSSSRDNRKTVRARSRTPSRRSRSHTPSRRRRSRSVGRRRSFSISPSRRSRTPSRRSRTPSRRSRTPSRRSRTPSRRSRTPSRRSRTPSRRRRSRSVVRRRSFSISPVRLRRSRTPLRRRFSRSPIRRKRSRSSERGRSPKRLTDLDKAQLLEIAKANAAAMCAKAGVPLPPNLKPAPPPTIEEKVAKKSGGATIEELTEKCKQIAQSKEDDDVIVNKPHVSDEEEEEPPFYHHPFKLSEPKPIFFNLNIAAAKPTPPKSQVTLTKEFPVSSGSQHRKKEADSVYGEWVPVEKNGEENKDDDNVFSSNLPSEPVDISTAMSERALAQKRLSENAFDLEAMSMLNRAQERIDAWAQLNSIPGQFTGSTGVQVLTQEQLANTGAQAWIKKDQFLRAAPVTGGMGAVLMRKMGWREGEGLGKNKEGNKEPILVDFKTDRKGLVAVGERAQKRSGNFSAAMKDLSGKHPVSALMEICNKRRWQPPEFLLVHDSGPDHRKHFLFRVLRNGALTRPNCMFFLNRY

>NP_060362.3

MCSGSGRRRSSLSPTMRPGTGAERGGLMMGHPGMHYAPMGMHPMGQRANMPPVPHGMMPQMMPPMGGPPMGQMPGMMSSVMPGMMMSHMSQASMQPALPPGVNSMDVAAGTASGAKSMWTEHKSPDGRTYYYNTETKQSTWEKPDDLKTPAEQLLSKCPWKEYKSDSGKPYYYNSQTKESRWAKPKELEDLEGYQNTIVAGSLITKSNLHAMIKAEESSKQEECTTTSTAPVPTTEIPTTMSTMAAAEAAAAVVAAAAAAAAAAAAANANASTSASNTVSGTVPVVPEPEVTSIVATVVDNENTVTISTEEQAQLTSTPAIQDQSVEVSSNTGEETSKQETVADFTPKKEEEESQPAKKTYTWNTKEEAKQAFKELLKEKRVPSNASWEQAMKMIINDPRYSALAKLSEKKQAFNAYKVQTEKEEKEEARSKYKEAKESFQRFLENHEKMTSTTRYKKAEQMFGEMEVWNAISERDRLEIYEDVLFFLSKKEKEQAKQLRKRNWEALKNILDNMANVTYSTTWSEAQQYLMDNPTFAEDEELQNMDKEDALICFEEHIRALEKEEEEEKQKSLLRERRRQRKNRESFQIFLDELHEHGQLHSMSSWMELYPTISSDIRFTNMLGQPGSTALDLFKFYVEDLKARYHDEKKIIKDILKDKGFVVEVNTTFEDFVAIISSTKRSTTLDAGNIKLAFNSLLEKAEAREREREKEEARKMKRKESAFKSMLKQAAPPIELDAVWEDIRERFVKEPAFEDITLESERKRIFKDFMHVLEHECQHHHSKNKKHSKKSKKHHRKRSRSRSGSDSDDDDSHSKKKRQRSESRSASEHSSSAESERSYKKSKKHKKKSKKRRHKSDSPESDAEREKDKKEKDRESEKDRTRQRSESKHKSPKKKTGKDSGNWDTSGSELSEGELEKRRRTLLEQLDDDQ

26 NP_061324.1 NP_001895.1

>NP_061324.1

MWIQQLLGLSSMSIRWPGRPLGSHAWILIAMFQLAVDLPACEALGPGPEFWLLPRSPPRPPRLWSFRSGQPARVPAPVWSPRPPRVERIHGQMQMPRARRAHRPRDQAAALVPKAGLAKPPAAAKSSPSLASSSSSSSSAVAGGAPEQQALLRRGKRHLQGDGLSSFDSRGSRPTTETEFIAWGPTGDEEALESNTFPGVYGPTTVSILQTRKTTVAATTTTTTTATPMTLQTKGFTESLDPRRRIPGGVSTTEPSTSPSNNGEVTQPPRILGEASGLAVHQIITITVSLIMVIAALITTLVLKNCCAQSGNTRRNSHQRKTNQQEESCQNLTDFPSARVPSSLDIFTAYNETLQCSHECVRASVPVYTDETLHSTTGEYKSTFNGNRPSSSDRHLIPVAFVSEKWFEISC

>NP_001895.1

MATQADLMELDMAMEPDRKAAVSHWQQQSYLDSGIHSGATTTAPSLSGKGNPEEEDVDTSQVLYEWEQGFSQSFTQEQVADIDGQYAMTRAQRVRAAMFPETLDEGMQIPSTQFDAAHPTNVQRLAEPSQMLKHAVVNLINYQDDAELATRAIPELTKLLNDEDQVVVNKAAVMVHQLSKKEASRHAIMRSPQMVSAIVRTMQNTNDVETARCTAGTLHNLSHHREGLLAIFKSGGIPALVKMLGSPVDSVLFYAITTLHNLLLHQEGAKMAVRLAGGLQKMVALLNKTNVKFLAITTDCLQILAYGNQESKLIILASGGPQALVNIMRTYTYEKLLWTTSRVLKVLSVCSSNKPAIVEAGGMQALGLHLTDPSQRLVQNCLWTLRNLSDAATKQEGMEGLLGTLVQLLGSDDINVVTCAAGILSNLTCNNYKNKMMVCQVGGIEALVRTVLRAGDREDITEPAICALRHLTSRHQEAEMAQNAVRLHYGLPVVVKLLHPPSHWPLIKATVGLIRNLALCPANHAPLREQGAIPRLVQLLVRAHQDTQRRTSMGGTQQQFVEGVRMEEIVEGCTGALHILARDVHNRIVIRGLNTIPLFVQLLYSPIENIQRVAAGVLCELAQDKEAAEAIEAEGATAPLTELLHSRNEGVATYAAAVLFRMSEDKPQDYKKRLSVELTSSLFRTEPMAWNETADLGLDIGAQGEPLGYRQDDPSYRSFHSGGYGQDALGMDPMMEHEMGGHHPGADYPVDGLPDLGHAQDLMDGLPPGDSNQLAWFDTDL

27 NP_006100.2 NP_001284.1

>NP_006100.2

MAAMAVGGAGGSRVSSGRDLNCVPEIADTLGAVAKQGFDFLCMPVFHPRFKREFIQEPAKNRPGPQTRSDLLLSGRDWNTLIVGKLSPWIRPDSKVEKIRRNSEAAMLQELNFGAYLGLPAFLLPLNQEDNTNLARVLTNHIHTGHHSSMFWMRVPLVAPEDLRDDIIENAPTTHTEEYSGEEKTWMWWHNFRTLCDYSKRIAVALEIGADLPSNHVIDRWLGEPIKAAILPTSIFLTNKKGFPVLSKMHQRLIFRLLKLEVQFIITGTNHHSEKEFCSYLQYLEYLSQNRPPPNAYELFAKGYEDYLQSPLQPLMDNLESQTYEVFEKDPIKYSQYQQAIYKCLLDRVPEEEKDTNVQVLMVLGAGRGPLVNASLRAAKQADRRIKLYAVEKNPNAVVTLENWQFEEWGSQVTVVSSDMREWVAPEKADIIVSELLGSFADNELSPECLDGAQHFLKDDGVSIPGEYTSFLAPISSSKLYNEVRACREKDRDPEAQFEMPYVVRLHNFHQLSAPQPCFTFSHPNRDPMIDNNRYCTLEFPVEVNTVLHGFAGYFETVLYQDITLSIRPETHSPGMFSWFPILFPIKQPITVREGQTICVRFWRCSNSKKVWYEWAVTAPVCSAIHNPTGRSYTIGL

>NP_001284.1

MSFLKSFPPPGPAEGLLRQQPDTEAVLNGKGLGTGTLYIAESRLSWLDGSGLGFSLEYPTISLHALSRDRSDCLGEHLYVMVNAKFEEESKEPVADEEEEDSDDDVEPITEFRFVPSDKSALEAMFTAMCECQALHPDPEDEDSDDYDGEEYDVEAHEQGQGDIPTFYTYEEGLSHLTAEGQATLERLEGMLSQSVSSQYNMAGVRTEDSIRDYEDGMEVDTTPTVAGQFEDADVDH

28 NP_006829.1 NP_001406.1

>NP_006829.1

MAGVEEVAASGSHLNGDLDPDDREEGAASTAEEAAKKKRRKKKKSKGPSAAGEQEPDKESGASVDEVARQLERSALEDKERDEDDEDGDGDGDGATGKKKKKKKKKRGPKVQTDPPSVPICDLYPNGVFPKGQECEYPPTQDGRTAAWRTTSEEKKALDQASEEIWNDFREAAEAHRQVRKYVMSWIKPGMTMIEICEKLEDCSRKLIKENGLNAGLAFPTGCSLNNCAAHYTPNAGDTTVLQYDDICKIDFGTHISGRIIDCAFTVTFNPKYDTLLKAVKDATNTGIKCAGIDVRLCDVGEAIQEVMESYEVEIDGKTYQVKPIRNLNGHSIGQYRIHAGKTVPIVKGGEATRMEEGEVYAIETFGSTGKGVVHDDMECSHYMKNFDVGHVPIRLPRTKHLLNVINENFGTLAFCRRWLDRLGESKYLMALKNLCDLGIVDPYPPLCDIKGSYTAQFEHTILLRPTCKEVVSRGDDY

>NP_001406.1

MAGGEAGVTLGQPHLSRQDLTTLDVTKLTPLSHEVISRQATINIGTIGHVAHGKSTVVKAISGVHTVRFKNELERNITIKLGYANAKIYKLDDPSCPRPECYRSCGSSTPDEFPTDIPGTKGNFKLVRHVSFVDCPGHDILMATMLNGAAVMDAALLLIAGNESCPQPQTSEHLAAIEIMKLKHILILQNKIDLVKESQAKEQYEQILAFVQGTVAEGAPIIPISAQLKYNIEVVCEYIVKKIPVPPRDFTSEPRLIVIRSFDVNKPGCEVDDLKGGVAGGSILKGVLKVGQEIEVRPGIVSKDSEGKLMCKPIFSKIVSLFAEHNDLQYAAPGGLIGVGTKIDPTLCRADRMVGQVLGAVGALPEIFTELEISYFLLRRLLGVRTEGDKKAAKVQKLSKNEVLMVNIGSLSTGGRVSAVKADLGKIVLTNPVCTEVGEKIALSRRVEKHWRLIGWGQIRRGVTIKPTVDDD

29 NP_005624.2 P15941

>NP_005624.2

MQAQQLPYEFFSEENAPKWRGLLVPALKKVQGQVHPTLESNDDALQYVEELILQLLNMLCQAQPRSASDVEERVQKSFPHPIDKWAIADAQSAIEKRKRRNPLSLPVEKIHPLLKEVLGYKIDHQVSVYIVAVLEYISADILKLVGNYVRNIRHYEITKQDIKVAMCADKVLMDMFHQDVEDINILSLTDEEPSTSGEQTYYDLVKAFMAEIRQYIRELNLIIKVFREPFVSNSKLFSANDVENIFSRIVDIHELSVKLLGHIEDTVEMTDEGSPHPLVGSCFEDLAEELAFDPYESYARDILRPGFHDRFLSQLSKPGAALYLQSIGEGFKEAVQYVLPRLLLAPVYHCLHYFELLKQLEEKSEDQEDKECLKQAITALLNVQSGMEKICSKSLAKRRLSESACRFYSQQMKGKQLAIKKMNEIQKNIDGWEGKDIGQCCNEFIMEGTLTRVGAKHERHIFLFDGLMICCKSNHGQPRLPGASNAEYRLKEKFFMRKVQINDKDDTNEYKHAFEIILKDENSVIFSAKSAEEKNNWMAALISLQYRSTLERMLDVTMLQEEKEEQMRLPSADVYRFAEPDSEENIIFEENMQPKAGIPIIKAGTVIKLIERLTYHMYADPNFVRTFLTTYRSFCKPQELLSLIIERFEIPEPEPTEADRIAIENGDQPLSAELKRFRKEYIQPVQLRVLNVCRHWVEHHFYDFERDAYLLQRMEEFIGTVRGKAMKKWVESITKIIQRKKIARDNGPGHNITFQSSPPTVEWHISRPGHIETFDLLTLHPIEIARQLTLLESDLYRAVQPSELVGSVWTKEDKEINSPNLLKMIRHTTNLTLWFEKCIVETENLEERVAVVSRIIEILQVFQELNNFNGVLEVVSAMNSSPVYRLDHTFEQIPSRQKKILEEAHELSEDHYKKYLAKLRSINPPCVPFFGIYLTNILKTEEGNPEVLKRHGKELINFSKRRKVAEITGEIQQYQNQPYCLRVESDIKRFFENLNPMGNSMEKEFTDYLFNKSLEIEPRNPKPLPRFPKKYSYPLKSPGVRPSNPRPGTMRHPTPLQQEPRKISYSRIPESETESTASAPNSPRTPLTPPPASGASSTTDVCSVFDSDHSSPFHSSNDTVFIQVTLPHGPRSASVSSISLTKGTDEVPVPPPVPPRRRPESAPAESSPSKIMSKHLDSPPAIPPRQPTSKAYSPRYSISDRTSISDPPESPPLLPPREPVRTPDVFSSSPLHLQPPPLGKKSDHGNAFFPNSPSPFTPPPPQTPSPHGTRRHLPSPPLTQEVDLHSIAGPPVPPRQSTSQHIPKLPPKTYKREHTHPSMHRDGPPLLENAHSS

>P15941

MTPGTQSPFFLLLLLTVLTVVTGSGHASSTPGGEKETSATQRSSVPSSTEKNAVSMTSSVLSSHSPGSGSSTTQGQDVTLAPATEPASGSAATWGQDVTSVPVTRPALGSTTPPAHDVTSAPDNKPAPGSTAPPAHGVTSAPDTRPAPGSTAPPAHGVTSAPDTRPAPGSTAPPAHGVTSAPDTRPAPGSTAPPAHGVTSAPDTRPAPGSTAPPAHGVTSAPDTRPAPGSTAPPAHGVTSAPDTRPAPGSTAPPAHGVTSAPDTRPAPGSTAPPAHGVTSAPDTRPAPGSTAPPAHGVTSAPDTRPAPGSTAPPAHGVTSAPDTRPAPGSTAPPAHGVTSAPDTRPAPGSTAPPAHGVTSAPDTRPAPGSTAPPAHGVTSAPDTRPAPGSTAPPAHGVTSAPDTRPAPGSTAPPAHGVTSAPDTRPAPGSTAPPAHGVTSAPDTRPAPGSTAPPAHGVTSAPDTRPAPGSTAPPAHGVTSAPDTRPAPGSTAPPAHGVTSAPDTRPAPGSTAPPAHGVTSAPDTRPAPGSTAPPAHGVTSAPDTRPAPGSTAPPAHGVTSAPDTRPAPGSTAPPAHGVTSAPDTRPAPGSTAPPAHGVTSAPDTRPAPGSTAPPAHGVTSAPDTRPAPGSTAPPAHGVTSAPDTRPAPGSTAPPAHGVTSAPDTRPAPGSTAPPAHGVTSAPDTRPAPGSTAPPAHGVTSAPDTRPAPGSTAPPAHGVTSAPDTRPAPGSTAPPAHGVTSAPDTRPAPGSTAPPAHGVTSAPDTRPAPGSTAPPAHGVTSAPDTRPAPGSTAPPAHGVTSAPDTRPAPGSTAPPAHGVTSAPDTRPAPGSTAPPAHGVTSAPDTRPAPGSTAPPAHGVTSAPDTRPAPGSTAPPAHGVTSAPDTRPAPGSTAPPAHGVTSAPDTRPAPGSTAPPAHGVTSAPDTRPAPGSTAPPAHGVTSAPDNRPALGSTAPPVHNVTSASGSASGSASTLVHNGTSARATTTPASKSTPFSIPSHHSDTPTTLASHSTKTDASSTHHSSVPPLTSSNHSTSPQLSTGVSFFFLSFHISNLQFNSSLEDPSTDYYQELQRDISEMFLQIYKQGGFLGLSNIKFRPGSVVVQLTLAFREGTINVHDVETQFNQYKTEAASRYNLTISDVSVSDVPFPFSAQSGAGVPGWGIALLVLVCVLVALAIVYLIALAVCQCRRKNYGQLDIFPARDTYHPMSEYPTYHTHGRYVPPSSTDRSPYEKVSAGNGGSSLSYTNPAVAAASANL

30 NP_055525.3 NP_005233.3

>NP_055525.3

MTGAEIESGAQVKPEKKPGEEVVGGAEIENDVPLVVRPKVRTQAQIMPGARPKNKSKVMPGASTKVETSAVGGARPKSKAKAIPVSRFKEEAQMWAQPRFGAERLSKTERNSQTNIIASPLVSTDSVLVAKTKYLSEDRELVNTDTESFPRRKAHYQAGFQPSFRSKEETNMGSWCCPRPTSKQEASPNSDFKWVDKSVSSLFWSGDEVTAKFHPGNRVKDSNRSMHMANQEANTMSRSQTNQELYIASSSGSEDESVKTPWFWARDKTNTWSGPREDPNSRSRFRSKKEVYVESSSGSEHEDHLESWFGAGKEAKFRSKMRAGKEANNRARHRAKREACIDFMPGSIDVIKKESCFWPEENANTFSRPMIKKEARARAMTKEEAKTKARARAKQEARSEEEALIGTWFWATDESSMADEASIESSLQVEDESIIGSWFWTEEEASMGTGASSKSRPRTDGERIGDSLFGAREKTSMKTGAEATSESILAADDEQVIIGSWFWAGEEVNQEAEEETIFGSWFWVIDAASVESGVGVSCESRTRSEEEEVIGPWFWSGEQVDIEAGIGEEARPGAEEETIFGSWFWAENQTYMDCRAETSCDTMQGAEEEEPIIGSWFWTRVEACVEGDVNSKSSLEDKEEAMIPCFGAKEEVSMKHGTGVRCRFMAGAEETNNKSCFWAEKEPCMYPAGGGSWKSRPEEEEDIVNSWFWSRKYTKPEAIIGSWLWATEESNIDGTGEKAKLLTEEETIINSWFWKEDEAISEATDREESRPEAEEGDIIGSWFWAGEEDRLEPAAETREEDRLAAEKEGIVGSWFGAREETIRREAGSCSKSSPKAEEEEVIIGSWFWEEEASPEAVAGVGFESKPGTEEEEITVGSWFWPEEEASIQAGSQAVEEMESETEEETIFGSWFWDGKEVSEEAGPCCVSKPEDDEEMIVESWFWSRDKAIKETGTVATCESKPENEEGAIVGSWFEAEDEVDNRTDNGSNCGSRTLADEDEAIVGSWFWAGDEAHFESNPSPVFRAICRSTCSVEQEPDPSRRPQSWEEVTVQFKPGPWGRVGFPSISPFRFPKEAASLFCEMFGGKPRNMVLSPEGEDQESLLQPDQPSPEFPFQYDPSYRSVQEIREHLRAKESTEPESSSCNCIQCELKIGSEEFEELLLLMEKIRDPFIHEISKIAMGMRSASQFTRDFIRDSGVVSLIETLLNYPSSRVRTSFLENMIRMAPPYPNLNIIQTYICKVCEETLAYSVDSPEQLSGIRMIRHLTTTTDYHTLVANYMSGFLSLLATGNAKTRFHVLKMLLNLSENLFMTKELLSAEAVSEFIGLFNREETNDNIQIVLAIFENIGNNIKKETVFSDDDFNIEPLISAFHKVEKFAKELQGKTDNQNDPEGDQEN

>NP_005233.3

MRSPSAAWLLGAAILLAASLSCSGTIQGTNRSSKGRSLIGKVDGTSHVTGKGVTVETVFSVDEFSASVLTGKLTTVFLPIVYTIVFVVGLPSNGMALWVFLFRTKKKHPAVIYMANLALADLLSVIWFPLKIAYHIHGNNWIYGEALCNVLIGFFYGNMYCSILFMTCLSVQRYWVIVNPMGHSRKKANIAIGISLAIWLLILLVTIPLYVVKQTIFIPALNITTCHDVLPEQLLVGDMFNYFLSLAIGVFLFPAFLTASAYVLMIRMLRSSAMDENSEKKRKRAIKLIVTVLAMYLICFTPSNLLLVVHYFLIKSQGQSHVYALYIVALCLSTLNSCIDPFVYYFVSHDFRDHAKNALLCRSVRTVKQMQVSLTSKKHSRKSSSYSSSSTTVKTSY

31 NP_000050.2 NP_149075.2

>NP_000050.2

MPIGSKERPTFFEIFKTRCNKADLGPISLNWFEELSSEAPPYNSEPAEESEHKNNNYEPNLFKTPQRKPSYNQLASTPIIFKEQGLTLPLYQSPVKELDKFKLDLGRNVPNSRHKSLRTVKTKMDQADDVSCPLLNSCLSESPVVLQCTHVTPQRDKSVVCGSLFHTPKFVKGRQTPKHISESLGAEVDPDMSWSSSLATPPTLSSTVLIVRNEEASETVFPHDTTANVKSYFSNHDESLKKNDRFIASVTDSENTNQREAASHGFGKTSGNSFKVNSCKDHIGKSMPNVLEDEVYETVVDTSEEDSFSLCFSKCRTKNLQKVRTSKTRKKIFHEANADECEKSKNQVKEKYSFVSEVEPNDTDPLDSNVANQKPFESGSDKISKEVVPSLACEWSQLTLSGLNGAQMEKIPLLHISSCDQNISEKDLLDTENKRKKDFLTSENSLPRISSLPKSEKPLNEETVVNKRDEEQHLESHTDCILAVKQAISGTSPVASSFQGIKKSIFRIRESPKETFNASFSGHMTDPNFKKETEASESGLEIHTVCSQKEDSLCPNLIDNGSWPATTTQNSVALKNAGLISTLKKKTNKFIYAIHDETSYKGKKIPKDQKSELINCSAQFEANAFEAPLTFANADSGLLHSSVKRSCSQNDSEEPTLSLTSSFGTILRKCSRNETCSNNTVISQDLDYKEAKCNKEKLQLFITPEADSLSCLQEGQCENDPKSKKVSDIKEEVLAAACHPVQHSKVEYSDTDFQSQKSLLYDHENASTLILTPTSKDVLSNLVMISRGKESYKMSDKLKGNNYESDVELTKNIPMEKNQDVCALNENYKNVELLPPEKYMRVASPSRKVQFNQNTNLRVIQKNQEETTSISKITVNPDSEELFSDNENNFVFQVANERNNLALGNTKELHETDLTCVNEPIFKNSTMVLYGDTGDKQATQVSIKKDLVYVLAEENKNSVKQHIKMTLGQDLKSDISLNIDKIPEKNNDYMNKWAGLLGPISNHSFGGSFRTASNKEIKLSEHNIKKSKMFFKDIEEQYPTSLACVEIVNTLALDNQKKLSKPQSINTVSAHLQSSVVVSDCKNSHITPQMLFSKQDFNSNHNLTPSQKAEITELSTILEESGSQFEFTQFRKPSYILQKSTFEVPENQMTILKTTSEECRDADLHVIMNAPSIGQVDSSKQFEGTVEIKRKFAGLLKNDCNKSASGYLTDENEVGFRGFYSAHGTKLNVSTEALQKAVKLFSDIENISEETSAEVHPISLSSSKCHDSVVSMFKIENHNDKTVSEKNNKCQLILQNNIEMTTGTFVEEITENYKRNTENEDNKYTAASRNSHNLEFDGSDSSKNDTVCIHKDETDLLFTDQHNICLKLSGQFMKEGNTQIKEDLSDLTFLEVAKAQEACHGNTSNKEQLTATKTEQNIKDFETSDTFFQTASGKNISVAKESFNKIVNFFDQKPEELHNFSLNSELHSDIRKNKMDILSYEETDIVKHKILKESVPVGTGNQLVTFQGQPERDEKIKEPTLLGFHTASGKKVKIAKESLDKVKNLFDEKEQGTSEITSFSHQWAKTLKYREACKDLELACETIEITAAPKCKEMQNSLNNDKNLVSIETVVPPKLLSDNLCRQTENLKTSKSIFLKVKVHENVEKETAKSPATCYTNQSPYSVIENSALAFYTSCSRKTSVSQTSLLEAKKWLREGIFDGQPERINTADYVGNYLYENNSNSTIAENDKNHLSEKQDTYLSNSSMSNSYSYHSDEVYNDSGYLSKNKLDSGIEPVLKNVEDQKNTSFSKVISNVKDANAYPQTVNEDICVEELVTSSSPCKNKNAAIKLSISNSNNFEVGPPAFRIASGKIVCVSHETIKKVKDIFTDSFSKVIKENNENKSKICQTKIMAGCYEALDDSEDILHNSLDNDECSTHSHKVFADIQSEEILQHNQNMSGLEKVSKISPCDVSLETSDICKCSIGKLHKSVSSANTCGIFSTASGKSVQVSDASLQNARQVFSEIEDSTKQVFSKVLFKSNEHSDQLTREENTAIRTPEHLISQKGFSYNVVNSSAFSGFSTASGKQVSILESSLHKVKGVLEEFDLIRTEHSLHYSPTSRQNVSKILPRVDKRNPEHCVNSEMEKTCSKEFKLSNNLNVEGGSSENNHSIKVSPYLSQFQQDKQQLVLGTKVSLVENIHVLGKEQASPKNVKMEIGKTETFSDVPVKTNIEVCSTYSKDSENYFETEAVEIAKAFMEDDELTDSKLPSHATHSLFTCPENEEMVLSNSRIGKRRGEPLILVGEPSIKRNLLNEFDRIIENQEKSLKASKSTPDGTIKDRRLFMHHVSLEPITCVPFRTTKERQEIQNPNFTAPGQEFLSKSHLYEHLTLEKSSSNLAVSGHPFYQVSATRNEKMRHLITTGRPTKVFVPPFKTKSHFHRVEQCVRNINLEENRQKQNIDGHGSDDSKNKINDNEIHQFNKNNSNQAAAVTFTKCEEEPLDLITSLQNARDIQDMRIKKKQRQRVFPQPGSLYLAKTSTLPRISLKAAVGGQVPSACSHKQLYTYGVSKHCIKINSKNAESFQFHTEDYFGKESLWTGKGIQLADGGWLIPSNDGKAGKEEFYRALCDTPGVDPKLISRIWVYNHYRWIIWKLAAMECAFPKEFANRCLSPERVLLQLKYRYDTEIDRSRRSAIKKIMERDDTAAKTLVLCVSDIISLSANISETSSNKTSSADTQKVAIIELTDGWYAVKAQLDPPLLAVLKNGRLTVGQKIILHGAELVGSPDACTPLEAPESLMLKISANSTRPARWYTKLGFFPDPRPFPLPLSSLFSDGGNVGCVDVIIQRAYPIQWMEKTSSGLYIFRNEREEEKEAAKYVEAQQKRLEALFTKIQEEFEEHEENTTKPYLPSRALTRQQVRALQDGAELYEAVKNAADPAYLEGYFSEEQLRALNNHRQMLNDKKQAQIQLEIRKAMESAEQKEQGLSRDVTTVWKLRIVSYSKKEKDSVILSIWRPSSDLYSLLTEGKRYRIYHLATSKSKSKSERANIQLAATKKTQYQQLPVSDEILFQIYQPREPLHFSKFLDPDFQPSCSEVDLIGFVVSVVKKTGLAPFVYLSDECYNLLAIKFWIDLNEDIIKPHMLIAASNLQWRPESKSGLLTLFAGDFSVFSASPKEGHFQETFNKMKNTVENIDILCNEAENKLMHILHANDPKWSTPTKDCTSGPYTAQIIPGTGNKLLMSSPNCEIYYQSPLSLCMAKRKSVSTPVSAQMTSKSCKGEKEIDDQKNCKKRRALDFLSRLPLPPPVSPICTFVSPAAQKAFQPPRSCGTKYETPIKKKELNSPQMTPFKKFNEISLLESNSIADEELALINTQALLSGSTGEKQFISVSESTRTAPTSSEDYLRLKRRCTTSLIKEQESSQASTEECEKNKQDTITTKKYI

>NP_149075.2

MVSKRRLSKSEDKESLTEDASKTRKQPLSKKTKKSHIANEVEENDSIFVKLLKISGIILKTGESQNQLAVDQIAFQKKLFQTLRRHPSYPKIIEEFVSGLESYIEDEDSFRNCLLSCERLQDEEASMGASYSKSLIKLLLGIDILQPAIIKTLFEKLPEYFFENKNSDEINIPRLIVSQLKWLDRVVDGKDLTTKIMQLISIAPENLQHDIITSLPEILGDSQHADVGKELSDLLIENTSLTVPILDVLSSLRLDPNFLLKVRQLVMDKLSSIRLEDLPVIIKFILHSVTAMDTLEVISELREKLDLQHCVLPSRLQASQVKLKSKGRASSSGNQESSGQSCIILLFDVIKSAIRYEKTISEAWIKAIENTASVSEHKVFDLVMLFIIYSTNTQTKKYIDRVLRNKIRSGCIQEQLLQSTFSVHYLVLKDMCSSILSLAQSLLHSLDQSIISFGSLLYKYAFKFFDTYCQQEVVGALVTHICSGNEAEVDTALDVLLELVVLNPSAMMMNAVFVKGILDYLDNISPQQIRKLFYVLSTLAFSKQNEASSHIQDDMHLVIRKQLSSTVFKYKLIGIIGAVTMAGIMAADRSESPSLTQERANLSDEQCTQVTSLLQLVHSCSEQSPQASALYYDEFANLIQHEKLDPKALEWVGHTICNDFQDAFVVDSCVVPEGDFPFPVKALYGLEEYDTQDGIAINLLPLLFSQDFAKDGGPVTSQESGQKLVSPLCLAPYFRLLRLCVERQHNGNLEEIDGLLDCPIFLTDLEPGEKLESMSAKERSFMCSLIFLTLNWFREIVNAFCQETSPEMKGKVLTRLKHIVELQIILEKYLAVTPDYVPPLGNFDVETLDITPHTVTAISAKIRKKGKIERKQKTDGSKTSSSDTLSEEKNSECDPTPSHRGQLNKEFTGKEEKTSLLLHNSHAFFRELDIEVFSILHCGLVTKFILDTEMHTEATEVVQLGPPELLFLLEDLSQKLESMLTPPIARRVPFLKNKGSRNIGFSHLQQRSAQEIVHCVFQLLTPMCNHLENIHNYFQCLAAENHGVVDGPGVKVQEYHIMSSCYQRLLQIFHGLFAWSGFSQPENQNLLYSALHVLSSRLKQGEHSQPLEELLSQSVHYLQNFHQSIPSFQCALYLIRLLMVILEKSTASAQNKEKIASLARQFLCRVWPSGDKEKSNISNDQLHALLCIYLEHTESILKAIEEIAGVGVPELINSPKDASSSTFPTLTRHTFVVFFRVMMAELEKTVKKIEPGTAADSQQIHEEKLLYWNMAVRDFSILINLIKVFDSHPVLHVCLKYGRLFVEAFLKQCMPLLDFSFRKHREDVLSLLETFQLDTRLLHHLCGHSKIHQDTRLTQHVPLLKKTLELLVCRVKAMLTLNNCREAFWLGNLKNRDLQGEEIKSQNSQESTADESEDDMSSQASKSKATEVSLQNPPESGTDGCILLIVLSWWSRTLPTYVYCQMLLCPFPFPP

32 NP_002816.1 NP_009171.1

>NP_002816.1

MQAQQYQQQRRKFAAAFLAFIFILAAVDTAEAGKKEKPEKKVKKSDCGEWQWSVCVPTSGDCGLGTREGTRTGAECKQTMKTQRCKIPCNWKKQFGAECKYQFQAWGECDLNTALKTRTGSLKRALHNAECQKTVTISKPCGKLTKPKPQAESKKKKKEGKKQEKMLD

>NP_009171.1

MLEAPGPSDGCELSNPSASRVSCAGQMLEVQPGLYFGGAAAVAEPDHLREAGITAVLTVDSEEPSFKAGPGVEDLWRLFVPALDKPETDLLSHLDRCVAFIGQARAEGRAVLVHCHAGVSRSVAIITAFLMKTDQLPFEKAYEKLQILKPEAKMNEGFEWQLKLYQAMGYEVDTSSAIYKQYRLQKVTEKYPELQNLPQELFAVDPTTVSQGLKDEVLYKCRKCRRSLFRSSSILDHREGSGPIAFAHKRMTPSSMLTTGRQAQCTSYFIEPVQWMESALLGVMDGQLLCPKCSAKLGSFNWYGEQCSCGRWITPAFQIHKNRVDEMKILPVLGSQTGKI

33 NP_597709.2 NP_002810.1

>NP_597709.2

MPLRPGSLVPEGAGFPKMAADVSVTHRPPLSPKSGAEVEAGDAAERRAPEEELPPLDPEEIRKRLEHTERQFRNRRKILIRGLPGDVTNQEVHDLLSDYELKYCFVDKYKGTAFVTLLNGEQAEAAINAFHQSRLRERELSVQLQPTDALLCVANLPPSLTQQQFEELVRPFGSLERCFLVYSERTGQSKGYGFAEYMKKDSAARAKSDLLGKPLGPRTLYVHWTDAGQLTPALLHSRCLCVDRLPPGFNDVDALCRALSAVHSPTFCQLACGQDGQLKGFAVLEYETAEMAEEAQQQADGLSLGGSHLRVSFCAPGPPGRSMLAALIAAQATALNRGKGLLPEPNILQLLNNLGPSASLQLLLNPLLHGSAGGKQGLLGAPPAMPLLNGPALSTALLQLALQTQGQKKPGILGDSPLGALQPGAQPANPLLGELPAGGGLPPELPPRRGKPPPLLPSVLGPAGGDREALGLGPPAAQLTPPPAPVGLRGSGLRGLQKDSGPLPTPPGVSLLGEPPKDYRIPLNPYLNLHSLLPASNLAGKEARGWGGAGRSRRPAEGPPTNPPAPGGGSSSSKAFQLKSRLLSPLSSARLPPEPGLSDSYSFDYPSDMGPRRLFSHPREPALGPHGPSRHKMSPPPSGFGERSSGGSGGGPLSHFYSGSPTSYFTSGLQAGLKQSHLSKAIGSSPLGSGEGLLGLSPGPNGHSHLLKTPLGGQKRSFAHLLPSPEPSPEGSYVGQHSQGLGGHYADSYLKRKRIF

>NP_002810.1

MDGIVPDIAVGTKRGSDELFSTCVTNGPFIMSSNSASAANGNDSKKFKGDSRSAGVPSRVIHIRKLPIDVTEGEVISLGLPFGKVTNLLMLKGKNQAFIEMNTEEAANTMVNYYTSVTPVLRGQPIYIQFSNHKELKTDSSPNQARAQAALQAVNSVQSGNLALAASAAAVDAGMAMAGQSPVLRIIVENLFYPVTLDVLHQIFSKFGTVLKIITFTKNNQFQALLQYADPVSAQHAKLSLDGQNIYNACCTLRIDFSKLTSLNVKYNNDKSRDYTRPDLPSGDSQPSLDQTMAAAFGAPGIISASPYAGAGFPPTFAIPQAAGLSVPNVHGALAPLAIPSAAAAAAAAGRIAIPGLAGAGNSVLLVSNLNPERVTPQSLFILFGVYGDVQRVKILFNKKENALVQMADGNQAQLAMSHLNGHKLHGKPIRITLSKHQNVQLPREGQEDQGLTKDYGNSPLHRFKKPGSKNFQNIFPPSATLHLSNIPPSVSEEDLKVLFSSNGGVVKGFKFFQKDRKMALIQMGSVEEAVQALIDLHNHDLGENHHLRVSFSKSTI

34 NP_006629.2 NP_055844.1

>NP_006629.2

MATLRRLREAPRHLLVCEKSNFGNHKSRHRHLVQTHYYNYRVSFLIPECGILSEELKNLVMNTGPYYFVKNLPLHELITPEFISTFIKKGSCYALTYNTHIDEDNTVALLPNGKLILSLDKDTYEETGLQGHPSQFSGRKIMKFIVSIDLMELSLNLDSKKYERISWSFKEKKPLKFDFLLAWHKTGSEESTMMSYFSKYQIQEHQPKVALSTLRDLQCPVLQSSELEGTPEVSCRALELFDWLGAVFSNVDLNNEPNNFISTYCCPEPSTVVAKAYLCTITGFILPEKICLLLEHLCHYFDEPKLAPWVTLSVQGFADSPVSWEKNEHGFRKGGEHLYNFVIFNNQDYWLQMAVGANDHCPP

>NP_055844.1

MSNAKERKHAKKMRNQPTNVTLSSGFVADRGVKHHSGGEKPFQAQKQEPHPGTSRQRQTRVNPHSLPDPEVNEQSSSKGMFRKKGGWKAGPEGTSQEIPKYITASTFAQARAAEISAMLKAVTQKSSNSLVFQTLPRHMRRRAMSHNVKRLPRRLQEIAQKEAEKAVHQKKEHSKNKCHKARRCHMNRTLEFNRRQKKNIWLETHIWHAKRFHMVKKWGYCLGERPTVKSHRACYRAMTNRCLLQDLSYYCCLELKGKEEEILKALSGMCNIDTGLTFAAVHCLSGKRQGSLVLYRVNKYPREMLGPVTFIWKSQRTPGDPSESRQLWIWLHPTLKQDILEEIKAACQCVEPIKSAVCIADPLPTPSQEKSQTELPDEKIGKKRKRKDDGENAKPIKKIIGDGTRDPCLPYSWISPTTGIIISDLTMEMNRFRLIGPLSHSILTEAIKAASVHTVGEDTEETPHRWWIETCKKPDSVSLHCRQEAIFELLGGITSPAEIPAGTILGLTVGDPRINLPQKKSNALPNPEKCQDNEKVRQLLLEGVPVECTHSFIWNQDICKSVTENKISDQDLNRMRSELLVPGSQLILGPHESKIPILLIQQPGKVTGEDRLGWGSGWDVLLPKGWGMAFWIPFIYRGVRVGGLKESAVHSQYKRSPNVPGDFPDCPAGMLFAEEQAKNLLEKYKRRPPAKRPNYVKLGTLAPFCCPWEQLTQDWESRVQAYEEPSVASSPNGKESDLRRSEVPCAPMPKKTHQPSDEVGTSIEHPREAEEVMDAGCQESAGPERITDQEASENHVAATGSHLCVLRSRKLLKQLSAWCGPSSEDSRGGRRAPGRGQQGLTREACLSILGHFPRALVWVSLSLLSKGSPEPHTMICVPAKEDFLQLHEDWHYCGPQESKHSDPFRSKILKQKEKKKREKRQKPGRASSDGPAGEEPVAGQEALTLGLWSGPLPRVTLHCSRTLLGFVTQGDFSMAVGCGEALGFVSLTGLLDMVSSQPAAQRGLVLLRPPASLQYRFARIAIEV

35 NP_055586.1 NP_000312.2

>NP_055586.1

MSGPGNKRAAGDGGSGPPEKKLSREEKTTTTLIEPIRLGGISSTEEMDLKVLQFKNKKLAERLEQRQACEDELRERIEKLEKRQATDDATLLIVNRYWAQLDETVEALLRCHESQGELSSAPEAPGTQEGPTCDGTPLPEPGTSELRDPLLMQLRPPLSEPALAFVVALGASSSEEVELELQGRMEFSKAAVSRVVEASDRLQRRVEELCQRVYSRGDSEPLSEAAQAHTRELGRENRRLQDLATQLQEKHHRISLEYSELQDKVTSAETKVLEMETTVEDLQWDIEKLRKREQKLNKHLAEALEQLNSGYYVSGSSSGFQGGQITLSMQKFEMLNAELEENQELANSRMAELEKLQAELQGAVRTNERLKVALRSLPEEVVRETGEYRMLQAQFSLLYNESLQVKTQLDEARGLLLATKNSHLRHIEHMESDELGLQKKLRTEVIQLEDTLAQVRKEYEMLRIEFEQNLAANEQAGPINREMRHLISSLQNHNHQLKGDAQRYKRKLREVQAEIGKLRAQASGSAHSTPNLGHPEDSGVSAPAPGKEEGGPGPVSTPDNRKEMAPVPGTTTTTTSVKKEELVPSEEDFQGITPGAQGPSSRGREPEARPKRELREREGPSLGPPPVASALSRADREKAKVEETKRKESELLKGLRAELKKAQESQKEMKLLLDMYKSAPKEQRDKVQLMAAERKAKAEVDELRSRIRELEERDRRESKKIADEDALRRIRQAEEQIEHLQRKLGATKQEEEALLSEMDVTGQAFEDMQEQNGRLLQQLREKDDANFKLMSERIKANQIHKLLREEKDELGEQVLGLKSQVDAQLLTVQKLEEKERALQGSLGGVEKELTLRSQALELNKRKAVEAAQLAEDLKVQLEHVQTRLREIQPCLAESRAAREKESFNLKRAQEDISRLRRKLEKQRKVEVYADADEILQEEIKEYKARLTCPCCNTRKKDAVLTKCFHVFCFECVRGRYEARQRKCPKCNAAFGAHDFHRIYIS

>NP_000312.2

MPPKTPRKTAATAAAAAAEPPAPPPPPPPEEDPEQDSGPEDLPLVRLEFEETEEPDFTALCQKLKIPDHVRERAWLTWEKVSSVDGVLGGYIQKKKELWGICIFIAAVDLDEMSFTFTELQKNIEISVHKFFNLLKEIDTSTKVDNAMSRLLKKYDVLFALFSKLERTCELIYLTQPSSSISTEINSALVLKVSWITFLLAKGEVLQMEDDLVISFQLMLCVLDYFIKLSPPMLLKEPYKTAVIPINGSPRTPRRGQNRSARIAKQLENDTRIIEVLCKEHECNIDEVKNVYFKNFIPFMNSLGLVTSNGLPEVENLSKRYEEIYLKNKDLDARLFLDHDKTLQTDSIDSFETQRTPRKSNLDEEVNVIPPHTPVRTVMNTIQQLMMILNSASDQPSENLISYFNNCTVNPKESILKRVKDIGYIFKEKFAKAVGQGCVEIGSQRYKLGVRLYYRVMESMLKSEEERLSIQNFSKLLNDNIFHMSLLACALEVVMATYSRSTSQNLDSGTDLSFPWILNVLNLKAFDFYKVIESFIKAEGNLTREMIKHLERCEHRIMESLAWLSDSPLFDLIKQSKDREGPTDHLESACPLNLPLQNNHTAADMYLSPVRSPKKKGSTTRVNSTANAETQATSAFQTQKPLKSTSLSLFYKKVYRLAYLRLNTLCERLLSEHPELEHIIWTLFQHTLQNEYELMRDRHLDQIMMCSMYGICKVKNIDLKFKIIVTAYKDLPHAVQETFKRVLIKEEEYDSIIVFYNSVFMQRLKTNILQYASTRPPTLSPIPHIPRSPYKFPSSPLRIPGGNIYISPLKSPYKISEGLPTPTKMTPRSRILVSIGESFGTSEKFQKINQMVCNSDRVLKRSAEGSNPPKPLKKLRFDIEGSDEADGSKHLPGESKFQQKLAEMTSTRTRMQKQKMNDSMDTSNKEEK

36 NP_001456.3 NP_057421.1

>NP_001456.3

MAKYNTGGNPTEDVSVNSRPFRVTGPNSSSGIQARKNLFNNQGNASPPAGPSNVPKFGSPKPPVAVKPSSEEKPDKEPKPPFLKPTGAGQRFGTPASLTTRDPEAKVGFLKPVGPKPINLPKEDSKPTFPWPPGNKPSLHSVNQDHDLKPLGPKSGPTPPTSENEQKQAFPKLTGVKGKFMSASQDLEPKPLFPKPAFGQKPPLSTENSHEDESPMKNVSSSKGSPAPLGVRSKSGPLKPAREDSENKDHAGEISSLPFPGVVLKPAASRGGPGLSKNGEEKKEDRKIDAAKNTFQSKINQEELASGTPPARFPKAPSKLTVGGPWGQSQEKEKGDKNSATPKQKPLPPLFTLGPPPPKPNRPPNVDLTKFHKTSSGNSTSKGQTSYSTTSLPPPPPSHPASQPPLPASHPSQPPVPSLPPRNIKPPFDLKSPVNEDNQDGVTHSDGAGNLDEEQDSEGETYEDIEASKEREKKREKEEKKRLELEKKEQKEKEKKEQEIKKKFKLTGPIQVIHLAKACCDVKGGKNELSFKQGEQIEIIRITDNPEGKWLGRTARGSYGYIKTTAVEIDYDSLKLKKDSLGAPSRPIEDDQEVYDDVAEQDDISSHSQSGSGGIFPPPPDDDIYDGIEEEDADDGSTLQVQEKSNTWSWGILKMLKGKDDRKKSIREKPKVSDSDNNEGSSFPAPPKQLDMGDEVYDDVDTSDFPVSSAEMSQGTNVGKAKTEEKDLKKLKKQEKEEKDFRKKFKYDGEIRVLYSTKVTTSITSKKWGTRDLQVKPGESLEVIQTTDDTKVLCRNEEGKYGYVLRSYLADNDGEIYDDIADGCIYDND

>NP_057421.1

MATSEQSICQARASVMVYDDTSKKWVPIKPGQQGFSRINIYHNTASNTFRVVGVKLQDQQVVINYSIVKGLKYNQATPTFHQWRDARQVYGLNFASKEEATTFSNAMLFALNIMNSQEGGPSSQRQVQNGPSPDEMDIQRRQVMEQHQQQRQESLERRTSATGPILPPGHPSSAASAPVSCSGPPPPPPPPVPPPPTGATPPPPPPLPAGGAQGSSHDESSMSGLAAAIAGAKLRRVQRPEDASGGSSPSGTSKSDANRASSGGGGGGLMEEMNKLLAKRRKAASQSDKPAEKKEDESQMEDPSTSPSPGTRAASQPPNSSEAGRKPWERSNSVEKPVSSILSRTPSVAKSPEAKSPLQSQPHSRMKPAGSVNDMALDAFDLDRMKQEILEEVVRELHKVKEEIIDAIRQELSGISTT

37 NP_065438.1 NP_068595.3

>NP_065438.1

MWGDLWLLPPASANPGTGTEAEFEKAAEEVRHLKTKPSDEEMLFIYGHYKQATVGDINTERPGMLDFTGKAKWDAWNELKGTSKEDAMKAYINKVEELKKKYGI

>NP_068595.3

MEPKASCPAAAPLMERKFHVLVGVTGSVAALKLPLLVSKLLDIPGLEVAVVTTERAKHFYSPQDIPVTLYSDADEWEIWKSRSDPVLHIDLRRWADLLLVAPLDANTLGKVASGICDNLLTCVMRAWDRSKPLLFCPAMNTAMWEHPITAQQVDQLKAFGYVEIPCVAKKLVCGDEGLGAMAEVGTIVDKVKEVLFQHSGFQQS

38 NP_004624.1 NP_000567.1

>NP_004624.1

MLRLYVLVMGVSAFTLQPAAHTGAARSCRFRGRHYKREFRLEGEPVALRCPQVPYWLWASVSPRINLTWHKNDSARTVPGEEETRMWAQDGALWLLPALQEDSGTYVCTTRNASYCDKMSIELRVFENTDAFLPFISYPQILTLSTSGVLVCPDLSEFTRDKTDVKIQWYKDSLLLDKDNEKFLSVRGTTHLLVHDVALEDAGYYRCVLTFAHEGQQYNITRSIELRIKKKKEETIPVIISPLKTISASLGSRLTIPCKVFLGTGTPLTTMLWWTANDTHIESAYPGGRVTEGPRQEYSENNENYIEVPLIFDPVTREDLHMDFKCVVHNTLSFQTLRTTVKEASSTFSWGIVLAPLSLAFLVLGGIWMHRRCKHRTGKADGLTVLWPHHQDFQSYPK

>NP_000567.1

MAEVPELASEMMAYYSGNEDDLFFEADGPKQMKCSFQDLDLCPLDGGIQLRISDHHYSKGFRQAASVVVAMDKLRKMLVPCPQTFQENDLSTFFPFIFEEEPIFFDTWDNEAYVHDAPVRSLNCTLRDSQQKSLVMSGPYELKALHLQGQDMEQQVVFSMSFVQGEESNDKIPVALGLKEKNLYLSCVLKDDKPTLQLESVDPKNYPKKKMEKRFVFNKIEINNKLEFESAQFPNWYISTSQAENMPVFLGGTKGGQDITDFTMQFVSS

39 NP_003874.2 NP_000312.2

>NP_003874.2

MAKTVAYFYDPDVGNFHYGAGHPMKPHRLALTHSLVLHYGLYKKMIVFKPYQASQHDMCRFHSEDYIDFLQRVSPTNMQGFTKSLNAFNVGDDCPVFPGLFEFCSRYTGASLQGATQLNNKICDIAINWAGGLHHAKKFEASGFCYVNDIVIGILELLKYHPRVLYIDIDIHHGDGVQEAFYLTDRVMTVSFHKYGNYFFPGTGDMYEVGAESGRYYCLNVPLRDGIDDQSYKHLFQPVINQVVDFYQPTCIVLQCGADSLGCDRLGCFNLSIRGHGECVEYVKSFNIPLLVLGGGGYTVRNVARCWTYETSLLVEEAISEELPYSEYFEYFAPDFTLHPDVSTRIENQNSRQYLDQIRQTIFENLKMLNHAPSVQIHDVPADLLTYDRTDEADAEERGPEENYSRPEAPNEFYDGDHDNDKESDVEI

>NP_000312.2

MPPKTPRKTAATAAAAAAEPPAPPPPPPPEEDPEQDSGPEDLPLVRLEFEETEEPDFTALCQKLKIPDHVRERAWLTWEKVSSVDGVLGGYIQKKKELWGICIFIAAVDLDEMSFTFTELQKNIEISVHKFFNLLKEIDTSTKVDNAMSRLLKKYDVLFALFSKLERTCELIYLTQPSSSISTEINSALVLKVSWITFLLAKGEVLQMEDDLVISFQLMLCVLDYFIKLSPPMLLKEPYKTAVIPINGSPRTPRRGQNRSARIAKQLENDTRIIEVLCKEHECNIDEVKNVYFKNFIPFMNSLGLVTSNGLPEVENLSKRYEEIYLKNKDLDARLFLDHDKTLQTDSIDSFETQRTPRKSNLDEEVNVIPPHTPVRTVMNTIQQLMMILNSASDQPSENLISYFNNCTVNPKESILKRVKDIGYIFKEKFAKAVGQGCVEIGSQRYKLGVRLYYRVMESMLKSEEERLSIQNFSKLLNDNIFHMSLLACALEVVMATYSRSTSQNLDSGTDLSFPWILNVLNLKAFDFYKVIESFIKAEGNLTREMIKHLERCEHRIMESLAWLSDSPLFDLIKQSKDREGPTDHLESACPLNLPLQNNHTAADMYLSPVRSPKKKGSTTRVNSTANAETQATSAFQTQKPLKSTSLSLFYKKVYRLAYLRLNTLCERLLSEHPELEHIIWTLFQHTLQNEYELMRDRHLDQIMMCSMYGICKVKNIDLKFKIIVTAYKDLPHAVQETFKRVLIKEEEYDSIIVFYNSVFMQRLKTNILQYASTRPPTLSPIPHIPRSPYKFPSSPLRIPGGNIYISPLKSPYKISEGLPTPTKMTPRSRILVSIGESFGTSEKFQKINQMVCNSDRVLKRSAEGSNPPKPLKKLRFDIEGSDEADGSKHLPGESKFQQKLAEMTSTRTRMQKQKMNDSMDTSNKEEK

40 NP_689846.1 NP_001984.1

>NP_689846.1

MTSKQAMSSNEQERLLCYNGEVLVFQLSKGNFADKEPTKTPILHVRRMVFDRGTKVFVQKSTGFFTIKEENSHLKIMCCNCVSDFRTGINLPYIVIEKNKKNNVFEYFLLILHSTNKFEMRLSFKLGYEMKDGLRVLNGPLILWRHVKAFFFISSQTGKVVSVSGNFSSIQWAGEIENLGMVLLGLKECCLSEEECTQEPSKSDYAIWNTKFCVYSLESQEVLSDIYIIPPAYSSVVTYVHICATEIIKNQLRISLIALTRKNQLISFQNGTPKNVCQLPFGDPCAVQLMDSGGGNLFFVVSFISNNACAVWKESFQVAAKWEKLSLVLIDDFIGSGTEQVLLLFKDSLNSDCLTSFKITDLGKINYSSEPSDCNEDDLFEDKQENRYLVVPPLETGLKVCFSSFRELRQHLLLKEKIISKSYKALINLVQGKDDNTSSAEEKECLVPLCGEEENSVHILDEKLSDNFQDSEQLVEKIWYRVIDDSLVVGVKTTSSLKLSLNDVTLSLLMDQAHDSRFRLLKCQNRVIKLSTNPFPAPYLMPCEIGLEAKRVTLTPDSKKEESFVCEHPSKKECVQIITAVTSLSPLLTFSKFCCTVLLQIMERESGNCPKDRYVVCGRVFLSLEDLSTGKYLLTFPKKKPIEHMEDLFALLAAFHKSCFQITSPGYALNSMKVWLLEHMKCEIIKEFPEVYFCERPGSFYGTLFTWKQRTPFEGILIIYSRNQTVMFQCLHNLIRILPINCFLKNLKSGSENFLIDNMAFTLEKELVTLSSLSSAIAKHESNFMQRCEVSKGKSSVVAAALSDRRENIHPYRKELQREKKKMLQTNLKVSGALYREITLKVAEVQLKSDFAAQKLSNL

>NP_001984.1

METPAWPRVPRPETAVARTLLLGWVFAQVAGASGTTNTVAAYNLTWKSTNFKTILEWEPKPVNQVYTVQISTKSGDWKSKCFYTTDTECDLTDEIVKDVKQTYLARVFSYPAGNVESTGSAGEPLYENSPEFTPYLETNLGQPTIQSFEQVGTKVNVTVEDERTLVRRNNTFLSLRDVFGKDLIYTLYYWKSSSSGKKTAKTNTNEFLIDVDKGENYCFSVQAVIPSRTVNRKSTDSPVECMGQEKGEFREIFYIIGAVVFVVIILVIILAISLHKCRKAGVGQSWKENSPLNVS

41 NP_001020128.1 NP_060842.3

>NP_001020128.1

MPVQPPSKDTEEMEAEGDSAAEMNGEEEESEEERSGSQTESEEESSEMDDEDYERRRSECVSEMLDLEKQFSELKEKLFRERLSQLRLRLEEVGAERAPEYTEPLGGLQRSLKIRIQVAGIYKGFCLDVIRNKYECELQGAKQHLESEKLLLYDTLQGELQERIQRLEEDRQSLDLSSEWWDDKLHARGSSRSWDSLPPSKRKKAPLVSGPYIVYMLQEIDILEDWTAIKKARAAVSPQKRKSDDRRTHRPLRVCPARLLWCCWALPLHLALAWTPPLPSSRPAQLWPWS

>NP_060842.3

MSNNLRRVFLKPAEENSGNASRCVSGCMYQVVQTIGSDGKNLLQLLPIPKSSGNLIPLVQSSVMSDALKGNTGKPVQVTFQTQISSSSTSASVQLPIFQPASSSNYFLTRTVDTSEKGRVTSVGTGNFSSSVSKVQSHGVKIDGLTMQTFAVPPSTQKDSSFIVVNTQSLPVTVKSPVLPSGHHLQIPAHAEVKSVPASSLPPSVQQKILATATTSTSGMVEASQMPTVIYVSPVNTVKNVVTKNFQNIYPKPVTEIAKPVILNTTQIPKNVATETQLKGGQHSQAAPVKWIFQDNLQPFTPSLVPVKSSNNVASKILKTFVDRKNLGDNTINMPPLSTIDPSGTRSKNMPIKDNALVMFNGKVYLLAKKGTDVLPSQIDQQNSVSPDTPVRKDTLQTVSSSPVTEISREVVNIVLAKSKSSQMETKSLSNTQLASMANLRAEKNKVEKPSPSTTNPHMNQSSNYLKQSKTLFTNPIFPVGFSTGHNAPRKVTAVIYARKGSVLQSIEKISSSVDATTVTSQQCVFRDQEPKIHNEMASTSDKGAQGRNDKKDSQGRSNKALHLKSDAEFKKIFGLTKDLRVCLTRIPDHLTSGEGFDSFSSLVKSGTYKETEFMVKEGERKQQNFDKKRKAKTNKKMDHIKKRKTENAYNAIINGEANVTGSQLLSSILPTSDVSQHNILTSHSKTRQEKRTEMEYYTHEKQEKGTLNSNAAYEQSHFFNKNYTEDIFPVTPPELEETIRDEKIRRLKQVLREKEAALEEMRKKMHQK

42 NP_057227.2 NP_003155.2

>NP_057227.2

MAAERQEALREFVAVTGAEEDRARFFLESAGWDLQIALASFYEDGGDEDIVTISQATPSSVSRGTAPSDNRVTSFRDLIHDQDEDEEEEEGQRFYAGGSERSGQQIVGPPRKKSPNELVDDLFKGAKEHGAVAVERVTKSPGETSKPRPFAGGGYRLGAAPEEESAYVAGEKRQHSSQDVHVVLKLWKSGFSLDNGELRSYQDPSNAQFLESIRRGEVPAELRRLAHGGQVNLDMEDHRDEDFVKPKGAFKAFTGEGQKLGSTAPQVLSTSSPAQQAENEAKASSSILIDESEPTTNIQIRLADGGRLVQKFNHSHRISDIRLFIVDARPAMAATSFILMTTFPNKELADESQTLKEANLLNAVIVQRLT

>NP_003155.2

MIPRKRYGSKNTDQGVYLGLSKTQVLSPATAGSSSSDIAPLPPPVTLVPPPPDTMSCRDRTQEFLSACKSLQTRQNGIQTNKPALRAVRQRSEFTLMAKRIGKDLSNTFAKLEKLTILAKRKSLFDDKAVEIEELTYIIKQDINSLNKQIAQLQDFVRAKGSQSGRHLQTHSNTIVVSLQSKLASMSNDFKSVLEVRTENLKQQRSRREQFSRAPVSALPLAPNHLGGGAVVLGAESHASKDVAIDMMDSRTSQQLQLIDEQDSYIQSRADTMQNIESTIVELGSIFQQLAHMVKEQEETIQRIDENVLGAQLDVEAAHSEILKYFQSVTSNRWLMVKIFLILIVFFIIFVVFLA

43 NP_110435.1 NP_001367.2

>NP_110435.1

MDGEDIPDFSSLKEETAYWKELSLKYKQSFQEARDELVEFQEGSRELEAELEAQLVQAEQRNRDLQADNQRLKYEVEALKEKLEHQYAQSYKQVSVLEDDLSQTRAIKEQLHKYVRELEQANDDLERAKRATIVSLEDFEQRLNQAIERNAFLESELDEKESLLVSVQRLKDEARDLRQELAVRERQQEVTRKSAPSSPTLDCEKMDSAVQASLSLPATPVGKGTENTFPSPKAIPNGFGTSPLTPSARISALNIVGDLLRKVGALESKLAACRNFAKDQASRKSYISGNVNCGVLNGNGTKFSRSGHTSFFDKGAVNGFDPAPPPPGLGSSRPSSAPGMLPLSV

>NP_001367.2

MSEPGGGGGEDGSAGLEVSAVQNVADVSVLQKHLRKLVPLLLEDGGEAPAALEAALEEKSALEQMRKFLSDPQVHTVLVERSTLKEDVGDEGEEEKEFISYNINIDIHYGVKSNSLAFIKRTPVIDADKPVSSQLRVLTLSEDSPYETLHSFISNAVAPFFKSYIRESGKADRDGDKMAPSVEKKIAELEMGLLHLQQNIEIPEISLPIHPMITNVAKQCYERGEKPKVTDFGDKVEDPTFLNQLQSGVNRWIREIQKVTKLDRDPASGTALQEISFWLNLERALYRIQEKRESPEVLLTLDILKHGKRFHATVSFDTDTGLKQALETVNDYNPLMKDFPLNDLLSATELDKIRQALVAIFTHLRKIRNTKYPIQRALRLVEAISRDLSSQLLKVLGTRKLMHVAYEEFEKVMVACFEVFQTWDDEYEKLQVLLRDIVKRKREENLKMVWRINPAHRKLQARLDQMRKFRRQHEQLRAVIVRVLRPQVTAVAQQNQGEVPEPQDMKVAEVLFDAADANAIEEVNLAYENVKEVDGLDVSKEGTEAWEAAMKRYDERIDRVETRITARLRDQLGTAKNANEMFRIFSRFNALFVRPHIRGAIREYQTQLIQRVKDDIESLHDKFKVQYPQSQACKMSHVRDLPPVSGSIIWAKQIDRQLTAYMKRVEDVLGKGWENHVEGQKLKQDGDSFRMKLNTQEIFDDWARKVQQRNLGVSGRIFTIESTRVRGRTGNVLKLKVNFLPEIITLSKEVRNLKWLGFRVPLAIVNKAHQANQLYPFAISLIESVRTYERTCEKVEERNTISLLVAGLKKEVQALIAEGIALVWESYKLDPYVQRLAETVFNFQEKVDDLLIIEEKIDLEVRSLETCMYDHKTFSEILNRVQKAVDDLNLHSYSNLPIWVNKLDMEIERILGVRLQAGLRAWTQVLLGQAEDKAEVDMDTDAPQVSHKPGGEPKIKNVVHELRITNQVIYLNPPIEECRYKLYQEMFAWKMVVLSLPRIQSQRYQVGVHYELTEEEKFYRNALTRMPDGPVALEESYSAVMGIVSEVEQYVKVWLQYQCLWDMQAENIYNRLGEDLNKWQALLVQIRKARGTFDNAETKKEFGPVVIDYGKVQSKVNLKYDSWHKEVLSKFGQMLGSNMTEFHSQISKSRQELEQHSVDTASTSDAVTFITYVQSLKRKIKQFEKQVELYRNGQRLLEKQRFQFPPSWLYIDNIEGEWGAFNDIMRRKDSAIQQQVANLQMKIVQEDRAVESRTTDLLTDWEKTKPVTGNLRPEEALQALTIYEGKFGRLKDDREKCAKAKEALELTDTGLLSGSEERVQVALEELQDLKGVWSELSKVWEQIDQMKEQPWVSVQPRKLRQNLDALLNQLKSFPARLRQYASYEFVQRLLKGYMKINMLVIELKSEALKDRHWKQLMKRLHVNWVVSELTLGQIWDVDLQKNEAIVKDVLLVAQGEMALEEFLKQIREVWNTYELDLVNYQNKCRLIRGWDDLFNKVKEHINSVSAMKLSPYYKVFEEDALSWEDKLNRIMALFDVWIDVQRRWVYLEGIFTGSADIKHLLPVETQRFQSISTEFLALMKKVSKSPLVMDVLNIQGVQRSLERLADLLGKIQKALGEYLERERSSFPRFYFVGDEDLLEIIGNSKNVAKLQKHFKKMFAGVSSIILNEDNSVVLGISSREGEEVMFKTPVSITEHPKINEWLTLVEKEMRVTLAKLLAESVTEVEIFGKATSIDPNTYITWIDKYQAQLVVLSAQIAWSENVETALSSMGGGGDAAPLHSVLSNVEVTLNVLADSVLMEQPPLRRRKLEHLITELVHQRDVTRSLIKSKIDNAKSFEWLSQMRFYFDPKQTDVLQQLSIQMANAKFNYGFEYLGVQDKLVQTPLTDRCYLTMTQALEARLGGSPFGPAGTGKTESVKALGHQLGRFVLVFNCDETFDFQAMGRIFVGLCQVGAWGCFDEFNRLEERMLSAVSQQVQCIQEALREHSNPNYDKTSAPITCELLNKQVKVSPDMAIFITMNPGYAGRSNLPDNLKKLFRSLAMTKPDRQLIAQVMLYSQGFRTAEVLANKIVPFFKLCDEQLSSQSHYDFGLRALKSVLVSAGNVKRERIQKIKREKEERGEAVDEGEIAENLPEQEILIQSVCETMVPKLVAEDIPLLFSLLSDVFPGVQYHRGEMTALREELKKVCQEMYLTYGDGEEVGGMWVEKVLQLYQITQINHGLMMVGPSGSGKSMAWRVLLKALERLEGVEGVAHIIDPKAISKDHLYGTLDPNTREWTDGLFTHVLRKIIDSVRGELQKRQWIVFDGDVDPEWVENLNSVLDDNKLLTLPNGERLSLPPNVRIMFEVQDLKYATLATVSRCGMVWFSEDVLSTDMIFNNFLARLRSIPLDEGEDEAQRRRKGKEDEGEEAASPMLQIQRDAATIMQPYFTSNGLVTKALEHAFQLEHIMDLTRLRCLGSLFSMLHQACRNVAQYNANHPDFPMQIEQLERYIQRYLVYAILWSLSGDSRLKMRAELGEYIRRITTVPLPTAPNIPIIDYEVSISGEWSPWQAKVPQIEVETHKVAAPDVVVPTLDTVRHEALLYTWLAEHKPLVLCGPPGSGKTMTLFSALRALPDMEVVGLNFSSATTPELLLKTFDHYCEYRRTPNGVVLAPVQLGKWLVLFCDEINLPDMDKYGTQRVISFIRQMVEHGGFYRTSDQTWVKLERIQFVGACNPPTDPGRKPLSHRFLRHVPVVYVDYPGPASLTQIYGTFNRAMLRLIPSLRTYAEPLTAAMVEFYTMSQERFTQDTQPHYIYSPREMTRWVRGIFEALRPLETLPVEGLIRIWAHEALRLFQDRLVEDEERRWTDENIDTVALKHFPNIDREKAMSRPILYSNWLSKDYIPVDQEELRDYVKARLKVFYEEELDVPLVLFNEVLDHVLRIDRIFRQPQGHLLLIGVSGAGKTTLSRFVAWMNGLSVYQIKVHRKYTGEDFDEDLRTVLRRSGCKNEKIAFIMDESNVLDSGFLERMNTLLANGEVPGLFEGDEYATLMTQCKEGAQKEGLMLDSHEELYKWFTSQVIRNLHVVFTMNPSSEGLKDRAATSPALFNRCVLNWFGDWSTEALYQVGKEFTSKMDLEKPNYIVPDYMPVVYDKLPQPPSHREAIVNSCVFVHQTLHQANARLAKRGGRTMAITPRHYLDFINHYANLFHEKRSELEEQQMHLNVGLRKIKETVDQVEELRRDLRIKSQELEVKNAAANDKLKKMVKDQQEAEKKKVMSQEIQEQLHKQQEVIADKQMSVKEDLDKVEPAVIEAQNAVKSIKKQHLVEVRSMANPPAAVKLALESICLLLGESTTDWKQIRSIIMRENFIPTIVNFSAEEISDAIREKMKKNYMSNPSYNYEIVNRASLACGPMVKWAIAQLNYADMLKRVEPLRNELQKLEDDAKDNQQKANEVEQMIRDLEASIARYKEEYAVLISEAQAIKADLAAVEAKVNRSTALLKSLSAERERWEKTSETFKNQMSTIAGDCLLSAAFIAYAGYFDQQMRQNLFTTWSHHLQQANIQFRTDIARTEYLSNADERLRWQASSLPADDLCTENAIMLKRFNRYPLIIDPSGQATEFIMNEYKDRKITRTSFLDDAFRKNLESALRFGNPLLVQDVESYDPVLNPVLNREVRRTGGRVLITLGDQDIDLSPSFVIFLSTRDPTVEFPPDLCSRVTFVNFTVTRSSLQSQCLNEVLKAERPDVDEKRSDLLKLQGEFQLRLRQLEKSLLQALNEVKGRILDDDTIITTLENLKREAAEVTRKVEETDIVMQEVETVSQQYLPLSTACSSIYFTMESLKQIHFLYQYSLQFFLDIYHNVLYENPNLKGVTDHTQRLSIITKDLFQVAFNRVARGMLHQDHITFAMLLARIKLKGTVGEPTYDAEFQHFLRGNEIVLSAGSTPRIQGLTVEQAEAVVRLSCLPAFKDLIAKVQADEQFGIWLDSSSPEQTVPYLWSEETPATPIGQAIHRLLLIQAFRPDRLLAMAHMFVSTNLGESFMSIMEQPLDLTHIVGTEVKPNTPVLMCSVPGYDASGHVEDLAAEQNTQITSIAIGSAEGFNQADKAINTAVKSGRWVMLKNVHLAPGWLMQLEKKLHSLQPHACFRLFLTMEINPKVPVNLLRAGRIFVFEPPPGVKANMLRTFSSIPVSRICKSPNERARLYFLLAWFHAIIQERLRYAPLGWSKKYEFGESDLRSACDTVDTWLDDTAKGRQNISPDKIPWSALKTLMAQSIYGGRVDNEFDQRLLNTFLERLFTTRSFDSEFKLACKVDGHKDIQMPDGIRREEFVQWVELLPDTQTPSWLGLPNNAERVLLTTQGVDMISKMLKMQMLEDEDDLAYAETEKKTRTDSTSDGRPAWMRTLHTTASNWLHLIPQTLSHLKRTVENIKDPLFRFFEREVKMGAKLLQDVRQDLADVVQVCEGKKKQTNYLRTLINELVKGILPRSWSHYTVPAGMTVIQWVSDFSERIKQLQNISLAAASGGAKELKNIHVCLGGLFVPEAYITATRQYVAQANSWSLEELCLEVNVTTSQGATLDACSFGVTGLKLQGATCNNNKLSLSNAISTALPLTQLRWVKQTNTEKKASVVTLPVYLNFTRADLIFTVDFEIATKEDPRSFYERGVAVLCTE

44 NP_061846.2 NP_003850.1

>NP_061846.2

MLSVGGLRLSLVRFSFLLLRGALLPSLAVTMTKLAQWLWGLAILGSTWVALTTGALGLELPLSCQEVLWPLPAYLLVSAGCYALGTVGYRVATFHDCEDAARELQSQIQEARADLARRGLRF

>NP_003850.1

MASLEVSRSPRRSRRELEVRSPRQNKYSVLLPTYNERENLPLIVWLLVKSFSESGINYEIIIIDDGSPDGTRDVAEQLEKIYGSDRILLRPREKKLGLGTAYIHGMKHATGNYIIIMDADLSHHPKFIPEFIRKQKEGNFDIVSGTRYKGNGGVYGWDLKRKIISRGANFLTQILLRPGASDLTGSFRLYRKEVLEKLIEKCVSKGYVFQMEMIVRARQLNYTIGEVPISFVDRVYGESKLGGNEIVSFLKGLLTLFATT

45 NP_005501.2 NP_001001998.1

>NP_005501.2

MDPRGTKRGAEKTEVAEPRNKLPRPAPSLPTDPALYSGPFPFYRRPSELGCFSLDAQRQYHGDARALRYYSPPPTNGPGPNFDLRDGYPDRYQPRDEEVQERLDHLLCWLLEHRGRLEGGPGWLAEAIVTWRGHLTKLLTTPYERQEGWQLAASRFQGTLYLSEVETPNARAQRLARPPLLRELMYMGYKFEQYMCADKPGSSPDPSGEVNTNVAFCSVLRSRLGSHPLLFSGEVDCTDPQAPSTQPPTCYVELKTSKEMHSPGQWRSFYRHKLLKWWAQSFLPGVPNVVAGFRNPDGFVSSLKTFPTMKMFEYVRNDRDGWNPSVCMNFCAAFLSFAQSTVVQDDPRLVHLFSWEPGGPVTVSVHQDAPYAFLPIWYVEAMTQDLPSPPKTPSPK

>NP_001001998.1

MAPPSTREPRVLSATSATKSDGEMVLPGFPDADSFVKFALGSVVAVTKASGGLPQFGDEYDFYRSFPGFQAFCETQGDRLLQCMSRVMQYHGCRSNIKDRSKVTELEDKFDLLVDANDVILERVGILLDEASGVNKNQQPVLPAGLQVPKTVVSSWNRKAAEYGKKAKSETFRLLHAKNIIRPQLKFREKIDNSNTPFLPKIFIKPNAQKPLPQALSKERRERPQDRPEDLDVPPALADFIHQQRTQQVEQDMFAHPYQYELNHFTPADAVLQKPQPQLYRPIEETPCHFISSLDELVELNEKLLNCQEFAVDLEHHSYRSFLGLTCLMQISTRTEDFIIDTLELRSDMYILNESLTDPAIVKVFHGADSDIEWLQKDFGLYVVNMFDTHQAARLLNLGRHSLDHLLKLYCNVDSNKQYQLADWRIRPLPEEMLSYARDDTHYLLYIYDKMRLEMWERGNGQPVQLQVVWQRSRDICLKKFIKPIFTDESYLELYRKQKKHLNTQQLTAFQLLFAWRDKTARREDESYGYVLPNHMMLKIAEELPKEPQGIIACCNPVPPLVRQQINEMHLLIQQAREMPLLKSEVAAGVKKSGPLPSAERLENVLFGPHDCSHAPPDGYPIIPTSGSVPVQKQASLFPDEKEDNLLGTTCLIATAVITLFNEPSAEDSKKGPLTVAQKKAQNIMESFENPFRMFLPSLGHRAPVSQAAKFDPSTKIYEISNRWKLAQVQVQKDSKEAVKKKAAEQTAAREQAKEACKAAAEQAISVRQQVVLENAAKKRERATSDPRTTEQKQEKKRLKISKKPKDPEPPEKEFTPYDYSQSDFKAFAGNSKSKVSSQFDPNKQTPSGKKCIAAKKIKQSVGNKSMSFPTGKSDRGFRYNWPQR

46 NP_055260.1 NP_001737.1

>NP_055260.1

MVAKQRIRMANEKHSKNITQRGNVAKTSRNAPEEKASVGPWLLALFIFVVCGSAIFQIIQSIRMGM

>NP_001737.1

MEGKWLLCMLLVLGTAIVEAHDGHDDDVIDIEDDLDDVIEEVEDSKPDTTAPPSSPKVTYKAPVPTGEVYFADSFDRGTLSGWILSKAKKDDTDDEIAKYDGKWEVEEMKESKLPGDKGLVLMSRAKHHAISAKLNKPFLFDTKPLIVQYEVNFQNGIECGGAYVKLLSKTPELNLDQFHDKTPYTIMFGPDKCGEDYKLHFIFRHKNPKTGIYEEKHAKRPDADLKTYFTDKKTHLYTLILNPDNSFEILVDQSVVNSGNLLNDMTPPVNPSREIEDPEDRKPEDWDERPKIPDPEAVKPDDWDEDAPAKIPDEEATKPEGWLDDEPEYVPDPDAEKPEDWDEDMDGEWEAPQIANPRCESAPGCGVWQRPVIDNPNYKGKWKPPMIDNPSYQGIWKPRKIPNPDFFEDLEPFRMTPFSAIGLELWSMTSDIFFDNFIICADRRIVDDWANDGWGLKKAADGAAEPGVVGQMIEAAEERPWLWVVYILTVALPVFLVILFCCSGKKQTSGMEYKKTDAPQPDVKEEEEEKEEEKDKGDEEEEGEEKLEEKQKSDAEEDGGTVSQEEEDRKPKAEEDEILNRSPRNRKPRRE

47 NP_005139.1 NP_000724.1

>NP_005139.1

MKVKKGGGGAGTATESAPGPSGQSVAPIPQPPAESESGSESEPDAGPGPRPGPLQRKQPIGPEDVLGLQRITGDYLCSPEENIYKIDFVRFKIRDMDSGTVLFEIKKPPVSERLPINRRDLDPNAGRFVRYQFTPAFLRLRQVGATVEFTVGDKPVNNFRMIERHYFRNQLLKSFDFHFGFCIPSSKNTCEHIYDFPPLSEELISEMIRHPYETQSDSFYFVDDRLVMHNKADYSYSGTP

>NP_000724.1

MQSGTHWRVLGLCLLSVGVWGQDGNEEMGGITQTPYKVSISGTTVILTCPQYPGSEILWQHNDKNIGGDEDDKNIGSDEDHLSLKEFSELEQSGYYVCYPRGSKPEDANFYLYLRARVCENCMEMDVMSVATIVIVDICITGGLLLLVYYWSKNRKAKAKPVTRGAGAGGRQRGQNKERPPPVPNPDYEPIRKGQRDLYSGLNQRRI

48 NP_003874.2 NP_003391.1

>NP_003874.2

MAKTVAYFYDPDVGNFHYGAGHPMKPHRLALTHSLVLHYGLYKKMIVFKPYQASQHDMCRFHSEDYIDFLQRVSPTNMQGFTKSLNAFNVGDDCPVFPGLFEFCSRYTGASLQGATQLNNKICDIAINWAGGLHHAKKFEASGFCYVNDIVIGILELLKYHPRVLYIDIDIHHGDGVQEAFYLTDRVMTVSFHKYGNYFFPGTGDMYEVGAESGRYYCLNVPLRDGIDDQSYKHLFQPVINQVVDFYQPTCIVLQCGADSLGCDRLGCFNLSIRGHGECVEYVKSFNIPLLVLGGGGYTVRNVARCWTYETSLLVEEAISEELPYSEYFEYFAPDFTLHPDVSTRIENQNSRQYLDQIRQTIFENLKMLNHAPSVQIHDVPADLLTYDRTDEADAEERGPEENYSRPEAPNEFYDGDHDNDKESDVEI

>NP_003391.1

MPAIMTMLADHAARQLLDFSQKLDINLLDNVVNCLYHGEGAQQRMAQEVLTHLKEHPDAWTRVDTILEFSQNMNTKYYGLQILENVIKTRWKILPRNQCEGIKKYVVGLIIKTSSDPTCVEKEKVYIGKLNMILVQILKQEWPKHWPTFISDIVGASRTSESLCQNNMVILKLLSEEVFDFSSGQITQVKSKHLKDSMCNEFSQIFQLCQFVMENSQNAPLVHATLETLLRFLNWIPLGYIFETKLISTLIYKFLNVPMFRNVSLKCLTEIAGVSVSQYEEQFVTLFTLTMMQLKQMLPLNTNIRLAYSNGKDDEQNFIQNLSLFLCTFLKEHDQLIEKRLNLRETLMEALHYMLLVSEVEETEIFKICLEYWNHLAAELYRESPFSTSASPLLSGSQHFDVPPRRQLYLPMLFKVRLLMVSRMAKPEEVLVVENDQGEVVREFMKDTDSINLYKNMRETLVYLTHLDYVDTERIMTEKLHNQVNGTEWSWKNLNTLCWAIGSISGAMHEEDEKRFLVTVIKDLLGLCEQKRGKDNKAIIASNIMYIVGQYPRFLRAHWKFLKTVVNKLFEFMHETHDGVQDMACDTFIKIAQKCRRHFVQVQVGEVMPFIDEILNNINTIICDLQPQQVHTFYEAVGYMIGAQTDQTVQEHLIEKYMLLPNQVWDSIIQQATKNVDILKDPETVKQLGSILKTNVRACKAVGHPFVIQLGRIYLDMLNVYKCLSENISAAIQANGEMVTKQPLIRSMRTVKRETLKLISGWVSRSNDPQMVAENFVPPLLDAVLIDYQRNVPAAREPEVLSTMAIIVNKLGGHITAEIPQIFDAVFECTLNMINKDFEEYPEHRTNFFLLLQAVNSHCFPAFLAIPPTQFKLVLDSIIWAFKHTMRNVADTGLQILFTLLQNVAQEEAAAQSFYQTYFCDILQHIFSVVTDTSHTAGLTMHASILAYMFNLVEEGKISTSLNPGNPVNNQIFLQEYVANLLKSAFPHLQDAQVKLFVTGLFSLNQDIPAFKEHLRDFLVQIKEFAGEDTSDLFLEEREIALRQADEEKHKRQMSVPGIFNPHEIPEEMCD

49 NP_003611.1 NP_000960.2

>NP_003611.1

MAGLYSLGVSVFSDQGGRKYMEDVTQIVVEPEPTAEEKPSPRRSLSQPLPPRPSPAALPGGEVSGKGPAVAAREARDPLPDAGASPAPSRCCRRRSSVAFFAVCDGHGGREAAQFAREHLWGFIKKQKGFTSSEPAKVCAAIRKGFLACHLAMWKKLAEWPKTMTGLPSTSGTTASVVIIRGMKMYVAHVGDSGVVLGIQDDPKDDFVRAVEVTQDHKPELPKERERIEGLGGSVMNKSGVNRVVWKRPRLTHNGPVRRSTVIDQIPFLAVARALGDLWSYDFFSGEFVVSPEPDTSVHTLDPQKHKYIILGSDGLWNMIPPQDAISMCQDQEEKKYLMGEHGQSCAKMLVNRALGRWRQRMLRADNTSAIVICISPEVDNQGNFTNEDELYLNLTDSPSYNSQETCVMTPSPCSTPPVKSLEEDPWPRVNSKDHIPALVRSNAFSENFLEVSAEIARENVQGVVIPSKDPEPLEENCAKALTLRIHDSLNNSLPIGLVPTNSTNTVMDQKNLKMSTPGQMKAQEIERTPPTNFKRTLEESNSGPLMKKHRRNGLSRSSGAQPASLPTTSQRKNSVKLTMRRRLRGQKKIGNPLLHQHRKTVCVC

>NP_000960.2

MGFVKVVKNKAYFKRYQVKFRRRREGKTDYYARKRLVIQDKNKYNTPKYRMIVRVTNRDIICQIAYARIEGDMIVCAAYAHELPKYGVKVGLTNYAAAYCTGLLLARRLLNRFGMDKIYEGQVEVTGDEYNVESIDGQPGAFTCYLDAGLARTTTGNKVFGALKGAVDGGLSIPHSTKRFPGYDSESKEFNAEVHRKHIMGQNVADYMRYLMEEDEDAYKKQFSQYIKNSVTPDMMEEMYKKAHAAIRENPVYEKKPKKEVKKKRWNRPKMSLAQKKDRVAQKKASFLRAQERAAES

50 NP_689926.1 BAD36741.1

>NP_689926.1

MTKFGFLRLSYEKQDTLLKLLILSMAAVLSFSTRLFAVLRFESVIHEFDPYFNYRTTRFLAEEGFYKFHNWFDDRAWYPLGRIIGGTIYPGLMITSAAIYHVLHFFHITIDIRNVCVFLAPLFSSFTTIVTYHLTKELKDAGAGLLAAAMIAVVPGYISRSVAGSYDNEGIAIFCMLLTYYMWIKAVKTGSICWAAKCALAYFYMVSSWGGYVFLINLIPLHVLVLMLTGRFSHRIYVAYCTVYCLGTILSMQISFVGFQPVLSSEHMAAFGVFGLCQIHAFVDYLRSKLNPQQFEVLFRSVISLVGFVLLTVGALLMLTGKISPWTGRFYSLLDPSYAKNNIPIIASVSEHQPTTWSSYYFDLQLLVFMFPVGLYYCFSNLSDARIFIIMYGVTSMYFSAVMVRLMLVLAPVMCILSGIGVSQVLSTYMKNLDISRPDKKSKKQQDSTYPIKNEVASGMILVMAFFLITYTFHSTWVTSEAYSSPSIVLSARGGDGSRIIFDDFREAYYWLRHNTPEDAKVMSWWDYGYQITAMANRTILVDNNTWNNTHISRVGQAMASTEEKAYEIMRELDVSYVLVIFGGLTGYSSDDINKFLWMVRIGGSTDTGKHIKENDYYTPTGEFRVDREGSPVLLNCLMYKMCYYRFGQVYTEAKRPPGFDRVRNAEIGNKDFELDVLEEAYTTEHWLVRIYKVKDLDNRGLSRT

>BAD36741.1

MLGTLRAMEGEDVEDDQLLQKLRASRRRFQRRMQRLIEKYNQPFEDTPVVQMATLTYETPQGLRIWGGRLIKERNKGEIQDSSMKPADRTDGSVQAAAWGPELPSHRTVLGADSKSGEVDATSDQEESVAWALAPAVPQSPLKNELRRKYLTQVDILLQGAEYFECAGNRAGRDVRVTPLPSLASPAVPAPGYCSRISGKSPGDPAKPASSPREWDPLHPSSTDMALVPRNDSLSLQETSSSSFLSSQPFEDDDICNVTISDLYAGMLHSMSRLLSTKPSSIISTKTFIMQNWNCRRRHRYKSRMNKTYCKGARRSQRSSKENFIPCSEPVKGTGALRDCKNVLDVSCRKTGLKLEKAFLEVNRPQIHKLDPSWKERKVTPSKYSSLIYFDSSATYNLDEENRFRTLKWLISPVKIVSRPTIRQGHGENRQREIEIRFDQLHREYCLSPRNQPRRMCLPDSWAMNMYRGGPASPGGLQGLETRRLSLPSSKAKAKSLSEAFENLGKRSLEAGRCLPKSDSSSSLPKTNPTHSATRPQQTSDLHVQGNSSGIFRKSVSPSKTLSVPDKEVPGHGRNRYDEIKEEFDKLHQKYCLKSPGQMTVPLCIGVSTDKASMEVRYQTEGFLGKLNPDPHFQGFQKLPSSPLGCRKSLLGSTAIEAPSSTCVARAITRDGTRDHQFPAKRPRLSEPQGSGRQGNSLGASDGVDNTVRPGDQGSSSQPNSEERGENTSYRMEEKSDFMLEKLETKSV

51 NP_116024.1 NP_066983.1

>NP_116024.1

MTLFHFGNCFALAYFPYFITYKCSGLSEYNAFWKCVQAGVTYLFVQLCKMLFLATFFPTWEGGIYDFIGEFMKASVDVADLIGLNLVMSRNAGKGEYKIMVAALGWATAELIMSRCIPLWVGARGIEFDWKYIQMSIDSNISLVHYIVASAQVWMITRYDLYHTFRPAVLLLMFLSVYKAFVMETFVHLCSLGSWAALLARAVVTGLLALSTLALYVAVVNVHS

>NP_066983.1

MAKLLSCVLGPRLYKIYRERDSERAPASVPETPTAVTAPHSSSWDTYYQPRALEKHADSILALASVFWSISYYSSPFAFFYLYRKGYLSLSKVVPFSHYAGTLLLLLAGVACLRGIGRWTNPQYRQFITILEATHRNQSSENKRQLANYNFDFRSWPVDFHWEEPSSRKESRGGPSRRGVALLRPEPLHRGTADTLLNRVKKLPCQITSYLVAHTLGRRMLYPGSVYLLQKALMPVLLQGQARLVEECNGRRAKLLACDGNEIDTMFVDRRGTAEPQGQKLVICCEGNAGFYEVGCVSTPLEAGYSVLGWNHPGFAGSTGVPFPQNEANAMDVVVQFAIHRLGFQPQDIIIYAWSIGGFTATWAAMSYPDVSAMILDASFDDLVPLALKVMPDSWRGLVTRTVRQHLNLNNAEQLCRYQGPVLLIRRTKDEIITTTVPEDIMSNRGNDLLLKLLQHRYPRVMAEEGLRVVRQWLEASSQLEEASIYSRWEVEEDWCLSVLRSYQAEHGPDFPWSVGEDMSADGRRQLALFLARKHLHNFEATHCTPLPAQNFQMPWHL

52 NP_002816.1 NP_060625.1

>NP_002816.1

MQAQQYQQQRRKFAAAFLAFIFILAAVDTAEAGKKEKPEKKVKKSDCGEWQWSVCVPTSGDCGLGTREGTRTGAECKQTMKTQRCKIPCNWKKQFGAECKYQFQAWGECDLNTALKTRTGSLKRALHNAECQKTVTISKPCGKLTKPKPQAESKKKKKEGKKQEKMLD

>NP_060625.1

MSQRDTLVHLFAGGCGGTVGAILTCPLEVVKTRLQSSSVTLYISEVQLNTMAGASVNRVVSPGPLHCLKVILEKEGPRSLFRGLGPNLVGVAPSRAIYFAAYSNCKEKLNDVFDPDSTQVHMISAAMAGFTAITATNPIWLIKTRLQLDARNRGERRMGAFECVRKVYQTDGLKGFYRGMSASYAGISETVIHFVIYESIKQKLLEYKTASTMENGEESVKEASDFVGMMLAAATSKTCATTIAYPHVVRTRLREEGTKYRSFFQTLSLLVQEEGYGSLYRGLTTHLVRQIPNTAIMMATYELVVYLLNG

53 NP_002961.1 NP_065853.2

>NP_002961.1

MAKFVIRPATAADCSDILRLIKELAKYEYMEEQVILTEKDLLEDGFGEHPFYHCLVAEVPKEHWTPEGHSIVGFAMYYFTYDPWIGKLLYLEDFFVMSDYRGFGIGSEILKNLSQVAMRCRCSSMHFLVAEWNEPSINFYKRRGASDLSSEEGWRLFKIDKEYLLKMATEE

>NP_065853.2

MLAGRPGTRSAVGELGTESSDNLDRAPLGPRESGGHHRPGSYLDMKIHLEKNLEEERQILLQQQKICRNRARKYFVESNRRKKAFEEKRKEQEEKEHQIREQILQQRKQKFEEVTEKFQRAHVPLSQRRKAVSRKPVPPLEEALKQIQESNLKSEVNLPFSRRPTINWRAIDSALPSALSKNDHKHQKQLLSKINCEKEMNENMRATLATSKNVFQLKLEETQKLLEDQHLSNLQKFGDEVNQITNSETLSSIDSLEATEHEEIYLTLNKEHSTSIQRNTISLKPANMQSTNLSCFDEDKLAFSKTQHINNWLTNLDASNTQNVTAFSDILSKSNVLPSWEYFNSKEQNPSPLNGTVERATNTANNSVPFVSSPPMFVLDKKCEKTSETSTMRTTDSTSGAFKRERPLVTESPTFKFSKSQSTSDSLTQEVATFPDQEKYSELNQENGTTSIPTSCVPVATPLVLPSNIQSARPSAKNSIHIKEIDAVQCSDKLDELKDGKEEEIKYFNCNKEELPLFSDSFQDAYIPHNPDSKDEKQKLAETSSLSNVTSNYDFVGQHKKMKYNIHERNGVRFLKSILKKESKYEHGYLKALIINQSFKFGNQKAAAIRDSIELTKEKGAEIPKTIKKLRWFDETSNIENNAENSHSLKNKTGTTQQHSQQFHIQSGAGSNIISVSTCAVNSADTKKSREDSISENVTTLGGSGADHMPLNCFIPSGYNFAKHAWPASKKEESKIPVHDDSKTKQGKPQRGRAKIIRKPGSAKVQSGFICTNRKGAVIQPQSASKVNIFTQAQGKLIIPCPPPQSTSNIRSGKNIQVSQCQPVTPENPQNIITHNSFNSKHVLPTEHSLNQWNQESSSPLSNACSDLVTVIPSLPSYCSSECQTFAKINHSNGTQAVARQDATLYCTQRSPVCEESYPSVTLRTAEEESVPLWKRGPNVLHQNKRATGSTVMRRKRIAETKRRNILEQKRQNPGSVGQKYSEQINNFGQSVLLSSSEPKQTTRGTSYIEEVSDSTSEFLMAENLVKASVPEDEILTVLNSKQIQKSNLPLNKTQQFNICTLSAEEQKILESLNDLSERLHYIQESICKNPSIKNTLQIIPLLEKREDRTSSCRDKR

54 NP_057403.1 NP_003644.2

>NP_057403.1

MSAEVKVTGQNQEQFLLLAKSAKGAALATLIHQVLEAPGVYVFGELLDMPNVRELAESDFASTFRLLTVFAYGTYADYLAEARNLPPLTEAQKNKLRHLSVVTLAAKVKCIPYAVLLEALALRNVRQLEDLVIEAVYADVLRGSLDQRNQRLEVDYSIGRDIQRQDLSAIARTLQEWCVGCEVVLSGIEEQVSRANQHKEQQLGLKQQIESEVANLKKTIKVTTAAAAAATSQDPEQHLTELREPAPGTNQRQPSKKASKGKGLRGSAKIWSKSN

>NP_003644.2

MASALEQFVNSVRQLSAQGQMTQLCELINKSGELLAKNLSHLDTVLGALDVQEHSLGVLAVLFVKFSMPSVPDFETLFSQVQLFISTCNGEHIRYATDTFAGLCHQLTNALVERKQPLRGIGILKQAIDKMQMNTNQLTSIHADLCQLCLLAKCFKPALPYLDVDMMDICKENGAYDAKHFLCYYYYGGMIYTGLKNFERALYFYEQAITTPAMAVSHIMLESYKKYILVSLILLGKVQQLPKYTSQIVGRFIKPLSNAYHELAQVYSTNNPSELRNLVNKHSETFTRDNNMGLVKQCLSSLYKKNIQRLTKTFLTLSLQDMASRVQLSGPQEAEKYVLHMIEDGEIFASINQKDGMVSFHDNPEKYNNPAMLHNIDQEMLKCIELDERLKAMDQEITVNPQFVQKSMGSQEDDSGNKPSSYS

55 NP_000312.2 NP_116090.2

>NP_000312.2

MPPKTPRKTAATAAAAAAEPPAPPPPPPPEEDPEQDSGPEDLPLVRLEFEETEEPDFTALCQKLKIPDHVRERAWLTWEKVSSVDGVLGGYIQKKKELWGICIFIAAVDLDEMSFTFTELQKNIEISVHKFFNLLKEIDTSTKVDNAMSRLLKKYDVLFALFSKLERTCELIYLTQPSSSISTEINSALVLKVSWITFLLAKGEVLQMEDDLVISFQLMLCVLDYFIKLSPPMLLKEPYKTAVIPINGSPRTPRRGQNRSARIAKQLENDTRIIEVLCKEHECNIDEVKNVYFKNFIPFMNSLGLVTSNGLPEVENLSKRYEEIYLKNKDLDARLFLDHDKTLQTDSIDSFETQRTPRKSNLDEEVNVIPPHTPVRTVMNTIQQLMMILNSASDQPSENLISYFNNCTVNPKESILKRVKDIGYIFKEKFAKAVGQGCVEIGSQRYKLGVRLYYRVMESMLKSEEERLSIQNFSKLLNDNIFHMSLLACALEVVMATYSRSTSQNLDSGTDLSFPWILNVLNLKAFDFYKVIESFIKAEGNLTREMIKHLERCEHRIMESLAWLSDSPLFDLIKQSKDREGPTDHLESACPLNLPLQNNHTAADMYLSPVRSPKKKGSTTRVNSTANAETQATSAFQTQKPLKSTSLSLFYKKVYRLAYLRLNTLCERLLSEHPELEHIIWTLFQHTLQNEYELMRDRHLDQIMMCSMYGICKVKNIDLKFKIIVTAYKDLPHAVQETFKRVLIKEEEYDSIIVFYNSVFMQRLKTNILQYASTRPPTLSPIPHIPRSPYKFPSSPLRIPGGNIYISPLKSPYKISEGLPTPTKMTPRSRILVSIGESFGTSEKFQKINQMVCNSDRVLKRSAEGSNPPKPLKKLRFDIEGSDEADGSKHLPGESKFQQKLAEMTSTRTRMQKQKMNDSMDTSNKEEK

>NP_116090.2

MGPDRVTARELCENDDLATSLVLDPYLGFRTHKMNVSPVPPLRRQQHLRSALETFLRQRDLEAAYRALTLGGWTARYFQSRGPRQEAALKTHVYRYLRAFLPESGFTILPCTRYSMETNGAKIVSTRAWKKNEKLELLVGCIAELREADEGLLRAGENDFSIMYSTRKRSAQLWLGPAAFINHDCKPNCKFVPADGNAACVKVLRDIEPGDEVTCFYGEGFFGEKNEHCECHTCERKGEGAFRTRPREPALPPRPLDKYQLRETKRRLQQGLDSGSRQGLLGPRACVHPSPLRRDPFCAACQPLRLPACSARPDTSPLWLQWLPQPQPRVRPRKRRRPRPRRAPVLSTHHAARVSLHRWGGCGPHCRLRGEALVALGQPPHARWAPQQDWHWARRYGLPYVVRVDLRRLAPAPPATPAPAGTPGPILIPKQALAFAPFSPPKRLRLVVSHGSIDLDVGGEEL

56 NP_003241.2 NP_003724.1

>NP_003241.2

MEQKPSKVECGSDPEENSARSPDGKRKRKNGQCSLKTSMSGYIPSYLDKDEQCVVCGDKATGYHYRCITCEGCKGFFRRTIQKNLHPTYSCKYDSCCVIDKITRNQCQLCRFKKCIAVGMAMDLVLDDSKRVAKRKLIEQNRERRRKEEMIRSLQQRPEPTPEEWDLIHIATEAHRSTNAQGSHWKQRRKFLPDDIGQSPIVSMPDGDKVDLEAFSEFTKIITPAITRVVDFAKKLPMFSELPCEDQIILLKGCCMEIMSLRAAVRYDPESDTLTLSGEMAVKREQLKNGGLGVVSDAIFELGKSLSAFNLDDTEVALLQAVLLMSTDRSGLLCVDKIEKSQEAYLLAFEHYVNHRKHNIPHFWPKLLMKEREVQSSILYKGAAAEGRPGGSLGVHPEGQQLLGMHVVQGPQVRQLEQQLGEAGSLQGPVLQHQSPKSPQQRLLELLHRSGILHARAVCGEDDSSEADSPSSSEEEPEVCEDLAGNAASP

>NP_003724.1

MALMQELYSTPASRLDSFVAQWLQPHREWKEEVLDAVRTVEEFLRQEHFQGKRGLDQDVRVLKVVKVGSFGNGTVLRSTREVELVAFLSCFHSFQEAAKHHKDVLRLIWKTMWQSQDLLDLGLEDLRMEQRVPDALVFTIQTRGTAEPITVTIVPAYRALGPSLPNSQPPPEVYVSLIKACGGPGNFCPSFSELQRNFVKHRPTKLKSLLRLVKHWYQQYVKARSPRANLPPLYALELLTIYAWEMGTEEDENFMLDEGFTTVMDLLLEYEVICIYWTKYYTLHNAIIEDCVRKQLKKERPIILDPADPTLNVAEGYRWDIVAQRASQCLKQDCCYDNRENPISSWNVKRARDIHLTVEQRGYPDFNLIVNPYEPIRKVKEKIRRTRGYSGLQRLSFQVPGSERQLLSSRCSLAKYGIFSHTHIYLLETIPSEIQVFVKNPDGGSYAYAINPNSFILGLKQQIEDQQGLPKKQQQLEFQGQVLQDWLGLGIYGIQDSDTLILSKKKGEALFPAS

57 NP_004851.1 NP_001026794.1

>NP_004851.1

MGGLASGGDVEPGLPVEVRGSNGAFYKGFVKDVHEDSVTIFFENNWQSERQIPFGDVRLPPPADYNKEITEGDEVEVYSRANEQEPCGWWLARVRMMKGDFYVIEYAACDATYNEIVTLERLRPVNPNPLATKGSFFKVTMAVPEDLREACSNENVHKEFKKALGANCIFLNITNSELFILSTTEAPVKRASLLGDMHFRSLRTKLLLMSRNEEATKHLETSKQLAAAFQEEFTVREDLMGLAIGTHGANIQQARKVPGVTAIELGEETCTFRIYGETPEACRQARSYLEFSEDSVQVPRNLVGKVIGKNGKVIQEIVDKSGVVRVRVEGDNDKKNPREEGMVPFIFVGTRENISNAQALLEYHLSYLQEVEQLRLERLQIDEQLRQIGLGFRPPGSGRGSGGSDKAGYSTDESSSSSLHATRTYGGSYGGRGRGRRTGGPAYGPSSDVSTASETESEKREEPNRAGPGDRDPPTRGEESRRRPTGGRGRGPPPAPRPTSRYNSSSISSVLKDPDSNPYSLLDTSEPEPPVDSEPGEPPPASARRRRSRRRRTDEDRTVMDGGLESDGPNMTENGLEDESRPQRRNRSRRRRNRGNRTDGSISGDRQPVTVADYISRAESQSRQSAPLERTKPSEDSLSGQKGDSVSKLPKGPSENGELSAPLELGSMVNGVS

>NP_001026794.1

MGDPGSEIIESVPPAGPEASESTTDENEDDIQFVSEGPLRPVLEYIDLVSSDDEEPSTSYTDENIKRKDHIDYQKDKVALTLARLARHVEVEKQQKEEKNRAFREKIDFQHAHGLQELEFIRGHSDTEAARLCVDQWLKMPGLKTGTINCGTKSSFRRGGHTWVSGKPILCPIMHCNKEFDNGHLLLGHLKRFDHSPCDPTITLHGPFFSSFACVVCYKKFVTQQQYRDHLFDKEATDDGHNNNLLPQIIQCFACPNCFLLFSRKEECSKHMSGKNHFHQSFKLGDNKGIAHPISFPSFAKKLLISLCKDVPFQVKCVACHKTLRSHMELTAHFRVHCRNAGPVAVAEKSITQVAEKFILRGYCPDCNQVFVDETSTQNHKQNSGHKVRVINSVEESVLLYCHSSEGNKDPSSDLHLLLDQSKFSSLKRTMSIKESSSLECIAIPKKKMNLKDKSHEGVACVQKEKSVVKTWFCECNQRFPSEDAVEKHVFSANTMGYKCVVCGKVCDDSGVIRLHMSRIHGGAHLNNFLFWCRTCKKELTRKDTIMAHVTEFHNGHRYFYEMDEVEGETLPSSSTTLDNLTANKPSSAITVIDHSPANSSPRGKWQCRICEDMFDSQEYVKQHCMSLASHKFHRYSCAHCRKPFHKIETLYRHCQDEHDNEIKIKYFCGLCDLIFNVEEAFLSHYEEHHSIDYVFVSEKTETSIKTEDDFPVIETSNQLTCGCRESYICKVNRKEDYSRCLQIMLDKGKLWFRCSLCSATAQNLTDMNTHIHQVHKEKSDEEEQQYVIKCGTCTKAFHDPESAQQHFHRKHCFLQKPSVAHFGSEKSNLYKFTASASHTERKLKQAINYSKSLDMEKGVENDLSYQNIEEEIVELPDLDYLRTMTHIVFVDFDNWSNFFGHLPGHLNQGTFIWGFQGGNTNWKPPLNCKIYNYLNRIGCFFLHPRCSKRKDAADFAICMHAGRLDEQLPKQIPFTILSGDQGFLELENQFKKTQRPAHILNPHHLEGDMMCALLNSISDTTKECDSDDNMGAKNTSIGEEFISTEDVELEEAIRRSLEEM

58 NP_001420.2 NP_000116.2

>NP_001420.2

MAENVVEPGPPSAKRPKLSSPALSASASDGTDFGSLFDLEHDLPDELINSTELGLTNGGDINQLQTSLGMVQDAASKHKQLSELLRSGSSPNLNMGVGGPGQVMASQAQQSSPGLGLINSMVKSPMTQAGLTSPNMGMGTSGPNQGPTQSTGMMNSPVNQPAMGMNTGMNAGMNPGMLAAGNGQGIMPNQVMNGSIGAGRGRQNMQYPNPGMGSAGNLLTEPLQQGSPQMGGQTGLRGPQPLKMGMMNNPNPYGSPYTQNPGQQIGASGLGLQIQTKTVLSNNLSPFAMDKKAVPGGGMPNMGQQPAPQVQQPGLVTPVAQGMGSGAHTADPEKRKLIQQQLVLLLHAHKCQRREQANGEVRQCNLPHCRTMKNVLNHMTHCQSGKSCQVAHCASSRQIISHWKNCTRHDCPVCLPLKNAGDKRNQQPILTGAPVGLGNPSSLGVGQQSAPNLSTVSQIDPSSIERAYAALGLPYQVNQMPTQPQVQAKNQQNQQPGQSPQGMRPMSNMSASPMGVNGGVGVQTPSLLSDSMLHSAINSQNPMMSENASVPSLGPMPTAAQPSTTGIRKQWHEDITQDLRNHLVHKLVQAIFPTPDPAALKDRRMENLVAYARKVEGDMYESANNRAEYYHLLAEKIYKIQKELEEKRRTRLQKQNMLPNAAGMVPVSMNPGPNMGQPQPGMTSNGPLPDPSMIRGSVPNQMMPRITPQSGLNQFGQMSMAQPPIVPRQTPPLQHHGQLAQPGALNPPMGYGPRMQQPSNQGQFLPQTQFPSQGMNVTNIPLAPSSGQAPVSQAQMSSSSCPVNSPIMPPGSQGSHIHCPQLPQPALHQNSPSPVPSRTPTPHHTPPSIGAQQPPATTIPAPVPTPPAMPPGPQSQALHPPPRQTPTPPTTQLPQQVQPSLPAAPSADQPQQQPRSQQSTAASVPTPTAPLLPPQPATPLSQPAVSIEGQVSNPPSTSSTEVNSQAIAEKQPSQEVKMEAKMEVDQPEPADTQPEDISESKVEDCKMESTETEERSTELKTEIKEEEDQPSTSATQSSPAPGQSKKKIFKPEELRQALMPTLEALYRQDPESLPFRQPVDPQLLGIPDYFDIVKSPMDLSTIKRKLDTGQYQEPWQYVDDIWLMFNNAWLYNRKTSRVYKYCSKLSEVFEQEIDPVMQSLGYCCGRKLEFSPQTLCCYGKQLCTIPRDATYYSYQNRYHFCEKCFNEIQGESVSLGDDPSQPQTTINKEQFSKRKNDTLDPELFVECTECGRKMHQICVLHHEIIWPAGFVCDGCLKKSARTRKENKFSAKRLPSTRLGTFLENRVNDFLRRQNHPESGEVTVRVVHASDKTVEVKPGMKARFVDSGEMAESFPYRTKALFAFEEIDGVDLCFFGMHVQEYGSDCPPPNQRRVYISYLDSVHFFRPKCLRTAVYHEILIGYLEYVKKLGYTTGHIWACPPSEGDDYIFHCHPPDQKIPKPKRLQEWYKKMLDKAVSERIVHDYKDIFKQATEDRLTSAKELPYFEGDFWPNVLEESIKELEQEEEERKREENTSNESTDVTKGDSKNAKKKNNKKTSKNKSSLSRGNKKKPGMPNVSNDLSQKLYATMEKHKEVFFVIRLIAGPAANSLPPIVDPDPLIPCDLMDGRDAFLTLARDKHLEFSSLRRAQWSTMCMLVELHTQSQDRFVYTCNECKHHVETRWHCTVCEDYDLCITCYNTKNHDHKMEKLGLGLDDESNNQQAAATQSPGDSRRLSIQRCIQSLVHACQCRNANCSLPSCQKMKRVVQHTKGCKRKTNGGCPICKQLIALCCYHAKHCQENKCPVPFCLNIKQKLRQQQLQHRLQQAQMLRRRMASMQRTGVVGQQQGLPSPTPATPTTPTGQQPTTPQTPQPTSQPQPTPPNSMPPYLPRTQAAGPVSQGKAAGQVTPPTPPQTAQPPLPGPPPAAVEMAMQIQRAAETQRQMAHVQIFQRPIQHQMPPMTPMAPMGMNPPPMTRGPSGHLEPGMGPTGMQQQPPWSQGGLPQPQQLQSGMPRPAMMSVAQHGQPLNMAPQPGLGQVGISPLKPGTVSQQALQNLLRTLRSPSSPLQQQQVLSILHANPQLLAAFIKQRAAKYANSNPQPIPGQPGMPQGQPGLQPPTMPGQQGVHSNPAMQNMNPMQAGVQRAGLPQQQPQQQLQPPMGGMSPQAQQMNMNHNTMPSQFRDILRRQQMMQQQQQQGAGPGIGPGMANHNQFQQPQGVGYPPQQQQRMQHHMQQMQQGNMGQIGQLPQALGAEAGASLQAYQQRLLQQQMGSPVQPNPMSPQQHMLPNQAQSPHLQGQQIPNSLSNQVRSPQPVPSPRPQSQPPHSSPSPRMQPQPSPHHVSPQTSSPHPGLVAAQANPMEQGHFASPDQNSMLSQLASNPGMANLHGASATDLGLSTDNSDLNSNLSQSTLDIH

>NP_000116.2

MTMTLHTKASGMALLHQIQGNELEPLNRPQLKIPLERPLGEVYLDSSKPAVYNYPEGAAYEFNAAAAANAQVYGQTGLPYGPGSEAAAFGSNGLGGFPPLNSVSPSPLMLLHPPPQLSPFLQPHGQQVPYYLENEPSGYTVREAGPPAFYRPNSDNRRQGGRERLASTNDKGSMAMESAKETRYCAVCNDYASGYHYGVWSCEGCKAFFKRSIQGHNDYMCPATNQCTIDKNRRKSCQACRLRKCYEVGMMKGGIRKDRRGGRMLKHKRQRDDGEGRGEVGSAGDMRAANLWPSPLMIKRSKKNSLALSLTADQMVSALLDAEPPILYSEYDPTRPFSEASMMGLLTNLADRELVHMINWAKRVPGFVDLTLHDQVHLLECAWLEILMIGLVWRSMEHPGKLLFAPNLLLDRNQGKCVEGMVEIFDMLLATSSRFRMMNLQGEEFVCLKSIILLNSGVYTFLSSTLKSLEEKDHIHRVLDKITDTLIHLMAKAGLTLQQQHQRLAQLLLILSHIRHMSNKGMEHLYSMKCKNVVPLYDLLLEMLDAHRLHAPTSRGGASVEETDQSHLATAGSTSSHSLQKYYITGEAEGFPATV

59 NP_001005743.1 AAH14214.1

>NP_001005743.1

MNKLRQSFRRKKDVYVPEASRPHQWQTDEEGVRTGKCSFPVKYLGHVEVDESRGMHICEDAVKRLKAERKFFKGFFGKTGKKAVKAVLWVSADGLRVVDEKTKDLIVDQTIEKVSFCAPDRNFDRAFSYICRDGTTRRWICHCFMAVKDTGERLSHAVGCAFAACLERKQKREKECGVTATFDASRTTFTREGSFRVTTATEQAEREEIMKQMQDAKKAETDKIVVGSSVAPGNTAPSPSSPTSPTSDATTSLEMNNPHAIPRRHAPIEQLARQGSFRGFPALSQKMSPFKRQLSLRINELPSTMQRKTDFPIKNAVPEVEGEAESISSLCSQITNAFSTPEDPFSSAPMTKPVTVVAPQSPTFQANGTDSAFHVLAKPAHTALAPVAMPVRETNPWAHAPDAANKEIAATCSGTEWGQSSGAASPGLFQAGHRRTPSEADRWLEEVSKSVRAQQPQASAAPLQPVLQPPPPTAISQPASPFQGNAFLTSQPVPVGVVPALQPAFVPAQSYPVANGMPYPAPNVPVVGITPSQMVANVFGTAGHPQAAHPHQSPSLVRQQTFPHYEASSATTSPFFKPPAQHLNGSAAFNGVDDGRLASADRHTEVPTGTCPVDPFEAQWAALENKSKQRTNPSPTNPFSSDLQKTFEIEL

>AAH14214.1

MPAVSKGDGMRGLAVFISDIRNCKSKEAEIKRINKELANIRSKFKGDKALDGYSKKKYVCKLLFIFLLGHDIDFGHMEAVNLLSSNKYTEKQIGYLFISVLVNSNSELIRLINNAIKNDLASRNPTFMCLALHCIANVGSREMGEAFAADIPRILVAGDSMDSVKQSAALCLLRLYKASPDLVPMGEWTARVVHLLNDQHMGVVTAAVSLITCLCKKNPDDFKTCVSLAVSRLSRIVSSASTDLQDYTYYFVPAPWLSVKLLRLLQCYPLPEDAAVKGRLVECLETVLNKAQEPPKSKKVQHSNAKNAILFETISLIIHYDSEPNLLVRACNQLGQFLQHRETNLRYLALESMCTLASSEFSHEAVKTHIDTVINALKTERDVSVRQRAADLLYAMCDRSNAKQIVSEMLRYLETADYAIREEIVLKVAILAEKYAVDYSWYVDTILNLIRIAGDYVSEEVWYRVLQIVTNRDDVQGYAAKTVFEALQAPACHENMVKVGGYILGEFGNLIAGDPRSSPPVQFSLLHSKFHLCSVATRALLLSTYIKFINLFPETKATIQGVLRAGSQLRNADVELQQRAVEYLTLSSVASTDVLATVLEEMPPFPERESSILAKLKRKKGPGAGSALDDGRRDPSSNDINGGMEPTPSTVSTPSPSADLLGLRAAPPPAAPPASAGAGNLLVDVFDGPAAQPSLGPTPEEAFLSPGPEDIGPPIPEADELLNKFVCKNNGVLFENQLLQIGVKSEFRQNLGRMYLFYGNKTSVQFQNFSPTVVHPGDLQTQLAVQTKRVAAQVDGGAQVQQVLNIECLRDFLTPPLLSVRFRYGGAPQALTLKLPVTINKFFQPTEMAAQDFFQRWKQLSLPQQEAQKIFKANHPMDAEVTKAKLLGFGSALLDNVDPNPGDREDTRVWGMPGTFLRPFVFLFLFICCCLHSGGLGGVPLPPFPPQAQRGEGPGKWMSPPLPPHPVVAPPTPSPSRGCVLL

60 NP_003631.2 NP_001547.1

>NP_003631.2

MRNLKLFRTLEFRDIQGPGNPQCFSLRTEQGTVLIGSEHGLIEVDPVSREVKNEVSLVAEGFLPEDGSGRIVGVQDLLDQESVCVATASGDVILCSLSTQQLECVGSVASGISVMSWSPDQELVLLATGQQTLIMMTKDFEPILEQQIHQDDFGESKFITVGWGRKETQFHGSEGRQAAFQMQMHESALPWDDHRPQVTWRGDGQFFAVSVVCPETGARKVRVWNREFALQSTSEPVAGLGPALAWKPSGSLIASTQDKPNQQDIVFFEKNGLLHGHFTLPFLKDEVKVNDLLWNADSSVLAVWLEDLQREESSIPKTCVQLWTVGNYHWYLKQSLSFSTCGKSKIVSLMWDPVTPYRLHVLCQGWHYLAYDWHWTTDRSVGDNSSDLSNVAVIDGNRVLVTVFRQTVVPPPMCTYQLLFPHPVNQVTFLAHPQKSNDLAVLDASNQISVYKCGDCPSADPTVKLGAVGGSGFKVCLRTPHLEKRYKIQFENNEDQDVNPLKLGLLTWIEEDVFLAVSHSEFSPRSVIHHLTAASSEMDEEHGQLNVSSSAAVDGVIISLCCNSKTKSVVLQLADGQIFKYLWESPSLAIKPWKNSGGFPVRFPYPCTQTELAMIGEEECVLGLTDRCRFFINDIEVASNITSFAVYDEFLLLTTHSHTCQCFCLRDASFKTLQAGLSSNHVSHGEVLRKVERGSRIVTVVPQDTKLVLQMPRGNLEVVHHRALVLAQIRKWLDKLMFKEAFECMRKLRINLNLIYDHNPKVFLGNVETFIKQIDSVNHINLFFTELKEEDVTKTMYPAPVTSSVYLSRDPDGNKIDLVCDAMRAVMESINPHKYCLSILTSHVKKTTPELEIVLQKVHELQGNAPSDPDAVSAEEALKYLLHLVDVNELYDHSLGTYDFDLVLMVAEKSQKDPKEYLPFLNTLKKMETNYQRFTIDKYLKRYEKAIGHLSKCGPEYFPECLNLIKDKNLYNEALKLYSPSSQQYQDISIAYGEHLMQEHMYEPAGLMFARCGAHEKALSAFLTCGNWKQALCVAAQLNFTKDQLVGLGRTLAGKLVEQRKHIDAAMVLEECAQDYEEAVLLLLEGAAWEEALRLVYKYNRLDIIETNVKPSILEAQKNYMAFLDSQTATFSRHKKRLLVVRELKEQAQQAGLDDEVPHGQESDLFSETSSVVSGSEMSGKYSHSNSRISARSSKNRRKAERKKHSLKEGSPLEDLALLEALSEVVQNTENLKDEVYHILKVLFLFEFDEQGRELQKAFEDTLQLMERSLPEIWTLTYQQNSATPVLGPNSTANSIMASYQQQKTSVPVLDAELFIPPKINRRTQWKLSLLD

>NP_001547.1

MSWSPSLTTQTCGAWEMKERLGTGGFGNVIRWHNQETGEQIAIKQCRQELSPRNRERWCLEIQIMRRLTHPNVVAARDVPEGMQNLAPNDLPLLAMEYCQGGDLRKYLNQFENCCGLREGAILTLLSDIASALRYLHENRIIHRDLKPENIVLQQGEQRLIHKIIDLGYAKELDQGSLCTSFVGTLQYLAPELLEQQKYTVTVDYWSFGTLAFECITGFRPFLPNWQPVQWHSKVRQKSEVDIVVSEDLNGTVKFSSSLPYPNNLNSVLAERLEKWLQLMLMWHPRQRGTDPTYGPNGCFKALDDILNLKLVHILNMVTGTIHTYPVTEDESLQSLKARIQQDTGIPEEDQELLQEAGLALIPDKPATQCISDGKLNEGHTLDMDLVFLFDNSKITYETQISPRPQPESVSCILQEPKRNLAFFQLRKVWGQVWHSIQTLKEDCNRLQQGQRAAMMNLLRNNSCLSKMKNSMASMSQQLKAKLDFFKTSIQIDLEKYSEQTEFGITSDKLLLAWREMEQAVELCGRENEVKLLVERMMALQTDIVDLQRSPMGRKQGGTLDDLEEQARELYRRLREKPRDQRTEGDSQEMVRLLLQAIQSFEKKVRVIYTQLSKTVVCKQKALELLPKVEEVVSLMNEDEKTVVRLQEKRQKELWNLLKIACSKVRGPVSGSPDSMNASRLSQPGQLMSQPSTASNSLPEPAKKSEELVAEAHNLCTLLENAIQDTVREQDQSFTALDWSWLQTEEEEHSCLEQAS

61 NP_002816.1 NP_055580.1

>NP_002816.1

MQAQQYQQQRRKFAAAFLAFIFILAAVDTAEAGKKEKPEKKVKKSDCGEWQWSVCVPTSGDCGLGTREGTRTGAECKQTMKTQRCKIPCNWKKQFGAECKYQFQAWGECDLNTALKTRTGSLKRALHNAECQKTVTISKPCGKLTKPKPQAESKKKKKEGKKQEKMLD

>NP_055580.1

MVGRNSAIAAGVCGALFIGYCIYFDRKRRSDPNFKNRLRERRKKQKLAKERAGLSKLPDLKDAEAVQKFFLEEIQLGEELLAQGEYEKGVDHLTNAIAVCGQPQQLLQVLQQTLPPPVFQMLLTKLPTISQRIVSAQSLAEDDVE

62 NP_001202.4 NP_001247.2

>NP_001202.4

MAAVKKEGGALSEAMSLEGDEWELSKENVQPLRQGRIMSTLQGALAQESACNNTLQQQKRAFEYEIRFYTGNDPLDVWDRYISWTEQNYPQGGKESNMSTLLERAVEALQGEKRYYSDPRFLNLWLKLGRLCNEPLDMYSYLHNQGIGVSLAQFYISWAEEYEARENFRKADAIFQEGIQQKAEPLERLQSQHRQFQARVSRQTLLALEKEEEEEVFESSVPQRSTLAELKSKGKKTARAPIIRVGGALKAPSQNRGLQNPFPQQMQNNSRITVFDENADEASTAELSKPTVQPWIAPPMPRAKENELQAGPWNTGRSLEHRPRGNTASLIAVPAVLPSFTPYVEETAQQPVMTPCKIEPSINHILSTRKPGKEEGDPLQRVQSHQQASEEKKEKMMYCKEKIYAGVGEFSFEEIRAEVFRKKLKEQREAELLTSAEKRAEMQKQIEEMEKKLKEIQTTQQERTGDQQEETMPTKETTKLQIASESQKIPGMTLSSSVCQVNCCARETSLAENIWQEQPHSKGPSVPFSIFDEFLLSEKKNKSPPADPPRVLAQRRPLAVLKTSESITSNEDVSPDVCDEFTGIEPLSEDAIITGFRNVTICPNPEDTCDFARAARFVSTPFHEIMSLKDLPSDPERLLPEEDLDVKTSEDQQTACGTIYSQTLSIKKLSPIIEDSREATHSSGFSGSSASVASTSSIKCLQIPEKLELTNETSENPTQSPWCSQYRRQLLKSLPELSASAELCIEDRPMPKLEIEKEIELGNEDYCIKREYLICEDYKLFWVAPRNSAELTVIKVSSQPVPWDFYINLKLKERLNEDFDHFCSCYQYQDGCIVWHQYINCFTLQDLLQHSEYITHEITVLIIYNLLTIVEMLHKAEIVHGDLSPRCLILRNRIHDPYDCNKNNQALKIVDFSYSVDLRVQLDVFTLSGFRTVQILEGQKILANCSSPYQVDLFGIADLAHLLLFKEHLQVFWDGSFWKLSQNISELKDGELWNKFFVRILNANDEATVSVLGELAAEMNGVFDTTFQSHLNKALWKVGKLTSPGALLFQ

>NP_001247.2

MTVLQEPVQAAIWQALNHYAYRDAVFLAERLYAEVHSEEALFLLATCYYRSGKAYKAYRLLKGHSCTTPQCKYLLAKCCVDLSKLAEGEQILSGGVFNKQKSHDDIVTEFGDSACFTLSLLGHVYCKTDRLAKGSECYQKSLSLNPFLWSPFESLCEIGEKPDPDQTFKFTSLQNFSNCLPNSCTTQVPNHSLSHRQPETVLTETPQDTIELNRLNLESSNSKYSLNTDSSVSYIDSAVISPDTVPLGTGTSILSKQVQNKPKTGRSLLGGPAALSPLTPSFGILPLETPSPGDGSYLQNYTNTPPVIDVPSTGAPSKKSVARIGQTGTKSVFSQSGNSREVTPILAQTQSSGPQTSTTPQVLSPTITSPPNALPRRSSRLFTSDSSTTKENSKKLKMKFPPKIPNRKTKSKTNKGGITQPNINDSLEITKLDSSIISEGKISTITPQIQAFNLQKAAAAGLMSLLREMGKGYLALCSYNCKEAINILSHLPSHHYNTGWVLCQIGRAYFELSEYMQAERIFSEVRRIENYRVEGMEIYSTTLWHLQKDVALSVLSKDLTDMDKNSPEAWCAAGNCFSLQREHDIAIKFFQRAIQVDPNYAYAYTLLGHEFVLTEELDKALACFRNAIRVNPRHYNAWYGLGMIYYKQEKFSLAEMHFQKALDINPQSSVLLCHIGVVQHALKKSEKALDTLNKAIVIDPKNPLCKFHRASVLFANEKYKSALQELEELKQIVPKESLVYFLIGKVYKKLGQTHLALMNFSWAMDLDPKGANNQIKEAIDKRYLPDDEEPITQEEQIMGTDESQESSMTDADDTQLHAAESDEF

63 NP_064581.2 NP_065976.2

>NP_064581.2

MVVMARLSRPERPDLVFEEEDLPYEEEIMRNQFSVKCWLRYIEFKQGAPKPRLNQLYERALKLLPCSYKLWYRYLKARRAQVKHRCVTDPAYEDVNNCHERAFVFMHKMPRLWLDYCQFLMDQGRVTHTRRTFDRALRALPITQHSRIWPLYLRFLRSHPLPETAVRGYRRFLKLSPESAEEYIEYLKSSDRLDEAAQRLATVVNDERFVSKAGKSNYQLWHELCDLISQNPDKVQSLNVDAIIRGGLTRFTDQLGKLWCSLADYYIRSGHFEKARDVYEEAIRTVMTVRDFTQVFDSYAQFEESMIAAKMETASELGREEEDDVDLELRLARFEQLISRRPLLLNSVLLRQNPHHVHEWHKRVALHQGRPREIINTYTEAVQTVDPFKATGKPHTLWVAFAKFYEDNGQLDDARVILEKATKVNFKQVDDLASVWCQCGELELRHENYDEALRLLRKATALPARRAEYFDGSEPVQNRVYKSLKVWSMLADLEESLGTFQSTKAVYDRILDLRIATPQIVINYAMFLEEHKYFEESFKAYERGISLFKWPNVSDIWSTYLTKFIARYGGRKLERARDLFEQALDGCPPKYAKTLYLLYAQLEEEWGLARHAMAVYERATRAVEPAQQYDMFNIYIKRAAEIYGVTHTRGIYQKAIEVLSDEHAREMCLRFADMECKLGEIDRARAIYSFCSQICDPRTTGAFWQTWKDFEVRHGNEDTIKEMLRIRRSVQATYNTQVNFMASQMLKVSGSATGTVSDLAPGQSGMDDMKLLEQRAEQLAAEAERDQPLRAQSKILFVRSDASREELAELAQQVNPEEIQLGEDEDEDEMDLEPNEVRLEQQSVPAAVFGSLKED

>NP_065976.2

MRRLAAEELGVVTMQRIFNSFVYTEKISNGESEVQQLAKKIREKFNRYLDVVNRNKQVVEASYTAHLTSPLTAIQDCCTIPPSMMEFDGNFNTNVSRTISCDRLSTTVNSRAFNPGRDLNSVLADNLKSNPGIKWQYFSSEEGIFTVFPAHKFRCKGSYEHRSRPIYVSTVRPQSKHIVVILDHGASVTDTQLQIAKDAAQVILSAIDEHDKISVLTVADTVRTCSLDQCYKTFLSPATSETKRKMSTFVSSVKSSDSPTQHAVGFQKAFQLIRSTNNNTKFQANTDMVIIYLSAGITSKDSSEEDKKATLQVINEENSFLNNSVMILTYALMNDGVTGLKELAFLRDLAEQNSGKYGVPDRMALPVIKGSMMVLNQLSNLETTVGRFYTNLPNRMIDEAVFSLPFSDEMGDGLIMTVSKPCYFGNLLLGIVGVDVNLAYILEDVTYYQDSLASYTFLIDDKGYTLMHPSLTRPYLLSEPPLHTDIIHYENIPKFELVRQNILSLPLGSQIIAVPVNSSLSWHINKLRETGKEAYNVSYAWKMVQDTSFILCIVVIQPEIPVKQLKNLNTVPSSKLLYHRLDLLGQPSACLHFKQLATLESPTIMLSAGSFSSPYEHLSQPETKRMVEHYTAYLSDNTRLIANPGLKFSVRNEVMATSHVTDEWMTQMEMSSLNTYIVRRYIATPNGVLRIYPGSLMDKAFDPTRRQWYLHAVANPGLISLTGPYLDVGGAGYVVTISHTIHSSSTQLSSGHTVAVMGIDFTLRYFYKVLMDLLPVCNQDGGNKIRCFIMEDRGYLVAHPTLIDPKGHAPVEQQHITHKEPLVANDILNHPNFVKKNLCNSFSDRTVQRFYKFNTSLAGDLTNLVHGSHCSKYRLARIPGTNAFVGIVNETCDSLAFCACSMVDRLCLNCHRMEQNECECPCECPLEVNECTGNLTNAENRNPSCEVHQEPVTYTAIDPGLQDALHQCVNSRCSQRLESGDCFGVLDCEWCMVDSDGKTHLDKPYCAPQKECFGGIVGAKSPYVDDMGAIGDEVITLNMIKSAPVGPVAGGIMGCIMVLVLAVYAYRHQIHRRSHQHMSPLAAQEMSVRMSNLENDRDERDDDSHEDRGIISNTRFIAAVIERHAHSPERRRRYWGRSGTESDHGYSTMSPQEDSENPPCNNDPLSAGVDVGNHDEDLDLDTPPQTAALLSHKFHHYRSHHPTLHHSHHLQAAVTVHTVDAEC

64 NP_055456.2 NP_005648.1

>NP_055456.2

MEDTQAIDWDVEEEEETEQSSESLRCNVEPVGRLHIFSGAHGPEKDFPLHLGKNVVGRMPDCSVALPFPSISKQHAEIEILAWDKAPILRDCGSLNGTQILRPPKVLSPGVSHRLRDQELILFADLLCQYHRLDVSLPFVSRGPLTVEETPRVQGETQPQRLLLAEDSEEEVDFLSERRMVKKSRTTSSSVIVPESDEEGHSPVLGGLGPPFAFNLNSDTDVEEGQQPATEEASSAARRGATVEAKQSEAEVVTEIQLEKDQPLVKERDNDTKVKRGAGNGVVPAGVILERSQPPGEDSDTDVDDDSRPPGRPAEVHLERAQPFGFIDSDTDAEEERIPATPVVIPMKKRKIFHGVGTRGPGAPGLAHLQESQAGSDTDVEEGKAPQAVPLEKSQASMVINSDTDDEEEVSAALTLAHLKESQPAIWNRDAEEDMPQRVVLLQRSQTTTERDSDTDVEEEELPVENREAVLKDHTKIRALVRAHSEKDQPPFGDSDDSVEADKSSPGIHLERSQASTTVDINTQVEKEVPPGSAIIHIKKHQVSVEGTNQTDVKAVGGPAKLLVVSLEEAWPLHGDCETDAEEGTSLTASVVADVRKSQLPAEGDAGAEWAAAVLKQERAHEVGAQGGPPVAQVEQDLPISRENLTDLVVDTDTLGESTQPQREGAQVPTGREREQHVGGTKDSEDNYGDSEDLDLQATQCFLENQGLEAVQSMEDEPTQAFMLTPPQELGPSHCSFQTTGTLDEPWEVLATQPFCLRESEDSETQPFDTHLEAYGPCLSPPRAIPGDQHPESPVHTEPMGIQGRGRQTVDKVMGIPKETAERVGPERGPLERETEKLLPERQTDVTGEEELTKGKQDREQKQLLARDTQRQESDKNGESASPERDRESLKVEIETSEEIQEKQVQKQTLPSKAFEREVERPVANRECDPAELEEKVPKVILERDTQRGEPEGGSQDQKGQASSPTPEPGVGAGDLPGPTSAPVPSGSQSGGRGSPVSPRRHQKGLLNCKMPPAEKASRIRAAEKVSRGDQESPDACLPPTVPEAPAPPQKPLNSQSQKHLAPPPLLSPLLPSIKPTVRKTRQDGSQEAPEAPLSSELEPFHPKPKIRTRKSSRMTPFPATSAAPEPHPSTSTAQPVTPKPTSQATRSRTNRSSVKTPEPVVPTAPELQPSTSTDQPVTSEPTSQVTRGRKSRSSVKTPETVVPTALELQPSTSTDRPVTSEPTSQATRGRKNRSSVKTPEPVVPTAPELQPSTSTDQPVTSEPTYQATRGRKNRSSVKTPEPVVPTAPELRPSTSTDRPVTPKPTSRTTRSRTNMSSVKTPETVVPTAPELQISTSTDQPVTPKPTSRTTRSRTNMSSVKNPESTVPIAPELPPSTSTEQPVTPEPTSRATRGRKNRSSGKTPETLVPTAPKLEPSTSTDQPVTPEPTSQATRGRTNRSSVKTPETVVPTAPELQPSTSTDQPVTPEPTSQATRGRTDRSSVKTPETVVPTAPELQASASTDQPVTSEPTSRTTRGRKNRSSVKTPETVVPAAPELQPSTSTDQPVTPEPTSRATRGRTNRSSVKTPESIVPIAPELQPSTSRNQLVTPEPTSRATRCRTNRSSVKTPEPVVPTAPEPHPTTSTDQPVTPKLTSRATRRKTNRSSVKTPKPVEPAASDLEPFTPTDQSVTPEAIAQGGQSKTLRSSTVRAMPVPTTPEFQSPVTTDQPISPEPITQPSCIKRQRAAGNPGSLAAPIDHKPCSAPLEPKSQASRNQRWGAVRAAESLTAIPEPASPQLLETPIHASQIQKVEPAGRSRFTPELQPKASQSRKRSLATMDSPPHQKQPQRGEVSQKTVIIKEEEEDTAEKPGKEEDVVTPKPGKRKRDQAEEEPNRIPSRSLRRTKLNQESTAPKVLFTGVVDARGERAVLALGGSLAGSAAEASHLVTDRIRRTVKFLCALGRGIPILSLDWLHQSRKAGFFLPPDEYVVTDPEQEKNFGFSLQDALSRARERRLLEGYEIYVTPGVQPPPPQMGEIISCCGGTYLPSMPRSYKPQRVVITCPQDFPHCSIPLRVGLPLLSPEFLLTGVLKQEAKPEAFVLSPLEMSST

>NP_005648.1

MDPTGSQLDSDFSQQDTPCLIIEDSQPESQVLEDDSGSHFSMLSRHLPNLQTHKENPVLDVVSNPEQTAGEERGDGNSGFNEHLKENKVADPVDSSNLDTCGSISQVIEQLPQPNRTSSVLGMSVESAPAVEEEKGEELEQKEKEKEEDTSGNTTHSLGAEDTASSQLGFGVLELSQSQDVEENTVPYEVDKEQLQSVTTNSGYTRLSDVDANTAIKHEEQSNEDIPIAEQSSKDIPVTAQPSKDVHVVKEQNPPPARSEDMPFSPKASVAAMEAKEQLSAQELMESGLQIQKSPEPEVLSTQEDLFDQSNKTVSSDGCSTPSREEGGCSLASTPATTLHLLQLSGQRSLVQDSLSTNSSDLVAPSPDAFRSTPFIVPSSPTEQEGRQDKPMDTSVLSEEGGEPFQKKLQSGEPVELENPPLLPESTVSPQASTPISQSTPVFPPGSLPIPSQPQFSHDIFIPSPSLEEQSNDGKKDGDMHSSSLTVECSKTSEIEPKNSPEDLGLSLTGDSCKLMLSTSEYSQSPKMESLSSHRIDEDGENTQIEDTEPMSPVLNSKFVPAENDSILMNPAQDGEVQLSQNDDKTKGDDTDTRDDISILATGCKGREETVAEDVCIDLTCDSGSQAVPSPATRSEALSSVLDQEEAMEIKEHHPEEGSSGSEVEEIPETPCESQGEELKEENMESVPLHLSLTETQSQGLCLQKEMPKKECSEAMEVETSVISIDSPQKLAILDQELEHKEQEAWEEATSEDSSVVIVDVKEPSPRVDVSCEPLEGVEKCSDSQSWEDIAPEIEPCAENRLDTKEEKSVEYEGDLKSGTAETEPVEQDSSQPSLPLVRADDPLRLDQELQQPQTQEKTSNSLTEDSKMANAKQLSSDAEAQKLGKPSAHASQSFCESSSETPFHFTLPKEGDIIPPLTGATPPLIGHLKLEPKRHSTPIGISNYPESTIATSDVMSESMVETHDPILGSGKGDSGAAPDVDDKLCLRMKLVSPETEASEESLQFNLEKPATGERKNGSTAVAESVASPQKTMSVLSCICEARQENEARSEDPPTTPIRGNLLHFPSSQGEEEKEKLEGDHTIRQSQQPMKPISPVKDPVSPASQKMVIQGPSSPQGEAMVTDVLEDQKEGRSTNKENPSKALIERPSQNNIGIQTMECSLRVPETVSAATQTIKNVCEQGTSTVDQNFGKQDATVQTERGSGEKPVSAPGDDTESLHSQGEEEFDMPQPPHGHVLHRHMRTIREVRTLVTRVITDVYYVDGTEVERKVTEETEEPIVECQECETEVSPSQTGGSSGDLGDISSFSSKASSLHRTSSGTSLSAMHSSGSSGKGAGPLRGKTSGTEPADFALPSSRGGPGKLSPRKGVSQTGTPVCEEDGDAGLGIRQGGKAPVTPRGRGRRGRPPSRTTGTRETAVPGPLGIEDISPNLSPDDKSFSRVVPRVPDSTRRTDVGAGALRRSDSPEIPFQAAAGPSDGLDASSPGNSFVGLRVVAKWSSNGYFYSGKITRDVGAGKYKLLFDDGYECDVLGKDILLCDPIPLDTEVTALSEDEYFSAGVVKGHRKESGELYYSIEKEGQRKWYKRMAVILSLEQGNRLREQYGLGPYEAVTPLTKAADISLDNLVEGKRKRRSNVSSPATPTASSSSSTTPTRKITESPRASMGVLSGKRKLITSEEERSPAKRGRKSATVKPGAVGAGEFVSPCESGDNTGEPSALEEQRGPLPLNKTLFLGYAFLLTMATTSDKLASRSKLPDGPTGSSEEEEEFLEIPPFNKQYTESQLRAGAGYILEDFNEAQCNTAYQCLLIADQHCRTRKYFLCLASGIPCVSHVWVHDSCHANQLQNYRNYLLPAGYSLEEQRILDWQPRENPFQNLKVLLVSDQQQNFLELWSEILMTGGAASVKQHHSSAHNKDIALGVFDVVVTDPSCPASVLKCAEALQLPVVSQEWVIQCLIVGERIGFKQHPKYKHDYVSH

65 NP_000061.1 NP_003485.1

>NP_000061.1

MPTVISASVAPRTAAEPRSPGPVPHPAQSKATEAGGGNPSGIYSAIISRNFPIIGVKEKTFEQLHKKCLEKKVLYVDPEFPPDETSLFYSQKFPIQFVWKRPPEICENPRFIIDGANRTDICQGELGDCWFLAAIACLTLNQHLLFRVIPHDQSFIENYAGIFHFQFWRYGEWVDVVIDDCLPTYNNQLVFTKSNHRNEFWSALLEKAYAKLHGSYEALKGGNTTEAMEDFTGGVAEFFEIRDAPSDMYKIMKKAIERGSLMGCSIDDGTNMTYGTSPSGLNMGELIARMVRNMDNSLLQDSDLDPRGSDERPTRTIIPVQYETRMACGLVRGHAYSVTGLDEVPFKGEKVKLVRLRNPWGQVEWNGSWSDRWKDWSFVDKDEKARLQHQVTEDGEFWMSYEDFIYHFTKLEICNLTADALQSDKLQTWTVSVNEGRWVRGCSAGGCRNFPDTFWTNPQYRLKLLEEDDDPDDSEVICSFLVALMQKNRRKDRKLGASLFTIGFAIYEVPKEMHGNKQHLQKDFFLYNASKARSKTYINMREVSQRFRLPPSEYVIVPSTYEPHQEGEFILRVFSEKRNLSEEVENTISVDRPVKKKKTKPIIFVSDRANSNKELGVDQESEEGKGKTSPDKQKQSPQPQPGSSDQESEEQQQFRNIFKQIAGDDMEICADELKKVLNTVVNKHKDLKTHGFTLESCRSMIALMDTDGSGKLNLQEFHHLWNKIKAWQKIFKHYDTDQSGTINSYEMRNAVNDAGFHLNNQLYDIITMRYADKHMNIDFDSFICCFVRLEGMFRAFHAFDKDGDGIIKLNVLEWLQLTMYA

>NP_003485.1

MLRVFILYAENVHTPDTDISDAYCSAVFAGVKKRTKVIKNSVNPVWNEGFEWDLKGIPLDQGSELHVVVKDHETMGRNRFLGEAKVPLREVLATPSLSASFNAPLLDTKKQPTGASLVLQVSYTPLPGAVPLFPPPTPLEPSPTLPDLDVVADTGGEEDTEDQGLTGDEAEPFLDQSGGPGAPTTPRKLPSRPPPHYPGIKRKRSAPTSRKLLSDKPQDFQIRVQVIEGRQLPGVNIKPVVKVTAAGQTKRTRIHKGNSPLFNETLFFNLFDSPGELFDEPIFITVVDSRSLRTDALLGEFRMDVGTIYREPRHAYLRKWLLLSDPDDFSAGARGYLKTSLCVLGPGDEAPLERKDPSEDKEDIESNLLRPTGVALRGAHFCLKVFRAEDLPQMDDAVMDNVKQIFGFESNKKNLVDPFVEVSFAGKMLCSKILEKTANPQWNQNITLPAMFPSMCEKMRIRIIDWDRLTHNDIVATTYLSMSKISAPGGEIEEEPAGAVKPSKASDLDDYLGFLPTFGPCYINLYGSPREFTGFPDPYTELNTGKGEGVAYRGRLLLSLETKLVEHSEQKVEDLPADDILRVEKYLRRRKYSLFAAFYSATMLQDVDDAIQFEVSIGNYGNKFDMTCLPLASTTQYSRAVFDGCHYYYLPWGNVKPVVVLSSYWEDISHRIETQNQLLGIADRLEAGLEQVHLALKAQCSTEDVDSLVAQLTDELIAGCSQPLGDIHETPSATHLDQYLYQLRTHHLSQITEAALALKLGHSELPAALEQAEDWLLRLRALAEEPQNSLPDIVIWMLQGDKRVAYQRVPAHQVLFSRRGANYCGKNCGKLQTIFLKYPMEKVPGARMPVQIRVKLWFGLSVDEKEFNQFAEGKLSVFAETYENETKLALVGNWGTTGLTYPKFSDVTGKIKLPKDSFRPSAGWTWAGDWFVCPEKTLLHDMDAGHLSFVEEVFENQTRLPGGQWIYMSDNYTDVNGEKVLPKDDIECPLGWKWEDEEWSTDLNRAVDEQGWEYSITIPPERKPKHWVPAEKMYYTHRRRRWVRLRRRDLSQMEALKRHRQAEAEGEGWEYASLFGWKFHLEYRKTDAFRRRRWRRRMEPLEKTGPAAVFALEGALGGVMDDKSEDSMSVSTLSFGVNRPTISCIFDYGNRYHLRCYMYQARDLAAMDKDSFSDPYAIVSFLHQSQKTVVVKNTLNPTWDQTLIFYEIEIFGEPATVAEQPPSIVVELYDHDTYGADEFMGRCICQPSLERMPRLAWFPLTRGSQPSGELLASFELIQREKPAIHHIPGFEVQETSRILDESEDTDLPYPPPQREANIYMVPQNIKPALQRTAIEILAWGLRNMKSYQLANISSPSLVVECGGQTVQSCVIRNLRKNPNFDICTLFMEVMLPREELYCPPITVKVIDNRQFGRRPVVGQCTIRSLESFLCDPYSAESPSPQGGPDDVSLLSPGEDVLIDIDDKEPLIPIQEEEFIDWWSKFFASIGEREKCGSYLEKDFDTLKVYDTQLENVEAFEGLSDFCNTFKLYRGKTQEETEDPSVIGEFKGLFKIYPLPEDPAIPMPPRQFHQLAAQGPQECLVRIYIVRAFGLQPKDPNGKCDPYIKISIGKKSVSDQDNYIPCTLEPVFGKMFELTCTLPLEKDLKITLYDYDLLSKDEKIGETVVDLENRLLSKFGARCGLPQTYCVSGPNQWRDQLRPSQLLHLFCQQHRVKAPVYRTDRVMFQDKEYSIEEIEAGRIPNPHLGPVEERLALHVLQQQGLVPEHVESRPLYSPLQPDIEQGKLQMWVDLFPKALGRPGPPFNITPRRARRFFLRCIIWNTRDVILDDLSLTGEKMSDIYVKGWMIGFEEHKQKTDVHYRSLGGEGNFNWRFIFPFDYLPAEQVCTIAKKDAFWRLDKTESKIPARVVFQIWDNDKFSFDDFLGSLQLDLNRMPKPAKTAKKCSLDQLDDAFHPEWFVSLFEQKTVKGWWPCVAEEGEKKILAGKLEMTLEIVAESEHEERPAGQGRDEPNMNPKLEDPRRPDTSFLWFTSPYKTMKFILWRRFRWAIILFIILFILLLFLAIFIYAFPNYAAMKLVKPFS

66 NP_071935.1 NP_056240.2

>NP_071935.1

MAAEVLPSARWQYCGAPDGSQRAVLVQFSNGKLQSPGNMRFTLYENKDSTNPRKRNQRILAAETDRLSYVGNNFGTGALKCNTLCRHFVGILNKTSGQMEVYDAELFNMQPLFSDVSVESELALESQTKTYREKMDSCIEAFGTTKQKRALNTRRMNRVGNESLNRAVAKAAETIIDTKGVTALVSDAIHNDLQDDSLYLPPCYDDAAKPEDVYKFEDLLSPAEYEALQSPSEAFRNVTSEEILKMIEENSHCTFVIEALKSLPSDVESRDRQARCIWFLDTLIKFRAHRVVKRKSALGPGVPHIINTKLLKHFTCLTYNNGRLRNLISDSMKAKITAYVIILALHIHDFQIDLTVLQRDLKLSEKRMMEIAKAMRLKISKRRVSVAAGSEEDHKLGTLSLPLPPAQTSDRLAKRRKIT

>NP_056240.2

MLISKNMPWRRLQGISFGMYSAEELKKLSVKSITNPRYLDSLGNPSANGLYDLALGPADSKEVCSTCVQDFSNCSGHLGHIELPLTVYNPLLFDKLYLLLRGSCLNCHMLTCPRAVIHLLLCQLRVLEVGALQAVYELERILNRFLEENPDPSASEIREELEQYTTEIVQNNLLGSQGAHVKNVCESKSKLIALFWKAHMNAKRCPHCKTGRSVVRKEHNSKLTITFPAMVHRTAGQKDSEPLGIEEAQIGKRGYLTPTSAREHLSALWKNEGFFLNYLFSGMDDDGMESRFNPSVFFLDFLVVPPSRYRPVSRLGDQMFTNGQTVNLQAVMKDVVLIRKLLALMAQEQKLPEEVATPTTDEEKDSLIAIDRSFLSTLPGQSLIDKLYNIWIRLQSHVNIVFDSEMDKLMMDKYPGIRQILEKKEGLFRKHMMGKRVDYAARSVICPDMYINTNEIGIPMVFATKLTYPQPVTPWNVQELRQAVINGPNVHPGASMVINEDGSRTALSAVDMTQREAVAKQLLTPATGAPKPQGTKIVCRHVKNGDILLLNRQPTLHRPSIQAHRARILPEEKVLRLHYANCKAYNADFDGDEMNAHFPQSELGRAEAYVLACTDQQYLVPKDGQPLAGLIQDHMVSGASMTTRGCFFTREHYMELVYRGLTDKVGRVKLLSPSILKPFPLWTGKQVVSTLLINIIPEDHIPLNLSGKAKITGKAWVKETPRSVPGFNPDSMCESQVIIREGELLCGVLDKAHYGSSAYGLVHCCYEIYGGETSGKVLTCLARLFTAYLQLYRGFTLGVEDILVKPKADVKRQRIIEESTHCGPQAVRAALNLPEAASYDEVRGKWQDAHLGKDQRDFNMIDLKFKEEVNHYSNEINKACMPFGLHRQFPENSLQMMVQSGAKGSTVNTMQISCLLGQIELEGRRPPLMASGKSLPCFEPYEFTPRAGGFVTGRFLTGIKPPEFFFHCMAGREGLVDTAVKTSRSGYLQRCIIKHLEGLVVQYDLTVRDSDGSVVQFLYGEDGLDIPKTQFLQPKQFPFLASNYEVIMKSQHLHEVLSRADPKKALHHFRAIKKWQSKHPNTLLRRGAFLSYSQKIQEAVKALKLESENRNGRSPGTQEMLRMWYELDEESRRKYQKKAAACPDPSLSVWRPDIYFASVSETFETKVDDYSQEWAAQTEKSYEKSELSLDRLRTLLQLKWQRSLCEPGEAVGLLAAQSIGEPSTQMTLNTFHFAGRGEMNVTLGIPRLREILMVASANIKTPMMSVPVLNTKKALKRVKSLKKQLTRVCLGEVLQKIDVQESFCMEEKQNKFQVYQLRFQFLPHAYYQQEKCLRPEDILRFMETRFFKLLMESIKKKNNKASAFRNVNTRRATQRDLDNAGELGRSRGEQEGDEEEEGHIVDAEAEEGDADASDAKRKEKQEEEVDYESEEEEEREGEENDDEDMQEERNPHREGARKTQEQDEEVGLGTEEDPSLPALLTQPRKPTHSQEPQGPEAMERRVQAVREIHPFIDDYQYDTEESLWCQVTVKLPLMKINFDMSSLVVSLAHGAVIYATKGITRCLLNETTNNKNEKELVLNTEGINLPELFKYAEVLDLRRLYSNDIHAIANTYGIEAALRVIEKEIKDVFAVYGIAVDPRHLSLVADYMCFEGVYKPLNRFGIRSNSSPLQQMTFETSFQFLKQATMLGSHDELRSPSACLVVGKVVRGGTGLFELKQPLR

67 NP_000536.4 NP_001895.1

>NP_000536.4

MVSKLSQLQTELLAALLESGLSKEALIQALGEPGPYLLAGEGPLDKGESCGGGRGELAELPNGLGETRGSEDETDDDGEDFTPPILKELENLSPEEAAHQKAVVETLLQEDPWRVAKMVKSYLQQHNIPQREVVDTTGLNQSHLSQHLNKGTPMKTQKRAALYTWYVRKQREVAQQFTHAGQGGLIEEPTGDELPTKKGRRNRFKWGPASQQILFQAYERQKNPSKEERETLVEECNRAECIQRGVSPSQAQGLGSNLVTEVRVYNWFANRRKEEAFRHKLAMDTYSGPPPGPGPGPALPAHSSPGLPPPALSPSKVHGVRYGQPATSETAEVPSSSGGPLVTVSTPLHQVSPTGLEPSHSLLSTEAKLVSAAGGPLPPVSTLTALHSLEQTSPGLNQQPQNLIMASLPGVMTIGPGEPASLGPTFTNTGASTLVIGLASTQAQSVPVINSMGSSLTTLQPVQFSQPLHPSYQQPLMPPVQSHVTQSPFMATMAQLQSPHALYSHKPEVAQYTHTGLLPQTMLITDTTNLSALASLTPTKQVFTSDTEASSESGLHTPASQATTLHVPSQDPASIQHLQPAHRLSASPTVSSSSLVLYQSSDSSNGQSHLLPSNHSVIETFISTQMASSSQ

>NP_001895.1

MATQADLMELDMAMEPDRKAAVSHWQQQSYLDSGIHSGATTTAPSLSGKGNPEEEDVDTSQVLYEWEQGFSQSFTQEQVADIDGQYAMTRAQRVRAAMFPETLDEGMQIPSTQFDAAHPTNVQRLAEPSQMLKHAVVNLINYQDDAELATRAIPELTKLLNDEDQVVVNKAAVMVHQLSKKEASRHAIMRSPQMVSAIVRTMQNTNDVETARCTAGTLHNLSHHREGLLAIFKSGGIPALVKMLGSPVDSVLFYAITTLHNLLLHQEGAKMAVRLAGGLQKMVALLNKTNVKFLAITTDCLQILAYGNQESKLIILASGGPQALVNIMRTYTYEKLLWTTSRVLKVLSVCSSNKPAIVEAGGMQALGLHLTDPSQRLVQNCLWTLRNLSDAATKQEGMEGLLGTLVQLLGSDDINVVTCAAGILSNLTCNNYKNKMMVCQVGGIEALVRTVLRAGDREDITEPAICALRHLTSRHQEAEMAQNAVRLHYGLPVVVKLLHPPSHWPLIKATVGLIRNLALCPANHAPLREQGAIPRLVQLLVRAHQDTQRRTSMGGTQQQFVEGVRMEEIVEGCTGALHILARDVHNRIVIRGLNTIPLFVQLLYSPIENIQRVAAGVLCELAQDKEAAEAIEAEGATAPLTELLHSRNEGVATYAAAVLFRMSEDKPQDYKKRLSVELTSSLFRTEPMAWNETADLGLDIGAQGEPLGYRQDDPSYRSFHSGGYGQDALGMDPMMEHEMGGHHPGADYPVDGLPDLGHAQDLMDGLPPGDSNQLAWFDTDL

68 NP_006459.3 NP_036336.1

>NP_006459.3

MTQAEIKLCSLLLQEHFGEIVEKIGVHLIRTGSQPLRVIAHDTGTSLDQVKKALCVLVQHNLVSYQVHKRGVVEYEAQCSRVLRMLRYPRYIYTTKTLYSDTGELIVEELLLNGKLTMSAVVKKVADRLTETMEDGKTMDYAEVSNTFVRLADTHFVQRCPSVPTTENSDPGPPPPAPTLVINEKDMYLVPKLSLIGKGKRRRSSDEDAAGEPKAKRPKYTTDNKEPIPDDGIYWQANLDRFHQHFRDQAIVSAVANRMDQTSSEIVRTMLRMSEITTSSSAPFTQPLSSNEIFRSLPVGYNISKQVLDQYLTLLADDPLEFVGKSGDSGGGMYVINLHKALASLATATLESVVQERFGSRCARIFRLVLQKKHIEQKQVEDFAMIPAKEAKDMLYKMLSENFMSLQEIPKTPDHAPSRTFYLYTVNILSAARMLLHRCYKSIANLIERRQFETKENKRLLEKSQRVEAIIASMQATGAEEAQLQEIEEMITAPERQQLETLKRNVNKLDASEIQVDETIFLLESYIECTMKRQ

>NP_036336.1

MNTADQARVGPADDGPAPSGEEEGEGGGEAGGKEPAADAAPGPSAAFRLMVTRREPAVKLQYAVSGLEPLAWSEDHRVSVSTARSIAVLELICDVHNPGQDLVIHRTSVPAPLNSCLLKVGSKTEVAECKEKFAASKDPTVSQTFMLDRVFNPEGKALPPMRGFKYTSWSPMGCDANGRCLLAALTMDNRLTIQANLNRLQWVQLVDLTEIYGERLYETSYRLSKNEAPEGNLGDFAEFQRRHSMQTPVRMEWSGICTTQQVKHNNESRDVGSVLLAVLFENGNIAVWQFQLPFVGKESISSCNTIESGITSPSVLFWWEYEHNNRKMSGLIVGSAFGPIKILPVNLKAVKGYFTLRQPVILWKEMDQLPVHSIKCVPLYHPYQKCSCSLVVAARGSYVFWRLLLISKAGLNLHNSHVTGLHSLPIVSMTADKQNGTVYTCSSDGKVRQVIPIFTDVALKFEHQLIKLSDVFGSVRTHGIAVKPCGAYLAIITTEGMINGLHPVNKNYQVQFVTLKTFEEAAAQLLESSVQNLFKQVDLIDLVRWKILKDKHIPQFLQEALEKKIESSGVTYFWRFKLFLLRILYQSMQKTPSEALWKPTHEDSKILLVDSPGMGNADDEQQEEGTSSKQVVKQGLQERSKEGDVEEPTDDSLPTTGDAGGREPMEEKLLEIQGKIEAVEMHLTREHMKPVLGEVYLHTWITENTSIPTRGLCNFLMSDEEYDDRTARVLIGHISKKMNKQTFPEHCSLCKEILPFTDRKQAVCSNGHIWLRCFLTYQSCQSLIYRRCLLHDSIARHPAPEDPDWIKRLLQSPCPFCDSPVF

69 NP_001311.3 NP_000116.2

>NP_001311.3

MSSSEEVSWISWFCGLRGNEFFCEVDEDYIQDKFNLTGLNEQVPHYRQALDMILDLEPDEELEDNPNQSDLIEQAAEMLYGLIHARYILTNRGIAQMLEKYQQGDFGYCPRVYCENQPMLPIGLSDIPGEAMVKLYCPKCMDVYTPKSSRHHHTDGAYFGTGFPHMLFMVHPEYRPKRPANQFVPRLYGFKIHPMAYQLQLQAASNFKSPVKTIR

>NP_000116.2

MTMTLHTKASGMALLHQIQGNELEPLNRPQLKIPLERPLGEVYLDSSKPAVYNYPEGAAYEFNAAAAANAQVYGQTGLPYGPGSEAAAFGSNGLGGFPPLNSVSPSPLMLLHPPPQLSPFLQPHGQQVPYYLENEPSGYTVREAGPPAFYRPNSDNRRQGGRERLASTNDKGSMAMESAKETRYCAVCNDYASGYHYGVWSCEGCKAFFKRSIQGHNDYMCPATNQCTIDKNRRKSCQACRLRKCYEVGMMKGGIRKDRRGGRMLKHKRQRDDGEGRGEVGSAGDMRAANLWPSPLMIKRSKKNSLALSLTADQMVSALLDAEPPILYSEYDPTRPFSEASMMGLLTNLADRELVHMINWAKRVPGFVDLTLHDQVHLLECAWLEILMIGLVWRSMEHPGKLLFAPNLLLDRNQGKCVEGMVEIFDMLLATSSRFRMMNLQGEEFVCLKSIILLNSGVYTFLSSTLKSLEEKDHIHRVLDKITDTLIHLMAKAGLTLQQQHQRLAQLLLILSHIRHMSNKGMEHLYSMKCKNVVPLYDLLLEMLDAHRLHAPTSRGGASVEETDQSHLATAGSTSSHSLQKYYITGEAEGFPATV

70 NP_071933.2 NP_006386.1

>NP_071933.2

MQNVINTVKGKALEVAEYLTPVLKESKFKETGVITPEEFVAAGDHLVHHCPTWQWATGEELKVKAYLPTGKQFLVTKNVPCYKRCKQMEYSDELEAIIEEDDGDGGWVDTYHNTGITGITEAVKEITLENKDNIRLQDCSALCEEEEDEDEGEAADMEEYEESGLLETDEATLDTRKIVEACKAKTDAGGEDAILQTRTYDLYITYDKYYQTPRLWLFGYDEQRQPLTVEHMYEDISQDHVKKTVTIENHPHLPPPPMCSVHPCRHAEVMKKIIETVAEGGGELGVHMYLLIFLKFVQAVIPTIEYDYTRHFTM

>NP_006386.1

MAAATGDPGLSKLQFAPFSSALDVGFWHELTQKKLNEYRLDEAPKDIKGYYYNGDSAGLPARLTLEFSAFDMSAPTPARCCPAIGTLYNTNTLESFKTADKKLLLEQAANEIWESIKSGTALENPVLLNKFLLLTFADLKKYHFYYWFCYPALCLPESLPLIQGPVGLDQRFSLKQIEALECAYDNLCQTEGVTALPYFLIKYDENMVLVSLLKHYSDFFQGQRTKITIGVYDPCNLAQYPGWPLRNFLVLAAHRWSSSFQSVEVVCFRDRTMQGARDVAHSIIFEVKLPEMAFSPDCPKAVGWEKNQKGGMGPRMVNLSECMDPKRLAESSVDLNLKLMCWRLVPTLDLDKVVSVKCLLLGAGTLGCNVARTLMGWGVRHITFVDNAKISYSNPVRQPLYEFEDCLGGGKPKALAAADRLQKIFPGVNARGFNMSIPMPGHPVNFSSVTLEQARRDVEQLEQLIESHDVVFLLMDTRESRWLPAVIAASKRKLVINAALGFDTFVVMRHGLKKPKQQGAGDLCPNHPVASADLLGSSLFANIPGYKLGCYFCNDVVAPGDSTRDRTLDQQCTVSRPGLAVIAGALAVELMVSVLQHPEGGYAIASSSDDRMNEPPTSLGLVPHQIRGFLSRFDNVLPVSLAFDKCTACSSKVLDQYEREGFNFLAKVFNSSHSFLEDLTGLTLLHQETQAAEIWDMSDDETI

71 NP_055217.2 NP_056347.1

>NP_055217.2

MGREFGNLTRMRHVISYSLSPFEQRAYPHVFTKGIPNVLRRIRESFFRVVPQFVVFYLIYTWGTEEFERSKRKNPAAYENDK

>NP_056347.1

MCSLPRGFEPQAPEDLAQRSLVELREMLKRQERLLRNEKFICKLPDKGKKIFDSFAKLKAAIAECEEVRRKSELFNPVSLDCKLRQKAIAEVDVGTDKAQNSDPILDTSSLVPGCSSVDNIKSSQTSQNQGLGRPTLEGDEETSEVEYTVNKGPASSNRDRVPPSSEASEHHPRHRVSSQAEDTSSSFDNLFIDRLQRITIADQGEQQSEENASTKNLTGLSSGTEKKPHYMEVLEMRAKNPVPQLRKFKTNVLPFRQNDSSSHCQKSGSPISSEERRRRDKQHLDDITAARLLPLHHMPTQLLSIEESLALQKQQKQNYEEMQAKLAAQKLAERLNIKMRSYNPEGESSGRYREVRDEDDDWSSDEF

72 NP_055191.2 NP_995314.1

>NP_055191.2

MTTHVTLEDALSNVDLLEELPLPDQQPCIEPPPSSIMYQANFDTNFEDRNAFVTGIARYIEQATVHSSMNEMLEEGHEYAVMLYTWRSCSRAIPQVKCNEQPNRVEIYEKTVEVLEPEVTKLMKFMYFQRKAIERFCSEVKRLCHAERRKDFVSEAYLLTLGKFINMFAVLDELKNMKCSVKNDHSAYKRAAQFLRKMADPQSIQESQNLSMFLANHNRITQCLHQQLEVIPGYEELLADIVNICVDYYENKMYLTPSEKHMLLKVMGFGLYLMDGNVSNIYKLDAKKRINLSKIDKFFKQLQVVPLFGDMQIELARYIKTSAHYEENKSKWTCTQSSISPQYNICEQMVQIRDDHIRFISELARYSNSEVVTGSGLDSQKSDEEYRELFDLALRGLQLLSKWSAHVMEVYSWKLVHPTDKFCNKDCPGTAEEYERATRYNYTSEEKFAFVEVIAMIKGLQVLMGRMESVFNQAIRNTIYAALQDFAQVTLREPLRQAVRKKKNVLISVLQAIRKTICDWEGGREPPNDPCLRGEKDPKGGFDIKVPRRAVGPSSTQLYMVRTMLESLIADKSGSKKTLRSSLDGPIVLAIEDFHKQSFFFTHLLNISEALQQCCDLSQLWFREFFLELTMGRRIQFPIEMSMPWILTDHILETKEPSMMEYVLYPLDLYNDSAYYALTKFKKQFLYDEIEAEVNLCFDQFVYKLADQIFAYYKAMAGSVLLDKRFRAECKNYGVIIPYPPSNRYETLLKQRHVQLLGRSIDLNRLITQRISAAMYKSLDQAISRFESEDLTSIVELEWLLEINRLTHRLLCKHMTLDSFDAMFREANHNVSAPYGRITLHVFWELNFDFLPNYCYNGSTNRFVRTAIPFTQEPQRDKPANVQPYYLYGSKPLNIAYSHIYSSYRNFVGPPHFKTICRLLGYQGIAVVMEELLKIVKSLLQGTILQYVKTLIEVMPKICRLPRHEYGSPGILEFFHHQLKDIIEYAELKTDVFQSLREVGNAILFCLLIEQALSQEEVCDLLHAAPFQNILPRVYIKEGERLEVRMKRLEAKYAPLHLVPLIERLGTPQQIAIAREGDLLTKERLCCGLSMFEVILTRIRSYLQDPIWRGPPPTNGVMHVDECVEFHRLWSAMQFVYCIPVGTNEFTAEQCFGDGLNWAGCSIIVLLGQQRRFDLFDFCYHLLKVQRQDGKDEIIKNVPLKKMADRIRKYQILNNEVFAILNKYMKSVETDSSTVEHVRCFQPPIHQSLATTC

>NP_995314.1

MSRSVLQPSQQKLAEKLTILNDRGVGMLTRLYNIKKQGQVWKACGDPKAKPSYLIDKNLESAVKFIVRKFPAVETRNNNQQLAQLQKEKSEILKNLALYYFTFVDVMEFKDHVCELLNTIDVCQVFFDITVNFDLTKNYLDLIITYTTLMILLSRIEERKAIIGLYNYAHEMTHGASDREYPRLGQMIVDYENPLKKMMEEFVPHSKSLSDALISLQMVYPRRNLSADQWRNAQLLSLISAPSTMLNPAQSDTMPCEYLSLDAMEKWIIFGFILCHGILNTDATALNLWKLALQSSSCLSLFRDEVFHIHKAAEDLFVNIRGYNKRINDIRECKEAAVSHAGSMHRERRKFLRSALKELATVLSDQPGLLGPKALFVFMALSFARDEIIWLLRHADNMPKKSADDFIDKHIAELIFYMEELRAHVRKYGPVMQRYYVQYLSGFDAVVLNELVQNLSVCPEDESIIMSSFVNTMTSLSVKQVEDGEVFDFRGMRLDWFRLQAYTSVSKASLGLADHRELGKMMNTIIFHTKMVDSLVEMLVETSDLSIFCFYSRAFEKMFQQCLELPSQSRYSIAFPLLCTHFMSCTHELCPEERHHIGDRSLSLCNMFLDEMAKQARNLITDICTEQCTLSDQLLPKHCAKTISQAVNKKSKKQTGKKGEPEREKPGVESMRKNRLVVTNLDKLHTALSELCFSINYVPNMVVWEHTFTPREYLTSHLEIRFTKSIVGMTMYNQATQEIAKPSELLTSVRAYMTVLQSIENYVQIDITRVFNNVLLQQTQHLDSHGEPTITSLYTNWYLETLLRQVSNGHIAYFPAMKAFVNLPTENELTFNAEEYSDISEMRSLSELLGPYGMKFLSESLMWHISSQVAELKKLVVENVDVLTQMRTSFDKPDQMAALFKRLSSVDSVLKRMTIIGVILSFRSLAQEALRDVLSYHIPFLVSSIEDFKDHIPRETDMKVAMNVYELSSAAGLPCEIDPALVVALSSQKSENISPEEEYKIACLLMVFVAVSLPTLASNVMSQYSPAIEGHCNNIHCLAKAINQIAAALFTIHKGSIEDRLKEFLALASSSLLKIGQETDKTTTRNRESVYLLLDMIVQESPFLTMDLLESCFPYVLLRNAYHAVYKQSVTSSA

73 NP_000356.1 NP_660198.1

>NP_000356.1

MAPSRKFFVGGNWKMNGRKQSLGELIGTLNAAKVPADTEVVCAPPTAYIDFARQKLDPKIAVAAQNCYKVTNGAFTGEISPGMIKDCGATWVVLGHSERRHVFGESDELIGQKVAHALAEGLGVIACIGEKLDEREAGITEKVVFEQTKVIADNVKDWSKVVLAYEPVWAIGTGKTATPQQAQEVHEKLRGWLKSNVSDAVAQSTRIIYGGSVTGATCKELASQPDVDGFLVGGASLKPEFVDIINAKQ

>NP_660198.1

MLIPFSMKNCFQLLCNCQVPAAGFKKTVKNGLILQSISNDVYQNLAVEDWIHDHMNLEGKPILFFWQNSPSVVIGRHQNPWQECNLNLMREEGIKLARRRSGGGTVYHDMGNINLTFFTTKKKYDRMENLKLIVRALNAVQPQLDVQATKRFDLLLDGQFKISGTASKIGRTTAYHHCTLLCSTDGTFLSSLLKSPYQGIRSNATASIPSLVKNLLEKDPTLTCEVLMNAVATEYAAYHQIDNHIHLINPTDETLFPGINSKAKELQTWEWIYGKTPKFSINTSFHVLYEQSHLEIKVFIDIKNGRIEICNIEAPDHWLPLEIRDKLNSSLIGSKFCPTETTMLTNILLRTCPQDHKLNSKWNILCEKIKGIM

74 NP_001420.2 NP_003202.3

>NP_001420.2

MAENVVEPGPPSAKRPKLSSPALSASASDGTDFGSLFDLEHDLPDELINSTELGLTNGGDINQLQTSLGMVQDAASKHKQLSELLRSGSSPNLNMGVGGPGQVMASQAQQSSPGLGLINSMVKSPMTQAGLTSPNMGMGTSGPNQGPTQSTGMMNSPVNQPAMGMNTGMNAGMNPGMLAAGNGQGIMPNQVMNGSIGAGRGRQNMQYPNPGMGSAGNLLTEPLQQGSPQMGGQTGLRGPQPLKMGMMNNPNPYGSPYTQNPGQQIGASGLGLQIQTKTVLSNNLSPFAMDKKAVPGGGMPNMGQQPAPQVQQPGLVTPVAQGMGSGAHTADPEKRKLIQQQLVLLLHAHKCQRREQANGEVRQCNLPHCRTMKNVLNHMTHCQSGKSCQVAHCASSRQIISHWKNCTRHDCPVCLPLKNAGDKRNQQPILTGAPVGLGNPSSLGVGQQSAPNLSTVSQIDPSSIERAYAALGLPYQVNQMPTQPQVQAKNQQNQQPGQSPQGMRPMSNMSASPMGVNGGVGVQTPSLLSDSMLHSAINSQNPMMSENASVPSLGPMPTAAQPSTTGIRKQWHEDITQDLRNHLVHKLVQAIFPTPDPAALKDRRMENLVAYARKVEGDMYESANNRAEYYHLLAEKIYKIQKELEEKRRTRLQKQNMLPNAAGMVPVSMNPGPNMGQPQPGMTSNGPLPDPSMIRGSVPNQMMPRITPQSGLNQFGQMSMAQPPIVPRQTPPLQHHGQLAQPGALNPPMGYGPRMQQPSNQGQFLPQTQFPSQGMNVTNIPLAPSSGQAPVSQAQMSSSSCPVNSPIMPPGSQGSHIHCPQLPQPALHQNSPSPVPSRTPTPHHTPPSIGAQQPPATTIPAPVPTPPAMPPGPQSQALHPPPRQTPTPPTTQLPQQVQPSLPAAPSADQPQQQPRSQQSTAASVPTPTAPLLPPQPATPLSQPAVSIEGQVSNPPSTSSTEVNSQAIAEKQPSQEVKMEAKMEVDQPEPADTQPEDISESKVEDCKMESTETEERSTELKTEIKEEEDQPSTSATQSSPAPGQSKKKIFKPEELRQALMPTLEALYRQDPESLPFRQPVDPQLLGIPDYFDIVKSPMDLSTIKRKLDTGQYQEPWQYVDDIWLMFNNAWLYNRKTSRVYKYCSKLSEVFEQEIDPVMQSLGYCCGRKLEFSPQTLCCYGKQLCTIPRDATYYSYQNRYHFCEKCFNEIQGESVSLGDDPSQPQTTINKEQFSKRKNDTLDPELFVECTECGRKMHQICVLHHEIIWPAGFVCDGCLKKSARTRKENKFSAKRLPSTRLGTFLENRVNDFLRRQNHPESGEVTVRVVHASDKTVEVKPGMKARFVDSGEMAESFPYRTKALFAFEEIDGVDLCFFGMHVQEYGSDCPPPNQRRVYISYLDSVHFFRPKCLRTAVYHEILIGYLEYVKKLGYTTGHIWACPPSEGDDYIFHCHPPDQKIPKPKRLQEWYKKMLDKAVSERIVHDYKDIFKQATEDRLTSAKELPYFEGDFWPNVLEESIKELEQEEEERKREENTSNESTDVTKGDSKNAKKKNNKKTSKNKSSLSRGNKKKPGMPNVSNDLSQKLYATMEKHKEVFFVIRLIAGPAANSLPPIVDPDPLIPCDLMDGRDAFLTLARDKHLEFSSLRRAQWSTMCMLVELHTQSQDRFVYTCNECKHHVETRWHCTVCEDYDLCITCYNTKNHDHKMEKLGLGLDDESNNQQAAATQSPGDSRRLSIQRCIQSLVHACQCRNANCSLPSCQKMKRVVQHTKGCKRKTNGGCPICKQLIALCCYHAKHCQENKCPVPFCLNIKQKLRQQQLQHRLQQAQMLRRRMASMQRTGVVGQQQGLPSPTPATPTTPTGQQPTTPQTPQPTSQPQPTPPNSMPPYLPRTQAAGPVSQGKAAGQVTPPTPPQTAQPPLPGPPPAAVEMAMQIQRAAETQRQMAHVQIFQRPIQHQMPPMTPMAPMGMNPPPMTRGPSGHLEPGMGPTGMQQQPPWSQGGLPQPQQLQSGMPRPAMMSVAQHGQPLNMAPQPGLGQVGISPLKPGTVSQQALQNLLRTLRSPSSPLQQQQVLSILHANPQLLAAFIKQRAAKYANSNPQPIPGQPGMPQGQPGLQPPTMPGQQGVHSNPAMQNMNPMQAGVQRAGLPQQQPQQQLQPPMGGMSPQAQQMNMNHNTMPSQFRDILRRQQMMQQQQQQGAGPGIGPGMANHNQFQQPQGVGYPPQQQQRMQHHMQQMQQGNMGQIGQLPQALGAEAGASLQAYQQRLLQQQMGSPVQPNPMSPQQHMLPNQAQSPHLQGQQIPNSLSNQVRSPQPVPSPRPQSQPPHSSPSPRMQPQPSPHHVSPQTSSPHPGLVAAQANPMEQGHFASPDQNSMLSQLASNPGMANLHGASATDLGLSTDNSDLNSNLSQSTLDIH

>NP_003202.3

MEAENAGSYSLQQAQAFYTFPFQQLMAEAPNMAVVNEQQMPEEVPAPAPAQEPVQEAPKGRKRKPRTTEPKQPVEPKKPVESKKSGKSAKSKEKQEKITDTFKVKRKVDRFNGVSEAELLTKTLPDILTFNLDIVIIGINPGLMAAYKGHHYPGPGNHFWKCLFMSGLSEVQLNHMDDHTLPGKYGIGFTNMVERTTPGSKDLSSKEFREGGRILVQKLQKYQPRIAVFNGKCIYEIFSKEVFGVKVKNLEFGLQPHKIPDTETLCYVMPSSSARCAQFPRAQDKVHYYIKLKDLRDQLKGIERNMDVQEVQYTFDLQLAQEDAKKMAVKEEKYDPGYEAAYGGAYGENPCSSEPCGFSSNGLIESVELRGESAFSGIPNGQWMTQSFTDQIPSFSNHCGTQEQEEESHA

75 NP_057213.2 NP_006839.2

>NP_057213.2

MAAAVRQDLAQLMNSSGSHKDLAGKYRQILEKAIQLSGAEQLEALKAFVEAMVNENVSLVISRQLLTDFCTHLPNLPDSTAKEIYHFTLEKIQPRVISFEEQVASIRQHLASIYEKEEDWRNAAQVLVGIPLETGQKQYNVDYKLETYLKIARLYLEDDDPVQAEAYINRASLLQNESTNEQLQIHYKVCYARVLDYRRKFIEAAQRYNELSYKTIVHESERLEALKHALHCTILASAGQQRSRMLATLFKDERCQQLAAYGILEKMYLDRIIRGNQLQEFAAMLMPHQKATTADGSSILDRAVIEHNLLSASKLYNNITFEELGALLEIPAAKAEKIASQMITEGRMNGFIDQIDGIVHFETREALPTWDKQIQSLCFQVNNLLEKISQTAPEWTAQAMEAQMAQ

>NP_006839.2

MEAEAGGLEELTDEEMAALGKEELVRRLRREEAARLAALVQRGRLMQEVNRQLQGHLGEIRELKQLNRRLQAENRELRDLCCFLDSERQRGRRAARQWQLFGTQASRAVREDLGGCWQKLAELEGRQEELLRENLALKELCLALGEEWGPRGGPSGAGGSGAGPAPELALPPCGPRDLGDGSSSTGSVGSPDQLPLACSPDD

76 NP_059523.1 NP_068751.4

>NP_059523.1

MAEDVSSAAPSPRRCADGRDADPTEEQMAETERNDEEQFECQELLECQVQVGAPEEEEEEEEDAGLVAEAEAVAAGWMLDFLCLSLCRAFRDGRSEDFRRTRNSAEAIIHGLSSLTACQLRTIYICQFLTRIAAGKTLDAQFENDERITPLESALMIWGSIEKEHDKLHEEIQNLIKIQAIAVCMENGNFKEAEEVFERIFGDPNSHMPFKSKLLMIISQKDTFHSFFQHFSYNHMMEKIKSYVNYVLSEKSSTFLMKAAAKVVESKRTRTITSQDKPSGNDVEMETEANLDTRKSVSDKQSAVTESSEGTVSLLRSHKNLFLSKLQHGTQQQDLNKKERRVGTPQSTKKKKESRRATESRIPVSKSQPVTPEKHRARKRQAWLWEEDKNLRSGVRKYGEGNWSKILLHYKFNNRTSVMLKDRWRTMKKLKLISSDSED

>NP_068751.4

MEPRAVAEAVETGEEDVIMEALRSYNQEHSQSFTFDDAQQEDRKRLAELLVSVLEQGLPPSHRVIWLQSVRILSRDRNCLDPFTSRQSLQALACYADISVSEGSVPESADMDVVLESLKCLCNLVLSSPVAQMLAAEARLVVKLTERVGLYRERSFPHDVQFFDLRLLFLLTALRTDVRQQLFQELKGVRLLTDTLELTLGVTPEGNPPPTLLPSQETERAMEILKVLFNITLDSIKGEVDEEDAALYRHLGTLLRHCVMIATAGDRTEEFHGHAVNLLGNLPLKCLDVLLTLEPHGDSTEFMGVNMDVIRALLIFLEKRLHKTHRLKESVAPVLSVLTECARMHRPARKFLKAQGWPPPQVLPPLRDVRTRPEVGEMLRNKLVRLMTHLDTDVKRVAAEFLFVLCSESVPRFIKYTGYGNAAGLLAARGLMAGGRPEGQYSEDEDTDTDEYKEAKASINPVTGRVEEKPPNPMEGMTEEQKEHEAMKLVTMFDKLSRNRVIQPMGMSPRGHLTSLQDAMCETMEQQLSSDPDSDPD

77 NP_000631.1 NP_000580.1

>NP_000631.1

MAFVCLAIGCLYTFLISTTFGCTSSSDTEIKVNPPQDFEIVDPGYLGYLYLQWQPPLSLDHFKECTVEYELKYRNIGSETWKTIITKNLHYKDGFDLNKGIEAKIHTLLPWQCTNGSEVQSSWAETTYWISPQGIPETKVQDMDCVYYNWQYLLCSWKPGIGVLLDTNYNLFYWYEGLDHALQCVDYIKADGQNIGCRFPYLEASDYKDFYICVNGSSENKPIRSSYFTFQLQNIVKPLPPVYLTFTRESSCEIKLKWSIPLGPIPARCFDYEIEIREDDTTLVTATVENETYTLKTTNETRQLCFVVRSKVNIYCSDDGIWSEWSDKQCWEGEDLSKKTLLRFWLPFGFILILVIFVTGLLLRKPNTYPKMIPEFFCDT

>NP_000580.1

MGLTSQLLPPLFFLLACAGNFVHGHKCDITLQEIIKTLNSLTEQKTLCTELTVTDIFAASKNTTEKETFCRAATVLRQFYSHHEKDTRCLGATAQQFHRHKQLIRFLKRLDRNLWGLAGLNSCPVKEANQSTLENFLERLKTIMREKYSKCSS

78 NP_000073.1 NP_001914.3

>NP_000073.1

MLGFLSARQTGLEDPLRLRRAESTRRVLGLELNKDRDVERIHGGGINTLDIEPVEGRYMLSGGSDGVIVLYDLENSSRQSYYTCKAVCSIGRDHPDVHRYSVETVQWYPHDTGMFTSSSFDKTLKVWDTNTLQTADVFNFEETVYSHHMSPVSTKHCLVAVGTRGPKVQLCDLKSGSCSHILQGHRQEILAVSWSPRYDYILATASADSRVKLWDVRRASGCLITLDQHNGKKSQAVESANTAHNGKVNGLCFTSDGLHLLTVGTDNRMRLWNSSNGENTLVNYGKVCNNSKKGLKFTVSCGCSSEFVFVPYGSTIAVYTVYSGEQITMLKGHYKTVDCCVFQSNFQELYSGSRDCNILAWVPSLYEPVPDDDETTTKSQLNPAFEDAWSSSDEEG

>NP_001914.3

MSYNYVVTAQKPTAVNGCVTGHFTSAEDLNLLIAKNTRLEIYVVTAEGLRPVKEVGMYGKIAVMELFRPKGESKDLLFILTAKYNACILEYKQSGESIDIITRAHGNVQDRIGRPSETGIIGIIDPECRMIGLRLYDGLFKVIPLDRDNKELKAFNIRLEELHVIDVKFLYGCQAPTICFVYQDPQGRHVKTYEVSLREKEFNKGPWKQENVEAEASMVIAVPEPFGGAIIIGQESITYHNGDKYLAIAPPIIKQSTIVCHNRVDPNGSRYLLGDMEGRLFMLLLEKEEQMDGTVTLKDLRVELLGETSIAECLTYLDNGVVFVGSRLGDSQLVKLNVDSNEQGSYVVAMETFTNLGPIVDMCVVDLERQGQGQLVTCSGAFKEGSLRIIRNGIGIHEHASIDLPGIKGLWPLRSDPNRETDDTLVLSFVGQTRVLMLNGEEVEETELMGFVDDQQTFFCGNVAHQQLIQITSASVRLVSQEPKALVSEWKEPQAKNISVASCNSSQVVVAVGRALYYLQIHPQELRQISHTEMEHEVACLDITPLGDSNGLSPLCAIGLWTDISARILKLPSFELLHKEMLGGEIIPRSILMTTFESSHYLLCALGDGALFYFGLNIETGLLSDRKKVTLGTQPTVLRTFRSLSTTNVFACSDRPTVIYSSNHKLVFSNVNLKEVNYMCPLNSDGYPDSLALANNSTLTIGTIDEIQKLHIRTVPLYESPRKICYQEVSQCFGVLSSRIEVQDTSGGTTALRPSASTQALSSSVSSSKLFSSSTAPHETSFGEEVEVHNLLIIDQHTFEVLHAHQFLQNEYALSLVSCKLGKDPNTYFIVGTAMVYPEEAEPKQGRIVVFQYSDGKLQTVAEKEVKGAVYSMVEFNGKLLASINSTVRLYEWTTEKELRTECNHYNNIMALYLKTKGDFILVGDLMRSVLLLAYKPMEGNFEEIARDFNPNWMSAVEILDDDNFLGAENAFNLFVCQKDSAATTDEERQHLQEVGLFHLGEFVNVFCHGSLVMQNLGETSTPTQGSVLFGTVNGMIGLVTSLSESWYNLLLDMQNRLNKVIKSVGKIEHSFWRSFHTERKTEPATGFIDGDLIESFLDISRPKMQEVVANLQYDDGSGMKREATADDLIKVVEELTRIH

79 NP_002148.1 NP_002147.2

>NP_002148.1

MAGQAFRKFLPLFDRVLVERSAAETVTKGGIMLPEKSQGKVLQATVVAVGSGSKGKGGEIQPVSVKVGDKVLLPEYGGTKVVLDDKDYFLFRDGDILGKYVD

>NP_002147.2

MLRLPTVFRQMRPVSRVLAPHLTRAYAKDVKFGADARALMLQGVDLLADAVAVTMGPKGRTVIIEQSWGSPKVTKDGVTVAKSIDLKDKYKNIGAKLVQDVANNTNEEAGDGTTTATVLARSIAKEGFEKISKGANPVEIRRGVMLAVDAVIAELKKQSKPVTTPEEIAQVATISANGDKEIGNIISDAMKKVGRKGVITVKDGKTLNDELEIIEGMKFDRGYISPYFINTSKGQKCEFQDAYVLLSEKKISSIQSIVPALEIANAHRKPLVIIAEDVDGEALSTLVLNRLKVGLQVVAVKAPGFGDNRKNQLKDMAIATGGAVFGEEGLTLNLEDVQPHDLGKVGEVIVTKDDAMLLKGKGDKAQIEKRIQEIIEQLDVTTSEYEKEKLNERLAKLSDGVAVLKVGGTSDVEVNEKKDRVTDALNATRAAVEEGIVLGGGCALLRCIPALDSLTPANEDQKIGIEIIKRTLKIPAMTIAKNAGVEGSLIVEKIMQSSSEVGYDAMAGDFVNMVEKGIIDPTKVVRTALLDAAGVASLLTTAEVVVTEIPKEEKDPGMGAMGGMGGGMGGGMF

80 NP_004856.1 NP_004483.1

>NP_004856.1

MDADSDVALDILITNVVCVFRTRCHLNLRKIALEGANVIYKRDVGKVLMKLRKPRITATIWSSGKIICTGATSEEEAKFGARRLARSLQKLGFQVIFTDFKVVNVLAVCNMPFEIRLPEFTKNNRPHASYEPELHPAVCYRIKSLRATLQIFSTGSITVTGPNVKAVATAVEQIYPFVFESRKEIL

>NP_004483.1

MAYQLYRNTTLGNSLQESLDELIQSQQITPQLALQVLLQFDKAINAALAQRVRNRVNFRGSLNTYRFCDNVWTFVLNDVEFREVTELIKVDKVKIVACDGKNTGSNTTE

81 AAA59925.1 NP_000475.1

>AAA59925.1

MAAHRPVEWVQAVVSRFDEQLPIKTGQQNTHTKVSTEHNKECLINISKYKFSLVISGLTTILKNVNNMRIFGEAAEKNLYLSQLIILDTLEKCLAGQPKDTMRLDETMLVKQLLPEICHFLHTCREGNQHAAELRNSASGVLFSLSCNNFNAVFSRISTRLQELTVCSEDNVDVHDIELLQYINVDCAKLKRLLKETAFKFKALKKVAQLAVINSLEKAFWNWVENYPDEFTKLYQIPQTDMAECAEKLFDLVDGFAESTKRKAAVWPLQIILLILCPEIIQDISKDVVDENNMNKKLFLDSLRKALAGHGGSRQLTESAAIACVKLCKASTYINWEDNSVIFLLVQSMVVDLKNLLFNPSKPFSRGSQPADVDLMIDCLVSCFRISPHNNQHFKICLAQNSPSTFHYVLVNSLHRIITNSALDWWPKIDAVYCHSVELRNMFGETLHKAVQGCGAHPAIRMAPSLTFKEKVTSLKFKEKPTDLETRSYKYLLLSMVKLIHADPKLLLCNPRKQGPETQGSTAELITGLVQLVPQSHMPEIAQEAMEALLVLHQLDSIDLWNPDAPVETFWEISSQMLFYICKKLTSHQMLSSTEILKWLREILICRNKFLLKNKQADRSSCHFLLFYGVGCDIPSSGNTSQMSMDHEELLRTPGASLRKGKGNSSMDSAAGCSGTPPICRQAQTKLEVALYMFLWNPDTEAVLVAMSCFRHLCEEADIRCGVDEVSVHNLLPNYNTFMEFASVSNMMSTGRAALQKRVMALLRRIEHPTAGNTEAWEDTHAKWEQATKLILNYPKAKMEDGQAAESLHKTIVKRRMSHVSGGGSIDLSDTDSLQEWINMTGFLCALGGVCLQQRSNSGLATYSPPMGPVSERKGSMISVMSSEGNADTPVSKFMDRLLSLMVCNHEKVGLQIRTNVKDLVGLELSPALYPMLFNKLKNTISKFFDSQGQVLLTDTNTQFVEQTIAIMKNLLDNHTEGSSEHLGQASIETMMLNLVRYVRVLGNMVHAIQIKTKLCQLVEVMMARRDDLSFCQEMKFRNKMVEYLTDWVMGTSNQAADDDVKCLTRDLDQASMEAVVSLLAGLPLQPEEGDGVELMEAKSQLFLKYFTLFMNLLNDCSEVEDESAQTGGRKRGMSRRLASLRHCTVLAMSNLLNANVDSGLMHSIGLGYHKDLQTRATFMEVLTKILQQGTEFDTLAETVLADRFERLVELVTMMGDQGELPIAMALANVVPCSQWDELARVLVTLFDSRHLLYQLLWNMFSKEVELADSMQTLFRGNSLASKIMTFCFKVYGATYLQKLLDPLLRIVITSSDWQHVSFEVDPTRLEPSESLEENQRNLLQMTEKFFHAIISSSSEFPPQLRSVCHCLYQATCHSLLNKATVKEKKENKKSVVSQRFPQNSIGAVGSAMFLRFINPAIVSPYEAGILDKKPPPRIERGLKLMSKILQSIANHVLFTKEEHMRPFNDFVKSNFDAARRFFLDIASDCPTSDAVNHSLSFISDGNVLALHRLLWNNQEKIGQYLSSNRDHKAVGRRPFDKMATLLAYLGPPEHKPVADTHWSSLNLTSSKFEEFMTRHQVHEKEEFKALKTLSIFYQAGTSKAGNPIFYYVARRFKTGQINGDLLIYHVLLTLKPYYAKPYEIVVDLTHTGPSNRFKTDFLSKWFVVFPGFAYDNVSAVYIYNCNSWVREYTKYHERLLTGLKGSKRLVFIDCPGKLAEHIEHEQQKLPAATLALEEDLKVFHNALKLAHKDTKVSIKVGSTAVQVTSAERTKVLGQSVFLNDIYYASEIEEICLVDENQFTLTIANQGTPLTFMHQECEAIVQSIIHIRTRWELSQPDSIPQHTKIRPKDVPGTLLNIALLNLGSSDPSLRSAAYNLLCALTCTFNLKIEGQLLETSGLCIPANNTLFIVSISKTLAANEPHLTLEFLEECISGFSKSSIELKHLCLEYMTPWLSNLVRFCKHNDDAKRQRVTAILDKLITMTINEKQMYPSIQAKIWGSLGQITDLLDVVLDSFIKTSATGGLGSIKAEVMADTAVALASGNVKLVSSKVIGRMCKIIDKTCLSPTPTLEQHLMWDDIAILARYMLMLSFNNSLDVAAHLPYLFHVVTFLVATGPLSLRASTHGLVINIIHSLCTCSQLHFSEETKQVLRLSLTEFSLPKFYLLFGISKVKSAAVIAFRSSYRDRSFSPGSYERETFALTSLETVTEALLEIMEACMRDIPTCKWLDQWTELAQRFAFQYNPSLQPRALVVFGCISKRVSHGQIKQIIRILSKALESCLKGPDTYNSQVLIEATVIALTKLQPLLNKDSPLHKALFWVAVAVLQLDEVNLYSAGTALLEQNLHTLDSLRIFNDKSPEEVFMAIRNPLEWHCKQMDHFVGLNFNSNFNFALVGHLLKGYRHPSPAIVARTVRILHTLLTLVNKHRNCDKFEVNTQSVAYLAALLTVSEEVRSRCSLKHRKSLLLTDISMENVPMDTYPIHHGDPSYRTLKETQPWSSPKGSEGYLAATYPTVGQTSPRARKSMSLDMGQPSQANTKKLLGTRKSFDHLISDTKAPKRQEMESGITTPPKMRRVAETDYEMETQRISSSQQHPHLRKVSVSESNVLLDEEVLTDPKIQALLLTVLATLVKYTTDEFDQRILYEYLAEASVVFPKVFPVVHNLLDSKINTLLSLCQDPNLLNPIHGIVQSVVYHEESPPQYQTSYLQSFGFNGLWRFAGPFSKQTQIPDYAELIVKFLDALIDTYLPGIDEETSEESLLTPTSPYPPALQSQLSITANLNLSNSMTSLATSQHSPGIDKENVELSPTTGHCNSGRTRHGSASQVQKQRSAGSFKRNSIKKIV

>NP_000475.1

MLPGLALLLLAAWTARALEVPTDGNAGLLAEPQIAMFCGRLNMHMNVQNGKWDSDPSGTKTCIDTKEGILQYCQEVYPELQITNVVEANQPVTIQNWCKRGRKQCKTHPHFVIPYRCLVGEFVSDALLVPDKCKFLHQERMDVCETHLHWHTVAKETCSEKSTNLHDYGMLLPCGIDKFRGVEFVCCPLAEESDNVDSADAEEDDSDVWWGGADTDYADGSEDKVVEVAEEEEVAEVEEEEADDDEDDEDGDEVEEEAEEPYEEATERTTSIATTTTTTTESVEEVVREVCSEQAETGPCRAMISRWYFDVTEGKCAPFFYGGCGGNRNNFDTEEYCMAVCGSAMSQSLLKTTQEPLARDPVKLPTTAASTPDAVDKYLETPGDENEHAHFQKAKERLEAKHRERMSQVMREWEEAERQAKNLPKADKKAVIQHFQEKVESLEQEAANERQQLVETHMARVEAMLNDRRRLALENYITALQAVPPRPRHVFNMLKKYVRAEQKDRQHTLKHFEHVRMVDPKKAAQIRSQVMTHLRVIYERMNQSLSLLYNVPAVAEEIQDEVDELLQKEQNYSDDVLANMISEPRISYGNDALMPSLTETKTTVELLPVNGEFSLDDLQPWHSFGADSVPANTENEVEPVDARPAADRGLTTRPGSGLTNIKTEEISEVKMDAEFRHDSGYEVHHQKLVFFAEDVGSNKGAIIGLMVGGVVIATVIVITLVMLKKKQYTSIHHGVVEVDAAVTPEERHLSKMQQNGYENPTYKFFEQMQN

82 NP_005220.1 NP_000336.1

>NP_005220.1

MDPSVTLWQFLLQLLREQGNGHIISWTSRDGGEFKLVDAEEVARLWGLRKNKTNMNYDKLSRALRYYYDKNIIRKVSGQKFVYKFVSYPEVAGCSTEDCPPQPEVSVTSTMPNVAPAAIHAAPGDTVSGKPGTPKGAGMAGPGGLARSSRNEYMRSGLYSTFTIQSLQPQPPPHPRPAVVLPNAAPAGAAAPPSGSRSTSPSPLEACLEAEEAGLPLQVILTPPEAPNLKSEELNVEPGLGRALPPEVKVEGPKEELEVAGERGFVPETTKAEPEVPPQEGVPARLPAVVMDTAGQAGGHAASSPEISQPQKGRKPRDLELPLSPSLLGGPGPERTPGSGSGSGLQAPGPALTPSLLPTHTLTPVLLTPSSLPPSIHFWSTLSPIAPRSPAKLSFQFPSSGSAQVHIPSISVDGLSTPVVLSPGPQKP

>NP_000336.1

MDVFMDVMKGLSKAKEGVVAAAEKTKQGVAEAAGKTKEGVLYVGSKTKEGVVHGVATVAEKTKEQVTNVGGAVVTGVTAVAQKTVEGAGSIAAATGFVKKDQLGKNEEGAPQEGILEDMPVDPDNEAYEMPSEEGYQDYEPEA

83 NP_056465.2 NP_061132.2

>NP_056465.2

MNAAVVRRTQEALGKVIRRPPLTEKLLSKPPFRYLHDIITEVIRMTGFMKGLYTDAEMKSDNVKDKDAKISFLQKAIDVVVMVSGEPLLAKPARIVAGHEPERTNELLQIIGKCCLNKLSSDDAVRRVLAGEKGEVKGRASLTSRSQELDNKNVREEESRVHKNTEDRGDAEIKERSTSRDRKQKEELKEDRKPREKDKDKEKAKENGGNRHREGERERAKARARPDNERQKDRGNRERDRDSERKKETERKSEGGKEKERLRDRDRERDRDKGKDRDRRRVKNGEHSWDLDREKNREHDKPEKKSASSGEMSKKLSDGTFKDSKAETETEISTRASKSLTTKTSKRRSKNSVEGRKEDNISAKSLDSIVSGINNEPNQETTTSEIGTKEANINSTSISDDNSASLRCENIQPNPTEKQKGDSTSDAEGDAGPAGQDKSEVPETPEIPNELSSNIRRIPRPGSARPAPPRVKRQDSMEALQMDRSGSGKTVSNVITESHNSDNEEDDQFVVEAAPQLSEMSEIEMVTAVELEEEEKHGGLVKKILETKKDYEKLQQSPKPGEKERSLFESAWKKEKDIVSKEIEKLRTSIQTLCKSALPLGKIMDYIQEDVDAMQNELQMWHSENRQHAEALQQEQRITDCAVEPLKAELAELEQLIKDQQDKICAVKANILKNEEKIQKMVYSINLTSRR

>NP_061132.2

MPGGGPQGAPAAAGGGGVSHRAGSRDCLPPAACFRRRRLARRPGYMRSSTGPGIGFLSPAVGTLFRFPGGVSGEESHHSESRARQCGLDSRGLLVRSPVSKSAAAPTVTSVRGTSAHFGIQLRGGTRLPDRLSWPCGPGSAGWQQEFAAMDSSETLDASWEAACSDGARRVRAAGSLPSAELSSNSCSPGCGPEVPPTPPGSHSAFTSSFSFIRLSLGSAGERGEAEGCPPSREAESHCQSPQEMGAKAASLDGPHEDPRCLSRPFSLLATRVSADLAQAARNSSRPERDMHSLPDMDPGSSSSLDPSLAGCGGDGSSGSGDAHSWDTLLRKWEPVLRDCLLRNRRQMEVISLRLKLQKLQEDAVENDDYDKAETLQQRLEDLEQEKISLHFQLPSRQPALSSFLGHLAAQVQAALRRGATQQASGDDTHTPLRMEPRLLEPTAQDSLHVSITRRDWLLQEKQQLQKEIEALQARMFVLEAKDQQLRREIEEQEQQLQWQGCDLTPLVGQLSLGQLQEVSKALQDTLASAGQIPFHAEPPETIRSLQERIKSLNLSLKEITTKVCMSEKFCSTLRKKVNDIETQLPALLEAKMHAISGNHFWTAKDLTEEIRSLTSEREGLEGLLSKLLVLSSRNVKKLGSVKEDYNRLRREVEHQETAYETSVKENTMKYMETLKNKLCSCKCPLLGKVWEADLEACRLLIQSLQLQEARGSLSVEDERQMDDLEGAAPPIPPRLHSEDKRKTPLKVLEEWKTHLIPSLHCAGGEQKEESYILSAELGEKCEDIGKKLLYLEDQLHTAIHSHDEDLIQSLRRELQMVKETLQAMILQLQPAKEAGEREAAASCMTAGVHEAQA

84 NP_001612.1 NP_006531.1

>NP_001612.1

MNSSSANITYASRKRRKPVQKTVKPIPAEGIKSNPSKRHRDRLNTELDRLASLLPFPQDVINKLDKLSVLRLSVSYLRAKSFFDVALKSSPTERNGGQDNCRAANFREGLNLQEGEFLLQALNGFVLVVTTDALVFYASSTIQDYLGFQQSDVIHQSVYELIHTEDRAEFQRQLHWALNPSQCTESGQGIEEATGLPQTVVCYNPDQIPPENSPLMERCFICRLRCLLDNSSGFLAMNFQGKLKYLHGQKKKGKDGSILPPQLALFAIATPLQPPSILEIRTKNFIFRTKHKLDFTPIGCDAKGRIVLGYTEAELCTRGSGYQFIHAADMLYCAESHIRMIKTGESGMIVFRLLTKNNRWTWVQSNARLLYKNGRPDYIIVTQRPLTDEEGTEHLRKRNTKLPFMFTTGEAVLYEATNPFPAIMDPLPLRTKNGTSGKDSATTSTLSKDSLNPSSLLAAMMQQDESIYLYPASSTSSTAPFENNFFNESMNECRNWQDNTAPMGNDTILKHEQIDQPQDVNSFAGGHPGLFQDSKNSDLYSIMKNLGIDFEDIRHMQNEKFFRNDFSGEVDFRDIDLTDEILTYVQDSLSKSPFIPSDYQQQQSLALNSSCMVQEHLHLEQQQQHHQKQVVVEPQQQLCQKMKHMQVNGMFENWNSNQFVPFNCPQQDPQQYNVFTDLHGISQEFPYKSEMDSMPYTQNFISCNQPVLPQHSKCTELDYPMGSFEPSPYPTTSSLEDFVTCLQLPENQKHGLNPQSAIITPQTCYAGAVSMYQCQPEPQHTHVGQMQYNPVLPGQQAFLNKFQNGVLNETYPAELNNINNTQTTTHLQPLHHPSEARPFPDLTSSGFL

>NP_006531.1

MSGMGENTSDPSRAETRKRKECPDQLGPSPKRNTEKRNREQENKYIEELAELIFANFNDIDNFNFKPDKCAILKETVKQIRQIKEQEKAAAANIDEVQKSDVSSTGQGVIDKDALGPMMLEALDGFFFVVNLEGNVVFVSENVTQYLRYNQEELMNKSVYSILHVGDHTEFVKNLLPKSIVNGGSWSGEPPRRNSHTFNCRMLVKPLPDSEEEGHDNQEAHQKYETMQCFAVSQPKSIKEEGEDLQSCLICVARRVPMKERPVLPSSESFTTRQDLQGKITSLDTSTMRAAMKPGWEDLVRRCIQKFHAQHEGESVSYAKRHHHEVLRQGLAFSQIYRFSLSDGTLVAAQTKSKLIRSQTTNEPQLVISLHMLHREQNVCVMNPDLTGQTMGKPLNPISSNSPAHQALCSGNPGQDMTLSSNINFPINGPKEQMGMPMGRFGGSGGMNHVSGMQATTPQGSNYALKMNSPSQSSPGMNPGQPTSMLSPRHRMSPGVAGSPRIPPSQFSPAGSLHSPVGVCSSTGNSHSYTNSSLNALQALSEGHGVSLGSSLASPDLKMGNLQNSPVNMNPPPLSKMGSLDSKDCFGLYGEPSEGTTGQAESSCHPGEQKETNDPNLPPAVSSERADGQSRLHDSKGQTKLLQLLTTKSDQMEPSPLASSLSDTNKDSTGSLPGSGSTHGTSLKEKHKILHRLLQDSSSPVDLAKLTAEATGKDLSQESSSTAPGSEVTIKQEPVSPKKKENALLRYLLDKDDTKDIGLPEITPKLERLDSKTDPASNTKLIAMKTEKEEMSFEPGDQPGSELDNLEEILDDLQNSQLPQLFPDTRPGAPAGSVDKQAIINDLMQLTAENSPVTPVGAQKTALRISQSTFNNPRPGQLGRLLPNQNLPLDITLQSPTGAGPFPPIRNSSPYSVIPQPGMMGNQGMIGNQGNLGNSSTGMIGNSASRPTMPSGEWAPQSSAVRVTCAATTSAMNRPVQGGMIRNPAASIPMRPSSQPGQRQTLQSQVMNIGPSELEMNMGGPQYSQQQAPPNQTAPWPESILPIDQASFASQNRQPFGSSPDDLLCPHPAAESPSDEGALLDQLYLALRNFDGLEEIDRALGIPELVSQSQAVDPEQFSSQDSNIMLEQKAPVFPQQYASQAQMAQGSYSPMQDPNFHTMGQRPSYATLRMQPRPGLRPTGLVQNQPNQLRLQLQHRLQAQQNRQPLMNQISNVSNVNLTLRPGVPTQAPINAQMLAQRQREILNQHLRQRQMHQQQQVQQRTLMMRGQGLNMTPSMVAPSGMPATMSNPRIPQANAQQFPFPPNYGISQQPDPGFTGATTPQSPLMSPRMAHTQSPMMQQSQANPAYQAPSDINGWAQGNMGGNSMFSQQSPPHFGQQANTSMYSNNMNINVSMATNTGGMSSMNQMTGQISMTSVTSVPTSGLSSMGPEQVNDPALRGGNLFPNQLPGMDMIKQEGDTTRKYC

85 NP_005094.1 NP_114064.1

>NP_005094.1

MEAPLVSLDEEFEDLRPSCSEDPEEKPQCFYGSSPHHLEDPSLSELENFSSEIISFKSMEDLVNEFDEKLNVCFRNYNAKTENLAPVKNQLQIQEEEETLQDEEVWDALTDNYIPSLSEDWRDPNIEALNGNCSDTEIHEKEEEEFNEKSENDSGINEEPLLTADQVIEEIEEMMQNSPDPEEEEEVLEEEDGGETSSQADSVLLQEMQALTQTFNNNWSYEGLRHMSGSELTELLDQVEGAIRDFSEELVQQLARRDELEFEKEVKNSFITVLIEVQNKQKEQRELMKKRRKEKGLSLQSSRIEKGNQMPLKRFSMEGISNILQSGIRQTFGSSGTDKQYLNTVIPYEKKASPPSVEDLQMLTNILFAMKEDNEKVPTLLTDYILKVLCPT

>NP_114064.1

MEPQVTLNVTFKNEIQSFLVSDPENTTWADIEAMVKVSFDLNTIQIKYLDEENEEVSINSQGEYEEALKMAVKQGNQLQMQVHEGHHVVDEAPPPVVGAKRLAARAGKKPLAHYSSLVRVLGSDMKTPEDPAVQSFPLVPCDTDQPQDKPPDWFTSYLETFREQVVNETVEKLEQKLHEKLVLQNPSLGSCPSEVSMPTSEETLFLPENQFSWHIACNNCQRRIVGVRYQCSLCPSYNICEDCEAGPYGHDTNHVLLKLRRPVVGSSEPFCHSKYSTPRLPAALEQVRLQKQVDKNFLKAEKQRLRAEKKQRKAEVKELKKQLKLHRKIHLWNSIHGLQSPKSPLGRPESLLQSNTLMLPLQPCTSVMPMLSAAFVDENLPDGTHLQPGTKFIKHWRMKNTGNVKWSADTKLKFMWGNLTLASTEKKDVLVPCLKAGHVGVVSVEFIAPALEGTYTSHWRLSHKGQQFGPRVWCSIIVDPFPSEESPDNIEKGMISSSKTDDLTCQQEETFLLAKEERQLGEVTEQTEGTAACIPQKAKNVASERELYIPSVDLLTAQDLLSFELLDINIVQELERVPHNTPVDVTPCMSPLPHDSPLIEKPGLGQIEEENEGAGFKALPDSMVSVKRKAENIASVEEAEEDLSGTQFVCETVIRSLTLDAAPDHNPPCRQKSLQMTFALPEGPLGNEKEEIIHIAEEEAVMEEEEDEEDEEEEDELKDEVQSQSSASSEDYIIILPECFDTSRPLGDSMYSSALSQPGLERGAEGKPGVEAGQEPAEAGERLPGGENQPQEHSISDILTTSQTLETVPLIPEVVELPPSLPRSSPCVHHHGSPGVDLPVTIPEVSSVPDQIRGEPRGSSGLVNSRQKSYDHSRHHHGSSIAGGLVKGALSVAASAYKALFAGPPVTAQPIISEDQTAALMAHLFEMGFCDRQLNLRLLKKHNYNILQVVTELLQLNNNDWYSQRY

86 NP_006863.2 NP_006704.2

>NP_006863.2

MACLNPVPKLYRSVIEDVIEGVRNLFAEEGIEEQVLKDLKQLWETKVLQSKATEDFFRNSIQSPLFTLQLPHSLHQTLQSSTASLVIPAGRTLPSFTTAELGTSNSSANFTFPGYPIHVPAGVTLQTVSGHLYKVNVPIMVTETSGRAGILQHPIQQVFQQLGQPSVIQTSVPQLNPWSLQATTEKSQRIETVLQQPAILPSGPVDRKHLENATSDILVSPGNEHKIVPEALLCHQESSHYISLPGVVFSPQVSQTNSNVESVLSGSASMAQNLHDESLSTSPHGALHQHVTDIQLHILKNRMYGCDSVKQPRNIEEPSNIPVSEKDSNSQVDLSIRVTDDDIGEIIQVDGSGDTSSNEEIGSTRDADENEFLGNIDGGDLKVPEEEADSISNEDSATNSSDNEDPQVNIVEEDPLNSGDDVSEQDVPDLFDTDNVIVCQYDKIHRSKNKWKFYLKDGVMCFGGRDYVFAKAIGDAEW

>NP_006704.2

MPKSKELVSSGSSGSDSDSEVDKKLKRKKQVAPEKPVKKQKTGETSRALSSSKQSSSSRDDNMFQIGKMRYVSVRDFKGKVLIDIREYWMDPEGEMKPGRKGISLNPEQWSQLKEQISDIDDAVRKL

87 NP_000578.2 NP_001726.2

>NP_000578.2

MKVISLFILVGFIGEFQSFSSASSPVNCQWDFYAPWSECNGCTKTQTRRRSVAVYGQYGGQPCVGNAFETQSCEPTRGCPTEEGCGERFRCFSGQCISKSLVCNGDSDCDEDSADEDRCEDSERRPSCDIDKPPPNIELTGNGYNELTGQFRNRVINTKSFGGQCRKVFSGDGKDFYRLSGNVLSYTFQVKINNDFNYEFYNSTWSYVKHTSTEHTSSSRKRSFFRSSSSSSRSYTSHTNEIHKGKSYQLLVVENTVEVAQFINNNPEFLQLAEPFWKELSHLPSLYDYSAYRRLIDQYGTHYLQSGSLGGEYRVLFYVDSEKLKQNDFNSVEEKKCKSSGWHFVVKFSSHGCKELENALKAASGTQNNVLRGEPFIRGGGAGFISGLSYLELDNPAGNKRRYSAWAESVTNLPQVIKQKLTPLYELVKEVPCASVKKLYLKWALEEYLDEFDPCHCRPCQNGGLATVEGTHCLCHCKPYTFGAACEQGVLVGNQAGGVDGGWSCWSSWSPCVQGKKTRSRECNNPPPSGGGRSCVGETTESTQCEDEELEHLRLLEPHCFPLSLVPTEFCPSPPALKDGFVQDEGTMFPVGKNVVYTCNEGYSLIGNPVARCGEDLRWLVGEMHCQKIACVLPVLMDGIQSHPQKPFYTVGEKVTVSCSGGMSLEGPSAFLCGSSLKWSPEMKNARCVQKENPLTQAVPKCQRWEKLQNSRCVCKMPYECGPSLDVCAQDERSKRILPLTVCKMHVLHCQGRNYTLTGRDSCTLPASAEKACGACPLWGKCDAESSKCVCREASECEEEGFSICVEVNGKEQTMSECEAGALRCRGQSISVTSIRPCAAETQ

>NP_001726.2

MGLLGILCFLIFLGKTWGQEQTYVISAPKIFRVGASENIVIQVYGYTEAFDATISIKSYPDKKFSYSSGHVHLSSENKFQNSAILTIQPKQLPGGQNPVSYVYLEVVSKHFSKSKRMPITYDNGFLFIHTDKPVYTPDQSVKVRVYSLNDDLKPAKRETVLTFIDPEGSEVDMVEEIDHIGIISFPDFKIPSNPRYGMWTIKAKYKEDFSTTGTAYFEVKEYVLPHFSVSIEPEYNFIGYKNFKNFEITIKARYFYNKVVTEADVYITFGIREDLKDDQKEMMQTAMQNTMLINGIAQVTFDSETAVKELSYYSLEDLNNKYLYIAVTVIESTGGFSEEAEIPGIKYVLSPYKLNLVATPLFLKPGIPYPIKVQVKDSLDQLVGGVPVTLNAQTIDVNQETSDLDPSKSVTRVDDGVASFVLNLPSGVTVLEFNVKTDAPDLPEENQAREGYRAIAYSSLSQSYLYIDWTDNHKALLVGEHLNIIVTPKSPYIDKITHYNYLILSKGKIIHFGTREKFSDASYQSINIPVTQNMVPSSRLLVYYIVTGEQTAELVSDSVWLNIEEKCGNQLQVHLSPDADAYSPGQTVSLNMATGMDSWVALAAVDSAVYGVQRGAKKPLERVFQFLEKSDLGCGAGGGLNNANVFHLAGLTFLTNANADDSQENDEPCKEILRPRRTLQKKIEEIAAKYKHSVVKKCCYDGACVNNDETCEQRAARISLGPRCIKAFTECCVVASQLRANISHKDMQLGRLHMKTLLPVSKPEIRSYFPESWLWEVHLVPRRKQLQFALPDSLTTWEIQGVGISNTGICVADTVKAKVFKDVFLEMNIPYSVVRGEQIQLKGTVYNYRTSGMQFCVKMSAVEGICTSESPVIDHQGTKSSKCVRQKVEGSSSHLVTFTVLPLEIGLHNINFSLETWFGKEILVKTLRVVPEGVKRESYSGVTLDPRGIYGTISRRKEFPYRIPLDLVPKTEIKRILSVKGLLVGEILSAVLSQEGINILTHLPKGSAEAELMSVVPVFYVFHYLETGNHWNIFHSDPLIEKQKLKKKLKEGMLSIMSYRNADYSYSVWKGGSASTWLTAFALRVLGQVNKYVEQNQNSICNSLLWLVENYQLDNGSFKENSQYQPIKLQGTLPVEARENSLYLTAFTVIGIRKAFDICPLVKIDTALIKADNFLLENTLPAQSTFTLAISAYALSLGDKTHPQFRSIVSALKREALVKGNPPIYRFWKDNLQHKDSSVPNTGTARMVETTAYALLTSLNLKDINYVNPVIKWLSEEQRYGGGFYSTQDTINAIEGLTEYSLLVKQLRLSMDIDVSYKHKGALHNYKMTDKNFLGRPVEVLLNDDLIVSTGFGSGLATVHVTTVVHKTSTSEEVCSFYLKIDTQDIEASHYRGYGNSDYKRIVACASYKPSREESSSGSSHAVMDISLPTGISANEEDLKALVEGVDQLFTDYQIKDGHVILQLNSIPSSDFLCVRFRIFELFEVGFLSPATFTVYEYHRPDKQCTMFYSTSNIKIQKVCEGAACKCVEADCGQMQEELDLTISAETRKQTACKPEIAYAYKVSITSITVENVFVKYKATLLDIYKTGEAVAEKDSEITFIKKVTCTNAELVKGRQYLIMGKEALQIKYNFSFRYIYPLDSLTWIEYWPRDTTCSSCQAFLANLDEFAEDIFLNGC

88 NP_000116.2 NP_009204.1

>NP_000116.2

MTMTLHTKASGMALLHQIQGNELEPLNRPQLKIPLERPLGEVYLDSSKPAVYNYPEGAAYEFNAAAAANAQVYGQTGLPYGPGSEAAAFGSNGLGGFPPLNSVSPSPLMLLHPPPQLSPFLQPHGQQVPYYLENEPSGYTVREAGPPAFYRPNSDNRRQGGRERLASTNDKGSMAMESAKETRYCAVCNDYASGYHYGVWSCEGCKAFFKRSIQGHNDYMCPATNQCTIDKNRRKSCQACRLRKCYEVGMMKGGIRKDRRGGRMLKHKRQRDDGEGRGEVGSAGDMRAANLWPSPLMIKRSKKNSLALSLTADQMVSALLDAEPPILYSEYDPTRPFSEASMMGLLTNLADRELVHMINWAKRVPGFVDLTLHDQVHLLECAWLEILMIGLVWRSMEHPGKLLFAPNLLLDRNQGKCVEGMVEIFDMLLATSSRFRMMNLQGEEFVCLKSIILLNSGVYTFLSSTLKSLEEKDHIHRVLDKITDTLIHLMAKAGLTLQQQHQRLAQLLLILSHIRHMSNKGMEHLYSMKCKNVVPLYDLLLEMLDAHRLHAPTSRGGASVEETDQSHLATAGSTSSHSLQKYYITGEAEGFPATV

>NP_009204.1

MAQNLKDLAGRLPAGPRGMGTALKLLLGAGAVAYGVRESVFTVEGGHRAIFFNRIGGVQQDTILAEGLHFRIPWFQYPIIYDIRARPRKISSPTGSKDLQMVNISLRVLSRPNAQELPSMYQRLGLDYEERVLPSIVNEVLKSVVAKFNASQLITQRAQVSLLIRRELTERAKDFSLILDDVAITELSFSREYTAAVEAKQVAQQEAQRAQFLVEKAKQEQRQKIVQAEGEAEAAKMLGEALSKNPGYIKLRKIRAAQNISKTIATSQNRIYLTADNLVLNLQDESFTRGSDSLIKGKK

89 NP_000244.1 NP_000375.2

>NP_000244.1

MILLAVLFLCFISSYSASVKGHTTGLSLNNDRLYKLTYSTEVLLDRGKGKLQDSVGYRISSNVDVALLWRNPDGDDDQLIQITMKDVNVENVNQQRGEKSIFKGKSPSKIMGKENLEALQRPTLLHLIHGKVKEFYSYQNEAVAIENIKRGLASLFQTQLSSGTTNEVDISGNCKVTYQAHQDKVIKIKALDSCKIARSGFTTPNQVLGVSSKATSVTTYKIEDSFVIAVLAEETHNFGLNFLQTIKGKIVSKQKLELKTTEAGPRLMSGKQAAAIIKAVDSKYTAIPIVGQVFQSHCKGCPSLSELWRSTRKYLQPDNLSKAEAVRNFLAFIQHLRTAKKEEILQILKMENKEVLPQLVDAVTSAQTSDSLEAILDFLDFKSDSSIILQERFLYACGFASHPNEELLRALISKFKGSIGSSDIRETVMIITGTLVRKLCQNEGCKLKAVVEAKKLILGGLEKAEKKEDTRMYLLALKNALLPEGIPSLLKYAEAGEGPISHLATTALQRYDLPFITDEVKKTLNRIYHQNRKVHEKTVRTAAAAIILNNNPSYMDVKNILLSIGELPQEMNKYMLAIVQDILRLEMPASKIVRRVLKEMVAHNYDRFSRSGSSSAYTGYIERSPRSASTYSLDILYSGSGILRRSNLNIFQYIGKAGLHGSQVVIEAQGLEALIAATPDEGEENLDSYAGMSAILFDVQLRPVTFFNGYSDLMSKMLSASGDPISVVKGLILLIDHSQELQLQSGLKANIEVQGGLAIDISGAMEFSLWYRESKTRVKNRVTVVITTDITVDSSFVKAGLETSTETEAGLEFISTVQFSQYPFLVCMQMDKDEAPFRQFEKKYERLSTGRGYVSQKRKESVLAGCEFPLHQENSEMCKVVFAPQPDSTSSGWF

>NP_000375.2

MDPPRPALLALLALPALLLLLLAGARAEEEMLENVSLVCPKDATRFKHLRKYTYNYEAESSSGVPGTADSRSATRINCKVELEVPQLCSFILKTSQCTLKEVYGFNPEGKALLKKTKNSEEFAAAMSRYELKLAIPEGKQVFLYPEKDEPTYILNIKRGIISALLVPPETEEAKQVLFLDTVYGNCSTHFTVKTRKGNVATEISTERDLGQCDRFKPIRTGISPLALIKGMTRPLSTLISSSQSCQYTLDAKRKHVAEAICKEQHLFLPFSYKNKYGMVAQVTQTLKLEDTPKINSRFFGEGTKKMGLAFESTKSTSPPKQAEAVLKTLQELKKLTISEQNIQRANLFNKLVTELRGLSDEAVTSLLPQLIEVSSPITLQALVQCGQPQCSTHILQWLKRVHANPLLIDVVTYLVALIPEPSAQQLREIFNMARDQRSRATLYALSHAVNNYHKTNPTGTQELLDIANYLMEQIQDDCTGDEDYTYLILRVIGNMGQTMEQLTPELKSSILKCVQSTKPSLMIQKAAIQALRKMEPKDKDQEVLLQTFLDDASPGDKRLAAYLMLMRSPSQADINKIVQILPWEQNEQVKNFVASHIANILNSEELDIQDLKKLVKEALKESQLPTVMDFRKFSRNYQLYKSVSLPSLDPASAKIEGNLIFDPNNYLPKESMLKTTLTAFGFASADLIEIGLEGKGFEPTLEALFGKQGFFPDSVNKALYWVNGQVPDGVSKVLVDHFGYTKDDKHEQDMVNGIMLSVEKLIKDLKSKEVPEARAYLRILGEELGFASLHDLQLLGKLLLMGARTLQGIPQMIGEVIRKGSKNDFFLHYIFMENAFELPTGAGLQLQISSSGVIAPGAKAGVKLEVANMQAELVAKPSVSVEFVTNMGIIIPDFARSGVQMNTNFFHESGLEAHVALKAGKLKFIIPSPKRPVKLLSGGNTLHLVSTTKTEVIPPLIENRQSWSVCKQVFPGLNYCTSGAYSNASSTDSASYYPLTGDTRLELELRPTGEIEQYSVSATYELQREDRALVDTLKFVTQAEGAKQTEATMTFKYNRQSMTLSSEVQIPDFDVDLGTILRVNDESTEGKTSYRLTLDIQNKKITEVALMGHLSCDTKEERKIKGVISIPRLQAEARSEILAHWSPAKLLLQMDSSATAYGSTVSKRVAWHYDEEKIEFEWNTGTNVDTKKMTSNFPVDLSDYPKSLHMYANRLLDHRVPQTDMTFRHVGSKLIVAMSSWLQKASGSLPYTQTLQDHLNSLKEFNLQNMGLPDFHIPENLFLKSDGRVKYTLNKNSLKIEIPLPFGGKSSRDLKMLETVRTPALHFKSVGFHLPSREFQVPTFTIPKLYQLQVPLLGVLDLSTNVYSNLYNWSASYSGGNTSTDHFSLRARYHMKADSVVDLLSYNVQGSGETTYDHKNTFTLSCDGSLRHKFLDSNIKFSHVEKLGNNPVSKGLLIFDASSSWGPQMSASVHLDSKKKQHLFVKEVKIDGQFRVSSFYAKGTYGLSCQRDPNTGRLNGESNLRFNSSYLQGTNQITGRYEDGTLSLTSTSDLQSGIIKNTASLKYENYELTLKSDTNGKYKNFATSNKMDMTFSKQNALLRSEYQADYESLRFFSLLSGSLNSHGLELNADILGTDKINSGAHKATLRIGQDGISTSATTNLKCSLLVLENELNAELGLSGASMKLTTNGRFREHNAKFSLDGKAALTELSLGSAYQAMILGVDSKNIFNFKVSQEGLKLSNDMMGSYAEMKFDHTNSLNIAGLSLDFSSKLDNIYSSDKFYKQTVNLQLQPYSLVTTLNSDLKYNALDLTNNGKLRLEPLKLHVAGNLKGAYQNNEIKHIYAISSAALSASYKADTVAKVQGVEFSHRLNTDIAGLASAIDMSTNYNSDSLHFSNVFRSVMAPFTMTIDAHTNGNGKLALWGEHTGQLYSKFLLKAEPLAFTFSHDYKGSTSHHLVSRKSISAALEHKVSALLTPAEQTGTWKLKTQFNNNEYSQDLDAYNTKDKIGVELTGRTLADLTLLDSPIKVPLLLSEPINIIDALEMRDAVEKPQEFTIVAFVKYDKNQDVHSINLPFFETLQEYFERNRQTIIVVLENVQRNLKHINIDQFVRKYRAALGKLPQQANDYLNSFNWERQVSHAKEKLTALTKKYRITENDIQIALDDAKINFNEKLSQLQTYMIQFDQYIKDSYDLHDLKIAIANIIDEIIEKLKSLDEHYHIRVNLVKTIHDLHLFIENIDFNKSGSSTASWIQNVDTKYQIRIQIQEKLQQLKRHIQNIDIQHLAGKLKQHIEAIDVRVLLDQLGTTISFERINDVLEHVKHFVINLIGDFEVAEKINAFRAKVHELIERYEVDQQIQVLMDKLVELAHQYKLKETIQKLSNVLQQVKIKDYFEKLVGFIDDAVKKLNELSFKTFIEDVNKFLDMLIKKLKSFDYHQFVDETNDKIREVTQRLNGEIQALELPQKAEALKLFLEETKATVAVYLESLQDTKITLIINWLQEALSSASLAHMKAKFRETLEDTRDRMYQMDIQQELQRYLSLVGQVYSTLVTYISDWWTLAAKNLTDFAEQYSIQDWAKRMKALVEQGFTVPEIKTILGTMPAFEVSLQALQKATFQTPDFIVPLTDLRIPSVQINFKDLKNIKIPSRFSTPEFTILNTFHIPSFTIDFVEMKVKIIRTIDQMLNSELQWPVPDIYLRDLKVEDIPLARITLPDFRLPEIAIPEFIIPTLNLNDFQVPDLHIPEFQLPHISHTIEVPTFGKLYSILKIQSPLFTLDANADIGNGTTSANEAGIAASITAKGESKLEVLNFDFQANAQLSNPKINPLALKESVKFSSKYLRTEHGSEMLFFGNAIEGKSNTVASLHTEKNTLELSNGVIVKINNQLTLDSNTKYFHKLNIPKLDFSSQADLRNEIKTLLKAGHIAWTSSGKGSWKWACPRFSDEGTHESQISFTIEGPLTSFGLSNKINSKHLRVNQNLVYESGSLNFSKLEIQSQVDSQHVGHSVLTAKGMALFGEGKAEFTGRHDAHLNGKVIGTLKNSLFFSAQPFEITASTNNEGNLKVRFPLRLTGKIDFLNNYALFLSPSAQQASWQVSARFNQYKYNQNFSAGNNENIMEAHVGINGEANLDFLNIPLTIPEMRLPYTIITTPPLKDFSLWEKTGLKEFLKTTKQSFDLSVKAQYKKNKHRHSITNPLAVLCEFISQSIKSFDRHFEKNRNNALDFVTKSYNETKIKFDKYKAEKSHDELPRTFQIPGYTVPVVNVEVSPFTIEMSAFGYVFPKAVSMPSFSILGSDVRVPSYTLILPSLELPVLHVPRNLKLSLPDFKELCTISHIFIPAMGNITYDFSFKSSVITLNTNAELFNQSDIVAHLLSSSSSVIDALQYKLEGTTRLTRKRGLKLATALSLSNKFVEGSHNSTVSLTTKNMEVSVATTTKAQIPILRMNFKQELNGNTKSKPTVSSSMEFKYDFNSSMLYSTAKGAVDHKLSLESLTSYFSIESSTKGDVKGSVLSREYSGTIASEANTYLNSKSTRSSVKLQGTSKIDDIWNLEVKENFAGEATLQRIYSLWEHSTKNHLQLEGLFFTNGEHTSKATLELSPWQMSALVQVHASQPSSFHDFPDLGQEVALNANTKNQKIRWKNEVRIHSGSFQSQVELSNDQEKAHLDIAGSLEGHLRFLKNIILPVYDKSLWDFLKLDVTTSIGRRQHLRVSTAFVYTKNPNGYSFSIPVKVLADKFIIPGLKLNDLNSVLVMPTFHVPFTDLQVPSCKLDFREIQIYKKLRTSSFALNLPTLPEVKFPEVDVLTKYSQPEDSLIPFFEITVPESQLTVSQFTLPKSVSDGIAALDLNAVANKIADFELPTIIVPEQTIEIPSIKFSVPAGIVIPSFQALTARFEVDSPVYNATWSASLKNKADYVETVLDSTCSSTVQFLEYELNVLGTHKIEDGTLASKTKGTFAHRDFSAEYEEDGKYEGLQEWEGKAHLNIKSPAFTDLHLRYQKDKKGISTSAASPAVGTVGMDMDEDDDFSKWNFYYSPQSSPDKKLTIFKTELRVRESDEETQIKVNWEEEAASGLLTSLKDNVPKATGVLYDYVNKYHWEHTGLTLREVSSKLRRNLQNNAEWVYQGAIRQIDDIDVRFQKAASGTTGTYQEWKDKAQNLYQELLTQEGQASFQGLKDNVFDGLVRVTQEFHMKVKHLIDSLIDFLNFPRFQFPGKPGIYTREELCTMFIREVGTVLSQVYSKVHNGSEILFSYFQDLVITLPFELRKHKLIDVISMYRELLKDLSKEAQEVFKAIQSLKTTEVLRNLQDLLQFIFQLIEDNIKQLKEMKFTYLINYIQDEINTIFSDYIPYVFKLLKENLCLNLHKFNEFIQNELQEASQELQQIHQYIMALREEYFDPSIVGWTVKYYELEEKIVSLIKNLLVALKDFHSEYIVSASNFTSQLSSQVEQFLHRNIQEYLSILTDPDGKGKEKIAELSATAQEIIKSQAIATKKIISDYHQQFRYKLQDFSDQLSDYYEKFIAESKRLIDLSIQNYHTFLIYITELLKKLQSTTVMNPYMKLAPGELTIIL

90 NP_000966.2 NP_002383.2

>NP_000966.2

MAQDQGEKENPMRELRIRKLCLNICVGESGDRLTRAAKVLEQLTGQTPVFSKARYTVRSFGIRRNEKIAVHCTVRGAKAEEILEKGLKVREYELRKNNFSDTGNFGFGIQEHIDLGIKYDPSIGIYGLDFYVVLGRPGFSIADKKRRTGCIGAKHRISKEEAMRWFQQKYDGIILPGK

>NP_002383.2

MVRSRQMCNTNMSVPTDGAVTTSQIPASEQETLVRPKPLLLKLLKSVGAQKDTYTMKEVLFYLGQYIMTKRLYDEKQQHIVYCSNDLLGDLFGVPSFSVKEHRKIYTMIYRNLVVVNQQESSDSGTSVSENRCHLEGGSDQKDLVQELQEEKPSSSHLVSRPSTSSRRRAISETEENSDELSGERQRKRHKSDSISLSFDESLALCVIREICCERSSSSESTGTPSNPDLDAGVSEHSGDWLDQDSVSDQFSVEFEVESLDSEDYSLSEEGQELSDEDDEVYQVTVYQAGESDTDSFEEDPEISLADYWKCTSCNEMNPPLPSHCNRCWALRENWLPEDKGKDKGEISEKAKLENSTQAEEGFDVPDCKKTIVNDSRESCVEENDDKITQASQSQESEDYSQPSTSSSIIYSSQEDVKEFEREETQDKEESVESSLPLNAIEPCVICQGRPKNGCIVHGKTGHLMACFTCAKKLKKRNKPCPVCRQPIQMIVLTYFP

91 NP_001026859.1 NP_006035.2

>NP_001026859.1

MTSGATRYRLSCSLRGHELDVRGLVCCAYPPGAFVSVSRDRTTRLWAPDSPNRSFTEMHCMSGHSNFVSCVCIIPSSDIYPHGLIATGGNDHNICIFSLDSPMPLYILKGHKNTVCSLSSGKFGTLLSGSWDTTAKVWLNDKCMMTLQGHTAAVWAVKILPEQGLMLTGSADKTVKLWKAGRCERTFSGHEDCVRGLAILSETEFLSCANDASIRRWQITGECLEVYYGHTNYIYSISVFPNCRDFVTTAEDRSLRIWKHGECAQTIRLPAQSIWCCCVLDNGDIVVGASDGIIRVFTESEDRTASAEEIKAFEKELSHATIDSKTGDLGDINAEQLPGREHLNEPGTREGQTRLIRDGEKVEAYQWSVSEGRWIKIGDVVGSSGANQQTSGKVLYEGKEFDYVFSIDVNEGGPSYKLPYNTSDDPWLTAYNFLQKNDLNPMFLDQVAKFIIDNTKGQMLGLGNPSFSDPFTGGGRYVPGSSGSSNTLPTADPFTGAGRYVPGSASMGTTMAGVDPFTGNSAYRSAASKTMNIYFPKKEAVTFDQANPTQILGKLKELNGTAPEEKKLTEDDLILLEKILSLICNSSSEKPTVQQLQILWKAINCPEDIVFPALDILRLSIKHPSVNENFCNEKEGAQFSSHLINLLNPKGKPANQLLALRTFCNCFVGQAGQKLMMSQRESLMSHAIELKSGSNKNIHIALATLALNYSVCFHKDHNIEGKAQCLSLISTILEVVQDLEATFRLLVALGTLISDDSNAVQLAKSLGVDSQIKKYSSVSEPAKVSECCRFILNLL

>NP_006035.2

MTSTGQDSTTTRQRRSRQNPQSPPQDSSVTSKRNIKKGAVPRSIPNLAEVKKKGKMKKLGQAMEEDLIVGLQGMDLNLEAEALAGTGLVLDEQLNEFHCLWDDSFPEGPERLHAIKEQLIQEGLLDRCVSFQARFAEKEELMLVHSLEYIDLMETTQYMNEGELRVLADTYDSVYLHPNSYSCACLASGSVLRLVDAVLGAEIRNGMAIIRPPGHHAQHSLMDGYCMFNHVAVAARYAQQKHRIRRVLIVDWDVHHGQGTQFTFDQDPSVLYFSIHRYEQGRFWPHLKASNWSTTGFGQGQGYTINVPWNQVGMRDADYIAAFLHVLLPVALEFQPQLVLVAAGFDALQGDPKGEMAATPAGFAQLTHLLMGLAGGKLILSLEGGYNLRALAEGVSASLHTLLGDPCPMLESPGAPCRSAQASVSCALEALEPFWEVLVRSTETVERDNMEEDNVEESEEEGPWEPPVLPILTWPVLQSRTGLVYDQNMMNHCNLWDSHHPEVPQRILRIMCRLEELGLAGRCLTLTPRPATEAELLTCHSAEYVGHLRATEKMKTRELHRESSNFDSIYICPSTFACAQLATGAACRLVEAVLSGEVLNGAAVVRPPGHHAEQDAACGFCFFNSVAVAARHAQTISGHALRILIVDWDVHHGNGTQHMFEDDPSVLYVSLHRYDHGTFFPMGDEGASSQIGRAAGTGFTVNVAWNGPRMGDADYLAAWHRLVLPIAYEFNPELVLVSAGFDAARGDPLGGCQVSPEGYAHLTHLLMGLASGRIILILEGGYNLTSISESMAACTRSLLGDPPPLLTLPRPPLSGALASITETIQVHRRYWRSLRVMKVEDREGPSSSKLVTKKAPQPAKPRLAERMTTREKKVLEAGMGKVTSASFGEESTPGQTNSETAVVALTQDQPSEAATGGATLAQTISEAAIGGAMLGQTTSEEAVGGATPDQTTSEETVGGAILDQTTSEDAVGGATLGQTTSEEAVGGATLAQTTSEAAMEGATLDQTTSEEAPGGTELIQTPLASSTDHQTPPTSPVQGTTPQISPSTLIGSLRTLELGSESQGASESQAPGEENLLGEAAGGQDMADSMLMQGSRGLTDQAIFYAVTPLPWCPHLVAVCPIPAAGLDVTQPCGDCGTIQENWVCLSCYQVYCGRYINGHMLQHHGNSGHPLVLSYIDLSAWCYYCQAYVHHQALLDVKNIAHQNKFGEDMPHPH

92 NP_006861.1 NP_001031.2

>NP_006861.1

MASGVQVADEVCRIFYDMKVRKCSTPEEIKKRKKAVIFCLSADKKCIIVEEGKEILVGDVGVTITDPFKHFVGMLPEKDCRYALYDASFETKESRKEELMFFLWAPELAPLKSKMIYASSKDAIKKKFQGIKHECQANGPEDLNRACIAEKLGGSLIVAFEGCPV

>NP_001031.2

MESRGPLATSRLLLLLLLLLLRHTRQGWALRPVLPTQSAHDPPAVHLSNGPGQEPIAVMTFDLTKITKTSSSFEVRTWDPEGVIFYGDTNPKDDWFMLGLRDGRPEIQLHNHWAQLTVGAGPRLDDGRWHQVEVKMEGDSVLLEVDGEEVLRLRQVSGPLTSKRHPIMRIALGGLLFPASNLRLPLVPALDGCLRRDSWLDKQAEISASAPTSLRSCDVESNPGIFLPPGTQAEFNLRDIPQPHAEPWAFSLDLGLKQAAGSGHLLALGTPENPSWLSLHLQDQKVVLSSGSGPGLDLPLVLGLPLQLKLSMSRVVLSQGSKMKALALPPLGLAPLLNLWAKPQGRLFLGALPGEDSSTSFCLNGLWAQGQRLDVDQALNRSHEIWTHSCPQSPGNGTDASH

93 NP_788276.1 NP_003876.1

>NP_788276.1

MEDHQHVPIDIQTSKLLDWLVDRRHCSLKWQSLVLTIREKINAAIQDMPESEEIAQLLSGSYIHYFHCLRILDLLKGTEASTKNIFGRYSSQRMKDWQEIIALYEKDNTYLVELSSLLVRNVNYEIPSLKKQIAKCQQLQQEYSRKEEECQAGAAEMREQFYHSCKQYGITGENVRGELLALVKDLPSQLAEIGAAAQQSLGEAIDVYQASVGFVCESPTEQVLPMLRFVQKRGNSTVYEWRTGTEPSVVERPHLEELPEQVAEDAIDWGDFGVEAVSEGTDSGISAEAAGIDWGIFPESDSKDPGGDGIDWGDDAVALQITVLEAGTQAPEGVARGPDALTLLEYTETRNQFLDELMELEIFLAQRAVELSEEADVLSVSQFQLAPAILQGQTKEKMVTMVSVLEDLIGKLTSLQLQHLFMILASPRYVDRVTEFLQQKLKQSQLLALKKELMVQKQQEALEEQAALEPKLDLLLEKTKELQKLIEADISKRYSGRPVNLMGTSL

>NP_003876.1

MGTVLSLSPSYRKATLFEDGAATVGHYTAVQNSKNAKDKNLKRHSIISVLPWKRIVAVSAKKKNSKKVQPNSSYQNNITHLNNENLKKSLSCANLSTFAQPPPAQPPAPPASQLSGSQTGGSSSVKKAPHPAVTSAGTPKRVIVQASTSELLRCLGEFLCRRCYRLKHLSPTDPVLWLRSVDRSLLLQGWQDQGFITPANVVFLYMLCRDVISSEVGSDHELQAVLLTCLYLSYSYMGNEISYPLKPFLVESCKEAFWDRCLSVINLMSSKMLQINADPHYFTQVFSDLKNESGQEDKKRLLLGLDR

94 NP_005327.1 NP_001348.2

>NP_005327.1

MSSVAVLTQESFAEHRSGLVPQQIKVATLNSEEESDPPTYKDAFPPLPEKAACLESAQEPAGAWGNKIRPIKASVITQVFHVPLEERKYKDMNQFGEGEQAKICLEIMQRTGAHLELSLAKDQGLSIMVSGKLDAVMKARKDIVARLQTQASATVAIPKEHHRFVIGKNGEKLQDLELKTATKIQIPRPDDPSNQIKITGTKEGIEKARHEVLLISAEQDKRAVERLEVEKAFHPFIAGPYNRLVGEIMQETGTRINIPPPSVNRTEIVFTGEKEQLAQAVARIKKIYEEKKKKTTTIAVEVKKSQHKYVIGPKGNSLQEILERTGVSVEIPPSDSISETVILRGEPEKLGQALTEVYAKANSFTVSSVAAPSWLHRFIIGKKGQNLAKITQQMPKVHIEFTEGEDKITLEGPTEDVNVAQEQIEGMVKDLINRMDYVEINIDHKFHRHLIGKSGANINRIKDQYKVSVRIPPDSEKSNLIRIEGDPQGVQQAKRELLELASRMENERTKDLIIEQRFHRTIIGQKGERIREIRDKFPEVIINFPDPAQKSDIVQLRGPKNEVEKCTKYMQKMVADLVENSYSISVPIFKQFHKNIIGKGGANIKKIREESNTKIDLPAENSNSETIIITGKRANCEAARSRILSIQKDLANIAEVEVSIPAKLHNSLIGTKGRLIRSIMEECGGVHIHFPVEGSGSDTVVIRGPSSDVEKAKKQLLHLAEEKQTKSFTVDIRAKPEYHKFLIGKGGGKIRKVRDSTGARVIFPAAEDKDQDLITIIGKEDAVREAQKELEALIQNLDNVVEDSMLVDPKHHRHFVIRRGQVLREIAEEYGGVMVSFPRSGTQSDKVTLKGAKDCVEAAKKRIQEIIEDLEAQVTLECAIPQKFHRSVMGPKGSRIQQITRDFSVQIKFPDREENAVHSTEPVVQENGDEAGEGREAKDCDPGSPRRCDIIIISGRKEKCEAAKEALEALVPVTIEVEVPFDLHRYVIGQKGSGIRKMMDEFEVNIHVPAPELQSDIIAITGLAANLDRAKAGLLERVKELQAEQEDRALRSFKLSVTVDPKYHPKIIGRKGAVITQIRLEHDVNIQFPDKDDGNQPQDQITITGYEKNTEAARDAILRIVGELEQMVSEDVPLDHRVHARIIGARGKAIRKIMDEFKVDIRFPQSGAPDPNCVTVTGLPENVEEAIDHILNLEEEYLADVVDSEALQVYMKPPAHEEAKAPSRGFVVRDAPWTASSSEKAPDMSSSEEFPSFGAQVAPKTLPWGPKR

>NP_001348.2

MGDVKNFLYAWCGKRKMTPSYEIRAVGNKNRQKFMCEVQVEGYNYTGMGNSTNKKDAQSNAARDFVNYLVRINEIKSEEVPAFGVASPPPLTDTPDTTANAEGDLPTTMGGPLPPHLALKAENNSEVGASGYGVPGPTWDRGANLKDYYSRKEEQEVQATLESEEVDLNAGLHGNWTLENAKARLNQYFQKEKIQGEYKYTQVGPDHNRSFIAEMTIYIKQLGRRIFAREHGSNKKLAAQSCALSLVRQLYHLGVVEAYSGLTKKKEGETVEPYKVNLSQDLEHQLQNIIQELNLEILPPPEDPSVPVALNIGKLAQFEPSQRQNQVGVVPWSPPQSNWNPWTSSNIDEGPLAFATPEQISMDLKNELMYQLEQDHDLQAILQERELLPVKKFESEILEAISQNSVVIIRGATGCGKTTQVPQFILDDFIQNDRAAECNIVVTQPRRISAVSVAERVAFERGEEPGKSCGYSVRFESILPRPHASIMFCTVGVLLRKLEAGIRGISHVIVDEIHERDINTDFLLVVLRDVVQAYPEVRIVLMSATIDTSMFCEYFFNCPIIEVYGRTYPVQEYFLEDCIQMTHFVPPPKDKKKKDKDDDGGEDDDANCNLICGDEYGPETRLSMSQLNEKETPFELIEALLKYIETLNVPGAVLVFLPGWNLIYTMQKHLEMNPHFGSHRYQILPLHSQIPREEQRKVFDPVPVGVTKVILSTNIAETSITINDVVYVIDSCKQKVKLFTAHNNMTNYATVWASKTNLEQRKGRAGRVRPGFCFHLCSRARFERLETHMTPEMFRTPLHEIALSIKLLRLGGIGQFLAKAIEPPPLDAVIEAEHTLRELDALDANDELTPLGRILAKLPIEPRFGKMMIMGCIFYVGDAICTIAAATCFPEPFINEGKRLGYIHRNFAGNRFSDHVALLSVFQAWDDARMGGEEAEIRFCEHKRLNMATLRMTWEAKVQLKEILINSGFPEDCLLTQVFTNTGPDNNLDVVISLLAFGVYPNVCYHKEKRKILTTEGRNALIHKSSVNCPFSSQDMKYPSPFFVFGEKIRTRAISAKGMTLVTPLQLLLFASKKVQSDGQIVLVDDWIKLQISHEAAACITGLRAAMEALVVEVTKQPAIISQLDPVNERMLNMIRQISRPSAAGINLMIGSTRYGDGPRPPKMARYDNGSGYRRGGSSYSGGGYGGGYSSGGYGSGGYGGSANSFRAGYGAGVGGGYRGVSRGGFRGNSGGDYRGPSGGYRGSGGFQRGGGRGAYGTGYFGQGRGGGGY

95 NP_002784.1 NP_002790.1

>NP_002784.1

MLSSTAMYSAPGRDLGMEPHRAAGPLQLRFSPYVFNGGTILAIAGEDFAIVASDTRLSEGFSIHTRDSPKCYKLTDKTVIGCSGFHGDCLTLTKIIEARLKMYKHSNNKAMTTGAIAAMLSTILYSRRFFPYYVYNIIGGLDEEGKGAVYSFDPVGSYQRDSFKAGGSASAMLQPLLDNQVGFKNMQNVEHVPLSLDRAMRLVKDVFISAAERDVYTGDALRICIVTKEGIREETVSLRKD

>NP_002790.1

MAAVSVYAPPVGGFSFDNCRRNAVLEADFAKRGYKLPKVRKTGTTIAGVVYKDGIVLGADTRATEGMVVADKNCSKIHFISPNIYCCGAGTAADTDMTTQLISSNLELHSLSTGRLPRVVTANRMLKQMLFRYQGYIGAALVLGGVDVTGPHLYSIYPHGSTDKLPYVTMGSGSLAAMAVFEDKFRPDMEEEEAKNLVSEAIAAGIFNDLGSGSNIDLCVISKNKLDFLRPYTVPNKKGTRLGRYRCEKGTTAVLTEKITPLEIEVLEETVQTMDTS

96 NP_000466.2 NP_036377.1

>NP_000466.2

MAGENHQWQGSILYNMLMSAKQTRAAPEAPETRLVDQCWGCSCGDEPGVGREGLLGGRNVALLYRCCFCGKDHPRQGSILYSMLTSAKQTYAAPKAPEATLGPCWGCSCGSDPGVGRAGLPGGRPVALLYRCCFCGEDHPRQGSILYSLLTSSKQTHVAPAAPEARPGGAWWDRSYFAQRPGGKEALPGGRATALLYRCCFCGEDHPQQGSTLYCVPTSTNQAQAAPEERPRAPWWDTSSGALRPVALKSPQVVCEAASAGLLKTLRFVKYLPCFQVLPLDQQLVLVRNCWASLLMLELAQDRLQFETVEVSEPSMLQKILTTRRRETGGNEPLPVPTLQHHLAPPAEARKVPSASQVQAIKCFLSKCWSLNISTKEYAYLKGTVLFNPDVPGLQCVKYIQGLQWGTQQILSEHTRMTHQGPHDRFIELNSTLFLLRFINANVIAELFFRPIIGTVSMDDMMLEMLCTKI

>NP_036377.1

MALTSFLPAPTQLSQDQLEAEEKARSQRSRQTSLVSSRREPPPYGYRKGWIPRLLEDFGDGGAFPEIHVAQYPLDMGRKKKMSNALAIQVDSEGKIKYDAIARQGQSKDKVIYSKYTDLVPKEVMNADDPDLQRPDEEAIKEITEKTRVALEKSVSQKVAAAMPVRAADKLAPAQYIRYTPSQQGVAFNSGAKQRVIRMVEMQKDPMEPPRFKINKKIPRGPPSPPAPVMHSPSRKMTVKEQQEWKIPPCISNWKNAKGYTIPLDKRLAADGRGLQTVHINENFAKLAEALYIADRKAREAVEMRAQVERKMAQKEKEKHEEKLREMAQKARERRAGIKTHVEKEDGEARERDEIRHDRRKERQHDRNLSRAAPDKRSKLQRNENRDISEVIALGVPNPRTSNEVQYDQRLFNQSKGMDSGFAGGEDEIYNVYDQAWRGGKDMAQSIYRPSKNLDKDMYGDDLEARIKTNRFVPDKEFSGSDRRQRGREGPVQFEEDPFGLDKFLEEAKQHGGSKRPSDSSRPKEHEHEGKKRRKE

97 NP_056468.1 NP_598399.1

>NP_056468.1

MRQNDKIMCILENRKKRDRKNLCRAINDFQQSFQKPETRREFDLSDPLALKKDLPARQSDNDVRNTISGMQKFMGEDLNFHERKKFQEEQNREWSLQQQREWKNARAEQKCAEALYTETRLQFDETAKHLQKLESTTRKAVCASVKDFNKSQAIESVERKKQEKKQEQEDNLAEITNLLRGDLLSENPQQAASSFGPHRVVPDRWKGMTQEQLEQIRLVQKQQIQEKLRLQEEKRQRDLDWDRRRIQGARATLLFERQQWRRQRDLRRALDSSNLSLAKEQHLQKKYMNEVYTNQPTGDYFTQFNTGSR

>NP_598399.1

MWRGLWTLAAQAARGPRRLCTRRSSGAPAPGSGATIFALSSGQGRCGIAVIRTSGPASGHALRILTAPRDLPLARHASLRLLSDPRSGEPLDRALVLWFPGPQSFTGEDCVEFHVHGGPAVVSGVLQALGSVPGLRPAEAGEFTRRAFANGKLNLTEVEGLADLIHAETEAQRRQALRQLDGELGHLCRGWAETLTKALAHVEAYIDFGEDDNLEEGVLEQGGSTWWWGRKTPHISPQRLPSLSLSACLLSPTADIEVRALQVALGAHLRDARRGQRLRSGAHVVVTGPPNAGKSSLVNLLSRKPVSIVSPEPGTTRDVLETPVDLAGFPVLLSDTAGLREGVGPVEQEGVRRARERLEQADLILAMLDASDLASPSSCNFLATVVASVGAQSPSDSSQRLLLVLNKSDLLSPEGPGPGPDLPPHLLLSCLTGEGLDGLLEALRKELAAVCGDPSTDPPLLTRARHQHHLQGCLDALGHYKQSKDLALAAEALRVARGHLTRLTGGGGTEEILDIIFQDFCVGK

98 NP_071757.2 NP_000116.2

>NP_071757.2

MAAAAAVEAAAPMGALWGLVHDFVVGQQEGPADQVAADVKSGNYTVLQVVEALGSSLENPEPRTRARGIQLLSQVLLHCHTLLLEKEVVHLILFYENRLKDHHLVIPSVLQGLKALSLCVALPPGLAVSVLKAIFQEVHVQSLPQVDRHTVYNIITNFMRTREEELKSLGADFTFGFIHVMDGEKDPRNLLVAFRIVHDLISRDYSLGPFVEELFEVTSCYFPIDFTPPPNDPHGIQREDLILSLRAVLASTPRFAEFLLPLLIEKVDSEVLSAKLDSLQTLNACCAVYGQKELKDFLPSLWASIRREVFQTASERVEAEGLAALHSLTACLSRSVLRADAEDLLDSFLSNILQDCRHHLCEPDMKLVWPSAKLLQAAAGASARACDSDTSNVPPLLLEQFHKHSQSSQRRTILEMLLGFLKLQQKWSYEDKDQRPLNGFKDQLCSLVFMALTDPSTQLQLVGIRTLTVLGAQPDLLSYEDLELAVGHLYRLSFLKEDSQSCRVAALEASGTLAALYPVAFSSHLVPKLAEELRVGESNLTNGDEPTQCSRHLCCLQALSAVSTHPSIVKETLPLLLQHLWQVNRGNMVAQSSDVIAVCQSLRQMAEKCQQDPESCWYFHQTAIPCLLALAVQASMPEKEPSVLRKVLLEDEVLAAMVSVIGTATTHLSPELAAQSVTHIVPLFLDGNVSFLPENSFPSRFQPFQDGSSGQRRLIALLMAFVCSLPRNVEIPQLNQLMREFLELSCCHSCPFSSTAAAKCFAGLLNKHPAGQQLDEFLQLAVDKVEAGLGSGPCRSQAFTLLLWVTKALVLRYHPLSSCLTARLMGLLSDPELGPAAADGFSLLMSDCTDVLTRAGHAEVRIMFRQRFFTDNVPALVQGFHAAPQDVKPNYLKGLSHVLNRLPKPVLLPELPTLLSLLLEALSCPDCVVQLSTLSCLQPLLLEAPQVMSLHVDTLVTKFLNLSSSPSMAVRIAALQCMHALTRLPTPVLLPYKPQVIRALAKPLDDKKRLVRKEAVSARGEWFLLGSPGS

>NP_000116.2

MTMTLHTKASGMALLHQIQGNELEPLNRPQLKIPLERPLGEVYLDSSKPAVYNYPEGAAYEFNAAAAANAQVYGQTGLPYGPGSEAAAFGSNGLGGFPPLNSVSPSPLMLLHPPPQLSPFLQPHGQQVPYYLENEPSGYTVREAGPPAFYRPNSDNRRQGGRERLASTNDKGSMAMESAKETRYCAVCNDYASGYHYGVWSCEGCKAFFKRSIQGHNDYMCPATNQCTIDKNRRKSCQACRLRKCYEVGMMKGGIRKDRRGGRMLKHKRQRDDGEGRGEVGSAGDMRAANLWPSPLMIKRSKKNSLALSLTADQMVSALLDAEPPILYSEYDPTRPFSEASMMGLLTNLADRELVHMINWAKRVPGFVDLTLHDQVHLLECAWLEILMIGLVWRSMEHPGKLLFAPNLLLDRNQGKCVEGMVEIFDMLLATSSRFRMMNLQGEEFVCLKSIILLNSGVYTFLSSTLKSLEEKDHIHRVLDKITDTLIHLMAKAGLTLQQQHQRLAQLLLILSHIRHMSNKGMEHLYSMKCKNVVPLYDLLLEMLDAHRLHAPTSRGGASVEETDQSHLATAGSTSSHSLQKYYITGEAEGFPATV

99 NP_004827.4 NP_001017963.1

>NP_004827.4

MERAISPGLLVRALLLLLLLLGLAARTVAAGRARGLPAPTAEAAFGLGAAAAPTSATRVPAAGAVAAAEVTVEDAEALPAAAGEQEPRGPEPDDETELRPRGRSLVIISTLDGRIAALDPENHGKKQWDLDVGSGSLVSSSLSKPEVFGNKMIIPSLDGALFQWDQDRESMETVPFTVESLLESSYKFGDDVVLVGGKSLTTYGLSAYSGKVRYICSALGCRQWDSDEMEQEEDILLLQRTQKTVRAVGPRSGNEKWNFSVGHFELRYIPDMETRAGFIESTFKPNENTEESKIISDVEEQEAAIMDIVIKVSVADWKVMAFSKKGGHLEWEYQFCTPIASAWLLKDGKVIPISLFDDTSYTSNDDVLEDEEDIVEAARGATENSVYLGMYRGQLYLQSSVRISEKFPSSPKALESVTNENAIIPLPTIKWKPLIHSPSRTPVLVGSDEFDKCLSNDKFSHEEYSNGALSILQYPYDNGYYLPYYKRERNKRSTQITVRFLDNPHYNKNIRKKDPVLLLHWWKEIVATILFCIIATTFIVRRLFHPHPHRQRKESETQCQTENKYDSVSGEANDSSWNDIKNSGYISRYLTDFEPIQCLGRGGFGVVFEAKNKVDDCNYAIKRIRLPNRELAREKVMREVKALAKLEHPGIVRYFNAWLEAPPEKWQEKMDEIWLKDESTDWPLSSPSPMDAPSVKIRRMDPFATKEHIEIIAPSPQRSRSFSVGISCDQTSSSESQFSPLEFSGMDHEDISESVDAAYNLQDSCLTDCDVEDGTMDGNDEGHSFELCPSEASPYVRSRERTSSSIVFEDSGCDNASSKEEPKTNRLHIGNHCANKLTAFKPTSSKSSSEATLSISPPRPTTLSLDLTKNTTEKLQPSSPKVYLYIQMQLCRKENLKDWMNGRCTIEERERSVCLHIFLQIAEAVEFLHSKGLMHRDLKPSNIFFTMDDVVKVGDFGLVTAMDQDEEEQTVLTPMPAYARHTGQVGTKLYMSPEQIHGNSYSHKVDIFSLGLILFELLYPFSTQMERVRTLTDVRNLKFPPLFTQKYPCEYVMVQDMLSPSPMERPEAINIIENAVFEDLDFPGKTVLRQRSRSLSSSGTKHSRQSNNSHSPLPSN

>NP_001017963.1

MPPCSGGDGSTPPGPSLRDRDCPAQSAEYPRDRLDPRPGSPSEASSPPFLRSRAPVNWYQEKAQVFLWHLLVSGSTTLLCLWKQPFHVSAFPVTASLAFRQSQGAGQHLYKDLQPFILLRLLMPEETQTQDQPMEEEEVETFAFQAEIAQLMSLIINTFYSNKEIFLRELISNSSDALDKIRYESLTDPSKLDSGRELHINLIPNKQGRTLTIVDTGIGMTKADLINNLGTIAKSGTKAFMEALQAGADISMIGQFGVGFYSAYLVAEKVTVITKHNDDEQYAWESSAGGSFTVRTDTGEPMGRGTKVILHLKEDQTEYLEERRIKEIVKKHSQFIGYPITLFVEKERDKEVSDDEAEEKEDKEEEKEKEEKESEDKPEIEDVGSDEEEEKKDGDKKKKKKIKEKYIDQEELNKTKPIWTRNPDDITNEEYGEFYKSLTNDWEDHLAVKHFSVEGQLEFRALLFVPRRAPFDLFENRKKKNNIKLYVRRVFIMDNCEELIPEYLNFIRGVVDSEDLPLNISREMLQQSKILKVIRKNLVKKCLELFTELAEDKENYKKFYEQFSKNIKLGIHEDSQNRKKLSELLRYYTSASGDEMVSLKDYCTRMKENQKHIYYITGETKDQVANSAFVERLRKHGLEVIYMIEPIDEYCVQQLKEFEGKTLVSVTKEGLELPEDEEEKKKQEEKKTKFENLCKIMKDILEKKVEKVVVSNRLVTSPCCIVTSTYGWTANMERIMKAQALRDNSTMGYMAAKKHLEINPDHSIIETLRQKAEADKNDKSVKDLVILLYETALLSSGFSLEDPQTHANRIYRMIKLGLGIDEDDPTADDTSAAVTEEMPPLEGDDDTSRMEEVD

100 NP_002937.1 NP_004517.2

>NP_002937.1

MWNSGFESYGSSSYGGAGGYTQSPGGFGSPAPSQAEKKSRARAQHIVPCTISQLLSATLVDEVFRIGNVEISQVTIVGIIRHAEKAPTNIVYKIDDMTAAPMDVRQWVDTDDTSSENTVVPPETYVKVAGHLRSFQNKKSLVAFKIMPLEDMNEFTTHILEVINAHMVLSKANSQPSAGRAPISNPGMSEAGNFGGNSFMPANGLTVAQNQVLNLIKACPRPEGLNFQDLKNQLKHMSVSSIKQAVDFLSNEGHIYSTVDDDHFKSTDAE

>NP_004517.2

MAESSESFTMASSPAQRRRGNDPLTSSPGRSSRRTDALTSSPGRDLPPFEDESEGLLGTEGPLEEEEDGEELIGDGMERDYRAIPELDAYEAEGLALDDEDVEELTASQREAAERAMRQRDREAGRGLGRMRRGLLYDSDEEDEERPARKRRQVERATEDGEEDEEMIESIENLEDLKGHSVREWVSMAGPRLEIHHRFKNFLRTHVDSHGHNVFKERISDMCKENRESLVVNYEDLAAREHVLAYFLPEAPAELLQIFDEAALEVVLAMYPKYDRITNHIHVRISHLPLVEELRSLRQLHLNQLIRTSGVVTSCTGVLPQLSMVKYNCNKCNFVLGPFCQSQNQEVKPGSCPECQSAGPFEVNMEETIYQNYQRIRIQESPGKVAAGRLPRSKDAILLADLVDSCKPGDEIELTGIYHNNYDGSLNTANGFPVFATVILANHVAKKDNKVAVGELTDEDVKMITSLSKDQQIGEKIFASIAPSIYGHEDIKRGLALALFGGEPKNPGGKHKVRGDINVLLCGDPGTAKSQFLKYIEKVSSRAIFTTGQGASAVGLTAYVQRHPVSREWTLEAGALVLADRGVCLIDEFDKMNDQDRTSIHEAMEQQSISISKAGIVTSLQARCTVIAAANPIGGRYDPSLTFSENVDLTEPIISRFDILCVVRDTVDPVQDEMLARFVVGSHVRHHPSNKEEEGLANGSAAEPAMPNTYGVEPLPQEVLKKYIIYAKERVHPKLNQMDQDKVAKMYSDLRKESMATGSIPITVRHIESMIRMAEAHARIHLRDYVIEDDVNMAIRVMLESFIDTQKFSVMRSMRKTFARYLSFRRDNNELLLFILKQLVAEQVTYQRNRFGAQQDTIEVPEKDLVDKARQINIHNLSAFYDSELFRMNKFSHDLKRKMILQQF

101 NP_000315.1 NP_002091.2

>NP_000315.1

MRFTFPLMAIVLEIAMIVLFGLFVEYETDQTVLEQLNITKPTDMGIFFELYPLFQDVHVMIFVGFGFLMTFLKKYGFSSVGINLLVAALGLQWGTIVQGILQSQGQKFNIGIKNMINADFSAATVLISFGAVLGKTSPTQMLIMTILEIVFFAHNEYLVSEIFKASDIGASMTIHAFGAYFGLAVAGILYRSGLRKGHENEESAYYSDLFAMIGTLFLWMFWPSFNSAIAEPGDKQCRAIVDTYFSLAACVLTAFAFSSLVEHRGKLNMVHIQNATLAGGVAVGTCADMAIHPFGSMIIGSIAGMVSVLGYKFLTPLFTTKLRIHDTCGVHNLHGLPGVVGGLAGIVAVAMGASNTSMAMQAAALGSSIGTAVVGGLMTGLILKLPLWGQPSDQNCYDDSVYWKVPKTR

>NP_002091.2

MYGKIIFVLLLSEIVSISALSTTEVAMHTSTSSSVTKSYISSQTNGETGQLVHRFTVPAPVVIILIILCVMAGIIGTILLISYSIRRLIKA

102 NP_002261.3 NP_005076.3

>NP_002261.3

MVWDRQTKMEYEWKPDEQGLQQILQLLKESQSPDTTIQRTVQQKLEQLNQYPDFNNYLIFVLTKLKSEDEPTRSLSGLILKNNVKAHFQNFPNGVTDFIKSECLNNIGDSSPLIRATVGILITTIASKGELQNWPDLLPKLCSLLDSEDYNTCEGAFGALQKICEDSAEILDSDVLDRPLNIMIPKFLQFFKHSSPKIRSHAVACVNQFIISRTQALMLHIDSFIENLFALAGDEEPEVRKNVCRALVMLLEVRMDRLLPHMHNIVEYMLQRTQDQDENVALEACEFWLTLAEQPICKDVLVRHLPKLIPVLVNGMKYSDIDIILLKGDVEEDETIPDSEQDIRPRFHRSRTVAQQHDEDGIEEEDDDDDEIDDDDTISDWNLRKCSAAALDVLANVYRDELLPHILPLLKELLFHHEWVVKESGILVLGAIAEGCMQGMIPYLPELIPHLIQCLSDKKALVRSITCWTLSRYAHWVVSQPPDTYLKPLMTELLKRILDSNKRVQEAACSAFATLEEEACTELVPYLAYILDTLVFAFSKYQHKNLLILYDAIGTLADSVGHHLNKPEYIQMLMPPLIQKWNMLKDEDKDLFPLLECLSSVATALQSGFLPYCEPVYQRCVNLVQKTLAQAMLNNAQPDQYEAPDKDFMIVALDLLSGLAEGLGGNIEQLVARSNILTLMYQCMQDKMPEVRQSSFALLGDLTKACFQHVKPCIADFMPILGTNLNPEFISVCNNATWAIGEISIQMGIEMQPYIPMVLHQLVEIINRPNTPKTLLENTAITIGRLGYVCPQEVAPMLQQFIRPWCTSLRNIRDNEEKDSAFRGICTMISVNPSGVIQDFIFFCDAVASWINPKDDLRDMFCKILHGFKNQVGDENWRRFSDQFPLPLKERLAAFYGV

>NP_005076.3

MGDEMDAMIPEREMKDFQFRALKKVRIFDSPEELPKERSSLLAVSNKYGLVFAGGASGLQIFPTKNLLIQNKPGDDPNKIVDKVQGLLVPMKFPIHHLALSCDNLTLSACMMSSEYGSIIAFFDVRTFSNEAKQQKRPFAYHKLLKDAGGMVIDMKWNPTVPSMVAVCLADGSIAVLQVTETVKVCATLPSTVAVTSVCWSPKGKQLAVGKQNGTVVQYLPTLQEKKVIPCPPFYESDHPVRVLDVLWIGTYVFAIVYAAADGTLETSPDVVMALLPKKEEKHPEIFVNFMEPCYGSCTERQHHYYLSYIEEWDLVLAASAASTEVSILARQSDQINWESWLLEDSSRAELPVTDKSDDSLPMGVVVDYTNQVEITISDEKTLPPAPVLMLLSTDGVLCPFYMINQNPGVKSLIKTPERLSLEGERQPKSPGSTPTTPTSSQAPQKLDASAAAAPASLPPSSPAAPIATFSLLPAGGAPTVFSFGSSSLKSSATVTGEPPSYSSGSDSSKAAPGPGPSTFSFVPPSKASLAPTPAASPVAPSAASFSFGSSGFKPTLESTPVPSVSAPNIAMKPSFPPSTSAVKVNLSEKFTAAATSTPVSSSQSAPPMSPFSSASKPAASGPLSHPTPLSAPPSSVPLKSSVLPSPSGRSAQGSSSPVPSMVQKSPRITPPAAKPGSPQAKSLQPAVAEKQGHQWKDSDPVMAGIGEEIAHFQKELEELKARTSKACFQVGTSEEMKMLRTESDDLHTFLLEIKETTESLHGDISSLKTTLLEGFAGVEEAREQNERNRDSGYLHLLYKRPLDPKSEAQLQEIRRLHQYVKFAVQDVNDVLDLEWDQHLEQKKKQRHLLVPERETLFNTLANNREIINQQRKRLNHLVDSLQQLRLYKQTSLWSLSSAVPSQSSIHSFDSDLESLCNALLKTTIESHTKSLPKVPAKLSPMKQAQLRNFLAKRKTPPVRSTAPASLSRSAFLSQRYYEDLDEVSSTSSVSQSLESEDARTSCKDDEAVVQAPRHAPVVRTPSIQPSLLPHAAPFAKSHLVHGSSPGVMGTSVATSASKIIPQGADSTMLATKTVKHGAPSPSHPISAPQAAAAAALRRQMASQAPAVNTLTESTLKNVPQVVNVQELKNNPATPSTAMGSSVPYSTAKTPHPVLTPVAANQAKQGSLINSLKPSGPTPASGQLSSGDKASGTAKIETAVTSTPSASGQFSKPFSFSPSGTGFNFGIITPTPSSNFTAAQGATPSTKESSQPDAFSSGGGSKPSYEAIPESSPPSGITSASNTTPGEPAASSSRPVAPSGTALSTTSSKLETPPSKLGELLFPSSLAGETLGSFSGLRVGQADDSTKPTNKASSTSLTSTQPTKTSGVPSGFNFTAPPVLGKHTEPPVTSSATTTSVAPPAATSTSSTAVFGSLPVTSAGSSGVISFGGTSLSAGKTSFSFGSQQTNSTVPPSAPPPTTAATPLPTSFPTLSFGSLLSSATTPSLPMSAGRSTEEATSSALPEKPGDSEVSASAASLLEEQQSAQLPQAPPQTSDSVKKEPVLAQPAVSNSGTAASSTSLVALSAEATPATTGVPDARTEAVPPASSFSVPGQTAVTAAAISSAGPVAVETSSTPIASSTTSIVAPGPSAEAAAFGTVTSGSSVFAQPPAASSSSAFNQLTNNTATAPSATPVFGQVAASTAPSLFGQQTGSTASTAAATPQVSSSGFSSPAFGTTAPGVFGQTTFGQASVFGQSASSAASVFSFSQPGFSSVPAFGQPASSTPTSTSGSVFGAASSTSSSSSFSFGQSSPNTGGGLFGQSNAPAFGQSPGFGQGGSVFGGTSAATTTAATSGFSFCQASGFGSSNTGSVFGQAASTGGIVFGQQSSSSSGSVFGSGNTGRGGGFFSGLGGKPSQDAANKNPFSSASGGFGSTATSNTSNLFGNSGAKTFGGFASSSFGEQKPTGTFSSGGGSVASQGFGFSSPNKTGGFGAAPVFGSPPTFGGSPGFGGVPAFGSAPAFTSPLGSTGGKVFGEGTAAASAGGFGFGSSSNTTSFGTLASQNAPTFGSLSQQTSGFGTQSSGFSGFGSGTGGFSFGSNNSSVQGFGGWRS

103 NP_665860.1 NP_071731.1

>NP_665860.1

MGLRTTKQIGRGTKAPGHQEDHMVKEPVEDTDPSTLSFNMSDKYPIQDTELPKAEECDTITLNCPRNSDMKNQGEENGFPDSTGDPLPEISKDNSCKENCTCSSCLLRAPTISDLLNDQDLLDVIRIKLDPCHPTVKNWRNFASKWGMSYDELCFLEQRPQSPTLEFLLRNSQRTVGQLMELCRLYHRADVEKVLRRWVDEEWPKRERGDPSRHF

>NP_071731.1

MAHVGDCTQTPWLPVLVVSLMCSARAEYSNCGENEYYNQTTGLCQECPPCGPGEEPYLSCGYGTKDEDYGCVPCPAEKFSKGGYQICRRHKDCEGFFRATVLTPGDMENDAECGPCLPGYYMLENRPRNIYGMVCYSCLLAPPNTKECVGATSGASANFPGTSGSSTLSPFQHAHKELSGQGHLATALIIAMSTIFIMAIAIVLIIMFYILKTKPSAPACCTSHPGKSVEAQVSKDEEKKEAPDNVVMFSEKDEFEKLTATPAKPTKSENDASSENEQLLSRSVDSDEEPAPDKQGSPELCLLSLVHLAREKSATSNKSAGIQSRRKKILDVYANVCGVVEGLSPTELPFDCLEKTSRMLSSTYNSEKAVVKTWRHLAESFGLKRDEIGGMTDGMQLFDRISTAGYSIPELLTKLVQIERLDAVESLCADILEWAGVVPPASQPHAAS

104 NP_004032.2 NP_061833.1

>NP_004032.2

MGDKGTRVFKKASPNGKLTVYLGKRDFVDHIDLVDPVDGVVLVDPEYLKERRVYVTLTCAFRYGREDLDVLGLTFRKDLFVANVQSFPPAPEDKKPLTRLQERLIKKLGEHAYPFTFEIPPNLPCSVTLQPGPEDTGKACGVDYEVKAFCAENLEEKIHKRNSVRLVIRKVQYAPERPGPQPTAETTRQFLMSDKPLHLEASLDKEIYYHGEPISVNVHVTNNTNKTVKKIKISVRQYADICLFNTAQYKCPVAMEEADDTVAPSSTFCKVYTLTPFLANNREKRGLALDGKLKHEDTNLASSTLLREGANREILGIIVSYKVKVKLVVSRGGLLGDLASSDVAVELPFTLMHPKPKEEPPHREVPENETPVDTNLIELDTNDDDIVFEDFARQRLKGMKDDKEEEEDGTGSPQLNNR

>NP_061833.1

MVDSVYRTRSLGVAAEGLPDQYADGEAARVWQLYIGDTRSRTAEYKAWLLGLLRQHGCQRVLDVACGTGVDSIMLVEEGFSVTSVDASDKMLKYALKERWNRRHEPAFDKWVIEEANWMTLDKDVPQSAEGGFDAVICLGNSFAHLPDCKGDQSEHRLALKNIASMVRAGGLLVIDHRNYDHILSTGCAPPGKNIYYKSDLTKDVTTSVLIVNNKAHMVTLDYTVQVPGAGQDGSPGLSKFRLSYYPHCLASFTELLQAAFGGKCQHSVLGDFKPYKPGQTYIPCYFIHVLKRTD

105 NP_002125.3 NP_006824.2

>NP_002125.3

MSAEVETSEGVDESEKKNSGALEKENQMRMADLSELLKEGTKEAHDRAENTQFVKDFLKGNIKKELFKLATTALYFTYSALEEEMERNKDHPAFAPLYFPMELHRKEALTKDMEYFFGENWEEQVQCPKAAQKYVERIHYIGQNEPELLVAHAYTRYMGDLSGGQVLKKVAQRALKLPSTGEGTQFYLFENVDNAQQFKQLYRARMNALDLNMKTKERIVEEANKAFEYNMQIFNELDQAGSTLARETLEDGFPVHDGKGDMRKCPFYAAEQDKGALEGSSCPFRTAMAVLRKPSLQFILAAGVALAAGLLAWYYM

>NP_006824.2

MAAAAAAAAATNGTGGSSGMEVDAAVVPSVMACGVTGSVSVALHPLVILNISDHWIRMRSQEGRPVQVIGALIGKQEGRNIEVMNSFELLSHTVEEKIIIDKEYYYTKEEQFKQVFKELEFLGWYTTGGPPDPSDIHVHKQVCEIIESPLFLKLNPMTKHTDLPVSVFESVIDIINGEATMLFAELTYTLATEEAERIGVDHVARMTATGSGENSTVAEHLIAQHSAIKMLHSRVKLILEYVKASEAGEVPFNHEILREAYALCHCLPVLSTDKFKTDFYDQCNDVGLMAYLGTITKTCNTMNQFVNKFNVLYDRQGIGRRMRGLFF

106 NP_004759.1 NP_009210.1

>NP_004759.1

MSNTTVVPSTAGPGPSGGPGGGGGGGGGGGGTEVIQVTNVSPSASSEQMRTLFGFLGKIDELRLFPPDDSPLPVSSRVCFVKFHDPDSAVVAQHLTNTVFVDRALIVVPYAEGVIPDEAKALSLLAPANAVAGLLPGGGLLPTPNPLTQIGAVPLAALGAPTLDPALAALGLPGANLNSQSLAADQLLKLMSTVDPKLNHVAAGLVSPSLKSDTSSKEIEEAMKRVREAQSLISAAIEPDKKEEKRRHSRSRSRSRRRRTPSSSRHRRSRSRSRRRSHSKSRSRRRSKSPRRRRSHSRERGRRSRSTSKTRDKKKEDKEKKRSKTPPKSYSTARRSRSASRERRRRRSRSGTRSPKKPRSPKRKLSRSPSPRRHKKEKKKDKDKERSRDERERSTSKKKKSKDKEKDRERKSESDKDVKQVTRDYDEEEQGYDSEKEKKEEKKPIETGSPKTKECSVEKGTGDSLRESKVNGDDHHEEDMDMSD

>NP_009210.1

MSDFDEFERQLNENKQERDKENRHRKRSHSRSRSRDRKRRSRSRDRRNRDQRSASRDRRRRSKPLTRGAKEEHGGLIRSPRHEKKKKVRKYWDVPPPGFEHITPMQYKAMQAAGQIPATALLPTMTPDGLAVTPTPVPVVGSQMTRQARRLYVGNIPFGITEEAMMDFFNAQMRLGGLTQAPGNPVLAVQINQDKNFAFLEFRSVDETTQAMAFDGIIFQGQSLKIRRPHDYQPLPGMSENPSVYVPGVVSTVVPDSAHKLFIGGLPNYLNDDQVKELLTSFGPLKAFNLVKDSATGLSKGYAFCEYVDINVTDQAIAGLNGMQLGDKKLLVQRASVGAKNATLVSPPSTINQTPVTLQVPGLMSSQVQMGGHPTEVLCLMNMVLPEELLDDEEYEEIVEDVRDECSKYGLVKSIEIPRPVDGVEVPGCGKIFVEFTSVFDCQKAMQGLTGRKFANRVVVTKYCDPDSYHRRDFW

107 NP_001612.1 NP_004119.1

>NP_001612.1

MNSSSANITYASRKRRKPVQKTVKPIPAEGIKSNPSKRHRDRLNTELDRLASLLPFPQDVINKLDKLSVLRLSVSYLRAKSFFDVALKSSPTERNGGQDNCRAANFREGLNLQEGEFLLQALNGFVLVVTTDALVFYASSTIQDYLGFQQSDVIHQSVYELIHTEDRAEFQRQLHWALNPSQCTESGQGIEEATGLPQTVVCYNPDQIPPENSPLMERCFICRLRCLLDNSSGFLAMNFQGKLKYLHGQKKKGKDGSILPPQLALFAIATPLQPPSILEIRTKNFIFRTKHKLDFTPIGCDAKGRIVLGYTEAELCTRGSGYQFIHAADMLYCAESHIRMIKTGESGMIVFRLLTKNNRWTWVQSNARLLYKNGRPDYIIVTQRPLTDEEGTEHLRKRNTKLPFMFTTGEAVLYEATNPFPAIMDPLPLRTKNGTSGKDSATTSTLSKDSLNPSSLLAAMMQQDESIYLYPASSTSSTAPFENNFFNESMNECRNWQDNTAPMGNDTILKHEQIDQPQDVNSFAGGHPGLFQDSKNSDLYSIMKNLGIDFEDIRHMQNEKFFRNDFSGEVDFRDIDLTDEILTYVQDSLSKSPFIPSDYQQQQSLALNSSCMVQEHLHLEQQQQHHQKQVVVEPQQQLCQKMKHMQVNGMFENWNSNQFVPFNCPQQDPQQYNVFTDLHGISQEFPYKSEMDSMPYTQNFISCNQPVLPQHSKCTELDYPMGSFEPSPYPTTSSLEDFVTCLQLPENQKHGLNPQSAIITPQTCYAGAVSMYQCQPEPQHTHVGQMQYNPVLPGQQAFLNKFQNGVLNETYPAELNNINNTQTTTHLQPLHHPSEARPFPDLTSSGFL

>NP_004119.1

MAERGELDLTGAKQNTGVWLVKVPKYLSQQWAKASGRGEVGKLRIAKTQGRTEVSFTLNEDLANIHDIGGKPASVSAPREHPFVLQSVGGQTLTVFTESSSDKLSLEGIVVQRAECRPAASENYMRLKRLQIEESSKPVRLSQQLDKVVTTNYKPVANHQYNIEYERKKKEDGKRARADKQHVLDMLFSAFEKHQYYNLKDLVDITKQPVVYLKEILKEIGVQNVKGIHKNTWELKPEYRHYQGEEKSD

108 NP_003073.1 NP_003075.1

>NP_003073.1

MGTPPGLQTDCEALLSRFQETDSVRFEDFTELWRNMKFGTIFCGRMRNLEKNMFTKEALALAWRYFLPPYTFQIRVGALYLLYGLYNTQLCQPKQKIRVALKDWDEVLKFQQDLVNAQHFDAAYIFRKLRLDRAFHFTAMPKLLSYRMKKKIHRAEVTEEFKDPSDRVMKLITSDVLEEMLNVHDHYQNMKHVISVDKSKPDKALSLIKDDFFDNIKNIVLEHQQWHKDRKNPSLKSKTNDGEEKMEGNSQETERCERAESLAKIKSKAFSVVIQASKSRRHRQVKLDSSDSDSASGQGQVKATRKKEKKERLKPAGRKMSLRNKGNVQNIHKEDKPLSLSMPVITEEEENESLSGTEFTASKKRRKH

>NP_003075.1

MAEGSRGGPTCSGVGGRQDPVSGSGGCNFPEYELPELNTRAFHVGAFGELWRGRLRGAGDLSLREPPASALPGSQAADSDREDAAVARDLDCSLEAAAELRAVCGLDKLKCLEDGEDPEVIPENTDLVTLGVRKRFLEHREETITIDRACRQETFVYEMESHAIGKKPENSADMIEEGELILSVNILYPVIFHKHKEHKPYQTMLVLGSQKLTQLRDSIRCVSDLQIGGEFSNTPDQAPEHISKDLYKSAFFYFEGTFYNDKRYPECRDLSRTIIEWSESHDRGYGKFQTARMEDFTFNDLCIKLGFPYLYCHQGDCEHVIVITDIRLVHHDDCLDRTLYPLLIKKHWLWTRKCFVCKMYTARWVTNNDSFAPEDPCFFCDVCFRMLHYDSEGNKLGEFLAYPYVDPGTFN

109 NP_009210.1 NP_006749.1

>NP_009210.1

MSDFDEFERQLNENKQERDKENRHRKRSHSRSRSRDRKRRSRSRDRRNRDQRSASRDRRRRSKPLTRGAKEEHGGLIRSPRHEKKKKVRKYWDVPPPGFEHITPMQYKAMQAAGQIPATALLPTMTPDGLAVTPTPVPVVGSQMTRQARRLYVGNIPFGITEEAMMDFFNAQMRLGGLTQAPGNPVLAVQINQDKNFAFLEFRSVDETTQAMAFDGIIFQGQSLKIRRPHDYQPLPGMSENPSVYVPGVVSTVVPDSAHKLFIGGLPNYLNDDQVKELLTSFGPLKAFNLVKDSATGLSKGYAFCEYVDINVTDQAIAGLNGMQLGDKKLLVQRASVGAKNATLVSPPSTINQTPVTLQVPGLMSSQVQMGGHPTEVLCLMNMVLPEELLDDEEYEEIVEDVRDECSKYGLVKSIEIPRPVDGVEVPGCGKIFVEFTSVFDCQKAMQGLTGRKFANRVVVTKYCDPDSYHRRDFW

>NP_006749.1

MAEYLASIFGTEKDKVNCSFYFKIGACRHGDRCSRLHNKPTFSQTIALLNIYRNPQNSSQSADGLRCAVSDVEMQEHYDEFFEEVFTEMEEKYGEVEEMNVCDNLGDHLVGNVYVKFRREEDAEKAVIDLNNRWFNGQPIHAELSPVTDFREACCRQYEMGECTRGGFCNFMHLKPISRELRRELYGRRRKKHRSRSRSRERRSRSRDRGRGGGGGGGGGGGGRERDRRRSRDRERSGRF

110 NP_000724.1 NP_001059.2

>NP_000724.1

MQSGTHWRVLGLCLLSVGVWGQDGNEEMGGITQTPYKVSISGTTVILTCPQYPGSEILWQHNDKNIGGDEDDKNIGSDEDHLSLKEFSELEQSGYYVCYPRGSKPEDANFYLYLRARVCENCMEMDVMSVATIVIVDICITGGLLLLVYYWSKNRKAKAKPVTRGAGAGGRQRGQNKERPPPVPNPDYEPIRKGQRDLYSGLNQRRI

>NP_001059.2

MAKSGGCGAGAGVGGGNGALTWVNNAAKKEESETANKNDSSKKLSVERVYQKKTQLEHILLRPDTYIGSVEPLTQFMWVYDEDVGMNCREVTFVPGLYKIFDEILVNAADNKQRDKNMTCIKVSIDPESNIISIWNNGKGIPVVEHKVEKVYVPALIFGQLLTSSNYDDDEKKVTGGRNGYGAKLCNIFSTKFTVETACKEYKHSFKQTWMNNMMKTSEAKIKHFDGEDYTCITFQPDLSKFKMEKLDKDIVALMTRRAYDLAGSCRGVKVMFNGKKLPVNGFRSYVDLYVKDKLDETGVALKVIHELANERWDVCLTLSEKGFQQISFVNSIATTKGGRHVDYVVDQVVGKLIEVVKKKNKAGVSVKPFQVKNHIWVFINCLIENPTFDSQTKENMTLQPKSFGSKCQLSEKFFKAASNCGIVESILNWVKFKAQTQLNKKCSSVKYSKIKGIPKLDDANDAGGKHSLECTLILTEGDSAKSLAVSGLGVIGRDRYGVFPLRGKILNVREASHKQIMENAEINNIIKIVGLQYKKSYDDAESLKTLRYGKIMIMTDQDQDGSHIKGLLINFIHHNWPSLLKHGFLEEFITPIVKASKNKQELSFYSIPEFDEWKKHIENQKAWKIKYYKGLGTSTAKEAKEYFADMERHRILFRYAGPEDDAAITLAFSKKKIDDRKEWLTNFMEDRRQRRLHGLPEQFLYGTATKHLTYNDFINKELILFSNSDNERSIPSLVDGFKPGQRKVLFTCFKRNDKREVKVAQLAGSVAEMSAYHHGEQALMMTIVNLAQNFVGSNNINLLQPIGQFGTRLHGGKDAASPRYIFTMLSTLARLLFPAVDDNLLKFLYDDNQRVEPEWYIPIIPMVLINGAEGIGTGWACKLPNYDAREIVNNVRRMLDGLDPHPMLPNYKNFKGTIQELGQNQYAVSGEIFVVDRNTVEITELPVRTWTQVYKEQVLEPMLNGTDKTPALISDYKEYHTDTTVKFVVKMTEEKLAQAEAAGLHKVFKLQTTLTCNSMVLFDHMGCLKKYETVQDILKEFFDLRLSYYGLRKEWLVGMLGAESTKLNNQARFILEKIQGKITIENRSKKDLIQMLVQRGYESDPVKAWKEAQEKAAEEDETQNQHDDSSSDSGTPSGPDFNYILNMSLWSLTKEKVEELIKQRDAKGREVNDLKRKSPSDLWKEDLAAFVEELDKVESQEREDVLAGMSGKAIKGKVGKPKVKKLQLEETMPSPYGRRIIPEITAMKADASKKLLKKKKGDLDTAAVKVEFDEEFSGAPVEGAGEEALTPSVPINKGPKPKREKKEPGTRVRKTPTSSGKPSAKKVKKRNPWSDDESKSESDLEETEPVVIPRDSLLRRAAAERPKYTFDFSEEEDDDADDDDDDNNDLEELKVKASPITNDGEDEFVPSDGLDKDEYTFSPGKSKATPEKSLHDKKSQDFGNLFSFPSYSQKSEDDSAKFDSNEEDSASVFSPSFGLKQTDKVPSKTVAAKKGKPSSDTVPKPKRAPKQKKVVEAVNSDSDSEFGIPKKTTTPKGKGRGAKKRKASGSENEGDYNPGRKTSKTTSKKPKKTSFDQDSDVDIFPSDFPTEPPSLPRTGRARKEVKYFAESDEEEDDVDFAMFN

111 NP_005556.1 NP_037446.1

>NP_005556.1

MALRNVPFRSEVLGWDPDSLADYFKKLNYKDCEKAVKKYHIDGARFLNLTENDIQKFPKLRVPILSKLSQEINKNEERRSIFTRKPQVPRFPEETESHEEDNGGWSSFEEDDYESPNDDQDGEDDGDYESPNEEEEAPVEDDADYEPPPSNDEEALQNSILPAKPFPNSNSMYIDRPPSGKTPQQPPVPPQRPMAALPPPPAGRNHSPLPPPQTNHEEPSRSRNHKTAKLPAPSIDRSTKPPLDRSLAPFDREPFTLGKKPPFSDKPSIPAGRSLGEHLPKIQKPPLPPTTERHERSSPLPGKKPPVPKHGWGPDRRENDEDDVHQRPLPQPALLPMSSNTFPSRSTKPSPMNPLPSSHMPGAFSESNSSFPQSASLPPYFSQGPSNRPPIRAEGRNFPLPLPNKPRPPSPAEEENSLNEEWYVSYITRPEAEAALRKINQDGTFLVRDSSKKTTTNPYVLMVLYKDKVYNIQIRYQKESQVYLLGTGLRGKEDFLSVSDIIDYFRKMPLLLIDGKNRGSRYQCTLTHAAGYP

>NP_037446.1

MDKLNKITVPASQKLRQLQKMVHDIKNNEGGIMNKIKKLKVKAPPSVPRRDYASESPADEEEQWSDDFDSDYENPDEHSDSEMYVMPAEENADDSYEPPPVEQETRPVHPALPFARGEYIDNRSSQRHSPPFSKTLPSKPSWPSEKARLTSTLPALTALQKPQVPPKPKGLLEDEADYVVPVEDNDENYIHPTESSSPPPEKAPMVNRSTKPNSSTPASPPGTASGRNSGAWETKSPPPAAPSPLPRAGKKPTTPLKTTPVASQQNASSVCEEKPIPAERHRGSSHRQEAVQSPVFPPAQKQIHQKPIPLPRFTEGGNPTVDGPLPSFSSNSTISEQEAGVLCKPWYAGACDRKSAEEALHRSNKDGSFLIRKSSGHDSKQPYTLVVFFNKRVYNIPVRFIEATKQYALGRKKNGEEYFGSVAEIIRNHQHSPLVLIDSQNNTKDSTRLKYAVKVS

112 NP_003854.1 NP_002632.1

>NP_003854.1

MATGTDQVVGLGLVAVSLIIFTYYTAWVILLPFIDSQHVIHKYFLPRAYAVAIPLAAGLLLLLFVGLFISYVMLKTKRVTKKAQ

>NP_002632.1

MACRGGAGNGHRASATLSRVSPGSLYTCRTRTHNICMVSDFFYPNMGGVESHIYQLSQCLIERGHKVIIVTHAYGNRKGIRYLTSGLKVYYLPLKVMYNQSTATTLFHSLPLLRYIFVRERVTIIHSHSSFSAMAHDALFHAKTMGLQTVFTDHSLFGFADVSSVLTNKLLTVSLCDTNHIICVSYTSKENTVLRAALNPEIVSVIPNAVDPTDFTPDPFRRHDSITIVVVSRLVYRKGIDLLSGIIPELCQKYPDLNFIIGGEGPKRIILEEVRERYQLHDRVRLLGALEHKDVRNVLVQGHIFLNTSLTEAFCMAIVEAASCGLQVVSTRVGGIPEVLPENLIILCEPSVKSLCEGLEKAIFQLKSGTLPAPENIHNIVKTFYTWRNVAERTEKVYDRVSVEAVLPMDKRLDRLISHCGPVTGYIFALLAVFNFLFLIFLRWMTPDSIIDVAIDATGPRGAWTNNYSHSKRGGENNEISETR

113 P15941 NP_000029.2

>P15941

MTPGTQSPFFLLLLLTVLTVVTGSGHASSTPGGEKETSATQRSSVPSSTEKNAVSMTSSVLSSHSPGSGSSTTQGQDVTLAPATEPASGSAATWGQDVTSVPVTRPALGSTTPPAHDVTSAPDNKPAPGSTAPPAHGVTSAPDTRPAPGSTAPPAHGVTSAPDTRPAPGSTAPPAHGVTSAPDTRPAPGSTAPPAHGVTSAPDTRPAPGSTAPPAHGVTSAPDTRPAPGSTAPPAHGVTSAPDTRPAPGSTAPPAHGVTSAPDTRPAPGSTAPPAHGVTSAPDTRPAPGSTAPPAHGVTSAPDTRPAPGSTAPPAHGVTSAPDTRPAPGSTAPPAHGVTSAPDTRPAPGSTAPPAHGVTSAPDTRPAPGSTAPPAHGVTSAPDTRPAPGSTAPPAHGVTSAPDTRPAPGSTAPPAHGVTSAPDTRPAPGSTAPPAHGVTSAPDTRPAPGSTAPPAHGVTSAPDTRPAPGSTAPPAHGVTSAPDTRPAPGSTAPPAHGVTSAPDTRPAPGSTAPPAHGVTSAPDTRPAPGSTAPPAHGVTSAPDTRPAPGSTAPPAHGVTSAPDTRPAPGSTAPPAHGVTSAPDTRPAPGSTAPPAHGVTSAPDTRPAPGSTAPPAHGVTSAPDTRPAPGSTAPPAHGVTSAPDTRPAPGSTAPPAHGVTSAPDTRPAPGSTAPPAHGVTSAPDTRPAPGSTAPPAHGVTSAPDTRPAPGSTAPPAHGVTSAPDTRPAPGSTAPPAHGVTSAPDTRPAPGSTAPPAHGVTSAPDTRPAPGSTAPPAHGVTSAPDTRPAPGSTAPPAHGVTSAPDTRPAPGSTAPPAHGVTSAPDTRPAPGSTAPPAHGVTSAPDTRPAPGSTAPPAHGVTSAPDTRPAPGSTAPPAHGVTSAPDTRPAPGSTAPPAHGVTSAPDTRPAPGSTAPPAHGVTSAPDTRPAPGSTAPPAHGVTSAPDNRPALGSTAPPVHNVTSASGSASGSASTLVHNGTSARATTTPASKSTPFSIPSHHSDTPTTLASHSTKTDASSTHHSSVPPLTSSNHSTSPQLSTGVSFFFLSFHISNLQFNSSLEDPSTDYYQELQRDISEMFLQIYKQGGFLGLSNIKFRPGSVVVQLTLAFREGTINVHDVETQFNQYKTEAASRYNLTISDVSVSDVPFPFSAQSGAGVPGWGIALLVLVCVLVALAIVYLIALAVCQCRRKNYGQLDIFPARDTYHPMSEYPTYHTHGRYVPPSSTDRSPYEKVSAGNGGSSLSYTNPAVAAASANL

>NP_000029.2

MAAASYDQLLKQVEALKMENSNLRQELEDNSNHLTKLETEASNMKEVLKQLQGSIEDEAMASSGQIDLLERLKELNLDSSNFPGVKLRSKMSLRSYGSREGSVSSRSGECSPVPMGSFPRRGFVNGSRESTGYLEELEKERSLLLADLDKEEKEKDWYYAQLQNLTKRIDSLPLTENFSLQTDMTRRQLEYEARQIRVAMEEQLGTCQDMEKRAQRRIARIQQIEKDILRIRQLLQSQATEAERSSQNKHETGSHDAERQNEGQGVGEINMATSGNGQGSTTRMDHETASVLSSSSTHSAPRRLTSHLGTKVEMVYSLLSMLGTHDKDDMSRTLLAMSSSQDSCISMRQSGCLPLLIQLLHGNDKDSVLLGNSRGSKEARARASAALHNIIHSQPDDKRGRREIRVLHLLEQIRAYCETCWEWQEAHEPGMDQDKNPMPAPVEHQICPAVCVLMKLSFDEEHRHAMNELGGLQAIAELLQVDCEMYGLTNDHYSITLRRYAGMALTNLTFGDVANKATLCSMKGCMRALVAQLKSESEDLQQVIASVLRNLSWRADVNSKKTLREVGSVKALMECALEVKKESTLKSVLSALWNLSAHCTENKADICAVDGALAFLVGTLTYRSQTNTLAIIESGGGILRNVSSLIATNEDHRQILRENNCLQTLLQHLKSHSLTIVSNACGTLWNLSARNPKDQEALWDMGAVSMLKNLIHSKHKMIAMGSAAALRNLMANRPAKYKDANIMSPGSSLPSLHVRKQKALEAELDAQHLSETFDNIDNLSPKASHRSKQRHKQSLYGDYVFDTNRHDDNRSDNFNTGNMTVLSPYLNTTVLPSSSSSRGSLDSSRSEKDRSLERERGIGLGNYHPATENPGTSSKRGLQISTTAAQIAKVMEEVSAIHTSQEDRSSGSTTELHCVTDERNALRRSSAAHTHSNTYNFTKSENSNRTCSMPYAKLEYKRSSNDSLNSVSSSDGYGKRGQMKPSIESYSEDDESKFCSYGQYPADLAHKIHSANHMDDNDGELDTPINYSLKYSDEQLNSGRQSPSQNERWARPKHIIEDEIKQSEQRQSRNQSTTYPVYTESTDDKHLKFQPHFGQQECVSPYRSRGANGSETNRVGSNHGINQNVSQSLCQEDDYEDDKPTNYSERYSEEEQHEEEERPTNYSIKYNEEKRHVDQPIDYSLKYATDIPSSQKQSFSFSKSSSGQSSKTEHMSSSSENTSTPSSNAKRQNQLHPSSAQSRSGQPQKAATCKVSSINQETIQTYCVEDTPICFSRCSSLSSLSSAEDEIGCNQTTQEADSANTLQIAEIKEKIGTRSAEDPVSEVPAVSQHPRTKSSRLQGSSLSSESARHKAVEFSSGAKSPSKSGAQTPKSPPEHYVQETPLMFSRCTSVSSLDSFESRSIASSVQSEPCSGMVSGIISPSDLPDSPGQTMPPSRSKTPPPPPQTAQTKREVPKNKAPTAEKRESGPKQAAVNAAVQRVQVLPDADTLLHFATESTPDGFSCSSSLSALSLDEPFIQKDVELRIMPPVQENDNGNETESEQPKESNENQEKEAEKTIDSEKDLLDDSDDDDIEILEECIISAMPTKSSRKAKKPAQTASKLPPPVARKPSQLPVYKLLPSQNRLQPQKHVSFTPGDDMPRVYCVEGTPINFSTATSLSDLTIESPPNELAAGEGVRGGAQSGEFEKRDTIPTEGRSTDEAQGGKTSSVTIPELDDNKAEEGDILAECINSAMPKGKSHKPFRVKKIMDQVQQASASSSAPNKNQLDGKKKKPTSPVKPIPQNTEYRTRVRKNADSKNNLNAERVFSDNKDSKKQNLKNNSKVFNDKLPNNEDRVRGSFAFDSPHHYTPIEGTPYCFSRNDSLSSLDFDDDDVDLSREKAELRKAKENKESEAKVTSHTELTSNQQSANKTQAIAKQPINRGQPKPILQKQSTFPQSSKDIPDRGAATDEKLQNFAIENTPVCFSHNSSLSSLSDIDQENNNKENEPIKETEPPDSQGEPSKPQASGYAPKSFHVEDTPVCFSRNSSLSSLSIDSEDDLLQECISSAMPKKKKPSRLKGDNEKHSPRNMGGILGEDLTLDLKDIQRPDSEHGLSPDSENFDWKAIQEGANSIVSSLHQAAAAACLSRQASSDSDSILSLKSGISLGSPFHLTPDQEEKPFTSNKGPRILKPGEKSTLETKKIESESKGIKGGKKVYKSLITGKVRSNSEISGQMKQPLQANMPSISRGRTMIHIPGVRNSSSSTSPVSKKGPPLKTPASKSPSEGQTATTSPRGAKPSVKSELSPVARQTSQIGGSSKAPSRSGSRDSTPSRPAQQPLSRPIQSPGRNSISPGRNGISPPNKLSQLPRTSSPSTASTKSSGSGKMSYTSPGRQMSQQNLTKQTGLSKNASSIPRSESASKGLNQMNNGNGANKKVELSRMSSTKSSGSESDRSERPVLVRQSTFIKEAPSPTLRRKLEESASFESLSPSSRPASPTRSQAQTPVLSPSLPDMSLSTHSSVQAGGWRKLPPNLSPTIEYNDGRPAKRHDIARSHSESPSRLPINRSGTWKREHSKHSSSLPRVSTWRRTGSSSSILSASSESSEKAKSEDEKHVNSISGTKQSKENQVSAKGTWRKIKENEFSPTNSTSQTVSSGATNGAESKTLIYQMAPAVSKTEDVWVRIEDCPINNPRSGRSPTGNTPPVIDSVSEKANPNIKDSKDNQAKQNVGNGSVPMRTVGLENRLNSFIQVDAPDQKGTEIKPGQNNPVPVSETNESSIVERTPFSSSSSSKHSSPSGTVAARVTPFNYNPSPRKSSADSTSARPSQIPTPVNNNTKKRDSKTDSTESSGTQSPKRHSGSYLVTSV

114 NP_003602.1 NP_065853.2

>NP_003602.1

MMAQSNMFTVADVLSQDELRKKLYQTFKDRGILDTLKTQLRNQLIHELMHPVLSGELQPRSISVEGSSLLIGASNSLVADHLQRCGYEYSLSVFFPESGLAKEKVFTMQDLLQLIKINPTSSLYKSLVSGSDKENQKGFLMHFLKELAEYHQAKESCNMETQTSSTFNRDSLAEKLQLIDDQFADAYPQRIKFESLEIKLNEYKREIEEQLRAEMCQKLKFFKDTEIAKIKMEAKKKYEKELTMFQNDFEKACQAKSEALVLREKSTLERIHKHQEIETKEIYAQRQLLLKDMDLLRGREAELKQRVEAFELNQKLQEEKHKSITEALRRQEQNIKSFEETYDRKLKNELLKYQLELKDDYIIRTNRLIEDERKNKEKAVHLQEELIAINSKKEELNQSVNRVKELELELESVKAQSLAITKQNHMLNEKVKEMSDYSLLKEEKLELLAQNKLLKQQLEESRNENLRLLNRLAQPAPELAVFQKELRKAEKAIVVEHEEFESCRQALHKQLQDEIEHSAQLKAQILGYKASVKSLTTQVADLKLQLKQTQTALENEVYCNPKQSVIDRSVNGLINGNVVPCNGEISGDFLNNPFKQENVLARMVASRITNYPTAWVEGSSPDSDLEFVANTKARVKELQQEAERLEKAFRSYHRRVIKNSAKSPLAAKSPPSLHLLEAFKNITSSSPERHIFGEDRVVSEQPQVGTLEERNDVVEALTGSAASRLRGGTSSRRLSSTPLPKAKRSLESEMYLEGLGRSHIASPSPCPDRMPLPSPTESRHSLSIPPVSSPPEQKVGLYRRQTELQDKSEFSDVDKLAFKDNEEFESSFESAGNMPRQLEMGGLSPAGDMSHVDAAAAAVPLSYQHPSVDQKQIEEQKEEEKIREQQVKERRQREERRQSNLQEVLERERRELEKLYQERKMIEESLKIKIKKELEMENELEMSNQEIKDKSAHSENPLEKYMKIIQQEQDQESADKSSKKMVQEGSLVDTLQSSDKVESLTGFSHEELDDSW

>NP_065853.2

MLAGRPGTRSAVGELGTESSDNLDRAPLGPRESGGHHRPGSYLDMKIHLEKNLEEERQILLQQQKICRNRARKYFVESNRRKKAFEEKRKEQEEKEHQIREQILQQRKQKFEEVTEKFQRAHVPLSQRRKAVSRKPVPPLEEALKQIQESNLKSEVNLPFSRRPTINWRAIDSALPSALSKNDHKHQKQLLSKINCEKEMNENMRATLATSKNVFQLKLEETQKLLEDQHLSNLQKFGDEVNQITNSETLSSIDSLEATEHEEIYLTLNKEHSTSIQRNTISLKPANMQSTNLSCFDEDKLAFSKTQHINNWLTNLDASNTQNVTAFSDILSKSNVLPSWEYFNSKEQNPSPLNGTVERATNTANNSVPFVSSPPMFVLDKKCEKTSETSTMRTTDSTSGAFKRERPLVTESPTFKFSKSQSTSDSLTQEVATFPDQEKYSELNQENGTTSIPTSCVPVATPLVLPSNIQSARPSAKNSIHIKEIDAVQCSDKLDELKDGKEEEIKYFNCNKEELPLFSDSFQDAYIPHNPDSKDEKQKLAETSSLSNVTSNYDFVGQHKKMKYNIHERNGVRFLKSILKKESKYEHGYLKALIINQSFKFGNQKAAAIRDSIELTKEKGAEIPKTIKKLRWFDETSNIENNAENSHSLKNKTGTTQQHSQQFHIQSGAGSNIISVSTCAVNSADTKKSREDSISENVTTLGGSGADHMPLNCFIPSGYNFAKHAWPASKKEESKIPVHDDSKTKQGKPQRGRAKIIRKPGSAKVQSGFICTNRKGAVIQPQSASKVNIFTQAQGKLIIPCPPPQSTSNIRSGKNIQVSQCQPVTPENPQNIITHNSFNSKHVLPTEHSLNQWNQESSSPLSNACSDLVTVIPSLPSYCSSECQTFAKINHSNGTQAVARQDATLYCTQRSPVCEESYPSVTLRTAEEESVPLWKRGPNVLHQNKRATGSTVMRRKRIAETKRRNILEQKRQNPGSVGQKYSEQINNFGQSVLLSSSEPKQTTRGTSYIEEVSDSTSEFLMAENLVKASVPEDEILTVLNSKQIQKSNLPLNKTQQFNICTLSAEEQKILESLNDLSERLHYIQESICKNPSIKNTLQIIPLLEKREDRTSSCRDKR

115 NP_061960.1 NP_068597.2

>NP_061960.1

MSDLRITEAFLYMDYLCFRALCCKGPPPARPEYDLVCIGLTGSGKTSLLSKLCSESPDNVVSTTGFSIKAVPFQNAILNVKELGGADNIRKYWSRYYQGSQGVIFVLDSASSEDDLEAARNELHSALQHPQLCTLPFLILANHQDKPAARSVQEIKKYFELEPLARGKRWILQPCSLDDMDALKDSFSQLINLLEEKDHEAVRM

>NP_068597.2

MNSRQAWRLFLSQGRGDRWVSRPRGHFSPALRREFFTTTTKEGYDRRPVDITPLEQRKLTFDTHALVQDLETHGFDKTQAETIVSALTALSNVSLDTIYKEMVTQAQQEITVQQLMAHLDAIRKDMVILEKSEFANLRAENEKMKIELDQVKQQLMHETSRIRADNKLDINLERSRVTDMFTDQEKQLMETTTEFTKKDTQTKSIISETSNKIDAEIASLKTLMESNKLETIRYLAASVFTCLAIALGFYRFWK

116 NP_066267.2 NP_950238.1

>NP_066267.2

MAHAASQLKKNRDLEINAEEEPEKKRKHRKRSRDRKKKSDANASYLRAARAGHLEKALDYIKNGVDINICNQNGLNALHLASKEGHVEVVSELLQREANVDAATKKGNTALHIASLAGQAEVVKVLVTNGANVNAQSQNGFTPLYMAAQENHLEVVKFLLDNGASQSLATEDGFTPLAVALQQGHDQVVSLLLENDTKGKVRLPALHIAARKDDTKAAALLLQNDNNADVESKSGFTPLHIAAHYGNINVATLLLNRAAAVDFTARNDITPLHVASKRGNANMVKLLLDRGAKIDAKTRDGLTPLHCGARSGHEQVVEMLLDRAAPILSKTKNGLSPLHMATQGDHLNCVQLLLQHNVPVDDVTNDYLTALHVAAHCGHYKVAKVLLDKKANPNAKALNGFTPLHIACKKNRIKVMELLLKHGASIQAVTESGLTPIHVAAFMGHVNIVSQLMHHGASPNTTNVRGETALHMAARSGQAEVVRYLVQDGAQVEAKAKDDQTPLHISARLGKADIVQQLLQQGASPNAATTSGYTPLHLSAREGHEDVAAFLLDHGASLSITTKKGFTPLHVAAKYGKLEVANLLLQKSASPDAAGKSGLTPLHVAAHYDNQKVALLLLDQGASPHAAAKNGYTPLHIAAKKNQMDIATTLLEYGADANAVTRQGIASVHLAAQEGHVDMVSLLLGRNANVNLSNKSGLTPLHLAAQEDRVNVAEVLVNQGAHVDAQTKMGYTPLHVGCHYGNIKIVNFLLQHSAKVNAKTKNGYTPLHQAAQQGHTHIINVLLQNNASPNELTVNGNTALGIARRLGYISVVDTLKIVTEETMTTTTVTEKHKMNVPETMNEVLDMSDDEVRKANAPEMLSDGEYISDVEEGEDAMTGDTDKYLGPQDLKELGDDSLPAEGYMGFSLGARSASLRSFSSDRSYTLNRSSYARDSMMIEELLVPSKEQHLTFTREFDSDSLRHYSWAADTLDNVNLVSSPIHSGFLVSFMVDARGGSMRGSRHHGMRIIIPPRKCTAPTRITCRLVKRHKLANPPPMVEGEGLASRLVEMGPAGAQFLGPVIVEIPHFGSMRGKERELIVLRSENGETWKEHQFDSKNEDLTELLNGMDEELDSPEELGKKRICRIITKDFPQYFAVVSRIKQESNQIGPEGGILSSTTVPLVQASFPEGALTKRIRVGLQAQPVPDEIVKKILGNKATFSPIVTVEPRRRKFHKPITMTIPVPPPSGEGVSNGYKGDTTPNLRLLCSITGGTSPAQWEDITGTTPLTFIKDCVSFTTNVSARFWLADCHQVLETVGLATQLYRELICVPYMAKFVVFAKMNDPVESSLRCFCMTDDKVDKTLEQQENFEEVARSKDIEVLEGKPIYVDCYGNLAPLTKGGQQLVFNFYSFKENRLPFSIKIRDTSQEPCGRLSFLKEPKTTKGLPQTAVCNLNITLPAHKKETESDQDDEIEKTDRRQSFASLALRKRYSYLTEPGMIERSTGATRSLPTTYSYKPFFSTRPYQSWTTAPITVPGPAKSGFTSLSSSSSNTPSASPLKSIWSVSTPSPIKSTLGASTTSSVKSISDVASPIRSFRTMSSPIKTVVSQSPYNIQVSSGTLARAPAVTEATPLKGLASNSTFSSRTSPVTTAGSLLERSSITMTPPASPKSNINMYSSSLPFKSIITSAAPLISSPLKSVVSPVKSAVDVISSAKITMASSLSSPVKQMPGHAEVALVNGSISPLKYPSSSTLINGCKATATLQEKISSATNSVSSVVSAATDTVEKVFSTTTAMPFSPLRSYVSAAPSAFQSLRTPSASALYTSLGSSISATTSSVTSSIITVPVYSVVNVLPEPALKKLPDSNSFTKSAAALLSPIKTLTTETHPQPHFSRTSSPVKSSLFLAPSALKLSTPSSLSSSQEILKDVAEMKEDLMRMTAILQTDVPEEKPFQPELPKEGRIDDEEPFKIVEKVKEDLVKVSEILKKDVCVDNKGSPKSPKSDKGHSPEDDWIEFSSEEIREARQQAAASQSPSLPERVQVKAKAASEKDYNLTKVIDYLTNDIGSSSLTNLKYKFEDAKKDGEERQKRVLKPAIALQEHKLKMPPASMRTSTSEKELCKMADSFFGTDTILESPDDFSQHDQDKSPLSDSGFETRSEKTPSAPQSAESTGPKPLFHEVPIPPVITETRTEVVHVIRSYDPSAGDVPQTQPEEPVSPKPSPTFMELEPKPTTSSIKEKVKAFQMKASSEEDDHNRVLSKGMRVKEETHITTTTRMVYHSPPGGEGASERIEETMSVHDIMKAFQSGRDPSKELAGLFEHKSAVSPDVHKSAAETSAQHAEKDNQMKPKLERIIEVHIEKGNQAEPTEVIIRETKKHPEKEMYVYQKDLSRGDINLKDFLPEKHDAFPCSEEQGQQEEEELTAEESLPSYLESSRVNTPVSQEEDSRPSSAQLISDDSYKTLKLLSQHSIEYHDDELSELRGESYRFAEKMLLSEKLDVSHSDTEESVTDHAGPPSSELQGSDKRSREKIATAPKKEILSKIYKDVSENGVGKVSKDEHFDKVTVLHYSGNVSSPKHAMWMRFTEDRLDRGREKLIYEDRVDRTVKEAEEKLTEVSQFFRDKTEKLNDELQSPEKKARPKNGKEYSSQSPTSSSPEKVLLTELLASNDEWVKARQHGPDGQGFPKAEEKAPSLPSSPEKMVLSQQTEDSKSTVEAKGSISQSKAPDGPQSGFQLKQSKLSSIRLKFEQGTHAKSKDMSQEDRKSDGQSRIPVKKIQESKLPVYQVFAREKQQKAIDLPDESVSVQKDFMVLKTKDEHAQSNEIVVNDSGSDNVKKQRTEMSSKAMPDSFSEQQAKDLACHITSDLATRGPWDKKVFRTWESSGATNNKSQKEKLSHVLVHDVRENHIGHPESKSVDQKNEFMSVTERERKLLTNGSLSEIKEMTVKSPSKKVLYREYVVKEGDHPGGLLDQPSRRSESSAVSHIPVRVADERRMLSSNIPDGFCEQSAFPKHELSQKLSQSSMSKETVETQHFNSIEDEKVTYSEISKVSKHQSYVGLCPPLEETETSPTKSPDSLEFSPGKESPSSDVFDHSPIDGLEKLAPLAQTEGGKEIKTLPVYVSFVQVGKQYEKEIQQGGVKKIISQECKTVQETRGTFYTTRQQKQPPSPQGSPEDDTLEQVSFLDSSGKSPLTPETPSSEEVSYEFTSKTPDSLIAYIPGKPSPIPEVSEESEEEEQAKSTSLKQTTVEETAVEREMPNDVSKDSNQRPKNNRVAYIEFPPPPPLDADQIESDKKHHYLPEKEVDMIEVNLQDEHDKYQLAEPVIRVQPPSPVPPGADVSDSSDDESIYQPVPVKKYTFKLKEVDDEQKEKPKASAEKASNQKELESNGSGKDNEFGLGLDSPQNEIAQNGNNDQSITECSIATTAEFSHDTDATEIDSLDGYDLQDEDDGLTESDSKLPIQAMEIKKDIWNTEGILKPADRSFSQSKLEVIEEEGKVGPDEDKPPSKSSSSEKTPDKTDQKSGAQFFTLEGRHPDRSVFPDTYFSYKVDEEFATPFKTVATKGLDFDPWSNNRGDDEVFDSKSREDETKPFGLAVEDRSPATTPDTTPARTPTDESTPTSEPNPFPFHEGKMFEMTRSGAIDMSKRDFVEERLQFFQIGEHTSEGKSGDQGEGDKSMVTATPQPQSGDTTVETNLERNVETPTVEPNPSIPTSGECQEGTSSSGSLEKSAAATNTSKVDPKLRTPIKMGISASTMTMKKEGPGEITDKIEAVMTSCQGLENETITMISNTANSQMGVRPHEKHDFQKDNFNNNNNLDSSTIQTDNIMSNIVLTEHSAPTCTTEKDNPVKVSSGKKTGVLQGHCVRDKQKVLGEQQKTKELIGIRQKSKLPIKATSPKDTFPPNHMSNTKASKMKQVSQSEKTKALTTSSCVDVKSRIPVKNTHRDNIIAVRKACATQKQGQPEKGKAKQLPSKLPVKVRSTCVTTTTTTATTTTTTTTTTTTSCTVKVRKSQLKEVCKHSIEYFKGISGETLKLVDRLSEEEKKMQSELSDEEESTSRNTSLSETSRGGQPSVTTKSARDKKTEAAPLKSKSEKAGSEKRSSRRTGPQSPCERTDIRMAIVADHLGLSWTELARELNFSVDEINQIRVENPNSLISQSFMLLKKWVTRDGKNATTDALTSVLTKINRIDIVTLLEGPIFDYGNISGTRSFADENNVFHDPVDGWQNETSSGNLESCAQARRVTGGLLDRLDDSPDQCRDSITSYLKGEAGKFEANGSHTEITPEAKTKSYFPESQNDVGKQSTKETLKPKIHGSGHVEEPASPLAAYQKSLEETSKLIIEETKPCVPVSMKKMSRTSPADGKPRLSLHEEEGSSGSEQKQGEGFKVKTKKEIRHVEKKSHS

>NP_950238.1

MGRLLALVVGAALVSSACGGCVEVDSETEAVYGMTFKILCISCKRRSETNAETFTEWTFRQKGTEEFVKILRYENEVLQLEEDERFEGRVVWNGSRGTKDLQDLSIFITNVTYNHSGDYECHVYRLLFFENYEHNTSVVKKIHIEVVDKGESGAACPFTVTHRRARWRDRWQAVDRTGWLCAWPANRPQQRAEGEGSSPSCPLQLWPLFLSSPRRGQSMPVPHRRSGYRTQLCHLCCMTSGRCLLSLSQRVVLGLPGIIIRCVSRGVV

117 NP_001886.1 NP_001960.2

>NP_001886.1

MSGPVPSRARVYTDVNTHRPREYWDYESHVVEWGNQDDYQLVRKLGRGKYSEVFEAINITNNEKVVVKILKPVKKKKIKREIKILENLRGGPNIITLADIVKDPVSRTPALVFEHVNNTDFKQLYQTLTDYDIRFYMYEILKALDYCHSMGIMHRDVKPHNVMIDHEHRKLRLIDWGLAEFYHPGQEYNVRVASRYFKGPELLVDYQMYDYSLDMWSLGCMLASMIFRKEPFFHGHDNYDQLVRIAKVLGTEDLYDYIDKYNIELDPRFNDILGRHSRKRWERFVHSENQHLVSPEALDFLDKLLRYDHQSRLTAREAMEHPYFYTVVKDQARMGSSSMPGGSTPVSSANMMSGISSVPTPSPLGPLAGSPVIAAANPLGMPVPAAAGAQQ

>NP_001960.2

MSVNVNRSVSDQFYRYKMPRLIAKVEGKGNGIKTVIVNMVDVAKALNRPPTYPTKYFGCELGAQTQFDVKNDRYIVNGSHEANKLQDMLDGFIKKFVLCPECENPETDLHVNPKKQTIGNSCKACGYRGMLDTHHKLCTFILKNPPENSDSGTGKKEKEKKNRKGKDKENGSVSSSETPPPPPPPNEINPPPHTMEEEEDDDWGEDTTEEAQRRRMDEISDHAKVLTLSDDLERTIEERVNILFDFVKKKKEEGVIDSSDKEIVAEAERLDVKAMGPLVLTEVLFNEKIREQIKKYRRHFLRFCHNNKKAQRYLLHGLECVVAMHQAQLISKIPHILKEMYDADLLEEEVIISWSEKASKKYVSKELAKEIRVKAEPFIKWLKEAEEESSGGEEEDEDENIEVVYSKAASVPKVETVKSDNKDDDIDIDAI

118 NP_000841.1 NP_060842.3

>NP_000841.1

MSMTLGYWDIRGLAHAIRLLLEYTDSSYEEKKYTMGDAPDYDRSQWLNEKFKLGLDFPNLPYLIDGAHKITQSNAILCYIARKHNLCGETEEEKIRVDILENQAMDVSNQLARVCYSPDFEKLKPEYLEELPTMMQHFSQFLGKRPWFVGDKITFVDFLAYDVLDLHRIFEPNCLDAFPNLKDFISRFEGLEKISAYMKSSRFLPKPLYTRVAVWGNK

>NP_060842.3

MSNNLRRVFLKPAEENSGNASRCVSGCMYQVVQTIGSDGKNLLQLLPIPKSSGNLIPLVQSSVMSDALKGNTGKPVQVTFQTQISSSSTSASVQLPIFQPASSSNYFLTRTVDTSEKGRVTSVGTGNFSSSVSKVQSHGVKIDGLTMQTFAVPPSTQKDSSFIVVNTQSLPVTVKSPVLPSGHHLQIPAHAEVKSVPASSLPPSVQQKILATATTSTSGMVEASQMPTVIYVSPVNTVKNVVTKNFQNIYPKPVTEIAKPVILNTTQIPKNVATETQLKGGQHSQAAPVKWIFQDNLQPFTPSLVPVKSSNNVASKILKTFVDRKNLGDNTINMPPLSTIDPSGTRSKNMPIKDNALVMFNGKVYLLAKKGTDVLPSQIDQQNSVSPDTPVRKDTLQTVSSSPVTEISREVVNIVLAKSKSSQMETKSLSNTQLASMANLRAEKNKVEKPSPSTTNPHMNQSSNYLKQSKTLFTNPIFPVGFSTGHNAPRKVTAVIYARKGSVLQSIEKISSSVDATTVTSQQCVFRDQEPKIHNEMASTSDKGAQGRNDKKDSQGRSNKALHLKSDAEFKKIFGLTKDLRVCLTRIPDHLTSGEGFDSFSSLVKSGTYKETEFMVKEGERKQQNFDKKRKAKTNKKMDHIKKRKTENAYNAIINGEANVTGSQLLSSILPTSDVSQHNILTSHSKTRQEKRTEMEYYTHEKQEKGTLNSNAAYEQSHFFNKNYTEDIFPVTPPELEETIRDEKIRRLKQVLREKEAALEEMRKKMHQK

119 NP_009103.2 NP_004055.1

>NP_009103.2

MAKRNAEKELTDRNWDQEDEAEEVGTFSMASEEVLKNRAIKKAKRRNVGFESDTGGAFKGFKGLVVPSGGGRFSGFGSGAGGKPLEGLSNGNNITSAPPFASAKAAADPKVAFGSLAANGPTTLVDKVSNPKTNGDSQQPSSSGLASSKACVGNAYHKQLAALNCSVRDWIVKHVNTNPLCDLTPIFKDYEKYLANIEQQHGNSGRNSESESNKVAAETQSPSLFGSTKLQQESTFLFHGNKTEDTPDKKMEVASEKKTDPSSLGATSASFNFGKKVDSSVLGSLSSVPLTGFSFSPGNSSLFGKDTTQSKPVSSPFPTKPLEGQAEGDSGECKGGDEEENDEPPKVVVTEVKEEDAFYSKKCKLFYKKDNEFKEKGIGTLHLKPTANQKTQLLVRADTNLGNILLNVLIPPNMPCTRTGKNNVLIVCVPNPPIDEKNATMPVTMLIRVKTSEDADELHKILLEKKDA

>NP_004055.1

MSNVRVSNGSPSLERMDARQAEHPKPSACRNLFGPVDHEELTRDLEKHCRDMEEASQRKWNFDFQNHKPLEGKYEWQEVEKGSLPEFYYRPPRPPKGACKVPAQESQDVSGSRPAAPLIGAPANSEDTHLVDPKTDPSDSQTGLAEQCAGIRKRPATDDSSTQNKRANRTEENVSDGSPNAGSVEQTPKKPGLRRRQT

120 NP_006839.2 NP_078919.2

>NP_006839.2

MEAEAGGLEELTDEEMAALGKEELVRRLRREEAARLAALVQRGRLMQEVNRQLQGHLGEIRELKQLNRRLQAENRELRDLCCFLDSERQRGRRAARQWQLFGTQASRAVREDLGGCWQKLAELEGRQEELLRENLALKELCLALGEEWGPRGGPSGAGGSGAGPAPELALPPCGPRDLGDGSSSTGSVGSPDQLPLACSPDD

>NP_078919.2

MAGLQRLASHLPVGVMLPHNTTEAPGPHSAKQDSYEQGDSSQQSLKGHLRNNFQKQLLSNKELILDKVYTHPKWNTQTKARSYSYPHCTGISQQDPESDSQGQGNGLFYSSGPQSWYPKANNQDFIPFTKKRVGVDRAFPLKPMVHRKSCSTGEAGTDGDHNVYPRPPEPREFSSRNFGVRNQGNFSVVGTVLAATQAEKAVANFDRTEWVQIRRLEAAGESLEEEIRRKQILLRGKLKKTEEELRRIQTQKEQAKENENGELQKIILPRSRVKGNKSNTMYKPIFSPEFEFEEEFSRDRREDETWGRSQQNSGPFQFSDYRIQRLKRERLVASNNKIRDPVSEPSVEKFSPPSETPVGALQGSARNSSLSMAPDSSGSSGSIEEPQLGECSHCGRKFLSFRLERHSNICSRMRGSKRKVFDSSRARAKGTELEQYLNWKGPASAKAEPPQKSNWR

121 NP_004437.2 NP_002878.2

>NP_004437.2

MATLSLTVNSGDPPLGALLAVEHVKDDVSISVEEGKENILHVSENVIFTDVNSILRYLARVATTAGLYGSNLMEHTEIDHWLEFSATKLSSCDSFTSTINELNHCLSLRTYLVGNSLSLADLCVWATLKGNAAWQEQLKQKKAPVHVKRWFGFLEAQQAFQSVGTKWDVSTTKARVAPEKKQDVGKFVELPGAEMGKVTVRFPPEASGYLHIGHAKAALLNQHYQVNFKGKLIMRFDDTNPEKEKEDFEKVILEDVAMLHIKPDQFTYTSDHFETIMKYAEKLIQEGKAYVDDTPAEQMKAEREQRIDSKHRKNPIEKNLQMWEEMKKGSQFGQSCCLRAKIDMSSNNGCMRDPTLYRCKIQPHPRTGNKYNVYPTYDFACPIVDSIEGVTHALRTTEYHDRDEQFYWIIEALGIRKPYIWEYSRLNLNNTVLSKRKLTWFVNEGLVDGWDDPRFPTVRGVLRRGMTVEGLKQFIAAQGSSRSVVNMEWDKIWAFNKKVIDPVAPRYVALLKKEVIPVNVPEAQEEMKEVAKHPKNPEVGLKPVWYSPKVFIEGADAETFSEGEMVTFINWGNLNITKIHKNADGKIISLDAKLNLENKDYKKTTKVTWLAETTHALPIPVICVTYEHLITKPVLGKDEDFKQYVNKNSKHEELMLGDPCLKDLKKGDIIQLQRRGFFICDQPYEPVSPYSCKEAPCVLIYIPDGHTKEMPTSGSKEKTKVEATKNETSAPFKERPTPSLNNNCTTSEDSLVLYNRVAVQGDVVRELKAKKAPKEDVDAAVKQLLSLKAEYKEKTGQEYKPGNPPAEIGQNISSNSSASILESKSLYDEVAAQGEVVRKLKAEKSPKAKINEAVECLLSLKAQYKEKTGKEYIPGQPPLSQSSDSSPTRNSEPAGLETPEAKVLFDKVASQGEVVRKLKTEKAPKDQVDIAVQELLQLKAQYKSLIGVEYKPVSATGAEDKDKKKKEKENKSEKQNKPQKQNDGQRKDPSKNQGGGLSSSGAGEGQGPKKQTRLGLEAKKEENLADWYSQVITKSEMIEYHDISGCYILRPWAYAIWEAIKDFFDAEIKKLGVENCYFPMFVSQSALEKEKTHVADFAPEVAWVTRSGKTELAEPIAIRPTSETVMYPAYAKWVQSHRDLPIKLNQWCNVVRWEFKHPQPFLRTREFLWQEGHSAFATMEEAAEEVLQILDLYAQVYEELLAIPVVKGRKTEKEKFAGGDYTTTIEAFISASGRAIQGGTSHHLGQNFSKMFEIVFEDPKIPGEKQFAYQNSWGLTTRTIGVMTMVHGDNMGLVLPPRVACVQVVIIPCGITNALSEEDKEALIAKCNDYRRRLLSVNIRVRADLRDNYSPGWKFNHWELKGVPIRLEVGPRDMKSCQFVAVRRDTGEKLTVAENEAETKLQAILEDIQVTLFTRASEDLKTHMVVANTMEDFQKILDSGKIVQIPFCGEIDCEDWIKKTTARDQDLEPGAPSMGAKSLCIPFKPLCELQPGAKCVCGKNPAKYYTLFGRSY

>NP_002878.2

MDVLVSECSARLLQQEEEIKSLTAEIDRLKNCGCLGASPNLEQLQEENLKLKYRLNILRKSLQAERNKPTKNMINIISRLQEVFGHAIKAAYPDLENPPLLVTPSQQAKFGDYQCNSAMGISQMLKTKEQKVNPREIAENITKHLPDNECIEKVEIAGPGFINVHLRKDFVSEQLTSLLVNGVQLPALGENKKVIVDFSSPNIAKEMHVGHLRSTIIGESISRLFEFAGYDVLRLNHVGDWGTQFGMLIAHLQDKFPDYLTVSPPIGDLQVFYKESKKRFDTEEEFKKRAYQCVVLLQGKNPDITKAWKLICDVSRQELNKIYDALDVSLIERGESFYQDRMNDIVKEFEDRGFVQVDDGRKIVFVPGCSIPLTIVKSDGGYTYDTSDLAAIKQRLFEEKADMIIYVVDNGQSVHFQTIFAAAQMIGWYDPKVTRVFHAGFGVVLGEDKKKFKTRSGETVRLMDLLGEGLKRSMDKLKEKERDKVLTAEELNAAQTSVAYGCIKYADLSHNRLNDYIFSFDKMLDDRGNTAAYLLYAFTRIRSIARLANIDEEMLQKAARETKILLDHEKEWKLGRCILRFPEILQKILDDLFLHTLCDYIYELATAFTEFYDSCYCVEKDRQTGKILKVNMWRMLLCEAVAAVMAKGFDILGIKPVQRM

122 NP_001006668.1 NP_055525.3

>NP_001006668.1

MRIMLLFTAILAFSLAQSFGAVCKEPQEEVVPGGGRSKRDPDLYQLLQRLFKSHSSLEGLLKALSQASTDPKESTSPEKRDMHDFFVGLMGKRSVQPEGKTGPFLPSVRVPRPLHPNQLGSTGKSSLGTEEQRPL

>NP_055525.3

MTGAEIESGAQVKPEKKPGEEVVGGAEIENDVPLVVRPKVRTQAQIMPGARPKNKSKVMPGASTKVETSAVGGARPKSKAKAIPVSRFKEEAQMWAQPRFGAERLSKTERNSQTNIIASPLVSTDSVLVAKTKYLSEDRELVNTDTESFPRRKAHYQAGFQPSFRSKEETNMGSWCCPRPTSKQEASPNSDFKWVDKSVSSLFWSGDEVTAKFHPGNRVKDSNRSMHMANQEANTMSRSQTNQELYIASSSGSEDESVKTPWFWARDKTNTWSGPREDPNSRSRFRSKKEVYVESSSGSEHEDHLESWFGAGKEAKFRSKMRAGKEANNRARHRAKREACIDFMPGSIDVIKKESCFWPEENANTFSRPMIKKEARARAMTKEEAKTKARARAKQEARSEEEALIGTWFWATDESSMADEASIESSLQVEDESIIGSWFWTEEEASMGTGASSKSRPRTDGERIGDSLFGAREKTSMKTGAEATSESILAADDEQVIIGSWFWAGEEVNQEAEEETIFGSWFWVIDAASVESGVGVSCESRTRSEEEEVIGPWFWSGEQVDIEAGIGEEARPGAEEETIFGSWFWAENQTYMDCRAETSCDTMQGAEEEEPIIGSWFWTRVEACVEGDVNSKSSLEDKEEAMIPCFGAKEEVSMKHGTGVRCRFMAGAEETNNKSCFWAEKEPCMYPAGGGSWKSRPEEEEDIVNSWFWSRKYTKPEAIIGSWLWATEESNIDGTGEKAKLLTEEETIINSWFWKEDEAISEATDREESRPEAEEGDIIGSWFWAGEEDRLEPAAETREEDRLAAEKEGIVGSWFGAREETIRREAGSCSKSSPKAEEEEVIIGSWFWEEEASPEAVAGVGFESKPGTEEEEITVGSWFWPEEEASIQAGSQAVEEMESETEEETIFGSWFWDGKEVSEEAGPCCVSKPEDDEEMIVESWFWSRDKAIKETGTVATCESKPENEEGAIVGSWFEAEDEVDNRTDNGSNCGSRTLADEDEAIVGSWFWAGDEAHFESNPSPVFRAICRSTCSVEQEPDPSRRPQSWEEVTVQFKPGPWGRVGFPSISPFRFPKEAASLFCEMFGGKPRNMVLSPEGEDQESLLQPDQPSPEFPFQYDPSYRSVQEIREHLRAKESTEPESSSCNCIQCELKIGSEEFEELLLLMEKIRDPFIHEISKIAMGMRSASQFTRDFIRDSGVVSLIETLLNYPSSRVRTSFLENMIRMAPPYPNLNIIQTYICKVCEETLAYSVDSPEQLSGIRMIRHLTTTTDYHTLVANYMSGFLSLLATGNAKTRFHVLKMLLNLSENLFMTKELLSAEAVSEFIGLFNREETNDNIQIVLAIFENIGNNIKKETVFSDDDFNIEPLISAFHKVEKFAKELQGKTDNQNDPEGDQEN

123 NP_036286.2 NP_803187.1

>NP_036286.2

MYSGAGPALAPPAPPPPIQGYAFKPPPRPDFGTSGRTIKLQANFFEMDIPKIDIYHYELDIKPEKCPRRVNREIVEHMVQHFKTQIFGDRKPVFDGRKNLYTAMPLPIGRDKVELEVTLPGEGKDRIFKVSIKWVSCVSLQALHDALSGRLPSVPFETIQALDVVMRHLPSMRYTPVGRSFFTASEGCSNPLGGGREVWFGFHQSVRPSLWKMMLNIDVSATAFYKAQPVIEFVCEVLDFKSIEEQQKPLTDSQRVKFTKEIKGLKVEITHCGQMKRKYRVCNVTRRPASHQTFPLQQESGQTVECTVAQYFKDRHKLVLRYPHLPCLQVGQEQKHTYLPLEVCNIVAGQRCIKKLTDNQTSTMIRATARSAPDRQEEISKLMRSASFNTDPYVREFGIMVKDEMTDVTGRVLQPPSILYGGRNKAIATPVQGVWDMRNKQFHTGIEIKVWAIACFAPQRQCTEVHLKSFTEQLRKISRDAGMPIQGQPCFCKYAQGADSVEPMFRHLKNTYAGLQLVVVILPGKTPVYAEVKRVGDTVLGMATQCVQMKNVQRTTPQTLSNLCLKINVKLGGVNNILLPQGRPPVFQQPVIFLGADVTHPPAGDGKKPSIAAVVGSMDAHPNRYCATVRVQQHRQEIIQDLAAMVRELLIQFYKSTRFKPTRIIFYRDGVSEGQFQQVLHHELLAIREACIKLEKDYQPGITFIVVQKRHHTRLFCTDKNERVGKSGNIPAGTTVDTKITHPTEFDFYLCSHAGIQGTSRPSHYHVLWDDNRFSSDELQILTYQLCHTYVRCTRSVSIPAPAYYAHLVAFRARYHLVDKEHDSAEGSHTSGQSNGRDHQALAKAVQVHQDTLRTMYFA

>NP_803187.1

MKSPALQPLSMAGLQLMTPASSPMGPFFGLPWQQEAIHDNIYTPRKYQVELLEAALDHNTIVCLNTGSGKTFIAVLLTKELSYQIRGDFSRNGKRTVFLVNSANQVAQQVSAVRTHSDLKVGEYSNLEVNASWTKERWNQEFTKHQVLIMTCYVALNVLKNGYLSLSDINLLVFDECHLAILDHPYREIMKLCENCPSCPRILGLTASILNGKCDPEELEEKIQKLEKILKSNAETATDLVVLDRYTSQPCEIVVDCGPFTDRSGLYERLLMELEEALNFINDCNISVHSKERDSTLISKQILSDCRAVLVVLGPWCADKVAGMMVRELQKYIKHEQEELHRKFLLFTDTFLRKIHALCEEHFSPASLDLKFVTPKVIKLLEILRKYKPYERQQFESVEWYNNRNQDNYVSWSDSEDDDEDEEIEEKEKPETNFPSPFTNILCGIIFVERRYTAVVLNRLIKEAGKQDPELAYISSNFITGHGIGKNQPRNKQMEAEFRKQEEVLRKFRAHETNLLIATSIVEEGVDIPKCNLVVRFDLPTEYRSYVQSKGRARAPISNYIMLADTDKIKSFEEDLKTYKAIEKILRNKCSKSVDTGETDIDPVMDDDDVFPPYVLRPDDGGPRVTINTAIGHINRYCARLPSDPFTHLAPKCRTRELPDGTFYSTLYLPINSPLRASIVGPPMSCVRLAERVVALICCEKLHKIGELDDHLMPVGKETVKYEEELDLHDEEETSVPGRPGSTKRRQCYPKAIPECLRDSYPRPDQPCYLYVIGMVLTTPLPDELNFRRRKLYPPEDTTRCFGILTAKPIPQIPHFPVYTRSGEVTISIELKKSGFMLSLQMLELITRLHQYIFSHILRLEKPALEFKPTDADSAYCVLPLNVVNDSSTLDIDFKFMEDIEKSEARIGIPSTKYTKETPFVFKLEDYQDAVIIPRYRNFDQPHRFYVADVYTDLTPLSKFPSPEYETFAEYYKTKYNLDLTNLNQPLLDVDHTSSRLNLLTPRHLNQKGKALPLSSAEKRKAKWESLQNKQILVPELCAIHPIPASLWRKAVCLPSILYRLHCLLTAEELRAQTASDAGVGVRSLPADFRYPNLDFGWKKSIDSKSFISISNSSSAENDNYCKHSTIVPENAAHQGANRTSSLENHDQMSVNCRTLLSESPGKLHVEVSADLTAINGLSYNQNLANGSYDLANRDFCQGNQLNYYKQEIPVQPTTSYSIQNLYSYENQPQPSDECTLLSNKYLDGNANKSTSDGSPVMAVMPGTTDTIQVLKGRMDSEQSPSIGYSSRTLGPNPGLILQALTLSNASDGFNLERLEMLGDSFLKHAITTYLFCTYPDAHEGRLSYMRSKKVSNCNLYRLGKKKGLPSRMVVSIFDPPVNWLPPGYVVNQDKSNTDKWEKDEMTKDCMLANGKLDEDYEEEDEEEESLMWRAPKEEADYEDDFLEYDQEHIRFIDNMLMGSGAFVKKISLSPFSTTDSAYEWKMPKKSSLGSMPFSSDFEDFDYSSWDAMCYLDPSKAVEEDDFVVGFWNPSEENCGVDTGKQSISYDLHTEQCIADKSIADCVEALLGCYLTSCGERAAQLFLCSLGLKVLPVIKRTDREKALCPTRENFNSQQKNLSVSCAAASVASSRSSVLKDSEYGCLKIPPRCMFDHPDADKTLNHLISGFENFEKKINYRFKNKAYLLQAFTHASYHYNTITDCYQRLEFLGDAILDYLITKHLYEDPRQHSPGVLTDLRSALVNNTIFASLAVKYDYHKYFKAVSPELFHVIDDFVQFQLEKNEMQGMDSELRRSEEDEEKEEDIEVPKAMGDIFESLAGAIYMDSGMSLETVWQVYYPMMRPLIEKFSANVPRSPVRELLEMEPETAKFSPAERTYDGKVRVTVEVVGKGKFKGVGRSYRIAKSAAARRALRSLKANQPQVPNS

124 NP_683877.1 NP_001003677.1

>NP_683877.1

MQLSKVKFRNQYDNDVTVWSPQGRIHQIEYAMEAVKQGSATVGLKSKTHAVLVALKRAQSELAAHQKKILHVDNHIGISIAGLTADARLLCNFMRQECLDSRFVFDRPLPVSRLVSLIGSKTQIPTQRYGRRPYGVGLLIAGYDDMGPHIFQTCPSANYFDCRAMSIGARSQSARTYLERHMSEFMECNLNELVKHGLRALRETLPAEQDLTTKNVSIGIVGKDLEFTIYDDDDVSPFLEGLEERPQRKAQPAQPADEPAEKADEPMEH

>NP_001003677.1

MLSPERLALPDYEYLAQRHVLTYMEDAVCQLLENREDISQYGIARFFTEYFNSVCQGTHILFREFSFVQATPHNRVSFLRAFWRCFRTVGKNGDLLTMKEYHCLLQLLCPDFPLELTQKAARIVLMDDAMDCLMSFSDFLFAFQIQFYYSEFLDSVAAIYEDLLSGKNPNTVIVPTSSSGQHRQRPALGGAGTLEGVEASLFYQCLENLCDRHKYSCPPPALVKEALSNVQRLTFYGFLMALSKHRGINQALGALPDKGDLMHDPAMDEELERLLLPFFRLAQVPGLVNSVTASPEASCLPSRTPPRVGSPWRPLHHSRKVDGESDGSTEETDESET

125 NP_036520.1 NP_115498.2

>NP_036520.1

MSVPGPSSPDGALTRPPYCLEAGEPTPGLSDTSPDEGLIEDLTIEDKAVEQLAEGLLSHYLPDLQRSKQALQELTQNQVVLLDTLEQEISKFKECHSMLDINALFAEAKHYHAKLVNIRKEMLMLHEKTSKLKKRALKLQQKRQKEELEREQQREKEFEREKQLTARPAKRM

>NP_115498.2

MLETLRERLLSVQQDFTSGLKTLSDKSREAKVKSKPRTVPFLPKYSAGLELLSRYEDTWAALHRRAKDCASAGELVDSEVVMLSAHWEKKKTSLVELQEQLQQLPALIADLESMTANLTHLEASFEEVENNLLHLEDLCGQCELERCKHMQSQQLENYKKNKRKELETFKAELDAEHAQKVLEMEHTQQMKLKERQKFFEEAFQQDMEQYLSTGYLQIAERREPIGSMSSMEVNVDMLEQMDLMDISDQEALDVFLNSGGEENTVLSPALGPESSTCQNEITLQVPNPSELRAKPPSSSSTCTDSATRDISEGGESPVVQSDEEEVQVDTALATSHTDREATPDGGEDSDS

126 NP_001001998.1 NP_055768.2

>NP_001001998.1

MAPPSTREPRVLSATSATKSDGEMVLPGFPDADSFVKFALGSVVAVTKASGGLPQFGDEYDFYRSFPGFQAFCETQGDRLLQCMSRVMQYHGCRSNIKDRSKVTELEDKFDLLVDANDVILERVGILLDEASGVNKNQQPVLPAGLQVPKTVVSSWNRKAAEYGKKAKSETFRLLHAKNIIRPQLKFREKIDNSNTPFLPKIFIKPNAQKPLPQALSKERRERPQDRPEDLDVPPALADFIHQQRTQQVEQDMFAHPYQYELNHFTPADAVLQKPQPQLYRPIEETPCHFISSLDELVELNEKLLNCQEFAVDLEHHSYRSFLGLTCLMQISTRTEDFIIDTLELRSDMYILNESLTDPAIVKVFHGADSDIEWLQKDFGLYVVNMFDTHQAARLLNLGRHSLDHLLKLYCNVDSNKQYQLADWRIRPLPEEMLSYARDDTHYLLYIYDKMRLEMWERGNGQPVQLQVVWQRSRDICLKKFIKPIFTDESYLELYRKQKKHLNTQQLTAFQLLFAWRDKTARREDESYGYVLPNHMMLKIAEELPKEPQGIIACCNPVPPLVRQQINEMHLLIQQAREMPLLKSEVAAGVKKSGPLPSAERLENVLFGPHDCSHAPPDGYPIIPTSGSVPVQKQASLFPDEKEDNLLGTTCLIATAVITLFNEPSAEDSKKGPLTVAQKKAQNIMESFENPFRMFLPSLGHRAPVSQAAKFDPSTKIYEISNRWKLAQVQVQKDSKEAVKKKAAEQTAAREQAKEACKAAAEQAISVRQQVVLENAAKKRERATSDPRTTEQKQEKKRLKISKKPKDPEPPEKEFTPYDYSQSDFKAFAGNSKSKVSSQFDPNKQTPSGKKCIAAKKIKQSVGNKSMSFPTGKSDRGFRYNWPQR

>NP_055768.2

MLKSKTFLKKTRAGGVMKIVREHYLRDDIGCGAPGCAACGGAHEGPALEPQPQDPASSVCPQPHYLLPDTNVLLHQIDVLEDPAIRNVIVLQTVLQEVRNRSAPVYKRIRDVTNNQEKHFYTFTNEHHRETYVEQEQGENANDRNDRAIRVAAKWYNEHLKKMSADNQLQVIFITNDRRNKEKAIEEGIPAFTCEEYVKSLTANPELIDRLACLSEEGNEIESGKIIFSEHLPLSKLQQGIKSGTYLQGTFRASRENYLEATVWIHGDSEENKEIILQGLKHLNRAVHEDIVAVELLPKSQWVAPSSVVLHDEGQNEEDVEKEEETERMLKTAVSEKMLKPTGRVVGIIKRNWRPYCGMLSKSDIKESRRHLFTPADKRIPRIRIETRQASTLEGRRIIVAIDGWPRNSRYPNGHFVRNLGDVGEKETETEVLLLEHDVPHQPFSQAVLSFLPKMPWSITEKDMKNREDLRHLCICSVDPPGCTDIDDALHCRELENGNLEVGVHIADVSHFIRPGNALDQESARRGTTVYLCEKRIDMVPELLSSNLCSLKCDVDRLAFSCIWEMNHNAEILKTKFTKSVINSKASLTYAEAQLRIDSANMNDDITTSLRGLNKLAKILKKRRIEKGALTLSSPEVRFHMDSETHDPIDLQTKELRETNSMVEEFMLLANISVAKKIHEEFSEHALLRKHPAPPPSNYEILVKAARSRNLEIKTDTAKSLAESLDQAESPTFPYLNTLLRILATRCMMQAVYFCSGMDNDFHHYGLASPIYTHFTSPIRRYADVIVHRLLAVAIGADCTYPELTDKHKLADICKNLNFRHKMAQYAQRASVAFHTQLFFKSKGIVSEEAYILFVRKNAIVVLIPKYGLEGTVFFEEKDKPNPQLIYDDEIPSLKIEDTVFHVFDKVKVKIMLDSSNLQHQKIRMSLVEPQIPGISIPTDTSNMDLNGPKKKKMKLGK

127 NP_079115.1 NP_006629.2

>NP_079115.1

MAGPVKDREAFQRLNFLYQAAHCVLAQDPENQALARFYCYTERTIAKRLVLRRDPSVKRTLCRGCSSLLVPGLTCTQRQRRCRGQRWTVQTCLTCQRSQRFLNDPGHLLWGDRPEAQLGSQADSKPLQPLPNTAHSISDRLPEEKMQTQGSSNQ

>NP_006629.2

MATLRRLREAPRHLLVCEKSNFGNHKSRHRHLVQTHYYNYRVSFLIPECGILSEELKNLVMNTGPYYFVKNLPLHELITPEFISTFIKKGSCYALTYNTHIDEDNTVALLPNGKLILSLDKDTYEETGLQGHPSQFSGRKIMKFIVSIDLMELSLNLDSKKYERISWSFKEKKPLKFDFLLAWHKTGSEESTMMSYFSKYQIQEHQPKVALSTLRDLQCPVLQSSELEGTPEVSCRALELFDWLGAVFSNVDLNNEPNNFISTYCCPEPSTVVAKAYLCTITGFILPEKICLLLEHLCHYFDEPKLAPWVTLSVQGFADSPVSWEKNEHGFRKGGEHLYNFVIFNNQDYWLQMAVGANDHCPP

128 NP_004636.1 NP_789839.1

>NP_004636.1

MAASETVRLRLQFDYPPPATPHCTAFWLLVDLNRCRVVTDLISLIRQRFGFSSGAFLGLYLEGGLLPPAESARLVRDNDCLRVKLEERGVAENSVVISNGDINLSLRKAKKRAFQLEEGEETEPDCKYSKKHWKSRENNNNNEKVLDLEPKAVTDQTVSKKNKRKNKATCGTVGDDNEEAKRKSPKKKEKCEYKKKAKNPKSPKVQAVKDWANQRCSSPKGSARNSLVKAKRKGSVSVCSKESPSSSSESESCDESISDGPSKVTLEARNSSEKLPTELSKEEPSTKNTTADKLAIKLGFSLTPSKGKTSGTTSSSSDSSAESDDQCLMSSSTPECAAGFLKTVGLFAGRGRPGPGLSSQTAGAAGWRRSGSNGGGQAPGASPSVSLPASLGRGWGREENLFSWKGAKGRGMRGRGRGRGHPVSCVVNRSTDNQRQQQLNDVVKNSSTIIQNPVETPKKDYSLLPLLAAAPQVGEKIAFKLLELTSSYSPDVSDYKEGRILSHNPETQQVDIEILSSLPALREPGKFDLVYHNENGAEVVEYAVTQESKITVFWKELIDPRLIIESPSNTSSTEPA

>NP_789839.1

MASLLKVDQEVKLKVDSFRERITSEAEDLVANFFPKKLLELDSFLKEPILNIHDLTQIHSDMNLPVPDPILLTNSHDGLDGPTYKKRRLDECEEAFQGTKVFVMPNGMLKSNQQLVDIIEKVKPEIRLLIEKCNTPSGKGPHICFDLQVKMWVQLLIPRIEDGNNFGVSIQEETVAELRTVESEAASYLDQISRYYITRAKLVSKIAKYPHVEDYRRTVTEIDEKEYISLRLIISELRNQYVTLHDMILKNIEKIKRPRSSNAETLY

129 NP_001810.1 NP_055048.1

>NP_001810.1

MQPTLLLSLLGAVGLAAVNSMPVDNRNHNEGMVTRCIIEVLSNALSKSSAPPITPECRQVLKTSRKDVKDKETTENENTKFEVRLLRDPADASEAHESSSRGEAGAPGEEDIQGPTKADTEKWAEGGGHSRERADEPQWSLYPSDSQVSEEVKTRHSEKSQREDEEEEEGENYQKGERGEDSSEEKHLEEPGETQNAFLNERKQASAIKKEELVARSETHAAGHSQEKTHSREKSSQESGEEAGSQENHPQESKGQPRSQEESEEGEEDATSEVDKRRTRPRHHHGRSRPDRSSQGGSLPSEEKGHPQEESEESNVSMASLGEKRDHHSTHYRASEEEPEYGEEIKGYPGVQAPEDLEWERYRGRGSEEYRAPRPQSEESWDEEDKRNYPSLELDKMAHGYGEESEEERGLEPGKGRHHRGRGGEPRAYFMSDTREEKRFLGEGHHRVQENQMDKARRHPQGAWKELDRNYLNYGEEGAPGKWQQQGDLQDTKENREEARFQDKQYSSHHTAEKRKRLGELFNPYYDPLQWKSSHFERRDNMNDNFLEGEEENELTLNEKNFFPEYNYDWWEKKPFSEDVNWGYEKRNLARVPKLDLKRQYDRVAQLDQLLHYRKKSAEFPDFYDSEEPVSTHQEAENEKDRADQTVLTEDEKKELENLAAMDLELQKIAEKFSQRG

>NP_055048.1

MNGEADCPTDLEMAAPKGQDRWSQEDMLTLLECMKNNLPSNDSSKFKTTESHMDWEKVAFKDFSGDMCKLKWVEISNEVRKFRTLTELILDAQEHVKNPYKGKKLKKHPDFPKKPLTPYFRFFMEKRAKYAKLHPEMSNLDLTKILSKKYKELPEKKKMKYIQDFQREKQEFERNLARFREDHPDLIQNAKKSDIPEKPKTPQQLWYTHEKKVYLKVRPDATTKEVKDSLGKQWSQLSDKKRLKWIHKALEQRKEYEEIMRDYIQKHPELNISEEGITKSTLTKAERQLKDKFDGRPTKPPPNSYSLYCAELMANMKDVPSTERMVLCSQQWKLLSQKEKDAYHKKCDQKKKDYEVELLRFLESLPEEEQQRVLGEEKMLNINKKQATSPASKKPAQEGGKGGSEKPKRPVSAMFIFSEEKRRQLQEERPELSESELTRLLARMWNDLSEKKKAKYKAREAALKAQSERKPGGEREERGKLPESPKRAEEIWQQSVIGDYLARFKNDRVKALKAMEMTWNNMEKKEKLMWIKKAAEDQKRYERELSEMRAPPAATNSSKKMKFQGEPKKPPMNGYQKFSQELLSNGELNHLPLKERMVEIGSRWQRISQSQKEHYKKLAEEQQKQYKVHLDLWVKSLSPQDRAAYKEYISNKRKSMTKLRGPNPKSSRTTLQSKSESEEDDEEDEDDEDEDEEEEDDENGDSSEDGGDSSESSSEDESEDGDENEEDDEDEDDDEDDDEDEDNESEGSSSSSSSSGDSSDSDSN

130 NP_061978.5 NP_001020537.2

>NP_061978.5

MMPAQYALTSSLVLLVLLSTARAGPFSSRSNVTLPAPRPPPQPGGHTVGAGVGSPSSQLYEHTVEGGEKQVVFTHRINLPPSTGCGCPPGTEPPVLASEVQALRVRLEILEELVKGLKEQCTGGCCPASAQAGTGQTDVRTLCSLHGVFDLSRCTCSCEPGWGGPTCSDPTDAEIPPSSPPSASGSCPDDCNDQGRCVRGRCVCFPGYTGPSCGWPSCPGDCQGRGRCVQGVCVCRAGFSGPDCSQRSCPRGCSQRGRCEGGRCVCDPGYTGDDCGMRSCPRGCSQRGRCENGRCVCNPGYTGEDCGVRSCPRGCSQRGRCKDGRCVCDPGYTGEDCGTRSCPWDCGEGGRCVDGRCVCWPGYTGEDCSTRTCPRDCRGRGRCEDGECICDTGYSGDDCGVRSCPGDCNQRGRCEDGRCVCWPGYTGTDCGSRACPRDCRGRGRCENGVCVCNAGYSGEDCGVRSCPGDCRGRGRCESGRCMCWPGYTGRDCGTRACPGDCRGRGRCVDGRCVCNPGFTGEDCGSRRCPGDCRGHGLCEDGVCVCDAGYSGEDCSTRSCPGGCRGRGQCLDGRCVCEDGYSGEDCGVRQCPNDCSQHGVCQDGVCICWEGYVSEDCSIRTCPSNCHGRGRCEEGRCLCDPGYTGPTCATRMCPADCRGRGRCVQGVCLCHVGYGGEDCGQEEPPASACPGGCGPRELCRAGQCVCVEGFRGPDCAIQTCPGDCRGRGECHDGSCVCKDGYAGEDCGEARVPSSASAYDQRGLAPGQEYQVTVRALRGTSWGLPASKTITTMIDGPQDLRVVAVTPTTLELGWLRPQAEVDRFVVSYVSAGNQRVRLEVPPEADGTLLTDLMPGVEYVVTVTAERGRAVSYPASVRANTEEREEESPPRPSLSQPPRRPWGNLTAELSRFRGTVQDLERHLRAHGYPLRANQTYTSVARHIHEYLQRQVLGSSADGALLVSLDGLRGQFERVVLRWRPQPPAEGPGGELTVPGTTRTVSLPDLRPGTTYHVEVHGVRAGQTSKSYAFITTTGPSTTQGAQAPLLQQRPQELGELRVLGRDETGRLRVVWTAQPDTFAYFQLRMRVPEGPGAHEEVLPGDVRQALVPPPPPGTPYELSLHGVPPGGKPSDPIIYQGIMDKDEEKPGKSSGPPRLGELTVTDRTSDSLLLRWTVPEGEFDSFVIQYKDRDGQPQVVPVEGPQRSAVITSLDPGRKYKFVLYGFVGKKRHGPLVAEAKILPQSDPSPGTPPHLGNLWVTDPTPDSLHLSWTVPEGQFDTFMVQYRDRDGRPQVVPVEGPERSFVVSSLDPDHKYRFTLFGIANKKRYGPLTADGTTAPERKEEPPRPEFLEQPLLGELTVTGVTPDSLRLSWTVAQGPFDSFMVQYKDAQGQPQAVPVAGDENEVTVPGLDPDRKYKMNLYGLRGRQRVGPESVVAKTAPQEDVDETPSPTELGTEAPESPEEPLLGELTVTGSSPDSLSLFWTVPQGSFDSFTVQYKDRDGRPRAVRVGGKESEVTVGGLEPGHKYKMHLYGLHEGQRVGPVSAVGVTAPQQEETPPATESPLEPRLGELTVTDVTPNSVGLSWTVPEGQFDSFIVQYKDKDGQPQVVPVAADQREVTVYNLEPERKYKMNMYGLHDGQRMGPLSVVIVTAPATEASKPPLEPRLGELTVTDITPDSVGLSWTVPEGEFDSFVVQYKDRDGQPQVVPVAADQREVTIPDLEPSRKYKFLLFGIQDGKRRSPVSVEAKTVARGDASPGAPPRLGELWVTDPTPDSLRLSWTVPEGQFDSFVVQFKDKDGPQVVPVEGHERSVTVTPLDAGRKYRFLLYGLLGKKRHGPLTADGTTEARSAMDDTGTKRPPKPRLGEELQVTTVTQNSVGLSWTVPEGQFDSFVVQYKDRDGQPQVVPVEGSLREVSVPGLDPAHRYKLLLYGLHHGKRVGPISAVAITAGREETETETTAPTPPAPEPHLGELTVEEATSHTLHLSWMVTEGEFDSFEIQYTDRDGQLQMVRIGGDRNDITLSGLESDHRYLVTLYGFSDGKHVGPVHVEALTVPEEEKPSEPPTATPEPPIKPRLGELTVTDATPDSLSLSWTVPEGQFDHFLVQYRNGDGQPKAVRVPGHEEGVTISGLEPDHKYKMNLYGFHGGQRMGPVSVVGVTEPSMEAPEPAEEPLLGELTVTGSSPDSLSLSWTVPQGRFDSFTVQYKDRDGRPQVVRVGGEESEVTVGGLEPGRKYKMHLYGLHEGRRVGPVSAVGVTAPEEESPDAPLAKLRLGQMTVRDITSDSLSLSWTVPEGQFDHFLVQFKNGDGQPKAVRVPGHEDGVTISGLEPDHKYKMNLYGFHGGQRVGPVSAVGLTASTEPPTPEPPIKPRLEELTVTDATPDSLSLSWTVPEGQFDHFLVQYKNGDGQPKATRVPGHEDRVTISGLEPDNKYKMNLYGFHGGQRVGPVSAIGVTEEETPSPTEPSMEAPEPPEEPLLGELTVTGSSPDSLSLSWTVPQGRFDSFTVQYKDRDGRPQVVRVGGEESEVTVGGLEPGRKYKMHLYGLHEGRRVGPVSTVGVTAPQEDVDETPSPTEPGTEAPGPPEEPLLGELTVTGSSPDSLSLSWTVPQGRFDSFTVQYKDRDGRPQAVRVGGQESKVTVRGLEPGRKYKMHLYGLHEGRRLGPVSAVGVTEDEAETTQAVPTMTPEPPIKPRLGELTMTDATPDSLSLSWTVPEGQFDHFLVQYRNGDGQPKAVRVPGHEDGVTISGLEPDHKYKMNLYGFHGGQRVGPISVIGVTEEETPSPTELSTEAPEPPEEPLLGELTVTGSSPDSLSLSWTIPQGHFDSFTVQYKDRDGRPQVMRVRGEESEVTVGGLEPGRKYKMHLYGLHEGRRVGPVSTVGVTVPTTTPEPPNKPRLGELTVTDATPDSLSLSWMVPEGQFDHFLVQYRNGDGQPKVVRVPGHEDGVTISGLEPDHKYKMNLYGFHGGQRVGPISVIGVTEEETPAPTEPSTEAPEPPEEPLLGELTVTGSSPDSLSLSWTIPQGRFDSFTVQYKDRDGRPQVVRVRGEESEVTVGGLEPGCKYKMHLYGLHEGQRVGPVSAVGVTAPKDEAETTQAVPTMTPEPPIKPRLGELTVTDATPDSLSLSWMVPEGQFDHFLVQYRNGDGQPKAVRVPGHEDGVTISGLEPDHKYKMNLYGFHGGQRVGPVSAIGVTEEETPSPTEPSTEAPEAPEEPLLGELTVTGSSPDSLSLSWTVPQGRFDSFTVQYKDRDGQPQVVRVRGEESEVTVGGLEPGRKYKMHLYGLHEGQRVGPVSTVGITAPLPTPLPVEPRLGELAVAAVTSDSVGLSWTVAQGPFDSFLVQYRDAQGQPQAVPVSGDLRAVAVSGLDPARKYKFLLFGLQNGKRHGPVPVEARTAPDTKPSPRLGELTVTDATPDSVGLSWTVPEGEFDSFVVQYKDKDGRLQVVPVAANQREVTVQGLEPSRKYRFLLYGLSGRKRLGPISADSTTAPLEKELPPHLGELTVAEETSSSLRLSWTVAQGPFDSFVVQYRDTDGQPRAVPVAADQRTVTVEDLEPGKKYKFLLYGLLGGKRLGPVSALGMTAPEEDTPAPELAPEAPEPPEEPRLGVLTVTDTTPDSMRLSWSVAQGPFDSFVVQYEDTNGQPQALLVDGDQSKILISGLEPSTPYRFLLYGLHEGKRLGPLSAEGTTGLAPAGQTSEESRPRLSQLSVTDVTTSSLRLNWEAPPGAFDSFLLRFGVPSPSTLEPHPRPLLQRELMVPGTRHSAVLRDLRSGTLYSLTLYGLRGPHKADSIQGTARTLSPVLESPRDLQFSEIRETSAKVNWMPPPSRADSFKVSYQLADGGEPQSVQVDGQARTQKLQGLIPGARYEVTVVSVRGFEESEPLTGFLTTVPDGPTQLRALNLTEGFAVLHWKPPQNPVDTYDVQVTAPGAPPLQAETPGSAVDYPLHDLVLHTNYTATVRGLRGPNLTSPASITFTTGLEAPRDLEAKEVTPRTALLTWTEPPVRPAGYLLSFHTPGGQNQEILLPGGITSHQLLGLFPSTSYNARLQAMWGQSLLPPVSTSFTTGGLRIPFPRDCGEEMQNGAGASRTSTIFLNGNRERPLNVFCDMETDGGGWLVFQRRMDGQTDFWRDWEDYAHGFGNISGEFWLGNEALHSLTQAGDYSMRVDLRAGDEAVFAQYDSFHVDSAAEYYRLHLEGYHGTAGDSMSYHSGSVFSARDRDPNSLLISCAVSYRGAWWYRNCHYANLNGLYGSTVDHQGVSWYHWKGFEFSVPFTEMKLRPRNFRSPAGGG

>NP_001020537.2

MTDRQTDTAPSPSYHLLPGRRRTVDAAASRGQGPEPAPGGGVEGVGARGVALKLFVQLLGCSRFGGAVVRAGEAEPSGAARSASSGREEPQPEEGEEEEEKEEERGPQWRLGARKPGSWTGEAAVCADSAPAARAPQALARASGRGGRVARRGAEESGPPHSPSRRGSASRAGPGRASETMNFLLSWVHWSLALLLYLHHAKWSQAAPMAEGGGQNHHEVVKFMDVYQRSYCHPIETLVDIFQEYPDEIEYIFKPSCVPLMRCGGCCNDEGLECVPTEESNITMQIMRIKPHQGQHIGEMSFLQHNKCECRPKKDRARQEKKSVRGKGKGQKRKRKKSRYKSWSVYVGARCCLMPWSLPGPHPCGPCSERRKHLFVQDPQTCKCSCKNTDSRCKARQLELNERTCRCDKPRR

131 NP_001427.2 NP_003945.2

>NP_001427.2

MKPGFSPRGGGFGGRGGFGDRGGRGGRGGFGGGRGRGGGFRGRGRGGGGGGGGGGGGGRGGGGFHSGGNRGRGRGGKRGNQSGKNVMVEPHRHEGVFICRGKEDALVTKNLVPGESVYGEKRVSISEGDDKIEYRAWNPFRSKLAAAILGGVDQIHIKPGAKVLYLGAASGTTVSHVSDIVGPDGLVYAVEFSHRSGRDLINLAKKRTNIIPVIEDARHPHKYRMLIAMVDVIFADVAQPDQTRIVALNAHTFLRNGGHFVISIKANCIDSTASAEAVFASEVKKMQQENMKPQEQLTLEPYERDHAVVVGVYRPPPKVKN

>NP_003945.2

MAVMEMACPGAPGSAVGQQKELPKAKEKTPPLGKKQSSVYKLEAVEKSPVFCGKWEILNDVITKGTAKEGSEAGPAAISIIAQAECENSQEFSPTFSERIFIAGSKQYSQSESLDQIPNNVAHATEGKMARVCWKGKRRSKARKKRKKKSSKSLAHAGVALAKPLPRTPEQESCTIPVQEDESPLGAPYVRNTPQFTKPLKEPGLGQLCFKQLGEGLRPALPRSELHKLISPLQCLNHVWKLHHPQDGGPLPLPTHPFPYSRLPHPFPFHPLQPWKPHPLESFLGKLACVDSQKPLPDPHLSKLACVDSPKPLPGPHLEPSCLSRGAHEKFSVEEYLVHALQGSVSSGQAHSLTSLAKTWAARGSRSREPSPKTEDNEGVLLTEKLKPVDYEYREEVHWATHQLRLGRGSFGEVHRMEDKQTGFQCAVKKVRLEVFRAEELMACAGLTSPRIVPLYGAVREGPWVNIFMELLEGGSLGQLVKEQGCLPEDRALYYLGQALEGLEYLHSRRILHGDVKADNVLLSSDGSHAALCDFGHAVCLQPDGLGKSLLTGDYIPGTETHMAPEVVLGRSCDAKVDVWSSCCMMLHMLNGCHPWTQFFRGPLCLKIASEPPPVREIPPSCAPLTAQAIQEGLRKEPIHRVSAAELGGKVNRALQQVGGLKSPWRGEYKEPRHPPPNQANYHQTLHAQPRELSPRAPGPRPAEETTGRAPKLQPPLPPEPPEPNKSPPLTLSKEESGMWEPLPLSSLEPAPARNPSSPERKATVPEQELQQLEIELFLNSLSQPFSLEEQEQILSCLSIDSLSLSDDSEKNPSKASQSSRDTLSSGVHSWSSQAEARSSSWNMVLARGRPTDTPSYFNGVKVQIQSLNGEHLHIREFHRVKVGDIATGISSQIPAAAFSLVTKDGQPVRYDMEVPDSGIDLQCTLAPDGSFAWSWRVKHGQLENRP

132 NP_001009944.1 NP_001894.2

>NP_001009944.1

MPPAAPARLALALGLGLWLGALAGGPGRGCGPCEPPCLCGPAPGAACRVNCSGRGLRTLGPALRIPADATALDVSHNLLRALDVGLLANLSALAELDISNNKISTLEEGIFANLFNLSEINLSGNPFECDCGLAWLPRWAEEQQVRVVQPEAATCAGPGSLAGQPLLGIPLLDSGCGEEYVACLPDNSSGTVAAVSFSAAHEGLLQPEACSAFCFSTGQGLAALSEQGWCLCGAAQPSSASFACLSLCSGPPPPPAPTCRGPTLLQHVFPASPGATLVGPHGPLASGQLAAFHIAAPLPVTATRWDFGDGSAEVDAAGPAASHRYVLPGRYHVTAVLALGAGSALLGTDVQVEAAPAALELVCPSSVQSDESLDLSIQNRGGSGLEAAYSIVALGEEPARAVHPLCPSDTEIFPGNGHCYRLVVEKAAWLQAQEQCQAWAGAALAMVDSPAVQRFLVSRVTRSLDVWIGFSTVQGVEVGPAPQGEAFSLESCQNWLPGEPHPATAEHCVRLGPTGWCNTDLCSAPHSYVCELQPGGPVQDAENLLVGAPSGDLQGPLTPLAQQDGLSAPHEPVEVMVFPGLRLSREAFLTTAEFGTQELRRPAQLRLQVYRLLSTAGTPENGSEPESRSPDNRTQLAPACMPGGRWCPGANICLPLDASCHPQACANGCTSGPGLPGAPYALWREFLFSVPAGPPAQYSVTLHGQDVLMLPGDLVGLQHDAGPGALLHCSPAPGHPGPRAPYLSANASSWLPHLPAQLEGTWACPACALRLLAATEQLTVLLGLRPNPGLRLPGRYEVRAEVGNGVSRHNLSCSFDVVSPVAGLRVIYPAPRDGRLYVPTNGSALVLQVDSGANATATARWPGGSVSARFENVCPALVATFVPGCPWETNDTLFSVVALPWLSEGEHVVDVVVENSASRANLSLRVTAEEPICGLRATPSPEARVLQGVLVRYSPVVEAGSDMVFRWTINDKQSLTFQNVVFNVIYQSAAVFKLSLTASNHVSNVTVNYNVTVERMNRMQGLQVSTVPAVLSPNATLALTAGVLVDSAVEVAFLWTFGDGEQALHQFQPPYNESFPVPDPSVAQVLVEHNVMHTYAAPGEYLLTVLASNAFENLTQQVPVSVRASLPSVAVGVSDGVLVAGRPVTFYPHPLPSPGGVLYTWDFGDGSPVLTQSQPAANHTYASRGTYHVRLEVNNTVSGAAAQADVRVFEELRGLSVDMSLAVEQGAPVVVSAAVQTGDNITWTFDMGDGTVLSGPEATVEHVYLRAQNCTVTVGAASPAGHLARSLHVLVFVLEVLRVEPAACIPTQPDARLTAYVTGNPAHYLFDWTFGDGSSNTTVRGCPTVTHNFTRSGTFPLALVLSSRVNRAHYFTSICVEPEVGNVTLQPERQFVQLGDEAWLVACAWPPFPYRYTWDFGTEEAAPTRARGPEVTFIYRDPGSYLVTVTASNNISAANDSALVEVQEPVLVTSIKVNGSLGLELQQPYLFSAVGRGRPASYLWDLGDGGWLEGPEVTHAYNSTGDFTVRVAGWNEVSRSEAWLNVTVKRRVRGLVVNASRTVVPLNGSVSFSTSLEAGSDVRYSWVLCDRCTPIPGGPTISYTFRSVGTFNIIVTAENEVGSAQDSIFVYVLQLIEGLQVVGGGRYFPTNHTVQLQAVVRDGTNVSYSWTAWRDRGPALAGSGKGFSLTVLEAGTYHVQLRATNMLGSAWADCTMDFVEPVGWLMVAASPNPAAVNTSVTLSAELAGGSGVVYTWSLEEGLSWETSEPFTTHSFPTPGLHLVTMTAGNPLGSANATVEVDVQVPVSGLSIRASEPGGSFVAAGSSVPFWGQLATGTNVSWCWAVPGGSSKRGPHVTMVFPDAGTFSIRLNASNAVSWVSATYNLTAEEPIVGLVLWASSKVVAPGQLVHFQILLAAGSAVTFRLQVGGANPEVLPGPRFSHSFPRVGDHVVSVRGKNHVSWAQAQVRIVVLEAVSGLQVPNCCEPGIATGTERNFTARVQRGSRVAYAWYFSLQKVQGDSLVILSGRDVTYTPVAAGLLEIQVRAFNALGSENRTLVLEVQDAVQYVALQSGPCFTNRSAQFEAATSPSPRRVAYHWDFGDGSPGQDTDEPRAEHSYLRPGDYRVQVNASNLVSFFVAQATVTVQVLACREPEVDVVLPLQVLMRRSQRNYLEAHVDLRDCVTYQTEYRWEVYRTASCQRPGRPARVALPGVDVSRPRLVLPRLALPVGHYCFVFVVSFGDTPLTQSIQANVTVAPERLVPIIEGGSYRVWSDTRDLVLDGSESYDPNLEDGDQTPLSFHWACVASTQREAGGCALNFGPRGSSTVTIPRERLAAGVEYTFSLTVWKAGRKEEATNQTVLIRSGRVPIVSLECVSCKAQAVYEVSRSSYVYLEGRCLNCSSGSKRGRWAARTFSNKTLVLDETTTSTGSAGMRLVLRRGVLRDGEGYTFTLTVLGRSGEEEGCASIRLSPNRPPLGGSCRLFPLGAVHALTTKVHFECTGWHDAEDAGAPLVYALLLRRCRQGHCEEFCVYKGSLSSYGAVLPPGFRPHFEVGLAVVVQDQLGAAVVALNRSLAITLPEPNGSATGLTVWLHGLTASVLPGLLRQADPQHVIEYSLALVTVLNEYERALDVAAEPKHERQHRAQIRKNITETLVSLRVHTVDDIQQIAAALAQCMGPSRELVCRSCLKQTLHKLEAMMLILQAETTAGTVTPTAIGDSILNITGDLIHLASSDVRAPQPSELGAESPSRMVASQAYNLTSALMRILMRSRVLNEEPLTLAGEEIVAQGKRSDPRSLLCYGGAPGPGCHFSIPEAFSGALANLSDVVQLIFLVDSNPFPFGYISNYTVSTKVASMAFQTQAGAQIPIERLASERAITVKVPNNSDWAARGHRSSANSANSVVVQPQASVGAVVTLDSSNPAAGLHLQLNYTLLDGHYLSEEPEPYLAVYLHSEPRPNEHNCSASRRIRPESLQGADHRPYTFFISPGSRDPAGSYHLNLSSHFRWSALQVSVGLYTSLCQYFSEEDMVWRTEGLLPLEETSPRQAVCLTRHLTAFGASLFVPPSHVRFVFPEPTADVNYIVMLTCAVCLVTYMVMAAILHKLDQLDASRGRAIPFCGQRGRFKYEILVKTGWGRGSGTTAHVGIMLYGVDSRSGHRHLDGDRAFHRNSLDIFRIATPHSLGSVWKIRVWHDNKGLSPAWFLQHVIVRDLQTARSAFFLVNDWLSVETEANGGLVEKEVLAASDAALLRFRRLLVAELQRGFFDKHIWLSIWDRPPRSRFTRIQRATCCVLLICLFLGANAVWYGAVGDSAYSTGHVSRLSPLSVDTVAVGLVSSVVVYPVYLAILFLFRMSRSKVAGSPSPTPAGQQVLDIDSCLDSSVLDSSFLTFSGLHAEQAFVGQMKSDLFLDDSKSLVCWPSGEGTLSWPDLLSDPSIVGSNLRQLARGQAGHGLGPEEDGFSLASPYSPAKSFSASDEDLIQQVLAEGVSSPAPTQDTHMETDLLSSLSSTPGEKTETLALQRLGELGPPSPGLNWEQPQAARLSRTGLVEGLRKRLLPAWCASLAHGLSLLLVAVAVAVSGWVGASFPPGVSVAWLLSSSASFLASFLGWEPLKVLLEALYFSLVAKRLHPDEDDTLVESPAVTPVSARVPRVRPPHGFALFLAKEEARKVKRLHGMLRSLLVYMLFLLVTLLASYGDASCHGHAYRLQSAIKQELHSRAFLAITRSEELWPWMAHVLLPYVHGNQSSPELGPPRLRQVRLQEALYPDPPGPRVHTCSAAGGFSTSDYDVGWESPHNGSGTWAYSAPDLLGAWSWGSCAVYDSGGYVQELGLSLEESRDRLRFLQLHNWLDNRSRAVFLELTRYSPAVGLHAAVTLRLEFPAAGRALAALSVRPFALRRLSAGLSLPLLTSVCLLLFAVHFAVAEARTWHREGRWRVLRLGAWARWLLVALTAATALVRLAQLGAADRQWTRFVRGRPRRFTSFDQVAQLSSAARGLAASLLFLLLVKAAQQLRFVRQWSVFGKTLCRALPELLGVTLGLVVLGVAYAQLAILLVSSCVDSLWSVAQALLVLCPGTGLSTLCPAESWHLSPLLCVGLWALRLWGALRLGAVILRWRYHALRGELYRPAWEPQDYEMVELFLRRLRLWMGLSKVKEFRHKVRFEGMEPLPSRSSRGSKVSPDVPPPSAGSDASHPSTSSSQLDGLSVSLGRLGTRCEPEPSRLQAVFEALLTQFDRLNQATEDVYQLEQQLHSLQGRRSSRAPAGSSRGPSPGLRPALPSRLARASRGVDLATGPSRTPLRAKNKVHPSST

>NP_001894.2

MTAVHAGNINFKWDPKSLEIRTLAVERLLEPLVTQVTTLVNTNSKGPSNKKRGRSKKAHVLAASVEQATENFLEKGDKIAKESQFLKEELVAAVEDVRKQGDLMKAAAGEFADDPCSSVKRGNMVRAARALLSAVTRLLILADMADVYKLLVQLKVVEDGILKLRNAGNEQDLGIQYKALKPEVDKLNIMAAKRQQELKDVGHRDQMAAARGILQKNVPILYTASQACLQHPDVAAYKANRDLIYKQLQQAVTGISNAAQATASDDASQHQGGGGGELAYALNNFDKQIIVDPLSFSEERFRPSLEERLESIISGAALMADSSCTRDDRRERIVAECNAVRQALQDLLSEYMGNAGRKERSDALNSAIDKMTKKTRDLRRQLRKAVMDHVSDSFLETNVPLLVLIEAAKNGNEKEVKEYAQVFREHANKLIEVANLACSISNNEEGVKLVRMSASQLEALCPQVINAALALAAKPQSKLAQENMDLFKEQWEKQVRVLTDAVDDITSIDDFLAVSENHILEDVNKCVIALQEKDVDGLDRTAGAIRGRAARVIHVVTSEMDNYEPGVYTEKVLEATKLLSNTVMPRFTEQVEAAVEALSSDPAQPMDENEFIDASRLVYDGIRDIRKAVLMIRTPEELDDSDFETEDFDVRSRTSVQTEDDQLIAGQSARAIMAQLPQEQKAKIAEQVASFQEEKSKLDAEVSKWDDSGNDIIVLAKQMCMIMMEMTDFTRGKGPLKNTSDVISAAKKIAEAGSRMDKLGRTIADHCPDSACKQDLLAYLQRIALYCHQLNICSKVKAEVQNLGGELVVSGVDSAMSLIQAAKNLMNAVVQTVKASYVASTKYQKSQGMASLNLPAVSWKMKAPEKKPLVKREKQDETQTKIKRASQKKHVNPVQALSEFKAMDSI

133 NP_004403.1 NP_065147.1

>NP_004403.1

MEPLRVLELYSGVGGMHHALRESCIPAQVVAAIDVNTVANEVYKYNFPHTQLLAKTIEGITLEEFDRLSFDMILMSPPCQPFTRIGRQGDMTDSRTNSFLHILDILPRLQKLPKYILLENVKGFEVSSTRDLLIQTIENCGFQYQEFLLSPTSLGIPNSRLRYFLIAKLQSEPLPFQAPGQVLMEFPKIESVHPQKYAMDVENKIQEKNVEPNISFDGSIQCSGKDAILFKLETAEEIHRKNQQDSDLSVKMLKDFLEDDTDVNQYLLPPKSLLRYALLLDIVQPTCRRSVCFTKGYGSYIEGTGSVLQTAEDVQVENIYKSLTNLSQEEQITKLLILKLRYFTPKEIANLLGFPPEFGFPEKITVKQRYRLLGNSLNVHVVAKLIKILYE

>NP_065147.1

MKLKDTKSRPKQSSCGKFQTKGIKVVGKWKEVKIDPNMFADGQMDDLVCFEELTDYQLVSPAKNPSSLFSKEAPKRKAQAVSEEEEEEEGKSSSPKKKIKLKKSKNVATEGTSTQKEFEVKDPELEAQGDDMVCDDPEAGEMTSENLVQTAPKKKKNKGKKGLEPSQSTAAKVPKKAKTWIPEVHDQKADVSAWKDLFVPRPVLRALSFLGFSAPTPIQALTLAPAIRDKLDILGAAETGSGKTLAFAIPMIHAVLQWQKRNAAPPPSNTEAPPGETRTEAGAETRSPGKAEAESDALPDDTVIESEALPSDIAAEARAKTGGTVSDQALLFGDDDAGEGPSSLIREKPVPKQNENEEENLDKEQTGNLKQELDDKSATCKAYPKRPLLGLVLTPTRELAVQVKQHIDAVARFTGIKTAILVGGMSTQKQQRMLNRRPEIVVATPGRLWELIKEKHYHLRNLRQLRCLVVDEADRMVEKGHFAELSQLLEMLNDSQYNPKRQTLVFSATLTLVHQAPARILHKKHTKKMDKTAKLDLLMQKIGMRGKPKVIDLTRNEATVETLTETKIHCETDEKDFYLYYFLMQYPGRSLVFANSISCIKRLSGLLKVLDIMPLTLHACMHQKQRLRNLEQFARLEDCVLLATDVAARGLDIPKVQHVIHYQVPRTSEIYVHRSGRTARATNEGLSLMLIGPEDVINFKKIYKTLKKDEDIPLFPVQTKYMDVVKERIRLARQIEKSEYRNFQACLHNSWIEQAAAALEIELEEDMYKGGKADQQEERRRQKQMKVLKKELRHLLSQPLFTESQKTKYPTQSGKPPLLVSAPSKSESALSCLSKQKKKKTKKPKEPQPEQPQPSTSAN

134 NP_003950.1 NP_009027.1

>NP_003950.1

MNSIKNVPARVLSRRPGHSLEAEREQFDKTQAISISKAINTQEAPVKEKHARRIILGTHHEKGAFTFWSYAIGLPLPSSSILSWKFCHVLHKVLRDGHPNVLHDCQRYRSNIREIGDLWGHLHDRYGQLVNVYTKLLLTKISFHLKHPQFPAGLEVTDEVLEKAAGTDVNNIFQLTVEMFDYMDCELKLSESVFRQLNTAIAVSQMSSGQCRLAPLIQVIQDCSHLYHYTVKLLFKLHSCLPADTLQGHRDRFHEQFHSLRNFFRRASDMLYFKRLIQIPRLPEGPPNFLRASALAEHIKPVVVIPEEAPEDEEPENLIEISTGPPAGEPVVVADLFDQTFGPPNGSVKDDRDLQIESLKREVEMLRSELEKIKLEAQRYIAQLKSQVNALEGELEEQRKQKQKALVDNEQLRHELAQLRAAQLEGERSQGLREEAERKASATEARYNKLKEKHSELVHVHAELLRKNADTAKQLTVTQQSQEEVARVKEQLAFQVEQVKRESELKLEEKSDQLEKLKRELEAKAGELARAQEALSHTEQSKSELSSRLDTLSAEKDALSGAVRQREADLLAAQSLVRETEAALSREQQRSSQEQGELQGRLAERESQEQGLRQRLLDEQFAVLRGAAAEAAGILQDAVSKLDDPLHLRCTSSPDYLVSRAQEALDAVSTLEEGHAQYLTSLADASALVAALTRFSHLAADTIINGGATSHLAPTDPADRLIDTCRECGARALELMGQLQDQQALRHMQASLVRTPLQGILQLGQELKPKSLDVRQEELGAVVDKEMAATSAAIEDAVRRIEDMMNQARHASSGVKLEVNERILNSCTDLMKAIRLLVTTSTSLQKEIVESGRGAATQQEFYAKNSRWTEGLISASKAVGWGATQLVEAADKVVLHTGKYEELIVCSHEIAASTAQLVAASKVKANKHSPHLSRLQECSRTVNERAANVVASTKSGQEQIEDRDTMDFSGLSLIKLKKQEMETQVRVLELEKTLEAERMRLGELRKQHYVLAGASGSPGEEVAIRPSTAPRSVTTKKPPLAQKPSVAPRQDHQLDKKDGIYPAQLVNY

>NP_009027.1

MAELDPFGAPAGAPGGPALGNGVAGAGEEDPAAAFLAQQESEIAGIENDEAFAILDGGAPGPQPHGEPPGGPDAVDGVMNGEYYQESNGPTDSYAAISQVDRLQSEPESIRKWREEQMERLEALDANSRKQEAEWKEKAIKELEEWYARQDEQLQKTKANNRVADEAFYKQPFADVIGYVTNINHPCYSLEQAAEEAFVNDIDESSPGTEWERVARLCDFNPKSSKQAKDVSRMRSVLISLKQAPLVH

135 NP_002892.1 NP_000323.2

>NP_002892.1

MARGGRGRRLGLALGLLLALVLAPRVLRAKPTVRKERVVRPDSELGERPPEDNQSFQYDHEAFLGKEDSKTFDQLTPDESKERLGKIVDRIDNDGDGFVTTEELKTWIKRVQKRYIFDNVAKVWKDYDRDKDDKISWEEYKQATYGYYLGNPAEFHDSSDHHTFKKMLPRDERRFKAADLNGDLTATREEFTAFLHPEEFEHMKEIVVLETLEDIDKNGDGFVDQDEYIADMFSHEENGPEPDWVLSEREQFNEFRDLNKDGKLDKDEIRHWILPQDYDHAQAEARHLVYESDKNKDEKLTKEEILENWNMFVGSQATNYGEDLTKNHDEL

>NP_000323.2

MKSNQERSNECLPPKKREIPATSRSSEEKAPTLPSDNHRVEGTAWLPGNPGGRGHGGGRHGPAGTSVELGLQQGIGLHKALSTGLDYSPPSAPRSVPVATTLPAAYATPQPGTPVSPVQYAHLPHTFQFIGSSQYSGTYASFIPSQLIPPTANPVTSAVASAAGATTPSQRSQLEAYSTLLANMGSLSQTPGHKAEQQQQQQQQQQQQHQHQQQQQQQQQQQQQQHLSRAPGLITPGSPPPAQQNQYVHISSSPQNTGRTASPPAIPVHLHPHQTMIPHTLTLGPPSQVVMQYADSGSHFVPREATKKAESSRLQQAIQAKEVLNGEMEKSRRYGAPSSADLGLGKAGGKSVPHPYESRHVVVHPSPSDYSSRDPSGVRASVMVLPNSNTPAADLEVQQATHREASPSTLNDKSGLHLGKPGHRSYALSPHTVIQTTHSASEPLPVGLPATAFYAGTQPPVIGYLSGQQQAITYAGSLPQHLVIPGTQPLLIPVGSTDMEASGAAPAIVTSSPQFAAVPHTFVTTALPKSENFNPEALVTQAAYPAMVQAQIHLPVVQSVASPAAAPPTLPPYFMKGSIIQLANGELKKVEDLKTEDFIQSAEISNDLKIDSSTVERIEDSHSPGVAVIQFAVGEHRAQVSVEVLVEYPFFVFGQGWSSCCPERTSQLFDLPCSKLSVGDVCISLTLKNLKNGSVKKGQPVDPASVLLKHSKADGLAGSRHRYAEQENGINQGSAQMLSENGELKFPEKMGLPAAPFLTKIEPSKPAATRKRRWSAPESRKLEKSEDEPPLTLPKPSLIPQEVKICIEGRSNVGK

136 NP_000362.1 NP_003631.2

>NP_000362.1

MASHRLLLLCLAGLVFVSEAGPTGTGESKCPLMVKVLDAVRGSPAINVAVHVFRKAADDTWEPFASGKTSESGELHGLTTEEEFVEGIYKVEIDTKSYWKALGISPFHEHAEVVFTANDSGPRRYTIAALLSPYSYSTTAVVTNPKE

>NP_003631.2

MRNLKLFRTLEFRDIQGPGNPQCFSLRTEQGTVLIGSEHGLIEVDPVSREVKNEVSLVAEGFLPEDGSGRIVGVQDLLDQESVCVATASGDVILCSLSTQQLECVGSVASGISVMSWSPDQELVLLATGQQTLIMMTKDFEPILEQQIHQDDFGESKFITVGWGRKETQFHGSEGRQAAFQMQMHESALPWDDHRPQVTWRGDGQFFAVSVVCPETGARKVRVWNREFALQSTSEPVAGLGPALAWKPSGSLIASTQDKPNQQDIVFFEKNGLLHGHFTLPFLKDEVKVNDLLWNADSSVLAVWLEDLQREESSIPKTCVQLWTVGNYHWYLKQSLSFSTCGKSKIVSLMWDPVTPYRLHVLCQGWHYLAYDWHWTTDRSVGDNSSDLSNVAVIDGNRVLVTVFRQTVVPPPMCTYQLLFPHPVNQVTFLAHPQKSNDLAVLDASNQISVYKCGDCPSADPTVKLGAVGGSGFKVCLRTPHLEKRYKIQFENNEDQDVNPLKLGLLTWIEEDVFLAVSHSEFSPRSVIHHLTAASSEMDEEHGQLNVSSSAAVDGVIISLCCNSKTKSVVLQLADGQIFKYLWESPSLAIKPWKNSGGFPVRFPYPCTQTELAMIGEEECVLGLTDRCRFFINDIEVASNITSFAVYDEFLLLTTHSHTCQCFCLRDASFKTLQAGLSSNHVSHGEVLRKVERGSRIVTVVPQDTKLVLQMPRGNLEVVHHRALVLAQIRKWLDKLMFKEAFECMRKLRINLNLIYDHNPKVFLGNVETFIKQIDSVNHINLFFTELKEEDVTKTMYPAPVTSSVYLSRDPDGNKIDLVCDAMRAVMESINPHKYCLSILTSHVKKTTPELEIVLQKVHELQGNAPSDPDAVSAEEALKYLLHLVDVNELYDHSLGTYDFDLVLMVAEKSQKDPKEYLPFLNTLKKMETNYQRFTIDKYLKRYEKAIGHLSKCGPEYFPECLNLIKDKNLYNEALKLYSPSSQQYQDISIAYGEHLMQEHMYEPAGLMFARCGAHEKALSAFLTCGNWKQALCVAAQLNFTKDQLVGLGRTLAGKLVEQRKHIDAAMVLEECAQDYEEAVLLLLEGAAWEEALRLVYKYNRLDIIETNVKPSILEAQKNYMAFLDSQTATFSRHKKRLLVVRELKEQAQQAGLDDEVPHGQESDLFSETSSVVSGSEMSGKYSHSNSRISARSSKNRRKAERKKHSLKEGSPLEDLALLEALSEVVQNTENLKDEVYHILKVLFLFEFDEQGRELQKAFEDTLQLMERSLPEIWTLTYQQNSATPVLGPNSTANSIMASYQQQKTSVPVLDAELFIPPKINRRTQWKLSLLD

137 NP_066964.1 NP_006181.1

>NP_066964.1

MVRSGNKAAVVLCMDVGFTMSNSIPGIESPFEQAKKVITMFVQRQVFAENKDEIALVLFGTDGTDNPLSGGDQYQNITVHRHLMLPDFDLLEDIESKIQPGSQQADFLDALIVSMDVIQHETIGKKFEKRHIEIFTDLSSRFSKSQLDIIIHSLKKCDISLQFFLPFSLGKEDGSGDRGDGPFRLGGHGPSFPLKGITEQQKEGLEIVKMVMISLEGEDGLDEIYSFSESLRKLCVFKKIERHSIHWPCRLTIGSNLSIRIAAYKSILQERVKKTWTVVDAKTLKKEDIQKETVYCLNDDDETEVLKEDIIQGFRYGSDIVPFSKVDEEQMKYKSEGKCFSVLGFCKSSQVQRRFFMGNQVLKVFAARDDEAAAVALSSLIHALDDLDMVAIVRYAYDKRANPQVGVAFPHIKHNYECLVYVQLPFMEDLRQYMFSSLKNSKKYAPTEAQLNAVDALIDSMSLAKKDEKTDTLEDLFPTTKIPNPRFQRLFQCLLHRALHPREPLPPIQQHIWNMLNPPAEVTTKSQIPLSKIKTLFPLIEAKKKDQVTAQEIFQDNHEDGPTAKKLKTEQGGAHFSVSSLAEGSVTSVGSVNPAENFRVLVKQKKASFEEASNQLINHIEQFLDTNETPYFMKSIDCIRAFREEAIKFSEEQRFNNFLKALQEKVEIKQLNHFWEIVVQDGITLITKEEASGSSVTAEEAKKFLAPKDKPSGDTAAVFEEGGDVDDLLDMI

>NP_006181.1

MSKPELKEDKMLEVHFVGDDDVLNHILDREGGAKLKKERAQLLVNPKKIIKKPEYDLEEDDQEVLKDQNYVEIMGRDVQESLKNGSATGGGNKVYSFQNRKHSEKMAKLASELAKTPQKSVSFSLKNDPEITINVPQSSKGHSASDKVQPKNNDKSEFLSTAPRSLRKRLIVPRSHSDSESEYSASNSEDDEGVAQEHEEDTNAVIFSQKIQAQNRVVSAPVGKETPSKRMKRDKTSDLVEEYFEAHSSSKVLTSDRTLQKLKRAKLDQQTLRNLLSKVSPSFSAELKQLNQQYEKLFHKWMLQLHLGFNIVLYGLGSKRDLLERFRTTMLQDSIHVVINGFFPGISVKSVLNSITEEVLDHMGTFRSILDQLDWIVNKFKEDSSLELFLLIHNLDSQMLRGEKSQQIIGQLSSLHNIYLIASIDHLNAPLMWDHAKQSLFNWLWYETTTYSPYTEETSYENSLLVKQSGSLPLSSLTHVLRSLTPNARGIFRLLIKYQLDNQDNPSYIGLSFQDFYQQCREAFLVNSDLTLRAQLTEFRDHKLIRTKKGTDGVEYLLIPVDNGTLTDFLEKEEEEA

138 NP_000050.2 NP_068741.1

>NP_000050.2

MPIGSKERPTFFEIFKTRCNKADLGPISLNWFEELSSEAPPYNSEPAEESEHKNNNYEPNLFKTPQRKPSYNQLASTPIIFKEQGLTLPLYQSPVKELDKFKLDLGRNVPNSRHKSLRTVKTKMDQADDVSCPLLNSCLSESPVVLQCTHVTPQRDKSVVCGSLFHTPKFVKGRQTPKHISESLGAEVDPDMSWSSSLATPPTLSSTVLIVRNEEASETVFPHDTTANVKSYFSNHDESLKKNDRFIASVTDSENTNQREAASHGFGKTSGNSFKVNSCKDHIGKSMPNVLEDEVYETVVDTSEEDSFSLCFSKCRTKNLQKVRTSKTRKKIFHEANADECEKSKNQVKEKYSFVSEVEPNDTDPLDSNVANQKPFESGSDKISKEVVPSLACEWSQLTLSGLNGAQMEKIPLLHISSCDQNISEKDLLDTENKRKKDFLTSENSLPRISSLPKSEKPLNEETVVNKRDEEQHLESHTDCILAVKQAISGTSPVASSFQGIKKSIFRIRESPKETFNASFSGHMTDPNFKKETEASESGLEIHTVCSQKEDSLCPNLIDNGSWPATTTQNSVALKNAGLISTLKKKTNKFIYAIHDETSYKGKKIPKDQKSELINCSAQFEANAFEAPLTFANADSGLLHSSVKRSCSQNDSEEPTLSLTSSFGTILRKCSRNETCSNNTVISQDLDYKEAKCNKEKLQLFITPEADSLSCLQEGQCENDPKSKKVSDIKEEVLAAACHPVQHSKVEYSDTDFQSQKSLLYDHENASTLILTPTSKDVLSNLVMISRGKESYKMSDKLKGNNYESDVELTKNIPMEKNQDVCALNENYKNVELLPPEKYMRVASPSRKVQFNQNTNLRVIQKNQEETTSISKITVNPDSEELFSDNENNFVFQVANERNNLALGNTKELHETDLTCVNEPIFKNSTMVLYGDTGDKQATQVSIKKDLVYVLAEENKNSVKQHIKMTLGQDLKSDISLNIDKIPEKNNDYMNKWAGLLGPISNHSFGGSFRTASNKEIKLSEHNIKKSKMFFKDIEEQYPTSLACVEIVNTLALDNQKKLSKPQSINTVSAHLQSSVVVSDCKNSHITPQMLFSKQDFNSNHNLTPSQKAEITELSTILEESGSQFEFTQFRKPSYILQKSTFEVPENQMTILKTTSEECRDADLHVIMNAPSIGQVDSSKQFEGTVEIKRKFAGLLKNDCNKSASGYLTDENEVGFRGFYSAHGTKLNVSTEALQKAVKLFSDIENISEETSAEVHPISLSSSKCHDSVVSMFKIENHNDKTVSEKNNKCQLILQNNIEMTTGTFVEEITENYKRNTENEDNKYTAASRNSHNLEFDGSDSSKNDTVCIHKDETDLLFTDQHNICLKLSGQFMKEGNTQIKEDLSDLTFLEVAKAQEACHGNTSNKEQLTATKTEQNIKDFETSDTFFQTASGKNISVAKESFNKIVNFFDQKPEELHNFSLNSELHSDIRKNKMDILSYEETDIVKHKILKESVPVGTGNQLVTFQGQPERDEKIKEPTLLGFHTASGKKVKIAKESLDKVKNLFDEKEQGTSEITSFSHQWAKTLKYREACKDLELACETIEITAAPKCKEMQNSLNNDKNLVSIETVVPPKLLSDNLCRQTENLKTSKSIFLKVKVHENVEKETAKSPATCYTNQSPYSVIENSALAFYTSCSRKTSVSQTSLLEAKKWLREGIFDGQPERINTADYVGNYLYENNSNSTIAENDKNHLSEKQDTYLSNSSMSNSYSYHSDEVYNDSGYLSKNKLDSGIEPVLKNVEDQKNTSFSKVISNVKDANAYPQTVNEDICVEELVTSSSPCKNKNAAIKLSISNSNNFEVGPPAFRIASGKIVCVSHETIKKVKDIFTDSFSKVIKENNENKSKICQTKIMAGCYEALDDSEDILHNSLDNDECSTHSHKVFADIQSEEILQHNQNMSGLEKVSKISPCDVSLETSDICKCSIGKLHKSVSSANTCGIFSTASGKSVQVSDASLQNARQVFSEIEDSTKQVFSKVLFKSNEHSDQLTREENTAIRTPEHLISQKGFSYNVVNSSAFSGFSTASGKQVSILESSLHKVKGVLEEFDLIRTEHSLHYSPTSRQNVSKILPRVDKRNPEHCVNSEMEKTCSKEFKLSNNLNVEGGSSENNHSIKVSPYLSQFQQDKQQLVLGTKVSLVENIHVLGKEQASPKNVKMEIGKTETFSDVPVKTNIEVCSTYSKDSENYFETEAVEIAKAFMEDDELTDSKLPSHATHSLFTCPENEEMVLSNSRIGKRRGEPLILVGEPSIKRNLLNEFDRIIENQEKSLKASKSTPDGTIKDRRLFMHHVSLEPITCVPFRTTKERQEIQNPNFTAPGQEFLSKSHLYEHLTLEKSSSNLAVSGHPFYQVSATRNEKMRHLITTGRPTKVFVPPFKTKSHFHRVEQCVRNINLEENRQKQNIDGHGSDDSKNKINDNEIHQFNKNNSNQAAAVTFTKCEEEPLDLITSLQNARDIQDMRIKKKQRQRVFPQPGSLYLAKTSTLPRISLKAAVGGQVPSACSHKQLYTYGVSKHCIKINSKNAESFQFHTEDYFGKESLWTGKGIQLADGGWLIPSNDGKAGKEEFYRALCDTPGVDPKLISRIWVYNHYRWIIWKLAAMECAFPKEFANRCLSPERVLLQLKYRYDTEIDRSRRSAIKKIMERDDTAAKTLVLCVSDIISLSANISETSSNKTSSADTQKVAIIELTDGWYAVKAQLDPPLLAVLKNGRLTVGQKIILHGAELVGSPDACTPLEAPESLMLKISANSTRPARWYTKLGFFPDPRPFPLPLSSLFSDGGNVGCVDVIIQRAYPIQWMEKTSSGLYIFRNEREEEKEAAKYVEAQQKRLEALFTKIQEEFEEHEENTTKPYLPSRALTRQQVRALQDGAELYEAVKNAADPAYLEGYFSEEQLRALNNHRQMLNDKKQAQIQLEIRKAMESAEQKEQGLSRDVTTVWKLRIVSYSKKEKDSVILSIWRPSSDLYSLLTEGKRYRIYHLATSKSKSKSERANIQLAATKKTQYQQLPVSDEILFQIYQPREPLHFSKFLDPDFQPSCSEVDLIGFVVSVVKKTGLAPFVYLSDECYNLLAIKFWIDLNEDIIKPHMLIAASNLQWRPESKSGLLTLFAGDFSVFSASPKEGHFQETFNKMKNTVENIDILCNEAENKLMHILHANDPKWSTPTKDCTSGPYTAQIIPGTGNKLLMSSPNCEIYYQSPLSLCMAKRKSVSTPVSAQMTSKSCKGEKEIDDQKNCKKRRALDFLSRLPLPPPVSPICTFVSPAAQKAFQPPRSCGTKYETPIKKKELNSPQMTPFKKFNEISLLESNSIADEELALINTQALLSGSTGEKQFISVSESTRTAPTSSEDYLRLKRRCTTSLIKEQESSQASTEECEKNKQDTITTKKYI

>NP_068741.1

MATPDAGLPGAEGVEPAPWAQLEAPARLLLQALQAGPEGARRGLGVLRALGSRGWEPFDWGRLLEALCREEPVVQGPDGRLELKPLLLRLPRICQRNLMSLLMAVRPSLPESGLLSVLQIAQQDLAPDPDAWLRALGELLRRDLGVGTSMEGASPLSERCQRQLQSLCRGLGLGGRRLKSPQAPDPEEEENRDSQQPGKRRKDSEEEAASPEGKRVPKRLRCWEEEEDHEKERPEHKSLESLADGGSASPIKDQPVMAVKTGEDGSNLDDAKGLAESLELPKAIQDQLPRLQQLLKTLEEGLEGLEDAPPVELQLLHECSPSQMDLLCAQLQLPQLSDLGLLRLCTWLLALSPDLSLSNATVLTRSLFLGRILSLTSSASRLLTTALTSFCAKYTYPVCSALLDPVLQAPGTGPAQTELLCCLVKMESLEPDAQVLMLGQILELPWKEETFLVLQSLLERQVEMTPEKFSVLMEKLCKKGLAATTSMAYAKLMLTVMTKYQANITETQRLGLAMALEPNTTFLRKSLKAALKHLGP

139 NP_054890.1 NP_004078.2

>NP_054890.1

MVCEKCEKKLGTVITPDTWKDGARNTTESGGRKLNENKALTSKKARFDPYGKNKFSTCRICKSSVHQPGSHYCQGCAYKKGICAMCGKKVLDTKNYKQTSV

>NP_004078.2

MPVRKQDTQRALHLLEEYRSKLSQTEDRQLRSSIERVINIFQSNLFQALIDIQEFYEVTLLDNPKCIDRSKPSEPIQPVNTWEISSLPSSTVTSETLPSSLSPSVEKYRYQDEDTPPQEHISPQITNEVIGPELVHVSEKNLSEIENVHGFVSHSHISPIKPTEAVLPSPPTVPVIPVLPVPAENTVILPTIPQANPPPVLVNTDSLETPTYVNGTDADYEYEEITLERGNSGLGFSIAGGTDNPHIGDDSSIFITKIITGGAAAQDGRLRVNDCILRVNEVDVRDVTHSKAVEALKEAGSIVRLYVKRRKPVSEKIMEIKLIKGPKGLGFSIAGGVGNQHIPGDNSIYVTKIIEGGAAHKDGKLQIGDKLLAVNNVCLEEVTHEEAVTALKNTSDFVYLKVAKPTSMYMNDGYAPPDITNSSSQPVDNHVSPSSFLGQTPASPARYSPVSKAVLGDDEITREPRKVVLHRGSTGLGFNIVGGEDGEGIFISFILAGGPADLSGELRKGDRIISVNSVDLRAASHEQAAAALKNAGQAVTIVAQYRPEEYSRFEAKIHDLREQMMNSSISSGSGSLRTSQKRSLYVRALFDYDKTKDSGLPSQGLNFKFGDILHVINASDDEWWQARQVTPDGESDEVGVIPSKRRVEKKERARLKTVKFNSKTRDKGQSFNDKRKKNLFSRKFPFYKNKDQSEQETSDADQHVTSNASDSESSYRGQEEYVLSYEPVNQQEVNYTRPVIILGPMKDRINDDLISEFPDKFGSCVPHTTRPKRDYEVDGRDYHFVTSREQMEKDIQEHKFIEAGQYNNHLYGTSVQSVREVAEKGKHCILDVSGNAIKRLQIAQLYPISIFIKPKSMENIMEMNKRLTEEQARKTFERAMKLEQEFTEHFTAIVQGDTLEDIYNQVKQIIEEQSGSYIWVPAKEKL

140 NP_653086.1 NP_062565.2

>NP_653086.1

MGTKAQVERKLLCLFILAILLCSLALGSVTVHSSEPEVRIPENNPVKLSCAYSGFSSPRVEWKFDQGDTTRLVCYNNKITASYEDRVTFLPTGITFKSVTREDTGTYTCMVSEEGGNSYGEVKVKLIVLVPPSKPTVNIPSSATIGNRAVLTCSEQDGSPPSEYTWFKDGIVMPTNPKSTRAFSNSSYVLNPTTGELVFDPLSASDTGEYSCEARNGYGTPMTSNAVRMEAVERNVGVIVAAVLVTLILLGILVFGIWFAYSRGHFDRTKKGTSSKKVIYSQPSARSEGEFKQTSSFLV

>NP_062565.2

MKVTVCFGRTRVVVPCGDGHMKVFSLIQQAVTRYRKAIAKDPNYWIQVHRLEHGDGGILDLDDILCDVADDKDRLVAVFDEQDPHHGGDGTSASSTGTQSPEIFGSELGTNNVSAFQPYQATSEIEVTPSVLRANMPLHVRRSSDPALIGLSTSVSDSNFSSEEPSRKNPTRWSTTAGFLKQNTAGSPKTCDRKKDENYRSLPRDTSNWSNQFQRDNARSSLSASHPMVGKWLEKQEQDEDGTEEDNSRVEPVGHADTGLEHIPNFSLDDMVKLVEVPNDGGPLGIHVVPFSARGGRTLGLLVKRLEKGGKAEHENLFRENDCIVRINDGDLRNRRFEQAQHMFRQAMRTPIIWFHVVPAANKEQYEQLSQSEKNNYYSSRFSPDSQYIDNRSVNSAGLHTVQRAPRLNHPPEQIDSHSRLPHSAHPSGKPPSAPASAPQNVFSTTVSSGYNTKKIGKRLNIQLKKGTEGLGFSITSRDVTIGGSAPIYVKNILPRGAAIQDGRLKAGDRLIEVNGVDLVGKSQEEVVSLLRSTKMEGTVSLLVFRQEDAFHPRELNAEPSQMQIPKETKAEDEDIVLTPDGTREFLTFEVPLNDSGSAGLGVSVKGNRSKENHADLGIFVKSIINGGAASKDGRLRVNDQLIAVNGESLLGKTNQDAMETLRRSMSTEGNKRGMIQLIVARRISKCNELKSPGSPPGPELPIETALDDRERRISHSLYSGIEGLDESPSRNAALSRIMGESGKYQLSPTVNMPQDDTVIIEDDRLPVLPPHLSDQSSSSSHDDVGFVTADAGTWAKAAISDSADCSLSPDVDPVLAFQREGFGRQSMSEKRTKQFSDASQLDFVKTRKSKSMDLGIADETKLNTVDDQKAGSPSRDVGPSLGLKKSSSLESLQTAVAEVTLNGDIPFHRPRPRIIRGRGCNESFRAAIDKSYDKPAVDDDDEGMETLEEDTEESSRSGRESVSTASDQPSHSLERQMNGNQEKGDKTDRKKDKTGKEKKKDRDKEKDKMKAKKGMLKGLGDMFRFGKHRKDDKIEKTGKIKIQESFTSEEERIRMKQEQERIQAKTREFRERQARERDYAEIQDFHRTFGCDDELMYGGVSSYEGSMALNARPQSPREGHMMDALYAQVKKPRNSKPSPVDSNRSTPSNHDRIQRLRQEFQQAKQDEDVEDRRRTYSFEQPWPNARPATQSGRHSVSVEVQMQRQRQEERESSQQAQRQYSSLPRQSRKNASSVSQDSWEQNYSPGEGFQSAKENPRYSSYQGSRNGYLGGHGFNARVMLETQELLRQEQRRKEQQMKKQPPSEGPSNYDSYKKVQDPSYAPPKGPFRQDVPPSPSQVARLNRLQTPEKGRPFYS

141 NP_006640.2 NP_060853.3

>NP_006640.2

MTANRLAESLLALSQQEELADLPKDYLLSESEDEGDNDGERKHQKLLEAISSLDGKNRRKLAERSEASLKVSEFNVSSEGSGEKLVLADLLEPVKTSSSLATVKKQLSRVKSKKTVELPLNKEEIERIHREVAFNKTAQVLSKWDPVVLKNRQAEQLVFPLEKEEPAIAPIEHVLSGWKARTPLEQEIFNLLHKNKQPVTDPLLTPVEKASLRAMSLEEAKMRRAELQRARALQSYYEAKARREKKIKSKKYHKVVKKGKAKKALKEFEQLRKVNPAAALEELEKIEKARMMERMSLKHQNSGKWAKSKAIMAKYDLEARQAMQEQLSKNKELTQKLQVASESEEEEGGTEDVEELLVPDVVNEVQMNADGPNPWMLRSCTSDTKEAATQEDPEQLPELEAHGVSESEGEERPVAEEEILLREFEERRSLRKRSELSQDAEPAGSQETKDSGSQEVLSELRVLSQKLKENHQSRKQKASSEGTIPQVQREEPAPEEEEPLLLQRPERVQTLEELEELGKEECFQNKELPRPVLEGQQSERTPNNRPDAPKEKKKKEQMIDLQNLLTTQSPSVKSLAVPTIEELEDEEERNHRQMIKEAFAGDDVIRDFLKEKREAVEASKPKDVDLTLPGWGEWGGVGLKPSAKKRRRFLIKAPEGPPRKDKNLPNVIINEKRNIHAAAHQVRVLPYPFTHHWQFERTIQTPIGSTWNTQRAFQKLTTPKVVTKPGHIINPIKAEDVGYRSSSRSDLSVIQRNPKRITTRHKKQLKKCSVD

>NP_060853.3

MATEIGSPPRFFHMPRFQHQAPRQLFYKRPDFAQQQAMQQLTFDGKRMRKAVNRKTIDYNPSVIKYLENRIWQRDQRDMRAIQPDAGYYNDLVPPIGMLNNPMNAVTTKFVRTSTNKVKCPVFVVRWTPEGRRLVTGASSGEFTLWNGLTFNFETILQAHDSPVRAMTWSHNDMWMLTADHGGYVKYWQSNMNNVKMFQAHKEAIREASFSPTDNKFATCSDDGTVRIWDFLRCHEERILRGHGADVKCVDWHPTKGLVVSGSKDSQQPIKFWDPKTGQSLATLHAHKNTVMEVKLNLNGNWLLTASRDHLCKLFDIRNLKEELQVFRGHKKEATAVAWHPVHEGLFASGGSDGSLLFWHVGVEKEVGGMEMAHEGMIWSLAWHPLGHILCSGSNDHTSKFWTRNRPGDKMRDRYNLNLLPGMSEDGVEYDDLEPNSLAVIPGMGIPEQLKLAMEQEQMGKDESNEIEMTIPGLDWGMEEVMQKDQKKVPQKKVPYAKPIPAQFQQAWMQNKVPIPAPNEVLNDRKEDIKLEEKKKTQAEIEQEMATLQYTNPQLLEQLKIERLAQKQVEQIQPPPSSGTPLLGPQPFPGQGPMSQIPQGFQQPHPSQQMPMNMAQMGPPGPQGQFRPPGPQGQMGPQGPPLHQGGGGPQGFMGPQGPQGPPQGLPRPQDMHGPQGMQRHPGPHGPLGPQGPPGPQGSSGPQGHMGPQGPPGPQGHIGPQGPPGPQGHLGPQGPPGTQGMQGPPGPRGMQGPPHPHGIQGGPGSQGIQGPVSQGPLMGLNPRGMQGPPGPRENQGPAPQGMIMGHPPQEMRGPHPPGGLLGHGPQEMRGPQEIRGMQGPPPQGSMLGPPQELRGPPGSQSQQGPPQGSLGPPPQGGMQGPPGPQGQQNPARGPHPSQGPIPFQQQKTPLLGDGPRAPFNQEGQSTGPPPLIPGLGQQGAQGRIPPLNPGQGPGPNKGDSRGPPNHHMGPMSERRHEQSGGPEHGPERGPFRGGQDCRGPPDRRGPHPDFPDDFSRPDDFHPDKRFGHRLREFEGRGGPLPQEEKWRRGGPGPPFPPDHREFSEGDGRGAARGPPGAWEGRRPGDERFPRDPEDPRFRGRREESFRRGAPPRHEGRAPPRGRDGFPGPEDFGPEENFDASEEAARGRDLRGRGRGTPRGGRKGLLPTPDEFPRFEGGRKPDSWDGNREPGPGHEHFRDTPRPDHPPHDGHSPASRERSSSLQGMDMASLPPRKRPWHDGPGTSEHREMEAPGGPSEDRGGKGRGGPGPAQRVPKSGRSSSLDGEHHDGYHRDEPFGGPPGSGTPSRGGRSGSNWGRGSNMNSGPPRRGASRGGGRGR

142 NP_006092.1 NP_000312.2

>NP_006092.1

MKRSSVSSGGAGRLSMQELRSQDVNKQGLYTPQTKEKPTFGKLSINKPTSERKVSLFGKRTSGHGSRNSQLGIFSSSEKIKDPRPLNDKAFIQQCIRQLCEFLTENGYAHNVSMKSLQAPSVKDFLKIFTFLYGFLCPSYELPDTKFEEEVPRIFKDLGYPFALSKSSMYTVGAPHTWPHIVAALVWLIDCIKIHTAMKESSPLFDDGQPWGEETEDGIMHNKLFLDYTIKCYESFMSGADSFDEMNAELQSKLKDLFNVDAFKLESLEAKNRALNEQIARLEQEREKEPNRLESLRKLKASLQGDVQKYQAYMSNLESHSAILDQKLNGLNEEIARVELECETIKQENTRLQNIIDNQKYSVADIERINHERNELQQTINKLTKDLEAEQQKLWNEELKYARGKEAIETQLAEYHKLARKLKLIPKGAENSKGYDFEIKFNPEAGANCLVKYRAQVYVPLKELLNETEEEINKALNKKMGLEDTLEQLNAMITESKRSVRTLKEEVQKLDDLYQQKIKEAEEEDEKCASELESLEKHKHLLESTVNQGLSEAMNELDAVQREYQLVVQTTTEERRKVGNNLQRLLEMVATHVGSVEKHLEEQIAKVDREYEECMSEDLSENIKEIRDKYEKKATLIKSSEE

>NP_000312.2

MPPKTPRKTAATAAAAAAEPPAPPPPPPPEEDPEQDSGPEDLPLVRLEFEETEEPDFTALCQKLKIPDHVRERAWLTWEKVSSVDGVLGGYIQKKKELWGICIFIAAVDLDEMSFTFTELQKNIEISVHKFFNLLKEIDTSTKVDNAMSRLLKKYDVLFALFSKLERTCELIYLTQPSSSISTEINSALVLKVSWITFLLAKGEVLQMEDDLVISFQLMLCVLDYFIKLSPPMLLKEPYKTAVIPINGSPRTPRRGQNRSARIAKQLENDTRIIEVLCKEHECNIDEVKNVYFKNFIPFMNSLGLVTSNGLPEVENLSKRYEEIYLKNKDLDARLFLDHDKTLQTDSIDSFETQRTPRKSNLDEEVNVIPPHTPVRTVMNTIQQLMMILNSASDQPSENLISYFNNCTVNPKESILKRVKDIGYIFKEKFAKAVGQGCVEIGSQRYKLGVRLYYRVMESMLKSEEERLSIQNFSKLLNDNIFHMSLLACALEVVMATYSRSTSQNLDSGTDLSFPWILNVLNLKAFDFYKVIESFIKAEGNLTREMIKHLERCEHRIMESLAWLSDSPLFDLIKQSKDREGPTDHLESACPLNLPLQNNHTAADMYLSPVRSPKKKGSTTRVNSTANAETQATSAFQTQKPLKSTSLSLFYKKVYRLAYLRLNTLCERLLSEHPELEHIIWTLFQHTLQNEYELMRDRHLDQIMMCSMYGICKVKNIDLKFKIIVTAYKDLPHAVQETFKRVLIKEEEYDSIIVFYNSVFMQRLKTNILQYASTRPPTLSPIPHIPRSPYKFPSSPLRIPGGNIYISPLKSPYKISEGLPTPTKMTPRSRILVSIGESFGTSEKFQKINQMVCNSDRVLKRSAEGSNPPKPLKKLRFDIEGSDEADGSKHLPGESKFQQKLAEMTSTRTRMQKQKMNDSMDTSNKEEK

143 NP_919307.1 NP_001013.1

>NP_919307.1

MSAALLRRGLELLAASEAPRDPPGQAKPRGAPVKRPRKTKAIQAQKLRNSAKGKVPKSALDEYRKRECRDHLRVNLKFLTRTRSTVAESVSQQILRQNRGRKACDRPVAKTKKKKAEGTVFTEEDFQKFQQEYFGS

>NP_001013.1

MPGVTVKDVNQQEFVRALAAFLKKSGKLKVPEWVDTVKLAKHKELAPYDENWFYTRAASTARHLYLRGGAGVGSMTKIYGGRQRNGVMPSHFSRGSKSVARRVLQALEGLKMVEKDQDGGRKLTPQGQRDLDRIAGQVAAANKKH

144 NP_036336.1 NP_001512.1

>NP_036336.1

MNTADQARVGPADDGPAPSGEEEGEGGGEAGGKEPAADAAPGPSAAFRLMVTRREPAVKLQYAVSGLEPLAWSEDHRVSVSTARSIAVLELICDVHNPGQDLVIHRTSVPAPLNSCLLKVGSKTEVAECKEKFAASKDPTVSQTFMLDRVFNPEGKALPPMRGFKYTSWSPMGCDANGRCLLAALTMDNRLTIQANLNRLQWVQLVDLTEIYGERLYETSYRLSKNEAPEGNLGDFAEFQRRHSMQTPVRMEWSGICTTQQVKHNNESRDVGSVLLAVLFENGNIAVWQFQLPFVGKESISSCNTIESGITSPSVLFWWEYEHNNRKMSGLIVGSAFGPIKILPVNLKAVKGYFTLRQPVILWKEMDQLPVHSIKCVPLYHPYQKCSCSLVVAARGSYVFWRLLLISKAGLNLHNSHVTGLHSLPIVSMTADKQNGTVYTCSSDGKVRQVIPIFTDVALKFEHQLIKLSDVFGSVRTHGIAVKPCGAYLAIITTEGMINGLHPVNKNYQVQFVTLKTFEEAAAQLLESSVQNLFKQVDLIDLVRWKILKDKHIPQFLQEALEKKIESSGVTYFWRFKLFLLRILYQSMQKTPSEALWKPTHEDSKILLVDSPGMGNADDEQQEEGTSSKQVVKQGLQERSKEGDVEEPTDDSLPTTGDAGGREPMEEKLLEIQGKIEAVEMHLTREHMKPVLGEVYLHTWITENTSIPTRGLCNFLMSDEEYDDRTARVLIGHISKKMNKQTFPEHCSLCKEILPFTDRKQAVCSNGHIWLRCFLTYQSCQSLIYRRCLLHDSIARHPAPEDPDWIKRLLQSPCPFCDSPVF

>NP_001512.1

MDTCGVGYVALGEAGPVGNMTVVDSPGQEVLNQLDVKTSSEMTSAEASVEMSLPTPLPGFEDSPDQRRLPPEQESLSRLEQPDLSSEMSKVSKPRASKPGRKRGGRTRKGPKRPQQPNPPSAPLVPGLLDQSNPLSTPMPKKRGRKSKAELLLLKLSKDLDRPESQSPKRPPEDFETPSGERPRRRAAQVALLYLQELAEELSTALPAPVSCPEGPKVSSPTKPKKIRQPAACPGGEEVDGAPRDEDFFLQVEAEDVEESEGPSESSSEPEPVVPRSTPRGSTSGKQKPHCRGMAPNGLPNHIMAPVWKCLHLTKDFREQKHSYWEFAEWIPLAWKWHLLSELEAAPYLPQEEKSPLFSVQREGLPEDGTLYRINRFSSITAHPERWDVSFFTGGPLWALDWCPVPEGAGASQYVALFSSPDMNETHPLSQLHSGPGLLQLWGLGTLQQESCPGNRAHFVYGIACDNGCIWDLKFCPSGAWELPGTPRKAPLLPRLGLLALACSDGKVLLFSLPHPEALLAQQPPDAVKPAIYKVQCVATLQVGSMQATDPSECGQCLSLAWMPTRPHQHLAAGYYNGMVVFWNLPTNSPLQRIRLSDGSLKLYPFQCFLAHDQAVRTLQWCKANSHFLVSAGSDRKIKFWDLRRPYEPINSIKRFLSTELAWLLPYNGVTVAQDNCYASYGLCGIHYIDAGYLGFKAYFTAPRKGTVWSLSGSDWLGTIAAGDISGELIAAILPDMALNPINVKRPVERRFPIYKADLIPYQDSPEGPDHSSASSGVPNPPKARTYTETVNHHYLLFQDTDLGSFHDLLRREPMLRMQEGEGHSQLCLDRLQLEAIHKVRFSPNLDSYGWLVSGGQSGLVRIHFVRGLASPLGHRMQLESRAHFNAMFQPSSPTRRPGFSPTSHRLLPTP

145 NP_004255.2 NP_003241.2

>NP_004255.2

MADRLTQLQDAVNSLADQFCNAIGVLQQCGPPASFNNIQTAINKDQPANPTEEYAQLFAALIARTAKDIDVLIDSLPSEESTAALQAASLYKLEEENHEAATCLEDVVYRGDMLLEKIQSALADIAQSQLKTRSGTHSQSLPDS

>NP_003241.2

MEQKPSKVECGSDPEENSARSPDGKRKRKNGQCSLKTSMSGYIPSYLDKDEQCVVCGDKATGYHYRCITCEGCKGFFRRTIQKNLHPTYSCKYDSCCVIDKITRNQCQLCRFKKCIAVGMAMDLVLDDSKRVAKRKLIEQNRERRRKEEMIRSLQQRPEPTPEEWDLIHIATEAHRSTNAQGSHWKQRRKFLPDDIGQSPIVSMPDGDKVDLEAFSEFTKIITPAITRVVDFAKKLPMFSELPCEDQIILLKGCCMEIMSLRAAVRYDPESDTLTLSGEMAVKREQLKNGGLGVVSDAIFELGKSLSAFNLDDTEVALLQAVLLMSTDRSGLLCVDKIEKSQEAYLLAFEHYVNHRKHNIPHFWPKLLMKEREVQSSILYKGAAAEGRPGGSLGVHPEGQQLLGMHVVQGPQVRQLEQQLGEAGSLQGPVLQHQSPKSPQQRLLELLHRSGILHARAVCGEDDSSEADSPSSSEEEPEVCEDLAGNAASP

146 NP_002125.3 NP_057144.1

>NP_002125.3

MSAEVETSEGVDESEKKNSGALEKENQMRMADLSELLKEGTKEAHDRAENTQFVKDFLKGNIKKELFKLATTALYFTYSALEEEMERNKDHPAFAPLYFPMELHRKEALTKDMEYFFGENWEEQVQCPKAAQKYVERIHYIGQNEPELLVAHAYTRYMGDLSGGQVLKKVAQRALKLPSTGEGTQFYLFENVDNAQQFKQLYRARMNALDLNMKTKERIVEEANKAFEYNMQIFNELDQAGSTLARETLEDGFPVHDGKGDMRKCPFYAAEQDKGALEGSSCPFRTAMAVLRKPSLQFILAAGVALAAGLLAWYYM

>NP_057144.1

MAAAVAMETDDAGNRLRFQLELEFVQCLANPNYLNFLAQRGYFKDKAFVNYLKYLLYWKDPEYAKYLKYPQCLHMLELLQYEHFRKELVNAQCAKFIDEQQILHWQHYSRKRMRLQQALAEQQQQNNTSGK

147 NP_008982.1 NP_001311.3

>NP_008982.1

MASNMDREMILADFQACTGIENIDEAITLLEQNNWDLVAAINGVIPQENGILQSEYGGETIPGPAFNPASHPASAPTSSSSSAFRPVMPSRQIVERQPRMLDFRVEYRDRNVDVVLEDTCTVGEIKQILENELQIPVSKMLLKGWKTGDVEDSTVLKSLHLPKNNSLYVLTPDLPPPSSSSHAGALQESLNQNFMLIITHREVQREYNLNFSGSSTIQEVKRNVYDLTSIPVRHQLWEGWPTSATDDSMCLAESGLSYPCHRLTVGRRSSPAQTREQSEEQITDVHMVSDSDGDDFEDATEFGVDDGEVFGMASSALRKSPMMPENAENEGDALLQFTAEFSSRYGDCHPVFFIGSLEAAFQEAFYVKARDRKLLAIYLHHDESVLTNVFCSQMLCAESIVSYLSQNFITWAWDLTKDSNRARFLTMCNRHFGSVVAQTIRTQKTDQFPLFLIIMGKRSSNEVLNVIQGNTTVDELMMRLMAAMEIFTAQQQEDIKDEDEREARENVKREQDEAYRLSLEADRAKREAHEREMAEQFRLEQIRKEQEEEREAIRLSLEQALPPEPKEENAEPVSKLRIRTPSGEFLERRFLASNKLQIVFDFVASKGFPWDEYKLLSTFPRRDVTQLDPNKSLLEVKLFPQETLFLEAKE

>NP_001311.3

MSSSEEVSWISWFCGLRGNEFFCEVDEDYIQDKFNLTGLNEQVPHYRQALDMILDLEPDEELEDNPNQSDLIEQAAEMLYGLIHARYILTNRGIAQMLEKYQQGDFGYCPRVYCENQPMLPIGLSDIPGEAMVKLYCPKCMDVYTPKSSRHHHTDGAYFGTGFPHMLFMVHPEYRPKRPANQFVPRLYGFKIHPMAYQLQLQAASNFKSPVKTIR

148 NP_078951.2 NP_000050.2

>NP_078951.2

MDEPPGKPLSCEEKEKLKEKLAFLKREYSKTLARLQRAQRAEKIKHSIKKTVEEQDCLSQQDLSPQLKHSEPKNKICVYDKLHIKTHLDEETGEKTSITLDVGPESFNPGDGPGGLPIQRTDDTQEHFPHRVSDPSGEQKQKLPSRRKKQQKRTFISQERDCVFGTDSLRLSGKRLKEQEEISSKNPARSPVTEIRTHLLSLKSELPDSPEPVTEINEDSVLIPPTAQPEKGVDTFLRRPNFTRATTVPLQTLSDSGSSQHLEHIPPKGSSELTTHDLKNIRFTSPVSLEAQGKKMTVSTDNLLVNKAISKSGQLPTSSNLEANISCSLNELTYNNLPANENQNLKEQNQTEKSLKSPSDTLDGRNENLQESEILSQPKSLSLEATSPLSAEKHSCTVPEGLLFPAEYYVRTTRSMSNCQRKVAVEAVIQSHLDVKKKGFKNKNKDASKNLNLSNEETDQSEIRMSGTCTGQPSSRTSQKLLSLTKVSSPAGPTEDNDLSRKAVAQAPGRRYTGKRKSACTPASDHCEPLLPTSSLSIVNRSKEEVTSHKYQHEKLFIQVKGKKSRHQKEDSLSWSNSAYLSLDDDAFTAPFHRDGMLSLKQLLSFLSITDFQLPDEDFGPLKLEKVKSCSEKPVEPFESKMFGERHLKEGSCIFPEELSPKRMDTEMEDLEEDLIVLPGKSHPKRPNSQSQHTKTGLSSSILLYTPLNTVAPDDNDRPTTDMCSPAFPILGTTPAFGPQGSYEKASTEVAGRTCCTPQLAHLKDSVCLASDTKQFDSSGSPAKPHTTLQVSGRQGQPTCDCDSVPPGTPPPIESFTFKENQLCRNTCQELHKHSVEQTETAELPASDSINPGNLQLVSELKNPSGSCSVDVSAMFWERAGCKEPCIITACEDVVSLWKALDAWQWEKLYTWHFAEVPVLQIVPVPDVYNLVCVALGNLEIREIRALFCSSDDESEKQVLLKSGNIKAVLGLTKRRLVSSSGTLSDQQVEVMTFAEDGGGKENQFLMPPEETILTFAEVQGMQEALLGTTIMNNIVIWNLKTGQLLKKMHIDDSYQASVCHKAYSEMGLLFIVLSHPCAKESESLRSPVFQLIVINPKTTLSVGVMLYCLPPGQAGRFLEGDVKDHCAAAILTSGTIAIWDLLLGQCTALLPPVSDQHWSFVKWSGTDSHLLAGQKDGNIFVYHYS

>NP_000050.2

MPIGSKERPTFFEIFKTRCNKADLGPISLNWFEELSSEAPPYNSEPAEESEHKNNNYEPNLFKTPQRKPSYNQLASTPIIFKEQGLTLPLYQSPVKELDKFKLDLGRNVPNSRHKSLRTVKTKMDQADDVSCPLLNSCLSESPVVLQCTHVTPQRDKSVVCGSLFHTPKFVKGRQTPKHISESLGAEVDPDMSWSSSLATPPTLSSTVLIVRNEEASETVFPHDTTANVKSYFSNHDESLKKNDRFIASVTDSENTNQREAASHGFGKTSGNSFKVNSCKDHIGKSMPNVLEDEVYETVVDTSEEDSFSLCFSKCRTKNLQKVRTSKTRKKIFHEANADECEKSKNQVKEKYSFVSEVEPNDTDPLDSNVANQKPFESGSDKISKEVVPSLACEWSQLTLSGLNGAQMEKIPLLHISSCDQNISEKDLLDTENKRKKDFLTSENSLPRISSLPKSEKPLNEETVVNKRDEEQHLESHTDCILAVKQAISGTSPVASSFQGIKKSIFRIRESPKETFNASFSGHMTDPNFKKETEASESGLEIHTVCSQKEDSLCPNLIDNGSWPATTTQNSVALKNAGLISTLKKKTNKFIYAIHDETSYKGKKIPKDQKSELINCSAQFEANAFEAPLTFANADSGLLHSSVKRSCSQNDSEEPTLSLTSSFGTILRKCSRNETCSNNTVISQDLDYKEAKCNKEKLQLFITPEADSLSCLQEGQCENDPKSKKVSDIKEEVLAAACHPVQHSKVEYSDTDFQSQKSLLYDHENASTLILTPTSKDVLSNLVMISRGKESYKMSDKLKGNNYESDVELTKNIPMEKNQDVCALNENYKNVELLPPEKYMRVASPSRKVQFNQNTNLRVIQKNQEETTSISKITVNPDSEELFSDNENNFVFQVANERNNLALGNTKELHETDLTCVNEPIFKNSTMVLYGDTGDKQATQVSIKKDLVYVLAEENKNSVKQHIKMTLGQDLKSDISLNIDKIPEKNNDYMNKWAGLLGPISNHSFGGSFRTASNKEIKLSEHNIKKSKMFFKDIEEQYPTSLACVEIVNTLALDNQKKLSKPQSINTVSAHLQSSVVVSDCKNSHITPQMLFSKQDFNSNHNLTPSQKAEITELSTILEESGSQFEFTQFRKPSYILQKSTFEVPENQMTILKTTSEECRDADLHVIMNAPSIGQVDSSKQFEGTVEIKRKFAGLLKNDCNKSASGYLTDENEVGFRGFYSAHGTKLNVSTEALQKAVKLFSDIENISEETSAEVHPISLSSSKCHDSVVSMFKIENHNDKTVSEKNNKCQLILQNNIEMTTGTFVEEITENYKRNTENEDNKYTAASRNSHNLEFDGSDSSKNDTVCIHKDETDLLFTDQHNICLKLSGQFMKEGNTQIKEDLSDLTFLEVAKAQEACHGNTSNKEQLTATKTEQNIKDFETSDTFFQTASGKNISVAKESFNKIVNFFDQKPEELHNFSLNSELHSDIRKNKMDILSYEETDIVKHKILKESVPVGTGNQLVTFQGQPERDEKIKEPTLLGFHTASGKKVKIAKESLDKVKNLFDEKEQGTSEITSFSHQWAKTLKYREACKDLELACETIEITAAPKCKEMQNSLNNDKNLVSIETVVPPKLLSDNLCRQTENLKTSKSIFLKVKVHENVEKETAKSPATCYTNQSPYSVIENSALAFYTSCSRKTSVSQTSLLEAKKWLREGIFDGQPERINTADYVGNYLYENNSNSTIAENDKNHLSEKQDTYLSNSSMSNSYSYHSDEVYNDSGYLSKNKLDSGIEPVLKNVEDQKNTSFSKVISNVKDANAYPQTVNEDICVEELVTSSSPCKNKNAAIKLSISNSNNFEVGPPAFRIASGKIVCVSHETIKKVKDIFTDSFSKVIKENNENKSKICQTKIMAGCYEALDDSEDILHNSLDNDECSTHSHKVFADIQSEEILQHNQNMSGLEKVSKISPCDVSLETSDICKCSIGKLHKSVSSANTCGIFSTASGKSVQVSDASLQNARQVFSEIEDSTKQVFSKVLFKSNEHSDQLTREENTAIRTPEHLISQKGFSYNVVNSSAFSGFSTASGKQVSILESSLHKVKGVLEEFDLIRTEHSLHYSPTSRQNVSKILPRVDKRNPEHCVNSEMEKTCSKEFKLSNNLNVEGGSSENNHSIKVSPYLSQFQQDKQQLVLGTKVSLVENIHVLGKEQASPKNVKMEIGKTETFSDVPVKTNIEVCSTYSKDSENYFETEAVEIAKAFMEDDELTDSKLPSHATHSLFTCPENEEMVLSNSRIGKRRGEPLILVGEPSIKRNLLNEFDRIIENQEKSLKASKSTPDGTIKDRRLFMHHVSLEPITCVPFRTTKERQEIQNPNFTAPGQEFLSKSHLYEHLTLEKSSSNLAVSGHPFYQVSATRNEKMRHLITTGRPTKVFVPPFKTKSHFHRVEQCVRNINLEENRQKQNIDGHGSDDSKNKINDNEIHQFNKNNSNQAAAVTFTKCEEEPLDLITSLQNARDIQDMRIKKKQRQRVFPQPGSLYLAKTSTLPRISLKAAVGGQVPSACSHKQLYTYGVSKHCIKINSKNAESFQFHTEDYFGKESLWTGKGIQLADGGWLIPSNDGKAGKEEFYRALCDTPGVDPKLISRIWVYNHYRWIIWKLAAMECAFPKEFANRCLSPERVLLQLKYRYDTEIDRSRRSAIKKIMERDDTAAKTLVLCVSDIISLSANISETSSNKTSSADTQKVAIIELTDGWYAVKAQLDPPLLAVLKNGRLTVGQKIILHGAELVGSPDACTPLEAPESLMLKISANSTRPARWYTKLGFFPDPRPFPLPLSSLFSDGGNVGCVDVIIQRAYPIQWMEKTSSGLYIFRNEREEEKEAAKYVEAQQKRLEALFTKIQEEFEEHEENTTKPYLPSRALTRQQVRALQDGAELYEAVKNAADPAYLEGYFSEEQLRALNNHRQMLNDKKQAQIQLEIRKAMESAEQKEQGLSRDVTTVWKLRIVSYSKKEKDSVILSIWRPSSDLYSLLTEGKRYRIYHLATSKSKSKSERANIQLAATKKTQYQQLPVSDEILFQIYQPREPLHFSKFLDPDFQPSCSEVDLIGFVVSVVKKTGLAPFVYLSDECYNLLAIKFWIDLNEDIIKPHMLIAASNLQWRPESKSGLLTLFAGDFSVFSASPKEGHFQETFNKMKNTVENIDILCNEAENKLMHILHANDPKWSTPTKDCTSGPYTAQIIPGTGNKLLMSSPNCEIYYQSPLSLCMAKRKSVSTPVSAQMTSKSCKGEKEIDDQKNCKKRRALDFLSRLPLPPPVSPICTFVSPAAQKAFQPPRSCGTKYETPIKKKELNSPQMTPFKKFNEISLLESNSIADEELALINTQALLSGSTGEKQFISVSESTRTAPTSSEDYLRLKRRCTTSLIKEQESSQASTEECEKNKQDTITTKKYI

149 NP_003876.1 NP_001895.1

>NP_003876.1

MGTVLSLSPSYRKATLFEDGAATVGHYTAVQNSKNAKDKNLKRHSIISVLPWKRIVAVSAKKKNSKKVQPNSSYQNNITHLNNENLKKSLSCANLSTFAQPPPAQPPAPPASQLSGSQTGGSSSVKKAPHPAVTSAGTPKRVIVQASTSELLRCLGEFLCRRCYRLKHLSPTDPVLWLRSVDRSLLLQGWQDQGFITPANVVFLYMLCRDVISSEVGSDHELQAVLLTCLYLSYSYMGNEISYPLKPFLVESCKEAFWDRCLSVINLMSSKMLQINADPHYFTQVFSDLKNESGQEDKKRLLLGLDR

>NP_001895.1

MATQADLMELDMAMEPDRKAAVSHWQQQSYLDSGIHSGATTTAPSLSGKGNPEEEDVDTSQVLYEWEQGFSQSFTQEQVADIDGQYAMTRAQRVRAAMFPETLDEGMQIPSTQFDAAHPTNVQRLAEPSQMLKHAVVNLINYQDDAELATRAIPELTKLLNDEDQVVVNKAAVMVHQLSKKEASRHAIMRSPQMVSAIVRTMQNTNDVETARCTAGTLHNLSHHREGLLAIFKSGGIPALVKMLGSPVDSVLFYAITTLHNLLLHQEGAKMAVRLAGGLQKMVALLNKTNVKFLAITTDCLQILAYGNQESKLIILASGGPQALVNIMRTYTYEKLLWTTSRVLKVLSVCSSNKPAIVEAGGMQALGLHLTDPSQRLVQNCLWTLRNLSDAATKQEGMEGLLGTLVQLLGSDDINVVTCAAGILSNLTCNNYKNKMMVCQVGGIEALVRTVLRAGDREDITEPAICALRHLTSRHQEAEMAQNAVRLHYGLPVVVKLLHPPSHWPLIKATVGLIRNLALCPANHAPLREQGAIPRLVQLLVRAHQDTQRRTSMGGTQQQFVEGVRMEEIVEGCTGALHILARDVHNRIVIRGLNTIPLFVQLLYSPIENIQRVAAGVLCELAQDKEAAEAIEAEGATAPLTELLHSRNEGVATYAAAVLFRMSEDKPQDYKKRLSVELTSSLFRTEPMAWNETADLGLDIGAQGEPLGYRQDDPSYRSFHSGGYGQDALGMDPMMEHEMGGHHPGADYPVDGLPDLGHAQDLMDGLPPGDSNQLAWFDTDL

150 NP_060676.2 NP_003090.2

>NP_060676.2

MPTTQQSPQDEQEKLLDEAIQAVKVQSFQMKRCLDKNKLMDALKHASNMLGELRTSMLSPKSYYELYMAISDELHYLEVYLTDEFAKGRKVADLYELVQYAGNIIPRLYLLITVGVVYVKSFPQSRKDILKDLVEMCRGVQHPLRGLFLRNYLLQCTRNILPDEGEPTDEETTGDISDSMDFVLLNFAEMNKLWVRMQHQGHSRDREKRERERQELRILVGTNLVRLSQLEGVNVERYKQIVLTGILEQVVNCRDALAQEYLMECIIQVFPDEFHLQTLNPFLRACAELHQNVNVKNIIIALIDRLALFAHREDGPGIPADIKLFDIFSQQVATVIQSRQDMPSEDVVSLQVSLINLAMKCYPDRVDYVDKVLETTVEIFNKLNLEHIATSSAVSKELTRLLKIPVDTYNNILTVLKLKHFHPLFEYFDYESRKSMSCYVLSNVLDYNTEIVSQDQVDSIMNLVSTLIQDQPDQPVEDPDPEDFADEQSLVGRFIHLLRSEDPDQQYLILNTARKHFGAGGNQRIRFTLPPLVFAAYQLAFRYKENSKVDDKWEKKCQKIFSFAHQTISALIKAELAELPLRLFLQGALAAGEIGFENHETVAYEFMSQAFSLYEDEISDSKAQLAAITLIIGTFERMKCFSEENHEPLRTQCALAASKLLKKPDQGRAVSTCAHLFWSGRNTDKNGEELHGGKRVMECLKKALKIANQCMDPSLQVQLFIEILNRYIYFYEKENDAVTIQVLNQLIQKIREDLPNLESSEETEQINKHFHNTLEHLRLRRESPESEGPIYEGLIL

>NP_003090.2

MASGGGGCSASERLPPPFPGLEPESEGAAGGSEPEAGDSDTEGEDIFTGAAVVSKHQSPKITTSLLPINNGSKENGIHEEQDQEPQDLFADATVELSLDSTQNNQKKVLAKTLISLPPQEATNSSKPQPTYEELEEEEQEDQFDLTVGITDPEKIGDGMNAYVAYKVTTQTSLPLFRSKQFAVKRRFSDFLGLYEKLSEKHSQNGFIVPPPPEKSLIGMTKVKVGKEDSSSAEFLEKRRAALERYLQRIVNHPTMLQDPDVREFLEKEELPRAVGTQTLSGAGLLKMFNKATDAVSKMTIKMNESDIWFEEKLQEVECEEQRLRKLHAVVETLVNHRKELALNTAQFAKSLAMLGSSEDNTALSRALSQLAEVEEKIEQLHQEQANNDFFLLAELLSDYIRLLAIVRAAFDQRMKTWQRWQDAQATLQKKREAEARLLWANKPDKLQQAKDEILEWESRVTQYERDFERISTVVRKEVIRFEKEKSKDFKNHVIKYLETLLYSQQQLAKYWEAFLPEAKAIS

151 NP_004827.4 NP_001547.1

>NP_004827.4

MERAISPGLLVRALLLLLLLLGLAARTVAAGRARGLPAPTAEAAFGLGAAAAPTSATRVPAAGAVAAAEVTVEDAEALPAAAGEQEPRGPEPDDETELRPRGRSLVIISTLDGRIAALDPENHGKKQWDLDVGSGSLVSSSLSKPEVFGNKMIIPSLDGALFQWDQDRESMETVPFTVESLLESSYKFGDDVVLVGGKSLTTYGLSAYSGKVRYICSALGCRQWDSDEMEQEEDILLLQRTQKTVRAVGPRSGNEKWNFSVGHFELRYIPDMETRAGFIESTFKPNENTEESKIISDVEEQEAAIMDIVIKVSVADWKVMAFSKKGGHLEWEYQFCTPIASAWLLKDGKVIPISLFDDTSYTSNDDVLEDEEDIVEAARGATENSVYLGMYRGQLYLQSSVRISEKFPSSPKALESVTNENAIIPLPTIKWKPLIHSPSRTPVLVGSDEFDKCLSNDKFSHEEYSNGALSILQYPYDNGYYLPYYKRERNKRSTQITVRFLDNPHYNKNIRKKDPVLLLHWWKEIVATILFCIIATTFIVRRLFHPHPHRQRKESETQCQTENKYDSVSGEANDSSWNDIKNSGYISRYLTDFEPIQCLGRGGFGVVFEAKNKVDDCNYAIKRIRLPNRELAREKVMREVKALAKLEHPGIVRYFNAWLEAPPEKWQEKMDEIWLKDESTDWPLSSPSPMDAPSVKIRRMDPFATKEHIEIIAPSPQRSRSFSVGISCDQTSSSESQFSPLEFSGMDHEDISESVDAAYNLQDSCLTDCDVEDGTMDGNDEGHSFELCPSEASPYVRSRERTSSSIVFEDSGCDNASSKEEPKTNRLHIGNHCANKLTAFKPTSSKSSSEATLSISPPRPTTLSLDLTKNTTEKLQPSSPKVYLYIQMQLCRKENLKDWMNGRCTIEERERSVCLHIFLQIAEAVEFLHSKGLMHRDLKPSNIFFTMDDVVKVGDFGLVTAMDQDEEEQTVLTPMPAYARHTGQVGTKLYMSPEQIHGNSYSHKVDIFSLGLILFELLYPFSTQMERVRTLTDVRNLKFPPLFTQKYPCEYVMVQDMLSPSPMERPEAINIIENAVFEDLDFPGKTVLRQRSRSLSSSGTKHSRQSNNSHSPLPSN

>NP_001547.1

MSWSPSLTTQTCGAWEMKERLGTGGFGNVIRWHNQETGEQIAIKQCRQELSPRNRERWCLEIQIMRRLTHPNVVAARDVPEGMQNLAPNDLPLLAMEYCQGGDLRKYLNQFENCCGLREGAILTLLSDIASALRYLHENRIIHRDLKPENIVLQQGEQRLIHKIIDLGYAKELDQGSLCTSFVGTLQYLAPELLEQQKYTVTVDYWSFGTLAFECITGFRPFLPNWQPVQWHSKVRQKSEVDIVVSEDLNGTVKFSSSLPYPNNLNSVLAERLEKWLQLMLMWHPRQRGTDPTYGPNGCFKALDDILNLKLVHILNMVTGTIHTYPVTEDESLQSLKARIQQDTGIPEEDQELLQEAGLALIPDKPATQCISDGKLNEGHTLDMDLVFLFDNSKITYETQISPRPQPESVSCILQEPKRNLAFFQLRKVWGQVWHSIQTLKEDCNRLQQGQRAAMMNLLRNNSCLSKMKNSMASMSQQLKAKLDFFKTSIQIDLEKYSEQTEFGITSDKLLLAWREMEQAVELCGRENEVKLLVERMMALQTDIVDLQRSPMGRKQGGTLDDLEEQARELYRRLREKPRDQRTEGDSQEMVRLLLQAIQSFEKKVRVIYTQLSKTVVCKQKALELLPKVEEVVSLMNEDEKTVVRLQEKRQKELWNLLKIACSKVRGPVSGSPDSMNASRLSQPGQLMSQPSTASNSLPEPAKKSEELVAEAHNLCTLLENAIQDTVREQDQSFTALDWSWLQTEEEEHSCLEQAS

152 NP_446464.1 NP_115580.2

>NP_446464.1

MSSKGSVVLAYSGGLDTSCILVWLKEQGYDVIAYLANIGQKEDFEEARKKALKLGAKKVFIEDVSREFVEEFIWPAIQSSALYEDRYLLGTSLARPCIARKQVEIAQREGAKYVSHGATGKGNDQVRFELSCYSLAPQIKVIAPWRMPEFYNRFKGRNDLMEYAKQHGIPIPVTPKNPWSMDENLMHISYEAGILENPKNQAPPGLYTKTQDPAKAPNTPDILEIEFKKGVPVKVTNVKDGTTHQTSLELFMYLNEVAGKHGVGRIDIVENRFIGMKSRGIYETPAGTILYHAHLDIEAFTMDREVRKIKQGLGLKFAELVYTGFWHSPECEFVRHCIAKSQERVEGKVQVSVLKGQVYILGRESPLSLYNEELVSMNVQGDYEPTDATGFININSLRLKEYHRLQSKVTAK

>NP_115580.2

MPALPLDQLQITHKDPKTGKLRTSPALHPEQKADRYFVLYKPPPKDNIPALVEEYLERATFVANDLDWLLALPHDKFWCQVIFDETLQKCLDSYLRYVPRKFDEGVASAPEVVDMQKRLHRSVFLTFLRMSTHKESKDHFISPSAFGEILYNNFLFDIPKILDLCVLFGKGNSPLLQKMIGNIFTQQPSYYSDLDETLPTILQVFSNILQHCGLQGDGANTTPQKLEERGRLTPSDMPLLELKDIVLYLCDTCTTLWAFLDIFPLACQTFQKHDFCYRLASFYEAAIPEMESAIKKRRLEDSKLLGDLWQRLSHSRKKLMEIFHIILNQICLLPILESSCDNIQGFIEEFLQIFSSLLQEKRFLRDYDALFPVAEDISLLQQASSVLDETRTAYILQAVESAWEGVDRRKATDAKDPSVIEEPNGEPNGVTVTAEAVSQASSHPENSEEEECMGAAAAVGPAMCGVELDSLISQVKDLLPDLGEGFILACLEYYHYDPEQVINNILEERLAPTLSQLDRNLDREMKPDPTPLLTSRHNVFQNDEFDVFSRDSVDLSRVHKGKSTRKEENTRSLLNDKRAVAAQRQRYEQYSVVVEEVPLQPGESLPYHSVYYEDEYDDTYDGNQVGANDADSDDELISRRPFTIPQVLRTKVPREGQEEDDDDEEDDADEEAPKPDHFVQDPAVLREKAEARRMAFLAKKGYRHDSSTAVAGSPRGHGQSRETTQERRKKEANKATRANHNRRTMADRKRSKGMIPS

153 AAA52539.1 NP_000588.2

>AAA52539.1

MGKISSLPTQLFKCCFCDFLKVKMHTMSSSHLFYLALCLLTFTSSATAGPETLCGAELVDALQFVCGDRGFYFNKPTGYGSSSRRAPQTGIVDECCFRSCDLRRLEMYCAPLKPAKSARSVRAQRHTDMPKTQKYQPPSTNKNTKSQRRKGWPKTHPGGEQKEGTEASLQIRGKKKEQRREIGSRNAECRGKKGK

>NP_000588.2

MLPRVGCPALPLPPPPLLPLLPLLLLLLGASGGGGGARAEVLFRCPPCTPERLAACGPPPVAPPAAVAAVAGGARMPCAELVREPGCGCCSVCARLEGEACGVYTPRCGQGLRCYPHPGSELPLQALVMGEGTCEKRRDAEYGASPEQVADNGDDHSEGGLVENHVDSTMNMLGGGGSAGRKPLKSGMKELAVFREKVTEQHRQMGKGGKHHLGLEEPKKLRPPPARTPCQQELDQVLERISTMRLPDERGPLEHLYSLHIPNCDKHGLYNLKQCKMSLNGQRGECWCVNPNTGKLIQGAPTIRGDPECHLFYNEQQEARGVHTQRMQ

154 NP_057326.2 NP_060277.1

>NP_057326.2

MELLQVTILFLLPSICSSNSTGVLEAANNSLVVTTTKPSITTPNTESLQKNVVTPTTGTTPKGTITNELLKMSLMSTATFLTSKDEGLKATTTDVRKNDSIISNVTVTSVTLPNAVSTLQSSKPKTETQSSIKTTEIPGSVLQPDASPSKTGTLTSIPVTIPENTSQSQVIGTEGGKNASTSATSRSYSSIILPVVIALIVITLSVFVLVGLYRMCWKADPGTPENGNDQPQSDKESVKLLTVKTISHESGEHSAQGKTKN

>NP_060277.1

MPAVLGFEGSANKIGVGVVRDGKVLANPRRTYVTPPGTGFLPGDTARHHRAVILDLLQEALTESGLTSQDIDCIAYTKGPGMGAPLVSVAVVARTVAQLWNKPLVGVNHCIGHIEMGRLITGATSPTVLYVSGGNTQVIAYSEHRYRIFGETIDIAVGNCLDRFARVLKISNDPSPGYNIEQMAKRGKKLVELPYTVKGMDVSFSGILSFIEDVAHRMLATGECTPEDLCFSLQETVFAMLVEITERAMAHCGSQEALIVGGVGCNVRLQEMMATMCQERGARLFATDERFCIDNGAMIAQAGWEMFRAGHRTPLSDSGVTQRYRTDEVEVTWRD

155 NP_001886.1 NP_690605.1

>NP_001886.1

MSGPVPSRARVYTDVNTHRPREYWDYESHVVEWGNQDDYQLVRKLGRGKYSEVFEAINITNNEKVVVKILKPVKKKKIKREIKILENLRGGPNIITLADIVKDPVSRTPALVFEHVNNTDFKQLYQTLTDYDIRFYMYEILKALDYCHSMGIMHRDVKPHNVMIDHEHRKLRLIDWGLAEFYHPGQEYNVRVASRYFKGPELLVDYQMYDYSLDMWSLGCMLASMIFRKEPFFHGHDNYDQLVRIAKVLGTEDLYDYIDKYNIELDPRFNDILGRHSRKRWERFVHSENQHLVSPEALDFLDKLLRYDHQSRLTAREAMEHPYFYTVVKDQARMGSSSMPGGSTPVSSANMMSGISSVPTPSPLGPLAGSPVIAAANPLGMPVPAAAGAQQ

>NP_690605.1

MTTPRNSVNGTFPAEPMKGPIAMQSGPKPLFRRMSSLVGPTQSFFMRESKTLGAVQIMNGLFHIALGGLLMIPAGIYAPICVTVWYPLWGGIMYIISGSLLAATEKNSRKCLVKGKMIMNSLSLFAAISGMILSIMDILNIKISHFLKMESLNFIRAHTPYINIYNCEPANPSEKNSPSTQYCYSIQSLFLGILSVMLIFAFFQELVIAGIVENEWKRTCSRPKSNIVLLSAEEKKEQTIEIKEEVVGLTETSSQPKNEEDIEIIPIQEEEEEETETNFPEPPQDQESSPIENDSSP

156 NP_006169.1 NP_065853.2

>NP_006169.1

MAGRSMQAARCPTDELSLTNCSVVNEKDFQSGQHVIVRTSPNHRYTFTLKTHPSVVPGSIAFSLPQRKWAGLSIGQEIEVSLYTFDKAKQCIGTMTIEIDFLQKKSNDSNPYDTDKMAAEFIQQFNNQAYSVGQQLVFSFNEKLFGLLVKDIESMDPSILKGEPATGKRQKIEVGLVVGNSQVAFEKAENSSLNLIGKAKTKENRQSIINPDWNFEKMGIGGLDKEFSDIFRRAFAFRVFPPEIVEQMGCIHVKGILLYGPPGCGKTLLARQIGKMLNAREPKVVNGPEILNKYVGESEANIRKLFADAEEEQRRLGANSGLHIIIFDEIDAICKQRGSMAGSTGVHDTVVNQLLSKIDGVEQLNNILVIGMTNRPDLIDEALLRPGRLEVKMEIGLPDEKGRLQILHIHTARMRGHQLLSADVDIKELAVETKNFSGAELEGLVRAAQSTAMNRHIKASTKVEVDMEKAESLQVTRGDFLASLENDIKPAFGTNQEDYASYIMNGIIKWGDPVTRVLDDGELLVQQTKNSDRTPLVSVLLEGPPHSGKTALAAKIAEESNFPFIKICSPDKMIGFSETAKCQAMKKIFDDAYKSQLSCVVVDDIERLLDYVPIGPRFSNLVLQALLVLLKKAPPQGRKLLIIGTTSRKDVLQEMEMLNAFSTTIHVPNIATGEQLLEALELLGNLKDKERTTIAQQVKGKKVWIGIKKLLMLIEMSLQMDPEYRVRKFLALLREEGASPLDFD

>NP_065853.2

MLAGRPGTRSAVGELGTESSDNLDRAPLGPRESGGHHRPGSYLDMKIHLEKNLEEERQILLQQQKICRNRARKYFVESNRRKKAFEEKRKEQEEKEHQIREQILQQRKQKFEEVTEKFQRAHVPLSQRRKAVSRKPVPPLEEALKQIQESNLKSEVNLPFSRRPTINWRAIDSALPSALSKNDHKHQKQLLSKINCEKEMNENMRATLATSKNVFQLKLEETQKLLEDQHLSNLQKFGDEVNQITNSETLSSIDSLEATEHEEIYLTLNKEHSTSIQRNTISLKPANMQSTNLSCFDEDKLAFSKTQHINNWLTNLDASNTQNVTAFSDILSKSNVLPSWEYFNSKEQNPSPLNGTVERATNTANNSVPFVSSPPMFVLDKKCEKTSETSTMRTTDSTSGAFKRERPLVTESPTFKFSKSQSTSDSLTQEVATFPDQEKYSELNQENGTTSIPTSCVPVATPLVLPSNIQSARPSAKNSIHIKEIDAVQCSDKLDELKDGKEEEIKYFNCNKEELPLFSDSFQDAYIPHNPDSKDEKQKLAETSSLSNVTSNYDFVGQHKKMKYNIHERNGVRFLKSILKKESKYEHGYLKALIINQSFKFGNQKAAAIRDSIELTKEKGAEIPKTIKKLRWFDETSNIENNAENSHSLKNKTGTTQQHSQQFHIQSGAGSNIISVSTCAVNSADTKKSREDSISENVTTLGGSGADHMPLNCFIPSGYNFAKHAWPASKKEESKIPVHDDSKTKQGKPQRGRAKIIRKPGSAKVQSGFICTNRKGAVIQPQSASKVNIFTQAQGKLIIPCPPPQSTSNIRSGKNIQVSQCQPVTPENPQNIITHNSFNSKHVLPTEHSLNQWNQESSSPLSNACSDLVTVIPSLPSYCSSECQTFAKINHSNGTQAVARQDATLYCTQRSPVCEESYPSVTLRTAEEESVPLWKRGPNVLHQNKRATGSTVMRRKRIAETKRRNILEQKRQNPGSVGQKYSEQINNFGQSVLLSSSEPKQTTRGTSYIEEVSDSTSEFLMAENLVKASVPEDEILTVLNSKQIQKSNLPLNKTQQFNICTLSAEEQKILESLNDLSERLHYIQESICKNPSIKNTLQIIPLLEKREDRTSSCRDKR

157 NP_001551.1 NP_056465.2

>NP_001551.1

MEWPARLCGLWALLLCAGGGGGGGGAAPTETQPPVTNLSVSVENLCTVIWTWNPPEGASSNCSLWYFSHFGDKQDKKIAPETRRSIEVPLNERICLQVGSQCSTNESEKPSILVEKCISPPEGDPESAVTELQCIWHNLSYMKCSWLPGRNTSPDTNYTLYYWHRSLEKIHQCENIFREGQYFGCSFDLTKVKDSSFEQHSVQIMVKDNAGKIKPSFNIVPLTSRVKPDPPHIKNLSFHNDDLYVQWENPQNFISRCLFYEVEVNNSQTETHNVFYVQEAKCENPEFERNVENTSCFMVPGVLPDTLNTVRIRVKTNKLCYEDDKLWSNWSQEMSIGKKRNSTLYITMLLIVPVIVAGAIIVLLLYLKRLKIIIFPPIPDPGKIFKEMFGDQNDDTLHWKKYDIYEKQTKEETDSVVLIENLKKASQ

>NP_056465.2

MNAAVVRRTQEALGKVIRRPPLTEKLLSKPPFRYLHDIITEVIRMTGFMKGLYTDAEMKSDNVKDKDAKISFLQKAIDVVVMVSGEPLLAKPARIVAGHEPERTNELLQIIGKCCLNKLSSDDAVRRVLAGEKGEVKGRASLTSRSQELDNKNVREEESRVHKNTEDRGDAEIKERSTSRDRKQKEELKEDRKPREKDKDKEKAKENGGNRHREGERERAKARARPDNERQKDRGNRERDRDSERKKETERKSEGGKEKERLRDRDRERDRDKGKDRDRRRVKNGEHSWDLDREKNREHDKPEKKSASSGEMSKKLSDGTFKDSKAETETEISTRASKSLTTKTSKRRSKNSVEGRKEDNISAKSLDSIVSGINNEPNQETTTSEIGTKEANINSTSISDDNSASLRCENIQPNPTEKQKGDSTSDAEGDAGPAGQDKSEVPETPEIPNELSSNIRRIPRPGSARPAPPRVKRQDSMEALQMDRSGSGKTVSNVITESHNSDNEEDDQFVVEAAPQLSEMSEIEMVTAVELEEEEKHGGLVKKILETKKDYEKLQQSPKPGEKERSLFESAWKKEKDIVSKEIEKLRTSIQTLCKSALPLGKIMDYIQEDVDAMQNELQMWHSENRQHAEALQQEQRITDCAVEPLKAELAELEQLIKDQQDKICAVKANILKNEEKIQKMVYSINLTSRR

158 NP_004636.1 NP_077817.2

>NP_004636.1

MAASETVRLRLQFDYPPPATPHCTAFWLLVDLNRCRVVTDLISLIRQRFGFSSGAFLGLYLEGGLLPPAESARLVRDNDCLRVKLEERGVAENSVVISNGDINLSLRKAKKRAFQLEEGEETEPDCKYSKKHWKSRENNNNNEKVLDLEPKAVTDQTVSKKNKRKNKATCGTVGDDNEEAKRKSPKKKEKCEYKKKAKNPKSPKVQAVKDWANQRCSSPKGSARNSLVKAKRKGSVSVCSKESPSSSSESESCDESISDGPSKVTLEARNSSEKLPTELSKEEPSTKNTTADKLAIKLGFSLTPSKGKTSGTTSSSSDSSAESDDQCLMSSSTPECAAGFLKTVGLFAGRGRPGPGLSSQTAGAAGWRRSGSNGGGQAPGASPSVSLPASLGRGWGREENLFSWKGAKGRGMRGRGRGRGHPVSCVVNRSTDNQRQQQLNDVVKNSSTIIQNPVETPKKDYSLLPLLAAAPQVGEKIAFKLLELTSSYSPDVSDYKEGRILSHNPETQQVDIEILSSLPALREPGKFDLVYHNENGAEVVEYAVTQESKITVFWKELIDPRLIIESPSNTSSTEPA

>NP_077817.2

MFPVAPKPQDSSQPSDRLMTEKQQEEAEWESINVLLMMHGLKPLSLVKRTDLKDLIIFDKQSSQRMRQNLKLLVEETSCQQNMIQELIETNQQLRNELQLEQSRAANQEQRANDLEQIMESVKSKIGELEDESLSRACHQQNKIKDLQKEQKTLQVKCQHYKKKRTEQEETIASLQMEVCRLKKEEEDRIVTQNRVFAYLCKRVPHTVLDRQLLCLIDYYESKIRKIHTQRQYKEDESQSEEENDYRNLDASPTYKGLLMSLQNQLKESKSKIDALSSEKLNLQKDLETRPTQHELRLYKQQVKKLEKALKKNVKLQELINHKKAEDTEKKDEPSKYNQQQALIDQRYFQVLCSINSIIHNPRAPVIIYKQTKGGVQNFNKDLVQDCGFEHLVPVIEMWADQLTSLKDLYKSLKTLSAELVPWLNLKKQDENEGIKVEDLLFIVDTMLEEVENKEKDSNMPHFQTLQAIVSHFQKLFDVPSLNGVYPRMNEVYTRLGEMNNAVRNLQELLELDSSSSLCVLVSTVGKLCRLINEDVNEQVMQVLGPEDLQSIIYKLEEHEEFFPAFQAFTNDLLEILEIDDLDAIVPAVKKLKVLSY

159 NP_612815.1 NP_000385.1

>NP_612815.1

MSQSNRELVVDFLSYKLSQKGYSWSQFSDVEENRTEAPEGTESEMETPSAINGNPSWHLADSPAVNGATGHSSSLDAREVIPMAAVKQALREAGDEFELRYRRAFSDLTSQLHITPGTAYQSFEQVVNELFRDGVNWGRIVAFFSFGGALCVESVDKEMQVLVSRIAAWMATYLNDHLEPWIQENGGWDTFVELYGNNAAAESRKGQERFNRWFLTGMTVAGVVLLGSLFSRK

>NP_000385.1

MDVTIQHPWFKRTLGPFYPSRLFDQFFGEGLFEYDLLPFLSSTISPYYRQSLFRTVLDSGISEVRSDRDKFVIFLDVKHFSPEDLTVKVQDDFVEIHGKHNERQDDHGYISREFHRRYRLPSNVDQSALSCSLSADGMLTFCGPKIQTGLDATHAERAIPVSREEKPTSAPSS

160 NP_714941.1 NP_055986.1

>NP_714941.1

MSGFNFGGTGAPTGGFTFGTAKTATTTPATGFSFSTSGTGGFNFGAPFQPATSTPSTGLFSLATQTPATQTTGFTFGTATLASGGTGFSLGIGASKLNLSNTAATPAMANPSGFGLGSSNLTNAISSTVTSSQGTAPTGFVFGPSTTSVAPATTSGGFSFTGGSTAQPSGFNIGSAGNSAQPTAPATLPFTPATPAATTAGATQPAAPTPTATITSTGPSLFASIATAPTSSATTGLSLCTPVTTAGAPTAGTQGFSLKAPGAASGTSTTTSTAATATATTTSSSSTTGFALNLKPLAPAGIPSNTAAAVTAPPGPGAAAGAAASSAMTYAQLESLINKWSLELEDQERHFLQQATQVNAWDRTLIENGEKITSLHREVEKVKLDQKRLDQELDFILSQQKELEDLLSPLEELVKEQSGTIYLQHADEEREKTYKLAENIDAQLKRMAQDLKDIIEHLNTSGAPADTSDPLQQICKILNAHMDSLQWIDQNSALLQRKVEEVTKVCEGRRKEQERSFRITFD

>NP_055986.1

MASEEASLRALESLMTEFFHDCTTNERKREIEELLNNFAQQIGAWRFCLYFLSSTRNDYVMMYSLTVFENLINKMWLGVPSQDKMEIRSCLPKLLLAHHKTLPYFIRNKLCKVIVDIGRQDWPMFYHDFFTNILQLIQSPVTTPLGLIMLKTTSEELACPREDLSVARKEELRKLLLDQVQTVLGLLTGILETVWDKHSVTAATPPPSPTSGESGDLLSNLLQSPSSAKLLNQPIPILDVESEYICSLALECLAHLFSWIPLSASITPSLLTTIFHFARFGCDIRARKMASVNGSSQNCVSGQERGRLGVLAMSCINELMSKNCVPMEFEEYLLRMFQQTFYLLQKITKDNNAHTVKSRLEELDESYIEKFTDFLRLFVSVHLRRIESYSQFPVVEFLTLLFKYTFHQPTHEGYFSCLDIWTLFLDYLTSKIKSRLGDKEAVLNRYEDALVLLLTEVLNRIQFRYNQAQLEELDDETLDDDQQTEWQRYLRQSLEVVAKVMELLPTHAFSTLFPVLQDNLEVYLGLQQFIVTSGSGHRLNITAENDCRRLHCSLRDLSSLLQAVGRLAEYFIGDVFAARFNDALTVVERLVKVTLYGSQIKLYNIETAVPSVLKPDLIDVHAQSLAALQAYSHWLAQYCSEVHRQNTQQFVTLISTTMDAITPLISTKVQDKLLLSACHLLVSLATTVRPVFLISIPAVQKVFNRITDASALRLVDKAQVLVCRALSNILLLPWPNLPENEQQWPVRSINHASLISALSRDYRNLKPSAVAPQRKMPLDDTKLIIHQTLSVLEDIVENISGESTKSRQICYQSLQESVQVSLALFPAFIHQSDVTDEMLSFFLTLFRGLRVQMGVPFTEQIIQTFLNMFTREQLAESILHEGSTGCRVVEKFLKILQVVVQEPGQVFKPFLPSIIALCMEQVYPIIAERPSPDVKAELFELLFRTLHHNWRYFFKSTVLASVQRGIAEEQMENEPQFSAIMQAFGQSFLQPDIHLFKQNLFYLETLNTKQKLYHKKIFRTAMLFQFVNVLLQVLVHKSHDLLQEEIGIAIYNMASVDFDGFFAAFLPEFLTSCDGVDANQKSVLGRNFKMDRDLPSFTQNVHRLVNDLRYYRLCNDSLPPGTVKL

161 NP_008937.1 NP_065853.2

>NP_008937.1

MSVVPPNRSQTGWPRGVTQFGNKYIQQTKPLTLERTINLYPLTNYTFGTKEPLYEKDSSVAARFQRMREEFDKIGMRRTVEGVLIVHEHRLPHVLLLQLGTTFFKLPGGELNPGEDEVEGLKRLMTEILGRQDGVLQDWVIDDCIGNWWRPNFEPPQYPYIPAHITKPKEHKKLFLVQLQEKALFAVPKNYKLVAAPLFELYDNAPGYGPIISSLPQLLSRFNFIYN

>NP_065853.2

MLAGRPGTRSAVGELGTESSDNLDRAPLGPRESGGHHRPGSYLDMKIHLEKNLEEERQILLQQQKICRNRARKYFVESNRRKKAFEEKRKEQEEKEHQIREQILQQRKQKFEEVTEKFQRAHVPLSQRRKAVSRKPVPPLEEALKQIQESNLKSEVNLPFSRRPTINWRAIDSALPSALSKNDHKHQKQLLSKINCEKEMNENMRATLATSKNVFQLKLEETQKLLEDQHLSNLQKFGDEVNQITNSETLSSIDSLEATEHEEIYLTLNKEHSTSIQRNTISLKPANMQSTNLSCFDEDKLAFSKTQHINNWLTNLDASNTQNVTAFSDILSKSNVLPSWEYFNSKEQNPSPLNGTVERATNTANNSVPFVSSPPMFVLDKKCEKTSETSTMRTTDSTSGAFKRERPLVTESPTFKFSKSQSTSDSLTQEVATFPDQEKYSELNQENGTTSIPTSCVPVATPLVLPSNIQSARPSAKNSIHIKEIDAVQCSDKLDELKDGKEEEIKYFNCNKEELPLFSDSFQDAYIPHNPDSKDEKQKLAETSSLSNVTSNYDFVGQHKKMKYNIHERNGVRFLKSILKKESKYEHGYLKALIINQSFKFGNQKAAAIRDSIELTKEKGAEIPKTIKKLRWFDETSNIENNAENSHSLKNKTGTTQQHSQQFHIQSGAGSNIISVSTCAVNSADTKKSREDSISENVTTLGGSGADHMPLNCFIPSGYNFAKHAWPASKKEESKIPVHDDSKTKQGKPQRGRAKIIRKPGSAKVQSGFICTNRKGAVIQPQSASKVNIFTQAQGKLIIPCPPPQSTSNIRSGKNIQVSQCQPVTPENPQNIITHNSFNSKHVLPTEHSLNQWNQESSSPLSNACSDLVTVIPSLPSYCSSECQTFAKINHSNGTQAVARQDATLYCTQRSPVCEESYPSVTLRTAEEESVPLWKRGPNVLHQNKRATGSTVMRRKRIAETKRRNILEQKRQNPGSVGQKYSEQINNFGQSVLLSSSEPKQTTRGTSYIEEVSDSTSEFLMAENLVKASVPEDEILTVLNSKQIQKSNLPLNKTQQFNICTLSAEEQKILESLNDLSERLHYIQESICKNPSIKNTLQIIPLLEKREDRTSSCRDKR

162 NP_005246.1 NP_003706.1

>NP_005246.1

MSLLQSALDFLAGPGSLGGASGRDQSDFVGQTVELGELRLRVRRVLAEGGFAFVYEAQDVGSGREYALKRLLSNEEEKNRAIIQEVCFMKKLSGHPNIVQFCSAASIGKEESDTGQAEFLLLTELCKGQLVEFLKKMESRGPLSCDTVLKIFYQTCRAVQHMHRQKPPIIHRDLKVENLLLSNQGTIKLCDFGSATTISHYPDYSWSAQRRALVEEEITRNTTPMYRTPEIIDLYSNFPIGEKQDIWALGCILYLLCFRQHPFEDGAKLRIVNGKYSIPPHDTQYTVFHSLIRAMLQVNPEERLSIAEVVHQLQEIAAARNVNPKSPITELLEQNGGYGSATLSRGPPPPVGPAGSGYSGGLALAEYDQPYGGFLDILRGGTERLFTNLKDTSSKVIQSVANYAKGDLDISYITSRIAVMSFPAEGVESALKNNIEDVRLFLDSKHPGHYAVYNLSPRTYRPSRFHNRVSECGWAARRAPHLHTLYNICRNMHAWLRQDHKNVCVVHCMDGRAASAVAVCSFLCFCRLFSTAEAAVYMFSMKRCPPGIWPSHKRYIEYMCDMVAEEPITPHSKPILVRAVVMTPVPLFSKQRSGCRPFCEVYVGDERVASTSQEYDKMRDFKIEDGKAVIPLGVTVQGDVLIVIYHARSTLGGRLQAKMASMKMFQIQFHTGFVPRNATTVKFAKYDLDACDIQEKYPDLFQVNLEVEVEPRDRPSREAPPWENSSMRGLNPKILFSSREEQQDILSKFGKPELPRQPGSTAQYDAGAGSPEAEPTDSDSPPSSSADASRFLHTLDWQEEKEAETGAENASSKESESALMEDRDESEVSDEGGSPISSEGQEPRADPEPPGLAAGLVQQDLVFEVETPAVLPEPVPQEDGVDLLGLHSEVGAGPAVPPQACKAPSSNTDLLSCLLGPPEAASQGPPEDLLSEDPLLLASPAPPLSVQSTPRGGPPAAADPFGPLLPSSGNNSQPCSNPDLFGEFLNSDSVTVPPSFPSAHSAPPPSCSADFLHLGDLPGEPSKMTASSSNPDLLGGWAAWTETAASAVAPTPATEGPLFSPGGQPAPCGSQASWTKSQNPDPFADLGDLSSGLQGSPAGFPPGGFIPKTATTAKGSSSWQTSRPPAQGASWPPQAKPPPKACTQPRPNYASNFSVIGAREERGVRAPSFAQKPKVSENDFEDLLSNQGFSSRSDKKGPKTIAEMRKQDLAKDTDPLKLKLLDWIEGKERNIRALLSTLHTVLWDGESRWTPVGMADLVAPEQVKKHYRRAVLAVHPDKAAGQPYEQHAKMIFMELNDAWSEFENQGSRPLF

>NP_003706.1

MNFLRGVMGGQSAGPQHTEAETIQKLCDRVASSTLLDDRRNAVRALKSLSKKYRLEVGIQAMEHLIHVLQTDRSDSEIIGYALDILYNIISNEEEEEVEENSTRQSEDLGSQFTEIFIKQQENVTLLLSLLEEFDFHVRWPGVKLLTSLLKQLGPQVQQIILVSPMGVSRLMDLLADSREVIRNDGVLLLQALTRSNGAIQKIVAFENAFERLLDIISEEGNSDGGIVVEDCLILLQNLLKNNNSNQNFFKEGSYIQRMKPWFEVGDENSGWSAQKVTNLHLMLQLVRVLVSPTNPPGATSSCQKAMFQCGLLQQLCTILMATGVPADILTETINTVSEVIRGCQVNQDYFASVNAPSNPPRPAIVVLLMSMVNERQPFVLRCAVLYCFQCFLYKNQKGQGEIVSTLLPSTIDATGNSVSAGQLLCGGLFSTDSLSNWCAAVALAHALQENATQKEQLLRVQLATSIGNPPVSLLQQCTNILSQGSKIQTRVGLLMLLCTWLSNCPIAVTHFLHNSANVPFLTGQIAENLGEEEQLVQGLCALLLGISIYFNDNSLESYMKEKLKQLIEKRIGKENFIEKLGFISKHELYSRASQKPQPNFPSPEYMIFDHEFTKLVKELEGVITKAIYKSSEEDKKEEEVKKTLEQHDNIVTHYKNMIREQDLQLEELRQQVSTLKCQNEQLQTAVTQQVSQIQQHKDQYNLLKIQLGKDNQHQGSYSEGAQMNGIQPEEIGRLREEIEELKRNQELLQSQLTEKDSMIENMKSSQTSGTNEQSSAIVSARDSEQVAELKQELATLKSQLNSQSVEITKLQTEKQELLQKTEAFAKSVEVQGETETIIATKTTDVEGRLSALLQETKELKNEIKALSEERTAIKEQLDSSNSTIAILQTEKDKLELEITDSKKEQDDLLVLLADQDQKILSLKNKLKDLGHPVEEEDELESGDQEDEDDESEDPGKDLDHI

163 NP_001020262.1 NP_852133.1

>NP_001020262.1

MPKAPKQQPPEPEWIGDGESTSPSDKVVKKGKKDKKIKKTFFEELAVEDKQAGEEEKVLKEKEQQQQQQQQQQKKKRDTRKGRRKKDVDDDGEEKELMERLKKLSVPTSDEEDEVPAPKPRGGKKTKGGNVFAALIQDQSEEEEEEEKHPPKPAKPEKNRINKAVSEEQQPALKGKKGKEEKSKGKAKPQNKFAALDNEEEDKEEEIIKEKEPPKQGKEKAKKAEQGSEEEGEGEEEEEEGGESKADDPYAHLSKKEKKKLKKQMEYERQVASLKAANAAENDFSVSQAEMSSRQAMLENASDIKLEKFSISAHGKELFVNADLYIVAGRRYGLVGPNGKGKTTLLKHIANRALSIPPNIDVLLCEQEVVADETPAVQAVLRADTKRLKLLEEERRLQGQLEQGDDTAAERLEKVYEELRATGAAAAEAKARRILAGLGFDPEMQNRPTQKFSGGWRMRVSLARALFMEPTLLMLDEPTNHLDLNAVIWLNNYLQGWRKTLLIVSHDQGFLDDVCTDIIHLDAQRLHYYRGNYMTFKKMYQQKQKELLKQYEKQEKKLKELKAGGKSTKQAEKQTKEALTRKQQKCRRKNQDEESQEAPELLKRPKEYTVRFTFPDPPPLSPPVLGLHGVTFGYQGQKPLFKNLDFGIDMDSRICIVGPNGVGKSTLLLLLTGKLTPTHGEMRKNHRLKIGFFNQQYAEQLRMEETPTEYLQRGFNLPYQDARKCLGRFGLESHAHTIQICKLSGGQKARVVFAELACREPDVLILDEPTNNLDIESIDALGEAINEYKGAVIVVSHDARLITETNCQLWVVEEQSVSQIDGDFEDYKREVLEALGEVMVSRPRE

>NP_852133.1

MAVRASFENNCEIGCFAKLTNTYCLVAIGGSENFYSVFEGELSDTIPVVHASIAGCRIIGRMCVGNRHGLLVPNNTTDQELQHIRNSLPDTVQIRRVEERLSALGNVTTCNDYVALVHPDLDRETEEILADVLKVEVFRQTVADQVLVGSYCVFSNQGGLVHPKTSIEDQDELSSLLQVPLVAGTVNRGSEVIAAGMVVNDWCAFCGLDTTSTELSVVESVFKLNEAQPSTIATSMRDSLIDSLT

164 NP_002816.1 NP_115557.1

>NP_002816.1

MQAQQYQQQRRKFAAAFLAFIFILAAVDTAEAGKKEKPEKKVKKSDCGEWQWSVCVPTSGDCGLGTREGTRTGAECKQTMKTQRCKIPCNWKKQFGAECKYQFQAWGECDLNTALKTRTGSLKRALHNAECQKTVTISKPCGKLTKPKPQAESKKKKKEGKKQEKMLD

>NP_115557.1

MRLPLSHSPEHVEMALLSNILAAYSFVSENPERAALYFVSGVCIGLVLTLAALVIRISCHTDCRRRPGKKFLQDRESSSDSSDSEDGSEDTVSDLSVRRHRRFERTLNKNVFTSAEELERAQRLEERERIIREIWMNGQPEVPGTRSLNRYY

165 NP_647593.1 AAH14214.1

>NP_647593.1

MAEMGSKGVTAGKIASNVQKKLTRAQEKVLQKLGKADETKDEQFEQCVQNFNKQLTEGTRLQKDLRTYLASVKAMHEASKKLNECLQEVYEPDWPGRDEANKIAENNDLLWMDYHQKLVDQALLTMDTYLGQFPDIKSRIAKRGRKLVDYDSARHHYESLQTAKKKDEAKIAKPVSLLEKAAPQWCQGKLQAHLVAQTNLLRNQAEEELIKAQKVFEEMNVDLQEELPSLWNSRVGFYVNTFQSIAGLEENFHKEMSKLNQNLNDVLVGLEKQHGSNTFTVKAQPSDNAPAKGNKSPSPPDGSPAATPEIRVNHEPEPAGGATPGATLPKSPSQLRKGPPVPPPPKHTPSKEVKQEQILSLFEDTFVPEISVTTPSQFEAPGPFSEQASLLDLDFDPLPPVTSPVKAPTPSGQSIPWDLWEPTESPAGSLPSGEPSAAEGTFAVSWPSQTAEPGPAQPAEASEVAGGTQPAAGAQEPGETAASEAASSSLPAVVVETFPATVNGTVEGGSGAGRLDLPPGFMFKVQAQHDYTATDTDELQLKAGDVVLVIPFQNPEEQDEGWLMGVKESDWNQHKELEKCRGVFPENFTERVP

>AAH14214.1

MPAVSKGDGMRGLAVFISDIRNCKSKEAEIKRINKELANIRSKFKGDKALDGYSKKKYVCKLLFIFLLGHDIDFGHMEAVNLLSSNKYTEKQIGYLFISVLVNSNSELIRLINNAIKNDLASRNPTFMCLALHCIANVGSREMGEAFAADIPRILVAGDSMDSVKQSAALCLLRLYKASPDLVPMGEWTARVVHLLNDQHMGVVTAAVSLITCLCKKNPDDFKTCVSLAVSRLSRIVSSASTDLQDYTYYFVPAPWLSVKLLRLLQCYPLPEDAAVKGRLVECLETVLNKAQEPPKSKKVQHSNAKNAILFETISLIIHYDSEPNLLVRACNQLGQFLQHRETNLRYLALESMCTLASSEFSHEAVKTHIDTVINALKTERDVSVRQRAADLLYAMCDRSNAKQIVSEMLRYLETADYAIREEIVLKVAILAEKYAVDYSWYVDTILNLIRIAGDYVSEEVWYRVLQIVTNRDDVQGYAAKTVFEALQAPACHENMVKVGGYILGEFGNLIAGDPRSSPPVQFSLLHSKFHLCSVATRALLLSTYIKFINLFPETKATIQGVLRAGSQLRNADVELQQRAVEYLTLSSVASTDVLATVLEEMPPFPERESSILAKLKRKKGPGAGSALDDGRRDPSSNDINGGMEPTPSTVSTPSPSADLLGLRAAPPPAAPPASAGAGNLLVDVFDGPAAQPSLGPTPEEAFLSPGPEDIGPPIPEADELLNKFVCKNNGVLFENQLLQIGVKSEFRQNLGRMYLFYGNKTSVQFQNFSPTVVHPGDLQTQLAVQTKRVAAQVDGGAQVQQVLNIECLRDFLTPPLLSVRFRYGGAPQALTLKLPVTINKFFQPTEMAAQDFFQRWKQLSLPQQEAQKIFKANHPMDAEVTKAKLLGFGSALLDNVDPNPGDREDTRVWGMPGTFLRPFVFLFLFICCCLHSGGLGGVPLPPFPPQAQRGEGPGKWMSPPLPPHPVVAPPTPSPSRGCVLL

166 NP_115516.1 NP_006839.2

>NP_115516.1

MCEGPSRISGPIPPDPTLCPDNYRRPTSAQGRLEGNALKLDLLTSDRALDTTAPRGPCIGPGAGEILERGQRGVGDVLLQLEGISLGPGASLKRKDPKDHEKENLRRIREIQKRFREQERSREQGQPRPLKALWRSPKYDKVESRVKAQLQEPGPASGTESAHFLRAHSRCGPGLPPPHVSSPQPTPPGPEAKEPGLGVDFIRHNARAAKRAPRRHSCSLQVLAQVLEQQRQAQEHYNATQKGHVPHYLLERRDLWRREAEARKQSQPDPAMPPGHTRMPENQRLETLTKLLQSQSQLLRELVLLPAGADSLRAQSHRAELDRKLVQVEEAIKIFSRPKVFVKMDD

>NP_006839.2

MEAEAGGLEELTDEEMAALGKEELVRRLRREEAARLAALVQRGRLMQEVNRQLQGHLGEIRELKQLNRRLQAENRELRDLCCFLDSERQRGRRAARQWQLFGTQASRAVREDLGGCWQKLAELEGRQEELLRENLALKELCLALGEEWGPRGGPSGAGGSGAGPAPELALPPCGPRDLGDGSSSTGSVGSPDQLPLACSPDD

167 NP_006557.1 NP_006496.2

>NP_006557.1

MDYPTLLLALLHVYRALCEEVLWHTSVPFAENMSLECVYPSMGILTQVEWFKIGTQQDSIAIFSPTHGMVIRKPYAERVYFLNSTMASNNMTLFFRNASEDDVGYYSCSLYTYPQGTWQKVIQVVQSDSFEAAVPSNSHIVSEPGKNVTLTCQPQMTWPVQAVRWEKIQPRQIDLLTYCNLVHGRNFTSKFPRQIVSNCSHGRWSVIVIPDVTVSDSGLYRCYLQASAGENETFVMRLTVAEGKTDNQYTLFVAGGTVLLLLFVISITTIIVIFLNRRRRRERRDLFTESWDTQKAPNNYRSPISTGQPTNQSMDDTREDIYVNYPTFSRRPKTRV

>NP_006496.2

MARAMAAAWPLLLVALLVLSWPPPGTGDVVVQAPTQVPGFLGDSVTLPCYLQVPNMEVTHVSQLTWARHGESGSMAVFHQTQGPSYSESKRLEFVAARLGAELRNASLRMFGLRVEDEGNYTCLFVTFPQGSRSVDIWLRVLAKPQNTAEVQKVQLTGEPVPMARCVSTGGRPPAQITWHSDLGGMPNTSQVPGFLSGTVTVTSLWILVPSSQVDGKNVTCKVEHESFEKPQLLTVNLTVYYPPEVSISGYDNNWYLGQNEATLTCDARSNPEPTGYNWSTTMGPLPPFAVAQGAQLLIRPVDKPINTTLICNVTNALGARQAELTVQVKEGPPSEHSGMSRNAIIFLVLGILVFLILLGIGIYFYWSKCSREVLWHCHLCPSSTEHASASANGHVSYSAVSRENSSSQDPQTEGTR

168 NP_055103.3 NP_003989.2

>NP_055103.3

MPVKRSLKLDGLLEENSFDPSKITRKKSVITYSPTTGTCQMSLFASPTSSEEQKHRNGLSNEKRKKLNHPSLTESKESTTKDNDEFMMLLSKVEKLSEEIMEIMQNLSSIQALEGSRELENLIGISCASHFLKREMQKTKELMTKVNKQKLFEKSTGLPHKASRHLDSYEFLKAILN

>NP_003989.2

MAEDDPYLGRPEQMFHLDPSLTHTIFNPEVFQPQMALPTADGPYLQILEQPKQRGFRFRYVCEGPSHGGLPGASSEKNKKSYPQVKICNYVGPAKVIVQLVTNGKNIHLHAHSLVGKHCEDGICTVTAGPKDMVVGFANLGILHVTKKKVFETLEARMTEACIRGYNPGLLVHPDLAYLQAEGGGDRQLGDREKELIRQAALQQTKEMDLSVVRLMFTAFLPDSTGSFTRRLEPVVSDAIYDSKAPNASNLKIVRMDRTAGCVTGGEEIYLLCDKVQKDDIQIRFYEEEENGGVWEGFGDFSPTDVHRQFAIVFKTPKYKDINITKPASVFVQLRRKSDLETSEPKPFLYYPEIKDKEEVQRKRQKLMPNFSDSFGGGSGAGAGGGGMFGSGGGGGGTGSTGPGYSFPHYGFPTYGGITFHPGTTKSNAGMKHGTMDTESKKDPEGCDKSDDKNTVNLFGKVIETTEQDQEPSEATVGNGEVTLTYATGTKEESAGVQDNLFLEKAMQLAKRHANALFDYAVTGDVKMLLAVQRHLTAVQDENGDSVLHLAIIHLHSQLVRDLLEVTSGLISDDIINMRNDLYQTPLHLAVITKQEDVVEDLLRAGADLSLLDRLGNSVLHLAAKEGHDKVLSILLKHKKAALLLDHPNGDGLNAIHLAMMSNSLPCLLLLVAAGADVNAQEQKSGRTALHLAVEHDNISLAGCLLLEGDAHVDSTTYDGTTPLHIAAGRGSTRLAALLKAAGADPLVENFEPLYDLDDSWENAGEDEGVVPGTTPLDMATSWQVFDILNGKPYEPEFTSDDLLAQGDMKQLAEDVKLQLYKLLEIPDPDKNWATLAQKLGLGILNNAFRLSPAPSKTLMDNYEVSGGTVRELVEALRQMGYTEAIEVIQAASSPVKTTSQAHSLPLSPASTRQQIDELRDSDSVCDSGVETSFRKLSFTESLTSGASLLTLNKMPHDYGQEGPLEGKI

169 NP_002937.1 NP_057632.2

>NP_002937.1

MWNSGFESYGSSSYGGAGGYTQSPGGFGSPAPSQAEKKSRARAQHIVPCTISQLLSATLVDEVFRIGNVEISQVTIVGIIRHAEKAPTNIVYKIDDMTAAPMDVRQWVDTDDTSSENTVVPPETYVKVAGHLRSFQNKKSLVAFKIMPLEDMNEFTTHILEVINAHMVLSKANSQPSAGRAPISNPGMSEAGNFGGNSFMPANGLTVAQNQVLNLIKACPRPEGLNFQDLKNQLKHMSVSSIKQAVDFLSNEGHIYSTVDDDHFKSTDAE

>NP_057632.2

MMGLGNGRRSMKSPPLVLAALVACIIVLGFNYWIASSRSVDLQTRIMELEGRVRRAAAERGAVELKKNEFQGELEKQREQLDKIQSSHNFQLESVNKLYQDEKAVLVNNITTGERLIRVLQDQLKTLQRNYGRLQQDVLQFQKNQTNLERKFSYDLSQCINQMKEVKEQCEERIEEVTKKGNEAVASRDLSENNDQRQQLQALSEPQPRLQAAGLPHTEVPQGKGNVLGNSKSQTPAPSSEVVLDSKRQVEKEETNEIQVVNEEPQRDRLPQEPGREQVVEDRPVGGRGFGGAGELGQTPQVQAALSVSQENPEMEGPERDQLVIPDGQEEEQEAAGEGRNQQKLRGEDDYNMDENEAESETDKQAALAGNDRNIDVFNVEDQKRDTINLLDQREKRNHTL

170 NP_006393.2 NP_060842.3

>NP_006393.2

MEPGAGHLDGHRAGSPSLRQALCDGSAVMFSSKERGRCTVINFVPLEAPLRSTPRSRQVTEACGGEGRAVPLGSEPEWSVGGMEATLEQHLEDTMKNPSIVGVLCTDSQGLNLGCRGTLSDEHAGVISVLAQQAAKLTSDPTDIPVVCLESDNGNIMIQKHDGITVAVHKMAS

>NP_060842.3

MSNNLRRVFLKPAEENSGNASRCVSGCMYQVVQTIGSDGKNLLQLLPIPKSSGNLIPLVQSSVMSDALKGNTGKPVQVTFQTQISSSSTSASVQLPIFQPASSSNYFLTRTVDTSEKGRVTSVGTGNFSSSVSKVQSHGVKIDGLTMQTFAVPPSTQKDSSFIVVNTQSLPVTVKSPVLPSGHHLQIPAHAEVKSVPASSLPPSVQQKILATATTSTSGMVEASQMPTVIYVSPVNTVKNVVTKNFQNIYPKPVTEIAKPVILNTTQIPKNVATETQLKGGQHSQAAPVKWIFQDNLQPFTPSLVPVKSSNNVASKILKTFVDRKNLGDNTINMPPLSTIDPSGTRSKNMPIKDNALVMFNGKVYLLAKKGTDVLPSQIDQQNSVSPDTPVRKDTLQTVSSSPVTEISREVVNIVLAKSKSSQMETKSLSNTQLASMANLRAEKNKVEKPSPSTTNPHMNQSSNYLKQSKTLFTNPIFPVGFSTGHNAPRKVTAVIYARKGSVLQSIEKISSSVDATTVTSQQCVFRDQEPKIHNEMASTSDKGAQGRNDKKDSQGRSNKALHLKSDAEFKKIFGLTKDLRVCLTRIPDHLTSGEGFDSFSSLVKSGTYKETEFMVKEGERKQQNFDKKRKAKTNKKMDHIKKRKTENAYNAIINGEANVTGSQLLSSILPTSDVSQHNILTSHSKTRQEKRTEMEYYTHEKQEKGTLNSNAAYEQSHFFNKNYTEDIFPVTPPELEETIRDEKIRRLKQVLREKEAALEEMRKKMHQK

171 NP_478102.1 NP_001002.1

>NP_478102.1

MGRGRCVGPSLQLRGQEWRCSPLVPKGGAAAAELGPGGGENMVRRFLVTLRIRRACGPPRVRVFVVHIPRLTGEWAAPGAPAAVALVLMLLRSQRLGQQPLPRRPGHDDGQRPSGGAAAAPRRGAQLRRPRHSHPTRARRCPGGLPGHAGGAAPGRGAAGRARCLGPSARGPG

>NP_001002.1

MFSSSAKIVKPNGEKPDEFESGISQALLELEMNSDLKAQLRELNITAAKEIEVGGGRKAIIIFVPVPQLKSFQKIQVRLVRELEKKFSGKHVVFIAQRRILPKPTRKSRTKNKQKRPRSRTLTAVHDAILEDLVFPSEIVGKRIRVKLDGSRLIKVHLDKAQQNNVEHKVETFSGVYKKLTGKDVNFEFPEFQL

172 NP_065116.2 NP_068749.3

>NP_065116.2

MPPKVTSELLRQLRQAMRNSEYVTEPIQAYIIPSGDAHQSEYIAPCDCRRAFVSGFDGSAGTAIITEEHAAMWTDGRYFLQAAKQMDSNWTLMKMGLKDTPTQEDWLVSVLPEGSRVGVDPLIIPTDYWKKMAKVLRSAGHHLIPVKENLVDKIWTDRPERPCKPLLTLGLDYTGISWKDKVADLRLKMAERNVMWFVVTALDEIAWLFNLRGSDVEHNPVFFSYAIIGLETIMLFIDGDRIDAPSVKEHLLLDLGLEAEYRIQVHPYKSILSELKALCADLSPREKVWVSDKASYAVSETIPKDHRCCMPYTPICIAKAVKNSAESEGMRRAHIKDAVALCELFNWLEKEVPKGGVTEISAADKAEEFRRQQADFVDLSFPTISSTGPNGAIIHYAPVPETNRTLSLDEVYLIDSGAQYKDGTTDVTRTMHFGTPTAYEKECFTYVLKGHIAVSAAVFPTGTKGHLLDSFARSALWDSGLDYLHGTGHGVGSFLNVHEGPCGISYKTFSDEPLEAGMIVTDEPGYYEDGAFGIRIENVVLVVPVKTKYNFNNRGSLTFEPLTLVPIQTKMIDVDSLTDKECDWLNNYHLTCRDVIGKELQKQGRQEALEWLIRETQPISKQH

>NP_068749.3

MLPAGEIGASPAAPCCSESGDERKNLEEKSDINVTVLIGSKQVSEGTDNGDLPSYVSAFIEKEVGNDLKSLKKLDKLIEQRTVSKMQLEEQVLTISSEIPKRIRSALKNAEESKQFLNQFLEQETHLFSAINSHLLTAQPWMDDLGTMISQIEEIERHLAYLKWISQIEELSDNIQQYLMTNNVPEAASTLVSMAELDIKLQESSCTHLLGFMRATVKFWHKILKDKLTSDFEEILAQLHWPFIAPPQSQTVGLSRPASAPEIYSYLETLFCQLLKLQTSDELLTEPKQLPEKYSLPASPSVILPIQVMLTPLQKRFRYHFRGNRQTNVLSKPEWYLAQVLMWIGNHTEFLDEKIQPILDKVGSLVNARLEFSRGLMMLVLEKLATDIPCLLYDDNLFCHLVDEVLLFERELHSVHGYPGTFASCMHILSEETCFQRWLTVERKFALQKMDSMLSSEAAWVSQYKDITDVDEMKVPDCAETFMTLLLVITDRYKNLPTASRKLQFLELQKDLVDDFRIRLTQVMKEETRASLGFRYCAILNAVNYISTVLADWADNVFFLQLQQAALEVFAENNTLSKLQLGQLASMESSVFDDMINLLERLKHDMLTRQVDHVFREVKDAAKLYKKERWLSLPSQSEQAVMSLSSSACPLLLTLRDHLLQLEQQLCFSLFKIFWQMLVEKLDVYIYQEIILANHFNEGGAAQLQFDMTRNLFPLFSHYCKRPENYFKHIKEACIVLNLNVGSALLLKDVLQSASGQLPATAALNEVGIYKLAQQDVEILLNLRTNWPNTGK

173 NP_683877.1 NP_001230.1

>NP_683877.1

MQLSKVKFRNQYDNDVTVWSPQGRIHQIEYAMEAVKQGSATVGLKSKTHAVLVALKRAQSELAAHQKKILHVDNHIGISIAGLTADARLLCNFMRQECLDSRFVFDRPLPVSRLVSLIGSKTQIPTQRYGRRPYGVGLLIAGYDDMGPHIFQTCPSANYFDCRAMSIGARSQSARTYLERHMSEFMECNLNELVKHGLRALRETLPAEQDLTTKNVSIGIVGKDLEFTIYDDDDVSPFLEGLEERPQRKAQPAQPADEPAEKADEPMEH

>NP_001230.1

MYHNSSQKRHWTFSSEEQLARLRADANRKFRCKAVANGKVLPNDPVFLEPHEEMTLCKYYEKRLLEFCSVFKPAMPRSVVGTACMYFKRFYLNNSVMEYHPRIIMLTCAFLACKVDEFNVSSPQFVGNLRESPLGQEKALEQILEYELLLIQQLNFHLIVHNPYRPFEGFLIDLKTRYPILENPEILRKTADDFLNRIALTDAYLLYTPSQIALTAILSSASRAGITMESYLSESLMLKENRTCLSQLLDIMKSMRNLVKKYEPPRSEEVAVLKQKLERCHSAELALNVITKKRKGYEDDDYVSKKSKHEEEEWTDDDLVESL

174 NP_005501.2 NP_061874.3

>NP_005501.2

MDPRGTKRGAEKTEVAEPRNKLPRPAPSLPTDPALYSGPFPFYRRPSELGCFSLDAQRQYHGDARALRYYSPPPTNGPGPNFDLRDGYPDRYQPRDEEVQERLDHLLCWLLEHRGRLEGGPGWLAEAIVTWRGHLTKLLTTPYERQEGWQLAASRFQGTLYLSEVETPNARAQRLARPPLLRELMYMGYKFEQYMCADKPGSSPDPSGEVNTNVAFCSVLRSRLGSHPLLFSGEVDCTDPQAPSTQPPTCYVELKTSKEMHSPGQWRSFYRHKLLKWWAQSFLPGVPNVVAGFRNPDGFVSSLKTFPTMKMFEYVRNDRDGWNPSVCMNFCAAFLSFAQSTVVQDDPRLVHLFSWEPGGPVTVSVHQDAPYAFLPIWYVEAMTQDLPSPPKTPSPK

>NP_061874.3

MGVPKFYRWISERYPCLSEVVKEHQIPEFDNLYLDMNGIIHQCSHPNDDDVHFRISDDKIFTDIFHYLEVLFRIIKPRKVFFMAVDGVAPRAKMNQQRGRRFRSAKEAEDKIKKAIEKGETLPTEARFDSNCITPGTEFMARLHEHLKYFVNMKISTDKSWQGVTIYFSGHETPGEGEHKIMEFIRSEKAKPDHDPNTRHCLYGLDADLIMLGLTSHEAHFSLLREEVRFGGKKTQRVCAPEETTFHLLHLSLMREYIDYEFSVLKEKITFKYDIERIIDDWILMGFLVGNDFIPHLPHLHINHDALPLLYGTYVTILPELGGYINESGHLNLPRFEKYLVKLSDFDREHFSEVFVDLKWFESKVGNKYLNEAAGVAAEEARNYKEKKKLKGQENSLCWTALDKNEGEMITSKDNLEDETEDDDLFETEFRQYKRTYYMTKMGVDVVSDDFLADQAACYVQAIQWILHYYYHGVQSWSWYYPYHYAPFLSDIHNISTLKIHFELGKPFKPFEQLLAVLPAASKNLLPACYQHLMTNEDSPIIEYYPPDFKTDLNGKQQEWEAVVLIPFIDEKRLLEAMETCNHSLKKEERKRNQHSECLMCWYDRDTEFIYPSPWPEKFPAIERCCTRYKIISLDAWRVDINKNKITRIDQKALYFCGFPTLKHIRHKFFLKKSGVQVFQQSSRGENMMLEILVDAESDELTVENVASSVLGKSVFVNWPHLEEARVVAVSDGETKFYLEEPPGTQKLYSGRTAPPSKVVHLGDKEQSNWAKEVQGISEHYLRRKGIIINETSAVVYAQLLTGRKYQINQNGEVRLEKQWSKQVVPFVYQTIVKDIRAFDSRFSNIKTLDDLFPLRSMVFMLGTPYYGCTGEVQDSGDVITEGRIRVIFSIPCEPNLDALIQNQHKYSIKYNPGYVLASRLGVSGYLVSRFTGSIFIGRGSRRNPHGDHKANVGLNLKFNKKNEEVPGYTKKVGSEWMYSSAAEQLLAEYLERAPELFSYIAKNSQEDVFYEDDIWPGENENGAEKVQEIITWLKGHPVSTLSRSSCDLQILDAAIVEKIEEEVEKCKQRKNNKKVRVTVKPHLLYRPLEQQHGVIPDRDAEFCLFDRVVNVRENFSVPVGLRGTIIGIKGANREADVLFEVLFDEEFPGGLTIRCSPGRGYRLPTSALVNLSHGSRSETGNQKLTAIVKPQPAVHQHSSSSSVSSGHLGALNHSPQSLFVPTQVPTKDDDEFCNIWQSLQGSGKMQYFQPTIQEKGAVLPQEISQVNQHHKSGFNDNSVKYQQRKHDPHRKFKEECKSPKAECWSQKMSNKQPNSGIENFLASLNISKENEVQSSHHGEPPSEEHLSPQSFAMGTRMLKEILKIDGSNTVDHKNEIKQIANEIPVSSNRRDEYGLPSQPKQNKKLASYMNKPHSANEYHNVQSMDNMCWPAPSQIPPVSTPVTELSRICSLVGMPQPDFSFLRMPQTMTVCQVKLSNGLLVHGPQCHSENEAKEKAALFALQQLGSLGMNFPLPSQVFANYPSAVPPGTIPPAFPPPTGWDHYGSNYALGAANIMPSSSHLFGSMPWGPSVPVPGKPFHHTLYSGTMPMAGGIPGGVHNQFIPLQVTKKRVANKKNFENKEAQSSQATPVQTSQPDSSNIVKVSPRESSSASLKSSPIAQPASSFQVETASQGHSISHHKSTPISSSRRKSRKLAVNFGVSKPSE

175 NP_001059.2 NP_001311.3

>NP_001059.2

MAKSGGCGAGAGVGGGNGALTWVNNAAKKEESETANKNDSSKKLSVERVYQKKTQLEHILLRPDTYIGSVEPLTQFMWVYDEDVGMNCREVTFVPGLYKIFDEILVNAADNKQRDKNMTCIKVSIDPESNIISIWNNGKGIPVVEHKVEKVYVPALIFGQLLTSSNYDDDEKKVTGGRNGYGAKLCNIFSTKFTVETACKEYKHSFKQTWMNNMMKTSEAKIKHFDGEDYTCITFQPDLSKFKMEKLDKDIVALMTRRAYDLAGSCRGVKVMFNGKKLPVNGFRSYVDLYVKDKLDETGVALKVIHELANERWDVCLTLSEKGFQQISFVNSIATTKGGRHVDYVVDQVVGKLIEVVKKKNKAGVSVKPFQVKNHIWVFINCLIENPTFDSQTKENMTLQPKSFGSKCQLSEKFFKAASNCGIVESILNWVKFKAQTQLNKKCSSVKYSKIKGIPKLDDANDAGGKHSLECTLILTEGDSAKSLAVSGLGVIGRDRYGVFPLRGKILNVREASHKQIMENAEINNIIKIVGLQYKKSYDDAESLKTLRYGKIMIMTDQDQDGSHIKGLLINFIHHNWPSLLKHGFLEEFITPIVKASKNKQELSFYSIPEFDEWKKHIENQKAWKIKYYKGLGTSTAKEAKEYFADMERHRILFRYAGPEDDAAITLAFSKKKIDDRKEWLTNFMEDRRQRRLHGLPEQFLYGTATKHLTYNDFINKELILFSNSDNERSIPSLVDGFKPGQRKVLFTCFKRNDKREVKVAQLAGSVAEMSAYHHGEQALMMTIVNLAQNFVGSNNINLLQPIGQFGTRLHGGKDAASPRYIFTMLSTLARLLFPAVDDNLLKFLYDDNQRVEPEWYIPIIPMVLINGAEGIGTGWACKLPNYDAREIVNNVRRMLDGLDPHPMLPNYKNFKGTIQELGQNQYAVSGEIFVVDRNTVEITELPVRTWTQVYKEQVLEPMLNGTDKTPALISDYKEYHTDTTVKFVVKMTEEKLAQAEAAGLHKVFKLQTTLTCNSMVLFDHMGCLKKYETVQDILKEFFDLRLSYYGLRKEWLVGMLGAESTKLNNQARFILEKIQGKITIENRSKKDLIQMLVQRGYESDPVKAWKEAQEKAAEEDETQNQHDDSSSDSGTPSGPDFNYILNMSLWSLTKEKVEELIKQRDAKGREVNDLKRKSPSDLWKEDLAAFVEELDKVESQEREDVLAGMSGKAIKGKVGKPKVKKLQLEETMPSPYGRRIIPEITAMKADASKKLLKKKKGDLDTAAVKVEFDEEFSGAPVEGAGEEALTPSVPINKGPKPKREKKEPGTRVRKTPTSSGKPSAKKVKKRNPWSDDESKSESDLEETEPVVIPRDSLLRRAAAERPKYTFDFSEEEDDDADDDDDDNNDLEELKVKASPITNDGEDEFVPSDGLDKDEYTFSPGKSKATPEKSLHDKKSQDFGNLFSFPSYSQKSEDDSAKFDSNEEDSASVFSPSFGLKQTDKVPSKTVAAKKGKPSSDTVPKPKRAPKQKKVVEAVNSDSDSEFGIPKKTTTPKGKGRGAKKRKASGSENEGDYNPGRKTSKTTSKKPKKTSFDQDSDVDIFPSDFPTEPPSLPRTGRARKEVKYFAESDEEEDDVDFAMFN

>NP_001311.3

MSSSEEVSWISWFCGLRGNEFFCEVDEDYIQDKFNLTGLNEQVPHYRQALDMILDLEPDEELEDNPNQSDLIEQAAEMLYGLIHARYILTNRGIAQMLEKYQQGDFGYCPRVYCENQPMLPIGLSDIPGEAMVKLYCPKCMDVYTPKSSRHHHTDGAYFGTGFPHMLFMVHPEYRPKRPANQFVPRLYGFKIHPMAYQLQLQAASNFKSPVKTIR

176 NP_004630.2 NP_076422.1

>NP_004630.2

MEPNDSTSTAVEEPDSLEVLVKTLDSQTRTFIVGAQMNVKEFKEHIAASVSIPSEKQRLIYQGRVLQDDKKLQEYNVGGKVIHLVERAPPQTHLPSGASSGTGSASATHGGGSPPGTRGPGASVHDRNANSYVMVGTFNLPSDGSAVDVHINMEQAPIQSEPRVRLVMAQHMIRDIQTLLSRMETLPYLQCRGGPQPQHSQPPPQPPAVTPEPVALSSQTSEPVESEAPPREPMEAEEVEERAPAQNPELTPGPAPAGPTPAPETNAPNHPSPAEYVEVLQELQRLESRLQPFLQRYYEVLGAAATTDYNNNHEGREEDQRLINLVGESLRLLGNTFVALSDLRCNLACTPPRHLHVVRPMSHYTTPMVLQQAAIPIQINVGTTVTMTGNGTRPPPTPNAEAPPPGPGQASSVAPSSTNVESSAEGAPPPGPAPPPATSHPRVIRISHQSVEPVVMMHMNIQDSGTQPGGVPSAPTGPLGPPGHGQTLGQQVPGFPTAPTRVVIARPTPPQARPSHPGGPPVSGTLQGAGLGTNASLAQMVSGLVGQLLMQPVLVAQGTPGMAPPPAPATASASAGTTNTATTAGPAPGGPAQPPPTPQPSMADLQFSQLLGNLLGPAGPGAGGPGVASPTITVAMPGVPAFLQGMTDFLQATQTAPPPPPPPPPPPPAPEQQTMPPPGSPSGGAGSPGGLGLESLSPEFFTSVVQGVLSSLLGSLGARAGSSESIAAFIQRLSGSSNIFEPGADGALGFFGALLSLLCQNFSMVDVVMLLHGHFQPLQRLQPQLRSFFHQHYLGGQEPTPSNIRMATHTLITGLEEYVRESFSLVQVQPGVDIIRTNLEFLQEQFNSIAAHVLHCTDSGFGARLLELCNQGLFECLALNLHCLGGQQMELAAVINGRIRRMSRGVNPSLVSWLTTMMGLRLQVVLEHMPVGPDAILRYVRRVGDPPQPLPEEPMEVQGAERASPEPQRENASPAPGTTAEEAMSRGPPPAPEGGSRDEQDGASAETEPWAAAVPPEWVPIIQQDIQSQRKVKPQPPLSDAYLSGMPAKRRKTMQGEGPQLLLSEAVSRAAKAAGARPLTSPESLSRDLEAPEVQESYRQQLRSDIQKRLQEDPNYSPQRFPNAQRAFADDP

>NP_076422.1

MEQDDPVEALTELRERRLGALELLQAAAGSGLAAYAVWALLLQPGFRRVPLRLQVPYVGASARQVEHVLSLLRGRPGKTVDLGSGDGRIVLAAHRCGLRPAVGYELNPWLVALARLHAWRAGCAGSVCYRRKDLWKVSLRDCRNVSVFLAPSVLPLLEDKLRTELPAGARVVSGRFPLPTWQPVTAVGEGLDRVWAYDVPEGGQAGEAASSRIPIQAAPGPSSAPIPGGLISQAS

177 NP_001886.1 NP_000917.3

>NP_001886.1

MSGPVPSRARVYTDVNTHRPREYWDYESHVVEWGNQDDYQLVRKLGRGKYSEVFEAINITNNEKVVVKILKPVKKKKIKREIKILENLRGGPNIITLADIVKDPVSRTPALVFEHVNNTDFKQLYQTLTDYDIRFYMYEILKALDYCHSMGIMHRDVKPHNVMIDHEHRKLRLIDWGLAEFYHPGQEYNVRVASRYFKGPELLVDYQMYDYSLDMWSLGCMLASMIFRKEPFFHGHDNYDQLVRIAKVLGTEDLYDYIDKYNIELDPRFNDILGRHSRKRWERFVHSENQHLVSPEALDFLDKLLRYDHQSRLTAREAMEHPYFYTVVKDQARMGSSSMPGGSTPVSSANMMSGISSVPTPSPLGPLAGSPVIAAANPLGMPVPAAAGAQQ

>NP_000917.3

MTELKAKGPRAPHVAGGPPSPEVGSPLLCRPAAGPFPGSQTSDTLPEVSAIPISLDGLLFPRPCQGQDPSDEKTQDQQSLSDVEGAYSRAEATRGAGGSSSSPPEKDSGLLDSVLDTLLAPSGPGQSQPSPPACEVTSSWCLFGPELPEDPPAAPATQRVLSPLMSRSGCKVGDSSGTAAAHKVLPRGLSPARQLLLPASESPHWSGAPVKPSPQAAAVEVEEEDGSESEESAGPLLKGKPRALGGAAAGGGAAAVPPGAAAGGVALVPKEDSRFSAPRVALVEQDAPMAPGRSPLATTVMDFIHVPILPLNHALLAARTRQLLEDESYDGGAGAASAFAPPRSSPCASSTPVAVGDFPDCAYPPDAEPKDDAYPLYSDFQPPALKIKEEEEGAEASARSPRSYLVAGANPAAFPDFPLGPPPPLPPRATPSRPGEAAVTAAPASASVSSASSSGSTLECILYKAEGAPPQQGPFAPPPCKAPGASGCLLPRDGLPSTSASAAAAGAAPALYPALGLNGLPQLGYQAAVLKEGLPQVYPPYLNYLRPDSEASQSPQYSFESLPQKICLICGDEASGCHYGVLTCGSCKVFFKRAMEGQHNYLCAGRNDCIVDKIRRKNCPACRLRKCCQAGMVLGGRKFKKFNKVRVVRALDAVALPQPVGVPNESQALSQRFTFSPGQDIQLIPPLINLLMSIEPDVIYAGHDNTKPDTSSSLLTSLNQLGERQLLSVVKWSKSLPGFRNLHIDDQITLIQYSWMSLMVFGLGWRSYKHVSGQMLYFAPDLILNEQRMKESSFYSLCLTMWQIPQEFVKLQVSQEEFLCMKVLLLLNTIPLEGLRSQTQFEEMRSSYIRELIKAIGLRQKGVVSSSQRFYQLTKLLDNLHDLVKQLHLYCLNTFIQSRALSVEFPEMMSEVIAAQLPKILAGMVKPLLFHKK

178 NP_005582.1 NP_001460.1

>NP_005582.1

MSTADALDDENTFKILVATDIHLGFMEKDAVRGNDTFVTLDEILRLAQENEVDFILLGGDLFHENKPSRKTLHTCLELLRKYCMGDRPVQFEILSDQSVNFGFSKFPWVNYQDGNLNISIPVFSIHGNHDDPTGADALCALDILSCAGFVNHFGRSMSVEKIDISPVLLQKGSTKIALYGLGSIPDERLYRMFVNKKVTMLRPKEDENSWFNLFVIHQNRSKHGSTNFIPEQFLDDFIDLVIWGHEHECKIAPTKNEQQLFYISQPGSSVVTSLSPGEAVKKHVGLLRIKGRKMNMHKIPLHTVRQFFMEDIVLANHPDIFNPDNPKVTQAIQSFCLEKIEEMLENAERERLGNSHQPEKPLVRLRVDYSGGFEPFSVLRFSQKFVDRVANPKDIIHFFRHREQKEKTGEEINFGKLITKPSEGTTLRVEDLVKQYFQTAEKNVQLSLLTERGMGEAVQEFVDKEEKDAIEELVKYQLEKTQRFLKERHIDALEDKIDEEVRRFRETRQKNTNEEDDEVREAMTRARALRSQSEESASAFSADDLMSIDLAEQMANDSDDSISAATNKGRGRGRGRRGGRGQNSASRGGSQRGRADTGLETSTRSRNSKTAVSASRNMSIIDAFKSTRQQPSRNVTTKNYSEVIEVDESDVEEDIFPTTSKTDQRWSSTSSSKIMSQSQVSKGVDFESSEDDDDDPFMNTSSLRRNRR

>NP_001460.1

MSGWESYYKTEGDEEAEEEQEENLEASGDYKYSGRDSLIFLVDASKAMFESQSEDELTPFDMSIQCIQSVYISKIISSDRDLLAVVFYGTEKDKNSVNFKNIYVLQELDNPGAKRILELDQFKGQQGQKRFQDMMGHGSDYSLSEVLWVCANLFSDVQFKMSHKRIMLFTNEDNPHGNDSAKASRARTKAGDLRDTGIFLDLMHLKKPGGFDISLFYRDIISIAEDEDLRVHFEESSKLEDLLRKVRAKETRKRALSRLKLKLNKDIVISVGIYNLVQKALKPPPIKLYRETNEPVKTKTRTFNTSTGGLLLPSDTKRSQIYGSRQIILEKEETEELKRFDDPGLMLMGFKPLVLLKKHHYLRPSLFVYPEESLVIGSSTLFSALLIKCLEKEVAALCRYTPRRNIPPYFVALVPQEEELDDQKIQVTPPGFQLVFLPFADDKRKMPFTEKIMATPEQVGKMKAIVEKLRFTYRSDSFENPVLQQHFRNLEALALDLMEPEQAVDLTLPKVEAMNKRLGSLVDEFKELVYPPDYNPEGKVTKRKHDNEGSGSKRPKVEYSEEELKTHISKGTLGKFTVPMLKEACRAYGLKSGLKKQELLEALTKHFQD

179 NP_000408.1 NP_000869.1

>NP_000408.1

MDSYLLMWGLLTFIMVPGCQAELCDDDPPEIPHATFKAMAYKEGTMLNCECKRGFRRIKSGSLYMLCTGNSSHSSWDNQCQCTSSATRNTTKQVTPQPEEQKERKTTEMQSPMQPVDQASLPGHCREPPPWENEATERIYHFVVGQMVYYQCVQGYRALHRGPAESVCKMTHGKTRWTQPQLICTGEMETSQFPGEEKPQASPEGRPESETSCLVTTTDFQIQTEMAATMETSIFTTEYQVAVAGCVFLLISVLLLSGLTWQRRQRKSRRTI

>NP_000869.1

MAAPALSWRLPLLILLLPLATSWASAAVNGTSQFTCFYNSRANISCVWSQDGALQDTSCQVHAWPDRRRWNQTCELLPVSQASWACNLILGAPDSQKLTTVDIVTLRVLCREGVRWRVMAIQDFKPFENLRLMAPISLQVVHVETHRCNISWEISQASHYFERHLEFEARTLSPGHTWEEAPLLTLKQKQEWICLETLTPDTQYEFQVRVKPLQGEFTTWSPWSQPLAFRTKPAALGKDTIPWLGHLLVGLSGAFGFIILVYLLINCRNTGPWLKKVLKCNTPDPSKFFSQLSSEHGGDVQKWLSSPFPSSSFSPGGLAPEISPLEVLERDKVTQLLLQQDKVPEPASLSSNHSLTSCFTNQGYFFFHLPDALEIEACQVYFTYDPYSEEDPDEGVAGAPTGSSPQPLQPLSGEDDAYCTFPSRDDLLLFSPSLLGGPSPPSTAPGGSGAGEERMPPSLQERVPRDWDPQPLGPPTPGVPDLVDFQPPPELVLREAGEEVPDAGPREGVSFPWSRPPGQGEFRALNARLPLNTDAYLSLQELQGQDPTHLV

180 NP_060780.2 NP_003077.2

>NP_060780.2

MPGRGRCPDCGSTELVEDSHYSQSQLVCSDCGCVVTEGVLTTTFSDEGNLREVTYSRSTGENEQVSRSQQRGLRRVRDLCRVLQLPPTFEDTAVAYYQQAYRHSGIRAARLQKKEVLVGCCVLITCRQHNWPLTMGAICTLLYADLDVFSSTYMQIVKLLGLDVPSLCLAELVKTYCSSFKLFQASPSVPAKYVEDKEKMLSRTMQLVELANETWLVTGRHPLPVITAATFLAWQSLQPADRLSCSLARFCKLANVDLPYPASSRLQELLAVLLRMAEQLAWLRVLRLDKRSVVKHIGDLLQHRQSLVRSAFRDGTAEVETREKEPPGWGQGQGEGEVGNNSLGLPQGKRPASPALLLPPCMLKSPKRICPVPPVSTVTGDENISDSEIEQYLRTPQEVRDFQRAQAARQAATSVPNPP

>NP_003077.2

MDVDAEREKITQEIKELERILDPGSSGSHVEISESSLESDSEADSLPSEDLDPADPPISEEERWGEASNDEDDPKDKTLPEDPETCLQLNMVYQEVIQEKLAEANLLLAQNREQQEELMRDLAGSKGTKVKDGKSLPPSTYMGHFMKPYFKDKVTGVGPPANEDTREKAAQGIKAFEELLVTKWKNWEKALLRKSVVSDRLQRLLQPKLLKLEYLHQKQSKVSSELERQALEKQGREAEKEIQDINQLPEEALLGNRLDSHDWEKISNINFEGSRSAEEIRKFWQNSEHPSINKQEWSREEEERLQAIAAAHGHLEWQKIAEELGTSRSAFQCLQKFQQHNKALKRKEWTEEEDRMLTQLVQEMRVGSHIPYRRIVYYMEGRDSMQLIYRWTKSLDPGLKKGYWAPEEDAKLLQAVAKYGEQDWFKIREEVPGRSDAQCRDRYLRRLHFSLKKGRWNLKEEEQLIELIEKYGVGHWAKIASELPHRSGSQCLSKWKIMMGKKQGLRRRRRRARHSVRWSSTSSSGSSSGSSGGSSSSSSSSSEEDEPEQAQAGEGDRALLSPQYMVPDMDLWVPARQSTSQPWRGGAGAWLGGPAASLSPPKGSSASQGGSKEASTTAAAPGEETSPVQVPARAHGPVPRSAQASHSADTRPAGAEKQALEGGRRLLTVPVETVLRVLRANTAARSCTQKEQLRQPPLPTSSPGVSSGDSVARSHVQWLRHRATQSGQRRWRHALHRRLLNRRLLLAVTPWVGDVVVPCTQASQRPAVVQTQADGLREQLQQARLASTPVFTLFTQLFHIDTAGCLEVVRERKALPPRLPQAGARDPPVHLLQASSSAQSTPGHLFPNVPAQEASKSASHKGSRRLASSRVERTLPQASLLASTGPRPKPKTVSELLQEKRLQEARAREATRGPVVLPSQLLVSSSVILQPPLPHTPHGRPAPGPTVLNVPLSGPGAPAAAKPGTSGSWQEAGTSAKDKRLSTMQALPLAPVFSEAEGTAPAASQAPALGPGQISVSCPESGLGQSQAPAASRKQGLPEAPPFLPAAPSPTPLPVQPLSLTHIGGPHVATSVPLPVTWVLTAQGLLPVPVPAVVSLPRPAGTPGPAGLLATLLPPLTETRAAQGPRAPALSSSWQPPANMNREPEPSCRTDTPAPPTHALSQSPAEADGSVAFVPGEAQVAREIPEPRTSSHADPPEAEPPWSGRLPAFGGVIPATEPRGTPGSPSGTQEPRGPLGLEKLPLRQPGPEKGALDLEKPPLPQPGPEKGALDLGLLSQEGEAATQQWLGGQRGVRVPLLGSRLPYQPPALCSLRALSGLLLHKKALEHKATSLVVGGEAERPAGALQASLGLVRGQLQDNPAYLLLRARFLAAFTLPALLATLAPQGVRTTLSVPSRVGSESEDEDLLSELELADRDGQPGCTTATCPIQGAPDSGKCSASSCLDTSNDPDDLDVLRTRHARHTRKRRRLV

181 NP_065908.1 NP_009190.2

>NP_065908.1

MASILDEYENSLSRSAVLQPGCPSVGIPHSGYVNAQLEKEVPIFTKQRIDFTPSERITSLVVSSNQLCMSLGKDTLLRIDLGKANEPNHVELGRKDDAKVHKMFLDHTGSHLLIALSSTEVLYVNRNGQKVRPLARWKGQLVESVGWNKALGTESSTGPILVGTAQGHIFEAELSASEGGLFGPAPDLYFRPLYVLNEEGGPAPVCSLEAERGPDGRSFVIATTRQRLFQFIGRAAEGAEAQGFSGLFAAYTDHPPPFREFPSNLGYSELAFYTPKLRSAPRAFAWMMGDGVLYGALDCGRPDSLLSEERVWEYPEGVGPGASPPLAIVLTQFHFLLLLADRVEAVCTLTGQVVLRDHFLEKFGPLKHMVKDSSTGQLWAYTERAVFRYHVQREARDVWRTYLDMNRFDLAKEYCRERPDCLDTVLAREADFCFRQRRYLESARCYALTQSYFEEIALKFLEARQEEALAEFLQRKLASLKPAERTQATLLTTWLTELYLSRLGALQGDPEALTLYRETKECFRTFLSSPRHKEWLFASRASIHELLASHGDTEHMVYFAVIMQDYERVVAYHCQHEAYEEALAVLARHRDPQLFYKFSPILIRHIPRQLVDAWIEMGSRLDARQLIPALVNYSQGGEVQQVSQAIRYMEFCVNVLGETEQAIHNYLLSLYARGRPDSLLAYLEQAGASPHRVHYDLKYALRLCAEHGHHRACVHVYKVLELYEEAVDLALQVDVDLAKQCADLPEEDEELRKKLWLKIARHVVQEEEDVQTAMACLASCPLLKIEDVLPFFPDFVTIDHFKEAICSSLKAYNHHIQELQREMEEATASAQRIRRDLQELRGRYGTVEPQDKCATCDFPLLNRPFYLFLCGHMFHADCLLQAVRPGLPAYKQARLEELQRKLGAAPPPAKGSARAKEAEGGAATAGPSREQLKADLDELVAAECVYCGELMIRSIDRPFIDPQRYEEEQLSWL

>NP_009190.2

MNVVFAVKQYISKMIEDSGPGMKVLLMDKETTGIVSMVYTQSEILQKEVYLFERIDSQNREIMKHLKAICFLRPTKENVDYIIQELRRPKYTIYFIYFSNVISKSDVKSLAEADEQEVVAEVQEFYGDYIAVNPHLFSLNILGCCQGRNWDPAQLSRTTQGLTALLLSLKKCPMIRYQLSSEAAKRLAECVKQVITKEYELFEFRRTEVPPLLLILDRCDDAITPLLNQWTYQAMVHELLGINNNRIDLSRVPGISKDLREVVLSAENDEFYANNMYLNFAEIGSNIKNLMEDFQKKKPKEQQKLESIADMKAFVENYPQFKKMSGTVSKHVTVVGELSRLVSERNLLEVSEVEQELACQNDHSSALQNIKRLLQNPKVTEFDAARLVMLYALHYERHSSNSLPGLMMDLRNKGVSEKYRKLVSAVVEYGGKRVRGSDLFSPKDAVAITKQFLKGLKGVENVYTQHQPFLHETLDHLIKGRLKENLYPYLGPSTLRDRPQDIIVFVIGGATYEEALTVYNLNRTTPGVRIVLGGTTVHNTKSFLEEVLASGLHSRSKESSQVTSRSASRR

182 NP_001914.3 NP_000098.1

>NP_001914.3

MSYNYVVTAQKPTAVNGCVTGHFTSAEDLNLLIAKNTRLEIYVVTAEGLRPVKEVGMYGKIAVMELFRPKGESKDLLFILTAKYNACILEYKQSGESIDIITRAHGNVQDRIGRPSETGIIGIIDPECRMIGLRLYDGLFKVIPLDRDNKELKAFNIRLEELHVIDVKFLYGCQAPTICFVYQDPQGRHVKTYEVSLREKEFNKGPWKQENVEAEASMVIAVPEPFGGAIIIGQESITYHNGDKYLAIAPPIIKQSTIVCHNRVDPNGSRYLLGDMEGRLFMLLLEKEEQMDGTVTLKDLRVELLGETSIAECLTYLDNGVVFVGSRLGDSQLVKLNVDSNEQGSYVVAMETFTNLGPIVDMCVVDLERQGQGQLVTCSGAFKEGSLRIIRNGIGIHEHASIDLPGIKGLWPLRSDPNRETDDTLVLSFVGQTRVLMLNGEEVEETELMGFVDDQQTFFCGNVAHQQLIQITSASVRLVSQEPKALVSEWKEPQAKNISVASCNSSQVVVAVGRALYYLQIHPQELRQISHTEMEHEVACLDITPLGDSNGLSPLCAIGLWTDISARILKLPSFELLHKEMLGGEIIPRSILMTTFESSHYLLCALGDGALFYFGLNIETGLLSDRKKVTLGTQPTVLRTFRSLSTTNVFACSDRPTVIYSSNHKLVFSNVNLKEVNYMCPLNSDGYPDSLALANNSTLTIGTIDEIQKLHIRTVPLYESPRKICYQEVSQCFGVLSSRIEVQDTSGGTTALRPSASTQALSSSVSSSKLFSSSTAPHETSFGEEVEVHNLLIIDQHTFEVLHAHQFLQNEYALSLVSCKLGKDPNTYFIVGTAMVYPEEAEPKQGRIVVFQYSDGKLQTVAEKEVKGAVYSMVEFNGKLLASINSTVRLYEWTTEKELRTECNHYNNIMALYLKTKGDFILVGDLMRSVLLLAYKPMEGNFEEIARDFNPNWMSAVEILDDDNFLGAENAFNLFVCQKDSAATTDEERQHLQEVGLFHLGEFVNVFCHGSLVMQNLGETSTPTQGSVLFGTVNGMIGLVTSLSESWYNLLLDMQNRLNKVIKSVGKIEHSFWRSFHTERKTEPATGFIDGDLIESFLDISRPKMQEVVANLQYDDGSGMKREATADDLIKVVEELTRIH

>NP_000098.1

MAPKKRPETQKTSEIVLRPRNKRSRSPLELEPEAKKLCAKGSGPSRRCDSDCLWVGLAGPQILPPCRSIVRTLHQHKLGRASWPSVQQGLQQSFLHTLDSYRILQKAAPFDRRATSLAWHPTHPSTVAVGSKGGDIMLWNFGIKDKPTFIKGIGAGGSITGLKFNPLNTNQFYASSMEGTTRLQDFKGNILRVFASSDTINIWFCSLDVSASSRMVVTGDNVGNVILLNMDGKELWNLRMHKKKVTHVALNPCCDWFLATASVDQTVKIWDLRQVRGKASFLYSLPHRHPVNAACFSPDGARLLTTDQKSEIRVYSASQWDCPLGLIPHPHRHFQHLTPIKAAWHPRYNLIVVGRYPDPNFKSCTPYELRTIDVFDGNSGKMMCQLYDPESSGISSLNEFNPMGDTLASAMGYHILIWSQEEARTRK

183 AAH53553.1 NP_001028229.1

>AAH53553.1

MEPELAAQKQPRPRRRSRRASGLSTEGATGPSADTSGSELDGRCSLRRGSSFTFLTPGPNWDFTLKRKRREKDDDVVSLSSLDLKEPSNKRVRPLARVTSLANLISPVRNGAVRRFGQTIQSFTLRGDHRSPASAQKFSSRSTVPTPAKRRSSALWSEMLDITMKESLTTREIRRQEAIYEMSRGEQDLIEDLKLARKAYHDPMLKLSIMSEEELTHIFGDLDSYIPLHEDLLTRIGEATKPDGTVEQIGHILVSWLPRLNAYRGYCSNQLAAKALLDQKKQDPRVQDFLQRCLESPFSRKLDLWSFLDIPRSRLVKYPLLLKEILKHTPKEHPDVQLLEDAILIIQGVLSDINLKKGESECQYYIDKLEYLDEKQRDPRIEASKVLLCHGELRSKSGHKLYIFLFQDILVLTRPVTRNERHSYQVYRQPIPVQELVLEDLQDGDVRMGGSFRGAFSNSEKAKNIFRIRFHDPSPAQSHTLQANDVFHKQQWFNCIRAAIAPFQSAGSPPELQGLPELHEECEGNHPSARKLTAQRRASTVSSVTQVEVDENAYRCGSGMQMAEDSKSLKTHQTQPGIRRARDKALSGGKRKETLV

>NP_001028229.1

MSKVIQKKNHWTSRVHECTVKRGPQGELGVTVLGGAEHGEFPYVGAVAAVEAAGLPGGGEGPRLGEGELLLEVQGVRVSGLPRYDVLGVIDSCKEAVTFKAVRQGGRLNKDLRHFLNQRFQKGSPDHELQQTIRDNLYRHAVPCTTRSPREGEVPGVDYNFLTVKEFLDLEQSGTLLEVGTYEGNYYGTPKPPSQPVSGKVITTDALHSLQSGSKQSTPKRTKSYNDMQNAGIVHAENEEEDDVPEMNSSFTADSGEQEEHTLQETALPPVNSSIIAAPITDPSQKFPQYLPLSAEDNLGPLPENWEMAYTENGEVYFIDHNTKTTSWLDPRCLNKQQKPLEECEDDEGVHTEELDSELELPAGWEKIEDPVYGIYYVDHINRKTQYENPVLEAKRKKQLEQQQQQQQQQQQQQQQQQQQQTEEWTEDHSALVPPVIPNHPPSNPEPAREVPLQGKPFFTRNPSELKGKFIHTKLRKSSRGFGFTVVGGDEPDEFLQIKSLVLDGPAALDGKMETGDVIVSVNDTCVLGHTHAQVVKIFQSIPIGASVDLELCRGYPLPFDPDDPNTSLVTSVAILDKEPIIVNGQETYDSPASHSSKTGKVNGMKDARPSSPADVASNSSHGYPNDTVSLASSIATQPELITVHIVKGPMGFGFTIADSPGGGGQRVKQIVDSPRCRGLKEGDLIVEVNKKNVQALTHNQVVDMLVECPKGSEVTLLVQRGGLPVPKKSPKSQPLERKDSQNSSQHSVSSHRSLHTASPSHSTQVLPEFPPAEAQAPDQTDSSGQKKPDPFKIWAQSRSMYENRLPDYQEQDIFLWRKETGFGFRILGGNEPGEPIYIGHIVPLGAADTDGRLRSGDELICVDGTPVIGKSHQLVVQLMQQAAKQGHVNLTVRRKVVFAVPKTENEVPSPASSHHSSNQPASLTEEKRTPQGSQNSLNTVSSGSGSTSGIGSGGGGGSGVVSTVVQPYDVEIRRGENEGFGFVIVSSVSRPEAGTTFGNACVAMPHKIGRIIEGSPADRCGKLKVGDRILAVNGCSITNKSHSDIVNLIKEAGNTVTLRIIPGDESSNATLLTNAEKIATITTTHTPSQQGTQETRNTTKPKQESQFEFKAPQATQEQDFYTVELERGAKGFGFSLRGGREYNMDLYVLRLAEDGPAERCGKMRIGDEILEINGETTKNMKHSRAIELIKNGGRRVRLFLKRGDGSVPEYDPSSDRHGPATGPQGVPEVRAGPDRRQHPSLESSYPPDLHKSSPHGEKRAHARDPKGSREYSRQPNEHHTWNGTSRKPDSGACRPKDRAPEGRRDAQAERAAAANGPKRRSPEKRREGTRSADNTLERREKHEKRRDVSPERRRERSPTRRRDGSPSRRRRSLERLLEQRRSPERRRGGSPERRAKSTDRRRARSPERRRERSLDKRNREDRASHREREEANLKQDAGRSSRHPPEQRRRPYKECSTDLSI

184 NP_060780.2 NP_005602.2

>NP_060780.2

MPGRGRCPDCGSTELVEDSHYSQSQLVCSDCGCVVTEGVLTTTFSDEGNLREVTYSRSTGENEQVSRSQQRGLRRVRDLCRVLQLPPTFEDTAVAYYQQAYRHSGIRAARLQKKEVLVGCCVLITCRQHNWPLTMGAICTLLYADLDVFSSTYMQIVKLLGLDVPSLCLAELVKTYCSSFKLFQASPSVPAKYVEDKEKMLSRTMQLVELANETWLVTGRHPLPVITAATFLAWQSLQPADRLSCSLARFCKLANVDLPYPASSRLQELLAVLLRMAEQLAWLRVLRLDKRSVVKHIGDLLQHRQSLVRSAFRDGTAEVETREKEPPGWGQGQGEGEVGNNSLGLPQGKRPASPALLLPPCMLKSPKRICPVPPVSTVTGDENISDSEIEQYLRTPQEVRDFQRAQAARQAATSVPNPP

>NP_005602.2

MPSGGDQSPPPPPPPPAAAASDEEEEDDGEAEDAAPSAESPTPQIQQRFDELCSRLNMDEAARPEAWDSYRSMSESYTLEGNDLHWLACALYVACRKSVPTVSKGTVEGNYVSLTRILKCSEQSLIEFFNKMKKWEDMANLPPHFRERTERLERNFTVSAVIFKKYEPIFQDIFKYPQEEQPRQQRGRKQRRQPCTVSEIFHFCWVLFIYAKGNFPMISDDLVNSYHLLLCALDLVYGNALQCSNRKELVNPNFKGLSEDFHAKDSKPSSDPPCIIEKLCSLHDGLVLEAKGIKEHFWKPYIRKLYEKKLLKGKEENLTGFLEPGNFGESFKAINKAYEEYVLSVGNLDERIFLGEDAEEEIGTLSRCLNAGSGTETAERVQMKNILQQHFDKSKALRISTPLTGVRYIKENSPCVTPVSTATHSLSRLHTMLTGLRNAPSEKLEQILRTCSRDPTQAIANRLKEMFEIYSQHFQPDEDFSNCAKEIASKHFRFAEMLYYKVLESVIEQEQKRLGDMDLSGILEQDAFHRSLLACCLEVVTFSYKPPGNFPFITEIFDVPLYHFYKVIEVFIRAEDGLCREVVKHLNQIEEQILDHLAWKPESPLWEKIRDNENRVPTCEEVMPPQNLERADEICIAGSPLTPRRVTEVRADTGGLGRSITSPTTLYDRYSSPPASTTRRRLFVENDSPSDGGTPGRMPPQPLVNAVPVQNVSGETVSVTPVPGQTLVTMATATVTANNGQTVTIPVQGIANENGGITFFPVQVNVGGQAQAVTGSIQPLSAQALAGSLSSQQVTGTTLQVPGQVAIQQISPGGQQQKQGQSVTSSSNRPRKTSSLSLFFRKVYHLAAVRLRDLCAKLDISDELRKKIWTCFEFSIIQCPELMMDRHLDQLLMCAIYVMAKVTKEDKSFQNIMRCYRTQPQARSQVYRSVLIKGKRKRRNSGSSDSRSHQNSPTELNKDRTSRDSSPVMRSSSTLPVPQPSSAPPTPTRLTGANSDMEEEERGDLIQFYNNIYIKQIKTFAMKYSQANMDAPPLSPYPFVRTGSPRRIQLSQNHPVYISPHKNETMLSPREKIFYYFSNSPSKRLREINSMIRTGETPTKKRGILLEDGSESPAKRICPENHSALLRRLQDVANDRGSH

185 NP_001078923.1 NP_060853.3

>NP_001078923.1

MSRKGPRAEVCADCSAPDPGWASISRGVLVCDECCSVHRSLGRHISIVKHLRHSAWPPTLLQMVHTLASNGANSIWEHSLLDPAQVQSGRRKANPQDKVHPIKSEFIRAKYQMLAFVHKLPCRDDDGVTAKDLSKQLHSSVRTGNLETCLRLLSLGAQANFFHPEKGTTPLHVAAKAGQTLQAELLVVYGADPGSPDVNGRTPIDYARQAGHHELAERLVECQYELTDRLAFYLCGRKPDHKNGHYIIPQMADRSRQKCMSQSLDLSELAKAAKKKLQALSNRLFEELAMDVYDEVDRRENDAVWLATQNHSTLVTERSAVPFLPVNPEYSATRNQGRQKLARFNAREFATLIIDILSEAKRRQQGKSLSSPTDNLELSLRSQSDLDDQHDYDSVASDEDTDQEPLRSTGATRSNRARSMDSSDLSDGAVTLQEYLELKKALATSEAKVQQLMKVNSSLSDELRRLQREIHKLQAENLQLRQPPGPVPTPPLPSERAEHTPMAPGGSTHRRDRQAFSMYEPGSALKPFGGPPGDELTTRLQPFHSTELEDDAIYSVHVPAGLYRIRKGVSASAVPFTPSSPLLSCSQEGSRHTSKLSRHGSGADSDYENTQSGDPLLGLEGKRFLELGKEEDFHPELESLDGDLDPGLPSTEDVILKTEQVTKNIQELLRAAQEFKHDSFVPCSEKIHLAVTEMASLFPKRPALEPVRSSLRLLNASAYRLQSECRKTVPPEPGAPVDFQLLTQQVIQCAYDIAKAAKQLVTITTREKKQ

>NP_060853.3

MATEIGSPPRFFHMPRFQHQAPRQLFYKRPDFAQQQAMQQLTFDGKRMRKAVNRKTIDYNPSVIKYLENRIWQRDQRDMRAIQPDAGYYNDLVPPIGMLNNPMNAVTTKFVRTSTNKVKCPVFVVRWTPEGRRLVTGASSGEFTLWNGLTFNFETILQAHDSPVRAMTWSHNDMWMLTADHGGYVKYWQSNMNNVKMFQAHKEAIREASFSPTDNKFATCSDDGTVRIWDFLRCHEERILRGHGADVKCVDWHPTKGLVVSGSKDSQQPIKFWDPKTGQSLATLHAHKNTVMEVKLNLNGNWLLTASRDHLCKLFDIRNLKEELQVFRGHKKEATAVAWHPVHEGLFASGGSDGSLLFWHVGVEKEVGGMEMAHEGMIWSLAWHPLGHILCSGSNDHTSKFWTRNRPGDKMRDRYNLNLLPGMSEDGVEYDDLEPNSLAVIPGMGIPEQLKLAMEQEQMGKDESNEIEMTIPGLDWGMEEVMQKDQKKVPQKKVPYAKPIPAQFQQAWMQNKVPIPAPNEVLNDRKEDIKLEEKKKTQAEIEQEMATLQYTNPQLLEQLKIERLAQKQVEQIQPPPSSGTPLLGPQPFPGQGPMSQIPQGFQQPHPSQQMPMNMAQMGPPGPQGQFRPPGPQGQMGPQGPPLHQGGGGPQGFMGPQGPQGPPQGLPRPQDMHGPQGMQRHPGPHGPLGPQGPPGPQGSSGPQGHMGPQGPPGPQGHIGPQGPPGPQGHLGPQGPPGTQGMQGPPGPRGMQGPPHPHGIQGGPGSQGIQGPVSQGPLMGLNPRGMQGPPGPRENQGPAPQGMIMGHPPQEMRGPHPPGGLLGHGPQEMRGPQEIRGMQGPPPQGSMLGPPQELRGPPGSQSQQGPPQGSLGPPPQGGMQGPPGPQGQQNPARGPHPSQGPIPFQQQKTPLLGDGPRAPFNQEGQSTGPPPLIPGLGQQGAQGRIPPLNPGQGPGPNKGDSRGPPNHHMGPMSERRHEQSGGPEHGPERGPFRGGQDCRGPPDRRGPHPDFPDDFSRPDDFHPDKRFGHRLREFEGRGGPLPQEEKWRRGGPGPPFPPDHREFSEGDGRGAARGPPGAWEGRRPGDERFPRDPEDPRFRGRREESFRRGAPPRHEGRAPPRGRDGFPGPEDFGPEENFDASEEAARGRDLRGRGRGTPRGGRKGLLPTPDEFPRFEGGRKPDSWDGNREPGPGHEHFRDTPRPDHPPHDGHSPASRERSSSLQGMDMASLPPRKRPWHDGPGTSEHREMEAPGGPSEDRGGKGRGGPGPAQRVPKSGRSSSLDGEHHDGYHRDEPFGGPPGSGTPSRGGRSGSNWGRGSNMNSGPPRRGASRGGGRGR

186 NP_006457.2 NP_542397.1

>NP_006457.2

MAEVKVKVQPPDADPVEIENRIIELCHQFPHGITDQVIQNEMPHIEAQQRAVAINRLLSMGQLDLLRSNTGLLYRIKDSQNAGKMKGSDNQEKLVYQIIEDAGNKGIWSRDIRYKSNLPLTEINKILKNLESKKLIKAVKSVAASKKKVYMLYNLQPDRSVTGGAWYSDQDFESEFVEVLNQQCFKFLQSKAETARESKQNPMIQRNSSFASSHEVWKYICELGISKVELSMEDIETILNTLIYDGKVEMTIIAAKEGTVGSVDGHMKLYRAVNPIIPPTGLVRAPCGLCPVFDDCHEGGEISPSNCIYMTEWLEF

>NP_542397.1

MEKIEEQFANLHIVKCSLGTKEPTYLLGIDTSKTVQAGKENLVAVLCSNGSIRIYDKERLNVLREFSGYPGLLNGVRFANSCDSVYSACTDGTVKCWDARVAREKPVQLFKGYPSNIFISFDINCNDHIICAGTEKVDDDALLVFWDARMNSQNLSTTKDSLGAYSETHSDDVTQVRFHPSNPNMVVSGSSDGLVNVFDINIDNEEDALVTTCNSISSVSCIGWSGKGYKQIYCMTHDEGFYWWDLNHLDTDEPVTRLNIQDVREVVNMKEDALDYLIGGLYHEKTDTLHVIGGTNKGRIHLMNCSMSGLTHVTSLQGGHAATVRSFCWNVQDDSLLTGGEDAQLLLWKPGAIEKTFTKKESMKIASSVHQRVRVHSNDSYKRRKKQ

187 NP_060853.3 NP_115496.2

>NP_060853.3

MATEIGSPPRFFHMPRFQHQAPRQLFYKRPDFAQQQAMQQLTFDGKRMRKAVNRKTIDYNPSVIKYLENRIWQRDQRDMRAIQPDAGYYNDLVPPIGMLNNPMNAVTTKFVRTSTNKVKCPVFVVRWTPEGRRLVTGASSGEFTLWNGLTFNFETILQAHDSPVRAMTWSHNDMWMLTADHGGYVKYWQSNMNNVKMFQAHKEAIREASFSPTDNKFATCSDDGTVRIWDFLRCHEERILRGHGADVKCVDWHPTKGLVVSGSKDSQQPIKFWDPKTGQSLATLHAHKNTVMEVKLNLNGNWLLTASRDHLCKLFDIRNLKEELQVFRGHKKEATAVAWHPVHEGLFASGGSDGSLLFWHVGVEKEVGGMEMAHEGMIWSLAWHPLGHILCSGSNDHTSKFWTRNRPGDKMRDRYNLNLLPGMSEDGVEYDDLEPNSLAVIPGMGIPEQLKLAMEQEQMGKDESNEIEMTIPGLDWGMEEVMQKDQKKVPQKKVPYAKPIPAQFQQAWMQNKVPIPAPNEVLNDRKEDIKLEEKKKTQAEIEQEMATLQYTNPQLLEQLKIERLAQKQVEQIQPPPSSGTPLLGPQPFPGQGPMSQIPQGFQQPHPSQQMPMNMAQMGPPGPQGQFRPPGPQGQMGPQGPPLHQGGGGPQGFMGPQGPQGPPQGLPRPQDMHGPQGMQRHPGPHGPLGPQGPPGPQGSSGPQGHMGPQGPPGPQGHIGPQGPPGPQGHLGPQGPPGTQGMQGPPGPRGMQGPPHPHGIQGGPGSQGIQGPVSQGPLMGLNPRGMQGPPGPRENQGPAPQGMIMGHPPQEMRGPHPPGGLLGHGPQEMRGPQEIRGMQGPPPQGSMLGPPQELRGPPGSQSQQGPPQGSLGPPPQGGMQGPPGPQGQQNPARGPHPSQGPIPFQQQKTPLLGDGPRAPFNQEGQSTGPPPLIPGLGQQGAQGRIPPLNPGQGPGPNKGDSRGPPNHHMGPMSERRHEQSGGPEHGPERGPFRGGQDCRGPPDRRGPHPDFPDDFSRPDDFHPDKRFGHRLREFEGRGGPLPQEEKWRRGGPGPPFPPDHREFSEGDGRGAARGPPGAWEGRRPGDERFPRDPEDPRFRGRREESFRRGAPPRHEGRAPPRGRDGFPGPEDFGPEENFDASEEAARGRDLRGRGRGTPRGGRKGLLPTPDEFPRFEGGRKPDSWDGNREPGPGHEHFRDTPRPDHPPHDGHSPASRERSSSLQGMDMASLPPRKRPWHDGPGTSEHREMEAPGGPSEDRGGKGRGGPGPAQRVPKSGRSSSLDGEHHDGYHRDEPFGGPPGSGTPSRGGRSGSNWGRGSNMNSGPPRRGASRGGGRGR

>NP_115496.2

MASSGGELGSLFDHHVQRAVCDTRAKYREGRRPRAVKVYTINLESQYLLIQGVPAVGVMKELVERFALYGAIEQYNALDEYPAEDFTEVYLIKFMNLQSARTAKRKMDEQSFFGGLLHVCYAPEFETVEETRKKLQMRKAYVVKTTENKDHYVTKKKLVTEHKDTEDFRQDFHSEMSGFCKAALNTSAGNSNPYLPYSCELPLCYFSSKCMCSSGGPVDRAPDSSKDGRNHHKTMGHYNHNDSLRKTQINSLKNSVACPGAQKAITSSEAVDRFMPRTTQLQERKRRREDDRKLGTFLQTNPTGNEIMIGPLLPDISKVDMHDDSLNTTANLIRHKLKEVISSVPKPPEDKPEDVHTSHPLKQRRRI

188 NP_004291.1 NP_001895.1

>NP_004291.1

MAEQATKSVLFVCLGNICRSPIAEAVFRKLVTDQNISENWRVDSAATSGYEIGNPPDYRGQSCMKRHGIPMSHVARQITKEDFATFDYILCMDESNLRDLNRKSNQVKTCKAKIELLGSYDPQKQLIIEDPYYGNDSDFETVYQQCVRCCRAFLEKAH

>NP_001895.1

MATQADLMELDMAMEPDRKAAVSHWQQQSYLDSGIHSGATTTAPSLSGKGNPEEEDVDTSQVLYEWEQGFSQSFTQEQVADIDGQYAMTRAQRVRAAMFPETLDEGMQIPSTQFDAAHPTNVQRLAEPSQMLKHAVVNLINYQDDAELATRAIPELTKLLNDEDQVVVNKAAVMVHQLSKKEASRHAIMRSPQMVSAIVRTMQNTNDVETARCTAGTLHNLSHHREGLLAIFKSGGIPALVKMLGSPVDSVLFYAITTLHNLLLHQEGAKMAVRLAGGLQKMVALLNKTNVKFLAITTDCLQILAYGNQESKLIILASGGPQALVNIMRTYTYEKLLWTTSRVLKVLSVCSSNKPAIVEAGGMQALGLHLTDPSQRLVQNCLWTLRNLSDAATKQEGMEGLLGTLVQLLGSDDINVVTCAAGILSNLTCNNYKNKMMVCQVGGIEALVRTVLRAGDREDITEPAICALRHLTSRHQEAEMAQNAVRLHYGLPVVVKLLHPPSHWPLIKATVGLIRNLALCPANHAPLREQGAIPRLVQLLVRAHQDTQRRTSMGGTQQQFVEGVRMEEIVEGCTGALHILARDVHNRIVIRGLNTIPLFVQLLYSPIENIQRVAAGVLCELAQDKEAAEAIEAEGATAPLTELLHSRNEGVATYAAAVLFRMSEDKPQDYKKRLSVELTSSLFRTEPMAWNETADLGLDIGAQGEPLGYRQDDPSYRSFHSGGYGQDALGMDPMMEHEMGGHHPGADYPVDGLPDLGHAQDLMDGLPPGDSNQLAWFDTDL

189 NP_733796.2 NP_060842.3

>NP_733796.2

MVLAQSRVSAGVGSPHCSGSGGGGSDSFPWPASHPGNPQCSFSTAFLASPRLSRGTLAYLPPAPWSSLATPSALLGSSCAPPPPPARCPQPRALSPELGTKAGPRRPHRWELPRSPSQGAQGPAPRRRLLETMKGIVAASGSETEDEDSMDIPLDLSSSAGSGKRRRRGNLPKESVQILRDWLYEHRYNAYPSEQEKALLSQQTHLSTLQVCNWFINARRRLLPDMLRKDGKDPNQFTISRRGAKISETSSVESVMGIKNFMPALEETPFHSCTAGPNPTLGRPLSPKPSSPGSVLARPSVICHTTVTALKDVPFSLCQSVGVGQNTDIQQIAAKNFTDTSLMYPEDTCKSGPSTNTQSGLFNTPPPTPPDLNQDFSGFQLLVDVALKRAAEMELQAKLTA

>NP_060842.3

MSNNLRRVFLKPAEENSGNASRCVSGCMYQVVQTIGSDGKNLLQLLPIPKSSGNLIPLVQSSVMSDALKGNTGKPVQVTFQTQISSSSTSASVQLPIFQPASSSNYFLTRTVDTSEKGRVTSVGTGNFSSSVSKVQSHGVKIDGLTMQTFAVPPSTQKDSSFIVVNTQSLPVTVKSPVLPSGHHLQIPAHAEVKSVPASSLPPSVQQKILATATTSTSGMVEASQMPTVIYVSPVNTVKNVVTKNFQNIYPKPVTEIAKPVILNTTQIPKNVATETQLKGGQHSQAAPVKWIFQDNLQPFTPSLVPVKSSNNVASKILKTFVDRKNLGDNTINMPPLSTIDPSGTRSKNMPIKDNALVMFNGKVYLLAKKGTDVLPSQIDQQNSVSPDTPVRKDTLQTVSSSPVTEISREVVNIVLAKSKSSQMETKSLSNTQLASMANLRAEKNKVEKPSPSTTNPHMNQSSNYLKQSKTLFTNPIFPVGFSTGHNAPRKVTAVIYARKGSVLQSIEKISSSVDATTVTSQQCVFRDQEPKIHNEMASTSDKGAQGRNDKKDSQGRSNKALHLKSDAEFKKIFGLTKDLRVCLTRIPDHLTSGEGFDSFSSLVKSGTYKETEFMVKEGERKQQNFDKKRKAKTNKKMDHIKKRKTENAYNAIINGEANVTGSQLLSSILPTSDVSQHNILTSHSKTRQEKRTEMEYYTHEKQEKGTLNSNAAYEQSHFFNKNYTEDIFPVTPPELEETIRDEKIRRLKQVLREKEAALEEMRKKMHQK

190 NP_001001683.1 NP_001001651.1

>NP_001001683.1

MATYSLANERLRALEDIEREIGAILQNAGTVILELSKEKTNERLLDRQAAAFTASVQHVEAELSAQIRYLTQVATGQPHEGSSYSSRKDCQMALKRVDYARLKLSDVARTCEQMLEN

>NP_001001651.1

MQREEKQLEASLDALLSQVADLKNSLGSFICKLENEYGRLTWPSVLDSFALLSGQLNTLNKVLKHEKTPLFRNQVIIPLVLSPDRDEDLMRQTEGRVPVFSHEVVPDHLRTKPDPEVEEQEKQLTTDAARIGADAAQKQIQSLNKMCSNLLEKISKEERESESGGLRPNKQTFNPTDTNALVAAVAFGKGLSNWRPSGSSGPGQAGQPGAGTILAGTSGLQQVQMAGAPSQQQPMLSGVQMAQAGQPGKMPSGIKTNIKSASMHPYQRPSCLGFILAIPLRRKVKKLLGQEGKKNAHLQLW

191 NP_001034679.2 NP_001895.1

>NP_001034679.2

MTATTRGSPVGGNDNQGQAPDGQSQPPLQQNQTSSPDSSNENSPATPPDEQGQGDAPPQLEDEEPAFPHTDLAKLDDMINRPRWVVPVLPKGELEVLLEAAIDLSKKGLDVKSEACQRFFRDGLTISFTKILTDEAVSGWKFEIHRCIINNTHRLVELCVAKLSQDWFPLLELLAMALNPHCKFHIYNGTRPCESVSSSVQLPEDELFARSPDPRSPKGWLVDLLNKFGTLNGFQILHDRFINGSALNVQIIAALIKPFGQCYEFLTLHTVKKYFLPIIEMVPQFLENLTDEELKKEAKNEAKNDALSMIIKSLKNLASRVPGQEETVKNLEIFRLKMILRLLQISSFNGKMNALNEVNKVISSVSYYTHRHGNPEEEEWLTAERMAEWIQQNNILSIVLRDSLHQPQYVEKLEKILRFVIKEKALTLQDLDNIWAAQAGKHEAIVKNVHDLLAKLAWDFSPEQLDHLFDCFKASWTNASKKQREKLLELIRRLAEDDKDGVMAHKVLNLLWNLAHSDDVPVDIMDLALSAHIKILDYSCSQDRDTQKIQWIDRFIEELRTNDKWVIPALKQIREICSLFGEAPQNLSQTQRSPHVFYRHDLINQLQHNHALVTLVAENLATYMESMRLYARDHEDYDPQTVRLGSRYSHVQEVQERLNFLRFLLKDGQLWLCAPQAKQIWKCLAENAVYLCDREACFKWYSKLMGDEPDLDPDINKDFFESNVLQLDPSLLTENGMKCFERFFKAVNCREGKLVAKRRAYMMDDLELIGLDYLWRVVIQSNDDIASRAIDLLKEIYTNLGPRLQVNQVVIHEDFIQSCFDRLKASYDTLCVLDGDKDSVNCARQEAVRMVRVLTVLREYINECDSDYHEERTILPMSRAFRGKHLSFVVRFPNQGRQVDDLEVWSHTNDTIGSVRRCILNRIKANVAHTKIELFVGGELIDPADDRKLIGQLNLKDKSLITAKLTQISSNMPSSPDSSSDSSTGSPGNHGNHYSDGPNPEVESCLPGVIMSLHPRYISFLWQVADLGSSLNMPPLRDGARVLMKLMPPDSTTIEKLRAICLDHAKLGESSLSPSLDSLFFGPSASQVLYLTEVVYALLMPAGAPLADDSSDFQFHFLKSGGLPLVLSMLTRNNFLPNADMETRRGAYLNALKIAKLLLTAIGYGHVRAVAEACQPGVEGVNPMTQINQVTHDQAVVLQSALQSIPNPSSECMLRNVSVRLAQQISDEASRYMPDICVIRAIQKIIWASGCGSLQLVFSPNEEITKIYEKTNAGNEPDLEDEQVCCEALEVMTLCFALIPTALDALSKEKAWQTFIIDLLLHCHSKTVRQVAQEQFFLMCTRCCMGHRPLLFFITLLFTVLGSTARERAKHSGDYFTLLRHLLNYAYNSNINVPNAEVLLNNEIDWLKRIRDDVKRTGETGIEETILEGHLGVTKELLAFQTSEKKFHIGCEKGGANLIKELIDDFIFPASNVYLQYMRNGELPAEQAIPVCGSPPTINAGFELLVALAVGCVRNLKQIVDSLTEMYYIGTAITTCEALTEWEYLPPVGPRPPKGFVGLKNAGATCYMNSVIQQLYMIPSIRNGILAIEGTGSDVDDDMSGDEKQDNESNVDPRDDVFGYPQQFEDKPALSKTEDRKEYNIGVLRHLQVIFGHLAASRLQYYVPRGFWKQFRLWGEPVNLREQHDALEFFNSLVDSLDEALKALGHPAMLSKVLGGSFADQKICQGCPHRYECEESFTTLNVDIRNHQNLLDSLEQYVKGDLLEGANAYHCEKCNKKVDTVKRLLIKKLPPVLAIQLKRFDYDWERECAIKFNDYFEFPRELDMEPYTVAGVAKLEGDNVNPESQLIQQSEQSESETAGSTKYRLVGVLVHSGQASGGHYYSYIIQRNGGDGERNRWYKFDDGDVTECKMDDDEEMKNQCFGGEYMGEVFDHMMKRMSYRRQKRWWNAYILFYERMDTIDQDDELIRYISELAITTRPHQIIMPSAIERSVRKQNVQFMHNRMQYSMEYFQFMKKLLTCNGVYLNPPPGQDHLLPEAEEITMISIQLAARFLFTTGFHTKKVVRGSASDWYDALCILLRHSKNVRFWFAHNVLFNVSNRFSEYLLECPSAEVRGAFAKLIVFIAHFSLQDGPCPSPFASPGPSSQAYDNLSLSDHLLRAVLNLLRREVSEHGRHLQQYFNLFVMYANLGVAEKTQLLKLSVPATFMLVSLDEGPGPPIKYQYAELGKLYSVVSQLIRCCNVSSRMQSSINGNPPLPNPFGDPNLSQPIMPIQQNVADILFVRTSYVKKIIEDCSNSEETVKLLRFCCWENPQFSSTVLSELLWQVAYSYTYELRPYLDLLLQILLIEDSWQTHRIHNALKGIPDDRDGLFDTIQRSKNHYQKRAYQCIKCMVALFSNCPVAYQILQGNGDLKRKWTWAVEWLGDELERRPYTGNPQYTYNNWSPPVQSNETSNGYFLERSHSARMTLAKACELCPEEVKKATSVQQIEMEESKEPDDQDAPDEHESPPPEDAPLYPHSPGSQYQQNNHVHGQPYTGPAAHHMNNPQRTGQRAQENYEGSEEVSPPQTKDQ

>NP_001895.1

MATQADLMELDMAMEPDRKAAVSHWQQQSYLDSGIHSGATTTAPSLSGKGNPEEEDVDTSQVLYEWEQGFSQSFTQEQVADIDGQYAMTRAQRVRAAMFPETLDEGMQIPSTQFDAAHPTNVQRLAEPSQMLKHAVVNLINYQDDAELATRAIPELTKLLNDEDQVVVNKAAVMVHQLSKKEASRHAIMRSPQMVSAIVRTMQNTNDVETARCTAGTLHNLSHHREGLLAIFKSGGIPALVKMLGSPVDSVLFYAITTLHNLLLHQEGAKMAVRLAGGLQKMVALLNKTNVKFLAITTDCLQILAYGNQESKLIILASGGPQALVNIMRTYTYEKLLWTTSRVLKVLSVCSSNKPAIVEAGGMQALGLHLTDPSQRLVQNCLWTLRNLSDAATKQEGMEGLLGTLVQLLGSDDINVVTCAAGILSNLTCNNYKNKMMVCQVGGIEALVRTVLRAGDREDITEPAICALRHLTSRHQEAEMAQNAVRLHYGLPVVVKLLHPPSHWPLIKATVGLIRNLALCPANHAPLREQGAIPRLVQLLVRAHQDTQRRTSMGGTQQQFVEGVRMEEIVEGCTGALHILARDVHNRIVIRGLNTIPLFVQLLYSPIENIQRVAAGVLCELAQDKEAAEAIEAEGATAPLTELLHSRNEGVATYAAAVLFRMSEDKPQDYKKRLSVELTSSLFRTEPMAWNETADLGLDIGAQGEPLGYRQDDPSYRSFHSGGYGQDALGMDPMMEHEMGGHHPGADYPVDGLPDLGHAQDLMDGLPPGDSNQLAWFDTDL

192 NP_060589.1 NP_001713.2

>NP_060589.1

MANEEDDPVVQEIDVYLAKSLAEKLYLFQYPVRPASMTYDDIPHLSAKIKPKQQKVELEMAIDTLNPNYCRSKGEQIALNVDGACADETSTYSSKLMDKQTFCSSQTTSNTSRYAAALYRQGELHLTPLHGILQLRPSFSYLDKADAKHREREAANEAGDSSQDEAEDDVKQITVRFSRPESEQARQRRVQSYEFLQKKHAEEPWVHLHYYGLRDSRSEHERQYLLCPGSSGVENTELVKSPSEYLMMLMPPSQEEEKDKPVAPSNVLSMAQLRTLPLADQIKILMKNVKVMPFANLMSLLGPSIDSVAVLRGIQKVAMLVQGNWVVKSDILYPKDSSSPHSGVPAEVLCRGRDFVMWKFTQSRWVVRKEVATVTKLCAEDVKDFLEHMAVVRINKGWEFILPYDGEFIKKHPDVVQRQHMLWTGIQAKLEKVYNLVKETMPKKPDAQSGPAGLVCGDQRIQVAKTKAQQNHALLERELQRRKEQLRVPAVPPGVRIKEEPVSEEGEEDEEQEAEEEPMDTSPSGLHSKLANGLPLGRAAGTDSFNGHPPQGCASTPVARELKAFVEATFQRQFVLTLSELKRLFNLHLASLPPGHTLFSGISDRMLQDTVLAAGCKQILVPFPPQTAASPDEQKVFALWESGDMSDQHRQVLLEIFSKNYRVRRNMIQSRLTQECGEDLSKQEVDKVLKDCCVSYGGMWYLKGTVQS

>NP_001713.2

MSEGNAAGEPSTPGGPRPLLTGARGLIGRRPAPPLTPGRLPSIRSRDLTLGGVKKKTFTPNIISRKIKEEPKEEVTVKKEKRERDRDRQREGHGRGRGRPEVIQSHSIFEQGPAEMMKKKGNWDKTVDVSDMGPSHIINIKKEKRETDEETKQILRMLEKDDFLDDPGLRNDTRNMPVQLPLAHSGWLFKEENDEPDVKPWLAGPKEEDMEVDIPAVKVKEEPRDEEEEAKMKAPPKAARKTPGLPKDVSVAELLRELSLTKEEELLFLQLPDTLPGQPPTQDIKPIKTEVQGEDGQVVLIKQEKDREAKLAENACTLADLTEGQVGKLLIRKSGRVQLLLGKVTLDVTMGTACSFLQELVSVGLGDSRTGEMTVLGHVKHKLVCSPDFESLLDHKHR

193 NP_478102.1 NP_000356.1

>NP_478102.1

MGRGRCVGPSLQLRGQEWRCSPLVPKGGAAAAELGPGGGENMVRRFLVTLRIRRACGPPRVRVFVVHIPRLTGEWAAPGAPAAVALVLMLLRSQRLGQQPLPRRPGHDDGQRPSGGAAAAPRRGAQLRRPRHSHPTRARRCPGGLPGHAGGAAPGRGAAGRARCLGPSARGPG

>NP_000356.1

MAPSRKFFVGGNWKMNGRKQSLGELIGTLNAAKVPADTEVVCAPPTAYIDFARQKLDPKIAVAAQNCYKVTNGAFTGEISPGMIKDCGATWVVLGHSERRHVFGESDELIGQKVAHALAEGLGVIACIGEKLDEREAGITEKVVFEQTKVIADNVKDWSKVVLAYEPVWAIGTGKTATPQQAQEVHEKLRGWLKSNVSDAVAQSTRIIYGGSVTGATCKELASQPDVDGFLVGGASLKPEFVDIINAKQ

194 NP_001027024.3 NP_062565.2

>NP_001027024.3

MRRPPGNGEAASEGPGGWGLWGVQESRRLCCAGHDRCKQALLQIGINMMALPGGRHLDSVTLPGQRLHLMQVDSVQRWMEDLKLMTECECMCVLQAKPISLEEDAQGDLILAGGPGPGDPLQLLLKRGWVISTELRRIGQKLAQDRWARVHSMSVRLTCHARSMVSEYSAVSRNSLKEMGEIEKLLMEKCSELSAVTERCLQVENEHVLKSMKACVSETLSMLGQHFGQLLELALTREVQALVRKIDASDNIYTTESTTGNLFSLTQEGAPLCRIIAKEGGVVALFKVCRQDSFRCLYPQALRTLASICCVEEGVHQLEKVDGVLCLADILTDNSHSEATRAEAAAVVAQVTSPHLPVTQHLSSFLESMEEIVTALVKLCQEASSGEVFLLASAALANITFFDTMACEMLLQLNAIRVLLEACSDKQRVDTPYTRDQIVTILANMSVLEQCASDIIQENGVQLIMGMLSEKPRSGTPAEVAACERVQQKAAVTLARLSRDPDVAREAVRLSCMSRLIELCRSPSERNSSDAVLVACLAALRRLAGVCPEGLQDSDFQQLVQPRLVDSFLLCSNMEESFV

>NP_062565.2

MKVTVCFGRTRVVVPCGDGHMKVFSLIQQAVTRYRKAIAKDPNYWIQVHRLEHGDGGILDLDDILCDVADDKDRLVAVFDEQDPHHGGDGTSASSTGTQSPEIFGSELGTNNVSAFQPYQATSEIEVTPSVLRANMPLHVRRSSDPALIGLSTSVSDSNFSSEEPSRKNPTRWSTTAGFLKQNTAGSPKTCDRKKDENYRSLPRDTSNWSNQFQRDNARSSLSASHPMVGKWLEKQEQDEDGTEEDNSRVEPVGHADTGLEHIPNFSLDDMVKLVEVPNDGGPLGIHVVPFSARGGRTLGLLVKRLEKGGKAEHENLFRENDCIVRINDGDLRNRRFEQAQHMFRQAMRTPIIWFHVVPAANKEQYEQLSQSEKNNYYSSRFSPDSQYIDNRSVNSAGLHTVQRAPRLNHPPEQIDSHSRLPHSAHPSGKPPSAPASAPQNVFSTTVSSGYNTKKIGKRLNIQLKKGTEGLGFSITSRDVTIGGSAPIYVKNILPRGAAIQDGRLKAGDRLIEVNGVDLVGKSQEEVVSLLRSTKMEGTVSLLVFRQEDAFHPRELNAEPSQMQIPKETKAEDEDIVLTPDGTREFLTFEVPLNDSGSAGLGVSVKGNRSKENHADLGIFVKSIINGGAASKDGRLRVNDQLIAVNGESLLGKTNQDAMETLRRSMSTEGNKRGMIQLIVARRISKCNELKSPGSPPGPELPIETALDDRERRISHSLYSGIEGLDESPSRNAALSRIMGESGKYQLSPTVNMPQDDTVIIEDDRLPVLPPHLSDQSSSSSHDDVGFVTADAGTWAKAAISDSADCSLSPDVDPVLAFQREGFGRQSMSEKRTKQFSDASQLDFVKTRKSKSMDLGIADETKLNTVDDQKAGSPSRDVGPSLGLKKSSSLESLQTAVAEVTLNGDIPFHRPRPRIIRGRGCNESFRAAIDKSYDKPAVDDDDEGMETLEEDTEESSRSGRESVSTASDQPSHSLERQMNGNQEKGDKTDRKKDKTGKEKKKDRDKEKDKMKAKKGMLKGLGDMFRFGKHRKDDKIEKTGKIKIQESFTSEEERIRMKQEQERIQAKTREFRERQARERDYAEIQDFHRTFGCDDELMYGGVSSYEGSMALNARPQSPREGHMMDALYAQVKKPRNSKPSPVDSNRSTPSNHDRIQRLRQEFQQAKQDEDVEDRRRTYSFEQPWPNARPATQSGRHSVSVEVQMQRQRQEERESSQQAQRQYSSLPRQSRKNASSVSQDSWEQNYSPGEGFQSAKENPRYSSYQGSRNGYLGGHGFNARVMLETQELLRQEQRRKEQQMKKQPPSEGPSNYDSYKKVQDPSYAPPKGPFRQDVPPSPSQVARLNRLQTPEKGRPFYS

195 NP_002175.2 NP_000556.1

>NP_002175.2

MLTLQTWLVQALFIFLTTESTGELLDPCGYISPESPVVQLHSNFTAVCVLKEKCMDYFHVNANYIVWKTNHFTIPKEQYTIINRTASSVTFTDIASLNIQLTCNILTFGQLEQNVYGITIISGLPPEKPKNLSCIVNEGKKMRCEWDGGRETHLETNFTLKSEWATHKFADCKAKRDTPTSCTVDYSTVYFVNIEVWVEAENALGKVTSDHINFDPVYKVKPNPPHNLSVINSEELSSILKLTWTNPSIKSVIILKYNIQYRTKDASTWSQIPPEDTASTRSSFTVQDLKPFTEYVFRIRCMKEDGKGYWSDWSEEASGITYEDRPSKAPSFWYKIDPSHTQGYRTVQLVWKTLPPFEANGKILDYEVTLTRWKSHLQNYTVNATKLTVNLTNDRYLATLTVRNLVGKSDAAVLTIPACDFQATHPVMDLKAFPKDNMLWVEWTTPRESVKKYILEWCVLSDKAPCITDWQQEDGTVHRTYLRGNLAESKCYLITVTPVYADGPGSPESIKAYLKQAPPSKGPTVRTKKVGKNEAVLEWDQLPVDVQNGFIRNYTIFYRTIIGNETAVNVDSSHTEYTLSSLTSDTLYMVRMAAYTDEGGKDGPEFTFTTPKFAQGEIEAIVVPVCLAFLLTTLLGVLFCFNKRDLIKKHIWPNVPDPSKSHIAQWSPHTPPRHNFNSKDQMYSDGNFTDVSVVEIEANDKKPFPEDLKSLDLFKKEKINTEGHSSGIGGSSCMSSSRPSISSSDENESSQNTSSTVQYSTVVHSGYRHQVPSVQVFSRSESTQPLLDSEERPEDLQLVDHVDGGDGILPRQQYFKQNCSQHESSPDISHFERSKQVSSVNEEDFVRLKQQISDHISQSCGSGQMKMFQEVSAADAFGPGTEGQVERFETVGMEAATDEGMPKSYLPQTVRQGGYMPQ

>NP_000556.1

MLAVGCALLAALLAAPGAALAPRRCPAQEVARGVLTSLPGDSVTLTCPGVEPEDNATVHWVLRKPAAGSHPSRWAGMGRRLLLRSVQLHDSGNYSCYRAGRPAGTVHLLVDVPPEEPQLSCFRKSPLSNVVCEWGPRSTPSLTTKAVLLVRKFQNSPAEDFQEPCQYSQESQKFSCQLAVPEGDSSFYIVSMCVASSVGSKFSKTQTFQGCGILQPDPPANITVTAVARNPRWLSVTWQDPHSWNSSFYRLRFELRYRAERSKTFTTWMVKDLQHHCVIHDAWSGLRHVVQLRAQEEFGQGEWSEWSPEAMGTPWTESRSPPAENEVSTPMQALTTNKDDDNILFRDSANATSLPVQDSSSVPLPTFLVAGGSLAFGTLLCIAIVLRFKKTWKLRALKEGKTSMHPPYSLGQLVPERPRPTPVLVPLISPPVSPSSLGSDNTSSHNRPDARDPRSPYDISNTDYFFPR

196 NP_002862.2 NP_006640.2

>NP_002862.2

MEPAEQPSELVSAEGRNRKAVLCQRCGSRVLQPGTALFSRRQLFLPSMRKKPALSDGSNPDGDLLQEHWLVEDMFIFENVGFTKDVGNIKFLVCADCEIGPIGWHCLDDKNSFYVALERVSHE

>NP_006640.2

MTANRLAESLLALSQQEELADLPKDYLLSESEDEGDNDGERKHQKLLEAISSLDGKNRRKLAERSEASLKVSEFNVSSEGSGEKLVLADLLEPVKTSSSLATVKKQLSRVKSKKTVELPLNKEEIERIHREVAFNKTAQVLSKWDPVVLKNRQAEQLVFPLEKEEPAIAPIEHVLSGWKARTPLEQEIFNLLHKNKQPVTDPLLTPVEKASLRAMSLEEAKMRRAELQRARALQSYYEAKARREKKIKSKKYHKVVKKGKAKKALKEFEQLRKVNPAAALEELEKIEKARMMERMSLKHQNSGKWAKSKAIMAKYDLEARQAMQEQLSKNKELTQKLQVASESEEEEGGTEDVEELLVPDVVNEVQMNADGPNPWMLRSCTSDTKEAATQEDPEQLPELEAHGVSESEGEERPVAEEEILLREFEERRSLRKRSELSQDAEPAGSQETKDSGSQEVLSELRVLSQKLKENHQSRKQKASSEGTIPQVQREEPAPEEEEPLLLQRPERVQTLEELEELGKEECFQNKELPRPVLEGQQSERTPNNRPDAPKEKKKKEQMIDLQNLLTTQSPSVKSLAVPTIEELEDEEERNHRQMIKEAFAGDDVIRDFLKEKREAVEASKPKDVDLTLPGWGEWGGVGLKPSAKKRRRFLIKAPEGPPRKDKNLPNVIINEKRNIHAAAHQVRVLPYPFTHHWQFERTIQTPIGSTWNTQRAFQKLTTPKVVTKPGHIINPIKAEDVGYRSSSRSDLSVIQRNPKRITTRHKKQLKKCSVD

197 NP_006452.3 NP_077721.1

>NP_006452.3

MWRVKKLSLSLSPSPQTGKPSMRTPLRELTLQPGALTNSGKRSPACSSLTPSLCKLGLQEGSNNSSPVDFVNNKRTDLSSEHFSHSSKWLETCQHESDEQPLDPIPQISSTPKTSEEAVDPLGNYMVKTIVLVPSPLGQQQDMIFEARLDTMAETNSISLNGPLRTDDLVREEVAPCMGDRFSEVAAVSEKPIFQESPSHLLEESPPNPCSEQLHCSKESLSSRTEAVREDLVPSESNAFLPSSVLWLSPSTALAADFRVNHVDPEEEIVEHGAMEEREMRFPTHPKESETEDQALVSSVEDILSTCLTPNLVEMESQEAPGPAVEDVGRILGSDTESWMSPLAWLEKGVNTSVMLENLRQSLSLPSMLRDAAIGTTPFSTCSVGTWFTPSAPQEKSTNTSQTGLVGTKHSTSETEQLLCGRPPDLTALSRHDLEDNLLSSLVILEVLSRQLRDWKSQLAVPHPETQDSSTQTDTSHSGITNKLQHLKESHEMGQALQQARNVMQSWVLISKELISLLHLSLLHLEEDKTTVSQESRRAETLVCCCFDLLKKLRAKLQSLKAEREEARHREEMALRGKDAAEIVLEAFCAHASQRISQLEQDLASMREFRGLLKDAQTQLVGLHAKQEELVQQTVSLTSTLQQDWRSMQLDYTTWTALLSRSRQLTEKLTVKSQQALQERDVAIEEKQEVSRVLEQVSAQLEECKGQTEQLELENSRLATDLRAQLQILANMDSQLKELQSQHTHCAQDLAMKDELLCQLTQSNEEQAAQWQKEEMALKHMQAELQQQQAVLAKEVRDLKETLEFADQENQVAHLELGQVECQLKTTLEVLRERSLQCENLKDTVENLTAKLASTIADNQEQDLEKTRQYSQKLGLLTEQLQSLTLFLQTKLKEKTEQETLLLSTACPPTQEHPLPNDRTFLGSILTAVADEEPESTPVPLLGSDKSAFTRVASMVSLQPAETPGMEESLAEMSIMTTELQSLCSLLQESKEEAIRTLQRKICELQARLQAQEEQHQEVQKAKEADIEKLNQALCLRYKNEKELQEVIQQQNEKILEQIDKSGELISLREEVTHLTRSLRRAETETKVLQEALAGQLDSNCQPMATNWIQEKVWLSQEVDKLRVMFLEMKNEKEKLMIKFQSHRNILEENLRRSDKELEKLDDIVQHIYKTLLSIPEVVRGCKELQGLLEFLS

>NP_077721.1

MAALSCLLDSVRRDIKKVDRELRQLRCIDEFSTRCLCDLYMHPYCCCDLHPYPYCLCYSKRSRSCGLCDLYPCCLCDYKLYCLRPSLRSLERKAIRAIEDEKRELAKLRRTTNRILASSCCSSNILGSVNVCGFEPDQVKVRVKDGKVCVSAERENRYDCLGSKKYSYMNICKEFSLPPCVDEKDVTYSYGLGSCVKIESPCYPCTSPCSPCSPCNPCNPCSPCNPCSPYDPCNPCYPCGSRFSCRKMIL

198 NP_001547.1 NP_037386.1

>NP_001547.1

MSWSPSLTTQTCGAWEMKERLGTGGFGNVIRWHNQETGEQIAIKQCRQELSPRNRERWCLEIQIMRRLTHPNVVAARDVPEGMQNLAPNDLPLLAMEYCQGGDLRKYLNQFENCCGLREGAILTLLSDIASALRYLHENRIIHRDLKPENIVLQQGEQRLIHKIIDLGYAKELDQGSLCTSFVGTLQYLAPELLEQQKYTVTVDYWSFGTLAFECITGFRPFLPNWQPVQWHSKVRQKSEVDIVVSEDLNGTVKFSSSLPYPNNLNSVLAERLEKWLQLMLMWHPRQRGTDPTYGPNGCFKALDDILNLKLVHILNMVTGTIHTYPVTEDESLQSLKARIQQDTGIPEEDQELLQEAGLALIPDKPATQCISDGKLNEGHTLDMDLVFLFDNSKITYETQISPRPQPESVSCILQEPKRNLAFFQLRKVWGQVWHSIQTLKEDCNRLQQGQRAAMMNLLRNNSCLSKMKNSMASMSQQLKAKLDFFKTSIQIDLEKYSEQTEFGITSDKLLLAWREMEQAVELCGRENEVKLLVERMMALQTDIVDLQRSPMGRKQGGTLDDLEEQARELYRRLREKPRDQRTEGDSQEMVRLLLQAIQSFEKKVRVIYTQLSKTVVCKQKALELLPKVEEVVSLMNEDEKTVVRLQEKRQKELWNLLKIACSKVRGPVSGSPDSMNASRLSQPGQLMSQPSTASNSLPEPAKKSEELVAEAHNLCTLLENAIQDTVREQDQSFTALDWSWLQTEEEEHSCLEQAS

>NP_037386.1

MQSTSNHLWLLSDILGQGATANVFRGRHKKTGDLFAIKVFNNISFLRPVDVQMREFEVLKKLNHKNIVKLFAIEEETTTRHKVLIMEFCPCGSLYTVLEEPSNAYGLPESEFLIVLRDVVGGMNHLRENGIVHRDIKPGNIMRVIGEDGQSVYKLTDFGAARELEDDEQFVSLYGTEEYLHPDMYERAVLRKDHQKKYGATVDLWSIGVTFYHAATGSLPFRPFEGPRRNKEVMYKIITGKPSGAISGVQKAENGPIDWSGDMPVSCSLSRGLQVLLTPVLANILEADQEKCWGFDQFFAETSDILHRMVIHVFSLQQMTAHKIYIHSYNTATIFHELVYKQTKIISSNQELIYEGRRLVLEPGRLAQHFPKTTEENPIFVVSREPLNTIGLIYEKISLPKVHPRYDLDGDASMAKAITGVVCYACRIASTLLLYQELMRKGIRWLIELIKDDYNETVHKKTEVVITLDFCIRNIEKTVKVYEKLMKINLEAAELGEISDIHTKLLRLSSSQGTIETSLQDIDSRLSPGGSLADAWAHQEGTHPKDRNVEKLQVLLNCMTEIYYQFKKDKAERRLAYNEEQIHKFDKQKLYYHATKAMTHFTDECVKKYEAFLNKSEEWIRKMLHLRKQLLSLTNQCFDIEEEVSKYQEYTNELQETLPQKMFTASSGIKHTMTPIYPSSNTLVEMTLGMKKLKEEMEGVVKELAENNHILERFGSLTMDGGLRNVDCL

199 NP_001001683.1 NP_598395.1

>NP_001001683.1

MATYSLANERLRALEDIEREIGAILQNAGTVILELSKEKTNERLLDRQAAAFTASVQHVEAELSAQIRYLTQVATGQPHEGSSYSSRKDCQMALKRVDYARLKLSDVARTCEQMLEN

>NP_598395.1

MAQQRALPQSKETLLQSYNKRLKDDIKSIMDNFTEIIKTAKIEDETQVSRATQGEQDNYEMHVRAANIVRAGESLMKLVSDLKQFLILNDFPSVNEAIDQRNQQLRTLQEECDRKLITLRDEISIDLYELEEEYYSSSSSLCEANDLPLCEAYGRLDLDTDSADGLSAPLLASPEPSAGPLQVAAPAHSHAGGPGPTEHA

200 NP_002937.1 NP_057144.1

>NP_002937.1

MWNSGFESYGSSSYGGAGGYTQSPGGFGSPAPSQAEKKSRARAQHIVPCTISQLLSATLVDEVFRIGNVEISQVTIVGIIRHAEKAPTNIVYKIDDMTAAPMDVRQWVDTDDTSSENTVVPPETYVKVAGHLRSFQNKKSLVAFKIMPLEDMNEFTTHILEVINAHMVLSKANSQPSAGRAPISNPGMSEAGNFGGNSFMPANGLTVAQNQVLNLIKACPRPEGLNFQDLKNQLKHMSVSSIKQAVDFLSNEGHIYSTVDDDHFKSTDAE

>NP_057144.1

MAAAVAMETDDAGNRLRFQLELEFVQCLANPNYLNFLAQRGYFKDKAFVNYLKYLLYWKDPEYAKYLKYPQCLHMLELLQYEHFRKELVNAQCAKFIDEQQILHWQHYSRKRMRLQQALAEQQQQNNTSGK

201 NP_005585.1 NP_068597.2

>NP_005585.1

MPGEATETVPATEQELPQPQAETGSGTESDSDESVPELEEQDSTQATTQQAQLAAAAEIDEEPVSKAKQSRSEKKARKAMSKLGLRQVTGVTRVTIRKSKNILFVITKPDVYKSPASDTYIVFGEAKIEDLSQQAQLAAAEKFKVQGEAVSNIQENTQTPTVQEESEEEEVDETGVEVKDIELVMSQANVSRAKAVRALKNNSNDIVNAIMELTM

>NP_068597.2

MNSRQAWRLFLSQGRGDRWVSRPRGHFSPALRREFFTTTTKEGYDRRPVDITPLEQRKLTFDTHALVQDLETHGFDKTQAETIVSALTALSNVSLDTIYKEMVTQAQQEITVQQLMAHLDAIRKDMVILEKSEFANLRAENEKMKIELDQVKQQLMHETSRIRADNKLDINLERSRVTDMFTDQEKQLMETTTEFTKKDTQTKSIISETSNKIDAEIASLKTLMESNKLETIRYLAASVFTCLAIALGFYRFWK

202 NP_006294.2 NP_004437.2

>NP_006294.2

MPMYQVKPYHGGGAPLRVELPTCMYRLPNVHGRSYGPAPGAGHVQEESNLSLQALESRQDDILKRLYELKAAVDGLSKMIQTPDADLDVTNIIQADEPTTLTTNALDLNSVLGKDYGALKDIVINANPASPPLSLLVLHRLLCEHFRVLSTVHTHSSVKSVPENLLKCFGEQNKKQPRQDYQLGFTLIWKNVPKTQMKFSIQTMCPIEGEGNIARFLFSLFGQKHNAVNATLIDSWVDIAIFQLKEGSSKEKAAVFRSMNSALGKSPWLAGNELTVADVVLWSVLQQIGGCSVTVPANVQRWMRSCENLAPFNTALKLLK

>NP_004437.2

MATLSLTVNSGDPPLGALLAVEHVKDDVSISVEEGKENILHVSENVIFTDVNSILRYLARVATTAGLYGSNLMEHTEIDHWLEFSATKLSSCDSFTSTINELNHCLSLRTYLVGNSLSLADLCVWATLKGNAAWQEQLKQKKAPVHVKRWFGFLEAQQAFQSVGTKWDVSTTKARVAPEKKQDVGKFVELPGAEMGKVTVRFPPEASGYLHIGHAKAALLNQHYQVNFKGKLIMRFDDTNPEKEKEDFEKVILEDVAMLHIKPDQFTYTSDHFETIMKYAEKLIQEGKAYVDDTPAEQMKAEREQRIDSKHRKNPIEKNLQMWEEMKKGSQFGQSCCLRAKIDMSSNNGCMRDPTLYRCKIQPHPRTGNKYNVYPTYDFACPIVDSIEGVTHALRTTEYHDRDEQFYWIIEALGIRKPYIWEYSRLNLNNTVLSKRKLTWFVNEGLVDGWDDPRFPTVRGVLRRGMTVEGLKQFIAAQGSSRSVVNMEWDKIWAFNKKVIDPVAPRYVALLKKEVIPVNVPEAQEEMKEVAKHPKNPEVGLKPVWYSPKVFIEGADAETFSEGEMVTFINWGNLNITKIHKNADGKIISLDAKLNLENKDYKKTTKVTWLAETTHALPIPVICVTYEHLITKPVLGKDEDFKQYVNKNSKHEELMLGDPCLKDLKKGDIIQLQRRGFFICDQPYEPVSPYSCKEAPCVLIYIPDGHTKEMPTSGSKEKTKVEATKNETSAPFKERPTPSLNNNCTTSEDSLVLYNRVAVQGDVVRELKAKKAPKEDVDAAVKQLLSLKAEYKEKTGQEYKPGNPPAEIGQNISSNSSASILESKSLYDEVAAQGEVVRKLKAEKSPKAKINEAVECLLSLKAQYKEKTGKEYIPGQPPLSQSSDSSPTRNSEPAGLETPEAKVLFDKVASQGEVVRKLKTEKAPKDQVDIAVQELLQLKAQYKSLIGVEYKPVSATGAEDKDKKKKEKENKSEKQNKPQKQNDGQRKDPSKNQGGGLSSSGAGEGQGPKKQTRLGLEAKKEENLADWYSQVITKSEMIEYHDISGCYILRPWAYAIWEAIKDFFDAEIKKLGVENCYFPMFVSQSALEKEKTHVADFAPEVAWVTRSGKTELAEPIAIRPTSETVMYPAYAKWVQSHRDLPIKLNQWCNVVRWEFKHPQPFLRTREFLWQEGHSAFATMEEAAEEVLQILDLYAQVYEELLAIPVVKGRKTEKEKFAGGDYTTTIEAFISASGRAIQGGTSHHLGQNFSKMFEIVFEDPKIPGEKQFAYQNSWGLTTRTIGVMTMVHGDNMGLVLPPRVACVQVVIIPCGITNALSEEDKEALIAKCNDYRRRLLSVNIRVRADLRDNYSPGWKFNHWELKGVPIRLEVGPRDMKSCQFVAVRRDTGEKLTVAENEAETKLQAILEDIQVTLFTRASEDLKTHMVVANTMEDFQKILDSGKIVQIPFCGEIDCEDWIKKTTARDQDLEPGAPSMGAKSLCIPFKPLCELQPGAKCVCGKNPAKYYTLFGRSY

203 NP_001505.1 NP_003241.2

>NP_001505.1

MASTSRLDALPRVTCPNHPDAILVEDYRAGDMICPECGLVVGDRVIDVGSEWRTFSNDKATKDPSRVGDSQNPLLSDGDLSTMIGKGTGAASFDEFGNSKYQNRRTMSSSDRAMMNAFKEITTMADRINLPRNIVDRTNNLFKQVYEQKSLKGRANDAIASACLYIACRQEGVPRTFKEICAVSRISKKEIGRCFKLILKALETSVDLITTGDFMSRFCSNLCLPKQVQMAATHIARKAVELDLVPGRSPISVAAAAIYMASQASAEKRTQKEIGDIAGVADVTIRQSYRLIYPRAPDLFPTDFKFDTPVDKLPQL

>NP_003241.2

MEQKPSKVECGSDPEENSARSPDGKRKRKNGQCSLKTSMSGYIPSYLDKDEQCVVCGDKATGYHYRCITCEGCKGFFRRTIQKNLHPTYSCKYDSCCVIDKITRNQCQLCRFKKCIAVGMAMDLVLDDSKRVAKRKLIEQNRERRRKEEMIRSLQQRPEPTPEEWDLIHIATEAHRSTNAQGSHWKQRRKFLPDDIGQSPIVSMPDGDKVDLEAFSEFTKIITPAITRVVDFAKKLPMFSELPCEDQIILLKGCCMEIMSLRAAVRYDPESDTLTLSGEMAVKREQLKNGGLGVVSDAIFELGKSLSAFNLDDTEVALLQAVLLMSTDRSGLLCVDKIEKSQEAYLLAFEHYVNHRKHNIPHFWPKLLMKEREVQSSILYKGAAAEGRPGGSLGVHPEGQQLLGMHVVQGPQVRQLEQQLGEAGSLQGPVLQHQSPKSPQQRLLELLHRSGILHARAVCGEDDSSEADSPSSSEEEPEVCEDLAGNAASP

204 NP_002490.1 NP_006172.1

>NP_002490.1

MAAERGARRLLSTPSFWLYCLLLLGRRAPGAAAARSGSAPQSPGASIRTFTPFYFLVEPVDTLSVRGSSVILNCSAYSEPSPKIEWKKDGTFLNLVSDDRRQLLPDGSLFISNVVHSKHNKPDEGYYQCVATVESLGTIISRTAKLIVAGLPRFTSQPEPSSVYAGNGAILNCEVNADLVPFVRWEQNRQPLLLDDRVIKLPSGMLVISNATEGDGGLYRCVVESGGPPKYSDEVELKVLPDPEVISDLVFLKQPSPLVRVIGQDVVLPCVASGLPTPTIKWMKNEEALDTESSERLVLLAGGSLEISDVTEDDAGTYFCIADNGNETIEAQAELTVQAQPEFLKQPTNIYAHESMDIVFECEVTGKPTPTVKWVKNGDMVIPSDYFKIVKEHNLQVLGLVKSDEGFYQCIAENDVGNAQAGAQLIILEHAPATTGPLPSAPRDVVASLVSTRFIKLTWRTPASDPHGDNLTYSVFYTKEGIARERVENTSHPGEMQVTIQNLMPATVYIFRVMAQNKHGSGESSAPLRVETQPEVQLPGPAPNLRAYAASPTSITVTWETPVSGNGEIQNYKLYYMEKGTDKEQDVDVSSHSYTINGLKKYTEYSFRVVAYNKHGPGVSTPDVAVRTLSDVPSAAPQNLSLEVRNSKSIMIHWQPPAPATQNGQITGYKIRYRKASRKSDVTETLVSGTQLSQLIEGLDRGTEYNFRVAALTINGTGPATDWLSAETFESDLDETRVPEVPSSLHVRPLVTSIVVSWTPPENQNIVVRGYAIGYGIGSPHAQTIKVDYKQRYYTIENLDPSSHYVITLKAFNNVGEGIPLYESAVTRPHTDTSEVDLFVINAPYTPVPDPTPMMPPVGVQASILSHDTIRITWADNSLPKHQKITDSRYYTVRWKTNIPANTKYKNANATTLSYLVTGLKPNTLYEFSVMVTKGRRSSTWSMTAHGTTFELVPTSPPKDVTVVSKEGKPKTIIVNWQPPSEANGKITGYIIYYSTDVNAEIHDWVIEPVVGNRLTHQIQELTLDTPYYFKIQARNSKGMGPMSEAVQFRTPKADSSDKMPNDQASGSGGKGSRLPDLGSDYKPPMSGSNSPHGSPTSPLDSNMLLVIIVSVGVITIVVVVIIAVFCTRRTTSHQKKKRAACKSVNGSHKYKGNSKDVKPPDLWIHHERLELKPIDKSPDPNPIMTDTPIPRNSQDITPVDNSMDSNIHQRRNSYRGHESEDSMSTLAGRRGMRPKMMMPFDSQPPQPVISAHPIHSLDNPHHHFHSSSLASPARSHLYHPGSPWPIGTSMSLSDRANSTESVRNTPSTDTMPASSSQTCCTDHQDPEGATSSSYLASSQEEDSGQSLPTAHVRPSHPLKSFAVPAIPPPGPPTYDPALPSTPLLSQQALNHHIHSVKTASIGTLGRSRPPMPVVVPSAPEVQETTRMLEDSESSYEPDELTKEMAHLEGLMKDLNAITTA

>NP_006172.1

MPGWPWGLLLTAGTLFAALSPGPPAPADPCHDEGGAPRGCVPGLVNAALGREVLASSTCGRPATRACDASDPRRAHSPALLTSPGGTASPLCWRSESLPRAPLNVTLTVPLGKAFELVFVSLRFCSAPPASVALLKSQDHGRSWAPLGFFSSHCDLDYGRLPAPANGPAGPGPEALCFPAPLAQPDGSGLLAFSMQDSSPPGLDLDSSPVLQDWVTATDVRVVLTRPSTAGDPRDMEAVVPYSYAATDLQVGGRCKCNGHASRCLLDTQGHLICDCRHGTEGPDCGRCKPFYCDRPWQRATARESHACLACSCNGHARRCRFNMELYRLSGRRSGGVCLNCRHNTAGRHCHYCREGFYRDPGRALSDRRACRACDCHPVGAAGKTCNQTTGQCPCKDGVTGLTCNRCAPGFQQSRSPVAPCVKTPIPGPTEDSSPVQPQDCDSHCKPARGSYRISLKKFCKKDYAVQVAVGARGEARGAWTRFPVAVLAVFRSGEERARRGSSALWVPAGDAACGCPRLLPGRRYLLLGGGPGAAAGGAGGRGPGLIAARGSLVLPWRDAWTRRLRRLQRRERRGRCSAA

205 NP_056536.2 NP_056280.1

>NP_056536.2

MDLGPLNICEEMTILHGGFLLAEQLFHPKALAELTKSDWERVGRPIVEALREISSAAAHSQPFAWKKKALIIIWAKVLQPHPVTPSDTETRWQEDLFFSVGNMIPTINHTILFELLKSLEASGLFIQLLMALPTTICHAELERFLEHVTVDTSAEDVAFFLDVWWEVMKHKGHPQDPLLSQFSAMAHKYLPALDEFPHPPKRLRSDPDACPTMPLLAMLLRGLTQIQSRILGPGRKCCALANLADMLTVFALTEDDPQEVSATVYLDKLATVISVWNSDTQNPYHQQALAEKVKEAERDVSLTSLAKLPSETIFVGCEFLHHLLREWGEELQAVLRSSQGTSYDSYRLCDSLTSFSQNATLYLNRTSLSKEDRQVVSELAECVRDFLRKTSTVLKNRALEDITASIAMAVIQQKMDRHMEVCYIFASEKKWAFSDEWVACLGSNRALFRQPDLVLRLLETVIDVSTADRAIPESQIRQVIHLILECYADLSLPGKNKVLAGILRSWGRKGLSEKLLAYVEGFQEDLNTTFNQLTQSASEQGLAKAVASVARLVIVHPEVTVKKMCSLAVVNLGTHKFLAQILTAFPALRFVEEQGPNSSATFMVSCLKETVWMKFSTPKEEKQFLELLNCLMSPVKPQGIPVAALLEPDEVLKEFVLPFLRLDVEEVDLSLRIFIQTLEANACREEYWLQTCSPFPLLFSLCQLLDRFSKYWQLPKEKRCLSLDRKDLAIHILELLCEIVSANAETFSPDVWIKSLSWLHRKLEQLDWTVGLRLKSFFEGHFKCEVPATLFEICKLSEDEWTSQAHPGYGAGTGLLAWMECCCVSSGISERMLSLLVVDVGNPEEVRLFSKGFLVALVQVMPWCSPQEWQRLHQLTRRLLEKQLLHVPYSLEYIQFVPLLNLKPFAQELQLSVLFLRTFQFLCSHSCRDWLPLEGWNHVVKLLCGSLTRLLDSVRAIQAAGPWVQGPEQDLTQEALFVYTQVFCHALHIMAMLHPEVCEPLYVLALETLTCYETLSKTNPSVSSLLQRAHEQRFLKSIAEGIGPEERRQTLLQKMSSF

>NP_056280.1

MGQEPRTLPPSPNWYCARCSDAVPGGLFGFAARTSVFLVRVGPGAGESPGTPPFRVIGELVGHTERVSGFTFSHHPGQYNLCATSSDDGTVKIWDVETKTVVTEHALHQHTISTLHWSPRVKDLIVSGDEKGVVFCYWFNRNDSQHLFIEPRTIFCLTCSPHHEDLVAIGYKDGIVVIIDISKKGEVIHRLRGHDDEIHSIAWCPLPGEDCLSINQEETSEEAEITNGNAVAQAPVTKGCYLATGSKDQTIRIWSCSRGRGVMILKLPFLKRRGGGIDPTVKERLWLTLHWPSNQPTQLVSSCFGGELLQWDLTQSWRRKYTLFSASSEGQNHSRIVFNLCPLQTEDDKQLLLSTSMDRDVKCWDIATLECSWTLPSLGGFAYSLAFSSVDIGSLAIGVGDGMIRVWNTLSIKNNYDVKNFWQGVKSKVTALCWHPTKEGCLAFGTDDGKVGLYDTYSNKPPQISSTYHKKTVYTLAWGPPVPPMSLGGEGDRPSLALYSCGGEGIVLQHNPWKLSGEAFDINKLIRDTNSIKYKLPVHTEISWKADGKIMALGNEDGSIEIFQIPNLKLICTIQQHHKLVNTISWHHEHGSQPELSYLMASGSNNAVIYVHNLKTVIESSPESPVTITEPYRTLSGHTAKITSVAWSPHHDGRLVSASYDGTAQVWDALREEPLCNFRGHQGRLLCVAWSPLDPDCIYSGADDFCVHKWLTSMQDHSRPPQGKKSIELEKKRLSQPKAKPKKKKKPTLRTPVKLESIDGNEEESMKENSGPVENGVSDQEGEEQAREPELPCGLAPAVSREPVICTPVSSGFEKSKVTINNKVILLKKEPPKEKPETLIKKRKARSLLPLSTSLDHRSKEELHQDCLVLATAKHSRELNEDVSADVEERFHLGLFTDRATLYRMIDIEGKGHLENGHPELFHQLMLWKGDLKGVLQTAAERGELTDNLVAMAPAAGYHVWLWAVEAFAKQLCFQDQYVKAASHLLSIHKVYEAVELLKSNHFYREAIAIAKARLRPEDPVLKDLYLSWGTVLERDGHYAVAAKCYLGATCAYDAAKVLAKKGDAASLRTAAELAAIVGEDELSASLALRCAQELLLANNWVGAQEALQLHESLQGQRLVFCLLELLSRHLEEKQLSEGKSSSSYHTWNTGTEGPFVERVTAVWKSIFSLDTPEQYQEAFQKLQNIKYPSATNNTPAKQLLLHICHDLTLAVLSQQMASWDEAVQALLRAVVRSYDSGSFTIMQEVYSAFLPDGCDHLRDKLGDHQSPATPAFKSLEAFFLYGRLYEFWWSLSRPCPNSSVWVRAGHRTLSVEPSQQLDTASTEETDPETSQPEPNRPSELDLRLTEEGERMLSTFKELFSEKHASLQNSQRTVAEVQETLAEMIRQHQKSQLCKSTANGPDKNEPEVEAEQPLCSSQSQCKEEKNEPLSLPELTKRLTEANQRMAKFPESIKAWPFPDVLECCLVLLLIRSHFPGCLAQEMQQQAQELLQKYGNTKTYRRHCQTFCM

206 NP_002125.3 NP_065853.2

>NP_002125.3

MSAEVETSEGVDESEKKNSGALEKENQMRMADLSELLKEGTKEAHDRAENTQFVKDFLKGNIKKELFKLATTALYFTYSALEEEMERNKDHPAFAPLYFPMELHRKEALTKDMEYFFGENWEEQVQCPKAAQKYVERIHYIGQNEPELLVAHAYTRYMGDLSGGQVLKKVAQRALKLPSTGEGTQFYLFENVDNAQQFKQLYRARMNALDLNMKTKERIVEEANKAFEYNMQIFNELDQAGSTLARETLEDGFPVHDGKGDMRKCPFYAAEQDKGALEGSSCPFRTAMAVLRKPSLQFILAAGVALAAGLLAWYYM

>NP_065853.2

MLAGRPGTRSAVGELGTESSDNLDRAPLGPRESGGHHRPGSYLDMKIHLEKNLEEERQILLQQQKICRNRARKYFVESNRRKKAFEEKRKEQEEKEHQIREQILQQRKQKFEEVTEKFQRAHVPLSQRRKAVSRKPVPPLEEALKQIQESNLKSEVNLPFSRRPTINWRAIDSALPSALSKNDHKHQKQLLSKINCEKEMNENMRATLATSKNVFQLKLEETQKLLEDQHLSNLQKFGDEVNQITNSETLSSIDSLEATEHEEIYLTLNKEHSTSIQRNTISLKPANMQSTNLSCFDEDKLAFSKTQHINNWLTNLDASNTQNVTAFSDILSKSNVLPSWEYFNSKEQNPSPLNGTVERATNTANNSVPFVSSPPMFVLDKKCEKTSETSTMRTTDSTSGAFKRERPLVTESPTFKFSKSQSTSDSLTQEVATFPDQEKYSELNQENGTTSIPTSCVPVATPLVLPSNIQSARPSAKNSIHIKEIDAVQCSDKLDELKDGKEEEIKYFNCNKEELPLFSDSFQDAYIPHNPDSKDEKQKLAETSSLSNVTSNYDFVGQHKKMKYNIHERNGVRFLKSILKKESKYEHGYLKALIINQSFKFGNQKAAAIRDSIELTKEKGAEIPKTIKKLRWFDETSNIENNAENSHSLKNKTGTTQQHSQQFHIQSGAGSNIISVSTCAVNSADTKKSREDSISENVTTLGGSGADHMPLNCFIPSGYNFAKHAWPASKKEESKIPVHDDSKTKQGKPQRGRAKIIRKPGSAKVQSGFICTNRKGAVIQPQSASKVNIFTQAQGKLIIPCPPPQSTSNIRSGKNIQVSQCQPVTPENPQNIITHNSFNSKHVLPTEHSLNQWNQESSSPLSNACSDLVTVIPSLPSYCSSECQTFAKINHSNGTQAVARQDATLYCTQRSPVCEESYPSVTLRTAEEESVPLWKRGPNVLHQNKRATGSTVMRRKRIAETKRRNILEQKRQNPGSVGQKYSEQINNFGQSVLLSSSEPKQTTRGTSYIEEVSDSTSEFLMAENLVKASVPEDEILTVLNSKQIQKSNLPLNKTQQFNICTLSAEEQKILESLNDLSERLHYIQESICKNPSIKNTLQIIPLLEKREDRTSSCRDKR

207 NP_116021.2 NP_057291.1

>NP_116021.2

MPFPVTTQGSQQTQPPQKHYGITSPISLAAPKETDCVLTQKLIETLKPFGVFEEEEELQRRILILGKLNNLVKEWIREISESKNLPQSVIENVGGKIFTFGSYRLGVHTKGADIDALCVAPRHVDRSDFFTSFYDKLKLQEEVKDLRAVEEAFVPVIKLCFDGIEIDILFARLALQTIPEDLDLRDDSLLKNLDIRCIRSLNGCRVTDEILHLVPNIDNFRLTLRAIKLWAKRHNIYSNILGFLGGVSWAMLVARTCQLYPNAIASTLVHKFFLVFSKWEWPNPVLLKQPEECNLNLPVWDPRVNPSDRYHLMPIITPAYPQQNSTYNVSVSTRMVMVEEFKQGLAITDEILLSKAEWSKLFEAPNFFQKYKHYIVLLASAPTEKQRLEWVGLVESKIRILVGSLEKNEFITLAHVNPQSFPAPKENPDKEEFRTMWVIGLVFKKTENSENLSVDLTYDIQSFTDTVYRQAINSKMFEVDMKIAAMHVKRKQLHQLLPNHVLQKKKKHSTEGVKLTALNDSSLDLSMDSDNSMSVPSPTSATKTSPLNSSGSSQGRNSPAPAVTAASVTNIQATEVSVPQVNSSESSGGTSSESIPQTATQPAISPPPKPTVSRVVSSTRLVNPPPRSSGNAATSGNAATKIPTPIVGVKRTSSPHKEESPKKTKTEEDETSEDANCLALSGHDKTEAKEQLDTETSTTQSETIQTAASLLASQKTSSTDLSDIPALPANPIPVIKNSIKLRLNR

>NP_057291.1

MSAIPAEESDQLLIRPLGAGQEVGRSCIILEFKGRKIMLDCGIHPGLEGMDALPYIDLIDPAEIDLLLISHFHLDHCGALPWFLQKTSFKGRTFMTHATKAIYRWLLSDYVKVSNISADDMLYTETDLEESMDKIETINFHEVKEVAGIKFWCYHAGHVLGAAMFMIEIAGVKLLYTGDFSRQEDRHLMAAEIPNIKPDILIIESTYGTHIHEKREEREARFCNTVHDIVNRGGRGLIPVFALGRAQELLLILDEYWQNHPELHDIPIYYASSLAKKCMAVYQTYVNAMNDKIRKQININNPFVFKHISNLKSMDHFDDIGPSVVMASPGMMQSGLSRELFESWCTDKRNGVIIAGYCVEGTLAKHIMSEPEEITTMSGQKLPLKMSVDYISFSAHTDYQQTSEFIRALKPPHVILVHGEQNEMARLKAALIREYEDNDEVHIEVHNPRNTEAVTLNFRGEKLAKVMGFLADKKPEQGQRVSGILVKRNFNYHILSPCDLSNYTDLAMSTVKQTQAIPYTGPFNLLCYQLQKLTGDVEELEIQEKPALKVFKNITVIQEPGMVVLEWLANPSNDMYADTVTTVILEVQSNPKIRKGAVQKVSKKLEMHVYSKRLEIMLQDIFGEDCVSVKDDSILSVTVDGKTANLNLETRTVECEEGSEDDESLREMVELAAQRLYEALTPVH

208 NP_005783.2 NP_002573.1

>NP_005783.2

MAAERKTRLSKNLLRMKFMQRGLDSETKKQLEEEEKKIISEEHWYLDLPELKEKESFIIEEQSFLLCEDLLYGRMSFRGFNPEVEKLMLQMNAKHKAEEVEDETVELDVSDEEMARRYETLVGTIGKKFARKRDHANYEEDENGDITPIKAKKMFLKPQD

>NP_002573.1

MEIIRSNFKSNLHKVYQAIEEADFFAIDGEFSGISDGPSVSALTNGFDTPEERYQKLKKHSMDFLLFQFGLCTFKYDYTDSKYITKSFNFYVFPKPFNRSSPDVKFVCQSSSIDFLASQGFDFNKVFRNGIPYLNQEEERQLREQYDEKRSQANGAGALSYVSPNTSKCPVTIPEDQKKFIDQVVEKIEDLLQSEENKNLDLEPCTGFQRKLIYQTLSWKYPKGIHVETLETEKKERYIVISKVDEEERKRREQQKHAKEQEELNDAVGFSRVIHAIANSGKLVIGHNMLLDVMHTVHQFYCPLPADLSEFKEMTTCVFPRLLDTKLMASTQPFKDIINNTSLAELEKRLKETPFNPPKVESAEGFPSYDTASEQLHEAGYDAYITGLCFISMANYLGSFLSPPKIHVSARSKLIEPFFNKLFLMRVMDIPYLNLEGPDLQPKRDHVLHVTFPKEWKTSDLYQLFSAFGNIQISWIDDTSAFVSLSQPEQVKIAVNTSKYAESYRIQTYAEYMGRKQEEKQIKRKWTEDSWKEADSKRLNPQCIPYTLQNHYYRNNSFTAPSTVGKRNLSPSQEEAGLEDGVSGEISDTELEQTDSCAEPLSEGRKKAKKLKRMKKELSPAGSISKNSPATLFEVPDTW

209 NP_891555.2 NP_391987.1

>NP_891555.2

MQRGAALCLRLWLCLGLLDGLVSGYSMTPPTLNITEESHVIDTGDSLSISCRGQHPLEWAWPGAQEAPATGDKDSEDTGVVRDCEGTDARPYCKVLLLHEVHANDTGSYVCYYKYIKARIEGTTAASSYVFVRDFEQPFINKPDTLLVNRKDAMWVPCLVSIPGLNVTLRSQSSVLWPDGQEVVWDDRRGMLVSTPLLHDALYLQCETTWGDQDFLSNPFLVHITGNELYDIQLLPRKSLELLVGEKLVLNCTVWAEFNSGVTFDWDYPGKQAERGKWVPERRSQQTHTELSSILTIHNVSQHDLGSYVCKANNGIQRFRESTEVIVHENPFISVEWLKGPILEATAGDELVKLPVKLAAYPPPEFQWYKDGKALSGRHSPHALVLKEVTEASTGTYTLALWNSAAGLRRNISLELVVNVPPQIHEKEASSPSIYSRHSRQALTCTAYGVPLPLSIQWHWRPWTPCKMFAQRSLRRRQQQDLMPQCRDWRAVTTQDAVNPIESLDTWTEFVEGKNKTVSKLVIQNANVSAMYKCVVSNKVGQDERLIYFYVTTIPDGFTIESKPSEELLEGQPVLLSCQADSYKYEHLRWYRLNLSTLHDAHGNPLLLDCKNVHLFATPLAASLEEVAPGARHATLSLSIPRVAPEHEGHYVCEVQDRRSHDKHCHKKYLSVQALEAPRLTQNLTDLLVNVSDSLEMQCLVAGAHAPSIVWYKDERLLEEKSGVDLADSNQKLSIQRVREEDAGRYLCSVCNAKGCVNSSASVAVEGSEDKGSMEIVILVGTGVIAVFFWVLLLLIFCNMRRPAHADIKTGYLSIIMDPGEVPLEEQCEYLSYDASQWEFPRERLHLGRVLGYGAFGKVVEASAFGIHKGSSCDTVAVKMLKEGATASEHRALMSELKILIHIGNHLNVVNLLGACTKPQGPLMVIVEFCKYGNLSNFLRAKRDAFSPCAEKSPEQRGRFRAMVELARLDRRRPGSSDRVLFARFSKTEGGARRASPDQEAEDLWLSPLTMEDLVCYSFQVARGMEFLASRKCIHRDLAARNILLSESDVVKICDFGLARDIYKDPDYVRKGSARLPLKWMAPESIFDKVYTTQSDVWSFGVLLWEIFSLGASPYPGVQINEEFCQRLRDGTRMRAPELATPAIRRIMLNCWSGDPKARPAFSELVEILGDLLQGRGLQEEEEVCMAPRSSQSSEEGSFSQVSTMALHIAQADAEDSPPSLQRHSLAARYYNWVSFPGCLARGAETRGSSRMKTFEEFPMTPTTYKGSVDNQTDSGMVLASEEFEQIESRHRQESGFSCKGPGQNVAVTRAHPDSQGRRRRPERGARGGQVFYNSEYGELSEPSEEDHCSPSARVTFFTDNSY

>NP_391987.1

MNLQPIFWIGLISSVCCVFAQTDENRCLKANAKSCGECIQAGPNCGWCTNSTFLQEGMPTSARCDDLEALKKKGCPPDDIENPRGSKDIKKNKNVTNRSKGTAEKLKPEDITQIQPQQLVLRLRSGEPQTFTLKFKRAEDYPIDLYYLMDLSYSMKDDLENVKSLGTDLMNEMRRITSDFRIGFGSFVEKTVMPYISTTPAKLRNPCTSEQNCTSPFSYKNVLSLTNKGEVFNELVGKQRISGNLDSPEGGFDAIMQVAVCGSLIGWRNVTRLLVFSTDAGFHFAGDGKLGGIVLPNDGQCHLENNMYTMSHYYDYPSIAHLVQKLSENNIQTIFAVTEEFQPVYKELKNLIPKSAVGTLSANSSNVIQLIIDAYNSLSSEVILENGKLSEGVTISYKSYCKNGVNGTGENGRKCSNISIGDEVQFEISITSNKCPKKDSDSFKIRPLGFTEEVEVILQYICECECQSEGIPESPKCHEGNGTFECGACRCNEGRVGRHCECSTDEVNSEDMDAYCRKENSSEICSNNGECVCGQCVCRKRDNTNEIYSGKFCECDNFNCDRSNGLICGGNGVCKCRVCECNPNYTGSACDCSLDTSTCEASNGQICNGRGICECGVCKCTDPKFQGQTCEMCQTCLGVCAEHKECVQCRAFNKGEKKDTCTQECSYFNITKVESRDKLPQPVQPDPVSHCKEKDVDDCWFYFTYSVNGNNEVMVHVVENPECPTGPDIIPIVAGVVAGIVLIGLALLLIWKLLMIIHDRREFAKFEKEKMNAKWDTSLSVAQPGVQWCDISSLQPLTSRFQQFSCLSLPSTWDYRVKILFIRVP

210 NP_714941.1 NP_060279.2

>NP_714941.1

MSGFNFGGTGAPTGGFTFGTAKTATTTPATGFSFSTSGTGGFNFGAPFQPATSTPSTGLFSLATQTPATQTTGFTFGTATLASGGTGFSLGIGASKLNLSNTAATPAMANPSGFGLGSSNLTNAISSTVTSSQGTAPTGFVFGPSTTSVAPATTSGGFSFTGGSTAQPSGFNIGSAGNSAQPTAPATLPFTPATPAATTAGATQPAAPTPTATITSTGPSLFASIATAPTSSATTGLSLCTPVTTAGAPTAGTQGFSLKAPGAASGTSTTTSTAATATATTTSSSSTTGFALNLKPLAPAGIPSNTAAAVTAPPGPGAAAGAAASSAMTYAQLESLINKWSLELEDQERHFLQQATQVNAWDRTLIENGEKITSLHREVEKVKLDQKRLDQELDFILSQQKELEDLLSPLEELVKEQSGTIYLQHADEEREKTYKLAENIDAQLKRMAQDLKDIIEHLNTSGAPADTSDPLQQICKILNAHMDSLQWIDQNSALLQRKVEEVTKVCEGRRKEQERSFRITFD

>NP_060279.2

MCSTLKKCGTYRTEVAECHDHGSTFQGRKKGGSSFRDNFDKRSCHYEHGGYERPPSHCQENDGSVEMRDVHKDQQLRHTPYSIRCERRMKWHSEDEIRITTWRNRKPPERKMSQNTQDGYTRNWFKVTIPYGIKYDKAWLMNSIQSHCSDRFTPVDFHYVRNRACFFVQDASAASALKDVSYKIYDDENQKICIFVNHSTAPYSVKNKLKPGQMEMLKLTMNKRYNVSQQALDLQNLRFDPDLMGRDIDIILNRRNCMAATLKIIERNFPELLSLNLCNNKLYQLDGLSDITEKAPKVKTLNLSKNKLESAWELGKVKGLKLEELWLEGNPLCSTFSDQSAYVSAIRDCFPKLLRLDGRELSAPVIVDIDSSETMKPCKENFTGSETLKHLVLQFLQQYYSIYDSGDRQGLLGAYHDEACFSLAIPFDPKDSAPSSLCKYFEDSRNMKTLKDPYLKGELLRRTKRDIVDSLSALPKTQHDLSSILVDVWCQTERMLCFSVNGVFKEVEGQSQGSVLAFTRTFIATPGSSSSLCIVNDELFVRDASPQETQSAFSIPVSTLSSSSEPSLSQEQQEMVQAFSAQSGMKLEWSQKCLQDNEWNYTRAGQAFTMLQTEGKIPAEAFKQIS

211 NP_065095.2 NP_078805.3

>NP_065095.2

MSHGHSHGGGGCRCAAEREEPPEQRGLAYGLYLRIDLERLQCLNESREGSGRGVFKPWEERTDRSKFVESDADEELLFNIPFTGNVKLKGIIIMGEDDDSHPSEMRLYKNIPQMSFDDTEREPDQTFSLNRDLTGELEYATKISRFSNVYHLSIHISKNFGADTTKVFYIGLRGEWTELRRHEVTICNYEASANPADHRVHQVTPQTHFIS

>NP_078805.3

MADVLSVLRQYNIQKKEIVVKGDEVIFGEFSWPKNVKTNYVVWGTGKEGQPREYYTLDSILFLLNNVHLSHPVYVRRAATENIPVVRRPDRKDLLGYLNGEASTSASIDRSAPLEIGLQRSTQVKRAADEVLAEAKKPRIEDEECVRLDKERLAARLEGHKEGIVQTEQIRSLSEAMSVEKIAAIKAKIMAKKRSTIKTDLDDDITALKQRSFVDAEVDVTRDIVSRERVWRTRTTILQSTGKNFSKNIFAILQSVKAREEGRAPEQRPAPNAAPVDPTLRTKQPIPAAYNRYDQERFKGKEETEGFKIDTMGTYHGMTLKSVTEGASARKTQTPAAQPVPRPVSQARPPPNQKKGSRTPIIIIPAATTSLITMLNAKDLLQDLKFVPSDEKKKQGCQRENETLIQRRKDQMQPGGTAISVTVPYRVVDQPLKLMPQDWDRVVAVFVQGPAWQFKGWPWLLPDGSPVDIFAKIKAFHLKYDEVRLDPNVQKWDVTVLELSYHKRHLDRPVFLRFWETLDRYMVKHKSHLRF

212 NP_001012300.1 NP_085136.1

>NP_001012300.1

MTRGTGGTAQRGRSGPGLSPDGIWMAKELYLKTSSVKEAGEGPRGLAGEGGWGGVPFAEALRILGGPNPTISLLARSQGLLDSSLMASGTASRSEDEESLAGQKRASSQALGTIPKRRSSSRFIKRKKFDDELVESSLAKSSTRAKGASGVEPGRCSGSEPSSSEKKKVSKAPSTPVPPSPAPAPGLTKRVKKSKQPLQVTKDLGRWKPADDLLLINAVLQTNDLTSVHLGVKFSCRFTLREVQERWYALLYDPVISKLACQAMRQLHPEAIAAIQSKALFSKAEEQLLSKVGSTSQPTLETFQDLLHRHPDAFYLARTAKALQAHWQLMKQYYLLEDQTVQPLPKGDQVLNFSDAEDLIDDSKLKDMRDEVLEHELMVADRRQKREIRQLEQELHKWQVLVDSITGMSSPDFDNQTLAVLRGRMVRYLMRSREITLGRATKDNQIDVDLSLEGPAWKISRKQGVIKLKNNGDFFIANEGRRPIYIDGRPVLCGSKWRLSNNSVVEIASLRFVFLINQDLIALIRAEAAKITPQ

>NP_085136.1

MATGDLKRSLRNLEQVLRLLNYPEEVDCVGLIKGDPAASLPIISYSFTSYSPYVTELIMESNVELIAKNDLRFIDAVYKLLRDQFNYKPILTKKQFIQCGFAEWKIQIVCDILNCVMKKHKELSSLQKIPSQQRKKISSGKSEPPLGNEKISAEAVGVDISGRFMTSGKKKAVVIRHLYNEDNVDISEDTLSPITDVNEAVDVSDLNATEIKMPEVKVPEIKAEQQDVNVNPEITALQTMLAECQENLKKLTSIEKRLDCLEQKMKGKVMVDENTWTNLLSRVTLLETEMLLSKKNDEFIEFNEVSEDYASCSDMDLLNPHRKSEVERPASIPLSSGYSTASSDSTPRASTVNYCGLNEISEETTIQKMERMKKMFEETAELLKCPNHYL

213 NP_110435.1 NP_006752.1

>NP_110435.1

MDGEDIPDFSSLKEETAYWKELSLKYKQSFQEARDELVEFQEGSRELEAELEAQLVQAEQRNRDLQADNQRLKYEVEALKEKLEHQYAQSYKQVSVLEDDLSQTRAIKEQLHKYVRELEQANDDLERAKRATIVSLEDFEQRLNQAIERNAFLESELDEKESLLVSVQRLKDEARDLRQELAVRERQQEVTRKSAPSSPTLDCEKMDSAVQASLSLPATPVGKGTENTFPSPKAIPNGFGTSPLTPSARISALNIVGDLLRKVGALESKLAACRNFAKDQASRKSYISGNVNCGVLNGNGTKFSRSGHTSFFDKGAVNGFDPAPPPPGLGSSRPSSAPGMLPLSV

>NP_006752.1

MDDREDLVYQAKLAEQAERYDEMVESMKKVAGMDVELTVEERNLLSVAYKNVIGARRASWRIISSIEQKEENKGGEDKLKMIREYRQMVETELKLICCDILDVLDKHLIPAANTGESKVFYYKMKGDYHRYLAEFATGNDRKEAAENSLVAYKAASDIAMTELPPTHPIRLGLALNFSVFYYEILNSPDRACRLAKAAFDDAIAELDTLSEESYKDSTLIMQLLRDNLTLWTSDMQGDGEEQNKEALQDVEDENQ

214 NP_055525.3 NP_006393.2

>NP_055525.3

MTGAEIESGAQVKPEKKPGEEVVGGAEIENDVPLVVRPKVRTQAQIMPGARPKNKSKVMPGASTKVETSAVGGARPKSKAKAIPVSRFKEEAQMWAQPRFGAERLSKTERNSQTNIIASPLVSTDSVLVAKTKYLSEDRELVNTDTESFPRRKAHYQAGFQPSFRSKEETNMGSWCCPRPTSKQEASPNSDFKWVDKSVSSLFWSGDEVTAKFHPGNRVKDSNRSMHMANQEANTMSRSQTNQELYIASSSGSEDESVKTPWFWARDKTNTWSGPREDPNSRSRFRSKKEVYVESSSGSEHEDHLESWFGAGKEAKFRSKMRAGKEANNRARHRAKREACIDFMPGSIDVIKKESCFWPEENANTFSRPMIKKEARARAMTKEEAKTKARARAKQEARSEEEALIGTWFWATDESSMADEASIESSLQVEDESIIGSWFWTEEEASMGTGASSKSRPRTDGERIGDSLFGAREKTSMKTGAEATSESILAADDEQVIIGSWFWAGEEVNQEAEEETIFGSWFWVIDAASVESGVGVSCESRTRSEEEEVIGPWFWSGEQVDIEAGIGEEARPGAEEETIFGSWFWAENQTYMDCRAETSCDTMQGAEEEEPIIGSWFWTRVEACVEGDVNSKSSLEDKEEAMIPCFGAKEEVSMKHGTGVRCRFMAGAEETNNKSCFWAEKEPCMYPAGGGSWKSRPEEEEDIVNSWFWSRKYTKPEAIIGSWLWATEESNIDGTGEKAKLLTEEETIINSWFWKEDEAISEATDREESRPEAEEGDIIGSWFWAGEEDRLEPAAETREEDRLAAEKEGIVGSWFGAREETIRREAGSCSKSSPKAEEEEVIIGSWFWEEEASPEAVAGVGFESKPGTEEEEITVGSWFWPEEEASIQAGSQAVEEMESETEEETIFGSWFWDGKEVSEEAGPCCVSKPEDDEEMIVESWFWSRDKAIKETGTVATCESKPENEEGAIVGSWFEAEDEVDNRTDNGSNCGSRTLADEDEAIVGSWFWAGDEAHFESNPSPVFRAICRSTCSVEQEPDPSRRPQSWEEVTVQFKPGPWGRVGFPSISPFRFPKEAASLFCEMFGGKPRNMVLSPEGEDQESLLQPDQPSPEFPFQYDPSYRSVQEIREHLRAKESTEPESSSCNCIQCELKIGSEEFEELLLLMEKIRDPFIHEISKIAMGMRSASQFTRDFIRDSGVVSLIETLLNYPSSRVRTSFLENMIRMAPPYPNLNIIQTYICKVCEETLAYSVDSPEQLSGIRMIRHLTTTTDYHTLVANYMSGFLSLLATGNAKTRFHVLKMLLNLSENLFMTKELLSAEAVSEFIGLFNREETNDNIQIVLAIFENIGNNIKKETVFSDDDFNIEPLISAFHKVEKFAKELQGKTDNQNDPEGDQEN

>NP_006393.2

MEPGAGHLDGHRAGSPSLRQALCDGSAVMFSSKERGRCTVINFVPLEAPLRSTPRSRQVTEACGGEGRAVPLGSEPEWSVGGMEATLEQHLEDTMKNPSIVGVLCTDSQGLNLGCRGTLSDEHAGVISVLAQQAAKLTSDPTDIPVVCLESDNGNIMIQKHDGITVAVHKMAS

215 NP_002175.2 NP_002301.1

>NP_002175.2

MLTLQTWLVQALFIFLTTESTGELLDPCGYISPESPVVQLHSNFTAVCVLKEKCMDYFHVNANYIVWKTNHFTIPKEQYTIINRTASSVTFTDIASLNIQLTCNILTFGQLEQNVYGITIISGLPPEKPKNLSCIVNEGKKMRCEWDGGRETHLETNFTLKSEWATHKFADCKAKRDTPTSCTVDYSTVYFVNIEVWVEAENALGKVTSDHINFDPVYKVKPNPPHNLSVINSEELSSILKLTWTNPSIKSVIILKYNIQYRTKDASTWSQIPPEDTASTRSSFTVQDLKPFTEYVFRIRCMKEDGKGYWSDWSEEASGITYEDRPSKAPSFWYKIDPSHTQGYRTVQLVWKTLPPFEANGKILDYEVTLTRWKSHLQNYTVNATKLTVNLTNDRYLATLTVRNLVGKSDAAVLTIPACDFQATHPVMDLKAFPKDNMLWVEWTTPRESVKKYILEWCVLSDKAPCITDWQQEDGTVHRTYLRGNLAESKCYLITVTPVYADGPGSPESIKAYLKQAPPSKGPTVRTKKVGKNEAVLEWDQLPVDVQNGFIRNYTIFYRTIIGNETAVNVDSSHTEYTLSSLTSDTLYMVRMAAYTDEGGKDGPEFTFTTPKFAQGEIEAIVVPVCLAFLLTTLLGVLFCFNKRDLIKKHIWPNVPDPSKSHIAQWSPHTPPRHNFNSKDQMYSDGNFTDVSVVEIEANDKKPFPEDLKSLDLFKKEKINTEGHSSGIGGSSCMSSSRPSISSSDENESSQNTSSTVQYSTVVHSGYRHQVPSVQVFSRSESTQPLLDSEERPEDLQLVDHVDGGDGILPRQQYFKQNCSQHESSPDISHFERSKQVSSVNEEDFVRLKQQISDHISQSCGSGQMKMFQEVSAADAFGPGTEGQVERFETVGMEAATDEGMPKSYLPQTVRQGGYMPQ

>NP_002301.1

MMDIYVCLKRPSWMVDNKRMRTASNFQWLLSTFILLYLMNQVNSQKKGAPHDLKCVTNNLQVWNCSWKAPSGTGRGTDYEVCIENRSRSCYQLEKTSIKIPALSHGDYEITINSLHDFGSSTSKFTLNEQNVSLIPDTPEILNLSADFSTSTLYLKWNDRGSVFPHRSNVIWEIKVLRKESMELVKLVTHNTTLNGKDTLHHWSWASDMPLECAIHFVEIRCYIDNLHFSGLEEWSDWSPVKNISWIPDSQTKVFPQDKVILVGSDITFCCVSQEKVLSALIGHTNCPLIHLDGENVAIKIRNISVSASSGTNVVFTTEDNIFGTVIFAGYPPDTPQQLNCETHDLKEIICSWNPGRVTALVGPRATSYTLVESFSGKYVRLKRAEAPTNESYQLLFQMLPNQEIYNFTLNAHNPLGRSQSTILVNITEKVYPHTPTSFKVKDINSTAVKLSWHLPGNFAKINFLCEIEIKKSNSVQEQRNVTIKGVENSSYLVALDKLNPYTLYTFRIRCSTETFWKWSKWSNKKQHLTTEASPSKGPDTWREWSSDGKNLIIYWKPLPINEANGKILSYNVSCSSDEETQSLSEIPDPQHKAEIRLDKNDYIISVVAKNSVGSSPPSKIASMEIPNDDLKIEQVVGMGKGILLTWHYDPNMTCDYVIKWCNSSRSEPCLMDWRKVPSNSTETVIESDEFRPGIRYNFFLYGCRNQGYQLLRSMIGYIEELAPIVAPNFTVEDTSADSILVKWEDIPVEELRGFLRGYLFYFGKGERDTSKMRVLESGRSDIKVKNITDISQKTLRIADLQGKTSYHLVLRAYTDGGVGPEKSMYVVTKENSVGLIIAILIPVAVAVIVGVVTSILCYRKREWIKETFYPDIPNPENCKALQFQKSVCEGSSALKTLEMNPCTPNNVEVLETRSAFPKIEDTEIISPVAERPEDRSDAEPENHVVVSYCPPIIEEEIPNPAADEAGGTAQVIYIDVQSMYQPQAKPEEEQENDPVGGAGYKPQMHLPINSTVEDIAAEEDLDKTAGYRPQANVNTWNLVSPDSPRSIDSNSEIVSFGSPCSINSRQFLIPPKDEDSPKSNGGGWSFTNFFQNKPND

216 NP_006087.2 NP_004850.1

>NP_006087.2

MSREMQDVDLAEVKPLVEKGETITGLLQEFDVQEQDIETLHGSVHVTLCGTPKGNRPVILTYHDIGMNHKTCYNPLFNYEDMQEITQHFAVCHVDAPGQQDGAASFPAGYMYPSMDQLAEMLPGVLQQFGLKSIIGMGTGAGAYILTRFALNNPEMVEGLVLINVNPCAEGWMDWAASKISGWTQALPDMVVSHLFGKEEMQSNVEVVHTYRQHIVNDMNPGNLHLFINAYNSRRDLEIERPMPGTHTVTLQCPALLVVGDSSPAVDAVVECNSKLDPTKTTLLKMADCGGLPQISQPAKLAEAFKYFVQGMGYMPSASMTRLMRSRTASGSSVTSLDGTRSRSHTSEGTRSRSHTSEGTRSRSHTSEGAHLDITPNSGAAGNSAGPKSMEVSC

>NP_004850.1

MAQILPIRFQEHLQLQNLGINPANIGFSTLTMESDKFICIREKVGEQAQVVIIDMNDPSNPIRRPISADSAIMNPASKVIALKAGKTLQIFNIEMKSKMKAHTMTDDVTFWKWISLNTVALVTDNAVYHWSMEGESQPVKMFDRHSSLAGCQIINYRTDAKQKWLLLTGISAQQNRVVGAMQLYSVDRKVSQPIEGHAASFAQFKMEGNAEESTLFCFAVRGQAGGKLHIIEVGTPPTGNQPFPKKAVDVFFPPEAQNDFPVAMQISEKHDVVFLITKYGYIHLYDLETGTCIYMNRISGETIFVTAPHEATAGIIGVNRKGQVLSVCVEEENIIPYITNVLQNPDLALRMAVRNNLAGAEELFARKFNALFAQGNYSEAAKVAANAPKGILRTPDTIRRFQSVPAQPGQTSPLLQYFGILLDQGQLNKYESLELCRPVLQQGRKQLLEKWLKEDKLECSEELGDLVKSVDPTLALSVYLRANVPNKVIQCFAETGQVQKIVLYAKKVGYTPDWIFLLRNVMRISPDQGQQFAQMLVQDEEPLADITQIVDVFMEYNLIQQCTAFLLDALKNNRPSEGPLQTRLLEMNLMHAPQVADAILGNQMFTHYDRAHIAQLCEKAGLLQRALEHFTDLYDIKRAVVHTHLLNPEWLVNYFGSLSVEDSLECLRAMLSANIRQNLQICVQVASKYHEQLSTQSLIELFESFKSFEGLFYFLGSIVNFSQDPDVHFKYIQAACKTGQIKEVERICRESNCYDPERVKNFLKEAKLTDQLPLIIVCDRFDFVHDLVLYLYRNNLQKYIEIYVQKVNPSRLPVVIGGLLDVDCSEDVIKNLILVVRGQFSTDELVAEVEKRNRLKLLLPWLEARIHEGCEEPATHNALAKIYIDSNNNPERFLRENPYYDSRVVGKYCEKRDPHLACVAYERGQCDLELINVCNENSLFKSLSRYLVRRKDPELWGSVLLESNPYRRPLIDQVVQTALSETQDPEEVSVTVKAFMTADLPNELIELLEKIVLDNSVFSEHRNLQNLLILTAIKADRTRVMEYINRLDNYDAPDIANIAISNELFEEAFAIFRKFDVNTSAVQVLIEHIGNLDRAYEFAERCNEPAVWSQLAKAQLQKGMVKEAIDSYIKADDPSSYMEVVQAANTSGNWEELVKYLQMARKKARESYVETELIFALAKTNRLAELEEFINGPNNAHIQQVGDRCYDEKMYDAAKLLYNNVSNFGRLASTLVHLGEYQAAVDGARKANSTRTWKEVCFACVDGKEFRLAQMCGLHIVVHADELEELINYYQDRGYFEELITMLEAALGLERAHMGMFTELAILYSKFKPQKMREHLELFWSRVNIPKVLRAAEQAHLWAELVFLYDKYEEYDNAIITMMNHPTDAWKEGQFKDIITKVANVELYYRAIQFYLEFKPLLLNDLLMVLSPRLDHTRAVNYFSKVKQLPLVKPYLRSVQNHNNKSVNESLNNLFITEEDYQALRTSIDAYDNFDNISLAQRLEKHELIEFRRIAAYLFKGNNRWKQSVELCKKDSLYKDAMQYASESKDTELAEELLQWFLQEEKRECFGACLFTCYDLLRPDVVLETAWRHNIMDFAMPYFIQVMKEYLTKVDKLDASESLRKEEEQATETQPIVYGQPQLMLTAGPSVAVPPQAPFGYGYTAPPYGQPQPGFGYSM

217 NP_004851.1 NP_001012300.1

>NP_004851.1

MGGLASGGDVEPGLPVEVRGSNGAFYKGFVKDVHEDSVTIFFENNWQSERQIPFGDVRLPPPADYNKEITEGDEVEVYSRANEQEPCGWWLARVRMMKGDFYVIEYAACDATYNEIVTLERLRPVNPNPLATKGSFFKVTMAVPEDLREACSNENVHKEFKKALGANCIFLNITNSELFILSTTEAPVKRASLLGDMHFRSLRTKLLLMSRNEEATKHLETSKQLAAAFQEEFTVREDLMGLAIGTHGANIQQARKVPGVTAIELGEETCTFRIYGETPEACRQARSYLEFSEDSVQVPRNLVGKVIGKNGKVIQEIVDKSGVVRVRVEGDNDKKNPREEGMVPFIFVGTRENISNAQALLEYHLSYLQEVEQLRLERLQIDEQLRQIGLGFRPPGSGRGSGGSDKAGYSTDESSSSSLHATRTYGGSYGGRGRGRRTGGPAYGPSSDVSTASETESEKREEPNRAGPGDRDPPTRGEESRRRPTGGRGRGPPPAPRPTSRYNSSSISSVLKDPDSNPYSLLDTSEPEPPVDSEPGEPPPASARRRRSRRRRTDEDRTVMDGGLESDGPNMTENGLEDESRPQRRNRSRRRRNRGNRTDGSISGDRQPVTVADYISRAESQSRQSAPLERTKPSEDSLSGQKGDSVSKLPKGPSENGELSAPLELGSMVNGVS

>NP_001012300.1

MTRGTGGTAQRGRSGPGLSPDGIWMAKELYLKTSSVKEAGEGPRGLAGEGGWGGVPFAEALRILGGPNPTISLLARSQGLLDSSLMASGTASRSEDEESLAGQKRASSQALGTIPKRRSSSRFIKRKKFDDELVESSLAKSSTRAKGASGVEPGRCSGSEPSSSEKKKVSKAPSTPVPPSPAPAPGLTKRVKKSKQPLQVTKDLGRWKPADDLLLINAVLQTNDLTSVHLGVKFSCRFTLREVQERWYALLYDPVISKLACQAMRQLHPEAIAAIQSKALFSKAEEQLLSKVGSTSQPTLETFQDLLHRHPDAFYLARTAKALQAHWQLMKQYYLLEDQTVQPLPKGDQVLNFSDAEDLIDDSKLKDMRDEVLEHELMVADRRQKREIRQLEQELHKWQVLVDSITGMSSPDFDNQTLAVLRGRMVRYLMRSREITLGRATKDNQIDVDLSLEGPAWKISRKQGVIKLKNNGDFFIANEGRRPIYIDGRPVLCGSKWRLSNNSVVEIASLRFVFLINQDLIALIRAEAAKITPQ

218 NP_078983.1 NP_079051.9

>NP_078983.1

MQTPVNIPVPVLRLPRGPDGFSRGFAPDGRRAPLRPEVPEIQECPIAQESLESQEQRARAALRERYLRSLLAMVGHQVSFTLHEGVRVAAHFGATDLDVANFYVSQLQTPIGVQAEALLRCSDIISYTFKP

>NP_079051.9

MSEWMKKGPLEWQDYIYKEVRVTASEKNEYKGWVLTTDPVSANIVLVNFLEDGSMSVTGIMGHAVQTVETMNEGDHRVREKLMHLFTSGDCKAYSPEDLEERKNSLKKWLEKNHIPITEQGDAPRTLCVAGVLTIDPPYGPENCSSSNEIILSRVQDLIEGHLTASQ

219 NP_003234.2 NP_002182.1

>NP_003234.2

MTSHYVIAIFALMSSCLATAGPEPGALCELSPVSASHPVQALMESFTVLSGCASRGTTGLPQEVHVLNLRTAGQGPGQLQREVTLHLNPISSVHIHHKSVVFLLNSPHPLVWHLKTERLATGVSRLFLVSEGSVVQFSSANFSLTAETEERNFPHGNEHLLNWARKEYGAVTSFTELKIARNIYIKVGEDQVFPPKCNIGKNFLSLNYLAEYLQPKAAEGCVMSSQPQNEEVHIIELITPNSNPYSAFQVDITIDIRPSQEDLEVVKNLILILKCKKSVNWVIKSFDVKGSLKIIAPNSIGFGKESERSMTMTKSIRDDIPSTQGNLVKWALDNGYSPITSYTMAPVANRFHLRLENNAEEMGDEEVHTIPPELRILLDPGALPALQNPPIRGGEGQNGGLPFPFPDISRRVWNEEGEDGLPRPKDPVIPSIQLFPGLREPEEVQGSVDIALSVKCDNEKMIVAVEKDSFQASGYSGMDVTLLDPTCKAKMNGTHFVLESPLNGCGTRPRWSALDGVVYYNSIVIQVPALGDSSGWPDGYEDLESGDNGFPGDMDEGDASLFTRPEIVVFNCSLQQVRNPSSFQEQPHGNITFNMELYNTDLFLVPSQGVFSVPENGHVYVEVSVTKAEQELGFAIQTCFISPYSNPDRMSHYTIIENICPKDESVKFYSPKRVHFPIPQADMDKKRFSFVFKPVFNTSLLFLQCELTLCTKMEKHPQKLPKCVPPDEACTSLDASIIWAMMQNKKTFTKPLAVIHHEAESKEKGPSMKEPNPISPPIFHGLDTLTVMGIAFAAFVIGALLTGALWYIYSHTGETAGRQQVPTSPPASENSSAAHSIGSTQSTPCSSSSTA

>NP_002182.1

MVLHLLLFLLLTPQGGHSCQGLELARELVLAKVRALFLDALGPPAVTREGGDPGVRRLPRRHALGGFTHRGSEPEEEEDVSQAILFPATDASCEDKSAARGLAQEAEEGLFRYMFRPSQHTRSRQVTSAQLWFHTGLDRQGTAASNSSEPLLGLLALSPGGPVAVPMSLGHAPPHWAVLHLATSALSLLTHPVLVLLLRCPLCTCSARPEATPFLVAHTRTRPPSGGERARRSTPLMSWPWSPSALRLLQRPPEEPAAHANCHRVALNISFQELGWERWIVYPPSFIFHYCHGGCGLHIPPNLSLPVPGAPPTPAQPYSLLPGAQPCCAALPGTMRPLHVRTTSDGGYSFKYETVPNLLTQHCACI

220 NP_001019.1 NP_055668.2

>NP_001019.1

MPPKDDKKKKDAGKSAKKDKDPVNKSGGKAKKKKWSKGKVRDKLNNLVLFDKATYDKLCKEVPNYKLITPAVVSERLKIRGSLARAALQELLSKGLIKLVSKHRAQVIYTRNTKGGDAPAAGEDA

>NP_055668.2

MGSAEDAVKEKLLWNVKKEVKQIMEEAVTRKFVHEDSSHIIALCGAVEACLLHQLRRRAAGFLRSDKMAALFTKVGKTCPVAGEICHKVQELQQQAEGRKPSGVSQEALRRQGSASGKAPALSPQALKHVWVRTALIEKVLDKVVQYLAENCSKYYEKEALLADPVFGPILASLLVGPCALEYTKLKTADHYWTDPSADELVQRHRIRGPPTRQDSPAKRPALGIRKRHSSGSASEDRLAACARECVESLHQNSRTRLLYGKNHVLVQPKEDMEAVPGYLSLHQSAESLTLKWTPNQLMNGTLGDSELEKSVYWDYALVVPFSQVVCIHCHQQKSGGTLVLVSQDGIQRPPLHFPQGGHLLSFLSCLENGLLPRGQLEPPLWTQQGKGKVFPKLRKRSSIRSVDMEEMGTGRATDYVFRIIYPGHRHEHITINYHHLAASRAASVDDDEEEEDKLHAMLSMICSRNLTAPNPMKDAGDMIEMQGFGPSLPAWHLEPLCSQGSSCLSCSSSSSPHATPSHCSCIPDRLPLRLLCESMKRQIVSRAFYGWLAHCRHLSTVRTHLSALVHHSVIPPDRPPGASAGLTKDVWSKYQKDKKNYKELELLRQVYYGGIEHEIRKDVWPFLLGHYKFGMSKKEMEQVDAVVAARYQQVLAEWKACEVVVRQREREAHPATRTKFSSGSSIDSHVQRLIHRDSTISNDVFISVDDLEPPEPQDPEDSRPKPEQEAGPGTPGTAVVEQQHSVEFDSPDSGLPSSRNYSVASGIQSSLDEGQSVGFEEEDGGGEEGSSGPGPAAHTLREPQDPSQEKPQAGELEAGEELAAVCAAAYTIELLDTVALNLHRIDKDVQRCDRNYWYFTPPNLERLRDVMCSYVWEHLDVGYVQGMCDLLAPLLVTLDNDQLAYSCFSHLMKRMSQNFPNGGAMDTHFANMRSLIQILDSELFELMHQNGDYTHFYFCYRWFLLDFKRELLYEDVFAVWEVIWAARHISSEHFVLFIALALVEAYREIIRDNNMDFTDIIKFFNERAEHHDAQEILRIARDLVHKVQMLIENK

221 NP_000030.1 NP_055580.1

>NP_000030.1

MKAAVLTLAVLFLTGSQARHFWQQDEPPQSPWDRVKDLATVYVDVLKDSGRDYVSQFEGSALGKQLNLKLLDNWDSVTSTFSKLREQLGPVTQEFWDNLEKETEGLRQEMSKDLEEVKAKVQPYLDDFQKKWQEEMELYRQKVEPLRAELQEGARQKLHELQEKLSPLGEEMRDRARAHVDALRTHLAPYSDELRQRLAARLEALKENGGARLAEYHAKATEHLSTLSEKAKPALEDLRQGLLPVLESFKVSFLSALEEYTKKLNTQ

>NP_055580.1

MVGRNSAIAAGVCGALFIGYCIYFDRKRRSDPNFKNRLRERRKKQKLAKERAGLSKLPDLKDAEAVQKFFLEEIQLGEELLAQGEYEKGVDHLTNAIAVCGQPQQLLQVLQQTLPPPVFQMLLTKLPTISQRIVSAQSLAEDDVE

222 NP_000116.2 NP_003241.2

>NP_000116.2

MTMTLHTKASGMALLHQIQGNELEPLNRPQLKIPLERPLGEVYLDSSKPAVYNYPEGAAYEFNAAAAANAQVYGQTGLPYGPGSEAAAFGSNGLGGFPPLNSVSPSPLMLLHPPPQLSPFLQPHGQQVPYYLENEPSGYTVREAGPPAFYRPNSDNRRQGGRERLASTNDKGSMAMESAKETRYCAVCNDYASGYHYGVWSCEGCKAFFKRSIQGHNDYMCPATNQCTIDKNRRKSCQACRLRKCYEVGMMKGGIRKDRRGGRMLKHKRQRDDGEGRGEVGSAGDMRAANLWPSPLMIKRSKKNSLALSLTADQMVSALLDAEPPILYSEYDPTRPFSEASMMGLLTNLADRELVHMINWAKRVPGFVDLTLHDQVHLLECAWLEILMIGLVWRSMEHPGKLLFAPNLLLDRNQGKCVEGMVEIFDMLLATSSRFRMMNLQGEEFVCLKSIILLNSGVYTFLSSTLKSLEEKDHIHRVLDKITDTLIHLMAKAGLTLQQQHQRLAQLLLILSHIRHMSNKGMEHLYSMKCKNVVPLYDLLLEMLDAHRLHAPTSRGGASVEETDQSHLATAGSTSSHSLQKYYITGEAEGFPATV

>NP_003241.2

MEQKPSKVECGSDPEENSARSPDGKRKRKNGQCSLKTSMSGYIPSYLDKDEQCVVCGDKATGYHYRCITCEGCKGFFRRTIQKNLHPTYSCKYDSCCVIDKITRNQCQLCRFKKCIAVGMAMDLVLDDSKRVAKRKLIEQNRERRRKEEMIRSLQQRPEPTPEEWDLIHIATEAHRSTNAQGSHWKQRRKFLPDDIGQSPIVSMPDGDKVDLEAFSEFTKIITPAITRVVDFAKKLPMFSELPCEDQIILLKGCCMEIMSLRAAVRYDPESDTLTLSGEMAVKREQLKNGGLGVVSDAIFELGKSLSAFNLDDTEVALLQAVLLMSTDRSGLLCVDKIEKSQEAYLLAFEHYVNHRKHNIPHFWPKLLMKEREVQSSILYKGAAAEGRPGGSLGVHPEGQQLLGMHVVQGPQVRQLEQQLGEAGSLQGPVLQHQSPKSPQQRLLELLHRSGILHARAVCGEDDSSEADSPSSSEEEPEVCEDLAGNAASP

223 NP_056453.1 NP_001056.1

>NP_056453.1

MAAAPVAAGSGAGRGRRSAATVAAWGGWGGRPRPGNILLQLRQGQLTGRGLVRAVQFTETFLTERDKQSKWSGIPQLLLKLHTTSHLHSDFVECQNILKEISPLLSMEAMAFVTEERKLTQETTYPNTYIFDLFGGVDLLVEILMRPTISIRGQKLKISDEMSKDCLSILYNTCVCTEGVTKRLAEKNDFVIFLFTLMTSKKTFLQTATLIEDILGVKKEMIRLDEVPNLSSLVSNFDQQQLANFCRILAVTISEMDTGNDDKHTLLAKNAQQKKSLSLGPSAAEINQAALLSIPGFVERLCKLATRKVSESTGTASFLQELEEWYTWLDNALVLDALMRVANEESEHNQASIVFPPPGASEENGLPHTSARTQLPQSMKIMHEIMYKLEVLYVLCVLLMGRQRNQVHRMIAEFKLIPGLNNLFDKLIWRKHSASALVLHGHNQNCDCSPDITLKIQFLRLLQSFSDHHENKYLLLNNQELNELSAISLKANIPEVEAVLNTDRSLVCDGKRGLLTRLLQVMKKEPAESSFRFWQARAVESFLRGTTSYADQMFLLKRGLLEHILYCIVDSECKSRDVLQSYFDLLGELMKFNVDAFKRFNKYINTDAKFQVFLKQINSSLVDSNMLVRCVTLSLDRFENQVDMKVAEVLSECRLLAYISQVPTQMSFLFRLINIIHVQTLTQENVSCLNTSLVILMLARRKERLPLYLRLLQRMEHSKKYPGFLLNNFHNLLRFWQQHYLHKDKDSTCLENSSCISFSYWKETVSILLNPDRQSPSALVSYIEEPYMDIDRDFTEE

>NP_001056.1

MGLSTVPDLLLPLVLLELLVGIYPSGVIGLVPHLGDREKRDSVCPQGKYIHPQNNSICCTKCHKGTYLYNDCPGPGQDTDCRECESGSFTASENHLRHCLSCSKCRKEMGQVEISSCTVDRDTVCGCRKNQYRHYWSENLFQCFNCSLCLNGTVHLSCQEKQNTVCTCHAGFFLRENECVSCSNCKKSLECTKLCLPQIENVKGTEDSGTTVLLPLVIFFGLCLLSLLFIGLMYRYQRWKSKLYSIVCGKSTPEKEGELEGTTTKPLAPNPSFSPTPGFTPTLGFSPVPSSTFTSSSTYTPGDCPNFAAPRREVAPPYQGADPILATALASDPIPNPLQKWEDSAHKPQSLDTDDPATLYAVVENVPPLRWKEFVRRLGLSDHEIDRLELQNGRCLREAQYSMLATWRRRTPRREATLELLGRVLRDMDLLGCLEDIEEALCGPAALPPAPSLLR

224 NP_002961.1 NP_115557.1

>NP_002961.1

MAKFVIRPATAADCSDILRLIKELAKYEYMEEQVILTEKDLLEDGFGEHPFYHCLVAEVPKEHWTPEGHSIVGFAMYYFTYDPWIGKLLYLEDFFVMSDYRGFGIGSEILKNLSQVAMRCRCSSMHFLVAEWNEPSINFYKRRGASDLSSEEGWRLFKIDKEYLLKMATEE

>NP_115557.1

MRLPLSHSPEHVEMALLSNILAAYSFVSENPERAALYFVSGVCIGLVLTLAALVIRISCHTDCRRRPGKKFLQDRESSSDSSDSEDGSEDTVSDLSVRRHRRFERTLNKNVFTSAEELERAQRLEERERIIREIWMNGQPEVPGTRSLNRYY

225 NP_006087.2 NP_004095.4

>NP_006087.2

MSREMQDVDLAEVKPLVEKGETITGLLQEFDVQEQDIETLHGSVHVTLCGTPKGNRPVILTYHDIGMNHKTCYNPLFNYEDMQEITQHFAVCHVDAPGQQDGAASFPAGYMYPSMDQLAEMLPGVLQQFGLKSIIGMGTGAGAYILTRFALNNPEMVEGLVLINVNPCAEGWMDWAASKISGWTQALPDMVVSHLFGKEEMQSNVEVVHTYRQHIVNDMNPGNLHLFINAYNSRRDLEIERPMPGTHTVTLQCPALLVVGDSSPAVDAVVECNSKLDPTKTTLLKMADCGGLPQISQPAKLAEAFKYFVQGMGYMPSASMTRLMRSRTASGSSVTSLDGTRSRSHTSEGTRSRSHTSEGTRSRSHTSEGAHLDITPNSGAAGNSAGPKSMEVSC

>NP_004095.4

MEEVVIAGMSGKLPESENLQEFWDNLIGGVDMVTDDDRRWKAGLYGLPRRSGKLKDLSRFDASFFGVHPKQAHTMDPQLRLLLEVTYEAIVDGGINPDSLRGTHTGVWVGVSGSETSEALSRDPETLVGYSMVGCQRAMMANRLSFFFDFRGPSIALDTACSSSLMALQNAYQAIHSGQCPAAIVGGINVLLKPNTSVQFLRLGMLSPEGTCKAFDTAGNGYCRSEGVVAVLLTKKSLARRVYATILNAGTNTDGFKEQGVTFPSGDIQEQLIRSLYQSAGVAPESFEYIEAHGTGTKVGDPQELNGITRALCATRQEPLLIGSTKSNMGHPEPASGLAALAKVLLSLEHGLWAPNLHFHSPNPEIPALLDGRLQVVDQPLPVRGGNVGINSFGFGGSNVHIILRPNTQPPPAPAPHATLPRLLRASGRTPEAVQKLLEQGLRHSQDLAFLSMLNDIAAVPATAMPFRGYAVLGGERGGPEVQQVPAGERPLWFICSGMGTQWRGMGLSLMRLDRFRDSILRSDEAVKPFGLKVSQLLLSTDESTFDDIVHSFVSLTAIQIGLIDLLSCMGLRPDGIVGHSLGEVACGYADGCLSQEEAVLAAYWRGQCIKEAHLPPGAMAAVGLSWEECKQRCPPGVVPACHNSKDTVTISGPQAPVFEFVEQLRKEGVFAKEVRTGGMAFHSYFMEAIAPPLLQELKKVIREPKPRSARWLSTSIPEAQWHSSLARTSSAEYNVNNLVSPVLFQEALWHVPEHAVVLEIAPHALLQAVLKRGLKPSCTIIPLMKKDHRDNLEFFLAGIGRLHLSGIDANPNALFPPVEFPAPRGTPLISPLIKWDHSLAWDVPAAEDFPNGSGSPSAAIYNIDTSSESPDHYLVDHTLDGRVLFPATGYLSIVWKTLARALGLGVEQLPVVFEDVVLHQATILPKTGTVSLEVRLLEASRAFEVSENGNLVVSGKVYQWDDPDPRLFDHPESPTPNPTEPLFLAQAEVYKELRLRGYDYGPHFQGILEASLEGDSGRLLWKDNWVSFMDTMLQMSILGSAKHGLYLPTRVTAIHIDPATHRQKLYTLQDKAQVADVVVSRWLRVTVAGGVHISGLHTESAPRRQQEQQVPILEKFCFTPHTEEGCLSERAALQEELQLCKGLVQALQTKVTQQGLKMVVPGLDGAQIPRDPSQQELPRLLSAACRLQLNGNLQLELAQVLAQERPKLPEDPLLSGLLDSPALKACLDTAVENMPSLKMKVVEVLAGHGHLYSRIPGLLSPHPLLQLSYTATDRHPQALEAAQAELQQHDVAQGQWDPADPAPSALGSADLLVCNCAVAALGDPASALSNMVAALREGGFLLLHTLLRGHPLGDIVAFLTSTEPQYGQGILSQDAWESLFSRVSLRLVGLKKSFYGSTLFLCRRPTPQDSPIFLPVDDTSFRWVESLKGILADEDSSRPVWLKAINCATSGVVGLVNCLRREPGGNRLRCVLLSNLSSTSHVPEVDPGSAELQKVLQGDLVMNVYRDGAWGAFRHFLLEEDKPEEPTAHAFVSTLTRGDLSSIRWVCSSLRHAQPTCPGAQLCTVYYASLNFRDIMLATGKLSPDAIPGKWTSQDSLLGMEFSGRDASGKRVMGLVPAKGLATSVLLSPDFLWDVPSNWTLEEAASVPVVYSTAYYALVVRGRVRPGETLLIHSGSGGVGQAAIAIALSLGCRVFTTVGSAEKRAYLQARFPQLDSTSFANSRDTSFEQHVLWHTGGKGVDLVLNSLAEEKLQASVRCLATHGRFLEIGKFDLSQNHPLGMAIFLKNVTFHGVLLDAFFNESSADWREVWALVQAGIRDGVVRPLKCTVFHGAQVEDAFRYMAQGKHIGKVVVQVLAEEPEAVLKGAKPKLMSAISKTFCPAHKSYIIAGGLGGFGLELAQWLIQRGVQKLVLTSRSGIRTGYQAKQVRRWRRQGVQVQVSTSNISSLEGARGLIAEAAQLGPVGGVFNLAVVLRDGLLENQTPEFFQDVCKPKYSGTLNLDRVTREACPELDYFVVFSSVSCGRGNAGQSNYGFANSAMERICEKRRHEGLPGLAVQWGAIGDVGILVETMSTNDTIVSGTLPQRMASCLEVLDLFLNQPHMVLSSFVLAEKAAAYRDRDSQRDLVEAVAHILGIRDLAAVNLDSSLADLGLDSLMSVEVRQTLERELNLVLSVREVRQLTLRKLQELSSKADEASELACPTPKEDGLAQQQTQLNLRSLLVNPEGPTLMRLNSVQSSERPLFLVHPIEGSTTVFHSLASRLSIPTYGLQCTRAAPLDSIHSLAAYYIDCIRQVQPEGPYRVAGYSYGACVAFEMCSQLQAQQSPAPTHNSLFLFDGSPTYVLAYTQSYRAKLTPGCEAEAETEAICFFVQQFTDMEHNRVLEALLPLKGLEERVAAAVDLIIKSHQGLDRQELSFAARSFYYKLRAAEQYTPKAKYHGNVMLLRAKTGGAYGEDLGADYNLSQVCDGKVSVHVIEGDHRTLLEGSGLESIISIIHSSLAEPRVSVREG

226 NP_002758.1 NP_775898.2

>NP_002758.1

MFCVTPPELETKMNITKGGLVLFSANSNSSCMELSKKIAERLGVEMGKVQVYQEPNRETRVQIQESVRGKDVFIIQTVSKDVNTTIMELLIMVYACKTSCAKSIIGVIPYFPYSKQCKMRKRGSIVSKLLASMMCKAGLTHLITMDLHQKEIQGFFNIPVDNLRASPFLLQYIQEEIPDYRNAVIVAKSPASAKRAQSFAERLRLGIAVIHGEAQDAESDLVDGRHSPPMVRSVAAIHPSLEIPMLIPKEKPPITVVGDVGGRIAIIVDDIIDDVDSFLAAAETLKERGAYKIFVMATHGLLSSDAPRRIEESAIDEVVVTNTIPHEVQKLQCPKIKTVDISMILSEAIRRIHNGESMSYLFRNIGLDD

>NP_775898.2

MALEAAGGPPEETLSLWKREQARLKAHVVDRDTEAWQRDPAFSGLQRVGGVDVSFVKGDSVRACASLVVLSFPELEVVYEESRMVSLTAPYVSGFLAFREVPFLLELVQQLREKEPGLMPQVLLVDGNGVLHHRGFGVACHLGVLTDLPCVGVAKKLLQVDGLENNALHKEKIRLLQTRGDSFPLLGDSGTVLGMALRSHDRSTRPLYISVGHRMSLEAAVRLTCCCCRFRIPEPVRQADICSREHIRKSLGLPGPPTPRSPKAQRPVACPKGDSGESSALC

227 NP_005403.2 NP_001311.3

>NP_005403.2

MLYFSLFWAARPLQRCGQLVRMAIRAQHSNAAQTQTGEANRGWTGQESLSDSDPEMWELLQREKDRQCRGLELIASENFCSRAALEALGSCLNNKYSEGYPGKRYYGGAEVVDEIELLCQRRALEAFDLDPAQWGVNVQPYSGSPANLAVYTALLQPHDRIMGLDLPDGGHLTHGYMSDVKRISATSIFFESMPYKLNPKTGLIDYNQLALTARLFRPRLIIAGTSAYARLIDYARMREVCDEVKAHLLADMAHISGLVAAKVIPSPFKHADIVTTTTHKTLRGARSGLIFYRKGVKAVDPKTGREIPYTFEDRINFAVFPSLQGGPHNHAIAAVAVALKQACTPMFREYSLQVLKNARAMADALLERGYSLVSGGTDNHLVLVDLRPKGLDGARAERVLELVSITANKNTCPGDRSAITPGGLRLGAPALTSRQFREDDFRRVVDFIDEGVNIGLEVKSKTAKLQDFKSFLLKDSETSQRLANLRQRVEQFARAFPMPGFDEH

>NP_001311.3

MSSSEEVSWISWFCGLRGNEFFCEVDEDYIQDKFNLTGLNEQVPHYRQALDMILDLEPDEELEDNPNQSDLIEQAAEMLYGLIHARYILTNRGIAQMLEKYQQGDFGYCPRVYCENQPMLPIGLSDIPGEAMVKLYCPKCMDVYTPKSSRHHHTDGAYFGTGFPHMLFMVHPEYRPKRPANQFVPRLYGFKIHPMAYQLQLQAASNFKSPVKTIR

228 NP_001886.1 NP_001035147.1

>NP_001886.1

MSGPVPSRARVYTDVNTHRPREYWDYESHVVEWGNQDDYQLVRKLGRGKYSEVFEAINITNNEKVVVKILKPVKKKKIKREIKILENLRGGPNIITLADIVKDPVSRTPALVFEHVNNTDFKQLYQTLTDYDIRFYMYEILKALDYCHSMGIMHRDVKPHNVMIDHEHRKLRLIDWGLAEFYHPGQEYNVRVASRYFKGPELLVDYQMYDYSLDMWSLGCMLASMIFRKEPFFHGHDNYDQLVRIAKVLGTEDLYDYIDKYNIELDPRFNDILGRHSRKRWERFVHSENQHLVSPEALDFLDKLLRYDHQSRLTAREAMEHPYFYTVVKDQARMGSSSMPGGSTPVSSANMMSGISSVPTPSPLGPLAGSPVIAAANPLGMPVPAAAGAQQ

>NP_001035147.1

MRIAVICFCLLGITCAIPVKQADSGSSEEKQLYNKYPDAVATWLNPDPSQKQNLLAPQNAVSSEETNDFKQETLPSKSNESHDHMDDMDDEDDDDHVDSQDSIDSNDSDDVDDTDDSHQSDESHHSDESDELVTDFPTDLPATEVFTPVVPTVDTYDGRGDSVVYGLRSKSKKFRRPDIQYPDATDEDITSHMESEELNGAYKAIPVAQDLNAPSDWDSRGKDSYETSQLDDQSAETHSHKQSRLYKRKANDESNEHSDVIDSQELSKVSREFHSHEFHSHEDMLVVDPKSKEEDKHLKFRISHELDSASSEVN

229 NP_001028229.1 NP_004516.2

>NP_001028229.1

MSKVIQKKNHWTSRVHECTVKRGPQGELGVTVLGGAEHGEFPYVGAVAAVEAAGLPGGGEGPRLGEGELLLEVQGVRVSGLPRYDVLGVIDSCKEAVTFKAVRQGGRLNKDLRHFLNQRFQKGSPDHELQQTIRDNLYRHAVPCTTRSPREGEVPGVDYNFLTVKEFLDLEQSGTLLEVGTYEGNYYGTPKPPSQPVSGKVITTDALHSLQSGSKQSTPKRTKSYNDMQNAGIVHAENEEEDDVPEMNSSFTADSGEQEEHTLQETALPPVNSSIIAAPITDPSQKFPQYLPLSAEDNLGPLPENWEMAYTENGEVYFIDHNTKTTSWLDPRCLNKQQKPLEECEDDEGVHTEELDSELELPAGWEKIEDPVYGIYYVDHINRKTQYENPVLEAKRKKQLEQQQQQQQQQQQQQQQQQQQQTEEWTEDHSALVPPVIPNHPPSNPEPAREVPLQGKPFFTRNPSELKGKFIHTKLRKSSRGFGFTVVGGDEPDEFLQIKSLVLDGPAALDGKMETGDVIVSVNDTCVLGHTHAQVVKIFQSIPIGASVDLELCRGYPLPFDPDDPNTSLVTSVAILDKEPIIVNGQETYDSPASHSSKTGKVNGMKDARPSSPADVASNSSHGYPNDTVSLASSIATQPELITVHIVKGPMGFGFTIADSPGGGGQRVKQIVDSPRCRGLKEGDLIVEVNKKNVQALTHNQVVDMLVECPKGSEVTLLVQRGGLPVPKKSPKSQPLERKDSQNSSQHSVSSHRSLHTASPSHSTQVLPEFPPAEAQAPDQTDSSGQKKPDPFKIWAQSRSMYENRLPDYQEQDIFLWRKETGFGFRILGGNEPGEPIYIGHIVPLGAADTDGRLRSGDELICVDGTPVIGKSHQLVVQLMQQAAKQGHVNLTVRRKVVFAVPKTENEVPSPASSHHSSNQPASLTEEKRTPQGSQNSLNTVSSGSGSTSGIGSGGGGGSGVVSTVVQPYDVEIRRGENEGFGFVIVSSVSRPEAGTTFGNACVAMPHKIGRIIEGSPADRCGKLKVGDRILAVNGCSITNKSHSDIVNLIKEAGNTVTLRIIPGDESSNATLLTNAEKIATITTTHTPSQQGTQETRNTTKPKQESQFEFKAPQATQEQDFYTVELERGAKGFGFSLRGGREYNMDLYVLRLAEDGPAERCGKMRIGDEILEINGETTKNMKHSRAIELIKNGGRRVRLFLKRGDGSVPEYDPSSDRHGPATGPQGVPEVRAGPDRRQHPSLESSYPPDLHKSSPHGEKRAHARDPKGSREYSRQPNEHHTWNGTSRKPDSGACRPKDRAPEGRRDAQAERAAAANGPKRRSPEKRREGTRSADNTLERREKHEKRRDVSPERRRERSPTRRRDGSPSRRRRSLERLLEQRRSPERRRGGSPERRAKSTDRRRARSPERRRERSLDKRNREDRASHREREEANLKQDAGRSSRHPPEQRRRPYKECSTDLSI

>NP_004516.2

MDRGPAAVACTLLLALVACLAPASGQECDSAHFRCGSGHCIPADWRCDGTKDCSDDADEIGCAVVTCQQGYFKCQSEGQCIPNSWVCDQDQDCDDGSDERQDCSQSTCSSHQITCSNGQCIPSEYRCDHVRDCPDGADENDCQYPTCEQLTCDNGACYNTSQKCDWKVDCRDSSDEINCTEICLHNEFSCGNGECIPRAYVCDHDNDCQDGSDEHACNYPTCGGYQFTCPSGRCIYQNWVCDGEDDCKDNGDEDGCESGPHDVHKCSPREWSCPESGRCISIYKVCDGILDCPGREDENNTSTGKYCSMTLCSALNCQYQCHETPYGGACFCPPGYIINHNDSRTCVEFDDCQIWGICDQKCESRPGRHLCHCEEGYILERGQYCKANDSFGEASIIFSNGRDLLIGDIHGRSFRILVESQNRGVAVGVAFHYHLQRVFWTDTVQNKVFSVDINGLNIQEVLNVSVETPENLAVDWVNNKIYLVETKVNRIDMVNLDGSYRVTLITENLGHPRGIAVDPTVGYLFFSDWESLSGEPKLERAFMDGSNRKDLVKTKLGWPAGVTLDMISKRVYWVDSRFDYIETVTYDGIQRKTVVHGGSLIPHPFGVSLFEGQVFFTDWTKMAVLKANKFTETNPQVYYQASLRPYGVTVYHSLRQPYATNPCKDNNGGCEQVCVLSHRTDNDGLGFRCKCTFGFQLDTDERHCIAVQNFLIFSSQVAIRGIPFTLSTQEDVMVPVSGNPSFFVGIDFDAQDSTIFFSDMSKHMIFKQKIDGTGREILAANRVENVESLAFDWISKNLYWTDSHYKSISVMRLADKTRRTVVQYLNNPRSVVVHPFAGYLFFTDWFRPAKIMRAWSDGSHLLPVINTTLGWPNGLAIDWAASRLYWVDAYFDKIEHSTFDGLDRRRLGHIEQMTHPFGLAIFGEHLFFTDWRLGAIIRVRKADGGEMTVIRSGIAYILHLKSYDVNIQTGSNACNQPTHPNGDCSHFCFPVPNFQRVCGCPYGMRLASNHLTCEGDPTNEPPTEQCGLFSFPCKNGRCVPNYYLCDGVDDCHDNSDEQLCGTLNNTCSSSAFTCGHGECIPAHWRCDKRNDCVDGSDEHNCPTHAPASCLDTQYTCDNHQCISKNWVCDTDNDCGDGSDEKNCNSTETCQPSQFNCPNHRCIDLSFVCDGDKDCVDGSDEVGCVLNCTASQFKCASGDKCIGVTNRCDGVFDCSDNSDEAGCPTRPPGMCHSDEFQCQEDGICIPNFWECDGHPDCLYGSDEHNACVPKTCPSSYFHCDNGNCIHRAWLCDRDNDCGDMSDEKDCPTQPFRCPSWQWQCLGHNICVNLSVVCDGIFDCPNGTDESPLCNGNSCSDFNGGCTHECVQEPFGAKCLCPLGFLLANDSKTCEDIDECDILGSCSQHCYNMRGSFRCSCDTGYMLESDGRTCKVTASESLLLLVASQNKIIADSVTSQVHNIYSLVENGSYIVAVDFDSISGRIFWSDATQGKTWSAFQNGTDRRVVFDSSIILTETIAIDWVGRNLYWTDYALETIEVSKIDGSHRTVLISKNLTNPRGLALDPRMNEHLLFWSDWGHHPRIERASMDGSMRTVIVQDKIFWPCGLTIDYPNRLLYFMDSYLDYMDFCDYNGHHRRQVIASDLIIRHPYALTLFEDSVYWTDRATRRVMRANKWHGGNQSVVMYNIQWPLGIVAVHPSKQPNSVNPCAFSRCSHLCLLSSQGPHFYSCVCPSGWSLSPDLLNCLRDDQPFLITVRQHIIFGISLNPEVKSNDAMVPIAGIQNGLDVEFDDAEQYIYWVENPGEIHRVKTDGTNRTVFASISMVGPSMNLALDWISRNLYSTNPRTQSIEVLTLHGDIRYRKTLIANDGTALGVGFPIGITVDPARGKLYWSDQGTDSGVPAKIASANMDGTSVKTLFTGNLEHLECVTLDIEEQKLYWAVTGRGVIERGNVDGTDRMILVHQLSHPWGIAVHDSFLYYTDEQYEVIERVDKATGANKIVLRDNVPNLRGLQVYHRRNAAESSNGCSNNMNACQQICLPVPGGLFSCACATGFKLNPDNRSCSPYNSFIVVSMLSAIRGFSLELSDHSETMVPVAGQGRNALHVDVDVSSGFIYWCDFSSSVASDNAIRRIKPDGSSLMNIVTHGIGENGVRGIAVDWVAGNLYFTNAFVSETLIEVLRINTTYRRVLLKVTVDMPRHIVVDPKNRYLFWADYGQRPKIERSFLDCTNRTVLVSEGIVTPRGLAVDRSDGYVYWVDDSLDIIARIRINGENSEVIRYGSRYPTPYGITVFENSIIWVDRNLKKIFQASKEPENTEPPTVIRDNINWLRDVTIFDKQVQPRSPAEVNNNPCLENNGGCSHLCFALPGLHTPKCDCAFGTLQSDGKNCAISTENFLIFALSNSLRSLHLDPENHSPPFQTINVERTVMSLDYDSVSDRIYFTQNLASGVGQISYATLSSGIHTPTVIASGIGTADGIAFDWITRRIYYSDYLNQMINSMAEDGSNRTVIARVPKPRAIVLDPCQGYLYWADWDTHAKIERATLGGNFRVPIVNSSLVMPSGLTLDYEEDLLYWVDASLQRIERSTLTGVDREVIVNAAVHAFGLTLYGQYIYWTDLYTQRIYRANKYDGSGQIAMTTNLLSQPRGINTVVKNQKQQCNNPCEQFNGGCSHICAPGPNGAECQCPHEGNWYLANNRKHCIVDNGERCGASSFTCSNGRCISEEWKCDNDNDCGDGSDEMESVCALHTCSPTAFTCANGRCVQYSYRCDYYNDCGDGSDEAGCLFRDCNATTEFMCNNRRCIPREFICNGVDNCHDNNTSDEKNCPDRTCQSGYTKCHNSNICIPRVYLCDGDNDCGDNSDENPTYCTTHTCSSSEFQCASGRCIPQHWYCDQETDCFDASDEPASCGHSERTCLADEFKCDGGRCIPSEWICDGDNDCGDMSDEDKRHQCQNQNCSDSEFLCVNDRPPDRRCIPQSWVCDGDVDCTDGYDENQNCTRRTCSENEFTCGYGLCIPKIFRCDRHNDCGDYSDERGCLYQTCQQNQFTCQNGRCISKTFVCDEDNDCGDGSDELMHLCHTPEPTCPPHEFKCDNGRCIEMMKLCNHLDDCLDNSDEKGCGINECHDPSISGCDHNCTDTLTSFYCSCRPGYKLMSDKRTCVDIDECTEMPFVCSQKCENVIGSYICKCAPGYLREPDGKTCRQNSNIEPYLIFSNRYYLRNLTIDGYFYSLILEGLDNVVALDFDRVEKRLYWIDTQRQVIERMFLNKTNKETIINHRLPAAESLAVDWVSRKLYWLDARLDGLFVSDLNGGHRRMLAQHCVDANNTFCFDNPRGLALHPQYGYLYWADWGHRAYIGRVGMDGTNKSVIISTKLEWPNGITIDYTNDLLYWADAHLGYIEYSDLEGHHRHTVYDGALPHPFAITIFEDTIYWTDWNTRTVEKGNKYDGSNRQTLVNTTHRPFDIHVYHPYRQPIVSNPCGTNNGGCSHLCLIKPGGKGFTCECPDDFRTLQLSGSTYCMPMCSSTQFLCANNEKCIPIWWKCDGQKDCSDGSDELALCPQRFCRLGQFQCSDGNCTSPQTLCNAHQNCPDGSDEDRLLCENHHCDSNEWQCANKRCIPESWQCDTFNDCEDNSDEDSSHCASRTCRPGQFRCANGRCIPQAWKCDVDNDCGDHSDEPIEECMSSAHLCDNFTEFSCKTNYRCIPKWAVCNGVDDCRDNSDEQGCEERTCHPVGDFRCKNHHCIPLRWQCDGQNDCGDNSDEENCAPRECTESEFRCVNQQCIPSRWICDHYNDCGDNSDERDCEMRTCHPEYFQCTSGHCVHSELKCDGSADCLDASDEADCPTRFPDGAYCQATMFECKNHVCIPPYWKCDGDDDCGDGSDEELHLCLDVPCNSPNRFRCDNNRCIYSHEVCNGVDDCGDGTDETEEHCRKPTPKPCTEYEYKCGNGHCIPHDNVCDDADDCGDWSDELGCNKGKERTCAENICEQNCTQLNEGGFICSCTAGFETNVFDRTSCLDINECEQFGTCPQHCRNTKGSYECVCADGFTSMSDRPGKRCAAEGSSPLLLLPDNVRIRKYNLSSERFSEYLQDEEYIQAVDYDWDPKDIGLSVVYYTVRGEGSRFGAIKRAYIPNFESGRNNLVQEVDLKLKYVMQPDGIAVDWVGRHIYWSDVKNKRIEVAKLDGRYRKWLISTDLDQPAAIAVNPKLGLMFWTDWGKEPKIESAWMNGEDRNILVFEDLGWPTGLSIDYLNNDRIYWSDFKEDVIETIKYDGTDRRVIAKEAMNPYSLDIFEDQLYWISKEKGEVWKQNKFGQGKKEKTLVVNPWLTQVRIFHQLRYNKSVPNLCKQICSHLCLLRPGGYSCACPQGSSFIEGSTTECDAAIELPINLPPPCRCMHGGNCYFDETDLPKCKCPSGYTGKYCEMAFSKGISPGTTAVAVLLTILLIVVIGALAIAGFFHYRRTGSLLPALPKLPSLSSLVKPSENGNGVTFRSGADLNMDIGVSGFGPETAIDRSMAMSEDFVMEMGKQPIIFENPMYSARDSAVKVVQPIQVTVSENVDNKNYGSPINPSEIVPETNPTSPAADGTQVTKWNLFKRKSKQTTNFENPIYAQMENEQKESVAATPPPSPSLPAKPKPPSRRDPTPTYSATEDTFKDTANLVKEDSEV

230 NP_000356.1 NP_065147.1

>NP_000356.1

MAPSRKFFVGGNWKMNGRKQSLGELIGTLNAAKVPADTEVVCAPPTAYIDFARQKLDPKIAVAAQNCYKVTNGAFTGEISPGMIKDCGATWVVLGHSERRHVFGESDELIGQKVAHALAEGLGVIACIGEKLDEREAGITEKVVFEQTKVIADNVKDWSKVVLAYEPVWAIGTGKTATPQQAQEVHEKLRGWLKSNVSDAVAQSTRIIYGGSVTGATCKELASQPDVDGFLVGGASLKPEFVDIINAKQ

>NP_065147.1

MKLKDTKSRPKQSSCGKFQTKGIKVVGKWKEVKIDPNMFADGQMDDLVCFEELTDYQLVSPAKNPSSLFSKEAPKRKAQAVSEEEEEEEGKSSSPKKKIKLKKSKNVATEGTSTQKEFEVKDPELEAQGDDMVCDDPEAGEMTSENLVQTAPKKKKNKGKKGLEPSQSTAAKVPKKAKTWIPEVHDQKADVSAWKDLFVPRPVLRALSFLGFSAPTPIQALTLAPAIRDKLDILGAAETGSGKTLAFAIPMIHAVLQWQKRNAAPPPSNTEAPPGETRTEAGAETRSPGKAEAESDALPDDTVIESEALPSDIAAEARAKTGGTVSDQALLFGDDDAGEGPSSLIREKPVPKQNENEEENLDKEQTGNLKQELDDKSATCKAYPKRPLLGLVLTPTRELAVQVKQHIDAVARFTGIKTAILVGGMSTQKQQRMLNRRPEIVVATPGRLWELIKEKHYHLRNLRQLRCLVVDEADRMVEKGHFAELSQLLEMLNDSQYNPKRQTLVFSATLTLVHQAPARILHKKHTKKMDKTAKLDLLMQKIGMRGKPKVIDLTRNEATVETLTETKIHCETDEKDFYLYYFLMQYPGRSLVFANSISCIKRLSGLLKVLDIMPLTLHACMHQKQRLRNLEQFARLEDCVLLATDVAARGLDIPKVQHVIHYQVPRTSEIYVHRSGRTARATNEGLSLMLIGPEDVINFKKIYKTLKKDEDIPLFPVQTKYMDVVKERIRLARQIEKSEYRNFQACLHNSWIEQAAAALEIELEEDMYKGGKADQQEERRRQKQMKVLKKELRHLLSQPLFTESQKTKYPTQSGKPPLLVSAPSKSESALSCLSKQKKKKTKKPKEPQPEQPQPSTSAN

231 NP_056075.1 NP_003541.2

>NP_056075.1

MAHAGGGSGGSGAGGPAGRGLSGARWGRSGSAGHEKLPVHVEDALTYLDQVKIRFGSDPATYNGFLEIMKEFKSQSIDTPGVIRRVSQLFHEHPDLIVGFNAFLPLGYRIDIPKNGKLNIQSPLTSQENSHNHGDGAEDFKQQVPYKEDKPQVPLESDSVEFNNAISYVNKIKTRFLDHPEIYRSFLEILHTYQKEQLNTRGRPFRGMSEEEVFTEVANLFRGQEDLLSEFGQFLPEAKRSLFTGNGPCEMHSVQKNEHDKTPEHSRKRSRPSLLRPVSAPAKKKMKLRGTKDLSIAAVGKYGTLQEFSFFDKVRRVLKSQEVYENFLRCIALFNQELVSGSELLQLVSPFLGKFPELFAQFKSFLGVKELSFAPPMSDRSGDGISREIDYASCKRIGSSYRALPKTYQQPKCSGRTAICKELDHWTLLQGSWTDDYCMSKFKNTCWIPGYSAGVLNDTWVSFPSWSEDSTFVSSKKTPYEEQLHRCEDERFELDVVLETNLATIRVLESVQKKLSRMAPEDQEKFRLDDSLGGTSEVIQRRAIYRIYGDKAPEIIESLKKNPVTAVPVVLKRLKAKEEEWREAQQGFNKIWREQYEKAYLKSLDHQAVNFKQNDTKALRSKSLLNEIESVYDEHQEQHSEGRSAPSSEPHLIFVYEDRQILEDAAALISYYVKRQPAIQKEDQGTIHQLLHQFVPSLFFSQQLDLGASEESADEDRDSPQGQTTDPSERKKPAPGPHSSPPEEKGAFGDAPATEQPPLPPPAPHKPLDDVYSLFFANNNWYFFLRLHQTLCSRLLKIYRQAQKQLLEYRTEKEREKLLCEGRREKGSDPAMELRLKQPSEVELEEYYPAFLDMVRSLLEGSIDPTQYEDTLREMFTIHAYVGFTMDKLVQNIARQLHHLVSDDVCLKVVELYLNEKKRGAAGGNLSSRCVRAARETSYQWKAERCMADENCFKVMFLQRKGQVIMTIELLDTEEAQTEDPVEVQHLARYVEQYVGTEGASSSPTEGFLLKPVFLQRNLKKFRRRWQSEQARALRGEARSSWKRLVGVESACDVDCRFKLSTHKMVFIVNSEDYMYRRGTLCRAKQVQPLVLLRHHQHFEEWHSRWLEDNVTVEAASLVQDWLMGEEDEDMVPCKTLCETVHVHGLPVTRYRVQYSRRPASP

>NP_003541.2

MEDLGENTMVLSTLRSLNNFISQRVEGGSGLDISTSAPGSLQMQYQQSMQLEERAEQIRSKSHLIQVEREKMQMELSHKRARVELERAASTSARNYEREVDRNQELLTRIRQLQEREAGAEEKMQEQLERNRQCQQNLDAASKRLREKEDSLAQAGETINALKGRISELQWSVMDQEMRVKRLESEKQELQEQLDLQHKKCQEANQKIQELQASQEARADHEQQIKDLEQKLSLQEQDAAIVKNMKSELVRLPRLERELKQLREESAHLREMRETNGLLQEELEGLQRKLGRQEKMQETLVGLELENERLLAKLQSWERLDQTMGLSIRTPEDLSRFVVELQQRELALKDKNSAVTSSARGLEKARQQLQEELRQVSGQLLEERKKRETHEALARRLQKRVLLLTKERDGMRAILGSYDSELTPAEYSPQLTRRMREAEDMVQKVHSHSAEMEAQLSQALEELGGQKQRADMLEMELKMLKSQSSSAEQSFLFSREEADTLRLKVEELEGERSRLEEEKRMLEAQLERRALQGDYDQSRTKVLHMSLNPTSVARQRLREDHSQLQAECERLRGLLRAMERGGTVPADLEAAAASLPSSKEVAELKKQVESAELKNQRLKEVFQTKIQEFRKACYTLTGYQIDITTENQYRLTSLYAEHPGDCLIFKATSPSGSKMQLLETEFSHTVGELIEVHLRRQDSIPAFLSSLTLELFSRQTVA

232 NP_001895.1 NP_001894.2

>NP_001895.1

MATQADLMELDMAMEPDRKAAVSHWQQQSYLDSGIHSGATTTAPSLSGKGNPEEEDVDTSQVLYEWEQGFSQSFTQEQVADIDGQYAMTRAQRVRAAMFPETLDEGMQIPSTQFDAAHPTNVQRLAEPSQMLKHAVVNLINYQDDAELATRAIPELTKLLNDEDQVVVNKAAVMVHQLSKKEASRHAIMRSPQMVSAIVRTMQNTNDVETARCTAGTLHNLSHHREGLLAIFKSGGIPALVKMLGSPVDSVLFYAITTLHNLLLHQEGAKMAVRLAGGLQKMVALLNKTNVKFLAITTDCLQILAYGNQESKLIILASGGPQALVNIMRTYTYEKLLWTTSRVLKVLSVCSSNKPAIVEAGGMQALGLHLTDPSQRLVQNCLWTLRNLSDAATKQEGMEGLLGTLVQLLGSDDINVVTCAAGILSNLTCNNYKNKMMVCQVGGIEALVRTVLRAGDREDITEPAICALRHLTSRHQEAEMAQNAVRLHYGLPVVVKLLHPPSHWPLIKATVGLIRNLALCPANHAPLREQGAIPRLVQLLVRAHQDTQRRTSMGGTQQQFVEGVRMEEIVEGCTGALHILARDVHNRIVIRGLNTIPLFVQLLYSPIENIQRVAAGVLCELAQDKEAAEAIEAEGATAPLTELLHSRNEGVATYAAAVLFRMSEDKPQDYKKRLSVELTSSLFRTEPMAWNETADLGLDIGAQGEPLGYRQDDPSYRSFHSGGYGQDALGMDPMMEHEMGGHHPGADYPVDGLPDLGHAQDLMDGLPPGDSNQLAWFDTDL

>NP_001894.2

MTAVHAGNINFKWDPKSLEIRTLAVERLLEPLVTQVTTLVNTNSKGPSNKKRGRSKKAHVLAASVEQATENFLEKGDKIAKESQFLKEELVAAVEDVRKQGDLMKAAAGEFADDPCSSVKRGNMVRAARALLSAVTRLLILADMADVYKLLVQLKVVEDGILKLRNAGNEQDLGIQYKALKPEVDKLNIMAAKRQQELKDVGHRDQMAAARGILQKNVPILYTASQACLQHPDVAAYKANRDLIYKQLQQAVTGISNAAQATASDDASQHQGGGGGELAYALNNFDKQIIVDPLSFSEERFRPSLEERLESIISGAALMADSSCTRDDRRERIVAECNAVRQALQDLLSEYMGNAGRKERSDALNSAIDKMTKKTRDLRRQLRKAVMDHVSDSFLETNVPLLVLIEAAKNGNEKEVKEYAQVFREHANKLIEVANLACSISNNEEGVKLVRMSASQLEALCPQVINAALALAAKPQSKLAQENMDLFKEQWEKQVRVLTDAVDDITSIDDFLAVSENHILEDVNKCVIALQEKDVDGLDRTAGAIRGRAARVIHVVTSEMDNYEPGVYTEKVLEATKLLSNTVMPRFTEQVEAAVEALSSDPAQPMDENEFIDASRLVYDGIRDIRKAVLMIRTPEELDDSDFETEDFDVRSRTSVQTEDDQLIAGQSARAIMAQLPQEQKAKIAEQVASFQEEKSKLDAEVSKWDDSGNDIIVLAKQMCMIMMEMTDFTRGKGPLKNTSDVISAAKKIAEAGSRMDKLGRTIADHCPDSACKQDLLAYLQRIALYCHQLNICSKVKAEVQNLGGELVVSGVDSAMSLIQAAKNLMNAVVQTVKASYVASTKYQKSQGMASLNLPAVSWKMKAPEKKPLVKREKQDETQTKIKRASQKKHVNPVQALSEFKAMDSI

233 NP_002583.1 NP_002907.1

>NP_002583.1

MFEARLVQGSILKKVLEALKDLINEACWDISSSGVNLQSMDSSHVSLVQLTLRSEGFDTYRCDRNLAMGVNLTSMSKILKCAGNEDIITLRAEDNADTLALVFEAPNQEKVSDYEMKLMDLDVEQLGIPEQEYSCVVKMPSGEFARICRDLSHIGDAVVISCAKDGVKFSASGELGNGNIKLSQTSNVDKEEEAVTIEMNEPVQLTFALRYLNFFTKATPLSSTVTLSMSADVPLVVEYKIADMGHLKYYLAPKIEDEEGS

>NP_002907.1

MQAFLKGTSISTKPPLTKDRGVAASAGSSGENKKAKPVPWVEKYRPKCVDEVAFQEEVVAVLKKSLEGADLPNLLFYGPPGTGKTSTILAAARELFGPELFRLRVLELNASDERGIQVVREKVKNFAQLTVSGSRSDGKPCPPFKIVILDEADSMTSAAQAALRRTMEKESKTTRFCLICNYVSRIIEPLTSRCSKFRFKPLSDKIQQQRLLDIAKKENVKISDEGIAYLVKVSEGDLRKAITFLQSATRLTGGKEITEKVITDIAGVIPAEKIDGVFAACQSGSFDKLEAVVKDLIDEGHAATQLVNQLHDVVVENNLSDKQKSIITEKLAEVDKCLADGADEHLQLISLCATVMQQLSQNC

234 NP_001744.2 NP_005624.2

>NP_001744.2

MSGGKYVDSEGHLYTVPIREQGNIYKPNNKAMADELSEKQVYDAHTKEIDLVNRDPKHLNDDVVKIDFEDVIAEPEGTHSFDGIWKASFTTFTVTKYWFYRLLSALFGIPMALIWGIYFAILSFLHIWAVVPCIKSFLIEIQCISRVYSIYVHTVCDPLFEAVGKIFSNVRINLQKEI

>NP_005624.2

MQAQQLPYEFFSEENAPKWRGLLVPALKKVQGQVHPTLESNDDALQYVEELILQLLNMLCQAQPRSASDVEERVQKSFPHPIDKWAIADAQSAIEKRKRRNPLSLPVEKIHPLLKEVLGYKIDHQVSVYIVAVLEYISADILKLVGNYVRNIRHYEITKQDIKVAMCADKVLMDMFHQDVEDINILSLTDEEPSTSGEQTYYDLVKAFMAEIRQYIRELNLIIKVFREPFVSNSKLFSANDVENIFSRIVDIHELSVKLLGHIEDTVEMTDEGSPHPLVGSCFEDLAEELAFDPYESYARDILRPGFHDRFLSQLSKPGAALYLQSIGEGFKEAVQYVLPRLLLAPVYHCLHYFELLKQLEEKSEDQEDKECLKQAITALLNVQSGMEKICSKSLAKRRLSESACRFYSQQMKGKQLAIKKMNEIQKNIDGWEGKDIGQCCNEFIMEGTLTRVGAKHERHIFLFDGLMICCKSNHGQPRLPGASNAEYRLKEKFFMRKVQINDKDDTNEYKHAFEIILKDENSVIFSAKSAEEKNNWMAALISLQYRSTLERMLDVTMLQEEKEEQMRLPSADVYRFAEPDSEENIIFEENMQPKAGIPIIKAGTVIKLIERLTYHMYADPNFVRTFLTTYRSFCKPQELLSLIIERFEIPEPEPTEADRIAIENGDQPLSAELKRFRKEYIQPVQLRVLNVCRHWVEHHFYDFERDAYLLQRMEEFIGTVRGKAMKKWVESITKIIQRKKIARDNGPGHNITFQSSPPTVEWHISRPGHIETFDLLTLHPIEIARQLTLLESDLYRAVQPSELVGSVWTKEDKEINSPNLLKMIRHTTNLTLWFEKCIVETENLEERVAVVSRIIEILQVFQELNNFNGVLEVVSAMNSSPVYRLDHTFEQIPSRQKKILEEAHELSEDHYKKYLAKLRSINPPCVPFFGIYLTNILKTEEGNPEVLKRHGKELINFSKRRKVAEITGEIQQYQNQPYCLRVESDIKRFFENLNPMGNSMEKEFTDYLFNKSLEIEPRNPKPLPRFPKKYSYPLKSPGVRPSNPRPGTMRHPTPLQQEPRKISYSRIPESETESTASAPNSPRTPLTPPPASGASSTTDVCSVFDSDHSSPFHSSNDTVFIQVTLPHGPRSASVSSISLTKGTDEVPVPPPVPPRRRPESAPAESSPSKIMSKHLDSPPAIPPRQPTSKAYSPRYSISDRTSISDPPESPPLLPPREPVRTPDVFSSSPLHLQPPPLGKKSDHGNAFFPNSPSPFTPPPPQTPSPHGTRRHLPSPPLTQEVDLHSIAGPPVPPRQSTSQHIPKLPPKTYKREHTHPSMHRDGPPLLENAHSS

235 NP_002125.3 NP_060842.3

>NP_002125.3

MSAEVETSEGVDESEKKNSGALEKENQMRMADLSELLKEGTKEAHDRAENTQFVKDFLKGNIKKELFKLATTALYFTYSALEEEMERNKDHPAFAPLYFPMELHRKEALTKDMEYFFGENWEEQVQCPKAAQKYVERIHYIGQNEPELLVAHAYTRYMGDLSGGQVLKKVAQRALKLPSTGEGTQFYLFENVDNAQQFKQLYRARMNALDLNMKTKERIVEEANKAFEYNMQIFNELDQAGSTLARETLEDGFPVHDGKGDMRKCPFYAAEQDKGALEGSSCPFRTAMAVLRKPSLQFILAAGVALAAGLLAWYYM

>NP_060842.3

MSNNLRRVFLKPAEENSGNASRCVSGCMYQVVQTIGSDGKNLLQLLPIPKSSGNLIPLVQSSVMSDALKGNTGKPVQVTFQTQISSSSTSASVQLPIFQPASSSNYFLTRTVDTSEKGRVTSVGTGNFSSSVSKVQSHGVKIDGLTMQTFAVPPSTQKDSSFIVVNTQSLPVTVKSPVLPSGHHLQIPAHAEVKSVPASSLPPSVQQKILATATTSTSGMVEASQMPTVIYVSPVNTVKNVVTKNFQNIYPKPVTEIAKPVILNTTQIPKNVATETQLKGGQHSQAAPVKWIFQDNLQPFTPSLVPVKSSNNVASKILKTFVDRKNLGDNTINMPPLSTIDPSGTRSKNMPIKDNALVMFNGKVYLLAKKGTDVLPSQIDQQNSVSPDTPVRKDTLQTVSSSPVTEISREVVNIVLAKSKSSQMETKSLSNTQLASMANLRAEKNKVEKPSPSTTNPHMNQSSNYLKQSKTLFTNPIFPVGFSTGHNAPRKVTAVIYARKGSVLQSIEKISSSVDATTVTSQQCVFRDQEPKIHNEMASTSDKGAQGRNDKKDSQGRSNKALHLKSDAEFKKIFGLTKDLRVCLTRIPDHLTSGEGFDSFSSLVKSGTYKETEFMVKEGERKQQNFDKKRKAKTNKKMDHIKKRKTENAYNAIINGEANVTGSQLLSSILPTSDVSQHNILTSHSKTRQEKRTEMEYYTHEKQEKGTLNSNAAYEQSHFFNKNYTEDIFPVTPPELEETIRDEKIRRLKQVLREKEAALEEMRKKMHQK

236 NP_060372.2 NP_003874.2

>NP_060372.2

MAATAAEAVASGSGEPREEAGALGPAWDESQLRSYSFPTRPIPRLSQSDPRAEELIENEEPVVLTDTNLVYPALKWDLEYLQENIGNGDFSVYSASTHKFLYYDEKKMANFQNFKPRSNREEMKFHEFVEKLQDIQQRGGEERLYLQQTLNDTVGRKIVMDFLGFNWNWINKQQGKRGWGQLTSNLLLIGMEGNVTPAHYDEQQNFFAQIKGYKRCILFPPDQFECLYPYPVHHPCDRQSQVDFDNPDYERFPNFQNVVGYETVVGPGDVLYIPMYWWHHIESLLNGGITITVNFWYKGAPTPKRIEYPLKAHQKVAIMRNIEKMLGEALGNPQEVGPLLNTMIKGRYN

>NP_003874.2

MAKTVAYFYDPDVGNFHYGAGHPMKPHRLALTHSLVLHYGLYKKMIVFKPYQASQHDMCRFHSEDYIDFLQRVSPTNMQGFTKSLNAFNVGDDCPVFPGLFEFCSRYTGASLQGATQLNNKICDIAINWAGGLHHAKKFEASGFCYVNDIVIGILELLKYHPRVLYIDIDIHHGDGVQEAFYLTDRVMTVSFHKYGNYFFPGTGDMYEVGAESGRYYCLNVPLRDGIDDQSYKHLFQPVINQVVDFYQPTCIVLQCGADSLGCDRLGCFNLSIRGHGECVEYVKSFNIPLLVLGGGGYTVRNVARCWTYETSLLVEEAISEELPYSEYFEYFAPDFTLHPDVSTRIENQNSRQYLDQIRQTIFENLKMLNHAPSVQIHDVPADLLTYDRTDEADAEERGPEENYSRPEAPNEFYDGDHDNDKESDVEI

237 NP_000917.3 NP_000116.2

>NP_000917.3

MTELKAKGPRAPHVAGGPPSPEVGSPLLCRPAAGPFPGSQTSDTLPEVSAIPISLDGLLFPRPCQGQDPSDEKTQDQQSLSDVEGAYSRAEATRGAGGSSSSPPEKDSGLLDSVLDTLLAPSGPGQSQPSPPACEVTSSWCLFGPELPEDPPAAPATQRVLSPLMSRSGCKVGDSSGTAAAHKVLPRGLSPARQLLLPASESPHWSGAPVKPSPQAAAVEVEEEDGSESEESAGPLLKGKPRALGGAAAGGGAAAVPPGAAAGGVALVPKEDSRFSAPRVALVEQDAPMAPGRSPLATTVMDFIHVPILPLNHALLAARTRQLLEDESYDGGAGAASAFAPPRSSPCASSTPVAVGDFPDCAYPPDAEPKDDAYPLYSDFQPPALKIKEEEEGAEASARSPRSYLVAGANPAAFPDFPLGPPPPLPPRATPSRPGEAAVTAAPASASVSSASSSGSTLECILYKAEGAPPQQGPFAPPPCKAPGASGCLLPRDGLPSTSASAAAAGAAPALYPALGLNGLPQLGYQAAVLKEGLPQVYPPYLNYLRPDSEASQSPQYSFESLPQKICLICGDEASGCHYGVLTCGSCKVFFKRAMEGQHNYLCAGRNDCIVDKIRRKNCPACRLRKCCQAGMVLGGRKFKKFNKVRVVRALDAVALPQPVGVPNESQALSQRFTFSPGQDIQLIPPLINLLMSIEPDVIYAGHDNTKPDTSSSLLTSLNQLGERQLLSVVKWSKSLPGFRNLHIDDQITLIQYSWMSLMVFGLGWRSYKHVSGQMLYFAPDLILNEQRMKESSFYSLCLTMWQIPQEFVKLQVSQEEFLCMKVLLLLNTIPLEGLRSQTQFEEMRSSYIRELIKAIGLRQKGVVSSSQRFYQLTKLLDNLHDLVKQLHLYCLNTFIQSRALSVEFPEMMSEVIAAQLPKILAGMVKPLLFHKK

>NP_000116.2

MTMTLHTKASGMALLHQIQGNELEPLNRPQLKIPLERPLGEVYLDSSKPAVYNYPEGAAYEFNAAAAANAQVYGQTGLPYGPGSEAAAFGSNGLGGFPPLNSVSPSPLMLLHPPPQLSPFLQPHGQQVPYYLENEPSGYTVREAGPPAFYRPNSDNRRQGGRERLASTNDKGSMAMESAKETRYCAVCNDYASGYHYGVWSCEGCKAFFKRSIQGHNDYMCPATNQCTIDKNRRKSCQACRLRKCYEVGMMKGGIRKDRRGGRMLKHKRQRDDGEGRGEVGSAGDMRAANLWPSPLMIKRSKKNSLALSLTADQMVSALLDAEPPILYSEYDPTRPFSEASMMGLLTNLADRELVHMINWAKRVPGFVDLTLHDQVHLLECAWLEILMIGLVWRSMEHPGKLLFAPNLLLDRNQGKCVEGMVEIFDMLLATSSRFRMMNLQGEEFVCLKSIILLNSGVYTFLSSTLKSLEEKDHIHRVLDKITDTLIHLMAKAGLTLQQQHQRLAQLLLILSHIRHMSNKGMEHLYSMKCKNVVPLYDLLLEMLDAHRLHAPTSRGGASVEETDQSHLATAGSTSSHSLQKYYITGEAEGFPATV

238 NP_065147.1 NP_001078923.1

>NP_065147.1

MKLKDTKSRPKQSSCGKFQTKGIKVVGKWKEVKIDPNMFADGQMDDLVCFEELTDYQLVSPAKNPSSLFSKEAPKRKAQAVSEEEEEEEGKSSSPKKKIKLKKSKNVATEGTSTQKEFEVKDPELEAQGDDMVCDDPEAGEMTSENLVQTAPKKKKNKGKKGLEPSQSTAAKVPKKAKTWIPEVHDQKADVSAWKDLFVPRPVLRALSFLGFSAPTPIQALTLAPAIRDKLDILGAAETGSGKTLAFAIPMIHAVLQWQKRNAAPPPSNTEAPPGETRTEAGAETRSPGKAEAESDALPDDTVIESEALPSDIAAEARAKTGGTVSDQALLFGDDDAGEGPSSLIREKPVPKQNENEEENLDKEQTGNLKQELDDKSATCKAYPKRPLLGLVLTPTRELAVQVKQHIDAVARFTGIKTAILVGGMSTQKQQRMLNRRPEIVVATPGRLWELIKEKHYHLRNLRQLRCLVVDEADRMVEKGHFAELSQLLEMLNDSQYNPKRQTLVFSATLTLVHQAPARILHKKHTKKMDKTAKLDLLMQKIGMRGKPKVIDLTRNEATVETLTETKIHCETDEKDFYLYYFLMQYPGRSLVFANSISCIKRLSGLLKVLDIMPLTLHACMHQKQRLRNLEQFARLEDCVLLATDVAARGLDIPKVQHVIHYQVPRTSEIYVHRSGRTARATNEGLSLMLIGPEDVINFKKIYKTLKKDEDIPLFPVQTKYMDVVKERIRLARQIEKSEYRNFQACLHNSWIEQAAAALEIELEEDMYKGGKADQQEERRRQKQMKVLKKELRHLLSQPLFTESQKTKYPTQSGKPPLLVSAPSKSESALSCLSKQKKKKTKKPKEPQPEQPQPSTSAN

>NP_001078923.1

MSRKGPRAEVCADCSAPDPGWASISRGVLVCDECCSVHRSLGRHISIVKHLRHSAWPPTLLQMVHTLASNGANSIWEHSLLDPAQVQSGRRKANPQDKVHPIKSEFIRAKYQMLAFVHKLPCRDDDGVTAKDLSKQLHSSVRTGNLETCLRLLSLGAQANFFHPEKGTTPLHVAAKAGQTLQAELLVVYGADPGSPDVNGRTPIDYARQAGHHELAERLVECQYELTDRLAFYLCGRKPDHKNGHYIIPQMADRSRQKCMSQSLDLSELAKAAKKKLQALSNRLFEELAMDVYDEVDRRENDAVWLATQNHSTLVTERSAVPFLPVNPEYSATRNQGRQKLARFNAREFATLIIDILSEAKRRQQGKSLSSPTDNLELSLRSQSDLDDQHDYDSVASDEDTDQEPLRSTGATRSNRARSMDSSDLSDGAVTLQEYLELKKALATSEAKVQQLMKVNSSLSDELRRLQREIHKLQAENLQLRQPPGPVPTPPLPSERAEHTPMAPGGSTHRRDRQAFSMYEPGSALKPFGGPPGDELTTRLQPFHSTELEDDAIYSVHVPAGLYRIRKGVSASAVPFTPSSPLLSCSQEGSRHTSKLSRHGSGADSDYENTQSGDPLLGLEGKRFLELGKEEDFHPELESLDGDLDPGLPSTEDVILKTEQVTKNIQELLRAAQEFKHDSFVPCSEKIHLAVTEMASLFPKRPALEPVRSSLRLLNASAYRLQSECRKTVPPEPGAPVDFQLLTQQVIQCAYDIAKAAKQLVTITTREKKQ

239 NP_001092903.1 NP_001461.1

>NP_001092903.1

MSKKGRSKGEKPEMETDAVQMANEELRAKLTSIQIEFQQEKSKVGKLRERLQEAKLEREQEQRRHTAYISELKAKLHEEKTKELQALREGLIRQHEQEAARTAKIKEGELQRLQATLNVLRDGAADKVKTALLTEAREEARRAFDGERLRLQQEILELKAARKQAEEALSNCMQADKTKAADLRAAYQAHQDEVHRIKRECERDIRRLMDEIKGKDRVILALEKELGVQAGQTQKLLLQKEALDEQLVQVKEAERHHSSPKRELPPGIGDMVELMGVQDQHMDERDVRRFQLKIAELNSVIRKLEDRNTLLADERNELLKRSRETEVQLKPLVEKNKRMNKKNEDLLQSIQRMEEKIKNLTRENVEMKEKLSAQASLKRHTSLNDLSLTRDEQEIEFLRLQVLEQQHVIDDLSLERERLLRSKRHRGKSLKPPKKHVVETFFGFDEESVDSETLSETSYNTDRTDRTPATPEEDLDDATAREEADLRFCQLTREYQALQRAYALLQEQVGGTLDAEREARTREQLQADLLRCQAKIEDLEKLLVEKGQDSKWVEEKQLLIRTNQDLLEKIYRLEMEENQLKNEMQDAKDQNELLEFRVLELEERERRSPAFNLQITTFPENHSSALQLFCHQEGVKDVNVSELMKKLDILGDNGNLRNEEQVAIIQAGTVLALCEKWLKQIEGTEAALTQKMLDLEKEKDLFSRQKGYLEEELDYRKQALDQAYLKIQDLEATLYTALQQEPGRRAGEALSEGQREDLQAAVEKVRRQILRQSREFDSQILRERMELLQQAQQRIRELEDKLEFQKRHLKELEEKFLFLFLFFSLAFILWP

>NP_001461.1

MLLLLLLAPLFLRPPGAGGAQTPNATSEGCQIIHPPWEGGIRYRGLTRDQVKAINFLPVDYEIEYVCRGEREVVGPKVRKCLANGSWTDMDTPSRCVRICSKSYLTLENGKVFLTGGDLPALDGARVDFRCDPDFHLVGSSRSICSQGQWSTPKPHCQVNRTPHSERRAVYIGALFPMSGGWPGGQACQPAVEMALEDVNSRRDILPDYELKLIHHDSKCDPGQATKYLYELLYNDPIKIILMPGCSSVSTLVAEAARMWNLIVLSYGSSSPALSNRQRFPTFFRTHPSATLHNPTRVKLFEKWGWKKIATIQQTTEVFTSTLDDLEERVKEAGIEITFRQSFFSDPAVPVKNLKRQDARIIVGLFYETEARKVFCEVYKERLFGKKYVWFLIGWYADNWFKIYDPSINCTVDEMTEAVEGHITTEIVMLNPANTRSISNMTSQEFVEKLTKRLKRHPEETGGFQEAPLAYDAIWALALALNKTSGGGGRSGVRLEDFNYNNQTITDQIYRAMNSSSFEGVSGHVVFDASGSRMAWTLIEQLQGGSYKKIGYYDSTKDDLSWSKTDKWIGGSPPADQTLVIKTFRFLSQKLFISVSVLSSLGIVLAVVCLSFNIYNSHVRYIQNSQPNLNNLTAVGCSLALAAVFPLGLDGYHIGRNQFPFVCQARLWLLGLGFSLGYGSMFTKIWWVHTVFTKKEEKKEWRKTLEPWKLYATVGLLVGMDVLTLAIWQIVDPLHRTIETFAKEEPKEDIDVSILPQLEHCSSRKMNTWLGIFYGYKGLLLLLGIFLAYETKSVSTEKINDHRAVGMAIYNVAVLCLITAPVTMILSSQQDAAFAFASLAIVFSSYITLVVLFVPKMRRLITRGEWQSEAQDTMKTGSSTNNNEEEKSRLLEKENRELEKIIAEKEERVSELRHQLQSRQQLRSRRHPPTPPEPSGGLPRGPPEPPDRLSCDGSRVHLLYK

240 NP_006753.1 NP_055779.1

>NP_006753.1

MDPRLSTVRQTCCCFNVRIATTALAIYHVIMSVLLFIEHSVEVAHGKASCKLSQMGYLRIADLISSFLLITMLFIISLSLLIGVVKNREKYLLPFLSLQIMDYLLCLLTLLGSYIELPAYLKLASRSRASSSKFPLMTLQLLDFCLSILTLCSSYMEVPTYLNFKSMNHMNYLPSQEDMPHNQFIKMMIIFSIAFITVLIFKVYMFKCVWRCYRLIKCMNSVEEKRNSKMLQKVVLPSYEEALSLPSKTPEGGPAPPPYSEV

>NP_055779.1

MTTSSIRRQMKNIVNNYSEAEIKVREATSNDPWGPSSSLMTEIADLTYNVVAFSEIMSMVWKRLNDHGKNWRHVYKALTLLDYLIKTGSERVAQQCRENIFAIQTLKDFQYIDRDGKDQGINVREKSKQLVALLKDEERLKAERAQALKTKERMAQVATGMGSNQITFGRGSSQPNLSTSHSEQEYGKAGGSPASYHGSPEASLCPQHRTGAPLGQSEELQPLSQRHPFLPHLGLASRPNGDWSQPCLTCDRAARATSPRVSSELEQARPQTSGEEELQLQLALAMSREVAEQEERLRRGDDLRLQMALEESRRDTVKIPKKKEHGSLPQQTTLLDLMDALPSSGPAAQKAEPWGPSASTNQTNPWGGPAAPASTSDPWPSFGTKPAASIDPWGVPTGATAQSVPKNSDPWAASQQPASSAGKRASDAWGAVSTTKPVSVSGSFELFSNLNGTIKDDFSEFDNLRTSKKTAESVTSLPSQNNGTTSPDPFESQPLTVASSKPSSARKTPESFLGPNAALVNLDSLVTRPAPPAQSLNPFLAPGAPATSAPVNPFQVNQPQPLTLNQLRGSPVLGTSTSFGPGPGVESMAVASMTSAAPQPALGATGSSLTPLGPAMMNMVGSVGIPPSAAQATGTTNPFLL

241 NP_001307.2 NP_000974.1

>NP_001307.2

MELSDANLQTLTEYLKKTLDPDPAIRRPAEKFLESVEGNQNYPLLLLTLLEKSQDNVIKVCASVTFKNYIKRNWRIVEDEPNKICEADRVAIKANIVHLMLSSPEQIQKQLSDAISIIGREDFPQKWPDLLTEMVNRFQSGDFHVINGVLRTAHSLFKRYRHEFKSNELWTEIKLVLDAFALPLTNLFKATIELCSTHANDASALRILFSSLILISKLFYSLNFQDLPEFFEDNMETWMNNFHTLLTLDNKLLQTDDEEEAGLLELLKSQICDNAALYAQKYDEEFQRYLPRFVTAIWNLLVTTGQEVKYDLLVSNAIQFLASVCERPHYKNLFEDQNTLTSICEKVIVPNMEFRAADEEAFEDNSEEYIRRDLEGSDIDTRRRAACDLVRGLCKFFEGPVTGIFSGYVNSMLQEYAKNPSVNWKHKDAAIYLVTSLASKAQTQKHGITQANELVNLTEFFVNHILPDLKSANVNEFPVLKADGIKYIMIFRNQVPKEHLLVSIPLLINHLQAESIVVHTYAAHALERLFTMRGPNNATLFTAAEIAPFVEILLTNLFKALTLPGSSENEYIMKAIMRSFSLLQEAIIPYIPTLITQLTQKLLAVSKNPSKPHFNHYMFEAICLSIRITCKANPAAVVNFEEALFLVFTEILQNDVQEFIPYVFQVMSLLLETHKNDIPSSYMALFPHLLQPVLWERTGNIPALVRLLQAFLERGSNTIASAAADKIPGLLGVFQKLIASKANDHQGFYLLNSIIEHMPPESVDQYRKQIFILLFQRLQNSKTTKFIKSFLVFINLYCIKYGALALQEIFDGIQPKMFGMVLEKIIIPEIQKVSGNVEKKICAVGITKLLTECPPMMDTEYTKLWTPLLQSLIGLFELPEDDTIPDEEHFIDIEDTPGYQTAFSQLAFAGKKEHDPVGQMVNNPKIHLAQSLHKLSTACPGRVPSMVSTSLNAEALQYLQGYLQAASVTLL

>NP_000974.1

MAPVKKLVVKGGKKKKQVLKFTLDCTHPVEDGIMDAANFEQFLQERIKVNGKAGNLGGGVVTIERSKSKITVTSEVPFSKRYLKYLTKKYLKKNNLRDWLRVVANSKESYELRYFQINQDEEEEEDED

242 NP_000061.1 NP_061938.2

>NP_000061.1

MPTVISASVAPRTAAEPRSPGPVPHPAQSKATEAGGGNPSGIYSAIISRNFPIIGVKEKTFEQLHKKCLEKKVLYVDPEFPPDETSLFYSQKFPIQFVWKRPPEICENPRFIIDGANRTDICQGELGDCWFLAAIACLTLNQHLLFRVIPHDQSFIENYAGIFHFQFWRYGEWVDVVIDDCLPTYNNQLVFTKSNHRNEFWSALLEKAYAKLHGSYEALKGGNTTEAMEDFTGGVAEFFEIRDAPSDMYKIMKKAIERGSLMGCSIDDGTNMTYGTSPSGLNMGELIARMVRNMDNSLLQDSDLDPRGSDERPTRTIIPVQYETRMACGLVRGHAYSVTGLDEVPFKGEKVKLVRLRNPWGQVEWNGSWSDRWKDWSFVDKDEKARLQHQVTEDGEFWMSYEDFIYHFTKLEICNLTADALQSDKLQTWTVSVNEGRWVRGCSAGGCRNFPDTFWTNPQYRLKLLEEDDDPDDSEVICSFLVALMQKNRRKDRKLGASLFTIGFAIYEVPKEMHGNKQHLQKDFFLYNASKARSKTYINMREVSQRFRLPPSEYVIVPSTYEPHQEGEFILRVFSEKRNLSEEVENTISVDRPVKKKKTKPIIFVSDRANSNKELGVDQESEEGKGKTSPDKQKQSPQPQPGSSDQESEEQQQFRNIFKQIAGDDMEICADELKKVLNTVVNKHKDLKTHGFTLESCRSMIALMDTDGSGKLNLQEFHHLWNKIKAWQKIFKHYDTDQSGTINSYEMRNAVNDAGFHLNNQLYDIITMRYADKHMNIDFDSFICCFVRLEGMFRAFHAFDKDGDGIIKLNVLEWLQLTMYA

>NP_061938.2

MCERAARLCRAGAHRLLREPPQQGRALGGLLRWVGARMGEPRESLAPAAPADPGPASPRGGTAVILDIFRRADKNDDGKLSLEEFQLFFADGVLNEKELEDLFHTIDSDNTNHVDTKELCDYFVDHMGDYEDVLASLETLNHSVLKAMGYTKKVYEGGSNVDQFVTRFLLKETANQIQSLLSSVESAVEAIEEQTSQLRQNHIKPSHSAAQTWCGSPTPASAPNHKLMAMEQGKTLPSATEDAKEEGLEAQISRLAELIGRLESKALWFDLQQRLSDEDGTNMHLQLVRQEMAVCPEQLSEFLDSLRQYLRGTTGVRNCFHITAVRLSDGFTFVIYEFWETEEAWKRHLQSPLCKAFRHVKVDTLSQPEALSRILVPAAWCTVGRD

243 NP_000544.2 NP_006221.1

>NP_000544.2

MSEKKLETTAQQRKCPEWMNVQNKRCAVEERKACVRKSVFEDDLPFLEFTGSIVYSYDASDCSFLSEDISMSLSDGDVVGFDMEWPPLYNRGKLGKVALIQLCVSESKCYLFHVSSMSVFPQGLKMLLENKAVKKAGVGIEGDQWKLLRDFDIKLKNFVELTDVANKKLKCTETWSLNSLVKHLLGKQLLKDKSIRCSNWSKFPLTEDQKLYAATDAYAGFIIYRNLEILDDTVQRFAINKEEEILLSDMNKQLTSISEEVMDLAKHLPHAFSKLENPRRVSILLKDISENLYSLRRMIIGSTNIETELRPSNNLNLLSFEDSTTGGVQQKQIREHEVLIHVEDETWDPTLDHLAKHDGEDVLGNKVERKEDGFEDGVEDNKLKENMERACLMSLDITEHELQILEQQSQEEYLSDIAYKSTEHLSPNDNENDTSYVIESDEDLEMEMLKHLSPNDNENDTSYVIESDEDLEMEMLKSLENLNSGTVEPTHSKCLKMERNLGLPTKEEEEDDENEANEGEEDDDKDFLWPAPNEEQVTCLKMYFGHSSFKPVQWKVIHSVLEERRDNVAVMATGYGKSLCFQYPPVYVGKIGLVISPLISLMEDQVLQLKMSNIPACFLGSAQSENVLTDIKLGKYRIVYVTPEYCSGNMGLLQQLEADIGITLIAVDEAHCISEWGHDFRDSFRKLGSLKTALPMVPIVALTATASSSIREDIVRCLNLRNPQITCTGFDRPNLYLEVRRKTGNILQDLQPFLVKTSSHWEFEGPTIIYCPSRKMTQQVTGELRKLNLSCGTYHAGMSFSTRKDIHHRFVRDEIQCVIATIAFGMGINKADIRQVIHYGAPKDMESYYQEIGRAGRDGLQSSCHVLWAPADINLNRHLLTEIRNEKFRLYKLKMMAKMEKYLHSSRCRRQIILSHFEDKQVQKASLGIMGTEKCCDNCRSRLDHCYSMDDSEDTSWDFGPQAFKLLSAVDILGEKFGIGLPILFLRGSNSQRLADQYRRHSLFGTGKDQTESWWKAFSRQLITEGFLVEVSRYNKFMKICALTKKGRNWLHKANTESQSLILQANEELCPKKLLLPSSKTVSSGTKEHCYNQVPVELSTEKKSNLEKLYSYKPCDKISSGSNISKKSIMVQSPEKAYSSSQPVISAQEQETQIVLYGKLVEARQKHANKMDVPPAILATNKILVDMAKMRPTTVENVKRIDGVSEGKAAMLAPLLEVIKHFCQTNSVQTDLFSSTKPQEEQKTSLVAKNKICTLSQSMAITYSLFQEKKMPLKSIAESRILPLMTIGMHLSQAVKAGCPLDLERAGLTPEVQKIIADVIRNPPVNSDMSKISLIRMLVPENIDTYLIHMAIEILKHGPDSGLQPSCDVNKRRCFPGSEEICSSSKRSKEEVGINTETSSAERKRRLPVWFAKGSDTSKKLMDKTKRGGLFS

>NP_006221.1

MFSEQAAQRAHTLLSPPSANNATFARVPVATYTNSSQPFRLGERSFSRQYAHIYATRLIQMRPFLENRAQQHWGSGVGVKKLCELQPEEKCCVVGTLFKAMPLQPSILREVSEEHNLLPQPPRSKYIHPDDELVLEDELQRIKLKGTIDVSKLVTGTVLAVFGSVRDDGKFLVEDYCFADLAPQKPAPPLDTDRFVLLVSGLGLGGGGGESLLGTQLLVDVVTGQLGDEGEQCSAAHVSRVILAGNLLSHSTQSRDSINKAKYLTKKTQAASVEAVKMLDEILLQLSASVPVDVMPGEFDPTNYTLPQQPLHPCMFPLATAYSTLQLVTNPYQATIDGVRFLGTSGQNVSDIFRYSSMEDHLEILEWTLRVRHISPTAPDTLGCYPFYKTDPFIFPECPHVYFCGNTPSFGSKIIRGPEDQTVLLVTVPDFSATQTACLVNLRSLACQPISFSGFGAEDDDLGGLGLGP

244 AAH20590.1 NP_000012.1

>AAH20590.1

MGAAVFFGCTFVAFGPAFALFLITVAGDPLRVIILVAGAFFWLVSLLLASVVWFILVHVTDRSDARLQYGLLIFGAAVSVLLQEVFRFAYYKLLKKADEGLASLSEDGRSPISIRQMAYVSGLSFGIISGVFSVINILADALGPGVVGIHGDSPYYFLTSAFLTAAIILLHTFWGVVFFDACERRRYWALGLVVGSHLLTSGLTFLNPWYEASLLPIYAVTVSMGLWAFITAGGSLRSIQRSLLCRRQEDSRVMVYSALRIPPED

>NP_000012.1

MTELPAPLSYFQNAQMSEDNHLSNTVRSQNDNRERQEHNDRRSLGHPEPLSNGRPQGNSRQVVEQDEEEDEELTLKYGAKHVIMLFVPVTLCMVVVVATIKSVSFYTRKDGQLIYTPFTEDTETVGQRALHSILNAAIMISVIVVMTILLVVLYKYRCYKVIHAWLIISSLLLLFFFSFIYLGEVFKTYNVAVDYITVALLIWNFGVVGMISIHWKGPLRLQQAYLIMISALMALVFIKYLPEWTAWLILAVISVYDLVAVLCPKGPLRMLVETAQERNETLFPALIYSSTMVWLVNMAEGDPEAQRRVSKNSKYNAESTERESQDTVAENDDGGFSEEWEAQRDSHLGPHRSTPESRAAVQELSSSILAGEDPEERGVKLGLGDFIFYSVLVGKASATASGDWNTTIACFVAILIGLCLTLLLLAIFKKALPALPISITFGLVFYFATDYLVQPFMDQLAFHQFYI

245 NP_478126.1 NP_001203.1

>NP_478126.1

MPGDHRRIRGPEESQPPQLYAADEEEAPGTRDPTRLRPVYARAGLLSQAKGSAYLEAGGTKVLCAVSGPRQAEGGERGGGPAGAGGEAPAALRGRLLCDFRRAPFAGRRRRAPPGGCEERELALALQEALEPAVRLGRYPRAQLEVSALLLEDGGSALAAALTAAALALADAGVEMYDLVVGCGLSLAPGPAPTWLLDPTRLEEERAAAGLTVALMPVLNQVAGLLGSGEGGLTESWAEAVRLGLEGCQRLYPVLQQSLVRAARRRGAAAQP

>NP_001203.1

MLPLLRCVPRVLGSSVAGLRAAAPASPFRQLLQPAPRLCTRPFGLLSVRAGSERRPGLLRPRGPCACGCGCGSLHTDGDKAFVDFLSDEIKEERKIQKHKTLPKMSGGWELELNGTEAKLVRKVAGEKITVTFNINNSIPPTFDGEEEPSQGQKVEEQEPELTSTPNFVVEVIKNDDGKKALVLDCHYPEDEVGQEDEAESDIFSIREVSFQSTGESEWKDTNYTLNTDSLDWALYDHLMDFLADRGVDNTFADELVELSTALEHQEYITFLEDLKSFVKSQ

246 NP_776170.2 NP_958437.1

>NP_776170.2

MAAAAEGVLATRSDEPARDDAAVETAEEAKEPAEADITELCRDMFSKMATYLTGELTATSEDYKLLENMNKLTSLKYLEMKDIAINISRNLKDLNQKYAGLQPYLDQINVIEEQVAALEQAAYKLDAYSKKLEAKYKKLEKR

>NP_958437.1

MSGGGTETPVGCEAAPGGGSKKRDSLGTAGSAHLIIKDLGEIHSRLLDHRPVIQGETRYFVKEFEEKRGLREMRVLENLKNMIHETNEHTLPKCRDTMRDSLSQVLQRLQAANDSVCRLQQREQERKKIHSDHLVASEKQHMLQWDNFMKEQPNKRAEVDEEHRKAMERLKEQYAEMEKDLAKFSTF

247 NP_005878.1 NP_065853.2

>NP_005878.1

MRPCIWIHVHLKPPCRLVELLPFSSALQGLSHLSLGTTLPVILPERNEEQNLQELSHNADKYQMGDCCKEEI

>NP_065853.2

MLAGRPGTRSAVGELGTESSDNLDRAPLGPRESGGHHRPGSYLDMKIHLEKNLEEERQILLQQQKICRNRARKYFVESNRRKKAFEEKRKEQEEKEHQIREQILQQRKQKFEEVTEKFQRAHVPLSQRRKAVSRKPVPPLEEALKQIQESNLKSEVNLPFSRRPTINWRAIDSALPSALSKNDHKHQKQLLSKINCEKEMNENMRATLATSKNVFQLKLEETQKLLEDQHLSNLQKFGDEVNQITNSETLSSIDSLEATEHEEIYLTLNKEHSTSIQRNTISLKPANMQSTNLSCFDEDKLAFSKTQHINNWLTNLDASNTQNVTAFSDILSKSNVLPSWEYFNSKEQNPSPLNGTVERATNTANNSVPFVSSPPMFVLDKKCEKTSETSTMRTTDSTSGAFKRERPLVTESPTFKFSKSQSTSDSLTQEVATFPDQEKYSELNQENGTTSIPTSCVPVATPLVLPSNIQSARPSAKNSIHIKEIDAVQCSDKLDELKDGKEEEIKYFNCNKEELPLFSDSFQDAYIPHNPDSKDEKQKLAETSSLSNVTSNYDFVGQHKKMKYNIHERNGVRFLKSILKKESKYEHGYLKALIINQSFKFGNQKAAAIRDSIELTKEKGAEIPKTIKKLRWFDETSNIENNAENSHSLKNKTGTTQQHSQQFHIQSGAGSNIISVSTCAVNSADTKKSREDSISENVTTLGGSGADHMPLNCFIPSGYNFAKHAWPASKKEESKIPVHDDSKTKQGKPQRGRAKIIRKPGSAKVQSGFICTNRKGAVIQPQSASKVNIFTQAQGKLIIPCPPPQSTSNIRSGKNIQVSQCQPVTPENPQNIITHNSFNSKHVLPTEHSLNQWNQESSSPLSNACSDLVTVIPSLPSYCSSECQTFAKINHSNGTQAVARQDATLYCTQRSPVCEESYPSVTLRTAEEESVPLWKRGPNVLHQNKRATGSTVMRRKRIAETKRRNILEQKRQNPGSVGQKYSEQINNFGQSVLLSSSEPKQTTRGTSYIEEVSDSTSEFLMAENLVKASVPEDEILTVLNSKQIQKSNLPLNKTQQFNICTLSAEEQKILESLNDLSERLHYIQESICKNPSIKNTLQIIPLLEKREDRTSSCRDKR

248 NP_000816.3 NP_000397.1

>NP_000816.3

MKPIQKLLAGLILLTWCVEGCSSQHWSYGLRPGGKRDAENLIDSFQEIVKEVGQLAETQRFECTTHQPRSPLRDLKGALESLIEEETGQKKI

>NP_000397.1

MANSASPEQNQNHCSAINNSIPLMQGNLPTLTLSGKIRVTVTFFLFLLSATFNASFLLKLQKWTQKKEKGKKLSRMKLLLKHLTLANLLETLIVMPLDGMWNITVQWYAGELLCKVLSYLKLFSMYAPAFMMVVISLDRSLAITRPLALKSNSKVGQSMVGLAWILSSVFAGPQLYIFRMIHLADSSGQTKVFSQCVTHCSFSQWWHQAFYNFFTFSCLFIIPLFIMLICNAKIIFTLTRVLHQDPHELQLNQSKNNIPRARLKTLKMTVAFATSFTVCWTPYYVLGIWYWFDPEMLNRLSDPVNHFFFLFAFLNPCFDPLIYGYFSL

249 NP_005709.1 NP_945327.1

>NP_005709.1

MTATLRPYLSAVRATLQAALCLENFSSQVVERHNKPEVEVRSSKELLLQPVTISRNEKEKVLIEGSINSVRVSIAVKQADEIEKILCHKFMRFMMMRAENFFILRRKPVEGYDISFLITNFHTEQMYKHKLVDFVIHFMEEIDKEISEMKLSVNARARIVAEEFLKNF

>NP_945327.1

MAGAVPGAIMDEDYYGSAAEWGDEADGGQQEDDSGEGEDDAEVQQECLHKFSTRDYIMEPSIFNTLKRYFQAGGSPENVIQLLSENYTAVAQTVNLLAEWLIQTGVEPVQVQETVENHLKSLLIKHFDPRKADSIFTEEGETPAWLEQMIAHTTWRDLFYKLAEAHPDCLMLNFTVKLISDAGYQGEITSVSTACQQLEVFSRVLRTSLATILDGGEENLEKNLPEFAKMVCHGEHTYLFAQAMMSVLAQEEQGGSAVRRIAQEVQRFAQEKGHDASQITLALGTAASYPRACQALGAMLSKGALNPADITVLFKMFTSMDPPPVELIRVPAFLDLFMQSLFKPGARINQDHKHKYIHILAYAASVVETWKKNKRVSINKDELKSTSKAVETVHNLCCNENKGASELVAELSTLYQCIRFPVVAMGVLKWVDWTVSEPRYFQLQTDHTPVHLALLDEISTCHQLLHPQVLQLLVKLFETEHSQLDVMEQLELKKTLLDRMVHLLSRGYVLPVVSYIRKCLEKLDTDISLIRYFVTEVLDVIAPPYTSDFVQLFLPILENDSIAGTIKTEGEHDPVTEFIAHCKSNFIMVN

250 NP_006182.2 NP_000312.2

>NP_006182.2

MSGEDEQQEQTIAEDLVVTKYKMGGDIANRVLRSLVEASSSGVSVLSLCEKGDAMIMEETGKIFKKEKEMKKGIAFPTSISVNNCVCHFSPLKSDQDYILKEGDLVKIDLGVHVDGFIANVAHTFVVDVAQGTQVTGRKADVIKAAHLCAEAALRLVKPGNQNTQVTEAWNKVAHSFNCTPIEGMLSHQLKQHVIDGEKTIIQNPTDQQKKDHEKAEFEVHEVYAVDVLVSSGEGKAKDAGQRTTIYKRDPSKQYGLKMKTSRAFFSEVERRFDAMPFTLRAFEDEKKARMGVVECAKHELLQPFNVLYEKEGEFVAQFKFTVLLMPNGPMRITSGPFEPDLYKSEMEVQDAELKALLQSSASRKTQKKKKKKASKTAENATSGETLEENEAGD

>NP_000312.2

MPPKTPRKTAATAAAAAAEPPAPPPPPPPEEDPEQDSGPEDLPLVRLEFEETEEPDFTALCQKLKIPDHVRERAWLTWEKVSSVDGVLGGYIQKKKELWGICIFIAAVDLDEMSFTFTELQKNIEISVHKFFNLLKEIDTSTKVDNAMSRLLKKYDVLFALFSKLERTCELIYLTQPSSSISTEINSALVLKVSWITFLLAKGEVLQMEDDLVISFQLMLCVLDYFIKLSPPMLLKEPYKTAVIPINGSPRTPRRGQNRSARIAKQLENDTRIIEVLCKEHECNIDEVKNVYFKNFIPFMNSLGLVTSNGLPEVENLSKRYEEIYLKNKDLDARLFLDHDKTLQTDSIDSFETQRTPRKSNLDEEVNVIPPHTPVRTVMNTIQQLMMILNSASDQPSENLISYFNNCTVNPKESILKRVKDIGYIFKEKFAKAVGQGCVEIGSQRYKLGVRLYYRVMESMLKSEEERLSIQNFSKLLNDNIFHMSLLACALEVVMATYSRSTSQNLDSGTDLSFPWILNVLNLKAFDFYKVIESFIKAEGNLTREMIKHLERCEHRIMESLAWLSDSPLFDLIKQSKDREGPTDHLESACPLNLPLQNNHTAADMYLSPVRSPKKKGSTTRVNSTANAETQATSAFQTQKPLKSTSLSLFYKKVYRLAYLRLNTLCERLLSEHPELEHIIWTLFQHTLQNEYELMRDRHLDQIMMCSMYGICKVKNIDLKFKIIVTAYKDLPHAVQETFKRVLIKEEEYDSIIVFYNSVFMQRLKTNILQYASTRPPTLSPIPHIPRSPYKFPSSPLRIPGGNIYISPLKSPYKISEGLPTPTKMTPRSRILVSIGESFGTSEKFQKINQMVCNSDRVLKRSAEGSNPPKPLKKLRFDIEGSDEADGSKHLPGESKFQQKLAEMTSTRTRMQKQKMNDSMDTSNKEEK

251 NP_775816.1 NP_002303.2

>NP_775816.1

MSGGFELQPRDGGPRVALAPGETVIGRGPLLGITDKRVSRRHAILEVAGGQLRIKPIHTNPCFYQSSEKSQLLPLKPNLWCYLNPGDSFSLLVDKYIFRILSIPSEVEMQCTLRNSQVLDEDNILNETPKSPVINLPHETTGASQLEGSTEIAKTQMTPTNSVSFLGENRDCNKQQPILAERKRILPTWMLAEHLSDQNLSVPAISGGNVIQGSGKEEICKDKSQLNTTQQGRRQLISSGSSENTSAEQDTGEECKNTDQEESTISSKEMPQSFSAITLSNTEMNNIKTNAQRNKLPIEELGKVSKHKIATKRTPHKEDEAMSCSENCSSAQGDSLQDESQGSHSESSSNPSNPETLHAKATDSVLQGSEGNKVKRTSCMYGANCYRKNPVHFQHFSHPGDSDYGGVQIVGQDETDDRPECPYGPSCYRKNPQHKIEYRHNTLPVRNVLDEDNDNVGQPNEYDLNDSFLDDEEEDYEPTDEDSDWEPGKEDEEKEDVEELLKEAKRFMKRK

>NP_002303.2

MAASQTSQTVASHVPFADLCSTLERIQKSKGRAEKIRHFREFLDSWRKFHDALHKNHKDVTDSFYPAMRLILPQLERERMAYGIKETMLAKLYIELLNLPRDGKDALKLLNYRTPTGTHGDAGDFAMIAYFVLKPRCLQKGSLTIQQVNDLLDSIASNNSAKRKDLIKKSLLQLITQSSALEQKWLIRMIIKDLKLGVSQQTIFSVFHNDAAELHNVTTDLEKVCRQLHDPSVGLSDISITLFSAFKPMLAAIADIEHIEKDMKHQSFYIETKLDGERMQMHKDGDVYKYFSRNGYNYTDQFGASPTEGSLTPFIHNAFKADIQICILDGEMMAYNPNTQTFMQKGTKFDIKRMVEDSDLQTCYCVFDVLMVNNKKLGHETLRKRYEILSSIFTPIPGRIEIVQKTQAHTKNEVIDALNEAIDKREEGIMVKQPLSIYKPDKRGEGWLKIKPEYVSGLMDELDILIVGGYWGKGSRGGMMSHFLCAVAEKPPPGEKPSVFHTLSRVGSGCTMKELYDLGLKLAKYWKPFHRKAPPSSILCGTEKPEVYIEPCNSVIVQIKAAEIVPSDMYKTGCTLRFPRIEKIRDDKEWHECMTLDDLEQLRGKASGKLASKHLYIGGDDEPQEKKRKAAPKMKKVIGIIEHLKAPNLTNVNKISNIFEDVEFCVMSGTDSQPKPDLENRIAEFGGYIVQNPGPDTYCVIAGSENIRVKNIILSNKHDVVKPAWLLECFKTKSFVPWQPRFMIHMCPSTKEHFAREYDCYGDSYFIDTDLNQLKEVFSGIKNSNEQTPEEMASLIADLEYRYSWDCSPLSMFRRHTVYLDSYAVINDLSTKNEGTRLAIKALELRFHGAKVVSCLAEGVSHVIIGEDHSRVADFKAFRRTFKRKFKILKESWVTDSIDKCELQEENQYLI

252 NP_001886.1 NP_000225.1

>NP_001886.1

MSGPVPSRARVYTDVNTHRPREYWDYESHVVEWGNQDDYQLVRKLGRGKYSEVFEAINITNNEKVVVKILKPVKKKKIKREIKILENLRGGPNIITLADIVKDPVSRTPALVFEHVNNTDFKQLYQTLTDYDIRFYMYEILKALDYCHSMGIMHRDVKPHNVMIDHEHRKLRLIDWGLAEFYHPGQEYNVRVASRYFKGPELLVDYQMYDYSLDMWSLGCMLASMIFRKEPFFHGHDNYDQLVRIAKVLGTEDLYDYIDKYNIELDPRFNDILGRHSRKRWERFVHSENQHLVSPEALDFLDKLLRYDHQSRLTAREAMEHPYFYTVVKDQARMGSSSMPGGSTPVSSANMMSGISSVPTPSPLGPLAGSPVIAAANPLGMPVPAAAGAQQ

>NP_000225.1

MQRSIMSFFHPKKEGKAKKPEKEASNSSRETEPPPKAALKEWNGVVSESDSPVKRPGRKAARVLGSEGEEEDEALSPAKGQKPALDCSQVSPPRPATSPENNASLSDTSPMDSSPSGIPKRRTARKQLPKRTIQEVLEEQSEDEDREAKRKKEEEEEETPKESLTEAEVATEKEGEDGDQPTTPPKPLKTSKAETPTESVSEPEVATKQELQEEEEQTKPPRRAPKTLSSFFTPRKPAVKKEVKEEEPGAPGKEGAAEGPLDPSGYNPAKNNYHPVEDACWKPGQKVPYLAVARTFEKIEEVSARLRMVETLSNLLRSVVALSPPDLLPVLYLSLNHLGPPQQGLELGVGDGVLLKAVAQATGRQLESVRAEAAEKGDVGLVAENSRSTQRLMLPPPPLTASGVFSKFRDIARLTGSASTAKKIDIIKGLFVACRHSEARFIARSLSGRLRLGLAEQSVLAALSQAVSLTPPGQEFPPAMVDAGKGKTAEARKTWLEEQGMILKQTFCEVPDLDRIIPVLLEHGLERLPEHCKLSPGIPLKPMLAHPTRGISEVLKRFEEAAFTCEYKYDGQRAQIHALEGGEVKIFSRNQEDNTGKYPDIISRIPKIKLPSVTSFILDTEAVAWDREKKQIQPFQVLTTRKRKEVDASEIQVQVCLYAFDLIYLNGESLVREPLSRRRQLLRENFVETEGEFVFATSLDTKDIEQIAEFLEQSVKDSCEGLMVKTLDVDATYEIAKRSHNWLKLKKDYLDGVGDTLDLVVIGAYLGRGKRAGRYGGFLLASYDEDSEELQAICKLGTGFSDEELEEHHQSLKALVLPSPRPYVRIDGAVIPDHWLDPSAVWEVKCADLSLSPIYPAARGLVDSDKGISLRFPRFIRVREDKQPEQATTSAQVACLYRKQSQIQNQQGEDSGSDPEDTY

253 AAH32474.1 NP_002459.1

>AAH32474.1

MASSTSLPAPGSRPKKPLGKMADWFRQTLLKKPKKRPNSPESTSSDASQPTSQDSPLPPSLSSVTSPSLPPTHASDSGSSRWSKDYDVCVCHSEEDLVAAQDLVSYLEGSTASLRCFLQLRDATPGGAIVSELCQALSSSHCRVLLITPGFLQDPWCKYQMLQALTEAPGAEGCTIPLLSGLSRAAYPPELRFMYYVDGRGPDGGFRQVKEAVMRYLQTLSWHLLYHGTPEIGVKLETENPCRASDSHKCDKRYRE

>NP_002459.1

MAAGGPGAGSAAPVSSTSSLPLAALNMRVRRRLSLFLNVRTQVAADWTALAEEMDFEYLEIRQLETQADPTGRLLDAWQGRPGASVGRLLELLTKLGRDDVLLELGPSIEEDCQKYILKQQQEEAEKPLQVAAVDSSVPRTAELAGITTLDDPLGHMPERFDAFICYCPSDIQFVQEMIRQLEQTNYRLKLCVSDRDVLPGTCVWSIASELIEKRCRRMVVVVSDDYLQSKECDFQTKFALSLSPGAHQKRLIPIKYKAMKKEFPSILRFITVCDYTNPCTKSWFWTRLAKALSLP

254 NP_004075.1 NP_002613.2

>NP_004075.1

MRTLAILAAILLVALQAQAEPLQARADEVAAAPEQIAADIPEVVVSLAWDESLAPKHPGSRKNMACYCRIPACIAGERRYGTCIYQGRLWAFCC

>NP_002613.2

MAAPVDLELKKAFTELQAKVIDTQQKVKLADIQIEQLNRTKKHAHLTDTEIMTLVDETNMYEGVGRMFILQSKEAIHSQLLEKQKIAEEKIKELEQKKSYLERSVKEAEDNIREMLMARRAQ

255 NP_055202.1 NP_005556.1

>NP_055202.1

MEEAILVPCVLGLLLLPILAMLMALCVHCHRLPGSYDSTSSDSLYPRGIQFKRPHTVAPWPPAYPPVTSYPPLSQPDLLPIPRSPQPLGGSHRTPSSRRDSDGANSVASYENEGASGIRGAQAGWGVWGPSWTRLTPVSLPPEPACEDADEDEDDYHNPGYLVVLPDSTPATSTAAPSAPALSTPGIRDSAFSMESIDDYVNVPESGESAEASLDGSREYVNVSQELHPGAAKTEPAALSSQEAEEVEEEGAPDYENLQELN

>NP_005556.1

MALRNVPFRSEVLGWDPDSLADYFKKLNYKDCEKAVKKYHIDGARFLNLTENDIQKFPKLRVPILSKLSQEINKNEERRSIFTRKPQVPRFPEETESHEEDNGGWSSFEEDDYESPNDDQDGEDDGDYESPNEEEEAPVEDDADYEPPPSNDEEALQNSILPAKPFPNSNSMYIDRPPSGKTPQQPPVPPQRPMAALPPPPAGRNHSPLPPPQTNHEEPSRSRNHKTAKLPAPSIDRSTKPPLDRSLAPFDREPFTLGKKPPFSDKPSIPAGRSLGEHLPKIQKPPLPPTTERHERSSPLPGKKPPVPKHGWGPDRRENDEDDVHQRPLPQPALLPMSSNTFPSRSTKPSPMNPLPSSHMPGAFSESNSSFPQSASLPPYFSQGPSNRPPIRAEGRNFPLPLPNKPRPPSPAEEENSLNEEWYVSYITRPEAEAALRKINQDGTFLVRDSSKKTTTNPYVLMVLYKDKVYNIQIRYQKESQVYLLGTGLRGKEDFLSVSDIIDYFRKMPLLLIDGKNRGSRYQCTLTHAAGYP

256 NP_003655.3 NP_001202.4

>NP_003655.3

MSSNSFPYNEQSGGGEATELGQEATSTISPSGAFGLFSSDLKKNEDLKQMLESNKDSAKLDAMKRIVGMIAKGKNASELFPAVVKNVASKNIEIKKLVYVYLVRYAEEQQDLALLSISTFQRALKDPNQLIRASALRVLSSIRVPIIVPIMMLAIKEASADLSPYVRKNAAHAIQKLYSLDPEQKEMLIEVIEKLLKDKSTLVAGSVVMAFEEVCPDRIDLIHKNYRKLCNLLVDVEEWGQVVIIHMLTRYARTQFVSPWKEGDELEDNGKNFYESDDDQKEKTDKKKKPYTMDPDHRLLIRNTKPLLQSRNAAVVMAVAQLYWHISPKSEAGIISKSLVRLLRSNREVQYIVLQNIATMSIQRKGMFEPYLKSFYVRSTDPTMIKTLKLEILTNLANEANISTLLREFQTYVKSQDKQFAAATIQTIGRCATNILEVTDTCLNGLVCLLSNRDEIVVAESVVVIKKLLQMQPAQHGEIIKHMAKLLDSITVPVARASILWLIGENCERVPKIAPDVLRKMAKSFTSEDDLVKLQILNLGAKLYLTNSKQTKLLTQYILNLGKYDQNYDIRDRTRFIRQLIVPNVKSGALSKYAKKIFLAQKPAPLLESPFKDRDHFQLGTLSHTLNIKATGYLELSNWPEVAPDPSVRNVEVIELAKEWTPAGKAKQENSAKKFYSESEEEEDSSDSSSDSESESGSESGEQGESGEEGDSNEDSSEDSSSEQDSESGRESGLENKRTAKRNSKAKGKSDSEDGEKENEKSKTSDSSNDESSSIEDSSSDSESESEPESESESRRVTKEKEKKTKQDRTPLTKDVSLLDLDDFNPVSTPVALPTPALSPSLMADLEGLHLSTSSSVISVSTPAFVPTKTHVLLHRMSGKGLAAHYFFPRQPCIFGDKMVSIQITLNNTTDRKIENIHIGEKKLPIGMKMHVFNPIDSLEPEGSITVSMGIDFCDSTQTASFQLCTKDDCFNVNIQPPVGELLLPVAMSEKDFKKEQGVLTGMNETSAVIIAAPQNFTPSVIFQKVVNVANVGAVPSGQDNIHRFAAKTVHSGSLMLVTVELKEGSTAQLIINTEKTVIGSVLLRELKPVLSQG

>NP_001202.4

MAAVKKEGGALSEAMSLEGDEWELSKENVQPLRQGRIMSTLQGALAQESACNNTLQQQKRAFEYEIRFYTGNDPLDVWDRYISWTEQNYPQGGKESNMSTLLERAVEALQGEKRYYSDPRFLNLWLKLGRLCNEPLDMYSYLHNQGIGVSLAQFYISWAEEYEARENFRKADAIFQEGIQQKAEPLERLQSQHRQFQARVSRQTLLALEKEEEEEVFESSVPQRSTLAELKSKGKKTARAPIIRVGGALKAPSQNRGLQNPFPQQMQNNSRITVFDENADEASTAELSKPTVQPWIAPPMPRAKENELQAGPWNTGRSLEHRPRGNTASLIAVPAVLPSFTPYVEETAQQPVMTPCKIEPSINHILSTRKPGKEEGDPLQRVQSHQQASEEKKEKMMYCKEKIYAGVGEFSFEEIRAEVFRKKLKEQREAELLTSAEKRAEMQKQIEEMEKKLKEIQTTQQERTGDQQEETMPTKETTKLQIASESQKIPGMTLSSSVCQVNCCARETSLAENIWQEQPHSKGPSVPFSIFDEFLLSEKKNKSPPADPPRVLAQRRPLAVLKTSESITSNEDVSPDVCDEFTGIEPLSEDAIITGFRNVTICPNPEDTCDFARAARFVSTPFHEIMSLKDLPSDPERLLPEEDLDVKTSEDQQTACGTIYSQTLSIKKLSPIIEDSREATHSSGFSGSSASVASTSSIKCLQIPEKLELTNETSENPTQSPWCSQYRRQLLKSLPELSASAELCIEDRPMPKLEIEKEIELGNEDYCIKREYLICEDYKLFWVAPRNSAELTVIKVSSQPVPWDFYINLKLKERLNEDFDHFCSCYQYQDGCIVWHQYINCFTLQDLLQHSEYITHEITVLIIYNLLTIVEMLHKAEIVHGDLSPRCLILRNRIHDPYDCNKNNQALKIVDFSYSVDLRVQLDVFTLSGFRTVQILEGQKILANCSSPYQVDLFGIADLAHLLLFKEHLQVFWDGSFWKLSQNISELKDGELWNKFFVRILNANDEATVSVLGELAAEMNGVFDTTFQSHLNKALWKVGKLTSPGALLFQ

257 NP_958437.1 NP_001478.1

>NP_958437.1

MSGGGTETPVGCEAAPGGGSKKRDSLGTAGSAHLIIKDLGEIHSRLLDHRPVIQGETRYFVKEFEEKRGLREMRVLENLKNMIHETNEHTLPKCRDTMRDSLSQVLQRLQAANDSVCRLQQREQERKKIHSDHLVASEKQHMLQWDNFMKEQPNKRAEVDEEHRKAMERLKEQYAEMEKDLAKFSTF

>NP_001478.1

MLSRLLKEHQAKQNERKELQEKRRREAITAATCLTEALVDHLNVGVAQAYMNQRKLDHEVKTLQVQAAQFAKQTGQWIGMVENFNQALKEIGDVENWARSIELDMRTIATALEYVYKGQLQSAPS

258 NP_002148.1 NP_005139.1

>NP_002148.1

MAGQAFRKFLPLFDRVLVERSAAETVTKGGIMLPEKSQGKVLQATVVAVGSGSKGKGGEIQPVSVKVGDKVLLPEYGGTKVVLDDKDYFLFRDGDILGKYVD

>NP_005139.1

MKVKKGGGGAGTATESAPGPSGQSVAPIPQPPAESESGSESEPDAGPGPRPGPLQRKQPIGPEDVLGLQRITGDYLCSPEENIYKIDFVRFKIRDMDSGTVLFEIKKPPVSERLPINRRDLDPNAGRFVRYQFTPAFLRLRQVGATVEFTVGDKPVNNFRMIERHYFRNQLLKSFDFHFGFCIPSSKNTCEHIYDFPPLSEELISEMIRHPYETQSDSFYFVDDRLVMHNKADYSYSGTP

259 NP_004585.1 NP_004032.2

>NP_004585.1

MLRAALSLLALPLAGAAEEPTQKPESPGEPPPGLELFRWQWHEVEAPYLVALWILVASLAKIVFHLSRKVTSLVPESCLLILLGLVLGGIVLAVAKKAEYQLEPGTFFLFLLPPIVLDSGYFMPSRLFFDNLGAILTYAVVGTLWNAFTTGAALWGLQQAGLVAPRVQAGLLDFLLFGSLISAVDPVAVLAVFEEVHVNETLFIIVFGESLLNDAVTVVLYKVCNSFVEMGSANVQATDYLKGVASLFVVSLGGAAVGLVFAFLLALTTRFTKRVRIIEPLLVFLLAYAAYLTAEMASLSAILAVTMCGLGCKKYVEANISHKSRTTVKYTMKTLASCAETVIFMLLGISAVDSSKWAWDSGLVLGTLIFILFFRALGVVLQTWVLNQFRLVPLDKIDQVVMSYGGLRGAVAFALVILLDRTKVPAKDYFVATTIVVVFFTVIVQGLTIKPLVKWLKVKRSEHHKPTLNQELHEHTFDHILAAVEDVVGHHGYHYWRDRWEQFDKKYLSQLLMRRSAYRIRDQIWDVYYRLNIRDAISFVDQGGHVLSSTGLTLPSMPSRNSVAETSVTNLLRESGSGACLDLQVIDTVRSGRDREDAVMHHLLCGGLYKPRRRYKASCSRHFISEDAQERQDKEVFQQNMKRRLESFKSTKHNICFTKSKPRPRKTGRRKKDGVANAEATNGKHRGLGFQDTAAVILTVESEEEEEESDSSETEKEDDEGIIFVARATSEVLQEGKVSGSLEVCPSPRIIPPSPTCAEKELPWKSGQGDLAVYVSSETTKIVPVDMQTGWNQSISSLESLASPPCNQAPILTCLPPHPRGTEEPQVPLHLPSDPRSSFAFPPSLAKAGRSRSESSADLPQQQELQPLMGHKDHTHLSPGTATSHWCIQFNRGSRL

>NP_004032.2

MGDKGTRVFKKASPNGKLTVYLGKRDFVDHIDLVDPVDGVVLVDPEYLKERRVYVTLTCAFRYGREDLDVLGLTFRKDLFVANVQSFPPAPEDKKPLTRLQERLIKKLGEHAYPFTFEIPPNLPCSVTLQPGPEDTGKACGVDYEVKAFCAENLEEKIHKRNSVRLVIRKVQYAPERPGPQPTAETTRQFLMSDKPLHLEASLDKEIYYHGEPISVNVHVTNNTNKTVKKIKISVRQYADICLFNTAQYKCPVAMEEADDTVAPSSTFCKVYTLTPFLANNREKRGLALDGKLKHEDTNLASSTLLREGANREILGIIVSYKVKVKLVVSRGGLLGDLASSDVAVELPFTLMHPKPKEEPPHREVPENETPVDTNLIELDTNDDDIVFEDFARQRLKGMKDDKEEEEDGTGSPQLNNR

260 NP_004476.1 NP_057278.2

>NP_004476.1

MKEGMSNNSTTSISQARKAVEQLKMEACMDRVKVSQAAADLLAYCEAHVREDPLIIPVPASENPFREKKFFCTIL

>NP_057278.2

MCDQTFLVNVFGSCDKCFKQRALRPVFKKSQQLSYCSTCAEIMATEGLHENETLASLKSEAESLKGKLEEERAKLHDVELHQVAERVEALGQFVMKTRRTLKGHGNKVLCMDWCKDKRRIVSSSQDGKVIVWDSFTTNKEHAVTMPCTWVMACAYAPSGCAIACGGLDNKCSVYPLTFDKNENMAAKKKSVAMHTNYLSACSFTNSDMQILTASGDGTCALWDVESGQLLQSFHGHGADVLCLDLAPSETGNTFVSGGCDKKAMVWDMRSGQCVQAFETHESDINSVRYYPSGDAFASGSDDATCRLYDLRADREVAIYSKESIIFGASSVDFSLSGRLLFAGYNDYTINVWDVLKGSRVSILFGHENRVSTLRVSPDGTAFCSGSWDHTLRVWA

261 NP_002176.2 NP_000871.1

>NP_002176.2

MTILGTTFGMVFSLLQVVSGESGYAQNGDLEDAELDDYSFSCYSQLEVNGSQHSLTCAFEDPDVNITNLEFEICGALVEVKCLNFRKLQEIYFIETKKFLLIGKSNICVKVGEKSLTCKKIDLTTIVKPEAPFDLSVVYREGANDFVVTFNTSHLQKKYVKVLMHDVAYRQEKDENKWTHVNLSSTKLTLLQRKLQPAAMYEIKVRSIPDHYFKGFWSEWSPSYYFRTPEINNSSGEMDPILLTISILSFFSVALLVILACVLWKKRIKPIVWPSLPDHKKTLEHLCKKPRKNLNVSFNPESFLDCQIHRVDDIQARDEVEGFLQDTFPQQLEESEKQRLGGDVQSPNCPSEDVVITPESFGRDSSLTCLAGNVSACDAPILSSSRSLDCRESGKNGPHVYQDLLLSLGTTNSTLPPPFSLQSGILTLNPVAQGQPILTSLGSNQEEAYVTMSSFYQNQ

>NP_000871.1

MFHVSFRYIFGLPPLILVLLPVASSDCDIEGKDGKQYESVLMVSIDQLLDSMKEIGSNCLNNEFNFFKRHICDANKEGMFLFRAARKLRQFLKMNSTGDFDLHLLKVSEGTTILLNCTGQVKGRKPAALGEAQPTKSLEENKSLKEQKKLNDLCFLKRLLQEIKTCWNKILMGTKEH

262 NP_071731.1 NP_001390.1

>NP_071731.1

MAHVGDCTQTPWLPVLVVSLMCSARAEYSNCGENEYYNQTTGLCQECPPCGPGEEPYLSCGYGTKDEDYGCVPCPAEKFSKGGYQICRRHKDCEGFFRATVLTPGDMENDAECGPCLPGYYMLENRPRNIYGMVCYSCLLAPPNTKECVGATSGASANFPGTSGSSTLSPFQHAHKELSGQGHLATALIIAMSTIFIMAIAIVLIIMFYILKTKPSAPACCTSHPGKSVEAQVSKDEEKKEAPDNVVMFSEKDEFEKLTATPAKPTKSENDASSENEQLLSRSVDSDEEPAPDKQGSPELCLLSLVHLAREKSATSNKSAGIQSRRKKILDVYANVCGVVEGLSPTELPFDCLEKTSRMLSSTYNSEKAVVKTWRHLAESFGLKRDEIGGMTDGMQLFDRISTAGYSIPELLTKLVQIERLDAVESLCADILEWAGVVPPASQPHAAS

>NP_001390.1

MGYPEVERRELLPAAAPRERGSQGCGCGGAPARAGEGNSCLLFLGFFGLSLALHLLTLCCYLELRSELRRERGAESRLGGSGTPGTSGTLSSLGGLDPDSPITSHLGQPSPKQQPLEPGEAALHSDSQDGHQMALLNFFFPDEKPYSEEESRRVRRNKRSKSNEGADGPVKNKKKGKKAGPPGPNGPPGPPGPPGPQGPPGIPGIPGIPGTTVMGPPGPPGPPGPQGPPGLQGPSGAADKAGTRENQPAVVHLQGQGSAIQVKNDLSGGVLNDWSRITMNPKVFKLHPRSGELEVLVDGTYFIYSQVEVYYINFTDFASYEVVVDEKPFLQCTRSIETGKTNYNTCYTAGVCLLKARQKIAVKMVHADISINMSKHTTFFGAIRLGEAPAS

263 NP_002816.1 NP_005451.2

>NP_002816.1

MQAQQYQQQRRKFAAAFLAFIFILAAVDTAEAGKKEKPEKKVKKSDCGEWQWSVCVPTSGDCGLGTREGTRTGAECKQTMKTQRCKIPCNWKKQFGAECKYQFQAWGECDLNTALKTRTGSLKRALHNAECQKTVTISKPCGKLTKPKPQAESKKKKKEGKKQEKMLD

>NP_005451.2

MEAPEYLDLDEIDFSDDISYSVTSLKTIPELCRRCDTQNEDRSVSSSSWNCGISTLITNTQKPTGIADVYSKFRPVKRVSPLKHQPETLENNESDDQKNQKVVEYQKGGESDLGPQPQELGPGDGVGGPPGKSSEPSTSLGELEHYDLDMDEILDVPYIKSSQQLASFTKVTSEKRILGLCTTINGLSGKACSTGSSESSSSNMAPFCVLSPVKSPHLRKASAVIHDQHKLSTEETEISPPLVKCGSAYEPENQSKDFLNKTFSDPHGRKVEKTTPDCQLRAFHLQSSAAESKPEEQVSGLNRTSSQGPEERSEYLKKVKSILNIVKEGQISLLPHLAADNLDKIHDENGNNLLHIAASQGHAECLQHLTSLMGEDCLNERNTEKLTPAGLAIKNGQLECVRWMVSETEAIAELSCSKDFPSLIHYAGCYGQEKILLWLLQFMQEQGISLDEVDQDGNSAVHVASQHGYLGCIQTLVEYGANVTMQNHAGEKPSQSAERQGHTLCSRYLVVVETCMSLASQVVKLTKQLKEQTVERVTLQNQLQQFLEAQKSEGKSLPSSPSSPSSPASRKSQWKSPDADDDSVAKSKPGVQEGIQVLGSLSASSRARPKAKDEDSDKILRQLLGKEISENVCTQEKLSLEFQDAQASSRNSKKIPLEKRELKLARLRQLMQRSLSESDTDSNNSEDPKTTPVRKADRPRPQPIVESVESMDSAESLHLMIKKHTLASGGRRFPFSIKASKSLDGHSPSPTSESSEPDLESQYPGSGSIPPNQPSGDPQQPSPDSTAAQKVATSPKSALKSPSSKRRTSQNLKLRVTFEEPVVQMEQPSLELNGEKDKDKGRTLQRTSTSNESGDQLKRPFGAFRSIMETLSGNQNNNNNYQAANQLKTSTLPLTSLGRKTDAKGNPASSASKGKNKAA

264 NP_002961.1 NP_006640.2

>NP_002961.1

MAKFVIRPATAADCSDILRLIKELAKYEYMEEQVILTEKDLLEDGFGEHPFYHCLVAEVPKEHWTPEGHSIVGFAMYYFTYDPWIGKLLYLEDFFVMSDYRGFGIGSEILKNLSQVAMRCRCSSMHFLVAEWNEPSINFYKRRGASDLSSEEGWRLFKIDKEYLLKMATEE

>NP_006640.2

MTANRLAESLLALSQQEELADLPKDYLLSESEDEGDNDGERKHQKLLEAISSLDGKNRRKLAERSEASLKVSEFNVSSEGSGEKLVLADLLEPVKTSSSLATVKKQLSRVKSKKTVELPLNKEEIERIHREVAFNKTAQVLSKWDPVVLKNRQAEQLVFPLEKEEPAIAPIEHVLSGWKARTPLEQEIFNLLHKNKQPVTDPLLTPVEKASLRAMSLEEAKMRRAELQRARALQSYYEAKARREKKIKSKKYHKVVKKGKAKKALKEFEQLRKVNPAAALEELEKIEKARMMERMSLKHQNSGKWAKSKAIMAKYDLEARQAMQEQLSKNKELTQKLQVASESEEEEGGTEDVEELLVPDVVNEVQMNADGPNPWMLRSCTSDTKEAATQEDPEQLPELEAHGVSESEGEERPVAEEEILLREFEERRSLRKRSELSQDAEPAGSQETKDSGSQEVLSELRVLSQKLKENHQSRKQKASSEGTIPQVQREEPAPEEEEPLLLQRPERVQTLEELEELGKEECFQNKELPRPVLEGQQSERTPNNRPDAPKEKKKKEQMIDLQNLLTTQSPSVKSLAVPTIEELEDEEERNHRQMIKEAFAGDDVIRDFLKEKREAVEASKPKDVDLTLPGWGEWGGVGLKPSAKKRRRFLIKAPEGPPRKDKNLPNVIINEKRNIHAAAHQVRVLPYPFTHHWQFERTIQTPIGSTWNTQRAFQKLTTPKVVTKPGHIINPIKAEDVGYRSSSRSDLSVIQRNPKRITTRHKKQLKKCSVD

265 NP_002936.1 NP_003935.2

>NP_002936.1

MVGQLSEGAIAAIMQKGDTNIKPILQVINIRPITTGNSPPRYRLLMSDGLNTLSSFMLATQLNPLVEEEQLSSNCVCQIHRFIVNTLKDGRRVVILMELEVLKSAEAVGVKIGNPVPYNEGLGQPQVAPPAPAASPAASSRPQPQNGSSGMGSTVSKAYGASKTFGKAAGPSLSHTSGGTQSKVVPIASLTPYQSKWTICARVTNKSQIRTWSNSRGEGKLFSLELVDESGEIRATAFNEQVDKFFPLIEVNKVYYFSKGTLKIANKQFTAVKNDYEMTFNNETSVMPCEDDHHLPTVQFDFTGIDDLENKSKDSLVDIIGICKSYEDATKITVRSNNREVAKRNIYLMDTSGKVVTATLWGEDADKFDGSRQPVLAIKGARVSDFGGRSLSVLSSSTIIANPDIPEAYKLRGWFDAEGQALDGVSISDLKSGGVGGSNTNWKTLYEVKSENLGQGDKPDYFSSVATVVYLRKENCMYQACPTQDCNKKVIDQQNGLYRCEKCDTEFPNFKYRMILSVNIADFQENQWVTCFQESAEAILGQNAAYLGELKDKNEQAFEEVFQNANFRSFIFRVRVKVETYNDESRIKATVMDVKPVDYREYGRRLVMSIRRSALM

>NP_003935.2

MATKCGNCGPGYSTPLEAMKGPREEIVYLPCIYRNTGTEAPDYLATVDVDPKSPQYCQVIHRLPMPNLKDELHHSGWNTCSSCFGDSTKSRTKLVLPSLISSRIYVVDVGSEPRAPKLHKVIEPKDIHAKCELAFLHTSHCLASGEVMISSLGDVKGNGKGGFVLLDGETFEVKGTWERPGGAAPLGYDFWYQPRHNVMISTEWAAPNVLRDGFNPADVEAGLYGSHLYVWDWQRHEIVQTLSLKDGLIPLEIRFLHNPDAAQGFVGCALSSTIQRFYKNEGGTWSVEKVIQVPPKKVKGWLLPEMPGLITDILLSLDDRFLYFSNWLHGDLRQYDISDPQRPRLTGQLFLGGSIVKGGPVQVLEDEELKSQPEPLVVKGKRVAGGPQMIQLSLDGKRLYITTSLYSAWDKQFYPDLIREGSVMLQVDVDTVKGGLKLNPNFLVDFGKEPLGPALAHELRYPGGDCSSDIWI

266 NP_005649.1 NP_001056.1

>NP_005649.1

MASSSGSSPRPAPDENEFPFGCPPTVCQDPKEPRALCCAGCLSENPRNGEDQICPKCRGEDLQSISPGSRLRTQEKAHPEVAEAGIGCPFAGVGCSFKGSPQSVQEHEVTSQTSHLNLLLGFMKQWKARLGCGLESGPMALEQNLSDLQLQAAVEVAGDLEVDCYRAPCSESQEELALQHFMKEKLLAELEGKLRVFENIVAVLNKEVEASHLALATSIHQSQLDRERILSLEQRVVELQQTLAQKDQALGKLEQSLRLMEEASFDGTFLWKITNVTRRCHESACGRTVSLFSPAFYTAKYGYKLCLRLYLNGDGTGKRTHLSLFIVIMRGEYDALLPWPFRNKVTFMLLDQNNREHAIDAFRPDLSSASFQRPQSETNVASGCPLFFPLSKLQSPKHAYVKDDTMFLKCIVETST

>NP_001056.1

MGLSTVPDLLLPLVLLELLVGIYPSGVIGLVPHLGDREKRDSVCPQGKYIHPQNNSICCTKCHKGTYLYNDCPGPGQDTDCRECESGSFTASENHLRHCLSCSKCRKEMGQVEISSCTVDRDTVCGCRKNQYRHYWSENLFQCFNCSLCLNGTVHLSCQEKQNTVCTCHAGFFLRENECVSCSNCKKSLECTKLCLPQIENVKGTEDSGTTVLLPLVIFFGLCLLSLLFIGLMYRYQRWKSKLYSIVCGKSTPEKEGELEGTTTKPLAPNPSFSPTPGFTPTLGFSPVPSSTFTSSSTYTPGDCPNFAAPRREVAPPYQGADPILATALASDPIPNPLQKWEDSAHKPQSLDTDDPATLYAVVENVPPLRWKEFVRRLGLSDHEIDRLELQNGRCLREAQYSMLATWRRRTPRREATLELLGRVLRDMDLLGCLEDIEEALCGPAALPPAPSLLR

267 NP_004171.2 NP_001547.1

>NP_004171.2

MDKNIGEQLNKAYEAFRQACMDRDSAVKELQQKTENYEQRIREQQEQLSLQQTIIDKLKSQLLLVNSTQDNNYGCVPLLEDSETRKNNLTLDQPQDKVISGIAREKLPKVRRQEVSSPRKETSARSLGSPLLHERGNIEKTFWDLKEEFHKICMLAKAQKDHLSKLNIPDTATETQCSVPIQCTDKTDKQEALFKPQAKDDINRGAPSITSVTPRGLCRDEEDTSFESLSKFNVKFPPMDNDSTFLHSTPERPGILSPATSEAVCQEKFNMEFRDNPGNFVKTEETLFEIQGIDPIASAIQNLKTTDKTKPSNLVNTCIRTTLDRAACLPPGDHNALYVNSFPLLDPSDAPFPSLDSPGKAIRGPQQPIWKPFPNQDSDSVVLSGTDSELHIPRVCEFCQAVFPPSITSRGDFLRHLNSHFNGET

>NP_001547.1

MSWSPSLTTQTCGAWEMKERLGTGGFGNVIRWHNQETGEQIAIKQCRQELSPRNRERWCLEIQIMRRLTHPNVVAARDVPEGMQNLAPNDLPLLAMEYCQGGDLRKYLNQFENCCGLREGAILTLLSDIASALRYLHENRIIHRDLKPENIVLQQGEQRLIHKIIDLGYAKELDQGSLCTSFVGTLQYLAPELLEQQKYTVTVDYWSFGTLAFECITGFRPFLPNWQPVQWHSKVRQKSEVDIVVSEDLNGTVKFSSSLPYPNNLNSVLAERLEKWLQLMLMWHPRQRGTDPTYGPNGCFKALDDILNLKLVHILNMVTGTIHTYPVTEDESLQSLKARIQQDTGIPEEDQELLQEAGLALIPDKPATQCISDGKLNEGHTLDMDLVFLFDNSKITYETQISPRPQPESVSCILQEPKRNLAFFQLRKVWGQVWHSIQTLKEDCNRLQQGQRAAMMNLLRNNSCLSKMKNSMASMSQQLKAKLDFFKTSIQIDLEKYSEQTEFGITSDKLLLAWREMEQAVELCGRENEVKLLVERMMALQTDIVDLQRSPMGRKQGGTLDDLEEQARELYRRLREKPRDQRTEGDSQEMVRLLLQAIQSFEKKVRVIYTQLSKTVVCKQKALELLPKVEEVVSLMNEDEKTVVRLQEKRQKELWNLLKIACSKVRGPVSGSPDSMNASRLSQPGQLMSQPSTASNSLPEPAKKSEELVAEAHNLCTLLENAIQDTVREQDQSFTALDWSWLQTEEEEHSCLEQAS

268 NP_068779.1 NP_277038.1

>NP_068779.1

MFGLKRNAVIGLNLYCGGAGLGAGSGGATRPGGRLLATEKEASARREIGGGEAGAVIGGSAGASPPSTLTPDSRRVARPPPIGAEVPDVTATPARLLFFAPTRRAAPLEEMEAPAADAIMSPEEELDGYEPEPLGKRPAVLPLLELVGESGNNTSTDGSLPSTPPPAEEEEDELYRQSLEIISRYLREQATGAKDTKPMGRSGATSRKALETLRRVGDGVQRNHETAFQGMLRKLDIKNEDDVKSLSRVMIHVFSDGVTNWGRIVTLISFGAFVAKHLKTINQESCIEPLAESITDVLVRTKRDWLVKQRGWDGFVEFFHVEDLEGGIRNVLLAFAGVAGVGAGLAYLIR

>NP_277038.1

MEPSQCVEELEDDVFQPEDGEPVTQPGSLLSADLFAQSLLDCPLSRLQLFPLTHCCGPGLRPTSQEDKATQTLSPASPSQGVMLPCGVTEEPQRLFYGNAGYRLPLPASFPAVLPIGEQPPEGQWQHQAEVQIARKLQCIADQFHRLHVQQHQQNQNRVWWQILLFLHNLALNGEENRNGAGPR

269 NP_057250.1 NP_003493.1

>NP_057250.1

MADSAELKQMVMSLRVSELQVLLGYAGRNKHGRKHELLTKALHLLKAGCSPAVQMKIKELYRRRFPQKIMTPADLSIPNVHSSPMPATLSPSTIPQLTYDGHPASSPLLPVSLLGPKHELELPHLTSALHPVHPDIKLQKLPFYDLLDELIKPTSLASDNSQRFRETCFAFALTPQQVQQISSSMDISGTKCDFTVQVQLRFCLSETSCPQEDHFPPNLCVKVNTKPCSLPGYLPPTKNGVEPKRPSRPINITSLVRLSTTVPNTIVVSWTAEIGRNYSMAVYLVKQLSSTVLLQRLRAKGIRNPDHSRALIKEKLTADPDSEIATTSLRVSLLCPLGKMRLTIPCRALTCSHLQCFDATLYIQMNEKKPTWVCPVCDKKAPYEHLIIDGLFMEILKYCTDCDEIQFKEDGTWAPMRSKKEVQEVSASYNGVDGCLSSTLEHQVASHHQSSNKNKKVEVIDLTIDSSSDEEEEEPSAKRTCPSLSPTSPLNNKGILSLPHQASPVSRTPSLPAVDTSYINTSLIQDYRHPFHMTPMPYDLQGLDFFPFLSGDNQHYNTSLLAAAAAAVSDDQDLLHSSRFFPYTSSQMFLDQLSAGGSTSLPTTNGSSSGSNSSLVSSNSLRESHSHTVTNRSSTDTASIFGIIPDIISLD

>NP_003493.1

MNIQEQGFPLDLGASFTEDAPRPPVPGEEGELVSTDPRPASYSFCSGKGVGIKGETSTATPRRSDLDLGYEPEGSASPTPPYLKWAESLHSLLDDQDGISLFRTFLKQEGCADLLDFWFACTGFRKLEPCDSNEEKRLKLARAIYRKYILDNNGIVSRQTKPATKSFIKGCIMKQLIDPAMFDQAQTEIQATMEENTYPSFLKSDIYLEYTRTGSESPKVCSDQSSGSGTGKGISGYLPTLNEDEEWKCDQDMDEDDGRDAAPPGRLPQKLLLETAAPRVSSSRRYSEGREFRYGSWREPVNPYYVNAGYALAPATSANDSEQQSLSSDADTLSLTDSSVDGIPPYRIRKQHRREMQESVQVNGRVPLPHIPRTYRVPKEVRVEPQKFAEELIHRLEAVQRTREAEEKLEERLKRVRMEEEGEDGDPSSGPPGPCHKLPPAPAWHHFPPRCVDMGCAGLRDAHEENPESILDEHVQRVLRTPGRQSPGPGHRSPDSGHVAKMPVALGGAASGHGKHVPKSGAKLDAAGLHHHRHVHHHVHHSTARPKEQVEAEATRRAQSSFAWGLEPHSHGARSRGYSESVGAAPNASDGLAHSGKVGVACKRNAKKAESGKSASTEVPGASEDAEKNQKIMQWIIEGEKEISRHRRTGHGSSGTRKPQPHENSRPLSLEHPWAGPQLRTSVQPSHLFIQDPTMPPHPAPNPLTQLEEARRRLEEEEKRASRAPSKQRYVQEVMRRGRACVRPACAPVLHVVPAVSDMELSETETRSQRKVGGGSAQPCDSIVVAYYFCGEPIPYRTLVRGRAVTLGQFKELLTKKGSYRYYFKKVSDEFDCGVVFEEVREDEAVLPVFEEKIIGKVEKVD

270 NP_001886.1 AAQ86961.1

>NP_001886.1

MSGPVPSRARVYTDVNTHRPREYWDYESHVVEWGNQDDYQLVRKLGRGKYSEVFEAINITNNEKVVVKILKPVKKKKIKREIKILENLRGGPNIITLADIVKDPVSRTPALVFEHVNNTDFKQLYQTLTDYDIRFYMYEILKALDYCHSMGIMHRDVKPHNVMIDHEHRKLRLIDWGLAEFYHPGQEYNVRVASRYFKGPELLVDYQMYDYSLDMWSLGCMLASMIFRKEPFFHGHDNYDQLVRIAKVLGTEDLYDYIDKYNIELDPRFNDILGRHSRKRWERFVHSENQHLVSPEALDFLDKLLRYDHQSRLTAREAMEHPYFYTVVKDQARMGSSSMPGGSTPVSSANMMSGISSVPTPSPLGPLAGSPVIAAANPLGMPVPAAAGAQQ

>AAQ86961.1

MESHSRAGKSRKSAKFRSISRSLMLCNAKTSDDGSSPDEKYPDPFEISLAQGKEGIFHSSVQLADTSEAGPSSVPDLALASEAAQLQAAGNDRGKTCRRIFFMKESSTASSREKPGKLEAQSSNFLFPKACHQRARSNSTSVNPYCTREIDFPMTKKSAAPTDRQPYSLCSNRKSLSQQLDCPAGKAAGTSRPTRSLSTAQLVQPSGGLQASVISNIVLMKGQAKGLGFSIVGGKDSIYGPIGIYVKTIFAGGAAAADGRLQEGDEILELNGGSMAGLTHQDALQKFKQAKKGLLTLTVRTRLTAPPSLCSHLSPPLCRSLSSSTCITKDSSSFALESPSAPISTAKPNYRIMVEVSLQKEAGVGLGIGLCSVPYFQCISGIFVHTLSPGSVAHLDGRLRCGDEIVEISDSPVHCLTLNEVYTILSHCDPGPVPIIVSRHPDPQVSEQQLKEAVAQAVENTKFGKERHQWSLEGVKRLESSWHGRPTLEKEREKNSAPPHRRAQKVMIRSSSDSSYMSGSPGGSPGSGSAEKPSSDVDISTHSPSLPLAREPVVLSIASSRLPQESPPLPESRDSHPPLRLKKSFEILVRKPMSSKPKPPPRKYFKSDSDPQKSLEERENSSCSSGHTPPTCGQEARELLPLLLPQEDTAGRSPSASAGCPGPGIGPQTKSSTEGEPGWRRASPVTQTSPIKHPLLKRQARMDYSFDTTAEDPWVRISDCIKNLFSPIMSENHGHMPLQPNASLNEEEGTQGHPDGTPPKLDTANGTPKVYKSADSSTVKKGPPVAPKPAWFRQSLKGLRNRASDPRGLPDPALSTQPAPASREHLGSHIRASSSSSSIRQRISSFETFGSSQLPDKGAQRLSLQPSSGEAAKPLGKHEEGRFSGLLGRGAAPTLVPQQPEQVLSSGSPAASEARDPGVSESPPPGRQPNQKTLPPGPDPLLRLLSTQAEESQGPVLKMPSQRARSFPLTRSQSCETKLLDEKTSKLYSISSQVSSAVMKSLLCLPSSISCAQTPCIPKEGASPTSSSNEDSAANGSAETSALDTGFSLNLSELREYTEGLTEAKEDDDGDHSSLQSGQSVISLLSSEELKKLIEEVKVLDEATLKQLDGIHVTILHKEEGAGLGFSLAGGADLENKVITVHRVFPNGLASQEGTIQKGNEVLSINGKSLKGTTHHDALAILRQAREPRQAVIVTRKLTPEAMPDLNSSTDSAASASAASDVSVESTAEATVCTVTLEKMSAGLGFSLEGGKGSLHGDKPLTINRIFKGAASEQSETVQPGDEILQLGGTAMQGLTRFEAWNIIKALPDGPVTIVIRRKSLQSKETTAAGDS

271 NP_005251.1 NP_066981.2

>NP_005251.1

MARPNKFLLWFCCFAWLCFPISLGSQASGGEAQIAASAELESGAMPWSLLQHIDERDRAGLLPALFKVLSVGRGGSPRLQPDSRALHYMKKLYKTYATKEGIPKSNRSHLYNTVRLFTPCTRHKQAPGDQVTGILPSVELLFNLDRITTVEHLLKSVLLYNINNSVSFSSAVKCVCNLMIKEPKSSSRTLGRAPYSFTFNSQFEFGKKHKWIQIDVTSLLQPLVASNKRSIHMSINFTCMKDQLEHPSAQNGLFNMTLVSPSLILYLNDTSAQAYHSWYSLHYKRRPSQGPDQERSLSAYPVGEEAAEDGRSSHHRHRRGQETVSSELKKPLGPASFNLSEYFRQFLLPQNECELHDFRLSFSQLKWDNWIVAPHRYNPRYCKGDCPRAVGHRYGSPVHTMVQNIIYEKLDSSVPRPSCVPAKYSPLSVLTIEPDGSIAYKEYEDMIATKCTCR

>NP_066981.2

MRATPLAAPAGSLSRKKRLELDDNLDTERPVQKRARSGPQPRLPPCLLPLSPPTAPDRATAVATASRLGPYVLLEPEEGGRAYQALHCPTGTEYTCKVYPVQEALAVLEPYARLPPHKHVARPTEVLAGTQLLYAFFTRTHGDMHSLVRSRHRIPEPEAAVLFRQMATALAHCHQHGLVLRDLKLCRFVFADRERKKLVLENLEDSCVLTGPDDSLWDKHACPAYVGPEILSSRASYSGKAADVWSLGVALFTMLAGHYPFQDSEPVLLFGKIRRGAYALPAGLSAPARCLVRCLLRREPAERLTATGILLHPWLRQDPMPLAPTRSHLWEAAQVVPDGLGLDEAREEEGDREVVLYG

272 NP_071767.3 NP_004949.1

>NP_071767.3

MNTSPGTVGSDPVILATAGYDHTVRFWQAHSGICTRTVQHQDSQVNALEVTPDRSMIAAAGYQHIRMYDLNSNNPNPIISYDGVNKNIASVGFHEDGRWMYTGGEDCTARIWDLRSRNLQCQRIFQVNAPINCVCLHPNQAELIVGDQSGAIHIWDLKTDHNEQLIPEPEVSITSAHIDPDASYMAAVNSTGNCYVWNLTGGIGDEVTQLIPKTKIPAHTRYALQCRFSPDSTLLATCSADQTCKIWRTSNFSLMTELSIKSGNPGESSRGWMWGCAFSGDSQYIVTASSDNLARLWCVETGEIKREYGGHQKAVVCLAFNDSVLG

>NP_004949.1

MLGTGPAAATTAATTSSNVSVLQQFASGLKSRNEETRAKAAKELQHYVTMELREMSQEESTRFYDQLNHHIFELVSSSDANERKGGILAIASLIGVEGGNATRIGRFANYLRNLLPSNDPVVMEMASKAIGRLAMAGDTFTAEYVEFEVKRALEWLGADRNEGRRHAAVLVLRELAISVPTFFFQQVQPFFDNIFVAVWDPKQAIREGAVAALRACLILTTQREPKEMQKPQWYRHTFEEAEKGFDETLAKEKGMNRDDRIHGALLILNELVRISSMEGERLREEMEEITQQQLVHDKYCKDLMGFGTKPRHITPFTSFQAVQPQQSNALVGLLGYSSHQGLMGFGTSPSPAKSTLVESRCCRDLMEEKFDQVCQWVLKCRNSKNSLIQMTILNLLPRLAAFRPSAFTDTQYLQDTMNHVLSCVKKEKERTAAFQALGLLSVAVRSEFKVYLPRVLDIIRAALPPKDFAHKRQKAMQVDATVFTCISMLARAMGPGIQQDIKELLEPMLAVGLSPALTAVLYDLSRQIPQLKKDIQDGLLKMLSLVLMHKPLRHPGMPKGLAHQLASPGLTTLPEASDVGSITLALRTLGSFEFEGHSLTQFVRHCADHFLNSEHKEIRMEAARTCSRLLTPSIHLISGHAHVVSQTAVQVVADVLSKLLVVGITDPDPDIRYCVLASLDERFDAHLAQAENLQALFVALNDQVFEIRELAICTVGRLSSMNPAFVMPFLRKMLIQILTELEHSGIGRIKEQSARMLGHLVSNAPRLIRPYMEPILKALILKLKDPDPDPNPGVINNVLATIGELAQVSGLEMRKWVDELFIIIMDMLQDSSLLAKRQVALWTLGQLVASTGYVVEPYRKYPTLLEVLLNFLKTEQNQGTRREAIRVLGLLGALDPYKHKVNIGMIDQSRDASAVSLSESKSSQDSSDYSTSEMLVNMGNLPLDEFYPAVSMVALMRIFRDQSLSHHHTMVVQAITFIFKSLGLKCVQFLPQVMPTFLNVIRVCDGAIREFLFQQLGMLVSFVKSHIRPYMDEIVTLMREFWVMNTSIQSTIILLIEQIVVALGGEFKLYLPQLIPHMLRVFMHDNSPGRIVSIKLLAAIQLFGANLDDYLHLLLPPIVKLFDAPEAPLPSRKAALETVDRLTESLDFTDYASRIIHPIVRTLDQSPELRSTAMDTLSSLVFQLGKKYQIFIPMVNKVLVRHRINHQRYDVLICRIVKGYTLADEEEDPLIYQHRMLRSGQGDALASGPVETGPMKKLHVSTINLQKAWGAARRVSKDDWLEWLRRLSLELLKDSSSPSLRSCWALAQAYNPMARDLFNAAFVSCWSELNEDQQDELIRSIELALTSQDIAEVTQTLLNLAEFMEHSDKGPLPLRDDNGIVLLGERAAKCRAYAKALHYKELEFQKGPTPAILESLISINNKLQQPEAAAGVLEYAMKHFGELEIQATWYEKLHEWEDALVAYDKKMDTNKDDPELMLGRMRCLEALGEWGQLHQQCCEKWTLVNDETQAKMARMAAAAAWGLGQWDSMEEYTCMIPRDTHDGAFYRAVLALHQDLFSLAQQCIDKARDLLDAELTAMAGESYSRAYGAMVSCHMLSELEEVIQYKLVPERREIIRQIWWERLQGCQRIVEDWQKILMVRSLVVSPHEDMRTWLKYASLCGKSGRLALAHKTLVLLLGVDPSRQLDHPLPTVHPQVTYAYMKNMWKSARKIDAFQHMQHFVQTMQQQAQHAIATEDQQHKQELHKLMARCFLKLGEWQLNLQGINESTIPKVLQYYSAATEHDRSWYKAWHAWAVMNFEAVLHYKHQNQARDEKKKLRHASGANITNATTAATTAATATTTASTEGSNSESEAESTENSPTPSPLQKKVTEDLSKTLLMYTVPAVQGFFRSISLSRGNNLQDTLRVLTLWFDYGHWPDVNEALVEGVKAIQIDTWLQVIPQLIARIDTPRPLVGRLIHQLLTDIGRYHPQALIYPLTVASKSTTTARHNAANKILKNMCEHSNTLVQQAMMVSEELIRVAILWHEMWHEGLEEASRLYFGERNVKGMFEVLEPLHAMMERGPQTLKETSFNQAYGRDLMEAQEWCRKYMKSGNVKDLTQAWDLYYHVFRRISKQLPQLTSLELQYVSPKLLMCRDLELAVPGTYDPNQPIIRIQSIAPSLQVITSKQRPRKLTLMGSNGHEFVFLLKGHEDLRQDERVMQLFGLVNTLLANDPTSLRKNLSIQRYAVIPLSTNSGLIGWVPHCDTLHALIRDYREKKKILLNIEHRIMLRMAPDYDHLTLMQKVEVFEHAVNNTAGDDLAKLLWLKSPSSEVWFDRRTNYTRSLAVMSMVGYILGLGDRHPSNLMLDRLSGKILHIDFGDCFEVAMTREKFPEKIPFRLTRMLTNAMEVTGLDGNYRITCHTVMEVLREHKDSVMAVLEAFVYDPLLNWRLMDTNTKGNKRSRTRTDSYSAGQSVEILDGVELGEPAHKKTGTTVPESIHSFIGDGLVKPEALNKKAIQIINRVRDKLTGRDFSHDDTLDVPTQVELLIKQATSHENLCQCYIGWCPFW

273 NP_002256.2 NP_006258.2

>NP_002256.2

MELITILEKTVSPDRLELEAAQKFLERAAVENLPTFLVELSRVLANPGNSQVARVAAGLQIKNSLTSKDPDIKAQYQQRWLAIDANARREVKNYVLQTLGTETYRPSSASQCVAGIACAEIPVNQWPELIPQLVANVTNPNSTEHMKESTLEAIGYICQDIDPEQLQDKSNEILTAIIQGMRKEEPSNNVKLAATNALLNSLEFTKANFDKESERHFIMQVVCEATQCPDTRVRVAALQNLVKIMSLYYQYMETYMGPALFAITIEAMKSDIDEVALQGIEFWSNVCDEEMDLAIEASEAAEQGRPPEHTSKFYAKGALQYLVPILTQTLTKQDENDDDDDWNPCKAAGVCLMLLATCCEDDIVPHVLPFIKEHIKNPDWRYRDAAVMAFGCILEGPEPSQLKPLVIQAMPTLIELMKDPSVVVRDTAAWTVGRICELLPEAAINDVYLAPLLQCLIEGLSAEPRVASNVCWAFSSLAEAAYEAADVADDQEEPATYCLSSSFELIVQKLLETTDRPDGHQNNLRSSAYESLMEIVKNSAKDCYPAVQKTTLVIMERLQQVLQMESHIQSTSDRIQFNDLQSLLCATLQNVLRKVQHQDALQISDVVMASLLRMFQSTAGSGGVQEDALMAVSTLVEVLGGEFLKYMEAFKPFLGIGLKNYAEYQVCLAAVGLVGDLCRALQSNIIPFCDEVMQLLLENLGNENVHRSVKPQILSVFGDIALAIGGEFKKYLEVVLNTLQQASQAQVDKSDYDMVDYLNELRESCLEAYTGIVQGLKGDQENVHPDVMLVQPRVEFILSFIDHIAGDEDHTDGVVACAAGLIGDLCTAFGKDVLKLVEARPMIHELLTEGRRSKTNKAKTLATWATKELRKLKNQA

>NP_006258.2

MRRSKADVERYIASVQGSTPSPRQKSMKGFYFAKLYYEAKEYDLAKKYICTYINVQERDPKAHRFLGLLYELEENTDKAVECYRRSVELNPTQKDLVLKIAELLCKNDVTDGRAKYWLERAAKLFPGSPAIYKLKEQLLDCEGEDGWNKLFDLIQSELYVRPDDVHVNIRLVEVYRSTKRLKDAVAHCHEAERNIALRSSLEWNSCVVQTLKEYLESLQCLESDKSDWRATNTDLLLAYANLMLLTLSTRDVQESRELLQSFDSALQSVKSLGGNDELSATFLEMKGHFYMHAGSLLLKMGQHSSNVQWRALSELAALCYLIAFQVPRPKIKLIKGEAGQNLLEMMACDRLSQSGHMLLNLSRGKQDFLKEIVETFANKSGQSALYDALFSSQSPKDTSFLGSDDIGNIDVREPELEDLTRYDVGAIRAHNGSLQHLTWLGLQWNSLPALPGIRKWLKQLFHHLPHETSRLETNAPESICILDLEVFLLGVVYTSHLQLKEKCNSHHSSYQPLCLPLPVCKQLCTERQKSWWDAVCTLIHRKAVPGNVAKLRLLVQHEINTLRAQEKHGLQPALLVHWAECLQKTGSGLNSFYDQREYIGRSVHYWKKVLPLLKIIKKKNSIPEPIDPLFKHFHSVDIQASEIVEYEEDAHITFAILDAVNGNIEDAVTAFESIKSVVSYWNLALIFHRKAEDIENDALSPEEQEECKNYLRKTRDYLIKIIDDSDSNLSVVKKLPVPLESVKEMLNSVMQELEDYSEGGPLYKNGSLRNADSEIKHSTPSPTKYSLSPSKSYKYSPKTPPRWAEDQNSLLKMICQQVEAIKKEMQELKLNSSNSASPHRWPTENYGPDSVPDGYQGSQTFHGAPLTVATTGPSVYYSQSPAYNSQYLLRPAANVTPTKGPVYGMNRLPPQQHIYAYPQQMHTPPVQSSSACMFSQEMYGPPALRFESPATGILSPRGDDYFNYNVQQTSTNPPLPEPGYFTKPPIAAHASRSAESKTIEFGKTNFVQPMPGEGLRPSLPTQAHTTQPTPFKFNSNFKSNDGDFTFSSPQVVTQPPPAAYSNSESLLGLLTSDKPLQGDGYSGAKPIPGGQTIGPRNTFNFGSKNVSGISFTENMGSSQQKNSGFRRSDDMFTFHGPGKSVFGTPTLETANKNHETDGGSAHGDDDDDGPHFEPVVPLPDKIEVKTGEEDEEEFFCNRAKLFRFDVESKEWKERGIGNVKILRHKTSGKIRLLMRREQVLKICANHYISPDMKLTPNAGSDRSFVWHALDYADELPKPEQLAIRFKTPEEAALFKCKFEEAQSILKAPGTNVAMASNQAVRIVKEPTSHDNKDICKSDAGNLNFEFQVAKKEGSWWHCNSCSLKNASTAKKCVSCQNLNPSNKELVGPPLAETVFTPKTSPENVQDRFALVTPKKEGHWDCSICLVRNEPTVSRCIACQNTKSANKSGSSFVHQASFKFGQGDLPKPINSDFRSVFSTKEGQWDCSACLVQNEGSSTKCAACQNPRKQSLPATSIPTPASFKFGTSETSKTLKSGFEDMFAKKEGQWDCSSCLVRNEANATRCVACQNPDKPSPSTSVPAPASFKFGTSETSKAPKSGFEGMFTKKEGQWDCSVCLVRNEASATKCIACQNPGKQNQTTSAVSTPASSETSKAPKSGFEGMFTKKEGQWDCSVCLVRNEASATKCIACQNPGKQNQTTSAVSTPASSETSKAPKSGFEGMFTKKEGQWDCSVCLVRNEASATKCIACQCPSKQNQTTAISTPASSEISKAPKSGFEGMFIRKGQWDCSVCCVQNESSSLKCVACDASKPTHKPIAEAPSAFTLGSEMKLHDSSGSQVGTGFKSNFSEKASKFGNTEQGFKFGHVDQENSPSFMFQGSSNTEFKSTKEGFSIPVSADGFKFGISEPGNQEKKSEKPLENGTGFQAQDISGQKNGRGVIFGQTSSTFTFADLAKSTSGEGFQFGKKDPNFKGFSGAGEKLFSSQYGKMANKANTSGDFEKDDDAYKTEDSDDIHFEPVVQMPEKVELVTGEEDEKVLYSQRVKLFRFDAEVSQWKERGLGNLKILKNEVNGKLRMLMRREQVLKVCANHWITTTMNLKPLSGSDRAWMWLASDFSDGDAKLEQLAAKFKTPELAEEFKQKFEECQRLLLDIPLQTPHKLVDTGRAAKLIQRAEEMKSGLKDFKTFLTNDQTKVTEEENKGSGTGAAGASDTTIKPNPENTGPTLEWDNYDLREDALDDSVSSSSVHASPLASSPVRKNLFRFGESTTGFNFSFKSALSPSKSPAKLNQSGTSVGTDEESDVTQEEERDGQYFEPVVPLPDLVEVSSGEENEQVVFSHRAKLYRYDKDVGQWKERGIGDIKILQNYDNKQVRIVMRRDQVLKLCANHRITPDMTLQNMKGTERVWLWTACDFADGERKVEHLAVRFKLQDVADSFKKIFDEAKTAQEKDSLITPHVSRSSTPRESPCGKIAVAVLEETTRERTDVIQGDDVADATSEVEVSSTSETTPKAVVSPPKFVFGSESVKSIFSSEKSKPFAFGNSSATGSLFGFSFNAPLKSNNSETSSVAQSGSESKVEPKKCELSKNSDIEQSSDSKVKNLFASFPTEESSINYTFKTPEKAKEKKKPEDSPSDDDVLIVYELTPTAEQKALATKLKLPPTFFCYKNRPDYVSEEEEDDEDFETAVKKLNGKLYLDGSEKCRPLEENTADNEKECIIVWEKKPTVEEKAKADTLKLPPTFFCGVCSDTDEDNGNGEDFQSELQKVQEAQKSQTEEITSTTDSVYTGGTEVMVPSFCKSEEPDSITKSISSPSVSSETMDKPVDLSTRKEIDTDSTSQGESKIVSFGFGSSTGLSFADLASSNSGDFAFGSKDKNFQWANTGAAVFGTQSVGTQSAGKVGEDEDGSDEEVVHNEDIHFEPIVSLPEVEVKSGEEDEEILFKERAKLYRWDRDVSQWKERGVGDIKILWHTMKNYYRILMRRDQVFKVCANHVITKTMELKPLNVSNNALVWTASDYADGEAKVEQLAVRFKTKEVADCFKKTFEECQQNLMKLQKGHVSLAAELSKETNPVVFFDVCADGEPLGRITMELFSNIVPRTAENFRALCTGEKGFGFKNSIFHRVIPDFVCQGGDITKHDGTGGQSIYGDKFEDENFDVKHTGPGLLSMANQGQNTNNSQFVITLKKAEHLDFKHVVFGFVKDGMDTVKKIESFGSPKGSVCRRITITECGQI

274 NP_055839.2 NP_009166.2

>NP_055839.2

MADHVQSLAQLENLCKQLYETTDTTTRLQAEKALVEFTNSPDCLSKCQLLLERGSSSYSQLLAATCLTKLVSRTNNPLPLEQRIDIRNYVLNYLATRPKLATFVTQALIQLYARITKLGWFDCQKDDYVFRNAITDVTRFLQDSVEYCIIGVTILSQLTNEINQADTTHPLTKHRKIASSFRDSSLFDIFTLSCNLLKQASGKNLNLNDESQHGLLMQLLKLTHNCLNFDFIGTSTDESSDDLCTVQIPTSWRSAFLDSSTLQLFFDLYHSIPPSFSPLVLSCLVQIASVRRSLFNNAERAKFLSHLVDGVKRILENPQSLSDPNNYHEFCRLLARLKSNYQLGELVKVENYPEVIRLIANFTVTSLQHWEFAPNSVHYLLSLWQRLAASVPYVKATDPHMLETYTPEVTKAYITSRLESVHIILRDGLEDPLEDTGLVQQQLDQLSTIGRCEYEKTCALLVQLFDQSAQSYQELLQSASASPMDIAVQEGRLTWLVYIIGAVIGGRVSFASTDEQDAMDGELVCRVLQLMNLTDSRLAQAGNEKLELAMLSFFEQFRKIYIGDQVQKSSKLYRRLSEVLGLNDETMVLSVFIGKIITNLKYWGRCEPITSKTLQLLNDLSIGYSSVRKLVKLSAVQFMLNNHTSEHFSFLGINNQSNLTDMRCRTTFYTALGRLLMVDLGEDEDQYEQFMLPLTAAFEAVAQMFSTNSFNEQEAKRTLVGLVRDLRGIAFAFNAKTSFMMLFEWIYPSYMPILQRAIELWYHDPACTTPVLKLMAELVHNRSQRLQFDVSSPNGILLFRETSKMITMYGNRILTLGEVPKDQVYALKLKGISIYFSMLKAALSGSYVNFGVFRLYGDDALDNALQTFIKLLLSIPHSDLLDYPKLSQSYYSLLEVLTQDHMNFIASLEPHVIMYILSSISEGLTALDTMVCTGCCSCLDHIVTYLFKQLSRSTKKRTTPLNQESDRFLHIMQQHPEMIQQMLSTVLNIIIFEDCRNQWSMSRPLLGLILLNEKYFSDLRNSIVNSQPPEKQQAMHLCFENLMEGIERNLLTKNRDRFTQNLSAFRREVNDSMKNSTYGVNSNDMMS

>NP_009166.2

MDEQALLGLNPNADSDFRQRALAYFEQLKISPDAWQVCAEALAQRTYSDDHVKFFCFQVLEHQVKYKYSELTTVQQQLIRETLISWLQAQMLNPQPEKTFIRNKAAQVFALLFVTEYLTKWPKFFFDILSVVDLNPRGVDLYLRILMAIDSELVDRDVVHTSEEARRNTLIKDTMREQCIPNLVESWYQILQNYQFTNSEVTCQCLEVVGAYVSWIDLSLIANDRFINMLLGHMSIEVLREEACDCLFEVVNKGMDPVDKMKLVESLCQVLQSAGFFSIDQEEDVDFLARFSKLVNGMGQSLIVSWSKLIKNGDIKNAQEALQAIETKVALMLQLLIHEDDDISSNIIGFCYDYLHILKQLTVLSDQQKANVEAIMLAVMKKLTYDEEYNFENEGEDEAMFVEYRKQLKLLLDRLAQVSPELLLASVRRVFSSTLQNWQTTRFMEVEVAIRLLYMLAEALPVSHGAHFSGDVSKASALQDMMRTLVTSGVSSYQHTSVTLEFFETVVRYEKFFTVEPQHIPCVLMAFLDHRGLRHSSAKVRSRTAYLFSRFVKSLNKQMNPFIEDILNRIQDLLELSPPENGHQSLLSSDDQLFIYETAGVLIVNSEYPAERKQALMRNLLTPLMEKFKILLEKLMLAQDEERQASLADCLNHAVGFASRTSKAFSNKQTVKQCGCSEVYLDCLQTFLPALSCPLQKDILRSGVRTFLHRMIICLEEEVLPFIPSASEHMLKDCEAKDLQEFIPLINQITAKFKIQVSPFLQQMFMPLLHAIFEVLLRPAEENDQSAALEKQMLRRSYFAFLQTVTGSGMSEVIANQGAENVERVLVTVIQGAVEYPDPIAQKTCFIILSKLVELWGGKDGPVGFADFVYKHIVPACFLAPLKQTFDLADAQTVLALSECAVTLKTIHLKRGPECVQYLQQEYLPSLQVAPEIIQEFCQALQQPDAKVFKNYLKVFFQRAKP

275 NP_002961.1 NP_009164.1

>NP_002961.1

MAKFVIRPATAADCSDILRLIKELAKYEYMEEQVILTEKDLLEDGFGEHPFYHCLVAEVPKEHWTPEGHSIVGFAMYYFTYDPWIGKLLYLEDFFVMSDYRGFGIGSEILKNLSQVAMRCRCSSMHFLVAEWNEPSINFYKRRGASDLSSEEGWRLFKIDKEYLLKMATEE

>NP_009164.1

MMLGSLAPDPGSRRHSGQAALRPRRYPTLWDRCRKRWLRPIFTQLLAAGLAYHTLLPIPSEPLFAAPGEHLHQCFVKESYCPPRVLAKEQ

276 NP_006040.1 NP_003077.2

>NP_006040.1

MLSRLQELRKEEETLLRLKAALHDQLNRLKVEELALQSMISSRRGDEMLSSHTVPEQSHDMLVHVDNEASINQTTLELSTKSHVTEEEEEEEEEESDS

>NP_003077.2

MDVDAEREKITQEIKELERILDPGSSGSHVEISESSLESDSEADSLPSEDLDPADPPISEEERWGEASNDEDDPKDKTLPEDPETCLQLNMVYQEVIQEKLAEANLLLAQNREQQEELMRDLAGSKGTKVKDGKSLPPSTYMGHFMKPYFKDKVTGVGPPANEDTREKAAQGIKAFEELLVTKWKNWEKALLRKSVVSDRLQRLLQPKLLKLEYLHQKQSKVSSELERQALEKQGREAEKEIQDINQLPEEALLGNRLDSHDWEKISNINFEGSRSAEEIRKFWQNSEHPSINKQEWSREEEERLQAIAAAHGHLEWQKIAEELGTSRSAFQCLQKFQQHNKALKRKEWTEEEDRMLTQLVQEMRVGSHIPYRRIVYYMEGRDSMQLIYRWTKSLDPGLKKGYWAPEEDAKLLQAVAKYGEQDWFKIREEVPGRSDAQCRDRYLRRLHFSLKKGRWNLKEEEQLIELIEKYGVGHWAKIASELPHRSGSQCLSKWKIMMGKKQGLRRRRRRARHSVRWSSTSSSGSSSGSSGGSSSSSSSSSEEDEPEQAQAGEGDRALLSPQYMVPDMDLWVPARQSTSQPWRGGAGAWLGGPAASLSPPKGSSASQGGSKEASTTAAAPGEETSPVQVPARAHGPVPRSAQASHSADTRPAGAEKQALEGGRRLLTVPVETVLRVLRANTAARSCTQKEQLRQPPLPTSSPGVSSGDSVARSHVQWLRHRATQSGQRRWRHALHRRLLNRRLLLAVTPWVGDVVVPCTQASQRPAVVQTQADGLREQLQQARLASTPVFTLFTQLFHIDTAGCLEVVRERKALPPRLPQAGARDPPVHLLQASSSAQSTPGHLFPNVPAQEASKSASHKGSRRLASSRVERTLPQASLLASTGPRPKPKTVSELLQEKRLQEARAREATRGPVVLPSQLLVSSSVILQPPLPHTPHGRPAPGPTVLNVPLSGPGAPAAAKPGTSGSWQEAGTSAKDKRLSTMQALPLAPVFSEAEGTAPAASQAPALGPGQISVSCPESGLGQSQAPAASRKQGLPEAPPFLPAAPSPTPLPVQPLSLTHIGGPHVATSVPLPVTWVLTAQGLLPVPVPAVVSLPRPAGTPGPAGLLATLLPPLTETRAAQGPRAPALSSSWQPPANMNREPEPSCRTDTPAPPTHALSQSPAEADGSVAFVPGEAQVAREIPEPRTSSHADPPEAEPPWSGRLPAFGGVIPATEPRGTPGSPSGTQEPRGPLGLEKLPLRQPGPEKGALDLEKPPLPQPGPEKGALDLGLLSQEGEAATQQWLGGQRGVRVPLLGSRLPYQPPALCSLRALSGLLLHKKALEHKATSLVVGGEAERPAGALQASLGLVRGQLQDNPAYLLLRARFLAAFTLPALLATLAPQGVRTTLSVPSRVGSESEDEDLLSELELADRDGQPGCTTATCPIQGAPDSGKCSASSCLDTSNDPDDLDVLRTRHARHTRKRRRLV

277 CAA45056.1 AAH71758.1

>CAA45056.1

MGIRLLCRVAFCFLAVGLVDVKVTQSSRYLVKRTGEKVFLECVQDMDHENMFWYRQDPGLGLRLIYFSYDVKMKEKGDIPEGYSVSREKKERFSLILESASTNQTSMYLCASSSTGLPYGYTFGSGTRLTVVEDLNKVFPPEVAVFEPSEAEISHTQKATLVCLATGFFPDHVELSWWVNGKEVHSGVSTDPQPLKEQPALNDSRYCLSSRLRVSATFWQNPRNHFRCQVQFYGLSENDEWTQDRAKPVTQIVSAEAWGRADCGFTSVSYQQGVLSATILYEILLGKATLYAVLVSALVLMAMVKRKDF

>AAH71758.1

MAMLLGASVLILWLQPDWVNSQQKNDDQQVKQNSPSLSVQEGRISILNCDYTNSMFDYFLWYKKYPAEGPTFLISISSIKDKNEDGRFTVFLNKSAKHLSLHIVPSQPGDSAVYFCAVRYNNNDMRFGAGTRLTVKPNIQNPDPAVYQLRDSKSSDKSVCLFTDFDSQTNVSQSKDSDVYITDKTVLDMRSMDFKSNSAVAWSNKSDFACANAFNNSIIPEDTFFPSPESSCDVKLVEKSFETDTNLNFQNLSVIGFRILLLKVAGFNLLMTLRLWSS

278 NP_003664.1 NP_005250.1

>NP_003664.1

MATSELSCEVSEENCERREAFWAEWKDLTLSTRPEEGCSLHEEDTQRHETYHQQGQCQVLVQRSPWLMMRMGILGRGLQEYQLPYQRVLPLPIFTPAKMGATKEEREDTPIQLQELLALETALGGQCVDRQEVAEITKQLPPVVPVSKPGALRRSLSRSMSQEAQRG

>NP_005250.1

MQKLQLCVYIYLFMLIVAGPVDLNENSEQKENVEKEGLCNACTWRQNTKSSRIEAIKIQILSKLRLETAPNISKDVIRQLLPKAPPLRELIDQYDVQRDDSSDGSLEDDDYHATTETIITMPTESDFLMQVDGKPKCCFFKFSSKIQYNKVVKAQLWIYLRPVETPTTVFVQILRLIKPMKDGTRYTGIRSLKLDMNPGTGIWQSIDVKTVLQNWLKQPESNLGIEIKALDENGHDLAVTFPGPGEDGLNPFLEVKVTDTPKRSRRDFGLDCDEHSTESRCCRYPLTVDFEAFGWDWIIAPKRYKANYCSGECEFVFLQKYPHTHLVHQANPRGSAGPCCTPTKMSPINMLYFNGKEQIIYGKIPAMVVDRCGCS

279 NP_001010844.1 NP_001560.2

>NP_001010844.1

MSLQKTPPTRVFVELVPWADRSRENNLASGRETLPGLRHPLSSTQAQTATREVQVSGTSEVSAGPDRAQVVVRVSSTKEAAAEAKKSVCRRLDYITQSLQQQGVQAENITVTKDFRRVENAYHMEAEVCITFTEFGKMQNICNFLVEKLDSSVVISPPQFYHTPGSVENLRRQACLVAVENAWRKAQEVCNLVGQTLGKPLLIKEEETKEWEGQIDDHQSSRLSSSLTVQQKIKSATIHAASKVFITFEVKGKEKRKKHL

>NP_001560.2

MAGGPGPGEPAAPGAQHFLYEVPPWVMCRFYKVMDALEPADWCQFAALIVRDQTELRLCERSGQRTASVLWPWINRNARVADLVHILTHLQLLRARDIITAWHPPAPLPSPGTTAPRPSSIPAPAEAEAWSPRKLPSSASTFLSPAFPGSQTHSGPELGLVPSPASLWPPPPSPAPSSTKPGPESSVSLLQGARPFPFCWPLCEISRGTHNFSEELKIGEGGFGCVYRAVMRNTVYAVKRLKENADLEWTAVKQSFLTEVEQLSRFRHPNIVDFAGYCAQNGFYCLVYGFLPNGSLEDRLHCQTQACPPLSWPQRLDILLGTARAIQFLHQDSPSLIHGDIKSSNVLLDERLTPKLGDFGLARFSRFAGSSPSQSSMVARTQTVRGTLAYLPEEYIKTGRLAVDTDTFSFGVVVLETLAGQRAVKTHGARTKYLKDLVEEEAEEAGVALRSTQSTLQAGLAADAWAAPIAMQIYKKHLDPRPGPCPPELGLGLGQLACCCLHRRAKRRPPMTQVYERLEKLQAVVAGVPGHSEAASCIPPSPQENSYVSSTGRAHSGAAPWQPLAAPSGASAQAAEQLQRGPNQPVESDESLGGLSAALRSWHLTPSCPLDPAPLREAGCPQGDTAGESSWGSGPGSRPTAVEGLALGSSASSSSEPPQIIINPARQKMVQKLALYEDGALDSLQLLSSSSLPGLGLEQDRQGPEESDEFQS

280 NP_001146.2 NP_078805.3

>NP_001146.2

MAKPAQGAKYRGSIHDFPGFDPNQDAEALYTAMKGFGSDKEAILDIITSRSNRQRQEVCQSYKSLYGKDLIADLKYELTGKFERLIVGLMRPPAYCDAKEIKDAISGIGTDEKCLIEILASRTNEQMHQLVAAYKDAYERDLEADIIGDTSGHFQKMLVVLLQGTREEDDVVSEDLVQQDVQDLYEAGELKWGTDEAQFIYILGNRSKQHLRLVFDEYLKTTGKPIEASIRGELSGDFEKLMLAVVKCIRSTPEYFAERLFKAMKGLGTRDNTLIRIMVSRSELDMLDIREIFRTKYEKSLYSMIKNDTSGEYKKTLLKLSGGDDDAAGQFFPEAAQVAYQMWELSAVARVELKGTVRPANDFNPDADAKALRKAMKGLGTDEDTIIDIITHRSNVQRQQIRQTFKSHFGRDLMTDLKSEISGDLARLILGLMMPPAHYDAKQLKKAMEGAGTDEKALIEILATRTNAEIRAINEAYKEDYHKSLEDALSSDTSGHFRRILISLATGHREEGGENLDQAREDAQVAAEILEIADTPSGDKTSLETRFMTILCTRSYPHLRRVFQEFIKMTNYDVEHTIKKEMSGDVRDAFVAIVQSVKNKPLFFADKLYKSMKGAGTDEKTLTRIMVSRSEIDLLNIRREFIEKYDKSLHQAIEGDTSGDFLKALLALCGGED

>NP_078805.3

MADVLSVLRQYNIQKKEIVVKGDEVIFGEFSWPKNVKTNYVVWGTGKEGQPREYYTLDSILFLLNNVHLSHPVYVRRAATENIPVVRRPDRKDLLGYLNGEASTSASIDRSAPLEIGLQRSTQVKRAADEVLAEAKKPRIEDEECVRLDKERLAARLEGHKEGIVQTEQIRSLSEAMSVEKIAAIKAKIMAKKRSTIKTDLDDDITALKQRSFVDAEVDVTRDIVSRERVWRTRTTILQSTGKNFSKNIFAILQSVKAREEGRAPEQRPAPNAAPVDPTLRTKQPIPAAYNRYDQERFKGKEETEGFKIDTMGTYHGMTLKSVTEGASARKTQTPAAQPVPRPVSQARPPPNQKKGSRTPIIIIPAATTSLITMLNAKDLLQDLKFVPSDEKKKQGCQRENETLIQRRKDQMQPGGTAISVTVPYRVVDQPLKLMPQDWDRVVAVFVQGPAWQFKGWPWLLPDGSPVDIFAKIKAFHLKYDEVRLDPNVQKWDVTVLELSYHKRHLDRPVFLRFWETLDRYMVKHKSHLRF

281 NP_055547.1 NP_291025.3

>NP_055547.1
[truncated: 5,295,721 more chars]
