# Supplementary material for: A novel matrix of sequence descriptors for predicting protein-protein interactions from amino acid sequences
Source: PLoS One. 2019 Jun 7;14(6):e0217312. doi: 10.1371/journal.pone.0217312 (PMC6555512; doi:10.1371/journal.pone.0217312)
Supplement: S4 File — There are 4262 protein-protein pairs from total 661 proteins, and the first column is protein ID from HPRD, the second column is the other protein ID and the two proteins constitute the positive Protein-protein interaction and protein identity of all the proteins from S4 file is below 25%. (DOC) [file pone.0217312.s004.doc]

**Online support information D**

There are 4262 protein-protein pairs from total 661 proteins, and the first column is protein ID from HPRD, the second column is the other protein ID and the two proteins constitute the positive Protein-protein interaction and protein identity of all the proteins from Supp-C and Supp-D is below 25%;

1 NP_003073.1 NP_000365.3

>NP_003073.1

MGTPPGLQTDCEALLSRFQETDSVRFEDFTELWRNMKFGTIFCGRMRNLEKNMFTKEALALAWRYFLPPYTFQIRVGALYLLYGLYNTQLCQPKQKIRVALKDWDEVLKFQQDLVNAQHFDAAYIFRKLRLDRAFHFTAMPKLLSYRMKKKIHRAEVTEEFKDPSDRVMKLITSDVLEEMLNVHDHYQNMKHVISVDKSKPDKALSLIKDDFFDNIKNIVLEHQQWHKDRKNPSLKSKTNDGEEKMEGNSQETERCERAESLAKIKSKAFSVVIQASKSRRHRQVKLDSSDSDSASGQGQVKATRKKEKKERLKPAGRKMSLRNKGNVQNIHKEDKPLSLSMPVITEEEENESLSGTEFTASKKRRKH

>NP_000365.3

MEANGLGPQGFPELKNDTFLRAAWGEETDYTPVWCMRQAGRYLPEFRETRAAQDFFSTCRSPEACCELTLQPLRRFPLDAAIIFSDILVVPQALGMEVTMVPGKGPSFPEPLREEQDLERLRDPEVVASELGYVFQAITLTRQRLAGRVPLIGFAGAPWTLMTYMVEGGGSSTMAQAKRWLYQRPQASHQLLRILTDALVPYLVGQVVAGAQALQLFESHAGHLGPQLFNKFALPYIRDVAKQVKARLREAGLAPVPMIIFAKDGHFALEELAQAGYEVVGLDWTVAPKKARECVGKTVTLQGNLDPCALYASEEEIGQLVKQMLDDFGPHRYIANLGHGLYPDMDPEHVGAFVDAVHKHSRLLRQN

2 NP_006707.1 NP_000476.1

>NP_006707.1

MNSGAMRIHSKGHFQGGIQVKNEKNRPSLKSLKTDNRPEKSKCKPLWGKVFYLDLPSVTISEKLQKDIKDLGGRVEEFLSKDISYLISNKKEAKFAQTLGRISPVPSPESAYTAETTSPHPSHDGSSFKSPDTVCLSRGKLLVEKAIKDHDFIPSNSILSNALSWGVKILHIDDIRYYIEQKKKELYLLKKSSTSVRDGGKRVGSGAQKTRTGRLKKPFVKVEDMSQLYRPFYLQLTNMPFINYSIQKPCSPFDVDKPSSMQKQTQVKLRIQTDGDKYGGTSIQLQLKEKKKKGYCECCLQKYEDLETHLLSEQHRNFAQSNQYQVVDDIVSKLVFDFVEYEKDTPKKKRIKYSVGSLSPVSASVLKKTEQKEKVELQHISQKDCQEDDTTVKEQNFLYKETQETEKKLLFISEPIPHPSNELRGLNEKMSNKCSMLSTAEDDIRQNFTQLPLHKNKQECILDISEHTLSENDLEELRVDHYKCNIQASVHVSDFSTDNSGSQPKQKSDTVLFPAKDLKEKDLHSIFTHDSGLITINSSQEHLTVQAKAPFHTPPEEPNECDFKNMDSLPSGKIHRKVKIILGRNRKENLEPNAEFDKRTEFITQEENRICSSPVQSLLDLFQTSEEKSEFLGFTSYTEKSGICNVLDIWEEENSDNLLTAFFSSPSTSTFTGF

>NP_000476.1

MADSELQLVEQRIRSFPDFPTPGVVFRDISPVLKDPASFRAAIGLLARHLKATHGGRIDYIAGLDSRGFLFGPSLAQELGLGCVLIRKRGKLPGPTLWASYSLEYGKAELEIQKDALEPGQRVVVVDDLLATGGTMNAACELLGRLQAEVLECVSLVELTSLKGREKLAPVPFFSLLQYE

3 Q5XKR4 NP_004171.2

>Q5XKR4

MLSHADLLDARLGMKDAAELLGHREAVKCRLGVGGSDPGGHPGDLAPNSDPVEGATLLPGEDITTVGSTPASLAVSAKDPDKQPGPQGGPNPSQAGQQQGQQKQKRHRTRFTPAQLNELERSFAKTHYPDIFMREELALRIGLTESRVQVWFQNRRAKWKKRKKTTNVFRAPGTLLPTPGLPQFPSAAAAAAAAMGDSLCSFHANDTRWAAAAMPGVSQLPLPPALGRQQAMAQSLSQCSLAAGPPPNSMGLSNSLAGSNGAGLQSHLYQPAFPGMVPASLPGPSNVSGSPQLCSSPDSSDVWRGTSIASLRRKALEHTVSMSFT

>NP_004171.2

MDKNIGEQLNKAYEAFRQACMDRDSAVKELQQKTENYEQRIREQQEQLSLQQTIIDKLKSQLLLVNSTQDNNYGCVPLLEDSETRKNNLTLDQPQDKVISGIAREKLPKVRRQEVSSPRKETSARSLGSPLLHERGNIEKTFWDLKEEFHKICMLAKAQKDHLSKLNIPDTATETQCSVPIQCTDKTDKQEALFKPQAKDDINRGAPSITSVTPRGLCRDEEDTSFESLSKFNVKFPPMDNDSTFLHSTPERPGILSPATSEAVCQEKFNMEFRDNPGNFVKTEETLFEIQGIDPIASAIQNLKTTDKTKPSNLVNTCIRTTLDRAACLPPGDHNALYVNSFPLLDPSDAPFPSLDSPGKAIRGPQQPIWKPFPNQDSDSVVLSGTDSELHIPRVCEFCQAVFPPSITSRGDFLRHLNSHFNGET

4 NP_996670.1 Q9Y238

>NP_996670.1

MAPKQDPKPKFQEGERVLCFHGPLLYEAKCVKVAIKDKQVKYFIHYSGWNKKSAVRPRRSEKSLKTHEDIVALFPVPEGAPSVHHPLLTSSWDEWVPESRVLKYVDTNLQKQRELQKANQEQYAEGKMRGAAPGKKTSGLQQKNVEVKTKKNKQKTPGNGDGGSTSETPQPPRKKRARVDPTVENEETFMNRVEVKVKIPEELKPWLVDDWDLITRQKQLFYLPAKKNVDSILEDYANYKKSRGNTDNKEYAVNEVVAGIKEYFNVMLGTQLLYKFERPQYAEILADHPDAPMSQVYGAPHLLRLFVRIGAMLAYTPLDEKSLALLLNYLHDFLKYLAKNSATLFSASDYEVAPPEYHRKAV

>Q9Y238

METRSSKTRRSLASRTNECQGTMWAPTSPPAGSSSPSQPTWKSSLYSSLAYSEAFHYSFAARPRRLTQLALAQRPEPQLLRLRPSSLRTQDISHLLTGVFRNLYSAEVIGDEVSASLIKARGSENERHEEFVDQLQQIRELYKQRLDEFEMLERHITQAQARAIAENERVMSQAGVQDLESLVRLPPVKSVSRWCIDSELLRKHHLISPEDYYTDTVPFHSAPKGISLPGCSKLTFSCEKRSVQKKELNKKLEDSCRKKLAEFEDELDHTVDSLTWNLTPKAKERTREPLKKASQPRNKNWMNHLRVPQRELDRLLLARMESRNHFLKNPRFFPPNTRYGGKSLVFPPKKPAPIGEFQSTEPEQSCADTPVFLAKPPIGFFTDYEIGPVYEMVIALQNTTTTSRYLRVLPPSTPYFALGLGMFPGKGGMVAPGMTCQYIVQFFPDCLGDFDDFILVETQSAHTLLIPLQARRPPPVLTLSPVLDCGYCLIGGVKMTRFICKNVGFSVGRFCIMPKTSWPPLSFKAIATVGFVEQPPFGILPSVFELAPGHAILVEVLFSPKSLGKAEQTFIIMCDNCQIKELVTIGIGQLIALDLIYISGEKSQPDPGELTDLTAQHFIRFEPENLRSTARKQLIIRNATHVELAFYWQIMKPNLQPLMPGETFSMDSIKCYPDKETAFSIMPRKGVLSPHTDHEFILSFSPHELRDFHSVLQMVLEEVPEPVSSEAESLGHSSYSVDDVIVLEIEVKGSVEPFQVLLEPYALIIPGENYIGINVKKAFKMWNNSKSPIRYLWGKISDCHIIEVEPGTGVIEPSEVGDFELNFTGGVPGPTSQDLLCEIEDSPSPVVLHIEAVFKGPALIINVSALQFGLLRLGQKATNSIQIRNVSQLPATWRMKESPVSLQERPEDVSPFDIEPSSGQLHSLGECRVDITLEALHCQHLETVLELEVENGAWSYLPVYAEVQKPHVYLQSSQVEVRNLYLGVPTKTTITLINGTLLPTQFHWGKLLGHQAEFCMVTVSPKHGLLGPSEECQLKLELTAHTQEELTHLALPCHVSGMKKPLVLGISGKPQGLQVAITISKESSDCSTEQWPGHPKELRLDFGSAVPLRTRVTRQLILTNRSPIRTRFSLKFEYFGSPQNSLSKKTSLPNMPPALLKTVRMQEHLAKREQLDFMESMLSHGKGAAFFPHFSQGMLGPYQQLCIDITGCANMWGEYWDNLICTVGDLLPEVIPVHMAAVGCPISSLRTTSYTIDQAQKEPAMRFGTQVSGGDTVTRTLRLNNSSPCDIRLDWETYVPEDKEDRLVELLVFYGPPFPLRDQAGNELVCPDTPEGGCLLWSPGPSSSSEFSHETDSSVEGSSSASNRVAQKLISVILQAHEGVPSGHLYCISPKQVVVPAGGSSTIYISFTPMVLSPEILHKVECTGYALGFMSLDSKVEREIPRKRHRLQDFAVGPLKLDLHSYVRPAQLSVELDYGGSMEFQCQASDLIPEQPCSGVLSELVTTHHLKLTNTTEIPHYFRLMVSRPFSVSQDGASQDHRAPGPGQKQECEEETASADKQLVLQAQENMLVNVSFSLSLELLSYQKLPADQTLPGVDIQQSASGEREMVFTQNLLLEYTNQTTQVVPLRAVVAVPELQLSTSWVDFGTCFVSQQRVREVYLMNLSGCRSYWTMLMGQQEPAKAAVAFRVSPNSGLLEARSANAPPTSIALQVFFTARSSELYESTMVVEGVLGEKSCTLRLRGQGSYDERYMLPHQP

5 NP_006222.2 NP_006750.3

>NP_006222.2

MSLRSGGRRRADPGADGEASRDDGATSSVSALKRLERSQWTDKMDLRFGFERLKEPGEKTGWLINMHPTEILDEDKRLGSAVDYYFIQDDGSRFKVALPYKPYFYIATRKGCEREVSSFLSKKFQGKIAKVETVPKEDLDLPNHLVGLKRNYIRLSFHTVEDLVKVRKEISPAVKKNREQDHASDAYTALLSSVLQRGGVITDEEETSKKIADQLDNIVDMREYDVPYHIRLSIDLKIHVAHWYNVRYRGNAFPVEITRRDDLVERPDPVVLAFDIETTKLPLKFPDAETDQIMMISYMIDGQGYLITNREIVSEDIEDFEFTPKPEYEGPFCVFNEPDEAHLIQRWFEHVQETKPTIMVTYNGDFFDWPFVEARAAVHGLSMQQEIGFQKDSQGEYKAPQCIHMDCLRWVKRDSYLPVGSHNLKAAAKAKLGYDPVELDPEDMCRMATEQPQTLATYSVSDAVATYYLYMKYVHPFIFALCTIIPMEPDEVLRKGSGTLCEALLMVQAFHANIIFPNKQEQEFNKLTDDGHVLDSETYVGGHVEALESGVFRSDIPCRFRMNPAAFDFLLQRVEKTLRHALEEEEKVPVEQVTNFEEVCDEIKSKLASLKDVPSRIECPLIYHLDVGAMYPNIILTNRLQPSAMVDEATCAACDFNKPGANCQRKMAWQWRGEFMPASRSEYHRIQHQLESEKFPPLFPEGPARAFHELSREEQAKYEKRRLADYCRKAYKKIHITKVEERLTTICQRENSFYVDTVRAFRDRRYEFKGLHKVWKKKLSAAVEVGDAAEVKRCKNMEVLYDSLQLAHKCILNSFYGYVMRKGARWYSMEMAGIVCFTGANIITQARELIEQIGRPLELDTDGIWCVLPNSFPENFVFKTTNVKKPKVTISYPGAMLNIMVKEGFTNDQYQELAEPSSLTYVTRSENSIFFEVDGPYLAMILPASKEEGKKLKKRYAVFNEDGSLAELKGFEVKRRGELQLIKIFQSSVFEAFLKGSTLEEVYGSVAKVADYWLDVLYSKAANMPDSELFELISENRSMSRKLEDYGEQKSTSISTAKRLAEFLGDQMVKDAGLSCRYIISRKPEGSPVTERAIPLAIFQAEPTVRKHFLRKWLKSSSLQDFDIRAILDWDYYIERLGSAIQKIITIPAALQQVKNPVPRVKHPDWLHKKLLEKNDVYKQKKISELFTLEGRRQVTMAEASEDSPRPSAPDMEDFGLVKLPHPAAPVTVKRKRVLWESQEESQDLTPTVPWQEILGQPPALGTSQEEWLVWLRFHKKKWQLQARQRLARRKRQRLESAEGVLRPGAIRDGPATGLGSFLRRTARSILDLPWQIVQISETSQAGLFRLWALVGSDLHCIRLSIPRVFYVNQRVAKAEEGASYRKVNRVLPRSNMVYNLYEYSVPEDMYQEHINEINAELSAPDIEGVYETQVPLLFRALVHLGCVCVVNKQLVRHLSGWEAETFALEHLEMRSLAQFSYLEPGSIRHIYLYHHAQAHKALFGIFIPSQRRASVFVLDTVRSNQMPSLGALYSAEHGLLLEKVGPELLPPPKHTFEVRAETDLKTICRAIQRFLLAYKEERRGPTLIAVQSSWELKRLASEIPVLEEFPLVPICVADKINYGVLDWQRHGARRMIRHYLNLDTCLSQAFEMSRYFHIPIGNLPEDISTFGSDLFFARHLQRHNHLLWLSPTARPDLGGKEADDNCLVMEFDDQATVEINSSGCYSTVCVELDLQNLAVNTILQSHHVNDMEGADSMGISFDVIQQASLEDMITGGQAASAPASYDETALCSNTFRILKSMVVGWVKEITQYHNIYADNQVMHFYRWLRSPSSLLHDPALHRTLHNMMKKLFLQLIAEFKRLGSSVIYANFNRIILCTKKRRVEDAIAYVEYITSSIHSKETFHSLTISFSRCWEFLLWMDPSNYGGIKGKVSSRIHCGLQDSQKAGGAEDEQENEDDEEERDGEEEEEAEESNVEDLLENNWNILQFLPQAASCQNYFLMIVSAYIVAVYHCMKDGLRRSAPGSTPVRRRGASQLSQEAEGAVGALPGMITFSQDYVANELTQSFFTITQKIQKKVTGSRNSTELSEMFPVLPGSHLLLNNPALEFIKYVCKVLSLDTNITNQVNKLNRDLLRLVDVGEFSEEAQFRDPCRSYVLPEVICRSCNFCRDLDLCKDSSFSEDGAVLPQWLCSNCQAPYDSSAIEMTLVEVLQKKLMAFTLQDLVCLKCRGVKETSMPVYCSCAGDFALTIHTQVFMEQIGIFRNIAQHYGMSYLLETLEWLLQKNPQLGH

>NP_006750.3

MSRFVQDLSKAMSQDGASQFQEVIRQELELSVKKELEKILTTASSHEFEHTKKDLDGFRKLFHRFLQEKGPSVDWGKIQRPPEDSIQPYEKIKARGLPDNISSVLNKLVVVKLNGGLGTSMGCKGPKSLIGVRNENTFLDLTVQQIEHLNKTYNTDVPLVLMNSFNTDEDTKKILQKYNHCRVKIYTFNQSRYPRINKESLLPVAKDVSYSGENTEAWYPPGHGDIYASFYNSGLLDTFIGEGKEYIFVSNIDNLGATVDLYILNHLMNPPNGKRCEFVMEVTNKTRADVKGGTLTQYEGKLRLVEIAQVPKAHVDEFKSVSKFKIFNTNNLWISLAAVKRLQEQNAIDMEIIVNAKTLDGGLNVIQLETAVGAAIKSFENSLGINVPRSRFLPVKTTSDLLLVMSNLYSLNAGSLTMSEKREFPTVPLVKLGSSFTKVQDYLRRFESIPDMLELDHLTVSGDVTFGKNVSLKGTVIIIANHGDRIDIPPGAVLENKIVSGNLRILDH

6 NP_009171.1 NP_003105.2

>NP_009171.1

MLEAPGPSDGCELSNPSASRVSCAGQMLEVQPGLYFGGAAAVAEPDHLREAGITAVLTVDSEEPSFKAGPGVEDLWRLFVPALDKPETDLLSHLDRCVAFIGQARAEGRAVLVHCHAGVSRSVAIITAFLMKTDQLPFEKAYEKLQILKPEAKMNEGFEWQLKLYQAMGYEVDTSSAIYKQYRLQKVTEKYPELQNLPQELFAVDPTTVSQGLKDEVLYKCRKCRRSLFRSSSILDHREGSGPIAFAHKRMTPSSMLTTGRQAQCTSYFIEPVQWMESALLGVMDGQLLCPKCSAKLGSFNWYGEQCSCGRWITPAFQIHKNRVDEMKILPVLGSQTGKI

>NP_003105.2

MTTKDYPSLWGFGTTKTFKIPIEHLDFKYIEKCSDVKHLEKILCVLRSGEEGYYPELTEFCEKHLQALAPESRALRKDKPAATAASFTAEEWEKIDGDIKSWVSEIKKEEDKMHFHETETFPAMKDNLPPVRGSNSCLHVGKEKYSKRPTKKKTPRDYAEWDKFDVEKECLKIDEDYKEKTVIDKSHLSKIETRIDTAGLTEKEKDFLATREKEKGNEAFNSGDYEEAVMYYTRSISALPTVVAYNNRAQAEIKLQNWNSAFQDCEKVLELEPGNVKALLRRATTYKHQNKLREATEDLSKVLDVEPDNDLAKKTLSEVERDLKNSEAASETQTKGKRMVIQEIENSEDEEGKSGRKHEDGGGDKKPAEPAGAARAAQPCVMGNIQKKLTGKAEGGKRPARGAPQRGQTPEAGADKRSPRRASAAAAAGGGATGHPGGGQGAENPAGLKSQGNELFRSGQFAEAAGKYSAAIALLEPAGSEIADDLSILYSNRAACYLKEGNCSGCIQDCNRALELHPFSMKPLLRRAMAYETLEQYGKAYVDYKTVLQIDCGLQLANDSVNRLSRILMELDGPNWREKLSPIPAVPASVPLQAWHPAKEMISKQAGDSSSHRQQGITDEKTFKALKEEGNQCVNDKNYKDALSKYSECLKINNKECAIYTNRALCYLKLCQFEEAKQDCDQALQLADGNVKAFYRRALAHKGLKNYQKSLIDLNKVILLDPSIIEAKMELEEVTRLLNLKDKTAPFNKEKERRKIEIQEVNEGKEEPGRPAGEVSMGCLASEKGGKSSRSPEDPEKLPIAKPNNAYEFGQIINALSTRKDKEACAHLLAITAPKDLPMFLSNKLEGDTFLLLIQSLKNNLIEKDPSLVYQHLLYLSKAERFKMMLTLISKGQKELIEQLFEDLSDTPNNHFTLEDIQALKRQYEL

7 O95766 Q12882

>O95766

MAAAAAGAGSGPWAAQEKQFPPALLSFFIYNPRFGPREGQEENKILFYHPNEVEKNEKIRNVGLCEAIVQFTRTFSPSKPAKSLHTQKNRQFFNEPEENFWMVMVVRNPIIEKQSKDGKPVIEYQEEELLDKVYSSVLRQCYSMYKLFNGTFLKAMEDGGVKLLKERLEKFFHRYLQTLHLQSCDLLDIFGGISFFPLDKMTYLKIQSFINRMEESLNIVKYTAFLYNDQLIWSGLEQDDMRILYKYLTTSLFPRHIEPELAGRDSPIRAEMPGNLQHYGRFLTGPLNLNDPDAKCRFPKIFVNTDDTYEELHLIVYKAMSAAVCFMIDASVHPTLDFCRRLDSIVGPQLTVLASDICEQFNINKRMSGSEKEPQFKFIYFNHMNLAEKSTVHMRKTPSVSLTSVHPDLMKILGDINSDFTRVDEDEEIIVKAMSDYWVVGKKSDRRELYVILNQKNANLIEVNEEVKKLCATQFNNIFFLD

>Q12882

MAPVLSKDSADIESILALNPRTQTHATLCSTSAKKLDKKHWKRNPDKNCFNCEKLENNFDDIKHTTLGERGALREAMRCLKCADAPCQKSCPTNLDIKSFITSIANKNYYGAAKMIFSDNPLGLTCGMVCPTSDLCVGGCNLYATEEGPINIGGLQQFATEVFKAMSIPQIRNPSLPPPEKMSEAYSAKIALFGAGPASISCASFLARLGYSDITIFEKQEYVGGLSTSEIPQFRLPYDVVNFEIELMKDLGVKIICGKSLSVNEMTLSTLKEKGYKAAFIGIGLPEPNKDAIFQGLTQDQGFYTSKDFLPLVAKGSKAGMCACHSPLPSIRGVVIVLGAGDTAFDCATSALRCGARRVFIVFRKGFVNIRAVPEEMELAKEEKCEFLPFLSPRKVIVKGGRIVAMQFVRTEQDETGKWNEDEDQMVHLKADVVISAFGSVLSDPKVKEALSPIKFNRWGLPEVDPETMQTSEAWVFAGGDVVGLANTTVESVNDGKQASWYIHKYVQSQYGASVSAKPELPLFYTPIDLVDISVEMAGLKFINPFGLASATPATSTSMIRRAFEAGWGFALTKTFSLDKDIVTNVSPRIIRGTTSGPMYGPGQSSFLNIELISEKTAAYWCQSVTELKADFPDNIVIASIMCSYNKNDWTELAKKSEDSGADALELNLSCPHGMGERGMGLACGQDPELVRNICRWVRQAVQIPFFAKLTPNVTDIVSIARAAKEGGANGVTATNTVSGLMGLKSDGTPWPAVGIAKRTTYGGVSGTAIRPIALRAVTSIARALPGFPILATGGIDSAESGLQFLHSGASVLQVCSAIQNQDFTVIEDYCTGLKALLYLKSIEELQDWDGQSPATVSHQKGKPVPRIAELMDKKLPSFGPYLEQRKKIIAENKIRLKEQNVAFSPLKRNCFIPKRPIPTIKDVIGKALQYLGTFGELSNVEQVVAMIDEEMCINCGKCYMTCNDSGYQAIQFDPETHLPTITDTCTGCTLCLSVCPIVDCIKMVSRTTPYEPKRGVPLSVNPVC

8 NP_058633.2 NP_003126.1

>NP_058633.2

MAPVHGDDSLSDSGSFVSSRARREKKSKKGRQEALERLKKAKAGEKYKYEVEDFTGVYEEVDEEQYSKLVQARQDDDWIVDDDGIGYVEDGREIFDDDLEDDALDADEKGKDGKARNKDKRNVKKLAVTKPNNIKSMFIACAGKKTADKAVDLSKDGLLGDILQDLNTETPQITPPPVMILKKKRSIGASPNPFSVHTATAVPSGKIASPVSRKEPPLTPVPLKRAEFAGDDVQVESTEEEQESGAMEFEDGDFDEPMEVEEVDLEPMAAKAWDKESEPAEEVKQEADSGKGTVSYLGSFLPDVSCWDIDQEGDSSFSVQEVQVDSSHLPLVKGADEEQVFHFYWLDAYEDQYNQPGVVFLFGKVWIESAETHVSCCVMVKNIERTLYFLPREMKIDLNTGKETGTPISMKDVYEEFDEKIATKYKIMKFKSKPVEKNYAFEIPDVPEKSEYLEVKYSAEMPQLPQDLKGETFSHVFGTNTSSLELFLMNRKIKGPCWLEVKSPQLLNQPVSWCKVEAMALKPDLVNVIKDVSPPPLVVMAFSMKTMQNAKNHQNEIIAMAALVHHSFALDKAAPKPPFQSHFCVVSKPKDCIFPYAFKEVIEKKNVKVEVAATERTLLGFFLAKVHKIDPDIIVGHNIYGFELEVLLQRINVCKAPHWSKIGRLKRSNMPKLGGRSGFGERNATCGRMICDVEISAKELIRCKSYHLSELVQQILKTERVVIPMENIQNMYSESSQLLYLLEHTWKDAKFILQIMCELNVLPLALQITNIAGNIMSRTLMGGRSERNEFLLLHAFYENNYIVPDKQIFRKPQQKLGDEDEEIDGDTNKYKKGRKKAAYAGGLVLDPKVGFYDKFILLLDFNSLYPSIIQEFNICFTTVQRVASEAQKVTEDGEQEQIPELPDPSLEMGILPREIRKLVERRKQVKQLMKQQDLNPDLILQYDIRQKALKLTANSMYGCLGFSYSRFYAKPLAALVTYKGREILMHTKEMVQKMNLEVIYGDTDSIMINTNSTNLEEVFKLGNKVKSEVNKLYKLLEIDIDGVFKSLLLLKKKKYAALVVEPTSDGNYVTKQELKGLDIVRRDWCDLAKDTGNFVIGQILSDQSRDTIVENIQKRLIEIGENVLNGSVPVSQFEINKALTKDPQDYPDKKSLPHVHVALWINSQGGRKVKAGDTVSYVICQDGSNLTASQRAYAPEQLQKQDNLTIDTQYYLAQQIHPVVARICEPIDGIDAVLIATWLGLDPTQFRVHHYHKDEENDALLGGPAQLTDEEKYRDCERFKCPCPTCGTENIYDNVFDGSGTDMEPSLYRCSNIDCKASPLTFTVQLSNKLIMDIRRFIKKYYDGWLICEEPTCRNRTRHLPLQFSRTGPLCPACMKATLQPEYSDKSLYTQLCFYRYIFDAECALEKLTTDHEKDKLKKQFFTPKVLQDYRKLKNTAEQFLSRSGYSEVNLSKLFAGCAVKS

>NP_003126.1

MACAAARSPADQDRFICIYPAYLNNKKTIAEGRRIPISKAVENPTATEIQDVCSAVGLNVFLEKNKMYSREWNRDVQYRGRVRVQLKQEDGSLCLVQFPSRKSVMLYAAEMIPKLKTRTQKTGGADQSLQQGEGSKKGKGKKKK

9 NP_004119.1 NP_115568.2

>NP_004119.1

MAERGELDLTGAKQNTGVWLVKVPKYLSQQWAKASGRGEVGKLRIAKTQGRTEVSFTLNEDLANIHDIGGKPASVSAPREHPFVLQSVGGQTLTVFTESSSDKLSLEGIVVQRAECRPAASENYMRLKRLQIEESSKPVRLSQQLDKVVTTNYKPVANHQYNIEYERKKKEDGKRARADKQHVLDMLFSAFEKHQYYNLKDLVDITKQPVVYLKEILKEIGVQNVKGIHKNTWELKPEYRHYQGEEKSD

>NP_115568.2

MDPKDRKKIQFSVPAPPSQLDPRQVEMIRRRRPTPAMLFRLSEHSSPEEEASPHQRASGEGHHLKSKRPNPCAYTPPSLKAVQRIAESHLQSISNLNENQASEEEDELGELRELGYPREEDEEEEEDDEEEEEEEDSQAEVLKVIRQSAGQKTTCGQGLEGPWERPPPLDESERDGGSEDQVEDPALSEPGEEPQRPSPSEPGT

10 Q5H9F3 Q06278

>Q5H9F3

MISTAPLYSGVHNWTSSDRIRMCGINEERRAPLSDEESTTGDCQHFGSQEFCVSSSFSKVELTAVGSGSNARGADPDGSATEKLGHKSEDKPDDPQPKMDYAGNVAEAEGFLVPLSSPGDGLKLPASDSAEASNSRADCSWTPLNTQMSKQVDCSPAGVKALDSRQGVGEKNTFILATLGTGVPVEGTLPLVTTNFSPLPAPICPPAPGSASVPHSVPDAFQVPLSVPAPVPHSGLVPVQVATSVPAPSPPLAPVPALAPAPPSVPTLISDSNPLSVSASVLVPVPASAPPSGPVPLSAPAPAPLSVPVSAPPLALIQAPVPPSAPTLVLAPVPTPVLAPMPASTPPAAPAPPSVPMPTPTPSSGPPSTPTLIPAFAPTPVPAPTPAPIFTPAPTPMPAATPAAIPTSAPIPASFSLSRVCFPAAQAPAMQKVPLSFQPGTVLTPSQPLVYIPPPSCGQPLSVATLPTTLGVSSTLTLPVLPSYLQDRCLPGVLASPELRSYPYAFSVARPLTSDSKLVSLEVNRLPCTSPSGSTTTQPAPDGVPGPLADTSLVTASAKVLPTPQPLLPAPSGSSAPPHPAKMPSGTEQQTEGTSVTFSPLKSPPQLEREMASPPECSEMPLDLSSKSNRQKLPLPNQRKTPPMPVLTPVHTSSKALLSTVLSRSQRTTQAAGGNVTSCLGSTSSPFVIFPEIVRNGDPSTWVKNSTALISTIPGTYVGVANPVPASLLLNKDPNLGLNRDPRHLPKQEPISIIDQGEPKGTGATCGKKGSQAGAEGQPSTVKRYTPARIAPGLPGCQTKELSLWKPTGPANIYPRCSVNGKPTSTQVLPVGWSPYHQASLLSIGISSAGQLTPSQGAPIRPTSVVSEFSGVPSLSSSEAVHGLPEGQPRPGGSFVPEQDPVTKNKTCRIAAKPYEEQVNPVLLTLSPQTGTLALSVQPSGGDIRMNQGPEESESHLCSDSTPKMEGPQGACGLKLAGDTKPKNQVLATYMSHELVLATPQNLPKMPELPLLPHDSHPKELILDVVPSSRRGSSTERPQLGSQVDLGRVKMEKVDGDVVFNLATCFRADGLPVAPQRGQAEVRAKAGQARVKQESVGVFACKNKWQPDDVTESLPPKKMKCGKEKDSEEQQLQPQAKAVVRSSHRPKCRKLPSDPQESTKKSPRGASDSGKEHNGVRGKHKHRKPTKPESQSPGKRADSHEEGSLEKKAKSSFRDFIPVVLSTRTRSQSGSICSSFAGMADSDMGSQEVFPTEEEEEVTPTPAKRRKVRKTQRDTQYRSHHAQDKSLLSQGRRHLWRAREMPWRTEAARQMWDTNEEEEEEEEEGLLKRKKRRRQKSRKYQTGEYLTEQEDEQRRKGRADLKARKQKTSSSQSLEHRLRNRNLLLPNKVQGISDSPNGFLPNNLEEPACLENSEKPSGKRKCKTKHMATVSEEAKDVVLYCLQKDSEDVNHRDNAGYTALHEACSRGWTDILNILLEHGANVNCSAQDGTRPVHDAVVNDNLETIWLLLSYGADPTLATYSGQTAMKLASSDTMKRFLSDHLSDLQGRAEGDPGVSWDFYSSSVLEEKDGFACDLLHNPPGSSDQEGDDPMEEDDFMFELSDKPLLPCYNLQVSVSRGPCNWFLFSDVLKRLKLSSRIFQARFPHFEITTMPKAEFYRQVASSQLLTPAERPGGLDDRSPPGSSETVELVRYEPDLLRLLGSEVEFQSCNS

>Q06278

MDRASELLFYVNGRKVIEKNVDPETMLLPYLRKKLRLTGTKYGCGGGGCGACTVMISRYNPITKRIRHHPANACLIPICSLYGAAVTTVEGIGSTHTRIHPVQERIAKCHGTQCGFCTPGMVMSIYTLLRNHPEPTLDQLTDALGGNLCRCTGYRPIIDACKTFCKTSGCCQSKENGVCCLDQGINGLPEFEEGSKTSPKLFAEEEFLPLDPTQELIFPPELMIMAEKQSQRTRVFGSERMMWFSPVTLKELLEFKFKYPQAPVIMGNTSVGPEVKFKGVFHPVIISPDRIEELSVVNHAYNGLTLGAGLSLAQVKDILADVVQKLPEEKTQMYHALLKHLGTLAGSQIRNMASLGGHIISRHPDSDLNPILAVGNCTLNLLSKEGKRQIPLNEQFLSKCPNADLKPQEILVSVNIPYSRKWEFVSAFRQAQRQENALAIVNSGMRVFFGEGDGIIRELCISYGGVGPATICAKNSCQKLIGRHWNEQMLDIACRLILNEVSLLGSAPGGKVEFKRTLIISFLFKFYLEVSQILKKMDPVHYPSLADKYESALEDLHSKHHCSTLKYQNIGPKQHPEDPIGHPIMHLSGVKHATGEAIYCDDMPLVDQELFLTFVTSSRAHAKIVSIDLSEALSMPGVVDIMTAEHLSDVNSFCFFTEAEKFLATDKVFCVGQLVCAVLADSEVQAKRAAKRVKIVYQDLEPLILTIEESIQHNSSFKPERKLEYGNVDEAFKVVDQILEGEIHMGGQEHFYMETQSMLVVPKGEDQEMDVYVSTQFPKYIQDIVASTLKLPANKVMCHVRRVGGAFGGKVLKTGIIAAVTAFAANKHGRAVRCVLERGEDMLITGGRHPYLGKYKAGFMNDGRILALDMEHYSNAGASLDESLFVIEMGLLKMDNAYKFPNLRCRGWACRTNLPSNTAFRGFGFPQAALITESCITEVAAKCGLSPEKVRIINMYKEIDQTPYKQEINAKNLIQCWRECMAMSSYSLRKVAVEKFNAENYWKKKGLAMVPLKFPVGLGSRAAGQAAALVHIYLDGSVLVTHGGIEMGQGVHTKMIQVVSRELRMPMSNVHLRGTSTETVPNANISGGSVVADLNGLAVKDACQTLLKRLEPIISKNPKGTWKDWAQTAFDESINLSAVGYFRGYESDMNWEKGEGQPFEYFVYGAACSEVEIDCLTGDHKNIRTDIVMDVGCSINPAIDIGQIEGAFIQGMGLYTIEELNYSPQGILHTRGPDQYKIPAICDMPTELHIALLPPSQNSNTLYSSKGLGESGVFLGCSVFFAIHDAVSAARQERGLHGPLTLNSPLTPEKIRMACEDKFTKMIPRDEPGSYVPWNVPI

11 NP_003070.3 Q6PRD7

>NP_003070.3

MSKRPSYAPPPTPAPATQMPSTPGFVGYNPYSHLAYNNYRLGGNPGTNSRVTASSGITIPKPPKPPDKPLMPYMRYSRKVWDQVKASNPDLKLWEIGKIIGGMWRDLTDEEKQEYLNEYEAEKIEYNESMKAYHNSPAYLAYINAKSRAEAALEEESRQRQSRMEKGEPYMSIQPAEDPDDYDDGFSMKHTATARFQRNHRLISEILSESVVPDVRSVVTTARMQVLKRQVQSLMVHQRKLEAELLQIEERHQEKKRKFLESTDSFNNELKRLCGLKVEVDMEKIAAEIAQAEEQARKRQEEREKEAAEQAERSQSSIVPEEEQAANKGEEKKDDENIPMETEETHLEETTESQQNGEEGTSTPEDKESGQEGVDSMAEEGTSDSNTGSESNSATVEEPPTDPIPEDEKKE

>Q6PRD7

MGTSSTDSQQAGHRRCSTSNTSAENLTCLSLPGSPGKTAPLPGPAQAGAGQPLPKGCAAVKAEVGIPAPHTSQEVRIHIRRLLSWAAPGACGLRSTPCALPQALPQARPCPGRWFFPGCSLPTGGAQTILSLWTWRHFLNWALQQREENSGRARRVPPVPRTAPVSKGEGSHPPQNSNGEKVKTITPDVGLHQSLTSDPTVAVLRAKRAPEAHPPRSCSGSLTARVCHMGVCQGQGDTEDGRMTLMG

12 NP_997001.1 NP_004332.2

>NP_997001.1

MVNVLKGVLIECDPAMKQFLLYLDESNALGKKFIIQDIDDTHVFVIAELVNVLQERVGELMDQNAFSLTQK

>NP_004332.2

MAALVLEDGSVLRGQPFGAAVSTAGEVVFQTGMVGYPEALTDPSYKAQILVLTYPLIGNYGIPPDEMDEFGLCKWFESSGIHVAALVVGECCPTPSHWSATRTLHEWLQQHGIPGLQGVDTRELTKKLREQGSLLGKLVQNGTEPSSLPFLDPNARPLVPEVSIKTPRVFNTGGAPRILALDCGLKYNQIRCLCQRGAEVTVVPWDHALDSQEYEGLFLSNGPGDPASYPSVVSTLSRVLSEPNPRPVFGICLGHQLLALAIGAKTYKMRYGNRGHNQPCLLVGSGRCFLTSQNHGFAVETDSLPADWAPLFTNANDGSNEGIVHNSLPFFSVQFHPEHQAGPSDMELLFDIFLETVKEATAGNPGGQTVRERLTERLCPPGIPTPGSGLPPPRKVLILGSGGLSIGQAGEFDYSGSQAIKALKEENIQTLLINPNIATVQTSQGLADKVYFLPITPHYVTQVIRNERPDGVLLTFGGQTALNCGVELTKAGVLARYGVRVLGTPVETIELTEDRRAFAARMAEIGEHVAPSEAANSLEQAQAAAERLGYPVLVRAAFALGGLGSGFASNREELSALVAPAFAHTSQVLVDKSLKGWKEIEYEVVRDAYGNCVTVCNMENLDPLGIHTGESIVVAPSQTLNDREYQLLRQTAIKVTQHLGIVGECNVQYALNPESEQYYIIEVNARLSRSSALASKATGYPLAYVAAKLALGIPLPELRNSVTGGTAAFEPSVDYCVVKIPRWDLSKFLRVSTKIGSCMKSVGEVMGIGRSFEEAFQKALRMVDENCVGFDHTVKPVSDMELETPTDKRIFVVAAALWAGYSVDRLYELTRIDRWFLHRMKRIIAHAQLLEQHRGQPLPPDLLQQAKCLGFSDKQIALAVLSTELAVRKLRQELGICPAVKQIDTVAAEWPAQTNYLYLTYWGTTHDLTFRTPHVLVLGSGVYRIGSSVEFDWCAVGCIQQLRKMGYKTIMVNYNPETVSTDYDMCDRLYFDEISFEVVMDIYELENPEGVILSMGGQLPNNMAMALHRQQCRVLGTSPEAIDSAENRFKFSRLLDTIGISQPQWRELSDLESARQFCQTVGYPCVVRPSYVLSGAAMNVAYTDGDLERFLSSAAAVSKEHPVVISKFIQEAKEIDVDAVASDGVVAAIAISEHVENAGVHSGDATLVTPPQDITAKTLERIKAIVHAVGQELQVTGPFNLQLIAKDDQLKVIECNVRVSRSFPFVSKTLGVDLVALATRVIMGEEVEPVGLMTGSGVVGVKVPQFSFSRLAGADVVLGVEMTSTGEVAGFGESRCEAYLKAMLSTGFKIPKKNILLTIGSYKNKSELLPTVRLLESLGYSLYASLGTADFYTEHGVKVTAVDWHFEEAVDGECPPQRSILEQLAEKNFELVINLSMRGAGGRRLSSFVTKGYRTRRLAADFSVPLIIDIKCTKLFVEALGQIGPAPPLKVHVDCMTSQKLVRLPGLIDVHVHLREPGGTHKEDFASGTAAALAGGITMVCAMPNTRPPIIDAPALALAQKLAEAGARCDFALFLGASSENAGTLGTVAGSAAGLKLYLNETFSELRLDSVVQWMEHFETWPSHLPIVAHAEQQTVAAVLMVAQLTQRSVHICHVARKEEILLIKAAKARGLPVTCEVAPHHLFLSHDDLERLGPGKGEVRPELGSRQDVEALWENMAVIDCFASDHAPHTLEEKCGSRPPPGFPGLETMLPLLLTAVSEGRLSLDDLLQRLHHNPRRIFHLPPQEDTYVEVDLEHEWTIPSHMPFSKAHWTPFEGQKVKGTVRRVVLRGEVAYIDGQVLVPPGYGQDVRKWPQGAVPQLPPSAPATSEMTTTPERPRRGIPGLPDGRFHLPPRIHRASDPGLPAEEPKEKSSRKVAEPELMGTPDGTCYPPPPVPRQASPQNLGTPGLLHPQTSPLLHSLVGQHILSVQQFTKDQMSHLFNVAHTLRMMVQKERSLDILKGKVMASMFYEVSTRTSSSFAAAMARLGGAVLSFSEATSSVQKGESLADSVQTMSCYADVVVLRHPQPGAVELAAKHCRRPVINAGDGVGEHPTQALLDIFTIREELGTVNGMTITMVGDLKHGRTVHSLACLLTQYRVSLRYVAPPSLRMPPTVRAFVASRGTKQEEFESIEEALPDTDVLYMTRIQKERFGSTQEYEACFGQFILTPHIMTRAKKKMVVMHPMPRVNEISVEVDSDPRAAYFRQAENGMYIRMALLATVLGRF

13 AAA93070.1 NP_660198.1

>AAA93070.1

MSGCRVFIGRLNPAAREKDVERFFKGYGRIRDIDLKRGFGFVEFEDPRDADDAVYELDGKELCSERVTIEHARARSRGGRGRGRYSDRFSSRRPRNDRRNAPPVRTENRLIVENLSSRVSWQDLKDFMRQAGEVTFADAHRPKLNEGVVEFASYGDLKNAIEKLSGKEINGRKIKLIEGSKRHSRSRSRSRSRTRSSSRSRSRSRSRSRKSYSRSRSRSRSRSRSKSRSVSRSPVPEKSQKRGSSSRSKSPASVDRQRSRSRSRSRSVDSGN

>NP_660198.1

MLIPFSMKNCFQLLCNCQVPAAGFKKTVKNGLILQSISNDVYQNLAVEDWIHDHMNLEGKPILFFWQNSPSVVIGRHQNPWQECNLNLMREEGIKLARRRSGGGTVYHDMGNINLTFFTTKKKYDRMENLKLIVRALNAVQPQLDVQATKRFDLLLDGQFKISGTASKIGRTTAYHHCTLLCSTDGTFLSSLLKSPYQGIRSNATASIPSLVKNLLEKDPTLTCEVLMNAVATEYAAYHQIDNHIHLINPTDETLFPGINSKAKELQTWEWIYGKTPKFSINTSFHVLYEQSHLEIKVFIDIKNGRIEICNIEAPDHWLPLEIRDKLNSSLIGSKFCPTETTMLTNILLRTCPQDHKLNSKWNILCEKIKGIM

14 NP_003784.2 NP_003977.1

>NP_003784.2

MAPWLQLLSLLGLLPGAVAAPAQPRAASFQAWGPPSPELLAPTRFALEMFNRGRAAGTRAVLGLVRGRVRRAGQGSLYSLEATLEEPPCNDPMVCRLPVSKKTLLCSFQVLDELGRHVLLRKDCGPVDTKVPGAGEPKSAFTQGSAMISSLSQNHPDNRNETFSSVISLLNEDPLSQDLPVKMASIFKNFVITYNRTYESKEEARWRLSVFVNNMVRAQKIQALDRGTAQYGVTKFSDLTEEEFRTIYLNTLLRKEPGNKMKQAKSVGDLAPPEWDWRSKGAVTKVKDQGMCGSCWAFSVTGNVEGQWFLNQGTLLSLSEQELLDCDKMDKACMGGLPSNAYSAIKNLGGLETEDDYSYQGHMQSCNFSAEKAKVYINDSVELSQNEQKLAAWLAKRGPISVAINAFGMQFYRHGISRPLRPLCSPWLIDHAVLLVGYGNRSDVPFWAIKNSWGTDWGEKGYYYLHRGSGACGVNTMASSAVVD

>NP_003977.1

MACTIQKAEALDGAHLMQILWYDEEESLYPAVWLRDNCPCSDCYLDSAKARKLLVEALDVNIGIKGLIFDRKKVYITWPDEHYSEFQADWLKKRCFSKQARAKLQRELFFPECQYWGSELQLPTLDFEDVLRYDEHAYKWLSTLKKVGIVRLTGASDKPGEVSKLGKRMGFLYLTFYGHTWQVQDKIDANNVAYTTGKLSFHTDYPALHHPPGVQLLHCIKQTVTGGDSEIVDGFNVCQKLKKNNPQAFQILSSTFVDFTDIGVDYCDFSVQSKHKIIELDDKGQVVRINFNNATRDTIFDVPVERVQPFYAALKEFVDLMNSKESKFTFKMNPGDVITFDNWRLLHGRRSYEAGTEISRHLEGAYADWDVVMSRLRILRQRVENGN

15 NP_000277.1 NP_003621.1

>NP_000277.1

MAEHGAHFTAASVADDQPSIFEVVAQDSLMTAVRPALQHVVKVLAESNPTHYGFLWRWFDEIFTLLDLLLQQHYLSRTSASFSENFYGLKRIVMGDTHKSQRLASAGLPKQQLWKSIMFLVLLPYLKVKLEKLVSSLREEDEYSIHPPSSRWKRFYRAFLAAYPFVNMAWEGWFLVQQLRYILGKAQHHSPLLRLAGVQLGRLTVQDIQALEHKPAKASMMQQPARSVSEKINSALKKAVGGVALSLSTGLSVGVFFLQFLDWWYSSENQETIKSLTALPTPPPPVHLDYNSDSPLLPKMKTVCPLCRKTRVNDTVLATSGYVFCYRCVFHYVRSHQACPITGYPTEVQHLIKLYSPEN

>NP_003621.1

MLRSVWNFLKRHKKKCIFLGTVLGGVYILGKYGQKKIREIQEREAAEYIAQARRQYHFESNQRTCNMTVLSMLPTLREALMQQLNSESLTALLKNRPSNKLEIWEDLKIISFTRSTVAVYSTCMLVVLLRVQLNIIGGYIYLDNAAVGKNGTTILAPPDVQQQYLSSIQHLLGDGLTELITVIKQAVQKVLGSVSLKHSLSLLDLEQKLKEIRNLVEQHKSSSWINKDGSKPLLCHYMMPDEETPLAVQACGLSPRDITTIKLLNETRDMLESPDFSTVLNTCLNRGFSRLLDNMAEFFRPTEQDLQHGNSMNSLSSVSLPLAKIIPIVNGQIHSVCSETPSHFVQDLLTMEQVKDFAANVYEAFSTPQQLEK

16 NP_004417.2 Q9UI17

>NP_004417.2

METESEQNSNSTNGSSSSGGSSRPQIAQMSLYERQAVQALQALQRQPNAAQYFHQFMLQQQLSNAQLHSLAAVQQATIAASRQASSPNTSTTQQQTTTTQASINLATTSAAQLISRSQSVSSPSATTLTQSVLLGNTTSPPLNQSQAQMYLRPQLGNLLQVNRTLGRNVPLASQLILMPNGAVAAVQQEVPSAQSPGVHADADQVQNLAVRNQQASAQGPQMQGSTQKAIPPGASPVSSLSQASSQALAVAQASSGATNQSLNLSQAGGGSGNSIPGSMGPGGGGQAHGGLGQLPSSGMGGGSCPRKGTGVVQPLPAAQTVTVSQGSQTEAESAAAKKAEADGSGQQNVGMNLTRTATPAPSQTLISSATYTQIQPHSLIQQQQQIHLQQKQVVIQQQIAIHHQQQFQHRQSQLLHTATHLQLAQQQQQQQQQQQQQQQPQATTLTAPQPPQVPPTQQVPPSQSQQQAQTLVVQPMLQSSPLSLPPDAAPKPPIPIQSKPPVAPIKPPQLGAAKMSAAQQPPPHIPVQVVGTRQPGTAQAQALGLAQLAAAVPTSRGMPGTVQSGQAHLASSPPSSQAPGALQECPPTLAPGMTLAPVQGTAHVVKGGATTSSPVVAQVPAAFYMQSVHLPGKPQTLAVKRKADSEEERDDVSTLGSMLPAKASPVAESPKVMDEKSSLGEKAESVANVNANTPSSELVALTPAPSVPPPTLAMVSRQMGDSKPPQAIVKPQILTHIIEGFVIQEGAEPFPVGCSQLLKESEKPLQTGLPTGLTENQSGGPLGVDSPSAELDKKANLLKCEYCGKYAPAEQFRGSKRFCSMTCAKRYNVSCSHQFRLKRKKMKEFQEANYARVRRRGPRRSSSDIARAKIQGKCHRGQEDSSRGSDNSSYDEALSPTSPGPLSVRAGHGERDLGNPNTAPPTPELHGINPVFLSSNPSRWSVEEVYEFIASLQGCQEIAEEFRSQEIDGQALLLLKEEHLMSAMNIKLGPALKICAKINVLKET

>Q9UI17

MLRPGAQLLRGLLLRSCPLQGSPGRPRSVCGREGEEKPPLSAETQWKDRAETVIIGGGCVGVSLAYHLAKAGMKDVVLLEKSELTAGSTWHAAGLTTYFHPGINLKKIHYDSIKLYEKLEEETGQVVGFHQPGSIRLATTPVRVDEFKYQMTRTGWHATEQYLIEPEKIQEMFPLLNMNKVLAGLYNPGDGHIDPYSLTMALAAGARKCGALLKYPAPVTSLKARSDGTWDVETPQGSMRANRIVNAAGFWAREVGKMIGLEHPLIPVQHQYVVTSTISEVKALKRELPVLRDLEGSYYLRQERDGLLFGPYESQEKMKVQDSWVTNGVPPGFGKELFESDLDRIMEHIKAAMEMVPVLKKADIINVVNGPITYSPDILPMVGPHQGVRNYWVAIGFGYGIIHAGGVGKYLSDWILHGEPPFDLIELDPNRYGKWTTTQYTEAKARESYGFNNIVGYPKEERFAGRPTQRVSGLYQRLESKCSMGFHAGWEQPHWFYKPGQDTQYRPSFRRTNWFEPVGSEYKQVMQRVGVTDLSPFGKFNIKGQDSIRLLDHLFANVIPKVGFTNISHMLTPKGRVYAELTVSHQSPGEFLLITGSGSELHDLRWIEEEAVKGGYDVEIKNITDELGVLGVAGPQARKVLQKLTPEDLSDDVFKFLQTKSLKVSNIPVTAIRISYTGELGWELYHRREDSVALYDAIMNAGQEEGIDNFGTYAMNALRLEKAFRAWGLEMNCDTNPLEAGLEYFVKLNKPADFIGKQALKQIKAKGLKRRLVCLTLATDDVDPEGNESIWYNGKVVGNTTSGSYSYSIQKSLAFAYVPVQLSEVGQQVEVELLGKNYPAVIIQEPLVLTEPTRNRLQKKGGKDKT

17 NP_055620.1 NP_689508.3

>NP_055620.1

MWMTPKRSKMEVDEALVFRPEWTQRYLVVEPPEGDGALCLVCRRLIVATRERDVRRHYEAEHEYYERYVADGERAALVERLRQGDLPVASFTPEERAARAGLGLCRLLALKGRGWGEGDFVYQCMEVLLREVLPEHVSVLQGVDLSPDITRQRILSIDRNLRNQLFNRARDFKAYSLALDDQAFVAYENYLLVFIRGVGPELEVQEDLLTIINLTHHFSVGALMSAILESLQTAGLSLQRMVGLTTTHTLRMIGENSGLVSYMREKAVSPNCWNVIHYSGFLHLELLSSYDVDVNQIINTISEWIVLIKTRGVRRPEFQTLLTESESEHGERVNGRCLNNWLRRGKTLKLIFSLRKEMEAFLVSVGATTVHFSDKQWLCDFGFLVDIMEHLRELSEELRVSKVFAAAAFDHICTFEVKLNLFQRHIEEKNLTDFPALREVVDELKQQNKEDEKIFDPDRYQMVICRLQKEFERHFKDLRFIKKDLELFSNPFNFKPEYAPISVRVELTKLQANTNLWNEYRIKDLGQFYAGLSAESYPIIKGVACKVASLFDSNQICEKAFSYLTRNQHTLSQPLTDEHLQALFRVATTEMEPGWDDLVRERNESNP

>NP_689508.3

MFEEKASSPSGKMGGEEKPIGAGEEKQKEGGKKKNKEGSGDGGRAELNPWPEYIYTRLEMYNILKAEHDSILAEKAEKDSKPIKVTLPDGKQVDAESWKTTPYQIACGISQGLADNTVIAKVNNVVWDLDRPLEEDCTLELLKFEDEEAQAVYWHSSAHIMGEAMERVYGGCLCYGPPIENGFYYDMYLEEGGVSSNDFSSLEALCKKIIKEKQAFERLEVKKETLLAMFKYNKFKCRILNEKVNTPTTTVYRCGPLIDLCRGPHVRHTGKIKALKIHKNSSTYWEGKADMETLQRIYGISFPDPKMLKEWEKFQEEAKNRDHRKIGRDQELYFFHELSPGSCFFLPKGAYIYNALIEFIRSEYRKRGFQEVVTPNIFNSRLWMTSGHWQHYSENMFSFEVEKELFALKPMNCPGHCLMFDHRPRSWRELPLRLADFGVLHRNELSGALTGLTRVRRFQQDDAHIFCAMEQIEDEIKGCLDFLRTVYSVFGFSFKLNLSTRPEKFLGDIEVWDQAEKQLENSLNEFGEKWELNSGDGAFYGPKIDIQIKDAIGRYHQCATIQLDFQLPIRFNLTYVSHDGDDKKRPVIVHRAILGSVERMIAILTENYGGKWPFWLSPRQVMVVPVGPTCDEYAQKVRQQFHDAKFMADIDLDPGCTLNKKIRNAQLAQYNFILVVGEKEKISGTVNIRTRDNKVHGERTISETIERLQQLKEFRSKQAEEEF

18 NP_005422.1 P28838

>NP_005422.1

MCSAFHRAESGTELLARLEGRSSLKEIEPNLFADEDSPVHGDILEFHGPEGTGKTEMLYHLTARCILPKSEGGLEVEVLFIDTDYHFDMLRLVTILEHRLSQSSEEIIKYCLGRFFLVYCSSSTHLLLTLYSLESMFCSHPSLCLLILDSLSAFYWIDRVNGGESVNLQESTLRKCSQCLEKLVNDYRLVLFATTQTIMQKASSSSEEPSHASRRLCDVDIDYRPYLCKAWQQLVKHRMFFSKQDDSQSSNQFSLVSRCLKSNSLKKHFFIIGESGVEFC

>P28838

MFLLPLPAAGRVVVRRLAVRRFGSRSLSTADMTKGLVLGIYSKEKEDDVPQFTSAGENFDKLLAGKLRETLNISGPPLKAGKTRTFYGLHQDFPSVVLVGLGKKAAGIDEQENWHEGKENIRAAVAAGCRQIQDLELSSVEVDPCGDAQAAAEGAVLGLYEYDDLKQKKKMAVSAKLYGSGDQEAWQKGVLFASGQNLARQLMETPANEMTPTRFAEIIEKNLKSASSKTEVHIRPKSWIEEQAMGSFLSVAKGSDEPPVFLEIHYKGSPNANEPPLVFVGKGITFDSGGISIKASANMDLMRADMGGAATICSAIVSAAKLNLPINIIGLAPLCENMPSGKANKPGDVVRAKNGKTIQVDNTDAEGRLILADALCYAHTFNPKVILNAATLTGAMDVALGSGATGVFTNSSWLWNKLFEASIETGDRVWRMPLFEHYTRQVVDCQLADVNNIGKYRSAGACTAAAFLKEFVTHPKWAHLDIAGVMTNKDEVPYLRKGMTGRPTRTLIEFLLRFSQDNA

19 O95926 Q9BXI3

>O95926

MAAIAASEVLVDSAEEGSLAAAAELAAQKREQRLRKFRELHLMRNEARKLNHQEVVEEDKRLKLPANWEAKKARLEWELKEEEKKKECAARGEDYEKVKLLEISAEDAERWERKKKRKNPDLGFSDYAAAQLRQYHRLTKQIKPDMETYERLREKHGEEFFPTSNSLLHGTHVPSTEEIDRMVIDLEKQIEKRDKYSRRRPYNDDADIDYINERNAKFNKKAERFYGKYTAEIKQNLERGTAV

>Q9BXI3

MEPGQPREPQEPREPGPGAETAAAPVWEEAKIFYDNLAPKKKPKSPKPQNAVTIAVSSRALFRMDEEQQIYTEQGVEEYVRYQLEHENEPFSPGPAFPFVKALEAVNRRLRELYPDSEDVFDIVLMTNNHAQVGVRLINSINHYDLFIERFCMTGGNSPICYLKAYHTNLYLSADAEKVREAIDEGIAAATIFSPSRDVVVSQSQLRVAFDGDAVLFSDESERIVKAHGLDRFFEHEKAHENKPLAQGPLKGFLEALGRLQKKFYSKGLRLECPIRTYLVTARSAASSGARALKTLRSWGLETDEALFLAGAPKGPLLEKIRPHIFFDDQMFHVAGAQEMGTVAAHVPYGVAQTPRRTAPAKQAPSAQ

20 NP_002157.2 P49753

>NP_002157.2

MKAFSPVRSVRKNSLSDHSLGISRSKTPVDDPMSLLYNMNDCYSKLKELVPSIPQNKKVSKMEILQHVIDYILDLQIALDSHPTIVSLHHQRPGQNQASRTPLTTLNTDISILSLQASEFPSELMSNDSKALCG

>P49753

MSNKLLSPHPHSVVLRSEFKMASSPAVLRASRLYQWSLKSSAQFLGSPQLRQVGQIIRVPARMAATLILEPAGRCCWDEPVRIAVRGLAPEQPVTLRASLRDEKGALFQAHARYRADTLGELDLERAPALGGSFAGLEPMGLLWALEPEKPLVRLVKRDVRTPLAVELEVLDGHDPDPGRLLCQTRHERYFLPPGVRREPVRVGRVRGTLFLPPEPGPFPGIVDMFGTGGGLLEYRASLLAGKGFAVMALAYYNYEDLPKTMETLHLEYFEEAMNYLLSHPEVKGPGVGLLGISKGGELCLSMASFLKGITAAVVINGSVANVGGTLRYKGETLPPVGVNRNRIKVTKDGYADIVDVLNSPLEGPDQKSFIPVERAESTFLFLVGQDDHNWKSEFYANEACKRLQAHGRRKPQIICYPETGHYIEPPYFPLCRASLHALVGSPIIWGGEPRAHAMAQVDAWKQLQTFFHKHLGGHEGTIPSKV

21 NP_066951.1 P0C7P0

>NP_066951.1

MIIPVRCFTCGKIVGNKWEAYLGLLQAEYTEGDALDALGLKRYCCRRMLLAHVDLIEKLLNYAPLEK

>P0C7P0

MRGAGAILRPAARGARDLNPRRDISSWLAQWFPRTPARSVVALKTPIKVELVAGKTYRWCVCGRSKKQPFCDGSHFFQRTGLSPLKFKAQETRMVALCTCKATQRPPYCDGTHRSERVQKAEVGSPL

22 NP_060612.2 Q9BRJ2

>NP_060612.2

MSAQGDCEFLVQRARELVPQDLWAAKAWLITARSLYPADFNIQYEMYTIERNAERTATAGRLLYDMFVNFPDQPVVWREISIITSALRNDSQDKQTQFLRSLFETLPGRVQCEMLLKVTEQCFNTLERSEMLLLLLRRFPETVVQHGVGLGEALLEAETIEEQESPVNCFRKLFVCDVLPLIINNHDVRLPANLLYKYLNKAAEFYINYVTRSTQIENQHQGAQDTSDLMSPSKRSSQKYIIEGLTEKSSQIVDPWERLFKILNVVGMRCEWQMDKGRRSYGDILHRMKDLCRYMNNFDSEAHAKYKNQVVYSTMLVFFKNAFQYVNSIQPSLFQGPNAPSQVPLVLLEDVSNVYGDVEIDRNKHIHKKRKLAEGREKTMSSDDEDCSAKGRNRHIVVNKAELANSTEVLESFKLARESWELLYSLEFLDKEFTRICLAWKTDTWLWLRIFLTDMIIYQGQYKKAIASLHHLAALQGSISQPQITGQGTLEHQRALIQLATCHFALGEYRMTCEKVLDLMCYMVLPIQDGGKSQEEPSKVKPKFRKGSDLKLLPCTSKAIMPYCLHLMLACFKLRAFTDNRDDMALGHVIVLLQQEWPRGENLFLKAVNKICQQGNFQYENFFNYVTNIDMLEEFAYLRTQEGGKIHLELLPNQGMLIKHHTVTRGITKGVKEDFRLAMERQVSRCGENLMVVLHRFCINEKILLLQTLT

>Q9BRJ2

MAAPIPQGFSCLSRFLGWWFRQPVLVTQSAAIVPVRTKKRFTPPIYQPKFKTEKEFMQHARKAGLVIPPEKSDRSIHLACTAGIFDAYVPPEGDARISSLSKEGLIERTERMKKTMASQVSIRRIKDYDANFKIKDFPEKAKDIFIEAHLCLNNSDHDRLHTLVTEHCFPDMTWDIKYKTVRWSFVESLEPSHVVQVRCSSMMNQGNVYGQITVRMHTRQTLAIYDRFGRLMYGQEDVPKDVLEYVVFEKQLTNPYGSWRMHTKIVPPWAPPKQPILKTVMIPGPQLKPEEEYEEAQGEAQKPQLA

23 P50897 NP_872578.1

>P50897

MASPGCLWLLAVALLPWTCASRALQHLDPPAPLPLVIWHGMGDSCCNPLSMGAIKKMVEKKIPGIYVLSLEIGKTLMEDVENSFFLNVNSQVTTVCQALAKDPKLQQGYNAMGFSQGGQFLRAVAQRCPSPPMINLISVGGQHQGVFGLPRCPGESSHICDFIRKTLNAGAYSKVVQERLVQAEYWHDPIKEDVYRNHSIFLADINQERGINESYKKNLMALKKFVMVKFLNDSIVDPVDSEWFGFYRSGQAKETIPLQETSLYTQDRLGLKEMDNAGQLVFLATEGDHLQLSEEWFYAHIIPFLG

>NP_872578.1

MAAPCVSYGGAVSYRLLLWGRGSLARKQGLWKTAAPELQTNVRSQILRLRHTAFVIPKKNVPTSKRETYTEDFIKKQIEEFNIGKRHLANMMGEDPETFTQEDIDRAIAYLFPSGLFEKRARPVMKHPEQIFPRQRAIQWGEDGRPFHYLFYTGKQSYYSLMHDVYGMLLNLEKHQSHLQAKSLLPEKTVTRDVIGSRWLIKEELEEMLVEKLSDLDYMQFIRLLEKLLTSQCGAAEEEFVQRFRRSVTLESKKQLIEPVQYDEQGMAFSKSEGKRKTAKAEAIVYKHGSGRIKVNGIDYQLYFPITQDREQLMFPFHFVDRLGKHDVTCTVSGGGRSAQAGAIRLAMAKALCSFVTEDEVEWMRQAGLLTTDPRVRERKKPGQEGARRKFTWKKR

24 NP_055048.1 NP_006535.1

>NP_055048.1

MNGEADCPTDLEMAAPKGQDRWSQEDMLTLLECMKNNLPSNDSSKFKTTESHMDWEKVAFKDFSGDMCKLKWVEISNEVRKFRTLTELILDAQEHVKNPYKGKKLKKHPDFPKKPLTPYFRFFMEKRAKYAKLHPEMSNLDLTKILSKKYKELPEKKKMKYIQDFQREKQEFERNLARFREDHPDLIQNAKKSDIPEKPKTPQQLWYTHEKKVYLKVRPDATTKEVKDSLGKQWSQLSDKKRLKWIHKALEQRKEYEEIMRDYIQKHPELNISEEGITKSTLTKAERQLKDKFDGRPTKPPPNSYSLYCAELMANMKDVPSTERMVLCSQQWKLLSQKEKDAYHKKCDQKKKDYEVELLRFLESLPEEEQQRVLGEEKMLNINKKQATSPASKKPAQEGGKGGSEKPKRPVSAMFIFSEEKRRQLQEERPELSESELTRLLARMWNDLSEKKKAKYKAREAALKAQSERKPGGEREERGKLPESPKRAEEIWQQSVIGDYLARFKNDRVKALKAMEMTWNNMEKKEKLMWIKKAAEDQKRYERELSEMRAPPAATNSSKKMKFQGEPKKPPMNGYQKFSQELLSNGELNHLPLKERMVEIGSRWQRISQSQKEHYKKLAEEQQKQYKVHLDLWVKSLSPQDRAAYKEYISNKRKSMTKLRGPNPKSSRTTLQSKSESEEDDEEDEDDEDEDEEEEDDENGDSSEDGGDSSESSSEDESEDGDENEEDDEDEDDDEDDDEDEDNESEGSSSSSSSSGDSSDSDSN

>NP_006535.1

MATTAELFEEPFVADEYIERLVWRTPGGGSRGGPEAFDPKRLLEEFVNHIQELQIMDERIQRKVEKLEQQCQKEAKEFAKKVQELQKSNQVAFQHFQELDEHISYVATKVCHLGDQLEGVNTPRQRAVEAQKLMKYFNEFLDGELKSDVFTNSEKIKEAADIIQKLHLIAQELPFDRFSEVKSKIASKYHDLECQLIQEFTSAQRRGEISRMREVAAVLLHFKGYSHCVDVYIKQCQEGAYLRNDIFEDAGILCQRVNKQVGDIFSNPETVLAKLIQNVFEIKLQSFVKEQLEECRKSDAEQYLKNLYDLYTRTTNLSSKLMEFNLGTDKQTFLSKLIKSIFISYLENYIEVETGYLKSRSAMILQRYYDSKNHQKRSIGTGGIQDLKERIRQRTNLPLGPSIDTHGETFLSQEVVVNLLQETKQAFERCHRLSDPSDLPRNAFRIFTILVEFLCIEHIDYALETGLAGIPSSDSRNANLYFLDVVQQANTIFHLFDKQFNDHLMPLISSSPKLSECLQKKKEIIEQMEMKLDTGIDRTLNCMIGQMKHILAAEQKKTDFKPEDENNVLIQYTNACVKVCAYVRKQVEKIKNSMDGKNVDTVLMELGVRFHRLIYEHLQQYSYSCMGGMLAICDVAEYRKCAKDFKIPMVLHLFDTLHALCNLLVVAPDNLKQVCSGEQLANLDKNILHSFVQLRADYRSARLARHFS

25 NP_110517.2 Q9UBR1

>NP_110517.2

MDVGELLSYQPNRGTKRPRDDEEEEQKMRRKQTGTRERGRYREEEMTVVEEADDDKKRLLQIIDRDGEEEEEEEEPLDESSVKKMILTFEKRSYKNQELRIKFPDNPEKFMESELDLNDIIQEMHVVATMPDLYHLLVELNAVQSLLGLLGHDNTDVSIAVVDLLQELTDIDTLHESEEGAEVLIDALVDGQVVALLVQNLERLDESVKEEADGVHNTLAIVENMAEFRPEMCTEGAQQGLLQWLLKRLKAKMPFDANKLYCSEVLAILLQDNDENRELLGELDGIDVLLQQLSVFKRHNPSTAEEQEMMENLFDSLCSCLMLSSNRERFLKGEGLQLMNLMLREKKISRSSALKVLDHAMIGPEGTDNCHKFVDILGLRTIFPLFMKSPRKIKKVGTTEKEHEEHVCSILASLLRNLRGQQRTRLLNKFTENDSEKVDRLMELHFKYLGAMQVADKKIEGEKHDMVRRGEIIDNDTEEEFYLRRLDAGLFVLQHICYIMAEICNANVPQIRQRVHQILNMRGSSIKIVRHIIKEYAENIGDGRSPEFRENEQKRILGLLENF

>Q9UBR1

MAGAEWKSLEECLEKHLPLPDLQEVKRVLYGKELRKLDLPREAFEAASREDFELQGYAFEAAEEQLRRPRIVHVGLVQNRIPLPANAPVAEQVSALHRRIKAIVEVAAMCGVNIICFQEAWTMPFAFCTREKLPWTEFAESAEDGPTTRFCQKLAKNHDMVVVSPILERDSEHGDVLWNTAVVISNSGAVLGKTRKNHIPRVGDFNESTYYMEGNLGHPVFQTQFGRIAVNICYGRHHPLNWLMYSINGAEIIFNPSATIGALSESLWPIEARNAAIANHCFTCAINRVGTEHFPNEFTSGDGKKAHQDFGYFYGSSYVAAPDSSRTPGLSRSRDGLLVAKLDLNLCQQVNDVWNFKMTGRYEMYARELAEAVKSNYSPTIVKE

26 NP_055884.2 NP_001001936.1

>NP_055884.2

MHKKRVEEGEASDFSLAWDSSVTAAGGLEGEPECDQKTSRALEDRNSVTSQEERNEDDEDMEDESIYTCDHCQQDFESLADLTDHRAHRCPGDGDDDPQLSWVASSPSSKDVASPTQMIGDGCDLGLGEEEGGTGLPYPCQFCDKSFIRLSYLKRHEQIHSDKLPFKCTYCSRLFKHKRSRDRHIKLHTGDKKYHCHECEAAFSRSDHLKIHLKTHSSSKPFKCTVCKRGFSSTSSLQSHMQAHKKNKEHLAKSEKEAKKDDFMCDYCEDTFSQTEELEKHVLTRHPQLSEKADLQCIHCPEVFVDENTLLAHIHQAHANQKHKCPMCPEQFSSVEGVYCHLDSHRQPDSSNHSVSPDPVLGSVASMSSATPDSSASVERGSTPDSTLKPLRGQKKMRDDGQGWTKVVYSCPYCSKRDFNSLAVLEIHLKTIHADKPQQSHTCQICLDSMPTLYNLNEHVRKLHKNHAYPVMQFGNISAFHCNYCPEMFADINSLQEHIRVSHCGPNANPSDGNNAFFCNQCSMGFLTESSLTEHIQQAHCSVGSAKLESPVVQPTQSFMEVYSCPYCTNSPIFGSILKLTKHIKENHKNIPLAHSKKSKAEQSPVSSDVEVSSPKRQRLSASANSISNGEYPCNQCDLKFSNFESFQTHLKLHLELLLRKQACPQCKEDFDSQESLLQHLTVHYMTTSTHYVCESCDKQFSSVDDLQKHLLDMHTFVLYHCTLCQEVFDSKVSIQVHLAVKHSNEKKMYRCTACNWDFRKEADLQVHVKHSHLGNPAKAHKCIFCGETFSTEVELQCHITTHSKKYNCKFCSKAFHAIILLEKHLREKHCVFDAATENGTANGVPPMATKKAEPADLQGMLLKNPEAPNSHEASEDDVDASEPMYGCDICGAAYTMEVLLQNHRLRDHNIRPGEDDGSRKKAEFIKGSHKCNVCSRTFFSENGLREHLQTHRGPAKHYMCPICGERFPSLLTLTEHKVTHSKSLDTGTCRICKMPLQSEEEFIEHCQMHPDLRNSLTGFRCVVCMQTVTSTLELKIHGTFHMQKLAGSSAASSPNGQGLQKLYKCALCLKEFRSKQDLVKLDVNGLPYGLCAGCMARSANGQVGGLAPPEPADRPCAGLRCPECSVKFESAEDLESHMQVDHRDLTPETSGPRKGTQTSPVPRKKTYQCIKCQMTFENEREIQIHVANHMIEEGINHECKLCNQMFDSPAKLLCHLIEHSFEGMGGTFKCPVCFTVFVQANKLQQHIFAVHGQEDKIYDCSQCPQKFFFQTELQNHTMSQHAQ

>NP_001001936.1

MERYKALEQLLTELDDFLKILDQENLSSTALVKKSCLAELLRLYTKSSSSDEEYIYMNKVTINKQQNAESQGKAPEEQGLLPNGEPSQHSSAPQKSLPDLPPPKMIPERKQLAIPKTESPEGYYEEAEPYDTSLNEDGEAVSSSYESYDEEDGSKGKSAPYQWPSPEAGIELMRDARICAFLWRKKWLGQWAKQLCVIKDNRLLCYKSSKDHSPQLDVNLLGSSVIHKEKQVRKKEHKLKITPMNADVIVLGLQSKDQAEQWLRVIQEVSGLPSEGASEGNQYTPDAQRFNCQKPDIAEKYLSASEYGSSVDGHPEVPETKDVKKKCSAGLKLSNLMNLGRKKSTSLEPVERSLETSSYLNVLVNSQWKSRWCSVRDNHLHFYQDRNRSKVAQQPLSLVGCEVVPDPSPDHLYSFRILHKGEELAKLEAKSSEEMGHWLGLLLSESGSKTDPEEFTYDYVDADRVSCIVSAAKNSLLLMQRKFSEPNTYIDGLPSQDRQEELYDDVDLSELTAAVEPTEEATPVADDPNERESDRVYLDLTPVKSFLHGPSSAQAQASSPTLSCLDNATEALPADSGPGPTPDEPCIKCPENLGEQQLESLEPEDPSLRITTVKIQTEQQRISFPPSCPDAVVATPPGASPPVKDRLRVTSAEIKLGKNRTEAEVKRYTEEKERLEKKKEEIRGHLAQLRKEKRELKETLLKCTDKEVLASLEQKLKEIDEECRGEESRRVDLELSIMEVKDNLKKAEAGPVTLGTTVDTTHLENVSPRPKAVTPASAPDCTPVNSATTLKNRPLSVVVTGKGTVLQKAKEWEKKGAS

27 NP_000512.1 P63302

>NP_000512.1

MELCGLGLPRPPMLLALLLATLLAAMLALLTQVALVVQVAEAARAPSVSAKPGPALWPLPLSVKMTPNLLHLAPENFYISHSPNSTAGPSCTLLEEAFRRYHGYIFGFYKWHHEPAEFQAKTQVQQLLVSITLQSECDAFPNISSDESYTLLVKEPVAVLKANRVWGALRGLETFSQLVYQDSYGTFTINESTIIDSPRFSHRGILIDTSRHYLPVKIILKTLDAMAFNKFNVLHWHIVDDQSFPYQSITFPELSNKGSYSLSHVYTPNDVRMVIEYARLRGIRVLPEFDTPGHTLSWGKGQKDLLTPCYSRQNKLDSFGPINPTLNTTYSFLTTFFKEISEVFPDQFIHLGGDEVEFKCWESNPKIQDFMRQKGFGTDFKKLESFYIQKVLDIIATINKGSIVWQEVFDDKAKLAPGTIVEVWKDSAYPEELSRVTASGFPVILSAPWYLDLISYGQDWRKYYKVEPLDFGGTQKQKQLFIGGEACLWGEYVDATNLTPRLWPRASAVGERLWSSKDVRDMDDAYDRLTRHRCRMVERGIAAQPLYAGYCNHENM

>P63302

MALAVRVVYCGAUGYKSKYLQLKKKLEDEFPGRLDICGEGTPQATGFFEVMVAGKLIHSKKKGDGYVDTESKFLKLVAAIKAALAQG

28 Q9UDW3 Q9BZQ8

>Q9UDW3

MGKRYFCDYCDRSFQDNLHNRKKHLNGLQHLKAKKVWYDMFRDAAAILLDEQNKRPCRKFLLTGQCDFGSNCRFSHMSERDLQELSIQVEEERRAREWLLDAPELPEGHLEDWLEKRAKRLSSAPSSRAEPIRTTVFQYPVGWPPVQELPPSLRAPPPGGWPLQPRVQWG

>Q9BZQ8

MGGSASSQLDEGKCAYIRGKTEAAIKNFSPYYSRQYSVAFCNHVRTEVEQQRDLTSQFLKTKPPLAPGTILYEAELSQFSEDIKKWKERYVVVKNDYAVESYENKEAYQRGAAPKCRILPAGGKVLTSEDEYNLLSDRHFPDPLASSEKENTQPFVVLPKEFPVYLWQPFFRHGYFCFHEAADQKRFSALLSDCVRHLNHDYMKQMTFEAQAFLEAVQFFRQEKGHYGSWEMITGDEIQILSNLVMEELLPTLQTDLLPKMKGKKNDRKRTWLGLLEEAYTLVQHQVSEGLSALKEECRALTKGLEGTIRSDMDQIVNSKNYLIGKIKAMVAQPAEKSCLESVQPFLASILEELMGPVSSGFSEVRVLFEKEVNEVSQNFQTTKDSVQLKEHLDRLMNLPLHSVKMEPCYTKVNLLHERLQDLKSRFRFPHIDLVVQRTQNYMQELMENAVFTFEQLLSPHLQGEASKTAVAIEKVKLRVLKQYDYDSSTIRKKIFQEALVQITLPTVQKALASTCKPELQKYEQFIFADHTNMIHVENVYEEILHQILLDETLKVIKEAAILKKHNLFEDNMALPSESVSSLTDLKPPTGSNQASPARRASAILPGVLGSETLSNEVFQESEEEKQPEVPSSLAKGESLSLPGPSPPPDGTEQVIISRVDDPVVNPVATEDTAGLPGTCSSELEFGGTLEDEEPAQEEPEPITASGSLKALRKLLTASVEVPVDSAPVMEEDTNGESHVPQENEEEEEKEPSQAAAIHPDNCEESEVSEREAQPPCPEAHGEELGGFPEVGSPASPPASGGLTEEPLGPMEGELPGEACTLTAHEGRGGKCTEEGDASQQEGCTLGSDPICLSESQVSEEQEEMGGQSSAAQATASVNAEEIKVARIHECQWVVEDAPNPDVLLSHKDDVKEGEGGQESFPELPSEE

29 NP_004261.1 NP_872578.1

>NP_004261.1

MGEPQQVSALPPPPMQYIKEYTDENIQEGLAPKPPPPIKDSYMMFGNQFQCDDLIIRPLESQGIERLHPMQFDHKKELRKLNMSILINFLDLLDILIRSPGSIKREEKLEDLKLLFVHVHHLINEYRPHQARETLRVMMEVQKRQRLETAERFQKHLERVIEMIQNCLASLPDDLPHSEAGMRVKTEPMDADDSNNCTGQNEHQRENSGHRRDQIIEKDAALCVLIDEMNERP

>NP_872578.1

MAAPCVSYGGAVSYRLLLWGRGSLARKQGLWKTAAPELQTNVRSQILRLRHTAFVIPKKNVPTSKRETYTEDFIKKQIEEFNIGKRHLANMMGEDPETFTQEDIDRAIAYLFPSGLFEKRARPVMKHPEQIFPRQRAIQWGEDGRPFHYLFYTGKQSYYSLMHDVYGMLLNLEKHQSHLQAKSLLPEKTVTRDVIGSRWLIKEELEEMLVEKLSDLDYMQFIRLLEKLLTSQCGAAEEEFVQRFRRSVTLESKKQLIEPVQYDEQGMAFSKSEGKRKTAKAEAIVYKHGSGRIKVNGIDYQLYFPITQDREQLMFPFHFVDRLGKHDVTCTVSGGGRSAQAGAIRLAMAKALCSFVTEDEVEWMRQAGLLTTDPRVRERKKPGQEGARRKFTWKKR

30 NP_002681.1 P28838

>NP_002681.1

MSKRKAPQETLNGGITDMLTELANFEKNVSQAIHKYNAYRKAASVIAKYPHKIKSGAEAKKLPGVGTKIAEKIDEFLATGKLRKLEKIRQDDTSSSINFLTRVSGIGPSAARKFVDEGIKTLEDLRKNEDKLNHHQRIGLKYFGDFEKRIPREEMLQMQDIVLNEVKKVDSEYIATVCGSFRRGAESSGDMDVLLTHPSFTSESTKQPKLLHQVVEQLQKVHFITDTLSKGETKFMGVCQLPSKNDEKEYPHRRIDIRLIPKDQYYCGVLYFTGSDIFNKNMRAHALEKGFTINEYTIRPLGVTGVAGEPLPVDSEKDIFDYIQWKYREPKDRSE

>P28838

MFLLPLPAAGRVVVRRLAVRRFGSRSLSTADMTKGLVLGIYSKEKEDDVPQFTSAGENFDKLLAGKLRETLNISGPPLKAGKTRTFYGLHQDFPSVVLVGLGKKAAGIDEQENWHEGKENIRAAVAAGCRQIQDLELSSVEVDPCGDAQAAAEGAVLGLYEYDDLKQKKKMAVSAKLYGSGDQEAWQKGVLFASGQNLARQLMETPANEMTPTRFAEIIEKNLKSASSKTEVHIRPKSWIEEQAMGSFLSVAKGSDEPPVFLEIHYKGSPNANEPPLVFVGKGITFDSGGISIKASANMDLMRADMGGAATICSAIVSAAKLNLPINIIGLAPLCENMPSGKANKPGDVVRAKNGKTIQVDNTDAEGRLILADALCYAHTFNPKVILNAATLTGAMDVALGSGATGVFTNSSWLWNKLFEASIETGDRVWRMPLFEHYTRQVVDCQLADVNNIGKYRSAGACTAAAFLKEFVTHPKWAHLDIAGVMTNKDEVPYLRKGMTGRPTRTLIEFLLRFSQDNA

31 NP_036558.3 O75880

>NP_036558.3

MFLYNLTLQRATGISFAIHGNFSGTKQQEIVVSRGKILELLRPDPNTGKVHTLLTVEVFGVIRSLMAFRLTGGTKDYIVVGSDSGRIVILEYQPSKNMFEKIHQETFGKSGCRRIVPGQFLAVDPKGRAVMISAIEKQKLVYILNRDAAARLTISSPLEAHKANTLVYHVVGVDVGFENPMFACLEMDYEEADNDPTGEAAANTQQTLTFYELDLGLNHVVRKYSEPLEEHGNFLITVPGGSDGPSGVLICSENYITYKNFGDQPDIRCPIPRRRNDLDDPERGMIFVCSATHKTKSMFFFLAQTEQGDIFKITLETDEDMVTEIRLKYFDTVPVAAAMCVLKTGFLFVASEFGNHYLYQIAHLGDDDEEPEFSSAMPLEEGDTFFFQPRPLKNLVLVDELDSLSPILFCQIADLANEDTPQLYVACGRGPRSSLRVLRHGLEVSEMAVSELPGNPNAVWTVRRHIEDEFDAYIIVSFVNATLVLSIGETVEEVTDSGFLGTTPTLSCSLLGDDALVQVYPDGIRHIRADKRVNEWKTPGKKTIVKCAVNQRQVVIALTGGELVYFEMDPSGQLNEYTERKEMSADVVCMSLANVPPGEQRSRFLAVGLVDNTVRIISLDPSDCLQPLSMQALPAQPESLCIVEMGGTEKQDELGERGSIGFLYLNIGLQNGVLLRTVLDPVTGDLSDTRTRYLGSRPVKLFRVRMQGQEAVLAMSSRSWLSYSYQSRFHLTPLSYETLEFASGFASEQCPEGIVAISTNTLRILALEKLGAVFNQVAFPLQYTPRKFVIHPESNNLIIIETDHNAYTEATKAQRKQQMAEEMVEAAGEDERELAAEMAAAFLNENLPESIFGAPKAGNGQWASVIRVMNPIQGNTLDLVQLEQNEAAFSVAVCRFSNTGEDWYVLVGVAKDLILNPRSVAGGFVYTYKLVNNGEKLEFLHKTPVEEVPAAIAPFQGRVLIGVGKLLRVYDLGKKKLLRKCENKHIANYISGIQTIGHRVIVSDVQESFIWVRYKRNENQLIIFADDTYPRWVTTASLLDYDTVAGADKFGNICVVRLPPNTNDEVDEDPTGNKALWDRGLLNGASQKAEVIMNYHVGETVLSLQKTTLIPGGSESLVYTTLSGGIGILVPFTSHEDHDFFQHVEMHLRSEHPPLCGRDHLSFRSYYFPVKNVIDGDLCEQFNSMEPNKQKNVSEELDRTPPEVSKKLEDIRTRYAF

>O75880

MAMLVLVPGRVMRPLGGQLWRFLPRGLEFWGPAEGTARVLLRQFCARQAEAWRASGRPGYCLGTRPLSTARPPPPWSQKGPGDSTRPSKPGPVSWKSLAITFAIGGALLAGMKHVKKEKAEKLEKERQRHIGKPLLGGPFSLTTHTGERKTDKDYLGQWLLIYFGFTHCPDVCPEELEKMIQVVDEIDSITTLPDLTPLFISIDPERDTKEAIANYVKEFSPKLVGLTGTREEVDQVARAYRVYYSPGPKDEDEDYIVDHTIIMYLIGPDGEFLDYFGQNKRKGEIAASIATHMRPYRKKS

32 NP_003855.1 NP_057564.3

>NP_003855.1

MNGFTPDEMSRGGDAAAAVAAVVAAAAAAASAGNGTGAGTGAEVPGAGAVSAAGPPGAAGPGPGQLCCLREDGERCGRAAGNASFSKRIQKSISQKKVKIELDKSARHLYICDYHKNLIQSVRNRRKRKGSDDDGGDSPVQDIDTPEVDLYQLQVNTLRRYKRHFKLPTRPGLNKAQLVEIVGCHFRSIPVNEKDTLTYFIYSVKNDKNKSDLKVDSGVH

>NP_057564.3

MKDPSRSSTSPSIINEDVIINGHSHEDDNPFAEYMWMENEEEFNRQIEEELWEEEFIERCFQEMLEEEEEHEWFIPARDLPQTMDQIQDQFNDLVISDGSSLEDLVVKSNLNPNAKEFVPGVKYGNI

33 NP_109377.1 NP_006635.2

>NP_109377.1

MQKATYYDSSAIYGGYPYQAANGFAYNANQQPYPASAALGADGEYHRPACSLQSPSSAGGHPKAHELSEACLRTLSAPPSQPPSLGEPPLHPPPPQAAPPAPQPPQPAPQPPAPTPAAPPPPSSASPPQNASNNPTPANAAKSPLLNSPTVAKQIFPWMKESRQNTKQKTSSSSSGESCAGDKSPPGQASSKRARTAYTSAQLVELEKEFHFNRYLCRPRRVEMANLLNLTERQIKIWFQNRRMKYKKDQKGKGMLTSSGGQSPSRSPVPPGAGGYLNSMHSLVNSVPYEPQSPPPFSKPPQGTYGLPPASYPASLPSCAPPPPPQKRYTAAGAGAGGTPDYDPHAHGLQGNGSYGTPHIQGSPVFVGGSYVEPMSNSGPALFGLTHLPHAASGAMDYGGAGPLGSGHHHGPGPGEPHPTYTDLTGHHPSQGRIQEAPKLTHL

>NP_006635.2

MSVVGLDVGSQSCYIAVARAGGIETIANEFSDRCTPSVISFGSKNRTIGVAAKNQQITHANNTVSNFKRFHGRAFNDPFIQKEKENLSYDLVPLKNGGVGIKVMYMGEEHLFSVEQITAMLLTKLKETAENSLKKPVTDCVISVPSFFTDAERRSVLDAAQIVGLNCLRLMNDMTAVALNYGIYKQDLPSLDEKPRIVVFVDMGHSAFQVSACAFNKGKLKVLGTAFDPFLGGKNFDEKLVEHFCAEFKTKYKLDAKSKIRALLRLYQECEKLKKLMSSNSTDLPLNIECFMNDKDVSGKMNRSQFEELCAELLQKIEVPLYSLLEQTHLKVEDVSAVEIVGGATRIPAVKERIAKFFGKDISTTLNADEAVARGCALQCAILSPAFKVREFSVTDAVPFPISLIWNHDSEDTEGVHEVFSRNHAAPFSKVLTFLRRGPFELEAFYSDPQGVPYPEAKIGRFVVQNVSAQKDGEKSRVKVKVRVNTHGIFTISTASMVEKVPTEENEMSSEADMECLNQRPPENPDTDKNVQQDNSEAGTQPQVQTDAQQTSQSPPSPELTSEENKIPDADKANEKKVDQPPEAKKPKIKVVNVELPIEANLVWQLGKDLLNMYIETEGKMIMQDKLEKERNDAKNAVEEYVYEFRDKLCGPYEKFICEQDHQNFLRLLTETEDWLYEEGEDQAKQAYVDKLEELMKIGTPVKVRFQEAEERPKMFEELGQRLQHYAKIAADFRNKDEKYNHIDESEMKKVEKSVNEVMEWMNNVMNAQAKKSLDQDPVVRAQEIKTKIKELNNTCEPVVTQPKPKIESPKLERTPNGPNIDKKEEDLEDKNNFGAEPPHQNGECYPNEKNSVNMDLD

34 NP_000160.1 NP_006635.2

>NP_000160.1

MQLRNPELHLGCALALRFLALVSWDIPGARALDNGLARTPTMGWLHWERFMCNLDCQEEPDSCISEKLFMEMAELMVSEGWKDAGYEYLCIDDCWMAPQRDSEGRLQADPQRFPHGIRQLANYVHSKGLKLGIYADVGNKTCAGFPGSFGYYDIDAQTFADWGVDLLKFDGCYCDSLENLADGYKHMSLALNRTGRSIVYSCEWPLYMWPFQKPNYTEIRQYCNHWRNFADIDDSWKSIKSILDWTSFNQERIVDVAGPGGWNDPDMLVIGNFGLSWNQQVTQMALWAIMAAPLFMSNDLRHISPQAKALLQDKDVIAINQDPLGKQGYQLRQGDNFEVWERPLSGLAWAVAMINRQEIGGPRSYTIAVASLGKGVACNPACFITQLLPVKRKLGFYEWTSRLRSHINPTGTVLLQLENTMQMSLKDLL

>NP_006635.2

MSVVGLDVGSQSCYIAVARAGGIETIANEFSDRCTPSVISFGSKNRTIGVAAKNQQITHANNTVSNFKRFHGRAFNDPFIQKEKENLSYDLVPLKNGGVGIKVMYMGEEHLFSVEQITAMLLTKLKETAENSLKKPVTDCVISVPSFFTDAERRSVLDAAQIVGLNCLRLMNDMTAVALNYGIYKQDLPSLDEKPRIVVFVDMGHSAFQVSACAFNKGKLKVLGTAFDPFLGGKNFDEKLVEHFCAEFKTKYKLDAKSKIRALLRLYQECEKLKKLMSSNSTDLPLNIECFMNDKDVSGKMNRSQFEELCAELLQKIEVPLYSLLEQTHLKVEDVSAVEIVGGATRIPAVKERIAKFFGKDISTTLNADEAVARGCALQCAILSPAFKVREFSVTDAVPFPISLIWNHDSEDTEGVHEVFSRNHAAPFSKVLTFLRRGPFELEAFYSDPQGVPYPEAKIGRFVVQNVSAQKDGEKSRVKVKVRVNTHGIFTISTASMVEKVPTEENEMSSEADMECLNQRPPENPDTDKNVQQDNSEAGTQPQVQTDAQQTSQSPPSPELTSEENKIPDADKANEKKVDQPPEAKKPKIKVVNVELPIEANLVWQLGKDLLNMYIETEGKMIMQDKLEKERNDAKNAVEEYVYEFRDKLCGPYEKFICEQDHQNFLRLLTETEDWLYEEGEDQAKQAYVDKLEELMKIGTPVKVRFQEAEERPKMFEELGQRLQHYAKIAADFRNKDEKYNHIDESEMKKVEKSVNEVMEWMNNVMNAQAKKSLDQDPVVRAQEIKTKIKELNNTCEPVVTQPKPKIESPKLERTPNGPNIDKKEEDLEDKNNFGAEPPHQNGECYPNEKNSVNMDLD

35 Q7Z5Q5 NP_001167.2

>Q7Z5Q5

MENYEALVGFDLCNTPLSSVAQKIMSAMHSGDLVDSKTWGKSTETMEVINKSSVKYSVQLEDRKTQSPEKKDLKSLRSQTSRGSAKLSPQSFSVRLTDQLSADQKQKSISSLTLSSCLIPQYNQEASVLQKKGHKRKHFLMENINNENKGSINLKRKHITYNNLSEKTSKQMALEEDTDDAEGYLNSGNSGALKKHFCDIRHLDDWAKSQLIEMLKQAAALVITVMYTDGSTQLGADQTPVSSVRGIVVLVKRQAEGGHGCPDAPACGPVLEGFVSDDPCIYIQIEHSAIWDQEQEAHQQFARNVLFQTMKCKCPVICFNAKDFVRIVLQFFGNDGSWKHVADFIGLDPRIAAWLIDPSDATPSFEDLVEKYCEKSITVKVNSTYGNSSRNIVNQNVRENLKTLYRLTMDLCSKLKDYGLWQLFRTLELPLIPILAVMESHAIQVNKEEMEKTSALLGARLKELEQEAHFVAGERFLITSNNQLREILFGKLKLHLLSQRNSLPRTGLQKYPSTSEAVLNALRDLHPLPKIILEYRQVHKIKSTFVDGLLACMKKGSISSTWNQTGTVTGRLSAKHPNIQGISKHPIQITTPKNFKGKEDKILTISPRAMFVSSKGHTFLAADFSQIELRILTHLSGDPELLKLFQESERDDVFSTLTSQWKDVPVEQVTHADREQTKKVVYAVVYGAGKERLAACLGVPIQEAAQFLESFLQKYKKIKDFARAAIAQCHQTGCVVSIMGRRRPLPRIHAHDQQLRAQAERQAVNFVVQGSAADLCKLAMIHVFTAVAASHTLTARLVAQIHDELLFEVEDPQIPECAALVRRTMESLEQVQALELQLQVPLKVSLSAGRSWGHLVPLQEAWGPPPGPCRTESPSNSLAAPGSPASTQPPPLHFSPSFCL

>NP_001167.2

MLGLDACELGAQLLELLRLALCARVLLADKEGGPPAVDEVLDEAVPEYRAPGRKSLLEIRQLDPDDRSLAKYKRVLLGPLPPAVDPSLPNVQVTRLTLLSEQAPGPVVMDLTGDLAVLKDQVFVLKEGVDYRVKISFKVHREIVSGLKCLHHTYRRGLRVDKTVYMVGSYGPSAQEYEFVTPVEEAPRGALVRGPYLVVSLFTDDDRTHHLSWEWGLCICQDWKD

36 Q9Y6W3 P50135

>Q9Y6W3

MDATALERDAVQFARLAVQRDHEGRYSEAVFYYKEAAQALIYAEMAGSSLENIQEKITEYLERVQALHSAVQSKSADPLKSKHQLDLERAHFLVTQAFDEDEKENVEDAIELYTEAVDLCLKTSYETADKVLQNKLKQLARQALDRAEALSEPLTKPVGKISSTSVKPKPPPVRAHFPLGANPFLERPQSFISPQSCDAQGQRYTAEEIEVLRTTSKINGIEYVPFMNVDLRERFAYPMPFCDRWGKLPLSPKQKTTFSKWVRPEDLTNNPTMIYTVSSFSIKQTIVSDCSFVASLAISAAYERRFNKKLITGIIYPQNKDGEPEYNPCGKYMVKLHLNGVPRKVIIDDQLPVDHKGELLCSYSNNKSELWVSLIEKAYMKVMGGYDFPGSNSNIDLHALTGWIPERIAMHSDSQTFSKDNSFRMLYQRFHKGDVLITASTGMMTEAEGEKWGLVPTHAYAVLDIREFKGLRFIQLKNPWSHLRWKGRYSENDVKNWTPELQKYLNFDPRTAQKIDNGIFWISWDDLCQYYDVIYLSWNPGLFKESTCIHSTWDAKQGPVKDAYSLANNPQYKLEVQCPQGGAAVWVLLSRHITDKDDFANNREFITMVVYKTDGKKVYYPADPPPYIDGIRINSPHYLTKIKLTTPGTHTFTLVVSQYEKQNTIHYTVRVYSACSFTFSKIPSPYTLSKRINGKWSGQSAGGCGNFQETHKNNPIYQFHIEKTGPLLIELRGPRQYSVGFEVVTVSTLGDPGPHGFLRKSSGDYRCGFCYLELENIPSGIFNIIPSTFLPKQEGPFFLDFNSIIPIKITQLQ

>P50135

MASSMRSLFSDHGKYVESFRRFLNHSTEHQCMQEFMDKKLPGIIGRIGDTKSEIKILSIGGGAGEIDLQILSKVQAQYPGVCINNEVVEPSAEQIAKYKELVAKTSNLENVKFAWHKETSSEYQSRMLEKKELQKWDFIHMIQMLYYVKDIPATLKFFHSLLGTNAKMLIIVVSGSSGWDKLWKKYGSRFPQDDLCQYITSDDLTQMLDNLGLKYECYDLLSTMDISDCFIDGNENGDLLWDFLTETCNFNATAPPDLRAELGKDLQEPEFSAKKEGKVLFNNTLSFIVIEA

37 Q9BSH3 Q8WUN7

>Q9BSH3

MSRVLVPCHVKGSVALQVGDVRTSQGRPGVLVIDVTFPSVAPFELQEITFKNYYTAFLSIRVRQYTSAHTPAKWVTCLRDYCLMPDPHSEEGAQEYVSLFKHQMLCDMARISELRLILRQPSPLWLSFTVEELQIYQQGPKSPSVTFPKWLSHPVPCEQPALLREGLPDPSRVSSEVQQMWALTEMIRASHTSARIGRFDVDGCYDLNLLSYT

>Q8WUN7

MGGCVGAQHDSSGSLNENSEGTGVALGRNQPLKKEKPKWKSDYPMTDGQLRSKRDEFWDTAPAFEGRKEIWDALKAAAHAFESNDHELAQAIIDGANITLPHGALTECYDELGNRYQLPVYCLAPPINMIEEKSDIETLDIPEPPPNSGYECQLRLRLSTGKDLKLVVRSTDTVFHMKRRLHAAEGVEPGSQRWFFSGRPLTDKMKFEELKIPKDYVVQVIVSQPVQNPTPVEN

38 NP_079224.1 NP_061833.1

>NP_079224.1

MSELTKELMELVWGTKSSPGLSDTIFCRWTQGFVFSESEGSALEQFEGGPCAVIAPVQAFLLKKLLFSSEKSSWRDCSEEEQKELLCHTLCDILESACCDHSGSYCLVSWLRGKTTEETASISGSPAESSCQVEHSSALAVEELGFERFHALIQKRSFRSLPELKDAVLDQYSMWGNKFGVLLFLYSVLLTKGIENIKNEIEDASEPLIDPVYGHGSQSLINLLLTGHAVSNVWDGDRECSGMKLLGIHEQAAVGFLTLMEALRYCKVGSYLKSPKFPIWIVGSETHLTVFFAKDMALVAPEAPSEQARRVFQTYDPEDNGFIPDSLLEDVMKALDLVSDPEYINLMKNKLDPEGLGIILLGPFLQEFFPDQGSSGPESFTVYHYNGLKQSNYNEKVMYVEGTAVVMGFEDPMLQTDDTPIKRCLQTKWPYIELLWTTDRSPSLN

>NP_061833.1

MVDSVYRTRSLGVAAEGLPDQYADGEAARVWQLYIGDTRSRTAEYKAWLLGLLRQHGCQRVLDVACGTGVDSIMLVEEGFSVTSVDASDKMLKYALKERWNRRHEPAFDKWVIEEANWMTLDKDVPQSAEGGFDAVICLGNSFAHLPDCKGDQSEHRLALKNIASMVRAGGLLVIDHRNYDHILSTGCAPPGKNIYYKSDLTKDVTTSVLIVNNKAHMVTLDYTVQVPGAGQDGSPGLSKFRLSYYPHCLASFTELLQAAFGGKCQHSVLGDFKPYKPGQTYIPCYFIHVLKRTD

39 Q9UJC5 Q96Q11

>Q9UJC5

MVIRVFIASSSGFVAIKKKQQDVVRFLEANKIEFEEVDITMSEEQRQWMYKNVPPEKKPTQGNPLPPQIFNGDRYCGDYDSFFESKESNTVFSFLGLKPRLASKAEP

>Q96Q11

MLRCLYHWHRPVLNRRWSRLCLLKQYLFTMKLQSPEFQSLFTEGLKSLTELFVKENHELRIAGGAVRDLLNGVKPQDIDFATTATPTQMKEMFQSAGIRMINNRGEKHGTITARLHEENFEITTLRIDVTTDGRHAEVEFTTDWQKDAERRDLTINSMFLGFDGTLFDYFNGYEDLKNKKVRFVGHAKQRIQEDYLRILRYFRFYGRIVDKPGDHDPETLEAIAENAKGLAGISGERIWVELKKILVGNHVNHLIHLIYDLDVAPYIGLPANASLEEFDKVSKNVDGFSPKPVTLLASLFKVQDDVTKLDLRLKIAKEEKNLGLFIVKNRKDLIKATDSSDPLKPYQDFIIDSREPDATTRVCELLKYQGEHCLLKEMQQWSIPPFPVSGHDIRKVGISSGKEIGALLQQLREQWKKSGYQMEKDELLSYIKKT

40 NP_006345.1 Q9H2W6

>NP_006345.1

MSELKDCPLQFHDFKSVDHLKVCPRYTAVLARSEDDGIGIEELDTLQLELETLLSSASRRLRVLEAETQILTDWQDKKGDRRFLKLGRDHELGAPPKHGKPKKQKLEGKAGHGPGPGPGRPKSKNLQPKIQEYEFTDDPIDVPRIPKNDAPNRFWASVEPYCADITSEEVRTLEELLKPPEDEAEHYKIPPLGKHYSQRWAQEDLLEEQKDGARAAAVADKKKGLMGPLTELDTKDVDALLKKSEAQHEQPEDGCPFGALTQRLLQALVEENIISPMEDSPIPDMSGKESGADGASTSPRNQNKPFSVPHTKSLESRIKEELIAQGLLESEDRPAEDSEDEVLAELRKRQAELKALSAHNRTKKHDLLRLAKEEVSRQELRQRVRMADNEVMDAFRKIMAARQKKRTPTKKEKDQAWKTLKERESILKLLDG

>Q9H2W6

MAAPVRRTLLGVAGGWRRFERLWAGSLSSRSLALAAAPSSNGSPWRLLGALCLQRPPVVSKPLTPLQEEMASLLQQIEIERSLYSDHELRALDENQRLAKKKADLHDEEDEQDILLAQDLEDMWEQKFLQFKLGARITEADEKNDRTSLNRKLDRNLVLLVREKFGDQDVWILPQAEWQPGETLRGTAERTLATLSENNMEAKFLGNAPCGHYTFKFPQAMRTESNLGAKVFFFKALLLTGDFSQAGNKGHHVWVTKDELGDYLKPKYLAQVRRFVSDL

41 Q12857 Q16698

>Q12857

MYSPLCLTQDEFHPFIEALLPHVRAFAYTWFNLQARKRKYFKKHEKRMSKEEERAVKDELLSEKPEVKQKWASRLLAKLRKDIRPEYREDFVLTVTGKKPPCCVLSNPDQKGKMRRIDCLRQADKVWRLDLVMVILFKGIPLESTDGERLVKSPQCSNPGLCVQPHHIGVSVKELDLYLAYFVHAADSSQSESPSQPSDADIKDQPENGHLGFQDSFVTSGVFSVTELVRVSQTPIAAGTGPNFSLSDLESSSYYSMSPGAMRRSLPSTSSTSSTKRLKSVEDEMDSPGEEPFYTGQGRSPGSGSQSSGWHEVEPGMPSPTTLKKSEKSGFSSPSPSQTSSLGTAFTQHHRPVITGPRASPHATPSTLHFPTSPIIQQPGPYFSHPAIRYHPQETLKEFVQLVCPDAGQQAGQVGFLNPNGSSQGKVHNPFLPTPMLPPPPPPPMARPVPLPVPDTKPPTTSTEGGAASPTSPTYSTPSTSPANRFVSVGPRDPSFVNIPQQTQSWYLG

>Q16698

MKLPARVFFTLGSRLPCGLAPRRFFSYGTKILYQNTEALQSKFFSPLQKAMLPPNSFQGKVAFITGGGTGLGKGMTTLLSSLGAQCVIASRKMDVLKATAEQISSQTGNKVHAIQCDVRDPDMVQNTVSELIKVAGHPNIVINNAAGNFISPTERLSPNAWKTITDIVLNGTAFVTLEIGKQLIKAQKGAAFLSITTIYAETGSGFVVPSASAKAGVEAMSKSLAAEWGKYGMRFNVIQPGPIKTKGAFSRLDPTGTFEKEMIGRIPCGRLGTVEELANLAAFLCSDYASWINGAVIKFDGGEEVLISGEFNDLRKVTKEQWDTIEELIRKTKGS

42 NP_003160.2 NP_005908.1

>NP_003160.2

MSDSEDSNFSEEEDSERSSDGEEAEVDEERRSAAGSEKEEEPEDEEEEEEEEEYDEEEEEEDDDRPPKKPRHGGFILDEADVDDEYEDEDQWEDGAEDILEKEEIEASNIDNVVLDEDRSGARRLQNLWRDQREEELGEYYMKKYAKSSVGETVYGGSDELSDDITQQQLLPGVKDPNLWTVKCKIGEERATAISLMRKFIAYQFTDTPLQIKSVVAPEHVKGYIYVEAYKQTHVKQAIEGVGNLRLGYWNQQMVPIKEMTDVLKVVKEVANLKPKSWVRLKRGIYKDDIAQVDYVEPSQNTISLKMIPRIDYDRIKARMSLKDWFAKRKKFKRPPQRLFDAEKIRSLGGDVASDGDFLIFEGNRYSRKGFLFKSFAMSAVITEGVKPTLSELEKFEDQPEGIDLEVVTESTGKEREHNFQPGDNVEVCEGELINLQGKILSVDGNKITIMPKHEDLKDMLEFPAQELRKYFKMGDHVKVIAGRFEGDTGLIVRVEENFVILFSDLTMHELKVLPRDLQLCSETASGVDVGGQHEWGELVQLDPQTVGVIVRLERETFQVLNMYGKVVTVRHQAVTRKKDNRFAVALDSEQNNIHVKDIVKVIDGPHSGREGEIRHLFRSFAFLHCKKLVENGGMFVCKTRHLVLAGGSKPRDVTNFTVGGFAPMSPRISSPMHPSAGGQRGGFGSPGGGSGGMSRGRGRRDNELIGQTVRISQGPYKGYIGVVKDATESTARVELHSTCQTISVDRQRLTTVGSRRPGGMTSTYGRTPMYGSQTPMYGSGSRTPMYGSQTPLQDGSRTPHYGSQTPLHDGSRTPAQSGAWDPNNPNTPSRAEEEYEYAFDDEPTPSPQAYGGTPNPQTPGYPDPSSPQVNPQYNPQTPGTPAMYNTDQFSPYAAPSPQGSYQPSPSPQSYHQVAPSPAGYQNTHSPASYHPTPSPMAYQASPSPSPVGYSPMTPGAPSPGGYNPHTPGSGIEQNSSDWVTTDIQVKVRDTYLDTQVVGQTGVIRSVTGGMCSVYLKDSEKVVSISSEHLEPITPTKNNKVKVILGEDREATGVLLSIDGEDGIVRMDLDEQLKILNLRFLGKLLEA

>NP_005908.1

MSEPIRVLVTGAAGQIAYSLLYSIGNGSVFGKDQPIILVLLDITPMMGVLDGVLMELQDCALPLLKDVIATDKEDVAFKDLDVAILVGSMPRREGMERKDLLKANVKIFKSQGAALDKYAKKSVKVIVVGNPANTNCLTASKSAPSIPKENFSCLTRLDHNRAKAQIALKLGVTANDVKNVIIWGNHSSTQYPDVNHAKVKLQGKEVGVYEALKDDSWLKGEFVTTVQQRGAAVIKARKLSSAMSAAKAICDHVRDIWFGTPEGEFVSMGVISDGNSYGVPDDLLYSFPVVIKNKTWKFVEGLPINDFSREKMDLTAKELTEEKESAFEFLSSA

43 NP_055596.3 Q6P1L8

>NP_055596.3

MKLYVFLVNTGTTLTFDTELTVQTVADLKHAIQSKYKIAIQHQVLVVNGGECMAADRRVCTYSAGTDTNPIFLFNKEMILCDRPPAIPKTTFSTENDMEIKVEESLMMPAVFHTVASRTQLALEMYEVAKKLCSFCEGLVHDEHLQHQGWAAIMANLEDCSNSYQKLLFKFESIYSNYLQSIEDIKLKLTHLGTAVSVMAKIPLLECLTRHSYRECLGRLDSLPEHEDSEKAEMKRSTELVLSPDMPRTTNESLLTSFPKSVEHVSPDTADAESGKEIRESCQSTVHQQDETTIDTKDGDLPFFNVSLLDWINVQDRPNDVESLVRKCFDSMSRLDPRIIRPFIAECRQTIAKLDNQNMKAIKGLEDRLYALDQMIASCGRLVNEQKELAQGFLANQKRAENLKDASVLPDLCLSHANQLMIMLQNHRKLLDIKQKCTTAKQELANNLHVRLKWCCFVMLHADQDGEKLQALLRLVIELLERVKIVEALSTVPQMYCLAVVEVVRRKMFIKHYREWAGALVKDGKRLYEAEKSKRESFGKLFRKSFLRNRLFRGLDSWPPSFCTQKPRKFDCELPDISLKDLQFLQSFCPSEVQPFLRVPLLCDFEPLHQHVLALHNLVKAAQSLDEMSQTITDLLSEQKASVSQTSPQSASSPRMESTAGITTTTSPRTPPPLTVQDPLCPAVCPLEELSPDSIDAHTFDFETIPHPNIEQTIHQVSLDLDSLAESPESDFMSAVNEFVIEENLSSPNPISDPQSPEMMVESLYSSVINAIDSRRMQDTNVCGKEDFGDHTSLNVQLERCRVVAQDSHFSIQTIKEDLCHFRTFVQKEQCDFSNSLKCTAVEIRNIIEKVKCSLEITLKEKHQKELLSLKNEYEGKLDGLIKETEENENKIKKLKGELVCLEEVLQNKDNEFALVKHEKEAVICLQNEKDQKLLEMENIMHSQNCEIKELKQSREIVLEDLKKLHVENDEKLQLLRAELQSLEQSHLKELEDTLQVRHIQEFEKVMTDHRVSLEELKKENQQIINQIQESHAEIIQEKEKQLQELKLKVSDLSDTRCKLEVELALKEAETDEIKILLEESRAQQKETLKSLLEQETENLRTEISKLNQKIQDNNENYQVGLAELRTLMTIEKDQCISELISRHEEESNILKAELNKVTSLHNQAFEIEKNLKEQIIELQSKLDSELSALERQKDEKITQQEEKYEAIIQNLEKDRQKLVSSQEQDREQLIQKLNCEKDEAIQTALKEFKLEREVVEKELLEKVKHLENQIAKSPAIDSTRGDSSSLVAELQEKLQEEKAKFLEQLEEQEKRKNEEMQNVRTSLIAEQQTNFNTVLTREKMRKENIINDLSDKLKSTMQQQERDKDLIESLSEDRARLLEEKKKLEEEVSKLRSSSFVPSPYVATAPELYGACAPELPGESDRSAVETADEGRVDSAMETSMMSVQENIHMLSEEKQRIMLLERTLQLKEEENKRLNQRLMSQSMSSVSSRHSEKIAIRDFQVGDLVLIILDERHDNYVLFTVSPTLYFLHSESLPALDLKPGEGASGASRRPWVLGKVMEKEYCQAKKAQNRFKVPLGTKFYRVKAVSWNKKV

>Q6P1L8

MAFFTGLWGPFTCVSRVLSHHCFSTTGSLSAIQKMTRVRVVDNSALGNSPYHRAPRCIHVYKKNGVGKVGDQILLAIKGQKKKALIVGHCMPGPRMTPRFDSNNVVLIEDNGNPVGTRIKTPIPTSLRKREGEYSKVLAIAQNFV

44 NP_006222.2 NP_000958.1

>NP_006222.2

MSLRSGGRRRADPGADGEASRDDGATSSVSALKRLERSQWTDKMDLRFGFERLKEPGEKTGWLINMHPTEILDEDKRLGSAVDYYFIQDDGSRFKVALPYKPYFYIATRKGCEREVSSFLSKKFQGKIAKVETVPKEDLDLPNHLVGLKRNYIRLSFHTVEDLVKVRKEISPAVKKNREQDHASDAYTALLSSVLQRGGVITDEEETSKKIADQLDNIVDMREYDVPYHIRLSIDLKIHVAHWYNVRYRGNAFPVEITRRDDLVERPDPVVLAFDIETTKLPLKFPDAETDQIMMISYMIDGQGYLITNREIVSEDIEDFEFTPKPEYEGPFCVFNEPDEAHLIQRWFEHVQETKPTIMVTYNGDFFDWPFVEARAAVHGLSMQQEIGFQKDSQGEYKAPQCIHMDCLRWVKRDSYLPVGSHNLKAAAKAKLGYDPVELDPEDMCRMATEQPQTLATYSVSDAVATYYLYMKYVHPFIFALCTIIPMEPDEVLRKGSGTLCEALLMVQAFHANIIFPNKQEQEFNKLTDDGHVLDSETYVGGHVEALESGVFRSDIPCRFRMNPAAFDFLLQRVEKTLRHALEEEEKVPVEQVTNFEEVCDEIKSKLASLKDVPSRIECPLIYHLDVGAMYPNIILTNRLQPSAMVDEATCAACDFNKPGANCQRKMAWQWRGEFMPASRSEYHRIQHQLESEKFPPLFPEGPARAFHELSREEQAKYEKRRLADYCRKAYKKIHITKVEERLTTICQRENSFYVDTVRAFRDRRYEFKGLHKVWKKKLSAAVEVGDAAEVKRCKNMEVLYDSLQLAHKCILNSFYGYVMRKGARWYSMEMAGIVCFTGANIITQARELIEQIGRPLELDTDGIWCVLPNSFPENFVFKTTNVKKPKVTISYPGAMLNIMVKEGFTNDQYQELAEPSSLTYVTRSENSIFFEVDGPYLAMILPASKEEGKKLKKRYAVFNEDGSLAELKGFEVKRRGELQLIKIFQSSVFEAFLKGSTLEEVYGSVAKVADYWLDVLYSKAANMPDSELFELISENRSMSRKLEDYGEQKSTSISTAKRLAEFLGDQMVKDAGLSCRYIISRKPEGSPVTERAIPLAIFQAEPTVRKHFLRKWLKSSSLQDFDIRAILDWDYYIERLGSAIQKIITIPAALQQVKNPVPRVKHPDWLHKKLLEKNDVYKQKKISELFTLEGRRQVTMAEASEDSPRPSAPDMEDFGLVKLPHPAAPVTVKRKRVLWESQEESQDLTPTVPWQEILGQPPALGTSQEEWLVWLRFHKKKWQLQARQRLARRKRQRLESAEGVLRPGAIRDGPATGLGSFLRRTARSILDLPWQIVQISETSQAGLFRLWALVGSDLHCIRLSIPRVFYVNQRVAKAEEGASYRKVNRVLPRSNMVYNLYEYSVPEDMYQEHINEINAELSAPDIEGVYETQVPLLFRALVHLGCVCVVNKQLVRHLSGWEAETFALEHLEMRSLAQFSYLEPGSIRHIYLYHHAQAHKALFGIFIPSQRRASVFVLDTVRSNQMPSLGALYSAEHGLLLEKVGPELLPPPKHTFEVRAETDLKTICRAIQRFLLAYKEERRGPTLIAVQSSWELKRLASEIPVLEEFPLVPICVADKINYGVLDWQRHGARRMIRHYLNLDTCLSQAFEMSRYFHIPIGNLPEDISTFGSDLFFARHLQRHNHLLWLSPTARPDLGGKEADDNCLVMEFDDQATVEINSSGCYSTVCVELDLQNLAVNTILQSHHVNDMEGADSMGISFDVIQQASLEDMITGGQAASAPASYDETALCSNTFRILKSMVVGWVKEITQYHNIYADNQVMHFYRWLRSPSSLLHDPALHRTLHNMMKKLFLQLIAEFKRLGSSVIYANFNRIILCTKKRRVEDAIAYVEYITSSIHSKETFHSLTISFSRCWEFLLWMDPSNYGGIKGKVSSRIHCGLQDSQKAGGAEDEQENEDDEEERDGEEEEEAEESNVEDLLENNWNILQFLPQAASCQNYFLMIVSAYIVAVYHCMKDGLRRSAPGSTPVRRRGASQLSQEAEGAVGALPGMITFSQDYVANELTQSFFTITQKIQKKVTGSRNSTELSEMFPVLPGSHLLLNNPALEFIKYVCKVLSLDTNITNQVNKLNRDLLRLVDVGEFSEEAQFRDPCRSYVLPEVICRSCNFCRDLDLCKDSSFSEDGAVLPQWLCSNCQAPYDSSAIEMTLVEVLQKKLMAFTLQDLVCLKCRGVKETSMPVYCSCAGDFALTIHTQVFMEQIGIFRNIAQHYGMSYLLETLEWLLQKNPQLGH

>NP_000958.1

MSHRKFSAPRHGSLGFLPRKRSSRHRGKVKSFPKDDPSKPVHLTAFLGYKAGMTHIVREVDRPGSKVNKKEVVEAVTIVETPPMVVVGIVGYVETPRGLRTFKTVFAEHISDECKRRFYKNWHKSKKKAFTKYCKKWQDEDGKKQLEKDFSSMKKYCQVIRVIAHTQMRLLPLRQKKAHLMEIQVNGGTVAEKLDWARERLEQQVPVNQVFGQDEMIDVIGVTKGKGYKGVTSRWHTKKLPRKTHRGLRKVACIGAWHPARVAFSVARAGQKGYHHRTEINKKIYKIGQGYLIKDGKLIKNNASTDYDLSDKSINPLGGFVHYGEVTNDFVMLKGCVVGTKKRVLTLRKSLLVQTKRRALEKIDLKFIDTTSKFGHGRFQTMEEKKAFMGPLKKDRIAKEEGA

45 NP_055136.1 NP_000846.1

>NP_055136.1

MGSELIGRLAPRLGLAEPDMLRKAEEYLRLSRVKCVGLSARTTETSSAVMCLDLAASWMKCPLDRAYLIKLSGLNKETYQSCLKSFECLLGLNSNIGIRDLAVQFSCIEAVNMASKILKSYESSLPQTQQVDLDLSRPLFTSAALLSACKILKLKVDKNKMVATSGVKKAIFDRLCKQLEKIGQQVDREPGDVATPPRKRKKIVVEAPAKEMEKVEEMPHKPQKDEDLTQDYEEWKRKILENAASAQKATAE

>NP_000846.1

MSRRKISSESFSSLGSDYLETSPEEEGECPLSRLCWNGSRSPPGPLEPSPAAAAAAAAPAPTPAASAAAAAATAGARRVQRRRRVNLDSLGESISRLTAPSPQTIQQTLKRTLQYYEHQVIGYRDAEKNFHNISNRCSYADHSNKEEIEDVSGILQCTANILGLKFEEIQKRFGEEFFNICFHENERVLRAVGGTLQDFFNGFDALLEHIRTSFGKQATLESPSFLCKELPEGTLMLHYFHPHHIVGFAMLGMIKAAGKKIYRLDVEVEQVANEKLCSDVSNPGNCSCLTFLIKECENTNIMKNLPQGTSQVPADLRISINTFCRAFPFHLMFDPSMSVLQLGEGLRKQLRCDTHKVLKFEDCFEIVSPKVNATFERVLLRLSTPFVIRTKPEASGSENKDKVMEVKGQMIHVPESNSILFLGSPCVDKLDELMGRGLHLSDIPIHDATRDVILVGEQAKAQDGLKKRMDKLKATLERTHQALEEEKKKTVDLLYSIFPGDVAQQLWQGQQVQARKFDDVTMLFSDIVGFTAICAQCTPMQVISMLNELYTRFDHQCGFLDIYKVETIGDAYCVAAGLHRKSLCHAKPIALMALKMMELSEEVLTPDGRPIQMRIGIHSGSVLAGVVGVRMPRYCLFGNNVTLASKFESGSHPRRINVSPTTYQLLKREESFTFIPRSREELPDNFPKEIPGICYFLEVRTGPKPPKPSLSSSRIKKVSYNIGTMFLRETSL

46 NP_006181.1 NP_002003.1

>NP_006181.1

MSKPELKEDKMLEVHFVGDDDVLNHILDREGGAKLKKERAQLLVNPKKIIKKPEYDLEEDDQEVLKDQNYVEIMGRDVQESLKNGSATGGGNKVYSFQNRKHSEKMAKLASELAKTPQKSVSFSLKNDPEITINVPQSSKGHSASDKVQPKNNDKSEFLSTAPRSLRKRLIVPRSHSDSESEYSASNSEDDEGVAQEHEEDTNAVIFSQKIQAQNRVVSAPVGKETPSKRMKRDKTSDLVEEYFEAHSSSKVLTSDRTLQKLKRAKLDQQTLRNLLSKVSPSFSAELKQLNQQYEKLFHKWMLQLHLGFNIVLYGLGSKRDLLERFRTTMLQDSIHVVINGFFPGISVKSVLNSITEEVLDHMGTFRSILDQLDWIVNKFKEDSSLELFLLIHNLDSQMLRGEKSQQIIGQLSSLHNIYLIASIDHLNAPLMWDHAKQSLFNWLWYETTTYSPYTEETSYENSLLVKQSGSLPLSSLTHVLRSLTPNARGIFRLLIKYQLDNQDNPSYIGLSFQDFYQQCREAFLVNSDLTLRAQLTEFRDHKLIRTKKGTDGVEYLLIPVDNGTLTDFLEKEEEEA

>NP_002003.1

MSFRFGQHLIKPSVVFLKTELSFALVNRKPVVPGHVLVCPLRPVERFHDLRPDEVADLFQTTQRVGTVVEKHFHGTSLTFSMQDGPEAGQTVKHVHVHVLPRKAGDFHRNDSIYEELQKHDKEDFPASWRSEEEMAAEAAALRVYFQ

47 NP_004950.2 NP_038203.2

>NP_004950.2

MDYSYDEDLDELCPVCGDKVSGYHYGLLTCESCKGFFKRTVQNNKHYTCTESQSCKIDKTQRKRCPFCRFQKCLTVGMRLEAVRADRMRGGRNKFGPMYKRDRALKQQKKAQIRANGFKLETGPPMGVPPPPPPAPDYVLPPSLHGPEPKGLAAGPPAGPLGDFGAPALPMAVPGAHGPLAGYLYPAFPGRAIKSEYPEPYASPPQPGLPYGYPEPFSGGPNVPELILQLLQLEPDEDQVRARILGCLQEPTKSRPDQPAAFGLLCRMADQTFISIVDWARRCMVFKELEVADQMTLLQNCWSELLVFDHIYRQVQHGKEGSILLVTGQEVELTTVATQAGSLLHSLVLRAQELVLQLLALQLDRQEFVCLKFIILFSLDLKFLNNHILVKDAQEKANAALLDYTLCHYPHCGDKFQQLLLCLVEVRALSMQAKEYLYHKHLGNEMPRNNLLIEMLQAKQT

>NP_038203.2

MLQQVPENINFPAEEEKILEFWTEFNCFQECLKQSKHKPKFTFYDGPPFATGLPHYGHILAGTIKDIVTRYAHQSGFHVDRRFGWDCHGLPVEYEIDKTLGIRGPEDVAKMGITEYNNQCRAIVMRYSAEWKSTVSRLGRWIDFDNDYKTLYPQFMESVWWVFKQLYDKGLVYRGVKVMPFSTACNTPLSNFESHQNYKDVQDPSVFVTFPLEEDETVSLVAWTTTPWTLPSNLAVCVNPEMQYVKIKDVARGRLLILMEARLSALYKLESDYEILERFPGAYLKGKKYRPLFDYFLKCKENGAFTVLVDNYVKEEEGTGVVHQAPYFGAEDYRVCMDFNIIRKDSLPVCPVDASGCFTTEVTDFAGQYVKDADKSIIRTLKEQGRLLVATTFTHSYPFCWRSDTPLIYKAVPSWFVRVENMVDQLLRNNDLCYWVPELVREKRFGNWLKDARDWTISRNRYWGTPIPLWVSDDFEEVVCIGSVAELEELSGAKISDLHRESVDHLTIPSRCGKGSLHRISEVFDCWFESGSMPYAQVHYPFENKREFEDAFPADFIAEGIDQTRGWFYTLLVLATALFGQPPFKNVIVNGLVLASDGQKMSKRKKNYPDPVSIIQKYGADALRLYLINSPVVRAENLRFKEEGVRDVLKDVLLPWYNAYRFLIQNVLRLQKEEEIEFLYNENTVRESPNITDRWILSFMQSLIGFFETEMAAYRLYTVVPRLVKFVDILTNWYVRMNRRRLKGENGMEDCVMALETLFSVLLSLCRLMAPYTPFLTELMYQNLKVLIDPVSVQDKDTLSIHYLMLPRVREELIDKKTESAVSQMQSVIELGRVIRDRKTIPIKYPLKEIVVIHQDPEALKDIKSLEKYIIEELNVRKVTLSTDKNKYGIRLRAEPDHMVLGKRLKGAFKAVMTSIKQLSSEELEQFQKTGTIVVEGHELHDEDIRLMYTFDQATGGTAQFEAHSDAQALVLLDVTPDQSMVDEGMAREVINRIQKLRKKCNLVPTDEITVYYKAKSEGTYLNSVIESHTEFIFTTIKAPLKPYPVSPSDKVLIQEKTQLKGSELEITLTRGSSLPGPACAYVNLNICANGSEQGGVLLLENPKGDNRLDLLKLKSVVTSIFGVKNTELAVFHDETEIQNQTDLLSLSGKTLCVTAGSAPSLINSSSTLLCQYINLQLLNAKPQECLMGTVGTLLLENPLGQNGLTHQGLLYEAAKVFGLRSRKLKLFLNETQTQEITEDIPVKTLNMKTVYVSVLPTTADF

48 NP_003073.1 Q9NVV4

>NP_003073.1

MGTPPGLQTDCEALLSRFQETDSVRFEDFTELWRNMKFGTIFCGRMRNLEKNMFTKEALALAWRYFLPPYTFQIRVGALYLLYGLYNTQLCQPKQKIRVALKDWDEVLKFQQDLVNAQHFDAAYIFRKLRLDRAFHFTAMPKLLSYRMKKKIHRAEVTEEFKDPSDRVMKLITSDVLEEMLNVHDHYQNMKHVISVDKSKPDKALSLIKDDFFDNIKNIVLEHQQWHKDRKNPSLKSKTNDGEEKMEGNSQETERCERAESLAKIKSKAFSVVIQASKSRRHRQVKLDSSDSDSASGQGQVKATRKKEKKERLKPAGRKMSLRNKGNVQNIHKEDKPLSLSMPVITEEEENESLSGTEFTASKKRRKH

>Q9NVV4

MAVPGVGLLTRLNLCARRRTRVQRPIVRLLSCPGTVAKDLRRDEQPSGSVETGFEDKIPKRRFSEMQNERREQAQRTVLIHCPEKISENKFLKYLSQFGPINNHFFYESFGLYAVVEFCQKESIGSLQNGTHTPSTAMETAIPFRSRFFNLKLKNQTSERSRVRSSNQLPRSNKQLFELLCYAESIDDQLNTLLKEFQLTEENTKLRYLTCSLIEDMAAAYFPDCIVRPFGSSVNTFGKLGCDLDMFLDLDETRNLSAHKISGNFLMEFQVKNVPSERIATQKILSVLGECLDHFGPGCVGVQKILNARCPLVRFSHQASGFQCDLTTNNRIALTSSELLYIYGALDSRVRALVFSVRCWARAHSLTSSIPGAWITNFSLTMMVIFFLQRRSPPILPTLDSLKTLADAEDKCVIEGNNCTFVRDLSRIKPSQNTETLELLLKEFFEYFGNFAFDKNSINIRQGREQNKPDSSPLYIQNPFETSLNISKNVSQSQLQKFVDLARESAWILQQEDTDRPSISSNRPWGLVSLLLPSAPNRKSFTKKKSNKFAIETVKNLLESLKGNRTENFTKTSGKRTISTQT

49 NP_056271.2 Q8N6F7

>NP_056271.2

MFAGLQDLGVANGEDLKETLTNCTEPLKAIEQFQTENGVLLPSLQSALPFLDLHGTPRLEFHQSVFDELRDKLLERVSAIASEGKAEERYKKLEDLLEKSFSLVKMPSLQPVVMCVMKHLPKVPEKKLKLVMADKELYRACAVEVKRQIWQDNQALFGDEVSPLLKQYILEKESALFSTELSVLHNFFSPSPKTRRQGEVVQRLTRMVGKNVKLYDMVLQFLRTLFLRTRNVHYCTLRAELLMSLHDLDVGEICTVDPCHKFTWCLDACIRERFVDSKRARELQGFLDGVKKGQEQVLGDLSMILCDPFAINTLALSTVRHLQELVGQETLPRDSPDLLLLLRLLALGQGAWDMIDSQVFKEPKMEVELITRFLPMLMSFLVDDYTFNVDQKLPAEEKAPVSYPNTLPESFTKFLQEQRMACEVGLYYVLHITKQRNKNALLRLLPGLVETFGDLAFGDIFLHLLTGNLALLADEFALEDFCSSLFDGFFLTASPRKENVHRHALRLLIHLHPRVAPSKLEALQKALEPTGQSGEAVKELYSQLGEKLEQLDHRKPSPAQAAETPALELPLPSVPAPAPL

>Q8N6F7

MGNSLLRENRRQQNTQEMPWNVRMQSPKQRTSRCWDHHIAEGCFCLPWKKILIFEKRQDSQNENERMSSTPIQDNVDQTYSEELCYTLINHRVLCTRPSGNSAEEYYENVPCKAERPRESLGGTETEYSLLHMPSTDPRHARSPEDEYELLMPHRISSHFLQQPRPLMAPSETQFSHL

50 NP_073622.2 Q9HBU6

>NP_073622.2

MCKMAIIPDWLRSHPHTRKFTHSRPHSSPCRVYSRNGSPNKFRSSSTTAVANPTLSSLDVKRILFQKITDRGDELQKAFQLLDTGQNLTVSKSELRRIITDFLMPLTREQFQDVLAQIPLSTSGTVPYLAFLSRFGGIDLYINGIKRGGGNEMNCCRTLRELEIQVGEKVFKNIKTVMKAFELIDVNKTGLVRPQELRRVLETFCMKLRDEEYEKFSKHYNIHKDTAVDYNVFLKNLSINNDLNLRYCMGNQEVSLENQQAKNSKKERLLGSASSEDIWRNYSLDEIERNFCLQLSKSYEKVEKALSAGDPCKGGYVSFNYLKIVLDTFVYQIPRRIFIQLMKRFGLKATTKINWKQFLTSFHEPQGLQVSSKGPLTKRNSINSRNESHKENIITKLFRHTEDHSASLKKALLIINTKPDGPITREEFRYILNCMAVKLSDSEFKELMQMLDPGDTGVVNTSMFIDLIEENCRMRKTSPCTDAKTPFLLAWDSVEEIVHDTITRNLQAFYNMLRSYDLGDTGRIGRNNFKKIMHVFCPFLTNAHFIKLCSKIQDIGSGRILYKKLLACIGIDGPPTVSPVLVPKDQLLSEHLQKDEQQQPDLSERTKLTEDKTTLTKKMTTEEVIEKFKKCIQQQDPAFKKRFLDFSKEPNGKINVHDFKKVLEDTGMPMDDDQYALLTTKIGFEKEGMSYLDFAAGFEDPPMRGPETTPPQPPTPSKSYVNSHFITAEECLKLFPRRLKESFRDPYSAFFKTDADRDGIINMHDLHRLLLHLLLNLKDDEFERFLGLLGLRLSVTLNFREFQNLCEKRPWRTDEAPQRLIRPKQKVADSELACEQAHQYLVTKAKNRWSDLSKNFLETDNEGNGILRRRDIKNALYGFDIPLTPREFEKLWARYDTEGKGHITYQEFLQKLGINYSPAVHRPCAEDYFNFMGHFTKPQQLQEEMKELQQSTEKAVAARDKLMDRHQDISKAFTKTDQSKTNYISICKMQEVLEECGCSLTEGELTHLLNSWGVSRHDNAINYLDFLRAVENSKSTGAQPKEKEESMPINFATLNPQEAVRKIQEVVESSQLALSTAFSALDKEDTGFVKATEFGQVLKDFCYKLTDNQYHYFLRKLRIHLTPYINWKYFLQNFSCFLEETADEWAEKMPKGPPPTSPKATADRDILARLHKAVTSHYHAITQEFENFDTMKTNTISREEFRAICNRRVQILTDEQFDRLWNEMPVNAKGRLKYPDFLSRFSSETAATPMATGDSAVAQRGSSVPDVSEGTRSALSLPTQELRPGSKSQSHPCTPASTTVIPGTPPLQNCDPIESRLRKRIQGCWRQLLKECKEKDVARQGDINASDFLALVEKFNLDISKEECQQLIIKYDLKSNGKFAYCDFIQSCVLLLKAKESSLMHRMKIQNAHKMKEAGAETPSFYSALLRIQPKIVHCWRPMRRTFKSYDEAGTGLLSVADFRTVLRQYSINLSEEEFFHILEYYDKTLSSKISYNDFLRAFLQ

>Q9HBU6

MLCGRPRSSSDNRNFLRERAGLSSAAVQTRIGNSAASRRSPAARPPVPAPPALPRGRPGTEGSTSLSAPAVLVVAVAVVVVVVSAVAWAMANYIHVPPGSPEVPKLNVTVQDQEEHRCREGALSLLQHLRPHWDPQEVTLQLFTDGITNKLIGCYVGNTMEDVVLVRIYGNKTELLVDRDEEVKSFRVLQAHGCAPQLYCTFNNGLCYEFIQGEALDPKHVCNPAIFRLIARQLAKIHAIHAHNGWIPKSNLWLKMGKYFSLIPTGFADEDINKRFLSDIPSSQILQEEMTWMKEILSNLGSPVVLCHNDLLCKNIIYNEKQGDVQFIDYEYSGYNYLAYDIGNHFNEFAGVSDVDYSLYPDRELQSQWLRAYLEAYKEFKGFGTEVTEKEVEILFIQVNQFALASHFFWGLWALIQAKYSTIEFDFLGYAIVRFNQYFKMKPEVTALKVPE

51 Q15562 NP_060383.2

>Q15562

MGEPRAGAALDDGSGWTGSEEGSEEGTGGSEGAGGDGGPDAEGVWSPDIEQSFQEALAIYPPCGRRKIILSDEGKMYGRNELIARYIKLRTGKTRTRKQVSSHIQVLARRKSREIQSKLKDQVSKDKAFQTMATMSSAQLISAPSLQAKLGPTGPQASELFQFWSGGSGPPWNVPDVKPFSQTPFTLSLTPPSTDLPGYEPPQALSPLPPPTPSPPAWQARGLGTARLQLVEFSAFVEPPDAVDSYQRHLFVHISQHCPSPGAPPLESVDVRQIYDKFPEKKGGLRELYDRGPPHAFFLVKFWADLNWGPSGEEAGAGGSISSGGFYGVSSQYESLEHMTLTCSSKVCSFGKQVVEKVETERAQLEDGRFVYRLLRSPMCEYLVNFLHKLRQLPERYMMNSVLENFTILQVVTNRDTQELLLCTAYVFEVSTSERGAQHHIYRLVRD

>NP_060383.2

MEQPWPPPGPWSLPRAEGEAEEESDFDVFPSSPRCPQLPGGGAQMYSHGIELACQKQKEFVKSSVACKWNLAEAQQKLGSLALHNSESLDQEHAKAQTAVSELRQREEEWRQKEEALVQREKMCLWSTDAISKDVFNKSFINQDKRKDTEDEDKSESFMQKYEQKIRHFGMLSRWDDSQRFLSDHPYLVCEETAKYLILWCFHLEAEKKGALMEQIAHQAVVMQFIMEMAKNCNVDPRGCFRLFFQKAKAEEEGYFEAFKNELEAFKSRVRLYSQSQSFQPMTVQNHVPHSGVGSIGLLESLPQNPDYLQYSISTALCSLNSVVHKEDDEPKMMDTV

52 Q9UI36 NP_660198.1

>Q9UI36

MAVPAALIPPTQLVPPQPPISTSASSSGTTTSTSSATSSPAPSIGPPASSGPTLFRPEPIASAAAAAATVTSTGGGGGGGGSGGGGGSSGNGGGGGGGGGGSNCNPNLAAASNGSGGGGGGISAGGGVASSTPINASTGSSSSSSSSSSSSSSSSSSSSSSSSCGPLPGKPVYSTPSPVENTPQNNECKMVDLRGAKVASFTVEGCELICLPQAFDLFLKHLVGGLHTVYTKLKRLEITPVVCNVEQVRILRGLGAIQPGVNRCKLISRKDFETLYNDCTNASSRPGRPPKRTQSVTSPENSHIMPHSVPGLMSPGIIPPTGLTAAAAAAAAATNAAIAEAMKVKKIKLEAMSNYHASNNQHGADSENGDMNSSVGSSDGSWDKETLPSSPSQGPQASITHPRMPGARSLPLSHPLNHLQQSHLLPNGLELPFMMMPHPLIPVSLPPASVTMAMSQMNHLSTIANMAAAAQVQSPPSRVETSVIKERVPDSPSPAPSLEEGRRPGSHPSSHRSSSVSSSPARTESSSDRIPVHQNGLSMNQMLMGLSPNVLPGPKEGDLAGHDMGHESKRMHIEKDETPLSTPTARDSLDKLSLTGHGQPLPPGFPSPFLFPDGLSSIETLLTNIQGLLKVAIDNARAQEKQVQLEKTELKMDFLRERELRETLEKQLAMEQKNRAIVQKRLKKEKKAKRKLQEALEFETKRREQAEQTLKQAASTDSLRVLNDSLTPEIEADRSGGRTDAERTIQDGRLYLKTTVMY

>NP_660198.1

MLIPFSMKNCFQLLCNCQVPAAGFKKTVKNGLILQSISNDVYQNLAVEDWIHDHMNLEGKPILFFWQNSPSVVIGRHQNPWQECNLNLMREEGIKLARRRSGGGTVYHDMGNINLTFFTTKKKYDRMENLKLIVRALNAVQPQLDVQATKRFDLLLDGQFKISGTASKIGRTTAYHHCTLLCSTDGTFLSSLLKSPYQGIRSNATASIPSLVKNLLEKDPTLTCEVLMNAVATEYAAYHQIDNHIHLINPTDETLFPGINSKAKELQTWEWIYGKTPKFSINTSFHVLYEQSHLEIKVFIDIKNGRIEICNIEAPDHWLPLEIRDKLNSSLIGSKFCPTETTMLTNILLRTCPQDHKLNSKWNILCEKIKGIM

53 NP_055586.1 Q6PRD7

>NP_055586.1

MSGPGNKRAAGDGGSGPPEKKLSREEKTTTTLIEPIRLGGISSTEEMDLKVLQFKNKKLAERLEQRQACEDELRERIEKLEKRQATDDATLLIVNRYWAQLDETVEALLRCHESQGELSSAPEAPGTQEGPTCDGTPLPEPGTSELRDPLLMQLRPPLSEPALAFVVALGASSSEEVELELQGRMEFSKAAVSRVVEASDRLQRRVEELCQRVYSRGDSEPLSEAAQAHTRELGRENRRLQDLATQLQEKHHRISLEYSELQDKVTSAETKVLEMETTVEDLQWDIEKLRKREQKLNKHLAEALEQLNSGYYVSGSSSGFQGGQITLSMQKFEMLNAELEENQELANSRMAELEKLQAELQGAVRTNERLKVALRSLPEEVVRETGEYRMLQAQFSLLYNESLQVKTQLDEARGLLLATKNSHLRHIEHMESDELGLQKKLRTEVIQLEDTLAQVRKEYEMLRIEFEQNLAANEQAGPINREMRHLISSLQNHNHQLKGDAQRYKRKLREVQAEIGKLRAQASGSAHSTPNLGHPEDSGVSAPAPGKEEGGPGPVSTPDNRKEMAPVPGTTTTTTSVKKEELVPSEEDFQGITPGAQGPSSRGREPEARPKRELREREGPSLGPPPVASALSRADREKAKVEETKRKESELLKGLRAELKKAQESQKEMKLLLDMYKSAPKEQRDKVQLMAAERKAKAEVDELRSRIRELEERDRRESKKIADEDALRRIRQAEEQIEHLQRKLGATKQEEEALLSEMDVTGQAFEDMQEQNGRLLQQLREKDDANFKLMSERIKANQIHKLLREEKDELGEQVLGLKSQVDAQLLTVQKLEEKERALQGSLGGVEKELTLRSQALELNKRKAVEAAQLAEDLKVQLEHVQTRLREIQPCLAESRAAREKESFNLKRAQEDISRLRRKLEKQRKVEVYADADEILQEEIKEYKARLTCPCCNTRKKDAVLTKCFHVFCFECVRGRYEARQRKCPKCNAAFGAHDFHRIYIS

>Q6PRD7

MGTSSTDSQQAGHRRCSTSNTSAENLTCLSLPGSPGKTAPLPGPAQAGAGQPLPKGCAAVKAEVGIPAPHTSQEVRIHIRRLLSWAAPGACGLRSTPCALPQALPQARPCPGRWFFPGCSLPTGGAQTILSLWTWRHFLNWALQQREENSGRARRVPPVPRTAPVSKGEGSHPPQNSNGEKVKTITPDVGLHQSLTSDPTVAVLRAKRAPEAHPPRSCSGSLTARVCHMGVCQGQGDTEDGRMTLMG

54 P54803 NP_004721.1

>P54803

MAEWLLSASWQRRAKAMTAAAGSAGRAAVPLLLCALLAPGGAYVLDDSDGLGREFDGIGAVSGGGATSRLLVNYPEPYRSQILDYLFKPNFGASLHILKVEIGGDGQTTDGTEPSHMHYALDENYFRGYEWWLMKEAKKRNPNITLIGLPWSFPGWLGKGFDWPYVNLQLTAYYVVTWIVGAKRYHDLDIDYIGIWNERSYNANYIKILRKMLNYQGLQRVKIIASDNLWESISASMLLDAELFKVVDVIGAHYPGTHSAKDAKLTGKKLWSSEDFSTLNSDMGAGCWGRILNQNYINGYMTSTIAWNLVASYYEQLPYGRCGLMTAQEPWSGHYVVESPVWVSAHTTQFTQPGWYYLKTVGHLEKGGSYVALTDGLGNLTIIIETMSHKHSKCIRPFLPYFNVSQQFATFVLKGSFSEIPELQVWYTKLGKTSERFLFKQLDSLWLLDSDGSFTLSLHEDELFTLTTLTTGRKGSYPLPPKSQPFPSTYKDDFNVDYPFFSEAPNFADQTGVFEYFTNIEDPGEHHFTLRQVLNQRPITWAADASNTISIIGDYNWTNLTIKCDVYIETPDTGGVFIAGRVNKGGILIRSARGIFFWIFANGSYRVTGDLAGWIIYALGRVEVTAKKWYTLTLTIKGHFASGMLNDKSLWTDIPVNFPKNGWAAIGTHSFEFAQFDNFLVEATR

>NP_004721.1

MADDPSAADRNVEIWKIKKLIKSLEAARGNGTSMISLIIPPKDQISRVAKMLADEFGTASNIKSRVNRLSVLGAITSVQQRLKLYNKVPPNGLVVYCGTIVTEEGKEKKVNIDFEPFKPINTSLYLCDNKFHTEALTALLSDDSKFGFIVIDGSGALFGTLQGNTREVLHKFTVDLPKKHGRGGQSALRFARLRMEKRHNYVRKVAETAVQLFISGDKVNVAGLVLAGSADFKTELSQSDMFDQRLQSKVLKLVDISYGGENGFNQAIELSTEVLSNVKFIQEKKLIGRYFDEISQDTGKYCFGVEDTLKALEMGAVEILIVYENLDIMRYVLHCQGTEEEKILYLTPEQEKDKSHFTDKETGQEHELIESMPLLEWFANNYKKFGATLEIVTDKSQEGSQFVKGFGGIGGILRYRVDFQGMEYQGGDDEFFDLDDY

55 Q6UXN9 Q9NZE8

>Q6UXN9

MKLTDSVLRSFRVAKVFRENSDKINCFDFSPNGETVISSSDDDSIVLYDCQEGKPKRTLYSKKYGVDLIRYTHAANTVVYSSNKIDDTIRYLSLHDNKYIRYFPGHSKRVVALSMSPVDDTFISGSLDKTIRLWDLRSPNCQGLMHLQGKPVCSFDPEGLIFAAGVNSEMVKLYDLRSFDKGPFATFKMQYDRTCEWTGLKFSNDGKLILISTNGSFIRLIDAFKGVVMHTFGGYANSKAVTLEASFTPDSQFIMIGSEDGKIHVWNGESGIKVAVLDGKHTGPITCLQFNPKFMTFASACSNMAFWLPTIDD

>Q9NZE8

MAASAFAGAVRAASGILRPLNILASSTYRNCVKNASLISALSTGRFSHIQTPVVSSTPRLTTSERNLTCGHTSVILNRMAPVLPSVLKLPVRSLTYFSARKGKRKTVKAVIDRFLRLHCGLWVRRKAGYKKKLWKKTPARKKRLREFVFCNKTQSKLLDKMTTSFWKRRNWYVDDPYQKYHDRTNLKV

56 NP_073624.2 NP_003134.1

>NP_073624.2

MENSEKTEVVLLACGSFNPITNMHLRLFELAKDYMNGTGRYTVVKGIISPVGDAYKKKGLIPAYHRVIMAELATKNSKWVEVDTWESLQKEWKETLKVLRHHQEKLEASDCDHQQNSPTLERPGRKRKWTETQDSSQKKSLEPKTKAVPKVKLLCGADLLESFAVPNLWKSEDITQIVANYGLICVTRAGNDAQKFIYESDVLWKHRSNIHVVNEWIANDISSTKIRRALRRGQSIRYLVPDLVQEYIEKHNLYSSESEDRNAGVILAPLQRNTAEAKT

>NP_003134.1

MFRRPVLQVLRQFVRHESETTTSLVLERSLNRVHLLGRVGQDPVLRQVEGKNPVTIFSLATNEMWRSGDSEVYQLGDVSQKTTWHRISVFRPGLRDVAYQYVKKGSRIYLEGKIDYGEYMDKNNVRRQATTIIADNIIFLSDQTKEKE

57 Q9UK76 NP_000894.1

>Q9UK76

MTTTTTFKGVDPNSRNSSRVLRPPGGGSNFSLGFDEPTEQPVRKNKMASNIFGTPEENQASWAKSAGAKSSGGREDLESSGLQRRNSSEASSGDFLDLKGEGDIHENVDTDLPGSLGQSEEKPVPAAPVPSPVAPAPVPSRRNPPGGKSSLVLG

>NP_000894.1

MVGRRALIVLAHSERTSFNYAMKEAAAAALKKKGWEVVESDLYAMNFNPIISRKDITGKLKDPANFQYPAESVLAYKEGHLSPDIVAEQKKLEAADLVIFQFPLQWFGVPAILKGWFERVFIGEFAYTYAAMYDKGPFRSKKAVLSITTGGSGSMYSLQGIHGDMNVILWPIQSGILHFCGFQVLEPQLTYSIGHTPADARIQILEGWKKRLENIWDETPLYFAPSSLFDLNFQAGFLMKKEVQDEEKNKKFGLSVGHHLGKSIPTDNQIKARK

58 NP_055586.1 NP_000476.1

>NP_055586.1

MSGPGNKRAAGDGGSGPPEKKLSREEKTTTTLIEPIRLGGISSTEEMDLKVLQFKNKKLAERLEQRQACEDELRERIEKLEKRQATDDATLLIVNRYWAQLDETVEALLRCHESQGELSSAPEAPGTQEGPTCDGTPLPEPGTSELRDPLLMQLRPPLSEPALAFVVALGASSSEEVELELQGRMEFSKAAVSRVVEASDRLQRRVEELCQRVYSRGDSEPLSEAAQAHTRELGRENRRLQDLATQLQEKHHRISLEYSELQDKVTSAETKVLEMETTVEDLQWDIEKLRKREQKLNKHLAEALEQLNSGYYVSGSSSGFQGGQITLSMQKFEMLNAELEENQELANSRMAELEKLQAELQGAVRTNERLKVALRSLPEEVVRETGEYRMLQAQFSLLYNESLQVKTQLDEARGLLLATKNSHLRHIEHMESDELGLQKKLRTEVIQLEDTLAQVRKEYEMLRIEFEQNLAANEQAGPINREMRHLISSLQNHNHQLKGDAQRYKRKLREVQAEIGKLRAQASGSAHSTPNLGHPEDSGVSAPAPGKEEGGPGPVSTPDNRKEMAPVPGTTTTTTSVKKEELVPSEEDFQGITPGAQGPSSRGREPEARPKRELREREGPSLGPPPVASALSRADREKAKVEETKRKESELLKGLRAELKKAQESQKEMKLLLDMYKSAPKEQRDKVQLMAAERKAKAEVDELRSRIRELEERDRRESKKIADEDALRRIRQAEEQIEHLQRKLGATKQEEEALLSEMDVTGQAFEDMQEQNGRLLQQLREKDDANFKLMSERIKANQIHKLLREEKDELGEQVLGLKSQVDAQLLTVQKLEEKERALQGSLGGVEKELTLRSQALELNKRKAVEAAQLAEDLKVQLEHVQTRLREIQPCLAESRAAREKESFNLKRAQEDISRLRRKLEKQRKVEVYADADEILQEEIKEYKARLTCPCCNTRKKDAVLTKCFHVFCFECVRGRYEARQRKCPKCNAAFGAHDFHRIYIS

>NP_000476.1

MADSELQLVEQRIRSFPDFPTPGVVFRDISPVLKDPASFRAAIGLLARHLKATHGGRIDYIAGLDSRGFLFGPSLAQELGLGCVLIRKRGKLPGPTLWASYSLEYGKAELEIQKDALEPGQRVVVVDDLLATGGTMNAACELLGRLQAEVLECVSLVELTSLKGREKLAPVPFFSLLQYE

59 NP_002884.1 Q16822

>NP_002884.1

MASKEMFEDTVEERVINEEYKIWKKNTPFLYDLVMTHALQWPSLTVQWLPEVTKPEGKDYALHWLVLGTHTSDEQNHLVVARVHIPNDDAQFDASHCDSDKGEFGGFGSVTGKIECEIKINHEGEVNRARYMPQNPHIIATKTPSSDVLVFDYTKHPAKPDPSGECNPDLRLRGHQKEGYGLSWNSNLSGHLLSASDDHTVCLWDINAGPKEGKIVDAKAIFTGHSAVVEDVAWHLLHESLFGSVADDQKLMIWDTRSNTTSKPSHLVDAHTAEVNCLSFNPYSEFILATGSADKTVALWDLRNLKLKLHTFESHKDEIFQVHWSPHNETILASSGTDRRLNVWDLSKIGEEQSAEDAEDGPPELLFIHGGHTAKISDFSWNPNEPWVICSVSEDNIMQIWQMAENIYNDEESDVTTSELEGQGS

>Q16822

MAALYRPGLRLNWHGLSPLGWPSCRSIQTLRVLSGDLGQLPTGIRDFVEHSARLCQPEGIHICDGTEAENTATLTLLEQQGLIRKLPKYNNCWLARTDPKDVARVESKTVIVTPSQRDTVPLPPGGARGQLGNWMSPADFQRAVDERFPGCMQGRTMYVLPFSMGPVGSPLSRIGVQLTDSAYVVASMRIMTRLGTPVLQALGDGDFVKCLHSVGQPLTGQGEPVSQWPCNPEKTLIGHVPDQREIISFGSGYGGNSLLGKKCFALRIASRLARDEGWLAEHMLILGITSPAGKKALCAAAFPSACGKTNLAMMRPALPGWKVECVGDDIAWMRFDSEGRLRAINPENGFFGVAPGTSATTNPNAMATIQSNTIFTNVAETSDGGVYWEGIDQPLPPGVTVTSWLGKPWKPGDKEPCAHPNSRFCAPARQCPIMDPAWEAPEGVPIDAIIFGGRRPKGVPLVYEAFNWRHGVFVGRAMRSESTAAAEHKGKIIMHDPFAMRPFFGYNFGHYLEHWLSMEGRKGAQLPRIFHVNWFRRDEAGHFLWPGFGENARVLDWICRRLEGEDSARETPIGLVPKEGALDLSGLRAIDTTQLFSLPKDFWEQEVRDIRSYLTEQVNQDLPKEVLAELEALERRVHKM

60 Q96JN0 Q9HD33

>Q96JN0

MQRMIQQFAAEYTSKNSSTQDPSQPNSTKNQSLPKASPVTTSPTAATTQNPVLSKLLMADQDSPLDLTVRKSQSEPSEQDGVLDLSTKKSPCAGSTSLSHSPGCSSTQGNGRPGRPSQYRPDGLRSGDGVPPRSLQDGTREGFGHSTSLKVPLARSLQISEELLSRNQLSTAASLGPSGLQNHGQHLILSREASWAKPHYEFNLSRMKFRGNGALSNISDLPFLAENSAFPKMALQAKQDGKKDVSHSSPVDLKIPQVRGMDLSWESRTGDQYSYSSLVMGSQTESALSKKLRAILPKQSRKSMLDAGPDSWGSDAEQSTSGQPYPTSDQEGDPGSKQPRKKRGRYRQYNSEILEEAISVVMSGKMSVSKAQSIYGIPHSTLEYKVKERLGTLKNPPKKKMKLMRSEGPDVSVKIELDPQGEAAQSANESKNE

>Q9HD33

MAAAGLALLCRRVSSALKSSRSLITPQVPACTGFFLSLLPKSTPNVTSFHQYRLLHTTLSRKGLEEFFDDPKNWGQEKVKSGAAWTCQQLRNKSNEDLHKLWYVLLKERNMLLTLEQEAKRQRLPMPSPERLDKVVDSMDALDKVVQEREDALRLLQTGQERARPGAWRRDIFGRIIWHKFKQWVIPWHLNKRYNRKRFFALPYVDHFLRLEREKRARIKARKENLERKKAKILLKKFPHLAEAQKSSLV

61 NP_055586.1 O75394

>NP_055586.1

MSGPGNKRAAGDGGSGPPEKKLSREEKTTTTLIEPIRLGGISSTEEMDLKVLQFKNKKLAERLEQRQACEDELRERIEKLEKRQATDDATLLIVNRYWAQLDETVEALLRCHESQGELSSAPEAPGTQEGPTCDGTPLPEPGTSELRDPLLMQLRPPLSEPALAFVVALGASSSEEVELELQGRMEFSKAAVSRVVEASDRLQRRVEELCQRVYSRGDSEPLSEAAQAHTRELGRENRRLQDLATQLQEKHHRISLEYSELQDKVTSAETKVLEMETTVEDLQWDIEKLRKREQKLNKHLAEALEQLNSGYYVSGSSSGFQGGQITLSMQKFEMLNAELEENQELANSRMAELEKLQAELQGAVRTNERLKVALRSLPEEVVRETGEYRMLQAQFSLLYNESLQVKTQLDEARGLLLATKNSHLRHIEHMESDELGLQKKLRTEVIQLEDTLAQVRKEYEMLRIEFEQNLAANEQAGPINREMRHLISSLQNHNHQLKGDAQRYKRKLREVQAEIGKLRAQASGSAHSTPNLGHPEDSGVSAPAPGKEEGGPGPVSTPDNRKEMAPVPGTTTTTTSVKKEELVPSEEDFQGITPGAQGPSSRGREPEARPKRELREREGPSLGPPPVASALSRADREKAKVEETKRKESELLKGLRAELKKAQESQKEMKLLLDMYKSAPKEQRDKVQLMAAERKAKAEVDELRSRIRELEERDRRESKKIADEDALRRIRQAEEQIEHLQRKLGATKQEEEALLSEMDVTGQAFEDMQEQNGRLLQQLREKDDANFKLMSERIKANQIHKLLREEKDELGEQVLGLKSQVDAQLLTVQKLEEKERALQGSLGGVEKELTLRSQALELNKRKAVEAAQLAEDLKVQLEHVQTRLREIQPCLAESRAAREKESFNLKRAQEDISRLRRKLEKQRKVEVYADADEILQEEIKEYKARLTCPCCNTRKKDAVLTKCFHVFCFECVRGRYEARQRKCPKCNAAFGAHDFHRIYIS

>O75394

MFLSAVFFAKSKSKNILVRMVSEAGTGFCFNTKRNRLREKLTLLHYDPVVKQRVLFVEKKKIRSL

62 NP_001460.1 P49753

>NP_001460.1

MSGWESYYKTEGDEEAEEEQEENLEASGDYKYSGRDSLIFLVDASKAMFESQSEDELTPFDMSIQCIQSVYISKIISSDRDLLAVVFYGTEKDKNSVNFKNIYVLQELDNPGAKRILELDQFKGQQGQKRFQDMMGHGSDYSLSEVLWVCANLFSDVQFKMSHKRIMLFTNEDNPHGNDSAKASRARTKAGDLRDTGIFLDLMHLKKPGGFDISLFYRDIISIAEDEDLRVHFEESSKLEDLLRKVRAKETRKRALSRLKLKLNKDIVISVGIYNLVQKALKPPPIKLYRETNEPVKTKTRTFNTSTGGLLLPSDTKRSQIYGSRQIILEKEETEELKRFDDPGLMLMGFKPLVLLKKHHYLRPSLFVYPEESLVIGSSTLFSALLIKCLEKEVAALCRYTPRRNIPPYFVALVPQEEELDDQKIQVTPPGFQLVFLPFADDKRKMPFTEKIMATPEQVGKMKAIVEKLRFTYRSDSFENPVLQQHFRNLEALALDLMEPEQAVDLTLPKVEAMNKRLGSLVDEFKELVYPPDYNPEGKVTKRKHDNEGSGSKRPKVEYSEEELKTHISKGTLGKFTVPMLKEACRAYGLKSGLKKQELLEALTKHFQD

>P49753

MSNKLLSPHPHSVVLRSEFKMASSPAVLRASRLYQWSLKSSAQFLGSPQLRQVGQIIRVPARMAATLILEPAGRCCWDEPVRIAVRGLAPEQPVTLRASLRDEKGALFQAHARYRADTLGELDLERAPALGGSFAGLEPMGLLWALEPEKPLVRLVKRDVRTPLAVELEVLDGHDPDPGRLLCQTRHERYFLPPGVRREPVRVGRVRGTLFLPPEPGPFPGIVDMFGTGGGLLEYRASLLAGKGFAVMALAYYNYEDLPKTMETLHLEYFEEAMNYLLSHPEVKGPGVGLLGISKGGELCLSMASFLKGITAAVVINGSVANVGGTLRYKGETLPPVGVNRNRIKVTKDGYADIVDVLNSPLEGPDQKSFIPVERAESTFLFLVGQDDHNWKSEFYANEACKRLQAHGRRKPQIICYPETGHYIEPPYFPLCRASLHALVGSPIIWGGEPRAHAMAQVDAWKQLQTFFHKHLGGHEGTIPSKV

63 NP_055675.1 Q9NX20

>NP_055675.1

MNLQRYWGEIPISSSQTNRSSFDLLPREFRLVEVHDPPLHQPSANKPKPPTMLDIPSEPCSLTIHTIQLIQHNRRLRNLIATAQAQNQQQTEGVKTEESEPLPSCPGSPPLPDDLLPLDCKNPNAPFQIRHSDPESDFYRGKGEPVTELSWHSCRQLLYQAVATILAHAGFDCANESVLETLTDVAHEYCLKFTKLLRFAVDREARLGQTPFPDVMEQVFHEVGIGSVLSLQKFWQHRIKDYHSYMLQISKQLSEEYERIVNPEKATEDAKPVKIKEEPVSDITFPVSEELEADLASGDQSLPMGVLGAQSERFPSNLEVEASPQASSAEVNASPLWNLAHVKMEPQESEEGNVSGHGVLGSDVFEEPMSGMSEAGIPQSPDDSDSSYGSHSTDSLMGSSPVFNQRCKKRMRKI

>Q9NX20

MWRLLARASAPLLRVPLSDSWALLPASAGVKTLLPVPSFEDVSIPEKPKLRFIERAPLVPKVRREPKNLSDIRGPSTEATEFTEGNFAILALGGGYLHWGHFEMMRLTINRSMDPKNMFAIWRVPAPFKPITRKSVGHRMGGGKGAIDHYVTPVKAGRLVVEMGGRCEFEEVQGFLDQVAHKLPFAAKAVSRGTLEKMRKDQEERERNNQNPWTFERIATANMLGIRKVLSPYDLTHKGKYWGKFYMPKRV

64 NP_055130.1 NP_056177.3

>NP_055130.1

MADGNEDLRADDLPGPAFESYESMELACPAERSGHVAVSDGRHMFVWGGYKSNQVRGLYDFYLPREELWIYNMETGRWKKINTEGDVPPSMSGSCAVCVDRVLYLFGGHHSRGNTNKFYMLDSRSTDRVLQWERIDCQGIPPSSKDKLGVWVYKNKLIFFGGYGYLPEDKVLGTFEFDETSFWNSSHPRGWNDHVHILDTETFTWSQPITTGKAPSPRAAHACATVGNRGFVFGGRYRDARMNDLHYLNLDTWEWNELIPQGICPVGRSWHSLTPVSSDHLFLFGGFTTDKQPLSDAWTYCISKNEWIQFNHPYTEKPRLWHTACASDEGEVIVFGGCANNLLVHHRAAHSNEILIFSVQPKSLVRLSLEAVICFKEMLANSWNCLPKHLLHSVNQRFGSNNTSGS

>NP_056177.3

MTPSEGARAGTGRELEMLDSLLALGGLVLLRDSVEWEGRSLLKALVKKSALCGEQVHILGCEVSEEEFREGFDSDINNRLVYHDFFRDPLNWSKTEEAFPGGPLGALRAMCKRTDPVPVTIALDSLSWLLLRLPCTTLCQVLHAVSHQDSCPGDSSSVGKVSVLGLLHEELHGPGPVGALSSLAQTEVTLGGTMGQASAHILCRRPRQRPTDQTQWFSILPDFSLDLQEGPSVESQPYSDPHIPPVDPTTHLTFNLHLSKKEREARDSLILPFQFSSEKQQALLRPRPGQATSHIFYEPDAYDDLDQEDPDDDLDI

65 NP_060740.1 NP_077268.1

>NP_060740.1

MGEAEVGGGGAAGDKGPGEAATSPAEETVVWSPEVEVCLFHAMLGHKPVGVNRHFHMICIRDKFSQNIGRQVPSKVIWDHLSTMYDMQALHESEILPFPNPERNFVLPEEIIQEVREGKVMIEEEMKEEMKEDVDPHNGADDVFSSSGSLGKASEKSSKDKEKNSSDLGCKEGADKRKRSRVTDKVLTANSNPSSPSAAKRRRT

>NP_077268.1

MIEVVCNDRLGKKVRVKCNTDDTIGDLKKLIAAQTGTRWNKIVLKKWYTIFKDHVSLGDYEIHDGMNLELYYQ

66 NP_004917.2 O43182

>NP_004917.2

MTTTLVSATIFDLSEVLCKGNKMLNYSAPSAGGCLLDRKAVGTPAGGGFPRRHSVTLPSSKFHQNQLLSSLKGEPAPALSSRDSRFRDRSFSEGGERLLPTQKQPGGGQVNSSRYKTELCRPFEENGACKYGDKCQFAHGIHELRSLTRHPKYKTELCRTFHTIGFCPYGPRCHFIHNAEERRALAGARDLSADRPRLQHSFSFAGFPSAAATAAATGLLDSPTSITPPPILSADDLLGSPTLPDGTNNPFAFSSQELASLFAPSMGLPGGGSPTTFLFRPMSESPHMFDSPPSPQDSLSDQEGYLSSSSSSHSGSDSPTLDNSRRLPIFSRLSISDD

>O43182

MSAQSLLHSVFSCSSPASSSAASAKGFSKRKLRQTRSLDPALIGGCGSDEAGAEGSARGATAGRLYSPSLPAESLGPRLASSSRGPPPRATRLPPPGPLCSSFSTPSTPQEKSPSGSFHFDYEVPLGRGGLKKSMAWDLPSVLAGPASSRSASSILCSSGGGPNGIFASPRRWLQQRKFQSPPDSRGHPYVVWKSEGDFTWNSMSGRSVRLRSVPIQSLSELERARLQEVAFYQLQQDCDLSCQITIPKDGQKRKKSLRKKLDSLGKEKNKDKEFIPQAFGMPLSQVIANDRAYKLKQDLQRDEQKDASDFVASLLPFGNKRQNKELSSSNSSLSSTSETPNESTSPNTPEPAPRARRRGAMSVDSITDLDDNQSRLLEALQLSLPAEAQSKKEKARDKKLSLNPIYRQVPRLVDSCCQHLEKHGLQTVGIFRVGSSKKRVRQLREEFDRGIDVSLEEEHSVHDVAALLKEFLRDMPDPLLTRELYTAFINTLLLEPEEQLGTLQLLIYLLPPCNCDTLHRLLQFLSIVARHADDNISKDGQEVTGNKMTSLNLATIFGPNLLHKQKSSDKEFSVQSSARAEESTAIIAVVQKMIENYEALFMVPPDLQNEVLISLLETDPDVVDYLLRRKASQSSSPDMLQSEVSFSVGGRHSSTDSNKASSGDISPYDNNSPVLSERSLLAMQEDAAPGGSEKLYRVPGQFMLVGHLSSSKSRESSPGPRLGKDLSEEPFDIWGTWHSTLKSGSKDPGMTGSSGDIFESSSLRAGPCSLSQGNLSPNWPRWQGSPAELDSDTQGARRTQAAAPATEGRAHPAVSRACSTPHVQVAGKAERPTARSEQYLTLSGAHDLSESELDVAGLQSRATPQCQRPHGSGRDDKRPPPPYPGPGKPAAAAAWIQGPPEGVETPTDQGGQAAEREQQVTQKKLSSANSLPAGEQDSPRLGDAGWLDWQRERWQIWELLSTDNPDALPETLV

67 NP_065988.1 NP_001893.2

>NP_065988.1

MSGRQRTLFQTWGSSISRSSGTPGCSSGTERPQSPGSSKAPLPAAAEAQLESDDDVLLVAAYEAERQLCLENGGFCTSAGALWIYPTNCPVRDYQLHISRAALFCNTLVCLPTGLGKTFIAAVVMYNFYRWFPSGKVVFMAPTKPLVTQQIEACYQVMGIPQSHMAEMTGSTQASTRKEIWCSKRVLFLTPQVMVNDLSRGACPAAEIKCLVIDEAHKALGNYAYCQVVRELVKYTNHFRILALSATPGSDIKAVQQVITNLLIGQIELRSEDSPDILTYSHERKVEKLIVPLGEELAAIQKTYIQILESFARSLIQRNVLMRRDIPNLTKYQIILARDQFRKNPSPNIVGIQQGIIEGEFAICISLYHGYELLQQMGMRSLYFFLCGIMDGTKGMTRSKNELGRNEDFMKLYNHLECMFARTRSTSANGISAIQQGDKNKKFVYSHPKLKKLEEVVIEHFKSWNAENTTEKKRDETRVMIFSSFRDSVQEIAEMLSQHQPIIRVMTFVGHASGKSTKGFTQKEQLEVVKQFRDGGYNTLVSTCVGEEGLDIGEVDLIICFDSQKSPIRLVQRMGRTGRKRQGRIVIILSEGREERIYNQSQSNKRSIYKAISSNRQVLHFYQRSPRMVPDGINPKLHKMFITHGVYEPEKPSRNLQRKSSIFSYRDGMRQSSLKKDWFLSEEEFKLWNRLYRLRDSDEIKEITLPQVQFSSLQNEENKPAQESTTGIHQLSLSEWRLWQDHPLPTHQVDHSDRCRHFIGLMQMIEGMRHEEGECSYELEVESYLQMEDVTSTFIAPRNESNNLASDTFITHKKSSFIKNINQGSSSSVIESDEECAEIVKQTHIKPTKIVSLKKKVSKEIKKDQLKKENNHGIIDSVDNDRNSTVENIFQEDLPNDKRTSDTDEIAATCTINENVIKEPCVLLTECQFTNKSTSSLAGNVLDSGYNSFNDEKSVSSNLFLPFEEELYIVRTDDQFYNCHSLTKEVLANVERFLSYSPPPLSGLSDLEYEIAKGTALENLLFLPCAEHLRSDKCTCLLSHSAVNSQQNLELNSLKCINYPSEKSCLYDIPNDNISDEPSLCDCDVHKHNQNENLVPNNRVQIHRSPAQNLVGENNHDVDNSDLPVLSTDQDESLLLFEDVNTEFDDVSLSPLNSKSESLPVSDKTAISETPLVSQFLISDELLLDNNSELQDQITRDANSFKSRDQRGVQEEKVKNHEDIFDCSRDLFSVTFDLGFCSPDSDDEILEHTSDSNRPLDDLYGRYLEIKEISDANYVSNQALIPRDHSKNFTSGTVIIPSNEDMQNPNYVHLPLSAAKNEELLSPGYSQFSLPVQKKVMSTPLSKSNTLNSFSKIRKEILKTPDSSKEKVNLQRFKEALNSTFDYSEFSLEKSKSSGPMYLHKSCHSVEDGQLLTSNESEDDEIFRRKVKRAKGNVLNSPEDQKNSEVDSPLHAVKKRRFPINRSELSSSDESENFPKPCSQLEDFKVCNGNARRGIKVPKRQSHLKHVARKFLDDEAELSEEDAEYVSSDENDESENEQDSSLLDFLNDETQLSQAINDSEMRAIYMKSLRSPMMNNKYKMIHKTHKNINIFSQIPEQDETYLEDSFCVDEEESCKGQSSEEEVCVDFNLITDDCFANSKKYKTRRAVMLKEMMEQNCAHSKKKLSRIILPDDSSEEENNVNDKRESNIAVNPSTVKKNKQQDHCLNSVPSGSSAQSKVRSTPRVNPLAKQSKQTSLNLKDTISEVSDFKPQNHNEVQSTTPPFTTVDSQKDCRKFPVPQKDGSALEDSSTSGASCSKSRPHLAGTHTSLRLPQEGKGTCILVGGHEITSGLEVISSLRAIHGLQVEVCPLNGCDYIVSNRMVVERRSQSEMLNSVNKNKFIEQIQHLQSMFERICVIVEKDREKTGDTSRMFRRTKSYDSLLTTLIGAGIRILFSSCQEETADLLKELSLVEQRKNVGIHVPTVVNSNKSEALQFYLSIPNISYITALNMCHQFSSVKRMANSSLQEISMYAQVTHQKAEEIYRYIHYVFDIQMLPNDLNQDRLKSDI

>NP_001893.2

MQEKDASSQGFLPHFQHFATQAIHVGQDPEQWTSRAVVPPISLSTTFKQGAPGQHSGFEYSRSGNPTRNCLEKAVAALDGAKYCLAFASGLAATVTITHLLKAGDQIICMDDVYGGTNRYFRQVASEFGLKISFVDCSKIKLLEAAITPETKLVWIETPTNPTQKVIDIEGCAHIVHKHGDIILVVDNTFMSPYFQRPLALGADISMYSATKYMNGHSDVVMGLVSVNCESLHNRLRFLQNSLGAVPSPIDCYLCNRGLKTLHVRMEKHFKNGMAVAQFLESNPWVEKVIYPGLPSHPQHELVKRQCTGCTGMVTFYIKGTLQHAEIFLKNLKLFTLAESLGGFESLAELPAIMTHASVLKNDRDVLGISDTLIRLSVGLEDEEDLLEDLDQALKAAHPPSGSHS

68 NP_000391.1 NP_000174.1

>NP_000391.1

MKLNVDGLLVYFPYDYIYPEQFSYMRELKRTLDAKGHGVLEMPSGTGKTVSLLALIMAYQRAYPLEVTKLIYCSRTVPEIEKVIEELRKLLNFYEKQEGEKLPFLGLALSSRKNLCIHPEVTPLRFGKDVDGKCHSLTASYVRAQYQHDTSLPHCRFYEEFDAHGREVPLPAGIYNLDDLKALGRRQGWCPYFLARYSILHANVVVYSYHYLLDPKIADLVSKELARKAVVVFDEAHNIDNVCIDSMSVNLTRRTLDRCQGNLETLQKTVLRIKETDEQRLRDEYRRLVEGLREASAARETDAHLANPVLPDEVLQEAVPGSIRTAEHFLGFLRRLLEYVKWRLRVQHVVQESPPAFLSGLAQRVCIQRKPLRFCAERLRSLLHTLEITDLADFSPLTLLANFATLVSTYAKGFTIIIEPFDDRTPTIANPILHFSCMDASLAIKPVFERFQSVIITSGTLSPLDIYPKILDFHPVTMATFTMTLARVCLCPMIIGRGNDQVAISSKFETREDIAVIRNYGNLLLEMSAVVPDGIVAFFTSYQYMESTVASWYEQGILENIQRNKLLFIETQDGAETSVALEKYQEACENGRGAILLSVARGKVSEGIDFVHHYGRAVIMFGVPYVYTQSRILKARLEYLRDQFQIRENDFLTFDAMRHAAQCVGRAIRGKTDYGLMVFADKRFARGDKRGKLPRWIQEHLTDANLNLTVDEGVQVAKYFLRQMAQPFHREDQLGLSLLSLEQLESEETLKRIEQIAQQL

>NP_000174.1

MTILTYPFKNLPTASKWALRFSIRPLSCSSQLRAAPAVQTKTKKTLAKPNIRNVVVVDGVRTPFLLSGTSYKDLMPHDLARAALTGLLHRTSVPKEVVDYIIFGTVIQEVKTSNVAREAALGAGFSDKTPAHTVTMACISANQAMTTGVGLIASGQCDVIVAGGVELMSDVPIRHSRKMRKLMLDLNKAKSMGQRLSLISKFRFNFLAPELPAVSEFSTSETMGHSADRLAAAFAVSRLEQDEYALRSHSLAKKAQDEGLLSDVVPFKVPGKDTVTKDNGIRPSSLEQMAKLKPAFIKPYGTVTAANSSFLTDGASAMLIMAEEKALAMGYKPKAYLRDFMYVSQDPKDQLLLGPTYATPKVLEKAGLTMNDIDAFEFHEAFSGQILANFKAMDSDWFAENYMGRKTKVGLPPLEKFNNWGGSLSLGHPFGATGCRLVMAAANRLRKEGGQYGLVAACAAGGQGHAMIVEAYPK

69 NP_004731.2 Q9UBR1

>NP_004731.2

MSSPSSPFREQSFLCAAGDAGEESRVQVLKNEVRRGSPVLLGWVEQAYADKCVCGPSAPPAPTPPSLSQRVMCNDLFKVNPFQLQQFRADPSTASLLLCPGGLDHKLNLRGKAWG

>Q9UBR1

MAGAEWKSLEECLEKHLPLPDLQEVKRVLYGKELRKLDLPREAFEAASREDFELQGYAFEAAEEQLRRPRIVHVGLVQNRIPLPANAPVAEQVSALHRRIKAIVEVAAMCGVNIICFQEAWTMPFAFCTREKLPWTEFAESAEDGPTTRFCQKLAKNHDMVVVSPILERDSEHGDVLWNTAVVISNSGAVLGKTRKNHIPRVGDFNESTYYMEGNLGHPVFQTQFGRIAVNICYGRHHPLNWLMYSINGAEIIFNPSATIGALSESLWPIEARNAAIANHCFTCAINRVGTEHFPNEFTSGDGKKAHQDFGYFYGSSYVAAPDSSRTPGLSRSRDGLLVAKLDLNLCQQVNDVWNFKMTGRYEMYARELAEAVKSNYSPTIVKE

70 NP_036377.1 NP_689953.1

>NP_036377.1

MALTSFLPAPTQLSQDQLEAEEKARSQRSRQTSLVSSRREPPPYGYRKGWIPRLLEDFGDGGAFPEIHVAQYPLDMGRKKKMSNALAIQVDSEGKIKYDAIARQGQSKDKVIYSKYTDLVPKEVMNADDPDLQRPDEEAIKEITEKTRVALEKSVSQKVAAAMPVRAADKLAPAQYIRYTPSQQGVAFNSGAKQRVIRMVEMQKDPMEPPRFKINKKIPRGPPSPPAPVMHSPSRKMTVKEQQEWKIPPCISNWKNAKGYTIPLDKRLAADGRGLQTVHINENFAKLAEALYIADRKAREAVEMRAQVERKMAQKEKEKHEEKLREMAQKARERRAGIKTHVEKEDGEARERDEIRHDRRKERQHDRNLSRAAPDKRSKLQRNENRDISEVIALGVPNPRTSNEVQYDQRLFNQSKGMDSGFAGGEDEIYNVYDQAWRGGKDMAQSIYRPSKNLDKDMYGDDLEARIKTNRFVPDKEFSGSDRRQRGREGPVQFEEDPFGLDKFLEEAKQHGGSKRPSDSSRPKEHEHEGKKRRKE

>NP_689953.1

MAASLRLLGAASGLRYWSRRLRPAAGSFAAVCSRSVASKTPVGFIGLGNMGNPMAKNLMKHGYPLIIYDVFPDACKEFQDAGEQVVSSPADVAEKADRIITMLPTSINAIEAYSGANGILKKVKKGSLLIDSSTIDPAVSKELAKEVEKMGAVFMDAPVSGGVGAARSGNLTFMVGGVEDEFAAAQELLGCMGSNVVYCGAVGTGQAAKICNNMLLAISMIGTAEAMNLGIRLGLDPKLLAKILNMSSGRCWSSDTYNPVPGVMDGVPSANNYQGGFGTTLMAKDLGLAQDSATSTKSPILLGSLAHQIYRMMCAKGYSKKDFSSVFQFLREEETF

71 NP_006345.1 Q8N142

>NP_006345.1

MSELKDCPLQFHDFKSVDHLKVCPRYTAVLARSEDDGIGIEELDTLQLELETLLSSASRRLRVLEAETQILTDWQDKKGDRRFLKLGRDHELGAPPKHGKPKKQKLEGKAGHGPGPGPGRPKSKNLQPKIQEYEFTDDPIDVPRIPKNDAPNRFWASVEPYCADITSEEVRTLEELLKPPEDEAEHYKIPPLGKHYSQRWAQEDLLEEQKDGARAAAVADKKKGLMGPLTELDTKDVDALLKKSEAQHEQPEDGCPFGALTQRLLQALVEENIISPMEDSPIPDMSGKESGADGASTSPRNQNKPFSVPHTKSLESRIKEELIAQGLLESEDRPAEDSEDEVLAELRKRQAELKALSAHNRTKKHDLLRLAKEEVSRQELRQRVRMADNEVMDAFRKIMAARQKKRTPTKKEKDQAWKTLKERESILKLLDG

>Q8N142

MSGTRASNDRPPGAGGVKRGRLQQEAAATGSRVTVVLGAQWGDEGKGKVVDLLATDADIISRCQGGNNAGHTVVVDGKEYDFHLLPSGIINTKAVSFIGNGVVIHLPGLFEEAEKNEKKGLKDWEKRLIISDRAHLVFDFHQAVDGLQEVQRQAQEGKNIGTTKKGIGPTYSSKAARTGLRICDLLSDFDEFSSRFKNLAHQHQSMFPTLEIDIEGQLKRLKGFAERIRPMVRDGVYFMYEALHGPPKKILVEGANAALLDIDFGTYPFVTSSNCTVGGVCTGLGIPPQNIGDVYGVVKAYTTRVGIGAFPTEQINEIGGLLQTRGHEWGVTTGRKRRCGWLDLMILRYAHMVNGFTALALTKLDILDVLGEVKVGVSYKLNGKRIPYFPANQEMLQKVEVEYETLPGWKADTTGARRWEDLPPQAQNYIRFVENHVGVAVKWVGVGKSRESMIQLF

72 NP_000512.1 Q9NVV4

>NP_000512.1

MELCGLGLPRPPMLLALLLATLLAAMLALLTQVALVVQVAEAARAPSVSAKPGPALWPLPLSVKMTPNLLHLAPENFYISHSPNSTAGPSCTLLEEAFRRYHGYIFGFYKWHHEPAEFQAKTQVQQLLVSITLQSECDAFPNISSDESYTLLVKEPVAVLKANRVWGALRGLETFSQLVYQDSYGTFTINESTIIDSPRFSHRGILIDTSRHYLPVKIILKTLDAMAFNKFNVLHWHIVDDQSFPYQSITFPELSNKGSYSLSHVYTPNDVRMVIEYARLRGIRVLPEFDTPGHTLSWGKGQKDLLTPCYSRQNKLDSFGPINPTLNTTYSFLTTFFKEISEVFPDQFIHLGGDEVEFKCWESNPKIQDFMRQKGFGTDFKKLESFYIQKVLDIIATINKGSIVWQEVFDDKAKLAPGTIVEVWKDSAYPEELSRVTASGFPVILSAPWYLDLISYGQDWRKYYKVEPLDFGGTQKQKQLFIGGEACLWGEYVDATNLTPRLWPRASAVGERLWSSKDVRDMDDAYDRLTRHRCRMVERGIAAQPLYAGYCNHENM

>Q9NVV4

MAVPGVGLLTRLNLCARRRTRVQRPIVRLLSCPGTVAKDLRRDEQPSGSVETGFEDKIPKRRFSEMQNERREQAQRTVLIHCPEKISENKFLKYLSQFGPINNHFFYESFGLYAVVEFCQKESIGSLQNGTHTPSTAMETAIPFRSRFFNLKLKNQTSERSRVRSSNQLPRSNKQLFELLCYAESIDDQLNTLLKEFQLTEENTKLRYLTCSLIEDMAAAYFPDCIVRPFGSSVNTFGKLGCDLDMFLDLDETRNLSAHKISGNFLMEFQVKNVPSERIATQKILSVLGECLDHFGPGCVGVQKILNARCPLVRFSHQASGFQCDLTTNNRIALTSSELLYIYGALDSRVRALVFSVRCWARAHSLTSSIPGAWITNFSLTMMVIFFLQRRSPPILPTLDSLKTLADAEDKCVIEGNNCTFVRDLSRIKPSQNTETLELLLKEFFEYFGNFAFDKNSINIRQGREQNKPDSSPLYIQNPFETSLNISKNVSQSQLQKFVDLARESAWILQQEDTDRPSISSNRPWGLVSLLLPSAPNRKSFTKKKSNKFAIETVKNLLESLKGNRTENFTKTSGKRTISTQT

73 Q8TCE9 Q9UHN1

>Q8TCE9

MSSLPVPYTLPVSLPVGSCVIITGTPILTFVKDPQLEVNFYTGMDEDSDIAFQFRLHFGHPAIMNSCVFGIWRYEEKCYYLPFEDGKPFELCIYVRHKEYKVMVNGQRIYNFAHRFPPASVKMLQVFRDISLTRVLISD

>Q9UHN1

MRSRVAVRACHKVCRCLLSGFGGRVDAGQPELLTERSSPKGGHVKSHAELEGNGEHPEAPGSGEGSEALLEICQRRHFLSGSKQQLSRDSLLSGCHPGFGPLGVELRKNLAAEWWTSVVVFREQVFPVDALHHKPGPLLPGDSAFRLVSAETLREILQDKELSKEQLVAFLENVLKTSGKLRENLLHGALEHYVNCLDLVNKRLPYGLAQIGVCFHPVFDTKQIRNGVKSIGEKTEASLVWFTPPRTSNQWLDFWLRHRLQWWRKFAMSPSNFSSSDCQDEEGRKGNKLYYNFPWGKELIETLWNLGDHELLHMYPGNVSKLHGRDGRKNVVPCVLSVNGDLDRGMLAYLYDSFQLTENSFTRKKNLHRKVLKLHPCLAPIKVALDVGRGPTLELRQVCQGLFNELLENGISVWPGYLETMQSSLEQLYSKYDEMSILFTVLVTETTLENGLIHLRSRDTTMKEMMHISKLKDFLIKYISSAKNV

74 NP_777584.1 Q9NY74

>NP_777584.1

MDLLWMPLLLVAACVSAVHSSPEVNAGVSSIHITKPVHILEERSLLVLTPAGLTQMLNQTRFLMVLFHNPSSKQSRNLAEELGKAVEIMGKGKNGIGFGKVDITIEKELQQEFGITKAPELKLFFEGNRSEPISCKGVVESAALVVWLRRQISQKAFLFNSSEQVAEFVISRPLVIVGFFQDLEEEVAELFYDVIKDFPELTFGVITIGNVIGRFHVTLDSVLVFKKGKIVNRQKLINDSTNKQELNRVIKQHLTDFVIEYNTENKDLISELHIMSHMLLFVSKSSESYGIIIQHYKLASKEFQNKILFILVDADEPRNGRVFKYFRVTEVDIPSVQILNLSSDARYKMPSDDITYESLKKFGRSFLSKNATKHQSSEEIPKYWDQGLVKQLVGKNFNVVVFDKEKDVFVMFYAPWSKKCKMLFPLLEELGRKYQNHSTIIIAKIDVTANDIQLMYLDRYPFFRLFPSGSQQAVLYKGEHTLKGFSDFLESHIKTKIEDEDELLSVEQNEVIEEEVLAEEKEVPMMRKGLPEQQSPELENMTKYVSKLEEPAGKKKTSEEVVVVVAKPKGPPVQKKKPKVKEEL

>Q9NY74

MSRRRKHDDSPSPKKTPHKTVAAEECGSVVEPGRRRLRSARGSWPCGAREGPPGPVRQREQPPTAALCSKSNPEERYETPKRALKMDSLSSSFSSPNDPDGQNDIFWDQNSPLTKQLGKGRKKQIYTTDSDEISHIVNRIAPQDEKPTTNSMLDMWIGETAIPCTPSVAKGKSRAKISCTKLKTQSQEEELMKLAKQFDKNMEELDVIQEQNKRNYDFTQMISETEILSNYKDNIQMWSLHNIVPEIDNATKKPIKGNTKISVANNQNSSQKPFDQIAEAAFNAIFDGSTQKCSGQLSQELPEAFWSTSNTTFVKTNALKEEKIITNETLVIEKLSNKTPRSLSSQVDTPIMTKSCVTSCTKEPETSNKYIDAFTTSDFEDDWENLLGSEPFAMQNIDMPELFPSKTAHVTDQKEICTFNSKTVKNTSRANTSPDARLGDSKVLQDLSSKTYDRELIDAEYRFSPNSNKSNKLSTGNKMKFENSSNKIVIQDEIQNCIVTSNLTKIKEDILTNSTEASERKSALNTRYSNEQKNKCILNQSIKAPVNTDLFGSANLGSKTSVSNPNQTSASKVGSFFDDWNDPSFANEIIKACHQLDNTWEADDVDDDLLYQACDDIERLTQQQDIRKDSKTSESICEINNNSEHGAKLTQQQDIRKDSKTSESICEINNNSEHGAKNMFAISKQGSNLVQSKHLNPGSISVQTSLTNSSQIDKPMKMEKGEMYGNSPRFLGATNLTMYSKISNCQINNLHVSYTNTDVPIQVNSSKLVLPGSSSLNVTSDHMNTEITTYKKKLSTNQPCHKTVTDEAQSNLNTTVGFSKFTFTRMKNSQILSQFNQNCITGSMSDTKITQGVEKKKGVNPLLEEAVGQQSLVKLSESLKQSSKEEEEKNRKCSPEEIQRKRQEALVRRMAKARASSVNAAPTSFL

75 NP_000170.1 Q16540

>NP_000170.1

MSRQSTLYSFFPKSPALSDANKASARASREGGRAAAAPGASPSPGGDAAWSEAGPGPRPLARSASPPKAKNLNGGLRRSVAPAAPTSCDFSPGDLVWAKMEGYPWWPCLVYNHPFDGTFIREKGKSVRVHVQFFDDSPTRGWVSKRLLKPYTGSKSKEAQKGGHFYSAKPEILRAMQRADEALNKDKIKRLELAVCDEPSEPEEEEEMEVGTTYVTDKSEEDNEIESEEEVQPKTQGSRRSSRQIKKRRVISDSESDIGGSDVEFKPDTKEEGSSDEISSGVGDSESEGLNSPVKVARKRKRMVTGNGSLKRKSSRKETPSATKQATSISSETKNTLRAFSAPQNSESQAHVSGGGDDSSRPTVWYHETLEWLKEEKRRDEHRRRPDHPDFDASTLYVPEDFLNSCTPGMRKWWQIKSQNFDLVICYKVGKFYELYHMDALIGVSELGLVFMKGNWAHSGFPEIAFGRYSDSLVQKGYKVARVEQTETPEMMEARCRKMAHISKYDRVVRREICRIITKGTQTYSVLEGDPSENYSKYLLSLKEKEEDSSGHTRAYGVCFVDTSLGKFFIGQFSDDRHCSRFRTLVAHYPPVQVLFEKGNLSKETKTILKSSLSCSLQEGLIPGSQFWDASKTLRTLLEEEYFREKLSDGIGVMLPQVLKGMTSESDSIGLTPGEKSELALSALGGCVFYLKKCLIDQELLSMANFEEYIPLDSDTVSTTRSGAIFTKAYQRMVLDAVTLNNLEIFLNGTNGSTEGTLLERVDTCHTPFGKRLLKQWLCAPLCNHYAINDRLDAIEDLMVVPDKISEVVELLKKLPDLERLLSKIHNVGSPLKSQNHPDSRAIMYEETTYSKKKIIDFLSALEGFKVMCKIIGIMEEVADGFKSKILKQVISLQTKNPEGRFPDLTVELNRWDTAFDHEKARKTGLITPKAGFDSDYDQALADIRENEQSLLEYLEKQRNRIGCRTIVYWGIGRNRYQLEIPENFTTRNLPEEYELKSTKKGCKRYWTKTIEKKLANLINAEERRDVSLKDCMRRLFYNFDKNYKDWQSAVECIAVLDVLLCLANYSRGGDGPMCRPVILLPEDTPPFLELKGSRHPCITKTFFGDDFIPNDILIGCEEEEQENGKAYCVLVTGPNMGGKSTLMRQAGLLAVMAQMGCYVPAEVCRLTPIDRVFTRLGASDRIMSGESTFFVELSETASILMHATAHSLVLVDELGRGTATFDGTAIANAVVKELAETIKCRTLFSTHYHSLVEDYSQNVAVRLGHMACMVENECEDPSQETITFLYKFIKGACPKSYGFNAARLANLPEEVIQKGHRKAREFEKMNQSLRLFREVCLASERSTVDAEAVHKLLTLIKEL

>Q16540

MARNVVYPLYRLGGPQLRVFRTNFFIQLVRPGVAQPEDTVQFRIPMEMTRVDLRNYLEGIYNVPVAAVRTRVQHGSNKRRDHRNVRIKKPDYKVAYVQLAHGQTFTFPDLFPEKDESPEGSAADDLYSMLEEERQQRQSSDPRRGGVPSWFGL

76 NP_055850.1 NP_005267.2

>NP_055850.1

MASKRKSTTPCMIPVKTVVLQDASMEAQPAETLPEGPQQDLPPEASAASSEAAQNPSSTDGSTLANGHRSTLDGYLYSCKYCDFRSHDMTQFVGHMNSEHTDFNKDPTFVCSGCSFLAKTPEGLSLHNATCHSGEASFVWNVAKPDNHVVVEQSIPESTSTPDLAGEPSAEGADGQAEIIITKTPIMKIMKGKAEAKKIHTLKENVPSQPVGEALPKLSTGEMEVREGDHSFINGAVPVSQASASSAKNPHAANGPLIGTVPVLPAGIAQFLSLQQQPPVHAQHHVHQPLPTAKALPKVMIPLSSIPTYNAAMDSNSFLKNSFHKFPYPTKAELCYLTVVTKYPEEQLKIWFTAQRLKQGISWSPEEIEDARKKMFNTVIQSVPQPTITVLNTPLVASAGNVQHLIQAALPGHVVGQPEGTGGGLLVTQPLMANGLQATSSPLPLTVTSVPKQPGVAPINTVCSNTTSAVKVVNAAQSLLTACPSITSQAFLDASIYKNKKSHEQLSALKGSFCRNQFPGQSEVEHLTKVTGLSTREVRKWFSDRRYHCRNLKGSRAMIPGDHSSIIIDSVPEVSFSPSSKVPEVTCIPTTATLATHPSAKRQSWHQTPDFTPTKYKERAPEQLRALESSFAQNPLPLDEELDRLRSETKMTRREIDSWFSERRKKVNAEETKKAEENASQEEEEAAEDEGGEEDLASELRVSGENGSLEMPSSHILAERKVSPIKINLKNLRVTEANGRNEIPGLGACDPEDDESNKLAEQLPGKVSCKKTAQQRHLLRQLFVQTQWPSNQDYDSIMAQTGLPRPEVVRWFGDSRYALKNGQLKWYEDYKRGNFPPGLLVIAPGNRELLQDYYMTHKMLYEEDLQNLCDKTQMSSQQVKQWFAEKMGEETRAVADTGSEDQGPGTGELTAVHKGMGDTYSEVSENSESWEPRVPEASSEPFDTSSPQAGRQLETD

>NP_005267.2

MASKKVCIVGSGNWGSAIAKIVGGNAAQLAQFDPRVTMWVFEEDIGGKKLTEIINTQHENVKYLPGHKLPPNVVAVPDVVQAAEDADILIFVVPHQFIGKICDQLKGHLKANATGISLIKGVDEGPNGLKLISEVIGERLGIPMSVLMGANIASEVADEKFCETTIGCKDPAQGQLLKELMQTPNFRITVVQEVDTVEICGALKNVVAVGAGFCDGLGFGDNTKAAVIRLGLMEMIAFAKLFCSGPVSSATFLESCGVADLITTCYGGRNRKVAEAFARTGKSIEQLEKELLNGQKLQGPETARELYSILQHKGLVDKFPLFMAVYKVCYEGQPVGEFIHCLQNHPEHM

77 NP_006222.2 NP_004291.1

>NP_006222.2

MSLRSGGRRRADPGADGEASRDDGATSSVSALKRLERSQWTDKMDLRFGFERLKEPGEKTGWLINMHPTEILDEDKRLGSAVDYYFIQDDGSRFKVALPYKPYFYIATRKGCEREVSSFLSKKFQGKIAKVETVPKEDLDLPNHLVGLKRNYIRLSFHTVEDLVKVRKEISPAVKKNREQDHASDAYTALLSSVLQRGGVITDEEETSKKIADQLDNIVDMREYDVPYHIRLSIDLKIHVAHWYNVRYRGNAFPVEITRRDDLVERPDPVVLAFDIETTKLPLKFPDAETDQIMMISYMIDGQGYLITNREIVSEDIEDFEFTPKPEYEGPFCVFNEPDEAHLIQRWFEHVQETKPTIMVTYNGDFFDWPFVEARAAVHGLSMQQEIGFQKDSQGEYKAPQCIHMDCLRWVKRDSYLPVGSHNLKAAAKAKLGYDPVELDPEDMCRMATEQPQTLATYSVSDAVATYYLYMKYVHPFIFALCTIIPMEPDEVLRKGSGTLCEALLMVQAFHANIIFPNKQEQEFNKLTDDGHVLDSETYVGGHVEALESGVFRSDIPCRFRMNPAAFDFLLQRVEKTLRHALEEEEKVPVEQVTNFEEVCDEIKSKLASLKDVPSRIECPLIYHLDVGAMYPNIILTNRLQPSAMVDEATCAACDFNKPGANCQRKMAWQWRGEFMPASRSEYHRIQHQLESEKFPPLFPEGPARAFHELSREEQAKYEKRRLADYCRKAYKKIHITKVEERLTTICQRENSFYVDTVRAFRDRRYEFKGLHKVWKKKLSAAVEVGDAAEVKRCKNMEVLYDSLQLAHKCILNSFYGYVMRKGARWYSMEMAGIVCFTGANIITQARELIEQIGRPLELDTDGIWCVLPNSFPENFVFKTTNVKKPKVTISYPGAMLNIMVKEGFTNDQYQELAEPSSLTYVTRSENSIFFEVDGPYLAMILPASKEEGKKLKKRYAVFNEDGSLAELKGFEVKRRGELQLIKIFQSSVFEAFLKGSTLEEVYGSVAKVADYWLDVLYSKAANMPDSELFELISENRSMSRKLEDYGEQKSTSISTAKRLAEFLGDQMVKDAGLSCRYIISRKPEGSPVTERAIPLAIFQAEPTVRKHFLRKWLKSSSLQDFDIRAILDWDYYIERLGSAIQKIITIPAALQQVKNPVPRVKHPDWLHKKLLEKNDVYKQKKISELFTLEGRRQVTMAEASEDSPRPSAPDMEDFGLVKLPHPAAPVTVKRKRVLWESQEESQDLTPTVPWQEILGQPPALGTSQEEWLVWLRFHKKKWQLQARQRLARRKRQRLESAEGVLRPGAIRDGPATGLGSFLRRTARSILDLPWQIVQISETSQAGLFRLWALVGSDLHCIRLSIPRVFYVNQRVAKAEEGASYRKVNRVLPRSNMVYNLYEYSVPEDMYQEHINEINAELSAPDIEGVYETQVPLLFRALVHLGCVCVVNKQLVRHLSGWEAETFALEHLEMRSLAQFSYLEPGSIRHIYLYHHAQAHKALFGIFIPSQRRASVFVLDTVRSNQMPSLGALYSAEHGLLLEKVGPELLPPPKHTFEVRAETDLKTICRAIQRFLLAYKEERRGPTLIAVQSSWELKRLASEIPVLEEFPLVPICVADKINYGVLDWQRHGARRMIRHYLNLDTCLSQAFEMSRYFHIPIGNLPEDISTFGSDLFFARHLQRHNHLLWLSPTARPDLGGKEADDNCLVMEFDDQATVEINSSGCYSTVCVELDLQNLAVNTILQSHHVNDMEGADSMGISFDVIQQASLEDMITGGQAASAPASYDETALCSNTFRILKSMVVGWVKEITQYHNIYADNQVMHFYRWLRSPSSLLHDPALHRTLHNMMKKLFLQLIAEFKRLGSSVIYANFNRIILCTKKRRVEDAIAYVEYITSSIHSKETFHSLTISFSRCWEFLLWMDPSNYGGIKGKVSSRIHCGLQDSQKAGGAEDEQENEDDEEERDGEEEEEAEESNVEDLLENNWNILQFLPQAASCQNYFLMIVSAYIVAVYHCMKDGLRRSAPGSTPVRRRGASQLSQEAEGAVGALPGMITFSQDYVANELTQSFFTITQKIQKKVTGSRNSTELSEMFPVLPGSHLLLNNPALEFIKYVCKVLSLDTNITNQVNKLNRDLLRLVDVGEFSEEAQFRDPCRSYVLPEVICRSCNFCRDLDLCKDSSFSEDGAVLPQWLCSNCQAPYDSSAIEMTLVEVLQKKLMAFTLQDLVCLKCRGVKETSMPVYCSCAGDFALTIHTQVFMEQIGIFRNIAQHYGMSYLLETLEWLLQKNPQLGH

>NP_004291.1

MAEQATKSVLFVCLGNICRSPIAEAVFRKLVTDQNISENWRVDSAATSGYEIGNPPDYRGQSCMKRHGIPMSHVARQITKEDFATFDYILCMDESNLRDLNRKSNQVKTCKAKIELLGSYDPQKQLIIEDPYYGNDSDFETVYQQCVRCCRAFLEKAH

78 NP_000391.1 Q12849

>NP_000391.1

MKLNVDGLLVYFPYDYIYPEQFSYMRELKRTLDAKGHGVLEMPSGTGKTVSLLALIMAYQRAYPLEVTKLIYCSRTVPEIEKVIEELRKLLNFYEKQEGEKLPFLGLALSSRKNLCIHPEVTPLRFGKDVDGKCHSLTASYVRAQYQHDTSLPHCRFYEEFDAHGREVPLPAGIYNLDDLKALGRRQGWCPYFLARYSILHANVVVYSYHYLLDPKIADLVSKELARKAVVVFDEAHNIDNVCIDSMSVNLTRRTLDRCQGNLETLQKTVLRIKETDEQRLRDEYRRLVEGLREASAARETDAHLANPVLPDEVLQEAVPGSIRTAEHFLGFLRRLLEYVKWRLRVQHVVQESPPAFLSGLAQRVCIQRKPLRFCAERLRSLLHTLEITDLADFSPLTLLANFATLVSTYAKGFTIIIEPFDDRTPTIANPILHFSCMDASLAIKPVFERFQSVIITSGTLSPLDIYPKILDFHPVTMATFTMTLARVCLCPMIIGRGNDQVAISSKFETREDIAVIRNYGNLLLEMSAVVPDGIVAFFTSYQYMESTVASWYEQGILENIQRNKLLFIETQDGAETSVALEKYQEACENGRGAILLSVARGKVSEGIDFVHHYGRAVIMFGVPYVYTQSRILKARLEYLRDQFQIRENDFLTFDAMRHAAQCVGRAIRGKTDYGLMVFADKRFARGDKRGKLPRWIQEHLTDANLNLTVDEGVQVAKYFLRQMAQPFHREDQLGLSLLSLEQLESEETLKRIEQIAQQL

>Q12849

MAGTRWVLGALLRGCGCNCSSCRRTGAACLPFYSAAGSIPSGVSGRRRLLLLLGAAAAAASQTRGLQTGPVPPGRLAGPPAVATSAAAAAAASYSALRASLLPQSLAAAAAVPTRSYSQESKTTYLEDLPPPPEYELAPSKLEEEVDDVFLIRAQGLPWSCTMEDVLNFFSDCRIRNGENGIHFLLNRDGKRRGDALIEMESEQDVQKALEKHRMYMGQRYVEVYEINNEDVDALMKSLQVKSSPVVNDGVVRLRGLPYSCNEKDIVDFFAGLNIVDITFVMDYRGRRKTGEAYVQFEEPEMANQALLKHREEIGNRYIEIFPSRRNEVRTHVGSYKGKKIASFPTAKYITEPEMVFEEHEVNEDIQPMTAFESEKEIELPKEVPEKLPEAADFGTTSSLHFVHMRGLPFQANAQDIINFFAPLKPVRITMEYSSSGKATGEADVHFETHEDAVAAMLKDRSHVHHRYIELFLNSCPKGK

79 NP_036601.2 Q9Y3B7

>NP_036601.2

MNKKKKPFLGMPAPLGYVPGLGRGATGFTTRSDIGPARDANDPVDDRHAPPGKRTVGDQMKKNQAADDDDEDLNDTNYDEFNGYAGSLFSSGPYEKDDEEADAIYAALDKRMDERRKERREQREKEEIEKYRMERPKIQQQFSDLKRKLAEVTEEEWLSIPEVGDARNKRQRNPRYEKLTPVPDSFFAKHLQTGENHTSVDPRQTQFGGLNTPYPGGLNTPYPGGMTPGLMTPGTGELDMRKIGQARNTLMDMRLSQVSDSVSGQTVVDPKGYLTDLNSMIPTHGGDINDIKKARLLLKSVRETNPHHPPAWIASARLEEVTGKLQVARNLIMKGTEMCPKSEDVWLEAARLQPGDTAKAVVAQAVRHLPQSVRIYIRAAELETDIRAKKRVLRKALEHVPNSVRLWKAAVELEEPEDARIMLSRAVECCPTSVELWLALARLETYENARKVLNKARENIPTDRHIWITAAKLEEANGNTQMVEKIIDRAITSLRANGVEINREQWIQDAEECDRAGSVATCQAVMRAVIGIGIEEEDRKHTWMEDADSCVAHNALECARAIYAYALQVFPSKKSVWLRAAYFEKNHGTRESLEALLQRAVAHCPKAEVLWLMGAKSKWLAGDVPAARSILALAFQANPNSEEIWLAAVKLESENDEYERARRLLAKARSSAPTARVFMKSVKLEWVQDNIRAAQDLCEEALRHYEDFPKLWMMKGQIEEQKEMMEKAREAYNQGLKKCPHSTPLWLLLSRLEEKIGQLTRARAILEKSRLKNPKNPGLWLESVRLEYRAGLKNIANTLMAKALQECPNSGILWSEAIFLEARPQRRTKSVDALKKCEHDPHVLLAVAKLFWSQRKITKAREWFHRTVKIDSDLGDAWAFFYKFELQHGTEEQQEEVRKRCESAEPRHGELWCAVSKDIANWQKKIGDILRLVAGRIKNTF

>Q9Y3B7

MSKLGRAARGLRKPEVGGVIRAIVRAGLAMPGPPLGPVLGQRGVSINQFCKEFNERTKDIKEGIPLPTKILVKPDRTFEIKIGQPTVSYFLKAAAGIEKGARQTGKEVAGLVTLKHVYEIARIKAQDEAFALQDVPLSSVVRSIIGSARSLGIRVVKDLSSEELAAFQKERAIFLAAQKEADLAAQEEAAKK

80 NP_000391.1 NP_001547.1

>NP_000391.1

MKLNVDGLLVYFPYDYIYPEQFSYMRELKRTLDAKGHGVLEMPSGTGKTVSLLALIMAYQRAYPLEVTKLIYCSRTVPEIEKVIEELRKLLNFYEKQEGEKLPFLGLALSSRKNLCIHPEVTPLRFGKDVDGKCHSLTASYVRAQYQHDTSLPHCRFYEEFDAHGREVPLPAGIYNLDDLKALGRRQGWCPYFLARYSILHANVVVYSYHYLLDPKIADLVSKELARKAVVVFDEAHNIDNVCIDSMSVNLTRRTLDRCQGNLETLQKTVLRIKETDEQRLRDEYRRLVEGLREASAARETDAHLANPVLPDEVLQEAVPGSIRTAEHFLGFLRRLLEYVKWRLRVQHVVQESPPAFLSGLAQRVCIQRKPLRFCAERLRSLLHTLEITDLADFSPLTLLANFATLVSTYAKGFTIIIEPFDDRTPTIANPILHFSCMDASLAIKPVFERFQSVIITSGTLSPLDIYPKILDFHPVTMATFTMTLARVCLCPMIIGRGNDQVAISSKFETREDIAVIRNYGNLLLEMSAVVPDGIVAFFTSYQYMESTVASWYEQGILENIQRNKLLFIETQDGAETSVALEKYQEACENGRGAILLSVARGKVSEGIDFVHHYGRAVIMFGVPYVYTQSRILKARLEYLRDQFQIRENDFLTFDAMRHAAQCVGRAIRGKTDYGLMVFADKRFARGDKRGKLPRWIQEHLTDANLNLTVDEGVQVAKYFLRQMAQPFHREDQLGLSLLSLEQLESEETLKRIEQIAQQL

>NP_001547.1

MSWSPSLTTQTCGAWEMKERLGTGGFGNVIRWHNQETGEQIAIKQCRQELSPRNRERWCLEIQIMRRLTHPNVVAARDVPEGMQNLAPNDLPLLAMEYCQGGDLRKYLNQFENCCGLREGAILTLLSDIASALRYLHENRIIHRDLKPENIVLQQGEQRLIHKIIDLGYAKELDQGSLCTSFVGTLQYLAPELLEQQKYTVTVDYWSFGTLAFECITGFRPFLPNWQPVQWHSKVRQKSEVDIVVSEDLNGTVKFSSSLPYPNNLNSVLAERLEKWLQLMLMWHPRQRGTDPTYGPNGCFKALDDILNLKLVHILNMVTGTIHTYPVTEDESLQSLKARIQQDTGIPEEDQELLQEAGLALIPDKPATQCISDGKLNEGHTLDMDLVFLFDNSKITYETQISPRPQPESVSCILQEPKRNLAFFQLRKVWGQVWHSIQTLKEDCNRLQQGQRAAMMNLLRNNSCLSKMKNSMASMSQQLKAKLDFFKTSIQIDLEKYSEQTEFGITSDKLLLAWREMEQAVELCGRENEVKLLVERMMALQTDIVDLQRSPMGRKQGGTLDDLEEQARELYRRLREKPRDQRTEGDSQEMVRLLLQAIQSFEKKVRVIYTQLSKTVVCKQKALELLPKVEEVVSLMNEDEKTVVRLQEKRQKELWNLLKIACSKVRGPVSGSPDSMNASRLSQPGQLMSQPSTASNSLPEPAKKSEELVAEAHNLCTLLENAIQDTVREQDQSFTALDWSWLQTEEEEHSCLEQAS

81 NP_079224.1 NP_006635.2

>NP_079224.1

MSELTKELMELVWGTKSSPGLSDTIFCRWTQGFVFSESEGSALEQFEGGPCAVIAPVQAFLLKKLLFSSEKSSWRDCSEEEQKELLCHTLCDILESACCDHSGSYCLVSWLRGKTTEETASISGSPAESSCQVEHSSALAVEELGFERFHALIQKRSFRSLPELKDAVLDQYSMWGNKFGVLLFLYSVLLTKGIENIKNEIEDASEPLIDPVYGHGSQSLINLLLTGHAVSNVWDGDRECSGMKLLGIHEQAAVGFLTLMEALRYCKVGSYLKSPKFPIWIVGSETHLTVFFAKDMALVAPEAPSEQARRVFQTYDPEDNGFIPDSLLEDVMKALDLVSDPEYINLMKNKLDPEGLGIILLGPFLQEFFPDQGSSGPESFTVYHYNGLKQSNYNEKVMYVEGTAVVMGFEDPMLQTDDTPIKRCLQTKWPYIELLWTTDRSPSLN

>NP_006635.2

MSVVGLDVGSQSCYIAVARAGGIETIANEFSDRCTPSVISFGSKNRTIGVAAKNQQITHANNTVSNFKRFHGRAFNDPFIQKEKENLSYDLVPLKNGGVGIKVMYMGEEHLFSVEQITAMLLTKLKETAENSLKKPVTDCVISVPSFFTDAERRSVLDAAQIVGLNCLRLMNDMTAVALNYGIYKQDLPSLDEKPRIVVFVDMGHSAFQVSACAFNKGKLKVLGTAFDPFLGGKNFDEKLVEHFCAEFKTKYKLDAKSKIRALLRLYQECEKLKKLMSSNSTDLPLNIECFMNDKDVSGKMNRSQFEELCAELLQKIEVPLYSLLEQTHLKVEDVSAVEIVGGATRIPAVKERIAKFFGKDISTTLNADEAVARGCALQCAILSPAFKVREFSVTDAVPFPISLIWNHDSEDTEGVHEVFSRNHAAPFSKVLTFLRRGPFELEAFYSDPQGVPYPEAKIGRFVVQNVSAQKDGEKSRVKVKVRVNTHGIFTISTASMVEKVPTEENEMSSEADMECLNQRPPENPDTDKNVQQDNSEAGTQPQVQTDAQQTSQSPPSPELTSEENKIPDADKANEKKVDQPPEAKKPKIKVVNVELPIEANLVWQLGKDLLNMYIETEGKMIMQDKLEKERNDAKNAVEEYVYEFRDKLCGPYEKFICEQDHQNFLRLLTETEDWLYEEGEDQAKQAYVDKLEELMKIGTPVKVRFQEAEERPKMFEELGQRLQHYAKIAADFRNKDEKYNHIDESEMKKVEKSVNEVMEWMNNVMNAQAKKSLDQDPVVRAQEIKTKIKELNNTCEPVVTQPKPKIESPKLERTPNGPNIDKKEEDLEDKNNFGAEPPHQNGECYPNEKNSVNMDLD

82 O95766 O75880

>O95766

MAAAAAGAGSGPWAAQEKQFPPALLSFFIYNPRFGPREGQEENKILFYHPNEVEKNEKIRNVGLCEAIVQFTRTFSPSKPAKSLHTQKNRQFFNEPEENFWMVMVVRNPIIEKQSKDGKPVIEYQEEELLDKVYSSVLRQCYSMYKLFNGTFLKAMEDGGVKLLKERLEKFFHRYLQTLHLQSCDLLDIFGGISFFPLDKMTYLKIQSFINRMEESLNIVKYTAFLYNDQLIWSGLEQDDMRILYKYLTTSLFPRHIEPELAGRDSPIRAEMPGNLQHYGRFLTGPLNLNDPDAKCRFPKIFVNTDDTYEELHLIVYKAMSAAVCFMIDASVHPTLDFCRRLDSIVGPQLTVLASDICEQFNINKRMSGSEKEPQFKFIYFNHMNLAEKSTVHMRKTPSVSLTSVHPDLMKILGDINSDFTRVDEDEEIIVKAMSDYWVVGKKSDRRELYVILNQKNANLIEVNEEVKKLCATQFNNIFFLD

>O75880

MAMLVLVPGRVMRPLGGQLWRFLPRGLEFWGPAEGTARVLLRQFCARQAEAWRASGRPGYCLGTRPLSTARPPPPWSQKGPGDSTRPSKPGPVSWKSLAITFAIGGALLAGMKHVKKEKAEKLEKERQRHIGKPLLGGPFSLTTHTGERKTDKDYLGQWLLIYFGFTHCPDVCPEELEKMIQVVDEIDSITTLPDLTPLFISIDPERDTKEAIANYVKEFSPKLVGLTGTREEVDQVARAYRVYYSPGPKDEDEDYIVDHTIIMYLIGPDGEFLDYFGQNKRKGEIAASIATHMRPYRKKS

83 NP_008882.2 NP_001893.2

>NP_008882.2

MAALAEEQTEVAVKLEPEGPPTLLPPQAGDGAGEGSGGTTNNGPNGGGGNVAASSSTGGDGGTPKPTVAVSAAAPAGAAPVPAAAPDAGAPHDRQTLLAVLQFLRQSKLREAEEALRREAGLLEEAVAGSGAPGEVDSAGAEVTSALLSRVTASAPGPAAPDPPGTGASGATVVSGSASGPAAPGKVGSVAVEDQPDVSAVLSAYNQQGDPTMYEEYYSGLKHFIECSLDCHRAELSQLFYPLFVHMYLELVYNQHENEAKSFFEKFHGDQECYYQDDLRVLSSLTKKEHMKGNETMLDFRTSKFVLRISRDSYQLLKRHLQEKQNNQIWNIVQEHLYIDIFDGMPRSKQQIDAMVGSLAGEAKREANKSKVFFGLLKEPEIEVPLDDEDEEGENEEGKPKKKKPKKDSIGSKSKKQDPNAPPQNRIPLPELKDSDKLDKIMNMKETTKRVRLGPDCLPSICFYTFLNAYQGLTAVDVTDDSSLIAGGFADSTVRVWSVTPKKLRSVKQASDLSLIDKESDDVLERIMDEKTASELKILYGHSGPVYGASFSPDRNYLLSSSEDGTVRLWSLQTFTCLVGYKGHNYPVWDTQFSPYGYYFVSGGHDRVARLWATDHYQPLRIFAGHLADVNCTRFHPNSNYVATGSADRTVRLWDVLNGNCVRIFTGHKGPIHSLTFSPNGRFLATGATDGRVLLWDIGHGLMVGELKGHTDTVCSLRFSRDGEILASGSMDNTVRLWDAIKAFEDLETDDFTTATGHINLPENSQELLLGTYMTKSTPVVHLHFTRRNLVLAAGAYSPQ

>NP_001893.2

MQEKDASSQGFLPHFQHFATQAIHVGQDPEQWTSRAVVPPISLSTTFKQGAPGQHSGFEYSRSGNPTRNCLEKAVAALDGAKYCLAFASGLAATVTITHLLKAGDQIICMDDVYGGTNRYFRQVASEFGLKISFVDCSKIKLLEAAITPETKLVWIETPTNPTQKVIDIEGCAHIVHKHGDIILVVDNTFMSPYFQRPLALGADISMYSATKYMNGHSDVVMGLVSVNCESLHNRLRFLQNSLGAVPSPIDCYLCNRGLKTLHVRMEKHFKNGMAVAQFLESNPWVEKVIYPGLPSHPQHELVKRQCTGCTGMVTFYIKGTLQHAEIFLKNLKLFTLAESLGGFESLAELPAIMTHASVLKNDRDVLGISDTLIRLSVGLEDEEDLLEDLDQALKAAHPPSGSHS

84 NP_071394.2 NP_001001936.1

>NP_071394.2

MTGEVGSEVHLEINDPNVISQEEADSPSDSGQGSYETIGPLSEGDSDEEIFVSKKLKNRKVLQDSDSETEDTNASPEKTTYDSAEEENKENLYAGKNTKIKRIYKTVADSDESYMEKSLYQENLEAQVKPCLELSLQSGNSTDFTTDRKSSKKHIHDKEGTAGKAKVKSKRRLEKEERKMEKIRQLKKKETKNQEDDVEQPFNDSGCLLVDKDLFETGLEDENNSPLEDEESLESIRAAVKNKVKKHKKKEPSLESGVHSFEEGSELSKGTTRKERKAARLSKEALKQLHSETQRLIRESALNLPYHMPENKTIHDFFKRKPRPTCHGNAMALLKSSKYQSSHHKEIIDTANTTEMNSDHHSKGSEQTTGAENEVETNALPVVSKETQIITGSDESCRKDLVKNEELEIQEKQKQSDIRPSPGDSSVLQQESNFLGNNHSEECQVGGLVAFEPHALEGEGPQNPEETDEKVEEPEQQNKSSAVGPPEKVRRFTLDRLKQLGVDVSIKPRLGADEDSFVILEPETNRELEALKQRFWKHANPAAKPRAGQTVNVNVIVKDMGTDGKEELKADVVPVTLAPKKLDGASHTKPGEKLQVLKAKLQEAMKLRRFEERQKRQALFKLDNEDGFEEEEEEEEEMTDESEEDGEEKVEKEEKEEELEEEEEKEEEEEEEGNQETAEFLLSSEEIETKDEKEMDKENNDGSSEIGKAVGFLSVPKSLSSDSTLLLFKDSSSKMGYFPTEEKSETDENSGKQPSKLDEDDSCSLLTKESSHNSSFELIGSTIPSYQPCNRQTGRGTSFFPTAGGFRSPSPGLFRASLVSSASKSSGKLSEPSLPIEDSQDLYNASPEPKTLFLGAGDFQFCLEDDTQSQLLDADGFLNVRNHRNQYQALKPRLPLASMDENAMDANMDELLDLCTGKFTSQAEKHLPRKSDKKENMEELLNLCSGKFTSQDASTPASSELNKQEKESSMGDPMEEALALCSGSFPTDKEEEDEEEEFGDFRLVSNDNEFDSDEDEHSDSGNDLALEDHEDDDEEELLKRSEKLKRQMRLRKYLEDEAEVSGSDVGSEDEYDGEEIDEYEEDVIDEVLPSDEELQSQIKKIHMKTMLDDDKRQLRLYQERYLADGDLHSDGPGRMRKFRWKNIDDASQMDLFHRDSDDDQTEEQLDESEARWRKERIEREQWLRDMAQQGKITAEEEEEIGEDSQFMILAKKVTAKALQKNASRPMVIQESKSLLRNPFEAIRPGSAQQVKTGSLLNQPKAVLQKLAALSDHNPSAPRNSRNFVFHTLSPVKAEAAKESSKSQVKKRGPSFMTSPSPKHLKTDDSTSGLTRSIFKYLES

>NP_001001936.1

MERYKALEQLLTELDDFLKILDQENLSSTALVKKSCLAELLRLYTKSSSSDEEYIYMNKVTINKQQNAESQGKAPEEQGLLPNGEPSQHSSAPQKSLPDLPPPKMIPERKQLAIPKTESPEGYYEEAEPYDTSLNEDGEAVSSSYESYDEEDGSKGKSAPYQWPSPEAGIELMRDARICAFLWRKKWLGQWAKQLCVIKDNRLLCYKSSKDHSPQLDVNLLGSSVIHKEKQVRKKEHKLKITPMNADVIVLGLQSKDQAEQWLRVIQEVSGLPSEGASEGNQYTPDAQRFNCQKPDIAEKYLSASEYGSSVDGHPEVPETKDVKKKCSAGLKLSNLMNLGRKKSTSLEPVERSLETSSYLNVLVNSQWKSRWCSVRDNHLHFYQDRNRSKVAQQPLSLVGCEVVPDPSPDHLYSFRILHKGEELAKLEAKSSEEMGHWLGLLLSESGSKTDPEEFTYDYVDADRVSCIVSAAKNSLLLMQRKFSEPNTYIDGLPSQDRQEELYDDVDLSELTAAVEPTEEATPVADDPNERESDRVYLDLTPVKSFLHGPSSAQAQASSPTLSCLDNATEALPADSGPGPTPDEPCIKCPENLGEQQLESLEPEDPSLRITTVKIQTEQQRISFPPSCPDAVVATPPGASPPVKDRLRVTSAEIKLGKNRTEAEVKRYTEEKERLEKKKEEIRGHLAQLRKEKRELKETLLKCTDKEVLASLEQKLKEIDEECRGEESRRVDLELSIMEVKDNLKKAEAGPVTLGTTVDTTHLENVSPRPKAVTPASAPDCTPVNSATTLKNRPLSVVVTGKGTVLQKAKEWEKKGAS

85 O95766 NP_036429.2

>O95766

MAAAAAGAGSGPWAAQEKQFPPALLSFFIYNPRFGPREGQEENKILFYHPNEVEKNEKIRNVGLCEAIVQFTRTFSPSKPAKSLHTQKNRQFFNEPEENFWMVMVVRNPIIEKQSKDGKPVIEYQEEELLDKVYSSVLRQCYSMYKLFNGTFLKAMEDGGVKLLKERLEKFFHRYLQTLHLQSCDLLDIFGGISFFPLDKMTYLKIQSFINRMEESLNIVKYTAFLYNDQLIWSGLEQDDMRILYKYLTTSLFPRHIEPELAGRDSPIRAEMPGNLQHYGRFLTGPLNLNDPDAKCRFPKIFVNTDDTYEELHLIVYKAMSAAVCFMIDASVHPTLDFCRRLDSIVGPQLTVLASDICEQFNINKRMSGSEKEPQFKFIYFNHMNLAEKSTVHMRKTPSVSLTSVHPDLMKILGDINSDFTRVDEDEEIIVKAMSDYWVVGKKSDRRELYVILNQKNANLIEVNEEVKKLCATQFNNIFFLD

>NP_036429.2

MVMEKPSPLLVGREFVRQYYTLLNKAPEYLHRFYGRNSSYVHGGVDASGKPQEAVYGQNDIHHKVLSLNFSECHTKIRHVDAHATLSDGVVVQVMGLLSNSGQPERKFMQTFVLAPEGSVPNKFYVHNDMFRYEDEVFGDSEPELDEESEDEVEEEQEERQPSPEPVQENANSGYYEAHPVTNGIEEPLEESSHEPEPEPESETKTEELKPQVEEKNLEELEEKSTTPPPAEPVSLPQEPPKAFSWASVTSKNLPPSGTVSSSGIPPHVKAPVSQPRVEAKPEVQSQPPRVREQRPRERPGFPPRGPRPGRGDMEQNDSDNRRIIRYPDSHQLFVGNLPHDIDENELKEFFMSFGNVVELRINTKGVGGKLPNFGFVVFDDSEPVQRILIAKPIMFRGEVRLNVEEKKTRAARERETRGGGDDRRDIRRNDRGPGGPRGIVGGGMMRDRDGRGPPPRGGMAQKLGSGRGTGQMEGRFTGQRR

86 NP_077021.1 NP_060383.2

>NP_077021.1

MDKSGIDSLDHVTSDAVELANRSDNSSDSSLFKTQCIPYSPKGEKRNPIRKFVRTPESVHASDSSSDSSFEPIPLTIKAIFERFKNRKKRYKKKKKRRYQPTGRPRGRPEGRRNPIYSLIDKKKQFRSRGSGFPFLESENEKNAPWRKILTFEQAVARGFFNYIEKLKYEHHLKESLKQMNVGEDLENEDFDSRRYKFLDDDGSISPIEESTAEDEDATHLEDNECDIKLAGDSFIVSSEFPVRLSVYLEEEDITEEAALSKKRATKAKNTGQRGLKM

>NP_060383.2

MEQPWPPPGPWSLPRAEGEAEEESDFDVFPSSPRCPQLPGGGAQMYSHGIELACQKQKEFVKSSVACKWNLAEAQQKLGSLALHNSESLDQEHAKAQTAVSELRQREEEWRQKEEALVQREKMCLWSTDAISKDVFNKSFINQDKRKDTEDEDKSESFMQKYEQKIRHFGMLSRWDDSQRFLSDHPYLVCEETAKYLILWCFHLEAEKKGALMEQIAHQAVVMQFIMEMAKNCNVDPRGCFRLFFQKAKAEEEGYFEAFKNELEAFKSRVRLYSQSQSFQPMTVQNHVPHSGVGSIGLLESLPQNPDYLQYSISTALCSLNSVVHKEDDEPKMMDTV

87 Q86WB0 NP_003977.1

>Q86WB0

MAAPCEGQAFAVGVEKNWGAVVRSPEGTPQKIRQLIDEGIAPEEGGVDAKDTSATSQSVNGSPQAEQPSLESTSKEAFFSRVETFSSLKWAGKPFELSPLVCAKYGWVTVECDMLKCSSCQAFLCASLQPAFDFDRYKQRCAELKKALCTAHEKFCFWPDSPSPDRFGMLPLDEPAILVSEFLDRFQSLCHLDLQLPSLRPEDLKTMCLTEDKISLLLHLLEDELDHRTDERKTTIKLGSDIQVHVTACILSVCGWACSSSLESMQLSLITCSQCMRKVGLWGFQQIESSMTDLDASFGLTSSPIPGLEGRPERLPLVPESPRRMMTRSQDATFSPGSEQAEKSPGPIVSRTRSWDSSSPVDRPEPEAASPTTRTRPVTRSMGTGDTPGLEVPSSPLRKAKRARLCSSSSSDTSSRSFFDPTSQHRDWCPWVNITLGKESRENGGTEPDASAPAEPGWKAVLTILLAHKQSSQPAETDSMSLSEKSRKVFRIFRQWESLCSC

>NP_003977.1

MACTIQKAEALDGAHLMQILWYDEEESLYPAVWLRDNCPCSDCYLDSAKARKLLVEALDVNIGIKGLIFDRKKVYITWPDEHYSEFQADWLKKRCFSKQARAKLQRELFFPECQYWGSELQLPTLDFEDVLRYDEHAYKWLSTLKKVGIVRLTGASDKPGEVSKLGKRMGFLYLTFYGHTWQVQDKIDANNVAYTTGKLSFHTDYPALHHPPGVQLLHCIKQTVTGGDSEIVDGFNVCQKLKKNNPQAFQILSSTFVDFTDIGVDYCDFSVQSKHKIIELDDKGQVVRINFNNATRDTIFDVPVERVQPFYAALKEFVDLMNSKESKFTFKMNPGDVITFDNWRLLHGRRSYEAGTEISRHLEGAYADWDVVMSRLRILRQRVENGN

88 Q86V15 NP_001547.1

>Q86V15

MDLGTAEGTRCTDPPAGKPAMAPKRKGGLKLNAICAKLSRQVVVEKRADAGSHTEGSPSQPRDQERSGPESGAARAPRSEEDKRRAVIEKWVNGEYSEEPAPTPVLGRIAREGLELPPEGVYMVQPQGCSDEEDHAEEPSKDGGALEEKDSDGAASKEDSGPSTRQASGEASSLRDYAASTMTEFLGMFGYDDQNTRDELARKISFEKLHAGSTPEAATSSMLPTSEDTLSKRARFSKYEEYIRKLKAGEQLSWPAPSTKTEERVGKEVVGTLPGLRLPSSTAHLETKATILPLPSHSSVQMQNLVARASKYDFFIQKLKTGENLRPQNGSTYKKPSKYDLENVKYLHLFKPGEGSPDMGGAIAFKTGKVGRPSKYDVRGIQKPGPAKVPPTPSLAPAPLASVPSAPSAPGPGPEPPASLSFNTPEYLKSTFSKTDSITTGTVSTVKNGLPTDKPAVTEDVNIYQKYIARFSGSQHCGHIHCAYQYREHYHCLDPECNYQRFTSKQDVIRHYNMHKKRDNSLQHGFMRFSPLDDCSVYYHGCHLNGKSTHYHCMQVGCNKVYTSTSDVMTHENFHKKNTQLINDGFQRFRATEDCGTADCQFYGQKTTHFHCRRPGCTFTFKNKCDIEKHKSYHIKDDAYAKDGFKKFYKYEECKYEGCVYSKATNHFHCIRAGCGFTFTSTSQMTSHKRKHERRHIRSSGALGLPPSLLGAKDTEHEESSNDDLVDFSALSSKNSSLSASPTSQQSSASLAAATAATEAGPSATKPPNSKISGLLPQGLPGSIPLALALSNSGLPTPTPYFPILAGRGSTSLPVGTPSLLGAVSSGSAASATPDTPTLVASGAGDSAPVAAASVPAPPASIMERISASKGLISPMMARLAAAALKPSATFDPGSGQQVTPARFPPAQVKPEPGESTGAPGPHEASQDRSLDLTVKEPSNESNGHAVPANSSLLSSLMNKMSQGNPGLGSLLNIKAEAEGSPAAEPSPFLGKAVKALVQEKLAEPWKVYLRRFGTKDFCDGQCDFLHKAHFHCVVEECGALFSTLDGAIKHANFHFRTEGGAAKGNTEAAFPASAAETKPPMAPSSPPVPPVTTATVSSLEGPAPSPASVPSTPTLLAWKQLASTIPQMPQIPASVPHLPASPLATTSLENAKPQVKPGFLQFQENDPCLATDCKYANKFHFHCLFGNCKYVCKTSGKAESHCLDHINPNNNLVNVRDQFAYYSLQCLCPNQHCEFRMRGHYHCLRTGCYFVTNITTKLPWHIKKHEKAERRAANGFKYFTKREECGRLGCKYNQVSSHFHCIREGCQFSFLLKHQMTSHARKHMRRMLGKNFDRVPPSQGPPGLMDAETDECMDYTGCSPGAMSSESSTMDRSCSSTPVGNESTAAGNTISMPTASGAKKRFWIIEDMSPFGKRRKTASSRKMLDEGMMLEGFRRFDLYEDCKDAACQFSLKVTHYHCTRENCGYKFCGRTHMYKHAQHHDRVDNLVLDDFKRFKASLSRHFADCPFSGTSTHFHCLRCRFRCTDSTKVTAHRKHHGKQDVISAAGFRQFSSSADCAVPDCKYKLKCSHFHCTFPGCRHTVVGMSQMDSHKRKHEKQERGEPAAEGPAPGPPISLDGSLSLGAEPGSLLFLQSAAAGLGLALGDAGDPGPPDAAAPGPREGAAAAAAAAGESSQEDEEEELELPEEEAEDDEDEDDDEDDDDEDDDEDDDDEDLRTDSEESLPEAAAEAAGAGARTPALAALAALGAPGPAPTAASSP

>NP_001547.1

MSWSPSLTTQTCGAWEMKERLGTGGFGNVIRWHNQETGEQIAIKQCRQELSPRNRERWCLEIQIMRRLTHPNVVAARDVPEGMQNLAPNDLPLLAMEYCQGGDLRKYLNQFENCCGLREGAILTLLSDIASALRYLHENRIIHRDLKPENIVLQQGEQRLIHKIIDLGYAKELDQGSLCTSFVGTLQYLAPELLEQQKYTVTVDYWSFGTLAFECITGFRPFLPNWQPVQWHSKVRQKSEVDIVVSEDLNGTVKFSSSLPYPNNLNSVLAERLEKWLQLMLMWHPRQRGTDPTYGPNGCFKALDDILNLKLVHILNMVTGTIHTYPVTEDESLQSLKARIQQDTGIPEEDQELLQEAGLALIPDKPATQCISDGKLNEGHTLDMDLVFLFDNSKITYETQISPRPQPESVSCILQEPKRNLAFFQLRKVWGQVWHSIQTLKEDCNRLQQGQRAAMMNLLRNNSCLSKMKNSMASMSQQLKAKLDFFKTSIQIDLEKYSEQTEFGITSDKLLLAWREMEQAVELCGRENEVKLLVERMMALQTDIVDLQRSPMGRKQGGTLDDLEEQARELYRRLREKPRDQRTEGDSQEMVRLLLQAIQSFEKKVRVIYTQLSKTVVCKQKALELLPKVEEVVSLMNEDEKTVVRLQEKRQKELWNLLKIACSKVRGPVSGSPDSMNASRLSQPGQLMSQPSTASNSLPEPAKKSEELVAEAHNLCTLLENAIQDTVREQDQSFTALDWSWLQTEEEEHSCLEQAS

89 NP_066951.1 Q9BU02

>NP_066951.1

MIIPVRCFTCGKIVGNKWEAYLGLLQAEYTEGDALDALGLKRYCCRRMLLAHVDLIEKLLNYAPLEK

>Q9BU02

MAQGLIEVERKFLPGPGTEERLQELGGTLEYRVTFRDTYYDTPELSLMQADHWLRRREDSGWELKCPGAAGVLGPHTEYKELTAEPTIVAQLCKVLRADGLGAGDVAAVLGPLGLQEVASFVTKRSAWKLVLLGADEEEPQLRVDLDTADFGYAVGEVEALVHEEAEVPTALEKIHRLSSMLGVPAQETAPAKLIVYLQRFRPQDYQRLLEVNSSRERPQETEDPDHCLG

90 P15586 Q6PRD7

>P15586

MRLLPLAPGRLRRGSPRHLPSCSPALLLLVLGGCLGVFGVAAGTRRPNVVLLLTDDQDEVLGGMTPLKKTKALIGEMGMTFSSAYVPSALCCPSRASILTGKYPHNHHVVNNTLEGNCSSKSWQKIQEPNTFPAILRSMCGYQTFFAGKYLNEYGAPDAGGLEHVPLGWSYWYALEKNSKYYNYTLSINGKARKHGENYSVDYLTDVLANVSLDFLDYKSNFEPFFMMIATPAPHSPWTAAPQYQKAFQNVFAPRNKNFNIHGTNKHWLIRQAKTPMTNSSIQFLDNAFRKRWQTLLSVDDLVEKLVKRLEFTGELNNTYIFYTSDNGYHTGQFSLPIDKRQLYEFDIKVPLLVRGPGIKPNQTSKMLVANIDLGPTILDIAGYDLNKTQMDGMSLLPILRGASNLTWRSDVLVEYQGEGRNVTDPTCPSLSPGVSQCFPDCVCEDAYNNTYACVRTMSALWNLQYCEFDDQEVFVEVYNLTADPDQITNIAKTIDPELLGKMNYRLMMLQSCSGPTCRTPGVFDPGYRFDPRLMFSNRGSVRTRRFSKHLL

>Q6PRD7

MGTSSTDSQQAGHRRCSTSNTSAENLTCLSLPGSPGKTAPLPGPAQAGAGQPLPKGCAAVKAEVGIPAPHTSQEVRIHIRRLLSWAAPGACGLRSTPCALPQALPQARPCPGRWFFPGCSLPTGGAQTILSLWTWRHFLNWALQQREENSGRARRVPPVPRTAPVSKGEGSHPPQNSNGEKVKTITPDVGLHQSLTSDPTVAVLRAKRAPEAHPPRSCSGSLTARVCHMGVCQGQGDTEDGRMTLMG

91 NP_001752.2 NP_550438.1

>NP_001752.2

MGSGGVVHCRCAKCFCYPTKRRIRRRPRNLTILSLPEDVLFHILKWLSVEDILAVRAVHSQLKDLVDNHASVWACASFQELWPSPGNLKLFERAAEKGNFEAAVKLGIAYLYNEGLSVSDEARAEVNGLKASRFFSLAERLNVGAAPFIWLFIRPPWSVSGSCCKAVVHESLRAECQLQRTHKASILHCLGRVLSLFEDEEKQQQAHDLFEEAAHQGCLTSSYLLWESDRRTDVSDPGRCLHSFRKLRDYAAKGCWEAQLSLAKACANANQLGLEVRASSEIVCQLFQASQAVSKQQVFSVQKGLNDTMRYILIDWLVEVATMKDFTSLCLHLTVECVDRYLRRRLVPRYRLQLLGIACMVICTRFISKEILTIREAVWLTDNTYKYEDLVRMMGEIVSALEGKIRVPTVVDYKEVLLTLVPVELRTQHLCSFLCELSLLHTSLSAYAPARLAAAALLLARLTHGQTQPWTTQLWDLTGFSYEDLIPCVLSLHKKCFHDDAPKDYRQVSLTAVKQRFEDKRYGEISQEEVLSYSQLCAALGVTQDSPDPPTFLSTGEIHAFLSSPSGRRTKRKRENSLQEDRGSFVTTPTAELSSQEETLLGSFLDWSLDCCSGYEGDQESEGEKEGDVTAPSGILDVTVVYLNPEQHCCQESSDEEACPEDKGPQDPQALALDTQIPATPGPKPLVRTSREPGKDVTTSGYSSVSTASPTSSVDGGLGALPQPTSVLSLDSDSHTQPCHHQARKSCLQCRPPSPPESSVPQQQVKRINLCIHSEEEDMNLGLVRL

>NP_550438.1

MAAGRLFLSRLRAPFSSMAKSPLEGVSSSRGLHAGRGPRRLSIEGNIAVGKSTFVKLLTKTYPEWHVATEPVATWQNIQAAGTQKACTAQSLGNLLDMMYREPARWSYTFQTFSFLSRLKVQLEPFPEKLLQARKPVQIFERSVYSDRYIFAKNLFENGSLSDIEWHIYQDWHSFLLWEFASRITLHGFIYLQASPQVCLKRLYQRAREEEKGIELAYLEQLHGQHEAWLIHKTTKLHFEALMNIPVLVLDVNDDFSEEVTKQEDLMREVNTFVKNL

92 NP_005307.1 O43182

>NP_005307.1

MATSSEEVLLIVKKVRQKKQDGALYLMAERIAWAPEGKDRFTISHMYADIKCQKISPEGKAKIQLQLVLHAGDTTNFHFSNESTAVKERDAVKDLLQQLLPKFKRKANKELEEKNRMLQEDPVLFQLYKDLVVSQVISAEEFWANRLNVNATDSSSTSNHKQDVGISAAFLADVRPQTDGCNGLRYNLTSDIIESIFRTYPAVKMKYAENVPHNMTEKEFWTRFFQSHYFHRDRLNTGSKDLFAECAKIDEKGLKTMVSLGVKNPLLDLTALEDKPLDEGYGISSVPSASNSKSIKENSNAAIIKRFNHHSAMVLAAGLRKQEAQNEQTSEPSNMDGNSGDADCFQPAVKRAKLQESIEYEDLGKNNSVKTIALNLKKSDRYYHGPTPIQSLQYATSQDIINSFQSIRQEMEAYTPKLTQVLSSSAASSTITALSPGGALMQGGTQQAINQMVPNDIQSELKHLYVAVGELLRHFWSCFPVNTPFLEEKVVKMKSNLERFQVTKLCPFQEKIRRQYLSTNLVSHIEEMLQTAYNKLHTWQSRRLMKKT

>O43182

MSAQSLLHSVFSCSSPASSSAASAKGFSKRKLRQTRSLDPALIGGCGSDEAGAEGSARGATAGRLYSPSLPAESLGPRLASSSRGPPPRATRLPPPGPLCSSFSTPSTPQEKSPSGSFHFDYEVPLGRGGLKKSMAWDLPSVLAGPASSRSASSILCSSGGGPNGIFASPRRWLQQRKFQSPPDSRGHPYVVWKSEGDFTWNSMSGRSVRLRSVPIQSLSELERARLQEVAFYQLQQDCDLSCQITIPKDGQKRKKSLRKKLDSLGKEKNKDKEFIPQAFGMPLSQVIANDRAYKLKQDLQRDEQKDASDFVASLLPFGNKRQNKELSSSNSSLSSTSETPNESTSPNTPEPAPRARRRGAMSVDSITDLDDNQSRLLEALQLSLPAEAQSKKEKARDKKLSLNPIYRQVPRLVDSCCQHLEKHGLQTVGIFRVGSSKKRVRQLREEFDRGIDVSLEEEHSVHDVAALLKEFLRDMPDPLLTRELYTAFINTLLLEPEEQLGTLQLLIYLLPPCNCDTLHRLLQFLSIVARHADDNISKDGQEVTGNKMTSLNLATIFGPNLLHKQKSSDKEFSVQSSARAEESTAIIAVVQKMIENYEALFMVPPDLQNEVLISLLETDPDVVDYLLRRKASQSSSPDMLQSEVSFSVGGRHSSTDSNKASSGDISPYDNNSPVLSERSLLAMQEDAAPGGSEKLYRVPGQFMLVGHLSSSKSRESSPGPRLGKDLSEEPFDIWGTWHSTLKSGSKDPGMTGSSGDIFESSSLRAGPCSLSQGNLSPNWPRWQGSPAELDSDTQGARRTQAAAPATEGRAHPAVSRACSTPHVQVAGKAERPTARSEQYLTLSGAHDLSESELDVAGLQSRATPQCQRPHGSGRDDKRPPPPYPGPGKPAAAAAWIQGPPEGVETPTDQGGQAAEREQQVTQKKLSSANSLPAGEQDSPRLGDAGWLDWQRERWQIWELLSTDNPDALPETLV

93 NP_002544.1 Q7RTR2

>NP_002544.1

MPHLENVVLCRESQVSILQSLFGERHHFSFPSIFIYGHTASGKTYVTQTLLKTLELPHVFVNCVECFTLRLLLEQILNKLNHLSSSEDGCSTEITCETFNDFVRLFKQVTTAENLKDQTVYIVLDKAEYLRDMEANLLPGFLRLQELADRNVTVLFLSEIVWEKFRPNTGCFEPFVLYFPDYSIGNLQKILSHDHPPEYSADFYAAYINILLGVFYTVCRDLKELRHLAVLNFPKYCEPVVKGEASERDTRKLWRNIEPHLKKAMQTVYLREISSSQWEKLQKDDTDPGQLKGLSAHTHVELPYYSKFILIAAYLASYNPARTDKRFFLKHHGKIKKTNFLKKHEKTSNHLLGPKPFPLDRLLAILYSIVDSRVAPTANIFSQITSLVTLQLLTLVGHDDQLDGPKYKCTVSLDFIRAIARTVNFDIIKYLYDFL

>Q7RTR2

MRKQEVRTGREAGQGHGTGSPAEQVKALMDLLAGKGSQGSQAPQALDRTPDAPLGPCSNDSRIQRHRKALLSKVGGGPELGGPWHRLASLLLVEGLTDLQLREHDFTQVEATRGGGHPARTVALDRLFLPLSRVSVPPRVSITIGVAGMGKTTLVRHFVRLWAHGQVGKDFSLVLPLTFRDLNTHEKLCADRLICSVFPHVGEPSLAVAVPARALLILDGLDECRTPLDFSNTVACTDPKKEIPVDHLITNIIRGNLFPEVSIWITSRPSASGQIPGGLVDRMTEIRGFNEEEIKVCLEQMFPEDQALLGWMLSQVQADRALYLMCTVPAFCRLTGMALGHLWRSRTGPQDAELWPPRTLCELYSWYFRMALSGEGQEKGKASPRIEQVAHGGRKMVGTLGRLAFHGLLKKKYVFYEQDMKAFGVDLALLQGAPCSCFLQREETLASSVAYCFTHLSLQEFVAAAYYYGASRRAIFDLFTESGVSWPRLGFLTHFRSAAQRAMQAEDGRLDVFLRFLSGLLSPRVNALLAGSLLAQGEHQAYRTQVAELLQGCLRPDAAVCARAINVLHCLHELQHTELARSVEEAMESGALARLTGPAHRAALAYLLQVSDACAQEANLSLSLSQGVLQSLLPQLLYCRKLRLDTNQFQDPVMELLGSVLSGKDCRIQKISLAENQISNKGAKALARSLLVNRSLTSLDLRGNSIGPQGAKALADALKINRTLTSLSLQGNTVRDDGARSMAEALASNRTLSMLHLQKNSIGPMGAQRMADALKQNRSLKELMFSSNSIGDGGAKALAEALKVNQGLESLDLQSNSISDAGVAALMGALCTNQTLLSLSLRENSISPEGAQAIAHALCANSTLKNLDLTANLLHDQGARAIAVAVRENRTLTSLHLQWNFIQAGAAQALGQALQLNRSLTSLDLQENAIGDDGACAVARALKVNTALTALYLQVASIGASGAQVLGEALAVNRTLEILDLRGNAIGVAGAKALANALKVNSSLRRLNLQENSLGMDGAICIATALSGNHRLQHINLQGNHIGDSGARMISEAIKTNAPTCTVEM

94 Q9UI36 Q6P1L8

>Q9UI36

MAVPAALIPPTQLVPPQPPISTSASSSGTTTSTSSATSSPAPSIGPPASSGPTLFRPEPIASAAAAAATVTSTGGGGGGGGSGGGGGSSGNGGGGGGGGGGSNCNPNLAAASNGSGGGGGGISAGGGVASSTPINASTGSSSSSSSSSSSSSSSSSSSSSSSSCGPLPGKPVYSTPSPVENTPQNNECKMVDLRGAKVASFTVEGCELICLPQAFDLFLKHLVGGLHTVYTKLKRLEITPVVCNVEQVRILRGLGAIQPGVNRCKLISRKDFETLYNDCTNASSRPGRPPKRTQSVTSPENSHIMPHSVPGLMSPGIIPPTGLTAAAAAAAAATNAAIAEAMKVKKIKLEAMSNYHASNNQHGADSENGDMNSSVGSSDGSWDKETLPSSPSQGPQASITHPRMPGARSLPLSHPLNHLQQSHLLPNGLELPFMMMPHPLIPVSLPPASVTMAMSQMNHLSTIANMAAAAQVQSPPSRVETSVIKERVPDSPSPAPSLEEGRRPGSHPSSHRSSSVSSSPARTESSSDRIPVHQNGLSMNQMLMGLSPNVLPGPKEGDLAGHDMGHESKRMHIEKDETPLSTPTARDSLDKLSLTGHGQPLPPGFPSPFLFPDGLSSIETLLTNIQGLLKVAIDNARAQEKQVQLEKTELKMDFLRERELRETLEKQLAMEQKNRAIVQKRLKKEKKAKRKLQEALEFETKRREQAEQTLKQAASTDSLRVLNDSLTPEIEADRSGGRTDAERTIQDGRLYLKTTVMY

>Q6P1L8

MAFFTGLWGPFTCVSRVLSHHCFSTTGSLSAIQKMTRVRVVDNSALGNSPYHRAPRCIHVYKKNGVGKVGDQILLAIKGQKKKALIVGHCMPGPRMTPRFDSNNVVLIEDNGNPVGTRIKTPIPTSLRKREGEYSKVLAIAQNFV

95 NP_997001.1 NP_054902.1

>NP_997001.1

MVNVLKGVLIECDPAMKQFLLYLDESNALGKKFIIQDIDDTHVFVIAELVNVLQERVGELMDQNAFSLTQK

>NP_054902.1

MAEVEETLKRLQSQKGVQGIIVVNTEGIPIKSTMDNPTTTQYASLMHSFILKARSTVRDIDPQNDLTFLRIRSKKNEIMVAPDKDYFLIVIQNPTE

96 NP_060853.3 Q96RP9

>NP_060853.3

MATEIGSPPRFFHMPRFQHQAPRQLFYKRPDFAQQQAMQQLTFDGKRMRKAVNRKTIDYNPSVIKYLENRIWQRDQRDMRAIQPDAGYYNDLVPPIGMLNNPMNAVTTKFVRTSTNKVKCPVFVVRWTPEGRRLVTGASSGEFTLWNGLTFNFETILQAHDSPVRAMTWSHNDMWMLTADHGGYVKYWQSNMNNVKMFQAHKEAIREASFSPTDNKFATCSDDGTVRIWDFLRCHEERILRGHGADVKCVDWHPTKGLVVSGSKDSQQPIKFWDPKTGQSLATLHAHKNTVMEVKLNLNGNWLLTASRDHLCKLFDIRNLKEELQVFRGHKKEATAVAWHPVHEGLFASGGSDGSLLFWHVGVEKEVGGMEMAHEGMIWSLAWHPLGHILCSGSNDHTSKFWTRNRPGDKMRDRYNLNLLPGMSEDGVEYDDLEPNSLAVIPGMGIPEQLKLAMEQEQMGKDESNEIEMTIPGLDWGMEEVMQKDQKKVPQKKVPYAKPIPAQFQQAWMQNKVPIPAPNEVLNDRKEDIKLEEKKKTQAEIEQEMATLQYTNPQLLEQLKIERLAQKQVEQIQPPPSSGTPLLGPQPFPGQGPMSQIPQGFQQPHPSQQMPMNMAQMGPPGPQGQFRPPGPQGQMGPQGPPLHQGGGGPQGFMGPQGPQGPPQGLPRPQDMHGPQGMQRHPGPHGPLGPQGPPGPQGSSGPQGHMGPQGPPGPQGHIGPQGPPGPQGHLGPQGPPGTQGMQGPPGPRGMQGPPHPHGIQGGPGSQGIQGPVSQGPLMGLNPRGMQGPPGPRENQGPAPQGMIMGHPPQEMRGPHPPGGLLGHGPQEMRGPQEIRGMQGPPPQGSMLGPPQELRGPPGSQSQQGPPQGSLGPPPQGGMQGPPGPQGQQNPARGPHPSQGPIPFQQQKTPLLGDGPRAPFNQEGQSTGPPPLIPGLGQQGAQGRIPPLNPGQGPGPNKGDSRGPPNHHMGPMSERRHEQSGGPEHGPERGPFRGGQDCRGPPDRRGPHPDFPDDFSRPDDFHPDKRFGHRLREFEGRGGPLPQEEKWRRGGPGPPFPPDHREFSEGDGRGAARGPPGAWEGRRPGDERFPRDPEDPRFRGRREESFRRGAPPRHEGRAPPRGRDGFPGPEDFGPEENFDASEEAARGRDLRGRGRGTPRGGRKGLLPTPDEFPRFEGGRKPDSWDGNREPGPGHEHFRDTPRPDHPPHDGHSPASRERSSSLQGMDMASLPPRKRPWHDGPGTSEHREMEAPGGPSEDRGGKGRGGPGPAQRVPKSGRSSSLDGEHHDGYHRDEPFGGPPGSGTPSRGGRSGSNWGRGSNMNSGPPRRGASRGGGRGR

>Q96RP9

MRLLGAAAVAALGRGRAPASLGWQRKQVNWKACRWSSSGVIPNEKIRNIGISAHIDSGKTTLTERVLYYTGRIAKMHEVKGKDGVGAVMDSMELERQRGITIQSAATYTMWKDVNINIIDTPGHVDFTIEVERALRVLDGAVLVLCAVGGVQCQTMTVNRQMKRYNVPFLTFINKLDRMGSNPARALQQMRSKLNHNAAFMQIPMGLEGNFKGIVDLIEERAIYFDGDFGQIVRYGEIPAELRAAATDHRQELIECVANSDEQLGEMFLEEKIPSISDLKLAIRRATLKRSFTPVFLGSALKNKGVQPLLDAVLEYLPNPSEVQNYAILNKEDDSKEKTKILMNSSRDNSHPFVGLAFKLEVGRFGQLTYVRSYQGELKKGDTIYNTRTRKKVRLQRLARMHADMMEDVEEVYAGDICALFGIDCASGDTFTDKANSGLSMESIHVPDPVISIAMKPSNKNDLEKFSKGIGRFTREDPTFKVYFDTENKETVISGMGELHLEIYAQRLEREYGCPCITGKPKVAFRETITAPVPFDFTHKKQSGGAGQYGKVIGVLEPLDPEDYTKLEFSDETFGSNIPKQFVPAVEKGFLDACEKGPLSGHKLSGLRFVLQDGAHHMVDSNEISFIRAGEGALKQALANATLCILEPIMAVEVVAPNEFQGQVIAGINRRHGVITGQDGVEDYFTLYADVPLNDMFGYSTELRSCTEGKGEYTMEYSRYQPCLPSTQEDVINKYLEATGQLPVKKGKAKN

97 Q7Z5Q5 NP_037386.1

>Q7Z5Q5

MENYEALVGFDLCNTPLSSVAQKIMSAMHSGDLVDSKTWGKSTETMEVINKSSVKYSVQLEDRKTQSPEKKDLKSLRSQTSRGSAKLSPQSFSVRLTDQLSADQKQKSISSLTLSSCLIPQYNQEASVLQKKGHKRKHFLMENINNENKGSINLKRKHITYNNLSEKTSKQMALEEDTDDAEGYLNSGNSGALKKHFCDIRHLDDWAKSQLIEMLKQAAALVITVMYTDGSTQLGADQTPVSSVRGIVVLVKRQAEGGHGCPDAPACGPVLEGFVSDDPCIYIQIEHSAIWDQEQEAHQQFARNVLFQTMKCKCPVICFNAKDFVRIVLQFFGNDGSWKHVADFIGLDPRIAAWLIDPSDATPSFEDLVEKYCEKSITVKVNSTYGNSSRNIVNQNVRENLKTLYRLTMDLCSKLKDYGLWQLFRTLELPLIPILAVMESHAIQVNKEEMEKTSALLGARLKELEQEAHFVAGERFLITSNNQLREILFGKLKLHLLSQRNSLPRTGLQKYPSTSEAVLNALRDLHPLPKIILEYRQVHKIKSTFVDGLLACMKKGSISSTWNQTGTVTGRLSAKHPNIQGISKHPIQITTPKNFKGKEDKILTISPRAMFVSSKGHTFLAADFSQIELRILTHLSGDPELLKLFQESERDDVFSTLTSQWKDVPVEQVTHADREQTKKVVYAVVYGAGKERLAACLGVPIQEAAQFLESFLQKYKKIKDFARAAIAQCHQTGCVVSIMGRRRPLPRIHAHDQQLRAQAERQAVNFVVQGSAADLCKLAMIHVFTAVAASHTLTARLVAQIHDELLFEVEDPQIPECAALVRRTMESLEQVQALELQLQVPLKVSLSAGRSWGHLVPLQEAWGPPPGPCRTESPSNSLAAPGSPASTQPPPLHFSPSFCL

>NP_037386.1

MQSTSNHLWLLSDILGQGATANVFRGRHKKTGDLFAIKVFNNISFLRPVDVQMREFEVLKKLNHKNIVKLFAIEEETTTRHKVLIMEFCPCGSLYTVLEEPSNAYGLPESEFLIVLRDVVGGMNHLRENGIVHRDIKPGNIMRVIGEDGQSVYKLTDFGAARELEDDEQFVSLYGTEEYLHPDMYERAVLRKDHQKKYGATVDLWSIGVTFYHAATGSLPFRPFEGPRRNKEVMYKIITGKPSGAISGVQKAENGPIDWSGDMPVSCSLSRGLQVLLTPVLANILEADQEKCWGFDQFFAETSDILHRMVIHVFSLQQMTAHKIYIHSYNTATIFHELVYKQTKIISSNQELIYEGRRLVLEPGRLAQHFPKTTEENPIFVVSREPLNTIGLIYEKISLPKVHPRYDLDGDASMAKAITGVVCYACRIASTLLLYQELMRKGIRWLIELIKDDYNETVHKKTEVVITLDFCIRNIEKTVKVYEKLMKINLEAAELGEISDIHTKLLRLSSSQGTIETSLQDIDSRLSPGGSLADAWAHQEGTHPKDRNVEKLQVLLNCMTEIYYQFKKDKAERRLAYNEEQIHKFDKQKLYYHATKAMTHFTDECVKKYEAFLNKSEEWIRKMLHLRKQLLSLTNQCFDIEEEVSKYQEYTNELQETLPQKMFTASSGIKHTMTPIYPSSNTLVEMTLGMKKLKEEMEGVVKELAENNHILERFGSLTMDGGLRNVDCL

98 NP_005850.1 Q9NX20

>NP_005850.1

MADRDSGSEQGGAALGSGGSLGHPGSGSGSGGGGGGGGGGGGSGGGGGGAPGGLQHETQELASKRVDIQNKRFYLDVKQNAKGRFLKIAEVGAGGNKSRLTLSMSVAVEFRDYLGDFIEHYAQLGPSQPPDLAQAQDEPRRALKSEFLVRENRKYYMDLKENQRGRFLRIRQTVNRGPGLGSTQGQTIALPAQGLIEFRDALAKLIDDYGVEEEPAELPEGTSLTVDNKRFFFDVGSNKYGVFMRVSEVKPTYRNSITVPYKVWAKFGHTFCKYSEEMKKIQEKQREKRAACEQLHQQQQQQQEETAAATLLLQGEEEGEED

>Q9NX20

MWRLLARASAPLLRVPLSDSWALLPASAGVKTLLPVPSFEDVSIPEKPKLRFIERAPLVPKVRREPKNLSDIRGPSTEATEFTEGNFAILALGGGYLHWGHFEMMRLTINRSMDPKNMFAIWRVPAPFKPITRKSVGHRMGGGKGAIDHYVTPVKAGRLVVEMGGRCEFEEVQGFLDQVAHKLPFAAKAVSRGTLEKMRKDQEERERNNQNPWTFERIATANMLGIRKVLSPYDLTHKGKYWGKFYMPKRV

99 NP_060370.1 O43182

>NP_060370.1

MLLGRLTSQLLRAVPWAGGRPPWPVSGVLGSRVCGPLYSTSPAGPGRAASLPRKGAQLELEEMLVPRKMSVSPLESWLTARCFLPRLDTGTAGTVAPPQSYQCPPSQIGEGAEQGDEGVADAPQIQCKNVLKIRRRKMNHHKYRKLVKKTRFLRRKVQEGRLRRKQIKFEKDLRRIWLKAGLKEAPEGWQTPKIYLRGK

>O43182

MSAQSLLHSVFSCSSPASSSAASAKGFSKRKLRQTRSLDPALIGGCGSDEAGAEGSARGATAGRLYSPSLPAESLGPRLASSSRGPPPRATRLPPPGPLCSSFSTPSTPQEKSPSGSFHFDYEVPLGRGGLKKSMAWDLPSVLAGPASSRSASSILCSSGGGPNGIFASPRRWLQQRKFQSPPDSRGHPYVVWKSEGDFTWNSMSGRSVRLRSVPIQSLSELERARLQEVAFYQLQQDCDLSCQITIPKDGQKRKKSLRKKLDSLGKEKNKDKEFIPQAFGMPLSQVIANDRAYKLKQDLQRDEQKDASDFVASLLPFGNKRQNKELSSSNSSLSSTSETPNESTSPNTPEPAPRARRRGAMSVDSITDLDDNQSRLLEALQLSLPAEAQSKKEKARDKKLSLNPIYRQVPRLVDSCCQHLEKHGLQTVGIFRVGSSKKRVRQLREEFDRGIDVSLEEEHSVHDVAALLKEFLRDMPDPLLTRELYTAFINTLLLEPEEQLGTLQLLIYLLPPCNCDTLHRLLQFLSIVARHADDNISKDGQEVTGNKMTSLNLATIFGPNLLHKQKSSDKEFSVQSSARAEESTAIIAVVQKMIENYEALFMVPPDLQNEVLISLLETDPDVVDYLLRRKASQSSSPDMLQSEVSFSVGGRHSSTDSNKASSGDISPYDNNSPVLSERSLLAMQEDAAPGGSEKLYRVPGQFMLVGHLSSSKSRESSPGPRLGKDLSEEPFDIWGTWHSTLKSGSKDPGMTGSSGDIFESSSLRAGPCSLSQGNLSPNWPRWQGSPAELDSDTQGARRTQAAAPATEGRAHPAVSRACSTPHVQVAGKAERPTARSEQYLTLSGAHDLSESELDVAGLQSRATPQCQRPHGSGRDDKRPPPPYPGPGKPAAAAAWIQGPPEGVETPTDQGGQAAEREQQVTQKKLSSANSLPAGEQDSPRLGDAGWLDWQRERWQIWELLSTDNPDALPETLV

100 NP_078805.3 Q9P0R6

>NP_078805.3

MADVLSVLRQYNIQKKEIVVKGDEVIFGEFSWPKNVKTNYVVWGTGKEGQPREYYTLDSILFLLNNVHLSHPVYVRRAATENIPVVRRPDRKDLLGYLNGEASTSASIDRSAPLEIGLQRSTQVKRAADEVLAEAKKPRIEDEECVRLDKERLAARLEGHKEGIVQTEQIRSLSEAMSVEKIAAIKAKIMAKKRSTIKTDLDDDITALKQRSFVDAEVDVTRDIVSRERVWRTRTTILQSTGKNFSKNIFAILQSVKAREEGRAPEQRPAPNAAPVDPTLRTKQPIPAAYNRYDQERFKGKEETEGFKIDTMGTYHGMTLKSVTEGASARKTQTPAAQPVPRPVSQARPPPNQKKGSRTPIIIIPAATTSLITMLNAKDLLQDLKFVPSDEKKKQGCQRENETLIQRRKDQMQPGGTAISVTVPYRVVDQPLKLMPQDWDRVVAVFVQGPAWQFKGWPWLLPDGSPVDIFAKIKAFHLKYDEVRLDPNVQKWDVTVLELSYHKRHLDRPVFLRFWETLDRYMVKHKSHLRF

>Q9P0R6

METDCNPMELSSMSGFEEGSELNGFEGTDMKDMRLEAEAVVNDVLFAVNNMFVSKSLRCADDVAYINVETKERNRYCLELTEAGLKVVGYAFDQVDDHLQTPYHETVYSLLDTLSPAYREAFGNALLQRLEALKRDGQS

101 NP_001507.2 NP_003493.1

>NP_001507.2

MVSDEDELNLLVIVVDANPIWWGKQALKESQFTLSKCIDAVMVLGNSHLFMNRSNKLAVIASHIQESRFLYPGKNGRLGDFFGDPGNPPEFNPSGSKDGKYELLTSANEVIVEEIKDLMTKSDIKGQHTETLLAGSLAKALCYIHRMNKEVKDNQEMKSRILVIKAAEDSALQYMNFMNVIFAAQKQNILIDACVLDSDSGLLQQACDITGGLYLKVPQMPSLLQYLLWVFLPDQDQRSQLILPPPVHVDYRAACFCHRNLIEIGYVCSVCLSIFCNFSPICTTCETAFKISLPPVLKAKKKKLKVSA

>NP_003493.1

MNIQEQGFPLDLGASFTEDAPRPPVPGEEGELVSTDPRPASYSFCSGKGVGIKGETSTATPRRSDLDLGYEPEGSASPTPPYLKWAESLHSLLDDQDGISLFRTFLKQEGCADLLDFWFACTGFRKLEPCDSNEEKRLKLARAIYRKYILDNNGIVSRQTKPATKSFIKGCIMKQLIDPAMFDQAQTEIQATMEENTYPSFLKSDIYLEYTRTGSESPKVCSDQSSGSGTGKGISGYLPTLNEDEEWKCDQDMDEDDGRDAAPPGRLPQKLLLETAAPRVSSSRRYSEGREFRYGSWREPVNPYYVNAGYALAPATSANDSEQQSLSSDADTLSLTDSSVDGIPPYRIRKQHRREMQESVQVNGRVPLPHIPRTYRVPKEVRVEPQKFAEELIHRLEAVQRTREAEEKLEERLKRVRMEEEGEDGDPSSGPPGPCHKLPPAPAWHHFPPRCVDMGCAGLRDAHEENPESILDEHVQRVLRTPGRQSPGPGHRSPDSGHVAKMPVALGGAASGHGKHVPKSGAKLDAAGLHHHRHVHHHVHHSTARPKEQVEAEATRRAQSSFAWGLEPHSHGARSRGYSESVGAAPNASDGLAHSGKVGVACKRNAKKAESGKSASTEVPGASEDAEKNQKIMQWIIEGEKEISRHRRTGHGSSGTRKPQPHENSRPLSLEHPWAGPQLRTSVQPSHLFIQDPTMPPHPAPNPLTQLEEARRRLEEEEKRASRAPSKQRYVQEVMRRGRACVRPACAPVLHVVPAVSDMELSETETRSQRKVGGGSAQPCDSIVVAYYFCGEPIPYRTLVRGRAVTLGQFKELLTKKGSYRYYFKKVSDEFDCGVVFEEVREDEAVLPVFEEKIIGKVEKVD

102 NP_008993.1 NP_060177.2

>NP_008993.1

MNRSRQVTCVAWVRCGVAKETPDKVELSKEEVKRLIAEAKEKLQEEGGGSDEEETGSPSEDGMQSARTQARPREPLEDGDPEDDRTLDDDELAEYDLDKYDEEGDPDAETLGESLLGLTVYGSNDQDPYVTLKDTEQYEREDFLIKPSDNLIVCGRAEQDQCNLEVHVYNQEEDSFYVHHDILLSAYPLSVEWLNFDPSPDDSTGNYIAVGNMTPVIEVWDLDIVDSLEPVFTLGSKLSKKKKKKGKKSSSAEGHTDAVLDLSWNKLIRNVLASASADNTVILWDMSLGKPAASLAVHTDKVQTLQFHPFEAQTLISGSYDKSVALYDCRSPDESHRMWRFSGQIERVTWNHFSPCHFLASTDDGFVYNLDARSDKPIFTLNAHNDEISGLDLSSQIKGCLVTASADKYVKIWDILGDRPSLVHSRDMKMGVLFCSSCCPDLPFIYAFGGQKEGLRVWDISTVSSVNEAFGRRERLVLGSARNSSISGPFGSRSSDTPMES

>NP_060177.2

MPEQFSVAEFLAVTAEDLSSPAGAAAFAAKMPRYRGAALAREEILEGDQAILQRIKKAVRAIHSSGLGHVENEEQYREAVESLGNSHLSQNSHELSTGFLNLAVFTREVAALFKNLIQNLNNIVSFPLDSLMKGQLRDGRQDSKKQLEKAWKDYEAKMAKLEKERDRARVTGGIPGEVAQDMQRERRIFQLHMCEYLLKAGESQMKQGPDFLQSLIKFFHAQHNFFQDGWKAAQSLFPFIEKLAASVHALHQAQEDELQKLTQLRDSLRGTLQLESREEHLSRKNSGCGYSIHQHQGNKQFGTEKVGFLYKKSDGIRRVWQKRKCGVKYGCLTISHSTINRPPVKLTLLTCQVRPNPEEKKCFDLVTHNRTYHFQAEDEHECEAWVSVLQNSKDEALSSAFLGEPSAGPGSWGSAGHDGEPHDLTKLLIAEVKSRPGNSQCCDCGAADPTWLSTNLGVLTCIQCSGVHRELGVRFSRMQSLTLDLLGPSELLLALNMGNTSFNEVMEAQLPSHGGPKPSAESDMGTRRDYIMAKYVEHRFARRCTPEPQRLWTAICNRDLLSVLEAFANGQDFGQPLPGPDAQAPEELVLHLAVKVANQASLPLVDFIIQNGGHLDAKAADGNTALHYAALYNQPDCLKLLLKGRALVGTVNEAGETALDIARKKHHKECEELLEQAQAGTFAFPLHVDYSWVISTEPGSDSEEDEEEKRCLLKLPAQAHWASGRLDISNKTYETVASLGAATPQGESEDCPPPLPVKNSSRTLVQGCARHASGDRSEVSSLSSEAPETPESLGSPASSSSLMSPLEPGDPSQAPPNSEEGLREPPGTSRPSLTSGTTPSEMYLPVRFSSESTRSYRRGARSPEDGPSARQPLPRRNVPVGITEGDGSRTGSLPASSVQLLQD

103 NP_002938.1 P30838

>NP_002938.1

MVDMMDLPRSRINAGMLAQFIDKPVCFVGRLEKIHPTGKMFILSDGEGKNGTIELMEPLDEEISGIVEVVGRVTAKATILCTSYVQFKEDSHPFDLGLYNEAVKIIHDFPQFYPLGIVQHD

>P30838

MSKISEAVKRARAAFSSGRTRPLQFRIQQLEALQRLIQEQEQELVGALAADLHKNEWNAYYEEVVYVLEEIEYMIQKLPEWAADEPVEKTPQTQQDELYIHSEPLGVVLVIGTWNYPFNLTIQPMVGAIAAGNAVVLKPSELSENMASLLATIIPQYLDKDLYPVINGGVPETTELLKERFDHILYTGSTGVGKIIMTAAAKHLTPVTLELGGKSPCYVDKNCDLDVACRRIAWGKFMNSGQTCVAPDYILCDPSIQNQIVEKLKKSLKEFYGEDAKKSRDYGRIISARHFQRVMGLIEGQKVAYGGTGDAATRYIAPTILTDVDPQSPVMQEEIFGPVLPIVCVRSLEEAIQFINQREKPLALYMFSSNDKVIKKMIAETSSGGVAANDVIVHITLHSLPFGGVGNSGMGSYHGKKSFETFSHRRSCLVRPLMNDEGLKVRYPPSPAKMTQH

104 NP_006181.1 Q9BYG8

>NP_006181.1

MSKPELKEDKMLEVHFVGDDDVLNHILDREGGAKLKKERAQLLVNPKKIIKKPEYDLEEDDQEVLKDQNYVEIMGRDVQESLKNGSATGGGNKVYSFQNRKHSEKMAKLASELAKTPQKSVSFSLKNDPEITINVPQSSKGHSASDKVQPKNNDKSEFLSTAPRSLRKRLIVPRSHSDSESEYSASNSEDDEGVAQEHEEDTNAVIFSQKIQAQNRVVSAPVGKETPSKRMKRDKTSDLVEEYFEAHSSSKVLTSDRTLQKLKRAKLDQQTLRNLLSKVSPSFSAELKQLNQQYEKLFHKWMLQLHLGFNIVLYGLGSKRDLLERFRTTMLQDSIHVVINGFFPGISVKSVLNSITEEVLDHMGTFRSILDQLDWIVNKFKEDSSLELFLLIHNLDSQMLRGEKSQQIIGQLSSLHNIYLIASIDHLNAPLMWDHAKQSLFNWLWYETTTYSPYTEETSYENSLLVKQSGSLPLSSLTHVLRSLTPNARGIFRLLIKYQLDNQDNPSYIGLSFQDFYQQCREAFLVNSDLTLRAQLTEFRDHKLIRTKKGTDGVEYLLIPVDNGTLTDFLEKEEEEA

>Q9BYG8

MPSMLERISKNLVKEIGSKDLTPVKYLLSATKLRQFVILRKKKDSRSSFWEQSDYVPVEFSLNDILEPSSSVLETVVTGPFHFSDIMIQKHKADMGVNVGIEVSVSGEASVDHGCSLEFQIVTIPSPNLEDFQKRKLLDPEPSFLKECRRRGDNLYVVTEAVELINNTVLYDSSSVNILGKIALWITYGKGQGQGESLRVKKKALTLQKGMVMAYKRKQLVIKEKAILISDDDEQRTFQDEYEISEMVGYCAARSEGLLPSFHTISPTLFNASSNDMKLKPELFLTQQFLSGHLPKYEQVHILPVGRIEEPFWQNFKHLQEEVFQKIKTLAQLSKDVQDVMFYSILAMLRDRGALQDLMNMLELDSSGHLDGPGGAILKKLQQDSNHAWFNPKDPILYLLEAIMVLSDFQHDLLACSMEKRILLQQQELVRSILEPNFRYPWSIPFTLKPELLAPLQSEGLAITYGLLEECGLRTELDNPRSTWDVEAKMPLSALYGTLSLLQQLAEA

105 NP_003855.1 NP_004291.1

>NP_003855.1

MNGFTPDEMSRGGDAAAAVAAVVAAAAAAASAGNGTGAGTGAEVPGAGAVSAAGPPGAAGPGPGQLCCLREDGERCGRAAGNASFSKRIQKSISQKKVKIELDKSARHLYICDYHKNLIQSVRNRRKRKGSDDDGGDSPVQDIDTPEVDLYQLQVNTLRRYKRHFKLPTRPGLNKAQLVEIVGCHFRSIPVNEKDTLTYFIYSVKNDKNKSDLKVDSGVH

>NP_004291.1

MAEQATKSVLFVCLGNICRSPIAEAVFRKLVTDQNISENWRVDSAATSGYEIGNPPDYRGQSCMKRHGIPMSHVARQITKEDFATFDYILCMDESNLRDLNRKSNQVKTCKAKIELLGSYDPQKQLIIEDPYYGNDSDFETVYQQCVRCCRAFLEKAH

106 NP_443082.2 P29144

>NP_443082.2

MAASVRQARSLLGVAATLAPGSRGYRARPPPRRRPGPRWPDPEDLLTPRWQLGPRYAAKQFARYGAASGVVPGSLWPSPEQLRELEAEEREWYPSLATMQESLRVKQLAEEQKRREREQHIAECMAKMPQMIVNWQQQQRENWEKAQADKERRARLQAEAQELLGYQVDPRSARFQELLQDLEKKERKRLKEEKQKRKKEARAAALAAAVAQDPAASGAPSS

>P29144

MATAATEEPFPFHGLLPKKETGAASFLCRYPEYDGRGVLIAVLDTGVDPGAPGMQVTTDGKPKIVDIIDTTGSGDVNTATEVEPKDGEIVGLSGRVLKIPASWTNPSGKYHIGIKNGYDFYPKALKERIQKERKEKIWDPVHRVALAEACRKQEEFDVANNGSSQANKLIKEELQSQVELLNSFEKKYSDPGPVYDCLVWHDGEVWRACIDSNEDGDLSKSTVLRNYKEAQEYGSFGTAEMLNYSVNIYDDGNLLSIVTSGGAHGTHVASIAAGHFPEEPERNGVAPGAQILSIKIGDTRLSTMETGTGLIRAMIEVINHKCDLVNYSYGEATHWPNSGRICEVINEAVWKHNIIYVSSAGNNGPCLSTVGCPGGTTSSVIGVGAYVSPDMMVAEYSLREKLPANQYTWSSRGPSADGALGVSISAPGGAIASVPNWTLRGTQLMNGTSMSSPNACGGIALILSGLKANNIDYTVHSVRRALENTAVKADNIEVFAQGHGIIQVDKAYDYLVQNTSFANKLGFTVTVGNNRGIYLRDPVQVAAPSDHGVGIEPVFPENTENSEKISLQLHLALTSNSSWVQCPSHLELMNQCRHINIRVDPRGLREGLHYTEVCGYDIASPNAGPLFRVPITAVIAAKVNESSHYDLAFTDVHFKPGQIRRHFIEVPEGATWAEVTVCSCSSEVSAKFVLHAVQLVKQRAYRSHEFYKFCSLPEKGTLTEAFPVLGGKAIEFCIARWWASLSDVNIDYTISFHGIVCTAPQLNIHASEGINRFDVQSSLKYEDLAPCITLKNWVQTLRPVSAKTKPLGSRDVLPNNRQLYEMVLTYNFHQPKSGEVTPSCPLLCELLYESEFDSQLWIIFDQNKRQMGSGDAYPHQYSLKLEKGDYTIRLQIRHEQISDLERLKDLPFIVSHRLSNTLSLDIHENHSFALLGKKKSSNLTLPPKYNQPFFVTSLPDDKIPKGAGPGCYLAGSLTLSKTELGKKADVIPVHYYLIPPPTKTKNGSKDKEKDSEKEKDLKEEFTEALRDLKIQWMTKLDSSDIYNELKETYPNYLPLYVARLHQLDAEKERMKRLNEIVDAANAVISHIDQTALAVYIAMKTDPRPDAATIKNDMDKQKSTLVDALCRKGCALADHLLHTQAQDGAISTDAEGKEEEGESPLDSLAETFWETTKWTDLFDNKVLTFAYKHALVNKMYGRGLKFATKLVEEKPTKENWKNCIQLMKLLGWTHCASFTENWLPIMYPPDYCVF

107 NP_002681.1 NP_003977.1

>NP_002681.1

MSKRKAPQETLNGGITDMLTELANFEKNVSQAIHKYNAYRKAASVIAKYPHKIKSGAEAKKLPGVGTKIAEKIDEFLATGKLRKLEKIRQDDTSSSINFLTRVSGIGPSAARKFVDEGIKTLEDLRKNEDKLNHHQRIGLKYFGDFEKRIPREEMLQMQDIVLNEVKKVDSEYIATVCGSFRRGAESSGDMDVLLTHPSFTSESTKQPKLLHQVVEQLQKVHFITDTLSKGETKFMGVCQLPSKNDEKEYPHRRIDIRLIPKDQYYCGVLYFTGSDIFNKNMRAHALEKGFTINEYTIRPLGVTGVAGEPLPVDSEKDIFDYIQWKYREPKDRSE

>NP_003977.1

MACTIQKAEALDGAHLMQILWYDEEESLYPAVWLRDNCPCSDCYLDSAKARKLLVEALDVNIGIKGLIFDRKKVYITWPDEHYSEFQADWLKKRCFSKQARAKLQRELFFPECQYWGSELQLPTLDFEDVLRYDEHAYKWLSTLKKVGIVRLTGASDKPGEVSKLGKRMGFLYLTFYGHTWQVQDKIDANNVAYTTGKLSFHTDYPALHHPPGVQLLHCIKQTVTGGDSEIVDGFNVCQKLKKNNPQAFQILSSTFVDFTDIGVDYCDFSVQSKHKIIELDDKGQVVRINFNNATRDTIFDVPVERVQPFYAALKEFVDLMNSKESKFTFKMNPGDVITFDNWRLLHGRRSYEAGTEISRHLEGAYADWDVVMSRLRILRQRVENGN

108 NP_008835.5 P82914

>NP_008835.5

MAGSGAGVRCSLLRLQETLSAADRCGAALAGHQLIRGLGQECVLSSSPAVLALQTSLVFSRDFGLLVFVRKSLNSIEFRECREEILKFLCIFLEKMGQKIAPYSVEIKNTCTSVYTKDRAAKCKIPALDLLIKLLQTFRSSRLMDEFKIGELFSKFYGELALKKKIPDTVLEKVYELLGLLGEVHPSEMINNAENLFRAFLGELKTQMTSAVREPKLPVLAGCLKGLSSLLCNFTKSMEEDPQTSREIFNFVLKAIRPQIDLKRYAVPSAGLRLFALHASQFSTCLLDNYVSLFEVLLKWCAHTNVELKKAALSALESFLKQVSNMVAKNAEMHKNKLQYFMEQFYGIIRNVDSNNKELSIAIRGYGLFAGPCKVINAKDVDFMYVELIQRCKQMFLTQTDTGDDRVYQMPSFLQSVASVLLYLDTVPEVYTPVLEHLVVMQIDSFPQYSPKMQLVCCRAIVKVFLALAAKGPVLRNCISTVVHQGLIRICSKPVVLPKGPESESEDHRASGEVRTGKWKVPTYKDYVDLFRHLLSSDQMMDSILADEAFFSVNSSSESLNHLLYDEFVKSVLKIVEKLDLTLEIQTVGEQENGDEAPGVWMIPTSDPAANLHPAKPKDFSAFINLVEFCREILPEKQAEFFEPWVYSFSYELILQSTRLPLISGFYKLLSITVRNAKKIKYFEGVSPKSLKHSPEDPEKYSCFALFVKFGKEVAVKMKQYKDELLASCLTFLLSLPHNIIELDVRAYVPALQMAFKLGLSYTPLAEVGLNALEEWSIYIDRHVMQPYYKDILPCLDGYLKTSALSDETKNNWEVSALSRAAQKGFNKVVLKHLKKTKNLSSNEAISLEEIRIRVVQMLGSLGGQINKNLLTVTSSDEMMKSYVAWDREKRLSFAVPFREMKPVIFLDVFLPRVTELALTASDRQTKVAACELLHSMVMFMLGKATQMPEGGQGAPPMYQLYKRTFPVLLRLACDVDQVTRQLYEPLVMQLIHWFTNNKKFESQDTVALLEAILDGIVDPVDSTLRDFCGRCIREFLKWSIKQITPQQQEKSPVNTKSLFKRLYSLALHPNAFKRLGASLAFNNIYREFREEESLVEQFVFEALVIYMESLALAHADEKSLGTIQQCCDAIDHLCRIIEKKHVSLNKAKKRRLPRGFPPSASLCLLDLVKWLLAHCGRPQTECRHKSIELFYKFVPLLPGNRSPNLWLKDVLKEEGVSFLINTFEGGGCGQPSGILAQPTLLYLRGPFSLQATLCWLDLLLAALECYNTFIGERTVGALQVLGTEAQSSLLKAVAFFLESIAMHDIIAAEKCFGTGAAGNRTSPQEGERYNYSKCTVVVRIMEFTTTLLNTSPEGWKLLKKDLCNTHLMRVLVQTLCEPASIGFNIGDVQVMAHLPDVCVNLMKALKMSPYKDILETHLREKITAQSIEELCAVNLYGPDAQVDRSRLAAVVSACKQLHRAGLLHNILPSQSTDLHHSVGTELLSLVYKGIAPGDERQCLPSLDLSCKQLASGLLELAFAFGGLCERLVSLLLNPAVLSTASLGSSQGSVIHFSHGEYFYSLFSETINTELLKNLDLAVLELMQSSVDNTKMVSAVLNGMLDQSFRERANQKHQGLKLATTILQHWKKCDSWWAKDSPLETKMAVLALLAKILQIDSSVSFNTSHGSFPEVFTTYISLLADTKLDLHLKGQAVTLLPFFTSLTGGSLEELRRVLEQLIVAHFPMQSREFPPGTPRFNNYVDCMKKFLDALELSQSPMLLELMTEVLCREQQHVMEELFQSSFRRIARRGSCVTQVGLLESVYEMFRKDDPRLSFTRQSFVDRSLLTLLWHCSLDALREFFSTIVVDAIDVLKSRFTKLNESTFDTQITKKMGYYKILDVMYSRLPKDDVHAKESKINQVFHGSCITEGNELTKTLIKLCYDAFTENMAGENQLLERRRLYHCAAYNCAISVICCVFNELKFYQGFLFSEKPEKNLLIFENLIDLKRRYNFPVEVEVPMERKKKYIEIRKEAREAANGDSDGPSYMSSLSYLADSTLSEEMSQFDFSTGVQSYSYSSQDPRPATGRFRRREQRDPTVHDDVLELEMDELNRHECMAPLTALVKHMHRSLGPPQGEEDSVPRDLPSWMKFLHGKLGNPIVPLNIRLFLAKLVINTEEVFRPYAKHWLSPLLQLAASENNGGEGIHYMVVEIVATILSWTGLATPTGVPKDEVLANRLLNFLMKHVFHPKRAVFRHNLEIIKTLVECWKDCLSIPYRLIFEKFSGKDPNSKDNSVGIQLLGIVMANDLPPYDPQCGIQSSEYFQALVNNMSFVRYKEVYAAAAEVLGLILRYVMERKNILEESLCELVAKQLKQHQNTMEDKFIVCLNKVTKSFPPLADRFMNAVFFLLPKFHGVLKTLCLEVVLCRVEGMTELYFQLKSKDFVQVMRHRDDERQKVCLDIIYKMMPKLKPVELRELLNPVVEFVSHPSTTCREQMYNILMWIHDNYRDPESETDNDSQEIFKLAKDVLIQGLIDENPGLQLIIRNFWSHETRLPSNTLDRLLALNSLYSPKIEVHFLSLATNFLLEMTSMSPDYPNPMFEHPLSECEFQEYTIDSDWRFRSTVLTPMFVETQASQGTLQTRTQEGSLSARWPVAGQIRATQQQHDFTLTQTADGRSSFDWLTGSSTDPLVDHTSPSSDSLLFAHKRSERLQRAPLKSVGPDFGKKRLGLPGDEVDNKVKGAAGRTDLLRLRRRFMRDQEKLSLMYARKGVAEQKREKEIKSELKMKQDAQVVLYRSYRHGDLPDIQIKHSSLITPLQAVAQRDPIIAKQLFSSLFSGILKEMDKFKTLSEKNNITQKLLQDFNRFLNTTFSFFPPFVSCIQDISCQHAALLSLDPAAVSAGCLASLQQPVGIRLLEEALLRLLPAELPAKRVRGKARLPPDVLRWVELAKLYRSIGEYDVLRGIFTSEIGTKQITQSALLAEARSDYSEAAKQYDEALNKQDWVDGEPTEAEKDFWELASLDCYNHLAEWKSLEYCSTASIDSENPPDLNKIWSEPFYQETYLPYMIRSKLKLLLQGEADQSLLTFIDKAMHGELQKAILELHYSQELSLLYLLQDDVDRAKYYIQNGIQSFMQNYSSIDVLLHQSRLTKLQSVQALTEIQEFISFISKQGNLSSQVPLKRLLNTWTNRYPDAKMDPMNIWDDIITNRCFFLSKIEEKLTPLPEDNSMNVDQDGDPSDRMEVQEQEEDISSLIRSCKFSMKMKMIDSARKQNNFSLAMKLLKELHKESKTRDDWLVSWVQSYCRLSHCRSRSQGCSEQVLTVLKTVSLLDENNVSSYLSKNILAFRDQNILLGTTYRIIANALSSEPACLAEIEEDKARRILELSGSSSEDSEKVIAGLYQRAFQHLSEAVQAAEEEAQPPSWSCGPAAGVIDAYMTLADFCDQQLRKEEENASVIDSAELQAYPALVVEKMLKALKLNSNEARLKFPRLLQIIERYPEETLSLMTKEISSVPCWQFISWISHMVALLDKDQAVAVQHSVEEITDNYPQAIVYPFIISSESYSFKDTSTGHKNKEFVARIKSKLDQGGVIQDFINALDQLSNPELLFKDWSNDVRAELAKTPVNKKNIEKMYERMYAALGDPKAPGLGAFRRKFIQTFGKEFDKHFGKGGSKLLRMKLSDFNDITNMLLLKMNKDSKPPGNLKECSPWMSDFKVEFLRNELEIPGQYDGRGKPLPEYHVRIAGFDERVTVMASLRRPKRIIIRGHDEREHPFLVKGGEDLRQDQRVEQLFQVMNGILAQDSACSQRALQLRTYSVVPMTSRLGLIEWLENTVTLKDLLLNTMSQEEKAAYLSDPRAPPCEYKDWLTKMSGKHDVGAYMLMYKGANRTETVTSFRKRESKVPADLLKRAFVRMSTSPEAFLALRSHFASSHALICISHWILGIGDRHLNNFMVAMETGGVIGIDFGHAFGSATQFLPVPELMPFRLTRQFINLMLPMKETGLMYSIMVHALRAFRSDPGLLTNTMDVFVKEPSFDWKNFEQKMLKKGGSWIQEINVAEKNWYPRQKICYAKRKLAGANPAVITCDELLLGHEKAPAFRDYVAVARGSKDHNIRAQEPESGLSEETQVKCLMDQATDPNILGRTWEGWEPWM

>P82914

MLRVAWRTLSLIRTRAVTQVLVPGLPGGGSAKFPFNQWGLQPRSLLLQAARGYVVRKPAQSRLDDDPPPSTLLKDYQNVPGIEKVDDVVKRLLSLEMANKKEMLKIKQEQFMKKIVANPEDTRSLEARIIALSVKIRSYEEHLEKHRKDKAHKRYLLMSIDQRKKMLKNLRNTNYDVFEKICWGLGIEYTFPPLYYRRAHRRFVTKKALCIRVFQETQKLKKRRRALKAAAAAQKQAKRRNPDSPAKAIPKTLKDSQ

109 Q15562 NP_055525.3

>Q15562

MGEPRAGAALDDGSGWTGSEEGSEEGTGGSEGAGGDGGPDAEGVWSPDIEQSFQEALAIYPPCGRRKIILSDEGKMYGRNELIARYIKLRTGKTRTRKQVSSHIQVLARRKSREIQSKLKDQVSKDKAFQTMATMSSAQLISAPSLQAKLGPTGPQASELFQFWSGGSGPPWNVPDVKPFSQTPFTLSLTPPSTDLPGYEPPQALSPLPPPTPSPPAWQARGLGTARLQLVEFSAFVEPPDAVDSYQRHLFVHISQHCPSPGAPPLESVDVRQIYDKFPEKKGGLRELYDRGPPHAFFLVKFWADLNWGPSGEEAGAGGSISSGGFYGVSSQYESLEHMTLTCSSKVCSFGKQVVEKVETERAQLEDGRFVYRLLRSPMCEYLVNFLHKLRQLPERYMMNSVLENFTILQVVTNRDTQELLLCTAYVFEVSTSERGAQHHIYRLVRD

>NP_055525.3

MTGAEIESGAQVKPEKKPGEEVVGGAEIENDVPLVVRPKVRTQAQIMPGARPKNKSKVMPGASTKVETSAVGGARPKSKAKAIPVSRFKEEAQMWAQPRFGAERLSKTERNSQTNIIASPLVSTDSVLVAKTKYLSEDRELVNTDTESFPRRKAHYQAGFQPSFRSKEETNMGSWCCPRPTSKQEASPNSDFKWVDKSVSSLFWSGDEVTAKFHPGNRVKDSNRSMHMANQEANTMSRSQTNQELYIASSSGSEDESVKTPWFWARDKTNTWSGPREDPNSRSRFRSKKEVYVESSSGSEHEDHLESWFGAGKEAKFRSKMRAGKEANNRARHRAKREACIDFMPGSIDVIKKESCFWPEENANTFSRPMIKKEARARAMTKEEAKTKARARAKQEARSEEEALIGTWFWATDESSMADEASIESSLQVEDESIIGSWFWTEEEASMGTGASSKSRPRTDGERIGDSLFGAREKTSMKTGAEATSESILAADDEQVIIGSWFWAGEEVNQEAEEETIFGSWFWVIDAASVESGVGVSCESRTRSEEEEVIGPWFWSGEQVDIEAGIGEEARPGAEEETIFGSWFWAENQTYMDCRAETSCDTMQGAEEEEPIIGSWFWTRVEACVEGDVNSKSSLEDKEEAMIPCFGAKEEVSMKHGTGVRCRFMAGAEETNNKSCFWAEKEPCMYPAGGGSWKSRPEEEEDIVNSWFWSRKYTKPEAIIGSWLWATEESNIDGTGEKAKLLTEEETIINSWFWKEDEAISEATDREESRPEAEEGDIIGSWFWAGEEDRLEPAAETREEDRLAAEKEGIVGSWFGAREETIRREAGSCSKSSPKAEEEEVIIGSWFWEEEASPEAVAGVGFESKPGTEEEEITVGSWFWPEEEASIQAGSQAVEEMESETEEETIFGSWFWDGKEVSEEAGPCCVSKPEDDEEMIVESWFWSRDKAIKETGTVATCESKPENEEGAIVGSWFEAEDEVDNRTDNGSNCGSRTLADEDEAIVGSWFWAGDEAHFESNPSPVFRAICRSTCSVEQEPDPSRRPQSWEEVTVQFKPGPWGRVGFPSISPFRFPKEAASLFCEMFGGKPRNMVLSPEGEDQESLLQPDQPSPEFPFQYDPSYRSVQEIREHLRAKESTEPESSSCNCIQCELKIGSEEFEELLLLMEKIRDPFIHEISKIAMGMRSASQFTRDFIRDSGVVSLIETLLNYPSSRVRTSFLENMIRMAPPYPNLNIIQTYICKVCEETLAYSVDSPEQLSGIRMIRHLTTTTDYHTLVANYMSGFLSLLATGNAKTRFHVLKMLLNLSENLFMTKELLSAEAVSEFIGLFNREETNDNIQIVLAIFENIGNNIKKETVFSDDDFNIEPLISAFHKVEKFAKELQGKTDNQNDPEGDQEN

110 NP_001230.1 NP_056520.2

>NP_001230.1

MYHNSSQKRHWTFSSEEQLARLRADANRKFRCKAVANGKVLPNDPVFLEPHEEMTLCKYYEKRLLEFCSVFKPAMPRSVVGTACMYFKRFYLNNSVMEYHPRIIMLTCAFLACKVDEFNVSSPQFVGNLRESPLGQEKALEQILEYELLLIQQLNFHLIVHNPYRPFEGFLIDLKTRYPILENPEILRKTADDFLNRIALTDAYLLYTPSQIALTAILSSASRAGITMESYLSESLMLKENRTCLSQLLDIMKSMRNLVKKYEPPRSEEVAVLKQKLERCHSAELALNVITKKRKGYEDDDYVSKKSKHEEEEWTDDDLVESL

>NP_056520.2

MSGSHTPACGPFSALTPSIWPQEILAKYTQKEESAEQPEFYYDEFGFRVYKEEGDEPGSSLLANSPLMEDAPQRLRWQAHLEFTHNHDVGDLTWDKIAVSLPRSEKLRSLVLAGIPHGMRPQLWMRLSGALQKKRNSELSYREIVKNSSNDETIAAKQIEKDLLRTMPSNACFASMGSIGVPRLRRVLRALAWLYPEIGYCQGTGMVAACLLLFLEEEDAFWMMSAIIEDLLPASYFSTTLLGVQTDQRVLRHLIVQYLPRLDKLLQEHDIELSLITLHWFLTAFASVVDIKLLLRIWDLFFYEGSRVLFQLTLGMLHLKEEELIQSENSASIFNTLSDIPSQMEDAELLLGVAMRLAGSLTDVAVETQRRKHLAYLIADQGQLLGAGTLTNLSQVVRRRTQRRKSTITALLFGEDDLEALKAKNIKQTELVADLREAILRVARHFQCTDPKNCSVELTPDYSMESHQRDHENYVACSRSHRRRAKALLDFERHDDDELGFRKNDIITIVSQKDEHCWVGELNGLRGWFPAKFVEVLDERSKEYSIAGDDSVTEGVTDLVRGTLCPALKALFEHGLKKPSLLGGACHPWLFIEEAAGREVERDFASVYSRLVLCKTFRLDEDGKVLTPEELLYRAVQSVNVTHDAVHAQMDVKLRSLICVGLNEQVLHLWLEVLCSSLPTVEKWYQPWSFLRSPGWVQIKCELRVLCCFAFSLSQDWELPAKREAQQPLKEGVRDMLVKHHLFSWDVDG

111 NP_001512.1 NP_004799.1

>NP_001512.1

MDTCGVGYVALGEAGPVGNMTVVDSPGQEVLNQLDVKTSSEMTSAEASVEMSLPTPLPGFEDSPDQRRLPPEQESLSRLEQPDLSSEMSKVSKPRASKPGRKRGGRTRKGPKRPQQPNPPSAPLVPGLLDQSNPLSTPMPKKRGRKSKAELLLLKLSKDLDRPESQSPKRPPEDFETPSGERPRRRAAQVALLYLQELAEELSTALPAPVSCPEGPKVSSPTKPKKIRQPAACPGGEEVDGAPRDEDFFLQVEAEDVEESEGPSESSSEPEPVVPRSTPRGSTSGKQKPHCRGMAPNGLPNHIMAPVWKCLHLTKDFREQKHSYWEFAEWIPLAWKWHLLSELEAAPYLPQEEKSPLFSVQREGLPEDGTLYRINRFSSITAHPERWDVSFFTGGPLWALDWCPVPEGAGASQYVALFSSPDMNETHPLSQLHSGPGLLQLWGLGTLQQESCPGNRAHFVYGIACDNGCIWDLKFCPSGAWELPGTPRKAPLLPRLGLLALACSDGKVLLFSLPHPEALLAQQPPDAVKPAIYKVQCVATLQVGSMQATDPSECGQCLSLAWMPTRPHQHLAAGYYNGMVVFWNLPTNSPLQRIRLSDGSLKLYPFQCFLAHDQAVRTLQWCKANSHFLVSAGSDRKIKFWDLRRPYEPINSIKRFLSTELAWLLPYNGVTVAQDNCYASYGLCGIHYIDAGYLGFKAYFTAPRKGTVWSLSGSDWLGTIAAGDISGELIAAILPDMALNPINVKRPVERRFPIYKADLIPYQDSPEGPDHSSASSGVPNPPKARTYTETVNHHYLLFQDTDLGSFHDLLRREPMLRMQEGEGHSQLCLDRLQLEAIHKVRFSPNLDSYGWLVSGGQSGLVRIHFVRGLASPLGHRMQLESRAHFNAMFQPSSPTRRPGFSPTSHRLLPTP

>NP_004799.1

MAEDSESAASQQSLELDDQDTCGIDGDNEEETEHAKGSPGGYLGAKKKKKKQKRKKEKPNSGGTKSDSASDSQEIKIQQPSKNPSVPMQKLQDIQRAMELLSACQGPARNIDEAAKHRYQFWDTQPVPKLDEVITSHGAIEPDKDNVRQEPYSLPQGFMWDTLDLSDAEVLKELYTLLNENYVEDDDNMFRFDYSPEFLLWALRPPGWLLQWHCGVRVSSNKKLVGFISAIPANIRIYDSVKKMVEINFLCVHKKLRSKRVAPVLIREITRRVNLEGIFQAVYTAGVVLPKPIATCRYWHRSLNPRKLVEVKFSHLSRNMTLQRTMKLYRLPDVTKTSGLRPMEPKDIKSVRELINTYLKQFHLAPVMDEEEVAHWFLPREHIIDTFVVESPNGKLTDFLSFYTLPSTVMHHPAHKSLKAAYSFYNIHTETPLLDLMSDALILAKSKGFDVFNALDLMENKTFLEKLKFGIGDGNLQYYLYNWRCPGTDSEKVGLVLQ

112 NP_003085.1 P51857

>NP_003085.1

MAYRGQGQKVQKVMVQPINLIFRYLQNRSRIQVWLYEQVNMRIEGCIIGFDEYMNLVLDDAEEIHSKTKSRKQLGRIMLKGDNITLLQSVSN

>P51857

MDLSAASHRIPLSDGNSIPIIGLGTYSEPKSTPKGACATSVKVAIDTGYRHIDGAYIYQNEHEVGEAIREKIAEGKVRREDIFYCGKLWATNHVPEMVRPTLERTLRVLQLDYVDLYIIEVPMAFKPGDEIYPRDENGKWLYHKSNLCATWEAMEACKDAGLVKSLGVSNFNRRQLELILNKPGLKHKPVSNQVECHPYFTQPKLLKFCQQHDIVITAYSPLGTSRNPIWVNVSSPPLLKDALLNSLGKRYNKTAAQIVLRFNIQRGVVVIPKSFNLERIKENFQIFDFSLTEEEMKDIEALNKNVRFVELLMWRDHPEYPFHDEY

113 Q9C002 NP_003945.2

>Q9C002

MSFFQLLMKRKELIPLVVFMTVAAGGASSFAVYSLWKTDVILDRKKNPEPWETVDPTVPQKLITINQQWKPIEELQNVQRVTK

>NP_003945.2

MAVMEMACPGAPGSAVGQQKELPKAKEKTPPLGKKQSSVYKLEAVEKSPVFCGKWEILNDVITKGTAKEGSEAGPAAISIIAQAECENSQEFSPTFSERIFIAGSKQYSQSESLDQIPNNVAHATEGKMARVCWKGKRRSKARKKRKKKSSKSLAHAGVALAKPLPRTPEQESCTIPVQEDESPLGAPYVRNTPQFTKPLKEPGLGQLCFKQLGEGLRPALPRSELHKLISPLQCLNHVWKLHHPQDGGPLPLPTHPFPYSRLPHPFPFHPLQPWKPHPLESFLGKLACVDSQKPLPDPHLSKLACVDSPKPLPGPHLEPSCLSRGAHEKFSVEEYLVHALQGSVSSGQAHSLTSLAKTWAARGSRSREPSPKTEDNEGVLLTEKLKPVDYEYREEVHWATHQLRLGRGSFGEVHRMEDKQTGFQCAVKKVRLEVFRAEELMACAGLTSPRIVPLYGAVREGPWVNIFMELLEGGSLGQLVKEQGCLPEDRALYYLGQALEGLEYLHSRRILHGDVKADNVLLSSDGSHAALCDFGHAVCLQPDGLGKSLLTGDYIPGTETHMAPEVVLGRSCDAKVDVWSSCCMMLHMLNGCHPWTQFFRGPLCLKIASEPPPVREIPPSCAPLTAQAIQEGLRKEPIHRVSAAELGGKVNRALQQVGGLKSPWRGEYKEPRHPPPNQANYHQTLHAQPRELSPRAPGPRPAEETTGRAPKLQPPLPPEPPEPNKSPPLTLSKEESGMWEPLPLSSLEPAPARNPSSPERKATVPEQELQQLEIELFLNSLSQPFSLEEQEQILSCLSIDSLSLSDDSEKNPSKASQSSRDTLSSGVHSWSSQAEARSSSWNMVLARGRPTDTPSYFNGVKVQIQSLNGEHLHIREFHRVKVGDIATGISSQIPAAAFSLVTKDGQPVRYDMEVPDSGIDLQCTLAPDGSFAWSWRVKHGQLENRP

114 NP_001460.1 NP_006432.1

>NP_001460.1

MSGWESYYKTEGDEEAEEEQEENLEASGDYKYSGRDSLIFLVDASKAMFESQSEDELTPFDMSIQCIQSVYISKIISSDRDLLAVVFYGTEKDKNSVNFKNIYVLQELDNPGAKRILELDQFKGQQGQKRFQDMMGHGSDYSLSEVLWVCANLFSDVQFKMSHKRIMLFTNEDNPHGNDSAKASRARTKAGDLRDTGIFLDLMHLKKPGGFDISLFYRDIISIAEDEDLRVHFEESSKLEDLLRKVRAKETRKRALSRLKLKLNKDIVISVGIYNLVQKALKPPPIKLYRETNEPVKTKTRTFNTSTGGLLLPSDTKRSQIYGSRQIILEKEETEELKRFDDPGLMLMGFKPLVLLKKHHYLRPSLFVYPEESLVIGSSTLFSALLIKCLEKEVAALCRYTPRRNIPPYFVALVPQEEELDDQKIQVTPPGFQLVFLPFADDKRKMPFTEKIMATPEQVGKMKAIVEKLRFTYRSDSFENPVLQQHFRNLEALALDLMEPEQAVDLTLPKVEAMNKRLGSLVDEFKELVYPPDYNPEGKVTKRKHDNEGSGSKRPKVEYSEEELKTHISKGTLGKFTVPMLKEACRAYGLKSGLKKQELLEALTKHFQD

>NP_006432.1

MAAAAVSSAKRSLRGELKQRLRAMSAEERLRQSRVLSQKVIAHSEYQKSKRISIFLSMQDEIETEEIIKDIFQRGKICFIPRYRFQSNHMDMVRIESPEEISLLPKTSWNIPQPGEGDVREEALSTGGLDLIFMPGLGFDKHGNRLGRGKGYYDAYLKRCLQHQEVKPYTLALAFKEQICLQVPVNENDMKVDEVLYEDSSTA

115 NP_490595.1 NP_002078.1

>NP_490595.1

MASGRGASSRWFFTREQLENTPSRRCGVEADKELSCRQQAANLIQEMGQRLNVSQLTINTAIVYMHRFYMHHSFTKFNKNIISSTALFLAAKVEEQARKLEHVIKVAHACLHPLEPLLDTKCDAYLQQTQELVILETIMLQTLGFEITIEHPHTDVVKCTQLVRASKDLAQTSYFMATNSLHLTTFCLQYKPTVIACVCIHLACKWSNWEIPVSTDGKHWWEYVDPTVTLELLDELTHEFLQILEKTPNRLKKIRNWRANQAARKPKVDGQVSETPLLGSSLVQNSILVDSVTGVPTNPSFQKPSTSAFPAPVPLNSGNISVQDSHTSDNLSMLATGMPSTSYGLSSHQEWPQHQDSARTEQLYSQKQETSLSGSQYNINFQQGPSISLHSGLHHRPDKISDHSSVKQEYTHKAGSSKHHGPISTTPGIIPQKMSLDKYREKRKLETLDLDVRDHYIAAQVEQQHKQGQSQAASSSSVTSPIKMKIPIANTEKYMADKKEKSGSLKLRIPIPPTDKSASKEELKMKIKVSSSERHSSSDEGSGKSKHSSPHISRDHKEKHKEHPSSRHHTSSHKHSHSHSGSSSGGSKHSADGIPPTVLRSPVGLSSDGISSSSSSSRKRLHVNDASHNHHSKMSKSSKSSGSSSSSSSSVKQYISSHNSVFNHPLPPPPPVTYQVGYGHLSTLVKLDKKPVETNGPDANHEYSTSSQHMDYKDTFDMLDSLLSAQGMNM

>NP_002078.1

MWTLVSWVALTAGLVAGTRCPDGQFCPVACCLDPGGASYSCCRPLLDKWPTTLSRHLGGPCQVDAHCSAGHSCIFTVSGTSSCCPFPEAVACGDGHHCCPRGFHCSADGRSCFQRSGNNSVGAIQCPDSQFECPDFSTCCVMVDGSWGCCPMPQASCCEDRVHCCPHGAFCDLVHTRCITPTGTHPLAKKLPAQRTNRAVALSSSVMCPDARSRCPDGSTCCELPSGKYGCCPMPNATCCSDHLHCCPQDTVCDLIQSKCLSKENATTDLLTKLPAHTVGDVKCDMEVSCPDGYTCCRLQSGAWGCCPFTQAVCCEDHIHCCPAGFTCDTQKGTCEQGPHQVPWMEKAPAHLSLPDPQALKRDVPCDNVSSCPSSDTCCQLTSGEWGCCPIPEAVCCSDHQHCCPQGYTCVAEGQCQRGSEIVAGLEKMPARRASLSHPRDIGCDQHTSCPVGQTCCPSLGGSWACCQLPHAVCCEDRQHCCPAGYTCNVKARSCEKEVVSAQPATFLARSPHVGVKDVECGEGHFCHDNQTCCRDNRQGWACCPYRQGVCCADRRHCCPAGFRCAARGTKCLRREAPRWDAPLRDPALRQLL

116 O00115 Q6PRD7

>O00115

MIPLLLAALLCVPAGALTCYGDSGQPVDWFVVYKLPALRGSGEAAQRGLQYKYLDESSGGWRDGRALINSPEGAVGRSLQPLYRSNTSQLAFLLYNDQPPQPSKAQDSSMRGHTKGVLLLDHDGGFWLVHSVPNFPPPASSAAYSWPHSACTYGQTLLCVSFPFAQFSKMGKQLTYTYPWVYNYQLEGIFAQEFPDLENVVKGHHVSQEPWNSSITLTSQAGAVFQSFAKFSKFGDDLYSGWLAAALGTNLQVQFWHKTVGILPSNCSDIWQVLNVNQIAFPGPAGPSFNSTEDHSKWCVSPKGPWTCVGDMNRNQGEEQRGGGTLCAQLPALWKAFQPLVKNYQPCNGMARKPSRAYKI

>Q6PRD7

MGTSSTDSQQAGHRRCSTSNTSAENLTCLSLPGSPGKTAPLPGPAQAGAGQPLPKGCAAVKAEVGIPAPHTSQEVRIHIRRLLSWAAPGACGLRSTPCALPQALPQARPCPGRWFFPGCSLPTGGAQTILSLWTWRHFLNWALQQREENSGRARRVPPVPRTAPVSKGEGSHPPQNSNGEKVKTITPDVGLHQSLTSDPTVAVLRAKRAPEAHPPRSCSGSLTARVCHMGVCQGQGDTEDGRMTLMG

117 NP_079058.1 NP_004835.2

>NP_079058.1

MEELEQGLLMQPWAWLQLAENSLLAKVFITKQGYALLVSDLQQVWHEQVDTSVVSQRAKELNKRLTAPPAAFLCHLDNLLRPLLKDAAHPSEATFSCDCVADALILRVRSELSGLPFYWNFHCMLASPSLVSQHLIRPLMGMSLALQCQVRELATLLHMKDLEIQDYQESGATLIRDRLKTEPFEENSFLEQFMIEKLPEACSIGDGKPFVMNLQDLYMAVTTQEVQVGQKHQGAGDPHTSNSASLQGIDSQCVNQPEQLVSSAPTLSAPEKESTGTSGPLQRPQLSKVKRKKPRGLFS

>NP_004835.2

MDAALKRSRSEEPAEILPPARDEEEEEEEGMEQGLEEEEEVDPRIQGELEKLNQSTDDINRRETELEDARQKFRSVLVEATVKLDELVKKIGKAVEDSKPYWEARRVARQAQLEAQKATQDFQRATEVLRAAKETISLAEQRLLEDDKRQFDSAWQEMLNHATQRVMEAEQTKTRSELVHKETAARYNAAMGRMRQLEKKLKRAINKSKPYFELKAKYYVQLEQLKKTVDDLQAKLTLAKGEYKMALKNLEMISDEIHERRRSSAMGPRGCGVGAEGSSTSVEDLPGSKPEPDAISVASEAFEDDSCSNFVSEDDSETQSVSSFSSGPTSPSEMPDQFPAVVRPGSLDLPSPVSLSEFGMMFPVLGPRSECSGASSPECEVERGDRAEGAENKTSDKANNNRGLSSSSGSGGSSKSQSSTSPEGQALENRMKQLSLQCSKGRDGIIADIKMVQIG

118 NP_055850.1 NP_000365.3

>NP_055850.1

MASKRKSTTPCMIPVKTVVLQDASMEAQPAETLPEGPQQDLPPEASAASSEAAQNPSSTDGSTLANGHRSTLDGYLYSCKYCDFRSHDMTQFVGHMNSEHTDFNKDPTFVCSGCSFLAKTPEGLSLHNATCHSGEASFVWNVAKPDNHVVVEQSIPESTSTPDLAGEPSAEGADGQAEIIITKTPIMKIMKGKAEAKKIHTLKENVPSQPVGEALPKLSTGEMEVREGDHSFINGAVPVSQASASSAKNPHAANGPLIGTVPVLPAGIAQFLSLQQQPPVHAQHHVHQPLPTAKALPKVMIPLSSIPTYNAAMDSNSFLKNSFHKFPYPTKAELCYLTVVTKYPEEQLKIWFTAQRLKQGISWSPEEIEDARKKMFNTVIQSVPQPTITVLNTPLVASAGNVQHLIQAALPGHVVGQPEGTGGGLLVTQPLMANGLQATSSPLPLTVTSVPKQPGVAPINTVCSNTTSAVKVVNAAQSLLTACPSITSQAFLDASIYKNKKSHEQLSALKGSFCRNQFPGQSEVEHLTKVTGLSTREVRKWFSDRRYHCRNLKGSRAMIPGDHSSIIIDSVPEVSFSPSSKVPEVTCIPTTATLATHPSAKRQSWHQTPDFTPTKYKERAPEQLRALESSFAQNPLPLDEELDRLRSETKMTRREIDSWFSERRKKVNAEETKKAEENASQEEEEAAEDEGGEEDLASELRVSGENGSLEMPSSHILAERKVSPIKINLKNLRVTEANGRNEIPGLGACDPEDDESNKLAEQLPGKVSCKKTAQQRHLLRQLFVQTQWPSNQDYDSIMAQTGLPRPEVVRWFGDSRYALKNGQLKWYEDYKRGNFPPGLLVIAPGNRELLQDYYMTHKMLYEEDLQNLCDKTQMSSQQVKQWFAEKMGEETRAVADTGSEDQGPGTGELTAVHKGMGDTYSEVSENSESWEPRVPEASSEPFDTSSPQAGRQLETD

>NP_000365.3

MEANGLGPQGFPELKNDTFLRAAWGEETDYTPVWCMRQAGRYLPEFRETRAAQDFFSTCRSPEACCELTLQPLRRFPLDAAIIFSDILVVPQALGMEVTMVPGKGPSFPEPLREEQDLERLRDPEVVASELGYVFQAITLTRQRLAGRVPLIGFAGAPWTLMTYMVEGGGSSTMAQAKRWLYQRPQASHQLLRILTDALVPYLVGQVVAGAQALQLFESHAGHLGPQLFNKFALPYIRDVAKQVKARLREAGLAPVPMIIFAKDGHFALEELAQAGYEVVGLDWTVAPKKARECVGKTVTLQGNLDPCALYASEEEIGQLVKQMLDDFGPHRYIANLGHGLYPDMDPEHVGAFVDAVHKHSRLLRQN

119 P09430 NP_002717.3

>P09430

MSTSRKLKSHGMRRSKSRSPHKGVKRGGSKRKYRKGNLKSRKRGDDANRNYRSHL

>NP_002717.3

MLSLQYPDVYRDETAVQDYHGHKICDPYAWLEDPDSEQTKAFVEAQNKITVPFLEQCPIRGLYKERMTELYDYPKYSCHFKKGKRYFYFYNTGLQNQRVLYVQDSLEGEARVFLDPNILSDDGTVALRGYAFSEDGEYFAYGLSASGSDWVTIKFMKVDGAKELPDVLERVKFSCMAWTHDGKGMFYNSYPQQDGKSDGTETSTNLHQKLYYHVLGTDQSEDILCAEFPDEPKWMGGAELSDDGRYVLLSIREGCDPVNRLWYCDLQQESSGIAGILKWVKLIDNFEGEYDYVTNEGTVFTFKTNRQSPNYRVINIDFRDPEESKWKVLVPEHEKDVLEWIACVRSNFLVLCYLHDVKNILQLHDLTTGALLKTFPLDVGSIVGYSGQKKDTEIFYQFTSFLSPGIIYHCDLTKEELEPRVFREVTVKGIDASDYQTVQIFYPSKDGTKIPMFIVHKKGIKLDGSHPAFLYGYGGFNISITPNYSVSRLIFVRHMGGILAVANIRGGGEYGETWHKGGILANKQNCFDDFQCAAEYLIKEGYTSPKRLTINGGSNGGLLVAACANQRPDLFGCVIAQVGVMDMLKFHKYTIGHAWTTDYGCSDSKQHFEWLVKYSPLHNVKLPEADDIQYPSMLLLTADHDDRVVPLHSLKFIATLQYIVGRSRKQSNPLLIHVDTKAGHGAGKPTAKVIEEVSDMFAFIARCLNVDWIP

120 Q9H0W9 P13995

>Q9H0W9

MACAEFSFHVPSLEELAGVMQKGLKDNFADVQVSVVDCPDLTKEPFTFPVKGICGKTRIAEVGGVPYLLPLVNQKKVYDLNKIAKEIKLPGAFILGAGAGPFQTLGFNSEFMPVIQTESEHKPPVNGSYFAHVNPADGGCLLEKYSEKCHDFQCALLANLFASEGQPGKVIEVKAKRRTGPLNFVTCMRETLEKHYGNKPIGMGGTFIIQKGKVKSHIMPAEFSSCPLNSDEEVNKWLHFYEMKAPLVCLPVFVSRDPGFDLRLEHTHFFSRHGEGGHYHYDTTPDIVEYLGYFLPAEFLYRIDQPKETHSIGRD

>P13995

MAATSLMSALAARLLQPAHSCSLRLRPFHLAAVRNEAVVISGRKLAQQIKQEVRQEVEEWVASGNKRPHLSVILVGENPASHSYVLNKTRAAAVVGINSETIMKPASISEEELLNLINKLNNDDNVDGLLVQLPLPEHIDERRICNAVSPDKDVDGFHVINVGRMCLDQYSMLPATPWGVWEIIKRTGIPTLGKNVVVAGRSKNVGMPIAMLLHTDGAHERPGGDATVTISHRYTPKEQLKKHTILADIVISAAGIPNLITADMIKEGAAVIDVGINRVHDPVTAKPKLVGDVDFEGVRQKAGYITPVPGGVGPMTVAMLMKNTIIAAKKVLRLEEREVLKSKELGVATN

121 Q9H0W9 NP_006180.1

>Q9H0W9

MACAEFSFHVPSLEELAGVMQKGLKDNFADVQVSVVDCPDLTKEPFTFPVKGICGKTRIAEVGGVPYLLPLVNQKKVYDLNKIAKEIKLPGAFILGAGAGPFQTLGFNSEFMPVIQTESEHKPPVNGSYFAHVNPADGGCLLEKYSEKCHDFQCALLANLFASEGQPGKVIEVKAKRRTGPLNFVTCMRETLEKHYGNKPIGMGGTFIIQKGKVKSHIMPAEFSSCPLNSDEEVNKWLHFYEMKAPLVCLPVFVSRDPGFDLRLEHTHFFSRHGEGGHYHYDTTPDIVEYLGYFLPAEFLYRIDQPKETHSIGRD

>NP_006180.1

MAEDRPQQPQLDMPLVLDQGLTRQMRLRVESLKQRGEKRQDGEKLLQPAESVYRLNFTQQQRLQFERWNVVLDKPGKVTITGTSQNWTPDLTNLMTRQLLDPTAIFWRKEDSDAIDWNEADALEFGERLSDLAKIRKVMYFLVTFGEGVEPANLKASVVFNQL

122 NP_005672.1 NP_116119.2

>NP_005672.1

MSDFSEELKGPVTDDEEVETSVLSGAGMHFPWLQTYVETVAIGGKRRKDFAQTTSACLSFIQEALLKHQWQQAAEYMYSYFQTLEDSDSYKRQAAPEIIWKLGSEILFYHPKSNMESFNTFANRMKNIGVMNYLKISLQHALYLLHHGMLKDAKRNLSEAETWRHGENTSSREILINLIQAYKGLLQYYTWSEKKMELSKLDKDDYAYNAVAQDVFNHSWKTSANISALIKIPGVWDPFVKSYVEMLEFYGDRDGAQEVLTNYAYDEKFPSNPNAHIYLYNFLKRQKAPRSKLISVLKILYQIVPSHKLMLEFHTLLRKSEKEEHRKLGLEVLFGVLDFAGCTKNITAWKYLAKYLKNILMGNHLAWVQEEWNSRKNWWPGFHFSYFWAKSDWKEDTALACEKAFVAGLLLGKGCRYFRYILKQDHQILGKKIKRMKRSVKKYSIVNPRL

>NP_116119.2

MEFLKTCVLRRNACTAVCFWRSKVVQKPSVRRISTTSPRSTVMPAWVIDKYGKNEVLRFTQNMMMPIIHYPNEVIVKVHAASVNPIDVNMRSGYGATALNMKRDPLHVKIKGEEFPLTLGRDVSGVVMECGLDVKYFKPGDEVWAAVPPWKQGTLSEFVVVSGNEVSHKPKSLTHTQAASLPYVALTAWSAINKVGGLNDKNCTGKRVLILGASGGVGTFAIQVMKAWDAHVTAVCSQDASELVRKLGADDVIDYKSGSVEEQLKSLKPFDFILDNVGGSTETWAPDFLKKWSGATYVTLVTPFLLNMDRLGIADGMLQTGVTVGSKALKHFWKGVHYRWAFFMASGPCLDDIAELVDAGKIRPVIEQTFPFSKVPEAFLKVERGHARGKTVINVV

123 Q9BXL8 NP_060441.2

>Q9BXL8

MFARGLKRKCVGHEEDVEGALAGLKTVSSYSLQRQSLLDMSLVKLQLCHMLVEPNLCRSVLIANTVRQIQEEMTQDGTWRTVAPQAAERAPLDRLVSTEILCRAAWGQEGAHPAPGLGDGHTQGPVSDLCPVTSAQAPRHLQSSAWEMDGPRENRGSFHKSLDQIFETLETKNPSCMEELFSDVDSPYYDLDTVLTGMMGGARPGPCEGLEGLAPATPGPSSSCKSDLGELDHVVEILVET

>NP_060441.2

MVFLTAQLWLRNRVTDRYFRIQEVLKHARHFRGRKNRCYRLAVRTVIRAFVKCTKARYLKKKNMRTLWINRITAASQEHGLKYPALIGNLVKCQVELNRKVLADLAIYEPKTFKSLAALASRRRHEGFAAALGDGKEPEGIFSRVVQYH

124 NP_068809.1 Q9NP74

>NP_068809.1

MSDNEDNFDGDDFDDVEEDEGLDDLENAEEEGQENVEILPSGERPQANQKRITTPYMTKYERARVLGTRALQIAMCAPVMVELEGETDPLLIAMKELKARKIPIIIRRYLPDGSYEDWGVDELIITD

>Q9NP74

MEEAELVKGRLQAITDKRKIQEEISQKRLKIEEDKLKHQHLKKKALREKWLLDGISSGKEQEEMKKQNQQDQHQIQVLEQSILRLEKEIQDLEKAELQISTKEEAILKKLKSIERTTEDIIRSVKVEREERAEESIEDIYANIPDLPKSYIPSRLRKEINEEKEDDEQNRKALYAMEIKVEKDLKTGESTVLSSIPLPSDDFKGTGIKVYDDGQKSVYAVSSNHSAAYNGTDGLAPVEVEELLRQASERNSKSPTEYHEPVYANPFYRPTTPQRETVTPGPNFQERIKIKTNGLGIGVNESIHNMGNGLSEERGNNFNHISPIPPVPHPRSVIQQAEEKLHTPQKRLMTPWEESNVMQDKDAPSPKPRLSPRETIFGKSEHQNSSPTCQEDEEDVRYNIVHSLPPDINDTEPVTMIFMGYQQAEDSEEDKKFLTGYDGIIHAELVVIDDEEEEDEGEAEKPSYHPIAPHSQVYQPAKPTPLPRKRSEASPHENTNHKSPHKNSISLKEQEESLGSPVHHSPFDAQTTGDGTEDPSLTALRMRMAKLGKKVI

125 NP_002936.1 NP_065723.1

>NP_002936.1

MVGQLSEGAIAAIMQKGDTNIKPILQVINIRPITTGNSPPRYRLLMSDGLNTLSSFMLATQLNPLVEEEQLSSNCVCQIHRFIVNTLKDGRRVVILMELEVLKSAEAVGVKIGNPVPYNEGLGQPQVAPPAPAASPAASSRPQPQNGSSGMGSTVSKAYGASKTFGKAAGPSLSHTSGGTQSKVVPIASLTPYQSKWTICARVTNKSQIRTWSNSRGEGKLFSLELVDESGEIRATAFNEQVDKFFPLIEVNKVYYFSKGTLKIANKQFTAVKNDYEMTFNNETSVMPCEDDHHLPTVQFDFTGIDDLENKSKDSLVDIIGICKSYEDATKITVRSNNREVAKRNIYLMDTSGKVVTATLWGEDADKFDGSRQPVLAIKGARVSDFGGRSLSVLSSSTIIANPDIPEAYKLRGWFDAEGQALDGVSISDLKSGGVGGSNTNWKTLYEVKSENLGQGDKPDYFSSVATVVYLRKENCMYQACPTQDCNKKVIDQQNGLYRCEKCDTEFPNFKYRMILSVNIADFQENQWVTCFQESAEAILGQNAAYLGELKDKNEQAFEEVFQNANFRSFIFRVRVKVETYNDESRIKATVMDVKPVDYREYGRRLVMSIRRSALM

>NP_065723.1

MGQCRSANAEDAQEFSDVERAIETLIKNFHQYSVEGGKETLTPSELRDLVTQQLPHLMPSNCGLEEKIANLGSCNDSKLEFRSFWELIGEAAKSVKLERPVRGH

126 NP_004587.1 Q8N183

>NP_004587.1

MAVPETRPNHTIYINNLNEKIKKDELKKSLYAIFSQFGQILDILVSRSLKMRGQAFVIFKEVSSATNALRSMQGFPFYDKPMRIQYAKTDSDIIAKMKGTFVERDRKREKRKPKSQETPATKKAVQGGGATPVVGAVQGPVPGMPPMTQAPRIMHHMPGQPPYMPPPGMIPPPGLAPGQIPPGAMPPQQLMPGQMPPAQPLSENPPNHILFLTNLPEETNELMLSMLFNQFPGFKEVRLVPGRHDIAFVEFDNEVQAGAARDALQGFKITQNNAMKISFAKK

>Q8N183

MGWSQDLFRALWRSLSREVKEHVGTDQFGNKYYYIPQYKNWRGQTIREKRIVEAANKKEVDYEAGDIPTEWEAWIRRTRKTPPTMEEILKNEKHREEIKIKSQDFYEKEKLLSKETSEELLPPPVQTQIKGHASAPYFGKEEPSVAPSSTGKTFQPGSWMPRDGKSHNQ

127 NP_003073.1 NP_075066.1

>NP_003073.1

MGTPPGLQTDCEALLSRFQETDSVRFEDFTELWRNMKFGTIFCGRMRNLEKNMFTKEALALAWRYFLPPYTFQIRVGALYLLYGLYNTQLCQPKQKIRVALKDWDEVLKFQQDLVNAQHFDAAYIFRKLRLDRAFHFTAMPKLLSYRMKKKIHRAEVTEEFKDPSDRVMKLITSDVLEEMLNVHDHYQNMKHVISVDKSKPDKALSLIKDDFFDNIKNIVLEHQQWHKDRKNPSLKSKTNDGEEKMEGNSQETERCERAESLAKIKSKAFSVVIQASKSRRHRQVKLDSSDSDSASGQGQVKATRKKEKKERLKPAGRKMSLRNKGNVQNIHKEDKPLSLSMPVITEEEENESLSGTEFTASKKRRKH

>NP_075066.1

MASGLVRLLQQGHRCLLAPVAPKLVPPVRGVKKGFRAAFRFQKELERQRLLRCPPPPVRRSEKPNWDYHAEIQAFGHRLQENFSLDLLKTAFVNSCYIKSEEAKRQQLGIEKEAVLLNLKSNQELSEQGTSFSQTCLTQFLEDEYPDMPTEGIKNLVDFLTGEEVVCHVARNLAVEQLTLSEEFPVPPAVLQQTFFAVIGALLQSSGPERTALFIRDFLITQMTGKELFEMWKIINPMGLLVEELKKRNVSAPESRLTRQSGGTTALPLYFVGLYCDKKLIAEGPGETVLVAEEEAARVALRKLYGFTENRRPWNYSKPKETLRAEKSITAS

128 NP_068809.1 Q8TCS8

>NP_068809.1

MSDNEDNFDGDDFDDVEEDEGLDDLENAEEEGQENVEILPSGERPQANQKRITTPYMTKYERARVLGTRALQIAMCAPVMVELEGETDPLLIAMKELKARKIPIIIRRYLPDGSYEDWGVDELIITD

>Q8TCS8

MAACRYCCSCLRLRPLSDGPFLLPRRDRALTQLQVRALWSSAGSRAVAVDLGNRKLEISSGKLARFADGSAVVQSGDTAVMVTAVSKTKPSPSQFMPLVVDYRQKAAAAGRIPTNYLRREIGTSDKEILTSRIIDRSIRPLFPAGYFYDTQVLCNLLAVDGVNEPDVLAINGASVALSLSDIPWNGPVGAVRIGIIDGEYVVNPTRKEMSSSTLNLVVAGAPKSQIVMLEASAENILQQDFCHAIKVGVKYTQQIIQGIQQLVKETGVTKRTPQKLFTPSPEIVKYTHKLAMERLYAVFTDYEHDKVSRDEAVNKIRLDTEEQLKEKFPEADPYEIIESFNVVAKEVFRSIVLNEYKRCDGRDLTSLRNVSCEVDMFKTLHGSALFQRGQTQVLCTVTFDSLESGIKSDQVITAINGIKDKNFMLHYEFPPYATNEIGKVTGLNRRELGHGALAEKALYPVIPRDFPFTIRVTSEVLESNGSSSMASACGGSLALMDSGVPISSAVAGVAIGLVTKTDPEKGEIEDYRLLTDILGIEDYNGDMDFKIAGTNKGITALQADIKLPGIPIKIVMEAIQQASVAKKEILQIMNKTISKPRASRKENGPVVETVQVPLSKRAKFVGPGGYNLKKLQAETGVTISQVDEETFSVFAPTPSAMHEARDFITEICKDDQEQQLEFGAVYTATITEIRDTGVMVKLYPNMTAVLLHNTQLDQRKIKHPTALGLEVGQEIQVKYFGRDPADGRMRLSRKVLQSPATTVVRTLNDRSSIVMGEPISQSSSNSQ

129 NP_006221.1 NP_060383.2

>NP_006221.1

MFSEQAAQRAHTLLSPPSANNATFARVPVATYTNSSQPFRLGERSFSRQYAHIYATRLIQMRPFLENRAQQHWGSGVGVKKLCELQPEEKCCVVGTLFKAMPLQPSILREVSEEHNLLPQPPRSKYIHPDDELVLEDELQRIKLKGTIDVSKLVTGTVLAVFGSVRDDGKFLVEDYCFADLAPQKPAPPLDTDRFVLLVSGLGLGGGGGESLLGTQLLVDVVTGQLGDEGEQCSAAHVSRVILAGNLLSHSTQSRDSINKAKYLTKKTQAASVEAVKMLDEILLQLSASVPVDVMPGEFDPTNYTLPQQPLHPCMFPLATAYSTLQLVTNPYQATIDGVRFLGTSGQNVSDIFRYSSMEDHLEILEWTLRVRHISPTAPDTLGCYPFYKTDPFIFPECPHVYFCGNTPSFGSKIIRGPEDQTVLLVTVPDFSATQTACLVNLRSLACQPISFSGFGAEDDDLGGLGLGP

>NP_060383.2

MEQPWPPPGPWSLPRAEGEAEEESDFDVFPSSPRCPQLPGGGAQMYSHGIELACQKQKEFVKSSVACKWNLAEAQQKLGSLALHNSESLDQEHAKAQTAVSELRQREEEWRQKEEALVQREKMCLWSTDAISKDVFNKSFINQDKRKDTEDEDKSESFMQKYEQKIRHFGMLSRWDDSQRFLSDHPYLVCEETAKYLILWCFHLEAEKKGALMEQIAHQAVVMQFIMEMAKNCNVDPRGCFRLFFQKAKAEEEGYFEAFKNELEAFKSRVRLYSQSQSFQPMTVQNHVPHSGVGSIGLLESLPQNPDYLQYSISTALCSLNSVVHKEDDEPKMMDTV

130 NP_003850.1 Q16822

>NP_003850.1

MASLEVSRSPRRSRRELEVRSPRQNKYSVLLPTYNERENLPLIVWLLVKSFSESGINYEIIIIDDGSPDGTRDVAEQLEKIYGSDRILLRPREKKLGLGTAYIHGMKHATGNYIIIMDADLSHHPKFIPEFIRKQKEGNFDIVSGTRYKGNGGVYGWDLKRKIISRGANFLTQILLRPGASDLTGSFRLYRKEVLEKLIEKCVSKGYVFQMEMIVRARQLNYTIGEVPISFVDRVYGESKLGGNEIVSFLKGLLTLFATT

>Q16822

MAALYRPGLRLNWHGLSPLGWPSCRSIQTLRVLSGDLGQLPTGIRDFVEHSARLCQPEGIHICDGTEAENTATLTLLEQQGLIRKLPKYNNCWLARTDPKDVARVESKTVIVTPSQRDTVPLPPGGARGQLGNWMSPADFQRAVDERFPGCMQGRTMYVLPFSMGPVGSPLSRIGVQLTDSAYVVASMRIMTRLGTPVLQALGDGDFVKCLHSVGQPLTGQGEPVSQWPCNPEKTLIGHVPDQREIISFGSGYGGNSLLGKKCFALRIASRLARDEGWLAEHMLILGITSPAGKKALCAAAFPSACGKTNLAMMRPALPGWKVECVGDDIAWMRFDSEGRLRAINPENGFFGVAPGTSATTNPNAMATIQSNTIFTNVAETSDGGVYWEGIDQPLPPGVTVTSWLGKPWKPGDKEPCAHPNSRFCAPARQCPIMDPAWEAPEGVPIDAIIFGGRRPKGVPLVYEAFNWRHGVFVGRAMRSESTAAAEHKGKIIMHDPFAMRPFFGYNFGHYLEHWLSMEGRKGAQLPRIFHVNWFRRDEAGHFLWPGFGENARVLDWICRRLEGEDSARETPIGLVPKEGALDLSGLRAIDTTQLFSLPKDFWEQEVRDIRSYLTEQVNQDLPKEVLAELEALERRVHKM

131 Q8N9N5 Q16822

>Q8N9N5

MMSEHDLADVVQIAVEDLSPDHPVVLENHVVTDEDEPALKRQRLEINCQDPSIKTICLRLDSIEAKLQALEATCKSLEEKLDLVTNKQHSPIQVPMVAGSPLGATQTCNKVRCVVPQTTVILNNDRQNAIVAKMEDPLSNRAPDSLENVISNAVPGRRQNTIVVKVPGQEDSHHEDGESGSEASDSVSSCGQAGSQSIGSNVTLITLNSEEDYPNGTWLGDENNPEMRVRCAIIPSDMLHISTNCRTAEKMALTLLDYLFHREVQAVSNLSGQGKHGKKQLDPLTIYGIRCHLFYKFGITESDWYRIKQSIDSKCRTAWRRKQRGQSLAVKSFSRRTPNSSSYCPSESMMSTPPPASELPQPQPQPQALHYALANAQQVQIHQIGEDGQVQVGHLHIAQVPQGEQVQITQDSEGNLQIHHVGQDGQLLEATRIPCLLAPSVFKASSGQVLQGAQLIAVASSDPAAAGVDGSPLQGSDIQVQYVQLAPVSDHTAGAQTAEALQPTLQPEMQLEHGAIQIQ

>Q16822

MAALYRPGLRLNWHGLSPLGWPSCRSIQTLRVLSGDLGQLPTGIRDFVEHSARLCQPEGIHICDGTEAENTATLTLLEQQGLIRKLPKYNNCWLARTDPKDVARVESKTVIVTPSQRDTVPLPPGGARGQLGNWMSPADFQRAVDERFPGCMQGRTMYVLPFSMGPVGSPLSRIGVQLTDSAYVVASMRIMTRLGTPVLQALGDGDFVKCLHSVGQPLTGQGEPVSQWPCNPEKTLIGHVPDQREIISFGSGYGGNSLLGKKCFALRIASRLARDEGWLAEHMLILGITSPAGKKALCAAAFPSACGKTNLAMMRPALPGWKVECVGDDIAWMRFDSEGRLRAINPENGFFGVAPGTSATTNPNAMATIQSNTIFTNVAETSDGGVYWEGIDQPLPPGVTVTSWLGKPWKPGDKEPCAHPNSRFCAPARQCPIMDPAWEAPEGVPIDAIIFGGRRPKGVPLVYEAFNWRHGVFVGRAMRSESTAAAEHKGKIIMHDPFAMRPFFGYNFGHYLEHWLSMEGRKGAQLPRIFHVNWFRRDEAGHFLWPGFGENARVLDWICRRLEGEDSARETPIGLVPKEGALDLSGLRAIDTTQLFSLPKDFWEQEVRDIRSYLTEQVNQDLPKEVLAELEALERRVHKM

132 NP_795361.1 Q93088

>NP_795361.1

MTHSLVCPETVSRVSSVLNRNTRQFGKKHLFDQDEETCWNSDQGPSQWVTLEFPQLIRVSQLQIQFQGGFSSRRGCLEGSQGTQALHKIVDFYPEDNNSLQTFPIPAAEVDRLKVTFEDATDFFGRVVIYHLRVLGEKV

>Q93088

MPPVGGKKAKKGILERLNAGEIVIGDGGFVFALEKRGYVKAGPWTPEAAVEHPEAVRQLHREFLRAGSNVMQTFTFYASEDKLENRGNYVLEKISGQEVNEAACDIARQVADEGDALVAGGVSQTPSYLSCKSETEVKKVFLQQLEVFMKKNVDFLIAEYFEHVEEAVWAVETLIASGKPVAATMCIGPEGDLHGVPPGECAVRLVKAGASIIGVNCHFDPTISLKTVKLMKEGLEAARLKAHLMSQPLAYHTPDCNKQGFIDLPEFPFGLEPRVATRWDIQKYAREAYNLGVRYIGGCCGFEPYHIRAIAEELAPERGFLPPASEKHGSWGSGLDMHTKPWVRARARKEYWENLRIASGRPYNPSMSKPDGWGVTKGTAELMQQKEATTEQQLKELFEKQKFKSQ

133 O95926 O43182

>O95926

MAAIAASEVLVDSAEEGSLAAAAELAAQKREQRLRKFRELHLMRNEARKLNHQEVVEEDKRLKLPANWEAKKARLEWELKEEEKKKECAARGEDYEKVKLLEISAEDAERWERKKKRKNPDLGFSDYAAAQLRQYHRLTKQIKPDMETYERLREKHGEEFFPTSNSLLHGTHVPSTEEIDRMVIDLEKQIEKRDKYSRRRPYNDDADIDYINERNAKFNKKAERFYGKYTAEIKQNLERGTAV

>O43182

MSAQSLLHSVFSCSSPASSSAASAKGFSKRKLRQTRSLDPALIGGCGSDEAGAEGSARGATAGRLYSPSLPAESLGPRLASSSRGPPPRATRLPPPGPLCSSFSTPSTPQEKSPSGSFHFDYEVPLGRGGLKKSMAWDLPSVLAGPASSRSASSILCSSGGGPNGIFASPRRWLQQRKFQSPPDSRGHPYVVWKSEGDFTWNSMSGRSVRLRSVPIQSLSELERARLQEVAFYQLQQDCDLSCQITIPKDGQKRKKSLRKKLDSLGKEKNKDKEFIPQAFGMPLSQVIANDRAYKLKQDLQRDEQKDASDFVASLLPFGNKRQNKELSSSNSSLSSTSETPNESTSPNTPEPAPRARRRGAMSVDSITDLDDNQSRLLEALQLSLPAEAQSKKEKARDKKLSLNPIYRQVPRLVDSCCQHLEKHGLQTVGIFRVGSSKKRVRQLREEFDRGIDVSLEEEHSVHDVAALLKEFLRDMPDPLLTRELYTAFINTLLLEPEEQLGTLQLLIYLLPPCNCDTLHRLLQFLSIVARHADDNISKDGQEVTGNKMTSLNLATIFGPNLLHKQKSSDKEFSVQSSARAEESTAIIAVVQKMIENYEALFMVPPDLQNEVLISLLETDPDVVDYLLRRKASQSSSPDMLQSEVSFSVGGRHSSTDSNKASSGDISPYDNNSPVLSERSLLAMQEDAAPGGSEKLYRVPGQFMLVGHLSSSKSRESSPGPRLGKDLSEEPFDIWGTWHSTLKSGSKDPGMTGSSGDIFESSSLRAGPCSLSQGNLSPNWPRWQGSPAELDSDTQGARRTQAAAPATEGRAHPAVSRACSTPHVQVAGKAERPTARSEQYLTLSGAHDLSESELDVAGLQSRATPQCQRPHGSGRDDKRPPPPYPGPGKPAAAAAWIQGPPEGVETPTDQGGQAAEREQQVTQKKLSSANSLPAGEQDSPRLGDAGWLDWQRERWQIWELLSTDNPDALPETLV

134 NP_000225.1 NP_077268.1

>NP_000225.1

MQRSIMSFFHPKKEGKAKKPEKEASNSSRETEPPPKAALKEWNGVVSESDSPVKRPGRKAARVLGSEGEEEDEALSPAKGQKPALDCSQVSPPRPATSPENNASLSDTSPMDSSPSGIPKRRTARKQLPKRTIQEVLEEQSEDEDREAKRKKEEEEEETPKESLTEAEVATEKEGEDGDQPTTPPKPLKTSKAETPTESVSEPEVATKQELQEEEEQTKPPRRAPKTLSSFFTPRKPAVKKEVKEEEPGAPGKEGAAEGPLDPSGYNPAKNNYHPVEDACWKPGQKVPYLAVARTFEKIEEVSARLRMVETLSNLLRSVVALSPPDLLPVLYLSLNHLGPPQQGLELGVGDGVLLKAVAQATGRQLESVRAEAAEKGDVGLVAENSRSTQRLMLPPPPLTASGVFSKFRDIARLTGSASTAKKIDIIKGLFVACRHSEARFIARSLSGRLRLGLAEQSVLAALSQAVSLTPPGQEFPPAMVDAGKGKTAEARKTWLEEQGMILKQTFCEVPDLDRIIPVLLEHGLERLPEHCKLSPGIPLKPMLAHPTRGISEVLKRFEEAAFTCEYKYDGQRAQIHALEGGEVKIFSRNQEDNTGKYPDIISRIPKIKLPSVTSFILDTEAVAWDREKKQIQPFQVLTTRKRKEVDASEIQVQVCLYAFDLIYLNGESLVREPLSRRRQLLRENFVETEGEFVFATSLDTKDIEQIAEFLEQSVKDSCEGLMVKTLDVDATYEIAKRSHNWLKLKKDYLDGVGDTLDLVVIGAYLGRGKRAGRYGGFLLASYDEDSEELQAICKLGTGFSDEELEEHHQSLKALVLPSPRPYVRIDGAVIPDHWLDPSAVWEVKCADLSLSPIYPAARGLVDSDKGISLRFPRFIRVREDKQPEQATTSAQVACLYRKQSQIQNQQGEDSGSDPEDTY

>NP_077268.1

MIEVVCNDRLGKKVRVKCNTDDTIGDLKKLIAAQTGTRWNKIVLKKWYTIFKDHVSLGDYEIHDGMNLELYYQ

135 NP_036377.1 NP_004799.1

>NP_036377.1

MALTSFLPAPTQLSQDQLEAEEKARSQRSRQTSLVSSRREPPPYGYRKGWIPRLLEDFGDGGAFPEIHVAQYPLDMGRKKKMSNALAIQVDSEGKIKYDAIARQGQSKDKVIYSKYTDLVPKEVMNADDPDLQRPDEEAIKEITEKTRVALEKSVSQKVAAAMPVRAADKLAPAQYIRYTPSQQGVAFNSGAKQRVIRMVEMQKDPMEPPRFKINKKIPRGPPSPPAPVMHSPSRKMTVKEQQEWKIPPCISNWKNAKGYTIPLDKRLAADGRGLQTVHINENFAKLAEALYIADRKAREAVEMRAQVERKMAQKEKEKHEEKLREMAQKARERRAGIKTHVEKEDGEARERDEIRHDRRKERQHDRNLSRAAPDKRSKLQRNENRDISEVIALGVPNPRTSNEVQYDQRLFNQSKGMDSGFAGGEDEIYNVYDQAWRGGKDMAQSIYRPSKNLDKDMYGDDLEARIKTNRFVPDKEFSGSDRRQRGREGPVQFEEDPFGLDKFLEEAKQHGGSKRPSDSSRPKEHEHEGKKRRKE

>NP_004799.1

MAEDSESAASQQSLELDDQDTCGIDGDNEEETEHAKGSPGGYLGAKKKKKKQKRKKEKPNSGGTKSDSASDSQEIKIQQPSKNPSVPMQKLQDIQRAMELLSACQGPARNIDEAAKHRYQFWDTQPVPKLDEVITSHGAIEPDKDNVRQEPYSLPQGFMWDTLDLSDAEVLKELYTLLNENYVEDDDNMFRFDYSPEFLLWALRPPGWLLQWHCGVRVSSNKKLVGFISAIPANIRIYDSVKKMVEINFLCVHKKLRSKRVAPVLIREITRRVNLEGIFQAVYTAGVVLPKPIATCRYWHRSLNPRKLVEVKFSHLSRNMTLQRTMKLYRLPDVTKTSGLRPMEPKDIKSVRELINTYLKQFHLAPVMDEEEVAHWFLPREHIIDTFVVESPNGKLTDFLSFYTLPSTVMHHPAHKSLKAAYSFYNIHTETPLLDLMSDALILAKSKGFDVFNALDLMENKTFLEKLKFGIGDGNLQYYLYNWRCPGTDSEKVGLVLQ

136 NP_005641.1 NP_002878.2

>NP_005641.1

MQSFREQSSYHGNQQSYPQEVHGSSRLEEFSPRQAQMFQNFGGTGGSSGSSGSGSGGGRRGAAAAAAAMASETSGHQGYQGFRKEAGDFYYMAGNKDPVTTGTPQPPQRRPSGPVQSYGPPQGSSFGNQYGSEGHVGQFQAQHSGLGGVSHYQQDYTGPFSPGSAQYQQQASSQQQQQQVQQLRQQLYQSHQPLPQATGQPASSSSHLQPMQRPSTLPSSAAGYQLRVGQFGQHYQSSASSSSSSSFPSPQRFSQSGQSYDGSYNVNAGSQYEGHNVGSNAQAYGTQSNYSYQPQSMKNFEQAKIPQGTQQGQQQQQPQQQQHPSQHVMQYTNAATKLPLQSQVGQYNQPEVPVRSPMQFHQNFSPISNPSPAASVVQSPSCSSTPSPLMQTGENLQCGQGSVPMGSRNRILQLMPQLSPTPSMMPSPNSHAAGFKGFGLEGVPEKRLTDPGLSSLSALSTQVANLPNTVQHMLLSDALTPQKKTSKRPSSSKKADSCTNSEGSSQPEEQLKSPMAESLDGGCSSSSEDQGERVRQLSGQSTSSDTTYKGGASEKAGSSPAQGAQNEPPRLNASPAAREEATSPGAKDMPLSSDGNPKVNEKTVGVIVSREAMTGRVEKPGGQDKGSQEDDPAATQRPPSNGGAKETSHASLPQPEPPGGGGSKGNKNGDNNSNHNGEGNGQSGHSAAGPGFTSRTEPSKSPGSLRYSYKDSFGSAVPRNVSGFPQYPTGQEKGDFTGHGERKGRNEKFPSLLQEVLQGYHHHPDRRYSRSTQEHQGMAGSLEGTTRPNVLVSQTNELASRGLLNKSIGSLLENPHWGPWERKSSSTAPEMKQINLTDYPIPRKFEIEPQSSAHEPGGSLSERRSVICDISPLRQIVRDPGAHSLGHMSADTRIGRNDRLNPTLSQSVILPGGLVSMETKLKSQSGQIKEEDFEQSKSQASFNNKKSGDHCHPPSIKHESYRGNASPGAATHDSLSDYGPQDSRPTPMRRVPGRVGGREGMRGRSPSQYHDFAEKLKMSPGRSRGPGGDPHHMNPHMTFSERANRSSLHTPFSPNSETLASAYHANTRAHAYGDPNAGLNSQLHYKRQMYQQQPEEYKDWSSGSAQGVIAAAQHRQEGPRKSPRQQQFLDRVRSPLKNDKDGMMYGPPVGTYHDPSAQEAGRCLMSSDGLPNKGMELKHGSQKLQESCWDLSRQTSPAKSSGPPGMSSQKRYGPPHETDGHGLAEATQSSKPGSVMLRLPGQEDHSSQNPLIMRRRVRSFISPIPSKRQSQDVKNSSTEDKGRLLHSSKEGADKAFNSYAHLSHSQDIKSIPKRDSSKDLPSPDSRNCPAVTLTSPAKTKILPPRKGRGLKLEAIVQKITSPNIRRSASSNSAEAGGDTVTLDDILSLKSGPPEGGSVAVQDADIEKRKGEVASDLVSPANQELHVEKPLPRSSEEWRGSVDDKVKTETHAETVTAGKEPPGAMTSTTSQKPGSNQGRPDGSLGGTAPLIFPDSKNVPPVGILAPEANPKAEEKENDTVTISPKQEGFPPKGYFPSGKKKGRPIGSVNKQKKQQQPPPPPPQPPQIPEGSADGEPKPKKQRQRRERRKPGAQPRKRKTKQAVPIVEPQEPEIKLKYATQPLDKTDAKNKSFYPYIHVVNKCELGAVCTIINAEEEEQTKLVRGRKGQRSLTPPPSSTESKALPASSFMLQGPVVTESSVMGHLVCCLCGKWASYRNMGDLFGPFYPQDYAATLPKNPPPKRATEMQSKVKVRHKSASNGSKTDTEEEEEQQQQQKEQRSLAAHPRFKRRHRSEDCGGGPRSLSRGLPCKKAATEGSSEKTVLDSKPSVPTTSEGGPELELQIPELPLDSNEFWVHEGCILWANGIYLVCGRLYGLQEALEIAREMKCSHCQEAGATLGCYNKGCSFRYHYPCAIDADCLLHEENFSVRCPKHKPPLPCPLPPLQNKTAKGSLSTEQSERG

>NP_002878.2

MDVLVSECSARLLQQEEEIKSLTAEIDRLKNCGCLGASPNLEQLQEENLKLKYRLNILRKSLQAERNKPTKNMINIISRLQEVFGHAIKAAYPDLENPPLLVTPSQQAKFGDYQCNSAMGISQMLKTKEQKVNPREIAENITKHLPDNECIEKVEIAGPGFINVHLRKDFVSEQLTSLLVNGVQLPALGENKKVIVDFSSPNIAKEMHVGHLRSTIIGESISRLFEFAGYDVLRLNHVGDWGTQFGMLIAHLQDKFPDYLTVSPPIGDLQVFYKESKKRFDTEEEFKKRAYQCVVLLQGKNPDITKAWKLICDVSRQELNKIYDALDVSLIERGESFYQDRMNDIVKEFEDRGFVQVDDGRKIVFVPGCSIPLTIVKSDGGYTYDTSDLAAIKQRLFEEKADMIIYVVDNGQSVHFQTIFAAAQMIGWYDPKVTRVFHAGFGVVLGEDKKKFKTRSGETVRLMDLLGEGLKRSMDKLKEKERDKVLTAEELNAAQTSVAYGCIKYADLSHNRLNDYIFSFDKMLDDRGNTAAYLLYAFTRIRSIARLANIDEEMLQKAARETKILLDHEKEWKLGRCILRFPEILQKILDDLFLHTLCDYIYELATAFTEFYDSCYCVEKDRQTGKILKVNMWRMLLCEAVAAVMAKGFDILGIKPVQRM

137 NP_005422.1 Q9Y2J8

>NP_005422.1

MCSAFHRAESGTELLARLEGRSSLKEIEPNLFADEDSPVHGDILEFHGPEGTGKTEMLYHLTARCILPKSEGGLEVEVLFIDTDYHFDMLRLVTILEHRLSQSSEEIIKYCLGRFFLVYCSSSTHLLLTLYSLESMFCSHPSLCLLILDSLSAFYWIDRVNGGESVNLQESTLRKCSQCLEKLVNDYRLVLFATTQTIMQKASSSSEEPSHASRRLCDVDIDYRPYLCKAWQQLVKHRMFFSKQDDSQSSNQFSLVSRCLKSNSLKKHFFIIGESGVEFC

>Q9Y2J8

MLRERTVRLQYGSRVEAVYVLGTYLWTDVYSAAPAGAQTFSLKHSEHVWVEVVRDGEAEEVATNGKQRWLLSPSTTLRVTMSQASTEASSDKVTVNYYDEEGSIPIDQAGLFLTAIEISLDVDADRDGVVEKNNPKKASWTWGPEGQGAILLVNCDRETPWLPKEDCRDEKVYSKEDLKDMSQMILRTKGPDRLPAGYEIVLYISMSDSDKVGVFYVENPFFGQRYIHILGRRKLYHVVKYTGGSAELLFFVEGLCFPDEGFSGLVSIHVSLLEYMAQDIPLTPIFTDTVIFRIAPWIMTPNILPPVSVFVCCMKDNYLFLKEVKNLVEKTNCELKVCFQYLNRGDRWIQDEIEFGYIEAPHKGFPVVLDSPRDGNLKDFPVKELLGPDFGYVTREPLFESVTSLDSFGNLEVSPPVTVNGKTYPLGRILIGSSFPLSGGRRMTKVVRDFLKAQQVQAPVELYSDWLTVGHVDEFMSFVPIPGTKKFLLLMASTSACYKLFREKQKDGHGEAIMFKGLGGMSSKRITINKILSNESLVQENLYFQRCLDWNRDILKKELGLTEQDIIDLPALFKMDEDHRARAFFPNMVNMIVLDKDLGIPKPFGPQVEEECCLEMHVRGLLEPLGLECTFIDDISAYHKFLGEVHCGTNVRRKPFTFKWWHMVP

138 Q96BP2 O75570

>Q96BP2

MATPSLRGRLARFGNPRKPVLKPNKPLILANRVGERRREKGEATCITEMSVMMACWKQNEFRDDACRKEIQGFLDCAARAQEARKMRSIQETLGESGSLLPNKLNKLLQRFPNKPYLS

>O75570

MNRHLCVWLFRHPSLNGYLQCHIQLHSHQFRQIHLDTRLQVFRQNRNCILHLLSKNWSRRYCHQDTKMLWKHKALQKYMENLSKEYQTLEQCLQHIPVNEENRRSLNRRHAELAPLAAIYQEIQETEQAIEELESMCKSLNKQDEKQLQELALEERQTIDQKINMLYNELFQSLVPKEKYDKNDVILEVTAGRTTGGDICQQFTREIFDMYQNYSCYKHWQFELLNYTPADYGGLHHAAARISGDGVYKHLKYEGGIHRVQRIPEVGLSSRMQRIHTGTMSVIVLPQPDEVDVKLDPKDLRIDTFRAKGAGGQHVNKTDSAVRLVHIPTGLVVECQQERSQIKNKEIAFRVLRARLYQQIIEKDKRQQQSARKLQVGTRAQSERIRTYNFTQDRVSDHRIAYEVRDIKEFLCGGKGLDQLIQRLLQSADEEAIAELLDEHLKSAK

139 NP_073598.1 NP_996895.1

>NP_073598.1

MAASQCLCCSKFLFQRQNLACFLTNPHCGSLVNADGHGEVWTDWNNMSKFFQYGWRCTTNENTYSNRTLMGNWNQERYDLRNIVQPKPLPSQFGHYFETTYDTSYNNKMPLSTHRFKREPHWFPGHQPELDPPRYKCTEKSTYMNSYSKP

>NP_996895.1

MIHSLFLINCSGDIFLEKHWKSVVSQSVCDYFFEAQEKAADVENVPPVISTPHHYLISIYRDKLFFVSVIQTEVPPLFVIEFLHRVADTFQDYFGECSEAAIKDNVVIVYELLEEMLDNGFPLATESNILKELIKPPTILRSVVNSITGSSNVGDTLPTGQLSNIPWRRAGVKYTNNEAYFDVVEEIDAIIDKSGSTVFAEIQGVIDACIKLSGMPDLSLSFMNPRLLDDVSFHPCIRFKRWESERVLSFIPPDGNFRLISYRVSSQNLVAIPVYVKHSISFKENSSCGRFDITIGPKQNMGKTIEGITVTVHMPKVVLNMNLTPTQGSYTFDPVTKVLTWDVGKITPQKLPSLKGLVNLQSGAPKPEENPSLNIQFKIQQLAISGLKVNRLDMYGEKYKPFKGVKYVTKAGKFQVRT

140 NP_066951.1 NP_110379.2

>NP_066951.1

MIIPVRCFTCGKIVGNKWEAYLGLLQAEYTEGDALDALGLKRYCCRRMLLAHVDLIEKLLNYAPLEK

>NP_110379.2

MEGPLSVFGDRSTGETIRSQNVMAAASIANIVKSSLGPVGLDKMLVDDIGDVTITNDGATILKLLEVEHPAAKVLCELADLQDKEVGDGTTSVVIIAAELLKNADELVKQKIHPTSVISGYRLACKEAVRYINENLIVNTDELGRDCLINAAKTSMSSKIIGINGDFFANMVVDAVLAIKYTDIRGQPRYPVNSVNILKAHGRSQMESMLISGYALNCVVGSQGMPKRIVNAKIACLDFSLQKTKMKLGVQVVITDPEKLDQIRQRESDITKERIQKILATGANVILTTGGIDDMCLKYFVEAGAMAVRRVLKRDLKRIAKASGATILSTLANLEGEETFEAAMLGQAEEVVQERICDDELILIKNTKARTSASIILRGANDFMCDEMERSLHDALCVVKRVLESKSVVPGGGAVEAALSIYLENYATSMGSREQLAIAEFARSLLVIPNTLAVNAAQDSTDLVAKLRAFHNEAQVNPERKNLKWIGLDLSNGKPRDNKQAGVFEPTIVKVKSLKFATEAAITILRIDDLIKLHPESKDDKHGSYEDAVHSGALND

141 NP_068809.1 NP_002684.1

>NP_068809.1

MSDNEDNFDGDDFDDVEEDEGLDDLENAEEEGQENVEILPSGERPQANQKRITTPYMTKYERARVLGTRALQIAMCAPVMVELEGETDPLLIAMKELKARKIPIIIRRYLPDGSYEDWGVDELIITD

>NP_002684.1

MSRLLWRKVAGATVGPGPVPAPGRWVSSSVPASDPSDGQRRRQQQQQQQQQQQQQPQQPQVLSSEGGQLRHNPLDIQMLSRGLHEQIFGQGGEMPGEAAVRRSVEHLQKHGLWGQPAVPLPDVELRLPPLYGDNLDQHFRLLAQKQSLPYLEAANLLLQAQLPPKPPAWAWAEGWTRYGPEGEAVPVAIPEERALVFDVEVCLAEGTCPTLAVAISPSAWYSWCSQRLVEERYSWTSQLSPADLIPLEVPTGASSPTQRDWQEQLVVGHNVSFDRAHIREQYLIQGSRMRFLDTMSMHMAISGLSSFQRSLWIAAKQGKHKVQPPTKQGQKSQRKARRGPAISSWDWLDISSVNSLAEVHRLYVGGPPLEKEPRELFVKGTMKDIRENFQDLMQYCAQDVWATHEVFQQQLPLFLERCPHPVTLAGMLEMGVSYLPVNQNWERYLAEAQGTYEELQREMKKSLMDLANDACQLLSGERYKEDPWLWDLEWDLQEFKQKKAKKVKKEPATASKLPIEGAGAPGDPMDQEDLGPCSEEEEFQQDVMARACLQKLKGTTELLPKRPQHLPGHPGWYRKLCPRLDDPAWTPGPSLLSLQMRVTPKLMALTWDGFPLHYSERHGWGYLVPGRRDNLAKLPTGTTLESAGVVCPYRAIESLYRKHCLEQGKQQLMPQEAGLAEEFLLTDNSAIWQTVEELDYLEVEAEAKMENLRAAVPGQPLALTARGGPKDTQPSYHHGNGPYNDVDIPGCWFFKLPHKDGNSCNVGSPFAKDFLPKMEDGTLQAGPGGASGPRALEINKMISFWRNAHKRISSQMVVWLPRSALPRAVIRHPDYDEEGLYGAILPQVVTAGTITRRAVEPTWLTASNARPDRVGSELKAMVQAPPGYTLVGADVDSQELWIAAVLGDAHFAGMHGCTAFGWMTLQGRKSRGTDLHSKTATTVGISREHAKIFNYGRIYGAGQPFAERLLMQFNHRLTQQEAAEKAQQMYAATKGLRWYRLSDEGEWLVRELNLPVDRTEGGWISLQDLRKVQRETARKSQWKKWEVVAERAWKGGTESEMFNKLESIATSDIPRTPVLGCCISRALEPSAVQEEFMTSRVNWVVQSSAVDYLHLMLVAMKWLFEEFAIDGRFCISIHDEVRYLVREEDRYRAALALQITNLLTRCMFAYKLGLNDLPQSVAFFSAVDIDRCLRKEVTMDCKTPSNPTGMERRYGIPQGEALDIYQIIELTKGSLEKRSQPGP

142 NP_054753.1 NP_001007554.1

>NP_054753.1

MAKVQVNNVVVLDNPSPFYNPFQFEITFECIEDLSEDLEWKIIYVGSAESEEYDQVLDSVLVGPVPAGRHMFVFQADAPNPGLIPDADAVGVTVVLITCTYRGQEFIRVGYYVNNEYTETELRENPPVKPDFSKLQRNILASNPRVTRFHINWEDNTEKLEDAESSNPNLQSLLSTDALPSASKGWSTSENSLNVMLESHMDCM

>NP_001007554.1

MSFDPNLLHNNGHNGYPNGTSAALRETGVIEKLLTSYGFIQCSERQARLFFHCSQYNGNLQDLKVGDDVEFEVSSDRRTGKPIAVKLVKIKQEILPEERMNGQVVCAVPHNLESKSPAAPGQSPTGSVCYERNGEVFYLTYTPEDVEGNVQLETGDKINFVIDNNKHTGAVSARNIMLLKKKQARCQGVVCAMKEAFGFIERGDVVKEIFFHYSEFKGDLETLQPGDDVEFTIKDRNGKEVATDVRLLPQGTVIFEDISIEHFEGTVTKVIPKVPSKNQNDPLPGRIKVDFVIPKELPFGDKDTKSKVTLLEGDHVRFNISTDRRDKLERATNIEVLSNTFQFTNEAREMGVIAAMRDGFGFIKCVDRDVRMFFHFSEILDGNQLHIADEVEFTVVPDMLSAQRNHAIRIKKLPKGTVSFHSHSDHRFLGTVEKEATFSNPKTTSPNKGKEKEAEDGIIAYDDCGVKLTIAFQAKDVEGSTSPQIGDKVEFSISDKQRPGQQVATCVRLLGRNSNSKRLLGYVATLKDNFGFIETANHDKEIFFHYSEFSGDVDSLELGDMVEYSLSKGKGNKVSAEKVNKTHSVNGITEEADPTIYSGKVIRPLRSVDPTQTEYQGMIEIVEEGDMKGEVYPFGIVGMANKGDCLQKGESVKFQLCVLGQNAQTMAYNITPLRRATVECVKDQFGFINYEVGDSKKLFFHVKEVQDGIELQAGDEVEFSVILNQRTGKCSACNVWRVCEGPKAVAAPRPDRLVNRLKNITLDDASAPRLMVLRQPRGPDNSMGFGAERKIRQAGVID

143 Q9UDW3 NP_004530.1

>Q9UDW3

MGKRYFCDYCDRSFQDNLHNRKKHLNGLQHLKAKKVWYDMFRDAAAILLDEQNKRPCRKFLLTGQCDFGSNCRFSHMSERDLQELSIQVEEERRAREWLLDAPELPEGHLEDWLEKRAKRLSSAPSSRAEPIRTTVFQYPVGWPPVQELPPSLRAPPPGGWPLQPRVQWG

>NP_004530.1

MVLAELYVSDREGSDATGDGTKEKPFKTGLKALMTVGKEPFPTIYVDSQKENERWNVISKSQLKNIKKMWHREQMKSESREKKEAEDSLRREKNLEEAKKITIKNDPSLPEPKCVKIGALEGYRGQRVKVFGWVHRLRRQGKNLMFLVLRDGTGYLQCVLADELCQCYNGVLLSTESSVAVYGMLNLTPKGKQAPGGHELSCDFWELIGLAPAGGADNLINEESDVDVQLNNRHMMIRGENMSKILKARSMVTRCFRDHFFDRGYYEVTPPTLVQTQVEGGATLFKLDYFGEEAFLTQSSQLYLETCLPALGDVFCIAQSYRAEQSRTRRHLAEYTHVEAECPFLTFDDLLNRLEDLVCDVVDRILKSPAGSIVHELNPNFQPPKRPFKRMNYSDAIVWLKEHDVKKEDGTFYEFGEDIPEAPERLMTDTINEPILLCRFPVEIKSFYMQRCPEDSRLTESVDVLMPNVGEIVGGSMRIFDSEEILAGYKREGIDPTPYYWYTDQRKYGTCPHGGYGLGLERFLTWILNRYHIRDVCLYPRFVQRCTP

144 NP_004119.1 Q2VPK5

>NP_004119.1

MAERGELDLTGAKQNTGVWLVKVPKYLSQQWAKASGRGEVGKLRIAKTQGRTEVSFTLNEDLANIHDIGGKPASVSAPREHPFVLQSVGGQTLTVFTESSSDKLSLEGIVVQRAECRPAASENYMRLKRLQIEESSKPVRLSQQLDKVVTTNYKPVANHQYNIEYERKKKEDGKRARADKQHVLDMLFSAFEKHQYYNLKDLVDITKQPVVYLKEILKEIGVQNVKGIHKNTWELKPEYRHYQGEEKSD

>Q2VPK5

MCQVGEDYGEPAPEEPPPAPRPSREQKCVKCKEAQPVVVIRAGDAFCRDCFKAFYVHKFRAMLGKNRLIFPGEKVLLAWSGGPSSSSMVWQVLEGLSQDSAKRLRFVAGVIFVDEGAACGQSLEERSKTLAEVKPILQATGFPWHVVALEEVFSLPPSVLWCSAQELVGSEGAYKAAVDSFLQQQHVLGAGGGPGPTQGEEQPPQPPLDPQNLARPPAPAQTEALSQLFCSVRTLTAKEELLQTLRTHLILHMARAHGYSKVMTGDSCTRLAIKLMTNLALGRGAFLAWDTGFSDERHGDVVVVRPMRDHTLKEVAFYNRLFSVPSVFTPAVDTKAPEKASIHRLMEAFILRLQTQFPSTVSTVYRTSEKLVKGPRDGPAAGDSGPRCLLCMCALDVDAADSATAFGAQTSSRLSQMQSPIPLTETRTPPGPCCSPGVGWAQRCGQGACRREDPQACIEEQLCYSCRVNMKDLPSLDPLPPYILAEAQLRTQRAWGLQEIRDCLIEDSDDEAGQS

145 Q9Y618 Q9Y2J8

>Q9Y618

MSGSTQPVAQTWRATEPRYPPHSLSYPVQIARTHTDVGLLEYQHHSRDYASHLSPGSIIQPQRRRPSLLSEFQPGNERSQELHLRPESHSYLPELGKSEMEFIESKRPRLELLPDPLLRPSPLLATGQPAGSEDLTKDRSLTGKLEPVSPPSPPHTDPELELVPPRLSKEELIQNMDRVDREITMVEQQISKLKKKQQQLEEEAAKPPEPEKPVSPPPIESKHRSLVQIIYDENRKKAEAAHRILEGLGPQVELPLYNQPSDTRQYHENIKINQAMRKKLILYFKRRNHARKQWEQKFCQRYDQLMEAWEKKVERIENNPRRRAKESKVREYYEKQFPEIRKQRELQERMQSRVGQRGSGLSMSAARSEHEVSEIIDGLSEQENLEKQMRQLAVIPPMLYDADQQRIKFINMNGLMADPMKVYKDRQVMNMWSEQEKETFREKFMQHPKNFGLIASFLERKTVAECVLYYYLTKKNENYKSLVRRSYRRRGKSQQQQQQQQQQQQQQQQQPMPRSSQEEKDEKEKEKEAEKEEEKPEVENDKEDLLKEKTDDTSGEDNDEKEAVASKGRKTANSQGRRKGRITRSMANEANSEEAITPQQSAELASMELNESSRWTEEEMETAKKGLLEHGRNWSAIARMVGSKTVSQCKNFYFNYKKRQNLDEILQQHKLKMEKERNARRKKKKAPAAASEEAAFPPVVEDEEMEASGVSGNEEEMVEEAEALHASGNEVPRGECSGPATVNNSSDTESIPSPHTEAAKDTGQNGPKPPATLGADGPPPGPPTPPPEDIPAPTEPTPASEATGAPTPPPAPPSPSAPPPVVPKEEKEEETAAAPPVEEGEEQKPPAAEELAVDTGKAEEPVKSECTEEAEEGPAKGKDAEAAEATAEGALKAEKKEGGSGRATTAKSSGAPQDSDSSATCSADEVDEAEGGDKNRLLSPRPSLLTPTGDPRANASPQKPLDLKQLKQRAAAIPPIQVTKVHEPPREDAAPTKPAPPAPPPPQNLQPESDAPQQPGSSPRGKSRSPAPPADKEAEKPVFFPAFAAEAQKLPGDPPCWTSGLPFPVPPREVIKASPHAPDPSAFSYAPPGHPLPLGLHDTARPVLPRPPTISNPPPLISSAKHPSVLERQIGAISQGMSVQLHVPYSEHAKAPVGPVTMGLPLPMDPKKLAPFSGVKQEQLSPRGQAGPPESLGVPTAQEASVLRGTALGSVPGGSITKGIPSTRVPSDSAITYRGSITHGTPADVLYKGTITRIIGEDSPSRLDRGREDSLPKGHVIYEGKKGHVLSYEGGMSVTQCSKEDGRSSSGPPHETAAPKRTYDMMEGRVGRAISSASIEGLMGRAIPPERHSPHHLKEQHHIRGSITQGIPRSYVEAQEDYLRREAKLLKREGTPPPPPPSRDLTEAYKTQALGPLKLKPAHEGLVATVKEAGRSIHEIPREELRHTPELPLAPRPLKEGSITQGTPLKYDTGASTTGSKKHDVRSLIGSPGRTFPPVHPLDVMADARALERACYEESLKSRPGTASSSGGSIARGAPVIVPELGKPRQSPLTYEDHGAPFAGHLPRGSPVTTREPTPRLQEGSLSSSKASQDRKLTSTPREIAKSPHSTVPEHHPHPISPYEHLLRGVSGVDLYRSHIPLAFDPTSIPRGIPLDAAAAYYLPRHLAPNPTYPHLYPPYLIRGYPDTAALENRQTIINDYITSQQMHHNAATAMAQRADMLRGLSPRESSLALNYAAGPRGIIDLSQVPHLPVLVPPTPGTPATAMDRLAYLPTAPQPFSSRHSSSPLSPGGPTHLTKPTTTSSSERERDRDRERDRDREREKSILTSTTTVEHAPIWRPGTEQSSGSSGSSGGGGGSSSRPASHSHAHQHSPISPRTQDALQQRPSVLHNTGMKGIITAVEPSTPTVLRSTSTSSPVRPAATFPPATHCPLGGTLDGVYPTLMEPVLLPKEAPRVARPERPRADTGHAFLAKPPARSGLEPASSPSKGSEPRPLVPPVSGHATIARTPAKNLAPHHASPDPPAPPASASDPHREKTQSKPFSIQELELRSLGYHGSSYSPEGVEPVSPVSSPSLTHDKGLPKHLEELDKSHLEGELRPKQPGPVKLGGEAAHLPHLRPLPESQPSSSPLLQTAPGVKGHQRVVTLAQHISEVITQDYTRHHPQQLSAPLPAPLYSFPGASCPVLDLRRPPSDLYLPPPDHGAPARGSPHSEGGKRSPEPNKTSVLGGGEDGIEPVSPPEGMTEPGHSRSAVYPLLYRDGEQTEPSRMGSKSPGNTSQPPAFFSKLTESNSAMVKSKKQEINKKLNTHNRNEPEYNISQPGTEIFNMPAITGTGLMTYRSQAVQEHASTNMGLEAIIRKALMGKYDQWEESPPLSANAFNPLNASASLPAAMPITAADGRSDHTLTSPGGGGKAKVSGRPSSRKAKSPAPGLASGDRPPSVSSVHSEGDCNRRTPLTNRVWEDRPSSAGSTPFPYNPLIMRLQAGVMASPPPPGLPAGSGPLAGPHHAWDEEPKPLLCSQYETLSDSE

>Q9Y2J8

MLRERTVRLQYGSRVEAVYVLGTYLWTDVYSAAPAGAQTFSLKHSEHVWVEVVRDGEAEEVATNGKQRWLLSPSTTLRVTMSQASTEASSDKVTVNYYDEEGSIPIDQAGLFLTAIEISLDVDADRDGVVEKNNPKKASWTWGPEGQGAILLVNCDRETPWLPKEDCRDEKVYSKEDLKDMSQMILRTKGPDRLPAGYEIVLYISMSDSDKVGVFYVENPFFGQRYIHILGRRKLYHVVKYTGGSAELLFFVEGLCFPDEGFSGLVSIHVSLLEYMAQDIPLTPIFTDTVIFRIAPWIMTPNILPPVSVFVCCMKDNYLFLKEVKNLVEKTNCELKVCFQYLNRGDRWIQDEIEFGYIEAPHKGFPVVLDSPRDGNLKDFPVKELLGPDFGYVTREPLFESVTSLDSFGNLEVSPPVTVNGKTYPLGRILIGSSFPLSGGRRMTKVVRDFLKAQQVQAPVELYSDWLTVGHVDEFMSFVPIPGTKKFLLLMASTSACYKLFREKQKDGHGEAIMFKGLGGMSSKRITINKILSNESLVQENLYFQRCLDWNRDILKKELGLTEQDIIDLPALFKMDEDHRARAFFPNMVNMIVLDKDLGIPKPFGPQVEEECCLEMHVRGLLEPLGLECTFIDDISAYHKFLGEVHCGTNVRRKPFTFKWWHMVP

146 NP_006006.3 NP_550438.1

>NP_006006.3

MAAQVAPAAASSLGNPPPPPPSELKKAEQQQREEAGGEAAAAAAAERGEMKAAAGQESEGPAVGPPQPLGKELQDGAESNGGGGGGGAGSGGGPGAEPDLKNSNGNAGPRPALNNNLTEPPGGGGGGSSDGVGAPPHSAAAALPPPAYGFGQPYGRSPSAVAAAAAAVFHQQHGGQQSPGLAALQSGGGGGLEPYAGPQQNSHDHGFPNHQYNSYYPNRSAYPPPAPAYALSSPRGGTPGSGAAAAAGSKPPPSSSASASSSSSSFAQQRFGAMGGGGPSAAGGGTPQPTATPTLNQLLTSPSSARGYQGYPGGDYSGGPQDGGAGKGPADMASQCWGAAAAAAAAAAASGGAQQRSHHAPMSPGSSGGGGQPLARTPQPSSPMDQMGKMRPQPYGGTNPYSQQQGPPSGPQQGHGYPGQPYGSQTPQRYPMTMQGRAQSAMGGLSYTQQIPPYGQQGPSGYGQQGQTPYYNQQSPHPQQQQPPYSQQPPSQTPHAQPSYQQQPQSQPPQLQSSQPPYSQQPSQPPHQQSPAPYPSQQSTTQQHPQSQPPYSQPQAQSPYQQQQPQQPAPSTLSQQAAYPQPQSQQSQQTAYSQQRFPPPQELSQDSFGSQASSAPSMTSSKGGQEDMNLSLQSRPSSLPDLSGSIDDLPMGTEGALSPGVSTSGISSSQGEQSNPAQSPFSPHTSPHLPGIRGPSPSPVGSPASVAQSRSGPLSPAAVPGNQMPPRPPSGQSDSIMHPSMNQSSIAQDRGYMQRNPQMPQYSSPQPGSALSPRQPSGGQIHTGMGSYQQNSMGSYGPQGGQYGPQGGYPRQPNYNALPNANYPSAGMAGGINPMGAGGQMHGQPGIPPYGTLPPGRMSHASMGNRPYGPNMANMPPQVGSGMCPPPGGMNRKTQETAVAMHVAANSIQNRPPGYPNMNQGGMMGTGPPYGQGINSMAGMINPQGPPYSMGGTMANNSAGMAASPEMMGLGDVKLTPATKMNNKADGTPKTESKSKKSSSSTTTNEKITKLYELGGEPERKMWVDRYLAFTEEKAMGMTNLPAVGRKPLDLYRLYVSVKEIGGLTQVNKNKKWRELATNLNVGTSSSAASSLKKQYIQCLYAFECKIERGEDPPPDIFAAADSKKSQPKIQPPSPAGSGSMQGPQTPQSTSSSMAEGGDLKPPTPASTPHSQIPPLPGMSRSNSVGIQDAFNDGSDSTFQKRNSMTPNPGYQPSMNTSDMMGRMSYEPNKDPYGSMRKAPGSDPFMSSGQGPNGGMGDPYSRAAGPGLGNVAMGPRQHYPYGGPYDRVRTEPGIGPEGNMSTGAPQPNLMPSNPDSGMYSPSRYPPQQQQQQQQRHDSYGNQFSTQGTPSGSPFPSQQTTMYQQQQQNYKRPMDGTYGPPAKRHEGEMYSVPYSTGQGQPQQQQLPPAQPQPASQQQAAQPSPQQDVYNQYGNAYPATATAATERRPAGGPQNQFPFQFGRDRVSAPPGTNAQQNMPPQMMGGPIQASAEVAQQGTMWQGRNDMTYNYANRQSTGSAPQGPAYHGVNRTDEMLHTDQRANHEGSWPSHGTRQPPYGPSAPVPPMTRPPPSNYQPPPSMQNHIPQVSSPAPLPRPMENRTSPSKSPFLHSGMKMQKAGPPVPASHIAPAPVQPPMIRRDITFPPGSVEATQPVLKQRRRLTMKDIGTPEAWRVMMSLKSGLLAESTWALDTINILLYDDNSIMTFNLSQLPGLLELLVEYFRRCLIEIFGILKEYEVGDPGQRTLLDPGRFSKVSSPAPMEGGEEEEELLGPKLEEEEEEEVVENDEEIAFSGKDKPASENSEEKLISKFDKLPVKIVQKNDPFVVDCSDKLGRVQEFDSGLLHWRIGGGDTTEHIQTHFESKTELLPSRPHAPCPPAPRKHVTTAEGTPGTTDQEGPPPDGPPEKRITATMDDMLSTRSSTLTEDGAKSSEAIKESSKFPFGISPAQSHRNIKILEDEPHSKDETPLCTLLDWQDSLAKRCVCVSNTIRSLSFVPGNDFEMSKHPGLLLILGKLILLHHKHPERKQAPLTYEKEEEQDQGVSCNKVEWWWDCLEMLRENTLVTLANISGQLDLSPYPESICLPVLDGLLHWAVCPSAEAQDPFSTLGPNAVLSPQRLVLETLSKLSIQDNNVDLILATPPFSRLEKLYSTMVRFLSDRKNPVCREMAVVLLANLAQGDSLAARAIAVQKGSIGNLLGFLEDSLAATQFQQSQASLLHMQNPPFEPTSVDMMRRAARALLALAKVDENHSEFTLYESRLLDISVSPLMNSLVSQVICDVLFLIGQS

>NP_550438.1

MAAGRLFLSRLRAPFSSMAKSPLEGVSSSRGLHAGRGPRRLSIEGNIAVGKSTFVKLLTKTYPEWHVATEPVATWQNIQAAGTQKACTAQSLGNLLDMMYREPARWSYTFQTFSFLSRLKVQLEPFPEKLLQARKPVQIFERSVYSDRYIFAKNLFENGSLSDIEWHIYQDWHSFLLWEFASRITLHGFIYLQASPQVCLKRLYQRAREEEKGIELAYLEQLHGQHEAWLIHKTTKLHFEALMNIPVLVLDVNDDFSEEVTKQEDLMREVNTFVKNL

147 CAA58827.1 NP_005787.1

>CAA58827.1

MADEEEDPTFEEENEEIGGGAEGGQGKRKRLFSKELRCMMYGFGDDQNPYTESVDILEDLVIEFITEMTHKAMSIGRQGRVQVEDIVFLIRKDPRKFARVKDLLTMNEELKRARKAFDEANYGS

>NP_005787.1

MGDKPIWEQIGSSFIQHYYQLFDNDRTQLGAIYIDASCLTWEGQQFQGKAAIVEKLSSLPFQKIQHSITAQDHQPTPDSCIISMVVGQLKADEDPIMGFHQMFLLKNINDAWVCTNDMFRLALHNFG

148 NP_005180.1 NP_659491.4

>NP_005180.1

MEELSSVGEQVFAAECILSKRLRKGKLEYLVKWRGWSSKHNSWEPEENILDPRLLLAFQKKEHEKEVQNRKRGKRPRGRPRKLTAMSSCSRRSKLKEPDAPSKSKSSSSSSSSTSSSSSSDEEDDSDLDAKRGPRGRETHPVPQKKAQILVAKPELKDPIRKKRGRKPLPPEQKATRRPVSLAKVLKTARKDLGAPASKLPPPLSAPVAGLAALKAHAKEACGGPSAMATPENLASLMKGMASSPGRGGISWQSSIVHYMNRMTQSQAQAASRLALKAQATNKCGLGLDLKVRTQKGELGMSPPGSKIPKAPSGGAVEQKVGNTGGPPHTHGASRVPAGCPGPQPAPTQELSLQVLDLQSVKNGMPGVGLLARHATATKGVPATNPAPGKGTGSGLIGASGATMPTDTSKSEKLASRAVAPPTPASKRDCVKGSATPSGQESRTAPGEARKAATLPEMSAGEESSSSDSDPDSASPPSTGQNPSVSVQTSQDWKPTRSLIEHVFVTDVTANLITVTVKESPTSVGFFNLRHY

>NP_659491.4

MDNKISPEAQVAELELDAVIGFNGHVPTGLKCHPDQEHMIYPLGCTVLIQAINTKEQNFLQGHGNNVSCLAISRSGEYIASGQVTFMGFKADIILWDYKNRELLARLSLHKGKIEALAFSPNDLYLVSLGGPDDGSVVVWSIAKRDAICGSPAAGLNVGNATNVIFSRCRDEMFMTAGNGTIRVWELDLPNRKIWPTECQTGQLKRIVMSIGVDDDDSFFYLGTTTGDILKMNPRTKLLTDVGPAKDKFSLGVSAIRCLKMGGLLVGSGAGLLVFCKSPGYKPIKKIQLQGGITSITLRGEGHQFLVGTEESHIYRVSFTDFKETLIATCHFDAVEDIVFPFGTAELFATCAKKDIRVWHTSSNRELLRITVPNMTCHGIDFMRDGKSIISAWNDGKIRAFAPETGRLMYVINNAHRIGVTAIATTSDCKRVISGGGEGEVRVWQIGCQTQKLEEALKEHKSSVSCIRVKRNNEECVTASTDGTCIIWDLVRLRRNQMILANTLFQCVCYHPEEFQIITSGTDRKIAYWEVFDGTVIRELEGSLSGSINGMDITQEGVHFVTGGNDHLVKVWDYNEGEVTHVGVGHSGNITRIRISPGNQYIVSVSADGAILRWKYPYTS

149 NP_000225.1 NP_659451.1

>NP_000225.1

MQRSIMSFFHPKKEGKAKKPEKEASNSSRETEPPPKAALKEWNGVVSESDSPVKRPGRKAARVLGSEGEEEDEALSPAKGQKPALDCSQVSPPRPATSPENNASLSDTSPMDSSPSGIPKRRTARKQLPKRTIQEVLEEQSEDEDREAKRKKEEEEEETPKESLTEAEVATEKEGEDGDQPTTPPKPLKTSKAETPTESVSEPEVATKQELQEEEEQTKPPRRAPKTLSSFFTPRKPAVKKEVKEEEPGAPGKEGAAEGPLDPSGYNPAKNNYHPVEDACWKPGQKVPYLAVARTFEKIEEVSARLRMVETLSNLLRSVVALSPPDLLPVLYLSLNHLGPPQQGLELGVGDGVLLKAVAQATGRQLESVRAEAAEKGDVGLVAENSRSTQRLMLPPPPLTASGVFSKFRDIARLTGSASTAKKIDIIKGLFVACRHSEARFIARSLSGRLRLGLAEQSVLAALSQAVSLTPPGQEFPPAMVDAGKGKTAEARKTWLEEQGMILKQTFCEVPDLDRIIPVLLEHGLERLPEHCKLSPGIPLKPMLAHPTRGISEVLKRFEEAAFTCEYKYDGQRAQIHALEGGEVKIFSRNQEDNTGKYPDIISRIPKIKLPSVTSFILDTEAVAWDREKKQIQPFQVLTTRKRKEVDASEIQVQVCLYAFDLIYLNGESLVREPLSRRRQLLRENFVETEGEFVFATSLDTKDIEQIAEFLEQSVKDSCEGLMVKTLDVDATYEIAKRSHNWLKLKKDYLDGVGDTLDLVVIGAYLGRGKRAGRYGGFLLASYDEDSEELQAICKLGTGFSDEELEEHHQSLKALVLPSPRPYVRIDGAVIPDHWLDPSAVWEVKCADLSLSPIYPAARGLVDSDKGISLRFPRFIRVREDKQPEQATTSAQVACLYRKQSQIQNQQGEDSGSDPEDTY

>NP_659451.1

MEELLPDGQIWANMDPEERMLAAATAFTHICAGQGEGDVRREAQSIQYDPYSKASVAPGKRPALPVQLQYPHVESNVPSETVSEASQRLRKPVMKRKVLRRKPDGEVLVTDESIISESESGTENDQDLWDLRQRLMNVQFQEDKESSFDVSQKFNLPHEYQGISQDQLICSLQREGMGSPAYEQDLIVASRPKSFILPKLDQLSRNRGKTDRVARYFEYKRDWDSIRLPGEDHRKELRWGVREQMLCRAEPQSKPQHIYVPNNYLVPTEKKRSALRWGVRCDLANGVIPRKLPFPLSPS

150 Q7Z591 Q8IXM3

>Q7Z591

MASSETEIRWAEPGLGKGPQRRRWAWAEDKRDVDRSSSQSWEEERLFPNATSPELLEDFRLAQQHLPPLEWDPHPQPDGHQDSESGETSGEEAEAEDVDSPASSHEPLAWLPQQGRQLDMTEEEPDGTLGSLEVEEAGESSSRLGYEAGLSLEGHGNTSPMALGHGQARGWVASGEQASGDKLSEHSEVNPSVELSPARSWSSGTVSLDHPSDSLDSTWEGETDGPQPTALAETLPEGPSHHLLSPDGRTGGSVARATPMEFQDSSAPPAQSPQHATDRWRRETTRFFCPQPKEHIWKQTKTSPKPLPSRFIGSISPLNPQPRPTRQGRPLPRQGATLAGRSSSNAPKYGRGQLNYPLPDFSKVGPRVRFPKDESYRPPKSRSHNRKPQAPARPLIFKSPAEIVQEVLLSSGEAALAKDTPPAHPITRVPQEFQTPEQATELVHQLQEDYHRLLTKYAEAENTIDQLRLGAKVNLFSDPPQPNHSIHTGMVPQGTKVLSFTIPQPRSAEWWPGPAEDPQASAASGWPSARGDLSPSSLTSMPTLGWLPENRDISEDQSSAEQTQALASQASQFLAKVESFERLIQAGRLMPQDQVKGFQRLKAAHAALEEEYLKACREQHPAQPLAGSKGTPGRFDPRRELEAEIYRLGSCLEELKEHIDQTQQEPEPPGSDSALDSTPALPCLHQPTHLPAPSGQAPMPAIKTSCPEPATTTAAASTGPCPLHVNVEVSSGNSEVEDRPQDPLARLRHKELQMEQVYHGLMERYLSVKSLPEAMRMEEEEEGEEEEEEEGGGDSLEVDGVAATPGKAEATRVLPRQCPVQAEKSHGAPLEEATEKMVSMKPPGFQASLARDGHMSGLGKAEAAPPGPGVPPHPPGTKSAASHQSSMTSLEGSGISERLPQKPLHRGGGPHLEETWMASPETDSGFVGSETSRVSPLTQTPEHRLSHISTAGTLAQPFAASVPRDGASYPKARGSLIPRRATEPSTPRSQAQRYLSSPSGPLRQRAPNFSLERTLAAEMAVPGSEFEGHKRISEQPLPNKTISPPPAPAPAAAPLPCGPTETIPSFLLTRAGRDQAICELQEEVSRLRLRLEDSLHQPLQGSPTRPASAFDRPARTRGRPADSPATWGSHYGSKSTERLPGEPRGEEQIVPPGRQRARSSSVPREVLRLSLSSESELPSLPLFSEKSKTTKDSPQAARDGKRGVGSAGWPDRVTFRGQYTGHEYHVLSPKAVPKGNGTVSCPHCRPIRTQDAGGAVTGDPLGPPPADTLQCPLCGQVGSPPEADGPGSATSGAEKATTRRKASSTPSPKQRSKQAGSSPRPPPGLWYLATAPPAPAPPAFAYISSVPIMPYPPAAVYYAPAGPTSAQPAAKWPPTASPPPARRHRHSIQLDLGDLEELNKALSRAVQAAESVRSTTRQMRSSLSADLRQAHSLRGSCLF

>Q8IXM3

MGVLAAAARCLVRGADRMSKWTSKRGPRSFRGRKGRGAKGIGFLTSGWRFVQIKEMVPEFVVPDLTGFKLKPYVSYLAPESEETPLTAAQLFSEAVAPAIEKDFKDGTFDPDNLEKYGFEPTQEGKLFQLYPRNFLR

151 NP_009210.1 P82664

>NP_009210.1

MSDFDEFERQLNENKQERDKENRHRKRSHSRSRSRDRKRRSRSRDRRNRDQRSASRDRRRRSKPLTRGAKEEHGGLIRSPRHEKKKKVRKYWDVPPPGFEHITPMQYKAMQAAGQIPATALLPTMTPDGLAVTPTPVPVVGSQMTRQARRLYVGNIPFGITEEAMMDFFNAQMRLGGLTQAPGNPVLAVQINQDKNFAFLEFRSVDETTQAMAFDGIIFQGQSLKIRRPHDYQPLPGMSENPSVYVPGVVSTVVPDSAHKLFIGGLPNYLNDDQVKELLTSFGPLKAFNLVKDSATGLSKGYAFCEYVDINVTDQAIAGLNGMQLGDKKLLVQRASVGAKNATLVSPPSTINQTPVTLQVPGLMSSQVQMGGHPTEVLCLMNMVLPEELLDDEEYEEIVEDVRDECSKYGLVKSIEIPRPVDGVEVPGCGKIFVEFTSVFDCQKAMQGLTGRKFANRVVVTKYCDPDSYHRRDFW

>P82664

MAARTAFGAVCRRLWQGLGNFSVNTSKGNTAKNGGLLLSTNMKWVQFSNLHVDVPKDLTKPVVTISDEPDILYKRLSVLVKGHDKAVLDSYEYFAVLAAKELGISIKVHEPPRKIERFTLLQSVHIYKKHRVQYEMRTLYRCLELEHLTGSTADVYLEYIQRNLPEGVAMEVTKTQLEQLPEHIKEPIWETLSEEKEESKS

152 NP_004526.1 NP_002717.3

>NP_004526.1

MSLENEDKRARTRSKALRGPPETTAADLSCPTPGCTGSGHVRGKYSRHRSLQSCPLAKKRKLEGAEAEHLVSKRKSHPLKLALDEGYGVDSDGSEDTEVKDASVSDESEGTLEGAEAETSGQDEIHRPETAEGRSPVKSHFGSNPIGSATASSKGSYSSYQGIIATSLLNLGQIAEETLVEEDLGQAAKPGPGIVHLLQEAAEGAASEEGEKGLFIQPEDAEEVVEVTTERSQDLCPQSLEDAASEESSKQKGILSHEEEDEEEEEEEEEEEEDEEEEEEEEEEEEEEEEEEEEEEEEEEEEEEEEAAPDVIFQEDTSHTSAQKAPELRGPESPSPKPEYSVIVEVRSDDDKDEDTHSRKSTVTDESEMQDMMTRGNLGLLEQAIALKAEQVRTVCEPGCPPAEQSQLGLGEPGKAAKPLDTVRKSYYSKDPSRAEKREIKCPTPGCDGTGHVTGLYPHHRSLSGCPHKDRIPPEILAMHENVLKCPTPGCTGQGHVNSNRNTHRSLSGCPIAAAEKLAKSHEKQQPQTGDPSKSSSNSDRILRPMCFVKQLEVPPYGSYRPNVAPATPRANLAKELEKFSKVTFDYASFDAQVFGKRMLAPKIQTSETSPKAFQCFDYSQDAEAAHMAATAILNLSTRCWEMPENLSTKPQDLPSKSVDIEVDENGTLDLSMHKHRKRENAFPSSSSCSSSPGVKSPDASQRHSSTSAPSSSMTSPQSSQASRQDEWDRPLDYTKPSRLREEEPEESEPAAHSFASSEADDQEVSEENFEERKYPGEVTLTNFKLKFLSKDIKKELLTCPTPGCDGSGHITGNYASHRSLSGCPLADKSLRNLMAAHSADLKCPTPGCDGSGHITGNYASHRSLSGCPRAKKSGVKVAPTKDDKEDPELMKCPVPGCVGLGHISGKYASHRSASGCPLAARRQKEGSLNGSSFSWKSLKNEGPTCPTPGCDGSGHANGSFLTHRSLSGCPRATFAGKKGKLSGDEVLSPKFKTSDVLENDEEIKQLNQEIRDLNESNSEMEAAMVQLQSQISSMEKNLKNIEEENKLIEEQNEALFLELSGLSQALIQSLANIRLPHMEPICEQNFDAYVSTLTDMYSNQDPENKDLLESIKQAVRGIQV

>NP_002717.3

MLSLQYPDVYRDETAVQDYHGHKICDPYAWLEDPDSEQTKAFVEAQNKITVPFLEQCPIRGLYKERMTELYDYPKYSCHFKKGKRYFYFYNTGLQNQRVLYVQDSLEGEARVFLDPNILSDDGTVALRGYAFSEDGEYFAYGLSASGSDWVTIKFMKVDGAKELPDVLERVKFSCMAWTHDGKGMFYNSYPQQDGKSDGTETSTNLHQKLYYHVLGTDQSEDILCAEFPDEPKWMGGAELSDDGRYVLLSIREGCDPVNRLWYCDLQQESSGIAGILKWVKLIDNFEGEYDYVTNEGTVFTFKTNRQSPNYRVINIDFRDPEESKWKVLVPEHEKDVLEWIACVRSNFLVLCYLHDVKNILQLHDLTTGALLKTFPLDVGSIVGYSGQKKDTEIFYQFTSFLSPGIIYHCDLTKEELEPRVFREVTVKGIDASDYQTVQIFYPSKDGTKIPMFIVHKKGIKLDGSHPAFLYGYGGFNISITPNYSVSRLIFVRHMGGILAVANIRGGGEYGETWHKGGILANKQNCFDDFQCAAEYLIKEGYTSPKRLTINGGSNGGLLVAACANQRPDLFGCVIAQVGVMDMLKFHKYTIGHAWTTDYGCSDSKQHFEWLVKYSPLHNVKLPEADDIQYPSMLLLTADHDDRVVPLHSLKFIATLQYIVGRSRKQSNPLLIHVDTKAGHGAGKPTAKVIEEVSDMFAFIARCLNVDWIP

153 NP_001036111.1 Q9NP92

>NP_001036111.1

MALVFQFGQPVRAQPLPGLCHGKLIRTNACDVCNSTDLPEVEIISLLEEQLPHYKLRADTIYGYDHDDWLHTPLISPDANIDLTTEQIEETLKYFLLCAERVGQMTKTYNDIDAVTRLLEEKERDLELAARIGQSLLKKNKTLTERNELLEEQVEHIREEVSQLRHELSMKDELLQFYTSAAEESEPESVCSTPLKRNESSSSVQNYFHLDSLQKKLKDLEEENVVLRSEASQLKTETITYEEKEQQLVNDCVKELRDANVQIASISEELAKKTEDAARQQEEITHLLSQIVDLQKKAKACAVENEELVQHLGAAKDAQRQLTAELRELEDKYAECMEMLHEAQEELKNLRNKTMPNTTSRRYHSLGLFPMDSLAAEIEGTMRKELQLEEAESPDITHQKRVFETVRNINQVVKQRSLTPSPMNIPGSNQSSAMNSLLSSCVSTPRSSFYGSDIGNVVLDNKTNSIILETEAADLGNDERSKKPGTPGTPGSHDLETALRRLSLRRENYLSERRFFEEEQERKLQELAEKGELRSGSLTPTESIMSLGTHSRFSEFTGFSGMSFSSRSYLPEKLQIVKPLEGSATLHHWQQLAQPHLGGILDPRPGVVTKGFRTLDVDLDEVYCLNDFEEDDTGDHISLPRLATSTPVQHPETSAHHPGKCMSQTNSTFTFTTCRILHPSDELTRVTPSLNSAPTPACGSTSHLKSTPVATPCTPRRLSLAESFTNTRESTTTMSTSLGLVWLLKERGISAAVYDPQSWDRAGRGSLLHSYTPKMAVIPSTPPNSPMQTPTSSPPSFEFKCTSPPYDNFLASKPASSILREVREKNVRSSESQTDVSVSNLNLVDKVRRFGVAKVVNSGRAHVPTLTEEQGPLLCGPPGPAPALVPRGLVPEGLPLRCPTVTSAIGGLQLNSGIRRNRSFPTMVGSSMQMKAPVTLTSGILMGAKLSKQTSLR

>Q9NP92

MAAARCWRPLLRGPRLSLHTAANAAATATETTCQDVAATPVARYPPIVASMTADSKAARLRRIERWQATVHAAESVDEKLRILTKMQFMKYMVYPQTFALNADRWYQYFTKTVFLSGLPPPPAEPEPEPEPEPEPALDLAALRAVACDCLLQEHFYLRRRRRVHRYEESEVISLPFLDQLVSTLVGLLSPHNPALAAAALDYRCPVHFYWVRGEEIIPRGHRRGRIDDLRYQIDDKPNNQIRISKQLAEFVPLDYSVPIEIPTIKCKPDKLPLFKRQYENHIFVGSKTADPCCYGHTQFHLLPDKLRRERLLRQNCADQIEVVFRANAIASLFAWTGAQAMYQGFWSEADVTRPFVSQAVITDGKYFSFFCYQLNTLALTTQADQNNPRKNICWGTQSKPLYETIEDNDVKGFNDDVLLQIVHFLLNRPKEEKSQLLEN

154 Q5XKR4 Q9NYK5

>Q5XKR4

MLSHADLLDARLGMKDAAELLGHREAVKCRLGVGGSDPGGHPGDLAPNSDPVEGATLLPGEDITTVGSTPASLAVSAKDPDKQPGPQGGPNPSQAGQQQGQQKQKRHRTRFTPAQLNELERSFAKTHYPDIFMREELALRIGLTESRVQVWFQNRRAKWKKRKKTTNVFRAPGTLLPTPGLPQFPSAAAAAAAAMGDSLCSFHANDTRWAAAAMPGVSQLPLPPALGRQQAMAQSLSQCSLAAGPPPNSMGLSNSLAGSNGAGLQSHLYQPAFPGMVPASLPGPSNVSGSPQLCSSPDSSDVWRGTSIASLRRKALEHTVSMSFT

>Q9NYK5

MEALAMGSRALRLWLVAPGGGIKWRFIATSPASQLSPTELTEMRNDLFNKEKARQLSLTPRTEKIEVKHVGKTDPGTVFVMNKNISTPYSCAMHLSEWYCRKSILALVDGQPWDMYKPLTKSCEIKFLTFKDCDPGEVNKAYWRSCAMMMGCVIERAFKDEYMVNLVRAPEVPVISGAFCYDVVLDSKLDEWMPTKENLRSFTKDAHALIYKDLPFETLEVEAKVALEIFQHSKYKVDFIEEKASQNPERIVKLHRIGDFIDVSEGPLIPRTSICFQYEVSAVHNLQPTQPSLIRRFQGVSLPVHLRAHFTIWDKLLERSRKMVTEDQSKATEECTST

155 NP_002884.1 NP_006651.2

>NP_002884.1

MASKEMFEDTVEERVINEEYKIWKKNTPFLYDLVMTHALQWPSLTVQWLPEVTKPEGKDYALHWLVLGTHTSDEQNHLVVARVHIPNDDAQFDASHCDSDKGEFGGFGSVTGKIECEIKINHEGEVNRARYMPQNPHIIATKTPSSDVLVFDYTKHPAKPDPSGECNPDLRLRGHQKEGYGLSWNSNLSGHLLSASDDHTVCLWDINAGPKEGKIVDAKAIFTGHSAVVEDVAWHLLHESLFGSVADDQKLMIWDTRSNTTSKPSHLVDAHTAEVNCLSFNPYSEFILATGSADKTVALWDLRNLKLKLHTFESHKDEIFQVHWSPHNETILASSGTDRRLNVWDLSKIGEEQSAEDAEDGPPELLFIHGGHTAKISDFSWNPNEPWVICSVSEDNIMQIWQMAENIYNDEESDVTTSELEGQGS

>NP_006651.2

MPSCGACTCGAAAVRLITSSLASAQRGISGGRIHMSVLGRLGTFETQILQRAPLRSFTETPAYFASKDGISKDGSGDGNKKSASEGSSKKSGSGNSGKGGNQLRCPKCGDLCTHVETFVSSTRFVKCEKCHHFFVVLSEADSKKSIIKEPESAAEAVKLAFQQKPPPPPKKIYNYLDKYVVGQSFAKKVLSVAVYNHYKRIYNNIPANLRQQAEVEKQTSLTPRELEIRRREDEYRFTKLLQIAGISPHGNALGASMQQQVNQQIPQEKRGGEVLDSSHDDIKLEKSNILLLGPTGSGKTLLAQTLAKCLDVPFAICDCTTLTQAGYVGEDIESVIAKLLQDANYNVEKAQQGIVFLDEVDKIGSVPGIHQLRDVGGEGVQQGLLKLLEGTIVNVPEKNSRKLRGETVQVDTTNILFVASGAFNGLDRIISRRKNEKYLGFGTPSNLGKGRRAAAAADLANRSGESNTHQDIEEKDRLLRHVEARDLIEFGMIPEFVGRLPVVVPLHSLDEKTLVQILTEPRNAVIPQYQALFSMDKCELNVTEDALKAIARLALERKTGARGLRSIMEKLLLEPMFEVPNSDIVCVEVDKEVVEGKKEPGYIRAPTKESSEEEYDSGVEEEGWPRQADAANS

156 NP_006581.2 NP_006436.3

>NP_006581.2

MSGRSKRESRGSTRGKRESESRGSSGRVKRERDREREPEAASSRGSPVRVKREFEPASAREAPASVVPFVRVKREREVDEDSEPEREVRAKNGRVDSEDRRSRHCPYLDTINRSVLDFDFEKLCSISLSHINAYACLVCGKYFQGRGLKSHAYIHSVQFSHHVFLNLHTLKFYCLPDNYEIIDSSLEDITYVLKPTFTKQQIANLDKQAKLSRAYDGTTYLPGIVGLNNIKANDYANAVLQALSNVPPLRNYFLEEDNYKNIKRPPGDIMFLLVQRFGELMRKLWNPRNFKAHVSPHEMLQAVVLCSKKTFQITKQGDGVDFLSWFLNALHSALGGTKKKKKTIVTDVFQGSMRIFTKKLPHPDLPAEEKEQLLHNDEYQETMVESTFMYLTLDLPTAPLYKDEKEQLIIPQVPLFNILAKFNGITEKEYKTYKENFLKRFQLTKLPPYLIFCIKRFTKNNFFVEKNPTIVNFPITNVDLREYLSEEVQAVHKNTTYDLIANIVHDGKPSEGSYRIHVLHHGTGKWYELQDLQVTDILPQMITLSEAYIQIWKRRDNDETNQQGA

>NP_006436.3

MAGVFPYRGPGNPVPGPLAPLPDYMSEEKLQEKARKWQQLQAKRYAEKRKFGFVDAQKEDMPPEHVRKIIRDHGDMTNRKFRHDKRVYLGALKYMPHAVLKLLENMPMPWEQIRDVPVLYHITGAISFVNEIPWVIEPVYISQWGSMWIMMRREKRDRRHFKRMRFPPFDDEEPPLDYADNILDVEPLEAIQLELDPEEDAPVLDWFYDHQPLRDSRKYVNGSTYQRWQFTLPMMSTLYRLANQLLTDLVDDNYFYLFDLKAFFTSKALNMAIPGGPKFEPLVRDINLQDEDWNEFNDINKIIIRQPIRTEYKIAFPYLYNNLPHHVHLTWYHTPNVVFIKTEDPDLPAFYFDPLINPISHRHSVKSQEPLPDDDEEFELPEFVEPFLKDTPLYTDNTANGIALLWAPRPFNLRSGRTRRALDIPLVKNWYREHCPAGQPVKVRVSYQKLLKYYVLNALKHRPPKAQKKRYLFRSFKATKFFQSTKLDWVEVGLQVCRQGYNMLNLLIHRKNLNYLHLDYNFNLKPVKTLTTKERKKSRFGNAFHLCREVLRLTKLVVDSHVQYRLGNVDAFQLADGLQYIFAHVGQLTGMYRYKYKLMRQIRMCKDLKHLIYYRFNTGPVGKGPGCGFWAAGWRVWLFFMRGITPLLERWLGNLLARQFEGRHSKGVAKTVTKQRVESHFDLELRAAVMHDILDMMPEGIKQNKARTILQHLSEAWRCWKANIPWKVPGLPTPIENMILRYVKAKADWWTNTAHYNRERIRRGATVDKTVCKKNLGRLTRLYLKAEQERQHNYLKDGPYITAEEAVAVYTTTVHWLESRRFSPIPFPPLSYKHDTKLLILALERLKEAYSVKSRLNQSQREELGLIEQAYDNPHEALSRIKRHLLTQRAFKEVGIEFMDLYSHLVPVYDVEPLEKITDAYLDQYLWYEADKRRLFPPWIKPADTEPPPLLVYKWCQGINNLQDVWETSEGECNVMLESRFEKMYEKIDLTLLNRLLRLIVDHNIADYMTAKNNVVINYKDMNHTNSYGIIRGLQFASFIVQYYGLVMDLLVLGLHRASEMAGPPQMPNDFLSFQDIATEAAHPIRLFCRYIDRIHIFFRFTADEARDLIQRYLTEHPDPNNENIVGYNNKKCWPRDARMRLMKHDVNLGRAVFWDIKNRLPRSVTTVQWENSFVSVYSKDNPNLLFNMCGFECRILPKCRTSYEEFTHKDGVWNLQNEVTKERTAQCFLRVDDESMQRFHNRVRQILMASGSTTFTKIVNKWNTALIGLMTYFREAVVNTQELLDLLVKCENKIQTRIKIGLNSKMPSRFPPVVFYTPKELGGLGMLSMGHVLIPQSDLRWSKQTDVGITHFRSGMSHEEDQLIPNLYRYIQPWESEFIDSQRVWAEYALKRQEAIAQNRRLTLEDLEDSWDRGIPRINTLFQKDRHTLAYDKGWRVRTDFKQYQVLKQNPFWWTHQRHDGKLWNLNNYRTDMIQALGGVEGILEHTLFKGTYFPTWEGLFWEKASGFEESMKWKKLTNAQRSGLNQIPNRRFTLWWSPTINRANVYVGFQVQLDLTGIFMHGKIPTLKISLIQIFRAHLWQKIHESIVMDLCQVFDQELDALEIETVQKETIHPRKSYKMNSSCADILLFASYKWNVSRPSLLADSKDVMDSTTTQKYWIDIQLRWGDYDSHDIERYARAKFLDYTTDNMSIYPSPTGVLIAIDLAYNLHSAYGNWFPGSKPLIQQAMAKIMKANPALYVLRERIRKGLQLYSSEPTEPYLSSQNYGELFSNQIIWFVDDTNVYRVTIHKTFEGNLTTKPINGAIFIFNPRTGQLFLKIIHTSVWAGQKRLGQLAKWKTAEEVAALIRSLPVEEQPKQIIVTRKGMLDPLEVHLLDFPNIVIKGSELQLPFQACLKVEKFGDLILKATEPQMVLFNLYDDWLKTISSYTAFSRLILILRALHVNNDRAKVILKPDKTTITEPHHIWPTLTDEEWIKVEVQLKDLILADYGKKNNVNVASLTQSEIRDIILGMEISAPSQQRQQIAEIEKQTKEQSQLTATQTRTVNKHGDEIITSTTSNYETQTFSSKTEWRVRAISAANLHLRTNHIYVSSDDIKETGYTYILPKNVLKKFICISDLRAQIAGYLYGVSPPDNPQVKEIRCIVMVPQWGTHQTVHLPGQLPQHEYLKEMEPLGWIHTQPNESPQLSPQDVTTHAKIMADNPSWDGEKTIIITCSFTPGSCTLTAYKLTPSGYEWGRQNTDKGNNPKGYLPSHYERVQMLLSDRFLGFFMVPAQSSWNYNFMGVRHDPNMKYELQLANPKEFYHEVHRPSHFLNFALLQEGEVYSADREDLYA

157 NP_005504.2 NP_000340.2

>NP_005504.2

MADPDVLTEVPAALKRLAKYVIRGFYGIEHALALDILIRNSCVKEEDMLELLKFDRKQLRSVLNNLKGDKFIKCRMRVETAADGKTTRHNYYFINYRTLVNVVKYKLDHMRRRIETDERDSTNRASFKCPVCSSTFTDLEANQLFDPMTGTFRCTFCHTEVEEDESAMPKKDARTLLARFNEQIEPIYALLRETEDVNLAYEILEPEPTEIPALKQSKDHAATTAGAASLAGGHHREAWATKGPSYEDLYTQNVVINMDDQEDLHRASLEGKSAKERPIWLRESTVQGAYGSEDMKEGGIDMDAFQEREEGHAGPDDNEEVMRALLIHEKKTSSAMAGSVGAAAPVTAANGSDSESETSESDDDSPPRPAAVAVHKREEDEEEDDEFEEVADDPIVMVAGRPFSYSEVSQRPELVAQMTPEEKEAYIAMGQRMFEDLFE

>NP_000340.2

MLLATFKLCAGSSYRHMRNMKGLRQQAVMAISQELNRRALGGPTPSTWINQVRRRSSLLGSRLEETLYSDQELAYLQQGEEAMQKALGILSNQEGWKKESQQDNGDKVMSKVVPDVGKVFRLEVVVDQPMERLYEELVERMEAMGEWNPNVKEIKVLQKIGKDTFITHELAAEAAGNLVGPRDFVSVRCAKRRGSTCVLAGMATDFGNMPEQKGVIRAEHGPTCMVLHPLAGSPSKTKLTWLLSIDLKGWLPKSIINQVLSQTQVDFANHLRKRLESHPASEARC

158 NP_000160.1 NP_001880.2

>NP_000160.1

MQLRNPELHLGCALALRFLALVSWDIPGARALDNGLARTPTMGWLHWERFMCNLDCQEEPDSCISEKLFMEMAELMVSEGWKDAGYEYLCIDDCWMAPQRDSEGRLQADPQRFPHGIRQLANYVHSKGLKLGIYADVGNKTCAGFPGSFGYYDIDAQTFADWGVDLLKFDGCYCDSLENLADGYKHMSLALNRTGRSIVYSCEWPLYMWPFQKPNYTEIRQYCNHWRNFADIDDSWKSIKSILDWTSFNQERIVDVAGPGGWNDPDMLVIGNFGLSWNQQVTQMALWAIMAAPLFMSNDLRHISPQAKALLQDKDVIAINQDPLGKQGYQLRQGDNFEVWERPLSGLAWAVAMINRQEIGGPRSYTIAVASLGKGVACNPACFITQLLPVKRKLGFYEWTSRLRSHINPTGTVLLQLENTMQMSLKDLL

>NP_001880.2

MATGQKLMRAVRVFEFGGPEVLKLRSDIAVPIPKDHQVLIKVHACGVNPVETYIRSGTYSRKPLLPYTPGSDVAGVIEAVGDNASAFKKGDRVFTSSTISGGYAEYALAADHTVYKLPEKLDFKQGAAIGIPYFTAYRALIHSACVKAGESVLVHGASGGVGLAACQIARAYGLKILGTAGTEEGQKIVLQNGAHEVFNHREVNYIDKIKKYVGEKGIDIIIEMLANVNLSKDLSLLSHGGRVIVVGSRGTIEINPRDTMAKESSIIGVTLFSSTKEEFQQYAAALQAGMEIGWLKPVIGSQYPLEKVAEAHENIIHGSGATGKMILLL

159 Q5H9F3 P82663

>Q5H9F3

MISTAPLYSGVHNWTSSDRIRMCGINEERRAPLSDEESTTGDCQHFGSQEFCVSSSFSKVELTAVGSGSNARGADPDGSATEKLGHKSEDKPDDPQPKMDYAGNVAEAEGFLVPLSSPGDGLKLPASDSAEASNSRADCSWTPLNTQMSKQVDCSPAGVKALDSRQGVGEKNTFILATLGTGVPVEGTLPLVTTNFSPLPAPICPPAPGSASVPHSVPDAFQVPLSVPAPVPHSGLVPVQVATSVPAPSPPLAPVPALAPAPPSVPTLISDSNPLSVSASVLVPVPASAPPSGPVPLSAPAPAPLSVPVSAPPLALIQAPVPPSAPTLVLAPVPTPVLAPMPASTPPAAPAPPSVPMPTPTPSSGPPSTPTLIPAFAPTPVPAPTPAPIFTPAPTPMPAATPAAIPTSAPIPASFSLSRVCFPAAQAPAMQKVPLSFQPGTVLTPSQPLVYIPPPSCGQPLSVATLPTTLGVSSTLTLPVLPSYLQDRCLPGVLASPELRSYPYAFSVARPLTSDSKLVSLEVNRLPCTSPSGSTTTQPAPDGVPGPLADTSLVTASAKVLPTPQPLLPAPSGSSAPPHPAKMPSGTEQQTEGTSVTFSPLKSPPQLEREMASPPECSEMPLDLSSKSNRQKLPLPNQRKTPPMPVLTPVHTSSKALLSTVLSRSQRTTQAAGGNVTSCLGSTSSPFVIFPEIVRNGDPSTWVKNSTALISTIPGTYVGVANPVPASLLLNKDPNLGLNRDPRHLPKQEPISIIDQGEPKGTGATCGKKGSQAGAEGQPSTVKRYTPARIAPGLPGCQTKELSLWKPTGPANIYPRCSVNGKPTSTQVLPVGWSPYHQASLLSIGISSAGQLTPSQGAPIRPTSVVSEFSGVPSLSSSEAVHGLPEGQPRPGGSFVPEQDPVTKNKTCRIAAKPYEEQVNPVLLTLSPQTGTLALSVQPSGGDIRMNQGPEESESHLCSDSTPKMEGPQGACGLKLAGDTKPKNQVLATYMSHELVLATPQNLPKMPELPLLPHDSHPKELILDVVPSSRRGSSTERPQLGSQVDLGRVKMEKVDGDVVFNLATCFRADGLPVAPQRGQAEVRAKAGQARVKQESVGVFACKNKWQPDDVTESLPPKKMKCGKEKDSEEQQLQPQAKAVVRSSHRPKCRKLPSDPQESTKKSPRGASDSGKEHNGVRGKHKHRKPTKPESQSPGKRADSHEEGSLEKKAKSSFRDFIPVVLSTRTRSQSGSICSSFAGMADSDMGSQEVFPTEEEEEVTPTPAKRRKVRKTQRDTQYRSHHAQDKSLLSQGRRHLWRAREMPWRTEAARQMWDTNEEEEEEEEEGLLKRKKRRRQKSRKYQTGEYLTEQEDEQRRKGRADLKARKQKTSSSQSLEHRLRNRNLLLPNKVQGISDSPNGFLPNNLEEPACLENSEKPSGKRKCKTKHMATVSEEAKDVVLYCLQKDSEDVNHRDNAGYTALHEACSRGWTDILNILLEHGANVNCSAQDGTRPVHDAVVNDNLETIWLLLSYGADPTLATYSGQTAMKLASSDTMKRFLSDHLSDLQGRAEGDPGVSWDFYSSSVLEEKDGFACDLLHNPPGSSDQEGDDPMEEDDFMFELSDKPLLPCYNLQVSVSRGPCNWFLFSDVLKRLKLSSRIFQARFPHFEITTMPKAEFYRQVASSQLLTPAERPGGLDDRSPPGSSETVELVRYEPDLLRLLGSEVEFQSCNS

>P82663

MPMKGRFPIRRTLQYLSQGNVVFKDSVKVMTVNYNTHGELGEGARKFVFFNIPQIQYKNPWVQIMMFKNMTPSPFLRFYLDSGEQVLVDVETKSNKEIMEHIRKILGKNEETLREEEEEKKQLSHPANFGPRKYCLRECICEVEGQVPCPSLVPLPKEMRGKYKAALKADAQD

160 NP_056975.1 NP_055433.2

>NP_056975.1

MSAAIAALAASYGSGSGSESDSDSESSRCPLPAADSLMHLTKSPSSKPSLAVAVDSAPEVAVKEDLETGVHLDPAVKEVQYNPTYETMFAPEFGPENPFRTQQMAAPRNMLSGYAEPAHINDFMFEQQRRTFATYGYALDPSLDNHQVSAKYIGSVEEAEKNQGLTVFETGQKKTEKRKKFKENDASNIDGFLGPWAKYVDEKDVAKPSEEEQKELDEITAKRQKKGKQEEEKPGEEKTILHVKEMYDYQGRSYLHIPQDVGVNLRSTMPPEKCYLPKKQIHVWSGHTKGVSAVRLFPLSGHLLLSCSMDCKIKLWEVYGERRCLRTFIGHSKAVRDICFNTAGTQFLSAAYDRYLKLWDTETGQCISRFTNRKVPYCVKFNPDEDKQNLFVAGMSDKKIVQWDIRSGEIVQEYDRHLGAVNTIVFVDENRRFVSTSDDKSLRVWEWDIPVDFKYIAEPSMHSMPAVTLSPNGKWLACQSMDNQILIFGAQNRFRLNKKKIFKGHMVAGYACQVDFSPDMSYVISGDGNGKLNIWDWKTTKLYSRFKAHDKVCIGAVWHPHETSKVITCGWDGLIKLWD

>NP_055433.2

MNWRFVELLYFLFIWGRISVQPSHQEPAGTDQHVSKEFDWLISDRGPFHHSRSYLSFVERHRQGFTTRYKIYREFARWKVRNTAIERRDLVRHPVPLMPEFQRSIRLLGRRPTTQQFIDTIIKKYGTHLLISATLGGEEALTMYMDKSRLDRKSGNATQSVEALHQLASSYFVDRDGTMRRLHEIQISTGAIKVTETRTGPLGCNSYDNLDSVSSVLLQSTESKLHLQGLQIIFPQYLQEKFVQSALSYIMCNGEGEYLCQNSQCRCQCAEEFPQCNCPITDIQIMEYTLANMAKSWAEAYKDLENSDEFKSFMKRLPSNHFLTIGSIHQHWGNDWDLQNRYKLLQSATEAQRQKIQRTARKLFGLSVRCRHNPNHQLPRERTIQQWLARVQSLLYCNENGFWGTFLESQRSCVCHGSTTLCQRPIPCVIGGNNSCAMCSLANISLCGSCNKGYKLYRGRCEPQNVDSERSEQFISFETDLDFQDLELKYLLQKMDSRLYVHTTFISNEIRLDTFFDPRWRKRMSLTLKSNKNRMDFIHMVIGMSMRICQMRNSSLDPMFFVYVNPFSGSHSEGWNMPFGEFGYPRWEKIRLQNSQCYNWTLLLGNRWKTFFETVHIYLRSRTRLPTLLRNETGQGPVDLSDPSKRQFYIKISDVQVFGYSLRFNADLLRSAVQQVNQSYTQGGQFYSSSSVMLLLLDIRDRINRLAPPVAPGKPQLDLFSCMLKHRLKLTNSEIIRVNHALDLYNTEILKQSDQMTAKLC

161 Q5H9F3 NP_001167.2

>Q5H9F3

MISTAPLYSGVHNWTSSDRIRMCGINEERRAPLSDEESTTGDCQHFGSQEFCVSSSFSKVELTAVGSGSNARGADPDGSATEKLGHKSEDKPDDPQPKMDYAGNVAEAEGFLVPLSSPGDGLKLPASDSAEASNSRADCSWTPLNTQMSKQVDCSPAGVKALDSRQGVGEKNTFILATLGTGVPVEGTLPLVTTNFSPLPAPICPPAPGSASVPHSVPDAFQVPLSVPAPVPHSGLVPVQVATSVPAPSPPLAPVPALAPAPPSVPTLISDSNPLSVSASVLVPVPASAPPSGPVPLSAPAPAPLSVPVSAPPLALIQAPVPPSAPTLVLAPVPTPVLAPMPASTPPAAPAPPSVPMPTPTPSSGPPSTPTLIPAFAPTPVPAPTPAPIFTPAPTPMPAATPAAIPTSAPIPASFSLSRVCFPAAQAPAMQKVPLSFQPGTVLTPSQPLVYIPPPSCGQPLSVATLPTTLGVSSTLTLPVLPSYLQDRCLPGVLASPELRSYPYAFSVARPLTSDSKLVSLEVNRLPCTSPSGSTTTQPAPDGVPGPLADTSLVTASAKVLPTPQPLLPAPSGSSAPPHPAKMPSGTEQQTEGTSVTFSPLKSPPQLEREMASPPECSEMPLDLSSKSNRQKLPLPNQRKTPPMPVLTPVHTSSKALLSTVLSRSQRTTQAAGGNVTSCLGSTSSPFVIFPEIVRNGDPSTWVKNSTALISTIPGTYVGVANPVPASLLLNKDPNLGLNRDPRHLPKQEPISIIDQGEPKGTGATCGKKGSQAGAEGQPSTVKRYTPARIAPGLPGCQTKELSLWKPTGPANIYPRCSVNGKPTSTQVLPVGWSPYHQASLLSIGISSAGQLTPSQGAPIRPTSVVSEFSGVPSLSSSEAVHGLPEGQPRPGGSFVPEQDPVTKNKTCRIAAKPYEEQVNPVLLTLSPQTGTLALSVQPSGGDIRMNQGPEESESHLCSDSTPKMEGPQGACGLKLAGDTKPKNQVLATYMSHELVLATPQNLPKMPELPLLPHDSHPKELILDVVPSSRRGSSTERPQLGSQVDLGRVKMEKVDGDVVFNLATCFRADGLPVAPQRGQAEVRAKAGQARVKQESVGVFACKNKWQPDDVTESLPPKKMKCGKEKDSEEQQLQPQAKAVVRSSHRPKCRKLPSDPQESTKKSPRGASDSGKEHNGVRGKHKHRKPTKPESQSPGKRADSHEEGSLEKKAKSSFRDFIPVVLSTRTRSQSGSICSSFAGMADSDMGSQEVFPTEEEEEVTPTPAKRRKVRKTQRDTQYRSHHAQDKSLLSQGRRHLWRAREMPWRTEAARQMWDTNEEEEEEEEEGLLKRKKRRRQKSRKYQTGEYLTEQEDEQRRKGRADLKARKQKTSSSQSLEHRLRNRNLLLPNKVQGISDSPNGFLPNNLEEPACLENSEKPSGKRKCKTKHMATVSEEAKDVVLYCLQKDSEDVNHRDNAGYTALHEACSRGWTDILNILLEHGANVNCSAQDGTRPVHDAVVNDNLETIWLLLSYGADPTLATYSGQTAMKLASSDTMKRFLSDHLSDLQGRAEGDPGVSWDFYSSSVLEEKDGFACDLLHNPPGSSDQEGDDPMEEDDFMFELSDKPLLPCYNLQVSVSRGPCNWFLFSDVLKRLKLSSRIFQARFPHFEITTMPKAEFYRQVASSQLLTPAERPGGLDDRSPPGSSETVELVRYEPDLLRLLGSEVEFQSCNS

>NP_001167.2

MLGLDACELGAQLLELLRLALCARVLLADKEGGPPAVDEVLDEAVPEYRAPGRKSLLEIRQLDPDDRSLAKYKRVLLGPLPPAVDPSLPNVQVTRLTLLSEQAPGPVVMDLTGDLAVLKDQVFVLKEGVDYRVKISFKVHREIVSGLKCLHHTYRRGLRVDKTVYMVGSYGPSAQEYEFVTPVEEAPRGALVRGPYLVVSLFTDDDRTHHLSWEWGLCICQDWKD

162 NP_001317.1 NP_057564.3

>NP_001317.1

MSGDGATEQAAEYVPEKVKKAEKKLEENPYDLDAWSILIREAQNQPIDKARKTYERLVAQFPSSGRFWKLYIEAEIKAKNYDKVEKLFQRCLMKVLHIDLWKCYLSYVRETKGKLPSYKEKMAQAYDFALDKIGMEIMSYQIWVDYINFLKGVEAVGSYAENQRITAVRRVYQRGCVNPMINIEQLWRDYNKYEEGINIHLAKKMIEDRSRDYMNARRVAKEYETVMKGLDRNAPSVPPQNTPQEAQQVDMWKKYIQWEKSNPLRTEDQTLITKRVMFAYEQCLLVLGHHPDIWYEAAQYLEQSSKLLAEKGDMNNAKLFSDEAANIYERAISTLLKKNMLLYFAYADYEESRMKYEKVHSIYNRLLAIEDIDPTLVYIQYMKFARRAEGIKSGRMIFKKAREDTRTRHHVYVTAALMEYYCSKDKSVAFKIFELGLKKYGDIPEYVLAYIDYLSHLNEDNNTRVLFERVLTSGSLPPEKSGEIWARFLAFESNIGDLASILKVEKRRFTAFKEEYEGKETALLVDRYKFMDLYPCSASELKALGYKDVSRAKLAAIIPDPVVAPSIVPVLKDEVDRKPEYPKPDTQQMIPFQPRHLAPPGLHPVPGGVFPVPPAAVVLMKLLPPPICFQGPFVQVDELMEIFRRCKIPNTVEEAVRIITGGAPELAVEGNGPVESNAVLTKAVKRPNEDSDEDEEKGAVVPPVHDIYRARQQKRIR

>NP_057564.3

MKDPSRSSTSPSIINEDVIINGHSHEDDNPFAEYMWMENEEEFNRQIEEELWEEEFIERCFQEMLEEEEEHEWFIPARDLPQTMDQIQDQFNDLVISDGSSLEDLVVKSNLNPNAKEFVPGVKYGNI

163 P0C665 NP_000282.1

>P0C665

MDRRRMALRPGSRRPTAFFFHSRWLVPNLLAFFLGLSGAGPIHLPMPWPNGRRHRVLDPHTQLSTHEAPGRWKPVAPRRMKACPQVLLEW

>NP_000282.1

MSLSNKLTLDKLDVKGKRVVMRVDFNVPMKNNQITNNQRIKAAVPSIKFCLDNGAKSVVLMSHLGRPDGVPMPDKYSLEPVAVELKSLLGKDVLFLKDCVGPEVEKACANPAAGSVILLENLRFHVEEEGKGKDASGNKVKAEPAKIEAFRASLSKLGDVYVNDAFGTAHRAHSSMVGVNLPQKAGGFLMKKELNYFAKALESPERPFLAILGGAKVADKIQLINNMLDKVNEMIIGGGMAFTFLKVLNNMEIGTSLFDEEGAKIVKDLMSKAEKNGVKITLPVDFVTADKFDENAKTGQATVASGIPAGWMGLDCGPESSKKYAEAVTRAKQIVWNGPVGVFEWEAFARGTKALMDEVVKATSRGCITIIGGGDTATCCAKWNTEDKVSHVSTGGGASLELLEGKVLPGVDALSNI

164 P15848 NP_001005.1

>P15848

MGPRGAASLPRGPGPRRLLLPVVLPLLLLLLLAPPGSGAGASRPPHLVFLLADDLGWNDVGFHGSRIRTPHLDALAAGGVLLDNYYTQPLCTPSRSQLLTGRYQIRTGLQHQIIWPCQPSCVPLDEKLLPQLLKEAGYTTHMVGKWHLGMYRKECLPTRRGFDTYFGYLLGSEDYYSHERCTLIDALNVTRCALDFRDGEEVATGYKNMYSTNIFTKRAIALITNHPPEKPLFLYLALQSVHEPLQVPEEYLKPYDFIQDKNRHHYAGMVSLMDEAVGNVTAALKSSGLWNNTVFIFSTDNGGQTLAGGNNWPLRGRKWSLWEGGVRGVGFVASPLLKQKGVKNRELIHISDWLPTLVKLARGHTNGTKPLDGFDVWKTISEGSPSPRIELLHNIDPNFVDSSPCPRNSMAPAKDDSSLPEYSAFNTSVHAAIRHGNWKLLTGYPGCGYWFPPPSQYNVSEIPSSDPPTKTLWLFDIDRDPEERHDLSREYPHIVTKLLSRLQFYHKHSVPVYFPAQDPRCDPKATGVWGPWM

>NP_001005.1

MLMPKKNRIAIYELLFKEGVMVAKKDVHMPKHPELADKNVPNLHVMKAMQSLKSRGYVKEQFAWRHFYWYLTNEGIQYLRDYLHLPPEIVPATLRRSRPETGRPRPKGLEGERPARLTRGEADRDTYRRSAVPPGADKKAEAGAGSATEFQFRGGFGRGRGQPPQ

165 P54803 Q93088

>P54803

MAEWLLSASWQRRAKAMTAAAGSAGRAAVPLLLCALLAPGGAYVLDDSDGLGREFDGIGAVSGGGATSRLLVNYPEPYRSQILDYLFKPNFGASLHILKVEIGGDGQTTDGTEPSHMHYALDENYFRGYEWWLMKEAKKRNPNITLIGLPWSFPGWLGKGFDWPYVNLQLTAYYVVTWIVGAKRYHDLDIDYIGIWNERSYNANYIKILRKMLNYQGLQRVKIIASDNLWESISASMLLDAELFKVVDVIGAHYPGTHSAKDAKLTGKKLWSSEDFSTLNSDMGAGCWGRILNQNYINGYMTSTIAWNLVASYYEQLPYGRCGLMTAQEPWSGHYVVESPVWVSAHTTQFTQPGWYYLKTVGHLEKGGSYVALTDGLGNLTIIIETMSHKHSKCIRPFLPYFNVSQQFATFVLKGSFSEIPELQVWYTKLGKTSERFLFKQLDSLWLLDSDGSFTLSLHEDELFTLTTLTTGRKGSYPLPPKSQPFPSTYKDDFNVDYPFFSEAPNFADQTGVFEYFTNIEDPGEHHFTLRQVLNQRPITWAADASNTISIIGDYNWTNLTIKCDVYIETPDTGGVFIAGRVNKGGILIRSARGIFFWIFANGSYRVTGDLAGWIIYALGRVEVTAKKWYTLTLTIKGHFASGMLNDKSLWTDIPVNFPKNGWAAIGTHSFEFAQFDNFLVEATR

>Q93088

MPPVGGKKAKKGILERLNAGEIVIGDGGFVFALEKRGYVKAGPWTPEAAVEHPEAVRQLHREFLRAGSNVMQTFTFYASEDKLENRGNYVLEKISGQEVNEAACDIARQVADEGDALVAGGVSQTPSYLSCKSETEVKKVFLQQLEVFMKKNVDFLIAEYFEHVEEAVWAVETLIASGKPVAATMCIGPEGDLHGVPPGECAVRLVKAGASIIGVNCHFDPTISLKTVKLMKEGLEAARLKAHLMSQPLAYHTPDCNKQGFIDLPEFPFGLEPRVATRWDIQKYAREAYNLGVRYIGGCCGFEPYHIRAIAEELAPERGFLPPASEKHGSWGSGLDMHTKPWVRARARKEYWENLRIASGRPYNPSMSKPDGWGVTKGTAELMQQKEATTEQQLKELFEKQKFKSQ

166 Q9P0K8 NP_006535.1

>Q9P0K8

MASDLESSLTSIDWLPQLTLRATIEKLGSASQAGPPGSSRKCSPGSPTDPNATLSKDEAAVHQDGKPRYSYATLITYAINSSPAKKMTLSEIYRWICDNFPYYKNAGIGWKNSIRHNLSLNKCFRKVPRPRDDPGKGSYWTIDTCPDISRKRRHPPDDDLSQDSPEQEASKSPRGGVAGSGEASLPPEGNPQMSLQSPTSIASYSQGTGSVDGGAVAAGASGRESAEGPPPLYNTNHDFKFSYSEINFQDLSWSFRNLYKSMLEKSSSSSQHGFSSLLGDIPPSNNYYMYQQQQPPPPQQQQQQQQPPQPPPQQSQPQQQQAPAQGPSAVGGAPPLHTPSTDGCTPPGGKQAGAEGYGPPPVMAMHPPPLQHGGYHPHQHHPHSHPAQQPPPPQPQAQGQAPINNTGFAFPSDWCSNIDSLKESFKMVNRLNWSSIEQSQFSELMESLRQAEQKNWTLDQHHIANLCDSLNHFLTQTGHVPPQGGTHRPPAPARIADSCALTSGKQESAMSQVNSYGHPQAPHLYPGPSPMYPIPTQDSAGYNRPAHHMVPRPSVPPPGANEEIPDDFDWDLIT

>NP_006535.1

MATTAELFEEPFVADEYIERLVWRTPGGGSRGGPEAFDPKRLLEEFVNHIQELQIMDERIQRKVEKLEQQCQKEAKEFAKKVQELQKSNQVAFQHFQELDEHISYVATKVCHLGDQLEGVNTPRQRAVEAQKLMKYFNEFLDGELKSDVFTNSEKIKEAADIIQKLHLIAQELPFDRFSEVKSKIASKYHDLECQLIQEFTSAQRRGEISRMREVAAVLLHFKGYSHCVDVYIKQCQEGAYLRNDIFEDAGILCQRVNKQVGDIFSNPETVLAKLIQNVFEIKLQSFVKEQLEECRKSDAEQYLKNLYDLYTRTTNLSSKLMEFNLGTDKQTFLSKLIKSIFISYLENYIEVETGYLKSRSAMILQRYYDSKNHQKRSIGTGGIQDLKERIRQRTNLPLGPSIDTHGETFLSQEVVVNLLQETKQAFERCHRLSDPSDLPRNAFRIFTILVEFLCIEHIDYALETGLAGIPSSDSRNANLYFLDVVQQANTIFHLFDKQFNDHLMPLISSSPKLSECLQKKKEIIEQMEMKLDTGIDRTLNCMIGQMKHILAAEQKKTDFKPEDENNVLIQYTNACVKVCAYVRKQVEKIKNSMDGKNVDTVLMELGVRFHRLIYEHLQQYSYSCMGGMLAICDVAEYRKCAKDFKIPMVLHLFDTLHALCNLLVVAPDNLKQVCSGEQLANLDKNILHSFVQLRADYRSARLARHFS

167 NP_000225.1 Q9UBR1

>NP_000225.1

MQRSIMSFFHPKKEGKAKKPEKEASNSSRETEPPPKAALKEWNGVVSESDSPVKRPGRKAARVLGSEGEEEDEALSPAKGQKPALDCSQVSPPRPATSPENNASLSDTSPMDSSPSGIPKRRTARKQLPKRTIQEVLEEQSEDEDREAKRKKEEEEEETPKESLTEAEVATEKEGEDGDQPTTPPKPLKTSKAETPTESVSEPEVATKQELQEEEEQTKPPRRAPKTLSSFFTPRKPAVKKEVKEEEPGAPGKEGAAEGPLDPSGYNPAKNNYHPVEDACWKPGQKVPYLAVARTFEKIEEVSARLRMVETLSNLLRSVVALSPPDLLPVLYLSLNHLGPPQQGLELGVGDGVLLKAVAQATGRQLESVRAEAAEKGDVGLVAENSRSTQRLMLPPPPLTASGVFSKFRDIARLTGSASTAKKIDIIKGLFVACRHSEARFIARSLSGRLRLGLAEQSVLAALSQAVSLTPPGQEFPPAMVDAGKGKTAEARKTWLEEQGMILKQTFCEVPDLDRIIPVLLEHGLERLPEHCKLSPGIPLKPMLAHPTRGISEVLKRFEEAAFTCEYKYDGQRAQIHALEGGEVKIFSRNQEDNTGKYPDIISRIPKIKLPSVTSFILDTEAVAWDREKKQIQPFQVLTTRKRKEVDASEIQVQVCLYAFDLIYLNGESLVREPLSRRRQLLRENFVETEGEFVFATSLDTKDIEQIAEFLEQSVKDSCEGLMVKTLDVDATYEIAKRSHNWLKLKKDYLDGVGDTLDLVVIGAYLGRGKRAGRYGGFLLASYDEDSEELQAICKLGTGFSDEELEEHHQSLKALVLPSPRPYVRIDGAVIPDHWLDPSAVWEVKCADLSLSPIYPAARGLVDSDKGISLRFPRFIRVREDKQPEQATTSAQVACLYRKQSQIQNQQGEDSGSDPEDTY

>Q9UBR1

MAGAEWKSLEECLEKHLPLPDLQEVKRVLYGKELRKLDLPREAFEAASREDFELQGYAFEAAEEQLRRPRIVHVGLVQNRIPLPANAPVAEQVSALHRRIKAIVEVAAMCGVNIICFQEAWTMPFAFCTREKLPWTEFAESAEDGPTTRFCQKLAKNHDMVVVSPILERDSEHGDVLWNTAVVISNSGAVLGKTRKNHIPRVGDFNESTYYMEGNLGHPVFQTQFGRIAVNICYGRHHPLNWLMYSINGAEIIFNPSATIGALSESLWPIEARNAAIANHCFTCAINRVGTEHFPNEFTSGDGKKAHQDFGYFYGSSYVAAPDSSRTPGLSRSRDGLLVAKLDLNLCQQVNDVWNFKMTGRYEMYARELAEAVKSNYSPTIVKE

168 NP_056076.1 P0C7P0

>NP_056076.1

MVALRGLGSGLQPWCPLDLRLEWVDTVWELDFTETEPLDPSIEAEIIETGLAAFTKLYESLLPFATGEHGSMESIWTFFIENNVSHSTLVALFYHFVQIVHKKNVSVQYREYGLHAAGLYFLLLEVPGSVANQVFHPVMFDKCIQTLKKSWPQESNLNRKRKKEQPKSSQANPGRHRKRGKPPRREDIEMDEIIEEQEDENICFSARDLSQIRNAIFHLLKNFLRLLPKFSLKEKPQCVQNCIEVFVSLTNFEPVLHECHVTQARALNQAKYIPELAYYGLYLLCSPIHGEGDKVISCVFHQMLSVILMLEVGEGSHRAPLAVTSQVINCRNQAVQFISALVDELKESIFPVVRILLQHICAKVVDKSEYRTFAAQSLVQLLSKLPCGEYAMFIAWLYKYSRSSKIPHRVFTLDVVLALLELPEREVDNTLSLEHQKFLKHKFLVQEIMFDRCLDKAPTVRSKALSSFAHCLELTVTSASESILELLINSPTFSVIESHPGTLLRNSSAFSYQRQTSNRSEPSGEINIDSSGETVGSGERCVMAMLRRRIRDEKTNVRKSALQVLVSILKHCDVSGMKEDLWILQDQCRDPAVSVRKQALQSLTELLMAQPRCVQIQKAWLRGVVPVVMDCESTVQEKALEFLDQLLLQNIRHHSHFHSGDDSQVLAWALLTLLTTESQELSRYLNKAFHIWSKKEKFSPTFINNVISHTGTEHSAPAWMLLSKIAGSSPRLDYSRIIQSWEKISSQQNPNSNTLGHILCVIGHIAKHLPKSTRDKVTDAVKCKLNGFQWSLEVISSAVDALQRLCRASAETPAEEQELLTQVCGDVLSTCEHRLSNIVLKENGTGNMDEDLLVKYIFTLGDIAQLCPARVEKRIFLLIQSVLASSADADHSPSSQGSSEAPASQPPPQVRGSVMPSVIRAHAIITLGKLCLQHEDLAKKSIPALVRELEVCEDVAVRNNVIIVMCDLCIRYTIMVDKYIPNISMCLKDSDPFIRKQTLILLTNLLQEEFVKWKGSLFFRFVSTLIDSHPDIASFGEFCLAHLLLKRNPVMFFQHFIECIFHFNNYEKHEKYNKFPQSEREKRLFSLKGKSNKERRMKIYKFLLEHFTDEQRFNITSKICLSILACFADGILPLDLDASELLSDTFEVLSSKEIKLLAMRSKPDKDLLMEEDDMALANVVMQEAQKKLISQVQKRNFIENIIPIIISLKTVLEKNKIPALRELMHYLREVMQDYRDELKDFFAVDKQLASELEYDMKKYQEQLVQEQELAKHADVAGTAGGAEVAPVAQVALCLETVPVPAGQENPAMSPAVSQPCTPRASAGHVAVSSPTPETGPLQRLLPKARPMSLSTIAILNSVKKAVESKSRHRSRSLGVLPFTLNSGSPEKTCSQVSSYSLEQESNGEIEHVTKRAISTPEKSISDVTFGAGVSYIGTPRTPSSAKEKIEGRSQGNDILCLSLPDKPPPQPQQWNVRSPARNKDTPACSRRSLRKTPLKTAN

>P0C7P0

MRGAGAILRPAARGARDLNPRRDISSWLAQWFPRTPARSVVALKTPIKVELVAGKTYRWCVCGRSKKQPFCDGSHFFQRTGLSPLKFKAQETRMVALCTCKATQRPPYCDGTHRSERVQKAEVGSPL

169 NP_003784.2 Q9BXL7

>NP_003784.2

MAPWLQLLSLLGLLPGAVAAPAQPRAASFQAWGPPSPELLAPTRFALEMFNRGRAAGTRAVLGLVRGRVRRAGQGSLYSLEATLEEPPCNDPMVCRLPVSKKTLLCSFQVLDELGRHVLLRKDCGPVDTKVPGAGEPKSAFTQGSAMISSLSQNHPDNRNETFSSVISLLNEDPLSQDLPVKMASIFKNFVITYNRTYESKEEARWRLSVFVNNMVRAQKIQALDRGTAQYGVTKFSDLTEEEFRTIYLNTLLRKEPGNKMKQAKSVGDLAPPEWDWRSKGAVTKVKDQGMCGSCWAFSVTGNVEGQWFLNQGTLLSLSEQELLDCDKMDKACMGGLPSNAYSAIKNLGGLETEDDYSYQGHMQSCNFSAEKAKVYINDSVELSQNEQKLAAWLAKRGPISVAINAFGMQFYRHGISRPLRPLCSPWLIDHAVLLVGYGNRSDVPFWAIKNSWGTDWGEKGYYYLHRGSGACGVNTMASSAVVD

>Q9BXL7

MPGGGPEMDDYMETLKDEEDALWENVECNRHMLSRYINPAKLTPYLRQCKVIDEQDEDEVLNAPMLPSKINRAGRLLDILHTKGQRGYVVFLESLEFYYPELYKLVTGKEPTRRFSTIVVEEGHEGLTHFLMNEVIKLQQQMKAKDLQRCELLARLRQLEDEKKQMTLTRVELLTFQERYYKMKEERDSYNDELVKVKDDNYNLAMRYAQLSEEKNMAVMRSRDLQLEIDQLKHRLNKMEEECKLERNQSLKLKNDIENRPKKEQVLELERENEMLKTKNQELQSIIQAGKRSLPDSDKAILDILEHDRKEALEDRQELVNRIYNLQEEARQAEELRDKYLEEKEDLELKCSTLGKDCEMYKHRMNTVMLQLEEVERERDQAFHSRDEAQTQYSQCLIEKDKYRKQIRELEEKNDEMRIEMVRREACIVNLESKLRRLSKDSNNLDQSLPRNLPVTIISQDFGDASPRTNGQEADDSSTSEESPEDSKYFLPYHPPQRRMNLKGIQLQRAKSPISLKRTSDFQAKGHEEEGTDASPSSCGSLPITNSFTKMQPPRSRSSIMSITAEPPGNDSIVRRYKEDAPHRSTVEEDNDSGGFDALDLDDDSHERYSFGPSSIHSSSSSHQSEGLDAYDLEQVNLMFRKFSLERPFRPSVTSVGHVRGPGPSVQHTTLNGDSLTSQLTLLGGNARGSFVHSVKPGSLAEKAGLREGHQLLLLEGCIRGERQSVPLDTCTKEEAHWTIQRCSGPVTLHYKVNHEGYRKLVKDMEDGLITSGDSFYIRLNLNISSQLDACTMSLKCDDVVHVRDTMYQDRHEWLCARVDPFTDHDLDMGTIPSYSRAQQLLLVKLQRLMHRGSREEVDGTHHTLRALRNTLQPEEALSTSDPRVSPRLSRASFLFGQLLQFVSRSENKYKRMNSNERVRIISGSPLGSLARSSLDATKLLTEKQEELDPESELGKNLSLIPYSLVRAFYCERRRPVLFTPTVLAKTLVQRLLNSGGAMEFTICKSDIVTRDEFLRRQKTETIIYSREKNPNAFECIAPANIEAVAAKNKHCLLEAGIGCTRDLIKSNIYPIVLFIRVCEKNIKRFRKLLPRPETEEEFLRVCRLKEKELEALPCLYATVEPDMWGSVEELLRVVKDKIGEEQRKTIWVDEDQL

170 NP_002583.1 NP_057461.1

>NP_002583.1

MFEARLVQGSILKKVLEALKDLINEACWDISSSGVNLQSMDSSHVSLVQLTLRSEGFDTYRCDRNLAMGVNLTSMSKILKCAGNEDIITLRAEDNADTLALVFEAPNQEKVSDYEMKLMDLDVEQLGIPEQEYSCVVKMPSGEFARICRDLSHIGDAVVISCAKDGVKFSASGELGNGNIKLSQTSNVDKEEEAVTIEMNEPVQLTFALRYLNFFTKATPLSSTVTLSMSADVPLVVEYKIADMGHLKYYLAPKIEDEEGS

>NP_057461.1

MSEEFEANTMDSLVDMPFATVDIQDDCGITDEPQINLKRSQENEWVKSDQVKKRKKKRKDYQPNYFLSIPITNKEIIKGIKILQNAIIQQDERLAKAMVSDGSFHITLLVMQLLNEDEVNIGIDALLELKPFIEELLQGKHLTLPFQGIGTFGNQVGFVKLAEGDHVNSLLEIAETANRTFQEKGILVGESRSFKPHLTFMKLSKSPWLRKNGVKKIDPDLYEKFISHRFGEEILYRIDLCSMLKKKQSNGYYHCESSIVIGEKNGGEPDDAELVRLSKRLVENAVLKAVQQYLEETQNKNKPGEGSSVKTEAADQNGNDNENNRK

171 NP_002544.1 Q9NVV4

>NP_002544.1

MPHLENVVLCRESQVSILQSLFGERHHFSFPSIFIYGHTASGKTYVTQTLLKTLELPHVFVNCVECFTLRLLLEQILNKLNHLSSSEDGCSTEITCETFNDFVRLFKQVTTAENLKDQTVYIVLDKAEYLRDMEANLLPGFLRLQELADRNVTVLFLSEIVWEKFRPNTGCFEPFVLYFPDYSIGNLQKILSHDHPPEYSADFYAAYINILLGVFYTVCRDLKELRHLAVLNFPKYCEPVVKGEASERDTRKLWRNIEPHLKKAMQTVYLREISSSQWEKLQKDDTDPGQLKGLSAHTHVELPYYSKFILIAAYLASYNPARTDKRFFLKHHGKIKKTNFLKKHEKTSNHLLGPKPFPLDRLLAILYSIVDSRVAPTANIFSQITSLVTLQLLTLVGHDDQLDGPKYKCTVSLDFIRAIARTVNFDIIKYLYDFL

>Q9NVV4

MAVPGVGLLTRLNLCARRRTRVQRPIVRLLSCPGTVAKDLRRDEQPSGSVETGFEDKIPKRRFSEMQNERREQAQRTVLIHCPEKISENKFLKYLSQFGPINNHFFYESFGLYAVVEFCQKESIGSLQNGTHTPSTAMETAIPFRSRFFNLKLKNQTSERSRVRSSNQLPRSNKQLFELLCYAESIDDQLNTLLKEFQLTEENTKLRYLTCSLIEDMAAAYFPDCIVRPFGSSVNTFGKLGCDLDMFLDLDETRNLSAHKISGNFLMEFQVKNVPSERIATQKILSVLGECLDHFGPGCVGVQKILNARCPLVRFSHQASGFQCDLTTNNRIALTSSELLYIYGALDSRVRALVFSVRCWARAHSLTSSIPGAWITNFSLTMMVIFFLQRRSPPILPTLDSLKTLADAEDKCVIEGNNCTFVRDLSRIKPSQNTETLELLLKEFFEYFGNFAFDKNSINIRQGREQNKPDSSPLYIQNPFETSLNISKNVSQSQLQKFVDLARESAWILQQEDTDRPSISSNRPWGLVSLLLPSAPNRKSFTKKKSNKFAIETVKNLLESLKGNRTENFTKTSGKRTISTQT

172 O00115 Q9NYK5

>O00115

MIPLLLAALLCVPAGALTCYGDSGQPVDWFVVYKLPALRGSGEAAQRGLQYKYLDESSGGWRDGRALINSPEGAVGRSLQPLYRSNTSQLAFLLYNDQPPQPSKAQDSSMRGHTKGVLLLDHDGGFWLVHSVPNFPPPASSAAYSWPHSACTYGQTLLCVSFPFAQFSKMGKQLTYTYPWVYNYQLEGIFAQEFPDLENVVKGHHVSQEPWNSSITLTSQAGAVFQSFAKFSKFGDDLYSGWLAAALGTNLQVQFWHKTVGILPSNCSDIWQVLNVNQIAFPGPAGPSFNSTEDHSKWCVSPKGPWTCVGDMNRNQGEEQRGGGTLCAQLPALWKAFQPLVKNYQPCNGMARKPSRAYKI

>Q9NYK5

MEALAMGSRALRLWLVAPGGGIKWRFIATSPASQLSPTELTEMRNDLFNKEKARQLSLTPRTEKIEVKHVGKTDPGTVFVMNKNISTPYSCAMHLSEWYCRKSILALVDGQPWDMYKPLTKSCEIKFLTFKDCDPGEVNKAYWRSCAMMMGCVIERAFKDEYMVNLVRAPEVPVISGAFCYDVVLDSKLDEWMPTKENLRSFTKDAHALIYKDLPFETLEVEAKVALEIFQHSKYKVDFIEEKASQNPERIVKLHRIGDFIDVSEGPLIPRTSICFQYEVSAVHNLQPTQPSLIRRFQGVSLPVHLRAHFTIWDKLLERSRKMVTEDQSKATEECTST

173 NP_006224.1 NP_002684.1

>NP_006224.1

MEPDGTYEPGFVGIRFCQECNNMLYPKEDKENRILLYACRNCDYQQEADNSCIYVNKITHEVDELTQIIADVSQDPTLPRTEDHPCQKCGHKEAVFFQSHSARAEDAMRLYYVCTAPHCGHRWTE

>NP_002684.1

MSRLLWRKVAGATVGPGPVPAPGRWVSSSVPASDPSDGQRRRQQQQQQQQQQQQQPQQPQVLSSEGGQLRHNPLDIQMLSRGLHEQIFGQGGEMPGEAAVRRSVEHLQKHGLWGQPAVPLPDVELRLPPLYGDNLDQHFRLLAQKQSLPYLEAANLLLQAQLPPKPPAWAWAEGWTRYGPEGEAVPVAIPEERALVFDVEVCLAEGTCPTLAVAISPSAWYSWCSQRLVEERYSWTSQLSPADLIPLEVPTGASSPTQRDWQEQLVVGHNVSFDRAHIREQYLIQGSRMRFLDTMSMHMAISGLSSFQRSLWIAAKQGKHKVQPPTKQGQKSQRKARRGPAISSWDWLDISSVNSLAEVHRLYVGGPPLEKEPRELFVKGTMKDIRENFQDLMQYCAQDVWATHEVFQQQLPLFLERCPHPVTLAGMLEMGVSYLPVNQNWERYLAEAQGTYEELQREMKKSLMDLANDACQLLSGERYKEDPWLWDLEWDLQEFKQKKAKKVKKEPATASKLPIEGAGAPGDPMDQEDLGPCSEEEEFQQDVMARACLQKLKGTTELLPKRPQHLPGHPGWYRKLCPRLDDPAWTPGPSLLSLQMRVTPKLMALTWDGFPLHYSERHGWGYLVPGRRDNLAKLPTGTTLESAGVVCPYRAIESLYRKHCLEQGKQQLMPQEAGLAEEFLLTDNSAIWQTVEELDYLEVEAEAKMENLRAAVPGQPLALTARGGPKDTQPSYHHGNGPYNDVDIPGCWFFKLPHKDGNSCNVGSPFAKDFLPKMEDGTLQAGPGGASGPRALEINKMISFWRNAHKRISSQMVVWLPRSALPRAVIRHPDYDEEGLYGAILPQVVTAGTITRRAVEPTWLTASNARPDRVGSELKAMVQAPPGYTLVGADVDSQELWIAAVLGDAHFAGMHGCTAFGWMTLQGRKSRGTDLHSKTATTVGISREHAKIFNYGRIYGAGQPFAERLLMQFNHRLTQQEAAEKAQQMYAATKGLRWYRLSDEGEWLVRELNLPVDRTEGGWISLQDLRKVQRETARKSQWKKWEVVAERAWKGGTESEMFNKLESIATSDIPRTPVLGCCISRALEPSAVQEEFMTSRVNWVVQSSAVDYLHLMLVAMKWLFEEFAIDGRFCISIHDEVRYLVREEDRYRAALALQITNLLTRCMFAYKLGLNDLPQSVAFFSAVDIDRCLRKEVTMDCKTPSNPTGMERRYGIPQGEALDIYQIIELTKGSLEKRSQPGP

174 NP_443082.2 Q96RP9

>NP_443082.2

MAASVRQARSLLGVAATLAPGSRGYRARPPPRRRPGPRWPDPEDLLTPRWQLGPRYAAKQFARYGAASGVVPGSLWPSPEQLRELEAEEREWYPSLATMQESLRVKQLAEEQKRREREQHIAECMAKMPQMIVNWQQQQRENWEKAQADKERRARLQAEAQELLGYQVDPRSARFQELLQDLEKKERKRLKEEKQKRKKEARAAALAAAVAQDPAASGAPSS

>Q96RP9

MRLLGAAAVAALGRGRAPASLGWQRKQVNWKACRWSSSGVIPNEKIRNIGISAHIDSGKTTLTERVLYYTGRIAKMHEVKGKDGVGAVMDSMELERQRGITIQSAATYTMWKDVNINIIDTPGHVDFTIEVERALRVLDGAVLVLCAVGGVQCQTMTVNRQMKRYNVPFLTFINKLDRMGSNPARALQQMRSKLNHNAAFMQIPMGLEGNFKGIVDLIEERAIYFDGDFGQIVRYGEIPAELRAAATDHRQELIECVANSDEQLGEMFLEEKIPSISDLKLAIRRATLKRSFTPVFLGSALKNKGVQPLLDAVLEYLPNPSEVQNYAILNKEDDSKEKTKILMNSSRDNSHPFVGLAFKLEVGRFGQLTYVRSYQGELKKGDTIYNTRTRKKVRLQRLARMHADMMEDVEEVYAGDICALFGIDCASGDTFTDKANSGLSMESIHVPDPVISIAMKPSNKNDLEKFSKGIGRFTREDPTFKVYFDTENKETVISGMGELHLEIYAQRLEREYGCPCITGKPKVAFRETITAPVPFDFTHKKQSGGAGQYGKVIGVLEPLDPEDYTKLEFSDETFGSNIPKQFVPAVEKGFLDACEKGPLSGHKLSGLRFVLQDGAHHMVDSNEISFIRAGEGALKQALANATLCILEPIMAVEVVAPNEFQGQVIAGINRRHGVITGQDGVEDYFTLYADVPLNDMFGYSTELRSCTEGKGEYTMEYSRYQPCLPSTQEDVINKYLEATGQLPVKKGKAKN

175 NP_004796.1 NP_005470.2

>NP_004796.1

MAAGGSDPRAGDVEEDASQLIFPKEFETAETLLNSEVHMLLEHRKQQNESAEDEQELSEVFMKTLNYTARFSRFKNRETIASVRSLLLQKKLHKFELACLANLCPETAEESKALIPSLEGRFEDEELQQILDDIQTKRSFQY

>NP_005470.2

MPCRREEEEEAGEEAEGEEEEEDSFLLLQQSVALGSSGEVDRLVAQIGETLQLDAAQHSPASPCGPPGAPLRAPGPLAAAVPADKARSPAVPLLLPPALAETVGPAPPGVLRCALGDRGRVRGRAAPYCVAELATGPSALSPLPPQADLDGPPGAGKQGIPQPLSGPCRRGWLRGAAASRRLQQRRGSQPETRTGDDDPHRLLQQLVLSGNLIKEAVRRLHSRRLQLRAKLPQRPLLGPLSAPVHEPPSPRSPRAACSDPGASGRAQLRTGDGVLVPGS

176 NP_005635.1 NP_002940.2

>NP_005635.1

MNQFGPSALINLSNFSSIKPEPASTPPQGSMANSTAVVKIPGTPGAGGRLSPENNQVLTKKKLQDLVREVDPNEQLDEDVEEMLLQIADDFIESVVTAACQLARHRKSSTLEVKDVQLHLERQWNMWIPGFGSEEIRPYKKACTTEAHKQRMALIRKTTKK

>NP_002940.2

MLPAAARPLWGPCLGLRAAAFRLARRQVPCVCAVRHMRSSGHQRCEALAGAPLDNAPKEYPPKIQQLVQDIASLTLLEISDLNELLKKTLKIQDVGLVPMGGVMSGAVPAAAAQEAVEEDIPIAKERTHFTVRLTEAKPVDKVKLIKEIKNYIQGINLVQAKKLVESLPQEIKANVAKAEAEKIKAALEAVGGTVVLE

177 AAH09210.1 NP_003215.1

>AAH09210.1

MAASRWARKAVVLLCASDLLLLLLLLPPPGSCAAEGSPGTPDESTPPPRKKKKDIRDYNDADMARLLEQWEKDDDIEEGDLPEHKRPSAPVDFSKIDPSKPESILKMTKKGKTLMMFVTVSGSPTEKETEEITSLWQGSLFNANYDVQRFIVGSDRAIFMLRDGSYAWEIKDFLVGQDRCADVTLEGQVYPGKGGGSKEKNKTKQDKGKKKKEGDLKSRSSKEENRAGNKREDL

>NP_003215.1

MYTLLSGLYKYMFQKDEYCILILGLDNAGKTTFLEQSKTRFNKNYKGMSLSKITTTVGLNIGTVDVGKARLMFWDLGGQEELQSLWDKYYAECHGVIYVIDSTDEERLAESKQAFEKVVTSEALCGVPVLVLANKQDVETCLSIPDIKTAFSDCTSKIGRRDCLTQACSALTGKGVREGIEWMVKCVVRNVHRPPRQRDIT

178 O00115 AAH07654.1

>O00115

MIPLLLAALLCVPAGALTCYGDSGQPVDWFVVYKLPALRGSGEAAQRGLQYKYLDESSGGWRDGRALINSPEGAVGRSLQPLYRSNTSQLAFLLYNDQPPQPSKAQDSSMRGHTKGVLLLDHDGGFWLVHSVPNFPPPASSAAYSWPHSACTYGQTLLCVSFPFAQFSKMGKQLTYTYPWVYNYQLEGIFAQEFPDLENVVKGHHVSQEPWNSSITLTSQAGAVFQSFAKFSKFGDDLYSGWLAAALGTNLQVQFWHKTVGILPSNCSDIWQVLNVNQIAFPGPAGPSFNSTEDHSKWCVSPKGPWTCVGDMNRNQGEEQRGGGTLCAQLPALWKAFQPLVKNYQPCNGMARKPSRAYKI

>AAH07654.1

MLSSCVRPVPTTVRFVDSLICNSSRSFMDLKALLSSLNDFASLSFAESWDNVGLLVEPSPPHTVNTLFLTNDLTEEVMEEVLQKKADLILSYHPPIFRPMKRITWNTWKERLVIRALENRVGIYSPHTAYDAAPQGVNNWLAKGLGACTSRPIHPSKAPNYPTEGNHRVEFNVNYTQDLDKVMSAVKGIDGVSVTSFSARTGNEEQTRINLNCTQKALMQVVDFLSRNKQLYQKTEILSLEKPLLLHTGMGRLCTLDESVSLATMIDRIKRHLKLSHIRLALGVGRTLESQVKVVALCAGSGSSVLQGVEADLYLTGEMSHHDTLDAASQGINVILCEHSNTERGFLSDLRDMLDSHLENKINIILSETDRDPLQVV

179 NP_005180.1 NP_004835.2

>NP_005180.1

MEELSSVGEQVFAAECILSKRLRKGKLEYLVKWRGWSSKHNSWEPEENILDPRLLLAFQKKEHEKEVQNRKRGKRPRGRPRKLTAMSSCSRRSKLKEPDAPSKSKSSSSSSSSTSSSSSSDEEDDSDLDAKRGPRGRETHPVPQKKAQILVAKPELKDPIRKKRGRKPLPPEQKATRRPVSLAKVLKTARKDLGAPASKLPPPLSAPVAGLAALKAHAKEACGGPSAMATPENLASLMKGMASSPGRGGISWQSSIVHYMNRMTQSQAQAASRLALKAQATNKCGLGLDLKVRTQKGELGMSPPGSKIPKAPSGGAVEQKVGNTGGPPHTHGASRVPAGCPGPQPAPTQELSLQVLDLQSVKNGMPGVGLLARHATATKGVPATNPAPGKGTGSGLIGASGATMPTDTSKSEKLASRAVAPPTPASKRDCVKGSATPSGQESRTAPGEARKAATLPEMSAGEESSSSDSDPDSASPPSTGQNPSVSVQTSQDWKPTRSLIEHVFVTDVTANLITVTVKESPTSVGFFNLRHY

>NP_004835.2

MDAALKRSRSEEPAEILPPARDEEEEEEEGMEQGLEEEEEVDPRIQGELEKLNQSTDDINRRETELEDARQKFRSVLVEATVKLDELVKKIGKAVEDSKPYWEARRVARQAQLEAQKATQDFQRATEVLRAAKETISLAEQRLLEDDKRQFDSAWQEMLNHATQRVMEAEQTKTRSELVHKETAARYNAAMGRMRQLEKKLKRAINKSKPYFELKAKYYVQLEQLKKTVDDLQAKLTLAKGEYKMALKNLEMISDEIHERRRSSAMGPRGCGVGAEGSSTSVEDLPGSKPEPDAISVASEAFEDDSCSNFVSEDDSETQSVSSFSSGPTSPSEMPDQFPAVVRPGSLDLPSPVSLSEFGMMFPVLGPRSECSGASSPECEVERGDRAEGAENKTSDKANNNRGLSSSSGSGGSSKSQSSTSPEGQALENRMKQLSLQCSKGRDGIIADIKMVQIG

180 NP_036363.2 NP_002684.1

>NP_036363.2

MNQNTTEPVAATETLAEVPEHVLRGLPEEVRLFPSAVDKTRIGVWATKPILKGKKFGPFVGDKKKRSQVKNNVYMWEVYYPNLGWMCIDATDPEKGNWLRYVNWACSGEEQNLFPLEINRAIYYKTLKPIAPGEELLVWYNGEDNPEIAAAIEEERASARSKRSSPKSRKGKKKSQENKNKGNKIQDIQLKTSEPDFTSANMRDSAEGPKEDEEKPSASALEQPATLQEVASQEVPPELATPAPAWEPQPEPDERLEAAACEVNDLGEEEEEEEEEDEEEEEDDDDDELEDEGEEEASMPNENSVKEPEIRCDEKPEDLLEEPKTTSEETLEDCSEVTPAMQIPRTKEEANGDVFETFMFPCQHCERKFTTKQGLERHMHIHISTVNHAFKCKYCGKAFGTQINRRRHERRHEAGLKRKPSQTLQPSEDLADGKASGENVASKDDSSPPSLGPDCLIMNSEKASQDTINSSVVEENGEVKELHPCKYCKKVFGTHTNMRRHQRRVHERHLIPKGVRRKGGLEEPQPPAEQAQATQNVYVPSTEPEEEGEADDVYIMDISSNISENLNYYIDGKIQTNNNTSNCDVIEMESASADLYGINCLLTPVTVEITQNIKTTQVPVTEDLPKEPLGSTNSEAKKRRTASPPALPKIKAETDSDPMVPSCSLSLPLSISTTEAVSFHKEKSVYLSSKLKQLLQTQDKLTPAGISATEIAKLGPVCVSAPASMLPVTSSRFKRRTSSPPSSPQHSPALRDFGKPSDGKAAWTDAGLTSKKSKLESHSDSPAWSLSGRDERETVSPPCFDEYKMSKEWTASSAFSSVCNQQPLDLSSGVKQKAEGTGKTPVQWESVLDLSVHKKHCSDSEGKEFKESHSVQPTCSAVKKRKPTTCMLQKVLLNEYNGIDLPVENPADGTRSPSPCKSLEAQPDPDLGPGSGFPAPTVESTPDVCPSSPALQTPSLSSGQLPPLLIPTDPSSPPPCPPVLTVATPPPPLLPTVPLPAPSSSASPHPCPSPLSNATAQSPLPILSPTVSPSPSPIPPVEPLMSAASPGPPTLSSSSSSSSSSSSFSSSSSSSSPSPPPLSAISSVVSSGDNLEASLPMISFKQEELENEGLKPREEPQSAAEQDVVVQETFNKNFVCNVCESPFLSIKDLTKHLSIHAEEWPFKCEFCVQLFKDKTDLSEHRFLLHGVGNIFVCSVCKKEFAFLCNLQQHQRDLHPDKVCTHHEFESGTLRPQNFTDPSKAHVEHMQSLPEDPLETSKEEEELNDSSEELYTTIKIMASGIKTKDPDVRLGLNQHYPSFKPPPFQYHHRNPMGIGVTATNFTTHNIPQTFTTAIRCTKCGKGVDNMPELHKHILACASASDKKRYTPKKNPVPLKQTVQPKNGVVVLDNSGKNAFRRMGQPKRLNFSVELSKMSSNKLKLNALKKKNQLVQKAILQKNKSAKQKADLKNACESSSHICPYCNREFTYIGSLNKHAAFSCPKKPLSPPKKKVSHSSKKGGHSSPASSDKNSNSNHRRRTADAEIKMQSMQTPLGKTRARSSGPTQVPLPSSSFRSKQNVKFAASVKSKKPSSSSLRNSSPIRMAKITHVEGKKPKAVAKNHSAQLSSKTSRSLHVRVQKSKAVLQSKSTLASKKRTDRFNIKSRERSGGPVTRSLQLAAAADLSENKREDGSAKQELKDFSYSLRLASRCSPPAAPYITRQYRKVKAPAAAQFQGPFFKE

>NP_002684.1

MSRLLWRKVAGATVGPGPVPAPGRWVSSSVPASDPSDGQRRRQQQQQQQQQQQQQPQQPQVLSSEGGQLRHNPLDIQMLSRGLHEQIFGQGGEMPGEAAVRRSVEHLQKHGLWGQPAVPLPDVELRLPPLYGDNLDQHFRLLAQKQSLPYLEAANLLLQAQLPPKPPAWAWAEGWTRYGPEGEAVPVAIPEERALVFDVEVCLAEGTCPTLAVAISPSAWYSWCSQRLVEERYSWTSQLSPADLIPLEVPTGASSPTQRDWQEQLVVGHNVSFDRAHIREQYLIQGSRMRFLDTMSMHMAISGLSSFQRSLWIAAKQGKHKVQPPTKQGQKSQRKARRGPAISSWDWLDISSVNSLAEVHRLYVGGPPLEKEPRELFVKGTMKDIRENFQDLMQYCAQDVWATHEVFQQQLPLFLERCPHPVTLAGMLEMGVSYLPVNQNWERYLAEAQGTYEELQREMKKSLMDLANDACQLLSGERYKEDPWLWDLEWDLQEFKQKKAKKVKKEPATASKLPIEGAGAPGDPMDQEDLGPCSEEEEFQQDVMARACLQKLKGTTELLPKRPQHLPGHPGWYRKLCPRLDDPAWTPGPSLLSLQMRVTPKLMALTWDGFPLHYSERHGWGYLVPGRRDNLAKLPTGTTLESAGVVCPYRAIESLYRKHCLEQGKQQLMPQEAGLAEEFLLTDNSAIWQTVEELDYLEVEAEAKMENLRAAVPGQPLALTARGGPKDTQPSYHHGNGPYNDVDIPGCWFFKLPHKDGNSCNVGSPFAKDFLPKMEDGTLQAGPGGASGPRALEINKMISFWRNAHKRISSQMVVWLPRSALPRAVIRHPDYDEEGLYGAILPQVVTAGTITRRAVEPTWLTASNARPDRVGSELKAMVQAPPGYTLVGADVDSQELWIAAVLGDAHFAGMHGCTAFGWMTLQGRKSRGTDLHSKTATTVGISREHAKIFNYGRIYGAGQPFAERLLMQFNHRLTQQEAAEKAQQMYAATKGLRWYRLSDEGEWLVRELNLPVDRTEGGWISLQDLRKVQRETARKSQWKKWEVVAERAWKGGTESEMFNKLESIATSDIPRTPVLGCCISRALEPSAVQEEFMTSRVNWVVQSSAVDYLHLMLVAMKWLFEEFAIDGRFCISIHDEVRYLVREEDRYRAALALQITNLLTRCMFAYKLGLNDLPQSVAFFSAVDIDRCLRKEVTMDCKTPSNPTGMERRYGIPQGEALDIYQIIELTKGSLEKRSQPGP

181 NP_002303.2 NP_002717.3

>NP_002303.2

MAASQTSQTVASHVPFADLCSTLERIQKSKGRAEKIRHFREFLDSWRKFHDALHKNHKDVTDSFYPAMRLILPQLERERMAYGIKETMLAKLYIELLNLPRDGKDALKLLNYRTPTGTHGDAGDFAMIAYFVLKPRCLQKGSLTIQQVNDLLDSIASNNSAKRKDLIKKSLLQLITQSSALEQKWLIRMIIKDLKLGVSQQTIFSVFHNDAAELHNVTTDLEKVCRQLHDPSVGLSDISITLFSAFKPMLAAIADIEHIEKDMKHQSFYIETKLDGERMQMHKDGDVYKYFSRNGYNYTDQFGASPTEGSLTPFIHNAFKADIQICILDGEMMAYNPNTQTFMQKGTKFDIKRMVEDSDLQTCYCVFDVLMVNNKKLGHETLRKRYEILSSIFTPIPGRIEIVQKTQAHTKNEVIDALNEAIDKREEGIMVKQPLSIYKPDKRGEGWLKIKPEYVSGLMDELDILIVGGYWGKGSRGGMMSHFLCAVAEKPPPGEKPSVFHTLSRVGSGCTMKELYDLGLKLAKYWKPFHRKAPPSSILCGTEKPEVYIEPCNSVIVQIKAAEIVPSDMYKTGCTLRFPRIEKIRDDKEWHECMTLDDLEQLRGKASGKLASKHLYIGGDDEPQEKKRKAAPKMKKVIGIIEHLKAPNLTNVNKISNIFEDVEFCVMSGTDSQPKPDLENRIAEFGGYIVQNPGPDTYCVIAGSENIRVKNIILSNKHDVVKPAWLLECFKTKSFVPWQPRFMIHMCPSTKEHFAREYDCYGDSYFIDTDLNQLKEVFSGIKNSNEQTPEEMASLIADLEYRYSWDCSPLSMFRRHTVYLDSYAVINDLSTKNEGTRLAIKALELRFHGAKVVSCLAEGVSHVIIGEDHSRVADFKAFRRTFKRKFKILKESWVTDSIDKCELQEENQYLI

>NP_002717.3

MLSLQYPDVYRDETAVQDYHGHKICDPYAWLEDPDSEQTKAFVEAQNKITVPFLEQCPIRGLYKERMTELYDYPKYSCHFKKGKRYFYFYNTGLQNQRVLYVQDSLEGEARVFLDPNILSDDGTVALRGYAFSEDGEYFAYGLSASGSDWVTIKFMKVDGAKELPDVLERVKFSCMAWTHDGKGMFYNSYPQQDGKSDGTETSTNLHQKLYYHVLGTDQSEDILCAEFPDEPKWMGGAELSDDGRYVLLSIREGCDPVNRLWYCDLQQESSGIAGILKWVKLIDNFEGEYDYVTNEGTVFTFKTNRQSPNYRVINIDFRDPEESKWKVLVPEHEKDVLEWIACVRSNFLVLCYLHDVKNILQLHDLTTGALLKTFPLDVGSIVGYSGQKKDTEIFYQFTSFLSPGIIYHCDLTKEELEPRVFREVTVKGIDASDYQTVQIFYPSKDGTKIPMFIVHKKGIKLDGSHPAFLYGYGGFNISITPNYSVSRLIFVRHMGGILAVANIRGGGEYGETWHKGGILANKQNCFDDFQCAAEYLIKEGYTSPKRLTINGGSNGGLLVAACANQRPDLFGCVIAQVGVMDMLKFHKYTIGHAWTTDYGCSDSKQHFEWLVKYSPLHNVKLPEADDIQYPSMLLLTADHDDRVVPLHSLKFIATLQYIVGRSRKQSNPLLIHVDTKAGHGAGKPTAKVIEEVSDMFAFIARCLNVDWIP

182 NP_073598.1 P51970

>NP_073598.1

MAASQCLCCSKFLFQRQNLACFLTNPHCGSLVNADGHGEVWTDWNNMSKFFQYGWRCTTNENTYSNRTLMGNWNQERYDLRNIVQPKPLPSQFGHYFETTYDTSYNNKMPLSTHRFKREPHWFPGHQPELDPPRYKCTEKSTYMNSYSKP

>P51970

MPGIVELPTLEELKVDEVKISSAVLKAAAHHYGAQCDKPNKEFMLCRWEEKDPRRCLEEGKLVNKCALDFFRQIKRHCAEPFTEYWTCIDYTGQQLFRHCRKQQAKFDECVLDKLGWVRPDLGELSKVTKVKTDRPLPENPYHSRPRPDPSPEIEGDLQPATHGSRFYFWTK

183 NP_904357.1 Q8WXI4

>NP_904357.1

MPFLGQDWRSPGWSWIKTEDGWKRCESCSQKLERENNRCNISHSIILNSEDGEIFNNEEHEYASKKRKKDHFRNDTNTQSFYREKWIYVHKESTKERHGYCTLGEAFNRLDFSSAIQDIRRFNYVVKLLQLIAKSQLTSLSGVAQKNYFNILDKIVQKVLDDHHNPRLIKDLLQDLSSTLCILIRGVGKSVLVGNINIWICRLETILAWQQQLQDLQMTKQVNNGLTLSDLPLHMLNNILYRFSDGWDIITLGQVTPTLYMLSEDRQLWKKLCQYHFAEKQFCRHLILSEKGHIEWKLMYFALQKHYPAKEQYGDTLHFCRHCSILFWKDYHLALLFKDSGHPCTAADPDSCFTPVSPQHFIDLFKF

>Q8WXI4

MIQNVGNHLRRGLASVFSNRTSRKSALRAGNDSAMADGEGYRNPTEVQMSQLVLPCHTNQRGELSVGQLLKWIDTTACLSAERHAGCPCVTASMDDIYFEHTISVGQVVNIKAKVNRAFNSSMEVGIQVASEDLCSEKQWNVCKALATFVARREITKVKLKQITPRTEEEKMEHSVAAERRRMRLVYADTIKDLLANCAIQGDLESRDCSRMVPAEKTRVESVELVLPPHANHQGNTFGGQIMAWMENVATIAASRLCRAHPTLKAIEMFHFRGPSQVGDRLVLKAIVNNAFKHSMEVGVCVEAYRQEAETHRRHINSAFMTFVVLDADDQPQLLPWIRPQPGDGERRYREASARKKIRLDRKYIVSCKQTEVPLSVPWDPSNQVYLSYNNVSSLKMLVAKDNWVLSSEISQVRLYTLEDDKFLSFHMEMVVHVDAAQAFLLLSDLRQRPEWDKHYRSVELVQQVDEDDAIYHVTSPALGGHTKPQDFVILASRRKPCDNGDPYVIALRSVTLPTHRETPEYRRGETLCSGFCLWREGDQLTKCCWVRVSLTELVSASGFYSWGLESRSKGRRSDGWNGKLAGGHLSTLKAIPVAKINSRFGYLQDT

184 O95766 NP_001025.1

>O95766

MAAAAAGAGSGPWAAQEKQFPPALLSFFIYNPRFGPREGQEENKILFYHPNEVEKNEKIRNVGLCEAIVQFTRTFSPSKPAKSLHTQKNRQFFNEPEENFWMVMVVRNPIIEKQSKDGKPVIEYQEEELLDKVYSSVLRQCYSMYKLFNGTFLKAMEDGGVKLLKERLEKFFHRYLQTLHLQSCDLLDIFGGISFFPLDKMTYLKIQSFINRMEESLNIVKYTAFLYNDQLIWSGLEQDDMRILYKYLTTSLFPRHIEPELAGRDSPIRAEMPGNLQHYGRFLTGPLNLNDPDAKCRFPKIFVNTDDTYEELHLIVYKAMSAAVCFMIDASVHPTLDFCRRLDSIVGPQLTVLASDICEQFNINKRMSGSEKEPQFKFIYFNHMNLAEKSTVHMRKTPSVSLTSVHPDLMKILGDINSDFTRVDEDEEIIVKAMSDYWVVGKKSDRRELYVILNQKNANLIEVNEEVKKLCATQFNNIFFLD

>NP_001025.1

MLSLRVPLAPITDPQQLQLSPLKGLSLVDKENTPPALSGTRVLASKTARRIFQEPTEPKTKAAAPGVEDEPLLRENPRRFVIFPIEYHDIWQMYKKAEASFWTAEEVDLSKDIQHWESLKPEERYFISHVLAFFAASDGIVNENLVERFSQEVQITEARCFYGFQIAMENIHSEMYSLLIDTYIKDPKEREFLFNAIETMPCVKKKADWALRWIGDKEATYGERVVAFAAVEGIFFSGSFASIFWLKKRGLMPGLTFSNELISRDEGLHCDFACLMFKHLVHKPSEERVREIIINAVRIEQEFLTEALPVKLIGMNCTLMKQYIEFVADRLMLELGFSKVFRVENPFDFMENISLEGKTNFFEKRVGEYQRMGVMSSPTENSFTLDADF

185 O75132 NP_003671.1

>O75132

MENNLKTCPKEDGDFVSDKIKFKIEEEDDDGIPPDSLERMDFKSEQEDMKQTDSGGERAGLGGTGCSCKPPGKYLSAESEDDYGALFSQYSSTLYDVAMEAVTQSLLSSRNMSSRKKSPAWKHFFISPRDSTKAICMYCVKEFSRGKNEKDLSTSCLMRHVRRAHPTVLIQENGSVSAVSSFPSPSLLLPPQPADAGDLSTILSPIKLVQKVASKIPSPDRITEESVSVVSSEEISSDMSVSEKCGREEALVGSSPHLPALHYDEPAENLAEKSLPLPKSTSGSRRRSAVWKHFYLSPLDNSKAVCIHCMNEFSRGKNGKDLGTSCLIRHMWRAHRAIVLQENGGTGIPPLYSTPPTLLPSLLPPEGELSSVSSSPVKPVRESPSASSSPDRLTEDLQSHLNPGDGLMEDVAAFSSSDDVGEASASSPEKQQADGLSPRLFESGAIFQQNKKVMKRLKSEVWHHFSLAPMDSLKAECRYCGCAISRGKKGDVGTSCLMRHLYRRHPEVVGSQKGFLGASLANSPYATLASAESSSSKLTDLPTVVTKNNQVMFPVNSKKTSKLWNHFSICSADSTKVVCLHCGRTISRGKKPTNLGTSCLLRHLQRFHSNVLKTEVSETARPSSPDTRVPRGTELSGASSFDDTNEKFYDSHPVAKKITSLIAEMIALDLQPYSFVDNVGFNRLLEYLKPQYSLPAPSYFSRTAIPGMYDNVKQIIMSHLKEAESGVIHFTSGIWMSNQTREYLTLTAHWVSFESPARPRCDDHHCSALLDVSQVDCDYSGNSIQKQLECWWEAWVTSTGLQVGITVTDNASIGKTLNEGEHSSVQCFSHTVNLIVSEAIKSQRMVQNLLSLARKICERVHRSPKAKEKLAELQREYALPQHHLIQDVPSKWSTSFHMLERLIEQKRAINEMSVECNFRELISCDQWEVMQSVCRALKPFEAASREMSTQMSTLSQVIPMVHILNRKVEMLFEETMGIDTMLRSLKEAMVSRLSATLHDPRYVFATLLDPRYKASLFTEEEAEQYKQDLIRELELMNSTSEDVAASHRCDAGSPSKDSAAEENLWSLVAKVKKKDPREKLPEAMVLAYLEEEVLEHSCDPLTYWNLKKASWPGLSALAVRFLGCPPSIVPSEKLFNTPTENGSLGQSRLMMEHFEKLIFLKVNLPLIYFQY

>NP_003671.1

MGDAPSPEEKLHLITRNLQEVLGEEKLKEILKERELKIYWGTATTGKPHVAYFVPMSKIADFLKAGCEVTILFADLHAYLDNMKAPWELLELRVSYYENVIKAMLESIGVPLEKLKFIKGTDYQLSKEYTLDVYRLSSVVTQHDSKKAGAEVVKQVEHPLLSGLLYPGLQALDEEYLKVDAQFGGIDQRKIFTFAEKYLPALGYSKRVHLMNPMVPGLTGSKMSSSEEESKIDLLDRKEDVKKKLKKAFCEPGNVENNGVLSFIKHVLFPLKSEFVILRDEKWGGNKTYTAYVDLEKDFAAEVVHPGDLKNSVEVALNKLLDPIREKFNTPALKKLASAAYPDPSKQKPMAKGPAKNSEPEEVIPSRLDIRVGKIITVEKHPDADSLYVEKIDVGEAEPRTVVSGLVQFVPKEELQDRLVVVLCNLKPQKMRGVESQGMLLCASIEGINRQVEPLDPPAGSAPGEHVFVKGYEKGQPDEELKPKKKVFEKLQADFKISEECIAQWKQTNFMTKLGSISCKSLKGGNIS

186 NP_079273.2 NP_076425.1

>NP_079273.2

MASPDRSKRKILKAKKTMPLSCRKQVEMLNKSRNVEALKTAIGSNVPSGNQSFSPSVITRTTEITKCSPSENGASSLDSNKNSISEKSKVFSQNCIKPVEEIVHSETKLEQVVCSYQKPSRTTESPSRVFTEEAKDSLNTSENDSEHQTNVTRSLFEHEGACSLKSSCCPPSVLSGVVQMPESTVTSTVGDKKTDQMVFHLETNSNSESHDKRQSDNILCSEDSGFVPVEKTPNLVNSVTSNNCADDILKTDECSRTSISNCESADSTWQSSLDTNNNSHYQKKRMFSENEENVKRMKTSEQINENICVSLERQTAFLEQVRHLIQQEIYSINYELFDKKLKELNQRIGKTECRNKHEGIADKLLAKIAKLQRRIKTVLLFQRNCLKPNMLSSNGASKVANSEAMILDKNLESVNSPIEKSSVNYEPSNPSEKGSKKINLSSDQNKSVSESNNDDVMLISVESPNLTTPITSNPTDTRKITSGNSSNSPNAEVMAVQKKLDSIIDLTKEGLSNCNTESPVSPLESHSKAASNSKETTPLAQNAVQVPESFEHLPPLPEPPAPLPELVDKTRDTLPPQKPELKVKRVFRPNGIALTWNITKINPKCAPVESYHLFLCHENSNNKLIWKKIGEIKALPLPMACTLSQFLASNRYYFTVQSKDIFGRYGPFCDIKSIPGFSENLT

>NP_076425.1

MARKKVRPRLIAELARRVRALREQLNRPRDSQLYAVDYETLTRPFSGRRLPVRAWADVRRESRLLQLLGRLPLFGLGRLVTRKSWLWQHDEPCYWRLTRVRPDYTAQNLDHGKAWGILTFKGKTESEAREIEHVMYHDWRLVPKHEEEAFTAFTPAPEDSLASVPYPPLLRAMIIAERQKNGDTSTEEPMLNVQRIRMEPWDYPAKQEDKGRAKGTPV

187 Q96JN0 NP_056177.3

>Q96JN0

MQRMIQQFAAEYTSKNSSTQDPSQPNSTKNQSLPKASPVTTSPTAATTQNPVLSKLLMADQDSPLDLTVRKSQSEPSEQDGVLDLSTKKSPCAGSTSLSHSPGCSSTQGNGRPGRPSQYRPDGLRSGDGVPPRSLQDGTREGFGHSTSLKVPLARSLQISEELLSRNQLSTAASLGPSGLQNHGQHLILSREASWAKPHYEFNLSRMKFRGNGALSNISDLPFLAENSAFPKMALQAKQDGKKDVSHSSPVDLKIPQVRGMDLSWESRTGDQYSYSSLVMGSQTESALSKKLRAILPKQSRKSMLDAGPDSWGSDAEQSTSGQPYPTSDQEGDPGSKQPRKKRGRYRQYNSEILEEAISVVMSGKMSVSKAQSIYGIPHSTLEYKVKERLGTLKNPPKKKMKLMRSEGPDVSVKIELDPQGEAAQSANESKNE

>NP_056177.3

MTPSEGARAGTGRELEMLDSLLALGGLVLLRDSVEWEGRSLLKALVKKSALCGEQVHILGCEVSEEEFREGFDSDINNRLVYHDFFRDPLNWSKTEEAFPGGPLGALRAMCKRTDPVPVTIALDSLSWLLLRLPCTTLCQVLHAVSHQDSCPGDSSSVGKVSVLGLLHEELHGPGPVGALSSLAQTEVTLGGTMGQASAHILCRRPRQRPTDQTQWFSILPDFSLDLQEGPSVESQPYSDPHIPPVDPTTHLTFNLHLSKKEREARDSLILPFQFSSEKQQALLRPRPGQATSHIFYEPDAYDDLDQEDPDDDLDI

188 NP_005641.1 Q9BXL7

>NP_005641.1

MQSFREQSSYHGNQQSYPQEVHGSSRLEEFSPRQAQMFQNFGGTGGSSGSSGSGSGGGRRGAAAAAAAMASETSGHQGYQGFRKEAGDFYYMAGNKDPVTTGTPQPPQRRPSGPVQSYGPPQGSSFGNQYGSEGHVGQFQAQHSGLGGVSHYQQDYTGPFSPGSAQYQQQASSQQQQQQVQQLRQQLYQSHQPLPQATGQPASSSSHLQPMQRPSTLPSSAAGYQLRVGQFGQHYQSSASSSSSSSFPSPQRFSQSGQSYDGSYNVNAGSQYEGHNVGSNAQAYGTQSNYSYQPQSMKNFEQAKIPQGTQQGQQQQQPQQQQHPSQHVMQYTNAATKLPLQSQVGQYNQPEVPVRSPMQFHQNFSPISNPSPAASVVQSPSCSSTPSPLMQTGENLQCGQGSVPMGSRNRILQLMPQLSPTPSMMPSPNSHAAGFKGFGLEGVPEKRLTDPGLSSLSALSTQVANLPNTVQHMLLSDALTPQKKTSKRPSSSKKADSCTNSEGSSQPEEQLKSPMAESLDGGCSSSSEDQGERVRQLSGQSTSSDTTYKGGASEKAGSSPAQGAQNEPPRLNASPAAREEATSPGAKDMPLSSDGNPKVNEKTVGVIVSREAMTGRVEKPGGQDKGSQEDDPAATQRPPSNGGAKETSHASLPQPEPPGGGGSKGNKNGDNNSNHNGEGNGQSGHSAAGPGFTSRTEPSKSPGSLRYSYKDSFGSAVPRNVSGFPQYPTGQEKGDFTGHGERKGRNEKFPSLLQEVLQGYHHHPDRRYSRSTQEHQGMAGSLEGTTRPNVLVSQTNELASRGLLNKSIGSLLENPHWGPWERKSSSTAPEMKQINLTDYPIPRKFEIEPQSSAHEPGGSLSERRSVICDISPLRQIVRDPGAHSLGHMSADTRIGRNDRLNPTLSQSVILPGGLVSMETKLKSQSGQIKEEDFEQSKSQASFNNKKSGDHCHPPSIKHESYRGNASPGAATHDSLSDYGPQDSRPTPMRRVPGRVGGREGMRGRSPSQYHDFAEKLKMSPGRSRGPGGDPHHMNPHMTFSERANRSSLHTPFSPNSETLASAYHANTRAHAYGDPNAGLNSQLHYKRQMYQQQPEEYKDWSSGSAQGVIAAAQHRQEGPRKSPRQQQFLDRVRSPLKNDKDGMMYGPPVGTYHDPSAQEAGRCLMSSDGLPNKGMELKHGSQKLQESCWDLSRQTSPAKSSGPPGMSSQKRYGPPHETDGHGLAEATQSSKPGSVMLRLPGQEDHSSQNPLIMRRRVRSFISPIPSKRQSQDVKNSSTEDKGRLLHSSKEGADKAFNSYAHLSHSQDIKSIPKRDSSKDLPSPDSRNCPAVTLTSPAKTKILPPRKGRGLKLEAIVQKITSPNIRRSASSNSAEAGGDTVTLDDILSLKSGPPEGGSVAVQDADIEKRKGEVASDLVSPANQELHVEKPLPRSSEEWRGSVDDKVKTETHAETVTAGKEPPGAMTSTTSQKPGSNQGRPDGSLGGTAPLIFPDSKNVPPVGILAPEANPKAEEKENDTVTISPKQEGFPPKGYFPSGKKKGRPIGSVNKQKKQQQPPPPPPQPPQIPEGSADGEPKPKKQRQRRERRKPGAQPRKRKTKQAVPIVEPQEPEIKLKYATQPLDKTDAKNKSFYPYIHVVNKCELGAVCTIINAEEEEQTKLVRGRKGQRSLTPPPSSTESKALPASSFMLQGPVVTESSVMGHLVCCLCGKWASYRNMGDLFGPFYPQDYAATLPKNPPPKRATEMQSKVKVRHKSASNGSKTDTEEEEEQQQQQKEQRSLAAHPRFKRRHRSEDCGGGPRSLSRGLPCKKAATEGSSEKTVLDSKPSVPTTSEGGPELELQIPELPLDSNEFWVHEGCILWANGIYLVCGRLYGLQEALEIAREMKCSHCQEAGATLGCYNKGCSFRYHYPCAIDADCLLHEENFSVRCPKHKPPLPCPLPPLQNKTAKGSLSTEQSERG

>Q9BXL7

MPGGGPEMDDYMETLKDEEDALWENVECNRHMLSRYINPAKLTPYLRQCKVIDEQDEDEVLNAPMLPSKINRAGRLLDILHTKGQRGYVVFLESLEFYYPELYKLVTGKEPTRRFSTIVVEEGHEGLTHFLMNEVIKLQQQMKAKDLQRCELLARLRQLEDEKKQMTLTRVELLTFQERYYKMKEERDSYNDELVKVKDDNYNLAMRYAQLSEEKNMAVMRSRDLQLEIDQLKHRLNKMEEECKLERNQSLKLKNDIENRPKKEQVLELERENEMLKTKNQELQSIIQAGKRSLPDSDKAILDILEHDRKEALEDRQELVNRIYNLQEEARQAEELRDKYLEEKEDLELKCSTLGKDCEMYKHRMNTVMLQLEEVERERDQAFHSRDEAQTQYSQCLIEKDKYRKQIRELEEKNDEMRIEMVRREACIVNLESKLRRLSKDSNNLDQSLPRNLPVTIISQDFGDASPRTNGQEADDSSTSEESPEDSKYFLPYHPPQRRMNLKGIQLQRAKSPISLKRTSDFQAKGHEEEGTDASPSSCGSLPITNSFTKMQPPRSRSSIMSITAEPPGNDSIVRRYKEDAPHRSTVEEDNDSGGFDALDLDDDSHERYSFGPSSIHSSSSSHQSEGLDAYDLEQVNLMFRKFSLERPFRPSVTSVGHVRGPGPSVQHTTLNGDSLTSQLTLLGGNARGSFVHSVKPGSLAEKAGLREGHQLLLLEGCIRGERQSVPLDTCTKEEAHWTIQRCSGPVTLHYKVNHEGYRKLVKDMEDGLITSGDSFYIRLNLNISSQLDACTMSLKCDDVVHVRDTMYQDRHEWLCARVDPFTDHDLDMGTIPSYSRAQQLLLVKLQRLMHRGSREEVDGTHHTLRALRNTLQPEEALSTSDPRVSPRLSRASFLFGQLLQFVSRSENKYKRMNSNERVRIISGSPLGSLARSSLDATKLLTEKQEELDPESELGKNLSLIPYSLVRAFYCERRRPVLFTPTVLAKTLVQRLLNSGGAMEFTICKSDIVTRDEFLRRQKTETIIYSREKNPNAFECIAPANIEAVAAKNKHCLLEAGIGCTRDLIKSNIYPIVLFIRVCEKNIKRFRKLLPRPETEEEFLRVCRLKEKELEALPCLYATVEPDMWGSVEELLRVVKDKIGEEQRKTIWVDEDQL

189 NP_006181.1 NP_777283.1

>NP_006181.1

MSKPELKEDKMLEVHFVGDDDVLNHILDREGGAKLKKERAQLLVNPKKIIKKPEYDLEEDDQEVLKDQNYVEIMGRDVQESLKNGSATGGGNKVYSFQNRKHSEKMAKLASELAKTPQKSVSFSLKNDPEITINVPQSSKGHSASDKVQPKNNDKSEFLSTAPRSLRKRLIVPRSHSDSESEYSASNSEDDEGVAQEHEEDTNAVIFSQKIQAQNRVVSAPVGKETPSKRMKRDKTSDLVEEYFEAHSSSKVLTSDRTLQKLKRAKLDQQTLRNLLSKVSPSFSAELKQLNQQYEKLFHKWMLQLHLGFNIVLYGLGSKRDLLERFRTTMLQDSIHVVINGFFPGISVKSVLNSITEEVLDHMGTFRSILDQLDWIVNKFKEDSSLELFLLIHNLDSQMLRGEKSQQIIGQLSSLHNIYLIASIDHLNAPLMWDHAKQSLFNWLWYETTTYSPYTEETSYENSLLVKQSGSLPLSSLTHVLRSLTPNARGIFRLLIKYQLDNQDNPSYIGLSFQDFYQQCREAFLVNSDLTLRAQLTEFRDHKLIRTKKGTDGVEYLLIPVDNGTLTDFLEKEEEEA

>NP_777283.1

MNTNDAKEYLARREIPQLFESLLNGLMCSKPEDPVEYLESCLQKVKELGGCDKVKWDTFVSQEKKTLPPLNGGQSRRSFLRNVMPENSNFPYRRYDRLPPIHQFSIESDTDLSETAELIEEYEVFDPTRPRPKIILVIGGPGSGKGTQSLKIAERYGFQYISVGELLRKKIHSTSSNRKWSLIAKIITTGELAPQETTITEIKQKLMQIPDEEGIVIDGFPRDVAQALSFEDQICTPDLVVFLACANQRLKERLLKRAEQQGRPDDNVKATQRRLMNFKQNAAPLVKYFQEKGLIMTFDADRDEDEVFYDISMAVDNKLFPNKEAAAGSSDLDPSMILDTGEIIDTGSDYEDQGDDQLNVFGEDTMGGFMEDLRKCKIIFIIGGPGSGKGTQCEKLVEKYGFTHLSTGELLREELASESERSKLIRDIMERGDLVPSGIVLELLKEAMVASLGDTRGFLIDGYPREVKQGEEFGRRIGDPQLVICMDCSADTMTNRLLQRSRSSLPVDDTTKTIAKRLEAYYRASIPVIAYYETKTQLHKINAEGTPEDVFLQLCTAIDSIF

190 P54803 NP_071761.1

>P54803

MAEWLLSASWQRRAKAMTAAAGSAGRAAVPLLLCALLAPGGAYVLDDSDGLGREFDGIGAVSGGGATSRLLVNYPEPYRSQILDYLFKPNFGASLHILKVEIGGDGQTTDGTEPSHMHYALDENYFRGYEWWLMKEAKKRNPNITLIGLPWSFPGWLGKGFDWPYVNLQLTAYYVVTWIVGAKRYHDLDIDYIGIWNERSYNANYIKILRKMLNYQGLQRVKIIASDNLWESISASMLLDAELFKVVDVIGAHYPGTHSAKDAKLTGKKLWSSEDFSTLNSDMGAGCWGRILNQNYINGYMTSTIAWNLVASYYEQLPYGRCGLMTAQEPWSGHYVVESPVWVSAHTTQFTQPGWYYLKTVGHLEKGGSYVALTDGLGNLTIIIETMSHKHSKCIRPFLPYFNVSQQFATFVLKGSFSEIPELQVWYTKLGKTSERFLFKQLDSLWLLDSDGSFTLSLHEDELFTLTTLTTGRKGSYPLPPKSQPFPSTYKDDFNVDYPFFSEAPNFADQTGVFEYFTNIEDPGEHHFTLRQVLNQRPITWAADASNTISIIGDYNWTNLTIKCDVYIETPDTGGVFIAGRVNKGGILIRSARGIFFWIFANGSYRVTGDLAGWIIYALGRVEVTAKKWYTLTLTIKGHFASGMLNDKSLWTDIPVNFPKNGWAAIGTHSFEFAQFDNFLVEATR

>NP_071761.1

MWIPVVGLPRRLRLSALAGAGRFCILGSEAATRKHLPARNHCGLSDSSPQLWPEPDFRNPPRKASKASLDFKRYVTDRRLAETLAQIYLGKPSRPPHLLLECNPGPGILTQALLEAGAKVVALESDKTFIPHLESLGKNLDGKLRVIHCDFFKLDPRSGGVIKPPAMSSRGLFKNLGIEAVPWTADIPLKVVGMFPSRGEKRALWKLAYDLYSCTSIYKFGRIEVNMFIGEKEFQKLMADPGNPDLYHVLSVIWQLACEIKVLHMEPWSSFDIYTRKGPLENPKRRELLDQLQQKLYLIQMIPRQNLFTKNLTPMNYNIFFHLLKHCFGRRSATVIDHLRSLTPLDARDILMQIGKQEDEKVVNMHPQDFKTLFETIERSKDCAYKWLYDETLEDR

191 NP_002583.1 NP_071761.1

>NP_002583.1

MFEARLVQGSILKKVLEALKDLINEACWDISSSGVNLQSMDSSHVSLVQLTLRSEGFDTYRCDRNLAMGVNLTSMSKILKCAGNEDIITLRAEDNADTLALVFEAPNQEKVSDYEMKLMDLDVEQLGIPEQEYSCVVKMPSGEFARICRDLSHIGDAVVISCAKDGVKFSASGELGNGNIKLSQTSNVDKEEEAVTIEMNEPVQLTFALRYLNFFTKATPLSSTVTLSMSADVPLVVEYKIADMGHLKYYLAPKIEDEEGS

>NP_071761.1

MWIPVVGLPRRLRLSALAGAGRFCILGSEAATRKHLPARNHCGLSDSSPQLWPEPDFRNPPRKASKASLDFKRYVTDRRLAETLAQIYLGKPSRPPHLLLECNPGPGILTQALLEAGAKVVALESDKTFIPHLESLGKNLDGKLRVIHCDFFKLDPRSGGVIKPPAMSSRGLFKNLGIEAVPWTADIPLKVVGMFPSRGEKRALWKLAYDLYSCTSIYKFGRIEVNMFIGEKEFQKLMADPGNPDLYHVLSVIWQLACEIKVLHMEPWSSFDIYTRKGPLENPKRRELLDQLQQKLYLIQMIPRQNLFTKNLTPMNYNIFFHLLKHCFGRRSATVIDHLRSLTPLDARDILMQIGKQEDEKVVNMHPQDFKTLFETIERSKDCAYKWLYDETLEDR

192 Q9BSH3 NP_002291.1

>Q9BSH3

MSRVLVPCHVKGSVALQVGDVRTSQGRPGVLVIDVTFPSVAPFELQEITFKNYYTAFLSIRVRQYTSAHTPAKWVTCLRDYCLMPDPHSEEGAQEYVSLFKHQMLCDMARISELRLILRQPSPLWLSFTVEELQIYQQGPKSPSVTFPKWLSHPVPCEQPALLREGLPDPSRVSSEVQQMWALTEMIRASHTSARIGRFDVDGCYDLNLLSYT

>NP_002291.1

MATLKEKLIAPVAEEEATVPNNKITVVGVGQVGMACAISILGKSLADELALVDVLEDKLKGEMMDLQHGSLFLQTPKIVADKDYSVTANSKIVVVTAGVRQQEGESRLNLVQRNVNVFKFIIPQIVKYSPDCIIIVVSNPVDILTYVTWKLSGLPKHRVIGSGCNLDSARFRYLMAEKLGIHPSSCHGWILGEHGDSSVAVWSGVNVAGVSLQELNPEMGTDNDSENWKEVHKMVVESAYEVIKLKGYTNWAIGLSVADLIESMLKNLSRIHPVSTMVKGMYGIENEVFLSLPCILNARGLTSVINQKLKDDEVAQLKKSADTLWDIQKDLKDL

193 NP_006707.1 Q14914

>NP_006707.1

MNSGAMRIHSKGHFQGGIQVKNEKNRPSLKSLKTDNRPEKSKCKPLWGKVFYLDLPSVTISEKLQKDIKDLGGRVEEFLSKDISYLISNKKEAKFAQTLGRISPVPSPESAYTAETTSPHPSHDGSSFKSPDTVCLSRGKLLVEKAIKDHDFIPSNSILSNALSWGVKILHIDDIRYYIEQKKKELYLLKKSSTSVRDGGKRVGSGAQKTRTGRLKKPFVKVEDMSQLYRPFYLQLTNMPFINYSIQKPCSPFDVDKPSSMQKQTQVKLRIQTDGDKYGGTSIQLQLKEKKKKGYCECCLQKYEDLETHLLSEQHRNFAQSNQYQVVDDIVSKLVFDFVEYEKDTPKKKRIKYSVGSLSPVSASVLKKTEQKEKVELQHISQKDCQEDDTTVKEQNFLYKETQETEKKLLFISEPIPHPSNELRGLNEKMSNKCSMLSTAEDDIRQNFTQLPLHKNKQECILDISEHTLSENDLEELRVDHYKCNIQASVHVSDFSTDNSGSQPKQKSDTVLFPAKDLKEKDLHSIFTHDSGLITINSSQEHLTVQAKAPFHTPPEEPNECDFKNMDSLPSGKIHRKVKIILGRNRKENLEPNAEFDKRTEFITQEENRICSSPVQSLLDLFQTSEEKSEFLGFTSYTEKSGICNVLDIWEEENSDNLLTAFFSSPSTSTFTGF

>Q14914

MVRTKTWTLKKHFVGYPTNSDFELKTAELPPLKNGEVLLEALFLTVDPYMRVAAKRLKEGDTMMGQQVAKVVESKNVALPKGTIVLASPGWTTHSISDGKDLEKLLTEWPDTIPLSLALGTVGMPGLTAYFGLLEICGVKGGETVMVNAAAGAVGSVVGQIAKLKGCKVVGAVGSDEKVAYLQKLGFDVVFNYKTVESLEETLKKASPDGYDCYFDNVGGEFSNTVIGQMKKFGRIAICGAISTYNRTGPLPPGPPPEIVIYQELRMEAFVVYRWQGDARQKALKDLLKWVLEGKIQYKEYIIEGFENMPAAFMGMLKGDNLGKTIVKA

194 O00462 NP_758958.3

>O00462

MRLHLLLLLALCGAGTTAAELSYSLRGNWSICNGNGSLELPGAVPGCVHSALFQQGLIQDSYYRFNDLNYRWVSLDNWTYSKEFKIPFEISKWQKVNLILEGVDTVSKILFNEVTIGETDNMFNRYSFDITNVVRDVNSIELRFQSAVLYAAQQSKAHTRYQVPPDCPPLVQKGECHVNFVRKEQCSFSWDWGPSFPTQGIWKDVRIEAYNICHLNYFTFSPIYDKSAQEWNLEIESTFDVVSSKPVGGQVIVAIPKLQTQQTYSIELQPGKRIVELFVNISKNITVETWWPHGHGNQTGYNMTVLFELDGGLNIEKSAKVYFRTVELIEEPIKGSPGLSFYFKINGFPIFLKGSNWIPADSFQDRVTSELLRLLLQSVVDANMNTLRVWGGGIYEQDEFYELCDELGIMVWQDFMFACALYPTDQGFLDSVTAEVAYQIKRLKSHPSIIIWSGNNENEEALMMNWYHISFTDRPIYIKDYVTLYVKNIRELVLAGDKSRPFITSSPTNGAETVAEAWVSQNPNSNYFGDVHFYDYISDCWNWKVFPKARFASEYGYQSWPSFSTLEKVSSTEDWSFNSKFSLHRQHHEGGNKQMLYQAGLHFKLPQSTDPLRTFKDTIYLTQVMQAQCVKTETEFYRRSRSEIVDQQGHTMGALYWQLNDIWQAPSWASLEYGGKWKMLHYFAQNFFAPLLPVGFENENTFYIYGVSDLHSDYSMTLSVRVHTWSSLEPVCSRVTERFVMKGGEAVCLYEEPVSELLRRCGNCTRESCVVSFYLSADHELLSPTNYHFLSSPKEAVGLCKAQITAIISQQGDIFVFDLETSAVAPFVWLDVGSIPGRFSDNGFLMTEKTRTILFYPWEPTSKNELEQSFHVTSLTDIY

>NP_758958.3

MLEKLMGADSLQLFRSRYTLGKIYFIGFQRSILLSKSENSLNSIAKETEEGRETVTRKEGWKRRHEDGYLEMAQRHLQRSLCPWVSYLPQPYAELEEVSSHVGKVFMARNYEFLAYEASKDRRQPLERMWTCNYNQQKDQSCNHKEITSTKAE

195 NP_056975.1 NP_000340.2

>NP_056975.1

MSAAIAALAASYGSGSGSESDSDSESSRCPLPAADSLMHLTKSPSSKPSLAVAVDSAPEVAVKEDLETGVHLDPAVKEVQYNPTYETMFAPEFGPENPFRTQQMAAPRNMLSGYAEPAHINDFMFEQQRRTFATYGYALDPSLDNHQVSAKYIGSVEEAEKNQGLTVFETGQKKTEKRKKFKENDASNIDGFLGPWAKYVDEKDVAKPSEEEQKELDEITAKRQKKGKQEEEKPGEEKTILHVKEMYDYQGRSYLHIPQDVGVNLRSTMPPEKCYLPKKQIHVWSGHTKGVSAVRLFPLSGHLLLSCSMDCKIKLWEVYGERRCLRTFIGHSKAVRDICFNTAGTQFLSAAYDRYLKLWDTETGQCISRFTNRKVPYCVKFNPDEDKQNLFVAGMSDKKIVQWDIRSGEIVQEYDRHLGAVNTIVFVDENRRFVSTSDDKSLRVWEWDIPVDFKYIAEPSMHSMPAVTLSPNGKWLACQSMDNQILIFGAQNRFRLNKKKIFKGHMVAGYACQVDFSPDMSYVISGDGNGKLNIWDWKTTKLYSRFKAHDKVCIGAVWHPHETSKVITCGWDGLIKLWD

>NP_000340.2

MLLATFKLCAGSSYRHMRNMKGLRQQAVMAISQELNRRALGGPTPSTWINQVRRRSSLLGSRLEETLYSDQELAYLQQGEEAMQKALGILSNQEGWKKESQQDNGDKVMSKVVPDVGKVFRLEVVVDQPMERLYEELVERMEAMGEWNPNVKEIKVLQKIGKDTFITHELAAEAAGNLVGPRDFVSVRCAKRRGSTCVLAGMATDFGNMPEQKGVIRAEHGPTCMVLHPLAGSPSKTKLTWLLSIDLKGWLPKSIINQVLSQTQVDFANHLRKRLESHPASEARC

196 NP_071394.2 NP_055433.2

>NP_071394.2

MTGEVGSEVHLEINDPNVISQEEADSPSDSGQGSYETIGPLSEGDSDEEIFVSKKLKNRKVLQDSDSETEDTNASPEKTTYDSAEEENKENLYAGKNTKIKRIYKTVADSDESYMEKSLYQENLEAQVKPCLELSLQSGNSTDFTTDRKSSKKHIHDKEGTAGKAKVKSKRRLEKEERKMEKIRQLKKKETKNQEDDVEQPFNDSGCLLVDKDLFETGLEDENNSPLEDEESLESIRAAVKNKVKKHKKKEPSLESGVHSFEEGSELSKGTTRKERKAARLSKEALKQLHSETQRLIRESALNLPYHMPENKTIHDFFKRKPRPTCHGNAMALLKSSKYQSSHHKEIIDTANTTEMNSDHHSKGSEQTTGAENEVETNALPVVSKETQIITGSDESCRKDLVKNEELEIQEKQKQSDIRPSPGDSSVLQQESNFLGNNHSEECQVGGLVAFEPHALEGEGPQNPEETDEKVEEPEQQNKSSAVGPPEKVRRFTLDRLKQLGVDVSIKPRLGADEDSFVILEPETNRELEALKQRFWKHANPAAKPRAGQTVNVNVIVKDMGTDGKEELKADVVPVTLAPKKLDGASHTKPGEKLQVLKAKLQEAMKLRRFEERQKRQALFKLDNEDGFEEEEEEEEEMTDESEEDGEEKVEKEEKEEELEEEEEKEEEEEEEGNQETAEFLLSSEEIETKDEKEMDKENNDGSSEIGKAVGFLSVPKSLSSDSTLLLFKDSSSKMGYFPTEEKSETDENSGKQPSKLDEDDSCSLLTKESSHNSSFELIGSTIPSYQPCNRQTGRGTSFFPTAGGFRSPSPGLFRASLVSSASKSSGKLSEPSLPIEDSQDLYNASPEPKTLFLGAGDFQFCLEDDTQSQLLDADGFLNVRNHRNQYQALKPRLPLASMDENAMDANMDELLDLCTGKFTSQAEKHLPRKSDKKENMEELLNLCSGKFTSQDASTPASSELNKQEKESSMGDPMEEALALCSGSFPTDKEEEDEEEEFGDFRLVSNDNEFDSDEDEHSDSGNDLALEDHEDDDEEELLKRSEKLKRQMRLRKYLEDEAEVSGSDVGSEDEYDGEEIDEYEEDVIDEVLPSDEELQSQIKKIHMKTMLDDDKRQLRLYQERYLADGDLHSDGPGRMRKFRWKNIDDASQMDLFHRDSDDDQTEEQLDESEARWRKERIEREQWLRDMAQQGKITAEEEEEIGEDSQFMILAKKVTAKALQKNASRPMVIQESKSLLRNPFEAIRPGSAQQVKTGSLLNQPKAVLQKLAALSDHNPSAPRNSRNFVFHTLSPVKAEAAKESSKSQVKKRGPSFMTSPSPKHLKTDDSTSGLTRSIFKYLES

>NP_055433.2

MNWRFVELLYFLFIWGRISVQPSHQEPAGTDQHVSKEFDWLISDRGPFHHSRSYLSFVERHRQGFTTRYKIYREFARWKVRNTAIERRDLVRHPVPLMPEFQRSIRLLGRRPTTQQFIDTIIKKYGTHLLISATLGGEEALTMYMDKSRLDRKSGNATQSVEALHQLASSYFVDRDGTMRRLHEIQISTGAIKVTETRTGPLGCNSYDNLDSVSSVLLQSTESKLHLQGLQIIFPQYLQEKFVQSALSYIMCNGEGEYLCQNSQCRCQCAEEFPQCNCPITDIQIMEYTLANMAKSWAEAYKDLENSDEFKSFMKRLPSNHFLTIGSIHQHWGNDWDLQNRYKLLQSATEAQRQKIQRTARKLFGLSVRCRHNPNHQLPRERTIQQWLARVQSLLYCNENGFWGTFLESQRSCVCHGSTTLCQRPIPCVIGGNNSCAMCSLANISLCGSCNKGYKLYRGRCEPQNVDSERSEQFISFETDLDFQDLELKYLLQKMDSRLYVHTTFISNEIRLDTFFDPRWRKRMSLTLKSNKNRMDFIHMVIGMSMRICQMRNSSLDPMFFVYVNPFSGSHSEGWNMPFGEFGYPRWEKIRLQNSQCYNWTLLLGNRWKTFFETVHIYLRSRTRLPTLLRNETGQGPVDLSDPSKRQFYIKISDVQVFGYSLRFNADLLRSAVQQVNQSYTQGGQFYSSSSVMLLLLDIRDRINRLAPPVAPGKPQLDLFSCMLKHRLKLTNSEIIRVNHALDLYNTEILKQSDQMTAKLC

197 P10071 NP_002291.1

>P10071

MEAQSHSSTTTEKKKVENSIVKCSTRTDVSEKAVASSTTSNEDESPGQTYHRERRNAITMQPQNVQGLSKVSEEPSTSSDERASLIKKEIHGSLPHVAEPSVPYRGTVFAMDPRNGYMEPHYHPPHLFPAFHPPVPIDARHHEGRYHYDPSPIPPLHMTSALSSSPTYPDLPFIRISPHRNPAAASESPFSPPHPYINPYMDYIRSLHSSPSLSMISATRGLSPTDAPHAGVSPAEYYHQMALLTGQRSPYADIIPSAATAGTGAIHMEYLHAMDSTRFSSPRLSARPSRKRTLSISPLSDHSFDLQTMIRTSPNSLVTILNNSRSSSSASGSYGHLSASAISPALSFTYSSAPVSLHMHQQILSRQQSLGSAFGHSPPLIHPAPTFPTQRPIPGIPTVLNPVQVSSGPSESSQNKPTSESAVSSTGDPMHNKRSKIKPDEDLPSPGARGQQEQPEGTTLVKEEGDKDESKQEPEVIYETNCHWEGCAREFDTQEQLVHHINNDHIHGEKKEFVCRWLDCSREQKPFKAQYMLVVHMRRHTGEKPHKCTFEGCTKAYSRLENLKTHLRSHTGEKPYVCEHEGCNKAFSNASDRAKHQNRTHSNEKPYVCKIPGCTKRYTDPSSLRKHVKTVHGPEAHVTKKQRGDIHPRPPPPRDSGSHSQSRSPGRPTQGALGEQQDLSNTTSKREECLQVKTVKAEKPMTSQPSPGGQSSCSSQQSPISNYSNSGLELPLTDGGSIGDLSAIDETPIMDSTISTATTALALQARRNPAGTKWMEHVKLERLKQVNGMFPRLNPILPPKAPAVSPLIGNGTQSNNTCSLGGPMTLLPGRSDLSGVDVTMLNMLNRRDSSASTISSAYLSSRRSSGISPCFSSRRSSEASQAEGRPQNVSVADSYDPISTDASRRSSEASQSDGLPSLLSLTPAQQYRLKAKYAAATGGPPPTPLPNMERMSLKTRLALLGDALEPGVALPPVHAPRRCSDGGAHGYGRRHLQPHDALGHGVRRASDPVRTGSEGLALPRVPRFSSLSSCNPPAMATSAEKRSLVLQNYTRPEGGQSRNFHSSPCPPSITENVTLESLTMDADANLNDEDFLPDDVVQYLNSQNQAGYEQHFPSALPDDSKVPHGPGDFDAPGLPDSHAGQQFHALEQPCPEGSKTDLPIQWNEVSSGSADLSSSKLKCGPRPAVPQTRAFGFCNGMVVHPQNPLRSGPAGGYQTLGENSNPYGGPEHLMLHNSPGSGTSGNAFHEQPCKAPQYGNCLNRQPVAPGALDGACGAGIQASKLKSTPMQGSGGQLNFGLPVAPNESAGSMVNGMQNQDPVGQGYLAHQLLGDSMQHPGAGRPGQQMLGQISATSHINIYQGPESCLPGAHGMGSQPSSLAVVRGYQPCASFGGSRRQAMPRDSLALQSGQLSDTSQTCRVNGIKMEMKGQPHPLCSNLQNYSGQFYDQTVGFSQQDTKAGSFSISDASCLLQGTSAKNSELLSPGANQVTSTVDSLDSHDLEGVQIDFDAIIDDGDHSSLMSGALSPSIIQNLSHSSSRLTTPRASLPFPALSMSTTNMAIGDMSSLLTSLAEESKFLAVMQ

>NP_002291.1

MATLKEKLIAPVAEEEATVPNNKITVVGVGQVGMACAISILGKSLADELALVDVLEDKLKGEMMDLQHGSLFLQTPKIVADKDYSVTANSKIVVVTAGVRQQEGESRLNLVQRNVNVFKFIIPQIVKYSPDCIIIVVSNPVDILTYVTWKLSGLPKHRVIGSGCNLDSARFRYLMAEKLGIHPSSCHGWILGEHGDSSVAVWSGVNVAGVSLQELNPEMGTDNDSENWKEVHKMVVESAYEVIKLKGYTNWAIGLSVADLIESMLKNLSRIHPVSTMVKGMYGIENEVFLSLPCILNARGLTSVINQKLKDDEVAQLKKSADTLWDIQKDLKDL

198 NP_000225.1 NP_006819.2

>NP_000225.1

MQRSIMSFFHPKKEGKAKKPEKEASNSSRETEPPPKAALKEWNGVVSESDSPVKRPGRKAARVLGSEGEEEDEALSPAKGQKPALDCSQVSPPRPATSPENNASLSDTSPMDSSPSGIPKRRTARKQLPKRTIQEVLEEQSEDEDREAKRKKEEEEEETPKESLTEAEVATEKEGEDGDQPTTPPKPLKTSKAETPTESVSEPEVATKQELQEEEEQTKPPRRAPKTLSSFFTPRKPAVKKEVKEEEPGAPGKEGAAEGPLDPSGYNPAKNNYHPVEDACWKPGQKVPYLAVARTFEKIEEVSARLRMVETLSNLLRSVVALSPPDLLPVLYLSLNHLGPPQQGLELGVGDGVLLKAVAQATGRQLESVRAEAAEKGDVGLVAENSRSTQRLMLPPPPLTASGVFSKFRDIARLTGSASTAKKIDIIKGLFVACRHSEARFIARSLSGRLRLGLAEQSVLAALSQAVSLTPPGQEFPPAMVDAGKGKTAEARKTWLEEQGMILKQTFCEVPDLDRIIPVLLEHGLERLPEHCKLSPGIPLKPMLAHPTRGISEVLKRFEEAAFTCEYKYDGQRAQIHALEGGEVKIFSRNQEDNTGKYPDIISRIPKIKLPSVTSFILDTEAVAWDREKKQIQPFQVLTTRKRKEVDASEIQVQVCLYAFDLIYLNGESLVREPLSRRRQLLRENFVETEGEFVFATSLDTKDIEQIAEFLEQSVKDSCEGLMVKTLDVDATYEIAKRSHNWLKLKKDYLDGVGDTLDLVVIGAYLGRGKRAGRYGGFLLASYDEDSEELQAICKLGTGFSDEELEEHHQSLKALVLPSPRPYVRIDGAVIPDHWLDPSAVWEVKCADLSLSPIYPAARGLVDSDKGISLRFPRFIRVREDKQPEQATTSAQVACLYRKQSQIQNQQGEDSGSDPEDTY

>NP_006819.2

MALPRLTGALRSFSNVTKQDNYNEEVADLKIKRSKLHEQVLDLGLTWKKIIKFLNEKLEKSKMQSINEDLKDILHAAKQIVGTDNGREAIESGAAFLFMTFHLKDSVGHKETKAIKQMFGPFPSSSATAACNATNRIISHFSQDDLTALVQMTEKEHGDRVFFGKNLAFSFDMHDLDHFDELPINGETQKTISLDYKKFLNEHLQEACTPELKPVEKTNGSFLWCEVEKYLNSTLKEMTEVPRVEDLCCTLYDMLASIKSGDELQDELFELLGPEGLELIEKLLQNRITIVDRFLNSSNDHRFQALQDNCKKILGENAKPNYGCQVTIQSEQEKQLMKQYRREEKRIARREKKAGEDLEVSEGLMCFDPKELRIQREQALLNARSVPILSRQRDADVEKIHYPHVYDSQAEAMKTSAFIAGAKMILPEGIQRENNKLYEEVRIPYSEPMPLSFEEKPVYIQDLDEIGQLAFKGMKRLNRIQSIVFETAYNTNENMLICAPTGAGKTNIAMLTVLHEIRQHFQQGVIKKNEFKIVYVAPMKALAAEMTDYFSRRLEPLGIIVKELTGDMQLSKSEILRTQMLVTTPEKWDVVTRKSVGDVALSQIVRLLILDEVHLLHEDRGPVLESIVARTLRQVESTQSMIRILGLSATLPNYLDVATFLHVNPYIGLFFFDGRFRPVPLGQTFLGIKCANKMQQLNNMDEVCYENVLKQVKAGHQVMVFVHARNATVRTAMSLIERAKNCGHIPFFFPTQGHDYVLAEKQVQRSRNKQVRELFPDGFSIHHAGMLRQDRNLVENLFSNGHIKVLVCTATLAWGVNLPAHAVIIKGTQIYAAKRGSFVDLGILDVMQIFGRAGRPQFDKFGEGIIITTHDKLSHYLTLLTQRNPIESQFLESLADNLNAEIALGTVTNVEEAVKWISYTYLYVRMRANPLAYGISHKAYQIDPTLRKHREQLVIEVGRKLDKAQMIRFEERTGYFSSTDLGRTASHYYIKYNTIETFNELFDAHKTEGDIFAIVSKAEEFDQIKVREEEIEELDTLLSNFCELSTPGGVENSYGKINILLQTYISRGEMDSFSLISDSAYVAQNAARIVRALFEIALRKRWPTMTYRLLNLSKVIDKRLWGWASPLRQFSILPPHILTRLEEKKLTVDKLKDMRKDEIGHILHHVNIGLKVKQCVHQIPSVMMEASIQPITRTVLRVTLSIYADFTWNDQVHGTVGEPWWIWVEDPTNDHIYHSEYFLALKKQVISKEAQLLVFTIPIFEPLPSQYYIRAVSDRWLGAEAVCIINFQHLILPERHPPHTELLDLQPLPITALGCKAYEALYNFSHFNPVQTQIFHTLYHTDCNVLLGAPTGSGKTVAAELAIFRVFNKYPTSKAVYIAPLKALVRERMDDWKVRIEEKLGKKVIELTGDVTPDMKSIAKADLIVTTPEKWDGVSRSWQNRNYVQQVTILIIDEIHLLGEERGPVLEVIVSRTNFISSHTEKPVRIVGLSTALANARDLADWLNIKQMGLFNFRPSVRPVPLEVHIQGFPGQHYCPRMASMNKPAFQAIRSHSPAKPVLIFVSSRRQTRLTALELIAFLATEEDPKQWLNMDEREMENIIATVRDSNLKLTLAFGIGMHHAGLHERDRKTVEELFVNCKVQVLIATSTLAWGVNFPAHLVIIKGTEYYDGKTRRYVDFPITDVLQMMGRAGRPQFDDQGKAVILVHDIKKDFYKKFLYEPFPVESSLLGVLSDHLNAEIAGGTITSKQDALDYITWTYFFRRLIMNPSYYNLGDVSHDSVNKFLSHLIEKSLIELELSYCIEIGEDNRSIEPLTYGRIASYYYLKHQTVKMFKDRLKPECSTEELLSILSDAEEYTDLPVRHNEDHMNSELAKCLPIESNPHSFDSPHTKAHLLLQAHLSRAMLPCPDYDTDTKTVLDQALRVCQAMLDVAANQGWLVTVLNITNLIQMVIQGRWLKDSSLLTLPNIENHHLHLFKKWKPIMKGPHARGRTSIESLPELIHACGGKDHVFSSMVESELHAAKTKQAWNFLSHLPVINVGISVKGSWDDLVEGHNELSVSTLTADKRDDNKWIKLHADQEYVLQVSLQRVHFGFHKGKPESCAVTPRFPKSKDEGWFLILGEVDKRELIALKRVGYIRNHHVASLSFYTPEIPGRYIYTLYFMSDCYLGLDQQYDIYLNVTQASLSAQVNTKVSDSLTDLALK

199 NP_006181.1 NP_659491.4

>NP_006181.1

MSKPELKEDKMLEVHFVGDDDVLNHILDREGGAKLKKERAQLLVNPKKIIKKPEYDLEEDDQEVLKDQNYVEIMGRDVQESLKNGSATGGGNKVYSFQNRKHSEKMAKLASELAKTPQKSVSFSLKNDPEITINVPQSSKGHSASDKVQPKNNDKSEFLSTAPRSLRKRLIVPRSHSDSESEYSASNSEDDEGVAQEHEEDTNAVIFSQKIQAQNRVVSAPVGKETPSKRMKRDKTSDLVEEYFEAHSSSKVLTSDRTLQKLKRAKLDQQTLRNLLSKVSPSFSAELKQLNQQYEKLFHKWMLQLHLGFNIVLYGLGSKRDLLERFRTTMLQDSIHVVINGFFPGISVKSVLNSITEEVLDHMGTFRSILDQLDWIVNKFKEDSSLELFLLIHNLDSQMLRGEKSQQIIGQLSSLHNIYLIASIDHLNAPLMWDHAKQSLFNWLWYETTTYSPYTEETSYENSLLVKQSGSLPLSSLTHVLRSLTPNARGIFRLLIKYQLDNQDNPSYIGLSFQDFYQQCREAFLVNSDLTLRAQLTEFRDHKLIRTKKGTDGVEYLLIPVDNGTLTDFLEKEEEEA

>NP_659491.4

MDNKISPEAQVAELELDAVIGFNGHVPTGLKCHPDQEHMIYPLGCTVLIQAINTKEQNFLQGHGNNVSCLAISRSGEYIASGQVTFMGFKADIILWDYKNRELLARLSLHKGKIEALAFSPNDLYLVSLGGPDDGSVVVWSIAKRDAICGSPAAGLNVGNATNVIFSRCRDEMFMTAGNGTIRVWELDLPNRKIWPTECQTGQLKRIVMSIGVDDDDSFFYLGTTTGDILKMNPRTKLLTDVGPAKDKFSLGVSAIRCLKMGGLLVGSGAGLLVFCKSPGYKPIKKIQLQGGITSITLRGEGHQFLVGTEESHIYRVSFTDFKETLIATCHFDAVEDIVFPFGTAELFATCAKKDIRVWHTSSNRELLRITVPNMTCHGIDFMRDGKSIISAWNDGKIRAFAPETGRLMYVINNAHRIGVTAIATTSDCKRVISGGGEGEVRVWQIGCQTQKLEEALKEHKSSVSCIRVKRNNEECVTASTDGTCIIWDLVRLRRNQMILANTLFQCVCYHPEEFQIITSGTDRKIAYWEVFDGTVIRELEGSLSGSINGMDITQEGVHFVTGGNDHLVKVWDYNEGEVTHVGVGHSGNITRIRISPGNQYIVSVSADGAILRWKYPYTS

200 NP_003790.1 NP_000476.1

>NP_003790.1

MANSAKAEEYEKMSLEQAKASVNSETESSFNINENTTASGTGLSEKTSVCRQVDIARKRKEFEDDLVKESSSCGKDTPSKKRKLDPEIVPEEKDCGDAEGNSKKRKRETEDVPKDKSSTGDGTQNKRKIALEDVPEKQKNLEEGHSSTVAAHYNELQEVGLEKRSQSRIFYLRNFNNWMKSVLIGEFLEKVRQKKKRDITVLDLGCGKGGDLLKWKKGRINKLVCTDIADVSVKQCQQRYEDMKNRRDSEYIFSAEFITADSSKELLIDKFRDPQMCFDICSCQFVCHYSFESYEQADMMLRNACERLSPGGYFIGTTPNSFELIRRLEASETESFGNEIYTVKFQKKGDYPLFGCKYDFNLEGVVDVPEFLVYFPLLNEMAKKYNMKLVYKKTFLEFYEEKIKNNENKMLLKRMQALEPYPANESSKLVSEKVDDYEHAAKYMKNSQVRLPLGTLSKSEWEATSIYLVFAFEKQQ

>NP_000476.1

MADSELQLVEQRIRSFPDFPTPGVVFRDISPVLKDPASFRAAIGLLARHLKATHGGRIDYIAGLDSRGFLFGPSLAQELGLGCVLIRKRGKLPGPTLWASYSLEYGKAELEIQKDALEPGQRVVVVDDLLATGGTMNAACELLGRLQAEVLECVSLVELTSLKGREKLAPVPFFSLLQYE

201 NP_003085.1 NP_009197.1

>NP_003085.1

MAYRGQGQKVQKVMVQPINLIFRYLQNRSRIQVWLYEQVNMRIEGCIIGFDEYMNLVLDDAEEIHSKTKSRKQLGRIMLKGDNITLLQSVSN

>NP_009197.1

MAASAAAAELQASGGPRHPVCLLVLGMAGSGKTTFVQRLTGHLHAQGTPPYVINLDPAVHEVPFPANIDIRDTVKYKEVMKQYGLGPNGGIVTSLNLFATRFDQVMKFIEKAQNMSKYVLIDTPGQIEVFTWSASGTIITEALASSFPTVVIYVMDTSRSTNPVTFMSNMLYACSILYKTKLPFIVVMNKTDIIDHSFAVEWMQDFEAFQDALNQETTYVSNLTRSMSLVLDEFYSSLRVVGVSAVLGTGLDELFVQVTSAAEEYEREYRPEYERLKKSLANAESQQQREQLERLRKDMGSVALDAGTAKDSLSPVLHPSDLILTRGTLDEEDEEADSDTDDIDHRVTEESHEEPAFQNFMQESMAQYWKRNNK

202 P22304 NP_036429.2

>P22304

MPPPRTGRGLLWLGLVLSSVCVALGSETQANSTTDALNVLLIIVDDLRPSLGCYGDKLVRSPNIDQLASHSLLFQNAFAQQAVCAPSRVSFLTGRRPDTTRLYDFNSYWRVHAGNFSTIPQYFKENGYVTMSVGKVFHPGISSNHTDDSPYSWSFPPYHPSSEKYENTKTCRGPDGELHANLLCPVDVLDVPEGTLPDKQSTEQAIQLLEKMKTSASPFFLAVGYHKPHIPFRYPKEFQKLYPLENITLAPDPEVPDGLPPVAYNPWMDIRQREDVQALNISVPYGPIPVDFQRKIRQSYFASVSYLDTQVGRLLSALDDLQLANSTIIAFTSDHGWALGEHGEWAKYSNFDVATHVPLIFYVPGRTASLPEAGEKLFPYLDPFDSASQLMEPGRQSMDLVELVSLFPTLAGLAGLQVPPRCPVPSFHVELCREGKNLLKHFRFRDLEEDPYLPGNPRELIAYSQYPRPSDIPQWNSDKPSLKDIKIMGYSIRTIDYRYTVWVGFNPDEFLANFSDIHAGELYFVDSDPLQDHNMYNDSQGGDLFQLLMP

>NP_036429.2

MVMEKPSPLLVGREFVRQYYTLLNKAPEYLHRFYGRNSSYVHGGVDASGKPQEAVYGQNDIHHKVLSLNFSECHTKIRHVDAHATLSDGVVVQVMGLLSNSGQPERKFMQTFVLAPEGSVPNKFYVHNDMFRYEDEVFGDSEPELDEESEDEVEEEQEERQPSPEPVQENANSGYYEAHPVTNGIEEPLEESSHEPEPEPESETKTEELKPQVEEKNLEELEEKSTTPPPAEPVSLPQEPPKAFSWASVTSKNLPPSGTVSSSGIPPHVKAPVSQPRVEAKPEVQSQPPRVREQRPRERPGFPPRGPRPGRGDMEQNDSDNRRIIRYPDSHQLFVGNLPHDIDENELKEFFMSFGNVVELRINTKGVGGKLPNFGFVVFDDSEPVQRILIAKPIMFRGEVRLNVEEKKTRAARERETRGGGDDRRDIRRNDRGPGGPRGIVGGGMMRDRDGRGPPPRGGMAQKLGSGRGTGQMEGRFTGQRR

203 NP_000225.1 NP_002717.3

>NP_000225.1

MQRSIMSFFHPKKEGKAKKPEKEASNSSRETEPPPKAALKEWNGVVSESDSPVKRPGRKAARVLGSEGEEEDEALSPAKGQKPALDCSQVSPPRPATSPENNASLSDTSPMDSSPSGIPKRRTARKQLPKRTIQEVLEEQSEDEDREAKRKKEEEEEETPKESLTEAEVATEKEGEDGDQPTTPPKPLKTSKAETPTESVSEPEVATKQELQEEEEQTKPPRRAPKTLSSFFTPRKPAVKKEVKEEEPGAPGKEGAAEGPLDPSGYNPAKNNYHPVEDACWKPGQKVPYLAVARTFEKIEEVSARLRMVETLSNLLRSVVALSPPDLLPVLYLSLNHLGPPQQGLELGVGDGVLLKAVAQATGRQLESVRAEAAEKGDVGLVAENSRSTQRLMLPPPPLTASGVFSKFRDIARLTGSASTAKKIDIIKGLFVACRHSEARFIARSLSGRLRLGLAEQSVLAALSQAVSLTPPGQEFPPAMVDAGKGKTAEARKTWLEEQGMILKQTFCEVPDLDRIIPVLLEHGLERLPEHCKLSPGIPLKPMLAHPTRGISEVLKRFEEAAFTCEYKYDGQRAQIHALEGGEVKIFSRNQEDNTGKYPDIISRIPKIKLPSVTSFILDTEAVAWDREKKQIQPFQVLTTRKRKEVDASEIQVQVCLYAFDLIYLNGESLVREPLSRRRQLLRENFVETEGEFVFATSLDTKDIEQIAEFLEQSVKDSCEGLMVKTLDVDATYEIAKRSHNWLKLKKDYLDGVGDTLDLVVIGAYLGRGKRAGRYGGFLLASYDEDSEELQAICKLGTGFSDEELEEHHQSLKALVLPSPRPYVRIDGAVIPDHWLDPSAVWEVKCADLSLSPIYPAARGLVDSDKGISLRFPRFIRVREDKQPEQATTSAQVACLYRKQSQIQNQQGEDSGSDPEDTY

>NP_002717.3

MLSLQYPDVYRDETAVQDYHGHKICDPYAWLEDPDSEQTKAFVEAQNKITVPFLEQCPIRGLYKERMTELYDYPKYSCHFKKGKRYFYFYNTGLQNQRVLYVQDSLEGEARVFLDPNILSDDGTVALRGYAFSEDGEYFAYGLSASGSDWVTIKFMKVDGAKELPDVLERVKFSCMAWTHDGKGMFYNSYPQQDGKSDGTETSTNLHQKLYYHVLGTDQSEDILCAEFPDEPKWMGGAELSDDGRYVLLSIREGCDPVNRLWYCDLQQESSGIAGILKWVKLIDNFEGEYDYVTNEGTVFTFKTNRQSPNYRVINIDFRDPEESKWKVLVPEHEKDVLEWIACVRSNFLVLCYLHDVKNILQLHDLTTGALLKTFPLDVGSIVGYSGQKKDTEIFYQFTSFLSPGIIYHCDLTKEELEPRVFREVTVKGIDASDYQTVQIFYPSKDGTKIPMFIVHKKGIKLDGSHPAFLYGYGGFNISITPNYSVSRLIFVRHMGGILAVANIRGGGEYGETWHKGGILANKQNCFDDFQCAAEYLIKEGYTSPKRLTINGGSNGGLLVAACANQRPDLFGCVIAQVGVMDMLKFHKYTIGHAWTTDYGCSDSKQHFEWLVKYSPLHNVKLPEADDIQYPSMLLLTADHDDRVVPLHSLKFIATLQYIVGRSRKQSNPLLIHVDTKAGHGAGKPTAKVIEEVSDMFAFIARCLNVDWIP

204 NP_002544.1 Q93088

>NP_002544.1

MPHLENVVLCRESQVSILQSLFGERHHFSFPSIFIYGHTASGKTYVTQTLLKTLELPHVFVNCVECFTLRLLLEQILNKLNHLSSSEDGCSTEITCETFNDFVRLFKQVTTAENLKDQTVYIVLDKAEYLRDMEANLLPGFLRLQELADRNVTVLFLSEIVWEKFRPNTGCFEPFVLYFPDYSIGNLQKILSHDHPPEYSADFYAAYINILLGVFYTVCRDLKELRHLAVLNFPKYCEPVVKGEASERDTRKLWRNIEPHLKKAMQTVYLREISSSQWEKLQKDDTDPGQLKGLSAHTHVELPYYSKFILIAAYLASYNPARTDKRFFLKHHGKIKKTNFLKKHEKTSNHLLGPKPFPLDRLLAILYSIVDSRVAPTANIFSQITSLVTLQLLTLVGHDDQLDGPKYKCTVSLDFIRAIARTVNFDIIKYLYDFL

>Q93088

MPPVGGKKAKKGILERLNAGEIVIGDGGFVFALEKRGYVKAGPWTPEAAVEHPEAVRQLHREFLRAGSNVMQTFTFYASEDKLENRGNYVLEKISGQEVNEAACDIARQVADEGDALVAGGVSQTPSYLSCKSETEVKKVFLQQLEVFMKKNVDFLIAEYFEHVEEAVWAVETLIASGKPVAATMCIGPEGDLHGVPPGECAVRLVKAGASIIGVNCHFDPTISLKTVKLMKEGLEAARLKAHLMSQPLAYHTPDCNKQGFIDLPEFPFGLEPRVATRWDIQKYAREAYNLGVRYIGGCCGFEPYHIRAIAEELAPERGFLPPASEKHGSWGSGLDMHTKPWVRARARKEYWENLRIASGRPYNPSMSKPDGWGVTKGTAELMQQKEATTEQQLKELFEKQKFKSQ

205 NP_001528.1 Q01581

>NP_001528.1

MAETDPKTVQDLTSVVQTLLQQMQDKFQTMSDQIIGRIDDMSSRIDDLEKNIADLMTQAGVEELESENKIPATQKS

>Q01581

MPGSLPLNAEACWPKDVGIVALEIYFPSQYVDQAELEKYDGVDAGKYTIGLGQAKMGFCTDREDINSLCMTVVQNLMERNNLSYDCIGRLEVGTETIIDKSKSVKTNLMQLFEESGNTDIEGIDTTNACYGGTAAVFNAVNWIESSSWDGRYALVVAGDIAVYATGNARPTGGVGAVALLIGPNAPLIFERGLRGTHMQHAYDFYKPDMLSEYPIVDGKLSIQCYLSALDRCYSVYCKKIHAQWQKEGNDKDFTLNDFGFMIFHSPYCKLVQKSLARMLLNDFLNDQNRDKNSIYSGLEAFGDVKLEDTYFDRDVEKAFMKASSELFSQKTKASLLVSNQNGNMYTSSVYGSLASVLAQYSPQQLAGKRIGVFSYGSGLAATLYSLKVTQDATPGSALDKITASLCDLKSRLDSRTGVAPDVFAENMKLREDTHHLVNYIPQGSIDSLFEGTWYLVRVDEKHRRTYARRPTPNDDTLDEGVGLVHSNIATEHIPSPAKKVPRLPATAAEPEAAVISNGEH

206 NP_877433.2 NP_002684.1

>NP_877433.2

MRHNQMCCETPPTVTVYVKSGSNRSHQPKKPITLKRPICKDNWQAFEKNTHNNNKSKRPKGPCLVIQRQDMTAFFKLFDDDLIQDFLWMDCCCKIADKYLLAMTFVYFKRAKFTISEHTRINFFIALYLANTVEEDEEETKYEIFPWALGKNWRKLFPNFLKLRDQLWDRIDYRAIVSRRCCEEVMAIAPTHYIWQRERSVHHSGAVRNYNRDEVQLPRGPSATPVDCSLCGKKRRYVRLGLSSSSSLSSHTAGVTEKHSQDSYNSLSMDIIGDPSQAYTGSEVVNDHQSNKGKKTNFLKKDKSMEWFTGSEE

>NP_002684.1

MSRLLWRKVAGATVGPGPVPAPGRWVSSSVPASDPSDGQRRRQQQQQQQQQQQQQPQQPQVLSSEGGQLRHNPLDIQMLSRGLHEQIFGQGGEMPGEAAVRRSVEHLQKHGLWGQPAVPLPDVELRLPPLYGDNLDQHFRLLAQKQSLPYLEAANLLLQAQLPPKPPAWAWAEGWTRYGPEGEAVPVAIPEERALVFDVEVCLAEGTCPTLAVAISPSAWYSWCSQRLVEERYSWTSQLSPADLIPLEVPTGASSPTQRDWQEQLVVGHNVSFDRAHIREQYLIQGSRMRFLDTMSMHMAISGLSSFQRSLWIAAKQGKHKVQPPTKQGQKSQRKARRGPAISSWDWLDISSVNSLAEVHRLYVGGPPLEKEPRELFVKGTMKDIRENFQDLMQYCAQDVWATHEVFQQQLPLFLERCPHPVTLAGMLEMGVSYLPVNQNWERYLAEAQGTYEELQREMKKSLMDLANDACQLLSGERYKEDPWLWDLEWDLQEFKQKKAKKVKKEPATASKLPIEGAGAPGDPMDQEDLGPCSEEEEFQQDVMARACLQKLKGTTELLPKRPQHLPGHPGWYRKLCPRLDDPAWTPGPSLLSLQMRVTPKLMALTWDGFPLHYSERHGWGYLVPGRRDNLAKLPTGTTLESAGVVCPYRAIESLYRKHCLEQGKQQLMPQEAGLAEEFLLTDNSAIWQTVEELDYLEVEAEAKMENLRAAVPGQPLALTARGGPKDTQPSYHHGNGPYNDVDIPGCWFFKLPHKDGNSCNVGSPFAKDFLPKMEDGTLQAGPGGASGPRALEINKMISFWRNAHKRISSQMVVWLPRSALPRAVIRHPDYDEEGLYGAILPQVVTAGTITRRAVEPTWLTASNARPDRVGSELKAMVQAPPGYTLVGADVDSQELWIAAVLGDAHFAGMHGCTAFGWMTLQGRKSRGTDLHSKTATTVGISREHAKIFNYGRIYGAGQPFAERLLMQFNHRLTQQEAAEKAQQMYAATKGLRWYRLSDEGEWLVRELNLPVDRTEGGWISLQDLRKVQRETARKSQWKKWEVVAERAWKGGTESEMFNKLESIATSDIPRTPVLGCCISRALEPSAVQEEFMTSRVNWVVQSSAVDYLHLMLVAMKWLFEEFAIDGRFCISIHDEVRYLVREEDRYRAALALQITNLLTRCMFAYKLGLNDLPQSVAFFSAVDIDRCLRKEVTMDCKTPSNPTGMERRYGIPQGEALDIYQIIELTKGSLEKRSQPGP

207 NP_000391.1 NP_055165.2

>NP_000391.1

MKLNVDGLLVYFPYDYIYPEQFSYMRELKRTLDAKGHGVLEMPSGTGKTVSLLALIMAYQRAYPLEVTKLIYCSRTVPEIEKVIEELRKLLNFYEKQEGEKLPFLGLALSSRKNLCIHPEVTPLRFGKDVDGKCHSLTASYVRAQYQHDTSLPHCRFYEEFDAHGREVPLPAGIYNLDDLKALGRRQGWCPYFLARYSILHANVVVYSYHYLLDPKIADLVSKELARKAVVVFDEAHNIDNVCIDSMSVNLTRRTLDRCQGNLETLQKTVLRIKETDEQRLRDEYRRLVEGLREASAARETDAHLANPVLPDEVLQEAVPGSIRTAEHFLGFLRRLLEYVKWRLRVQHVVQESPPAFLSGLAQRVCIQRKPLRFCAERLRSLLHTLEITDLADFSPLTLLANFATLVSTYAKGFTIIIEPFDDRTPTIANPILHFSCMDASLAIKPVFERFQSVIITSGTLSPLDIYPKILDFHPVTMATFTMTLARVCLCPMIIGRGNDQVAISSKFETREDIAVIRNYGNLLLEMSAVVPDGIVAFFTSYQYMESTVASWYEQGILENIQRNKLLFIETQDGAETSVALEKYQEACENGRGAILLSVARGKVSEGIDFVHHYGRAVIMFGVPYVYTQSRILKARLEYLRDQFQIRENDFLTFDAMRHAAQCVGRAIRGKTDYGLMVFADKRFARGDKRGKLPRWIQEHLTDANLNLTVDEGVQVAKYFLRQMAQPFHREDQLGLSLLSLEQLESEETLKRIEQIAQQL

>NP_055165.2

MHSEAEESKEVATDVFNSKNLAVQAQKKILGKMVSKSIATTLIDDTSSEVLDELYRVTREYTQNKKEAEKIIKNLIKTVIKLAILYRNNQFNQDELALMEKFKKKVHQLAMTVVSFHQVDYTFDRNVLSRLLNECREMLHQIIQRHLTAKSHGRVNNVFDHFSDCEFLAALYNPFGNFKPHLQKLCDGINKMLDEENI

208 NP_006749.1 Q9UDX5

>NP_006749.1

MAEYLASIFGTEKDKVNCSFYFKIGACRHGDRCSRLHNKPTFSQTIALLNIYRNPQNSSQSADGLRCAVSDVEMQEHYDEFFEEVFTEMEEKYGEVEEMNVCDNLGDHLVGNVYVKFRREEDAEKAVIDLNNRWFNGQPIHAELSPVTDFREACCRQYEMGECTRGGFCNFMHLKPISRELRRELYGRRRKKHRSRSRSRERRSRSRDRGRGGGGGGGGGGGGRERDRRRSRDRERSGRF

>Q9UDX5

MSEPQPRGAERDLYRDTWVRYLGYANEVGEAFRSLVPAAVVWLSYGVASSYVLADAIDKGKKAGEVPSPEAGRSARVTVAVVDTFVWQALASVAIPGFTINRVCAASLYVLGTATRWPLAVRKWTTTALGLLTIPIIIHPIDRSVDFLLDSSLRKLYPTVGKPSSS

209 NP_006029.1 NP_036232.1

>NP_006029.1

MGKPSSMDTKFKDDLFRKYVQFHESKVDTTTSRQRPGSDECLRVAASTLLSLHKVDPFYRFRLIQFYEVVESSLRSLSSSSLRALHGAFSMLETVGINLFLYPWKKEFRSIKTYTGPFVYYVKSTLLEEDIRAILSCMGYTPELGTAYKLRELVETLQVKMVSFELFLAKVECEQMLEIHSQVKDKGYSELDIVSERKSSAEDVRGCSDALRRRAEGREHLTASMSRVALQKSASERAAKDYYKPRVTKPSRSVDAYDSYWESRKPPLKASLSLRKEPVATDVGDDLKDEIIRPSPSLLTMASSPHGSPDVLPPASPSNGPALLRGTYFSTQDDVDLYTDSEPRATYRRQDALRPDVWLLRNDAHSLYHKRSPPAKESALSKCQSCGLSCSSSLCQRCDSLLTCPPASKPSAFPSKASTHDSLAHGASLREKYPGQTQGLDRLPHLHSKSKPSTTPTSRCGFCNRPGATNTCTQCSKVSCDACLSAYHYDPCYKKSELHKFMPNNQLNYKSTQLSHLVYR

>NP_036232.1

MQVAMNGKARKEAVQTAAKELLKFVNRSPSPFHAVAECRNRLLQAGFSELKETEKWNIKPESKYFMTRNSSTIIAFAVGGQYVPGNGFSLIGAHTDSPCLRVKRRSRRSQVGFQQVGVETYGGGIWSTWFDRDLTLAGRVIVKCPTSGRLEQQLVHVERPILRIPHLAIHLQRNINENFGPNTEMHLVPILATAIQEELEKGTPEPGPLNAVDERHHSVLMSLLCAHLGLSPKDIVEMELCLADTQPAVLGGAYDEFIFAPRLDNLHSCFCALQALIDSCAGPGSLATEPHVRMVTLYDNEEVGSESAQGAQSLLTELVLRRISASCQHPTAFEEAIPKSFMISADMAHAVHPNYLDKHEENHRPLFHKGPVIKVNSKQRYASNAVSEALIREVANKVKVPLQDLMVRNDTPCGTTIGPILASRLGLRVLDLGSPQLAMHSIREMACTTGVLQTLTLFKGFFELFPSLSHNLLVD

210 NP_003654.3 P82921

>NP_003654.3

MERFVVTAPPARNRSKTALYVTPLDRVTEFGGELHEDGGKLFCTSCNVVLNHVRKSAISDHLKSKTHTKRKAEFEEQNVRKKQRPLTASLQCNSTAQTEKVSVIQDFVKMCLEANIPLEKADHPAVRAFLSRHVKNGGSIPKSDQLRRAYLPDGYENENQLLNSQDC

>P82921

MAKHLKFIARTVMVQEGNVESAYRTLNRILTMDGLIEDIKHRRYYEKPCRRRQRESYERCRRIYNMEMARKINFLMRKNRADPWQGC

211 NP_055596.3 NP_001001936.1

>NP_055596.3

MKLYVFLVNTGTTLTFDTELTVQTVADLKHAIQSKYKIAIQHQVLVVNGGECMAADRRVCTYSAGTDTNPIFLFNKEMILCDRPPAIPKTTFSTENDMEIKVEESLMMPAVFHTVASRTQLALEMYEVAKKLCSFCEGLVHDEHLQHQGWAAIMANLEDCSNSYQKLLFKFESIYSNYLQSIEDIKLKLTHLGTAVSVMAKIPLLECLTRHSYRECLGRLDSLPEHEDSEKAEMKRSTELVLSPDMPRTTNESLLTSFPKSVEHVSPDTADAESGKEIRESCQSTVHQQDETTIDTKDGDLPFFNVSLLDWINVQDRPNDVESLVRKCFDSMSRLDPRIIRPFIAECRQTIAKLDNQNMKAIKGLEDRLYALDQMIASCGRLVNEQKELAQGFLANQKRAENLKDASVLPDLCLSHANQLMIMLQNHRKLLDIKQKCTTAKQELANNLHVRLKWCCFVMLHADQDGEKLQALLRLVIELLERVKIVEALSTVPQMYCLAVVEVVRRKMFIKHYREWAGALVKDGKRLYEAEKSKRESFGKLFRKSFLRNRLFRGLDSWPPSFCTQKPRKFDCELPDISLKDLQFLQSFCPSEVQPFLRVPLLCDFEPLHQHVLALHNLVKAAQSLDEMSQTITDLLSEQKASVSQTSPQSASSPRMESTAGITTTTSPRTPPPLTVQDPLCPAVCPLEELSPDSIDAHTFDFETIPHPNIEQTIHQVSLDLDSLAESPESDFMSAVNEFVIEENLSSPNPISDPQSPEMMVESLYSSVINAIDSRRMQDTNVCGKEDFGDHTSLNVQLERCRVVAQDSHFSIQTIKEDLCHFRTFVQKEQCDFSNSLKCTAVEIRNIIEKVKCSLEITLKEKHQKELLSLKNEYEGKLDGLIKETEENENKIKKLKGELVCLEEVLQNKDNEFALVKHEKEAVICLQNEKDQKLLEMENIMHSQNCEIKELKQSREIVLEDLKKLHVENDEKLQLLRAELQSLEQSHLKELEDTLQVRHIQEFEKVMTDHRVSLEELKKENQQIINQIQESHAEIIQEKEKQLQELKLKVSDLSDTRCKLEVELALKEAETDEIKILLEESRAQQKETLKSLLEQETENLRTEISKLNQKIQDNNENYQVGLAELRTLMTIEKDQCISELISRHEEESNILKAELNKVTSLHNQAFEIEKNLKEQIIELQSKLDSELSALERQKDEKITQQEEKYEAIIQNLEKDRQKLVSSQEQDREQLIQKLNCEKDEAIQTALKEFKLEREVVEKELLEKVKHLENQIAKSPAIDSTRGDSSSLVAELQEKLQEEKAKFLEQLEEQEKRKNEEMQNVRTSLIAEQQTNFNTVLTREKMRKENIINDLSDKLKSTMQQQERDKDLIESLSEDRARLLEEKKKLEEEVSKLRSSSFVPSPYVATAPELYGACAPELPGESDRSAVETADEGRVDSAMETSMMSVQENIHMLSEEKQRIMLLERTLQLKEEENKRLNQRLMSQSMSSVSSRHSEKIAIRDFQVGDLVLIILDERHDNYVLFTVSPTLYFLHSESLPALDLKPGEGASGASRRPWVLGKVMEKEYCQAKKAQNRFKVPLGTKFYRVKAVSWNKKV

>NP_001001936.1

MERYKALEQLLTELDDFLKILDQENLSSTALVKKSCLAELLRLYTKSSSSDEEYIYMNKVTINKQQNAESQGKAPEEQGLLPNGEPSQHSSAPQKSLPDLPPPKMIPERKQLAIPKTESPEGYYEEAEPYDTSLNEDGEAVSSSYESYDEEDGSKGKSAPYQWPSPEAGIELMRDARICAFLWRKKWLGQWAKQLCVIKDNRLLCYKSSKDHSPQLDVNLLGSSVIHKEKQVRKKEHKLKITPMNADVIVLGLQSKDQAEQWLRVIQEVSGLPSEGASEGNQYTPDAQRFNCQKPDIAEKYLSASEYGSSVDGHPEVPETKDVKKKCSAGLKLSNLMNLGRKKSTSLEPVERSLETSSYLNVLVNSQWKSRWCSVRDNHLHFYQDRNRSKVAQQPLSLVGCEVVPDPSPDHLYSFRILHKGEELAKLEAKSSEEMGHWLGLLLSESGSKTDPEEFTYDYVDADRVSCIVSAAKNSLLLMQRKFSEPNTYIDGLPSQDRQEELYDDVDLSELTAAVEPTEEATPVADDPNERESDRVYLDLTPVKSFLHGPSSAQAQASSPTLSCLDNATEALPADSGPGPTPDEPCIKCPENLGEQQLESLEPEDPSLRITTVKIQTEQQRISFPPSCPDAVVATPPGASPPVKDRLRVTSAEIKLGKNRTEAEVKRYTEEKERLEKKKEEIRGHLAQLRKEKRELKETLLKCTDKEVLASLEQKLKEIDEECRGEESRRVDLELSIMEVKDNLKKAEAGPVTLGTTVDTTHLENVSPRPKAVTPASAPDCTPVNSATTLKNRPLSVVVTGKGTVLQKAKEWEKKGAS

212 Q9BWT1 NP_005116.1

>Q9BWT1

MDARRVPQKDLRVKKNLKKFRYVKLISMETSSSSDDSCDSFASDNFANTRLQSVREGCRTRSQCRHSGPLRVAMKFPARSTRGATNKKAESRQPSENSVTDSNSDSEDESGMNFLEKRALNIKQNKAMLAKLMSELESFPGSFRGRHPLPGSDSQSRRPRRRTFPGVASRRNPERRARPLTRSRSRILGSLDALPMEEEEEEDKYMLVRKRKTVDGYMNEDDLPRSRRSRSSVTLPHIIRPVEEITEEELENVCSNSREKIYNRSLGSTCHQCRQKTIDTKTNCRNPDCWGVRGQFCGPCLRNRYGEEVRDALLDPNWHCPPCRGICNCSFCRQRDGRCATGVLVYLAKYHGFGNVHAYLKSLKQEFEMQA

>NP_005116.1

MASDSGNQGTLCTLEFAVQMTCQSCVDAVRKSLQGVAGVQDVEVHLEDQMVLVHTTLPSQEVQALLEGTGRQAVLKGMGSGQLQNLGAAVAILGGPGTVQGVVRFLQLTPERCLIEGTIDGLEPGLHGLHVHQYGDLTNNCNSCGNHFNPDGASHGGPQDSDRHRGDLGNVRADADGRAIFRMEDEQLKVWDVIGRSLIIDEGEDDLGRGGHPLSKITGNSGERLACGIIARSAGLFQNPKQICSCDGLTIWEERGRPIAGKGRKESAQPPAHL

213 Q96AV8 NP_001880.2

>Q96AV8

MEVNCLTLKDLISPRQPRLDFAVEDGENAQKENIFVDRSRMAPKTPIKNEPIDLSKQKKFTPERNPITPVKLVDRQQAEPWTPTANLKMLISAASPDIRDREKKKGLFRPIENKDDAFTDSLQLDVVGDSAVDEFEKQRPSRKQKSLGLLCQKFLARYPSYPLSTEKTTISLDEVAVSLGVERRRIYDIVNVLESLHLVSRVAKNQYGWHGRHSLPKTLRNLQRLGEEQKYEEQMAYLQQKELDLIDYKFGERKKDGDPDSQEQQLLDFSEPDCPSSSANSRKDKSLRIMSQKFVMLFLVSKTKIVTLDVAAKILIEESQDAPDHSKFKTKVRRLYDIANVLTSLALIKKVHVTEERGRKPAFKWIGPVDFSSSDEELVDVSASVLPELKRETYGQIQVCAKQKLARHGSFNTVQASERIQRKVNSEPSSPYREEQGSGGYSLEIGSLAAVYRQKIEDNSQGKAFASKRVVPPSSSLDPVAPFPVLSVDPEYCVNPLAHPVFSVAQTDLQAFSMQNGLNGQVDVSLASAASAVESLKPALLAGQPLVYVPSASLFMLYGSLQEGPASGSGSERDDRSSEAPATVELSSAPSAQKRLCEERKPQEEDEPATKRQSREYEDGPLSLVMPKKPSDSTDLASPKTMGNRASIPLKDIHVNGQLPAAEEISGKATANSLVSSEWGNPSRNTDVEKPSKENESTKEPSLLQYLCVQSPAGLNGFNVLLSGSQTPPTVGPSSGQLPSFSVPCMVLPSPPLGPFPVLYSPAMPGPVSSTLGALPNTGPVNFSLPGLGSIAQLLVGPTAVVNPKSSTLPSADPQLQSQPSLNLSPVMSRSHSVVQQPESPVYVGHPVSVVKLQQSPVPVTPKSIQRTHRETFFKTPGSLGDPVLKRRERNQSRNTSSAQRRLEIPSGGAD

>NP_001880.2

MATGQKLMRAVRVFEFGGPEVLKLRSDIAVPIPKDHQVLIKVHACGVNPVETYIRSGTYSRKPLLPYTPGSDVAGVIEAVGDNASAFKKGDRVFTSSTISGGYAEYALAADHTVYKLPEKLDFKQGAAIGIPYFTAYRALIHSACVKAGESVLVHGASGGVGLAACQIARAYGLKILGTAGTEEGQKIVLQNGAHEVFNHREVNYIDKIKKYVGEKGIDIIIEMLANVNLSKDLSLLSHGGRVIVVGSRGTIEINPRDTMAKESSIIGVTLFSSTKEEFQQYAAALQAGMEIGWLKPVIGSQYPLEKVAEAHENIIHGSGATGKMILLL

214 P04066 NP_000476.1

>P04066

MRAPGMRSRPAGPALLLLLLFLGAAESVRRAQPPRRYTPDWPSLDSRPLPAWFDEAKFGVFIHWGVFSVPAWGSEWFWWHWQGEGRPQYQRFMRDNYPPGFSYADFGPQFTARFFHPEEWADLFQAAGAKYVVLTTKHHEGFTNWPSPVSWNWNSKDVGPHRDLVGELGTALRKRNIRYGLYHSLLEWFHPLYLLDKKNGFKTQHFVSAKTMPELYDLVNSYKPDLIWSDGEWECPDTYWNSTNFLSWLYNDSPVKDEVVVNDRWGQNCSCHHGGYYNCEDKFKPQSLPDHKWEMCTSIDKFSWGYRRDMALSDVTEESEIISELVQTVSLGGNYLLNIGPTKDGLIVPIFQERLLAVGKWLSINGEAIYASKPWRVQWEKNTTSVWYTSKGSAVYAIFLHWPENGVLNLESPITTSTTKITMLGIQGDLKWSTDPDKGLFISLPQLPPSAVPAEFAWTIKLTGVK

>NP_000476.1

MADSELQLVEQRIRSFPDFPTPGVVFRDISPVLKDPASFRAAIGLLARHLKATHGGRIDYIAGLDSRGFLFGPSLAQELGLGCVLIRKRGKLPGPTLWASYSLEYGKAELEIQKDALEPGQRVVVVDDLLATGGTMNAACELLGRLQAEVLECVSLVELTSLKGREKLAPVPFFSLLQYE

215 NP_996670.1 P30838

>NP_996670.1

MAPKQDPKPKFQEGERVLCFHGPLLYEAKCVKVAIKDKQVKYFIHYSGWNKKSAVRPRRSEKSLKTHEDIVALFPVPEGAPSVHHPLLTSSWDEWVPESRVLKYVDTNLQKQRELQKANQEQYAEGKMRGAAPGKKTSGLQQKNVEVKTKKNKQKTPGNGDGGSTSETPQPPRKKRARVDPTVENEETFMNRVEVKVKIPEELKPWLVDDWDLITRQKQLFYLPAKKNVDSILEDYANYKKSRGNTDNKEYAVNEVVAGIKEYFNVMLGTQLLYKFERPQYAEILADHPDAPMSQVYGAPHLLRLFVRIGAMLAYTPLDEKSLALLLNYLHDFLKYLAKNSATLFSASDYEVAPPEYHRKAV

>P30838

MSKISEAVKRARAAFSSGRTRPLQFRIQQLEALQRLIQEQEQELVGALAADLHKNEWNAYYEEVVYVLEEIEYMIQKLPEWAADEPVEKTPQTQQDELYIHSEPLGVVLVIGTWNYPFNLTIQPMVGAIAAGNAVVLKPSELSENMASLLATIIPQYLDKDLYPVINGGVPETTELLKERFDHILYTGSTGVGKIIMTAAAKHLTPVTLELGGKSPCYVDKNCDLDVACRRIAWGKFMNSGQTCVAPDYILCDPSIQNQIVEKLKKSLKEFYGEDAKKSRDYGRIISARHFQRVMGLIEGQKVAYGGTGDAATRYIAPTILTDVDPQSPVMQEEIFGPVLPIVCVRSLEEAIQFINQREKPLALYMFSSNDKVIKKMIAETSSGGVAANDVIVHITLHSLPFGGVGNSGMGSYHGKKSFETFSHRRSCLVRPLMNDEGLKVRYPPSPAKMTQH

216 NP_612211.1 Q9BZQ8

>NP_612211.1

MFVLVEMVDTVRIPPWQFERKLNDSIAEELNKKLANKVVYNVGLCICLFDITKLEDAYVFPGDGASHTKVHFRCVVFHPFLDEILIGKIKGCSPEGVHVSLGFFDDILIPPESLQQPAKFDEAEQVWVWEYETEEGAHDLYMDTGEEIRFRVVDESFVDTSPTGPSSADATTSSEELPKKEAPYTLVGSISEPGLGLLSWWTSN

>Q9BZQ8

MGGSASSQLDEGKCAYIRGKTEAAIKNFSPYYSRQYSVAFCNHVRTEVEQQRDLTSQFLKTKPPLAPGTILYEAELSQFSEDIKKWKERYVVVKNDYAVESYENKEAYQRGAAPKCRILPAGGKVLTSEDEYNLLSDRHFPDPLASSEKENTQPFVVLPKEFPVYLWQPFFRHGYFCFHEAADQKRFSALLSDCVRHLNHDYMKQMTFEAQAFLEAVQFFRQEKGHYGSWEMITGDEIQILSNLVMEELLPTLQTDLLPKMKGKKNDRKRTWLGLLEEAYTLVQHQVSEGLSALKEECRALTKGLEGTIRSDMDQIVNSKNYLIGKIKAMVAQPAEKSCLESVQPFLASILEELMGPVSSGFSEVRVLFEKEVNEVSQNFQTTKDSVQLKEHLDRLMNLPLHSVKMEPCYTKVNLLHERLQDLKSRFRFPHIDLVVQRTQNYMQELMENAVFTFEQLLSPHLQGEASKTAVAIEKVKLRVLKQYDYDSSTIRKKIFQEALVQITLPTVQKALASTCKPELQKYEQFIFADHTNMIHVENVYEEILHQILLDETLKVIKEAAILKKHNLFEDNMALPSESVSSLTDLKPPTGSNQASPARRASAILPGVLGSETLSNEVFQESEEEKQPEVPSSLAKGESLSLPGPSPPPDGTEQVIISRVDDPVVNPVATEDTAGLPGTCSSELEFGGTLEDEEPAQEEPEPITASGSLKALRKLLTASVEVPVDSAPVMEEDTNGESHVPQENEEEEEKEPSQAAAIHPDNCEESEVSEREAQPPCPEAHGEELGGFPEVGSPASPPASGGLTEEPLGPMEGELPGEACTLTAHEGRGGKCTEEGDASQQEGCTLGSDPICLSESQVSEEQEEMGGQSSAAQATASVNAEEIKVARIHECQWVVEDAPNPDVLLSHKDDVKEGEGGQESFPELPSEE

217 NP_003075.1 NP_000958.1

>NP_003075.1

MAEGSRGGPTCSGVGGRQDPVSGSGGCNFPEYELPELNTRAFHVGAFGELWRGRLRGAGDLSLREPPASALPGSQAADSDREDAAVARDLDCSLEAAAELRAVCGLDKLKCLEDGEDPEVIPENTDLVTLGVRKRFLEHREETITIDRACRQETFVYEMESHAIGKKPENSADMIEEGELILSVNILYPVIFHKHKEHKPYQTMLVLGSQKLTQLRDSIRCVSDLQIGGEFSNTPDQAPEHISKDLYKSAFFYFEGTFYNDKRYPECRDLSRTIIEWSESHDRGYGKFQTARMEDFTFNDLCIKLGFPYLYCHQGDCEHVIVITDIRLVHHDDCLDRTLYPLLIKKHWLWTRKCFVCKMYTARWVTNNDSFAPEDPCFFCDVCFRMLHYDSEGNKLGEFLAYPYVDPGTFN

>NP_000958.1

MSHRKFSAPRHGSLGFLPRKRSSRHRGKVKSFPKDDPSKPVHLTAFLGYKAGMTHIVREVDRPGSKVNKKEVVEAVTIVETPPMVVVGIVGYVETPRGLRTFKTVFAEHISDECKRRFYKNWHKSKKKAFTKYCKKWQDEDGKKQLEKDFSSMKKYCQVIRVIAHTQMRLLPLRQKKAHLMEIQVNGGTVAEKLDWARERLEQQVPVNQVFGQDEMIDVIGVTKGKGYKGVTSRWHTKKLPRKTHRGLRKVACIGAWHPARVAFSVARAGQKGYHHRTEINKKIYKIGQGYLIKDGKLIKNNASTDYDLSDKSINPLGGFVHYGEVTNDFVMLKGCVVGTKKRVLTLRKSLLVQTKRRALEKIDLKFIDTTSKFGHGRFQTMEEKKAFMGPLKKDRIAKEEGA

218 NP_003160.2 Q9BU02

>NP_003160.2

MSDSEDSNFSEEEDSERSSDGEEAEVDEERRSAAGSEKEEEPEDEEEEEEEEEYDEEEEEEDDDRPPKKPRHGGFILDEADVDDEYEDEDQWEDGAEDILEKEEIEASNIDNVVLDEDRSGARRLQNLWRDQREEELGEYYMKKYAKSSVGETVYGGSDELSDDITQQQLLPGVKDPNLWTVKCKIGEERATAISLMRKFIAYQFTDTPLQIKSVVAPEHVKGYIYVEAYKQTHVKQAIEGVGNLRLGYWNQQMVPIKEMTDVLKVVKEVANLKPKSWVRLKRGIYKDDIAQVDYVEPSQNTISLKMIPRIDYDRIKARMSLKDWFAKRKKFKRPPQRLFDAEKIRSLGGDVASDGDFLIFEGNRYSRKGFLFKSFAMSAVITEGVKPTLSELEKFEDQPEGIDLEVVTESTGKEREHNFQPGDNVEVCEGELINLQGKILSVDGNKITIMPKHEDLKDMLEFPAQELRKYFKMGDHVKVIAGRFEGDTGLIVRVEENFVILFSDLTMHELKVLPRDLQLCSETASGVDVGGQHEWGELVQLDPQTVGVIVRLERETFQVLNMYGKVVTVRHQAVTRKKDNRFAVALDSEQNNIHVKDIVKVIDGPHSGREGEIRHLFRSFAFLHCKKLVENGGMFVCKTRHLVLAGGSKPRDVTNFTVGGFAPMSPRISSPMHPSAGGQRGGFGSPGGGSGGMSRGRGRRDNELIGQTVRISQGPYKGYIGVVKDATESTARVELHSTCQTISVDRQRLTTVGSRRPGGMTSTYGRTPMYGSQTPMYGSGSRTPMYGSQTPLQDGSRTPHYGSQTPLHDGSRTPAQSGAWDPNNPNTPSRAEEEYEYAFDDEPTPSPQAYGGTPNPQTPGYPDPSSPQVNPQYNPQTPGTPAMYNTDQFSPYAAPSPQGSYQPSPSPQSYHQVAPSPAGYQNTHSPASYHPTPSPMAYQASPSPSPVGYSPMTPGAPSPGGYNPHTPGSGIEQNSSDWVTTDIQVKVRDTYLDTQVVGQTGVIRSVTGGMCSVYLKDSEKVVSISSEHLEPITPTKNNKVKVILGEDREATGVLLSIDGEDGIVRMDLDEQLKILNLRFLGKLLEA

>Q9BU02

MAQGLIEVERKFLPGPGTEERLQELGGTLEYRVTFRDTYYDTPELSLMQADHWLRRREDSGWELKCPGAAGVLGPHTEYKELTAEPTIVAQLCKVLRADGLGAGDVAAVLGPLGLQEVASFVTKRSAWKLVLLGADEEEPQLRVDLDTADFGYAVGEVEALVHEEAEVPTALEKIHRLSSMLGVPAQETAPAKLIVYLQRFRPQDYQRLLEVNSSRERPQETEDPDHCLG

219 Q9Y675 NP_001631.3

>Q9Y675

MERARDRLHLRRTTEQHVPEVEVQVKRRRTASLSNQECQLYPRRSQQQQVPVVDFQAELRQAFLAETPRGG

>NP_001631.3

MERQVLLSEPEEAAALYRGLSRQPALSAACLGPEVTTQYGGQYRTVHTEWTQRDLERMENIRFCRQYLVFHDGDSVVFAGPAGNSVETRGELLSRESPSGTMKAVLRKAGGTGPGEEKQFLEVWEKNRKLKSFNLSALEKHGPVYEDDCFGCLSWSHSETHLLYVAEKKRPKAESFFQTKALDVSASDDEIARLKKPDQAIKGDQFVFYEDWGENMVSKSIPVLCVLDVESGNISVLEGVPENVSPGQAFWAPGDAGVVFVGWWHEPFRLGIRFCTNRRSALYYVDLIGGKCELLSDDSLAVSSPRLSPDQCRIVYLQYPSLIPHHQCSQLCLYDWYTKVTSVVVDVVPRQLGENFSGIYCSLLPLGCWSADSQRVVFDSAQRSRQDLFAVDTQVGTVTSLTAGGSGGSWKLLTIDQDLMVAQFSTPSLPPTLKVGFLPSAGKEQSVLWVSLEEAEPIPDIHWGIRVLQPPPEQENVQYAGLDFEAILLQPGSPPDKTQVPMVVMPHGGPHSSFVTAWMLFPAMLCKMGFAVLLVNYRGSTGFGQDSILSLPGNVGHQDVKDVQFAVEQVLQEEHFDASHVALMGGSHGGFISCHLIGQYPETYRACVARNPVINIASMLGSTDIPDWCVVEAGFPFSSDCLPDLSVWAEMLDKSPIRYIPQVKTPLLLMLGQEDRRVPFKQGMEYYRALKTRNVPVRLLLYPKSTHALSEVEVESDSFMNAVLWLRTHLGS

220 NP_036354.1 NP_000404.1

>NP_036354.1

MTPLVSRLSRLWAIMRKPRAAVGSGHRKQAASQEGRQKHAKNNSQAKPSACDGMIAECPGAPAGLARQPEEVVLQASVSSYHLFRDVAEVTAFRGSLLSWYDQEKRDLPWRRRAEDEMDLDRRAYAVWVSEVMLQQTQVATVINYYTGWMQKWPTLQDLASASLEEVNQLWAGLGYYSRGRRLQEGARKVVEELGGHMPRTAETLQQLLPGVGRYTAGAIASIAFGQATGVVDGNVARVLCRVRAIGADPSSTLVSQQLWGLAQQLVDPARPGDFNQAAMELGATVCTPQRPLCSQCPVESLCRARQRVEQEQLLASGSLSGSPDVEECAPNTGQCHLCLPPSEPWDQTLGVVNFPRKASRKPPREESSATCVLEQPGALGAQILLVQRPNSGLLAGLWEFPSVTWEPSEQLQRKALLQELQRWAGPLPATHLRHLGEVVHTFSHIKLTYQVYGLALEGQTPVTTVPPGARWLTQEEFHTAAVSTAMKKVFRVYQGQQPGTCMGSKRSQVSSPCSRKKPRMGQQVLDNFFRSHISTDAHSLNSAAQ

>NP_000404.1

MARTVVLITGCSSGIGLHLAVRLASDPSQSFKVYATLRDLKTQGRLWEAARALACPPGSLETLQLDVRDSKSVAAARERVTEGRVDVLVCNAGLGLLGPLEALGEDAVASVLDVNVVGTVRMLQAFLPDMKRRGSGRVLVTGSVGGLMGLPFNDVYCASKFALEGLCESLAVLLLPFGVHLSLIECGPVHTAFMEKVLGSPEEVLDRTDIHTFHRFYQYLAHSKQVFREAAQNPEEVAEVFLTALRAPKPTLRYFTTERFLPLLRMRLDDPSGSNYVTAMHREVFGDVPAKAEAGAEAGGGAGPGAEDEAGRSAVGDPELGDPPAAPQ

221 NP_073624.2 NP_004171.2

>NP_073624.2

MENSEKTEVVLLACGSFNPITNMHLRLFELAKDYMNGTGRYTVVKGIISPVGDAYKKKGLIPAYHRVIMAELATKNSKWVEVDTWESLQKEWKETLKVLRHHQEKLEASDCDHQQNSPTLERPGRKRKWTETQDSSQKKSLEPKTKAVPKVKLLCGADLLESFAVPNLWKSEDITQIVANYGLICVTRAGNDAQKFIYESDVLWKHRSNIHVVNEWIANDISSTKIRRALRRGQSIRYLVPDLVQEYIEKHNLYSSESEDRNAGVILAPLQRNTAEAKT

>NP_004171.2

MDKNIGEQLNKAYEAFRQACMDRDSAVKELQQKTENYEQRIREQQEQLSLQQTIIDKLKSQLLLVNSTQDNNYGCVPLLEDSETRKNNLTLDQPQDKVISGIAREKLPKVRRQEVSSPRKETSARSLGSPLLHERGNIEKTFWDLKEEFHKICMLAKAQKDHLSKLNIPDTATETQCSVPIQCTDKTDKQEALFKPQAKDDINRGAPSITSVTPRGLCRDEEDTSFESLSKFNVKFPPMDNDSTFLHSTPERPGILSPATSEAVCQEKFNMEFRDNPGNFVKTEETLFEIQGIDPIASAIQNLKTTDKTKPSNLVNTCIRTTLDRAACLPPGDHNALYVNSFPLLDPSDAPFPSLDSPGKAIRGPQQPIWKPFPNQDSDSVVLSGTDSELHIPRVCEFCQAVFPPSITSRGDFLRHLNSHFNGET

222 NP_001460.1 NP_005495.2

>NP_001460.1

MSGWESYYKTEGDEEAEEEQEENLEASGDYKYSGRDSLIFLVDASKAMFESQSEDELTPFDMSIQCIQSVYISKIISSDRDLLAVVFYGTEKDKNSVNFKNIYVLQELDNPGAKRILELDQFKGQQGQKRFQDMMGHGSDYSLSEVLWVCANLFSDVQFKMSHKRIMLFTNEDNPHGNDSAKASRARTKAGDLRDTGIFLDLMHLKKPGGFDISLFYRDIISIAEDEDLRVHFEESSKLEDLLRKVRAKETRKRALSRLKLKLNKDIVISVGIYNLVQKALKPPPIKLYRETNEPVKTKTRTFNTSTGGLLLPSDTKRSQIYGSRQIILEKEETEELKRFDDPGLMLMGFKPLVLLKKHHYLRPSLFVYPEESLVIGSSTLFSALLIKCLEKEVAALCRYTPRRNIPPYFVALVPQEEELDDQKIQVTPPGFQLVFLPFADDKRKMPFTEKIMATPEQVGKMKAIVEKLRFTYRSDSFENPVLQQHFRNLEALALDLMEPEQAVDLTLPKVEAMNKRLGSLVDEFKELVYPPDYNPEGKVTKRKHDNEGSGSKRPKVEYSEEELKTHISKGTLGKFTVPMLKEACRAYGLKSGLKKQELLEALTKHFQD

>NP_005495.2

MKDCSNGCSAECTGEGGSKEVVGTFKAKDLIVTPATILKEKPDPNNLVFGTVFTDHMLTVEWSSEFGWEKPHIKPLQNLSLHPGSSALHYAVELFEGLKAFRGVDNKIRLFQPNLNMDRMYRSAVRATLPVFDKEELLECIQQLVKLDQEWVPYSTSASLYIRPTFIGTEPSLGVKKPTKALLFVLLSPVGPYFSSGTFNPVSLWANPKYVRAWKGGTGDCKMGGNYGSSLFAQCEAVDNGCQQVLWLYGEDHQITEVGTMNLFLYWINEDGEEELATPPLDGIILPGVTRRCILDLAHQWGEFKVSERYLTMDDLTTALEGNRVREMFGSGTACVVCPVSDILYKGETIHIPTMENGPKLASRILSKLTDIQYGREESDWTIVLS

223 NP_055136.1 Q06278

>NP_055136.1

MGSELIGRLAPRLGLAEPDMLRKAEEYLRLSRVKCVGLSARTTETSSAVMCLDLAASWMKCPLDRAYLIKLSGLNKETYQSCLKSFECLLGLNSNIGIRDLAVQFSCIEAVNMASKILKSYESSLPQTQQVDLDLSRPLFTSAALLSACKILKLKVDKNKMVATSGVKKAIFDRLCKQLEKIGQQVDREPGDVATPPRKRKKIVVEAPAKEMEKVEEMPHKPQKDEDLTQDYEEWKRKILENAASAQKATAE

>Q06278

MDRASELLFYVNGRKVIEKNVDPETMLLPYLRKKLRLTGTKYGCGGGGCGACTVMISRYNPITKRIRHHPANACLIPICSLYGAAVTTVEGIGSTHTRIHPVQERIAKCHGTQCGFCTPGMVMSIYTLLRNHPEPTLDQLTDALGGNLCRCTGYRPIIDACKTFCKTSGCCQSKENGVCCLDQGINGLPEFEEGSKTSPKLFAEEEFLPLDPTQELIFPPELMIMAEKQSQRTRVFGSERMMWFSPVTLKELLEFKFKYPQAPVIMGNTSVGPEVKFKGVFHPVIISPDRIEELSVVNHAYNGLTLGAGLSLAQVKDILADVVQKLPEEKTQMYHALLKHLGTLAGSQIRNMASLGGHIISRHPDSDLNPILAVGNCTLNLLSKEGKRQIPLNEQFLSKCPNADLKPQEILVSVNIPYSRKWEFVSAFRQAQRQENALAIVNSGMRVFFGEGDGIIRELCISYGGVGPATICAKNSCQKLIGRHWNEQMLDIACRLILNEVSLLGSAPGGKVEFKRTLIISFLFKFYLEVSQILKKMDPVHYPSLADKYESALEDLHSKHHCSTLKYQNIGPKQHPEDPIGHPIMHLSGVKHATGEAIYCDDMPLVDQELFLTFVTSSRAHAKIVSIDLSEALSMPGVVDIMTAEHLSDVNSFCFFTEAEKFLATDKVFCVGQLVCAVLADSEVQAKRAAKRVKIVYQDLEPLILTIEESIQHNSSFKPERKLEYGNVDEAFKVVDQILEGEIHMGGQEHFYMETQSMLVVPKGEDQEMDVYVSTQFPKYIQDIVASTLKLPANKVMCHVRRVGGAFGGKVLKTGIIAAVTAFAANKHGRAVRCVLERGEDMLITGGRHPYLGKYKAGFMNDGRILALDMEHYSNAGASLDESLFVIEMGLLKMDNAYKFPNLRCRGWACRTNLPSNTAFRGFGFPQAALITESCITEVAAKCGLSPEKVRIINMYKEIDQTPYKQEINAKNLIQCWRECMAMSSYSLRKVAVEKFNAENYWKKKGLAMVPLKFPVGLGSRAAGQAAALVHIYLDGSVLVTHGGIEMGQGVHTKMIQVVSRELRMPMSNVHLRGTSTETVPNANISGGSVVADLNGLAVKDACQTLLKRLEPIISKNPKGTWKDWAQTAFDESINLSAVGYFRGYESDMNWEKGEGQPFEYFVYGAACSEVEIDCLTGDHKNIRTDIVMDVGCSINPAIDIGQIEGAFIQGMGLYTIEELNYSPQGILHTRGPDQYKIPAICDMPTELHIALLPPSQNSNTLYSSKGLGESGVFLGCSVFFAIHDAVSAARQERGLHGPLTLNSPLTPEKIRMACEDKFTKMIPRDEPGSYVPWNVPI

224 NP_006707.1 NP_000365.3

>NP_006707.1

MNSGAMRIHSKGHFQGGIQVKNEKNRPSLKSLKTDNRPEKSKCKPLWGKVFYLDLPSVTISEKLQKDIKDLGGRVEEFLSKDISYLISNKKEAKFAQTLGRISPVPSPESAYTAETTSPHPSHDGSSFKSPDTVCLSRGKLLVEKAIKDHDFIPSNSILSNALSWGVKILHIDDIRYYIEQKKKELYLLKKSSTSVRDGGKRVGSGAQKTRTGRLKKPFVKVEDMSQLYRPFYLQLTNMPFINYSIQKPCSPFDVDKPSSMQKQTQVKLRIQTDGDKYGGTSIQLQLKEKKKKGYCECCLQKYEDLETHLLSEQHRNFAQSNQYQVVDDIVSKLVFDFVEYEKDTPKKKRIKYSVGSLSPVSASVLKKTEQKEKVELQHISQKDCQEDDTTVKEQNFLYKETQETEKKLLFISEPIPHPSNELRGLNEKMSNKCSMLSTAEDDIRQNFTQLPLHKNKQECILDISEHTLSENDLEELRVDHYKCNIQASVHVSDFSTDNSGSQPKQKSDTVLFPAKDLKEKDLHSIFTHDSGLITINSSQEHLTVQAKAPFHTPPEEPNECDFKNMDSLPSGKIHRKVKIILGRNRKENLEPNAEFDKRTEFITQEENRICSSPVQSLLDLFQTSEEKSEFLGFTSYTEKSGICNVLDIWEEENSDNLLTAFFSSPSTSTFTGF

>NP_000365.3

MEANGLGPQGFPELKNDTFLRAAWGEETDYTPVWCMRQAGRYLPEFRETRAAQDFFSTCRSPEACCELTLQPLRRFPLDAAIIFSDILVVPQALGMEVTMVPGKGPSFPEPLREEQDLERLRDPEVVASELGYVFQAITLTRQRLAGRVPLIGFAGAPWTLMTYMVEGGGSSTMAQAKRWLYQRPQASHQLLRILTDALVPYLVGQVVAGAQALQLFESHAGHLGPQLFNKFALPYIRDVAKQVKARLREAGLAPVPMIIFAKDGHFALEELAQAGYEVVGLDWTVAPKKARECVGKTVTLQGNLDPCALYASEEEIGQLVKQMLDDFGPHRYIANLGHGLYPDMDPEHVGAFVDAVHKHSRLLRQN

225 NP_003911.1 Q16822

>NP_003911.1

MDLHMMNCELLATCSALGYLEGDTYHKEPDCLESVKDLIRYLRHEDETRDVRQQLGAAQILQSDLLPILTQHHQDKPLFDAVIRLMVNLTQPALLCFGNLPKEPSFRHHFLQVLTYLQAYKEAFASEKAFGVLSETLYELLQLGWEERQEEDNLLIERILLLVRNILHVPADLDQEKKIDDDASAHDQLLWAIHLSGLDDLLLFLASSSAEEQWSLHVLEIVSLMFRDQNPEQLAGVGQGRLAQERSADFAELEVLRQREMAEKKTRALQRGNRHSRFGGSYIVQGLKSIGERDLIFHKGLHNLRNYSSDLGKQPKKVPKRRQAARELSIQRRSALNVRLFLRDFCSEFLENCYNRLMGSVKDHLLREKAQQHDETYYMWALAFFMAFNRAASFRPGLVSETLSVRTFHFIEQNLTNYYEMMLTDRKEAASWARRMHLALKAYQELLATVNEMDLSPDEAVRESSRIIKNNIFYVMEYRELFLALFRKFDERCQPRSFLRDLVETTHLFLKMLERFCRSRGNLVVQNKQKKRRKKKKKVLDQAIVSGNVPSSPEEVEAVWPALAEQLQCCAQNSELSMDSVVPFDAASEVPVEEQRAEAMVRIQDCLLAGQAPQALTLLRSAREVWPEGDVFGSQDISPEEEIQLLKQILSAPLPRQQGPEERGAEEEEEEEEEEEEELQVVQVSEKEFNFLDYLKRFACSTVVRAYVLLLRSYQQNSAHTNHCIVKMLHRLAHDLKMEALLFQLSVFCLFNRLLSDPAAGAYKELVTFAKYILGKFFALAAVNQKAFVELLFWKNTAVVREMTEGYGSLDDRSSSRRAPTWSPEEEAHLQELYLANKDVEGQDVVEAILAHLNTVPRTRKQIIHHLVQMGLADSVKDFQRKGTHIVLWTGDQELELQRLFEEFRDSDDVLGHIMKNITAKRSRARIVDKLLALGLVAERRELYKKRQKKLASSILPNGAESLKDFCQEDLEEEENLPEEDSEEEEEGGSEAEQVQGSLVLSNENLGQSLHQEGFSIPLLWLQNCLIRAADDREEDGCSQAVPLVPLTEENEEAMENEQFQQLLRKLGVRPPASGQETFWRIPAKLSPTQLRRAAASLSQPEEEQKLQPELQPKVPGEQGSDEEHCKEHRAQALRALLLAHKKKAGLASPEEEDAVGKEPLKAAPKKRQLLDSDEEQEEDEGRNRAPELGAPGIQKKKRYQIEDDEDD

>Q16822

MAALYRPGLRLNWHGLSPLGWPSCRSIQTLRVLSGDLGQLPTGIRDFVEHSARLCQPEGIHICDGTEAENTATLTLLEQQGLIRKLPKYNNCWLARTDPKDVARVESKTVIVTPSQRDTVPLPPGGARGQLGNWMSPADFQRAVDERFPGCMQGRTMYVLPFSMGPVGSPLSRIGVQLTDSAYVVASMRIMTRLGTPVLQALGDGDFVKCLHSVGQPLTGQGEPVSQWPCNPEKTLIGHVPDQREIISFGSGYGGNSLLGKKCFALRIASRLARDEGWLAEHMLILGITSPAGKKALCAAAFPSACGKTNLAMMRPALPGWKVECVGDDIAWMRFDSEGRLRAINPENGFFGVAPGTSATTNPNAMATIQSNTIFTNVAETSDGGVYWEGIDQPLPPGVTVTSWLGKPWKPGDKEPCAHPNSRFCAPARQCPIMDPAWEAPEGVPIDAIIFGGRRPKGVPLVYEAFNWRHGVFVGRAMRSESTAAAEHKGKIIMHDPFAMRPFFGYNFGHYLEHWLSMEGRKGAQLPRIFHVNWFRRDEAGHFLWPGFGENARVLDWICRRLEGEDSARETPIGLVPKEGALDLSGLRAIDTTQLFSLPKDFWEQEVRDIRSYLTEQVNQDLPKEVLAELEALERRVHKM

226 NP_997001.1 NP_001007554.1

>NP_997001.1

MVNVLKGVLIECDPAMKQFLLYLDESNALGKKFIIQDIDDTHVFVIAELVNVLQERVGELMDQNAFSLTQK

>NP_001007554.1

MSFDPNLLHNNGHNGYPNGTSAALRETGVIEKLLTSYGFIQCSERQARLFFHCSQYNGNLQDLKVGDDVEFEVSSDRRTGKPIAVKLVKIKQEILPEERMNGQVVCAVPHNLESKSPAAPGQSPTGSVCYERNGEVFYLTYTPEDVEGNVQLETGDKINFVIDNNKHTGAVSARNIMLLKKKQARCQGVVCAMKEAFGFIERGDVVKEIFFHYSEFKGDLETLQPGDDVEFTIKDRNGKEVATDVRLLPQGTVIFEDISIEHFEGTVTKVIPKVPSKNQNDPLPGRIKVDFVIPKELPFGDKDTKSKVTLLEGDHVRFNISTDRRDKLERATNIEVLSNTFQFTNEAREMGVIAAMRDGFGFIKCVDRDVRMFFHFSEILDGNQLHIADEVEFTVVPDMLSAQRNHAIRIKKLPKGTVSFHSHSDHRFLGTVEKEATFSNPKTTSPNKGKEKEAEDGIIAYDDCGVKLTIAFQAKDVEGSTSPQIGDKVEFSISDKQRPGQQVATCVRLLGRNSNSKRLLGYVATLKDNFGFIETANHDKEIFFHYSEFSGDVDSLELGDMVEYSLSKGKGNKVSAEKVNKTHSVNGITEEADPTIYSGKVIRPLRSVDPTQTEYQGMIEIVEEGDMKGEVYPFGIVGMANKGDCLQKGESVKFQLCVLGQNAQTMAYNITPLRRATVECVKDQFGFINYEVGDSKKLFFHVKEVQDGIELQAGDEVEFSVILNQRTGKCSACNVWRVCEGPKAVAAPRPDRLVNRLKNITLDDASAPRLMVLRQPRGPDNSMGFGAERKIRQAGVID

227 Q9H582 NP_009197.1

>Q9H582

MRSFLQQDVNKTKSRLNVLNGLANNMDDLKINTDITGAKEELLDDNNFISDKESGVHKPKDCQTSFQKNNTLTLPEELSKDKSENALSGGQSSLFIHAGAPTVSSENFILPKGAAVNGPVSHSSLTKTSNMNKGSVSLTTGQPVDQPTTESCSTLKVAADLQLSTPQKASQHQVLFLLSDVAHAKNPTHSNKKLPTSASVGCDIQNSVGSNIKSDGTLINQVEVGEDGEDLLVKDDCVNTVTGISSGTDGFRSENDTNWDPQKEFIQFLMTNEETVDKAPPHSKIGLEKKRKRKMDVSKITRYTEDCFSDSNCVPNKSKMQEVDFLEQNEELQAVDSQKYALSKVKPESTDEDLESVDAFQHLIYNPDKCGEESSPVHTSTFLSNTLKKKCEESDSESPATFSTEEPSFYPCTKCNVNFREKKHLHRHMMYHLDGNSHFRHLNVPRPYACRECGRTFRDRNSLLKHMIIHQERRQKLMEEIRELKELQDEGRSARLQCPQCVFGTNCPKTFVQHAKTHEKDKRYYCCEECNFMAVTENELECHRGIAHGAVVKCPMVTSDIAQRKTQKKTFMKDSVVGSSKKSATYICKMCPFTTSAKSVLKKHTEYLHSSSCVDSFGSPLGLDKRKNDILEEPVDSDSTKTLTKQQSTTFPKNSALKQDVKRTFGSTSQSSSFSKIHKRPHRIQKARKSIAQSGVNMCNQNSSPHKNVTIKSSVDQKPKYFHQAAKEKSNAKANSHYLYRHKYENYRMIKKSGESYPVHFKKEEASSLNSLHLFSSSSNSHNNFISDPHKPDAKRPESFKDHRRVAVKRVIKESKKESSVGGEDLDSYPDFLHKMTVVVLQKLNSAEKKDSYETEDESSWDNVELGDYTTQAIEDETYSDINQEHVNLFPLFKSKVEGQEPGENATLSYDQNDGFYFEYYEDTGSNNFLHEIHDPQHLETADASLSKHSSVFHWTDLSLEKKSCPYCPATFETGVGLSNHVRGHLHRAGLSYEARHVVSPEQIATSDKMQHFKRTGTGTPVKRVRKAIEKSETTSEHTCQLCGGWFDTKIGLSNHVRGHLKRLGKTKWDAHKSPICVLNEMMQNEEKYEKILKALNSRRIIPRPFVAQKLASSDDFISQNVIPLEAYRNGLKTEALSVSASEEEGLNFLNEYDETKPELPSGKKNQSLTLIELLKNKRMGEERNSAISPQKIHNQTARKRFVQKCVLPLNEDSPLMYQPQKMDLTMHSALDCKQKKSRSRSGSKKKMLTLPHGADEVYILRCRFCGLVFRGPLSVQEDWIKHLQRHIVNANLPRTGAGMVEVTSLLKKPASITETSFSLLMAEAAS

>NP_009197.1

MAASAAAAELQASGGPRHPVCLLVLGMAGSGKTTFVQRLTGHLHAQGTPPYVINLDPAVHEVPFPANIDIRDTVKYKEVMKQYGLGPNGGIVTSLNLFATRFDQVMKFIEKAQNMSKYVLIDTPGQIEVFTWSASGTIITEALASSFPTVVIYVMDTSRSTNPVTFMSNMLYACSILYKTKLPFIVVMNKTDIIDHSFAVEWMQDFEAFQDALNQETTYVSNLTRSMSLVLDEFYSSLRVVGVSAVLGTGLDELFVQVTSAAEEYEREYRPEYERLKKSLANAESQQQREQLERLRKDMGSVALDAGTAKDSLSPVLHPSDLILTRGTLDEEDEEADSDTDDIDHRVTEESHEEPAFQNFMQESMAQYWKRNNK

228 NP_872363.1 NP_005495.2

>NP_872363.1

MRILANKTRLPHPRRREAPGSPPLSPRGHCPPAPAKPMHPENKLTNHGKTGNGGAQSQHQNVNQGPTCNVGSKGVGAGNHGAKANQISPSNSSLKNPQAGVPPFSSLKGKVKRDRSVSVDSGEQREAGTPSLDSEAKEVAPRSKRRCVLERKQPYSGDEWCSGPDSEEDDKPIGATHNCNVADPAMAAPQLGPGQTTQLPLSESSVPGAPHGPPPGLRPDAPGGGGGGGGVPGKPPSQFVYVFTTHLANTAAEAVLQGRADSILAYHQQNVPRAKLDQAPKVPPTPEPLPLSTPSAGTPQSQPPPLPPPPPPAPGSAPPALPPEGPPEDSSQDLAPNSVGAASTGGGTGGTHPNTPTATTANNPLPPGGDPSSAPGPALLGEAAAPGNGQRSLVGSEGLSKEQLEHRERSLQTLRDIERLLLRSGETEPFLKGPPGGAGEGGPPAQAPPPPQQPPTAPPSGLKKYEEPLQSMISQTQSLGGPPLEHEVPGHPPGGDMGQQMNMMIQRLGQDSLTPEQVAWRKLQEEYYEEKRRKEEQIGLHGSRPLQDMMGMGGMMVRGPPPPYHSKPGDQWPPGMGAQLRGPMDVQDPMQLRGGPPFPGPRFPGNQIQRVPGFGGMQSMPMEVPMNAMQRPVRPGMGWTEDLPPMGGPSNFAQNTMPYPGGQGEAERFMTPRVREELLRHQLLEKRSMGMQRPLGMAGSGMGQSMEMERMMQAHRQMDPAMFPGQMAGGEGLAGTPMGMEFGGGRGLLSPPMGQSGLREVDPPMGPGNLNMNMNVNMNMNMNLNVQMTPQQQMLMSQKMRGPGDLMGPQGLSPEEMARVRAQNSSGVMGGPQKMLMPSQFPNQGQQGFSGGQGPYQAMSQDMGNTQDMFSPDQSSMPMSNVGTTRLSHMPLPPASNPPGTVHSAPNRGLGRRPSDLTISINQMGSPGMGHLKSPTLSQVHSPLVTSPSANLKSPQTPSQMVPLPSANPPGPLKSPQVLGSSLSVRSPTGSPSRLKSPSMAVPSPGWVASPKTAMPSPGVSQNKQPPLNMNSSTTLSNMEQGTLPPSGPRSSSSAPPANPPSGLMNPSLPFTSSPDPTPSQNPLSLMMTQMSKYAMPSSTPLYHNAIKTIATSDDELLPDRPLLPPPPPPQGSGPGISNSQPSQMHLNSAAAQSPMGMNLPGQQPLSHEPPPAMLPSPTPLGSNIPLHPNAQGTGGPPQNSMMMAPGGPDSLNAPCGPVPSSSQMMPFPPRLQQPHGAMAPTGGGGGGPGLQQHYPSGMALPPEDLPNQPPGPMPPQQHLMGKAMAGRMGDAYPPGVLPGVASVLNDPELSEVIRPTPTGIPEFDLSRIIPSEKPSSTLQYFPKSENQPPKAQPPNLHLMNLQNMMAEQTPSRPPNLPGQQGVQRGLNMSMCHPGQMSLLGRTGVPPQQGMVPHGLHQGVMSPPQGLMTQQNFMLMKQRGVGGEVYSQPPHMLSPQGSLMGPPPQQNLMVSHPLRQRSVSLDSQMGYLPAPGGMANLPF

>NP_005495.2

MKDCSNGCSAECTGEGGSKEVVGTFKAKDLIVTPATILKEKPDPNNLVFGTVFTDHMLTVEWSSEFGWEKPHIKPLQNLSLHPGSSALHYAVELFEGLKAFRGVDNKIRLFQPNLNMDRMYRSAVRATLPVFDKEELLECIQQLVKLDQEWVPYSTSASLYIRPTFIGTEPSLGVKKPTKALLFVLLSPVGPYFSSGTFNPVSLWANPKYVRAWKGGTGDCKMGGNYGSSLFAQCEAVDNGCQQVLWLYGEDHQITEVGTMNLFLYWINEDGEEELATPPLDGIILPGVTRRCILDLAHQWGEFKVSERYLTMDDLTTALEGNRVREMFGSGTACVVCPVSDILYKGETIHIPTMENGPKLASRILSKLTDIQYGREESDWTIVLS

229 CAA58827.1 P49753

>CAA58827.1

MADEEEDPTFEEENEEIGGGAEGGQGKRKRLFSKELRCMMYGFGDDQNPYTESVDILEDLVIEFITEMTHKAMSIGRQGRVQVEDIVFLIRKDPRKFARVKDLLTMNEELKRARKAFDEANYGS

>P49753

MSNKLLSPHPHSVVLRSEFKMASSPAVLRASRLYQWSLKSSAQFLGSPQLRQVGQIIRVPARMAATLILEPAGRCCWDEPVRIAVRGLAPEQPVTLRASLRDEKGALFQAHARYRADTLGELDLERAPALGGSFAGLEPMGLLWALEPEKPLVRLVKRDVRTPLAVELEVLDGHDPDPGRLLCQTRHERYFLPPGVRREPVRVGRVRGTLFLPPEPGPFPGIVDMFGTGGGLLEYRASLLAGKGFAVMALAYYNYEDLPKTMETLHLEYFEEAMNYLLSHPEVKGPGVGLLGISKGGELCLSMASFLKGITAAVVINGSVANVGGTLRYKGETLPPVGVNRNRIKVTKDGYADIVDVLNSPLEGPDQKSFIPVERAESTFLFLVGQDDHNWKSEFYANEACKRLQAHGRRKPQIICYPETGHYIEPPYFPLCRASLHALVGSPIIWGGEPRAHAMAQVDAWKQLQTFFHKHLGGHEGTIPSKV

230 Q8N9N5 O75880

>Q8N9N5

MMSEHDLADVVQIAVEDLSPDHPVVLENHVVTDEDEPALKRQRLEINCQDPSIKTICLRLDSIEAKLQALEATCKSLEEKLDLVTNKQHSPIQVPMVAGSPLGATQTCNKVRCVVPQTTVILNNDRQNAIVAKMEDPLSNRAPDSLENVISNAVPGRRQNTIVVKVPGQEDSHHEDGESGSEASDSVSSCGQAGSQSIGSNVTLITLNSEEDYPNGTWLGDENNPEMRVRCAIIPSDMLHISTNCRTAEKMALTLLDYLFHREVQAVSNLSGQGKHGKKQLDPLTIYGIRCHLFYKFGITESDWYRIKQSIDSKCRTAWRRKQRGQSLAVKSFSRRTPNSSSYCPSESMMSTPPPASELPQPQPQPQALHYALANAQQVQIHQIGEDGQVQVGHLHIAQVPQGEQVQITQDSEGNLQIHHVGQDGQLLEATRIPCLLAPSVFKASSGQVLQGAQLIAVASSDPAAAGVDGSPLQGSDIQVQYVQLAPVSDHTAGAQTAEALQPTLQPEMQLEHGAIQIQ

>O75880

MAMLVLVPGRVMRPLGGQLWRFLPRGLEFWGPAEGTARVLLRQFCARQAEAWRASGRPGYCLGTRPLSTARPPPPWSQKGPGDSTRPSKPGPVSWKSLAITFAIGGALLAGMKHVKKEKAEKLEKERQRHIGKPLLGGPFSLTTHTGERKTDKDYLGQWLLIYFGFTHCPDVCPEELEKMIQVVDEIDSITTLPDLTPLFISIDPERDTKEAIANYVKEFSPKLVGLTGTREEVDQVARAYRVYYSPGPKDEDEDYIVDHTIIMYLIGPDGEFLDYFGQNKRKGEIAASIATHMRPYRKKS

231 Q9BQ83 NP_000476.1

>Q9BQ83

MGPAGVAARPGRFFGVYLLYCLNPRYRGRVYVGFTVNTARRVQQHNGGRKKGGAWRTSGRGPWEMVLVVHGFPSSVAALRFEWAWQHPHASRRLAHVGPRLRGETAFAFHLRVLAHMLRAPPWARLPLTLRWVRPDLRQDLCLPPPPHVPLAFGPPPPQAPAPRRRAGPFDDAEPEPDQGDPGACCSLCAQTIQDEEGPLCCPHPGCLLRAHVICLAEEFLQEEPGQLLPLEGQCPCCEKSLLWGDLIWLCQMDTEKEVEDSELEEAHWTDLLET

>NP_000476.1

MADSELQLVEQRIRSFPDFPTPGVVFRDISPVLKDPASFRAAIGLLARHLKATHGGRIDYIAGLDSRGFLFGPSLAQELGLGCVLIRKRGKLPGPTLWASYSLEYGKAELEIQKDALEPGQRVVVVDDLLATGGTMNAACELLGRLQAEVLECVSLVELTSLKGREKLAPVPFFSLLQYE

232 Q9UL03 NP_001087.2

>Q9UL03

MPILLFLIDTSASMNQRSHLGTTYLDTAKGAVETFMKLRARDPASRGDRYMLVTFEEPPYAIKAGWKENHATFMNELKNLQAEGLTTLGQSLRTAFDLLNLNRLVTGIDNYGQGRNPFFLEPAIIITITDGSKLTTTSGVQDELHLPLNSPLPGSELTKEPFRWDQRLFALVLRLPGTMSVESEQLTGVPLDDSAITPMCEVTGGRSYSVCSPRMLNQCLESLVQKVQSGVVINFEKAGPDPSPVEDGQPDISRPFGSQPWHSCHKLIYVRPNPKTGVPIGHWPVPESFWPDQNSPTLPPRTSHPVVKFSCTDCEPMVIDKLPFDKYELEPSPLTQFILERKSPQTCWQVYVSNSAKYSELGHPFGYLKASTALNCVNLFVMPYNYPVLLPLLDDLFKVHKAKPTLKWRQSFESYLKTMPPYYLGPLKKAVRMMGAPNLIADSMEYGLSYSVISYLKKLSQQAKIESDRVIGSVGKKVVQETGIKVRSRSHGLSMAYRKDFQQLLQGISEDVPHRLLDLNMKEYTGFQVALLNKDLKPQTFRNAYDIPRRNLLDHLTRMRSNLLKSTRRFLKGQDEDQVHSVPIAQMGNYQEYLKQVPSPLRELDPDQPRRLHTFGNPFKLDKKGMMIDEADEFVAGPQNKHKRPGEPNMQGIPKRRRCMSPLLRGRQQNPVVNNHIGGKGPPAPTTQAQPDLIKPLPLHKISETTNDSIIHDVVENHVADQLSSDITPNAMDTEFSASSPASLLERPTNHMEALGHDHLGTNDLTVGGFLENHEEPRDKEQCAEENIPASSLNKGKKLMHCRSHEEVNTELKAQIMKEIRKPGRKYERIFTLLKHVQGSLQTRLIFLQNVIKEASRFKKRMLIEQLENFLDEIHRRANQINHINSN

>NP_001087.2

MSAKAISEQTGKELLYKFICTTSAIQNRFKYARVTPDTDWARLLQDHPWLLSQNLVVKPDQLIKRRGKLGLVGVNLTLDGVKSWLKPRLGQEATVGKATGFLKNFLIEPFVPHSQAEEFYVCIYATREGDYVLFHHEGGVDVGDVDAKAQKLLVGVDEKLNPEDIKKHLLVHAPEDKKEILASFISGLFNFYEDLYFTYLEINPLVVTKDGVYVLDLAAKVDATADYICKVKWGDIEFPPPFGREAYPEEAYIADLDAKSGASLKLTLLNPKGRIWTMVAGGGASVVYSDTICDLGGVNELANYGEYSGAPSEQQTYDYAKTILSLMTREKHPDGKILIIGGSIANFTNVAATFKGIVRAIRDYQGPLKEHEVTIFVRRGGPNYQEGLRVMGEVGKTTGIPIHVFGTETHMTAIVGMALGHRPIPNQPPTAAHTANFLLNASGSTSTPAPSRTASFSESRADEVAPAKKAKPAMPQDSVPSPRSLQGKSTTLFSRHTKAIVWGMQTRAVQGMLDFDYVCSRDEPSVAAMVYPFTGDHKQKFYWGHKEILIPVFKNMADAMRKHPEVDVLINFASLRSAYDSTMETMNYAQIRTIAIIAEGIPEALTRKLIKKADQKGVTIIGPATVGGIKPGCFKIGNTGGMLDNILASKLYRPGSVAYVSRSGGMSNELNNIISRTTDGVYEGVAIGGDRYPGSTFMDHVLRYQDTPGVKMIVVLGEIGGTEEYKICRGIKEGRLTKPIVCWCIGTCATMFSSEVQFGHAGACANQASETAVAKNQALKEAGVFVPRSFDELGEIIQSVYEDLVANGVIVPAQEVPPPTVPMDYSWARELGLIRKPASFMTSICDERGQELIYAGMPITEVFKEEMGIGGVLGLLWFQKRLPKYSCQFIEMCLMVTADHGPAVSGAHNTIICARAGKDLVSSLTSGLLTIGDRFGGALDAAAKMFSKAFDSGIIPMEFVNKMKKEGKLIMGIGHRVKSINNPDMRVQILKDYVRQHFPATPLLDYALEVEKITTSKKPNLILNVDGLIGVAFVDMLRNCGSFTREEADEYIDIGALNGIFVLGRSMGFIGHYLDQKRLKQGLYRHPWDDISYVLPEHMSM

233 Q9C002 NP_076425.1

>Q9C002

MSFFQLLMKRKELIPLVVFMTVAAGGASSFAVYSLWKTDVILDRKKNPEPWETVDPTVPQKLITINQQWKPIEELQNVQRVTK

>NP_076425.1

MARKKVRPRLIAELARRVRALREQLNRPRDSQLYAVDYETLTRPFSGRRLPVRAWADVRRESRLLQLLGRLPLFGLGRLVTRKSWLWQHDEPCYWRLTRVRPDYTAQNLDHGKAWGILTFKGKTESEAREIEHVMYHDWRLVPKHEEEAFTAFTPAPEDSLASVPYPPLLRAMIIAERQKNGDTSTEEPMLNVQRIRMEPWDYPAKQEDKGRAKGTPV

234 Q96KF2 NP_006397.1

>Q96KF2

MLCAHFSDQGPAHLTTSKSAFLSNKKTSTLKHLLGETRSDGSACNSGISGGRGRKIP

>NP_006397.1

MEALPLLAATTPDHGRHRRLLLLPLLLFLLPAGAVQGWETEERPRTREEECHFYAGGQVYPGEASRVSVADHSLHLSKAKISKPAPYWEGTAVIDGEFKELKLTDYRGKYLVFFFYPLDFTFVCPTEIIAFGDRLEEFRSINTEVVACSVDSQFTHLAWINTPRRQGGLGPIRIPLLSDLTHQISKDYGVYLEDSGHTLRGLFIIDDKGILRQITLNDLPVGRSVDETLRLVQAFQYTDKHGEVCPAGWKPGSETIIPDPAGKLKYFDKLN

235 NP_073624.2 Q9P032

>NP_073624.2

MENSEKTEVVLLACGSFNPITNMHLRLFELAKDYMNGTGRYTVVKGIISPVGDAYKKKGLIPAYHRVIMAELATKNSKWVEVDTWESLQKEWKETLKVLRHHQEKLEASDCDHQQNSPTLERPGRKRKWTETQDSSQKKSLEPKTKAVPKVKLLCGADLLESFAVPNLWKSEDITQIVANYGLICVTRAGNDAQKFIYESDVLWKHRSNIHVVNEWIANDISSTKIRRALRRGQSIRYLVPDLVQEYIEKHNLYSSESEDRNAGVILAPLQRNTAEAKT

>Q9P032

MGALVIRGIRNFNLENRAEREISKMKPSVAPRHPSTNSLLREQISLYPEVKGEIARKDEKLLSFLKDVYVDSKDPVSSLQVKAAETCQEPKEFRLPKDHHFDMINIKSIPKGKISIVEALTLLNNHKLFPETWTAEKIMQEYQLEQKDVNSLLKYFVTFEVEIFPPEDKKAIRSK

236 NP_542380.1 NP_064586.1

>NP_542380.1

MPKRGKKGAVAEDGDELRTEPEAKKSKTAAKKNDKEAAGEGPALYEDPPDQKTSPSGKPATLKICSWNVDGLRAWIKKKGLDWVKEEAPDILCLQETKCSENKLPAELQELPGLSHQYWSAPSDKEGYSGVGLLSRQCPLKVSYGIGDEEHDQEGRVIVAEFDSFVLVTAYVPNAGRGLVRLEYRQRWDEAFRKFLKGLASRKPLVLCGDLNVAHEEIDLRNPKGNKKNAGFTPQERQGFGELLQAVPLADSFRHLYPNTPYAYTFWTYMMNARSKNVGWRLDYFLLSHSLLPALCDSKIRSKALGSDHCPITLYLAL

>NP_064586.1

MIRLGGWCARRLCSAAVPAGRRGAAGGLGLAGGRALRVLVDMDGVLADFEGGFLRKFRARFPDQPFIALEDRRGFWVSEQYGRLRPGLSEKAISIWESKNFFFELEPLPGAVEAVKEMASLQNTDVFICTSPIKMFKYCPYEKYAWVEKYFGPDFLEQIVLTRDKTVVSADLLIDDRPDITGAEPTPSWEHVLFTACHNQHLQLQPPRRRLHSWADDWKAILDSKRPC

237 NP_689574.1 Q16698

>NP_689574.1

MAEPTGLLEMSELPGDSSVPQVGTASGVSDVLRGAVGGGVRVQEAREGPVAEAARSMARMPGPVPGPIPSSVPGLASAPDPHQQLAFLEINRQLLFREYLDGSSMIPVRLLRDFEERRRLFVEGCKAREAAFDADPPQMDFAAVAFTVALTASEALSPLAD

>Q16698

MKLPARVFFTLGSRLPCGLAPRRFFSYGTKILYQNTEALQSKFFSPLQKAMLPPNSFQGKVAFITGGGTGLGKGMTTLLSSLGAQCVIASRKMDVLKATAEQISSQTGNKVHAIQCDVRDPDMVQNTVSELIKVAGHPNIVINNAAGNFISPTERLSPNAWKTITDIVLNGTAFVTLEIGKQLIKAQKGAAFLSITTIYAETGSGFVVPSASAKAGVEAMSKSLAAEWGKYGMRFNVIQPGPIKTKGAFSRLDPTGTFEKEMIGRIPCGRLGTVEELANLAAFLCSDYASWINGAVIKFDGGEEVLISGEFNDLRKVTKEQWDTIEELIRKTKGS

238 NP_073562.1 NP_004754.1

>NP_073562.1

MESLLQHLDRFSELLAVSSTTYVSTWDPATVRRALQWARYLRHIHRRFGRHGPIRTALERRLHNQWRQEGGFGRGPVPGLANFQALGHCDVLLSLRLLENRALGDAARYHLVQQLFPGPGVRDADEETLQESLARLARRRSAVHMLRFNGYRENPNLQEDSLMKTQAELLLERLQEVGKAEAERPARFLSSLWERLPQNNFLKVIAVALLQPPLSRRPQEELEPGIHKSPGEGSQVLVHWLLGNSEVFAAFCRALPAGLLTLVTSRHPALSPVYLGLLTDWGQRLHYDLQKGIWVGTESQDVPWEELHNRFQSLCQAPPPLKDKVLTALETCKAQDGDFEVPGLSIWTDLLLALRSGAFRKRQVLGLSAGLSSV

>NP_004754.1

MFRKGKKRHSSSSSQSSEISTKSKSVDSSLGGLSRSSTVASLDTDSTKSSGQSNNNSDTCAEFRIKYVGAIEKLKLSEGKGLEGPLDLINYIDVAQQDGKLPFVPPEEEFIMGVSKYGIKVSTSDQYDVLHRHALYLIIRMVCYDDGLGAGKSLLALKTTDASNEEYSLWVYQCNSLEQAQAICKVLSTAFDSVLTSEKP

239 NP_073557.3 NP_065801.1

>NP_073557.3

METDESPSPLPCGPAGEAVMESRARPFQALPREQSPPPPLQTSSGAEVMDVGSGGDGQSELPAEDPFNFYGASLLSKGSFSKGRLLIDPNCSGHSPRTARHAPAVRKFSPDLKLLKDVKISVSFTESCRSKDRKVLYTGAERDVRAECGLLLSPVSGDVHACPFGGSVGDGVGIGGESADKKDEENELDQEKRVEYAVLDELEDFTDNLELDEEGAGGFTAKAIVQRDRVDEEALNFPYEDDFDNDVDALLEEGLCAPKKRRTEEKYGGDSDHPSDGETSVQPMMTKIKTVLKSRGRPPTEPLPDGWIMTFHNSGVPVYLHRESRVVTWSRPYFLGTGSIRKHDPPLSSIPCLHYKKMKDNEEREQSSDLTPSGDVSPVKPLSRSAELEFPLDEPDSMGADPGPPDEKDPLGAEAAPGALGQVKAKVEVCKDESVDLEEFRSYLEKRFDFEQVTVKKFRTWAERRQFNREMKRKQAESERPILPANQKLITLSVQDAPTKKEFVINPNGKSEVCILHEYMQRVLKVRPVYNFFECENPSEPFGASVTIDGVTYGSGTASSKKLAKNKAARATLEILIPDFVKQTSEEKPKDSEELEYFNHISIEDSRVYELTSKAGLLSPYQILHECLKRNHGMGDTSIKFEVVPGKNQKSEYVMACGKHTVRGWCKNKRVGKQLASQKILQLLHPHVKNWGSLLRMYGRESSKMVKQETSDKSVIELQQYAKKNKPNLHILSKLQEEMKRLAEEREETRKKPKMSIVASAQPGGEPLCTVDV

>NP_065801.1

MAMDQVNALCEQLVKAVTVMMDPNSTQRYRLEALKFCEEFKEKCPICVPCGLRLAEKTQVAIVRHFGLQILEHVVKFRWNGMSRLEKVYLKNSVMELIANGTLNILEEENHIKDALSRIVVEMIKREWPQHWPDMLIELDTLSKQGETQTELVMFILLRLAEDVVTFQTLPPQRRRDIQQTLTQNMERIFSFLLNTLQENVNKYQQVKTDTSQESKAQANCRVGVAALNTLAGYIDWVSMSHITAENCKLLEILCLLLNEQELQLGAAECLLIAVSRKGKLEDRKPLMVLFGDVAMHYILSAAQTADGGGLVEKHYVFLKRLCQVLCALGNQLCALLGADSDVETPSNFGKYLESFLAFTTHPSQFLRSSTQMTWGALFRHEILSRDPLLLAIIPKYLRASMTNLVKMGFPSKTDSPSCEYSRFDFDSDEDFNAFFNSSRAQQGEVMRLACRLDPKTSFQMAGEWLKYQLSTFLDAGSVNSCSAVGTGEGSLCSVFSPSFVQWEAMTLFLESVITQMFRTLNREEIPVNDGIELLQMVLNFDTKDPLILSCVLTNVSALFPFVTYRPEFLPQVFSKLFSSVTFETVEESKAPRTRAVRNVRRHACSSIIKMCRDYPQLVLPNFDMLYNHVKQLLSNELLLTQMEKCALMEALVLISNQFKNYERQKVFLEELMAPVASIWLSQDMHRVLSDVDAFIAYVGTDQKSCDPGLEDPCGLNRARMSFCVYSILGVVKRTCWPTDLEEAKAGGFVVGYTSSGNPIFRNPCTEQILKLLDNLLALIRTHNTLYAPEMLAKMAEPFTKALDMLDAEKSAILGLPQPLLELNDSPVFKTVLERMQRFFSTLYENCFHILGKAGPSMQQDFYTVEDLATQLLSSAFVNLNNIPDYRLRPMLRVFVKPLVLFCPPEHYEALVSPILGPLFTYLHMRLSQKWQVINQRSLLCGEDEAADENPESQEMLEEQLVRMLTREVMDLITVCCVSKKGADHSSAPPADGDDEEMMATEVTPSAMAELTDLGKCLMKHEDVCTALLITAFNSLAWKDTLSCQRTTSQLCWPLLKQVLSGTLLADAVTWLFTSVLKGLQMHGQHDGCMASLVHLAFQIYEALRPRYLEIRAVMEQIPEIQKDSLDQFDCKLLNPSLQKVADKRRKDQFKRLIAGCIGKPLGEQFRKEVHIKNLPSLFKKTKPMLETEVLDNDGGGLATIFEP

240 NP_003381.1 Q9H9P8

>NP_003381.1

MSFLSRQQPPPPRRAGAACTLRQKLIFSPCSDCEEEEEEEEEEGSGHSTGEDSAFQEPDSPLPPARSPTEPGPERRRSPGPAPGSPGELEEDLLLPGACPGADEAGGGAEGDSWEEEGFGSSSPVKSPAAPYFLGSSFSPVRCGGPGDASPRGCGARRAGEGRRSPRPDHPGTPPHKTFRKLRLFDTPHTPKSLLSKARGIDSSSVKLRGSSLFMDTEKSGKREFDVRQTPQVNINPFTPDSLLLHSSGQCRRRKRTYWNDSCGEDMEASDYELEDETRPAKRITITESNMKSRYTTEFHELEKIGSGEFGSVFKCVKRLDGCIYAIKRSKKPLAGSVDEQNALREVYAHAVLGQHSHVVRYFSAWAEDDHMLIQNEYCNGGSLADAISENYRIMSYFKEAELKDLLLQVGRGLRYIHSMSLVHMDIKPSNIFISRTSIPNAASEEGDEDDWASNKVMFKIGDLGHVTRISSPQVEEGDSRFLANEVLQENYTHLPKADIFALALTVVCAAGAEPLPRNGDQWHEIRQGRLPRIPQVLSQEFTELLKVMIHPDPERRPSAMALVKHSVLLSASRKSAEQLRIELNAEKFKNSLLQKELKKAQMAKAAAEERALFTDRMATRSTTQSNRTSRLIGKKMNRSVSLTIY

>Q9H9P8

MVPALRYLVGACGRARGRFAGGSPGACGFASGRPRPLCGGSRSASTSSFDIVIVGGGIVGLASARALILRHPSLSIGVLEKEKDLAVHQTGHNSGVIHSGIYYKPESLKAKLCVQGAALLYEYCQQKGISYKQCGKLIVAVEQEEIPRLQALYEKGLQNGVPGLRLIQQEDIKKKEPYCRGLMAIDCPHTGIVDYRQVALSFAQDFQEAGGSVLTNFEVKGIEMAKESPSRSIDGMQYPIVIKNTKGEEIRCQYVVTCAGLYSDRISELSGCTPDPRIVPFRGDYLLLKPEKCYLVKGNIYPVPDSRFPFLGVHFTPRMDGSIWLGPNAVLAFKREGYRPFDFSATDVMDIIINSGLIKLASQNFSYGVTEMYKACFLGATVKYLQKFIPEITISDILRGPAGVRAQALDRDGNLVEDFVFDAGVGDIGNRILHVRNAPSPAATSSIAISGMIADEVQQRFEL

241 NP_004731.2 Q9BU02

>NP_004731.2

MSSPSSPFREQSFLCAAGDAGEESRVQVLKNEVRRGSPVLLGWVEQAYADKCVCGPSAPPAPTPPSLSQRVMCNDLFKVNPFQLQQFRADPSTASLLLCPGGLDHKLNLRGKAWG

>Q9BU02

MAQGLIEVERKFLPGPGTEERLQELGGTLEYRVTFRDTYYDTPELSLMQADHWLRRREDSGWELKCPGAAGVLGPHTEYKELTAEPTIVAQLCKVLRADGLGAGDVAAVLGPLGLQEVASFVTKRSAWKLVLLGADEEEPQLRVDLDTADFGYAVGEVEALVHEEAEVPTALEKIHRLSSMLGVPAQETAPAKLIVYLQRFRPQDYQRLLEVNSSRERPQETEDPDHCLG

242 NP_001611.1 Q16698

>NP_001611.1

MEKEETTRELLLPNWQGSGSHGLTIAQRDDGVFVQEVTQNSPAARTGVVKEGDQIVGATIYFDNLQSGEVTQLLNTMGHHTVGLKLHRKGDRSPEPGQTWTREVFSSCSSEVVLSGDDEEYQRIYTTKIKPRLKSEDGVEGDLGETQSRTITVTRRVTAYTVDVTGREGAKDIDISSPEFKIKIPRHELTEISNVDVETQSGKTVIRLPSGSGAASPTGSAVDIRAGAISASGPELQGAGHSKLQVTMPGIKVGGSGVNVNAKGLDLGGRGGVQVPAVDISSSLGGRAVEVQGPSLESGDHGKIKFPTMKVPKFGVSTGREGQTPKAGLRVSAPEVSVGHKGGKPGLTIQAPQLEVSVPSANIEGLEGKLKGPQITGPSLEGDLGLKGAKPQGHIGVDASAPQIGGSITGPSVEVQAPDIDVQGPGSKLNVPKMKVPKFSVSGAKGEETGIDVTLPTGEVTVPGVSGDVSLPEIATGGLEGKMKGTKVKTPEMIIQKPKISMQDVDLSLGSPKLKGDIKVSAPGVQGDVKGPQVALKGSRVDIETPNLEGTLTGPRLGSPSGKTGTCRISMSEVDLNVAAPKVKGGVDVTLPRVEGKVKVPEVDVRGPKVDVSAPDVEAHGPEWNLKMPKMKMPTFSTPGAKGEGPDVHMTLPKGDISISGPKVNVEAPDVNLEGLGGKLKGPDVKLPDMSVKTPKISMPDVDLHVKGTKVKGEYDVTVPKLEGELKGPKVDIDAPDVDVHGPDWHLKMPKMKMPKFSVPGFKAEGPEVDVNLPKADVDISGPKIDVTAPDVSIEEPEGKLKGPKFKMPEMNIKVPKISMPDVDLHLKGPNVKGEYDVTMPKVESEIKVPDVELKSAKMDIDVPDVEVQGPDWHLKMPKMKMPKFSMPGFKAEGPEVDVNLPKADVDISGPKVGVEVPDVNIEGPEGKLKGPKFKMPEMNIKAPKISMPDVDLHMKGPKVKGEYDMTVPKLEGDLKGPKVDVSAPDVEMQGPDWNLKMPKIKMPKFSMPSLKGEGPEFDVNLSKANVDISAPKVDTNAPDLSLEGPEGKLKGPKFKMPEMHFRAPKMSLPDVDLDLKGPKMKGNVDISAPKIEGEMQVPDVDIRGPKVDIKAPDVEGQGLDWSLKIPKMKMPKFSMPSLKGEGPEVDVNLPKADVVVSGPKVDIEAPDVSLEGPEGKLKGPKFKMPEMHFKTPKISMPDVDLHLKGPKVKGDVDVSVPKVEGEMKVPDVEIKGPKMDIDAPDVEVQGPDWHLKMPKMKMPKFSMPGFKGEGREVDVNLPKADIDVSGPKVDVEVPDVSLEGPEGKLKGPKFKMPEMHFKAPKISMPDVDLNLKGPKLKGDVDVSLPEVEGEMKVPDVDIKGPKVDISAPDVDVHGPDWHLKMPKVKMPKFSMPGFKGEGPEVDVKLPKADVDVSGPKMDAEVPDVNIEGPDAKLKGPKFKMPEMSIKPQKISIPDVGLHLKGPKMKGDYDVTVPKVEGEIKAPDVDIKGPKVDINAPDVEVHGPDWHLKMPKVKMPKFSMPGFKGEGPEVDMNLPKADLGVSGPKVDIDVPDVNLEAPEGKLKGPKFKMPSMNIQTHKISMPDVGLNLKAPKLKTDVDVSLPKVEGDLKGPEIDVKAPKMDVNVGDIDIEGPEGKLKGPKFKMPEMHFKAPKISMPDVDLHLKGPKVKGDMDVSVPKVEGEMKVPDVDIKGPKVDIDAPDVEVHDPDWHLKMPKMKMPKFSMPGFKAEGPEVDVNLPKADIDVSGPSVDTDAPDLDIEGPEGKLKGSKFKMPKLNIKAPKVSMPDVDLNLKGPKLKGEIDASVPELEGDLRGPQVDVKGPFVEAEVPDVDLECPDAKLKGPKFKMPEMHFKAPKISMPDVDLHLKGPKVKGDADVSVPKLEGDLTGPSVGVEVPDVELECPDAKLKGPKFKMPDMHFKAPKISMPDVDLHLKGPKVKGDVDVSVPKLEGDLTGPSVGVEVPDVELECPDAKLKGPKFKMPEMHFKTPKISMPDVDLHLKGPKVKGDMDVSVPKVEGEMKVPDVDIKGPKMDIDAPDVDVHGPDWHLKMPKMKMPKFSMPGFKAEGPEVDVNLPKADVVVSGPKVDVEVPDVSLEGPEGKLKGPKLKMPEMHFKAPKISMPDVDLHLKGPKVKGDVDVSLPKLEGDLTGPSVDVEVPDVELECPDAKLKGPKFKMPEMHFKTPKISMPDVNLNLKGPKVKGDMDVSVPKVEGEMKVPDVDIRGPKVDIDAPDVDVHGPDWHLKMPKMKMPKFSMPGFKGEGPEVDVNLPKADVDVSGPKVDVEVPDVSLEGPEGKLKGPKFKMPEMHFKTPKISMPDVDFNLKGPKIKGDVDVSAPKLEGELKGPELDVKGPKLDADMPEVAVEGPNGKWKTPKFKMPDMHFKAPKISMPDLDLHLKSPKAKGEVDVDVPKLEGDLKGPHVDVSGPDIDIEGPEGKLKGPKFKMPDMHFKAPNISMPDVDLNLKGPKIKGDVDVSVPEVEGKLEVPDMNIRGPKVDVNAPDVQAPDWHLKMPKMKMPKFSMPGFKAEGPEVDVNLPKADVDISGPKVDIEGPDVNIEGPEGKLKGPKLKMPEMNIKAPKISMPDFDLHLKGPKVKGDVDVSLPKVEGDLKGPEVDIKGPKVDINAPDVGVQGPDWHLKMPKVKMPKFSMPGFKGEGPDGDVKLPKADIDVSGPKVDIEGPDVNIEGPEGKLKGPKFKMPEMNIKAPKISMPDIDLNLKGPKVKGDVDVSLPKVEGDLKGPEVDIKGPKVDIDAPDVDVHGPDWHLKMPKIKMPKISMPGFKGEGPDVDVNLPKADIDVSGPKVDVECPDVNIEGPEGKWKSPKFKMPEMHFKTPKISMPDIDLNLTGPKIKGDVDVTGPKVEGDLKGPEVDLKGPKVDIDVPDVNVQGPDWHLKMPKMKMPKFSMPGFKAEGPEVDVNLPKADVDVSGPKVDVEGPDVNIEGPEGKLKGPKFKMPEMNIKAPKIPMPDFDLHLKGPKVKGDVDISLPKVEGDLKGPEVDIRGPQVDIDVPDVGVQGPDWHLKMPKVKMPKFSMPGFKGEGPDVDVNLPKADLDVSGPKVDIDVPDVNIEGPEGKLKGPKFKMPEMNIKAPKISMPDIDLNLKGPKVKGDMDVSLPKVEGDMKVPDVDIKGPKVDINAPDVDVQGPDWHLKMPKIKMPKISMPGFKGEGPEVDVNLPKADLDVSGPKVDVDVPDVNIEGPDAKLKGPKFKMPEMNIKAPKISMPDLDLNLKGPKMKGEVDVSLANVEGDLKGPALDIKGPKIDVDAPDIDIHGPDAKLKGPKLKMPDMHVNMPKISMPEIDLNLKGSKLKGDVDVSGPKLEGDIKAPSLDIKGPEVDVSGPKLNIEGKSKKSRFKLPKFNFSGSKVQTPEVDVKGKKPDIDITGPKVDINAPDVEVQGKVKGSKFKMPFLSISSPKVSMPDVELNLKSPKVKGDLDIAGPNLEGDFKGPKVDIKAPEVNLNAPDVDVHGPDWNLKMPKMKMPKFSVSGLKAEGPDVAVDLPKGDINIEGPSMNIEGPDLNVEGPEGGLKGPKFKMPDMNIKAPKISMPDIDLNLKGPKVKGDVDISLPKLEGDLKGPEVDIKGPKVDINAPDVDVHGPDWHLKMPKVKMPKFSMPGFKGEGPEVDVTLPKADIDISGPNVDVDVPDVNIEGPDAKLKGPKFKMPEMNIKAPKISMPDFDLNLKGPKMKGDVVVSLPKVEGDLKGPEVDIKGPKVDIDTPDINIEGSEGKFKGPKFKIPEMHLKAPKISMPDIDLNLKGPKVKGDVDVSLPKMEGDLKGPEVDIKGPKVDINAPDVDVQGPDWHLKMPKVKMPKFSMPGFKGEGPDVDVNLPKADLDVSGPKVDIDVPDVNIEGPEGKLKGPKFKMPEMNIKAPKISMPDIDLNLKGPKVKGDMDVSLPKVEGDMQVPDLDIKGPKVDINAPDVDVRGPDWHLKMPKIKMPKISMPGFKGEGPEVDVNLPKADLDVSGPKVDVDVPDVNIEGPDAKLKGPKFKMPEMNIKAPKISMPDFDLHLKGPKVKGDVDVSLPKMEGDLKAPEVDIKGPKVDIDAPDVDVHGPDWHLKMPKVKMPKFSMPGFKGEGPEVDVNLPKADIDVSGPKVDIDTPDIDIHGPEGKLKGPKFKMPDLHLKAPKISMPEVDLNLKGPKMKGDVDVSLPKVEGDLKGPEVDIKGPKVDIDVPDVDVQGPDWHLKMPKVKMPKFSMPGFKGEGPDVDVNLPKADLDVSGPKVDIDVPDVNIEGPDAKLKGPKFKMPEMNIKAPKISMPDFDLHLKGPKVKGDVDVSLPKVEGDLKGPEVDIKGPKVDIDAPDVDVHGPDWHLKMPKVKMPKFSMPGFKGEGPDVDVTLPKADIEISGPKVDIDAPDVSIEGPDAKLKGPKFKMPEMNIKAPKISMPDIDFNLKGPKVKGDVDVSLPKVEGDLKGPEIDIKGPSLDIDTPDVNIEGPEGKLKGPKFKMPEMNIKAPKISMPDFDLHLKGPKVKGDVDVSLPKVESDLKGPEVDIEGPEGKLKGPKFKMPDVHFKSPQISMSDIDLNLKGPKIKGDMDISVPKLEGDLKGPKVDVKGPKVGIDTPDIDIHGPEGKLKGPKFKMPDLHLKAPKISMPEVDLNLKGPKVKGDMDISLPKVEGDLKGPEVDIRDPKVDIDVPDVDVQGPDWHLKMPKVKMPKFSMPGFKGEGPDVDVNLPKADIDVSGPKVDVDVPDVNIEGPDAKLKGPKFKMPEMSIKAPKISMPDIDLNLKGPKVKGDVDVTLPKVEGDLKGPEADIKGPKVDINTPDVDVHGPDWHLKMPKVKMPKFSMPGFKGEGPDVDVSLPKADIDVSGPKVDVDIPDVNIEGPDAKLKGPKFKMPEINIKAPKISIPDVDLDLKGPKVKGDFDVSVPKVEGTLKGPEVDLKGPRLDFEGPDAKLSGPSLKMPSLEISAPKVTAPDVDLHLKAPKIGFSGPKLEGGEVDLKGPKVEAPSLDVHMDSPDINIEGPDVKIPKFKKPKFGFGAKSPKADIKSPSLDVTVPEAELNLETPEISVGGKGKKSKFKMPKIHMSGPKIKAKKQGFDLNVPGGEIDASLKAPDVDVNIAGPDAALKVDVKSPKTKKTMFGKMYFPDVEFDIKSPKFKAEAPLPSPKLEGELQAPDLELSLPAIHVEGLDIKAKAPKVKMPDVDISVPKIEGDLKGPKVQANLGAPDINIEGLDAKVKTPSFGISAPQVSIPDVNVNLKGPKIKGDVPSVGLEGPDVDLQGPEAKIKFPKFSMPKIGIPGVKMEGGGAEVHAQLPSLEGDLRGPDVKLEGPDVSLKGPGVDLPSVNLSMPKVSGPDLDLNLKGPSLKGDLDASVPSMKVHAPGLNLSGVGGKMQVGGDGVKVPGIDATTKLNVGAPDVTLRGPSLQGDLAVSGDIKCPKVSVGAPDLSLEASEGSIKLPKMKLPQFGISTPGSDLHVNAKGPQVSGELKGPGVDVNLKGPRISAPNVDFNLEGPKVKGSLGATGEIKGPTVGGGLPGIGVQGLEGNLQMPGIKSSGCDVNLPGVNVKLPTGQISGPEIKGGLKGSEVGFHGAAPDISVKGPAFNMASPESDFGINLKGPKIKGGADVSGGVSAPDISLGEGHLSVKGSGGEWKGPQVSSALNLDTSKFAGGLHFSGPKVEGGVKGGQIGLQAPGLSVSGPQGHLESGSGKVTFPKMKIPKFTFSGRELVGREMGVDVHFPKAEASIQAGAGDGEWEESEVKLKKSKIKMPKFNFSKPKGKGGVTGSPEASISGSKGDLKSSKASLGSLEGEAEAEASSPKGKFSLFKSKKPRHRSNSFSDEREFSGPSTPTGTLEFEGGEVSLEGGKVKGKHGKLKFGTFGGLGSKSKGHYEVTGSDDETGKLQGSGVSLASKKSRLSSSSSNDSGNKVGIQLPEVELSVSTKKE

>Q16698

MKLPARVFFTLGSRLPCGLAPRRFFSYGTKILYQNTEALQSKFFSPLQKAMLPPNSFQGKVAFITGGGTGLGKGMTTLLSSLGAQCVIASRKMDVLKATAEQISSQTGNKVHAIQCDVRDPDMVQNTVSELIKVAGHPNIVINNAAGNFISPTERLSPNAWKTITDIVLNGTAFVTLEIGKQLIKAQKGAAFLSITTIYAETGSGFVVPSASAKAGVEAMSKSLAAEWGKYGMRFNVIQPGPIKTKGAFSRLDPTGTFEKEMIGRIPCGRLGTVEELANLAAFLCSDYASWINGAVIKFDGGEEVLISGEFNDLRKVTKEQWDTIEELIRKTKGS

243 NP_005002.3 Q96Q11

>NP_005002.3

MEEHGVTQTEHMATIEAHAVAQQVQQVHVATYTEHSMLSADEDSPSSPEDTSYDDSDILNSTAADEVTAHLAAAGPVGMAAAAAVATGKKRKRPHVFESNPSIRKRQQTRLLRKLRATLDEYTTRVGQQAIVLCISPSKPNPVFKVFGAAPLENVVRKYKSMILEDLESALAEHAPAPQEVNSELPPLTIDGIPVSVDKMTQAQLRAFIPEMLKYSTGRGKPGWGKESCKPIWWPEDIPWANVRSDVRTEEQKQRVSWTQALRTIVKNCYKQHGREDLLYAFEDQQTQTQATATHSIAHLVPSQTVVQTFSNPDGTVSLIQVGTGATVATLADASELPTTVTVAQVNYSAVADGEVEQNWATLQGGEMTIQTTQASEATQAVASLAEAAVAASQEMQQGATVTMALNSEAAAHAVATLAEATLQGGGQIVLSGETAAAVGALTGVQDANGLVQIPVSMYQTVVTSLAQGNGPVQVAMAPVTTRISDSAVTMDGQAVEVVTLEQ

>Q96Q11

MLRCLYHWHRPVLNRRWSRLCLLKQYLFTMKLQSPEFQSLFTEGLKSLTELFVKENHELRIAGGAVRDLLNGVKPQDIDFATTATPTQMKEMFQSAGIRMINNRGEKHGTITARLHEENFEITTLRIDVTTDGRHAEVEFTTDWQKDAERRDLTINSMFLGFDGTLFDYFNGYEDLKNKKVRFVGHAKQRIQEDYLRILRYFRFYGRIVDKPGDHDPETLEAIAENAKGLAGISGERIWVELKKILVGNHVNHLIHLIYDLDVAPYIGLPANASLEEFDKVSKNVDGFSPKPVTLLASLFKVQDDVTKLDLRLKIAKEEKNLGLFIVKNRKDLIKATDSSDPLKPYQDFIIDSREPDATTRVCELLKYQGEHCLLKEMQQWSIPPFPVSGHDIRKVGISSGKEIGALLQQLREQWKKSGYQMEKDELLSYIKKT

244 NP_001315.1 NP_000365.3

>NP_001315.1

MYRTKVGLKDRQQLYKLIISQLLYDGYISIANGLINEIKPQSVCAPSEQLLHLIKLGMENDDTAVQYAIGRSDTVAPGTGIDLEFDADVQTMSPEASEYETCYVTSHKGPCRVATYSRDGQLIATGSADASIKILDTERMLAKSAMPIEVMMNETAQQNMENHPVIRTLYDHVDEVTCLAFHPTEQILASGSRDYTLKLFDYSKPSAKRAFKYIQEAEMLRSISFHPSGDFILVGTQHPTLRLYDINTFQCFVSCNPQDQHTDAICSVNYNSSANMYVTGSKDGCIKLWDGVSNRCITTFEKAHDGAEVCSAIFSKNSKYILSSGKDSVAKLWEISTGRTLVRYTGAGLSGRQVHRTQAVFNHTEDYVLLPDERTISLCCWDSRTAERRNLLSLGHNNIVRCIVHSPTNPGFMTCSDDFRARFWYRRSTTD

>NP_000365.3

MEANGLGPQGFPELKNDTFLRAAWGEETDYTPVWCMRQAGRYLPEFRETRAAQDFFSTCRSPEACCELTLQPLRRFPLDAAIIFSDILVVPQALGMEVTMVPGKGPSFPEPLREEQDLERLRDPEVVASELGYVFQAITLTRQRLAGRVPLIGFAGAPWTLMTYMVEGGGSSTMAQAKRWLYQRPQASHQLLRILTDALVPYLVGQVVAGAQALQLFESHAGHLGPQLFNKFALPYIRDVAKQVKARLREAGLAPVPMIIFAKDGHFALEELAQAGYEVVGLDWTVAPKKARECVGKTVTLQGNLDPCALYASEEEIGQLVKQMLDDFGPHRYIANLGHGLYPDMDPEHVGAFVDAVHKHSRLLRQN

245 B2CW77 NP_054902.1

>B2CW77

MDRPGPGSARPGRTVHVWGYRVEWKVRNGRKLQPSEWAGRGDLGGFKRRWKDTRATVGTTFRRRSRVSLVGELSKFPLPSDSSGGKSSSSFARGALAWCRQRNPNPSCAAAETGARTSLPKERCRGWRLGNWLHKHPHPNTCPRLPACWLPPILTERGERVPKLVPLLACYPKSKPKD

>NP_054902.1

MAEVEETLKRLQSQKGVQGIIVVNTEGIPIKSTMDNPTTTQYASLMHSFILKARSTVRDIDPQNDLTFLRIRSKKNEIMVAPDKDYFLIVIQNPTE

246 P22304 P51857

>P22304

MPPPRTGRGLLWLGLVLSSVCVALGSETQANSTTDALNVLLIIVDDLRPSLGCYGDKLVRSPNIDQLASHSLLFQNAFAQQAVCAPSRVSFLTGRRPDTTRLYDFNSYWRVHAGNFSTIPQYFKENGYVTMSVGKVFHPGISSNHTDDSPYSWSFPPYHPSSEKYENTKTCRGPDGELHANLLCPVDVLDVPEGTLPDKQSTEQAIQLLEKMKTSASPFFLAVGYHKPHIPFRYPKEFQKLYPLENITLAPDPEVPDGLPPVAYNPWMDIRQREDVQALNISVPYGPIPVDFQRKIRQSYFASVSYLDTQVGRLLSALDDLQLANSTIIAFTSDHGWALGEHGEWAKYSNFDVATHVPLIFYVPGRTASLPEAGEKLFPYLDPFDSASQLMEPGRQSMDLVELVSLFPTLAGLAGLQVPPRCPVPSFHVELCREGKNLLKHFRFRDLEEDPYLPGNPRELIAYSQYPRPSDIPQWNSDKPSLKDIKIMGYSIRTIDYRYTVWVGFNPDEFLANFSDIHAGELYFVDSDPLQDHNMYNDSQGGDLFQLLMP

>P51857

MDLSAASHRIPLSDGNSIPIIGLGTYSEPKSTPKGACATSVKVAIDTGYRHIDGAYIYQNEHEVGEAIREKIAEGKVRREDIFYCGKLWATNHVPEMVRPTLERTLRVLQLDYVDLYIIEVPMAFKPGDEIYPRDENGKWLYHKSNLCATWEAMEACKDAGLVKSLGVSNFNRRQLELILNKPGLKHKPVSNQVECHPYFTQPKLLKFCQQHDIVITAYSPLGTSRNPIWVNVSSPPLLKDALLNSLGKRYNKTAAQIVLRFNIQRGVVVIPKSFNLERIKENFQIFDFSLTEEEMKDIEALNKNVRFVELLMWRDHPEYPFHDEY

247 NP_002403.1 P30838

>NP_002403.1

MDKDCEMKRTTLDSPLGKLELSGCEQGLHEIKLLGKGTSAADAVEVPAPAAVLGGPEPLMQCTAWLNAYFHQPEAIEEFPVPALHHPVFQQESFTRQVLWKLLKVVKFGEVISYQQLAALAGNPKAARAVGGAMRGNPVPILIPCHRVVCSSGAVGNYSGGLAVKEWLLAHEGHRLGKPGLGGSSGLAGAWLKGAGATSGSPPAGRN

>P30838

MSKISEAVKRARAAFSSGRTRPLQFRIQQLEALQRLIQEQEQELVGALAADLHKNEWNAYYEEVVYVLEEIEYMIQKLPEWAADEPVEKTPQTQQDELYIHSEPLGVVLVIGTWNYPFNLTIQPMVGAIAAGNAVVLKPSELSENMASLLATIIPQYLDKDLYPVINGGVPETTELLKERFDHILYTGSTGVGKIIMTAAAKHLTPVTLELGGKSPCYVDKNCDLDVACRRIAWGKFMNSGQTCVAPDYILCDPSIQNQIVEKLKKSLKEFYGEDAKKSRDYGRIISARHFQRVMGLIEGQKVAYGGTGDAATRYIAPTILTDVDPQSPVMQEEIFGPVLPIVCVRSLEEAIQFINQREKPLALYMFSSNDKVIKKMIAETSSGGVAANDVIVHITLHSLPFGGVGNSGMGSYHGKKSFETFSHRRSCLVRPLMNDEGLKVRYPPSPAKMTQH

248 Q8N9N5 NP_006592.3

>Q8N9N5

MMSEHDLADVVQIAVEDLSPDHPVVLENHVVTDEDEPALKRQRLEINCQDPSIKTICLRLDSIEAKLQALEATCKSLEEKLDLVTNKQHSPIQVPMVAGSPLGATQTCNKVRCVVPQTTVILNNDRQNAIVAKMEDPLSNRAPDSLENVISNAVPGRRQNTIVVKVPGQEDSHHEDGESGSEASDSVSSCGQAGSQSIGSNVTLITLNSEEDYPNGTWLGDENNPEMRVRCAIIPSDMLHISTNCRTAEKMALTLLDYLFHREVQAVSNLSGQGKHGKKQLDPLTIYGIRCHLFYKFGITESDWYRIKQSIDSKCRTAWRRKQRGQSLAVKSFSRRTPNSSSYCPSESMMSTPPPASELPQPQPQPQALHYALANAQQVQIHQIGEDGQVQVGHLHIAQVPQGEQVQITQDSEGNLQIHHVGQDGQLLEATRIPCLLAPSVFKASSGQVLQGAQLIAVASSDPAAAGVDGSPLQGSDIQVQYVQLAPVSDHTAGAQTAEALQPTLQPEMQLEHGAIQIQ

>NP_006592.3

MQPASAKWYDRRDYVFIEFCVEDSKDVNVNFEKSKLTFSCLGGSDNFKHLNEIDLFHCIDPNDSKHKRTDRSILCCLRKGESGQSWPRLTKERAKLNWLSVDFNNWKDWEDDSDEDMSNFDRFSEMMNNMGGDEDVDLPEVDGADDDSQDSDDEKMPDLE

249 Q5H9F3 NP_550438.1

>Q5H9F3

MISTAPLYSGVHNWTSSDRIRMCGINEERRAPLSDEESTTGDCQHFGSQEFCVSSSFSKVELTAVGSGSNARGADPDGSATEKLGHKSEDKPDDPQPKMDYAGNVAEAEGFLVPLSSPGDGLKLPASDSAEASNSRADCSWTPLNTQMSKQVDCSPAGVKALDSRQGVGEKNTFILATLGTGVPVEGTLPLVTTNFSPLPAPICPPAPGSASVPHSVPDAFQVPLSVPAPVPHSGLVPVQVATSVPAPSPPLAPVPALAPAPPSVPTLISDSNPLSVSASVLVPVPASAPPSGPVPLSAPAPAPLSVPVSAPPLALIQAPVPPSAPTLVLAPVPTPVLAPMPASTPPAAPAPPSVPMPTPTPSSGPPSTPTLIPAFAPTPVPAPTPAPIFTPAPTPMPAATPAAIPTSAPIPASFSLSRVCFPAAQAPAMQKVPLSFQPGTVLTPSQPLVYIPPPSCGQPLSVATLPTTLGVSSTLTLPVLPSYLQDRCLPGVLASPELRSYPYAFSVARPLTSDSKLVSLEVNRLPCTSPSGSTTTQPAPDGVPGPLADTSLVTASAKVLPTPQPLLPAPSGSSAPPHPAKMPSGTEQQTEGTSVTFSPLKSPPQLEREMASPPECSEMPLDLSSKSNRQKLPLPNQRKTPPMPVLTPVHTSSKALLSTVLSRSQRTTQAAGGNVTSCLGSTSSPFVIFPEIVRNGDPSTWVKNSTALISTIPGTYVGVANPVPASLLLNKDPNLGLNRDPRHLPKQEPISIIDQGEPKGTGATCGKKGSQAGAEGQPSTVKRYTPARIAPGLPGCQTKELSLWKPTGPANIYPRCSVNGKPTSTQVLPVGWSPYHQASLLSIGISSAGQLTPSQGAPIRPTSVVSEFSGVPSLSSSEAVHGLPEGQPRPGGSFVPEQDPVTKNKTCRIAAKPYEEQVNPVLLTLSPQTGTLALSVQPSGGDIRMNQGPEESESHLCSDSTPKMEGPQGACGLKLAGDTKPKNQVLATYMSHELVLATPQNLPKMPELPLLPHDSHPKELILDVVPSSRRGSSTERPQLGSQVDLGRVKMEKVDGDVVFNLATCFRADGLPVAPQRGQAEVRAKAGQARVKQESVGVFACKNKWQPDDVTESLPPKKMKCGKEKDSEEQQLQPQAKAVVRSSHRPKCRKLPSDPQESTKKSPRGASDSGKEHNGVRGKHKHRKPTKPESQSPGKRADSHEEGSLEKKAKSSFRDFIPVVLSTRTRSQSGSICSSFAGMADSDMGSQEVFPTEEEEEVTPTPAKRRKVRKTQRDTQYRSHHAQDKSLLSQGRRHLWRAREMPWRTEAARQMWDTNEEEEEEEEEGLLKRKKRRRQKSRKYQTGEYLTEQEDEQRRKGRADLKARKQKTSSSQSLEHRLRNRNLLLPNKVQGISDSPNGFLPNNLEEPACLENSEKPSGKRKCKTKHMATVSEEAKDVVLYCLQKDSEDVNHRDNAGYTALHEACSRGWTDILNILLEHGANVNCSAQDGTRPVHDAVVNDNLETIWLLLSYGADPTLATYSGQTAMKLASSDTMKRFLSDHLSDLQGRAEGDPGVSWDFYSSSVLEEKDGFACDLLHNPPGSSDQEGDDPMEEDDFMFELSDKPLLPCYNLQVSVSRGPCNWFLFSDVLKRLKLSSRIFQARFPHFEITTMPKAEFYRQVASSQLLTPAERPGGLDDRSPPGSSETVELVRYEPDLLRLLGSEVEFQSCNS

>NP_550438.1

MAAGRLFLSRLRAPFSSMAKSPLEGVSSSRGLHAGRGPRRLSIEGNIAVGKSTFVKLLTKTYPEWHVATEPVATWQNIQAAGTQKACTAQSLGNLLDMMYREPARWSYTFQTFSFLSRLKVQLEPFPEKLLQARKPVQIFERSVYSDRYIFAKNLFENGSLSDIEWHIYQDWHSFLLWEFASRITLHGFIYLQASPQVCLKRLYQRAREEEKGIELAYLEQLHGQHEAWLIHKTTKLHFEALMNIPVLVLDVNDDFSEEVTKQEDLMREVNTFVKNL

250 NP_004260.2 P13995

>NP_004260.2

MADVINVSVNLEAFSQAISAIQALRSSVSRVFDCLKDGMRNKETLEGREKAFIAHFQDNLHSVNRDLNELERLSNLVGKPSENHPLHNSGLLSLDPVQDKTPLYSQLLQAYKWSNKLQYHAGLASGLLNQQSLKRSANQMGVSAKRRPKAQPTTLVLPPQYVDDVISRIDRMFPEMSIHLSRPNGTSAMLLVTLGKVLKVIVVMRSLFIDRTIVKGYNENVYTEDGKLDIWSKSNYQVFQKVTDHATTALLHYQLPQMPDVVVRSFMTWLRSYIKLFQAPCQRCGKFLQDGLPPTWRDFRTLEAFHDTCRQ

>P13995

MAATSLMSALAARLLQPAHSCSLRLRPFHLAAVRNEAVVISGRKLAQQIKQEVRQEVEEWVASGNKRPHLSVILVGENPASHSYVLNKTRAAAVVGINSETIMKPASISEEELLNLINKLNNDDNVDGLLVQLPLPEHIDERRICNAVSPDKDVDGFHVINVGRMCLDQYSMLPATPWGVWEIIKRTGIPTLGKNVVVAGRSKNVGMPIAMLLHTDGAHERPGGDATVTISHRYTPKEQLKKHTILADIVISAAGIPNLITADMIKEGAAVIDVGINRVHDPVTAKPKLVGDVDFEGVRQKAGYITPVPGGVGPMTVAMLMKNTIIAAKKVLRLEEREVLKSKELGVATN

251 Q96BN2 NP_071933.2

>Q96BN2

MATFVSELEAAKKNLSEALGDNVKQYWANLKLWFKQKISKEEFDLEAHRLLTQDNVHSHNDFLLAILTRCQILVSTPDGAGSLPWPGGSAAKPGKPKGKKKLSSVRQKFDHRFQPQNPLSGAQQFVAKDPQDDDDLKLCSHTMMLPTRGQLEGRMIVTAYEHGLDNVTEEAVSAVVYAVENHLKDILTSVVSRRKAYRLRDGHFKYAFGSNVTPQPYLKNSVVAYNNLIESPPAFTAPCAGQNPASHPPPDDAEQQAALLLACSGDTLPASLPPVNMYDLFEALQVHREVIPTHTVYALNIERIITKLWHPNHEELQQDKVHRQRLAAKEGLLLC

>NP_071933.2

MQNVINTVKGKALEVAEYLTPVLKESKFKETGVITPEEFVAAGDHLVHHCPTWQWATGEELKVKAYLPTGKQFLVTKNVPCYKRCKQMEYSDELEAIIEEDDGDGGWVDTYHNTGITGITEAVKEITLENKDNIRLQDCSALCEEEEDEDEGEAADMEEYEESGLLETDEATLDTRKIVEACKAKTDAGGEDAILQTRTYDLYITYDKYYQTPRLWLFGYDEQRQPLTVEHMYEDISQDHVKKTVTIENHPHLPPPPMCSVHPCRHAEVMKKIIETVAEGGGELGVHMYLLIFLKFVQAVIPTIEYDYTRHFTM

252 NP_004483.1 Q9NX20

>NP_004483.1

MAYQLYRNTTLGNSLQESLDELIQSQQITPQLALQVLLQFDKAINAALAQRVRNRVNFRGSLNTYRFCDNVWTFVLNDVEFREVTELIKVDKVKIVACDGKNTGSNTTE

>Q9NX20

MWRLLARASAPLLRVPLSDSWALLPASAGVKTLLPVPSFEDVSIPEKPKLRFIERAPLVPKVRREPKNLSDIRGPSTEATEFTEGNFAILALGGGYLHWGHFEMMRLTINRSMDPKNMFAIWRVPAPFKPITRKSVGHRMGGGKGAIDHYVTPVKAGRLVVEMGGRCEFEEVQGFLDQVAHKLPFAAKAVSRGTLEKMRKDQEERERNNQNPWTFERIATANMLGIRKVLSPYDLTHKGKYWGKFYMPKRV

253 NP_005504.2 NP_055433.2

>NP_005504.2

MADPDVLTEVPAALKRLAKYVIRGFYGIEHALALDILIRNSCVKEEDMLELLKFDRKQLRSVLNNLKGDKFIKCRMRVETAADGKTTRHNYYFINYRTLVNVVKYKLDHMRRRIETDERDSTNRASFKCPVCSSTFTDLEANQLFDPMTGTFRCTFCHTEVEEDESAMPKKDARTLLARFNEQIEPIYALLRETEDVNLAYEILEPEPTEIPALKQSKDHAATTAGAASLAGGHHREAWATKGPSYEDLYTQNVVINMDDQEDLHRASLEGKSAKERPIWLRESTVQGAYGSEDMKEGGIDMDAFQEREEGHAGPDDNEEVMRALLIHEKKTSSAMAGSVGAAAPVTAANGSDSESETSESDDDSPPRPAAVAVHKREEDEEEDDEFEEVADDPIVMVAGRPFSYSEVSQRPELVAQMTPEEKEAYIAMGQRMFEDLFE

>NP_055433.2

MNWRFVELLYFLFIWGRISVQPSHQEPAGTDQHVSKEFDWLISDRGPFHHSRSYLSFVERHRQGFTTRYKIYREFARWKVRNTAIERRDLVRHPVPLMPEFQRSIRLLGRRPTTQQFIDTIIKKYGTHLLISATLGGEEALTMYMDKSRLDRKSGNATQSVEALHQLASSYFVDRDGTMRRLHEIQISTGAIKVTETRTGPLGCNSYDNLDSVSSVLLQSTESKLHLQGLQIIFPQYLQEKFVQSALSYIMCNGEGEYLCQNSQCRCQCAEEFPQCNCPITDIQIMEYTLANMAKSWAEAYKDLENSDEFKSFMKRLPSNHFLTIGSIHQHWGNDWDLQNRYKLLQSATEAQRQKIQRTARKLFGLSVRCRHNPNHQLPRERTIQQWLARVQSLLYCNENGFWGTFLESQRSCVCHGSTTLCQRPIPCVIGGNNSCAMCSLANISLCGSCNKGYKLYRGRCEPQNVDSERSEQFISFETDLDFQDLELKYLLQKMDSRLYVHTTFISNEIRLDTFFDPRWRKRMSLTLKSNKNRMDFIHMVIGMSMRICQMRNSSLDPMFFVYVNPFSGSHSEGWNMPFGEFGYPRWEKIRLQNSQCYNWTLLLGNRWKTFFETVHIYLRSRTRLPTLLRNETGQGPVDLSDPSKRQFYIKISDVQVFGYSLRFNADLLRSAVQQVNQSYTQGGQFYSSSSVMLLLLDIRDRINRLAPPVAPGKPQLDLFSCMLKHRLKLTNSEIIRVNHALDLYNTEILKQSDQMTAKLC

254 Q9NQ92 NP_550438.1

>Q9NQ92

MDLQAAGAQAQGAAEPSRGPPLPSARGAPPSPEAGFATADHSSQERETEKAMDRLARGTQSIPNDSPARGEGTHSEEEGFAMDEEDSDGELNTWELSEGTNCPPKEQPGDLFNEDWDSELKADQGNPYDADDIQESISQELKPWVCCAPQGDMIYDPSWHHPPPLIPYYSKMVFETGQFDDAED

>NP_550438.1

MAAGRLFLSRLRAPFSSMAKSPLEGVSSSRGLHAGRGPRRLSIEGNIAVGKSTFVKLLTKTYPEWHVATEPVATWQNIQAAGTQKACTAQSLGNLLDMMYREPARWSYTFQTFSFLSRLKVQLEPFPEKLLQARKPVQIFERSVYSDRYIFAKNLFENGSLSDIEWHIYQDWHSFLLWEFASRITLHGFIYLQASPQVCLKRLYQRAREEEKGIELAYLEQLHGQHEAWLIHKTTKLHFEALMNIPVLVLDVNDDFSEEVTKQEDLMREVNTFVKNL

255 O95766 Q9GZZ1

>O95766

MAAAAAGAGSGPWAAQEKQFPPALLSFFIYNPRFGPREGQEENKILFYHPNEVEKNEKIRNVGLCEAIVQFTRTFSPSKPAKSLHTQKNRQFFNEPEENFWMVMVVRNPIIEKQSKDGKPVIEYQEEELLDKVYSSVLRQCYSMYKLFNGTFLKAMEDGGVKLLKERLEKFFHRYLQTLHLQSCDLLDIFGGISFFPLDKMTYLKIQSFINRMEESLNIVKYTAFLYNDQLIWSGLEQDDMRILYKYLTTSLFPRHIEPELAGRDSPIRAEMPGNLQHYGRFLTGPLNLNDPDAKCRFPKIFVNTDDTYEELHLIVYKAMSAAVCFMIDASVHPTLDFCRRLDSIVGPQLTVLASDICEQFNINKRMSGSEKEPQFKFIYFNHMNLAEKSTVHMRKTPSVSLTSVHPDLMKILGDINSDFTRVDEDEEIIVKAMSDYWVVGKKSDRRELYVILNQKNANLIEVNEEVKKLCATQFNNIFFLD

>Q9GZZ1

MKGSRIELGDVTPHNIKQLKRLNQVIFPVSYNDKFYKDVLEVGELAKLAYFNDIAVGAVCCRVDHSQNQKRLYIMTLGCLAPYRRLGIGTKMLNHVLNICEKDGTFDNIYLHVQISNESAIDFYRKFGFEIIETKKNYYKRIEPADAHVLQKNLKVPSGQNADVQKTDN

256 NP_000170.1 NP_055300.1

>NP_000170.1

MSRQSTLYSFFPKSPALSDANKASARASREGGRAAAAPGASPSPGGDAAWSEAGPGPRPLARSASPPKAKNLNGGLRRSVAPAAPTSCDFSPGDLVWAKMEGYPWWPCLVYNHPFDGTFIREKGKSVRVHVQFFDDSPTRGWVSKRLLKPYTGSKSKEAQKGGHFYSAKPEILRAMQRADEALNKDKIKRLELAVCDEPSEPEEEEEMEVGTTYVTDKSEEDNEIESEEEVQPKTQGSRRSSRQIKKRRVISDSESDIGGSDVEFKPDTKEEGSSDEISSGVGDSESEGLNSPVKVARKRKRMVTGNGSLKRKSSRKETPSATKQATSISSETKNTLRAFSAPQNSESQAHVSGGGDDSSRPTVWYHETLEWLKEEKRRDEHRRRPDHPDFDASTLYVPEDFLNSCTPGMRKWWQIKSQNFDLVICYKVGKFYELYHMDALIGVSELGLVFMKGNWAHSGFPEIAFGRYSDSLVQKGYKVARVEQTETPEMMEARCRKMAHISKYDRVVRREICRIITKGTQTYSVLEGDPSENYSKYLLSLKEKEEDSSGHTRAYGVCFVDTSLGKFFIGQFSDDRHCSRFRTLVAHYPPVQVLFEKGNLSKETKTILKSSLSCSLQEGLIPGSQFWDASKTLRTLLEEEYFREKLSDGIGVMLPQVLKGMTSESDSIGLTPGEKSELALSALGGCVFYLKKCLIDQELLSMANFEEYIPLDSDTVSTTRSGAIFTKAYQRMVLDAVTLNNLEIFLNGTNGSTEGTLLERVDTCHTPFGKRLLKQWLCAPLCNHYAINDRLDAIEDLMVVPDKISEVVELLKKLPDLERLLSKIHNVGSPLKSQNHPDSRAIMYEETTYSKKKIIDFLSALEGFKVMCKIIGIMEEVADGFKSKILKQVISLQTKNPEGRFPDLTVELNRWDTAFDHEKARKTGLITPKAGFDSDYDQALADIRENEQSLLEYLEKQRNRIGCRTIVYWGIGRNRYQLEIPENFTTRNLPEEYELKSTKKGCKRYWTKTIEKKLANLINAEERRDVSLKDCMRRLFYNFDKNYKDWQSAVECIAVLDVLLCLANYSRGGDGPMCRPVILLPEDTPPFLELKGSRHPCITKTFFGDDFIPNDILIGCEEEEQENGKAYCVLVTGPNMGGKSTLMRQAGLLAVMAQMGCYVPAEVCRLTPIDRVFTRLGASDRIMSGESTFFVELSETASILMHATAHSLVLVDELGRGTATFDGTAIANAVVKELAETIKCRTLFSTHYHSLVEDYSQNVAVRLGHMACMVENECEDPSQETITFLYKFIKGACPKSYGFNAARLANLPEEVIQKGHRKAREFEKMNQSLRLFREVCLASERSTVDAEAVHKLLTLIKEL

>NP_055300.1

MPNYKLTYFNMRGRAEIIRYIFAYLDIQYEDHRIEQADWPEIKSTLPFGKIPILEVDGLTLHQSLAIARYLTKNTDLAGNTEMEQCHVDAIVDTLDDFMSCFPWAEKKQDVKEQMFNELLTYNAPHLMQDLDTYLGGREWLIGNSVTWADFYWEICSTTLLVFKPDLLDNHPRLVTLRKKVQAIPAVANWIKRRPQTKL

257 NP_004647.1 O60783

>NP_004647.1

MNKGWLELESDPGLFTLLVEDFGVKGVQVEEIYDLQSKCQGPVYGFIFLFKWIEERRSRRKVSTLVDDTSVIDDDIVNNMFFAHQLIPNSCATHALLSVLLNCSSVDLGPTLSRMKDFTKGFSPESKGYAIGNAPELAKAHNSHARPEPRHLPEKQNGLSAVRTMEAFHFVSYVPITGRLFELDGLKVYPIDHGPWGEDEEWTDKARRVIMERIGLATAGEPYHDIRFNLMAVVPDRRIKYEARLHVLKVNRQTVLEALQQLIRVTQPELIQTHKSQESQLPEESKSASNKSPLVLEANRAPAASEGNHTDGAEEAAGSCAQAPSHSPPNKPKLVVKPPGSSLNGVHPNPTPIVQRLPAFLDNHNYAKSPMQEEEDLAAGVGRSRVPVRPPQQYSDDEDDYEDDEEDDVQNTNSALRYKGKGTGKPGALSGSADGQLSVLQPNTINVLAEKLKESQKDLSIPLSIKTSSGAGSPAVAVPTHSQPSPTPSNESTDTASEIGSAFNSPLRSPIRSANPTRPSSPVTSHISKVLFGEDDSLLRVDCIRYNRAVRDLGPVISTGLLHLAEDGVLSPLALTEGGKGSSPSIRPIQGSQGSSSPVEKEVVEATDSREKTGMVRPGEPLSGEKYSPKELLALLKCVEAEIANYEACLKEEVEKRKKFKIDDQRRTHNYDEFICTFISMLAQEGMLANLVEQNISVRRRQGVSIGRLHKQRKPDRRKRSRPYKAKRQ

>O60783

MAAFMLGSLLRTFKQMVPSSASGQVRSHYVDWRMWRDVKRRKMAYEYADERLRINSLRKNTILPKILQDVADEEIAALPRDSCPVRIRNRCVMTSRPRGVKRRWRLSRIVFRHLADHGQLSGIQRATW

258 AAA93070.1 Q9H0U6

>AAA93070.1

MSGCRVFIGRLNPAAREKDVERFFKGYGRIRDIDLKRGFGFVEFEDPRDADDAVYELDGKELCSERVTIEHARARSRGGRGRGRYSDRFSSRRPRNDRRNAPPVRTENRLIVENLSSRVSWQDLKDFMRQAGEVTFADAHRPKLNEGVVEFASYGDLKNAIEKLSGKEINGRKIKLIEGSKRHSRSRSRSRSRTRSSSRSRSRSRSRSRKSYSRSRSRSRSRSRSKSRSVSRSPVPEKSQKRGSSSRSKSPASVDRQRSRSRSRSRSVDSGN

>Q9H0U6

MALRSRFWGLFSVCRNPGCRFAALSTSSEPAAKPEVDPVENEAVAPEFTNRNPRNLELLSVARKERGWRTVFPSREFWHRLRVIRTQHHVEALVEHQNGKVVVSASTREWAIKKHLYSTRNVVACESIGRVLAQRCLEAGINFMVYQPTPWEAASDSMKRLQSAMTEGGVVLREPQRIYE

259 B2CW77 NP_110379.2

>B2CW77

MDRPGPGSARPGRTVHVWGYRVEWKVRNGRKLQPSEWAGRGDLGGFKRRWKDTRATVGTTFRRRSRVSLVGELSKFPLPSDSSGGKSSSSFARGALAWCRQRNPNPSCAAAETGARTSLPKERCRGWRLGNWLHKHPHPNTCPRLPACWLPPILTERGERVPKLVPLLACYPKSKPKD

>NP_110379.2

MEGPLSVFGDRSTGETIRSQNVMAAASIANIVKSSLGPVGLDKMLVDDIGDVTITNDGATILKLLEVEHPAAKVLCELADLQDKEVGDGTTSVVIIAAELLKNADELVKQKIHPTSVISGYRLACKEAVRYINENLIVNTDELGRDCLINAAKTSMSSKIIGINGDFFANMVVDAVLAIKYTDIRGQPRYPVNSVNILKAHGRSQMESMLISGYALNCVVGSQGMPKRIVNAKIACLDFSLQKTKMKLGVQVVITDPEKLDQIRQRESDITKERIQKILATGANVILTTGGIDDMCLKYFVEAGAMAVRRVLKRDLKRIAKASGATILSTLANLEGEETFEAAMLGQAEEVVQERICDDELILIKNTKARTSASIILRGANDFMCDEMERSLHDALCVVKRVLESKSVVPGGGAVEAALSIYLENYATSMGSREQLAIAEFARSLLVIPNTLAVNAAQDSTDLVAKLRAFHNEAQVNPERKNLKWIGLDLSNGKPRDNKQAGVFEPTIVKVKSLKFATEAAITILRIDDLIKLHPESKDDKHGSYEDAVHSGALND

260 NP_001505.1 Q8WUN7

>NP_001505.1

MASTSRLDALPRVTCPNHPDAILVEDYRAGDMICPECGLVVGDRVIDVGSEWRTFSNDKATKDPSRVGDSQNPLLSDGDLSTMIGKGTGAASFDEFGNSKYQNRRTMSSSDRAMMNAFKEITTMADRINLPRNIVDRTNNLFKQVYEQKSLKGRANDAIASACLYIACRQEGVPRTFKEICAVSRISKKEIGRCFKLILKALETSVDLITTGDFMSRFCSNLCLPKQVQMAATHIARKAVELDLVPGRSPISVAAAAIYMASQASAEKRTQKEIGDIAGVADVTIRQSYRLIYPRAPDLFPTDFKFDTPVDKLPQL

>Q8WUN7

MGGCVGAQHDSSGSLNENSEGTGVALGRNQPLKKEKPKWKSDYPMTDGQLRSKRDEFWDTAPAFEGRKEIWDALKAAAHAFESNDHELAQAIIDGANITLPHGALTECYDELGNRYQLPVYCLAPPINMIEEKSDIETLDIPEPPPNSGYECQLRLRLSTGKDLKLVVRSTDTVFHMKRRLHAAEGVEPGSQRWFFSGRPLTDKMKFEELKIPKDYVVQVIVSQPVQNPTPVEN

261 NP_002687.1 NP_005495.2

>NP_002687.1

MFYHISLEHEILLHPRYFGPNLLNTVKQKLFTEVEGTCTGKYGFVIAVTTIDNIGAGVIQPGRGFVLYPVKYKAIVFRPFKGEVVDAVVTQVNKVGLFTEIGPMSCFISRHSIPSEMEFDPNSNPPCYKTMDEDIVIQQDDEIRLKIVGTRVDKNDIFAIGSLMDDYLGLVS

>NP_005495.2

MKDCSNGCSAECTGEGGSKEVVGTFKAKDLIVTPATILKEKPDPNNLVFGTVFTDHMLTVEWSSEFGWEKPHIKPLQNLSLHPGSSALHYAVELFEGLKAFRGVDNKIRLFQPNLNMDRMYRSAVRATLPVFDKEELLECIQQLVKLDQEWVPYSTSASLYIRPTFIGTEPSLGVKKPTKALLFVLLSPVGPYFSSGTFNPVSLWANPKYVRAWKGGTGDCKMGGNYGSSLFAQCEAVDNGCQQVLWLYGEDHQITEVGTMNLFLYWINEDGEEELATPPLDGIILPGVTRRCILDLAHQWGEFKVSERYLTMDDLTTALEGNRVREMFGSGTACVVCPVSDILYKGETIHIPTMENGPKLASRILSKLTDIQYGREESDWTIVLS

262 NP_079224.1 P40939

>NP_079224.1

MSELTKELMELVWGTKSSPGLSDTIFCRWTQGFVFSESEGSALEQFEGGPCAVIAPVQAFLLKKLLFSSEKSSWRDCSEEEQKELLCHTLCDILESACCDHSGSYCLVSWLRGKTTEETASISGSPAESSCQVEHSSALAVEELGFERFHALIQKRSFRSLPELKDAVLDQYSMWGNKFGVLLFLYSVLLTKGIENIKNEIEDASEPLIDPVYGHGSQSLINLLLTGHAVSNVWDGDRECSGMKLLGIHEQAAVGFLTLMEALRYCKVGSYLKSPKFPIWIVGSETHLTVFFAKDMALVAPEAPSEQARRVFQTYDPEDNGFIPDSLLEDVMKALDLVSDPEYINLMKNKLDPEGLGIILLGPFLQEFFPDQGSSGPESFTVYHYNGLKQSNYNEKVMYVEGTAVVMGFEDPMLQTDDTPIKRCLQTKWPYIELLWTTDRSPSLN

>P40939

MVACRAIGILSRFSAFRILRSRGYICRNFTGSSALLTRTHINYGVKGDVAVVRINSPNSKVNTLSKELHSEFSEVMNEIWASDQIRSAVLISSKPGCFIAGADINMLAACKTLQEVTQLSQEAQRIVEKLEKSTKPIVAAINGSCLGGGLEVAISCQYRIATKDRKTVLGTPEVLLGALPGAGGTQRLPKMVGVPAALDMMLTGRSIRADRAKKMGLVDQLVEPLGPGLKPPEERTIEYLEEVAITFAKGLADKKISPKRDKGLVEKLTAYAMTIPFVRQQVYKKVEEKVRKQTKGLYPAPLKIIDVVKTGIEQGSDAGYLCESQKFGELVMTKESKALMGLYHGQVLCKKNKFGAPQKDVKHLAILGAGLMGAGIAQVSVDKGLKTILKDATLTALDRGQQQVFKGLNDKVKKKALTSFERDSIFSNLTGQLDYQGFEKADMVIEAVFEDLSLKHRVLKEVEAVIPDHCIFASNTSALPISEIAAVSKRPEKVIGMHYFSPVDKMQLLEIITTEKTSKDTSASAVAVGLKQGKVIIVVKDGPGFYTTRCLAPMMSEVIRILQEGVDPKKLDSLTTSFGFPVGAATLVDEVGVDVAKHVAEDLGKVFGERFGGGNPELLTQMVSKGFLGRKSGKGFYIYQEGVKRKDLNSDMDSILASLKLPPKSEVSSDEDIQFRLVTRFVNEAVMCLQEGILATPAEGDIGAVFGLGFPPCLGGPFRFVDLYGAQKIVDRLKKYEAAYGKQFTPCQLLADHANSPNKKFYQ

263 NP_071378.1 NP_002684.1

>NP_071378.1

MEENEVESSSDAAPGPGRPEEPSESGLGVGTSEAVSADSSDAAAAPGQAEADDSGVGQSSDRGSRSQEEVSESSSSADPLPNSYLPDSSSVSHGPVAGVTGGPPALVHSSALPDPNMLVSDCTASSSDLGSAIDKIIESTIGPDLIQNCITVTSAEDGGAETTRYLILQGPDDGAPMTSPMSSSTLAHSLAAIEALADGPTSTSTCLEAQGGPSSPVQLPPASGAEEPDLQSLEAMMEVVVVQQFKCKMCQYRSSTKATLLRHMRERHFRPVAAAAAAAGKKGRLRKWSTSTKSQEEEGPEEEDDDDIVDAGAIDDLEEDSDYNPAEDEPRGRQLRLQRPTPSTPRPRRRPGRPRKLPRLEISDLPDGVEGEPLVSSQSGQSPPEPQDPEAPSSSGPGHLVAMGKVSRTPVEAGVSQSDAENAAPSCPDEHDTLPRRRGRPSRRFLGKKYRKYYYKSPKPLLRPFLCRICGSRFLSHEDLRFHVNSHEAGDPQLFKCLQCSYRSRRWSSLKEHMFNHVGSKPYKCDECSYTSVYRKDVIRHAAVHSRDRKKRPDPTPKLSSFPCPVCGRVYPMQKRLTQHMKTHSTEKPHMCDKCGKSFKKRYTFKMHLLTHIQAVANRRFKCEFCEFVCEDKKALLNHQLSHVSDKPFKCSFCPYRTFREDFLLSHVAVKHTGAKPFACEYCHFSTRHKKNLRLHVRCRHASSFEEWGRRHPEEPPSRRRPFFSLQQIEELKQQHSAAPGPPPSSPGPPEIPPEATTFQSSEAPSLLCSDTLGGATIIYQQGAEESTAMATQTALDLLLNMSAQRELGGTALQVAVVKSEDVEAGLASPGGQPSPEGATPQVVTLHVAEPGGGAAAESQLGPPDLPQITLAPGPFGGTGYSVITAPPMEEGTSAPGTPYSEEPAGEAAQAVVVSDTLKEAGTHYIMATDGTQLHHIELTADGSISFPSPDALASGAKWPLLQCGGLPRDGPEPPSPAKTHCVGDSQSSASSPPATSKALGLAVPPSPPSAATAASKKFSCKICAEAFPGRAEMESHKRAHAGPGAFKCPDCPFSARQWPEVRAHMAQHSSLRPHQCSQCSFASKNKKDLRRHMLTHTKEKPFACHLCGQRFNRNGHLKFHIQRLHSPDGRKSGTPTARAPTQTPTQTIILNSDDETLATLHTALQSSHGVLGPERLQQALSQEHIIVAQEQTVTNQEEAAYIQEITTADGQTVQHLVTSDNQVQYIISQDGVQHLLPQEYVVVPEGHHIQVQEGQITHIQYEQGAPFLQESQIQYVPVSPGQQLVTQAQLEAAAHSAVTAVADAAMAQAQGLFGTDETVPEHIQQLQHQGIEYDVITLADD

>NP_002684.1

MSRLLWRKVAGATVGPGPVPAPGRWVSSSVPASDPSDGQRRRQQQQQQQQQQQQQPQQPQVLSSEGGQLRHNPLDIQMLSRGLHEQIFGQGGEMPGEAAVRRSVEHLQKHGLWGQPAVPLPDVELRLPPLYGDNLDQHFRLLAQKQSLPYLEAANLLLQAQLPPKPPAWAWAEGWTRYGPEGEAVPVAIPEERALVFDVEVCLAEGTCPTLAVAISPSAWYSWCSQRLVEERYSWTSQLSPADLIPLEVPTGASSPTQRDWQEQLVVGHNVSFDRAHIREQYLIQGSRMRFLDTMSMHMAISGLSSFQRSLWIAAKQGKHKVQPPTKQGQKSQRKARRGPAISSWDWLDISSVNSLAEVHRLYVGGPPLEKEPRELFVKGTMKDIRENFQDLMQYCAQDVWATHEVFQQQLPLFLERCPHPVTLAGMLEMGVSYLPVNQNWERYLAEAQGTYEELQREMKKSLMDLANDACQLLSGERYKEDPWLWDLEWDLQEFKQKKAKKVKKEPATASKLPIEGAGAPGDPMDQEDLGPCSEEEEFQQDVMARACLQKLKGTTELLPKRPQHLPGHPGWYRKLCPRLDDPAWTPGPSLLSLQMRVTPKLMALTWDGFPLHYSERHGWGYLVPGRRDNLAKLPTGTTLESAGVVCPYRAIESLYRKHCLEQGKQQLMPQEAGLAEEFLLTDNSAIWQTVEELDYLEVEAEAKMENLRAAVPGQPLALTARGGPKDTQPSYHHGNGPYNDVDIPGCWFFKLPHKDGNSCNVGSPFAKDFLPKMEDGTLQAGPGGASGPRALEINKMISFWRNAHKRISSQMVVWLPRSALPRAVIRHPDYDEEGLYGAILPQVVTAGTITRRAVEPTWLTASNARPDRVGSELKAMVQAPPGYTLVGADVDSQELWIAAVLGDAHFAGMHGCTAFGWMTLQGRKSRGTDLHSKTATTVGISREHAKIFNYGRIYGAGQPFAERLLMQFNHRLTQQEAAEKAQQMYAATKGLRWYRLSDEGEWLVRELNLPVDRTEGGWISLQDLRKVQRETARKSQWKKWEVVAERAWKGGTESEMFNKLESIATSDIPRTPVLGCCISRALEPSAVQEEFMTSRVNWVVQSSAVDYLHLMLVAMKWLFEEFAIDGRFCISIHDEVRYLVREEDRYRAALALQITNLLTRCMFAYKLGLNDLPQSVAFFSAVDIDRCLRKEVTMDCKTPSNPTGMERRYGIPQGEALDIYQIIELTKGSLEKRSQPGP

264 Q15562 Q9NP74

>Q15562

MGEPRAGAALDDGSGWTGSEEGSEEGTGGSEGAGGDGGPDAEGVWSPDIEQSFQEALAIYPPCGRRKIILSDEGKMYGRNELIARYIKLRTGKTRTRKQVSSHIQVLARRKSREIQSKLKDQVSKDKAFQTMATMSSAQLISAPSLQAKLGPTGPQASELFQFWSGGSGPPWNVPDVKPFSQTPFTLSLTPPSTDLPGYEPPQALSPLPPPTPSPPAWQARGLGTARLQLVEFSAFVEPPDAVDSYQRHLFVHISQHCPSPGAPPLESVDVRQIYDKFPEKKGGLRELYDRGPPHAFFLVKFWADLNWGPSGEEAGAGGSISSGGFYGVSSQYESLEHMTLTCSSKVCSFGKQVVEKVETERAQLEDGRFVYRLLRSPMCEYLVNFLHKLRQLPERYMMNSVLENFTILQVVTNRDTQELLLCTAYVFEVSTSERGAQHHIYRLVRD

>Q9NP74

MEEAELVKGRLQAITDKRKIQEEISQKRLKIEEDKLKHQHLKKKALREKWLLDGISSGKEQEEMKKQNQQDQHQIQVLEQSILRLEKEIQDLEKAELQISTKEEAILKKLKSIERTTEDIIRSVKVEREERAEESIEDIYANIPDLPKSYIPSRLRKEINEEKEDDEQNRKALYAMEIKVEKDLKTGESTVLSSIPLPSDDFKGTGIKVYDDGQKSVYAVSSNHSAAYNGTDGLAPVEVEELLRQASERNSKSPTEYHEPVYANPFYRPTTPQRETVTPGPNFQERIKIKTNGLGIGVNESIHNMGNGLSEERGNNFNHISPIPPVPHPRSVIQQAEEKLHTPQKRLMTPWEESNVMQDKDAPSPKPRLSPRETIFGKSEHQNSSPTCQEDEEDVRYNIVHSLPPDINDTEPVTMIFMGYQQAEDSEEDKKFLTGYDGIIHAELVVIDDEEEEDEGEAEKPSYHPIAPHSQVYQPAKPTPLPRKRSEASPHENTNHKSPHKNSISLKEQEESLGSPVHHSPFDAQTTGDGTEDPSLTALRMRMAKLGKKVI

265 NP_003073.1 NP_001880.2

>NP_003073.1

MGTPPGLQTDCEALLSRFQETDSVRFEDFTELWRNMKFGTIFCGRMRNLEKNMFTKEALALAWRYFLPPYTFQIRVGALYLLYGLYNTQLCQPKQKIRVALKDWDEVLKFQQDLVNAQHFDAAYIFRKLRLDRAFHFTAMPKLLSYRMKKKIHRAEVTEEFKDPSDRVMKLITSDVLEEMLNVHDHYQNMKHVISVDKSKPDKALSLIKDDFFDNIKNIVLEHQQWHKDRKNPSLKSKTNDGEEKMEGNSQETERCERAESLAKIKSKAFSVVIQASKSRRHRQVKLDSSDSDSASGQGQVKATRKKEKKERLKPAGRKMSLRNKGNVQNIHKEDKPLSLSMPVITEEEENESLSGTEFTASKKRRKH

>NP_001880.2

MATGQKLMRAVRVFEFGGPEVLKLRSDIAVPIPKDHQVLIKVHACGVNPVETYIRSGTYSRKPLLPYTPGSDVAGVIEAVGDNASAFKKGDRVFTSSTISGGYAEYALAADHTVYKLPEKLDFKQGAAIGIPYFTAYRALIHSACVKAGESVLVHGASGGVGLAACQIARAYGLKILGTAGTEEGQKIVLQNGAHEVFNHREVNYIDKIKKYVGEKGIDIIIEMLANVNLSKDLSLLSHGGRVIVVGSRGTIEINPRDTMAKESSIIGVTLFSSTKEEFQQYAAALQAGMEIGWLKPVIGSQYPLEKVAEAHENIIHGSGATGKMILLL

266 Q5XKR4 NP_071906.1

>Q5XKR4

MLSHADLLDARLGMKDAAELLGHREAVKCRLGVGGSDPGGHPGDLAPNSDPVEGATLLPGEDITTVGSTPASLAVSAKDPDKQPGPQGGPNPSQAGQQQGQQKQKRHRTRFTPAQLNELERSFAKTHYPDIFMREELALRIGLTESRVQVWFQNRRAKWKKRKKTTNVFRAPGTLLPTPGLPQFPSAAAAAAAAMGDSLCSFHANDTRWAAAAMPGVSQLPLPPALGRQQAMAQSLSQCSLAAGPPPNSMGLSNSLAGSNGAGLQSHLYQPAFPGMVPASLPGPSNVSGSPQLCSSPDSSDVWRGTSIASLRRKALEHTVSMSFT

>NP_071906.1

MDALVEDDICILNHEKAHKRDTVTPVSIYSGDESVASHFALVTAYEDIKKRLKDSEKENSLLKKRIRFLEEKLIARFEEETSSVGREQVNKAYHAYREVCIDRDNLKSKLDKMNKDNSESLKVLNEQLQSKEVELLQLRTEVETQQVMRNLNPPSSNWEVEKLSCDLKIHGLEQELELMRKECSDLKIELQKAKQTDPYQEDNLKSRDLQKLSISSDNMQHAYWELKREMSNLHLVTQVQAELLRKLKTSTAIKKACAPVGCSEDLGRDSTKLHLMNFTATYTRHPPLLPNGKALCHTTSSPLPGDVKVLSEKAILQSWTDNERSIPNDGTCFQEHSSYGRNSLEDNSWVFPSPPKSSETAFGETKTKTLPLPNLPPLHYLDQHNQNCLYKN

267 P13284 NP_038203.2

>P13284

MDSRHTFAPAAMTLSPLLLFLPPLLLLLDVPTAAVQASPLQALDFFGNGPPVNYKTGNLYLRGPLKKSNAPLVNVTLYYEALCGGCRAFLIRELFPTWLLVMEILNVTLVPYGNAQEQNVSGRWEFKCQHGEEECKFNKVEACVLDELDMELAFLTIVCMEEFEDMERSLPLCLQLYAPGLSPDTIMECAMGDRGMQLMHANAQRTDALQPPHEYVPWVTVNGKPLEDQTQLLTLVCQLYQGKKPDVCPSSTSSLRSVCFK

>NP_038203.2

MLQQVPENINFPAEEEKILEFWTEFNCFQECLKQSKHKPKFTFYDGPPFATGLPHYGHILAGTIKDIVTRYAHQSGFHVDRRFGWDCHGLPVEYEIDKTLGIRGPEDVAKMGITEYNNQCRAIVMRYSAEWKSTVSRLGRWIDFDNDYKTLYPQFMESVWWVFKQLYDKGLVYRGVKVMPFSTACNTPLSNFESHQNYKDVQDPSVFVTFPLEEDETVSLVAWTTTPWTLPSNLAVCVNPEMQYVKIKDVARGRLLILMEARLSALYKLESDYEILERFPGAYLKGKKYRPLFDYFLKCKENGAFTVLVDNYVKEEEGTGVVHQAPYFGAEDYRVCMDFNIIRKDSLPVCPVDASGCFTTEVTDFAGQYVKDADKSIIRTLKEQGRLLVATTFTHSYPFCWRSDTPLIYKAVPSWFVRVENMVDQLLRNNDLCYWVPELVREKRFGNWLKDARDWTISRNRYWGTPIPLWVSDDFEEVVCIGSVAELEELSGAKISDLHRESVDHLTIPSRCGKGSLHRISEVFDCWFESGSMPYAQVHYPFENKREFEDAFPADFIAEGIDQTRGWFYTLLVLATALFGQPPFKNVIVNGLVLASDGQKMSKRKKNYPDPVSIIQKYGADALRLYLINSPVVRAENLRFKEEGVRDVLKDVLLPWYNAYRFLIQNVLRLQKEEEIEFLYNENTVRESPNITDRWILSFMQSLIGFFETEMAAYRLYTVVPRLVKFVDILTNWYVRMNRRRLKGENGMEDCVMALETLFSVLLSLCRLMAPYTPFLTELMYQNLKVLIDPVSVQDKDTLSIHYLMLPRVREELIDKKTESAVSQMQSVIELGRVIRDRKTIPIKYPLKEIVVIHQDPEALKDIKSLEKYIIEELNVRKVTLSTDKNKYGIRLRAEPDHMVLGKRLKGAFKAVMTSIKQLSSEELEQFQKTGTIVVEGHELHDEDIRLMYTFDQATGGTAQFEAHSDAQALVLLDVTPDQSMVDEGMAREVINRIQKLRKKCNLVPTDEITVYYKAKSEGTYLNSVIESHTEFIFTTIKAPLKPYPVSPSDKVLIQEKTQLKGSELEITLTRGSSLPGPACAYVNLNICANGSEQGGVLLLENPKGDNRLDLLKLKSVVTSIFGVKNTELAVFHDETEIQNQTDLLSLSGKTLCVTAGSAPSLINSSSTLLCQYINLQLLNAKPQECLMGTVGTLLLENPLGQNGLTHQGLLYEAAKVFGLRSRKLKLFLNETQTQEITEDIPVKTLNMKTVYVSVLPTTADF

268 NP_006070.2 NP_004763.1

>NP_006070.2

MADHMMAMNHGRFPDGTNGLHHHPAHRMGMGQFPSPHHHQQQQPQHAFNALMGEHIHYGAGNMNATSGIRHAMGPGTVNGGHPPSALAPAARFNNSQFMGPPVASQGGSLPASMQLQKLNNQYFNHHPYPHNHYMPDLHPAAGHQMNGTNQHFRDCNPKHSGGSSTPGGSGGSSTPGGSGSSSGGGAGSSNSGGGSGSGNMPASVAHVPAAMLPPNVIDTDFIDEEVLMSLVIEMGLDRIKELPELWLGQNEFDFMTDFVCKQQPSRVSC

>NP_004763.1

MVYYPELFVWVSQEPFPNKDMEGRLPKGRLPVPKEVNRKKNDETNAASLTPLGSSELRSPRISYLHFF

269 O00462 Q9BYC8

>O00462

MRLHLLLLLALCGAGTTAAELSYSLRGNWSICNGNGSLELPGAVPGCVHSALFQQGLIQDSYYRFNDLNYRWVSLDNWTYSKEFKIPFEISKWQKVNLILEGVDTVSKILFNEVTIGETDNMFNRYSFDITNVVRDVNSIELRFQSAVLYAAQQSKAHTRYQVPPDCPPLVQKGECHVNFVRKEQCSFSWDWGPSFPTQGIWKDVRIEAYNICHLNYFTFSPIYDKSAQEWNLEIESTFDVVSSKPVGGQVIVAIPKLQTQQTYSIELQPGKRIVELFVNISKNITVETWWPHGHGNQTGYNMTVLFELDGGLNIEKSAKVYFRTVELIEEPIKGSPGLSFYFKINGFPIFLKGSNWIPADSFQDRVTSELLRLLLQSVVDANMNTLRVWGGGIYEQDEFYELCDELGIMVWQDFMFACALYPTDQGFLDSVTAEVAYQIKRLKSHPSIIIWSGNNENEEALMMNWYHISFTDRPIYIKDYVTLYVKNIRELVLAGDKSRPFITSSPTNGAETVAEAWVSQNPNSNYFGDVHFYDYISDCWNWKVFPKARFASEYGYQSWPSFSTLEKVSSTEDWSFNSKFSLHRQHHEGGNKQMLYQAGLHFKLPQSTDPLRTFKDTIYLTQVMQAQCVKTETEFYRRSRSEIVDQQGHTMGALYWQLNDIWQAPSWASLEYGGKWKMLHYFAQNFFAPLLPVGFENENTFYIYGVSDLHSDYSMTLSVRVHTWSSLEPVCSRVTERFVMKGGEAVCLYEEPVSELLRRCGNCTRESCVVSFYLSADHELLSPTNYHFLSSPKEAVGLCKAQITAIISQQGDIFVFDLETSAVAPFVWLDVGSIPGRFSDNGFLMTEKTRTILFYPWEPTSKNELEQSFHVTSLTDIY

>Q9BYC8

MALAMLVLVVSPWSAARGVLRNYWERLLRKLPQSRPGFPSPPWGPALAVQGPAMFTEPANDTSGSKENSSLLDSIFWMAAPKNRRTIEVNRCRRRNPQKLIKVKNNIDVCPECGHLKQKHVLCAYCYEKVCKETAEIRRQIGKQEGGPFKAPTIETVVLYTGETPSEQDQGKRIIERDRKRPSWFTQN

270 NP_002403.1 NP_036337.1

>NP_002403.1

MDKDCEMKRTTLDSPLGKLELSGCEQGLHEIKLLGKGTSAADAVEVPAPAAVLGGPEPLMQCTAWLNAYFHQPEAIEEFPVPALHHPVFQQESFTRQVLWKLLKVVKFGEVISYQQLAALAGNPKAARAVGGAMRGNPVPILIPCHRVVCSSGAVGNYSGGLAVKEWLLAHEGHRLGKPGLGGSSGLAGAWLKGAGATSGSPPAGRN

>NP_036337.1

MERRLGVRAWVKENRGSFQPPVCNKLMHQEQLKVMFVGGPNTRKDYHIEEGEEVFYQLEGDMVLRVLEQGKHRDVVIRQGEIFLLPARVPHSPQRFANTVGLVVERRRLETELDGLRYYVGDTMDVLFEKWFYCKDLGTQLAPIIQEFFSSEQYRTGKPIPDQLLKEPPFPLSTRSIMEPMSLDAWLDSHHRELQAGTPLSLFGDTYETQVIAYGQGSSEGLRQNVDVWLWQLEGSSVVTMGGRRLSLAPDDSLLVLAGTSYAWERTQGSVALSVTQDPACKKPLG

271 NP_054753.1 P83881

>NP_054753.1

MAKVQVNNVVVLDNPSPFYNPFQFEITFECIEDLSEDLEWKIIYVGSAESEEYDQVLDSVLVGPVPAGRHMFVFQADAPNPGLIPDADAVGVTVVLITCTYRGQEFIRVGYYVNNEYTETELRENPPVKPDFSKLQRNILASNPRVTRFHINWEDNTEKLEDAESSNPNLQSLLSTDALPSASKGWSTSENSLNVMLESHMDCM

>P83881

MVNVPKTRRTFCKKCGKHQPHKVTQYKKGKDSLYAQGKRRYDRKQSGYGGQTKPIFRKKAKTTKKIVLRLECVEPNCRSKRMLAIKRCKHFELGGDKKRKGQVIQF

272 NP_055586.1 NP_036361.1

>NP_055586.1

MSGPGNKRAAGDGGSGPPEKKLSREEKTTTTLIEPIRLGGISSTEEMDLKVLQFKNKKLAERLEQRQACEDELRERIEKLEKRQATDDATLLIVNRYWAQLDETVEALLRCHESQGELSSAPEAPGTQEGPTCDGTPLPEPGTSELRDPLLMQLRPPLSEPALAFVVALGASSSEEVELELQGRMEFSKAAVSRVVEASDRLQRRVEELCQRVYSRGDSEPLSEAAQAHTRELGRENRRLQDLATQLQEKHHRISLEYSELQDKVTSAETKVLEMETTVEDLQWDIEKLRKREQKLNKHLAEALEQLNSGYYVSGSSSGFQGGQITLSMQKFEMLNAELEENQELANSRMAELEKLQAELQGAVRTNERLKVALRSLPEEVVRETGEYRMLQAQFSLLYNESLQVKTQLDEARGLLLATKNSHLRHIEHMESDELGLQKKLRTEVIQLEDTLAQVRKEYEMLRIEFEQNLAANEQAGPINREMRHLISSLQNHNHQLKGDAQRYKRKLREVQAEIGKLRAQASGSAHSTPNLGHPEDSGVSAPAPGKEEGGPGPVSTPDNRKEMAPVPGTTTTTTSVKKEELVPSEEDFQGITPGAQGPSSRGREPEARPKRELREREGPSLGPPPVASALSRADREKAKVEETKRKESELLKGLRAELKKAQESQKEMKLLLDMYKSAPKEQRDKVQLMAAERKAKAEVDELRSRIRELEERDRRESKKIADEDALRRIRQAEEQIEHLQRKLGATKQEEEALLSEMDVTGQAFEDMQEQNGRLLQQLREKDDANFKLMSERIKANQIHKLLREEKDELGEQVLGLKSQVDAQLLTVQKLEEKERALQGSLGGVEKELTLRSQALELNKRKAVEAAQLAEDLKVQLEHVQTRLREIQPCLAESRAAREKESFNLKRAQEDISRLRRKLEKQRKVEVYADADEILQEEIKEYKARLTCPCCNTRKKDAVLTKCFHVFCFECVRGRYEARQRKCPKCNAAFGAHDFHRIYIS

>NP_036361.1

MSTSWSDRLQNAADMPANMDKHALKKYRREAYHRVFVNRSLAMEKIKCFGFDMDYTLAVYKSPEYESLGFELTVERLVSIGYPQELLSFAYDSTFPTRGLVFDTLYGNLLKVDAYGNLLVCAHGFNFIRGPETREQYPNKFIQRDDTERFYILNTLFNLPETYLLACLVDFFTNCPRYTSCETGFKDGDLFMSYRSMFQDVRDAVDWVHYKGSLKEKTVENLEKYVVKDGKLPLLLSRMKEVGKVFLATNSDYKYTDKIMTYLFDFPHGPKPGSSHRPWQSYFDLILVDARKPLFFGEGTVLRQVDTKTGKLKIGTYTGPLQHGIVYSGGSSDTICDLLGAKGKDILYIGDHIFGDILKSKKRQGWRTFLVIPELAQELHVWTDKSSLFEELQSLDIFLAELYKHLDSSSNERPDISSIQRRIKKVTHDMDMCYGMMGSLFRSGSRQTLFASQVMRYADLYAASFINLLYYPFSYLFRAAHVLMPHESTVEHTHVDINEMESPLATRNRTSVDFKDTDYKRHQLTRSISEIKPPNLFPLAPQEITHCHDEDDDEEEEEEEE

273 NP_002303.2 P49753

>NP_002303.2

MAASQTSQTVASHVPFADLCSTLERIQKSKGRAEKIRHFREFLDSWRKFHDALHKNHKDVTDSFYPAMRLILPQLERERMAYGIKETMLAKLYIELLNLPRDGKDALKLLNYRTPTGTHGDAGDFAMIAYFVLKPRCLQKGSLTIQQVNDLLDSIASNNSAKRKDLIKKSLLQLITQSSALEQKWLIRMIIKDLKLGVSQQTIFSVFHNDAAELHNVTTDLEKVCRQLHDPSVGLSDISITLFSAFKPMLAAIADIEHIEKDMKHQSFYIETKLDGERMQMHKDGDVYKYFSRNGYNYTDQFGASPTEGSLTPFIHNAFKADIQICILDGEMMAYNPNTQTFMQKGTKFDIKRMVEDSDLQTCYCVFDVLMVNNKKLGHETLRKRYEILSSIFTPIPGRIEIVQKTQAHTKNEVIDALNEAIDKREEGIMVKQPLSIYKPDKRGEGWLKIKPEYVSGLMDELDILIVGGYWGKGSRGGMMSHFLCAVAEKPPPGEKPSVFHTLSRVGSGCTMKELYDLGLKLAKYWKPFHRKAPPSSILCGTEKPEVYIEPCNSVIVQIKAAEIVPSDMYKTGCTLRFPRIEKIRDDKEWHECMTLDDLEQLRGKASGKLASKHLYIGGDDEPQEKKRKAAPKMKKVIGIIEHLKAPNLTNVNKISNIFEDVEFCVMSGTDSQPKPDLENRIAEFGGYIVQNPGPDTYCVIAGSENIRVKNIILSNKHDVVKPAWLLECFKTKSFVPWQPRFMIHMCPSTKEHFAREYDCYGDSYFIDTDLNQLKEVFSGIKNSNEQTPEEMASLIADLEYRYSWDCSPLSMFRRHTVYLDSYAVINDLSTKNEGTRLAIKALELRFHGAKVVSCLAEGVSHVIIGEDHSRVADFKAFRRTFKRKFKILKESWVTDSIDKCELQEENQYLI

>P49753

MSNKLLSPHPHSVVLRSEFKMASSPAVLRASRLYQWSLKSSAQFLGSPQLRQVGQIIRVPARMAATLILEPAGRCCWDEPVRIAVRGLAPEQPVTLRASLRDEKGALFQAHARYRADTLGELDLERAPALGGSFAGLEPMGLLWALEPEKPLVRLVKRDVRTPLAVELEVLDGHDPDPGRLLCQTRHERYFLPPGVRREPVRVGRVRGTLFLPPEPGPFPGIVDMFGTGGGLLEYRASLLAGKGFAVMALAYYNYEDLPKTMETLHLEYFEEAMNYLLSHPEVKGPGVGLLGISKGGELCLSMASFLKGITAAVVINGSVANVGGTLRYKGETLPPVGVNRNRIKVTKDGYADIVDVLNSPLEGPDQKSFIPVERAESTFLFLVGQDDHNWKSEFYANEACKRLQAHGRRKPQIICYPETGHYIEPPYFPLCRASLHALVGSPIIWGGEPRAHAMAQVDAWKQLQTFFHKHLGGHEGTIPSKV

274 Q9BWT1 Q9P2W6

>Q9BWT1

MDARRVPQKDLRVKKNLKKFRYVKLISMETSSSSDDSCDSFASDNFANTRLQSVREGCRTRSQCRHSGPLRVAMKFPARSTRGATNKKAESRQPSENSVTDSNSDSEDESGMNFLEKRALNIKQNKAMLAKLMSELESFPGSFRGRHPLPGSDSQSRRPRRRTFPGVASRRNPERRARPLTRSRSRILGSLDALPMEEEEEEDKYMLVRKRKTVDGYMNEDDLPRSRRSRSSVTLPHIIRPVEEITEEELENVCSNSREKIYNRSLGSTCHQCRQKTIDTKTNCRNPDCWGVRGQFCGPCLRNRYGEEVRDALLDPNWHCPPCRGICNCSFCRQRDGRCATGVLVYLAKYHGFGNVHAYLKSLKQEFEMQA

>Q9P2W6

MGRTWCGMWRRRRPGRRSAVPRWPHLSSQSGVEPPDRWTGTPGWPSRDQEAPGSMMPPAAAQPSAHGALVPPATAHEPVDHPALHWLACCCCLSLPGQLPLAIRLGWDLDLEAGPSSGKLCPRARRWQPLPS

275 NP_055048.1 Q14894

>NP_055048.1

MNGEADCPTDLEMAAPKGQDRWSQEDMLTLLECMKNNLPSNDSSKFKTTESHMDWEKVAFKDFSGDMCKLKWVEISNEVRKFRTLTELILDAQEHVKNPYKGKKLKKHPDFPKKPLTPYFRFFMEKRAKYAKLHPEMSNLDLTKILSKKYKELPEKKKMKYIQDFQREKQEFERNLARFREDHPDLIQNAKKSDIPEKPKTPQQLWYTHEKKVYLKVRPDATTKEVKDSLGKQWSQLSDKKRLKWIHKALEQRKEYEEIMRDYIQKHPELNISEEGITKSTLTKAERQLKDKFDGRPTKPPPNSYSLYCAELMANMKDVPSTERMVLCSQQWKLLSQKEKDAYHKKCDQKKKDYEVELLRFLESLPEEEQQRVLGEEKMLNINKKQATSPASKKPAQEGGKGGSEKPKRPVSAMFIFSEEKRRQLQEERPELSESELTRLLARMWNDLSEKKKAKYKAREAALKAQSERKPGGEREERGKLPESPKRAEEIWQQSVIGDYLARFKNDRVKALKAMEMTWNNMEKKEKLMWIKKAAEDQKRYERELSEMRAPPAATNSSKKMKFQGEPKKPPMNGYQKFSQELLSNGELNHLPLKERMVEIGSRWQRISQSQKEHYKKLAEEQQKQYKVHLDLWVKSLSPQDRAAYKEYISNKRKSMTKLRGPNPKSSRTTLQSKSESEEDDEEDEDDEDEDEEEEDDENGDSSEDGGDSSESSSEDESEDGDENEEDDEDEDDDEDDDEDEDNESEGSSSSSSSSGDSSDSDSN

>Q14894

MSRVPAFLSAAEVEEHLRSSSLLIPPLETALANFSSGPEGGVMQPVRTVVPVTKHRGYLGVMPAYSAAEDALTTKLVTFYEDRGITSVVPSHQATVLLFEPSNGTLLAVMDGNVITAKRTAAVSAIATKFLKPPSSEVLCILGAGVQAYSHYEIFTEQFSFKEVRIWNRTKENAEKFADTVQGEVRVCSSVQEAVAGADVIITVTLATEPILFGEWVKPGAHINAVGASRPDWRELDDELMKEAVLYVDSQEAALKESGDVLLSGAEIFAELGEVIKGVKPAHCEKTTVFKSLGMAVEDTVAAKLIYDSWSSGK

276 Q9BSH3 O00764

>Q9BSH3

MSRVLVPCHVKGSVALQVGDVRTSQGRPGVLVIDVTFPSVAPFELQEITFKNYYTAFLSIRVRQYTSAHTPAKWVTCLRDYCLMPDPHSEEGAQEYVSLFKHQMLCDMARISELRLILRQPSPLWLSFTVEELQIYQQGPKSPSVTFPKWLSHPVPCEQPALLREGLPDPSRVSSEVQQMWALTEMIRASHTSARIGRFDVDGCYDLNLLSYT

>O00764

MEEECRVLSIQSHVIRGYVGNRAATFPLQVLGFEIDAVNSVQFSNHTGYAHWKGQVLNSDELQELYEGLRLNNMNKYDYVLTGYTRDKSFLAMVVDIVQELKQQNPRLVYVCDPVLGDKWDGEGSMYVPEDLLPVYKEKVVPLADIITPNQFEAELLSGRKIHSQEEALRVMDMLHSMGPDTVVITSSDLPSPQGSNYLIVLGSQRRRNPAGSVVMERIRMDIRKVDAVFVGTGDLFAAMLLAWTHKHPNNLKVACEKTVSTLHHVLQRTIQCAKAQAGEGVRPSPMQLELRMVQSKRDIEDPEIVVQATVL

277 NP_109377.1 P08319

>NP_109377.1

MQKATYYDSSAIYGGYPYQAANGFAYNANQQPYPASAALGADGEYHRPACSLQSPSSAGGHPKAHELSEACLRTLSAPPSQPPSLGEPPLHPPPPQAAPPAPQPPQPAPQPPAPTPAAPPPPSSASPPQNASNNPTPANAAKSPLLNSPTVAKQIFPWMKESRQNTKQKTSSSSSGESCAGDKSPPGQASSKRARTAYTSAQLVELEKEFHFNRYLCRPRRVEMANLLNLTERQIKIWFQNRRMKYKKDQKGKGMLTSSGGQSPSRSPVPPGAGGYLNSMHSLVNSVPYEPQSPPPFSKPPQGTYGLPPASYPASLPSCAPPPPPQKRYTAAGAGAGGTPDYDPHAHGLQGNGSYGTPHIQGSPVFVGGSYVEPMSNSGPALFGLTHLPHAASGAMDYGGAGPLGSGHHHGPGPGEPHPTYTDLTGHHPSQGRIQEAPKLTHL

>P08319

MGTKGKVIKCKAAIAWEAGKPLCIEEVEVAPPKAHEVRIQIIATSLCHTDATVIDSKFEGLAFPVIVGHEAAGIVESIGPGVTNVKPGDKVIPLYAPLCRKCKFCLSPLTNLCGKISNLKSPASDQQLMEDKTSRFTCKGKPVYHFFGTSTFSQYTVVSDINLAKIDDDANLERVCLLGCGFSTGYGAAINNAKVTPGSTCAVFGLGGVGLSAVMGCKAAGASRIIGIDINSEKFVKAKALGATDCLNPRDLHKPIQEVIIELTKGGVDFALDCAGGSETMKAALDCTTAGWGSCTFIGVAAGSKGLTVFPEELIIGRTINGTFFGGWKSVDSIPKLVTDYKNKKFNLDALVTHTLPFDKISEAFDLMNQGKSIRTILIF

278 Q96KF2 Q9NYK5

>Q96KF2

MLCAHFSDQGPAHLTTSKSAFLSNKKTSTLKHLLGETRSDGSACNSGISGGRGRKIP

>Q9NYK5

MEALAMGSRALRLWLVAPGGGIKWRFIATSPASQLSPTELTEMRNDLFNKEKARQLSLTPRTEKIEVKHVGKTDPGTVFVMNKNISTPYSCAMHLSEWYCRKSILALVDGQPWDMYKPLTKSCEIKFLTFKDCDPGEVNKAYWRSCAMMMGCVIERAFKDEYMVNLVRAPEVPVISGAFCYDVVLDSKLDEWMPTKENLRSFTKDAHALIYKDLPFETLEVEAKVALEIFQHSKYKVDFIEEKASQNPERIVKLHRIGDFIDVSEGPLIPRTSICFQYEVSAVHNLQPTQPSLIRRFQGVSLPVHLRAHFTIWDKLLERSRKMVTEDQSKATEECTST

279 NP_114432.1 P51970

>NP_114432.1

MSSMWSEYTIGGVKIYFPYKAYPSQLAMMNSILRGLNSKQHCLLESPTGSGKSLALLCSALAWQQSLSGKPADEGVSEKAEVQLSCCCACHSKDFTNNDMNQGTSRHFNYPSTPPSERNGTSSTCQDSPEKTTLAAKLSAKKQASIYRDENDDFQVEKKRIRPLETTQQIRKRHCFGTEVHNLDAKVDSGKTVKLNSPLEKINSFSPQKPPGHCSRCCCSTKQGNSQESSNTIKKDHTGKSKIPKIYFGTRTHKQIAQITRELRRTAYSGVPMTILSSRDHTCVHPEVVGNFNRNEKCMELLDGKNGKSCYFYHGVHKISDQHTLQTFQGMCKAWDIEELVSLGKKLKACPYYTARELIQDADIIFCPYNYLLDAQIRESMDLNLKEQVVILDEAHNIEDCARESASYSVTEVQLRFARDELDSMVNNNIRKKDHEPLRAVCCSLINWLEANAEYLVERDYESACKIWSGNEMLLTLHKMGITTATFPILQGHFSAVLQKEEKISPIYGKEEAREVPVISASTQIMLKGLFMVLDYLFRQNSRFADDYKIAIQQTYSWTNQIDISDKNGLLVLPKNKKRSRQKTAVHVLNFWCLNPAVAFSDINGKVQTIVLTSGTLSPMKSFSSELGVTFTIQLEANHIIKNSQVWVGTIGSGPKGRNLCATFQNTETFEFQDEVGALLLSVCQTVSQGILCFLPSYKLLEKLKERWLSTGLWHNLELVKTVIVEPQGGEKTNFDELLQVYYDAIKYKGEKDGALLVAVCRGKVSEGLDFSDDNARAVITIGIPFPNVKDLQVELKRQYNDHHSKLRGLLPGRQWYEIQAYRALNQALGRCIRHRNDWGALILVDDRFRNNPSRYISGLSKWVRQQIQHHSTFESALESLAEFSKKHQKVLNVSIKDRTNIQDNESTLEVTSLKYSTPPYLLEAASHLSPENFVEDEAKICVQELQCPKIITKNSPLPSSIISRKEKNDPVFLEEAGKAEKIVISRSTSPTFNKQTKRVSWSSFNSLGQYFTGKIPKATPELGSSENSASSPPRFKTEKMESKTVLPFTDKCESSNLTVNTSFGSCPQSETIISSLKIDATLTRKNHSEHPLCSEEALDPDIELSLVSEEDKQSTSNRDFETEAEDESIYFTPELYDPEDTDEEKNDLAETDRGNRLANNSDCILAKDLFEIRTIKEVDSAREVKAEDCIDTKLNGILHIEESKIDDIDGNVKTTWINELELGKTHEIEIKNFKPSPSKNKGMFPGFK

>P51970

MPGIVELPTLEELKVDEVKISSAVLKAAAHHYGAQCDKPNKEFMLCRWEEKDPRRCLEEGKLVNKCALDFFRQIKRHCAEPFTEYWTCIDYTGQQLFRHCRKQQAKFDECVLDKLGWVRPDLGELSKVTKVKTDRPLPENPYHSRPRPDPSPEIEGDLQPATHGSRFYFWTK

280 AAA93070.1 Q9H2W6

>AAA93070.1

MSGCRVFIGRLNPAAREKDVERFFKGYGRIRDIDLKRGFGFVEFEDPRDADDAVYELDGKELCSERVTIEHARARSRGGRGRGRYSDRFSSRRPRNDRRNAPPVRTENRLIVENLSSRVSWQDLKDFMRQAGEVTFADAHRPKLNEGVVEFASYGDLKNAIEKLSGKEINGRKIKLIEGSKRHSRSRSRSRSRTRSSSRSRSRSRSRSRKSYSRSRSRSRSRSRSKSRSVSRSPVPEKSQKRGSSSRSKSPASVDRQRSRSRSRSRSVDSGN

>Q9H2W6

MAAPVRRTLLGVAGGWRRFERLWAGSLSSRSLALAAAPSSNGSPWRLLGALCLQRPPVVSKPLTPLQEEMASLLQQIEIERSLYSDHELRALDENQRLAKKKADLHDEEDEQDILLAQDLEDMWEQKFLQFKLGARITEADEKNDRTSLNRKLDRNLVLLVREKFGDQDVWILPQAEWQPGETLRGTAERTLATLSENNMEAKFLGNAPCGHYTFKFPQAMRTESNLGAKVFFFKALLLTGDFSQAGNKGHHVWVTKDELGDYLKPKYLAQVRRFVSDL

281 NP_004483.1 NP_001995.1

>NP_004483.1

MAYQLYRNTTLGNSLQESLDELIQSQQITPQLALQVLLQFDKAINAALAQRVRNRVNFRGSLNTYRFCDNVWTFVLNDVEFREVTELIKVDKVKIVACDGKNTGSNTTE

>NP_001995.1

MPLSRWLRSVGVFLLPAPYWAPRERWLGSLRRPSLVHGYPVLAWHSARCWCQAWTEEPRALCSSLRMNGDQNSDVYAQEKQDFVQHFSQIVRVLTEDEMGHPEIGDAIARLKEVLEYNAIGGKYNRGLTVVVAFRELVEPRKQDADSLQRAWTVGWCVELLQAFFLVADDIMDSSLTRRGQICWYQKPGVGLDAINDANLLEACIYRLLKLYCREQPYYLNLIELFLQSSYQTEIGQTLDLLTAPQGNVDLVRFTEKRYKSIVKYKTAFYSFYLPIAAAMYMAGIDGEKEHANAKKILLEMGEFFQIQDDYLDLFGDPSVTGKIGTDIQDNKCSWLVVQCLQRATPEQYQILKENYGQKEAEKVARVKALYEELDLPAVFLQYEEDSYSHIMALIEQYAAPLPPAVFLGLARKIYKRRK

282 NP_004292.1 Q8IUQ0

>NP_004292.1

MSGGVYGGDEVGALVFDIGSYTVRAGYAGEDCPKVDFPTAIGMVVERDDGSTLMEIDGDKGKQGGPTYYIDTNALRVPRENMEAISPLKNGMVEDWDSFQAILDHTYKMHVKSEASLHPVLMSEAPWNTRAKREKLTELMFEHYNIPAFFLCKTAVLTAFANGRSTGLILDSGATHTTAIPVHDGYVLQQGIVKSPLAGDFITMQCRELFQEMNIELVPPYMIASKEAVREGSPANWKRKEKLPQVTRSWHNYMCNCVIQDFQASVLQVSDSTYDEQVAAQMPTVHYEFPNGYNCDFGAERLKIPEGLFDPSNVKGLSGNTMLGVSHVVTTSVGMCDIDIRPGLYGSVIVAGGNTLIQSFTDRLNRELSQKTPPSMRLKLIANNTTVERRFSSWIGGSILASLGTFQQMWISKQEYEEGGKQCVERKCP

>Q8IUQ0

MGPVSLLPKYQKLNTWNGDLAKMTHLQAGLSPETIEKARLELNENPDVLHQDIQQVRDMIITRPDIGFLRTDDAFILRFLRARKFHQADAFRLLAQYFQYRQLNLDMFKNFKADDPGIKRALIDGFPGVLENRDHYGRKILLLFAANWDQSRNSFTDILRAILLSLEVLIEDPELQINGFILIIDWSNFSFKQASKLTPSILKLAIEGLQDSFPARFGGVHFVNQPWYIHALYTLIKPFLKDKTRKRIFLHGNNLNSLHQLIHPEFLPSEFGGTLPPYDMGTWARTLLGPDYSDENDYTHTSYNAMHVKHTSSNLERECSPKLMKRSQSVVEAGTLKHEEKGENENTQPLLALD

283 NP_001460.1 Q9BZQ8

>NP_001460.1

MSGWESYYKTEGDEEAEEEQEENLEASGDYKYSGRDSLIFLVDASKAMFESQSEDELTPFDMSIQCIQSVYISKIISSDRDLLAVVFYGTEKDKNSVNFKNIYVLQELDNPGAKRILELDQFKGQQGQKRFQDMMGHGSDYSLSEVLWVCANLFSDVQFKMSHKRIMLFTNEDNPHGNDSAKASRARTKAGDLRDTGIFLDLMHLKKPGGFDISLFYRDIISIAEDEDLRVHFEESSKLEDLLRKVRAKETRKRALSRLKLKLNKDIVISVGIYNLVQKALKPPPIKLYRETNEPVKTKTRTFNTSTGGLLLPSDTKRSQIYGSRQIILEKEETEELKRFDDPGLMLMGFKPLVLLKKHHYLRPSLFVYPEESLVIGSSTLFSALLIKCLEKEVAALCRYTPRRNIPPYFVALVPQEEELDDQKIQVTPPGFQLVFLPFADDKRKMPFTEKIMATPEQVGKMKAIVEKLRFTYRSDSFENPVLQQHFRNLEALALDLMEPEQAVDLTLPKVEAMNKRLGSLVDEFKELVYPPDYNPEGKVTKRKHDNEGSGSKRPKVEYSEEELKTHISKGTLGKFTVPMLKEACRAYGLKSGLKKQELLEALTKHFQD

>Q9BZQ8

MGGSASSQLDEGKCAYIRGKTEAAIKNFSPYYSRQYSVAFCNHVRTEVEQQRDLTSQFLKTKPPLAPGTILYEAELSQFSEDIKKWKERYVVVKNDYAVESYENKEAYQRGAAPKCRILPAGGKVLTSEDEYNLLSDRHFPDPLASSEKENTQPFVVLPKEFPVYLWQPFFRHGYFCFHEAADQKRFSALLSDCVRHLNHDYMKQMTFEAQAFLEAVQFFRQEKGHYGSWEMITGDEIQILSNLVMEELLPTLQTDLLPKMKGKKNDRKRTWLGLLEEAYTLVQHQVSEGLSALKEECRALTKGLEGTIRSDMDQIVNSKNYLIGKIKAMVAQPAEKSCLESVQPFLASILEELMGPVSSGFSEVRVLFEKEVNEVSQNFQTTKDSVQLKEHLDRLMNLPLHSVKMEPCYTKVNLLHERLQDLKSRFRFPHIDLVVQRTQNYMQELMENAVFTFEQLLSPHLQGEASKTAVAIEKVKLRVLKQYDYDSSTIRKKIFQEALVQITLPTVQKALASTCKPELQKYEQFIFADHTNMIHVENVYEEILHQILLDETLKVIKEAAILKKHNLFEDNMALPSESVSSLTDLKPPTGSNQASPARRASAILPGVLGSETLSNEVFQESEEEKQPEVPSSLAKGESLSLPGPSPPPDGTEQVIISRVDDPVVNPVATEDTAGLPGTCSSELEFGGTLEDEEPAQEEPEPITASGSLKALRKLLTASVEVPVDSAPVMEEDTNGESHVPQENEEEEEKEPSQAAAIHPDNCEESEVSEREAQPPCPEAHGEELGGFPEVGSPASPPASGGLTEEPLGPMEGELPGEACTLTAHEGRGGKCTEEGDASQQEGCTLGSDPICLSESQVSEEQEEMGGQSSAAQATASVNAEEIKVARIHECQWVVEDAPNPDVLLSHKDDVKEGEGGQESFPELPSEE

284 NP_079224.1 NP_003126.1

>NP_079224.1

MSELTKELMELVWGTKSSPGLSDTIFCRWTQGFVFSESEGSALEQFEGGPCAVIAPVQAFLLKKLLFSSEKSSWRDCSEEEQKELLCHTLCDILESACCDHSGSYCLVSWLRGKTTEETASISGSPAESSCQVEHSSALAVEELGFERFHALIQKRSFRSLPELKDAVLDQYSMWGNKFGVLLFLYSVLLTKGIENIKNEIEDASEPLIDPVYGHGSQSLINLLLTGHAVSNVWDGDRECSGMKLLGIHEQAAVGFLTLMEALRYCKVGSYLKSPKFPIWIVGSETHLTVFFAKDMALVAPEAPSEQARRVFQTYDPEDNGFIPDSLLEDVMKALDLVSDPEYINLMKNKLDPEGLGIILLGPFLQEFFPDQGSSGPESFTVYHYNGLKQSNYNEKVMYVEGTAVVMGFEDPMLQTDDTPIKRCLQTKWPYIELLWTTDRSPSLN

>NP_003126.1

MACAAARSPADQDRFICIYPAYLNNKKTIAEGRRIPISKAVENPTATEIQDVCSAVGLNVFLEKNKMYSREWNRDVQYRGRVRVQLKQEDGSLCLVQFPSRKSVMLYAAEMIPKLKTRTQKTGGADQSLQQGEGSKKGKGKKKK

285 NP_005002.3 P51970

>NP_005002.3

MEEHGVTQTEHMATIEAHAVAQQVQQVHVATYTEHSMLSADEDSPSSPEDTSYDDSDILNSTAADEVTAHLAAAGPVGMAAAAAVATGKKRKRPHVFESNPSIRKRQQTRLLRKLRATLDEYTTRVGQQAIVLCISPSKPNPVFKVFGAAPLENVVRKYKSMILEDLESALAEHAPAPQEVNSELPPLTIDGIPVSVDKMTQAQLRAFIPEMLKYSTGRGKPGWGKESCKPIWWPEDIPWANVRSDVRTEEQKQRVSWTQALRTIVKNCYKQHGREDLLYAFEDQQTQTQATATHSIAHLVPSQTVVQTFSNPDGTVSLIQVGTGATVATLADASELPTTVTVAQVNYSAVADGEVEQNWATLQGGEMTIQTTQASEATQAVASLAEAAVAASQEMQQGATVTMALNSEAAAHAVATLAEATLQGGGQIVLSGETAAAVGALTGVQDANGLVQIPVSMYQTVVTSLAQGNGPVQVAMAPVTTRISDSAVTMDGQAVEVVTLEQ

>P51970

MPGIVELPTLEELKVDEVKISSAVLKAAAHHYGAQCDKPNKEFMLCRWEEKDPRRCLEEGKLVNKCALDFFRQIKRHCAEPFTEYWTCIDYTGQQLFRHCRKQQAKFDECVLDKLGWVRPDLGELSKVTKVKTDRPLPENPYHSRPRPDPSPEIEGDLQPATHGSRFYFWTK

286 NP_004261.1 NP_005403.2

>NP_004261.1

MGEPQQVSALPPPPMQYIKEYTDENIQEGLAPKPPPPIKDSYMMFGNQFQCDDLIIRPLESQGIERLHPMQFDHKKELRKLNMSILINFLDLLDILIRSPGSIKREEKLEDLKLLFVHVHHLINEYRPHQARETLRVMMEVQKRQRLETAERFQKHLERVIEMIQNCLASLPDDLPHSEAGMRVKTEPMDADDSNNCTGQNEHQRENSGHRRDQIIEKDAALCVLIDEMNERP

>NP_005403.2

MLYFSLFWAARPLQRCGQLVRMAIRAQHSNAAQTQTGEANRGWTGQESLSDSDPEMWELLQREKDRQCRGLELIASENFCSRAALEALGSCLNNKYSEGYPGKRYYGGAEVVDEIELLCQRRALEAFDLDPAQWGVNVQPYSGSPANLAVYTALLQPHDRIMGLDLPDGGHLTHGYMSDVKRISATSIFFESMPYKLNPKTGLIDYNQLALTARLFRPRLIIAGTSAYARLIDYARMREVCDEVKAHLLADMAHISGLVAAKVIPSPFKHADIVTTTTHKTLRGARSGLIFYRKGVKAVDPKTGREIPYTFEDRINFAVFPSLQGGPHNHAIAAVAVALKQACTPMFREYSLQVLKNARAMADALLERGYSLVSGGTDNHLVLVDLRPKGLDGARAERVLELVSITANKNTCPGDRSAITPGGLRLGAPALTSRQFREDDFRRVVDFIDEGVNIGLEVKSKTAKLQDFKSFLLKDSETSQRLANLRQRVEQFARAFPMPGFDEH

287 Q5XKR4 P83881

>Q5XKR4

MLSHADLLDARLGMKDAAELLGHREAVKCRLGVGGSDPGGHPGDLAPNSDPVEGATLLPGEDITTVGSTPASLAVSAKDPDKQPGPQGGPNPSQAGQQQGQQKQKRHRTRFTPAQLNELERSFAKTHYPDIFMREELALRIGLTESRVQVWFQNRRAKWKKRKKTTNVFRAPGTLLPTPGLPQFPSAAAAAAAAMGDSLCSFHANDTRWAAAAMPGVSQLPLPPALGRQQAMAQSLSQCSLAAGPPPNSMGLSNSLAGSNGAGLQSHLYQPAFPGMVPASLPGPSNVSGSPQLCSSPDSSDVWRGTSIASLRRKALEHTVSMSFT

>P83881

MVNVPKTRRTFCKKCGKHQPHKVTQYKKGKDSLYAQGKRRYDRKQSGYGGQTKPIFRKKAKTTKKIVLRLECVEPNCRSKRMLAIKRCKHFELGGDKKRKGQVIQF

288 NP_008835.5 NP_001025.1

>NP_008835.5

MAGSGAGVRCSLLRLQETLSAADRCGAALAGHQLIRGLGQECVLSSSPAVLALQTSLVFSRDFGLLVFVRKSLNSIEFRECREEILKFLCIFLEKMGQKIAPYSVEIKNTCTSVYTKDRAAKCKIPALDLLIKLLQTFRSSRLMDEFKIGELFSKFYGELALKKKIPDTVLEKVYELLGLLGEVHPSEMINNAENLFRAFLGELKTQMTSAVREPKLPVLAGCLKGLSSLLCNFTKSMEEDPQTSREIFNFVLKAIRPQIDLKRYAVPSAGLRLFALHASQFSTCLLDNYVSLFEVLLKWCAHTNVELKKAALSALESFLKQVSNMVAKNAEMHKNKLQYFMEQFYGIIRNVDSNNKELSIAIRGYGLFAGPCKVINAKDVDFMYVELIQRCKQMFLTQTDTGDDRVYQMPSFLQSVASVLLYLDTVPEVYTPVLEHLVVMQIDSFPQYSPKMQLVCCRAIVKVFLALAAKGPVLRNCISTVVHQGLIRICSKPVVLPKGPESESEDHRASGEVRTGKWKVPTYKDYVDLFRHLLSSDQMMDSILADEAFFSVNSSSESLNHLLYDEFVKSVLKIVEKLDLTLEIQTVGEQENGDEAPGVWMIPTSDPAANLHPAKPKDFSAFINLVEFCREILPEKQAEFFEPWVYSFSYELILQSTRLPLISGFYKLLSITVRNAKKIKYFEGVSPKSLKHSPEDPEKYSCFALFVKFGKEVAVKMKQYKDELLASCLTFLLSLPHNIIELDVRAYVPALQMAFKLGLSYTPLAEVGLNALEEWSIYIDRHVMQPYYKDILPCLDGYLKTSALSDETKNNWEVSALSRAAQKGFNKVVLKHLKKTKNLSSNEAISLEEIRIRVVQMLGSLGGQINKNLLTVTSSDEMMKSYVAWDREKRLSFAVPFREMKPVIFLDVFLPRVTELALTASDRQTKVAACELLHSMVMFMLGKATQMPEGGQGAPPMYQLYKRTFPVLLRLACDVDQVTRQLYEPLVMQLIHWFTNNKKFESQDTVALLEAILDGIVDPVDSTLRDFCGRCIREFLKWSIKQITPQQQEKSPVNTKSLFKRLYSLALHPNAFKRLGASLAFNNIYREFREEESLVEQFVFEALVIYMESLALAHADEKSLGTIQQCCDAIDHLCRIIEKKHVSLNKAKKRRLPRGFPPSASLCLLDLVKWLLAHCGRPQTECRHKSIELFYKFVPLLPGNRSPNLWLKDVLKEEGVSFLINTFEGGGCGQPSGILAQPTLLYLRGPFSLQATLCWLDLLLAALECYNTFIGERTVGALQVLGTEAQSSLLKAVAFFLESIAMHDIIAAEKCFGTGAAGNRTSPQEGERYNYSKCTVVVRIMEFTTTLLNTSPEGWKLLKKDLCNTHLMRVLVQTLCEPASIGFNIGDVQVMAHLPDVCVNLMKALKMSPYKDILETHLREKITAQSIEELCAVNLYGPDAQVDRSRLAAVVSACKQLHRAGLLHNILPSQSTDLHHSVGTELLSLVYKGIAPGDERQCLPSLDLSCKQLASGLLELAFAFGGLCERLVSLLLNPAVLSTASLGSSQGSVIHFSHGEYFYSLFSETINTELLKNLDLAVLELMQSSVDNTKMVSAVLNGMLDQSFRERANQKHQGLKLATTILQHWKKCDSWWAKDSPLETKMAVLALLAKILQIDSSVSFNTSHGSFPEVFTTYISLLADTKLDLHLKGQAVTLLPFFTSLTGGSLEELRRVLEQLIVAHFPMQSREFPPGTPRFNNYVDCMKKFLDALELSQSPMLLELMTEVLCREQQHVMEELFQSSFRRIARRGSCVTQVGLLESVYEMFRKDDPRLSFTRQSFVDRSLLTLLWHCSLDALREFFSTIVVDAIDVLKSRFTKLNESTFDTQITKKMGYYKILDVMYSRLPKDDVHAKESKINQVFHGSCITEGNELTKTLIKLCYDAFTENMAGENQLLERRRLYHCAAYNCAISVICCVFNELKFYQGFLFSEKPEKNLLIFENLIDLKRRYNFPVEVEVPMERKKKYIEIRKEAREAANGDSDGPSYMSSLSYLADSTLSEEMSQFDFSTGVQSYSYSSQDPRPATGRFRRREQRDPTVHDDVLELEMDELNRHECMAPLTALVKHMHRSLGPPQGEEDSVPRDLPSWMKFLHGKLGNPIVPLNIRLFLAKLVINTEEVFRPYAKHWLSPLLQLAASENNGGEGIHYMVVEIVATILSWTGLATPTGVPKDEVLANRLLNFLMKHVFHPKRAVFRHNLEIIKTLVECWKDCLSIPYRLIFEKFSGKDPNSKDNSVGIQLLGIVMANDLPPYDPQCGIQSSEYFQALVNNMSFVRYKEVYAAAAEVLGLILRYVMERKNILEESLCELVAKQLKQHQNTMEDKFIVCLNKVTKSFPPLADRFMNAVFFLLPKFHGVLKTLCLEVVLCRVEGMTELYFQLKSKDFVQVMRHRDDERQKVCLDIIYKMMPKLKPVELRELLNPVVEFVSHPSTTCREQMYNILMWIHDNYRDPESETDNDSQEIFKLAKDVLIQGLIDENPGLQLIIRNFWSHETRLPSNTLDRLLALNSLYSPKIEVHFLSLATNFLLEMTSMSPDYPNPMFEHPLSECEFQEYTIDSDWRFRSTVLTPMFVETQASQGTLQTRTQEGSLSARWPVAGQIRATQQQHDFTLTQTADGRSSFDWLTGSSTDPLVDHTSPSSDSLLFAHKRSERLQRAPLKSVGPDFGKKRLGLPGDEVDNKVKGAAGRTDLLRLRRRFMRDQEKLSLMYARKGVAEQKREKEIKSELKMKQDAQVVLYRSYRHGDLPDIQIKHSSLITPLQAVAQRDPIIAKQLFSSLFSGILKEMDKFKTLSEKNNITQKLLQDFNRFLNTTFSFFPPFVSCIQDISCQHAALLSLDPAAVSAGCLASLQQPVGIRLLEEALLRLLPAELPAKRVRGKARLPPDVLRWVELAKLYRSIGEYDVLRGIFTSEIGTKQITQSALLAEARSDYSEAAKQYDEALNKQDWVDGEPTEAEKDFWELASLDCYNHLAEWKSLEYCSTASIDSENPPDLNKIWSEPFYQETYLPYMIRSKLKLLLQGEADQSLLTFIDKAMHGELQKAILELHYSQELSLLYLLQDDVDRAKYYIQNGIQSFMQNYSSIDVLLHQSRLTKLQSVQALTEIQEFISFISKQGNLSSQVPLKRLLNTWTNRYPDAKMDPMNIWDDIITNRCFFLSKIEEKLTPLPEDNSMNVDQDGDPSDRMEVQEQEEDISSLIRSCKFSMKMKMIDSARKQNNFSLAMKLLKELHKESKTRDDWLVSWVQSYCRLSHCRSRSQGCSEQVLTVLKTVSLLDENNVSSYLSKNILAFRDQNILLGTTYRIIANALSSEPACLAEIEEDKARRILELSGSSSEDSEKVIAGLYQRAFQHLSEAVQAAEEEAQPPSWSCGPAAGVIDAYMTLADFCDQQLRKEEENASVIDSAELQAYPALVVEKMLKALKLNSNEARLKFPRLLQIIERYPEETLSLMTKEISSVPCWQFISWISHMVALLDKDQAVAVQHSVEEITDNYPQAIVYPFIISSESYSFKDTSTGHKNKEFVARIKSKLDQGGVIQDFINALDQLSNPELLFKDWSNDVRAELAKTPVNKKNIEKMYERMYAALGDPKAPGLGAFRRKFIQTFGKEFDKHFGKGGSKLLRMKLSDFNDITNMLLLKMNKDSKPPGNLKECSPWMSDFKVEFLRNELEIPGQYDGRGKPLPEYHVRIAGFDERVTVMASLRRPKRIIIRGHDEREHPFLVKGGEDLRQDQRVEQLFQVMNGILAQDSACSQRALQLRTYSVVPMTSRLGLIEWLENTVTLKDLLLNTMSQEEKAAYLSDPRAPPCEYKDWLTKMSGKHDVGAYMLMYKGANRTETVTSFRKRESKVPADLLKRAFVRMSTSPEAFLALRSHFASSHALICISHWILGIGDRHLNNFMVAMETGGVIGIDFGHAFGSATQFLPVPELMPFRLTRQFINLMLPMKETGLMYSIMVHALRAFRSDPGLLTNTMDVFVKEPSFDWKNFEQKMLKKGGSWIQEINVAEKNWYPRQKICYAKRKLAGANPAVITCDELLLGHEKAPAFRDYVAVARGSKDHNIRAQEPESGLSEETQVKCLMDQATDPNILGRTWEGWEPWM

>NP_001025.1

MLSLRVPLAPITDPQQLQLSPLKGLSLVDKENTPPALSGTRVLASKTARRIFQEPTEPKTKAAAPGVEDEPLLRENPRRFVIFPIEYHDIWQMYKKAEASFWTAEEVDLSKDIQHWESLKPEERYFISHVLAFFAASDGIVNENLVERFSQEVQITEARCFYGFQIAMENIHSEMYSLLIDTYIKDPKEREFLFNAIETMPCVKKKADWALRWIGDKEATYGERVVAFAAVEGIFFSGSFASIFWLKKRGLMPGLTFSNELISRDEGLHCDFACLMFKHLVHKPSEERVREIIINAVRIEQEFLTEALPVKLIGMNCTLMKQYIEFVADRLMLELGFSKVFRVENPFDFMENISLEGKTNFFEKRVGEYQRMGVMSSPTENSFTLDADF

289 NP_079100.2 Q9NZD2

>NP_079100.2

MEIGTEISRKIRSAIKGKLQELGAYVDEELPDYIMVMVANKKSQDQMTEDLSLFLGNNTIRFTVWLHGVLDKLRSVTTEPSSLKSSDTNIFDSNVPSNKSNFSRGDERRHEAAVPPLAIPSARPEKRDSRVSTSSQESKTTNVRQTYDDGAATRLMSTVKPLREPAPSEDVIDIKPEPDDLIDEDLNFVQENPLSQKKPTVTLTYGSSRPSIEIYRPPASRNADSGVHLNRLQFQQQQNSIHAAKQLDMQSSWVYETGRLCEPEVLNSLEETYSPFFRNNSEKMSMEDENFRKRKLPVVSSVVKVKKFNHDGEEEEEDDDYGSRTGSISSSVSVPAKPERRPSLPPSKQANKNLILKAISEAQESVTKTTNYSTVPQKQTLPVAPRTRTSQEELLAEVVQGQSRTPRISPPIKEEETKGDSVEKNQGTQQRQLLSRLQIDPVMAETLQMSQDYYDMESMVHADTRSFILKKPKLSEEVVVAPNQESGMKTADSLRVLSGHLMQTRDLVQPDKPASPKFIVTLDGVPSPPGYMSDQEEDMCFEGMKPVNQTAASNKGLRGLLHPQQLHLLSRQLEDPNGSFSNAEMSELSVAQKPEKLLERCKYWPACKNGDECAYHHPISPCKAFPNCKFAEKCLFVHPNCKYDAKCTKPDCPFTHVSRRIPVLSPKPAVAPPAPPSSSQLCRYFPACKKMECPFYHPKHCRFNTQCTRPDCTFYHPTINVPPRHALKWIRPQTSE

>Q9NZD2

MALLAEHLLKPLPADKQIETGPFLEAVSHLPPFFDCLGSPVFTPIKADISGNITKIKAVYDTNPAKFRTLQNILEVEKEMYGAEWPKVGATLALMWLKRGLRFIQVFLQSICDGERDENHPNLIRVNATKAYEMALKKYHGWIVQKIFQAALYAAPYKSDFLKALSKGQNVTEEECLEKIRLFLVNYTATIDVIYEMYTQMNAELNYKV

290 NP_443082.2 Q9BUE6

>NP_443082.2

MAASVRQARSLLGVAATLAPGSRGYRARPPPRRRPGPRWPDPEDLLTPRWQLGPRYAAKQFARYGAASGVVPGSLWPSPEQLRELEAEEREWYPSLATMQESLRVKQLAEEQKRREREQHIAECMAKMPQMIVNWQQQQRENWEKAQADKERRARLQAEAQELLGYQVDPRSARFQELLQDLEKKERKRLKEEKQKRKKEARAAALAAAVAQDPAASGAPSS

>Q9BUE6

MSASLVRATVRAVSKRKLQPTRAALTLTPSAVNKIKQLLKDKPEHVGVKVGVRTRGCNGLSYTLEYTKTKGDSDEEVIQDGVRVFIEKKAQLTLLGTEMDYVEDKLSSEFVFNNPNIKGTCGCGESFNI

291 NP_005307.1 NP_002961.1

>NP_005307.1

MATSSEEVLLIVKKVRQKKQDGALYLMAERIAWAPEGKDRFTISHMYADIKCQKISPEGKAKIQLQLVLHAGDTTNFHFSNESTAVKERDAVKDLLQQLLPKFKRKANKELEEKNRMLQEDPVLFQLYKDLVVSQVISAEEFWANRLNVNATDSSSTSNHKQDVGISAAFLADVRPQTDGCNGLRYNLTSDIIESIFRTYPAVKMKYAENVPHNMTEKEFWTRFFQSHYFHRDRLNTGSKDLFAECAKIDEKGLKTMVSLGVKNPLLDLTALEDKPLDEGYGISSVPSASNSKSIKENSNAAIIKRFNHHSAMVLAAGLRKQEAQNEQTSEPSNMDGNSGDADCFQPAVKRAKLQESIEYEDLGKNNSVKTIALNLKKSDRYYHGPTPIQSLQYATSQDIINSFQSIRQEMEAYTPKLTQVLSSSAASSTITALSPGGALMQGGTQQAINQMVPNDIQSELKHLYVAVGELLRHFWSCFPVNTPFLEEKVVKMKSNLERFQVTKLCPFQEKIRRQYLSTNLVSHIEEMLQTAYNKLHTWQSRRLMKKT

>NP_002961.1

MAKFVIRPATAADCSDILRLIKELAKYEYMEEQVILTEKDLLEDGFGEHPFYHCLVAEVPKEHWTPEGHSIVGFAMYYFTYDPWIGKLLYLEDFFVMSDYRGFGIGSEILKNLSQVAMRCRCSSMHFLVAEWNEPSINFYKRRGASDLSSEEGWRLFKIDKEYLLKMATEE

292 Q5TB30 O00764

>Q5TB30

MESQGVPPGPYRATKLWNEVTTSFRAGMPLRKHRQHFKKYGNCFTAGEAVDWLYDLLRNNSNFGPEVTRQQTIQLLRKFLKNHVIEDIKGRWGSENVDDNNQLFRFPATSPLKTLPRRYPELRKNNIENFSKDKDSIFKLRNLSRRTPKRHGLHLSQENGEKIKHEIINEDQENAIDNRELSQEDVEEVWRYVILIYLQTILGVPSLEEVINPKQVIPQYIMYNMANTSKRGVVILQNKSDDLPHWVLSAMKCLANWPRSNDMNNPTYVGFERDVFRTIADYFLDLPEPLLTFEYYELFVNILVVCGYITVSDRSSGIHKIQDDPQSSKFLHLNNLNSFKSTECLLLSLLHREKNKEESDSTERLQISNPGFQERCAKKMQLVNLRNRRVSANDIMGGSCHNLIGLSNMHDLSSNSKPRCCSLEGIVDVPGNSSKEASSVFHQSFPNIEGQNNKLFLESKPKQEFLLNLHSEENIQKPFSAGFKRTSTLTVQDQEELCNGKCKSKQLCRSQSLLLRSSTRRNSYINTPVAEIIMKPNVGQGSTSVQTAMESELGESSATINKRLCKSTIELSENSLLPASSMLTGTQSLLQPHLERVAIDALQLCCLLLPPPNRRKLQLLMRMISRMSQNVDMPKLHDAMGTRSLMIHTFSRCVLCCAEEVDLDELLAGRLVSFLMDHHQEILQVPSYLQTAVEKHLDYLKKGHVSIVSLKILCNSQLQVANSSQNSLIKKGDFISSHKQPLKVRNVDRIWDSNSYMTLSIPPSLISVLILCLTMAPSHDEECG

>O00764

MEEECRVLSIQSHVIRGYVGNRAATFPLQVLGFEIDAVNSVQFSNHTGYAHWKGQVLNSDELQELYEGLRLNNMNKYDYVLTGYTRDKSFLAMVVDIVQELKQQNPRLVYVCDPVLGDKWDGEGSMYVPEDLLPVYKEKVVPLADIITPNQFEAELLSGRKIHSQEEALRVMDMLHSMGPDTVVITSSDLPSPQGSNYLIVLGSQRRRNPAGSVVMERIRMDIRKVDAVFVGTGDLFAAMLLAWTHKHPNNLKVACEKTVSTLHHVLQRTIQCAKAQAGEGVRPSPMQLELRMVQSKRDIEDPEIVVQATVL

293 NP_002543.2 NP_057717.1

>NP_002543.2

MSSRKSKSNSLIHTECLSQVQRILRERFCRQSPHSNLFGVQVQYKHLSELLKRTALHGESNSVLIIGPRGSGKTMLINHALKELMEIEEVSENVLQVHLNGLLQINDKIALKEITRQLNLENVVGDKVFGSFAENLSFLLEALKKGDRTSSCPVIFILDEFDLFAHHKNQTLLYNLFDISQSAQTPIAVIGLTCRLDILELLEKRVKSRFSHRQIHLMNSFGFPQYVKIFKEQLSLPAEFPDKVFAEKWNENVQYLSEDRSVQEVLQKHFNISKNLRSLHMLLMLALNRVTASHPFMTAVDLMEASQLCSMDSKANIVHGLSVLEICLIIAMKHLNDIYEEEPFNFQMVYNEFQKFVQRKAHSVYNFEKPVVMKAFEHLQQLELIKPMERTSGNSQREYQLMKLLLDNTQIMNALQKYPNCPTDVRQWATSSLSWL

>NP_057717.1

MALLKANKDLISAGLKEFSVLLNQQVFNDPLVSEEDMVTVVEDWMNFYINYYRQQVTGEPQERDKALQELRQELNTLANPFLAKYRDFLKSHELPSHPPPSS

294 Q7Z591 NP_004591.2

>Q7Z591

MASSETEIRWAEPGLGKGPQRRRWAWAEDKRDVDRSSSQSWEEERLFPNATSPELLEDFRLAQQHLPPLEWDPHPQPDGHQDSESGETSGEEAEAEDVDSPASSHEPLAWLPQQGRQLDMTEEEPDGTLGSLEVEEAGESSSRLGYEAGLSLEGHGNTSPMALGHGQARGWVASGEQASGDKLSEHSEVNPSVELSPARSWSSGTVSLDHPSDSLDSTWEGETDGPQPTALAETLPEGPSHHLLSPDGRTGGSVARATPMEFQDSSAPPAQSPQHATDRWRRETTRFFCPQPKEHIWKQTKTSPKPLPSRFIGSISPLNPQPRPTRQGRPLPRQGATLAGRSSSNAPKYGRGQLNYPLPDFSKVGPRVRFPKDESYRPPKSRSHNRKPQAPARPLIFKSPAEIVQEVLLSSGEAALAKDTPPAHPITRVPQEFQTPEQATELVHQLQEDYHRLLTKYAEAENTIDQLRLGAKVNLFSDPPQPNHSIHTGMVPQGTKVLSFTIPQPRSAEWWPGPAEDPQASAASGWPSARGDLSPSSLTSMPTLGWLPENRDISEDQSSAEQTQALASQASQFLAKVESFERLIQAGRLMPQDQVKGFQRLKAAHAALEEEYLKACREQHPAQPLAGSKGTPGRFDPRRELEAEIYRLGSCLEELKEHIDQTQQEPEPPGSDSALDSTPALPCLHQPTHLPAPSGQAPMPAIKTSCPEPATTTAAASTGPCPLHVNVEVSSGNSEVEDRPQDPLARLRHKELQMEQVYHGLMERYLSVKSLPEAMRMEEEEEGEEEEEEEGGGDSLEVDGVAATPGKAEATRVLPRQCPVQAEKSHGAPLEEATEKMVSMKPPGFQASLARDGHMSGLGKAEAAPPGPGVPPHPPGTKSAASHQSSMTSLEGSGISERLPQKPLHRGGGPHLEETWMASPETDSGFVGSETSRVSPLTQTPEHRLSHISTAGTLAQPFAASVPRDGASYPKARGSLIPRRATEPSTPRSQAQRYLSSPSGPLRQRAPNFSLERTLAAEMAVPGSEFEGHKRISEQPLPNKTISPPPAPAPAAAPLPCGPTETIPSFLLTRAGRDQAICELQEEVSRLRLRLEDSLHQPLQGSPTRPASAFDRPARTRGRPADSPATWGSHYGSKSTERLPGEPRGEEQIVPPGRQRARSSSVPREVLRLSLSSESELPSLPLFSEKSKTTKDSPQAARDGKRGVGSAGWPDRVTFRGQYTGHEYHVLSPKAVPKGNGTVSCPHCRPIRTQDAGGAVTGDPLGPPPADTLQCPLCGQVGSPPEADGPGSATSGAEKATTRRKASSTPSPKQRSKQAGSSPRPPPGLWYLATAPPAPAPPAFAYISSVPIMPYPPAAVYYAPAGPTSAQPAAKWPPTASPPPARRHRHSIQLDLGDLEELNKALSRAVQAAESVRSTTRQMRSSLSADLRQAHSLRGSCLF

>NP_004591.2

MEESVNQMQPLNEKQIANSQDGYVWQVTDMNRLHRFLCFGSEGGTYYIKEQKLGLENAEALIRLIEDGRGCEVIQEIKSFSQEGRTTKQEPMLFALAICSQCSDISTKQAAFKAVSEVCRIPTHLFTFIQFKKDLKESMKCGMWGRALRKAIADWYNEKGGMALALAVTKYKQRNGWSHKDLLRLSHLKPSSEGLAIVTKYITKGWKEVHELYKEKALSVETEKLLKYLEAVEKVKRTRDELEVIHLIEEHRLVREHLLTNHLKSKEVWKALLQEMPLTALLRNLGKMTANSVLEPGNSEVSLVCEKLCNEKLLKKARIHPFHILIALETYKTGHGLRGKLKWRPDEEILKALDAAFYKTFKTVEPTGKRFLLAVDVSASMNQRVLGSILNASTVAAAMCMVVTRTEKDSYVVAFSDEMVPCPVTTDMTLQQVLMAMSQIPAGGTDCSLPMIWAQKTNTPADVFIVFTDNETFAGGVHPAIALREYRKKMDIPAKLIVCGMTSNGFTIADPDDRGMLDMCGFDTGALDVIRNFTLDMI

295 Q9Y6W3 NP_000365.3

>Q9Y6W3

MDATALERDAVQFARLAVQRDHEGRYSEAVFYYKEAAQALIYAEMAGSSLENIQEKITEYLERVQALHSAVQSKSADPLKSKHQLDLERAHFLVTQAFDEDEKENVEDAIELYTEAVDLCLKTSYETADKVLQNKLKQLARQALDRAEALSEPLTKPVGKISSTSVKPKPPPVRAHFPLGANPFLERPQSFISPQSCDAQGQRYTAEEIEVLRTTSKINGIEYVPFMNVDLRERFAYPMPFCDRWGKLPLSPKQKTTFSKWVRPEDLTNNPTMIYTVSSFSIKQTIVSDCSFVASLAISAAYERRFNKKLITGIIYPQNKDGEPEYNPCGKYMVKLHLNGVPRKVIIDDQLPVDHKGELLCSYSNNKSELWVSLIEKAYMKVMGGYDFPGSNSNIDLHALTGWIPERIAMHSDSQTFSKDNSFRMLYQRFHKGDVLITASTGMMTEAEGEKWGLVPTHAYAVLDIREFKGLRFIQLKNPWSHLRWKGRYSENDVKNWTPELQKYLNFDPRTAQKIDNGIFWISWDDLCQYYDVIYLSWNPGLFKESTCIHSTWDAKQGPVKDAYSLANNPQYKLEVQCPQGGAAVWVLLSRHITDKDDFANNREFITMVVYKTDGKKVYYPADPPPYIDGIRINSPHYLTKIKLTTPGTHTFTLVVSQYEKQNTIHYTVRVYSACSFTFSKIPSPYTLSKRINGKWSGQSAGGCGNFQETHKNNPIYQFHIEKTGPLLIELRGPRQYSVGFEVVTVSTLGDPGPHGFLRKSSGDYRCGFCYLELENIPSGIFNIIPSTFLPKQEGPFFLDFNSIIPIKITQLQ

>NP_000365.3

MEANGLGPQGFPELKNDTFLRAAWGEETDYTPVWCMRQAGRYLPEFRETRAAQDFFSTCRSPEACCELTLQPLRRFPLDAAIIFSDILVVPQALGMEVTMVPGKGPSFPEPLREEQDLERLRDPEVVASELGYVFQAITLTRQRLAGRVPLIGFAGAPWTLMTYMVEGGGSSTMAQAKRWLYQRPQASHQLLRILTDALVPYLVGQVVAGAQALQLFESHAGHLGPQLFNKFALPYIRDVAKQVKARLREAGLAPVPMIIFAKDGHFALEELAQAGYEVVGLDWTVAPKKARECVGKTVTLQGNLDPCALYASEEEIGQLVKQMLDDFGPHRYIANLGHGLYPDMDPEHVGAFVDAVHKHSRLLRQN

296 NP_060740.1 Q96RP9

>NP_060740.1

MGEAEVGGGGAAGDKGPGEAATSPAEETVVWSPEVEVCLFHAMLGHKPVGVNRHFHMICIRDKFSQNIGRQVPSKVIWDHLSTMYDMQALHESEILPFPNPERNFVLPEEIIQEVREGKVMIEEEMKEEMKEDVDPHNGADDVFSSSGSLGKASEKSSKDKEKNSSDLGCKEGADKRKRSRVTDKVLTANSNPSSPSAAKRRRT

>Q96RP9

MRLLGAAAVAALGRGRAPASLGWQRKQVNWKACRWSSSGVIPNEKIRNIGISAHIDSGKTTLTERVLYYTGRIAKMHEVKGKDGVGAVMDSMELERQRGITIQSAATYTMWKDVNINIIDTPGHVDFTIEVERALRVLDGAVLVLCAVGGVQCQTMTVNRQMKRYNVPFLTFINKLDRMGSNPARALQQMRSKLNHNAAFMQIPMGLEGNFKGIVDLIEERAIYFDGDFGQIVRYGEIPAELRAAATDHRQELIECVANSDEQLGEMFLEEKIPSISDLKLAIRRATLKRSFTPVFLGSALKNKGVQPLLDAVLEYLPNPSEVQNYAILNKEDDSKEKTKILMNSSRDNSHPFVGLAFKLEVGRFGQLTYVRSYQGELKKGDTIYNTRTRKKVRLQRLARMHADMMEDVEEVYAGDICALFGIDCASGDTFTDKANSGLSMESIHVPDPVISIAMKPSNKNDLEKFSKGIGRFTREDPTFKVYFDTENKETVISGMGELHLEIYAQRLEREYGCPCITGKPKVAFRETITAPVPFDFTHKKQSGGAGQYGKVIGVLEPLDPEDYTKLEFSDETFGSNIPKQFVPAVEKGFLDACEKGPLSGHKLSGLRFVLQDGAHHMVDSNEISFIRAGEGALKQALANATLCILEPIMAVEVVAPNEFQGQVIAGINRRHGVITGQDGVEDYFTLYADVPLNDMFGYSTELRSCTEGKGEYTMEYSRYQPCLPSTQEDVINKYLEATGQLPVKKGKAKN

297 NP_005048.2 Q8N983

>NP_005048.2

MNLELLESFGQNYPEEADGTLDCISMALTCTFNRWGTLLAVGCNDGRIVIWDFLTRGIAKIISAHIHPVCSLCWSRDGHKLVSASTDNIVSQWDVLSGDCDQRFRFPSPILKVQYHPRDQNKVLVCPMKSAPVMLTLSDSKHVVLPVDDDSDLNVVASFDRRGEYIYTGNAKGKILVLKTDSQDLVASFRVTTGTSNTTAIKSIEFARKGSCFLINTADRIIRVYDGREILTCGRDGEPEPMQKLQDLVNRTPWKKCCFSGDGEYIVAGSARQHALYIWEKSIGNLVKILHGTRGELLLDVAWHPVRPIIASISSGVVSIWAQNQVENWSAFAPDFKELDENVEYEERESEFDIEDEDKSEPEQTGADAAEDEEVDVTSVDPIAAFCSSDEELEDSKALLYLPIAPEVEDPEENPYGPPPDAVQTSLMDEGASSEKKRQSSADGSQPPKKKPKTTNIELQGVPNDEVHPLLGVKGDGKSKKKQAGRPKGSKGKEKDSPFKPKLYKGDRGLPLEGSAKGKVQAELSQPLTAGGAISELL

>Q8N983

MTARGTPSRFLASVLHNGLGRYVQQLQRLSFSVSRDGASSRGAREFVEREVIDFARRNPGVVIYVNSRPCCVPRVVAEYLNGAVREESIHCKSVEEISTLVQKLADQSGLDVIRIRKPFHTDNPSIQGQWHPFTNKPTTFRGLRPREVQDPAPAQDTGLRLSAVAPQILLPGWPDPPDLPTVDPISSSLTSAPAPMLSAVSCLPIVPALTTVCSA

298 NP_004796.1 Q9BYN0

>NP_004796.1

MAAGGSDPRAGDVEEDASQLIFPKEFETAETLLNSEVHMLLEHRKQQNESAEDEQELSEVFMKTLNYTARFSRFKNRETIASVRSLLLQKKLHKFELACLANLCPETAEESKALIPSLEGRFEDEELQQILDDIQTKRSFQY

>Q9BYN0

MGLRAGGTLGRAGAGRGAPEGPGPSGGAQGGSIHSGRIAAVHNVPLSVLIRPLPSVLDPAKVQSLVDTIREDPDSVPPIDVLWIKGAQGGDYFYSFGGCHRYAAYQQLQRETIPAKLVQSTLSDLRVYLGASTPDLQ

299 NP_005850.1 P50135

>NP_005850.1

MADRDSGSEQGGAALGSGGSLGHPGSGSGSGGGGGGGGGGGGSGGGGGGAPGGLQHETQELASKRVDIQNKRFYLDVKQNAKGRFLKIAEVGAGGNKSRLTLSMSVAVEFRDYLGDFIEHYAQLGPSQPPDLAQAQDEPRRALKSEFLVRENRKYYMDLKENQRGRFLRIRQTVNRGPGLGSTQGQTIALPAQGLIEFRDALAKLIDDYGVEEEPAELPEGTSLTVDNKRFFFDVGSNKYGVFMRVSEVKPTYRNSITVPYKVWAKFGHTFCKYSEEMKKIQEKQREKRAACEQLHQQQQQQQEETAAATLLLQGEEEGEED

>P50135

MASSMRSLFSDHGKYVESFRRFLNHSTEHQCMQEFMDKKLPGIIGRIGDTKSEIKILSIGGGAGEIDLQILSKVQAQYPGVCINNEVVEPSAEQIAKYKELVAKTSNLENVKFAWHKETSSEYQSRMLEKKELQKWDFIHMIQMLYYVKDIPATLKFFHSLLGTNAKMLIIVVSGSSGWDKLWKKYGSRFPQDDLCQYITSDDLTQMLDNLGLKYECYDLLSTMDISDCFIDGNENGDLLWDFLTETCNFNATAPPDLRAELGKDLQEPEFSAKKEGKVLFNNTLSFIVIEA

300 NP_003085.1 NP_659491.4

>NP_003085.1

MAYRGQGQKVQKVMVQPINLIFRYLQNRSRIQVWLYEQVNMRIEGCIIGFDEYMNLVLDDAEEIHSKTKSRKQLGRIMLKGDNITLLQSVSN

>NP_659491.4

MDNKISPEAQVAELELDAVIGFNGHVPTGLKCHPDQEHMIYPLGCTVLIQAINTKEQNFLQGHGNNVSCLAISRSGEYIASGQVTFMGFKADIILWDYKNRELLARLSLHKGKIEALAFSPNDLYLVSLGGPDDGSVVVWSIAKRDAICGSPAAGLNVGNATNVIFSRCRDEMFMTAGNGTIRVWELDLPNRKIWPTECQTGQLKRIVMSIGVDDDDSFFYLGTTTGDILKMNPRTKLLTDVGPAKDKFSLGVSAIRCLKMGGLLVGSGAGLLVFCKSPGYKPIKKIQLQGGITSITLRGEGHQFLVGTEESHIYRVSFTDFKETLIATCHFDAVEDIVFPFGTAELFATCAKKDIRVWHTSSNRELLRITVPNMTCHGIDFMRDGKSIISAWNDGKIRAFAPETGRLMYVINNAHRIGVTAIATTSDCKRVISGGGEGEVRVWQIGCQTQKLEEALKEHKSSVSCIRVKRNNEECVTASTDGTCIIWDLVRLRRNQMILANTLFQCVCYHPEEFQIITSGTDRKIAYWEVFDGTVIRELEGSLSGSINGMDITQEGVHFVTGGNDHLVKVWDYNEGEVTHVGVGHSGNITRIRISPGNQYIVSVSADGAILRWKYPYTS

301 Q8TF76 NP_001367.2

>Q8TF76

MAASLPGPGSRLFRTYGAADGRRQRRPGREAAQWFPPQDRRRFFNSSGSSDASIGDPSQSDDPDDPDDPDFPGSPVRRRRRCPGGRVPKDRPSLTVTPKRWKLRARPSLTVTPRRLGLRARPPQKCSTPCGPLRLPPFPSRDSGRLSPDLSVCGQPRDGDELGISASLFSSLASPCPGSPTPRDSVISIGTSACLVAASAVPSDLHLPEVSLDRASLPCSQEEATGGAKDTRMVHQTRASLRSVLFGLMNSGTPEDSEFRADGKNMRESCCKRKLVVGNGPEGPGLSSTGKRRATGQDSCQERGLQEAVRREHQEASVPKGRIVPRGTDRLERTRSSRKSKHQEATETSLLHSHRFKKGQKLGKDSFPTQDLTPLQNACFWTKTRASFSFHKKKIVTDVSEVCSIYTTATSLSGSLLSECSNRPVMNRTSGAPSSWHSSSMYLLSPLNTLSISNKKASDAEKVYGECSQKGPVPFSHCLPTEKLQRCEKIGEGVFGEVFQTIADHTPVAIKIIAIEGPDLVNGSHQKTFEEILPEIIISKELSLLSGEVCNRTEGFIGLNSVHCVQGSYPPLLLKAWDHYNSTKGSANDRPDFFKDDQLFIVLEFEFGGIDLEQMRTKLSSLATAKSILHQLTASLAVAEASLRFEHRDLHWGNVLLKKTSLKKLHYTLNGKSSTIPSCGLQVSIIDYTLSRLERDGIVVFCDVSMDEDLFTGDGDYQFDIYRLMKKENNNRWGEYHPYSNVLWLHYLTDKMLKQMTFKTKCNTPAMKQIKRKIQEFHRTMLNFSSATDLLCQHSLFK

>NP_001367.2

MSEPGGGGGEDGSAGLEVSAVQNVADVSVLQKHLRKLVPLLLEDGGEAPAALEAALEEKSALEQMRKFLSDPQVHTVLVERSTLKEDVGDEGEEEKEFISYNINIDIHYGVKSNSLAFIKRTPVIDADKPVSSQLRVLTLSEDSPYETLHSFISNAVAPFFKSYIRESGKADRDGDKMAPSVEKKIAELEMGLLHLQQNIEIPEISLPIHPMITNVAKQCYERGEKPKVTDFGDKVEDPTFLNQLQSGVNRWIREIQKVTKLDRDPASGTALQEISFWLNLERALYRIQEKRESPEVLLTLDILKHGKRFHATVSFDTDTGLKQALETVNDYNPLMKDFPLNDLLSATELDKIRQALVAIFTHLRKIRNTKYPIQRALRLVEAISRDLSSQLLKVLGTRKLMHVAYEEFEKVMVACFEVFQTWDDEYEKLQVLLRDIVKRKREENLKMVWRINPAHRKLQARLDQMRKFRRQHEQLRAVIVRVLRPQVTAVAQQNQGEVPEPQDMKVAEVLFDAADANAIEEVNLAYENVKEVDGLDVSKEGTEAWEAAMKRYDERIDRVETRITARLRDQLGTAKNANEMFRIFSRFNALFVRPHIRGAIREYQTQLIQRVKDDIESLHDKFKVQYPQSQACKMSHVRDLPPVSGSIIWAKQIDRQLTAYMKRVEDVLGKGWENHVEGQKLKQDGDSFRMKLNTQEIFDDWARKVQQRNLGVSGRIFTIESTRVRGRTGNVLKLKVNFLPEIITLSKEVRNLKWLGFRVPLAIVNKAHQANQLYPFAISLIESVRTYERTCEKVEERNTISLLVAGLKKEVQALIAEGIALVWESYKLDPYVQRLAETVFNFQEKVDDLLIIEEKIDLEVRSLETCMYDHKTFSEILNRVQKAVDDLNLHSYSNLPIWVNKLDMEIERILGVRLQAGLRAWTQVLLGQAEDKAEVDMDTDAPQVSHKPGGEPKIKNVVHELRITNQVIYLNPPIEECRYKLYQEMFAWKMVVLSLPRIQSQRYQVGVHYELTEEEKFYRNALTRMPDGPVALEESYSAVMGIVSEVEQYVKVWLQYQCLWDMQAENIYNRLGEDLNKWQALLVQIRKARGTFDNAETKKEFGPVVIDYGKVQSKVNLKYDSWHKEVLSKFGQMLGSNMTEFHSQISKSRQELEQHSVDTASTSDAVTFITYVQSLKRKIKQFEKQVELYRNGQRLLEKQRFQFPPSWLYIDNIEGEWGAFNDIMRRKDSAIQQQVANLQMKIVQEDRAVESRTTDLLTDWEKTKPVTGNLRPEEALQALTIYEGKFGRLKDDREKCAKAKEALELTDTGLLSGSEERVQVALEELQDLKGVWSELSKVWEQIDQMKEQPWVSVQPRKLRQNLDALLNQLKSFPARLRQYASYEFVQRLLKGYMKINMLVIELKSEALKDRHWKQLMKRLHVNWVVSELTLGQIWDVDLQKNEAIVKDVLLVAQGEMALEEFLKQIREVWNTYELDLVNYQNKCRLIRGWDDLFNKVKEHINSVSAMKLSPYYKVFEEDALSWEDKLNRIMALFDVWIDVQRRWVYLEGIFTGSADIKHLLPVETQRFQSISTEFLALMKKVSKSPLVMDVLNIQGVQRSLERLADLLGKIQKALGEYLERERSSFPRFYFVGDEDLLEIIGNSKNVAKLQKHFKKMFAGVSSIILNEDNSVVLGISSREGEEVMFKTPVSITEHPKINEWLTLVEKEMRVTLAKLLAESVTEVEIFGKATSIDPNTYITWIDKYQAQLVVLSAQIAWSENVETALSSMGGGGDAAPLHSVLSNVEVTLNVLADSVLMEQPPLRRRKLEHLITELVHQRDVTRSLIKSKIDNAKSFEWLSQMRFYFDPKQTDVLQQLSIQMANAKFNYGFEYLGVQDKLVQTPLTDRCYLTMTQALEARLGGSPFGPAGTGKTESVKALGHQLGRFVLVFNCDETFDFQAMGRIFVGLCQVGAWGCFDEFNRLEERMLSAVSQQVQCIQEALREHSNPNYDKTSAPITCELLNKQVKVSPDMAIFITMNPGYAGRSNLPDNLKKLFRSLAMTKPDRQLIAQVMLYSQGFRTAEVLANKIVPFFKLCDEQLSSQSHYDFGLRALKSVLVSAGNVKRERIQKIKREKEERGEAVDEGEIAENLPEQEILIQSVCETMVPKLVAEDIPLLFSLLSDVFPGVQYHRGEMTALREELKKVCQEMYLTYGDGEEVGGMWVEKVLQLYQITQINHGLMMVGPSGSGKSMAWRVLLKALERLEGVEGVAHIIDPKAISKDHLYGTLDPNTREWTDGLFTHVLRKIIDSVRGELQKRQWIVFDGDVDPEWVENLNSVLDDNKLLTLPNGERLSLPPNVRIMFEVQDLKYATLATVSRCGMVWFSEDVLSTDMIFNNFLARLRSIPLDEGEDEAQRRRKGKEDEGEEAASPMLQIQRDAATIMQPYFTSNGLVTKALEHAFQLEHIMDLTRLRCLGSLFSMLHQACRNVAQYNANHPDFPMQIEQLERYIQRYLVYAILWSLSGDSRLKMRAELGEYIRRITTVPLPTAPNIPIIDYEVSISGEWSPWQAKVPQIEVETHKVAAPDVVVPTLDTVRHEALLYTWLAEHKPLVLCGPPGSGKTMTLFSALRALPDMEVVGLNFSSATTPELLLKTFDHYCEYRRTPNGVVLAPVQLGKWLVLFCDEINLPDMDKYGTQRVISFIRQMVEHGGFYRTSDQTWVKLERIQFVGACNPPTDPGRKPLSHRFLRHVPVVYVDYPGPASLTQIYGTFNRAMLRLIPSLRTYAEPLTAAMVEFYTMSQERFTQDTQPHYIYSPREMTRWVRGIFEALRPLETLPVEGLIRIWAHEALRLFQDRLVEDEERRWTDENIDTVALKHFPNIDREKAMSRPILYSNWLSKDYIPVDQEELRDYVKARLKVFYEEELDVPLVLFNEVLDHVLRIDRIFRQPQGHLLLIGVSGAGKTTLSRFVAWMNGLSVYQIKVHRKYTGEDFDEDLRTVLRRSGCKNEKIAFIMDESNVLDSGFLERMNTLLANGEVPGLFEGDEYATLMTQCKEGAQKEGLMLDSHEELYKWFTSQVIRNLHVVFTMNPSSEGLKDRAATSPALFNRCVLNWFGDWSTEALYQVGKEFTSKMDLEKPNYIVPDYMPVVYDKLPQPPSHREAIVNSCVFVHQTLHQANARLAKRGGRTMAITPRHYLDFINHYANLFHEKRSELEEQQMHLNVGLRKIKETVDQVEELRRDLRIKSQELEVKNAAANDKLKKMVKDQQEAEKKKVMSQEIQEQLHKQQEVIADKQMSVKEDLDKVEPAVIEAQNAVKSIKKQHLVEVRSMANPPAAVKLALESICLLLGESTTDWKQIRSIIMRENFIPTIVNFSAEEISDAIREKMKKNYMSNPSYNYEIVNRASLACGPMVKWAIAQLNYADMLKRVEPLRNELQKLEDDAKDNQQKANEVEQMIRDLEASIARYKEEYAVLISEAQAIKADLAAVEAKVNRSTALLKSLSAERERWEKTSETFKNQMSTIAGDCLLSAAFIAYAGYFDQQMRQNLFTTWSHHLQQANIQFRTDIARTEYLSNADERLRWQASSLPADDLCTENAIMLKRFNRYPLIIDPSGQATEFIMNEYKDRKITRTSFLDDAFRKNLESALRFGNPLLVQDVESYDPVLNPVLNREVRRTGGRVLITLGDQDIDLSPSFVIFLSTRDPTVEFPPDLCSRVTFVNFTVTRSSLQSQCLNEVLKAERPDVDEKRSDLLKLQGEFQLRLRQLEKSLLQALNEVKGRILDDDTIITTLENLKREAAEVTRKVEETDIVMQEVETVSQQYLPLSTACSSIYFTMESLKQIHFLYQYSLQFFLDIYHNVLYENPNLKGVTDHTQRLSIITKDLFQVAFNRVARGMLHQDHITFAMLLARIKLKGTVGEPTYDAEFQHFLRGNEIVLSAGSTPRIQGLTVEQAEAVVRLSCLPAFKDLIAKVQADEQFGIWLDSSSPEQTVPYLWSEETPATPIGQAIHRLLLIQAFRPDRLLAMAHMFVSTNLGESFMSIMEQPLDLTHIVGTEVKPNTPVLMCSVPGYDASGHVEDLAAEQNTQITSIAIGSAEGFNQADKAINTAVKSGRWVMLKNVHLAPGWLMQLEKKLHSLQPHACFRLFLTMEINPKVPVNLLRAGRIFVFEPPPGVKANMLRTFSSIPVSRICKSPNERARLYFLLAWFHAIIQERLRYAPLGWSKKYEFGESDLRSACDTVDTWLDDTAKGRQNISPDKIPWSALKTLMAQSIYGGRVDNEFDQRLLNTFLERLFTTRSFDSEFKLACKVDGHKDIQMPDGIRREEFVQWVELLPDTQTPSWLGLPNNAERVLLTTQGVDMISKMLKMQMLEDEDDLAYAETEKKTRTDSTSDGRPAWMRTLHTTASNWLHLIPQTLSHLKRTVENIKDPLFRFFEREVKMGAKLLQDVRQDLADVVQVCEGKKKQTNYLRTLINELVKGILPRSWSHYTVPAGMTVIQWVSDFSERIKQLQNISLAAASGGAKELKNIHVCLGGLFVPEAYITATRQYVAQANSWSLEELCLEVNVTTSQGATLDACSFGVTGLKLQGATCNNNKLSLSNAISTALPLTQLRWVKQTNTEKKASVVTLPVYLNFTRADLIFTVDFEIATKEDPRSFYERGVAVLCTE

302 NP_057640.1 NP_002055.1

>NP_057640.1

MSKGRAEAAAGAAGILLRYLQEQNRPYSSQDVFGNLQREHGLGKAVVVKTLEQLAQQGKIKEKMYGKQKIYFADQDQFDMVSDADLQVLDGKIVALTAKVQSLQQSCRYMEAELKELSSALTTPEMQKEIQELKKECAGYRERLKNIKAATNHVTPEEKEQVYRERQKYCKEWRKRKRMATELSDAILEGYPKSKKQFFEEVGIETDEDYNVTLPDP

>NP_002055.1

MAQEFVNCKIQPGKVVVFIKPTCPYCRRAQEILSQLPIKQGLLEFVDITATNHTNEIQDYLQQLTGARTVPRVFIGKDCIGGCSDLVSLQQSGELLTRLKQIGALQ

303 NP_002575.1 NP_689508.3

>NP_002575.1

MAALPGTVPRMMRPAPGQNYPRTGFPLEVSTPLGQGRVNQLGGVFINGRPLPNHIRHKIVEMAHHGIRPCVISRQLRVSHGCVSKILCRYQETGSIRPGAIGGSKPRQVATPDVEKKIEEYKRENPGMFSWEIRDRLLKDGHCDRSTVPSGLVSSISRVLRIKFGKKEEEDEADKKEDDGEKKAKHSIDGILGDKGNRLDEGSDVESEPDLPLKRKQRRSRTTFTAEQLEELEKAFERTHYPDIYTREELAQRTKLTEARVQVWFSNRRARWRKQAGANQLAAFNHLLPGGFPPTGMPTLPPYQLPDSTYPTTTISQDGGSTVHRPQPLPPSTMHQGGLAAAAAAADTSSAYGARHSFSSYSDSFMNPAAPSNHMNPVSNGLSPQVMSILGNPSAVPPQPQADFSISPLHGGLDSATSISASCSQRADSIKPGDSLPTSQAYCPPTYSTTGYSVDPVAGYQYGQYGQSECLVPWASPVPIPSPTPRASCLFMESYKVVSGWGMSISQMEKLKSSQMEQFT

>NP_689508.3

MFEEKASSPSGKMGGEEKPIGAGEEKQKEGGKKKNKEGSGDGGRAELNPWPEYIYTRLEMYNILKAEHDSILAEKAEKDSKPIKVTLPDGKQVDAESWKTTPYQIACGISQGLADNTVIAKVNNVVWDLDRPLEEDCTLELLKFEDEEAQAVYWHSSAHIMGEAMERVYGGCLCYGPPIENGFYYDMYLEEGGVSSNDFSSLEALCKKIIKEKQAFERLEVKKETLLAMFKYNKFKCRILNEKVNTPTTTVYRCGPLIDLCRGPHVRHTGKIKALKIHKNSSTYWEGKADMETLQRIYGISFPDPKMLKEWEKFQEEAKNRDHRKIGRDQELYFFHELSPGSCFFLPKGAYIYNALIEFIRSEYRKRGFQEVVTPNIFNSRLWMTSGHWQHYSENMFSFEVEKELFALKPMNCPGHCLMFDHRPRSWRELPLRLADFGVLHRNELSGALTGLTRVRRFQQDDAHIFCAMEQIEDEIKGCLDFLRTVYSVFGFSFKLNLSTRPEKFLGDIEVWDQAEKQLENSLNEFGEKWELNSGDGAFYGPKIDIQIKDAIGRYHQCATIQLDFQLPIRFNLTYVSHDGDDKKRPVIVHRAILGSVERMIAILTENYGGKWPFWLSPRQVMVVPVGPTCDEYAQKVRQQFHDAKFMADIDLDPGCTLNKKIRNAQLAQYNFILVVGEKEKISGTVNIRTRDNKVHGERTISETIERLQQLKEFRSKQAEEEF

304 NP_109377.1 NP_004127.1

>NP_109377.1

MQKATYYDSSAIYGGYPYQAANGFAYNANQQPYPASAALGADGEYHRPACSLQSPSSAGGHPKAHELSEACLRTLSAPPSQPPSLGEPPLHPPPPQAAPPAPQPPQPAPQPPAPTPAAPPPPSSASPPQNASNNPTPANAAKSPLLNSPTVAKQIFPWMKESRQNTKQKTSSSSSGESCAGDKSPPGQASSKRARTAYTSAQLVELEKEFHFNRYLCRPRRVEMANLLNLTERQIKIWFQNRRMKYKKDQKGKGMLTSSGGQSPSRSPVPPGAGGYLNSMHSLVNSVPYEPQSPPPFSKPPQGTYGLPPASYPASLPSCAPPPPPQKRYTAAGAGAGGTPDYDPHAHGLQGNGSYGTPHIQGSPVFVGGSYVEPMSNSGPALFGLTHLPHAASGAMDYGGAGPLGSGHHHGPGPGEPHPTYTDLTGHHPSQGRIQEAPKLTHL

>NP_004127.1

MDAPKAGYAFEYLIETLNDSSHKKFFDVSKLGTKYDVLPYSIRVLLEAAVRNCDGFLMKKEDVMNILDWKTKQSNVEVPFFPARVLLQDFTGIPAMVDFAAMREAVKTLGGDPEKVHPACPTDLTVDHSLQIDFSKCAIQNAPNPGGGDLQKAGKLSPLKVQPKKLPCRGQTTCRGSCDSGELGRNSGTFSSQIENTPILCPFHLQPVPEPETVLKNQEVEFGRNRERLQFFKWSSRVFKNVAVIPPGTGMAHQINLEYLSRVVFEEKDLLFPDSVVGTDSHITMVNGLGILGWGVGGIETEAVMLGLPVSLTLPEVVGCELTGSSNPFVTSIDVVLGITKHLRQVGVAGKFVEFFGSGVSQLSIVDRTTIANMCPEYGAILSFFPVDNVTLKHLEHTGFSKAKLESMETYLKAVKLFRNDQNSSGEPEYSQVIQINLNSIVPSVSGPKRPQDRVAVTDMKSDFQACLNEKVGFKGFQIAAEKQKDIVSIHYEGSEYKLSHGSVVIAAVISCTNNCNPSVMLAAGLLAKKAVEAGLRVKPYIRTSLSPGSGMVTHYLSSSGVLPYLSKLGFEIVGYGCSTCVGNTAPLSDAVLNAVKQGDLVTCGILSGNKNFEGRLCDCVRANYLASPPLVVAYAIAGTVNIDFQTEPLGTDPTGKNIYLHDIWPSREEVHRVEEEHVILSMFKALKDKIEMGNKRWNSLEAPDSVLFPWDLKSTYIRCPSFFDKLTKEPIALQAIENAHVLLYLGDSVTTDHISPAGSIARNSAAAKYLTNRGLTPREFNSYGARRGNDAVMTRGTFANIKLFNKFIGKPAPKTIHFPSGQTLDVFEAAELYQKEGIPLIILAGKKYGSGNSRDWAAKGPYLLGVKAVLAESYEKIHKDHLIGIGIAPLQFLPGENADSLGLSGRETFSLTFPEELSPGITLNIQTSTGKVFSVIASFEDDVEITLYKHGGLLNFVARKFS

305 Q05952 Q9Y3D3

>Q05952

MDTQTHSLPITHTQLHSNSQPQSRTCTRHCQTFSQSCRQSHRGSRSQSSSQSPASHRNPTGAHSSSGHQSQSPNTSPPPKRHKKTMNSHHSPMRPTILHCRCPKNRKNLEGKLKKKKMAKRIQQVYKTKTRSSGWKSN

>Q9Y3D3

MVHLTTLLCKAYRGGHLTIRLALGGCTNRPFYRIVAAHNKCPRDGRFVEQLGSYDPLPNSHGEKLVALNLDRIRHWIGCGAHLSKPMEKLLGLAGFFPLHPMMITNAERLRRKRAREVLLASQKTDAEATDTEATET

306 NP_003359.3 NP_004763.1

>NP_003359.3

MPGVIPSESNGLSRGSPSKKNRLSLKFFQKKETKRALDFTDSQENEEKASEYRASEIDQVVPAAQSSPINCEKRENLLPFVGLNNLGNTCYLNSILQVLYFCPGFKSGVKHLFNIISRKKEALKDEANQKDKGNCKEDSLASYELICSLQSLIISVEQLQASFLLNPEKYTDELATQPRRLLNTLRELNPMYEGYLQHDAQEVLQCILGNIQETCQLLKKEEVKNVAELPTKVEEIPHPKEEMNGINSIEMDSMRHSEDFKEKLPKGNGKRKSDTEFGNMKKKVKLSKEHQSLEENQRQTRSKRKATSDTLESPPKIIPKYISENESPRPSQKKSRVKINWLKSATKQPSILSKFCSLGKITTNQGVKGQSKENECDPEEDLGKCESDNTTNGCGLESPGNTVTPVNVNEVKPINKGEEQIGFELVEKLFQGQLVLRTRCLECESLTERREDFQDISVPVQEDELSKVEESSEISPEPKTEMKTLRWAISQFASVERIVGEDKYFCENCHHYTEAERSLLFDKMPEVITIHLKCFAASGLEFDCYGGGLSKINTPLLTPLKLSLEEWSTKPTNDSYGLFAVVMHSGITISSGHYTASVKVTDLNSLELDKGNFVVDQMCEIGKPEPLNEEEARGVVENYNDEEVSIRVGGNTQPSKVLNKKNVEAIGLLGGQKSKADYELYNKASNPDKVASTAFAENRNSETSDTTGTHESDRNKESSDQTGINISGFENKISYVVQSLKEYEGKWLLFDDSEVKVTEEKDFLNSLSPSTSPTSTPYLLFYKKL

>NP_004763.1

MVYYPELFVWVSQEPFPNKDMEGRLPKGRLPVPKEVNRKKNDETNAASLTPLGSSELRSPRISYLHFF

307 NP_110517.2 Q9P0R6

>NP_110517.2

MDVGELLSYQPNRGTKRPRDDEEEEQKMRRKQTGTRERGRYREEEMTVVEEADDDKKRLLQIIDRDGEEEEEEEEPLDESSVKKMILTFEKRSYKNQELRIKFPDNPEKFMESELDLNDIIQEMHVVATMPDLYHLLVELNAVQSLLGLLGHDNTDVSIAVVDLLQELTDIDTLHESEEGAEVLIDALVDGQVVALLVQNLERLDESVKEEADGVHNTLAIVENMAEFRPEMCTEGAQQGLLQWLLKRLKAKMPFDANKLYCSEVLAILLQDNDENRELLGELDGIDVLLQQLSVFKRHNPSTAEEQEMMENLFDSLCSCLMLSSNRERFLKGEGLQLMNLMLREKKISRSSALKVLDHAMIGPEGTDNCHKFVDILGLRTIFPLFMKSPRKIKKVGTTEKEHEEHVCSILASLLRNLRGQQRTRLLNKFTENDSEKVDRLMELHFKYLGAMQVADKKIEGEKHDMVRRGEIIDNDTEEEFYLRRLDAGLFVLQHICYIMAEICNANVPQIRQRVHQILNMRGSSIKIVRHIIKEYAENIGDGRSPEFRENEQKRILGLLENF

>Q9P0R6

METDCNPMELSSMSGFEEGSELNGFEGTDMKDMRLEAEAVVNDVLFAVNNMFVSKSLRCADDVAYINVETKERNRYCLELTEAGLKVVGYAFDQVDDHLQTPYHETVYSLLDTLSPAYREAFGNALLQRLEALKRDGQS

308 NP_055136.1 O60783

>NP_055136.1

MGSELIGRLAPRLGLAEPDMLRKAEEYLRLSRVKCVGLSARTTETSSAVMCLDLAASWMKCPLDRAYLIKLSGLNKETYQSCLKSFECLLGLNSNIGIRDLAVQFSCIEAVNMASKILKSYESSLPQTQQVDLDLSRPLFTSAALLSACKILKLKVDKNKMVATSGVKKAIFDRLCKQLEKIGQQVDREPGDVATPPRKRKKIVVEAPAKEMEKVEEMPHKPQKDEDLTQDYEEWKRKILENAASAQKATAE

>O60783

MAAFMLGSLLRTFKQMVPSSASGQVRSHYVDWRMWRDVKRRKMAYEYADERLRINSLRKNTILPKILQDVADEEIAALPRDSCPVRIRNRCVMTSRPRGVKRRWRLSRIVFRHLADHGQLSGIQRATW

309 NP_006157.1 NP_005908.1

>NP_006157.1

MTMDGDSSTTDASQLGISADYIGGSHYVIQPHDDTEDSMNDHEDTNGSKESFREQDIYLPIANVARIMKNAIPQTGKIAKDAKECVQECVSEFISFITSEASERCHQEKRKTINGEDILFAMSTLGFDSYVEPLKLYLQKFREAMKGEKGIGGAVTATDGLSEELTEEAFTNQLPAGLITTDGQQQNVMVYTTSYQQISGVQQIQFS

>NP_005908.1

MSEPIRVLVTGAAGQIAYSLLYSIGNGSVFGKDQPIILVLLDITPMMGVLDGVLMELQDCALPLLKDVIATDKEDVAFKDLDVAILVGSMPRREGMERKDLLKANVKIFKSQGAALDKYAKKSVKVIVVGNPANTNCLTASKSAPSIPKENFSCLTRLDHNRAKAQIALKLGVTANDVKNVIIWGNHSSTQYPDVNHAKVKLQGKEVGVYEALKDDSWLKGEFVTTVQQRGAAVIKARKLSSAMSAAKAICDHVRDIWFGTPEGEFVSMGVISDGNSYGVPDDLLYSFPVVIKNKTWKFVEGLPINDFSREKMDLTAKELTEEKESAFEFLSSA

310 NP_004119.1 Q6V1X1

>NP_004119.1

MAERGELDLTGAKQNTGVWLVKVPKYLSQQWAKASGRGEVGKLRIAKTQGRTEVSFTLNEDLANIHDIGGKPASVSAPREHPFVLQSVGGQTLTVFTESSSDKLSLEGIVVQRAECRPAASENYMRLKRLQIEESSKPVRLSQQLDKVVTTNYKPVANHQYNIEYERKKKEDGKRARADKQHVLDMLFSAFEKHQYYNLKDLVDITKQPVVYLKEILKEIGVQNVKGIHKNTWELKPEYRHYQGEEKSD

>Q6V1X1

MWKRSEQMKIKSGKCNMAAAMETEQLGVEIFETADCEENIESQDRPKLEPFYVERYSWSQLKKLLADTRKYHGYMMAKAPHDFMFVKRNDPDGPHSDRIYYLAMSGENRENTLFYSEIPKTINRAAVLMLSWKPLLDLFQATLDYGMYSREEELLRERKRIGTVGIASYDYHQGSGTFLFQAGSGIYHVKDGGPQGFTQQPLRPNLVETSCPNIRMDPKLCPADPDWIAFIHSNDIWISNIVTREERRLTYVHNELANMEEDARSAGVATFVLQEEFDRYSGYWWCPKAETTPSGGKILRILYEENDESEVEIIHVTSPMLETRRADSFRYPKTGTANPKVTFKMSEIMIDAEGRIIDVIDKELIQPFEILFEGVEYIARAGWTPEGKYAWSILLDRSQTRLQIVLISPELFIPVEDDVMERQRLIESVPDSVTPLIIYEETTDIWINIHDIFHVFPQSHEEEIEFIFASECKTGFRHLYKITSILKESKYKRSSGGLPAPSDFKCPIKEEIAITSGEWEVLGRHGSNIQVDEVRRLVYFEGTKDSPLEHHLYVVSYVNPGEVTRLTDRGYSHSCCISQHCDFFISKYSNQKNPHCVSLYKLSSPEDDPTCKTKEFWATILDSAGPLPDYTPPEIFSFESTTGFTLYGMLYKPHDLQPGKKYPTVLFIYGGPQVQLVNNRFKGVKYFRLNTLASLGYVVVVIDNRGSCHRGLKFEGAFKYKMGQIEIDDQVEGLQYLASRYDFIDLDRVGIHGWSYGGYLSLMALMQRSDIFRVAIAGAPVTLWIFYDTGYTERYMGHPDQNEQGYYLGSVAMQAEKFPSEPNRLLLLHGFLDENVHFAHTSILLSFLVRAGKPYDLQIYPQERHSIRVPESGEHYELHLLHYLQENLGSRIAALKVI

311 NP_036354.1 Q7L0Y3

>NP_036354.1

MTPLVSRLSRLWAIMRKPRAAVGSGHRKQAASQEGRQKHAKNNSQAKPSACDGMIAECPGAPAGLARQPEEVVLQASVSSYHLFRDVAEVTAFRGSLLSWYDQEKRDLPWRRRAEDEMDLDRRAYAVWVSEVMLQQTQVATVINYYTGWMQKWPTLQDLASASLEEVNQLWAGLGYYSRGRRLQEGARKVVEELGGHMPRTAETLQQLLPGVGRYTAGAIASIAFGQATGVVDGNVARVLCRVRAIGADPSSTLVSQQLWGLAQQLVDPARPGDFNQAAMELGATVCTPQRPLCSQCPVESLCRARQRVEQEQLLASGSLSGSPDVEECAPNTGQCHLCLPPSEPWDQTLGVVNFPRKASRKPPREESSATCVLEQPGALGAQILLVQRPNSGLLAGLWEFPSVTWEPSEQLQRKALLQELQRWAGPLPATHLRHLGEVVHTFSHIKLTYQVYGLALEGQTPVTTVPPGARWLTQEEFHTAAVSTAMKKVFRVYQGQQPGTCMGSKRSQVSSPCSRKKPRMGQQVLDNFFRSHISTDAHSLNSAAQ

>Q7L0Y3

MAAFLKMSVSVNFFRPFTRFLVPFTLHRKRNNLTILQRYMSSKIPAVTYPKNESTPPSEELELDKWKTTMKSSVQEECVSTISSSKDEDPLAATREFIEMWRLLGREVPEHITEEELKTLMECVSNTAKKKYLKYLYTKEKVKKARQIKKEMKAAAREEAKNIKLLETTEEDKQKNFLFLRLWDRNMDIAMGWKGAQAMQFGQPLVFDMAYENYMKRKELQNTVSQLLESEGWNRRNVDPFHIYFCNLKIDGALHRELVKRYQEKWDKLLLTSTEKSHVDLFPKDSIIYLTADSPNVMTTFRHDKVYVIGSFVDKSMQPGTSLAKAKRLNLATECLPLDKYLQWEIGNKNLTLDQMIRILLCLKNNGNWQEALQFVPKRKHTGFLEISQHSQEFINRLKKAKT

312 NP_006749.1 NP_066950.1

>NP_006749.1

MAEYLASIFGTEKDKVNCSFYFKIGACRHGDRCSRLHNKPTFSQTIALLNIYRNPQNSSQSADGLRCAVSDVEMQEHYDEFFEEVFTEMEEKYGEVEEMNVCDNLGDHLVGNVYVKFRREEDAEKAVIDLNNRWFNGQPIHAELSPVTDFREACCRQYEMGECTRGGFCNFMHLKPISRELRRELYGRRRKKHRSRSRSRERRSRSRDRGRGGGGGGGGGGGGRERDRRRSRDRERSGRF

>NP_066950.1

MPGKKARKNAQPSPARAPAELEVECATQLRRFGDKLNFRQKLLNLISKLFCSGT

313 Q9Y6W3 P82663

>Q9Y6W3

MDATALERDAVQFARLAVQRDHEGRYSEAVFYYKEAAQALIYAEMAGSSLENIQEKITEYLERVQALHSAVQSKSADPLKSKHQLDLERAHFLVTQAFDEDEKENVEDAIELYTEAVDLCLKTSYETADKVLQNKLKQLARQALDRAEALSEPLTKPVGKISSTSVKPKPPPVRAHFPLGANPFLERPQSFISPQSCDAQGQRYTAEEIEVLRTTSKINGIEYVPFMNVDLRERFAYPMPFCDRWGKLPLSPKQKTTFSKWVRPEDLTNNPTMIYTVSSFSIKQTIVSDCSFVASLAISAAYERRFNKKLITGIIYPQNKDGEPEYNPCGKYMVKLHLNGVPRKVIIDDQLPVDHKGELLCSYSNNKSELWVSLIEKAYMKVMGGYDFPGSNSNIDLHALTGWIPERIAMHSDSQTFSKDNSFRMLYQRFHKGDVLITASTGMMTEAEGEKWGLVPTHAYAVLDIREFKGLRFIQLKNPWSHLRWKGRYSENDVKNWTPELQKYLNFDPRTAQKIDNGIFWISWDDLCQYYDVIYLSWNPGLFKESTCIHSTWDAKQGPVKDAYSLANNPQYKLEVQCPQGGAAVWVLLSRHITDKDDFANNREFITMVVYKTDGKKVYYPADPPPYIDGIRINSPHYLTKIKLTTPGTHTFTLVVSQYEKQNTIHYTVRVYSACSFTFSKIPSPYTLSKRINGKWSGQSAGGCGNFQETHKNNPIYQFHIEKTGPLLIELRGPRQYSVGFEVVTVSTLGDPGPHGFLRKSSGDYRCGFCYLELENIPSGIFNIIPSTFLPKQEGPFFLDFNSIIPIKITQLQ

>P82663

MPMKGRFPIRRTLQYLSQGNVVFKDSVKVMTVNYNTHGELGEGARKFVFFNIPQIQYKNPWVQIMMFKNMTPSPFLRFYLDSGEQVLVDVETKSNKEIMEHIRKILGKNEETLREEEEEKKQLSHPANFGPRKYCLRECICEVEGQVPCPSLVPLPKEMRGKYKAALKADAQD

314 NP_005002.3 NP_000161.2

>NP_005002.3

MEEHGVTQTEHMATIEAHAVAQQVQQVHVATYTEHSMLSADEDSPSSPEDTSYDDSDILNSTAADEVTAHLAAAGPVGMAAAAAVATGKKRKRPHVFESNPSIRKRQQTRLLRKLRATLDEYTTRVGQQAIVLCISPSKPNPVFKVFGAAPLENVVRKYKSMILEDLESALAEHAPAPQEVNSELPPLTIDGIPVSVDKMTQAQLRAFIPEMLKYSTGRGKPGWGKESCKPIWWPEDIPWANVRSDVRTEEQKQRVSWTQALRTIVKNCYKQHGREDLLYAFEDQQTQTQATATHSIAHLVPSQTVVQTFSNPDGTVSLIQVGTGATVATLADASELPTTVTVAQVNYSAVADGEVEQNWATLQGGEMTIQTTQASEATQAVASLAEAAVAASQEMQQGATVTMALNSEAAAHAVATLAEATLQGGGQIVLSGETAAAVGALTGVQDANGLVQIPVSMYQTVVTSLAQGNGPVQVAMAPVTTRISDSAVTMDGQAVEVVTLEQ

>NP_000161.2

MQSCARAWGLRLGRGVGGGRRLAGGSGPCWAPRSRDSSSGGGDSAAAGASRLLERLLPRHDDFARRHIGPGDKDQREMLQTLGLASIDELIEKTVPANIRLKRPLKMEDPVCENEILATLHAISSKNQIWRSYIGMGYYNCSVPQTILRNLLENSGWITQYTPYQPEVSQGRLESLLNYQTMVCDITGLDMANASLLDEGTAAAEALQLCYRHNKRRKFLVDPRCHPQTIAVVQTRAKYTGVLTELKLPCEMDFSGKDVSGVLFQYPDTEGKVEDFTELVERAHQSGSLACCATDLLALCILRPPGEFGVDIALGSSQRFGVPLGYGGPHAAFFAVRESLVRMMPGRMVGVTRDATGKEVYRLALQTREQHIRRDKATSNICTAQALLANMAAMFAIYHGSHGLEHIARRVHNATLILSEGLKRAGHQLQHDLFFDTLKIQCGCSVKEVLGRAAQRQINFRLFEDGTLGISLDETVNEKDLDDLLWIFGCESSAELVAESMGEECRGIPGSVFKRTSPFLTHQVFNSYHSETNIVRYMKKLENKDISLVHSMIPLGSCTMKLNSSSELAPITWKEFANIHPFVPLDQAQGYQQLFRELEKDLCELTGYDQVCFQPNSGAQGEYAGLATIRAYLNQKGEGHRTVCLIPKSAHGTNPASAHMAGMKIQPVEVDKYGNIDAVHLKAMVDKHKENLAAIMITYPSTNGVFEENISDVCDLIHQHGGQVYLDGANMNAQVGICRPGDFGSDVSHLNLHKTFCIPHGGGGPGMGPIGVKKHLAPFLPNHPVISLKRNEDACPVGTVSAAPWGSSSILPISWAYIKMMGGKGLKQATETAILNANYMAKRLETHYRILFRGARGYVGHEFILDTRPFKKSANIEAVDVAKRLQDYGFHAPTMSWPVAGTLMVEPTESEDKAELDRFCDAMISIRQEIADIEEGRIDPRVNPLKMSPHSLTCVTSSHWDRPYSREVAAFPLPFVKPENKFWPTIARIDDIYGDQHLVCTCPPMEVYESPFSEQKRASS

315 Q7Z5Q5 NP_076425.1

>Q7Z5Q5

MENYEALVGFDLCNTPLSSVAQKIMSAMHSGDLVDSKTWGKSTETMEVINKSSVKYSVQLEDRKTQSPEKKDLKSLRSQTSRGSAKLSPQSFSVRLTDQLSADQKQKSISSLTLSSCLIPQYNQEASVLQKKGHKRKHFLMENINNENKGSINLKRKHITYNNLSEKTSKQMALEEDTDDAEGYLNSGNSGALKKHFCDIRHLDDWAKSQLIEMLKQAAALVITVMYTDGSTQLGADQTPVSSVRGIVVLVKRQAEGGHGCPDAPACGPVLEGFVSDDPCIYIQIEHSAIWDQEQEAHQQFARNVLFQTMKCKCPVICFNAKDFVRIVLQFFGNDGSWKHVADFIGLDPRIAAWLIDPSDATPSFEDLVEKYCEKSITVKVNSTYGNSSRNIVNQNVRENLKTLYRLTMDLCSKLKDYGLWQLFRTLELPLIPILAVMESHAIQVNKEEMEKTSALLGARLKELEQEAHFVAGERFLITSNNQLREILFGKLKLHLLSQRNSLPRTGLQKYPSTSEAVLNALRDLHPLPKIILEYRQVHKIKSTFVDGLLACMKKGSISSTWNQTGTVTGRLSAKHPNIQGISKHPIQITTPKNFKGKEDKILTISPRAMFVSSKGHTFLAADFSQIELRILTHLSGDPELLKLFQESERDDVFSTLTSQWKDVPVEQVTHADREQTKKVVYAVVYGAGKERLAACLGVPIQEAAQFLESFLQKYKKIKDFARAAIAQCHQTGCVVSIMGRRRPLPRIHAHDQQLRAQAERQAVNFVVQGSAADLCKLAMIHVFTAVAASHTLTARLVAQIHDELLFEVEDPQIPECAALVRRTMESLEQVQALELQLQVPLKVSLSAGRSWGHLVPLQEAWGPPPGPCRTESPSNSLAAPGSPASTQPPPLHFSPSFCL

>NP_076425.1

MARKKVRPRLIAELARRVRALREQLNRPRDSQLYAVDYETLTRPFSGRRLPVRAWADVRRESRLLQLLGRLPLFGLGRLVTRKSWLWQHDEPCYWRLTRVRPDYTAQNLDHGKAWGILTFKGKTESEAREIEHVMYHDWRLVPKHEEEAFTAFTPAPEDSLASVPYPPLLRAMIIAERQKNGDTSTEEPMLNVQRIRMEPWDYPAKQEDKGRAKGTPV

316 NP_000312.2 NP_004754.1

>NP_000312.2

MPPKTPRKTAATAAAAAAEPPAPPPPPPPEEDPEQDSGPEDLPLVRLEFEETEEPDFTALCQKLKIPDHVRERAWLTWEKVSSVDGVLGGYIQKKKELWGICIFIAAVDLDEMSFTFTELQKNIEISVHKFFNLLKEIDTSTKVDNAMSRLLKKYDVLFALFSKLERTCELIYLTQPSSSISTEINSALVLKVSWITFLLAKGEVLQMEDDLVISFQLMLCVLDYFIKLSPPMLLKEPYKTAVIPINGSPRTPRRGQNRSARIAKQLENDTRIIEVLCKEHECNIDEVKNVYFKNFIPFMNSLGLVTSNGLPEVENLSKRYEEIYLKNKDLDARLFLDHDKTLQTDSIDSFETQRTPRKSNLDEEVNVIPPHTPVRTVMNTIQQLMMILNSASDQPSENLISYFNNCTVNPKESILKRVKDIGYIFKEKFAKAVGQGCVEIGSQRYKLGVRLYYRVMESMLKSEEERLSIQNFSKLLNDNIFHMSLLACALEVVMATYSRSTSQNLDSGTDLSFPWILNVLNLKAFDFYKVIESFIKAEGNLTREMIKHLERCEHRIMESLAWLSDSPLFDLIKQSKDREGPTDHLESACPLNLPLQNNHTAADMYLSPVRSPKKKGSTTRVNSTANAETQATSAFQTQKPLKSTSLSLFYKKVYRLAYLRLNTLCERLLSEHPELEHIIWTLFQHTLQNEYELMRDRHLDQIMMCSMYGICKVKNIDLKFKIIVTAYKDLPHAVQETFKRVLIKEEEYDSIIVFYNSVFMQRLKTNILQYASTRPPTLSPIPHIPRSPYKFPSSPLRIPGGNIYISPLKSPYKISEGLPTPTKMTPRSRILVSIGESFGTSEKFQKINQMVCNSDRVLKRSAEGSNPPKPLKKLRFDIEGSDEADGSKHLPGESKFQQKLAEMTSTRTRMQKQKMNDSMDTSNKEEK

>NP_004754.1

MFRKGKKRHSSSSSQSSEISTKSKSVDSSLGGLSRSSTVASLDTDSTKSSGQSNNNSDTCAEFRIKYVGAIEKLKLSEGKGLEGPLDLINYIDVAQQDGKLPFVPPEEEFIMGVSKYGIKVSTSDQYDVLHRHALYLIIRMVCYDDGLGAGKSLLALKTTDASNEEYSLWVYQCNSLEQAQAICKVLSTAFDSVLTSEKP

317 NP_002543.2 P82675

>NP_002543.2

MSSRKSKSNSLIHTECLSQVQRILRERFCRQSPHSNLFGVQVQYKHLSELLKRTALHGESNSVLIIGPRGSGKTMLINHALKELMEIEEVSENVLQVHLNGLLQINDKIALKEITRQLNLENVVGDKVFGSFAENLSFLLEALKKGDRTSSCPVIFILDEFDLFAHHKNQTLLYNLFDISQSAQTPIAVIGLTCRLDILELLEKRVKSRFSHRQIHLMNSFGFPQYVKIFKEQLSLPAEFPDKVFAEKWNENVQYLSEDRSVQEVLQKHFNISKNLRSLHMLLMLALNRVTASHPFMTAVDLMEASQLCSMDSKANIVHGLSVLEICLIIAMKHLNDIYEEEPFNFQMVYNEFQKFVQRKAHSVYNFEKPVVMKAFEHLQQLELIKPMERTSGNSQREYQLMKLLLDNTQIMNALQKYPNCPTDVRQWATSSLSWL

>P82675

MATAVRAVGCLPVLCSGTAGHLLGRQCSLNTLPAASILAWKSVLGNGHLSSLGTRDTHPYASLSRALQTQCCISSPSHLMSQQYRPYSFFTKLTADELWKGALAETGAGAKKGRGKRTKKKKRKDLNRGQIIGEGRYGFLWPGLNVPLMKNGAVQTIAQRSKEEQEKVEADMIQQREEWDRKKKMKVKRERGWSGNSWGGISLGPPDPGPCGETYEDFDTRILEVRNVFTMTAKEGRKKSIRVLVAVGNGKGAAGFSIGKATDRMDAFRKAKNRAVHHLHYIERYEDHTIFHDISLRFKRTHIKMKKQPKGYGLRCHRAIITICRLIGIKDMYAKVSGSINMLSLTQGLFRGLSRQETHQQLADKKGLHVVEIREECGPLPIVVASPRGPLRKDPEPEDEVPDVKLDWEDVKTAQGMKRSVWSNLKRAAT

318 NP_002938.1 NP_005495.2

>NP_002938.1

MVDMMDLPRSRINAGMLAQFIDKPVCFVGRLEKIHPTGKMFILSDGEGKNGTIELMEPLDEEISGIVEVVGRVTAKATILCTSYVQFKEDSHPFDLGLYNEAVKIIHDFPQFYPLGIVQHD

>NP_005495.2

MKDCSNGCSAECTGEGGSKEVVGTFKAKDLIVTPATILKEKPDPNNLVFGTVFTDHMLTVEWSSEFGWEKPHIKPLQNLSLHPGSSALHYAVELFEGLKAFRGVDNKIRLFQPNLNMDRMYRSAVRATLPVFDKEELLECIQQLVKLDQEWVPYSTSASLYIRPTFIGTEPSLGVKKPTKALLFVLLSPVGPYFSSGTFNPVSLWANPKYVRAWKGGTGDCKMGGNYGSSLFAQCEAVDNGCQQVLWLYGEDHQITEVGTMNLFLYWINEDGEEELATPPLDGIILPGVTRRCILDLAHQWGEFKVSERYLTMDDLTTALEGNRVREMFGSGTACVVCPVSDILYKGETIHIPTMENGPKLASRILSKLTDIQYGREESDWTIVLS

319 NP_003194.3 NP_002055.1

>NP_003194.3

MAHRPKRTFRQRAADSSDSDGAEESPAEPGAPRELPVPGSAEEEPPSGGGRAQVAGLPHRVRGPRGRGRVWASSRRATKAAPRADEGSESRTLDVSTDEEDKIHHSSESKDDQGLSSDSSSSLGEKELSSTVKIPDAAFIQAARRKRELARAQDDYISLDVQHTSSISGMKRESEDDPESEPDDHEKRIPFTLRPQTLRQRMAEESISRNEETSEESQEDEKQDTWEQQQMRKAVKIIEERDIDLSCGNGSSKVKKFDTSISFPPVNLEIIKKQLNTRLTLLQETHRSHLREYEKYVQDVKSSKSTIQNLESSSNQALNCKFYKSMKIYVENLIDCLNEKIINIQEIESSMHALLLKQAMTFMKRRQDELKHESTYLQQLSRKDETSTSGNFSVDEKTQWILEEIESRRTKRRQARVLSGNCNHQEGTSSDDELPSAEMIDFQKSQGDILQKQKKVFEEVQDDFCNIQNILLKFQQWREKFPDSYYEAFISLCIPKLLNPLIRVQLIDWNPLKLESTGLKEMPWFKSVEEFMDSSVEDSKKESSSDKKVLSAIINKTIIPRLTDFVEFLWDPLSTSQTTSLITHCRVILEEHSTCENEVSKSRQDLLKSIVSRMKKAVEDDVFIPLYPKSAVENKTSPHSKFQERQFWSGLKLFRNILLWNGLLTDDTLQELGLGKLLNRYLIIALLNATPGPDVVKKCNQVAACLPEKWFENSAMRTSIPQLENFIQFLLQSAHKLSRSEFRDEVEEIILILVKIKALNQAESFIGEHHLDHLKSLIKED

>NP_002055.1

MAQEFVNCKIQPGKVVVFIKPTCPYCRRAQEILSQLPIKQGLLEFVDITATNHTNEIQDYLQQLTGARTVPRVFIGKDCIGGCSDLVSLQQSGELLTRLKQIGALQ

320 Q96T37 Q9Y3E5

>Q96T37

MRTAGRDPVPRRSPRWRRAVPLCETSAGRRVTQLRGDDLRRPATMKGKERSPVKAKRSRGGEDSTSRGERSKKLGGSGGSNGSSSGKTDSGGGSRRSLHLDKSSSRGGSREYDTGGGSSSSRLHSYSSPSTKNSSGGGESRSSSRGGGGESRSSGAASSAPGGGDGAEYKTLKISELGSQLSDEAVEDGLFHEFKRFGDVSVKISHLSGSGSGDERVAFVNFRRPEDARAAKHARGRLVLYDRPLKIEAVYVSRRRSRSPLDKDTYPPSASVVGASVGGHRHPPGGGGGQRSLSPGGAALGYRDYRLQQLALGRLPPPPPPPLPRDLERERDYPFYERVRPAYSLEPRVGAGAGAAPFREVDEISPEDDQRANRTLFLGNLDITVTESDLRRAFDRFGVITEVDIKRPSRGQTSTYGFLKFENLDMSHRAKLAMSGKIIIRNPIKIGYGKATPTTRLWVGGLGPWVPLAALAREFDRFGTIRTIDYRKGDSWAYIQYESLDAAHAAWTHMRGFPLGGPDRRLRVDFADTEHRYQQQYLQPLPLTHYELVTDAFGHRAPDPLRGARDRTPPLLYRDRDRDLYPDSDWVPPPPPVRERSTRTAATSVPAYEPLDSLDRRRDGWSLDRDRGDRDLPSSRDQPRKRRLPEESGGRHLDRSPESDRPRKRHCAPSPDRSPELSSSRDRYNSDNDRSSRLLLERPSPIRDRRGSLEKSQGDKRDRKNSASAERDRKHRTTAPTEGKSPLKKEDRSDGSAPSTSTASSKLKSPSQKQDGGTAPVASASPKLCLAWQGMLLLKNSNFPSNMHLLQGDLQVASSLLVEGSTGGKVAQLKITQRLRLDQPKLDEVTRRIKVAGPNGYAILLAVPGSSDSRSSSSSAASDTATSTQRPLRNLVSYLKQKQAAGVISLPVGGNKDKENTGVLHAFPPCEFSQQFLDSPAKALAKSEEDYLVMIIVRGFGFQIGVRYENKKRENLALTLL

>Q9Y3E5

MPSKSLVMEYLAHPSTLGLAVGVACGMCLGWSLRVCFGMLPKSKTSKTHTDTESEASILGDSGEYKMILVVRNDLKMGKGKVAAQCSHAAVSAYKQIQRRNPEMLKQWEYCGQPKVVVKAPDEETLIALLAHAKMLGLTVSLIQDAGRTQIAPGSQTVLGIGPGPADLIDKVTGHLKLY

321 NP_490595.1 NP_004754.1

>NP_490595.1

MASGRGASSRWFFTREQLENTPSRRCGVEADKELSCRQQAANLIQEMGQRLNVSQLTINTAIVYMHRFYMHHSFTKFNKNIISSTALFLAAKVEEQARKLEHVIKVAHACLHPLEPLLDTKCDAYLQQTQELVILETIMLQTLGFEITIEHPHTDVVKCTQLVRASKDLAQTSYFMATNSLHLTTFCLQYKPTVIACVCIHLACKWSNWEIPVSTDGKHWWEYVDPTVTLELLDELTHEFLQILEKTPNRLKKIRNWRANQAARKPKVDGQVSETPLLGSSLVQNSILVDSVTGVPTNPSFQKPSTSAFPAPVPLNSGNISVQDSHTSDNLSMLATGMPSTSYGLSSHQEWPQHQDSARTEQLYSQKQETSLSGSQYNINFQQGPSISLHSGLHHRPDKISDHSSVKQEYTHKAGSSKHHGPISTTPGIIPQKMSLDKYREKRKLETLDLDVRDHYIAAQVEQQHKQGQSQAASSSSVTSPIKMKIPIANTEKYMADKKEKSGSLKLRIPIPPTDKSASKEELKMKIKVSSSERHSSSDEGSGKSKHSSPHISRDHKEKHKEHPSSRHHTSSHKHSHSHSGSSSGGSKHSADGIPPTVLRSPVGLSSDGISSSSSSSRKRLHVNDASHNHHSKMSKSSKSSGSSSSSSSSVKQYISSHNSVFNHPLPPPPPVTYQVGYGHLSTLVKLDKKPVETNGPDANHEYSTSSQHMDYKDTFDMLDSLLSAQGMNM

>NP_004754.1

MFRKGKKRHSSSSSQSSEISTKSKSVDSSLGGLSRSSTVASLDTDSTKSSGQSNNNSDTCAEFRIKYVGAIEKLKLSEGKGLEGPLDLINYIDVAQQDGKLPFVPPEEEFIMGVSKYGIKVSTSDQYDVLHRHALYLIIRMVCYDDGLGAGKSLLALKTTDASNEEYSLWVYQCNSLEQAQAICKVLSTAFDSVLTSEKP

322 Q96T37 NP_003126.1

>Q96T37

MRTAGRDPVPRRSPRWRRAVPLCETSAGRRVTQLRGDDLRRPATMKGKERSPVKAKRSRGGEDSTSRGERSKKLGGSGGSNGSSSGKTDSGGGSRRSLHLDKSSSRGGSREYDTGGGSSSSRLHSYSSPSTKNSSGGGESRSSSRGGGGESRSSGAASSAPGGGDGAEYKTLKISELGSQLSDEAVEDGLFHEFKRFGDVSVKISHLSGSGSGDERVAFVNFRRPEDARAAKHARGRLVLYDRPLKIEAVYVSRRRSRSPLDKDTYPPSASVVGASVGGHRHPPGGGGGQRSLSPGGAALGYRDYRLQQLALGRLPPPPPPPLPRDLERERDYPFYERVRPAYSLEPRVGAGAGAAPFREVDEISPEDDQRANRTLFLGNLDITVTESDLRRAFDRFGVITEVDIKRPSRGQTSTYGFLKFENLDMSHRAKLAMSGKIIIRNPIKIGYGKATPTTRLWVGGLGPWVPLAALAREFDRFGTIRTIDYRKGDSWAYIQYESLDAAHAAWTHMRGFPLGGPDRRLRVDFADTEHRYQQQYLQPLPLTHYELVTDAFGHRAPDPLRGARDRTPPLLYRDRDRDLYPDSDWVPPPPPVRERSTRTAATSVPAYEPLDSLDRRRDGWSLDRDRGDRDLPSSRDQPRKRRLPEESGGRHLDRSPESDRPRKRHCAPSPDRSPELSSSRDRYNSDNDRSSRLLLERPSPIRDRRGSLEKSQGDKRDRKNSASAERDRKHRTTAPTEGKSPLKKEDRSDGSAPSTSTASSKLKSPSQKQDGGTAPVASASPKLCLAWQGMLLLKNSNFPSNMHLLQGDLQVASSLLVEGSTGGKVAQLKITQRLRLDQPKLDEVTRRIKVAGPNGYAILLAVPGSSDSRSSSSSAASDTATSTQRPLRNLVSYLKQKQAAGVISLPVGGNKDKENTGVLHAFPPCEFSQQFLDSPAKALAKSEEDYLVMIIVRGFGFQIGVRYENKKRENLALTLL

>NP_003126.1

MACAAARSPADQDRFICIYPAYLNNKKTIAEGRRIPISKAVENPTATEIQDVCSAVGLNVFLEKNKMYSREWNRDVQYRGRVRVQLKQEDGSLCLVQFPSRKSVMLYAAEMIPKLKTRTQKTGGADQSLQQGEGSKKGKGKKKK

323 P54803 NP_061821.1

>P54803

MAEWLLSASWQRRAKAMTAAAGSAGRAAVPLLLCALLAPGGAYVLDDSDGLGREFDGIGAVSGGGATSRLLVNYPEPYRSQILDYLFKPNFGASLHILKVEIGGDGQTTDGTEPSHMHYALDENYFRGYEWWLMKEAKKRNPNITLIGLPWSFPGWLGKGFDWPYVNLQLTAYYVVTWIVGAKRYHDLDIDYIGIWNERSYNANYIKILRKMLNYQGLQRVKIIASDNLWESISASMLLDAELFKVVDVIGAHYPGTHSAKDAKLTGKKLWSSEDFSTLNSDMGAGCWGRILNQNYINGYMTSTIAWNLVASYYEQLPYGRCGLMTAQEPWSGHYVVESPVWVSAHTTQFTQPGWYYLKTVGHLEKGGSYVALTDGLGNLTIIIETMSHKHSKCIRPFLPYFNVSQQFATFVLKGSFSEIPELQVWYTKLGKTSERFLFKQLDSLWLLDSDGSFTLSLHEDELFTLTTLTTGRKGSYPLPPKSQPFPSTYKDDFNVDYPFFSEAPNFADQTGVFEYFTNIEDPGEHHFTLRQVLNQRPITWAADASNTISIIGDYNWTNLTIKCDVYIETPDTGGVFIAGRVNKGGILIRSARGIFFWIFANGSYRVTGDLAGWIIYALGRVEVTAKKWYTLTLTIKGHFASGMLNDKSLWTDIPVNFPKNGWAAIGTHSFEFAQFDNFLVEATR

>NP_061821.1

MSIAGVAAQEIRVPLKTGFLHNGRAMGNMRKTYWSSRSEFKNNFLNIDPITMAYSLNSSAQERLIPLGHASKSAPMNGHCFAENGPSQKSSLPPLLIPPSENLGPHEEDQVVCGFKKLTVNGVCASTPPLTPIKNSPSLFPCAPLCERGSRPLPPLPISEALSLDDTDCEVEFLTSSDTDFLLEDSTLSDFKYDVPGRRSFRGCGQINYAYFDTPAVSAADLSYVSDQNGGVPDPNPPPPQTHRRLRRSHSGPAGSFNKPAIRISNCCIHRASPNSDEDKPEVPPRVPIPPRPVKPDYRRWSAEVTSSTYSDEDRPPKVPPREPLSPSNSRTPSPKSLPSYLNGVMPPTQSFAPDPKYVSSKALQRQNSEGSASKVPCILPIIENGKKVSSTHYYLLPERPPYLDKYEKFFREAEETNGGAQIQPLPADCGISSATEKPDSKTKMDLGGHVKRKHLSYVVSP

324 NP_001713.2 NP_005026.3

>NP_001713.2

MSEGNAAGEPSTPGGPRPLLTGARGLIGRRPAPPLTPGRLPSIRSRDLTLGGVKKKTFTPNIISRKIKEEPKEEVTVKKEKRERDRDRQREGHGRGRGRPEVIQSHSIFEQGPAEMMKKKGNWDKTVDVSDMGPSHIINIKKEKRETDEETKQILRMLEKDDFLDDPGLRNDTRNMPVQLPLAHSGWLFKEENDEPDVKPWLAGPKEEDMEVDIPAVKVKEEPRDEEEEAKMKAPPKAARKTPGLPKDVSVAELLRELSLTKEEELLFLQLPDTLPGQPPTQDIKPIKTEVQGEDGQVVLIKQEKDREAKLAENACTLADLTEGQVGKLLIRKSGRVQLLLGKVTLDVTMGTACSFLQELVSVGLGDSRTGEMTVLGHVKHKLVCSPDFESLLDHKHR

>NP_005026.3

MSALCWGRGAAGLKRALRPCGRPGLPGKEGTAGGVCGPRRSSSASPQEQDQDRRKDWGHVELLEVLQARVRQLQAESVSEVVVNRVDVARLPECGSGDGSLQPPRKVQMGAKDATPVPCGRWAKILEKDKRTQQMRMQRLKAKLQMPFQSGEFKALTRRLQVEPRLLSKQMAGCLEDCTRQAPESPWEEQLARLLQEAPGKLSLDVEQAPSGQHSQAQLSGQQQRLLAFFKCCLLTDQLPLAHHLLVVHHGQRQKRKLLTLDMYNAVMLGWARQGAFKELVYVLFMVKDAGLTPDLLSYAAALQCMGRQDQDAGTIERCLEQMSQEGLKLQALFTAVLLSEEDRATVLKAVHKVKPTFSLPPQLPPPVNTSKLLRDVYAKDGRVSYPKLHLPLKTLQCLFEKQLHMELASRVCVVSVEKPTLPSKEVKHARKTLKTLRDQWEKALCRALRETKNRLEREVYEGRFSLYPFLCLLDEREVVRMLLQVLQALPAQGESFTTLARELSARTFSRHVVQRQRVSGQVQALQNHYRKYLCLLASDAEVPEPCLPRQYWEELGAPEALREQPWPLPVQMELGKLLAEMLVQATQMPCSLDKPHRSSRLVPVLYHVYSFRNVQQIGILKPHPAYVQLLEKAAEPTLTFEAVDVPMLCPPLPWTSPHSGAFLLSPTKLMRTVEGATQHQELLETCPPTALHGALDALTQLGNCAWRVNGRVLDLVLQLFQAKGCPQLGVPAPPSEAPQPPEAHLPHSAAPARKAELRRELAHCQKVAREMHSLRAEALYRLSLAQHLRDRVFWLPHNMDFRGRTYPCPPHFNHLGSDVARALLEFAQGRPLGPHGLDWLKIHLVNLTGLKKREPLRKRLAFAEEVMDDILDSADQPLTGRKWWMGAEEPWQTLACCMEVANAVRASDPAAYVSHLPVHQDGSCNGLQHYAALGRDSVGAASVNLEPSDVPQDVYSGVAAQVEVFRRQDAQRGMRVAQVLEGFITRKVVKQTVMTVVYGVTRYGGRLQIEKRLRELSDFPQEFVWEASHYLVRQVFKSLQEMFSGTRAIQHWLTESARLISHMGSVVEWVTPLGVPVIQPYRLDSKVKQIGGGIQSITYTHNGDISRKPNTRKQKNGFPPNFIHSLDSSHMMLTALHCYRKGLTFVSVHDCYWTHAADVSVMNQVCREQFVRLHSEPILQDLSRFLVKRFCSEPQKILEASQLKETLQAVPKPGAFDLEQVKRSTYFFS

325 NP_002963.1 NP_110379.2

>NP_002963.1

MARLADYFVLVAFGPHPRGSGEGQGQILQRFPEKDWEDNPFPQGIELFCQPSGWQLCPERNPPTFFVAVLTDINSERHYCACLTFWEPAEPSQETTRVEDATEREEEGDEGGQTHLSPTAPAPSAQLFAPKTLVLVSRLDHTEVFRNSLGLIYAIHVEGLNVCLENVIGNLLTCTVPLAGGSQRTISLGAGDRQVIQTPLADSLPVSRCSVALLFRQLGITNVLSLFCAALTEHKVLFLSRSYQRLADACRGLLALLFPLRYSFTYVPILPAQLLEVLSTPTPFIIGVNAAFQAETQELLDVIVADLDGGTVTIPECVHIPPLPEPLQSQTHSVLSMVLDPELELADLAFPPPTTSTSSLKMQDKELRAVFLRLFAQLLQGYRWCLHVVRIHPEPVIRFHKAAFLGQRGLVEDDFLMKVLEGMAFAGFVSERGVPYRPTDLFDELVAHEVARMRADENHPQRVLRHVQELAEQLYKNENPYPAVAMHKVQRPGESSHLRRVPRPFPRLDEGTVQWIVDQAAAKMQGAPPAVKAERRTTVPSGPPMTAILERCSGLHVNSARRLEVVRNCISYVFEGKMLEAKKLLPAVLRALKGRAARRCLAQELHLHVQQNRAVLDHQQFDFVVRMMNCCLQDCTSLDEHGIAAALLPLVTAFCRKLSPGVTQFAYSCVQEHVVWSTPQFWEAMFYGDVQTHIRALYLEPTEDLAPAQEVGEAPSQEDERSALDVASEQRRLWPTLSREKQQELVQKEESTVFSQAIHYANRMSYLLLPLDSSKSRLLRERAGLGDLESASNSLVTNSMAGSVAESYDTESGFEDAETCDVAGAVVRFINRFVDKVCTESGVTSDHLKGLHVMVPDIVQMHIETLEAVQRESRRLPPIQKPKLLRPRLLPGEECVLDGLRVYLLPDGREEGAGGSAGGPALLPAEGAVFLTTYRVIFTGMPTDPLVGEQVVVRSFPVAALTKEKRISVQTPVDQLLQDGLQLRSCTFQLLKMAFDEEVGSDSAELFRKQLHKLRYPPDIRATFAFTLGSAHTPGRPPRVTKDKGPSLRTLSRNLVKNAKKTIGRQHVTRKKYNPPSWEHRGQPPPEDQEDEISVSEELEPSTLTPSSALKPSDRMTMSSLVERACCRDYQRLGLGTLSSSLSRAKSEPFRISPVNRMYAICRSYPGLLIVPQSVQDNALQRVSRCYRQNRFPVVCWRSGRSKAVLLRSGGLHGKGVVGLFKAQNAPSPGQSQADSSSLEQEKYLQAVVSSMPRYADASGRNTLSGFSSAHMGSHGKWGSVRTSGRSSGLGTDVGSRLAGRDALAPPQANGGPPDPGFLRPQRAALYILGDKAQLKGVRSDPLQQWELVPIEVFEARQVKASFKKLLKACVPGCPAAEPSPASFLRSLEDSEWLIQIHKLLQVSVLVVELLDSGSSVLVGLEDGWDITTQVVSLVQLLSDPFYRTLEGFRLLVEKEWLSFGHRFSHRGAHTLAGQSSGFTPVFLQFLDCVHQVHLQFPMEFEFSQFYLKFLGYHHVSRRFRTFLLDSDYERIELGLLYEEKGERRGQVPCRSVWEYVDRLSKRTPVFHNYMYAPEDAEVLRPYSNVSNLKVWDFYTEETLAEGPPYDWELAQGPPEPPEEERSDGGAPQSRRRVVWPCYDSCPRAQPDAISRLLEELQRLETELGQPAERWKDTWDRVKAAQRLEGRPDGRGTPSSLLVSTAPHHRRSLGVYLQEGPVGSTLSLSLDSDQSSGSTTSGSRQAARRSTSTLYSQFQTAESENRSYEGTLYKKGAFMKPWKARWFVLDKTKHQLRYYDHRVDTECKGVIDLAEVEAVAPGTPTMGAPKTVDEKAFFDVKTTRRVYNFCAQDVPSAQQWVDRIQSCLSDA

>NP_110379.2

MEGPLSVFGDRSTGETIRSQNVMAAASIANIVKSSLGPVGLDKMLVDDIGDVTITNDGATILKLLEVEHPAAKVLCELADLQDKEVGDGTTSVVIIAAELLKNADELVKQKIHPTSVISGYRLACKEAVRYINENLIVNTDELGRDCLINAAKTSMSSKIIGINGDFFANMVVDAVLAIKYTDIRGQPRYPVNSVNILKAHGRSQMESMLISGYALNCVVGSQGMPKRIVNAKIACLDFSLQKTKMKLGVQVVITDPEKLDQIRQRESDITKERIQKILATGANVILTTGGIDDMCLKYFVEAGAMAVRRVLKRDLKRIAKASGATILSTLANLEGEETFEAAMLGQAEEVVQERICDDELILIKNTKARTSASIILRGANDFMCDEMERSLHDALCVVKRVLESKSVVPGGGAVEAALSIYLENYATSMGSREQLAIAEFARSLLVIPNTLAVNAAQDSTDLVAKLRAFHNEAQVNPERKNLKWIGLDLSNGKPRDNKQAGVFEPTIVKVKSLKFATEAAITILRIDDLIKLHPESKDDKHGSYEDAVHSGALND

326 Q8IXF0 P50135

>Q8IXF0

MAPTKPSFQQDPSRRERITAQHPLPNQSECRKIYRYDGIYCESTYQNLQALRKEKSRDAARSRRGKENFEFYELAKLLPLPAAITSQLDKASIIRLTISYLKMRDFANQGDPPWNLRMEGPPPNTSVKVIGAQRRRSPSALAIEVFEAHLGSHILQSLDGFVFALNQEGKFLYISETVSIYLGLSQVELTGSSVFDYVHPGDHVEMAEQLGMKLPPGRGLLSQGTAEDGASSASSSSQSETPEPVESTSPSLLTTDNTLERSFFIRMKSTLTKRGVHIKSSGYKVIHITGRLRLRVSLSHGRTVPSQIMGLVVVAHALPPPTINEVRIDCHMFVTRVNMDLNIIYCENRISDYMDLTPVDIVGKRCYHFIHAEDVEGIRHSHLDLLNKGQCVTKYYRWMQKNGGYIWIQSSATIAINAKNANEKNIIWVNYLLSNPEYKDTPMDIAQLPHLPEKTSESSETSDSESDSKDTSGITEDNENSKSDEKGNQSENSEDPEPDRKKSGNACDNDMNCNDDGHSSSNPDSRDSDDSFEHSDFENPKAGEDGFGALGAMQIKVERYVESESDLRLQNCESLTSDSAKDSDSAGEAGAQASSKHQKRKKRRKRQKGGSASRRRLSSASSPGGLDAGLVEPPRLLSSPNSASVLKIKTEISEPINFDNDSSIWNYPPNREISRNESPYSMTKPPSSEHFPSPQGGGGGGGGGGGLHVAIPDSVLTPPGADGAAARKTQFGASATAALAPVASDPLSPPLSASPRDKHPGNGGGGGGGGGGAGGGGPSASNSLLYTGDLEALQRLQAGNVVLPLVHRVTGTLAATSTAAQRVYTTGTIRYAPAEVTLAMQSNLLPNAHAVNFVDVNSPGFGLDPKTPMEMLYHHVHRLNMSGPFGGAVSAASLTQMPAGNVFTTAEGLFSTLPFPVYSNGIHAAQTLERKED

>P50135

MASSMRSLFSDHGKYVESFRRFLNHSTEHQCMQEFMDKKLPGIIGRIGDTKSEIKILSIGGGAGEIDLQILSKVQAQYPGVCINNEVVEPSAEQIAKYKELVAKTSNLENVKFAWHKETSSEYQSRMLEKKELQKWDFIHMIQMLYYVKDIPATLKFFHSLLGTNAKMLIIVVSGSSGWDKLWKKYGSRFPQDDLCQYITSDDLTQMLDNLGLKYECYDLLSTMDISDCFIDGNENGDLLWDFLTETCNFNATAPPDLRAELGKDLQEPEFSAKKEGKVLFNNTLSFIVIEA

327 NP_055675.1 NP_002056.2

>NP_055675.1

MNLQRYWGEIPISSSQTNRSSFDLLPREFRLVEVHDPPLHQPSANKPKPPTMLDIPSEPCSLTIHTIQLIQHNRRLRNLIATAQAQNQQQTEGVKTEESEPLPSCPGSPPLPDDLLPLDCKNPNAPFQIRHSDPESDFYRGKGEPVTELSWHSCRQLLYQAVATILAHAGFDCANESVLETLTDVAHEYCLKFTKLLRFAVDREARLGQTPFPDVMEQVFHEVGIGSVLSLQKFWQHRIKDYHSYMLQISKQLSEEYERIVNPEKATEDAKPVKIKEEPVSDITFPVSEELEADLASGDQSLPMGVLGAQSERFPSNLEVEASPQASSAEVNASPLWNLAHVKMEPQESEEGNVSGHGVLGSDVFEEPMSGMSEAGIPQSPDDSDSSYGSHSTDSLMGSSPVFNQRCKKRMRKI

>NP_002056.2

MTTSASSHLNKGIKQVYMSLPQGEKVQAMYIWIDGTGEGLRCKTRTLDSEPKCVEELPEWNFDGSSTLQSEGSNSDMYLVPAAMFRDPFRKDPNKLVLCEVFKYNRRPAETNLRHTCKRIMDMVSNQHPWFGMEQEYTLMGTDGHPFGWPSNGFPGPQGPYYCGVGADRAYGRDIVEAHYRACLYAGVKIAGTNAEVMPAQWEFQIGPCEGISMGDHLWVARFILHRVCEDFGVIATFDPKPIPGNWNGAGCHTNFSTKAMREENGLKYIEEAIEKLSKRHQYHIRAYDPKGGLDNARRLTGFHETSNINDFSAGVANRSASIRIPRTVGQEKKGYFEDRRPSANCDPFSVTEALIRTCLLNETGDEPFQYKN

328 NP_872363.1 Q9NVV4

>NP_872363.1

MRILANKTRLPHPRRREAPGSPPLSPRGHCPPAPAKPMHPENKLTNHGKTGNGGAQSQHQNVNQGPTCNVGSKGVGAGNHGAKANQISPSNSSLKNPQAGVPPFSSLKGKVKRDRSVSVDSGEQREAGTPSLDSEAKEVAPRSKRRCVLERKQPYSGDEWCSGPDSEEDDKPIGATHNCNVADPAMAAPQLGPGQTTQLPLSESSVPGAPHGPPPGLRPDAPGGGGGGGGVPGKPPSQFVYVFTTHLANTAAEAVLQGRADSILAYHQQNVPRAKLDQAPKVPPTPEPLPLSTPSAGTPQSQPPPLPPPPPPAPGSAPPALPPEGPPEDSSQDLAPNSVGAASTGGGTGGTHPNTPTATTANNPLPPGGDPSSAPGPALLGEAAAPGNGQRSLVGSEGLSKEQLEHRERSLQTLRDIERLLLRSGETEPFLKGPPGGAGEGGPPAQAPPPPQQPPTAPPSGLKKYEEPLQSMISQTQSLGGPPLEHEVPGHPPGGDMGQQMNMMIQRLGQDSLTPEQVAWRKLQEEYYEEKRRKEEQIGLHGSRPLQDMMGMGGMMVRGPPPPYHSKPGDQWPPGMGAQLRGPMDVQDPMQLRGGPPFPGPRFPGNQIQRVPGFGGMQSMPMEVPMNAMQRPVRPGMGWTEDLPPMGGPSNFAQNTMPYPGGQGEAERFMTPRVREELLRHQLLEKRSMGMQRPLGMAGSGMGQSMEMERMMQAHRQMDPAMFPGQMAGGEGLAGTPMGMEFGGGRGLLSPPMGQSGLREVDPPMGPGNLNMNMNVNMNMNMNLNVQMTPQQQMLMSQKMRGPGDLMGPQGLSPEEMARVRAQNSSGVMGGPQKMLMPSQFPNQGQQGFSGGQGPYQAMSQDMGNTQDMFSPDQSSMPMSNVGTTRLSHMPLPPASNPPGTVHSAPNRGLGRRPSDLTISINQMGSPGMGHLKSPTLSQVHSPLVTSPSANLKSPQTPSQMVPLPSANPPGPLKSPQVLGSSLSVRSPTGSPSRLKSPSMAVPSPGWVASPKTAMPSPGVSQNKQPPLNMNSSTTLSNMEQGTLPPSGPRSSSSAPPANPPSGLMNPSLPFTSSPDPTPSQNPLSLMMTQMSKYAMPSSTPLYHNAIKTIATSDDELLPDRPLLPPPPPPQGSGPGISNSQPSQMHLNSAAAQSPMGMNLPGQQPLSHEPPPAMLPSPTPLGSNIPLHPNAQGTGGPPQNSMMMAPGGPDSLNAPCGPVPSSSQMMPFPPRLQQPHGAMAPTGGGGGGPGLQQHYPSGMALPPEDLPNQPPGPMPPQQHLMGKAMAGRMGDAYPPGVLPGVASVLNDPELSEVIRPTPTGIPEFDLSRIIPSEKPSSTLQYFPKSENQPPKAQPPNLHLMNLQNMMAEQTPSRPPNLPGQQGVQRGLNMSMCHPGQMSLLGRTGVPPQQGMVPHGLHQGVMSPPQGLMTQQNFMLMKQRGVGGEVYSQPPHMLSPQGSLMGPPPQQNLMVSHPLRQRSVSLDSQMGYLPAPGGMANLPF

>Q9NVV4

MAVPGVGLLTRLNLCARRRTRVQRPIVRLLSCPGTVAKDLRRDEQPSGSVETGFEDKIPKRRFSEMQNERREQAQRTVLIHCPEKISENKFLKYLSQFGPINNHFFYESFGLYAVVEFCQKESIGSLQNGTHTPSTAMETAIPFRSRFFNLKLKNQTSERSRVRSSNQLPRSNKQLFELLCYAESIDDQLNTLLKEFQLTEENTKLRYLTCSLIEDMAAAYFPDCIVRPFGSSVNTFGKLGCDLDMFLDLDETRNLSAHKISGNFLMEFQVKNVPSERIATQKILSVLGECLDHFGPGCVGVQKILNARCPLVRFSHQASGFQCDLTTNNRIALTSSELLYIYGALDSRVRALVFSVRCWARAHSLTSSIPGAWITNFSLTMMVIFFLQRRSPPILPTLDSLKTLADAEDKCVIEGNNCTFVRDLSRIKPSQNTETLELLLKEFFEYFGNFAFDKNSINIRQGREQNKPDSSPLYIQNPFETSLNISKNVSQSQLQKFVDLARESAWILQQEDTDRPSISSNRPWGLVSLLLPSAPNRKSFTKKKSNKFAIETVKNLLESLKGNRTENFTKTSGKRTISTQT

329 NP_002681.1 NP_803877.2

>NP_002681.1

MSKRKAPQETLNGGITDMLTELANFEKNVSQAIHKYNAYRKAASVIAKYPHKIKSGAEAKKLPGVGTKIAEKIDEFLATGKLRKLEKIRQDDTSSSINFLTRVSGIGPSAARKFVDEGIKTLEDLRKNEDKLNHHQRIGLKYFGDFEKRIPREEMLQMQDIVLNEVKKVDSEYIATVCGSFRRGAESSGDMDVLLTHPSFTSESTKQPKLLHQVVEQLQKVHFITDTLSKGETKFMGVCQLPSKNDEKEYPHRRIDIRLIPKDQYYCGVLYFTGSDIFNKNMRAHALEKGFTINEYTIRPLGVTGVAGEPLPVDSEKDIFDYIQWKYREPKDRSE

>NP_803877.2

MERIEGASVGRCAASPYLRPLTLHYRQNGAQKSWDFMKTHDSVTVLLFNSSRRSLVLVKQFRPAVYAGEVERRFPGSLAAVDQDGPRELQPALPGSAGVTVELCAGLVDQPGLSLEEVACKEAWEECGYHLAPSDLRRVATYWSGVGLTGSRQTMFYTEVTDAQRSGPGGGLVEEGELIEVVHLPLEGAQAFADDPDIPKTLGVIFGVSWFLSQVAPNLDLQ

330 NP_002403.1 NP_006819.2

>NP_002403.1

MDKDCEMKRTTLDSPLGKLELSGCEQGLHEIKLLGKGTSAADAVEVPAPAAVLGGPEPLMQCTAWLNAYFHQPEAIEEFPVPALHHPVFQQESFTRQVLWKLLKVVKFGEVISYQQLAALAGNPKAARAVGGAMRGNPVPILIPCHRVVCSSGAVGNYSGGLAVKEWLLAHEGHRLGKPGLGGSSGLAGAWLKGAGATSGSPPAGRN

>NP_006819.2

MALPRLTGALRSFSNVTKQDNYNEEVADLKIKRSKLHEQVLDLGLTWKKIIKFLNEKLEKSKMQSINEDLKDILHAAKQIVGTDNGREAIESGAAFLFMTFHLKDSVGHKETKAIKQMFGPFPSSSATAACNATNRIISHFSQDDLTALVQMTEKEHGDRVFFGKNLAFSFDMHDLDHFDELPINGETQKTISLDYKKFLNEHLQEACTPELKPVEKTNGSFLWCEVEKYLNSTLKEMTEVPRVEDLCCTLYDMLASIKSGDELQDELFELLGPEGLELIEKLLQNRITIVDRFLNSSNDHRFQALQDNCKKILGENAKPNYGCQVTIQSEQEKQLMKQYRREEKRIARREKKAGEDLEVSEGLMCFDPKELRIQREQALLNARSVPILSRQRDADVEKIHYPHVYDSQAEAMKTSAFIAGAKMILPEGIQRENNKLYEEVRIPYSEPMPLSFEEKPVYIQDLDEIGQLAFKGMKRLNRIQSIVFETAYNTNENMLICAPTGAGKTNIAMLTVLHEIRQHFQQGVIKKNEFKIVYVAPMKALAAEMTDYFSRRLEPLGIIVKELTGDMQLSKSEILRTQMLVTTPEKWDVVTRKSVGDVALSQIVRLLILDEVHLLHEDRGPVLESIVARTLRQVESTQSMIRILGLSATLPNYLDVATFLHVNPYIGLFFFDGRFRPVPLGQTFLGIKCANKMQQLNNMDEVCYENVLKQVKAGHQVMVFVHARNATVRTAMSLIERAKNCGHIPFFFPTQGHDYVLAEKQVQRSRNKQVRELFPDGFSIHHAGMLRQDRNLVENLFSNGHIKVLVCTATLAWGVNLPAHAVIIKGTQIYAAKRGSFVDLGILDVMQIFGRAGRPQFDKFGEGIIITTHDKLSHYLTLLTQRNPIESQFLESLADNLNAEIALGTVTNVEEAVKWISYTYLYVRMRANPLAYGISHKAYQIDPTLRKHREQLVIEVGRKLDKAQMIRFEERTGYFSSTDLGRTASHYYIKYNTIETFNELFDAHKTEGDIFAIVSKAEEFDQIKVREEEIEELDTLLSNFCELSTPGGVENSYGKINILLQTYISRGEMDSFSLISDSAYVAQNAARIVRALFEIALRKRWPTMTYRLLNLSKVIDKRLWGWASPLRQFSILPPHILTRLEEKKLTVDKLKDMRKDEIGHILHHVNIGLKVKQCVHQIPSVMMEASIQPITRTVLRVTLSIYADFTWNDQVHGTVGEPWWIWVEDPTNDHIYHSEYFLALKKQVISKEAQLLVFTIPIFEPLPSQYYIRAVSDRWLGAEAVCIINFQHLILPERHPPHTELLDLQPLPITALGCKAYEALYNFSHFNPVQTQIFHTLYHTDCNVLLGAPTGSGKTVAAELAIFRVFNKYPTSKAVYIAPLKALVRERMDDWKVRIEEKLGKKVIELTGDVTPDMKSIAKADLIVTTPEKWDGVSRSWQNRNYVQQVTILIIDEIHLLGEERGPVLEVIVSRTNFISSHTEKPVRIVGLSTALANARDLADWLNIKQMGLFNFRPSVRPVPLEVHIQGFPGQHYCPRMASMNKPAFQAIRSHSPAKPVLIFVSSRRQTRLTALELIAFLATEEDPKQWLNMDEREMENIIATVRDSNLKLTLAFGIGMHHAGLHERDRKTVEELFVNCKVQVLIATSTLAWGVNFPAHLVIIKGTEYYDGKTRRYVDFPITDVLQMMGRAGRPQFDDQGKAVILVHDIKKDFYKKFLYEPFPVESSLLGVLSDHLNAEIAGGTITSKQDALDYITWTYFFRRLIMNPSYYNLGDVSHDSVNKFLSHLIEKSLIELELSYCIEIGEDNRSIEPLTYGRIASYYYLKHQTVKMFKDRLKPECSTEELLSILSDAEEYTDLPVRHNEDHMNSELAKCLPIESNPHSFDSPHTKAHLLLQAHLSRAMLPCPDYDTDTKTVLDQALRVCQAMLDVAANQGWLVTVLNITNLIQMVIQGRWLKDSSLLTLPNIENHHLHLFKKWKPIMKGPHARGRTSIESLPELIHACGGKDHVFSSMVESELHAAKTKQAWNFLSHLPVINVGISVKGSWDDLVEGHNELSVSTLTADKRDDNKWIKLHADQEYVLQVSLQRVHFGFHKGKPESCAVTPRFPKSKDEGWFLILGEVDKRELIALKRVGYIRNHHVASLSFYTPEIPGRYIYTLYFMSDCYLGLDQQYDIYLNVTQASLSAQVNTKVSDSLTDLALK

331 NP_060323.1 NP_803877.2

>NP_060323.1

MSFLLPKLTSKKEVDQAIKSTAEKVLVLRFGRDEDPVCLQLDDILSKTSSDLSKMAAIYLVDVDQTAVYTQYFDISYIPSTVFFFNGQHMKVDYGSPDHTKFVGSFKTKQDFIDLIEVIYRGAMRGKLIVQSPIDPKNIPKYDLLYQDI

>NP_803877.2

MERIEGASVGRCAASPYLRPLTLHYRQNGAQKSWDFMKTHDSVTVLLFNSSRRSLVLVKQFRPAVYAGEVERRFPGSLAAVDQDGPRELQPALPGSAGVTVELCAGLVDQPGLSLEEVACKEAWEECGYHLAPSDLRRVATYWSGVGLTGSRQTMFYTEVTDAQRSGPGGGLVEEGELIEVVHLPLEGAQAFADDPDIPKTLGVIFGVSWFLSQVAPNLDLQ

332 NP_055675.1 NP_055129.2

>NP_055675.1

MNLQRYWGEIPISSSQTNRSSFDLLPREFRLVEVHDPPLHQPSANKPKPPTMLDIPSEPCSLTIHTIQLIQHNRRLRNLIATAQAQNQQQTEGVKTEESEPLPSCPGSPPLPDDLLPLDCKNPNAPFQIRHSDPESDFYRGKGEPVTELSWHSCRQLLYQAVATILAHAGFDCANESVLETLTDVAHEYCLKFTKLLRFAVDREARLGQTPFPDVMEQVFHEVGIGSVLSLQKFWQHRIKDYHSYMLQISKQLSEEYERIVNPEKATEDAKPVKIKEEPVSDITFPVSEELEADLASGDQSLPMGVLGAQSERFPSNLEVEASPQASSAEVNASPLWNLAHVKMEPQESEEGNVSGHGVLGSDVFEEPMSGMSEAGIPQSPDDSDSSYGSHSTDSLMGSSPVFNQRCKKRMRKI

>NP_055129.2

MTTEQRRSLQAFQDYIRKTLDPTYILSYMAPWFREEEVQYIQAEKNNKGPMEAATLFLKFLLELQEEGWFRGFLDALDHAGYSGLYEAIESWDFKKIEKLEEYRLLLKRLQPEFKTRIIPTDIISDLSECLINQECEEILQICSTKGMMAGAEKLVECLLRSDKENWPKTLKLALEKERNKFSELWIVEKGIKDVETEDLEDKMETSDIQIFYQEDPECQNLSENSCPPSEVSDTNLYSPFKPRNYQLELALPAMKGKNTIICAPTGCGKTFVSLLICEHHLKKFPQGQKGKVVFFANQIPVYEQQKSVFSKYFERHGYRVTGISGATAENVPVEQIVENNDIIILTPQILVNNLKKGTIPSLSIFTLMIFDECHNTSKQHPYNMIMFNYLDQKLGGSSGPLPQVIGLTASVGVGDAKNTDEALDYICKLCASLDASVIATVKHNLEELEQVVYKPQKFFRKVESRISDKFKYIIAQLMRDTESLAKRICKDLENLSQIQNREFGTQKYEQWIVTVQKACMVFQMPDKDEESRICKALFLYTSHLRKYNDALIISEHARMKDALDYLKDFFSNVRAAGFDEIEQDLTQRFEEKLQELESVSRDPSNENPKLEDLCFILQEEYHLNPETITILFVKTRALVDALKNWIEGNPKLSFLKPGILTGRGKTNQNTGMTLPAQKCILDAFKASGDHNILIATSVADEGIDIAQCNLVILYEYVGNVIKMIQTRGRGRARGSKCFLLTSNAGVIEKEQINMYKEKMMNDSILRLQTWDEAVFREKILHIQTHEKFIRDSQEKPKPVPDKENKKLLCRKCKALACYTADVRVIEECHYTVLGDAFKECFVSRPHPKPKQFSSFEKRAKIFCARQNCSHDWGIHVKYKTFEIPVIKIESFVVEDIATGVQTLYSKWKDFHFEKIPFDPAEMSK

333 NP_004917.2 P13995

>NP_004917.2

MTTTLVSATIFDLSEVLCKGNKMLNYSAPSAGGCLLDRKAVGTPAGGGFPRRHSVTLPSSKFHQNQLLSSLKGEPAPALSSRDSRFRDRSFSEGGERLLPTQKQPGGGQVNSSRYKTELCRPFEENGACKYGDKCQFAHGIHELRSLTRHPKYKTELCRTFHTIGFCPYGPRCHFIHNAEERRALAGARDLSADRPRLQHSFSFAGFPSAAATAAATGLLDSPTSITPPPILSADDLLGSPTLPDGTNNPFAFSSQELASLFAPSMGLPGGGSPTTFLFRPMSESPHMFDSPPSPQDSLSDQEGYLSSSSSSHSGSDSPTLDNSRRLPIFSRLSISDD

>P13995

MAATSLMSALAARLLQPAHSCSLRLRPFHLAAVRNEAVVISGRKLAQQIKQEVRQEVEEWVASGNKRPHLSVILVGENPASHSYVLNKTRAAAVVGINSETIMKPASISEEELLNLINKLNNDDNVDGLLVQLPLPEHIDERRICNAVSPDKDVDGFHVINVGRMCLDQYSMLPATPWGVWEIIKRTGIPTLGKNVVVAGRSKNVGMPIAMLLHTDGAHERPGGDATVTISHRYTPKEQLKKHTILADIVISAAGIPNLITADMIKEGAAVIDVGINRVHDPVTAKPKLVGDVDFEGVRQKAGYITPVPGGVGPMTVAMLMKNTIIAAKKVLRLEEREVLKSKELGVATN

334 NP_060323.1 NP_000365.3

>NP_060323.1

MSFLLPKLTSKKEVDQAIKSTAEKVLVLRFGRDEDPVCLQLDDILSKTSSDLSKMAAIYLVDVDQTAVYTQYFDISYIPSTVFFFNGQHMKVDYGSPDHTKFVGSFKTKQDFIDLIEVIYRGAMRGKLIVQSPIDPKNIPKYDLLYQDI

>NP_000365.3

MEANGLGPQGFPELKNDTFLRAAWGEETDYTPVWCMRQAGRYLPEFRETRAAQDFFSTCRSPEACCELTLQPLRRFPLDAAIIFSDILVVPQALGMEVTMVPGKGPSFPEPLREEQDLERLRDPEVVASELGYVFQAITLTRQRLAGRVPLIGFAGAPWTLMTYMVEGGGSSTMAQAKRWLYQRPQASHQLLRILTDALVPYLVGQVVAGAQALQLFESHAGHLGPQLFNKFALPYIRDVAKQVKARLREAGLAPVPMIIFAKDGHFALEELAQAGYEVVGLDWTVAPKKARECVGKTVTLQGNLDPCALYASEEEIGQLVKQMLDDFGPHRYIANLGHGLYPDMDPEHVGAFVDAVHKHSRLLRQN

335 Q96AV8 Q2M3C7

>Q96AV8

MEVNCLTLKDLISPRQPRLDFAVEDGENAQKENIFVDRSRMAPKTPIKNEPIDLSKQKKFTPERNPITPVKLVDRQQAEPWTPTANLKMLISAASPDIRDREKKKGLFRPIENKDDAFTDSLQLDVVGDSAVDEFEKQRPSRKQKSLGLLCQKFLARYPSYPLSTEKTTISLDEVAVSLGVERRRIYDIVNVLESLHLVSRVAKNQYGWHGRHSLPKTLRNLQRLGEEQKYEEQMAYLQQKELDLIDYKFGERKKDGDPDSQEQQLLDFSEPDCPSSSANSRKDKSLRIMSQKFVMLFLVSKTKIVTLDVAAKILIEESQDAPDHSKFKTKVRRLYDIANVLTSLALIKKVHVTEERGRKPAFKWIGPVDFSSSDEELVDVSASVLPELKRETYGQIQVCAKQKLARHGSFNTVQASERIQRKVNSEPSSPYREEQGSGGYSLEIGSLAAVYRQKIEDNSQGKAFASKRVVPPSSSLDPVAPFPVLSVDPEYCVNPLAHPVFSVAQTDLQAFSMQNGLNGQVDVSLASAASAVESLKPALLAGQPLVYVPSASLFMLYGSLQEGPASGSGSERDDRSSEAPATVELSSAPSAQKRLCEERKPQEEDEPATKRQSREYEDGPLSLVMPKKPSDSTDLASPKTMGNRASIPLKDIHVNGQLPAAEEISGKATANSLVSSEWGNPSRNTDVEKPSKENESTKEPSLLQYLCVQSPAGLNGFNVLLSGSQTPPTVGPSSGQLPSFSVPCMVLPSPPLGPFPVLYSPAMPGPVSSTLGALPNTGPVNFSLPGLGSIAQLLVGPTAVVNPKSSTLPSADPQLQSQPSLNLSPVMSRSHSVVQQPESPVYVGHPVSVVKLQQSPVPVTPKSIQRTHRETFFKTPGSLGDPVLKRRERNQSRNTSSAQRRLEIPSGGAD

>Q2M3C7

MDGNSLLSVPSNLESSRMYDVLEPQQGRGCGSSGSGPGNSITACKKVLRSNSLLESTDYWLQNQRMPCQIGFVEDKSENCASVCFVNLDVNKDECSTEHLQQKLVNVSPDLPKLISSMNVQQPKENEIVVLSGLASGNLQADFEVSQCPWLPDICLVQCARGNRPNSTNCIIFEINKFLIGLELVQERQLHLETNILKLEDDTNCSLSSIEEDFLTASEHLEEESEVDESRNDYENINVSANVLESKQLKGATQVEWNCNKEKWLYALEDKYINKYPTPLIKTERSPENLTKNTALQSLDPSAKPSQWKREAVGNGRQATHYYHSEAFKGQMEKSQALYIPKDAYFSMMDKDVPSACAVAEQRSNLNPGDHEDTRNALPPRQDGEVTTGKYATNLAESVLQDAFIRLSQSQSTLPQESAVSVSVGSSLLPSCYSTKDTVVSRSWNELPKIVVVQSPDGSDAAPQPGISSWPEMEVSVETSSILSGENSSRQPQSALEVALACAATVIGTISSPQATERLKMEQVVSNFPPGSSGALQTQAPQGLKEPSINEYSFPSALCGMTQVASAVAVCGLGEREEVTCSVAPSGSLPPAAEASEAMPPLCGLASMELGKEAIAKGLLKEAALVLTRPNTYSSIGDFLDSMNRRIMETASKSQTLCSENVVRNELAHTLSNVILRHSIDEVHHKNMIIDPNDNRHSSEILDTLMESTNQLLLDVICFTFKKMSHIVRLGECPAVLSKETIRRRETEPSCQPSDPGASQAWTKATESSSSSPLSNSHNTSLVINNLVDGMYSKQDKGGVRPGLFKNPTLQSQLSRSHRVPDSSTATTSSKEIYLKGIAGEDTKSPHHSENECRASSEGQRSPTVSQSRSGSQEAEESIHPNTQEKYNCATSRINEVQVNLSLLGDDLLLPAQSTLQTKHPDIYCITDFAEELADTVVSMATEIAAICLDNSSGKQPWFCAWKRGSEFLMTPNVPCRSLKRKKESQGSGTAVRKHKPPRLSEIKRKTDEHPELKEKLMNRVVDESMNLEDVPDSVNLFANEVAAKIMNLTEFSMVDGMWQAQGYPRNRLLSGDRWSRLKASSCESIPEEDSEARAYVNSLGLMSTLSQPVSRASSVSKQSSCESITDEFSRFMVNQMENEGRGFELLLDYYAGKNASSILNSAMQQACRKSDHLSVRPSCPSKQSSTESITEEFYRYMLRDIERDSRESASSRRSSQDWTAGLLSPSLRSPVCHRQSSMPDSRSPCSRLTVNVPIKANSLDGFAQNCPQDFLSVQPVSSASSSGLCKSDSCLYRRGGTDHITNMLIHETWASSIEALMRKNKIIVDDAEEADTEPVSGGSPSQAEKCANRLAASRMCSGPTLLVQESLDCPRKDSVTECKQPPVSSLSKTASLTNHSPLDSKKETSSCQDPVPINHKRRSLCSREVPLIQIETDQREACAGEPEPFLSKSSLLEEAEGHSNDKNIPDVVRGGDTAVSACQIHSDSLDTRDVPEAEASTEARAPDEAPNPPSSSEESTGSWTQLANEEDNPDDTSSFLQLSERSMSNGNSSATSSLGIMDLDIYQESMPSSPMINELVEEKKILKGQSESTEAPASGPPTGTASPQRSLLVINFDLEPECPDAELRATLQWIAASELGIPTIYFKKSQENRIEKFLDVVQLVHRKSWKVGDIFHAVVQYCKMHEEQKDGRLSLFDWLLELG

336 Q7Z5Q5 Q9GZZ1

>Q7Z5Q5

MENYEALVGFDLCNTPLSSVAQKIMSAMHSGDLVDSKTWGKSTETMEVINKSSVKYSVQLEDRKTQSPEKKDLKSLRSQTSRGSAKLSPQSFSVRLTDQLSADQKQKSISSLTLSSCLIPQYNQEASVLQKKGHKRKHFLMENINNENKGSINLKRKHITYNNLSEKTSKQMALEEDTDDAEGYLNSGNSGALKKHFCDIRHLDDWAKSQLIEMLKQAAALVITVMYTDGSTQLGADQTPVSSVRGIVVLVKRQAEGGHGCPDAPACGPVLEGFVSDDPCIYIQIEHSAIWDQEQEAHQQFARNVLFQTMKCKCPVICFNAKDFVRIVLQFFGNDGSWKHVADFIGLDPRIAAWLIDPSDATPSFEDLVEKYCEKSITVKVNSTYGNSSRNIVNQNVRENLKTLYRLTMDLCSKLKDYGLWQLFRTLELPLIPILAVMESHAIQVNKEEMEKTSALLGARLKELEQEAHFVAGERFLITSNNQLREILFGKLKLHLLSQRNSLPRTGLQKYPSTSEAVLNALRDLHPLPKIILEYRQVHKIKSTFVDGLLACMKKGSISSTWNQTGTVTGRLSAKHPNIQGISKHPIQITTPKNFKGKEDKILTISPRAMFVSSKGHTFLAADFSQIELRILTHLSGDPELLKLFQESERDDVFSTLTSQWKDVPVEQVTHADREQTKKVVYAVVYGAGKERLAACLGVPIQEAAQFLESFLQKYKKIKDFARAAIAQCHQTGCVVSIMGRRRPLPRIHAHDQQLRAQAERQAVNFVVQGSAADLCKLAMIHVFTAVAASHTLTARLVAQIHDELLFEVEDPQIPECAALVRRTMESLEQVQALELQLQVPLKVSLSAGRSWGHLVPLQEAWGPPPGPCRTESPSNSLAAPGSPASTQPPPLHFSPSFCL

>Q9GZZ1

MKGSRIELGDVTPHNIKQLKRLNQVIFPVSYNDKFYKDVLEVGELAKLAYFNDIAVGAVCCRVDHSQNQKRLYIMTLGCLAPYRRLGIGTKMLNHVLNICEKDGTFDNIYLHVQISNESAIDFYRKFGFEIIETKKNYYKRIEPADAHVLQKNLKVPSGQNADVQKTDN

337 NP_073624.2 NP_000846.1

>NP_073624.2

MENSEKTEVVLLACGSFNPITNMHLRLFELAKDYMNGTGRYTVVKGIISPVGDAYKKKGLIPAYHRVIMAELATKNSKWVEVDTWESLQKEWKETLKVLRHHQEKLEASDCDHQQNSPTLERPGRKRKWTETQDSSQKKSLEPKTKAVPKVKLLCGADLLESFAVPNLWKSEDITQIVANYGLICVTRAGNDAQKFIYESDVLWKHRSNIHVVNEWIANDISSTKIRRALRRGQSIRYLVPDLVQEYIEKHNLYSSESEDRNAGVILAPLQRNTAEAKT

>NP_000846.1

MSRRKISSESFSSLGSDYLETSPEEEGECPLSRLCWNGSRSPPGPLEPSPAAAAAAAAPAPTPAASAAAAAATAGARRVQRRRRVNLDSLGESISRLTAPSPQTIQQTLKRTLQYYEHQVIGYRDAEKNFHNISNRCSYADHSNKEEIEDVSGILQCTANILGLKFEEIQKRFGEEFFNICFHENERVLRAVGGTLQDFFNGFDALLEHIRTSFGKQATLESPSFLCKELPEGTLMLHYFHPHHIVGFAMLGMIKAAGKKIYRLDVEVEQVANEKLCSDVSNPGNCSCLTFLIKECENTNIMKNLPQGTSQVPADLRISINTFCRAFPFHLMFDPSMSVLQLGEGLRKQLRCDTHKVLKFEDCFEIVSPKVNATFERVLLRLSTPFVIRTKPEASGSENKDKVMEVKGQMIHVPESNSILFLGSPCVDKLDELMGRGLHLSDIPIHDATRDVILVGEQAKAQDGLKKRMDKLKATLERTHQALEEEKKKTVDLLYSIFPGDVAQQLWQGQQVQARKFDDVTMLFSDIVGFTAICAQCTPMQVISMLNELYTRFDHQCGFLDIYKVETIGDAYCVAAGLHRKSLCHAKPIALMALKMMELSEEVLTPDGRPIQMRIGIHSGSVLAGVVGVRMPRYCLFGNNVTLASKFESGSHPRRINVSPTTYQLLKREESFTFIPRSREELPDNFPKEIPGICYFLEVRTGPKPPKPSLSSSRIKKVSYNIGTMFLRETSL

338 NP_055675.1 Q9BYN0

>NP_055675.1

MNLQRYWGEIPISSSQTNRSSFDLLPREFRLVEVHDPPLHQPSANKPKPPTMLDIPSEPCSLTIHTIQLIQHNRRLRNLIATAQAQNQQQTEGVKTEESEPLPSCPGSPPLPDDLLPLDCKNPNAPFQIRHSDPESDFYRGKGEPVTELSWHSCRQLLYQAVATILAHAGFDCANESVLETLTDVAHEYCLKFTKLLRFAVDREARLGQTPFPDVMEQVFHEVGIGSVLSLQKFWQHRIKDYHSYMLQISKQLSEEYERIVNPEKATEDAKPVKIKEEPVSDITFPVSEELEADLASGDQSLPMGVLGAQSERFPSNLEVEASPQASSAEVNASPLWNLAHVKMEPQESEEGNVSGHGVLGSDVFEEPMSGMSEAGIPQSPDDSDSSYGSHSTDSLMGSSPVFNQRCKKRMRKI

>Q9BYN0

MGLRAGGTLGRAGAGRGAPEGPGPSGGAQGGSIHSGRIAAVHNVPLSVLIRPLPSVLDPAKVQSLVDTIREDPDSVPPIDVLWIKGAQGGDYFYSFGGCHRYAAYQQLQRETIPAKLVQSTLSDLRVYLGASTPDLQ

339 NP_004517.2 Q8TF40

>NP_004517.2

MAESSESFTMASSPAQRRRGNDPLTSSPGRSSRRTDALTSSPGRDLPPFEDESEGLLGTEGPLEEEEDGEELIGDGMERDYRAIPELDAYEAEGLALDDEDVEELTASQREAAERAMRQRDREAGRGLGRMRRGLLYDSDEEDEERPARKRRQVERATEDGEEDEEMIESIENLEDLKGHSVREWVSMAGPRLEIHHRFKNFLRTHVDSHGHNVFKERISDMCKENRESLVVNYEDLAAREHVLAYFLPEAPAELLQIFDEAALEVVLAMYPKYDRITNHIHVRISHLPLVEELRSLRQLHLNQLIRTSGVVTSCTGVLPQLSMVKYNCNKCNFVLGPFCQSQNQEVKPGSCPECQSAGPFEVNMEETIYQNYQRIRIQESPGKVAAGRLPRSKDAILLADLVDSCKPGDEIELTGIYHNNYDGSLNTANGFPVFATVILANHVAKKDNKVAVGELTDEDVKMITSLSKDQQIGEKIFASIAPSIYGHEDIKRGLALALFGGEPKNPGGKHKVRGDINVLLCGDPGTAKSQFLKYIEKVSSRAIFTTGQGASAVGLTAYVQRHPVSREWTLEAGALVLADRGVCLIDEFDKMNDQDRTSIHEAMEQQSISISKAGIVTSLQARCTVIAAANPIGGRYDPSLTFSENVDLTEPIISRFDILCVVRDTVDPVQDEMLARFVVGSHVRHHPSNKEEEGLANGSAAEPAMPNTYGVEPLPQEVLKKYIIYAKERVHPKLNQMDQDKVAKMYSDLRKESMATGSIPITVRHIESMIRMAEAHARIHLRDYVIEDDVNMAIRVMLESFIDTQKFSVMRSMRKTFARYLSFRRDNNELLLFILKQLVAEQVTYQRNRFGAQQDTIEVPEKDLVDKARQINIHNLSAFYDSELFRMNKFSHDLKRKMILQQF

>Q8TF40

MAPTLFQKLFSKRTGLGAPGRDARDPDCGFSWPLPEFDPSQIRLIVYQDCERRGRNVLFDSSVKRRNEDISVSKLCSDAQVKVFGKCCQLKPGGDSSSSLDSSVTSSSDIKDQCLKYQGSRCSSDANMLGEMMFGSVAMSYKGSTLKIHQIRSPPQLMLSKVFTARTGSSICGSLNTLQDSLEFINQDNNTLKADNNTVINGLLGNIGLSQFCSPRRAFSEQGPLRLIRSASFFAVHSNPMDMPGRELNEDRDSGIARSASLSSLLITPFPSPNSSLTRSCASSYQRRWRRSQTTSLENGVFPRWSIEESFNLSDESCGPNPGIVRKKKIAIGVIFSLSKDEDENNKFNEFFFSHFPLFESHMNKLKSAIEQAMKMSRRSADASQRSLAYNRIVDALNEFRTTICNLYTMPRIGEPVWLTMMSGTPEKNHLCYRFMKEFTFLMENASKNQFLPALITAVLTNHLAWVPTVMPNGQPPIKIFLEKHSSQSVDMLAKTHPYNPLWAQLGDLYGAIGSPVRLARTVVVGKRQDMVQRLLYFLTYFIRCSELQETHLLENGEDEAIVMPGTVITTTLEKGEIEESEYVLVTMHRNKSSLLFKESEEIRTPNCNCKYCSHPLLGQNVENISQQEREDIQNSSKELLGISDECQMISPSDCQEENAVDVKQYRDKLRTCFDAKLETVVCTGSVPVDKCALSESGLESTEETWQSEKLLDSDSHTGKAMRSTGMVVEKKPPDKIVPASFSCEAAQTKVTFLIGDSMSPDSDTELRSQAVVDQITRHHTKPLKEERGAIDQHQETKQTTKDQSGESDTQNMVSEEPCELPCWNHSDPESMSLFDEYFNDDSIETRTIDDVPFKTSTDSKDHCCMLEFSKILCTKNNKQNNEFCKCIETVPQDSCKTCFPQQDQRDTLSILVPHGDKESSDKKIAVGTEWDIPRNESSDSALGDSESEDTGHDMTRQVSSYYGGEQEDWAEEDEIPFPGSKLIEVSAVQPNIANFGRSLLGGYCSSYVPDFVLQGIGSDERFRQCLMSDLSHAVQHPVLDEPIAEAVCIIADMDKWTVQVASSQRRVTDNKLGKEVLVSSLVSNLLHSTLQLYKHNLSPNFCVMHLEDRLQELYFKSKMLSEYLRGQMRVHVKELGVVLGIESSDLPLLAAVASTHSPYVAQILL

340 NP_004119.1 NP_000377.1

>NP_004119.1

MAERGELDLTGAKQNTGVWLVKVPKYLSQQWAKASGRGEVGKLRIAKTQGRTEVSFTLNEDLANIHDIGGKPASVSAPREHPFVLQSVGGQTLTVFTESSSDKLSLEGIVVQRAECRPAASENYMRLKRLQIEESSKPVRLSQQLDKVVTTNYKPVANHQYNIEYERKKKEDGKRARADKQHVLDMLFSAFEKHQYYNLKDLVDITKQPVVYLKEILKEIGVQNVKGIHKNTWELKPEYRHYQGEEKSD

>NP_000377.1

MSSSGLNSEKVAALIQKLNSDPQFVLAQNVGTTHDLLDICLKRATVQRAQHVFQHAVPQEGKPITNQKSSGRCWIFSCLNVMRLPFMKKLNIEEFEFSQSYLFFWDKVERCYFFLSAFVDTAQRKEPEDGRLVQFLLMNPANDGGQWDMLVNIVEKYGVIPKKCFPESYTTEATRRMNDILNHKMREFCIRLRNLVHSGATKGEISATQDVMMEEIFRVVCICLGNPPETFTWEYRDKDKNYQKIGPITPLEFYREHVKPLFNMEDKICLVNDPRPQHKYNKLYTVEYLSNMVGGRKTLYNNQPIDFLKKMVAASIKDGEAVWFGCDVGKHFNSKLGLSDMNLYDHELVFGVSLKNMNKAERLTFGESLMTHAMTFTAVSEKDDQDGAFTKWRVENSWGEDHGHKGYLCMTDEWFSEYVYEVVVDRKHVPEEVLAVLEQEPIILPAWDPMGALAE

341 NP_006822.1 Q16698

>NP_006822.1

MGEEANDDKKPTTKFELERETELRFEVEASQSVQLELLTGMAEIFGTELTRNKKFTFDAGAKVAVFTWHGCSVQLSGRTEVAYVSKDTPMLLYLNTHTALEQMRRQAEKEEERGPRVMVVGPTDVGKSTVCRLLLNYAVRLGRRPTYVELDVGQGSVSIPGTMGALYIERPADVEEGFSIQAPLVYHFGSTTPGTNIKLYNKITSRLADVFNQRCEVNRRASVSGCVINTCGWVKGSGYQALVHAASAFEVDVVVVLDQERLYNELKRDLPHFVRTVLLPKSGGVVERSKDFRRECRDERIREYFYGFRGCFYPHAFNVKFSDVKIYKVGAPTIPDSCLPLGMSQEDNQLKLVPVTPGRDMVHHLLSVSTAEGTEENLSETSVAGFIVVTSVDLEHQVFTVLSPAPRPLPKNFLLIMDIRFMDLK

>Q16698

MKLPARVFFTLGSRLPCGLAPRRFFSYGTKILYQNTEALQSKFFSPLQKAMLPPNSFQGKVAFITGGGTGLGKGMTTLLSSLGAQCVIASRKMDVLKATAEQISSQTGNKVHAIQCDVRDPDMVQNTVSELIKVAGHPNIVINNAAGNFISPTERLSPNAWKTITDIVLNGTAFVTLEIGKQLIKAQKGAAFLSITTIYAETGSGFVVPSASAKAGVEAMSKSLAAEWGKYGMRFNVIQPGPIKTKGAFSRLDPTGTFEKEMIGRIPCGRLGTVEELANLAAFLCSDYASWINGAVIKFDGGEEVLISGEFNDLRKVTKEQWDTIEELIRKTKGS

342 NP_004587.1 P82914

>NP_004587.1

MAVPETRPNHTIYINNLNEKIKKDELKKSLYAIFSQFGQILDILVSRSLKMRGQAFVIFKEVSSATNALRSMQGFPFYDKPMRIQYAKTDSDIIAKMKGTFVERDRKREKRKPKSQETPATKKAVQGGGATPVVGAVQGPVPGMPPMTQAPRIMHHMPGQPPYMPPPGMIPPPGLAPGQIPPGAMPPQQLMPGQMPPAQPLSENPPNHILFLTNLPEETNELMLSMLFNQFPGFKEVRLVPGRHDIAFVEFDNEVQAGAARDALQGFKITQNNAMKISFAKK

>P82914

MLRVAWRTLSLIRTRAVTQVLVPGLPGGGSAKFPFNQWGLQPRSLLLQAARGYVVRKPAQSRLDDDPPPSTLLKDYQNVPGIEKVDDVVKRLLSLEMANKKEMLKIKQEQFMKKIVANPEDTRSLEARIIALSVKIRSYEEHLEKHRKDKAHKRYLLMSIDQRKKMLKNLRNTNYDVFEKICWGLGIEYTFPPLYYRRAHRRFVTKKALCIRVFQETQKLKKRRRALKAAAAAQKQAKRRNPDSPAKAIPKTLKDSQ

343 NP_002544.1 O75570

>NP_002544.1

MPHLENVVLCRESQVSILQSLFGERHHFSFPSIFIYGHTASGKTYVTQTLLKTLELPHVFVNCVECFTLRLLLEQILNKLNHLSSSEDGCSTEITCETFNDFVRLFKQVTTAENLKDQTVYIVLDKAEYLRDMEANLLPGFLRLQELADRNVTVLFLSEIVWEKFRPNTGCFEPFVLYFPDYSIGNLQKILSHDHPPEYSADFYAAYINILLGVFYTVCRDLKELRHLAVLNFPKYCEPVVKGEASERDTRKLWRNIEPHLKKAMQTVYLREISSSQWEKLQKDDTDPGQLKGLSAHTHVELPYYSKFILIAAYLASYNPARTDKRFFLKHHGKIKKTNFLKKHEKTSNHLLGPKPFPLDRLLAILYSIVDSRVAPTANIFSQITSLVTLQLLTLVGHDDQLDGPKYKCTVSLDFIRAIARTVNFDIIKYLYDFL

>O75570

MNRHLCVWLFRHPSLNGYLQCHIQLHSHQFRQIHLDTRLQVFRQNRNCILHLLSKNWSRRYCHQDTKMLWKHKALQKYMENLSKEYQTLEQCLQHIPVNEENRRSLNRRHAELAPLAAIYQEIQETEQAIEELESMCKSLNKQDEKQLQELALEERQTIDQKINMLYNELFQSLVPKEKYDKNDVILEVTAGRTTGGDICQQFTREIFDMYQNYSCYKHWQFELLNYTPADYGGLHHAAARISGDGVYKHLKYEGGIHRVQRIPEVGLSSRMQRIHTGTMSVIVLPQPDEVDVKLDPKDLRIDTFRAKGAGGQHVNKTDSAVRLVHIPTGLVVECQQERSQIKNKEIAFRVLRARLYQQIIEKDKRQQQSARKLQVGTRAQSERIRTYNFTQDRVSDHRIAYEVRDIKEFLCGGKGLDQLIQRLLQSADEEAIAELLDEHLKSAK

344 P10071 NP_112176.1

>P10071

MEAQSHSSTTTEKKKVENSIVKCSTRTDVSEKAVASSTTSNEDESPGQTYHRERRNAITMQPQNVQGLSKVSEEPSTSSDERASLIKKEIHGSLPHVAEPSVPYRGTVFAMDPRNGYMEPHYHPPHLFPAFHPPVPIDARHHEGRYHYDPSPIPPLHMTSALSSSPTYPDLPFIRISPHRNPAAASESPFSPPHPYINPYMDYIRSLHSSPSLSMISATRGLSPTDAPHAGVSPAEYYHQMALLTGQRSPYADIIPSAATAGTGAIHMEYLHAMDSTRFSSPRLSARPSRKRTLSISPLSDHSFDLQTMIRTSPNSLVTILNNSRSSSSASGSYGHLSASAISPALSFTYSSAPVSLHMHQQILSRQQSLGSAFGHSPPLIHPAPTFPTQRPIPGIPTVLNPVQVSSGPSESSQNKPTSESAVSSTGDPMHNKRSKIKPDEDLPSPGARGQQEQPEGTTLVKEEGDKDESKQEPEVIYETNCHWEGCAREFDTQEQLVHHINNDHIHGEKKEFVCRWLDCSREQKPFKAQYMLVVHMRRHTGEKPHKCTFEGCTKAYSRLENLKTHLRSHTGEKPYVCEHEGCNKAFSNASDRAKHQNRTHSNEKPYVCKIPGCTKRYTDPSSLRKHVKTVHGPEAHVTKKQRGDIHPRPPPPRDSGSHSQSRSPGRPTQGALGEQQDLSNTTSKREECLQVKTVKAEKPMTSQPSPGGQSSCSSQQSPISNYSNSGLELPLTDGGSIGDLSAIDETPIMDSTISTATTALALQARRNPAGTKWMEHVKLERLKQVNGMFPRLNPILPPKAPAVSPLIGNGTQSNNTCSLGGPMTLLPGRSDLSGVDVTMLNMLNRRDSSASTISSAYLSSRRSSGISPCFSSRRSSEASQAEGRPQNVSVADSYDPISTDASRRSSEASQSDGLPSLLSLTPAQQYRLKAKYAAATGGPPPTPLPNMERMSLKTRLALLGDALEPGVALPPVHAPRRCSDGGAHGYGRRHLQPHDALGHGVRRASDPVRTGSEGLALPRVPRFSSLSSCNPPAMATSAEKRSLVLQNYTRPEGGQSRNFHSSPCPPSITENVTLESLTMDADANLNDEDFLPDDVVQYLNSQNQAGYEQHFPSALPDDSKVPHGPGDFDAPGLPDSHAGQQFHALEQPCPEGSKTDLPIQWNEVSSGSADLSSSKLKCGPRPAVPQTRAFGFCNGMVVHPQNPLRSGPAGGYQTLGENSNPYGGPEHLMLHNSPGSGTSGNAFHEQPCKAPQYGNCLNRQPVAPGALDGACGAGIQASKLKSTPMQGSGGQLNFGLPVAPNESAGSMVNGMQNQDPVGQGYLAHQLLGDSMQHPGAGRPGQQMLGQISATSHINIYQGPESCLPGAHGMGSQPSSLAVVRGYQPCASFGGSRRQAMPRDSLALQSGQLSDTSQTCRVNGIKMEMKGQPHPLCSNLQNYSGQFYDQTVGFSQQDTKAGSFSISDASCLLQGTSAKNSELLSPGANQVTSTVDSLDSHDLEGVQIDFDAIIDDGDHSSLMSGALSPSIIQNLSHSSSRLTTPRASLPFPALSMSTTNMAIGDMSSLLTSLAEESKFLAVMQ

>NP_112176.1

MAAPLSVEVEFGGGAELLFDGIKKHRVTLPGQEEPWDIRNLLIWIKKNLLKERPELFIQGDSVRPGILVLINDADWELLGELDYQLQDQDSVLFISTLHGG

345 NP_006181.1 NP_001547.1

>NP_006181.1

MSKPELKEDKMLEVHFVGDDDVLNHILDREGGAKLKKERAQLLVNPKKIIKKPEYDLEEDDQEVLKDQNYVEIMGRDVQESLKNGSATGGGNKVYSFQNRKHSEKMAKLASELAKTPQKSVSFSLKNDPEITINVPQSSKGHSASDKVQPKNNDKSEFLSTAPRSLRKRLIVPRSHSDSESEYSASNSEDDEGVAQEHEEDTNAVIFSQKIQAQNRVVSAPVGKETPSKRMKRDKTSDLVEEYFEAHSSSKVLTSDRTLQKLKRAKLDQQTLRNLLSKVSPSFSAELKQLNQQYEKLFHKWMLQLHLGFNIVLYGLGSKRDLLERFRTTMLQDSIHVVINGFFPGISVKSVLNSITEEVLDHMGTFRSILDQLDWIVNKFKEDSSLELFLLIHNLDSQMLRGEKSQQIIGQLSSLHNIYLIASIDHLNAPLMWDHAKQSLFNWLWYETTTYSPYTEETSYENSLLVKQSGSLPLSSLTHVLRSLTPNARGIFRLLIKYQLDNQDNPSYIGLSFQDFYQQCREAFLVNSDLTLRAQLTEFRDHKLIRTKKGTDGVEYLLIPVDNGTLTDFLEKEEEEA

>NP_001547.1

MSWSPSLTTQTCGAWEMKERLGTGGFGNVIRWHNQETGEQIAIKQCRQELSPRNRERWCLEIQIMRRLTHPNVVAARDVPEGMQNLAPNDLPLLAMEYCQGGDLRKYLNQFENCCGLREGAILTLLSDIASALRYLHENRIIHRDLKPENIVLQQGEQRLIHKIIDLGYAKELDQGSLCTSFVGTLQYLAPELLEQQKYTVTVDYWSFGTLAFECITGFRPFLPNWQPVQWHSKVRQKSEVDIVVSEDLNGTVKFSSSLPYPNNLNSVLAERLEKWLQLMLMWHPRQRGTDPTYGPNGCFKALDDILNLKLVHILNMVTGTIHTYPVTEDESLQSLKARIQQDTGIPEEDQELLQEAGLALIPDKPATQCISDGKLNEGHTLDMDLVFLFDNSKITYETQISPRPQPESVSCILQEPKRNLAFFQLRKVWGQVWHSIQTLKEDCNRLQQGQRAAMMNLLRNNSCLSKMKNSMASMSQQLKAKLDFFKTSIQIDLEKYSEQTEFGITSDKLLLAWREMEQAVELCGRENEVKLLVERMMALQTDIVDLQRSPMGRKQGGTLDDLEEQARELYRRLREKPRDQRTEGDSQEMVRLLLQAIQSFEKKVRVIYTQLSKTVVCKQKALELLPKVEEVVSLMNEDEKTVVRLQEKRQKELWNLLKIACSKVRGPVSGSPDSMNASRLSQPGQLMSQPSTASNSLPEPAKKSEELVAEAHNLCTLLENAIQDTVREQDQSFTALDWSWLQTEEEEHSCLEQAS

346 NP_060780.2 NP_077268.1

>NP_060780.2

MPGRGRCPDCGSTELVEDSHYSQSQLVCSDCGCVVTEGVLTTTFSDEGNLREVTYSRSTGENEQVSRSQQRGLRRVRDLCRVLQLPPTFEDTAVAYYQQAYRHSGIRAARLQKKEVLVGCCVLITCRQHNWPLTMGAICTLLYADLDVFSSTYMQIVKLLGLDVPSLCLAELVKTYCSSFKLFQASPSVPAKYVEDKEKMLSRTMQLVELANETWLVTGRHPLPVITAATFLAWQSLQPADRLSCSLARFCKLANVDLPYPASSRLQELLAVLLRMAEQLAWLRVLRLDKRSVVKHIGDLLQHRQSLVRSAFRDGTAEVETREKEPPGWGQGQGEGEVGNNSLGLPQGKRPASPALLLPPCMLKSPKRICPVPPVSTVTGDENISDSEIEQYLRTPQEVRDFQRAQAARQAATSVPNPP

>NP_077268.1

MIEVVCNDRLGKKVRVKCNTDDTIGDLKKLIAAQTGTRWNKIVLKKWYTIFKDHVSLGDYEIHDGMNLELYYQ

347 NP_005369.2 O14561

>NP_005369.2

MPSCSTSTMPGMICKNPDLEFDSLQPCFYPDEDDFYFGGPDSTPPGEDIWKKFELLPTPPLSPSRGFAEHSSEPPSWVTEMLLENELWGSPAEEDAFGLGGLGGLTPNPVILQDCMWSGFSAREKLERAVSEKLQHGRGPPTAGSTAQSPGAGAASPAGRGHGGAAGAGRAGAALPAELAHPAAECVDPAVVFPFPVNKREPAPVPAAPASAPAAGPAVASGAGIAAPAGAPGVAPPRPGGRQTSGGDHKALSTSGEDTLSDSDDEDDEEEDEEEEIDVVTVEKRRSSSNTKAVTTFTITVRPKNAALGPGRAQSSELILKRCLPIHQQHNYAAPSPYVESEDAPPQKKIKSEASPRPLKSVIPPKAKSLSPRNSDSEDSERRRNHNILERQRRNDLRSSFLTLRDHVPELVKNEKAAKVVILKKATEYVHSLQAEEHQLLLEKEKLQARQQQLLKKIEHARTC

>O14561

MASRVLSAYVSRLPAAFAPLPRVRMLAVARPLSTALCSAGTQTRLGTLQPALVLAQVPGRVTQLCRQYSDMPPLTLEGIQDRVLYVLKLYDKIDPEKLSVNSHFMKDLGLDSLDQVEIIMAMEDEFGFEIPDIDAEKLMCPQEIVDYIADKKDVYE

348 NP_060740.1 O75880

>NP_060740.1

MGEAEVGGGGAAGDKGPGEAATSPAEETVVWSPEVEVCLFHAMLGHKPVGVNRHFHMICIRDKFSQNIGRQVPSKVIWDHLSTMYDMQALHESEILPFPNPERNFVLPEEIIQEVREGKVMIEEEMKEEMKEDVDPHNGADDVFSSSGSLGKASEKSSKDKEKNSSDLGCKEGADKRKRSRVTDKVLTANSNPSSPSAAKRRRT

>O75880

MAMLVLVPGRVMRPLGGQLWRFLPRGLEFWGPAEGTARVLLRQFCARQAEAWRASGRPGYCLGTRPLSTARPPPPWSQKGPGDSTRPSKPGPVSWKSLAITFAIGGALLAGMKHVKKEKAEKLEKERQRHIGKPLLGGPFSLTTHTGERKTDKDYLGQWLLIYFGFTHCPDVCPEELEKMIQVVDEIDSITTLPDLTPLFISIDPERDTKEAIANYVKEFSPKLVGLTGTREEVDQVARAYRVYYSPGPKDEDEDYIVDHTIIMYLIGPDGEFLDYFGQNKRKGEIAASIATHMRPYRKKS

349 NP_002681.1 Q9NVV4

>NP_002681.1

MSKRKAPQETLNGGITDMLTELANFEKNVSQAIHKYNAYRKAASVIAKYPHKIKSGAEAKKLPGVGTKIAEKIDEFLATGKLRKLEKIRQDDTSSSINFLTRVSGIGPSAARKFVDEGIKTLEDLRKNEDKLNHHQRIGLKYFGDFEKRIPREEMLQMQDIVLNEVKKVDSEYIATVCGSFRRGAESSGDMDVLLTHPSFTSESTKQPKLLHQVVEQLQKVHFITDTLSKGETKFMGVCQLPSKNDEKEYPHRRIDIRLIPKDQYYCGVLYFTGSDIFNKNMRAHALEKGFTINEYTIRPLGVTGVAGEPLPVDSEKDIFDYIQWKYREPKDRSE

>Q9NVV4
[truncated: 4,606,508 more chars]
