# Supplementary material for: A novel matrix of sequence descriptors for predicting protein-protein interactions from amino acid sequences
Source: PLoS One. 2019 Jun 7;14(6):e0217312. doi: 10.1371/journal.pone.0217312 (PMC6555512; doi:10.1371/journal.pone.0217312)
Supplement: S1 File — There are 36,630 protein-protein pairs from total 9476 proteins, and the first column is protein ID from HPRD, the second column is the other protein ID and the two proteins constitute the positive Protein-protein interaction. (DOC) [file pone.0217312.s001.doc]

**Online support information A**

There are 36,630 protein-protein pairs from total 9476 proteins, and the first column is protein ID from HPRD, the second column is the other protein ID and the two proteins constitute the positive Protein-protein interaction.

Index	Protein_1_ID	protein_2_ID

1 NP_663777.1 NP_001233.1

>NP_663777.1

MESSKKMDSPGALQTNPPLKLHTDRSAGTPVFVPEQGGYKEKFVKTVEDKYKCEKCHLVLCSPKQTECGHRFCESCMAALLSSSSPKCTACQESIVKDKVFKDNCCKREILALQIYCRNESRGCAEQLMLGHLLVHLKNDCHFEELPCVRPDCKEKVLRKDLRDHVEKACKYREATCSHCKSQVPMIALQKHEDTDCPCVVVSCPHKCSVQTLLRSELSAHLSECVNAPSTCSFKRYGCVFQGTNQQIKAHEASSAVQHVNLLKEWSNSLEKKVSLLQNESVEKNKSIQSLHNQICSFEIEIERQKEMLRNNESKILHLQRVIDSQAEKLKELDKEIRPFRQNWEEADSMKSSVESLQNRVTELESVDKSAGQVARNTGLLESQLSRHDQMLSVHDIRLADMDLRFQVLETASYNGVLIWKIRDYKRRKQEAVMGKTLSLYSQPFYTGYFGYKMCARVYLNGDGMGKGTHLSLFFVIMRGEYDALLPWPFKQKVTLMLMDQGSSRRHLGDAFKPDPNSSSFKKPTGEMNIASGCPVFVAQTVLENGTYIKDDTIFIKVIVDTSDLPDP

>NP_001233.1

MARPHPWWLCVLGTLVGLSATPAPKSCPERHYWAQGKLCCQMCEPGTFLVKDCDQHRKAAQCDPCIPGVSFSPDHHTRPHCESCRHCNSGLLVRNCTITANAECACRNGWQCRDKECTECDPLPNPSLTARSSQALSPHPQPTHLPYVSEMLEARTAGHMQTLADFRQLPARTLSTHWPPQRSLCSSDFIRILVIFSGMFLVFTLAGALFLHQRRKYRSNKGESPVEPAEPCRYSCPREEEGSTIPIQEDYRKPEPACSP

2 NP_055436.2 NP_001420.2

>NP_055436.2

MVMSSYMVNSKYVDPKFPPCEEYLQGGYLGEQGADYYGGGAQGADFQPPGLYPRPDFGEQPFGGSGPGPGSALPARGHGQEPGGPGGHYAAPGEPCPAPPAPPPAPLPGARAYSQSDPKQPPSGTALKQPAVVYPWMKKVHVNSVNPNYTGGEPKRSRTAYTRQQVLELEKEFHFNRYLTRRRRIEIAHTLCLSERQIKIWFQNRRMKWKKDHKLPNTKGRSSSSSSSSSCSSSVAPSQHLQPMAKDHHTDLTTL

>NP_001420.2

MAENVVEPGPPSAKRPKLSSPALSASASDGTDFGSLFDLEHDLPDELINSTELGLTNGGDINQLQTSLGMVQDAASKHKQLSELLRSGSSPNLNMGVGGPGQVMASQAQQSSPGLGLINSMVKSPMTQAGLTSPNMGMGTSGPNQGPTQSTGMMNSPVNQPAMGMNTGMNAGMNPGMLAAGNGQGIMPNQVMNGSIGAGRGRQNMQYPNPGMGSAGNLLTEPLQQGSPQMGGQTGLRGPQPLKMGMMNNPNPYGSPYTQNPGQQIGASGLGLQIQTKTVLSNNLSPFAMDKKAVPGGGMPNMGQQPAPQVQQPGLVTPVAQGMGSGAHTADPEKRKLIQQQLVLLLHAHKCQRREQANGEVRQCNLPHCRTMKNVLNHMTHCQSGKSCQVAHCASSRQIISHWKNCTRHDCPVCLPLKNAGDKRNQQPILTGAPVGLGNPSSLGVGQQSAPNLSTVSQIDPSSIERAYAALGLPYQVNQMPTQPQVQAKNQQNQQPGQSPQGMRPMSNMSASPMGVNGGVGVQTPSLLSDSMLHSAINSQNPMMSENASVPSLGPMPTAAQPSTTGIRKQWHEDITQDLRNHLVHKLVQAIFPTPDPAALKDRRMENLVAYARKVEGDMYESANNRAEYYHLLAEKIYKIQKELEEKRRTRLQKQNMLPNAAGMVPVSMNPGPNMGQPQPGMTSNGPLPDPSMIRGSVPNQMMPRITPQSGLNQFGQMSMAQPPIVPRQTPPLQHHGQLAQPGALNPPMGYGPRMQQPSNQGQFLPQTQFPSQGMNVTNIPLAPSSGQAPVSQAQMSSSSCPVNSPIMPPGSQGSHIHCPQLPQPALHQNSPSPVPSRTPTPHHTPPSIGAQQPPATTIPAPVPTPPAMPPGPQSQALHPPPRQTPTPPTTQLPQQVQPSLPAAPSADQPQQQPRSQQSTAASVPTPTAPLLPPQPATPLSQPAVSIEGQVSNPPSTSSTEVNSQAIAEKQPSQEVKMEAKMEVDQPEPADTQPEDISESKVEDCKMESTETEERSTELKTEIKEEEDQPSTSATQSSPAPGQSKKKIFKPEELRQALMPTLEALYRQDPESLPFRQPVDPQLLGIPDYFDIVKSPMDLSTIKRKLDTGQYQEPWQYVDDIWLMFNNAWLYNRKTSRVYKYCSKLSEVFEQEIDPVMQSLGYCCGRKLEFSPQTLCCYGKQLCTIPRDATYYSYQNRYHFCEKCFNEIQGESVSLGDDPSQPQTTINKEQFSKRKNDTLDPELFVECTECGRKMHQICVLHHEIIWPAGFVCDGCLKKSARTRKENKFSAKRLPSTRLGTFLENRVNDFLRRQNHPESGEVTVRVVHASDKTVEVKPGMKARFVDSGEMAESFPYRTKALFAFEEIDGVDLCFFGMHVQEYGSDCPPPNQRRVYISYLDSVHFFRPKCLRTAVYHEILIGYLEYVKKLGYTTGHIWACPPSEGDDYIFHCHPPDQKIPKPKRLQEWYKKMLDKAVSERIVHDYKDIFKQATEDRLTSAKELPYFEGDFWPNVLEESIKELEQEEEERKREENTSNESTDVTKGDSKNAKKKNNKKTSKNKSSLSRGNKKKPGMPNVSNDLSQKLYATMEKHKEVFFVIRLIAGPAANSLPPIVDPDPLIPCDLMDGRDAFLTLARDKHLEFSSLRRAQWSTMCMLVELHTQSQDRFVYTCNECKHHVETRWHCTVCEDYDLCITCYNTKNHDHKMEKLGLGLDDESNNQQAAATQSPGDSRRLSIQRCIQSLVHACQCRNANCSLPSCQKMKRVVQHTKGCKRKTNGGCPICKQLIALCCYHAKHCQENKCPVPFCLNIKQKLRQQQLQHRLQQAQMLRRRMASMQRTGVVGQQQGLPSPTPATPTTPTGQQPTTPQTPQPTSQPQPTPPNSMPPYLPRTQAAGPVSQGKAAGQVTPPTPPQTAQPPLPGPPPAAVEMAMQIQRAAETQRQMAHVQIFQRPIQHQMPPMTPMAPMGMNPPPMTRGPSGHLEPGMGPTGMQQQPPWSQGGLPQPQQLQSGMPRPAMMSVAQHGQPLNMAPQPGLGQVGISPLKPGTVSQQALQNLLRTLRSPSSPLQQQQVLSILHANPQLLAAFIKQRAAKYANSNPQPIPGQPGMPQGQPGLQPPTMPGQQGVHSNPAMQNMNPMQAGVQRAGLPQQQPQQQLQPPMGGMSPQAQQMNMNHNTMPSQFRDILRRQQMMQQQQQQGAGPGIGPGMANHNQFQQPQGVGYPPQQQQRMQHHMQQMQQGNMGQIGQLPQALGAEAGASLQAYQQRLLQQQMGSPVQPNPMSPQQHMLPNQAQSPHLQGQQIPNSLSNQVRSPQPVPSPRPQSQPPHSSPSPRMQPQPSPHHVSPQTSSPHPGLVAAQANPMEQGHFASPDQNSMLSQLASNPGMANLHGASATDLGLSTDNSDLNSNLSQSTLDIH

3 NP_003630.1 NP_001073594.1

>NP_003630.1

MNRHLWKSQLCEMVQPSGGPAADQDVLGEESPLGKPAMLHLPSEQGAPETLQRCLEENQELRDAIRQSNQILRERCEELLHFQASQREEKEFLMCKFQEARKLVERLGLEKLDLKRQKEQALREVEHLKRCQQQMAEDKASVKAQVTSLLGELQESQSRLEAATKECQALEGRARAASEQARQLESEREALQQQHSVQVDQLRMQGQSVEAALRMERQAASEEKRKLAQLQVAYHQLFQEYDNHIKSSVVGSERKRGMQLEDLKQQLQQAEEALVAKQEVIDKLKEEAEQHKIVMETVPVLKAQADIYKADFQAERQAREKLAEKKELLQEQLEQLQREYSKLKASCQESARIEDMRKRHVEVSQAPLPPAPAYLSSPLALPSQRRSPPEEPPDFCCPKCQYQAPDMDTLQIHVMECIE

>NP_001073594.1

MEGGRRARVVIESKRNFFLGAFPTPFPAEHVELGRLGDSETAMVPGKGGADYILLPFKKMDFSRNLYDIGEQLDSEDLASLKFLSLDYIPQRKQEPIKDALMLFQRLQEKRMLEESNLSFLKELLFRINRLDLLITYLNTRKEEMERELQTPGRAQISAYRVMLYQISEEVSRSELRSFKFLLQEEISKCKLDDDMNLLDIFIEMEKRVILGEGKLDILKRVCAQINKSLLKIINDYEEFSKERSSSLEGSPDEFSNGEELCGVMTISDSPREQDSESQTLDKVYQMKSKPRGYCLIINNHNFAKAREKVPKLHSIRDRNGTHLDAGALTTTFEELHFEIKPHDDCTVEQIYEILKIYQLMDHSNMDCFICCILSHGDKGIIYGTDGQEAPIYELTSQFTGLKCPSLAGKPKVFFIQACQGDNYQKGIPVETDSEEQPYLEMDLSSPQTRYIPDEADFLLGMATVNNCVSYRNPAEGTWYIQSLCQSLRERCPRGDDILTILTEVNYEVSNKDDKKNMGKQMPQPTFTLRKKLVFPSD

4 NP_001001998.1 NP_067000.1

>NP_001001998.1

MAPPSTREPRVLSATSATKSDGEMVLPGFPDADSFVKFALGSVVAVTKASGGLPQFGDEYDFYRSFPGFQAFCETQGDRLLQCMSRVMQYHGCRSNIKDRSKVTELEDKFDLLVDANDVILERVGILLDEASGVNKNQQPVLPAGLQVPKTVVSSWNRKAAEYGKKAKSETFRLLHAKNIIRPQLKFREKIDNSNTPFLPKIFIKPNAQKPLPQALSKERRERPQDRPEDLDVPPALADFIHQQRTQQVEQDMFAHPYQYELNHFTPADAVLQKPQPQLYRPIEETPCHFISSLDELVELNEKLLNCQEFAVDLEHHSYRSFLGLTCLMQISTRTEDFIIDTLELRSDMYILNESLTDPAIVKVFHGADSDIEWLQKDFGLYVVNMFDTHQAARLLNLGRHSLDHLLKLYCNVDSNKQYQLADWRIRPLPEEMLSYARDDTHYLLYIYDKMRLEMWERGNGQPVQLQVVWQRSRDICLKKFIKPIFTDESYLELYRKQKKHLNTQQLTAFQLLFAWRDKTARREDESYGYVLPNHMMLKIAEELPKEPQGIIACCNPVPPLVRQQINEMHLLIQQAREMPLLKSEVAAGVKKSGPLPSAERLENVLFGPHDCSHAPPDGYPIIPTSGSVPVQKQASLFPDEKEDNLLGTTCLIATAVITLFNEPSAEDSKKGPLTVAQKKAQNIMESFENPFRMFLPSLGHRAPVSQAAKFDPSTKIYEISNRWKLAQVQVQKDSKEAVKKKAAEQTAAREQAKEACKAAAEQAISVRQQVVLENAAKKRERATSDPRTTEQKQEKKRLKISKKPKDPEPPEKEFTPYDYSQSDFKAFAGNSKSKVSSQFDPNKQTPSGKKCIAAKKIKQSVGNKSMSFPTGKSDRGFRYNWPQR

>NP_067000.1

MLFYSFFKSLVGKDVVVELKNDLSICGTLHSVDQYLNIKLTDISVTDPEKYPHMLSVKNCFIRGSVVRYVQLPADEVDTQLLQDAARKEALQQKQ

5 NP_001888.2 NP_001782.1

>NP_001888.2

MQSGPRPPLPAPGLALALTLTMLARLASAASFFGENHLEVPVATALTDIDLQLQFSTSQPEALLLLAAGPADHLLLQLYSGRLQVRLVLGQEELRLQTPAETLLSDSIPHTVVLTVVEGWATLSVDGFLNASSAVPGAPLEVPYGLFVGGTGTLGLPYLRGTSRPLRGCLHAATLNGRSLLRPLTPDVHEGCAEEFSASDDVALGFSGPHSLAAFPAWGTQDEGTLEFTLTTQSRQAPLAFQAGGRRGDFIYVDIFEGHLRAVVEKGQGTVLLHNSVPVADGQPHEVSVHINAHRLEISVDQYPTHTSNRGVLSYLEPRGSLLLGGLDAEASRHLQEHRLGLTPEATNASLLGCMEDLSVNGQRRGLREALLTRNMAAGCRLEEEEYEDDAYGHYEAFSTLAPEAWPAMELPEPCVPEPGLPPVFANFTQLLTISPLVVAEGGTAWLEWRHVQPTLDLMEAELRKSQVLFSVTRGARHGELELDIPGAQARKMFTLLDVVNRKARFIHDGSEDTSDQLVLEVSVTARVPMPSCLRRGQTYLLPIQVNPVNDPPHIIFPHGSLMVILEHTQKPLGPEVFQAYDPDSACEGLTFQVLGTSSGLPVERRDQPGEPATEFSCRELEAGSLVYVHRGGPAQDLTFRVSDGLQASPPATLKVVAIRPAIQIHRSTGLRLAQGSAMPILPANLSVETNAVGQDVSVLFRVTGALQFGELQKQGAGGVEGAEWWATQAFHQRDVEQGRVRYLSTDPQHHAYDTVENLALEVQVGQEILSNLSFPVTIQRATVWMLRLEPLHTQNTQQETLTTAHLEATLEEAGPSPPTFHYEVVQAPRKGNLQLQGTRLSDGQGFTQDDIQAGRVTYGATARASEAVEDTFRFRVTAPPYFSPLYTFPIHIGGDPDAPVLTNVLLVVPEGGEGVLSADHLFVKSLNSASYLYEVMERPRHGRLAWRGTQDKTTMVTSFTNEDLLRGRLVYQHDDSETTEDDIPFVATRQGESSGDMAWEEVRGVFRVAIQPVNDHAPVQTISRIFHVARGGRRLLTTDDVAFSDADSGFADAQLVLTRKDLLFGSIVAVDEPTRPIYRFTQEDLRKRRVLFVHSGADRGWIQLQVSDGQHQATALLEVQASEPYLRVANGSSLVVPQGGQGTIDTAVLHLDTNLDIRSGDEVHYHVTAGPRWGQLVRAGQPATAFSQQDLLDGAVLYSHNGSLSPRDTMAFSVEAGPVHTDATLQVTIALEGPLAPLKLVRHKKIYVFQGEAAEIRRDQLEAAQEAVPPADIVFSVKSPPSAGYLVMVSRGALADEPPSLDPVQSFSQEAVDTGRVLYLHSRPEAWSDAFSLDVASGLGAPLEGVLVELEVLPAAIPLEAQNFSVPEGGSLTLAPPLLRVSGPYFPTLLGLSLQVLEPPQHGALQKEDGPQARTLSAFSWRMVEEQLIRYVHDGSETLTDSFVLMANASEMDRQSHPVAFTVTVLPVNDQPPILTTNTGLQMWEGATAPIPAEALRSTDGDSGSEDLVYTIEQPSNGRVVLRGAPGTEVRSFTQAQLDGGLVLFSHRGTLDGGFRFRLSDGEHTSPGHFFRVTAQKQVLLSLKGSQTLTVCPGSVQPLSSQTLRASSSAGTDPQLLLYRVVRGPQLGRLFHAQQDSTGEALVNFTQAEVYAGNILYEHEMPPEPFWEAHDTLELQLSSPPARDVAATLAVAVSFEAACPQRPSHLWKNKGLWVPEGQRARITVAALDASNLLASVPSPQRSEHDVLFQVTQFPSRGQLLVSEEPLHAGQPHFLQSQLAAGQLVYAHGGGGTQQDGFHFRAHLQGPAGASVAGPQTSEAFAITVRDVNERPPQPQASVPLRLTRGSRAPISRAQLSVVDPDSAPGEIEYEVQRAPHNGFLSLVGGGLGPVTRFTQADVDSGRLAFVANGSSVAGIFQLSMSDGASPPLPMSLAVDILPSAIEVQLRAPLEVPQALGRSSLSQQQLRVVSDREEPEAAYRLIQGPQYGHLLVGGRPTSAFSQFQIDQGEVVFAFTNFSSSHDHFRVLALARGVNASAVVNVTVRALLHVWAGGPWPQGATLRLDPTVLDAGELANRTGSVPRFRLLEGPRHGRVVRVPRARTEPGGSQLVEQFTQQDLEDGRLGLEVGRPEGRAPGPAGDSLTLELWAQGVPPAVASLDFATEPYNAARPYSVALLSVPEAARTEAGKPESSTPTGEPGPMASSPEPAVAKGGFLSFLEANMFSVIIPMCLVLLLLALILPLLFYLRKRNKTGKHDVQVLTAKPRNGLAGDTETFRKVEPGQAIPLTAVPGQGPPPGGQPDPELLQFCRTPNPALKNGQYWV

>NP_001782.1

MQTIKCVVVGDGAVGKTCLLISYTTNKFPSEYVPTVFDNYAVTVMIGGEPYTLGLFDTAGQEDYDRLRPLSYPQTDVFLVCFSVVSPSSFENVKEKWVPEITHHCPKTPFLLVGTQIDLRDDPSTIEKLAKNKQKPITPETAEKLARDLKAVKYVECSALTQKGLKNVFDEAILAALEPPEPKKSRRCVLL

6 NP_001033.1 NP_002037.2

>NP_001033.1

MPSGFQQIGSEDGEPPQQRVTGTLVLAVFSAVLGSLQFGYNIGVINAPQKVIEQSYNETWLGRQGPEGPSSIPPGTLTTLWALSVAIFSVGGMISSFLIGIISQWLGRKRAMLVNNVLAVLGGSLMGLANAAASYEMLILGRFLIGAYSGLTSGLVPMYVGEIAPTHLRGALGTLNQLAIVIGILIAQVLGLESLLGTASLWPLLLGLTVLPALLQLVLLPFCPESPRYLYIIQNLEGPARKSLKRLTGWADVSGVLAELKDEKRKLERERPLSLLQLLGSRTHRQPLIIAVVLQLSQQLSGINAVFYYSTSIFETAGVGQPAYATIGAGVVNTVFTLVSVLLVERAGRRTLHLLGLAGMCGCAILMTVALLLLERVPAMSYVSIVAIFGFVAFFEIGPGPIPWFIVAELFSQGPRPAAMAVAGFSNWTSNFIIGMGFQYVAEAMGPYVFLLFAVLLLGFFIFTFLRVPETRGRTFDQISAAFHRTPSLLEQEVKPSTELEYLGPDEND

>NP_002037.2

MGKVKVGVNGFGRIGRLVTRAAFNSGKVDIVAINDPFIDLNYMVYMFQYDSTHGKFHGTVKAENGKLVINGNPITIFQERDPSKIKWGDAGAEYVVESTGVFTTMEKAGAHLQGGAKRVIISAPSADAPMFVMGVNHEKYDNSLKIISNASCTTNCLAPLAKVIHDNFGIVEGLMTTVHAITATQKTVDGPSGKLWRDGRGALQNIIPASTGAAKAVGKVIPELDGKLTGMAFRVPTANVSVVDLTCRLEKPAKYDDIKKVVKQASEGPLKGILGYTEHQVVSSDFNSDTHSSTFDAGAGIALNDHFVKLISWYDNEFGYSNRVVDLMAHMASKE

7 NP_003001.1 NP_006292.2

>NP_003001.1

MAAPSPSGGGGSGGGSGSGTPGPVGSPAPGHPAVSSMQGKRKALKLNFANPPFKSTARFTLNPNPTGVQNPHIERLRTHSIESSGKLKISPEQHWDFTAEDLKDLGEIGRGAYGSVNKMVHKPSGQIMAVKRIRSTVDEKEQKQLLMDLDVVMRSSDCPYIVQFYGALFREGDCWICMELMSTSFDKFYKYVYSVLDDVIPEEILGKITLATVKALNHLKENLKIIHRDIKPSNILLDRSGNIKLCDFGISGQLVDSIAKTRDAGCRPYMAPERIDPSASRQGYDVRSDVWSLGITLYELATGRFPYPKWNSVFDQLTQVVKGDPPQLSNSEEREFSPSFINFVNLCLTKDESKRPKYKELLKHPFILMYEERAVEVACYVCKILDQMPATPSSPMYVD

>NP_006292.2

MACLHETRTPSPSFGGFVSTLSEASMRKLDPDTSDCTPEKDLTPTHVLQLHEQDAGGPGGAAGSPESRASRVRADEVRLQCQSGSGFLEGLFGCLRPVWTMIGKAYSTEHKQQQEDLWEVPFEEILDLQWVGSGAQGAVFLGRFHGEEVAVKKVRDLKETDIKHLRKLKHPNIITFKGVCTQAPCYCILMEFCAQGQLYEVLRAGRPVTPSLLVDWSMGIAGGMNYLHLHKIIHRDLKSPNMLITYDDVVKISDFGTSKELSDKSTKMSFAGTVAWMAPEVIRNEPVSEKVDIWSFGVVLWELLTGEIPYKDVDSSAIIWGVGSNSLHLPVPSSCPDGFKILLRQCWNSKPRNRPSFRQILLHLDIASADVLSTPQETYFKSQAEWREEVKLHFEKIKSEGTCLHRLEEELVMRRREELRHALDIREHYERKLERANNLYMELNALMLQLELKERELLRREQALERRCPGLLKPHPSRGLLHGNTMEKLIKKRNVPQKLSPHSKRPDILKTESLLPKLDAALSGVGLPGCPKAPPSPGRSRRGKTRHRKASAKGSCGDLPGLRTAVPPHEPGGPGSPGGLGGGPSAWEACPPALRGLHHDLLLRKMSSSSPDLLSAALGSRGRGATGGAGDPGSPPPARGDTPPSEGSAPGSTSPDSPGGAKGEPPPPVGPGEGVGLLGTGREGTSGRGGSRAGSQHLTPAALLYRAAVTRSQKRGISSEEEEGEVDSEVELTSSQRWPQSLNMRQSLSTFSSENPSDGEEGTASEPSPSGTPEVGSTNTDERPDERSDDMCSQGSEIPLDPPPSEVIPGPEPSSLPIPHQELLRERGPPNSEDSDCDSTELDNSNSVDALRPPASLPP

8 NP_060901.2 NP_000199.2

>NP_060901.2

MASNFNDIVKQGYVRIRSRRLGIYQRCWLVFKKASSKGPKRLEKFSDERAAYFRCYHKVTELNNVKNVARLPKSTKKHAIGIYFNDDTSKTFACESDLEADEWCKVLQMECVGTRINDISLGEPDLLATGVEREQSERFNVYLMPSPNLDVHGECALQITYEYICLWDVQNPRVKLISWPLSALRRYGRDTTWFTFEAGRMCETGEGLFIFQTRDGEAIYQKVHSAALAIAEQHERLLQSVKNSMLQMKMSERAASLSTMVPLPRSAYWQHITRQHSTGQLYRLQDVSSPLKLHRTETFPAYRSEH

>NP_000199.2

MATGGRRGAAAAPLLVAVAALLLGAAGHLYPGEVCPGMDIRNNLTRLHELENCSVIEGHLQILLMFKTRPEDFRDLSFPKLIMITDYLLLFRVYGLESLKDLFPNLTVIRGSRLFFNYALVIFEMVHLKELGLYNLMNITRGSVRIEKNNELCYLATIDWSRILDSVEDNYIVLNKDDNEECGDICPGTAKGKTNCPATVINGQFVERCWTHSHCQKVCPTICKSHGCTAEGLCCHSECLGNCSQPDDPTKCVACRNFYLDGRCVETCPPPYYHFQDWRCVNFSFCQDLHHKCKNSRRQGCHQYVIHNNKCIPECPSGYTMNSSNLLCTPCLGPCPKVCHLLEGEKTIDSVTSAQELRGCTVINGSLIINIRGGNNLAAELEANLGLIEEISGYLKIRRSYALVSLSFFRKLRLIRGETLEIGNYSFYALDNQNLRQLWDWSKHNLTITQGKLFFHYNPKLCLSEIHKMEEVSGTKGRQERNDIALKTNGDQASCENELLKFSYIRTSFDKILLRWEPYWPPDFRDLLGFMLFYKEAPYQNVTEFDGQDACGSNSWTVVDIDPPLRSNDPKSQNHPGWLMRGLKPWTQYAIFVKTLVTFSDERRTYGAKSDIIYVQTDATNPSVPLDPISVSNSSSQIILKWKPPSDPNGNITHYLVFWERQAEDSELFELDYCLKGLKLPSRTWSPPFESEDSQKHNQSEYEDSAGECCSCPKTDSQILKELEESSFRKTFEDYLHNVVFVPRKTSSGTGAEDPRPSRKRRSLGDVGNVTVAVPTVAAFPNTSSTSVPTSPEEHRPFEKVVNKESLVISGLRHFTGYRIELQACNQDTPEERCSVAAYVSARTMPEAKADDIVGPVTHEIFENNVVHLMWQEPKEPNGLIVLYEVSYRRYGDEELHLCVSRKHFALERGCRLRGLSPGNYSVRIRATSLAGNGSWTEPTYFYVTDYLDVPSNIAKIIIGPLIFVFLFSVVIGSIYLFLRKRQPDGPLGPLYASSNPEYLSASDVFPCSVYVPDEWEVSREKITLLRELGQGSFGMVYEGNARDIIKGEAETRVAVKTVNESASLRERIEFLNEASVMKGFTCHHVVRLLGVVSKGQPTLVVMELMAHGDLKSYLRSLRPEAENNPGRPPPTLQEMIQMAAEIADGMAYLNAKKFVHRDLAARNCMVAHDFTVKIGDFGMTRDIYETDYYRKGGKGLLPVRWMAPESLKDGVFTTSSDMWSFGVVLWEITSLAEQPYQGLSNEQVLKFVMDGGYLDQPDNCPERVTDLMRMCWQFNPKMRPTFLEIVNLLKDDLHPSFPEVSFFHSEENKAPESEELEMEFEDMENVPLDRSSHCQREEAGGRDGGSSLGFKRSYEEHIPYTHMNGGKKNGRILTLPRSNPS

9 NP_001020276.1 NP_001895.1

>NP_001020276.1

MASSSGSKAEFIVGGKYKLVRKIGSGSFGDIYLAINITNGEEVAVKLESQKARHPQLLYESKLYKILQGGVGIPHIRWYGQEKDYNVLVMDLLGPSLEDLFNFCSRRFTMKTVLMLADQMISRIEYVHTKNFIHRDIKPDNFLMGIGRHCNKCLESPVGKRKRSMTVSTSQDPSFSGLNQLFLIDFGLAKKYRDNRTRQHIPYREDKNLTGTARYASINAHLGIEQSRRDDMESLGYVLMYFNRTSLPWQGLKAATKKQKYEKISEKKMSTPVEVLCKGFPAEFAMYLNYCRGLRFEEAPDYMYLRQLFRILFRTLNHQYDYTFDWTMLKQKAAQQAASSSGQGQQAQTPTGKQTDKTKSNMKGF

>NP_001895.1

MATQADLMELDMAMEPDRKAAVSHWQQQSYLDSGIHSGATTTAPSLSGKGNPEEEDVDTSQVLYEWEQGFSQSFTQEQVADIDGQYAMTRAQRVRAAMFPETLDEGMQIPSTQFDAAHPTNVQRLAEPSQMLKHAVVNLINYQDDAELATRAIPELTKLLNDEDQVVVNKAAVMVHQLSKKEASRHAIMRSPQMVSAIVRTMQNTNDVETARCTAGTLHNLSHHREGLLAIFKSGGIPALVKMLGSPVDSVLFYAITTLHNLLLHQEGAKMAVRLAGGLQKMVALLNKTNVKFLAITTDCLQILAYGNQESKLIILASGGPQALVNIMRTYTYEKLLWTTSRVLKVLSVCSSNKPAIVEAGGMQALGLHLTDPSQRLVQNCLWTLRNLSDAATKQEGMEGLLGTLVQLLGSDDINVVTCAAGILSNLTCNNYKNKMMVCQVGGIEALVRTVLRAGDREDITEPAICALRHLTSRHQEAEMAQNAVRLHYGLPVVVKLLHPPSHWPLIKATVGLIRNLALCPANHAPLREQGAIPRLVQLLVRAHQDTQRRTSMGGTQQQFVEGVRMEEIVEGCTGALHILARDVHNRIVIRGLNTIPLFVQLLYSPIENIQRVAAGVLCELAQDKEAAEAIEAEGATAPLTELLHSRNEGVATYAAAVLFRMSEDKPQDYKKRLSVELTSSLFRTEPMAWNETADLGLDIGAQGEPLGYRQDDPSYRSFHSGGYGQDALGMDPMMEHEMGGHHPGADYPVDGLPDLGHAQDLMDGLPPGDSNQLAWFDTDL

10 NP_005973.1 NP_005068.2

>NP_005973.1

MSMLPSFGFTQEQVACVCEVLQQGGNLERLGRFLWSLPACDHLHKNESVLKAKAVVAFHRGNFRELYKILESHQFSPHNHPKLQQLWLKAHYVEAEKLRGRPLGAVGKYRVRRKFPLPRTIWDGEETSYCFKEKSRGVLREWYAHNPYPSPREKRELAEATGLTTTQVSNWFKNRRQRDRAAEAKERENTENNNSSSNKQNQLSPLEGGKPLMSSSEEEFSPPQSPDQNSVLLLQGNMGHARSSNYSLPGLTASQPSHGLQTHQHQLQDSLLGPLTSSLVDLGS

>NP_005068.2

MFPQSRHPTPHQAAGQPFKFTIPESLDRIKEEFQFLQAQYHSLKLECEKLASEKTEMQRHYVMYYEMSYGLNIEMHKQTEIAKRLNTICAQVIPFLSQEHQQQVAQAVERAKQVTMAELNAIIGQQQLQAQHLSHGHGPPVPLTPHPSGLQPPGIPPLGGSAGLLALSSALSGQSHLAIKDDKKHHDAEHHRDREPGTSNSLLVPDSLRGTDKRRNGPEFSNDIKKRKVDDKDSSHYDSDGDKSDDNLVVDVSNEDPSSPRASPAHSPRENGIDKNRLLKKDASSSPASTASSASSTSLKSKEMSLHEKASTPVLKSSTPTPRSDMPTPGTSATPGLRPGLGKPPAIDPLVNQAAAGLRTPLAVPGPYPAPFGMVPHAGMNGELTSPGAAYASLHNMSPQMSAAAAAAAVVAYGRSPMVGFDPPPHMRVPTIPPNLAGIPGGKPAYSFHVTADGQMQPVPFPPDALIGPGIPRHARQINTLNHGEVVCAVTISNPTRHVYTGGKGCVKVWDISHPGNKSPVSQLDCLNRDNYIRSCKLLPDGCTLIVGGEASTLSIWDLAAPTPRIKAELTSSAPACYALAISPDSKVCFSCCSDGNIAVWDLHNQTLVRQFQGHTDGASCIDISNDGTKLWTGGLDNTVRSWDLREGRQLQQHDFTSQIFSLGYCPTGEWLAVGMESSNVEVLHVNKPDKYQLHLHESCVLSLKFAYCGKWFVSTGKDNLLNAWRTPYGASIFQSKESSSVLSCDISVDDKYIVTGSGDKKATVYEVIY

11 NP_036377.1 NP_005602.2

>NP_036377.1

MALTSFLPAPTQLSQDQLEAEEKARSQRSRQTSLVSSRREPPPYGYRKGWIPRLLEDFGDGGAFPEIHVAQYPLDMGRKKKMSNALAIQVDSEGKIKYDAIARQGQSKDKVIYSKYTDLVPKEVMNADDPDLQRPDEEAIKEITEKTRVALEKSVSQKVAAAMPVRAADKLAPAQYIRYTPSQQGVAFNSGAKQRVIRMVEMQKDPMEPPRFKINKKIPRGPPSPPAPVMHSPSRKMTVKEQQEWKIPPCISNWKNAKGYTIPLDKRLAADGRGLQTVHINENFAKLAEALYIADRKAREAVEMRAQVERKMAQKEKEKHEEKLREMAQKARERRAGIKTHVEKEDGEARERDEIRHDRRKERQHDRNLSRAAPDKRSKLQRNENRDISEVIALGVPNPRTSNEVQYDQRLFNQSKGMDSGFAGGEDEIYNVYDQAWRGGKDMAQSIYRPSKNLDKDMYGDDLEARIKTNRFVPDKEFSGSDRRQRGREGPVQFEEDPFGLDKFLEEAKQHGGSKRPSDSSRPKEHEHEGKKRRKE

>NP_005602.2

MPSGGDQSPPPPPPPPAAAASDEEEEDDGEAEDAAPSAESPTPQIQQRFDELCSRLNMDEAARPEAWDSYRSMSESYTLEGNDLHWLACALYVACRKSVPTVSKGTVEGNYVSLTRILKCSEQSLIEFFNKMKKWEDMANLPPHFRERTERLERNFTVSAVIFKKYEPIFQDIFKYPQEEQPRQQRGRKQRRQPCTVSEIFHFCWVLFIYAKGNFPMISDDLVNSYHLLLCALDLVYGNALQCSNRKELVNPNFKGLSEDFHAKDSKPSSDPPCIIEKLCSLHDGLVLEAKGIKEHFWKPYIRKLYEKKLLKGKEENLTGFLEPGNFGESFKAINKAYEEYVLSVGNLDERIFLGEDAEEEIGTLSRCLNAGSGTETAERVQMKNILQQHFDKSKALRISTPLTGVRYIKENSPCVTPVSTATHSLSRLHTMLTGLRNAPSEKLEQILRTCSRDPTQAIANRLKEMFEIYSQHFQPDEDFSNCAKEIASKHFRFAEMLYYKVLESVIEQEQKRLGDMDLSGILEQDAFHRSLLACCLEVVTFSYKPPGNFPFITEIFDVPLYHFYKVIEVFIRAEDGLCREVVKHLNQIEEQILDHLAWKPESPLWEKIRDNENRVPTCEEVMPPQNLERADEICIAGSPLTPRRVTEVRADTGGLGRSITSPTTLYDRYSSPPASTTRRRLFVENDSPSDGGTPGRMPPQPLVNAVPVQNVSGETVSVTPVPGQTLVTMATATVTANNGQTVTIPVQGIANENGGITFFPVQVNVGGQAQAVTGSIQPLSAQALAGSLSSQQVTGTTLQVPGQVAIQQISPGGQQQKQGQSVTSSSNRPRKTSSLSLFFRKVYHLAAVRLRDLCAKLDISDELRKKIWTCFEFSIIQCPELMMDRHLDQLLMCAIYVMAKVTKEDKSFQNIMRCYRTQPQARSQVYRSVLIKGKRKRRNSGSSDSRSHQNSPTELNKDRTSRDSSPVMRSSSTLPVPQPSSAPPTPTRLTGANSDMEEEERGDLIQFYNNIYIKQIKTFAMKYSQANMDAPPLSPYPFVRTGSPRRIQLSQNHPVYISPHKNETMLSPREKIFYYFSNSPSKRLREINSMIRTGETPTKKRGILLEDGSESPAKRICPENHSALLRRLQDVANDRGSH

12 NP_001702.1 NP_000079.2

>NP_001702.1

MWPLWRLVSLLALSQALPFEQRGFWDFTLDDGPFMMNDEEASGADTSGVLDPDSVTPTYSAMCPFGCHCHLRVVQCSDLGLKSVPKEISPDTTLLDLQNNDISELRKDDFKGLQHLYALVLVNNKISKIHEKAFSPLRKLQKLYISKNHLVEIPPNLPSSLVELRIHDNRIRKVPKGVFSGLRNMNCIEMGGNPLENSGFEPGAFDGLKLNYLRISEAKLTGIPKDLPETLNELHLDHNKIQAIELEDLLRYSKLYRLGLGHNQIRMIENGSLSFLPTLRELHLDNNKLARVPSGLPDLKLLQVVYLHSNNITKVGVNDFCPMGFGVKRAYYNGISLFNNPVPYWEVQPATFRCVTDRLAIQFGNYKK

>NP_000079.2

MFSFVDLRLLLLLAATALLTHGQEEGQVEGQDEDIPPITCVQNGLRYHDRDVWKPEPCRICVCDNGKVLCDDVICDETKNCPGAEVPEGECCPVCPDGSESPTDQETTGVEGPKGDTGPRGPRGPAGPPGRDGIPGQPGLPGPPGPPGPPGPPGLGGNFAPQLSYGYDEKSTGGISVPGPMGPSGPRGLPGPPGAPGPQGFQGPPGEPGEPGASGPMGPRGPPGPPGKNGDDGEAGKPGRPGERGPPGPQGARGLPGTAGLPGMKGHRGFSGLDGAKGDAGPAGPKGEPGSPGENGAPGQMGPRGLPGERGRPGAPGPAGARGNDGATGAAGPPGPTGPAGPPGFPGAVGAKGEAGPQGPRGSEGPQGVRGEPGPPGPAGAAGPAGNPGADGQPGAKGANGAPGIAGAPGFPGARGPSGPQGPGGPPGPKGNSGEPGAPGSKGDTGAKGEPGPVGVQGPPGPAGEEGKRGARGEPGPTGLPGPPGERGGPGSRGFPGADGVAGPKGPAGERGSPGPAGPKGSPGEAGRPGEAGLPGAKGLTGSPGSPGPDGKTGPPGPAGQDGRPGPPGPPGARGQAGVMGFPGPKGAAGEPGKAGERGVPGPPGAVGPAGKDGEAGAQGPPGPAGPAGERGEQGPAGSPGFQGLPGPAGPPGEAGKPGEQGVPGDLGAPGPSGARGERGFPGERGVQGPPGPAGPRGANGAPGNDGAKGDAGAPGAPGSQGAPGLQGMPGERGAAGLPGPKGDRGDAGPKGADGSPGKDGVRGLTGPIGPPGPAGAPGDKGESGPSGPAGPTGARGAPGDRGEPGPPGPAGFAGPPGADGQPGAKGEPGDAGAKGDAGPPGPAGPAGPPGPIGNVGAPGAKGARGSAGPPGATGFPGAAGRVGPPGPSGNAGPPGPPGPAGKEGGKGPRGETGPAGRPGEVGPPGPPGPAGEKGSPGADGPAGAPGTPGPQGIAGQRGVVGLPGQRGERGFPGLPGPSGEPGKQGPSGASGERGPPGPMGPPGLAGPPGESGREGAPGAEGSPGRDGSPGAKGDRGETGPAGPPGAPGAPGAPGPVGPAGKSGDRGETGPAGPAGPVGPVGARGPAGPQGPRGDKGETGEQGDRGIKGHRGFSGLQGPPGPPGSPGEQGPSGASGPAGPRGPPGSAGAPGKDGLNGLPGPIGPPGPRGRTGDAGPVGPPGPPGPPGPPGPPSAGFDFSFLPQPPQEKAHDGGRYYRADDANVVRDRDLEVDTTLKSLSQQIENIRSPEGSRKNPARTCRDLKMCHSDWKSGEYWIDPNQGCNLDAIKVFCNMETGETCVYPTQPSVAQKNWYISKNPKDKRHVWFGESMTDGFQFEYGGQGSDPADVAIQLTFLRLMSTEASQNITYHCKNSVAYMDQQTGNLKKALLLQGSNEIEIRAEGNSRFTYSVTVDGCTSHTGAWGKTVIEYKTTKTSRLPIIDVAPLDVGAPDQEFGFDVGPVCFL

13 NP_003334.2 NP_000453.2

>NP_003334.2

MAGTALKRLMAVYKQLTLNPPEGIVAGPMNEENFFEWEALIMGPEDTCFEFGVFPAILSFPLDYPLSPPKMRFTCEMFHPNIYPDGRVCISILHAPGDDPHGLREQPERWSPVQSVEKILLSVVSMLAEPNDESGANVDASKMWRDDREQFYKIAKQIVQKSLGL

>NP_000453.2

MEKLHQCYWKSGEPQSDDIEASRMKRAAAKHLIERYYHQLTEGCGNEACTNEFCASCPTFLRMDNNAAAIKALELYKINAKLCDPHPSKKGASSAYLENSKGAPNNSCSEIKMNKKGARIDFKDVTYLTEEKVYEILELCREREDYSPLIRVIGRVFSSAEALVQSFRKVKQHTKEELKSLQAKDEDKDEDEKEKAACSAAAMEEDSEASSSRIGDSSQGDNNLQKLGPDDVSVDIDAIRRVYTRLLSNEKIETAFLNALVYLSPNVECDLTYHNVYSRDPNYLNLFIIVMENRNLHSPEYLEMALPLFCKAMSKLPLAAQGKLIRLWSKYNADQIRRMMETFQQLITYKVISNEFNSRNLVNDDDAIVAASKCLKMVYYANVVGGEVDTNHNEEDDEEPIPESSELTLQELLGEERRNKKGPRVDPLETELGVKTLDCRKPLIPFEEFINEPLNEVLEMDKDYTFFKVETENKFSFMTCPFILNAVTKNLGLYYDNRIRMYSERRITVLYSLVQGQQLNPYLRLKVRRDHIIDDALVRLEMIAMENPADLKKQLYVEFEGEQGVDEGGVSKEFFQLVVEEIFNPDIGMFTYDESTKLFWFNPSSFETEGQFTLIGIVLGLAIYNNCILDVHFPMVVYRKLMGKKGTFRDLGDSHPVLYQSLKDLLEYEGNVEDDMMITFQISQTDLFGNPMMYDLKENGDKIPITNENRKEFVNLYSDYILNKSVEKQFKAFRRGFHMVTNESPLKYLFRPEEIELLICGSRNLDFQALEETTEYDGGYTRDSVLIREFWEIVHSFTDEQKRLFLQFTTGTDRAPVGGLGKLKMIIAKNGPDTERLPTSHTCFNVLLLPEYSSKEKLKERLLKAITYAKGFGML

14 NP_000124.1 NP_000123.1

>NP_000124.1

MQRVNMIMAESPGLITICLLGYLLSAECTVFLDHENANKILNRPKRYNSGKLEEFVQGNLERECMEEKCSFEEAREVFENTERTTEFWKQYVDGDQCESNPCLNGGSCKDDINSYECWCPFGFEGKNCELDVTCNIKNGRCEQFCKNSADNKVVCSCTEGYRLAENQKSCEPAVPFPCGRVSVSQTSKLTRAETVFPDVDYVNSTEAETILDNITQSTQSFNDFTRVVGGEDAKPGQFPWQVVLNGKVDAFCGGSIVNEKWIVTAAHCVETGVKITVVAGEHNIEETEHTEQKRNVIRIIPHHNYNAAINKYNHDIALLELDEPLVLNSYVTPICIADKEYTNIFLKFGSGYVSGWGRVFHKGRSALVLQYLRVPLVDRATCLRSTKFTIYNNMFCAGFHEGGRDSCQGDSGGPHVTEVEGTSFLTGIISWGEECAMKGKYGIYTKVSRYVNWIKEKTKLT

>NP_000123.1

MQIELSTCFFLCLLRFCFSATRRYYLGAVELSWDYMQSDLGELPVDARFPPRVPKSFPFNTSVVYKKTLFVEFTDHLFNIAKPRPPWMGLLGPTIQAEVYDTVVITLKNMASHPVSLHAVGVSYWKASEGAEYDDQTSQREKEDDKVFPGGSHTYVWQVLKENGPMASDPLCLTYSYLSHVDLVKDLNSGLIGALLVCREGSLAKEKTQTLHKFILLFAVFDEGKSWHSETKNSLMQDRDAASARAWPKMHTVNGYVNRSLPGLIGCHRKSVYWHVIGMGTTPEVHSIFLEGHTFLVRNHRQASLEISPITFLTAQTLLMDLGQFLLFCHISSHQHDGMEAYVKVDSCPEEPQLRMKNNEEAEDYDDDLTDSEMDVVRFDDDNSPSFIQIRSVAKKHPKTWVHYIAAEEEDWDYAPLVLAPDDRSYKSQYLNNGPQRIGRKYKKVRFMAYTDETFKTREAIQHESGILGPLLYGEVGDTLLIIFKNQASRPYNIYPHGITDVRPLYSRRLPKGVKHLKDFPILPGEIFKYKWTVTVEDGPTKSDPRCLTRYYSSFVNMERDLASGLIGPLLICYKESVDQRGNQIMSDKRNVILFSVFDENRSWYLTENIQRFLPNPAGVQLEDPEFQASNIMHSINGYVFDSLQLSVCLHEVAYWYILSIGAQTDFLSVFFSGYTFKHKMVYEDTLTLFPFSGETVFMSMENPGLWILGCHNSDFRNRGMTALLKVSSCDKNTGDYYEDSYEDISAYLLSKNNAIEPRSFSQNSRHPSTRQKQFNATTIPENDIEKTDPWFAHRTPMPKIQNVSSSDLLMLLRQSPTPHGLSLSDLQEAKYETFSDDPSPGAIDSNNSLSEMTHFRPQLHHSGDMVFTPESGLQLRLNEKLGTTAATELKKLDFKVSSTSNNLISTIPSDNLAAGTDNTSSLGPPSMPVHYDSQLDTTLFGKKSSPLTESGGPLSLSEENNDSKLLESGLMNSQESSWGKNVSSTESGRLFKGKRAHGPALLTKDNALFKVSISLLKTNKTSNNSATNRKTHIDGPSLLIENSPSVWQNILESDTEFKKVTPLIHDRMLMDKNATALRLNHMSNKTTSSKNMEMVQQKKEGPIPPDAQNPDMSFFKMLFLPESARWIQRTHGKNSLNSGQGPSPKQLVSLGPEKSVEGQNFLSEKNKVVVGKGEFTKDVGLKEMVFPSSRNLFLTNLDNLHENNTHNQEKKIQEEIEKKETLIQENVVLPQIHTVTGTKNFMKNLFLLSTRQNVEGSYDGAYAPVLQDFRSLNDSTNRTKKHTAHFSKKGEEENLEGLGNQTKQIVEKYACTTRISPNTSQQNFVTQRSKRALKQFRLPLEETELEKRIIVDDTSTQWSKNMKHLTPSTLTQIDYNEKEKGAITQSPLSDCLTRSHSIPQANRSPLPIAKVSSFPSIRPIYLTRVLFQDNSSHLPAASYRKKDSGVQESSHFLQGAKKNNLSLAILTLEMTGDQREVGSLGTSATNSVTYKKVENTVLPKPDLPKTSGKVELLPKVHIYQKDLFPTETSNGSPGHLDLVEGSLLQGTEGAIKWNEANRPGKVPFLRVATESSAKTPSKLLDPLAWDNHYGTQIPKEEWKSQEKSPEKTAFKKKDTILSLNACESNHAIAAINEGQNKPEIEVTWAKQGRTERLCSQNPPVLKRHQREITRTTLQSDQEEIDYDDTISVEMKKEDFDIYDEDENQSPRSFQKKTRHYFIAAVERLWDYGMSSSPHVLRNRAQSGSVPQFKKVVFQEFTDGSFTQPLYRGELNEHLGLLGPYIRAEVEDNIMVTFRNQASRPYSFYSSLISYEEDQRQGAEPRKNFVKPNETKTYFWKVQHHMAPTKDEFDCKAWAYFSDVDLEKDVHSGLIGPLLVCHTNTLNPAHGRQVTVQEFALFFTIFDETKSWYFTENMERNCRAPCNIQMEDPTFKENYRFHAINGYIMDTLPGLVMAQDQRIRWYLLSMGSNENIHSIHFSGHVFTVRKKEEYKMALYNLYPGVFETVEMLPSKAGIWRVECLIGEHLHAGMSTLFLVYSNKCQTPLGMASGHIRDFQITASGQYGQWAPKLARLHYSGSINAWSTKEPFSWIKVDLLAPMIIHGIKTQGARQKFSSLYISQFIIMYSLDGKKWQTYRGNSTGTLMVFFGNVDSSGIKHNIFNPPIIARYIRLHPTHYSIRSTLRMELMGCDLNSCSMPLGMESKAISDAQITASSYFTNMFATWSPSKARLHLQGRSNAWRPQVNNPKEWLQVDFQKTMKVTGVTTQGVKSLLTSMYVKEFLISSSQDGHQWTLFFQNGKVKVFQGNQDSFTPVVNSLDPPLLTRYLRIHPQSWVHQIALRMEVLGCEAQDLY

15 NP_003712.1 NP_000529.1

>NP_003712.1

MELTQPAEDLIQTQQTPASELGDPEDPGEEAADGSDTVVLSLFPCTPEPVNPEPDASVSSPQAGSSLKHSTTLTNRQRGNEVSALPATLDSLSIHQLAAQGELDQLKEHLRKGDNLVNKPDERGFTPLIWASAFGEIETVRFLLEWGADPHILAKERESALSLASTGGYTDIVGLLLERDVDINIYDWNGGTPLLYAVRGNHVKCVEALLARGADLTTEADSGYTPMDLAVALGYRKVQQVIENHILKLFQSNLVPADPE

>NP_000529.1

MEAQGVAEGAGPGAASGVPHPAALAPAAAPTLAPASVAAAASQFTLLVMQPCAGQDEAAAPGGSVGAGKPVRYLCEGAGDGEEEAGEDEADLLDTSDPPGGGESAASLEDLEDEETHSGGEGSSGGARRRGSGGGSMSKTCTYEGCSETTSQVAKQRKPWMCKKHRNKMYKDKYKKKKSDQALNCGGTASTGSAGNVKLEESADNILSIVKQRTGSFGDRPARPTLLEQVLNQKRLSLLRSPEVVQFLQKQQQLLNQQVLEQRQQQFPGTSM

16 NP_003391.1 NP_006485.1

>NP_003391.1

MPAIMTMLADHAARQLLDFSQKLDINLLDNVVNCLYHGEGAQQRMAQEVLTHLKEHPDAWTRVDTILEFSQNMNTKYYGLQILENVIKTRWKILPRNQCEGIKKYVVGLIIKTSSDPTCVEKEKVYIGKLNMILVQILKQEWPKHWPTFISDIVGASRTSESLCQNNMVILKLLSEEVFDFSSGQITQVKSKHLKDSMCNEFSQIFQLCQFVMENSQNAPLVHATLETLLRFLNWIPLGYIFETKLISTLIYKFLNVPMFRNVSLKCLTEIAGVSVSQYEEQFVTLFTLTMMQLKQMLPLNTNIRLAYSNGKDDEQNFIQNLSLFLCTFLKEHDQLIEKRLNLRETLMEALHYMLLVSEVEETEIFKICLEYWNHLAAELYRESPFSTSASPLLSGSQHFDVPPRRQLYLPMLFKVRLLMVSRMAKPEEVLVVENDQGEVVREFMKDTDSINLYKNMRETLVYLTHLDYVDTERIMTEKLHNQVNGTEWSWKNLNTLCWAIGSISGAMHEEDEKRFLVTVIKDLLGLCEQKRGKDNKAIIASNIMYIVGQYPRFLRAHWKFLKTVVNKLFEFMHETHDGVQDMACDTFIKIAQKCRRHFVQVQVGEVMPFIDEILNNINTIICDLQPQQVHTFYEAVGYMIGAQTDQTVQEHLIEKYMLLPNQVWDSIIQQATKNVDILKDPETVKQLGSILKTNVRACKAVGHPFVIQLGRIYLDMLNVYKCLSENISAAIQANGEMVTKQPLIRSMRTVKRETLKLISGWVSRSNDPQMVAENFVPPLLDAVLIDYQRNVPAAREPEVLSTMAIIVNKLGGHITAEIPQIFDAVFECTLNMINKDFEEYPEHRTNFFLLLQAVNSHCFPAFLAIPPTQFKLVLDSIIWAFKHTMRNVADTGLQILFTLLQNVAQEEAAAQSFYQTYFCDILQHIFSVVTDTSHTAGLTMHASILAYMFNLVEEGKISTSLNPGNPVNNQIFLQEYVANLLKSAFPHLQDAQVKLFVTGLFSLNQDIPAFKEHLRDFLVQIKEFAGEDTSDLFLEEREIALRQADEEKHKRQMSVPGIFNPHEIPEEMCD

>NP_006485.1

MKTPADTGFAFPDWAYKPESSPGSRQIQLWHFILELLRKEEYQGVIAWQGDYGEFVIKDPDEVARLWGVRKCKPQMNYDKLSRALRYYYNKRILHKTKGKRFTYKFNFNKLVLVNYPFIDVGLAGGAVPQSAPPVPSGGSHFRFPPSTPSEVLSPTEDPRSPPACSSSSSSLFSAVVARRLGRGSVSDCSDGTSELEEPLGEDPRARPPGPPDLGAFRGPPLARLPHDPGVFRVYPRPRGGPEPLSPFPVSPLAGPGSLLPPQLSPALPMTPTHLAYTPSPTLSPMYPSGGGGPSGSGGGSHFSFSPEDMKRYLQAHTQSVYNYHLSPRAFLHYPGLVVPQPQRPDKCPLPPMAPETPPVPSSASSSSSSSSSPFKFKLQRPPLGRRQRAAGEKAVAAADKSGGSAGGLAEGAGALAPPPPPPQIKVEPISEGESEEVEVTDISDEDEEDGEVFKTPRAPPAPPKPEPGEAPGASQCMPLKLRFKRRWSEDCRLEGGGGPAGGFEDEGEDKKVRGEGPGEAGGPLTPRRVSSDLQHATAQLSLEHRDS

17 NP_002801.1 NP_038466.2

>NP_002801.1

MVLESTMVCVDNSEYMRNGDFLPTRLQAQQDAVNIVCHSKTRSNPENNVGLITLANDCEVLTTLTPDTGRILSKLHTVQPKGKITFCTGIRVAHLALKHRQGKNHKMRIIAFVGSPVEDNEKDLVKLAKRLKKEKVNVDIINFGEEEVNTEKLTAFVNTLNGKDGTGSHLVTVPPGPSLADALISSPILAGEGGAMLGLGASDFEFGVDPSADPELALALRVSMEEQRQRQEEEARRAAAASAAEAGIATTGTEDSDDALLKMTISQQEFGRTGLPDLSSMTEEEQIAYAMQMSLQGAEFGQAESADIDASSAMDTSEPAKEEDDYDVMQDPEFLQSVLENLPGVDPNNEAIRNAMGSLASQATKDGKKDKKEEDKK

>NP_038466.2

MAESGESGGPPGSQDSAAGAEGAGAPAAAASAEPKIMKVTVKTPKEKEEFAVPENSSVQQFKEEISKRFKSHTDQLVLIFAGKILKDQDTLSQHGIHDGLTVHLVIKTQNRPQDHSAQQTNTAGSNVTTSSTPNSNSTSGSATSNPFGLGGLGGLAGLSSLGLNTTNFSELQSQMQRQLLSNPEMMVQIMENPFVQSMLSNPDLMRQLIMANPQMQQLIQRNPEISHMLNNPDIMRQTLELARNPAMMQEMMRNQDRALSNLESIPGGYNALRRMYTDIQEPMLSAAQEQFGGNPFASLVSNTSSGEGSQPSRTENRDPLPNPWAPQTSQSSSASSGTASTVGGTTGSTASGTSGQSTTAPNLVPGVGASMFNTPGMQSLLQQITENPQLMQNMLSAPYMRSMMQSLSQNPDLAAQMMLNNPLFAGNPQLQEQMRQQLPTFLQQMQNPDTLSAMSNPRAMQALLQIQQGLQTLATEAPGLIPGFTPGLGALGSTGGSSGTNGSNATPSENTSPTAGTTEPGHQQFIQQMLQALAGVNPQLQNPEVRFQQQLEQLSAMGFLNREANLQALIATGGDINAAIERLLGSQPS

18 NP_542166.1 Q92900

>NP_542166.1

MPAERKKPASMEEKDSLPNNKEKDCSERRTVSSKERPKDDIKLTAKKEVSKAPEDKKKRLEDDKRKKEDKERKKKDEEKVKAEEESKKKEEEEKKKHQEEERKKQEEQAKRQQEEEAAAQMKEKEESIQLHQEAWERHHLRKELRSKNQNAPDSRPEENFFSRLDSSLKKNTAFVKKLKTITEQQRDSLSHDFNGLNLSKYIAEAVASIVEAKLKISDVNCAVHLCSLFHQRYADFAPSLLQVWKKHFEARKEEKTPNITKLRTDLRFIAELTIVGIFTDKEGLSLIYEQLKNIINADRESHTHVSVVISFCRHCGDDIAGLVPRKVKSAAEKFNLSFPPSEIISPEKQQPFQNLLKEYFTSLTKHLKRDHRELQNTERQNRRILHSKGELSEDRHKQYEEFAMSYQKLLANSQSLADLLDENMPDLPQDKPTPEEHGPGIDIFTPGKPGEYDLEGGIWEDEDARNFYENLIDLKAFVPAILFKDNEKSCQNKESNKDDTKEAKESKENKEVSSPDDLELELENLEINDDTLELEGGDEAEDLTKKLLDEQEQEDEEASTGSHLKLIVDAFLQQLPNCVNRDLIDKAAMDFCMNMNTKANRKKLVRALFIVPRQRLDLLPFYARLVATLHPCMSDVAEDLCSMLRGDFRFHVRKKDQINIETKNKTVRFIGELTKFKMFTKNDTLHCLKMLLSDFSHHHIEMACTLLETCGRFLFRSPESHLRTSVLLEQMMRKKQAMHLDARYVTMVENAYYYCNPPPAEKTVKKKRPPLQEYVRKLLYKDLSKVTTEKVLRQMRKLPWQDQEVKDYVICCMINIWNVKYNSIHCVANLLAGLVLYQEDVGIHVVDGVLEDIRLGMEVNQPKFNQRRISSAKFLGELYNYRMVESAVIFRTLYSFTSFGVNPDGSPSSLDPPEHLFRIRLVCTILDTCGQYFDRGSSKRKLDCFLVYFQRYVWWKKSLEVWTKDHPFPIDIDYMISDTLELLRPKIKLCNSLEESIRQVQDLEREFLIKLGLVNDKDSKDSMTEGENLEEDEEEEEGGAETEEQSGNESEVNEPEEEEGSDNDDDEGEEEEEENTDYLTDSNKENETDEENTEVMIKGGGLKHVPCVEDEDFIQALDKMMLENLQQRSGESVKVHQLDVAIPLHLKSQLRKGPPLGGGEGEAESADTMPFVMLTRKGNKQQFKILNVPMSSQLAANHWNQQQAEQEERMRMKKLTLDINERQEQEDYQEMLQSLAQRPAPANTNRERRPRYQHPKGAPNADLIFKTGGRRR

>Q92900

MSVEAYGPSSQTLTFLDTEEAELLGADTQGSEFEFTDFTLPSQTQTPPGGPGGPGGGGAGGPGGAGAGAAAGQLDAQVGPEGILQNGAVDDSVAKTSQLLAELNFEEDEEDTYYTKDLPIHACSYCGIHDPACVVYCNTSKKWFCNGRGNTSGSHIVNHLVRAKCKEVTLHKDGPLGETVLECYNCGCRNVFLLGFIPAKADSVVVLLCRQPCASQSSLKDINWDSSQWQPLIQDRCFLSWLVKIPSEQEQLRARQITAQQINKLEELWKENPSATLEDLEKPGVDEEPQHVLLRYEDAYQYQNIFGPLVKLEADYDKKLKESQTQDNITVRWDLGLNKKRIAYFTLPKTDSGNEDLVIIWLRDMRLMQGDEICLRYKGDLAPLWKGIGHVIKVPDNYGDEIAIELRSSVGAPVEVTHNFQVDFVWKSTSFDRMQSALKTFAVDETSVSGYIYHKLLGHEVEDVIIKCQLPKRFTAQGLPDLNHSQVYAVKTVLQRPLSLIQGPPGTGKTVTSATIVYHLARQGNGPVLVCAPSNIAVDQLTEKIHQTGLKVVRLCAKSREAIDSPVSFLALHNQIRNMDSMPELQKLQQLKDETGELSSADEKRYRALKRTAERELLMNADVICCTCVGAGDPRLAKMQFRSILIDESTQATEPECMVPVVLGAKQLILVGDHCQLGPVVMCKKAAKAGLSQSLFERLVVLGIRPIRLQVQYRMHPALSAFPSNIFYEGSLQNGVTAADRVKKGFDFQWPQPDKPMFFYVTQGQEEIASSGTSYLNRTEAANVEKITTKLLKAGAKPDQIGIITPYEGQRSYLVQYMQFSGSLHTKLYQEVEIASVDAFQGREKDFIILSCVRANEHQGIGFLNDPRRLNVALTRARYGVIIVGNPKALSKQPLWNHLLNYYKEQKVLVEGPLNNLRESLMQFSKPRKLVNTINPGARFMTTAMYDAREAIIPGSVYDRSSQGRPSSMYFQTHDQIGMISAGPSHVAAMNIPIPFNLVMPPMPPPGYFGQANGPAAGRGTPKGKTGRGGRQKNRFGLPGPSQTNLPNSQASQDVASQPFSQGALTQGYISMSQPSQMSQPGLSQPELSQDSYLGDEFKSQIDVALSQDSTYQGERAYQHGGVTGLSQY

19 NP_005493.2 NP_937879.1

>NP_005493.2

MACWPQLRLLLWKNLTFRRRQTCQLLLEVAWPLFIFLILISVRLSYPPYEQHECHFPNKAMPSAGTLPWVQGIICNANNPCFRYPTPGEAPGVVGNFNKSIVARLFSDARRLLLYSQKDTSMKDMRKVLRTLQQIKKSSSNLKLQDFLVDNETFSGFLYHNLSLPKSTVDKMLRADVILHKVFLQGYQLHLTSLCNGSKSEEMIQLGDQEVSELCGLPREKLAAAERVLRSNMDILKPILRTLNSTSPFPSKELAEATKTLLHSLGTLAQELFSMRSWSDMRQEVMFLTNVNSSSSSTQIYQAVSRIVCGHPEGGGLKIKSLNWYEDNNYKALFGGNGTEEDAETFYDNSTTPYCNDLMKNLESSPLSRIIWKALKPLLVGKILYTPDTPATRQVMAEVNKTFQELAVFHDLEGMWEELSPKIWTFMENSQEMDLVRMLLDSRDNDHFWEQQLDGLDWTAQDIVAFLAKHPEDVQSSNGSVYTWREAFNETNQAIRTISRFMECVNLNKLEPIATEVWLINKSMELLDERKFWAGIVFTGITPGSIELPHHVKYKIRMDIDNVERTNKIKDGYWDPGPRADPFEDMRYVWGGFAYLQDVVEQAIIRVLTGTEKKTGVYMQQMPYPCYVDDIFLRVMSRSMPLFMTLAWIYSVAVIIKGIVYEKEARLKETMRIMGLDNSILWFSWFISSLIPLLVSAGLLVVILKLGNLLPYSDPSVVFVFLSVFAVVTILQCFLISTLFSRANLAAACGGIIYFTLYLPYVLCVAWQDYVGFTLKIFASLLSPVAFGFGCEYFALFEEQGIGVQWDNLFESPVEEDGFNLTTSVSMMLFDTFLYGVMTWYIEAVFPGQYGIPRPWYFPCTKSYWFGEESDEKSHPGSNQKRISEICMEEEPTHLKLGVSIQNLVKVYRDGMKVAVDGLALNFYEGQITSFLGHNGAGKTTTMSILTGLFPPTSGTAYILGKDIRSEMSTIRQNLGVCPQHNVLFDMLTVEEHIWFYARLKGLSEKHVKAEMEQMALDVGLPSSKLKSKTSQLSGGMQRKLSVALAFVGGSKVVILDEPTAGVDPYSRRGIWELLLKYRQGRTIILSTHHMDEADVLGDRIAIISHGKLCCVGSSLFLKNQLGTGYYLTLVKKDVESSLSSCRNSSSTVSYLKKEDSVSQSSSDAGLGSDHESDTLTIDVSAISNLIRKHVSEARLVEDIGHELTYVLPYEAAKEGAFVELFHEIDDRLSDLGISSYGISETTLEEIFLKVAEESGVDAETSDGTLPARRNRRAFGDKQSCLRPFTEDDAADPNDSDIDPESRETDLLSGMDGKGSYQVKGWKLTQQQFVALLWKRLLIARRSRKGFFAQIVLPAVFVCIALVFSLIVPPFGKYPSLELQPWMYNEQYTFVSNDAPEDTGTLELLNALTKDPGFGTRCMEGNPIPDTPCQAGEEEWTTAPVPQTIMDLFQNGNWTMQNPSPACQCSSDKIKKMLPVCPPGAGGLPPPQRKQNTADILQDLTGRNISDYLVKTYVQIIAKSLKNKIWVNEFRYGGFSLGVSNTQALPPSQEVNDAIKQMKKHLKLAKDSSADRFLNSLGRFMTGLDTKNNVKVWFNNKGWHAISSFLNVINNAILRANLQKGENPSHYGITAFNHPLNLTKQQLSEVALMTTSVDVLVSICVIFAMSFVPASFVVFLIQERVSKAKHLQFISGVKPVIYWLSNFVWDMCNYVVPATLVIIIFICFQQKSYVSSTNLPVLALLLLLYGWSITPLMYPASFVFKIPSTAYVVLTSVNLFIGINGSVATFVLELFTDNKLNNINDILKSVFLIFPHFCLGRGLIDMVKNQAMADALERFGENRFVSPLSWDLVGRNLFAMAVEGVVFFLITVLIQYRFFIRPRPVNAKLSPLNDEDEDVRRERQRILDGGGQNDILEIKELTKIYRRKRKPAVDRICVGIPPGECFGLLGVNGAGKSSTFKMLTGDTTVTRGDAFLNKNSILSNIHEVHQNMGYCPQFDAITELLTGREHVEFFALLRGVPEKEVGKVGEWAIRKLGLVKYGEKYAGNYSGGNKRKLSTAMALIGGPPVVFLDEPTTGMDPKARRFLWNCALSVVKEGRSVVLTSHSMEECEALCTRMAIMVNGRFRCLGSVQHLKNRFGDGYTIVVRIAGSNPDLKPVQDFFGLAFPGSVLKEKHRNMLQYQLPSSLSSLARIFSILSQSKKRLHIEDYSVSQTTLDQVFVNFAKDQSDDDHLKDLSLHKNQTVVDVAVLTSFLQDEKVKESYV

>NP_937879.1

MSVRLPQSIDRLSSLSSLGDSAPERKSPSHHRQPSDASETTGLVQRCVIIQKDQHGFGFTVSGDRIVLVQSVRPGGAAMKAGVKEGDRIIKVNGTMVTNSSHLEVVKLIKSGAYVALTLLGSSPSSMGISGLQQDPSPAGAPRITSVIPSPPPPPPLPPPQRITGPKPLQDPEVQKHATQILRNMLRQEEKELQRICEVYSRNPASLLEEQIEGARRRVTQLQLKIQQETGGSVDILPLYGDTSQRPSEGRLSLDSQEGDSGLDSGTERFPSLSESLMNRNSVLSDPGLDSPRTSPVIMARVAQHHRRQGSDAAVPSTGDQGVDQSPKPLIIGPEEDYDPGYFNNESDIIFQDLEKLKSRPAHLGVFLRYIFSQADPSPLLFYLCAEVYQQASPKDSRSLGKDIWNIFLEKNAPLRVKIPEMLQAEIDSRLRNSEDARGVLCEAQEAAMPEIQEQIHDYRTKRTLGLGSLYGENDLLDLDGDPLRERQVAEKQLAALGDILSKYEEDRSAPMDFALNTYMSHAGIRLREARPSNTAEKAQSAPDKDKWLPFFPKTKKSSNSKKEKDALEDKKRNPILKYIGKPKSSSQSTFHIPLSPVEVKPGNVRNIIQHFENNQQYDAPEPGTQRLSTGSFPEDLLESDSSRSEIRLGRSESLKGREEMKRSRKAENVPRSRSDVDMDAAAEATRLHQSASSSTSSLSTRSLENPTPPFTPKMGRRSIESPSLGFCTDTLLPHLLEDDLGQLSDLEPEPDAQNWQHTVGKDVVAGLTQREIDRQEVINELFVTEASHLRTLRVLDLIFYQRMKKENLMPREELARLFPNLPELIEIHNSWCEAMKKLREEGPIIKEISDLMLARFDGPAREELQQVAAQFCSYQSIALELIKTKQRKESRFQLFMQEAESHPQCRRLQLRDLIISEMQRLTKYPLLLESIIKHTEGGTSEHEKLCRARDQCREILKYVNEAVKQTENRHRLEGYQKRLDATALERASNPLAAEFKSLDLTTRKMIHEGPLTWRISKDKTLDLHVLLLEDLLVLLQKQDEKLLLKCHSKTAVGSSDSKQTFSPVLKLNAVLIRSVATDKRAFFIICTSKLGPPQIYELVALTSSDKNTWMELLEEAVRNATRHPGAAPMPVHPPPPGPREPAQQGPTPSRVELDDSDVFHGEPEPEELPGGTGSQQRVQGKHQVLLEDPEQEGSAEEEELGVLPCPSTSLDGENRGIRTRNPIHLAFPGPLFMEGLADSALEDVENLRHLILWSLLPGHTMETQAAQEPEDDLTPTPSVISVTSHPWDPGSPGQAPPGGEGDNTQLAGLEGERPEQEDMGLCSLEHLPPRTRNSGIWESPELDRNLAEDASSTEAAGGYKVVRKAEVAGSKVVPALPESGQSEPGPPEVEGGTKATGNCFYVSMPSGPPDSSTDHSEAPMSPPQPDSLPAGQTEPQPQLQGGNDDPRRPSRSPPSLALRDVGMIFHTIEQLTLKLNRLKDMELAHRELLKSLGGESSGGTTPVGSFHTEAARWTDGSLSPPAKEPLASDSRNSHELGPCPEDGSDAPLEDSTADAAASPGP

20 NP_004214.1 NP_004553.1

>NP_004214.1

MMASMRVVKELEDLQKKPPPYLRNLSSDDANVLVWHALLLPDQPPYHLKAFNLRISFPPEYPFKPPMIKFTTKIYHPNVDENGQICLPIISSENWKPCTKTCQVLEALNVLVNRPNIREPLRMDLADLLTQNPELFRKNAEEFTLRFGVDRPS

>NP_004553.1

MIVFVRFNSSHGFPVEVDSDTSIFQLKEVVAKRQGVPADQLRVIFAGKELRNDWTVQNCDLDQQSIVHIVQRPWRKGQEMNATGGDDPRNAAGGCEREPQSLTRVDLSSSVLPGDSVGLAVILHTDSRKDSPPAGSPAGRSIYNSFYVYCKGPCQRVQPGKLRVQCSTCRQATLTLTQGPSCWDDVLIPNRMSGECQSPHCPGTSAEFFFKCGAHPTSDKETPVALHLIATNSRNITCITCTDVRSPVLVFQCNSRHVICLDCFHLYCVTRLNDRQFVHDPQLGYSLPCVAGCPNSLIKELHHFRILGEEQYNRYQQYGAEECVLQMGGVLCPRPGCGAGLLPEPDQRKVTCEGGNGLGCGFAFCRECKEAYHEGECSAVFEASGTTTQAYRVDERAAEQARWEAASKETIKKTTKPCPRCHVPVEKNGGCMHMKCPQPQCRLEWCWNCGCEWNRVCMGDHWFDV

21 NP_061828.1 NP_004055.1

>NP_061828.1

MQIFVKTLTGKTITLEVEPSDTIENVKAKIQDKEGIPPDQQRLIFAGKQLEDGRTLSDYNIQKESTLHLVLRLRGGMQIFVKTLTGKTITLEVEPSDTIENVKAKIQDKEGIPPDQQRLIFAGKQLEDGRTLSDYNIQKESTLHLVLRLRGGMQIFVKTLTGKTITLEVEPSDTIENVKAKIQDKEGIPPDQQRLIFAGKQLEDGRTLSDYNIQKESTLHLVLRLRGGC

>NP_004055.1

MSNVRVSNGSPSLERMDARQAEHPKPSACRNLFGPVDHEELTRDLEKHCRDMEEASQRKWNFDFQNHKPLEGKYEWQEVEKGSLPEFYYRPPRPPKGACKVPAQESQDVSGSRPAAPLIGAPANSEDTHLVDPKTDPSDSQTGLAEQCAGIRKRPATDDSSTQNKRANRTEENVSDGSPNAGSVEQTPKKPGLRRRQT

22 NP_001737.1 NP_009108.1

>NP_001737.1

MEGKWLLCMLLVLGTAIVEAHDGHDDDVIDIEDDLDDVIEEVEDSKPDTTAPPSSPKVTYKAPVPTGEVYFADSFDRGTLSGWILSKAKKDDTDDEIAKYDGKWEVEEMKESKLPGDKGLVLMSRAKHHAISAKLNKPFLFDTKPLIVQYEVNFQNGIECGGAYVKLLSKTPELNLDQFHDKTPYTIMFGPDKCGEDYKLHFIFRHKNPKTGIYEEKHAKRPDADLKTYFTDKKTHLYTLILNPDNSFEILVDQSVVNSGNLLNDMTPPVNPSREIEDPEDRKPEDWDERPKIPDPEAVKPDDWDEDAPAKIPDEEATKPEGWLDDEPEYVPDPDAEKPEDWDEDMDGEWEAPQIANPRCESAPGCGVWQRPVIDNPNYKGKWKPPMIDNPSYQGIWKPRKIPNPDFFEDLEPFRMTPFSAIGLELWSMTSDIFFDNFIICADRRIVDDWANDGWGLKKAADGAAEPGVVGQMIEAAEERPWLWVVYILTVALPVFLVILFCCSGKKQTSGMEYKKTDAPQPDVKEEEEEKEEEKDKGDEEEEGEEKLEEKQKSDAEEDGGTVSQEEEDRKPKAEEDEILNRSPRNRKPRRE

>NP_009108.1

MYSEIQRERADIGGLMARPEYREWNPELIKPKKLLNPVKASRSHQELHRELLMNHRRGLGVDSKPELQRVLEHRRRNQLIKKKKEELEAKRLQCPFEQELLRRQQRLNQLEKPPEKEEDHAPEFIKVRENLRRIATLTSEEREL

23 NP_057661.3 NP_004603.1

>NP_057661.3

MSAGGDFGNPLRKFKLVFLGEQSVGKTSLITRFMYDSFDNTYQATIGIDFLSKTMYLEDRTVRLQLWDTAGQERFRSLIPSYIRDSTVAVVVYDITNLNSFQQTSKWIDDVRTERGSDVIIMLVGNKTDLADKRQITIEEGEQRAKELSVMFIETSAKTGYNVKQLFRRVASALPGMENVQEKSKEGMIDIKLDKPQEPPASEGGCSC

>NP_004603.1

MEAAVAAPRPRLLLLVLAAAAAAAAALLPGATALQCFCHLCTKDNFTCVTDGLCFVSVTETTDKVIHNSMCIAEIDLIPRDRPFVCAPSSKTGSVTTTYCCNQDHCNKIELPTTVKSSPGLGPVELAAVIAGPVCFVCISLMLMVYICHNRTVIHHRVPNEEDPSLDRPFISEGTTLKDLIYDMTTSGSGSGLPLLVQRTIARTIVLQESIGKGRFGEVWRGKWRGEEVAVKIFSSREERSWFREAEIYQTVMLRHENILGFIAADNKDNGTWTQLWLVSDYHEHGSLFDYLNRYTVTVEGMIKLALSTASGLAHLHMEIVGTQGKPAIAHRDLKSKNILVKKNGTCCIADLGLAVRHDSATDTIDIAPNHRVGTKRYMAPEVLDDSINMKHFESFKRADIYAMGLVFWEIARRCSIGGIHEDYQLPYYDLVPSDPSVEEMRKVVCEQKLRPNIPNRWQSCEALRVMAKIMRECWYANGAARLTALRIKKTLSQLSQQEGIKM

24 NP_002816.1 NP_715638.1

>NP_002816.1

MQAQQYQQQRRKFAAAFLAFIFILAAVDTAEAGKKEKPEKKVKKSDCGEWQWSVCVPTSGDCGLGTREGTRTGAECKQTMKTQRCKIPCNWKKQFGAECKYQFQAWGECDLNTALKTRTGSLKRALHNAECQKTVTISKPCGKLTKPKPQAESKKKKKEGKKQEKMLD

>NP_715638.1

MADSENQGPAEPSQAAAAAEAAAEEVMAEGGAQGGDCDSAAGDPDSAAGQMAEEPQTPAENAPKPKNDFIESLPNSVKCRVLALKKLQKRCDKIEAKFDKEFQALEKKYNDIYKPLLAKIQELTGEMEGCAWTLEGEEEEEEEYEDDEEEGEDEEEEEAAAEAAAGAKHDDAHAEMPDDAKK

25 NP_000917.3 NP_056986.2

>NP_000917.3

MTELKAKGPRAPHVAGGPPSPEVGSPLLCRPAAGPFPGSQTSDTLPEVSAIPISLDGLLFPRPCQGQDPSDEKTQDQQSLSDVEGAYSRAEATRGAGGSSSSPPEKDSGLLDSVLDTLLAPSGPGQSQPSPPACEVTSSWCLFGPELPEDPPAAPATQRVLSPLMSRSGCKVGDSSGTAAAHKVLPRGLSPARQLLLPASESPHWSGAPVKPSPQAAAVEVEEEDGSESEESAGPLLKGKPRALGGAAAGGGAAAVPPGAAAGGVALVPKEDSRFSAPRVALVEQDAPMAPGRSPLATTVMDFIHVPILPLNHALLAARTRQLLEDESYDGGAGAASAFAPPRSSPCASSTPVAVGDFPDCAYPPDAEPKDDAYPLYSDFQPPALKIKEEEEGAEASARSPRSYLVAGANPAAFPDFPLGPPPPLPPRATPSRPGEAAVTAAPASASVSSASSSGSTLECILYKAEGAPPQQGPFAPPPCKAPGASGCLLPRDGLPSTSASAAAAGAAPALYPALGLNGLPQLGYQAAVLKEGLPQVYPPYLNYLRPDSEASQSPQYSFESLPQKICLICGDEASGCHYGVLTCGSCKVFFKRAMEGQHNYLCAGRNDCIVDKIRRKNCPACRLRKCCQAGMVLGGRKFKKFNKVRVVRALDAVALPQPVGVPNESQALSQRFTFSPGQDIQLIPPLINLLMSIEPDVIYAGHDNTKPDTSSSLLTSLNQLGERQLLSVVKWSKSLPGFRNLHIDDQITLIQYSWMSLMVFGLGWRSYKHVSGQMLYFAPDLILNEQRMKESSFYSLCLTMWQIPQEFVKLQVSQEEFLCMKVLLLLNTIPLEGLRSQTQFEEMRSSYIRELIKAIGLRQKGVVSSSQRFYQLTKLLDNLHDLVKQLHLYCLNTFIQSRALSVEFPEMMSEVIAAQLPKILAGMVKPLLFHKK

>NP_056986.2

MTSIHFVVHPLPGTEDQLNDRLREVSEKLNKYNLNSHPPLNVLEQATIKQCVVGPNHAAFLLEDGRVCRIGFSVQPDRLELGKPDNNDGSKLNSNSGAGRTSRPGRTSDSPWFLSGSETLGRLAGNTLGSRWSSGVGGSGGGSSGRSSAGARDSRRQTRVIRTGRDRGSGLLGSQPQPVIPASVIPEELISQAQVVLQGKSRSVIIRELQRTNLDVNLAVNNLLSRDDEDGDDGDDTASESYLPGEDLMSLLDADIHSAHPSVIIDADAMFSEDISYFGYPSFRRSSLSRLGSSRVLLLPLERDSELLRERESVLRLRERRWLDGASFDNERGSTSKEGEPNLDKKNTPVQSPVSLGEDLQWWPDKDGTKFICIGALYSELLAVSSKGELYQWKWSESEPYRNAQNPSLHHPRATFLGLTNEKIVLLSANSIRATVATENNKVATWVDETLSSVASKLEHTAQTYSELQGERIVSLHCCALYTCAQLENSLYWWGVVPFSQRKKMLEKARAKNKKPKSSAGISSMPNITVGTQVCLRNNPLYHAGAVAFSISAGIPKVGVLMESVWNMNDSCRFQLRSPESLKNMEKASKTTEAKPESKQEPVKTEMGPPPSPASTCSDASSIASSASMPYKRRRSTPAPKEEEKVNEEQWSLREVVFVEDVKNVPVGKVLKVDGAYVAVKFPGTSSNTNCQNSSGPDADPSSLLQDCRLLRIDELQVVKTGGTPKVPDCFQRTPKKLCIPEKTEILAVNVDSKGVHAVLKTGNWVRYCIFDLATGKAEQENNFPTSSIAFLGQNERNVAIFTAGQESPIILRDGNGTIYPMAKDCMGGIRDPDWLDLPPISSLGMGVHSLINLPANSTIKKKAAVIIMAVEKQTLMQHILRCDYEACRQYLMNLEQAVVLEQNLQMLQTFISHRCDGNRNILHACVSVCFPTSNKETKEEEEAERSERNTFAERLSAVEAIANAISVVSSNGPGNRAGSSSSRSLRLREMMRRSLRAAGLGRHEAGASSSDHQDPVSPPIAPPSWVPDPPAMDPDGDIDFILAPAVGSLTTAATGTGQGPSTSTIPGPSTEPSVVESKDRKANAHFILKLLCDSVVLQPYLRELLSAKDARGMTPFMSAVSGRAYPAAITILETAQKIAKAEISSSEKEEDVFMGMVCPSGTNPDDSPLYVLCCNDTCSFTWTGAEHINQDIFECRTCGLLESLCCCTECARVCHKGHDCKLKRTSPTAYCDCWEKCKCKTLIAGQKSARLDLLYRLLTATNLVTLPNSRGEHLLLFLVQTVARQTVEHCQYRPPRIREDRNRKTASPEDSDMPDHDLEPPRFAQLALERVLQDWNALKSMIMFGSQENKDPLSASSRIGHLLPEEQVYLNQQSGTIRLDCFTHCLIVKCTADILLLDTLLGTLVKELQNKYTPGRREEAIAVTMRFLRSVARVFVILSVEMASSKKKNNFIPQPIGKCKRVFQALLPYAVEELCNVAESLIVPVRMGIARPTAPFTLASTSIDAMQGSEELFSVEPLPPRPSSDQSSSSSQSQSSYIIRNPQQRRISQSQPVRGRDEEQDDIVSADVEEVEVVEGVAGEEDHHDEQEEHGEENAEAEGQHDEHDEDGSDMELDLLAAAETESDSESNHSNQDNASGRRSVVTAATAGSEAGASSVPAFFSEDDSQSNDSSDSDSSSSQSDDIEQETFMLDEPLERTTNSSHANGAAQAPRSMQWAVRNTQHQRAASTAPSSTSTPAASSAGLIYIDPSNLRRSGTISTSAAAAAAALEASNASSYLTSASSLARAYSIVIRQISDLMGLIPKYNHLVYSQIPAAVKLTYQDAVNLQNYVEEKLIPTWNWMVSIMDSTEAQLRYGSALASAGDPGHPNHPLHASQNSARRERMTAREEASLRTLEGRRRATLLSARQGMMSARGDFLNYALSLMRSHNDEHSDVLPVLDVCSLKHVAYVFQALIYWIKAMNQQTTLDTPQLERKRTRELLELGIDNEDSEHENDDDTNQSATLNDKDDDSLPAETGQNHPFFRRSDSMTFLGCIPPNPFEVPLAEAIPLADQPHLLQPNARKEDLFGRPSQGLYSSSASSGKCLMEVTVDRNCLEVLPTKMSYAANLKNVMNMQNRQKKEGEEQPVLPEETESSKPGPSAHDLAAQLKSSLLAEIGLTESEGPPLTSFRPQCSFMGMVISHDMLLGRWRLSLELFGRVFMEDVGAEPGSILTELGGFEVKESKFRREMEKLRNQQSRDLSLEVDRDRDLLIQQTMRQLNNHFGRRCATTPMAVHRVKVTFKDEPGEGSGVARSFYTAIAQAFLSNEKLPNLECIQNANKGTHTSLMQRLRNRGERDREREREREMRRSSGLRAGSRRDRDRDFRRQLSIDTRPFRPASEGNPSDDPEPLPAHRQALGERLYPRVQAMQPAFASKITGMLLELSPAQLLLLLASEDSLRARVDEAMELIIAHGRENGADSILDLGLVDSSEKVQQENRKRHGSSRSVVDMDLDDTDDGDDNAPLFYQPGKRGFYTPRPGKNTEARLNCFRNIGRILGLCLLQNELCPITLNRHVIKVLLGRKVNWHDFAFFDPVMYESLRQLILASQSSDADAVFSAMDLAFAIDLCKEEGGGQVELIPNGVNIPVTPQNVYEYVRKYAEHRMLVVAEQPLHAMRKGLLDVLPKNSLEDLTAEDFRLLVNGCGEVNVQMLISFTSFNDESGENAEKLLQFKRWFWSIVEKMSMTERQDLVYFWTSSPSLPASEEGFQPMPSITIRPPDDQHLPTANTCISRLYVPLYSSKQILKQKLLLAIKTKNFGFV

26 AAF91084.1 NP_003591.2

>AAF91084.1

MERPEEGKQSPPPQPWGRLLRLGAEEGEPHVLLRKREWTIGRRRGCDLSFPSNKLVSGDHCRIVVDEKSGQVTLEDTSTSGTVINKLKVVKKQTCPLQTGDVIYLVYRKNEPEHNVAYLYESLSEKQGMTQESFEANKENVFHGTKDTSGAGAGRGADPRVPPSSPATQVCFEEPQPSTSTSDLFPTASASSTEPSPAGRERSSSCGSGGGGISPKGSGPSVASDEVSSFASALPDRKTASFSSLEPQDQEDLEPVKKKMRGDGDLDLNGQLLVAQPRRNAQTVHEDVRAAAGKPDKMEETLTCIICQDLLHDCVSLQPCMHTFCAACYSGWMERSSLCPTCRCPVERICKNHILNNLVEAYLIQHPDKSRSEEDVQSMDARNKITQDMLQPKVRRSFSDEEGSSEDLLELSDVDSESSDISQPYVVCRQCPEYRRQAAQPPHCPAPEGEPGAPQALGDAPSTSVSLTTAVQDYVCPLQGSHALCTCCFQPMPDRRAEREQDPRVAPQQCAVCLQPFCHLYWGCTRTGCYGCLAPFCELNLGDKCLDGVLNNNSYESDILKNYLATRGLTWKNMLTESLVALQRGVFLLSDYRVTGDTVLCYCCGLRSFRELTYQYRQNIPASELPVAVTSRPDCYWGRNCRTQVKAHHAMKFNHICEQTRFKN

>NP_003591.2

MDRSKENCISGPVKATAPVGGPKRVLVTQQFPCQNPLPVNSGQAQRVLCPSNSSQRIPLQAQKLVSSHKPVQNQKQKQLQATSVPHPVSRPLNNTQKSKQPLPSAPENNPEEELASKQKNEESKKRQWALEDFEIGRPLGKGKFGNVYLAREKQSKFILALKVLFKAQLEKAGVEHQLRREVEIQSHLRHPNILRLYGYFHDATRVYLILEYAPLGTVYRELQKLSKFDEQRTATYITELANALSYCHSKRVIHRDIKPENLLLGSAGELKIADFGWSVHAPSSRRTTLCGTLDYLPPEMIEGRMHDEKVDLWSLGVLCYEFLVGKPPFEANTYQETYKRISRVEFTFPDFVTEGARDLISRLLKHNPSQRPMLREVLEHPWITANSSKPSNCQNKESASKQS

27 AAH07560.1 NP_005150.1

>AAH07560.1

MNPNCARCGKIVYPTEKVNCLDKFWHKACFHCETCKMTLNMKNYKGYEKKPYCNAHYPKQSFTMVADTPENLRLKQQSELQSQVRYKEEFEKNKGKGFSVVADTPELQRIKKTQDQISNIKYHEEFEKSRMGPSGGEGMEPERRDSQDGSSYRRPLEQQQPHHIPTSAPVYQQPQQQPVAQSYGGYKEPAAPVSIQRSAPICLQHIPRHRIRPGRDPSILQCLCFLKPATACDSYPSSSFFCQLKPSSATSAGSLLWQASPLIDFLVFSLDGTGMGLSGGGRGPWGRAGMGDLLACGPHLPLCSLPSHPPAQLLTYPHIPGLG

>NP_005150.1

MCDDEETTALVCDNGSGLVKAGFAGDDAPRAVFPSIVGRPRHQGVMVGMGQKDSYVGDEAQSKRGILTLKYPIEHGIITNWDDMEKIWHHTFYNELRVAPEEHPTLLTEAPLNPKANREKMTQIMFETFNVPAMYVAIQAVLSLYASGRTTGIVLDSGDGVTHNVPIYEGYALPHAIMRLDLAGRDLTDYLMKILTERGYSFVTTAEREIVRDIKEKLCYVALDFENEMATAASSSSLEKSYELPDGQVITIGNERFRCPETLFQPSFIGMESAGIHETTYNSIMKCDIDIRKDLYANNVLSGGTTMYPGIADRMQKEITALAPSTMKIKIIAPPERKYSVWIGGSILASLSTFQQMWISKQEYDEAGPSIVHRKCF

28 NP_056318.1 NP_002077.1

>NP_056318.1

MNGAPSPEDGASPSSPPLPPPPPPSWREFCESHARAAALDFARRFRLYLASHPQYAGPGAEAAFSRRFAELFLQHFEAEVARASGSLSPPILAPLSPGAEISPHDLSLESCRVGGPLAVLGPSRSSEDLAGPLPSSVSSSSTTSSKPKLKKRFSLRSVGRSVRGSVRGILQWRGTVDPPSSAGPLETSSGPPVLGGNSNSNSSGGAGTVGRGLVSDGTSPGERWTHRFERLRLSRGGGALKDGAGMVQREELLSFMGAEEAAPDPAGVGRGGGVAGPPSGGGGQPQWQKCRLLLRSEGEGGGGSRLEFFVPPKASRPRLSIPCSSITDVRTTTALEMPDRENTFVVKVEGPSEYIMETVDAQHVKAWVSDIQECLSPGPCPATSPRPMTLPLAPGTSFLTRENTDSLELSCLNHSESLPSQDLLLGPSESNDRLSQGAYGGLSDRPSASISPSSASIAASHFDSMELLPPELPPRIPIEEGPPAGTVHPLSAPYPPLDTPETATGSFLFQGEPEGGEGDQPLSGYPWFHGMLSRLKAAQLVLTGGTGSHGVFLVRQSETRRGEYVLTFNFQGKAKHLRLSLNEEGQCRVQHLWFQSIFDMLEHFRVHPIPLESGGSSDVVLVSYVPSSQRQQGREQAGSHAGVCEGDGCHPDASCTLMPFGASDCVTDHLP

>NP_002077.1

MEAIAKYDFKATADDELSFKRGDILKVLNEECDQNWYKAELNGKDGFIPKNYIEMKPHPWFFGKIPRAKAEEMLSKQRHDGAFLIRESESAPGDFSLSVKFGNDVQHFKVLRDGAGKYFLWVVKFNSLNELVDYHRSTSVSRNQQIFLRDIEQVPQQPTYVQALFDFDPQEDGELGFRRGDFIHVMDNSDPNWWKGACHGQTGMFPRNYVTPVNRNV

29 NP_038466.2 NP_000741.1

>NP_038466.2

MAESGESGGPPGSQDSAAGAEGAGAPAAAASAEPKIMKVTVKTPKEKEEFAVPENSSVQQFKEEISKRFKSHTDQLVLIFAGKILKDQDTLSQHGIHDGLTVHLVIKTQNRPQDHSAQQTNTAGSNVTTSSTPNSNSTSGSATSNPFGLGGLGGLAGLSSLGLNTTNFSELQSQMQRQLLSNPEMMVQIMENPFVQSMLSNPDLMRQLIMANPQMQQLIQRNPEISHMLNNPDIMRQTLELARNPAMMQEMMRNQDRALSNLESIPGGYNALRRMYTDIQEPMLSAAQEQFGGNPFASLVSNTSSGEGSQPSRTENRDPLPNPWAPQTSQSSSASSGTASTVGGTTGSTASGTSGQSTTAPNLVPGVGASMFNTPGMQSLLQQITENPQLMQNMLSAPYMRSMMQSLSQNPDLAAQMMLNNPLFAGNPQLQEQMRQQLPTFLQQMQNPDTLSAMSNPRAMQALLQIQQGLQTLATEAPGLIPGFTPGLGALGSTGGSSGTNGSNATPSENTSPTAGTTEPGHQQFIQQMLQALAGVNPQLQNPEVRFQQQLEQLSAMGFLNREANLQALIATGGDINAAIERLLGSQPS

>NP_000741.1

MRRAPSLVLFFLVALCGRGNCRVANAEEKLMDDLLNKTRYNNLIRPATSSSQLISIKLQLSLAQLISVNEREQIMTTNVWLKQEWTDYRLTWNSSRYEGVNILRIPAKRIWLPDIVLYNNADGTYEVSVYTNLIVRSNGSVLWLPPAIYKSACKIEVKYFPFDQQNCTLKFRSWTYDHTEIDMVLMTPTASMDDFTPSGEWDIVALPGRRTVNPQDPSYVDVTYDFIIKRKPLFYTINLIIPCVLTTLLAILVFYLPSDCGEKMTLCISVLLALTFFLLLISKIVPPTSLDVPLIGKYLMFTMVLVTFSIVTSVCVLNVHHRSPSTHTMAPWVKRCFLHKLPTFLFMKRPGPDSSPARAFPPSKSCVTKPEATATSTSPSNFYGNSMYFVNPASAASKSPAGSTPVAIPRDFWLRSSGRFRQDVQEALEGVSFIAQHMKNDDEDQSVVEDWKYVAMVVDRLFLWVFMFVCVLGTVGLFLPPLFQTHAASEGPYAAQRD

30 NP_004679.1 NP_005524.1

>NP_004679.1

MEADKDDTQQILKEHSPDEFIKDEQNKGLIDEITKKNIQLKKEIQKLETELQEATKEFQIKEDIPETKMKFLSVETPENDSQLSNISCSFQVSSKVPYEIQKGQALITFEKEEVAQNVVSMSKHHVQIKDVNLEVTAKPVPLNSGVRFQVYVEVSKMKINVTEIPDTLREDQMRDKLELSFSKFRNGGGEVDRVDYDRQSGSAVITFVEIGVADKILKKKEYPLYINQTCHRVTVSPYTEIHLKKYQIFSGTSKRTVLLTGMEGIQMDEEIVEDLINIHFQRAKNGGGEVDVVKCSLGQPHIAYFEE

>NP_005524.1

MSAPLDAALHALQEEQARLKMRLWDLQQLRKELGDSPKDKVPFSVPKIPLVFRGHTQQDPEVPKSLVSNLRIHCPLLAGSALITFDDPKVAEQVLQQKEHTINMEECRLRVQVQPLELPMVTTIQVMVSSQLSGRRVLVTGFPASLRLSEEELLDKLEIFFGKTRNGGGDVDVRELLPGSVMLGFARDGVAQRLCQIGQFTVPLGGQQVPLRVSPYVNGEIQKAEIRSQPVPRSVLVLNIPDILDGPELHDVLEIHFQKPTRGGGEVEALTVVPQGQQGLAVFTSESG

31 NP_055191.2 NP_003922.1

>NP_055191.2

MTTHVTLEDALSNVDLLEELPLPDQQPCIEPPPSSIMYQANFDTNFEDRNAFVTGIARYIEQATVHSSMNEMLEEGHEYAVMLYTWRSCSRAIPQVKCNEQPNRVEIYEKTVEVLEPEVTKLMKFMYFQRKAIERFCSEVKRLCHAERRKDFVSEAYLLTLGKFINMFAVLDELKNMKCSVKNDHSAYKRAAQFLRKMADPQSIQESQNLSMFLANHNRITQCLHQQLEVIPGYEELLADIVNICVDYYENKMYLTPSEKHMLLKVMGFGLYLMDGNVSNIYKLDAKKRINLSKIDKFFKQLQVVPLFGDMQIELARYIKTSAHYEENKSKWTCTQSSISPQYNICEQMVQIRDDHIRFISELARYSNSEVVTGSGLDSQKSDEEYRELFDLALRGLQLLSKWSAHVMEVYSWKLVHPTDKFCNKDCPGTAEEYERATRYNYTSEEKFAFVEVIAMIKGLQVLMGRMESVFNQAIRNTIYAALQDFAQVTLREPLRQAVRKKKNVLISVLQAIRKTICDWEGGREPPNDPCLRGEKDPKGGFDIKVPRRAVGPSSTQLYMVRTMLESLIADKSGSKKTLRSSLDGPIVLAIEDFHKQSFFFTHLLNISEALQQCCDLSQLWFREFFLELTMGRRIQFPIEMSMPWILTDHILETKEPSMMEYVLYPLDLYNDSAYYALTKFKKQFLYDEIEAEVNLCFDQFVYKLADQIFAYYKAMAGSVLLDKRFRAECKNYGVIIPYPPSNRYETLLKQRHVQLLGRSIDLNRLITQRISAAMYKSLDQAISRFESEDLTSIVELEWLLEINRLTHRLLCKHMTLDSFDAMFREANHNVSAPYGRITLHVFWELNFDFLPNYCYNGSTNRFVRTAIPFTQEPQRDKPANVQPYYLYGSKPLNIAYSHIYSSYRNFVGPPHFKTICRLLGYQGIAVVMEELLKIVKSLLQGTILQYVKTLIEVMPKICRLPRHEYGSPGILEFFHHQLKDIIEYAELKTDVFQSLREVGNAILFCLLIEQALSQEEVCDLLHAAPFQNILPRVYIKEGERLEVRMKRLEAKYAPLHLVPLIERLGTPQQIAIAREGDLLTKERLCCGLSMFEVILTRIRSYLQDPIWRGPPPTNGVMHVDECVEFHRLWSAMQFVYCIPVGTNEFTAEQCFGDGLNWAGCSIIVLLGQQRRFDLFDFCYHLLKVQRQDGKDEIIKNVPLKKMADRIRKYQILNNEVFAILNKYMKSVETDSSTVEHVRCFQPPIHQSLATTC

>NP_003922.1

MPLVKRNIDPRHLCHTALPRGIKNELECVTNISLANIIRQLSSLSKYAEDIFGELFNEAHSFSFRVNSLQERVDRLSVSVTQLDPKEEELSLQDITMRKAFRSSTIQDQQLFDRKTLPIPLQETYDVCEQPPPLNILTPYRDDGKEGLKFYTNPSYFFDLWKEKMLQDTEDKRKEKRKQKQKNLDRPHEPEKVPRAPHDRRREWQKLAQGPELAEDDANLLHKHIEVANGPASHFETRPQTYVDHMDGSYSLSALPFSQMSELLTRAEERVLVRPHEPPPPPPMHGAGDAKPIPTCISSATGLIENRPQSPATGRTPVFVSPTPPPPPPPLPSALSTSSLRASMTSTPPPPVPPPPPPPATALQAPAVPPPPAPLQIAPGVLHPAPPPIAPPLVQPSPPVARAAPVCETVPVHPLPQGEVQGLPPPPPPPPLPPPGIRPSSPVTVTALAHPPSGLHPTPSTAPGPHVPLMPPSPPSQVIPASEPKRHPSTLPVISDARSVLLEAIRKGIQLRKVEEQREQEAKHERIENDVATILSRRIAVEYSDSEDDSEFDEVDWLE

32 NP_001033.1 NP_001077362.1

>NP_001033.1

MPSGFQQIGSEDGEPPQQRVTGTLVLAVFSAVLGSLQFGYNIGVINAPQKVIEQSYNETWLGRQGPEGPSSIPPGTLTTLWALSVAIFSVGGMISSFLIGIISQWLGRKRAMLVNNVLAVLGGSLMGLANAAASYEMLILGRFLIGAYSGLTSGLVPMYVGEIAPTHLRGALGTLNQLAIVIGILIAQVLGLESLLGTASLWPLLLGLTVLPALLQLVLLPFCPESPRYLYIIQNLEGPARKSLKRLTGWADVSGVLAELKDEKRKLERERPLSLLQLLGSRTHRQPLIIAVVLQLSQQLSGINAVFYYSTSIFETAGVGQPAYATIGAGVVNTVFTLVSVLLVERAGRRTLHLLGLAGMCGCAILMTVALLLLERVPAMSYVSIVAIFGFVAFFEIGPGPIPWFIVAELFSQGPRPAAMAVAGFSNWTSNFIIGMGFQYVAEAMGPYVFLLFAVLLLGFFIFTFLRVPETRGRTFDQISAAFHRTPSLLEQEVKPSTELEYLGPDEND

>NP_001077362.1

MDELAGGGGGGPGMAAPPRQQQGPGGNLGLSPGGNGAAGGGGPPASEGAGPAAGPELSRPQQYTIPGILHYIQHEWARFEMERAHWEVERAELQARIAFLQGERKGQENLKKDLVRRIKMLEYALKQERAKYHKLKYGTELNQGDLKMPTFESEETKDTEAPTAPQNSQLTWKQGRQLLRQYLQEVGYTDTILDVRSQRVRSLLGLSNSEPNGSVETKNLEQILNGGESPKQKGQEIKRSSGDVLETFNFLENADDSDEDEENDMIEGIPEGKDKHRMNKHKIGNEGLAADLTDDPDTEEALKEFDFLVTAEDGEGAGEARSSGDGTEWDKDDLSPTAEVWDVDQGLISKLKEQYKKERKGKKGVKRANRTKLYDMIADLGDDELPHIPSGIINQSRSASTRMTDHEGARAEEAEPITFPSGGGKSFIMGSDDVLLSVLGLGDLADLTVTNDADYSYDLPANKDAFRKTWNPKYTLRSHFDGVRALAFHPVEPVLVTASEDHTLKLWNLQKTVPAKKSASLDVEPIYTFRAHIGPVLSLAISSNGEQCFSGGIDATIQWWNMPSPSVDPYDTYEPNVLAGTLVGHTDAVWGLAYSGIKNQLLSCSADGTVRLWNPQEKLPCICTYNGDKKHGIPTSVDFIGCDPAHMVTSFNTGSAVIYDLETSQSLVILSSQVDSGLQSNNHINRVVSHPTLPVTITAHEDRHIKFFDNKTGKMIHSMVAHLDAVTSLAVDPNGIYLMSGSHDCSIRLWNLDSKTCVQEITAHRKKLDESIYDVAFHSSKAYIASAGADALAKVFV

33 NP_008830.2 NP_002964.2

>NP_008830.2

MAALSGVRWLTRALVSAGNPGAWRGLSTSAAAHAASRSQAEDVRVEGSFPVTMLPGDGVGPELMHAVKEVFKAAAVPVEFQEHHLSEVQNMASEEKLEQVLSSMKENKVAIIGKIHTPMEYKGELASYDMRLRRKLDLFANVVHVKSLPGYMTRHNNLDLVIIREQTEGEYSSLEHESARGVIECLKIVTRAKSQRIAKFAFDYATKKGRGKVTAVHKANIMKLGDGLFLQCCEEVAELYPKIKFETMIIDNCCMQLVQNPYQFDVLVMPNLYGNIIDNLAAGLVGGAGVVPGESYSAEYAVFETGARHPFAQAVGRNIANPTAMLLSASNMLRHLNLEYHSSMIADAVKKVIKVGKVRTRDMGGYSTTTDFIKSVIGHLQTKGS

>NP_002964.2

MRSAAAAPRSPAVATESRRFAAARWPGWRSLQRPARRSGRGGGGAAPGPYPSAAPPPPGPGPPPSRQSSPPSASDCFGSNGNGGGAFRPGSRRLLGLGGPPRPFVVVLLPLASPGAPPAAPTRASPLGARASPPRSGVSLARPAPGCPRPACEPVYGPLTMSLKPQQQQQQQQQQQQQQQQQQQQQQQPPPAAANVRKPGGSGLLASPAAAPSPSSSSVSSSSATAPSSVVAATSGGGRPGLGRGRNSNKGLPQSTISFDGIYANMRMVHILTSVVGSKCEVQVKNGGIYEGVFKTYSPKCDLVLDAAHEKSTESSSGPKREEIMESILFKCSDFVVVQFKDMDSSYAKRDAFTDSAISAKVNGEHKEKDLEPWDAGELTANEELEALENDVSNGWDPNDMFRYNEENYGVVSTYDSSLSSYTVPLERDNSEEFLKREARANQLAEEIESSAQYKARVALENDDRSEEEKYTAVQRNSSEREGHSINTRENKYIPPGQRNREVISWGSGRQNSPRMGQPGSGSMPSRSTSHTSDFNPNSGSDQRVVNGGVPWPSPCPSPSSRPPSRYQSGPNSLPPRAATPTRPPSRPPSRPSRPPSHPSAHGSPAPVSTMPKRMSSEGPPRMSPKAQRHPRNHRVSAGRGSISSGLEFVSHNPPSEAATPPVARTSPSGGTWSSVVSGVPRLSPKTHRPRSPRQNSIGNTPSGPVLASPQAGIIPTEAVAMPIPAASPTPASPASNRAVTPSSEAKDSRLQDQRQNSPAGNKENIKPNETSPSFSKAENKGISPVVSEHRKQIDDLKKFKNDFRLQPSSTSESMDQLLNKNREGEKSRDLIKDKIEPSAKDSFIENSSSNCTSGSSKPNSPSISPSILSNTEHKRGPEVTSQGVQTSSPACKQEKDDKEEKKDAAEQVRKSTLNPNAKEFNPRSFSQPKPSTTPTSPRPQAQPSPSMVGHQQPTPVYTQPVCFAPNMMYPVPVSPGVQPLYPIPMTPMPVNQAKTYRAVPNMPQQRQDQHHQSAMMHPASAAGPPIAATPPAYSTQYVAYSPQQFPNQPLVQHVPHYQSQHPHVYSPVIQGNARMMAPPTHAQPGLVSSSATQYGAHEQTHAMYACPKLPYNKETSPSFYFAISTGSLAQQYAHPNATLHPHTPHPQPSATPTGQQQSQHGGSHPAPSPVQHHQHQAAQALHLASPQQQSAIYHAGLAPTPPSMTPASNTQSPQNSFPAAQQTVFTIHPSHVQPAYTNPPHMAHVPQAHVQSGMVPSHPTAHAPMMLMTTQPPGGPQAALAQSALQPIPVSTTAHFPYMTHPSVQAHHQQQL

34 NP_150634.1 NP_003211.1

>NP_150634.1

MADKVLKEKRKLFIRSMGEGTINGLLDELLQTRVLNKEEMEKVKRENATVMDKTRALIDSVIPKGAQACQICITYICEEDSYLAGTLGLSADQTSGNYLNMQDSQGVLSSFPAPQAVQDNPAMPTSSGSEGNVKLCSLEEAQRIWKQKSAEIYPIMDKSSRTRLALIICNEEFDSIPRRTGAEVDITGMTMLLQNLGYSVDVKKNLTASDMTTELEAFAHRPEHKTSDSTFLVFMSHGIREGICGKKHSEQVPDILQLNAIFNMLNTKNCPSLKDKPKVIIIQACRGDSPGVVWFKDSVGVSGNLSLPTTEEFEDDAIKKAHIEKDFIAFCSSTPDNVSWRHPTMGSVFIGRLIEHMQEYACSCDVEEIFRKVRFSFEQPDGRAQMPTTERVTLTRCFYLFPGH

>NP_003211.1

MLWKLTDNIKYEDCEDRHDGTSNGTARLPQLGTVGQSPYTSAPPLSHTPNADFQPPYFPPPYQPIYPQSQDPYSHVNDPYSLNPLHAQPQPQHPGWPGQRQSQESGLLHTHRGLPHQLSGLDPRRDYRRHEDLLHGPHALSSGLGDLSIHSLPHAIEEVPHVEDPGINIPDQTVIKKGPVSLSKSNSNAVSAIPINKDNLFGGVVNPNEVFCSVPGRLSLLSSTSKYKVTVAEVQRRLSPPECLNASLLGGVLRRAKSKNGGRSLREKLDKIGLNLPAGRRKAANVTLLTSLVEGEAVHLARDFGYVCETEFPAKAVAEFLNRQHSDPNEQVTRKNMLLATKQICKEFTDLLAQDRSPLGNSRPNPILEPGIQSCLTHFNLISHGFGSPAVCAAVTALQNYLTEALKAMDKMYLSNNPNSHTDNNAKSSDKEEKHRK

35 NP_647593.1 NP_009297.2

>NP_647593.1

MAEMGSKGVTAGKIASNVQKKLTRAQEKVLQKLGKADETKDEQFEQCVQNFNKQLTEGTRLQKDLRTYLASVKAMHEASKKLNECLQEVYEPDWPGRDEANKIAENNDLLWMDYHQKLVDQALLTMDTYLGQFPDIKSRIAKRGRKLVDYDSARHHYESLQTAKKKDEAKIAKPVSLLEKAAPQWCQGKLQAHLVAQTNLLRNQAEEELIKAQKVFEEMNVDLQEELPSLWNSRVGFYVNTFQSIAGLEENFHKEMSKLNQNLNDVLVGLEKQHGSNTFTVKAQPSDNAPAKGNKSPSPPDGSPAATPEIRVNHEPEPAGGATPGATLPKSPSQLRKGPPVPPPPKHTPSKEVKQEQILSLFEDTFVPEISVTTPSQFEAPGPFSEQASLLDLDFDPLPPVTSPVKAPTPSGQSIPWDLWEPTESPAGSLPSGEPSAAEGTFAVSWPSQTAEPGPAQPAEASEVAGGTQPAAGAQEPGETAASEAASSSLPAVVVETFPATVNGTVEGGSGAGRLDLPPGFMFKVQAQHDYTATDTDELQLKAGDVVLVIPFQNPEEQDEGWLMGVKESDWNQHKELEKCRGVFPENFTERVP

>NP_009297.2

MGQQPGKVLGDQRRPSLPALHFIKGAGKKESSRHGGPHCNVFVEHEALQRPVASDFEPQGLSEAARWNSKENLLAGPSENDPNLFVALYDFVASGDNTLSITKGEKLRVLGYNHNGEWCEAQTKNGQGWVPSNYITPVNSLEKHSWYHGPVSRNAAEYLLSSGINGSFLVRESESSPGQRSISLRYEGRVYHYRINTASDGKLYVSSESRFNTLAELVHHHSTVADGLITTLHYPAPKRNKPTVYGVSPNYDKWEMERTDITMKHKLGGGQYGEVYEGVWKKYSLTVAVKTLKEDTMEVEEFLKEAAVMKEIKHPNLVQLLGVCTREPPFYIITEFMTYGNLLDYLRECNRQEVNAVVLLYMATQISSAMEYLEKKNFIHRDLAARNCLVGENHLVKVADFGLSRLMTGDTYTAHAGAKFPIKWTAPESLAYNKFSIKSDVWAFGVLLWEIATYGMSPYPGIDLSQVYELLEKDYRMERPEGCPEKVYELMRACWQWNPSDRPSFAEIHQAFETMFQESSISDEVEKELGKQGVRGAVSTLLQAPELPTKTRTSRRAAEHRDTTDVPEMPHSKGQGESDPLDHEPAVSPLLPRKERGPPEGGLNEDERLLPKDKKTNLFSALIKKKKKTAPTPPKRSSSFREMDGQPERRGAGEEEGRDISNGALAFTPLDTADPAKSPKPSNGAGVPNGALRESGGSGFRSPHLWKKSSTLTSSRLATGEEEGGGSSSKRFLRSCSASCVPHGAKDTEWRSVTLPRDLQSTGRQFDSSTFGGHKSEKPALPRKRAGENRSDQVTRGTVTPPPRLVKKNEEAADEVFKDIMESSPGSSPPNLTPKPLRRQVTVAPASGLPHKEEAGKGSALGTPAAAEPVTPTSKAGSGAPGGTSKGPAEESRVRRHKHSSESPGRDKGKLSRLKPAPPPPPAASAGKAGGKPSQSPSQEAAGEAVLGAKTKATSLVDAVNSDAAKPSQPGEGLKKPVLPATPKPQSAKPSGTPISPAPVPSTLPSASSALAGDQPSSTAFIPLISTRVSLRKTRQPPERIASGAITKGVVLDSTEALCLAISRNSEQMASHSAVLEAGKNLYTFCVSYVDSIQQMRNKFAFREAINKLENNLRELQICPATAGSGPAATQDFSKLLSSVKEISDIVQR

36 NP_057065.2 NP_000215.1

>NP_057065.2

MATITCTRFTEEYQLFEELGKGAFSVVRRCVKVLAGQEYAAKIINTKKLSARDHQKLEREARICRLLKHPNIVRLHDSISEEGHHYLIFDLVTGGELFEDIVAREYYSEADASHCIQQILEAVLHCHQMGVVHRDLKPENLLLASKLKGAAVKLADFGLAIEVEGEQQAWFGFAGTPGYLSPEVLRKDPYGKPVDLWACGVILYILLVGYPPFWDEDQHRLYQQIKAGAYDFPSPEWDTVTPEAKDLINKMLTINPSKRITAAEALKHPWISHRSTVASCMHRQETVDCLKKFNARRKLKGAILTTMLATRNFSGGKSGGNKKSDGVKKRKSSSSVQLMESSESTNTTIEDEDTKVRKQEIIKVTEQLIEAISNGDFESYTKMCDPGMTAFEPEALGNLVEGLDFHRFYFENLWSRNSKPVHTTILNPHIHLMGDESACIAYIRITQYLDAGGIPRTAQSEETRVWHRRDGKWQIVHFHRSGAPSVLPH

>NP_000215.1

MSFTTRSTFSTNYRSLGSVQAPSYGARPVSSAASVYAGAGGSGSRISVSRSTSFRGGMGSGGLATGIAGGLAGMGGIQNEKETMQSLNDRLASYLDRVRSLETENRRLESKIREHLEKKGPQVRDWSHYFKIIEDLRAQIFANTVDNARIVLQIDNARLAADDFRVKYETELAMRQSVENDIHGLRKVIDDTNITRLQLETEIEALKEELLFMKKNHEEEVKGLQAQIASSGLTVEVDAPKSQDLAKIMADIRAQYDELARKNREELDKYWSQQIEESTTVVTTQSAEVGAAETTLTELRRTVQSLEIDLDSMRNLKASLENSLREVEARYALQMEQLNGILLHLESELAQTRAEGQRQAQEYEALLNIKVKLEAEIATYRRLLEDGEDFNLGDALDSSNSMQTIQKTTTRRIVDGKVVSETNDTKVLRH

37 NP_003945.2 NP_000960.2

>NP_003945.2

MAVMEMACPGAPGSAVGQQKELPKAKEKTPPLGKKQSSVYKLEAVEKSPVFCGKWEILNDVITKGTAKEGSEAGPAAISIIAQAECENSQEFSPTFSERIFIAGSKQYSQSESLDQIPNNVAHATEGKMARVCWKGKRRSKARKKRKKKSSKSLAHAGVALAKPLPRTPEQESCTIPVQEDESPLGAPYVRNTPQFTKPLKEPGLGQLCFKQLGEGLRPALPRSELHKLISPLQCLNHVWKLHHPQDGGPLPLPTHPFPYSRLPHPFPFHPLQPWKPHPLESFLGKLACVDSQKPLPDPHLSKLACVDSPKPLPGPHLEPSCLSRGAHEKFSVEEYLVHALQGSVSSGQAHSLTSLAKTWAARGSRSREPSPKTEDNEGVLLTEKLKPVDYEYREEVHWATHQLRLGRGSFGEVHRMEDKQTGFQCAVKKVRLEVFRAEELMACAGLTSPRIVPLYGAVREGPWVNIFMELLEGGSLGQLVKEQGCLPEDRALYYLGQALEGLEYLHSRRILHGDVKADNVLLSSDGSHAALCDFGHAVCLQPDGLGKSLLTGDYIPGTETHMAPEVVLGRSCDAKVDVWSSCCMMLHMLNGCHPWTQFFRGPLCLKIASEPPPVREIPPSCAPLTAQAIQEGLRKEPIHRVSAAELGGKVNRALQQVGGLKSPWRGEYKEPRHPPPNQANYHQTLHAQPRELSPRAPGPRPAEETTGRAPKLQPPLPPEPPEPNKSPPLTLSKEESGMWEPLPLSSLEPAPARNPSSPERKATVPEQELQQLEIELFLNSLSQPFSLEEQEQILSCLSIDSLSLSDDSEKNPSKASQSSRDTLSSGVHSWSSQAEARSSSWNMVLARGRPTDTPSYFNGVKVQIQSLNGEHLHIREFHRVKVGDIATGISSQIPAAAFSLVTKDGQPVRYDMEVPDSGIDLQCTLAPDGSFAWSWRVKHGQLENRP

>NP_000960.2

MGFVKVVKNKAYFKRYQVKFRRRREGKTDYYARKRLVIQDKNKYNTPKYRMIVRVTNRDIICQIAYARIEGDMIVCAAYAHELPKYGVKVGLTNYAAAYCTGLLLARRLLNRFGMDKIYEGQVEVTGDEYNVESIDGQPGAFTCYLDAGLARTTTGNKVFGALKGAVDGGLSIPHSTKRFPGYDSESKEFNAEVHRKHIMGQNVADYMRYLMEEDEDAYKKQFSQYIKNSVTPDMMEEMYKKAHAAIRENPVYEKKPKKEVKKKRWNRPKMSLAQKKDRVAQKKASFLRAQERAAES

38 NP_036413.1 AAQ86961.1

>NP_036413.1

MAAGVAAWLPFARAAAIGWMPVASGPMPAPPRQERKRTQDALIVLNVSGTRFQTWQDTLERYPDTLLGSSERDFFYHPETQQYFFDRDPDIFRHILNFYRTGKLHYPRHECISAYDEELAFFGLIPEIIGDCCYEEYKDRRRENAERLQDDADTDTAGESALPTMTARQRVWRAFENPHTSTMALVFYYVTGFFIAVSVIANVVETVPCGSSPGHIKELPCGERYAVAFFCLDTACVMIFTVEYLLRLAAAPSRYRFVRSVMSIIDVVAILPYYIGLVMTDNEDVSGAFVTLRVFRVFRIFKFSRHSQGLRILGYTLKSCASELGFLLFSLTMAIIIFATVMFYAEKGSSASKFTSIPAAFWYTIVTMTTLGYGDMVPKTIAGKIFGSICSLSGVLVIALPVPVIVSNFSRIYHQNQRADKRRAQKKARLARIRAAKSGSANAYMQSKRNGLLSNQLQSSEDEQAFVSKSGSSFETQHHHLLHCLEKTTNHEFVDEQVFEESCMEVATVNRPSSHSPSLSSQQGVTSTCCSRRHKKTFRIPNANVSGSHQGSIQELSTIQIRCVERTPLSNSRSSLNAKMEECVKLNCEQPYVTTAIISIPTPPVTTPEGDDRPESPEYSGGNIVRVSAL

>AAQ86961.1

MESHSRAGKSRKSAKFRSISRSLMLCNAKTSDDGSSPDEKYPDPFEISLAQGKEGIFHSSVQLADTSEAGPSSVPDLALASEAAQLQAAGNDRGKTCRRIFFMKESSTASSREKPGKLEAQSSNFLFPKACHQRARSNSTSVNPYCTREIDFPMTKKSAAPTDRQPYSLCSNRKSLSQQLDCPAGKAAGTSRPTRSLSTAQLVQPSGGLQASVISNIVLMKGQAKGLGFSIVGGKDSIYGPIGIYVKTIFAGGAAAADGRLQEGDEILELNGGSMAGLTHQDALQKFKQAKKGLLTLTVRTRLTAPPSLCSHLSPPLCRSLSSSTCITKDSSSFALESPSAPISTAKPNYRIMVEVSLQKEAGVGLGIGLCSVPYFQCISGIFVHTLSPGSVAHLDGRLRCGDEIVEISDSPVHCLTLNEVYTILSHCDPGPVPIIVSRHPDPQVSEQQLKEAVAQAVENTKFGKERHQWSLEGVKRLESSWHGRPTLEKEREKNSAPPHRRAQKVMIRSSSDSSYMSGSPGGSPGSGSAEKPSSDVDISTHSPSLPLAREPVVLSIASSRLPQESPPLPESRDSHPPLRLKKSFEILVRKPMSSKPKPPPRKYFKSDSDPQKSLEERENSSCSSGHTPPTCGQEARELLPLLLPQEDTAGRSPSASAGCPGPGIGPQTKSSTEGEPGWRRASPVTQTSPIKHPLLKRQARMDYSFDTTAEDPWVRISDCIKNLFSPIMSENHGHMPLQPNASLNEEEGTQGHPDGTPPKLDTANGTPKVYKSADSSTVKKGPPVAPKPAWFRQSLKGLRNRASDPRGLPDPALSTQPAPASREHLGSHIRASSSSSSIRQRISSFETFGSSQLPDKGAQRLSLQPSSGEAAKPLGKHEEGRFSGLLGRGAAPTLVPQQPEQVLSSGSPAASEARDPGVSESPPPGRQPNQKTLPPGPDPLLRLLSTQAEESQGPVLKMPSQRARSFPLTRSQSCETKLLDEKTSKLYSISSQVSSAVMKSLLCLPSSISCAQTPCIPKEGASPTSSSNEDSAANGSAETSALDTGFSLNLSELREYTEGLTEAKEDDDGDHSSLQSGQSVISLLSSEELKKLIEEVKVLDEATLKQLDGIHVTILHKEEGAGLGFSLAGGADLENKVITVHRVFPNGLASQEGTIQKGNEVLSINGKSLKGTTHHDALAILRQAREPRQAVIVTRKLTPEAMPDLNSSTDSAASASAASDVSVESTAEATVCTVTLEKMSAGLGFSLEGGKGSLHGDKPLTINRIFKGAASEQSETVQPGDEILQLGGTAMQGLTRFEAWNIIKALPDGPVTIVIRRKSLQSKETTAAGDS

39 NP_006316.1 NP_001020272.1

>NP_006316.1

MAAQGEPQVQFKLVLVGDGGTGKTTFVKRHLTGEFEKKYVATLGVEVHPLVFHTNRGPIKFNVWDTAGQEKFGGLRDGYYIQAQCAIIMFDVTSRVTYKNVPNWHRDLVRVCENIPIVLCGNKVDIKDRKVKAKSIVFHRKKNLQYYDISAKSNYNFEKPFLWLARKLIGDPNLEFVAMPALAPPEVVMDPALAAQYEHDLEVAQTTALPDEDDDL

>NP_001020272.1

MGNHAGKRELNAEKASTNSETNRGESEKKRNLGELSRTTSEDNEVFGEADANQNNGTSSQDTAVTDSKRTADPKNAWQDAHPADPGSRPHLIRLFSRDAPGREDNTFKDRPSESDELQTIQEDSAATSESLDVMASQKRPSQRHGSKYLATASTMDHARHGFLPRHRDTGILDSIGRFFGGDRGAPKRGSGKDSHHPARTAHYGSLPQKSHGRTQDENPVVHFFKNIVTPRTPPPSQGKGRGLSLSRFSWGAEGQRPGFGYGGRASDYKSAHKGFKGVDAQGTLSKIFKLGGRDSRSGSPMARR

40 NP_002743.3 NP_065853.2

>NP_002743.3

MSDSKCDSQFYSVQVADSTFTVLKRYQQLKPIGSGAQGIVCAAFDTVLGINVAVKKLSRPFQNQTHAKRAYRELVLLKCVNHKNIISLLNVFTPQKTLEEFQDVYLVMELMDANLCQVIHMELDHERMSYLLYQMLCGIKHLHSAGIIHRDLKPSNIVVKSDCTLKILDFGLARTACTNFMMTPYVVTRYYRAPEVILGMGYKENVDIWSVGCIMGELVKGCVIFQGTDHIDQWNKVIEQLGTPSAEFMKKLQPTVRNYVENRPKYPGIKFEELFPDWIFPSESERDKIKTSQARDLLSKMLVIDPDKRISVDEALRHPYITVWYDPAEAEAPPPQIYDAQLEEREHAIEEWKELIYKEVMDWEERSKNGVVKDQPSDAAVSSNATPSQSSSINDISSMSTEQTLASDTDSSLDASTGPLEGCR

>NP_065853.2

MLAGRPGTRSAVGELGTESSDNLDRAPLGPRESGGHHRPGSYLDMKIHLEKNLEEERQILLQQQKICRNRARKYFVESNRRKKAFEEKRKEQEEKEHQIREQILQQRKQKFEEVTEKFQRAHVPLSQRRKAVSRKPVPPLEEALKQIQESNLKSEVNLPFSRRPTINWRAIDSALPSALSKNDHKHQKQLLSKINCEKEMNENMRATLATSKNVFQLKLEETQKLLEDQHLSNLQKFGDEVNQITNSETLSSIDSLEATEHEEIYLTLNKEHSTSIQRNTISLKPANMQSTNLSCFDEDKLAFSKTQHINNWLTNLDASNTQNVTAFSDILSKSNVLPSWEYFNSKEQNPSPLNGTVERATNTANNSVPFVSSPPMFVLDKKCEKTSETSTMRTTDSTSGAFKRERPLVTESPTFKFSKSQSTSDSLTQEVATFPDQEKYSELNQENGTTSIPTSCVPVATPLVLPSNIQSARPSAKNSIHIKEIDAVQCSDKLDELKDGKEEEIKYFNCNKEELPLFSDSFQDAYIPHNPDSKDEKQKLAETSSLSNVTSNYDFVGQHKKMKYNIHERNGVRFLKSILKKESKYEHGYLKALIINQSFKFGNQKAAAIRDSIELTKEKGAEIPKTIKKLRWFDETSNIENNAENSHSLKNKTGTTQQHSQQFHIQSGAGSNIISVSTCAVNSADTKKSREDSISENVTTLGGSGADHMPLNCFIPSGYNFAKHAWPASKKEESKIPVHDDSKTKQGKPQRGRAKIIRKPGSAKVQSGFICTNRKGAVIQPQSASKVNIFTQAQGKLIIPCPPPQSTSNIRSGKNIQVSQCQPVTPENPQNIITHNSFNSKHVLPTEHSLNQWNQESSSPLSNACSDLVTVIPSLPSYCSSECQTFAKINHSNGTQAVARQDATLYCTQRSPVCEESYPSVTLRTAEEESVPLWKRGPNVLHQNKRATGSTVMRRKRIAETKRRNILEQKRQNPGSVGQKYSEQINNFGQSVLLSSSEPKQTTRGTSYIEEVSDSTSEFLMAENLVKASVPEDEILTVLNSKQIQKSNLPLNKTQQFNICTLSAEEQKILESLNDLSERLHYIQESICKNPSIKNTLQIIPLLEKREDRTSSCRDKR

41 NP_000210.2 NP_000229.1

>NP_000210.2

MILSNTTAVTPFLTKLWQETVQQGGNMSGLARRSPRSSDGKLEALYVLMVLGFFGFFTLGIMLSYIRSKKLEHSNDPFNVYIESDAWQEKDKAYVQARVLESYRSCYVVENHLAIEQPNTHLPETKPSP

>NP_000229.1

MPVRRGHVAPQNTFLDTIIRKFEGQSRKFIIANARVENCAVIYCNDGFCELCGYSRAEVMQRPCTCDFLHGPRTQRRAAAQIAQALLGAEERKVEIAFYRKDGSCFLCLVDVVPVKNEDGAVIMFILNFEVVMEKDMVGSPAHDTNHRGPPTSWLAPGRAKTFRLKLPALLALTARESSVRSGGAGGAGAPGAVVVDVDLTPAAPSSESLALDEVTAMDNHVAGLGPAEERRALVGPGSPPRSAPGQLPSPRAHSLNPDASGSSCSLARTRSRESCASVRRASSADDIEAMRAGVLPPPPRHASTGAMHPLRSGLLNSTSDSDLVRYRTISKIPQITLNFVDLKGDPFLASPTSDREIIAPKIKERTHNVTEKVTQVLSLGADVLPEYKLQAPRIHRWTILHYSPFKAVWDWLILLLVIYTAVFTPYSAAFLLKETEEGPPATECGYACQPLAVVDLIVDIMFIVDILINFRTTYVNANEEVVSHPGRIAVHYFKGWFLIDMVAAIPFDLLIFGSGSEELIGLLKTARLLRLVRVARKLDRYSEYGAAVLFLLMCTFALIAHWLACIWYAIGNMEQPHMDSRIGWLHNLGDQIGKPYNSSGLGGPSIKDKYVTALYFTFSSLTSVGFGNVSPNTNSEKIFSICVMLIGSLMYASIFGNVSAIIQRLYSGTARYHTQMLRVREFIRFHQIPNPLRQRLEEYFQHAWSYTNGIDMNAVLKGFPECLQADICLHLNRSLLQHCKPFRGATKGCLRALAMKFKTTHAPPGDTLVHAGDLLTALYFISRGSIEILRGDVVVAILGKNDIFGEPLNLYARPGKSNGDVRALTYCDLHKIHRDDLLEVLDMYPEFSDHFWSSLEITFNLRDTNMIPGSPGSTELEGGFSRQRKRKLSFRRRTDKDTEQPGEVSALGPGRAGAGPSSRGRPGGPWGESPSSGPSSPESSEDEGPGRSSSPLRLVPFSSPRPPGEPPGGEPLMEDCEKSSDTCNPLSGAFSGVSNIFSFWGDSRGRQYQELPRCPAPTPSLLNIPLSSPGRRPRGDVESRLDALQRQLNRLETRLSADMATVLQLLQRQMTLVPPAYSAVTTPGPGPTSTSPLLPVSPLPTLTLDSLSQVSQFMACEELPPGAPELPQEGPTRRLSLPGQLGALTSQPLHRHGSDPGS

42 NP_001757.1 CAA45056.1

>NP_001757.1

MGCLLFLLLWALLQAWGSAEVPQRLFPLRCLQISSFANSSWTRTDGLAWLGELQTHSWSNDSDTVRSLKPWSQGTFSDQQWETLQHIFRVYRSSFTRDVKEFAKMLRLSYPLELQVSAGCEVHPGNASNNFFHVAFQGKDILSFQGTSWEPTQEAPLWVNLAIQVLNQDKWTRETVQWLLNGTCPQFVSGLLESGKSELKKQVKPKAWLSRGPSPGPGRLLLVCHVSGFYPKPVWVKWMRGEQEQQGTQPGDILPNADETWYLRATLDVVAGEAAGLSCRVKHSSLEGQDIVLYWGGSYTSMGLIALAVLACLLFLLIVGFTSRFKRQTSYQGVL

>CAA45056.1

MGIRLLCRVAFCFLAVGLVDVKVTQSSRYLVKRTGEKVFLECVQDMDHENMFWYRQDPGLGLRLIYFSYDVKMKEKGDIPEGYSVSREKKERFSLILESASTNQTSMYLCASSSTGLPYGYTFGSGTRLTVVEDLNKVFPPEVAVFEPSEAEISHTQKATLVCLATGFFPDHVELSWWVNGKEVHSGVSTDPQPLKEQPALNDSRYCLSSRLRVSATFWQNPRNHFRCQVQFYGLSENDEWTQDRAKPVTQIVSAEAWGRADCGFTSVSYQQGVLSATILYEILLGKATLYAVLVSALVLMAMVKRKDF

43 NP_002960.2 NP_002077.1

>NP_002960.2

MSSPPPARSGFYRQEVTKTAWEVRAVYRDLQPVGSGAYGAVCSAVDGRTGAKVAIKKLYRPFQSELFAKRAYRELRLLKHMRHENVIGLLDVFTPDETLDDFTDFYLVMPFMGTDLGKLMKHEKLGEDRIQFLVYQMLKGLRYIHAAGIIHRDLKPGNLAVNEDCELKILDFGLARQADSEMTGYVVTRWYRAPEVILNWMRYTQTVDIWSVGCIMAEMITGKTLFKGSDHLDQLKEIMKVTGTPPAEFVQRLQSDEAKNYMKGLPELEKKDFASILTNASPLAVNLLEKMLVLDAEQRVTAGEALAHPYFESLHDTEDEPQVQKYDDSFDDVDRTLDEWKRVTYKEVLSFKPPRQLGARVSKETPL

>NP_002077.1

MEAIAKYDFKATADDELSFKRGDILKVLNEECDQNWYKAELNGKDGFIPKNYIEMKPHPWFFGKIPRAKAEEMLSKQRHDGAFLIRESESAPGDFSLSVKFGNDVQHFKVLRDGAGKYFLWVVKFNSLNELVDYHRSTSVSRNQQIFLRDIEQVPQQPTYVQALFDFDPQEDGELGFRRGDFIHVMDNSDPNWWKGACHGQTGMFPRNYVTPVNRNV

44 NP_002537.3 NP_000629.3

>NP_002537.3

MNNLLCCALVFLDISIKWTTQETFPPKYLHYDEETSHQLLCDKCPPGTYLKQHCTAKWKTVCAPCPDHYYTDSWHTSDECLYCSPVCKELQYVKQECNRTHNRVCECKEGRYLEIEFCLKHRSCPPGFGVVQAGTPERNTVCKRCPDGFFSNETSSKAPCRKHTNCSVFGLLLTQKGNATHDNICSGNSESTQKCGIDVTLCEEAFFRFAVPTKFTPNWLSVLVDNLPGTKVNAESVERIKRQHSSQEQTFQLLKLWKHQNKDQDIVKKIIQDIDLCENSVQRHIGHANLTFEQLRSLMESLPGKKVGAEDIEKTIKACKPSDQILKLLSLWRIKNGDQDTLKGLMHALKHSKTYHFPKTVTQSLKKTIRFLHSFTMYKLYQKLFLEMIGNQVQSVKISCL

>NP_000629.3

MAPLRPLLILALLAWVALADQESCKGRCTEGFNVDKKCQCDELCSYYQSCCTDYTAECKPQVTRGDVFTMPEDEYTVYDDGEEKNNATVHEQVGGPSLTSDLQAQSKGNPEQTPVLKPEEEAPAPEVGASKPEGIDSRPETLHPGRPQPPAEEELCSGKPFDAFTDLKNGSLFAFRGQYCYELDEKAVRPGYPKLIRDVWGIEGPIDAAFTRINCQGKTYLFKGSQYWRFEDGVLDPDYPRNISDGFDGIPDNVDAALALPAHSYSGRERVYFFKGKQYWEYQFQHQPSQEECEGSSLSAVFEHFAMMQRDSWEDIFELLFWGRTSAGTRQPQFISRDWHGVPGQVDAAMAGRIYISGMAPRPSLAKKQRFRHRNRKGYRSQRGHSRGRNQNSRRPSRATWLSLFSSEESNLGANNYDDYRMDWLVPATCEPIQSVFFFSGDKYYRVNLRTRRVDTVDPPYPRSIAQYWLGCPAPGHL

45 NP_001296.1 NP_003248.3

>NP_001296.1

MASMGLQVMGIALAVLGWLAVMLCCALPMWRVTAFIGSNIVTSQTIWEGLWMNCVVQSTGQMQCKVYDSLLALPQDLQAARALVIISIIVAALGVLLSVVGGKCTNCLEDESAKAKTMIVAGVVFLLAGLMVIVPVSWTAHNIIQDFYNPLVASGQKREMGASLYVGWAASGLLLLGGGLLCCNCPPRTDKPYSAKYSAARSAAASNYV

>NP_003248.3

MSARAAAAKSTAMEETAIWEQHTVTLHRAPGFGFGIAISGGRDNPHFQSGETSIVISDVLKGGPAEGQLQENDRVAMVNGVSMDNVEHAFAVQQLRKSGKNAKITIRRKKKVQIPVSRPDPEPVSDNEEDSYDEEIHDPRSGRSGVVNRRSEKIWPRDRSASRERSLSPRSDRRSVASSQPAKPTKVTLVKSRKNEEYGLRLASHIFVKEISQDSLAARDGNIQEGDVVLKINGTVTENMSLTDAKTLIERSKGKLKMVVQRDERATLLNVPDLSDSIHSANASERDDISEIQSLASDHSGRSHDRPPRRSRSRSPDQRSEPSDHSRHSPQQPSNGSLRSRDEERISKPGAVSTPVKHADDHTPKTVEEVTVERNEKQTPSLPEPKPVYAQVGQPDVDLPVSPSDGVLPNSTHEDGILRPSMKLVKFRKGDSVGLRLAGGNDVGIFVAGVLEDSPAAKEGLEEGDQILRVNNVDFTNIIREEAVLFLLDLPKGEEVTILAQKKKDVYRRIVESDVGDSFYIRTHFEYEKESPYGLSFNKGEVFRVVDTLYNGKLGSWLAIRIGKNHKEVERGIIPNKNRAEQLASVQYTLPKTAGGDRADFWRFRGLRSSKRNLRKSREDLSAQPVQTKFPAYERVVLREAGFLRPVTIFGPIADVAREKLAREEPDIYQIAKSEPRDAGTDQRSSGIIRLHTIKQIIDQDKHALLDVTPNAVDRLNYAQWYPIVVFLNPDSKQGVKTMRMRLCPESRKSARKLYERSHKLRKNNHHLFTTTINLNSMNDGWYGALKEAIQQQQNQLVWVSEGKADGATSDDLDLHDDRLSYLSAPGSEYSMYSTDSRHTSDYEDTDTEGGAYTDQELDETLNDEVGTPPESAITRSSEPVREDSSGMHHENQTYPPYSPQAQPQPIHRIDSPGFKPASQQKAEASSPVPYLSPETNPASSTSAVNHNVNLTNVRLEEPTPAPSTSYSPQADSLRTPSTEAAHIMLRDQEPSLSSHVDPTKVYRKDPYPEEMMRQNHVLKQPAVSHPGHRPDKEPNLTYEPQLPYVEKQASRDLEQPTYRYESSSYTDQFSRNYEHRLRYEDRVPMYEEQWSYYDDKQPYPSRPPFDNQHSQDLDSRQHPEESSERGYFPRFEEPAPLSYDSRPRYEQAPRASALRHEEQPAPGYDTHGRLRPEAQPHPSAGPKPAESKQYFEQYSRSYEQVPPQGFTSRAGHFEPLHGAAAVPPLIPSSQHKPEALPSNTKPLPPPPTQTEEEEDPAMKPQSVLTRVKMFENKRSASLETKKDVNDTGSFKPPEVASKPSGAPIIGPKPTSQNQFSEHDKTLYRIPEPQKPQLKPPEDIVRSNHYDPEEDEEYYRKQLSYFDRRSFENKPPAHIAASHLSEPAKPAHSQNQSNFSSYSSKGKPPEADGVDRSFGEKRYEPIQATPPPPPLPSQYAQPSQPVTSASLHIHSKGAHGEGNSVSLDFQNSLVSKPDPPPSQNKPATFRPPNREDTAQAAFYPQKSFPDKAPVNGTEQTQKTVTPAYNRFTPKPYTSSARPFERKFESPKFNHNLLPSETAHKPDLSSKTPTSPKTLVKSHSLAQPPEFDSGVETFSIHAEKPKYQINNISTVPKAIPVSPSAVEEDEDEDGHTVVATARGIFNSNGGVLSSIETGVSIIIPQGAIPEGVEQEIYFKVCRDNSILPPLDKEKGETLLSPLVMCGPHGLKFLKPVELRLPHCDPKTWQNKCLPGDPNYLVGANCVSVLIDHF

46 NP_061120.2 NP_114034.1

>NP_061120.2

MSARTPLPTVNERDTENHTSVDGYTEPHIQPTKSSSRQNIPRCRNSITSATDEQPHIGNYRLQKTIGKGNFAKVKLARHVLTGREVAVKIIDKTQLNPTSLQKLFREVRIMKILNHPNIGEVFDYLVAHGRMKEKEARAKFRQIVSAVQYCHQKYIVHRDLKAENLLLDGDMNIKIADFGFSNEFTVGNKLDTFCGSPPYAAPELFQGKKYDGPEVDVWSLGVILYTLVSGSLPFDGQNLKELRERVLRGKYRIPFYMSTDCENLLKKLLVLNPIKRGSLEQIMKDRWMNVGHEEEELKPYTEPDPDFNDTKRIDIMVTMGFARDEINDALINQKYDEVMATYILLGRKPPEFEGGESLSSGNLCQRSRPSSDLNNSTLQSPAHLKVQRSISANQKQRRFSDHAGPSIPPAVSYTKRPQANSVESEQKEEWDKDVARKLGSTTVGSKSEMTASPLVGPERKKSSTIPSNNVYSGGSMARRNTYVCERTTDRYVALQNGKDSSLTEMSVSSISSAGSSVASAVPSARPRHQKSMSTSGHPIKVTLPTIKDGSEAYRPGTTQRVPAASPSAHSISTATPDRTRFPRGSSSRSTFHGEQLRERRSVAYNGPPASPSHETGAFAHARRGTSTGIISKITSKFVRRSTSGEPKERDKEEGKDSKPRSLRFTWSMKTTSSMDPNDMMREIRKVLDANNCDYEQKERFLLFCVHGDARQDSLVQWEMEVCKLPRLSLNGVRFKRISGTSIAFKNIASKIANELML

>NP_114034.1

MADERKDEAKAPHWTSAPLTEASAHSHPPEIKDQGGAGEGLVRSANGFPYREDEEGAFGEHGSQGTYSNTKENGINGELTSADRETAEEVSARIVQVVTAEAVAVLKGEQEKEAQHKDQTAALPLAAEETANLPPSPPPSPASEQTVTVEEDLLTASKMEFHDQQELTPSTAEPSDQKEKESEKQSKPGEDLKHAALVSQPETTKTYPDKKDMQGTEEEKAPLALFGHTLVASLEDMKQKTEPSLVVPGIDLPKEPPTPKEQKDWFIEMPTEAKKDEWGLVAPISPGPLTPMREKDVFDDIPKWEGKQFDSPMPSPFQGGSFTLPLDVMKNEIVTETSPFAPAFLQPDDKKSLQQTSGPATAKDSFKIEEPHEAKPDKMAEAPPSEAMTLPKDAHIPVVEEHVMGKVLEEEKEAINQETVQQRDTFTPSGQEPILTEKETELKLEEKTTISDKEAVPKESKPPKPADEEIGIIQTSTEHTFSEQKDQEPTTDMLKQDSFPVSLEQAVTDSAMTSKTLEKAMTEPSALIEKSSIQELFEMRVDDKDKIEGVGAATSAELDMPFYEDKSGMSKYFETSALKEEATKSIEPGSDYYELSDTRESVHESIDTMSPMHKNGDKEFQTGKESQPSPPAQEAGYSTLAQSYPSDLPEEPSSPQERMFTIDPKVYGEKRDLHSKNKDDLTLSRSLGLGGRSAIEQRSMSINLPMSCLDSIALGFNFGRGHDLSPLASDILTNTSGSMDEGDDYLPATTPALEKAPCFPVESKEEEQIEKVKATGEESTQAEISCESPFLAKDFYKNGTVMAPDLPEMLDLAGTRSRLASVSADAEVARRKSVPSETVVEDSRTGLPPVTDENHVIVKTDSQLEDLGYCVFNKYTVPLPSPVQDSENLSGESGTFYEGTDDKVRRDLATDLSLIEVKLAAAGRVKDEFSVDKEASAHISGDKSGLSKEFDQEKKANDRLDTVLEKSEEHADSKEHAKKTEEAGDEIETFGLGVTYEQALAKDLSIPTDASSEKAEKGLSSVPEIAEVEPSKKVEQGLDFAVQGQLDVKISDFGQMASGLNIDDRRATELKLEATQDMTPSSKAPQEADAFMGVESGHMKEGTKVSETEVKQKVAKPDLVHQEAVDKEESYESSGEHESLTMESLKADEGKKETSPESSLIQDEIAVKLSVEIPCPPAVSEADLATDERADVQMEFIQGPKEESKETPDISITPSDVAEPLHETIVSEPAEIQSEEEEIEAQGEYDKLLFRSDTLQITDLGVSGAREEFVETCPSEHKGVIESVVTIEDDFITVVQTTTDEGESGSHSVRFAALEQPEVERRPSPHDEEEFEVEEAAEAQAEPKDGSPEAPASPEREEVALSEYKTETYDDYKDETTIDDSIMDADSLWVDTQDDDRSIMTEQLETIPKEEKAEKEARRSSLEKHRKEKPFKTGRGRISTPERKVAKKEPSTVSRDEVRRKKAVYKKAELAKKTEVQAHSPSRKFILKPAIKYTRPTHLSCVKRKTTAAGGESALAPSVFKQAKDKVSDGVTKSPEKRSSLPRPSSILPPRRGVSGDRDENSFSLNSSISSSARRTTRSEPIRRAGKSGTSTPTTPGSTAITPGTPPSYSSRTPGTPGTPSYPRTPHTPGTPKSAILVPSEKKVAIIRTPPKSPATPKQLRLINQPLPDLKNVKSKIGSTDNIKYQPKGGQVRILNKKIDFSKVHSRSGSKDNIKHSAGGGNVQIVTKKIDLSHVTSKCGSLKNIGHRPGGGRVKIESVKLDFKEKAQAKVGSLDNAHHVPGGGNVKIDSQKLNFREHAKARVDHGAEIITQSPGRSSVASPRRLSNVSSSGSINLLESPQLATLAEDVTAALAKQGL

47 NP_058633.2 NP_000537.3

>NP_058633.2

MAPVHGDDSLSDSGSFVSSRARREKKSKKGRQEALERLKKAKAGEKYKYEVEDFTGVYEEVDEEQYSKLVQARQDDDWIVDDDGIGYVEDGREIFDDDLEDDALDADEKGKDGKARNKDKRNVKKLAVTKPNNIKSMFIACAGKKTADKAVDLSKDGLLGDILQDLNTETPQITPPPVMILKKKRSIGASPNPFSVHTATAVPSGKIASPVSRKEPPLTPVPLKRAEFAGDDVQVESTEEEQESGAMEFEDGDFDEPMEVEEVDLEPMAAKAWDKESEPAEEVKQEADSGKGTVSYLGSFLPDVSCWDIDQEGDSSFSVQEVQVDSSHLPLVKGADEEQVFHFYWLDAYEDQYNQPGVVFLFGKVWIESAETHVSCCVMVKNIERTLYFLPREMKIDLNTGKETGTPISMKDVYEEFDEKIATKYKIMKFKSKPVEKNYAFEIPDVPEKSEYLEVKYSAEMPQLPQDLKGETFSHVFGTNTSSLELFLMNRKIKGPCWLEVKSPQLLNQPVSWCKVEAMALKPDLVNVIKDVSPPPLVVMAFSMKTMQNAKNHQNEIIAMAALVHHSFALDKAAPKPPFQSHFCVVSKPKDCIFPYAFKEVIEKKNVKVEVAATERTLLGFFLAKVHKIDPDIIVGHNIYGFELEVLLQRINVCKAPHWSKIGRLKRSNMPKLGGRSGFGERNATCGRMICDVEISAKELIRCKSYHLSELVQQILKTERVVIPMENIQNMYSESSQLLYLLEHTWKDAKFILQIMCELNVLPLALQITNIAGNIMSRTLMGGRSERNEFLLLHAFYENNYIVPDKQIFRKPQQKLGDEDEEIDGDTNKYKKGRKKAAYAGGLVLDPKVGFYDKFILLLDFNSLYPSIIQEFNICFTTVQRVASEAQKVTEDGEQEQIPELPDPSLEMGILPREIRKLVERRKQVKQLMKQQDLNPDLILQYDIRQKALKLTANSMYGCLGFSYSRFYAKPLAALVTYKGREILMHTKEMVQKMNLEVIYGDTDSIMINTNSTNLEEVFKLGNKVKSEVNKLYKLLEIDIDGVFKSLLLLKKKKYAALVVEPTSDGNYVTKQELKGLDIVRRDWCDLAKDTGNFVIGQILSDQSRDTIVENIQKRLIEIGENVLNGSVPVSQFEINKALTKDPQDYPDKKSLPHVHVALWINSQGGRKVKAGDTVSYVICQDGSNLTASQRAYAPEQLQKQDNLTIDTQYYLAQQIHPVVARICEPIDGIDAVLIATWLGLDPTQFRVHHYHKDEENDALLGGPAQLTDEEKYRDCERFKCPCPTCGTENIYDNVFDGSGTDMEPSLYRCSNIDCKASPLTFTVQLSNKLIMDIRRFIKKYYDGWLICEEPTCRNRTRHLPLQFSRTGPLCPACMKATLQPEYSDKSLYTQLCFYRYIFDAECALEKLTTDHEKDKLKKQFFTPKVLQDYRKLKNTAEQFLSRSGYSEVNLSKLFAGCAVKS

>NP_000537.3

MEEPQSDPSVEPPLSQETFSDLWKLLPENNVLSPLPSQAMDDLMLSPDDIEQWFTEDPGPDEAPRMPEAAPPVAPAPAAPTPAAPAPAPSWPLSSSVPSQKTYQGSYGFRLGFLHSGTAKSVTCTYSPALNKMFCQLAKTCPVQLWVDSTPPPGTRVRAMAIYKQSQHMTEVVRRCPHHERCSDSDGLAPPQHLIRVEGNLRVEYLDDRNTFRHSVVVPYEPPEVGSDCTTIHYNYMCNSSCMGGMNRRPILTIITLEDSSGNLLGRNSFEVRVCACPGRDRRTEEENLRKKGEPHHELPPGSTKRALPNNTSSSPQPKKKPLDGEYFTLQIRGRERFEMFRELNEALELKDAQAGKEPGGSRAHSSHLKSKKGQSTSRHKKLMFKTEGPDSD

48 NP_001246.2 NP_001202.4

>NP_001246.2

MAQFAFESDLHSLLQLDAPIPNAPPARWQRKAKEAAGPAPSPMRAANRSHSAGRTPGRTPGKSSSKVQTTPSKPGGDRYIPHRSAAQMEVASFLLSKENQPENSQTPTKKEHQKAWALNLNGFDVEEAKILRLSGKPQNAPEGYQNRLKVLYSQKATPGSSRKTCRYIPSLPDRILDAPEIRNDYYLNLVDWSSGNVLAVALDNSVYLWSASSGDILQLLQMEQPGEYISSVAWIKEGNYLAVGTSSAEVQLWDVQQQKRLRNMTSHSARVGSLSWNSYILSSGSRSGHIHHHDVRVAEHHVATLSGHSQEVCGLRWAPDGRHLASGGNDNLVNVWPSAPGEGGWVPLQTFTQHQGAVKAVAWCPWQSNVLATGGGTSDRHIRIWNVCSGACLSAVDAHSQVCSILWSPHYKELISGHGFAQNQLVIWKYPTMAKVAELKGHTSRVLSLTMSPDGATVASAAADETLRLWRCFELDPARRREREKASAAKSSLIHQGIR

>NP_001202.4

MAAVKKEGGALSEAMSLEGDEWELSKENVQPLRQGRIMSTLQGALAQESACNNTLQQQKRAFEYEIRFYTGNDPLDVWDRYISWTEQNYPQGGKESNMSTLLERAVEALQGEKRYYSDPRFLNLWLKLGRLCNEPLDMYSYLHNQGIGVSLAQFYISWAEEYEARENFRKADAIFQEGIQQKAEPLERLQSQHRQFQARVSRQTLLALEKEEEEEVFESSVPQRSTLAELKSKGKKTARAPIIRVGGALKAPSQNRGLQNPFPQQMQNNSRITVFDENADEASTAELSKPTVQPWIAPPMPRAKENELQAGPWNTGRSLEHRPRGNTASLIAVPAVLPSFTPYVEETAQQPVMTPCKIEPSINHILSTRKPGKEEGDPLQRVQSHQQASEEKKEKMMYCKEKIYAGVGEFSFEEIRAEVFRKKLKEQREAELLTSAEKRAEMQKQIEEMEKKLKEIQTTQQERTGDQQEETMPTKETTKLQIASESQKIPGMTLSSSVCQVNCCARETSLAENIWQEQPHSKGPSVPFSIFDEFLLSEKKNKSPPADPPRVLAQRRPLAVLKTSESITSNEDVSPDVCDEFTGIEPLSEDAIITGFRNVTICPNPEDTCDFARAARFVSTPFHEIMSLKDLPSDPERLLPEEDLDVKTSEDQQTACGTIYSQTLSIKKLSPIIEDSREATHSSGFSGSSASVASTSSIKCLQIPEKLELTNETSENPTQSPWCSQYRRQLLKSLPELSASAELCIEDRPMPKLEIEKEIELGNEDYCIKREYLICEDYKLFWVAPRNSAELTVIKVSSQPVPWDFYINLKLKERLNEDFDHFCSCYQYQDGCIVWHQYINCFTLQDLLQHSEYITHEITVLIIYNLLTIVEMLHKAEIVHGDLSPRCLILRNRIHDPYDCNKNNQALKIVDFSYSVDLRVQLDVFTLSGFRTVQILEGQKILANCSSPYQVDLFGIADLAHLLLFKEHLQVFWDGSFWKLSQNISELKDGELWNKFFVRILNANDEATVSVLGELAAEMNGVFDTTFQSHLNKALWKVGKLTSPGALLFQ

49 NP_001900.1 NP_954642.1

>NP_001900.1

MQPSSLLPLALCLLAAPASALVRIPLHKFTSIRRTMSEVGGSVEDLIAKGPVSKYSQAVPAVTEGPIPEVLKNYMDAQYYGEIGIGTPPQCFTVVFDTGSSNLWVPSIHCKLLDIACWIHHKYNSDKSSTYVKNGTSFDIHYGSGSLSGYLSQDTVSVPCQSASSASALGGVKVERQVFGEATKQPGITFIAAKFDGILGMAYPRISVNNVLPVFDNLMQQKLVDQNIFSFYLSRDPDAQPGGELMLGGTDSKYYKGSLSYLNVTRKAYWQVHLDQVEVASGLTLCKEGCEAIVDTGTSLMVGPVDEVRELQKAIGAVPLIQGEYMIPCEKVSTLPAITLKLGGKGYKLSPEDYTLKVSQAGKTLCLSGFMGMDIPPPSGPLWILGDVFIGRYYTVFDRDNNRVGFAEAARL

>NP_954642.1

MRALTLLALLALAALCIAGQAGAKPSGAESSKGAAFVSKQEGSEVVKRPRRYLYQWLGAPVPYPDPLEPRREVCELNPDCDELADHIGFQEAYRRFYGPV

50 NP_004916.1 NP_002654.2

>NP_004916.1

MGRQKELVSRCGEMLHIRYRLLRQALAECLGTLILVMFGCGSVAQVVLSRGTHGGFLTINLAFGFAVTLGILIAGQVSGAHLNPAVTFAMCFLAREPWIKLPIYTLAQTLGAFLGAGIVFGLYYDAIWHFADNQLFVSGPNGTAGIFATYPSGHLDMINGFFDQFIGTASLIVCVLAIVDPYNNPVPRGLEAFTVGLVVLVIGTSMGFNSGYAVNPARDFGPRLFTALAGWGSAVFTTGQHWWWVPIVSPLLGSIAGVFVYQLMIGCHLEQPPPSNEEENVKLAHVKHKEQI

>NP_002654.2

MTATPESLFPTGDELDSSQLQMESDEVDTLKEGEDPADRMHPFLAIYELQSLKVHPLVFAPGVPVTAQVVGTERYTSGSKVGTCTLYSVRLTHGDFSWTTKKKYRHFQELHRDLLRHKVLMSLLPLARFAVAYSPARDAGNREMPSLPRAGPEGSTRHAASKQKYLENYLNCLLTMSFYRNYHAMTEFLEVSQLSFIPDLGRKGLEGMIRKRSGGHRVPGLTCCGRDQVCYRWSKRWLVVKDSFLLYMCLETGAISFVQLFDPGFEVQVGKRSTEARHGVRIDTSHRSLILKCSSYRQARWWAQEITELAQGPGRDFLQLHRHDSYAPPRPGTLARWFVNGAGYFAAVADAILRAQEEIFITDWWLSPEVYLKRPAHSDDWRLDIMLKRKAEEGVRVSILLFKEVELALGINSGYSKRALMLLHPNIKVMRHPDQVTLWAHHEKLLVVDQVVAFLGGLDLAYGRWDDLHYRLTDLGDSSESAASQPPTPRPDSPATPDLSHNQFFWLGKDYSNLITKDWVQLDRPFEDFIDRETTPRMPWRDVGVVVHGLPARDLARHFIQRWNFTKTTKAKYKTPIYPYLLPKSTSTANQLPFTLPGGQCTTVQVLRSVDRWSAGTLENSILNAYLHTIRESQHFLYIENQFFISCSDGRTVLNKVGDEIVDRILKAHKQGWCYRVYVLLPLLPGFEGDISTGGGNSIQAILHFTYRTLCRGEYSILHRLKAAMGTAWRDYISICGLRTHGELGGHPVSELIYIHSKVLIADDRTVIIGSANINDRSLLGKRDSELAVLIEDTETEPSLMNGAEYQAGRFALSLRKHCFGVILGANTRPDLDLRDPICDDFFQLWQDMAESNANIYEQIFRCLPSNATRSLRTLREYVAVEPLATVSPPLARSELTQVQGHLVHFPLKFLEDESLLPPLGSKEGMIPLEVWT

51 NP_003450.2 NP_001275.1

>NP_003450.2

MEPSPAAGGLETTRLVSPRDRGGAGGSLRLKSLFTEPSEPLPEESKPVEMPFHHCHRDPLPPPGLTPERLHARRQLYAACAVCFVFMAGEVVGGYLAHSLAIMTDAAHLLADVGSMMGSLFSLWLSTRPATRTMTFGWHRSETLGALASVVSLWMVTGILLYLAFVRLLHSDYHIEGGAMLLTASIAVCANLLMAFVLHQAGPPHSHGSRGAEYAPLEEGPEEPLPLGNTSVRAAFVHVLGDLLQSFGVLAASILIYFKPQYKAADPISTFLFSICALGSTAPTLRDVLRILMEGTPRNVGFEPVRDTLLSVPGVRATHELHLWALTLTYHVASAHLAIDSTADPEAVLAEASSRLYSRFGFSSCTLQVEQYQPEMAQCLRCQEPPQA

>NP_001275.1

MIKAILIFNNHGKPRLSKFYQPYSEDTQQQIIRETFHLVSKRDENVCNFLEGGLLIGGSDNKLIYRHYATLYFVFCVDSSESELGILDLIQVFVETLDKCFENVCELDLIFHVDKVHNILAEMVMGGMVLETNMNEIVTQIDAQNKLEKSEAGLAGAPARAVSAVKNMNLPEIPRNINIGDISIKVPNLPSFK

52 NP_892017.2 NP_002190.1

>NP_892017.2

MAAAVAVAAASRRQSCYLCDLPRMPWAMIWDFTEPVCRGCVNYEGADRVEFVIETARQLKRAHGCFPEGRSPPGAAASAAAKPPPLSAKDILLQQQQQLGHGGPEAAPRAPQALERYPLAAAAERPPRLGSDFGSSRPAASLAQPPTPQPPPVNGILVPNGFSKLEEPPELNRQSPNPRRGHAVPPTLVPLMNGSATPLPTALGLGGRAAASLAAVSGTAAASLGSAQPTDLGAHKRPASVSSSAAVEHEQREAAAKEKQPPPPAHRGPADSLSTAAGAAELSAEGAGKSRGSGEQDWVNRPKTVRDTLLALHQHGHSGPFESKFKKEPALTAGRLLGFEANGANGSKAVARTARKRKPSPEPEGEVGPPKINGEAQPWLSTSTEGLKIPMTPTSSFVSPPPPTASPHSNRTTPPEAAQNGQSPMAALILVADNAGGSHASKDANQVHSTTRRNSNSPPSPSSMNQRRLGPREVGGQGAGNTGGLEPVHPASLPDSSLATSAPLCCTLCHERLEDTHFVQCPSVPSHKFCFPCSRQSIKQQGASGEVYCPSGEKCPLVGSNVPWAFMQGEIATILAGDVKVKKERDS

>NP_002190.1

MPVERMRMRPWLEEQINSNTIPGLKWLNKEKKIFQIPWMHAARHGWDVEKDAPLFRNRAIHTGKHQPGVDKPDPKTWKANFRCAMNSLPDIEEVKDKSIKKGNNAFRVYRMLPLSERPSKKGKKPKTEKEDKVKHIKQEPVESSLGLSNGVSDLSPEYAVLTSTIKNEVDSTVNIIVVGQSHLDSNIENQEIVTNPPDICQVVEVTTESDEQPVSMSELYPLQISPVSSYAESETTDSVPSDEESAEGRPHWRKRNIEGKQYLSNMGTRGSYLLPGMASFVTSNKPDLQVTIKEESNPVPYNSSWPPFQDLPLSSSMTPASSSSRPDRETRASVIKKTSDITQARVKSC

53 NP_001092.1 NP_060649.3

>NP_001092.1

MDDDIAALVVDNGSGMCKAGFAGDDAPRAVFPSIVGRPRHQGVMVGMGQKDSYVGDEAQSKRGILTLKYPIEHGIVTNWDDMEKIWHHTFYNELRVAPEEHPVLLTEAPLNPKANREKMTQIMFETFNTPAMYVAIQAVLSLYASGRTTGIVMDSGDGVTHTVPIYEGYALPHAILRLDLAGRDLTDYLMKILTERGYSFTTTAEREIVRDIKEKLCYVALDFEQEMATAASSSSLEKSYELPDGQVITIGNERFRCPEALFQPSFLGMESCGIHETTFNSIMKCDVDIRKDLYANTVLSGGTTMYPGIADRMQKEITALAPSTMKIKIIAPPERKYSVWIGGSILASLSTFQQMWISKQEYDESGPSIVHRKCF

>NP_060649.3

MDSLEEPQKKVFKARKTMRVSDRQQLEAVYKVKEELLKTDVKLLNGNHENGDLDPTSPLENMDYIKDKEEVNGIEEICFDPEGSKAEWKETPCILSVNVKNKQDDDLNCEPLSPHNITPEPVSKLPAEPVSGDPAPGDLDAGDPASGVLASGDSTSGDPTSSEPSSSDAASGDATSGDAPSGDVSPGDATSGDATADDLSSGDPTSSDPIPGEPVPVEPISGDCAADDIASSEITSVDLASGAPASTDPASDDLASGDLSSSELASDDLATGELASDELTSESTFDRTFEPKSVPVCEPVPEIDNIEPSSNKDDDFLEKNGADEKLEQIQSKDSLDEKNKADNNIDANEETLETDDTTICSDRPPENEKKVEEDIITELALGEDAISSSMEIDQGEKNEDETSADLVETINENVIEDNKSENILENTDSMETDEIIPILEKLAPSEDELTCFSKTSLLPIDETNPDLEEKMESSFGSPSKQESSESLPKEAFLVLSDEEDISGEKDESEVISQNETCSPAEVESNEKDNKPEEEEQVIHEDDERPSEKNEFSRRKRSKSEDMDNVQSKRRRYMEEEYEAEFQVKITAKGDINQKLQKVIQWLLEEKLCALQCAVFDKTLAELKTRVEKIECNKRHKTVLTELQAKIARLTKRFEAAKEDLKKRHEHPPNPPVSPGKTVNDVNSNNNMSYRNAGTVRQMLESKRNVSESAPPSFQTPVNTVSSTNLVTPPAVVSSQPKLQTPVTSGSLTATSVLPAPNTATVVATTQVPSGNPQPTISLQPLPVILHVPVAVSSQPQLLQSHPGTLVTNQPSGNVEFISVQSPPTVSGLTKNPVSLPSLPNPTKPNNVPSVPSPSIQRNPTASAAPLGTTLAVQAVPTAHSIVQATRTSLPTVGPSGLYSPSTNRGPIQMKIPISAFSTSSAAEQNSNTTPRIENQTNKTIDASVSKKAADSTSQCGKATGSDSSGVIDLTMDDEESGASQDPKKLNHTPVSTMSSSQPVSRPLQPIQPAPPLQPSGVPTSGPSQTTIHLLPTAPTTVNVTHRPVTQVTTRLPVPRAPANHQVVYTTLPAPPAQAPLRGTVMQAPAVRQVNPQNSVTVRVPQTTTYVVNNGLTLGSTGPQLTVHHRPPQVHTEPPRPVHPAPLPEAPQPQRLPPEAASTSLPQKPHLKLARVQSQNGIVLSWSVLEVDRSCATVDSYHLYAYHEEPSATVPSQWKKIGEVKALPLPMACTLTQFVSGSKYYFAVRAKDIYGRFGPFCDPQSTDVISSTQSS

54 NP_003617.1 NP_002831.2

>NP_003617.1

MMCEVMPTISEAEGPPGGGGGHGSGSPSQPDADSHFEQLMVSMLEERDRLLDTLRETQETLALTQGKLHEVGHERDSLQRQLNTALPQEFAALTKELNVCREQLLEREEEIAELKAERNNTRLLLEHLECLVSRHERSLRMTVVKRQAQSPAGVSSEVEVLKALKSLFEHHKALDEKVRERLRVALERCSLLEEELGATHKELMILKEQNNQKKTLTDGVLDINHEQENTPSTSGKRSSDGSLSHEEDLAKVIELQEIISKQSREQSQMKERLASLSSHVTELEEDLDTARKDLIKSEEMNTKLQRDVREAMAQKEDMEERITTLEKRYLAAQREATSVHDLNDKLENEIANKDSMHRQTEDKNRQLQERLELAEQKLQQTLRKAETLPEVEAELAQRVAALSKAEERHGNIEERLRQMEAQLEEKNQELQRARQREKMNEEHNKRLSDTVDKLLSESNERLQLHLKERMAALEDKNSLLREVESAKKQLEETQHDKDQLVLNIEALRAELDHMRLRGASLHHGRPHLGSVPDFRFPMADGHTDSYSTSAVLRRPQKGRLAALRDEPSKVQTLNEQDWERAQQASVLANVAQAFESDADVSDGEDDRDTLLSSVDLLSPSGQADAHTLAMMLQEQLDAINKEIRLIQEEKENTEQRAEEIESRVGSGSLDNLGRFRSMSSIPPYPASSLASSSPPGSGRSTPRRIPHSPAREVDRLGVMTLLPPSREEVRDDKTTIKCETSPPSSPRALRLDRLHKGALHTVSHEDIRDIRNSTGSQDGPVSNPSSSNSSQDSLHKAPKKKGIKSSIGRLFGKKEKGRPGQTGKEALGQAGVSETDNSSQDALGLSKLGGQAEKNRKLQKKHELLEEARRQGLPFAQWDGPTVVVWLELWVGMPAWYVAACRANVKSGAIMSALSDTEIQREIGISNPLHRLKLRLAIQEIMSLTSPSAPPTSRTTLAYGDMNHEWIGNEWLPSLGLPQYRSYFMECLVDARMLDHLTKKDLRGQLKMVDSFHRNSFQCGIMCLRRLNYDRKELERKREESQSEIKDVLVWSNDRVIRWILSIGLKEYANNLIESGVHGALLALDETFDFSALALLLQIPTQNTQARAVLEREFNNLLVMGTDRRFDEDDDKSFRRAPSWRKKFRPKDIRGLAAGSAETLPANFRVTSSMSSPSMQPKKMQMDGNVSGTQRLDSATVRTYSC

>NP_002831.2

MAPEPAPGRTMVPLVPALVMLGLVAGAHGDSKPVFIKVPEDQTGLSGGVASFVCQATGEPKPRITWMKKGKKVSSQRFEVIEFDDGAGSVLRIQPLRVQRDEAIYECTATNSLGEINTSAKLSVLEEEQLPPGFPSIDMGPQLKVVEKARTATMLCAAGGNPDPEISWFKDFLPVDPATSNGRIKQLRSGALQIESSEESDQGKYECVATNSAGTRYSAPANLYVRVRRVAPRFSIPPSSQEVMPGGSVNLTCVAVGAPMPYVKWMMGAEELTKEDEMPVGRNVLELSNVVRSANYTCVAISSLGMIEATAQVTVKALPKPPIDLVVTETTATSVTLTWDSGNSEPVTYYGIQYRAAGTEGPFQEVDGVATTRYSIGGLSPFSEYAFRVLAVNSIGRGPPSEAVRARTGEQAPSSPPRRVQARMLSASTMLVQWEPPEEPNGLVRGYRVYYTPDSRRPPNAWHKHNTDAGLLTTVGSLLPGITYSLRVLAFTAVGDGPPSPTIQVKTQQGVPAQPADFQAEVESDTRIQLSWLLPPQERIIMYELVYWAAEDEDQQHKVTFDPTSSYTLEDLKPDTLYRFQLAARSDMGVGVFTPTIEARTAQSTPSAPPQKVMCVSMGSTTVRVSWVPPPADSRNGVITQYSVAYEAVDGEDRGRHVVDGISREHSSWDLVGLEKWTEYRVWVRAHTDVGPGPESSPVLVRTDEDVPSGPPRKVEVEPLNSTAVHVYWKLPVPSKQHGQIRGYQVTYVRLENGEPRGLPIIQDVMLAEAQWRPEESEDYETTISGLTPETTYSVTVAAYTTKGDGARSKPKIVTTTGAVPGRPTMMISTTAMNTALLQWHPPKELPGELLGYRLQYCRADEARPNTIDFGKDDQHFTVTGLHKGTTYIFRLAAKNRAGLGEEFEKEIRTPEDLPSGFPQNLHVTGLTTSTTELAWDPPVLAERNGRIISYTVVFRDINSQQELQNITTDTRFTLTGLKPDTTYDIKVRAWTSKGSGPLSPSIQSRTMPVEQVFAKNFRVAAAMKTSVLLSWEVPDSYKSAVPFKILYNGQSVEVDGHSMRKLIADLQPNTEYSFVLMNRGSSAGGLQHLVSIRTAPDLLPHKPLPASAYIEDGRFDLSMPHVQDPSLVRWFYIVVVPIDRVGGSMLTPRWSTPEELELDELLEAIEQGGEEQRRRRRQAERLKPYVAAQLDVLPETFTLGDKKNYRGFYNRPLSPDLSYQCFVLASLKEPMDQKRYASSPYSDEIVVQVTPAQQQEEPEMLWVTGPVLAVILIILIVIAILLFKRKRTHSPSSKDEQSIGLKDSLLAHSSDPVEMRRLNYQTPGMRDHPPIPITDLADNIERLKANDGLKFSQEYESIDPGQQFTWENSNLEVNKPKNRYANVIAYDHSRVILTSIDGVPGSDYINANYIDGYRKQNAYIATQGPLPETMGDFWRMVWEQRTATVVMMTRLEEKSRVKCDQYWPARGTETCGLIQVTLLDTVELATYTVRTFALHKSGSSEKRELRQFQFMAWPDHGVPEYPTPILAFLRRVKACNPLDAGPMVVHCSAGVGRTGCFIVIDAMLERMKHEKTVDIYGHVTCMRSQRNYMVQTEDQYVFIHEALLEAATCGHTEVPARNLYAHIQKLGQVPPGESVTAMELEFKLLASSKAHTSRFISANLPCNKFKNRLVNIMPYELTRVCLQPIRGVEGSDYINASFLDGYRQQKAYIATQGPLAESTEDFWRMLWEHNSTIIVMLTKLREMGREKCHQYWPAERSARYQYFVVDPMAEYNMPQYILREFKVTDARDGQSRTIRQFQFTDWPEQGVPKTGEGFIDFIGQVHKTKEQFGQDGPITVHCSAGVGRTGVFITLSIVLERMRYEGVVDMFQTVKTLRTQRPAMVQTEDQYQLCYRAALEYLGSFDHYAT

55 NP_000336.1 NP_005900.1

>NP_000336.1

MDVFMDVMKGLSKAKEGVVAAAEKTKQGVAEAAGKTKEGVLYVGSKTKEGVVHGVATVAEKTKEQVTNVGGAVVTGVTAVAQKTVEGAGSIAAATGFVKKDQLGKNEEGAPQEGILEDMPVDPDNEAYEMPSEEGYQDYEPEA

>NP_005900.1

MATVVVEATEPEPSGSIANPAASTSPSLSHRFLDSKFYLLVVVGEIVTEEHLRRAIGNIELGIRSWDTNLIECNLDQELKLFVSRHSARFSPEVPGQKILHHRSDVLETVVLINPSDEAVSTEVRLMITDAARHKLLVLTGQCFENTGELILQSGSFSFQNFIEIFTDQEIGELLSTTHPANKASLTLFCPEEGDWKNSNLDRHNLQDFINIKLNSASILPEMEGLSEFTEYLSESVEVPSPFDILEPPTSGGFLKLSKPCCYIFPGGRGDSALFAVNGFNMLINGGSERKSCFWKLIRHLDRVDSILLTHIGDDNLPGINSMLQRKIAELEEEQSQGSTTNSDWMKNLISPDLGVVFLNVPENLKNPEPNIKMKRSIEEACFTLQYLNKLSMKPEPLFRSVGNTIDPVILFQKMGVGKLEMYVLNPVKSSKEMQYFMQQWTGTNKDKAEFILPNGQEVDLPISYLTSVSSLIVWHPANPAEKIIRVLFPGNSTQYNILEGLEKLKHLDFLKQPLATQKDLTGQVPTPVVKQTKLKQRADSRESLKPAAKPLPSKSVRKESKEETPEVTKVNHVEKPPKVESKEKVMVKKDKPVKTETKPSVTEKEVPSKEEPSPVKAEVAEKQATDVKPKAAKEKTVKKETKVKPEDKKEEKEKPKKEVAKKEDKTPIKKEEKPKKEEVKKEVKKEIKKEEKKEPKKEVKKETPPKEVKKEVKKEEKKEVKKEEKEPKKEIKKLPKDAKKSSTPLSEAKKPAALKPKVPKKEESVKKDSVAAGKPKEKGKIKVIKKEGKAAEAVAAAVGTGATTAAVMAAAGIAAIGPAKELEAERSLMSSPEDLTKDFEELKAEEVDVTKDIKPQLELIEDEEKLKETEPVEAYVIQKEREVTKGPAESPDEGITTTEGEGECEQTPEELEPVEKQGVDDIEKFEDEGAGFEESSETGDYEEKAETEEAEEPEEDGEEHVCVSASKHSPTEDEESAKAEADAYIREKRESVASGDDRAEEDMDEAIEKGEAEQSEEEADEEDKAEDAREEEYEPEKMEAEDYVMAVVDKAAEAGGAEEQYGFLTTPTKQLGAQSPGREPASSIHDETLPGGSESEATASDEENREDQPEEFTATSGYTQSTIEISSEPTPMDEMSTPRDVMSDETNNEETESPSQEFVNITKYESSLYSQEYSKPADVTPLNGFSEGSKTDATDGKDYNASASTISPPSSMEEDKFSRSALRDAYCSEVKASTTLDIKDSISAVSSEKVSPSKSPSLSPSPPSPLEKTPLGERSVNFSLTPNEIKVSAEAEVAPVSPEVTQEVVEEHCASPEDKTLEVVSPSQSVTGSAGHTPYYQSPTDEKSSHLPTEVIEKPPAVPVSFEFSDAKDENERASVSPMDEPVPDSESPIEKVLSPLRSPPLIGSESAYESFLSADDKASGRGAESPFEEKSGKQGSPDQVSPVSEMTSTSLYQDKQEGKSTDFAPIKEDFGQEKKTDDVEAMSSQPALALDERKLGDVSPTQIDVSQFGSFKEDTKMSISEGTVSDKSATPVDEGVAEDTYSHMEGVASVSTASVATSSFPEPTTDDVSPSLHAEVGSPHSTEVDDSLSVSVVQTPTTFQETEMSPSKEECPRPMSISPPDFSPKTAKSRTPVQDHRSEQSSMSIEFGQESPEQSLAMDFSRQSPDHPTVGAGVLHITENGPTEVDYSPSDMQDSSLSHKIPPMEEPSYTQDNDLSELISVSQVEASPSTSSAHTPSQIASPLQEDTLSDVAPPRDMSLYASLTSEKVQSLEGEKLSPKSDISPLTPRESSPLYSPTFSDSTSAVKEKTATCHSSSSPPIDAASAEPYGFRASVLFDTMQHHLALNRDLSTPGLEKDSGGKTPGDFSYAYQKPEETTRSPDEEDYDYESYEKTTRTSDVGGYYYEKIERTTKSPSDSGYSYETIGKTTKTPEDGDYSYEIIEKTTRTPEEGGYSYDISEKTTSPPEVSGYSYEKTERSRRLLDDISNGYDDSEDGGHTLGDPSYSYETTEKITSFPESEGYSYETSTKTTRTPDTSTYCYETAEKITRTPQASTYSYETSDLCYTAEKKSPSEARQDVDLCLVSSCEYKHPKTELSPSFINPNPLEWFASEEPTEESEKPLTQSGGAPPPPGGKQQGRQCDETPPTSVSESAPSQTDSDVPPETEECPSITADANIDSEDESETIPTDKTVTYKHMDPPPAPVQDRSPSPRHPDVSMVDPEALAIEQNLGKALKKDLKEKTKTKKPGTKTKSSSPVKKSDGKSKPLAASPKPAGLKESSDKVSRVASPKKKESVEKAAKPTTTPEVKAARGEEKDKETKNAANASASKSAKTATAGPGTTKTTKSSAVPPGLPVYLDLCYIPNHSNSKNVDVEFFKRVRSSYYVVSGNDPAAEEPSRAVLDALLEGKAQWGSNMQVTLIPTHDSEVMREWYQETHEKQQDLNIMVLASSSTVVMQDESFPACKIEL

56 NP_056477.1 NP_203129.1

>NP_056477.1

MHLKHLRTLLSPQDGAAKVTCMAWSQNNAKFAVCTVDRVVLLYDEHGERRDKFSTKPADMKYGRKSYMVKGMAFSPDSTKIAIGQTDNIIYVYKIGEDWGDKKVICNKFIQTSAVTCLQWPAEYIIVFGLAEGKVRLANTKTNKSSTIYGTESYVVSLTTNCSGKGILSGHADGTIVRYFFDDEGSGESQGKLVNHPCPPYALAWATNSIVAAGCDRKIVAYGKEGHMLQTFDYSRDPQEREFTTAVSSPGGQSVVLGSYDRLRVFNWIPRRSIWEEAKPKEITNLYTITALAWKRDGSRLCVGTLCGGVEQFDCCLRRSIYKNKFELTYVGPSQVIVKNLSSGTRVVLKSHYGYEVEEVKILGKERYLVAHTSETLLLGDLNTNRLSEIAWQGSGGNEKYFFENENVCMIFNAGELTLVEYGNNDTLGSVRTEFMNPHLISVRINERCQRGTEDNKKLAYLIDIKTIAIVDLIGGYNIGTVSHESRVDWLELNETGHKLLFRDRKLRLHLYDIESCSKTMILNFCSYMQWVPGSDVLVAQNRNSLCVWYNIEAPERVTMFTIRGDVIGLERGGGKTEVMVMEGVTTVAYTLDEGLIEFGTAIDDGNYIRATAFLETLEMTPETEAMWKTLSKLALEARQLHIAERCFSALGQVAKARFLHETNEIADQVSREYGGEGTDFYQVRARLAMLEKNYKLAEMIFLEQNAVEEAMGMYQELHRWDECIAVAEAKGHPALEKLRRSYYQWLMDTQQEERAGELQESQGDGLAAISLYLKAGLPAKAARLVLTREELLANTELVEHITAALIKGELYERAGDLFEKIHNPQKALECYRKGNAFMKAVELARLAFPVEVVKLEEAWGDHLVQQKQLDAAINHYIEARCSIKAIEAALGARQWKKAIYILDLQDRNTASKYYPLVAQHYASLQEYEIAEELYTKGDRTKDAIDMYTQAGRWEQAHKLAMKCMRPEDVSVLYITQAQEMEKQGKYREAERLYVTVQEPDLAITMYKKHKLYDDMIRLVGKHHPDLLSDTHLHLGKELEAEGRLQEAEYHYLEAQEWKATVNMYRASGLWEEAYRVARTQGGANAHKHVAYLWAKSLGGEAAVRLLNKLGLLEAAVDHAADNCSFEFAFELSRLALKHKTPEVHLKYAMFLEDEGKFEEAEAEFIRAGKPKEAVLMFVHNQDWEAAQRVAEAHDPDSVAEVLVGQARGALEEKDFQKAEGLLLRAQRPGLALNYYKEAGLWSDALRICKDYVPSQLEALQEEYEREATKKGARGVEGFVEQARHWEQAGEYSRAVDCYLKVRDSGNSGLAEKCWMKAAELSIKFLPPQRNMEVVLAVGPQLIGIGKHSAAAELYLNLDLVKEAIDAFIEGEEWNKAKRVAKELDPRYEDYVDQHYKEFLKNQGKVDSLVGVDVIAALDLYVEQGQWDKCIETATKQNYKILHKYVALYATHLIREGSSAQALALYVQHGAPANPQNFNIYKRIFTDMVSSPGTNCAEAYHSWADLRDVLFNLCENLVKSSEANSPAHEEFKTMLLIAHYYATRSAAQSVKQLETVAARLSVSLLRHTQLLPVDKAFYEAGIAAKAVGWDNMAFIFLNRFLDLTDAIEEGTLDGLDHSDFQDTDIPFEVPLPAKQHVPEAEREEVRDWVLTVSMDQRLEQVLPRDERGAYEASLVAASTGVRALPCLITGYPILRNKIEFKRPGKAANKDNWNKFLMAIKTSHSPVCQDVLKFISQWCGGLPSTSFSFQ

>NP_203129.1

MMQSATVPAEGAVKGLPEMLGVPMQQIPQCAGCNQHILDKFILKVLDRHWHSSCLKCADCQMQLADRCFSRAGSVYCKEDFFKRFGTKCTACQQGIPPTQVVRKAQDFVYHLHCFACIICNRQLATGDEFYLMEDGRLVCKEDYETAKQNDDSEAGAKRPRTTITAKQLETLKNAYKNSPKPARHVREQLSSETGLDMRVVQVWFQNRRAKEKRLKKDAGRHRWGQFYKSVKRSRGSSKQEKESSAEDCGVSDSELSFREDQILSELGHTNRIYGNVGDVTGGQLMNGSFSMDGTGQSYQDLRDGSPYGIPQSPSSISSLPSHAPLLNGLDYTVDSNLGIIAHAGQGVSQTLRAMAGGPTSDISTGSSVGYPDFPTSPGSWLDEMDHPPF

57 NP_001090.2 NP_004439.2

>NP_001090.2

MRAAPLLLARAASLSLGFLFLLFFWLDRSVLAKELKFVTLVFRHGDRSPIDTFPTDPIKESSWPQGFGQLTQLGMEQHYELGEYIRKRYRKFLNESYKHEQVYIRSTDVDRTLMSAMTNLAALFPPEGVSIWNPILLWQPIPVHTVPLSEDQLLYLPFRNCPRFQELESETLKSEEFQKRLHPYKDFIATLGKLSGLHGQDLFGIWSKVYDPLYCESVHNFTLPSWATEDTMTKLRELSELSLLSLYGIHKQKEKSRLQGGVLVNEILNHMKRATQIPSYKKLIMYSAHDTTVSGLQMALDVYNGLLPPYASCHLTELYFEKGEYFVEMYYRNETQHEPYPLMLPGCSPSCPLERFAELVGPVIPQDWSTECMTTNSHQGTEDSTD

>NP_004439.2

MELAALCRWGLLLALLPPGAASTQVCTGTDMKLRLPASPETHLDMLRHLYQGCQVVQGNLELTYLPTNASLSFLQDIQEVQGYVLIAHNQVRQVPLQRLRIVRGTQLFEDNYALAVLDNGDPLNNTTPVTGASPGGLRELQLRSLTEILKGGVLIQRNPQLCYQDTILWKDIFHKNNQLALTLIDTNRSRACHPCSPMCKGSRCWGESSEDCQSLTRTVCAGGCARCKGPLPTDCCHEQCAAGCTGPKHSDCLACLHFNHSGICELHCPALVTYNTDTFESMPNPEGRYTFGASCVTACPYNYLSTDVGSCTLVCPLHNQEVTAEDGTQRCEKCSKPCARVCYGLGMEHLREVRAVTSANIQEFAGCKKIFGSLAFLPESFDGDPASNTAPLQPEQLQVFETLEEITGYLYISAWPDSLPDLSVFQNLQVIRGRILHNGAYSLTLQGLGISWLGLRSLRELGSGLALIHHNTHLCFVHTVPWDQLFRNPHQALLHTANRPEDECVGEGLACHQLCARGHCWGPGPTQCVNCSQFLRGQECVEECRVLQGLPREYVNARHCLPCHPECQPQNGSVTCFGPEADQCVACAHYKDPPFCVARCPSGVKPDLSYMPIWKFPDEEGACQPCPINCTHSCVDLDDKGCPAEQRASPLTSIISAVVGILLVVVLGVVFGILIKRRQQKIRKYTMRRLLQETELVEPLTPSGAMPNQAQMRILKETELRKVKVLGSGAFGTVYKGIWIPDGENVKIPVAIKVLRENTSPKANKEILDEAYVMAGVGSPYVSRLLGICLTSTVQLVTQLMPYGCLLDHVRENRGRLGSQDLLNWCMQIAKGMSYLEDVRLVHRDLAARNVLVKSPNHVKITDFGLARLLDIDETEYHADGGKVPIKWMALESILRRRFTHQSDVWSYGVTVWELMTFGAKPYDGIPAREIPDLLEKGERLPQPPICTIDVYMIMVKCWMIDSECRPRFRELVSEFSRMARDPQRFVVIQNEDLGPASPLDSTFYRSLLEDDDMGDLVDAEEYLVPQQGFFCPDPAPGAGGMVHHRHRSSSTRSGGGDLTLGLEPSEEEAPRSPLAPSEGAGSDVFDGDLGMGAAKGLQSLPTHDPSPLQRYSEDPTVPLPSETDGYVAPLTCSPQPEYVNQPDVRPQPPSPREGPLPAARPAGATLERPKTLSPGKNGVVKDVFAFGGAVENPEYLTPQGGAAPQPHPPPAFSPAFDNLYYWDQDPPERGAPPSTFKGTPTAENPEYLGLDVPV

58 NP_004105.1 NP_055006.1

>NP_004105.1

MAAAIASSLIRQKRQAREREKSNACKCVSSPSKGKTSCDKNKLNVFSRVKLFGSKKRRRRRPEPQLKGIVTKLYSRQGYHLQLQADGTIDGTKDEDSTYTLFNLIPVGLRVVAIQGVQTKLYLAMNSEGYLYTSELFTPECKFKESVFENYYVTYSSMIYRQQQSGRGWYLGLNKEGEIMKGNHVKKNKPAAHFLPKPLKVAMYKEPSLHDLTEFSRSGSGTPTKSRSVSGVLNGGKSMSHNEST

>NP_055006.1

MAARLLAPPGPDSFKPFTPESLANIERRIAESKLKKPPKADGSHREDDEDSKPKPNSDLEAGKSLPFIYGDIPQGLVAVPLEDFDPYYLTQKTFVVLNRGKTLFRFSATPALYILSPFNLIRRIAIKILIHSVFSMIIMCTILTNCVFMTFSNPPDWSKNVEYTFTGIYTFESLVKIIARGFCIDGFTFLRDPWNWLDFSVIMMAYITEFVNLGNVSALRTFRVLRALKTISVIPGLKTIVGALIQSVKKLSDVMILTVFCLSVFALIGLQLFMGNLRNKCVVWPINFNESYLENGTKGFDWEEYINNKTNFYTVPGMLEPLLCGNSSDAGQCPEGYQCMKAGRNPNYGYTSFDTFSWAFLALFRLMTQDYWENLYQLTLRAAGKTYMIFFVLVIFVGSFYLVNLILAVVAMAYEEQNQATLEEAEQKEAEFKAMLEQLKKQQEEAQAAAMATSAGTVSEDAIEEEGEEGGGSPRSSSEISKLSSKSAKERRNRRKKRKQKELSEGEEKGDPEKVFKSESEDGMRRKAFRLPDNRIGRKFSIMNQSLLSIPGSPFLSRHNSKSSIFSFRGPGRFRDPGSENEFADDEHSTVEESEGRRDSLFIPIRARERRSSYSGYSGYSQGSRSSRIFPSLRRSVKRNSTVDCNGVVSLIGGPGSHIGGRLLPEATTEVEIKKKGPGSLLVSMDQLASYGRKDRINSIMSVVTNTLVEELEESQRKCPPCWYKFANTFLIWECHPYWIKLKEIVNLIVMDPFVDLAITICIVLNTLFMAMEHHPMTPQFEHVLAVGNLVFTGIFTAEMFLKLIAMDPYYYFQEGWNIFDGFIVSLSLMELSLADVEGLSVLRSFRLLRVFKLAKSWPTLNMLIKIIGNSVGALGNLTLVLAIIVFIFAVVGMQLFGKSYKECVCKINQDCELPRWHMHDFFHSFLIVFRVLCGEWIETMWDCMEVAGQAMCLIVFMMVMVIGNLVVLNLFLALLLSSFSADNLAATDDDGEMNNLQISVIRIKKGVAWTKLKVHAFMQAHFKQREADEVKPLDELYEKKANCIANHTGADIHRNGDFQKNGNGTTSGIGSSVEKYIIDEDHMSFINNPNLTVRVPIAVGESDFENLNTEDVSSESDPEGSKDKLDDTSSSEGSTIDIKPEVEEVPVEQPEEYLDPDACFTEGCVQRFKCCQVNIEEGLGKSWWILRKTCFLIVEHNWFETFIIFMILLSSGALAFEDIYIEQRKTIRTILEYADKVFTYIFILEMLLKWTAYGFVKFFTNAWCWLDFLIVAVSLVSLIANALGYSELGAIKSLRTLRALRPLRALSRFEGMRVVVNALVGAIPSIMNVLLVCLIFWLIFSIMGVNLFAGKYHYCFNETSEIRFEIEDVNNKTECEKLMEGNNTEIRWKNVKINFDNVGAGYLALLQVATFKGWMDIMYAAVDSRKPDEQPKYEDNIYMYIYFVIFIIFGSFFTLNLFIGVIIDNFNQQKKKFGGQDIFMTEEQKKYYNAMKKLGSKKPQKPIPRPLNKIQGIVFDFVTQQAFDIVIMMLICLNMVTMMVETDTQSKQMENILYWINLVFVIFFTCECVLKMFALRHYYFTIGWNIFDFVVVILSIVGMFLADIIEKYFVSPTLFRVIRLARIGRILRLIKGAKGIRTLLFALMMSLPALFNIGLLLFLVMFIFSIFGMSNFAYVKHEAGIDDMFNFETFGNSMICLFQITTSAGWDGLLLPILNRPPDCSLDKEHPGSGFKGDCGNPSVGIFFFVSYIIISFLIVVNMYIAIILENFSVATEESADPLSEDDFETFYEIWEKFDPDATQFIEYCKLADFADALEHPLRVPKPNTIELIAMDLPMVSGDRIHCLDILFAFTKRVLGDSGELDILRQQMEERFVASNPSKVSYEPITTTLRRKQEEVSAVVLQRAYRGHLARRGFICKKTTSNKLENGGTHREKKESTPSTASLPSYDSVTKPEKEKQQRAEEGRRERAKRQKEVRESKC

59 NP_006249.1 NP_002914.1

>NP_006249.1

MGTLRDLQYALQEKIEELRQRDALIDELELELDQKDELIQKLQNELDKYRSVIRPATQQAQKQSASTLQGEPRTKRQAISAEPTAFDIQDLSHVTLPFYPKSPQSKDLIKEAILDNDFMKNLELSQIQEIVDCMYPVEYGKDSCIIKEGDVGSLVYVMEDGKVEVTKEGVKLCTMGPGKVFGELAILYNCTRTATVKTLVNVKLWAIDRQCFQTIMMRTGLIKHTEYMEFLKSVPTFQSLPEEILSKLADVLEETHYENGEYIIRQGARGDTFFIISKGTVNVTREDSPSEDPVFLRTLGKGDWFGEKALQGEDVRTANVIAAEAVTCLVIDRDSFKHLIGGLDDVSNKAYEDAEAKAKYEAEAAFFANLKLSDFNIIDTLGVGGFGRVELVQLKSEESKTFAMKILKKRHIVDTRQQEHIRSEKQIMQGAHSDFIVRLYRTFKDSKYLYMLMEACLGGELWTILRDRGSFEDSTTRFYTACVVEAFAYLHSKGIIYRDLKPENLILDHRGYAKLVDFGFAKKIGFGKKTWTFCGTPEYVAPEIILNKGHDISADYWSLGILMYELLTGSPPFSGPDPMKTYNIILRGIDMIEFPKKIAKNAANLIKKLCRDNPSERLGNLKNGVKDIQKHKWFEGFNWEGLRKGTLTPPIIPSVASPTDTSNFDSFPEDNDEPPPDDNSGWDIDF

>NP_002914.1

MQSAMFLAVQHDCRPMDKSAGSGHKSEEKREKMKRTLLKDWKTRLSYFLQNSSTPGKPKTGKKSKQQAFIKPSPEEAQLWSEAFDELLASKYGLAAFRAFLKSEFCEENIEFWLACEDFKKTKSPQKLSSKARKIYTDFIEKEAPKEINIDFQTKTLIAQNIQEATSGCFTTAQKRVYSLMENNSYPRFLESEFYQDLCKKPQITTEPHAT

60 NP_057542.2 NP_060116.2

>NP_057542.2

MGEAGAGAGASGGPEASPEAEVVKLLPFLAPGARADLQAAAVRHVLALTGCGPGRALLAGQAALLQALMELAPASAPARDAARALVNLAADPGLHETLLAADPGLPARLMGRALDPQWPWAEEAAAALANLSREPAPCAALMAALAAAEPADSGLERLVRALCTPGYNARAPLHYLAPLLSNLSQRPAARAFLLDPDRCVVQRLLPLTQYPDSSVRRGGVVGTLRNCCFEHRHHEWLLGPEVDILPFLLLPLAGPEDFSEEEMERLPVDLQYLPPDKQREPDADIRKMLVEAIMLLTATAPGRQQVRDQGAYLILRELHSWEPEPDVRTACEKLIQVLIGDEPERGMENLLEVQVPEDVEQQLQQLDCREQEQLERELAPEPWVERATPT

>NP_060116.2

MASVAAARAVPVGSGLRGLQRTLPLVVILGATGTGKSTLALQLGQRLGGEIVSADSMQVYEGLDIITNKVSAQEQRICRHHMISFVDPLVTNYTVVDFRNRATALIEDIFARDKIPIVVGGTNYYIESLLWKVLVNTKPQEMGTEKVIDRKVELEKEDGLVLHKRLSQVDPEMAAKLHPHDKRKVARSLQVFEETGISHSEFLHRQHTEEGGGPLGGPLKFSNPCILWLHADQAVLDERLDKRVDDMLAAGLLEELRDFHRRYNQKNVSENSQDYQHGIFQSIGFKEFHEYLITEGKCTLETSNQLLKKGIEALKQVTKRYARKQNRWVKNRFLSRPGPIVPPVYGLEVSDVSKWEESVLEPALEIVQSFIQGHKPTATPIKMPYNEAENKRSYHLCDLCDRIIIGDREWAAHIKSKSHLNQLKKRRRLDSDAVNTIESQSVSPDHNKEPKEKGSPGQNDQELKCSV

61 NP_004594.1 NP_006169.1

>NP_004594.1

MKDRTQELRTAKDSDDDDDVAVTVDRDRFMDEFFEQVEEIRGFIDKIAENVEEVKRKHSAILASPNPDEKTKEELEELMSDIKKTANKVRSKLKSIEQSIEQEEGLNRSSADLRIRKTQHSTLSRKFVEVMSEYNATQSDYRERCKGRIQRQLEITGRTTTSEELEDMLESGNPAIFASGIIMDSSISKQALSEIETRHSEIIKLENSIRELHDMFMDMAMLVESQGEMIDRIEYNVEHAVDYVERAVSDTKKAVKYQSKARRKKIMIIICCVILGIVIASTVGGIFA

>NP_006169.1

MAGRSMQAARCPTDELSLTNCSVVNEKDFQSGQHVIVRTSPNHRYTFTLKTHPSVVPGSIAFSLPQRKWAGLSIGQEIEVSLYTFDKAKQCIGTMTIEIDFLQKKSNDSNPYDTDKMAAEFIQQFNNQAYSVGQQLVFSFNEKLFGLLVKDIESMDPSILKGEPATGKRQKIEVGLVVGNSQVAFEKAENSSLNLIGKAKTKENRQSIINPDWNFEKMGIGGLDKEFSDIFRRAFAFRVFPPEIVEQMGCIHVKGILLYGPPGCGKTLLARQIGKMLNAREPKVVNGPEILNKYVGESEANIRKLFADAEEEQRRLGANSGLHIIIFDEIDAICKQRGSMAGSTGVHDTVVNQLLSKIDGVEQLNNILVIGMTNRPDLIDEALLRPGRLEVKMEIGLPDEKGRLQILHIHTARMRGHQLLSADVDIKELAVETKNFSGAELEGLVRAAQSTAMNRHIKASTKVEVDMEKAESLQVTRGDFLASLENDIKPAFGTNQEDYASYIMNGIIKWGDPVTRVLDDGELLVQQTKNSDRTPLVSVLLEGPPHSGKTALAAKIAEESNFPFIKICSPDKMIGFSETAKCQAMKKIFDDAYKSQLSCVVVDDIERLLDYVPIGPRFSNLVLQALLVLLKKAPPQGRKLLIIGTTSRKDVLQEMEMLNAFSTTIHVPNIATGEQLLEALELLGNLKDKERTTIAQQVKGKKVWIGIKKLLMLIEMSLQMDPEYRVRKFLALLREEGASPLDFD

62 NP_005602.2 NP_002625.1

>NP_005602.2

MPSGGDQSPPPPPPPPAAAASDEEEEDDGEAEDAAPSAESPTPQIQQRFDELCSRLNMDEAARPEAWDSYRSMSESYTLEGNDLHWLACALYVACRKSVPTVSKGTVEGNYVSLTRILKCSEQSLIEFFNKMKKWEDMANLPPHFRERTERLERNFTVSAVIFKKYEPIFQDIFKYPQEEQPRQQRGRKQRRQPCTVSEIFHFCWVLFIYAKGNFPMISDDLVNSYHLLLCALDLVYGNALQCSNRKELVNPNFKGLSEDFHAKDSKPSSDPPCIIEKLCSLHDGLVLEAKGIKEHFWKPYIRKLYEKKLLKGKEENLTGFLEPGNFGESFKAINKAYEEYVLSVGNLDERIFLGEDAEEEIGTLSRCLNAGSGTETAERVQMKNILQQHFDKSKALRISTPLTGVRYIKENSPCVTPVSTATHSLSRLHTMLTGLRNAPSEKLEQILRTCSRDPTQAIANRLKEMFEIYSQHFQPDEDFSNCAKEIASKHFRFAEMLYYKVLESVIEQEQKRLGDMDLSGILEQDAFHRSLLACCLEVVTFSYKPPGNFPFITEIFDVPLYHFYKVIEVFIRAEDGLCREVVKHLNQIEEQILDHLAWKPESPLWEKIRDNENRVPTCEEVMPPQNLERADEICIAGSPLTPRRVTEVRADTGGLGRSITSPTTLYDRYSSPPASTTRRRLFVENDSPSDGGTPGRMPPQPLVNAVPVQNVSGETVSVTPVPGQTLVTMATATVTANNGQTVTIPVQGIANENGGITFFPVQVNVGGQAQAVTGSIQPLSAQALAGSLSSQQVTGTTLQVPGQVAIQQISPGGQQQKQGQSVTSSSNRPRKTSSLSLFFRKVYHLAAVRLRDLCAKLDISDELRKKIWTCFEFSIIQCPELMMDRHLDQLLMCAIYVMAKVTKEDKSFQNIMRCYRTQPQARSQVYRSVLIKGKRKRRNSGSSDSRSHQNSPTELNKDRTSRDSSPVMRSSSTLPVPQPSSAPPTPTRLTGANSDMEEEERGDLIQFYNNIYIKQIKTFAMKYSQANMDAPPLSPYPFVRTGSPRRIQLSQNHPVYISPHKNETMLSPREKIFYYFSNSPSKRLREINSMIRTGETPTKKRGILLEDGSESPAKRICPENHSALLRRLQDVANDRGSH

>NP_002625.1

MAAKVFESIGKFGLALAVAGGVVNSALYNVDAGHRAVIFDRFRGVQDIVVGEGTHFLIPWVQKPIIFDCRSRPRNVPVITGSKDLQNVNITLRILFRPVASQLPRIFTSIGEDYDERVLPSITTEILKSVVARFDAGELITQRELVSRQVSDDLTERAATFGLILDDVSLTHLTFGKEFTEAVEAKQVAQQEAERARFVVEKAEQQKKAAIISAEGDSKAAELIANSLATAGDGLIELRKLEAAEDIAYQLSRSRNITYLPAGQSVLLQLPQ

63 NP_116764.2 NP_932070.1

>NP_116764.2

MAAPSAGSWSTFQHKELMAADRGRRILGVCGMHPHHQETLKKNRVVLAKQLLLSELLEHLLEKDIITLEMRELIQAKVGSFSQNVELLNLLPKRGPQAFDAFCEALRETKQGHLEDMLLTTLSGLQHVLPPLSCDYDLSLPFPVCESCPLYKKLRLSTDTVEHSLDNKDGPVCLQVKPCTPEFYQTHFQLAYRLQSRPRGLALVLSNVHFTGEKELEFRSGGDVDHSTLVTLFKLLGYDVHVLCDQTAQEMQEKLQNFAQLPAHRVTDSCIVALLSHGVEGAIYGVDGKLLQLQEVFQLFDNANCPSLQNKPKMFFIQACRGDETDRGVDQQDGKNHAGSPGCEESDAGKEKLPKMRLPTRSDMICGYACLKGTAAMRNTKRGSWYIEALAQVFSERACDMHVADMLVKVNALIKDREGYAPGTEFHRCKEMSEYCSTLCRHLYLFPGHPPT

>NP_932070.1

MCSGAGVMMARWAARGRAGWRSTVRILSPLGHCEPGVSRSCRAAQAMDCEVNNGSSLRDECITNLLVFGFLQSCSDNSFRRELDALGHELPVLAPQWEGYDELQTDGNRSSHSRLGRIEADSESQEDIIRNIARHLAQVGDSMDRSIPPGLVNGLALQLRNTSRSEEDRNRDLATALEQLLQAYPRDMEKEKTMLVLALLLAKKVASHTPSLLRDVFHTTVNFINQNLRTYVRSLARNGMD

64 NP_004228.1 NP_001078837.1

>NP_004228.1

MDEAVGDLKQALPCVAESPTVHVEVHQRGSSTAKKEDINLSVRKLLNRHNIVFGDYTWTEFDEPFLTRNVQSVSIIDTELKVKDSQPIDLSACTVALHIFQLNEDGPSSENLEEETENIIAANHWVLPAAEFHGLWDSLVYDVEVKSHLLDYVMTTLLFSDKNVNSNLITWNRVVLLHGPPGTGKTSLCKALAQKLTIRLSSRYRYGQLIEINSHSLFSKWFSESGKLVTKMFQKIQDLIDDKDALVFVLIDEVESLTAARNACRAGTEPSDAIRVVNAVLTQIDQIKRHSNVVILTTSNITEKIDVAFVDRADIKQYIGPPSAAAIFKIYLSCLEELMKCQIIYPRQQLLTLRELEMIGFIENNVSKLSLLLNDISRKSEGLSGRVLRKLPFLAHALYVQAPTVTIEGFLQALSLAVDKQFEERKKLAAYI

>NP_001078837.1

MEEIPAQEAAGSPRVQFQSLETQSECLSPEPQFVQDTDMEQGLTGGFPISKPDGISQLEQDLQVFDLETKTREVLRDDCSDGETREENKLLIPKQKISEEVHSYKVRVGRLKHDITQVPETREVYKSEDRLERLQEILRKFLYLEREFRQITISKETFTSEKNNECHEPEKSFSLDSTIDADQRVLRIQNTDDNDKYDMSFNQNSASGKHEHLNLTEDFQSSECKESLMDLSHLNKWESIPNTEKSYKCDVCGKIFHQSSALTRHQRIHTREKPYKCKECEKSFSQSSSLSRHKRIHTREKPYKCEASDKSCEASDKSCSPSSGIIQHKKIHTRAKSYKCSSCERVFSRSVHLTQHQKIHKEMPCKCTVCGSDFCHTSYLLEHQRVHHEEKAYEYDEYGLAYIKQQGIHFREKPYTCSECGKDFRLNSHLIQHQRIHTGEKAHECNECGKAFSQTSCLIQHHKMHRKEKSYECNEYEGSFSHSSDLILQQEVLTRQKAFDCDVWEKNSSQRAHLVQHQSIHTKENS

65 NP_036437.1 NP_001274.1

>NP_036437.1

MPAVSKGDGMRGLAVFISDIRNCKSKEAEIKRINKELANIRSKFKGDKALDGYSKKKYVCKLLFIFLLGHDIDFGHMEAVNLLSSNRYTEKQIGYLFISVLVNSNSELIRLINNAIKNDLASRNPTFMGLALHCIASVGSREMAEAFAGEIPKVLVAGDTMDSVKQSAALCLLRLYRTSPDLVPMGDWTSRVVHLLNDQHLGVVTAATSLITTLAQKNPEEFKTSVSLAVSRLSRIVTSASTDLQDYTYYFVPAPWLSVKLLRLLQCYPPPDPAVRGRLTECLETILNKAQEPPKSKKVQHSNAKNAVLFEAISLIIHHDSEPNLLVRACNQLGQFLQHRETNLRYLALESMCTLASSEFSHEAVKTHIETVINALKTERDVSVRQRAVDLLYAMCDRSNAPQIVAEMLSYLETADYSIREEIVLKVAILAEKYAVDYTWYVDTILNLIRIAGDYVSEEVWYRVIQIVINRDDVQGYAAKTVFEALQAPACHENLVKVGGYILGEFGNLIAGDPRSSPLIQFHLLHSKFHLCSVPTRALLLSTYIKFVNLFPEVKPTIQDVLRSDSQLRNADVELQQRAVEYLRLSTVASTDILATVLEEMPPFPERESSILAKLKKKKGPSTVTDLEDTKRDRSVDVNGGPEPAPASTSAVSTPSPSADLLGLGAAPPAPAGPPPSSGGSGLLVDVFSDSASVVAPLAPGSEDNFARFVCKNNGVLFENQLLQIGLKSEFRQNLGRMFIFYGNKTSTQFLNFTPTLICSDDLQPNLNLQTKPVDPTVEGGAQVQQVVNIECVSDFTEAPVLNIQFRYGGTFQNVSVQLPITLNKFFQPTEMASQDFFQRWKQLSNPQQEVQNIFKAKHPMDTEVTKAKIIGFGSALLEEVDPNPANFVGAGIIHTKTTQIGCLLRLEPNLQAQMYRLTLRTSKEAVSQRLCELLSAQF

>NP_001274.1

MMRFMLLFSRQGKLRLQKWYLATSDKERKKMVRELMQVVLARKPKMCSFLEWRDLKVVYKRYASLYFCCAIEGQDNELITLELIHRYVELLDKYFGSVCELDIIFNFEKAYFILDEFLMGGDVQDTSKKSVLKAIEQADLLQEEDESPRSVLEEMGLA

66 NP_588612.1 AAH28049.1

>NP_588612.1

MIPKEQKGPVMAAMGDLTEPVPTLDLGKKLSVPQDLMMEELSLRNNRGSLLFQKRQRRVQKFTFELAASQRAMLAGSARRKVTGTAESGTVANANGPEGPNYRSELHIFPASPGASLGGPEGAHPAAAPAGCVPSPSALAPGYAEPLKGVPPEKFNHTAIPKGYRCPWQEFVSYRDYQSDGRSHTPSPNDYRNFNKTPVPFGGPLVGGTFPRPGTPFIPEPLSGLELLRLRPSFNRVAQGWVRNLPESEEL

>AAH28049.1

MAAPEPARAAPPPPPPPPPPPGADRVVKAVPFPPTHRLTSEEVFDLDGIPRVDVLKNHLVKEGRVDEEIALRIINEGAAILRREKTMIEVEAPITVCGDIHGQFFDLMKLFEVGGSPANTRYLFLGDYVDRGYFSIECVLYLWVLKILYPSTLFLLRGNHECRHLTEYFTFKQECKIKYSERVYEACMEAFDSLPLAALLNQQFLCVHGGLSPEIHTLDDIRRLDRFKEPPAFGPMCDLLWSDPSEDFGNEKSQEHFSHNTVRGCSYFYNYPAVCEFLQNNNLLSIIRAHEAQDAGYRMYRKSQTTGFPSLITIFSAPNYLDVYNNKAAVLKYENNVMNIRQFNCSPHPYWLPNFMDVFTWSLPFVGEKVTEMLVNVLSICSDDELMTEGEDQFDVGSAAARKEIIRNKIRAIGKMARVFSVLREESESVLTLKGLTPTGMLPSGVLAGGRQTLQSATVEAIEAEKAIRGFSPPHRICSFEEAKGLDRINERMPPRKDAVQQDGFNSLNTAHATENHGTGNHTAQ

67 NP_005391.1 NP_542437.2

>NP_005391.1

MVVFNGLLKIKICEAVSLKPTAWSLRHAVGPRPQTFLLDPYIALNVDDSRIGQTATKQKTNSPAWHDEFVTDVCNGRKIELAVFHDAPIGYDDFVANCTIQFEELLQNGSRHFEDWIDLEPEGRVYVIIDLSGSSGEAPKDNEERVFRERMRPRKRQGAVRRRVHQVNGHKFMATYLRQPTYCSHCRDFIWGVIGKQGYQCQVCTCVVHKRCHELIITKCAGLKKQETPDQVGSQRFSVNMPHKFGIHNYKVPTFCDHCGSLLWGLLRQGLQCKVCKMNVHRRCETNVAPNCGVDARGIAKVLADLGVTPDKITNSGQRRKKLIAGAESPQPASGSSPSEEDRSKSAPTSPCDQEIKELENNIRKALSFDNRGEEHRAASSPDGQLMSPGENGEVRQGQAKRLGLDEFNFIKVLGKGSFGKVMLAELKGKDEVYAVKVLKKDVILQDDDVDCTMTEKRILALARKHPYLTQLYCCFQTKDRLFFVMEYVNGGDLMFQIQRSRKFDEPRSRFYAAEVTSALMFLHQHGVIYRDLKLDNILLDAEGHCKLADFGMCKEGILNGVTTTTFCGTPDYIAPEILQELEYGPSVDWWALGVLMYEMMAGQPPFEADNEDDLFESILHDDVLYPVWLSKEAVSILKAFMTKNPHKRLGCVASQNGEDAIKQHPFFKEIDWVLLEQKKIKPPFKPRIKTKRDVNNFDQDFTREEPVLTLVDEAIVKQINQEEFKGFSYFGEDLMP

>NP_542437.2

MKKWSSTDLGAAADPLQKDTCPDPLDGDPNSRPPPAKPQLSTAKSRTRLFGKGDSEEAFPVDCPHEEGELDSCPTITVSPVITIQRPGDGPTGARLLSQDSVAASTEKTLRLYDRRSIFEAVAQNNCQDLESLLLFLQKSKKHLTDNEFKDPETGKTCLLKAMLNLHDGQNTTIPLLLEIARQTDSLKELVNASYTDSYYKGQTALHIAIERRNMALVTLLVENGADVQAAAHGDFFKKTKGRPGFYFGELPLSLAACTNQLGIVKFLLQNSWQTADISARDSVGNTVLHALVEVADNTADNTKFVTSMYNEILMLGAKLHPTLKLEELTNKKGMTPLALAAGTGKIGVLAYILQREIQEPECRHLSRKFTEWAYGPVHSSLYDLSCIDTCEKNSVLEVIAYSSSETPNRHDMLLVEPLNRLLQDKWDRFVKRIFYFNFLVYCLYMIIFTMAAYYRPVDGLPPFKMEKTGDYFRVTGEILSVLGGVYFFFRGIQYFLQRRPSMKTLFVDSYSEMLFFLQSLFMLATVVLYFSHLKEYVASMVFSLALGWTNMLYYTRGFQQMGIYAVMIEKMILRDLCRFMFVYIVFLFGFSTAVVTLIEDGKNDSLPSESTSHRWRGPACRPPDSSYNSLYSTCLELFKFTIGMGDLEFTENYDFKAVFIILLLAYVILTYILLLNMLIALMGETVNKIAQESKNIWKLQRAITILDTEKSFLKCMRKAFRSGKLLQVGYTPDGKDDYRWCFRVDEVNWTTWNTNVGIINEDPGNCEGVKRTLSFSLRSSRVSGRHWKNFALVPLLREASARDRQSAQPEEVYLRQFSGSLKPEDAEVFKSPAASGEK

68 NP_009298.1 NP_058431.2

>NP_009298.1

MGQQVGRVGEAPGLQQPQPRGIRGSSAARPSGRRRDPAGRTTETGFNIFTQHDHFASCVEDGFEGDKTGGSSPEALHRPYGCDVEPQALNEAIRWSSKENLLGATESDPNLFVALYDFVASGDNTLSITKGEKLRVLGYNQNGEWSEVRSKNGQGWVPSNYITPVNSLEKHSWYHGPVSRSAAEYLLSSLINGSFLVRESESSPGQLSISLRYEGRVYHYRINTTADGKVYVTAESRFSTLAELVHHHSTVADGLVTTLHYPAPKCNKPTVYGVSPIHDKWEMERTDITMKHKLGGGQYGEVYVGVWKKYSLTVAVKTLKEDTMEVEEFLKEAAVMKEIKHPNLVQLLGVCTLEPPFYIVTEYMPYGNLLDYLRECNREEVTAVVLLYMATQISSAMEYLEKKNFIHRDLAARNCLVGENHVVKVADFGLSRLMTGDTYTAHAGAKFPIKWTAPESLAYNTFSIKSDVWAFGVLLWEIATYGMSPYPGIDLSQVYDLLEKGYRMEQPEGCPPKVYELMRACWKWSPADRPSFAETHQAFETMFHDSSISEEVAEELGRAASSSSVVPYLPRLPILPSKTRTLKKQVENKENIEGAQDATENSASSLAPGFIRGAQASSGSPALPRKQRDKSPSSLLEDAKETCFTRDRKGGFFSSFMKKRNAPTPPKRSSSFREMENQPHKKYELTGNFSSVASLQHADGFSFTPAQQEANLVPPKCYGGSFAQRNLCNDDGGGGGGSGTAGGGWSGITGFFTPRLIKKTLGLRAGKPTASDDTSKPFPRSNSTSSMSSGLPEQDRMAMTLPRNCQRSKLQLERTVSTSSQPEENVDRANDMLPKKSEESAAPSRERPKAKLLPRGATALPLRTPSGDLAITEKDPPGVGVAGVAAAPKGKEKNGGARLGMAGVPEDGEQPGWPSPAKAAPVLPTTHNHKVPVLISPTLKHTPADVQLIGTDSQGNKFKLLSEHQVTSSGDKDRPRRVKPKCAPPPPPVMRLLQHPSICSDPTEEPTALTAGQSTSETQEGGKKAALGAVPISGKAGRPVMPPPQVPLPTSSISPAKMANGTAGTKVALRKTKQAAEKISADKISKEALLECADLLSSALTEPVPNSQLVDTGHQLLDYCSGYVDCIPQTRNKFAFREAVSKLELSLQELQVSSAAAGVPGTNPVLNNLLSCVQEISDVVQR

>NP_058431.2

MAGNFDSEERSSWYWGRLSRQEAVALLQGQRHGVFLVRDSSTSPGDYVLSVSENSRVSHYIINSSGPRPPVPPSPAQPPPGVSPSRLRIGDQEFDSLPALLEFYKIHYLDTTTLIEPVSRSRQGSGVILRQEEAEYVRALFDFNGNDEEDLPFKKGDILRIRDKPEEQWWNAEDSEGKRGMIPVPYVEKYRPASASVSALIGGNQEGSHPQPLGGPEPGPYAQPSVNTPLPNLQNGPIYARVIQKRVPNAYDKTALALEVGELVKVTKINVSGQWEGECNGKRGHFPFTHVRLLDQQNPDEDFS

69 NP_006353.2 NP_003601.1

>NP_006353.2

MADEGKSYSEHDDERVNFPQRKKKGRGPFRWKYGEGNRRSGRGGSGIRSSRLEEDDGDVAMSDAQDGPRVRYNPYTTRPNRRGDTWHDRDRIHVTVRRDRAPPERGGAGTSQDGTSKNWFKITIPYGRKYDKAWLLSMIQSKCSVPFTPIEFHYENTRAQFFVEDASTASALKAVNYKILDRENRRISIIINSSAPPHTILNELKPEQVEQLKLIMSKRYDGSQQALDLKGLRSDPDLVAQNIDVVLNRRSCMAATLRIIEENIPELLSLNLSNNRLYRLDDMSSIVQKAPNLKILNLSGNELKSERELDKIKGLKLEELWLDGNSLCDTFRDQSTYISAIRERFPKLLRLDGHELPPPIAFDVEAPTTLPPCKGSYFGTENLKSLVLHFLQQYYAIYDSGDRQGLLDAYHDGACCSLSIPFIPQNPARSSLAEYFKDSRNVKKLKDPTLRFRLLKHTRLNVVAFLNELPKTQHDVNSFVVDISAQTSTLLCFSVNGVFKEVDGKSRDSLRAFTRTFIAVPASNSGLCIVNDELFVRNASSEEIQRAFAMPAPTPSSSPVPTLSPEQQEMLQAFSTQSGMNLEWSQKCLQDNNWDYTRSAQAFTHLKAKGEIPEVAFMK

>NP_003601.1

MSLFGTTSGFGTSGTSMFGSATTDNHNPMKDIEVTSSPDDSIGCLSFSPPTLPGNFLIAGSWANDVRCWEVQDSGQTIPKAQQMHTGPVLDVCWSDDGSKVFTASCDKTAKMWDLSSNQAIQIAQHDAPVKTIHWIKAPNYSCVMTGSWDKTLKFWDTRSSNPMMVLQLPERCYCADVIYPMAVVATAERGLIVYQLENQPSEFRRIESPLKHQHRCVAIFKDKQNKPTGFALGSIEGRVAIHYINPPNPAKDNFTFKCHRSNGTNTSAPQDIYAVNGIAFHPVHGTLATVGSDGRFSFWDKDARTKLKTSEQLDQPISACCFNHNGNIFAYASSYDWSKGHEFYNPQKKNYIFLRNAAEELKPRNKK

70 NP_001020276.1 NP_000537.3

>NP_001020276.1

MASSSGSKAEFIVGGKYKLVRKIGSGSFGDIYLAINITNGEEVAVKLESQKARHPQLLYESKLYKILQGGVGIPHIRWYGQEKDYNVLVMDLLGPSLEDLFNFCSRRFTMKTVLMLADQMISRIEYVHTKNFIHRDIKPDNFLMGIGRHCNKCLESPVGKRKRSMTVSTSQDPSFSGLNQLFLIDFGLAKKYRDNRTRQHIPYREDKNLTGTARYASINAHLGIEQSRRDDMESLGYVLMYFNRTSLPWQGLKAATKKQKYEKISEKKMSTPVEVLCKGFPAEFAMYLNYCRGLRFEEAPDYMYLRQLFRILFRTLNHQYDYTFDWTMLKQKAAQQAASSSGQGQQAQTPTGKQTDKTKSNMKGF

>NP_000537.3

MEEPQSDPSVEPPLSQETFSDLWKLLPENNVLSPLPSQAMDDLMLSPDDIEQWFTEDPGPDEAPRMPEAAPPVAPAPAAPTPAAPAPAPSWPLSSSVPSQKTYQGSYGFRLGFLHSGTAKSVTCTYSPALNKMFCQLAKTCPVQLWVDSTPPPGTRVRAMAIYKQSQHMTEVVRRCPHHERCSDSDGLAPPQHLIRVEGNLRVEYLDDRNTFRHSVVVPYEPPEVGSDCTTIHYNYMCNSSCMGGMNRRPILTIITLEDSSGNLLGRNSFEVRVCACPGRDRRTEEENLRKKGEPHHELPPGSTKRALPNNTSSSPQPKKKPLDGEYFTLQIRGRERFEMFRELNEALELKDAQAGKEPGGSRAHSSHLKSKKGQSTSRHKKLMFKTEGPDSD

71 NP_002341.1 NP_000213.1

>NP_002341.1

MGCIKSKGKDSLSDDGVDLKTQPVRNTERTIYVRDPTSNKQQRPVPESQLLPGQRFQTKDPEEQGDIVVALYPYDGIHPDDLSFKKGEKMKVLEEHGEWWKAKSLLTKKEGFIPSNYVAKLNTLETEEWFFKDITRKDAERQLLAPGNSAGAFLIRESETLKGSFSLSVRDFDPVHGDVIKHYKIRSLDNGGYYISPRITFPCISDMIKHYQKQADGLCRRLEKACISPKPQKPWDKDAWEIPRESIKLVKRLGAGQFGEVWMGYYNNSTKVAVKTLKPGTMSVQAFLEEANLMKTLQHDKLVRLYAVVTREEPIYIITEYMAKGSLLDFLKSDEGGKVLLPKLIDFSAQIAEGMAYIERKNYIHRDLRAANVLVSESLMCKIADFGLARVIEDNEYTAREGAKFPIKWTAPEAINFGCFTIKSDVWSFGILLYEIVTYGKIPYPGRTNADVMTALSQGYRMPRVENCPDELYDIMKMCWKEKAEERPTFDYLQSVLDDFYTATEGQYQQQP

>NP_000213.1

MRGARGAWDFLCVLLLLLRVQTGSSQPSVSPGEPSPPSIHPGKSDLIVRVGDEIRLLCTDPGFVKWTFEILDETNENKQNEWITEKAEATNTGKYTCTNKHGLSNSIYVFVRDPAKLFLVDRSLYGKEDNDTLVRCPLTDPEVTNYSLKGCQGKPLPKDLRFIPDPKAGIMIKSVKRAYHRLCLHCSVDQEGKSVLSEKFILKVRPAFKAVPVVSVSKASYLLREGEEFTVTCTIKDVSSSVYSTWKRENSQTKLQEKYNSWHHGDFNYERQATLTISSARVNDSGVFMCYANNTFGSANVTTTLEVVDKGFINIFPMINTTVFVNDGENVDLIVEYEAFPKPEHQQWIYMNRTFTDKWEDYPKSENESNIRYVSELHLTRLKGTEGGTYTFLVSNSDVNAAIAFNVYVNTKPEILTYDRLVNGMLQCVAAGFPEPTIDWYFCPGTEQRCSASVLPVDVQTLNSSGPPFGKLVVQSSIDSSAFKHNGTVECKAYNDVGKTSAYFNFAFKGNNKEQIHPHTLFTPLLIGFVIVAGMMCIIVMILTYKYLQKPMYEVQWKVVEEINGNNYVYIDPTQLPYDHKWEFPRNRLSFGKTLGAGAFGKVVEATAYGLIKSDAAMTVAVKMLKPSAHLTEREALMSELKVLSYLGNHMNIVNLLGACTIGGPTLVITEYCCYGDLLNFLRRKRDSFICSKQEDHAEAALYKNLLHSKESSCSDSTNEYMDMKPGVSYVVPTKADKRRSVRIGSYIERDVTPAIMEDDELALDLEDLLSFSYQVAKGMAFLASKNCIHRDLAARNILLTHGRITKICDFGLARDIKNDSNYVVKGNARLPVKWMAPESIFNCVYTFESDVWSYGIFLWELFSLGSSPYPGMPVDSKFYKMIKEGFRMLSPEHAPAEMYDIMKTCWDADPLKRPTFKQIVQLIEKQISESTNHIYSNLANCSPNRQKPVVDHSVRINSVGSTASSSQPLLVHDDV

72 NP_003870.3 NP_002871.1

>NP_003870.3

MSAEVIHQVEEALDTDEKEMLLFLCRDVAIDVVPPNVRDLLDILRERGKLSVGDLAELLYRVRRFDLLKRILKMDRKAVETHLLRNPHLVSDYRVLMAEIGEDLDKSDVSSLIFLMKDYMGRGKISKEKSFLDLVVELEKLNLVAPDQLDLLEKCLKNIHRIDLKTKIQKYKQSVQGAGTSYRNVLQAAIQKSLKDPSNNFRLHNGRSKEQRLKEQLGAQQEPVKKSIQESEAFLPQSIPEERYKMKSKPLGICLIIDCIGNETELLRDTFTSLGYEVQKFLHLSMHGISQILGQFACMPEHRDYDSFVCVLVSRGGSQSVYGVDQTHSGLPLHHIRRMFMGDSCPYLAGKPKMFFIQNYVVSEGQLENSSLLEVDGPAMKNVEFKAQKRGLCTVHREADFFWSLCTADMSLLEQSHSSPSLYLQCLSQKLRQERKRPLLDLHIELNGYMYDWNSRVSAKEKYYVWLQHTLRKKLILSYT

>NP_002871.1

MEHIQGAWKTISNGFGFKDAVFDGSSCISPTIVQQFGYQRRASDDGKLTDPSKTSNTIRVFLPNKQRTVVNVRNGMSLHDCLMKALKVRGLQPECCAVFRLLHEHKGKKARLDWNTDAASLIGEELQVDFLDHVPLTTHNFARKTFLKLAFCDICQKFLLNGFRCQTCGYKFHEHCSTKVPTMCVDWSNIRQLLLFPNSTIGDSGVPALPSLTMRRMRESVSRMPVSSQHRYSTPHAFTFNTSSPSSEGSLSQRQRSTSTPNVHMVSTTLPVDSRMIEDAIRSHSESASPSALSSSPNNLSPTGWSQPKTPVPAQRERAPVSGTQEKNKIRPRGQRDSSYYWEIEASEVMLSTRIGSGSFGTVYKGKWHGDVAVKILKVVDPTPEQFQAFRNEVAVLRKTRHVNILLFMGYMTKDNLAIVTQWCEGSSLYKHLHVQETKFQMFQLIDIARQTAQGMDYLHAKNIIHRDMKSNNIFLHEGLTVKIGDFGLATVKSRWSGSQQVEQPTGSVLWMAPEVIRMQDNNPFSFQSDVYSYGIVLYELMTGELPYSHINNRDQIIFMVGRGYASPDLSKLYKNCPKAMKRLVADCVKKVKEERPLFPQILSSIELLQHSLPKINRSASEPSLHRAAHTEDINACTLTTSPRLPVF

73 NP_056097.1 NP_036457.1

>NP_056097.1

MEPRMESCLAQVLQKDVGKRLQVGQELIDYFSDKQKSADLEHDQTMLDKLVDGLATSWVNSSNYKVVLLGMDILSALVTRLQDRFKAQIGTVLPSLIDRLGDAKDSVREQDQTLLLKIMDQAANPQYVWDRMLGGFKHKNFRTREGICLCLIATLNASGAQTLTLSKIVPHICNLLGDPNSQVRDAAINSLVEIYRHVGERVRADLSKKGLPQSRLNVIFTKFDEVQKSGNMIQSANDKNFDDEDSVDGNRPSSASSTSSKAPPSSRRNVGMGTTRRLGSSTLGSKSSAAKEGAGAVDEEDFIKAFDDVPVVQIYSSRDLEESINKIREILSDDKHDWEQRVNALKKIRSLLLAGAAEYDNFFQHLRLLDGAFKLSAKDLRSQVVREACITLGHLSSVLGNKFDHGAEAIMPTIFNLIPNSAKIMATSGVVAVRLIIRHTHIPRLIPVITSNCTSKSVAVRRRCFEFLDLLLQEWQTHSLERHISVLAETIKKGIHDADSEARIEARKCYWGFHSHFSREAEHLYHTLESSYQKALQSHLKNSDSIVSLPQSDRSSSSSQESLNRPLSAKRSPTGSTTSRASTVSTKSVSTTGSLQRSRSDIDVNAAASAKSKVSSSSGTTPFSSAAALPPGSYASLGRIRTRRQSSGSATNVASTPDNRGRSRAKVVSQSQRSRSANPAGAGSRSSSPGKLLGSGYGGLTGGSSRGPPVTPSSEKRSKIPRSQGCSRETSPNRIGLARSSRIPRPSMSQGCSRDTSRESSRDTSPARGFPPLDRFGLGQPGRIPGSVNAMRVLSTSTDLEAAVADALKKPVRRRYEPYGMYSDDDANSDASSVCSERSYGSRNGGIPHYLRQTEDVAEVLNHCASSNWSERKEGLLGLQNLLKSQRTLSRVELKRLCEIFTRMFADPHSKRVFSMFLETLVDFIIIHKDDLQDWLFVLLTQLLKKMGADLLGSVQAKVQKALDVTRDSFPFDQQFNILMRFIVDQTQTPNLKVKVAILKYIESLARQMDPTDFVNSSETRLAVSRIITWTTEPKSSDVRKAAQIVLISLFELNTPEFTMLLGALPKTFQDGATKLLHNHLKNSSNTSVGSPSNTIGRTPSRHTSSRTSPLTSPTNCSHGGLSPSRLWGWSADGLAKHPPPFSQPNSIPTAPSHKALRRSYSPSMLDYDTENLNSEEIYSSLRGVTEAIEKFSFRSQEDLNEPIKRDGKKECDIVSRDGGAASPATEGRGGSEVEGGRTALDNKTSLLNTQPPRAFPGPRARDYNPYPYSDAINTYDKTALKEAVFDDDMEQLRDVPIDHSDLVADLLKELSNHNERVEERKGALLELLKITREDSLGVWEEHFKTILLLLLETLGDKDHSIRALALRVLREILRNQPARFKNYAELTIMKTLEAHKDSHKEVVRAAEEAASTLASSIHPEQCIKVLCPIIQTADYPINLAAIKMQTKVVERIAKESLLQLLVDIIPGLLQGYDNTESSVRKASVFCLVAIYSVIGEDLKPHLAQLTGSKMKLLNLYIKRAQTTNSNSSSSSDVSTHS

>NP_036457.1

MAVNVYSTSVTSDNLSRHDMLAWINESLQLNLTKIEQLCSGAAYCQFMDMLFPGSIALKKVKFQAKLEHEYIQNFKILQAGFKRMGVDKIIPVDKLVKGKFQDNFEFVQWFKKFFDANYDGKDYDPVAARQGQETAVAPSLVAPALNKPKKPLTSSSAAPQRPISTQRTAAAPKAGPGVVRKNPGVGNGDDEAAELMQQVNVLKLTVEDLEKERDFYFGKLRNIELICQENEGENDPVLQRIVDILYATDEGFVIPDEGGPQEEQEEY

74 NP_115822.1 NP_006268.1

>NP_115822.1

MVISLNSCLSFICLLLCHWIGTASPLNLEDPNVCSHWESYSVTVQESYPHPFDQIYYTSCTDILNWFKCTRHRVSYRTAYRHGEKTMYRRKSQCCPGFYESGEMCVPHCADKCVHGRCIAPNTCQCEPGWGGTNCSSACDGDHWGPHCTSRCQCKNGALCNPITGACHCAAGFRGWRCEDRCEQGTYGNDCHQRCQCQNGATCDHVTGECRCPPGYTGAFCEDLCPPGKHGPQCEQRCPCQNGGVCHHVTGECSCPSGWMGTVCGQPCPEGRFGKNCSQECQCHNGGTCDAATGQCHCSPGYTGERCQDECPVGTYGVLCAETCQCVNGGKCYHVSGACLCEAGFAGERCEARLCPEGLYGIKCDKRCPCHLENTHSCHPMSGECACKPGWSGLYCNETCSPGFYGEACQQICSCQNGADCDSVTGKCTCAPGFKGIDCSTPCPLGTYGINCSSRCGCKNDAVCSPVDGSCTCKAGWHGVDCSIRCPSGTWGFGCNLTCQCLNGGACNTLDGTCTCAPGWRGEKCELPCQDGTYGLNCAERCDCSHADGCHPTTGHCRCLPGWSGVHCDSVCAEGRWGPNCSLPCYCKNGASCSPDDGICECAPGFRGTTCQRICSPGFYGHRCSQTCPQCVHSSGPCHHITGLCDCLPGFTGALCNEVCPSGRFGKNCAGICTCTNNGTCNPIDRSCQCYPGWIGSDCSQPCPPAHWGPNCIHTCNCHNGAFCSAYDGECKCTPGWTGLYCTQRCPLGFYGKDCALICQCQNGADCDHISGQCTCRTGFMGRHCEQKCPSGTYGYGCRQICDCLNNSTCDHITGTCYCSPGWKGARCDQAGVIIVGNLNSLSRTSTALPADSYQIGAIAGIIILVLVVLFLLALFIIYRHKQKGKESSMPAVTYTPAMRVVNADYTISGTLPHSNGGNANSHYFTNPSYHTLTQCATSPHVNNRDRMTVTKSKNNQLFVNLKNVNPGKRGPVGDCTGTLPADWKHGGYLNELGAFGLDRSYMGKSLKDLGKNSEYNSSNCSLSSSENPYATIKDPPVLIPKSSECGYVEMKSPARRDSPYAEINNSTSANRNVYEVEPTVSVVQGVFSNNGRLSQDPYDLPKNSHIPCHYDLLPVRDSSSSPKQEDSGGSSSNSSSSSE

>NP_006268.1

MAQFPTAMNGGPNMWAITSEERTKHDRQFDNLKPSGGYITGDQARNFFLQSGLPAPVLAEIWALSDLNKDGKMDQQEFSIAMKLIKLKLQGQQLPVVLPPIMKQPPMFSPLISARFGMGSMPNLSIPQPLPPAAPITSLSSATSGTNLPPLMMPTPLVPSVSTSSLPNGTASLIQPLPIPYSSSTLPHGSSYSLMMGGFGGASIQKAQSLIDLGSSSSTSSTASLSGNSPKTGTSEWAVPQPTRLKYRQKFNTLDKSMSGYLSGFQARNALLQSNLSQTQLATIWTLADVDGDGQLKAEEFILAMHLTDMAKAGQPLPLTLPPELVPPSFRGGKQIDSINGTLPSYQKMQEEEPQKKLPVTFEDKRKANYERGNMELEKRRQALMEQQQREAERKAQKEKEEWERKQRELQEQEWKKQLELEKRLEKQRELERQREEERRKDIERREAAKQELERQRRLEWERIRRQELLNQKNREQEEIVRLNSKKKNLHLELEALNGKHQQISGRLQDVRLKKQTQKTELEVLDKQCDLEIMEIKQLQQELQEYQNKLIYLVPEKQLLNERIKNMQFSNTPDSGVSLLHKKSLEKEELCQRLKEQLDALEKETASKLSEMDSFNNQLKCGNMDDSVLQCLLSLLSCLNNLFLLLKELRETYNTQQLALEQLYKIKRDKLKEIERKRLELMQKKKLEDEAARKAKQGKENLWKENLRKEEEEKQKRLQEEKTQEKIQEEERKAEEKQRKDKDTLKAEEKKRETASVLVNYRALYPFEARNHDEMSFNSGDIIQVDEKTVGEPGWLYGSFQGNFGWFPCNYVEKMPSSENEKAVSPKKALLPPTVSLSATSTSSEPLSSNQPASVTDYQNVSFSNLTVNTSWQKKSAFTRTVSPGSVSPIHGQGQVVENLKAQALCSWTAKKDNHLNFSKHDIITVLEQQENWWFGEVHGGRGWFPKSYVKIIPGSEVKREEPEALYAAVNKKPTSAAYSVGEEYIALYPYSSVEPGDLTFTEGEEILVTQKDGEWWTGSIGDRSGIFPSNYVKPKDQESFGSASKSGASNKKPEIAQVTSAYVASGSEQLSLAPGQLILILKKNTSGWWQGELQARGKKRQKGWFPASHVKLLGPSSERATPAFHPVCQVIAMYDYAANNEDELSFSKGQLINVMNKDDPDWWQGEINGVTGLFPSNYVKMTTDSDPSQQWCADLQTLDTMQPIERKRQGYIHELIQTEERYMADLQLVVEVFQKRMAESGFLTEGEMALIFVNWKELIMSNTKLLKALRVRKKTGGEKMPVQMIGDILAAELSHMQAYIRFCSCQLNGAALLQQKTDEDTDFKEFLKKLASDPRCKGMPLSSFLLKPMQRITRYPLLIRSILENTPESHADHSSLKLALERAEELCSQVNEGVREKENSDRLEWIQAHVQCEGLAEQLIFNSLTNCLGPRKLLHSGKLYKTKSNKELHGFLFNDFLLLTYMVKQFAVSSGSEKLFSSKSNAQFKMYKTPIFLNEVLVKLPTDPSSDEPVFHISHIDRVYTLRTDNINERTAWVQKIKAASEQYIDTEKKKREKAYQARSQKTSGIGRLMVHVIEATELKACKPNGKSNPYCEISMGSQSYTTRTIQDTLNPKWNFNCQFFIKDLYQDVLCLTLFDRDQFSPDDFLGRTEIPVAKIRTEQESKGPMTRRLLLHEVPTGEVWVRFDLQLFEQKTLL

75 NP_005334.1 AAA59925.1

>NP_005334.1

MTEYKLVVVGAGGVGKSALTIQLIQNHFVDEYDPTIEDSYRKQVVIDGETCLLDILDTAGQEEYSAMRDQYMRTGEGFLCVFAINNTKSFEDIHQYREQIKRVKDSDDVPMVLVGNKCDLAARTVESRQAQDLARSYGIPYIETSAKTRQGVEDAFYTLVREIRQHKLRKLNPPDESGPGCMSCKCVLS

>AAA59925.1

MAAHRPVEWVQAVVSRFDEQLPIKTGQQNTHTKVSTEHNKECLINISKYKFSLVISGLTTILKNVNNMRIFGEAAEKNLYLSQLIILDTLEKCLAGQPKDTMRLDETMLVKQLLPEICHFLHTCREGNQHAAELRNSASGVLFSLSCNNFNAVFSRISTRLQELTVCSEDNVDVHDIELLQYINVDCAKLKRLLKETAFKFKALKKVAQLAVINSLEKAFWNWVENYPDEFTKLYQIPQTDMAECAEKLFDLVDGFAESTKRKAAVWPLQIILLILCPEIIQDISKDVVDENNMNKKLFLDSLRKALAGHGGSRQLTESAAIACVKLCKASTYINWEDNSVIFLLVQSMVVDLKNLLFNPSKPFSRGSQPADVDLMIDCLVSCFRISPHNNQHFKICLAQNSPSTFHYVLVNSLHRIITNSALDWWPKIDAVYCHSVELRNMFGETLHKAVQGCGAHPAIRMAPSLTFKEKVTSLKFKEKPTDLETRSYKYLLLSMVKLIHADPKLLLCNPRKQGPETQGSTAELITGLVQLVPQSHMPEIAQEAMEALLVLHQLDSIDLWNPDAPVETFWEISSQMLFYICKKLTSHQMLSSTEILKWLREILICRNKFLLKNKQADRSSCHFLLFYGVGCDIPSSGNTSQMSMDHEELLRTPGASLRKGKGNSSMDSAAGCSGTPPICRQAQTKLEVALYMFLWNPDTEAVLVAMSCFRHLCEEADIRCGVDEVSVHNLLPNYNTFMEFASVSNMMSTGRAALQKRVMALLRRIEHPTAGNTEAWEDTHAKWEQATKLILNYPKAKMEDGQAAESLHKTIVKRRMSHVSGGGSIDLSDTDSLQEWINMTGFLCALGGVCLQQRSNSGLATYSPPMGPVSERKGSMISVMSSEGNADTPVSKFMDRLLSLMVCNHEKVGLQIRTNVKDLVGLELSPALYPMLFNKLKNTISKFFDSQGQVLLTDTNTQFVEQTIAIMKNLLDNHTEGSSEHLGQASIETMMLNLVRYVRVLGNMVHAIQIKTKLCQLVEVMMARRDDLSFCQEMKFRNKMVEYLTDWVMGTSNQAADDDVKCLTRDLDQASMEAVVSLLAGLPLQPEEGDGVELMEAKSQLFLKYFTLFMNLLNDCSEVEDESAQTGGRKRGMSRRLASLRHCTVLAMSNLLNANVDSGLMHSIGLGYHKDLQTRATFMEVLTKILQQGTEFDTLAETVLADRFERLVELVTMMGDQGELPIAMALANVVPCSQWDELARVLVTLFDSRHLLYQLLWNMFSKEVELADSMQTLFRGNSLASKIMTFCFKVYGATYLQKLLDPLLRIVITSSDWQHVSFEVDPTRLEPSESLEENQRNLLQMTEKFFHAIISSSSEFPPQLRSVCHCLYQATCHSLLNKATVKEKKENKKSVVSQRFPQNSIGAVGSAMFLRFINPAIVSPYEAGILDKKPPPRIERGLKLMSKILQSIANHVLFTKEEHMRPFNDFVKSNFDAARRFFLDIASDCPTSDAVNHSLSFISDGNVLALHRLLWNNQEKIGQYLSSNRDHKAVGRRPFDKMATLLAYLGPPEHKPVADTHWSSLNLTSSKFEEFMTRHQVHEKEEFKALKTLSIFYQAGTSKAGNPIFYYVARRFKTGQINGDLLIYHVLLTLKPYYAKPYEIVVDLTHTGPSNRFKTDFLSKWFVVFPGFAYDNVSAVYIYNCNSWVREYTKYHERLLTGLKGSKRLVFIDCPGKLAEHIEHEQQKLPAATLALEEDLKVFHNALKLAHKDTKVSIKVGSTAVQVTSAERTKVLGQSVFLNDIYYASEIEEICLVDENQFTLTIANQGTPLTFMHQECEAIVQSIIHIRTRWELSQPDSIPQHTKIRPKDVPGTLLNIALLNLGSSDPSLRSAAYNLLCALTCTFNLKIEGQLLETSGLCIPANNTLFIVSISKTLAANEPHLTLEFLEECISGFSKSSIELKHLCLEYMTPWLSNLVRFCKHNDDAKRQRVTAILDKLITMTINEKQMYPSIQAKIWGSLGQITDLLDVVLDSFIKTSATGGLGSIKAEVMADTAVALASGNVKLVSSKVIGRMCKIIDKTCLSPTPTLEQHLMWDDIAILARYMLMLSFNNSLDVAAHLPYLFHVVTFLVATGPLSLRASTHGLVINIIHSLCTCSQLHFSEETKQVLRLSLTEFSLPKFYLLFGISKVKSAAVIAFRSSYRDRSFSPGSYERETFALTSLETVTEALLEIMEACMRDIPTCKWLDQWTELAQRFAFQYNPSLQPRALVVFGCISKRVSHGQIKQIIRILSKALESCLKGPDTYNSQVLIEATVIALTKLQPLLNKDSPLHKALFWVAVAVLQLDEVNLYSAGTALLEQNLHTLDSLRIFNDKSPEEVFMAIRNPLEWHCKQMDHFVGLNFNSNFNFALVGHLLKGYRHPSPAIVARTVRILHTLLTLVNKHRNCDKFEVNTQSVAYLAALLTVSEEVRSRCSLKHRKSLLLTDISMENVPMDTYPIHHGDPSYRTLKETQPWSSPKGSEGYLAATYPTVGQTSPRARKSMSLDMGQPSQANTKKLLGTRKSFDHLISDTKAPKRQEMESGITTPPKMRRVAETDYEMETQRISSSQQHPHLRKVSVSESNVLLDEEVLTDPKIQALLLTVLATLVKYTTDEFDQRILYEYLAEASVVFPKVFPVVHNLLDSKINTLLSLCQDPNLLNPIHGIVQSVVYHEESPPQYQTSYLQSFGFNGLWRFAGPFSKQTQIPDYAELIVKFLDALIDTYLPGIDEETSEESLLTPTSPYPPALQSQLSITANLNLSNSMTSLATSQHSPGIDKENVELSPTTGHCNSGRTRHGSASQVQKQRSAGSFKRNSIKKIV

76 NP_000681.2 NP_060942.2

>NP_000681.2

MLRAAARFGPRLGRRLLSAAATQAVPAPNQQPEVFCNQIFINNEWHDAVSRKTFPTVNPSTGEVICQVAEGDKEDVDKAVKAARAAFQLGSPWRRMDASHRGRLLNRLADLIERDRTYLAALETLDNGKPYVISYLVDLDMVLKCLRYYAGWADKYHGKTIPIDGDFFSYTRHEPVGVCGQIIPWNFPLLMQAWKLGPALATGNVVVMKVAEQTPLTALYVANLIKEAGFPPGVVNIVPGFGPTAGAAIASHEDVDKVAFTGSTEIGRVIQVAAGSSNLKRVTLELGGKSPNIIMSDADMDWAVEQAHFALFFNQGQCCCAGSRTFVQEDIYDEFVERSVARAKSRVVGNPFDSKTEQGPQVDETQFKKILGYINTGKQEGAKLLCGGGIAADRGYFIQPTVFGDVQDGMTIAKEEIFGPVMQILKFKTIEEVVGRANNSTYGLAAAVFTKDLDKANYLSQALQAGTVWVNCYDVFGAQSPFGGYKMSGSGRELGEYGLQAYTEVKTVTVKVPQKNS

>NP_060942.2

MAAAELADTQLMLGVGLIEKDTNGEVLWVWCYPSTTATLRNLLLRKCCLTDENKLLHPFVFGQYRRTWFYITIIEVPDSSILKKVTHFSIVLTAKDFNPEKYAAFTRILCRMYLKHGSPVKMMESYIAVLTKGICQSEENGSFLSKDFDARKAYPAGSIKDIVSQFGMETVILHTALMLKKRIVVYHPKIEAVQEFTRTLPALVWHRQDWTILHSYVHLNADELEALQMCTGYVAGFVDLEVSNRPDLYDVFVNLAESEITIAPLAKEAMAMGKLHKEMGQLIVQSAEDPEKSESQVIQDIALKTREIFTNLAPFSEVSADGEKRVLNLEALKQKRFPPATENFLYHLAAAEQMLKI

77 NP_008848.1 NP_612370.3

>NP_008848.1

MYGNYSHFMKFPAGYGGSPGHTGSTSMSPSAALSTGKPMDSHPSYTDTPVSAPRTLSAVGTPLNALGSPYRVITSAMGPPSGALAAPPGINLVAPPSSQLNVVNSVSSSEDIKPLPGLPGIGNMNYPSTSPGSLVKHICAICGDRSSGKHYGVYSCEGCKGFFKRTIRKDLIYTCRDNKDCLIDKRQRNRCQYCRYQKCLVMGMKREAVQEERQRSRERAESEAECATSGHEDMPVERILEAELAVEPKTESYGDMNMENSTNDPVTNICHAADKQLFTLVEWAKRIPHFSDLTLEDQVILLRAGWNELLIASFSHRSVSVQDGILLATGLHVHRSSAHSAGVGSIFDRVLTELVSKMKDMQMDKSELGCLRAIVLFNPDAKGLSNPSEVETLREKVYATLEAYTKQKYPEQPGRFAKLLLRLPALRSIGLKCLEHLFFFKLIGDTPIDTFLMEMLETPLQIT

>NP_612370.3

MPLFFRKRKPSEEARKRLEYQMCLAKEAGADDILDISKCELSEIPFGAFATCKVLQKKVLIVHTNHLTSLLPKSCSLLSLATIKVLDLHDNQLTALPDDLGQLTALQVLNVERNQLMQLPRSIGNLTQLQTLNVKDNKLKELPDTVGELRSLRTLNISGNEIQRLPQMLAHVRTLEMLSLDASAMVYPPREVCGAGTAAILQFLCKESGLEYYPPSQYLLPILEQDGIENSRDSPDGPTDRFSREELEWQNRFSDYEKRKEQKMLEKLEFERRLELGQREHTQLLQQSSSQKDEILQTVKEEQSRLEQGLSEHQRHLNAERQRLQEQLKQTEQNISSRIQKLLQDNQRQKKSSEILKSLENERIRMEQLMSITQEETESLRRRDVASAMQQMLTESCKNRLIQMAYESQRQNLVQQACSSMAEMDERFQQILSWQQMDQNKAISQILQESAMQKAAFEALQVKKDLMHRQIRSQIKLIETELLQLTQLELKRKSLDTESLQEMISEQRWALSSLLQQLLKEKQQREEELREILTELEAKSETRQENYWLIQYQRLLNQKPLSLKLQEEGMERQLVALLEELSAEHYLPIFAHHRLSLDLLSQMSPGDLAKVGVSEAGLQHEILRRVQELLDAARIQPELKPPMGEVVTPTAPQEPPESVRPSAPPAELEVQASECVVCLEREAQMIFLNCGHVCCCQQCCQPLRTCPLCRQDIAQRLRIYHSS

78 NP_005612.1 NP_001127.1

>NP_005612.1

MTKLEEHLEGIVNIFHQYSVRKGHFDTLSKGELKQLLTKELANTIKNIKDKAVIDEIFQGLDANQDEQVDFQEFISLVAIALKAAHYHTHKE

>NP_001127.1

MAAGTAVGAWVLVLSLWGAVVGAQNITARIGEPLVLKCKGAPKKPPQRLEWKLNTGRTEAWKVLSPQGGGPWDSVARVLPNGSLFLPAVGIQDEGIFRCQAMNRNGKETKSNYRVRVYQIPGKPEIVDSASELTAGVPNKVGTCVSEGSYPAGTLSWHLDGKPLVPNEKGVSVKEQTRRHPETGLFTLQSELMVTPARGGDPRPTFSCSFSPGLPRHRALRTAPIQPRVWEPVPLEEVQLVVEPEGGAVAPGGTVTLTCEVPAQPSPQIHWMKDGVPLPLPPSPVLILPEIGPQDQGTYSCVATHSSHGPQESRAVSISIIEPGEEGPTAGSVGGSGLGTLALALGILGGLGTAALLIGVILWQRRQRRGEERKAPENQEEEEERAELNQSEEPEAGESSTGGP

79 NP_005513.1 NP_003293.2

>NP_005513.1

MDNARMNSFLEYPILSSGDSGTCSARAYPSDHRITTFQSCAVSANSCGGDDRFLVGRGVQIGSPHHHHHHHHHHPQPATYQTSGNLGVSYSHSSCGPSYGSQNFSAPYSPYALNQEADVSGGYPQCAPAVYSGNLSSPMVQHHHHHQGYAGGAVGSPQYIHHSYGQEHQSLALATYNNSLSPLHASHQEACRSPASETSSPAQTFDWMKVKRNPPKTGKVGEYGYLGQPNAVRTNFTTKQLTELEKEFHFNKYLTRARRVEIAASLQLNETQVKIWFQNRRMKQKKREKEGLLPISPATPPGNDEKAEESSEKSSSSPCVPSPGSSTSDTLTTSH

>NP_003293.2

MSGPTWLPPKQPEPARAPQGRAIPRGTPGPPPAHGAALQPHPRVNFCPLPSEQCYQAPGGPEDRGPAWVGSHGVLQHTQGLPADRGGLRPGSLDAEIDLLSSTLAELNGGRGHASRRPDRQAYEPPPPPAYRTGSLKPNPASPLPASPYGGPTPASYTTASTPAGPAFPVQVKVAQPVRGCGPPRRGASQASGPLPGPHFPLPGRGEVWGPGYRSQREPGPGAKEEAAGVSGPAGRGRGGEHGPQVPLSQPPEDELDRLTKKLVHDMNHPPSGEYFGQCGGCGEDVVGDGAGVVALDRVFHVGCFVCSTCRAQLRGQHFYAVERRAYCEGCYVATLEKCATCSQPILDRILRAMGKAYHPGCFTCVVCHRGLDGIPFTVDATSQIHCIEDFHRKFAPRCSVCGGAIMPEPGQEETVRIVALDRSFHIGCYKCEECGLLLSSEGECQGCYPLDGHILCKACSAWRIQELSATVTTDC

80 NP_003759.1 NP_060686.1

>NP_003759.1

MAEYGTLLQDLTNNITLEDLEQLKSACKEDIPSEKSEEITTGSAWFSFLESHNKLDKDNLSYIEHIFEISRRPDLLTMVVDYRTRVLKISEEDELDTKLTRIPSAKKYKDIIRQPSEEEIIKLAPPPKKA

>NP_060686.1

MAECGASGSGSSGDSLDKSITLPPDEIFRNLENAKRFAIDIGGSLTKLAYYSTVQHKVAKVRSFDHSGKDTEREHEPPYEISVQEEITARLHFIKFENTYIEACLDFIKDHLVNTETKVIQATGGGAYKFKDLIEEKLRLKVDKEDVMTCLIKGCNFVLKNIPHEAFVYQKDSDPEFRFQTNHPHIFPYLLVNIGSGVSIVKVETEDRFEWVGGSSIGGGTFWGLGALLTKTKKFDELLHLASRGQHSNVDMLVRDVYGGAHQTLGLSGNLIASSFGKSATADQEFSKEDMAKSLLHMISNDIGQLACLHARLHSLDRVYFGGFFIRGHPVTMRTITYSINFFSKGEVQALFLRHEGYLGAIGAFLKGAEQDNPNQYSWGENYAGSSGLMSASPELGPAQRARSGTFDLLEMDRLERPLVDLPLLLDPPSYVPDTVDLTDDALARKYWLTCFEEALDGVVKRAVASQPDSVDAAERAEKFRQKYWNKLQTLRQQPFAYGTLTVRSLLDTREHCLNEFNFPDPYSKVKQRENGVALRCFPGVVRSLDALGWEERQLALVKGLLAGNVFDWGAKAVSAVLESDPYFGFEEAKRKLQERPWLVDSYSEWLQRLKGPPHKCALIFADNSGIDIILGVFPFVRELLLRGTEVILACNSGPALNDVTHSESLIVAERIAGMDPVVHSALQEERLLLVQTGSSSPCLDLSRLDKGLAALVRERGADLVVIEGMGRAVHTNYHAALRCESLKLAVIKNAWLAERLGGRLFSVIFKYEVPAE

81 NP_015561.1 NP_001041659.1

>NP_015561.1

MADLANEEKPAIAPPVFVFQKDKGQKSPAEQKNLSDSGEEPRGEAEAPHHGTGHPESAGEHALEPPAPAGASASTPPPPAPEAQLPPFPRELAGRSAGGSSPEGGEDSDREDGNYCPPVKRERTSSLTQFPPSQSEERSSGFRLKPPTLIHGQAPSAGLPSQKPKEQQRSVLRPAVLQAPQPKALSQTVPSSGTNGVSLPADCTGAVPAASPDTAAWRSPSEAADEVCALEEKEPQKNESSNASEEEACEKKDPATQQAFVFGQNLRDRVKLINESVDEADMENAGHPSADTPTATNYFLQYISSSLENSTNSADASSNKFVFGQNMSERVLSPPKLNEVSSDANRENAAAESGSESSSQEATPEKESLAESAAAYTKATARKCLLEKVEVITGEEAESNVLQMQCKLFVFDKTSQSWVERGRGLLRLNDMASTDDGTLQSRLVMRTQGSLRLILNTKLWAQMQIDKASEKSIRITAMDTEDQGVKVFLISASSKDTGQLYAALHHRILALRSRVEQEQEAKMPAPEPGAAPSNEEDDSDDDDVLAPSGATAAGAGDEGDGQTTGST

>NP_001041659.1

MSPKRIAKRRSPPADAIPKSKKVKDTRAAASRRVPGARSCQGACGPSPPDQKTRPVSHRSHSTEPGLVLTLGQGDVGQLGLGENVMERKKPALVSIPEDVVQAEAGGMHTVCLSKSGQVYSFGCNDEGALGRDTSVEGSEMVPGKVELQEKVVQVSAGDSHTAALTDDGRVFLWGSFRDNNGVIGLLEPMKKSMVPVQVQLDVPVVKVASGNDHLVMLTADGDLYTLGCGEQGQLGRVPELFANRGGRQGLERLLVPKCVMLKSRGSRGHVRFQDAFCGAYFTFAISHEGHVYGFGLSNYHQLGTPGTESCFIPQNLTSFKNSTKSWVGFSGGQHHTVCMDSEGKAYSLGRAEYGRLGLGEGAEEKSIPTLISRLPAVSSVACGASVGYAVTKDGRVFAWGMGTNYQLGTGQDEDAWSPVEMMGKQLENRVVLSVSSGGQHTVLLVKDKEQS

82 NP_000052.1 NP_002728.1

>NP_000052.1

MAAVILESIFLKRSQQKKKTSPLNFKKRLFLLTVHKLSYYEYDFERGRRGSKKGSIDVEKITCVETVVPEKNPPPERQIPRRGEESSEMEQISIIERFPYPFQVVYDEGPLYVFSPTEELRKRWIHQLKNVIRYNSDLVQKYHPCFWIDGQYLCCSQTAKNAMGCQILENRNGSLKPGSSHRKTKKPLPPTPEEDQILKKPLPPEPAAAPVSTSELKKVVALYDYMPMNANDLQLRKGDEYFILEESNLPWWRARDKNGQEGYIPSNYVTEAEDSIEMYEWYSKHMTRSQAEQLLKQEGKEGGFIVRDSSKAGKYTVSVFAKSTGDPQGVIRHYVVCSTPQSQYYLAEKHLFSTIPELINYHQHNSAGLISRLKYPVSQQNKNAPSTAGLGYGSWEIDPKDLTFLKELGTGQFGVVKYGKWRGQYDVAIKMIKEGSMSEDEFIEEAKVMMNLSHEKLVQLYGVCTKQRPIFIITEYMANGCLLNYLREMRHRFQTQQLLEMCKDVCEAMEYLESKQFLHRDLAARNCLVNDQGVVKVSDFGLSRYVLDDEYTSSVGSKFPVRWSPPEVLMYSKFSSKSDIWAFGVLMWEIYSLGKMPYERFTNSETAEHIAQGLRLYRPHLASEKVYTIMYSCWHEKADERPTFKILLSNILDVMDEES

>NP_002728.1

MADVFPGNDSTASQDVANRFARKGALRQKNVHEVKDHKFIARFFKQPTFCSHCTDFIWGFGKQGFQCQVCCFVVHKRCHEFVTFSCPGADKGPDTDDPRSKHKFKIHTYGSPTFCDHCGSLLYGLIHQGMKCDTCDMNVHKQCVINVPSLCGMDHTEKRGRIYLKAEVADEKLHVTVRDAKNLIPMDPNGLSDPYVKLKLIPDPKNESKQKTKTIRSTLNPQWNESFTFKLKPSDKDRRLSVEIWDWDRTTRNDFMGSLSFGVSELMKMPASGWYKLLNQEEGEYYNVPIPEGDEEGNMELRQKFEKAKLGPAGNKVISPSEDRKQPSNNLDRVKLTDFNFLMVLGKGSFGKVMLADRKGTEELYAIKILKKDVVIQDDDVECTMVEKRVLALLDKPPFLTQLHSCFQTVDRLYFVMEYVNGGDLMYHIQQVGKFKEPQAVFYAAEISIGLFFLHKRGIIYRDLKLDNVMLDSEGHIKIADFGMCKEHMMDGVTTRTFCGTPDYIAPEIIAYQPYGKSVDWWAYGVLLYEMLAGQPPFDGEDEDELFQSIMEHNVSYPKSLSKEAVSICKGLMTKHPAKRLGCGPEGERDVREHAFFRRIDWEKLENREIQPPFKPKVCGKGAENFDKFFTRGQPVLTPPDQLVIANIDQSDFEGFSYVNPQFVHPILQSAV

83 NP_005609.3 NP_036433.2

>NP_005609.3

MGSRCALALAVLSALLCQVWSSGVFELKLQEFVNKKGLLGNRNCCRGGAGPPPCACRTFFRVCLKHYQASVSPEPPCTYGSAVTPVLGVDSFSLPDGGGADSAFSNPIRFPFGFTWPGTFSLIIEALHTDSPDDLATENPERLISRLATQRHLTVGEEWSQDLHSSGRTDLKYSYRFVCDEHYYGEGCSVFCRPRDDAFGHFTCGERGEKVCNPGWKGPYCTEPICLPGCDEQHGFCDKPGECKCRVGWQGRYCDECIRYPGCLHGTCQQPWQCNCQEGWGGLFCNQDLNYCTHHKPCKNGATCTNTGQGSYTCSCRPGYTGATCELGIDECDPSPCKNGGSCTDLENSYSCTCPPGFYGKICELSAMTCADGPCFNGGRCSDSPDGGYSCRCPVGYSGFNCEKKIDYCSSSPCSNGAKCVDLGDAYLCRCQAGFSGRHCDDNVDDCASSPCANGGTCRDGVNDFSCTCPPGYTGRNCSAPVSRCEHAPCHNGATCHERGHRYVCECARGYGGPNCQFLLPELPPGPAVVDLTEKLEGQGGPFPWVAVCAGVILVLMLLLGCAAVVVCVRLRLQKHRPPADPCRGETETMNNLANCQREKDISVSIIGATQIKNTNKKADFHGDHSADKNGFKARYPAVDYNLVQDLKGDDTAVRDAHSKRDTKCQPQGSSGEEKGTPTTLRGGEASERKRPDSGCSTSKDTKYQSVYVISEEKDECVIATEV

>NP_036433.2

MSKSLKKKSHWTSKVHESVIGRNPEGQLGFELKGGAENGQFPYLGEVKPGKVAYESGSKLVSEELLLEVNETPVAGLTIRDVLAVIKHCKDPLRLKCVKQGGIVDKDLRHYLNLRFQKGSVDHELQQIIRDNLYLRTVPCTTRPHKEGEVPGVDYIFITVEDFMELEKSGALLESGTYEDNYYGTPKPPAEPAPLLLNVTDQILPGATPSAEGKRKRNKSVSNMEKASIEPPEEEEEERPVVNGNGVVVTPESSEHEDKSAGASGEMPSQPYPAPVYSQPEELKEQMDDTKPTKPEDNEEPDPLPDNWEMAYTEKGEVYFIDHNTKTTSWLDPRLAKKAKPPEECKENELPYGWEKIDDPIYGTYYVDHINRRTQFENPVLEAKRKLQQHNMPHTELGTKPLQAPGFREKPLFTRDASQLKGTFLSTTLKKSNMGFGFTIIGGDEPDEFLQVKSVIPDGPAAQDGKMETGDVIVYINEVCVLGHTHADVVKLFQSVPIGQSVNLVLCRGYPLPFDPEDPANSMVPPLAIMERPPPVMVNGRHNYETYLEYISRTSQSVPDITDRPPHSLHSMPTDGQLDGTYPPPVHDDNVSMASSGATQAELMTLTIVKGAQGFGFTIADSPTGQRVKQILDIQGCPGLCEGDLIVEINQQNVQNLSHTEVVDILKDCPIGSETSLIIHRGGFFSPWKTPKPIMDRWENQGSPQTSLSAPAIPQNLPFPPALHRSSFPDSTEAFDPRKPDPYELYEKSRAIYESRQQVPPRTSFRMDSSGPDYKELDVHLRRMESGFGFRILGGDEPGQPILIGAVIAMGSADRDGRLHPGDELVYVDGIPVAGKTHRYVIDLMHHAARNGQVNLTVRRKVLCGGEPCPENGRSPGSVSTHHSSPRSDYATYTNSNHAAPSSNASPPEGFASHSLQTSDVVIHRKENEGFGFVIISSLNRPESGSTITVPHKIGRIIDGSPADRCAKLKVGDRILAVNGQSIINMPHADIVKLIKDAGLSVTLRIIPQEELNSPTSAPSSEKQSPMAQQSPLAQQSPLAQPSPATPNSPIAQPAPPQPLQLQGHENSYRSEVKARQDVKPDIRQPPFTDYRQPPLDYRQPPGGDYQQPPPLDYRQPPLLDYRQHSPDTRQYPLSDYRQPQDFDYFTVDMEKGAKGFGFSIRGGREYKMDLYVLRLAEDGPAIRNGRMRVGDQIIEINGESTRDMTHARAIELIKSGGRRVRLLLKRGTGQVPEYDEPAPWSSPAAAAPGLPEVGVSLDDGLAPFSPSHPAPPSDPSHQISPGPTWDIKREHDVRKPKELSACGQKKQRLGEQRERSASPQRAARPRLEEAPGGQGRPEAGRPASEARAPGLAAADAADAARAGGKEAPRAAAGSELCRREGPGAAPAFAGPGGGGSGALEAEGRAGARAGPRPGPRPPGGAPARKAAVAPGPWKVPGSDKLPSVLKPGASAASR

84 NP_006564.1 NP_001183.2

>NP_006564.1

MDDSTEREQSRLTSCLKKREEMKLKECVSILPRKESPSVRSSKDGKLLAATLLLALLSCCLTVVSFYQVAALQGDLASLRAELQGHHAEKLPAGAGAPKAGLEEAPAVTAGLKIFEPPAPGEGNSSQNSRNKRAVQGPEETVTQDCLQLIADSETPTIQKGSYTFVPWLLSFKRGSALEEKENKILVKETGYFFIYGQVLYTDKTYAMGHLIQRKKVHVFGDELSLVTLFRCIQNMPETLPNNSCYSAGIAKLEEGDELQLAIPRENAQISLDGDVTFFGALKLL

>NP_001183.2

MLQMAGQCSQNEYFDSLLHACIPCQLRCSSNTPPLTCQRYCNASVTNSVKGTNAILWTCLGLSLIISLAVFVLMFLLRKINSEPLKDEFKNTGSGLLGMANIDLEKSRTGDEIILPRGLEYTVEECTCEDCIKSKPKVDSDHCFPLPAMEEGATILVTTKTNDYCKSLPAALSATEIEKSISAR

85 NP_073576.1 AAH13362.1

>NP_073576.1

MSNPGGRRNGPVKLRLTVLCAKNLVKKDFFRLPDPFAKVVVDGSGQCHSTDTVKNTLDPKWNQHYDLYIGKSDSVTISVWNHKKIHKKQGAGFLGCVRLLSNAINRLKDTGYQRLDLCKLGPNDNDTVRGQIVVSLQSRDRIGTGGQVVDCSRLFDNDLPDGWEERRTASGRIQYLNHITRTTQWERPTRPASEYSSPGRPLSCFVDENTPISGTNGATCGQSSDPRLAERRVRSQRHRNYMSRTHLHTPPDLPEGYEQRTTQQGQVYFLHTQTGVSTWHDPRVPRDLSNINCEELGPLPPGWEIRNTATGRVYFVDHNNRTTQFTDPRLSANLHLVLNRQNQLKDQQQQQVVSLCPDDTECLTVPRYKRDLVQKLKILRQELSQQQPQAGHCRIEVSREEIFEESYRQVMKMRPKDLWKRLMIKFRGEEGLDYGGVAREWLYLLSHEMLNPYYGLFQYSRDDIYTLQINPDSAVNPEHLSYFHFVGRIMGMAVFHGHYIDGGFTLPFYKQLLGKSITLDDMELVDPDLHNSLVWILENDITGVLDHTFCVEHNAYGEIIQHELKPNGKSIPVNEENKKEYVRLYVNWRFLRGIEAQFLALQKGFNEVIPQHLLKTFDEKELELIICGLGKIDVNDWKVNTRLKHCTPDSNIVKWFWKAVEFFDEERRARLLQFVTGSSRVPLQGFKALQGAAGPRLFTIHQIDACTNNLPKAHTCFNRIDIPPYESYEKLYEKLLTAIEETCGFAVE

>AAH13362.1

MASNSIFDSFPTYSPTFIRDPSTSRRFTPPSPAFPCGGGGGKMGENSGALSAQAAVGPGGRARPEVRSMVDVLADHAGELVRTDSPNFLCSVLPSHWRCNKTLPVAFKVVALGDVPDGTVVTVMAGNDENYSAELRNASAVMKNQVARFNDLRFVGRSGRGKSFTLTITVFTNPTQVATYHRAIKVTVDGPREPRRHRQKLEDQTKPFPDRFGDLERLRMRVTPSTPSPRGSLSTTSHFSSQPQTPIQGTSELNPFSDPRQFDRSFPTLPTLTESRFPDPRMHYPGAMSAAFPYSATPSGTSISSLSVAGMPATSRFHHTYLPPPYPGAPQNQSGPFQANPSPYHLYYGTSSGSYQFSMVAGSSSGGDRSPTRMLASCTSSAASVAAGNLMNPSLGGQSDGVEADGSHSNSPTALSTPGRMDEAVWRPY

86 NP_000827.1 NP_002730.1

>NP_000827.1

MRGAGGPRGPRGPAKMLLLLALACASPFPEEAPGPGGAGGPGGGLGGARPLNVALVFSGPAYAAEAARLGPAVAAAVRSPGLDVRPVALVLNGSDPRSLVLQLCDLLSGLRVHGVVFEDDSRAPAVAPILDFLSAQTSLPIVAVHGGAALVLTPKEKGSTFLQLGSSTEQQLQVIFEVLEEYDWTSFVAVTTRAPGHRAFLSYIEVLTDGSLVGWEHRGALTLDPGAGEAVLSAQLRSVSAQIRLLFCAREEAEPVFRAAEEAGLTGSGYVWFMVGPQLAGGGGSGAPGEPPLLPGGAPLPAGLFAVRSAGWRDDLARRVAAGVAVVARGAQALLRDYGFLPELGHDCRAQNRTHRGESLHRYFMNITWDNRDYSFNEDGFLVNPSLVVISLTRDRTWEVVGSWEQQTLRLKYPLWSRYGRFLQPVDDTQHLTVATLEERPFVIVEPADPISGTCIRDSVPCRSQLNRTHSPPPDAPRPEKRCCKGFCIDILKRLAHTIGFSYDLYLVTNGKHGKKIDGVWNGMIGEVFYQRADMAIGSLTINEERSEIVDFSVPFVETGISVMVARSNGTVSPSAFLEPYSPAVWVMMFVMCLTVVAVTVFIFEYLSPVGYNRSLATGKRPGGSTFTIGKSIWLLWALVFNNSVPVENPRGTTSKIMVLVWAFFAVIFLASYTANLAAFMIQEEYVDTVSGLSDRKFQRPQEQYPPLKFGTVPNGSTEKNIRSNYPDMHSYMVRYNQPRVEEALTQLKAGKLDAFIYDAAVLNYMARKDEGCKLVTIGSGKVFATTGYGIALHKGSRWKRPIDLALLQFLGDDEIEMLERLWLSGICHNDKIEVMSSKLDIDNMAGVFYMLLVAMGLSLLVFAWEHLVYWRLRHCLGPTHRMDFLLAFSRGMYSCCSAEAAPPPAKPPPPPQPLPSPAYPAPGPAPGPAPFVPRERASVDRWRRTKGAGPPGGAGLADGFHRYYGPIEPQGLGLGLGEARAAPRGAAGRPLSPPAAQPPQKPPASYFAIVRDKEPAEPPAGAFPGFPSPPAPPAAAATAVGPPLCRLAFEDESPPAPARWPRSDPESQPLLGPGAGGAGGTGGAGGGAPAAPPPCCAAPPPCPYLDLEPSPSDSEDSESLGGASLGGLDPWWFADFPYPYAERLGPPPGRYWSVDKLGGWRAGSWDYLPPRSGPAAWHCRHCASLELLPPPRHLSCSHDGLDGGWWAPPPPPWAAGPLPRRRARCGCPRSHPHRPRASHRTPAAAAPHHHRHRRAAGGWDLPPPAPTSRSLEDLSSCPRAAPARRLTGPSRHARRCPHAAHWGPPLPTASHRRHRGGDLGTRRGSAHFSSLESEV

>NP_002730.1

MAGLGPGVGDSEGGPRPLFCRKGALRQKVVHEVKSHKFTARFFKQPTFCSHCTDFIWGIGKQGLQCQVCSFVVHRRCHEFVTFECPGAGKGPQTDDPRNKHKFRLHSYSSPTFCDHCGSLLYGLVHQGMKCSCCEMNVHRRCVRSVPSLCGVDHTERRGRLQLEIRAPTADEIHVTVGEARNLIPMDPNGLSDPYVKLKLIPDPRNLTKQKTRTVKATLNPVWNETFVFNLKPGDVERRLSVEVWDWDRTSRNDFMGAMSFGVSELLKAPVDGWYKLLNQEEGEYYNVPVADADNCSLLQKFEACNYPLELYERVRMGPSSSPIPSPSPSPTDPKRCFFGASPGRLHISDFSFLMVLGKGSFGKVMLAERRGSDELYAIKILKKDVIVQDDDVDCTLVEKRVLALGGRGPGGRPHFLTQLHSTFQTPDRLYFVMEYVTGGDLMYHIQQLGKFKEPHAAFYAAEIAIGLFFLHNQGIIYRDLKLDNVMLDAEGHIKITDFGMCKENVFPGTTTRTFCGTPDYIAPEIIAYQPYGKSVDWWSFGVLLYEMLAGQPPFDGEDEEELFQAIMEQTVTYPKSLSREAVAICKGFLTKHPGKRLGSGPDGEPTIRAHGFFRWIDWERLERLEIPPPFRPRPCGRSGENFDKFFTRAAPALTPPDRLVLASIDQADFQGFTYVNPDFVHPDARSPTSPVPVPVM

87 NP_058521.1 NP_058633.2

>NP_058521.1

MGKVWKQQMYPQYATYYYPQYLQAKQSLVPAHPMAPPSPSTTSSNNNSSSSSNSGWDQLSKTNLYIRGLPPHTTDQDLVKLCQPYGKIVSTKAILDKTTNKCKGYGFVDFDSPAAAQKAVSALKASGVQAQMAKQQEQDPTNLYISNLPLSMDEQELENMLKPFGQVISTRILRDSSGTSRGVGFARMESTEKCEAVIGHFNGKFIKTPPGVSAPTEPLLCKFADGGQKKRQNPNKYIPNGRPWHREGEAGMTLTYDPTTAAIQNGFYPSPYSIATNRMITQTSITPYIASPVSAYQVAKETRENKYRGSAIKVQSPSWMQPQPYILQHPGAVLTPSMEHTMSLQPASMISPLAQQMSHLSLGSTGTYMPATSAMQGAYLPQYAHMQTTAVPVEEASGQQQVAVETSNDHSPYTFQPNK

>NP_058633.2

MAPVHGDDSLSDSGSFVSSRARREKKSKKGRQEALERLKKAKAGEKYKYEVEDFTGVYEEVDEEQYSKLVQARQDDDWIVDDDGIGYVEDGREIFDDDLEDDALDADEKGKDGKARNKDKRNVKKLAVTKPNNIKSMFIACAGKKTADKAVDLSKDGLLGDILQDLNTETPQITPPPVMILKKKRSIGASPNPFSVHTATAVPSGKIASPVSRKEPPLTPVPLKRAEFAGDDVQVESTEEEQESGAMEFEDGDFDEPMEVEEVDLEPMAAKAWDKESEPAEEVKQEADSGKGTVSYLGSFLPDVSCWDIDQEGDSSFSVQEVQVDSSHLPLVKGADEEQVFHFYWLDAYEDQYNQPGVVFLFGKVWIESAETHVSCCVMVKNIERTLYFLPREMKIDLNTGKETGTPISMKDVYEEFDEKIATKYKIMKFKSKPVEKNYAFEIPDVPEKSEYLEVKYSAEMPQLPQDLKGETFSHVFGTNTSSLELFLMNRKIKGPCWLEVKSPQLLNQPVSWCKVEAMALKPDLVNVIKDVSPPPLVVMAFSMKTMQNAKNHQNEIIAMAALVHHSFALDKAAPKPPFQSHFCVVSKPKDCIFPYAFKEVIEKKNVKVEVAATERTLLGFFLAKVHKIDPDIIVGHNIYGFELEVLLQRINVCKAPHWSKIGRLKRSNMPKLGGRSGFGERNATCGRMICDVEISAKELIRCKSYHLSELVQQILKTERVVIPMENIQNMYSESSQLLYLLEHTWKDAKFILQIMCELNVLPLALQITNIAGNIMSRTLMGGRSERNEFLLLHAFYENNYIVPDKQIFRKPQQKLGDEDEEIDGDTNKYKKGRKKAAYAGGLVLDPKVGFYDKFILLLDFNSLYPSIIQEFNICFTTVQRVASEAQKVTEDGEQEQIPELPDPSLEMGILPREIRKLVERRKQVKQLMKQQDLNPDLILQYDIRQKALKLTANSMYGCLGFSYSRFYAKPLAALVTYKGREILMHTKEMVQKMNLEVIYGDTDSIMINTNSTNLEEVFKLGNKVKSEVNKLYKLLEIDIDGVFKSLLLLKKKKYAALVVEPTSDGNYVTKQELKGLDIVRRDWCDLAKDTGNFVIGQILSDQSRDTIVENIQKRLIEIGENVLNGSVPVSQFEINKALTKDPQDYPDKKSLPHVHVALWINSQGGRKVKAGDTVSYVICQDGSNLTASQRAYAPEQLQKQDNLTIDTQYYLAQQIHPVVARICEPIDGIDAVLIATWLGLDPTQFRVHHYHKDEENDALLGGPAQLTDEEKYRDCERFKCPCPTCGTENIYDNVFDGSGTDMEPSLYRCSNIDCKASPLTFTVQLSNKLIMDIRRFIKKYYDGWLICEEPTCRNRTRHLPLQFSRTGPLCPACMKATLQPEYSDKSLYTQLCFYRYIFDAECALEKLTTDHEKDKLKKQFFTPKVLQDYRKLKNTAEQFLSRSGYSEVNLSKLFAGCAVKS

88 NP_003118.1 NP_005697.2

>NP_003118.1

MDPSGVKVLETAEDIQERRQQVLDRYHRFKELSTLRRQKLEDSYRFQFFQRDAEELEKWIQEKLQIASDENYKDPTNLQGKLQKHQAFEAEVQANSGAIVKLDETGNLMISEGHFASETIRTRLMELHRQWELLLEKMREKGIKLLQAQNLVQYLRECEDVMDWINDKEAIVTSEELGQDLEHVEVLQKKFEEFQTDMAAHEERVNEVNQFAAKLIQEQHPEEELIKTKQDEVNAAWQRLKGLALQRQGKLFGAAEVQRFNRDVDETISWIKEKEQLMASDDFGRDLASVQALLRKHEGLERDLAALEDKVKALCAEADRLQQSHPLSATQIQVKREELITNWEQIRTLAAERHARLNDSYRLQRFLADFRDLTSWVTEMKALINADELASDVAGAEALLDRHQEHKGEIDAHEDSFKSADESGQALLAAGHYASDEVREKLTVLSEERAALLELWELRRQQYEQCMDLQLFYRDTEQVDNWMSKQEAFLLNEDLGDFLDSVEALLKKHEDFEKSLSAQEEKITALDEFATKLIQNNHYAMEDVATRRDALLSRRNALHERAMRRRAQLADSFHLQQFFRDSDELKSWVNEKMKTATDEAYKDPSNLQGKVQKHQAFEAELSANQSRIDALEKAGQKLIDVNHYAKDEVAARMNEVISLWKKLLEATELKGIKLREANQQQQFNRNVEDIELWLYEVEGHLASDDYGKDLTNVQNLQKKHALLEADVAAHQDRIDGVTIQARQFQDAGHFDAENIKKKQEALVARYEALKEPMVARKQKLADSLRLQQLFRDVEDEETWIREKEPIAASTNRGKDLIGVQNLLKKHQALQAEIAGHEPRIKAVTQKGNAMVEEGHFAAEDVKAKLHELNQKWEALKAKASQRRQDLEDSLQAQQYFADANEAESWMREKEPIVGSTDYGKDEDSAEALLKKHEALMSDLSAYGSSIQALREQAQSCRQQVAPTDDETGKELVLALYDYQEKSPREVTMKKGDILTLLNSTNKDWWKVEVNDRQGFVPAAYVKKLDPAQSASRENLLEEQGSIALRQEQIDNQTRITKEAGSVSLRMKQVEELYHSLLELGEKRKGMLEKSCKKFMLFREANELQQWINEKEAALTSEEVGADLEQVEVLQKKFDDFQKDLKANESRLKDINKVAEDLESEGLMAEEVQAVQQQEVYGMMPRDETDSKTASPWKSARLMVHTVATFNSIKELNERWRSLQQLAEERSQLLGSAHEVQRFHRDADETKEWIEEKNQALNTDNYGHDLASVQALQRKHEGFERDLAALGDKVNSLGETAERLTQSHPESAEDLQEKCTELNQAWSSLGKRADQRKAKLGDSHDLQRFLSDFRDLMSWINGIRGLVSSDELAKDVTGAEALLERHQEHRTEIDARAGTFQAFEQFGQQLLAHGHYASPEIKQKLDILDQERADLEKAWVQRRMMLDQCLELQLFHRDCEQAENWMAAREAFLNTEDKGDSLDSVEALIKKHEDFDKAINVQEEKIAALQAFADQLIAAGHYAKGDISSRRNEVLDRWRRLKAQMIEKRSKLGESQTLQQFSRDVDEIEAWISEKLQTASDESYKDPTNIQSKHQKHQAFEAELHANADRIRGVIDMGNSLIERGACAGNEDAVKARLAALADQWQFLVQKSAEKSQKLKEANKQQNFNTGIKDIAFWLSEVEALLASEDYGKDLASVNNLLKKHQLLEADISAHEDRLKDLNSQADSLMTSSAFDTSQVKDKRDTINGRFQKIKSMAASRRAKLNESHRLHQFFRDMDDEESWIKEKKLLVGSEDYGRDLTGVQNLRKKHKRLEAELAAHEPAIQGVLDTGKKLSDDNTIGKEEIQQRLAQFVEHWKELKQLAAARGQRLEESLEYQQFVANVEEEEAWINEKMTLVASEDYGDTLAAIQGLLKKHEAFETAFTVHKDRVNDVCTNGQDLIKKNNHHEENISSKMKGLNGKVSDLEKAAAQRKANVDENSAFLQFNWKADVVESWIGEKENSLKTDDYGRDLSSVQTLLTKQETFDAGLQAFQQEGIANITALKDQLLAAKHVQSKAIEARHASLMKRWSQLLANSAARKKKLLEAQSHFRKVEDLFLTFAKKASAFNSWFENAEEDLTDPVRCNSLEEIKALREAHDAFRSSLSSAQADFNQLAELDRQIKSFRVASNPYTWFTMEALEETWRNLQKIIKERELELQKEQRRQEENDKLRQEFAQHANAFHQWIQETRTYLLDGSCMVEESGTLESQLEATKRKHQEIRAMRSQLKKIEDLGAAMEEALILDNKYTEHSTVGLAQQWDQLDQLGMRMQHNLEQQIQARNTTGVTEEALKEFSMMFKHFDKDKSGRLNHQDGKSCLRSLGYDLPMVEEGEPDPEFEAILDTVDPNRDGHVSLQEYMAFMISRETENVKSSEEIESAFRALSSEGKPYVTKEELYQNLTREQADYCVSHMKPIVDGKGRELPTAFDYVEFTRSLFVN

>NP_005697.2

MAEAGTGEPSPSVEGEHGTEYDTLPSDTVSLSDSDSDLSLPGGAEVEALSPMGLPGEEDSGPDEPPSPPSGLLPATVQPFHLRGMSSTFSQRSRDIFDCLEGAARRAPSSVAHTSMSDNGGFKRPLAPSGRSPVEGLGRAHRSPASPRVPPVPDYVAHPERWTKYSLEDVTEVSEQSNQATALAFLGSQSLAAPTDCVSSFNQDPSSCGEGRVIFTKPVRGVEARHERKRVLGKVGEPGRGGLGNPATDRGEGPVELAHLAGPGSPEAEEWGSHHGGLQEVEALSGSVHSGSVPGLPPVETVGFHGSRKRSRDHFRNKSSSPEDPGAEV

89 NP_003122.1 NP_073207.1

>NP_003122.1

MLPTQAGAAAALGRGSALGGSLNRTPTGRPGGGGGTRGANGGRVPGNGAGLGPGRLEREAAAAAATTPAPTAGALYSGSEGDSESGEEEELGAERRGLKRSLSEMEIGMVVGGPEASAAATGGYGPVSGAVSGAKPGKKTRGRVKIKMEFIDNKLRRYTTFSKRKTGIMKKAYELSTLTGTQVLLLVASETGHVYTFATRKLQPMITSETGKALIQTCLNSPDSPPRSDPTTDQRMSATGFEETDLTYQVSESDSSGETKDTLKPAFTVTNLPGTTSTIQTAPSTSTTMQVSSGPSFPITNYLAPVSASVSPSAVSSANGTVLKSTGSGPVSSGGLMQLPTSFTLMPGGAVAQQVPVQAIQVHQAPQQASPSRDSSTDLTQTSSSGTVTLPATIMTSSVPTTVGGHMMYPSPHAVMYAPTSGLGDGSLTVLNAFSQAPSTMQVSHSQVQEPGGVPQVFLTASSGTVQIPVSAVQLHQMAVIGQQAGSSSNLTELQVVNLDTAHSTKSE

>NP_073207.1

MTSSYGHVLERQPALGGRLDSPGNLDTLQAKKNFSVSHLLDLEEAGDMVAAQADENVGEAGRSLLESPGLTSGSDTPQQDNDQLNSEEKKKRKQRRNRTTFNSSQLQALERVFERTHYPDAFVREDLARRVNLTEARVQVWFQNRRAKFRRNERAMLANKNASLLKSYSGDVTAVEQPIVPRPAPRPTDYLSWGTASPYSAMATYSATCANNSPAQGINMANSIANLRLKAKEYSLQRNQVPTVN

90 NP_004955.2 NP_612482.2

>NP_004955.2

MAQTQGTRRKVCYYYDGDVGNYYYGQGHPMKPHRIRMTHNLLLNYGLYRKMEIYRPHKANAEEMTKYHSDDYIKFLRSIRPDNMSEYSKQMQRFNVGEDCPVFDGLFEFCQLSTGGSVASAVKLNKQQTDIAVNWAGGLHHAKKSEASGFCYVNDIVLAILELLKYHQRVLYIDIDIHHGDGVEEAFYTTDRVMTVSFHKYGEYFPGTGDLRDIGAGKGKYYAVNYPLRDGIDDESYEAIFKPVMSKVMEMFQPSAVVLQCGSDSLSGDRLGCFNLTIKGHAKCVEFVKSFNLPMLMLGGGGYTIRNVARCWTYETAVALDTEIPNELPYNDYFEYFGPDFKLHISPSNMTNQNTNEYLEKIKQRLFENLRMLPHAPGVQMQAIPEDAIPEESGDEDEDDPDKRISICSSDKRIACEEEFSDSEEEGEGGRKNSSNFKKAKRVKTEDEKEKDPEEKKEVTEEEKTKEEKPEAKGVKEEVKLA

>NP_612482.2

MSDQDHSMDEMTAVVKIEKGVGGNNGGNGNGGGAFSQARSSSTGSSSSTGGGGQESQPSPLALLAATCSRIESPNENSNNSQGPSQSGGTGELDLTATQLSQGANGWQIISSSSGATPTSKEQSGSSTNGSNGSESSKNRTVSGGQYVVAAAPNLQNQQVLTGLPGVMPNIQYQVIPQFQTVDGQQLQFAATGAQVQQDGSGQIQIIPGANQQIITNRGSGGNIIAAMPNLLQQAVPLQGLANNVLSGQTQYVTNVPVALNGNITLLPVNSVSAATLTPSSQAVTISSSGSQESGSQPVTSGTTISSASLVSSQASSSSFFTNANSYSTTTTTSNMGIMNFTTSGSSGTNSQGQTPQRVSGLQGSDALNIQQNQTSGGSLQAGQQKEGEQNQQTQQQQILIQPQLVQGGQALQALQAAPLSGQTFTTQAISQETLQNLQLQAVPNSGPIIIRTPTVGPNGQVSWQTLQLQNLQVQNPQAQTITLAPMQGVSLGQTSSSNTTLTPIASAASIPAGTVTVNAAQLSSMPGLQTINLSALGTSGIQVHPIQGLPLAIANAPGDHGAQLGLHGAGGDGIHDDTAGGEEGENSPDAQPQAGRRTRREACTCPYCKDSEGRGSGDPGKKKQHICHIQGCGKVYGKTSHLRAHLRWHTGERPFMCTWSYCGKRFTRSDELQRHKRTHTGEKKFACPECPKRFMRSDHLSKHIKTHQNKKGGPGVALSVGTLPLDSGAGSEGSGTATPSALITTNMVAMEAICPEGIARLANSGINVMQVADLQSINISGNGF

91 NP_000211.1 NP_004776.3

>NP_000211.1

MNASSRNVFDTLIRVLTESMFKHLRKWVVTRFFGHSRQRARLVSKDGRCNIEFGNVEAQSRFIFFVDIWTTVLDLKWRYKMTIFITAFLGSWFFFGLLWYAVAYIHKDLPEFHPSANHTPCVENINGLTSAFLFSLETQVTIGYGFRCVTEQCATAIFLLIFQSILGVIINSFMCGAILAKISRPKKRAKTITFSKNAVISKRGGKLCLLIRVANLRKSLLIGSHIYGKLLKTTVTPEGETIILDQININFVVDAGNENLFFISPLTIYHVIDHNSPFFHMAAETLLQQDFELVVFLDGTVESTSATCQVRTSYVPEEVLWGYRFAPIVSKTKEGKYRVDFHNFSKTVEVETPHCAMCLYNEKDVRARMKRGYDNPNFILSEVNETDDTKM

>NP_004776.3

MAAPEPLRPRLCRLVRGEQGYGFHLHGEKGRRGQFIRRVEPGSPAEAAALRAGDRLVEVNGVNVEGETHHQVVQRIKAVEGQTRLLVVDQETDEELRRRQLTCTEEMAQRGLPPAHDPWEPKPDWAHTGSHSSEAGKKDVSGPLRELRPRLCHLRKGPQGYGFNLHSDKSRPGQYIRSVDPGSPAARSGLRAQDRLIEVNGQNVEGLRHAEVVASIKAREDEARLLVVDPETDEHFKRLRVTPTEEHVEGPLPSPVTNGTSPAQLNGGSACSSRSDLPGSDKDTEESGLHLSPTAAEAKEKARAMRVNKRAPQMDWNRKREIFSNF

92 NP_036229.1 NP_002257.1

>NP_036229.1

MGILFTRIWRLFNHQEHKVIIVGLDNAGKTTILYQFSMNEVVHTSPTIGSNVEEIVINNTRFLMWDIGGQESLRSSWNTYYTNTEFVIVVVDSTDRERISVTREELYKMLAHEDLRKAGLLIFANKQDVKECMTVAEISQFLKLTSIKDHQWHIQACCALTGEGLCQGLEWMMSRLKIR

>NP_002257.1

MSTNENANTPAARLHRFKNKGKDSTEMRRRRIEVNVELRKAKKDDQMLKRRNVSSFPDDATSPLQENRNNQGTVNWSVDDIVKGINSSNVENQLQATQAARKLLSREKQPPIDNIIRAGLIPKFVSFLGRTDCSPIQFESAWALTNIASGTSEQTKAVVDGGAIPAFISLLASPHAHISEQAVWALGNIAGDGSVFRDLVIKYGAVDPLLALLAVPDMSSLACGYLRNLTWTLSNLCRNKNPAPPIDAVEQILPTLVRLLHHDDPEVLADTCWAISYLTDGPNERIGMVVKTGVVPQLVKLLGASELPIVTPALRAIGNIVTGTDEQTQVVIDAGALAVFPSLLTNPKTNIQKEATWTMSNITAGRQDQIQQVVNHGLVPFLVSVLSKADFKTQKEAVWAVTNYTSGGTVEQIVYLVHCGIIEPLMNLLTAKDTKIILVILDAISNIFQAAEKLGETEKLSIMIEECGGLDKIEALQNHENESVYKASLSLIEKYFSVEEEEDQNVVPETTSEGYTFQVQDGAPGTFNF

93 NP_002768.3 NP_068810.2

>NP_002768.3

MAHRPPSPALASVLLALLLSGAARAAEIVGGHEAQPHSRPYMASLQMRGNPGSHFCGGTLIHPSFVLTAAHCLRDIPQRLVNVVLGAHNVRTQEPTQQHFSVAQVFLNNYDAENKLNDVLLIQLSSPANLSASVATVQLPQQDQPVPHGTQCLAMGWGRVGAHDPPAQVLQELNVTVVTFFCRPHNICTFVPRRKAGICFGDSGGPLICDGIIQGIDSFVIWGCATRLFPDFFTRVALYVDWIRSTLRRVEAKGRP

>NP_068810.2

MDELFPLIFPAEPAQASGPYVEIIEQPKQRGMRFRYKCEGRSAGSIPGERSTDTTKTHPTIKINGYTGPGTVRISLVTKDPPHRPHPHELVGKDCRDGFYEAELCPDRCIHSFQNLGIQCVKKRDLEQAISQRIQTNNNPFQVPIEEQRGDYDLNAVRLCFQVTVRDPSGRPLRLPPVLPHPIFDNRAPNTAELKICRVNRNSGSCLGGDEIFLLCDKVQKEDIEVYFTGPGWEARGSFSQADVHRQVAIVFRTPPYADPSLQAPVRVSMQLRRPSDRELSEPMEFQYLPDTDDRHRIEEKRKRTYETFKSIMKKSPFSGPTDPRPPPRRIAVPSRSSASVPKPAPQPYPFTSSLSTINYDEFPTMVFPSGQISQASALAPAPPQVLPQAPAPAPAPAMVSALAQAPAPVPVLAPGPPQAVAPPAPKPTQAGEGTLSEALLQLQFDDEDLGALLGNSTDPAVFTDLASVDNSEFQQLLNQGIPVAPHTTEPMLMEYPEAITRLVTGAQRPPDPAPAPLGAPGLPNGLLSGDEDFSSIADMDFSALLSQISS

94 NP_005251.1 NP_057137.1

>NP_005251.1

MARPNKFLLWFCCFAWLCFPISLGSQASGGEAQIAASAELESGAMPWSLLQHIDERDRAGLLPALFKVLSVGRGGSPRLQPDSRALHYMKKLYKTYATKEGIPKSNRSHLYNTVRLFTPCTRHKQAPGDQVTGILPSVELLFNLDRITTVEHLLKSVLLYNINNSVSFSSAVKCVCNLMIKEPKSSSRTLGRAPYSFTFNSQFEFGKKHKWIQIDVTSLLQPLVASNKRSIHMSINFTCMKDQLEHPSAQNGLFNMTLVSPSLILYLNDTSAQAYHSWYSLHYKRRPSQGPDQERSLSAYPVGEEAAEDGRSSHHRHRRGQETVSSELKKPLGPASFNLSEYFRQFLLPQNECELHDFRLSFSQLKWDNWIVAPHRYNPRYCKGDCPRAVGHRYGSPVHTMVQNIIYEKLDSSVPRPSCVPAKYSPLSVLTIEPDGSIAYKEYEDMIATKCTCR

>NP_057137.1

MDEDGLPLMGSGIDLTKVPAIQQKRTVAFLNQFVVHTVQFLNRFSTVCEEKLADLSLRIQQIETTLNILDAKLSSIPGLDDVTVEVSPLNVTSVTNGAHPEATSEQPQQNSTQDSGLQESEVSAENILTVAKDPRYARYLKMVQVGVPVMAIRNKMISEGLDPDLLERPDAPVPDGESEKTVEESSDSESSFSD

95 NP_000204.3 NP_899236.1

>NP_000204.3

MAGPRPSPWARLLLAALISVSLSGTLANRCKKAPVKSCTECVRVDKDCAYCTDEMFRDRRCNTQAELLAAGCQRESIVVMESSFQITEETQIDTTLRRSQMSPQGLRVRLRPGEERHFELEVFEPLESPVDLYILMDFSNSMSDDLDNLKKMGQNLARVLSQLTSDYTIGFGKFVDKVSVPQTDMRPEKLKEPWPNSDPPFSFKNVISLTEDVDEFRNKLQGERISGNLDAPEGGFDAILQTAVCTRDIGWRPDSTHLLVFSTESAFHYEADGANVLAGIMSRNDERCHLDTTGTYTQYRTQDYPSVPTLVRLLAKHNIIPIFAVTNYSYSYYEKLHTYFPVSSLGVLQEDSSNIVELLEEAFNRIRSNLDIRALDSPRGLRTEVTSKMFQKTRTGSFHIRRGEVGIYQVQLRALEHVDGTHVCQLPEDQKGNIHLKPSFSDGLKMDAGIICDVCTCELQKEVRSARCSFNGDFVCGQCVCSEGWSGQTCNCSTGSLSDIQPCLREGEDKPCSGRGECQCGHCVCYGEGRYEGQFCEYDNFQCPRTSGFLCNDRGRCSMGQCVCEPGWTGPSCDCPLSNATCIDSNGGICNGRGHCECGRCHCHQQSLYTDTICEINYSAIHPGLCEDLRSCVQCQAWGTGEKKGRTCEECNFKVKMVDELKRAEEVVVRCSFRDEDDDCTYSYTMEGDGAPGPNSTVLVHKKKDCPPGSFWWLIPLLLLLLPLLALLLLLCWKYCACCKACLALLPCCNRGHMVGFKEDHYMLRENLMASDHLDTPMLRSGNLKGRDVVRWKVTNNMQRPGFATHAASINPTELVPYGLSLRLARLCTENLLKPDTRECAQLRQEVEENLNEVYRQISGVHKLQQTKFRQQPNAGKKQDHTIVDTVLMAPRSAKPALLKLTEKQVEQRAFHDLKVAPGYYTLTADQDARGMVEFQEGVELVDVRVPLFIRPEDDDEKQLLVEAIDVPAGTATLGRRLVNITIIKEQARDVVSFEQPEFSVSRGDQVARIPVIRRVLDGGKSQVSYRTQDGTAQGNRDYIPVEGELLFQPGEAWKELQVKLLELQEVDSLLRGRQVRRFHVQLSNPKFGAHLGQPHSTTIIIRDPDELDRSFTSQMLSSQPPPHGDLGAPQNPNAKAAGSRKIHFNWLPPSGKPMGYRVKYWIQGDSESEAHLLDSKVPSVELTNLYPYCDYEMKVCAYGAQGEGPYSSLVSCRTHQEVPSEPGRLAFNVVSSTVTQLSWAEPAETNGEITAYEVCYGLVNDDNRPIGPMKKVLVDNPKNRMLLIENLRESQPYRYTVKARNGAGWGPEREAIINLATQPKRPMSIPIIPDIPIVDAQSGEDYDSFLMYSDDVLRSPSGSQRPSVSDDTGCGWKFEPLLGEELDLRRVTWRLPPELIPRLSASSGRSSDAEAPHGPPDDGGAGGKGGSLPRSATPGPPGEHLVNGRMDFAFPGSTNSLHRMTTTSAAAYGTHLSPHVPHRVLSTSSTLTRDYNSLTRSEHSHSTTLPRDYSTLTSVSSHDSRLTAGVPDTPTRLVFSALGPTSLRVSWQEPRCERPLQGYSVEYQLLNGGELHRLNIPNPAQTSVVVEDLLPNHSYVFRVRAQSQEGWGREREGVITIESQVHPQSPLCPLPGSAFTLSTPSAPGPLVFTALSPDSLQLSWERPRRPNGDIVGYLVTCEMAQGGGPATAFRVDGDSPESRLTVPGLSENVPYKFKVQARTTEGFGPEREGIITIESQDGGPFPQLGSRAGLFQHPLQSEYSSITTTHTSATEPFLVDGLTLGAQHLEAGGSLTRHVTQEFVSRTLTTSGTLSTHMDQQFFQT

>NP_899236.1

MAGYLSPAAYLYVEEQEYLQAYEDVLERYKDERDKVQKKTFTKWINQHLMKVRKHVNDLYEDLRDGHNLISLLEVLSGDTLPREKGRMRFHRLQNVQIALDYLKRRQVKLVNIRNDDITDGNPKLTLGLIWTIILHFQISDIHVTGESEDMSAKERLLLWTQQATEGYAGIRCENFTTCWRDGKLFNAIIHKYRPDLIDMNTVAVQSNLANLEHAFYVAEKIGVIRLLDPEDVDVSSPDEKSVITYVSSLYDAFPKVPEGGEGIGANDVEVKWIEYQNMVNYLIQWIRHHVTTMSERTFPNNPVELKALYNQYLQFKETEIPPKETEKSKIKRLYKLLEIWIEFGRIKLLQGYHPNDIEKEWGKLIIAMLEREKALRPEVERLEMLQQIANRVQRDSVICEDKLILAGNALQSDSKRLESGVQFQNEAEIAGYILECENLLRQHVIDVQILIDGKYYQADQLVQRVAKLRDEIMALRNECSSVYSKGRILTTEQTKLMISGITQSLNSGFAQTLHPSLTSGLTQSLTPSLTSSSMTSGLSSGMTSRLTPSVTPAYTPGFPSGLVPNFSSGVEPNSLQTLKLMQIRKPLLKSSLLDQNLTEEEINMKFVQDLLNWVDEMQVQLDRTEWGSDLPSVESHLENHKNVHRAIEEFESSLKEAKISEIQMTAPLKLTYAEKLHRLESQYAKLLNTSRNQERHLDTLHNFVSRATNELIWLNEKEEEEVAYDWSERNTNIARKKDYHAELMRELDQKEENIKSVQEIAEQLLLENHPARLTIEAYRAAMQTQWSWILQLCQCVEQHIKENTAYFEFFNDAKEATDYLRNLKDAIQRKYSCDRSSSIHKLEDLVQESMEEKEELLQYKSTIANLMGKAKTIIQLKPRNSDCPLKTSIPIKAICDYRQIEITIYKDDECVLANNSHRAKWKVISPTGNEAMVPSVCFTVPPPNKEAVDLANRIEQQYQNVLTLWHESHINMKSVVSWHYLINEIDRIRASNVASIKTMLPGEHQQVLSNLQSRFEDFLEDSQESQVFSGSDITQLEKEVNVCKQYYQELLKSAEREEQEESVYNLYISEVRNIRLRLENCEDRLIRQIRTPLERDDLHESVFRITEQEKLKKELERLKDDLGTITNKCEEFFSQAAASSSVPTLRSELNVVLQNMNQVYSMSSTYIDKLKTVNLVLKNTQAAEALVKLYETKLCEEEAVIADKNNIENLISTLKQWRSEVDEKRQVFHALEDELQKAKAISDEMFKTYKERDLDFDWHKEKADQLVERWQNVHVQIDNRLRDLEGIGKSLKYYRDTYHPLDDWIQQVETTQRKIQENQPENSKTLATQLNQQKMLVSEIEMKQSKMDECQKYAEQYSATVKDYELQTMTYRAMVDSQQKSPVKRRRMQSSADLIIQEFMDLRTRYTALVTLMTQYIKFAGDSLKRLEEEEKSLEEEKKEHVEKAKELQKWVSNISKTLKDAEKAGKPPFSKQKISSEEISTKKEQLSEALQTIQLFLAKHGDKMTDEERNELEKQVKTLQESYNLLFSESLKQLQESQTSGDVKVEEKIVAERQQEYKEKLQGICDLLTQTENRLIGHQEAFMIGDGTVELKKYQSKQEELQKDMQGSAQALAEVVKNTENFLKENGEKLSQEDKALIEQKLNEAKIKCEQLNLKAEQSKKELDKVVTTAIKEETEKVAAVKQLEESKTKIENLLDWLSNVDKDSERAGTKHKQVIEQNGTHFQEGDGKSAIGEEDEVNGNLLETDVDGQVGTTQENLNQQYQKVKAQHEKIISQHQAVIIATQSAQVLLEKQGQYLSPEEKEKLQKNMKELKVHYETALAESEKKMKLTHSLQEELEKFDADYTEFEHWLQQSEQELENLEAGADDINGLMTKLKRQKSFSEDVISHKGDLRYITISGNRVLEAAKSCSKRDGGKVDTSATHREVQRKLDHATDRFRSLYSKCNVLGNNLKDLVDKYQHYEDASCGLLAGLQACEATASKHLSEPIAVDPKNLQRQLEETKALQGQISSQQVAVEKLKKTAEVLLDARGSLLPAKNDIQKTLDDIVGRYEDLSKSVNERNEKLQITLTRSLSVQDGLDEMLDWMGNVESSLKEQGQVPLNSTALQDIISKNIMLEQDIAGRQSSINAMNEKVKKFMETTDPSTASSLQAKMKDLSARFSEASHKHKETLAKMEELKTKVELFENLSEKLQTFLETKTQALTEVDVPGKDVTELSQYMQESTSEFLEHKKHLEVLHSLLKEISSHGLPSDKALVLEKTNNLSKKFKEMEDTIKEKKEAVTSCQEQLDAFQVLVKSLKSWIKETTKKVPIVQPSFGAEDLGKSLEDTKKLQEKWSLKTPEIQKVNNSGISLCNLISAVTTPAKAIAAVKSGGAVLNGEGTATNTEEFWANKGLTSIKKDMTDISHGYEDLGLLLKDKIAELNTKLSKLQKAQEESSAMMQWLQKMNKTATKWQQTPAPTDTEAVKTQVEQNKSFEAELKQNVNKVQELKDKLTELLEENPDTPEAPRWKQMLTEIDSKWQELNQLTIDRQQKLEESSNNLTQFQTVEAQLKQWLVEKELMVSVLGPLSIDPNMLNTQRQQVQILLQEFATRKPQYEQLTAAGQGILSRPGEDPSLRGIVKEQLAAVTQKWDSLTGQLSDRCDWIDQAIVKSTQYQSLLRSLSDKLSDLDNKLSSSLAVSTHPDAMNQQLETAQKMKQEIQQEKKQIKVAQALCEDLSALVKEEYLKAELSRQLEGILKSFKDVEQKAENHVQHLQSACASSHQFQQMSRDFQAWLDTKKEEQNKSHPISAKLDVLESLIKDHKDFSKTLTAQSHMYEKTIAEGENLLLKTQGSEKAALQLQLNTIKTNWDTFNKQVKERENKLKESLEKALKYKEQVETLWPWIDKCQNNLEEIKFCLDPAEGENSIAKLKSLQKEMDQHFGMVELLNNTANSLLSVCEIDKEVVTDENKSLIQKVDMVTEQLHSKKFCLENMTQKFKEFQEVSKESKRQLQCAKEQLDIHDSLGSQAYSNKYLTMLQTQQKSLQALKHQVDLAKRLAQDLVVEASDSKGTSDVLLQVETIAQEHSTLSQQVDEKCSFLETKLQGIGHFQNTIREMFSQFAEFDDELDSMAPVGRDAETLQKQKETIKAFLKKLEALMASNDNANKTCKMMLATEETSPDLVGIKRDLEALSKQCNKLLDRAQAREEQVEGTIKRLEEFYSKLKEFSILLQKAEEHEESQGPVGMETETINQQLNMFKVFQKEEIEPLQGKQQDVNWLGQGLIQSAAKSTSTQGLEHDLDDVNARWKTLNKKVAQRAAQLQEALLHCGRFQDALESLLSWMVDTEELVANQKPPSAEFKVVKAQIQEQKLLQRLLDDRKSTVEVIKREGEKIATTAEPADKVKILKQLSLLDSRWEALLNKAETRNRQLEGISVVAQQFHETLEPLNEWLTTIEKRLVNCEPIGTQASKLEEQIAQHKALEDDIINHNKHLHQAVSIGQSLKVLSSREDKDMVQSKLDFSQVWYIEIQEKSHSRSELLQQALCNAKIFGEDEVELMNWLNEVHDKLSKLSVQDYSTEGLWKQQSELRVLQEDILLRKQNVDQALLNGLELLKQTTGDEVLIIQDKLEAIKARYKDITKLSTDVAKTLEQALQLARRLHSTHEELCTWLDKVEVELLSYETQVLKGEEASQAQMRPKELKKEAKNNKALLDSLNEVSSALLELVPWRAREGLEKMVAEDNERYRLVSDTITQKVEEIDAAILRSQQFDQAADAELSWITETEKKLMSLGDIRLEQDQTSAQLQVQKTFTMEILRHKDIIDDLVKSGHKIMTACSEEEKQSMKKKLDKVLKNYDTICQINSERYLQLERAQSLVNQFWETYEELWPWLTETQSIISQLPAPALEYETLRQQQEEHRQLRELIAEHKPHIDKMNKTGPQLLELSPGEGFSIQEKYVAADTLYSQIKEDVKKRAVALDEAISQSTQFHDKIDQILESLERIVERLRQPPSISAEVEKIKEQISENKNVSVDMEKLQPLYETLKQRGEEMIARSGGTDKDISAKAVQDKLDQMVFIWENIHTLVEEREAKLLDVMELAEKFWCDHMSLIVTIKDTQDFIRDLEDPGIDPSVVKQQQEAAETIREEIDGLQEELDIVINLGSELIAACGEPDKPIVKKSIDELNSAWDSLNKAWKDRIDKLEEAMQAAVQYQDGLQAVFDWVDIAGGKLASMSPIGTDLETVKQQIEELKQFKSEAYQQQIEMERLNHQAELLLKKVTEESDKHTVQDPLMELKLIWDSLEERIINRQHKLEGALLALGQFQHALDELLAWLTHTEGLLSEQKPVGGDPKAIEIELAKHHVLQNDVLAHQSTVEAVNKAGNDLIESSAGEEASNLQNKLEVLNQRWQNVLEKTEQRKQQLDGALRQAKGFHGEIEDLQQWLTDTERHLLASKPLGGLPETAKEQLNVHMEVCAAFEAKEETYKSLMQKGQQMLARCPKSAETNIDQDINNLKEKWESVETKLNERKTKLEEALNLAMEFHNSLQDFINWLTQAEQTLNVASRPSLILDTVLFQIDEHKVFANEVNSHREQIIELDKTGTHLKYFSQKQDVVLIKNLLISVQSRWEKVVQRLVERGRSLDDARKRAKQFHEAWSKLMEWLEESEKSLDSELEIANDPDKIKTQLAQHKEFQKSLGAKHSVYDTTNRTGRSLKEKTSLADDNLKLDDMLSELRDKWDTICGKSVERQNKLEEALLFSGQFTDALQALIDWLYRVEPQLAEDQPVHGDIDLVMNLIDNHKAFQKELGKRTSSVQALKRSARELIEGSRDDSSWVKVQMQELSTRWETVCALSISKQTRLEAALRQAEEFHSVVHALLEWLAEAEQTLRFHGVLPDDEDALRTLIDQHKEFMKKLEEKRAELNKATTMGDTVLAICHPDSITTIKHWITIIRARFEEVLAWAKQHQQRLASALAGLIAKQELLEALLAWLQWAETTLTDKDKEVIPQEIEEVKALIAEHQTFMEEMTRKQPDVDKVTKTYKRRAADPSSLQSHIPVLDKGRAGRKRFPASSLYPSGSQTQIETKNPRVNLLVSKWQQVWLLALERRRKLNDALDRLEELREFANFDFDIWRKKYMRWMNHKKSRVMDFFRRIDKDQDGKITRQEFIDGILSSKFPTSRLEMSAVADIFDRDGDGYIDYYEFVAALHPNKDAYKPITDADKIEDEVTRQVAKCKCAKRFQVEQIGDNKYRFFLGNQFGDSQQLRLVRILRSTVMVRVGGGWMALDEFLVKNDPCRAKGRTNMELREKFILADGASQGMAAFRPRGRRSRPSSRGASPNRSTSVSSQAAQAASPQVPATTTPKILHPLTRNYGKPWLTNSKMSTPCKAAECSDFPVPSAEGTPIQGSKLRLPGYLSGKGFHSGEDSGLITTAAARVRTQFADSKKTPSRPGSRAGSKAGSRASSRRGSDASDFDISEIQSVCSDVETVPQTHRPTPRAGSRPSTAKPSKIPTPQRKSPASKLDKSSKR

96 NP_001547.1 NP_001073594.1

>NP_001547.1

MSWSPSLTTQTCGAWEMKERLGTGGFGNVIRWHNQETGEQIAIKQCRQELSPRNRERWCLEIQIMRRLTHPNVVAARDVPEGMQNLAPNDLPLLAMEYCQGGDLRKYLNQFENCCGLREGAILTLLSDIASALRYLHENRIIHRDLKPENIVLQQGEQRLIHKIIDLGYAKELDQGSLCTSFVGTLQYLAPELLEQQKYTVTVDYWSFGTLAFECITGFRPFLPNWQPVQWHSKVRQKSEVDIVVSEDLNGTVKFSSSLPYPNNLNSVLAERLEKWLQLMLMWHPRQRGTDPTYGPNGCFKALDDILNLKLVHILNMVTGTIHTYPVTEDESLQSLKARIQQDTGIPEEDQELLQEAGLALIPDKPATQCISDGKLNEGHTLDMDLVFLFDNSKITYETQISPRPQPESVSCILQEPKRNLAFFQLRKVWGQVWHSIQTLKEDCNRLQQGQRAAMMNLLRNNSCLSKMKNSMASMSQQLKAKLDFFKTSIQIDLEKYSEQTEFGITSDKLLLAWREMEQAVELCGRENEVKLLVERMMALQTDIVDLQRSPMGRKQGGTLDDLEEQARELYRRLREKPRDQRTEGDSQEMVRLLLQAIQSFEKKVRVIYTQLSKTVVCKQKALELLPKVEEVVSLMNEDEKTVVRLQEKRQKELWNLLKIACSKVRGPVSGSPDSMNASRLSQPGQLMSQPSTASNSLPEPAKKSEELVAEAHNLCTLLENAIQDTVREQDQSFTALDWSWLQTEEEEHSCLEQAS

>NP_001073594.1

MEGGRRARVVIESKRNFFLGAFPTPFPAEHVELGRLGDSETAMVPGKGGADYILLPFKKMDFSRNLYDIGEQLDSEDLASLKFLSLDYIPQRKQEPIKDALMLFQRLQEKRMLEESNLSFLKELLFRINRLDLLITYLNTRKEEMERELQTPGRAQISAYRVMLYQISEEVSRSELRSFKFLLQEEISKCKLDDDMNLLDIFIEMEKRVILGEGKLDILKRVCAQINKSLLKIINDYEEFSKERSSSLEGSPDEFSNGEELCGVMTISDSPREQDSESQTLDKVYQMKSKPRGYCLIINNHNFAKAREKVPKLHSIRDRNGTHLDAGALTTTFEELHFEIKPHDDCTVEQIYEILKIYQLMDHSNMDCFICCILSHGDKGIIYGTDGQEAPIYELTSQFTGLKCPSLAGKPKVFFIQACQGDNYQKGIPVETDSEEQPYLEMDLSSPQTRYIPDEADFLLGMATVNNCVSYRNPAEGTWYIQSLCQSLRERCPRGDDILTILTEVNYEVSNKDDKKNMGKQMPQPTFTLRKKLVFPSD

97 NP_002728.1 NP_000825.1

>NP_002728.1

MADVFPGNDSTASQDVANRFARKGALRQKNVHEVKDHKFIARFFKQPTFCSHCTDFIWGFGKQGFQCQVCCFVVHKRCHEFVTFSCPGADKGPDTDDPRSKHKFKIHTYGSPTFCDHCGSLLYGLIHQGMKCDTCDMNVHKQCVINVPSLCGMDHTEKRGRIYLKAEVADEKLHVTVRDAKNLIPMDPNGLSDPYVKLKLIPDPKNESKQKTKTIRSTLNPQWNESFTFKLKPSDKDRRLSVEIWDWDRTTRNDFMGSLSFGVSELMKMPASGWYKLLNQEEGEYYNVPIPEGDEEGNMELRQKFEKAKLGPAGNKVISPSEDRKQPSNNLDRVKLTDFNFLMVLGKGSFGKVMLADRKGTEELYAIKILKKDVVIQDDDVECTMVEKRVLALLDKPPFLTQLHSCFQTVDRLYFVMEYVNGGDLMYHIQQVGKFKEPQAVFYAAEISIGLFFLHKRGIIYRDLKLDNVMLDSEGHIKIADFGMCKEHMMDGVTTRTFCGTPDYIAPEIIAYQPYGKSVDWWAYGVLLYEMLAGQPPFDGEDEDELFQSIMEHNVSYPKSLSKEAVSICKGLMTKHPAKRLGCGPEGERDVREHAFFRRIDWEKLENREIQPPFKPKVCGKGAENFDKFFTRGQPVLTPPDQLVIANIDQSDFEGFSYVNPQFVHPILQSAV

>NP_000825.1

MKPRAECCSPKFWLVLAVLAVSGSRARSQKSPPSIGIAVILVGTSDEVAIKDAHEKDDFHHLSVVPRVELVAMNETDPKSIITRICDLMSDRKIQGVVFADDTDQEAIAQILDFISAQTLTPILGIHGGSSMIMADKDESSMFFQFGPSIEQQASVMLNIMEEYDWYIFSIVTTYFPGYQDFVNKIRSTIENSFVGWELEEVLLLDMSLDDGDSKIQNQLKKLQSPIILLYCTKEEATYIFEVANSVGLTGYGYTWIVPSLVAGDTDTVPAEFPTGLISVSYDEWDYGLPARVRDGIAIITTAASDMLSEHSFIPEPKSSCYNTHEKRIYQSNMLNRYLINVTFEGRNLSFSEDGYQMHPKLVIILLNKERKWERVGKWKDKSLQMKYYVWPRMCPETEEQEDDHLSIVTLEEAPFVIVESVDPLSGTCMRNTVPCQKRIVTENKTDEEPGYIKKCCKGFCIDILKKISKSVKFTYDLYLVTNGKHGKKINGTWNGMIGEVVMKRAYMAVGSLTINEERSEVVDFSVPFIETGISVMVSRSNGTVSPSAFLEPFSADVWVMMFVMLLIVSAVAVFVFEYFSPVGYNRCLADGREPGGPSFTIGKAIWLLWGLVFNNSVPVQNPKGTTSKIMVSVWAFFAVIFLASYTANLAAFMIQEEYVDQVSGLSDKKFQRPNDFSPPFRFGTVPNGSTERNIRNNYAEMHAYMGKFNQRGVDDALLSLKTGKLDAFIYDAAVLNYMAGRDEGCKLVTIGSGKVFASTGYGIAIQKDSGWKRQVDLAILQLFGDGEMEELEALWLTGICHNEKNEVMSSQLDIDNMAGVFYMLGAAMALSLITFICEHLFYWQFRHCFMGVCSGKPGMVFSISRGIYSCIHGVAIEERQSVMNSPTATMNNTHSNILRLLRTAKNMANLSGVNGSPQSALDFIRRESSVYDISEHRRSFTHSDCKSYNNPPCEENLFSDYISEVERTFGNLQLKDSNVYQDHYHHHHRPHSIGSASSIDGLYDCDNPPFTTQSRSISKKPLDIGLPSSKHSQLSDLYGKFSFKSDRYSGHDDLIRSDVSDISTHTVTYGNIEGNAAKRRKQQYKDSLKKRPASAKSRREFDEIELAYRRRPPRSPDHKRYFRDKEGLRDFYLDQFRTKENSPHWEHVDLTDIYKERSDDFKRDSISGGGPCTNRSHIKHGTGDKHGVVSGVPAPWEKNLTNVEWEDRSGGNFCRSCPSKLHNYSTTVTGQNSGRQACIRCEACKKAGNLYDISEDNSLQELDQPAAPVAVTSNASTTKYPQSPTNSKAQKKNRNKLRRQHSYDTFVDLQKEEAALAPRSVSLKDKGRFMDGSPYAHMFEMSAGESTFANNKSSVPTAGHHHHNNPGGGYMLSKSLYPDRVTQNPFIPTFGDDQCLLHGSKSYFFRQPTVAGASKARPDFRALVTNKPVVSALHGAVPARFQKDICIGNQSNPCVPNNKNPRAFNGSSNGHVYEKLSSIESDV

98 NP_005347.3 NP_644805.1

>NP_005347.3

MGCGCSSHPEDDWMENIDVCENCHYPIVPLDGKGTLLIRNGSEVRDPLVTYEGSNPPASPLQDNLVIALHSYEPSHDGDLGFEKGEQLRILEQSGEWWKAQSLTTGQEGFIPFNFVAKANSLEPEPWFFKNLSRKDAERQLLAPGNTHGSFLIRESESTAGSFSLSVRDFDQNQGEVVKHYKIRNLDNGGFYISPRITFPGLHELVRHYTNASDGLCTRLSRPCQTQKPQKPWWEDEWEVPRETLKLVERLGAGQFGEVWMGYYNGHTKVAVKSLKQGSMSPDAFLAEANLMKQLQHQRLVRLYAVVTQEPIYIITEYMENGSLVDFLKTPSGIKLTINKLLDMAAQIAEGMAFIEERNYIHRDLRAANILVSDTLSCKIADFGLARLIEDNEYTAREGAKFPIKWTAPEAINYGTFTIKSDVWSFGILLTEIVTHGRIPYPGMTNPEVIQNLERGYRMVRPDNCPEELYQLMRLCWKERPEDRPTFDYLRSVLEDFFTATEGQYQPQP

>NP_644805.1

MAQWNQLQQLDTRYLEQLHQLYSDSFPMELRQFLAPWIESQDWAYAASKESHATLVFHNLLGEIDQQYSRFLQESNVLYQHNLRRIKQFLQSRYLEKPMEIARIVARCLWEESRLLQTAATAAQQGGQANHPTAAVVTEKQQMLEQHLQDVRKRVQDLEQKMKVVENLQDDFDFNYKTLKSQGDMQDLNGNNQSVTRQKMQQLEQMLTALDQMRRSIVSELAGLLSAMEYVQKTLTDEELADWKRRQQIACIGGPPNICLDRLENWITSLAESQLQTRQQIKKLEELQQKVSYKGDPIVQHRPMLEERIVELFRNLMKSAFVVERQPCMPMHPDRPLVIKTGVQFTTKVRLLVKFPELNYQLKIKVCIDKDSGDVAALRGSRKFNILGTNTKVMNMEESNNGSLSAEFKHLTLREQRCGNGGRANCDASLIVTEELHLITFETEVYHQGLKIDLETHSLPVVVISNICQMPNAWASILWYNMLTNNPKNVNFFTKPPIGTWDQVAEVLSWQFSSTTKRGLSIEQLTTLAEKLLGPGVNYSGCQITWAKFCKENMAGKGFSFWVWLDNIIDLVKKYILALWNEGYIMGFISKERERAILSTKPPGTFLLRFSESSKEGGVTFTWVEKDISGKTQIQSVEPYTKQQLNNMSFAEIIMGYKIMDATNILVSPLVYLYPDIPKEEAFGKYCRPESQEHPEADPGSAAPYLKTKFICVTPTTCSNTIDLPMSPRTLDSLMQFGNNGEGAEPSAGGQFESLTFDMELTSECATSPM

99 NP_001003811.1 NP_061938.2

>NP_001003811.1

MISAHCNLRLLCSSDSSASASQVAGTTEVVENLVTNDNSPNIPEAIDRLFSDIANINRESMAEITDIQIEEMAVNLWNWALTIGGGWLVNEEQKIRLHYVACKLLSMCEASFASEQSIQRLIMMNMRIGKEWLDAGNFLIADECFQAAVASLEQLYVKLIQRSSPEADLTMEKITVESDHFRVLSYQAESAVAQGDFQRASMCVLQCKDMLMRLPQMTSSLHHLCYNFGVETQKNNKYEESSFWLSQSYDIGKMDKKSTGPEMLAKVLRLLATNYLDWDDTKYYDKALNAVNLANKEHLSSPGLFLKMKILLKGETSNEELLEAVMEILHLDMPLDFCLNIAKLLMDHERESVGFHFLTIIHERFKSSENIGKVLILHTDMLLQRKEELLAKEKIEEIFLAHQTGRQLTAESMNWLHNILWRQAASSFEVQNYTDALQWYYYSLRFYSTDEMDLDFTKLQRNMACCYLNLQQLDKAKEAVAEAERHDPRNVFTQFYIFKIAVIEGNSERALQAIITLENILTDEESEDNDLVAERGSPTMLLSLAAQFALENGQQIVAEKALEYLAQHSEDQEQVLTAVKCLLRFLLPKIAEMPESEDKKKEMDRLLTCLNRAFVKLSQPFGEEALSLESRANEAQWFRKTAWNLAVQCDKDPVMMREFFILSYKMSQFCPSDQVILIARKTCLLMAVAVDLEQGRKASTAFEQTMFLSRALEEIQTCNDIHNFLKQTGTFSNDSCEKLLLLYEFEVRAKLNDPLLESFLESVWELPHLETKTFETIAIIAMEKPAHYPLIALKALKKALLLYKKEEPIDISQYSKCMHNLVNLSVPDGASNVELCPLEEVWGYFEDALSHISRTKDYPEMEILWLMVKSWNTGVLMFSRSKYASAEKWCGLALRFLNHLTSFKESYETQMNMLYSQLVEALSNNKGPVFHEHGYWSKSD

>NP_061938.2

MCERAARLCRAGAHRLLREPPQQGRALGGLLRWVGARMGEPRESLAPAAPADPGPASPRGGTAVILDIFRRADKNDDGKLSLEEFQLFFADGVLNEKELEDLFHTIDSDNTNHVDTKELCDYFVDHMGDYEDVLASLETLNHSVLKAMGYTKKVYEGGSNVDQFVTRFLLKETANQIQSLLSSVESAVEAIEEQTSQLRQNHIKPSHSAAQTWCGSPTPASAPNHKLMAMEQGKTLPSATEDAKEEGLEAQISRLAELIGRLESKALWFDLQQRLSDEDGTNMHLQLVRQEMAVCPEQLSEFLDSLRQYLRGTTGVRNCFHITAVRLSDGFTFVIYEFWETEEAWKRHLQSPLCKAFRHVKVDTLSQPEALSRILVPAAWCTVGRD

100 NP_892113.3 NP_000116.2

>NP_892113.3

MNLLPPKPKYNPLRNESLSSMEEGASGSTPPEELPSPPASSLGPILPPLPGDDSPTTLCSFFPRMSNLRLANPAGGRPGSKGEPGRAADDGEGIVGAAMPDSGPLPLLQDMNKLSGGGGRRTRVEGGQLGGEEWTRHGSFVNKPTRGWLHPNDKVMGPGVSYLVRYMGCVEVLQSMRALDFNTRTQVTREAISLVCEAVPGAKGATRRRKPCSRPLSSILGRSNLKFAGMPITLTVSTSSLNLMAADCKQIIANHHMQSISFASGGDPDTAEYVAYVAKDPVNQRACHILECPEGLAQDVISTIGQAFELRFKQYLRNPPKLVTPHDRMAGFDGSAWDEEEEEPPDHQYYNDFPGKEPPLGGVVDMRLREGAAPGAARPTAPNAQTPSHLGATLPVGQPVGGDPEVRKQMPPPPPCPGRELFDDPSYVNVQNLDKARQAVGGAGPPNPAINGSAPRDLFDMKPFEDALRVPPPPQSVSMAEQLRGEPWFHGKLSRREAEALLQLNGDFLVRESTTTPGQYVLTGLQSGQPKHLLLVDPEGVVRTKDHRFESVSHLISYHMDNHLPIISAGSELCLQQPVERKL

>NP_000116.2

MTMTLHTKASGMALLHQIQGNELEPLNRPQLKIPLERPLGEVYLDSSKPAVYNYPEGAAYEFNAAAAANAQVYGQTGLPYGPGSEAAAFGSNGLGGFPPLNSVSPSPLMLLHPPPQLSPFLQPHGQQVPYYLENEPSGYTVREAGPPAFYRPNSDNRRQGGRERLASTNDKGSMAMESAKETRYCAVCNDYASGYHYGVWSCEGCKAFFKRSIQGHNDYMCPATNQCTIDKNRRKSCQACRLRKCYEVGMMKGGIRKDRRGGRMLKHKRQRDDGEGRGEVGSAGDMRAANLWPSPLMIKRSKKNSLALSLTADQMVSALLDAEPPILYSEYDPTRPFSEASMMGLLTNLADRELVHMINWAKRVPGFVDLTLHDQVHLLECAWLEILMIGLVWRSMEHPGKLLFAPNLLLDRNQGKCVEGMVEIFDMLLATSSRFRMMNLQGEEFVCLKSIILLNSGVYTFLSSTLKSLEEKDHIHRVLDKITDTLIHLMAKAGLTLQQQHQRLAQLLLILSHIRHMSNKGMEHLYSMKCKNVVPLYDLLLEMLDAHRLHAPTSRGGASVEETDQSHLATAGSTSSHSLQKYYITGEAEGFPATV

101 NP_004746.2 NP_004947.2

>NP_004746.2

MEEEGGSSGGAAGTSADGGDGGEQLLTVKHELRTANLTGHAEKVGIENFELLKVLGTGAYGKVFLVRKISGHDTGKLYAMKVLKKATIVQKAKTTEHTRTERQVLEHIRQSPFLVTLHYAFQTETKLHLILDYINGGELFTHLSQRERFTEHEVQIYVGEIVLALEHLHKLGIIYRDIKLENILLDSNGHVVLTDFGLSKEFVADETERAYSFCGTIEYMAPDIVRGGDSGHDKAVDWWSLGVLMYELLTGASPFTVDGEKNSQAEISRRILKSEPPYPQEMSALAKDLIQRLLMKDPKKRLGCGPRDADEIKEHLFFQKINWDDLAAKKVPAPFKPVIRDELDVSNFAEEFTEMDPTYSPAALPQSSEKLFQGYSFVAPSILFKRNAAVIDPLQFHMGVERPGVTNVARSAMMKDSPFYQHYDLDLKDKPLGEGSFSICRKCVHKKSNQAFAVKIISKRMEANTQKEITALKLCEGHPNIVKLHEVFHDQLHTFLVMELLNGGELFERIKKKKHFSETEASYIMRKLVSAVSHMHDVGVVHRDLKPENLLFTDENDNLEIKIIDFGFARLKPPDNQPLKTPCFTLHYAAPELLNQNGYDESCDLWSLGVILYTMLSGQVPFQSHDRSLTCTSAVEIMKKIKKGDFSFEGEAWKNVSQEAKDLIQGLLTVDPNKRLKMSGLRYNEWLQDGSQLSSNPLMTPDILGSSGAAVHTCVKATFHAFNKYKREGFCLQNVDKAPLAKRRKMKKTSTSTETRSSSSESSHSSSSHSHGKTTPTKTLQPSNPADSNNPETLFQFSDSVA

>NP_004947.2

MDGFYDQQVPYMVTNSQRGRNCNEKPTNVRKRKFINRDLAHDSEELFQDLSQLQETWLAEAQVPDNDEQFVPDYQAESLAFHGLPLKIKKEPHSPCSEISSACSQEQPFKFSYGEKCLYNVSAYDQKPQVGMRPSNPPTPSSTPVSPLHHASPNSTHTPKPDRAFPAHLPPSQSIPDSSYPMDHRFRRQLSEPCNSFPPLPTMPREGRPMYQRQMSEPNIPFPPQGFKQEYHDPVYEHNTMVGSAASQSFPPPLMIKQEPRDFAYDSEVPSCHSIYMRQEGFLAHPSRTEGCMFEKGPRQFYDDTCVVPEKFDGDIKQEPGMYREGPTYQRRGSLQLWQFLVALLDDPSNSHFIAWTGRGMEFKLIEPEEVARRWGIQKNRPAMNYDKLSRSLRYYYEKGIMQKVAGERYVYKFVCDPEALFSMAFPDNQRPLLKTDMERHINEEDTVPLSHFDESMAYMPEGGCCNPHPYNEGYVY

102 NP_006473.1 NP_036547.1

>NP_006473.1

MLTRKPSAAAPAAYPTGRGGDSAVRQLQASPGLGAGPTRSGVGTGPPSPIALPPLRASNAAAAAHTIGGSKHTMNDHLHVGSHAHGQIQVQQLFEDNSNKRTVLTTQPNGLTTVGKTGLPVVPERQLDSIHRRQGSSTSLKSMEGMGKVKATPMTPEQAMKQYMQKLTAFEHHEIFSYPEIYFLGLNAKKRQGMTGGPNNGGYDDDQGSYVQVPHDHVAYRYEVLKVIGKGSFGQVVKAYDHKVHQHVALKMVRNEKRFHRQAAEEIRILEHLRKQDKDNTMNVIHMLENFTFRNHICMTFELLSMNLYELIKKNKFQGFSLPLVRKFAHSILQCLDALHKNRIIHCDLKPENILLKQQGRSGIKVIDFGSSCYEHQRVYTYIQSRFYRAPEVILGARYGMPIDMWSLGCILAELLTGYPLLPGEDEGDQLACMIELLGMPSQKLLDASKRAKNFVSSKGYPRYCTVTTLSDGSVVLNGGRSRRGKLRGPPESREWGNALKGCDDPLFLDFLKQCLEWDPAVRMTPGQALRHPWLRRRLPKPPTGEKTSVKRITESTGAITSISKLPPPSSSASKLRTNLAQMTDANGNIQQRTVLPKLVS

>NP_036547.1

MRRSAAPSQLQGNSFKKPKFIPPGRSNPGLNEEITKLNPDIKLFEGVAINNTFLPSQNDLRICSLNLPSEESTREINNRDNCSGKYCFEAPTLATLDPPHTVHSAPKEVAVSKEQEEKSDSLVKYFSVVWCKPSKKKHKKWEGDAVLIVKGKSFILKNLEGKDIGRGIGYKFKELEKIEEGQTLMICGKEIEVMGVISPDDFSSGRCFQLGGGSTAISHSSQVARKCFSNPFKSVCKPSSKENRQNDFQNCKPRHDPYTPNSLVMPRPDKNHQWVFNKNCFPLVDVVIDPYLVYHLRPHQKEGIIFLYECVMGMRMNGRCGAILADEMGLGKTLQCISLIWTLQCQGPYGGKPVIKKTLIVTPGSLVNNWKKEFQKWLGSERIKIFTVDQDHKVEEFIKSIFYSVLIISYEMLLRSLDQIKNIKFDLLICDEGHRLKNSAIKTTTALISLSCEKRIILTGTPIQNDLQEFFALIDFVNPGILGSLSSYRKIYEEPIILSREPSASEEEKELGERRAAELTCLTGLFILRRTQEIINKYLPPKIENVVFCRPGALQIELYRKLLNSQVVRFCLQGLLENSPHLICIGALKKLCNHPCLLFNSIKEKECSSTCDKNEEKSLYKGLLSVFPADYNPLLFTEKESGKLQVLSKLLAVIHELRPTEKVVLVSNYTQTLNILQEVCKRHGYAYTRLDGQTPISQRQQIVDGFNSQHSSFFIFLLSSKAGGVGLNLIGGSHLILYDIDWNPATDIQAMSRVWRDGQKYPVHIYRLLTTGTIEEKIYQRQISKQGLCGAVVDLTKTSEHIQFSVEELKNLFTLHESSDCVTHDLLDCECTGEEVHTGDSLEKFIVSRDCQLGPHHQKSNSLKPLSMSQLKQWKHFSGDHLNLTDPFLERITENVSFIFQNITTQATGT

103 NP_006739.1 NP_005179.2

>NP_006739.1

MGNSMKSTPAPAERPLPNPEGLDSDFLAVLSDYPSPDISPPIFRRGEKLRVISDEGGWWKAISLSTGRESYIPGICVARVYHGWLFEGLGRDKAEELLQLPDTKVGSFMIRESETKKGFYSLSVRHRQVKHYRIFRLPNNWYYISPRLTFQCLEDLVNHYSEVADGLCCVLTTPCLTQSTAAPAVRASSSPVTLRQKTVDWRRVSRLQEDPEGTENPLGVDESLFSYGLRESIASYLSLTSEDNTSFDRKKKSISLMYGGSKRKSSFFSSPPYFED

>NP_005179.2

MAGNVKKSSGAGGGSGSGGSGSGGLIGLMKDAFQPHHHHHHHLSPHPPGTVDKKMVEKCWKLMDKVVRLCQNPKLALKNSPPYILDLLPDTYQHLRTILSRYEGKMETLGENEYFRVFMENLMKKTKQTISLFKEGKERMYEENSQPRRNLTKLSLIFSHMLAELKGIFPSGLFQGDTFRITKADAAEFWRKAFGEKTIVPWKSFRQALHEVHPISSGLEAMALKSTIDLTCNDYISVFEFDIFTRLFQPWSSLLRNWNSLAVTHPGYMAFLTYDEVKARLQKFIHKPGSYIFRLSCTRLGQWAIGYVTADGNILQTIPHNKPLFQALIDGFREGFYLFPDGRNQNPDLTGLCEPTPQDHIKVTQEQYELYCEMGSTFQLCKICAENDKDVKIEPCGHLMCTSCLTSWQESEGQGCPFCRCEIKGTEPIVVDPFDPRGSGSLLRQGAEGAPSPNYDDDDDERADDTLFMMKELAGAKVERPPSPFSMAPQASLPPVPPRLDLLPQRVCVPSSASALGTASKAASGSLHKDKPLPVPPTLRDLPPPPPPDRPYSVGAESRPQRRPLPCTPGDCPSRDKLPPVPSSRLGDSWLPRPIPKVPVSAPSSSDPWTGRELTNRHSLPFSLPSQMEPRPDVPRLGSTFSLDTSMSMNSSPLVGPECDHPKIKPSSSANAIYSLAARPLPVPKLPPGEQCEGEEDTEYMTPSSRPLRPLDTSQSSRACDCDQQIDSCTYEAMYNIQSQAPSITESSTFGEGNLAAAHANTGPEESENEDDGYDVPKPPVPAVLARRTLSDISNASSSFGWLSLDGDPTTNVTEGSQVPERPPKPFPRRINSERKAGSCQQGSGPAASAATASPQLSSEIENLMSQGYSYQDIQKALVIAQNNIEMAKNILREFVSISSPAHVAT

104 NP_001460.1 NP_055435.2

>NP_001460.1

MSGWESYYKTEGDEEAEEEQEENLEASGDYKYSGRDSLIFLVDASKAMFESQSEDELTPFDMSIQCIQSVYISKIISSDRDLLAVVFYGTEKDKNSVNFKNIYVLQELDNPGAKRILELDQFKGQQGQKRFQDMMGHGSDYSLSEVLWVCANLFSDVQFKMSHKRIMLFTNEDNPHGNDSAKASRARTKAGDLRDTGIFLDLMHLKKPGGFDISLFYRDIISIAEDEDLRVHFEESSKLEDLLRKVRAKETRKRALSRLKLKLNKDIVISVGIYNLVQKALKPPPIKLYRETNEPVKTKTRTFNTSTGGLLLPSDTKRSQIYGSRQIILEKEETEELKRFDDPGLMLMGFKPLVLLKKHHYLRPSLFVYPEESLVIGSSTLFSALLIKCLEKEVAALCRYTPRRNIPPYFVALVPQEEELDDQKIQVTPPGFQLVFLPFADDKRKMPFTEKIMATPEQVGKMKAIVEKLRFTYRSDSFENPVLQQHFRNLEALALDLMEPEQAVDLTLPKVEAMNKRLGSLVDEFKELVYPPDYNPEGKVTKRKHDNEGSGSKRPKVEYSEEELKTHISKGTLGKFTVPMLKEACRAYGLKSGLKKQELLEALTKHFQD

>NP_055435.2

MIMSSYLMDSNYIDPKFPPCEEYSQNSYIPEHSPEYYGRTRESGFQHHHQELYPPPPPRPSYPERQYSCTSLQGPGNSRGHGPAQAGHHHPEKSQSLCEPAPLSGASASPSPAPPACSQPAPDHPSSAASKQPIVYPWMKKIHVSTVNPNYNGGEPKRSRTAYTRQQVLELEKEFHYNRYLTRRRRIEIAHSLCLSERQIKIWFQNRRMKWKKDHRLPNTKVRSAPPAGAAPSTLSAATPGTSEDHSQSATPPEQQRAEDITRL

105 NP_003336.1 NP_003191.1

>NP_003336.1

MSGIALSRLAQERKAWRKDHPFGFVAVPTKNPDGTMNLMNWECAIPGKKGTPWEGGLFKLRMLFKDDYPSSPPKCKFEPPLFHPNVYPSGTVCLSILEEDKDWRPAITIKQILLGIQELLNEPNIQDPAQAEAYTIYCQNRVEYEKRVRAQAKKFAPS

>NP_003191.1

MNQPQRMAPVGTDKELSDLLDFSMMFPLPVTNGKGRPASLAGAQFGGSGLEDRPSSGSWGSGDQSSSSFDPSRTFSEGTHFTESHSSLSSSTFLGPGLGGKSGERGAYASFGRDAGVGGLTQAGFLSGELALNSPGPLSPSGMKGTSQYYPSYSGSSRRRAADGSLDTQPKKVRKVPPGLPSSVYPPSSGEDYGRDATAYPSAKTPSSTYPAPFYVADGSLHPSAELWSPPGQAGFGPMLGGGSSPLPLPPGSGPVGSSGSSSTFGGLHQHERMGYQLHGAEVNGGLPSASSFSSAPGATYGGVSSHTPPVSGADSLLGSRGTTAGSSGDALGKALASIYSPDHSSNNFSSSPSTPVGSPQGLAGTSQWPRAGAPGALSPSYDGGLHGLQSKIEDHLDEAIHVLRSHAVGTAGDMHTLLPGHGALASGFTGPMSLGGRHAGLVGGSHPEDGLAGSTSLMHNHAALPSQPGTLPDLSRPPDSYSGLGRAGATAAASEIKREEKEDEENTSAADHSEEEKKELKAPRARTSPDEDEDDLLPPEQKAEREKERRVANNARERLRVRDINEAFKELGRMCQLHLNSEKPQTKLLILHQAVSVILNLEQQVRERNLNPKAACLKRREEEKVSGVVGDPQMVLSAPHPGLSEAHNPAGHM

106 NP_000456.1 NP_000242.1

>NP_000456.1

MPDNRQPRNRQPRIRSGNEPRSAPAMEPDGRGAWAHSRAALDRLEKLLRCSRCTNILREPVCLGGCEHIFCSNCVSDCIGTGCPVCYTPAWIQDLKINRQLDSMIQLCSKLRNLLHDNELSDLKEDKPRKSLFNDAGNKKNSIKMWFSPRSKKVRYVVSKASVQTQPAIKKDASAQQDSYEFVSPSPPADVSERAKKASARSGKKQKKKTLAEINQKWNLEAEKEDGEFDSKEESKQKLVSFCSQPSVISSPQINGEIDLLASGSLTESECFGSLTEVSLPLAEQIESPDTKSRNEVVTPEKVCKNYLTSKKSLPLENNGKRGHHNRLSSPISKRCRTSILSTSGDFVKQTVPSENIPLPECSSPPSCKRKVGGTSGRKNSNMSDEFISLSPGTPPSTLSSSSYRQVMSSPSAMKLLPNMAVKRNHRGETLLHIASIKGDIPSVEYLLQNGSDPNVKDHAGWTPLHEACNHGHLKVVELLLQHKALVNTTGYQNDSPLHDAAKNGHVDIVKLLLSYGASRNAVNIFGLRPVDYTDDESMKSLLLLPEKNESSSASHCSVMNTGQRRDGPLVLIGSGLSSEQQKMLSELAVILKAKKYTEFDSTVTHVVVPGDAVQSTLKCMLGILNGCWILKFEWVKACLRRKVCEQEEKYEIPEGPRRSRLNREQLLPKLFDGCYFYLWGTFKHHPKDNLIKLVTAGGGQILSRKPKPDSDVTQTINTVAYHARPDSDQRFCTQYIIYEDLCNYHPERVRQGKVWKAPSSWFIDCVMSFELLPLDS

>NP_000242.1

MAVQPKETLQLESAAEVGFVRFFQGMPEKPTTTVRLFDRGDFYTAHGEDALLAAREVFKTQGVIKYMGPAGAKNLQSVVLSKMNFESFVKDLLLVRQYRVEVYKNRAGNKASKENDWYLAYKASPGNLSQFEDILFGNNDMSASIGVVGVKMSAVDGQRQVGVGYVDSIQRKLGLCEFPDNDQFSNLEALLIQIGPKECVLPGGETAGDMGKLRQIIQRGGILITERKKADFSTKDIYQDLNRLLKGKKGEQMNSAVLPEMENQVAVSSLSAVIKFLELLSDDSNFGQFELTTFDFSQYMKLDIAAVRALNLFQGSVEDTTGSQSLAALLNKCKTPQGQRLVNQWIKQPLMDKNRIEERLNLVEAFVEDAELRQTLQEDLLRRFPDLNRLAKKFQRQAANLQDCYRLYQGINQLPNVIQALEKHEGKHQKLLLAVFVTPLTDLRSDFSKFQEMIETTLDMDQVENHEFLVKPSFDPNLSELREIMNDLEKKMQSTLISAARDLGLDPGKQIKLDSSAQFGYYFRVTCKEEKVLRNNKNFSTVDIQKNGVKFTNSKLTSLNEEYTKNKTEYEEAQDAIVKEIVNISSGYVEPMQTLNDVLAQLDAVVSFAHVSNGAPVPYVRPAILEKGQGRIILKASRHACVEVQDEIAFIPNDVYFEKDKQMFHIITGPNMGGKSTYIRQTGVIVLMAQIGCFVPCESAEVSIVDCILARVGAGDSQLKGVSTFMAEMLETASILRSATKDSLIIIDELGRGTSTYDGFGLAWAISEYIATKIGAFCMFATHFHELTALANQIPTVNNLHVTALTTEETLTMLYQVKKGVCDQSFGIHVAELANFPKHVIECAKQKALELEEFQYIGESQGYDIMEPAAKKCYLEREQGEKIIQEFLSKVKQMPFTEMSEENITIKLKQLKAEVIAKNNSFVNEIISRIKVTT

107 NP_001666.2 NP_004119.1

>NP_001666.2

MTEMSFLSSEVLVGDLMSPFDQSGLGAEESLGLLDDYLEVAKHFKPHGFSSDKAKAGSSEWLAVDGLVSPSNNSKEDAFSGTDWMLEKMDLKEFDLDALLGIDDLETMPDDLLTTLDDTCDLFAPLVQETNKQPPQTVNPIGHLPESLTKPDQVAPFTFLQPLPLSPGVLSSTPDHSFSLELGSEVDITEGDRKPDYTAYVAMIPQCIKEEDTPSDNDSGICMSPESYLGSPQHSPSTRGSPNRSLPSPGVLCGSARPKPYDPPGEKMVAAKVKGEKLDKKLKKMEQNKTAATRYRQKKRAEQEALTGECKELEKKNEALKERADSLAKEIQYLKDLIEEVRKARGKKRVP

>NP_004119.1

MAERGELDLTGAKQNTGVWLVKVPKYLSQQWAKASGRGEVGKLRIAKTQGRTEVSFTLNEDLANIHDIGGKPASVSAPREHPFVLQSVGGQTLTVFTESSSDKLSLEGIVVQRAECRPAASENYMRLKRLQIEESSKPVRLSQQLDKVVTTNYKPVANHQYNIEYERKKKEDGKRARADKQHVLDMLFSAFEKHQYYNLKDLVDITKQPVVYLKEILKEIGVQNVKGIHKNTWELKPEYRHYQGEEKSD

108 NP_001639.1 NP_000005.2

>NP_001639.1

MWVPVVFLTLSVTWIGAAPLILSRIVGGWECEKHSQPWQVLVASRGRAVCGGVLVHPQWVLTAAHCIRNKSVILLGRHSLFHPEDTGQVFQVSHSFPHPLYDMSLLKNRFLRPGDDSSHDLMLLRLSEPAELTDAVKVMDLPTQEPALGTTCYASGWGSIEPEEFLTPKKLQCVDLHVISNDVCAQVHPQKVTKFMLCAGRWTGGKSTCSGDSGGPLVCNGVLQGITSWGSEPCALPERPSLYTKVVHYRKWIKDTIVANP

>NP_000005.2

MGKNKLLHPSLVLLLLVLLPTDASVSGKPQYMVLVPSLLHTETTEKGCVLLSYLNETVTVSASLESVRGNRSLFTDLEAENDVLHCVAFAVPKSSSNEEVMFLTVQVKGPTQEFKKRTTVMVKNEDSLVFVQTDKSIYKPGQTVKFRVVSMDENFHPLNELIPLVYIQDPKGNRIAQWQSFQLEGGLKQFSFPLSSEPFQGSYKVVVQKKSGGRTEHPFTVEEFVLPKFEVQVTVPKIITILEEEMNVSVCGLYTYGKPVPGHVTVSICRKYSDASDCHGEDSQAFCEKFSGQLNSHGCFYQQVKTKVFQLKRKEYEMKLHTEAQIQEEGTVVELTGRQSSEITRTITKLSFVKVDSHFRQGIPFFGQVRLVDGKGVPIPNKVIFIRGNEANYYSNATTDEHGLVQFSINTTNVMGTSLTVRVNYKDRSPCYGYQWVSEEHEEAHHTAYLVFSPSKSFVHLEPMSHELPCGHTQTVQAHYILNGGTLLGLKKLSFYYLIMAKGGIVRTGTHGLLVKQEDMKGHFSISIPVKSDIAPVARLLIYAVLPTGDVIGDSAKYDVENCLANKVDLSFSPSQSLPASHAHLRVTAAPQSVCALRAVDQSVLLMKPDAELSASSVYNLLPEKDLTGFPGPLNDQDDEDCINRHNVYINGITYTPVSSTNEKDMYSFLEDMGLKAFTNSKIRKPKMCPQLQQYEMHGPEGLRVGFYESDVMGRGHARLVHVEEPHTETVRKYFPETWIWDLVVVNSAGVAEVGVTVPDTITEWKAGAFCLSEDAGLGISSTASLRAFQPFFVELTMPYSVIRGEAFTLKATVLNYLPKCIRVSVQLEASPAFLAVPVEKEQAPHCICANGRQTVSWAVTPKSLGNVNFTVSAEALESQELCGTEVPSVPEHGRKDTVIKPLLVEPEGLEKETTFNSLLCPSGGEVSEELSLKLPPNVVEESARASVSVLGDILGSAMQNTQNLLQMPYGCGEQNMVLFAPNIYVLDYLNETQQLTPEIKSKAIGYLNTGYQRQLNYKHYDGSYSTFGERYGRNQGNTWLTAFVLKTFAQARAYIFIDEAHITQALIWLSQRQKDNGCFRSSGSLLNNAIKGGVEDEVTLSAYITIALLEIPLTVTHPVVRNALFCLESAWKTAQEGDHGSHVYTKALLAYAFALAGNQDKRKEVLKSLNEEAVKKDNSVHWERPQKPKAPVGHFYEPQAPSAEVEMTSYVLLAYLTAQPAPTSEDLTSATNIVKWITKQQNAQGGFSSTQDTVVALHALSKYGAATFTRTGKAAQVTIQSSGTFSSKFQVDNNNRLLLQQVSLPELPGEYSMKVTGEGCVYLQTSLKYNILPEKEEFPFALGVQTLPQTCDEPKAHTSFQISLSVSYTGSRSASNMAIVDVKMVSGFIPLKPTVKMLERSNHVSRTEVSSNHVLIYLDKVSNQTLSLFFTVLQDVPVRDLKPAIVKVYDYYETDEFAIAEYNAPCSKDLGNA

109 NP_000323.2 NP_056077.1

>NP_000323.2

MKSNQERSNECLPPKKREIPATSRSSEEKAPTLPSDNHRVEGTAWLPGNPGGRGHGGGRHGPAGTSVELGLQQGIGLHKALSTGLDYSPPSAPRSVPVATTLPAAYATPQPGTPVSPVQYAHLPHTFQFIGSSQYSGTYASFIPSQLIPPTANPVTSAVASAAGATTPSQRSQLEAYSTLLANMGSLSQTPGHKAEQQQQQQQQQQQQHQHQQQQQQQQQQQQQQHLSRAPGLITPGSPPPAQQNQYVHISSSPQNTGRTASPPAIPVHLHPHQTMIPHTLTLGPPSQVVMQYADSGSHFVPREATKKAESSRLQQAIQAKEVLNGEMEKSRRYGAPSSADLGLGKAGGKSVPHPYESRHVVVHPSPSDYSSRDPSGVRASVMVLPNSNTPAADLEVQQATHREASPSTLNDKSGLHLGKPGHRSYALSPHTVIQTTHSASEPLPVGLPATAFYAGTQPPVIGYLSGQQQAITYAGSLPQHLVIPGTQPLLIPVGSTDMEASGAAPAIVTSSPQFAAVPHTFVTTALPKSENFNPEALVTQAAYPAMVQAQIHLPVVQSVASPAAAPPTLPPYFMKGSIIQLANGELKKVEDLKTEDFIQSAEISNDLKIDSSTVERIEDSHSPGVAVIQFAVGEHRAQVSVEVLVEYPFFVFGQGWSSCCPERTSQLFDLPCSKLSVGDVCISLTLKNLKNGSVKKGQPVDPASVLLKHSKADGLAGSRHRYAEQENGINQGSAQMLSENGELKFPEKMGLPAAPFLTKIEPSKPAATRKRRWSAPESRKLEKSEDEPPLTLPKPSLIPQEVKICIEGRSNVGK

>NP_056077.1

MMNRTTPDQELVPASEPVWERPWSVEEIRRSSQSWSLAADAGLLQFLQEFSQQTISRTHEIKKQVDGLIRETKATDCRLHNVFNDFLMLSNTQFIENRVYDEEVEEPVLKAEAEKTEQEKTREQKEVDLIPKVQEAVNYGLQVLDSAFEQLDIKAGNSDSEEDDANGRVELILEPKDLYIDRPLPYLIGSKLFMEQEDVGLGELSSEEGSVGSDRGSIVDTEEEKEEEESDEDFAHHSDNEQNQHTTQMSDEEEDDDGCDLFADSEKEEEDIEDIEENTRPKRSRPTSFADELAARIKGDAMGRVDEEPTTLPSGEAKPRKTLKEKKERRTPSDDEEDNLFAPPKLTDEDFSPFGSGGGLFSGGKGLFDDEDEESDLFTEASQDRQAGASVKEESSSSKPGKKIPAGAVSVFLGDTDVFGAASVPSLKEPQKPEQPTPRKSPYGPPPTGLFDDDDGDDDDDFFSAPHSKPSKTRKVQSTADIFGDEEGDLFKEKAVASPEATVSQTDENKARAEKKVTLSYSKNLKPSSETKTQKGLFSDEEDSEDLFSSQSASNLKGASLLPGKLPTSVSLFDDEDEEDNLFGGTAAKKQTLSLQAQREEKAKASELSKKKASALLFSSDEEWNIPASQTHLASDSRSKGEPRDSGTLQSQEAKAVKKTSLFEEDKEDDLFAIAKDSQKKTQRVSLLFEDDVDSGGSLFGSPPTSVPPATKKKETVSEAPPLLFSDEEEKEAQLGVKSVDKKVESAKESLKFGRTDVAESEKEGLLTRSAQETVKHSDLFSSSSPWDKGTKPRTKTVLSLFDEEEDKMEDQNIIQAPQKEVGKGCDPDAHPKSTGVFQDEELLFSHKLQKDNDPDVDLFAGTKKTKLLEPSVGSLFGDDEDDDLFSSAKSQPLVQEKKRVVKKDHSVNSFKNQKHPESIQGSKEKGIWKPETPQANLAINPAALLPTAASQISEVKPVLPELAFPSSEHRRSHGLESVPVLPGSGEAGVSFDLPAQADTLHSANKSRVKMRGKRRPQTRAARRLAAQESSEAEDMSVPRGPIAQWADGAISPNGHRPQLRAASGEDSTEEALAAAAAPWEGGPVPGVDTSPFAKSLGHSRGEADLFDSGDIFSTGTGSQSVERTKPKAKIAENPANPPVGGKAKSPMFPALGEASSDDDLFQSAKPKPAKKTNPFPLLEDEDDLFTDQKVKKNETKSSSQQDVILTTQDIFEDDIFATEAIKPSQKTREKEKTLESNLFDDNIDIFADLTVKPKEKSKKKVEAKSIFDDDMDDIFSTGIQAKTTKPKSRSAQAAPEPRFEHKVSNIFDDPLNAFGGQ

110 NP_002222.1 NP_061984.2

>NP_002222.1

MGSACIKVTKYFLFLFNLIFFILGAVILGFGVWILADKSSFISVLQTSSSSLRMGAYVFIGVGAVTMLMGFLGCIGAVNEVRCLLGLYFAFLLLILIAQVTAGALFYFNMGKLKQEMGGIVTELIRDYNSSREDSLQDAWDYVQAQVKCCGWVSFYNWTDNAELMNRPEVTYPCSCEVKGEEDNSLSVRKGFCEAPGNRTQSGNHPEDWPVYQEGCMEKVQAWLQENLGIILGVGVGVAIIELLGMVLSICLCRHVHSEDYSKVPKY

>NP_061984.2

MAISGVPVLGFFIIAVLMSAQESWAIKEEHVIIQAEFYLNPDQSGEFMFDFDGDEIFHVDMAKKETVWRLEEFGRFASFEAQGALANIAVDKANLEIMTKRSNYTPITNVPPEVTVLTNSPVELREPNVLICFIDKFTPPVVNVTWLRNGKPVTTGVSETVFLPREDHLFRKFHYLPFLPSTEDVYDCRVEHWGLDEPLLKHWEFDAPSPLPETTENVVCALGLTVGLVGIIIGTIFIIKGLRKSNAAERRGPL

111 NP_997006.1 NP_005198.1

>NP_997006.1

MSGGEVVCSGWLRKSPPEKKLKRYAWKRRWFVLRSGRLTGDPDVLEYYKNDHAKKPIRIIDLNLCQQVDAGLTFNKKEFENSYIFDINTIDRIFYLVADSEEEMNKWVRCICDICGFNPTEEDPVKPPGSSLQAPADLPLAINTAPPSTQADSSSATLPPPYQLINVPPHLETLGIQEDPQDYLLLINCQSKKPEPTRTHADSAKSTSSETDCNDNVPSHKNPASSQSKHGMNGFFQQQMIYDSPPSRAPSASVDSSLYNLPRSYSHDVLPKVSPSSTEADGELYVFNTPSGTSSVETQMRHVSISYDIPPTPGNTYQIPRTFPEGTLGQTSKLDTIPDIPPPRPPKPHPAHDRSPVETCSIPRTASDTDSSYCIPTAGMSPSRSNTISTVDLNKLRKDASSQDCYDIPRAFPSDRSSSLEGFHNHFKVKNVLTVGSVSSEELDENYVPMNPNSPPRQHSSSFTEPIQEANYVPMTPGTFDFSSFGMQVPPPAHMGFRSSPKTPPRRPVPVADCEPPPVDRNLKPDRKGQSPKILRLKPHGLERTDSQTIGDFATRRKVKPAPLEIKPLPEWEELQAPVRSPITRSFARDSSRFPMSPRPDSVHSTTSSSDSHDSEENYVPMNPNLSSEDPNLFGSNSLDGGSSPMIKPKGDKQVEYLDLDLDSGKSTPPRKQKSSGSGSSVADERVDYVVVDQQKTLALKSTREAWTDGRQSTESETPAKSVK

>NP_005198.1

MSSARFDSSDRSAWYMGPVSRQEAQTRLQGQRHGMFLVRDSSTCPGDYVLSVSENSRVSHYIINSLPNRRFKIGDQEFDHLPALLEFYKIHYLDTTTLIEPAPRYPSPPMGSVSAPNLPTAEDNLEYVRTLYDFPGNDAEDLPFKKGEILVIIEKPEEQWWSARNKDGRVGMIPVPYVEKLVRSSPHGKHGNRNSNSYGIPEPAHAYAQPQTTTPLPAVSGSPGAAITPLPSTQNGPVFAKAIQKRVPCAYDKTALALEVGDIVKVTRMNINGQWEGEVNGRKGLFPFTHVKIFDPQNPDENE

112 NP_733779.1 NP_060908.1

>NP_733779.1

MPSIKLQSSDGEIFEVDVEIAKQSVTIKTMLEDLGMDDEGDDDPVPLPNVNAAILKKVIQWCTHHKDDPPPPEDDENKEKRTDDIPVWDQEFLKVDQGTLFELILAANYLDIKGLLDVTCKTVANMIKGKTPEEIRKTFNIKNDFTEEEEAQVRKENQWCEEK

>NP_060908.1

MDAPHSKAALDSINELPENILLELFTHVPARQLLLNCRLVCSLWRDLIDLMTLWKRKCLREGFITKDWDQPVADWKIFYFLRSLHRNLLRNPCAEEDMFAWQIDFNGGDRWKVESLPGAHGTDFPDPKVKKYFVTSYEMCLKSQLVDLVAEGYWEELLDTFRPDIVVKDWFAARADCGCTYQLKVQLASADYFVLASFEPPPVTIQQWNNATWTEVSYTFSDYPRGVRYILFQHGGRDTQYWAGWYGPRVTNSSIVVSPKMTRNQASSEAQPGQKHGQEEAAQSPYRAVVQIF

113 NP_115528.3 NP_002348.1

>NP_115528.3

MAHHLPAAMESHQDFRSIKAKFQASQPEPSDLPKKPPKPEFGKLKKFSQPELSEHPKKAPLPEFGAVSLKPPPPEVTDLPKKPPPPEVTDLPKKPPPPEVTDLPKKPPPPEVTDLPKKPSKLELSDLSKKFPQLGATPFPRKPLQPEVGEAPLKASLPEPGAPARKPLQPDELSHPARPPSEPKSGAFPRKLWQPEAGEATPRSPQPELSTFPKKPAQPEFNVYPKKPPQPQVGGLPKKSVPQPEFSEAAQTPLWKPQSSEPKRDSSAFPKKASQPPLSDFPKKPPQPELGDLTRTSSEPEVSVLPKRPRPAEFKALSKKPPQPELGGLPRTSSEPEFNSLPRKLLQPERRGPPRKFSQPEPSAVLKRHPQPEFFGDLPRKPPLPSSASESSLPAAVAGFSSRHPLSPGFGAAGTPRWRSGGLVHSGGARPGLRPSHPPRRRPLPPASSLGHPPAKPPLPPGPVDMQSFRRPSAASIDLRRTRSAAGLHFQDRQPEDIPVPDEIYELYDDVEPRDDSSPSPKGRDEAPSVQQAARRPPQDPALRKEKDPQPQQLPPMDPKLLKQLRKAEKAEREFRKKFKFEGEIVVHTKMMIDPNAKTRRGGGKHLGIRRGEILEVIEFTSNEEMLCRDPKGKYGYVPRTALLPLETEVYDDVDFCDPLENQPLPLGR

>NP_002348.1

MAAAVRMNIQMLLEAADYLERREREAEHGYASMLPYNNKDRDALKRRNKSKKNNSSSRSTHNEMEKNRRAHLRLCLEKLKGLVPLGPESSRHTTLSLLTKAKLHIKKLEDCDRKAVHQIDQLQREQRHLKRQLEKLGIERIRMDSIGSTVSSERSDSDREEIDVDVESTDYLTGDLDWSSSSVSDSDERGSMQSLGSDEGYSSTSIKRIKLQDSHKACLGL

114 NP_003185.1 NP_005504.2

>NP_003185.1

MDQNNSLPPYAQGLASPQGAMTPGIPIFSPMMPYGTGLTPQPIQNTNSLSILEEQQRQQQQQQQQQQQQQQQQQQQQQQQQQQQQQQQQQQQQQQAVAAAAVQQSTSQQATQGTSGQAPQLFHSQTLTTAPLPGTTPLYPSPMTPMTPITPATPASESSGIVPQLQNIVSTVNLGCKLDLKTIALRARNAEYNPKRFAAVIMRIREPRTTALIFSSGKMVCTGAKSEEQSRLAARKYARVVQKLGFPAKFLDFKIQNMVGSCDVKFPIRLEGLVLTHQQFSSYEPELFPGLIYRMIKPRIVLLIFVSGKVVLTGAKVRAEIYEAFENIYPILKGFRKTT

>NP_005504.2

MADPDVLTEVPAALKRLAKYVIRGFYGIEHALALDILIRNSCVKEEDMLELLKFDRKQLRSVLNNLKGDKFIKCRMRVETAADGKTTRHNYYFINYRTLVNVVKYKLDHMRRRIETDERDSTNRASFKCPVCSSTFTDLEANQLFDPMTGTFRCTFCHTEVEEDESAMPKKDARTLLARFNEQIEPIYALLRETEDVNLAYEILEPEPTEIPALKQSKDHAATTAGAASLAGGHHREAWATKGPSYEDLYTQNVVINMDDQEDLHRASLEGKSAKERPIWLRESTVQGAYGSEDMKEGGIDMDAFQEREEGHAGPDDNEEVMRALLIHEKKTSSAMAGSVGAAAPVTAANGSDSESETSESDDDSPPRPAAVAVHKREEDEEEDDEFEEVADDPIVMVAGRPFSYSEVSQRPELVAQMTPEEKEAYIAMGQRMFEDLFE

115 CAA64246.1 NP_000923.1

>CAA64246.1

MGGPARSPSASTNCLLCLRAPKPLLRAHNLGSNPKLAGTTDQLQPPQPRDHFRTPRPPGTSAQGTLQPETRVQSGREATALPASRSTTRAEPSASGSLPSLCLHRASPRPRTLSLQRAPAWAAGLSGTARDDPLSSPQKGRASVPGTPGPPPPPDSVGIQSPGVWDARAMTVERAVVAKPEVWYREGRAGAPAPPAARKPPYSYIRRHAMAIGSPRLTLGGIYKFITEGFPFYPDNPKKWQNSIRHNLTINDCFLKIPREAGRRRKGNYWALDPNAEDMFESGSFLRRRKASSVGLSTYPAYMQDAAAAAAAAAAAAIFPGAVPPRAPPNRAPSIQAKRAAVAGRPPHLLPAESPGHFRVFGLVPERPLKQELGPAPWGPGGSFAFSSDGAPATTNGYQPRQASPGPVRPTPSYAAAYAGPDGSTPREKAVRYFADAGRVGGTPCPQRAAAVAGGDHGGLLRRTSPGQFGALEPATTGGQLGGPVQAPTMLAMLPLIPVG

>NP_000923.1

MAGAQPGVHALQLEPPTVVETLRRGSKFIKWDEETSSRNLVTLRVDPNGFFLYWTGPNMEVDTLDISSIRDTRTGRYARLPKDPKIREVLGFGGPDARLEEKLMTVVSGPDPVNTVFLNFMAVQDDTAKVWSEELFKLAMNILAQNASRNTFLRKAYTKLKLQVNQDGRIPVKNILKMFSADKKRVETALESCGLKFNRSESIRPDEFSLEIFERFLNKLCLRPDIDKILLEIGAKGKPYLTLEQLMDFINQKQRDPRLNEVLYPPLRPSQARLLIEKYEPNQQFLERDQMSMEGFSRYLGGEENGILPLEALDLSTDMTQPLSAYFINSSHNTYLTAGQLAGTSSVEMYRQALLWGCRCVELDVWKGRPPEEEPFITHGFTMTTEVPLRDVLEAIAETAFKTSPYPVILSFENHVDSAKQQAKMAEYCRSIFGDALLIEPLDKYPLAPGVPLPSPQDLMGRILVKNKKRHRPSAGGPDSAGRKRPLEQSNSALSESSAATEPSSPQLGSPSSDSCPGLSNGEEVGLEKPSLEPQKSLGDEGLNRGPYVLGPADREDEEEDEEEEEQTDPKKPTTDEGTASSEVNATEEMSTLVNYIEPVKFKSFEAARKRNKCFEMSSFVETKAMEQLTKSPMEFVEYNKQQLSRIYPKGTRVDSSNYMPQLFWNVGCQLVALNFQTLDVAMQLNAGVFEYNGRSGYLLKPEFMRRPDKSFDPFTEVIVDGIVANALRVKVISGQFLSDRKVGIYVEVDMFGLPVDTRRKYRTRTSQGNSFNPVWDEEPFDFPKVVLPTLASLRIAAFEEGGKFVGHRILPVSAIRSGYHYVCLRNEANQPLCLPALLIYTEASDYIPDDHQDYAEALINPIKHVSLMDQRARQLAALIGESEAQAGQETCQDTQSQQLGSQPSSNPTPSPLDASPRRPPGPTTSPASTSLSSPGQRDDLIASILSEVAPTPLDELRGHKALVKLRSRQERDLRELRKKHQRKAVTLTRRLLDGLAQAQAEGRCRLRPGALGGAADVEDTKEGEDEAKRYQEFQNRQVQSLLELREAQVDAEAQRRLEHLRQALQRLREVVLDANTTQFKRLKEMNEREKKELQKILDRKRHNSISEAKMRDKHKKEAELTEINRRHITESVNSIRRLEEAQKQRHDRLVAGQQQVLQQLAEEEPKLLAQLAQECQEQRARLPQEIRRSLLGEMPEGLGDGPLVACASNGHAPGSSGHLSGADSESQEENTQL

116 NP_002653.1 NP_001092.1

>NP_002653.1

MSLKNEPRVNTSALQKIAADMSNIIENLDTRELHFEGEEVDYDVSPSDPKIQEVYIPFSAIYNTQGFKEPNIQTYLSGCPIKAQVLEVERFTSTTRVPSINLYTIELTHGEFKWQVKRKFKHFQEFHRELLKYKAFIRIPIPTRRHTFRRQNVREEPREMPSLPRSSENMIREEQFLGRRKQLEDYLTKILKMPMYRNYHATTEFLDISQLSFIHDLGPKGIEGMIMKRSGGHRIPGLNCCGQGRACYRWSKRWLIVKDSFLLYMKPDSGAIAFVLLVDKEFKIKVGKKETETKYGIRIDNLSRTLILKCNSYRHARWWGGAIEEFIQKHGTNFLKDHRFGSYAAIQENALAKWYVNAKGYFEDVANAMEEANEEIFITDWWLSPEIFLKRPVVEGNRWRLDCILKRKAQQGVRIFIMLYKEVELALGINSEYTKRTLMRLHPNIKVMRHPDHVSSTVYLWAHHEKLVIIDQSVAFVGGIDLAYGRWDDNEHRLTDVGSVKRVTSGPSLGSLPPAAMESMESLRLKDKNEPVQNLPIQKSIDDVDSKLKGIGKPRKFSKFSLYKQLHRHHLHDADSISSIDSTSSYFNHYRSHHNLIHGLKPHFKLFHPSSESEQGLTRPHADTGSIRSLQTGVGELHGETRFWHGKDYCNFVFKDWVQLDKPFADFIDRYSTPRMPWHDIASAVHGKAARDVARHFIQRWNFTKIMKSKYRSLSYPFLLPKSQTTAHELRYQVPGSVHANVQLLRSAADWSAGIKYHEESIHAAYVHVIENSRHYIYIENQFFISCADDKVVFNKIGDAIAQRILKAHRENQKYRVYVVIPLLPGFEGDISTGGGNALQAIMHFNYRTMCRGENSILGQLKAELGNQWINYISFCGLRTHAELEGNLVTELIYVHSKLLIADDNTVIIGSANINDRSMLGKRDSEMAVIVQDTETVPSVMDGKEYQAGRFARGLRLQCFRVVLGYLDDPSEDIQDPVSDKFFKEVWVSTAARNATIYDKVFRCLPNDEVHNLIQLRDFINKPVLAKEDPIRAEEELKKIRGFLVQFPFYFLSEESLLPSVGTKEAIVPMEVWT

>NP_001092.1

MDDDIAALVVDNGSGMCKAGFAGDDAPRAVFPSIVGRPRHQGVMVGMGQKDSYVGDEAQSKRGILTLKYPIEHGIVTNWDDMEKIWHHTFYNELRVAPEEHPVLLTEAPLNPKANREKMTQIMFETFNTPAMYVAIQAVLSLYASGRTTGIVMDSGDGVTHTVPIYEGYALPHAILRLDLAGRDLTDYLMKILTERGYSFTTTAEREIVRDIKEKLCYVALDFEQEMATAASSSSLEKSYELPDGQVITIGNERFRCPEALFQPSFLGMESCGIHETTFNSIMKCDVDIRKDLYANTVLSGGTTMYPGIADRMQKEITALAPSTMKIKIIAPPERKYSVWIGGSILASLSTFQQMWISKQEYDESGPSIVHRKCF

117 NP_056036.1 NP_995314.1

>NP_056036.1

MEAGSVVRAIFDFCPSVSEELPLFVGDIIEVLAVVDEFWLLGKKEDVTGQFPSSFVEIVTIPSLKEGERLFVCICEFTSQELDNLPLHRGDLVILDGIPTAGWLQGRSCWGARGFFPSSCVRELCLSSQSRQWHSQSALFQIPEYSMGQARALMGLSAQLDEELDFREGDVITIIGVPEPGWFEGELEGRRGIFPEGFVELLGPLRTVDESVSSGNQDDCIVNGEVDTPVGEEEIGPDEDEEEPGTYGVALYRFQALEPNELDFEVGDKIRILATLEDGWLEGSLKGRTGIFPYRFVKLCPDTRVEETMALPQEGSLARIPETSLDCLENTLGVEEQRHETSDHEAEEPDCIISEAPTSPLGHLTSEYDTDRNSYQDEDTAGGPPRSPGVEWEMPLATDSPTSDPTEVVNGISSQPQVPFHPNLQKSQYYSTVGGSHPHSEQYPDLLPLEARTRDYASLPPKRMYSQLKTLQKPVLPLYRGSSVSASRVVKPRQSSPQLHNLASYTKKHHTSSVYSISERLEMKPGPQAQGLVMEAATHSQGDGSTDLDSKLTQQLIEFEKSLAGPGTEPDKILRHFSIMDFNSEKDIVRGSSKLITEQELPERRKALRPPPPRPCTPVSTSPHLLVDQNLKPAPPLVVRPSRPAPLPPSAQQRTNAVSPKLLSRHRPTCETLEKEGPGHMGRSLDQTSPCPLVLVRIEEMERDLDMYSRAQEELNLMLEEKQDESSRAETLEDLKFCESNIESLNMELQQLREMTLLSSQSSSLVAPSGSVSAENPEQRMLEKRAKVIEELLQTERDYIRDLEMCIERIMVPMQQAQVPNIDFEGLFGNMQMVIKVSKQLLAALEISDAVGPVFLGHRDELEGTYKIYCQNHDEAIALLEIYEKDEKIQKHLQDSLADLKSLYNEWGCTNYINLGSFLIKPVQRVMRYPLLLMELLNSTPESHPDKVPLTNAVLAVKEINVNINEYKRRKDLVLKYRKGDEDSLMEKISKLNIHSIIKKSNRVSSHLKHLTGFAPQIKDEVFEETEKNFRMQERLIKSFIRDLSLYLQHIRESACVKVVAAVSMWDVCMERGHRDLEQFERVHRYISDQLFTNFKERTERLVISPLNQLLSMFTGPHKLVQKRFDKLLDFYNCTERAEKLKDKKTLEELQSARNNYEALNAQLLDELPKFHQYAQGLFTNCVHGYAEAHCDFVHQALEQLKPLLSLLKVAGREGNLIAIFHEEHSRVLQQLQVFTFFPESLPATKKPFERKTIDRQSARKPLLGLPSYMLQSEELRASLLARYPPEKLFQAERNFNAAQDLDVSLLEGDLVGVIKKKDPMGSQNRWLIDNGVTKGFVYSSFLKPYNPRRSHSDASVGSHSSTESEHGSSSPRFPRQNSGSTLTFNPSSMAVSFTSGSCQKQPQDASPPPKECDQGTLSASLNPSNSESSPSRCPSDPDSTSQPRSGDSADVARDVKQPTATPRSYRNFRHPEIVGYSVPGRNGQSQDLVKGCARTAQAPEDRSTEPDGSEAEGNQVYFAVYTFKARNPNELSVSANQKLKILEFKDVTGNTEWWLAEVNGKKGYVPSNYIRKTEYT

>NP_995314.1

MSRSVLQPSQQKLAEKLTILNDRGVGMLTRLYNIKKQGQVWKACGDPKAKPSYLIDKNLESAVKFIVRKFPAVETRNNNQQLAQLQKEKSEILKNLALYYFTFVDVMEFKDHVCELLNTIDVCQVFFDITVNFDLTKNYLDLIITYTTLMILLSRIEERKAIIGLYNYAHEMTHGASDREYPRLGQMIVDYENPLKKMMEEFVPHSKSLSDALISLQMVYPRRNLSADQWRNAQLLSLISAPSTMLNPAQSDTMPCEYLSLDAMEKWIIFGFILCHGILNTDATALNLWKLALQSSSCLSLFRDEVFHIHKAAEDLFVNIRGYNKRINDIRECKEAAVSHAGSMHRERRKFLRSALKELATVLSDQPGLLGPKALFVFMALSFARDEIIWLLRHADNMPKKSADDFIDKHIAELIFYMEELRAHVRKYGPVMQRYYVQYLSGFDAVVLNELVQNLSVCPEDESIIMSSFVNTMTSLSVKQVEDGEVFDFRGMRLDWFRLQAYTSVSKASLGLADHRELGKMMNTIIFHTKMVDSLVEMLVETSDLSIFCFYSRAFEKMFQQCLELPSQSRYSIAFPLLCTHFMSCTHELCPEERHHIGDRSLSLCNMFLDEMAKQARNLITDICTEQCTLSDQLLPKHCAKTISQAVNKKSKKQTGKKGEPEREKPGVESMRKNRLVVTNLDKLHTALSELCFSINYVPNMVVWEHTFTPREYLTSHLEIRFTKSIVGMTMYNQATQEIAKPSELLTSVRAYMTVLQSIENYVQIDITRVFNNVLLQQTQHLDSHGEPTITSLYTNWYLETLLRQVSNGHIAYFPAMKAFVNLPTENELTFNAEEYSDISEMRSLSELLGPYGMKFLSESLMWHISSQVAELKKLVVENVDVLTQMRTSFDKPDQMAALFKRLSSVDSVLKRMTIIGVILSFRSLAQEALRDVLSYHIPFLVSSIEDFKDHIPRETDMKVAMNVYELSSAAGLPCEIDPALVVALSSQKSENISPEEEYKIACLLMVFVAVSLPTLASNVMSQYSPAIEGHCNNIHCLAKAINQIAAALFTIHKGSIEDRLKEFLALASSSLLKIGQETDKTTTRNRESVYLLLDMIVQESPFLTMDLLESCFPYVLLRNAYHAVYKQSVTSSA

118 NP_064543.3 NP_653294.1

>NP_064543.3

MEEETHTDAKIRAENGTGSSPRGPGCSLRHFACEQNLLSRPDGSASFLQGDTSVLAGVYGPAEVKVSKEIFNKATLEVILRPKIGLPGVAEKSRERLIRNTCEAVVLGTLHPRTSITVVLQVVSDAGSLLACCLNAACMALVDAGVPMRALFCGVACALDSDGTLVLDPTSKQEKEARAVLTFALDSVERKLLMSSTKGLYSDTELQQCLAAAQAASQHVFRFYRESLQRRYSKS

>NP_653294.1

MAAVILPSTAAPSSLFPASQQKGHTQGGELVNELLTSWLRGLVTFEDVAVEFTQEEWALLDPAQRTLYRDVMLENCRNLASLGCRVNKPSLISQLEQDKKVVTEERGILPSTCPDLETLLKAKWLTPKKNVFRKEQSKGVKTERSHRGVKLNECNQCFKVFSTKSNLTQHKRIHTGEKPYDCSQCGKSFSSRSYLTIHKRIHNGEKPYECNHCGKAFSDPSSLRLHLRIHTGEKPYECNQCFHVFRTSCNLKSHKRIHTGENHHECNQCGKAFSTRSSLTGHNSIHTGEKPYECHDCGKTFRKSSYLTQHVRTHTGEKPYECNECGKSFSSSFSLTVHKRIHTGEKPYECSDCGKAFNNLSAVKKHLRTHTGEKPYECNHCGKSFTSNSYLSVHKRIHNRWI

119 NP_002650.1 NP_059973.2

>NP_002650.1

MGHPPLLPLLLLLHTCVPASWGLRCMQCKTNGDCRVEECALGQDLCRTTIVRLWEEGEELELVEKSCTHSEKTNRTLSYRTGLKITSLTEVVCGLDLCNQGNSGRAVTYSRSRYLECISCGSSDMSCERGRHQSLQCRSPEEQCLDVVTHWIQEGEEGRPKDDRHLRGCGYLPGCPGSNGFHNNDTFHFLKCCNTTKCNEGPILELENLPQNGRQCYSCKGNSTHGCSSEETFLIDCRGPMNQCLVATGTHEPKNQSYMVRGCATASMCQHAHLGDAFSMNHIDVSCCTKSGCNHPDLDVQYRSGAAPQPGPAHLSLTITLLMTARLWGGTLLWT

>NP_059973.2

MSELPGDVRAFLREHPSLRLQTDARKVRCILTGHELPCRLPELQVYTRGKKYQRLVRASPAFDYAEFEPHIVPSTKNPHQLFCKLTLRHINKCPEHVLRHTQGRRYQRALCKYEECQKQGVEYVPACLVHRRRRREDQMDGDGPRPREAFWEPTSSDEGGAASDDSMTDLYPPELFTRKDLGSTEDGDGTDDFLTDKEDEKAKPPREKATDEGRRETTVYRGLVQKRGKKQLGSLKKKFKSHHRKPKSFSSCKQPG

120 NP_005824.2 NP_004242.1

>NP_005824.2

MKQLPVLEPGDKPRKATWYTLTVPGDSPCARVGHSCSYLPPVGNAKRGKVFIVGGANPNRSFSDVHTMDLGKHQWDLDTCKGLLPRYEHASFIPSCTPDRIWVFGGANQSGNRNCLQVLNPETRTWTTPEVTSPPPSPRTFHTSSAAIGNQLYVFGGGERGAQPVQDTKLHVFDANTLTWSQPETLGNPPSPRHGHVMVAAGTKLFIHGGLAGDRFYDDLHCIDISDMKWQKLNPTGAAPAGCAAHSAVAMGKHVYIFGGMTPAGALDTMYQYHTEEQHWTLLKFDTLLPPGRLDHSMCIIPWPVTCASEKEDSNSLTLNHEAEKEDSADKVMSHSGDSHEESQTATLLCLVFGGMNTEGEIYDDCIVTVVD

>NP_004242.1

MAGKSSLFKVILLGDGGVGKSSLMNRYVTNKFDTQLFHTIGVEFLNKDLEVDGHFVTMQIWDTAGQERFRSLRTPFYRGSDCCLLTFSVDDSQSFQNLSNWKKEFIYYADVKEPESFPFVILGNKIDISERQVSTEEAQAWCRDNGDYPYFETSAKDATNVAAAFEEAVRRVLATEDRSDHLIQTDTVNLHRKPKPSSSCC

121 NP_002718.2 NP_003012.1

>NP_002718.2

MMQKLLKCSRLVLALALILVLESSVQGYPTRRARYQWVRCNPDSNSANCLEEKGPMFELLPGESNKIPRLRTDLFPKTRIQDLNRIFPLSEDYSGSGFGSGSGSGSGSGSGFLTEMEQDYQLVDESDAFHDNLRSLDRNLPSDSQDLGQHGLEEDFML

>NP_003012.1

MDNKKRLAYAIIQFLHDQLRHGGLSSDAQESLEVAIQCLETAFGVTVEDSDLALPQTLPEIFEAAATGKEMPQDLRSPARTPPSEEDSAEAERLKTEGNEQMKVENFEAAVHFYGKAIELNPANAVYFCNRAAAYSKLGNYAGAVQDCERAICIDPAYSKAYGRMGLALSSLNKHVEAVAYYKKALELDPDNETYKSNLKIAELKLREAPSPTGGVGSFDIAGLLNNPGFMSMASNLMNNPQIQQLMSGMISGGNNPLGTPGTSPSQNDLASLIQAGQQFAQQMQQQNPELIEQLRSQIRSRTPSASNDDQQE

122 NP_060277.1 NP_055174.1

>NP_060277.1

MPAVLGFEGSANKIGVGVVRDGKVLANPRRTYVTPPGTGFLPGDTARHHRAVILDLLQEALTESGLTSQDIDCIAYTKGPGMGAPLVSVAVVARTVAQLWNKPLVGVNHCIGHIEMGRLITGATSPTVLYVSGGNTQVIAYSEHRYRIFGETIDIAVGNCLDRFARVLKISNDPSPGYNIEQMAKRGKKLVELPYTVKGMDVSFSGILSFIEDVAHRMLATGECTPEDLCFSLQETVFAMLVEITERAMAHCGSQEALIVGGVGCNVRLQEMMATMCQERGARLFATDERFCIDNGAMIAQAGWEMFRAGHRTPLSDSGVTQRYRTDEVEVTWRD

>NP_055174.1

MRLLAFLSLLALVLQETGTASLPRKERKRREEQMPREGDSFEVLPLRNDVLNPDNYGEVIDLSNYEELTDYGDQLPEVKVTSLAPATSISPAKSTTAPGTPSSNPTMTRPTTAGLLLSSQPNHGLPTCLVCVCLGSSVYCDDIDLEDIPPLPRRTAYLYARFNRISRIRAEDFKGLTKLKRIDLSNNLISSIDNDAFRLLHALQDLILPENQLEALPVLPSGIEFLDVRLNRLQSSGIQPAAFRAMEKLQFLYLSDNLLDSIPGPLPLSLRSVHLQNNLIETMQRDVFCDPEEHKHTRRQLEDIRLDGNPINLSLFPSAYFCLPRLPIGRFT

123 NP_006766.1 NP_001008712.1

>NP_006766.1

MVGEMETKEKPKPTPDYLMQLMNDKKLMSSLPNFCGIFNHLERLLDEEISRVRKDMYNDTLNGSTEKRSAELPDAVGPIVQLQEKLYVPVKEYPDFNFVGRILGPRGLTAKQLEAETGCKIMVRGKGSMRDKKKEEQNRGKPNWEHLNEDLHVLITVEDAQNRAEIKLKRAVEEVKKLLVPAAEGEDSLKKMQLMELAILNGTYRDANIKSPALAFSLAATAQAAPRIITGPAPVLPPAALRTPTPAGPTIMPLIRQIQTAVMPNGTPHPTAAIVPPGPEAGLIYTPYEYPYTLAPATSILEYPIEPSGVLGAVATKVRRHDMRVHPYQRIVTADRAATGN

>NP_001008712.1

MNNGGKAEKENTPSEANLQEEEVRTLFVSGLPLDIKPRELYLLFRPFKGYEGSLIKLTSKQPVGFVSFDSRSEAEAAKNALNGIRFDPEIPQTLRLEFAKANTKMAKNKLVGTPNPSTPLPNTVPQFIAREPYELTVPALYPSSPEVWAPYPLYPAELAPALPPPAFTYPASLHAQCFSPEAKPNTPVFCPLLQQIRFVSGNVFVTYQPTADQQRELPC

124 NP_001026855.2 NP_060441.2

>NP_001026855.2

MRFGSKMMPMFLTVYLSNNEQHFTEVPVTPETICRDVVDLCKEPGESDCHLAEVWCGSERPVADNERMFDVLQRFGSQRNEVRFFLRHERPPGRDIVSGPRSQDPSLKRNGVKVPGEYRRKENGVNSPRMDLTLAELQEMASRQQQQIEAQQQLLATKEQRLKFLKQQDQRQQQQVAEQEKLKRLKEIAENQEAKLKKVRALKGHVEQKRLSNGKLVEEIEQMNNLFQQKQRELVLAVSKVEELTRQLEMLKNGRIDSHHDNQSAVAELDRLYKELQLRNKLNQEQNAKLQQQRECLNKRNSEVAVMDKRVNELRDRLWKKKAALQQKENLPVSSDGNLPQQAASAPSRVAAVGPYIQSSTMPRMPSRPELLVKPALPDGSLVIQASEGPMKIQTLPNMRSGAASQTKGSKIHPVGPDWSPSNADLFPSQGSASVPQSTGNALDQVDDGEVPLREKEKKVRPFSMFDAVDQSNAPPSFGTLRKNQSSEDILRDAQVANKNVAKVPPPVPTKPKQINLPYFGQTNQPPSDIKPDGSSQQLSTVVPSMGTKPKPAGQQPRVLLSPSIPSVGQDQTLSPGSKQESPPAAAVRPFTPQPSKDTLLPPFRKPQTVAASSIYSMYTQQQAPGKNFQQAVQSALTKTHTRGPHFSSVYGKPVIAAAQNQQQHPENIYSNSQGKPGSPEPETEPVSSVQENHENERIPRPLSPTKLLPFLSNPYRNQSDADLEALRKKLSNAPRPLKKRSSITEPEGPNGPNIQKLLYQRTTIAAMETISVPSYPSKSASVTASSESPVEIQNPYLHVEPEKEVVSLVPESLSPEDVGNASTENSDMPAPSPGLDYEPEGVPDNSPNLQNNPEEPNPEAPHVLDVYLEEYPPYPPPPYPSGEPEGPGEDSVSMRPPEITGQVSLPPGKRTNLRKTGSERIAHGMRVKFNPLALLLDSSLEGEFDLVQRIIYEVDDPSLPNDEGITALHNAVCAGHTEIVKFLVQFGVNVNAADSDGWTPLHCAASCNNVQVCKFLVESGAAVFAMTYSDMQTAADKCEEMEEGYTQCSQFLYGVQEKMGIMNKGVIYALWDYEPQNDDELPMKEGDCMTIIHREDEDEIEWWWARLNDKEGYVPRNLLGLYPRIKPRQRSLA

>NP_060441.2

MVFLTAQLWLRNRVTDRYFRIQEVLKHARHFRGRKNRCYRLAVRTVIRAFVKCTKARYLKKKNMRTLWINRITAASQEHGLKYPALIGNLVKCQVELNRKVLADLAIYEPKTFKSLAALASRRRHEGFAAALGDGKEPEGIFSRVVQYH

125 Q92900 NP_115887.1

>Q92900

MSVEAYGPSSQTLTFLDTEEAELLGADTQGSEFEFTDFTLPSQTQTPPGGPGGPGGGGAGGPGGAGAGAAAGQLDAQVGPEGILQNGAVDDSVAKTSQLLAELNFEEDEEDTYYTKDLPIHACSYCGIHDPACVVYCNTSKKWFCNGRGNTSGSHIVNHLVRAKCKEVTLHKDGPLGETVLECYNCGCRNVFLLGFIPAKADSVVVLLCRQPCASQSSLKDINWDSSQWQPLIQDRCFLSWLVKIPSEQEQLRARQITAQQINKLEELWKENPSATLEDLEKPGVDEEPQHVLLRYEDAYQYQNIFGPLVKLEADYDKKLKESQTQDNITVRWDLGLNKKRIAYFTLPKTDSGNEDLVIIWLRDMRLMQGDEICLRYKGDLAPLWKGIGHVIKVPDNYGDEIAIELRSSVGAPVEVTHNFQVDFVWKSTSFDRMQSALKTFAVDETSVSGYIYHKLLGHEVEDVIIKCQLPKRFTAQGLPDLNHSQVYAVKTVLQRPLSLIQGPPGTGKTVTSATIVYHLARQGNGPVLVCAPSNIAVDQLTEKIHQTGLKVVRLCAKSREAIDSPVSFLALHNQIRNMDSMPELQKLQQLKDETGELSSADEKRYRALKRTAERELLMNADVICCTCVGAGDPRLAKMQFRSILIDESTQATEPECMVPVVLGAKQLILVGDHCQLGPVVMCKKAAKAGLSQSLFERLVVLGIRPIRLQVQYRMHPALSAFPSNIFYEGSLQNGVTAADRVKKGFDFQWPQPDKPMFFYVTQGQEEIASSGTSYLNRTEAANVEKITTKLLKAGAKPDQIGIITPYEGQRSYLVQYMQFSGSLHTKLYQEVEIASVDAFQGREKDFIILSCVRANEHQGIGFLNDPRRLNVALTRARYGVIIVGNPKALSKQPLWNHLLNYYKEQKVLVEGPLNNLRESLMQFSKPRKLVNTINPGARFMTTAMYDAREAIIPGSVYDRSSQGRPSSMYFQTHDQIGMISAGPSHVAAMNIPIPFNLVMPPMPPPGYFGQANGPAAGRGTPKGKTGRGGRQKNRFGLPGPSQTNLPNSQASQDVASQPFSQGALTQGYISMSQPSQMSQPGLSQPELSQDSYLGDEFKSQIDVALSQDSTYQGERAYQHGGVTGLSQY

>NP_115887.1

MEPPDQCSQYMTSLLSPAVDDEKELQDMNAMVLSLTEEVKEEEEDAQPEPEQGTAAGEKLKSAGAQGGEEKDGGGEEKDGGGAGVPGHLWEGDLEGTSGSDGNVEDSDQSEKEPGQQYSRPQGAVGGLEPGNAQQPNVHAFTPLQLQELERIFQREQFPSEFLRRRLARSMNVTELAVQIWFENRRAKWRRHQRALMARNMLPFMAVGQPVMVTAAEAITAPLFISGMRDDYFWDHSHSSSLCFPMPPFPPPSLPLPLMLLPPMPPAGQAEFGPFPFVIVPSFTFPNV

126 NP_002505.1 AAA52539.1

>NP_002505.1

MQSVQSTSFCLRKQCLCLTFLLLHLLGQVAATQRCPPQCPGRCPATPPTCAPGVRAVLDGCSCCLVCARQRGESCSDLEPCDESSGLYCDRSADPSNQTGICTAVEGDNCVFDGVIYRSGEKFQPSCKFQCTCRDGQIGCVPRCQLDVLLPEPNCPAPRKVEVPGECCEKWICGPDEEDSLGGLTLAAYRPEATLGVEVSDSSVNCIEQTTEWTACSKSCGMGFSTRVTNRNRQCEMLKQTRLCMVRPCEQEPEQPTDKKGKKCLRTKKSLKAIHLQFKNCTSLHTYKPRFCGVCSDGRCCTPHNTKTIQAEFQCSPGQIVKKPVMVIGTCTCHTNCPKNNEAFLQELELKTTRGKM

>AAA52539.1

MGKISSLPTQLFKCCFCDFLKVKMHTMSSSHLFYLALCLLTFTSSATAGPETLCGAELVDALQFVCGDRGFYFNKPTGYGSSSRRAPQTGIVDECCFRSCDLRRLEMYCAPLKPAKSARSVRAQRHTDMPKTQKYQPPSTNKNTKSQRRKGWPKTHPGGEQKEGTEASLQIRGKKKEQRREIGSRNAECRGKKGK

127 NP_000236.2 NP_644805.1

>NP_000236.2

MKAPAVLAPGILVLLFTLVQRSNGECKEALAKSEMNVNMKYQLPNFTAETPIQNVILHEHHIFLGATNYIYVLNEEDLQKVAEYKTGPVLEHPDCFPCQDCSSKANLSGGVWKDNINMALVVDTYYDDQLISCGSVNRGTCQRHVFPHNHTADIQSEVHCIFSPQIEEPSQCPDCVVSALGAKVLSSVKDRFINFFVGNTINSSYFPDHPLHSISVRRLKETKDGFMFLTDQSYIDVLPEFRDSYPIKYVHAFESNNFIYFLTVQRETLDAQTFHTRIIRFCSINSGLHSYMEMPLECILTEKRKKRSTKKEVFNILQAAYVSKPGAQLARQIGASLNDDILFGVFAQSKPDSAEPMDRSAMCAFPIKYVNDFFNKIVNKNNVRCLQHFYGPNHEHCFNRTLLRNSSGCEARRDEYRTEFTTALQRVDLFMGQFSEVLLTSISTFIKGDLTIANLGTSEGRFMQVVVSRSGPSTPHVNFLLDSHPVSPEVIVEHTLNQNGYTLVITGKKITKIPLNGLGCRHFQSCSQCLSAPPFVQCGWCHDKCVRSEECLSGTWTQQICLPAIYKVFPNSAPLEGGTRLTICGWDFGFRRNNKFDLKKTRVLLGNESCTLTLSESTMNTLKCTVGPAMNKHFNMSIIISNGHGTTQYSTFSYVDPVITSISPKYGPMAGGTLLTLTGNYLNSGNSRHISIGGKTCTLKSVSNSILECYTPAQTISTEFAVKLKIDLANRETSIFSYREDPIVYEIHPTKSFISGGSTITGVGKNLNSVSVPRMVINVHEAGRNFTVACQHRSNSEIICCTTPSLQQLNLQLPLKTKAFFMLDGILSKYFDLIYVHNPVFKPFEKPVMISMGNENVLEIKGNDIDPEAVKGEVLKVGNKSCENIHLHSEAVLCTVPNDLLKLNSELNIEWKQAISSTVLGKVIVQPDQNFTGLIAGVVSISTALLLLLGFFLWLKKRKQIKDLGSELVRYDARVHTPHLDRLVSARSVSPTTEMVSNESVDYRATFPEDQFPNSSQNGSCRQVQYPLTDMSPILTSGDSDISSPLLQNTVHIDLSALNPELVQAVQHVVIGPSSLIVHFNEVIGRGHFGCVYHGTLLDNDGKKIHCAVKSLNRITDIGEVSQFLTEGIIMKDFSHPNVLSLLGICLRSEGSPLVVLPYMKHGDLRNFIRNETHNPTVKDLIGFGLQVAKGMKYLASKKFVHRDLAARNCMLDEKFTVKVADFGLARDMYDKEYYSVHNKTGAKLPVKWMALESLQTQKFTTKSDVWSFGVLLWELMTRGAPPYPDVNTFDITVYLLQGRRLLQPEYCPDPLYEVMLKCWHPKAEMRPSFSELVSRISAIFSTFIGEHYVHVNATYVNVKCVAPYPSLLSSEDNADDEVDTRPASFWETS

>NP_644805.1

MAQWNQLQQLDTRYLEQLHQLYSDSFPMELRQFLAPWIESQDWAYAASKESHATLVFHNLLGEIDQQYSRFLQESNVLYQHNLRRIKQFLQSRYLEKPMEIARIVARCLWEESRLLQTAATAAQQGGQANHPTAAVVTEKQQMLEQHLQDVRKRVQDLEQKMKVVENLQDDFDFNYKTLKSQGDMQDLNGNNQSVTRQKMQQLEQMLTALDQMRRSIVSELAGLLSAMEYVQKTLTDEELADWKRRQQIACIGGPPNICLDRLENWITSLAESQLQTRQQIKKLEELQQKVSYKGDPIVQHRPMLEERIVELFRNLMKSAFVVERQPCMPMHPDRPLVIKTGVQFTTKVRLLVKFPELNYQLKIKVCIDKDSGDVAALRGSRKFNILGTNTKVMNMEESNNGSLSAEFKHLTLREQRCGNGGRANCDASLIVTEELHLITFETEVYHQGLKIDLETHSLPVVVISNICQMPNAWASILWYNMLTNNPKNVNFFTKPPIGTWDQVAEVLSWQFSSTTKRGLSIEQLTTLAEKLLGPGVNYSGCQITWAKFCKENMAGKGFSFWVWLDNIIDLVKKYILALWNEGYIMGFISKERERAILSTKPPGTFLLRFSESSKEGGVTFTWVEKDISGKTQIQSVEPYTKQQLNNMSFAEIIMGYKIMDATNILVSPLVYLYPDIPKEEAFGKYCRPESQEHPEADPGSAAPYLKTKFICVTPTTCSNTIDLPMSPRTLDSLMQFGNNGEGAEPSAGGQFESLTFDMELTSECATSPM

128 NP_061874.3 NP_112420.1

>NP_061874.3

MGVPKFYRWISERYPCLSEVVKEHQIPEFDNLYLDMNGIIHQCSHPNDDDVHFRISDDKIFTDIFHYLEVLFRIIKPRKVFFMAVDGVAPRAKMNQQRGRRFRSAKEAEDKIKKAIEKGETLPTEARFDSNCITPGTEFMARLHEHLKYFVNMKISTDKSWQGVTIYFSGHETPGEGEHKIMEFIRSEKAKPDHDPNTRHCLYGLDADLIMLGLTSHEAHFSLLREEVRFGGKKTQRVCAPEETTFHLLHLSLMREYIDYEFSVLKEKITFKYDIERIIDDWILMGFLVGNDFIPHLPHLHINHDALPLLYGTYVTILPELGGYINESGHLNLPRFEKYLVKLSDFDREHFSEVFVDLKWFESKVGNKYLNEAAGVAAEEARNYKEKKKLKGQENSLCWTALDKNEGEMITSKDNLEDETEDDDLFETEFRQYKRTYYMTKMGVDVVSDDFLADQAACYVQAIQWILHYYYHGVQSWSWYYPYHYAPFLSDIHNISTLKIHFELGKPFKPFEQLLAVLPAASKNLLPACYQHLMTNEDSPIIEYYPPDFKTDLNGKQQEWEAVVLIPFIDEKRLLEAMETCNHSLKKEERKRNQHSECLMCWYDRDTEFIYPSPWPEKFPAIERCCTRYKIISLDAWRVDINKNKITRIDQKALYFCGFPTLKHIRHKFFLKKSGVQVFQQSSRGENMMLEILVDAESDELTVENVASSVLGKSVFVNWPHLEEARVVAVSDGETKFYLEEPPGTQKLYSGRTAPPSKVVHLGDKEQSNWAKEVQGISEHYLRRKGIIINETSAVVYAQLLTGRKYQINQNGEVRLEKQWSKQVVPFVYQTIVKDIRAFDSRFSNIKTLDDLFPLRSMVFMLGTPYYGCTGEVQDSGDVITEGRIRVIFSIPCEPNLDALIQNQHKYSIKYNPGYVLASRLGVSGYLVSRFTGSIFIGRGSRRNPHGDHKANVGLNLKFNKKNEEVPGYTKKVGSEWMYSSAAEQLLAEYLERAPELFSYIAKNSQEDVFYEDDIWPGENENGAEKVQEIITWLKGHPVSTLSRSSCDLQILDAAIVEKIEEEVEKCKQRKNNKKVRVTVKPHLLYRPLEQQHGVIPDRDAEFCLFDRVVNVRENFSVPVGLRGTIIGIKGANREADVLFEVLFDEEFPGGLTIRCSPGRGYRLPTSALVNLSHGSRSETGNQKLTAIVKPQPAVHQHSSSSSVSSGHLGALNHSPQSLFVPTQVPTKDDDEFCNIWQSLQGSGKMQYFQPTIQEKGAVLPQEISQVNQHHKSGFNDNSVKYQQRKHDPHRKFKEECKSPKAECWSQKMSNKQPNSGIENFLASLNISKENEVQSSHHGEPPSEEHLSPQSFAMGTRMLKEILKIDGSNTVDHKNEIKQIANEIPVSSNRRDEYGLPSQPKQNKKLASYMNKPHSANEYHNVQSMDNMCWPAPSQIPPVSTPVTELSRICSLVGMPQPDFSFLRMPQTMTVCQVKLSNGLLVHGPQCHSENEAKEKAALFALQQLGSLGMNFPLPSQVFANYPSAVPPGTIPPAFPPPTGWDHYGSNYALGAANIMPSSSHLFGSMPWGPSVPVPGKPFHHTLYSGTMPMAGGIPGGVHNQFIPLQVTKKRVANKKNFENKEAQSSQATPVQTSQPDSSNIVKVSPRESSSASLKSSPIAQPASSFQVETASQGHSISHHKSTPISSSRRKSRKLAVNFGVSKPSE

>NP_112420.1

MSKSESPKEPEQLRKLFIGGLSFETTDESLRSHFEQWGTLTDCVVMRDPNTKRSRGFGFVTYATVEEVDAAMNARPHKVDGRVVEPKRAVSREDSQRPGAHLTVKKIFVGGIKEDTEEHHLRDYFEQYGKIEVIEIMTDRGSGKKRGFAFVTFDDHDSVDKIVIQKYHTVNGHNCEVRKALSKQEMASASSSQRGRSGSGNFGGGRGGGFGGNDNFGRGGNFSGRGGFGGSRGGGGYGGSGDGYNGFGNDGGYGGGGPGYSGGSRGYGSGGQGYGNQGSGYGGSGSYDSYNNGGGGGFGGGSGSNFGGGGSYNDFGNYNNQSSNFGPMKGGNFGGRSSGPYGGGGQYFAKPRNQGGYGGSSSSSSYGSGRRF

129 NP_000108.1 NP_149076.1

>NP_000108.1

MDNYADLSDTELTTLLRRYNIPHGPVVGSTRRLYEKKIFEYETQRRRLSPPSSSAASSYSFSDLNSTRGDADMYDLPKKEDALLYQSKGYNDDYYEESYFTTRTYGEPESAGPSRAVRQSVTSFPDADAFHHQVHDDDLLSSSEEECKDRERPMYGRDSAYQSITHYRPVSASRSSLDLSYYPTSSSTSFMSSSSSSSSWLTRRAIRPENRAPGAGLGQDRQVPLWGQLLLFLVFVIVLFFIYHFMQAEEGNPF

>NP_149076.1

MAGGPPNTKAEMEMSLAEELNHGRQGENQEHLVIAEMMELGSRSRGASQKKQKLEQKAAGSASAKRVWNMTATRPKKMGSQLPKPRMLRESGHGDAHLQEYAGNFQGIRFHYDRNPGTDAVAQTSLEEFNVLEMEVMRRQLYAVNRRLRALEEQGATWRHRETLIIAVLVSASIANLWLWMNQ

130 NP_003874.2 NP_002219.1

>NP_003874.2

MAKTVAYFYDPDVGNFHYGAGHPMKPHRLALTHSLVLHYGLYKKMIVFKPYQASQHDMCRFHSEDYIDFLQRVSPTNMQGFTKSLNAFNVGDDCPVFPGLFEFCSRYTGASLQGATQLNNKICDIAINWAGGLHHAKKFEASGFCYVNDIVIGILELLKYHPRVLYIDIDIHHGDGVQEAFYLTDRVMTVSFHKYGNYFFPGTGDMYEVGAESGRYYCLNVPLRDGIDDQSYKHLFQPVINQVVDFYQPTCIVLQCGADSLGCDRLGCFNLSIRGHGECVEYVKSFNIPLLVLGGGGYTVRNVARCWTYETSLLVEEAISEELPYSEYFEYFAPDFTLHPDVSTRIENQNSRQYLDQIRQTIFENLKMLNHAPSVQIHDVPADLLTYDRTDEADAEERGPEENYSRPEAPNEFYDGDHDNDKESDVEI

>NP_002219.1

MTAKMETTFYDDALNASFLPSESGPYGYSNPKILKQSMTLNLADPVGSLKPHLRAKNSDLLTSPDVGLLKLASPELERLIIQSSNGHITTTPTPTQFLCPKNVTDEQEGFAEGFVRALAELHSQNTLPSVTSAAQPVNGAGMVAPAVASVAGGSGSGGFSASLHSEPPVYANLSNFNPGALSSGGGAPSYGAAGLAFPAQPQQQQQPPHHLPQQMPVQHPRLQALKEEPQTVPEMPGETPPLSPIDMESQERIKAERKRMRNRIAASKCRKRKLERIARLEEKVKTLKAQNSELASTANMLREQVAQLKQKVMNHVNSGCQLMLTQQLQTF

131 NP_006414.1 NP_037438.2

>NP_006414.1

MAAQKDQQKDAEAEGLSGTTLLPKLIPSGAGREWLERRRATIRPWSTFVDQQRFSRPRNLGELCQRLVRNVEYYQSNYVFVFLGLILYCVVTSPMLLVALAVFFGACYILYLRTLESKLVLFGREVSPEHQYALAGGISFPFFWLAGAGSAVFWVLGATLVVIGSHAAFHQIEAVDGEELQMEPV

>NP_037438.2

MSRQAKDDFLRHYTVSDPRTHPKGYTEYKVTAQFISKKDPEDVKEVVVWKRYSDFRKLHGDLAYTHRNLFRRLEEFPAFPRAQVFGRFEASVIEERRKGAEDLLRFTVHIPALNNSPQLKEFFRGGEVTRPLEVSRDLHILPPPLIPTPPPDDPRLSQLLPAERRGLEELEVPVDPPPSSPAQEALDLLFNCESTEEASGSPARGPLTEAELALFDPFSKEEGAAPSPTHVAELATMEVESARLDQEPWEPGGQEEEEDGEGGPTPAYLSQATELITQALRDEKAGAYAAALQGYRDGVHVLLQGVPSDPLPARQEGVKKKAAEYLKRAEEILRLHLSQLPP

132 CAA89989.1 NP_612482.2

>CAA89989.1

MRLSKTLVDMDMADYSAALDPAYTTLEFENVQVLTMGNDLLPLRLARLRHPLRHHWSISGGVDSSPQGDTSPSEGTNLNAPNSLGVSALCAICGDRATGKHYGASSCDGCKGFFRRSVRKNHMYSCRFSRQCVVDKDKRNQCRYCRLKKCFRAGMKKEAVQNERDRISTRRSSYEDSSLPSINALLQAEVLSRQITSPVSGINGDIRAKKIASIADVCESMKEQLLVLVEWAKYIPAFCELPLDDQVALLRAHAGEHLLLGATKRSMVFKDVLLLGNDYIVPRHCPELAEMSRVSIRILDELVLPFQELQIDDNEYAYLKAIIFFDPDAKGLSDPGKIKRLRSQVQVSLEDYINDRQYDSRGRFGELLLLLPTLQSITWQMIEQIQFIKLFGMAKIDNLLQEMLLGGSPSDAPHAHHPLHPHLMQEHMGTNVIVANTMPTHLSNGQMCEWPRPRGQAATPETPQPSPPGGSGSEPYKLLPGAVATIVKPLSAIPQPTITKQEVI

>NP_612482.2

MSDQDHSMDEMTAVVKIEKGVGGNNGGNGNGGGAFSQARSSSTGSSSSTGGGGQESQPSPLALLAATCSRIESPNENSNNSQGPSQSGGTGELDLTATQLSQGANGWQIISSSSGATPTSKEQSGSSTNGSNGSESSKNRTVSGGQYVVAAAPNLQNQQVLTGLPGVMPNIQYQVIPQFQTVDGQQLQFAATGAQVQQDGSGQIQIIPGANQQIITNRGSGGNIIAAMPNLLQQAVPLQGLANNVLSGQTQYVTNVPVALNGNITLLPVNSVSAATLTPSSQAVTISSSGSQESGSQPVTSGTTISSASLVSSQASSSSFFTNANSYSTTTTTSNMGIMNFTTSGSSGTNSQGQTPQRVSGLQGSDALNIQQNQTSGGSLQAGQQKEGEQNQQTQQQQILIQPQLVQGGQALQALQAAPLSGQTFTTQAISQETLQNLQLQAVPNSGPIIIRTPTVGPNGQVSWQTLQLQNLQVQNPQAQTITLAPMQGVSLGQTSSSNTTLTPIASAASIPAGTVTVNAAQLSSMPGLQTINLSALGTSGIQVHPIQGLPLAIANAPGDHGAQLGLHGAGGDGIHDDTAGGEEGENSPDAQPQAGRRTRREACTCPYCKDSEGRGSGDPGKKKQHICHIQGCGKVYGKTSHLRAHLRWHTGERPFMCTWSYCGKRFTRSDELQRHKRTHTGEKKFACPECPKRFMRSDHLSKHIKTHQNKKGGPGVALSVGTLPLDSGAGSEGSGTATPSALITTNMVAMEAICPEGIARLANSGINVMQVADLQSINISGNGF

133 NP_005698.1 NP_060362.3

>NP_005698.1

MALPPFFGQGRPGPPPPQPPPPAPFGCPPPPLPSPAFPPPLPQRPGPFPGASAPFLQPPLALQPRASAEASRGGGGAGAFYPVPPPPLPPPPPQCRPFPGTDAGERPRPPPPGPGPPWSPRWPEAPPPPADVLGDAALQRLRDRQWLEAVFGTPRRAGCPVPQRTHAGPSLGEVRARLLRALRLVRRLRGLSQALREAEADGAAWVLLYSQTAPLRAELAERLQPLTQAAYVGEARRRLERVRRRRLRLRERAREREAEREAEAARAVEREQEIDRWRVKCVQEVEEKKREQELKAAADGVLSEVRKKQADTKRMVDILRALEKLRKLRKEAAARKGVCPPASADETFTHHLQRLRKLIKKRSELYEAEERALRVMLEGEQEEERKRELEKKQRKEKEKILLQKREIESKLFGDPDEFPLAHLLEPFRQYYLQAEHSLPALIQIRHDWDQYLVPSDHPKGNFVPQGWVLPPLPSNDIWATAVKLH

>NP_060362.3

MCSGSGRRRSSLSPTMRPGTGAERGGLMMGHPGMHYAPMGMHPMGQRANMPPVPHGMMPQMMPPMGGPPMGQMPGMMSSVMPGMMMSHMSQASMQPALPPGVNSMDVAAGTASGAKSMWTEHKSPDGRTYYYNTETKQSTWEKPDDLKTPAEQLLSKCPWKEYKSDSGKPYYYNSQTKESRWAKPKELEDLEGYQNTIVAGSLITKSNLHAMIKAEESSKQEECTTTSTAPVPTTEIPTTMSTMAAAEAAAAVVAAAAAAAAAAAAANANASTSASNTVSGTVPVVPEPEVTSIVATVVDNENTVTISTEEQAQLTSTPAIQDQSVEVSSNTGEETSKQETVADFTPKKEEEESQPAKKTYTWNTKEEAKQAFKELLKEKRVPSNASWEQAMKMIINDPRYSALAKLSEKKQAFNAYKVQTEKEEKEEARSKYKEAKESFQRFLENHEKMTSTTRYKKAEQMFGEMEVWNAISERDRLEIYEDVLFFLSKKEKEQAKQLRKRNWEALKNILDNMANVTYSTTWSEAQQYLMDNPTFAEDEELQNMDKEDALICFEEHIRALEKEEEEEKQKSLLRERRRQRKNRESFQIFLDELHEHGQLHSMSSWMELYPTISSDIRFTNMLGQPGSTALDLFKFYVEDLKARYHDEKKIIKDILKDKGFVVEVNTTFEDFVAIISSTKRSTTLDAGNIKLAFNSLLEKAEAREREREKEEARKMKRKESAFKSMLKQAAPPIELDAVWEDIRERFVKEPAFEDITLESERKRIFKDFMHVLEHECQHHHSKNKKHSKKSKKHHRKRSRSRSGSDSDDDDSHSKKKRQRSESRSASEHSSSAESERSYKKSKKHKKKSKKRRHKSDSPESDAEREKDKKEKDRESEKDRTRQRSESKHKSPKKKTGKDSGNWDTSGSELSEGELEKRRRTLLEQLDDDQ

134 NP_000675.1 NP_004032.2

>NP_000675.1

MGAGVLVLGASEPGNLSSAAPLPDGAATAARLLVPASPPASLLPPASESPEPLSQQWTAGMGLLMALIVLLIVAGNVLVIVAIAKTPRLQTLTNLFIMSLASADLVMGLLVVPFGATIVVWGRWEYGSFFCELWTSVDVLCVTASIETLCVIALDRYLAITSPFRYQSLLTRARARGLVCTVWAISALVSFLPILMHWWRAESDEARRCYNDPKCCDFVTNRAYAIASSVVSFYVPLCIMAFVYLRVFREAQKQVKKIDSCERRFLGGPARPPSPSPSPVPAPAPPPGPPRPAAAAATAPLANGRAGKRRPSRLVALREQKALKTLGIIMGVFTLCWLPFFLANVVKAFHRELVPDRLFVFFNWLGYANSAFNPIIYCRSPDFRKAFQGLLCCARRAARRRHATHGDRPRASGCLARPGPPPSPGAASDDDDDDVVGATPPARLLEPWAGCNGGAAADSDSSLDEPCRPGFASESKV

>NP_004032.2

MGDKGTRVFKKASPNGKLTVYLGKRDFVDHIDLVDPVDGVVLVDPEYLKERRVYVTLTCAFRYGREDLDVLGLTFRKDLFVANVQSFPPAPEDKKPLTRLQERLIKKLGEHAYPFTFEIPPNLPCSVTLQPGPEDTGKACGVDYEVKAFCAENLEEKIHKRNSVRLVIRKVQYAPERPGPQPTAETTRQFLMSDKPLHLEASLDKEIYYHGEPISVNVHVTNNTNKTVKKIKISVRQYADICLFNTAQYKCPVAMEEADDTVAPSSTFCKVYTLTPFLANNREKRGLALDGKLKHEDTNLASSTLLREGANREILGIIVSYKVKVKLVVSRGGLLGDLASSDVAVELPFTLMHPKPKEEPPHREVPENETPVDTNLIELDTNDDDIVFEDFARQRLKGMKDDKEEEEDGTGSPQLNNR

135 NP_066961.2 NP_085050.2

>NP_066961.2

MAAASVTPPGSLELLQPGFSKTLLGTKLEAKYLCSACRNVLRRPFQAQCGHRYCSFCLASILSSGPQNCAACVHEGIYEEGISILESSSAFPDNAARREVESLPAVCPSDGCTWKGTLKEYESCHEGRCPLMLTECPACKGLVRLGEKERHLEHECPERSLSCRHCRAPCCGADVKAHHEVCPKFPLTCDGCGKKKIPREKFQDHVKTCGKCRVPCRFHAIGCLETVEGEKQQEHEVQWLREHLAMLLSSVLEAKPLLGDQSHAGSELLQRCESLEKKTATFENIVCVLNREVERVAMTAEACSRQHRLDQDKIEALSSKVQQLERSIGLKDLAMADLEQKVLEMEASTYDGVFIWKISDFARKRQEAVAGRIPAIFSPAFYTSRYGYKMCLRIYLNGDGTGRGTHLSLFFVVMKGPNDALLRWPFNQKVTLMLLDQNNREHVIDAFRPDVTSSSFQRPVNDMNIASGCPLFCPVSKMEAKNSYVRDDAIFIKAIVDLTGL

>NP_085050.2

MPRHCSAAGCCTRDTRETRNRGISFHRLPKKDNPRRGLWLANCQRLDPSGQGLWDPASEYIYFCSKHFEEDCFELVGISGYHRLKEGAVPTIFESFSKLRRTTKTKGHSYPPGPAEVSRLRRCRKRCSEGRGPTTPFSPPPPADVTCFPVEEASAPATLPASPAGRLEPGLSSPFSDLLGPLGAQADEAGCSAQPSPERQPSPLEPRPVSPSAYMLRLPPPAGAYIQNEHSYQVGSALLWKRRAEAALDALDKAQRQLQACKRREQRLRLRLTKLQQERAREKRAQADARQTLKEHVQDFAMQLSSSMA

136 NP_077720.1 NP_003986.2

>NP_077720.1

MHLSQLLACALLLTLLSLRPSEAKPGAPPKVPRTPPAEELAEPQAAGGGQKKGDKAPGGGGANLKGDRSRLLRDLRVDTKSRAAWARLLQEHPNARKYKGANKKGLSKGCFGLKLDRIGSMSGLGC

>NP_003986.2

MALPSLLLLVAALAGGVRPPGARNLTLAVVLPEHNLSYAWAWPRVGPAVALAVEALGRALPVDLRFVSSELEGACSEYLAPLSAVDLKLYHDPDLLLGPGCVYPAASVARFASHWRLPLLTAGAVASGFSAKNDHYRTLVRTGPSAPKLGEFVVTLHGHFNWTARAALLYLDARTDDRPHYFTIEGVFEALQGSNLSVQHQVYAREPGGPEQATHFIRANGRIVYICGPLEMLHEILLQAQRENLTNGDYVFFYLDVFGESLRAGPTRATGRPWQDNRTREQAQALREAFQTVLVITYREPPNPEYQEFQNRLLIRAREDFGVELGPSLMNLIAGCFYDGILLYAEVLNETIQEGGTREDGLRIVEKMQGRRYHGVTGLVVMDKNNDRETDFVLWAMGDLDSGDFQPAAHYSGAEKQIWWTGRPIPWVKGAPPSDNPPCAFDLDDPSCDKTPLSTLAIVALGTGITFIMFGVSSFLIFRKLMLEKELASMLWRIRWEELQFGNSERYHKGAGSRLTLSLRGSSYGSLMTAHGKYQIFANTGHFKGNVVAIKHVNKKRIELTRQVLFELKHMRDVQFNHLTRFIGACIDPPNICIVTEYCPRGSLQDILENDSINLDWMFRYSLINDLVKGMAFLHNSIISSHGSLKSSNCVVDSRFVLKITDYGLASFRSTAEPDDSHALYAKKLWTAPELLSGNPLPTTGMQKADVYSFGIILQEIALRSGPFYLEGLDLSPKEIVQKVRNGQRPYFRPSIDRTQLNEELVLLMERCWAQDPAERPDFGQIKGFIRRFNKEGGTSILDNLLLRMEQYANNLEKLVEERTQAYLEEKRKAEALLYQILPHSVAEQLKRGETVQAEAFDSVTIYFSDIVGFTALSAESTPMQVVTLLNDLYTCFDAIIDNFDVYKVETIGDAYMVVSGLPGRNGQRHAPEIARMALALLDAVSSFRIRHRPHDQLRLRIGVHTGPVCAGVVGLKMPRYCLFGDTVNTASRMESNGQALKIHVSSTTKDALDELGCFQLELRGDVEMKGKGKMRTYWLLGERKGPPGLL

137 NP_006191.2 NP_002665.2

>NP_006191.2

MGWGSRCCCPGRLDLLCVLALLGGCLLPVCRTRVYTNHWAVKIAGGFPEANRIASKYGFINIGQIGALKDYYHFYHSRTIKRSVISSRGTHSFISMEPKVEWIQQQVVKKRTKRDYDFSRAQSTYFNDPKWPSMWYMHCSDNTHPCQSDMNIEGAWKRGYTGKNIVVTILDDGIERTHPDLMQNYDALASCDVNGNDLDPMPRYDASNENKHGTRCAGEVAAAANNSHCTVGIAFNAKIGGVRMLDGDVTDMVEAKSVSFNPQHVHIYSASWGPDDDGKTVDGPAPLTRQAFENGVRMGRRGLGSVFVWASGNGGRSKDHCSCDGYTNSIYTISISSTAESGKKPWYLEECSSTLATTYSSGESYDKKIITTDLRQRCTDNHTGTSASAPMAAGIIALALEANPFLTWRDVQHVIVRTSRAGHLNANDWKTNAAGFKVSHLYGFGLMDAEAMVMEAEKWTTVPRQHVCVESTDRQIKTIRPNSAVRSIYKASGCSDNPNRHVNYLEHVVVRITITHPRRGDLAIYLTSPSGTRSQLLANRLFDHSMEGFKNWEFMTIHCWGERAAGDWVLEVYDTPSQLRNFKTPGKLKEWSLVLYGTSVQPYSPTNEFPKVERFRYSRVEDPTDDYGTEDYAGPCDPECSEVGCDGPGPDHCNDCLHYYYKLKNNTRICVSSCPPGHYHADKKRCRKCAPNCESCFGSHGDQCMSCKYGYFLNEETNSCVTHCPDGSYQDTKKNLCRKCSENCKTCTEFHNCTECRDGLSLQGSRCSVSCEDGRYFNGQDCQPCHRFCATCAGAGADGCINCTEGYFMEDGRCVQSCSISYYFDHSSENGYKSCKKCDISCLTCNGPGFKNCTSCPSGYLLDLGMCQMGAICKDATEESWAEGGFCMLVKKNNLCQRKVLQQLCCKTCTFQG

>NP_002665.2

MAKMNLSSYILILTFSLFSQGILLSASKSIRNLDDDMVFNTFRLGKGFQKEDTAEKSVIAPSLEQYKNDESSFMNEEENKVSKNTGSKHNFLNHGLPLNLAIKPYLALKGSVAFPAENGVQNTESTQEKREIGDEENSAKFPIGRRDFDMLRCMLGRVYRPCWQV

138 NP_006563.2 NP_005217.2

>NP_006563.2

MADFLPSRSVLSVCFPGCLLTSGEAEQQRKSKEIDKCLSREKTYVKRLVKILLLGAGESGKSTFLKQMRIIHGQDFDQRAREEFRPTIYSNVIKGMRVLVDAREKLHIPWGDNSNQQHGDKMMSFDTRAPMAAQGMVETRVFLQYLPAIRALWADSGIQNAYDRRREFQLGESVKYFLDNLDKLGEPDYIPSQQDILLARRPTKGIHEYDFEIKNVPFKMVDVGGQRSERKRWFECFDSVTSILFLVSSSEFDQVLMEDRLTNRLTESLNIFETIVNNRVFSNVSIILFLNKTDLLEEKVQIVSIKDYFLEFEGDPHCLRDVQKFLVECFRNKRRDQQQKPLYHHFTTAINTENIRLVFRDVKDTILHDNLKQLMLQ

>NP_005217.2

MATALPPRLQPVRGNETLREHYQYVGKLAGRLKEASEGSTLTTVLFLVICSFIVLENLMVLIAIWKNNKFHNRMYFFIGNLALCDLLAGIAYKVNILMSGKKTFSLSPTVWFLREGSMFVALGASTCSLLAIAIERHLTMIKMRPYDANKRHRVFLLIGMCWLIAFTLGALPILGWNCLHNLPDCSTILPLYSKKYIAFCISIFTAILVTIVILYARIYFLVKSSSRKVANHNNSERSMALLRTVVIVVSVFIACWSPLFILFLIDVACRVQACPILFKAQWFIVLAVLNSAMNPVIYTLASKEMRRAFFRLVCNCLVRGRGARASPIQPALDPSRSKSSSSNNSSHSPKVKEDLPHTAPSSCIMDKNAALQNGIFCN

139 NP_002253.1 NP_031359.2

>NP_002253.1

MAVFKTTLWRLISGTLGIICLSLMATLGILLKNSFTKLSIEPAFTPGPNIELQKDSDCCSCQEKWVGYRCNCYFISSEQKTWNESRHLCASQKSSLLQLQNTDELDFMSSSQQFYWIGLSYSEEHTAWLWENGSALSQYLFPSFETFNTKNCIAYNPNGNALDESCEDKNRYICKQQLI

>NP_031359.2

MSKQRGTFSEVSLAQDPKWQQRKPKGNKSSISGTEQEIFQVELNLQNASLNHQGIDKIYDCQGLLPPPEKLTAEVLGIICIVLMATVLKTIVLIPFLEQNNSSPNARTQKARHCGHCPEEWITYSNSCYYIGKERRTWEESLQACASKNSSSLLCIDNEEEMKFLASILPSSWIGVFRNSSHHPWVTINGLAFKHEIKDSDHAERNCAMLHVRGLISDQCGSSRIIVSISFRIKALELAVHQIKFYICSNRNDIMIA

140 NP_005045.2 NP_006316.1

>NP_005045.2

MRRSKADVERYVASVLGLTPSPRQKSMKGFYFAKLYYEAKEYDLAKKYICTYINVQERDPKAHRFLGLLYELEENTEKAVECYRRSVELNPTQKDLVLKIAELLCKNDVTDGRAKYWVERAAKLFPGSPAIYKLKEQLLDCEGEDGWNKLFDLIQSELYVRPDDVHVNIRLVELYRSTKRLKDAVAHCHEAERNIALRSSLEWNSCVVQTLKEYLESLQCLESDKSDWQATNTDLLLAYANLMLLTLSTRDVQENRELLESFDSALQSAKSSLGGNDELSATFLEMKGHFYMYAGSLLLKMGQHGNNVQWRALSELAALCYLIAFQVPRPKIKLREGKAGQNLLEMMACDRLSQSGHMLLSLSRGKQDFLKEVVETFANKIGQSALYDALFSSQSPKDTSFLGSDDIGKIDVQEPELEDLARYDVGAIRAHNGSLQHLTWLGLQWNSLPALPGIRKWLKQLFHRLPHETSRLETNAPESICILDLEVFLLGVVYTSHLQLKEKCNSHHSSYQPLCLPFPVCKQLCTERQKSWWDAVCTLIHRKAVPGNLAKLRLLVQHEINTLRAQEKHGLQPALLVHWAKYLQKTGSGLNSFYGQLEYIGRSVHYWKKVLPLLKIIKKNSIPEPIDPLFKHFHSVDIQASEIVEYEEDAHITFAMLDAVNGNIEDAVTAFESIKSVVSYWNLALIFHRKAEDIENDALSPEEQEECRNYLTKTRDYLIKIIDDGDSNLSVVKKLPVPLESVKQMLNSVMQELEDYSEGGPLYKNGSLRNADSEIKHSTPSPTKYSLSPSKSYKYSPETPPRWTEDRNSLLNMICQQVEAIKKEMQELKLNSSKSASRHRWPTENYGPDSVPDGYQGSQTFHGAPLTVATTGPSVYYSQSPAYNSQYLLRPAANVTPTKGSSNTEFKSTKEGFSIPVSADGFKFGISEPGNQEKKREKPLENDTGFQAQDISGRKKGRGVIFGQTSSTFTFADVAKSTSGEGFQFGKKDLNFKGFSGAGEKLFSSRYGKMANKANTSGDFEKDDDAYKTEDSDDIHFEPVVQMPEKVELVTGEEGEKVLYSQGVKLFRFDAEVRQWKERGLGNLKILKNEVNGKLRMLMRREQVLKVCANHWITTTMNLKPLSGSDRAWMWSASDFSDGDAKLERLAAKFKTPELAEEFKQKFEECQRLLLDIPLQTPHKLVDTGRAAKLIQRAEEMKSGLKDFKTFLTNDQTKVTEEENKGSGTGAAGASDTTIKPNAENTGPTLEWDNYDLREDALDDSVSSSSVHASPLASSPVRKNLFRFDESTTGSNFSFKSALSLSKSPAKLNQSGTSVGTDEESVVTQEEERDGQYFEPVVPLPDLVEVSSGEENEQVVFSHRAEIYRYDKDVGQWKERGIGDIKILQNYDNKQVRIVMRRDQVLKLCANHRITPDMSLQNMKGTERVWVWTACDFADGERKVEHLAVRFKLQDVADSFKKIFDEAKTAQEKDSLITPHVSRSSTPRESPCGKIAVAILEETTRERTDVIQGDDVADAASEVEVSSTSETTTKAVVSPPKFVFVSESVKRIFSSEKSKPFVFGNSSATGSLFGFSFNAPLKSNNSETSSVAQSGSESKVEPKKCELSKNSDIEQSSDSKVKNLSASFPTEESSINYTFKTPEKEPPLWHAEFTKEELVQKLRSTTKSADHLNGLLREIEATNAVLMEQIKLLKSEIRRLERNQEREKSAANLEYLKNVLLQFIFLKPGSERERLLPVINTMLQLSPEEKGKLAAVAQDEEENASRSSG

>NP_006316.1

MAAQGEPQVQFKLVLVGDGGTGKTTFVKRHLTGEFEKKYVATLGVEVHPLVFHTNRGPIKFNVWDTAGQEKFGGLRDGYYIQAQCAIIMFDVTSRVTYKNVPNWHRDLVRVCENIPIVLCGNKVDIKDRKVKAKSIVFHRKKNLQYYDISAKSNYNFEKPFLWLARKLIGDPNLEFVAMPALAPPEVVMDPALAAQYEHDLEVAQTTALPDEDDDL

141 NP_201585.1 NP_001605.1

>NP_201585.1

MTLSVLSRKDKERVIRRLLLQAPPGEFVNAFDDLCLLIRDEKLMHHQGECAGHQHCQKYSVPLCIDGNPVLLSHHNVMGDYRFFDHQSKLSFKYDLLQNQLKDIQSHGIIQNEAEYLRVVLLCALKLYVNDHYPKGNCNMLRKTVKSKEYLIACIEDHNYETGECWNGLWKSKWIFQVNPFLTQVTGRIFVQAHFFRCVNLHIEISKDLKESLEIVNQAQLALSFARLVEEQENKFQAAVLEELQELSNEALRKILRRDLPVTRTLIDWHRILSDLNLVMYPKLGYVIYSRSVLCNWII

>NP_001605.1

MEEEIAALVIDNGSGMCKAGFAGDDAPRAVFPSIVGRPRHQGVMVGMGQKDSYVGDEAQSKRGILTLKYPIEHGIVTNWDDMEKIWHHTFYNELRVAPEEHPVLLTEAPLNPKANREKMTQIMFETFNTPAMYVAIQAVLSLYASGRTTGIVMDSGDGVTHTVPIYEGYALPHAILRLDLAGRDLTDYLMKILTERGYSFTTTAEREIVRDIKEKLCYVALDFEQEMATAASSSSLEKSYELPDGQVITIGNERFRCPEALFQPSFLGMESCGIHETTFNSIMKCDVDIRKDLYANTVLSGGTTMYPGIADRMQKEITALAPSTMKIKIIAPPERKYSVWIGGSILASLSTFQQMWISKQEYDESGPSIVHRKCF

142 NP_003946.3 NP_000112.1

>NP_003946.3

MVTHSKFPAAGMSRPLDTSLRLKTFSSKSEYQLVVNAVRKLQESGFYWSAVTGGEANLLLSAEPAGTFLIRDSSDQRHFFTLSVKTQSGTKNLRIQCEGGSFSLQSDPRSTQPVPRFDCVLKLVHHYMPPPGAPSFPSPPTEPSSEVPEQPSAQPLPGSPPRRAYYIYSGGEKIPLVLSRPLSSNVATLQHLCRKTVNGHLDSYEKVTQLPGPIREFLDQYDAPL

>NP_000112.1

MDHLGASLWPQVGSLCLLLAGAAWAPPPNLPDPKFESKAALLAARGPEELLCFTERLEDLVCFWEEAASAGVGPGNYSFSYQLEDEPWKLCRLHQAPTARGAVRFWCSLPTADTSSFVPLELRVTAASGAPRYHRVIHINEVVLLDAPVGLVARLADESGHVVLRWLPPPETPMTSHIRYEVDVSAGNGAGSVQRVEILEGRTECVLSNLRGRTRYTFAVRARMAEPSFGGFWSAWSEPVSLLTPSDLDPLILTLSLILVVILVLLTVLALLSHRRALKQKIWPGIPSPESEFEGLFTTHKGNFQLWLYQNDGCLWWSPCTPFTEDPPASLEVLSERCWGTMQAVEPGTDDEGPLLEPVGSEHAQDTYLVLDKWLLPRNPPSEDLPGPGGSVDIVAMDEGSEASSCSSALASKPSPEGASAASFEYTILDPSSQLLRPWTLCPELPPTPPHLKYLYLVVSDSGISTDYSSGDSQGAQGGLSDGPYSNPYENSLIPAAEPLPPSYVACS

143 NP_003452.1 NP_001093.1

>NP_003452.1

MAAPRPSPAISVSVSAPAFYAPQKKFGPVVAPKPKVNPFRPGDSEPPPAPGAQRAQMGRVGEIPPPPPEDFPLPPPPLAGDGDDAEGALGGAFPPPPPPIEESFPPAPLEEEIFPSPPPPPEEEGGPEAPIPPPPQPREKVSSIDLEIDSLSSLLDDMTKNDPFKARVSSGYVPPPVATPFSSKSSTKPAAGGTAPLPPWKSPSSSQPLPQVPAPAQSQTQFHVQPQPQPKPQVQLHVQSQTQPVSLANTQPRGPPASSPAPAPKFSPVTPKFTPVASKFSPGAPGGSGSQPNQKLGHPEALSAGTGSPQPPSFTYAQQREKPRVQEKQHPVPPPAQNQNQVRSPGAPGPLTLKEVEELEQLTQQLMQDMEHPQRQNVAVNELCGRCHQPLARAQPAVRALGQLFHIACFTCHQCAQQLQGQQFYSLEGAPYCEGCYTDTLEKCNTCGEPITDRMLRATGKAYHPHCFTCVVCARPLEGTSFIVDQANRPHCVPDYHKQYAPRCSVCSEPIMPEPGRDETVRVVALDKNFHMKCYKCEDCGKPLSIEADDNGCFPLDGHVLCRKCHTARAQT

>NP_001093.1

MDHYDSQQTNDYMQPEEDWDRDLLLDPAWEKQQRKTFTAWCNSHLRKAGTQIENIEEDFRDGLKLMLLLEVISGERLAKPERGKMRVHKISNVNKALDFIASKGVKLVSIGAEEIVDGNVKMTLGMIWTIILRFAIQDISVEETSAKEGLLLWCQRKTAPYKNVNIQNFHISWKDGLGFCALIHRHRPELIDYGKLRKDDPLTNLNTAFDVAEKYLDIPKMLDAEDIVGTARPDEKAIMTYVSSFYHAFSGAQKAETAANRICKVLAVNQENEQLMEDYEKLASDLLEWIRRTIPWLENRVPENTMHAMQQKLEDFRDYRRLHKPPKVQEKCQLEINFNTLQTKLRLSNRPAFMPSEGRMVSDINNAWGCLEQVEKGYEEWLLNEIRRLERLDHLAEKFRQKASIHEAWTDGKEAMLRQKDYETATLSEIKALLKKHEAFESDLAAHQDRVEQIAAIAQELNELDYYDSPSVNARCQKICDQWDNLGALTQKRREALERTEKLLETIDQLYLEYAKRAAPFNNWMEGAMEDLQDTFIVHTIEEIQGLTTAHEQFKATLPDADKERLAILGIHNEVSKIVQTYHVNMAGTNPYTTITPQEINGKWDHVRQLVPRRDQALTEEHARQQHNERLRKQFGAQANVIGPWIQTKMEEIGRISIEMHGTLEDQLSHLRQYEKSIVNYKPKIDQLEGDHQLIQEALIFDNKHTNYTMEHIRVGWEQLLTTIARTINEVENQILTRDAKGISQEQMNEFRASFNHFDRDHSGTLGPEEFKACLISLGYDIGNDPQGEAEFARIMSIVDPNRLGVVTFQAFIDFMSRETADTDTADQVMASFKILAGDKNYITMDELRRELPPDQAEYCIARMAPYTGPDSVPGALDYMSFSTALYGESDL

144 NP_000220.1 NP_000030.1

>NP_000220.1

MGPPGSPWQWVTLLLGLLLPPAAPFWLLNVLFPPHTTPKAELSNHTRPVILVPGCLGNQLEAKLDKPDVVNWMCYRKTEDFFTIWLDLNMFLPLGVDCWIDNTRVVYNRSSGLVSNAPGVQIRVPGFGKTYSVEYLDSSKLAGYLHTLVQNLVNNGYVRDETVRAAPYDWRLEPGQQEEYYRKLAGLVEEMHAAYGKPVFLIGHSLGCLHLLYFLLRQPQAWKDRFIDGFISLGAPWGGSIKPMLVLASGDNQGIPIMSSIKLKEEQRITTTSPWMFPSRMAWPEDHVFISTPSFNYTGRDFQRFFADLHFEEGWYMWLQSRDLLAGLPAPGVEVYCLYGVGLPTPRTYIYDHGFPYTDPVGVLYEDGDDTVATRSTELCGLWQGRQPQPVHLLPLHGIQHLNMVFSNLTLEHINAILLGAYRQGPPASPTASPEPPPPE

>NP_000030.1

MKAAVLTLAVLFLTGSQARHFWQQDEPPQSPWDRVKDLATVYVDVLKDSGRDYVSQFEGSALGKQLNLKLLDNWDSVTSTFSKLREQLGPVTQEFWDNLEKETEGLRQEMSKDLEEVKAKVQPYLDDFQKKWQEEMELYRQKVEPLRAELQEGARQKLHELQEKLSPLGEEMRDRARAHVDALRTHLAPYSDELRQRLAARLEALKENGGARLAEYHAKATEHLSTLSEKAKPALEDLRQGLLPVLESFKVSFLSALEEYTKKLNTQ

145 NP_001744.2 NP_000116.2

>NP_001744.2

MSGGKYVDSEGHLYTVPIREQGNIYKPNNKAMADELSEKQVYDAHTKEIDLVNRDPKHLNDDVVKIDFEDVIAEPEGTHSFDGIWKASFTTFTVTKYWFYRLLSALFGIPMALIWGIYFAILSFLHIWAVVPCIKSFLIEIQCISRVYSIYVHTVCDPLFEAVGKIFSNVRINLQKEI

>NP_000116.2

MTMTLHTKASGMALLHQIQGNELEPLNRPQLKIPLERPLGEVYLDSSKPAVYNYPEGAAYEFNAAAAANAQVYGQTGLPYGPGSEAAAFGSNGLGGFPPLNSVSPSPLMLLHPPPQLSPFLQPHGQQVPYYLENEPSGYTVREAGPPAFYRPNSDNRRQGGRERLASTNDKGSMAMESAKETRYCAVCNDYASGYHYGVWSCEGCKAFFKRSIQGHNDYMCPATNQCTIDKNRRKSCQACRLRKCYEVGMMKGGIRKDRRGGRMLKHKRQRDDGEGRGEVGSAGDMRAANLWPSPLMIKRSKKNSLALSLTADQMVSALLDAEPPILYSEYDPTRPFSEASMMGLLTNLADRELVHMINWAKRVPGFVDLTLHDQVHLLECAWLEILMIGLVWRSMEHPGKLLFAPNLLLDRNQGKCVEGMVEIFDMLLATSSRFRMMNLQGEEFVCLKSIILLNSGVYTFLSSTLKSLEEKDHIHRVLDKITDTLIHLMAKAGLTLQQQHQRLAQLLLILSHIRHMSNKGMEHLYSMKCKNVVPLYDLLLEMLDAHRLHAPTSRGGASVEETDQSHLATAGSTSSHSLQKYYITGEAEGFPATV

146 NP_005089.2 NP_996919.1

>NP_005089.2

MSTGSVSDPEEMELRGLQREYPVPASKRPPLRGVERSYASPSDNSSAEEEDPDGEEERCALGTAGSAEGCKRKRPRVAGGGGAGGSAGGGGKKPLPAKGSAAECKQSQRNAANARERARMRVLSKAFSRLKTSLPWVPPDTKLSKLDTLRLASSYIAHLRQLLQEDRYENGYVHPVNLTWPFVVSGRPDSDTKEVSAANRLCGTTA

>NP_996919.1

MNPQQQRMAAIGTDKELSDLLDFSAMFSPPVNSGKTRPTTLGSSQFSGSGIDERGGTTSWGTSGQPSPSYDSSRGFTDSPHYSDHLNDSRLGAHEGLSPTPFMNSNLMGKTSERGSFSLYSRDTGLPGCQSSLLRQDLGLGSPAQLSSSGKPGTAYYSFSATSSRRRPLHDSAALDPLQAKKVRKVPPGLPSSVYAPSPNSDDFNRESPSYPSPKPPTSMFASTFFMQDGTHNSSDLWSSSNGMSQPGFGGILGTSTSHMSQSSSYGNLHSHDRLSYPPHSVSPTDINTSLPPMSSFHRGSTSSSPYVAASHTPPINGSDSILGTRGNAAGSSQTGDALGKALASIYSPDHTSSSFPSNPSTPVGSPSPLTGTSQWPRPGGQAPSSPSYENSLHSLKNRVEQQLHEHLQDAMSFLKDVCEQSRMEDRLDRLDDAIHVLRNHAVGPSTSLPAGHSDIHSLLGPSHNAPIGSLNSNYGGSSLVASSRSASMVGTHREDSVSLNGNHSVLSSTVTTSSTDLNHKTQENYRGGLQSQSGTVVTTEIKTENKEKDENLHEPPSSDDMKSDDESSQKDIKVSSRGRTSSTNEDEDLNPEQKIEREKERRMANNARERLRVRDINEAFKELGRMCQLHLKSEKPQTKLLILHQAVAVILSLEQQVRERNLNPKAACLKRREEEKVSAVSAEPPTTLPGTHPGLSETTNPMGHM

147 NP_001722.1 NP_003241.2

>NP_001722.1

MHPFYTRAATMIGEIAAAVSFISKFLRTKGLTSERQLQTFSQSLQELLAEHYKHHWFPEKPCKGSGYRCIRINHKMDPLIGQAAQRIGLSSQELFRLLPSELTLWVDPYEVSYRIGEDGSICVLYEASPAGGSTQNSTNVQMVDSRISCKEELLLGRTSPSKNYNMMTVSG

>NP_003241.2

MEQKPSKVECGSDPEENSARSPDGKRKRKNGQCSLKTSMSGYIPSYLDKDEQCVVCGDKATGYHYRCITCEGCKGFFRRTIQKNLHPTYSCKYDSCCVIDKITRNQCQLCRFKKCIAVGMAMDLVLDDSKRVAKRKLIEQNRERRRKEEMIRSLQQRPEPTPEEWDLIHIATEAHRSTNAQGSHWKQRRKFLPDDIGQSPIVSMPDGDKVDLEAFSEFTKIITPAITRVVDFAKKLPMFSELPCEDQIILLKGCCMEIMSLRAAVRYDPESDTLTLSGEMAVKREQLKNGGLGVVSDAIFELGKSLSAFNLDDTEVALLQAVLLMSTDRSGLLCVDKIEKSQEAYLLAFEHYVNHRKHNIPHFWPKLLMKEREVQSSILYKGAAAEGRPGGSLGVHPEGQQLLGMHVVQGPQVRQLEQQLGEAGSLQGPVLQHQSPKSPQQRLLELLHRSGILHARAVCGEDDSSEADSPSSSEEEPEVCEDLAGNAASP

148 NP_001015053.1 NP_620309.1

>NP_001015053.1

MNSPNESADGMSGREPSLEILPRTSLHSIPVTVEVKPVLPRAMPSSMGGGGGGSPSPVELRGALVGSVDPTLREQQLQQELLALKQQQQLQKQLLFAEFQKQHDHLTRQHEVQLQKHLKQQQEMLAAKQQQEMLAAKRQQELEQQRQREQQRQEELEKQRLEQQLLILRNKEKSKESAIASTEVKLRLQEFLLSKSKEPTPGGLNHSLPQHPKCWGAHHASLDQSSPPQSGPPGTPPSYKLPLPGPYDSRDDFPLRKTASEPNLKVRSRLKQKVAERRSSPLLRRKDGTVISTFKKRAVEITGAGPGASSVCNSAPGSGPSSPNSSHSTIAENGFTGSVPNIPTEMLPQHRALPLDSSPNQFSLYTSPSLPNISLGLQATVTVTNSHLTASPKLSTQQEAERQALQSLRQGGTLTGKFMSTSSIPGCLLGVALEGDGSPHGHASLLQHVLLLEQARQQSTLIAVPLHGQSPLVTGERVATSMRTVGKLPRHRPLSRTQSSPLPQSPQALQQLVMQQQHQQFLEKQKQQQLQLGKILTKTGELPRQPTTHPEETEEELTEQQEVLLGEGALTMPREGSTESESTQEDLEEEDEEDDGEEEEDCIQVKDEEGESGAEEGPDLEEPGAGYKKLFSDAQPLQPLQVYQAPLSLATVPHQALGRTQSSPAAPGGMKSPPDQPVKHLFTTGVVYDTFMLKHQCMCGNTHVHPEHAGRIQSIWSRLQETGLLSKCERIRGRKATLDEIQTVHSEYHTLLYGTSPLNRQKLDSKKLLGPISQKMYAVLPCGGIGVDSDTVWNEMHSSSAVRMAVGCLLELAFKVAAGELKNGFAIIRPPGHHAEESTAMGFCFFNSVAITAKLLQQKLNVGKVLIVDWDIHHGNGTQQAFYNDPSVLYISLHRYDNGNFFPGSGAPEEVGGGPGVGYNVNVAWTGGVDPPIGDVEYLTAFRTVVMPIAHEFSPDVVLVSAGFDAVEGHLSPLGGYSVTARCFGHLTRQLMTLAGGRVVLALEGGHDLTAICDASEACVSALLSVELQPLDEAVLQQKPNINAVATLEKVIEIQSKHWSCVQKFAAGLGRSLREAQAGETEEAETVSAMALLSVGAEQAQAAAAREHSPRPAEEPMEQEPAL

>NP_620309.1

MASPADSCIQFTRHASDVLLNLNRLRSRDILTDVVIVVSREQFRAHKTVLMACSGLFYSIFTDQLKCNLSVINLDPEINPEGFCILLDFMYTSRLNLREGNIMAVMATAMYLQMEHVVDTCRKFIKASEAEMVSAIKPPREEFLNSRMLMPQDIMAYRGREVVENNLPLRSAPGCESRAFAPSLYSGLSTPPASYSMYSHLPVSSLLFSDEEFRDVRMPVANPFPKERALPCDSARPVPGEYSRPTLEVSPNVCHSNIYSPKETIPEEARSDMHYSVAEGLKPAAPSARNAPYFPCDKASKEEERPSSEDEIALHFEPPNAPLNRKGLVSPQSPQKSDCQPNSPTESCSSKNACILQASGSPPAKSPTDPKACNWKKYKFIVLNSLNQNAKPEGPEQAELGRLSPRAYTAPPACQPPMEPENLDLQSPTKLSASGEDSTIPQASRLNNIVNRSMTGSPRSSSESHSPLYMHPPKCTSCGSQSPQHAEMCLHTAGPTFPEEMGETQSEYSDSSCENGAFFCNECDCRFSEEASLKRHTLQTHSDKPYKCDRCQASFRYKGNLASHKTVHTGEKPYRCNICGAQFNRPANLKTHTRIHSGEKPYKCETCGARFVQVAHLRAHVLIHTGEKPYPCEICGTRFRHLQTLKSHLRIHTGEKPYHCEKCNLHFRHKSQLRLHLRQKHGAITNTKVQYRVSATDLPPELPKAC

149 NP_003177.2 NP_001091.1

>NP_003177.2

MANKGPSYGMSREVQSKIEKKYDEELEERLVEWIIVQCGPDVGRPDRGRLGFQVWLKNGVILSKLVNSLYPDGSKPVKVPENPPSMVFKQMEQVAQFLKAAEDYGVIKTDMFQTVDLFEGKDMAAVQRTLMALGSLAVTKNDGHYRGDPNWFMKKAQEHKREFTESQLQEGKHVIGLQMGSNRGASQAGMTGYGRPRQIIS

>NP_001091.1

MCDEDETTALVCDNGSGLVKAGFAGDDAPRAVFPSIVGRPRHQGVMVGMGQKDSYVGDEAQSKRGILTLKYPIEHGIITNWDDMEKIWHHTFYNELRVAPEEHPTLLTEAPLNPKANREKMTQIMFETFNVPAMYVAIQAVLSLYASGRTTGIVLDSGDGVTHNVPIYEGYALPHAIMRLDLAGRDLTDYLMKILTERGYSFVTTAEREIVRDIKEKLCYVALDFENEMATAASSSSLEKSYELPDGQVITIGNERFRCPETLFQPSFIGMESAGIHETTYNSIMKCDIDIRKDLYANNVMSGGTTMYPGIADRMQKEITALAPSTMKIKIIAPPERKYSVWIGGSILASLSTFQQMWITKQEYDEAGPSIVHRKCF

150 NP_005202.2 NP_000748.3

>NP_005202.2

MGPGVLLLLLVATAWHGQGIPVIEPSVPELVVKPGATVTLRCVGNGSVEWDGPPSPHWTLYSDGSSSILSTNNATFQNTGTYRCTEPGDPLGGSAAIHLYVKDPARPWNVLAQEVVVFEDQDALLPCLLTDPVLEAGVSLVRVRGRPLMRHTNYSFSPWHGFTIHRAKFIQSQDYQCSALMGGRKVMSISIRLKVQKVIPGPPALTLVPAELVRIRGEAAQIVCSASSVDVNFDVFLQHNNTKLAIPQQSDFHNNRYQKVLTLNLDQVDFQHAGNYSCVASNVQGKHSTSMFFRVVESAYLNLSSEQNLIQEVTVGEGLNLKVMVEAYPGLQGFNWTYLGPFSDHQPEPKLANATTKDTYRHTFTLSLPRLKPSEAGRYSFLARNPGGWRALTFELTLRYPPEVSVIWTFINGSGTLLCAASGYPQPNVTWLQCSGHTDRCDEAQVLQVWDDPYPEVLSQEPFHKVTVQSLLTVETLEHNQTYECRAHNSVGSGSWAFIPISAGAHTHPPDEFLFTPVVVACMSIMALLLLLLLLLLYKYKQKPKYQVRWKIIESYEGNSYTFIDPTQLPYNEKWEFPRNNLQFGKTLGAGAFGKVVEATAFGLGKEDAVLKVAVKMLKSTAHADEKEALMSELKIMSHLGQHENIVNLLGACTHGGPVLVITEYCCYGDLLNFLRRKAEAMLGPSLSPGQDPEGGVDYKNIHLEKKYVRRDSGFSSQGVDTYVEMRPVSTSSNDSFSEQDLDKEDGRPLELRDLLHFSSQVAQGMAFLASKNCIHRDVAARNVLLTNGHVAKIGDFGLARDIMNDSNYIVKGNARLPVKWMAPESIFDCVYTVQSDVWSYGILLWEIFSLGLNPYPGILVNSKFYKLVKDGYQMAQPAFAPKNIYSIMQACWALEPTHRPTFQQICSFLQEQAQEDRRERDYTNLPSSSRSGGSGSSSSELEEESSSEHLTCCEQGDIAQPLLQPNNYQFC

>NP_000748.3

MTAPGAAGRCPPTTWLGSLLLLVCLLASRSITEEVSEYCSHMIGSGHLQSLQRLIDSQMETSCQITFEFVDQEQLKDPVCYLKKAFLLVQDIMEDTMRFRDNTPNAIAIVQLQELSLRLKSCFTKDYEEHDKACVRTFYETPLQLLEKVKNVFNETKNLLDKDWNIFSKNCNNSFAECSSQDVVTKPDCNCLYPKAIPSSDPASVSPHQPLAPSMAPVAGLTWEDSEGTEGSSLLPGEQPLHTVDPGSAKQRPPRSTCQSFEPPETPVVKDSTIGGSPQPRPSVGAFNPGMEDILDSAMGTNWVPEEASGEASEIPVPQGTELSPSRPGGGSMQTEPARPSNFLSASSPLPASAKGQQPADVTGTALPRVGPVRPTGQDWNHTPQKTDHPSALLRDPPEPGSPRISSLRPQGLSNPSTLSAQPQLSRSHSSGSVLPLGELEGRRSTRDRRSPAEPEGGPASEGAARPLPRFNSVPLTDTGHERQSEGSSSPQLQESVFHLLVPSVILVLLAVGGLLFYRWRRRSHQEPQRADSPLEQPEGSPLTQDDRQVELPV

151 NP_002458.2 NP_612482.2

>NP_002458.2

MDFFRVVENQQPPATMPLNVSFTNRNYDLDYDSVQPYFYCDEEENFYQQQQQSELQPPAPSEDIWKKFELLPTPPLSPSRRSGLCSPSYVAVTPFSLRGDNDGGGGSFSTADQLEMVTELLGGDMVNQSFICDPDDETFIKNIIIQDCMWSGFSAAAKLVSEKLASYQAARKDSGSPNPARGHSVCSTSSLYLQDLSAAASECIDPSVVFPYPLNDSSSPKSCASQDSSAFSPSSDSLLSSTESSPQGSPEPLVLHEETPPTTSSDSEEEQEDEEEIDVVSVEKRQAPGKRSESGSPSAGGHSKPPHSPLVLKRCHVSTHQHNYAAPPSTRKDYPAAKRVKLDSVRVLRQISNNRKCTSPRSSDTEENVKRRTHNVLERQRRNELKRSFFALRDQIPELENNEKAPKVVILKKATAYILSVQAEEQKLISEEDLLRKRREQLKHKLEQLRNSCA

>NP_612482.2

MSDQDHSMDEMTAVVKIEKGVGGNNGGNGNGGGAFSQARSSSTGSSSSTGGGGQESQPSPLALLAATCSRIESPNENSNNSQGPSQSGGTGELDLTATQLSQGANGWQIISSSSGATPTSKEQSGSSTNGSNGSESSKNRTVSGGQYVVAAAPNLQNQQVLTGLPGVMPNIQYQVIPQFQTVDGQQLQFAATGAQVQQDGSGQIQIIPGANQQIITNRGSGGNIIAAMPNLLQQAVPLQGLANNVLSGQTQYVTNVPVALNGNITLLPVNSVSAATLTPSSQAVTISSSGSQESGSQPVTSGTTISSASLVSSQASSSSFFTNANSYSTTTTTSNMGIMNFTTSGSSGTNSQGQTPQRVSGLQGSDALNIQQNQTSGGSLQAGQQKEGEQNQQTQQQQILIQPQLVQGGQALQALQAAPLSGQTFTTQAISQETLQNLQLQAVPNSGPIIIRTPTVGPNGQVSWQTLQLQNLQVQNPQAQTITLAPMQGVSLGQTSSSNTTLTPIASAASIPAGTVTVNAAQLSSMPGLQTINLSALGTSGIQVHPIQGLPLAIANAPGDHGAQLGLHGAGGDGIHDDTAGGEEGENSPDAQPQAGRRTRREACTCPYCKDSEGRGSGDPGKKKQHICHIQGCGKVYGKTSHLRAHLRWHTGERPFMCTWSYCGKRFTRSDELQRHKRTHTGEKKFACPECPKRFMRSDHLSKHIKTHQNKKGGPGVALSVGTLPLDSGAGSEGSGTATPSALITTNMVAMEAICPEGIARLANSGINVMQVADLQSINISGNGF

152 NP_596869.3 NP_002464.1

>NP_596869.3

MTTQAPTFTQPLQSVVVLEGSTATFEAHISGFPVPEVSWFRDGQVISTSTLPGVQISFSDGRAKLTIPAVTKANSGRYSLKATNGSGQATSTAELLVKAETAPPNFVQRLQSMTVRQGSQVRLQVRVTGIPTPVVKFYRDGAEIQSSLDFQISQEGDLYSLLIAEAYPEDSGTYSVNATNSVGRATSTAELLVQGEEEVPAKKTKTIVSTAQISESRQTRIEKKIEAHFDARSIATVEMVIDGAAGQQLPHKTPPRIPPKPKSRSPTPPSIAAKAQLARQQSPSPIRHSPSPVRHVRAPTPSPVRSVSPAARISTSPIRSVRSPLLMRKTQASTVATGPEVPPPWKQEGYVASSSEAEMRETTLTTSTQIRTEERWEGRYGVQEQVTISGAAGAAASVSASASYAAEAVATGAKEVKQDADKSAAVATVVAAVDMARVREPVISAVEQTAQRTTTTAVHIQPAQEQVRKEAEKTAVTKVVVAADKAKEQELKSRTKEVITTKQEQMHVTHEQIRKETEKTFVPKVVISAAKAKEQETRISEEITKKQKQVTQEAIRQETEITAASMVVVATAKSTKLETVPGAQEETTTQQDQMHLSYEKIMKETRKTVVPKVIVATPKVKEQDLVSRGREGITTKREQVQITQEKMRKEAEKTALSTIAVATAKAKEQETILRTRETMATRQEQIQVTHGKVDVGKKAEAVATVVAAVDQARVREPREPGHLEESYAQQTTLEYGYKERISAAKVAEPPQRPASEPHVVPKAVKPRVIQAPSETHIKTTDQKGMHISSQIKKTTDLTTERLVHVDKRPRTASPHFTVSKISVPKTEHGYEASIAGSAIATLQKELSATSSAQKITKSVKAPTVKPSETRVRAEPTPLPQFPFADTPDTYKSEAGVEVKKEVGVSITGTTVREERFEVLHGREAKVTETARVPAPVEIPVTPPTLVSGLKNVTVIEGESVTLECHISGYPSPTVTWYREDYQIESSIDFQITFQSGIARLMIREAFAEDSGRFTCSAVNEAGTVSTSCYLAVQVSEEFEKETTAVTEKFTTEEKRFVESRDVVMTDTSLTEEQAGPGEPAAPYFITKPVVQKLVEGGSVVFGCQVGGNPKPHVYWKKSGVPLTTGYRYKVSYNKQTGECKLVISMTFADDAGEYTIVVRNKHGETSASASLLEEADYELLMKSQQEMLYQTQVTAFVQEPKVGETAPGFVYSEYEKEYEKEQALIRKKMAKDTVVVRTYVEDQEFHISSFEERLIKEIEYRIIKTTLEELLEEDGEEKMAVDISESEAVESGFDSRIKNYRILEGMGVTFHCKMSGYPLPKIAWYKDGKRIKHGERYQMDFLQDGRASLRIPVVLPEDEGIYTAFASNIKGNAICSGKLYVEPAAPLGAPTYIPTLEPVSRIRSLSPRSVSRSPIRMSPARMSPARMSPARMSPARMSPGRRLEETDESQLERLYKPVFVLKPVSFKCLEGQTARFDLKVVGRPMPETFWFHDGQQIVNDYTHKVVIKEDGTQSLIIVPATPSDSGEWTVVAQNRAGRSSISVILTVEAVEHQVKPMFVEKLKNVNIKEGSRLEMKVRATGNPNPDIVWLKNSDIIVPHKYPKIRIEGTKGEAALKIDSTVSQDSAWYTATAINKAGRDTTRCKVNVEVEFAEPEPERKLIIPRGTYRAKEIAAPELEPLHLRYGQEQWEEGDLYDKEKQQKPFFKKKLTSLRLKRFGPAHFECRLTPIGDPTMVVEWLHDGKPLEAANRLRMINEFGYCSLDYGVAYSRDSGIITCRATNKYGTDHTSATLIVKDEKSLVEESQLPEGRKGLQRIEELERMAHEGALTGVTTDQKEKQKPDIVLYPEPVRVLEGETARFRCRVTGYPQPKVNWYLNGQLIRKSKRFRVRYDGIHYLDIVDCKSYDTGEVKVTAENPEGVIEHKVKLEIQQREDFRSVLRRAPEPRPEFHVHEPGKLQFEVQKVDRPVDTTETKEVVKLKRAERITHEKVPEESEELRSKFKRRTEEGYYEAITAVELKSRKKDESYEELLRKTKDELLHWTKELTEEEKKALAEEGKITIPTFKPDKIELSPSMEAPKIFERIQSQTVGQGSDAHFRVRVVGKPDPECEWYKNGVKIERSDRIYWYWPEDNVCELVIRDVTAEDSASIMVKAINIAGETSSHAFLLVQAKQLITFTQELQDVVAKEKDTMATFECETSEPFVKVKWYKDGMEVHEGDKYRMHSDRKVHFLSILTIDTSDAEDYSCVLVEDENVKTTAKLIVEGAVVEFVKELQDIEVPESYSGELECIVSPENIEGKWYHNDVELKSNGKYTITSRRGRQNLTVKDVTKEDQGEYSFVIDGKKTTCKLKMKPRPIAILQGLSDQKVCEGDIVQLEVKVSLESVEGVWMKDGQEVQPSDRVHIVIDKQSHMLLIEDMTKEDAGNYSFTIPALGLSTSGRVSVYSVDVITPLKDVNVIEGTKAVLECKVSVPDVTSVKWYLNDEQIKPDDRVQAIVKGTKQRLVINRTHASDEGPYKLIVGRVETNCNLSVEKIKIIRGLRDLTCTETQNVVFEVELSHSGIDVLWNFKDKEIKPSSKYKIEAHGKIYKLTVLNMMKDDEGKYTFYAGENMTSGKLTVAGGAISKPLTDQTVAESQEAVFECEVANPDSKGEWLRDGKHLPLTNNIRSESDGHKRRLIIAATKLDDIGEYTYKVATSKTSAKLKVEAVKIKKTLKNLTVTETQDAVFTVELTHPNVKGVQWIKNGVVLESNEKYAISVKGTIYSLRIKNCAIVDESVYGFRLGRLGASARLHVETVKIIKKPKDVTALENATVAFEVSVSHDTVPVKWFHKSVEIKPSDKHRLVSERKVHKLMLQNISPSDAGEYTAVVGQLECKAKLFVETLHITKTMKNIEVPETKTASFECEVSHFNVPSMWLKNGVEIEMSEKFKIVVQGKLHQLIIMNTSTEDSAEYTFVCGNDQVSATLTVTPIMITSMLKDINAEEKDTITFEVTVNYEGISYKWLKNGVEIKSTDKCQMRTKKLTHSLNIRNVHFGDAADYTFVAGKATSTATLYVEARHIEFRKHIKDIKVLEKKRAMFECEVSEPDITVQWMKDDQELQITDRIKIQKEKYVHRLLIPSTRMSDAGKYTVVAGGNVSTAKLFVEGRDVRIRSIKKEVQVIEKQRAVVEFEVNEDDVDAHWYKDGIEINFQVQERHKYVVERRIHRMFISETRQSDAGEYTFVAGRNRSSVTLYVNAPEPPQVLQELQPVTVQSGKPARFCAVISGRPQPKISWYKEEQLLSTGFKCKFLHDGQEYTLLLIEAFPEDAAVYTCEAKNDYGVATTSASLSVEVPEVVSPDQEMPVYPPAIITPLQDTVTSEGQPARFQCRVSGTDLKVSWYSKDKKIKPSRFFRMTQFEDTYQLEIAEAYPEDEGTYTFVASNAVGQVSSTANLSLEAPESILHERIEQEIEMEMKAAPVIKRKIEPLEVALGHLAKFTCEIQSAPNVRFQWFKAGREIYESDKCSIRSSKYISSLEILRTQVVDCGEYTCKASNEYGSVSCTATLTVTEAYPPTFLSRPKSLTTFVGKAAKFICTVTGTPVIETIWQKDGAALSPSPNWRISDAENKHILELSNLTIQDRGVYSCKASNKFGADICQAELIIIDKPHFIKELEPVQSAINKKVHLECQVDEDRKVTVTWSKDGQKLPPGKDYKICFEDKIATLEIPLAKLKDSGTYVCTASNEAGSSSCSATVTVREPPSFVKKVDPSYLMLPGESARLHCKLKGSPVIQVTWFKNNKELSESNTVRMYFVNSEAILDITDVKVEDSGSYSCEAVNDVGSDSCSTEIVIKEPPSFIKTLEPADIVRGTNALLQCEVSGTGPFEISWFKDKKQIRSSKKYRLFSQKSLVCLEIFSFNSADVGEYECVVANEVGKCGCMATHLLKEPPTFVKKVDDLIALGGQTVTLQAAVRGSEPISVTWMKGQEVIREDGKIKMSFSNGVAVLIIPDVQISFGGKYTCLAENEAGSQTSVGELIVKEPAKIIERAELIQVTAGDPATLEYTVAGTPELKPKWYKDGRPLVASKKYRISFKNNVAQLKFYSAELHDSGQYTFEISNEVGSSSCETTFTVLDRDIAPFFTKPLRNVDSVVNGTCRLDCKIAGSLPMRVSWFKDGKEIAASDRYRIAFVEGTASLEIIRVDMNDAGNFTCRATNSVGSKDSSGALIVQEPPSFVTKPGSKDVLPGSAVCLKSTFQGSTPLTIRWFKGNKELVSGGSCYITKEALESSLELYLVKTSDSGTYTCKVSNVAGGVECSANLFVKEPATFVEKLEPSQLLKKGDATQLACKVTGTPPIKITWFANDREIKESSKHRMSFVESTAVLRLTDVGIEDSGEYMCEAQNEAGSDHCSSIVIVKESPYFTKEFKPIEVLKEYDVMLLAEVAGTPPFEITWFKDNTILRSGRKYKTFIQDHLVSLQILKFVAADAGEYQCRVTNEVGSSICSARVTLREPPSFIKKIESTSSLRGGTAAFQATLKGSLPITVTWLKDSDEITEDDNIRMTFENNVASLYLSGIEVKHDGKYVCQAKNDAGIQRCSALLSVKEPATITEEAVSIDVTQGDPATLQVKFSGTKEITAKWFKDGQELTLGSKYKISVTDTVSILKIISTEKKDSGEYTFEVQNDVGRSSCKARINVLDLIIPPSFTKKLKKMDSIKGSFIDLECIVAGSHPISIQWFKDDQEISASEKYKFSFHDNTAFLEISQLEGTDSGTYTCSATNKAGHNQCSGHLTVKEPPYFVEKPQSQDVNPNTRVQLKALVGGTAPMTIKWFKDNKELHSGAARSVWKDDTSTSLELFAAKATDSGTYICQLSNDVGTATSKATLFVKEPPQFIKKPSPVLVLRNGQSTTFECQITGTPKIRVSWYLDGNEITAIQKHGISFIDGLATFQISGARVENSGTYVCEARNDAGTASCSIELKVKEPPTFIRELKPVEVVKYSDVELECEVTGTPPFEVTWLKNNREIRSSKKYTLTDRVSVFNLHITKCDPSDTGEYQCIVSNEGGSCSCSTRVALKEPPSFIKKIENTTTVLKSSATFQSTVAGSPPISITWLKDDQILDEDDNVYISFVDSVATLQIRSVDNGHSGRYTCQAKNESGVERCYAFLLVQEPAQIVEKAKSVDVTEKDPMTLECVVAGTPELKVKWLKDGKQIVPSRYFSMSFENNVASFRIQSVMKQDSGQYTFKVENDFGSSSCDAYLRVLDQNIPPSFTKKLTKMDKVLGSSIHMECKVSGSLPISAQWFKDGKEISTSAKYRLVCHERSVSLEVNNLELEDTANYTCKVSNVAGDDACSGILTVKEPPSFLVKPGRQQAIPDSTVEFKAILKGTPPFKIKWFKDDVELVSGPKCFIGLEGSTSFLNLYSVDASKTGQYTCHVTNDVGSDSCTTMLLVTEPPKFVKKLEASKIVKAGDSSRLECKIAGSPEIRVVWFRNEHELPASDKYRMTFIDSVAVIQMNNLSTEDSGDFICEAQNPAGSTSCSTKVIVKEPPVFSSFPPIVETLKNAEVSLECELSGTPPFEVVWYKDKRQLRSSKKYKIASKNFHTSIHILNVDTSDIGEYHCKAQNEVGSDTCVCTVKLKEPPRFVSKLNSLTVVAGEPAELQASIEGAQPIFVQWLKEKEEVIRESENIRITFVENVATLQFAKAEPANAGKYICQIKNDGGMRENMATLMVLEPAVIVEKAGPMTVTVGETCTLECKVAGTPELSVEWYKDGKLLTSSQKHKFSFYNKISSLRILSVERQDAGTYTFQVQNNVGKSSCTAVVDVSDRAVPPSFTRRLKNTGGVLGASCILECKVAGSSPISVAWFHEKTKIVSGAKYQTTFSDNVCTLQLNSLDSSDMGNYTCVAANVAGSDECRAVLTVQEPPSFVKEPEPLEVLPGKNVTFTSVIRGTPPFKVNWFRGARELVKGDRCNIYFEDTVAELELFNIDISQSGEYTCVVSNNAGQASCTTRLFVKEPAAFLKRLSDHSVEPGKSIILESTYTGTLPISVTWKKDGFNITTSEKCNIVTTEKTCILEILNSTKRDAGQYSCEIENEAGRDVCGALVSTLEPPYFVTELEPLEAAVGDSVSLQCQVAGTPEITVSWYKGDTKLRPTPEYRTYFTNNVATLVFNKVNINDSGEYTCKAENSIGTASSKTVFRIQERQLPPSFARQLKDIEQTVGLPVTLTCRLNGSAPIQVCWYRDGVLLRDDENLQTSFVDNVATLKILQTDLSHSGQYSCSASNPLGTASSSARLTAREPKKSPFFDIKPVSIDVIAGESADFECHVTGAQPMRITWSKDNKEIRPGGNYTITCVGNTPHLRILKVGKGDSGQYTCQATNDVGKDMCSAQLSVKEPPKFVKKLEASKVAKQGESIQLECKISGSPEIKVSWFRNDSELHESWKYNMSFINSVALLTINEASAEDSGDYICEAHNGVGDASCSTALTVKAPPVFTQKPSPVGALKGSDVILQCEISGTPPFEVVWVKDRKQVRNSKKFKITSKHFDTSLHILNLEASDVGEYHCKATNEVGSDTCSCSVKFKEPPRFVKKLSDTSTLIGDAVELRAIVEGFQPISVVWLKDRGEVIRESENTRISFIDNIATLQLGSPEASNSGKYICQIKNDAGMRECSAVLTVLEPARIIEKPEPMTVTTGNPFALECVVTGTPELSAKWFKDGRELSADSKHHITFINKVASLKIPCAEMSDKGLYSFEVKNSVGKSNCTVSVHVSDRIVPPSFIRKLKDVNAILGASVVLECRVSGSAPISVGWFQDGNEIVSGPKCQSSFSENVCTLNLSLLEPSDTGIYTCVAANVAGSDECSAVLTVQEPPSFEQTPDSVEVLPGMSLTFTSVIRGTPPFKVKWFKGSRELVPGESCNISLEDFVTELELFEVQPLESGDYSCLVTNDAGSASCTTHLFVKEPATFVKRLADFSVETGSPIVLEATYTGTPPISVSWIKDEYLISQSERCSITMTEKSTILEILESTIEDYAQYSCLIENEAGQDICEALVSVLEPPYFIEPLEHVEAVIGEPATLQCKVDGTPEIRISWYKEHTKLRSAPAYKMQFKNNVASLVINKVDHSDVGEYSCKADNSVGAVASSAVLVIKARKLPPFFARKLKDVHETLGFPVAFECRINGSEPLQVSWYKDGVLLKDDANLQTSFVHNVATLQILQTDQSHIGQYNCSASNPLGTASSSAKLILSEHEVPPFFDLKPVSVDLALGESGTFKCHVTGTAPIKITWAKDNREIRPGGNYKMTLVENTATLTVLKVGKGDAGQYTCYASNIAGKDSCSAQLGVQEPPRFIKKLEPSRIVKQDEFTRYECKIGGSPEIKVLWYKDETEIQESSKFRMSFVDSVAVLEMHNLSVEDSGDYTCEAHNAAGSASSSTSLKVKEPPIFRKKPHPIETLKGADVHLECELQGTPPFHVSWYKDKRELRSGKKYKIMSENFLTSIHILNVDAADIGEYQCKATNDVGSDTCVGSIALKAPPRFVKKLSDISTVVGKEVQLQTTIEGAEPISVVWFKDKGEIVRESDNIWISYSENIATLQFSRVEPANAGKYTCQIKNDAGMQECFATLSVLEPATIVEKPESIKVTTGDTCTLECTVAGTPELSTKWFKDGKELTSDNKYKISFFNKVSGLKIINVAPSDSGVYSFEVQNPVGKDSCTASLQVSDRTVPPSFTRKLKETNGLSGSSVVMECKVYGSPPISVSWFHEGNEISSGRKYQTTLTDNTCALTVNMLEESDSGDYTCIATNMAGSDECSAPLTVREPPSFVQKPDPMDVLTGTNVTFTSIVKGTPPFSVSWFKGSSELVPGDRCNVSLEDSVAELELFDVDTSQSGEYTCIVSNEAGKASCTTHLYIKAPAKFVKRLNDYSIEKGKPLILEGTFTGTPPISVTWKKNGINVTPSQRCNITTTEKSAILEIPSSTVEDAGQYNCYIENASGKDSCSAQILILEPPYFVKQLEPVKVSVGDSASLQCQLAGTPEIGVSWYKGDTKLRPTTTYKMHFRNNVATLVFNQVDINDSGEYICKAENSVGEVSASTFLTVQEQKLPPSFSRQLRDVQETVGLPVVFDCAISGSEPISVSWYKDGKPLKDSPNVQTSFLDNTATLNIFKTDRSLAGQYSCTATNPIGSASSSARLILTEGKNPPFFDIRLAPVDAVVGESADFECHVTGTQPIKVSWAKDSREIRSGGKYQISYLENSAHLTVLKVDKGDSGQYTCYAVNEVGKDSCTAQLNIKERLIPPSFTKRLSETVEETEGNSFKLEGRVAGSQPITVAWYKNNIEIQPTSNCEITFKNNTLVLQVRKAGMNDAGLYTCKVSNDAGSALCTSSIVIKEPKKPPVFDQHLTPVTVSEGEYVQLSCHVQGSEPIRIQWLKAGREIKPSDRCSFSFASGTAVLELRDVAKADSGDYVCKASNVAGSDTTKSKVTIKDKPAVAPATKKAAVDGRLFFVSEPQSIRVVEKTTATFIAKVGGDPIPNVKWTKGKWRQLNQGGRVFIHQKGDEAKLEIRDTTKTDSGLYRCVAFNEHGEIESNVNLQVDERKKQEKIEGDLRAMLKKTPILKKGAGEEEEIDIMELLKNVDPKEYEKYARMYGITDFRGLLQAFELLKQSQEEETHRLEIEEIERSERDEKEFEELVSFIQQRLSQTEPVTLIKDIENQTVLKDNDAVFEIDIKINYPEIKLSWYKGTEKLEPSDKFEISIDGDRHTLRVKNCQLKDQGNYRLVCGPHIASAKLTVIEPAWERHLQDVTLKEGQTCTMTCQFSVPNVKSEWFRNGRILKPQGRHKTEVEHKVHKLTIADVRAEDQGQYTCKYEDLETSAELRIEAEPIQFTKRIQNIVVSEHQSATFECEVSFDDAIVTWYKGPTELTESQKYNFRNDGRCHYMTIHNVTPDDEGVYSVIARLEPRGEARSTAELYLTTKEIKLELKPPDIPDSRVPIPTMPIRAVPPEEIPPVVAPPIPLLLPTPEEKKPPPKRIEVTKKAVKKDAKKVVAKPKEMTPREEIVKKPPPPTTLIPAKAPEIIDVSSKAEEVKIMTITRKKEVQKEKEAVYEKKQAVHKEKRVFIESFEEPYDELEVEPYTEPFEQPYYEEPDEDYEEIKVEAKKEVHEEWEEDFEEGQEYYEREEGYDEGEEEWEEAYQEREVIQVQKEVYEESHERKVPAKVPEKKAPPPPKVIKKPVIEKIEKTSRRMEEEKVQVTKVPEVSKKIVPQKPSRTPVQEEVIEVKVPAVHTKKMVISEEKMFFASHTEEEVSVTVPEVQKEIVTEEKIHVAISKRVEPPPKVPELPEKPAPEEVAPVPIPKKVEPPAPKVPEVPKKPVPEEKKPVPVPKKEPAAPPKVPEVPKKPVPEEKIPVPVAKKKEAPPAKVPEVQKGVVTEEKITIVTQREESPPPAVPEIPKKKVPEERKPVPRKEEEVPPPPKVPALPKKPVPEEKVAVPVPVAKKAPPPRAEVSKKTVVEEKRFVAEEKLSFAVPQRVEVTRHEVSAEEEWSYSEEEEGVSISVYREEEREEEEEAEVTEYEVMEEPEEYVVEEKLHIISKRVEAEPAEVTERQEKKIVLKPKIPAKIEEPPPAKVPEAPKKIVPEKKVPAPVPKKEKVPPPKVPEEPKKPVPEKKVPPKVIKMEEPLPAKVTERHMQITQEEKVLVAVTKKEAPPKARVPEEPKRAVPEEKVLKLKPKREEEPPAKVTEFRKRVVKEEKVSIEAPKREPQPIKEVTIMEEKERAYTLEEEAVSVQREEEYEEYEEYDYKEFEEYEPTEEYDQYEEYEEREYERYEEHEEYITEPEKPIPVKPVPEEPVPTKPKAPPAKVLKKAVPEEKVPVPIPKKLKPPPPKVPEEPKKVFEEKIRISITKREKEQVTEPAAKVPMKPKRVVAEEKVPVPRKEVAPPVRVPEVPKELEPEEVAFEEEVVTHVEEYLVEEEEEYIHEEEEFITEEEVVPVIPVKVPEVPRKPVPEEKKPVPVPKKKEAPPAKVPEVPKKPEEKVPVLIPKKEKPPPAKVPEVPKKPVPEEKVPVPVPKKVEAPPAKVPEVPKKPVPEKKVPVPAPKKVEAPPAKVPEVPKKLIPEEKKPTPVPKKVEAPPPKVPKKREPVPVPVALPQEEEVLFEEEIVPEEEVLPEEEEVLPEEEEVLPEEEEVLPEEEEIPPEEEEVPPEEEYVPEEEEFVPEEEVLPEVKPKVPVPAPVPEIKKKVTEKKVVIPKKEEAPPAKVPEVPKKVEEKRIILPKEEEVLPVEVTEEPEEEPISEEEIPEEPPSIEEVEEVAPPRVPEVIKKAVPEAPTPVPKKVEAPPAKVSKKIPEEKVPVPVQKKEAPPAKVPEVPKKVPEKKVLVPKKEAVPPAKGRTVLEEKVSVAFRQEVVVKERLELEVVEAEVEEIPEEEEFHEVEEYFEEGEFHEVEEFIKLEQHRVEEEHRVEKVHRVIEVFEAEEVEVFEKPKAPPKGPEISEKIIPPKKPPTKVVPRKEPPAKVPEVPKKIVVEEKVRVPEEPRVPPTKVPEVLPPKEVVPEKKVPVPPAKKPEAPPPKVPEAPKEVVPEKKVPVPPPKKPEVPPTKVPEVPKAAVPEKKVPEAIPPKPESPPPEVPEAPKEVVPEKKVPAAPPKKPEVTPVKVPEAPKEVVPEKKVPVPPPKKPEVPPTKVPEVPKVAVPEKKVPEAIPPKPESPPPEVFEEPEEVALEEPPAEVVEEPEPAAPPQVTVPPKKPVPEKKAPAVVAKKPELPPVKVPEVPKEVVPEKKVPLVVPKKPEAPPAKVPEVPKEVVPEKKVAVPKKPEVPPAKVPEVPKKPVLEEKPAVPVPERAESPPPEVYEEPEEIAPEEEIAPEEEKPVPVAEEEEPEVPPPAVPEEPKKIIPEKKVPVIKKPEAPPPKEPEPEKVIEKPKLKPRPPPPPPAPPKEDVKEKIFQLKAIPKKKVPEKPQVPEKVELTPLKVPGGEKKVRKLLPERKPEPKEEVVLKSVLRKRPEEEEPKVEPKKLEKVKKPAVPEPPPPKPVEEVEVPTVTKRERKIPEPTKVPEIKPAIPLPAPEPKPKPEAEVKTIKPPPVEPEPTPIAAPVTVPVVGKKAEAKAPKEEAAKPKGPIKGVPKKTPSPIEAERRKLRPGSGGEKPPDEAPFTYQLKAVPLKFVKEIKDIILTESEFVGSSAIFECLVSPSTAITTWMKDGSNIRESPKHRFIADGKDRKLHIIDVQLSDAGEYTCVLRLGNKEKTSTAKLVVEELPVRFVKTLEEEVTVVKGQPLYLSCELNKERDVVWRKDGKIVVEKPGRIVPGVIGLMRALTINDADDTDAGTYTVTVENANNLECSSCVKVVEVIRDWLVKPIRDQHVKPKGTAIFACDIAKDTPNIKWFKGYDEIPAEPNDKTEILRDGNHLYLKIKNAMPEDIAEYAVEIEGKRYPAKLTLGEREVELLKPIEDVTIYEKESASFDAEISEADIPGQWKLKGELLRPSPTCEIKAEGGKRFLTLHKVKLDQAGEVLYQALNAITTAILTVKEIELDFAVPLKDVTVPERRQARFECVLTREANVIWSKGPDIIKSSDKFDIIADGKKHILVINDSQFDDEGVYTAEVEGKKTSARLFVTGIRLKFMSPLEDQTVKEGETATFVCELSHEKMHVVWFKNDAKLHTSRTVLISSEGKTHKLEMKEVTLDDISQIKAQVKELSSTAQLKVLEADPYFTVKLHDKTAVEKDEITLKCEVSKDVPVKWFKDGEEIVPSPKYSIKADGLRRILKIKKADLKDKGEYVCDCGTDKTKANVTVEARLIKVEKPLYGVEVFVGETAHFEIELSEPDVHGQWKLKGQPLTASPDCEIIEDGKKHILILHNCQLGMTGEVSFQAANAKSAANLKVKELPLIFITPLSDVKVFEKDEAKFECEVSREPKTFRWLKGTQEITGDDRFELIKDGTKHSMVIKSAAFEDEAKYMFEAEDKHTSGKLIIEGIRLKFLTPLKDVTAKEKESAVFTVELSHDNIRVKWFKNDQRLHTTRSVSMQDEGKTHSITFKDLSIDDTSQIRVEAMGMSSEAKLTVLEGDPYFTGKLQDYTGVEKDEVILQCEISKADAPVKWFKDGKEIKPSKNAVIKADGKKRMLILKKALKSDIGQYTCDCGTDKTSGKLDIEDREIKLVRPLHSVEVMETETARFETEISEDDIHANWKLKGEALLQTPDCEIKEEGKIHSLVLHNCRLDQTGGVDFQAANVKSSAHLRVKPRVIGLLRPLKDVTVTAGETATFDCELSYEDIPVEWYLKGKKLEPSDKVVPRSEGKVHTLTLRDVKLEDAGEVQLTAKDFKTHANLFVKEPPVEFTKPLEDQTVEEGATAVLECEVSRENAKVKWFKNGTEILKSKKYEIVADGRVRKLVIHDCTPEDIKTYTCDAKDFKTSCNLNVVPPHVEFLRPLTDLQVREKEMARFECELSRENAKVKWFKDGAEIKKGKKYDIISKGAVRILVINKCLLDDEAEYSCEVRTARTSGMLTVLEEEAVFTKNLANIEVSETDTIKLVCEVSKPGAEVIWYKGDEEIIETGRYEILTEGRKRILVIQNAHLEDAGNYNCRLPSSRTDGKVKVHELAAEFISKPQNLEILEGEKAEFVCSISKESFPVQWKRDDKTLESGDKYDVIADGKKRVLVVKDATLQDMGTYVVMVGAARAAAHLTVIEKLRIVVPLKDTRVKEQQEVVFNCEVNTEGAKAKWFRNEEAIFDSSKYIILQKDLVYTLRIRDAHLDDQANYNVSLTNHRGENVKSAANLIVEEEDLRIVEPLKDIETMEKKSVTFWCKVNRLNVTLKWTKNGEEVPFDNRVSYRVDKYKHMLTIKDCGFPDEGEYIVTAGQDKSVAELLIIEAPTEFVEHLEDQTVTEFDDAVFSCQLSREKANVKWYRNGREIKEGKKYKFEKDGSIHRLIIKDCRLDDECEYACGVEDRKSRARLFVEEIPVEIIRPPQDILEAPGADVVFLAELNKDKVEVQWLRNNMVVVQGDKHQMMSEGKIHRLQICDIKPRDQGEYRFIAKDKEARAKLELAAAPKIKTADQDLVVDVGKPLTMVVPYDAYPKAEAEWFKENEPLSTKTIDTTAEQTSFRILEAKKGDKGRYKIVLQNKHGKAEGFINLKVIDVPGPVRNLEVTETFDGEVSLAWEEPLTDGGSKIIGYVVERRDIKRKTWVLATDRAESCEFTVTGLQKGGVEYLFRVSARNRVGTGEPVETDNPVEARSKYDVPGPPLNVTITDVNRFGVSLTWEPPEYDGGAEITNYVIELRDKTSIRWDTAMTVRAEDLSATVTDVVEGQEYSFRVRAQNRIGVGKPSAATPFVKVADPIERPSPPVNLTSSDQTQSSVQLKWEPPLKDGGSPILGYIIERCEEGKDNWIRCNMKLVPELTYKVTGLEKGNKYLYRVSAENKAGVSDPSEILGPLTADDAFVEPTMDLSAFKDGLEVIVPNPITILVPSTGYPRPTATWCFGDKVLETGDRVKMKTLSAYAELVISPSERSDKGIYTLKLENRVKTISGEIDVNVIARPSAPKELKFGDITKDSVHLTWEPPDDDGGSPLTGYVVEKREVSRKTWTKVMDFVTDLEFTVPDLVQGKEYLFKVCARNKCGPGEPAYVDEPVNMSTPATVPDPPENVKWRDRTANSIFLTWDPPKNDGGSRIKGYIVERCPRGSDKWVACGEPVAETKMEVTGLEEGKWYAYRVKALNRQGASKPSRPTEEIQAVDTQEAPEIFLDVKLLAGLTVKAGTKIELPATVTGKPEPKITWTKADMILKQDKRITIENVPKKSTVTIVDSKRSDTGTYIIEAVNVCGRATAVVEVNVLDKPGPPAAFDITDVTNESCLLTWNPPRDDGGSKITNYVVERRATDSEVWHKLSSTVKDTNFKATKLIPNKEYIFRVAAENMYGVGEPVQASPITAKYQFDPPGPPTRLEPSDITKDAVTLTWCEPDDDGGSPITGYWVERLDPDTDKWVRCNKMPVKDTTYRVKGLTNKKKYRFRVLAENLAGPGKPSKSTEPILIKDPIDPPWPPGKPTVKDVGKTSVRLNWTKPEHDGGAKIESYVIEMLKTGTDEWVRVAEGVPTTQHLLPGLMEGQEYSFRVRAVNKAGESEPSEPSDPVLCREKLYPPSPPRWLEVINITKNTADLKWTVPEKDGGSPITNYIVEKRDVRRKGWQTVDTTVKDTKCTVTPLTEGSLYVFRVAAENAIGQSDYTEIEDSVLAKDTFTTPGPPYALAVVDVTKRHVDLKWEPPKNDGGRPIQRYVIEKKERLGTRWVKAGKTAGPDCNFRVTDVIEGTEVQFQVRAENEAGVGHPSEPTEILSIEDPTSPPSPPLDLHVTDAGRKHIAIAWKPPEKNGGSPIIGYHVEMCPVGTEKWMRVNSRPIKDLKFKVEEGVVPDKEYVLRVRAVNAIGVSEPSEISENVVAKDPDCKPTIDLETHDIIVIEGEKLSIPVPFRAVPVPTVSWHKDGKEVKASDRLTMKNDHISAHLEVPKSVRADAGIYTITLENKLGSATASINVKVIGLPGPCKDIKASDITKSSCKLTWEPPEFDGGTPILHYVLERREAGRRTYIPVMSGENKLSWTVKDLIPNGEYFFRVKAVNKVGGGEYIELKNPVIAQDPKQPPDPPVDVEVHNPTAEAMTITWKPPLYDGGSKIMGYIIEKIAKGEERWKRCNEHLVPILTYTAKGLEEGKEYQFRVRAENAAGISEPSRATPPTKAVDPIDAPKVILRTSLEVKRGDEIALDASISGSPYPTITWIKDENVIVPEEIKKRAAPLVRRRKGEVQEEEPFVLPLTQRLSIDNSKKGESQLRVRDSLRPDHGLYMIKVENDHGIAKAPCTVSVLDTPGPPINFVFEDIRKTSVLCKWEPPLDDGGSEIINYTLEKKDKTKPDSEWIVVTSTLRHCKYSVTKLIEGKEYLFRVRAENRFGPGPPCVSKPLVAKDPFGPPDAPDKPIVEDVTSNSMLVKWNEPKDNGSPILGYWLEKREVNSTHWSRVNKSLLNALKANVDGLLEGLTYVFRVCAENAAGPGKFSPPSDPKTAHDPISPPGPPIPRVTDTSSTTIELEWEPPAFNGGGEIVGYFVDKQLVGTNEWSRCTEKMIKVRQYTVKEIREGADYKLRVSAVNAAGEGPPGETQPVTVAEPQEPPAVELDVSVKGGIQIMAGKTLRIPAVVTGRPVPTKVWTKEEGELDKDRVVIDNVGTKSELIIKDALRKDHGRYVITATNSCGSKFAAARVEVFDVPGPVLDLKPVVTNRKMCLLNWSDPEDDGGSEITGFIIERKDAKMHTWRQPIETERSKCDITGLLEGQEYKFRVIAKNKFGCGPPVEIGPILAVDPLGPPTSPERLTYTERTKSTITLDWKEPRSNGGSPIQGYIIEKRRHDKPDFERVNKRLCPTTSFLVENLDEHQMYEFRVKAVNEIGESEPSLPLNVVIQDDEVPPTIKLRLSVRGDTIKVKAGEPVHIPADVTGLPMPKIEWSKNETVIEKPTDALQITKEEVSRSEAKTELSIPKAVREDKGTYTVTASNRLGSVFRNVHVEVYDRPSPPRNLAVTDIKAESCYLTWDAPLDNGGSEITHYVIDKRDASRKKAEWEEVTNTAVEKRYGIWKLIPNGQYEFRVRAVNKYGISDECKSDKVVIQDPYRLPGPPGKPKVLARTKGSMLVSWTPPLDNGGSPITGYWLEKREEGSPYWSRVSRAPITKVGLKGVEFNVPRLLEGVKYQFRAMAINAAGIGPPSEPSDPEVAGDPIFPPGPPSCPEVKDKTKSSISLGWKPPAKDGGSPIKGYIVEMQEEGTTDWKRVNEPDKLITTCECVVPNLKELRKYRFRVKAVNEAGESEPSDTTGEIPATDIQEEPEVFIDIGAQDCLVCKAGSQIRIPAVIKGRPTPKSSWEFDGKAKKAMKDGVHDIPEDAQLETAENSSVIIIPECKRSHTGKYSITAKNKAGQKTANCRVKVMDVPGPPKDLKVSDITRGSCRLSWKMPDDDGGDRIKGYVIEKRTIDGKAWTKVNPDCGSTTFVVPDLLSEQQYFFRVRAENRFGIGPPVETIQRTTARDPIYPPDPPIKLKIGLITKNTVHLSWKPPKNDGGSPVTHYIVECLAWDPTGTKKEAWRQCNKRDVEELQFTVEDLVEGGEYEFRVKAVNAAGVSKPSATVGPCDCQRPDMPPSIDLKEFMEVEEGTNVNIVAKIKGVPFPTLTWFKAPPKKPDNKEPVLYDTHVNKLVVDDTCTLVIPQSRRSDTGLYTITAVNNLGTASKEMRLNVLGRPGPPVGPIKFESVSADQMTLSWFPPKDDGGSKITNYVIEKREANRKTWVHVSSEPKECTYTIPKLLEGHEYVFRIMAQNKYGIGEPLDSEPETARNLFSVPGAPDKPTVSSVTRNSMTVNWEEPEYDGGSPVTGYWLEMKDTTSKRWKRVNRDPIKAMTLGVSYKVTGLIEGSDYQFRVYAINAAGVGPASLPSDPATARDPIAPPGPPFPKVTDWTKSSADLEWSPPLKDGGSKVTGYIVEYKEEGKEEWEKGKDKEVRGTKLVVTGLKEGAFYKFRVRAVNIAGIGEPGEVTDVIEMKDRLVSPDLQLDASVRDRIVVHAGGVIRIIAYVSGKPPPTVTWNMNERTLPQEATIETTAISSSMVIKNCQRSHQGVYSLLAKNEAGERKKTIIVDVLDVPGPVGTPFLAHNLTNESCKLTWFSPEDDGGSPITNYVIEKRESDRRAWTPVTYTVTRQNATVQGLIQGKAYFFRIAAENSIGMGPFVETSEALVIREPITVPERPEDLEVKEVTKNTVTLTWNPPKYDGGSEIINYVLESRLIGTEKFHKVTNDNLLSRKYTVKGLKEGDTYEYRVSAVNIVGQGKPSFCTKPITCKDELAPPTLHLDFRDKLTIRVGEAFALTGRYSGKPKPKVSWFKDEADVLEDDRTHIKTTPATLALEKIKAKRSDSGKYCVVVENSTGSRKGFCQVNVVDRPGPPVGPVSFDEVTKDYMVISWKPPLDDGGSKITNYIIEKKEVGKDVWMPVTSASAKTTCKVSKLLEGKDYIFRIHAENLYGISDPLVSDSMKAKDRFRVPDAPDQPIVTEVTKDSALVTWNKPHDGGKPITNYILEKRETMSKRWARVTKDPIHPYTKFRVPDLLEGCQYEFRVSAENEIGIGDPSPPSKPVFAKDPIAKPSPPVNPEAIDTTCNSVDLTWQPPRHDGGSKILGYIVEYQKVGDEEWRRANHTPESCPETKYKVTGLRDGQTYKFRVLAVNAAGESDPAHVPEPVLVKDRLEPPELILDANMAREQHIKVGDTLRLSAIIKGVPFPKVTWKKEDRDAPTKARIDVTPVGSKLEIRNAAHEDGGIYSLTVENPAGSKTVSVKVLVLDKPGPPRDLEVSEIRKDSCYLTWKEPLDDGGSVITNYVVERRDVASAQWSPLSATSKKKSHFAKHLNEGNQYLFRVAAENQYGRGPFVETPKPIKALDPLHPPGPPKDLHHVDVDKTEVSLVWNKPDRDGGSPITGYLVEYQEEGTQDWIKFKTVTNLECVVTGLQQGKTYRFRVKAENIVGLGLPDTTIPIECQEKLVPPSVELDVKLIEGLVVKAGTTVRFPAIIRGVPVPTAKWTTDGSEIKTDEHYTVETDNFSSVLTIKNCLRRDTGEYQITVSNAAGSKTVAVHLTVLDVPGPPTGPINILDVTPEHMTISWQPPKDDGGSPVINYIVEKQDTRKDTWGVVSSGSSKTKLKIPHLQKGCEYVFRVRAENKIGVGPPLDSTPTVAKHKFSPPSPPGKPVVTDITENAATVSWTLPKSDGGSPITGYYMERREVTGKWVRVNKTPIADLKFRVTGLYEGNTYEFRVFAENLAGLSKPSPSSDPIKACRPIKPPGPPINPKLKDKSRETADLVWTKPLSDGGSPILGYVVECQKPGTAQWNRINKDELIRQCAFRVPGLIEGNEYRFRIKAANIVGEGEPRELAESVIAKDILHPPEVELDVTCRDVITVRVGQTIRILARVKGRPEPDITWTKEGKVLVREKRVDLIQDLPRVELQIKEAVRADHGKYIISAKNSSGHAQGSAIVNVLDRPGPCQNLKVTNVTKENCTISWENPLDNGGSEITNFIVEYRKPNQKGWSIVASDVTKRLIKANLLANNEYYFRVCAENKVGVGPTIETKTPILAINPIDRPGEPENLHIADKGKTFVYLKWRRPDYDGGSPNLSYHVERRLKGSDDWERVHKGSIKETHYMVDRCVENQIYEFRVQTKNEGGESDWVKTEEVVVKEDLQKPVLDLKLSGVLTVKAGDTIRLEAGVRGKPFPEVAWTKDKDATDLTRSPRVKIDTRADSSKFSLTKAKRSDGGKYVVTATNTAGSFVAYATVNVLDKPGPVRNLKIVDVSSDRCTVCWDPPEDDGGCEIQNYILEKCETKRMVWSTYSATVLTPGTTVTRLIEGNEYIFRVRAENKIGTGPPTESKPVIAKTKYDKPGRPDPPEVTKVSKEEMTVVWNPPEYDGGKSITGYFLEKKEKHSTRWVPVNKSAIPERRMKVQNLLPDHEYQFRVKAENEIGIGEPSLPSRPVVAKDPIEPPGPPTNFRVVDTTKHSITLGWGKPVYDGGAPIIGYVVEMRPKIADASPDEGWKRCNAAAQLVRKEFTVTSLDENQEYEFRVCAQNQVGIGRPAELKEAIKPKEILEPPEIDLDASMRKLVIVRAGCPIRLFAIVRGRPAPKVTWRKVGIDNVVRKGQVDLVDTMAFLVIPNSTRDDSGKYSLTLVNPAGEKAVFVNVRVLDTPGPVSDLKVSDVTKTSCHVSWAPPENDGGSQVTHYIVEKREADRKTWSTVTPEVKKTSFHVTNLVPGNEYYFRVTAVNEYGPGVPTDVPKPVLASDPLSEPDPPRKLEVTEMTKNSATLAWLPPLRDGGAKIDGYITSYREEEQPADRWTEYSVVKDLSLVVTGLKEGKKYKFRVAARNAVGVSLPREAEGVYEAKEQLLPPKILMPEQITIKAGKKLRIEAHVYGKPHPTCKWKKGEDEVVTSSHLAVHKADSSSILIIKDVTRKDSGYYSLTAENSSGTDTQKIKVVVMDAPGPPQPPFDISDIDADACSLSWHIPLEDGGSNITNYIVEKCDVSRGDWVTALASVTKTSCRVGKLIPGQEYIFRVRAENRFGISEPLTSPKMVAQFPFGVPSEPKNARVTKVNKDCIFVAWDRPDSDGGSPIIGYLIERKERNSLLWVKANDTLVRSTEYPCAGLVEGLEYSFRIYALNKAGSSPPSKPTEYVTARMPVDPPGKPEVIDVTKSTVSLIWARPKHDGGSKIIGYFVEACKLPGDKWVRCNTAPHQIPQEEYTATGLEEKAQYQFRAIARTAVNISPPSEPSDPVTILAENVPPRIDLSVAMKSLLTVKAGTNVCLDATVFGKPMPTVSWKKDGTLLKPAEGIKMAMQRNLCTLELFSVNRKDSGDYTITAENSSGSKSATIKLKVLDKPGPPASVKINKMYSDRAMLSWEPPLEDGGSEITNYIVDKRETSRPNWAQVSATVPITSCSVEKLIEGHEYQFRICAENKYGVGDPVFTEPAIAKNPYDPPGRCDPPVISNITKDHMTVSWKPPADDGGSPITGYLLEKRETQAVNWTKVNRKPIIERTLKATGLQEGTEYEFRVTAINKAGPGKPSDASKAAYARDPQYPPGPPAFPKVYDTTRSSVSLSWGKPAYDGGSPIIGYLVEVKRADSDNWVRCNLPQNLQKTRFEVTGLMEDTQYQFRVYAVNKIGYSDPSDVPDKHYPKDILIPPEGELDADLRKTLILRAGVTMRLYVPVKGRPPPKITWSKPNVNLRDRIGLDIKSTDFDTFLRCENVNKYDAGKYILTLENSCGKKEYTIVVKVLDTPGPPVNVTVKEISKDSAYVTWEPPIIDGGSPIINYVVQKRDAERKSWSTVTTECSKTSFRVANLEEGKSYFFRVFAENEYGIGDPGETRDAVKASQTPGPVVDLKVRSVSKSSCSIGWKKPHSDGGSRIIGYVVDFLTEENKWQRVMKSLSLQYSAKDLTEGKEYTFRVSAENENGEGTPSEITVVARDDVVAPDLDLKGLPDLCYLAKENSNFRLKIPIKGKPAPSVSWKKGEDPLATDTRVSVESSAVNTTLIVYDCQKSDAGKYTITLKNVAGTKEGTISIKVVGKPGIPTGPIKFDEVTAEAMTLKWAPPKDDGGSEITNYILEKRDSVNNKWVTCASAVQKTTFRVTRLHEGMEYTFRVSAENKYGVGEGLKSEPIVARHPFDVPDAPPPPNIVDVRHDSVSLTWTDPKKTGGSPITGYHLEFKERNSLLWKRANKTPIRMRDFKVTGLTEGLEYEFRVMAINLAGVGKPSLPSEPVVALDPIDPPGKPEVINITRNSVTLIWTEPKYDGGHKLTGYIVEKRDLPSKSWMKANHVNVPECAFTVTDLVEGGKYEFRIRAKNTAGAISAPSESTETIICKDEYEAPTIVLDPTIKDGLTIKAGDTIVLNAISILGKPLPKSSWSKAGKDIRPSDITQITSTPTSSMLTIKYATRKDAGEYTITATNPFGTKVEHVKVTVLDVPGPPGPVEISNVSAEKATLTWTPPLEDGGSPIKSYILEKRETSRLLWTVVSEDIQSCRHVATKLIQGNEYIFRVSAVNHYGKGEPVQSEPVKMVDRFGPPGPPEKPEVSNVTKNTATVSWKRPVDDGGSEITGYHVERREKKSLRWVRAIKTPVSDLRCKVTGLQEGSTYEFRVSAENRAGIGPPSEASDSVLMKDAAYPPGPPSNPHVTDTTKKSASLAWGKPHYDGGLEITGYVVEHQKVGDEAWIKDTTGTALRITQFVVPDLQTKEKYNFRISAINDAGVGEPAVIPDVEIVEREMAPDFELDAELRRTLVVRAGLSIRIFVPIKGRPAPEVTWTKDNINLKNRANIENTESFTLLIIPECNRYDTGKFVMTIENPAGKKSGFVNVRVLDTPGPVLNLRPTDITKDSVTLHWDLPLIDGGSRITNYIVEKREATRKSYSTATTKCHKCTYKVTGLSEGCEYFFRVMAENEYGIGEPTETTEPVKASEAPSPPDSLNIMDITKSTVSLAWPKPKHDGGSKITGYVIEAQRKGSDQWTHITTVKGLECVVRNLTEGEEYTFQVMAVNSAGRSAPRESRPVIVKEQTMLPELDLRGIYQKLVIAKAGDNIKVEIPVLGRPKPTVTWKKGDQILKQTQRVNFETTATSTILNINECVRSDSGPYPLTARNIVGEVGDVITIQVHDIPGPPTGPIKFDEVSSDFVTFSWDPPENDGGVPISNYVVEMRQTDSTTWVELATTVIRTTYKATRLTTGLEYQFRVKAQNRYGVGPGITSACIVANYPFKVPGPPGTPQVTAVTKDSMTISWHEPLSDGGSPILGYHVERKERNGILWQTVSKALVPGNIFKSSGLTDGIAYEFRVIAENMAGKSKPSKPSEPMLALDPIDPPGKPVPLNITRHTVTLKWAKPEYTGGFKITSYIVEKRDLPNGRWLKANFSNILENEFTVSGLTEDAAYEFRVIAKNAAGAISPPSEPSDAITCRDDVEAPKIKVDVKFKDTVILKAGEAFRLEADVSGRPPPTMEWSKDGKELEGTAKLEIKIADFSTNLVNKDSTRRDSGAYTLTATNPGGFAKHIFNVKVLDRPGPPEGPLAVTEVTSEKCVLSWFPPLDDGGAKIDHYIVQKRETSRLAWTNVASEVQVTKLKVTKLLKGNEYIFRVMAVNKYGVGEPLESEPVLAVNPYGPPDPPKNPEVTTITKDSMVVCWGHPDSDGGSEIINYIVERRDKAGQRWIKCNKKTLTDLRYKVSGLTEGHEYEFRIMAENAAGISAPSPTSPFYKACDTVFKPGPPGNPRVLDTSRSSISIAWNKPIYDGGSEITGYMVEIALPEEDEWQIVTPPAGLKATSYTITGLTENQEYKIRIYAMNSEGLGEPALVPGTPKAEDRMLPPEIELDADLRKVVTIRACCTLRLFVPIKGRPAPEVKWARDHGESLDKASIESTSSYTLLIVGNVNRFDSGKYILTVENSSGSKSAFVNVRVLDTPGPPQDLKVKEVTKTSVTLTWDPPLLDGGSKIKNYIVEKRESTRKAYSTVATNCHKTSWKVDQLQEGCSYYFRVLAENEYGIGLPAETAESVKASERPLPPGKITLMDVTRNSVSLSWEKPEHDGGSRILGYIVEMQTKGSDKWATCATVKVTEATITGLIQGEEYSFRVSAQNEKGISDPRQLSVPVIAKDLVIPPAFKLLFNTFTVLAGEDLKVDVPFIGRPTPAVTWHKDNVPLKQTTRVNAESTENNSLLTIKDACREDVGHYVVKLTNSAGEAIETLNVIVLDKPGPPTGPVKMDEVTADSITLSWGPPKYDGGSSINNYIVEKRDTSTTTWQIVSATVARTTIKACRLKTGCEYQFRIAAENRYGKSTYLNSEPTVAQYPFKVPGPPGTPVVTLSSRDSMEVQWNEPISDGGSRVIGYHLERKERNSILWVKLNKTPIPQTKFKTTGLEEGVEYEFRVSAENIVGIGKPSKVSECYVARDPCDPPGRPEAIIVTRNSVTLQWKKPTYDGGSKITGYIVEKKELPEGRWMKASFTNIIDTHFEVTGLVEDHRYEFRVIARNAAGVFSEPSESTGAITARDEVDPPRISMDPKYKDTIVVHAGESFKVDADIYGKPIPTIQWIKGDQELSNTARLEIKSTDFATSLSVKDAVRVDSGNYILKAKNVAGERSVTVNVKVLDRPGPPEGPVVISGVTAEKCTLAWKPPLQDGGSDIINYIVERRETSRLVWTVVDANVQTLSCKVTKLLEGNEYTFRIMAVNKYGVGEPLESEPVVAKNPFVVPDAPKAPEVTTVTKDSMIVVWERPASDGGSEILGYVLEKRDKEGIRWTRCHKRLIGELRLRVTGLIENHDYEFRVSAENAAGLSEPSPPSAYQKACDPIYKPGPPNNPKVIDITRSSVFLSWSKPIYDGGCEIQGYIVEKCDVSVGEWTMCTPPTGINKTNIEVEKLLEKHEYNFRICAINKAGVGEHADVPGPIIVEEKLEAPDIDLDLELRKIINIRAGGSLRLFVPIKGRPTPEVKWGKVDGEIRDAAIIDVTSSFTSLVLDNVNRYDSGKYTLTLENSSGTKSAFVTVRVLDTPSPPVNLKVTEITKDSVSITWEPPLLDGGSKIKNYIVEKREATRKSYAAVVTNCHKNSWKIDQLQEGCSYYFRVTAENEYGIGLPAQTADPIKVAEVPQPPGKITVDDVTRNSVSLSWTKPEHDGGSKIIQYIVEMQAKHSEKWSECARVKSLQAVITNLTQGEEYLFRVVAVNEKGRSDPRSLAVPIVAKDLVIEPDVKPAFSSYSVQVGQDLKIEVPISGRPKPTITWTKDGLPLKQTTRINVTDSLDLTTLSIKETHKDDGGQYGITVANVVGQKTASIEIVTLDKPDPPKGPVKFDDVSAESITLSWNPPLYTGGCQITNYIVQKRDTTTTVWDVVSATVARTTLKVTKLKTGTEYQFRIFAENRYGQSFALESDPIVAQYPYKEPGPPGTPFATAISKDSMVIQWHEPVNNGGSPVIGYHLERKERNSILWTKVNKTIIHDTQFKAQNLEEGIEYEFRVYAENIVGVGKASKNSECYVARDPCDPPGTPEPIMVKRNEITLQWTKPVYDGGSMITGYIVEKRDLPDGRWMKASFTNVIETQFTVSGLTEDQRYEFRVIAKNAAGAISKPSDSTGPITAKDEVELPRISMDPKFRDTIVVNAGETFRLEADVHGKPLPTIEWLRGDKEIEESARCEIKNTDFKALLIVKDAIRIDGGQYILRASNVAGSKSFPVNVKVLDRPGPPEGPVQVTGVTSEKCSLTWSPPLQDGGSDISHYVVEKRETSRLAWTVVASEVVTNSLKVTKLLEGNEYVFRIMAVNKYGVGEPLESAPVLMKNPFVLPGPPKSLEVTNIAKDSMTVCWNRPDSDGGSEIIGYIVEKRDRSGIRWIKCNKRRITDLRLRVTGLTEDHEYEFRVSAENAAGVGEPSPATVYYKACDPVFKPGPPTNAHIVDTTKNSITLAWGKPIYDGGSEILGYVVEICKADEEEWQIVTPQTGLRVTRFEISKLTEHQEYKIRVCALNKVGLGEATSVPGTVKPEDKLEAPELDLDSELRKGIVVRAGGSARIHIPFKGRPTPEITWSREEGEFTDKVQIEKGVNYTQLSIDNCDRNDAGKYILKLENSSGSKSAFVTVKVLDTPGPPQNLAVKEVRKDSAFLVWEPPIIDGGAKVKNYVIDKRESTRKAYANVSSKCSKTSFKVENLTEGAIYYFRVMAENEFGVGVPVETVDAVKAAEPPSPPGKVTLTDVSQTSASLMWEKPEHDGGSRVLGYVVEMQPKGTEKWSIVAESKVCNAVVTGLSSGQEYQFRVKAYNEKGKSDPRVLGVPVIAKDLTIQPSLKLPFNTYSIQAGEDLKIEIPVIGRPRPNISWVKDGEPLKQTTRVNVEETATSTVLHIKEGNKDDFGKYTVTATNSAGTATENLSVIVLEKPGPPVGPVRFDEVSADFVVISWEPPAYTGGCQISNYIVEKRDTTTTTWHMVSATVARTTIKITKLKTGTEYQFRIFAENRYGKSAPLDSKAVIVQYPFKEPGPPGTPFVTSISKDQMLVQWHEPVNDGGTKIIGYHLEQKEKNSILWVKLNKTPIQDTKFKTTGLDEGLEYEFKVSAENIVGIGKPSKVSECFVARDPCDPPGRPEAIVITRNNVTLKWKKPAYDGGSKITGYIVEKKDLPDGRWMKASFTNVLETEFTVSGLVEDQRYEFRVIARNAAGNFSEPSDSSGAITARDEIDAPNASLDPKYKDVIVVHAGETFVLEADIRGKPIPDVVWSKDGKELEETAARMEIKSTIQKTTLVVKDCIRTDGGQYILKLSNVGGTKSIPITVKVLDRPGPPEGPLKVTGVTAEKCYLAWNPPLQDGGANISHYIIEKRETSRLSWTQVSTEVQALNYKVTKLLPGNEYIFRVMAVNKYGIGEPLESGPVTACNPYKPPGPPSTPEVSAITKDSMVVTWARPVDDGGTEIEGYILEKRDKEGVRWTKCNKKTLTDLRLRVTGLTEGHSYEFRVAAENAAGVGEPSEPSVFYRACDALYPPGPPSNPKVTDTSRSSVSLAWSKPIYDGGAPVKGYVVEVKEAAADEWTTCTPPTGLQGKQFTVTKLKENTEYNFRICAINSEGVGEPATLPGSVVAQERIEPPEIELDADLRKVVVLRASATLRLFVTIKGRPEPEVKWEKAEGILTDRAQIEVTSSFTMLVIDNVTRFDSGRYNLTLENNSGSKTAFVNVRVLDSPSAPVNLTIREVKKDSVTLSWEPPLIDGGAKITNYIVEKRETTRKAYATITNNCTKTTFRIENLQEGCSYYFRVLASNEYGIGLPAETTEPVKVSEPPLPPGRVTLVDVTRNTATIKWEKPESDGGSKITGYVVEMQTKGSEKWSTCTQVKTLEATISGLTAGEEYVFRVAAVNEKGRSDPRQLGVPVIARDIEIKPSVELPFHTFNVKAREQLKIDVPFKGRPQATVNWRKDGQTLKETTRVNVSSSKTVTSLSIKEASKEDVGTYELCVSNSAGSITVPITIIVLDRPGPPGPIRIDEVSCDSITISWNPPEYDGGCQISNYIVEKKETTSTTWHIVSQAVARTSIKIVRLTTGSEYQFRVCAENRYGKSSYSESSAVVAEYPFSPPGPPGTPKVVHATKSTMLVTWQVPVNDGGSRVIGYHLEYKERSSILWSKANKILIADTQMKVSGLDEGLMYEYRVYAENIAGIGKCSKSCEPVPARDPCDPPGQPEVTNITRKSVSLKWSKPHYDGGAKITGYIVERRELPDGRWLKCNYTNIQETYFEVTELTEDQRYEFRVFARNAADSVSEPSESTGPIIVKDDVEPPRVMMDVKFRDVIVVKAGEVLKINADIAGRPLPVISWAKDGIEIEERARTEIISTDNHTLLTVKDCIRRDTGQYVLTLKNVAGTRSVAVNCKVLDKPGPPAGPLEINGLTAEKCSLSWGRPQEDGGADIDYYIVEKRETSHLAWTICEGELQMTSCKVTKLLKGNEYIFRVTGVNKYGVGEPLESVAIKALDPFTVPSPPTSLEITSVTKESMTLCWSRPESDGGSEISGYIIERREKNSLRWVRVNKKPVYDLRVKSTGLREGCEYEYRVYAENAAGLSLPSETSPLIRAEDPVFLPSPPSKPKIVDSGKTTITIAWVKPLFDGGAPITGYTVEYKKSDDTDWKTSIQSLRGTEYTISGLTTGAEYVFRVKSVNKVGASDPSDSSDPQIAKEREEEPLFDIDSEMRKTLIVKAGASFTMTVPFRGRPVPNVLWSKPDTDLRTRAYVDTTDSRTSLTIENANRNDSGKYTLTIQNVLSAASLTLVVKVLDTPGPPTNITVQDVTKESAVLSWDVPENDGGAPVKNYHIEKREASKKAWVSVTNNCNRLSYKVTNLQEGAIYYFRVSGENEFGVGIPAETKEGVKITEKPSPPEKLGVTSISKDSVSLTWLKPEHDGGSRIVHYVVEALEKGQKNWVKCAVAKSTHHVVSGLRENSEYFFRVFAENQAGLSDPRELLLPVLIKEQLEPPEIDMKNFPSHTVYVRAGSNLKVDIPISGKPLPKVTLSRDGVPLKATMRFNTEITAENLTINLKESVTADAGRYEITAANSSGTTKAFINIVVLDRPGPPTGPVVISDITEESVTLKWEPPKYDGGSQVTNYILLKRETSTAVWTEVSATVARTMMKVMKLTTGEEYQFRIKAENRFGISDHIDSACVTVKLPYTTPGPPSTPWVTNVTRESITVGWHEPVSNGGSAVVGYHLEMKDRNSILWQKANKLVIRTTHFKVTTISAGLIYEFRVYAENAAGVGKPSHPSEPVLAIDACEPPRNVRITDISKNSVSLSWQQPAFDGGSKITGYIVERRDLPDGRWTKASFTNVTETQFIISGLTQNSQYEFRVFARNAVGSISNPSEVVGPITCIDSYGGPVIDLPLEYTEVVKYRAGTSVKLRAGISGKPAPTIEWYKDDKELQTNALVCVENTTDLASILIKDADRLNSGCYELKLRNAMGSASATIRVQILDKPGPPGGPIEFKTVTAEKITLLWRPPADDGGAKITHYIVEKRETSRVVWSMVSEHLEECIITTTKIIKGNEYIFRVRAVNKYGIGEPLESDSVVAKNAFVTPGPPGIPEVTKITKNSMTVVWSRPIADGGSDISGYFLEKRDKKSLGWFKVLKETIRDTRQKVTGLTENSDYQYRVCAVNAAGQGPFSEPSEFYKAADPIDPPGPPAKIRIADSTKSSITLGWSKPVYDGGSAVTGYVVEIRQGEEEEWTTVSTKGEVRTTEYVVSNLKPGVNYYFRVSAVNCAGQGEPIEMNEPVQAKDILEAPEIDLDVALRTSVIAKAGEDVQVLIPFKGRPPPTVTWRKDEKNLGSDARYSIENTDSSSLLTIPQVTRNDTGKYILTIENGVGEPKSSTVSVKVLDTPAACQKLQVKHVSRGTVTLLWDPPLIDGGSPIINYVIEKRDATKRTWSVVSHKCSSTSFKLIDLSEKTPFFFRVLAENEIGIGEPCETTEPVKAAEVPAPIRDLSMKDSTKTSVILSWTKPDFDGGSVITEYVVERKGKGEQTWSHAGISKTCEIEVSQLKEQSVLEFRVFAKNEKGLSDPVTIGPITVKELIITPEVDLSDIPGAQVTVRIGHNVHLELPYKGKPKPSISWLKDGLPLKESEFVRFSKTENKITLSIKNAKKEHGGKYTVILDNAVCRIAVPITVITLGPPSKPKGPIRFDEIKADSVILSWDVPEDNGGGEITCYSIEKRETSQTNWKMVCSSVARTTFKVPNLVKDAEYQFRVRAENRYGVSQPLVSSIIVAKHQFRIPGPPGKPVIYNVTSDGMSLTWDAPVYDGGSEVTGFHVEKKERNSILWQKVNTSPISGREYRATGLVEGLDYQFRVYAENSAGLSSPSDPSKFTLAVSPVDPPGTPDYIDVTRETITLKWNPPLRDGGSKIVGYSIEKRQGNERWVRCNFTDVSECQYTVTGLSPGDRYEFRIIARNAVGTISPPSQSSGIIMTRDENVPPIVEFGPEYFDGLIIKSGESLRIKALVQGRPVPRVTWFKDGVEIEKRMNMEITDVLGSTSLFVRDATRDHRGVYTVEAKNASGSAKAEIKVKVQDTPGKVVGPIRFTNITGEKMTLWWDAPLNDGCAPITHYIIEKRETSRLAWALIEDKCEAQSYTAIKLINGNEYQFRVSAVNKFGVGRPLDSDPVVAQIQYTVPDAPGIPEPSNITGNSITLTWARPESDGGSEIQQYILERREKKSTRWVKVISKRPISETRFKVTGLTEGNEYEFHVMAENAAGVGPASGISRLIKCREPVNPPGPPTVVKVTDTSKTTVSLEWSKPVFDGGMEIIGYIIEMCKADLGDWHKVNAEACVKTRYTVTDLQAGEEYKFRVSAINGAGKGDSCEVTGTIKAVDRLTAPELDIDANFKQTHVVRAGASIRLFIAYQGRPTPTAVWSKPDSNLSLRADIHTTDSFSTLTVENCNRNDAGKYTLTVENNSGSKSITFTVKVLDTPGPPGPITFKDVTRGSATLMWDAPLLDGGARIHHYVVEKREASRRSWQVISEKCTRQIFKVNDLAEGVPYYFRVSAVNEYGVGEPYEMPEPIVATEQPAPPRRLDVVDTSKSSAVLAWLKPDHDGGSRITGYLLEMRQKGSDFWVEAGHTKQLTFTVERLVEKTEYEFRVKAKNDAGYSEPREAFSSVIIKEPQIEPTADLTGITNQLITCKAGSPFTIDVPISGRPAPKVTWKLEEMRLKETDRVSITTTKDRTTLTVKDSMRGDSGRYFLTLENTAGVKTFSVTVVVIGRPGPVTGPIEVSSVSAESCVLSWGEPKDGGGTEITNYIVEKRESGTTAWQLVNSSVKRTQIKVTHLTKYMEYSFRVSSENRFGVSKPLESAPIIAEHPFVPPSAPTRPEVYHVSANAMSIRWEEPYHDGGSKIIGYWVEKKERNTILWVKENKVPCLECNYKVTGLVEGLEYQFRTYALNAAGVSKASEASRPIMAQNPVDAPGRPEVTDVTRSTVSLIWSAPAYDGGSKVVGYIIERKPVSEVGDGRWLKCNYTIVSDNFFTVTALSEGDTYEFRVLAKNAAGVISKGSESTGPVTCRDEYAPPKAELDARLHGDLVTIRAGSDLVLDAAVGGKPEPKIIWTKGDKELDLCEKVSLQYTGKRATAVIKFCDRSDSGKYTLTVKNASGTKAVSVMVKVLDSPGPCGKLTVSRVTQEKCTLAWSLPQEDGGAEITHYIVERRETSRLNWVIVEGECPTLSYVVTRLIKNNEYIFRVRAVNKYGPGVPVESEPIVARNSFTIPSPPGIPEEVGTGKEHIIIQWTKPESDGGNEISNYLVDKREKKSLRWTRVNKDYVVYDTRLKVTSLMEGCDYQFRVTAVNAAGNSEPSEASNFISCREPSYTPGPPSAPRVVDTTKHSISLAWTKPMYDGGTDIVGYVLEMQEKDTDQWYRVHTNATIRNTEFTVPDLKMGQKYSFRVAAVNVKGMSEYSESIAEIEPVERIEIPDLELADDLKKTVTIRAGASLRLMVSVSGRPPPVITWSKQGIDLASRAIIDTTESYSLLIVDKVNRYDAGKYTIEAENQSGKKSATVLVKVYDTPGPCPSVKVKEVSRDSVTITWEIPTIDGGAPVNNYIVEKREAAMRAFKTVTTKCSKTLYRISGLVEGTMYYFRVLPENIYGIGEPCETSDAVLVSEVPLVPAKLEVVDVTKSTVTLAWEKPLYDGGSRLTGYVLEACKAGTERWMKVVTLKPTVLEHTVTSLNEGEQYLFRIRAQNEKGVSEPRETVTAVTVQDLRVLPTIDLSTMPQKTIHVPAGRPVELVIPIAGRPPPAASWFFAGSKLRESERVTVETHTKVAKLTIRETTIRDTGEYTLELKNVTGTTSETIKVIILDKPGPPTGPIKIDEIDATSITISWEPPELDGGAPLSGYVVEQRDAHRPGWLPVSESVTRSTFKFTRLTEGNEYVFRVAATNRFGIGSYLQSEVIECRSSIRIPGPPETLQIFDVSRDGMTLTWYPPEDDGGSQVTGYIVERKEVRADRWVRVNKVPVTMTRYRSTGLTEGLEYEHRVTAINARGSGKPSRPSKPIVAMDPIAPPGKPQNPRVTDTTRTSVSLAWSVPEDEGGSKVTGYLIEMQKVDQHEWTKCNTTPTKIREYTLTHLPQGAEYRFRVLACNAGGPGEPAEVPGTVKVTEMLEYPDYELDERYQEGIFVRQGGVIRLTIPIKGKPFPICKWTKEGQDISKRAMIATSETHTELVIKEADRGDSGTYDLVLENKCGKKAVYIKVRVIGSPNSPEGPLEYDDIQVRSVRVSWRPPADDGGADILGYILERREVPKAAWYTIDSRVRGTSLVVKGLKENVEYHFRVSAENQFGISKPLKSEEPVTPKTPLNPPEPPSNPPEVLDVTKSSVSLSWSRPKDDGGSRVTGYYIERKETSTDKWVRHNKTQITTTMYTVTGLVPDAEYQFRIIAQNDVGLSETSPASEPVVCKDPFDKPSQPGELEILSISKDSVTLQWEKPECDGGKEILGYWVEYRQSGDSAWKKSNKERIKDKQFTIGGLLEATEYEFRVFAENETGLSRPRRTAMSIKTKLTSGEAPGIRKEMKDVTTKLGEAAQLSCQIVGRPLPDIKWYRFGKELIQSRKYKMSSDGRTHTLTVMTEEQEDEGVYTCIATNEVGEVETSSKLLLQATPQFHPGYPLKEKYYGAVGSTLRLHVMYIGRPVPAMTWFHGQKLLQNSENITIENTEHYTHLVMKNVQRKTHAGKYKVQLSNVFGTVDAILDVEIQDKPDKPTGPIVIEALLKNSAVISWKPPADDGGSWITNYVVEKCEAKEGAEWQLVSSAISVTTCRIVNLTENAGYYFRVSAQNTFGISDPLEVSSVVIIKSPFEKPGAPGKPTITAVTKDSCVVAWKPPASDGGAKIRNYYLEKREKKQNKWISVTTEEIRETVFSVKNLIEGLEYEFRVKCENLGGESEWSEISEPITPKSDVPIQAPHFKEELRNLNVRYQSNATLVCKVTGHPKPIVKWYRQGKEIIADGLKYRIQEFKGGYHQLIIASVTDDDATVYQVRATNQGGSVSGTASLEVEVPAKIHLPKTLEGMGAVHALRGEVVSIKIPFSGKPDPVITWQKGQDLIDNNGHYQVIVTRSFTSLVFPNGVERKDAGFYVVCAKNRFGIDQKTVELDVADVPDPPRGVKVSDVSRDSVNLTWTEPASDGGSKITNYIVEKCATTAERWLRVGQARETRYTVINLFGKTSYQFRVIAENKFGLSKPSEPSEPTITKEDKTRAMNYDEEVDETREVSMTKASHSSTKELYEKYMIAEDLGRGEFGIVHRCVETSSKKTYMAKFVKVKGTDQVLVKKEISILNIARHRNILHLHESFESMEELVMIFEFISGLDIFERINTSAFELNEREIVSYVHQVCEALQFLHSHNIGHFDIRPENIIYQTRRSSTIKIIEFGQARQLKPGDNFRLLFTAPEYYAPEVHQHDVVSTATDMWSLGTLVYVLLSGINPFLAETNQQIIENIMNAEYTFDEEAFKEISIEAMDFVDRLLVKERKSRMTASEALQHPWLKQKIERVSTKVIRTLKHRRYYHTLIKKDLNMVVSAARISCGGAIRSQKGVSVAKVKVASIEIGPVSGQIMHAVGEEGGHVKYVCKIENYDQSTQVTWYFGVRQLENSEKYEITYEDGVAILYVKDITKLDDGTYRCKVVNDYGEDSSYAELFVKGVREVYDYYCRRTMKKIKRRTDTMRLLERPPEFTLPLYNKTAYVGENVRFGVTITVHPEPHVTWYKSGQKIKPGDNDKKYTFESDKGLYQLTINSVTTDDDAEYTVVARNKYGEDSCKAKLTVTLHPPPTDSTLRPMFKRLLANAECQEGQSVCFEIRVSGIPPPTLKWEKDGQPLSLGPNIEIIHEGLDYYALHIRDTLPEDTGYYRVTATNTAGSTSCQAHLQVERLRYKKQEFKSKEEHERHVQKQIDKTLRMAEILSGTESVPLTQVAKEALREAAVLYKPAVSTKTVKGEFRLEIEEKKEERKLRMPYDVPEPRKYKQTTIEEDQRIKQFVPMSDMKWYKKIRDQYEMPGKLDRVVQKRPKRIRLSRWEQFYVMPLPRITDQYRPKWRIPKLSQDDLEIVRPARRRTPSPDYDFYYRPRRRSLGDISDEELLLPIDDYLAMKRTEEERLRLEEELELGFSASPPSRSPPHFELSSLRYSSPQAHVKVEETRKDFRYSTYHIPTKAEASTSYAELRERHAQAAYRQPKQRQRIMAEREDEELLRPVTTTQHLSEYKSELDFMSKEEKSRKKSRRQREVTEITEIEEEYEISKHAQRESSSSASRLLRRRRSLSPTYIELMRPVSELIRSRPQPAEEYEDDTERRSPTPERTRPRSPSPVSSERSLSRFERSARFDIFSRYESMKAALKTQKTSERKYEVLSQQPFTLDHAPRITLRMRSHRVPCGQNTRFILNVQSKPTAEVKWYHNGVELQESSKIHYTNTSGVLTLEILDCHTDDSGTYRAVCTNYKGEASDYATLDVTGGDYTTYASQRRDEEVPRSVFPELTRTEAYAVSSFKKTSEMEASSSVREVKSQMTETRESLSSYEHSASAEMKSAALEEKSLEEKSTTRKIKTTLAARILTKPRSMTVYEGESARFSCDTDGEPVPTVTWLRKGQVLSTSARHQVTTTKYKSTFEISSVQASDEGNYSVVVENSEGKQEAEFTLTIQKARVTEKAVTSPPRVKSPEPRVKSPEAVKSPKRVKSPEPSHPKAVSPTETKPTPTEKVQHLPVSAPPKITQFLKAEASKEIAKLTCVVESSVLRAKEVTWYKDGKKLKENGHFQFHYSADGTYELKINNLTESDQGEYVCEISGEGGTSKTNLQFMGQAFKSIHEKVSKISETKKSDQKTTESTVTRKTEPKAPEPISSKPVIVTGLQDTTVSSDSVAKFAVKATGEPRPTAIWTKDGKAITQGGKYKLSEDKGGFFLEIHKTDTSDSGLYTCTVKNSAGSVSSSCKLTIKAIKDTEAQKVSTQKTSEITPQKKAVVQEEISQKALRSEEIKMSEAKSQEKLALKEEASKVLISEEVKKSAATSLEKSIVHEEITKTSQASEEVRTHAEIKAFSTQMSINEGQRLVLKANIAGATDVKWVLNGVELTNSEEYRYGVSGSDQTLTIKQASHRDEGILTCISKTKEGIVKCQYDLTLSKELSDAPAFISQPRSQNINEGQNVLFTCEISGEPSPEIEWFKNNLPISISSNVSISRSRNVYSLEIRNASVSDSGKYTIKAKNFRGQCSATASLMVLPLVEEPSREVVLRTSGDTSLQGSFSSQSVQMSASKQEASFSSFSSSSASSMTEMKFASMSAQSMSSMQESFVEMSSSSFMGISNMTQLESSTSKMLKAGIRGIPPKIEALPSDISIDEGKVLTVACAFTGEPTPEVTWSCGGRKIHSQEQGRFHIENTDDLTTLIIMDVQKQDGGLYTLSLGNEFGSDSATVNIHIRSI

>NP_002464.1

MAQQAADKYLYVDKNFINNPLAQADWAAKKLVWVPSDKSGFEPASLKEEVGEEAIVELVENGKKVKVNKDDIQKMNPPKFSKVEDMAELTCLNEASVLHNLKERYYSGLIYTYSGLFCVVINPYKNLPIYSEEIVEMYKGKKRHEMPPHIYAITDTAYRSMMQDREDQSILCTGESGAGKTENTKKVIQYLAYVASSHKSKKDQGELERQLLQANPILEAFGNAKTVKNDNSSRFGKFIRINFDVNGYIVGANIETYLLEKSRAIRQAKEERTFHIFYYLLSGAGEHLKTDLLLEPYNKYRFLSNGHVTIPGQQDKDMFQETMEAMRIMGIPEEEQMGLLRVISGVLQLGNIVFKKERNTDQASMPDNTAAQKVSHLLGINVTDFTRGILTPRIKVGRDYVQKAQTKEQADFAIEALAKATYERMFRWLVLRINKALDKTKRQGASFIGILDIAGFEIFDLNSFEQLCINYTNEKLQQLFNHTMFILEQEEYQREGIEWNFIDFGLDLQPCIDLIEKPAGPPGILALLDEECWFPKATDKSFVEKVMQEQGTHPKFQKPKQLKDKADFCIIHYAGKVDYKADEWLMKNMDPLNDNIATLLHQSSDKFVSELWKDVDRIIGLDQVAGMSETALPGAFKTRKGMFRTVGQLYKEQLAKLMATLRNTNPNFVRCIIPNHEKKAGKLDPHLVLDQLRCNGVLEGIRICRQGFPNRVVFQEFRQRYEILTPNSIPKGFMDGKQACVLMIKALELDSNLYRIGQSKVFFRAGVLAHLEEERDLKITDVIIGFQACCRGYLARKAFAKRQQQLTAMKVLQRNCAAYLKLRNWQWWRLFTKVKPLLQVSRQEEEMMAKEEELVKVREKQLAAENRLTEMETLQSQLMAEKLQLQEQLQAETELCAEAEELRARLTAKKQELEEICHDLEARVEEEEERCQHLQAEKKKMQQNIQELEEQLEEEESARQKLQLEKVTTEAKLKKLEEEQIILEDQNCKLAKEKKLLEDRIAEFTTNLTEEEEKSKSLAKLKNKHEAMITDLEERLRREEKQRQELEKTRRKLEGDSTDLSDQIAELQAQIAELKMQLAKKEEELQAALARVEEEAAQKNMALKKIRELESQISELQEDLESERASRNKAEKQKRDLGEELEALKTELEDTLDSTAAQQELRSKREQEVNILKKTLEEEAKTHEAQIQEMRQKHSQAVEELAEQLEQTKRVKANLEKAKQTLENERGELANEVKVLLQGKGDSEHKRKKVEAQLQELQVKFNEGERVRTELADKVTKLQVELDNVTGLLSQSDSKSSKLTKDFSALESQLQDTQELLQEENRQKLSLSTKLKQVEDEKNSFREQLEEEEEAKHNLEKQIATLHAQVADMKKKMEDSVGCLETAEEVKRKLQKDLEGLSQRHEEKVAAYDKLEKTKTRLQQELDDLLVDLDHQRQSACNLEKKQKKFDQLLAEEKTISAKYAEERDRAEAEAREKETKALSLARALEEAMEQKAELERLNKQFRTEMEDLMSSKDDVGKSVHELEKSKRALEQQVEEMKTQLEELEDELQATEDAKLRLEVNLQAMKAQFERDLQGRDEQSEEKKKQLVRQVREMEAELEDERKQRSMAVAARKKLEMDLKDLEAHIDSANKNRDEAIKQLRKLQAQMKDCMRELDDTRASREEILAQAKENEKKLKSMEAEMIQLQEELAAAERAKRQAQQERDELADEIANSSGKGALALEEKRRLEARIAQLEEELEEEQGNTELINDRLKKANLQIDQINTDLNLERSHAQKNENARQQLERQNKELKVKLQEMEGTVKSKYKASITALEAKIAQLEEQLDNETKERQAACKQVRRTEKKLKDVLLQVDDERRNAEQYKDQADKASTRLKQLKRQLEEAEEEAQRANASRRKLQRELEDATETADAMNREVSSLKNKLRRGDLPFVVPRRMARKGAGDGSDEEVDGKADGAEAKPAE

153 NP_150241.2 NP_001518.2

>NP_150241.2

MEPAPARSPRPQQDPARPQEPTMPPPETPSEGRQPSPSPSPTERAPASEEEFQFLRCQQCQAEAKCPKLLPCLHTLCSGCLEASGMQCPICQAPWPLGADTPALDNVFFESLQRRLSVYRQIVDAQAVCTRCKESADFWCFECEQLLCAKCFEAHQWFLKHEARPLAELRNQSVREFLDGTRKTNNIFCSNPNHRTPTLTSIYCRGCSKPLCCSCALLDSSHSELKCDISAEIQQRQEELDAMTQALQEQDSAFGAVHAQMHAAVGQLGRARAETEELIRERVRQVVAHVRAQERELLEAVDARYQRDYEEMASRLGRLDAVLQRIRTGSALVQRMKCYASDQEVLDMHGFLRQALCRLRQEEPQSLQAAVRTDGFDEFKVRLQDLSSCITQGKDAAVSKKASPEAASTPRDPIDVDLPEEAERVKAQVQALGLAEAQPMAVVQSVPGAHPVPVYAFSIKGPSYGEDVSNTTTAQKRKCSQTQCPRKVIKMESEEGKEARLARSSPEQPRPSTSKAVSPPHLDGPPSPRSPVIGSEVFLPNSNHVASGAGEAEERVVVISSSEDSDAENSSSRELDDSSSESSDLQLEGPSTLRVLDENLADPQAEDRPLVFFDLKIDNETQKISQLAAVNRESKFRVVIQPEAFFSIYSKAVSLEVGLQHFLSFLSSMRRPILACYKLWGPGLPNFFRALEDINRLWEFQEAISGFLAALPLIRERVPGASSFKLKNLAQTYLARNMSERSAMAAVLAMRDLCRLLEVSPGPQLAQHVYPFSSLQCFASLQPLVQAAVLPRAEARLLALHNVSFMELLSAHRRDRQGGLKKYSRYLSLQTTTLPPAQPAFNLQALGTYFEGLLEGPALARAEGVSTPLAGRGLAERASQQS

>NP_001518.2

MRSPPCGLLRWFGGPLLASWCRCHLRFRAFGTSAGWYRAFPAPPPLLPPACPSPRDYRPHVSLSPFLSRPSRGGSSSSSSSRRRSPVAAVAGEPMAYSQGGGKKKVCYYYDGDIGNYYYGQGHPMKPHRIRMTHNLLLNYGLYRKMEIYRPHKATAEEMTKYHSDEYIKFLRSIRPDNMSEYSKQMQRFNVGEDCPVFDGLFEFCQLSTGGSVAGAVKLNRQQTDMAVNWAGGLHHAKKSEASGFCYVNDIVLAILELLKYHQRVLYIDIDIHHGDGVEEAFYTTDRVMTVSFHKYGEYFPGTGDLRDIGAGKGKYYAVNFPMRDGIDDESYGQIFKPIISKVMEMYQPSAVVLQCGADSLSGDRLGCFNLTVKGHAKCVEVVKTFNLPLLMLGGGGYTIRNVARCWTYETAVALDCEIPNELPYNDYFEYFGPDFKLHISPSNMTNQNTPEYMEKIKQRLFENLRMLPHAPGVQMQAIPEDAVHEDSGDEDGEDPDKRISIRASDKRIACDEEFSDSEDEGEGGRRNVADHKKGAKKARIEEDKKETEDKKTDVKEEDKSKDNSGEKTDTKGTKSEQLSNP

154 NP_006816.2 NP_115580.2

>NP_006816.2

MPSAKQRGSKGGHGAASPSEKGAHPSGGADDVAKKPPPAPQQPPPPPAPHPQQHPQQHPQNQAHGKGGHRGGGGGGGKSSSSSSASAAAAAAAASSSASCSRRLGRALNFLFYLALVAAAAFSGWCVHHVLEEVQQVRRSHQDFSRQREELGQGLQGVEQKVQSLQATFGTFESILRSSQHKQDLTEKAVKQGESEVSRISEVLQKLQNEILKDLSDGIHVVKDARERDFTSLENTVEERLTELTKSINDNIAIFTEVQKRSQKEINDMKAKVASLEESEGNKQDLKALKEAVKEIQTSAKSREWDMEALRSTLQTMESDIYTEVRELVSLKQEQQAFKEAADTERLALQALTEKLLRSEESVSRLPEEIRRLEEELRQLKSDSHGPKEDGGFRHSEAFEALQQKSQGLDSRLQHVEDGVLSMQVASARQTESLESLLSKSQEHEQRLAALQGRLEGLGSSEADQDGLASTVRSLGETQLVLYGDVEELKRSVGELPSTVESLQKVQEQVHTLLSQDQAQAARLPPQDFLDRLSSLDNLKASVSQVEADLKMLRTAVDSLVAYSVKIETNENNLESAKGLLDDLRNDLDRLFVKVEKIHEKV

>NP_115580.2

MPALPLDQLQITHKDPKTGKLRTSPALHPEQKADRYFVLYKPPPKDNIPALVEEYLERATFVANDLDWLLALPHDKFWCQVIFDETLQKCLDSYLRYVPRKFDEGVASAPEVVDMQKRLHRSVFLTFLRMSTHKESKDHFISPSAFGEILYNNFLFDIPKILDLCVLFGKGNSPLLQKMIGNIFTQQPSYYSDLDETLPTILQVFSNILQHCGLQGDGANTTPQKLEERGRLTPSDMPLLELKDIVLYLCDTCTTLWAFLDIFPLACQTFQKHDFCYRLASFYEAAIPEMESAIKKRRLEDSKLLGDLWQRLSHSRKKLMEIFHIILNQICLLPILESSCDNIQGFIEEFLQIFSSLLQEKRFLRDYDALFPVAEDISLLQQASSVLDETRTAYILQAVESAWEGVDRRKATDAKDPSVIEEPNGEPNGVTVTAEAVSQASSHPENSEEEECMGAAAAVGPAMCGVELDSLISQVKDLLPDLGEGFILACLEYYHYDPEQVINNILEERLAPTLSQLDRNLDREMKPDPTPLLTSRHNVFQNDEFDVFSRDSVDLSRVHKGKSTRKEENTRSLLNDKRAVAAQRQRYEQYSVVVEEVPLQPGESLPYHSVYYEDEYDDTYDGNQVGANDADSDDELISRRPFTIPQVLRTKVPREGQEEDDDDEEDDADEEAPKPDHFVQDPAVLREKAEARRMAFLAKKGYRHDSSTAVAGSPRGHGQSRETTQERRKKEANKATRANHNRRTMADRKRSKGMIPS

155 NP_003734.3 NP_068556.1

>NP_003734.3

MSGLGDSSSDPANPDSHKRKGSPCDTLASSTEKRRREQENKYLEELAELLSANISDIDSLSVKPDKCKILKKTVDQIQLMKRMEQEKSTTDDDVQKSDISSSSQGVIEKESLGPLLLEALDGFFFVVNCEGRIVFVSENVTSYLGYNQEELMNTSVYSILHVGDHAEFVKNLLPKSLVNGVPWPQEATRRNSHTFNCRMLIHPPDEPGTENQEACQRYEVMQCFTVSQPKSIQEDGEDFQSCLICIARRLPRPPAITGVESFMTKQDTTGKIISIDTSSLRAAGRTGWEDLVRKCIYAFFQPQGREPSYARQLFQEVMTRGTASSPSYRFILNDGTMLSAHTKCKLCYPQSPDMQPFIMGIHIIDREHSGLSPQDDTNSGMSIPRVNPSVNPSISPAHGVARSSTLPPSNSNMVSTRINRQQSSDLHSSSHSNSSNSQGSFGCSPGSQIVANVALNQGQASSQSSNPSLNLNNSPMEGTGISLAQFMSPRRQVTSGLATRPRMPNNSFPPNISTLSSPVGMTSSACNNNNRSYSNIPVTSLQGMNEGPNNSVGFSASSPVLRQMSSQNSPSRLNIQPAKAESKDNKEIASILNEMIQSDNSSSDGKPLDSGLLHNNDRLSDGDSKYSQTSHKLVQLLTTTAEQQLRHADIDTSCKDVLSCTGTSNSASANSSGGSCPSSHSSLTERHKILHRLLQEGSPSDITTLSVEPDKKDSASTSVSVTGQVQGNSSIKLELDASKKKESKDHQLLRYLLDKDEKDLRSTPNLSLDDVKVKVEKKEQMDPCNTNPTPMTKPTPEEIKLEAQSQFTADLDQFDQLLPTLEKAAQLPGLCETDRMDGAVTSVTIKSEILPASLQSATARPTSRLNRLPELELEAIDNQFGQPGTGDQIPWTNNTVTAINQSKSEDQCISSQLDELLCPPTTVEGRNDEKALLEQLVSFLSGKDETELAELDRALGIDKLVQGGGLDVLSERFPPQQATPPLIMEERPNLYSQPYSSPSPTANLPSPFQGMVRQKPSLGTMPVQVTPPRGAFSPGMGMQPRQTLNRPPAAPNQLRLQLQQRLQGQQQLIHQNRQAILNQFAATAPVGINMRSGMQQQITPQPPLNAQMLAQRQRELYSQQHRQRQLIQQQRAMLMRQQSFGNNLPPSSGLPVQMGNPRLPQGAPQQFPYPPNYGTNPGTPPASTSPFSQLAANPEASLANRNSMVSRGMTGNIGGQFGTGINPQMQQNVFQYPGAGMVPQGEANFAPSLSPGSSMVPMPIPPPQSSLLQQTPPASGYQSPDMKAWQQGAIGNNNVFSQAVQNQPTPAQPGVYNNMSITVSMAGGNTNVQNMNPMMAQMQMSSLQMPGMNTVCPEQINDPALRHTGLYCNQLSSTDLLKTEADGTQQVQQVQVFADVQCTVNLVGGDPYLNQPGPLGTQKPTSGPQTPQAQQKSLLQQLLTE

>NP_068556.1

MLGAVKMEGHEPSDWSSYYAEPEGYSSVSNMNAGLGMNGMNTYMSMSAAAMGSGSGNMSAGSMNMSSYVGAGMSPSLAGMSPGAGAMAGMGGSAGAAGVAGMGPHLSPSLSPLGGQAAGAMGGLAPYANMNSMSPMYGQAGLSRARDPKTYRRSYTHAKPPYSYISLITMAIQQSPNKMLTLSEIYQWIMDLFPFYRQNQQRWQNSIRHSLSFNDCFLKVPRSPDKPGKGSFWTLHPDSGNMFENGCYLRRQKRFKCEKQLALKEAAGAAGSGKKAAAGAQASQAQLGEAAGPASETPAGTESPHSSASPCQEHKRGGLGELKGTPAAALSPPEPAPSPGQQQQAAAHLLGPPHHPGLPPEAHLKPEHHYAFNHPFSINNLMSSEQQHHHSHHHHQPHKMDLKAYEQVMHYPGYGSPMPGSLAMGPVTNKTGLDASPLAADTSYYQGVYSRPIMNSS

156 NP_378663.1 NP_001895.1

>NP_378663.1

MDPAEAVLQEKALKFMCSMPRSLWLGCSSLADSMPSLRCLYNPGTGALTAFQNSSEREDCNNGEPPRKIIPEKNSLRQTYNSCARLCLNQETVCLASTAMKTENCVAKTKLANGTSSMIVPKQRKLSASYEKEKELCVKYFEQWSESDQVEFVEHLISQMCHYQHGHINSYLKPMLQRDFITALPARGLDHIAENILSYLDAKSLCAAELVCKEWYRVTSDGMLWKKLIERMVRTDSLWRGLAERRGWGQYLFKNKPPDGNAPPNSFYRALYPKIIQDIETIESNWRCGRHSLQRIHCRSETSKGVYCLQYDDQKIVSGLRDNTIKIWDKNTLECKRILTGHTGSVLCLQYDERVIITGSSDSTVRVWDVNTGEMLNTLIHHCEAVLHLRFNNGMMVTCSKDRSIAVWDMASPTDITLRRVLVGHRAAVNVVDFDDKYIVSASGDRTIKVWNTSTCEFVRTLNGHKRGIACLQYRDRLVVSGSSDNTIRLWDIECGACLRVLEGHEELVRCIRFDNKRIVSGAYDGKIKVWDLVAALDPRAPAGTLCLRTLVEHSGRVFRLQFDEFQIVSSSHDDTILIWDFLNDPAAQAEPPRSPSRTYTYISR

>NP_001895.1

MATQADLMELDMAMEPDRKAAVSHWQQQSYLDSGIHSGATTTAPSLSGKGNPEEEDVDTSQVLYEWEQGFSQSFTQEQVADIDGQYAMTRAQRVRAAMFPETLDEGMQIPSTQFDAAHPTNVQRLAEPSQMLKHAVVNLINYQDDAELATRAIPELTKLLNDEDQVVVNKAAVMVHQLSKKEASRHAIMRSPQMVSAIVRTMQNTNDVETARCTAGTLHNLSHHREGLLAIFKSGGIPALVKMLGSPVDSVLFYAITTLHNLLLHQEGAKMAVRLAGGLQKMVALLNKTNVKFLAITTDCLQILAYGNQESKLIILASGGPQALVNIMRTYTYEKLLWTTSRVLKVLSVCSSNKPAIVEAGGMQALGLHLTDPSQRLVQNCLWTLRNLSDAATKQEGMEGLLGTLVQLLGSDDINVVTCAAGILSNLTCNNYKNKMMVCQVGGIEALVRTVLRAGDREDITEPAICALRHLTSRHQEAEMAQNAVRLHYGLPVVVKLLHPPSHWPLIKATVGLIRNLALCPANHAPLREQGAIPRLVQLLVRAHQDTQRRTSMGGTQQQFVEGVRMEEIVEGCTGALHILARDVHNRIVIRGLNTIPLFVQLLYSPIENIQRVAAGVLCELAQDKEAAEAIEAEGATAPLTELLHSRNEGVATYAAAVLFRMSEDKPQDYKKRLSVELTSSLFRTEPMAWNETADLGLDIGAQGEPLGYRQDDPSYRSFHSGGYGQDALGMDPMMEHEMGGHHPGADYPVDGLPDLGHAQDLMDGLPPGDSNQLAWFDTDL

157 NP_057126.2 NP_061910.1

>NP_057126.2

MAEPASVAAESLAGSRARAARTVLGQVVLPGEELLLPEQEDAEGPGGAVERPLSLNARACSRVRVVCGPGLRRCGDRLLVTKCGRLRHKEPGSGSGGGVYWVDSQQKRYVPVKGDHVIGIVTAKSGDIFKVDVGGSEPASLSYLSFEGATKRNRPNVQVGDLIYGQFVVANKDMEPEMVCIDSCGRANGMGVIGQDGLLFKVTLGLIRKLLAPDCEIIQEVGKLYPLEIVFGMNGRIWVKAKTIQQTLILANILEACEHMTSDQRKQIFSRLAES

>NP_061910.1

MAGLELLSDQGYRVDGRRAGELRKIQARMGVFAQADGSAYIEQGNTKALAVVYGPHEIRGSRARALPDRALVNCQYSSATFSTGERKRRPHGDRKSCEMGLQLRQTFEAAILTQLHPRSQIDIYVQVLQADGGTYAACVNAATLAVLDAGIPMRDFVCACSAGFVDGTALADLSHVEEAAGGPQLALALLPASGQIALLEMDARLHEDHLERVLEAAAQAARDVHTLLDRVVRQHVREASILLGD

158 NP_005169.1 NP_003874.2

>NP_005169.1

MDEGPVDLRTRPKAAGLPGAALPLRKRPLRAPSPEPAAPRGAAGLVVPLDPLRGGCDLPAVPGPPHGLARPEALYYPGALLPLYPTRAMGSPFPLVNLPTPLYPMMCPMEHPLSADIAMATRADEDGDTPLHIAVVQGNLPAVHRLVNLFQQGGRELDIYNNLRQTPLHLAVITTLPSVVRLLVTAGASPMALDRHGQTAAHLACEHRSPTCLRALLDSAAPGTLDLEARNYDGLTALHVAVNTECQETVQLLLERGADIDAVDIKSGRSPLIHAVENNSLSMVQLLLQHGANVNAQMYSGSSALHSASGRGLLPLVRTLVRSGADSSLKNCHNDTPLMVARSRRVIDILRGKATRPASTSQPDPSPDRSANTSPESSSRLSSNGLLSASPSSSPSQSPPRDPPGFPMAPPNFFLPSPSPPAFLPFAGVLRGPGRPVPPSPAPGGS

>NP_003874.2

MAKTVAYFYDPDVGNFHYGAGHPMKPHRLALTHSLVLHYGLYKKMIVFKPYQASQHDMCRFHSEDYIDFLQRVSPTNMQGFTKSLNAFNVGDDCPVFPGLFEFCSRYTGASLQGATQLNNKICDIAINWAGGLHHAKKFEASGFCYVNDIVIGILELLKYHPRVLYIDIDIHHGDGVQEAFYLTDRVMTVSFHKYGNYFFPGTGDMYEVGAESGRYYCLNVPLRDGIDDQSYKHLFQPVINQVVDFYQPTCIVLQCGADSLGCDRLGCFNLSIRGHGECVEYVKSFNIPLLVLGGGGYTVRNVARCWTYETSLLVEEAISEELPYSEYFEYFAPDFTLHPDVSTRIENQNSRQYLDQIRQTIFENLKMLNHAPSVQIHDVPADLLTYDRTDEADAEERGPEENYSRPEAPNEFYDGDHDNDKESDVEI

159 NP_003237.2 NP_000063.2

>NP_003237.2

MGLAWGLGVLFLMHVCGTNRIPESGGDNSVFDIFELTGAARKGSGRRLVKGPDPSSPAFRIEDANLIPPVPDDKFQDLVDAVRAEKGFLLLASLRQMKKTRGTLLALERKDHSGQVFSVVSNGKAGTLDLSLTVQGKQHVVSVEEALLATGQWKSITLFVQEDRAQLYIDCEKMENAELDVPIQSVFTRDLASIARLRIAKGGVNDNFQGVLQNVRFVFGTTPEDILRNKGCSSSTSVLLTLDNNVVNGSSPAIRTNYIGHKTKDLQAICGISCDELSSMVLELRGLRTIVTTLQDSIRKVTEENKELANELRRPPLCYHNGVQYRNNEEWTVDSCTECHCQNSVTICKKVSCPIMPCSNATVPDGECCPRCWPSDSADDGWSPWSEWTSCSTSCGNGIQQRGRSCDSLNNRCEGSSVQTRTCHIQECDKRFKQDGGWSHWSPWSSCSVTCGDGVITRIRLCNSPSPQMNGKPCEGEARETKACKKDACPINGGWGPWSPWDICSVTCGGGVQKRSRLCNNPTPQFGGKDCVGDVTENQICNKQDCPIDGCLSNPCFAGVKCTSYPDGSWKCGACPPGYSGNGIQCTDVDECKEVPDACFNHNGEHRCENTDPGYNCLPCPPRFTGSQPFGQGVEHATANKQVCKPRNPCTDGTHDCNKNAKCNYLGHYSDPMYRCECKPGYAGNGIICGEDTDLDGWPNENLVCVANATYHCKKDNCPNLPNSGQEDYDKDGIGDACDDDDDNDKIPDDRDNCPFHYNPAQYDYDRDDVGDRCDNCPYNHNPDQADTDNNGEGDACAADIDGDGILNERDNCQYVYNVDQRDTDMDGVGDQCDNCPLEHNPDQLDSDSDRIGDTCDNNQDIDEDGHQNNLDNCPYVPNANQADHDKDGKGDACDHDDDNDGIPDDKDNCRLVPNPDQKDSDGDGRGDACKDDFDHDSVPDIDDICPENVDISETDFRRFQMIPLDPKGTSQNDPNWVVRHQGKELVQTVNCDPGLAVGYDEFNAVDFSGTFFINTERDDDYAGFVFGYQSSSRFYVVMWKQVTQSYWDTNPTRAQGYSGLSVKVVNSTTGPGEHLRNALWHTGNTPGQVRTLWHDPRHIGWKDFTAYRWRLSHRPKTGFIRVVMYEGKKIMADSGPIYDKTYAGGRLGLFVFSQEMVFFSDLKYECRDP

>NP_000063.2

MGCDRNCGLIAGAVIGAVLAVFGGILMPVGDLLIQKTIKKQVVLEEGTIAFKNWVKTGTEVYRQFWIFDVQNPQEVMMNSSNIQVKQRGPYTYRVRFLAKENVTQDAEDNTVSFLQPNGAIFEPSLSVGTEADNFTVLNLAVAAASHIYQNQFVQMILNSLINKSKSSMFQVRTLRELLWGYRDPFLSLVPYPVTTTVGLFYPYNNTADGVYKVFNGKDNISKVAIIDTYKGKRNLSYWESHCDMINGTDAASFPPFVEKSQVLQFFSSDICRSIYAVFESDVNLKGIPVYRFVLPSKAFASPVENPDNYCFCTEKIISKNCTSYGVLDISKCKEGRPVYISLPHFLYASPDVSEPIDGLNPNEEEHRTYLDIEPITGFTLQFAKRLQVNLLVKPSEKIQVLKNLKRNYIVPILWLNETGTIGDEKANMFRSQVTGKINLLGLIEMILLSVGVVMFVAFMISYCACRSKTIK

160 NP_001921.1 NP_001961.1

>NP_001921.1

MEGSLEREAPAGALAAVLKHSSTLPPESTQVRGYDFNRGVNYRALLEAFGTTGFQATNFGRAVQQVNAMIEKKLEPLSQDEDQHADLTQSRRPLTSCTIFLGYTSNLISSGIRETIRYLVQHNMVDVLVTTAGGVEEDLIKCLAPTYLGEFSLRGKELRENGINRIGNLLVPNENYCKFEDWLMPILDQMVMEQNTEGVKWTPSKMIARLGKEINNPESVYYWAQKNHIPVFSPALTDGSLGDMIFFHSYKNPGLVLDIVEDLRLINTQAIFAKCTGMIILGGGVVKHHIANANLMRNGADYAVYINTAQEFDGSDSGARPDEAVSWGKIRVDAQPVKVYADASLVFPLLVAETFAQKMDAFMHEKNED

>NP_001961.1

MADDLDFETGDAGASATFPMQCSALRKNGFVVLKGRPCKIVEMSTSKTGKHGHAKVHLVGIDIFTGKKYEDICPSTHNMDVPNIKRNDFQLIGIQDGYLSLLQDSGEVREDLRLPEGDLGKEIEQKYDCGEEILITVLSAMTEEAAVAIKAMAK

161 NP_001887.1 NP_006635.2

>NP_001887.1

MPGPAAGSRARVYAEVNSLRSREYWDYEAHVPSWGNQDDYQLVRKLGRGKYSEVFEAINITNNERVVVKILKPVKKKKIKREVKILENLRGGTNIIKLIDTVKDPVSKTPALVFEYINNTDFKQLYQILTDFDIRFYMYELLKALDYCHSKGIMHRDVKPHNVMIDHQQKKLRLIDWGLAEFYHPAQEYNVRVASRYFKGPELLVDYQMYDYSLDMWSLGCMLASMIFRREPFFHGQDNYDQLVRIAKVLGTEELYGYLKKYHIDLDPHFNDILGQHSRKRWENFIHSENRHLVSPEALDLLDKLLRYDHQQRLTAKEAMEHPYFYPVVKEQSQPCADNAVLSSGLTAAR

>NP_006635.2

MSVVGLDVGSQSCYIAVARAGGIETIANEFSDRCTPSVISFGSKNRTIGVAAKNQQITHANNTVSNFKRFHGRAFNDPFIQKEKENLSYDLVPLKNGGVGIKVMYMGEEHLFSVEQITAMLLTKLKETAENSLKKPVTDCVISVPSFFTDAERRSVLDAAQIVGLNCLRLMNDMTAVALNYGIYKQDLPSLDEKPRIVVFVDMGHSAFQVSACAFNKGKLKVLGTAFDPFLGGKNFDEKLVEHFCAEFKTKYKLDAKSKIRALLRLYQECEKLKKLMSSNSTDLPLNIECFMNDKDVSGKMNRSQFEELCAELLQKIEVPLYSLLEQTHLKVEDVSAVEIVGGATRIPAVKERIAKFFGKDISTTLNADEAVARGCALQCAILSPAFKVREFSVTDAVPFPISLIWNHDSEDTEGVHEVFSRNHAAPFSKVLTFLRRGPFELEAFYSDPQGVPYPEAKIGRFVVQNVSAQKDGEKSRVKVKVRVNTHGIFTISTASMVEKVPTEENEMSSEADMECLNQRPPENPDTDKNVQQDNSEAGTQPQVQTDAQQTSQSPPSPELTSEENKIPDADKANEKKVDQPPEAKKPKIKVVNVELPIEANLVWQLGKDLLNMYIETEGKMIMQDKLEKERNDAKNAVEEYVYEFRDKLCGPYEKFICEQDHQNFLRLLTETEDWLYEEGEDQAKQAYVDKLEELMKIGTPVKVRFQEAEERPKMFEELGQRLQHYAKIAADFRNKDEKYNHIDESEMKKVEKSVNEVMEWMNNVMNAQAKKSLDQDPVVRAQEIKTKIKELNNTCEPVVTQPKPKIESPKLERTPNGPNIDKKEEDLEDKNNFGAEPPHQNGECYPNEKNSVNMDLD

162 NP_002201.1 NP_001034221.1

>NP_002201.1

MAFPPRRRLRLGPRGLPLLLSGLLLPLCRAFNLDVDSPAEYSGPEGSYFGFAVDFFVPSASSRMFLLVGAPKANTTQPGIVEGGQVLKCDWSSTRRCQPIEFDATGNRDYAKDDPLEFKSHQWFGASVRSKQDKILACAPLYHWRTEMKQEREPVGTCFLQDGTKTVEYAPCRSQDIDADGQGFCQGGFSIDFTKADRVLLGGPGSFYWQGQLISDQVAEIVSKYDPNVYSIKYNNQLATRTAQAIFDDSYLGYSVAVGDFNGDGIDDFVSGVPRAARTLGMVYIYDGKNMSSLYNFTGEQMAAYFGFSVAATDINGDDYADVFIGAPLFMDRGSDGKLQEVGQVSVSLQRASGDFQTTKLNGFEVFARFGSAIAPLGDLDQDGFNDIAIAAPYGGEDKKGIVYIFNGRSTGLNAVPSQILEGQWAARSMPPSFGYSMKGATDIDKNGYPDLIVGAFGVDRAILYRARPVITVNAGLEVYPSILNQDNKTCSLPGTALKVSCFNVRFCLKADGKGVLPRKLNFQVELLLDKLKQKGAIRRALFLYSRSPSHSKNMTISRGGLMQCEELIAYLRDESEFRDKLTPITIFMEYRLDYRTAADTTGLQPILNQFTPANISRQAHILLDCGEDNVCKPKLEVSVDSDQKKIYIGDDNPLTLIVKAQNQGEGAYEAELIVSIPLQADFIGVVRNNEALARLSCAFKTENQTRQVVCDLGNPMKAGTQLLAGLRFSVHQQSEMDTSVKFDLQIQSSNLFDKVSPVVSHKVDLAVLAAVEIRGVSSPDHIFLPIPNWEHKENPETEEDVGPVVQHIYELRNNGPSSFSKAMLHLQWPYKYNNNTLLYILHYDIDGPMNCTSDMEINPLRIKISSLQTTEKNDTVAGQGERDHLITKRDLALSEGDIHTLGCGVAQCLKIVCQVGRLDRGKSAILYVKSLLWTETFMNKENQNHSYSLKSSASFNVIEFPYKNLPIEDITNSTLVTTNVTWGIQPAPMPVPVWVIILAVLAGLLLLAVLVFVMYRMGFFKRVRPPQEEQEREQLQPHENGEGNSET

>NP_001034221.1

MGSLFPLSLLFFLAAAYPGVGSALGRRTKRAQSPKGSPLAPSGTSVPFWVRMSPEFVAVQPGKSVQLNCSNSCPQPQNSSLRTPLRQGKTLRGPGWVSYQLLDVRAWSSLAHCLVTCAGKTRWATSRITAYSVPGGLLGGDPEAWKPGHLFRKPGALHRPGSGQRDLDLRVCCWTPRLLAARDLPRAPQSRRPGGPQQLGTHYTDARLEPRAHSFGLRFHRCPCRDPPHCGRCVPMQVPSYEVPGVKGDVLCRLSEKKRNMKQSGEMAIHGG

163 NP_001002760.1 NP_000453.2

>NP_001002760.1

MMTLVPRARTRAGQDHYSHPCPRFSQVLLTEGIMTYCLTKNLSDVNILHRLLKNGNVRNTLLQSKVGLLTYYVKLYPGEVTLLTRPSIQMRLCCITGSVSRPRSQK

>NP_000453.2

MEKLHQCYWKSGEPQSDDIEASRMKRAAAKHLIERYYHQLTEGCGNEACTNEFCASCPTFLRMDNNAAAIKALELYKINAKLCDPHPSKKGASSAYLENSKGAPNNSCSEIKMNKKGARIDFKDVTYLTEEKVYEILELCREREDYSPLIRVIGRVFSSAEALVQSFRKVKQHTKEELKSLQAKDEDKDEDEKEKAACSAAAMEEDSEASSSRIGDSSQGDNNLQKLGPDDVSVDIDAIRRVYTRLLSNEKIETAFLNALVYLSPNVECDLTYHNVYSRDPNYLNLFIIVMENRNLHSPEYLEMALPLFCKAMSKLPLAAQGKLIRLWSKYNADQIRRMMETFQQLITYKVISNEFNSRNLVNDDDAIVAASKCLKMVYYANVVGGEVDTNHNEEDDEEPIPESSELTLQELLGEERRNKKGPRVDPLETELGVKTLDCRKPLIPFEEFINEPLNEVLEMDKDYTFFKVETENKFSFMTCPFILNAVTKNLGLYYDNRIRMYSERRITVLYSLVQGQQLNPYLRLKVRRDHIIDDALVRLEMIAMENPADLKKQLYVEFEGEQGVDEGGVSKEFFQLVVEEIFNPDIGMFTYDESTKLFWFNPSSFETEGQFTLIGIVLGLAIYNNCILDVHFPMVVYRKLMGKKGTFRDLGDSHPVLYQSLKDLLEYEGNVEDDMMITFQISQTDLFGNPMMYDLKENGDKIPITNENRKEFVNLYSDYILNKSVEKQFKAFRRGFHMVTNESPLKYLFRPEEIELLICGSRNLDFQALEETTEYDGGYTRDSVLIREFWEIVHSFTDEQKRLFLQFTTGTDRAPVGGLGKLKMIIAKNGPDTERLPTSHTCFNVLLLPEYSSKEKLKERLLKAITYAKGFGML

164 NP_003121.1 NP_005822.1

>NP_003121.1

MAYPGHPGAGGGYYPGGYGGAPGGPAFPGQTQDPLYGYFAAVAGQDGQIDADELQRCLTQSGIAGGYKPFNLETCRLMVSMLDRDMSGTMGFNEFKELWAVLNGWRQHFISFDTDRSGTVDPQELQKALTTMGFRLSPQAVNSIAKRYSTNGKITFDDYIACCVKLRALTDSFRRRDTAQQGVVNFPYDDFIQCVMSV

>NP_005822.1

MEETIKDPPTSAVLLDHCHFSQVIFNSVEKFYIPGGDVTCHYTFTQHFIPRRKDWIGIFRVGWKTTREYYTFMWVTLPIDLNNKSAKQQEVQFKAYYLPKDDEYYQFCYVDEDGVVRGASIPFQFRPENEEDILVVTTQGEVEEIEQHNKELCKENQELKDSCISLQKQNSDMQAELQKKQEELETLQSINKKLELKVKEQKDYWETELLQLKEQNQKMSSENEKMGIRVDQLQAQLSTQEKEMEKLVQGDQDKTEQLEQLKKENDHLFLSLTEQRKDQKKLEQTVEQMKQNETTAMKKQQELMDENFDLSKRLSENEIICNALQRQKERLEGENDLLKRENSRLLSYMGLDFNSLPYQVPTSDEGGARQNPGLAYGNPYSGIQESSSPSPLSIKKCPICKADDICDHTLEQQQMQPLCFNCPICDKIFPATEKQIFEDHVFCHSL

165 NP_002982.2 NP_001828.1

>NP_002982.2

MAGLMTIVTSLLFLGVCAHHIIPTGSVVIPSPCCMFFVSKRIPENRVVSYQLSSRSTCLKAGVIFTTKKGQQFCGDPKQEWVQRYMKNLDAKQKKASPRARAVAVKGPVQRYPGNQTTC

>NP_001828.1

MTTSLDTVETFGTTSYYDDVGLLCEKADTRALMAQFVPPLYSLVFTVGLLGNVVVVMILIKYRRLRIMTNIYLLNLAISDLLFLVTLPFWIHYVRGHNWVFGHGMCKLLSGFYHTGLYSEIFFIILLTIDRYLAIVHAVFALRARTVTFGVITSIVTWGLAVLAALPEFIFYETEELFEETLCSALYPEDTVYSWRHFHTLRMTIFCLVLPLLVMAICYTGIIKTLLRCPSKKKYKAIRLIFVIMAVFFIFWTPYNVAILLSSYQSILFGNDCERSKHLDLVMLVTEVIAYSHCCMNPVIYAFVGERFRKYLRHFFHRHLLMHLGRYIPFLPSEKLERTSSVSPSTAEPELSIVF

166 NP_005577.1 NP_056207.2

>NP_005577.1

MYQVSGQRPSGCDAPYGAPSAAPGPAQTLSLLPGLEVVTGSTHPAEAAPEEGSLEEAATPMPQGNGPGIPQGLDSTDLDVPTEAVTCQPQGNPLGCTPLLPNDSGHPSELGGTRRAGNGALGGPKAHRKLQTHPSLASQGSKKSKSSSKSTTSQIPLQAQEDCCVHCILSCLFCEFLTLCNIVLDCATCGSCSSEDSCLCCCCCGSGECADCDLPCDLDCGILDACCESADCLEICMECCGLCFSS

>NP_056207.2

MATPLPPPSPRHLRLLRLLLSGLVLGAALRGAAAGHPDVAACPGSLDCALKRRARCPPGAHACGPCLQPFQEDQQGLCVPRMRRPPGGGRPQPRLEDEIDFLAQELARKESGHSTPPLPKDRQRLPEPATLGFSARGQGLELGLPSTPGTPTPTPHTSMGSPVSSDPVHMSPLEPRGGQGDGLALVLILAFCVAGAAALSVASLCWCRLHREIRLTQKADYATAKAPGSPAAPRISPGDQRLAQSAEMYHYQHQRQQMLCLERHKEPPKELDTASSDEENEDGDFTVYECPGLAPTGEMEVRNPLFDHAALSAPLPAPSSPPALP

167 NP_068799.2 NP_000537.3

>NP_068799.2

MNIDDKLEGLFLKCGGIDEMQSSRTMVVMGGVSGQSTVSGELQDSVLQDRSMPHQEILAADEVLQESEMRQQDMISHDELMVHEETVKNDEEQMETHERLPQGLQYALNVPISVKQEITFTDVSEQLMRDKKQIREPVDLQKKKKRKQRSPAKILTINEDGSLGLKTPKSHVCEHCNAAFRTNYHLQRHVFIHTGEKPFQCSQCDMRFIQKYLLQRHEKIHTGEKPFRCDECGMRFIQKYHMERHKRTHSGEKPYQCEYCLQYFSRTDRVLKHKRMCHENHDKKLNRCAIKGGLLTSEEDSGFSTSPKDNSLPKKKRQKTEKKSSGMDKESALDKSDLKKDKNDYLPLYSSSTKVKDEYMVAEYAVEMPHSSVGGSHLEDASGEIHPPKLVLKKINSKRSLKQPLEQNQTISPLSTYEESKVSKYAFELVDKQALLDSEGNADIDQVDNLQEGPSKPVHSSTNYDDAMQFLKKKRYLQAASNNSREYALNVGTIASQPSVTQAAVASVIDESTTASILESQALNVEIKSNHDKNVIPDEVLQTLLDHYSHKANGQHEISFSVADTEVTSSISINSSEVPEVTPSENVGSSSQASSSDKANMLQEYSKFLQQALDRTSQNDAYLNSPSLNFVTDNQTLPNQPAFSSIDKQVYATMPINSFRSGMNSPLRTTPDKSHFGLIVGDSQHSFPFSGDETNHASATSTQDFLDQVTSQKKAEAQPVHQAYQMSSFEQPFRAPYHGSRAGIATQFSTANGQVNLRGPGTSAEFSEFPLVNVNDNRAGMTSSPDATTGQTFG

>NP_000537.3

MEEPQSDPSVEPPLSQETFSDLWKLLPENNVLSPLPSQAMDDLMLSPDDIEQWFTEDPGPDEAPRMPEAAPPVAPAPAAPTPAAPAPAPSWPLSSSVPSQKTYQGSYGFRLGFLHSGTAKSVTCTYSPALNKMFCQLAKTCPVQLWVDSTPPPGTRVRAMAIYKQSQHMTEVVRRCPHHERCSDSDGLAPPQHLIRVEGNLRVEYLDDRNTFRHSVVVPYEPPEVGSDCTTIHYNYMCNSSCMGGMNRRPILTIITLEDSSGNLLGRNSFEVRVCACPGRDRRTEEENLRKKGEPHHELPPGSTKRALPNNTSSSPQPKKKPLDGEYFTLQIRGRERFEMFRELNEALELKDAQAGKEPGGSRAHSSHLKSKKGQSTSRHKKLMFKTEGPDSD

168 NP_937859.1 NP_009107.1

>NP_937859.1

MTVGKSSKMLQHIDYRMRCILQDGRIFIGTFKAFDKHMNLILCDCDEFRKIKPKNSKQAEREEKRVLGLVLLRGENLVSMTVEGPPPKDTGIARVPLAGAAGGPGIGRAAGRGIPAGVPMPQAPAGLAGPVRGVGGPSQQVMTPQGRGTVAAAAAAATASIAGAPTQYPPGRGGPPPPMGRGAPPPGMMGPPPGMRPPMGPPMGIPPGRGTPMGMPPPGMRPPPPGMRGPPPPGMRPPRP

>NP_009107.1

MSRFLNVLRSWLVMVSIIAMGNTLQSFRDHTFLYEKLYTGKPNLVNGLQARTFGIWTLLSSVIRCLCAIDIHNKTLYHITLWTFLLALGHFLSELFVYGTAAPTIGVLAPLMVASFSILGMLVGLRYLEVEPVSRQKKRN

169 NP_005634.1 NP_004597.2

>NP_005634.1

MDDAHESPSDKGGETGESDETAAVPGDPGATDTDGIPEETDGDADVDLKEAAAEEGELESQDVSDLTTVEREDSSLLNPAAKKLKIDTKEKKEKKQKVDEDEIQKMQILVSSFSEEQLNRYEMYRRSAFPKAAIKRLIQSITGTSVSQNVVIAMSGISKVFVGEVVEEALDVCEKWGEMPPLQPKHMREAVRRLKSKGQIPNSKHKKIIFF

>NP_004597.2

MGPGCDLLLRTAATITAAAIMSDTDSDEDSAGGGPFSLAGFLFGNINGAGQLEGESVLDDECKKHLAGLGALGLGSLITELTANEELTGTDGALVNDEGWVRSTEDAVDYSDINEVAEDESRRYQQTMGSLQPLCHSDYDEDDYDADCEDIDCKLMPPPPPPPGPMKKDKDQDSITGVSENGEGIILPSIIAPSSLASEKVDFSSSSDSESEMGPQEATQAESEDGKLTLPLAGIMQHDATKLLPSVTELFPEFRPGKVLRFLRLFGPGKNVPSVWRSARRKRKKKHRELIQEEQIQEVECSVESEVSQKSLWNYDYAPPPPPEQCLSDDEITMMAPVESKFSQSTGDIDKVTDTKPRVAEWRYGPARLWYDMLGVPEDGSGFDYGFKLRKTEHEPVIKSRMIEEFRKLEENNGTDLLADENFLMVTQLHWEDDIIWDGEDVKHKGTKPQRASLAGWLPSSMTRNAMAYNVQQGFAATLDDDKPWYSIFPIDNEDLVYGRWEDNIIWDAQAMPRLLEPPVLTLDPNDENLILEIPDEKEEATSNSPSKESKKESSLKKSRILLGKTGVIKEEPQQNMSQPEVKDPWNLSNDEYYYPKQQGLRGTFGGNIIQHSIPAVELRQPFFPTHMGPIKLRQFHRPPLKKYSFGALSQPGPHSVQPLLKHIKKKAKMREQERQASGGGEMFFMRTPQDLTGKDGDLILAEYSEENGPLMMQVGMATKIKNYYKRKPGKDPGAPDCKYGETVYCHTSPFLGSLHPGQLLQAFENNLFRAPIYLHKMPETDFLIIRTRQGYYIRELVDIFVVGQQCPLFEVPGPNSKRANTHIRDFLQVFIYRLFWKSKDRPRRIRMEDIKKAFPSHSESSIRKRLKLCADFKRTGMDSNWWVLKSDFRLPTEEEIRAMVSPEQCCAYYSMIAAEQRLKDAGYGEKSFFAPEEENEEDFQMKIDDEVRTAPWNTTRAFIAAMKGKCLLEVTGVADPTGCGEGFSYVKIPNKPTQQKDDKEPQPVKKTVTGTDADLRRLSLKNAKQLLRKFGVPEEEIKKLSRWEVIDVVRTMSTEQARSGEGPMSKFARGSRFSVAEHQERYKEECQRIFDLQNKVLSSTEVLSTDTDSSSAEDSDFEEMGKNIENMLQNKKTSSQLSREREEQERKELQRMLLAAGSAASGNNHRDDDTASVTSLNSSATGRCLKIYRTFRDEEGKEYVRCETVRKPAVIDAYVRIRTTKDEEFIRKFALFDEQHREEMRKERRRIQEQLRRLKRNQEKEKLKGPPEKKPKKMKERPDLKLKCGACGAIGHMRTNKFCPLYYQTNAPPSNPVAMTEEQEEELEKTVIHNDNEELIKVEGTKIVLGKQLIESADEVRRKSLVLKFPKQQLPPKKKRRVGTTVHCDYLNRPHKSIHRRRTDPMVTLSSILESIINDMRDLPNTYPFHTPVNAKVVKDYYKIITRPMDLQTLRENVRKRLYPSREEFREHLELIVKNSATYNGPKHSLTQISQSMLDLCDEKLKEKEDKLARLEKAINPLLDDDDQVAFSFILDNIVTQKMMAVPDSWPFHHPVNKKFVPDYYKVIVNPMDLETIRKNISKHKYQSRESFLDDVNLILANSVKYNGPESQYTKTAQEIVNVCYQTLTEYDEHLTQLEKDICTAKEAALEEAELESLDPMTPGPYTPQPPDLYDTNTSLSMSRDASVFQDESNMSVLDIPSATPEKQVTQEGEDGDGDLADEEEGTVQQPQASVLYEDLLMSEGEDDEEDAGSDEEGDNPFSAIQLSESGSDSDVGSGGIRPKQPRMLQENTRMDMENEESMMSYEGDGGEASHGLEDSNISYGSYEEPDPKSNTQDTSFSSIGGYEVSEEEEDEEEEEQRSGPSVLSQVHLSEDEEDSEDFHSIAGDSDLDSDE

170 NP_000154.1 NP_072051.1

>NP_000154.1

MDLWQLLLTLALAGSSDAFSGSEATAAILSRAPWSLQSVNPGLKTNSSKEPKFTKCRSPERETFSCHWTDEVHHGTKNLGPIQLFYTRRNTQEWTQEWKECPDYVSAGENSCYFNSSFTSIWIPYCIKLTSNGGTVDEKCFSVDEIVQPDPPIALNWTLLNVSLTGIHADIQVRWEAPRNADIQKGWMVLEYELQYKEVNETKWKMMDPILTTSVPVYSLKVDKEYEVRVRSKQRNSGNYGEFSEVLYVTLPQMSQFTCEEDFYFPWLLIIIFGIFGLTVMLFVFLFSKQQRIKMLILPPVPVPKIKGIDPDLLKEGKLEEVNTILAIHDSYKPEFHSDDSWVEFIELDIDEPDEKTEESDTDRLLSSDHEKSHSNLGVKDGDSGRTSCCEPDILETDFNANDIHEGTSEVAQPQRLKGEADLLCLDQKNQNNSPYHDACPATQQPSVIQAEKNKPQPLPTEGAESTHQAAHIQLSNPSSLSNIDFYAQVSDITPAGSVVLSPGQKNKAGMSQCDMHPEMVSLCQENFLMDNAYFCEADAKKCIPVAPHIKVESHIQPSLNQEDIYITTESLTTAAGRPGTGEHVPGSEMPVPDYTSIHIVQSPQGLILNATALPLPDKEFLSSCGYVSTDQLNKIMP

>NP_072051.1

MAAGSRTSLLLAFGLLCLSWLQEGSAFPTIPLSRLFDNAMLRARRLYQLAYDTYQEFEEAYILKEQKYSFLQNPQTSLCFSESIPTPSNRVKTQQKSNLELLRISLLLIQSWLEPVQLLRSVFANSLVYGASDSNVYRHLKDLEEGIQTLMWVRVAPGIPNPGAPLASRDWGEKHCCPLFSSQALTQENSPYSSFPLVNPPGLSLQPGGEGGKWMNERGREQCPSAWPLLLFLHFAEAGRWQPPDWADLQSVLQQV

171 NP_001229.1 NP_001780.2

>NP_001229.1

MPRERRERDAKERDTMKEDGGAEFSARSRKRKANVTVFLQDPDEEMAKIDRTARDQCGSQPWDNNAVCADPCSLIPTPDKEDDDRVYPNSTCKPRIIAPSRGSPLPVLSWANREEVWKIMLNKEKTYLRDQHFLEQHPLLQPKMRAILLDWLMEVCEVYKLHRETFYLAQDFFDRYMATQENVVKTLLQLIGISSLFIAAKLEEIYPPKLHQFAYVTDGACSGDEILTMELMIMKALKWRLSPLTIVSWLNVYMQVAYLNDLHEVLLPQYPQQIFIQIAELLDLCVLDVDCLEFPYGILAASALYHFSSSELMQKVSGYQWCDIENCVKWMVPFAMVIRETGSSKLKHFRGVADEDAHNIQTHRDSLDLLDKARAKKAMLSEQNRASPLPSGLLTPPQSGKKQSSGPEMA

>NP_001780.2

MELGPEPPHRRRLLFACSPPPASQPVVKALFGASAAGGLSPVTNLTVTMDQLQGLGSDYEQPLEVKNNSNLQRMGSSESTDSGFCLDSPGPLDSKENLENPMRRIHSLPQKLLGCSPALKRSHSDSLDHDIFQLIDPDENKENEAFEFKKPVRPVSRGCLHSHGLQEGKDLFTQRQNSAPARMLSSNERDSSEPGNFIPLFTPQSPVTATLSDEDDGFVDLLDGENLKNEEETPSCMASLWTAPLVMRTTNLDNRCKLFDSPSLCSSSTRSVLKRPERSQEESPPGSTKRRKSMSGASPKESTNPEKAHETLHQSLSLASSPKGTIENILDNDPRDLIGDFSKGYLFHTVAGKHQDLKYISPEIMASVLNGKFANLIKEFVIIDCRYPYEYEGGHIKGAVNLHMEEEVEDFLLKKPIVPTDGKRVIVVFHCEFSSERGPRMCRYVRERDRLGNEYPKLHYPELYVLKGGYKEFFMKCQSYCEPPSYRPMHHEDFKEDLKKFRTKSRTWAGEKSKREMYSRLKKL

172 NP_000057.1 NP_001822.2

>NP_000057.1

MKNSRTWAWRAPVELFLLCAALGCLSLPGSRGERPHSFGSNAVNKSFAKSRQMRSVDVTLMPIDCELSSWSSWTTCDPCQKKRYRYAYLLQPSQFHGEPCNFSDKEVEDCVTNRPCGSQVRCEGFVCAQTGRCVNRRLLCNGDNDCGDQSDEANCRRIYKKCQHEMDQYWGIGSLASGINLFTNSFEGPVLDHRYYAGGCSPHYILNTRFRKPYNVESYTPQTQGKYEFILKEYESYSDFERNVTEKMASKSGFSFGFKIPGIFELGISSQSDRGKHYIRRTKRFSHTKSVFLHARSDLEVAHYKLKPRSLMLHYEFLQRVKRLPLEYSYGEYRDLFRDFGTHYITEAVLGGIYEYTLVMNKEAMERGDYTLNNVHACAKNDFKIGGAIEEVYVSLGVSVGKCRGILNEIKDRNKRDTMVEDLVVLVRGGASEHITTLAYQELPTADLMQEWGDAVQYNPAIIKVKVEPLYELVTATDFAYSSTVRQNMKQALEEFQKEVSSCHCAPCQGNGVPVLKGSRCDCICPVGSQGLACEVSYRKNTPIDGKWNCWSNWSSCSGRRKTRQRQCNNPPPQNGGSPCSGPASETLDCS

>NP_001822.2

MQVCSQPQRGCVREQSAINTAPPSAHNAASPGGARGHRVPLTEACKDSRIGGMMKTLLLFVGLLLTWESGQVLGDQTVSDNELQEMSNQGSKYVNKEIQNAVNGVKQIKTLIEKTNEERKTLLSNLEEAKKKKEDALNETRESETKLKELPGVCNETMMALWEECKPCLKQTCMKFYARVCRSGSGLVGRQLEEFLNQSSPFYFWMNGDRIDSLLENDRQQTHMLDVMQDHFSRASSIIDELFQDRFFTREPQDTYHYLPFSLPHRRPHFFFPKSRIVRSLMPFSPYEPLNFHAMFQPFLEMIHEAQQAMDIHFHSPAFQHPPTEFIREGDDDRTVCREIRHNSTGCLRMKDQCDKCREILSVDCSTNNPSQAKLRRELDESLQVAERLTRKYNELLKSYQWKMLNTSSLLEQLNEQFNWVSRLANLTQGEDQYYLRVTTVASHTSDSDVPSGVTEVVVKLFDSDPITVTVPVEVSRKNPKFMETVAEKALQEYRKKHREE

173 NP_001014809.1 NP_788276.1

>NP_001014809.1

MADRRRAWNTEDDLPVYLARPGSAAQTPRQKYGGMFAAVEGAYENKTIDFDAYSVGRRGSARTPRSAGRPDAVGLPGPGGSEDTASDVSEPSGSAVSSPGERDERPPTLRIRRPAPRDLPLGRDNGQSDRLLIKGGRIINDDQSLYADVYLEDGLIKQIGENLIVPGGVKTIEANGRMVIPGGIDVNTYLQKPSQGMTAADDFFQGTRAALVGGTTMIIDHVVPEPGSSLLTSFEKWHEAADTKSCCDYSLHVDITSWYDGVREELEVLVQDKGVNSFQVYMAYKDVYQMSDSQLYEAFTFLKGLGAVILVHAENGDLIAQEQKRILEMGITGPEGHALSRPEELEAEAVFRAITIAGRINCPVYITKVMSKSAADIIALARKKGPLVFGEPIAASLGTDGTHYWSKNWAKAAAFVTSPPLSPDPTTPDYLTSLLACGDLQVTGSGHCPYSTAQKAVGKDNFTLIPEGVNGIEERMTVVWDKAVATGKMDENQFVAVTSTNAAKIFNLYPRKGRIAVGSDADVVIWDPDKLKTITAKSHKSAVEYNIFEGMECHGSPLVVISQGKIVFEDGNINVNKGMGRFIPRKAFPEHLYQRVKIRNKVFGLQGVSRGMYDGPVYEVPATPKYATPAPSAKSSPSKHQPPPIRNLHQSNFSLSGAQIDDNNPRRTGHRIVAPPGGRSNITSLG

>NP_788276.1

MEDHQHVPIDIQTSKLLDWLVDRRHCSLKWQSLVLTIREKINAAIQDMPESEEIAQLLSGSYIHYFHCLRILDLLKGTEASTKNIFGRYSSQRMKDWQEIIALYEKDNTYLVELSSLLVRNVNYEIPSLKKQIAKCQQLQQEYSRKEEECQAGAAEMREQFYHSCKQYGITGENVRGELLALVKDLPSQLAEIGAAAQQSLGEAIDVYQASVGFVCESPTEQVLPMLRFVQKRGNSTVYEWRTGTEPSVVERPHLEELPEQVAEDAIDWGDFGVEAVSEGTDSGISAEAAGIDWGIFPESDSKDPGGDGIDWGDDAVALQITVLEAGTQAPEGVARGPDALTLLEYTETRNQFLDELMELEIFLAQRAVELSEEADVLSVSQFQLAPAILQGQTKEKMVTMVSVLEDLIGKLTSLQLQHLFMILASPRYVDRVTEFLQQKLKQSQLLALKKELMVQKQQEALEEQAALEPKLDLLLEKTKELQKLIEADISKRYSGRPVNLMGTSL

174 NP_858045.1 NP_000035.2

>NP_858045.1

MSGLGENLDPLASDSRKRKLPCDTPGQGLTCSGEKRRREQESKYIEELAELISANLSDIDNFNVKPDKCAILKETVRQIRQIKEQGKTISNDDDVQKADVSSTGQGVIDKDSLGPLLLQALDGFLFVVNRDGNIVFVSENVTQYLQYKQEDLVNTSVYNILHEEDRKDFLKNLPKSTVNGVSWTNETQRQKSHTFNCRMLMKTPHDILEDINASPEMRQRYETMQCFALSQPRAMMEEGEDLQSCMICVARRITTGERTFPSNPESFITRHDLSGKVVNIDTNSLRSSMRPGFEDIIRRCIQRFFSLNDGQSWSQKRHYQEAYLNGHAETPVYRFSLADGTIVTAQTKSKLFRNPVTNDRHGFVSTHFLQREQNGYRPNPNPVGQGIRPPMAGCNSSVGGMSMSPNQGLQMPSSRAYGLADPSTTGQMSGARYGGSSNIASLTPGPGMQSPSSYQNNNYGLNMSSPPHGSPGLAPNQQNIMISPRNRGSPKIASHQFSPVAGVHSPMASSGNTGNHSFSSSSLSALQAISEGVGTSLLSTLSSPGPKLDNSPNMNITQPSKVSNQDSKSPLGFYCDQNPVESSMCQSNSRDHLSDKESKESSVEGAENQRGPLESKGHKKLLQLLTCSSDDRGHSSLTNSPLDSSCKESSVSVTSPSGVSSSTSGGVSSTSNMHGSLLQEKHRILHKLLQNGNSPAEVAKITAEATGKDTSSITSCGDGNVVKQEQLSPKKKENNALLRYLLDRDDPSDALSKELQPQVEGVDNKMSQCTSSTIPSSSQEKDPKIKTETSEEGSGDLDNLDAILGDLTSSDFYNNSISSNGSHLGTKQQVFQGTNSLGLKSSQSVQSIRPPYNRAVSLDSPVSVGSSPPVKNISAFPMLPKQPMLGGNPRMMDSQENYGSSMGGPNRNVTVTQTPSSGDWGLPNSKAGRMEPMNSNSMGRPGGDYNTSLPRPALGGSIPTLPLRSNSIPGARPVLQQQQQMLQMRPGEIPMGMGANPYGQAAASNQLGSWPDGMLSMEQVSHGTQNRPLLRNSLDDLVGPPSNLEGQSDERALLDQLHTLLSNTDATGLEEIDRALGIPELVNQGQALEPKQDAFQGQEAAVMMDQKAGLYGQTYPAQGPPMQGGFHLQGQSPSFNSMMNQMNQQGNFPLQGMHPRANIMRPRTNTPKQLRMQLQQRLQGQQFLNQSRQALELKMENPTAGGAAVMRPMMQPQVSSQQGFLNAQMVAQRSRELLSHHFRQQRVAMMMQQQQQQQQQQQQQQQQQQQQQQQQQQQQQTQAFSPPPNVTASPSMDGLLAGPTMPQAPPQQFPYQPNYGMGQQPDPAFGRVSSPPNAMMSSRMGPSQNPMMQHPQAASIYQSSEMKGWPSGNLARNSSFSQQQFAHQGNPAVYSMVHMNGSSGHMGQMNMNPMPMSGMPMGPDQKYC

>NP_000035.2

MEVQLGLGRVYPRPPSKTYRGAFQNLFQSVREVIQNPGPRHPEAASAAPPGASLLLLQQQQQQQQQQQQQQQQQQQQQQQETSPRQQQQQQGEDGSPQAHRRGPTGYLVLDEEQQPSQPQSALECHPERGCVPEPGAAVAASKGLPQQLPAPPDEDDSAAPSTLSLLGPTFPGLSSCSADLKDILSEASTMQLLQQQQQEAVSEGSSSGRAREASGAPTSSKDNYLGGTSTISDNAKELCKAVSVSMGLGVEALEHLSPGEQLRGDCMYAPLLGVPPAVRPTPCAPLAECKGSLLDDSAGKSTEDTAEYSPFKGGYTKGLEGESLGCSGSAAAGSSGTLELPSTLSLYKSGALDEAAAYQSRDYYNFPLALAGPPPPPPPPHPHARIKLENPLDYGSAWAAAAAQCRYGDLASLHGAGAAGPGSGSPSAAASSSWHTLFTAEEGQLYGPCGGGGGGGGGGGGGGGGGGGGGGGEAGAVAPYGYTRPPQGLAGQESDFTAPDVWYPGGMVSRVPYPSPTCVKSEMGPWMDSYSGPYGDMRLETARDHVLPIDYYFPPQKTCLICGDEASGCHYGALTCGSCKVFFKRAAEGKQKYLCASRNDCTIDKFRRKNCPSCRLRKCYEAGMTLGARKLKKLGNLKLQEEGEASSTTSPTEETTQKLTVSHIEGYECQPIFLNVLEAIEPGVVCAGHDNNQPDSFAALLSSLNELGERQLVHVVKWAKALPGFRNLHVDDQMAVIQYSWMGLMVFAMGWRSFTNVNSRMLYFAPDLVFNEYRMHKSRMYSQCVRMRHLSQEFGWLQITPQEFLCMKALLLFSIIPVDGLKNQKFFDELRMNYIKELDRIIACKRKNPTSCSRRFYQLTKLLDSVQPIARELHQFTFDLLIKSHMVSVDFPEMMAEIISVQVPKILSGKVKPIYFHTQ

175 NP_054726.1 NP_877428.1

>NP_054726.1

MEPGTNSFRVEFPDFSSTILQKLNQQRQQGQLCDVSIVVQGHIFRAHKAVLAASSPYFCDQVLLKNSRRIVLPDVMNPRVFENILLSSYTGRLVMPAPEIVSYLTAASFLQMWHVVDKCTEVLEGNPTVLCQKLNHGSDHQSPSSSSYNGLVESFELGSGGHTDFPKAQELRDGENEEESTKDELSSQLTEHEYLPSNSSTEHDRLSTEMASQDGEEGASDSAEFHYTRPMYSKPSIMAHKRWIHVKPERLEQACEGMDVHATYDEHQVTESINTVQTEHTVQPSGVEEDFHIGEKKVEAEFDEQADESNYDEQVDFYGSSMEEFSGERSDGNLIGHRQEAALAAGYSENIEMVTGIKEEASHLGFSATDKLYPCQCGKSFTHKSQRDRHMSMHLGLRPYGCGVCGKKFKMKHHLVGHMKIHTGIKPYECNICAKRFMWRDSFHRHVTSCTKSYEAAKAEQNTTEAN

>NP_877428.1

MDEEEDNLSLLTALLEENESALDCNSEENNFLTRENGEPDAFDELFDADGDGESYTEEADDGETGETRDEKENLATLFGDMEDLTDEEEVPASQSTENRVLPAPAPRREKTNEELQEELRNLQEQMKALQEQLKVTTIKQTASPARLQKSPVEKSPRPPLKERRVQRIQESTCFSAELDVPALPRTKRVARTPKASPPDPKSSSSRMTSAPSQPLQTISRNKPSGITRGQIVGTPGSSGETTQPICVEAFSGLRLRRPRVSSTEMNKKMTGRKLIRLSQIKEKMAREKLEEIDWVTFGVILKKVTPQSVNSGKTFSIWKLNDLRDLTQCVSLFLFGEVHKALWKTEQGTVVGILNANPMKPKDGSEEVCLSIDHPQKVLIMGEALDLGTCKAKKKNGEPCTQTVNLRDCEYCQYHVQAQYKKLSAKRADLQSTFSGGRIPKKFARRGTSLKERLCQDGFYYGGVSSASYAASIAAAVAPKKKIQTTLSNLVVKGTNLIIQETRQKLGIPQKSLSCSEEFKELMDLPTCGARNLKQHLAKATASGIMGSPKPAIKSISASALLKQQKQRMLEMRRRKSEEIQKRFLQSSSEVESPAVPSSSRQPPAQPPRTGSEFPRLEGAPATMTPKLGRGVLEGDDVLFYDESPPPRPKLSALAEAKKLAAITKLRAKGQVLTKTNPNSIKKKQKDPQDILEVKERVEKNTMFSSQAEDELEPARKKRREQLAYLESEEFQKILKAKSKHTGILKEAEAEMQERYFEPLVKKEQMEEKMRNIREVKCRVVTCKTCAYTHFKLLETCVSEQHEYHWHDGVKRFFKCPCGNRSISLDRLPNKHCSNCGLYKWERDGMLKEKTGPKIGGETLLPRGEEHAKFLNSLK

176 NP_056981.2 NP_000456.1

>NP_056981.2

MAAELVEAKNMVMSFRVSDLQMLLGFVGRSKSGLKHELVTRALQLVQFDCSPELFKKIKELYETRYAKKNSEPAPQPHRPLDPLTMHSTYDRAGAVPRTPLAGPNIDYPVLYGKYLNGLGRLPAKTLKPEVRLVKLPFFNMLDELLKPTELVPQNNEKLQESPCIFALTPRQVELIRNSRELQPGVKAVQVVLRICYSDTSCPQEDQYPPNIAVKVNHSYCSVPGYYPSNKPGVEPKRPCRPINLTHLMYLSSATNRITVTWGNYGKSYSVALYLVRQLTSSELLQRLKTIGVKHPELCKALVKEKLRLDPDSEIATTGVRVSLICPLVKMRLSVPCRAETCAHLQCFDAVFYLQMNEKKPTWMCPVCDKPAPYDQLIIDGLLSKILSECEDADEIEYLVDGSWCPIRAEKERSCSPQGAILVLGPSDANGLLPAPSVNGSGALGSTGGGGPVGSMENGKPGADVVDLTLDSSSSSEDEEEEEEEEEDEDEEGPRPKRRCPFQKGLVPAC

>NP_000456.1

MPDNRQPRNRQPRIRSGNEPRSAPAMEPDGRGAWAHSRAALDRLEKLLRCSRCTNILREPVCLGGCEHIFCSNCVSDCIGTGCPVCYTPAWIQDLKINRQLDSMIQLCSKLRNLLHDNELSDLKEDKPRKSLFNDAGNKKNSIKMWFSPRSKKVRYVVSKASVQTQPAIKKDASAQQDSYEFVSPSPPADVSERAKKASARSGKKQKKKTLAEINQKWNLEAEKEDGEFDSKEESKQKLVSFCSQPSVISSPQINGEIDLLASGSLTESECFGSLTEVSLPLAEQIESPDTKSRNEVVTPEKVCKNYLTSKKSLPLENNGKRGHHNRLSSPISKRCRTSILSTSGDFVKQTVPSENIPLPECSSPPSCKRKVGGTSGRKNSNMSDEFISLSPGTPPSTLSSSSYRQVMSSPSAMKLLPNMAVKRNHRGETLLHIASIKGDIPSVEYLLQNGSDPNVKDHAGWTPLHEACNHGHLKVVELLLQHKALVNTTGYQNDSPLHDAAKNGHVDIVKLLLSYGASRNAVNIFGLRPVDYTDDESMKSLLLLPEKNESSSASHCSVMNTGQRRDGPLVLIGSGLSSEQQKMLSELAVILKAKKYTEFDSTVTHVVVPGDAVQSTLKCMLGILNGCWILKFEWVKACLRRKVCEQEEKYEIPEGPRRSRLNREQLLPKLFDGCYFYLWGTFKHHPKDNLIKLVTAGGGQILSRKPKPDSDVTQTINTVAYHARPDSDQRFCTQYIIYEDLCNYHPERVRQGKVWKAPSSWFIDCVMSFELLPLDS

177 NP_009097.2 AAH14214.1

>NP_009097.2

MSGQSLTDRITAAQHSVTGSAVSKTVCKATTHEIMGPKKKHLDYLIQCTNEMNVNIPQLADSLFERTTNSSWVVVFKSLITTHHLMVYGNERFIQYLASRNTLFNLSNFLDKSGLQGYDMSTFIRRYSRYLNEKAVSYRQVAFDFTKVKRGADGVMRTMNTEKLLKTVPIIQNQMDALLDFNVNSNELTNGVINAAFMLLFKDAIRLFAAYNEGIINLLEKYFDMKKNQCKEGLDIYKKFLTRMTRISEFLKVAEQVGIDRGDIPDLSQAPSSLLDALEQHLASLEGKKIKDSTAASRATTLSNAVSSLASTGLSLTKVDEREKQAALEEEQARLKALKEQRLKELAKKPHTSLTTAASPVSTSAGGIMTAPAIDIFSTPSSSNSTSKLPNDLLDLQQPTFHPSVHPMSTASQVASTWGDPFSATVDAVDDAIPSLNPFLTKSSGDVHLSISSDVSTFTTRTPTHEMFVGFTPSPVAQPHPSAGLNVDFESVFGNKSTNVIVDSGGFDELGGLLKPTVASQNQNLPVAKLPPSKLVSDDLDSSLANLVGNLGIGNGTTKNDVNWSQPGEKKLTGGSNWQPKVAPTTAWNAATMAPPVMAYPATTPTGMIGYGIPPQMGSVPVMTQPTLIYSQPVMRPPNPFGPVSGAQIQFM

>AAH14214.1

MPAVSKGDGMRGLAVFISDIRNCKSKEAEIKRINKELANIRSKFKGDKALDGYSKKKYVCKLLFIFLLGHDIDFGHMEAVNLLSSNKYTEKQIGYLFISVLVNSNSELIRLINNAIKNDLASRNPTFMCLALHCIANVGSREMGEAFAADIPRILVAGDSMDSVKQSAALCLLRLYKASPDLVPMGEWTARVVHLLNDQHMGVVTAAVSLITCLCKKNPDDFKTCVSLAVSRLSRIVSSASTDLQDYTYYFVPAPWLSVKLLRLLQCYPLPEDAAVKGRLVECLETVLNKAQEPPKSKKVQHSNAKNAILFETISLIIHYDSEPNLLVRACNQLGQFLQHRETNLRYLALESMCTLASSEFSHEAVKTHIDTVINALKTERDVSVRQRAADLLYAMCDRSNAKQIVSEMLRYLETADYAIREEIVLKVAILAEKYAVDYSWYVDTILNLIRIAGDYVSEEVWYRVLQIVTNRDDVQGYAAKTVFEALQAPACHENMVKVGGYILGEFGNLIAGDPRSSPPVQFSLLHSKFHLCSVATRALLLSTYIKFINLFPETKATIQGVLRAGSQLRNADVELQQRAVEYLTLSSVASTDVLATVLEEMPPFPERESSILAKLKRKKGPGAGSALDDGRRDPSSNDINGGMEPTPSTVSTPSPSADLLGLRAAPPPAAPPASAGAGNLLVDVFDGPAAQPSLGPTPEEAFLSPGPEDIGPPIPEADELLNKFVCKNNGVLFENQLLQIGVKSEFRQNLGRMYLFYGNKTSVQFQNFSPTVVHPGDLQTQLAVQTKRVAAQVDGGAQVQQVLNIECLRDFLTPPLLSVRFRYGGAPQALTLKLPVTINKFFQPTEMAAQDFFQRWKQLSLPQQEAQKIFKANHPMDAEVTKAKLLGFGSALLDNVDPNPGDREDTRVWGMPGTFLRPFVFLFLFICCCLHSGGLGGVPLPPFPPQAQRGEGPGKWMSPPLPPHPVVAPPTPSPSRGCVLL

178 NP_004283.2 NP_003397.1

>NP_004283.2

MESPGESGAGSPGAPSPSSFTTGHLAREKPAQDPLYDVPNASGGQAGGPQRPGRVVSLRERLLLTRPVWLQLQANAAAALHMLRTEPPGTFLVRKSNTRQCQALCMRLPEASGPSFVSSHYILESPGGVSLEGSELMFPDLVQLICAYCHTRDILLLPLQLPRAIHHAATHKELEAISHLGIEFWSSSLNIKAQRGPAGGPVLPQLKARSPQELDQGTGAALCFFNPLFPGDLGPTKREKFKRSFKVRVSTETSSPLSPPAVPPPPVPVLPGAVPSQTERLPPCQLLRRESSVGYRVPAGSGPSLPPMPSLQEVDCGSPSSSEEEGVPGSRGSPATSPHLGRRRPLLRSMSAAFCSLLAPERQVGRAAAALMQDRHTAAGQLVQDLLTQVRAGPEPQELQGIRQALSRARAMLSAELGPEKLLSPKRLEHVLEKSLHCSVLKPLRPILAARLRRRLAADGSLGRLAEGLRLARAQGPGAFGSHLSLPSPVELEQVRQKLLQLLRTYSPSAQVKRLLQACKLLYMALRTQEGEGAGADEFLPLLSLVLAHCDLPELLLEAEYMSELLEPSLLTGEGGYYLTSLSASLALLSGLGQAHTLPLSPVQELRRSLSLWEQRRLPATHCFQHLLRVAYQDPSSGCTSKTLAVPPEASIATLNQLCATKFRVTQPNTFGLFLYKEQGYHRLPPGALAHRLPTTGYLVYRRAEWPETQGAVTEEEGSGQSEARSRGEEQGCQGDGDAGVKASPRDIREQSETTAEGGQGQAQEGPAQPGEPEAEGSRAAEE

>NP_003397.1

MDKNELVQKAKLAEQAERYDDMAACMKSVTEQGAELSNEERNLLSVAYKNVVGARRSSWRVVSSIEQKTEGAEKKQQMAREYREKIETELRDICNDVLSLLEKFLIPNASQAESKVFYLKMKGDYYRYLAEVAAGDDKKGIVDQSQQAYQEAFEISKKEMQPTHPIRLGLALNFSVFYYEILNSPEKACSLAKTAFDEAIAELDTLSEESYKDSTLIMQLLRDNLTLWTSDTQGDEAEAGEGGEN

179 NP_009330.1 NP_000407.1

>NP_009330.1

MSQWYELQQLDSKFLEQVHQLYDDSFPMEIRQYLAQWLEKQDWEHAANDVSFATIRFHDLLSQLDDQYSRFSLENNFLLQHNIRKSKRNLQDNFQEDPIQMSMIIYSCLKEERKILENAQRFNQAQSGNIQSTVMLDKQKELDSKVRNVKDKVMCIEHEIKSLEDLQDEYDFKCKTLQNREHETNGVAKSDQKQEQLLLKKMYLMLDNKRKEVVHKIIELLNVTELTQNALINDELVEWKRRQQSACIGGPPNACLDQLQNWFTIVAESLQQVRQQLKKLEELEQKYTYEHDPITKNKQVLWDRTFSLFQQLIQSSFVVERQPCMPTHPQRPLVLKTGVQFTVKLRLLVKLQELNYNLKVKVLFDKDVNERNTVKGFRKFNILGTHTKVMNMEESTNGSLAAEFRHLQLKEQKNAGTRTNEGPLIVTEELHSLSFETQLCQPGLVIDLETTSLPVVVISNVSQLPSGWASILWYNMLVAEPRNLSFFLTPPCARWAQLSEVLSWQFSSVTKRGLNVDQLNMLGEKLLGPNASPDGLIPWTRFCKENINDKNFPFWLWIESILELIKKHLLPLWNDGCIMGFISKERERALLKDQQPGTFLLRFSESSREGAITFTWVERSQNGGEPDFHAVEPYTKKELSAVTFPDIIRNYKVMAAENIPENPLKYLYPNIDKDHAFGKYYSRPKEAPEPMELDGPKGTGYIKTELISVSEVHPSRLQTTDNLLPMSPEEFDEVSRIVGSVEFDSMMNTV

>NP_000407.1

MALLFLLPLVMQGVSRAEMGTADLGPSSVPTPTNVTIESYNMNPIVYWEYQIMPQVPVFTVEVKNYGVKNSEWIDACINISHHYCNISDHVGDPSNSLWVRVKARVGQKESAYAKSEEFAVCRDGKIGPPKLDIRKEEKQIMIDIFHPSVFVNGDEQEVDYDPETTCYIRVYNVYVRMNGSEIQYKILTQKEDDCDEIQCQLAIPVSSLNSQYCVSAEGVLHVWGVTTEKSKEVCITIFNSSIKGSLWIPVVAALLLFLVLSLVFICFYIKKINPLKEKSIILPKSLISVVRSATLETKPESKYVSLITSYQPFSLEKEVVCEEPLSPATVPGMHTEDNPGKVEHTEELSSITEVVTTEENIPDVVPGSHLTPIERESSSPLSSNQSEPGSIALNSYHSRNCSESDHSRNGFDTDSSCLESHSSLSDSEFPPNNKGEIKTEGQELITVIKAPTSFGYDKPHVLVDLLVDDSGKESLIGYRPTEDSKEFS

180 NP_004347.1 NP_000213.1

>NP_004347.1

MGVEGCTKCIKYLLFVFNFVFWLAGGVILGVALWLRHDPQTTNLLYLELGDKPAPNTFYVGIYILIAVGAVMMFVGFLGCYGAIQESQCLLGTFFTCLVILFACEVAAGIWGFVNKDQIAKDVKQFYDQALQQAVVDDDANNAKAVVKTFHETLDCCGSSTLTALTTSVLKNNLCPSGSNIISNLFKEDCHQKIDDLFSGKLYLIGIAAIVVAVIMIFEMILSMVLCCGIRNSSVY

>NP_000213.1

MRGARGAWDFLCVLLLLLRVQTGSSQPSVSPGEPSPPSIHPGKSDLIVRVGDEIRLLCTDPGFVKWTFEILDETNENKQNEWITEKAEATNTGKYTCTNKHGLSNSIYVFVRDPAKLFLVDRSLYGKEDNDTLVRCPLTDPEVTNYSLKGCQGKPLPKDLRFIPDPKAGIMIKSVKRAYHRLCLHCSVDQEGKSVLSEKFILKVRPAFKAVPVVSVSKASYLLREGEEFTVTCTIKDVSSSVYSTWKRENSQTKLQEKYNSWHHGDFNYERQATLTISSARVNDSGVFMCYANNTFGSANVTTTLEVVDKGFINIFPMINTTVFVNDGENVDLIVEYEAFPKPEHQQWIYMNRTFTDKWEDYPKSENESNIRYVSELHLTRLKGTEGGTYTFLVSNSDVNAAIAFNVYVNTKPEILTYDRLVNGMLQCVAAGFPEPTIDWYFCPGTEQRCSASVLPVDVQTLNSSGPPFGKLVVQSSIDSSAFKHNGTVECKAYNDVGKTSAYFNFAFKGNNKEQIHPHTLFTPLLIGFVIVAGMMCIIVMILTYKYLQKPMYEVQWKVVEEINGNNYVYIDPTQLPYDHKWEFPRNRLSFGKTLGAGAFGKVVEATAYGLIKSDAAMTVAVKMLKPSAHLTEREALMSELKVLSYLGNHMNIVNLLGACTIGGPTLVITEYCCYGDLLNFLRRKRDSFICSKQEDHAEAALYKNLLHSKESSCSDSTNEYMDMKPGVSYVVPTKADKRRSVRIGSYIERDVTPAIMEDDELALDLEDLLSFSYQVAKGMAFLASKNCIHRDLAARNILLTHGRITKICDFGLARDIKNDSNYVVKGNARLPVKWMAPESIFNCVYTFESDVWSYGIFLWELFSLGSSPYPGMPVDSKFYKMIKEGFRMLSPEHAPAEMYDIMKTCWDADPLKRPTFKQIVQLIEKQISESTNHIYSNLANCSPNRQKPVVDHSVRINSVGSTASSSQPLLVHDDV

181 NP_005632.1 NP_005633.2

>NP_005632.1

MAEEKKLKLSNTVLPSESMKVVAESMGIAQIQEETCQLLTDEVSYRIKEIAQDALKFMHMGKRQKLTTSDIDYALKLKNVEPLYGFHAQEFIPFRFASGGGRELYFYEEKEVDLSDIINTPLPRVPLDVCLKAHWLSIEGCQPAIPENPPPAPKEQQKAEATEPLKSAKPGQEEDGPLKGKGQGATTADGKGKEKKAPPLLEGAPLRLKPRSIHELSVEQQLYYKEITEACVGSCEAKRAEALQSIATDPGLYQMLPRFSTFISEGVRVNVVQNNLALLIYLMRMVKALMDNPTLYLEKYVHELIPAVMTCIVSRQLCLRPDVDNHWALRDFAARLVAQICKHFSTTTNNIQSRITKTFTKSWVDEKTPWTTRYGSIAGLAELGHDVIKTLILPRLQQEGERIRSVLDGPVLSNIDRIGADHVQSLLLKHCAPVLAKLRPPPDNQDAYRAEFGSLGPLLCSQVVKARAQAALQAQQVNRTTLTITQPRPTLTLSQAPQPGPRTPGLLKVPGSIALPVQTLVSARAAAPPQPSPPPTKFIVMSSSSSAPSTQQVLSLSTSAPGSGSTTTSPVTTTVPSVQPIVKLVSTATTAPPSTAPSGPGSVQKYIVVSLPPTGEGKGGPTSHPSPVPPPASSPSPLSGSALCGGKQEAGDSPPPAPGTPKANGSQPNSGSPQPAP

>NP_005633.2

MSKSKDDAPHELESQFILRLPPEYASTVRRAVQSGHVNLKDRLTIELHPDGRHGIVRVDRVPLASKLVDLPCVMESLKTIDKKTFYKTADICQMLVSTVDGDLYPPVEEPVASTDPKASKKKDKDKEKKFIWNHGITLPLKNVRKRRFRKTAKKKYIESPDVEKEVKRLLSTDAEAVSTRWEIIAEDETKEAENQGLDISSPGMSGHRQGHDSLEHDELREIFNDLSSSSEDEDETQHQDEEDINIIDTEEDLERQLQDKLNESDEQHQENEGTNQLVMGIQKQIDNMKGKLQETQDRAKRQEDLIMKVENLALKNRFQAVLDELKQKEDREKEQLSSLQEELESLLEK

182 NP_001886.1 NP_612482.2

>NP_001886.1

MSGPVPSRARVYTDVNTHRPREYWDYESHVVEWGNQDDYQLVRKLGRGKYSEVFEAINITNNEKVVVKILKPVKKKKIKREIKILENLRGGPNIITLADIVKDPVSRTPALVFEHVNNTDFKQLYQTLTDYDIRFYMYEILKALDYCHSMGIMHRDVKPHNVMIDHEHRKLRLIDWGLAEFYHPGQEYNVRVASRYFKGPELLVDYQMYDYSLDMWSLGCMLASMIFRKEPFFHGHDNYDQLVRIAKVLGTEDLYDYIDKYNIELDPRFNDILGRHSRKRWERFVHSENQHLVSPEALDFLDKLLRYDHQSRLTAREAMEHPYFYTVVKDQARMGSSSMPGGSTPVSSANMMSGISSVPTPSPLGPLAGSPVIAAANPLGMPVPAAAGAQQ

>NP_612482.2

MSDQDHSMDEMTAVVKIEKGVGGNNGGNGNGGGAFSQARSSSTGSSSSTGGGGQESQPSPLALLAATCSRIESPNENSNNSQGPSQSGGTGELDLTATQLSQGANGWQIISSSSGATPTSKEQSGSSTNGSNGSESSKNRTVSGGQYVVAAAPNLQNQQVLTGLPGVMPNIQYQVIPQFQTVDGQQLQFAATGAQVQQDGSGQIQIIPGANQQIITNRGSGGNIIAAMPNLLQQAVPLQGLANNVLSGQTQYVTNVPVALNGNITLLPVNSVSAATLTPSSQAVTISSSGSQESGSQPVTSGTTISSASLVSSQASSSSFFTNANSYSTTTTTSNMGIMNFTTSGSSGTNSQGQTPQRVSGLQGSDALNIQQNQTSGGSLQAGQQKEGEQNQQTQQQQILIQPQLVQGGQALQALQAAPLSGQTFTTQAISQETLQNLQLQAVPNSGPIIIRTPTVGPNGQVSWQTLQLQNLQVQNPQAQTITLAPMQGVSLGQTSSSNTTLTPIASAASIPAGTVTVNAAQLSSMPGLQTINLSALGTSGIQVHPIQGLPLAIANAPGDHGAQLGLHGAGGDGIHDDTAGGEEGENSPDAQPQAGRRTRREACTCPYCKDSEGRGSGDPGKKKQHICHIQGCGKVYGKTSHLRAHLRWHTGERPFMCTWSYCGKRFTRSDELQRHKRTHTGEKKFACPECPKRFMRSDHLSKHIKTHQNKKGGPGVALSVGTLPLDSGAGSEGSGTATPSALITTNMVAMEAICPEGIARLANSGINVMQVADLQSINISGNGF

183 NP_005408.1 NP_067651.2

>NP_005408.1

MGSNKSKPKDASQRRRSLEPAENVHGAGGGAFPASQTPSKPASADGHRGPSAAFAPAAAEPKLFGGFNSSDTVTSPQRAGPLAGGVTTFVALYDYESRTETDLSFKKGERLQIVNNTEGDWWLAHSLSTGQTGYIPSNYVAPSDSIQAEEWYFGKITRRESERLLLNAENPRGTFLVRESETTKGAYCLSVSDFDNAKGLNVKHYKIRKLDSGGFYITSRTQFNSLQQLVAYYSKHADGLCHRLTTVCPTSKPQTQGLAKDAWEIPRESLRLEVKLGQGCFGEVWMGTWNGTTRVAIKTLKPGTMSPEAFLQEAQVMKKLRHEKLVQLYAVVSEEPIYIVTEYMSKGSLLDFLKGETGKYLRLPQLVDMAAQIASGMAYVERMNYVHRDLRAANILVGENLVCKVADFGLARLIEDNEYTARQGAKFPIKWTAPEAALYGRFTIKSDVWSFGILLTELTTKGRVPYPGMVNREVLDQVERGYRMPCPPECPESLHDLMCQCWRKEPEERPTFEYLQAFLEDYFTSTEPQYQPGENL

>NP_067651.2

MEELIVELRLFLELLDHEYLTSTVREKKAVITNILLRIQSSKGFDVKDHAQKQETANSLPAPPQMPLPEIPQPWLPPDSGPPPLPTSSLPEGYYEEAVPLSPGKAPEYITSNYDSDAMSSSYESYDEEEEDGKGKKTRHQWPSEEASMDLVKDAKICAFLLRKKRFGQWTKLLCVIKDTKLLCYKSSKDQQPQMELPLQGCNITYIPKDSKKKKHELKITQQGTDPLVLAVQSKEQAEQWLKVIKEAYSGCSGPVDSECPPPPSSPVHKAELEKKLSSERPSSDGEGVVENGITTCNGKEQVKRKKSSKSEAKGTVSKVTGKKITKIISLGKKKPSTDEQTSSAEEDVPTCGYLNVLSNSRWRERWCRVKDNKLIFHKDRTDLKTHIVSIPLRGCEVIPGLDSKHPLTFRLLRNGQEVAVLEASSSEDMGRWIGILLAETGSSTDPEALHYDYIDVEMSASVIQTAKQTFCFMNRRVISANPYLGGTSNGYAHPSGTALHYDDVPCINGSLKGKKPPVASNGVTGKGKTLSSQPKKADPAAVVKRTGSNAAQYKYGKNRVEADAKRLQTKEEELLKRKEALRNRLAQLRKERKDLRAAIEVNAGRKPQAILEEKLKQLEEECRQKEAERVSLELELTEVKESLKKALAGGVTLGLAIEPKSGTSSPQSPVFRHRTLENSPISSCDTSDTEGPVPVNSAAVLKKSQAAPGSSPCRGHVLRKAKEWELKNGT

184 NP_004248.2 NP_004320.2

>NP_004248.2

MMSIKAFTLVSAVERELLMGDKERVNIECVECCGRDLYVGTNDCFVYHFLLEERPVPAGPATFTATKQLQRHLGFKKPVNELRAASALNRLLVLCDNSISLVNMLNLEPVPSGARIKGAATFALNENPVSGDPFCVEVCIISVKRRTIQMFLVYEDRVQIVKEVSTAEQPLAVAVDGHFLCLALTTQYIIHNYSTGVSQDLFPYCSEERPPIVKRIGRQEFLLAGPGGLGMFATVAGISQRAPVHWSENVIGAAVSFPYVIALDDEFITVHSMLDQQQKQTLPFKEGHILQDFEGRVIVATSKGVYILVPLPLEKQIQDLLASRRVEEALVLAKGARRNIPKEKFQVMYRRILQQAGFIQFAQLQFLEAKELFRSGQLDVRELISLYPFLLPTSSSFTRSHPPLHEYADLNQLTQGDQEKMAKCKRFLMSYLNEVRSTEVANGYKEDIDTALLKLYAEADHDSLLDLLVTENFCLLTDSAAWLEKHKKYFALGLLYHYNNQDAAAVQLWVNIVNGDVQDSTRSDLYEYIVDFLTYCLDEELVWAYADWVLQKSEEVGVQVFTKRPLDEQQKNSFNPDDIINCLKKYPKALVKYLEHLVIDKRLQKEEYHTHLAVLYLEEVLLQRASASGKGAEATETQAKLRRLLQKSDLYRVHFLLERLQGAGLPMESAILHGKLGEHEKALHILVHELQDFAAAEDYCLWCSEGRDPPHRQQLFHTLLAIYLHAGPTAHELAVAAVDLLNRHATEFDAAQVLQMLPDTWSVQLLCPFLMGAMRDSIHARRTMQVALGLARSENLIYTYDKMKLKGSSIQLSDKKLCQICQNPFCEPVFVRYPNGGLVHTHCAASRHTNPSSSSPGTRT

>NP_004320.2

MPQLYIYIRLLGAYLFIISRVQGQNLDSMLHGTGMKSDSDQKKSENGVTLAPEDTLPFLKCYCSGHCPDDAINNTCITNGHCFAIIEEDDQGETTLASGCMKYEGSDFQCKDSPKAQLRRTIECCRTNLCNQYLQPTLPPVVIGPFFDGSIRWLVLLISMAVCIIAMIIFSSCFCYKHYCKSISSRRRYNRDLEQDEAFIPVGESLKDLIDQSQSSGSGSGLPLLVQRTIAKQIQMVRQVGKGRYGEVWMGKWRGEKVAVKVFFTTEEASWFRETEIYQTVLMRHENILGFIAADIKGTGSWTQLYLITDYHENGSLYDFLKCATLDTRALLKLAYSAACGLCHLHTEIYGTQGKPAIAHRDLKSKNILIKKNGSCCIADLGLAVKFNSDTNEVDVPLNTRVGTKRYMAPEVLDESLNKNHFQPYIMADIYSFGLIIWEMARRCITGGIVEEYQLPYYNMVPSDPSYEDMREVVCVKRLRPIVSNRWNSDECLRAVLKLMSECWAHNPASRLTALRIKKTLAKMVESQDVKI

185 NP_005154.2 NP_001633.1

>NP_005154.2

MSDVAIVKEGWLHKRGEYIKTWRPRYFLLKNDGTFIGYKERPQDVDQREAPLNNFSVAQCQLMKTERPRPNTFIIRCLQWTTVIERTFHVETPEEREEWTTAIQTVADGLKKQEEEEMDFRSGSPSDNSGAEEMEVSLAKPKHRVTMNEFEYLKLLGKGTFGKVILVKEKATGRYYAMKILKKEVIVAKDEVAHTLTENRVLQNSRHPFLTALKYSFQTHDRLCFVMEYANGGELFFHLSRERVFSEDRARFYGAEIVSALDYLHSEKNVVYRDLKLENLMLDKDGHIKITDFGLCKEGIKDGATMKTFCGTPEYLAPEVLEDNDYGRAVDWWGLGVVMYEMMCGRLPFYNQDHEKLFELILMEEIRFPRTLGPEAKSLLSGLLKKDPKQRLGGGSEDAKEIMQHRFFAGIVWQHVYEKKLSPPFKPQVTSETDTRYFDEEFTAQMITITPPDQDDSMECVDSERRPHFPQFSYSASGTA

>NP_001633.1

MAATGTAAAAATGRLLLLLLVGLTAPALALAGYIEALAANAGTGFAVAEPQIAMFCGKLNMHVNIQTGKWEPDPTGTKSCFETKEEVLQYCQEMYPELQITNVMEANQRVSIDNWCRRDKKQCKSRFVTPFKCLVGEFVSDVLLVPEKCQFFHKERMEVCENHQHWHTVVKEACLTQGMTLYSYGMLLPCGVDQFHGTEYVCCPQTKIIGSVSKEEEEEDEEEEEEEDEEEDYDVYKSEFPTEADLEDFTEAAVDEDDEDEEEGEEVVEDRDYYYDTFKGDDYNEENPTEPGSDGTMSDKEITHDVKAVCSQEAMTGPCRAVMPRWYFDLSKGKCVRFIYGGCGGNRNNFESEDYCMAVCKAMIPPTPLPTNDVDVYFETSADDNEHARFQKAKEQLEIRHRNRMDRVKKEWEEAELQAKNLPKAERQTLIQHFQAMVKALEKEAASEKQQLVETHLARVEAMLNDRRRMALENYLAALQSDPPRPHRILQALRRYVRAENKDRLHTIRHYQHVLAVDPEKAAQMKSQVMTHLHVIEERRNQSLSLLYKVPYVAQEIQEEIDELLQEQRADMDQFTASISETPVDVRVSSEESEEIPPFHPFHPFPALPENEDTQPELYHPMKKGSGVGEQDGGLIGAEEKVINSKNKVDENMVIDETLDVKEMIFNAERVGGLEEERESVGPLREDFSLSSSALIGLLVIAVAIATVIVISLVMLRKRQYGTISHGIVEVDPMLTPEERHLNKMQNHGYENPTYKYLEQMQI

186 NP_003347.1 NP_036611.2

>NP_003347.1

MVGLKPSDVPPTMAVKFLGAGTAACFADLVTFPLDTAKVRLQIQGENQAVQTARLVQYRGVLGTILTMVRTEGPCSPYNGLVAGLQRQMSFASIRIGLYDSVKQVYTPKGADNSSLTTRILAGCTTGAMAVTCAQPTDVVKVRFQASIHLGPSRSDRKYSGTMDAYRTIAREEGVRGLWKGTLPNIMRNAIVNCAEVVTYDILKEKLLDYHLLTDNFPCHFVSAFGAGFCATVVASPVDVVKTRYMNSPPGQYFSPLDCMIKMVAQEGPTAFYKGFTPSFLRLGSWNVVMFVTYEQLKRALMKVQMLRESPF

>NP_036611.2

MVDREQLVQKARLAEQAERYDDMAAAMKNVTELNEPLSNEERNLLSVAYKNVVGARRSSWRVISSIEQKTSADGNEKKIEMVRAYREKIEKELEAVCQDVLSLLDNYLIKNCSETQYESKVFYLKMKGDYYRYLAEVATGEKRATVVESSEKAYSEAHEISKEHMQPTHPIRLGLALNYSVFYYEIQNAPEQACHLAKTAFDDAIAELDTLNEDSYKDSTLIMQLLRDNLTLWTSDQQDDDGGEGNN

187 NP_113618.2 NP_004628.4

>NP_113618.2

MEPRRAAPGVPGWGSREAAGSASAAELVYHLAGALGTELQDLARRFGPEAAAGLVPLVVRALELLEQAAVGPAPDSLQVSAQPAEQELRRLREENERLRRELRAGPQEERALLRQLKEVTDRQRDELRAHNRDLRQRGQETEALQEQLQRLLLVNAELRHKLAAMQTQLRAAQDRERERQQPGEAATPQAKERARGQAGRPGHQHGQEPEWATAGAGAPGNPEDPAEAAQQLGRPSEAGQCRFSREEFEQILQERNELKAKVFLLKEELAYFQRELLTDHRVPGLLLEAMKVAVRKQRKKIKAKMLGTPEEAESSEDEAGPWILLSDDKGDHPPPPESKIQSFFGLWYRGKAESSEDETSSPAPSKLGGEEEAQPQSPAPDPPCSALHEHLCLGASAAPEA

>NP_004628.4

MTSRKKVLLKVIILGDSGVGKTSLMNQYVNKKFSNQYKATIGADFLTKEVMVDDRLVTMQIWDTAGQERFQSLGVAFYRGADCCVLVFDVTAPNTFKTLDSWRDEFLIQASPRDPENFPFVVLGNKIDLENRQVATKRAQAWCYSKNNIPYFETSAKEAINVEQAFQTIARNALKQETEVELYNEFPEPIKLDKNDRAKASAESCSC

188 NP_060876.3 NP_004439.2

>NP_060876.3

MKGARWRRVPWVSLSCLCLCLLPHVVPGTTEDTLITGSKTPAPVTSTGSTTATLEGQSTAASSRTSNQDISASSQNHQTKSTETTSKAQTDTLTQMMTSTLFSSPSVHNVMETVTQETAPPDEMTTSFPSSVTNTLMMTSKTITMTTSTDSTLGNTEETSTAGTESSTPVTSAVSITAGQEGQSRTTSWRTSIQDTSASSQNHWTRSTQTTRESQTSTLTHRTTSTPSFSPSVHNVTGTVSQKTSPSGETATSSLCSVTNTSMMTSEKITVTTSTGSTLGNPGETSSVPVTGSLMPVTSAALVTVDPEGQSPATFSRTSTQDTTAFSKNHQTQSVETTRVSQINTLNTLTPVTTSTVLSSPSGFNPSGTVSQETFPSGETTISSPSSVSNTFLVTSKVFRMPISRDSTLGNTEETSLSVSGTISAITSKVSTIWWSDTLSTALSPSSLPPKISTAFHTQQSEGAETTGRPHERSSFSPGVSQEIFTLHETTTWPSSFSSKGHTTWSQTELPSTSTGAATRLVTGNPSTRAAGTIPRVPSKVSAIGEPGEPTTYSSHSTTLPKTTGAGAQTQWTQETGTTGEALLSSPSYSVIQMIKTATSPSSSPMLDRHTSQQITTAPSTNHSTIHSTSTSPQESPAVSQRGHTRAPQTTQESQTTRSVSPMTDTKTVTTPGSSFTASGHSPSEIVPQDAPTISAATTFAPAPTGNGHTTQAPTTALQAAPSSHDATLGPSGGTSLSKTGALTLANSVVSTPGGPEGQWTSASASTSPDTAAAMTHTHQAESTEASGQTQTSEPASSGSRTTSAGTATPSSSGASGTTPSGSEGISTSGETTRFSSNPSRDSHTTQSTTELLSASASHGAIPVSTGMASSIVPGTFHPTLSEASTAGRPTGQSSPTSPSASPQETAAISRMAQTQRTGTSRGSDTISLASQATDTFSTVPPTPPSITSSGLTSPQTQTHTLSPSGSGKTFTTALISNATPLPVTSTSSASTGHATPLAVSSATSASTVSSDSPLKMETSGMTTPSLKTDGGRRTATSPPPTTSQTIISTIPSTAMHTRSTAAPIPILPERGVSLFPYGAGAGDLEFVRRTVDFTSPLFKPATGFPLGSSLRDSLYFTDNGQIIFPESDYQIFSYPNPLPTGFTGRDPVALVAPFWDDADFSTGRGTTFYQEYETFYGEHSLLVQQAESWIRKMTNNGGYKARWALKVTWVNAHAYPAQWTLGSNTYQAILSTDGSRSYALFLYQSGGMQWDVAQRSGNPVLMGFSSGDGYFENSPLMSQPVWERYRPDRFLNSNSGLQGLQFYRLHREERPNYRLECLQWLKSQPRWPSWGWNQVSCPCSWQQGRRDLRFQPVSIGRWGLGSRQLCSFTSWRGGVCCSYGPWGEFREGWHVQRPWQLAQELEPQSWCCRWNDKPYLCALYQQRRPHVGCATYRPPQPAWMFGDPHITTLDGVSYTFNGLGDFLLVGAQDGNSSFLLQGRTAQTGSAQATNFIAFAAQYRSSSLGPVTVQWLLEPHDAIRVLLDNQTVTFQPDHEDGGGQETFNATGVLLSRNGSEVSASFDGWATVSVIALSNILHASASLPPEYQNRTEGLLGVWNNNPEDDFRMPNGSTIPPGSPEEMLFHFGMTWQINGTGLLGKRNDQLPSNFTPVFYSQLQKNSSWAEHLISNCDGDSSCIYDTLALRNASIGLHTREVSKNYEQANATLNQYPPSINGGRVIEAYKGQTTLIQYTSNAEDANFTLRDSCTDLELFENGTLLWTPKSLEPFTLEILARSAKIGLASALQPRTVVCHCNAESQCLYNQTSRVGNSSLEVAGCKCDGGTFGRYCEGSEDACEEPCFPSVHCVPGKGCEACPPNLTGDGRHCAALGSSFLCQNQSCPVNYCYNQGHCYISQTLGCQPMCTCPPAFTDSRCFLAGNNFSPTVNLELPLRVIQLLLSEEENASMAEVNASVAYRLGTLDMRAFLRNSQVERIDSAAPASGSPIQHWMVISEFQYRPRGPVIDFLNNQLLAAVVEAFLYHVPRRSEEPRNDVVFQPISGEDVRDVTALNVSTLKAYFRCDGYKGYDLVYSPQSGFTCVSPCSRGYCDHGGQCQHLPSGPRCSCVSFSIYTAWGEHCEHLSMKLDAFFGIFFGALGGLLLLGVGTFVVLRFWGCSGARFSYFLNSAEALP

>NP_004439.2

MELAALCRWGLLLALLPPGAASTQVCTGTDMKLRLPASPETHLDMLRHLYQGCQVVQGNLELTYLPTNASLSFLQDIQEVQGYVLIAHNQVRQVPLQRLRIVRGTQLFEDNYALAVLDNGDPLNNTTPVTGASPGGLRELQLRSLTEILKGGVLIQRNPQLCYQDTILWKDIFHKNNQLALTLIDTNRSRACHPCSPMCKGSRCWGESSEDCQSLTRTVCAGGCARCKGPLPTDCCHEQCAAGCTGPKHSDCLACLHFNHSGICELHCPALVTYNTDTFESMPNPEGRYTFGASCVTACPYNYLSTDVGSCTLVCPLHNQEVTAEDGTQRCEKCSKPCARVCYGLGMEHLREVRAVTSANIQEFAGCKKIFGSLAFLPESFDGDPASNTAPLQPEQLQVFETLEEITGYLYISAWPDSLPDLSVFQNLQVIRGRILHNGAYSLTLQGLGISWLGLRSLRELGSGLALIHHNTHLCFVHTVPWDQLFRNPHQALLHTANRPEDECVGEGLACHQLCARGHCWGPGPTQCVNCSQFLRGQECVEECRVLQGLPREYVNARHCLPCHPECQPQNGSVTCFGPEADQCVACAHYKDPPFCVARCPSGVKPDLSYMPIWKFPDEEGACQPCPINCTHSCVDLDDKGCPAEQRASPLTSIISAVVGILLVVVLGVVFGILIKRRQQKIRKYTMRRLLQETELVEPLTPSGAMPNQAQMRILKETELRKVKVLGSGAFGTVYKGIWIPDGENVKIPVAIKVLRENTSPKANKEILDEAYVMAGVGSPYVSRLLGICLTSTVQLVTQLMPYGCLLDHVRENRGRLGSQDLLNWCMQIAKGMSYLEDVRLVHRDLAARNVLVKSPNHVKITDFGLARLLDIDETEYHADGGKVPIKWMALESILRRRFTHQSDVWSYGVTVWELMTFGAKPYDGIPAREIPDLLEKGERLPQPPICTIDVYMIMVKCWMIDSECRPRFRELVSEFSRMARDPQRFVVIQNEDLGPASPLDSTFYRSLLEDDDMGDLVDAEEYLVPQQGFFCPDPAPGAGGMVHHRHRSSSTRSGGGDLTLGLEPSEEEAPRSPLAPSEGAGSDVFDGDLGMGAAKGLQSLPTHDPSPLQRYSEDPTVPLPSETDGYVAPLTCSPQPEYVNQPDVRPQPPSPREGPLPAARPAGATLERPKTLSPGKNGVVKDVFAFGGAVENPEYLTPQGGAAPQPHPPPAFSPAFDNLYYWDQDPPERGAPPSTFKGTPTAENPEYLGLDVPV

189 NP_002779.1 NP_542417.1

>NP_002779.1

MSSIGTGYDLSASTFSPDGRVFQVEYAMKAVENSSTAIGIRCKDGVVFGVEKLVLSKLYEEGSNKRLFNVDRHVGMAVAGLLADARSLADIAREEASNFRSNFGYNIPLKHLADRVAMYVHAYTLYSAVRPFGCSFMLGSYSVNDGAQLYMIDPSGVSYGYWGCAIGKARQAAKTEIEKLQMKEMTCRDIVKEVAKIIYIVHDEVKDKAFELELSWVGELTNGRHEIVPKDIREEAEKYAKESLKEEDESDDDNM

>NP_542417.1

MSDRSGPTAKGKDGKKYSSLNLFDTYKGKSLEIQKPAVAPRHGLQSLGKVAIARRMPPPANLPSLKAENKGNDPNVSLVPKDGTGWASKQEQSDPKSSDASTAQPPESQPLPASQTPASNQPKRPPAAPENTPLVPSGVKSWAQASVTHGAHGDGGRASSLLSRFSREEFPTLQAAGDQDKAAKERESAEQSSGPGPSLRPQNSTTWRDGGGRGPDELEGPDSKLHHGHDPRGGLQPSGPPQFPPYRGMMPPFMYPPYLPFPPPYGPQGPYRYPTPDGPSRFPRVAGPRGSGPPMRLVEPVGRPSILKEDNLKEFDQLDQENDDGWAGAHEEVDYTEKLKFSDEEDGRDSDEEGAEGHRDSQSASGEERPPEADGKKGNSPNSEPPTPKTAWAETSRPPETEPGPPAPKPPLPPPHRGPAGNWGPPGDYPDRGGPPCKPPAPEDEDEAWRQRRKQSSSEISLAVERARRRREEEERRMQEERRAACAEKLKRLDEKFGAPDKRLKAEPAAPPAAPSTPAPPPAVPKELPAPPAPPPASAPTPEKEPEEPAQAPPAQSTPTPGVAAAPTLVSGGGSTSSTSSGSFEASPVEPQLPSKEGPEPPEEVPPPTTPPVPKVEPKGDGIGPTRQPPSQGLGYPKYQKSLPPRFQRQQQEQLLKQQQQHQWQQHQQGSAPPTPVPPSPPQPVTLGAVPAPQAPPPPPKALYPGALGRPPPMPPMNFDPRWMMIPPYVDPRLLQGRPPLDFYPPGVHPSGLVPRERSDSGGSSSEPFDRHAPAMLRERGTPPVDPKLAWVGDVFTATPAEPRPLTSPLRQAADEDDKGMRSETPPVPPPPPYLASYPGFPENGAPGPPISRFPLEEPGPRPLPWPPGSDEVAKIQTPPPKKEPPKEETAQLTGPEAGRKPARGVGSGGQGPPPPRRESRTETRWGPRPGSSRRGIPPEEPGAPPRRAGPIKKPPPPTKVEELPPKPLEQGDETPKPPKPDPLKITKGKLGGPKETPPNGNLSPAPRLRRDYSYERVGPTSCRGRGRGEYFARGRGFRGTYGGRGRGARSREFRSYREFRGDDGRGGGTGGPNHPPAPRGRTASETRSEGSEYEEIPKRRRQRGSETGSETHESDLAPSDKEAPTPKEGTLTQVPLAPPPPGAPPSPAPARFTARGGRVFTPRGVPSRRGRGGGRPPPQVCPGWSPPAKSLAPKKPPTGPLPPSKEPLKEKLIPGPLSPVARGGSNGGSNVGMEDGERPRRRRHGRAQQQDKPPRFRRLKQERENAARGSEGKPSLTLPASAPAPEEALTTVTVAPAPRRAAAKSPDLSNQNSDQANEEWETASESSDFTSERRGDKEAPPPVLLTPKAVGTPGGGGGGAVPGISAMSRGDLSQRAKDLSKRSFSSQRPGMERQNRRPGPGGKAGSSGSSSGGGGGGPGGRTGPGRGDKRSWPSPKNRSRPPEERPPGLPLPPPPPSSSAVFRLDQVIHSNPAGIQQALAQLSSRQGSVTAPGGHPRHKPGPPQAPQGPSPRPPTRYEPQRVNSGLSSDPHFEEPGPMVRGVGGTPRDSAGVSPFPPKRRERPPRKPELLQEESLPPPHSSGFLGSKPEGPGPQAESRDTGTEALTPHIWNRLHTATSRKSYRPSSMEPWMEPLSPFEDVAGTEMSQSDSGVDLSGDSQVSSGPCSQRSSPDGGLKGAAEGPPKRPGGSSPLNAVPCEGPPGSEPPRRPPPAPHDGDRKELPREQPLPPGPIGTERSQHTDRGTEPGPIRPSHRPGPPVQFGTSDKDSDLRLVVGDSLKAEKELTASVTEAIPVSRDWELLPSAAASAEPQSKNLDSGHCVPEPSSSGQRLYPEVFYGSAGPSSSQISGGAMDSQLHPNSGGFRPGTPSLHPYRSQPLYLPPGPAPPSALLSGVALKGQFLDFSTMQATELGKLPAGGVLYPPPSFLYSPAFCPSPLPDTSLLQVRQDLPSPSDFYSTPLQPGGQSGFLPSGAPAQQMLLPMVDSQLPVVNFGSLPPAPPPAPPPLSLLPVGPALQPPSLAVRPPPAPATRVLPSPARPFPASLGRAELHPVELKPFQDYQKLSSNLGGPGSSRTPPTGRSFSGLNSRLKATPSTYSGVFRTQRVDLYQQASPPDALRWIPKPWERTGLPPREGPSRRAEEPGSRGDKEPGLPPPR

190 NP_005640.2 NP_003737.1

>NP_005640.2

MAAGQREARPQVSLTFEDVAVLFTRDEWRKLAPSQRNLYRDVMLENYRNLVSLGLPFTKPKVISLLQQGEDPWEVEKDGSGVSSLGSKSSHKTTKSTQTQDSSFQGLILKRSNRNVPWDLKLEKPYIYEGRLEKKQDKKGSFQIVSATHKKIPTIERSHKNTELSQNFSPKSVLIRQQILPREKTPPKCEIQGNSLKQNSQLLNQPKITADKRYKCSLCEKTFINTSSLRKHEKNHSGEKLFKCKECSKAFSQSSALIQHQITHTGEKPYICKECGKAFTLSTSLYKHLRTHTVEKSYRCKECGKSFSRRSGLFIHQKIHAEENPCKYNPGRKASSCSTSLSGCQRIHSRKKSYLCNECGNTFKSSSSLRYHQRIHTGEKPFKCSECGRAFSQSASLIQHERIHTGEKPYRCNECGKGFTSISRLNRHRIIHTGEKFYNCNECGKALSSHSTLIIHERIHTGEKPCKCKVCGKAFRQSSALIQHQRMHTGERPYKCNECGKTFRCNSSLSNHQRIHTGEKPYRCEECGISFGQSSALIQHRRIHTGEKPFKCNTCGKTFRQSSSRIAHQRIHTGEKPYECNTCGKLFNHRSSLTNHYKIHIEEDP

>NP_003737.1

MCDRKAVIKNADMSEEMQQDSVECATQALEKYNIEKDIAAHIKKEFDKKYNPTWHCIVGRNFGSYVTHETKHFIYFYLGQVAILLFKSG

191 NP_001886.1 NP_068660.1

>NP_001886.1

MSGPVPSRARVYTDVNTHRPREYWDYESHVVEWGNQDDYQLVRKLGRGKYSEVFEAINITNNEKVVVKILKPVKKKKIKREIKILENLRGGPNIITLADIVKDPVSRTPALVFEHVNNTDFKQLYQTLTDYDIRFYMYEILKALDYCHSMGIMHRDVKPHNVMIDHEHRKLRLIDWGLAEFYHPGQEYNVRVASRYFKGPELLVDYQMYDYSLDMWSLGCMLASMIFRKEPFFHGHDNYDQLVRIAKVLGTEDLYDYIDKYNIELDPRFNDILGRHSRKRWERFVHSENQHLVSPEALDFLDKLLRYDHQSRLTAREAMEHPYFYTVVKDQARMGSSSMPGGSTPVSSANMMSGISSVPTPSPLGPLAGSPVIAAANPLGMPVPAAAGAQQ

>NP_068660.1

MEVPQPEPAPGSALSPAGVCGGAQRPGHLPGLLLGSHGLLGSPVRAAASSPVTTLTQTMHDLAGLGSRSRLTHLSLSRRASESSLSSESSESSDAGLCMDSPSPMDPHMAEQTFEQAIQAASRIIRNEQFAIRRFQSMPVRLLGHSPVLRNITNSQAPDGRRKSEAGSGAASSSGEDKENVRFWKAGVGALREEEGACWGGSLACEDPPLPSWLQDGFVFKMPWKPTHPSSTHALAEWASRREAFAQRPSSAPDLMCLSPDRKMEVEELSPLALGRFSLTPAEGDTEEDDGFVDILESDLKDDDAVPPGMESLISAPLVKTLEKEEEKDLVMYSKCQRLFRSPSMPCSVIRPILKRLERPQDRDTPVQNKRRRSVTPPEEQQEAEEPKARVLRSKSLCHDEIENLLDSDHRELIGDYSKAFLLQTVDGKHQDLKYISPETMVALLTGKFSNIVDKFVIVDCRYPYEYEGGHIKTAVNLPLERDAESFLLKSPIAPCSLDKRVILIFHCEFSSERGPRMCRFIRERDRAVNDYPSLYYPEMYILKGGYKEFFPQHPNFCEPQDYRPMNHEAFKDELKTFRLKTRSWAGERSRRELCSRLQDQ

192 NP_066361.1 NP_002871.1

>NP_066361.1

MREYKVVVLGSGGVGKSALTVQFVTGTFIEKYDPTIEDFYRKEIEVDSSPSVLEILDTAGTEQFASMRDLYIKNGQGFILVYSLVNQQSFQDIKPMRDQIIRVKRYEKVPVILVGNKVDLESEREVSSSEGRALAEEWGCPFMETSAKSKTMVDELFAEIVRQMNYAAQPDKDDPCCSACNIQ

>NP_002871.1

MEHIQGAWKTISNGFGFKDAVFDGSSCISPTIVQQFGYQRRASDDGKLTDPSKTSNTIRVFLPNKQRTVVNVRNGMSLHDCLMKALKVRGLQPECCAVFRLLHEHKGKKARLDWNTDAASLIGEELQVDFLDHVPLTTHNFARKTFLKLAFCDICQKFLLNGFRCQTCGYKFHEHCSTKVPTMCVDWSNIRQLLLFPNSTIGDSGVPALPSLTMRRMRESVSRMPVSSQHRYSTPHAFTFNTSSPSSEGSLSQRQRSTSTPNVHMVSTTLPVDSRMIEDAIRSHSESASPSALSSSPNNLSPTGWSQPKTPVPAQRERAPVSGTQEKNKIRPRGQRDSSYYWEIEASEVMLSTRIGSGSFGTVYKGKWHGDVAVKILKVVDPTPEQFQAFRNEVAVLRKTRHVNILLFMGYMTKDNLAIVTQWCEGSSLYKHLHVQETKFQMFQLIDIARQTAQGMDYLHAKNIIHRDMKSNNIFLHEGLTVKIGDFGLATVKSRWSGSQQVEQPTGSVLWMAPEVIRMQDNNPFSFQSDVYSYGIVLYELMTGELPYSHINNRDQIIFMVGRGYASPDLSKLYKNCPKAMKRLVADCVKKVKEERPLFPQILSSIELLQHSLPKINRSASEPSLHRAAHTEDINACTLTTSPRLPVF

193 NP_006171.2 NP_733930.1

>NP_006171.2

MSSWIRWHGPAMARLWGFCWLVVGFWRAAFACPTSCKCSASRIWCSDPSPGIVAFPRLEPNSVDPENITEIFIANQKRLEIINEDDVEAYVGLRNLTIVDSGLKFVAHKAFLKNSNLQHINFTRNKLTSLSRKHFRHLDLSELILVGNPFTCSCDIMWIKTLQEAKSSPDTQDLYCLNESSKNIPLANLQIPNCGLPSANLAAPNLTVEEGKSITLSCSVAGDPVPNMYWDVGNLVSKHMNETSHTQGSLRITNISSDDSGKQISCVAENLVGEDQDSVNLTVHFAPTITFLESPTSDHHWCIPFTVKGNPKPALQWFYNGAILNESKYICTKIHVTNHTEYHGCLQLDNPTHMNNGDYTLIAKNEYGKDEKQISAHFMGWPGIDDGANPNYPDVIYEDYGTAANDIGDTTNRSNEIPSTDVTDKTGREHLSVYAVVVIASVVGFCLLVMLFLLKLARHSKFGMKDFSWFGFGKVKSRQGVGPASVISNDDDSASPLHHISNGSNTPSSSEGGPDAVIIGMTKIPVIENPQYFGITNSQLKPDTFVQHIKRHNIVLKRELGEGAFGKVFLAECYNLCPEQDKILVAVKTLKDASDNARKDFHREAELLTNLQHEHIVKFYGVCVEGDPLIMVFEYMKHGDLNKFLRAHGPDAVLMAEGNPPTELTQSQMLHIAQQIAAGMVYLASQHFVHRDLATRNCLVGENLLVKIGDFGMSRDVYSTDYYRVGGHTMLPIRWMPPESIMYRKFTTESDVWSLGVVLWEIFTYGKQPWYQLSNNEVIECITQGRVLQRPRTCPQEVYELMLGCWQREPHMRKNIKGIHTLLQNLAKASPVYLDILG

>NP_733930.1

MQSREEEWFHQVRRVMTILFLTMVISYFGCMKAAPMKEANIRGQGGLAYPGVRTHGTLESVNGPKAGSRGLTSLADTFEHVIEELLDEDQKVRPNEENNKDADLYTSRVMLSSQVPLEPPLLFLLEEYKNYLDAANMSMRVRRHSDPARRGELSVCDSISEWVTAADKKTAVDMSGGTVTVLEKVPVSKGQLKQYFYETKCNPMGYTKEGCRGIDKRHWNSQCRTTQSYVRALTMDSKKRIGWRFIRIDTSCVCTLTIKRGR

194 NP_945320.1 NP_009209.1

>NP_945320.1

MCHKNGFPQEGGITAAFLQKRKLRLSKNHRPARAKVTEHVRGTRPGRATAGPAASTRAAGSLFFDRWGNRGPAGCRGSSHLPQQLKFTTSDSCDRIKDEFQLLQAQYHSLKLECDKLASEKSEMQRHYVMYYEMSYGLNIEMHKQAEIVKRLNGICAQVLPYLSQEHQQQVLGAIERAKQVTAPELNSIIRQQLQAHQLSQLQALALPLTPLPVGLQPPSLPAVSAGTGLLSLSALGSQAHLSKEDKNGHDGDTHQEDDGEKSD

>NP_009209.1

MKFVYKEEHPFEKRRSEGEKIRKKYPDRVPVIVEKAPKARIGDLDKKKYLVPSDLTVGQFYFLIRKRIHLRAEDALFFFVNNVIPPTSATMGQLYQEHHEEDFFLYIAYSDESVYGL

195 NP_002341.1 NP_009112.1

>NP_002341.1

MGCIKSKGKDSLSDDGVDLKTQPVRNTERTIYVRDPTSNKQQRPVPESQLLPGQRFQTKDPEEQGDIVVALYPYDGIHPDDLSFKKGEKMKVLEEHGEWWKAKSLLTKKEGFIPSNYVAKLNTLETEEWFFKDITRKDAERQLLAPGNSAGAFLIRESETLKGSFSLSVRDFDPVHGDVIKHYKIRSLDNGGYYISPRITFPCISDMIKHYQKQADGLCRRLEKACISPKPQKPWDKDAWEIPRESIKLVKRLGAGQFGEVWMGYYNNSTKVAVKTLKPGTMSVQAFLEEANLMKTLQHDKLVRLYAVVTREEPIYIITEYMAKGSLLDFLKSDEGGKVLLPKLIDFSAQIAEGMAYIERKNYIHRDLRAANVLVSESLMCKIADFGLARVIEDNEYTAREGAKFPIKWTAPEAINFGCFTIKSDVWSFGILLYEIVTYGKIPYPGRTNADVMTALSQGYRMPRVENCPDELYDIMKMCWKEKAEERPTFDYLQSVLDDFYTATEGQYQQQP

>NP_009112.1

MDVVDPDIFNRDPRDHYDLLQRLGGGTYGEVFKARDKVSGDLVALKMVKMEPDDDVSTLQKEILILKTCRHANIVAYHGSYLWLQKLWICMEFCGAGSLQDIYQVTGSLSELQISYVCREVLQGLAYLHSQKKIHRDIKGANILINDAGEVRLADFGISAQIGATLARRLSFIGTPYWMAPEVAAVALKGGYNELCDIWSLGITAIELAELQPPLFDVHPLRVLFLMTKSGYQPPRLKEKGKWSAAFHNFIKVTLTKSPKKRPSATKMLSHQLVSQPGLNRGLILDLLDKLKNPGKGPSIGDIEDEEPELPPAIPRRIRSTHRSSSLGIPDADCCRRHMEFRKLRGMETRPPANTARLQPPRDLRSSSPRKQLSESSDDDYDDVDIPTPAEDTPPPLPPKPKFRSPSDEGPGSMGDDGQLSPGVLVRCASGPPPNSPRPGPPPSTSSPHLTAHSEPSLWNPPSRELDKPPLLPPKKEKMKRKGCALLVKLFNGCPLRIHSTAAWTHPSTKDQHLLLGAEEGIFILNRNDQEATLEMLFPSRTTWVYSINNVLMSLSGKTPHLYSHSILGLLERKETRAGNPIAHISPHRLLARKNMVSTKIQDTKGCRACCVAEGASSGGPFLCGALETSVVLLQWYQPMNKFLLVRQVLFPLPTPLSVFALLTGPGSELPAVCIGVSPGRPGKSVLFHTVRFGALSCWLGEMSTEHRGPVQVTQVEEDMVMVLMDGSVKLVTPEGSPVRGLRTPEIPMTEAVEAVAMVGGQLQAFWKHGVQVWALGSDQLLQELRDPTLTFRLLGSPRLECSGTISPHCNLLLPGSSNSPASASRVAGITGL

196 CAA41990.1 NP_115754.2

>CAA41990.1

MSTLYVSPHPDAFPSLRALIAARYGEAGEGPGWGGAHPRICLQPPPTSRTSFPPPRLPALEQGPGGLWVWGATAVAQLLWPAGLGGPGGSRAAVLVQQWVSYADTELIPAACGATLPALGLRSSAQDPQAVLGALGRALSPLEEWLRLHTYLAGEAPTLADLAAVTALLLPFRYVLDPPARRIWNNVTRWFVTCVRQPEFRAVLGEVVLYSGARPLSHQPGPEAPALPKTAAQLKKEAKKREKLEKFQQKQKIQQQQPPPGEKKPKPEKREKRDPGVITYDLPTPPGEKKDVSGPMPDSYSPRYVEAAWYPWWEQQGFFKPEYGRPNVSAANPRGVFMMCIPPPNVTGSLHLGHALTNAIQDSLTRWHRMRGETTLWNPGCDHAGIATQVVVEKKLWREQGLSRHQLGREAFLQEVWKWKEEKGDRIYHQLKKLGSSLDWDRACFTMDPKLSAAVTEAFVRLHEEGIIYRSTRLVNWSCTLNSAISDIEVDKKELTGRTLLSVPGYKEKVEFGVLVSFAYKVQGSDSDEEVVVATTRIETMLGDVAVAVHPKDTRYQHLKGKNVIHPFLSRSLPIVFDEFVDMDFGTGAGKITPAHDQNDYEVGQRHGLEAISIMDSRGGPHQCASAFPGPAQVLRPGKRCLVALKERGLFRGIEDNPMVVPLCNRSKDVVEPLLRPQWYVRCGEMAQAASAAVTRGDLRILPEAHQRTWHAWMDNIREWCISRQLWWGHRIPAYFVTVSDPAVPPGEDPDGRYWVSGRNEAEAREKAAKEFGVSPDKISLQQDEDVLDTWFFSGLFPLSILGWPNQSEDLSVFYPGTLLETGHDILFFWVARMVMLGLKLTGRLPFREVYLHAIVRDAHGRKMSKSLGNVIDPLDVIYGISLQGLHNQLLNSNLDPSEVEKAKEGQKADFPAGIPECGTDALRFGLCAYMSQGRDINLDVNRILGYRHFCNKLWNATKFALRGLGKGFVPSPTSQPGGHESLVDRWIRSRLTEAVRLSNQGFQAYDFPAVTTAQYSFWLYELCDVYLECLKPVLNGVDQVAAECARQTLYTCLDVGLRLLSPFMPFVTEELFQRLPRRMPQAPPSLCVTPYPEPSECSWKDPEAEAALELALSITRAVRSLRADYNLTRIRPDCFLEVADEATGALASAVSGYVQALASAGVVAVLALGAPAPQGCAVALASDRCSIHLQLQGLVDPARELGKLQAKRVEAQRQAQRLRERRAASGYPVKVPLEVQEADEAKLQQTEAELRKVDEAIALFQKML

>NP_115754.2

MRSGKASCTLETVWEDKHKYEEAERRFYEHEATQAAASAQQLPAEGPAMNGPGQDDPEDADEAEAPDGGSRRDPRKSQDSRKPLQKKRKRSPKSGLGPADLALLGLSAERVWLDKSLFDQAESSYRQKLADVAAQAAWPPALAPWGLCTHGNQVACHHVTWGIWVNKSSFDQAERAFVEWSQALLLAPDGSRRQGTPNTGQQVAVPDLAHQPSPPVNGQPPLGSLQALVREVWLEKPRYDAAERGFYEALFDGHPPGKVRLQERAGLAEGARRGRRDRRGRNILGNKRAGLRRADGEAPSALPYCYFLQKDAEAPWLSKPAYDSAECRHHAAEALRVAWCLEAASLSHRPGPRSGLSVSSLRPNRKMATNFLAHEKIWFDKFKYDDAERRFYEQMNGPVAGASRQENGASVILRDIARARENIQKSLAGSSGPGASSGTSGDHGELVVRIASLEVENQSLRGVVQELQQAISKLEARLNVLEKSSPGHRATAPQTQHVSPMRQVEPPAKKPATPAEDDEDDDIDLFGSDNEEEDKEAAQLREERLRQYAEKKAKKPALVAKSSILLDVKPWDDETDMAQLEACVRSIQLDGLVWGASKLVPVGYGIRKLQIQCVVEDDKVGTDLLEEEITKFEEHVQSVDIAAFNKI

197 NP_001096.1 NP_002945.1

>NP_001096.1

MVDGVMILPVLIMIALPSPSMEDEKPKVNPKLYMCVCEGLSCGNEDHCEGQQCFSSLSINDGFHVYQKGCFQVYEQGKMTCKTPPSPGQAVECCQGDWCNRNITAQLPTKGKSFPGTQNFHLEVGLIILSVVFAVCLLACLLGVALRKFKRRNQERLNPRDVEYGTIEGLITTNVGDSTLADLLDHSCTSGSGSGLPFLVQRTVARQITLLECVGKGRYGEVWRGSWQGENVAVKIFSSRDEKSWFRETELYNTVMLRHENILGFIASDMTSRHSSTQLWLITHYHEMGSLYDYLQLTTLDTVSCLRIVLSIASGLAHLHIEIFGTQGKPAIAHRDLKSKNILVKKNGQCCIADLGLAVMHSQSTNQLDVGNNPRVGTKRYMAPEVLDETIQVDCFDSYKRVDIWAFGLVLWEVARRMVSNGIVEDYKPPFYDVVPNDPSFEDMRKVVCVDQQRPNIPNRWFSDPTLTSLAKLMKECWYQNPSARLTALRIKKTLTKIDNSLDKLKTDC

>NP_002945.1

MQIFVKTLTGKTITLEVEPSDTIENVKAKIQDKEGIPPDQQRLIFAGKQLEDGRTLSDYNIQKESTLHLVLRLRGGAKKRKKKSYTTPKKNKHKRKKVKLAVLKYYKVDENGKISRLRRECPSDECGAGVFMASHFDRHYCGKCCLTYCFNKPEDK

198 NP_631961.1 NP_004597.2

>NP_631961.1

MSDSGSYGQSGGEQQSYSTYGNPGSQGYGQASQSYSGYGQTTDSSYGQNYSGYSSYGQSQSGYSQSYGGYENQKQSSYSQQPYNNQGQQQNMESSGSQGGRAPSYDQPDYGQQDSYDQQSGYDQHQGSYDEQSNYDQQHDSYSQNQQSYHSQRENYSHHTQDDRRDVSRYGEDNRGYGGSQGGGRGRGGYDKDGRGPMTGSSGGDRGGFKNFGGHRDYGPRTDADSESDNSDNNTIFVQGLGEGVSTDQVGEFFKQIGIIKTNKKTGKPMINLYTDKDTGKPKGEATVSFDDPPSAKAAIDWFDGKEFHGNIIKVSFATRRPEFMRGGGSGGGRRGRGGYRGRGGFQGRGGDPKSGDWVCPNPSCGNMNFARRNSCNQCNEPRPEDSRPSGGDFRGRGYGGERGYRGRGGRGGDRGGYGGDRSGGGYGGDRSSGGGYSGDRSGGGYGGDRSGGGYGGDRGGGYGGDRGGGYGGDRGGGYGGDRGGYGGDRGGGYGGDRGGYGGDRGGYGGDRGGYGGDRGGYGGDRSRGGYGGDRGGGSGYGGDRSGGYGGDRSGGGYGGDRGGGYGGDRGGYGGKMGGRNDYRNDQRNRPY

>NP_004597.2

MGPGCDLLLRTAATITAAAIMSDTDSDEDSAGGGPFSLAGFLFGNINGAGQLEGESVLDDECKKHLAGLGALGLGSLITELTANEELTGTDGALVNDEGWVRSTEDAVDYSDINEVAEDESRRYQQTMGSLQPLCHSDYDEDDYDADCEDIDCKLMPPPPPPPGPMKKDKDQDSITGVSENGEGIILPSIIAPSSLASEKVDFSSSSDSESEMGPQEATQAESEDGKLTLPLAGIMQHDATKLLPSVTELFPEFRPGKVLRFLRLFGPGKNVPSVWRSARRKRKKKHRELIQEEQIQEVECSVESEVSQKSLWNYDYAPPPPPEQCLSDDEITMMAPVESKFSQSTGDIDKVTDTKPRVAEWRYGPARLWYDMLGVPEDGSGFDYGFKLRKTEHEPVIKSRMIEEFRKLEENNGTDLLADENFLMVTQLHWEDDIIWDGEDVKHKGTKPQRASLAGWLPSSMTRNAMAYNVQQGFAATLDDDKPWYSIFPIDNEDLVYGRWEDNIIWDAQAMPRLLEPPVLTLDPNDENLILEIPDEKEEATSNSPSKESKKESSLKKSRILLGKTGVIKEEPQQNMSQPEVKDPWNLSNDEYYYPKQQGLRGTFGGNIIQHSIPAVELRQPFFPTHMGPIKLRQFHRPPLKKYSFGALSQPGPHSVQPLLKHIKKKAKMREQERQASGGGEMFFMRTPQDLTGKDGDLILAEYSEENGPLMMQVGMATKIKNYYKRKPGKDPGAPDCKYGETVYCHTSPFLGSLHPGQLLQAFENNLFRAPIYLHKMPETDFLIIRTRQGYYIRELVDIFVVGQQCPLFEVPGPNSKRANTHIRDFLQVFIYRLFWKSKDRPRRIRMEDIKKAFPSHSESSIRKRLKLCADFKRTGMDSNWWVLKSDFRLPTEEEIRAMVSPEQCCAYYSMIAAEQRLKDAGYGEKSFFAPEEENEEDFQMKIDDEVRTAPWNTTRAFIAAMKGKCLLEVTGVADPTGCGEGFSYVKIPNKPTQQKDDKEPQPVKKTVTGTDADLRRLSLKNAKQLLRKFGVPEEEIKKLSRWEVIDVVRTMSTEQARSGEGPMSKFARGSRFSVAEHQERYKEECQRIFDLQNKVLSSTEVLSTDTDSSSAEDSDFEEMGKNIENMLQNKKTSSQLSREREEQERKELQRMLLAAGSAASGNNHRDDDTASVTSLNSSATGRCLKIYRTFRDEEGKEYVRCETVRKPAVIDAYVRIRTTKDEEFIRKFALFDEQHREEMRKERRRIQEQLRRLKRNQEKEKLKGPPEKKPKKMKERPDLKLKCGACGAIGHMRTNKFCPLYYQTNAPPSNPVAMTEEQEEELEKTVIHNDNEELIKVEGTKIVLGKQLIESADEVRRKSLVLKFPKQQLPPKKKRRVGTTVHCDYLNRPHKSIHRRRTDPMVTLSSILESIINDMRDLPNTYPFHTPVNAKVVKDYYKIITRPMDLQTLRENVRKRLYPSREEFREHLELIVKNSATYNGPKHSLTQISQSMLDLCDEKLKEKEDKLARLEKAINPLLDDDDQVAFSFILDNIVTQKMMAVPDSWPFHHPVNKKFVPDYYKVIVNPMDLETIRKNISKHKYQSRESFLDDVNLILANSVKYNGPESQYTKTAQEIVNVCYQTLTEYDEHLTQLEKDICTAKEAALEEAELESLDPMTPGPYTPQPPDLYDTNTSLSMSRDASVFQDESNMSVLDIPSATPEKQVTQEGEDGDGDLADEEEGTVQQPQASVLYEDLLMSEGEDDEEDAGSDEEGDNPFSAIQLSESGSDSDVGSGGIRPKQPRMLQENTRMDMENEESMMSYEGDGGEASHGLEDSNISYGSYEEPDPKSNTQDTSFSSIGGYEVSEEEEDEEEEEQRSGPSVLSQVHLSEDEEDSEDFHSIAGDSDLDSDE

199 NP_057457.1 NP_000537.3

>NP_057457.1

MAALRYAGLDDTDSEDELPPGWEERTTKDGWVYYANHTEEKTQWEHPKTGKRKRVAGDLPYGWEQETDENGQVFFVDHINKRTTYLDPRLAFTVDDNPTKPTTRQRYDGSTTAMEILQGRDFTGKVVVVTGANSGIGFETAKSFALHGAHVILACRNMARASEAVSRILEEWHKAKVEAMTLDLALLRSVQHFAEAFKAKNVPLHVLVCNAATFALPWSLTKDGLETTFQVNHLGHFYLVQLLQDVLCRSAPARVIVVSSESHRFTDINDSLGKLDFSRLSPTKNDYWAMLAYNRSKLCNILFSNELHRRLSPRGVTSNAVHPGNMMYSNIHRSWWVYTLLFTLARPFTKSMQQGAATTVYCAAVPELEGLGGMYFNNCCRCMPSPEAQSEETARTLWALSERLIQERLGSQSG

>NP_000537.3

MEEPQSDPSVEPPLSQETFSDLWKLLPENNVLSPLPSQAMDDLMLSPDDIEQWFTEDPGPDEAPRMPEAAPPVAPAPAAPTPAAPAPAPSWPLSSSVPSQKTYQGSYGFRLGFLHSGTAKSVTCTYSPALNKMFCQLAKTCPVQLWVDSTPPPGTRVRAMAIYKQSQHMTEVVRRCPHHERCSDSDGLAPPQHLIRVEGNLRVEYLDDRNTFRHSVVVPYEPPEVGSDCTTIHYNYMCNSSCMGGMNRRPILTIITLEDSSGNLLGRNSFEVRVCACPGRDRRTEEENLRKKGEPHHELPPGSTKRALPNNTSSSPQPKKKPLDGEYFTLQIRGRERFEMFRELNEALELKDAQAGKEPGGSRAHSSHLKSKKGQSTSRHKKLMFKTEGPDSD

200 Q01892 NP_002219.1

>Q01892

MLALEAAQLDGPHFSCLYPDGVFYDLDSCKHSSYPDSEGAPDSLWDWTVAPPVPATPYEAFDPAAAAFSHPQAAQLCYEPPTYSPAGNLELAPSLEAPGPGLPAYPTENFASQTLVPPAYAPYPSPVLSEEEDLPLDSPALEVSDSESDEALVAGPEGKGSEAGTRKKLRLYQFLLGLLTRGDMRECVWWVEPGAGVFQFSSKHKELLARRWGQQKGNRKRMTYQKLARALRNYAKTGEIRKVKRKLTYQFDSALLPAVRRA

>NP_002219.1

MTAKMETTFYDDALNASFLPSESGPYGYSNPKILKQSMTLNLADPVGSLKPHLRAKNSDLLTSPDVGLLKLASPELERLIIQSSNGHITTTPTPTQFLCPKNVTDEQEGFAEGFVRALAELHSQNTLPSVTSAAQPVNGAGMVAPAVASVAGGSGSGGFSASLHSEPPVYANLSNFNPGALSSGGGAPSYGAAGLAFPAQPQQQQQPPHHLPQQMPVQHPRLQALKEEPQTVPEMPGETPPLSPIDMESQERIKAERKRMRNRIAASKCRKRKLERIARLEEKVKTLKAQNSELASTANMLREQVAQLKQKVMNHVNSGCQLMLTQQLQTF

201 NP_003812.1 NP_005649.1

>NP_003812.1

MNGEAICSALPTIPYHKLADLRYLSRGASGTVSSARHADWRVQVAVKHLHIHTPLLDSERKDVLREAEILHKARFSYILPILGICNEPEFLGIVTEYMPNGSLNELLHRKTEYPDVAWPLRFRILHEIALGVNYLHNMTPPLLHHDLKTQNILLDNEFHVKIADFGLSKWRMMSLSQSRSSKSAPEGGTIIYMPPENYEPGQKSRASIKHDIYSYAVITWEVLSRKQPFEDVTNPLQIMYSVSQGHRPVINEESLPYDIPHRARMISLIESGWAQNPDERPSFLKCLIELEPVLRTFEEITFLEAVIQLKKTKLQSVSSAIHLCDKKKMELSLNIPVNHGPQEESCGSSQLHENSGSPETSRSLPAPQDNDFLSRKAQDCYFMKLHHCPGNHSWDSTISGSQRAAFCDHKTTPCSSAIINPLSTAGNSERLQPGIAQQWIQSKREDIVNQMTEACLNQSLDALLSRDLIMKEDYELVSTKPTRTSKVRQLLDTTDIQGEEFAKVIVQKLKDNKQMGLQPYPEILVVSRSPSLNLLQNKSM

>NP_005649.1

MASSSGSSPRPAPDENEFPFGCPPTVCQDPKEPRALCCAGCLSENPRNGEDQICPKCRGEDLQSISPGSRLRTQEKAHPEVAEAGIGCPFAGVGCSFKGSPQSVQEHEVTSQTSHLNLLLGFMKQWKARLGCGLESGPMALEQNLSDLQLQAAVEVAGDLEVDCYRAPCSESQEELALQHFMKEKLLAELEGKLRVFENIVAVLNKEVEASHLALATSIHQSQLDRERILSLEQRVVELQQTLAQKDQALGKLEQSLRLMEEASFDGTFLWKITNVTRRCHESACGRTVSLFSPAFYTAKYGYKLCLRLYLNGDGTGKRTHLSLFIVIMRGEYDALLPWPFRNKVTFMLLDQNNREHAIDAFRPDLSSASFQRPQSETNVASGCPLFFPLSKLQSPKHAYVKDDTMFLKCIVETST

202 NP_000475.1 NP_036456.1

>NP_000475.1

MLPGLALLLLAAWTARALEVPTDGNAGLLAEPQIAMFCGRLNMHMNVQNGKWDSDPSGTKTCIDTKEGILQYCQEVYPELQITNVVEANQPVTIQNWCKRGRKQCKTHPHFVIPYRCLVGEFVSDALLVPDKCKFLHQERMDVCETHLHWHTVAKETCSEKSTNLHDYGMLLPCGIDKFRGVEFVCCPLAEESDNVDSADAEEDDSDVWWGGADTDYADGSEDKVVEVAEEEEVAEVEEEEADDDEDDEDGDEVEEEAEEPYEEATERTTSIATTTTTTTESVEEVVREVCSEQAETGPCRAMISRWYFDVTEGKCAPFFYGGCGGNRNNFDTEEYCMAVCGSAMSQSLLKTTQEPLARDPVKLPTTAASTPDAVDKYLETPGDENEHAHFQKAKERLEAKHRERMSQVMREWEEAERQAKNLPKADKKAVIQHFQEKVESLEQEAANERQQLVETHMARVEAMLNDRRRLALENYITALQAVPPRPRHVFNMLKKYVRAEQKDRQHTLKHFEHVRMVDPKKAAQIRSQVMTHLRVIYERMNQSLSLLYNVPAVAEEIQDEVDELLQKEQNYSDDVLANMISEPRISYGNDALMPSLTETKTTVELLPVNGEFSLDDLQPWHSFGADSVPANTENEVEPVDARPAADRGLTTRPGSGLTNIKTEEISEVKMDAEFRHDSGYEVHHQKLVFFAEDVGSNKGAIIGLMVGGVVIATVIVITLVMLKKKQYTSIHHGVVEVDAAVTPEERHLSKMQQNGYENPTYKFFEQMQN

>NP_036456.1

MADRAEMFSLSTFHSLSPPGCRPPQDISLEEFDDEDLSEITDDCGLGLSYDSDHCEKDSLSLGRSEQPHPICSFQDDFQEFEMIDDNEEEDDEDEEEEEEEEEGDGEGQEGGDPGSEAPAPGPLIPSPSVEEPHKHRPTTLRLTTLGAQDSLNNNGGFDLVRPASWQETALCSPAPEALRELPGPLPATDTGPGGAQSPVRPGCDCEGNRPAEPPAPGGTSPSSDPGIEADLRSRSSGGRGGRRSSQELSSPGSDSEDAGGARLGRMISSISETELELSSDGGSSSSGRSSHLTNSIEEASSPASEPEPPREPPRRPAFLPVGPDDTNSEYESGSESEPDLSEDADSPWLLSNLVSRMISEGSSPIRCPGQCLSPAPRPPGEPVSPAGGAAQDSQDPEAAAGPGGVELVDMETLCAPPPPAPAAPRPGPAQPGPCLFLSNPTRDTITPLWAAPGRAARPGRACSAACSEEEDEEDDEEEEDAEDSAGSPGGRGTGPSAPRDASLVYDAVKYTLVVDEHTQLELVSLRRCAGLGHDSEEDSGGEASEEEAGAALLGGGQVSGDTSPDSPDLTFSKKFLNVFVNSTSRSSSTESFGLFSCLVNGEEREQTHRAVFRFIPRHPDELELDVDDPVLVEAEEDDFWFRGFNMRTGERGVFPAFYAHAVPGPAKDLLGSKRSPCWVERFDVQFLGSVEVPCHQGNGILCAAMQKIATARKLTVHLRPPASCDLEISLRGVKLSLSGGGPEFQRCSHFFQMKNISFCGCHPRNSCYFGFITKHPLLSRFACHVFVSQESMRPVAQSVGRAFLEYYQEHLAYACPTEDIYLE

203 NP_002897.1 NP_000359.1

>NP_002897.1

MPKPINVRVTTMDAELEFAIQPNTTGKQLFDQVVKTVGLREVWFFGLQYVDSKGYSTWLKLNKKVTQQDVKKENPLQFKFRAKFFPEDVSEELIQEITQRLFFLQVKEAILNDEIYCPPETAVLLASYAVQAKYGDYNKEIHKPGYLANDRLLPQRVLEQHKLTKEQWEERIQNWHEEHRGMLREDSMMEYLKIAQDLEMYGVNYFEIKNKKGTELWLGVDALGLNIYEHDDKLTPKIGFPWSEIRNISFNDKKFVIKPIDKKAPDFVFYAPRLRINKRILALCMGNHELYMRRRKPDTIEVQQMKAQAREEKHQKQLERAQLENEKKKREIAEKEKERIEREKEELMERLKQIEEQTIKAQKELEEQTRKALELDQERKRAKEEAERLEKERRAAEEAKSAIAKQAADQMKNQEQLAAELAEFTAKIALLEEAKKKKEEEATEWQHKAFAAQEDLEKTKEELKTVMSAPPPPPPPPVIPPTENEHDEHDENNAEASAELSNEGVMNHRSEEERVTETQKNERVKKQLQALSSELAQARDETKKTQNDVLHAENVKAGRDKYKTLRQIRQGNTKQRIDEFEAM

>NP_000359.1

MAQQANVGELLAMLDSPMLGVRDDVTAVFKENLNSDRGPMLVNTLVDYYLETSSQPALHILTTLQEPHDKHLLDRINEYVGKAATRLSILSLLGHVIRLQPSWKHKLSQAPLLPSLLKCLKMDTDVVVLTTGVLVLITMLPMIPQSGKQHLLDFFDIFGRLSSWCLKKPGHVAEVYLVHLHASVYALFHRLYGMYPCNFVSFLRSHYSMKENLETFEEVVKPMMEHVRIHPELVTGSKDHELDPRRWKRLETHDVVIECAKISLDPTEASYEDGYSVSHQISARFPHRSADVTTSPYADTQNSYGCATSTPYSTSRLMLLNMPGQLPQTLSSPSTRLITEPPQATLWSPSMVCGMTTPPTSPGNVPPDLSHPYSKVFGTTAGGKGTPLGTPATSPPPAPLCHSDDYVHISLPQATVTPPRKEERMDSARPCLHRQHHLLNDRGSEEPPGSKGSVTLSDLPGFLGDLASEEDSIEKDKEEAAISRELSEITTAEAEPVVPRGGFDSPFYRDSLPGSQRKTHSAASSSQGASVNPEPLHSSLDKLGPDTPKQAFTPIDLPCGSADESPAGDRECQTSLETSIFTPSPCKIPPPTRVGFGSGQPPPYDHLFEVALPKTAHHFVIRKTEELLKKAKGNTEEDGVPSTSPMEVLDRLIQQGADAHSKELNKLPLPSKSVDWTHFGGSPPSDEIRTLRDQLLLLHNQLLYERFKRQQHALRNRRLLRKVIKAAALEEHNAAMKDQLKLQEKDIQMWKVSLQKEQARYNQLQEQRDTMVTKLHSQIRQLQHDREEFYNQSQELQTKLEDCRNMIAELRIELKKANNKVCHTELLLSQVSQKLSNSESVQQQMEFLNRQLLVLGEVNELYLEQLQNKHSDTTKEVEMMKAAYRKELEKNRSHVLQQTQRLDTSQKRILELESHLAKKDHLLLEQKKYLEDVKLQARGQLQAAESRYEAQKRITQVFELEILDLYGRLEKDGLLKKLEEEKAEAAEAAEERLDCCNDGCSDSMVGHNEEASGHNGETKTPRPSSARGSSGSRGGGGSSSSSSELSTPEKPPHQRAGPFSSRWETTMGEASASIPTTVGSLPSSKSFLGMKARELFRNKSESQCDEDGMTSSLSESLKTELGKDLGVEAKIPLNLDGPHPSPPTPDSVGQLHIMDYNETHHEHS

204 NP_005484.2 NP_002954.2

>NP_005484.2

MSGQPPPPPPQQQQQQQQLSPPPPAALAPVSGVVLPAPPAVSAGSSPAGSPGGGAGGEGLGAAAAALLLHPPPPPPPATAAPPPPPPPPPPPASAAAPASGPPAPPGLAAGPGPAGGAPTPALVAGSSAAAPFPHGDSALNEQEKELQRRLKRLYPAVDEQETPLPRSWSPKDKFSYIGLSQNNLRVHYKGHGKTPKDAASVRATHPIPAACGIYYFEVKIVSKGRDGYMGIGLSAQGVNMNRLPGWDKHSYGYHGDDGHSFCSSGTGQPYGPTFTTGDVIGCCVNLINNTCFYTKNGHSLGIAFTDLPPNLYPTVGLQTPGEVVDANFGQHPFVFDIEDYMREWRTKIQAQIDRFPIGDREGEWQTMIQKMVSSYLVHHGYCATAEAFARSTDQTVLEELASIKNRQRIQKLVLAGRMGEAIETTQQLYPSLLERNPNLLFTLKVRQFIEMVNGTDSEVRCLGGRSPKSQDSYPVSPRPFSSPSMSPSHGMNIHNLASGKGSTAHFSGFESCSNGVISNKAHQSYCHSNKHQSSNLNVPELNSINMSRSQQVNNFTSNDVDMETDHYSNGVGETSSNGFLNGSSKHDHEMEDCDTEMEVDSSQLRRQLCGGSQAAIERMIHFGRELQAMSEQLRRDCGKNTANKKMLKDAFSLLAYSDPWNSPVGNQLDPIQREPVCSALNSAILETHNLPKQPPLALAMGQATQCLGLMARSGIGSCAFATVEDYLH

>NP_002954.2

MSNTQAERSIIGMIDMFHKYTRRDDKIEKPSLLTMMKENFPNFLSACDKKGTNYLADVFEKKDKNEDKKIDFSEFLSLLGDIATDYHKQSHGAAPCSGGSQ

205 NP_065082.2 NP_003664.1

>NP_065082.2

MAKAPSWAGVGALAYKAPEALWPAEAVMDGTMEDSEAVQRATALIEQRLAQEEENEKLRGDARQKLPMDLLVLEDEKHHGAQSAALQKVKGQERVRKTSLDLRREIIDVGGIQNLIELRKKRKQKKRDALAASHEPPPEPEEITGPVDEETFLKAAVEGKMKVIEKFLADGGSADTCDQFRRTALHRASLEGHMEILEKLLDNGATVDFQDRLDCTAMHWACRGGHLEVVKLLQSHGADTNVRDKLLSTPLHVAVRTGQVEIVEHFLSLGLEINARDREGDTALHDAVRLNRYKIIKLLLLHGADMMTKNLAGKTPTDLVQLWQADTRHALEHPEPGAEHNGLEGPNDSGRETPQPVPAQ

>NP_003664.1

MATSELSCEVSEENCERREAFWAEWKDLTLSTRPEEGCSLHEEDTQRHETYHQQGQCQVLVQRSPWLMMRMGILGRGLQEYQLPYQRVLPLPIFTPAKMGATKEEREDTPIQLQELLALETALGGQCVDRQEVAEITKQLPPVVPVSKPGALRRSLSRSMSQEAQRG

206 NP_004801.1 NP_000213.1

>NP_004801.1

MEAVAKFDFTASGEDELSFHTGDVLKILSNQEEWFKAELGSQEGYVPKNFIDIQFPKWFHEGLSRHQAENLLMGKEVGFFIIRASQSSPGDFSISVRHEDDVQHFKVMRDNKGNYFLWTEKFPSLNKLVDYYRTNSISRQKQIFLRDRTREDQGHRGNSLDRRSQGGPHLSGAVGEEIRPSMNRKLSDHPPTLPLQQHQHQPQPPQYAPAPQQLQQPPQQRYLQHHHFHQERRGGSLDINDGHCGTGLGSEMNAALMHRRHTDPVQLQAAGRVRWARALYDFEALEDDELGFHSGEVVEVLDSSNPSWWTGRLHNKLGLFPANYVAPMTR

>NP_000213.1

MRGARGAWDFLCVLLLLLRVQTGSSQPSVSPGEPSPPSIHPGKSDLIVRVGDEIRLLCTDPGFVKWTFEILDETNENKQNEWITEKAEATNTGKYTCTNKHGLSNSIYVFVRDPAKLFLVDRSLYGKEDNDTLVRCPLTDPEVTNYSLKGCQGKPLPKDLRFIPDPKAGIMIKSVKRAYHRLCLHCSVDQEGKSVLSEKFILKVRPAFKAVPVVSVSKASYLLREGEEFTVTCTIKDVSSSVYSTWKRENSQTKLQEKYNSWHHGDFNYERQATLTISSARVNDSGVFMCYANNTFGSANVTTTLEVVDKGFINIFPMINTTVFVNDGENVDLIVEYEAFPKPEHQQWIYMNRTFTDKWEDYPKSENESNIRYVSELHLTRLKGTEGGTYTFLVSNSDVNAAIAFNVYVNTKPEILTYDRLVNGMLQCVAAGFPEPTIDWYFCPGTEQRCSASVLPVDVQTLNSSGPPFGKLVVQSSIDSSAFKHNGTVECKAYNDVGKTSAYFNFAFKGNNKEQIHPHTLFTPLLIGFVIVAGMMCIIVMILTYKYLQKPMYEVQWKVVEEINGNNYVYIDPTQLPYDHKWEFPRNRLSFGKTLGAGAFGKVVEATAYGLIKSDAAMTVAVKMLKPSAHLTEREALMSELKVLSYLGNHMNIVNLLGACTIGGPTLVITEYCCYGDLLNFLRRKRDSFICSKQEDHAEAALYKNLLHSKESSCSDSTNEYMDMKPGVSYVVPTKADKRRSVRIGSYIERDVTPAIMEDDELALDLEDLLSFSYQVAKGMAFLASKNCIHRDLAARNILLTHGRITKICDFGLARDIKNDSNYVVKGNARLPVKWMAPESIFNCVYTFESDVWSYGIFLWELFSLGSSPYPGMPVDSKFYKMIKEGFRMLSPEHAPAEMYDIMKTCWDADPLKRPTFKQIVQLIEKQISESTNHIYSNLANCSPNRQKPVVDHSVRINSVGSTASSSQPLLVHDDV

207 NP_055816.2 NP_006229.1

>NP_055816.2

MVRETRHLWVGNLPENVREEKIIEHFKRYGRVESVKILPKRGSEGGVAAFVDFVDIKSAQKAHNSVNKMGDRDLRTDYNEPGTIPSAARGLDDTVSIASRSREVSGFRGGGGGPAYGPPPSLHAREGRYERRLDGASDNRERAYEHSAYGHHERGTGGFDRTRHYDQDYYRDPRERTLQHGLYYASRSRSPNRFDAHDPRYEPRAREQFTLPSVVHRDIYRDDITREVRGRRPERNYQHSRSRSPHSSQSRNQSPQRLASQASRPTRSPSGSGSRSRSSSSDSISSSSSTSSDSSDSSSSSSDDSPARSVQSAAVPAPTSQLLSSLEKDEPRKSFGIKVQNLPVRSTDTSLKDGLFHEFKKFGKVTSVQIHGTSEERYGLVFFRQQEDQEKALTASKGKLFFGMQIEVTAWIGPETESENEFRPLDERIDEFHPKATRTLFIGNLEKTTTYHDLRNIFQRFGEIVDIDIKKVNGVPQYAFLQYCDIASVCKAIKKMDGEYLGNNRLKLGFGKSMPTNCVWLDGLSSNVSDQYLTRHFCRYGPVVKVVFDRLKGMALVLYNEIEYAQAAVKETKGRKIGGNKIKVDFANRESQLAFYHCMEKSGQDIRDFYEMLAERREERRASYDYNQDRTYYESVRTPGTYPEDSRRDYPARGREFYSEWETYQGDYYESRYYDDPREYRDYRNDPYEQDIREYSYRQRERERERERFESDRDRDHERRPIERSQSPVHLRRPQSPGASPSQAERLPSDSERRLYSRSSDRSGSCSSLSPPRYEKLDKSRLERYTKNEKTDKERTFDPERVERERRLIRKEKVEKDKTDKQKRKGKVHSPSSQSSETDQENEREQSPEKPRSCNKLSREKADKEGIAKNRLELMPCVVLTRVKEKEGKVIDHTPVEKLKAKLDNDTVKSSALDQKLQVSQTEPAKSDLSKLESVRMKVPKEKGLSSHVEVVEKEGRLKARKHLKPEQPADGVSAVDLEKLEARKRRFADSNLKAEKQKPEVKKSSPEMEDARVLSKKQPDVSSREVILLREGEAERKPVRKEILKRESKKIKLDRLNTVASPKDCQELASISVGSGSRPSSDLQARLGELAGESVENQEVQSKKPIPSKPQLKQLQVLDDQGPEREDVRKNYCSLRDETPERKSGQEKSHSVNTEEKIGIDIDHTQSYRKQMEQSRRKQQMEMEIAKSEKFGSPKKDVDEYERRSLVHEVGKPPQDVTDDSPPSKKKRMDHVDFDICTKRERNYRSSRQISEDSERTGGSPSVRHGSFHEDEDPIGSPRLLSVKGSPKVDEKVLPYSNITVREESLKFNPYDSSRREQMADMAKIKLSVLNSEDELNRWDSQMKQDAGRFDVSFPNSIIKRDSLRKRSVRDLEPGEVPSDSDEDGEHKSHSPRASALYESSRLSFLLRDREDKLRERDERLSSSLERNKFYSFALDKTITPDTKALLERAKSLSSSREENWSFLDWDSRFANFRNNKDKEKVDSAPRPIPSWYMKKKKIRTDSEGKMDDKKEDHKEEEQERQELFASRFLHSSIFEQDSKRLQHLERKEEDSDFISGRIYGKQTSEGANSTTDSIQEPVVLFHSRFMELTRMQQKEKEKDQKPKEVEKQEDTENHPKTPESAPENKDSELKTPPSVGPPSVTVVTLESAPSALEKTTGDKTVEAPLVTEEKTVEPATVSEEAKPASEPAPAPVEQLEQVDLPPGADPDKEAAMMPAGVEEGSSGDQPPYLDAKPPTPGASFSQAESNVDPEPDSTQPLSKPAQKSEEANEPKAEKPDATADAEPDANQKAEAAPESQPPASEDLEVDPPVAAKDKKPNKSKRSKTPVQAAAVSIVEKPVTRKSERIDREKLKRSNSPRGEAQKLLELKMEAEKITRTASKNSAADLEHPEPSLPLSRTRRRNVRSVYATMGDHENRSPVKEPVEQPRVTRKRLERELQEAAAVPTTPRRGRPPKTRRRADEEEENEAKEPAETLKPPEGWRSPRSQKTAAGGGPQGKKGKNEPKVDATRPEATTEVGPQIGVKESSMEPKAAEEEAGSEQKRDRKDAGTDKNPPETAPVEVVEKKPAPEKNSKSKRGRSRNSRLAVDKSASLKNVDAAVSPRGAAAQAGERESGVVAVSPEKSESPQKEDGLSSQLKSDPVDPDKEPEKEDVSASGPSPEATQLAKQMELEQAVEHIAKLAEASASAAYKADAPEGLAPEDRDKPAHQASETELAAAIGSIINDISGEPENFPAPPPYPGESQTDLQPPAGAQALQPSEEGMETDEAVSGILETEAATESSRPPVNAPDPSAGPTDTKEARGNSSETSHSVPEAKGSKEVEVTLVRKDKGRQKTTRSRRKRNTNKKVVAPVESHVPESNQAQGESPAANEGTTVQHPEAPQEEKQSEKPHSTPPQSCTSDLSKIPSTENSSQEISVEERTPTKASVPPDLPPPPQPAPVDEEPQARFRVHSIIESDPVTPPSDPSIPIPTLPSVTAAKLSPPVASGGIPHQSPPTKVTEWITRQEEPRAQSTPSPALPPDTKASDVDTSSSTLRKILMDPKYVSATSVTSTSVTTAIAEPVSAAPCLHEAPPPPVDSKKPLEEKTAPPVTNNSEIQASEVLVAADKEKVAPVIAPKITSVISRMPVSIDLENSQKITLAKPAPQTLTGLVSALTGLVNVSLVPVNALKGPVKGSVTTLKSLVSTPAGPVNVLKGPVNVLTGPVNVLTTPVNATVGTVNAAPGTVNAAASAVNATASAVTVTAGAVTAASGGVTATTGTVTMAGAVIAPSTKCKQRASANENSRFHPGSMPVIDDRPADAGSGAGLRVNTSEGVVLLSYSGQKTEGPQRISAKISQIPPASAMDIEFQQSVSKSQVKPDSVTASQPPSKGPQAPAGYANVATHSTLVLTAQTYNASPVISSVKADRPSLEKPEPIHLSVSTPVTQGGTVKVLTQGINTPPVLVHNQLVLTPSIVTTNKKLADPVTLKIETKVLQPANLGSTLTPHHPPALPSKLPTEVNHVPSGPSIPADRTVSHLAAAKLDAHSPRPSGPGPSSFPRASHPSSTASTALSTNATVMLAAGIPVPQFISSIHPEQSVIMPPHSITQTVSLSHLSQGEVRMNTPTLPSITYSIRPEALHSPRAPLQPQQIEVRAPQRASTPQPAPAGVPALASQHPPEEEVHYHLPVARATAPVQSEVLVMQSEYRLHPYTVPRDVRIMVHPHVTAVSEQPRAADGVVKVPPASKAPQQPGKEAAKTPDAKAAPTPTPAPVPVPVPLPAPAPAPHGEARILTVTPSNQLQGLPLTPPVVVTHGVQIVHSSGELFQEYRYGDIRTYHPPAQLTHTQFPAASSVGLPSRTKTAAQGPPPEGEPLQPPQPVQSTQPAQPAPPCPPSQLGQPGQPPSSKMPQVSQEAKGTQTGVEQPRLPAGPANRPPEPHTQVQRAQAETGPTSFPSPVSVSMKPDLPVSLPTQTAPKQPLFVPTTSGPSTPPGLVLPHTEFQPAPKQDSSPHLTSQRPVDMVQLLKKYPIVWQGLLALKNDTAAVQLHFVSGNNVLAHRSLPLSEGGPPLRIAQRMRLEATQLEGVARRMTVETDYCLLLALPCGRDQEDVVSQTESLKAAFITYLQAKQAAGIINVPNPGSNQPAYVLQIFPPCEFSESHLSRLAPDLLASISNISPHLMIVIASV

>NP_006229.1

MEQPQEEAPEVREEEEKEEVAEAEGAPELNGGPQHALPSSSYTDLSRSSSPPSLLDQLQMGCDGASCGSLNMECRVCGDKASGFHYGVHACEGCKGFFRRTIRMKLEYEKCERSCKIQKKNRNKCQYCRFQKCLALGMSHNAIRFGRMPEAEKRKLVAGLTANEGSQYNPQVADLKAFSKHIYNAYLKNFNMTKKKARSILTGKASHTAPFVIHDIETLWQAEKGLVWKQLVNGLPPYKEISVHVFYRCQCTTVETVRELTEFAKSIPSFSSLFLNDQVTLLKYGVHEAIFAMLASIVNKDGLLVANGSGFVTREFLRSLRKPFSDIIEPKFEFAVKFNALELDDSDLALFIAAIILCGDRPGLMNVPRVEAIQDTILRALEFHLQANHPDAQYLFPKLLQKMADLRQLVTEHAQMMQRIKKTETETSLHPLLQEIYKDMY

208 NP_003438.1 NP_659456.2

>NP_003438.1

MATEPKKAAAQNSPEDEGLLIVKIEEEEFIHGQDTCLQRSELLKQELCRQLFRQFCYQDSPGPREALSRLRELCCQWLKPEIHTKEQILELLVLEQFLTILPGDLQAWVHEHYPESGEEAVTILEDLERGTDEAVLQVQAHEHGQEIFQKKVSPPGPALNVKLQPVETKAHFDSSEPQLLWDCDNESENSRSMPKLEIFEKIESQRIISGRISGYISEASGESQDICKSAGRVKRQWEKESGESQRLSSAQDEGFGKILTHKNTVRGEIISHDGCERRLNLNSNEFTHQKSCKHGTCDQSFKWNSDFINHQIIYAGEKNHQYGKSFKSPKLAKHAAVFSGDKTHQCNECGKAFRHSSKLARHQRIHTGERCYECNECGKSFAESSDLTRHRRIHTGERPFGCKECGRAFNLNSHLIRHQRIHTREKPYECSECGKTFRVSSHLIRHFRIHTGEKPYECSECGRAFSQSSNLSQHQRIHMRENLLM

>NP_659456.2

MDPKAGGGGEEDDCVDSGAETGGSDYSHLSSTSNGFSTWRMPCCLFLYCTHGDKEKGRWSLQVEHTWPSGELSVEEAQDPFLVSIHIIADPGESQPLQEAIDNVLAWIHPDLPLFRVSERRASRRRRKPPKGAQPALAVVLFLQEEYGEEQILQLHRTLQQPPWRHHHTEQVHGRFLPYLPCSQDFFTLAPGTPLWAIRPVHYGKEIVRFTVYCRYDNYADSLRFYQLILRRNPSQKKADFCIFPIFSNLDVDIQFSLKRLPCDQCPVPTDSSVLEFRVRDIGELVPLLPNPCSPISEGRWQTEDHDGNKILLQAQRVHKKFPKPGRVHHASEKKRHSTPLPSTAVPSHTPGSSQQSPLNSPHPGPIRTGLPPGHQQEFAGRANSTPNPPWSFQRSKSLFCLPTGGPSLASSAEPQWFSNTGAPGHRASEWRHGHLLSIDDLEGAQETDVDTGLRLSSSDLSVVSAYSAPSRFCSTVETPLPSERCSSHWAAHKDSREGPLPTVSRVTTEASWASLPFFTKRSSSSSATARAAPPAPSTSTLTDSSPQLPCDTPKVKQTDGDMPPPPGSAGPGDNDMEEFYI

209 NP_002266.2 NP_787072.2

>NP_002266.2

MTTTFLQTSSSTFGGGSTRGGSLLAGGGGFGGGSLSGGGGSRSISASSARFVSSGSGGGYGGGMRVCGFGGGAGSVFGGGFGGGVGGGFGGGFGGGDGGLLSGNEKITMQNLNDRLASYLDKVRALEEANADLEVKIHDWYQKQTPASPECDYSQYFKTIEELRDKIMATTIDNSRVILEIDNARLAADDFRLKYENELALRQGVEADINGLRRVLDELTLARTDLEMQIEGLNEELAYLKKNHEEEMKEFSSQLAGQVNVEMDAAPGVDLTRVLAEMREQYEAMAEKNRRDVEAWFFSKTEELNKEVASNTEMIQTSKTEITDLRRTMQELEIELQSQLSMKAGLENSLAETECRYATQLQQIQGLIGGLEAQLSELRCEMEAQNQEYKMLLDIKTRLEQEIATYRSLLEGQDAKMAGIGIREASSGGGGSSSNFHINVEESVDGQVVSSHKREI

>NP_787072.2

MAMAMSDSGASRLRRQLESGGFEARLYVKQLSQQSDGDRDLQEHRQRIQALAEETAQNLKRNVYQNYRQFIETAREISYLESEMYQLSHLLTEQKSSLESIPLTLLPAAAAAGAAAASGGEEGVGGAGGRDHLRGQAGFFSTPGGASRDGSGPGEEGKQRTLTTLLEKVEGCRHLLETPGQYLVYNGDLVEYDADHMAQLQRVHGFLMNDCLLVATWLPQRRGMYRYNALYSLDGLAVVNVKDNPPMKDMFKLLMFPESRIFQAENAKIKREWLEVLEDTKRALSEKRRREQEEAAAPRGPPQVTSKATNPFEDDEEEEPAVPEVEEEKVDLSMEWIQELPEDLDVCIAQRDFEGAVDLLDKLNHYLEDKPSPPPVKELRAKVEERVRQLTEVLVFELSPDRSLRGGPKATRRAVSQLIRLGQCTKACELFLRNRAAAVHTAIRQLRIEGATLLYIHKLCHVFFTSLLETAREFEIDFAGTDSGCYSAFVVWARSAMGMFVDAFSKQVFDSKESLSTAAECVKVAKEHCQQLGDIGLDLTFIIHALLVKDIQGALHSYKEIIIEATKHRNSEEMWRRMNLMTPEALGKLKEEMKSCGVSNFEQYTGDDCWVNLSYTVVAFTKQTMGFLEEALKLYFPELHMVLLESLVEIILVAVQHVDYSLRCEQDPEKKAFIRQNASFLYETVLPVVEKRFEEGVGKPAKQLQDLRNASRLIRVNPESTTSVV

210 NP_003391.1 NP_001961.1

>NP_003391.1

MPAIMTMLADHAARQLLDFSQKLDINLLDNVVNCLYHGEGAQQRMAQEVLTHLKEHPDAWTRVDTILEFSQNMNTKYYGLQILENVIKTRWKILPRNQCEGIKKYVVGLIIKTSSDPTCVEKEKVYIGKLNMILVQILKQEWPKHWPTFISDIVGASRTSESLCQNNMVILKLLSEEVFDFSSGQITQVKSKHLKDSMCNEFSQIFQLCQFVMENSQNAPLVHATLETLLRFLNWIPLGYIFETKLISTLIYKFLNVPMFRNVSLKCLTEIAGVSVSQYEEQFVTLFTLTMMQLKQMLPLNTNIRLAYSNGKDDEQNFIQNLSLFLCTFLKEHDQLIEKRLNLRETLMEALHYMLLVSEVEETEIFKICLEYWNHLAAELYRESPFSTSASPLLSGSQHFDVPPRRQLYLPMLFKVRLLMVSRMAKPEEVLVVENDQGEVVREFMKDTDSINLYKNMRETLVYLTHLDYVDTERIMTEKLHNQVNGTEWSWKNLNTLCWAIGSISGAMHEEDEKRFLVTVIKDLLGLCEQKRGKDNKAIIASNIMYIVGQYPRFLRAHWKFLKTVVNKLFEFMHETHDGVQDMACDTFIKIAQKCRRHFVQVQVGEVMPFIDEILNNINTIICDLQPQQVHTFYEAVGYMIGAQTDQTVQEHLIEKYMLLPNQVWDSIIQQATKNVDILKDPETVKQLGSILKTNVRACKAVGHPFVIQLGRIYLDMLNVYKCLSENISAAIQANGEMVTKQPLIRSMRTVKRETLKLISGWVSRSNDPQMVAENFVPPLLDAVLIDYQRNVPAAREPEVLSTMAIIVNKLGGHITAEIPQIFDAVFECTLNMINKDFEEYPEHRTNFFLLLQAVNSHCFPAFLAIPPTQFKLVLDSIIWAFKHTMRNVADTGLQILFTLLQNVAQEEAAAQSFYQTYFCDILQHIFSVVTDTSHTAGLTMHASILAYMFNLVEEGKISTSLNPGNPVNNQIFLQEYVANLLKSAFPHLQDAQVKLFVTGLFSLNQDIPAFKEHLRDFLVQIKEFAGEDTSDLFLEEREIALRQADEEKHKRQMSVPGIFNPHEIPEEMCD

>NP_001961.1

MADDLDFETGDAGASATFPMQCSALRKNGFVVLKGRPCKIVEMSTSKTGKHGHAKVHLVGIDIFTGKKYEDICPSTHNMDVPNIKRNDFQLIGIQDGYLSLLQDSGEVREDLRLPEGDLGKEIEQKYDCGEEILITVLSAMTEEAAVAIKAMAK

211 NP_001791.1 NP_078958.1

>NP_001791.1

MLLEEVRAGDRLSGAAARGDVQEVRRLLHRELVHPDALNRFGKTALQVMMFGSTAIALELLKQGASPNVQDTSGTSPVHDAARTGFLDTLKVLVEHGADVNVPDGTGALPIHLAVQEGHTAVVSFLAAESDLHRRDARGLTPLELALQRGAQDLVDILQGHMVAPL

>NP_078958.1

MEGAGYRVVFEKGGVYLHTSAKKYQDRDSLIAGVIRVVEKDNDVLLHWAPVEEAGDSTQILFSKKDSSGGDSCASEEEPTFDPGYEPDWAVISTVRPQPCHSEPTRGAEPSCPQGSWAFSVSLGELKSIRRSKPGLSWAYLVLVTQAGGSLPALHFHRGGTRALLRVLSRYLLLASSPQDSRLYLVFPHDSSALSNSFHHLQLFDQDSSNVVSRFLQDPYSTTFSSFSRVTNFFRGALQPQPEGAASDLPPPPDDEPEPGFEVISCVELGPRPTVERGPPVTEEEWARHVGPEGRLQQVPELKNRIFSGGLSPSLRREAWKFLLGYLSWEGTAEEHKAHIRKKTDEYFRMKLQWKSVSPEQERRNSLLHGYRSLIERDVSRTDRTNKFYEGPENPGLGLLNDILLTYCMYHFDLGYVQGMSDLLSPILYVIQNEVDAFWCFCGFMELVQGNFEESQETMKRQLGRLLLLLRVLDPLLCDFLDSQDSGSLCFCFRWLLIWFKREFPFPDVLRLWEVLWTGLPGPNLHLLVACAILDMERDTLMLSGFGSNEILKHINELTMKLSVEDVLTRAEALHRQLTACPELPHNVQEILGLAPPAEPHSPSPTASPLPLSPTRAPPTPPPSTDTAPQPDSSLEILPEEEDEGADS

212 NP_004737.2 NP_036441.1

>NP_004737.2

MKGLSGSRSHHHGVTCDSACDSLSHHSDRKPYLLSPVEHHPADHPYYTQRNSFQAECVGPFSDPLASSTFPRRHYTSQQELKDECALVPRTLATKANRIPANLLDQFERQLPLSRDGYHTLQYKRTAVEHRSDSPGRIRHLVHSVQKLFTKSHSLEGPSKGSVNGGKASPDEAQAARYGKRSKSKERRAEPKARPSTSPGWWSSDDNLDGDMCIYHAPSGVMTMGRCPDRSASQYFLEAYNTISEQAVKASRSNNDVKCSTCANLPVSLDTPLLKKSAWSSTLTVSRAREVYQKASVNMDQAMVKSESCQQERSCQYLQVPQDEWTGYTPRGKDDEIPCRRMRSGSYIKAMGDEDSGDSDTSPKPSPKVAARRESYLKATQPSLTELTTLKISNEHSPKLQIRSHSYLRAVSEVSINRSLDSLDPAGLLTSPKFRSRNESYMRAMSTISQVSEMEVNGQFESVCESVFSELESQAVEALDLPMPGCFRMRSHSYVRAIEKGCSQDDECVSLRSSSPPRTTTTVRTIQSSTVSSCITTYKKTPPPVPPRTTTKPFISITAQSSTESAQDAYMDGQGQRGDIISQSGLSNSTESLDSMKALTAAIEAANAQIHGPASQHMGNNTATVTTTTTIATVTTEDRKKDHFKKNRCLSIGIQVDDAEEPDKTGENKAPSKFQSVGVQVEEEKCFRRFTRSNSVTTAVQADLDFHDNLENSLESIEDNSCPGPMARQFSRDASTSTVSIQGSGNHYHACAADDDFDTDFDPSILPPPDPWIDSITEDPLEAVQRSVCHRDGHWFLKLLQAERDRMEGWCQQMEREERENNLPEDILGKIRTAVGSAQLLMAQKFYQFRELCEENLNPNAHPRPTSQDLAGFWDMLQLSIENISMKFDELHQLKANNWKQMDPLDKKERRAPPPVPKKPAKGPAPLIRERSLESSQRQEARKRLMAAKRAASVRQNSATESAESIEIYIPEAQTRL

>NP_036441.1

MMMNVPGGGAAAVMMTGYNNGRCPRNSLYSDCIIEEKTVVLQKKDNEGFGFVLRGAKADTPIEEFTPTPAFPALQYLESVDEGGVAWQAGLRTGDFLIEVNNENVVKVGHRQVVNMIRQGGNHLVLKVVTVTRNLDPDDTARKKAPPPPKRAPTTALTLRSKSMTSELEELVDKDKPEEIVPASKPSRAAENMAVEPRVATIKQRPSSRCFPAGSDMNSVYERQGIAVMTPTVPGSPKAPFLGIPRGTMRRQKSIDSRIFLSGITEEERQFLAPPMLKFTRSLSMPDTSEDIPPPPQSVPPSPPPPSPTTYNCPKSPTPRVYGTIKPAFNQNSAAKVSPATRSDTVATMMREKGMYFRRELDRYSLDSEDLYSRNAGPQANFRNKRGQMPENPYSEVGKIASKAVYVPAKPARRKGMLVKQSNVEDSPEKTCSIPIPTIIVKEPSTSSSGKSSQGSSMEIDPQAPEPPSQLRPDESLTVSSPFAAAIAGAVRDREKRLEARRNSPAFLSTDLGDEDVGLGPPAPRTRPSMFPEEGDFADEDSAEQLSSPMPSATPREPENHFVGGAEASAPGEAGRPLNSTSKAQGPESSPAVPSASSGTAGPGNYVHPLTGRLLDPSSPLALALSARDRAMKESQQGPKGEAPKADLNKPLYIDTKMRPSLDAGFPTVTRQNTRGPLRRQETENKYETDLGRDRKGDDKKNMLIDIMDTSQQKSAGLLMVHTVDATKLDNALQEEDEKAEVEMKPDSSPSEVPEGVSETEGALQISAAPEPTTVPGRTIVAVGSMEEAVILPFRIPPPPLASVDLDEDFIFTEPLPPPLEFANSFDIPDDRAASVPALSDLVKQKKSDTPQSPSLNSSQPTNSADSKKPASLSNCLPASFLPPPESFDAVADSGIEEVDSRSSSDHHLETTSTISTVSSISTLSSEGGENVDTCTVYADGQAFMVDKPPVPPKPKMKPIIHKSNALYQDALVEEDVDSFVIPPPAPPPPPGSAQPGMAKVLQPRTSKLWGDVTEIKSPILSGPKANVISELNSILQQMNREKLAKPGEGLDSPMGAKSASLAPRSPEIMSTISGTRSTTVTFTVRPGTSQPITLQSRPPDYESRTSGTRRAPSPVVSPTEMNKETLPAPLSAATASPSPALSDVFSLPSQPPSGDLFGLNPAGRSRSPSPSILQQPISNKPFTTKPVHLWTKPDVADWLESLNLGEHKEAFMDNEIDGSHLPNLQKEDLIDLGVTRVGHRMNIERALKQLLDR

213 AAD39742.1 NP_037365.2

>AAD39742.1

MHPPETTTKMASVRFMVTPTKIDDIPGLSDTSPDLSSRSSSRVRFSSRESVPETSRSEPMSEMSGATTSLATVALDPPSDRTSHPQDVIEDLSQNSITGEHSQLLDDGHKKARNAYLNNSNYEEGDEYFDKNLALFEEEMDTRPKVSSLLNRMANYTNLTQGAKEHEEAENITEGKKKPTKTPQMGTFMGVYLPCLQNIFGVILFLRLTWVVGTAGVLQAFAIVLICCCCTMLTAISMSAIATNGVVPAGGSYFMISRALGPEFGGAVGLCFYLGTTFAAAMYILGAIEIFLVYIVPRAAIFHSDDALKESAAMLNNMRVYGTAFLVLMVLVVFIGVRYVNKFASLFLACVIVSILAIYAGAIKSSFAPPHFPVCMLGNRTLSSRHIDVCSKTKEINNMTVPSKLWGFFCNSSQFFNATCDEYFVHNNVTSIQGIPGLASGIITENLWSNYLPKGEIIEKPSAKSSDVLGSLNHEYVLVDITTSFTLLVGIFFPSVTGIMAGSNRSGDLKDAQKSIPIGTILAILTTSFVYLSNVVLFGACIEGVVLRDKFGDAVKGNLVVGTLSWPSPWVIVIGSFFSTCGAGLQSLTGAPRLLQAIAKDNIIPFLRVFGHSKANGEPTWALLLTAAIAELGILIASLDLVAPILSMFFLMCYLFVNLACALQTLLRTPNWRPRFRYYHWALSFMGMSICLALMFISSWYYAIVAMVIAGMIYKYIEYQGAEKEWGDGIRGLSLSAARFALLRLEEGPPHTKNWRPQLLVLLKLDEDLHVKHPRLLTFASQLKAGKGLTIVGSVIVGNFLENYGEALAAEQTIKHLMEAEKVKGFCQLVVAAKLREGISHLIQSCGLGGMKHNTVVMGWPNGWRQSEDARAWKTFIGTVRVTTAAHLALLVAKNISFFPSNVEQFSEGNIDVWWIVHDGGMLMLLPFLLKQHKVWRKCSIRIFTVAQLEDNSIQMKKDLATFLYHLRIEAEVEVVEMHDSDISAYTYERTLMMEQRSQMLRHMRLSKTERDREAQLVKDRNSMLRLTSIGSDEDEETETYQEKVHMTWTKDKYMASRGQKAKSMEGFQDLLNMRPDQSNVRRMHTAVKLNEVIVNKSHEAKLVLLNMPGPPRNPEGDENYMEFLEVLTEGLERVLLVRGGGSEVITIYS

>NP_037365.2

MAEPSGSPVHVQLPQQAAPVTAAAAAAPAAATAAPAPAAPAAPAPAPAPAAQAVGWPICRDAYELQEVIGSGATAVVQAALCKPRQERVAIKRINLEKCQTSMDELLKEIQAMSQCSHPNVVTYYTSFVVKDELWLVMKLLSGGSMLDIIKYIVNRGEHKNGVLEEAIIATILKEVLEGLDYLHRNGQIHRDLKAGNILLGEDGSVQIADFGVSAFLATGGDVTRNKVRKTFVGTPCWMAPEVMEQVRGYDFKADMWSFGITAIELATGAAPYHKYPPMKVLMLTLQNDPPTLETGVEDKEMMKKYGKSFRKLLSLCLQKDPSKRPTAAELLKCKFFQKAKNREYLIEKLLTRTPDIAQRAKKVRRVPGSSGHLHKTEDGDWEWSDDEMDEKSEEGKAAFSQEKSRRVKEENPEIAVSASTIPEQIQSLSVHDSQGPPNANEDYREASSCAVNLVLRLRNSRKELNDIRFEFTPGRDTADGVSQELFSAGLVDGHDVVIVAANLQKIVDDPKALKTLTFKLASGCDGSEIPDEVKLIGFAQLSVS

214 NP_002266.2 NP_612423.1

>NP_002266.2

MTTTFLQTSSSTFGGGSTRGGSLLAGGGGFGGGSLSGGGGSRSISASSARFVSSGSGGGYGGGMRVCGFGGGAGSVFGGGFGGGVGGGFGGGFGGGDGGLLSGNEKITMQNLNDRLASYLDKVRALEEANADLEVKIHDWYQKQTPASPECDYSQYFKTIEELRDKIMATTIDNSRVILEIDNARLAADDFRLKYENELALRQGVEADINGLRRVLDELTLARTDLEMQIEGLNEELAYLKKNHEEEMKEFSSQLAGQVNVEMDAAPGVDLTRVLAEMREQYEAMAEKNRRDVEAWFFSKTEELNKEVASNTEMIQTSKTEITDLRRTMQELEIELQSQLSMKAGLENSLAETECRYATQLQQIQGLIGGLEAQLSELRCEMEAQNQEYKMLLDIKTRLEQEIATYRSLLEGQDAKMAGIGIREASSGGGGSSSNFHINVEESVDGQVVSSHKREI

>NP_612423.1

MALVSADSRIAELLTELHQLIKQTQEERSRSEHNLVNIQKTHERMQTENKISPYYRTKLRGLYTTAKADAEAECNILRKALDKIAEIKSLLEERRIAAKIAGLYNDSEPPRKTMRRGVLMTLLQQSAMTLPLWIGKPGDKPPPLCGAIPASGDYVARPGDKVAARVKAVDGDEQWILAEVVSYSHATNKYEVDDIDEEGKERHTLSRRRVIPLPQWKANPETDPEALFQKEQLVLALYPQTTCFYRALIHAPPQRPQDDYSVLFEDTSYADGYSPPLNVAQRYVVACKEPKKK

215 NP_000116.2 NP_001527.2

>NP_000116.2

MTMTLHTKASGMALLHQIQGNELEPLNRPQLKIPLERPLGEVYLDSSKPAVYNYPEGAAYEFNAAAAANAQVYGQTGLPYGPGSEAAAFGSNGLGGFPPLNSVSPSPLMLLHPPPQLSPFLQPHGQQVPYYLENEPSGYTVREAGPPAFYRPNSDNRRQGGRERLASTNDKGSMAMESAKETRYCAVCNDYASGYHYGVWSCEGCKAFFKRSIQGHNDYMCPATNQCTIDKNRRKSCQACRLRKCYEVGMMKGGIRKDRRGGRMLKHKRQRDDGEGRGEVGSAGDMRAANLWPSPLMIKRSKKNSLALSLTADQMVSALLDAEPPILYSEYDPTRPFSEASMMGLLTNLADRELVHMINWAKRVPGFVDLTLHDQVHLLECAWLEILMIGLVWRSMEHPGKLLFAPNLLLDRNQGKCVEGMVEIFDMLLATSSRFRMMNLQGEEFVCLKSIILLNSGVYTFLSSTLKSLEEKDHIHRVLDKITDTLIHLMAKAGLTLQQQHQRLAQLLLILSHIRHMSNKGMEHLYSMKCKNVVPLYDLLLEMLDAHRLHAPTSRGGASVEETDQSHLATAGSTSSHSLQKYYITGEAEGFPATV

>NP_001527.2

MENFVATLANGMSLQPPLEEVSCGQAESSEKPNAEDMTSKDYYFDSYAHFGIHEEMLKDEVRTLTYRNSMFHNRHLFKDKVVLDVGSGTGILCMFAAKAGARKVIGIVCSSISDYAVKIVKANKLDHVVTIIKGKVEEVELPVEKVDIIISEWMGYCLFYESMLNTVLYARDKWLAPDGLIFPDRATLYVTAIEDRQYKDYKIHWWENVYGFDMSCIKDVAIKEPLVDVVDPKQLVTNACLIKEVDIYTVKVEDLTFTSPFCLQVKRNDYVHALVAYFNIEFTRCHKRTGFSTSPESPYTHWKQTVFYMEDYLTVKTGEEIFGTIGMRPNAKNNRDLDFTIDLDFKGQLCELSCSTDYRMR

216 NP_005498.1 NP_036611.2

>NP_005498.1

MASGVAVSDGVIKVFNDMKVRKSSTPEEVKKRKKAVLFCLSEDKKNIILEEGKEILVGDVGQTVDDPYATFVKMLPDKDCRYALYDATYETKESKKEDLVFIFWAPESAPLKSKMIYASSKDAIKKKLTGIKHELQANCYEEVKDRCTLAEKLGGSAVISLEGKPL

>NP_036611.2

MVDREQLVQKARLAEQAERYDDMAAAMKNVTELNEPLSNEERNLLSVAYKNVVGARRSSWRVISSIEQKTSADGNEKKIEMVRAYREKIEKELEAVCQDVLSLLDNYLIKNCSETQYESKVFYLKMKGDYYRYLAEVATGEKRATVVESSEKAYSEAHEISKEHMQPTHPIRLGLALNYSVFYYEIQNAPEQACHLAKTAFDDAIAELDTLNEDSYKDSTLIMQLLRDNLTLWTSDQQDDDGGEGNN

217 NP_056345.3 NP_003227.1

>NP_056345.3

MGSSQSVEIPGGGTEGYHVLRVQENSPGHRAGLEPFFDFIVSINGSRLNKDNDTLKDLLKANVEKPVKMLIYSSKTLELRETSVTPSNLWGGQGLLGVSIRFCSFDGANENVWHVLEVESNSPAALAGLRPHSDYIIGADTVMNESEDLFSLIETHEAKPLKLYVYNTDTDNCREVIITPNSAWGGEGSLGCGIGYGYLHRIPTRPFEEGKKISLPGQMAGTPITPLKDGFTEVQLSSVNPPSLSPPGTTGIEQSLTGLSISSTPPAVSSVLSTGVPTVPLLPPQVNQSLTSVPPMNPATTLPGLMPLPAGLPNLPNLNLNLPAPHIMPGVGLPELVNPGLPPLPSMPPRNLPGIAPLPLPSEFLPSFPLVPESSSAASSGELLSSLPPTSNAPSDPATTTAKADAASSLTVDVTPPTAKAPTTVEDRVGDSTPVSEKPVSAAVDANASESP

>NP_003227.1

MVPSAGQLALFALGIVLAACQALENSTSPLSADPPVAAAVVSHFNDCPDSHTQFCFHGTCRFLVQEDKPACVCHSGYVGARCEHADLLAVVAASQKKQAITALVVVSIVALAVLIITCVLIHCCQVRKHCEWCRALICRHEKPSALLKGRTACCHSETVV

218 NP_055567.1 NP_056978.2

>NP_055567.1

MHPFPESKPVLALCVISYFVMMGILTIYTSYKEKSIFLVAHRKDPTGMDPDDIWQLSSSLKRFDDKYTLKLTFISGRTKQQREAEFTKSIAKFFDHSGTLVMDAYEPEISRLHDSLAIERKIK

>NP_056978.2

MASTISAYKEKMKELSVLSLICSCFYTQPHPNTVYQYGDMEVKQLDKRASGQSFEVILKSPSDLSPESPMLSSPPKKKDTSLEELQKRLEAAEERRKTQEAQVLKQLAERREHEREVLHKALEENNNFSRQAEEKLNYKMELSKEIREAHLAALRERLREKELHAAEVRRNKEQREEMSG

219 NP_005237.2 NP_002221.1

>NP_005237.2

MGFGSDLKNSHEAVLKLQDWELRLLETVKKFMALRIKSDKEYASTLQNLCNQVDKESTVQMNYVSNVSKSWLLMIQQTEQLSRIMKTHAEDLNSGPLHRLTMMIKDKQQVKKSYIGVHQQIEAEMIKVTKTELEKLKCSYRQLIKEMNSAKEKYKEALAKGKETEKAKERYDKATMKLHMLHNQYVLALKGAQLHQNQYYDITLPLLLDSLQKMQEEMIKALKGIFDEYSQITSLVTEEIVNVHKEIQMSVEQIDPSTEYNNFIDVHRTTAAKEQEIEFDTSLLEENENLQANEIMWNNLTAESLQVMLKTLAEELMQTQQMLLNKEEAVLELEKRIEESSETCEKKSDIVLLLSQKQALEELKQSVQQLRCTEAKFSAQKELLEQKVQENDGKEPPPVVNYEEDARSVTSMERKERLSKFESIRHSIAGIIRSPKSALGSSALSDMISISEKPLAEQDWYHGAIPRIEAQELLKKQGDFLVRESHGKPGEYVLSVYSDGQRRHFIIQYVDNMYRFEGTGFSNIPQLIDHHYTTKQVITKKSGVVLLNPIPKDKKWILSHEDVILGELLGKGNFGEVYKGTLKDKTSVAVKTCKEDLPQELKIKFLQEAKILKQYDHPNIVKLIGVCTQRQPVYIIMELVSGGDFLTFLRRKKDELKLKQLVKFSLDAAAGMLYLESKNCIHRDLAARNCLVGENNVLKISDFGMSRQEDGGVYSSSGLKQIPIKWTAPEALNYGRYSSESDVWSFGILLWETFSLGVCPYPGMTNQQAREQVERGYRMSAPQHCPEDISKIMMKCWDYKPENRPKFSELQKELTIIKRKLT

>NP_002221.1

MEVMNLMEQPIKVTEWQQTYTYDSGIHSGANTCVPSVSSKGIMEEDEACGRQYTLKKTTTYTQGVPPSQGDLEYQMSTTARAKRVREAMCPGVSGEDSSLLLATQVEGQATNLQRLAEPSQLLKSAIVHLINYQDDAELATRALPELTKLLNDEDPVVVTKAAMIVNQLSKKEASRRALMGSPQLVAAVVRTMQNTSDLDTARCTTSILHNLSHHREGLLAIFKSGGIPALVRMLSSPVESVLFYAITTLHNLLLYQEGAKMAVRLADGLQKMVPLLNKNNPKFLAITTDCLQLLAYGNQESKLIILANGGPQALVQIMRNYSYEKLLWTTSRVLKVLSVCPSNKPAIVEAGGMQALGKHLTSNSPRLVQNCLWTLRNLSDVATKQEGLESVLKILVNQLSVDDVNVLTCATGTLSNLTCNNSKNKTLVTQNSGVEALIHAILRAGDKDDITEPAVCALRHLTSRHPEAEMAQNSVRLNYGIPAIVKLLNQPNQWPLVKATIGLIRNLALCPANHAPLQEAAVIPRLVQLLVKAHQDAQRHVAAGTQQPYTDGVRMEEIVEGCTGALHILARDPMNRMEIFRLNTIPLFVQLLYSSVENIQRVAAGVLCELAQDKEAADAIDAEGASAPLMELLHSRNEGTATYAAAVLFRISEDKNPDYRKRVSVELTNSLFKHDPAAWEAAQSMIPINEPYGDDMDATYRPMYSSDVPLDPLEMHMDMDGDYPIDTYSDGLRPPYPTADHMLA

220 NP_001017963.1 NP_000375.2

>NP_001017963.1

MPPCSGGDGSTPPGPSLRDRDCPAQSAEYPRDRLDPRPGSPSEASSPPFLRSRAPVNWYQEKAQVFLWHLLVSGSTTLLCLWKQPFHVSAFPVTASLAFRQSQGAGQHLYKDLQPFILLRLLMPEETQTQDQPMEEEEVETFAFQAEIAQLMSLIINTFYSNKEIFLRELISNSSDALDKIRYESLTDPSKLDSGRELHINLIPNKQGRTLTIVDTGIGMTKADLINNLGTIAKSGTKAFMEALQAGADISMIGQFGVGFYSAYLVAEKVTVITKHNDDEQYAWESSAGGSFTVRTDTGEPMGRGTKVILHLKEDQTEYLEERRIKEIVKKHSQFIGYPITLFVEKERDKEVSDDEAEEKEDKEEEKEKEEKESEDKPEIEDVGSDEEEEKKDGDKKKKKKIKEKYIDQEELNKTKPIWTRNPDDITNEEYGEFYKSLTNDWEDHLAVKHFSVEGQLEFRALLFVPRRAPFDLFENRKKKNNIKLYVRRVFIMDNCEELIPEYLNFIRGVVDSEDLPLNISREMLQQSKILKVIRKNLVKKCLELFTELAEDKENYKKFYEQFSKNIKLGIHEDSQNRKKLSELLRYYTSASGDEMVSLKDYCTRMKENQKHIYYITGETKDQVANSAFVERLRKHGLEVIYMIEPIDEYCVQQLKEFEGKTLVSVTKEGLELPEDEEEKKKQEEKKTKFENLCKIMKDILEKKVEKVVVSNRLVTSPCCIVTSTYGWTANMERIMKAQALRDNSTMGYMAAKKHLEINPDHSIIETLRQKAEADKNDKSVKDLVILLYETALLSSGFSLEDPQTHANRIYRMIKLGLGIDEDDPTADDTSAAVTEEMPPLEGDDDTSRMEEVD

>NP_000375.2

MDPPRPALLALLALPALLLLLLAGARAEEEMLENVSLVCPKDATRFKHLRKYTYNYEAESSSGVPGTADSRSATRINCKVELEVPQLCSFILKTSQCTLKEVYGFNPEGKALLKKTKNSEEFAAAMSRYELKLAIPEGKQVFLYPEKDEPTYILNIKRGIISALLVPPETEEAKQVLFLDTVYGNCSTHFTVKTRKGNVATEISTERDLGQCDRFKPIRTGISPLALIKGMTRPLSTLISSSQSCQYTLDAKRKHVAEAICKEQHLFLPFSYKNKYGMVAQVTQTLKLEDTPKINSRFFGEGTKKMGLAFESTKSTSPPKQAEAVLKTLQELKKLTISEQNIQRANLFNKLVTELRGLSDEAVTSLLPQLIEVSSPITLQALVQCGQPQCSTHILQWLKRVHANPLLIDVVTYLVALIPEPSAQQLREIFNMARDQRSRATLYALSHAVNNYHKTNPTGTQELLDIANYLMEQIQDDCTGDEDYTYLILRVIGNMGQTMEQLTPELKSSILKCVQSTKPSLMIQKAAIQALRKMEPKDKDQEVLLQTFLDDASPGDKRLAAYLMLMRSPSQADINKIVQILPWEQNEQVKNFVASHIANILNSEELDIQDLKKLVKEALKESQLPTVMDFRKFSRNYQLYKSVSLPSLDPASAKIEGNLIFDPNNYLPKESMLKTTLTAFGFASADLIEIGLEGKGFEPTLEALFGKQGFFPDSVNKALYWVNGQVPDGVSKVLVDHFGYTKDDKHEQDMVNGIMLSVEKLIKDLKSKEVPEARAYLRILGEELGFASLHDLQLLGKLLLMGARTLQGIPQMIGEVIRKGSKNDFFLHYIFMENAFELPTGAGLQLQISSSGVIAPGAKAGVKLEVANMQAELVAKPSVSVEFVTNMGIIIPDFARSGVQMNTNFFHESGLEAHVALKAGKLKFIIPSPKRPVKLLSGGNTLHLVSTTKTEVIPPLIENRQSWSVCKQVFPGLNYCTSGAYSNASSTDSASYYPLTGDTRLELELRPTGEIEQYSVSATYELQREDRALVDTLKFVTQAEGAKQTEATMTFKYNRQSMTLSSEVQIPDFDVDLGTILRVNDESTEGKTSYRLTLDIQNKKITEVALMGHLSCDTKEERKIKGVISIPRLQAEARSEILAHWSPAKLLLQMDSSATAYGSTVSKRVAWHYDEEKIEFEWNTGTNVDTKKMTSNFPVDLSDYPKSLHMYANRLLDHRVPQTDMTFRHVGSKLIVAMSSWLQKASGSLPYTQTLQDHLNSLKEFNLQNMGLPDFHIPENLFLKSDGRVKYTLNKNSLKIEIPLPFGGKSSRDLKMLETVRTPALHFKSVGFHLPSREFQVPTFTIPKLYQLQVPLLGVLDLSTNVYSNLYNWSASYSGGNTSTDHFSLRARYHMKADSVVDLLSYNVQGSGETTYDHKNTFTLSCDGSLRHKFLDSNIKFSHVEKLGNNPVSKGLLIFDASSSWGPQMSASVHLDSKKKQHLFVKEVKIDGQFRVSSFYAKGTYGLSCQRDPNTGRLNGESNLRFNSSYLQGTNQITGRYEDGTLSLTSTSDLQSGIIKNTASLKYENYELTLKSDTNGKYKNFATSNKMDMTFSKQNALLRSEYQADYESLRFFSLLSGSLNSHGLELNADILGTDKINSGAHKATLRIGQDGISTSATTNLKCSLLVLENELNAELGLSGASMKLTTNGRFREHNAKFSLDGKAALTELSLGSAYQAMILGVDSKNIFNFKVSQEGLKLSNDMMGSYAEMKFDHTNSLNIAGLSLDFSSKLDNIYSSDKFYKQTVNLQLQPYSLVTTLNSDLKYNALDLTNNGKLRLEPLKLHVAGNLKGAYQNNEIKHIYAISSAALSASYKADTVAKVQGVEFSHRLNTDIAGLASAIDMSTNYNSDSLHFSNVFRSVMAPFTMTIDAHTNGNGKLALWGEHTGQLYSKFLLKAEPLAFTFSHDYKGSTSHHLVSRKSISAALEHKVSALLTPAEQTGTWKLKTQFNNNEYSQDLDAYNTKDKIGVELTGRTLADLTLLDSPIKVPLLLSEPINIIDALEMRDAVEKPQEFTIVAFVKYDKNQDVHSINLPFFETLQEYFERNRQTIIVVLENVQRNLKHINIDQFVRKYRAALGKLPQQANDYLNSFNWERQVSHAKEKLTALTKKYRITENDIQIALDDAKINFNEKLSQLQTYMIQFDQYIKDSYDLHDLKIAIANIIDEIIEKLKSLDEHYHIRVNLVKTIHDLHLFIENIDFNKSGSSTASWIQNVDTKYQIRIQIQEKLQQLKRHIQNIDIQHLAGKLKQHIEAIDVRVLLDQLGTTISFERINDVLEHVKHFVINLIGDFEVAEKINAFRAKVHELIERYEVDQQIQVLMDKLVELAHQYKLKETIQKLSNVLQQVKIKDYFEKLVGFIDDAVKKLNELSFKTFIEDVNKFLDMLIKKLKSFDYHQFVDETNDKIREVTQRLNGEIQALELPQKAEALKLFLEETKATVAVYLESLQDTKITLIINWLQEALSSASLAHMKAKFRETLEDTRDRMYQMDIQQELQRYLSLVGQVYSTLVTYISDWWTLAAKNLTDFAEQYSIQDWAKRMKALVEQGFTVPEIKTILGTMPAFEVSLQALQKATFQTPDFIVPLTDLRIPSVQINFKDLKNIKIPSRFSTPEFTILNTFHIPSFTIDFVEMKVKIIRTIDQMLNSELQWPVPDIYLRDLKVEDIPLARITLPDFRLPEIAIPEFIIPTLNLNDFQVPDLHIPEFQLPHISHTIEVPTFGKLYSILKIQSPLFTLDANADIGNGTTSANEAGIAASITAKGESKLEVLNFDFQANAQLSNPKINPLALKESVKFSSKYLRTEHGSEMLFFGNAIEGKSNTVASLHTEKNTLELSNGVIVKINNQLTLDSNTKYFHKLNIPKLDFSSQADLRNEIKTLLKAGHIAWTSSGKGSWKWACPRFSDEGTHESQISFTIEGPLTSFGLSNKINSKHLRVNQNLVYESGSLNFSKLEIQSQVDSQHVGHSVLTAKGMALFGEGKAEFTGRHDAHLNGKVIGTLKNSLFFSAQPFEITASTNNEGNLKVRFPLRLTGKIDFLNNYALFLSPSAQQASWQVSARFNQYKYNQNFSAGNNENIMEAHVGINGEANLDFLNIPLTIPEMRLPYTIITTPPLKDFSLWEKTGLKEFLKTTKQSFDLSVKAQYKKNKHRHSITNPLAVLCEFISQSIKSFDRHFEKNRNNALDFVTKSYNETKIKFDKYKAEKSHDELPRTFQIPGYTVPVVNVEVSPFTIEMSAFGYVFPKAVSMPSFSILGSDVRVPSYTLILPSLELPVLHVPRNLKLSLPDFKELCTISHIFIPAMGNITYDFSFKSSVITLNTNAELFNQSDIVAHLLSSSSSVIDALQYKLEGTTRLTRKRGLKLATALSLSNKFVEGSHNSTVSLTTKNMEVSVATTTKAQIPILRMNFKQELNGNTKSKPTVSSSMEFKYDFNSSMLYSTAKGAVDHKLSLESLTSYFSIESSTKGDVKGSVLSREYSGTIASEANTYLNSKSTRSSVKLQGTSKIDDIWNLEVKENFAGEATLQRIYSLWEHSTKNHLQLEGLFFTNGEHTSKATLELSPWQMSALVQVHASQPSSFHDFPDLGQEVALNANTKNQKIRWKNEVRIHSGSFQSQVELSNDQEKAHLDIAGSLEGHLRFLKNIILPVYDKSLWDFLKLDVTTSIGRRQHLRVSTAFVYTKNPNGYSFSIPVKVLADKFIIPGLKLNDLNSVLVMPTFHVPFTDLQVPSCKLDFREIQIYKKLRTSSFALNLPTLPEVKFPEVDVLTKYSQPEDSLIPFFEITVPESQLTVSQFTLPKSVSDGIAALDLNAVANKIADFELPTIIVPEQTIEIPSIKFSVPAGIVIPSFQALTARFEVDSPVYNATWSASLKNKADYVETVLDSTCSSTVQFLEYELNVLGTHKIEDGTLASKTKGTFAHRDFSAEYEEDGKYEGLQEWEGKAHLNIKSPAFTDLHLRYQKDKKGISTSAASPAVGTVGMDMDEDDDFSKWNFYYSPQSSPDKKLTIFKTELRVRESDEETQIKVNWEEEAASGLLTSLKDNVPKATGVLYDYVNKYHWEHTGLTLREVSSKLRRNLQNNAEWVYQGAIRQIDDIDVRFQKAASGTTGTYQEWKDKAQNLYQELLTQEGQASFQGLKDNVFDGLVRVTQEFHMKVKHLIDSLIDFLNFPRFQFPGKPGIYTREELCTMFIREVGTVLSQVYSKVHNGSEILFSYFQDLVITLPFELRKHKLIDVISMYRELLKDLSKEAQEVFKAIQSLKTTEVLRNLQDLLQFIFQLIEDNIKQLKEMKFTYLINYIQDEINTIFSDYIPYVFKLLKENLCLNLHKFNEFIQNELQEASQELQQIHQYIMALREEYFDPSIVGWTVKYYELEEKIVSLIKNLLVALKDFHSEYIVSASNFTSQLSSQVEQFLHRNIQEYLSILTDPDGKGKEKIAELSATAQEIIKSQAIATKKIISDYHQQFRYKLQDFSDQLSDYYEKFIAESKRLIDLSIQNYHTFLIYITELLKKLQSTTVMNPYMKLAPGELTIIL

221 NP_002793.2 NP_006839.2

>NP_002793.2

MGQSQSGGHGPGGGKKDDKDKKKKYEPPVPTRVGKKKKKTKGPDAASKLPLVTPHTQCRLKLLKLERIKDYLLMEEEFIRNQEQMKPLEEKQEEERSKVDDLRGTPMSVGTLEEIIDDNHAIVSTSVGSEHYVSILSFVDKDLLEPGCSVLLNHKVHAVIGVLMDDTDPLVTVMKVEKAPQETYADIGGLDNQIQEIKESVELPLTHPEYYEEMGIKPPKGVILYGPPGTGKTLLAKAVANQTSATFLRVVGSELIQKYLGDGPKLVRELFRVAEEHAPSIVFIDEIDAIGTKRYDSNSGGEREIQRTMLELLNQLDGFDSRGDVKVIMATNRIETLDPALIRPGRIDRKIEFPLPDEKTKKRIFQIHTSRMTLADDVTLDDLIMAKDDLSGADIKAICTEAGLMALRERRMKVTNEDFKKSKENVLYKKQEGTPEGLYL

>NP_006839.2

MEAEAGGLEELTDEEMAALGKEELVRRLRREEAARLAALVQRGRLMQEVNRQLQGHLGEIRELKQLNRRLQAENRELRDLCCFLDSERQRGRRAARQWQLFGTQASRAVREDLGGCWQKLAELEGRQEELLRENLALKELCLALGEEWGPRGGPSGAGGSGAGPAPELALPPCGPRDLGDGSSSTGSVGSPDQLPLACSPDD

222 NP_005892.1 NP_000772.1

>NP_005892.1

MSSILPFTPPVVKRLLGWKKSAGGSGGAGGGEQNGQEEKWCEKAVKSLVKKLKKTGRLDELEKAITTQNCNTKCVTIPSTCSEIWGLSTPNTIDQWDTTGLYSFSEQTRSLDGRLQVSHRKGLPHVIYCRLWRWPDLHSHHELKAIENCEYAFNLKKDEVCVNPYHYQRVETPVLPPVLVPRHTEILTELPPLDDYTHSIPENTNFPAGIEPQSNYIPETPPPGYISEDGETSDQQLNQSMDTGSPAELSPTTLSPVNHSLDLQPVTYSEPAFWCSIAYYELNQRVGETFHASQPSLTVDGFTDPSNSERFCLGLLSNVNRNATVEMTRRHIGRGVRLYYIGGEVFAECLSDSAIFVQSPNCNQRYGWHPATVCKIPPGCNLKIFNNQEFAALLAQSVNQGFEAVYQLTRMCTIRMSFVKGWGAEYRRQTVTSTPCWIELHLNGPLQWLDKVLTQMGSPSVRCSSMS

>NP_000772.1

MLAKGLPPRSVLVKGYQTFLSAPREGLGRLRVPTGEGAGISTRSPRPFNEIPSPGDNGWLNLYHFWRETGTHKVHLHHVQNFQKYGPIYREKLGNVESVYVIDPEDVALLFKSEGPNPERFLIPPWVAYHQYYQRPIGVLLKKSAAWKKDRVALNQEVMAPEATKNFLPLLDAVSRDFVSVLHRRIKKAGSGNYSGDISDDLFRFAFESITNVIFGERQGMLEEVVNPEAQRFIDAIYQMFHTSVPMLNLPPDLFRLFRTKTWKDHVAAWDVIFSKADIYTQNFYWELRQKGSVHHDYRGMLYRLLGDSKMSFEDIKANVTEMLAGGVDTTSMTLQWHLYEMARNLKVQDMLRAEVLAARHQAQGDMATMLQLVPLLKASIKETLRLHPISVTLQRYLVNDLVLRDYMIPAKTLVQVAIYALGREPTFFFDPENFDPTRWLSKDKNITYFRNLGFGWGVRQCLGRRIAELEMTIFLINMLENFRVEIQHLSDVGTTFNLILMPEKPISFTFWPFNQEATQQ

223 NP_005044.1 NP_002941.1

>NP_005044.1

MAVTITLKTLQQQTFKIRMEPDETVKVLKEKIEAEKGRDAFPVAGQKLIYAGKILSDDVPIRDYRIDEKNFVVVMVTKTKAGQGTSAPPEASPTAAPESSTSFPPAPTSGMSHPPPAAREDKSPSEESAPTTSPESVSGSVPSSGSSGREEDAASTLVTGSEYETMLTEIMSMGYERERVVAALRASYNNPHRAVEYLLTGIPGSPEPEHGSVQESQVSEQPATEAAGENPLEFLRDQPQFQNMRQVIQQNPALLPALLQQLGQENPQLLQQISRHQEQFIQMLNEPPGELADISDVEGEVGAIGEEAPQMNYIQVTPQEKEAIERLKALGFPESLVIQAYFACEKNENLAANFLLSQNFDDE

>NP_002941.1

MEAPAAGLFLLLLLGTWAPAPGSASSEAPPLINEDVKRTVDLSSHLAKVTAEVVLAHLGGGSTSRATSFLLALEPELEARLAHLGVQVKGEDEEENNLEVRETKIKGKSGRFFTVKLPVALDPGAKISVIVETVYTHVLHPYPTQITQSEKQFVVFEGNHYFYSPYPTKTQTMRVKLASRNVESYTKLGNPTRSEDLLDYGPFRDVPAYSQDTFKVHYENNSPFLTITSMTRVIEVSHWGNIAVEENVDLKHTGAVLKGPFSRYDYQRQPDSGISSIRSFKTILPAAAQDVYYRDEIGNVSTSHLLILDDSVEMEIRPRFPLFGGWKTHYIVGYNLPSYEYLYNLGDQYALKMRFVDHVFDEQVIDSLTVKIILPEGAKNIEIDSPYEISRAPDELHYTYLDTFGRPVIVAYKKNLVEQHIQDIVVHYTFNKVLMLQEPLLVVAAFYILFFTVIIYVRLDFSITKDPAAEARMKVACITEQVLTLVNKRIGLYRHFDETVNRYKQSRDISTLNSGKKSLETEHKALTSEIALLQSRLKTEGSDLCDRVSEMQKLDAQVKELVLKSAVEAERLVAGKLKKDTYIENEKLISGKRQELVTKIDHILDAL

224 NP_057625.1 NP_071448.1

>NP_057625.1

MEEWDVPQMKKEVESLKYQLAFQREMASKTIPELLKWIEDGIPKDPFLNPDLMKNNPWVEKGKCTIL

>NP_071448.1

MAALVEPLGLERDVSRAVELLERLQRSGELPPQKLQALQRVLQSRFCSAIREVYEQLYDTLDITGSAEIRAHATAKATVAAFTASEGHAHPRVVELPKTDEGLGFNIMGGKEQNSPIYISRVIPGGVADRHGGLKRGDQLLSVNGVSVEGEQHEKAVELLKAAQGSVKLVVRYTPRVLEEMEARFEKMRSARRRQQHQSYSSLESRG

225 NP_003185.1 NP_006090.2

>NP_003185.1

MDQNNSLPPYAQGLASPQGAMTPGIPIFSPMMPYGTGLTPQPIQNTNSLSILEEQQRQQQQQQQQQQQQQQQQQQQQQQQQQQQQQQQQQQQQQQAVAAAAVQQSTSQQATQGTSGQAPQLFHSQTLTTAPLPGTTPLYPSPMTPMTPITPATPASESSGIVPQLQNIVSTVNLGCKLDLKTIALRARNAEYNPKRFAAVIMRIREPRTTALIFSSGKMVCTGAKSEEQSRLAARKYARVVQKLGFPAKFLDFKIQNMVGSCDVKFPIRLEGLVLTHQQFSSYEPELFPGLIYRMIKPRIVLLIFVSGKVVLTGAKVRAEIYEAFENIYPILKGFRKTT

>NP_006090.2

MAELGELKHMVMSFRVSELQVLLGFAGRNKSGRKHELLAKALHLLKSSCAPSVQMKIKELYRRRFPRKTLGPSDLSLLSLPPGTSPVGSPGPLAPIPPTLLAPGTLLGPKREVDMHPPLPQPVHPDVTMKPLPFYEVYGELIRPTTLASTSSQRFEEAHFTFALTPQQVQQILTSREVLPGAKCDYTIQVQLRFCLCETSCPQEDYFPPNLFVKVNGKLCPLPGYLPPTKNGAEPKRPSRPINITPLARLSATVPNTIVVNWSSEFGRNYSLSVYLVRQLTAGTLLQKLRAKGIRNPDHSRALIKEKLTADPDSEVATTSLRVSLMCPLGKMRLTVPCRALTCAHLQSFDAALYLQMNEKKPTWTCPVCDKKAPYESLIIDGLFMEILSSCSDCDEIQFMEDGSWCPMKPKKEASEVCPPPGYGLDGLQYSPVQGGDPSENKKKVEVIDLTIESSSDEEDLPPTKKHCSVTSAAIPALPGSKGVLTSGHQPSSVLRSPAMGTLGGDFLSSLPLHEYPPAFPLGADIQGLDLFSFLQTESQHYGPSVITSLDEQDALGHFFQYRGTPSHFLGPLAPTLGSSHCSATPAPPPGRVSSIVAPGGALREGHGGPLPSGPSLTGCRSDIISLD

226 NP_005139.1 NP_060791.3

>NP_005139.1

MKVKKGGGGAGTATESAPGPSGQSVAPIPQPPAESESGSESEPDAGPGPRPGPLQRKQPIGPEDVLGLQRITGDYLCSPEENIYKIDFVRFKIRDMDSGTVLFEIKKPPVSERLPINRRDLDPNAGRFVRYQFTPAFLRLRQVGATVEFTVGDKPVNNFRMIERHYFRNQLLKSFDFHFGFCIPSSKNTCEHIYDFPPLSEELISEMIRHPYETQSDSFYFVDDRLVMHNKADYSYSGTP

>NP_060791.3

MAATKRKRRGGFAVQAKKPKRNEIDAEPPAKRHATAEEVEEEERDRIPGPVCKGKWKNKERILIFSSRGINFRTRHLMQDLRMLMPHSKADTKMDRKDKLFVINEVCEMKNCNKCIYFEAKKKQDLYMWLSNSPHGPSAKFLVQNIHTLAELKMTGNCLKGSRPLLSFDPAFDELPHYALLKELLIQIFSTPRYHPKSQPFVDHVFTFTILDNRIWFRNFQIIEEDAALVEIGPRFVLNLIKIFQGSFGGPTLYENPHYQSPNMHRRVIRSITAAKYREKQQVKDVQKLRKKEPKTLLPHDPTADVFVTPAEEKPIEIQWVKPEPKVDLKARKKRIYKRQRKMKQRMDSGKTK

227 NP_005391.1 NP_963868.1

>NP_005391.1

MVVFNGLLKIKICEAVSLKPTAWSLRHAVGPRPQTFLLDPYIALNVDDSRIGQTATKQKTNSPAWHDEFVTDVCNGRKIELAVFHDAPIGYDDFVANCTIQFEELLQNGSRHFEDWIDLEPEGRVYVIIDLSGSSGEAPKDNEERVFRERMRPRKRQGAVRRRVHQVNGHKFMATYLRQPTYCSHCRDFIWGVIGKQGYQCQVCTCVVHKRCHELIITKCAGLKKQETPDQVGSQRFSVNMPHKFGIHNYKVPTFCDHCGSLLWGLLRQGLQCKVCKMNVHRRCETNVAPNCGVDARGIAKVLADLGVTPDKITNSGQRRKKLIAGAESPQPASGSSPSEEDRSKSAPTSPCDQEIKELENNIRKALSFDNRGEEHRAASSPDGQLMSPGENGEVRQGQAKRLGLDEFNFIKVLGKGSFGKVMLAELKGKDEVYAVKVLKKDVILQDDDVDCTMTEKRILALARKHPYLTQLYCCFQTKDRLFFVMEYVNGGDLMFQIQRSRKFDEPRSRFYAAEVTSALMFLHQHGVIYRDLKLDNILLDAEGHCKLADFGMCKEGILNGVTTTTFCGTPDYIAPEILQELEYGPSVDWWALGVLMYEMMAGQPPFEADNEDDLFESILHDDVLYPVWLSKEAVSILKAFMTKNPHKRLGCVASQNGEDAIKQHPFFKEIDWVLLEQKKIKPPFKPRIKTKRDVNNFDQDFTREEPVLTLVDEAIVKQINQEEFKGFSYFGEDLMP

>NP_963868.1

MANGVIPPPGGASPLPQVRVPLEEPPLSPDVEEEDDDLGKTLAVSRFGDLISKPPAWDPEKPSRSYSERDFEFHRHTSHHTHHPLSARLPPPHKLRRLPPTSARHTRRKRKKEKTSAPPSEGTPPIQEEGGAGVDEEEEEEEEEEGESEAEPVEPPPSGTPQKAKFSIGSDEDDSPGLPGRAAVTKPLPSVGPHTDKSPQHSSRPCSELRDGDGTTDLALSSPRLLCCLPSSPSPRARASRLAGEKSRPWSPSASYDLRERLCPGSALGNPGGPEQQVPTDEAEAQMLGSADLDDMKSHRLEDNPGVRRHLVKKPSRTQGGRGSPSGLAPILRRKKKKKKLDRRPHEVFVELNELMLDRSQEPHWRETARWIKFEEDVEEETERWGKPHVASLSFRSLLELRRTIAHGAALLDLEQTTLPGIAHLVVETMIVSDQIRPEDRASVLRTLLLKHSHPNDDKDSGFFPRNPSSSSMNSVLGNHHPTPSHGPDGAVPTMADDLGEPAPLWPHDPDAKEKPLHMPGGDGHRGKSLKLLEKIPEDAEATVVLVGCVPFLEQPAAAFVRLNEAVLLESVLEVPVPVRFLFVMLGPSHTSTDYHELGRSIATLMSDKLFHEAAYKADDRQDLLSAISEFLDGSIVIPPSEVEGRDLLRSVAAFQRELLRKRREREQTKVEMTTRGGYTAPGKELSLELGGSEATPEDDPLLRTGSVFGGLVRDVRRRYPHYPSDLRDALHSQCVAAVLFIYFAALSPAITFGGLLGEKTEGLMGVSELIVSTAVLGVLFSLLGAQPLLVVGFSGPLLVFEEAFFKFCRAQDLEYLTGRVWVGLWLVVFVLALVAAEGSFLVRYISPFTQEIFAFLISLIFIYETFYKLYKVFTERPLLPFYPPEGALEGSLDAGLEPNGSALPPTEGPPSPRNQPNTALLSLILMLGTFFIAFFLRKFRNSRFLGGKARRIIGDFGIPISILVMVLVDYSITDTYTQKLTVPTGLSVTSPDKRSWFIPPLGSARPFPPWMMVAAAVPALLVLILIFMETQITALIVSQKARRLLKGSGFHLDLLLIGSLGGLCGLFGLPWLTAATVRSITHVNALTVMRTAIAPGDKPQIQEVREQRVTGVLIASLVGLSIVMGAVLRRIPLAVLFGIFLYMGVTSLSGIQLSQRLLLILMPAKHHPEQPYVTKVKTWRMHLFTCIQLGCIALLWVVKSTAASLAFPFLLLLTVPLRHCLLPRLFQDRELQALDSEDAEPNFDEDGQDEYNELHMPV

228 NP_006262.1 NP_005971.1

>NP_006262.1

MGSELETAMETLINVFHAHSGKEGDKYKLSKKELKELLQTELSGFLDAQKDVDAVDKVMKELDENGDGEVDFQEYVVLVAALTVACNNFFWENS

>NP_005971.1

MTELETAMGMIIDVFSRYSGSEGSTQTLTKGELKVLMEKELPGFLQSGKDKDAVDKLLKDLDANGDAQVDFSEFIVFVAAITSACHKYFEKAGLK

229 NP_005755.1 NP_006824.2

>NP_005755.1

MSALSLLILGLLTAVPPASCQQGLGNLQPWMQGLIAVAVFLVLVAIAFAVNHFWCQEEPEPAHMILTVGNKADGVLVGTDGRYSSMAASFRSSEHENAYENVPEEEGKVRSTPM

>NP_006824.2

MAAAAAAAAATNGTGGSSGMEVDAAVVPSVMACGVTGSVSVALHPLVILNISDHWIRMRSQEGRPVQVIGALIGKQEGRNIEVMNSFELLSHTVEEKIIIDKEYYYTKEEQFKQVFKELEFLGWYTTGGPPDPSDIHVHKQVCEIIESPLFLKLNPMTKHTDLPVSVFESVIDIINGEATMLFAELTYTLATEEAERIGVDHVARMTATGSGENSTVAEHLIAQHSAIKMLHSRVKLILEYVKASEAGEVPFNHEILREAYALCHCLPVLSTDKFKTDFYDQCNDVGLMAYLGTITKTCNTMNQFVNKFNVLYDRQGIGRRMRGLFF

230 NP_955631.1 NP_056132.1

>NP_955631.1

MEAFPWAPRSPRRGRAPPPMALVPSARYVSAPGPAHPQPFSSWNDYLGLATLITKAVDGEPRFGCARGGNGGGGSPPSSSSSSCCSPHTGAGPGALGPALGPPDYDEDDDDDSDEPGSRGRYLGSALELRALELCAGPAEAGLLEERFAELSPFAGRAAAVLLGCAPAAAAAATTTSEATPREERAPAWAAEPRLHAASGAAAARLLKPELQVCVFCRNNKEAMALYTTHILKGPDGRVLCPVLRRYTCPLCGASGDNAHTIKYCPLSKVPPPPARPPPRSARDGPPGKKLR

>NP_056132.1

MNHDFQALALESRGMGELLPTKKFWEPDDSTKDGQKGIFLGDDEWRETAWGASHHSMSQPIMVQRRSGQGFHGNSEVNAILSPRSESGGLGVSMVEYVLSSSPADKLDSRFRKGNFGTRDAETDGPEKGDQKGKASPFEEDQNRDLKQGDDDDSKINGRGLPNGMDADCKDFNRTPGSRQASPTEVVERLGPNTNPSEGLGPLPNPTANKPLVEEFSNPETQNLDAMEQVGLESLQFDYPGNQVPMDSSGATVGLFDYNSQQQLFQRTNALTVQQLTAAQQQQYALAAAQQPHIAGVFSAGLAPAAFVPNPYIISAAPPGTDPYTAAGLAAAATLAGPAVVPPQYYGVPWGVYPANLFQQQAAAAANNTASQQAASQAQPGQQQVLRAGAGQRPLTPNQGQQGQQAESLAAAAAANPTLAFGQGLATGMPGYQVLAPTAYYDQTGALVVGPGARTGLGAPVRLMAPTPVLISSAAAQAAAAAAAGGTASSLTGSTNGLFRPIGTQPPQQQQQQPSTNLQSNSFYGSSSLTNSSQSSSLFSHGPGQPGSTSLGFGSGNSLGAAIGSALSGFGSSVGSSASSSATRRESLSTSSDLYKRSSSSLAPIGQPFYNSLGFSSSPSPIGMPLPSQTPGHSLTPPPSLSSHGSSSSLHLGGLTNGSGRYISAAPGAEAKYRSASSTSSLFSSSSQLFPPSRLRYNRSDIMPSGRSRLLEDFRNNRFPNLQLRDLIGHIVEFSQDQHGSRFIQQKLERATPAERQMVFNEILQAAYQLMTDVFGNYVIQKFFEFGSLDQKLALATRIRGHVLPLALQMYGCRVIQKALESISSDQQSEMVKELDGHVLKCVKDQNGNHVVQKCIECVQPQSLQFIIDAFKGQVFVLSTHPYGCRVIQRILEHCTAEQTLPILEELHQHTEQLVQDQYGNYVIQHVLEHGRPEDKSKIVSEIRGKVLALSQHKFASNVVEKCVTHASRAERALLIDEVCCQNDGPHSALYTMMKDQYANYVVQKMIDMAEPAQRKIIMHKIRPHITTLRKYTYGKHILAKLEKYYLKNSPDLGPIGGPPNGML

231 NP_067080.1 NP_066268.1

>NP_067080.1

MERPEPELIRQSWRAVSRSPLEHGTVLFARLFALEPDLLPLFQYNCRQFSSPEDCLSSPEFLDHIRKVMLVIDAAVTNVEDLSSLEEYLASLGRKHRAVGVKLSSFSTVGESLLYMLEKCLGPAFTPATRAAWSQLYGAVVQAMSRGWDGE

>NP_066268.1

MGCTLSAEERAALERSKAIEKNLKEDGISAAKDVKLLLLGAGESGKSTIVKQMKIIHEDGFSGEDVKQYKPVVYSNTIQSLAAIVRAMDTLGIEYGDKERKADAKMVCDVVSRMEDTEPFSAELLSAMMRLWGDSGIQECFNRSREYQLNDSAKYYLDSLDRIGAADYQPTEQDILRTRVKTTGIVETHFTFKNLHFRLFDVGGQRSERKKWIHCFEDVTAIIFCVALSGYDQVLHEDETTNRMHESLMLFDSICNNKFFIDTSIILFLNKKDLFGEKIKKSPLTICFPEYTGPNTYEDAAAYIQAQFESKNRSPNKEIYCHMTCATDTNNIQVVFDAVTDIIIANNLRGCGLY

232 NP_009107.1 NP_659451.1

>NP_009107.1

MSRFLNVLRSWLVMVSIIAMGNTLQSFRDHTFLYEKLYTGKPNLVNGLQARTFGIWTLLSSVIRCLCAIDIHNKTLYHITLWTFLLALGHFLSELFVYGTAAPTIGVLAPLMVASFSILGMLVGLRYLEVEPVSRQKKRN

>NP_659451.1

MEELLPDGQIWANMDPEERMLAAATAFTHICAGQGEGDVRREAQSIQYDPYSKASVAPGKRPALPVQLQYPHVESNVPSETVSEASQRLRKPVMKRKVLRRKPDGEVLVTDESIISESESGTENDQDLWDLRQRLMNVQFQEDKESSFDVSQKFNLPHEYQGISQDQLICSLQREGMGSPAYEQDLIVASRPKSFILPKLDQLSRNRGKTDRVARYFEYKRDWDSIRLPGEDHRKELRWGVREQMLCRAEPQSKPQHIYVPNNYLVPTEKKRSALRWGVRCDLANGVIPRKLPFPLSPS

233 NP_000892.1 NP_001091.1

>NP_000892.1

METKGYHSLPEGLDMERRWGQVSQAVERSSLGPTERTDENNYMEIVNVSCVSGAIPNNSTQGSSKEKQELLPCLQQDNNRPGILTSDIKTELESKELSATVAESMGLYMDSVRDADYSYEQQNQQGSMSPAKIYQNVEQLVKFYKGNGHRPSTLSCVNTPLRSFMSDSGSSVNGGVMRAIVKSPIMCHEKSPSVCSPLNMTSSVCSPAGINSVSSTTASFGSFPVHSPITQGTPLTCSPNAENRGSRSHSPAHASNVGSPLSSPLSSMKSSISSPPSHCSVKSPVSSPNNVTLRSSVSSPANINNSRCSVSSPSNTNNRSTLSSPAASTVGSICSPVNNAFSYTASGTSAGSSTLRDVVPSPDTQEKGAQEVPFPKTEEVESAISNGVTGQLNIVQYIKPEPDGAFSSSCLGGNSKINSDSSFSVPIKQESTKHSCSGTSFKGNPTVNPFPFMDGSYFSFMDDKDYYSLSGILGPPVPGFDGNCEGSGFPVGIKQEPDDGSYYPEASIPSSAIVGVNSGGQSFHYRIGAQGTISLSRSARDQSFQHLSSFPPVNTLVESWKSHGDLSSRRSDGYPVLEYIPENVSSSTLRSVSTGSSRPSKICLVCGDEASGCHYGVVTCGSCKVFFKRAVEGQHNYLCAGRNDCIIDKIRRKNCPACRLQKCLQAGMNLGARKSKKLGKLKGIHEEQPQQQQPPPPPPPPQSPEEGTTYIAPAKEPSVNTALVPQLSTISRALTPSPVMVLENIEPEIVYAGYDSSKPDTAENLLSTLNRLAGKQMIQVVKWAKVLPGFKNLPLEDQITLIQYSWMCLSSFALSWRSYKHTNSQFLYFAPDLVFNEEKMHQSAMYELCQGMHQISLQFVRLQLTFEEYTIMKVLLLLSTIPKDGLKSQAAFEEMRTNYIKELRKMVTKCPNNSGQSWQRFYQLTKLLDSMHDLVSDLLEFCFYTFRESHALKVEFPAMLVEIISDQLPKVESGNAKPLYFHRK

>NP_001091.1

MCDEDETTALVCDNGSGLVKAGFAGDDAPRAVFPSIVGRPRHQGVMVGMGQKDSYVGDEAQSKRGILTLKYPIEHGIITNWDDMEKIWHHTFYNELRVAPEEHPTLLTEAPLNPKANREKMTQIMFETFNVPAMYVAIQAVLSLYASGRTTGIVLDSGDGVTHNVPIYEGYALPHAIMRLDLAGRDLTDYLMKILTERGYSFVTTAEREIVRDIKEKLCYVALDFENEMATAASSSSLEKSYELPDGQVITIGNERFRCPETLFQPSFIGMESAGIHETTYNSIMKCDIDIRKDLYANNVMSGGTTMYPGIADRMQKEITALAPSTMKIKIIAPPERKYSVWIGGSILASLSTFQQMWITKQEYDEAGPSIVHRKCF

234 NP_004413.1 NP_001008712.1

>NP_004413.1

MAGSSTGGGGVGETKVIYHLDEEETPYLVKIPVPAERITLGDFKSVLQRPAGAKYFFKSMDQDFGVVKEEISDDNARLPCFNGRVVSWLVSSDNPQPEMAPPVHEPRAELAPPAPPLPPLPPERTSGIGDSRPPSFHPNVSSSHENLEPETETESVVSLRRERPRRRDSSEHGAGGHRTGGPSRLERHLAGYESSSTLMTSELESTSLGDSDEEDTMSRFSSSTEQSSASRLLKRHRRRRKQRPPRLERTSSFSSVTDSTMSLNIITVTLNMEKYNFLGISIVGQSNERGDGGIYIGSIMKGGAVAADGRIEPGDMLLQVNDMNFENMSNDDAVRVLRDIVHKPGPIVLTVAKCWDPSPQAYFTLPRNEPIQPIDPAAWVSHSAALTGTFPAYPGSSSMSTITSGSSLPDGCEGRGLSVHTDMASVTKAMAAPESGLEVRDRMWLKITIPNAFLGSDVVDWLYHHVEGFPERREARKYASGLLKAGLIRHTVNKITFSEQCYYVFGDLSGGCESYLVNLSLNDNDGSSGASDQDTLAPLPGATPWPLLPTFSYQYPAPHPYSPQPPPYHELSSYTYGGGSASSQHSEGSRSSGSTRSDGGAGRTGRPEERAPESKSGSGSESEPSSRGGSLRRGGEASGTSDGGPPPSRGSTGGAPNLRAHPGLHPYGPPPGMALPYNPMMVVMMPPPPPPVPPAVQPPGAPPVRDLGSVPPELTASRQSFHMAMGNPSEFFVDVM

>NP_001008712.1

MNNGGKAEKENTPSEANLQEEEVRTLFVSGLPLDIKPRELYLLFRPFKGYEGSLIKLTSKQPVGFVSFDSRSEAEAAKNALNGIRFDPEIPQTLRLEFAKANTKMAKNKLVGTPNPSTPLPNTVPQFIAREPYELTVPALYPSSPEVWAPYPLYPAELAPALPPPAFTYPASLHAQCFSPEAKPNTPVFCPLLQQIRFVSGNVFVTYQPTADQQRELPC

235 NP_008945.2 NP_001029.1

>NP_008945.2

MASASSSRAGVALPFEKSQLTLKVVSAKPKVHNRQPRINSYVEVAVDGLPSETKKTGKRIGSSELLWNEIIILNVTAQSHLDLKVWSCHTLRNELLGTASVNLSNVLKNNGGKMENMQLTLNLQTENKGSVVSGGELTIFLDGPTVDLGNVPNGSALTDGSQLPSRDSSGTAVAPENRHQPPSTNCFGGRSRTHRHSGASARTTPATGEQSPGARSRHRQPVKNSGHSGLANGTVNDEPTTATDPEEPSVVGVTSPPAAPLSVTPNPNTTSLPAPATPAEGEEPSTSGTQQLPAAAQAPDALPAGWEQRELPNGRVYYVDHNTKTTTWERPLPPGWEKRTDPRGRFYYVDHNTRTTTWQRPTAEYVRNYEQWQSQRNQLQGAMQHFSQRFLYQSSSASTDHDPLGPLPPGWEKRQDNGRVYYVNHNTRTTQWEDPRTQGMIQEPALPPGWEMKYTSEGVRYFVDHNTRTTTFKDPRPGFESGTKQGSPGAYDRSFRWKYHQFRFLCHSNALPSHVKISVSRQTLFEDSFQQIMNMKPYDLRRRLYIIMRGEEGLDYGGIAREWFFLLSHEVLNPMYCLFEYAGKNNYCLQINPASSINPDHLTYFRFIGRFIAMALYHGKFIDTGFTLPFYKRMLNKRPTLKDLESIDPEFYNSIVWIKENNLEECGLELYFIQDMEILGKVTTHELKEGGESIRVTEENKEEYIMLLTDWRFTRGVEEQTKAFLDGFNEVAPLEWLRYFDEKELELMLCGMQEIDMSDWQKSTIYRHYTKNSKQIQWFWQVVKEMDNEKRIRLLQFVTGTCRLPVGGFAELIGSNGPQKFCIDKVGKETWLPRSHTCFNRLDLPPYKSYEQLREKLLYAIEETEGFGQE

>NP_001029.1

MEGNKLEEQDSSPPQSTPGLMKGNKREEQGLGPEPAAPQQPTAEEEALIEFHRSYRELFEFFCNNTTIHGAIRLVCSQHNRMKTAFWAVLWLCTFGMMYWQFGLLFGEYFSYPVSLNINLNSDKLVFPAVTICTLNPYRYPEIKEELEELDRITEQTLFDLYKYSSFTTLVAGSRSRRDLRGTLPHPLQRLRVPPPPHGARRARSVASSLRDNNPQVDWKDWKIGFQLCNQNKSDCFYQTYSSGVDAVREWYRFHYINILSRLPETLPSLEEDTLGNFIFACRFNQVSCNQANYSHFHHPMYGNCYTFNDKNNSNLWMSSMPGINNGLSLMLRAEQNDFIPLLSTVTGARVMVHGQDEPAFMDDGGFNLRPGVETSISMRKETLDRLGGDYGDCTKNGSDVPVENLYPSKYTQQVCIHSCFQESMIKECGCAYIFYPRPQNVEYCDYRKHSSWGYCYYKLQVDFSSDHLGCFTKCRKPCSVTSYQLSAGYSRWPSVTSQEWVFQMLSRQNNYTVNNKRNGVAKVNIFFKELNYKTNSESPSVTMVTLLSNLGSQWSLWFGSSVLSVVEMAELVFDLLVIMFLMLLRRFRSRYWSPGRGGRGAQEVASTLASSPPSHFCPHPMSLSLSQPGPAPSPALTAPPPAYATLGPRPSPGGSAGASSSTCPLGGP

236 NP_004752.1 NP_056461.1

>NP_004752.1

MLPRPLRLLLDTSPPGGVVLSSFRSRDPEEGGGPGGLVVGGGQEEEEEEEEEAPVSVWDEEEDGAVFTVTSRQYRPLDPLVPMPPPRSSRRLRAGTLEALVRHLLDTRTSGTDVSFMSAFLATHRAFTSTPALLGLMADRLEALESHPTDELERTTEVAISVLSTWLASHPEDFGSEAKGQLDRLESFLLQTGYAAGKGVGGGSADLIRNLRSRVDPQAPDLPKPLALPGDPPADPTDVLVFLADHLAEQLTLLDAELFLNLIPSQCLGGLWGHRDRPGHSHLCPSVRATVTQFNKVAGAVVSSVLGATSTGEGPGEVTIRPLRPPQRARLLEKWIRVAEECRLLRNFSSVYAVVSALQSSPIHRLRAAWGEATRDSLRVFSSLCQIFSEEDNYSQSRELLVQEVKLQSPLEPHSKKAPRSGSRGGGVVPYLGTFLKDLVMLDAASKDELENGYINFDKRRKEFAVLSELRRLQNECRGYNLQPDHDIQRWLQGLRPLTEAQSHRVSCEVEPPGSSDPPAPRVLRPTLVISQWTEVLGSVGVPTPLVSCDRPSTGGDEAPTTPAPLLTRLAQHMKWPSVSSLDSALESSPSLHSPADPSHLSPPASSPRPSRGHRRSASCGSPLSGGAEEASGGTGYGGEGSGPGASDCRIIRVQMELGEDGSVYKSILVTSQDKAPSVISRVLKKNNRDSAVASEYELVQLLPGERELTIPASANVFYAMDGASHDFLLRQRRRSSTATPGVTSGPSASGTPPSEGGGGSFPRIKATGRKIARALF

>NP_056461.1

MREYKLVVLGSGGVGKSALTVQFVQGIFVEKYDPTIEDSYRKQVEVDAQQCMLEILDTAGTEQFTAMRDLYMKNGQGFALVYSITAQSTFNDLQDLREQILRVKDTDDVPMILVGNKCDLEDERVVGKEQGQNLARQWNNCAFLESSAKSKINVNEIFYDLVRQINRKTPVPGKARKKSSCQLL

237 NP_001012271.1 NP_004337.2

>NP_001012271.1

MGAPTLPPAWQPFLKDHRISTFKNWPFLEGCACTPERMAEAGFIHCPTENEPDLAQCFFCFKELEGWEPDDDPIGPGTVAYACNTSTLGGRGGRITREEHKKHSSGCAFLSVKKQFEELTLGEFLKLDRERAKNKIAKETNNKKKEFEETAEKVRRAIEQLAAMD

>NP_004337.2

MENTENSVDSKSIKNLEPKIIHGSESMDSGISLDNSYKMDYPEMGLCIIINNKNFHKSTGMTSRSGTDVDAANLRETFRNLKYEVRNKNDLTREEIVELMRDVSKEDHSKRSSFVCVLLSHGEEGIIFGTNGPVDLKKITNFFRGDRCRSLTGKPKLFIIQACRGTELDCGIETDSGVDDDMACHKIPVEADFLYAYSTAPGYYSWRNSKDGSWFIQSLCAMLKQYADKLEFMHILTRVNRKVATEFESFSFDATFHAKKQIPCIVSMLTKELYFYH

238 NP_005892.1 NP_002725.1

>NP_005892.1

MSSILPFTPPVVKRLLGWKKSAGGSGGAGGGEQNGQEEKWCEKAVKSLVKKLKKTGRLDELEKAITTQNCNTKCVTIPSTCSEIWGLSTPNTIDQWDTTGLYSFSEQTRSLDGRLQVSHRKGLPHVIYCRLWRWPDLHSHHELKAIENCEYAFNLKKDEVCVNPYHYQRVETPVLPPVLVPRHTEILTELPPLDDYTHSIPENTNFPAGIEPQSNYIPETPPPGYISEDGETSDQQLNQSMDTGSPAELSPTTLSPVNHSLDLQPVTYSEPAFWCSIAYYELNQRVGETFHASQPSLTVDGFTDPSNSERFCLGLLSNVNRNATVEMTRRHIGRGVRLYYIGGEVFAECLSDSAIFVQSPNCNQRYGWHPATVCKIPPGCNLKIFNNQEFAALLAQSVNQGFEAVYQLTRMCTIRMSFVKGWGAEYRRQTVTSTPCWIELHLNGPLQWLDKVLTQMGSPSVRCSSMS

>NP_002725.1

MESGSTAASEEARSLRECELYVQKHNIQALLKDSIVQLCTARPERPMAFLREYFERLEKEEAKQIQNLQKAGTRTDSREDEISPPPPNPVVKGRRRRGAISAEVYTEEDAASYVRKVIPKDYKTMAALAKAIEKNVLFSHLDDNERSDIFDAMFSVSFIAGETVIQQGDEGDNFYVIDQGETDVYVNNEWATSVGEGGSFGELALIYGTPRAATVKAKTNVKLWGIDRDSYRRILMGSTLRKRKMYEEFLSKVSILESLDKWERLTVADALEPVQFEDGQKIVVQGEPGDEFFIILEGSAAVLQRRSENEEFVEVGRLGPSDYFGEIALLMNRPRAATVVARGPLKCVKLDRPRFERVLGPCSDILKRNIQQYNSFVSLSV

239 NP_003833.3 NP_003780.1

>NP_003833.3

MEQRGQNAPAASGARKRHGPGPREARGARPGLRVPKTLVLVVAAVLLLVSAESALITQQDLAPQQRAAPQQKRSSPSEGLCPPGHHISEDGRDCISCKYGQDYSTHWNDLLFCLRCTRCDSGEVELSPCTTTRNTVCQCEEGTFREEDSPEMCRKCRTGCPRGMVKVGDCTPWSDIECVHKESGTKHSGEAPAVEETVTSSPGTPASPCSLSGIIIGVTVAAVVLIVAVFVCKSLLWKKVLPYLKGICSGGGGDPERVDRSSQRPGAEDNVLNEIVSILQPTQVPEQEMEVQEPAEPTGVNMLSPGESEHLLEPAEAERSQRRRLLVPANEGDPTETLRQCFDDFADLVPFDSWEPLMRKLGLMDNEIKVAKAEAAGHRDTLYTMLIKWVNKTGRDASVHTLLDALETLGERLAKQKIEDHLLSSGKFMYLEGNADSAMS

>NP_003780.1

MAAGQNGHEEWVGSAYLFVESSLDKVVLSDAYAHPQQKVAVYRALQAALAESGGSPDVLQMLKIHRSDPQLIVQLRFCGRQPCGRFLRAYREGALRAALQRSLAAALAQHSVPLQLELRAGAERLDALLADEERCLSCILAQQPDRLRDEELAELEDALRNLKCGSGARGGDGEVASAPLQPPVPSLSEVKPPPPPPPAQTFLFQGQPVVNRPLSLKDQQTFARSVGLKWRKVGRSLQRGCRALRDPALDSLAYEYEREGLYEQAFQLLRRFVQAEGRRATLQRLVEALEENELTSLAEDLLGLTDPNGGLA

240 NP_002058.2 NP_000614.1

>NP_002058.2

MTLESMMACCLSDEVKESKRINAEIEKQLRRDKRDARRELKLLLLGTGESGKSTFIKQMRIIHGAGYSEEDKRGFTKLVYQNIFTAMQAMIRAMETLKILYKYEQNKANALLIREVDVEKVTTFEHQYVSAIKTLWEDPGIQECYDRRREYQLSDSAKYYLTDVDRIATLGYLPTQQDVLRVRVPTTGIIEYPFDLENIIFRMVDVGGQRSERRKWIHCFENVTSIMFLVALSEYDQVLVESDNENRMEESKALFRTIITYPWFQNSSVILFLNKKDLLEDKILYSHLVDYFPEFDGPQRDAQAAREFILKMFVDLNPDSDKIIYSHFTCATDTENIRFVFAAVKDTILQLNLKEYNLV

>NP_000614.1

MFSPWKISMFLSVREDSVPTTASFSADMLNVTLQGPTLNGTFAQSKCPQVEWLGWLNTIQPPFLWVLFVLATLENIFVLSVFCLHKSSCTVAEIYLGNLAAADLILACGLPFWAITISNNFDWLFGETLCRVVNAIISMNLYSSICFLMLVSIDRYLALVKTMSMGRMRGVRWAKLYSLVIWGCTLLLSSPMLVFRTMKEYSDEGHNVTACVISYPSLIWEVFTNMLLNVVGFLLPLSVITFCTMQIMQVLRNNEMQKFKEIQTERRATVLVLVVLLLFIICWLPFQISTFLDTLHRLGILSSCQDERIIDVITQIASFMAYSNSCLNPLVYVIVGKRFRKKSWEVYQGVCQKGGCRSEPIQMENSMGTLRTSISVERQIHKLQDWAGSRQ

241 NP_624302.1 NP_000583.2

>NP_624302.1

MRWLLLYYALCFSLSKASAHTVELNNMFGQIQSPGYPDSYPSDSEVTWNITVPDGFRIKLYFMHFNLESSYLCEYDYVKVETEDQVLATFCGRETTDTEQTPGQEVVLSPGSFMSITFRSDFSNEERFTGFDAHYMAVDVDECKEREDEELSCDHYCHNYIGGYYCSCRFGYILHTDNRTCRVECSDNLFTQRTGVITSPDFPNPYPKSSECLYTIELEEGFMVNLQFEDIFDIEDHPEVPCPYDYIKIKVGPKVLGPFCGEKAPEPISTQSHSVLILFHSDNSGENRGWRLSYRAAGNECPELQPPVHGKIEPSQAKYFFKDQVLVSCDTGYKVLKDNVEMDTFQIECLKDGTWSNKIPTCKIVDCRAPGELEHGLITFSTRNNLTTYKSEIKYSCQEPYYKMLNNNTGIYTCSAQGVWMNKVLGRSLPTCLPECGQPSRSLPSLVKRIIGGRNAEPGLFPWQALIVVEDTSRVPNDKWFGSGALLSASWILTAAHVLRSQRRDTTVIPVSKEHVTVYLGLHDVRDKSGAVNSSAARVVLHPDFNIQNYNHDIALVQLQEPVPLGPHVMPVCLPRLEPEGPAPHMLGLVAGWGISNPNVTVDEIISSGTRTLSDVLQYVKLPVVPHAECKTSYESRSGNYSVTENMFCAGYYEGGKDTCLGDSGGAFVIFDDLSQRWVVQGLVSWGGPEECGSKQVYGVYTKVSNYVDWVWEQMGLPQSVVEPQVER

>NP_000583.2

MRLLWGLIWASSFFTLSLQKPRLLLFSPSVVHLGVPLSVGVQLQDVPRGQVVKGSVFLRNPSRNNVPCSPKVDFTLSSERDFALLSLQVPLKDAKSCGLHQLLRGPEVQLVAHSPWLKDSLSRTTNIQGINLLFSSRRGHLFLQTDQPIYNPGQRVRYRVFALDQKMRPSTDTITVMVENSHGLRVRKKEVYMPSSIFQDDFVIPDISEPGTWKISARFSDGLESNSSTQFEVKKYVLPNFEVKITPGKPYILTVPGHLDEMQLDIQARYIYGKPVQGVAYVRFGLLDEDGKKTFFRGLESQTKLVNGQSHISLSKAEFQDALEKLNMGITDLQGLRLYVAAAIIESPGGEMEEAELTSWYFVSSPFSLDLSKTKRHLVPGAPFLLQALVREMSGSPASGIPVKVSATVSSPGSVPEVQDIQQNTDGSGQVSIPIIIPQTISELQLSVSAGSPHPAIARLTVAAPPSGGPGFLSIERPDSRPPRVGDTLNLNLRAVGSGATFSHYYYMILSRGQIVFMNREPKRTLTSVSVFVDHHLAPSFYFVAFYYHGDHPVANSLRVDVQAGACEGKLELSVDGAKQYRNGESVKLHLETDSLALVALGALDTALYAAGSKSHKPLNMGKVFEAMNSYDLGCGPGGGDSALQVFQAAGLAFSDGDQWTLSRKRLSCPKEKTTRKKRNVNFQKAINEKLGQYASPTAKRCCQDGVTRLPMMRSCEQRAARVQQPDCREPFLSCCQFAESLRKKSRDKGQAGLQRALEILQEEDLIDEDDIPVRSFFPENWLWRVETVDRFQILTLWLPDSLTTWEIHGLSLSKTKGLCVATPVQLRVFREFHLHLRLPMSVRRFEQLELRPVLYNYLDKNLTVSVHVSPVEGLCLAGGGGLAQQVLVPAGSARPVAFSVVPTAATAVSLKVVARGSFEFPVGDAVSKVLQIEKEGAIHREELVYELNPLDHRGRTLEIPGNSDPNMIPDGDFNSYVRVTASDPLDTLGSEGALSPGGVASLLRLPRGCGEQTMIYLAPTLAASRYLDKTEQWSTLPPETKDHAVDLIQKGYMRIQQFRKADGSYAAWLSRGSSTWLTAFVLKVLSLAQEQVGGSPEKLQETSNWLLSQQQADGSFQDLSPVIHRSMQGGLVGNDETVALTAFVTIALHHGLAVFQDEGAEPLKQRVEASISKASSFLGEKASAGLLGAHAAAITAYALTLTKAPADLRGVAHNNLMAMAQETGDNLYWGSVTGSQSNAVSPTPAPRNPSDPMPQAPALWIETTAYALLHLLLHEGKAEMADQAAAWLTRQGSFQGGFRSTQDTVIALDALSAYWIASHTTEERGLNVTLSSTGRNGFKSHALQLNNRQIRGLEEELQFSLGSKINVKVGGNSKGTLKVLRTYNVLDMKNTTCQDLQIEVTVKGHVEYTMEANEDYEDYEYDELPAKDDPDAPLQPVTPLQLFEGRRNRRRREAPKVVEEQESRVHYTVCIWRNGKVGLSGMAIADVTLLSGFHALRADLEKLTSLSDRYVSHFETEGPHVLLYFDSVPTSRECVGFEAVQEVPVGLVQPASATLYDYYNPERRCSVFYGAPSKSRLLATLCSAEVCQCAEGKCPRQRRALERGLQDEDGYRMKFACYYPRVEYGFQVKVLREDSRAAFRLFETKITQVLHFTKDVKAAANQMRNFLVRASCRLRLEPGKEYLIMGLDGATYDLEGHPQYLLDSNSWIEEMPSERLCRSTRQRAACAQLNDFLQEYGTQGCQV

242 NP_006384.1 NP_003452.1

>NP_006384.1

MRVPVFEDIKDETEEEKIGEEENEEDQVFYKPVIEDLSMELARKCTELISDIRYKEEFKKSKDKCTFVTDSPMLNHVKNIGAFISEAKYKGTIKADLSNSLYKRMPATIDSVFAGEVTQLQSEVAYKQKHDAAKGFSDYAHMKEPPEVKHAMEVNKHQSNISYRKDVQDTHTYSAELDRPDIKMATQISKIISNAEYKKGQGIMNKEPAVIGRPDFEHAVEASKLSSQIKYKEKFDNEMKDKKHHYNPLESASFRQNQLAATLASNVKYKKDIQNMHDPVSDLPNLLFLDHVLKASKMLSGREYKKLFEENKGMYHFDADAVEHLHHKGNAVLQSQVKYKEEYEKNKGKPMLEFVETPSYQASKEAQKMQSEKVYKEDFEKEIKGRSSLDLDKTPEFLHVKYITNLLREKEYKKDLENEIKGKGMELNSEVLDIQRAKRASEMASEKEYKKDLESIIKGKGMQAGTDTLEMQHAKKAAEIASEKDYKRDLETEIKGKGMQVSTDTLDVQRAKKASEMASQKQYKKDLENEIKGKGMQVSMDIPDILRAKRTSEIYSQRKYKDEAEKMLSNYSTIADTPEIQRIKTTQQNISAVFYKKEVGAGTAVKDSPEIERVKKNQQNISSVKYKEEIKHATAISDPPELKRVKENQKNISNLQYKEQNYKATPVSMTPEIERVRRNQEQLSAVKYKGELQRGTAISDPPELKRAKENQKNISNVYYRGQLGRATTLSVTPEMERVKKNQENISSVKYTQDHKQMKGRPSLILDTPAMRHVKEAQNHISMVKYHEDFEKTKGRGFTPVVDDPVTERVRKNTQVVSDAAYKGVHPHIVEMDRRPGIIVDLKVWRTDPGSIFDLDPLEDNIQSRSLHMLSEKASHYRRHWSRSHSSSTFGTGLGDDRSEISEIYPSFSCCSEVTRPSDEGAPVLPGAYQQSHSQGYGYMHQTSVSSMRSMQHSPNLRTYRAMYDYSAQDEDEVSFRDGDYIVNVQPIDDGWMYGTVQRTGRTGMLPANYIEFVN

>NP_003452.1

MAAPRPSPAISVSVSAPAFYAPQKKFGPVVAPKPKVNPFRPGDSEPPPAPGAQRAQMGRVGEIPPPPPEDFPLPPPPLAGDGDDAEGALGGAFPPPPPPIEESFPPAPLEEEIFPSPPPPPEEEGGPEAPIPPPPQPREKVSSIDLEIDSLSSLLDDMTKNDPFKARVSSGYVPPPVATPFSSKSSTKPAAGGTAPLPPWKSPSSSQPLPQVPAPAQSQTQFHVQPQPQPKPQVQLHVQSQTQPVSLANTQPRGPPASSPAPAPKFSPVTPKFTPVASKFSPGAPGGSGSQPNQKLGHPEALSAGTGSPQPPSFTYAQQREKPRVQEKQHPVPPPAQNQNQVRSPGAPGPLTLKEVEELEQLTQQLMQDMEHPQRQNVAVNELCGRCHQPLARAQPAVRALGQLFHIACFTCHQCAQQLQGQQFYSLEGAPYCEGCYTDTLEKCNTCGEPITDRMLRATGKAYHPHCFTCVVCARPLEGTSFIVDQANRPHCVPDYHKQYAPRCSVCSEPIMPEPGRDETVRVVALDKNFHMKCYKCEDCGKPLSIEADDNGCFPLDGHVLCRKCHTARAQT

243 NP_002564.1 NP_005997.2

>NP_002564.1

MSGEENPASKPTPVQDVQGDGRWMSLHHRFVADSKDKEPEVVFIGDSLVQLMHQCEIWRELFSPLHALNFGIGGDGTQHVLWRLENGELEHIRPKIVVVWVGTNNHGHTAEQVTGGIKAIVQLVNERQPQARVVVLGLLPRGQHPNPLREKNRQVNELVRAALAGHPRAHFLDADPGFVHSDGTISHHDMYDYLHLSRLGYTPVCRALHSLLLRLLAQDQGQGAPLLEPAP

>NP_005997.2

MDLTKMGMIQLQNPSHPTGLLCKANQMRLAGTLCDVVIMVDSQEFHAHRTVLACTSKMFEILFHRNSQHYTLDFLSPKTFQQILEYAYTATLQAKAEDLDDLLYAAEILEIEYLEEQCLKMLETIQASDDNDTEATMADGGAEEEEDRKARYLKNIFISKHSSEESGYASVAGQSLPGPMVDQSPSVSTSFGLSAMSPTKAAVDSLMTIGQSLLQGTLQPPAGPEEPTLAGGGRHPGVAEVKTEMMQVDEVPSQDSPGAAESSISGGMGDKVEERGKEGPGTPTRSSVITSARELHYGREESAEQVPPPAEAGQAPTGRPEHPAPPPEKHLGIYSVLPNHKADAVLSMPSSVTSGLHVQPALAVSMDFSTYGGLLPQGFIQRELFSKLGELAVGMKSESRTIGEQCSVCGVELPDNEAVEQHRKLHSGMKTYGCELCGKRFLDSLRLRMHLLAHSAGAKAFVCDQCGAQFSKEDALETHRQTHTGTDMAVFCLLCGKRFQAQSALQQHMEVHAGVRSYICSECNRTFPSHTALKRHLRSHTGDHPYECEFCGSCFRDESTLKSHKRIHTGEKPYECNGCGKKFSLKHQLETHYRVHTGEKPFECKLCHQRSRDYSAMIKHLRTHNGASPYQCTICTEYCPSLSSMQKHMKGHKPEEIPPDWRIEKTYLYLCYV

244 NP_002125.3 NP_055525.3

>NP_002125.3

MSAEVETSEGVDESEKKNSGALEKENQMRMADLSELLKEGTKEAHDRAENTQFVKDFLKGNIKKELFKLATTALYFTYSALEEEMERNKDHPAFAPLYFPMELHRKEALTKDMEYFFGENWEEQVQCPKAAQKYVERIHYIGQNEPELLVAHAYTRYMGDLSGGQVLKKVAQRALKLPSTGEGTQFYLFENVDNAQQFKQLYRARMNALDLNMKTKERIVEEANKAFEYNMQIFNELDQAGSTLARETLEDGFPVHDGKGDMRKCPFYAAEQDKGALEGSSCPFRTAMAVLRKPSLQFILAAGVALAAGLLAWYYM

>NP_055525.3

MTGAEIESGAQVKPEKKPGEEVVGGAEIENDVPLVVRPKVRTQAQIMPGARPKNKSKVMPGASTKVETSAVGGARPKSKAKAIPVSRFKEEAQMWAQPRFGAERLSKTERNSQTNIIASPLVSTDSVLVAKTKYLSEDRELVNTDTESFPRRKAHYQAGFQPSFRSKEETNMGSWCCPRPTSKQEASPNSDFKWVDKSVSSLFWSGDEVTAKFHPGNRVKDSNRSMHMANQEANTMSRSQTNQELYIASSSGSEDESVKTPWFWARDKTNTWSGPREDPNSRSRFRSKKEVYVESSSGSEHEDHLESWFGAGKEAKFRSKMRAGKEANNRARHRAKREACIDFMPGSIDVIKKESCFWPEENANTFSRPMIKKEARARAMTKEEAKTKARARAKQEARSEEEALIGTWFWATDESSMADEASIESSLQVEDESIIGSWFWTEEEASMGTGASSKSRPRTDGERIGDSLFGAREKTSMKTGAEATSESILAADDEQVIIGSWFWAGEEVNQEAEEETIFGSWFWVIDAASVESGVGVSCESRTRSEEEEVIGPWFWSGEQVDIEAGIGEEARPGAEEETIFGSWFWAENQTYMDCRAETSCDTMQGAEEEEPIIGSWFWTRVEACVEGDVNSKSSLEDKEEAMIPCFGAKEEVSMKHGTGVRCRFMAGAEETNNKSCFWAEKEPCMYPAGGGSWKSRPEEEEDIVNSWFWSRKYTKPEAIIGSWLWATEESNIDGTGEKAKLLTEEETIINSWFWKEDEAISEATDREESRPEAEEGDIIGSWFWAGEEDRLEPAAETREEDRLAAEKEGIVGSWFGAREETIRREAGSCSKSSPKAEEEEVIIGSWFWEEEASPEAVAGVGFESKPGTEEEEITVGSWFWPEEEASIQAGSQAVEEMESETEEETIFGSWFWDGKEVSEEAGPCCVSKPEDDEEMIVESWFWSRDKAIKETGTVATCESKPENEEGAIVGSWFEAEDEVDNRTDNGSNCGSRTLADEDEAIVGSWFWAGDEAHFESNPSPVFRAICRSTCSVEQEPDPSRRPQSWEEVTVQFKPGPWGRVGFPSISPFRFPKEAASLFCEMFGGKPRNMVLSPEGEDQESLLQPDQPSPEFPFQYDPSYRSVQEIREHLRAKESTEPESSSCNCIQCELKIGSEEFEELLLLMEKIRDPFIHEISKIAMGMRSASQFTRDFIRDSGVVSLIETLLNYPSSRVRTSFLENMIRMAPPYPNLNIIQTYICKVCEETLAYSVDSPEQLSGIRMIRHLTTTTDYHTLVANYMSGFLSLLATGNAKTRFHVLKMLLNLSENLFMTKELLSAEAVSEFIGLFNREETNDNIQIVLAIFENIGNNIKKETVFSDDDFNIEPLISAFHKVEKFAKELQGKTDNQNDPEGDQEN

245 NP_002592.1 NP_067075.1

>NP_002592.1

MSAKDERAREILRGFKLNWMNLRDAETGKILWQGTEDLSVPGVEHEARVPKKILKCKAVSRELNFSSTEQMEKFRLEQKVYFKGQCLEEWFFEFGFVIPNSTNTWQSLIEAAPESQMMPASVLTGNVIIETKFFDDDLLVSTSRVRLFYV

>NP_067075.1

MDEDVLTTLKILIIGESGVGKSSLLLRFTDDTFDPELAATIGVDFKVKTISVDGNKAKLAIWDTAGQERFRTLTPSYYRGAQGVILVYDVTRRDTFVKLDNWLNELETYCTRNDIVNMLVGNKIDKENREVDRNEGLKFARKHSMLFIEASAKTCDGVQCAFEELVEKIIQTPGLWESENQNKGVKLSHREEGQGGGACGGYCSVL

246 P07910 NP_006839.2

>P07910

MASNVTNKTDPRSMNSRVFIGNLNTLVVKKSDVEAIFSKYGKIVGCSVHKGFAFVQYVNERNARAAVAGEDGRMIAGQVLDINLAAEPKVNRGKAGVKRSAAEMYGSVTEHPSPSPLLSSSFDLDYDFQRDYYDRMYSYPARVPPPPPIARAVVPSKRQRVSGNTSRRGKSGFNSKSGQRGSSKSGKLKGDDLQAIKKELTQIKQKVDSLLENLEKMEKEQSKQAVEMKNDKSEEEQSSSSVKKDETNVKMESEGGADDSAEEGDLLDDDDNEDRGDDQLELIKDDEKEAEEGEDDRDSANGEDDS

>NP_006839.2

MEAEAGGLEELTDEEMAALGKEELVRRLRREEAARLAALVQRGRLMQEVNRQLQGHLGEIRELKQLNRRLQAENRELRDLCCFLDSERQRGRRAARQWQLFGTQASRAVREDLGGCWQKLAELEGRQEELLRENLALKELCLALGEEWGPRGGPSGAGGSGAGPAPELALPPCGPRDLGDGSSSTGSVGSPDQLPLACSPDD

247 NP_003884.1 NP_796372.1

>NP_003884.1

MLDRDVGPTPMYPPTYLEPGIGRHTPYGNQTDYRIFELNKRLQNWTEECDNLWWDAFTTEFFEDDAMLTITFCLEDGPKRYTIGRTLIPRYFRSIFEGGATELYYVLKHPKEAFHSNFVSLDCDQGSMVTQHGKPMFTQVCVEGRLYLEFMFDDMMRIKTWHFSIRQHRELIPRSILAMHAQDPQMLDQLSKNITRCGLSNSTLNYLRLCVILEPMQELMSRHKTYSLSPRDCLKTCLFQKWQRMVAPPAEPTRQQPSKRRKRKMSGGSTMSSGGGNTNNSNSKKKSPASTFALSSQVPDVMVVGEPTLMGGEFGDEDERLITRLENTQFDAANGIDDEDSFNNSPALGANSPWNSKPPSSQESKSENPTSQASQ

>NP_796372.1

MLDGLKMEENFQSAIDTSASFSSLLGRAVSPKSVCEGCQRVILDRFLLRLNDSFWHEQCVQCASCKEPLETTCFYRDKKLYCKYDYEKLFAVKCGGCFEAIAPNEFVMRAQKSVYHLSCFCCCVCERQLQKGDEFVLKEGQLLCKGDYEKERELLSLVSPAASDSGKSDDEESLCKSAHGAGKGTAEEGKDHKRPKRPRTILTTQQRRAFKASFEVSSKPCRKVRETLAAETGLSVRVVQVWFQNQRAKMKKLARRQQQQQQDQQNTQRLSSAQTNGGGSAGMEGIMNPYTALPTPQQLLAIEQSVYSSDPFRQGLTPPQMPGDHMHPYGAEPLFHDLDSDDTSLSNLGDCFLATSEAGPLQSRVGNPIDHLYSMQNSYFTS

248 NP_002730.1 NP_003156.1

>NP_002730.1

MAGLGPGVGDSEGGPRPLFCRKGALRQKVVHEVKSHKFTARFFKQPTFCSHCTDFIWGIGKQGLQCQVCSFVVHRRCHEFVTFECPGAGKGPQTDDPRNKHKFRLHSYSSPTFCDHCGSLLYGLVHQGMKCSCCEMNVHRRCVRSVPSLCGVDHTERRGRLQLEIRAPTADEIHVTVGEARNLIPMDPNGLSDPYVKLKLIPDPRNLTKQKTRTVKATLNPVWNETFVFNLKPGDVERRLSVEVWDWDRTSRNDFMGAMSFGVSELLKAPVDGWYKLLNQEEGEYYNVPVADADNCSLLQKFEACNYPLELYERVRMGPSSSPIPSPSPSPTDPKRCFFGASPGRLHISDFSFLMVLGKGSFGKVMLAERRGSDELYAIKILKKDVIVQDDDVDCTLVEKRVLALGGRGPGGRPHFLTQLHSTFQTPDRLYFVMEYVTGGDLMYHIQQLGKFKEPHAAFYAAEIAIGLFFLHNQGIIYRDLKLDNVMLDAEGHIKITDFGMCKENVFPGTTTRTFCGTPDYIAPEIIAYQPYGKSVDWWSFGVLLYEMLAGQPPFDGEDEEELFQAIMEQTVTYPKSLSREAVAICKGFLTKHPGKRLGSGPDGEPTIRAHGFFRWIDWERLERLEIPPPFRPRPCGRSGENFDKFFTRAAPALTPPDRLVLASIDQADFQGFTYVNPDFVHPDARSPTSPVPVPVM

>NP_003156.1

MAPIGLKAVVGEKIMHDVIKKVKKKGEWKVLVVDQLSMRMLSSCCKMTDIMTEGITIVEDINKRREPLPSLEAVYLITPSEKSVHSLISDFKDPPTAKYRAAHVFFTDSCPDALFNELVKSRAAKVIKTLTEINIAFLPYESQVYSLDSADSFQSFYSPHKAQMKNPILERLAEQIATLCATLKEYPAVRYRGEYKDNALLAQLIQDKLDAYKADDPTMGEGPDKARSQLLILDRGFDPSSPVLHELTFQAMSYDLLPIENDVYKYETSGIGEARVKEVLLDEDDDLWIALRHKHIAEVSQEVTRSLKDFSSSKRMNTGEKTTMRDLSQMLKKMPQYQKELSKYSTHLHLAEDCMKHYQGTVDKLCRVEQDLAMGTDAEGEKIKDPMRAIVPILLDANVSTYDKIRIILLYIFLKNGITEENLNKLIQHAQIPPEDSEIITNMAHLGVPIVTDSTLRRRSKPERKERISEQTYQLSRWTPIIKDIMEDTIEDKLDTKHYPYISTRSSASFSTTAVSARYGHWHKNKAPGEYRSGPRLIIFILGGVSLNEMRCAYEVTQANGKWEVLIGSTHILTPTKFLMDLRHPDFRESSRVSFEDQAPTME

249 NP_002383.2 NP_003461.1

>NP_002383.2

MVRSRQMCNTNMSVPTDGAVTTSQIPASEQETLVRPKPLLLKLLKSVGAQKDTYTMKEVLFYLGQYIMTKRLYDEKQQHIVYCSNDLLGDLFGVPSFSVKEHRKIYTMIYRNLVVVNQQESSDSGTSVSENRCHLEGGSDQKDLVQELQEEKPSSSHLVSRPSTSSRRRAISETEENSDELSGERQRKRHKSDSISLSFDESLALCVIREICCERSSSSESTGTPSNPDLDAGVSEHSGDWLDQDSVSDQFSVEFEVESLDSEDYSLSEEGQELSDEDDEVYQVTVYQAGESDTDSFEEDPEISLADYWKCTSCNEMNPPLPSHCNRCWALRENWLPEDKGKDKGEISEKAKLENSTQAEEGFDVPDCKKTIVNDSRESCVEENDDKITQASQSQESEDYSQPSTSSSIIYSSQEDVKEFEREETQDKEESVESSLPLNAIEPCVICQGRPKNGCIVHGKTGHLMACFTCAKKLKKRNKPCPVCRQPIQMIVLTYFP

>NP_003461.1

MNHQQQQQQQKAGEQQLSEPEDMEMEAGDTDDPPRITQNPVINGNVALSDGHNTAEEDMEDDTSWRSEATFQFTVERFSRLSESVLSPPCFVRNLPWKIMVMPRFYPDRPHQKSVGFFLQCNAESDSTSWSCHAQAVLKIINYRDDEKSFSRRISHLFFHKENDWGFSNFMAWSEVTDPEKGFIDDDKVTFEVFVQADAPHGVAWDSKKHTGYVGLKNQGATCYMNSLLQTLFFTNQLRKAVYMMPTEGDDSSKSVPLALQRVFYELQHSDKPVGTKKLTKSFGWETLDSFMQHDVQELCRVLLDNVENKMKGTCVEGTIPKLFRGKMVSYIQCKEVDYRSDRREDYYDIQLSIKGKKNIFESFVDYVAVEQLDGDNKYDAGEHGLQEAEKGVKFLTLPPVLHLQLMRFMYDPQTDQNIKINDRFEFPEQLPLDEFLQKTDPKDPANYILHAVLVHSGDNHGGHYVVYLNPKGDGKWCKFDDDVVSRCTKEEAIEHNYGGHDDDLSVRHCTNAYMLVYIRESKLSEVLQAVTDHDIPQQLVERLQEEKRIEAQKRKERQEAHLYMQVQIVAEDQFCGHQGNDMYDEEKVKYTVFKVLKNSSLAEFVQSLSQTMGFPQDQIRLWPMQARSNGTKRPAMLDNEADGNKTMIELSDNENPWTIFLETVDPELAASGATLPKFDKDHDVMLFLKMYDPKTRSLNYCGHIYTPISCKIRDLLPVMCDRAGFIQDTSLILYEEVKPNLTERIQDYDVSLDKALDELMDGDIIVFQKDDPENDNSELPTAKEYFRDLYHRVDVIFCDKTIPNDPGFVVTLSNRMNYFQVAKTVAQRLNTDPMLLQFFKSQGYRDGPGNPLRHNYEGTLRDLLQFFKPRQPKKLYYQQLKMKITDFENRRSFKCIWLNSQFREEEITLYPDKHGCVRDLLEECKKAVELGEKASGKLRLLEIVSYKIIGVHQEDELLECLSPATSRTFRIEEIPLDQVDIDKENEMLVTVAHFHKEVFGTFGIPFLLRIHQGEHFREVMKRIQSLLDIQEKEFEKFKFAIVMTGRHQYINEDEYEVNLKDFEPQPGNMSHPRPWLGLDHFNKAPKRSRYTYLEKAIKIHN

250 NP_005634.1 NP_003185.1

>NP_005634.1

MDDAHESPSDKGGETGESDETAAVPGDPGATDTDGIPEETDGDADVDLKEAAAEEGELESQDVSDLTTVEREDSSLLNPAAKKLKIDTKEKKEKKQKVDEDEIQKMQILVSSFSEEQLNRYEMYRRSAFPKAAIKRLIQSITGTSVSQNVVIAMSGISKVFVGEVVEEALDVCEKWGEMPPLQPKHMREAVRRLKSKGQIPNSKHKKIIFF

>NP_003185.1

MDQNNSLPPYAQGLASPQGAMTPGIPIFSPMMPYGTGLTPQPIQNTNSLSILEEQQRQQQQQQQQQQQQQQQQQQQQQQQQQQQQQQQQQQQQQQAVAAAAVQQSTSQQATQGTSGQAPQLFHSQTLTTAPLPGTTPLYPSPMTPMTPITPATPASESSGIVPQLQNIVSTVNLGCKLDLKTIALRARNAEYNPKRFAAVIMRIREPRTTALIFSSGKMVCTGAKSEEQSRLAARKYARVVQKLGFPAKFLDFKIQNMVGSCDVKFPIRLEGLVLTHQQFSSYEPELFPGLIYRMIKPRIVLLIFVSGKVVLTGAKVRAEIYEAFENIYPILKGFRKTT

251 NP_005630.1 NP_001019265.1

>NP_005630.1

MVSESHHEALAAPPVTTVATVLPSNATEPASPGEGKEDAFSKLKEKFMNELHKIPLPPWALIAIAIVAVLLVLTCCFCICKKCLFKKKNKKKGKEKGGKNAINMKDVKDLGKTMKDQALKDDDAETGLTDGEEKEEPKEEEKLGKLQYSLDYDFQNNQLLVGIIQAAELPALDMGGTSDPYVKVFLLPDKKKKFETKVHRKTLNPVFNEQFTFKVPYSELGGKTLVMAVYDFDRFSKHDIIGEFKVPMNTVDFGHVTEEWRDLQSAEKEEQEKLGDICFSLRYVPTAGKLTVVILEAKNLKKMDVGGLSDPYVKIHLMQNGKRLKKKKTTIKKNTLNPYYNESFSFEVPFEQIQKVQVVVTVLDYDKIGKNDAIGKVFVGYNSTGAELRHWSDMLANPRRPIAQWHTLQVEEEVDAMLAVKK

>NP_001019265.1

MDSKESLTPGREENPSSVLAQERGDVMDFYKTLRGGATVKVSASSPSLAVASQSDSKQRRLLVDFPKGSVSNAQQPDLSKAVSLSMGLYMGETETKVMGNDLGFPQQGQISLSSGETDLKLLEESIANLNRSTSVPENPKSSASTAVSAAPTEKEFPKTHSDVSSEQQHLKGQTGTNGGNVKLYTTDQSTFDILQDLEFSSGSPGKETNESPWRSDLLIDENCLLSPLAGEDDSFLLEGNSNEDCKPLILPDTKPKIKDNGDLVLSSPSNVTLPQVKTEKEDFIELCTPGVIKQEKLGTVYCQASFPGANIIGNKMSAISVHGVSTSGGQMYHYDMNTASLSQQQDQKPIFNVIPPIPVGSENWNRCQGSGDDNLTSLGTLNFPGRTVFSNGYSSPSMRPDVSSPPSSSSTATTGPPPKLCLVCSDEASGCHYGVLTCGSCKVFFKRAVEGRQHNYLCAGRNDCIIDKIRRKNCPACRYRKCLQAGMNLEARKTKKKIKGIQQATTGVSQETSENPGNKTIVPATLPQLTPTLVSLLEVIEPEVLYAGYDSSVPDSTWRIMTTLNMLGGRQVIAAVKWAKAIPGFRNLHLDDQMTLLQYSWMFLMAFALGWRSYRQSSANLLCFAPDLIINEQRMTLPCMYDQCKHMLYVSSELHRLQVSYEEYLCMKTLLLLSSVPKDGLKSQELFDEIRMTYIKELGKAIVKREGNSSQNWQRFYQLTKLLDSMHEVVENLLNYCFQTFLDKTMSIEFPEMLAEIITNQIPKYSNGNIKKLLFHQK

252 NP_005391.1 NP_000433.3

>NP_005391.1

MVVFNGLLKIKICEAVSLKPTAWSLRHAVGPRPQTFLLDPYIALNVDDSRIGQTATKQKTNSPAWHDEFVTDVCNGRKIELAVFHDAPIGYDDFVANCTIQFEELLQNGSRHFEDWIDLEPEGRVYVIIDLSGSSGEAPKDNEERVFRERMRPRKRQGAVRRRVHQVNGHKFMATYLRQPTYCSHCRDFIWGVIGKQGYQCQVCTCVVHKRCHELIITKCAGLKKQETPDQVGSQRFSVNMPHKFGIHNYKVPTFCDHCGSLLWGLLRQGLQCKVCKMNVHRRCETNVAPNCGVDARGIAKVLADLGVTPDKITNSGQRRKKLIAGAESPQPASGSSPSEEDRSKSAPTSPCDQEIKELENNIRKALSFDNRGEEHRAASSPDGQLMSPGENGEVRQGQAKRLGLDEFNFIKVLGKGSFGKVMLAELKGKDEVYAVKVLKKDVILQDDDVDCTMTEKRILALARKHPYLTQLYCCFQTKDRLFFVMEYVNGGDLMFQIQRSRKFDEPRSRFYAAEVTSALMFLHQHGVIYRDLKLDNILLDAEGHCKLADFGMCKEGILNGVTTTTFCGTPDYIAPEILQELEYGPSVDWWALGVLMYEMMAGQPPFEADNEDDLFESILHDDVLYPVWLSKEAVSILKAFMTKNPHKRLGCVASQNGEDAIKQHPFFKEIDWVLLEQKKIKPPFKPRIKTKRDVNNFDQDFTREEPVLTLVDEAIVKQINQEEFKGFSYFGEDLMP

>NP_000433.3

MQPRWAQGATMWLGVLLTLLLCSSLEGQENSFTINSVDMKSLPDWTVQNGKNLTLQCFADVSTTSHVKPQHQMLFYKDDVLFYNISSMKSTESYFIPEVRIYDSGTYKCTVIVNNKEKTTAEYQLLVEGVPSPRVTLDKKEAIQGGIVRVNCSVPEEKAPIHFTIEKLELNEKMVKLKREKNSRDQNFVILEFPVEEQDRVLSFRCQARIISGIHMQTSESTKSELVTVTESFSTPKFHISPTGMIMEGAQLHIKCTIQVTHLAQEFPEIIIQKDKAIVAHNRHGNKAVYSVMAMVEHSGNYTCKVESSRISKVSSIVVNITELFSKPELESSFTHLDQGERLNLSCSIPGAPPANFTIQKEDTIVSQTQDFTKIASKSDSGTYICTAGIDKVVKKSNTVQIVVCEMLSQPRISYDAQFEVIKGQTIEVRCESISGTLPISYQLLKTSKVLENSTKNSNDPAVFKDNPTEDVEYQCVADNCHSHAKMLSEVLRVKVIAPVDEVQISILSSKVVESGEDIVLQCAVNEGSGPITYKFYREKEGKPFYQMTSNATQAFWTKQKASKEQEGEYYCTAFNRANHASSVPRSKILTVRVILAPWKKGLIAVVIIGVIIALLIIAAKCYFLRKAKAKQMPVEMSRPAVPLLNSNNEKMSDPNMEANSHYGHNDDVRNHAMKPINDNKEPLNSDVQYTEVQVSSAESHKDLGKKDTETVYSEVRKAVPDAVESRYSRTEGSLDGT

253 NP_055521.1 NP_000035.2

>NP_055521.1

MATAAETSASEPEAESKAGPKADGEEDEVKAARTRRKVLSRAVAAATYKTMGPAWDQQEEGVSESDGDEYAMASSAESSPGEYEWEYDEEEEKNQLEIERLEEQLSINVYDYNCHVDLIRLLRLEGELTKVRMARQKMSEIFPLTEELWLEWLHDEISMAQDGLDREHVYDLFEKAVKDYICPNIWLEYGQYSVGGIGQKGGLEKVRSVFERALSSVGLHMTKGLALWEAYREFESAIVEAARLEKVHSLFRRQLAIPLYDMEATFAEYEEWSEDPIPESVIQNYNKALQQLEKYKPYEEALLQAEAPRLAEYQAYIDFEMKIGDPARIQLIFERALVENCLVPDLWIRYSQYLDRQLKVKDLVLSVHNRAIRNCPWTVALWSRYLLAMERHGVDHQVISVTFEKALNAGFIQATDYVEIWQAYLDYLRRRVDFKQDSSKELEELRAAFTRALEYLKQEVEERFNESGDPSCVIMQNWARIEARLCNNMQKARELWDSIMTRGNAKYANMWLEYYNLERAHGDTQHCRKALHRAVQCTSDYPEHVCEVLLTMERTEGSLEDWDIAVQKTETRLARVNEQRMKAAEKEAALVQQEEEKAEQRKRARAEKKALKKKKKIRGPEKRGADEDDEKEWGDDEEEQPSKRRRVENSIPAAGETQNVEVAAGPAGKCAAVDVEPPSKQKEKAASLKRDMPKVLHDSSKDSITVFVSNLPYSMQEPDTKLRPLFEACGEVVQIRPIFSNRGDFRGYCYVEFKEEKSALQALEMDRKSVEGRPMFVSPCVDKSKNPDFKVFRYSTSLEKHKLFISGLPFSCTKEELEEICKAHGTVKDLRLVTNRAGKPKGLAYVEYENESQASQAVMKMDGMTIKENIIKVAISNPPQRKVPEKPETRKAPGGPMLLPQTYGARGKGRTQLSLLPRALQRPSAAAPQAENGPAAAPAVAAPAATEAPKMSNADFAKLFLRK

>NP_000035.2

MEVQLGLGRVYPRPPSKTYRGAFQNLFQSVREVIQNPGPRHPEAASAAPPGASLLLLQQQQQQQQQQQQQQQQQQQQQQQETSPRQQQQQQGEDGSPQAHRRGPTGYLVLDEEQQPSQPQSALECHPERGCVPEPGAAVAASKGLPQQLPAPPDEDDSAAPSTLSLLGPTFPGLSSCSADLKDILSEASTMQLLQQQQQEAVSEGSSSGRAREASGAPTSSKDNYLGGTSTISDNAKELCKAVSVSMGLGVEALEHLSPGEQLRGDCMYAPLLGVPPAVRPTPCAPLAECKGSLLDDSAGKSTEDTAEYSPFKGGYTKGLEGESLGCSGSAAAGSSGTLELPSTLSLYKSGALDEAAAYQSRDYYNFPLALAGPPPPPPPPHPHARIKLENPLDYGSAWAAAAAQCRYGDLASLHGAGAAGPGSGSPSAAASSSWHTLFTAEEGQLYGPCGGGGGGGGGGGGGGGGGGGGGGGEAGAVAPYGYTRPPQGLAGQESDFTAPDVWYPGGMVSRVPYPSPTCVKSEMGPWMDSYSGPYGDMRLETARDHVLPIDYYFPPQKTCLICGDEASGCHYGALTCGSCKVFFKRAAEGKQKYLCASRNDCTIDKFRRKNCPSCRLRKCYEAGMTLGARKLKKLGNLKLQEEGEASSTTSPTEETTQKLTVSHIEGYECQPIFLNVLEAIEPGVVCAGHDNNQPDSFAALLSSLNELGERQLVHVVKWAKALPGFRNLHVDDQMAVIQYSWMGLMVFAMGWRSFTNVNSRMLYFAPDLVFNEYRMHKSRMYSQCVRMRHLSQEFGWLQITPQEFLCMKALLLFSIIPVDGLKNQKFFDELRMNYIKELDRIIACKRKNPTSCSRRFYQLTKLLDSVQPIARELHQFTFDLLIKSHMVSVDFPEMMAEIISVQVPKILSGKVKPIYFHTQ

254 NP_060614.2 NP_000375.2

>NP_060614.2

MGIKFLEVIKPFCAVLPEIQKPERKIQFREKVLWTAITLFIFLVCCQIPLFGIMSSDSADPFYWMRVILASNRGTLMELGISPIVTSGLIMQLLAGAKIIEVGDTPKDRALFNGAQKLFGMIITIGQAIVYVMTGMYGDPAEMGAGICLLIIIQLFVAGLIVLLLDELLQKGYGLGSGISLFIATNICETIVWKAFSPTTINTGRGTEFEGAVIALFHLLATRTDKVRALREAFYRQNLPNLMNLIATVFVFAVVIYFQGFRVDLPIKSARYRGQYSSYPIKLFYTSNIPIILQSALVSNLYVISQMLSVRFSGNFLVNLLGQWADVSGGGPARSYPVGGLCYYLSPPESMGAIFEDPVHVVVYIIFMLGSCAFFSKTWIEVSGSSAKDVAKQLKEQQMVMRGHRDTSMVHELNRYIPTAAAFGGLCIGALSVLADFLGAIGSGTGILLAVTIIYQYFEIFVKEQAEVGGMGALFF

>NP_000375.2

MDPPRPALLALLALPALLLLLLAGARAEEEMLENVSLVCPKDATRFKHLRKYTYNYEAESSSGVPGTADSRSATRINCKVELEVPQLCSFILKTSQCTLKEVYGFNPEGKALLKKTKNSEEFAAAMSRYELKLAIPEGKQVFLYPEKDEPTYILNIKRGIISALLVPPETEEAKQVLFLDTVYGNCSTHFTVKTRKGNVATEISTERDLGQCDRFKPIRTGISPLALIKGMTRPLSTLISSSQSCQYTLDAKRKHVAEAICKEQHLFLPFSYKNKYGMVAQVTQTLKLEDTPKINSRFFGEGTKKMGLAFESTKSTSPPKQAEAVLKTLQELKKLTISEQNIQRANLFNKLVTELRGLSDEAVTSLLPQLIEVSSPITLQALVQCGQPQCSTHILQWLKRVHANPLLIDVVTYLVALIPEPSAQQLREIFNMARDQRSRATLYALSHAVNNYHKTNPTGTQELLDIANYLMEQIQDDCTGDEDYTYLILRVIGNMGQTMEQLTPELKSSILKCVQSTKPSLMIQKAAIQALRKMEPKDKDQEVLLQTFLDDASPGDKRLAAYLMLMRSPSQADINKIVQILPWEQNEQVKNFVASHIANILNSEELDIQDLKKLVKEALKESQLPTVMDFRKFSRNYQLYKSVSLPSLDPASAKIEGNLIFDPNNYLPKESMLKTTLTAFGFASADLIEIGLEGKGFEPTLEALFGKQGFFPDSVNKALYWVNGQVPDGVSKVLVDHFGYTKDDKHEQDMVNGIMLSVEKLIKDLKSKEVPEARAYLRILGEELGFASLHDLQLLGKLLLMGARTLQGIPQMIGEVIRKGSKNDFFLHYIFMENAFELPTGAGLQLQISSSGVIAPGAKAGVKLEVANMQAELVAKPSVSVEFVTNMGIIIPDFARSGVQMNTNFFHESGLEAHVALKAGKLKFIIPSPKRPVKLLSGGNTLHLVSTTKTEVIPPLIENRQSWSVCKQVFPGLNYCTSGAYSNASSTDSASYYPLTGDTRLELELRPTGEIEQYSVSATYELQREDRALVDTLKFVTQAEGAKQTEATMTFKYNRQSMTLSSEVQIPDFDVDLGTILRVNDESTEGKTSYRLTLDIQNKKITEVALMGHLSCDTKEERKIKGVISIPRLQAEARSEILAHWSPAKLLLQMDSSATAYGSTVSKRVAWHYDEEKIEFEWNTGTNVDTKKMTSNFPVDLSDYPKSLHMYANRLLDHRVPQTDMTFRHVGSKLIVAMSSWLQKASGSLPYTQTLQDHLNSLKEFNLQNMGLPDFHIPENLFLKSDGRVKYTLNKNSLKIEIPLPFGGKSSRDLKMLETVRTPALHFKSVGFHLPSREFQVPTFTIPKLYQLQVPLLGVLDLSTNVYSNLYNWSASYSGGNTSTDHFSLRARYHMKADSVVDLLSYNVQGSGETTYDHKNTFTLSCDGSLRHKFLDSNIKFSHVEKLGNNPVSKGLLIFDASSSWGPQMSASVHLDSKKKQHLFVKEVKIDGQFRVSSFYAKGTYGLSCQRDPNTGRLNGESNLRFNSSYLQGTNQITGRYEDGTLSLTSTSDLQSGIIKNTASLKYENYELTLKSDTNGKYKNFATSNKMDMTFSKQNALLRSEYQADYESLRFFSLLSGSLNSHGLELNADILGTDKINSGAHKATLRIGQDGISTSATTNLKCSLLVLENELNAELGLSGASMKLTTNGRFREHNAKFSLDGKAALTELSLGSAYQAMILGVDSKNIFNFKVSQEGLKLSNDMMGSYAEMKFDHTNSLNIAGLSLDFSSKLDNIYSSDKFYKQTVNLQLQPYSLVTTLNSDLKYNALDLTNNGKLRLEPLKLHVAGNLKGAYQNNEIKHIYAISSAALSASYKADTVAKVQGVEFSHRLNTDIAGLASAIDMSTNYNSDSLHFSNVFRSVMAPFTMTIDAHTNGNGKLALWGEHTGQLYSKFLLKAEPLAFTFSHDYKGSTSHHLVSRKSISAALEHKVSALLTPAEQTGTWKLKTQFNNNEYSQDLDAYNTKDKIGVELTGRTLADLTLLDSPIKVPLLLSEPINIIDALEMRDAVEKPQEFTIVAFVKYDKNQDVHSINLPFFETLQEYFERNRQTIIVVLENVQRNLKHINIDQFVRKYRAALGKLPQQANDYLNSFNWERQVSHAKEKLTALTKKYRITENDIQIALDDAKINFNEKLSQLQTYMIQFDQYIKDSYDLHDLKIAIANIIDEIIEKLKSLDEHYHIRVNLVKTIHDLHLFIENIDFNKSGSSTASWIQNVDTKYQIRIQIQEKLQQLKRHIQNIDIQHLAGKLKQHIEAIDVRVLLDQLGTTISFERINDVLEHVKHFVINLIGDFEVAEKINAFRAKVHELIERYEVDQQIQVLMDKLVELAHQYKLKETIQKLSNVLQQVKIKDYFEKLVGFIDDAVKKLNELSFKTFIEDVNKFLDMLIKKLKSFDYHQFVDETNDKIREVTQRLNGEIQALELPQKAEALKLFLEETKATVAVYLESLQDTKITLIINWLQEALSSASLAHMKAKFRETLEDTRDRMYQMDIQQELQRYLSLVGQVYSTLVTYISDWWTLAAKNLTDFAEQYSIQDWAKRMKALVEQGFTVPEIKTILGTMPAFEVSLQALQKATFQTPDFIVPLTDLRIPSVQINFKDLKNIKIPSRFSTPEFTILNTFHIPSFTIDFVEMKVKIIRTIDQMLNSELQWPVPDIYLRDLKVEDIPLARITLPDFRLPEIAIPEFIIPTLNLNDFQVPDLHIPEFQLPHISHTIEVPTFGKLYSILKIQSPLFTLDANADIGNGTTSANEAGIAASITAKGESKLEVLNFDFQANAQLSNPKINPLALKESVKFSSKYLRTEHGSEMLFFGNAIEGKSNTVASLHTEKNTLELSNGVIVKINNQLTLDSNTKYFHKLNIPKLDFSSQADLRNEIKTLLKAGHIAWTSSGKGSWKWACPRFSDEGTHESQISFTIEGPLTSFGLSNKINSKHLRVNQNLVYESGSLNFSKLEIQSQVDSQHVGHSVLTAKGMALFGEGKAEFTGRHDAHLNGKVIGTLKNSLFFSAQPFEITASTNNEGNLKVRFPLRLTGKIDFLNNYALFLSPSAQQASWQVSARFNQYKYNQNFSAGNNENIMEAHVGINGEANLDFLNIPLTIPEMRLPYTIITTPPLKDFSLWEKTGLKEFLKTTKQSFDLSVKAQYKKNKHRHSITNPLAVLCEFISQSIKSFDRHFEKNRNNALDFVTKSYNETKIKFDKYKAEKSHDELPRTFQIPGYTVPVVNVEVSPFTIEMSAFGYVFPKAVSMPSFSILGSDVRVPSYTLILPSLELPVLHVPRNLKLSLPDFKELCTISHIFIPAMGNITYDFSFKSSVITLNTNAELFNQSDIVAHLLSSSSSVIDALQYKLEGTTRLTRKRGLKLATALSLSNKFVEGSHNSTVSLTTKNMEVSVATTTKAQIPILRMNFKQELNGNTKSKPTVSSSMEFKYDFNSSMLYSTAKGAVDHKLSLESLTSYFSIESSTKGDVKGSVLSREYSGTIASEANTYLNSKSTRSSVKLQGTSKIDDIWNLEVKENFAGEATLQRIYSLWEHSTKNHLQLEGLFFTNGEHTSKATLELSPWQMSALVQVHASQPSSFHDFPDLGQEVALNANTKNQKIRWKNEVRIHSGSFQSQVELSNDQEKAHLDIAGSLEGHLRFLKNIILPVYDKSLWDFLKLDVTTSIGRRQHLRVSTAFVYTKNPNGYSFSIPVKVLADKFIIPGLKLNDLNSVLVMPTFHVPFTDLQVPSCKLDFREIQIYKKLRTSSFALNLPTLPEVKFPEVDVLTKYSQPEDSLIPFFEITVPESQLTVSQFTLPKSVSDGIAALDLNAVANKIADFELPTIIVPEQTIEIPSIKFSVPAGIVIPSFQALTARFEVDSPVYNATWSASLKNKADYVETVLDSTCSSTVQFLEYELNVLGTHKIEDGTLASKTKGTFAHRDFSAEYEEDGKYEGLQEWEGKAHLNIKSPAFTDLHLRYQKDKKGISTSAASPAVGTVGMDMDEDDDFSKWNFYYSPQSSPDKKLTIFKTELRVRESDEETQIKVNWEEEAASGLLTSLKDNVPKATGVLYDYVNKYHWEHTGLTLREVSSKLRRNLQNNAEWVYQGAIRQIDDIDVRFQKAASGTTGTYQEWKDKAQNLYQELLTQEGQASFQGLKDNVFDGLVRVTQEFHMKVKHLIDSLIDFLNFPRFQFPGKPGIYTREELCTMFIREVGTVLSQVYSKVHNGSEILFSYFQDLVITLPFELRKHKLIDVISMYRELLKDLSKEAQEVFKAIQSLKTTEVLRNLQDLLQFIFQLIEDNIKQLKEMKFTYLINYIQDEINTIFSDYIPYVFKLLKENLCLNLHKFNEFIQNELQEASQELQQIHQYIMALREEYFDPSIVGWTVKYYELEEKIVSLIKNLLVALKDFHSEYIVSASNFTSQLSSQVEQFLHRNIQEYLSILTDPDGKGKEKIAELSATAQEIIKSQAIATKKIISDYHQQFRYKLQDFSDQLSDYYEKFIAESKRLIDLSIQNYHTFLIYITELLKKLQSTTVMNPYMKLAPGELTIIL

255 NP_005891.1 NP_001619.1

>NP_005891.1

MNVTSLFSFTSPAVKRLLGWKQGDEEEKWAEKAVDALVKKLKKKKGAMEELEKALSCPGQPSNCVTIPRSLDGRLQVSHRKGLPHVIYCRVWRWPDLQSHHELKPLECCEFPFGSKQKEVCINPYHYKRVESPVLPPVLVPRHSEYNPQHSLLAQFRNLGQNEPHMPLNATFPDSFQQPNSHPFPHSPNSSYPNSPGSSSSTYPHSPTSSDPGSPFQMPADTPPPAYLPPEDPMTQDGSQPMDTNMMAPPLPSEINRGDVQAVAYEEPKHWCSIVYYELNNRVGEAFHASSTSVLVDGFTDPSNNKNRFCLGLLSNVNRNSTIENTRRHIGKGVHLYYVGGEVYAECLSDSSIFVQSRNCNYHHGFHPTTVCKIPSGCSLKIFNNQEFAQLLAQSVNHGFETVYELTKMCTIRMSFVKGWGAEYHRQDVTSTPCWIEIHLHGPLQWLDKVLTQMGSPHNPISSVS

>NP_001619.1

MASRLLLNNGAKMPILGLGTWKSPPGQVTEAVKVAIDVGYRHIDCAHVYQNENEVGVAIQEKLREQVVKREELFIVSKLWCTYHEKGLVKGACQKTLSDLKLDYLDLYLIHWPTGFKPGKEFFPLDESGNVVPSDTNILDTWAAMEELVDEGLVKAIGISNFNHLQVEMILNKPGLKYKPAVNQIECHPYLTQEKLIQYCQSKGIVVTAYSPLGSPDRPWAKPEDPSLLEDPRIKAIAAKHNKTTAQVLIRFPMQRNLVVIPKSVTPERIAENFKVFDFELSSQDMTTLLSYNRNWRVCALLSCTSHKDYPFHEEF

256 NP_004963.1 NP_003143.2

>NP_004963.1

MGMACLTMTEMEGTSTSSIYQNGDISGNANSMKQIDPVLQVYLYHSLGKSEADYLTFPSGEYVAEEICIAASKACGITPVYHNMFALMSETERIWYPPNHVFHIDESTRHNVLYRIRFYFPRWYCSGSNRAYRHGISRGAEAPLLDDFVMSYLFAQWRHDFVHGWIKVPVTHETQEECLGMAVLDMMRIAKENDQTPLAIYNSISYKTFLPKCIRAKIQDYHILTRKRIRYRFRRFIQQFSQCKATARNLKLKYLINLETLQSAFYTEKFEVKEPGSGPSGEEIFATIIITGNGGIQWSRGKHKESETLTEQDLQLYCDFPNIIDVSIKQANQEGSNESRVVTIHKQDGKNLEIELSSLREALSFVSLIDGYYRLTADAHHYLCKEVAPPAVLENIQSNCHGPISMDFAISKLKKAGNQTGLYVLRCSPKDFNKYFLTFAVERENVIEYKHCLITKNENEEYNLSGTKKNFSSLKDLLNCYQMETVRSDNIIFQFTKCCPPKPKDKSNLLVFRTNGVSDVPTSPTLQRPTHMNQMVFHKIRNEDLIFNESLGQGTFTKIFKGVRREVGDYGQLHETEVLLKVLDKAHRNYSESFFEAASMMSKLSHKHLVLNYGVCVCGDENILVQEFVKFGSLDTYLKKNKNCINILWKLEVAKQLAWAMHFLEENTLIHGNVCAKNILLIREEDRKTGNPPFIKLSDPGISITVLPKDILQERIPWVPPECIENPKNLNLATDKWSFGTTLWEICSGGDKPLSALDSQRKLQFYEDRHQLPAPKWAELANLINNCMDYEPDFRPSFRAIIRDLNSLFTPDYELLTENDMLPNMRIGALGFSGAFEDRDPTQFEERHLKFLQQLGKGNFGSVEMCRYDPLQDNTGEVVAVKKLQHSTEEHLRDFEREIEILKSLQHDNIVKYKGVCYSAGRRNLKLIMEYLPYGSLRDYLQKHKERIDHIKLLQYTSQICKGMEYLGTKRYIHRDLATRNILVENENRVKIGDFGLTKVLPQDKEYYKVKEPGESPIFWYAPESLTESKFSVASDVWSFGVVLYELFTYIEKSKSPPAEFMRMIGNDKQGQMIVFHLIELLKNNGRLPRPDGCPDEIYMIMTECWNNNVNQRPSFRDLALRVDQIRDNMAG

>NP_003143.2

MAGWIQAQQLQGDALRQMQVLYGQHFPIEVRHYLAQWIESQPWDAIDLDNPQDRAQATQLLEGLVQELQKKAEHQVGEDGFLLKIKLGHYATQLQKTYDRCPLELVRCIRHILYNEQRLVREANNCSSPAGILVDAMSQKHLQINQTFEELRLVTQDTENELKKLQQTQEYFIIQYQESLRIQAQFAQLAQLSPQERLSRETALQQKQVSLEAWLQREAQTLQQYRVELAEKHQKTLQLLRKQQTIILDDELIQWKRRQQLAGNGGPPEGSLDVLQSWCEKLAEIIWQNRQQIRRAEHLCQQLPIPGPVEEMLAEVNATITDIISALVTSTFIIEKQPPQVLKTQTKFAATVRLLVGGKLNVHMNPPQVKATIISEQQAKSLLKNENTRNECSGEILNNCCVMEYHQATGTLSAHFRNMSLKRIKRADRRGAESVTEEKFTVLFESQFSVGSNELVFQVKTLSLPVVVIVHGSQDHNATATVLWDNAFAEPGRVPFAVPDKVLWPQLCEALNMKFKAEVQSNRGLTKENLVFLAQKLFNNSSSHLEDYSGLSVSWSQFNRENLPGWNYTFWQWFDGVMEVLKKHHKPHWNDGAILGFVNKQQAHDLLINKPDGTFLLRFSDSEIGGITIAWKFDSPERNLWNLKPFTTRDFSIRSLADRLGDLSYLIYVFPDRPKDEVFSKYYTPVLAKAVDGYVKPQIKQVVPEFVNASADAGGSSATYMDQAPSPAVCPQAPYNMYPQNPDHVLDQDGEFDLDETMDVARHVEELLRRPMDSLDSRLSPPAGLFTSARGSLS

257 NP_002736.3 NP_066554.2

>NP_002736.3

MAAAAAAGAGPEMVRGQVFDVGPRYTNLSYIGEGAYGMVCSAYDNVNKVRVAIKKISPFEHQTYCQRTLREIKILLRFRHENIIGINDIIRAPTIEQMKDVYIVQDLMETDLYKLLKTQHLSNDHICYFLYQILRGLKYIHSANVLHRDLKPSNLLLNTTCDLKICDFGLARVADPDHDHTGFLTEYVATRWYRAPEIMLNSKGYTKSIDIWSVGCILAEMLSNRPIFPGKHYLDQLNHILGILGSPSQEDLNCIINLKARNYLLSLPHKNKVPWNRLFPNADSKALDLLDKMLTFNPHKRIEVEQALAHPYLEQYYDPSDEPIAEAPFKFDMELDDLPKEKLKELIFEETARFQPGYRS

>NP_066554.2

MMSFGGADALLGAPFAPLHGGGSLHYALARKGGAGGTRSAAGSSSGFHSWTRTSVSSVSASPSRFRGAGAASSTDSLDTLSNGPEGCMVAVATSRSEKEQLQALNDRFAGYIDKVRQLEAHNRSLEGEAAALRQQQAGRSAMGELYEREVREMRGAVLRLGAARGQLRLEQEHLLEDIAHVRQRLDDEARQREEAEAAARALARFAQEAEAARVDLQKKAQALQEECGYLRRHHQEEVGELLGQIQGSGAAQAQMQAETRDALKCDVTSALREIRAQLEGHAVQSTLQSEEWFRVRLDRLSEAAKVNTDAMRSAQEEITEYRRQLQARTTELEALKSTKDSLERQRSELEDRHQADIASYQEAIQQLDAELRNTKWEMAAQLREYQDLLNVKMALDIEIAAYRKLLEGEECRIGFGPIPFSLPEGLPKIPSVSTHIKVKSEEKIKVVEKSEKETVIVEEQTEETQVTEEVTEEEEKEAKEEEGKEEEGGEEEEAEGGEEETKSPPAEEAASPEKEAKSPVKEEAKSPAEAKSPEKEEAKSPAEVKSPEKAKSPAKEEAKSPPEAKSPEKEEAKSPAEVKSPEKAKSPAKEEAKSPAEAKSPEKAKSPVKEEAKSPAEAKSPVKEEAKSPAEVKSPEKAKSPTKEEAKSPEKAKSPEKEEAKSPEKAKSPVKAEAKSPEKAKSPVKAEAKSPEKAKSPVKEEAKSPEKAKSPVKEEAKSPEKAKSPVKEEAKTPEKAKSPVKEEAKSPEKAKSPEKAKTLDVKSPEAKTPAKEEARSPADKFPEKAKSPVKEEVKSPEKAKSPLKEDAKAPEKEIPKKEEVKSPVKEEEKPQEVKVKEPPKKAEEEKAPATPKTEEKKDSKKEEAPKKEAPKPKVEEKKEPAVEKPKESKVEAKKEEAEDKKKVPTPEKEAPAKVEVKEDAKPKEKTEVAKKEPDDAKAKEPSKPAEKKEAAPEKKDTKEEKAKKPEEKPKTEAKAKEDDKTLSKEPSKPKAEKAEKSSSTDQKDSKPPEKATEDKAAKGK

258 NP_003178.1 NP_005924.2

>NP_003178.1

MESGKTASPKSMPKDAQMMAQILKDMGITEYEPRVINQMLEFAFRYVTTILDDAKIYSSHAKKATVDADDVRLAIQCRADQSFTSPPPRDFLLDIARQRNQTPLPLIKPYSGPRLPPDRYCLTAPNYRLKSLQKKASTSAGRITVPRLSVGSVTSRPSTPTLGTPTPQTMSVSTKVGTPMSLTGQRFTVQMPTSQSPAVKASIPATSAVQNVLINPSLIGSKNILITTNMMSSQNTANESSNALKRKREDDDDDDDDDDDYDNL

>NP_005924.2

MAHSCRWRFPARPGTTGGGGGGGRRGLGGAPRQRVPALLLPPGPPVGGGGPGAPPSPPAVAAAAAAAGSSGAGVPGGAAAASAASSSSASSSSSSSSSASSGPALLRVGPGFDAALQVSAAIGTNLRRFRAVFGESGGGGGSGEDEQFLGFGSDEEVRVRSPTRSPSVKTSPRKPRGRPRSGSDRNSAILSDPSVFSPLNKSETKSGDKIKKKDSKSIEKKRGRPPTFPGVKIKITHGKDISELPKGNKEDSLKKIKRTPSATFQQATKIKKLRAGKLSPLKSKFKTGKLQIGRKGVQIVRRRGRPPSTERIKTPSGLLINSELEKPQKVRKDKEGTPPLTKEDKTVVRQSPRRIKPVRIIPSSKRTDATIAKQLLQRAKKGAQKKIEKEAAQLQGRKVKTQVKNIRQFIMPVVSAISSRIIKTPRRFIEDEDYDPPIKIARLESTPNSRFSAPSCGSSEKSSAASQHSSQMSSDSSRSSSPSVDTSTDSQASEEIQVLPEERSDTPEVHPPLPISQSPENESNDRRSRRYSVSERSFGSRTTKKLSTLQSAPQQQTSSSPPPPLLTPPPPLQPASSISDHTPWLMPPTIPLASPFLPASTAPMQGKRKSILREPTFRWTSLKHSRSEPQYFSSAKYAKEGLIRKPIFDNFRPPPLTPEDVGFASGFSASGTAASARLFSPLHSGTRFDMHKRSPLLRAPRFTPSEAHSRIFESVTLPSNRTSAGTSSSGVSNRKRKRKVFSPIRSEPRSPSHSMRTRSGRLSSSELSPLTPPSSVSSSLSISVSPLATSALNPTFTFPSHSLTQSGESAEKNQRPRKQTSAPAEPFSSSSPTPLFPWFTPGSQTERGRNKDKAPEELSKDRDADKSVEKDKSRERDREREKENKRESRKEKRKKGSEIQSSSALYPVGRVSKEKVVGEDVATSSSAKKATGRKKSSSHDSGTDITSVTLGDTTAVKTKILIKKGRGNLEKTNLDLGPTAPSLEKEKTLCLSTPSSSTVKHSTSSIGSMLAQADKLPMTDKRVASLLKKAKAQLCKIEKSKSLKQTDQPKAQGQESDSSETSVRGPRIKHVCRRAAVALGRKRAVFPDDMPTLSALPWEEREKILSSMGNDDKSSIAGSEDAEPLAPPIKPIKPVTRNKAPQEPPVKKGRRSRRCGQCPGCQVPEDCGVCTNCLDKPKFGGRNIKKQCCKMRKCQNLQWMPSKAYLQKQAKAVKKKEKKSKTSEKKDSKESSVVKNVVDSSQKPTPSAREDPAPKKSSSEPPPRKPVEEKSEEGNVSAPGPESKQATTPASRKSSKQVSQPALVIPPQPPTTGPPRKEVPKTTPSEPKKKQPPPPESGPEQSKQKKVAPRPSIPVKQKPKEKEKPPPVNKQENAGTLNILSTLSNGNSSKQKIPADGVHRIRVDFKEDCEAENVWEMGGLGILTSVPITPRVVCFLCASSGHVEFVYCQVCCEPFHKFCLEENERPLEDQLENWCCRRCKFCHVCGRQHQATKQLLECNKCRNSYHPECLGPNYPTKPTKKKKVWICTKCVRCKSCGSTTPGKGWDAQWSHDFSLCHDCAKLFAKGNFCPLCDKCYDDDDYESKMMQCGKCDRWVHSKCENLSDEMYEILSNLPESVAYTCVNCTERHPAEWRLALEKELQISLKQVLTALLNSRTTSHLLRYRQAAKPPDLNPETEESIPSRSSPEGPDPPVLTEVSKQDDQQPLDLEGVKRKMDQGNYTSVLEFSDDIVKIIQAAINSDGGQPEIKKANSMVKSFFIRQMERVFPWFSVKKSRFWEPNKVSSNSGMLPNAVLPPSLDHNYAQWQEREENSHTEQPPLMKKIIPAPKPKGPGEPDSPTPLHPPTPPILSTDRSREDSPELNPPPGIEDNRQCALCLTYGDDSANDAGRLLYIGQNEWTHVNCALWSAEVFEDDDGSLKNVHMAVIRGKQLRCEFCQKPGATVGCCLTSCTSNYHFMCSRAKNCVFLDDKKVYCQRHRDLIKGEVVPENGFEVFRRVFVDFEGISLRRKFLNGLEPENIHMMIGSMTIDCLGILNDLSDCEDKLFPIGYQCSRVYWSTTDARKRCVYTCKIVECRPPVVEPDINSTVEHDENRTIAHSPTSFTESSSKESQNTAEIISPPSPDRPPHSQTSGSCYYHVISKVPRIRTPSYSPTQRSPGCRPLPSAGSPTPTTHEIVTVGDPLLSSGLRSIGSRRHSTSSLSPQRSKLRIMSPMRTGNTYSRNNVSSVSTTGTATDLESSAKVVDHVLGPLNSSTSLGQNTSTSSNLQRTVVTVGNKNSHLDGSSSSEMKQSSASDLVSKSSSLKGEKTKVLSSKSSEGSAHNVAYPGIPKLAPQVHNTTSRELNVSKIGSFAEPSSVSFSSKEALSFPHLHLRGQRNDRDQHTDSTQSANSSPDEDTEVKTLKLSGMSNRSSIINEHMGSSSRDRRQKGKKSCKETFKEKHSSKSFLEPGQVTTGEEGNLKPEFMDEVLTPEYMGQRPCNNVSSDKIGDKGLSMPGVPKAPPMQVEGSAKELQAPRKRTVKVTLTPLKMENESQSKNALKESSPASPLQIESTSPTEPISASENPGDGPVAQPSPNNTSCQDSQSNNYQNLPVQDRNLMLPDGPKPQEDGSFKRRYPRRSARARSNMFFGLTPLYGVRSYGEEDIPFYSSSTGKKRGKRSAEGQVDGADDLSTSDEDDLYYYNFTRTVISSGGEERLASHNLFREEEQCDLPKISQLDGVDDGTESDTSVTATTRKSSQIPKRNGKENGTENLKIDRPEDAGEKEHVTKSSVGHKNEPKMDNCHSVSRVKTQGQDSLEAQLSSLESSRRVHTSTPSDKNLLDTYNTELLKSDSDNNNSDDCGNILPSDIMDFVLKNTPSMQALGESPESSSSELLNLGEGLGLDSNREKDMGLFEVFSQQLPTTEPVDSSVSSSISAEEQFELPLELPSDLSVLTTRSPTVPSQNPSRLAVISDSGEKRVTITEKSVASSESDPALLSPGVDPTPEGHMTPDHFIQGHMDADHISSPPCGSVEQGHGNNQDLTRNSSTPGLQVPVSPTVPIQNQKYVPNSTDSPGPSQISNAAVQTTPPHLKPATEKLIVVNQNMQPLYVLQTLPNGVTQKIQLTSSVSSTPSVMETNTSVLGPMGGGLTLTTGLNPSLPTSQSLFPSASKGLLPMSHHQHLHSFPAATQSSFPPNISNPPSGLLIGVQPPPDPQLLVSESSQRTDLSTTVATPSSGLKKRPISRLQTRKNKKLAPSSTPSNIAPSDVVSNMTLINFTPSQLPNHPSLLDLGSLNTSSHRTVPNIIKRSKSSIMYFEPAPLLPQSVGGTAATAAGTSTISQDTSHLTSGSVSGLASSSSVLNVVSMQTTTTPTSSASVPGHVTLTNPRLLGTPDIGSISNLLIKASQQSLGIQDQPVALPPSSGMFPQLGTSQTPSTAAITAASSICVLPSTQTTGITAASPSGEADEHYQLQHVNQLLASKTGIHSSQRDLDSASGPQVSNFTQTVDAPNSMGLEQNKALSSAVQASPTSPGGSPSSPSSGQRSASPSVPGPTKPKPKTKRFQLPLDKGNGKKHKVSHLRTSSSEAHIPDQETTSLTSGTGTPGAEAEQQDTASVEQSSQKECGQPAGQVAVLPEVQVTQNPANEQESAEPKTVEEEESNFSSPLMLWLQQEQKRKESITEKKPKKGLVFEISSDDGFQICAESIEDAWKSLTDKVQEARSNARLKQLSFAGVNGLRMLGILHDAVVFLIEQLSGAKHCRNYKFRFHKPEEANEPPLNPHGSARAEVHLRKSAFDMFNFLASKHRQPPEYNPNDEEEEEVQLKSARRATSMDLPMPMRFRHLKKTSKEAVGVYRSPIHGRGLFCKRNIDAGEMVIEYAGNVIRSIQTDKREKYYDSKGIGCYMFRIDDSEVVDATMHGNAARFINHSCEPNCYSRVINIDGQKHIVIFAMRKIYRGEELTYDYKFPIEDASNKLPCNCGAKKCRKFLN

259 NP_001319.1 NP_003480.2

>NP_001319.1

MGSSHLLNKGLPLGVRPPIMNGPLHPRPLVALLDGRDCTVEMPILKDVATVAFCDAQSTQEIHEKVLNEAVGALMYHTITLTREDLEKFKALRIIVRIGSGFDNIDIKSAGDLGIAVCNVPAASVEETADSTLCHILNLYRRATWLHQALREGTRVQSVEQIREVASGAARIRGETLGIIGLGRVGQAVALRAKAFGFNVLFYDPYLSDGVERALGLQRVSTLQDLLFHSDCVTLHCGLNEHNHHLINDFTVKQMRQGAFLVNTARGGLVDEKALAQALKEGRIRGAALDVHESEPFSFSQGPLKDAPNLICTPHAAWYSEQASIEMREEAAREIRRAITGRIPDSLKNCVNKDHLTAATHWASMDPAVVHPELNGAAYRYPPGVVGVAPTGIPAAVEGIVPSAMSLSHGLPPVAHPPHAPSPGQTVKPEADRDHASDQL

>NP_003480.2

MTHGEELGSDVHQDSIVLTYLEGLLMHQAAGGSGTAVDKKSAGHNEEDQNFNISGSAFPTCQSNGPVLNTHTYQGSGMLHLKKARLLQSSEDWNAAKRKRLSDSIMNLNVKKEALLAGMVDSVPKGKQDSTLLASLLQSFSSRLQTVALSQQIRQSLKEQGYALSHDSLKVEKDLRCYGVASSHLKTLLKKSKVKDQKPDTNLPDVTKNLIRDRFAESPHHVGQSGTKVMSEPLSCAARLQAVASMVEKRASPATSPKPSVACSQLALLLSSEAHLQQYSREHALKTQNANQAASERLAAMARLQENGQKDVGSYQLPKGMSSHLNGQARTSSSKLMASKSSATVFQNPMGIIPSSPKNAGYKNSLERNNIKQAANNSLLLHLLKSQTIPKPMNGHSHSERGSIFEESSTPTTIDEYSDNNPSFTDDSSGDESSYSNCVPIDLSCKHRTEKSESDQPVSLDNFTQSLLNTWDPKVPDVDIKEDQDTSKNSKLNSHQKVTLLQLLLGHKNEENVEKNTSPQGVHNDVSKFNTQNYARTSVIESPSTNRTTPVSTPPLLTSSKAGSPINLSQHSLVIKWNSPPYVCSTQSEKLTNTASNHSMDLTKSKDPPGEKPAQNEGAQNSATFSASKLLQNLAQCGMQSSMSVEEQRPSKQLLTGNTDKPIGMIDRLNSPLLSNKTNAVEENKAFSSQPTGPEPGLSGSEIENLLERRTVLQLLLGNPNKGKSEKKEKTPLRDESTQEHSERALSEQILMVKIKSEPCDDLQIPNTNVHLSHDAKSAPFLGMAPAVQRSAPALPVSEDFKSEPVSPQDFSFSKNGLLSRLLRQNQDSYLADDSDRSHRNNEMALLESKNLCMVPKKRKLYTEPLENPFKKMKNNIVDAANNHSAPEVLYGSLLNQEELKFSRNDLEFKYPAGHGSASESEHRSWARESKSFNVLKQLLLSENCVRDLSPHRSNSVADSKKKGHKNNVTNSKPEFSISSLNGLMYSSTQPSSCMDNRTFSYPGVVKTPVSPTFPEHLGCAGSRPESGLLNGCSMPSEKGPIKWVITDAEKNEYEKDSPRLTKTNPILYYMLQKGGNSVTSRETQDKDIWREASSAESVSQVTAKEELLPTAETKASFFNLRSPYNSHMGNNASRPHSANGEVYGLLGSVLTIKKESE

260 NP_005499.1 NP_002974.1

>NP_005499.1

MNPTDIADTTLDESIYSNYYLYESIPKPCTKEGIKAFGELFLPPLYSLVFVFGLLGNSVVVLVLFKYKRLRSMTDVYLLNLAISDLLFVFSLPFWGYYAADQWVFGLGLCKMISWMYLVGFYSGIFFVMLMSIDRYLAIVHAVFSLRARTLTYGVITSLATWSVAVFASLPGFLFSTCYTERNHTYCKTKYSLNSTTWKVLSSLEINILGLVIPLGIMLFCYSMIIRTLQHCKNEKKNKAVKMIFAVVVLFLGFWTPYNIVLFLETLVELEVLQDCTFERYLDYAIQATETLAFVHCCLNPIIYFFLGEKFRKYILQLFKTCRGLFVLCQYCGLLQIYSADTPSSSYTQSTMDHDLHDAL

>NP_002974.1

MQVSTAALAVLLCTMALCNQFSASLAADTPTACCFSYTSRQIPQNFIADYFETSSQCSKPGVIFLTKRSRQVCADPSEEWVQKYVSDLELSA

261 NP_064589.2 NP_005892.1

>NP_064589.2

MEIVGCRAEDNSCPFRPPAMLFHGISGGHIQGIMEEMERRSKTEARLAKGAQLNGRDAGMPPLSPEKPALCAGCGGKISDRYYLLAVDKQWHLRCLKCCECKLALESELTCFAKDGSIYCKEDYYRRFSVQRCARCHLGISASEMVMRARDSVYHLSCFTCSTCNKTLTTGDHFGMKDSLVYCRAHFETLLQGEYPPQLSYTELAAKSGGLALPYFNGTGTVQKGRPRKRKSPALGVDIVNYNSGCNENEADHLDRDQQPYPPSQKTKRMRTSFKHHQLRTMKSYFAINHNPDAKDLKQLAQKTGLTKRVLQVWFQNARAKFRRNLLRQENGGVDKADGTSLPAPPSADSGALTPPGTATTLTDLTNPTITVVTSVTSNMDSHESGSPSQTTLTNLF

>NP_005892.1

MSSILPFTPPVVKRLLGWKKSAGGSGGAGGGEQNGQEEKWCEKAVKSLVKKLKKTGRLDELEKAITTQNCNTKCVTIPSTCSEIWGLSTPNTIDQWDTTGLYSFSEQTRSLDGRLQVSHRKGLPHVIYCRLWRWPDLHSHHELKAIENCEYAFNLKKDEVCVNPYHYQRVETPVLPPVLVPRHTEILTELPPLDDYTHSIPENTNFPAGIEPQSNYIPETPPPGYISEDGETSDQQLNQSMDTGSPAELSPTTLSPVNHSLDLQPVTYSEPAFWCSIAYYELNQRVGETFHASQPSLTVDGFTDPSNSERFCLGLLSNVNRNATVEMTRRHIGRGVRLYYIGGEVFAECLSDSAIFVQSPNCNQRYGWHPATVCKIPPGCNLKIFNNQEFAALLAQSVNQGFEAVYQLTRMCTIRMSFVKGWGAEYRRQTVTSTPCWIELHLNGPLQWLDKVLTQMGSPSVRCSSMS

262 NP_062535.2 NP_001008709.1

>NP_062535.2

MYPNWGRYGGSSHYPPPPVPPPPPVALPEASPGPGYSSSTTPAAPSSSGFMSFREQHLAQLQQLQQMHQKQMQCVLQPHHLPPPPLPPPPVMPGGGYGDWQPPPPPMPPPPGPALSYQKQQQYKHQMLHHQRDGPPGLVPMELESPPESPPVPPGSYMPPSQSYMPPPQPPPSYYPPTSSQPYLPPAQPSPSQSPPSQSYLAPTPSYSSSSSSSQSYLSHSQSYLPSSQASPSRPSQGHSKSQLLAPPPPSAPPGNKTTVQQEPLESGAKNKSTEQQQAAPEPDPSTMTPQEQQQYWYRQHLLSLQQRTKVHLPGHKKGPVVAKDTPEPVKEEVTVPATSQVPESPSSEEPPLPPPNEEVPPPLPPEEPQSEDPEEDARLKQLQAAAAHWQQHQQHRVGFQYQGIMQKHTQLQQILQQYQQIIQPPPHIQTMSVDMQLRHYEMQQQQFQHLYQEWEREFQLWEEQLHSYPHKDQLQEYEKQWKTWQGHMKATQSYLQEKVNSFQNMKNQYMGNMSMPPPFVPYSQMPPPLPTMPPPVLPPSLPPPVMPPALPATVPPPGMPPPVMPPSLPTSVPPPGMPPSLSSAGPPPVLPPPSLSSAGPPPVLPPPSLSSTAPPPVMPLPPLSSATPPPGIPPPGVPQGIPPQLTAAPVPPASSSQSSQVPEKPRPALLPTPVSFGSAPPTTYHPPLQSAGPSEQVNSKAPLSKSALPYSSFSSDQGLGESSAAPSQPITAVKDMPVRSGGLLPDPPRSSYLESPRGPRFDGPRRFEDLGSRCEGPRPKGPRFEGNRPDGPRPRYEGHPAEGTKSKWGMIPRGPASQFYITPSTSLSPRQSGPQWKGPKPAFGQQHQQQPKSQAEPLSGNKEPLADTSSNQQKNFKMQSAAFSIAADVKDVKAAQSNENLSDSQQEPPKSEVSEGPVEPSNWDQNVQSMETQIDKAQAVTQPVPLANKPVPAQSTFPSKTGGMEGGTAVATSSLTADNDFKPVGIGLPHSENNQDKGLPRPDNRDNRLEGNRGNSSSYRGPGQSRMEDTRDKGLVNRGRGQAISRGPGLVKQEDFRDKMMGRREDSREKMNRGEGSRDRGLVRPGSSREKVPGGLQGSQDRGAAGSRERGPPRRAGSQERGPLRRAGSRERIPPRRAGSRERGPPRGPGSRERGLGRSDFGRDRGPFRPEPGDGGEKMYPYHRDEPPRAPWNHGEERGHEEFPLDGRNAPMERERLDDWDRERYWRECERDYQDDTLELYNREDRFSAPPSRSHDGDRRGPWWDDWERDQDMDEDYNREMERDMDRDVDRISRPMDMYDRSLDNEWDRDYGRPLDEQESQFRERDIPSLPPLPPLPPLPPLDRYRDDRWREERNREHGYDRDFRDRGELRIREYPERGDTWREKRDYVPDRMDWERERLSDRWYPSDVDRHSPMAEHMPSSHHSSEMMGSDASLDSDQGLGGVMVLSQRQHEIILKAAQELKMLREQKEQLQKMKDFGSEPQMADHLPPQESRLQNTSSRPGMYPPPGSYRPPPPMGKPPGSIVRPSAPPARSSVPVTRPPVPIPPPPPPPPLPPPPPVIKPQTSAVEQERWDEDSFYGLWDTNDEQGLNSEFKSETAAIPSAPVLPPPPVHSSIPPPGPVPMGMPPMSKPPPVQQTVDYGHGRDISTNKVEQIPYGERITLRPDPLPERSTFETEHAGQRDRYDRERDREPYFDRQSNVIADHRDFKRDRETHRDRDRDRGVIDYDRDRFDRERRPRDDRAQSYRDKKDHSSSRRGGFDRPSYDRKSDRPVYEGPSMFGGERRTYPEERMPLPAPSLSHQPPPAPRVEKKPESKNVDDILKPPGRESRPERIVVIMRGLPGSGKTHVAKLIRDKEVEFGGPAPRVLSLDDYFITEVEKEEKDPDSGKKVKKKVMEYEYEAEMEETYRTSMFKTFKKTLDDGFFPFIILDAINDRVRHFDQFWSAAKTKGFEVYLAEMSADNQTCGKRNIHGRKLKEINKMADHWETAPRHMMRLDIRSLLQDAAIEEVEMEDFDANIEEQKEEKKDAEEEESELGYIPKSKWEMDTSEAKLDKLDGLRTGTKRKRDWEAIASRMEDYLQLPDDYDTRASEPGKKRVRWADLEEKKDADRKRAIGFVVGQTDWEKITDESGHLAEKALNRTKYI

>NP_001008709.1

MSDSEKLNLDSIIGRLLEGSRVLTPHCAPVQGSRPGKNVQLTENEIRGLCLKSREIFLSQPILLELEAPLKICGDIHGQYYDLLRLFEYGGFPPESNYLFLGDYVDRGKQSLETICLLLAYKIKYPENFFLLRGNHECASINRIYGFYDECKRRYNIKLWKTFTDCFNCLPIAAIVDEKIFCCHGGLSPDLQSMEQIRRIMRPTDVPDQGLLCDLLWSDPDKDVQGWGENDRGVSFTFGAEVVAKFLHKHDLDLICRAHQVVEDGYEFFAKRQLVTLFSAPNYCGEFDNAGAMMSVDETLMCSFQILKPADKNKGKYGQFSGLNPGGRPITPPRNSAKAKK

263 NP_002367.4 NP_068375.3

>NP_002367.4

MSTRTPLPTVNERDTENHTSHGDGRQEVTSRTSRSGARCRNSIASCADEQPHIGNYRLLKTIGKGNFAKVKLARHILTGREVAIKIIDKTQLNPTSLQKLFREVRIMKILNHPNIVKLFEVIETEKTLYLIMEYASGGEVFDYLVAHGRMKEKEARSKFRQIVSAVQYCHQKRIVHRDLKAENLLLDADMNIKIADFGFSNEFTVGGKLDTFCGSPPYAAPELFQGKKYDGPEVDVWSLGVILYTLVSGSLPFDGQNLKELRERVLRGKYRIPFYMSTDCENLLKRFLVLNPIKRGTLEQIMKDRWINAGHEEDELKPFVEPELDISDQKRIDIMVGMGYSQEEIQESLSKMKYDEITATYLLLGRKSSELDASDSSSSSNLSLAKVRPSSDLNNSTGQSPHHKVQRSVFSSQKQRRYSDHAGPAIPSVVAYPKRSQTSTADSDLKEDGISSRKSSGSAVGGKGIAPASPMLGNASNPNKADIPERKKSSTVPSSNTASGGMTRRNTYVCSERTTADRHSVIQNGKENSTIPDQRTPVASTHSISSAATPDRIRFPRGTASRSTFHGQPRERRTATYNGPPASPSLSHEATPLSQTRSRGSTNLFSKLTSKLTRSRNVSAEQKDENKEAKPRSLRFTWSMKTTSSMDPGDMMREIRKVLDANNCDYEQRERFLLFCVHGDGHAENLVQWEMEVCKLPRLSLNGVRFKRISGTSIAFKNIASKIANELKL

>NP_068375.3

MAAYLQWRRFVFFDKELVKEPLSNDGAAPGATPASGSAASKFLCLPPGITVCDSGRGSLVFGDMEGQIWFLPRSLQLTGFQAYKLRVTHLYQLKQHNILASVGEDEEGINPLVKIWNLEKRDGGNPLCTRIFPAIPGTEPTVVSCLTVHENLNFMAIGFTDGSVTLNKGDITRDRHSKTQILHKGNYPVTGLAFRQAGKTTHLFVVTTENVQSYIVSGKDYPRVELDTHGCGLRCSALSDPSQDLQFIVAGDECVYLYQPDERGPCFAFEGHKLIAHWFRGYLIIVSRDRKVSPKSEFTSRDSQSSDKQILNIYDLCNKFIAYSTVFEDVVDVLAEWGSLYVLTRDGRVHALQEKDTQTKLEMLFKKNLFEMAINLAKSQHLDSDGLAQIFMQYGDHLYSKGNHDGAVQQYIRTIGKLEPSYVIRKFLDAQRIHNLTAYLQTLHRQSLANADHTTLLLNCYTKLKDSSKLEEFIKKKSESEVHFDVETAIKVLRQAGYYSHALYLAENHAHHEWYLKIQLEDIKNYQEALRYIGKLPFEQAESNMKRYGKILMHHIPEQTTQLLKGLCTDYRPSLEGRSDREAPGCRANSEEFIPIFANNPRELKAFLEHMSEVQPDSPQGIYDTLLELRLQNWAHEKDPQVKEKLHAEAISLLKSGRFCDVFDKALVLCQMHDFQDGVLYLYEQGKLFQQIMHYHMQHEQYRQVISVCERHGEQDPSLWEQALSYFARKEEDCKEYVAAVLKHIENKNLMPPLLVVQTLAHNSTATLSVIRDYLVQKLQKQSQQIAQDELRVRRYREETTRIRQEIQELKASPKIFQKTKCSICNSALELPSVHFLCGHSFHQHCFESYSESDADCPTCLPENRKVMDMIRAQEQKRDLHDQFQHQLKCSNDSFSVIADYFGRGVFNKLTLLTDPPTARLTSSLEAGLQRDLLMHSRRGT

264 NP_001008656.1 NP_001887.1

>NP_001008656.1

MAEARKRRELLPLIYHHLLRAGYVRAAREVKEQSGQKCFLAQPVTLLDIYTHWQQTSELGRKRKAEEDAALQAKKTRVSDPISTSESSEEEEEAEAETAKATPRLASTNSSVLGADLPSSMKEKAKAETEKAGKTGNSMPHPATGKTVANLLSGKSPRKSAEPSANTTLVSETEEEGSVPAFGAAAKPGMVSAGQADSSSEDTSSSSDETDVEGKPSVKPAQVKASSVSTKESPARKAAPAPGKVGDVTPQVKGGALPPAKRAKKPEEESESSEEGSESEEEAPAGTRSQVKASEKILQVRAASAPAKGTPGKGATPAPPGKAGAVASQTKAGKPEEDSESSSEESSDSEEETPAAKALLQAKASGKTSQVGAASAPAKESPRKGAAPAPPGKTGPAVAKAQAGKREEDSQSSSEESDSEEEAPAQAKPSGKAPQVRAASAPAKESPRKGAAPAPPRKTGPAAAQVQVGKQEEDSRSSSEESDSDREALAAMNAAQVKPLGKSPQVKPASTMGMGPLGKGAGPVPPGKVGPATPSAQVGKWEEDSESSSEESSDSSDGEVPTAVAPAQEKSLGNILQAKPTSSPAKGPPQKAGPVAVQVKAEKPMDNSESSEESSDSADSEEAPAAMTAAQAKPALKIPQTKACPKKTNTTASAKVAPVRVGTQAPRKAGTATSPAGSSPAVAGGTQRPAEDSSSSEESDSEEEKTGLAVTVGQAKSVGKGLQVKAASVPVKGSLGQGTAPVLPGKTGPTVTQVKAEKQEDSESSEEESDSEEAAASPAQVKTSVKKTQAKANPAAARAPSAKGTISAPGKVVTAAAQAKQRSPSKVKPPVRNPQNSTVLARGPASVPSVGKAVATAAQAQTGPEEDSGSSEEESDSEEEAETLAQVKPSGKTHQIRAALAPAKESPRKGAAPTPPGKTGPSAAQAGKQDDSGSSSEESDSDGEAPAAVTSAQVIKPPLIFVDPNRSPAGPAATPAQAQAASTPRKARASESTARSSSSESEDEDVIPATQCLTPGIRTNVVTMPTAHPRIAPKASMAGASSSKESSRISDGKKQEGPATQVDSAVGTLPATSPQSTSVQAKGTNKLRKPKLPEVQQATKAPESSDDSEDSSDSSSGSEEDGEGPQGAKSAHTLGPTPSRTETLVEETAAESSEDDVVAPSQSLLSGYMTPGLTPANSQASKATPKLDSSPSVSSTLAAKDDPDGKQEAKPQQAAGMLSPKTGGKEAASGTTPQKSRKPKKGAGNPQASTLALQSNITQCLLGQPWPLNEAQVQASVVKVLTELLEQERKKVVDTTKESSRKGWESRKRKLSGDQPAARTPRSKKKKKLGAGEGGEASVSPEKTSTTSKGKAKRDKASGDVKEKKGKGSLGSQGAKDEPEEELQKGMGTVEGGDQSNPKSKKEKKKSDKRKKDKEKKEKKKKAKKASTKDSESPSQKKKKKKKKTAEQTV

>NP_001887.1

MPGPAAGSRARVYAEVNSLRSREYWDYEAHVPSWGNQDDYQLVRKLGRGKYSEVFEAINITNNERVVVKILKPVKKKKIKREVKILENLRGGTNIIKLIDTVKDPVSKTPALVFEYINNTDFKQLYQILTDFDIRFYMYELLKALDYCHSKGIMHRDVKPHNVMIDHQQKKLRLIDWGLAEFYHPAQEYNVRVASRYFKGPELLVDYQMYDYSLDMWSLGCMLASMIFRREPFFHGQDNYDQLVRIAKVLGTEELYGYLKKYHIDLDPHFNDILGQHSRKRWENFIHSENRHLVSPEALDLLDKLLRYDHQQRLTAKEAMEHPYFYPVVKEQSQPCADNAVLSSGLTAAR

265 NP_000072.2 NP_008819.1

>NP_000072.2

MSTDSNSLAREFLTDVNRLCNAVVQRVEAREEEEEETHMATLGQYLVHGRGFLLLTKLNSIIDQALTCREELLTLLLSLLPLVWKIPVQEEKATDFNLPLSADIILTKEKNSSSQRSTQEKLHLEGSALSSQVSAKVNVFRKSRRQRKITHRYSVRDARKTQLSTSDSEANSDEKGIAMNKHRRPHLLHHFLTSFPKQDHPKAKLDRLATKEQTPPDAMALENSREIIPRQGSNTDILSEPAALSVISNMNNSPFDLCHVLLSLLEKVCKFDVTLNHNSPLAASVVPTLTEFLAGFGDCCSLSDNLESRVVSAGWTEEPVALIQRMLFRTVLHLLSVDVSTAEMMPENLRKNLTELLRAALKIRICLEKQPDPFAPRQKKTLQEVQEDFVFSKYRHRALLLPELLEGVLQILICCLQSAASNPFYFSQAMDLVQEFIQHHGFNLFETAVLQMEWLVLRDGVPPEASEHLKALINSVMKIMSTVKKVKSEQLHHSMCTRKRHRRCEYSHFMHHHRDLSGLLVSAFKNQVSKNPFEETADGDVYYPERCCCIAVCAHQCLRLLQQASLSSTCVQILSGVHNIGICCCMDPKSVIIPLLHAFKLPALKNFQQHILNILNKLILDQLGGAEISPKIKKAACNICTVDSDQLAQLEETLQGNLCDAELSSSLSSPSYRFQGILPSSGSEDLLWKWDALKAYQNFVFEEDRLHSIQIANHICNLIQKGNIVVQWKLYNYIFNPVLQRGVELAHHCQHLSVTSAQSHVCSHHNQCLPQDVLQIYVKTLPILLKSRVIRDLFLSCNGVSQIIELNCLNGIRSHSLKAFETLIISLGEQQKDASVPDIDGIDIEQKELSSVHVGTSFHHQQAYSDSPQSLSKFYAGLKEAYPKRRKTVNQDVHINTINLFLCVAFLCVSKEAESDRESANDSEDTSGYDSTASEPLSHMLPCISLESLVLPSPEHMHQAADIWSMCRWIYMLSSVFQKQFYRLGGFRVCHKLIFMIIQKLFRSHKEEQGKKEGDTSVNENQDLNRISQPKRTMKEDLLSLAIKSDPIPSELGSLKKSADSLGKLELQHISSINVEEVSATEAAPEEAKLFTSQESETSLQSIRLLEALLAICLHGARTSQQKMELELPNQNLSVESILFEMRDHLSQSKVIETQLAKPLFDALLRVALGNYSADFEHNDAMTEKSHQSAEELSSQPGDFSEEAEDSQCCSFKLLVEEEGYEADSESNPEDGETQDDGVDLKSETEGFSASSSPNDLLENLTQGEIIYPEICMLELNLLSASKAKLDVLAHVFESFLKIIRQKEKNVFLLMQQGTVKNLLGGFLSILTQDDSDFQACQRVLVDLLVSLMSSRTCSEELTLLLRIFLEKSPCTKILLLGILKIIESDTTMSPSQYLTFPLLHAPNLSNGVSSQKYPGILNSKAMGLLRRARVSRSKKEADRESFPHRLLSSWHIAPVHLPLLGQNCWPHLSEGFSVSLWFNVECIHEAESTTEKGKKIKKRNKSLILPDSSFDGTESDRPEGAEYINPGERLIEEGCIHIISLGSKALMIQVWADPHNATLIFRVCMDSNDDMKAVLLAQVESQENIFLPSKWQHLVLTYLQQPQGKRRIHGKISIWVSGQRKPDVTLDFMLPRKTSLSSDSNKTFCMIGHCLSSQEEFLQLAGKWDLGNLLLFNGAKVGSQEAFYLYACGPNHTSVMPCKYGKPVNDYSKYINKEILRCEQIRELFMTKKDVDIGLLIESLSVVYTTYCPAQYTIYEPVIRLKGQMKTQLSQRPFSSKEVQSILLEPHHLKNLQPTEYKTIQGILHEIGGTGIFVFLFARVVELSSCEETQALALRVILSLIKYNQQRVHELENCNGLSMIHQVLIKQKCIVGFYILKTLLEGCCGEDIIYMNENGEFKLDVDSNAIIQDVKLLEELLLDWKIWSKAEQGVWETLLAALEVLIRADHHQQMFNIKQLLKAQVVHHFLLTCQVLQEYKEGQLTPMPREVCRSFVKIIAEVLGSPPDLELLTIIFNFLLAVHPPTNTYVCHNPTNFYFSLHIDGKIFQEKVRSIMYLRHSSSGGRSLMSPGFMVISPSGFTASPYEGENSSNIIPQQMAAHMLRSRSLPAFPTSSLLTQSQKLTGSLGCSIDRLQNIADTYVATQSKKQNSLGSSDTLKKGKEDAFISSCESAKTVCEMEAVLSAQVSVSDVPKGVLGFPVVKADHKQLGAEPRSEDDSPGDESCPRRPDYLKGLASFQRSHSTIASLGLAFPSQNGSAAVGRWPSLVDRNTDDWENFAYSLGYEPNYNRTASAHSVTEDCLVPICCGLYELLSGVLLILPDVLLEDVMDKLIQADTLLVLVNHPSPAIQQGVIKLLDAYFARASKEQKDKFLKNRGFSLLANQLYLHRGTQELLECFIEMFFGRHIGLDEEFDLEDVRNMGLFQKWSVIPILGLIETSLYDNILLHNALLLLLQILNSCSKVADMLLDNGLLYVLCNTVAALNGLEKNIPMSEYKLLACDIQQLFIAVTIHACSSSGSQYFRVIEDLIVMLGYLQNSKNKRTQNMAVALQLRVLQAAMEFIRTTANHDSENLTDSLQSPSAPHHAVVQKRKSIAGPRKFPLAQTESLLMKMRSVANDELHVMMQRRMSQENPSQATETELAQRLQRLTVLAVNRIIYQEFNSDIIDILRTPENVTQSKTSVFQTEISEENIHHEQSSVFNPFQKEIFTYLVEGFKVSIGSSKASGSKQQWTKILWSCKETFRMQLGRLLVHILSPAHAAQERKQIFEIVHEPNHQEILRDCLSPSLQHGAKLVLYLSELIHNHQGELTEEELGTAELLMNALKLCGHKCIPPSASTKADLIKMIKEEQKKYETEEGVNKAAWQKTVNNNQQSLFQRLDSKSKDISKIAADITQAVSLSQGNERKKVIQHIRGMYKVDLSASRHWQELIQQLTHDRAVWYDPIYYPTSWQLDPTEGPNRERRRLQRCYLTIPNKYLLRDRQKSEDVVKPPLSYLFEDKTHSSFSSTVKDKAASESIRVNRRCISVAPSRETAGELLLGKCGMYFVEDNASDTVESSSLQGELEPASFSWTYEEIKEVHKRWWQLRDNAVEIFLTNGRTLLLAFDNTKVRDDVYHNILTNNLPNLLEYGNITALTNLWYTGQITNFEYLTHLNKHAGRSFNDLMQYPVFPFILADYVSETLDLNDLLIYRNLSKPIAVQYKEKEDRYVDTYKYLEEEYRKGAREDDPMPPVQPYHYGSHYSNSGTVLHFLVRMPPFTKMFLAYQDQSFDIPDRTFHSTNTTWRLSSFESMTDVKELIPEFFYLPEFLVNREGFDFGVRQNGERVNHVNLPPWARNDPRLFILIHRQALESDYVSQNICQWIDLVFGYKQKGKASVQAINVFHPATYFGMDVSAVEDPVQRRALETMIKTYGQTPRQLFHMAHVSRPGAKLNIEGELPAAVGLLVQFAFRETREQVKEITYPSPLSWIKGLKWGEYVGSPSAPVPVVCFSQPHGERFGSLQALPTRAICGLSRNFCLLMTYSKEQGVRSMNSTDIQWSAILSWGYADNILRLKSKQSEPPVNFIQSSQQYQVTSCAWVPDSCQLFTGSKCGVITAYTNRFTSSTPSEIEMETQIHLYGHTEEITSLFVCKPYSILISVSRDGTCIIWDLNRLCYVQSLAGHKSPVTAVSASETSGDIATVCDSAGGGSDLRLWTVNGDLVGHVHCREIICSVAFSNQPEGVSINVIAGGLENGIVRLWSTWDLKPVREITFPKSNKPIISLTFSCDGHHLYTANSDGTVIAWCRKDQQRLKQPMFYSFLSSYAAG

>NP_008819.1

MADQLTEEQIAEFKEAFSLFDKDGDGTITTKELGTVMRSLGQNPTEAELQDMINEVDADGNGTIDFPEFLTMMARKMKDTDSEEEIREAFRVFDKDGNGYISAAELRHVMTNLGEKLTDEEVDEMIREADIDGDGQVNYEEFVQMMTAK

266 NP_001918.3 NP_001031.2

>NP_001918.3

MSQAYSSSQRVSSYRRTFGGAPGFPLGSPLSSPVFPRAGFGSKGSSSSVTSRVYQVSRTSGGAGGLGSLRASRLGTTRTPSSYGAGELLDFSLADAVNQEFLTTRTNEKVELQELNDRFANYIEKVRFLEQQNAALAAEVNRLKGREPTRVAELYEEELRELRRQVEVLTNQRARVDVERDNLLDDLQRLKAKLQEEIQLKEEAENNLAAFRADVDAATLARIDLERRIESLNEEIAFLKKVHEEEIRELQAQLQEQQVQVEMDMSKPDLTAALRDIRAQYETIAAKNISEAEEWYKSKVSDLTQAANKNNDALRQAKQEMMEYRHQIQSYTCEIDALKGTNDSLMRQMRELEDRFASEASGYQDNIARLEEEIRHLKDEMARHLREYQDLLNVKMALDVEIATYRKLLEGEESRINLPIQTYSALNFRETSPEQRGSEVHTKKTVMIKTIETRDGEVVSEATQQQHEVL

>NP_001031.2

MESRGPLATSRLLLLLLLLLLRHTRQGWALRPVLPTQSAHDPPAVHLSNGPGQEPIAVMTFDLTKITKTSSSFEVRTWDPEGVIFYGDTNPKDDWFMLGLRDGRPEIQLHNHWAQLTVGAGPRLDDGRWHQVEVKMEGDSVLLEVDGEEVLRLRQVSGPLTSKRHPIMRIALGGLLFPASNLRLPLVPALDGCLRRDSWLDKQAEISASAPTSLRSCDVESNPGIFLPPGTQAEFNLRDIPQPHAEPWAFSLDLGLKQAAGSGHLLALGTPENPSWLSLHLQDQKVVLSSGSGPGLDLPLVLGLPLQLKLSMSRVVLSQGSKMKALALPPLGLAPLLNLWAKPQGRLFLGALPGEDSSTSFCLNGLWAQGQRLDVDQALNRSHEIWTHSCPQSPGNGTDASH

267 NP_005695.3 NP_002060.4

>NP_005695.3

MARAQALVLALTFQLCAPETETPAAGCTFEEASDPAVPCEYSQAQYDDFQWEQVRIHPGTRAPADLPHGSYLMVNTSQHAPGQRAHVIFQSLSENDTHCVQFSYFLYSRDGHSPGTLGVYVRVNGGPLGSAVWNMTGSHGRQWHQAELAVSTFWPNEYQVLFEALISPDRRGYMGLDDILLLSYPCAKAPHFSRLGDVEVNAGQNASFQCMAAGRAAEAERFLLQRQSGALVPAAGVRHISHRRFLATFPLAAVSRAEQDLYRCVSQAPRGAGVSNFAELIVKEPPTPIAPPQLLRAGPTYLIIQLNTNSIIGDGPIVRKEIEYRMARGPWAEVHAVSLQTYKLWHLDPDTEYEISVLLTRPGDGGTGRPGPPLISRTKCAEPMRAPKGLAFAEIQARQLTLQWEPLGYNVTRCHTYTVSLCYHYTLGSSHNQTIRECVKTEQGVSRYTIKNLLPYRNVHVRLVLTNPEGRKEGKEVTFQTDEDVPSGIAAESLTFTPLEDMIFLKWEEPQEPNGLITQYEISYQSIESSDPAVNVPGPRRTISKLRNETYHVFSNLHPGTTYLFSVRARTGKGFGQAALTEITTNISAPSFDYADMPSPLGESENTITVLLRPAQGRGAPISVYQVIVEEERARRLRREPGGQDCFPVPLTFEAALARGLVHYFGAELAASSLPEAMPFTVGDNQTYRGFWNPPLEPRKAYLIYFQAASHLKGETRLNCIRIARKAACKESKRPLEVSQRSEEMGLILGICAGGLAVLILLLGAIIVIIRKGRDHYAYSYYPKPVNMTKATVNYRQEKTHMMSAVDRSFTDQSTLQEDERLGLSFMDTHGYSTRGDQRSGGVTEASSLLGGSPRRPCGRKGSPYHTGQLHPAVRVADLLQHINQMKTAEGYGFKQEYESFFEGWDATKKKDKVKGSRQEPMPAYDRHRVKLHPMLGDPNADYINANYIDGYHRSNHFIATQGPKPEMVYDFWRMVWQEHCSSIVMITKLVEVGRVKCSRYWPEDSDTYGDIKIMLVKTETLAEYVVRTFALERRGYSARHEVRQFHFTAWPEHGVPYHATGLLAFIRRVKASTPPDAGPIVIHCSAGTGRTGCYIVLDVMLDMAECEGVVDIYNCVKTLCSRRVNMIQTEEQYIFIHDAILEACLCGETTIPVSEFKATYKEMIRIDPQSNSSQLREEFQTLNSVTPPLDVEECSIALLPRNRDKNRSMDVLPPDRCLPFLISTDGDSNNYINAALTDSYTRSAAFIVTLHPLQSTTPDFWRLVYDYGCTSIVMLNQLNQSNSAWPCLQYWPEPGRQQYGLMEVEFMSGTADEDLVARVFRVQNISRLQEGHLLVRHFQFLRWSAYRDTPDSKKAFLHLLAEVDKWQAESGDGRTIVHCLNGGGRSGTFCACATVLEMIRCHNLVDVFFAAKTLRNYKPNMVETMDQYHFCYDVALEYLEGLESR

>NP_002060.4

MGCTLSAEDKAAVERSKMIDRNLREDGEKAAREVKLLLLGAGESGKSTIVKQMKIIHEAGYSEEECKQYKAVVYSNTIQSIIAIIRAMGRLKIDFGDSARADDARQLFVLAGAAEEGFMTAELAGVIKRLWKDSGVQACFNRSREYQLNDSAAYYLNDLDRIAQPNYIPTQQDVLRTRVKTTGIVETHFTFKDLHFKMFDVGGQRSERKKWIHCFEGVTAIIFCVALSDYDLVLAEDEEMNRMHESMKLFDSICNNKWFTDTSIILFLNKKDLFEEKIKKSPLTICYPEYAGSNTYEEAAAYIQCQFEDLNKRKDTKEIYTHFTCATDTKNVQFVFDAVTDVIIKNNLKDCGLF

268 NP_001026897.1 NP_060649.3

>NP_001026897.1

MTLEAIRYSRGSLQILDQLLLPKQSRYEAVGSVHQAWEAIRAMKVRGAPAIALVGCLSLAVELQAGAGGPGLAALVAFVRDKLSFLVTARPTAVNMARAARDLADVAAREAEREGATEEAVRERVICCTEDMLEKDLRDNRSIGDLGARHLLERVAPSGGKVTVLTHCNTGALATAGYGTALGVIRSLHSLGRLEHAFCTETRPYNQGARLTAFELVYEQIPATLITDSMVAAAMAHRGVSAVVVGADRVVANGDTANKVGTYQLAIVAKHHGIPFYVAAPSSSCDLRLETGKEIIIEERPGQELTDVNGVRIAAPGIGVWNPAFDVTPHDLITGGIITELGVFAPEELRTALTTTISSRDGTLDGPQM

>NP_060649.3

MDSLEEPQKKVFKARKTMRVSDRQQLEAVYKVKEELLKTDVKLLNGNHENGDLDPTSPLENMDYIKDKEEVNGIEEICFDPEGSKAEWKETPCILSVNVKNKQDDDLNCEPLSPHNITPEPVSKLPAEPVSGDPAPGDLDAGDPASGVLASGDSTSGDPTSSEPSSSDAASGDATSGDAPSGDVSPGDATSGDATADDLSSGDPTSSDPIPGEPVPVEPISGDCAADDIASSEITSVDLASGAPASTDPASDDLASGDLSSSELASDDLATGELASDELTSESTFDRTFEPKSVPVCEPVPEIDNIEPSSNKDDDFLEKNGADEKLEQIQSKDSLDEKNKADNNIDANEETLETDDTTICSDRPPENEKKVEEDIITELALGEDAISSSMEIDQGEKNEDETSADLVETINENVIEDNKSENILENTDSMETDEIIPILEKLAPSEDELTCFSKTSLLPIDETNPDLEEKMESSFGSPSKQESSESLPKEAFLVLSDEEDISGEKDESEVISQNETCSPAEVESNEKDNKPEEEEQVIHEDDERPSEKNEFSRRKRSKSEDMDNVQSKRRRYMEEEYEAEFQVKITAKGDINQKLQKVIQWLLEEKLCALQCAVFDKTLAELKTRVEKIECNKRHKTVLTELQAKIARLTKRFEAAKEDLKKRHEHPPNPPVSPGKTVNDVNSNNNMSYRNAGTVRQMLESKRNVSESAPPSFQTPVNTVSSTNLVTPPAVVSSQPKLQTPVTSGSLTATSVLPAPNTATVVATTQVPSGNPQPTISLQPLPVILHVPVAVSSQPQLLQSHPGTLVTNQPSGNVEFISVQSPPTVSGLTKNPVSLPSLPNPTKPNNVPSVPSPSIQRNPTASAAPLGTTLAVQAVPTAHSIVQATRTSLPTVGPSGLYSPSTNRGPIQMKIPISAFSTSSAAEQNSNTTPRIENQTNKTIDASVSKKAADSTSQCGKATGSDSSGVIDLTMDDEESGASQDPKKLNHTPVSTMSSSQPVSRPLQPIQPAPPLQPSGVPTSGPSQTTIHLLPTAPTTVNVTHRPVTQVTTRLPVPRAPANHQVVYTTLPAPPAQAPLRGTVMQAPAVRQVNPQNSVTVRVPQTTTYVVNNGLTLGSTGPQLTVHHRPPQVHTEPPRPVHPAPLPEAPQPQRLPPEAASTSLPQKPHLKLARVQSQNGIVLSWSVLEVDRSCATVDSYHLYAYHEEPSATVPSQWKKIGEVKALPLPMACTLTQFVSGSKYYFAVRAKDIYGRFGPFCDPQSTDVISSTQSS

269 NP_055130.1 NP_001034966.1

>NP_055130.1

MADGNEDLRADDLPGPAFESYESMELACPAERSGHVAVSDGRHMFVWGGYKSNQVRGLYDFYLPREELWIYNMETGRWKKINTEGDVPPSMSGSCAVCVDRVLYLFGGHHSRGNTNKFYMLDSRSTDRVLQWERIDCQGIPPSSKDKLGVWVYKNKLIFFGGYGYLPEDKVLGTFEFDETSFWNSSHPRGWNDHVHILDTETFTWSQPITTGKAPSPRAAHACATVGNRGFVFGGRYRDARMNDLHYLNLDTWEWNELIPQGICPVGRSWHSLTPVSSDHLFLFGGFTTDKQPLSDAWTYCISKNEWIQFNHPYTEKPRLWHTACASDEGEVIVFGGCANNLLVHHRAAHSNEILIFSVQPKSLVRLSLEAVICFKEMLANSWNCLPKHLLHSVNQRFGSNNTSGS

>NP_001034966.1

MMEERAAAAVAAAASSCRPLGSGAGPGPTGAAPVSAPAPGPGPAGKGGGGGGSPGPTAGPEPLSLPGILHFIQHEWARFEAEKARWEAERAELQAQVAFLQGERKGQENLKTDLVRRIKMLEYALKQERAKYHKLKFGTDLNQGEKKADVSEQVSNGPVESVTLENSPLVWKEGRQLLRQYLEEVGYTDTILDMRSKRVRSLLGRSLELNGAVEPSEGAPRAPPGPAGLSGGESLLVKQIEEQIKRNAAGKDGKERLGGSVLGQIPFLQNCEDEDSDEDDELDSVQHKKQRVKLPSKALVPEMEDEDEEDDSEDAINEFDFLGSGEDGEGAPDPRRCTVDGSPHELESRRVKLQGILADLRDVDGLPPKVTGPPPGTPQPRPHEGSFGFSSDVFIMDTIGGGEVSLGDLADLTVTNDNDLSCDLSDSKDAFKKTWNPKFTLRSHYDGIRSLAFHHSQSALLTASEDGTLKLWNLQKAVTAKKNAALDVEPIHAFRAHRGPVLAVAMGSNSEYCYSGGADACIHSWKIPDLSMDPYDGYDPSVLSHVLEGHGDAVWGLAFSPTSQRLASCSADGTVRIWDPSSSSPACLCTFPTASEHGVPTSVAFTSTEPAHIVASFRSGDTVLYDMEVGSALLTLESRGSSGPTQINQVVSHPNQPLTITAHDDRGIRFLDNRTGKPVHSMVAHLDAVTCLAVDPNGAFLMSGSHDCSLRLWSLDNKTCVQEITAHRKKHEEAIHAVACHPSKALIASAGADALAKVFV

270 NP_002969.2 NP_001030.2

>NP_002969.2

MAEHRSMDGRMEAATRGGSHLQAAAQTPPRPGPPSAPPPPPKEGHQEGLVELPASFRELLTFFCTNATIHGAIRLVCSRGNRLKTTSWGLLSLGALVALCWQLGLLFERHWHRPVLMAVSVHSERKLLPLVTLCDGNPRRPSPVLRHLELLDEFARENIDSLYNVNLSKGRAALSATVPRHEPPFHLDREIRLQRLSHSGSRVRVGFRLCNSTGGDCFYRGYTSGVAAVQDWYHFHYVDILALLPAAWEDSHGSQDGHFVLSCSYDGLDCQARQFRTFHHPTYGSCYTVDGVWTAQRPGITHGVGLVLRVEQQPHLPLLSTLAGIRVMVHGRNHTPFLGHHSFSVRPGTEATISIREDEVHRLGSPYGHCTAGGEGVEVELLHNTSYTRQACLVSCFQQLMVETCSCGYYLHPLPAGAEYCSSARHPAWGHCFYRLYQDLETHRLPCTSRCPRPCRESAFKLSTGTSRWPSAKSAGWTLATLGEQGLPHQSHRQRSSLAKINIVYQELNYRSVEEAPVYSVPQLLSAMGSLCSLWFGASVLSLLELLELLLDASALTLVLGGRRLRRAWFSWPRASPASGASSIKPEASQMPPPAGGTSDDPEPSGPHLPRVMLPGVLAGVSAEESWAGPQPLETLDT

>NP_001030.2

MAPGEKIKAKIKKNLPVTGPQAPTIKELMRWYCLNTNTHGCRRIVVSRGRLRRLLWIGFTLTAVALILWQCALLVFSFYTVSVSIKVHFRKLDFPAVTICNINPYKYSTVRHLLADLEQETREALKSLYGFPESRKRREAESWNSVSEGKQPRFSHRIPLLIFDQDEKGKARDFFTGRKRKVGGSIIHKASNVMHIESKQVVGFQLCSNDTSDCATYTFSSGINAIQEWYKLHYMNIMAQVPLEKKINMSYSAEELLVTCFFDGVSCDARNFTLFHHPMHGNCYTFNNRENETILSTSMGGSEYGLQVILYINEEEYNPFLVSSTGAKVIIHRQDEYPFVEDVGTEIETAMVTSIGMHLTESFKLSEPYSQCTEDGSDVPIRNIYNAAYSLQICLHSCFQTKMVEKCGCAQYSQPLPPAANYCNYQQHPNWMYCYYQLHRAFVQEELGCQSVCKEACSFKEWTLTTSLAQWPSVVSEKWLLPVLTWDQGRQVNKKLNKTDLAKLLIFYKDLNQRSIMESPANSIEMLLSNFGGQLGLWMSCSVVCVIEIIEVFFIDFFSIIARRQWQKAKEWWAWKQAPPCPEAPRSPQGQDNPALDIDDDLPTFNSALHLPPALGTQVPGTPPPKYNTLRLERAFSNQLTDTQMLDEL

271 NP_005611.1 NP_006263.1

>NP_005611.1

MAKISSPTETERCIESLIAVFQKYAGKDGYNYTLSKTEFLSFMNTELAAFTKNQKDPGVLDRMMKKLDTNSDGQLDFSEFLNLIGGLAMACHDSFLKAVPSQKRT

>NP_006263.1

MSELEKAMVALIDVFHQYSGREGDKHKLKKSELKELINNELSHFLEEIKEQEVVDKVMETLDNDGDGECDFQEFMAFVAMVTTACHEFFEHE

272 NP_000591.1 NP_005997.2

>NP_000591.1

MNSFSTSAFGPVAFSLGLLLVLPAAFPAPVPPGEDSKDVAAPHRQPLTSSERIDKQIRYILDGISALRKETCNKSNMCESSKEALAENNLNLPKMAEKDGCFQSGFNEETCLVKIITGLLEFEVYLEYLQNRFESSEEQARAVQMSTKVLIQFLQKKAKNLDAITTPDPTTNASLLTKLQAQNQWLQDMTTHLILRSFKEFLQSSLRALRQM

>NP_005997.2

MDLTKMGMIQLQNPSHPTGLLCKANQMRLAGTLCDVVIMVDSQEFHAHRTVLACTSKMFEILFHRNSQHYTLDFLSPKTFQQILEYAYTATLQAKAEDLDDLLYAAEILEIEYLEEQCLKMLETIQASDDNDTEATMADGGAEEEEDRKARYLKNIFISKHSSEESGYASVAGQSLPGPMVDQSPSVSTSFGLSAMSPTKAAVDSLMTIGQSLLQGTLQPPAGPEEPTLAGGGRHPGVAEVKTEMMQVDEVPSQDSPGAAESSISGGMGDKVEERGKEGPGTPTRSSVITSARELHYGREESAEQVPPPAEAGQAPTGRPEHPAPPPEKHLGIYSVLPNHKADAVLSMPSSVTSGLHVQPALAVSMDFSTYGGLLPQGFIQRELFSKLGELAVGMKSESRTIGEQCSVCGVELPDNEAVEQHRKLHSGMKTYGCELCGKRFLDSLRLRMHLLAHSAGAKAFVCDQCGAQFSKEDALETHRQTHTGTDMAVFCLLCGKRFQAQSALQQHMEVHAGVRSYICSECNRTFPSHTALKRHLRSHTGDHPYECEFCGSCFRDESTLKSHKRIHTGEKPYECNGCGKKFSLKHQLETHYRVHTGEKPFECKLCHQRSRDYSAMIKHLRTHNGASPYQCTICTEYCPSLSSMQKHMKGHKPEEIPPDWRIEKTYLYLCYV

273 NP_001008493.1 NP_003452.1

>NP_001008493.1

MSEQSICQARAAVMVYDDANKKWVPAGGSTGFSRVHIYHHTGNNTFRVVGRKIQDHQVVINCAIPKGLKYNQATQTFHQWRDARQVYGLNFGSKEDANVFASAMMHALEVLNSQETGPTLPRQNSQLPAQVQNGPSQEELEIQRRQLQEQQRQKELERERLERERMERERLERERLERERLERERLEQEQLERERQERERQERLERQERLERQERLERQERLDRERQERQERERLERLERERQERERQEQLEREQLEWERERRISSAAAPASVETPLNSVLGDSSASEPGLQAASQPAETPSQQGIVLGPLAPPPPPPLPPGPAQASVALPPPPGPPPPPPLPSTGPPPPPPPPPLPNQVPPPPPPPPAPPLPASGFFLASMSEDNRPLTGLAAAIAGAKLRKVSRMEDTSFPSGGNAIGVNSASSKTDTGRGNGPLPLGGSGLMEEMSALLARRRRIAEKGSTIETEQKEDKGEDSEPVTSKASSTSTPEPTRKPWERTNTMNGSKSPVISRRDSPRKNQIVFDNRSYDSLHRPKSTPLSQPSANGVQTEGLDYDRLKQDILDEMRKELTKLKEELIDAIRQELSKSNTA

>NP_003452.1

MAAPRPSPAISVSVSAPAFYAPQKKFGPVVAPKPKVNPFRPGDSEPPPAPGAQRAQMGRVGEIPPPPPEDFPLPPPPLAGDGDDAEGALGGAFPPPPPPIEESFPPAPLEEEIFPSPPPPPEEEGGPEAPIPPPPQPREKVSSIDLEIDSLSSLLDDMTKNDPFKARVSSGYVPPPVATPFSSKSSTKPAAGGTAPLPPWKSPSSSQPLPQVPAPAQSQTQFHVQPQPQPKPQVQLHVQSQTQPVSLANTQPRGPPASSPAPAPKFSPVTPKFTPVASKFSPGAPGGSGSQPNQKLGHPEALSAGTGSPQPPSFTYAQQREKPRVQEKQHPVPPPAQNQNQVRSPGAPGPLTLKEVEELEQLTQQLMQDMEHPQRQNVAVNELCGRCHQPLARAQPAVRALGQLFHIACFTCHQCAQQLQGQQFYSLEGAPYCEGCYTDTLEKCNTCGEPITDRMLRATGKAYHPHCFTCVVCARPLEGTSFIVDQANRPHCVPDYHKQYAPRCSVCSEPIMPEPGRDETVRVVALDKNFHMKCYKCEDCGKPLSIEADDNGCFPLDGHVLCRKCHTARAQT

274 NP_002816.1 NP_064516.2

>NP_002816.1

MQAQQYQQQRRKFAAAFLAFIFILAAVDTAEAGKKEKPEKKVKKSDCGEWQWSVCVPTSGDCGLGTREGTRTGAECKQTMKTQRCKIPCNWKKQFGAECKYQFQAWGECDLNTALKTRTGSLKRALHNAECQKTVTISKPCGKLTKPKPQAESKKKKKEGKKQEKMLD

>NP_064516.2

MAEPSGAETRPPIRVTVKTPKDKEEIVICDRASVKEFKEEISRRFKAQQDQLVLIFAGKILKDGDTLNQHGIKDGLTVHLVIKTPQKAQDPAAATASSPSTPDPASAPSTTPASPATPAQPSTSGSASSDAGSGSRRSSGGGPSPGAGEGSPSATASILSGFGGILGLGSLGLGSANFMELQQQMQRQLMSNPEMLSQIMENPLVQDMMSNPDLMRHMIMANPQMQQLMERNPEISHMLNNPELMRQTMELARNPAMMQEMMRNQDRALSNLESIPGGYNALRRMYTDIQEPMFSAAREQFGNNPFSSLAGNSDSSSSQPLRTENREPLPNPWSPSPPTSQAPGSGGEGTGGSGTSQVHPTVSNPFGINAASLGSGMFNSPEMQALLQQISENPQLMQNVISAPYMRSMMQTLAQNPDFAAQMMVNVPLFAGNPQLQEQLRLQLPVFLQQMQNPESLSILTNPRAMQALLQIQQGLQTLQTEAPGLVPSLGSFGISRTPAPSAGSNAGSTPEAPTSSPATPATSSPTGASSAQQQLMQQMIQLLAGSGNSQVQTPEVRFQQQLEQLNSMGFINREANLQALIATGGDINAAIERLLGSQLS

275 NP_055512.1 NP_008881.2

>NP_055512.1

MPSKTKYNLVDDGHDLRIPLHNEDAFQHGICFEAKYVGSLDVPRPNSRVEIVAAMRRIRYEFKAKNIKKKKVSIMVSVDGVKVILKKKKKLLLLQKKEWTWDESKMLVMQDPIYRIFYVSHDSQDLKIFSYIARDGASNIFRCNVFKSKKKSQAMRIVRTVGQAFEVCHKLSLQHTQQNADGQEDGESERNSNSSGDPGRQLTGAERASTATAEETDIDAVEVPLPGNDVLEFSRGVTDLDAVGKEGGSHTGSKVSHPQEPMLTASPRMLLPSSSSKPPGLGTETPLSTHHQMQLLQQLLQQQQQQTQVAVAQVHLLKDQLAAEAAARLEAQARVHQLLLQNKDMLQHISLLVKQVQELELKLSGQNAMGSQDSLLEITFRSGALPVLCDPTTPKPEDLHSPPLGAGLADFAHPAGSPLGRRDCLVKLECFRFLPPEDTPPPAQGEALLGGLELIKFRESGIASEYESNTDESEERDSWSQEELPRLLNVLQRQELGDGLDDEIAV

>NP_008881.2

MNYLRRRLSDSNFMANLPNGYMTDLQRPQPPPPPPGAHSPGATPGPGTATAERSSGVAPAASPAAPSPGSSGGGGFFSSLSNAVKQTTAAAAATFSEQVGGGSGGAGRGGAASRVLLVIDEPHTDWAKYFKGKKIHGEIDIKVEQAEFSDLNLVAHANGGFSVDMEVLRNGVKVVRSLKPDFVLIRQHAFSMARNGDYRSLVIGLQYAGIPSVNSLHSVYNFCDKPWVFAQMVRLHKKLGTEEFPLIDQTFYPNHKEMLSSTTYPVVVKMGHAHSGMGKVKVDNQHDFQDIASVVALTKTYATAEPFIDAKYDVRVQKIGQNYKAYMRTSVSGNWKTNTGSAMLEQIAMSDRYKLWVDTCSEIFGGLDICAVEALHGKDGRDHIIEVVGSSMPLIGDHQDEDKQLIVELVVNKMAQALPRQRQRDASPGRGSHGQTPSPGALPLGRQTSQQPAGPPAQQRPPPQGGPPQPGPGPQRQGPPLQQRPPPQGQQHLSGLGPPAGSPLPQRLPSPTSAPQQPASQAAPPTQGQGRQSRPVAGGPGAPPAARPPASPSPQRQAGPPQATRQTSVSGPAPPKASGAPPGGQQRQGPPQKPPGPAGPTRQASQAGPVPRTGPPTTQQPRPSGPGPAGRPKPQLAQKPSQDVPPPATAAAGGPPHPQLNKSQSLTNAFNLPEPAPPRPSLSQDEVKAETIRSLRKSFASLFSD

276 NP_066970.2 NP_002583.1

>NP_066970.2

MVTPCPTSPSSPAARAGRRDNDQNLRAPVKKSRRPRLRRKQPLHPLNPCPLPGDSGICDLFESPSSGSDGAESPSAARGGSPLPGPAQPVAQLDLQTFRDYGQSCYAFRKAQESHFHPREALARQPQVTAESRCKLLSWLIPVHRQFGLSFESLCLTVNTLDRFLTTTPVAADCFQLLGVTSLLIACKQVEVHPPRVKQLLALCCGAFSRQQLCNLECIVLHKLHFTLGAPTISFFLEHFTHARVGAGQAEASEALEAQALARGVAELSLADYAFTSYSPSLLAICCLALADRMLRVSRPVDLRLGDHPEAALEDCMGKLQLLVAINSTSLTHMLPVQICEKCSLPPSSK

>NP_002583.1

MFEARLVQGSILKKVLEALKDLINEACWDISSSGVNLQSMDSSHVSLVQLTLRSEGFDTYRCDRNLAMGVNLTSMSKILKCAGNEDIITLRAEDNADTLALVFEAPNQEKVSDYEMKLMDLDVEQLGIPEQEYSCVVKMPSGEFARICRDLSHIGDAVVISCAKDGVKFSASGELGNGNIKLSQTSNVDKEEEAVTIEMNEPVQLTFALRYLNFFTKATPLSSTVTLSMSADVPLVVEYKIADMGHLKYYLAPKIEDEEGS

277 NP_001969.1 NP_006395.2

>NP_001969.1

MERLQKQPLTSPGSVSPSRDSSVPGSPSSIVAKMDNQVLGYKDLAAIPKDKAILDIERPDLMIYEPHFTYSLLEHVELPRQRERSLSPKSTSPPPSPEVWADSRSPGIISQASAPRTTGTPRTSLPHFHHPETSRPDSNIYKKPPIYKQRESVGGSPQTKHLIEDLIIESSKFPAAQPPDPNQPAKIETDYWPCPPSLAVVETEWRKRKASRRGAEEEEEEEDDDSGEEMKALRERQREELSKVTSNLGKMILKEEMEKSLPIRRKTRSLPDRTPFHTSLHQGTSKSSSLPRYGRTTLSRLQSTEFSPSGSETGSPGLQNGEGQRGRMDRGNSLPCVLEQKIYPYEMLVVTNKGRTKLPPGVDRMRLERHLSAEDFSRVFAMSPEEFGKLALWKRNELKKKASLF

>NP_006395.2

MLTTLLPILLLSGWAFCSQDASDGLQRLHMLQISYFRDPYHVWYQGNASLGGHLTHVLEGPDTNTTIIQLQPLQEPESWARTQSGLQSYLLQFHGLVRLVHQERTLAFPLTIRCFLGCELPPEGSRAHVFFEVAVNGSSFVSFRPERALWQADTQVTSGVVTFTLQQLNAYNRTRYELREFLEDTCVQYVQKHISAENTKGSQTSRSYTSLVLGVLVGSFIIAGVAVGIFLCTGGRRC

278 NP_054782.2 NP_009112.1

>NP_054782.2

MAANLSRNGPALQEAYVRVVTEKSPTDWALFTYEGNSNDIRVAGTGEGGLEEMVEELNSGKVMYAFCRVKDPNSGLPKFVLINWTGEGVNDVRKGACASHVSTMASFLKGAHVTINARAEEDVEPECIMEKVAKASGANYSFHKESGRFQDVGPQAPVGSVYQKTNAVSEIKRVGKDSFWAKAEKEEENRRLEEKRRAEEAQRQLEQERRERELREAARREQRYQEQGGEASPQSRTWEQQQEVVSRNRNEQESAVHPREIFKQKERAMSTTSISSPQPGKLRSPFLQKQLTQPETHFGREPAAAISRPRADLPAEEPAPSTPPCLVQAEEEAVYEEPPEQETFYEQPPLVQQQGAGSEHIDHHIQGQGLSGQGLCARALYDYQAADDTEISFDPENLITGIEVIDEGWWRGYGPDGHFGMFPANYVELIE

>NP_009112.1

MDVVDPDIFNRDPRDHYDLLQRLGGGTYGEVFKARDKVSGDLVALKMVKMEPDDDVSTLQKEILILKTCRHANIVAYHGSYLWLQKLWICMEFCGAGSLQDIYQVTGSLSELQISYVCREVLQGLAYLHSQKKIHRDIKGANILINDAGEVRLADFGISAQIGATLARRLSFIGTPYWMAPEVAAVALKGGYNELCDIWSLGITAIELAELQPPLFDVHPLRVLFLMTKSGYQPPRLKEKGKWSAAFHNFIKVTLTKSPKKRPSATKMLSHQLVSQPGLNRGLILDLLDKLKNPGKGPSIGDIEDEEPELPPAIPRRIRSTHRSSSLGIPDADCCRRHMEFRKLRGMETRPPANTARLQPPRDLRSSSPRKQLSESSDDDYDDVDIPTPAEDTPPPLPPKPKFRSPSDEGPGSMGDDGQLSPGVLVRCASGPPPNSPRPGPPPSTSSPHLTAHSEPSLWNPPSRELDKPPLLPPKKEKMKRKGCALLVKLFNGCPLRIHSTAAWTHPSTKDQHLLLGAEEGIFILNRNDQEATLEMLFPSRTTWVYSINNVLMSLSGKTPHLYSHSILGLLERKETRAGNPIAHISPHRLLARKNMVSTKIQDTKGCRACCVAEGASSGGPFLCGALETSVVLLQWYQPMNKFLLVRQVLFPLPTPLSVFALLTGPGSELPAVCIGVSPGRPGKSVLFHTVRFGALSCWLGEMSTEHRGPVQVTQVEEDMVMVLMDGSVKLVTPEGSPVRGLRTPEIPMTEAVEAVAMVGGQLQAFWKHGVQVWALGSDQLLQELRDPTLTFRLLGSPRLECSGTISPHCNLLLPGSSNSPASASRVAGITGL

279 NP_004122.1 AAD02233.1

>NP_004122.1

MQPILLLLAFLLLPRADAGEIIGGHEAKPHSRPYMAYLMIWDQKSLKRCGGFLIQDDFVLTAAHCWGSSINVTLGAHNIKEQEPTQQFIPVKRPIPHPAYNPKNFSNDIMLLQLERKAKRTRAVQPLRLPSNKAQVKPGQTCSVAGWGQTAPLGKHSHTLQEVKMTVQEDRKCESDLRHYYDSTIELCVGDPEIKKTSFKGDSGGPLVCNKVAQGIVSYGRNNGMPPRACTKVSSFVHWIKKTMKRY

>AAD02233.1

MEELSADEIRRRRLARLAGGQTSQPTTPLTSPQRENPPGPPIAASAPGPSQSLGLNVHNMTPATSPIGASGVAHRSQSSEGVSSLSSSPSNSLETQSQSLSRSQSMDIDGVSCEKSMSQVDVDSGIENMEVDENDRREKRSLSDKEPSSGPEVSEEQALQLVCKIFRVSWKDRDRDVIFLSSLSAQFKQNPKEVFSDFKDLIGQILMEVLMMSTQTRDENPFASLTATSQPIAAAARSPDRNLLLNTGSNPGTSPMFCSVASFGASSLSSLYESSPAPTPSFWSSVPVMGPSLASPSRAASQLAVPSTPLSPHSAASGTAAGSQPSSPRYRPYTVTHPWASSGVSILSSSPSPPALASSPQAVPASSSRQRPSSTGPPLPPASPSATSRRPSSLRISPSLGASGGASNWDSYSDHFTIETCKETDMLNYLIECFDRVGIEEKKAPKMCSQPAVSQLLSNIRSQCISHTALVLQGSLTQPRSLQQPSFLVPYMLCRNLPYGFIQELVRTTHQDEEVFKQIFIPILQGLALAAKECSLDSDYFKYPLMALGELCETKFGKTHPVCNLVASLRLWLPKSLSPGCGRELQRLSYLGAFFSFSVFAEDDVKVVEKYFSGPAITLENTRVVSQSLQHYLELGRQELFKILHSILLNGETREAALSYMAAVVNANMKKAQMQTDDRLVSTDGFMLNFLWVLQQLSTKIKLETVDPTYIFHPRCRITLPNDETRVNATMEDVNDWLTELYGDQPPFSEPKFPTECFFLTLHAHHLSILPSCRRYIRRLRAIRELNRTVEDLKNNESQWKDSPLATRHREMLKRCKTQLKKLVRCKACADAGLLDESFLRRCLNFYGLLIQLLLRILDPAYPDITLPLNSDVPKVFAALPEFYVEDVAEFLFFIVQYSPQALYEPCTQDIVMFLVVMLCNQNYIRNPYLVAKLVEVMFMTNPAVQPRTQKFFEMIENHPLSTKLLVPSLMKFYTDVEHTGATSEFYDKFTIRYHISTIFKSLWQNIAHHGTFMEEFNSGKQFVRYINMLINDTTFLLDESLESLKRIHEVQEEMKNKEQWDQLPRDQQQARQSQLAQDERVSRSYLALATETVDMFHILTKQVQKPFLRPELGPRLAAMLNFNLQQLCGPKCRDLKVENPEKYGFEPKKLLDQLTDIYLQLDCARFAKAIADDQRSYSKELFEEVISKMRKAGIKSTIAIEKFKLLAEKVEEIVAKNARAEIDYSDAPDEFRDPLMDTLMTDPVRLPSGTIMDRSIILRHLLNSPTDPFNRQTLTESMLEPVPELKEQIQAWMREKQNSDH

280 NP_006097.1 NP_005418.1

>NP_006097.1

MDPGQQPPPQPAPQGQGQPPSQPPQGQGPPSGPGQPAPAATQAAPQAPPAGHQIVHVRGDSETDLEALFNAVMNPKTANVPQTVPMRLRKLPDSFFKPPEPKSHSRQASTDAGTAGALTPQHVRAHSSPASLQLGAVSPGTLTPTGVVSGPAATPTAQHLRQSSFEIPDDVPLPAGWEMAKTSSGQRYFLNHIDQTTTWQDPRKAMLSQMNVTAPTSPPVQQNMMNSASAMNQRISQSAPVKQPPPLAPQSPQGGVMGGSNSNQQQQMRLQQLQMEKERLRLKQQELLRQVRPQELALRSQLPTLEQDGGTQNPVSSPGMSQELRTMTTNSSDPFLNSGTYHSRDESTDSGLSMSSYSVPRTPDDFLNSVDEMDTGDTINQSTLPSQQNRFPDYLEAIPGTNVDLGTLEGDGMNIEGEELMPSLQEALSSDILNDMESVLAATKLDKESFLTWL

>NP_005418.1

MAQSTATSPDGGTTFEHLWSSLEPDSTYFDLPQSSRGNNEVVGGTDSSMDVFHLEGMTTSVMAQFNLLSSTMDQMSSRAASASPYTPEHAASVPTHSPYAQPSSTFDTMSPAPVIPSNTDYPGPHHFEVTFQQSSTAKSATWTYSPLLKKLYCQIAKTCPIQIKVSTPPPPGTAIRAMPVYKKAEHVTDVVKRCPNHELGRDFNEGQSAPASHLIRVEGNNLSQYVDDPVTGRQSVVVPYEPPQVGTEFTTILYNFMCNSSCVGGMNRRPILIIITLEMRDGQVLGRRSFEGRICACPGRDRKADEDHYREQQALNESSAKNGAASKRAFKQSPPAVPALGAGVKKRRHGDEDTYYLQVRGRENFEILMKLKESLELMELVPQPLVDSYRQQQQLLQRPSHLQPPSYGPVLSPMNKVHGGMNKLPSVNQLVGQPPPHSSAATPNLGPVGPGMLNNHGHAVPANGEMSSSHSAQSMVSGSHCTPPPPYHADPSLVSFLTGLGCPNCIEYFTSQGLQSIYHLQNLTIEDLGALKIPEQYRMTIWRGLQDLKQGHDYSTAQQLLRSSNAATISIGGSGELQRQRVMEAVHFRVRHTITIPNRGGPGGGPDEWADFGFDLPDCKARKQPIKEEFTEAEIH

281 NP_002505.1 NP_006477.2

>NP_002505.1

MQSVQSTSFCLRKQCLCLTFLLLHLLGQVAATQRCPPQCPGRCPATPPTCAPGVRAVLDGCSCCLVCARQRGESCSDLEPCDESSGLYCDRSADPSNQTGICTAVEGDNCVFDGVIYRSGEKFQPSCKFQCTCRDGQIGCVPRCQLDVLLPEPNCPAPRKVEVPGECCEKWICGPDEEDSLGGLTLAAYRPEATLGVEVSDSSVNCIEQTTEWTACSKSCGMGFSTRVTNRNRQCEMLKQTRLCMVRPCEQEPEQPTDKKGKKCLRTKKSLKAIHLQFKNCTSLHTYKPRFCGVCSDGRCCTPHNTKTIQAEFQCSPGQIVKKPVMVIGTCTCHTNCPKNNEAFLQELELKTTRGKM

>NP_006477.2

MERAAPSRRVPLPLLLLGGLALLAAGVDADVLLEACCADGHRMATHQKDCSLPYATESKECRMVQEQCCHSQLEELHCATGISLANEQDRCATPHGDNASLEATFVKRCCHCCLLGRAAQAQGQSCEYSLMVGYQCGQVFRACCVKSQETGDLDVGGLQETDKIIEVEEEQEDPYLNDRCRGGGPCKQQCRDTGDEVVCSCFVGYQLLSDGVSCEDVNECITGSHSCRLGESCINTVGSFRCQRDSSCGTGYELTEDNSCKDIDECESGIHNCLPDFICQNTLGSFRCRPKLQCKSGFIQDALGNCIDINECLSISAPCPIGHTCINTEGSYTCQKNVPNCGRGYHLNEEGTRCVDVDECAPPAEPCGKGHRCVNSPGSFRCECKTGYYFDGISRMCVDVNECQRYPGRLCGHKCENTLGSYLCSCSVGFRLSVDGRSCEDINECSSSPCSQECANVYGSYQCYCRRGYQLSDVDGVTCEDIDECALPTGGHICSYRCINIPGSFQCSCPSSGYRLAPNGRNCQDIDECVTGIHNCSINETCFNIQGGFRCLAFECPENYRRSAATLQQEKTDTVRCIKSCRPNDVTCVFDPVHTISHTVISLPTFREFTRPEEIIFLRAITPPHPASQANIIFDITEGNLRDSFDIIKRYMDGMTVGVVRQVRPIVGPFHAVLKLEMNYVVGGVVSHRNVVNVHIFVSEYWF

282 NP_056485.2 NP_001420.2

>NP_056485.2

MKETIQGTGSWGPEPPGPGIPPAYSSPRRERLRWPPPPKPRLKSGGGFGPDPGSGTTVPARRLPVPRPSFDASASEEEEEEEEEEDEDEEEEVAAWRLPPRWSQLGTSQRPRPSRPTHRKTCSQRRRRAMRAFRMLLYSKSTSLTFHWKLWGRHRGRRRGLAHPKNHLSPQQGGATPQVPSPCCRFDSPRGPPPPRLGLLGALMAEDGVRGSPPVPSGPPMEEDGLRWTPKSPLDPDSGLLSCTLPNGFGGQSGPEGERSLAPPDASILISNVCSIGDHVAQELFQGSDLGMAEEAERPGEKAGQHSPLREEHVTCVQSILDEFLQTYGSLIPLSTDEVVEKLEDIFQQEFSTPSRKGLVLQLIQSYQRMPGNAMVRGFRVAYKRHVLTMDDLGTLYGQNWLNDQVMNMYGDLVMDTVPEKVHFFNSFFYDKLRTKGYDGVKRWTKNVDIFNKELLLIPIHLEVHWSLISVDVRRRTITYFDSQRTLNRRCPKHIAKYLQAEAVKKDRLDFHQGWKGYFKMNVARQNNDSDCGAFVLQYCKHLALSQPFSFTQQDMPKLRRQIYKELCHCKLTV

>NP_001420.2

MAENVVEPGPPSAKRPKLSSPALSASASDGTDFGSLFDLEHDLPDELINSTELGLTNGGDINQLQTSLGMVQDAASKHKQLSELLRSGSSPNLNMGVGGPGQVMASQAQQSSPGLGLINSMVKSPMTQAGLTSPNMGMGTSGPNQGPTQSTGMMNSPVNQPAMGMNTGMNAGMNPGMLAAGNGQGIMPNQVMNGSIGAGRGRQNMQYPNPGMGSAGNLLTEPLQQGSPQMGGQTGLRGPQPLKMGMMNNPNPYGSPYTQNPGQQIGASGLGLQIQTKTVLSNNLSPFAMDKKAVPGGGMPNMGQQPAPQVQQPGLVTPVAQGMGSGAHTADPEKRKLIQQQLVLLLHAHKCQRREQANGEVRQCNLPHCRTMKNVLNHMTHCQSGKSCQVAHCASSRQIISHWKNCTRHDCPVCLPLKNAGDKRNQQPILTGAPVGLGNPSSLGVGQQSAPNLSTVSQIDPSSIERAYAALGLPYQVNQMPTQPQVQAKNQQNQQPGQSPQGMRPMSNMSASPMGVNGGVGVQTPSLLSDSMLHSAINSQNPMMSENASVPSLGPMPTAAQPSTTGIRKQWHEDITQDLRNHLVHKLVQAIFPTPDPAALKDRRMENLVAYARKVEGDMYESANNRAEYYHLLAEKIYKIQKELEEKRRTRLQKQNMLPNAAGMVPVSMNPGPNMGQPQPGMTSNGPLPDPSMIRGSVPNQMMPRITPQSGLNQFGQMSMAQPPIVPRQTPPLQHHGQLAQPGALNPPMGYGPRMQQPSNQGQFLPQTQFPSQGMNVTNIPLAPSSGQAPVSQAQMSSSSCPVNSPIMPPGSQGSHIHCPQLPQPALHQNSPSPVPSRTPTPHHTPPSIGAQQPPATTIPAPVPTPPAMPPGPQSQALHPPPRQTPTPPTTQLPQQVQPSLPAAPSADQPQQQPRSQQSTAASVPTPTAPLLPPQPATPLSQPAVSIEGQVSNPPSTSSTEVNSQAIAEKQPSQEVKMEAKMEVDQPEPADTQPEDISESKVEDCKMESTETEERSTELKTEIKEEEDQPSTSATQSSPAPGQSKKKIFKPEELRQALMPTLEALYRQDPESLPFRQPVDPQLLGIPDYFDIVKSPMDLSTIKRKLDTGQYQEPWQYVDDIWLMFNNAWLYNRKTSRVYKYCSKLSEVFEQEIDPVMQSLGYCCGRKLEFSPQTLCCYGKQLCTIPRDATYYSYQNRYHFCEKCFNEIQGESVSLGDDPSQPQTTINKEQFSKRKNDTLDPELFVECTECGRKMHQICVLHHEIIWPAGFVCDGCLKKSARTRKENKFSAKRLPSTRLGTFLENRVNDFLRRQNHPESGEVTVRVVHASDKTVEVKPGMKARFVDSGEMAESFPYRTKALFAFEEIDGVDLCFFGMHVQEYGSDCPPPNQRRVYISYLDSVHFFRPKCLRTAVYHEILIGYLEYVKKLGYTTGHIWACPPSEGDDYIFHCHPPDQKIPKPKRLQEWYKKMLDKAVSERIVHDYKDIFKQATEDRLTSAKELPYFEGDFWPNVLEESIKELEQEEEERKREENTSNESTDVTKGDSKNAKKKNNKKTSKNKSSLSRGNKKKPGMPNVSNDLSQKLYATMEKHKEVFFVIRLIAGPAANSLPPIVDPDPLIPCDLMDGRDAFLTLARDKHLEFSSLRRAQWSTMCMLVELHTQSQDRFVYTCNECKHHVETRWHCTVCEDYDLCITCYNTKNHDHKMEKLGLGLDDESNNQQAAATQSPGDSRRLSIQRCIQSLVHACQCRNANCSLPSCQKMKRVVQHTKGCKRKTNGGCPICKQLIALCCYHAKHCQENKCPVPFCLNIKQKLRQQQLQHRLQQAQMLRRRMASMQRTGVVGQQQGLPSPTPATPTTPTGQQPTTPQTPQPTSQPQPTPPNSMPPYLPRTQAAGPVSQGKAAGQVTPPTPPQTAQPPLPGPPPAAVEMAMQIQRAAETQRQMAHVQIFQRPIQHQMPPMTPMAPMGMNPPPMTRGPSGHLEPGMGPTGMQQQPPWSQGGLPQPQQLQSGMPRPAMMSVAQHGQPLNMAPQPGLGQVGISPLKPGTVSQQALQNLLRTLRSPSSPLQQQQVLSILHANPQLLAAFIKQRAAKYANSNPQPIPGQPGMPQGQPGLQPPTMPGQQGVHSNPAMQNMNPMQAGVQRAGLPQQQPQQQLQPPMGGMSPQAQQMNMNHNTMPSQFRDILRRQQMMQQQQQQGAGPGIGPGMANHNQFQQPQGVGYPPQQQQRMQHHMQQMQQGNMGQIGQLPQALGAEAGASLQAYQQRLLQQQMGSPVQPNPMSPQQHMLPNQAQSPHLQGQQIPNSLSNQVRSPQPVPSPRPQSQPPHSSPSPRMQPQPSPHHVSPQTSSPHPGLVAAQANPMEQGHFASPDQNSMLSQLASNPGMANLHGASATDLGLSTDNSDLNSNLSQSTLDIH

283 NP_003091.2 NP_004293.1

>NP_003091.2

MAAEREPPPLGDGKPTDFEDLEDGEDLFTSTVSTLESSPSSPEPASLPAEDISANSNGPKPTEVVLDDDREDLFAEATEEVSLDSPEREPILSSEPSPAVTPVTPTTLIAPRIESKSMSAPVIFDRSREEIEEEANGDIFDIEIGVSDPEKVGDGMNAYMAYRVTTKTSLSMFSKSEFSVKRRFSDFLGLHSKLASKYLHVGYIVPPAPEKSIVGMTKVKVGKEDSSSTEFVEKRRAALERYLQRTVKHPTLLQDPDLRQFLESSELPRAVNTQALSGAGILRMVNKAADAVNKMTIKMNESDAWFEEKQQQFENLDQQLRKLHVSVEALVCHRKELSANTAAFAKSAAMLGNSEDHTALSRALSQLAEVEEKIDQLHQEQAFADFYMFSELLSDYIRLIAAVKGVFDHRMKCWQKWEDAQITLLKKREAEAKMMVANKPDKIQQAKNEIREWEAKVQQGERDFEQISKTIRKEVGRFEKERVKDFKTVIIKYLESLVQTQQQLIKYWEAFLPEAKAIA

>NP_004293.1

MAESAGASSFFPLVVLLLAGSGGSGPRGVQALLCACTSCLQANYTCETDGACMVSIFNLDGMEHHVRTCIPKVELVPAGKPFYCLSSEDLRNTHCCYTDYCNRIDLRVPSGHLKEPEHPSMWGPVELVGIIAGPVFLLFLIIIIVFLVINYHQRVYHNRQRLDMEDPSCEMCLSKDKTLQDLVYDLSTSGSGSGLPLFVQRTVARTIVLQEIIGKGRFGEVWRGRWRGGDVAVKIFSSREERSWFREAEIYQTVMLRHENILGFIAADNKDNGTWTQLWLVSDYHEHGSLFDYLNRYTVTIEGMIKLALSAASGLAHLHMEIVGTQGKPGIAHRDLKSKNILVKKNGMCAIADLGLAVRHDAVTDTIDIAPNQRVGTKRYMAPEVLDETINMKHFDSFKCADIYALGLVYWEIARRCNSGGVHEEYQLPYYDLVPSDPSIEEMRKVVCDQKLRPNIPNWWQSYEALRVMGKMMRECWYANGAARLTALRIKKTLSQLSVQEDVKI

284 NP_001559.1 NP_065147.1

>NP_001559.1

MAEYDLTTRIAHFLDRHLVFPLLEFLSVKEIYNEKELLQGKLDLLSDTNMVDFAMDVYKNLYSDDIPHALREKRTTVVAQLKQLQAETEPIVKMFEDPETTRQMQSTRDGRMLFDYLADKHGFRQEYLDTLYRYAKFQYECGNYSGAAEYLYFFRVLVPATDRNALSSLWGKLASEILMQNWDAAMEDLTRLKETIDNNSVSSPLQSLQQRTWLIHWSLFVFFNHPKGRDNIIDLFLYQPQYLNAIQTMCPHILRYLTTAVITNKDVRKRRQVLKDLVKVIQQESYTYKDPITEFVECLYVNFDFDGAQKKLRECESVLVNDFFLVACLEDFIENARLFIFETFCRIHQCISINMLADKLNMTPEEAERWIVNLIRNARLDAKIDSKLGHVVMGNNAVSPYQQVIEKTKSLSFRSQMLAMNIEKKLNQNSRSEAPNWATQDSGFY

>NP_065147.1

MKLKDTKSRPKQSSCGKFQTKGIKVVGKWKEVKIDPNMFADGQMDDLVCFEELTDYQLVSPAKNPSSLFSKEAPKRKAQAVSEEEEEEEGKSSSPKKKIKLKKSKNVATEGTSTQKEFEVKDPELEAQGDDMVCDDPEAGEMTSENLVQTAPKKKKNKGKKGLEPSQSTAAKVPKKAKTWIPEVHDQKADVSAWKDLFVPRPVLRALSFLGFSAPTPIQALTLAPAIRDKLDILGAAETGSGKTLAFAIPMIHAVLQWQKRNAAPPPSNTEAPPGETRTEAGAETRSPGKAEAESDALPDDTVIESEALPSDIAAEARAKTGGTVSDQALLFGDDDAGEGPSSLIREKPVPKQNENEEENLDKEQTGNLKQELDDKSATCKAYPKRPLLGLVLTPTRELAVQVKQHIDAVARFTGIKTAILVGGMSTQKQQRMLNRRPEIVVATPGRLWELIKEKHYHLRNLRQLRCLVVDEADRMVEKGHFAELSQLLEMLNDSQYNPKRQTLVFSATLTLVHQAPARILHKKHTKKMDKTAKLDLLMQKIGMRGKPKVIDLTRNEATVETLTETKIHCETDEKDFYLYYFLMQYPGRSLVFANSISCIKRLSGLLKVLDIMPLTLHACMHQKQRLRNLEQFARLEDCVLLATDVAARGLDIPKVQHVIHYQVPRTSEIYVHRSGRTARATNEGLSLMLIGPEDVINFKKIYKTLKKDEDIPLFPVQTKYMDVVKERIRLARQIEKSEYRNFQACLHNSWIEQAAAALEIELEEDMYKGGKADQQEERRRQKQMKVLKKELRHLLSQPLFTESQKTKYPTQSGKPPLLVSAPSKSESALSCLSKQKKKKTKKPKEPQPEQPQPSTSAN

285 NP_060923.2 NP_064516.2

>NP_060923.2

MNRLPDDYDPYAVEEPSDEEPALSSSEDEVDVLLHGTPDQKRKLIRECLTGESESSSEDEFEKEMEAELNSTMKTMEDKLSSLGTGSSSGNGKVATAPTRYYDDIYFDSDSEDEDRAVQVTKKKKKKQHKIPTNDELLYDPEKDNRDQAWVDAQRRGYHGLGPQRSRQQQPVPNSDAVLNCPACMTTLCLDCQRHESYKTQYRAMFVMNCSINKEEVLRYKASENRKKRRVHKKMRSNREDAAEKAETDVEEIYHPVMCTECSTEVAVYDKDEVFHFFNVLASHS

>NP_064516.2

MAEPSGAETRPPIRVTVKTPKDKEEIVICDRASVKEFKEEISRRFKAQQDQLVLIFAGKILKDGDTLNQHGIKDGLTVHLVIKTPQKAQDPAAATASSPSTPDPASAPSTTPASPATPAQPSTSGSASSDAGSGSRRSSGGGPSPGAGEGSPSATASILSGFGGILGLGSLGLGSANFMELQQQMQRQLMSNPEMLSQIMENPLVQDMMSNPDLMRHMIMANPQMQQLMERNPEISHMLNNPELMRQTMELARNPAMMQEMMRNQDRALSNLESIPGGYNALRRMYTDIQEPMFSAAREQFGNNPFSSLAGNSDSSSSQPLRTENREPLPNPWSPSPPTSQAPGSGGEGTGGSGTSQVHPTVSNPFGINAASLGSGMFNSPEMQALLQQISENPQLMQNVISAPYMRSMMQTLAQNPDFAAQMMVNVPLFAGNPQLQEQLRLQLPVFLQQMQNPESLSILTNPRAMQALLQIQQGLQTLQTEAPGLVPSLGSFGISRTPAPSAGSNAGSTPEAPTSSPATPATSSPTGASSAQQQLMQQMIQLLAGSGNSQVQTPEVRFQQQLEQLNSMGFINREANLQALIATGGDINAAIERLLGSQLS

286 NP_078918.3 P15941

>NP_078918.3

MWGRTARRRCPRELRRGREALLVLLALLALAGLGSVLRAQRGAGAGAAEPGPPRTPRPGRREPVMPRPPVPANALGARGEAVRLQLQGEELRLQEESVRLHQINIYLSDRISLHRRLPERWNPLCKEKKYDYDNLPRTSVIIAFYNEAWSTLLRTVYSVLETSPDILLEEVILVDDYSDREHLKERLANELSGLPKVRLIRANKREGLVRARLLGASAARGDVLTFLDCHCECHEGWLEPLLQRIHEEESAVVCPVIDVIDWNTFEYLGNSGEPQIGGFDWRLVFTWHTVPERERIRMQSPVDVIRSPTMAGGLFAVSKKYFEYLGSYDTGMEVWGGENLEFSFRIWQCGGVLETHPCSHVGHVFPKQAPYSRNKALANSVRAAEVWMDEFKELYYHRNPRARLEPFGDVTERKQLRDKLQCKDFKWFLETVYPELHVPEDRPGFFGMLQNKGLTDYCFDYNPPDENQIVGHQVILYLCHGMGQNQFFEYTSQKEIRYNTHQPEGCIAVEAGMDTLIMHLCEETAPENQKFILQEDGSLFHEQSKKCVQAARKESSDSFVPLLRDCTNSDHQKWFFKERML

>P15941

MTPGTQSPFFLLLLLTVLTVVTGSGHASSTPGGEKETSATQRSSVPSSTEKNAVSMTSSVLSSHSPGSGSSTTQGQDVTLAPATEPASGSAATWGQDVTSVPVTRPALGSTTPPAHDVTSAPDNKPAPGSTAPPAHGVTSAPDTRPAPGSTAPPAHGVTSAPDTRPAPGSTAPPAHGVTSAPDTRPAPGSTAPPAHGVTSAPDTRPAPGSTAPPAHGVTSAPDTRPAPGSTAPPAHGVTSAPDTRPAPGSTAPPAHGVTSAPDTRPAPGSTAPPAHGVTSAPDTRPAPGSTAPPAHGVTSAPDTRPAPGSTAPPAHGVTSAPDTRPAPGSTAPPAHGVTSAPDTRPAPGSTAPPAHGVTSAPDTRPAPGSTAPPAHGVTSAPDTRPAPGSTAPPAHGVTSAPDTRPAPGSTAPPAHGVTSAPDTRPAPGSTAPPAHGVTSAPDTRPAPGSTAPPAHGVTSAPDTRPAPGSTAPPAHGVTSAPDTRPAPGSTAPPAHGVTSAPDTRPAPGSTAPPAHGVTSAPDTRPAPGSTAPPAHGVTSAPDTRPAPGSTAPPAHGVTSAPDTRPAPGSTAPPAHGVTSAPDTRPAPGSTAPPAHGVTSAPDTRPAPGSTAPPAHGVTSAPDTRPAPGSTAPPAHGVTSAPDTRPAPGSTAPPAHGVTSAPDTRPAPGSTAPPAHGVTSAPDTRPAPGSTAPPAHGVTSAPDTRPAPGSTAPPAHGVTSAPDTRPAPGSTAPPAHGVTSAPDTRPAPGSTAPPAHGVTSAPDTRPAPGSTAPPAHGVTSAPDTRPAPGSTAPPAHGVTSAPDTRPAPGSTAPPAHGVTSAPDTRPAPGSTAPPAHGVTSAPDTRPAPGSTAPPAHGVTSAPDTRPAPGSTAPPAHGVTSAPDTRPAPGSTAPPAHGVTSAPDTRPAPGSTAPPAHGVTSAPDTRPAPGSTAPPAHGVTSAPDNRPALGSTAPPVHNVTSASGSASGSASTLVHNGTSARATTTPASKSTPFSIPSHHSDTPTTLASHSTKTDASSTHHSSVPPLTSSNHSTSPQLSTGVSFFFLSFHISNLQFNSSLEDPSTDYYQELQRDISEMFLQIYKQGGFLGLSNIKFRPGSVVVQLTLAFREGTINVHDVETQFNQYKTEAASRYNLTISDVSVSDVPFPFSAQSGAGVPGWGIALLVLVCVLVALAIVYLIALAVCQCRRKNYGQLDIFPARDTYHPMSEYPTYHTHGRYVPPSSTDRSPYEKVSAGNGGSSLSYTNPAVAAASANL

287 NP_001096.1 NP_112214.1

>NP_001096.1

MVDGVMILPVLIMIALPSPSMEDEKPKVNPKLYMCVCEGLSCGNEDHCEGQQCFSSLSINDGFHVYQKGCFQVYEQGKMTCKTPPSPGQAVECCQGDWCNRNITAQLPTKGKSFPGTQNFHLEVGLIILSVVFAVCLLACLLGVALRKFKRRNQERLNPRDVEYGTIEGLITTNVGDSTLADLLDHSCTSGSGSGLPFLVQRTVARQITLLECVGKGRYGEVWRGSWQGENVAVKIFSSRDEKSWFRETELYNTVMLRHENILGFIASDMTSRHSSTQLWLITHYHEMGSLYDYLQLTTLDTVSCLRIVLSIASGLAHLHIEIFGTQGKPAIAHRDLKSKNILVKKNGQCCIADLGLAVMHSQSTNQLDVGNNPRVGTKRYMAPEVLDETIQVDCFDSYKRVDIWAFGLVLWEVARRMVSNGIVEDYKPPFYDVVPNDPSFEDMRKVVCVDQQRPNIPNRWFSDPTLTSLAKLMKECWYQNPSARLTALRIKKTLTKIDNSLDKLKTDC

>NP_112214.1

MESLVFARRSGPTPSAAELARPLAEGLIKSPKPLMKKQAVKRHHHKHNLRHRYEFLETLGKGTYGKVKKARESSGRLVAIKSIRKDKIKDEQDLMHIRREIEIMSSLNHPHIIAIHEVFENSSKIVIVMEYASRGDLYDYISERQQLSEREARHFFRQIVSAVHYCHQNRVVHRDLKLENILLDANGNIKIADFGLSNLYHQGKFLQTFCGSPLYASPEIVNGKPYTGPEVDSWSLGVLLYILVHGTMPFDGHDHKILVKQISNGAYREPPKPSDACGLIRWLLMVNPTRRATLEDVASHWWVNWGYATRVGEQEAPHEGGHPGSDSARASMADWLRRSSRPLLENGAKVCSFFKQHAPGGGSTTPGLERQHSLKKSRKENDMAQSLHSDTADDTAHRPGKSNLKLPKGILKKKVSASAEGVQEDPPELSPIPASPGQAAPLLPKKGILKKPRQRESGYYSSPEPSESGELLDAGDVFVSGDPKEQKPPQASGLLLHRKGILKLNGKFSQTALELAAPTTFGSLDELAPPRPLARASRPSGAVSEDSILSSESFDQLDLPERLPEPPLRGCVSVDNLTGLEEPPSEGPGSCLRRWRQDPLGDSCFSLTDCQEVTATYRQALRVCSKLT

288 NP_620777.1 NP_002736.3

>NP_620777.1

MSEESDMDKAIKETSILEEYSINWTQKLGAGISGPVRVCVKKSTQERFALKILLDRPKARNEVRLHMMCATHPNIVQIIEVFANSVQFPHESSPRARLLIVMEMMEGGELFHRISQHRHFTEKQASQVTKQIALALRHCHLLNIAHRDLKPENLLFKDNSLDAPVKLCDFGFAKIDQGDLMTPQFTPYYVAPQVLEAQRRHQKEKSGIIPTSPTPYTYNKSCDLWSLGVIIYVMLCGYPPFYSKHHSRTIPKDMRRKIMTGSFEFPEEEWSQISEMAKDVVRKLLKVKPEERLTIEGVLDHPWLNSTEALDNVLPSAQLMMDKAVVAGIQQAHAEQLANMRIQDLKVSLKPLHSVNNPILRKRKLLGTKPKDSVYIHDHENGAEDSNVALEKLRDVIAQCILPQAGKGENEDEKLNEVMQEAWKYNRECKLLRDTLQSFSWNGRGFTDKVDRLKLAEIVKQVIEEQTTSHESQ

>NP_002736.3

MAAAAAAGAGPEMVRGQVFDVGPRYTNLSYIGEGAYGMVCSAYDNVNKVRVAIKKISPFEHQTYCQRTLREIKILLRFRHENIIGINDIIRAPTIEQMKDVYIVQDLMETDLYKLLKTQHLSNDHICYFLYQILRGLKYIHSANVLHRDLKPSNLLLNTTCDLKICDFGLARVADPDHDHTGFLTEYVATRWYRAPEIMLNSKGYTKSIDIWSVGCILAEMLSNRPIFPGKHYLDQLNHILGILGSPSQEDLNCIINLKARNYLLSLPHKNKVPWNRLFPNADSKALDLLDKMLTFNPHKRIEVEQALAHPYLEQYYDPSDEPIAEAPFKFDMELDDLPKEKLKELIFEETARFQPGYRS

289 NP_112226.2 NP_002871.1

>NP_112226.2

MLSPLPTGPLEACFSVQSRTSSPMEPPIPQSAPLTPNSVMVQPLLDSRMSHSRLQHPLTILPIDQVKTSHVENDYIDNPSLALTTGPKRTRGGAPELAPTPARCDQDVTHHWISFSGRPSSVSSSSSTSSDQRLLDHMAPPPVADQASPRAVRIQPKVVHCQPLDLKGPAVPPELDKHFLLCEACGKCKCKECASPRTLPSCWVCNQECLCSAQTLVNYGTCMCLVQGIFYHCTNEDDEGSCADHPCSCSRSNCCARWSFMGALSVVLPCLLCYLPATGCVKLAQRGYDRLRRPGCRCKHTNSVICKAASGDAKTSRPDKPF

>NP_002871.1

MEHIQGAWKTISNGFGFKDAVFDGSSCISPTIVQQFGYQRRASDDGKLTDPSKTSNTIRVFLPNKQRTVVNVRNGMSLHDCLMKALKVRGLQPECCAVFRLLHEHKGKKARLDWNTDAASLIGEELQVDFLDHVPLTTHNFARKTFLKLAFCDICQKFLLNGFRCQTCGYKFHEHCSTKVPTMCVDWSNIRQLLLFPNSTIGDSGVPALPSLTMRRMRESVSRMPVSSQHRYSTPHAFTFNTSSPSSEGSLSQRQRSTSTPNVHMVSTTLPVDSRMIEDAIRSHSESASPSALSSSPNNLSPTGWSQPKTPVPAQRERAPVSGTQEKNKIRPRGQRDSSYYWEIEASEVMLSTRIGSGSFGTVYKGKWHGDVAVKILKVVDPTPEQFQAFRNEVAVLRKTRHVNILLFMGYMTKDNLAIVTQWCEGSSLYKHLHVQETKFQMFQLIDIARQTAQGMDYLHAKNIIHRDMKSNNIFLHEGLTVKIGDFGLATVKSRWSGSQQVEQPTGSVLWMAPEVIRMQDNNPFSFQSDVYSYGIVLYELMTGELPYSHINNRDQIIFMVGRGYASPDLSKLYKNCPKAMKRLVADCVKKVKEERPLFPQILSSIELLQHSLPKINRSASEPSLHRAAHTEDINACTLTTSPRLPVF

290 NP_002858.2 NP_005891.1

>NP_002858.2

MASVTDGKTGVKDASDQNFDYMFKLLIIGNSSVGKTSFLFRYADDTFTPAFVSTVGIDFKVKTVYRHEKRVKLQIWDTAGQERYRTITTAYYRGAMGFILMYDITNEESFNAVQDWATQIKTYSWDNAQVILVGNKCDMEEERVVPTEKGQLLAEQLGFDFFEASAKENISVRQAFERLVDAICDKMSDSLDTDPSMLGSSKNTRLSDTPPLLQQNCSC

>NP_005891.1

MNVTSLFSFTSPAVKRLLGWKQGDEEEKWAEKAVDALVKKLKKKKGAMEELEKALSCPGQPSNCVTIPRSLDGRLQVSHRKGLPHVIYCRVWRWPDLQSHHELKPLECCEFPFGSKQKEVCINPYHYKRVESPVLPPVLVPRHSEYNPQHSLLAQFRNLGQNEPHMPLNATFPDSFQQPNSHPFPHSPNSSYPNSPGSSSSTYPHSPTSSDPGSPFQMPADTPPPAYLPPEDPMTQDGSQPMDTNMMAPPLPSEINRGDVQAVAYEEPKHWCSIVYYELNNRVGEAFHASSTSVLVDGFTDPSNNKNRFCLGLLSNVNRNSTIENTRRHIGKGVHLYYVGGEVYAECLSDSSIFVQSRNCNYHHGFHPTTVCKIPSGCSLKIFNNQEFAQLLAQSVNHGFETVYELTKMCTIRMSFVKGWGAEYHRQDVTSTPCWIEIHLHGPLQWLDKVLTQMGSPHNPISSVS

291 NP_001194.1 NP_002945.1

>NP_001194.1

MLLRSAGKLNVGTKKEDGESTAPTPRPKVLRCKCHHHCPEDSVNNICSTDGYCFTMIEEDDSGLPVVTSGCLGLEGSDFQCRDTPIPHQRRSIECCTERNECNKDLHPTLPPLKNRDFVDGPIHHRALLISVTVCSLLLVLIILFCYFRYKRQETRPRYSIGLEQDETYIPPGESLRDLIEQSQSSGSGSGLPLLVQRTIAKQIQMVKQIGKGRYGEVWMGKWRGEKVAVKVFFTTEEASWFRETEIYQTVLMRHENILGFIAADIKGTGSWTQLYLITDYHENGSLYDYLKSTTLDAKSMLKLAYSSVSGLCHLHTEIFSTQGKPAIAHRDLKSKNILVKKNGTCCIADLGLAVKFISDTNEVDIPPNTRVGTKRYMPPEVLDESLNRNHFQSYIMADMYSFGLILWEVARRCVSGGIVEEYQLPYHDLVPSDPSYEDMREIVCIKKLRPSFPNRWSSDECLRQMGKLMTECWAHNPASRLTALRVKKTLAKMSESQDIKL

>NP_002945.1

MQIFVKTLTGKTITLEVEPSDTIENVKAKIQDKEGIPPDQQRLIFAGKQLEDGRTLSDYNIQKESTLHLVLRLRGGAKKRKKKSYTTPKKNKHKRKKVKLAVLKYYKVDENGKISRLRRECPSDECGAGVFMASHFDRHYCGKCCLTYCFNKPEDK

292 NP_003255.2 NP_003290.1

>NP_003255.2

MPHTLWMVWVLGVIISLSKEESSNQASLSCDRNGICKGSSGSLNSIPSGLTEAVKSLDLSNNRITYISNSDLQRCVNLQALVLTSNGINTIEEDSFSSLGSLEHLDLSYNYLSNLSSSWFKPLSSLTFLNLLGNPYKTLGETSLFSHLTKLQILRVGNMDTFTKIQRKDFAGLTFLEELEIDASDLQSYEPKSLKSIQNVSHLILHMKQHILLLEIFVDVTSSVECLELRDTDLDTFHFSELSTGETNSLIKKFTFRNVKITDESLFQVMKLLNQISGLLELEFDDCTLNGVGNFRASDNDRVIDPGKVETLTIRRLHIPRFYLFYDLSTLYSLTERVKRITVENSKVFLVPCLLSQHLKSLEYLDLSENLMVEEYLKNSACEDAWPSLQTLILRQNHLASLEKTGETLLTLKNLTNIDISKNSFHSMPETCQWPEKMKYLNLSSTRIHSVTGCIPKTLEILDVSNNNLNLFSLNLPQLKELYISRNKLMTLPDASLLPMLLVLKISRNAITTFSKEQLDSFHTLKTLEAGGNNFICSCEFLSFTQEQQALAKVLIDWPANYLCDSPSHVRGQQVQDVRLSVSECHRTALVSGMCCALFLLILLTGVLCHRFHGLWYMKMMWAWLQAKRKPRKAPSRNICYDAFVSYSERDAYWVENLMVQELENFNPPFKLCLHKRDFIPGKWIIDNIIDSIEKSHKTVFVLSENFVKSEWCKYELDFSHFRLFDENNDAAILILLEPIEKKAIPQRFCKLRKIMNTKTYLEWPMDEAQREGFWVNLRAAIKS

>NP_003290.1

MRALWVLGLCCVLLTFGSVRADDEVDVDGTVEEDLGKSREGSRTDDEVVQREEEAIQLDGLNASQIRELREKSEKFAFQAEVNRMMKLIINSLYKNKEIFLRELISNASDALDKIRLISLTDENALSGNEELTVKIKCDKEKNLLHVTDTGVGMTREELVKNLGTIAKSGTSEFLNKMTEAQEDGQSTSELIGQFGVGFYSAFLVADKVIVTSKHNNDTQHIWESDSNEFSVIADPRGNTLGRGTTITLVLKEEASDYLELDTIKNLVKKYSQFINFPIYVWSSKTETVEEPMEEEEAAKEEKEESDDEAAVEEEEEEKKPKTKKVEKTVWDWELMNDIKPIWQRPSKEVEEDEYKAFYKSFSKESDDPMAYIHFTAEGEVTFKSILFVPTSAPRGLFDEYGSKKSDYIKLYVRRVFITDDFHDMMPKYLNFVKGVVDSDDLPLNVSRETLQQHKLLKVIRKKLVRKTLDMIKKIADDKYNDTFWKEFGTNIKLGVIEDHSNRTRLAKLLRFQSSHHPTDITSLDQYVERMKEKQDKIYFMAGSSRKEAESSPFVERLLKKGYEVIYLTEPVDEYCIQALPEFDGKRFQNVAKEGVKFDESEKTKESREAVEKEFEPLLNWMKDKALKDKIEKAVVSQRLTESPCALVASQYGWSGNMERIMKAQAYQTGKDISTNYYASQKKTFEINPRHPLIRDMLRRIKEDEDDKTVLDLAVVLFETATLRSGYLLPDTKAYGDRIERMLRLSLNIDPDAKVEEEPEEEPEETAEDTTEDTEQDEDEEMDVGTDEEEETAKESTAEKDEL

293 NP_733745.1 NP_115791.2

>NP_733745.1

MTEGTKKTSKKFKFFKFKGFGSLSNLPRSFTLRRSSASISRQSHLEPDTFEATQDDMVTVPKSPPAYARSSDMYSHMGTMPRPSIKKAQNSQAARQAQEAGPKPNLVPGGVPDPPGLEAAKEVMVKATGPLEDTPAMEPNPSAVEVDPIRKPEVPTGDVEEERPPRDVHSERAAGEPEAGSDYVKFSKEKYILDSSPEKLHKELEEELKLSSTDLRSHAWYHGRIPREVSETLVQRNGDFLIRDSLTSLGDYVLTCRWRNQALHFKINKVVVKAGESYTHIQYLFEQESFDHVPALVRYHVGSRKAVSEQSGAIIYCPVNRTFPLRYLEASYGLGQGSSKPASPVSPSGPKGSHMKRRSVTMTDGLTADKVTRSDGCPTSTSLPRPRDSIRSCALSMDQIPDLHSPMSPISESPSSPAYSTVTRVHAAPAAPSATALPASPVARRSSEPQLCPGSAPKTHGESDKGPHTSPSHTLGKASPSPSLSSYSDPDSGHYCQLQPPVRGSREWAATETSSQQARSYGERLKELSENGAPEGDWGKTFTVPIVEVTSSFNPATFQSLLIPRDNRPLEVGLLRKVKELLAEVDARTLARHVTKVDCLVARILGVTKEMQTLMGVRWGMELLTLPHGRQLRLDLLERFHTMSIMLAVDILGCTGSAEERAALLHKTIQLAAELRGTMGNMFSFAAVMGALDMAQISRLEQTWVTLRQRHTEGAILYEKKLKPFLKSLNEGKEGPPLSNTTFPHVLPLITLLECDSAPPEGPEPWGSTEHGVEVVLAHLEAARTVAHHGGLYHTNAEVKLQGFQARPELLEVFSTEFQMRLLWGSQGASSSQARRYEKFDKVLTALSHKLEPAVRSSEL

>NP_115791.2

MDDYMETLKDEEDALWENVECNRHMLSRYINPAKLTPYLRQCKVIDEQDEDEVLNAPMLPSKINRAGRLLDILHTKGQRGYVVFLESLEFYYPELYKLVTGKEPTRRFSTIVVEEGHEGLTHFLMNEVIKLQQQMKAKDLQRCELLARLRQLEDEKKQMTLTRVELLTFQERYYKMKEERDSYNDELVKVKDDNYNLAMRYAQLSEEKNMAVMRSRDLQLEIDQLKHRLNKMEEECKLERNQSLKLKNDIENRPKKEQVLELERENEMLKTKNQELQSIIQAGKRSLPDSDKAILDILEHDRKEALEDRQELVNRIYNLQEEARQAEELRDKYLEEKEDLELKCSTLGKDCEMYKHRMNTVMLQLEEVERERDQAFHSRDEAQTQYSQCLIEKDKYRKQIRELEEKNDEMRIEMVRREACIVNLESKLRRLSKDSNNLDQSLPRNLPVTIISQDFGDASPRTNGQEADDSSTSEESPEDSKYFLPYHPPQRRMNLKGIQLQRAKSPISLKRTSDFQAKGHEEEGTDASPSSCGSLPITNSFTKMQPPRSRSSIMSITAEPPGNDSIVRRYKEDAPHRSTVEEDNDSGGFDALDLDDDSHERYSFGPSSIHSSSSSHQSEGLDAYDLEQVNLMFRKFSLERPFRPSVTSVGHVRGPGPSVQHTTLNGDSLTSQLTLLGGNARGSFVHSVKPGSLAEKAGLREGHQLLLLEGCIRGERQSVPLDTCTKEEAHWTIQRCSGPVTLHYKVNHEGYRKLVKDMEDGLITSGDSFYIRLNLNISSQLDACTMSLKCDDVVHVRDTMYQDRHEWLCARVDPFTDHDLDMGTIPSYSRAQQLLLVKLQRLMHRGSREEVDGTHHTLRALRNTLQPEEALSTSDPRVSPRLSRASFLFGQLLQFVSRSENKYKRMNSNERVRIISGSPLGSLARSSLDATKLLTEKQEELDPESELGKNLSLIPYSLVRAFYCERRRPVLFTPTVLAKTLVQRLLNSGGAMEFTICKSDIVTRDEFLRRQKTETIIYSREKNPNAFECIAPANIEAVAAKNKHCLLEAGIGCTRDLIKSNIYPIVLFIRVCEKNIKRFRKLLPRPETEEEFLRVCRLKEKELEALPCLYATVEPDMWGSVEELLRVVKDKIGEEQRKTIWVDEDQL

294 NP_997246.2 NP_006144.1

>NP_997246.2

MEGKRQLEKRDFGKRLSLDSSLVEYMDSNKYIEHLLTQLEEQHRSLWREKLAVARLQREVAQRTSEGAMHEKLIHELEEERHLRLQSEKRLQEVTLESERNRIQMRSLQQQFSRMEETVRNLLQSQGSPEQKKEETVNIMVYQEKLSEEERKHKEALEDLHMVVDEDSRSESSSTDEGKEKTKLLLERLKALEAENSALALENENQREQYERCLDEVANQVVQALLTQKDLREECVKLKTRVFDLEQQNRTLSILFQQRVRPTSDLLLQKLHSRLLDLSSGDLLSEVERNRSLTQSRTDAEVHEHQLNTKSALKCPGLGAVIPGHLCPRNSYSSSSELSLSSTCSEYSSGSSYTWHDGKNLRKRQSSQNWDKRLSIDSSLPSGFASPTNELPPTRIKESHILEGLRKLQKRKVLLEPPSVITKWGYKDCMNSNEGIYSPGIKSSSLKEYPPCKTADLGSPCKEPHKTFVYDLDSHVDADDDPSTLALLQAVPNQSCRPHGSKLTHSVSDSLFGWETNRKHFLEGTSSVYPKERPEKLTSCASSCPLEMKLCPSVQTPQVQRERGPQGQGHGRMALNLQLSDTDDNETFDELHIESSDEKSPSDVSLAADTDKSVENLDVLVGFGKSLCGSPEEEEKQVPIPSETRPKTFSFIKQQRVVKRTSSEECVTVIFDAEDGEPIEFSSHQTGVVTVTRNEISINSTPAGPKAEHTELLPQGIACLQPRAAARDYTFFKRSEEDTEKNIPKDNVDNVPRVSTESFSSRTVTQNPQQQKLVKPTHNISCQSNSRSSAPMGIYQKQNLTKIPPRGKSSPQKSKLMEPEATTLLPSSGLVTLEKSPALAPGKLSRFMKTESSGPLFELRSDPHIPKHSAQLPHSSRMPSRRDWVQCPKSQTPGSRSRPAIESSDSGEPPTRDEHCGSGPEAGVKSPSPPPPPGRSVSLLARPSYDYSPAPSSTKSETRVPSETARTPFKSPLLKGISAPVISSNPATTEVQRKKPSVAFKKPIFTHPMPSPEAVIQTRCPAHAPSSSFTVMALGPPKVSPKRGVPKTSPRQTLGTPQRDIGLQTPRISPSTHEPLEMTSSKSVSPGRKGQLNDSASTPPKPSFLGVNESPSSQVSSSSSSSSPAKSHNSPHGCQSAHEKGLKTRLPVGLKVLMKSPQLLRKSSTVPGKHEKDSLNEASKSSVAVNKSKPEDSKNPASMEITAGERNVTLPDSQAQGSLADGLPLETALQEPLESSIPGSDGRDGVDNRSMRRSLSSSKPHLKPALGMNGAKARSHSFSTHSGDKPSTPPIEGSGKVRTQIITNTAERGNSLTRQNSSTESSPNKAPSAPMLESLPSVGRPSGHPSSGKGSLGSSGSFSSQHGSPSKLPLRIPPKSEGLLIPPGKEDQQAFTQGECPSANVAVLGEPGSDRRSCPPTPTDCPEALQSPGRTQHPSTFETSSTSKLETSGRHPDASATATDAVSSEAPLSPTIEEKVMLCIQENVEKGQVQTKPTSVEAKQKPGPSFASWFGFRKSRLPALSSRKMDISKTKVEKKDAKVLGFGNRQLKSERKKEKKKPELQCETENELIKDTKSADNPDGGLQSKNNRRTPQDIYNQLKIEPRNRHSPVACSTKDTFMTELLNRVDKKAAPQTESGSSNASCRNVLKGSSQGSCLIGSSISTQGNHKKNMKIKADMEVPKDSLVKEANENLQEDEDDAVADSVFQSHIIESNCQMRTLDSGIGTFPLPDSGNRSTGRYLCQPDSPEDAEPLLPLQSALSAVSSMRAQTLEREVPSSTDGQRPADSAIVHSTSDPIMTARGMRPLQSRLPKPASSGKVSSQKQNEAEPRPQTCSSFGYAEDPMASQPLPDWGSEVAATGTQDKAPRMCTYSASGGSNSDSDLDYGDNGFGAGRGQLVKALKSAAPEIETT

>NP_006144.1

MAEEVVVVAKFDYVAQQEQELDIKKNERLWLLDDSKSWWRVRNSMNKTGFVPSNYVERKNSARKASIVKNLKDTLGIGKVKRKPSVPDSASPADDSFVDPGERLYDLNMPAYVKFNYMAEREDELSLIKGTKVIVMEKCSDGWWRGSYNGQVGWFPSNYVTEEGDSPLGDHVGSLSEKLAAVVNNLNTGQVLHVVQALYPFSSSNDEELNFEKGDVMDVIEKPENDPEWWKCRKINGMVGLVPKNYVTVMQNNPLTSGLEPSPPQCDYIRPSLTGKFAGNPWYYGKVTRHQAEMALNERGHEGDFLIRDSESSPNDFSVSLKAQGKNKHFKVQLKETVYCIGQRKFSTMEELVEHYKKAPIFTSEQGEKLYLVKHLS

295 NP_000367.1 NP_068811.1

>NP_000367.1

MEAMAASTSLPDPGDFDRNVPRICGVCGDRATGFHFNAMTCEGCKGFFRRSMKRKALFTCPFNGDCRITKDNRRHCQACRLKRCVDIGMMKEFILTDEEVQRKREMILKRKEEEALKDSLRPKLSEEQQRIIAILLDAHHKTYDPTYSDFCQFRPPVRVNDGGGSHPSRPNSRHTPSFSGDSSSSCSDHCITSSDMMDSSSFSNLDLSEEDSDDPSVTLELSQLSMLPHLADLVSYSIQKVIGFAKMIPGFRDLTSEDQIVLLKSSAIEVIMLRSNESFTMDDMSWTCGNQDYKYRVSDVTKAGHSLELIEPLIKFQVGLKKLNLHEEEHVLLMAICIVSPDRPGVQDAALIEAIQDRLSNTLQTYIRCRHPPPGSHLLYAKMIQKLADLRSLNEEHSKQYRCLSFQPECSMKLTPLVLEVFGNEIS

>NP_068811.1

MSWAARPPFLPQRHAAGQCGPVGVRKEMHCGVASRWRRRRPWLDPAAAAAAAVAGGEQQTPEPEPGEAGRDGMGDSGRDSRSPDSSSPNPLPQGVPPPSPPGPPLPPSTAPSLGGSGAPPPPPMPPPPLGSPFPVISSSMGSPGLPPPAPPGFSGPVSSPQINSTVSLPGGGSGPPEDVKPPVLGVRGLHCPPPPGGPGAGKRLCAICGDRSSGKHYGVYSCEGCKGFFKRTIRKDLTYSCRDNKDCTVDKRQRNRCQYCRYQKCLATGMKREAVQEERQRGKDKDGDGEGAGGAPEEMPVDRILEAELAVEQKSDQGVEGPGGTGGSGSSPNDPVTNICQAADKQLFTLVEWAKRIPHFSSLPLDDQVILLRAGWNELLIASFSHRSIDVRDGILLATGLHVHRNSAHSAGVGAIFDRVLTELVSKMRDMRMDKTELGCLRAIILFNPDAKGLSNPSEVEVLREKVYASLETYCKQKYPEQQGRFAKLLLRLPALRSIGLKCLEHLFFFKLIGDTPIDTFLMEMLEAPHQLA

296 NP_009225.1 NP_003487.1

>NP_009225.1

MDLSALRVEEVQNVINAMQKILECPICLELIKEPVSTKCDHIFCKFCMLKLLNQKKGPSQCPLCKNDITKRSLQESTRFSQLVEELLKIICAFQLDTGLEYANSYNFAKKENNSPEHLKDEVSIIQSMGYRNRAKRLLQSEPENPSLQETSLSVQLSNLGTVRTLRTKQRIQPQKTSVYIELGSDSSEDTVNKATYCSVGDQELLQITPQGTRDEISLDSAKKAACEFSETDVTNTEHHQPSNNDLNTTEKRAAERHPEKYQGSSVSNLHVEPCGTNTHASSLQHENSSLLLTKDRMNVEKAEFCNKSKQPGLARSQHNRWAGSKETCNDRRTPSTEKKVDLNADPLCERKEWNKQKLPCSENPRDTEDVPWITLNSSIQKVNEWFSRSDELLGSDDSHDGESESNAKVADVLDVLNEVDEYSGSSEKIDLLASDPHEALICKSERVHSKSVESNIEDKIFGKTYRKKASLPNLSHVTENLIIGAFVTEPQIIQERPLTNKLKRKRRPTSGLHPEDFIKKADLAVQKTPEMINQGTNQTEQNGQVMNITNSGHENKTKGDSIQNEKNPNPIESLEKESAFKTKAEPISSSISNMELELNIHNSKAPKKNRLRRKSSTRHIHALELVVSRNLSPPNCTELQIDSCSSSEEIKKKKYNQMPVRHSRNLQLMEGKEPATGAKKSNKPNEQTSKRHDSDTFPELKLTNAPGSFTKCSNTSELKEFVNPSLPREEKEEKLETVKVSNNAEDPKDLMLSGERVLQTERSVESSSISLVPGTDYGTQESISLLEVSTLGKAKTEPNKCVSQCAAFENPKGLIHGCSKDNRNDTEGFKYPLGHEVNHSRETSIEMEESELDAQYLQNTFKVSKRQSFAPFSNPGNAEEECATFSAHSGSLKKQSPKVTFECEQKEENQGKNESNIKPVQTVNITAGFPVVGQKDKPVDNAKCSIKGGSRFCLSSQFRGNETGLITPNKHGLLQNPYRIPPLFPIKSFVKTKCKKNLLEENFEEHSMSPEREMGNENIPSTVSTISRNNIRENVFKEASSSNINEVGSSTNEVGSSINEIGSSDENIQAELGRNRGPKLNAMLRLGVLQPEVYKQSLPGSNCKHPEIKKQEYEEVVQTVNTDFSPYLISDNLEQPMGSSHASQVCSETPDDLLDDGEIKEDTSFAENDIKESSAVFSKSVQKGELSRSPSPFTHTHLAQGYRRGAKKLESSEENLSSEDEELPCFQHLLFGKVNNIPSQSTRHSTVATECLSKNTEENLLSLKNSLNDCSNQVILAKASQEHHLSEETKCSASLFSSQCSELEDLTANTNTQDPFLIGSSKQMRHQSESQGVGLSDKELVSDDEERGTGLEENNQEEQSMDSNLGEAASGCESETSVSEDCSGLSSQSDILTTQQRDTMQHNLIKLQQEMAELEAVLEQHGSQPSNSYPSIISDSSALEDLRNPEQSTSEKAVLTSQKSSEYPISQNPEGLSADKFEVSADSSTSKNKEPGVERSSPSKCPSLDDRWYMHSCSGSLQNRNYPSQEELIKVVDVEEQQLEESGPHDLTETSYLPRQDLEGTPYLESGISLFSDDPESDPSEDRAPESARVGNIPSSTSALKVPQLKVAESAQSPAAAHTTDTAGYNAMEESVSREKPELTASTERVNKRMSMVVSGLTPEEFMLVYKFARKHHITLTNLITEETTHVVMKTDAEFVCERTLKYFLGIAGGKWVVSYFWVTQSIKERKMLNEHDFEVRGDVVNGRNHQGPKRARESQDRKIFRGLEICCYGPFTNMPTDQLEWMVQLCGASVVKELSSFTLGTGVHPIVVVQPDAWTEDNGFHAIGQMCEAPVVTREWVLDSVALYQCQELDTYLIPQIPHSHY

>NP_003487.1

MAFVATQGATVVDQTTLMKKYLQFVAALTDVNTPDETKLKMMQEVSENFENVTSSPQYSTFLEHIIPRFLTFLQDGEVQFLQEKPAQQLRKLVLEIIHRIPTNEHLRPHTKNVLSVMFRFLETENEENVLICLRIIIELHKQFRPPITQEIHHFLDFVKQIYKELPKVVNRYFENPQVIPENTVPPPEMVGMITTIAVKVNPEREDSETRTHSIIPRGSLSLKVLAELPIIVVLMYQLYKLNIHNVVAEFVPLIMNTIAIQVSAQARQHKLYNKELYADFIAAQIKTLSFLAYIIRIYQELVTKYSQQMVKGMLQLLSNCPAETAHLRKELLIAAKHILTTELRNQFIPCMDKLFDESILIGSGYTARETLRPLAYSTLADLVHHVRQHLPLSDLSLAVQLFAKNIDDESLPSSIQTMSCKLLLNLVDCIRSKSEQESGNGRDVLMRMLEVFVLKFHTIARYQLSAIFKKCKPQSELGAVEAALPGVPTAPAAPGPAPSPAPVPAPPPPPPPPPPATPVTPAPVPPFEKQGEKDKEDKQTFQVTDCRSLVKTLVCGVKTITWGITSCKAPGEAQFIPNKQLQPKETQIYIKLVKYAMQALDIYQVQIAGNGQTYIRVANCQTVRMKEEKEVLEHFAGVFTMMNPLTFKEIFQTTVPYMVERISKNYALQIVANSFLANPTTSALFATILVEYLLDRLPEMGSNVELSNLYLKLFKLVFGSVSLFAAENEQMLKPHLHKIVNSSMELAQTAKEPYNYFLLLRALFRSIGGGSHDLLYQEFLPLLPNLLQGLNMLQSGLHKQHMKDLFVELCLTVPVRLSSLLPYLPMLMDPLVSALNGSQTLVSQGLRTLELCVDNLQPDFLYDHIQPVRAELMQALWRTLRNPADSISHVAYRVLGKFGGSNRKMLKESQKLHYVVTEVQGPSITVEFSDCKASLQLPMEKAIETALDCLKSANTEPYYRRQAWEVIKCFLVAMMSLEDNKHALYQLLAHPNFTEKTIPNVIISHRYKAQDTPARKTFEQALTGAFMSAVIKDLRPSALPFVASLIRHYTMVAVAQQCGPFLLPCYQVGSQPSTAMFHSEENGSKGMDPLVLIDAIAICMAYEEKELCKIGEVALAVIFDVASIILGSKERACQLPLFSYIVERLCACCYEQAWYAKLGGVVSIKFLMERLPLTWVLQNQQTFLKALLFVMMDLTGEVSNGAVAMAKTTLEQLLMRCATPLKDEERAEEIVAAQEKSFHHVTHDLVREVTSPNSTVRKQAMHSLQVLAQVTGKSVTVIMEPHKEVLQDMVPPKKHLLRHQPANAQIGLMEGNTFCTTLQPRLFTMDLNVVEHKVFYTELLNLCEAEDSALTKLPCYKSLPSLVPLRIAALNALAACNYLPQSREKIIAALFKALNSTNSELQEAGEACMRKFLEGATIEVDQIHTHMRPLLMMLGDYRSLTLNVVNRLTSVTRLFPNSFNDKFCDQMMQHLRKWMEVVVITHKGGQRSDGNEMKICSAIINLFHLIPAAPQTLVKPLLEVVMKTERAMLIEAGSPFREPLIKFLTRHPSQTVELFMMEATLNDPQWSRMFMSFLKHKDARPLRDVLAANPNRFITLLLPGGAQTAVRPGSPSTSTMRLDLQFQAIKIISIIVKNDDSWLASQHSLVSQLRRVWVSENFQERHRKENMAATNWKEPKLLAYCLLNYCKRNYGDIELLFQLLRAFTGRFLCNMTFLKEYMEEEIPKNYSIAQKRALFFRFVDFNDPNFGDELKAKVLQHILNPAFLYSFEKGEGEQLLGPPNPEGDNPESITSVFITKVLDPEKQADMLDSLRIYLLQYATLLVEHAPHHIHDNNKNRNSKLRRLMTFAWPCLLSKACVDPACKYSGHLLLAHIIAKFAIHKKIVLQVFHSLLKAHAMEARAIVRQAMAILTPAVPARMEDGHQMLTHWTRKIIVEEGHTVPQLVHILHLIVQHFKVYYPVRHHLVQHMVSAMQRLGFTPSVTIEQRRLAVDLSEVVIKWELQRIKDQQPDSDMDPNSSGEGVNSVSSSIKRGLSVDSAQEVKRFRTATGAISAVFGRSQSLPGADSLLAKPIDKQHTDTVVNFLIRVACQVNDNTNTAGSPGEVLSRRCVNLLKTALRPDMWPKSELKLQWFDKLLMTVEQPNQVNYGNICTGLEVLSFLLTVLQSPAILSSFKPLQRGIAACMTCGNTKVLRAVHSLLSRLMSIFPTEPSTSSVASKYEELECLYAAVGKVIYEGLTNYEKATNANPSQLFGTLMILKSACSNNPSYIDRLISVFMRSLQKMVREHLNPQAASGSTEATSGTSELVMLSLELVKTRLAVMSMEMRKNFIQAILTSLIEKSPDAKILRAVVKIVEEWVKNNSPMAANQTPTLREKSILLVKMMTYIEKRFPEDLELNAQFLDLVNYVYRDETLSGSELTAKLEPAFLSGLRCAQPLIRAKFFEVFDNSMKRRVYERLLYVTCSQNWEAMGNHFWIKQCIELLLAVCEKSTPIGTSCQGAMLPSITNVINLADSHDRAAFAMVTHVKQEPRERENSESKEEDVEIDIELAPGDQTSTPKTKELSEKDIGNQLHMLTNRHDKFLDTLREVKTGALLSAFVQLCHISTTLAEKTWVQLFPRLWKILSDRQQHALAGEISPFLCSGSHQVQRDCQPSALNCFVEAMSQCVPPIPIRPCVLKYLGKTHNLWFRSTLMLEHQAFEKGLSLQIKPKQTTEFYEQESITPPQQEILDSLAELYSLLQEEDMWAGLWQKRCKYSETATAIAYEQHGFFEQAQESYEKAMDKAKKEHERSNASPAIFPEYQLWEDHWIRCSKELNQWEALTEYGQSKGHINPYLVLECAWRVSNWTAMKEALVQVEVSCPKEMAWKVNMYRGYLAICHPEEQQLSFIERLVEMASSLAIREWRRLPHVVSHVHTPLLQAAQQIIELQEAAQINAGLQPTNLGRNNSLHDMKTVVKTWRNRLPIVSDDLSHWSSIFMWRQHHYQAIVTAYENSSQHDPSSNNAMLGVHASASAIIQYGKIARKQGLVNVALDILSRIHTIPTVPIVDCFQKIRQQVKCYLQLAGVMGKNECMQGLEVIESTNLKYFTKEMTAEFYALKGMFLAQINKSEEANKAFSAAVQMHDVLVKAWAMWGDYLENIFVKERQLHLGVSAITCYLHACRHQNESKSRKYLAKVLWLLSFDDDKNTLADAVDKYCIGVPPIQWLAWIPQLLTCLVGSEGKLLLNLISQVGRVYPQAVYFPIRTLYLTLKIEQRERYKSDPGPIRATAPMWRCSRIMHMQRELHPTLLSSLEGIVDQMVWFRENWHEEVLRQLQQGLAKCYSVAFEKSGAVSDAKITPHTLNFVKKLVSTFGVGLENVSNVSTMFSSAASESLARRAQATAQDPVFQKLKGQFTTDFDFSVPGSMKLHNLISKLKKWIKILEAKTKQLPKFFLIEEKCRFLSNFSAQTAEVEIPGEFLMPKPTHYYIKIARFMPRVEIVQKHNTAARRLYIRGHNGKIYPYLVMNDACLTESRREERVLQLLRLLNPCLEKRKETTKRHLFFTVPRVVAVSPQMRLVEDNPSSLSLVEIYKQRCAKKGIEHDNPISRYYDRLATVQARGTQASHQVLRDILKEVQSNMVPRSMLKEWALHTFPNATDYWTFRKMFTIQLALIGFAEFVLHLNRLNPEMLQIAQDTGKLNVAYFRFDINDATGDLDANRPVPFRLTPNISEFLTTIGVSGPLTASMIAVARCFAQPNFKVDGILKTVLRDEIIAWHKKTQEDTSSPLSAAGQPENMDSQQLVSLVQKAVTAIMTRLHNLAQFEGGESKVNTLVAAANSLDNLCRMDPAWHPWL

297 NP_115498.2 NP_001381.2

>NP_115498.2

MLETLRERLLSVQQDFTSGLKTLSDKSREAKVKSKPRTVPFLPKYSAGLELLSRYEDTWAALHRRAKDCASAGELVDSEVVMLSAHWEKKKTSLVELQEQLQQLPALIADLESMTANLTHLEASFEEVENNLLHLEDLCGQCELERCKHMQSQQLENYKKNKRKELETFKAELDAEHAQKVLEMEHTQQMKLKERQKFFEEAFQQDMEQYLSTGYLQIAERREPIGSMSSMEVNVDMLEQMDLMDISDQEALDVFLNSGGEENTVLSPALGPESSTCQNEITLQVPNPSELRAKPPSSSSTCTDSATRDISEGGESPVVQSDEEEVQVDTALATSHTDREATPDGGEDSDS

>NP_001381.2

MIEDSGKRGNTMAERRQLFAEMRAQDLDRIRLSTYRTACKLRFVQKKCNLHLVDIWNVIEALRENALNNLDPNTELNVSRLEAVLSTIFYQLNKRMPTTHQIHVEQSISLLLNFLLAAFDPEGHGKISVFAVKMALATLCGGKIMDKLRYIFSMISDSSGVMVYGRYDQFLREVLKLPTAVFEGPSFGYTEQSARSCFSQQKKVTLNGFLDTLMSDPPPQCLVWLPLLHRLANVENVFHPVECSYCHSESMMGFRYRCQQCHNYQLCQDCFWRGHAGGSHSNQHQMKEYTSWKSPAKKLTNALSKSLSCASSREPLHPMFPDQPEKPLNLAHIVDTWPPRPVTSMNDTLFSHSVPSSGSPFITRSSPPKDSEVEQNKLLARAAPAFLKGKGIQYSLNVADRLADEHVLIGLYVNMLRNNPSCMLESSNRLDEEHRLIARYAARLAAESSSSQPPQQRSAPDISFTIDANKQQRQLIAELENKNREILQEIQRLRLEHEQASQPTPEKAQQNPTLLAELRLLRQRKDELEQRMSALQESRRELMVQLEGLMKLLKTQGAGSPRSSPSHTISRPIPMPIRSASACSTPTHTPQDSLTGVGGDVQEAFAQSSRRNLRNDLLVAADSITNTMSSLVKELNSEVGSETESNVDSEFARTQFEDLVPSPTSEKAFLAQIHARKPGYIHSGATTSTMRGDMVTEDADPYVQPEDENYENDSVRQLENELQMEEYLKQKLQDEAYQVSLQG

298 NP_004279.3 NP_059431.1

>NP_004279.3

MSLQRLLQHSSNGNLADFCAGPAYSSYSTLTGSLTMDDNRRIQMLADTVATLPRGRKQLALTRSSSLSDFSWSQRKLVTVEKQDNETFGFEIQSYRPQNQNACSSEMFTLICKIQEDSPAHCAGLQAGDVLANINGVSTEGFTYKQVVDLIRSSGNLLTIETLNGTMILKRTELEAKLQVLKQTLKQKWVEYRSLQLQEHRLLHGDAANCPSLENMDLDELSLFGPLPGPGPALVDRNRLSSESSCKSWLSSMTMDSEDGYQTCVSEDSSRGAFSRQTSTDDECFIPKEGDDFLRRSSSRRNRSISNTSSGSMSPLWEGNLSSMFGTLPRKSRKGSVRKQLLKFIPGLHRAVEEEESRF

>NP_059431.1

MEDGVYEPPDLTPEERMELENIRRRKQELLVEIQRLREELSEAMSEVEGLEANEGSKTLQRNRKMAMGRKKFNMDPKKGIQFLVENELLQNTPEEIARFLYKGEGLNKTAIGDYLGEREELNLAVLHAFVDLHEFTDLNLVQALRQFLWSFRLPGEAQKIDRMMEAFAQRYCLCNPGVFQSTDTCYVLSFAVIMLNTSLHNPNVRDKPGLERFVAMNRGINEGGDLPEELLRNLYDSIRNEPFKIPEDDGNDLTHTFFNPDREGWLLKLGGGRVKTWKRRWFILTDNCLYYFEYTTDKEPRGIIPLENLSIREVDDPRKPNCFELYIPNNKGQLIKACKTEADGRVVEGNHMVYRISAPTQEEKDEWIKSIQAAVSVDPFYEMLAARKKRISVKKKQEQP

299 NP_001649.1 NP_065875.2

>NP_001649.1

MGNIFANLFKGLFGKKEMRILMVGLDAAGKTTILYKLKLGEIVTTIPTIGFNVETVEYKNISFTVWDVGGQDKIRPLWRHYFQNTQGLIFVVDSNDRERVNEAREELMRMLAEDELRDAVLLVFANKQDLPNAMNAAEITDKLGLHSLRHRNWYIQATCATSGDGLYEGLDWLSNQLRNQK

>NP_065875.2

MATRRTGLSEGDGDKLKACEVSKNKDGKEQSETVSLSEDETFSWPGPKTVTLKRTSQGFGFTLRHFIVYPPESAIQFSYKDEENGNRGGKQRNRLEPMDTIFVKQVKEGGPAFEAGLCTGDRIIKVNGESVIGKTYSQVIALIQNSDTTLELSVMPKDEDILQVLQFTKDVTALAYSQDAYLKGNEAYSGNARNIPEPPPICYPWLPSAPSAMAQPVEISPPDSSLSKQQTSTPVLTQPGRAYRMEIQVPPSPTDVAKSNTAVCVCNESVRTVIVPSEKVVDLLSNRNNHTGPSHRTEEVRYGVSEQTSLKTVSRTTSPPLSIPTTHLIHQPAGSRSLEPSGILLKSGNYSGHSDGISSSRSQAVEAPSVSVNHYSPNSHQHIDWKNYKTYKEYIDNRRLHIGCRTIQERLDSLRAASQSTTDYNQVVPNRTTLQGRRRSTSHDRVPQSVQIRQRSVSQERLEDSVLMKYCPRSASQGALTSPSVSFSNHRTRSWDYIEGQDETLENVNSGTPIPDSNGEKKQTYKWSGFTEQDDRRGICERPRQQEIHKSFRGSNFTVAPSVVNSDNRRMSGRGVGSVSQFKKIPPDLKTLQSNRNFQTTCGMSLPRGISQDRSPLVKVRSNSLKAPSTHVTKPSFSQKSFVSIKDQRPVNHLHQNSLLNQQTWVRTDSAPDQQVETGKSPSLSGASAKPAPQSSENAGTSDLELPVSQRNQDLSLQEAETEQSDTLDNKEAVILREKPPSGRQTPQPLRHQSYILAVNDQETGSDTTCWLPNDARREVHIKRMEERKASSTSPPGDSLASIPFIDEPTSPSIDHDIAHIPASAVISASTSQVPSIATVPPCLTTSAPLIRRQLSHDHESVGPPSLDAQPNSKTERSKSYDEGLDDYREDAKLSFKHVSSLKGIKIADSQKSSEDSGSRKDSSSEVFSDAAKEGWLHFRPLVTDKGKRVGGSIRPWKQMYVVLRGHSLYLYKDKREQTTPSEEEQPISVNACLIDISYSETKRKNVFRLTTSDCECLFQAEDRDDMLAWIKTIQESSNLNEEDTGVTNRDLISRRIKEYNNLMSKAEQLPKTPRQSLSIRQTLLGAKSEPKTQSPHSPKEESERKLLSKDDTSPPKDKGTWRKGIPSIMRKTFEKKPTATGTFGVRLDDCPPAHTNRYIPLIVDICCKLVEERGLEYTGIYRVPGNNAAISSMQEELNKGMADIDIQDDKWRDLNVISSLLKSFFRKLPEPLFTNDKYADFIEANRKEDPLDRLKTLKRLIHDLPEHHYETLKFLSAHLKTVAENSEKNKMEPRNLAIVFGPTLVRTSEDNMTHMVTHMPDQYKIVETLIQHHDWFFTEEGAEEPLTTVQEESTVDSQPVPNIDHLLTNIGRTGVSPGDVSDSATSDSTKSKGSWGSGKDQYSRELLVSSIFAAASRKRKKPKEKAQPSSSEDELDNVFFKKENVEQCHNDTKEESKKESETLGRKQKIIIAKENSTRKDPSTTKDEKISLGKESTPSEEPSPPHNSKHNKSPTLSCRFAILKESPRSLLAQKSSHLEETGSDSGTLLSTSSQASLARFSMKKSTSPETKHSEFLANVSTITSDYSTTSSATYLTSLDSSRLSPEVQSVAESKGDEADDERSELISEGRPVETDSESEFPVFPTALTSERLFRGKLQEVTKSSRRNSEGSELSCTEGSLTSSLDSRRQLFSSHKLIECDTLSRKKSARFKSDSGSLGDAKNEKEAPSLTKVFDVMKKGKSTGSLLTPTRGESEKQEPTWKTKIADRLKLRPRAPADDMFGVGNHKVNAETAKRKSIRRRHTLGGHRDATEISVLNFWKVHEQSGERESELSAVNRLKPKCSAQDLSISDWLARERLRTSTSDLSRGEIGDPQTENPSTREIATTDTPLSLHCNTGSSSSTLASTNRPLLSIPPQSPDQINGESFQNVSKNASSAANAQPHKLSETPGSKAEFHPCL

300 NP_002695.1 NP_001034966.1

>NP_002695.1

MSLRLDTTPSCNSARPLHALQVLLLLSLLLTALASSTKGQTKRNLAKGKEESLDSDLYAELRCMCIKTTSGIHPKNIQSLEVIGKGTHCNQVEVIATLKDGRKICLDPDAPRIKKIVQKKLAGDESAD

>NP_001034966.1

MMEERAAAAVAAAASSCRPLGSGAGPGPTGAAPVSAPAPGPGPAGKGGGGGGSPGPTAGPEPLSLPGILHFIQHEWARFEAEKARWEAERAELQAQVAFLQGERKGQENLKTDLVRRIKMLEYALKQERAKYHKLKFGTDLNQGEKKADVSEQVSNGPVESVTLENSPLVWKEGRQLLRQYLEEVGYTDTILDMRSKRVRSLLGRSLELNGAVEPSEGAPRAPPGPAGLSGGESLLVKQIEEQIKRNAAGKDGKERLGGSVLGQIPFLQNCEDEDSDEDDELDSVQHKKQRVKLPSKALVPEMEDEDEEDDSEDAINEFDFLGSGEDGEGAPDPRRCTVDGSPHELESRRVKLQGILADLRDVDGLPPKVTGPPPGTPQPRPHEGSFGFSSDVFIMDTIGGGEVSLGDLADLTVTNDNDLSCDLSDSKDAFKKTWNPKFTLRSHYDGIRSLAFHHSQSALLTASEDGTLKLWNLQKAVTAKKNAALDVEPIHAFRAHRGPVLAVAMGSNSEYCYSGGADACIHSWKIPDLSMDPYDGYDPSVLSHVLEGHGDAVWGLAFSPTSQRLASCSADGTVRIWDPSSSSPACLCTFPTASEHGVPTSVAFTSTEPAHIVASFRSGDTVLYDMEVGSALLTLESRGSSGPTQINQVVSHPNQPLTITAHDDRGIRFLDNRTGKPVHSMVAHLDAVTCLAVDPNGAFLMSGSHDCSLRLWSLDNKTCVQEITAHRKKHEEAIHAVACHPSKALIASAGADALAKVFV

301 NP_002515.1 NP_057425.3

>NP_002515.1

MTEYKLVVVGAGGVGKSALTIQLIQNHFVDEYDPTIEDSYRKQVVIDGETCLLDILDTAGQEEYSAMRDQYMRTGEGFLCVFAINNSKSFADINLYREQIKRVKDSDDVPMVLVGNKCDLPTRTVDTKQAHELAKSYGIPFIETSAKTRQGVEDAFYTLVREIRQYRMKKLNSSDDGTQGCMGLPCVVM

>NP_057425.3

MTSEEMTASVLIPVTQRKVVSAQSAADESSEKVSDINISKAHTVRRSGETSHTISQLNKLKEEPSGSNLPKILSIAREKIVSDENSNEKCWEKIMPDSAKNLNINCNNILRNHQHGLPQRQFYEMYNSVAEEDLCLETGIPSPLERKVFPGIQLELDRPSMGISPLGNQSVIIETGRAHPDSRRAVFHFHYEVDRRMSDTFCTLSENLILDDCGNCVPLPGGEEKQKKNYVAYTCKLMELAKNCDNKNEQLQCDHCDTLNDKYFCFEGSCEKVDMVYSGDSFCRKDFTDSQAAKTFLSHFEDFPDNCDDVEEDAFKSKKERSTLLVRRFCKNDREVKKSVYTGTRAIVRTLPSGHIGLTAWSYIDQKRNGPLLPCGRVMEPPSTVEIRQDGSQRLSEAQWYPIYNAVRREETENTVGSLLHFLTKLPASETAHGRISVGPCLKQCVRDTVCEYRATLQRTSISQYITGSLLEATTSLGARSGLLSTFGGSTGRMMLKERQPGPSVANSNALPSSSAGISKELIDLQPLIQFPEEVASILMEQEQTIYRRVLPVDYLCFLTRDLGTPECQSSLPCLKASISASILTTQNGEHNALEDLVMRFNEVSSWVTWLILTAGSMEEKREVFSYLVHVAKCCWNMGNYNAVMEFLAGLRSRKVLKMWQFMDQSDIETMRSLKDAMAQHESSCEYRKVVTRALHIPGCKVVPFCGVFLKELCEVLDGASGLMKLCPRYNSQEETLEFVADYSGQDNFLQRVGQNGLKNSEKESTVNSIFQVIRSCNRSLETDEEDSPSEGNSSRKSSLKDKSRWQFIIGDLLDSDNDIFEQSKEYDSHGSEDSQKAFDHGTELIPWYVLSIQADVHQFLLQGATVIHYDQDTHLSARCFLQLQPDNSTLTWVKPTTASPASSKAKLGVLNNTAEPGKFPLLGNAGLSSLTEGVLDLFAVKAVYMGHPGIDIHTVCVQNKLGSMFLSETGVTLLYGLQTTDNRLLHFVAPKHTAKMLFSGLLELTRAVRKMRKFPDQRQQWLRKQYVSLYQEDGRYEGPTLAHAVELFGGRRWSARNPSPGTSAKNAEKPNMQRNNTLGISTTKKKKKILMRGESGEVTDDEMATRKAKMHKECRSRSGSDPQDINEQEESEVNAIANPPNPLPSRRAHSLTTAGSPNLAAGTSSPIRPVSSPVLSSSNKSPSSAWSSSSWHGRIKGGMKGFQSFMVSDSNMSFVEFVELFKSFSVRSRKDLKDLFDVYAVPCNRSGSESAPLYTNLTIDENTSDLQPDLDLLTRNVSDLGLFIKSKQQLSDNQRQISDAIAAASIVTNGTGIESTSLGIFGVGILQLNDFLVNCQGEHCTYDEILSIIQKFEPSISMCHQGLMSFEGFARFLMDKENFASKNDESQENIKELQLPLSYYYIESSHNTYLTGHQLKGESSVELYSQVLLQGCRSVELDCWDGDDGMPIIYHGHTLTTKIPFKEVVEAIDRSAFINSDLPIIISIENHCSLPQQRKMAEIFKTVFGEKLVTKFLFETDFSDDPMLPSPDQLRKKVLLKNKKLKAHQTPVDILKQKAHQLASMQVQAYNGGNANPRPANNEEEEDEEDEYDYDYESLSDDNILEDRPENKSCNDKLQFEYNEEIPKRIKKADNSACNKGKVYDMELGEEFYLDQNKKESRQIAPELSDLVIYCQAVKFPGLSTLNASGSSRGKERKSRKSIFGNNPGRMSPGETASFNKTSGKSSCEGIRQTWEESSSPLNPTTSLSAIIRTPKCYHISSLNENAAKRLCRRYSQKLTQHTACQLLRTYPAATRIDSSNPNPLMFWLHGIQLVALNYQTDDLPLHLNAAMFEANGGCGYVLKPPVLWDKNCPMYQKFSPLERDLDSMDPAVYSLTIVSGQNVCPSNSMGSPCIEVDVLGMPLDSCHFRTKPIHRNTLNPMWNEQFLFHVHFEDLVFLRFAVVENNSSAVTAQRIIPLKALKRGYRHLQLRNLHNEVLEISSLFINSRRMEENSSGNTMSASSMFNTEERKCLQTHRVTVHGVPGPEPFTVFTINGGTKAKQLLQQILTNEQDIKPVTTDYFLMEEKYFISKEKNECRKQPFQRAIGPEEEIMQILSSWFPEEGYMGRIVLKTQQENLEEKNIVQDDKEVILSSEEESFFVQVHDVSPEQPRTVIKAPRVSTAQDVIQQTLCKAKYSYSILSNPNPSDYVLLEEVVKDTTNKKTTTPKSSQRVLLDQECVFQAQSKWKGAGKFILKLKEQVQASREDKKKGISFASELKKLTKSTKQPRGLTSPSQLLTSESIQTKEEKPVGGLSSSDTMDYRQ

302 NP_005997.2 NP_060231.1

>NP_005997.2

MDLTKMGMIQLQNPSHPTGLLCKANQMRLAGTLCDVVIMVDSQEFHAHRTVLACTSKMFEILFHRNSQHYTLDFLSPKTFQQILEYAYTATLQAKAEDLDDLLYAAEILEIEYLEEQCLKMLETIQASDDNDTEATMADGGAEEEEDRKARYLKNIFISKHSSEESGYASVAGQSLPGPMVDQSPSVSTSFGLSAMSPTKAAVDSLMTIGQSLLQGTLQPPAGPEEPTLAGGGRHPGVAEVKTEMMQVDEVPSQDSPGAAESSISGGMGDKVEERGKEGPGTPTRSSVITSARELHYGREESAEQVPPPAEAGQAPTGRPEHPAPPPEKHLGIYSVLPNHKADAVLSMPSSVTSGLHVQPALAVSMDFSTYGGLLPQGFIQRELFSKLGELAVGMKSESRTIGEQCSVCGVELPDNEAVEQHRKLHSGMKTYGCELCGKRFLDSLRLRMHLLAHSAGAKAFVCDQCGAQFSKEDALETHRQTHTGTDMAVFCLLCGKRFQAQSALQQHMEVHAGVRSYICSECNRTFPSHTALKRHLRSHTGDHPYECEFCGSCFRDESTLKSHKRIHTGEKPYECNGCGKKFSLKHQLETHYRVHTGEKPFECKLCHQRSRDYSAMIKHLRTHNGASPYQCTICTEYCPSLSSMQKHMKGHKPEEIPPDWRIEKTYLYLCYV

>NP_060231.1

MGGGERYNIPAPQSRNVSKNQQQLNRQKTKEQNSQMKIVHKKKERGHGYNSSAAAWQAMQNGGKNKNFPNNQSWNSSLSGPRLLFKSQANQNYAGAKFSEPPSPSVLPKPPSHWVPVSFNPSDKEIMTFQLKTLLKVQV

303 NP_006245.2 NP_006209.2

>NP_006245.2

MAPFLRIAFNSYELGSLQAEDEANQPFCAVKMKEALSTERGKTLVQKKPTMYPEWKSTFDAHIYEGRVIQIVLMRAAEEPVSEVTVGVSVLAERCKKNNGKAEFWLDLQPQAKVLMSVQYFLEDVDCKQSMRSEDEAKFPTMNRRGAIKQAKIHYIKNHEFIATFFGQPTFCSVCKDFVWGLNKQGYKCRQCNAAIHKKCIDKIIGRCTGTAANSRDTIFQKERFNIDMPHRFKVHNYMSPTFCDHCGSLLWGLVKQGLKCEDCGMNVHHKCREKVANLCGINQKLLAEALNQVTQRASRRSDSASSEPVGIYQGFEKKTGVAGEDMQDNSGTYGKIWEGSSKCNINNFIFHKVLGKGSFGKVLLGELKGRGEYFAIKALKKDVVLIDDDVECTMVEKRVLTLAAENPFLTHLICTFQTKDHLFFVMEFLNGGDLMYHIQDKGRFELYRATFYAAEIMCGLQFLHSKGIIYRDLKLDNVLLDRDGHIKIADFGMCKENIFGESRASTFCGTPDYIAPEILQGLKYTFSVDWWSFGVLLYEMLIGQSPFHGDDEDELFESIRVDTPHYPRWITKESKDILEKLFEREPTKRLGVTGNIKIHPFFKTINWTLLEKRRLEPPFRPKVKSPRDYSNFDQEFLNEKARLSYSDKNLIDSMDQSAFAGFSFVNPKFEHLLED

>NP_006209.2

MPPRPSSGELWGIHLMPPRILVECLLPNGMIVTLECLREATLITIKHELFKEARKYPLHQLLQDESSYIFVSVTQEAEREEFFDETRRLCDLRLFQPFLKVIEPVGNREEKILNREIGFAIGMPVCEFDMVKDPEVQDFRRNILNVCKEAVDLRDLNSPHSRAMYVYPPNVESSPELPKHIYNKLDKGQIIVVIWVIVSPNNDKQKYTLKINHDCVPEQVIAEAIRKKTRSMLLSSEQLKLCVLEYQGKYILKVCGCDEYFLEKYPLSQYKYIRSCIMLGRMPNLMLMAKESLYSQLPMDCFTMPSYSRRISTATPYMNGETSTKSLWVINSALRIKILCATYVNVNIRDIDKIYVRTGIYHGGEPLCDNVNTQRVPCSNPRWNEWLNYDIYIPDLPRAARLCLSICSVKGRKGAKEEHCPLAWGNINLFDYTDTLVSGKMALNLWPVPHGLEDLLNPIGVTGSNPNKETPCLELEFDWFSSVVKFPDMSVIEEHANWSVSREAGFSYSHAGLSNRLARDNELRENDKEQLKAISTRDPLSEITEQEKDFLWSHRHYCVTIPEILPKLLLSVKWNSRDEVAQMYCLVKDWPPIKPEQAMELLDCNYPDPMVRGFAVRCLEKYLTDDKLSQYLIQLVQVLKYEQYLDNLLVRFLLKKALTNQRIGHFFFWHLKSEMHNKTVSQRFGLLLESYCRACGMYLKHLNRQVEAMEKLINLTDILKQEKKDETQKVQMKFLVEQMRRPDFMDALQGFLSPLNPAHQLGNLRLEECRIMSSAKRPLWLNWENPDIMSELLFQNNEIIFKNGDDLRQDMLTLQIIRIMENIWQNQGLDLRMLPYGCLSIGDCVGLIEVVRNSHTIMQIQCKGGLKGALQFNSHTLHQWLKDKNKGEIYDAAIDLFTRSCAGYCVATFILGIGDRHNSNIMVKDDGQLFHIDFGHFLDHKKKKFGYKRERVPFVLTQDFLIVISKGAQECTKTREFERFQEMCYKAYLAIRQHANLFINLFSMMLGSGMPELQSFDDIAYIRKTLALDKTEQEALEYFMKQMNDAHHGGWTTKMDWIFHTIKQHALN

304 NP_057472.2 NP_002341.1

>NP_057472.2

MSGISGCPFFLWGLLALLGLALVISLIFNISHYVEKQRQDKMYSYSSDHTRVDEYYIEDTPIYGNLDDMISEPMDENCYEQMKARPEKSVNKMQEATPSAQATNETQMCYASLDHSVKGKRRKPRKQNTHFSDKDGDEQLHAIDASVSKTTLVDSFSPESQAVEENIHDDPIRLFGLIRAKREPIN

>NP_002341.1

MGCIKSKGKDSLSDDGVDLKTQPVRNTERTIYVRDPTSNKQQRPVPESQLLPGQRFQTKDPEEQGDIVVALYPYDGIHPDDLSFKKGEKMKVLEEHGEWWKAKSLLTKKEGFIPSNYVAKLNTLETEEWFFKDITRKDAERQLLAPGNSAGAFLIRESETLKGSFSLSVRDFDPVHGDVIKHYKIRSLDNGGYYISPRITFPCISDMIKHYQKQADGLCRRLEKACISPKPQKPWDKDAWEIPRESIKLVKRLGAGQFGEVWMGYYNNSTKVAVKTLKPGTMSVQAFLEEANLMKTLQHDKLVRLYAVVTREEPIYIITEYMAKGSLLDFLKSDEGGKVLLPKLIDFSAQIAEGMAYIERKNYIHRDLRAANVLVSESLMCKIADFGLARVIEDNEYTAREGAKFPIKWTAPEAINFGCFTIKSDVWSFGILLYEIVTYGKIPYPGRTNADVMTALSQGYRMPRVENCPDELYDIMKMCWKEKAEERPTFDYLQSVLDDFYTATEGQYQQQP

305 NP_000312.2 NP_009225.1

>NP_000312.2

MPPKTPRKTAATAAAAAAEPPAPPPPPPPEEDPEQDSGPEDLPLVRLEFEETEEPDFTALCQKLKIPDHVRERAWLTWEKVSSVDGVLGGYIQKKKELWGICIFIAAVDLDEMSFTFTELQKNIEISVHKFFNLLKEIDTSTKVDNAMSRLLKKYDVLFALFSKLERTCELIYLTQPSSSISTEINSALVLKVSWITFLLAKGEVLQMEDDLVISFQLMLCVLDYFIKLSPPMLLKEPYKTAVIPINGSPRTPRRGQNRSARIAKQLENDTRIIEVLCKEHECNIDEVKNVYFKNFIPFMNSLGLVTSNGLPEVENLSKRYEEIYLKNKDLDARLFLDHDKTLQTDSIDSFETQRTPRKSNLDEEVNVIPPHTPVRTVMNTIQQLMMILNSASDQPSENLISYFNNCTVNPKESILKRVKDIGYIFKEKFAKAVGQGCVEIGSQRYKLGVRLYYRVMESMLKSEEERLSIQNFSKLLNDNIFHMSLLACALEVVMATYSRSTSQNLDSGTDLSFPWILNVLNLKAFDFYKVIESFIKAEGNLTREMIKHLERCEHRIMESLAWLSDSPLFDLIKQSKDREGPTDHLESACPLNLPLQNNHTAADMYLSPVRSPKKKGSTTRVNSTANAETQATSAFQTQKPLKSTSLSLFYKKVYRLAYLRLNTLCERLLSEHPELEHIIWTLFQHTLQNEYELMRDRHLDQIMMCSMYGICKVKNIDLKFKIIVTAYKDLPHAVQETFKRVLIKEEEYDSIIVFYNSVFMQRLKTNILQYASTRPPTLSPIPHIPRSPYKFPSSPLRIPGGNIYISPLKSPYKISEGLPTPTKMTPRSRILVSIGESFGTSEKFQKINQMVCNSDRVLKRSAEGSNPPKPLKKLRFDIEGSDEADGSKHLPGESKFQQKLAEMTSTRTRMQKQKMNDSMDTSNKEEK

>NP_009225.1

MDLSALRVEEVQNVINAMQKILECPICLELIKEPVSTKCDHIFCKFCMLKLLNQKKGPSQCPLCKNDITKRSLQESTRFSQLVEELLKIICAFQLDTGLEYANSYNFAKKENNSPEHLKDEVSIIQSMGYRNRAKRLLQSEPENPSLQETSLSVQLSNLGTVRTLRTKQRIQPQKTSVYIELGSDSSEDTVNKATYCSVGDQELLQITPQGTRDEISLDSAKKAACEFSETDVTNTEHHQPSNNDLNTTEKRAAERHPEKYQGSSVSNLHVEPCGTNTHASSLQHENSSLLLTKDRMNVEKAEFCNKSKQPGLARSQHNRWAGSKETCNDRRTPSTEKKVDLNADPLCERKEWNKQKLPCSENPRDTEDVPWITLNSSIQKVNEWFSRSDELLGSDDSHDGESESNAKVADVLDVLNEVDEYSGSSEKIDLLASDPHEALICKSERVHSKSVESNIEDKIFGKTYRKKASLPNLSHVTENLIIGAFVTEPQIIQERPLTNKLKRKRRPTSGLHPEDFIKKADLAVQKTPEMINQGTNQTEQNGQVMNITNSGHENKTKGDSIQNEKNPNPIESLEKESAFKTKAEPISSSISNMELELNIHNSKAPKKNRLRRKSSTRHIHALELVVSRNLSPPNCTELQIDSCSSSEEIKKKKYNQMPVRHSRNLQLMEGKEPATGAKKSNKPNEQTSKRHDSDTFPELKLTNAPGSFTKCSNTSELKEFVNPSLPREEKEEKLETVKVSNNAEDPKDLMLSGERVLQTERSVESSSISLVPGTDYGTQESISLLEVSTLGKAKTEPNKCVSQCAAFENPKGLIHGCSKDNRNDTEGFKYPLGHEVNHSRETSIEMEESELDAQYLQNTFKVSKRQSFAPFSNPGNAEEECATFSAHSGSLKKQSPKVTFECEQKEENQGKNESNIKPVQTVNITAGFPVVGQKDKPVDNAKCSIKGGSRFCLSSQFRGNETGLITPNKHGLLQNPYRIPPLFPIKSFVKTKCKKNLLEENFEEHSMSPEREMGNENIPSTVSTISRNNIRENVFKEASSSNINEVGSSTNEVGSSINEIGSSDENIQAELGRNRGPKLNAMLRLGVLQPEVYKQSLPGSNCKHPEIKKQEYEEVVQTVNTDFSPYLISDNLEQPMGSSHASQVCSETPDDLLDDGEIKEDTSFAENDIKESSAVFSKSVQKGELSRSPSPFTHTHLAQGYRRGAKKLESSEENLSSEDEELPCFQHLLFGKVNNIPSQSTRHSTVATECLSKNTEENLLSLKNSLNDCSNQVILAKASQEHHLSEETKCSASLFSSQCSELEDLTANTNTQDPFLIGSSKQMRHQSESQGVGLSDKELVSDDEERGTGLEENNQEEQSMDSNLGEAASGCESETSVSEDCSGLSSQSDILTTQQRDTMQHNLIKLQQEMAELEAVLEQHGSQPSNSYPSIISDSSALEDLRNPEQSTSEKAVLTSQKSSEYPISQNPEGLSADKFEVSADSSTSKNKEPGVERSSPSKCPSLDDRWYMHSCSGSLQNRNYPSQEELIKVVDVEEQQLEESGPHDLTETSYLPRQDLEGTPYLESGISLFSDDPESDPSEDRAPESARVGNIPSSTSALKVPQLKVAESAQSPAAAHTTDTAGYNAMEESVSREKPELTASTERVNKRMSMVVSGLTPEEFMLVYKFARKHHITLTNLITEETTHVVMKTDAEFVCERTLKYFLGIAGGKWVVSYFWVTQSIKERKMLNEHDFEVRGDVVNGRNHQGPKRARESQDRKIFRGLEICCYGPFTNMPTDQLEWMVQLCGASVVKELSSFTLGTGVHPIVVVQPDAWTEDNGFHAIGQMCEAPVVTREWVLDSVALYQCQELDTYLIPQIPHSHY

306 NP_874369.1 NP_001420.2

>NP_874369.1

MAEVVSPVPGAGRREPGEVGRARGPPVADPGVALSPQGEIIEGCRLPVLRRNQDNEDEWPLAEILSVKDISGRKLFYVHYIDFNKRLDEWVTHERLDLKKIQFPKKEAKTPTKNGLPGSRPGSPEREVPASAQASGKTLPIPVQITLRFNLPKEREAIPGGEPDQPLSSSSCLQPNHRSTKRKVEVVSPATPVPSETAPASVFPQNGAARRAVAAQPGRKRKSNCLGTDEDSQDSSDGIPSAPRMTGSLVSDRSHDDIVTRMKNIECIELGRHRLKPWYFSPYPQELTTLPVLYLCEFCLKYGRSLKCLQRHLTKCDLRHPPGNEIYRKGTISFFEIDGRKNKSYSQNLCLLAKCFLDHKTLYYDTDPFLFYVMTEYDCKGFHIVGYFSKEKESTEDYNVACILTLPPYQRRGYGKLLIEFSYELSKVEGKTGTPEKPLSDLGLLSYRSYWSQTILEILMGLKSESGERPQITINEISEITSIKKEDVISTLQYLNLINYYKGQYILTLSEDIVDGHERAMLKRLLRIDSKCLHFTPKDWSKRGKW

>NP_001420.2

MAENVVEPGPPSAKRPKLSSPALSASASDGTDFGSLFDLEHDLPDELINSTELGLTNGGDINQLQTSLGMVQDAASKHKQLSELLRSGSSPNLNMGVGGPGQVMASQAQQSSPGLGLINSMVKSPMTQAGLTSPNMGMGTSGPNQGPTQSTGMMNSPVNQPAMGMNTGMNAGMNPGMLAAGNGQGIMPNQVMNGSIGAGRGRQNMQYPNPGMGSAGNLLTEPLQQGSPQMGGQTGLRGPQPLKMGMMNNPNPYGSPYTQNPGQQIGASGLGLQIQTKTVLSNNLSPFAMDKKAVPGGGMPNMGQQPAPQVQQPGLVTPVAQGMGSGAHTADPEKRKLIQQQLVLLLHAHKCQRREQANGEVRQCNLPHCRTMKNVLNHMTHCQSGKSCQVAHCASSRQIISHWKNCTRHDCPVCLPLKNAGDKRNQQPILTGAPVGLGNPSSLGVGQQSAPNLSTVSQIDPSSIERAYAALGLPYQVNQMPTQPQVQAKNQQNQQPGQSPQGMRPMSNMSASPMGVNGGVGVQTPSLLSDSMLHSAINSQNPMMSENASVPSLGPMPTAAQPSTTGIRKQWHEDITQDLRNHLVHKLVQAIFPTPDPAALKDRRMENLVAYARKVEGDMYESANNRAEYYHLLAEKIYKIQKELEEKRRTRLQKQNMLPNAAGMVPVSMNPGPNMGQPQPGMTSNGPLPDPSMIRGSVPNQMMPRITPQSGLNQFGQMSMAQPPIVPRQTPPLQHHGQLAQPGALNPPMGYGPRMQQPSNQGQFLPQTQFPSQGMNVTNIPLAPSSGQAPVSQAQMSSSSCPVNSPIMPPGSQGSHIHCPQLPQPALHQNSPSPVPSRTPTPHHTPPSIGAQQPPATTIPAPVPTPPAMPPGPQSQALHPPPRQTPTPPTTQLPQQVQPSLPAAPSADQPQQQPRSQQSTAASVPTPTAPLLPPQPATPLSQPAVSIEGQVSNPPSTSSTEVNSQAIAEKQPSQEVKMEAKMEVDQPEPADTQPEDISESKVEDCKMESTETEERSTELKTEIKEEEDQPSTSATQSSPAPGQSKKKIFKPEELRQALMPTLEALYRQDPESLPFRQPVDPQLLGIPDYFDIVKSPMDLSTIKRKLDTGQYQEPWQYVDDIWLMFNNAWLYNRKTSRVYKYCSKLSEVFEQEIDPVMQSLGYCCGRKLEFSPQTLCCYGKQLCTIPRDATYYSYQNRYHFCEKCFNEIQGESVSLGDDPSQPQTTINKEQFSKRKNDTLDPELFVECTECGRKMHQICVLHHEIIWPAGFVCDGCLKKSARTRKENKFSAKRLPSTRLGTFLENRVNDFLRRQNHPESGEVTVRVVHASDKTVEVKPGMKARFVDSGEMAESFPYRTKALFAFEEIDGVDLCFFGMHVQEYGSDCPPPNQRRVYISYLDSVHFFRPKCLRTAVYHEILIGYLEYVKKLGYTTGHIWACPPSEGDDYIFHCHPPDQKIPKPKRLQEWYKKMLDKAVSERIVHDYKDIFKQATEDRLTSAKELPYFEGDFWPNVLEESIKELEQEEEERKREENTSNESTDVTKGDSKNAKKKNNKKTSKNKSSLSRGNKKKPGMPNVSNDLSQKLYATMEKHKEVFFVIRLIAGPAANSLPPIVDPDPLIPCDLMDGRDAFLTLARDKHLEFSSLRRAQWSTMCMLVELHTQSQDRFVYTCNECKHHVETRWHCTVCEDYDLCITCYNTKNHDHKMEKLGLGLDDESNNQQAAATQSPGDSRRLSIQRCIQSLVHACQCRNANCSLPSCQKMKRVVQHTKGCKRKTNGGCPICKQLIALCCYHAKHCQENKCPVPFCLNIKQKLRQQQLQHRLQQAQMLRRRMASMQRTGVVGQQQGLPSPTPATPTTPTGQQPTTPQTPQPTSQPQPTPPNSMPPYLPRTQAAGPVSQGKAAGQVTPPTPPQTAQPPLPGPPPAAVEMAMQIQRAAETQRQMAHVQIFQRPIQHQMPPMTPMAPMGMNPPPMTRGPSGHLEPGMGPTGMQQQPPWSQGGLPQPQQLQSGMPRPAMMSVAQHGQPLNMAPQPGLGQVGISPLKPGTVSQQALQNLLRTLRSPSSPLQQQQVLSILHANPQLLAAFIKQRAAKYANSNPQPIPGQPGMPQGQPGLQPPTMPGQQGVHSNPAMQNMNPMQAGVQRAGLPQQQPQQQLQPPMGGMSPQAQQMNMNHNTMPSQFRDILRRQQMMQQQQQQGAGPGIGPGMANHNQFQQPQGVGYPPQQQQRMQHHMQQMQQGNMGQIGQLPQALGAEAGASLQAYQQRLLQQQMGSPVQPNPMSPQQHMLPNQAQSPHLQGQQIPNSLSNQVRSPQPVPSPRPQSQPPHSSPSPRMQPQPSPHHVSPQTSSPHPGLVAAQANPMEQGHFASPDQNSMLSQLASNPGMANLHGASATDLGLSTDNSDLNSNLSQSTLDIH

307 NP_009297.2 NP_001460.1

>NP_009297.2

MGQQPGKVLGDQRRPSLPALHFIKGAGKKESSRHGGPHCNVFVEHEALQRPVASDFEPQGLSEAARWNSKENLLAGPSENDPNLFVALYDFVASGDNTLSITKGEKLRVLGYNHNGEWCEAQTKNGQGWVPSNYITPVNSLEKHSWYHGPVSRNAAEYLLSSGINGSFLVRESESSPGQRSISLRYEGRVYHYRINTASDGKLYVSSESRFNTLAELVHHHSTVADGLITTLHYPAPKRNKPTVYGVSPNYDKWEMERTDITMKHKLGGGQYGEVYEGVWKKYSLTVAVKTLKEDTMEVEEFLKEAAVMKEIKHPNLVQLLGVCTREPPFYIITEFMTYGNLLDYLRECNRQEVNAVVLLYMATQISSAMEYLEKKNFIHRDLAARNCLVGENHLVKVADFGLSRLMTGDTYTAHAGAKFPIKWTAPESLAYNKFSIKSDVWAFGVLLWEIATYGMSPYPGIDLSQVYELLEKDYRMERPEGCPEKVYELMRACWQWNPSDRPSFAEIHQAFETMFQESSISDEVEKELGKQGVRGAVSTLLQAPELPTKTRTSRRAAEHRDTTDVPEMPHSKGQGESDPLDHEPAVSPLLPRKERGPPEGGLNEDERLLPKDKKTNLFSALIKKKKKTAPTPPKRSSSFREMDGQPERRGAGEEEGRDISNGALAFTPLDTADPAKSPKPSNGAGVPNGALRESGGSGFRSPHLWKKSSTLTSSRLATGEEEGGGSSSKRFLRSCSASCVPHGAKDTEWRSVTLPRDLQSTGRQFDSSTFGGHKSEKPALPRKRAGENRSDQVTRGTVTPPPRLVKKNEEAADEVFKDIMESSPGSSPPNLTPKPLRRQVTVAPASGLPHKEEAGKGSALGTPAAAEPVTPTSKAGSGAPGGTSKGPAEESRVRRHKHSSESPGRDKGKLSRLKPAPPPPPAASAGKAGGKPSQSPSQEAAGEAVLGAKTKATSLVDAVNSDAAKPSQPGEGLKKPVLPATPKPQSAKPSGTPISPAPVPSTLPSASSALAGDQPSSTAFIPLISTRVSLRKTRQPPERIASGAITKGVVLDSTEALCLAISRNSEQMASHSAVLEAGKNLYTFCVSYVDSIQQMRNKFAFREAINKLENNLRELQICPATAGSGPAATQDFSKLLSSVKEISDIVQR

>NP_001460.1

MSGWESYYKTEGDEEAEEEQEENLEASGDYKYSGRDSLIFLVDASKAMFESQSEDELTPFDMSIQCIQSVYISKIISSDRDLLAVVFYGTEKDKNSVNFKNIYVLQELDNPGAKRILELDQFKGQQGQKRFQDMMGHGSDYSLSEVLWVCANLFSDVQFKMSHKRIMLFTNEDNPHGNDSAKASRARTKAGDLRDTGIFLDLMHLKKPGGFDISLFYRDIISIAEDEDLRVHFEESSKLEDLLRKVRAKETRKRALSRLKLKLNKDIVISVGIYNLVQKALKPPPIKLYRETNEPVKTKTRTFNTSTGGLLLPSDTKRSQIYGSRQIILEKEETEELKRFDDPGLMLMGFKPLVLLKKHHYLRPSLFVYPEESLVIGSSTLFSALLIKCLEKEVAALCRYTPRRNIPPYFVALVPQEEELDDQKIQVTPPGFQLVFLPFADDKRKMPFTEKIMATPEQVGKMKAIVEKLRFTYRSDSFENPVLQQHFRNLEALALDLMEPEQAVDLTLPKVEAMNKRLGSLVDEFKELVYPPDYNPEGKVTKRKHDNEGSGSKRPKVEYSEEELKTHISKGTLGKFTVPMLKEACRAYGLKSGLKKQELLEALTKHFQD

308 NP_002816.1 NP_002806.2

>NP_002816.1

MQAQQYQQQRRKFAAAFLAFIFILAAVDTAEAGKKEKPEKKVKKSDCGEWQWSVCVPTSGDCGLGTREGTRTGAECKQTMKTQRCKIPCNWKKQFGAECKYQFQAWGECDLNTALKTRTGSLKRALHNAECQKTVTISKPCGKLTKPKPQAESKKKKKEGKKQEKMLD

>NP_002806.2

MAAAAVVEFQRAQSLLSTDREASIDILHSIVKRDIQENDEEAVQVKEQSILELGSLLAKTGQAAELGGLLKYVRPFLNSISKAKAARLVRSLLDLFLDMEAATGQEVELCLECIEWAKSEKRTFLRQALEARLVSLYFDTKRYQEALHLGSQLLRELKKMDDKALLVEVQLLESKTYHALSNLPKARAALTSARTTANAIYCPPKLQATLDMQSGIIHAAEEKDWKTAYSYFYEAFEGYDSIDSPKAITSLKYMLLCKIMLNTPEDVQALVSGKLALRYAGRQTEALKCVAQASKNRSLADFEKALTDYRAELRDDPIISTHLAKLYDNLLEQNLIRVIEPFSRVQIEHISSLIKLSKADVERKLSQMILDKKFHGILDQGEGVLIIFDEPPVDKTYEAALETIQNMSKVVDSLYNKAKKLT

309 NP_001253.1 NP_068597.2

>NP_001253.1

MAEPWGNELASAAARGDLEQLTSLLQNNVNVNAQNGFGRTALQVMKLGNPEIARRLLLRGANPDLKDRTGFAVIHDAARAGFLDTLQTLLEFQADVNIEDNEGNLPLHLAAKEGHLRVVEFLVKHTASNVGHRNHKGDTACDLARLYGRNEVVSLMQANGAGGATNLQ

>NP_068597.2

MNSRQAWRLFLSQGRGDRWVSRPRGHFSPALRREFFTTTTKEGYDRRPVDITPLEQRKLTFDTHALVQDLETHGFDKTQAETIVSALTALSNVSLDTIYKEMVTQAQQEITVQQLMAHLDAIRKDMVILEKSEFANLRAENEKMKIELDQVKQQLMHETSRIRADNKLDINLERSRVTDMFTDQEKQLMETTTEFTKKDTQTKSIISETSNKIDAEIASLKTLMESNKLETIRYLAASVFTCLAIALGFYRFWK

310 NP_001009944.1 NP_001006611.1

>NP_001009944.1

MPPAAPARLALALGLGLWLGALAGGPGRGCGPCEPPCLCGPAPGAACRVNCSGRGLRTLGPALRIPADATALDVSHNLLRALDVGLLANLSALAELDISNNKISTLEEGIFANLFNLSEINLSGNPFECDCGLAWLPRWAEEQQVRVVQPEAATCAGPGSLAGQPLLGIPLLDSGCGEEYVACLPDNSSGTVAAVSFSAAHEGLLQPEACSAFCFSTGQGLAALSEQGWCLCGAAQPSSASFACLSLCSGPPPPPAPTCRGPTLLQHVFPASPGATLVGPHGPLASGQLAAFHIAAPLPVTATRWDFGDGSAEVDAAGPAASHRYVLPGRYHVTAVLALGAGSALLGTDVQVEAAPAALELVCPSSVQSDESLDLSIQNRGGSGLEAAYSIVALGEEPARAVHPLCPSDTEIFPGNGHCYRLVVEKAAWLQAQEQCQAWAGAALAMVDSPAVQRFLVSRVTRSLDVWIGFSTVQGVEVGPAPQGEAFSLESCQNWLPGEPHPATAEHCVRLGPTGWCNTDLCSAPHSYVCELQPGGPVQDAENLLVGAPSGDLQGPLTPLAQQDGLSAPHEPVEVMVFPGLRLSREAFLTTAEFGTQELRRPAQLRLQVYRLLSTAGTPENGSEPESRSPDNRTQLAPACMPGGRWCPGANICLPLDASCHPQACANGCTSGPGLPGAPYALWREFLFSVPAGPPAQYSVTLHGQDVLMLPGDLVGLQHDAGPGALLHCSPAPGHPGPRAPYLSANASSWLPHLPAQLEGTWACPACALRLLAATEQLTVLLGLRPNPGLRLPGRYEVRAEVGNGVSRHNLSCSFDVVSPVAGLRVIYPAPRDGRLYVPTNGSALVLQVDSGANATATARWPGGSVSARFENVCPALVATFVPGCPWETNDTLFSVVALPWLSEGEHVVDVVVENSASRANLSLRVTAEEPICGLRATPSPEARVLQGVLVRYSPVVEAGSDMVFRWTINDKQSLTFQNVVFNVIYQSAAVFKLSLTASNHVSNVTVNYNVTVERMNRMQGLQVSTVPAVLSPNATLALTAGVLVDSAVEVAFLWTFGDGEQALHQFQPPYNESFPVPDPSVAQVLVEHNVMHTYAAPGEYLLTVLASNAFENLTQQVPVSVRASLPSVAVGVSDGVLVAGRPVTFYPHPLPSPGGVLYTWDFGDGSPVLTQSQPAANHTYASRGTYHVRLEVNNTVSGAAAQADVRVFEELRGLSVDMSLAVEQGAPVVVSAAVQTGDNITWTFDMGDGTVLSGPEATVEHVYLRAQNCTVTVGAASPAGHLARSLHVLVFVLEVLRVEPAACIPTQPDARLTAYVTGNPAHYLFDWTFGDGSSNTTVRGCPTVTHNFTRSGTFPLALVLSSRVNRAHYFTSICVEPEVGNVTLQPERQFVQLGDEAWLVACAWPPFPYRYTWDFGTEEAAPTRARGPEVTFIYRDPGSYLVTVTASNNISAANDSALVEVQEPVLVTSIKVNGSLGLELQQPYLFSAVGRGRPASYLWDLGDGGWLEGPEVTHAYNSTGDFTVRVAGWNEVSRSEAWLNVTVKRRVRGLVVNASRTVVPLNGSVSFSTSLEAGSDVRYSWVLCDRCTPIPGGPTISYTFRSVGTFNIIVTAENEVGSAQDSIFVYVLQLIEGLQVVGGGRYFPTNHTVQLQAVVRDGTNVSYSWTAWRDRGPALAGSGKGFSLTVLEAGTYHVQLRATNMLGSAWADCTMDFVEPVGWLMVAASPNPAAVNTSVTLSAELAGGSGVVYTWSLEEGLSWETSEPFTTHSFPTPGLHLVTMTAGNPLGSANATVEVDVQVPVSGLSIRASEPGGSFVAAGSSVPFWGQLATGTNVSWCWAVPGGSSKRGPHVTMVFPDAGTFSIRLNASNAVSWVSATYNLTAEEPIVGLVLWASSKVVAPGQLVHFQILLAAGSAVTFRLQVGGANPEVLPGPRFSHSFPRVGDHVVSVRGKNHVSWAQAQVRIVVLEAVSGLQVPNCCEPGIATGTERNFTARVQRGSRVAYAWYFSLQKVQGDSLVILSGRDVTYTPVAAGLLEIQVRAFNALGSENRTLVLEVQDAVQYVALQSGPCFTNRSAQFEAATSPSPRRVAYHWDFGDGSPGQDTDEPRAEHSYLRPGDYRVQVNASNLVSFFVAQATVTVQVLACREPEVDVVLPLQVLMRRSQRNYLEAHVDLRDCVTYQTEYRWEVYRTASCQRPGRPARVALPGVDVSRPRLVLPRLALPVGHYCFVFVVSFGDTPLTQSIQANVTVAPERLVPIIEGGSYRVWSDTRDLVLDGSESYDPNLEDGDQTPLSFHWACVASTQREAGGCALNFGPRGSSTVTIPRERLAAGVEYTFSLTVWKAGRKEEATNQTVLIRSGRVPIVSLECVSCKAQAVYEVSRSSYVYLEGRCLNCSSGSKRGRWAARTFSNKTLVLDETTTSTGSAGMRLVLRRGVLRDGEGYTFTLTVLGRSGEEEGCASIRLSPNRPPLGGSCRLFPLGAVHALTTKVHFECTGWHDAEDAGAPLVYALLLRRCRQGHCEEFCVYKGSLSSYGAVLPPGFRPHFEVGLAVVVQDQLGAAVVALNRSLAITLPEPNGSATGLTVWLHGLTASVLPGLLRQADPQHVIEYSLALVTVLNEYERALDVAAEPKHERQHRAQIRKNITETLVSLRVHTVDDIQQIAAALAQCMGPSRELVCRSCLKQTLHKLEAMMLILQAETTAGTVTPTAIGDSILNITGDLIHLASSDVRAPQPSELGAESPSRMVASQAYNLTSALMRILMRSRVLNEEPLTLAGEEIVAQGKRSDPRSLLCYGGAPGPGCHFSIPEAFSGALANLSDVVQLIFLVDSNPFPFGYISNYTVSTKVASMAFQTQAGAQIPIERLASERAITVKVPNNSDWAARGHRSSANSANSVVVQPQASVGAVVTLDSSNPAAGLHLQLNYTLLDGHYLSEEPEPYLAVYLHSEPRPNEHNCSASRRIRPESLQGADHRPYTFFISPGSRDPAGSYHLNLSSHFRWSALQVSVGLYTSLCQYFSEEDMVWRTEGLLPLEETSPRQAVCLTRHLTAFGASLFVPPSHVRFVFPEPTADVNYIVMLTCAVCLVTYMVMAAILHKLDQLDASRGRAIPFCGQRGRFKYEILVKTGWGRGSGTTAHVGIMLYGVDSRSGHRHLDGDRAFHRNSLDIFRIATPHSLGSVWKIRVWHDNKGLSPAWFLQHVIVRDLQTARSAFFLVNDWLSVETEANGGLVEKEVLAASDAALLRFRRLLVAELQRGFFDKHIWLSIWDRPPRSRFTRIQRATCCVLLICLFLGANAVWYGAVGDSAYSTGHVSRLSPLSVDTVAVGLVSSVVVYPVYLAILFLFRMSRSKVAGSPSPTPAGQQVLDIDSCLDSSVLDSSFLTFSGLHAEQAFVGQMKSDLFLDDSKSLVCWPSGEGTLSWPDLLSDPSIVGSNLRQLARGQAGHGLGPEEDGFSLASPYSPAKSFSASDEDLIQQVLAEGVSSPAPTQDTHMETDLLSSLSSTPGEKTETLALQRLGELGPPSPGLNWEQPQAARLSRTGLVEGLRKRLLPAWCASLAHGLSLLLVAVAVAVSGWVGASFPPGVSVAWLLSSSASFLASFLGWEPLKVLLEALYFSLVAKRLHPDEDDTLVESPAVTPVSARVPRVRPPHGFALFLAKEEARKVKRLHGMLRSLLVYMLFLLVTLLASYGDASCHGHAYRLQSAIKQELHSRAFLAITRSEELWPWMAHVLLPYVHGNQSSPELGPPRLRQVRLQEALYPDPPGPRVHTCSAAGGFSTSDYDVGWESPHNGSGTWAYSAPDLLGAWSWGSCAVYDSGGYVQELGLSLEESRDRLRFLQLHNWLDNRSRAVFLELTRYSPAVGLHAAVTLRLEFPAAGRALAALSVRPFALRRLSAGLSLPLLTSVCLLLFAVHFAVAEARTWHREGRWRVLRLGAWARWLLVALTAATALVRLAQLGAADRQWTRFVRGRPRRFTSFDQVAQLSSAARGLAASLLFLLLVKAAQQLRFVRQWSVFGKTLCRALPELLGVTLGLVVLGVAYAQLAILLVSSCVDSLWSVAQALLVLCPGTGLSTLCPAESWHLSPLLCVGLWALRLWGALRLGAVILRWRYHALRGELYRPAWEPQDYEMVELFLRRLRLWMGLSKVKEFRHKVRFEGMEPLPSRSSRGSKVSPDVPPPSAGSDASHPSTSSSQLDGLSVSLGRLGTRCEPEPSRLQAVFEALLTQFDRLNQATEDVYQLEQQLHSLQGRRSSRAPAGSSRGPSPGLRPALPSRLARASRGVDLATGPSRTPLRAKNKVHPSST

>NP_001006611.1

MTGKATPPSLYSWRGVLFTCLPAARTRKRKEMSRQTATALPTGTSKCPPSQRVPALTGTTASNNDLASLFECPVCFDYVLPPILQCQSGHLVCSNCRPKLTCCPTCRGPLGSIRNLAMEKVANSVLFPCKYASSGCEITLPHTEKADHEELCEFRPYSCPCPGASCKWQGSLDAVMPHLMHQHKSITTLQGEDIVFLATDINLPGAVDWVMMQSCFGFHFMLVLEKQEKYDGHQQFFAIVQLIGTRKQAENFAYRLELNGHRRRLTWEATPRSIHEGIATAIMNSDCLVFDTSIAQLFAENGNLGINVTISMC

311 NP_008881.2 NP_002651.2

>NP_008881.2

MNYLRRRLSDSNFMANLPNGYMTDLQRPQPPPPPPGAHSPGATPGPGTATAERSSGVAPAASPAAPSPGSSGGGGFFSSLSNAVKQTTAAAAATFSEQVGGGSGGAGRGGAASRVLLVIDEPHTDWAKYFKGKKIHGEIDIKVEQAEFSDLNLVAHANGGFSVDMEVLRNGVKVVRSLKPDFVLIRQHAFSMARNGDYRSLVIGLQYAGIPSVNSLHSVYNFCDKPWVFAQMVRLHKKLGTEEFPLIDQTFYPNHKEMLSSTTYPVVVKMGHAHSGMGKVKVDNQHDFQDIASVVALTKTYATAEPFIDAKYDVRVQKIGQNYKAYMRTSVSGNWKTNTGSAMLEQIAMSDRYKLWVDTCSEIFGGLDICAVEALHGKDGRDHIIEVVGSSMPLIGDHQDEDKQLIVELVVNKMAQALPRQRQRDASPGRGSHGQTPSPGALPLGRQTSQQPAGPPAQQRPPPQGGPPQPGPGPQRQGPPLQQRPPPQGQQHLSGLGPPAGSPLPQRLPSPTSAPQQPASQAAPPTQGQGRQSRPVAGGPGAPPAARPPASPSPQRQAGPPQATRQTSVSGPAPPKASGAPPGGQQRQGPPQKPPGPAGPTRQASQAGPVPRTGPPTTQQPRPSGPGPAGRPKPQLAQKPSQDVPPPATAAAGGPPHPQLNKSQSLTNAFNLPEPAPPRPSLSQDEVKAETIRSLRKSFASLFSD

>NP_002651.2

MAGAASPCANGCGPGAPSDAEVLHLCRSLEVGTVMTLFYSKKSQRPERKTFQVKLETRQITWSRGADKIEGAIDIREIKEIRPGKTSRDFDRYQEDPAFRPDQSHCFVILYGMEFRLKTLSLQATSEDEVNMWIKGLTWLMEDTLQAPTPLQIERWLRKQFYSVDRNREDRISAKDLKNMLSQVNYRVPNMRFLRERLTDLEQRSGDITYGQFAQLYRSLMYSAQKTMDLPFLEASTLRAGERPELCRVSLPEFQQFLLDYQGELWAVDRLQVQEFMLSFLRDPLREIEEPYFFLDEFVTFLFSKENSVWNSQLDAVCPDTMNNPLSHYWISSSHNTYLTGDQFSSESSLEAYARCLRMGCRCIELDCWDGPDGMPVIYHGHTLTTKIKFSDVLHTIKEHAFVASEYPVILSIEDHCSIAQQRNMAQYFKKVLGDTLLTKPVEISADGLPSPNQLKRKILIKHKKLAEGSAYEEVPTSMMYSENDISNSIKNGILYLEDPVNHEWYPHYFVLTSSKIYYSEETSSDQGNEDEEEPKEVSSSTELHSNEKWFHGKLGAGRDGRHIAERLLTEYCIETGAPDGSFLVRESETFVGDYTLSFWRNGKVQHCRIHSRQDAGTPKFFLTDNLVFDSLYDLITHYQQVPLRCNEFEMRLSEPVPQTNAHESKEWYHASLTRAQAEHMLMRVPRDGAFLVRKRNEPNSYAISFRAEGKIKHCRVQQEGQTVMLGNSEFDSLVDLISYYEKHPLYRKMKLRYPINEEALEKIGTAEPDYGALYEGRNPGFYVEANPMPTFKCAVKALFDYKAQREDELTFIKSAIIQNVEKQEGGWWRGDYGGKKQLWFPSNYVEEMVNPVALEPEREHLDENSPLGDLLRGVLDVPACQIAIRPEGKNNRLFVFSISMASVAHWSLDVAADSQEELQDWVKKIREVAQTADARLTEGKIMERRKKIALELSELVVYCRPVPFDEEKIGTERACYRDMSSFPETKAEKYVNKAKGKKFLQYNRLQLSRIYPKGQRLDSSNYDPLPMWICGSQLVALNFQTPDKPMQMNQALFMTGRHCGYVLQPSTMRDEAFDPFDKSSLRGLEPCAISIEVLGARHLPKNGRGIVCPFVEIEVAGAEYDSTKQKTEFVVDNGLNPVWPAKPFHFQISNPEFAFLRFVVYEEDMFSDQNFLAQATFPVKGLKTGYRAVPLKNNYSEDLELASLLIKIDIFPAKENGDLSPFSGTSLRERGSDASGQLFHGRAREGSFESRYQQPFEDFRISQEHLADHFDSRERRAPRRTRVNGDNRL

312 NP_056036.1 NP_543157.1

>NP_056036.1

MEAGSVVRAIFDFCPSVSEELPLFVGDIIEVLAVVDEFWLLGKKEDVTGQFPSSFVEIVTIPSLKEGERLFVCICEFTSQELDNLPLHRGDLVILDGIPTAGWLQGRSCWGARGFFPSSCVRELCLSSQSRQWHSQSALFQIPEYSMGQARALMGLSAQLDEELDFREGDVITIIGVPEPGWFEGELEGRRGIFPEGFVELLGPLRTVDESVSSGNQDDCIVNGEVDTPVGEEEIGPDEDEEEPGTYGVALYRFQALEPNELDFEVGDKIRILATLEDGWLEGSLKGRTGIFPYRFVKLCPDTRVEETMALPQEGSLARIPETSLDCLENTLGVEEQRHETSDHEAEEPDCIISEAPTSPLGHLTSEYDTDRNSYQDEDTAGGPPRSPGVEWEMPLATDSPTSDPTEVVNGISSQPQVPFHPNLQKSQYYSTVGGSHPHSEQYPDLLPLEARTRDYASLPPKRMYSQLKTLQKPVLPLYRGSSVSASRVVKPRQSSPQLHNLASYTKKHHTSSVYSISERLEMKPGPQAQGLVMEAATHSQGDGSTDLDSKLTQQLIEFEKSLAGPGTEPDKILRHFSIMDFNSEKDIVRGSSKLITEQELPERRKALRPPPPRPCTPVSTSPHLLVDQNLKPAPPLVVRPSRPAPLPPSAQQRTNAVSPKLLSRHRPTCETLEKEGPGHMGRSLDQTSPCPLVLVRIEEMERDLDMYSRAQEELNLMLEEKQDESSRAETLEDLKFCESNIESLNMELQQLREMTLLSSQSSSLVAPSGSVSAENPEQRMLEKRAKVIEELLQTERDYIRDLEMCIERIMVPMQQAQVPNIDFEGLFGNMQMVIKVSKQLLAALEISDAVGPVFLGHRDELEGTYKIYCQNHDEAIALLEIYEKDEKIQKHLQDSLADLKSLYNEWGCTNYINLGSFLIKPVQRVMRYPLLLMELLNSTPESHPDKVPLTNAVLAVKEINVNINEYKRRKDLVLKYRKGDEDSLMEKISKLNIHSIIKKSNRVSSHLKHLTGFAPQIKDEVFEETEKNFRMQERLIKSFIRDLSLYLQHIRESACVKVVAAVSMWDVCMERGHRDLEQFERVHRYISDQLFTNFKERTERLVISPLNQLLSMFTGPHKLVQKRFDKLLDFYNCTERAEKLKDKKTLEELQSARNNYEALNAQLLDELPKFHQYAQGLFTNCVHGYAEAHCDFVHQALEQLKPLLSLLKVAGREGNLIAIFHEEHSRVLQQLQVFTFFPESLPATKKPFERKTIDRQSARKPLLGLPSYMLQSEELRASLLARYPPEKLFQAERNFNAAQDLDVSLLEGDLVGVIKKKDPMGSQNRWLIDNGVTKGFVYSSFLKPYNPRRSHSDASVGSHSSTESEHGSSSPRFPRQNSGSTLTFNPSSMAVSFTSGSCQKQPQDASPPPKECDQGTLSASLNPSNSESSPSRCPSDPDSTSQPRSGDSADVARDVKQPTATPRSYRNFRHPEIVGYSVPGRNGQSQDLVKGCARTAQAPEDRSTEPDGSEAEGNQVYFAVYTFKARNPNELSVSANQKLKILEFKDVTGNTEWWLAEVNGKKGYVPSNYIRKTEYT

>NP_543157.1

MAGHPWHGTAALASSQAWRDGRERQALVSCRALYTYEDGSDDLKLAASGEGGLQELSGHFENQKVMYGFCSVKDSQAALPKYVLINWVGEDVPDARKCACASHVAKVAEFFQGVDVIVNASSVEDIDAGAIGQRLSNGLARLSSPVLHRLRLREDENAEPVGTTYQKTDAAVEMKRINREQFWEQAKKEEELRKEEERKKALDERLRFEQERMEQERQEQEERERRYREREQQIEEHRRKQQTLEAEEAKRRLKEQSIFGDHRDEEEETHMKKSESEVEEAAAIIAQRPDNPREFFKQQERVASASAGSCDVPSPFNHRPGSHLDSHRRMAPTPIPTRSPSDSSTASTPVAEQIERALDEVTSSQPPPLPPPPPPAQETQEPSPILDSEETRAAAPQAWAGPMEEPPQAQAPPRGPGSPAEDLMFMESAEQAVLAAPVEPATADATEIHDAADTIETDTATADTTVANNVPPAATSLIDLWPGNGEGASTLQGEPRAPTPPSGTEVTLAEVPLLDEVAPEPLLPAGEGCATLLNFDELPEPPATFCDPEEVEGEPLAAPQTPTLPSALEELEQEQEPEPHLLTNGETTQKEGTQASEGYFSQSQEEEFAQSEELCAKAPPPVFYNKPPEIDITCWDADPVPEEEEGFEGGD

313 NP_003465.3 NP_001954.1

>NP_003465.3

MAARPLPVSPARALLLALAGALLAPCEARGVSLWNQGRADEVVSASVGSGDLWIPVKSFDSKNHPEVLNIRLQRESKELIINLERNEGLIASSFTETHYLQDGTDVSLARNYTVILGHCYYHGHVRGYSDSAVSLSTCSGLRGLIVFENESYVLEPMKSATNRYKLFPAKKLKSVRGSCGSHHNTPNLAAKNVFPPPSQTWARRHKRETLKATKYVELVIVADNREFQRQGKDLEKVKQRLIEIANHVDKFYRPLNIRIVLVGVEVWNDMDKCSVSQDPFTSLHEFLDWRKMKLLPRKSHDNAQLVSGVYFQGTTIGMAPIMSMCTADQSGGIVMDHSDNPLGAAVTLAHELGHNFGMNHDTLDRGCSCQMAVEKGGCIMNASTGYPFPMVFSSCSRKDLETSLEKGMGVCLFNLPEVRESFGGQKCGNRFVEEGEECDCGEPEECMNRCCNATTCTLKPDAVCAHGLCCEDCQLKPAGTACRDSSNSCDLPEFCTGASPHCPANVYLHDGHSCQDVDGYCYNGICQTHEQQCVTLWGPGAKPAPGICFERVNSAGDPYGNCGKVSKSSFAKCEMRDAKCGKIQCQGGASRPVIGTNAVSIETNIPLQQGGRILCRGTHVYLGDDMPDPGLVLAGTKCADGKICLNRQCQNISVFGVHECAMQCHGRGVCNNRKNCHCEAHWAPPFCDKFGFGGSTDSGPIRQADNQGLTIGILVTILCLLAAGFVVYLKRKTLIRLLFTNKKTTIEKLRCVRPSRPPRGFQPCQAHLGHLGKGLMRKPPDSYPPKDNPRRLLQCQNVDISRPLNGLNVPQPQSTQRVLPPLHRAPRAPSVPARPLPAKPALRQAQGTCKPNPPQKPLPADPLARTTRLTHALARTPGQWETGLRLAPLRPAPQYPHQVPRSTHTAYIK

>NP_001954.1

MLLTLIILLPVVSKFSFVSLSAPQHWSCPEGTLAGNGNSTCVGPAPFLIFSHGNSIFRIDTEGTNYEQLVVDAGVSVIMDFHYNEKRIYWVDLERQLLQRVFLNGSRQERVCNIEKNVSGMAINWINEEVIWSNQQEGIITVTDMKGNNSHILLSALKYPANVAVDPVERFIFWSSEVAGSLYRADLDGVGVKALLETSEKITAVSLDVLDKRLFWIQYNREGSNSLICSCDYDGGSVHISKHPTQHNLFAMSLFGDRIFYSTWKMKTIWIANKHTGKDMVRINLHSSFVPLGELKVVHPLAQPKAEDDTWEPEQKLCKLRKGNCSSTVCGQDLQSHLCMCAEGYALSRDRKYCEDVNECAFWNHGCTLGCKNTPGSYYCTCPVGFVLLPDGKRCHQLVSCPRNVSECSHDCVLTSEGPLCFCPEGSVLERDGKTCSGCSSPDNGGCSQLCVPLSPVSWECDCFPGYDLQLDEKSCAASGPQPFLLFANSQDIRHMHFDGTDYGTLLSQQMGMVYALDHDPVENKIYFAHTALKWIERANMDGSQRERLIEEGVDVPEGLAVDWIGRRFYWTDRGKSLIGRSDLNGKRSKIITKENISQPRGIAVHPMAKRLFWTDTGINPRIESSSLQGLGRLVIASSDLIWPSGITIDFLTDKLYWCDAKQSVIEMANLDGSKRRRLTQNDVGHPFAVAVFEDYVWFSDWAMPSVIRVNKRTGKDRVRLQGSMLKPSSLVVVHPLAKPGADPCLYQNGGCEHICKKRLGTAWCSCREGFMKASDGKTCLALDGHQLLAGGEVDLKNQVTPLDILSKTRVSEDNITESQHMLVAEIMVSDQDDCAPVGCSMYARCISEGEDATCQCLKGFAGDGKLCSDIDECEMGVPVCPPASSKCINTEGGYVCRCSEGYQGDGIHCLDIDECQLGVHSCGENASCTNTEGGYTCMCAGRLSEPGLICPDSTPPPHLREDDHHYSVRNSDSECPLSHDGYCLHDGVCMYIEALDKYACNCVVGYIGERCQYRDLKWWELRHAGHGQQQKVIVVAVCVVVLVMLLLLSLWGAHYYRTQKLLSKNPKNPYEESSRDVRSRRPADTEDGMSSCPQPWFVVIKEHQDLKNGGQPVAGEDGQAADGSMQPTSWRQEPQLCGMGTEQGCWIPVSSDKGSCPQVMERSFHMPSYGTQTLEGGVEKPHSLLSANPLWQQRALDPPHQMELTQ

314 NP_001943.1 NP_005216.1

>NP_001943.1

MSQQRPARKLPSLLLDPTEETVRRRCRDPINVEGLLPSKIRINLEDNVQYVSMRKALKVKRPRFDVSLVYLTRKFMDLVRSAPGGILDLNKVATKLGVRKRRVYDITNVLDGIDLVEKKSKNHIRWIGSDLSNFGAVPQQKKLQEELSDLSAMEDALDELIKDCAQQLFELTDDKENERLAYVTYQDIHSIQAFHEQIVIAVKAPAETRLDVPAPREDSITVHIRSTNGPIDVYLCEVEQGQTSNKRSEGVGTSSSESTHPEGPEEEENPQQSEELLEVSN

>NP_005216.1

MALAGAPAGGPCAPALEALLGAGALRLLDSSQIVIISAAQDASAPPAPTGPAAPAAGPCDPDLLLFATPQAPRPTPSAPRPALGRPPVKRRLDLETDHQYLAESSGPARGRGRHPGKGVKSPGEKSRYETSLNLTTKRFLELLSHSADGVVDLNWAAEVLKVQKRRIYDITNVLEGIQLIAKKSKNHIQWLGSHTTVGVGGRLEGLTQDLRQLQESEQQLDHLMNICTTQLRLLSEDTDSQRLAYVTCQDLRSIADPAEQMVMVIKAPPETQLQAVDSSENFQISLKSKQGPIDVFLCPEETVGGISPGKTPSQEVTSEEENRATDSATIVSPPPSSPPSSLTTDPSQSLLSLEQEPLLSRMGSLRAPVDEDRLSPLVAADSLLEHVREDFSGLLPEEFISLSPPHEALDYHFGLEEGEGIRDLFDCDFGDLTPLDF

315 NP_620603.1 NP_660144.1

>NP_620603.1

MAEPLKEEDGEDGSAEPPGPVKVEPAHTAASVAAKNLALLKARSFDVTFDVGDEYEIIETIGNGAYGVVSSARRRLTGQQVAIKKIPNAFDVVTNAKRTLRELKILKHFKHDNIIAIKDILRPTVPYGEFKSVYVVLDLMESDLHQIIHSSQPLTLEHVRYFLYQLLRGLKYMHSAQVIHRDLKPSNLLVNENCELKIGDFGMARGLCTSPAEHQYFMTEYVATRWYRAPELMLSLHEYTQAIDLWSVGCIFGEMLARRQLFPGKNYVHQLQLIMMVLGTPSPAVIQAVGAERVRAYIQSLPPRQPVPWETVYPGADRQALSLLGRMLRFEPSARISAAAALRHPFLAKYHDPDDEPDCAPPFDFAFDREALTRERIKEAIVAEIEDFHARREGIRQQIRFQPSLQPVASEPGCPDVEMPSPWAPSGDCAMESPPPAPPPCPGPAPDTIDLTLQPPPPVSEPAPPKKDGAISDNTKAALKAALLKSLRSRLRDGPSAPLEAPEPRKPVTAQERQREREEKRRRRQERAKEREKRRQERERKERGAGASGGPSTDPLAGLVLSDNDRSLLERWTRMARPAAPALTSVPAPAPAPTPTPTPVQPTSPPPGPVAQPTGPQPQSAGSTSGPVPQPACPPPGPAPHPTGPPGPIPVPAPPQIATSTSLLAAQSLVPPPGLPGSSTPGVLPYFPPGLPPPDAGGAPQSSMSESPDVNLVTQQLSKSQVEDPLPPVFSGTPKGSGAGYGVGFDLEEFLNQSFDMGVADGPQDGQADSASLSASLLADWLEGHGMNPADIESLQREIQMDSPMLLADLPDLQDP

>NP_660144.1

MLWLALGPFPAMENQVLVIRIKIPNSGAVDWTVHSGPQLLFRDVLDVIGQVLPEATTTAFEYEDEDGDRITVRSDEEMKAMLSYYYSTVMEQQVNGQLIEPLQIFPRACKPPGERNIHGLKVNTRAGPSQHSSPAVSDSLPSNSLKKSSAELKKILANGQMNEQDIRYRDTLGHGNGGTVYKAYHVPSGKILAVKVILLDITLELQKQIMSELEILYKCDSSYIIGFYGAFFVENRISICTEFMDGGSLDVYRKMPEHVLGRIAVAVVKGLTYLWSLKILHRDVKPSNMLVNTRGQVKLCDFGVSTQLVNSIAKTYVGTNAYMAPERISGEQYGIHSDVWSLGISFMEIQKNQGSLMPLQLLQCIVDEDSPVLPVGEFSEPFVHFITQCMRKQPKERPAPEELMGHPFIVQFNDGNAAVVSMWVCRALEERRSLASLPSPSPLSAGGLAWGAPCVAHPTRPSPYLLV

316 NP_002706.1 NP_006245.2

>NP_002706.1

MDEKVFTKELDQWIEQLNECKQLSESQVKSLCEKAKEILTKESNVQEVRCPVTVCGDVHGQFHDLMELFRIGGKSPDTNYLFMGDYVDRGYYSVETVTLLVALKVRYRERITILRGNHESRQITQVYGFYDECLRKYGNANVWKYFTDLFDYLPLTALVDGQIFCLHGGLSPSIDTLDHIRALDRLQEVPHEGPMCDLLWSDPDDRGGWGISPRGAGYTFGQDISETFNHANGLTLVSRAHQLVMEGYNWCHDRNVVTIFSAPNYCYRCGNQAAIMELDDTLKYSFLQFDPAPRRGEPHVTRRTPDYFL

>NP_006245.2

MAPFLRIAFNSYELGSLQAEDEANQPFCAVKMKEALSTERGKTLVQKKPTMYPEWKSTFDAHIYEGRVIQIVLMRAAEEPVSEVTVGVSVLAERCKKNNGKAEFWLDLQPQAKVLMSVQYFLEDVDCKQSMRSEDEAKFPTMNRRGAIKQAKIHYIKNHEFIATFFGQPTFCSVCKDFVWGLNKQGYKCRQCNAAIHKKCIDKIIGRCTGTAANSRDTIFQKERFNIDMPHRFKVHNYMSPTFCDHCGSLLWGLVKQGLKCEDCGMNVHHKCREKVANLCGINQKLLAEALNQVTQRASRRSDSASSEPVGIYQGFEKKTGVAGEDMQDNSGTYGKIWEGSSKCNINNFIFHKVLGKGSFGKVLLGELKGRGEYFAIKALKKDVVLIDDDVECTMVEKRVLTLAAENPFLTHLICTFQTKDHLFFVMEFLNGGDLMYHIQDKGRFELYRATFYAAEIMCGLQFLHSKGIIYRDLKLDNVLLDRDGHIKIADFGMCKENIFGESRASTFCGTPDYIAPEILQGLKYTFSVDWWSFGVLLYEMLIGQSPFHGDDEDELFESIRVDTPHYPRWITKESKDILEKLFEREPTKRLGVTGNIKIHPFFKTINWTLLEKRRLEPPFRPKVKSPRDYSNFDQEFLNEKARLSYSDKNLIDSMDQSAFAGFSFVNPKFEHLLED

317 NP_006799.1 NP_055109.1

>NP_006799.1

MPGPTPSGTNVGSSGRSPSKAVAARAAGSTVRQRKNASCGTRSAGRTTSAGTGGMWRFYTEDSPGLKVGPVPVLVMSLLFIASVFMLHIWGKYTRS

>NP_055109.1

MAIRKKSTKSPPVLSHEFVLQNHADIVSCVAMVFLLGLMFEITAKASIIFVTLQYNVTLPATEEQATESVSLYYYGIKDLATVFFYMLVAIIIHAVIQEYMLDKINRRMHFSKTKHSKFNESGQLSAFYLFACVWGTFILISENYISDPTILWRAYPHNLMTFQMKFFYISQLAYWLHAFPELYFQKTKKEDIPRQLVYIGLYLFHIAGAYLLNLNHLGLVLLVLHYFVEFLFHISRLFYFSNEKYQKGFSLWAVLFVLGRLLTLILSVLTVGFGLARAENQKLDFSTGNFNVLAVRIAVLASICVTQAFMMWKFINFQLRRWREHSAFQAPAVKKKPTVTKGRSSKKGTENGVNGTLTSNVADSPRNKKEKSS

318 NP_001020330.1 NP_001903.1

>NP_001020330.1

MHRRRSRSCREDQKPVMDDQRDLISNNEQLPMLGRRPGAPESKCSRGALYTGFSILVTLLLAGQATTAYFLYQQQGRLDKLTVTSQNLQLENLRMKLPKPPKPVSKMRMATPLLMQALPMGALPQGPMQNATKYGNMTEDHVMHLLQNADPLKVYPPLKGSFPENLRHLKNTMETIDWKVFESWMHHWLLFEMSRHSLEQKPTDAPPKVLTKCQEEVSHIPAVHPGSFRPKCDENGNYLPLQCYGSIGYCWCVFPNGTEVPNTRSRGHHNCSESLELEDPSSGLGVTKQDLGPVPM

>NP_001903.1

MNPTLILAAFCLGIASATLTFDHSLEAQWTKWKAMHNRLYGMNEEGWRRAVWEKNMKMIELHNQEYREGKHSFTMAMNAFGDMTSEEFRQVMNGFQNRKPRKGKVFQEPLFYEAPRSVDWREKGYVTPVKNQGQCGSCWAFSATGALEGQMFRKTGRLISLSEQNLVDCSGPQGNEGCNGGLMDYAFQYVQDNGGLDSEESYPYEATEESCKYNPKYSVANDTGFVDIPKQEKALMKAVATVGPISVAIDAGHESFLFYKEGIYFEPDCSSEDMDHGVLVVGYGFESTESDNNKYWLVKNSWGEEWGMGGYVKMAKDRRNHCGIASAASYPTV

319 NP_003585.3 NP_112533.1

>NP_003585.3

MEEVRCPEHGTFCFLKTGVRDGPNKGKSFYVCRADTCSFVRATDIPVSHCLLHEDFVVELQGLLLPQDKKEYRLFFRCIRSKAEGKRWCGSIPWQDPDSKEHSVSNKSQHASETFHHSSNWLRNPFKVLDKNQEPALWKQLIKGEGEEKKADKKQREKGDQLFDQKKEQKPEMMEKDLSSGLVPKKKQSVVQEKKQEEGAEIQCEAETGGTHKRDFSEIKSQQCQGNELTRPSASSQEKSSGKSQDVQRESEPLREKVTQLLPQNVHSHNSISKPQKGGPLNKEYTNWEAKETKAKDGPSIQATQKSLPQGHFQERPETHSVPAPGGPAAQAAPAAPGLSLGEGREAATSSDDEEEDDVVFVSSKPGSPLLFDSTLDLETKENLQFPDRSVQRKVSPASGVSKKVEPSDPVARRVYLTTQLKQKKSTLASVNIQALPDKGQKLIKQIQELEEVLSGLTLSPEQGTNEKSNSQVPQQSHFTKTTTGPPHLVPPQPLPRRGTQPVGSLELKSACQVTAGGSSQCYRGHTNQDHVHAVWKITSEAIGQLHRSLESCPGETVVAEDPAGLKVPLLLHQKQALAWLLWRESQKPQGGILADDMGLGKTLTMIALILTQKNQEKKEEKEKSTALTWLSKDDSCDFTSHGTLIICPASLIHHWKNEVEKRVNSNKLRVYLYHGPNRDSRARVLSTYDIVITTYSLVAKEIPTNKQEAEIPGANLNVEGTSTPLLRIAWARIILDEAHNVKNPRVQTSIAVCKLQACARWAVTGTPIQNNLLDMYSLLKFLRCSPFDEFNLWRSQVDNGSKKGGERLSILTKSLLLRRTKDQLDSTGRPLVILPQRKFQLHHLKLSEDEETVYNVFFARSRSALQSYLKRHESRGNQSGRSPNNPFSRVALEFGSEEPRHSEAADSPRSSTVHILSQLLRLRQCCCHLSLLKSALDPMELKGEGLVLSLEEQLSALTLSELRDSEPSSTVSLNGTFFKMELFEGMRESTKISSLLAELEAIQRNSASQKSVIVSQWTNMLKVVALHLKKHGLTYATIDGSVNPKQRMDLVEAFNHSRGPQVMLISLLAGGVGLNLTGGNHLFLLDMHWNPSLEDQACDRIYRVGQQKDVVIHRFVCEGTVEEKILQLQEKKKDLAKQVLSGSGESVTKLTLADLRVLFGI

>NP_112533.1

MEKTLETVPLERKKREKEQFRKLFIGGLSFETTEESLRNYYEQWGKLTDCVVMRDPASKRSRGFGFVTFSSMAEVDAAMAARPHSIDGRVVEPKRAVAREESGKPGAHVTVKKLFVGGIKEDTEEHHLRDYFEEYGKIDTIEIITDRQSGKKRGFGFVTFDDHDPVDKIVLQKYHTINGHNAEVRKALSRQEMQEVQSSRSGRGGNFGFGDSRGGGGNFGPGPGSNFRGGSDGYGSGRGFGDGYNGYGGGPGGGNFGGSPGYGGGRGGYGGGGPGYGNQGGGYGGGYDNYGGGNYGSGNYNDFGNYNQQPSNYGPMKSGNFGGSRNMGGPYGGGNYGPGGSGGSGGYGGRSRY

320 CAC69380.1 NP_000824.1

>CAC69380.1

MEFVRALWLGLALALGPGSAGGHPQPCGVLARLGGSVRLGALLPRAPLARARARAALARAALAPRLPHNLSLELVVAAPPARDPASLTRGLCQALVPPGVAALLAFPEARPELLQLHFLAAATETPVLSLLRREARAPLGAPNPFHLQLHWASPLETLLDVLVAVLQAHAWEDVGLALCRTQDPGGLVALWTSRAGRPPQLVLDLSRRDTGDAGLRARLAPMAAPVGGEAPVPAAVLLGCDIARARRVLEAVPPGPHWLLGTPLPPKALPTAGLPPGLLALGEVARPPLEAAIHDIVQLVARALGSAAQVQPKRALLPAPVNCGDLQPAGPESPGRFLARFLANTSFQGRTGPVWVTGSSQVHMSRHFKVWSLRRDPRGAPAWATVGSWRDGQLDLEPGGASARPPPPQGAQVWPKLRVVTLLEHPFVFARDPDEDGQCPAGQLCLDPGTNDSATLDALFAALANGSAPRALRKCCYGYCIDLLERLAEDTPFDFELYLVGDGKYGALRDGRWTGLVGDLLAGRAHMAVTSFSINSARSQVVDFTSPFFSTSLGIMVRARDTASPIGAFMWPLHWSTWLGVFAALHLTALFLTVYEWRSPYGLTPRGRNRSTVFSYSSALNLCYAILFRRTVSSKTPKCPTGRLLMNLWAIFCLLVLSSYTANLAAVMVGDKTFEELSGIHDPKLHHPAQGFRFGTVWESSAEAYIKKSFPDMHAHMRRHSAPTTPRGVAMLTSDPPKLNAFIMDKSLLDYEVSIDADCKLLTVGKPFAIEGYGIGLPQNSPLTSNLSEFISRYKSSGFIDLLHDKWYKMVPCGKRVFAVTETLQMSIYHFAGLFVLLCLGLGSALLSSLGEHAFFRLALPRIRKGSRLQYWLHTSQKIHRALNTEPPEGSKEETAEAEPSGPEVEQQQQQQDQPTAPEGWKRARRAVDKERRVRFLLEPAVVVAPEADAEAEAAPREGPVWLCSYGRPPAARPTGAPQPGELQELERRIEVARERLRQALVRRGQLLAQLGDSARHRPRRLLQARAAPAEAPPHSGRPGSQE

>NP_000824.1

MGRVGYWTLLVLPALLVWRGPAPSAAAEKGPPALNIAVMLGHSHDVTERELRTLWGPEQAAGLPLDVNVVALLMNRTDPKSLITHVCDLMSGARIHGLVFGDDTDQEAVAQMLDFISSHTFVPILGIHGGASMIMADKDPTSTFFQFGASIQQQATVMLKIMQDYDWHVFSLVTTIFPGYREFISFVKTTVDNSFVGWDMQNVITLDTSFEDAKTQVQLKKIHSSVILLYCSKDEAVLILSEARSLGLTGYDFFWIVPSLVSGNTELIPKEFPSGLISVSYDDWDYSLEARVRDGIGILTTAASSMLEKFSYIPEAKASCYGQMERPEVPMHTLHPFMVNVTWDGKDLSFTEEGYQVHPRLVVIVLNKDREWEKVGKWENHTLSLRHAVWPRYKSFSDCEPDDNHLSIVTLEEAPFVIVEDIDPLTETCVRNTVPCRKFVKINNSTNEGMNVKKCCKGFCIDILKKLSRTVKFTYDLYLVTNGKHGKKVNNVWNGMIGEVVYQRAVMAVGSLTINEERSEVVDFSVPFVETGISVMVSRSNGTVSPSAFLEPFSASVWVMMFVMLLIVSAIAVFVFEYFSPVGYNRNLAKGKAPHGPSFTIGKAIWLLWGLVFNNSVPVQNPKGTTSKIMVSVWAFFAVIFLASYTANLAAFMIQEEFVDQVTGLSDKKFQRPHDYSPPFRFGTVPNGSTERNIRNNYPYMHQYMTKFNQKGVEDALVSLKTGKLDAFIYDAAVLNYKAGRDEGCKLVTIGSGYIFATTGYGIALQKGSPWKRQIDLALLQFVGDGEMEELETLWLTGICHNEKNEVMSSQLDIDNMAGVFYMLAAAMALSLITFIWEHLFYWKLRFCFTGVCSDRPGLLFSISRGIYSCIHGVHIEEKKKSPDFNLTGSQSNMLKLLRSAKNISSMSNMNSSRMDSPKRAADFIQRGSLIMDMVSDKGNLMYSDNRSFQGKESIFGDNMNELQTFVANRQKDNLNNYVFQGQHPLTLNESNPNTVEVAVSTESKANSRPRQLWKKSVDSIRQDSLSQNPVSQRDEATAENRTHSLKSPRYLPEEMAHSDISETSNRATCHREPDNSKNHKTKDNFKRSVASKYPKDCSEVERTYLKTKSSSPRDKIYTIDGEKEPGFHLDPPQFVENVTLPENVDFPDPYQDPSENFRKGDSTLPMNRNPLHNEEGLSNNDQYKLYSKHFTLKDKGSPHSETSERYRQNSTHCRSCLSNMPTYSGHFTMRSPFKCDACLRMGNLYDIDEDQMLQETGNPATGEQVYQQDWAQNNALQLQKNKLRISRQHSYDNIVDKPRELDLSRPSRSISLKDRERLLEGNFYGSLFSVPSSKLSGKKSSLFPQGLEDSKRSKSLLPDHTSDNPFLHSHRDDQRLVIGRCPSDPYKHSLPSQAVNDSYLRSSLRSTASYCSRDSRGHNDVYISEHVMPYAANKNNMYSTPRVLNSCSNRRVYKKMPSIESDV

321 NP_066288.2 NP_001073922.1

>NP_066288.2

MEDSDSAAKQLGLAEAAAVAAAAAVAAAAAAAAGGEAEEPVLSRDEDSEEDADSEAERETPRVTAVAVMAAEPGHMDMGAEALPGPDEAAAAAAFAEVTTVTVANVGAAADNVFTTSVANAASISGHVLSGRTALQIGDSLNTEKATLIVVHTDGSIVETTGLKGPAAPLTPGPQSPPTPLAPGQEKGGTKYNWDPSVYDSELPVRCRNISGTLYKNRLGSGGRGRCIKQGENWYSPTEFEAMAGRASSKDWKRSIRYAGRPLQCLIQDGILNPHAASCTCAACCDDMTLSGPVRLFVPYKRRKKENELPTTPVKKDSPKNITLLPATAATTFTVTPSGQITTSGALTFDRASTVEATAVISESPAQGDVFAGATVQEASVQPPCRASHPEPHYPGYQDSCQIAPFPEAALPTSHPKIVLTSLPALAVPPPTPTKAAPPALVNGLELSEPRSWLYLEEMVNSLLNTAQQLKTLFEQAKHASTYREAATNQAKIHADAERKEQSCVNCGREAMSECTGCHKVNYCSTFCQRKDWKDHQHICGQSAAVTVQADEVHVAESVMEKVTV

>NP_001073922.1

MGDGATAPPVNGEASACPAPPTHLDRTTCARRRPARLPPPWGAAGIRPAPRASRASFWARFPPHCFPSGCSGSRVLEGGEIGFRFRGGRVAAAALRSRWARGDAAAAGSREGRGRMNRAKPTTVRRPSAAAKPSGHPPPGDFIALGSKGQANESKTASTLLKPAPSGLPSERKRDAAAALSSASALTGLTKRPKLSSTPPLSALGRLAEAAVAEKRAISPSIKEPSVVPIEVLPTVLLDEIEAAELEGNDDRIEGVLCGAVKQLKVTRAKPDSTLYLSLMYLAKIKPNIFATEGVIEALCSLLRRDASINFKAKGNSLVSVLACNLLMAAYEEDENWPEIFVKVYIEDSLGERIWVDSPHCKTFVDNIQTAFNTRMPPRSVLLQGEAGRVAGDLGAGSSPHPSLTEEEDSQTELLIAEEKLSPEQEGQLMPRYEELAESVEEYVLDMLRDQLNRRQPIDNVSRNLLRLLTSTCGYKEVRLLAVQKLEMWLQNPKLTRPAQDLLMSVCMNCNTHGSEDMDVISHLIKIRLKPKVLLNHFMLCIRELLSAHKDNLGTTIKLVIFNELSSARNPNNMQVLYTALQHSSELAPKFLAMVFQDLLTNKDDYLRASRALLREIIKQTKHEINFQAFCLGLMQERKEPQYLEMEFKERFVVHITDVLAVSMMLGITAQVKEAGIAWDKGEKRNLEVLRSFQNQIAAIQRDAVWWLHTVVPSISKLAPKDYVHCLHKVLFTEQPETYYKWDNWPPESDRNFFLRLCSEVPILEDTLMRILVIGLSRELPLGPADAMELADHLVKRAAAVQADDVEVLKVGRTQLIDAVLNLCTYHHPENIQLPPGYQPPNLAISTLYWKAWPLLLVVAAFNPENIGLAAWEEYPTLKMLMEMVMTNNYSYPPCTLTDEETRTEMLNRELQTAQREKQEILAFEGHLAAASTKQTITESSSLLLSQLTSLDPQYAAHTAPPCACRRDLGPPRRPPPHILDQVKSLNQSLRLGHLLCRSRNPDFLLHIIQRQASSQSMPWLADLVQSSEGSLDVLPVQCLCEFLLHDAVDDAASGEEDDEGESKEQKAKKRQRQQKQRQLLGRLQDLLLGPKADEQTTCEVLDYFLRRLGSSQVASRVLAMKGETLPALPKAGPAHECLPQGLSLVLSEGSLRDGEEKEPPMEEDVGDTDVLQGYQWLLRDLPRLPLFDSVRSTTALALQQAIHMETDPQTISAYLIYLSQHTPVEEQAQHSDLALDVARLVVERSTIMSHLFSKLSPSAASDAVLSALLSIFSRYVRRMRQSKEGEEVYSWSESQDQVFLRWSSGETATMHILVVHAMVILLTLGPPRADDSEFQALLDIWFPEEKPLPTAFLVDTSEEALLLPDWLKLRMIRSEVLRLVDAALQDLEPQQLLLFVQSFGIPVSSMSKLLQFLDQAVAHDPQTLEQNIMDKSKMPGTCGVGSHTLTEPQPGQAFGAWVRAGVTLLRFADYMAHLVEVQHERGASGGQTFHSLLTASLPPRRDSTEAPKPKSSPEQPIGQGRIRVGTQLRVLGPEDDLAGMFLQIFPLSPDPRWQSSSPRPVALALQQALGQELARVVQGSPEVPGITVRVLQALATLLSSPHGGALVMSMHRSHFLACPLLRQLCQYQRCVPQDTGFSSLFLKVLLQMLQWLDSPGVEGGPLRAQLRMLASQASAGRRLSDVRGGLLRLAEALAFRQDLEVVSSTVRAVIATLRSGEQCSVEPDLISKVLQGLIEVRSPHLEELLTAFFSATADAASPFPACKPVVVVSSLLLQEEEPLAGGKPGADGGSLEAVRLGPSSGLLVDWLEMLDPEVVSSCPDLQLRLLFSRRKGKGQAQVPSFRPYLLTLFTHQSSWPTLHQCIRVLLGKSREQRFDPSASLDFLWACIHVPRIWQGRDQRTPQAGLGRKRREELVLRVQGPELISLVELILAEAETRSQDGDTAACSLIQARLPLLLSCCCGDDESVRKVTEHLSGCIQQWGDSVLGRRCRDLLLQLYLQRPELRVPVPEVLLHSEGAASSSVCKLDGLIHRFITLLADTSDSRALENRGADASMACRKLAVAHPLLLLRHLPMIAALLHGRTHLNFQEFRQQNHLSCFLHVLGLLELLQPHVFRSEHQGALWDCLLSFIRLLLNYRKSSRHLAAFINKFVQFIHKYITYNAPAAISFLQKHADPLHDLSFDNSDLVMLKSLLAGLSLPSRDDRTDRGLDEEGEEESSAGSLPLVSVSLFTPLTAAEMAPYMKRLSRGQTVEDLLEVLSDIDEMSRRRPEILSFFSTNLQRLMSSAEECCRNLAFSLALRSMQNSPSIAAAFLPTFMYCLGSQDFEVVQTALRNLPEYALLCQEHAAVLLHRAFLVGMYGQMDPSAQISEALRILHMEAVM

322 NP_000452.2 NP_004228.1

>NP_000452.2

MTPNSMTENGLTAWDKPKHCPDREHDWKLVGMSEACLHRKSHSERRSTLKNEQSSPHLIQTTWTSSIFHLDHDDVNDQSVSSAQTFQTEEKKCKGYIPSYLDKDELCVVCGDKATGYHYRCITCEGCKGFFRRTIQKNLHPSYSCKYEGKCVIDKVTRNQCQECRFKKCIYVGMATDLVLDDSKRLAKRKLIEENREKRRREELQKSIGHKPEPTDEEWELIKTVTEAHVATNAQGSHWKQKRKFLPEDIGQAPIVNAPEGGKVDLEAFSHFTKIITPAITRVVDFAKKLPMFCELPCEDQIILLKGCCMEIMSLRAAVRYDPESETLTLNGEMAVTRGQLKNGGLGVVSDAIFDLGMSLSSFNLDDTEVALLQAVLLMSSDRPGLACVERIEKYQDSFLLAFEHYINYRKHHVTHFWPKLLMKVTDLRMIGACHASRFLHMKVECPTELFPPLFLEVFED

>NP_004228.1

MDEAVGDLKQALPCVAESPTVHVEVHQRGSSTAKKEDINLSVRKLLNRHNIVFGDYTWTEFDEPFLTRNVQSVSIIDTELKVKDSQPIDLSACTVALHIFQLNEDGPSSENLEEETENIIAANHWVLPAAEFHGLWDSLVYDVEVKSHLLDYVMTTLLFSDKNVNSNLITWNRVVLLHGPPGTGKTSLCKALAQKLTIRLSSRYRYGQLIEINSHSLFSKWFSESGKLVTKMFQKIQDLIDDKDALVFVLIDEVESLTAARNACRAGTEPSDAIRVVNAVLTQIDQIKRHSNVVILTTSNITEKIDVAFVDRADIKQYIGPPSAAAIFKIYLSCLEELMKCQIIYPRQQLLTLRELEMIGFIENNVSKLSLLLNDISRKSEGLSGRVLRKLPFLAHALYVQAPTVTIEGFLQALSLAVDKQFEERKKLAAYI

323 NP_005558.1 NP_000582.1

>NP_005558.1

MTPPRLFWVWLLVAGTQGVNDGDMRLADGGATNQGRVEIFYRGQWGTVCDNLWDLTDASVVCRALGFENATQALGRAAFGQGSGPIMLDEVQCTGTEASLADCKSLGWLKSNCRHERDAGVVCTNETRSTHTLDLSRELSEALGQIFDSQRGCDLSISVNVQGEDALGFCGHTVILTANLEAQALWKEPGSNVTMSVDAECVPMVRDLLRYFYSRRIDITLSSVKCFHKLASAYGARQLQGYCASLFAILLPQDPSFQMPLDLYAYAVATGDALLEKLCLQFLAWNFEALTQAEAWPSVPTDLLQLLLPRSDLAVPSELALLKAVDTWSWGERASHEEVEGLVEKIRFPMMLPEELFELQFNLSLYWSHEALFQKKTLQALEFHTVPFQLLARYKGLNLTEDTYKPRIYTSPTWSAFVTDSSWSARKSQLVYQSRRGPLVKYSSDYFQAPSDYRYYPYQSFQTPQHPSFLFQDKRVSWSLVYLPTIQSCWNYGFSCSSDELPVLGLTKSGGSDRTIAYENKALMLCEGLFVADVTDFEGWKAAIPSALDTNSSKSTSSFPCPAGHFNGFRTVIRPFYLTNSSGVD

>NP_000582.1

MERASCLLLLLLPLVHVSATTPEPCELDDEDFRCVCNFSEPQPDWSEAFQCVSAVEVEIHAGGLNLEPFLKRVDADADPRQYADTVKALRVRRLTVGAAQVPAQLLVGALRVLAYSRLKELTLEDLKITGTMPPLPLEATGLALSSLRLRNVSWATGRSWLAELQQWLKPGLKVLSIAQAHSPAFSCEQVRAFPALTSLDLSDNPGLGERGLMAALCPHKFPAIQNLALRNTGMETPTGVCAALAAAGVQPHSLDLSHNSLRATVNPSAPRCMWSSALNSLNLSFAGLEQVPKGLPAKLRVLDLSCNRLNRAPQPDELPEVDNLTLDGNPFLVPGTALPHEGSMNSGVVPACARSTLSVGVSGTLVLLQGARGFA

324 NP_001972.1 Q8NFH8

>NP_001972.1

MAAAAQLSLTQLSSGNPVYEKYYRQVDTGNTGRVLASDAAAFLKKSGLPDLILGKIWDLADTDGKGILNKQEFFVALRLVACAQNGLEVSLSSLNLAVPPPRFHDTSSPLLISGTSAAELPWAVKPEDKAKYDAIFDSLSPVNGFLSGDKVKPVLLNSKLPVDILGRVWELSDIDHDGMLDRDEFAVAMFLVYCALEKEPVPMSLPPALVPPSKRKTWVVSPAEKAKYDEIFLKTDKDMDGFVSGLEVREIFLKTGLPSTLLAHIWSLCDTKDCGKLSKDQFALAFHLISQKLIKGIDPPHVLTPEMIPPSDRASLQKNIIGSSPVADFSAIKELDTLNNEIVDLQREKNNVEQDLKEKEDTIKQRTSEVQDLQDEVQRENTNLQKLQAQKQQVQELLDELDEQKAQLEEQLKEVRKKCAEEAQLISSLKAELTSQESQISTYEEELAKAREELSRLQQETAELEESVESGKAQLEPLQQHLQDSQQEISSMQMKLMEMKDLENHNSQLNWCSSPHSILVNGATDYCSLSTSSSETANLNEHVEGQSNLESEPIHQESPARSSPELLPSGVTDENEVTTAVTEKVCSELDNNRHSKEEDPFNVDSSSLTGPVADTNLDFFQSDPFVGSDPFKDDPFGKIDPFGGDPFKGSDPFASDCFFRQSTDPFATSSTDPFSAANNSSITSVETLKHNDPFAPGGTVVAASDSATDPFASVFGNESFGGGFADFSTLSKVNNEDPFRSATSSSVSNVVITKNVFEETSVKSEDEPPALPPKIGTPTRPCPLPPGKRSINKLDSPDPFKLNDPFQPFPGNDSPKEKDPEIFCDPFTSATTTTNKEADPSNFANFSAYPSEEDMIEWAKRESEREEEQRLARLNQQEQEDLELAIALSKSEISEA

>Q8NFH8

MEAAAAAAAAAAAAAAAGGGCGSGPPPLLLSEGEQQCYSELFARCAGAAGGGPGSGPPEAARVAPGTATAAAGPVADLFRASQLPAETLHQITELCGAKRVGYFGPTQFYIALKLIAAAQSGLPVRIESIKCELPLPRFMMSKNDGEIRFGNPAELHGTKVQIPYLTTEKNSFKRMDDEDKQQETQSPTMSPLASPPSSPPHYQRVPLSHGYSKLRSSAEQMHPAPYEARQPLVQPEGSSSGGPGTKPLRHQASLIRSFSVERELQDNSSYPDEPWRITEEQREYYVNQFRSLQPDPSSFISGSVAKNFFTKSKLSIPELSYIWELSDADCDGALTLPEFCAAFHLIVARKNGYPLPEGLPPTLQPEYLQAAFPKPKWDCQLFDSYSESLPANQQPRDLNRMEKTSVKDMADLPVPNQDVTSDDKQALKSTINEALPKDVSEDPATPKDSNSLKARPRSRSYSSTSIEEAMKRGEDPPTPPPRPQKTHSRASSLDLNKVFQPSVPATKSGLLPPPPALPPRPCPSQSEQVSEAELLPQLSRAPSQAAESSPAKKDVLYSQPPSKPIRRKFRPENQATENQEPSTAASGPASAATMKPHPTVQKQSSKQKKAIQTAIRKNKEANAVLARLNSELQQQLKEVHQERIALENQLEQLRPVTVL

325 NP_203124.1 NP_004406.2

>NP_203124.1

MDCVGWPPGRKWHLEKNTSCGGSSGICASYVTQMADDQGCIEEQGVEDSANEDSVDAKPDRSSFVPSLFSKKKKNVTMRSIKTTRDRVPTYQYNMNFEKLGKCIIINNKNFDKVTGMGVRNGTDKDAEALFKCFRSLGFDVIVYNDCSCAKMQDLLKKASEEDHTNAACFACILLSHGEENVIYGKDGVTPIKDLTAHFRGDRCKTLLEKPKLFFIQACRGTELDDGIQADSGPINDTDANPRYKIPVEADFLFAYSTVPGYYSWRSPGRGSWFVQALCSILEEHGKDLEIMQILTRVNDRVARHFESQSDDPHFHEKKQIPCVVSMLTKELYFSQ

>NP_004406.2

MSCNGGSHPRINTLGRMIRAESGPDLRYEVTSGGGGTSRMYYSRRGVITDQNSDGYCQTGTMSRHQNQNTIQELLQNCSDCLMRAELIVQPELKYGDGIQLTRSRELDECFAQANDQMEILDSLIREMRQMGQPCDAYQKRLLQLQEQMRALYKAISVPRVRRASSKGGGGYTCQSGSGWDEFTKHVTSECLGWMRQQRAEMDMVAWGVDLASVEQHINSHRGIHNSIGDYRWQLDKIKADLREKSAIYQLEEEYENLLKASFERMDHLRQLQNIIQATSREIMWINDCEEEELLYDWSDKNTNIAQKQEAFSIRMSQLEVKEKELNKLKQESDQLVLNQHPASDKIEAYMDTLQTQWSWILQITKCIDVHLKENAAYFQFFEEAQSTEAYLKGLQDSIRKKYPCDKNMPLQHLLEQIKELEKEREKILEYKRQVQNLVNKSKKIVQLKPRNPDYRSNKPIILRALCDYKQDQKIVHKGDECILKDNNERSKWYVTGPGGVDMLVPSVGLIIPPPNPLAVDLSCKIEQYYEAILALWNQLYINMKSLVSWHYCMIDIEKIRAMTIAKLKTMRQEDYMKTIADLELHYQEFIRNSQGSEMFGDDDKRKIQSQFTDAQKHYQTLVIQLPGYPQHQTVTTTEITHHGTCQDVNHNKVIETNRENDKQETWMLMELQKIRRQIEHCEGRMTLKNLPLADQGSSHHITVKINELKSVQNDSQAIAEVLNQLKDMLANFRGSEKYCYLQNEVFGLFQKLENINGVTDGYLNSLCTVRALLQAILQTEDMLKVYEARLTEEETVCLDLDKVEAYRCGLKKIKNDLNLKKSLLATMKTELQKAQQIHSQTSQQYPLYDLDLGKFGEKVTQLTDRWQRIDKQIDFRLWDLEKQIKQLRNYRDNYQAFCKWLYDAKRRQDSLESMKFGDSNTVMRFLNEQKNLHSEISGKRDKSEEVQKIAELCANSIKDYELQLASYTSGLETLLNIPIKRTMIQSPSGVILQEAADVHARYIELLTRSGDYYRFLSEMLKSLEDLKLKNTKIEVLEEELRLARDANSENCNKNKFLDQNLQKYQAECSQFKAKLASLEELKRQAELDGKSAKQNLDKCYGQIKELNEKITRLTYEIEDEKRRRKSVEDRFDQQKNDYDQLQKARQCEKENLGWQKLESEKAIKEKEYEIERLRVLLQEEGTRKREYENELAKVRNHYNEEMSNLRNKYETEINITKTTIKEISMQKEDDSKNLRNQLDRLSRENRDLKDEIVRLNDSILQATEQRRRAEENALQQKACGSEIMQKKQHLEIELKQVMQQRSEDNARHKQSLEEAAKTIQDKNKEIERLKAEFQEEAKRRWEYENELSKVRNNYDEEIISLKNQFETEINITKTTIHQLTMQKEEDTSGYRAQIDNLTRENRSLSEEIKRLKNTLTQTTENLRRVEEDIQQQKATGSEVSQRKQQLEVELRQVTQMRTEESVRYKQSLDDAAKTIQDKNKEIERLKQLIDKETNDRKCLEDENARLQRVQYDLQKANSSATETINKLKVQEQELTRLRIDYERVSQERTVKDQDITRFQNSLKELQLQKQKVEEELNRLKRTASEDSCKRKKLEEELEGMRRSLKEQAIKITNLTQQLEQASIVKKRSEDDLRQQRDVLDGHLREKQRTQEELRRLSSEVEALRRQLLQEQESVKQAHLRNEHFQKAIEDKSRSLNESKIEIERLQSLTENLTKEHLMLEEELRNLRLEYDDLRRGRSEADSDKNATILELRSQLQISNNRTLELQGLINDLQRERENLRQEIEKFQKQALEASNRIQESKNQCTQVVQERESLLVKIKVLEQDKARLQRLEDELNRAKSTLEAETRVKQRLECEKQQIQNDLNQWKTQYSRKEEAIRKIESEREKSEREKNSLRSEIERLQAEIKRIEERCRRKLEDSTRETQSQLETERSRYQREIDKLRQRPYGSHRETQTECEWTVDTSKLVFDGLRKKVTAMQLYECQLIDKTTLDKLLKGKKSVEEVASEIQPFLRGAGSIAGASASPKEKYSLVEAKRKKLISPESTVMLLEAQAATGGIIDPHRNEKLTVDSAIARDLIDFDDRQQIYAAEKAITGFDDPFSGKTVSVSEAIKKNLIDRETGMRLLEAQIASGGVVDPVNSVFLPKDVALARGLIDRDLYRSLNDPRDSQKNFVDPVTKKKVSYVQLKERCRIEPHTGLLLLSVQKRSMSFQGIRQPVTVTELVDSGILRPSTVNELESGQISYDEVGERIKDFLQGSSCIAGIYNETTKQKLGIYEAMKIGLVRPGTALELLEAQAATGFIVDPVSNLRLPVEEAYKRGLVGIEFKEKLLSAERAVTGYNDPETGNIISLFQAMNKELIEKGHGIRLLEAQIATGGIIDPKESHRLPVDIAYKRGYFNEELSEILSDPSDDTKGFFDPNTEENLTYLQLKERCIKDEETGLCLLPLKEKKKQVQTSQKNTLRKRRVVIVDPETNKEMSVQEAYKKGLIDYETFKELCEQECEWEEITITGSDGSTRVVLVDRKTGSQYDIQDAIDKGLVDRKFFDQYRSGSLSLTQFADMISLKNGVGTSSSMGSGVSDDVFSSSRHESVSKISTISSVRNLTIRSSSFSDTLEESSPIAAIFDTENLEKISITEGIERGIVDSITGQRLLEAQACTGGIIHPTTGQKLSLQDAVSQGVIDQDMATRLKPAQKAFIGFEGVKGKKKMSAAEAVKEKWLPYEAGQRFLEFQYLTGGLVDPEVHGRISTEEAIRKGFIDGRAAQRLQDTSSYAKILTCPKTKLKISYKDAINRSMVEDITGLRLLEAASVSSKGLPSPYNMSSAPGSRSGSRSGSRSGSRSGSRSGSRRGSFDATGNSSYSYSYSFSSSSIGH

326 NP_001194.1 NP_071732.1

>NP_001194.1

MLLRSAGKLNVGTKKEDGESTAPTPRPKVLRCKCHHHCPEDSVNNICSTDGYCFTMIEEDDSGLPVVTSGCLGLEGSDFQCRDTPIPHQRRSIECCTERNECNKDLHPTLPPLKNRDFVDGPIHHRALLISVTVCSLLLVLIILFCYFRYKRQETRPRYSIGLEQDETYIPPGESLRDLIEQSQSSGSGSGLPLLVQRTIAKQIQMVKQIGKGRYGEVWMGKWRGEKVAVKVFFTTEEASWFRETEIYQTVLMRHENILGFIAADIKGTGSWTQLYLITDYHENGSLYDYLKSTTLDAKSMLKLAYSSVSGLCHLHTEIFSTQGKPAIAHRDLKSKNILVKKNGTCCIADLGLAVKFISDTNEVDIPPNTRVGTKRYMPPEVLDESLNRNHFQSYIMADMYSFGLILWEVARRCVSGGIVEEYQLPYHDLVPSDPSYEDMREIVCIKKLRPSFPNRWSSDECLRQMGKLMTECWAHNPASRLTALRVKKTLAKMSESQDIKL

>NP_071732.1

MQAPHKEHLYKLLVIGDLGVGKTSIIKRYVHQNFSSHYRATIGVDFALKVLHWDPETVVRLQLWDIAGQERFGNMTRVYYREAMGAFIVFDVTRPATFEAVAKWKNDLDSKLSLPNGKPVSVVLLANKCDQGKDVLMNNGLKMDQFCKEHGFVGWFETSAKENINIDEASRCLVKHILANECDLMESIEPDVVKPHLTSTKVASCSGCAKS

327 NP_005822.1 NP_443156.2

>NP_005822.1

MEETIKDPPTSAVLLDHCHFSQVIFNSVEKFYIPGGDVTCHYTFTQHFIPRRKDWIGIFRVGWKTTREYYTFMWVTLPIDLNNKSAKQQEVQFKAYYLPKDDEYYQFCYVDEDGVVRGASIPFQFRPENEEDILVVTTQGEVEEIEQHNKELCKENQELKDSCISLQKQNSDMQAELQKKQEELETLQSINKKLELKVKEQKDYWETELLQLKEQNQKMSSENEKMGIRVDQLQAQLSTQEKEMEKLVQGDQDKTEQLEQLKKENDHLFLSLTEQRKDQKKLEQTVEQMKQNETTAMKKQQELMDENFDLSKRLSENEIICNALQRQKERLEGENDLLKRENSRLLSYMGLDFNSLPYQVPTSDEGGARQNPGLAYGNPYSGIQESSSPSPLSIKKCPICKADDICDHTLEQQQMQPLCFNCPICDKIFPATEKQIFEDHVFCHSL

>NP_443156.2

MILEERPDGAGAGEESPRLQGCDSLTQIQCGQLQSRRAQIHQQIDKELQMRTGAENLYRATSNNRVRETVALELSYVNSNLQLLKEELEELSGGVDPGRHGSEAVTVPMIPLGLKETKELDWSTPLKELISVHFGEDGASYEAEIRELEALRQAMRTPSRNESGLELLTAYYNQLCFLDARFLTPARSLGLFFHWYDSLTGVPAQQRALAFEKGSVLFNIGALHTQIGARQDRSCTEGARRAMEAFQRAAGAFSLLRENFSHAPSPDMSAASLCALEQLMMAQAQECVFEGLSPPASMAPQDCLAQLRLAQEAAQVAAEYRLVHRTMAQPPVHDYVPVSWTALVHVKAEYFRSLAHYHVAMALCDGSPATEGELPTHEQVFLQPPTSSKPRGPVLPQELEERRQLGKAHLKRAILGQEEALRLHALCRVLREVDLLRAVISQTLQRSLAKYAELDREDDFCEAAEAPDIQPKTHQKPEARMPRLSQGKGPDIFHRLGPLSVFSAKNRWRLVGPVHLTRGEGGFGLTLRGDSPVLIAAVIPGSQAAAAGLKEGDYIVSVNGQPCRWWRHAEVVTELKAAGEAGASLQVVSLLPSSRLPSLGDRRPVLLGPRGLLRSQREHGCKTPASTWASPRPLLNWSRKAQQGKTGGCPQPCAPVKPAPPSSLKHPGWP

328 NP_003572.2 NP_005624.2

>NP_003572.2

MTEEVIVIAKWDYTAQQDQELDIKKNERLWLLDDSKTWWRVRNAANRTGYVPSNYVERKNSLKKGSLVKNLKDTLGLGKTRRKTSARDASPTPSTDAEYPANGSGADRIYDLNIPAFVKFAYVAEREDELSLVKGSRVTVMEKCSDGWWRGSYNGQIGWFPSNYVLEEVDEAAAESPSFLSLRKGASLSNGQGSRVLHVVQTLYPFSSVTEEELNFEKGETMEVIEKPENDPEWWKCKNARGQVGLVPKNYVVVLSDGPALHPAHAPQISYTGPSSSGRFAGREWYYGNVTRHQAECALNERGVEGDFLIRDSESSPSDFSVSLKASGKNKHFKVQLVDNVYCIGQRRFHTMDELVEHYKKAPIFTSEHGEKLYLVRALQ

>NP_005624.2

MQAQQLPYEFFSEENAPKWRGLLVPALKKVQGQVHPTLESNDDALQYVEELILQLLNMLCQAQPRSASDVEERVQKSFPHPIDKWAIADAQSAIEKRKRRNPLSLPVEKIHPLLKEVLGYKIDHQVSVYIVAVLEYISADILKLVGNYVRNIRHYEITKQDIKVAMCADKVLMDMFHQDVEDINILSLTDEEPSTSGEQTYYDLVKAFMAEIRQYIRELNLIIKVFREPFVSNSKLFSANDVENIFSRIVDIHELSVKLLGHIEDTVEMTDEGSPHPLVGSCFEDLAEELAFDPYESYARDILRPGFHDRFLSQLSKPGAALYLQSIGEGFKEAVQYVLPRLLLAPVYHCLHYFELLKQLEEKSEDQEDKECLKQAITALLNVQSGMEKICSKSLAKRRLSESACRFYSQQMKGKQLAIKKMNEIQKNIDGWEGKDIGQCCNEFIMEGTLTRVGAKHERHIFLFDGLMICCKSNHGQPRLPGASNAEYRLKEKFFMRKVQINDKDDTNEYKHAFEIILKDENSVIFSAKSAEEKNNWMAALISLQYRSTLERMLDVTMLQEEKEEQMRLPSADVYRFAEPDSEENIIFEENMQPKAGIPIIKAGTVIKLIERLTYHMYADPNFVRTFLTTYRSFCKPQELLSLIIERFEIPEPEPTEADRIAIENGDQPLSAELKRFRKEYIQPVQLRVLNVCRHWVEHHFYDFERDAYLLQRMEEFIGTVRGKAMKKWVESITKIIQRKKIARDNGPGHNITFQSSPPTVEWHISRPGHIETFDLLTLHPIEIARQLTLLESDLYRAVQPSELVGSVWTKEDKEINSPNLLKMIRHTTNLTLWFEKCIVETENLEERVAVVSRIIEILQVFQELNNFNGVLEVVSAMNSSPVYRLDHTFEQIPSRQKKILEEAHELSEDHYKKYLAKLRSINPPCVPFFGIYLTNILKTEEGNPEVLKRHGKELINFSKRRKVAEITGEIQQYQNQPYCLRVESDIKRFFENLNPMGNSMEKEFTDYLFNKSLEIEPRNPKPLPRFPKKYSYPLKSPGVRPSNPRPGTMRHPTPLQQEPRKISYSRIPESETESTASAPNSPRTPLTPPPASGASSTTDVCSVFDSDHSSPFHSSNDTVFIQVTLPHGPRSASVSSISLTKGTDEVPVPPPVPPRRRPESAPAESSPSKIMSKHLDSPPAIPPRQPTSKAYSPRYSISDRTSISDPPESPPLLPPREPVRTPDVFSSSPLHLQPPPLGKKSDHGNAFFPNSPSPFTPPPPQTPSPHGTRRHLPSPPLTQEVDLHSIAGPPVPPRQSTSQHIPKLPPKTYKREHTHPSMHRDGPPLLENAHSS

329 NP_003737.1 NP_861460.1

>NP_003737.1

MCDRKAVIKNADMSEEMQQDSVECATQALEKYNIEKDIAAHIKKEFDKKYNPTWHCIVGRNFGSYVTHETKHFIYFYLGQVAILLFKSG

>NP_861460.1

MRTDSSKMTDVESGVANFASSARAGRRNALPDIQSSAATDGTSDLPLKLEALSVKEDAKEKDEKTTQDQLEKPQNEEK

330 NP_858045.1 NP_003343.1

>NP_858045.1

MSGLGENLDPLASDSRKRKLPCDTPGQGLTCSGEKRRREQESKYIEELAELISANLSDIDNFNVKPDKCAILKETVRQIRQIKEQGKTISNDDDVQKADVSSTGQGVIDKDSLGPLLLQALDGFLFVVNRDGNIVFVSENVTQYLQYKQEDLVNTSVYNILHEEDRKDFLKNLPKSTVNGVSWTNETQRQKSHTFNCRMLMKTPHDILEDINASPEMRQRYETMQCFALSQPRAMMEEGEDLQSCMICVARRITTGERTFPSNPESFITRHDLSGKVVNIDTNSLRSSMRPGFEDIIRRCIQRFFSLNDGQSWSQKRHYQEAYLNGHAETPVYRFSLADGTIVTAQTKSKLFRNPVTNDRHGFVSTHFLQREQNGYRPNPNPVGQGIRPPMAGCNSSVGGMSMSPNQGLQMPSSRAYGLADPSTTGQMSGARYGGSSNIASLTPGPGMQSPSSYQNNNYGLNMSSPPHGSPGLAPNQQNIMISPRNRGSPKIASHQFSPVAGVHSPMASSGNTGNHSFSSSSLSALQAISEGVGTSLLSTLSSPGPKLDNSPNMNITQPSKVSNQDSKSPLGFYCDQNPVESSMCQSNSRDHLSDKESKESSVEGAENQRGPLESKGHKKLLQLLTCSSDDRGHSSLTNSPLDSSCKESSVSVTSPSGVSSSTSGGVSSTSNMHGSLLQEKHRILHKLLQNGNSPAEVAKITAEATGKDTSSITSCGDGNVVKQEQLSPKKKENNALLRYLLDRDDPSDALSKELQPQVEGVDNKMSQCTSSTIPSSSQEKDPKIKTETSEEGSGDLDNLDAILGDLTSSDFYNNSISSNGSHLGTKQQVFQGTNSLGLKSSQSVQSIRPPYNRAVSLDSPVSVGSSPPVKNISAFPMLPKQPMLGGNPRMMDSQENYGSSMGGPNRNVTVTQTPSSGDWGLPNSKAGRMEPMNSNSMGRPGGDYNTSLPRPALGGSIPTLPLRSNSIPGARPVLQQQQQMLQMRPGEIPMGMGANPYGQAAASNQLGSWPDGMLSMEQVSHGTQNRPLLRNSLDDLVGPPSNLEGQSDERALLDQLHTLLSNTDATGLEEIDRALGIPELVNQGQALEPKQDAFQGQEAAVMMDQKAGLYGQTYPAQGPPMQGGFHLQGQSPSFNSMMNQMNQQGNFPLQGMHPRANIMRPRTNTPKQLRMQLQQRLQGQQFLNQSRQALELKMENPTAGGAAVMRPMMQPQVSSQQGFLNAQMVAQRSRELLSHHFRQQRVAMMMQQQQQQQQQQQQQQQQQQQQQQQQQQQQQTQAFSPPPNVTASPSMDGLLAGPTMPQAPPQQFPYQPNYGMGQQPDPAFGRVSSPPNAMMSSRMGPSQNPMMQHPQAASIYQSSEMKGWPSGNLARNSSFSQQQFAHQGNPAVYSMVHMNGSSGHMGQMNMNPMPMSGMPMGPDQKYC

>NP_003343.1

MSDQEAKPSTEDLGDKKEGEYIKLKVIGQDSSEIHFKVKMTTHLKKLKESYCQRQGVPMNSLRFLFEGQRIADNHTPKELGMEEEDVIEVYQEQTGGHSTV

331 NP_115584.1 NP_612815.1

>NP_115584.1

MATAERRALGIGFQWLSLATLVLICAGQGGRREDGGPACYGGFDLYFILDKSGSVLHHWNEIYYFVEQLAHKFISPQLRMSFIVFSTRGTTLMKLTEDREQIRQGLEELQKVLPGGDTYMHEGFERASEQIYYENRQGYRTASVIIALTDGELHEDLFFYSEREANRSRDLGAIVYCVGVKDFNETQLARIADSKDHVFPVNDGFQALQGIIHSILKKSCIEILAAEPSTICAGESFQVVVRGNGFRHARNVDRVLCSFKINDSVTLNEKPFSVEDTYLLCPAPILKEVGMKAALQVSMNDGLSFISSSVIITTTHCSDGSILAIALLILFLLLALALLWWFWPLCCTVIIKEVPPPPAEESEEEDDDGLPKKKWPTVDASYYGGRGVGGIKRMEVRWGEKGSTEEGAKLEKAKNARVKMPEQEYEFPEPRNLNNNMRRPSSPRKWYSPIKGKLDALWVLLRKGYDRVSVMRPQPGDTGRCINFTRVKNNQPAKYPLNNAYHTSSPPPAPIYTPPPPAPHCPPPPPSAPTPPIPSPPSTLPPPPQAPPPNRAPPPSRPPPRPSV

>NP_612815.1

MSQSNRELVVDFLSYKLSQKGYSWSQFSDVEENRTEAPEGTESEMETPSAINGNPSWHLADSPAVNGATGHSSSLDAREVIPMAAVKQALREAGDEFELRYRRAFSDLTSQLHITPGTAYQSFEQVVNELFRDGVNWGRIVAFFSFGGALCVESVDKEMQVLVSRIAAWMATYLNDHLEPWIQENGGWDTFVELYGNNAAAESRKGQERFNRWFLTGMTVAGVVLLGSLFSRK

332 NP_054829.2 NP_009107.1

>NP_054829.2

MAAAANSGSSLPLFDCPTWAGKPPPGLHLDVVKGDKLIEKLIIDEKKYYLFGRNPDLCDFTIDHQSCSRVHAALVYHKHLKRVFLIDLNSTHGTFLGHIRLEPHKPQQIPIDSTVSFGASTRAYTLREKPQTLPSAVKGDEKMGGEDDELKGLLGLPEEETELDNLTEFNTAHNKRISTLTIEEGNLDIQRPKRKRKNSRVTFSEDDEIINPEDVDPSVGRFRNMVQTAVVPVKKKRVEGPGSLGLEESGSRRMQNFAFSGGLYGGLPPTHSEAGSQPHGIHGTALIGGLPMPYPNLAPDVDLTPVVPSAVNMNPAPNPAVYNPEAVNEPKKKKYAKEAWPGKKPTPSLLI

>NP_009107.1

MSRFLNVLRSWLVMVSIIAMGNTLQSFRDHTFLYEKLYTGKPNLVNGLQARTFGIWTLLSSVIRCLCAIDIHNKTLYHITLWTFLLALGHFLSELFVYGTAAPTIGVLAPLMVASFSILGMLVGLRYLEVEPVSRQKKRN

333 NP_001869.1 NP_001751.1

>NP_001869.1

MPNFSGNWKIIRSENFEELLKVLGVNVMLRKIAVAAASKPAVEIKQEGDTFYIKTSTTVRTTEINFKVGEEFEEQTVDGRPCKSLVKWESENKMVCEQKLLKGEGPKTSWTRELTNDGELILTMTADDVVCTRVYVRE

>NP_001751.1

MELLCCEGTRHAPRAGPDPRLLGDQRVLQSLLRLEERYVPRASYFQCVQREIKPHMRKMLAYWMLEVCEEQRCEEEVFPLAMNYLDRYLSCVPTRKAQLQLLGAVCMLLASKLRETTPLTIEKLCIYTDHAVSPRQLRDWEVLVLGKLKWDLAAVIAHDFLAFILHRLSLPRDRQALVKKHAQTFLALCATDYTFAMYPPSMIATGSIGAAVQGLGACSMSGDELTELLAGITGTEVDCLRACQEQIEAALRESLREASQTSSSPAPKAPRGSSSQGPSQTSTPTDVTAIHL

334 AAB94503.1 NP_444277.2

>AAB94503.1

MRGPSGPPGLLYVPHLLLCLLCLLPPPLQYAAGQSQMPKDQPLWALLEQYCHTIMTLTNLSGPYSYCNTTLDQIGTCWPRSAAGALVERPCPEYFNGVKYNTTRNAYRECLENGTWASKINYSQCEPILDDKQRKYDLHYRIALVVNYLGHCVSVAALVAAFLLFLALRSIRCLRNVIHWNLITTFILRNVMWFLLQLVDHEVHESNEVWCRCITTIFNYFVVTNFFWMFVEGCYLHTAIVMTYSTERLRKCLFLFIGWCIPFPIIVAWAIGKLYYENEQCWFGKEPGDLVDYIYQGPIILVLLINFVFLFNIVRILMTKLRASTTSETIQYRKAVKATLVLLPLLGITYMLFFVNPGEDDLSQIMFIYFNSFLQSFQGFFVSVFYCFFNGEVRSAVRKRWHRWQDHHSLRVPMARAMSIPTSPTRISFHSIKQTAAV

>NP_444277.2

MLMPVHFLLLLLLLLGGPRTGLPHKFYKAKPIFSCLNTALSEAEKGQWEDASLLSKRSFHYLRSRDASSGEEEEGKEKKTFPISGARGGARGTRYRYVSQAQPRGKPRQDTAKSPHRTKFTLSLDVPTNIMNLLFNIAKAKNLRAQAAANAHLMAQIGRKK

335 NP_004243.1 NP_003051.1

>NP_004243.1

MSADAAAGAPLPRLCCLEKGPNGYGFHLHGEKGKLGQYIRLVEPGSPAEKAGLLAGDRLVEVNGENVEKETHQQVVSRIRAALNAVRLLVVDPETDEQLQKLGVQVREELLRAQEAPGQAEPPAAAEVQGAGNENEPREADKSHPEQRELRPRLCTMKKGPSGYGFNLHSDKSKPGQFIRSVDPDSPAEASGLRAQDRIVEVNGVCMEGKQHGDVVSAIRAGGDETKLLVVDRETDEFFKKCRVIPSQEHLNGPLPVPFTNGEIQKENSREALAEAALESPRPALVRSASSDTSEELNSQDSPPKQDSTAPSSTSSSDPILDFNISLAMAKERAHQKRSSKRAPQMDWSKKNELFSNL

>NP_003051.1

MRDYDEVTAFLGEWGPFQRLIFFLLSASIIPNGFTGLSSVFLIATPEHRCRVPDAANLSSAWRNHTVPLRLRDGREVPHSCRRYRLATIANFSALGLEPGRDVDLGQLEQESCLDGWEFSQDVYLSTIVTEWNLVCEDDWKAPLTISLFFVGVLLGSFISGQLSDRFGRKNVLFVTMGMQTGFSFLQIFSKNFEMFVVLFVLVGMGQISNYVAAFVLGTEILGKSVRIIFSTLGVCIFYAFGYMVLPLFAYFIRDWRMLLVALTMPGVLCVALWWFIPESPRWLISQGRFEEAEVIIRKAAKANGIVVPSTIFDPSELQDLSSKKQQSHNILDLLRTWNIRMVTIMSIMLWMTISVGYFGLSLDTPNLHGDIFVNCFLSAMVEVPAYVLAWLLLQYLPRRYSMATALFLGGSVLLFMQLVPPDLYYLATVLVMVGKFGVTAAFSMVYVYTAELYPTVVRNMGVGVSSTASRLGSILSPYFVYLGAYDRFLPYILMGSLTILTAILTLFLPESFGTPLPDTIDQMLRVKGMKHRKTPSHTRMLKDGQERPTILKSTAF

336 NP_001036068.1 NP_002886.2

>NP_001036068.1

MAGVGPGGYAAEFVPPPECPVFEPSWEEFTDPLSFIGRIRPLAEKTGICKIRPPKDWQPPFACEVKSFRFTPRVQRLNELEAMTRVRLDFLDQLAKFWELQGSTLKIPVVERKILDLYALSKIVASKGGFEMVTKEKKWSKVGSRLGYLPGKGTGSLLKSHYERILYPYELFQSGVSLMGVQMPNLDLKEKVEPEVLSTDTQTSPEPGTRMNILPKRTRRVKTQSESGDVSRNTELKKLQIFGAGPKVVGLAMGTKDKEDEVTRRRKVTNRSDAFNMQMRQRKGTLSVNFVDLYVCMFCGRGNNEDKLLLCDGCDDSYHTFCLIPPLPDVPKGDWRCPKCVAEECSKPREAFGFEQAVREYTLQSFGEMADNFKSDYFNMPVHMVPTELVEKEFWRLVSSIEEDVIVEYGADISSKDFGSGFPVKDGRRKILPEEEEYALSGWNLNNMPVLEQSVLAHINVDISGMKVPWLYVGMCFSSFCWHIEDHWSYSINYLHWGEPKTWYGVPSHAAEQLEEVMRELAPELFESQPDLLHQLVTIMNPNVLMEHGVPVYRTNQCAGEFVVTFPRAYHSGFNQGYNFAEAVNFCTADWLPIGRQCVNHYRRLRRHCVFSHEELIFKMAADPECLDVGLAAMVCKELTLMTEEETRLRESVVQMGVLMSEEEVFELVPDDERQCSACRTTCFLSALTCSCNPERLVCLYHPTDLCPCPMQKKCLRYRYPLEDLPSLLYGVKVRAQSYDTWVSRVTEALSANFNHKKDLIELRVMLEDAEDRKYPENDLFRKLRDAVKEAETCASVAQLLLSKKQKHRQSPDSGRTRTKLTVEELKAFVQQLFSLPCVISQARQVKNLLDDVEEFHERAQEAMMDETPDSSKLQMLIDMGSSLYVELPELPRLKQELQQARWLDEVRLTLSDPQQVTLDVMKKLIDSGVGLAPHHAVEKAMAELQELLTVSERWEEKAKVCLQARPRHSVASLESIVNEAKNIPAFLPNVLSLKEALQKAREWTAKVEAIQSGSNYAYLEQLESLSAKGRPIPVRLEALPQVESQVAAARAWRERTGRTFLKKNSSHTLLQVLSPRTDIGVYGSGKNRRKKVKELIEKEKEKDLDLEPLSDLEEGLEETRDTAMVVAVFKEREQKEIEAMHSLRAANLAKMTMVDRIEEVKFCICRKTASGFMLQCELCKDWFHNSCVPLPKSSSQKKGSSWQAKEVKFLCPLCMRSRRPRLETILSLLVSLQKLPVRLPEGEALQCLTERAMSWQDRARQALATDELSSALAKLSVLSQRMVEQAAREKTEKIISAELQKAAANPDLQGHLPSFQQSAFNRVVSSVSSSPRQTMDYDDEETDSDEDIRETYGYDMKDTASVKSSSSLEPNLFCDEEIPIKSEEVVTHMWTAPSFCAEHAYSSASKSCSQGSSTPRKQPRKSPLVPRSLEPPVLELSPGAKAQLEELMMVGDLLEVSLDETQHIWRILQATHPPSEDRFLHIMEDDSMEEKPLKVKGKDSSEKKRKRKLEKVEQLFGEGKQKSKELKKMDKPRKKKLKLGADKSKELNKLAKKLAKEEERKKKKEKAAAAKVELVKESTEKKREKKVLDIPSKYDWSGAEESDDENAVCAAQNCQRPCKDKVDWVQCDGGCDEWFHQVCVGVSPEMAENEDYICINCAKKQGPVSPGPAPPPSFIMSYKLPMEDLKETS

>NP_002886.2

MFEDKPHAEGAAVVAAAGEALQALCQELNLDEGSAAEALDDFTAIRGNYSLEGEVTHWLACSLYVACRKSIIPTVGKGIMEGNCVSLTRILRSAKLSLIQFFSKMKKWMDMSNLPQEFRERIERLERNFEVSTVIFKKYEPIFLDIFQNPYEEPPKLPRSRKQRRIPCSVKDLFNFCWTLFVYTKGNFRMIGDDLVNSYHLLLCCLDLIFANAIMCPNRQDLLNPSFKGLPSDFHTADFTASEEPPCIIAVLCELHDGLLVEAKGIKEHYFKPYISKLFDRKILKGECLLDLSSFTDNSKAVNKEYEEYVLTVGDFDERIFLGADAEEEIGTPRKFTRDTPLGKLTAQANVEYNLQQHFEKKRSFAPSTPLTGRRYLREKEAVITPVASATQSVSRLQSIVAGLKNAPSDQLINIFESCVRNPVENIMKILKGIGETFCQHYTQSTDEQPGSHIDFAVNRLKLAEILYYKILETVMVQETRRLHGMDMSVLLEQDIFHRSLMACCLEIVLFAYSSPRTFPWIIEVLNLQPFYFYKVIEVVIRSEEGLSRDMVKHLNSIEEQILESLAWSHDSALWEALQVSANKVPTCEEVIFPNNFETGNGGNVQGHLPLMPMSPLMHPRVKEVRTDSGSLRRDMQPLSPISVHERYSSPTAGSAKRRLFGEDPPKEMLMDKIITEGTKLKIAPSSSITAENVSILPGQTLLTMATAPVTGTTGHKVTIPLHGVANDAGEITLIPLSMNTNQESKVKSPVSLTAHSLIGASPKQTNLTKAQEVHSTGINRPKRTGSLALFYRKVYHLASVRLRDLCLKLDVSNELRRKIWTCFEFTLVHCPDLMKDRHLDQLLLCAFYIMAKVTKEERTFQEIMKSYRNQPQANSHVYRSVLLKSIPREVVAYNKNINDDFEMIDCDLEDATKTPDCSSGPVKEERGDLIKFYNTIYVGRVKSFALKYDLANQDHMMDAPPLSPFPHIKQQPGSPRRISQQHSIYISPHKNGSGLTPRSALLYKFNGSPSKSLKDINNMIRQGEQRTKKRVIAIDSDAESPAKRVCQENDDVLLKRLQDVVSERANH

337 NP_114120.2 NP_694994.2

>NP_114120.2

MVLAMASQDVQNFFQPFSSWISRVYEALQQAGDMLSASLVNISKQDSKLSDKLDQDLDNIQIQETYFEDEEQDNDWSQEDANSLFLEVDHFSCCNSDLQDSAQNSSPSLSQHAKDSCSTMSQWPNWASDDRKLPHVLSSIAEEEHHLEKQRSGLQHGFDSQLPGTLETVNGKKQVNSFGDDEELSTSSDSDEEVIKQFEISVSRSQSFRSVTSEKGKQTGLEQKPKFSRSLLTHGEDGTEVSACEDLDGASQRRYSENLSYGEDDHIPAHSQSPCERGDAKHHGTSHQESSVVQSLRRQSTEGSLEMETAFNSRGFEDSYATDSSSMWSPEEQDRTNLQVPSGVSEPISKCGDLDVIFEYRAASQKLTVTIVRAQGLPDKDRSGVNSWQVHVVLLPGKKHRGRTNIQRGPNPVFREKVTFAKLEPRDVAACAVRFRLYAARKMTRERMMGEKLFYLSHLHPEGEMKVTLVLEPRSNISSGGSPLSPSAVSHSDSTSSTQSLSHGGAPELLVGLSYNATTGRLSVEMIKGSHFRNLAVNRAPDTYGKLFLLNSVGQEMSRCKTSIRRGQPNPVYKETFVFQVALFQLSDVTLMISVYNRRTMKRKEMIGWIALGQNSSGEEEQDHWEEMKETKGQQICRWHTLLES

>NP_694994.2

MAIEGGERTCGVHELICIRKVSPEAVGFLSAVGVFIILMLLLFLYINKKFCFENVGGFPDLGSEYSTRKNSQDKIYNSYMDKDEHGSSSESEDEALGKYHEALSRTHNSRLPLADSRQRNYAWETRQKYSPLSAEYDGYSSEASIDEGNCIQRMRRTPPLDELQPPPYQDDSGSPHLSCTPSEIGDSKCEFSHCSNSPRCSYNKCPSEGSTGHEIESFHNKGYEEDVPSDSTAVLSPEDMSAQGSSSQLPKPFDPEPEAKYGTLDVTFDYDSQEQKLLVTVTAVTDIPTYNRTGGNSWQVHLVLLPIKKQRAKTSIQRGPCPVFTETFKFNHVESEMIGNYAVRFRLYGVHRMKKEKIVGEKIFYLTKLNLQGKMSLPVILEPSYNHSGCDSQMSVSEMSCSESTSSCQSLEHGSVPEILIGLLYNATTGRLSAEVIKGSHFKNLAANRPPNTYVKLTLLNSMGQEMSKCKTSIRRGQPNPVYKETFVFQVALFQLSDVTLILSVYNKRSMKRKEMIGWISLGLNSSGEEELNHWTEMKESKGQQVCRWHALLES

338 NP_004746.2 NP_005162.1

>NP_004746.2

MEEEGGSSGGAAGTSADGGDGGEQLLTVKHELRTANLTGHAEKVGIENFELLKVLGTGAYGKVFLVRKISGHDTGKLYAMKVLKKATIVQKAKTTEHTRTERQVLEHIRQSPFLVTLHYAFQTETKLHLILDYINGGELFTHLSQRERFTEHEVQIYVGEIVLALEHLHKLGIIYRDIKLENILLDSNGHVVLTDFGLSKEFVADETERAYSFCGTIEYMAPDIVRGGDSGHDKAVDWWSLGVLMYELLTGASPFTVDGEKNSQAEISRRILKSEPPYPQEMSALAKDLIQRLLMKDPKKRLGCGPRDADEIKEHLFFQKINWDDLAAKKVPAPFKPVIRDELDVSNFAEEFTEMDPTYSPAALPQSSEKLFQGYSFVAPSILFKRNAAVIDPLQFHMGVERPGVTNVARSAMMKDSPFYQHYDLDLKDKPLGEGSFSICRKCVHKKSNQAFAVKIISKRMEANTQKEITALKLCEGHPNIVKLHEVFHDQLHTFLVMELLNGGELFERIKKKKHFSETEASYIMRKLVSAVSHMHDVGVVHRDLKPENLLFTDENDNLEIKIIDFGFARLKPPDNQPLKTPCFTLHYAAPELLNQNGYDESCDLWSLGVILYTMLSGQVPFQSHDRSLTCTSAVEIMKKIKKGDFSFEGEAWKNVSQEAKDLIQGLLTVDPNKRLKMSGLRYNEWLQDGSQLSSNPLMTPDILGSSGAAVHTCVKATFHAFNKYKREGFCLQNVDKAPLAKRRKMKKTSTSTETRSSSSESSHSSSSHSHGKTTPTKTLQPSNPADSNNPETLFQFSDSVA

>NP_005162.1

MEDSHKSTTSETAPQPGSAVQGAHISHIAQQVSSLSESEESQDSSDSIGSSQKAHGILARRPSYRKILKDLSSEDTRGRKGDGENSGVSAAVTSMSVPTPIYQTSSGQYIAIAPNGALQLASPGTDGVQGLQTLTMTNSGSTQQGTTILQYAQTSDGQQILVPSNQVVVQTASGDMQTYQIRTTPSATSLPQTVVMTSPVTLTSQTTKTDDPQLKREIRLMKNREAARECRRKKKEYVKCLENRVAVLENQNKTLIEELKTLKDLYSNKSV

339 NP_003484.1 NP_001915.1

>NP_003484.1

MARTKQTARKSTGGKAPRKQLATKVARKSAPATGGVKKPHRYRPGTVALREIRRYQKSTELLIRKLPFQRLMREIAQDFKTDLRFQSSAVMALQEACESYLVGLFEDTNLCVIHAKRVTIMPKDIQLARRIRGERA

>NP_001915.1

MTLEEFSAGEQKTERMDKVGDALEEVLSKALSQRTITVGVYEAAKLLNVDPDNVVLCLLAADEDDDRDVALQIHFTLIQAFCCENDINILRVSNPGRLAELLLLETDAGPAASEGAEQPPDLHCVLVTNPHSSQWKDPALSQLICFCRESRYMDQWVPVINLPER

340 NP_005616.2 NP_000818.1

>NP_005616.2

MSLYPSLEDLKVDKVIQAQTAFSANPANPAILSEASAPIPHDGNLYPRLYPELSQYMGLSLNEEEIRANVAVVSGAPLQGQLVARPSSINYMVAPVTGNDVGIRRAEIKQGIREVILCKDQDGKIGLRLKSIDNGIFVQLVQANSPASLVGLRFGDQVLQINGENCAGWSSDKAHKVLKQAFGEKITMTIRDRPFERTITMHKDSTGHVGFIFKNGKITSIVKDSSAARNGLLTEHNICEINGQNVIGLKDSQIADILSTSGTVVTITIMPAFIFEHIIKRMAPSIMKSLMDHTIPEV

>NP_000818.1

MQHIFAFFCTGFLGAVVGANFPNNIQIGGLFPNQQSQEHAAFRFALSQLTEPPKLLPQIDIVNISDSFEMTYRFCSQFSKGVYAIFGFYERRTVNMLTSFCGALHVCFITPSFPVDTSNQFVLQLRPELQDALISIIDHYKWQKFVYIYDADRGLSVLQKVLDTAAEKNWQVTAVNILTTTEEGYRMLFQDLEKKKERLVVVDCESERLNAILGQIIKLEKNGIGYHYILANLGFMDIDLNKFKESGANVTGFQLVNYTDTIPAKIMQQWKNSDARDHTRVDWKRPKYTSALTYDGVKVMAEAFQSLRRQRIDISRRGNAGDCLANPAVPWGQGIDIQRALQQVRFEGLTGNVQFNEKGRRTNYTLHVIEMKHDSIRKIGYWNEDDKFVPAATDAQAGGDNSSVQNRTYIVTTILEDPYVMLKKNANQFEGNDRYEGYCVELAAEIAKHVGYSYRLEIVSDGKYGARDPDTKAWNGMVGELVYGRADVAVAPLTITLVREEVIDFSKPFMSLGISIMIKKPQKSKPGVFSFLDPLAYEIWMCIVFAYIGVSVVLFLVSRFSPYEWHSEEFEEGRDQTTSDQSNEFGIFNSLWFSLGAFMQQGCDISPRSLSGRIVGGVWWFFTLIIISSYTANLAAFLTVERMVSPIESAEDLAKQTEIAYGTLEAGSTKEFFRRSKIAVFEKMWTYMKSAEPSVFVRTTEEGMIRVRKSKGKYAYLLESTMNEYIEQRKPCDTMKVGGNLDSKGYGIATPKGSALRNPVNLAVLKLNEQGLLDKLKNKWWYDKGECGSGGGDSKDKTSALSLSNVAGVFYILIGGLGLAMLVALIEFCYKSRSESKRMKGFCLIPQQSINEAIRTSTLPRNSGAGASSGGSGENGRVVSHDFPKSMQSIPCMSHSSGMPLGATGL

341 NP_115955.1 NP_078994.2

>NP_115955.1

MKITGGLLLLCTVVYFCSSSEAASLSPKKVDCSIYKKYPVVAIPCPITYLPVCGSDYITYGNECHLCTESLKSNGRVQFLHDGSC

>NP_078994.2

MKIVIRGDRNTGKTALWHRLQGRPFVEEYIPTQEIQVTSIHWSYKTTDDIVKVEVWDVVDKGKCKKRGDGLKMENDPQEAESEMALDAEFLDVYKNCNGVVMMFDITKQWTFNYILRELPKVPTHVPVCVLGNYRDMGEHRVILPDDVRDFIDNLDRPPGSSYFRYAESSMKNSFGLKYLHKFFNIPFLQLQRETLLRQLETNQLDMDATLEELSVQQETEDQNYGIFLEMMEARSRGHASPLAANGQSPSPGSQSPVVPAGAVSTGSSSPGTPQPAPQLPLNAAPPSSVPPVPPSEALPPPACPSAPAPRRSIISRLFGTSPATEAAPPPPEPVPAAEGPATVQSVEDFVPDDRLDRSFLEDTTPARDEKKVGAKAAQQDSDSDGEALGGNPMVAGFQDDVDLEDQPRGSPPLPAGPVPSQDITLSSEEEAEVAAPTKGPAPAPQQCSEPETKWSSIPASKPRRGTAPTRTAAPPWPGGVSVRTGPEKRSSTRPPAEMEPGKGEQASSSESDPEGPIAAQMLSFVMDDPDFESEGSDTQRRADDFPVRDDPSDVTDEDEGPAEPPPPPKLPLPAFRLKNDSDLFGLGLEEAGPKESSEEGKEGKTPSKEKKKKKKKGKEEEEKAAKKKSKHKKSKDKEEGKEERRRRQQRPPRSRERTAADELEAFLGGGAPGGRHPGGGDYEEL

342 NP_003241.2 NP_004951.1

>NP_003241.2

MEQKPSKVECGSDPEENSARSPDGKRKRKNGQCSLKTSMSGYIPSYLDKDEQCVVCGDKATGYHYRCITCEGCKGFFRRTIQKNLHPTYSCKYDSCCVIDKITRNQCQLCRFKKCIAVGMAMDLVLDDSKRVAKRKLIEQNRERRRKEEMIRSLQQRPEPTPEEWDLIHIATEAHRSTNAQGSHWKQRRKFLPDDIGQSPIVSMPDGDKVDLEAFSEFTKIITPAITRVVDFAKKLPMFSELPCEDQIILLKGCCMEIMSLRAAVRYDPESDTLTLSGEMAVKREQLKNGGLGVVSDAIFELGKSLSAFNLDDTEVALLQAVLLMSTDRSGLLCVDKIEKSQEAYLLAFEHYVNHRKHNIPHFWPKLLMKEREVQSSILYKGAAAEGRPGGSLGVHPEGQQLLGMHVVQGPQVRQLEQQLGEAGSLQGPVLQHQSPKSPQQRLLELLHRSGILHARAVCGEDDSSEADSPSSSEEEPEVCEDLAGNAASP

>NP_004951.1

MASNDYTQQATQSYGAYPTQPGQGYSQQSSQPYGQQSYSGYSQSTDTSGYGQSSYSSYGQSQNTGYGTQSTPQGYGSTGGYGSSQSSQSSYGQQSSYPGYGQQPAPSSTSGSYGSSSQSSSYGQPQSGSYSQQPSYGGQQQSYGQQQSYNPPQGYGQQNQYNSSSGGGGGGGGGGNYGQDQSSMSSGGGSGGGYGNQDQSGGGGSGGYGQQDRGGRGRGGSGGGGGGGGGGYNRSSGGYEPRGRGGGRGGRGGMGGSDRGGFNKFGGPRDQGSRHDSEQDNSDNNTIFVQGLGENVTIESVADYFKQIGIIKTNKKTGQPMINLYTDRETGKLKGEATVSFDDPPSAKAAIDWFDGKEFSGNPIKVSFATRRADFNRGGGNGRGGRGRGGPMGRGGYGGGGSGGGGRGGFPSGGGGGGGQQRAGDWKCPNPTCENMNFSWRNECNQCKAPKPDGPGGGPGGSHMGGNYGDDRRGGRGGYDRGGYRGRGGDRGGFRGGRGGGDRGGFGPGKMDSRGEHRQDRRERPY

343 NP_066305.2 NP_002389.1

>NP_066305.2

MEAPASAQTPHPHEPISFGIDQILNSPDQDSAPAPRGPDGASYLGGPPGGRPGATYPSLPASFAGLGAPFEDAGSYSVNLSLAPAGVIRVPAHRPLPGAVPPPLPSALPAMPSVPTVSSLGGLNFPWMESSRRFVKDRFTAAAALTPFTVTRRIGHPYQNRTPPKRKKPRTSFSRVQICELEKRFHRQKYLASAERAALAKSLKMTDAQVKTWFQNRRTKWRRQTAEEREAERQQASRLMLQLQHDAFQKSLNDSIQPDPLCLHNSSLFALQNLQPWEEDSSKVPAVTSLV

>NP_002389.1

MAQRYDDLPHYGGMDGVGIPSTMYGDPHAARSMQPVHHLNHGPPLHSHQYPHTAHTNAMAPSMGSSVNDALKRDKDAIYGHPLFPLLALIFEKCELATCTPREPGVAGGDVCSSESFNEDIAVFAKQIRAEKPLFSSNPELDNLMIQAIQVLRFHLLELEKVHELCDNFCHRYISCLKGKMPIDLVIDDREGGSKSDSEDITRSANLTDQPSWNRDHDDTASTRSGGTPGPSSGGHTSHSGDNSSEQGDGLDNSVASPSTGDDDDPDKDKKRHKKRGIFPKVATNIMRAWLFQHLTHPYPSEEQKKQLAQDTGLTILQVNNWFINARRRIVQPMIDQSNRAVSQGTPYNPDGQPMGGFVMDGQQHMGIRAPGPMSGMGMNMGMEGQWHYM

344 NP_009297.2 NP_001744.2

>NP_009297.2

MGQQPGKVLGDQRRPSLPALHFIKGAGKKESSRHGGPHCNVFVEHEALQRPVASDFEPQGLSEAARWNSKENLLAGPSENDPNLFVALYDFVASGDNTLSITKGEKLRVLGYNHNGEWCEAQTKNGQGWVPSNYITPVNSLEKHSWYHGPVSRNAAEYLLSSGINGSFLVRESESSPGQRSISLRYEGRVYHYRINTASDGKLYVSSESRFNTLAELVHHHSTVADGLITTLHYPAPKRNKPTVYGVSPNYDKWEMERTDITMKHKLGGGQYGEVYEGVWKKYSLTVAVKTLKEDTMEVEEFLKEAAVMKEIKHPNLVQLLGVCTREPPFYIITEFMTYGNLLDYLRECNRQEVNAVVLLYMATQISSAMEYLEKKNFIHRDLAARNCLVGENHLVKVADFGLSRLMTGDTYTAHAGAKFPIKWTAPESLAYNKFSIKSDVWAFGVLLWEIATYGMSPYPGIDLSQVYELLEKDYRMERPEGCPEKVYELMRACWQWNPSDRPSFAEIHQAFETMFQESSISDEVEKELGKQGVRGAVSTLLQAPELPTKTRTSRRAAEHRDTTDVPEMPHSKGQGESDPLDHEPAVSPLLPRKERGPPEGGLNEDERLLPKDKKTNLFSALIKKKKKTAPTPPKRSSSFREMDGQPERRGAGEEEGRDISNGALAFTPLDTADPAKSPKPSNGAGVPNGALRESGGSGFRSPHLWKKSSTLTSSRLATGEEEGGGSSSKRFLRSCSASCVPHGAKDTEWRSVTLPRDLQSTGRQFDSSTFGGHKSEKPALPRKRAGENRSDQVTRGTVTPPPRLVKKNEEAADEVFKDIMESSPGSSPPNLTPKPLRRQVTVAPASGLPHKEEAGKGSALGTPAAAEPVTPTSKAGSGAPGGTSKGPAEESRVRRHKHSSESPGRDKGKLSRLKPAPPPPPAASAGKAGGKPSQSPSQEAAGEAVLGAKTKATSLVDAVNSDAAKPSQPGEGLKKPVLPATPKPQSAKPSGTPISPAPVPSTLPSASSALAGDQPSSTAFIPLISTRVSLRKTRQPPERIASGAITKGVVLDSTEALCLAISRNSEQMASHSAVLEAGKNLYTFCVSYVDSIQQMRNKFAFREAINKLENNLRELQICPATAGSGPAATQDFSKLLSSVKEISDIVQR

>NP_001744.2

MSGGKYVDSEGHLYTVPIREQGNIYKPNNKAMADELSEKQVYDAHTKEIDLVNRDPKHLNDDVVKIDFEDVIAEPEGTHSFDGIWKASFTTFTVTKYWFYRLLSALFGIPMALIWGIYFAILSFLHIWAVVPCIKSFLIEIQCISRVYSIYVHTVCDPLFEAVGKIFSNVRINLQKEI

345 NP_000669.1 NP_066301.1

>NP_000669.1

MTFRDLLSVSFEGPRPDSSAGGSSAGGGGGSAGGAAPSEGPAVGGVPGGAGGGGGVVGAGSGEDNRSSAGEPGSAGAGGDVNGTAAVGGLVVSAQGVGVGVFLAAFILMAVAGNLLVILSVACNRHLQTVTNYFIVNLAVADLLLSATVLPFSATMEVLGFWAFGRAFCDVWAAVDVLCCTASILSLCTISVDRYVGVRHSLKYPAIMTERKAAAILALLWVVALVVSVGPLLGWKEPVPPDERFCGITEEAGYAVFSSVCSFYLPMAVIVVMYCRVYVVARSTTRSLEAGVKRERGKASEVVLRIHCRGAATGADGAHGMRSAKGHTFRSSLSVRLLKFSREKKAAKTLAIVVGVFVLCWFPFFFVLPLGSLFPQLKPSEGVFKVIFWLGYFNSCVNPLIYPCSSREFKRAFLRLLRCQCRRRRRRRPLWRVYGHHWRASTSGLRQDCAPSSGDAPPGAPLALTALPDPDPEPPGTPEMQAPVASRRKPPSAFREWRLLGPFRRPTTQLRAKVSSLSHKIRAGGAQRAEAACAQRSEVEAVSLGVPHEVAEGATCQAYELADYSNLRETDI

>NP_066301.1

MAVAAAAAAAGPAGAGGGRAQRSGLLEVLVRDRWHKVLVNLSEDALVLSSEEGAAAYNGIGTATNGSFCRGAGAGHPGAGGAQPPDSPAGVRTAFTDLPEQVPESISNQKRGVKVLKQELGGLGISIKGGKENKMPILISKIFKGLAADQTQALYVGDAILSVNGADLRDATHDEAVQALKRAGKEVLLEVKYMREATPYVKKGSPVSEIGWETPPPESPRLGGSTSDPPSSQSFSFHRDRKSIPLKMCYVTRSMALADPENRQLEIHSPDAKHTVILRSKDSATAQAWFSAIHSNVNDLLTRVIAEVREQLGKTGIAGSREIRHLGWLAEKVPGESKKQWKPALVVLTEKDLLIYDSMPRRKEAWFSPVHTYPLLATRLVHSGPGKGSPQAGVDLSFATRTGTRQGIETHLFRAETSRDLSHWTRSIVQGCHNSAELIAEISTACTYKNQECRLTIHYENGFSITTEPQEGAFPKTIIQSPYEKLKMSSDDGIRMLYLDFGGKDGEIQLDLHSCPKPIVFIIHSFLSAKITRLGLVA

346 NP_058644.1 NP_003396.1

>NP_058644.1

MARPQRTPARSPDSIVEVKSKFDAEFRRFALPRASVSGFQEFSRLLRAVHQIPGLDVLLGYTDAHGDLLPLTNDDSLHRALASGPPPLRLLVQKRAEADSSGLAFASNSLQRRKKGLLLRPVAPLRTRPPLLISLPQDFRQVSSVIDVDLLPETHRRVRLHKHGSDRPLGFYIRDGMSVRVAPQGLERVPGIFISRLVRGGLAESTGLLAVSDEILEVNGIEVAGKTLDQVTDMMVANSHNLIVTVKPANQRNNVVRGASGRLTGPPSAGPGPAEPDSDDDSSDLVIENRQPPSSNGLSQGPPCWDLHPGCRHPGTRSSLPSLDDQEQASSGWGSRIRGDGSGFSL

>NP_003396.1

MGDREQLLQRARLAEQAERYDDMASAMKAVTELNEPLSNEDRNLLSVAYKNVVGARRSSWRVISSIEQKTMADGNEKKLEKVKAYREKIEKELETVCNDVLSLLDKFLIKNCNDFQYESKVFYLKMKGDYYRYLAEVASGEKKNSVVEASEAAYKEAFEISKEQMQPTHPIRLGLALNFSVFYYEIQNAPEQACLLAKQAFDDAIAELDTLNEDSYKDSTLIMQLLRDNLTLWTSDQQDEEAGEGN

347 NP_002680.2 NP_114406.1

>NP_002680.2

MSASAQQLAEELQIFGLDCEEALIEKLVELCVQYGQNEEGMVGELIAFCTSTHKVGLTSEILNSFEHEFLSKRLSKARHSTCKDSGHAGARDIVSIQELIEVEEEEEILLNSYTTPSKGSQKRAISTPETPLTKRSVSTRSPHQLLSPSSFSPSATPSQKYNSRSNRGEVVTSFGLAQGVSWSGRGGAGNISLKVLGCPEALTGSYKSMFQKLPDIREVLTCKIEELGSELKEHYKIEAFTPLLAPAQEPVTLLGQIGCDSNGKLNNKSVILEGDREHSSGAQIPVDLSELKEYSLFPGQVVIMEGINTTGRKLVATKLYEGVPLPFYQPTEEDADFEQSMVLVACGPYTTSDSITYDPLLDLIAVINHDRPDVCILFGPFLDAKHEQVENCLLTSPFEDIFKQCLRTIIEGTRSSGSHLVFVPSLRDVHHEPVYPQPPFSYSDLSREDKKQVQFVSEPCSLSINGVIFGLTSTDLLFHLGAEEISSSSGTSDRFSRILKHILTQRSYYPLYPPQEDMAIDYESFYVYAQLPVTPDVLIIPSELRYFVKDVLGCVCVNPGRLTKGQVGGTFARLYLRRPAADGAERQSPCIAVQVVRI

>NP_114406.1

MKRRASDRGAGETSARAKALGSGISGNNAKRAGPFILGPRLGNSPVPSIVQCLARKDGTDDFYQLKILTLEERGDQGIESQEERQGKMLLHTEYSLLSLLHTQDGVVHHHGLFQDRTCEIVEDTESSRMVKKMKKRICLVLDCLCAHDFSDKTADLINLQHYVIKEKRLSERETVVIFYDVVRVVEALHQKNIVHRDLKLGNMVLNKRTHRITITNFCLGKHLVSEGDLLKDQRGSPAYISPDVLSGRPYRGKPSDMWALGVVLFTMLYGQFPFYDSIPQELFRKIKAAEYTIPEDGRVSENTVCLIRKLLVLDPQQRLAAADVLEALSAIIASWQSLSSLSGPLQVVPDIDDQMSNADSSQEAKVTEECSQYEFENYMRQQLLLAEEKSSIHDARSWVPKRQFGSAPPVRRLGHDAQPMTSLDTAILAQRYLRK

348 NP_005234.1 NP_057682.1

>NP_005234.1

MASTDYSTYSQAAAQQGYSAYTAQPTQGYAQTTQAYGQQSYGTYGQPTDVSYTQAQTTATYGQTAYATSYGQPPTGYTTPTAPQAYSQPVQGYGTGAYDTTTATVTTTQASYAAQSAYGTQPAYPAYGQQPAATAPTRPQDGNKPTETSQPQSSTGGYNQPSLGYGQSNYSYPQVPGSYPMQPVTAPPSYPPTSYSSTQPTSYDQSSYSQQNTYGQPSSYGQQSSYGQQSSYGQQPPTSYPPQTGSYSQAPSQYSQQSSSYGQQSSFRQDHPSSMGVYGQESGGFSGPGENRSMSGPDNRGRGRGGFDRGGMSRGGRGGGRGGMGSAGERGGFNKPGGPMDEGPDLDLGPPVDPDEDSDNSAIYVQGLNDSVTLDDLADFFKQCGVVKMNKRTGQPMIHIYLDKETGKPKGDATVSYEDPPTAKAAVEWFDGKDFQGSKLKVSLARKKPPMNSMRGGLPPREGRGMPPPLRGGPGGPGGPGGPMGRMGGRGGDRGGFPPRGPRGSRGNPSGGGNVQHRAGDWQCPNPGCGNQNFAWRTECNQCKAPKPEGFLPPPFPPPGGDRGRGGPGGMRGGRGGLMDRGGPGGMFRGGRGGDRGGFRGGRGMDRGGFGGGRRGGPGGPPGPLMEQMGGRRGGRGGPGKMDKGEHRQERRDRPY

>NP_057682.1

MMLIPTHHFRNIERKPEYLQPEKCVPPPYPGPVGTMWFIRDGCGIACAIVTWFLVLYAEFVVLFVMLIPSRDYVYSIINGIVFNLLAFLALASHCRAMLTDPGAVPKGNATKEFIESLQLKPGQVVYKCPKCCSIKPDRAHHCSVCKRCIRKMDHHCPWVNNCVGENNQKYFVLFTMYIALISLHALIMVGFHFLHCFEEDWTTYGLNREEMAETGISLHEKMQPLNFSSTECSSFSPPTTVILLILLCFEGLLFLIFTSVMFGTQVHSICTDETGIEQLKKEERRWAKKTKWMNMKAVFGHPFSLGWASPFATPDQGKADPYQYVV

349 NP_002266.2 NP_003067.3

>NP_002266.2

MTTTFLQTSSSTFGGGSTRGGSLLAGGGGFGGGSLSGGGGSRSISASSARFVSSGSGGGYGGGMRVCGFGGGAGSVFGGGFGGGVGGGFGGGFGGGDGGLLSGNEKITMQNLNDRLASYLDKVRALEEANADLEVKIHDWYQKQTPASPECDYSQYFKTIEELRDKIMATTIDNSRVILEIDNARLAADDFRLKYENELALRQGVEADINGLRRVLDELTLARTDLEMQIEGLNEELAYLKKNHEEEMKEFSSQLAGQVNVEMDAAPGVDLTRVLAEMREQYEAMAEKNRRDVEAWFFSKTEELNKEVASNTEMIQTSKTEITDLRRTMQELEIELQSQLSMKAGLENSLAETECRYATQLQQIQGLIGGLEAQLSELRCEMEAQNQEYKMLLDIKTRLEQEIATYRSLLEGQDAKMAGIGIREASSGGGGSSSNFHINVEESVDGQVVSSHKREI

>NP_003067.3

MAARAGFQSVAPSGGAGASGGAGAAAALGPGGTPGPPVRMGPAPGQGLYRSPMPGAAYPRPGMLPGSRMTPQGPSMGPPGYGGNPSVRPGLAQSGMDQSRKRPAPQQIQQVQQQAVQNRNHNAKKKKMADKILPQRIRELVPESQAYMDLLAFERKLDQTIMRKRLDIQEALKRPIKQKRKLRIFISNTFNPAKSDAEDGEGTVASWELRVEGRLLEDSALSKYDATKQKRKFSSFFKSLVIELDKDLYGPDNHLVEWHRTATTQETDGFQVKRPGDVNVRCTVLLMLDYQPPQFKLDPRLARLLGIHTQTRPVIIQALWQYIKTHKLQDPHEREFVICDKYLQQIFESQRMKFSEIPQRLHALLMPPEPIIINHVISVDPNDQKKTACYDIDVEVDDTLKTQMNSFLLSTASQQEIATLDNKIHETIETINQLKTQREFMLSFARDPQGFINDWLQSQCRDLKTMTDVVGNPEEERRAEFYFQPWAQEAVCRYFYSKVQQRRQELEQALGIRNT

350 NP_000045.1 NP_000481.2

>NP_000045.1

MLMASTTSAVPGHPSLPSLPSNSSQERPLDTRDPLLARAELALLSIVFVAVALSNGLVLAALARRGRRGHWAPIHVFIGHLCLADLAVALFQVLPQLAWKATDRFRGPDALCRAVKYLQMVGMYASSYMILAMTLDRHRAICRPMLAYRHGSGAHWNRPVLVAWAFSLLLSLPQLFIFAQRNVEGGSGVTDCWACFAEPWGRRTYVTWIALMVFVAPTLGIAACQVLIFREIHASLVPGPSERPGGRRRGRRTGSPGEGAHVSAAVAKTVRMTLVIVVVYVLCWAPFFLVQLWAAWDPEAPLEGAPFVLLMLLASLNSCTNPWIYASFSSSVSSELRSLLCCARGRTPPSLGPQDESCTTASSSLAKDTSS

>NP_000481.2

MPDTMLPACFLGLLAFSSACYFQNCPRGGKRAMSDLELRQCLPCGPGGKGRCFGPSICCADELGCFVGTAEALRCQEENYLPSPCQSGQKACGSGGRCAAFGVCCNDESCVTEPECREGFHRRARASDRSNATQLDGPAGALLLRLVQLAGAPEPFEPAQPDAY

351 NP_000817.2 NP_001073892.1

>NP_000817.2

MQKIMHISVLLSPVLWGLIFGVSSNSIQIGGLFPRGADQEYSAFRVGMVQFSTSEFRLTPHIDNLEVANSFAVTNAFCSQFSRGVYAIFGFYDKKSVNTITSFCGTLHVSFITPSFPTDGTHPFVIQMRPDLKGALLSLIEYYQWDKFAYLYDSDRGLSTLQAVLDSAAEKKWQVTAINVGNINNDKKDEMYRSLFQDLELKKERRVILDCERDKVNDIVDQVITIGKHVKGYHYIIANLGFTDGDLLKIQFGGANVSGFQIVDYDDSLVSKFIERWSTLEEKEYPGAHTTTIKYTSALTYDAVQVMTEAFRNLRKQRIEISRRGNAGDCLANPAVPWGQGVEIERALKQVQVEGLSGNIKFDQNGKRINYTINIMELKTNGPRKIGYWSEVDKMVVTLTELPSGNDTSGLENKTVVVTTILESPYVMMKKNHEMLEGNERYEGYCVDLAAEIAKHCGFKYKLTIVGDGKYGARDADTKIWNGMVGELVYGKADIAIAPLTITLVREEVIDFSKPFMSLGISIMIKKPQKSKPGVFSFLDPLAYEIWMCIVFAYIGVSVVLFLVSRFSPYEWHTEEFEDGRETQSSESTNEFGIFNSLWFSLGAFMRQGCDISPRSLSGRIVGGVWWFFTLIIISSYTANLAAFLTVERMVSPIESAEDLSKQTEIAYGTLDSGSTKEFFRRSKIAVFDKMWTYMRSAEPSVFVRTTAEGVARVRKSKGKYAYLLESTMNEYIEQRKPCDTMKVGGNLDSKGYGIATPKGSSLRTPVNLAVLKLSEQGVLDKLKNKWWYDKGECGAKDSGSKEKTSALSLSNVAGVFYILVGGLGLAMLVALIEFCYKSRAEAKRMKVAKNAQNINPSSSQNSQNFATYKEGYNVYGIESVKI

>NP_001073892.1

MRLSSLTEEAVDGGNGGEWGEDLLEQFTCIPSRVVEAKTAPEWPLCPGEGQGLHGEPLPDHCQGTGGQIDPSLGRGGRAKQRGSRGSWWAAGETIANMLCGLSRETPGEADDGPYSKGGKDAGGADVSLACRRQSIPEEFRGITVVELIKKEGSTLGLTISGGTDKDGKPRVSNLRPGGLAARSDLLNIGDYIRSVNGIHLTRLRHDEIITLLKNVGERVVLEVEYELPPPAPENNPRIISKTVDVSLYKEGNSFGFVLRGGAHEDGHKSRPLVLTYVRPGGPADREGSLKVGDRLLSVDGIPLHGASHATALATLRQCSHEALFQVEYDVATPDTVANASGPLMVEIVKTPGSALGISLTTTSLRNKSVITIDRIKPASVVDRSGALHPGDHILSIDGTSMEHCSLLEATKLLASISEKVRLEILPVPQSQRPLRPQRQVVKVQRSEQLHRWDPCVPSCHSPRPGHCRMPTWATPAGQDQSRSLSSTPFSSPTLNHAFSCNNPSTLPRGSQPMSPRTTMGRRRQRRREHKSSLSLASSTVGPGGQIVHTETTEVVLCGDPLSGFGLQLQGGIFATETLSSPPLVCFIEPDSPAERCGLLQVGDRVLSINGIATEDGTMEEANQLLRDAALAHKVVLEVEFDVAESVIPSSGTFHVKLPKKRSVELGITISSASRKRGEPLIISDIKKGSVAHRTGTLEPGDKLLAIDNIRLDNCPMEDAVQILRQCEDLVKLKIRKDEDNSDELETTGAVSYTVELKRYGGPLGITISGTEEPFDPIVISGLTKRGLAERTGAIHVGDRILAINNVSLKGRPLSEAIHLLQVAGETVTLKIKKQLDRPLLPRKSGSLSETSDADEDPADALKGGLPAARFSPAVPSVDSAVESWDSSATEGGFGGPGSYTPQAAARGTTPQERRPGWLRGSPPPTEPRRTSYTPTPADESFPEEEEEDDWEPPTSPAPGPAREEGFWRMFGEALEDLESCGQSELLRELEASIMTGTVQRVALEGRPGHRPWQRGREVRASPAEMEELLLPTPLEMHKVTLHKDPMRHDFGFSVSDGLLEKGVYVHTVRPDGPAHRGGLQPFDRVLQVNHVRTRDFDCCLAVPLLAEAGDVLELIISRKPHTAHSSRAPRSPGPSSPRML

352 NP_005891.1 NP_003324.1

>NP_005891.1

MNVTSLFSFTSPAVKRLLGWKQGDEEEKWAEKAVDALVKKLKKKKGAMEELEKALSCPGQPSNCVTIPRSLDGRLQVSHRKGLPHVIYCRVWRWPDLQSHHELKPLECCEFPFGSKQKEVCINPYHYKRVESPVLPPVLVPRHSEYNPQHSLLAQFRNLGQNEPHMPLNATFPDSFQQPNSHPFPHSPNSSYPNSPGSSSSTYPHSPTSSDPGSPFQMPADTPPPAYLPPEDPMTQDGSQPMDTNMMAPPLPSEINRGDVQAVAYEEPKHWCSIVYYELNNRVGEAFHASSTSVLVDGFTDPSNNKNRFCLGLLSNVNRNSTIENTRRHIGKGVHLYYVGGEVYAECLSDSSIFVQSRNCNYHHGFHPTTVCKIPSGCSLKIFNNQEFAQLLAQSVNHGFETVYELTKMCTIRMSFVKGWGAEYHRQDVTSTPCWIEIHLHGPLQWLDKVLTQMGSPHNPISSVS

>NP_003324.1

MQIFVKTLTGKTITLEVEPSDTIENVKAKIQDKEGIPPDQQRLIFAGKQLEDGRTLSDYNIQKESTLHLVLRLRGGIIEPSLRQLAQKYNCDKMICRKCYARLHPRAVNCRKKKCGHTNNLRPKKKVK

353 NP_006210.1 NP_000677.2

>NP_006210.1

MCFSFIMPPAMADILDIWAVDSQIASDGSIPVDFLLPTGIYIQLEVPREATISYIKQMLWKQVHNYPMFNLLMDIDSYMFACVNQTAVYEELEDETRRLCDVRPFLPVLKLVTRSCDPGEKLDSKIGVLIGKGLHEFDSLKDPEVNEFRRKMRKFSEEKILSLVGLSWMDWLKQTYPPEHEPSIPENLEDKLYGGKLIVAVHFENCQDVFSFQVSPNMNPIKVNELAIQKRLTIHGKEDEVSPYDYVLQVSGRVEYVFGDHPLIQFQYIRNCVMNRALPHFILVECCKIKKMYEQEMIAIEAAINRNSSNLPLPLPPKKTRIISHVWENNNPFQIVLVKGNKLNTEETVKVHVRAGLFHGTELLCKTIVSSEVSGKNDHIWNEPLEFDINICDLPRMARLCFAVYAVLDKVKTKKSTKTINPSKYQTIRKAGKVHYPVAWVNTMVFDFKGQLRTGDIILHSWSSFPDELEEMLNPMGTVQTNPYTENATALHVKFPENKKQPYYYPPFDKIIEKAAEIASSDSANVSSRGGKKFLPVLKEILDRDPLSQLCENEMDLIWTLRQDCREIFPQSLPKLLLSIKWNKLEDVAQLQALLQIWPKLPPREALELLDFNYPDQYVREYAVGCLRQMSDEELSQYLLQLVQVLKYEPFLDCALSRFLLERALGNRRIGQFLFWHLRSEVHIPAVSVQFGVILEAYCRGSVGHMKVLSKQVEALNKLKTLNSLIKLNAVKLNRAKGKEAMHTCLKQSAYREALSDLQSPLNPCVILSELYVEKCKYMDSKMKPLWLVYNNKVFGEDSVGVIFKNGDDLRQDMLTLQMLRLMDLLWKEAGLDLRMLPYGCLATGDRSGLIEVVSTSETIADIQLNSSNVAAAAAFNKDALLNWLKEYNSGDDLDRAIEEFTLSCAGYCVASYVLGIGDRHSDNIMVKKTGQLFHIDFGHILGNFKSKFGIKRERVPFILTYDFIHVIQQGKTGNTEKFGRFRQCCEDAYLILRRHGNLFITLFALMLTAGLPELTSVKDIQYLKDSLALGKSEEEALKQFKQKFDEALRESWTTKVNWMAHTVRKDYRS

>NP_000677.2

MKGNSTLATTSKNITSGLHFGLVNISGNNESTLNCSQKPSDKHLDAIPILYYIIFVIGFLVNIVVVTLFCCQKGPKKVSSIYIFNLAVADLLLLATLPLWATYYSYRYDWLFGPVMCKVFGSFLTLNMFASIFFITCMSVDRYQSVIYPFLSQRRNPWQASYIVPLVWCMACLSSLPTFYFRDVRTIEYLGVNACIMAFPPEKYAQWSAGIALMKNILGFIIPLIFIATCYFGIRKHLLKTNSYGKNRITRDQVLKMAAAVVLAFIICWLPFHVLTFLDALAWMGVINSCEVIAVIDLALPFAILLGFTNSCVNPFLYCFVGNRFQQKLRSVFRVPITWLQGKRESMSCRKSSSLREMETFVS

354 NP_004908.3 NP_001031.2

>NP_004908.3

MATAGNPWGWFLGYLILGVAGSLVSGSCSQIINGEDCSPHSQPWQAALVMENELFCSGVLVHPQWVLSAAHCFQNSYTIGLGLHSLEADQEPGSQMVEASLSVRHPEYNRPLLANDLMLIKLDESVSESDTIRSISIASQCPTAGNSCLVSGWGLLANGRMPTVLQCVNVSVVSEEVCSKLYDPLYHPSMFCAGGGHDQKDSCNGDSGGPLICNGYLQGLVSFGKAPCGQVGVPGVYTNLCKFTEWIEKTVQAS

>NP_001031.2

MESRGPLATSRLLLLLLLLLLRHTRQGWALRPVLPTQSAHDPPAVHLSNGPGQEPIAVMTFDLTKITKTSSSFEVRTWDPEGVIFYGDTNPKDDWFMLGLRDGRPEIQLHNHWAQLTVGAGPRLDDGRWHQVEVKMEGDSVLLEVDGEEVLRLRQVSGPLTSKRHPIMRIALGGLLFPASNLRLPLVPALDGCLRRDSWLDKQAEISASAPTSLRSCDVESNPGIFLPPGTQAEFNLRDIPQPHAEPWAFSLDLGLKQAAGSGHLLALGTPENPSWLSLHLQDQKVVLSSGSGPGLDLPLVLGLPLQLKLSMSRVVLSQGSKMKALALPPLGLAPLLNLWAKPQGRLFLGALPGEDSSTSFCLNGLWAQGQRLDVDQALNRSHEIWTHSCPQSPGNGTDASH

355 NP_001017963.1 NP_003945.2

>NP_001017963.1

MPPCSGGDGSTPPGPSLRDRDCPAQSAEYPRDRLDPRPGSPSEASSPPFLRSRAPVNWYQEKAQVFLWHLLVSGSTTLLCLWKQPFHVSAFPVTASLAFRQSQGAGQHLYKDLQPFILLRLLMPEETQTQDQPMEEEEVETFAFQAEIAQLMSLIINTFYSNKEIFLRELISNSSDALDKIRYESLTDPSKLDSGRELHINLIPNKQGRTLTIVDTGIGMTKADLINNLGTIAKSGTKAFMEALQAGADISMIGQFGVGFYSAYLVAEKVTVITKHNDDEQYAWESSAGGSFTVRTDTGEPMGRGTKVILHLKEDQTEYLEERRIKEIVKKHSQFIGYPITLFVEKERDKEVSDDEAEEKEDKEEEKEKEEKESEDKPEIEDVGSDEEEEKKDGDKKKKKKIKEKYIDQEELNKTKPIWTRNPDDITNEEYGEFYKSLTNDWEDHLAVKHFSVEGQLEFRALLFVPRRAPFDLFENRKKKNNIKLYVRRVFIMDNCEELIPEYLNFIRGVVDSEDLPLNISREMLQQSKILKVIRKNLVKKCLELFTELAEDKENYKKFYEQFSKNIKLGIHEDSQNRKKLSELLRYYTSASGDEMVSLKDYCTRMKENQKHIYYITGETKDQVANSAFVERLRKHGLEVIYMIEPIDEYCVQQLKEFEGKTLVSVTKEGLELPEDEEEKKKQEEKKTKFENLCKIMKDILEKKVEKVVVSNRLVTSPCCIVTSTYGWTANMERIMKAQALRDNSTMGYMAAKKHLEINPDHSIIETLRQKAEADKNDKSVKDLVILLYETALLSSGFSLEDPQTHANRIYRMIKLGLGIDEDDPTADDTSAAVTEEMPPLEGDDDTSRMEEVD

>NP_003945.2

MAVMEMACPGAPGSAVGQQKELPKAKEKTPPLGKKQSSVYKLEAVEKSPVFCGKWEILNDVITKGTAKEGSEAGPAAISIIAQAECENSQEFSPTFSERIFIAGSKQYSQSESLDQIPNNVAHATEGKMARVCWKGKRRSKARKKRKKKSSKSLAHAGVALAKPLPRTPEQESCTIPVQEDESPLGAPYVRNTPQFTKPLKEPGLGQLCFKQLGEGLRPALPRSELHKLISPLQCLNHVWKLHHPQDGGPLPLPTHPFPYSRLPHPFPFHPLQPWKPHPLESFLGKLACVDSQKPLPDPHLSKLACVDSPKPLPGPHLEPSCLSRGAHEKFSVEEYLVHALQGSVSSGQAHSLTSLAKTWAARGSRSREPSPKTEDNEGVLLTEKLKPVDYEYREEVHWATHQLRLGRGSFGEVHRMEDKQTGFQCAVKKVRLEVFRAEELMACAGLTSPRIVPLYGAVREGPWVNIFMELLEGGSLGQLVKEQGCLPEDRALYYLGQALEGLEYLHSRRILHGDVKADNVLLSSDGSHAALCDFGHAVCLQPDGLGKSLLTGDYIPGTETHMAPEVVLGRSCDAKVDVWSSCCMMLHMLNGCHPWTQFFRGPLCLKIASEPPPVREIPPSCAPLTAQAIQEGLRKEPIHRVSAAELGGKVNRALQQVGGLKSPWRGEYKEPRHPPPNQANYHQTLHAQPRELSPRAPGPRPAEETTGRAPKLQPPLPPEPPEPNKSPPLTLSKEESGMWEPLPLSSLEPAPARNPSSPERKATVPEQELQQLEIELFLNSLSQPFSLEEQEQILSCLSIDSLSLSDDSEKNPSKASQSSRDTLSSGVHSWSSQAEARSSSWNMVLARGRPTDTPSYFNGVKVQIQSLNGEHLHIREFHRVKVGDIATGISSQIPAAAFSLVTKDGQPVRYDMEVPDSGIDLQCTLAPDGSFAWSWRVKHGQLENRP

356 NP_055296.2 NP_005822.1

>NP_055296.2

MLRVVSWNINGIRRPLQGVANQEPSNCAAVAVGRILDELDADIVCLQETKVTRDALTEPLAIVEGYNSYFSFSRNRSGYSGVATFCKDNATPVAAEEGLSGLFATQNGDVGCYGNMDEFTQEELRALDSEGRALLTQHKIRTWEGKEKTLTLINVYCPHADPGRPERLVFKMRFYRLLQIRAEALLAAGSHVIILGDLNTAHRPIDHWDAVNLECFEEDPGRKWMDSLLSNLGCQSASHVGPFIDSYRCFQPKQEGAFTCWSAVTGARHLNYGSRLDYVLGDRTLVIDTFQASFLLPEVMGSDHCPVGAVLSVSSVPAKQCPPLCTRFLPEFAGTQLKILRFLVPLEQSPVLEQSTLQHNNQTRVQTCQNKAQVRSTRPQPSQVGSSRGQKNLKSYFQPSPSCPQASPDIELPSLPLMSALMTPKTPEEKAVAKVVKGQAKTSEAKDEKELRTSFWKSVLAGPLRTPLCGGHREPCVMRTVKKPGPNLGRRFYMCARPRGPPTDPSSRCNFFLWSRPS

>NP_005822.1

MEETIKDPPTSAVLLDHCHFSQVIFNSVEKFYIPGGDVTCHYTFTQHFIPRRKDWIGIFRVGWKTTREYYTFMWVTLPIDLNNKSAKQQEVQFKAYYLPKDDEYYQFCYVDEDGVVRGASIPFQFRPENEEDILVVTTQGEVEEIEQHNKELCKENQELKDSCISLQKQNSDMQAELQKKQEELETLQSINKKLELKVKEQKDYWETELLQLKEQNQKMSSENEKMGIRVDQLQAQLSTQEKEMEKLVQGDQDKTEQLEQLKKENDHLFLSLTEQRKDQKKLEQTVEQMKQNETTAMKKQQELMDENFDLSKRLSENEIICNALQRQKERLEGENDLLKRENSRLLSYMGLDFNSLPYQVPTSDEGGARQNPGLAYGNPYSGIQESSSPSPLSIKKCPICKADDICDHTLEQQQMQPLCFNCPICDKIFPATEKQIFEDHVFCHSL

357 NP_057016.1 NP_005540.1

>NP_057016.1

MNARGLGSELKDSIPVTELSASGPFESHDLLRKGFSCVKNELLPSHPLELSEKNFQLNQDKMNFSTLRNIQGLFAPLKLQMEFKAVQQVQRLPFLSSSNLSLDVLRGNDETIGFEDILNDPSQSEVMGEPHLMVEYKLGLL

>NP_005540.1

MDVCGWKEMEVALVNFDNSDEIQEEPGYATDFDSTSPKGRPGGSSFSNGKILISESTNHETAFSKLPGDYADPPGPEPVVLNEGNQRVIINIAGLRFETQLRTLSQFPETLLGDREKRMQFFDSMRNEYFFDRNRPSFDGILYYYQSGGKIRRPANVPIDIFADEISFYELGSEAMDQFREDEGFIKDPETLLPTNDIHRQFWLLFEYPESSSAARAVAVVSVLVVVISITIFCLETLPEFREDRELKVVRDPNLNMSKTVLSQTMFTDPFFMVESTCIVWFTFELVLRFVVCPSKTDFFRNIMNIIDIISIIPYFATLITELVQETEPSAQQNMSLAILRIIRLVRVFRIFKLSRHSKGLQILGQTLKASMRELGLLIFFLFIGVILFSSAVYFAEVDEPESHFSSIPDGFWWAVVTMTTVGYGDMCPTTPGGKIVGTLCAIAGVLTIALPVPVIVSNFNYFYHRETENEEKQNIPGEIERILNSVGSRMGSTDSLNKTNGGCSTEKSRK

358 NP_001005735.1 NP_006234.1

>NP_001005735.1

MSRESDVEAQQSHGSSACSQPHGSVTQSQGSSSQSQGISSSSTSTMPNSSQSSHSSSGTLSSLETVSTQELYSIPEDQEPEDQEPEEPTPAPWARLWALQDGFANLETESGHVTQSDLELLLSSDPPASASQSAGIRGVRHHPRPVCSLKCVNDNYWFGRDKSCEYCFDEPLLKRTDKYRTYSKKHFRIFREVGPKNSYIAYIEDHSGNGTFVNTELVGKGKRRPLNNNSEIALSLSRNKVFVFFDLTVDDQSVYPKALRDEYIMSKTLGSGACGEVKLAFERKTCKKVAIKIISKRKFAIGSAREADPALNVETEIEILKKLNHPCIIKIKNFFDAEDYYIVLELMEGGELFDKVVGNKRLKEATCKLYFYQMLLAVQYLHENGIIHRDLKPENVLLSSQEEDCLIKITDFGHSKILGETSLMRTLCGTPTYLAPEVLVSVGTAGYNRAVDCWSLGVILFICLSGYPPFSEHRTQVSLKDQITSGKYNFIPEVWAEVSEKALDLVKKLLVVDPKARFTTEEALRHPWLQDEDMKRKFQDLLSEENESTALPQVLAQPSTSRKRPREGEAEGAETTKRPAVCAAVL

>NP_006234.1

MSSSSPPAGAASAAISASEKVDGFTRKSVRKAQRQKRSQGSSQFRSQGSQAELHPLPQLKDATSNEQQELFCQKLQQCCILFDFMDSVSDLKSKEIKRATLNELVEYVSTNRGVIVESAYSDIVKMISANIFRTLPPSDNPDFDPEEDEPTLEASWPHIQLVYEFFLRFLESPDFQPSIAKRYIDQKFVQQLLELFDSEDPRERDFLKTVLHRIYGKFLGLRAFIRKQINNIFLRFIYETEHFNGVAELLEILGSIINGFALPLKAEHKQFLMKVLIPMHTAKGLALFHAQLAYCVVQFLEKDTTLTEPVIRGLLKFWPKTCSQKEVMFLGEIEEILDVIEPTQFKKIEEPLFKQISKCVSSSHFQVAERALYFWNNEYILSLIEENIDKILPIMFASLYKISKEHWNPTIVALVYNVLKTLMEMNGKLFDDLTSSYKAERQREKKKELEREELWKKLEELKLKKALEKQNSAYNMHSILSNTSAE

359 NP_077006.1 NP_004571.2

>NP_077006.1

MGKKLDLSKLTDEEAQHVLEVVQRDFDLRRKEEERLEALKGKIKKESSKRELLSDTAHLNETHCARCLQPYQLLVNSKRQCLECGLFTCKSCGRVHPEEQGWICDPCHLARVVKIGSLEWYYEHVKARFKRFGSAKVIRSLHGRLQGGAGPELISEERSGDSDQTDEDGEPGSEAQAQAQPFGSKKKRLLSVHDFDFEGDSDDSTQPQGHSLHLSSVPEARDSPQSLTDESCSEKAAPHKAEGLEEADTGASGCHSHPEEQPTSISPSRHGALAELCPPGGSHRMALGTAAALGSNVIRNEQLPLQYLADVDTSDEESIRAHVMASHHSKRRGRASSESQIFELNKHISAVECLLTYLENTVVPPLAKGLGAGVRTEADVEEEALRRKLEELTSNVSDQETSSEEEEAKDEKAEPNRDKSVGPLPQADPEVGTAAHQTNRQEKSPQDPGDPVQYNRTTDEELSELEDRVAVTASEVQQAESEVSDIESRIAALRAAGLTVKPSGKPRRKSNLPIFLPRVAGKLGKRPEDPNADPSSEAKAMAVPYLLRRKFSNSLKSQGKDDDSFDRKSVYRGSLTQRNPNARKGMASHTFAKPVVAHQS

>NP_004571.2

MSDGDYDYLIKFLALGDSGVGKTSVLYQYTDGKFNSKFITTVGIDFREKRVVYRASGPDGATGRGQRIHLQLWDTAGQERFRSLTTAFFRDAMGFLLLFDLTNEQSFLNVRNWISQLQMHAYCENPDIVLCGNKSDLEDQRVVKEEEAIALAEKYGIPYFETSAANGTNISQAIEMLLDLIMKRMERCVDKSWIPEGVVRSNGHASTDQLSEEKEKGACGC

360 NP_002064.1 NP_000789.1

>NP_002064.1

MGCRQSSEEKEAARRSRRIDRHLRSESQRQRREIKLLLLGTSNSGKSTIVKQMKIIHSGGFNLEACKEYKPLIIYNAIDSLTRIIRALAALRIDFHNPDRAYDAVQLFALTGPAESKGEITPELLGVMRRLWADPGAQACFSRSSEYHLEDNAAYYLNDLERIAAADYIPTVEDILRSRDMTTGIVENKFTFKELTFKMVDVGGQRSERKKWIHCFEGVTAIIFCVELSGYDLKLYEDNQTSRMAESLRLFDSICNNNWFINTSLILFLNKKDLLAEKIRRIPLTICFPEYKGQNTYEEAAVYIQRQFEDLNRNKETKEIYSHFTCATDTSNIQFVFDAVTDVIIQNNLKYIGLC

>NP_000789.1

MLPPGSNGTAYPGQFALYQQLAQGNAVGGSAGAPPLGPSQVVTACLLTLLIIWTLLGNVLVCAAIVRSRHLRANMTNVFIVSLAVSDLFVALLVMPWKAVAEVAGYWPFGAFCDVWVAFDIMCSTASILNLCVISVDRYWAISRPFRYKRKMTQRMALVMVGLAWTLSILISFIPVQLNWHRDQAASWGGLDLPNNLANWTPWEEDFWEPDVNAENCDSSLNRTYAISSSLISFYIPVAIMIVTYTRIYRIAQVQIRRISSLERAAEHAQSCRSSAACAPDTSLRASIKKETKVLKTLSVIMGVFVCCWLPFFILNCMVPFCSGHPEGPPAGFPCVSETTFDVFVWFGWANSSLNPVIYAFNADFQKVFAQLLGCSHFCSRTPVETVNISNELISYNQDIVFHKEIAAAYIHMMPNAVTPGNREVDNDEEEGPFDRMFQIYQTSPDGDPVAESVWELDCEGEISLDKITPFTPNGFH

361 NP_000652.2 NP_066547.1

>NP_000652.2

MKTILSNQTVDIPENVDITLKGRTVIVKGPRGTLRRDFNHINVELSLLGKKKKRLRVDKWWGNRKELATVRTICSHVQNMIKGVTLGFRYKMRSVYAHFPINVVIQENGSLVEIRNFLGEKYIRRVRMRPGVACSVSQAQKDELILEGNDIELVSNSAALIQQATTVKNKDIRKFLDGIYVSEKGTVQQADE

>NP_066547.1

MSYYQRPFSPSAYSLPASLNSSIVMQHGTSLDSTDTYPQHAQSLDGTTSSSIPLYRSSEEEKRVTVIKAPHYPGIGPVDESGIPTAIRTTVDRPKDWYKTMFKQIHMVHKPDDDTDMYNTPYTYNAGLYNPPYSAQSHPAAKTQTYRPLSKSHSDNSPNAFKDASSPVPPPHVPPPVPPLRPRDRSSTEKHDWDPPDRKVDTRKFRSEPRSIFEYEPGKSSILQHERPASLYQSSIDRSLERPMSSASMASDFRKRRKSEPAVGPPRGLGDQSASRTSPGRVDLPGSSTTLTKSFTSSSPSSPSRAKGGDDSKICPSLCSYSGLNGNPSSELDYCSTYRQHLDVPRDSPRAISFKNGWQMARQNAEIWSSTEETVSPKIKSRSCDDLLNDDCDSFPDPKVKSESMGSLLCEEDSKESCPMAWGSPYVPEVRSNGRSRIRHRSARNAPGFLKMYKKMHRINRKDLMNSEVICSVKSRILQYESEQQHKDLLRAWSQCSTEEVPRDMVPTRISEFEKLIQKSKSMPNLGDDMLSPVTLEPPQNGLCPKRRFSIEYLLEEENQSGPPARGRRGCQSNALVPIHIEVTSDEQPRAHVEFSDSDQDGVVSDHSDYIHLEGSSFCSESDFDHFSFTSSESFYGSSHHHHHHHHHHHRHLISSCKGRCPASYTRFTTMLKHERARHENTEEPRRQEMDPGLSKLAFLVSPVPFRRKKNSAPKKQTEKAKCKASVFEALDSALKDICDQIKAEKKRGSLPDNSILHRLISELLPDVPERNSSLRALRRSPLHQPLHPLPPDGAIHCPPYQNDCGRMPRSASFQDVDTANSSCHHQDRGGALQDRESPRSYSSTLTDMGRSAPRERRGTPEKEKLPAKAVYDFKAQTSKELSFKKGDTVYILRKIDQNWYEGEHHGRVGIFPISYVEKLTPPEKAQPARPPPPAQPGEIGEAIAKYNFNADTNVELSLRKGDRVILLKRVDQNWYEGKIPGTNRQGIFPVSYVEVVKKNTKGAEDYPDPPIPHSYSSDRIHSLSSNKPQRPVFTHENIQGGGEPFQALYNYTPRNEDELELRESDVIDVMEKCDDGWFVGTSRRTKFFGTFPGNYVKRL

362 NP_001943.1 NP_009042.1

>NP_001943.1

MSQQRPARKLPSLLLDPTEETVRRRCRDPINVEGLLPSKIRINLEDNVQYVSMRKALKVKRPRFDVSLVYLTRKFMDLVRSAPGGILDLNKVATKLGVRKRRVYDITNVLDGIDLVEKKSKNHIRWIGSDLSNFGAVPQQKKLQEELSDLSAMEDALDELIKDCAQQLFELTDDKENERLAYVTYQDIHSIQAFHEQIVIAVKAPAETRLDVPAPREDSITVHIRSTNGPIDVYLCEVEQGQTSNKRSEGVGTSSSESTHPEGPEEEENPQQSEELLEVSN

>NP_009042.1

MAKDAGLIEANGELKVFIDQNLSPGKGVVSLVAVHPSTVNPLGKQLLPKTFGQSNVNIAQQVVIGTPQRPAASNTLVVGSPHTPSTHFASQNQPSDSSPWSAGKRNRKGEKNGKGLRHFSMKVCEKVQRKGTTSYNEVADELVAEFSAADNHILPNESAYDQKNIRRRVYDALNVLMAMNIISKEKKEIKWIGLPTNSAQECQNLEVERQRRLERIKQKQSQLQELILQQIAFKNLVQRNRHAEQQASRPPPPNSVIHLPFIIVNTSKKTVIDCSISNDKFEYLFNFDNTFEIHDDIEVLKRMGMACGLESGSCSAEDLKMARSLVPKALEPYVTEMAQGTVGGVFITTAGSTSNGTRFSASDLTNGADGMLATSSNGSQYSGSRVETPVSYVGEDDEEDDDFNENDEDD

363 NP_003395.1 NP_689543.1

>NP_003395.1

MTMDKSELVQKAKLAEQAERYDDMAAAMKAVTEQGHELSNEERNLLSVAYKNVVGARRSSWRVISSIEQKTERNEKKQQMGKEYREKIEAELQDICNDVLELLDKYLIPNATQPESKVFYLKMKGDYFRYLSEVASGDNKQTTVSNSQQAYQEAFEISKKEMQPTHPIRLGLALNFSVFYYEILNSPEKACSLAKTAFDEAIAELDTLNEESYKDSTLIMQLLRDNLTLWTSENQGDEGDAGEGEN

>NP_689543.1

MNKLNFHNNRVMQDRRSVCIFLPNDESLNIIINVKILCHQLLVQVCDLLRLKDCHLFGLSVIQNNEHVYMELSQKLYKYCPKEWKKEASKGIDQFGPPMIIHFRVQYYVENGRLISDRAARYYYYWHLRKQVLHSQCVLREEAYFLLAAFALQADLGNFKRNKHYGKYFEPEAYFPSWVVSKRGKDYILKHIPNMHKDQFALTASEAHLKYIKEAVRLDDVAVHYYRLYKDKREIEASLTLGLTMRGIQIFQNLDEEKQLLYDFPWTNVGKLVFVGKKFEILPDGLPSARKLIYYTGCPMRSRHLLQLLSNSHRLYMNLQPVLRHIRKLEENEEKKQYRESYISDNLDLDMDQLEKRSRASGSSAGSMKHKRLSRHSTASHSSSHTSGIEADTKPRDTGPEDSYSSSAIHRKLKTCSSMTSHGSSHTSGVESGGKDRLEEDLQDDEIEMLVDDPRDLEQMNEESLEVSPDMCIYITEDMLMSRKLNGHSGLIVKEIGSSTSSSSETVVKLRGQSTDSLPQTICRKPKTSTDRHSLSLDDIRLYQKDFLRIAGLCQDTAQSYTFGCGHELDEEGLYCNSCLAQQCINIQDAFPVKRTSKYFSLDLTHDEVPEFVV

364 NP_001217.2 NP_003371.2

>NP_001217.2

MSSASGLRRGHPAGGEENMTETDAFYKREMFDPAEKYKMDHRRRGIALIFNHERFFWHLTLPERRGTCADRDNLTRRFSDLGFEVKCFNDLKAEELLLKIHEVSTVSHADADCFVCVFLSHGEGNHIYAYDAKIEIQTLTGLFKGDKCHSLVGKPKIFIIQACRGNQHDVPVIPLDVVDNQTEKLDTNITEVDAASVYTLPAGADFLMCYSVAEGYYSHRETVNGSWYIQDLCEMLGKYGSSLEFTELLTLVNRKVSQRRVDFCKDPSAIGKKQVPCFASMLTKKLHFFPKSN

>NP_003371.2

MSTRSVSSSSYRRMFGGPGTASRPSSSRSYVTTSTRTYSLGSALRPSTSRSLYASSPGGVYATRSSAVRLRSSVPGVRLLQDSVDFSLADAINTEFKNTRTNEKVELQELNDRFANYIDKVRFLEQQNKILLAELEQLKGQGKSRLGDLYEEEMRELRRQVDQLTNDKARVEVERDNLAEDIMRLREKLQEEMLQREEAENTLQSFRQDVDNASLARLDLERKVESLQEEIAFLKKLHEEEIQELQAQIQEQHVQIDVDVSKPDLTAALRDVRQQYESVAAKNLQEAEEWYKSKFADLSEAANRNNDALRQAKQESTEYRRQVQSLTCEVDALKGTNESLERQMREMEENFAVEAANYQDTIGRLQDEIQNMKEEMARHLREYQDLLNVKMALDIEIATYRKLLEGEESRISLPLPNFSSLNLRETNLDSLPLVDTHSKRTLLIKTVETRDGQVINETSQHHDDLE

365 NP_758958.3 NP_001908.3

>NP_758958.3

MLEKLMGADSLQLFRSRYTLGKIYFIGFQRSILLSKSENSLNSIAKETEEGRETVTRKEGWKRRHEDGYLEMAQRHLQRSLCPWVSYLPQPYAELEEVSSHVGKVFMARNYEFLAYEASKDRRQPLERMWTCNYNQQKDQSCNHKEITSTKAE

>NP_001908.3

MRVVVIGAGVIGLSTALCIHERYHSVLQPLDIKVYADRFTPLTTTDVAAGLWQPYLSDPNNPQEADWSQQTFDYLLSHVHSPNAENLGLFLISGYNLFHEAIPDPSWKDTVLGFRKLTPRELDMFPDYGYGWFHTSLILEGKNYLQWLTERLTERGVKFFQRKVESFEEVAREGADVIVNCTGVWAGALQRDPLLQPGRGQIMKVDAPWMKHFILTHDPERGIYNSPYIIPGTQTVTLGGIFQLGNWSELNNIQDHNTIWEGCCRLEPTLKNARIIGERTGFRPVRPQIRLEREQLRTGPSNTEVIHNYGHGGYGLTIHWGCALEAAKLFGRILEEKKLSRMPPSHL

366 NP_005577.1 NP_057669.1

>NP_005577.1

MYQVSGQRPSGCDAPYGAPSAAPGPAQTLSLLPGLEVVTGSTHPAEAAPEEGSLEEAATPMPQGNGPGIPQGLDSTDLDVPTEAVTCQPQGNPLGCTPLLPNDSGHPSELGGTRRAGNGALGGPKAHRKLQTHPSLASQGSKKSKSSSKSTTSQIPLQAQEDCCVHCILSCLFCEFLTLCNIVLDCATCGSCSSEDSCLCCCCCGSGECADCDLPCDLDCGILDACCESADCLEICMECCGLCFSS

>NP_057669.1

MGDSRRRSLGNQPSSEAAGRSEREQDGDPRGLQSSVYESRRVTDPERQDLDNAELGPEDPEEELPPEEVAGEEFPETLDPKEALSELERVLDKDLEEDIPEISRLSISQKLPSTTMTKARKRRRRRRLMELAEPKINWQVLKDRKGRCGKGYAWISPCKMSLHFCLCWPSVYWTERFLEDTTLTITVPAVSRRVEELSRPKRFYLEYYNNNRTTPVWPIPRSSLEYRASSRLKELAAPKIRDNFWSMPMSEVSQVSRAAQMAVPSSRILQLSKPKAPATLLEEWDPVPKPKPHVSDHNRLLHLARPKAQSDKCVPDRDPRWEVLDVTKKVVASPRIISLAKPKVRKGLNEGYDRRPLASMSLPPPKASPEKCDQPRPGL

367 NP_002101.2 NP_060708.1

>NP_002101.2

MGGRSSCEDPGCPRDEERAPRMGCMKSKFLQVGGNTFSKTETSASPHCPVYVPDPTSTIKPGPNSHNSNTPGIREAGSEDIIVVALYDYEAIHHEDLSFQKGDQMVVLEESGEWWKARSLATRKEGYIPSNYVARVDSLETEEWFFKGISRKDAERQLLAPGNMLGSFMIRDSETTKGSYSLSVRDYDPRQGDTVKHYKIRTLDNGGFYISPRSTFSTLQELVDHYKKGNDGLCQKLSVPCMSSKPQKPWEKDAWEIPRESLKLEKKLGAGQFGEVWMATYNKHTKVAVKTMKPGSMSVEAFLAEANVMKTLQHDKLVKLHAVVTKEPIYIITEFMAKGSLLDFLKSDEGSKQPLPKLIDFSAQIAEGMAFIEQRNYIHRDLRAANILVSASLVCKIADFGLARVIEDNEYTAREGAKFPIKWTAPEAINFGSFTIKSDVWSFGILLMEIVTYGRIPYPGMSNPEVIRALERGYRMPRPENCPEELYNIMMRCWKNRPEERPTFEYIQSVLDDFYTATESQYQQQP

>NP_060708.1

MTVFFKTLRNHWKKTTAGLCLLTWGGHWLYGKHCDNLLRRAACQEAQVFGNQLIPPNAQVKKATVFLNPAACKGKARTLFEKNAAPILHLSGMDVTIVKTDYEGQAKKLLELMENTDVIIVAGGDGTLQEVVTGVLRRTDEATFSKIPIGFIPLGETSSLSHTLFAESGNKVQHITDATLAIVKGETVPLDVLQIKGEKEQPVFAMTGLRWGSFRDAGVKVSKYWYLGPLKIKAAHFFSTLKEWPQTHQASISYTGPTERPPNEPEETPVQRPSLYRRILRRLASYWAQPQDALSQEVSPEVWKDVQLSTIELSITTRNNQLDPTSKEDFLNICIEPDTISKGDFITIGSRKVRNPKLHVEGTECLQASQCTLLIPEGAGGSFSIDSEEYEAMPVEVKLLPRKLQFFCDPRKREQMLTSPTQ

368 NP_858045.1 NP_444284.1

>NP_858045.1

MSGLGENLDPLASDSRKRKLPCDTPGQGLTCSGEKRRREQESKYIEELAELISANLSDIDNFNVKPDKCAILKETVRQIRQIKEQGKTISNDDDVQKADVSSTGQGVIDKDSLGPLLLQALDGFLFVVNRDGNIVFVSENVTQYLQYKQEDLVNTSVYNILHEEDRKDFLKNLPKSTVNGVSWTNETQRQKSHTFNCRMLMKTPHDILEDINASPEMRQRYETMQCFALSQPRAMMEEGEDLQSCMICVARRITTGERTFPSNPESFITRHDLSGKVVNIDTNSLRSSMRPGFEDIIRRCIQRFFSLNDGQSWSQKRHYQEAYLNGHAETPVYRFSLADGTIVTAQTKSKLFRNPVTNDRHGFVSTHFLQREQNGYRPNPNPVGQGIRPPMAGCNSSVGGMSMSPNQGLQMPSSRAYGLADPSTTGQMSGARYGGSSNIASLTPGPGMQSPSSYQNNNYGLNMSSPPHGSPGLAPNQQNIMISPRNRGSPKIASHQFSPVAGVHSPMASSGNTGNHSFSSSSLSALQAISEGVGTSLLSTLSSPGPKLDNSPNMNITQPSKVSNQDSKSPLGFYCDQNPVESSMCQSNSRDHLSDKESKESSVEGAENQRGPLESKGHKKLLQLLTCSSDDRGHSSLTNSPLDSSCKESSVSVTSPSGVSSSTSGGVSSTSNMHGSLLQEKHRILHKLLQNGNSPAEVAKITAEATGKDTSSITSCGDGNVVKQEQLSPKKKENNALLRYLLDRDDPSDALSKELQPQVEGVDNKMSQCTSSTIPSSSQEKDPKIKTETSEEGSGDLDNLDAILGDLTSSDFYNNSISSNGSHLGTKQQVFQGTNSLGLKSSQSVQSIRPPYNRAVSLDSPVSVGSSPPVKNISAFPMLPKQPMLGGNPRMMDSQENYGSSMGGPNRNVTVTQTPSSGDWGLPNSKAGRMEPMNSNSMGRPGGDYNTSLPRPALGGSIPTLPLRSNSIPGARPVLQQQQQMLQMRPGEIPMGMGANPYGQAAASNQLGSWPDGMLSMEQVSHGTQNRPLLRNSLDDLVGPPSNLEGQSDERALLDQLHTLLSNTDATGLEEIDRALGIPELVNQGQALEPKQDAFQGQEAAVMMDQKAGLYGQTYPAQGPPMQGGFHLQGQSPSFNSMMNQMNQQGNFPLQGMHPRANIMRPRTNTPKQLRMQLQQRLQGQQFLNQSRQALELKMENPTAGGAAVMRPMMQPQVSSQQGFLNAQMVAQRSRELLSHHFRQQRVAMMMQQQQQQQQQQQQQQQQQQQQQQQQQQQQQTQAFSPPPNVTASPSMDGLLAGPTMPQAPPQQFPYQPNYGMGQQPDPAFGRVSSPPNAMMSSRMGPSQNPMMQHPQAASIYQSSEMKGWPSGNLARNSSFSQQQFAHQGNPAVYSMVHMNGSSGHMGQMNMNPMPMSGMPMGPDQKYC

>NP_444284.1

MEHQLLCCEVETIRRAYPDANLLNDRVLRAMLKAEETCAPSVSYFKCVQKEVLPSMRKIVATWMLEVCEEQKCEEEVFPLAMNYLDRFLSLEPVKKSRLQLLGATCMFVASKMKETIPLTAEKLCIYTDNSIRPEELLQMELLLVNKLKWNLAAMTPHDFIEHFLSKMPEAEENKQIIRKHAQTFVALCATDVKFISNPPSMVAAGSVVAAVQGLNLRSPNNFLSYYRLTRFLSRVIKCDPDCLRACQEQIEALLESSLRQAQQNMDPKAAEEEEEEEEEVDLACTPTDVRDVDI

369 NP_443732.2 NP_068379.1

>NP_443732.2

MDDATVLRKKGYIVGINLGKGSYAKVKSAYSERLKFNVAVKIIDRKKTPTDFVERFLPREMDILATVNHGSIIKTYEIFETSDGRIYIIMELGVQGDLLEFIKCQGALHEDVARKMFRQLSSAVKYCHDLDIVHRDLKCENLLLDKDFNIKLSDFGFSKRCLRDSNGRIILSKTFCGSAAYAAPEVLQSIPYQPKVYDIWSLGVILYIMVCGSMPYDDSDIRKMLRIQKEHRVDFPRSKNLTCECKDLIYRMLQPDVSQRLHIDEILSHSWLQPPKPKATSSASFKREGEGKYRAECKLDTKTDLRPDHRPDHKLGAKTQHRLLVVPENENRMEDRLAETSRAKDHHISGAEVGKAST

>NP_068379.1

MASVVVKTIWQSKEIHEAGDTPTGVESCSQLVPEAPRRVTSRAKGIPKKKKAVSFHGVEPQMSHQPMHWCLNLKRSSACTNVSLLNLAAMEPTDSTGTDSTVEDLSGQLTLAGPPASPTLPWDPDDADITEILSGVNSGLVRAKDSITSLKEKTNRVNQHVQSLQSECSVLSENLERRRQEAEELEGYCIQLKENCWKVTRSVEDAEIKTNVLKQNSALLEEKLRYLQQQLQDETPRRQEAELQEPEEKQEPEEKQEPEEKQKPEAGLSWNSLGPAATSQGCPGPPGSPDKPSRPHGLVPAGWGMGPRAGEGPYVSEQELQKLFTGIEELRREVSSLTARWHQEEGAVQEALRLLGGLGGRVDGFLGQWERAQREQAQTARDLQELRGRADELCTMVERSAVSVASLRSELEGLGPLKPILEEFGRQFQNSRRGPDLSMNLDRSHQGNCARCASQGSQLSTESLQQLLDRALTSLVDEVKQRGLTPACPSCQRLHKKILELERQALAKHVRAEALSSTLRLAQDEALRAKNLLLTDKMKPEEKMATLDHLHLKMCSLHDHLSNLPLEGSTGTMGGGSSAGTPPKQGGSAPEQ

370 NP_001950.1 NP_002100.2

>NP_001950.1

MGFGDLKSPAGLQVLNDYLADKSYIEGYVPSQADVAVFEAVSSPPPADLCHALRWYNHIKSYEKEKASLPGVKKALGKYGPADVEDTTGSGATDSKDDDDIDLFGSDDEEESEEAKRLREERLAQYESKKAKKPALVAKSSILLDVKPWDDETDMAKLEECVRSIQADGLVWGSSKLVPVGYGIKKLQIQCVVEDDKVGTDMLEEQITAFEDYVQSMDVAAFNKI

>NP_002100.2

MAERAALEELVKLQGERVRGLKQQKASAELIEEEVAKLLKLKAQLGPDESKQKFVLKTPKGTRDYSPRQMAVREKVFDVIIRCFKRHGAEVIDTPVFELKETLMGKYGEDSKLIYDLKDQGGELLSLRYDLTVPFARYLAMNKLTNIKRYHIAKVYRRDNPAMTRGRYREFYQCDFDIAGNFDPMIPDAECLKIMCEILSSLQIGDFLVKVNDRRILDGMFAICGVSDSKFRTICSSVDKLDKVSWEEVKNEMVGEKGLAPEVADRIGDYVQQHGGVSLVEQLLQDPKLSQNKQALEGLGDLKLLFEYLTLFGIDDKISFDLSLARGLDYYTGVIYEAVLLQTPAQAGEEPLGVGSVAAGGRYDGLVGMFDPKGRKVPCVGLSIGVERIFSIVEQRLEALEEKIRTTETQVLVASAQKKLLEERLKLVSELWDAGIKAELLYKKNPKLLNQLQYCEEAGIPLVAIIGEQELKDGVIKLRSVTSREEVDVRREDLVEEIKRRTGQPLCIC

371 NP_001356.1 NP_000882.1

>NP_001356.1

MSQRPRAPRSALWLLAPPLLRWAPPLLTVLHSDLFQALLDILDYYEASLSESQKYRYQDEDTPPLEHSPAHLPNQANSPPVIVNTDTLEAPGYELQVNGTEGEMEYEEITLERGNSGLGFSIAGGTDNPHIGDDPSIFITKIIPGGAAAQDGRLRVNDSILFVNEVDVREVTHSAAVEALKEAGSIVRLYVMRRKPPAEKVMEIKLIKGPKGLGFSIAGGVGNQHIPGDNSIYVTKIIEGGAAHKDGRLQIGDKILAVNSVGLEDVMHEDAVAALKNTYDVVYLKVAKPSNAYLSDSYAPPDITTSYSQHLDNEISHSSYLGTDYPTAMTPTSPRRYSPVAKDLLGEEDIPREPRRIVIHRGSTGLGFNIVGGEDGEGIFISFILAGGPADLSGELRKGDQILSVNGVDLRNASHEQAAIALKNAGQTVTIIAQYKPEEYSRFEAKIHDLREQLMNSSLGSGTASLRSNPKRGFYIRALFDYDKTKDCGFLSQALSFRFGDVLHVIDASDEEWWQARRVHSDSETDDIGFIPSKRRVERREWSRLKAKDWGSSSGSQGREDSVLSYETVTQMEVHYARPIIILGPTKDRANDDLLSEFPDKFGSCVPHTTRPKREYEIDGRDYHFVSSREKMEKDIQAHKFIEAGQYNSHLYGTSVQSVREVAEQGKHCILDVSANAVRRLQAAHLHPIAIFIRPRSLENVLEINKRITEEQARKAFDRATKLEQEFTECFSAIVEGDSFEEIYHKVKRVIEDLSGPYIWVPARERL

>NP_000882.1

MGSVRTNRYSIVSSEEDGMKLATMAVANGFGNGKSKVHTRQQCRSRFVKKDGHCNVQFINVGEKGQRYLADIFTTCVDIRWRWMLVIFCLAFVLSWLFFGCVFWLIALLHGDLDASKEGKACVSEVNSFTAAFLFSIETQTTIGYGFRCVTDECPIAVFMVVFQSIVGCIIDAFIIGAVMAKMAKPKKRNETLVFSHNAVIAMRDGKLCLMWRVGNLRKSHLVEAHVRAQLLKSRITSEGEYIPLDQIDINVGFDSGIDRIFLVSPITIVHEIDEDSPLYDLSKQDIDNADFEIVVILEGMVEATAMTTQCRSSYLANEILWGHRYEPVLFEEKHYYKVDYSRFHKTYEVPNTPLCSARDLAEKKYILSNANSFCYENEVALTSKEEDDSENGVPESTSTDTPPDIDLHNQASVPLEPRPLRRESEI

372 NP_003067.3 NP_005822.1

>NP_003067.3

MAARAGFQSVAPSGGAGASGGAGAAAALGPGGTPGPPVRMGPAPGQGLYRSPMPGAAYPRPGMLPGSRMTPQGPSMGPPGYGGNPSVRPGLAQSGMDQSRKRPAPQQIQQVQQQAVQNRNHNAKKKKMADKILPQRIRELVPESQAYMDLLAFERKLDQTIMRKRLDIQEALKRPIKQKRKLRIFISNTFNPAKSDAEDGEGTVASWELRVEGRLLEDSALSKYDATKQKRKFSSFFKSLVIELDKDLYGPDNHLVEWHRTATTQETDGFQVKRPGDVNVRCTVLLMLDYQPPQFKLDPRLARLLGIHTQTRPVIIQALWQYIKTHKLQDPHEREFVICDKYLQQIFESQRMKFSEIPQRLHALLMPPEPIIINHVISVDPNDQKKTACYDIDVEVDDTLKTQMNSFLLSTASQQEIATLDNKIHETIETINQLKTQREFMLSFARDPQGFINDWLQSQCRDLKTMTDVVGNPEEERRAEFYFQPWAQEAVCRYFYSKVQQRRQELEQALGIRNT

>NP_005822.1

MEETIKDPPTSAVLLDHCHFSQVIFNSVEKFYIPGGDVTCHYTFTQHFIPRRKDWIGIFRVGWKTTREYYTFMWVTLPIDLNNKSAKQQEVQFKAYYLPKDDEYYQFCYVDEDGVVRGASIPFQFRPENEEDILVVTTQGEVEEIEQHNKELCKENQELKDSCISLQKQNSDMQAELQKKQEELETLQSINKKLELKVKEQKDYWETELLQLKEQNQKMSSENEKMGIRVDQLQAQLSTQEKEMEKLVQGDQDKTEQLEQLKKENDHLFLSLTEQRKDQKKLEQTVEQMKQNETTAMKKQQELMDENFDLSKRLSENEIICNALQRQKERLEGENDLLKRENSRLLSYMGLDFNSLPYQVPTSDEGGARQNPGLAYGNPYSGIQESSSPSPLSIKKCPICKADDICDHTLEQQQMQPLCFNCPICDKIFPATEKQIFEDHVFCHSL

373 NP_009138.1 NP_620448.1

>NP_009138.1

MPPSPLDDRVVVALSRPVRPQDLNLCLDSSYLGSANPGSNSHPPVIATTVVSLKAANLTYMPSSSGSARSLNCGCSSASCCTVATYDKDNQAQTQAIAAGTTTTAIGTSTTCPANQMVNNNENTGSLSPSSGVGSPVSGTPKQLASIKIIYPNDLAKKMTKCSKSHLPSQGPVIIDCRPFMEYNKSHIQGAVHINCADKISRRRLQQGKITVLDLISCREGKDSFKRIFSKEIIVYDENTNEPSRVMPSQPLHIVLESLKREGKEPLVLKGGLSSFKQNHENLCDNSLQLQECREVGGGASAASSLLPQPIPTTPDIENAELTPILPFLFLGNEQDAQDLDTMQRLNIGYVINVTTHLPLYHYEKGLFNYKRLPATDSNKQNLRQYFEEAFEFIEEAHQCGKGLLIHCQAGVSRSATIVIAYLMKHTRMTMTDAYKFVKGKRPIISPNLNFMGQLLEFEEDLNNGVTPRILTPKLMGVETVV

>NP_620448.1

MSLHFLYYCSEPTLDVKIAFCQGFDKQVDVSYIAKHYNMSKSKVDNQFYSVEVGDSTFTVLKRYQNLKPIGSGAQGIVCAAYDAVLDRNVAIKKLSRPFQNQTHAKRAYRELVLMKCVNHKNIISLLNVFTPQKTLEEFQDVYLVMELMDANLCQVIQMELDHERMSYLLYQMLCGIKHLHSAGIIHRDLKPSNIVVKSDCTLKILDFGLARTAGTSFMMTPYVVTRYYRAPEVILGMGYKENVDIWSVGCIMGEMVRHKILFPGRDYIDQWNKVIEQLGTPCPEFMKKLQPTVRNYVENRPKYAGLTFPKLFPDSLFPADSEHNKLKASQARDLLSKMLVIDPAKRISVDDALQHPYINVWYDPAEVEAPPPQIYDKQLDEREHTIEEWKELIYKEVMNSEEKTKNGVVKGQPSPSGAAVNSSESLPPSSSVNDISSMSTDQTLASDTDSSLEASAGPLGCCR

374 NP_002733.2 NP_001421.2

>NP_002733.2

MSAPPVLRPPSPLLPVAAAAAAAAAALVPGSGPGPAPFLAPVAAPVGGISFHLQIGLSREPVLLLQDSSGDYSLAHVREMACSIVDQKFPECGFYGMYDKILLFRHDPTSENILQLVKAASDIQEGDLIEVVLSASATFEDFQIRPHALFVHSYRAPAFCDHCGEMLWGLVRQGLKCEGCGLNYHKRCAFKIPNNCSGVRRRRLSNVSLTGVSTIRTSSAELSTSAPDEPLLQKSPSESFIGREKRSNSQSYIGRPIHLDKILMSKVKVPHTFVIHSYTRPTVCQYCKKLLKGLFRQGLQCKDCRFNCHKRCAPKVPNNCLGEVTINGDLLSPGAESDVVMEEGSDDNDSERNSGLMDDMEEAMVQDAEMAMAECQNDSGEMQDPDPDHEDANRTISPSTSNNIPLMRVVQSVKHTKRKSSTVMKEGWMVHYTSKDTLRKRHYWRLDSKCITLFQNDTGSRYYKEIPLSEILSLEPVKTSALIPNGANPHCFEITTANVVYYVGENVVNPSSPSPNNSVLTSGVGADVARMWEIAIQHALMPVIPKGSSVGTGTNLHRDISVSISVSNCQIQENVDISTVYQIFPDEVLGSGQFGIVYGGKHRKTGRDVAIKIIDKLRFPTKQESQLRNEVAILQNLHHPGVVNLECMFETPERVFVVMEKLHGDMLEMILSSEKGRLPEHITKFLITQILVALRHLHFKNIVHCDLKPENVLLASADPFPQVKLCDFGFARIIGEKSFRRSVVGTPAYLAPEVLRNKGYNRSLDMWSVGVIIYVSLSGTFPFNEDEDIHDQIQNAAFMYPPNPWKEISHEAIDLINNLLQVKMRKRYSVDKTLSHPWLQDYQTWLDLRELECKIGERYITHESDDLRWEKYAGEQGLQYPTHLINPSASHSDTPETEETEMKALGERVSIL

>NP_001421.2

MTADKEKKRSSSERRKEKSRDAARCRRSKETEVFYELAHELPLPHSVSSHLDKASIMRLAISFLRTHKLLSSVCSENESEAEADQQMDNLYLKALEGFIAVVTQDGDMIFLSENISKFMGLTQVELTGHSIFDFTHPCDHEEIRENLSLKNGSGFGKKSKDMSTERDFFMRMKCTVTNRGRTVNLKSATWKVLHCTGQVKVYNNCPPHNSLCGYKEPLLSCLIIMCEPIQHPSHMDIPLDSKTFLSRHSMDMKFTYCDDRITELIGYHPEELLGRSAYEFYHALDSENMTKSHQNLCTKGQVVSGQYRMLAKHGGYVWLETQGTVIYNPRNLQPQCIMCVNYVLSEIEKNDVVFSMDQTESLFKPHLMAMNSIFDSSGKGAVSEKSNFLFTKLKEEPEELAQLAPTPGDAIISLDFGNQNFEESSAYGKAILPPSQPWATELRSHSTQSEAGSLPAFTVPQAAAPGSTTPSATSSSSSCSTPNSPEDYYTSLDNDLKIEVIEKLFAMDTEAKDQCSTQTDFNELDLETLAPYIPMDGEDFQLSPICPEERLLAENPQSTPQHCFSAMTNIFQPLAPVAPHSPFLLDKFQQQLESKKTEPEHRPMSSIFFDAGSKASLPPCCGQASTPLSSMGGRSNTQWPPDPPLHFGPTKWAVGDQRTEFLGAAPLGPPVSPPHVSTFKTRSAKGFGARGPDVLSPAMVALSNKLKLKRQLEYEEQAFQDLSGGDPPGGSTSHLMWKRMKNLRGGSCPLMPDKPLSANVPNDKFTQNPMRGLGHPLRHLPLPQPPSAISPGENSKSRFPPQCYATQYQDYSLSSAHKVSGMASRLLGPSFESYLLPELTRYDCEVNVPVLGSSTLLQGGDLLRALDQAT

375 NP_009330.1 NP_004371.2

>NP_009330.1

MSQWYELQQLDSKFLEQVHQLYDDSFPMEIRQYLAQWLEKQDWEHAANDVSFATIRFHDLLSQLDDQYSRFSLENNFLLQHNIRKSKRNLQDNFQEDPIQMSMIIYSCLKEERKILENAQRFNQAQSGNIQSTVMLDKQKELDSKVRNVKDKVMCIEHEIKSLEDLQDEYDFKCKTLQNREHETNGVAKSDQKQEQLLLKKMYLMLDNKRKEVVHKIIELLNVTELTQNALINDELVEWKRRQQSACIGGPPNACLDQLQNWFTIVAESLQQVRQQLKKLEELEQKYTYEHDPITKNKQVLWDRTFSLFQQLIQSSFVVERQPCMPTHPQRPLVLKTGVQFTVKLRLLVKLQELNYNLKVKVLFDKDVNERNTVKGFRKFNILGTHTKVMNMEESTNGSLAAEFRHLQLKEQKNAGTRTNEGPLIVTEELHSLSFETQLCQPGLVIDLETTSLPVVVISNVSQLPSGWASILWYNMLVAEPRNLSFFLTPPCARWAQLSEVLSWQFSSVTKRGLNVDQLNMLGEKLLGPNASPDGLIPWTRFCKENINDKNFPFWLWIESILELIKKHLLPLWNDGCIMGFISKERERALLKDQQPGTFLLRFSESSREGAITFTWVERSQNGGEPDFHAVEPYTKKELSAVTFPDIIRNYKVMAAENIPENPLKYLYPNIDKDHAFGKYYSRPKEAPEPMELDGPKGTGYIKTELISVSEVHPSRLQTTDNLLPMSPEEFDEVSRIVGSVEFDSMMNTV

>NP_004371.2

MAENLLDGPPNPKRAKLSSPGFSANDSTDFGSLFDLENDLPDELIPNGGELGLLNSGNLVPDAASKHKQLSELLRGGSGSSINPGIGNVSASSPVQQGLGGQAQGQPNSANMASLSAMGKSPLSQGDSSAPSLPKQAASTSGPTPAASQALNPQAQKQVGLATSSPATSQTGPGICMNANFNQTHPGLLNSNSGHSLINQASQGQAQVMNGSLGAAGRGRGAGMPYPTPAMQGASSSVLAETLTQVSPQMTGHAGLNTAQAGGMAKMGITGNTSPFGQPFSQAGGQPMGATGVNPQLASKQSMVNSLPTFPTDIKNTSVTNVPNMSQMQTSVGIVPTQAIATGPTADPEKRKLIQQQLVLLLHAHKCQRREQANGEVRACSLPHCRTMKNVLNHMTHCQAGKACQVAHCASSRQIISHWKNCTRHDCPVCLPLKNASDKRNQQTILGSPASGIQNTIGSVGTGQQNATSLSNPNPIDPSSMQRAYAALGLPYMNQPQTQLQPQVPGQQPAQPQTHQQMRTLNPLGNNPMNIPAGGITTDQQPPNLISESALPTSLGATNPLMNDGSNSGNIGTLSTIPTAAPPSSTGVRKGWHEHVTQDLRSHLVHKLVQAIFPTPDPAALKDRRMENLVAYAKKVEGDMYESANSRDEYYHLLAEKIYKIQKELEEKRRSRLHKQGILGNQPALPAPGAQPPVIPQAQPVRPPNGPLSLPVNRMQVSQGMNSFNPMSLGNVQLPQAPMGPRAASPMNHSVQMNSMGSVPGMAISPSRMPQPPNMMGAHTNNMMAQAPAQSQFLPQNQFPSSSGAMSVGMGQPPAQTGVSQGQVPGAALPNPLNMLGPQASQLPCPPVTQSPLHPTPPPASTAAGMPSLQHTTPPGMTPPQPAAPTQPSTPVSSSGQTPTPTPGSVPSATQTQSTPTVQAAAQAQVTPQPQTPVQPPSVATPQSSQQQPTPVHAQPPGTPLSQAAASIDNRVPTPSSVASAETNSQQPGPDVPVLEMKTETQAEDTEPDPGESKGEPRSEMMEEDLQGASQVKEETDIAEQKSEPMEVDEKKPEVKVEVKEEEESSSNGTASQSTSPSQPRKKIFKPEELRQALMPTLEALYRQDPESLPFRQPVDPQLLGIPDYFDIVKNPMDLSTIKRKLDTGQYQEPWQYVDDVWLMFNNAWLYNRKTSRVYKFCSKLAEVFEQEIDPVMQSLGYCCGRKYEFSPQTLCCYGKQLCTIPRDAAYYSYQNRYHFCEKCFTEIQGENVTLGDDPSQPQTTISKDQFEKKKNDTLDPEPFVDCKECGRKMHQICVLHYDIIWPSGFVCDNCLKKTGRPRKENKFSAKRLQTTRLGNHLEDRVNKFLRRQNHPEAGEVFVRVVASSDKTVEVKPGMKSRFVDSGEMSESFPYRTKALFAFEEIDGVDVCFFGMHVQEYGSDCPPPNTRRVYISYLDSIHFFRPRCLRTAVYHEILIGYLEYVKKLGYVTGHIWACPPSEGDDYIFHCHPPDQKIPKPKRLQEWYKKMLDKAFAERIIHDYKDIFKQATEDRLTSAKELPYFEGDFWPNVLEESIKELEQEEEERKKEESTAASETTEGSQGDSKNAKKKNNKKTNKNKSSISRANKKKPSMPNVSNDLSQKLYATMEKHKEVFFVIHLHAGPVINTLPPIVDPDPLLSCDLMDGRDAFLTLARDKHWEFSSLRRSKWSTLCMLVELHTQGQDRFVYTCNECKHHVETRWHCTVCEDYDLCINCYNTKSHAHKMVKWGLGLDDEGSSQGEPQSKSPQESRRLSIQRCIQSLVHACQCRNANCSLPSCQKMKRVVQHTKGCKRKTNGGCPVCKQLIALCCYHAKHCQENKCPVPFCLNIKHKLRQQQIQHRLQQAQLMRRRMATMNTRNVPQQSLPSPTSAPPGTPTQQPSTPQTPQPPAQPQPSPVSMSPAGFPSVARTQPPTTVSTGKPTSQVPAPPPPAQPPPAAVEAARQIEREAQQQQHLYRVNINNSMPPGRTGMGTPGSQMAPVSLNVPRPNQVSGPVMPSMPPGQWQQAPLPQQQPMPGLPRPVISMQAQAAVAGPRMPSVQPPRSISPSALQDLLRTLKSPSSPQQQQQVLNILKSNPQLMAAFIKQRTAKYVANQPGMQPQPGLQSQPGMQPQPGMHQQPSLQNLNAMQAGVPRPGVPPQQQAMGGLNPQGQALNIMNPGHNPNMASMNPQYREMLRRQLLQQQQQQQQQQQQQQQQQQGSAGMAGGMAGHGQFQQPQGPGGYPPAMQQQQRMQQHLPLQGSSMGQMAAQMGQLGQMGQPGLGADSTPNIQQALQQRILQQQQMKQQIGSPGQPNPMSPQQHMLSGQPQASHLPGQQIATSLSNQVRSPAPVQSPRPQSQPPHSSPSPRIQPQPSPHHVSPQTGSPHPGLAVTMASSIDQGHLGNPEQSAMLPQLNTPSRSALSSELSLVGDTTGDTLEKFVEGL

376 NP_006644.1 NP_000595.1

>NP_006644.1

MGSCCSCLNRDSVPDNHPTKFKVTNVDDEGVELGSGVMELTQSELVLHLHRREAVRWPYLCLRRYGYDSNLFSFESGRRCQTGQGIFAFKCSRAEEIFNLLQDLMQCNSINVMEEPVIITRNSHPAELDLPRAPQPPNALGYTVSSFSNGCPGEGPRFSAPRRLSTSSLRHPSLGEESTHALIAPDEQSHTYVNTPASEDDHRRGRHCLQPLPEGQAPFLPQARGPDQRDPQVFLQPGQVKFVLGPTPARRHMVKCQGLCPSLHDPPHHNNNNEAPSECPAQPKCTYENVTGGLWRGAGWRLSPEEPGWNGLAHRRAALLHYENLPPLPPVWESQAQQLGGEAGDDGDSRDGLTPSSNGFPDGEEDETPLQKPTSTRAAIRSHGSFPVPLTRRRGSPRVFNFDFRRPGPEPPRQLNYIQVELKGWGGDRPKGPQNPSSPQAPMPTTHPARSSDSYAVIDLKKTVAMSNLQRALPRDDGTARKTRHNSTDLPL

>NP_000595.1

MWSWKCLLFWAVLVTATLCTARPSPTLPEQAQPWGAPVEVESFLVHPGDLLQLRCRLRDDVQSINWLRDGVQLAESNRTRITGEEVEVQDSVPADSGLYACVTSSPSGSDTTYFSVNVSDALPSSEDDDDDDDSSSEEKETDNTKPNRMPVAPYWTSPEKMEKKLHAVPAAKTVKFKCPSSGTPNPTLRWLKNGKEFKPDHRIGGYKVRYATWSIIMDSVVPSDKGNYTCIVENEYGSINHTYQLDVVERSPHRPILQAGLPANKTVALGSNVEFMCKVYSDPQPHIQWLKHIEVNGSKIGPDNLPYVQILKTAGVNTTDKEMEVLHLRNVSFEDAGEYTCLAGNSIGLSHHSAWLTVLEALEERPAVMTSPLYLEIIIYCTGAFLISCMVGSVIVYKMKSGTKKSDFHSQMAVHKLAKSIPLRRQVTVSADSSASMNSGVLLVRPSRLSSSGTPMLAGVSEYELPEDPRWELPRDRLVLGKPLGEGCFGQVVLAEAIGLDKDKPNRVTKVAVKMLKSDATEKDLSDLISEMEMMKMIGKHKNIINLLGACTQDGPLYVIVEYASKGNLREYLQARRPPGLEYCYNPSHNPEEQLSSKDLVSCAYQVARGMEYLASKKCIHRDLAARNVLVTEDNVMKIADFGLARDIHHIDYYKKTTNGRLPVKWMAPEALFDRIYTHQSDVWSFGVLLWEIFTLGGSPYPGVPVEELFKLLKEGHRMDKPSNCTNELYMMMRDCWHAVPSQRPTFKQLVEDLDRIVALTSNQEYLDLSMPLDQYSPSFPDTRSSTCSSGEDSVFSHEPLPEEPCLPRHPAQLANGGLKRR

377 NP_789782.1 NP_689497.1

>NP_789782.1

MSVFGKLFGAGGGKAGKGGPTPQEAIQRLRDTEEMLSKKQEFLEKKIEQELTAAKKHGTKNKRAALQALKRKKRYEKQLAQIDGTLSTIEFQREALENANTNTEVLKNMGYAAKAMKAAHDNMDIDKVDELMQDIADQQELAEEISTAISKPVGFGEEFDEDELMAELEELEQEELDKNLLEISGPETVPLPNVPSIALPSKPAKKKEEEDDDMKELENWAGSM

>NP_689497.1

MSKLGKFFKGGGSSKSRAAPSPQEALVRLRETEEMLGKKQEYLENRIQREIALAKKHGTQNKRAALQALKRKKRFEKQLTQIDGTLSTIEFQREALENSHTNTEVLRNMGFAAKAMKSVHENMDLNKIDDLMQEITEQQDIAQEISEAFSQRVGFGDDFDEDELMAELEELEQEELNKKMTNIRLPNVPSSSLPAQPNRKPGMSSTARRSRAASSQRAEEEDDDIKQLAAWAT

378 NP_000925.2 NP_001897.4

>NP_000925.2

MALLWGLLVLSWSCLQGPCSVFSPVSAMEPLGRQLTSGPNQEQVSPLTLLKLGNQEPGGQTALKSPPGVCSRDPTPEQTHRLARAMMAFTADLFSLVAQTSTCPNLILSPLSVALALSHLALGAQNHTLQRLQQVLHAGSGPCLPHLLSRLCQDLGPGAFRLAARMYLQKGFPIKEDFLEQSEQLFGAKPVSLTGKQEDDLANINQWVKEATEGKIQEFLSGLPEDTVLLLLNAIHFQGFWRNKFDPSLTQRDSFHLDEQFTVPVEMMQARTYPLRWFLLEQPEIQVAHFPFKNNMSFVVLVPTHFEWNVSQVLANLSWDTLHPPLVWERPTKVRLPKLYLKHQMDLVATLSQLGLQELFQAPDLRGISEQSLVVSGVQHQSTLELSEVGVEAAAATSIAMSRMSLSSFSVNRPFLFFIFEDTTGLPLFVGSVRNPNPSAPRELKEQQDSPGNKDFLQSLKGFPRGDKLFGPDLKLVPPMEEDYPQFGSPK

>NP_001897.4

MASLWLLSCFSLVGAAFGCGVPAIHPVLSGLSRIVNGEDAVPGSWPWQVSLQDKTGFHFCGGSLISEDWVVTAAHCGVRTSDVVVAGEFDQGSDEENIQVLKIAKVFKNPKFSILTVNNDITLLKLATPARFSQTVSAVCLPSADDDFPAGTLCATTGWGKTKYNANKTPDKLQQAALPLLSNAECKKSWGRRITDVMICAGASGVSSCMGDSGGPLVCQKDGAWTLVGIVSWGSDTCSTSSPGVYARVTKLIPWVQKILAAN

379 NP_000956.2 NP_149052.1

>NP_000956.2

MFDCMDVLSVSPGQILDFYTASPSSCMLQEKALKACFSGLTQTEWQHRHTAQSIETQSTSSEELVPSPPSPLPPPRVYKPCFVCQDKSSGYHYGVSACEGCKGFFRRSIQKNMIYTCHRDKNCVINKVTRNRCQYCRLQKCFEVGMSKESVRNDRNKKKKETSKQECTESYEMTAELDDLTEKIRKAHQETFPSLCQLGKYTTNSSADHRVRLDLGLWDKFSELATKCIIKIVEFAKRLPGFTGLTIADQITLLKAACLDILILRICTRYTPEQDTMTFSDGLTLNRTQMHNAGFGPLTDLVFTFANQLLPLEMDDTETGLLSAICLICGDRQDLEEPTKVDKLQEPLLEALKIYIRKRRPSKPHMFPKILMKITDLRSISAKGAERVITLKMEIPGSMPPLIQEMLENSEGHEPLTPSSSGNTAEHSPSISPSSVENSGVSQSPLVQ

>NP_149052.1

MAWPCITRACCIARFWNQLDKADIAVPLVFTKYSEATEHPGAPPQPPPPQQQAQPALAPPSARAVAIETQPAQGELDAVARATGPAPGPTGEREPAAGPGRSGPGPGLGSGSTSGPADSVMRQDYRAWKVQRPEPSCRPRSEYQPSDAPFERETQYQKDFRAWPLPRRGDHPWIPKPVQISAASQASAPILGAPKRRPQSQERWPVQAAAEAREQEAAPGGAGGLAAGKASGADERDTRRKAGPAWIVRRAEGLGHEQTPLPAAQAQVQATGPEAGRGRAAADALNRQIREEVASAVSSSYRNEFRAWTDIKPVKPIKAKPQYKPPDDKMVHETSYSAQFKGEASKPTTADNKVIDRRRIRSLYSEPFKEPPKVEKPSVQSSKPKKTSASHKPTRKAKDKQAVSGQAAKKKSAEGPSTTKPDDKEQSKEMNNKLAEAKESLAQPVSDSSKTQGPVATEPDKDQGSVVPGLLKGQGPMVQEPLKKQGSVVPGPPKDLGPMIPLPVKDQDHTVPEPLKNESPVISAPVKDQGPSVPVPPKNQSPMVPAKVKDQGSVVPESLKDQGPRIPEPVKNQAPMVPAPVKDEGPMVSASVKDQGPMVSAPVKDQGPIVPAPVKGEGPIVPAPVKDEGPMVSAPIKDQDPMVPEHPKDESAMATAPIKNQGSMVSEPVKNQGLVVSGPVKDQDVVVPEHAKVHDSAVVAPVKNQGPVVPESVKNQDPILPVLVKDQGPTVLQPPKNQGRIVPEPLKNQVPIVPVPLKDQDPLVPVPAKDQGPAVPEPLKTQGPRDPQLPTVSPLPRVMIPTAPHTEYIESSP

380 NP_055717.2 NP_000827.1

>NP_055717.2

MKGLGDSRPRHLSDSLDPPHEPLFAGTDRNPYLLSPTEAFAREARFPGQNTLPGDGLFPLNNQLPPPSSTFPRIHYNSHFEVPEESPFPSHAQATKINRLPANLLDQFEKQLPIHRDGFSTLQFPRGEAKARGESPGRIRHLVHSVQRLFFTKAPSLEGTAGKVGGNGSKKGGMEDGKGRRAKSKERAKAGEPKRRSRSNISGWWSSDDNLDGEAGAFRSSGPASGLMTLGRQAERSQPRYFMHAYNTISGHMLKTTKNNTTELTAPPPPPAPPATCPSLGVGTDTNYVKRGSWSTLTLSHAHEVCQKTSATLDKSLLKSKSCHQGLAYHYLQVPGGGGEWSTTLLSPRETDAAAEGPIPCRRMRSGSYIKAMGDEDSDESGGSPKPSPKTAARRQSYLRATQQSLGEQSNPRRSLDRLDSVDMLLPSKCPSWEEDYTPVSDSLNDSSCISQIFGQASLIPQLFGHEQQVREAELSDQYEAACESACSEAESTAAETLDLPLPSYFRSRSHSYLRAIQAGCSQEEDSVSLQSLSPPPSTGSLSNSRTLPSSSCLVAYKKTPPPVPPRTTSKPFISVTVQSSTESAQDTYLDSQDHKSEVTSQSGLSNSSDSLDSSTRPPSVTRGGVAPAPEAPEPPPKHAALKSEQGTLTSSESHPEAAPKRKLSSIGIQERTRRNGSHLSEDNGPKAIDVMAPSSESSVPSHSMSSRRDTDSDTQDANDSSCKSSERSLPDCTPHPNSISIDAGPRQAPKIAQIKRNLSYGDNSDPALEASSLPPPDPWLETSSSSPAEPAQPGACRRDGYWFLKLLQAETERLEGWCCQMDKETKENNLSEEVLGKVLSAVGSAQLLMSQKFQQFRGLCEQNLNPDANPRPTAQDLAGFWDLLQLSIEDISMKFDELYHLKANSWQLVETPEKRKEEKKPPPPVPKKPAKSKPAVSRDKASDASDKQRQEARKRLLAAKRAASVRQNSATESADSIEIYVPEAQTRL

>NP_000827.1

MRGAGGPRGPRGPAKMLLLLALACASPFPEEAPGPGGAGGPGGGLGGARPLNVALVFSGPAYAAEAARLGPAVAAAVRSPGLDVRPVALVLNGSDPRSLVLQLCDLLSGLRVHGVVFEDDSRAPAVAPILDFLSAQTSLPIVAVHGGAALVLTPKEKGSTFLQLGSSTEQQLQVIFEVLEEYDWTSFVAVTTRAPGHRAFLSYIEVLTDGSLVGWEHRGALTLDPGAGEAVLSAQLRSVSAQIRLLFCAREEAEPVFRAAEEAGLTGSGYVWFMVGPQLAGGGGSGAPGEPPLLPGGAPLPAGLFAVRSAGWRDDLARRVAAGVAVVARGAQALLRDYGFLPELGHDCRAQNRTHRGESLHRYFMNITWDNRDYSFNEDGFLVNPSLVVISLTRDRTWEVVGSWEQQTLRLKYPLWSRYGRFLQPVDDTQHLTVATLEERPFVIVEPADPISGTCIRDSVPCRSQLNRTHSPPPDAPRPEKRCCKGFCIDILKRLAHTIGFSYDLYLVTNGKHGKKIDGVWNGMIGEVFYQRADMAIGSLTINEERSEIVDFSVPFVETGISVMVARSNGTVSPSAFLEPYSPAVWVMMFVMCLTVVAVTVFIFEYLSPVGYNRSLATGKRPGGSTFTIGKSIWLLWALVFNNSVPVENPRGTTSKIMVLVWAFFAVIFLASYTANLAAFMIQEEYVDTVSGLSDRKFQRPQEQYPPLKFGTVPNGSTEKNIRSNYPDMHSYMVRYNQPRVEEALTQLKAGKLDAFIYDAAVLNYMARKDEGCKLVTIGSGKVFATTGYGIALHKGSRWKRPIDLALLQFLGDDEIEMLERLWLSGICHNDKIEVMSSKLDIDNMAGVFYMLLVAMGLSLLVFAWEHLVYWRLRHCLGPTHRMDFLLAFSRGMYSCCSAEAAPPPAKPPPPPQPLPSPAYPAPGPAPGPAPFVPRERASVDRWRRTKGAGPPGGAGLADGFHRYYGPIEPQGLGLGLGEARAAPRGAAGRPLSPPAAQPPQKPPASYFAIVRDKEPAEPPAGAFPGFPSPPAPPAAAATAVGPPLCRLAFEDESPPAPARWPRSDPESQPLLGPGAGGAGGTGGAGGGAPAAPPPCCAAPPPCPYLDLEPSPSDSEDSESLGGASLGGLDPWWFADFPYPYAERLGPPPGRYWSVDKLGGWRAGSWDYLPPRSGPAAWHCRHCASLELLPPPRHLSCSHDGLDGGWWAPPPPPWAAGPLPRRRARCGCPRSHPHRPRASHRTPAAAAPHHHRHRRAAGGWDLPPPAPTSRSLEDLSSCPRAAPARRLTGPSRHARRCPHAAHWGPPLPTASHRRHRGGDLGTRRGSAHFSSLESEV

381 NP_055525.3 NP_001050.1

>NP_055525.3

MTGAEIESGAQVKPEKKPGEEVVGGAEIENDVPLVVRPKVRTQAQIMPGARPKNKSKVMPGASTKVETSAVGGARPKSKAKAIPVSRFKEEAQMWAQPRFGAERLSKTERNSQTNIIASPLVSTDSVLVAKTKYLSEDRELVNTDTESFPRRKAHYQAGFQPSFRSKEETNMGSWCCPRPTSKQEASPNSDFKWVDKSVSSLFWSGDEVTAKFHPGNRVKDSNRSMHMANQEANTMSRSQTNQELYIASSSGSEDESVKTPWFWARDKTNTWSGPREDPNSRSRFRSKKEVYVESSSGSEHEDHLESWFGAGKEAKFRSKMRAGKEANNRARHRAKREACIDFMPGSIDVIKKESCFWPEENANTFSRPMIKKEARARAMTKEEAKTKARARAKQEARSEEEALIGTWFWATDESSMADEASIESSLQVEDESIIGSWFWTEEEASMGTGASSKSRPRTDGERIGDSLFGAREKTSMKTGAEATSESILAADDEQVIIGSWFWAGEEVNQEAEEETIFGSWFWVIDAASVESGVGVSCESRTRSEEEEVIGPWFWSGEQVDIEAGIGEEARPGAEEETIFGSWFWAENQTYMDCRAETSCDTMQGAEEEEPIIGSWFWTRVEACVEGDVNSKSSLEDKEEAMIPCFGAKEEVSMKHGTGVRCRFMAGAEETNNKSCFWAEKEPCMYPAGGGSWKSRPEEEEDIVNSWFWSRKYTKPEAIIGSWLWATEESNIDGTGEKAKLLTEEETIINSWFWKEDEAISEATDREESRPEAEEGDIIGSWFWAGEEDRLEPAAETREEDRLAAEKEGIVGSWFGAREETIRREAGSCSKSSPKAEEEEVIIGSWFWEEEASPEAVAGVGFESKPGTEEEEITVGSWFWPEEEASIQAGSQAVEEMESETEEETIFGSWFWDGKEVSEEAGPCCVSKPEDDEEMIVESWFWSRDKAIKETGTVATCESKPENEEGAIVGSWFEAEDEVDNRTDNGSNCGSRTLADEDEAIVGSWFWAGDEAHFESNPSPVFRAICRSTCSVEQEPDPSRRPQSWEEVTVQFKPGPWGRVGFPSISPFRFPKEAASLFCEMFGGKPRNMVLSPEGEDQESLLQPDQPSPEFPFQYDPSYRSVQEIREHLRAKESTEPESSSCNCIQCELKIGSEEFEELLLLMEKIRDPFIHEISKIAMGMRSASQFTRDFIRDSGVVSLIETLLNYPSSRVRTSFLENMIRMAPPYPNLNIIQTYICKVCEETLAYSVDSPEQLSGIRMIRHLTTTTDYHTLVANYMSGFLSLLATGNAKTRFHVLKMLLNLSENLFMTKELLSAEAVSEFIGLFNREETNDNIQIVLAIFENIGNNIKKETVFSDDDFNIEPLISAFHKVEKFAKELQGKTDNQNDPEGDQEN

>NP_001050.1

MATLPAAETWIDGGGGVGADAVNLTASLAAGAATGAVETGWLQLLDQAGNLSSSPSALGLPVASPAPSQPWANLTNQFVQPSWRIALWSLAYGVVVAVAVLGNLIVIWIILAHKRMRTVTNYFLVNLAFSDASMAAFNTLVNFIYALHSEWYFGANYCRFQNFFPITAVFASIYSMTAIAVDRYMAIIDPLKPRLSATATKIVIGSIWILAFLLAFPQCLYSKTKVMPGRTLCFVQWPEGPKQHFTYHIIVIILVYCFPLLIMGITYTIVGITLWGGEIPGDTCDKYHEQLKAKRKVVKMMIIVVMTFAICWLPYHIYFILTAIYQQLNRWKYIQQVYLASFWLAMSSTMYNPIIYCCLNKRFRAGFKRAFRWCPFIKVSSYDELELKTTRFHPNRQSSMYTVTRMESMTVVFDPNDADTTRSSRKKRATPRDPSFNGCSRRNSKSASATSSFISSPYTSVDEYS

382 NP_036364.2 NP_031370.2

>NP_036364.2

MEDPTLYIVERPLPGYPDAEAPEPSSAGAQAAEEPSGAGSEELIKSDQVNGVLVLSLLDKIIGAVDQIQLTQAQLEERQAEMEGAVQSIQGELSKLGKAHATTSNTVSKLLEKVRKVSVNVKTVRGSLERQAGQIKKLEVNEAELLRRRNFKVMIYQDEVKLPAKLSISKSLKESEALPEKEGEELGEGERPEEDAAALELSSDEAVEVEEVIEESRAERIKRSGLRRVDDFKKAFSKEKMEKTKVRTRENLEKTRLKTKENLEKTRHTLEKRMNKLGTRLVPAERREKLKTSRDKLRKSFTPDHVVYARSKTAVYKVPPFTFHVKKIREGQVEVLKATEMVEVGADDDEGGAERGEAGDLRRGSSPDVHALLEITEESDAVLVDKSDSD

>NP_031370.2

MEGESSRFEIHTPVSDKKKKKCSIHKERPQKHSHEIFRDSSLVNEQSQITRRKKRKKDFQHLISSPLKKSRICDETANATSTLKKRKKRRYSALEVDEEAGVTVVLVDKENINNTPKHFRKDVDVVCVDMSIEQKLPRKPKTDKFQVLAKSHAHKSEALHSKVREKKNKKHQRKAASWESQRARDTLPQSESHQEESWLSVGPGGEITELPASAHKNKSKKKKKKSSNREYETLAMPEGSQAGREAGTDMQESQPTVGLDDETPQLLGPTHKKKSKKKKKKKSNHQEFEALAMPEGSQVGSEVGADMQESRPAVGLHGETAGIPAPAYKNKSKKKKKKSNHQEFEAVAMPESLESAYPEGSQVGSEVGTVEGSTALKGFKESNSTKKKSKKRKLTSVKRARVSGDDFSVPSKNSESTLFDSVEGDGAMMEEGVKSRPRQKKTQACLASKHVQEAPRLEPANEEHNVETAEDSEIRYLSADSGDADDSDADLGSAVKQLQEFIPNIKDRATSTIKRMYRDDLERFKEFKAQGVAIKFGKFSVKENKQLEKNVEDFLALTGIESADKLLYTDRYPEEKSVITNLKRRYSFRLHIGRNIARPWKLIYYRAKKMFDVNNYKGRYSEGDTEKLKMYHSLLGNDWKTIGEMVARSSLSVALKFSQISSQRNRGAWSKSETRKLIKAVEEVILKKMSPQELKEVDSKLQENPESCLSIVREKLYKGISWVEVEAKVQTRNWMQCKSKWTEILTKRMTNGRRIYYGMNALRAKVSLIERLYEINVEDTNEIDWEDLASAIGDVPPSYVQTKFSRLKAVYVPFWQKKTFPEIIDYLYETTLPLLKEKLEKMMEKKGTKIQTPAAPKQVFPFRDIFYYEDDSEGEDIEKESEGQAPCMAHACNSSTLGGQGRWII

383 NP_003861.1 BAA88957.1

>NP_003861.1

MSAADEVDGLGVARPHYGSVLDNERLTAEEMDERRRQNVAYEYLCHLEEAKRWMEACLGEDLPPTTELEEGLRNGVYLAKLGNFFSPKVVSLKKIYDREQTRYKATGLHFRHTDNVIQWLNAMDEIGLPKIFYPETTDIYDRKNMPRCIYCIHALSLYLFKLGLAPQIQDLYGKVDFTEEEINNMKTELEKYGIQMPAFSKIGGILANELSVDEAALHAAVIAINEAIDRRIPADTFAALKNPNAMLVNLEEPLASTYQDILYQAKQDKMTNAKNRTENSERERDVYEELLTQAEIQGNINKVNTFSALANIDLALEQGDALALFRALQSPALGLRGLQQQNSDWYLKQLLSDKQQKRQSGQTDPLQKEELQSGVDAANSAAQQYQRRLAAVALINAAIQKGVAEKTVLELMNPEAQLPQVYPFAADLYQKELATLQRQSPEHNLTHPELSVAVEMLSSVALINRALESGDVNTVWKQLSSSVTGLTNIEEENCQRYLDELMKLKAQAHAENNEFITWNDIQACVDHVNLVVQEEHERILAIGLINEALDEGDAQKTLQALQIPAAKLEGVLAEVAQHYQDTLIRAKREKAQEIQDESAVLWLDEIQGGIWQSNKDTQEAQKFALGIFAINEAVESGDVGKTLSALRSPDVGLYGVIPECGETYHSDLAEAKKKKLAVGDNNSKWVKHWVKGGYYYYHNLETQEGGWDEPPNFVQNSMQLSREEIQSSISGVTAAYNREQLWLANEGLITRLQARCRGYLVRQEFRSRMNFLKKQIPAITCIQSQWRGYKQKKAYQDRLAYLRSHKDEVVKIQSLARMHQARKRYRDRLQYFRDHINDIIKIQAFIRANKARDDYKTLINAEDPPMVVVRKFVHLLDQSDQDFQEELDLMKMREEVITLIRSNQQLENDLNLMDIKIGLLVKNKITLQDVVSHSKKLTKKNKEQLSDMMMINKQKGGLKALSKEKREKLEAYQHLFYLLQTNPTYLAKLIFQMPQNKSTKFMDSVIFTLYNYASNQREEYLLLRLFKTALQEEIKSKVDQIQEIVTGNPTVIKMVVSFNRGARGQNALRQILAPVVKEIMDDKSLNIKTDPVDIYKSWVNQMESQTGEASKLPYDVTPEQALAHEEVKTRLDSSIRNMRAVTDKFLSAIVSSVDKIPYGMRFIAKVLKDSLHEKFPDAGEDELLKIIGNLLYYRYMNPAIVAPDAFDIIDLSAGGQLTTDQRRNLGSIAKMLQHAASNKMFLGDNAHLSIINEYLSQSYQKFRRFFQTACDVPELQDKFNVDEYSDLVTLTKPVIYISIGEIINTHTLLLDHQDAIAPEHNDPIHELLDDLGEVPTIESLIGESSGNLNDPNKEALAKTEVSLTLTNKFDVPGDENAEMDARTILLNTKRLIVDVIRFQPGETLTEILETPATSEQEAEHQRAMQRRAIRDAKTPDKMKKSKSVKEDSNLTLQEKKEKIQTGLKKLTELGTVDPKNKYQELINDIARDIRNQRRYRQRRKAELVKLQQTYAALNSKATFYGEQVDYYKSYIKTCLDNLASKGKVSKKPREMKGKKSKKISLKYTAARLHEKGVLLEIEDLQVNQFKNVIFEISPTEEVGDFEVKAKFMGVQMETFMLHYQDLLQLQYEGVAVMKLFDRAKVNVNLLIFLLNKKFYGK

>BAA88957.1

MGPWSRSLSALLLLLQVSSWLCQEPEPCHPGFDAESYTFTVPRRHLERGRVLGRVNFEDCTGRQRTAYFSLDTRFKVGTDGVITVKRPLRFHNPQIHFLVYAWDSTYRKFSTKVTLNTVGHHHRPPPHQASVSGIQAELLTFPNSSPGLRRQKRDWVIPPISCPENEKGPFPKNLVQIKSNKDKEGKVFYSITGQGADTPPVGVFIIERETGWLKVTEPLDRERIATYTLFSHAVSSNGNAVEDPMEILITVTDQNDNKPEFTQEVFKGSVMEGALPGTSVMEVTATDADDDVNTYNAAIAYTILSQDPELPDKNMFTINRNTGVISVVTTGLDRESFPTYTLVVQAADLQGEGLSTTATAVITVTDTNDNPPIFNPTTYKGQVPENEANVVITTLKVTDADAPNTPAWEAVYTILNDDGGQFVVTTNPVNNDGILKTAKDLARCRNWHPHSCSYPCPLGLDFEAKQQYILHVAVTNVVPFEVSLTTSTATVTVDVLDVNEAPIFVPPEKRVEVSEDFGVGQEITSYTAQEPDTFMEQKITYRIWRDTANWLEINPDTGAISTRAELDREDFEHVKNSTYTALIIATDNGSPVATGTGTLLLILSDVNDNAPIPEPRTIFFCERNPKPQVINIIDADLPPNTSPFTAELTHGASANWTIQYNDPTQESIILKPKMALEVGDYKINLKLMDNQNKDQVTTLEVSVCDCEGAAGVCRKAQPVEAGLQIPAILGILGGILALLILILLLLLFLRRRAVVKEPLLPPEDDTRDNVYYYDEEGGGEEDQDFDLSQLHRGLDARPEVTRNDVAPTLMSVPRYLPRPANPDEIGNFIDENLKAADTDPTAPPYDSLLVFDYEGSGSEAASLSSLNSSESDKDQDYDYLNEWGNRFKKLADMYGGGEDD

384 NP_036426.3 NP_002877.2

>NP_036426.3

MAIGVWQLLLDMGIMLSVDQHLYFQDTYVFYQFSSDECSYLYCEFEREEEWQNGVKLLLQLVPLIPARGGICELSHQKIEDSEESSDEILVRLTSAVQRELAAVIALKARKSAIEQDEENNDKHVAVTEAESVPDSQAGVMCKLQERDEIGRIELVQKLAKENYQFLQTDKKEQEKSEHQDDEVTTVQVKEQDQSVLVLKKVQCCGPAPTAGSAESHWRYVVVSGTPEKILEHLLNDLHLEEVQDKETETLLDDFLLTYTVFMTTDDLCQALLRHYSAKKYQGKEENSDVPRRKRKVLHLVSQWIALYKDWLPEDEHSKMFLKTIYRNVLDDVYEYPILEKELKEFQKILGMHRRHTVDEYSPQKKNKALFHQFSLKENWLQHRGTVTETEEIFCHVYITEHSYVSVKAKVSSIAQEILKVVAEKIQYAEEDLALVAITFSGEKHELQPNDLVISKSLEASGRIYVYRKDLADTLNPFAENEESQQRSMRILGMNTWDLALELMNFDWSLFNSIHEQELIYFTFSRQGSGEHTANLSLLLQRCNEVQLWVATEILLCSQLGKRVQLVKKFIKIAAHCKAQRNLNSFFAIVMGLNTASVSRLSQTWEKIPGKFKKLFSELESLTDPSLNHKAYRDAFKKMKPPKIPFMPLLLKDVTFIHEGNKTFLDNLVNFEKLHMIADTVRTLRHCRTNQFGDLSPKEHQELKSYVNHLYVIDSQQALFELSHRIEPRV

>NP_002877.2

MREYKVVVLGSGGVGKSALTVQFVTGSFIEKYDPTIEDFYRKEIEVDSSPSVLEILDTAGTEQFASMRDLYIKNGQGFILVYSLVNQQSFQDIKPMRDQIIRVKRYERVPMILVGNKVDLEGEREVSYGEGKALAEEWSCPFMETSAKNKASVDELFAEIVRQMNYAAQPNGDEGCCSACVIL

385 NP_000723.1 NP_004059.2

>NP_000723.1

MEHSTFLSGLVLATLLSQVSPFKIPIEELEDRVFVNCNTSITWVEGTVGTLLSDITRLDLGKRILDPRGIYRCNGTDIYKDKESTVQVHYRMCQSCVELDPATVAGIIVTDVIATLLLALGVFCFAGHETGRLSGAADTQALLRNDQVYQPLRDRDDAQYSHLGGNWARNK

>NP_004059.2

MIGGLFIYNHKGEVLISRVYRDDIGRNAVDAFRVNVIHARQQVRSPVTNIARTSFFHVKRSNIWLAAVTKQNVNAAMVFEFLYKMCDVMAAYFGKISEENIKNNFVLIYELLDEILDFGYPQNSETGALKTFITQQGIKSQHQTKEEQSQITSQVTGQIGWRREGIKYRRNELFLDVLESVNLLMSPQGQVLSAHVSGRVVMKSYLSGMPECKFGMNDKIVIEKQGKGTADETSKSGKQSIAIDDCTFHQCVRLSKFDSERSISFIPPDGEFELMRYRTTKDIILPFRVIPLVREVGRTKLEVKVVIKSNFKPSLLAQKIEVRIPTPLNTSGVQVICMKGKAKYKASENAIVWKIKRMAGMKESQISAEIELLPTNDKKKWARPPISMNFEVPFAPSGLKVRYLKVFEPKLNYSDHDVIKWVRYIGRSGIYETRC

386 NP_055102.3 NP_064516.2

>NP_055102.3

MLVGQGAGPLGPAVVTAAVVLLLSGVGPAHGSEDIVVGCGGFVKSDVEINYSLIEIKLYTKHGTLKYQTDCAPNNGYFMIPLYDKGDFILKIEPPLGWSFEPTTVELHVDGVSDICTKGGDINFVFTGFSVNGKVLSKGQPLGPAGVQVSLRNTGTEAKIQSTVTQPGGKFAFFKVLPGDYEILATHPTWALKEASTTVRVTNSNANAASPLIVAGYNVSGSVRSDGEPMKGVKFLLFSSLVTKEDVLGCNVSPVPGFQPQDESLVYLCYTVSREDGSFSFYSLPSGGYTVIPFYRGERITFDVAPSRLDFTVEHDSLKIEPVFHVMGFSVTGRVLNGPEGDGVPEAVVTLNNQIKVKTKADGSFRLENITTGTYTIHAQKEHLYFETVTIKIAPNTPQLADIIATGFSVCGQISIIRFPDTVKQMNKYKVVLSSQDKDKSLVTVETDAHGSFCFKAKPGTYKVQVMVPEAETRAGLTLKPQTFPLTVTNRPMMDVAFVQFLASVSGKVSCLDTCGDLLVTLQSLSRQGEKRSLQLSGKVNAMTFTFDNVLPGKYKISIMHEDWCWKNKSLEVEVLEDDMSAVEFRQTGYMLRCSLSHAITLEFYQDGNGRENVGIYNLSKGVNRFCLSKPGVYKVTPRSCHRFEQAFYTYDTSSPSILTLTAIRHHVLGTITTDKMMDVTVTIKSSIDSEPALVLGPLKSVQELRREQQLAEIEARRQEREKNGNEEGEERMTKPPVQEMVDELQGPFSYDFSYWARSGEKITVTPSSKELLFYPPSMEAVVSGESCPGKLIEIHGKAGLFLEGQIHPELEGVEIVISEKGASSPLITVFTDDKGAYSVGPLHSDLEYTVTSQKEGYVLTAVEGTIGDFKAYALAGVSFEIKAEDDQPLPGVLLSLSGGLFRSNLLTQDNGILTFSNLSPGQYYFKPMMKEFRFEPSSQMIEVQEGQNLKITITGYRTAYSCYGTVSSLNGEPEQGVAMEAVGQNDCSIYGEDTVTDEEGKFRLRGLLPGCVYHVQLKAEGNDHIERALPHHRVIEVGNNDIDDVNIIVFRQINQFDLSGNVITSSEYLPTLWVKLYKSENLDNPIQTVSLGQSLFFHFPPLLRDGENYVVLLDSTLPRSQYDYILPQVSFTAVGYHKHITLIFNPTRKLPEQDIAQGSYIALPLTLLVLLAGYNHDKLIPLLLQLTSRLQGVRALGQAASDNSGPEDAKRQAKKQKTRRT

>NP_064516.2

MAEPSGAETRPPIRVTVKTPKDKEEIVICDRASVKEFKEEISRRFKAQQDQLVLIFAGKILKDGDTLNQHGIKDGLTVHLVIKTPQKAQDPAAATASSPSTPDPASAPSTTPASPATPAQPSTSGSASSDAGSGSRRSSGGGPSPGAGEGSPSATASILSGFGGILGLGSLGLGSANFMELQQQMQRQLMSNPEMLSQIMENPLVQDMMSNPDLMRHMIMANPQMQQLMERNPEISHMLNNPELMRQTMELARNPAMMQEMMRNQDRALSNLESIPGGYNALRRMYTDIQEPMFSAAREQFGNNPFSSLAGNSDSSSSQPLRTENREPLPNPWSPSPPTSQAPGSGGEGTGGSGTSQVHPTVSNPFGINAASLGSGMFNSPEMQALLQQISENPQLMQNVISAPYMRSMMQTLAQNPDFAAQMMVNVPLFAGNPQLQEQLRLQLPVFLQQMQNPESLSILTNPRAMQALLQIQQGLQTLQTEAPGLVPSLGSFGISRTPAPSAGSNAGSTPEAPTSSPATPATSSPTGASSAQQQLMQQMIQLLAGSGNSQVQTPEVRFQQQLEQLNSMGFINREANLQALIATGGDINAAIERLLGSQLS

387 NP_000630.1 NP_003969.2

>NP_000630.1

MQQPFNYPYPQIYWVDSSASSPWAPPGTVLPCPTSVPRRPGQRRPPPPPPPPPLPPPPPPPPLPPLPLPPLKKRGNHSTGLCLLVMFFMVLVALVGLGLGMFQLFHLQKELAELRESTSQMHTASSLEKQIGHPSPPPEKKELRKVAHLTGKSNSRSMPLEWEDTYGIVLLSGVKYKKGGLVINETGLYFVYSKVYFRGQSCNNLPLSHKVYMRNSKYPQDLVMMEGKMMSYCTTGQMWARSSYLGAVFNLTSADHLYVNVSELSLVNFEESQTFFGLYKL

>NP_003969.2

MMPQLQFKDAFWCRDFTAHTGYEVLLQRLLDGRKMCKDMEELLRQRAQAEERYGKELVQIARKAGGQTEINSLRASFDSLKQQMENVGSSHIQLALTLREELRSLEEFRERQKEQRKKYEAVMDRVQKSKLSLYKKAMESKKTYEQKCRDADDAEQAFERISANGHQKQVEKSQNKARQCKDSATEAERVYRQSIAQLEKVRAEWEQEHRTTCEAFQLQEFDRLTILRNALWVHSNQLSMQCVKDDELYEEVRLTLEGCSIDADIDSFIQAKSTGTEPPAPVPYQNYYDREVTPLTSSPGIQPSCGMIKRFSGLLHGSPKTTSLAASAASTETLTPTPERNEGVYTAIAVQEIQGNPASPAQEYRALYDYTAQNPDELDLSAGDILEVILEGEDGWWTVERNGQRGFVPGSYLEKL

388 NP_000359.1 NP_006817.1

>NP_000359.1

MAQQANVGELLAMLDSPMLGVRDDVTAVFKENLNSDRGPMLVNTLVDYYLETSSQPALHILTTLQEPHDKHLLDRINEYVGKAATRLSILSLLGHVIRLQPSWKHKLSQAPLLPSLLKCLKMDTDVVVLTTGVLVLITMLPMIPQSGKQHLLDFFDIFGRLSSWCLKKPGHVAEVYLVHLHASVYALFHRLYGMYPCNFVSFLRSHYSMKENLETFEEVVKPMMEHVRIHPELVTGSKDHELDPRRWKRLETHDVVIECAKISLDPTEASYEDGYSVSHQISARFPHRSADVTTSPYADTQNSYGCATSTPYSTSRLMLLNMPGQLPQTLSSPSTRLITEPPQATLWSPSMVCGMTTPPTSPGNVPPDLSHPYSKVFGTTAGGKGTPLGTPATSPPPAPLCHSDDYVHISLPQATVTPPRKEERMDSARPCLHRQHHLLNDRGSEEPPGSKGSVTLSDLPGFLGDLASEEDSIEKDKEEAAISRELSEITTAEAEPVVPRGGFDSPFYRDSLPGSQRKTHSAASSSQGASVNPEPLHSSLDKLGPDTPKQAFTPIDLPCGSADESPAGDRECQTSLETSIFTPSPCKIPPPTRVGFGSGQPPPYDHLFEVALPKTAHHFVIRKTEELLKKAKGNTEEDGVPSTSPMEVLDRLIQQGADAHSKELNKLPLPSKSVDWTHFGGSPPSDEIRTLRDQLLLLHNQLLYERFKRQQHALRNRRLLRKVIKAAALEEHNAAMKDQLKLQEKDIQMWKVSLQKEQARYNQLQEQRDTMVTKLHSQIRQLQHDREEFYNQSQELQTKLEDCRNMIAELRIELKKANNKVCHTELLLSQVSQKLSNSESVQQQMEFLNRQLLVLGEVNELYLEQLQNKHSDTTKEVEMMKAAYRKELEKNRSHVLQQTQRLDTSQKRILELESHLAKKDHLLLEQKKYLEDVKLQARGQLQAAESRYEAQKRITQVFELEILDLYGRLEKDGLLKKLEEEKAEAAEAAEERLDCCNDGCSDSMVGHNEEASGHNGETKTPRPSSARGSSGSRGGGGSSSSSSELSTPEKPPHQRAGPFSSRWETTMGEASASIPTTVGSLPSSKSFLGMKARELFRNKSESQCDEDGMTSSLSESLKTELGKDLGVEAKIPLNLDGPHPSPPTPDSVGQLHIMDYNETHHEHS

>NP_006817.1

MEKTELIQKAKLAEQAERYDDMATCMKAVTEQGAELSNEERNLLSVAYKNVVGGRRSAWRVISSIEQKTDTSDKKLQLIKDYREKVESELRSICTTVLELLDKYLIANATNPESKVFYLKMKGDYFRYLAEVACGDDRKQTIDNSQGAYQEAFDISKKEMQPTHPIRLGLALNFSVFYYEILNNPELACTLAKTAFDEAIAELDTLNEDSYKDSTLIMQLLRDNLTLWTSDSAGEECDAAEGAEN

389 NP_003241.2 NP_056989.2

>NP_003241.2

MEQKPSKVECGSDPEENSARSPDGKRKRKNGQCSLKTSMSGYIPSYLDKDEQCVVCGDKATGYHYRCITCEGCKGFFRRTIQKNLHPTYSCKYDSCCVIDKITRNQCQLCRFKKCIAVGMAMDLVLDDSKRVAKRKLIEQNRERRRKEEMIRSLQQRPEPTPEEWDLIHIATEAHRSTNAQGSHWKQRRKFLPDDIGQSPIVSMPDGDKVDLEAFSEFTKIITPAITRVVDFAKKLPMFSELPCEDQIILLKGCCMEIMSLRAAVRYDPESDTLTLSGEMAVKREQLKNGGLGVVSDAIFELGKSLSAFNLDDTEVALLQAVLLMSTDRSGLLCVDKIEKSQEAYLLAFEHYVNHRKHNIPHFWPKLLMKEREVQSSILYKGAAAEGRPGGSLGVHPEGQQLLGMHVVQGPQVRQLEQQLGEAGSLQGPVLQHQSPKSPQQRLLELLHRSGILHARAVCGEDDSSEADSPSSSEEEPEVCEDLAGNAASP

>NP_056989.2

MEVAVEKAVAAAAAASAAASGGPSAAPSGENEAESRQGPDSERGGEAARLNLLDTCAVCHQNIQSRAPKLLPCLHSFCQRCLPAPQRYLMLPAPMLGSAETPPPVPAPGSPVSGSSPFATQVGVIRCPVCSQECAERHIIDNFFVKDTTEVPSSTVEKSNQVCTSCEDNAEANGFCVECVEWLCKTCIRAHQRVKFTKDHTVRQKEEVSPEAVGVTSQRPVFCPFHKKEQLKLYCETCDKLTCRDCQLLEHKEHRYQFIEEAFQNQKVIIDTLITKLMEKTKYIKFTGNQIQNRIIEVNQNQKQVEQDIKVAIFTLMVEINKKGKALLHQLESLAKDHRMKLMQQQQEVAGLSKQLEHVMHFSKWAVSSGSSTALLYSKRLITYRLRHLLRARCDASPVTNNTIQFHCDPSFWAQNIINLGSLVIEDKESQPQMPKQNPVVEQNSQPPSGLSSNQLSKFPTQISLAQLRLQHMQQQVMAQRQQVQRRPAPVGLPNPRMQGPIQQPSISHQQPPPRLINFQNHSPKPNGPVLPPHPQQLRYPPNQNIPRQAIKPNPLQMAFLAQQAIKQWQISSGQGTPSTTNSTSSTPSSPTITSAAGYDGKAFGSPMIDLSSPVGGSYNLPSLPDIDCSSTIMLDNIVRKDTNIDHGQPRPPSNRTVQSPNSSVPSPGLAGPVTMTSVHPPIRSPSASSVGSRGSSGSSSKPAGADSTHKVPVVMLEPIRIKQENSGPPENYDFPVVIVKQESDEESRPQNANYPRSILTSLLLNSSQSSTSEETVLRSDAPDSTGDQPGLHQDNSSNGKSEWLDPSQKSPLHVGETRKEDDPNEDWCAVCQNGGELLCCEKCPKVFHLSCHVPTLTNFPSGEWICTFCRDLSKPEVEYDCDAPSHNSEKKKTEGLVKLTPIDKRKCERLLLFLYCHEMSLAFQDPVPLTVPDYYKIIKNPMDLSTIKKRLQEDYSMYSKPEDFVADFRLIFQNCAEFNEPDSEVANAGIKLENYFEELLKNLYPEKRFPKPEFRNESEDNKFSDDSDDDFVQPRKKRLKSIEERQLLK

390 NP_852478.1 NP_391987.1

>NP_852478.1

MAPRPRARPGVAVACCWLLTVVLRCCVSFNVDVKNSMTFSGPVEDMFGYTVQQYENEEGKWVLIGSPLVGQPKNRTGDVYKCPVGRGESLPCVKLDLPVNTSIPNVTEVKENMTFGSTLVTNPNGGFLACGPLYAYRCGHLHYTTGICSDVSPTFQVVNSIAPVQECSTQLDIVIVLDGSNSIYPWDSVTAFLNDLLERMDIGPKQTQVGIVQYGENVTHEFNLNKYSSTEEVLVAAKKIVQRGGRQTMTALGIDTARKEAFTEARGARRGVKKVMVIVTDGESHDNHRLKKVIQDCEDENIQRFSIAILGSYNRGNLSTEKFVEEIKSIASEPTEKHFFNVSDELALVTIVKTLGERIFALEATADQSAASFEMEMSQTGFSAHYSQDWVMLGAVGAYDWNGTVVMQKASQIIIPRNTTFNVESTKKNEPLASYLGYTVNSATASSGDVLYIAGQPRYNHTGQVIIYRMEDGNIKILQTLSGEQIGSYFGSILTTTDIDKDSNTDILLVGAPMYMGTEKEEQGKVYVYALNQTRFEYQMSLEPIKQTCCSSRQHNSCTTENKNEPCGARFGTAIAAVKDLNLDGFNDIVIGAPLEDDHGGAVYIYHGSGKTIRKEYAQRIPSGGDGKTLKFFGQSIHGEMDLNGDGLTDVTIGGLGGAALFWSRDVAVVKVTMNFEPNKVNIQKKNCHMEGKETVCINATVCFDVKLKSKEDTIYEADLQYRVTLDSLRQISRSFFSGTQERKVQRNITVRKSECTKHSFYMLDKHDFQDSVRITLDFNLTDPENGPVLDDSLPNSVHEYIPFAKDCGNKEKCISDLSLHVATTEKDLLIVRSQNDKFNVSLTVKNTKDSAYNTRTIVHYSPNLVFSGIEAIQKDSCESNHNITCKVGYPFLRRGEMVTFKILFQFNTSYLMENVTIYLSATSDSEEPPETLSDNVVNISIPVKYEVGLQFYSSASEYHISIAANETVPEVINSTEDIGNEINIFYLIRKSGSFPMPELKLSISFPNMTSNGYPVLYPTGLSSSENANCRPHIFEDPFSINSGKKMTTSTDHLKRGTILDCNTCKFATITCNLTSSDISQVNVSLILWKPTFIKSYFSSLNLTIRGELRSENASLVLSSSNQKRELAIQISKDGLPGRVPLWVILLSAFAGLLLLMLLILALWKIGFFKRPLKKKMEK

>NP_391987.1

MNLQPIFWIGLISSVCCVFAQTDENRCLKANAKSCGECIQAGPNCGWCTNSTFLQEGMPTSARCDDLEALKKKGCPPDDIENPRGSKDIKKNKNVTNRSKGTAEKLKPEDITQIQPQQLVLRLRSGEPQTFTLKFKRAEDYPIDLYYLMDLSYSMKDDLENVKSLGTDLMNEMRRITSDFRIGFGSFVEKTVMPYISTTPAKLRNPCTSEQNCTSPFSYKNVLSLTNKGEVFNELVGKQRISGNLDSPEGGFDAIMQVAVCGSLIGWRNVTRLLVFSTDAGFHFAGDGKLGGIVLPNDGQCHLENNMYTMSHYYDYPSIAHLVQKLSENNIQTIFAVTEEFQPVYKELKNLIPKSAVGTLSANSSNVIQLIIDAYNSLSSEVILENGKLSEGVTISYKSYCKNGVNGTGENGRKCSNISIGDEVQFEISITSNKCPKKDSDSFKIRPLGFTEEVEVILQYICECECQSEGIPESPKCHEGNGTFECGACRCNEGRVGRHCECSTDEVNSEDMDAYCRKENSSEICSNNGECVCGQCVCRKRDNTNEIYSGKFCECDNFNCDRSNGLICGGNGVCKCRVCECNPNYTGSACDCSLDTSTCEASNGQICNGRGICECGVCKCTDPKFQGQTCEMCQTCLGVCAEHKECVQCRAFNKGEKKDTCTQECSYFNITKVESRDKLPQPVQPDPVSHCKEKDVDDCWFYFTYSVNGNNEVMVHVVENPECPTGPDIIPIVAGVVAGIVLIGLALLLIWKLLMIIHDRREFAKFEKEKMNAKWDTSLSVAQPGVQWCDISSLQPLTSRFQQFSCLSLPSTWDYRVKILFIRVP

391 AAH00632.1 NP_005630.1

>AAH00632.1

MAAEPLTELEESIETVVTTFFTFARQEGRKDSLSVNEFKELVTQQLPHLLKDVGSLDEKMKSLDVNQDSELKFNEYWRLIGELAKEIRKKKDLKIRKK

>NP_005630.1

MVSESHHEALAAPPVTTVATVLPSNATEPASPGEGKEDAFSKLKEKFMNELHKIPLPPWALIAIAIVAVLLVLTCCFCICKKCLFKKKNKKKGKEKGGKNAINMKDVKDLGKTMKDQALKDDDAETGLTDGEEKEEPKEEEKLGKLQYSLDYDFQNNQLLVGIIQAAELPALDMGGTSDPYVKVFLLPDKKKKFETKVHRKTLNPVFNEQFTFKVPYSELGGKTLVMAVYDFDRFSKHDIIGEFKVPMNTVDFGHVTEEWRDLQSAEKEEQEKLGDICFSLRYVPTAGKLTVVILEAKNLKKMDVGGLSDPYVKIHLMQNGKRLKKKKTTIKKNTLNPYYNESFSFEVPFEQIQKVQVVVTVLDYDKIGKNDAIGKVFVGYNSTGAELRHWSDMLANPRRPIAQWHTLQVEEEVDAMLAVKK

392 NP_002266.2 NP_005545.1

>NP_002266.2

MTTTFLQTSSSTFGGGSTRGGSLLAGGGGFGGGSLSGGGGSRSISASSARFVSSGSGGGYGGGMRVCGFGGGAGSVFGGGFGGGVGGGFGGGFGGGDGGLLSGNEKITMQNLNDRLASYLDKVRALEEANADLEVKIHDWYQKQTPASPECDYSQYFKTIEELRDKIMATTIDNSRVILEIDNARLAADDFRLKYENELALRQGVEADINGLRRVLDELTLARTDLEMQIEGLNEELAYLKKNHEEEMKEFSSQLAGQVNVEMDAAPGVDLTRVLAEMREQYEAMAEKNRRDVEAWFFSKTEELNKEVASNTEMIQTSKTEITDLRRTMQELEIELQSQLSMKAGLENSLAETECRYATQLQQIQGLIGGLEAQLSELRCEMEAQNQEYKMLLDIKTRLEQEIATYRSLLEGQDAKMAGIGIREASSGGGGSSSNFHINVEESVDGQVVSSHKREI

>NP_005545.1

MASTSTTIRSHSSSRRGFSANSARLPGVSRSGFSSVSVSRSRGSGGLGGACGGAGFGSRSLYGLGGSKRISIGGGSCAISGGYGSRAGGSYGFGGAGSGFGFGGGAGIGFGLGGGAGLAGGFGGPGFPVCPPGGIQEVTVNQSLLTPLNLQIDPTIQRVRAEEREQIKTLNNKFASFIDKVRFLEQQNKVLETKWTLLQEQGTKTVRQNLEPLFEQYINNLRRQLDSIVGERGRLDSELRGMQDLVEDFKNKYEDEINKRTAAENEFVTLKKDVDAAYMNKVELQAKADTLTDEINFLRALYDAELSQMQTHISDTSVVLSMDNNRNLDLDSIIAEVKAQYEEIAQRSRAEAESWYQTKYEELQVTAGRHGDDLRNTKQEIAEINRMIQRLRSEIDHVKKQCANLQAAIADAEQRGEMALKDAKNKLEGLEDALQKAKQDLARLLKEYQELMNVKLALDVEIATYRKLLEGEECRLNGEGVGQVNISVVQSTVSSGYGGASGVGSGLGLGGGSSYSYGSGLGVGGGFSSSSGRAIGGGLSSVGGGSSTIKYTTTSSSSRKSYKH

393 NP_006394.1 NP_065574.2

>NP_006394.1

MKYKNLMARALYDNVPECAEELAFRKGDILTVIEQNTGGLEGWWLCSLHGRQGIVPGNRVKLLIGPMQETASSHEQPASGLMQQTFGQQKLYQVPNPQAAPRDTIYQVPPSYQNQGIYQVPTGHGTQEQEVYQVPPSVQRSIGGTSGPHVGKKVITPVRTGHGYVYEYPSRYQKDVYDIPPSHTTQGVYDIPPSSAKGPVFSVPVGEIKPQGVYDIPPTKGVYAIPPSACRDEAGLREKDYDFPPPMRQAGRPDLRPEGVYDIPPTCTKPAGKDLHVKYNCDIPGAAEPVARRHQSLSPNHPPPQLGQSVGSQNDAYDVPRGVQFLEPPAETSEKANPQERDGVYDVPLHNPPDAKGSRDLVDGINRLSFSSTGSTRSNMSTSSTSSKESSLSASPAQDKRLFLDPDTAIERLQRLQQALEMGVSSLMALVTTDWRCYGYMERHINEIRTAVDKVELFLKEYLHFVKGAVANAACLPELILHNKMKRELQRVEDSHQILSQTSHDLNECSWSLNILAINKPQNKCDDLDRFVMVAKTVPDDAKQLTTTINTNAEALFRPGPGSLHLKNGPESIMNSTEYPHGGSQGQLLHPGDHKAQAHNKALPPGLSKEQAPDCSSSDGSERSWMDDYDYVHLQGKEEFERQQKELLEKENIMKQNKMQLEHHQLSQFQLLEQEITKPVENDISKWKPSQSLPTTNSGVSAQDRQLLCFYYDQCETHFISLLNAIDALFSCVSSAQPPRIFVAHSKFVILSAHKLVFIGDTLTRQVTAQDIRNKVMNSSNQLCEQLKTIVMATKMAALHYPSTTALQEMVHQVTDLSRNAQLFKRSLLEMATF

>NP_065574.2

MGLRTAKKRGLGGGGKWKREEGGGTRGRREVRPACFLQSGGRGDPGDVGGPAGNPGCSPHPRAATRPPPLPAHTPAHTPEWCGAASAEAAEPRRAGPHLCIPAPGLTKTPILEKVPRKMAAKTPSSEESGLPKLPVPPLQQTLATYLQCMRHLVSEEQFRKSQAIVQQFGAPGGLGETLQQKLLERQEKTANWVSEYWLNDMYLNNRLALPVNSSPAVIFARQHFPGTDDQLRFAASLISGVLSYKALLDSHSIPTDCAKGQLSGQPLCMKQYYGLFSSYRLPGHTQDTLVAQNSSIMPEPEHVIVACCNQFFVLDVVINFRRLSEGDLFTQLRKIVKMASNEDERLPPIGLLTSDGRSEWAEARTVLVKDSTNRDSLDMIERCICLVCLDAPGGVELSDTHRALQLLHGGGYSKNGANRWYDKSLQFVVGRDGTCGVVCEHSPFDGIVLVQCTEHLLKHVTQSSRKLIRADSVSELPAPRRLRWKCSPEIQGHLASSAEKLQRIVKNLDFIVYKFDNYGKTFIKKQKCSPDAFIQVALQLAFYRLHRRLVPTYESASIRRFQEGRVDNIRSATPEALAFVRAVTDHKAAVPASEKLLLLKDAIRAQTAYTVMAITGMAIDNHLLALRELARAMCKELPEMFMDETYLMSNRFVLSTSQVPTTTEMFCCYGPVVPNGYGACYNPQPETILFCISSFHSCKETSSSKFAKAVEESLIDMRDLCSLLPPTESKPLATKEKATRPSQGHQP

394 NP_008998.1 NP_002681.1

>NP_008998.1

MPRRKRNAGSSSDGTEDSDFSTDLEHTDSSESDGTSRRSARVTRSSARLSQSSQDSSPVRNLQSFGTEEPAYSTRRVTRSQQQPTPVTPKKYPLRQTRSSGSETEQVVDFSDRETKNTADHDESPPRTPTGNAPSSESDIDISSPNVSHDESIAKDMSLKDSGSDLSHRPKRRRFHESYNFNMKCPTPGCNSLGHLTGKHERHFSISGCPLYHNLSADECKVRAQSRDKQIEERMLSHRQDDNNRHATRHQAPTERQLRYKEKVAELRKKRNSGLSKEQKEKYMEHRQTYGNTREPLLENLTSEYDLDLFRRAQARASEDLEKLRLQGQITEGSNMIKTIAFGRYELDTWYHSPYPEEYARLGRLYMCEFCLKYMKSQTILRRHMAKCVWKHPPGDEIYRKGSISVFEVDGKKNKIYCQNLCLLAKLFLDHKTLYYDVEPFLFYVMTEADNTGCHLIGYFSKEKNSFLNYNVSCILTMPQYMRQGYGKMLIDFSYLLSKVEEKVGSPERPLSDLGLISYRSYWKEVLLRYLHNFQGKEISIKEISQETAVNPVDIVSTLQALQMLKYWKGKHLVLKRQDLIDEWIAKEAKRSNSNKTMDPSCLKWTPPKGT

>NP_002681.1

MSKRKAPQETLNGGITDMLTELANFEKNVSQAIHKYNAYRKAASVIAKYPHKIKSGAEAKKLPGVGTKIAEKIDEFLATGKLRKLEKIRQDDTSSSINFLTRVSGIGPSAARKFVDEGIKTLEDLRKNEDKLNHHQRIGLKYFGDFEKRIPREEMLQMQDIVLNEVKKVDSEYIATVCGSFRRGAESSGDMDVLLTHPSFTSESTKQPKLLHQVVEQLQKVHFITDTLSKGETKFMGVCQLPSKNDEKEYPHRRIDIRLIPKDQYYCGVLYFTGSDIFNKNMRAHALEKGFTINEYTIRPLGVTGVAGEPLPVDSEKDIFDYIQWKYREPKDRSE

395 NP_004447.2 NP_077084.1

>NP_004447.2

MGQTGKKSEKGPVCWRKRVKSEYMRLRQLKRFRRADEVKSMFSSNRQKILERTEILNQEWKQRRIQPVHILTSVSSLRGTRECSVTSDLDFPTQVIPLKTLNAVASVPIMYSWSPLQQNFMVEDETVLHNIPYMGDEVLDQDGTFIEELIKNYDGKVHGDRECGFINDEIFVELVNALGQYNDDDDDDDGDDPEEREEKQKDLEDHRDDKESRPPRKFPSDKIFEAISSMFPDKGTAEELKEKYKELTEQQLPGALPPECTPNIDGPNAKSVQREQSLHSFHTLFCRRCFKYDCFLHRKCNYSFHATPNTYKRKNTETALDNKPCGPQCYQHLEGAKEFAAALTAERIKTPPKRPGGRRRGRLPNNSSRPSTPTINVLESKDTDSDREAGTETGGENNDKEEEEKKDETSSSSEANSRCQTPIKMKPNIEPPENVEWSGAEASMFRVLIGTYYDNFCAIARLIGTKTCRQVYEFRVKESSIIAPAPAEDVDTPPRKKKRKHRLWAAHCRKIQLKKDGSSNHVYNYQPCDHPRQPCDSSCPCVIAQNFCEKFCQCSSECQNRFPGCRCKAQCNTKQCPCYLAVRECDPDLCLTCGAADHWDSKNVSCKNCSIQRGSKKHLLLAPSDVAGWGIFIKDPVQKNEFISEYCGEIISQDEADRRGKVYDKYMCSFLFNLNNDFVVDATRKGNKIRFANHSVNPNCYAKVMMVNGDHRIGIFAKRAIQTGEELFFDYRYSQADALKYVGIEREMEIP

>NP_077084.1

MAQPPRLSRSGASSLWDPASPAPTSGPRPRLWEGQDVLARWTDGLLYLGTIKKVDSAREVCLVQFEDDSQFLVLWKDISPAALPGEELLCCVCRSETVVPGNRLVSCEKCRHAYHQDCHVPRAPAPGEGEGTSWVCRQCVFAIATKRGGALKKGPYARAMLGMKLSLPYGLKGLDWDAGHLSNRQQSYCYCGGPGEWNLKMLQCRSCLQWFHEACTQCLSKPLLYGDRFYEFECCVCRGGPEKVRRLQLRWVDVAHLVLYHLSVCCKKKYFDFDREILPFTSENWDSLLLGELSDTPKGERSSKLLSALNSHKDRFISGREIKKRKCLFGLHARMPPPVEPPTGDGALTSFPSGQGPGGGVSRPLGKRRRPEPEPLRRRQKGKVEELGPPSAVRNQPEPQEQRERAHLQRALQASVSPPSPSPNQSYQGSSGYNFRPTDARCLPSSPIRMFASFHPSASTAGTSGDSGPPDRSPLELHIGFPTDIPKSAPHSMTASSSSVSSPSPGLPRRSAPPSPLCRSLSPGTGGGVRGGVGYLSRGDPVRVLARRVRPDGSVQYLVEWGGGGIF

396 NP_758957.2 NP_057075.1

>NP_758957.2

MDVGPSSLPHLGLKLLLLLLLLPLRGQANTGCYGIPGMPGLPGAPGKDGYDGLPGPKGEPGIPAIPGIRGPKGQKGEPGLPGHPGKNGPMGPPGMPGVPGPMGIPGEPGEEGRYKQKFQSVFTVTRQTHQPPAPNSLIRFNAVLTNPQGDYDTSTGKFTCKVPGLYYFVYHASHTANLCVLLYRSGVKVVTFCGHTSKTNQVNSGGVLLRLQVGEEVWLAVNDYYDMVGIQGSDSVFSGFLLFPD

>NP_057075.1

MEGPRGWLVLCVLAISLASMVTEDLCRAPDGKKGEAGRPGRRGRPGLKGEQGEPGAPGIRTGIQGLKGDQGEPGPSGNPGKVGYPGPSGPLGARGIPGIKGTKGSPGNIKDQPRPAFSAIRRNPPMGGNVVIFDTVITNQEEPYQNHSGRFVCTVPGYYYFTFQVLSQWEICLSIVSSSRGQVRRSLGFCDTTNKGLFQVVSGGMVLQLQQGDQVWVEKDPKKGHIYQGSEADSVFSGFLIFPSA

397 CAA89989.1 NP_006804.1

>CAA89989.1

MRLSKTLVDMDMADYSAALDPAYTTLEFENVQVLTMGNDLLPLRLARLRHPLRHHWSISGGVDSSPQGDTSPSEGTNLNAPNSLGVSALCAICGDRATGKHYGASSCDGCKGFFRRSVRKNHMYSCRFSRQCVVDKDKRNQCRYCRLKKCFRAGMKKEAVQNERDRISTRRSSYEDSSLPSINALLQAEVLSRQITSPVSGINGDIRAKKIASIADVCESMKEQLLVLVEWAKYIPAFCELPLDDQVALLRAHAGEHLLLGATKRSMVFKDVLLLGNDYIVPRHCPELAEMSRVSIRILDELVLPFQELQIDDNEYAYLKAIIFFDPDAKGLSDPGKIKRLRSQVQVSLEDYINDRQYDSRGRFGELLLLLPTLQSITWQMIEQIQFIKLFGMAKIDNLLQEMLLGGSPSDAPHAHHPLHPHLMQEHMGTNVIVANTMPTHLSNGQMCEWPRPRGQAATPETPQPSPPGGSGSEPYKLLPGAVATIVKPLSAIPQPTITKQEVI

>NP_006804.1

MTVVSVPQREPLVLGGRLAPLGFSSRGYFGALPMVTTAPPPLPRIPDPRALPPTLFLPHFLGGDGPCLTPQPRAPAALPNRSLAVAGGTPRAAPKKRRKKKVRASPAGQLPSRFHQYQQHRPSLEGGRSPATGPSGAQEVPGPAAALAPSPAAAAGTEGASPDLAPLRPAAPGQTPLRKEVLKSKMGKSEKIALPHGQLVHGIHLYEQPKINRQKSKYNLPLTKITSAKRNENNFWQDSVSSDRIQKQEKKPFKNTENIKNSHLKKSAFLTEVSQKENYAGAKFSDPPSPSVLPKPPSHWMGSTVENSNQNRELMAVHLKTLLKVQT

398 NP_115822.1 NP_003578.1

>NP_115822.1

MVISLNSCLSFICLLLCHWIGTASPLNLEDPNVCSHWESYSVTVQESYPHPFDQIYYTSCTDILNWFKCTRHRVSYRTAYRHGEKTMYRRKSQCCPGFYESGEMCVPHCADKCVHGRCIAPNTCQCEPGWGGTNCSSACDGDHWGPHCTSRCQCKNGALCNPITGACHCAAGFRGWRCEDRCEQGTYGNDCHQRCQCQNGATCDHVTGECRCPPGYTGAFCEDLCPPGKHGPQCEQRCPCQNGGVCHHVTGECSCPSGWMGTVCGQPCPEGRFGKNCSQECQCHNGGTCDAATGQCHCSPGYTGERCQDECPVGTYGVLCAETCQCVNGGKCYHVSGACLCEAGFAGERCEARLCPEGLYGIKCDKRCPCHLENTHSCHPMSGECACKPGWSGLYCNETCSPGFYGEACQQICSCQNGADCDSVTGKCTCAPGFKGIDCSTPCPLGTYGINCSSRCGCKNDAVCSPVDGSCTCKAGWHGVDCSIRCPSGTWGFGCNLTCQCLNGGACNTLDGTCTCAPGWRGEKCELPCQDGTYGLNCAERCDCSHADGCHPTTGHCRCLPGWSGVHCDSVCAEGRWGPNCSLPCYCKNGASCSPDDGICECAPGFRGTTCQRICSPGFYGHRCSQTCPQCVHSSGPCHHITGLCDCLPGFTGALCNEVCPSGRFGKNCAGICTCTNNGTCNPIDRSCQCYPGWIGSDCSQPCPPAHWGPNCIHTCNCHNGAFCSAYDGECKCTPGWTGLYCTQRCPLGFYGKDCALICQCQNGADCDHISGQCTCRTGFMGRHCEQKCPSGTYGYGCRQICDCLNNSTCDHITGTCYCSPGWKGARCDQAGVIIVGNLNSLSRTSTALPADSYQIGAIAGIIILVLVVLFLLALFIIYRHKQKGKESSMPAVTYTPAMRVVNADYTISGTLPHSNGGNANSHYFTNPSYHTLTQCATSPHVNNRDRMTVTKSKNNQLFVNLKNVNPGKRGPVGDCTGTLPADWKHGGYLNELGAFGLDRSYMGKSLKDLGKNSEYNSSNCSLSSSENPYATIKDPPVLIPKSSECGYVEMKSPARRDSPYAEINNSTSANRNVYEVEPTVSVVQGVFSNNGRLSQDPYDLPKNSHIPCHYDLLPVRDSSSSPKQEDSGGSSSNSSSSSE

>NP_003578.1

MATPAGLERWVQDELHSVLGLSERHVAQFLIGTAQRCTSAEEFVQRLRDTDTLDLSGPARDFALRLWNKVPRKAVVEKPARAAEREARALLEKNRSYRLLEDSEESSEETVSRAGSSLQKKRKKRKHLRKKREEEEEEEASEKGKKKTGGSKQQTEKPESEDEWERTERERLQDLEERDAFAERVRQRDKDRTRNVLERSDKKAYEEAQKRLKMAEEDRKAMIPELRKKSRREYLAKREREKLEDLEAELADEEFLFGDVELSRHERQELKYKRRVRDLAREYRAAGEQEKLEATNRYHMPKETRGQPARAVDLVEEESGAPGEEQRRWEEARLGAASLKFGARDAASQEPKYQLVLEEEETIEFVRATQLQGDEEPSAPPTSTQAQQKESIQAVRRSLPVFPFREELLAAIANHQVLIIEGETGSGKTTQIPQYLFEEGYTNKGMKIACTQPRRVAAMSVAARVAREMGVKLGNEVGYSIRFEDCTSERTVLRYMTDGMLLREFLSEPDLASYSVVMVDEAHERTLHTDILFGLIKDVARFRPELKVLVASATMDTARFSTFFDDAPVFRIPGRRFPVDIFYTKAPEADYLEACVVSVLQIHVTQPPGDILVFLTGQEEIEAACEMLQDPCRRLGSKIRELLVLPIYANLPSDMQARIFQPTPPGARKVVVATNIAETSPTIEGIIYVLDPGFCKQKSYNPRTGMESLTVTPCSKASANQRAGRAGRVAAGKCFRLYTAWAYQHELEETTVPEIQRTSLGNVVLLLKSLGIHDLMHFDFLDPPPYETLLLTLEQLYALGALNHLGELTTSGRKMAELPVDPMLSKMILASEKYSCSEEILTVAAMLSVNNSIFYRPKDKVVHADNARVNFFLPGGDHLVLLNVYTQWAESGYSSQWCCENFVQFRSMRRARDVREQLEGLLERVEVGLSSCQGDYIRVRKAITAGYFYHTARLTRSGYRTVKQQQTVFIHPNSSLFEQQPRWLLYHELVLTTKEFMRQVLEIESSWLLEVAPHYYKAKELEDPHAKKMPKKIGKTREELG

399 NP_003681.1 NP_006824.2

>NP_003681.1

MSQSRHRAEAPPLEREDSGTFSLGKMITAKPGKTPIQVLHEYGMKTKNIPVYECERSDVQIHVPTFTFRVTVGDITCTGEGTSKKLAKHRAAEAAINILKANASICFAVPDPLMPDPSKQPKNQLNPIGSLQELAIHHGWRLPEYTLSQEGGPAHKREYTTICRLESFMETGKGASKKQAKRNAAEKFLAKFSNISPENHISLTNVVGHSLGCTWHSLRNSPGEKINLLKRSLLSIPNTDYIQLLSEIAKEQGFNITYLDIDELSANGQYQCLAELSTSPITVCHGSGISCGNAQSDAAHNALQYLKIIAERK

>NP_006824.2

MAAAAAAAAATNGTGGSSGMEVDAAVVPSVMACGVTGSVSVALHPLVILNISDHWIRMRSQEGRPVQVIGALIGKQEGRNIEVMNSFELLSHTVEEKIIIDKEYYYTKEEQFKQVFKELEFLGWYTTGGPPDPSDIHVHKQVCEIIESPLFLKLNPMTKHTDLPVSVFESVIDIINGEATMLFAELTYTLATEEAERIGVDHVARMTATGSGENSTVAEHLIAQHSAIKMLHSRVKLILEYVKASEAGEVPFNHEILREAYALCHCLPVLSTDKFKTDFYDQCNDVGLMAYLGTITKTCNTMNQFVNKFNVLYDRQGIGRRMRGLFF

400 NP_003152.1 NP_115984.3

>NP_003152.1

MRRRRRRDGFYPAPDFRDREAEDMAGVFDIDLDQPEDAGSEDELEEGGQLNESMDHGGVGPYELGMEHCEKFEISETSVNRGPEKIRPECFELLRVLGKGGYGKVFQVRKVTGANTGKIFAMKVLKKAMIVRNAKDTAHTKAERNILEEVKHPFIVDLIYAFQTGGKLYLILEYLSGGELFMQLEREGIFMEDTACFYLAEISMALGHLHQKGIIYRDLKPENIMLNHQGHVKLTDFGLCKESIHDGTVTHTFCGTIEYMAPEILMRSGHNRAVDWWSLGALMYDMLTGAPPFTGENRKKTIDKILKCKLNLPPYLTQEARDLLKKLLKRNAASRLGAGPGDAGEVQAHPFFRHINWEELLARKVEPPFKPLLQSEEDVSQFDSKFTRQTPVDSPDDSTLSESANQVFLGFTYVAPSVLESVKEKFSFEPKIRSPRRFIGSPRTPVSPVKFSPGDFWGRGASASTANPQTPVEYPMETSGIEQMDVTMSGEASAPLPIRQPNSGPYKKQAFPMISKRPEHLRMNL

>NP_115984.3

MMKTEPRGPGGPLRSASPHRSAYEAGIQALKPPDAPGPDEAPKGAHHKKYGSNVHRIKSMFLQMGTTAGPSGEAGGGAGLAEAPRASERGVRLSLPRASSLNENVDHSALLKLGTSVSERVSRFDSKPAPSAQPAPPPHPPSRLQETRKLFERSAPAAAGGDKEAAARRLLRQERAGLQDRKLDVVVRFNGSTEALDKLDADAVSPTVSQLSAVFEKADSRTGLHRGPGLPRAAGVPQVNSKLVSKRSRVFQPPPPPPPAPSGDAPAEKERCPAGQQPPQHRVAPARPPPKPREVRKIKPVEVEESGESEAESAPGEVIQAEVTVHAALENGSTVATAASPAPEEPKAQAAPEKEAAAVAPPERGVGNGRAPDVAPEEVDESKKEDFSEADLVDVSAYSGLGEDSAGSALEEDDEDDEEDGEPPYEPESGCVEIPGLSEEEDPAPSRKIHFSTAPIQVFSTYSNEDYDRRNEDVDPMAASAEYELEKRVERLELFPVELEKDSEGLGISIIGMGAGADMGLEKLGIFVKTVTEGGAAHRDGRIQVNDLLVEVDGTSLVGVTQSFAASVLRNTKGRVRFMIGRERPGEQSEVAQLIQQTLEQERWQREMMEQRYAQYGEDDEETGEYATDEDEELSPTFPGGEMAIEVFELAENEDALSPVDMEPEKLVHKFKELQIKHAVTEAEIQQLKRKLQSLEQEKGRWRVEKAQLEQSVEENKERMEKLEGYWGEAQSLCQAVDEHLRETQAQYQALERKYSKAKRLIKDYQQKEIEFLKKETAQRRVLEESELARKEEMDKLLDKISELEGNLQTLRNSNST

401 NP_002383.2 NP_003327.2

>NP_002383.2

MVRSRQMCNTNMSVPTDGAVTTSQIPASEQETLVRPKPLLLKLLKSVGAQKDTYTMKEVLFYLGQYIMTKRLYDEKQQHIVYCSNDLLGDLFGVPSFSVKEHRKIYTMIYRNLVVVNQQESSDSGTSVSENRCHLEGGSDQKDLVQELQEEKPSSSHLVSRPSTSSRRRAISETEENSDELSGERQRKRHKSDSISLSFDESLALCVIREICCERSSSSESTGTPSNPDLDAGVSEHSGDWLDQDSVSDQFSVEFEVESLDSEDYSLSEEGQELSDEDDEVYQVTVYQAGESDTDSFEEDPEISLADYWKCTSCNEMNPPLPSHCNRCWALRENWLPEDKGKDKGEISEKAKLENSTQAEEGFDVPDCKKTIVNDSRESCVEENDDKITQASQSQESEDYSQPSTSSSIIYSSQEDVKEFEREETQDKEESVESSLPLNAIEPCVICQGRPKNGCIVHGKTGHLMACFTCAKKLKKRNKPCPVCRQPIQMIVLTYFP

>NP_003327.2

MSTPARRRLMRDFKRLQEDPPAGVSGAPSENNIMVWNAVIFGPEGTPFEDGTFKLTIEFTEEYPNKPPTVRFVSKMFHPNVYADGSICLDILQNRWSPTYDVSSILTSIQSLLDEPNPNSPANSQAAQLYQENKREYEKRVSAIVEQSWRDC

402 NP_005334.1 NP_055393.1

>NP_005334.1

MTEYKLVVVGAGGVGKSALTIQLIQNHFVDEYDPTIEDSYRKQVVIDGETCLLDILDTAGQEEYSAMRDQYMRTGEGFLCVFAINNTKSFEDIHQYREQIKRVKDSDDVPMVLVGNKCDLAARTVESRQAQDLARSYGIPYIETSAKTRQGVEDAFYTLVREIRQHKLRKLNPPDESGPGCMSCKCVLS

>NP_055393.1

MTAAQAAGEEAPPGVRSVKVVLVGDGGCGKTSLLMVFADGAFPESYTPTVFERYMVNLQVKGKPVHLHIWDTAGQDDYDRLRPLFYPDASVLLLCFDVTSPNSFDNIFNRWYPEVNHFCKKVPIIVVGCKTDLRKDKSLVNKLRRNGLEPVTYHRGQEMARSVGAVAYLECSARLHDNVHAVFQEAAEVALSSRGRNFWRRITQGFCVVT

403 NP_000724.1 NP_003168.2

>NP_000724.1

MQSGTHWRVLGLCLLSVGVWGQDGNEEMGGITQTPYKVSISGTTVILTCPQYPGSEILWQHNDKNIGGDEDDKNIGSDEDHLSLKEFSELEQSGYYVCYPRGSKPEDANFYLYLRARVCENCMEMDVMSVATIVIVDICITGGLLLLVYYWSKNRKAKAKPVTRGAGAGGRQRGQNKERPPPVPNPDYEPIRKGQRDLYSGLNQRRI

>NP_003168.2

MASSGMADSANHLPFFFGNITREEAEDYLVQGGMSDGLYLLRQSRNYLGGFALSVAHGRKAHHYTIERELNGTYAIAGGRTHASPADLCHYHSQESDGLVCLLKKPFNRPQGVQPKTGPFEDLKENLIREYVKQTWNLQGQALEQAIISQKPQLEKLIATTAHEKMPWFHGKISREESEQIVLIGSKTNGKFLIRARDNNGSYALCLLHEGKVLHYRIDKDKTGKLSIPEGKKFDTLWQLVEHYSYKADGLLRVLTVPCQKIGTQGNVNFGGRPQLPGSHPATWSAGGIISRIKSYSFPKPGHRKSSPAQGNRQESTVSFNPYEPELAPWAADKGPQREALPMDTEVYESPYADPEEIRPKEVYLDRKLLTLEDKELGSGNFGTVKKGYYQMKKVVKTVAVKILKNEANDPALKDELLAEANVMQQLDNPYIVRMIGICEAESWMLVMEMAELGPLNKYLQQNRHVKDKNIIELVHQVSMGMKYLEESNFVHRDLAARNVLLVTQHYAKISDFGLSKALRADENYYKAQTHGKWPVKWYAPECINYYKFSSKSDVWSFGVLMWEAFSYGQKPYRGMKGSEVTAMLEKGERMGCPAGCPREMYDLMNLCWTYDVENRPGFAAVELRLRNYYYDVVN

404 NP_003940.3 NP_115705.2

>NP_003940.3

MRPKRLGRCCAGSRLGPGDPAALTCAPSPSASPAPEPSAQPQARGTGQRVGSRATSGSQFLSEARTGARPASEAGAKAGARRPSAFSAIQGDVRSMPDNSDAPWTRFVFQGPFGSRATGRGTGKAAGIWKTPAAYVGRRPGVSGPERAAFIRELEEALCPNLPPPVKKITQEDVKVMLYLLEELLPPVWESVTYGMVLQRERDLNTAARIGQSLVKQNSVLMEENSKLEALLGSAKEEILYLRHQVNLRDELLQLYSDSDEEDEDEEEEEEEKEAEEEQEEEEAEEDLQCAHPCDAPKLISQEALLHQHHCPQLEALQEKLRLLEEENHQLREEASQLDTLEDEEQMLILECVEQFSEASQQMAELSEVLVLRLENYERQQQEVARLQAQVLKLQQRCRMYGAETEKLQKQLASEKEIQMQLQEESVWVGSQLQDLREKYMDCGGMLIEMQEEVKTLRQQPPVSTGSATHYPYSVPLETLPGFQETLAEELRTSLRRMISDPVYFMERNYEMPRGDTSSLRYDFRYSEDREQVRGFEAEEGLMLAADIMRGEDFTPAEEFVPQEELGAAKKVPAEEGVMEEAELVSEETEGWEEVELELDEATRMNVVTSALEASGLGPSHLDMNYVLQQLANWQDAHYRRQLRWKMLQKGECPHGALPAASRTSCRSSCR

>NP_115705.2

MATAMYLEHYLDSIENLPCELQRNFQLMRELDQRTEDKKAEIDILAAEYISTVKTLSPDQRVERLQKIQNAYSKCKEYSDDKVQLAMQTYEMVDKHIRRLDADLARFEADLKDKMEGSDFESSGGRGLKKGRGQKEKRGSRGRGRRTSEEDTPKKKKHKGGSEFTDTILSVHPSDVLDMPVDPNEPTYCLCHQVSYGEMIGCDNPDCPIEWFHFACVDLTTKPKGKWFCPRCVQEKRKKK

405 NP_003740.2 NP_002825.3

>NP_003740.2

MASPPRHGPPGPASGDGPNLNNNNNNNNHSVRKCGYLRKQKHGHKRFFVLRGPGAGGDEATAGGGSAPQPPRLEYYESEKKWRSKAGAPKRVIALDCCLNINKRADAKHKYLIALYTKDEYFAVAAENEQEQEGWYRALTDLVSEGRAAAGDAPPAAAPAASCSASLPGALGGSAGAAGAEDSYGLVAPATAAYREVWQVNLKPKGLGQSKNLTGVYRLCLSARTIGFVKLNCEQPSVTLQLMNIRRCGHSDSFFFIEVGRSAVTGPGELWMQADDSVVAQNIHETILEAMKALKELFEFRPRSKSQSSGSSATHPISVPGARRHHHLVNLPPSQTGLVRRSRTDSLAATPPAAKCSSCRVRTASEGDGGAAAGAAAAGARPVSVAGSPLSPGPVRAPLSRSHTLSGGCGGRGSKVALLPAGGALQHSRSMSMPVAHSPPAATSPGSLSSSSGHGSGSYPPPPGPHPPLPHPLHHGPGQRPSSGSASASGSPSDPGFMSLDEYGSSPGDLRAFCSHRSNTPESIAETPPARDGGGGGEFYGYMTMDRPLSHCGRSYRRVSGDAAQDLDRGLRKRTYSLTTPARQRPVPQPSSASLDEYTLMRATFSGSAGRLCPSCPASSPKVAYHPYPEDYGDIEIGSHRSSSSNLGADDGYMPMTPGAALAGSGSGSCRSDDYMPMSPASVSAPKQILQPRAAAAAAAAVPSAGPAGPAPTSAAGRTFPASGGGYKASSPAESSPEDSGYMRMWCGSKLSMEHADGKLLPNGDYLNVSPSDAVTTGTPPDFFSAALHPGGEPLRGVPGCCYSSLPRSYKAPYTCGGDSDQYVLMSSPVGRILEEERLEPQATPGPSQAASAFGAGPTQPPHPVVPSPVRPSGGRPEGFLGQRGRAVRPTRLSLEGLPSLPSMHEYPLPPEPKSPGEYINIDFGEPGARLSPPAPPLLASAASSSSLLSASSPASSLGSGTPGTSSDSRQRSPLSDYMNLDFSSPKSPKPGAPSGHPVGSLDGLLSPEASSPYPPLPPRPSASPSSSLQPPPPPPAPGELYRLPPASAVATAQGPGAASSLSSDTGDNGDYTEMAFGVAATPPQPIAAPPKPEAARVASPTSGVKRLSLMEQVSGVEAFLQASQPPDPHRGAKVIRADPQGGRRRHSSETFSSTTTVTPVSPSFAHNPKRHNSASVENVSLRKSSEGGVGVGPGGGDEPPTSPRQLQPAPPLAPQGRPWTPGQPGGLVGCPGSGGSPMRRETSAGFQNGLNYIAIDVREEPGLPPQPQPPPPPLPQPGDKSSWGRTRSLGGLISAVGVGSTGGGCGGPGPGALPPANTYASIDFLSHHLKEATIVKE

>NP_002825.3

MTSRRWFHPNITGVEAENLLLTRGVDGSFLARPSKSNPGDFTLSVRRNGAVTHIKIQNTGDYYDLYGGEKFATLAELVQYYMEHHGQLKEKNGDVIELKYPLNCADPTSERWFHGHLSGKEAEKLLTEKGKHGSFLVRESQSHPGDFVLSVRTGDDKGESNDGKSKVTHVMIRCQELKYDVGGGERFDSLTDLVEHYKKNPMVETLGTVLQLKQPLNTTRINAAEIESRVRELSKLAETTDKVKQGFWEEFETLQQQECKLLYSRKEGQRQENKNKNRYKNILPFDHTRVVLHDGDPNEPVSDYINANIIMPEFETKCNNSKPKKSYIATQGCLQNTVNDFWRMVFQENSRVIVMTTKEVERGKSKCVKYWPDEYALKEYGVMRVRNVKESAAHDYTLRELKLSKVGQGNTERTVWQYHFRTWPDHGVPSDPGGVLDFLEEVHHKQESIMDAGPVVVHCSAGIGRTGTFIVIDILIDIIREKGVDCDIDVPKTIQMVRSQRSGMVQTEAQYRFIYMAVQHYIETLQRRIEEEQKSKRKGHEYTNIKYSLADQTSGDQSPLPPCTPTPPCAEMREDSARVYENVGLMQQQKSFR

406 NP_116170.3 NP_002028.1

>NP_116170.3

MNYEGARSERENHAADDSEGGALDMCCSERLPGLPQPIVMEALDEAEGLQDSQREMPPPPPPSPPSDPAQKPPPRGAGSHSLTVRSSLCLFAASQFLLACGVLWFSGYGHIWSQNATNLVSSLLTLLKQLEPTAWLDSGTWGVPSLLLVFLSVGLVLVTTLVWHLLRTPPEPPTPLPPEDRRQSVSRQPSFTYSEWMEEKIEDDFLDLDPVPETPVFDCVMDIKPEADPTSLTVKSMGLQERRGSNVSLTLDMCTPGCNEEGFGYLMSPREESAREYLLSASRVLQAEELHEKALDPFLLQAEFFEIPMNFVDPKEYDIPGLVRKNRYKTILPNPHSRVCLTSPDPDDPLSSYINANYIRGYGGEEKVYIATQGPIVSTVADFWRMVWQEHTPIIVMITNIEEMNEKCTEYWPEEQVAYDGVEITVQKVIHTEDYRLRLISLKSGTEERGLKHYWFTSWPDQKTPDRAPPLLHLVREVEEAAQQEGPHCAPIIVHCSAGIGRTGCFIATSICCQQLRQEGVVDILKTTCQLRQDRGGMIQTCEQYQFVHHVMSLYEKQLSHQSPE

>NP_002028.1

MGCVQCKDKEATKLTEERDGSLNQSSGYRYGTDPTPQHYPSFGVTSIPNYNNFHAAGGQGLTVFGGVNSSSHTGTLRTRGGTGVTLFVALYDYEARTEDDLSFHKGEKFQILNSSEGDWWEARSLTTGETGYIPSNYVAPVDSIQAEEWYFGKLGRKDAERQLLSFGNPRGTFLIRESETTKGAYSLSIRDWDDMKGDHVKHYKIRKLDNGGYYITTRAQFETLQQLVQHYSERAAGLCCRLVVPCHKGMPRLTDLSVKTKDVWEIPRESLQLIKRLGNGQFGEVWMGTWNGNTKVAIKTLKPGTMSPESFLEEAQIMKKLKHDKLVQLYAVVSEEPIYIVTEYMNKGSLLDFLKDGEGRALKLPNLVDMAAQVAAGMAYIERMNYIHRDLRSANILVGNGLICKIADFGLARLIEDNEYTARQGAKFPIKWTAPEAALYGRFTIKSDVWSFGILLTELVTKGRVPYPGMNNREVLEQVERGYRMPCPQDCPISLHELMIHCWKKDPEERPTFEYLQSFLEDYFTATEPQYQPGENL

407 NP_000409.1 NP_006312.1

>NP_000409.1

MGWLCSGLLFPVSCLVLLQVASSGNMKVLQEPTCVSDYMSISTCEWKMNGPTNCSTELRLLYQLVFLLSEAHTCIPENNGGAGCVCHLLMDDVVSADNYTLDLWAGQQLLWKGSFKPSEHVKPRAPGNLTVHTNVSDTLLLTWSNPYPPDNYLYNHLTYAVNIWSENDPADFRIYNVTYLEPSLRIAASTLKSGISYRARVRAWAQCYNTTWSEWSPSTKWHNSYREPFEQHLLLGVSVSCIVILAVCLLCYVSITKIKKEWWDQIPNPARSRLVAIIIQDAQGSQWEKRSRGQEPAKCPHWKNCLTKLLPCFLEHNMKRDEDPHKAAKEMPFQGSGKSAWCPVEISKTVLWPESISVVRCVELFEAPVECEEEEEVEEEKGSFCASPESSRDDFQEGREGIVARLTESLFLDLLGEENGGFCQQDMGESCLLPPSGSTSAHMPWDEFPSAGPKEAPPWGKEQPLHLEPSPPASPTQSPDNLTCTETPLVIAGNPAYRSFSNSLSQSPCPRELGPDPLLARHLEEVEPEMPCVPQLSEPTTVPQPEPETWEQILRRNVLQHGAAAAPVSAPTSGYQEFVHAVEQGGTQASAVVGLGPPGEAGYKAFSSLLASSAVSPEKCGFGASSGEEGYKPFQDLIPGCPGDPAPVPVPLFTFGLDREPPRSPQSSHLPSSSPEHLGLEPGEKVEDMPKPPLPQEQATDPLVDSLGSGIVYSALTCHLCGHLKQCHGQEDGGQTPVMASPCCGCCCGDRSSPPTTPLRAPDPSPGGVPLEASLCPASLAPSGISEKSKSSSSFHPAPGNAQSSSQTPKIVNFVSVGPTYMRVS

>NP_006312.1

MSVDMNSQGSDSNEEDYDPNCEEEEEEEEDDPGDIEDYYVGVASDVEQQGADAFDPEEYQFTCLTYKESEGALNEHMTSLASVLKVSHSVAKLILVNFHWQVSEILDRYKSNSAQLLVEARVQPNPSKHVPTSHPPHHCAVCMQFVRKENLLSLACQHQFCRSCWEQHCSVLVKDGVGVGVSCMAQDCPLRTPEDFVFPLLPNEELREKYRRYLFRDYVESHYQLQLCPGADCPMVIRVQEPRARRVQCNRCNEVFCFKCRQMYHAPTDCATIRKWLTKCADDSETANYISAHTKDCPKCNICIEKNGGCNHMQCSKCKHDFCWMCLGDWKTHGSEYYECSRYKENPDIVNQSQQAQAREALKKYLFYFERWENHNKSLQLEAQTYQRIHEKIQERVMNNLGTWIDWQYLQNAAKLLAKCRYTLQYTYPYAYYMESGPRKKLFEYQQAQLEAEIENLSWKVERADSYDRGDLENQMHIAEQRRRTLLKDFHDT

408 NP_001410.2 NP_003002.1

>NP_001410.2

MSNGYEDHMAEDCRGDIGRTNLIVNYLPQNMTQDELRSLFSSIGEVESAKLIRDKVAGHSLGYGFVNYVTAKDAERAINTLNGLRLQSKTIKVSYARPSSEVIKDANLYISGLPRTMTQKDVEDMFSRFGRIINSRVLVDQTTGLSRGVAFIRFDKRSEAEEAITSFNGHKPPGSSEPITVKFAANPNQNKNVALLSQLYHSPARRFGGPVHHQAQRFRFSPMGVDHMSGLSGVNVPGNASSGWCIFIYNLGQDADEGILWQMFGPFGAVTNVKVIRDFNTNKCKGFGFVTMTNYEEAAMAIASLNGYRLGDKILQVSFKTNKSHK

>NP_003002.1

MSAQAAKVSKKELNSNHDGADETSEKEQQEAIEHIDEVQNEIDRLNEQASEEILKVEQKYNKLRQPFFQKRSELIAKIPNFWVTTFVNHPQVSALLGEEDEEALHYLTRVEVTEFEDIKSGYRIDFYFDENPYFENKVLSKEFHLNESGDPSSKSTEIKWKSGKDLTKRSSQTQNKASRKRQHEEPESFFTWFTDHSDAGADELGEVIKDDIWPNPLQYYLVPDMDDEEGEGEEDDDDDEEEEGLEDIDEEGDEDEGEEDEDDDEGEEGEEDEGEDD

409 NP_004880.1 NP_001009999.1

>NP_004880.1

MASVGECPAPVPVKDKKLLEVKLGELPSWILMRDFSPSGIFGAFQRGYYRYYNKYINVKKGSISGITMVLACYVLFSYSFSYKHLKHERLRKYH

>NP_001009999.1

MLSGKKAAAAAAAAAAAATGTEAGPGTAGGSENGSEVAAQPAGLSGPAEVGPGAVGERTPRKKEPPRASPPGGLAEPPGSAGPQAGPTVVPGSATPMETGIAETPEGRRTSRRKRAKVEYREMDESLANLSEDEYYSEEERNAKAEKEKKLPPPPPQAPPEEENESEPEEPSGQAGGLQDDSSGGYGDGQASGVEGAAFQSRLPHDRMTSQEAACFPDIISGPQQTQKVFLFIRNRTLQLWLDNPKIQLTFEATLQQLEAPYNSDTVLVHRVHSYLERHGLINFGIYKRIKPLPTKKTGKVIIIGSGVSGLAAARQLQSFGMDVTLLEARDRVGGRVATFRKGNYVADLGAMVVTGLGGNPMAVVSKQVNMELAKIKQKCPLYEANGQADTVKVPKEKDEMVEQEFNRLLEATSYLSHQLDFNVLNNKPVSLGQALEVVIQLQEKHVKDEQIEHWKKIVKTQEELKELLNKMVNLKEKIKELHQQYKEASEVKPPRDITAEFLVKSKHRDLTALCKEYDELAETQGKLEEKLQELEANPPSDVYLSSRDRQILDWHFANLEFANATPLSTLSLKHWDQDDDFEFTGSHLTVRNGYSCVPVALAEGLDIKLNTAVRQVRYTASGCEVIAVNTRSTSQTFIYKCDAVLCTLPLGVLKQQPPAVQFVPPLPEWKTSAVQRMGFGNLNKVVLCFDRVFWDPSVNLFGHVGSTTASRGELFLFWNLYKAPILLALVAGEAAGIMENISDDVIVGRCLAILKGIFGSSAVPQPKETVVSRWRADPWARGSYSYVAAGSSGNDYDLMAQPITPGPSIPGAPQPIPRLFFAGEHTIRNYPATVHGALLSGLREAGRIADQFLGAMYTLPRQATPGVPAQQSPSM

410 NP_003946.3 NP_005202.2

>NP_003946.3

MVTHSKFPAAGMSRPLDTSLRLKTFSSKSEYQLVVNAVRKLQESGFYWSAVTGGEANLLLSAEPAGTFLIRDSSDQRHFFTLSVKTQSGTKNLRIQCEGGSFSLQSDPRSTQPVPRFDCVLKLVHHYMPPPGAPSFPSPPTEPSSEVPEQPSAQPLPGSPPRRAYYIYSGGEKIPLVLSRPLSSNVATLQHLCRKTVNGHLDSYEKVTQLPGPIREFLDQYDAPL

>NP_005202.2

MGPGVLLLLLVATAWHGQGIPVIEPSVPELVVKPGATVTLRCVGNGSVEWDGPPSPHWTLYSDGSSSILSTNNATFQNTGTYRCTEPGDPLGGSAAIHLYVKDPARPWNVLAQEVVVFEDQDALLPCLLTDPVLEAGVSLVRVRGRPLMRHTNYSFSPWHGFTIHRAKFIQSQDYQCSALMGGRKVMSISIRLKVQKVIPGPPALTLVPAELVRIRGEAAQIVCSASSVDVNFDVFLQHNNTKLAIPQQSDFHNNRYQKVLTLNLDQVDFQHAGNYSCVASNVQGKHSTSMFFRVVESAYLNLSSEQNLIQEVTVGEGLNLKVMVEAYPGLQGFNWTYLGPFSDHQPEPKLANATTKDTYRHTFTLSLPRLKPSEAGRYSFLARNPGGWRALTFELTLRYPPEVSVIWTFINGSGTLLCAASGYPQPNVTWLQCSGHTDRCDEAQVLQVWDDPYPEVLSQEPFHKVTVQSLLTVETLEHNQTYECRAHNSVGSGSWAFIPISAGAHTHPPDEFLFTPVVVACMSIMALLLLLLLLLLYKYKQKPKYQVRWKIIESYEGNSYTFIDPTQLPYNEKWEFPRNNLQFGKTLGAGAFGKVVEATAFGLGKEDAVLKVAVKMLKSTAHADEKEALMSELKIMSHLGQHENIVNLLGACTHGGPVLVITEYCCYGDLLNFLRRKAEAMLGPSLSPGQDPEGGVDYKNIHLEKKYVRRDSGFSSQGVDTYVEMRPVSTSSNDSFSEQDLDKEDGRPLELRDLLHFSSQVAQGMAFLASKNCIHRDVAARNVLLTNGHVAKIGDFGLARDIMNDSNYIVKGNARLPVKWMAPESIFDCVYTVQSDVWSYGILLWEIFSLGLNPYPGILVNSKFYKLVKDGYQMAQPAFAPKNIYSIMQACWALEPTHRPTFQQICSFLQEQAQEDRRERDYTNLPSSSRSGGSGSSSSELEEESSSEHLTCCEQGDIAQPLLQPNNYQFC

411 NP_002560.1 NP_001013049.1

>NP_002560.1

MELRPWLLWVVAATGTLVLLAADAQGQKVFTNTWAVRIPGGPAVANSVARKHGFLNLGQIFGDYYHFWHRGVTKRSLSPHRPRHSRLQREPQVQWLEQQVAKRRTKRDVYQEPTDPKFPQQWYLSGVTQRDLNVKAAWAQGYTGHGIVVSILDDGIEKNHPDLAGNYDPGASFDVNDQDPDPQPRYTQMNDNRHGTRCAGEVAAVANNGVCGVGVAYNARIGGVRMLDGEVTDAVEARSLGLNPNHIHIYSASWGPEDDGKTVDGPARLAEEAFFRGVSQGRGGLGSIFVWASGNGGREHDSCNCDGYTNSIYTLSISSATQFGNVPWYSEACSSTLATTYSSGNQNEKQIVTTDLRQKCTESHTGTSASAPLAAGIIALTLEANKNLTWRDMQHLVVQTSKPAHLNANDWATNGVGRKVSHSYGYGLLDAGAMVALAQNWTTVAPQRKCIIDILTEPKDIGKRLEVRKTVTACLGEPNHITRLEHAQARLTLSYNRRGDLAIHLVSPMGTRSTLLAARPHDYSADGFNDWAFMTTHSWDEDPSGEWVLEIENTSEANNYGTLTKFTLVLYGTAPEGLPVPPESSGCKTLTSSQACVVCEEGFSLHQKSCVQHCPPGFAPQVLDTHYSTENDVETIRASVCAPCHASCATCQGPALTDCLSCPSHASLDPVEQTCSRQSQSSRESPPQQQPPRLPPEVEAGQRLRAGLLPSHLPEVVAGLSCAFIVLVFVTVFLVLQLRSGFSFRGVKVYTMDRGLISYKGLPPEAWQEECPSDSEEDEGRGERTAFIKDQSAL

>NP_001013049.1

MGKVGAGGGSQARLSALLAGAGLLILCAPGVCGGGSCCPSPHPSSAPRSASTPRGFSHQGRPGRAPATPLPLVVRPLFSVAPGDRALSLERARGTGASMAVAARSGRRRRSGADQEKAERGEGASRSPRGVLRDGGQQEPGTRERDPDKATRFRMEELRLTSTTFALTGDSAHNQAMVHWSGHNSSVILILTKLYDYNLGSITESSLWRSTDYGTTYEKLNDKVGLKTILSYLYVCPTNKRKIMLLTDPEIESSLLISSDEGATYQKYRLNFYIQSLLFHPKQEDWILAYSQDQKLYSSAEFGRRWQLIQEGVVPNRFYWSVMGSNKEPDLVHLEARTVDGHSHYLTCRMQNCTEANRNQPFPGYIDPDSLIVQDHYVFVQLTSGGRPHYYVSYRRNAFAQMKLPKYALPKDMHVISTDENQVFAAVQEWNQNDTYNLYISDTRGVYFTLALENVQSSRGPEGNIMIDLYEVAGIKGMFLANKKIDNQVKTFITYNKGRDWRLLQAPDTDLRGDPVHCLLPYCSLHLHLKVSENPYTSGIIASKDTAPSIIVASGNIGSELSDTDISMFVSSDAGNTWRQIFEEEHSVLYLDQGGVLVAMKHTSLPIRHLWLSFDEGRSWSKYSFTSIPLFVDGVLGEPGEETLIMTVFGHFSHRSEWQLVKVDYKSIFDRRCAEEDYRPWQLHSQGEACIMGAKRIYKKRKSERKCMQGKYAGAMESEPCVCTEADFDCDYGYERHSNGQCLPAFWFNPSSLSKDCSLGQSYLNSTGYRKVVSNNCTDGVREQYTAKPQKCPGKAPRGLRIVTADGKLTAEQGHNVTLMVQLEEGDVQRTLIQVDFGDGIAVSYVNLSSMEDGIKHVYQNVGIFRVTVQVDNSLGSDSAVLYLHVTCPLEHVHLSLPFVTTKNKEVNATAVLWPSQVGTLTYVWWYGNNTEPLITLEGSISFRFTSEGMNTITVQVSAGNAILQDTKTIAVYEEFRSLRLSFSPNLDDYNPDIPEWRRDIGRVIKKSLVEATGVPGQHILVAVLPGLPTTAELFVLPYQDPAGENKRSTDDLEQISELLIHTLNQNSVHFELKPGVRVLVHAAHLTAAPLVDLTPTHSGSAMLMLLSVVFVGLAVFVIYKFKRKIPGINVYAQMQNEKEQEMISPVSHSESRPNVPQTELRRPGQLIDEKVESQLIGSISIVAENQSTKEIPTYVNV

412 NP_005094.1 NP_000456.1

>NP_005094.1

MEAPLVSLDEEFEDLRPSCSEDPEEKPQCFYGSSPHHLEDPSLSELENFSSEIISFKSMEDLVNEFDEKLNVCFRNYNAKTENLAPVKNQLQIQEEEETLQDEEVWDALTDNYIPSLSEDWRDPNIEALNGNCSDTEIHEKEEEEFNEKSENDSGINEEPLLTADQVIEEIEEMMQNSPDPEEEEEVLEEEDGGETSSQADSVLLQEMQALTQTFNNNWSYEGLRHMSGSELTELLDQVEGAIRDFSEELVQQLARRDELEFEKEVKNSFITVLIEVQNKQKEQRELMKKRRKEKGLSLQSSRIEKGNQMPLKRFSMEGISNILQSGIRQTFGSSGTDKQYLNTVIPYEKKASPPSVEDLQMLTNILFAMKEDNEKVPTLLTDYILKVLCPT

>NP_000456.1

MPDNRQPRNRQPRIRSGNEPRSAPAMEPDGRGAWAHSRAALDRLEKLLRCSRCTNILREPVCLGGCEHIFCSNCVSDCIGTGCPVCYTPAWIQDLKINRQLDSMIQLCSKLRNLLHDNELSDLKEDKPRKSLFNDAGNKKNSIKMWFSPRSKKVRYVVSKASVQTQPAIKKDASAQQDSYEFVSPSPPADVSERAKKASARSGKKQKKKTLAEINQKWNLEAEKEDGEFDSKEESKQKLVSFCSQPSVISSPQINGEIDLLASGSLTESECFGSLTEVSLPLAEQIESPDTKSRNEVVTPEKVCKNYLTSKKSLPLENNGKRGHHNRLSSPISKRCRTSILSTSGDFVKQTVPSENIPLPECSSPPSCKRKVGGTSGRKNSNMSDEFISLSPGTPPSTLSSSSYRQVMSSPSAMKLLPNMAVKRNHRGETLLHIASIKGDIPSVEYLLQNGSDPNVKDHAGWTPLHEACNHGHLKVVELLLQHKALVNTTGYQNDSPLHDAAKNGHVDIVKLLLSYGASRNAVNIFGLRPVDYTDDESMKSLLLLPEKNESSSASHCSVMNTGQRRDGPLVLIGSGLSSEQQKMLSELAVILKAKKYTEFDSTVTHVVVPGDAVQSTLKCMLGILNGCWILKFEWVKACLRRKVCEQEEKYEIPEGPRRSRLNREQLLPKLFDGCYFYLWGTFKHHPKDNLIKLVTAGGGQILSRKPKPDSDVTQTINTVAYHARPDSDQRFCTQYIIYEDLCNYHPERVRQGKVWKAPSSWFIDCVMSFELLPLDS

413 NP_004834.1 NP_009330.1

>NP_004834.1

MRGGRGAPFWLWPLPKLALLPLLWVLFQRTRPQGSAGPLQCYGVGPLGDLNCSWEPLGDLGAPSELHLQSQKYRSNKTQTVAVAAGRSWVAIPREQLTMSDKLLVWGTKAGQPLWPPVFVNLETQMKPNAPRLGPDVDFSEDDPLEATVHWAPPTWPSHKVLICQFHYRRCQEAAWTLLEPELKTIPLTPVEIQDLELATGYKVYGRCRMEKEEDLWGEWSPILSFQTPPSAPKDVWVSGNLCGTPGGEEPLLLWKAPGPCVQVSYKVWFWVGGRELSPEGITCCCSLIPSGAEWARVSAVNATSWEPLTNLSLVCLDSASAPRSVAVSSIAGSTELLVTWQPGPGEPLEHVVDWARDGDPLEKLNWVRLPPGNLSALLPGNFTVGVPYRITVTAVSASGLASASSVWGFREELAPLVGPTLWRLQDAPPGTPAIAWGEVPRHQLRGHLTHYTLCAQSGTSPSVCMNVSGNTQSVTLPDLPWGPCELWVTASTIAGQGPPGPILRLHLPDNTLRWKVLPGILFLWGLFLLGCGLSLATSGRCYHLRHKVLPRWVWEKVPDPANSSSGQPHMEQVPEAQPLGDLPILEVEEMEPPPVMESSQPAQATAPLDSGYEKHFLPTPEELGLLGPPRPQVLA

>NP_009330.1

MSQWYELQQLDSKFLEQVHQLYDDSFPMEIRQYLAQWLEKQDWEHAANDVSFATIRFHDLLSQLDDQYSRFSLENNFLLQHNIRKSKRNLQDNFQEDPIQMSMIIYSCLKEERKILENAQRFNQAQSGNIQSTVMLDKQKELDSKVRNVKDKVMCIEHEIKSLEDLQDEYDFKCKTLQNREHETNGVAKSDQKQEQLLLKKMYLMLDNKRKEVVHKIIELLNVTELTQNALINDELVEWKRRQQSACIGGPPNACLDQLQNWFTIVAESLQQVRQQLKKLEELEQKYTYEHDPITKNKQVLWDRTFSLFQQLIQSSFVVERQPCMPTHPQRPLVLKTGVQFTVKLRLLVKLQELNYNLKVKVLFDKDVNERNTVKGFRKFNILGTHTKVMNMEESTNGSLAAEFRHLQLKEQKNAGTRTNEGPLIVTEELHSLSFETQLCQPGLVIDLETTSLPVVVISNVSQLPSGWASILWYNMLVAEPRNLSFFLTPPCARWAQLSEVLSWQFSSVTKRGLNVDQLNMLGEKLLGPNASPDGLIPWTRFCKENINDKNFPFWLWIESILELIKKHLLPLWNDGCIMGFISKERERALLKDQQPGTFLLRFSESSREGAITFTWVERSQNGGEPDFHAVEPYTKKELSAVTFPDIIRNYKVMAAENIPENPLKYLYPNIDKDHAFGKYYSRPKEAPEPMELDGPKGTGYIKTELISVSEVHPSRLQTTDNLLPMSPEEFDEVSRIVGSVEFDSMMNTV

414 NP_000042.3 NP_002384.2

>NP_000042.3

MSLVLNDLLICCRQLEHDRATERKKEVEKFKRLIRDPETIKHLDRHSDSKQGKYLNWDAVFRFLQKYIQKETECLRIAKPNVSASTQASRQKKMQEISSLVKYFIKCANRRAPRLKCQELLNYIMDTVKDSSNGAIYGADCSNILLKDILSVRKYWCEISQQQWLELFSVYFRLYLKPSQDVHRVLVARIIHAVTKGCCSQTDGLNSKFLDFFSKAIQCARQEKSSSGLNHILAALTIFLKTLAVNFRIRVCELGDEILPTLLYIWTQHRLNDSLKEVIIELFQLQIYIHHPKGAKTQEKGAYESTKWRSILYNLYDLLVNEISHIGSRGKYSSGFRNIAVKENLIELMADICHQVFNEDTRSLEISQSYTTTQRESSDYSVPCKRKKIELGWEVIKDHLQKSQNDFDLVPWLQIATQLISKYPASLPNCELSPLLMILSQLLPQQRHGERTPYVLRCLTEVALCQDKRSNLESSQKSDLLKLWNKIWCITFRGISSEQIQAENFGLLGAIIQGSLVEVDREFWKLFTGSACRPSCPAVCCLTLALTTSIVPGTVKMGIEQNMCEVNRSFSLKESIMKWLLFYQLEGDLENSTEVPPILHSNFPHLVLEKILVSLTMKNCKAAMNFFQSVPECEHHQKDKEELSFSEVEELFLQTTFDKMDFLTIVRECGIEKHQSSIGFSVHQNLKESLDRCLLGLSEQLLNNYSSEITNSETLVRCSRLLVGVLGCYCYMGVIAEEEAYKSELFQKAKSLMQCAGESITLFKNKTNEEFRIGSLRNMMQLCTRCLSNCTKKSPNKIASGFFLRLLTSKLMNDIADICKSLASFIKKPFDRGEVESMEDDTNGNLMEVEDQSSMNLFNDYPDSSVSDANEPGESQSTIGAINPLAEEYLSKQDLLFLDMLKFLCLCVTTAQTNTVSFRAADIRRKLLMLIDSSTLEPTKSLHLHMYLMLLKELPGEEYPLPMEDVLELLKPLSNVCSLYRRDQDVCKTILNHVLHVVKNLGQSNMDSENTRDAQGQFLTVIGAFWHLTKERKYIFSVRMALVNCLKTLLEADPYSKWAILNVMGKDFPVNEVFTQFLADNHHQVRMLAAESINRLFQDTKGDSSRLLKALPLKLQQTAFENAYLKAQEGMREMSHSAENPETLDEIYNRKSVLLTLIAVVLSCSPICEKQALFALCKSVKENGLEPHLVKKVLEKVSETFGYRRLEDFMASHLDYLVLEWLNLQDTEYNLSSFPFILLNYTNIEDFYRSCYKVLIPHLVIRSHFDEVKSIANQIQEDWKSLLTDCFPKILVNILPYFAYEGTRDSGMAQQRETATKVYDMLKSENLLGKQIDHLFISNLPEIVVELLMTLHEPANSSASQSTDLCDFSGDLDPAPNPPHFPSHVIKATFAYISNCHKTKLKSILEILSKSPDSYQKILLAICEQAAETNNVYKKHRILKIYHLFVSLLLKDIKSGLGGAWAFVLRDVIYTLIHYINQRPSCIMDVSLRSFSLCCDLLSQVCQTAVTYCKDALENHLHVIVGTLIPLVYEQVEVQKQVLDLLKYLVIDNKDNENLYITIKLLDPFPDHVVFKDLRITQQKIKYSRGPFSLLEEINHFLSVSVYDALPLTRLEGLKDLRRQLELHKDQMVDIMRASQDNPQDGIMVKLVVNLLQLSKMAINHTGEKEVLEAVGSCLGEVGPIDFSTIAIQHSKDASYTKALKLFEDKELQWTFIMLTYLNNTLVEDCVKVRSAAVTCLKNILATKTGHSFWEIYKMTTDPMLAYLQPFRTSRKKFLEVPRFDKENPFEGLDDINLWIPLSENHDIWIKTLTCAFLDSGGTKCEILQLLKPMCEVKTDFCQTVLPYLIHDILLQDTNESWRNLLSTHVQGFFTSCLRHFSQTSRSTTPANLDSESEHFFRCCLDKKSQRTMLAVVDYMRRQKRPSSGTIFNDAFWLDLNYLEVAKVAQSCAAHFTALLYAEIYADKKSMDDQEKRSLAFEEGSQSTTISSLSEKSKEETGISLQDLLLEIYRSIGEPDSLYGCGGGKMLQPITRLRTYEHEAMWGKALVTYDLETAIPSSTRQAGIIQALQNLGLCHILSVYLKGLDYENKDWCPELEELHYQAAWRNMQWDHCTSVSKEVEGTSYHESLYNALQSLRDREFSTFYESLKYARVKEVEEMCKRSLESVYSLYPTLSRLQAIGELESIGELFSRSVTHRQLSEVYIKWQKHSQLLKDSDFSFQEPIMALRTVILEILMEKEMDNSQRECIKDILTKHLVELSILARTFKNTQLPERAIFQIKQYNSVSCGVSEWQLEEAQVFWAKKEQSLALSILKQMIKKLDASCAANNPSLKLTYTECLRVCGNWLAETCLENPAVIMQTYLEKAVEVAGNYDGESSDELRNGKMKAFLSLARFSDTQYQRIENYMKSSEFENKQALLKRAKEEVGLLREHKIQTNRYTVKVQRELELDELALRALKEDRKRFLCKAVENYINCLLSGEEHDMWVFRLCSLWLENSGVSEVNGMMKRDGMKIPTYKFLPLMYQLAARMGTKMMGGLGFHEVLNNLISRISMDHPHHTLFIILALANANRDEFLTKPEVARRSRITKNVPKQSSQLDEDRTEAANRIICTIRSRRPQMVRSVEALCDAYIILANLDATQWKTQRKGINIPADQPITKLKNLEDVVVPTMEIKVDHTGEYGNLVTIQSFKAEFRLAGGVNLPKIIDCVGSDGKERRQLVKGRDDLRQDAVMQQVFQMCNTLLQRNTETRKRKLTICTYKVVPLSQRSGVLEWCTGTVPIGEFLVNNEDGAHKRYRPNDFSAFQCQKKMMEVQKKSFEEKYEVFMDVCQNFQPVFRYFCMEKFLDPAIWFEKRLAYTRSVATSSIVGYILGLGDRHVQNILINEQSAELVHIDLGVAFEQGKILPTPETVPFRLTRDIVDGMGITGVEGVFRRCCEKTMEVMRNSQETLLTIVEVLLYDPLFDWTMNPLKALYLQQRPEDETELHPTLNADDQECKRNLSDIDQSFNKVAERVLMRLQEKLKGVEEGTVLSVGGQVNLLIQQAIDPKNLSRLFPGWKAWV

>NP_002384.2

MTSFSTSAQCSTSDSACRISPGQINQVRPKLPLLKILHAAGAQGEMFTVKEVMHYLGQYIMVKQLYDQQEQHMVYCGGDLLGELLGRQSFSVKDPSPLYDMLRKNLVTLATATTDAAQTLALAQDHSMDIPSQDQLKQSAEESSTSRKRTTEDDIPTLPTSEHKCIHSREDEDLIENLAQDETSRLDLGFEEWDVAGLPWWFLGNLRSNYTPRSNGSTDLQTNQDVGTAIVSDTTDDLWFLNESVSEQLGVGIKVEAADTEQTSEEVGKVSDKKVIEVGKNDDLEDSKSLSDDTDVEVTSEDEWQCTECKKFNSPSKRYCFRCWALRKDWYSDCSKLTHSLSTSDITAIPEKENEGNDVPDCRRTISAPVVRPKDAYIKKENSKLFDPCNSVEFLDLAHSSESQETISSMGEQLDNLSEQRTDTENMEDCQNLLKPCSLCEKRPRDGNIIHGRTGHLVTCFHCARRLKKAGASCPICKKEIQLVIKVFIA

415 NP_002933.1 NP_003137.1

>NP_002933.1

MSLLMFTQLLLCGFLYVRVDGSRLRQEDFPPRIVEHPSDVIVSKGEPTTLNCKAEGRPTPTIEWYKDGERVETDKDDPRSHRMLLPSGSLFFLRIVHGRRSKPDEGSYVCVARNYLGEAVSRNASLEVALLRDDFRQNPTDVVVAAGEPAILECQPPRGHPEPTIYWKKDKVRIDDKEERISIRGGKLMISNTRKSDAGMYTCVGTNMVGERDSDPAELTVFERPTFLRRPINQVVLEEEAVEFRCQVQGDPQPTVRWKKDDADLPRGRYDIKDDYTLRIKKTMSTDEGTYMCIAENRVGKMEASATLTVRAPPQFVVRPRDQIVAQGRTVTFPCETKGNPQPAVFWQKEGSQNLLFPNQPQQPNSRCSVSPTGDLTITNIQRSDAGYYICQALTVAGSILAKAQLEVTDVLTDRPPPIILQGPANQTLAVDGTALLKCKATGDPLPVISWLKEGFTFPGRDPRATIQEQGTLQIKNLRISDTGTYTCVATSSSGETSWSAVLDVTESGATISKNYDLSDLPGPPSKPQVTDVTKNSVTLSWQPGTPGTLPASAYIIEAFSQSVSNSWQTVANHVKTTLYTVRGLRPNTIYLFMVRAINPQGLSDPSPMSDPVRTQDISPPAQGVDHRQVQKELGDVLVRLHNPVVLTPTTVQVTWTVDRQPQFIQGYRVMYRQTSGLQATSSWQNLDAKVPTERSAVLVNLKKGVTYEIKVRPYFNEFQGMDSESKTVRTTEEAPSAPPQSVTVLTVGSYNSTSISVSWDPPPPDHQNGIIQEYKIWCLGNETRFHINKTVDAAIRSVIIGGLFPGIQYRVEVAASTSAGVGVKSEPQPIIIGRRNEVVITENNNSITEQITDVVKQPAFIAGIGGACWVILMGFSIWLYWRRKKRKGLSNYAVTFQRGDGGLMSNGSRPGLLNAGDPSYPWLADSWPATSLPVNNSNSGPNEIGNFGRGDVLPPVPGQGDKTATMLSDGAIYSSIDFTTKTSYNSSSQITQATPYATTQILHSNSIHELAVDLPDPQWKSSIQQKTDLMGFGYSLPDQNKGNNGGKGGKKKKNKNSSKPQKNNGSTWANVPLPPPPVQPLPGTELEHYAVEQQENGYDSDSWCPPLPVQTYLHQGLEDELEEDDDRVPTPPVRGVASSPAISFGQQSTATLTPSPREEMQPMLQAHLDELTRAYQFDIAKQTWHIQSNNQPPQPPVPPLGYVSGALISDLETDVADDDADDEEEALEIPRPLRALDQTPGSSMDNLDSSVTGKAFTSSQRPRPTSPFSTDSNTSAALSQSQRPRPTKKHKGGRMDQQPALPHRREGMTDEEALVPYSKPSFPSPGGHSSSGTASSKGSTGPRKTEVLRAGHQRNASDLLDIGYMGSNSQGQFTGEL

>NP_003137.1

MAETLEFNDVYQEVKGSMNDGRLRLSRQGIIFKNSKTGKVDNIQAGELTEGIWRRVALGHGLKLLTKNGHVYKYDGFRESEFEKLSDFFKTHYRLELMEKDLCVKGWNWGTVKFGGQLLSFDIGDQPVFEIPLSNVSQCTTGKNEVTLEFHQNDDAEVSLMEVRFYVPPTQEDGVDPVEAFAQNVLSKADVIQATGDAICIFRELQCLTPRGRYDIRIYPTFLHLHGKTFDYKIPYTTVLRLFLLPHKDQRQMFFVISLDPPIKQGQTRYHFLILLFSKDEDISLTLNMNEEEVEKRFEGRLTKNMSGSLYEMVSRVMKALVNRKITVPGNFQGHSGAQCITCSYKASSGLLYPLERGFIYVHKPPVHIRFDEISFVNFARGTTTTRSFDFEIETKQGTQYTFSSIEREEYGKLFDFVNAKKLNIKNRGLKEGMNPSYDEYADSDEDQHDAYLERMKEEGKIREENANDSSDDSGEETDESFNPGEEEEDVAEEFDSNASASSSSNEGDSDRDEKKRKQLKKAKMAKDRKSRKKPVEVKKGKDPNAPKRPMSAYMLWLNASREKIKSDHPGISITDLSKKAGEIWKGMSKEKKEEWDRKAEDARRDYEKAMKEYEGGRGESSKRDKSKKKKKVKVKMEKKSTPSRGSSSKSSSRQLSESFKSKEFVSSDESSSGENKSKKKRRRSEDSEEEELASTPPSSEDSASGSDE

416 NP_000323.2 NP_003884.1

>NP_000323.2

MKSNQERSNECLPPKKREIPATSRSSEEKAPTLPSDNHRVEGTAWLPGNPGGRGHGGGRHGPAGTSVELGLQQGIGLHKALSTGLDYSPPSAPRSVPVATTLPAAYATPQPGTPVSPVQYAHLPHTFQFIGSSQYSGTYASFIPSQLIPPTANPVTSAVASAAGATTPSQRSQLEAYSTLLANMGSLSQTPGHKAEQQQQQQQQQQQQHQHQQQQQQQQQQQQQQHLSRAPGLITPGSPPPAQQNQYVHISSSPQNTGRTASPPAIPVHLHPHQTMIPHTLTLGPPSQVVMQYADSGSHFVPREATKKAESSRLQQAIQAKEVLNGEMEKSRRYGAPSSADLGLGKAGGKSVPHPYESRHVVVHPSPSDYSSRDPSGVRASVMVLPNSNTPAADLEVQQATHREASPSTLNDKSGLHLGKPGHRSYALSPHTVIQTTHSASEPLPVGLPATAFYAGTQPPVIGYLSGQQQAITYAGSLPQHLVIPGTQPLLIPVGSTDMEASGAAPAIVTSSPQFAAVPHTFVTTALPKSENFNPEALVTQAAYPAMVQAQIHLPVVQSVASPAAAPPTLPPYFMKGSIIQLANGELKKVEDLKTEDFIQSAEISNDLKIDSSTVERIEDSHSPGVAVIQFAVGEHRAQVSVEVLVEYPFFVFGQGWSSCCPERTSQLFDLPCSKLSVGDVCISLTLKNLKNGSVKKGQPVDPASVLLKHSKADGLAGSRHRYAEQENGINQGSAQMLSENGELKFPEKMGLPAAPFLTKIEPSKPAATRKRRWSAPESRKLEKSEDEPPLTLPKPSLIPQEVKICIEGRSNVGK

>NP_003884.1

MLDRDVGPTPMYPPTYLEPGIGRHTPYGNQTDYRIFELNKRLQNWTEECDNLWWDAFTTEFFEDDAMLTITFCLEDGPKRYTIGRTLIPRYFRSIFEGGATELYYVLKHPKEAFHSNFVSLDCDQGSMVTQHGKPMFTQVCVEGRLYLEFMFDDMMRIKTWHFSIRQHRELIPRSILAMHAQDPQMLDQLSKNITRCGLSNSTLNYLRLCVILEPMQELMSRHKTYSLSPRDCLKTCLFQKWQRMVAPPAEPTRQQPSKRRKRKMSGGSTMSSGGGNTNNSNSKKKSPASTFALSSQVPDVMVVGEPTLMGGEFGDEDERLITRLENTQFDAANGIDDEDSFNNSPALGANSPWNSKPPSSQESKSENPTSQASQ

417 NP_003143.2 NP_000206.2

>NP_003143.2

MAGWIQAQQLQGDALRQMQVLYGQHFPIEVRHYLAQWIESQPWDAIDLDNPQDRAQATQLLEGLVQELQKKAEHQVGEDGFLLKIKLGHYATQLQKTYDRCPLELVRCIRHILYNEQRLVREANNCSSPAGILVDAMSQKHLQINQTFEELRLVTQDTENELKKLQQTQEYFIIQYQESLRIQAQFAQLAQLSPQERLSRETALQQKQVSLEAWLQREAQTLQQYRVELAEKHQKTLQLLRKQQTIILDDELIQWKRRQQLAGNGGPPEGSLDVLQSWCEKLAEIIWQNRQQIRRAEHLCQQLPIPGPVEEMLAEVNATITDIISALVTSTFIIEKQPPQVLKTQTKFAATVRLLVGGKLNVHMNPPQVKATIISEQQAKSLLKNENTRNECSGEILNNCCVMEYHQATGTLSAHFRNMSLKRIKRADRRGAESVTEEKFTVLFESQFSVGSNELVFQVKTLSLPVVVIVHGSQDHNATATVLWDNAFAEPGRVPFAVPDKVLWPQLCEALNMKFKAEVQSNRGLTKENLVFLAQKLFNNSSSHLEDYSGLSVSWSQFNRENLPGWNYTFWQWFDGVMEVLKKHHKPHWNDGAILGFVNKQQAHDLLINKPDGTFLLRFSDSEIGGITIAWKFDSPERNLWNLKPFTTRDFSIRSLADRLGDLSYLIYVFPDRPKDEVFSKYYTPVLAKAVDGYVKPQIKQVVPEFVNASADAGGSSATYMDQAPSPAVCPQAPYNMYPQNPDHVLDQDGEFDLDETMDVARHVEELLRRPMDSLDSRLSPPAGLFTSARGSLS

>NP_000206.2

MAPPSEETPLIPQRSCSLLSTEAGALHVLLPARGPGPPQRLSFSFGDHLAEDLCVQAAKASGILPVYHSLFALATEDLSCWFPPSHIFSVEDASTQVLLYRIRFYFPNWFGLEKCHRFGLRKDLASAILDLPVLEHLFAQHRSDLVSGRLPVGLSLKEQGECLSLAVLDLARMAREQAQRPGELLKTVSYKACLPPSLRDLIQGLSFVTRRRIRRTVRRALRRVAACQADRHSLMAKYIMDLERLDPAGAAETFHVGLPGALGGHDGLGLLRVAGDGGIAWTQGEQEVLQPFCDFPEIVDISIKQAPRVGPAGEHRLVTVTRTDNQILEAEFPGLPEALSFVALVDGYFRLTTDSQHFFCKEVAPPRLLEEVAEQCHGPITLDFAINKLKTGGSRPGSYVLRRSPQDFDSFLLTVCVQNPLGPDYKGCLIRRSPTGTFLLVGLSRPHSSLRELLATCWDGGLHVDGVAVTLTSCCIPRPKEKSNLIVVQRGHSPPTSSLVQPQSQYQLSQMTFHKIPADSLEWHENLGHGSFTKIYRGCRHEVVDGEARKTEVLLKVMDAKHKNCMESFLEAASLMSQVSYRHLVLLHGVCMAGDSTMVQEFVHLGAIDMYLRKRGHLVPASWKLQVVKQLAYALNYLEDKGLPHGNVSARKVLLAREGADGSPPFIKLSDPGVSPAVLSLEMLTDRIPWVAPECLREAQTLSLEADKWGFGATVWEVFSGVTMPISALDPAKKLQFYEDRQQLPAPKWTELALLIQQCMAYEPVQRPSFRAVIRDLNSLISSDYELLSDPTPGALAPRDGLWNGAQLYACQDPTIFEERHLKYISQLGKGNFGSVELCRYDPLGDNTGALVAVKQLQHSGPDQQRDFQREIQILKALHSDFIVKYRGVSYGPGRQSLRLVMEYLPSGCLRDFLQRHRARLDASRLLLYSSQICKGMEYLGSRRCVHRDLAARNILVESEAHVKIADFGLAKLLPLDKDYYVVREPGQSPIFWYAPESLSDNIFSRQSDVWSFGVVLYELFTYCDKSCSPSAEFLRMMGCERDVPALCRLLELLEEGQRLPAPPACPAEVHELMKLCWAPSPQDRPSFSALGPQLDMLWSGSRGCETHAFTAHPEGKHHSLSFS

418 AAC08964.1 NP_000108.1

>AAC08964.1

MTTSQKHRDFVAEPMGEKPVGSLAGIGEVLGKKLEERGFDKAYVVLGQFLVLKKDEDLFREWLKDTCGANAKQSRDCFGCLREWCDPSCDALWELSIPSLIQSLQPSRDSSPVLYEGKDCYCRTHLRRTPGSFGSFLP

>NP_000108.1

MDNYADLSDTELTTLLRRYNIPHGPVVGSTRRLYEKKIFEYETQRRRLSPPSSSAASSYSFSDLNSTRGDADMYDLPKKEDALLYQSKGYNDDYYEESYFTTRTYGEPESAGPSRAVRQSVTSFPDADAFHHQVHDDDLLSSSEEECKDRERPMYGRDSAYQSITHYRPVSASRSSLDLSYYPTSSSTSFMSSSSSSSSWLTRRAIRPENRAPGAGLGQDRQVPLWGQLLLFLVFVIVLFFIYHFMQAEEGNPF

419 NP_004679.1 NP_002458.2

>NP_004679.1

MEADKDDTQQILKEHSPDEFIKDEQNKGLIDEITKKNIQLKKEIQKLETELQEATKEFQIKEDIPETKMKFLSVETPENDSQLSNISCSFQVSSKVPYEIQKGQALITFEKEEVAQNVVSMSKHHVQIKDVNLEVTAKPVPLNSGVRFQVYVEVSKMKINVTEIPDTLREDQMRDKLELSFSKFRNGGGEVDRVDYDRQSGSAVITFVEIGVADKILKKKEYPLYINQTCHRVTVSPYTEIHLKKYQIFSGTSKRTVLLTGMEGIQMDEEIVEDLINIHFQRAKNGGGEVDVVKCSLGQPHIAYFEE

>NP_002458.2

MDFFRVVENQQPPATMPLNVSFTNRNYDLDYDSVQPYFYCDEEENFYQQQQQSELQPPAPSEDIWKKFELLPTPPLSPSRRSGLCSPSYVAVTPFSLRGDNDGGGGSFSTADQLEMVTELLGGDMVNQSFICDPDDETFIKNIIIQDCMWSGFSAAAKLVSEKLASYQAARKDSGSPNPARGHSVCSTSSLYLQDLSAAASECIDPSVVFPYPLNDSSSPKSCASQDSSAFSPSSDSLLSSTESSPQGSPEPLVLHEETPPTTSSDSEEEQEDEEEIDVVSVEKRQAPGKRSESGSPSAGGHSKPPHSPLVLKRCHVSTHQHNYAAPPSTRKDYPAAKRVKLDSVRVLRQISNNRKCTSPRSSDTEENVKRRTHNVLERQRRNELKRSFFALRDQIPELENNEKAPKVVILKKATAYILSVQAEEQKLISEEDLLRKRREQLKHKLEQLRNSCA

420 NP_003891.1 NP_005044.1

>NP_003891.1

MASLTVKAYLLGKEDAAREIRRFSFCCSPEPEAEAEAAAGPGPCERLLSRVAALFPALRPGGFQAHYRDEDGDLVAFSSDEELTMAMSYVKDDIFRIYIKEKKECRRDHRPPCAQEAPRNMVHPNVICDGCNGPVVGTRYKCSVCPDYDLCSVCEGKGLHRGHTKLAFPSPFGHLSEGFSHSRWLRKVKHGHFGWPGWEMGPPGNWSPRPPRAGEARPGPTAESASGPSEDPSVNFLKNVGESVAAALSPLGIEVDIDVEHGGKRSRLTPVSPESSSTEEKSSSQPSSCCSDPSKPGGNVEGATQSLAEQMRKIALESEGRPEEQMESDNCSGGDDDWTHLSSKEVDPSTGELQSLQMPESEGPSSLDPSQEGPTGLKEAALYPHLPPEADPRLIESLSQMLSMGFSDEGGWLTRLLQTKNYDIGAALDTIQYSKHPPPL

>NP_005044.1

MAVTITLKTLQQQTFKIRMEPDETVKVLKEKIEAEKGRDAFPVAGQKLIYAGKILSDDVPIRDYRIDEKNFVVVMVTKTKAGQGTSAPPEASPTAAPESSTSFPPAPTSGMSHPPPAAREDKSPSEESAPTTSPESVSGSVPSSGSSGREEDAASTLVTGSEYETMLTEIMSMGYERERVVAALRASYNNPHRAVEYLLTGIPGSPEPEHGSVQESQVSEQPATEAAGENPLEFLRDQPQFQNMRQVIQQNPALLPALLQQLGQENPQLLQQISRHQEQFIQMLNEPPGELADISDVEGEVGAIGEEAPQMNYIQVTPQEKEAIERLKALGFPESLVIQAYFACEKNENLAANFLLSQNFDDE

421 NP_003630.1 NP_001269.3

>NP_003630.1

MNRHLWKSQLCEMVQPSGGPAADQDVLGEESPLGKPAMLHLPSEQGAPETLQRCLEENQELRDAIRQSNQILRERCEELLHFQASQREEKEFLMCKFQEARKLVERLGLEKLDLKRQKEQALREVEHLKRCQQQMAEDKASVKAQVTSLLGELQESQSRLEAATKECQALEGRARAASEQARQLESEREALQQQHSVQVDQLRMQGQSVEAALRMERQAASEEKRKLAQLQVAYHQLFQEYDNHIKSSVVGSERKRGMQLEDLKQQLQQAEEALVAKQEVIDKLKEEAEQHKIVMETVPVLKAQADIYKADFQAERQAREKLAEKKELLQEQLEQLQREYSKLKASCQESARIEDMRKRHVEVSQAPLPPAPAYLSSPLALPSQRRSPPEEPPDFCCPKCQYQAPDMDTLQIHVMECIE

>NP_001269.3

MERPPGLRPGAGGPWEMRERLGTGGFGNVCLYQHRELDLKIAIKSCRLELSTKNRERWCHEIQIMKKLNHANVVKACDVPEELNILIHDVPLLAMEYCSGGDLRKLLNKPENCCGLKESQILSLLSDIGSGIRYLHENKIIHRDLKPENIVLQDVGGKIIHKIIDLGYAKDVDQGSLCTSFVGTLQYLAPELFENKPYTATVDYWSFGTMVFECIAGYRPFLHHLQPFTWHEKIKKKDPKCIFACEEMSGEVRFSSHLPQPNSLCSLVVEPMENWLQLMLNWDPQQRGGPVDLTLKQPRCFVLMDHILNLKIVHILNMTSAKIISFLLPPDESLHSLQSRIERETGINTGSQELLSETGISLDPRKPASQCVLDGVRGCDSYMVYLFDKSKTVYEGPFASRSLSDCVNYIVQDSKIQLPIIQLRKVWAEAVHYVSGLKEDYSRLFQGQRAAMLSLLRYNANLTKMKNTLISASQQLKAKLEFFHKSIQLDLERYSEQMTYGISSEKMLKAWKEMEEKAIHYAEVGVIGYLEDQIMSLHAEIMELQKSPYGRRQGDLMESLEQRAIDLYKQLKHRPSDHSYSDSTEMVKIIVHTVQSQDRVLKELFGHLSKLLGCKQKIIDLLPKVEVALSNIKEADNTVMFMQGKRQKEIWHLLKIACTQSSARSLVGSSLEGAVTPQTSAWLPPTSAEHDHSLSCVVTPQDGETSAQMIEENLNCLGHLSTIIHEANEEQGNSMMNLDWSWLTE

422 NP_006753.1 NP_149112.1

>NP_006753.1

MDPRLSTVRQTCCCFNVRIATTALAIYHVIMSVLLFIEHSVEVAHGKASCKLSQMGYLRIADLISSFLLITMLFIISLSLLIGVVKNREKYLLPFLSLQIMDYLLCLLTLLGSYIELPAYLKLASRSRASSSKFPLMTLQLLDFCLSILTLCSSYMEVPTYLNFKSMNHMNYLPSQEDMPHNQFIKMMIIFSIAFITVLIFKVYMFKCVWRCYRLIKCMNSVEEKRNSKMLQKVVLPSYEEALSLPSKTPEGGPAPPPYSEV

>NP_149112.1

MSSACDAGDHYPLHLLVWKNDYRQLEKELQGQNVEAVDPRGRTLLHLAVSLGHLESARVLLRHKADVTKENRQGWTVLHEAVSTGDPEMVYTVLQHRDYHNTSMALEGVPELLQKILEAPDFYVQMKWEFTSWVPLVSRICPNDVCRIWKSGAKLRVDITLLGFENMSWIRGRRSFIFKGEDNWAELMEVNHDDKVVTTERFDLSQEMERLTLDLMKPKSREVERRLTSPVINTSLDTKNIAFERTKSGFWGWRTDKAEVVNGYEAKVYTVNNVNVITKIRTEHLTEEEKKRYKADRNPLESLLGTVEHQFGAQGDLTTECATANNPTAITPDEYFNEEFDLKDRDIGRPKELTIRTQKFKAMLWMCEEFPLSLVEQVIPIIDLMARTSAHFARLRDFIKLEFPPGFPVKIEIPLFHVLNARITFGNVNGCSTAEESVSQNVEGTQADSASHITNFEVDQSVFEIPESYYVQDNGRNVHLQDEDYEIMQFAIQQSLLESSRSQELSGPASNGGISQTNTYDAQYERAIQESLLTSTEGLCPSALSETSRFDNDLQLAMELSAKELEEWELRLQEEEAELQQVLQLSLTDK

423 NP_002358.1 NP_060117.2

>NP_002358.1

MPRGQKSKLRAREKRQRTRGQTQDLKVGQPTAAEKEESPSSSSSVLRDTASSSLAFGIPQEPQREPPTTSAAAAMSCTGSDKGDESQDEENASSSQASTSTERSLKDSLTRKTKMLVQFLLYKYKMKEPTTKAEMLKIISKKYKEHFPEIFRKVSQRTELVFGLALKEVNPTTHSYILVSMLGPNDGNQSSAWTLPRNGLLMPLLSVIFLNGNCAREEEIWEFLNMLGIYDGKRHLIFGEPRKLITQDLVQEKYLEYQQVPNSDPPRYQFLWGPRAHAETSKMKVLEFLAKVNDTTPNNFPLLYEEALRDEEERAGARPRVAARRGTTAMTSAYSRATSSSSSQPM

>NP_060117.2

MGKKGKVGKSRRDKFYHLAKETGYRSRSAFKLIQLNRRFQFLQKARALLDLCAAPGGWLQVAAKFMPVSSLIVGVDLVPIKPLPNVVTLQEDITTERCRQALRKELKTWKVDVVLNDGAPNVGASWVHDAYSQAHLTLMALRLACDFLARGGSFITKVFRSRDYQPLLWIFQQLFRRVQATKPQASRHESAEIFVVCQGFLAPDKVDSKFFDPKFAFKEVEVQAKTVTELVTKKKPKAEGYAEGDLTLYHRTSVTDFLRAANPVDFLSKASEIMVDDEELAQHPATTEDIRVCCQDIRVLGRKELRSLLNWRTKLRRYVAKKLKEQAKALDISLSSGEEDEGDEEDSTAGTTKQPSKEEEEEEEEEQLNQTLAEMKAQEVAELKRKKKKLLREQRKQRERVELKMDLPGVSIADEGETGMFSLCTIRGHQLLEEVTQGDMSAADTFLSDLPRDDIYVSDVEDDGDDTSLDSDLDPEELAGVRGHQGLRDQKRMRLTEVQDDKEEEEEENPLLVPLEEKAVLQEEQANLWFSKGSFAGIEDDADEALEISQAQLLFENRRKGRQQQQKQQLPQTPPSCLKTEIMSPLYQDEAPKGTEASSGTEAATGLEGEEKDGISDSDSSTSSEEEESWEPLRGKKRSRGPKSDDDGFEIVPIEDPAKHRILDPEGLALGAVIASSKKAKRDLIDNSFNRYTFNEDEGELPEWFVQEEKQHRIRQLPVGKKEVEHYRKRWREINARPIKKVAEAKARKKRRMLKRLEQTRKKAEAVVNTVDISEREKVAQLRSLYKKAGLGKEKRHVTYVVAKKGVGRKVRRPAGVRGHFKVVDSRMKKDQRAQQRKEQKKKHKRK

424 NP_005891.1 NP_000035.2

>NP_005891.1

MNVTSLFSFTSPAVKRLLGWKQGDEEEKWAEKAVDALVKKLKKKKGAMEELEKALSCPGQPSNCVTIPRSLDGRLQVSHRKGLPHVIYCRVWRWPDLQSHHELKPLECCEFPFGSKQKEVCINPYHYKRVESPVLPPVLVPRHSEYNPQHSLLAQFRNLGQNEPHMPLNATFPDSFQQPNSHPFPHSPNSSYPNSPGSSSSTYPHSPTSSDPGSPFQMPADTPPPAYLPPEDPMTQDGSQPMDTNMMAPPLPSEINRGDVQAVAYEEPKHWCSIVYYELNNRVGEAFHASSTSVLVDGFTDPSNNKNRFCLGLLSNVNRNSTIENTRRHIGKGVHLYYVGGEVYAECLSDSSIFVQSRNCNYHHGFHPTTVCKIPSGCSLKIFNNQEFAQLLAQSVNHGFETVYELTKMCTIRMSFVKGWGAEYHRQDVTSTPCWIEIHLHGPLQWLDKVLTQMGSPHNPISSVS

>NP_000035.2

MEVQLGLGRVYPRPPSKTYRGAFQNLFQSVREVIQNPGPRHPEAASAAPPGASLLLLQQQQQQQQQQQQQQQQQQQQQQQETSPRQQQQQQGEDGSPQAHRRGPTGYLVLDEEQQPSQPQSALECHPERGCVPEPGAAVAASKGLPQQLPAPPDEDDSAAPSTLSLLGPTFPGLSSCSADLKDILSEASTMQLLQQQQQEAVSEGSSSGRAREASGAPTSSKDNYLGGTSTISDNAKELCKAVSVSMGLGVEALEHLSPGEQLRGDCMYAPLLGVPPAVRPTPCAPLAECKGSLLDDSAGKSTEDTAEYSPFKGGYTKGLEGESLGCSGSAAAGSSGTLELPSTLSLYKSGALDEAAAYQSRDYYNFPLALAGPPPPPPPPHPHARIKLENPLDYGSAWAAAAAQCRYGDLASLHGAGAAGPGSGSPSAAASSSWHTLFTAEEGQLYGPCGGGGGGGGGGGGGGGGGGGGGGGEAGAVAPYGYTRPPQGLAGQESDFTAPDVWYPGGMVSRVPYPSPTCVKSEMGPWMDSYSGPYGDMRLETARDHVLPIDYYFPPQKTCLICGDEASGCHYGALTCGSCKVFFKRAAEGKQKYLCASRNDCTIDKFRRKNCPSCRLRKCYEAGMTLGARKLKKLGNLKLQEEGEASSTTSPTEETTQKLTVSHIEGYECQPIFLNVLEAIEPGVVCAGHDNNQPDSFAALLSSLNELGERQLVHVVKWAKALPGFRNLHVDDQMAVIQYSWMGLMVFAMGWRSFTNVNSRMLYFAPDLVFNEYRMHKSRMYSQCVRMRHLSQEFGWLQITPQEFLCMKALLLFSIIPVDGLKNQKFFDELRMNYIKELDRIIACKRKNPTSCSRRFYQLTKLLDSVQPIARELHQFTFDLLIKSHMVSVDFPEMMAEIISVQVPKILSGKVKPIYFHTQ

425 NP_976226.1 NP_061852.2

>NP_976226.1

MDEQEALNSIMNDLVALQMNRRHRMPGYETMKNKDTGHSNRQKKHNSSSSALLNSPTVTTSSCAGASEKKKFLSDVRIKFEHNGERRIIAFSRPVKYEDVEHKVTTVFGQPLDLHYMNNELSILLKNQDDLDKAIDILDRSSSMKSLRILLLSQDRNHNSSSPHSGVSRQVRIKASQSAGDINTIYQPPEPRSRHLSVSSQNPGRSSPPPGYVPERQQHIARQGSYTSINSEGEFIPETSEQCMLDPLSSAENSLSGSCQSLDRSADSPSFRKSRMSRAQSFPDNRQEYSDRETQLYDKGVKGGTYPRRYHVSVHHKDYSDGRRTFPRIRRHQGNLFTLVPSSRSLSTNGENMGLAVQYLDPRGRLRSADSENALSVQERNVPTKSPSAPINWRRGKLLGQGAFGRVYLCYDVDTGRELASKQVQFDPDSPETSKEVSALECEIQLLKNLQHERIVQYYGCLRDRAEKTLTIFMEYMPGGSVKDQLKAYGALTESVTRKYTRQILEGMSYLHSNMIVHRDIKGANILRDSAGNVKLGDFGASKRLQTICMSGTGMRSVTGTPYWMSPEVISGEGYGRKADVWSLGCTVVEMLTEKPPWAEYEAMAAIFKIATQPTNPQLPSHISEHGRDFLRRIFVEARQRPSAEELLTHHFAQLMY

>NP_061852.2

MSGGAAEKQSSTPGSLFLSPPAPAPKNGSSSDSSVGEKLGAAAADAVTGRTEEYRRRRHTMDKDSRGAAATTTTTEHRFFRRSVICDSNATALELPGLPLSLPQPSIPAAVPQSAPPEPHREETVTATATSQVAQQPPAAAAPGEQAVAGPAPSTVPSSTSKDRPVSQPSLVGSKEEPPPARSGSGGGSAKEPQEERSQQQDDIEELETKAVGMSNDGRFLKFDIEIGRGSFKTVYKGLDTETTVEVAWCELQDRKLTKSERQRFKEEAEMLKGLQHPNIVRFYDSWESTVKGKKCIVLVTELMTSGTLKTYLKRFKVMKIKVLRSWCRQILKGLQFLHTRTPPIIHRDLKCDNIFITGPTGSVKIGDLGLATLKRASFAKSVIGTPEFMAPEMYEEKYDESVDVYAFGMCMLEMATSEYPYSECQNAAQIYRRVTSGVKPASFDKVAIPEVKEIIEGCIRQNKDERYSIKDLLNHAFFQEETGVRVELAEEDDGEKIAIKLWLRIEDIKKLKGKYKDNEAIEFSFDLERDVPEDVAQEMVESGYVCEGDHKTMAKAIKDRVSLIKRKREQRQLVREEQEKKKQEESSLKQQVEQSSASQTGIKQLPSASTGIPTASTTSASVSTQVEPEEPEADQHQQLQYQQPSISVLSDGTVDSGQGSSVFTESRVSSQQTVSYGSQHEQAHSTGTVPGHIPSTVQAQSQPHGVYPPSSVAQGQSQGQPSSSSLTGVSSSQPIQHPQQQQGIQQTAPPQQTVQYSLSQTSTSSEATTAQPVSQPQAPQVLPQVSAGKQLPVSQPVPTIQGEPQIPVATQPSVVPVHSGAHFLPVGQPLPTPLLPQYPVSQIPISTPHVSTAQTGFSSLPITMAAGITQPLLTLASSATTAAIPGVSTVVPSQLPTLLQPVTQLPSQVHPQLLQPAVQSMGIPANLGQAAEVPLSSGDVLYQGFPPRLPPQYPGDSNIAPSSNVASVCIHSTVLSPPMPTEVLATPGYFPTVVQPYVESNLLVPMGGVGGQVQVSQPGGSLAQAPTTSSQQAVLESTQGVSQVAPAEPVAVAQTQATQPTTLASSVDSAHSDVASGMSDGNENVPSSSGRHEGRTTKRHYRKSVRSRSRHEKTSRPKLRILNVSNKGDRVVECQLETHNRKMVTFKFDLDGDNPEEIATIMVNNDFILAIERESFVDQVREIIEKADEMLSEDVSVEPEGDQGLESLQGKDDYGFSGSQKLEGEFKQPIPASSMPQQIGIPTSSLTQVVHSAGRRFIVSPVPESRLRESKVFPSEITDTVAASTAQSPGMNLSHSASSLSLQQAFSELRRAQMTEGPNTAPPNFSHTGPTFPVVPPFLSSIAGVPTTAAATAPVPATSSPPNDISTSVIQSEVTVPTEEGIAGVATSTGVVTSGGLPIPPVSESPVLSSVVSSITIPAVVSISTTSPSLQVPTSTSEIVVSSTALYPSVTVSATSASAGGSTATPGPKPPAVVSQQAAGSTTVGATLTSVSTTTSFPSTASQLSIQLSSSTSTPTLAETVVVSAHSLDKTSHSSTTGLAFSLSAPSSSSSPGAGVSSYISQPGGLHPLVIPSVIASTPILPQAAGPTSTPLLPQVPSIPPLVQPVANVPAVQQTLIHSQPQPALLPNQPHTHCPEVDSDTQPKAPGIDDIKTLEEKLRSLFSEHSSSGAQHASVSLETSLVIESTVTPGIPTTAVAPSKLLTSTTSTCLPPTNLPLGTVALPVTPVVTPGQVSTPVSTTTSGVKPGTAPSKPPLTKAPVLPVGTELPAGTLPSEQLPPFPGPSLTQSQQPLEDLDAQLRRTLSPEMITVTSAVGPVSMAAPTAITEAGTQPQKGVSQVKEGPVLATSSGAGVFKMGRFQVSVAADGAQKEGKNKSEDAKSVHFESSTSESSVLSSSSPESTLVKPEPNGITIPGISSDVPESAHKTTASEAKSDTGQPTKVGRFQVTTTANKVGRFSVSKTEDKITDTKKEGPVASPPFMDLEQAVLPAVIPKKEKPELSEPSHLNGPSSDPEAAFLSRDVDDGSGSPHSPHQLSSKSLPSQNLSQSLSNSFNSSYMSSDNESDIEDEDLKLELRRLRDKHLKEIQDLQSRQKHEIESLYTKLGKVPPAVIIPPAAPLSGRRRRPTKSKGSKSSRSSSLGNKSPQLSGNLSGQSAASVLHPQQTLHPPGNIPESGQNQLLQPLKPSPSSDNLYSAFTSDGAISVPSLSAPGQGTSSTNTVGATVNSQAAQAQPPAMTSSRKGTFTDDLHKLVDNWARDAMNLSGRRGSKGHMNYEGPGMARKFSAPGQLCISMTSNLGGSAPISAASATSLGHFTKSMCPPQQYGFPATPFGAQWSGTGGPAPQPLGQFQPVGTASLQNFNISNLQKSISNPPGSNLRTT

426 NP_060921.3 NP_002277.3

>NP_060921.3

MFLMPTSSELNSGQNFLTQWMTNPSRAGVILNRGFPILEADKEKRAAVDISTSFPIKGTHFSDSFSFINEEDSLLEEQKLESNNPYKPQSDKSETHTAFPCIKKGPQVAACHSAPGHQEENKNDFIPDLASEFKEGAYKDPLFKKLEQLKEVQQKKQEQLKRQQLEQLQRLMEEQEKLLTMVSGQCTLPGLSLLPDDQSQKHRSPGNTTTGERATCCFPSYVYPDPTQEETYPSNILSHEQSNFCRTAHGDFVLTSKRASPNLFSEAQYQEAPVEKNNLKEENRNHPTGESILCWEKVTEQIQEANDKNLQKHDDSSEVANIEERPIKAAIGERKQTFEDYLEEQIQLEEQELKQKQLKEAEGPLPIKAKPKQPFLKRGEGLARFTNAKSKFQKGKESKLVTNQSTSEDQPLFKMDRQQLQRKTALKNKELCADNPILKKDSKARTKSGSVTLSQKPKMLKCSNRKSLSPSGLKIQTGKKCDGQFRDQIKFENKVTSNNKENVTECPKPCDTGCTGWNKTQGKDRLPLSTGPASRLAAKSPIRETMKESESSLDVSLQKKLETWEREKEKENLELDEFLFLEQAADEISFSSNSSFVLKILERDQQICKGHRMSSTPVKAVPQKTNPADPISHCNRSEDLDHTAREKESECEVAPKQLHSLSSADELREQPCKIRKAVQKSTSENQTEWNARDDEGVPNSDSSTDSEEQLDVTIKPSTEDRERGISSREDSPQVCDDKGPFKDTRTQEDKRRDVDLDLSDKDYSSDESIMESIKHKVSEPSRSSSLSLSKMDFDDERTWTDLEENLCNHDVVLGNESTYGTPQTCYPNNEIGILDKTIKRKIAPVKRGEDLSKSRRSRSPPTSELMMKFFPSLKPKPKSDSHLGNELKLNISQDQPPGDNARSQVLREKIIELETEIEKFKAENASLAKLRIERESALEKLRKEIADFEQQKAKELARIEEFKKEEMRKLQKERKVFEKYTTAARTFPDKKEREEIQTLKQQIADLREDLKRKETKWSSTHSRLRSQIQMLVRENTDLREEIKVMERFRLDAWKRAEAIESSLEVEKKDKLANTSVRFQNSQISSGTQVEKYKKNYLPMQGNPPRRSKSAPPRDLGNLDKGQAASPREPLEPLNFPDPEYKEEEEDQDIQGEISHPDGKVEKVYKNGCRVILFPNGTRKEVSADGKTITVTFFNGDVKQVMPDQRVIYYYAAAQTTHTTYPEGLEVLHFSSGQIEKHYPDGRKEITFPDQTVKNLFPDGQEESIFPDGTIVRVQRDGNKLIEFNNGQRELHTAQFKRREYPDGTVKTVYANGHQETKYRSGRIRVKDKEGNVLMDTEL

>NP_002277.3

MWEAQFLGLLFLQPLWVAPVKPLQPGAEVPVVWAQEGAPAQLPCSPTIPLQDLSLLRRAGVTWQHQPDSGPPAAAPGHPLAPGPHPAAPSSWGPRPRRYTVLSVGPGGLRSGRLPLQPRVQLDERGRQRGDFSLWLRPARRADAGEYRAAVHLRDRALSCRLRLRLGQASMTASPPGSLRASDWVILNCSFSRPDRPASVHWFRNRGQGRVPVRESPHHHLAESFLFLPQVSPMDSGPWGCILTYRDGFNVSIMYNLTVLGLEPPTPLTVYAGAGSRVGLPCRLPAGVGTRSFLTAKWTPPGGGPDLLVTGDNGDFTLRLEDVSQAQAGTYTCHIHLQEQQLNATVTLAIITVTPKSFGSPGSLGKLLCEVTPVSGQERFVWSSLDTPSQRSFSGPWLEAQEAQLLSQPWQCQLYQGERLLGAAVYFTELSSPGAQRSGRAPGALPAGHLLLFLTLGVLSLLLLVTGAFGFHLWRRQWRPRRFSALEQGIHPPQAQSKIEELEQEPEPEPEPEPEPEPEPEPEQL

427 NP_004029.2 NP_005849.1

>NP_004029.2

MKLFWLLFTIGFCWAQYSSNTQQGRTSIVHLFEWRWVDIALECERYLAPKGFGGVQVSPPNENVAIHNPFRPWWERYQPVSYKLCTRSGNEDEFRNMVTRCNNVGVRIYVDAVINHMCGNAVSAGTSSTCGSYFNPGSRDFPAVPYSGWDFNDGKCKTGSGDIENYNDATQVRDCRLSGLLDLALGKDYVRSKIAEYMNHLIDIGVAGFRIDASKHMWPGDIKAILDKLHNLNSNWFPEGSKPFIYQEVIDLGGEPIKSSDYFGNGRVTEFKYGAKLGTVIRKWNGEKMSYLKNWGEGWGFMPSDRALVFVDNHDNQRGHGAGGASILTFWDARLYKMAVGFMLAHPYGFTRVMSSYRWPRYFENGKDVNDWVGPPNDNGVTKEVTINPDTTCGNDWVCEHRWRQIRNMVNFRNVVDGQPFTNWYDNGSNQVAFGRGNRGFIVFNNDDWTFSLTLQTGLPAGTYCDVISGDKINGNCTGIKIYVSDDGKAHFSISNSAEDPFIAIHAESKL

>NP_005849.1

MDQGYGGYGAWSAGPANTQGAYGTGVASWQGYENYNYYGAQNTSVTTGATYSYGPASWEAAKANDGGLAAGAPAMHMASYGPEPCTDNSDSLIAKINQRLDMMSKEGGRGGSGGGGEGIQDRESSFRFQPFESYDSRPCLPEHNPYRPSYSYDYEFDLGSDRNGSFGGQYSECRDPARERGSLDGFMRGRGQGRFQDRSNPGTFMRSDPFVPPAASSEPLSTPWNELNYVGGRGLGGPSPSRPPPSLFSQSMAPDYGVMGMQGAGGYDSTMPYGCGRSQPRMRDRDRPKRRGFDRFGPDGTGRKRKQFQLYEEPDTKLARVDSEGDFSENDDAAGDFRSGDEEFKGEDELCDSGRQRGEKEDEDEDVKKRREKQRRRDRTRDRAADRIQFACSVCKFRSFDDEEIQKHLQSKFHKETLRFISTKLPDKTVEFLQEYIVNRNKKIEKRRQELMEKETAKPKPDPFKGIGQEHFFKKIEAAHCLACDMLIPAQPQLLQRHLHSVDHNHNRRLAAEQFKKTSLHVAKSVLNNRHIVKMLEKYLKGEDPFTSETVDPEMEGDDNLGGEDKKETPEEVAADVLAEVITAAVRAVDGEGAPAPESSGEPAEDEGPTDTAEAGSDPQAEQLLEEQVPCGTAHEKGVPKARSEAAEAGNGAETMAAEAESAQTRVAPAPAAADAEVEQTDAESKDAVPTE

428 NP_055390.1 NP_005243.1

>NP_055390.1

MERSGQRVTTWDCDQGKHSDSDYREDGMDLGSDAGSSSSSSRASSQSNSTKVTPCSECKSSSSPGGSLDLVSALEDYEEPFPVYQKKVIDEWAPEEDGEEEEEEDERDQRGYRDDRSPAREPGDVSARTRSGGGGGRSATTAMPPPVPNGNLHQHDPQDLRHNGNVVVAGRPSCSRGPRRAIQKPQPAGGRRSGRGPAAGGLCLQPPDGGTCVPEEPPVPPMDWEALEKHLAGLQFREQEVRNQGQARTNSTSAQKNERESIRQKLALGSFFDDGPGIYTSCSKSGKPSLSSRLQSGMNLQICFVNDSGSDKDSDADDSKTETSLDTPLSPMSKQSSSYSDRDTTEEESESLDDMDFLTRQKKLQAEAKMALAMAKPMAKMQVEVEKQNRKKSPVADLLPHMPHISECLMKRSLKPTDLRDMTIGQLQVIVNDLHSQIESLNEELVQLLLIRDELHTEQDAMLVDIEDLTRHAESQQKHMAEKMPAK

>NP_005243.1

MMFSGFNADYEASSSRCSSASPAGDSLSYYHSPADSFSSMGSPVNAQDFCTDLAVSSANFIPTVTAISTSPDLQWLVQPALVSSVAPSQTRAPHPFGVPAPSAGAYSRAGVVKTMTGGRAQSIGRRGKVEQLSPEEEEKRRIRRERNKMAAAKCRNRRRELTDTLQAETDQLEDEKSALQTEIANLLKEKEKLEFILAAHRPACKIPDDLGFPEEMSVASLDLTGGLPEVATPESEEAFTLPLLNDPEPKPSVEPVKSISSMELKTEPFDDFLFPASSRPSGSETARSVPDMDLSGSFYAADWEPLHSGSLGMGPMATELEPLCTPVVTCTPSCTAYTSSFVFTYPEADSFPSCAAAHRKGSSSNEPSSDSLSSPTLLAL

429 NP_631946.1 NP_077309.1

>NP_631946.1

MADIDNKEQSELDQDLDDVEEVEEEETGEETKLKARQLTVQMMQNPQILAALQERLDGLVETPTGYIESLPRVVKRRVNALKNLQVKCAQIEAKFYEEVHDLERKYAVLYQPLFDKRFEIINAIYEPTEEECEWKPDEEDEISEELKEKAKIEDEKKDEEKEDPKGIPEFWLTVFKNVDLLSDMVQEHDEPILKHLKDIKVKFSDAGQPMSFVLEFHFEPNEYFTNEVLTKTYRMRSEPDDSDPFSFDGPEIMGCTGCQIDWKKGKNVTLKTIKKKQKHKGRGTVRTVTKTVSNDSFFNFFAPPEVPESGDLDDDAEAILAADFEIGHFLRERIIPRSVLYFTGEAIEDDDDDYDEEGEEADEEGEEEGDEENDPDYDPKKDQNPAECKQQ

>NP_077309.1

MEEQREALRKIIKTLAVKNEEIQSFIYSLKQMLLNVEANSAKVQEDLEAEFQSLFSLLEELKEGMLMKIKQDRASRTYELQNQLAACTRALESSEELLETANQTLQAMDSEDFPQAAKQIKDGVTMAPAFRLSLKAKVSDNMSHLMVDFAQERQMLQALKFLPVPSAPVIDLAESLVADNCVTLVWRMPDEDSKIDHYVLEYRRTNFEGPPRLKEDQPWMVIEGIRQTEYTLTGLKFDMKYMNFRVKACNKAVAGEFSEPVTLETPAFMFRLDASTSHQNLRVDDLSVEWDAMGGKVQDIKAREKDGKGRTASPINSPARGTPSPKRMPSGRGGRDRFTAESYTVLGDTLIDGGEHYWEVRYEPDSKAFGVGVAYRSLGRFEQLGKTAASWCLHVNNWLQVSFTAKHANKVKVLDAPVPDCLGVHCDFHQGLLSFYNARTKQVLHTFKTRFTQPLLPAFTVWCGSFQVTTGLQVPSAVRCLQKRGSATSSSNTSLT

430 NP_004516.2 NP_000032.1

>NP_004516.2

MDRGPAAVACTLLLALVACLAPASGQECDSAHFRCGSGHCIPADWRCDGTKDCSDDADEIGCAVVTCQQGYFKCQSEGQCIPNSWVCDQDQDCDDGSDERQDCSQSTCSSHQITCSNGQCIPSEYRCDHVRDCPDGADENDCQYPTCEQLTCDNGACYNTSQKCDWKVDCRDSSDEINCTEICLHNEFSCGNGECIPRAYVCDHDNDCQDGSDEHACNYPTCGGYQFTCPSGRCIYQNWVCDGEDDCKDNGDEDGCESGPHDVHKCSPREWSCPESGRCISIYKVCDGILDCPGREDENNTSTGKYCSMTLCSALNCQYQCHETPYGGACFCPPGYIINHNDSRTCVEFDDCQIWGICDQKCESRPGRHLCHCEEGYILERGQYCKANDSFGEASIIFSNGRDLLIGDIHGRSFRILVESQNRGVAVGVAFHYHLQRVFWTDTVQNKVFSVDINGLNIQEVLNVSVETPENLAVDWVNNKIYLVETKVNRIDMVNLDGSYRVTLITENLGHPRGIAVDPTVGYLFFSDWESLSGEPKLERAFMDGSNRKDLVKTKLGWPAGVTLDMISKRVYWVDSRFDYIETVTYDGIQRKTVVHGGSLIPHPFGVSLFEGQVFFTDWTKMAVLKANKFTETNPQVYYQASLRPYGVTVYHSLRQPYATNPCKDNNGGCEQVCVLSHRTDNDGLGFRCKCTFGFQLDTDERHCIAVQNFLIFSSQVAIRGIPFTLSTQEDVMVPVSGNPSFFVGIDFDAQDSTIFFSDMSKHMIFKQKIDGTGREILAANRVENVESLAFDWISKNLYWTDSHYKSISVMRLADKTRRTVVQYLNNPRSVVVHPFAGYLFFTDWFRPAKIMRAWSDGSHLLPVINTTLGWPNGLAIDWAASRLYWVDAYFDKIEHSTFDGLDRRRLGHIEQMTHPFGLAIFGEHLFFTDWRLGAIIRVRKADGGEMTVIRSGIAYILHLKSYDVNIQTGSNACNQPTHPNGDCSHFCFPVPNFQRVCGCPYGMRLASNHLTCEGDPTNEPPTEQCGLFSFPCKNGRCVPNYYLCDGVDDCHDNSDEQLCGTLNNTCSSSAFTCGHGECIPAHWRCDKRNDCVDGSDEHNCPTHAPASCLDTQYTCDNHQCISKNWVCDTDNDCGDGSDEKNCNSTETCQPSQFNCPNHRCIDLSFVCDGDKDCVDGSDEVGCVLNCTASQFKCASGDKCIGVTNRCDGVFDCSDNSDEAGCPTRPPGMCHSDEFQCQEDGICIPNFWECDGHPDCLYGSDEHNACVPKTCPSSYFHCDNGNCIHRAWLCDRDNDCGDMSDEKDCPTQPFRCPSWQWQCLGHNICVNLSVVCDGIFDCPNGTDESPLCNGNSCSDFNGGCTHECVQEPFGAKCLCPLGFLLANDSKTCEDIDECDILGSCSQHCYNMRGSFRCSCDTGYMLESDGRTCKVTASESLLLLVASQNKIIADSVTSQVHNIYSLVENGSYIVAVDFDSISGRIFWSDATQGKTWSAFQNGTDRRVVFDSSIILTETIAIDWVGRNLYWTDYALETIEVSKIDGSHRTVLISKNLTNPRGLALDPRMNEHLLFWSDWGHHPRIERASMDGSMRTVIVQDKIFWPCGLTIDYPNRLLYFMDSYLDYMDFCDYNGHHRRQVIASDLIIRHPYALTLFEDSVYWTDRATRRVMRANKWHGGNQSVVMYNIQWPLGIVAVHPSKQPNSVNPCAFSRCSHLCLLSSQGPHFYSCVCPSGWSLSPDLLNCLRDDQPFLITVRQHIIFGISLNPEVKSNDAMVPIAGIQNGLDVEFDDAEQYIYWVENPGEIHRVKTDGTNRTVFASISMVGPSMNLALDWISRNLYSTNPRTQSIEVLTLHGDIRYRKTLIANDGTALGVGFPIGITVDPARGKLYWSDQGTDSGVPAKIASANMDGTSVKTLFTGNLEHLECVTLDIEEQKLYWAVTGRGVIERGNVDGTDRMILVHQLSHPWGIAVHDSFLYYTDEQYEVIERVDKATGANKIVLRDNVPNLRGLQVYHRRNAAESSNGCSNNMNACQQICLPVPGGLFSCACATGFKLNPDNRSCSPYNSFIVVSMLSAIRGFSLELSDHSETMVPVAGQGRNALHVDVDVSSGFIYWCDFSSSVASDNAIRRIKPDGSSLMNIVTHGIGENGVRGIAVDWVAGNLYFTNAFVSETLIEVLRINTTYRRVLLKVTVDMPRHIVVDPKNRYLFWADYGQRPKIERSFLDCTNRTVLVSEGIVTPRGLAVDRSDGYVYWVDDSLDIIARIRINGENSEVIRYGSRYPTPYGITVFENSIIWVDRNLKKIFQASKEPENTEPPTVIRDNINWLRDVTIFDKQVQPRSPAEVNNNPCLENNGGCSHLCFALPGLHTPKCDCAFGTLQSDGKNCAISTENFLIFALSNSLRSLHLDPENHSPPFQTINVERTVMSLDYDSVSDRIYFTQNLASGVGQISYATLSSGIHTPTVIASGIGTADGIAFDWITRRIYYSDYLNQMINSMAEDGSNRTVIARVPKPRAIVLDPCQGYLYWADWDTHAKIERATLGGNFRVPIVNSSLVMPSGLTLDYEEDLLYWVDASLQRIERSTLTGVDREVIVNAAVHAFGLTLYGQYIYWTDLYTQRIYRANKYDGSGQIAMTTNLLSQPRGINTVVKNQKQQCNNPCEQFNGGCSHICAPGPNGAECQCPHEGNWYLANNRKHCIVDNGERCGASSFTCSNGRCISEEWKCDNDNDCGDGSDEMESVCALHTCSPTAFTCANGRCVQYSYRCDYYNDCGDGSDEAGCLFRDCNATTEFMCNNRRCIPREFICNGVDNCHDNNTSDEKNCPDRTCQSGYTKCHNSNICIPRVYLCDGDNDCGDNSDENPTYCTTHTCSSSEFQCASGRCIPQHWYCDQETDCFDASDEPASCGHSERTCLADEFKCDGGRCIPSEWICDGDNDCGDMSDEDKRHQCQNQNCSDSEFLCVNDRPPDRRCIPQSWVCDGDVDCTDGYDENQNCTRRTCSENEFTCGYGLCIPKIFRCDRHNDCGDYSDERGCLYQTCQQNQFTCQNGRCISKTFVCDEDNDCGDGSDELMHLCHTPEPTCPPHEFKCDNGRCIEMMKLCNHLDDCLDNSDEKGCGINECHDPSISGCDHNCTDTLTSFYCSCRPGYKLMSDKRTCVDIDECTEMPFVCSQKCENVIGSYICKCAPGYLREPDGKTCRQNSNIEPYLIFSNRYYLRNLTIDGYFYSLILEGLDNVVALDFDRVEKRLYWIDTQRQVIERMFLNKTNKETIINHRLPAAESLAVDWVSRKLYWLDARLDGLFVSDLNGGHRRMLAQHCVDANNTFCFDNPRGLALHPQYGYLYWADWGHRAYIGRVGMDGTNKSVIISTKLEWPNGITIDYTNDLLYWADAHLGYIEYSDLEGHHRHTVYDGALPHPFAITIFEDTIYWTDWNTRTVEKGNKYDGSNRQTLVNTTHRPFDIHVYHPYRQPIVSNPCGTNNGGCSHLCLIKPGGKGFTCECPDDFRTLQLSGSTYCMPMCSSTQFLCANNEKCIPIWWKCDGQKDCSDGSDELALCPQRFCRLGQFQCSDGNCTSPQTLCNAHQNCPDGSDEDRLLCENHHCDSNEWQCANKRCIPESWQCDTFNDCEDNSDEDSSHCASRTCRPGQFRCANGRCIPQAWKCDVDNDCGDHSDEPIEECMSSAHLCDNFTEFSCKTNYRCIPKWAVCNGVDDCRDNSDEQGCEERTCHPVGDFRCKNHHCIPLRWQCDGQNDCGDNSDEENCAPRECTESEFRCVNQQCIPSRWICDHYNDCGDNSDERDCEMRTCHPEYFQCTSGHCVHSELKCDGSADCLDASDEADCPTRFPDGAYCQATMFECKNHVCIPPYWKCDGDDDCGDGSDEELHLCLDVPCNSPNRFRCDNNRCIYSHEVCNGVDDCGDGTDETEEHCRKPTPKPCTEYEYKCGNGHCIPHDNVCDDADDCGDWSDELGCNKGKERTCAENICEQNCTQLNEGGFICSCTAGFETNVFDRTSCLDINECEQFGTCPQHCRNTKGSYECVCADGFTSMSDRPGKRCAAEGSSPLLLLPDNVRIRKYNLSSERFSEYLQDEEYIQAVDYDWDPKDIGLSVVYYTVRGEGSRFGAIKRAYIPNFESGRNNLVQEVDLKLKYVMQPDGIAVDWVGRHIYWSDVKNKRIEVAKLDGRYRKWLISTDLDQPAAIAVNPKLGLMFWTDWGKEPKIESAWMNGEDRNILVFEDLGWPTGLSIDYLNNDRIYWSDFKEDVIETIKYDGTDRRVIAKEAMNPYSLDIFEDQLYWISKEKGEVWKQNKFGQGKKEKTLVVNPWLTQVRIFHQLRYNKSVPNLCKQICSHLCLLRPGGYSCACPQGSSFIEGSTTECDAAIELPINLPPPCRCMHGGNCYFDETDLPKCKCPSGYTGKYCEMAFSKGISPGTTAVAVLLTILLIVVIGALAIAGFFHYRRTGSLLPALPKLPSLSSLVKPSENGNGVTFRSGADLNMDIGVSGFGPETAIDRSMAMSEDFVMEMGKQPIIFENPMYSARDSAVKVVQPIQVTVSENVDNKNYGSPINPSEIVPETNPTSPAADGTQVTKWNLFKRKSKQTTNFENPIYAQMENEQKESVAATPPPSPSLPAKPKPPSRRDPTPTYSATEDTFKDTANLVKEDSEV

>NP_000032.1

MKVLWAALLVTFLAGCQAKVEQAVETEPEPELRQQTEWQSGQRWELALGRFWDYLRWVQTLSEQVQEELLSSQVTQELRALMDETMKELKAYKSELEEQLTPVAEETRARLSKELQAAQARLGADMEDVCGRLVQYRGEVQAMLGQSTEELRVRLASHLRKLRKRLLRDADDLQKRLAVYQAGAREGAERGLSAIRERLGPLVEQGRVRAATVGSLAGQPLQERAQAWGERLRARMEEMGSRTRDRLDEVKEQVAEVRAKLEEQAQQIRLQAEAFQARLKSWFEPLVEDMQRQWAGLVEKVQAAVGTSAAPVPSDNH

431 NP_005139.1 NP_005347.3

>NP_005139.1

MKVKKGGGGAGTATESAPGPSGQSVAPIPQPPAESESGSESEPDAGPGPRPGPLQRKQPIGPEDVLGLQRITGDYLCSPEENIYKIDFVRFKIRDMDSGTVLFEIKKPPVSERLPINRRDLDPNAGRFVRYQFTPAFLRLRQVGATVEFTVGDKPVNNFRMIERHYFRNQLLKSFDFHFGFCIPSSKNTCEHIYDFPPLSEELISEMIRHPYETQSDSFYFVDDRLVMHNKADYSYSGTP

>NP_005347.3

MGCGCSSHPEDDWMENIDVCENCHYPIVPLDGKGTLLIRNGSEVRDPLVTYEGSNPPASPLQDNLVIALHSYEPSHDGDLGFEKGEQLRILEQSGEWWKAQSLTTGQEGFIPFNFVAKANSLEPEPWFFKNLSRKDAERQLLAPGNTHGSFLIRESESTAGSFSLSVRDFDQNQGEVVKHYKIRNLDNGGFYISPRITFPGLHELVRHYTNASDGLCTRLSRPCQTQKPQKPWWEDEWEVPRETLKLVERLGAGQFGEVWMGYYNGHTKVAVKSLKQGSMSPDAFLAEANLMKQLQHQRLVRLYAVVTQEPIYIITEYMENGSLVDFLKTPSGIKLTINKLLDMAAQIAEGMAFIEERNYIHRDLRAANILVSDTLSCKIADFGLARLIEDNEYTAREGAKFPIKWTAPEAINYGTFTIKSDVWSFGILLTEIVTHGRIPYPGMTNPEVIQNLERGYRMVRPDNCPEELYQLMRLCWKERPEDRPTFDYLRSVLEDFFTATEGQYQPQP

432 NP_057253.2 NP_665906.1

>NP_057253.2

MAELRPSGAPGPTAPPAPGPTAPPAFASLFPPGLHAIYGECRRLYPDQPNPLQVTAIVKYWLGGPDPLDYVSMYRNVGSPSANIPEHWHYISFGLSDLYGDNRVHEFTGTDGPSGFGFELTFRLKRETGESAPPTWPAELMQGLARYVFQSENTFCSGDHVSWHSPLDNSESRIQHMLLTEDPQMQPVQTPFGVVTFLQIVGVCTEELHSAQQWNGQGILELLRTVPIAGGPWLITDMRRGETIFEIDPHLQERVDKGIETDGSNLSGVSAKCAWDDLSRPPEDDEDSRSICIGTQPRRLSGKDTEQIRETLRRGLEINSKPVLPPINPQRQNGLAHDRAPSRKDSLESDSSTAIIPHELIRTRQLESVHLKFNQESGALIPLCLRGRLLHGRHFTYKSITGDMAITFVSTGVEGAFATEEHPYAAHGPWLQILLTEEFVEKMLEDLEDLTSPEEFKLPKEYSWPEKKLKVSILPDVVFDSPLH

>NP_665906.1

MSESSSKSSQPLASKQEKDGTEKRGRGRPRKQPPVSPGTALVGSQKEPSEVPTPKRPRGRPKGSKNKGAAKTRKTTTTPGRKPRGRPKKLEKEEEEGISQESSEEEQ

433 NP_002078.1 NP_005513.1

>NP_002078.1

MWTLVSWVALTAGLVAGTRCPDGQFCPVACCLDPGGASYSCCRPLLDKWPTTLSRHLGGPCQVDAHCSAGHSCIFTVSGTSSCCPFPEAVACGDGHHCCPRGFHCSADGRSCFQRSGNNSVGAIQCPDSQFECPDFSTCCVMVDGSWGCCPMPQASCCEDRVHCCPHGAFCDLVHTRCITPTGTHPLAKKLPAQRTNRAVALSSSVMCPDARSRCPDGSTCCELPSGKYGCCPMPNATCCSDHLHCCPQDTVCDLIQSKCLSKENATTDLLTKLPAHTVGDVKCDMEVSCPDGYTCCRLQSGAWGCCPFTQAVCCEDHIHCCPAGFTCDTQKGTCEQGPHQVPWMEKAPAHLSLPDPQALKRDVPCDNVSSCPSSDTCCQLTSGEWGCCPIPEAVCCSDHQHCCPQGYTCVAEGQCQRGSEIVAGLEKMPARRASLSHPRDIGCDQHTSCPVGQTCCPSLGGSWACCQLPHAVCCEDRQHCCPAGYTCNVKARSCEKEVVSAQPATFLARSPHVGVKDVECGEGHFCHDNQTCCRDNRQGWACCPYRQGVCCADRRHCCPAGFRCAARGTKCLRREAPRWDAPLRDPALRQLL

>NP_005513.1

MDNARMNSFLEYPILSSGDSGTCSARAYPSDHRITTFQSCAVSANSCGGDDRFLVGRGVQIGSPHHHHHHHHHHPQPATYQTSGNLGVSYSHSSCGPSYGSQNFSAPYSPYALNQEADVSGGYPQCAPAVYSGNLSSPMVQHHHHHQGYAGGAVGSPQYIHHSYGQEHQSLALATYNNSLSPLHASHQEACRSPASETSSPAQTFDWMKVKRNPPKTGKVGEYGYLGQPNAVRTNFTTKQLTELEKEFHFNKYLTRARRVEIAASLQLNETQVKIWFQNRRMKQKKREKEGLLPISPATPPGNDEKAEESSEKSSSSPCVPSPGSSTSDTLTTSH

434 NP_005685.1 NP_006824.2

>NP_005685.1

MPGLVDSNPAPPESQEKKPLKPCCACPETKKARDACIIEKGEEHCGHLIEAHKECMRALGFKI

>NP_006824.2

MAAAAAAAAATNGTGGSSGMEVDAAVVPSVMACGVTGSVSVALHPLVILNISDHWIRMRSQEGRPVQVIGALIGKQEGRNIEVMNSFELLSHTVEEKIIIDKEYYYTKEEQFKQVFKELEFLGWYTTGGPPDPSDIHVHKQVCEIIESPLFLKLNPMTKHTDLPVSVFESVIDIINGEATMLFAELTYTLATEEAERIGVDHVARMTATGSGENSTVAEHLIAQHSAIKMLHSRVKLILEYVKASEAGEVPFNHEILREAYALCHCLPVLSTDKFKTDFYDQCNDVGLMAYLGTITKTCNTMNQFVNKFNVLYDRQGIGRRMRGLFF

435 NP_937762.1 NP_002989.2

>NP_937762.1

MAAAARPRGRALGPVLPPTPLLLLVLRVLPACGATARDPGAAAGLSLHPTYFNLAEAARIWATATCGERGPGEGRPQPELYCKLVGGPTAPGSGHTIQGQFCDYCNSEDPRKAHPVTNAIDGSERWWQSPPLSSGTQYNRVNLTLDLGQLFHVAYILIKFANSPRPDLWVLERSVDFGSTYSPWQYFAHSKVDCLKEFGREANMAVTRDDDVLCVTEYSRIVPLENGEVVVSLINGRPGAKNFTFSHTLREFTKATNIRLRFLRTNTLLGHLISKAQRDPTVTRRYYYSIKDISIGGQCVCNGHAEVCNINNPEKLFRCECQHHTCGETCDRCCTGYNQRRWRPAAWEQSHECEACNCHGHASNCYYDPDVERQQASLNTQGIYAGGGVCINCQHNTAGVNCEQCAKGYYRPYGVPVDAPDGCIPCSCDPEHADGCEQGSGRCHCKPNFHGDNCEKCAIGYYNFPFCLRIPIFPVSTPSSEDPVAGDIKGCDCNLEGVLPEICDAHGRCLCRPGVEGPRCDTCRSGFYSFPICQACWCSALGSYQMPCSSVTGQCECRPGVTGQRCDRCLSGAYDFPHCQGSSSACDPAGTINSNLGYCQCKLHVEGPTCSRCKLLYWNLDKENPSGCSECKCHKAGTVSGTGECRQGDGDCHCKSHVGGDSCDTCEDGYFALEKSNYFGCQGCQCDIGGALSSMCSGPSGVCQCREHVVGKVCQRPENNYYFPDLHHMKYEIEDGSTPNGRDLRFGFDPLAFPEFSWRGYAQMTSVQNDVRITLNVGKSSGSLFRVILRYVNPGTEAVSGHITIYPSWGAAQSKEIIFLPSKEPAFVTVPGNGFADPFSITPGIWVACIKAEGVLLDYLVLLPRDYYEASVLQLPVTEPCAYAGPPQENCLLYQHLPVTRFPCTLACEARHFLLDGEPRPVAVRQPTPAHPVMVDLSGREVELHLRLRIPQVGHYVVVVEYSTEAAQLFVVDVNVKSSGSVLAGQVNIYSCNYSVLCRSAVIDHMSRIAMYELLADADIQLKGHMARFLLHQVCIIPIEEFSAEYVRPQVHCIASYGRFVNQSATCVSLAHETPPTALILDVLSGRPFPHLPQQSSPSVDVLPGVTLKAPQNQVTLRGRVPHLGRYVFVIHFYQAAHPTFPAQVSVDGGWPRAGSFHASFCPHVLGCRDQVIAEGQIEFDISEPEVAATVKVPEGKSLVLVRVLVVPAENYDYQILHKKSMDKSLEFITNCGKNSFYLDPQTASRFCKNSARSLVAFYHKGALPCECHPTGATGPHCSPEGGQCPCQPNVIGRQCTRCATGHYGFPRCKPCSCGRRLCEEMTGQCRCPPRTVRPQCEVCETHSFSFHPMAGCEGCNCSRRGTIEAAMPECDRDSGQCRCKPRITGRQCDRCASGFYRFPECVPCNCNRDGTEPGVCDPGTGACLCKENVEGTECNVCREGSFHLDPANLKGCTSCFCFGVNNQCHSSHKRRTKFVDMLGWHLETADRVDIPVSFNPGSNSMVADLQELPATIHSASWVAPTSYLGDKVSSYGGYLTYQAKSFGLPGDMVLLEKKPDVQLTGQHMSIIYEETNTPRPDRLHHGRVHVVEGNFRHASSRAPVSREELMTVLSRLADVRIQGLYFTETQRLTLSEVGLEEASDTGSGRIALAVEICACPPAYAGDSCQGCSPGYYRDHKGLYTGRCVPCNCNGHSNQCQDGSGICVNCQHNTAGEHCERCQEGYYGNAVHGSCRACPCPHTNSFATGCVVNGGDVRCSCKAGYTGTQCERCAPGYFGNPQKFGGSCQPCSCNSNGQLGSCHPLTGDCINQEPKDSSPAEECDDCDSCVMTLLNDLATMGEQLRLVKSQLQGLSASAGLLEQMRHMETQAKDLRNQLLNYRSAISNHGSKIEGLERELTDLNQEFETLQEKAQVNSRKAQTLNNNVNRATQSAKELDVKIKNVIRNVHILLKQISGTDGEGNNVPSGDFSREWAEAQRMMRELRNRNFGKHLREAEADKRESQLLLNRIRTWQKTHQGENNGLANSIRDSLNEYEAKLSDLRARLQEAAAQAKQANGLNQENERALGAIQRQVKEINSLQSDFTKYLTTADSSLLQTNIALQLMEKSQKEYEKLAASLNEARQELSDKVRELSRSAGKTSLVEEAEKHARSLQELAKQLEEIKRNASGDELVRCAVDAATAYENILNAIKAAEDAANRAASASESALQTVIKEDLPRKAKTLSSNSDKLLNEAKMTQKKLKQEVSPALNNLQQTLNIVTVQKEVIDTNLTTLRDGLHGIQRGDIDAMISSAKSMVRKANDITDEVLDGLNPIQTDVERIKDTYGRTQNEDFKKALTDADNSVNKLTNKLPDLWRKIESINQQLLPLGNISDNMDRIRELIQQARDAASKVAVPMRFNGKSGVEVRLPNDLEDLKGYTSLSLFLQRPNSRENGGTENMFVMYLGNKDASRDYIGMAVVDGQLTCVYNLGDREAELQVDQILTKSETKEAVMDRVKFQRIYQFARLNYTKGATSSKPETPGVYDMDGRNSNTLLNLDPENVVFYVGGYPPDFKLPSRLSFPPYKGCIELDDLNENVLSLYNFKKTFNLNTTEVEPCRRRKEESDKNYFEGTGYARVPTQPHAPIPTFGQTIQTTVDRGLLFFAENGDRFISLNIEDGKLMVRYKLNSELPKERGVGDAINNGRDHSIQIKIGKLQKRMWINVDVQNTIIDGEVFDFSTYYLGGIPIAIRERFNISTPAFRGCMKNLKKTSGVVRLNDTVGVTKKCSEDWKLVRSASFSRGGQLSFTDLGLPPTDHLQASFGFQTFQPSGILLDHQTWTRNLQVTLEDGYIELSTSDSGSPIFKSPQTYMDGLLHYVSVISDNSGLRLLIDDQLLRNSKRLKHISSSRQSLRLGGSNFEGCISNVFVQRLSLSPEVLDLTSNSLKRDVSLGGCSLNKPPFLMLLKGSTRFNKTKTFRINQLLQDTPVASPRSVKVWQDACSPLPKTQANHGALQFGDIPTSHLLFKLPQELLKPRSQFAVDMQTTSSRGLVFHTGTKNSFMALYLSKGRLVFALGTDGKKLRIKSKEKCNDGKWHTVVFGHDGEKGRLVVDGLRAREGSLPGNSTISIRAPVYLGSPPSGKPKSLPTNSFVGCLKNFQLDSKPLYTPSSSFGVSSCLGGPLEKGIYFSEEGGHVVLAHSVLLGPEFKLVFSIRPRSLTGILIHIGSQPGKHLCVYLEAGKVTASMDSGAGGTSTSVTPKQSLCDGQWHSVAVTIKQHILHLELDTDSSYTAGQIPFPPASTQEPLHLGGAPANLTTLRIPVWKSFFGCLRNIHVNHIPVPVTEALEVQGPVSLNGCPDQ

>NP_002989.2

MRRAWILLTLGLVACVSAESRAELTSDKDMYLDNSSIEEASGVYPIDDDDYASASGSGADEDVESPELTTSRPLPKILLTSAAPKVETTTLNIQNKIPAQTKSPEETDKEKVHLSDSERKMDPAEEDTNVYTEKHSDSLFKRTEVLAAVIAGGVIGFLFAIFLILLLVYRMRKKDEGSYDLGERKPSSAAYQKAPTKEFYA

436 NP_647478.1 NP_001220.2

>NP_647478.1

MGPKDSAKCLHRGPQPSHWAAGDGPTQERCGPRSLGSPVLGLDTCRAWDHVDGQILGQLRPLTEEEEEEGAGATLSRGPAFPGMGSEELRLASFYDWPLTAEVPPELLAAAGFFHTGHQDKVRCFFCYGGLQSWKRGDDPWTEHAKWFPSCQFLLRSKGRDFVHSVQETHSQLLGSWDPWEEPEDAAPVAPSVPASGYPELPTPRREVQSESAQEPGGVSPAEAQRAWWVLEPPGARDVEAQLRRLQEERTCKVCLDRAVSIVFVPCGHLVCAECAPGLQLCPICRAPVRSRVRTFLS

>NP_001220.2

MDEADRRLLRRCRLRLVEELQVDQLWDALLSRELFRPHMIEDIQRAGSGSRRDQARQLIIDLETRGSQALPLFISCLEDTGQDMLASFLRTNRQAAKLSKPTLENLTPVVLRPEIRKPEVLRPETPRPVDIGSGGFGDVGALESLRGNADLAYILSMEPCGHCLIINNVNFCRESGLRTRTGSNIDCEKLRRRFSSLHFMVEVKGDLTAKKMVLALLELAQQDHGALDCCVVVILSHGCQASHLQFPGAVYGTDGCPVSVEKIVNIFNGTSCPSLGGKPKLFFIQACGGEQKDHGFEVASTSPEDESPGSNPEPDATPFQEGLRTFDQLDAISSLPTPSDIFVSYSTFPGFVSWRDPKSGSWYVETLDDIFEQWAHSEDLQSLLLRVANAVSVKGIYKQMPGCFNFLRKKLFFKTS

437 NP_005139.1 NP_002113.2

>NP_005139.1

MKVKKGGGGAGTATESAPGPSGQSVAPIPQPPAESESGSESEPDAGPGPRPGPLQRKQPIGPEDVLGLQRITGDYLCSPEENIYKIDFVRFKIRDMDSGTVLFEIKKPPVSERLPINRRDLDPNAGRFVRYQFTPAFLRLRQVGATVEFTVGDKPVNNFRMIERHYFRNQLLKSFDFHFGFCIPSSKNTCEHIYDFPPLSEELISEMIRHPYETQSDSFYFVDDRLVMHNKADYSYSGTP

>NP_002113.2

MILNKALLLGALALTTVMSPCGGEDIVADHVASCGVNLYQFYGPSGQYTHEFDGDEQFYVDLERKETAWRWPEFSKFGGFDPQGALRNMAVAKHNLNIMIKRYNSTAATNEVPEVTVFSKSPVTLGQPNTLICLVDNIFPPVVNITWLSNGQSVTEGVSETSFLSKSDHSFFKISYLTFLPSADEIYDCKVEHWGLDQPLLKHWEPEIPAPMSELTETVVCALGLSVGLMGIVVGTVFIIQGLRSVGASRHQGPL

438 NP_003737.1 NP_000025.1

>NP_003737.1

MCDRKAVIKNADMSEEMQQDSVECATQALEKYNIEKDIAAHIKKEFDKKYNPTWHCIVGRNFGSYVTHETKHFIYFYLGQVAILLFKSG

>NP_000025.1

MPYQYPALTPEQKKELSDIAHRIVAPGKGILAADESTGSIAKRLQSIGTENTEENRRFYRQLLLTADDRVNPCIGGVILFHETLYQKADDGRPFPQVIKSKGGVVGIKVDKGVVPLAGTNGETTTQGLDGLSERCAQYKKDGADFAKWRCVLKIGEHTPSALAIMENANVLARYASICQQNGIVPIVEPEILPDGDHDLKRCQYVTEKVLAAVYKALSDHHIYLEGTLLKPNMVTPGHACTQKFSHEEIAMATVTALRRTVPPAVTGITFLSGGQSEEEASINLNAINKCPLLKPWALTFSYGRALQASALKAWGGKKENLKAAQEEYVKRALANSLACQGKYTPSGQAGAAASESLFVSNHAY

439 NP_066961.2 NP_005997.2

>NP_066961.2

MAAASVTPPGSLELLQPGFSKTLLGTKLEAKYLCSACRNVLRRPFQAQCGHRYCSFCLASILSSGPQNCAACVHEGIYEEGISILESSSAFPDNAARREVESLPAVCPSDGCTWKGTLKEYESCHEGRCPLMLTECPACKGLVRLGEKERHLEHECPERSLSCRHCRAPCCGADVKAHHEVCPKFPLTCDGCGKKKIPREKFQDHVKTCGKCRVPCRFHAIGCLETVEGEKQQEHEVQWLREHLAMLLSSVLEAKPLLGDQSHAGSELLQRCESLEKKTATFENIVCVLNREVERVAMTAEACSRQHRLDQDKIEALSSKVQQLERSIGLKDLAMADLEQKVLEMEASTYDGVFIWKISDFARKRQEAVAGRIPAIFSPAFYTSRYGYKMCLRIYLNGDGTGRGTHLSLFFVVMKGPNDALLRWPFNQKVTLMLLDQNNREHVIDAFRPDVTSSSFQRPVNDMNIASGCPLFCPVSKMEAKNSYVRDDAIFIKAIVDLTGL

>NP_005997.2

MDLTKMGMIQLQNPSHPTGLLCKANQMRLAGTLCDVVIMVDSQEFHAHRTVLACTSKMFEILFHRNSQHYTLDFLSPKTFQQILEYAYTATLQAKAEDLDDLLYAAEILEIEYLEEQCLKMLETIQASDDNDTEATMADGGAEEEEDRKARYLKNIFISKHSSEESGYASVAGQSLPGPMVDQSPSVSTSFGLSAMSPTKAAVDSLMTIGQSLLQGTLQPPAGPEEPTLAGGGRHPGVAEVKTEMMQVDEVPSQDSPGAAESSISGGMGDKVEERGKEGPGTPTRSSVITSARELHYGREESAEQVPPPAEAGQAPTGRPEHPAPPPEKHLGIYSVLPNHKADAVLSMPSSVTSGLHVQPALAVSMDFSTYGGLLPQGFIQRELFSKLGELAVGMKSESRTIGEQCSVCGVELPDNEAVEQHRKLHSGMKTYGCELCGKRFLDSLRLRMHLLAHSAGAKAFVCDQCGAQFSKEDALETHRQTHTGTDMAVFCLLCGKRFQAQSALQQHMEVHAGVRSYICSECNRTFPSHTALKRHLRSHTGDHPYECEFCGSCFRDESTLKSHKRIHTGEKPYECNGCGKKFSLKHQLETHYRVHTGEKPFECKLCHQRSRDYSAMIKHLRTHNGASPYQCTICTEYCPSLSSMQKHMKGHKPEEIPPDWRIEKTYLYLCYV

440 NP_004979.3 NP_036377.1

>NP_004979.3

MSLEQRSLHCKPEEALEAQQEALGLVCVQAATSSSSPLVLGTLEEVPTAGSTDPPQSPQGASAFPTTINFTRQRQPSEGSSSREEEGPSTSCILESLFRAVITKKVADLVGFLLLKYRAREPVTKAEMLESVIKNYKHCFPEIFGKASESLQLVFGIDVKEADPTGHSYVLVTCLGLSYDGLLGDNQIMPKTGFLIIVLVMIAMEGGHAPEEEIWEELSVMEVYDGREHSAYGEPRKLLTQDLVQEKYLEYRQVPDSDPARYEFLWGPRALAETSYVKVLEYVIKVSARVRFFFPSLREAALREEEEGV

>NP_036377.1

MALTSFLPAPTQLSQDQLEAEEKARSQRSRQTSLVSSRREPPPYGYRKGWIPRLLEDFGDGGAFPEIHVAQYPLDMGRKKKMSNALAIQVDSEGKIKYDAIARQGQSKDKVIYSKYTDLVPKEVMNADDPDLQRPDEEAIKEITEKTRVALEKSVSQKVAAAMPVRAADKLAPAQYIRYTPSQQGVAFNSGAKQRVIRMVEMQKDPMEPPRFKINKKIPRGPPSPPAPVMHSPSRKMTVKEQQEWKIPPCISNWKNAKGYTIPLDKRLAADGRGLQTVHINENFAKLAEALYIADRKAREAVEMRAQVERKMAQKEKEKHEEKLREMAQKARERRAGIKTHVEKEDGEARERDEIRHDRRKERQHDRNLSRAAPDKRSKLQRNENRDISEVIALGVPNPRTSNEVQYDQRLFNQSKGMDSGFAGGEDEIYNVYDQAWRGGKDMAQSIYRPSKNLDKDMYGDDLEARIKTNRFVPDKEFSGSDRRQRGREGPVQFEEDPFGLDKFLEEAKQHGGSKRPSDSSRPKEHEHEGKKRRKE

441 NP_055187.1 NP_003913.3

>NP_055187.1

MGNCLKSPTSDDISLLHESQSDRASFGEGTEPDQEPPPPYQEQVPVPVYHPTPSQTRLATQLTEEEQIRIAQRIGLIQHLPKGVYDPGRDGSEKKIRECVICMMDFVYGDPIRFLPCMHIYHLDCIDDWLMRSFTCPSCMEPVDAALLSSYETN

>NP_003913.3

MATMIPPVKLKWLEHLNSSWITEDSESIATREGVAVLYSKLVSNKEVVPLPQQVLCLKGPQLPDFERESLSSDEQDHYLDALLSSQLALAKMVCSDSPFAGALRKRLLVLQRVFYALSNKYHDKGKVKQQQHSPESSSGSADVHSVSERPRSSTDALIEMGVRTGLSLLFALLRQSWMMPVSGPGLSLCNDVIHTAIEVVSSLPPLSLANESKIPPMGLDCLSQVTTFLKGVTIPNSGADTLGRRLASELLLGLAAQRGSLRYLLEWIEMALGASAVVHTMEKGKLLSSQEGMISFDCFMTILMQMRRSLGSSADRSQWREPTRTSDGLCSLYEAALCLFEEVCRMASDYSRTCASPDSIQTGDAPIVSETCEVYVWGSNSSHQLVEGTQEKILQPKLAPSFSDAQTIEAGQYCTFVISTDGSVRACGKGSYGRLGLGDSNNQSTLKKLTFEPHRSIKKVSSSKGSDGHTLAFTTEGEVFSWGDGDYGKLGHGNSSTQKYPKLIQGPLQGKVVVCVSAGYRHSAAVTEDGELYTWGEGDFGRLGHGDSNSRNIPTLVKDISNVGEVSCGSSHTIALSKDGRTVWSFGGGDNGKLGHGDTNRVYKPKVIEALQGMFIRKVCAGSQSSLALTSTGQVYAWGCGACLGCGSSEATALRPKLIEELAATRIVDVSIGDSHCLALSHDNEVYAWGNNSMGQCGQGNSTGPITKPKKVSGLDGIAIQQISAGTSHSLAWTALPRDRQVVAWHRPYCVDLEESTFSHLRSFLERYCDKINSEIPPLPFPSSREHHSFLKLCLKLLSNHLALALAGGVATSILGRQAGPLRNLLFRLMDSTVPDEIQEVVIETLSVGATMLLPPLRERMELLHSLLPQGPDRWESLSKGQRMQLDIILTSLQDHTHVASLLGYSSPSDAADLSSVCTGYGNLSDQPYGTQSCHPDTHLAEILMKTLLRNLGFYTDQAFGELEKNSDKFLLGTSSSENSQPAHLHELLCSLQKQLLAFCHINNISENSSSVALLHKHLQLLLPHATDIYSRSANLLKESPWNGSVGEKLRDVIYVSAAGSMLCQIVNSLLLLPVSVARPLLSYLLDLLPPLDCLNRLLPAADLLEDQELQWPLHGGPELIDPAGLPLPQPAQSWVWLVDLERTIALLIGRCLGGMLQGSPVSPEEQDTAYWMKTPLFSDGVEMDTPQLDKCMSCLLEVALSGNEEQKPFDYKLRPEIAVYVDLALGCSKEPARSLWISMQDYAVSKDWDSATLSNESLLDTVSRFVLAALLKHTNLLSQACGESRYQPGKHLSEVYRCVYKVRSRLLACKNLELIQTRSSSRDRWISENQDSADVDPQEHSFTRTIDEEAEMEEQAERDREEGHPEPEDEEEEREHEVMTAGKIFQCFLSAREVARSRDRDRMNSGAGSGARADDPPPQSQQERRVSTDLPEGQDVYTAACNSVIHRCALLILGVSPVIDELQKRREEGQLQQPSTSASEGGGLMTRSESLTAESRLVHTSPNYRLIKSRSESDLSQPESDEEGYALSGRRNVDLDLAASHRKRGPMHSQLESLSDSWARLKHSRDWLCNSSYSFESDFDLTKSLGVHTLIENVVSFVSGDVGNAPGFKEPEESMSTSPQASIIAMEQQQLRAELRLEALHQILVLLSGMEEKGSISLAGSRLSSGFQSSTLLTSVRLQFLAGCFGLGTVGHTGGKGESGRLHHYQDGIRAAKRNIQIEIQVAVHKIYQQLSATLERALQANKHHIEAQQRLLLVTVFALSVHYQPVDVSLAISTGLLNVLSQLCGTDTMLGQPLQLLPKTGVSQLSTALKVASTRLLQILAITTGTYADKLSPKVVQSLLDLLCSQLKNLLSQTGVLHMASFGEGEQEDGEEEEKKVDSSGETEKKDFRAALRKQHAAELHLGDFLVFLRRVVSSKAIQSKMASPKWTEVLLNIASQKCSSGIPLVGNLRTRLLALHVLEAVLPACESGVEDDQMAQIVERLFSLLSDCMWETPIAQAKHAIQIKEKEQEIKLQKQGELEEEDENLPIQEVSFDPEKAQCCLVENGQILTHGSGGKGYGLASTGVTSGCYQWKFYIVKENRGNEGTCVGVSRWPVHDFNHRTTSDMWLYRAYSGNLYHNGEQTLTLSSFTQGDFITCVLDMEARTISFGKNGEEPKLAFEDVDAAELYPCVMFYSSNPGEKVKICDMQMRGTPRDLLPGDPICSPVAAVLAEATIQLIRILHRTDRWTYCINKKMMERLHKIKICIKESGQKLKKSRSVQSREENEMREEKESKEEEKGKHTRHGLADLSELQLRTLCIEVWPVLAVIGGVDAGLRVGGRCVHKQTGRHATLLGVVKEGSTSAKVQWDEAEITISFPTFWSPSDTPLYNLEPCEPLPFDVARFRGLTASVLLDLTYLTGVHEDMGKQSTKRHEKKHRHESEEKGDVEQKPESESALDMRTGLTSDDVKSQSTTSSKSENEIASFSLDPTLPSVESQHQITEGKRKNHEHMSKNHDVAQSEIRAVQLSYLYLGAMKSLSALLGCSKYAELLLIPKVLAENGHNSDCASSPVVHEDVEMRAALQFLMRHMVKRAVMRSPIKRALGLADLERAQAMIYKLVVHGLLEDQFGGKIKQEIDQQAEESDPAQQAQTPVTTSPSASSTTSFMSSSLEDTTTATTPVTDTETVPASESPGVMPLSLLRQMFSSYPTTTVLPTRRAQTPPISSLPTSPSDEVGRRQSLTSPDSQSARPANRTALSDPSSRLSTSPPPPAIAVPLLEMGFSLRQIAKAMEATGARGEADAQNITVLAMWMIEHPGHEDEEEPQSGSTADSRPGAAVLGSGGKSNDPCYLQSPGDIPSADAAEMEEGFSESPDNLDHTENAASGSGPSARGRSAVTRRHKFDLAARTLLARAAGLYRSVQAHRNQSRREGISLQQDPGALYDFNLDEELEIDLDDEAMEAMFGQDLTSDNDILGMWIPEVLDWPTWHVCESEDREEVVVCELCECSVVSFNQHMKRNHPGCGRSANRQGYRSNGSYVDGWFGGECGSGNPYYLLCGTCREKYLAMKTKSKSTSSERYKGQAPDLIGKQDSVYEEDWDMLDVDEDEKLTGEEEFELLAGPLGLNDRRIVPEPVQFPDSDPLGASVAMVTATNSMEETLMQIGCHGSVEKSSSGRITLGEQAAALANPHDRVVALRRVTAAAQVLLARTMVMRALSLLSVSGSSCSLAAGLESLGLTDIRTLVRLMCLAAAGRAGLSTSPSAMASTSERSRGGHSKANKPISCLAYLSTAVGCLASNAPSAAKLLVQLCTQNLISAATGVNLTTVDDSIQRKFLPSFLRGIAEENKLVTSPNFVVTQALVALLADKGAKLRPNYDKSEVEKKGPLELANALAACCLSSRLSSQHRQWAAQQLVRTLAAHDRDNQTTLQTLADMGGDLRKCSFIKLEAHQNRVMTCVWCNKKGLLATSGNDGTIRVWNVTKKQYSLQQTCVFNRLEGDAEESLGSPSDPSFSPVSWSISGKYLAGALEKMVNIWQVNGGKGLVDIQPHWVSALAWPEEGPATAWSGESPELLLVGRMDGSLGLIEVVDVSTMHRRELEHCYRKDVSVTCIAWFSEDRPFAVGYFDGKLLLGTKEPLEKGGIVLIDAHKDTLISMKWDPTGHILMTCAKEDSVKLWGSISGCWCCLHSLCHPSIVNGIAWCRLPGKGSKLQLLMATGCQSGLVCVWRIPQDTTQTNVTSAEGWWEQESNCQDGYRKSSGAKCVYQLRGHITPVRTVAFSSDGLALVSGGLGGLMNIWSLRDGSVLQTVVIGSGAIQTTVWIPEVGVAACSNRSKDVLVVNCTAEWAAANHVLATCRTALKQQGVLGLNMAPCMRAFLERLPMMLQEQYAYEKPHVVCGDQLVHSPYMQCLASLAVGLHLDQLLCNPPVPPHHQNCLPDPASWNPNEWAWLECFSTTIKAAEALTNGAQFPESFTVPDLEPVPEDELVFLMDNSKWINGMDEQIMSWATSRPEDWHLGGKCDVYLWGAGRHGQLAEAGRNVMVPAAAPSFSQAQQVICGQNCTFVIQANGTVLACGEGSYGRLGQGNSDDLHVLTVISALQGFVVTQLVTSCGSDGHSMALTESGEVFSWGDGDYGKLGHGNSDRQRRPRQIEALQGEEVVQMSCGFKHSAVVTSDGKLFTFGNGDYGRLGLGNTSNKKLPERVTALEGYQIGQVACGLNHTLAVSADGSMVWAFGDGDYGKLGLGNSTAKSSPQKIDVLCGIGIKKVACGTQFSVALTKDGHVYTFGQDRLIGLPEGRARNHNRPQQIPVLAGVIIEDVAVGAEHTLALASNGDVYAWGSNSEGQLGLGHTNHVREPTLVTGLQGKNVRQISAGRCHSAAWTAPPVPPRAPGVSVPLQLGLPDTVPPQYGALREVSIHTVRARLRLLYHFSDLMYSSWRLLNLSPNNQNSTSHYNAGTWGIVQGQLRPLLAPRVYTLPMVRSIGKTMVQGKNYGPQITVKRISTRGRKCKPIFVQIARQVVKLNASDLRLPSRAWKVKLVGEGADDAGGVFDDTITEMCQELETGIVDLLIPSPNATAEVGYNRDRFLFNPSACLDEHLMQFKFLGILMGVAIRTKKPLDLHLAPLVWKQLCCVPLTLEDLEEVDLLYVQTLNSILHIEDSGITEESFHEMIPLDSFVGQSADGKMVPIIPGGNSIPLTFSNRKEYVERAIEYRLHEMDRQVAAVREGMSWIVPVPLLSLLTAKQLEQMVCGMPEISVEVLKKVVRYREVDEQHQLVQWFWHTLEEFSNEERVLFMRFVSGRSRLPANTADISQRFQIMKVDRPYDSLPTSQTCFFQLRLPPYSSQLVMAERLRYAINNCRSIDMDNYMLSRNVDNAEGSDTDY

442 NP_036228.1 NP_005206.2

>NP_036228.1

MPGIDKLPIEETLEDSPQTRSLLGVFEEDATAISNYMNQLYQAMHRIYDAQNELSAATHLTSKLLKEYEKQRFPLGGDDEVMSSTLQQFSKVIDELSSCHAVLSTQLADAMMFPITQFKERDLKEILTLKEVFQIASNDHDAAINRYSRLSKKRENDKVKYEVTEDVYTSRKKQHQTMMHYFCALNTLQYKKKIALLEPLLGYMQAQISFFKMGSENLNEQLEEFLANIGTSVQNVRREMDSDIETMQQTIEDLEVASDPLYVPDPDPTKFPVNRNLTRKAGYLNARNKTGLVSSTWDRQFYFTQGGNLMSQARGDVAGGLAMDIDNCSVMAVDCEDRRYCFQITSFDGKKSSILQAESKKDHEEWICTINNISKQIYLSENPEETAARVNQSALEAVTPSPSFQQRHESLRPAAGQSRPPTARTSSSGSLGSESTNLAALSLDSLVAPDTPIQFDIISPVCEDQPGQAKAFGQGGRRTNPFGESGGSTKSETEDSILHQLFIVRFLGSMEVKSDDHPDVVYETMRQILAARAIHNIFRMTESHLLVTCDCLKLIDPQTQVTRLTFPLPCVVLYATHQENKRLFGFVLRTSSGRSESNLSSVCYIFESNNEGEKICDSVGLAKQIALHAELDRRASEKQKEIERVKEKQQKELNKQKQIEKDLEEQSRLIAASSRPNQASSEGQFVVLSSSQSEESDLGEGGKKRESEA

>NP_005206.2

MENSLRCVWVPKLAFVLFGASLFSAHLQVTGFQIKAFTALRFLSEPSDAVTMRGGNVLLDCSAESDRGVPVIKWKKDGIHLALGMDERKQQLSNGSLLIQNILHSRHHKPDEGLYQCEASLGDSGSIISRTAKVAVAGPLRFLSQTESVTAFMGDTVLLKCEVIGEPMPTIHWQKNQQDLTPIPGDSRVVVLPSGALQISRLQPGDIGIYRCSARNPASSRTGNEAEVRILSDPGLHRQLYFLQRPSNVVAIEGKDAVLECCVSGYPPPSFTWLRGEEVIQLRSKKYSLLGGSNLLISNVTDDDSGMYTCVVTYKNENISASAELTVLVPPWFLNHPSNLYAYESMDIEFECTVSGKPVPTVNWMKNGDVVIPSDYFQIVGGSNLRILGVVKSDEGFYQCVAENEAGNAQTSAQLIVPKPAIPSSSVLPSAPRDVVPVLVSSRFVRLSWRPPAEAKGNIQTFTVFFSREGDNRERALNTTQPGSLQLTVGNLKPEAMYTFRVVAYNEWGPGESSQPIKVATQPELQVPGPVENLQAVSTSPTSILITWEPPAYANGPVQGYRLFCTEVSTGKEQNIEVDGLSYKLEGLKKFTEYSLRFLAYNRYGPGVSTDDITVVTLSDVPSAPPQNVSLEVVNSRSIKVSWLPPPSGTQNGFITGYKIRHRKTTRRGEMETLEPNNLWYLFTGLEKGSQYSFQVSAMTVNGTGPPSNWYTAETPENDLDESQVPDQPSSLHVRPQTNCIIMSWTPPLNPNIVVRGYIIGYGVGSPYAETVRVDSKQRYYSIERLESSSHYVISLKAFNNAGEGVPLYESATTRSITDPTDPVDYYPLLDDFPTSVPDLSTPMLPPVGVQAVALTHDAVRVSWADNSVPKNQKTSEVRLYTVRWRTSFSASAKYKSEDTTSLSYTATGLKPNTMYEFSVMVTKNRRSSTWSMTAHATTYEAAPTSAPKDLTVITREGKPRAVIVSWQPPLEANGKITAYILFYTLDKNIPIDDWIMETISGDRLTHQIMDLNLDTMYYFRIQARNSKGVGPLSDPILFRTLKVEHPDKMANDQGRHGDGGYWPVDTNLIDRSTLNEPPIGQMHPPHGSVTPQKNSNLLVIIVVTVGVITVLVVVIVAVICTRRSSAQQRKKRATHSAGKRKGSQKDLRPPDLWIHHEEMEMKNIEKPSGTDPAGRDSPIQSCQDLTPVSHSQSETQLGSKSTSHSGQDTEEAGSSMSTLERSLAARRAPRAKLMIPMDAQSNNPAVVSAIPVPTLESAQYPGILPSPTCGYPHPQFTLRPVPFPTLSVDRGFGAGRSQSVSEGPTTQQPPMLPPSQPEHSSSEEAPSRTIPTACVRPTHPLRSFANPLLPPPMSAIEPKVPYTPLLSQPGPTLPKTHVKTASLGLAGKARSPLLPVSVPTAPEVSEESHKPTEDSANVYEQDDLSEQMASLEGLMKQLNAITGSAF

443 AAH00566.1 AAQ03994.1

>AAH00566.1

MVKLAAKCILAGDPAVGKTALAQIFRSDGAHFQKSYTLTTGMDLVVKTVPVPDTGDSVELFIFDSAGKELFSEMLDKLWESPNVLCLVYDVTNEESFNNCSKWLEKARSQAPGISLPGVLVGNKTDLAGRRAVDSAEARAWALGQGLECFETSVKEMENFEAPFHCLAKQFHQLYREKVEVFRALA

>AAQ03994.1

MRKIDLCLSSEGSEVILATSSDEKHPPENIIDGNPETFWTTTGMFPQEFIICFHKHVRIERLVIQSYFVQTLKIEKSTSKEPVDFEQWIEKDLVHTEGQLQNEEIVAHDGSATYLRFIIVSAFDHFASVHSVSAEGTVVSNLSS

444 NP_036233.2 NP_660215.1

>NP_036233.2

MEAADASRSNGSSPEARDARSPSGPSGSLENGTKADGKDAKTTNGHGGEAAEGKSLGSALKPGEGRSALFAGNEWRRPIIQFVESGDDKNSNYFSMDSMEGKRSPYAGLQLGAAKKPPVTFAEKGELRKSIFSESRKPTVSIMEPGETRRNSYPRADTGLFSRSKSGSEEVLCDSCIGNKQKAVKSCLVCQASFCELHLKPHLEGAAFRDHQLLEPIRDFEARKCPVHGKTMELFCQTDQTCICYLCMFQEHKNHSTVTVEEAKAEKETELSLQKEQLQLKIIEIEDEAEKWQKEKDRIKSFTTNEKAILEQNFRDLVRDLEKQKEEVRAALEQREQDAVDQVKVIMDALDERAKVLHEDKQTREQLHSISDSVLFLQEFGALMSNYSLPPPLPTYHVLLEGEGLGQSLGNFKDDLLNVCMRHVEKMCKADLSRNFIERNHMENGGDHRYVNNYTNSFGGEWSAPDTMKRYSMYLTPKGGVRTSYQPSSPGRFTKETTQKNFNNLYGTKGNYTSRVWEYSSSIQNSDNDLPVVQGSSSFSLKGYPSLMRSQSPKAQPQTWKSGKQTMLSHYRPFYVNKGNGIGSNEAP

>NP_660215.1

MAAPDLSTNLQEEATCAICLDYFTDPVMTDCGHNFCRECIRRCWGQPEGPYACPECRELSPQRNLRPNRPLAKMAEMARRLHPPSPVPQGVCPAHREPLAAFCGDELRLLCAACERSGEHWAHRVRPLQDAAEDLKAKLEKSLEHLRKQMQDALLFQAQADETCVLWQKMVESQRQNVLGEFERLRRLLAEEEQQLLQRLEEEELEVLPRLREGAAHLGQQSAHLAELIAELEGRCQLPALGLLQDIKDALRRVQDVKLQPPEVVPMELRTVCRVPGLVETLRRFRGDVTLDPDTANPELILSEDRRSVQRGDLRQALPDSPERFDPGPCVLGQERFTSGRHYWEVEVGDRTSWALGVCRENVNRKEKGELSAGNGFWILVFLGSYYNSSERALAPLRDPPRRVGIFLDYEAGHLSFYSATDGSLLFIFPEIPFSGTLRPLFSPLSSSPTPMTICRPKGGSGDTLAPQ

445 NP_055062.1 NP_001895.1

>NP_055062.1

MKPLAIPANHGVMGQQEKHSLPADFTKLHLTDSLHPQVTHVSSSHSGCSITSDSGSSSLSDIYQATESEAGDMDLSGLPETAVDSEDDDDEEDIERASDPLMSRDIVRDCLEKDPIDRTDDDIEQLLEFMHQLPAFANMTMSVRRELCAVMVFAVVERAGTIVLNDGEELDSWSVILNGSVEVTYPDGKAEILCMGNSFGVSPTMDKEYMKGVMRTKVDDCQFVCIAQQDYCRILNQVEKNMQKVEEEGEIVMVKEHRELDRTGTRKGHIVIKGTSERLTMHLVEEHSVVDPTFIEDFLLTYRTFLSSPMEVGKKLLEWFNDPSLRDKVTRVVLLWVNNHFNDFEGDPAMTRFLEEFENNLEREKMGGHLRLLNIACAAKAKRRLMTLTKPSREAPLPFILLGGSEKGFGIFVDSVDSGSKATEAGLKRGDQILEVNGQNFENIQLSKAMEILRNNTHLSITVKTNLFVFKELLTRLSEEKRNGAPHLPKIGDIKKASRYSIPDLAVDVEQVIGLEKVNKKSKANTVGGRNKLKKILDKTRISILPQKPYNDIGIGQSQDDSIVGLRQTKHIPTALPVSGTLSSSNPDLLQSHHRILDFSATPDLPDQVLRVFKADQQSRYIMISKDTTAKEVVIQAIREFAVTATPDQYSLCEVSVTPEGVIKQRRLPDQLSKLADRIQLSGRYYLKNNMETETLCSDEDAQELLRESQISLLQLSTVEVATQLSMRNFELFRNIEPTEYIDDLFKLRSKTSCANLKRFEEVINQETFWVASEILRETNQLKRMKIIKHFIKIALHCRECKNFNSMFAIISGLNLAPVARLRTTWEKLPNKYEKLFQDLQDLFDPSRNMAKYRNVLNSQNLQPPIIPLFPVIKKDLTFLHEGNDSKVDGLVNFEKLRMIAKEIRHVGRMASVNMDPALMFRTRKKKWRSLGSLSQGSTNATVLDVAQTGGHKKRVRRSSFLNAKKLYEDAQMARKVKQYLSNLELEMDEESLQTLSLQCEPATNTLPKNPGDKKPVKSETSPVAPRAGSQQKAQSLPQPQQQPPPAHKINQGLQVPAVSLYPSRKKVPVKDLPPFGINSPQALKKILSLSEEGSLERHKKQAEDTISNASSQLSSPPTSPQSSPRKGYTLAPSGTVDNFSDSGHSEISSRSSIVSNSSFDSVPVSLHDERRQRHSVSIVETNLGMGRMERRTMIEPDQYSLGSYAPMSEGRGLYATATVISSPSTEELSQDQGDRASLDAADSGRGSWTSCSSGSHDNIQTIQHQRSWETLPFGHTHFDYSGDPAGLWASSSHMDQIMFSDHSTKYNRQNQSRESLEQAQSRASWASSTGYWGEDSEGDTGTIKRRGGKDVSIEAESSSLTSVTTEETKPVPMPAHIAVASSTTKGLIARKEGRYREPPPTPPGYIGIPITDFPEGHSHPARKPPDYNVALQRSRMVARSSDTAGPSSVQQPHGHPTSSRPVNKPQWHKPNESDPRLAPYQSQGFSTEEDEDEQVSAV

>NP_001895.1

MATQADLMELDMAMEPDRKAAVSHWQQQSYLDSGIHSGATTTAPSLSGKGNPEEEDVDTSQVLYEWEQGFSQSFTQEQVADIDGQYAMTRAQRVRAAMFPETLDEGMQIPSTQFDAAHPTNVQRLAEPSQMLKHAVVNLINYQDDAELATRAIPELTKLLNDEDQVVVNKAAVMVHQLSKKEASRHAIMRSPQMVSAIVRTMQNTNDVETARCTAGTLHNLSHHREGLLAIFKSGGIPALVKMLGSPVDSVLFYAITTLHNLLLHQEGAKMAVRLAGGLQKMVALLNKTNVKFLAITTDCLQILAYGNQESKLIILASGGPQALVNIMRTYTYEKLLWTTSRVLKVLSVCSSNKPAIVEAGGMQALGLHLTDPSQRLVQNCLWTLRNLSDAATKQEGMEGLLGTLVQLLGSDDINVVTCAAGILSNLTCNNYKNKMMVCQVGGIEALVRTVLRAGDREDITEPAICALRHLTSRHQEAEMAQNAVRLHYGLPVVVKLLHPPSHWPLIKATVGLIRNLALCPANHAPLREQGAIPRLVQLLVRAHQDTQRRTSMGGTQQQFVEGVRMEEIVEGCTGALHILARDVHNRIVIRGLNTIPLFVQLLYSPIENIQRVAAGVLCELAQDKEAAEAIEAEGATAPLTELLHSRNEGVATYAAAVLFRMSEDKPQDYKKRLSVELTSSLFRTEPMAWNETADLGLDIGAQGEPLGYRQDDPSYRSFHSGGYGQDALGMDPMMEHEMGGHHPGADYPVDGLPDLGHAQDLMDGLPPGDSNQLAWFDTDL

446 NP_002778.1 NP_002783.1

>NP_002778.1

MAERGYSFSLTTFSPSGKLVQIEYALAAVAGGAPSVGIKAANGVVLATEKKQKSILYDERSVHKVEPITKHIGLVYSGMGPDYRVLVHRARKLAQQYYLVYQEPIPTAQLVQRVASVMQEYTQSGGVRPFGVSLLICGWNEGRPYLFQSDPSGAYFAWKATAMGKNYVNGKTFLEKRYNEDLELEDAIHTAILTLKESFEGQMTEDNIEVGICNEAGFRRLTPTEVKDYLAAIA

>NP_002783.1

MSYDRAITVFSPDGHLFQVEYAQEAVKKGSTAVGVRGRDIVVLGVEKKSVAKLQDERTVRKICALDDNVCMAFAGLTADARIVINRARVECQSHRLTVEDPVTVEYITRYIASLKQRYTQSNGRRPFGISALIVGFDFDGTPRLYQTDPSGTYHAWKANAIGRGAKSVREFLEKNYTDEAIETDDLTIKLVIKALLEVVQSGGKNIELAVMRRDQSLKILNPEEIEKYVAEIEKEKEENEKKKQKKAS

447 NP_003011.1 NP_002585.2

>NP_003011.1

MVSRMVSTMLSGLLFWLASGWTPAFAYSPRTPDRVSEADIQRLLHGVMEQLGIARPRVEYPAHQAMNLVGPQSIEGGAHEGLQHLGPFGNIPNIVAELTGDNIPKDFSEDQGYPDPPNPCPVGKTDDGCLENTPDTAEFSREFQLHQHLFDPEHDYPGLGKWNKKLLYEKMKGGERRKRRSVNPYLQGQRLDNVVAKKSVPHFSDEDKDPE

>NP_002585.2

MKGGCVSQWKAAAGFLFCVMVFASAERPVFTNHFLVELHKGGEDKARQVAAEHGFGVRKLPFAEGLYHFYHNGLAKAKRRRSLHHKQQLERDPRVKMALQQEGFDRKKRGYRDINEIDINMNDPLFTKQWYLINTGQADGTPGLDLNVAEAWELGYTGKGVTIGIMDDGIDYLHPDLASNYNAEASYDFSSNDPYPYPRYTDDWFNSHGTRCAGEVSAAANNNICGVGVAYNSKVAGIRMLDQPFMTDIIEASSISHMPQLIDIYSASWGPTDNGKTVDGPRELTLQAMADGVNKGRGGKGSIYVWASGDGGSYDDCNCDGYASSMWTISINSAINDGRTALYDESCSSTLASTFSNGRKRNPEAGVATTDLYGNCTLRHSGTSAAAPEAAGVFALALEANLGLTWRDMQHLTVLTSKRNQLHDEVHQWRRNGVGLEFNHLFGYGVLDAGAMVKMAKDWKTVPERFHCVGGSVQDPEKIPSTGKLVLTLTTDACEGKENFVRYLEHVQAVITVNATRRGDLNINMTSPMGTKSILLSRRPRDDDSKVGFDKWPFMTTHTWGEDARGTWTLELGFVGSAPQKGVLKEWTLMLHGTQSAPYIDQVVRDYQSKLAMSKKEELEEELDEAVERSLKSILNKN

448 NP_003499.1 NP_005421.1

>NP_003499.1

MAVAPLRGALLLWQLLAAGGAALEIGRFDPERGRGAAPCQAVEIPMCRGIGYNLTRMPNLLGHTSQGEAAAELAEFAPLVQYGCHSHLRFFLCSLYAPMCTDQVSTPIPACRPMCEQARLRCAPIMEQFNFGWPDSLDCARLPTRNDPHALCMEAPENATAGPAEPHKGLGMLPVAPRPARPPGDLGPGAGGSGTCENPEKFQYVEKSRSCAPRCGPGVEVFWSRRDKDFALVWMAVWSALCFFSTAFTVLTFLLEPHRFQYPERPIIFLSMCYNVYSLAFLIRAVAGAQSVACDQEAGALYVIQEGLENTGCTLVFLLLYYFGMASSLWWVVLTLTWFLAAGKKWGHEAIEAHGSYFHMAAWGLPALKTIVILTLRKVAGDELTGLCYVASTDAAALTGFVLVPLSGYLVLGSSFLLTGFVALFHIRKIMKTGGTNTEKLEKLMVKIGVFSILYTVPATCVIVCYVYERLNMDFWRLRATEQPCAAAAGPGGRRDCSLPGGSVPTVAVFMLKIFMSLVVGITSGVWVWSSKTFQTWQSLCYRKIAAGRARAKACRAPGSYGRGTHCHYKAPTVVLHMTKTDPSLENPTHL

>NP_005421.1

MGLWALLPGWVSATLLLALAALPAALAANSSGRWWGIVNVASSTNLLTDSKSLQLVLEPSLQLLSRKQRRLIRQNPGILHSVSGGLQSAVRECKWQFRNRRWNCPTAPGPHLFGKIVNRGCRETAFIFAITSAGVTHSVARSCSEGSIESCTCDYRRRGPGGPDWHWGGCSDNIDFGRLFGREFVDSGEKGRDLRFLMNLHNNEAGRTTVFSEMRQECKCHGMSGSCTVRTCWMRLPTLRAVGDVLRDRFDGASRVLYGNRGSNRASRAELLRLEPEDPAHKPPSPHDLVYFEKSPNFCTYSGRLGTAGTAGRACNSSSPALDGCELLCCGRGHRTRTQRVTERCNCTFHWCCHVSCRNCTHTRVLHECL

449 NP_002988.3 NP_002816.1

>NP_002988.3

MRRAALWLWLCALALSLQPALPQIVATNLPPEDQDGSGDDSDNFSGSGAGALQDITLSQQTPSTWKDTQLLTAIPTSPEPTGLEATAASTSTLPAGEGPKEGEAVVLPEVEPGLTAREQEATPRPRETTQLPTTHQASTTTATTAQEPATSHPHRDMQPGHHETSTPAGPSQADLHTPHTEDGGPSATERAAEDGASSQLPAAEGSGEQDFTFETSGENTAVVAVEPDRRNQSPVDQGATGASQGLLDRKEVLGGVIAGGLVGLIFAVCLVGFMLYRMKKKDEGSYSLEEPKQANGGAYQKPTKQEEFYA

>NP_002816.1

MQAQQYQQQRRKFAAAFLAFIFILAAVDTAEAGKKEKPEKKVKKSDCGEWQWSVCVPTSGDCGLGTREGTRTGAECKQTMKTQRCKIPCNWKKQFGAECKYQFQAWGECDLNTALKTRTGSLKRALHNAECQKTVTISKPCGKLTKPKPQAESKKKKKEGKKQEKMLD

450 NP_000112.1 NP_005179.2

>NP_000112.1

MDHLGASLWPQVGSLCLLLAGAAWAPPPNLPDPKFESKAALLAARGPEELLCFTERLEDLVCFWEEAASAGVGPGNYSFSYQLEDEPWKLCRLHQAPTARGAVRFWCSLPTADTSSFVPLELRVTAASGAPRYHRVIHINEVVLLDAPVGLVARLADESGHVVLRWLPPPETPMTSHIRYEVDVSAGNGAGSVQRVEILEGRTECVLSNLRGRTRYTFAVRARMAEPSFGGFWSAWSEPVSLLTPSDLDPLILTLSLILVVILVLLTVLALLSHRRALKQKIWPGIPSPESEFEGLFTTHKGNFQLWLYQNDGCLWWSPCTPFTEDPPASLEVLSERCWGTMQAVEPGTDDEGPLLEPVGSEHAQDTYLVLDKWLLPRNPPSEDLPGPGGSVDIVAMDEGSEASSCSSALASKPSPEGASAASFEYTILDPSSQLLRPWTLCPELPPTPPHLKYLYLVVSDSGISTDYSSGDSQGAQGGLSDGPYSNPYENSLIPAAEPLPPSYVACS

>NP_005179.2

MAGNVKKSSGAGGGSGSGGSGSGGLIGLMKDAFQPHHHHHHHLSPHPPGTVDKKMVEKCWKLMDKVVRLCQNPKLALKNSPPYILDLLPDTYQHLRTILSRYEGKMETLGENEYFRVFMENLMKKTKQTISLFKEGKERMYEENSQPRRNLTKLSLIFSHMLAELKGIFPSGLFQGDTFRITKADAAEFWRKAFGEKTIVPWKSFRQALHEVHPISSGLEAMALKSTIDLTCNDYISVFEFDIFTRLFQPWSSLLRNWNSLAVTHPGYMAFLTYDEVKARLQKFIHKPGSYIFRLSCTRLGQWAIGYVTADGNILQTIPHNKPLFQALIDGFREGFYLFPDGRNQNPDLTGLCEPTPQDHIKVTQEQYELYCEMGSTFQLCKICAENDKDVKIEPCGHLMCTSCLTSWQESEGQGCPFCRCEIKGTEPIVVDPFDPRGSGSLLRQGAEGAPSPNYDDDDDERADDTLFMMKELAGAKVERPPSPFSMAPQASLPPVPPRLDLLPQRVCVPSSASALGTASKAASGSLHKDKPLPVPPTLRDLPPPPPPDRPYSVGAESRPQRRPLPCTPGDCPSRDKLPPVPSSRLGDSWLPRPIPKVPVSAPSSSDPWTGRELTNRHSLPFSLPSQMEPRPDVPRLGSTFSLDTSMSMNSSPLVGPECDHPKIKPSSSANAIYSLAARPLPVPKLPPGEQCEGEEDTEYMTPSSRPLRPLDTSQSSRACDCDQQIDSCTYEAMYNIQSQAPSITESSTFGEGNLAAAHANTGPEESENEDDGYDVPKPPVPAVLARRTLSDISNASSSFGWLSLDGDPTTNVTEGSQVPERPPKPFPRRINSERKAGSCQQGSGPAASAATASPQLSSEIENLMSQGYSYQDIQKALVIAQNNIEMAKNILREFVSISSPAHVAT

451 NP_002728.1 NP_003370.2

>NP_002728.1

MADVFPGNDSTASQDVANRFARKGALRQKNVHEVKDHKFIARFFKQPTFCSHCTDFIWGFGKQGFQCQVCCFVVHKRCHEFVTFSCPGADKGPDTDDPRSKHKFKIHTYGSPTFCDHCGSLLYGLIHQGMKCDTCDMNVHKQCVINVPSLCGMDHTEKRGRIYLKAEVADEKLHVTVRDAKNLIPMDPNGLSDPYVKLKLIPDPKNESKQKTKTIRSTLNPQWNESFTFKLKPSDKDRRLSVEIWDWDRTTRNDFMGSLSFGVSELMKMPASGWYKLLNQEEGEYYNVPIPEGDEEGNMELRQKFEKAKLGPAGNKVISPSEDRKQPSNNLDRVKLTDFNFLMVLGKGSFGKVMLADRKGTEELYAIKILKKDVVIQDDDVECTMVEKRVLALLDKPPFLTQLHSCFQTVDRLYFVMEYVNGGDLMYHIQQVGKFKEPQAVFYAAEISIGLFFLHKRGIIYRDLKLDNVMLDSEGHIKIADFGMCKEHMMDGVTTRTFCGTPDYIAPEIIAYQPYGKSVDWWAYGVLLYEMLAGQPPFDGEDEDELFQSIMEHNVSYPKSLSKEAVSICKGLMTKHPAKRLGCGPEGERDVREHAFFRRIDWEKLENREIQPPFKPKVCGKGAENFDKFFTRGQPVLTPPDQLVIANIDQSDFEGFSYVNPQFVHPILQSAV

>NP_003370.2

MPKPINVRVTTMDAELEFAIQPNTTGKQLFDQVVKTIGLREVWYFGLHYVDNKGFPTWLKLDKKVSAQEVRKENPLQFKFRAKFYPEDVAEELIQDITQKLFFLQVKEGILSDEIYCPPETAVLLGSYAVQAKFGDYNKEVHKSGYLSSERLIPQRVMDQHKLTRDQWEDRIQVWHAEHRGMLKDNAMLEYLKIAQDLEMYGINYFEIKNKKGTDLWLGVDALGLNIYEKDDKLTPKIGFPWSEIRNISFNDKKFVIKPIDKKAPDFVFYAPRLRINKRILQLCMGNHELYMRRRKPDTIEVQQMKAQAREEKHQKQLERQQLETEKKRRETVEREKEQMMREKEELMLRLQDYEEKTKKAERELSEQIQRALQLEEERKRAQEEAERLEADRMAALRAKEELERQAVDQIKSQEQLAAELAEYTAKIALLEEARRRKEDEVEEWQHRAKEAQDDLVKTKEELHLVMTAPPPPPPPVYEPVSYHVQESLQDEGAEPTGYSAELSSEGIRDDRNEEKRITEAEKNERVQRQLLTLSSELSQARDENKRTHNDIIHNENMRQGRDKYKTLRQIRQGNTKQRIDEFEAL

452 NP_001226.2 NP_055048.1

>NP_001226.2

MRSLLLLSAFCLLEAALAAEVKKPAAAAAPGTAEKLSPKAATLAERSAGLAFSLYQAMAKDQAVENILVSPVVVASSLGLVSLGGKATTASQAKAVLSAEQLRDEEVHAGLGELLRSLSNSTARNVTWKLGSRLYGPSSVSFADDFVRSSKQHYNCEHSKINFRDKRSALQSINEWAAQTTDGKLPEVTKDVERTDGALLVNAMFFKPHWDEKFHHKMVDNRGFMVTRSYTVGVMMMHRTGLYNYYDDEKEKLQIVEMPLAHKLSSLIILMPHHVEPLERLEKLLTKEQLKIWMGKMQKKAVAISLPKGVVEVTHDLQKHLAGLGLTEAIDKNKADLSRMSGKKDLYLASVFHATAFELDTDGNPFDQDIYGREELRSPKLFYADHPFIFLVRDTQSGSLLFIGRLVRPKGDKMRDEL

>NP_055048.1

MNGEADCPTDLEMAAPKGQDRWSQEDMLTLLECMKNNLPSNDSSKFKTTESHMDWEKVAFKDFSGDMCKLKWVEISNEVRKFRTLTELILDAQEHVKNPYKGKKLKKHPDFPKKPLTPYFRFFMEKRAKYAKLHPEMSNLDLTKILSKKYKELPEKKKMKYIQDFQREKQEFERNLARFREDHPDLIQNAKKSDIPEKPKTPQQLWYTHEKKVYLKVRPDATTKEVKDSLGKQWSQLSDKKRLKWIHKALEQRKEYEEIMRDYIQKHPELNISEEGITKSTLTKAERQLKDKFDGRPTKPPPNSYSLYCAELMANMKDVPSTERMVLCSQQWKLLSQKEKDAYHKKCDQKKKDYEVELLRFLESLPEEEQQRVLGEEKMLNINKKQATSPASKKPAQEGGKGGSEKPKRPVSAMFIFSEEKRRQLQEERPELSESELTRLLARMWNDLSEKKKAKYKAREAALKAQSERKPGGEREERGKLPESPKRAEEIWQQSVIGDYLARFKNDRVKALKAMEMTWNNMEKKEKLMWIKKAAEDQKRYERELSEMRAPPAATNSSKKMKFQGEPKKPPMNGYQKFSQELLSNGELNHLPLKERMVEIGSRWQRISQSQKEHYKKLAEEQQKQYKVHLDLWVKSLSPQDRAAYKEYISNKRKSMTKLRGPNPKSSRTTLQSKSESEEDDEEDEDDEDEDEEEEDDENGDSSEDGGDSSESSSEDESEDGDENEEDDEDEDDDEDDDEDEDNESEGSSSSSSSSGDSSDSDSN

453 NP_004413.1 NP_009126.1

>NP_004413.1

MAGSSTGGGGVGETKVIYHLDEEETPYLVKIPVPAERITLGDFKSVLQRPAGAKYFFKSMDQDFGVVKEEISDDNARLPCFNGRVVSWLVSSDNPQPEMAPPVHEPRAELAPPAPPLPPLPPERTSGIGDSRPPSFHPNVSSSHENLEPETETESVVSLRRERPRRRDSSEHGAGGHRTGGPSRLERHLAGYESSSTLMTSELESTSLGDSDEEDTMSRFSSSTEQSSASRLLKRHRRRRKQRPPRLERTSSFSSVTDSTMSLNIITVTLNMEKYNFLGISIVGQSNERGDGGIYIGSIMKGGAVAADGRIEPGDMLLQVNDMNFENMSNDDAVRVLRDIVHKPGPIVLTVAKCWDPSPQAYFTLPRNEPIQPIDPAAWVSHSAALTGTFPAYPGSSSMSTITSGSSLPDGCEGRGLSVHTDMASVTKAMAAPESGLEVRDRMWLKITIPNAFLGSDVVDWLYHHVEGFPERREARKYASGLLKAGLIRHTVNKITFSEQCYYVFGDLSGGCESYLVNLSLNDNDGSSGASDQDTLAPLPGATPWPLLPTFSYQYPAPHPYSPQPPPYHELSSYTYGGGSASSQHSEGSRSSGSTRSDGGAGRTGRPEERAPESKSGSGSESEPSSRGGSLRRGGEASGTSDGGPPPSRGSTGGAPNLRAHPGLHPYGPPPGMALPYNPMMVVMMPPPPPPVPPAVQPPGAPPVRDLGSVPPELTASRQSFHMAMGNPSEFFVDVM

>NP_009126.1

MELADVGAAASSQGVHDQVLPTPNASSRVIVHVDLDCFYAQVEMISNPELKDKPLGVQQKYLVVTCNYEARKLGVKKLMNVRDAKEKCPQLVLVNGEDLTRYREMSYKVTELLEEFSPVVERLGFDENFVDLTEMVEKRLQQLQSDELSAVTVSGHVYNNQSINLLDVLHIRLLVGSQIAAEMREAMYNQLGLTGCAGVASNKLLAKLVSGVFKPNQQTVLLPESCQHLIHSLNHIKEIPGIGYKTAKCLEALGINSVRDLQTFSPKILEKELGISVAQRIQKLSFGEDNSPVILSGPPQSFSEEDSFKKCSSEVEAKNKIEELLASLLNRVCQDGRKPHTVRLIIRRYSSEKHYGRESRQCPIPSHVIQKLGTGNYDVMTPMVDILMKLFRNMVNVKMPFHLTLLSVCFCNLKALNTAKKGLIDYYLMPSLSTTSRSGKHSFKMKDTHMEDFPKDKETNRDFLPSGRIESTRTRESPLDTTNFSKEKDINEFPLCSLPEGVDQEVFKQLPVDIQEEILSGKSREKFQGKGSVSCPLHASRGVLSFFSKKQMQDIPINPRDHLSSSKQVSSVSPCEPGTSGFNSSSSSYMSSQKDYSYYLDNRLKDERISQGPKEPQGFHFTNSNPAVSAFHSFPNLQSEQLFSRNHTTDSHKQTVATDSHEGLTENREPDSVDEKITFPSDIDPQVFYELPEAVQKELLAEWKRTGSDFHIGHK

454 NP_056292.1 NP_899196.1

>NP_056292.1

MKRRLDDQESPVYAAQQRRIPGSTEAFPHQHRVLAPAPPVYEAVSETMQSATGIQYSVTPSYQVSAMPQSSGSHGPAIAAVHSSHHHPTAVQPHGGQVVQSHAHPAPPVAPVQGQQQFQRLKVEDALSYLDQVKLQFGSQPQVYNDFLDIMKEFKSQSIDTPGVISRVSQLFKGHPDLIMGFNTFLPPGYKIEVQTNDMVNVTTPGQVHQIPTHGIQPQPQPPPQHPSQPSAQSAPAPAQPAPQPPPAKVSKPSQLQAHTPASQQTPPLPPYASPRSPPVQPHTPVTISLGTAPSLQNNQPVEFNHAINYVNKIKNRFQGQPDIYKAFLEILHTYQKEQRNAKEAGGNYTPALTEQEVYAQVARLFKNQEDLLSEFGQFLPDANSSVLLSKTTAEKVDSVRNDHGGTVKKPQLNNKPQRPSQNGCQIRRHPTGTTPPVKKKPKLLNLKDSSMADASKHGGGTESLFFDKVRKALRSAEAYENFLRCLVIFNQEVISRAELVQLVSPFLGKFPELFNWFKNFLGYKESVHLETYPKERATEGIAMEIDYASCKRLGSSYRALPKSYQQPKCTGRTPLCKEVLNDTWVSFPSWSEDSTFVSSKKTQYEEHIYRCEDERFELDVVLETNLATIRVLEAIQKKLSRLSAEEQAKFRLDNTLGGTSEVIHRKALQRIYADKAADIIDGLRKNPSIAVPIVLKRLKMKEEEWREAQRGFNKVWREQNEKYYLKSLDHQGINFKQNDTKVLRSKSLLNEIESIYDERQEQATEENAGVPVGPHLSLAYEDKQILEDAAALIIHHVKRQTGIQKEDKYKIKQIMHHFIPDLLFAQRGDLSDVEEEEEEEMDVDEATGAVKKHNGVGGSPPKSKLLFSNTAAQKLRGMDEVYNLFYVNNNWYIFMRLHQILCLRLLRICSQAERQIEEENREREWEREVLGIKRDKSDSPAIQLRLKEPMDVDVEDYYPAFLDMVRSLLDGNIDSSQYEDSLREMFTIHAYIAFTMDKLIQSIVRQLQHIVSDEICVQVTDLYLAENNNGATGGQLNTQNSRSLLESTYQRKAEQLMSDENCFKLMFIQSQGQVQLTIELLDTEEENSDDPVEAERWSDYVERYMNSDTTSPELREHLAQKPVFLPRNLRRIRKCQRGREQQEKEGKEGNSKKTMENVDSLDKLECRFKLNSYKMVYVIKSEDYMYRRTALLRAHQSHERVSKRLHQRFQAWVDKWTKEHVPREMAAETSKWLMGEGLEGLVPCTTTCDTETLHFVSINKYRVKYGTVFKAP

>NP_899196.1

MENSDSNDKGSGDQSAAQRRSQMDRLDREEAFYQFVNNLSEEDYRLMRDNNLLGTPGESTEEELLRRLQQIKEGPPPQNSDENRGGDSSDDVSNGDSIIDWLNSVRQTGNTTRSGQRGNQSWRAVSRTNPNSGDFRFSLEINVNRNNGSQNSENENEPSARRSSGENVENNSQRQVENPRSESTSARPSRSERNSTEALTEVPPTRGQRRARSRSPDHRRTRARAERSRSPLHPMSEIPRRSHHSISSQTFEHPLVNETEGSSRTRHHVTLRQQISGPELLSRGLFAASGTRNASQGAGSSDTAASGESTGSGQRPPTIVLDLQVRRVRPGEYRQRDSIASRTRSRSQTPNNTVTYESERGGFRRTFSRSERAGVRTYVSTIRIPIRRILNTGLSETTSVAIQTMLRQIMTGFGELSYFMYSDSDSEPTGSVSNRNMERAESRSGRGGSGGGSSSGSSSSSSSSSSSSSSSSSSSSPSSSSGGESSETSSDLFEGSNEGSSSSGSSGARREGRHRAPVTFDESGSLPFLSLAQFFLLNEDDDDQPRGLTKEQIDNLAMRSFGENDALKTCSVCITEYTEGNKLRKLPCSHEYHVHCIDRWLSENSTCPICRRAVLASGNRESVV

455 NP_075067.2 NP_072097.2

>NP_075067.2

MAAHLSYGRVNLNVLREAVRRELREFLDKCAGSKAIVWDEYLTGPFGLIAQYSLLKEHEVEKMFTLKGNRLPAADVKNIIFFVRPRLELMDIIAENVLSEDRRGPTRDFHILFVPRRSLLCEQRLKDLGVLGSFIHREEYSLDLIPFDGDLLSMESEGAFKECYLEGDQTSLYHAAKGLMTLQALYGTIPQIFGKGECARQVANMMIRMKREFTGSQNSIFPVFDNLLLLDRNVDLLTPLATQLTYEGLIDEIYGIQNSYVKLPPEKFAPKKQGDGGKDLPTEAKKLQLNSAEELYAEIRDKNFNAVGSVLSKKAKIISAAFEERHNAKTVGEIKQFVSQLPHMQAARGSLANHTSIAELIKDVTTSEDFFDKLTVEQEFMSGIDTDKVNNYIEDCIAQKHSLIKVLRLVCLQSVCNSGLKQKVLDYYKREILQTYGYEHILTLHNLEKAGLLKPQTGGRNNYPTIRKTLRLWMDDVNEQNPTDISYVYSGYAPLSVRLAQLLSRPGWRSIEEVLRILPGPHFEERQPLPTGLQKKRQPGENRVTLIFFLGGVTFAEIAALRFLSQLEDGGTEYVIATTKLMNGTSWIEALMEKPF

>NP_072097.2

MDCYTANWNPLGDSAFYRKYELYSMDWDLKEELRDCLVAAAPYGGPIALLRNPWRKEKAASVRPVLDIYSASGMPLASLLWKSGPVVSLGWSAEEELLCVQEDGAVLVYGLHGDFRRHFSMGNEVLQNRVLDARIFHTEFGSGVAILTGAHRFTLSANVGDLKLRRMPEVPGLQSAPSCWTVLCQDRVAHILLAVGPDLYLLDHAACSAVTPPGLAPGVSSFLQMAVSFTYRHLALFTDTGYIWMGTASLKEKLCEFNCNIRAPPKQMVWCSRPRSKERAVVVAWERRLMVVGDAPESIQFVLDEDSYLVPELDGVRIFSRSTHEFLHEVPAASEEIFKIASMAPGALLLEAQKEYEKESQKADEYLREIQELGQLTQAVQQCIEAAGHEHQPDMQKSLLRAASFGKCFLDRFPPDSFVHMCQDLRVLNAVRDYHIGIPLTYSQYKQLTIQVLLDRLVLRRLYPLAIQICEYLRLPEVQGVSRILAHWACYKVQQKDVSDEDVARAINQKLGDTPGVSYSDIAARAYGCGRTELAIKLLEYEPRSGEQVPLLLKMKRSKLALSKAIESGDTDLVFTVLLHLKNELNRGDFFMTLRNQPMALSLYRQFCKHQELETLKDLYNQDDNHQELGSFHIRASYAAEERIEGRVAALQTAADAFYKAKNEFAAKATEDQMRLLRLQRRLEDELGGQFLDLSLHDTVTTLILGGHNKRAEQLARDFRIPDKRLWWLKLTALADLEDWEELEKFSKSKKSPIGYLPFVEICMKQHNKYEAKKYASRVGPEQKVKALLLVGDVAQAADVAIEHRNEAELSLVLSHCTGATDGATADKIQRARAQAQKK

456 NP_055048.1 NP_001886.1

>NP_055048.1

MNGEADCPTDLEMAAPKGQDRWSQEDMLTLLECMKNNLPSNDSSKFKTTESHMDWEKVAFKDFSGDMCKLKWVEISNEVRKFRTLTELILDAQEHVKNPYKGKKLKKHPDFPKKPLTPYFRFFMEKRAKYAKLHPEMSNLDLTKILSKKYKELPEKKKMKYIQDFQREKQEFERNLARFREDHPDLIQNAKKSDIPEKPKTPQQLWYTHEKKVYLKVRPDATTKEVKDSLGKQWSQLSDKKRLKWIHKALEQRKEYEEIMRDYIQKHPELNISEEGITKSTLTKAERQLKDKFDGRPTKPPPNSYSLYCAELMANMKDVPSTERMVLCSQQWKLLSQKEKDAYHKKCDQKKKDYEVELLRFLESLPEEEQQRVLGEEKMLNINKKQATSPASKKPAQEGGKGGSEKPKRPVSAMFIFSEEKRRQLQEERPELSESELTRLLARMWNDLSEKKKAKYKAREAALKAQSERKPGGEREERGKLPESPKRAEEIWQQSVIGDYLARFKNDRVKALKAMEMTWNNMEKKEKLMWIKKAAEDQKRYERELSEMRAPPAATNSSKKMKFQGEPKKPPMNGYQKFSQELLSNGELNHLPLKERMVEIGSRWQRISQSQKEHYKKLAEEQQKQYKVHLDLWVKSLSPQDRAAYKEYISNKRKSMTKLRGPNPKSSRTTLQSKSESEEDDEEDEDDEDEDEEEEDDENGDSSEDGGDSSESSSEDESEDGDENEEDDEDEDDDEDDDEDEDNESEGSSSSSSSSGDSSDSDSN

>NP_001886.1

MSGPVPSRARVYTDVNTHRPREYWDYESHVVEWGNQDDYQLVRKLGRGKYSEVFEAINITNNEKVVVKILKPVKKKKIKREIKILENLRGGPNIITLADIVKDPVSRTPALVFEHVNNTDFKQLYQTLTDYDIRFYMYEILKALDYCHSMGIMHRDVKPHNVMIDHEHRKLRLIDWGLAEFYHPGQEYNVRVASRYFKGPELLVDYQMYDYSLDMWSLGCMLASMIFRKEPFFHGHDNYDQLVRIAKVLGTEDLYDYIDKYNIELDPRFNDILGRHSRKRWERFVHSENQHLVSPEALDFLDKLLRYDHQSRLTAREAMEHPYFYTVVKDQARMGSSSMPGGSTPVSSANMMSGISSVPTPSPLGPLAGSPVIAAANPLGMPVPAAAGAQQ

457 NP_001252.1 NP_006212.1

>NP_001252.1

MAKQYDSVECPFCDEVSKYEKLAKIGQGTFGEVFKARHRKTGQKVALKKVLMENEKEGFPITALREIKILQLLKHENVVNLIEICRTKASPYNRCKGSIYLVFDFCEHDLAGLLSNVLVKFTLSEIKRVMQMLLNGLYYIHRNKILHRDMKAANVLITRDGVLKLADFGLARAFSLAKNSQPNRYTNRVVTLWYRPPELLLGERDYGPPIDLWGAGCIMAEMWTRSPIMQGNTEQHQLALISQLCGSITPEVWPNVDNYELYEKLELVKGQKRKVKDRLKAYVRDPYALDLIDKLLVLDPAQRIDSDDALNHDFFWSDPMPSDLKGMLSTHLTSMFEYLAPPRRKGSQITQQSTNQSRNPATTNQTEFERVF

>NP_006212.1

MADEEKLPPGWEKRMSRSSGRVYYFNHITNASQWERPSGNSSSGGKNGQGEPARVRCSHLLVKHSQSRRPSSWRQEKITRTKEEALELINGYIQKIKSGEEDFESLASQFSDCSSAKARGDLGAFSRGQMQKPFEDASFALRTGEMSGPVFTDSGIHIILRTE

458 NP_006411.1 NP_002725.1

>NP_006411.1

MQESQTKSMFVSRALEKILADKEVKRPQHSQLRRACQVALDEIKAEIEKQRLGTAAPPKANFIEADKYFLPFELACQSKSPRVVSTSLDCLQKLIAYGHITGNAPDSGAPGKRLIDRIVETICSCFQGPQTDEGVQLQIIKALLTAVTSPHIEIHEGTILQTVRTCYNIYLASKNLINQTTAKATLTQMLNVIFTRMENQVLQEARRLEKPIQSKPQSPVIQAAAVSPKFVRLKHSQAQSKPTTPEKTDLTNGEHARSDSGKVSTENGDAPRERGSSLSGTDDGAQEVVKDILEDVVTSAIKEAAEKHGLTEPERVLGELECQECAIPPGVDENSQTNGIADDRQSLSSADNLESDAQGHQVAARFSHVLQKDAFLVFRSLCKLSMKPLGEGPPDPKSHELRSKVVSLQLLLSVLQNAGPVFRTHEMFINAIKQYLCVALSKNGVSSVPDVFELSLAIFLTLLSNFKMHLKMQIEVFFKEIFLNILETSTSSFEHRWMVIQTLTRICADAQCVVDIYVNYDCDLNAANIFERLVNDLSKIAQGRSGHELGMTPLQELSLRKKGLECLVSILKCMVEWSKDLYVNPNHQTSLGQERLTDQEIGDGKGLDMARRCSVTSMESTVSSGTQTTVQDDPEQFEVIKQQKEIIEHGIELFNKKPKRGIQFLQEQGMLGTSVEDIAQFLHQEERLDSTQVGDFLGDSARFNKEVMYAYVDQLDFCEKEFVSALRTFLEGFRLPGEAQKIDRLMEKFAARYIECNQGQTLFASADTAYVLAYSIIMLTTDLHSPQVKNKMTKEQYIKMNRGINDSKDLPEEYLSSIYEEIEGKKIAMKETKELTIATKSTKQNVASEKQRRLLYNLEMEQMAKTAKALMEAVSHAKAPFTSATHLDHVRPMFKLVWTPLLAAYSIGLQNCDDTEVASLCLEGIRCAIRIACIFGMQLERDAYVQALARFSLLTASSSITKMKQKNIDTIKTLITVAHTDGNYLGNSWHEILKCISQLELAQLIGTGVKTRYLSGSGREREGSLKGHTLAGEEFMGLGLGNLVSGGVNKRQMASFQESVGETSSQSVVVAVDRIFTGSTRLDGNAIVDFVRWLCAVSMDELASPHHPRMFSLQKIVEISYYNMNRIRLQWSRIWHVIGDHFNKVGCNPNEDVAIFAVDSLRQLSMKFLEKGELANFRFQKDFLRPFEHIMKKNRSPTIRDMAIRCIAQMVNSQAANIRSGWKNIFAVFHQAASDHDGNIVELAFQTTCHIVTTIFQHHFPAAIDSFQDAVKCLSEFACNAAFPDTSMEAIRLIRFCGKYVSERPRVLQEYTSDDMNVAPGDRVWVRGWFPILFELSCIINRCKLDVRTRGLTVMFEIMKSYGHTFEKHWWQDLFRIVFRIFDNMKLPEQLSEKSEWMTTTCNHALYAICDVFTQFYEALNEVLLSDVFAQLQWCVKQDNEQLARSGTNCLENLVISNGEKFSPEVWDETCNCMLDIFKTTIPHVLLTWRPVGMEEDSSEKHLDVDLDRQSLSSIDKNPSERGQSQLSNPTDDSWKGRPYANQKLFASLLIKCVVQLELIQTIDNIVFYPATSKKEDAEHMVAAQQDTLDADIHIETEDQGMYKYMSSQHLFKLLDCLQESHSFSKAFNSNYEQRTVLWRAGFKGKSKPNLLKQETSSLACCLRILFRMYVDENRRDSWEEIQQRLLTVCSEALAYFITVNSESHREAWTSLLLLLLTKTLKINDEKFKAHASMYYPYLCEIMQFDLIPELRAVLRKFFLRISVVYKIWIPEEPSQVPAALSPVW

>NP_002725.1

MESGSTAASEEARSLRECELYVQKHNIQALLKDSIVQLCTARPERPMAFLREYFERLEKEEAKQIQNLQKAGTRTDSREDEISPPPPNPVVKGRRRRGAISAEVYTEEDAASYVRKVIPKDYKTMAALAKAIEKNVLFSHLDDNERSDIFDAMFSVSFIAGETVIQQGDEGDNFYVIDQGETDVYVNNEWATSVGEGGSFGELALIYGTPRAATVKAKTNVKLWGIDRDSYRRILMGSTLRKRKMYEEFLSKVSILESLDKWERLTVADALEPVQFEDGQKIVVQGEPGDEFFIILEGSAAVLQRRSENEEFVEVGRLGPSDYFGEIALLMNRPRAATVVARGPLKCVKLDRPRFERVLGPCSDILKRNIQQYNSFVSLSV

459 NP_001005271.1 AAH17247.1

>NP_001005271.1

MVVSEEEEEEEEEGDEEEEEEVEAADEDDEEDDDEGVLGRGPGHDRGRDRHSPPGCHLFPPPPPPPPPLPPPPPPPPPDKDDIRLLPSALGVKKRKRGPKKQKENKPGKPRKRKKRDSEEEFGSERDEYREKSESGGSEYGTGPGRKRRRKHREKKEKKTKRRKKGEGDGGQKQVEQKSSATLLLTWGLEDVEHVFSEEDYHTLTNYKAFSQFMRPLIAKKNPKIPMSKMMTILGAKWREFSANNPFKGSAAAVAAAAAAAAAAVAEQVSAAVSSATPIAPSGPPALPPPPAADIQPPPIRRAKTKEGKGPGHKRRSKSPRVPDGRKKLRGKKMAPLKIKLGLLGGKRKKGGSYVFQSDEGPEPEAEESDLDSGSVHSASGRPDGPVRTKKLKRGRPGRKKKKVLGCPAVAGEEEVDGYETDHQDYCEVCQQGGEIILCDTCPRAYHLVCLDPELDRAPEGKWSCPHCEKEGVQWEAKEEEEEYEEEGEEEGEKEEEDDHMEYCRVCKDGGELLCCDACISSYHIHCLNPPLPDIPNGEWLCPRCTCPVLKGRVQKILHWRWGEPPVAVPAPQQADGNPDVPPPRPLQGRSEREFFVKWVGLSYWHCSWAKELQLEIFHLVMYRNYQRKNDMDEPPPLDYGSGEDDGKSDKRKVKDPHYAEMEEKYYRFGIKPEWMTVHRIINHSVDKKGNYHYLVKWRDLPYDQSTWEEDEMNIPEYEEHKQSYWRHRELIMGEDPAQPRKYKKKKKELQGDGPPSSPTNDPTVKYETQPRFITATGGTLHMYQLEGLNWLRFSWAQGTDTILADEMGLGKTIQTIVFLYSLYKEGHTKGPFLVSAPLSTIINWEREFQMWAPKFYVVTYTGDKDSRAIIRENEFSFEDNAIKGGKKAFKMKREAQVKFHVLLTSYELITIDQAALGSIRWACLVVDEAHRLKNNQSKFFRVLNGYKIDHKLLLTGTPLQNNLEELFHLLNFLTPERFNNLEGFLEEFADISKEDQIKKLHDLLGPHMLRRLKADVFKNMPAKTELIVRVELSPMQKKYYKYILTRNFEALNSRGGGNQVSLLNIMMDLKKCCNHPYLFPVAAMESPKLPSGAYEGGALIKSSGKLMLLQKMLRKLKEQGHRVLIFSQMTKMLDLLEDFLDYEGYKYERIDGGITGALRQEAIDRFNAPGAQQFCFLLSTRAGGLGINLATADTVIIFDSDWNPHNDIQAFSRAHRIGQANKVMIYRFVTRASVEERITQVAKRKMMLTHLVVRPGLGSKAGSMSKQELDDILKFGTEELFKDENEGENKEEDSSVIHYDNEAIARLLDRNQDATEDTDVQNMNEYLSSFKVAQYVVREEDKIEEIEREIIKQEENVDPDYWEKLLRHHYEQQQEDLARNLGKGKRVRKQVNYNDAAQEDQDNQSEYSVGSEEEDEDFDERPEGRRQSKRQLRNEKDKPLPPLLARVGGNIEVLGFNTRQRKAFLNAVMRWGMPPQDAFTTQWLVRDLRGKTEKEFKAYVSLFMRHLCEPGADGSETFADGVPREGLSRQQVLTRIGVMSLVKKKVQEFEHINGRWSMPELMPDPSADSKRSSRASSPTKTSPTTPEASATNSPCTSKPATPAPSEKGEGIRTPLEKEEAENQEEKPEKNSRIGEKMETEADAPSPAPSLGERLEPRKIPLEDEVPGVPGEMEPEPGYRGDREKSATESTPGERGEEKPLDGQEHRERPEGETGDLGKREDVKGDRELRPGPRDEPRSNGRREEKTEKPRFMFNIADGGFTELHTLWQNEERAAISSGKLNEIWHRRHDYWLLAGIVLHGYARWQDIQNDAQFAIINEPFKTEANKGNFLEMKNKFLARRFKLLEQALVIEEQLRRAAYLNLSQEPAHPAMALHARFAEAECLAESHQHLSKESLAGNKPANAVLHKVLNQLEELLSDMKADVTRLPATLSRIPPIAARLQMSERSILSRLASKGTEPHPTPAYPPGPYATPPGYGAAFSAAPVGALAAAGANYSQMPAGSFITAATNGPPVLVKKEKEMVGALVSDGLDRKEPRAGEVICIDD

>AAH17247.1

MADYSTVPPPSSGSAGGGGGGGGGGGVNDAFKDALQRARQIAAKIGGDAGTSLNSNDYGYGGQKRPLEDGDQPDAKKVAPQNDSFGTQLPPMHQQQRSVMTEEYKVPDGMVGFIIGRGGEQISRIQQESGCKIQIAPDSGGLPERSCMLTGTPESVQSAKRLLDQIVEKGRPAPGFHHGDGPGNAVQEIMIPASKAGLVIGKGGETIKQLQERAGVKMVMIQDGPQNTGADKPLRITGDPYKVQQAKEMVLELIRDQGGFREVRNEYGSRIGGNEGIDVPIPRFAVGIVIGRNGEMIKKIQNDAGVRIQFKPDDGTTPERIAQITGPPDRCQHAAEIITDLLRSVQAGNPGGPGPGGRGRGRGQGNWNMGPPGGLQEFNFIVPTGKTGLIIGKGGETIKSISQQSGARIELQRNPPPNADPNMKLFTIRGTPQQIDYARQLIEEKIGGPVNPLGPPVPHGPHGVPGPHGPPGPPGPGTPMGPYNPAPYNPGPPGPAPHGPPAPYAPQGWGNAYPHWQQQAPPDPAKAGTDPNSAAWAAYYAHYYQQQAQPPPAAPAGAPTTTQTNGQGDQQNPAPAGQVDYTKAWEEYYKKMGQAVPAPTGAPPGGQPDYSAAWAEYYRQQAAYYAQTSPQGMPQHPPAPQCRFDPASIELAL

460 NP_001060.1 NP_055469.2

>NP_001060.1

MREIVHIQAGQCGNQIGAKFWEVISDEHGIDPTGSYHGDSDLQLERINVYYNEAAGNKYVPRAILVDLEPGTMDSVRSGPFGQIFRPDNFVFGQSGAGNNWAKGHYTEGAELVDSVLDVVRKESESCDCLQGFQLTHSLGGGTGSGMGTLLISKIREEYPDRIMNTFSVMPSPKVSDTVVEPYNATLSVHQLVENTDETYSIDNEALYDICFRTLKLTTPTYGDLNHLVSATMSGVTTCLRFPGQLNADLRKLAVNMVPFPRLHFFMPGFAPLTSRGSQQYRALTVPELTQQMFDSKNMMAACDPRHGRYLTVAAIFRGRMSMKEVDEQMLNVQNKNSSYFVEWIPNNVKTAVCDIPPRGLKMSATFIGNSTAIQELFKRISEQFTAMFRRKAFLHWYTGEGMDEMEFTEAESNMNDLVSEYQQYQDATADEQGEFEEEEGEDEA

>NP_055469.2

MKPGPPHRAGAAHGAGAGAGAAAGPGARGLLLPPLLLLLLAGRAAGAQRWRSENFERPVDLEGSGDDDSFPDDELDDLYSGSGSGYFEQESGIETAMRFSPDVALAVSTTPAVLPTTNIQPVGTPFEELPSERPTLEPATSPLVVTEVPEEPSQRATTVSTTMATTAATSTGDPTVATVPATVATATPSTPAAPPFTATTAVIRTTGVRRLLPLPLTTVATARATTPEAPSPPTTAAVLDTEAPTPRLVSTATSRPRALPRPATTQEPDIPERSTLPLGTTAPGPTEVAQTPTPETFLTTIRNEPEVPVSGGPSGDFELPEEETTQPDTANEVVAVGGAAAKASSPPGTLPKGARPGPGLLDNAIDSGSSAAQLPQKSILERKEVLVAVIVGGVVGALFAAFLVTLLIYRMKKKDEGSYTLEEPKQASVTYQKPDKQEEFYA

461 NP_002014.2 AAA50404.1

>NP_002014.2

MQWTSLLLLAGLFSLSQAQYEDDPHWWFHYLRSQQSTYYDPYDPYPYETYEPYPYGVDEGPAYTYGSPSPPDPRDCPQECDCPPNFPTAMYCDNRNLKYLPFVPSRMKYVYFQNNQITSIQEGVFDNATGLLWIALHGNQITSDKVGRKVFSKLRHLERLYLDHNNLTRMPGPLPRSLRELHLDHNQISRVPNNALEGLENLTALYLQHNEIQEVGSSMRGLRSLILLDLSYNHLRKVPDGLPSALEQLYMEHNNVYTVPDSYFRGAPKLLYVRLSHNSLTNNGLASNTFNSSSLLELDLSYNQLQKIPPVNTNLENLYLQGNRINEFSISSFCTVVDVVNFSKLQVLRLDGNEIKRSAMPADAPLCLRLASLIEI

>AAA50404.1

MHYCVLSAFLILHLVTVALSLSTCSTLDMDQFMRKRIEAIRGQILSKLKLTSPPEDYPEPEEVPPEVISIYNSTRDLLQEKASRRAAACERERSDEEYYAKEVYKIDMPPFFPSETVCPVVTTPSGSVGSLCSRQSQVLCGYLDAIPPTFYRPYFRIVRFDVSAMEKNASNLVKAEFRVFRLQNPKARVPEQRIELYQILKSKDLTSPTQRYIDSKVVKTRAEGEWLSFDVTDAVHEWLHHKDRNLGFKISLHCPCCTFVPSNNYIIPNKSEELEARFAGIDGTSTYTSGDQKTIKSTRKKNSGKTPHLLLMLLPSYRLESQQTNRRKKRALDAAYCFRNVQDNCCLRPLYIDFKRDLGWKWIHEPKGYNANFCAGACPYLWSSDTQHSRVLSLYNTINPEASASPCCVSQDLEPLTILYYIGKTPKIEQLSNMIVKSCKCS

462 NP_003752.2 Q15531

>NP_003752.2

MEEASEGGGNDRVRNLQSEVEGVKNIMTQNVERILARGENLEHLRNKTEDLEATSEHFKTTSQKVARKFWWKNVKMIVLICVIVFIIILFIVLFATGAFS

>Q15531

MKDRTQVLRTRRNSDDKEEVVHVDRDHFMDEFFEQEEEIRGCIEKLSEDVEQVKKQHSAILAAPNPDERTKQELEDLTADIKKTANKVRSKLKAIEQSIEQEEGSTAPRPILRIRKTQHSTLSRKFVEVMTEYNATQSKYRDRCKDRIQRQLEITGRTTTNEELEDMLESGKLPIFTDDIKMDSQMTKQALNEIETRHNEIIKLETSIRELHDMFVDMAMLVESQGEMIDRIEYNVEHSVDYVERAVSDTKKAVKYQSKARRKKIIIIICCVVLGVVLASSIGCTLGL

463 NP_783552.1 NP_057512.1

>NP_783552.1

MISVKRNTWRALSLVIGDCRKKGNFEYCQDRTEKHSTMPDSPVDVKTQSRLTPPTMPPPPTTQGAPRTSSFTPTTLTNGTSHSPTALNGAPSPPNGFSNGPSSSSSSSLANQQLPPACGARQLSKLKRFLTTLQQFGNDISPEIGERVRTLVLGLVNSTLTIEEFHSKLQEATNFPLRPFVIPFLKANLPLLQRELLHCARLAKQNPAQYLAQHEQLLLDASTTSPVDSSELLLDVNENGKRRTPDRTKENGFDREPLHSEHPSKRPCTISPGQRYSPNNGLSYQPNGLPHPTPPPPQHYRLDDMAIAHHYRDSYRHPSHRDLRDRNRPMGLHGTRQEEMIDHRLTDREWAEEWKHLDHLLNCIMDMVEKTRRSLTVLRRCQEADREELNYWIRRYSDAEDLKKGGGSSSSHSRQQSPVNPDPVALDAHREFLHRPASGYVPEEIWKKAEEAVNEVKRQAMTELQKAVSEAERKAHDMITTERAKMERTVAEAKRQAAEDALAVINQQEDSSESCWNCGRKASETCSGCNTARYCGSFCQHKDWEKHHHICGQTLQAQQQGDTPAVSSSVTPNSGAGSPMDTPPAATPRSTTPGTPSTIETTPR

>NP_057512.1

MAELQQLQEFEIPTGREALRGNHSALLRVADYCEDNYVQATDKRKALEETMAFTTQALASVAYQVGNLAGHTLRMLDLQGAALRQVEARVSTLGQMVNMHMEKVARREIGTLATVQRLPPGQKVIAPENLPPLTPYCRRPLNFGCLDDIGHGIKDLSTQLSRTGTLSRKSIKAPATPASATLGRPPRIPEPVHLPVVPDGRLSAASSASSLASAGSAEGVGGAPTPKGQAAPPAPPLPSSLDPPPPPAAVEVFQRPPTLEELSPPPPDEELPLPLDLPPPPPLDGDELGLPPPPPGFGPDEPSWVPASYLEKVVTLYPYTSQKDNELSFSEGTVICVTRRYSDGWCEGVSSEGTGFFPGNYVEPSC

464 NP_005852.2 NP_570722.2

>NP_005852.2

MKGKEEKEGGARLGAGGGSPEKSPSAQELKEQGNRLFVGRKYPEAAACYGRAITRNPLVAVYYTNRALCYLKMQQHEQALADCRRALELDGQSVKAHFFLGQCQLEMESYDEAIANLQRAYSLAKEQRLNFGDDIPSALRIAKKKRWNSIEERRIHQESELHSYLSRLIAAERERELEECQRNHEGDEDDSHVRAQQACIEAKHDKYMADMDELFSQVDEKRKKRDIPDYLCGKISFELMREPCITPSGITYDRKDIEEHLQRVGHFDPVTRSPLTQEQLIPNLAMKEVIDAFISENGWVEDY

>NP_570722.2

MPLQVSDYSWQQTKTAVFLSLPLKGVCVRDTDVFCTENYLKVNFPPFLFEAFLYAPIDDESSKAKIGNDTIVFTLYKKEAAMWETLSVTGVDKEMMQRIREKSILQAQERAKEATEAKAAAKREDQKYALSVMMKIEEEERKKIEDMKENERIKATKALEAWKEYQRKAEEQKKIQREEKLCQKEKQIKEERKKIKYKSLTRNLASRNLAPKGRNSENIFTEKLKEDSIPAPRSVGSIKINFTPRVFPTALRESQVAEEEEWLHKQAEARRAMNTDIAELCDLKEEEKNPEWLKDKGNKLFATENYLAAINAYNLAIRLNNKMPLLYLNRAACHLKLKNLHKAIEDSSKALELLMPPVTDNANARMKAHVRRGTAFCQLELYVEGLQDYEAALKIDPSNKIVQIDAEKIRNVIQGTELKS

465 NP_002258.2 NP_004636.1

>NP_002258.2

MAENPSLENHRIKSFKNKGRDVETMRRHRNEVTVELRKNKRDEHLLKKRNVPQEESLEDSDVDADFKAQNVTLEAILQNATSDNPVVQLSAVQAARKLLSSDRNPPIDDLIKSGILPILVKCLERDDNPSLQFEAAWALTNIASGTSAQTQAVVQSNAVPLFLRLLRSPHQNVCEQAVWALGNIIGDGPQCRDYVISLGVVKPLLSFISPSIPITFLRNVTWVIVNLCRNKDPPPPMETVQEILPALCVLIYHTDINILVDTVWALSYLTDGGNEQIQMVIDSGVVPFLVPLLSHQEVKVQTAALRAVGNIVTGTDEQTQVVLNCDVLSHFPNLLSHPKEKINKEAVWFLSNITAGNQQQVQAVIDAGLIPMIIHQLAKGDFGTQKEAAWAISNLTISGRKDQVEYLVQQNVIPPFCNLLSVKDSQVVQVVLDGLKNILIMAGDEASTIAEIIEECGGLEKIEVLQQHENEDIYKLAFEIIDQYFSGDDIDEDPCLIPEATQGGTYNFDPTANLQTKEFNF

>NP_004636.1

MAASETVRLRLQFDYPPPATPHCTAFWLLVDLNRCRVVTDLISLIRQRFGFSSGAFLGLYLEGGLLPPAESARLVRDNDCLRVKLEERGVAENSVVISNGDINLSLRKAKKRAFQLEEGEETEPDCKYSKKHWKSRENNNNNEKVLDLEPKAVTDQTVSKKNKRKNKATCGTVGDDNEEAKRKSPKKKEKCEYKKKAKNPKSPKVQAVKDWANQRCSSPKGSARNSLVKAKRKGSVSVCSKESPSSSSESESCDESISDGPSKVTLEARNSSEKLPTELSKEEPSTKNTTADKLAIKLGFSLTPSKGKTSGTTSSSSDSSAESDDQCLMSSSTPECAAGFLKTVGLFAGRGRPGPGLSSQTAGAAGWRRSGSNGGGQAPGASPSVSLPASLGRGWGREENLFSWKGAKGRGMRGRGRGRGHPVSCVVNRSTDNQRQQQLNDVVKNSSTIIQNPVETPKKDYSLLPLLAAAPQVGEKIAFKLLELTSSYSPDVSDYKEGRILSHNPETQQVDIEILSSLPALREPGKFDLVYHNENGAEVVEYAVTQESKITVFWKELIDPRLIIESPSNTSSTEPA

466 NP_001777.1 NP_060962.2

>NP_001777.1

MEDYTKIEKIGEGTYGVVYKGRHKTTGQVVAMKKIRLESEEEGVPSTAIREISLLKELRHPNIVSLQDVLMQDSRLYLIFEFLSMDLKKYLDSIPPGQYMDSSLVKSYLYQILQGIVFCHSRRVLHRDLKPQNLLIDDKGTIKLADFGLARAFGIPIRVYTHEVVTLWYRSPEVLLGSARYSTPVDIWSIGTIFAELATKKPLFHGDSEIDQLFRIFRALGTPNNEVWPEVESLQDYKNTFPKWKPGSLASHVKNLDENGLDLLSKMLIYDPAKRISGKMALNHPYFNDLDNQIKKM

>NP_060962.2

MEGISNFKTPSKLSEKKKSVLCSTPTINIPASPFMQKLGFGTGVNVYLMKRSPRGLSHSPWAVKKINPICNDHYRSVYQKRLMDEAKILKSLHHPNIVGYRAFTEANDGSLCLAMEYGGEKSLNDLIEERYKASQDPFPAAIILKVALNMARGLKYLHQEKKLLHGDIKSSNVVIKGDFETIKICDVGVSLPLDENMTVTDPEACYIGTEPWKPKEAVEENGVITDKADIFAFGLTLWEMMTLSIPHINLSNDDDDEDKTFDESDFDDEAYYAALGTRPPINMEELDESYQKVIELFSVCTNEDPKDRPSAAHIVEALETDV

467 NP_997080.1 NP_005424.1

>NP_997080.1

MRLALLWALGLLGAGSPLPSWPLPNIGGTEEQQAESEKAPREPLEPQVLQDDLPISLKKVLQTSLPEPLRIKLELDGDSHILELLQNRELVPGRPTLVWYQPDGTRVVSEGHTLENCCYQGRVRGYAGSWVSICTCSGLRGLVVLTPERSYTLEQGPGDLQGPPIISRIQDLHLPGHTCALSWRESVHTQKPPEHPLGQRHIRRRRDVVTETKTVELVIVADHSEAQKYRDFQHLLNRTLEVALLLDTFFRPLNVRVALVGLEAWTQRDLVEISPNPAVTLENFLHWRRAHLLPRLPHDSAQLVTGTSFSGPTVGMAIQNSICSPDFSGGVNMDHSTSILGVASSIAHELGHSLGLDHDLPGNSCPCPGPAPAKTCIMEASTDFLPGLNFSNCSRRALEKALLDGMGSCLFERLPSLPPMAAFCGNMFVEPGEQCDCGFLDDCVDPCCDSLTCQLRPGAQCASDGPCCQNCQLRPSGWQCRPTRGDCDLPEFCPGDSSQCPPDVSLGDGEPCAGGQAVCMHGRCASYAQQCQSLWGPGAQPAAPLCLQTANTRGNAFGSCGRNPSGSYVSCTPRDAICGQLQCQTGRTQPLLGSIRDLLWETIDVNGTELNCSWVHLDLGSDVAQPLLTLPGTACGPGLVCIDHRCQRVDLLGAQECRSKCHGHGVCDSNRHCYCEEGWAPPDCTTQLKATSSLTTGLLLSLLVLLVLVMLGASYWYRARLHQRLCQLKGPTCQYRAAQSGPSERPGPPQRALLARGTKQASALSFPAPPSRPLPPDPVSKRLQAELADRPNPPTRPLPADPVVRSPKSQGPAKPPPPRKPLPADPQGRCPSGDLPGPGAGIPPLVVPSRPAPPPPTVSSLYL

>NP_005424.1

MGCIKSKENKSPAIKYRPENTPEPVSTSVSHYGAEPTTVSPCPSSSAKGTAVNFSSLSMTPFGGSSGVTPFGGASSSFSVVPSSYPAGLTGGVTIFVALYDYEARTTEDLSFKKGERFQIINNTEGDWWEARSIATGKNGYIPSNYVAPADSIQAEEWYFGKMGRKDAERLLLNPGNQRGIFLVRESETTKGAYSLSIRDWDEIRGDNVKHYKIRKLDNGGYYITTRAQFDTLQKLVKHYTEHADGLCHKLTTVCPTVKPQTQGLAKDAWEIPRESLRLEVKLGQGCFGEVWMGTWNGTTKVAIKTLKPGTMMPEAFLQEAQIMKKLRHDKLVPLYAVVSEEPIYIVTEFMSKGSLLDFLKEGDGKYLKLPQLVDMAAQIADGMAYIERMNYIHRDLRAANILVGENLVCKIADFGLARLIEDNEYTARQGAKFPIKWTAPEAALYGRFTIKSDVWSFGILQTELVTKGRVPYPGMVNREVLEQVERGYRMPCPQGCPESLHELMNLCWKKDPDERPTFEYIQSFLEDYFTATEPQYQPGENL

468 NP_005893.1 NP_004646.2

>NP_005893.1

MSSILPFTPPIVKRLLGWKKGEQNGQEEKWCEKAVKSLVKKLKKTGQLDELEKAITTQNVNTKCITIPRSLDGRLQVSHRKGLPHVIYCRLWRWPDLHSHHELRAMELCEFAFNMKKDEVCVNPYHYQRVETPVLPPVLVPRHTEIPAEFPPLDDYSHSIPENTNFPAGIEPQSNIPETPPPGYLSEDGETSDHQMNHSMDAGSPNLSPNPMSPAHNNLDLQPVTYCEPAFWCSISYYELNQRVGETFHASQPSMTVDGFTDPSNSERFCLGLLSNVNRNAAVELTRRHIGRGVRLYYIGGEVFAECLSDSAIFVQSPNCNQRYGWHPATVCKIPPGCNLKIFNNQEFAALLAQSVNQGFEAVYQLTRMCTIRMSFVKGWGAEYRRQTVTSTPCWIELHLNGPLQWLDKVLTQMGSPSIRCSSVS

>NP_004646.2

MSSAMLVTCLPDPSSSFREDAPRPPVPGEEGETPPCQPGVGKGQVTKPMSVSSNTRRNEDGLGEPEGRASPDSPLTRWTKSLHSLLGDQDGAYLFRTFLEREKCVDTLDFWFACNGFRQMNLKDTKTLRVAKAIYKRYIENNSIVSKQLKPATKTYIRDGIKKQQIDSIMFDQAQTEIQSVMEENAYQMFLTSDIYLEYVRSGGENTAYMSNGGLGSLKVVCGYLPTLNEEEEWTCADFKCKLSPTVVGLSSKTLRATASVRSTETVDSGYRSFKRSDPVNPYHIGSGYVFAPATSANDSEISSDALTDDSMSMTDSSVDGIPPYRVGSKKQLQREMHRSVKANGQVSLPHFPRTHRLPKEMTPVEPATFAAELISRLEKLKLELESRHSLEERLQQIREDEEREGSELTLNSREGAPTQHPLSLLPSGSYEEDPQTILDDHLSRVLKTPGCQSPGVGRYSPRSRSPDHHHHHHSQYHSLLPPGGKLPPAAASPGACPLLGGKGFVTKQTTKHVHHHYIHHHAVPKTKEEIEAEATQRVHCFCPGGSEYYSYSKCKSHSKAPETMPSEQFGGSRGSTLPKRNGKGTEPGLALPAREGGAPGGAGALQLPREEGDRSQDVWQWMLESERQSKPKPHSAQSTKKAYPLESARSSPGERASRHHLWGGNSGHPRTTPRAHLFTQDPAMPPLTPPNTLAQLEEACRRLAEVSKPPKQRCCVASQQRDRNHSATVQTGATPFSNPSLAPEDHKEPKKLAGVHALQASELVVTYFFCGEEIPYRRMLKAQSLTLGHFKEQLSKKGNYRYYFKKASDEFACGAVFEEIWEDETVLPMYEGRILGKVERID

469 NP_001054.1 NP_001737.1

>NP_001054.1

MRLAVGALLVCAVLGLCLAVPDKTVRWCAVSEHEATKCQSFRDHMKSVIPSDGPSVACVKKASYLDCIRAIAANEADAVTLDAGLVYDAYLAPNNLKPVVAEFYGSKEDPQTFYYAVAVVKKDSGFQMNQLRGKKSCHTGLGRSAGWNIPIGLLYCDLPEPRKPLEKAVANFFSGSCAPCADGTDFPQLCQLCPGCGCSTLNQYFGYSGAFKCLKDGAGDVAFVKHSTIFENLANKADRDQYELLCLDNTRKPVDEYKDCHLAQVPSHTVVARSMGGKEDLIWELLNQAQEHFGKDKSKEFQLFSSPHGKDLLFKDSAHGFLKVPPRMDAKMYLGYEYVTAIRNLREGTCPEAPTDECKPVKWCALSHHERLKCDEWSVNSVGKIECVSAETTEDCIAKIMNGEADAMSLDGGFVYIAGKCGLVPVLAENYNKSDNCEDTPEAGYFAVAVVKKSASDLTWDNLKGKKSCHTAVGRTAGWNIPMGLLYNKINHCRFDEFFSEGCAPGSKKDSSLCKLCMGSGLNLCEPNNKEGYYGYTGAFRCLVEKGDVAFVKHQTVPQNTGGKNPDPWAKNLNEKDYELLCLDGTRKPVEEYANCHLARAPNHAVVTRKDKEACVHKILRQQQHLFGSNVTDCSGNFCLFRSETKDLLFRDDTVCLAKLHDRNTYEKYLGEEYVKAVGNLRKCSTSSLLEACTFRRP

>NP_001737.1

MEGKWLLCMLLVLGTAIVEAHDGHDDDVIDIEDDLDDVIEEVEDSKPDTTAPPSSPKVTYKAPVPTGEVYFADSFDRGTLSGWILSKAKKDDTDDEIAKYDGKWEVEEMKESKLPGDKGLVLMSRAKHHAISAKLNKPFLFDTKPLIVQYEVNFQNGIECGGAYVKLLSKTPELNLDQFHDKTPYTIMFGPDKCGEDYKLHFIFRHKNPKTGIYEEKHAKRPDADLKTYFTDKKTHLYTLILNPDNSFEILVDQSVVNSGNLLNDMTPPVNPSREIEDPEDRKPEDWDERPKIPDPEAVKPDDWDEDAPAKIPDEEATKPEGWLDDEPEYVPDPDAEKPEDWDEDMDGEWEAPQIANPRCESAPGCGVWQRPVIDNPNYKGKWKPPMIDNPSYQGIWKPRKIPNPDFFEDLEPFRMTPFSAIGLELWSMTSDIFFDNFIICADRRIVDDWANDGWGLKKAADGAAEPGVVGQMIEAAEERPWLWVVYILTVALPVFLVILFCCSGKKQTSGMEYKKTDAPQPDVKEEEEEKEEEKDKGDEEEEGEEKLEEKQKSDAEEDGGTVSQEEEDRKPKAEEDEILNRSPRNRKPRRE

470 NP_000537.3 NP_002281.1

>NP_000537.3

MEEPQSDPSVEPPLSQETFSDLWKLLPENNVLSPLPSQAMDDLMLSPDDIEQWFTEDPGPDEAPRMPEAAPPVAPAPAAPTPAAPAPAPSWPLSSSVPSQKTYQGSYGFRLGFLHSGTAKSVTCTYSPALNKMFCQLAKTCPVQLWVDSTPPPGTRVRAMAIYKQSQHMTEVVRRCPHHERCSDSDGLAPPQHLIRVEGNLRVEYLDDRNTFRHSVVVPYEPPEVGSDCTTIHYNYMCNSSCMGGMNRRPILTIITLEDSSGNLLGRNSFEVRVCACPGRDRRTEEENLRKKGEPHHELPPGSTKRALPNNTSSSPQPKKKPLDGEYFTLQIRGRERFEMFRELNEALELKDAQAGKEPGGSRAHSSHLKSKKGQSTSRHKKLMFKTEGPDSD

>NP_002281.1

MALSSAWRSVLPLWLLWSAACSRAASGDDNAFPFDIEGSSAVGRQDPPETSEPRVALGRLPPAAEKCNAGFFHTLSGECVPCDCNGNSNECLDGSGYCVHCQRNTTGEHCEKCLDGYIGDSIRGAPQFCQPCPCPLPHLANFAESCYRKNGAVRCICNENYAGPNCERCAPGYYGNPLLIGSTCKKCDCSGNSDPNLIFEDCDEVTGQCRNCLRNTTGFKCERCAPGYYGDARIAKNCAVCNCGGGPCDSVTGECLEEGFEPPTGCDKCVWDLTDDLRLAALSIEEGKSGVLSVSSGAAAHRHVNEINATIYLLKTKLSERENQYALRKIQINNAENTMKSLLSDVEELVEKENQASRKGQLVQKESMDTINHASQLVEQAHDMRDKIQEINNKMLYYGEEHELSPKEISEKLVLAQKMLEEIRSRQPFFTQRELVDEEADEAYELLSQAESWQRLHNETRTLFPVVLEQLDDYNAKLSDLQEALDQALNHVRDAEDMNRATAARQRDHEKQQERVREQMEVVNMSLSTSADSLTTPRLTLSELDDIIKNASGIYAEIDGAKSELQVKLSNLSNLSHDLVQEAIDHAQDLQQEANELSRKLHSSDMNGLVQKALDASNVYENIVNYVSEANETAEFALNTTDRIYDAVSGIDTQIIYHKDESENLLNQARELQAKAESSSDEAVADTSRRVGGALARKSALKTRLSDAVKQLQAAERGDAQQRLGQSRLITEEANRTTMEVQQATAPMANNLTNWSQNLQHFDSSAYNTAVNSARDAVRNLTEVVPQLLDQLRTVEQKRPASNVSASIQRIRELIAQTRSVASKIQVSMMFDGQSAVEVHSRTSMDDLKAFTSLSLYMKPPVKRPELTETADQFILYLGSKNAKKEYMGLAIKNDNLVYVYNLGTKDVEIPLDSKPVSSWPAYFSIVKIERVGKHGKVFLTVPSLSSTAEEKFIKKGEFSGDDSLLDLDPEDTVFYVGGVPSNFKLPTSLNLPGFVGCLELATLNNDVISLYNFKHIYNMDPSTSVPCARDKLAFTQSRAASYFFDGSGYAVVRDITRRGKFGQVTRFDIEVRTPADNGLILLMVNGSMFFRLEMRNGYLHVFYDFGFSSGRVHLEDTLKKAQINDAKYHEISIIYHNDKKMILVVDRRHVKSMDNEKMKIPFTDIYIGGAPPEILQSRALRAHLPLDINFRGCMKGFQFQKKDFNLLEQTETLGVGYGCPEDSLISRRAYFNGQSFIASIQKISFFDGFEGGFNFRTLQPNGLLFYYASGSDVFSISLDNGTVIMDVKGIKVQSVDKQYNDGLSHFVISSVSPTRYELIVDKSRVGSKNPTKGKIEQTQASEKKFYFGGSPISAQYANFTGCISNAYFTRVDRDVEVEDFQRYTEKVHTSLYECPIESSPLFLLHKKGKNLSKPKASQNKKGGKSKDAPSWDPVALKLPERNTPRNSHCHLSNSPRAIEHAYQYGGTANSRQEFEHLKGDFGAKSQFSIRLRTRSSHGMIFYVSDQEENDFMTLFLAHGRLVYMFNVGHKKLKIRSQEKYNDGLWHDVIFIRERSSGRLVIDGLRVLEESLPPTEATWKIKGPIYLGGVAPGKAVKNVQINSIYSFSGCLSNLQLNGASITSASQTFSVTPCFEGPMETGTYFSTEGGYVVLDESFNIGLKFEIAFEVRPRSSSGTLVHGHSVNGEYLNVHMKNGQVIVKVNNGIRDFSTSVTPKQSLCDGRWHRITVIRDSNVVQLDVDSEVNHVVGPLNPKPIDHREPVFVGGVPESLLTPRLAPSKPFTGCIRHFVIDGHPVSFSKAALVSGAVSINSCPAA

471 NP_057661.3 NP_829884.1

>NP_057661.3

MSAGGDFGNPLRKFKLVFLGEQSVGKTSLITRFMYDSFDNTYQATIGIDFLSKTMYLEDRTVRLQLWDTAGQERFRSLIPSYIRDSTVAVVVYDITNLNSFQQTSKWIDDVRTERGSDVIIMLVGNKTDLADKRQITIEEGEQRAKELSVMFIETSAKTGYNVKQLFRRVASALPGMENVQEKSKEGMIDIKLDKPQEPPASEGGCSC

>NP_829884.1

MYGSARSVGKVEPSSQSPGRSPRLPRSPRLGHRRTNSTGGSSGSSVGGGSGKTLSMENIQSLNAAYATSGPMYLSDHENVGSETPKSTMTLGRSGGRLPYGVRMTAMGSSPNIASSGVASDTIAFGEHHLPPVSMASTVPHSLRQARDNTIMDLQTQLKEVLRENDLLRKDVEVKESKLSSSMNSIKTFWSPELKKERALRKDEASKITIWKEQYRVVQEENQHMQMTIQALQDELRIQRDLNQLFQQDSSSRTGEPCVAELTEENFQRLHAEHERQAKELFLLRKTLEEMELRIETQKQTLNARDESIKKLLEMLQSKGLSAKATEEDHERTRRLAEAEMHVHHLESLLEQKEKENSMLREEMHRRFENAPDSAKTKALQTVIEMKDSKISSMERGLRDLEEEIQMLKSNGALSTEEREEEMKQMEVYRSHSKFMKNKVEQLKEELSSKEAQWEELKKKAAGLQAEIGQVKQELSRKDTELLALQTKLETLTNQFSDSKQHIEVLKESLTAKEQRAAILQTEVDALRLRLEEKETMLNKKTKQIQDMAEEKGTQAGEIHDLKDMLDVKERKVNVLQKKIENLQEQLRDKEKQMSSLKERVKSLQADTTNTDTALTTLEEALAEKERTIERLKEQRDRDEREKQEEIDNYKKDLKDLKEKVSLLQGDLSEKEASLLDLKEHASSLASSGLKKDSRLKTLEIALEQKKEECLKMESQLKKAHEAALEARASPEMSDRIQHLEREITRYKDESSKAQAEVDRLLEILKEVENEKNDKDKKIAELERQVKDQNKKVANLKHKEQVEKKKSAQMLEEARRREDNLNDSSQQLQDSLRKKDDRIEELEEALRESVQITAEREMVLAQEESARTNAEKQVEELLMAMEKVKQELESMKAKLSSTQQSLAEKETHLTNLRAERRKHLEEVLEMKQEALLAAISEKDANIALLELSSSKKKTQEEVAALKREKDRLVQQLKQQTQNRMKLMADNYEDDHFKSSHSNQTNHKPSPDQIIQPLLELDQNRSKLKLYIGHLTTLCHDRDPLILRGLTPPASYNLDDDQAAWENELQKMTRGQLQDELEKGERDNAELQEFANAILQQIADHCPDILEQVVNALEESS

472 NP_002255.2 NP_057353.1

>NP_002255.2

MTTPGKENFRLKSYKNKSLNPDEMRRRREEEGLQLRKQKREEQLFKRRNVATAEEETEEEVMSDGGFHEAQINNMEMAPGGVITSDMIEMIFSKSPEQQLSATQKFRKLLSKEPNPPIDEVISTPGVVARFVEFLKRKENCTLQFESAWVLTNIASGNSLQTRIVIQAGAVPIFIELLSSEFEDVQEQAVWALGNIAGDSTMCRDYVLDCNILPPLLQLFSKQNRLTMTRNAVWALSNLCRGKSPPPEFAKVSPCLNVLSWLLFVSDTDVLADACWALSYLSDGPNDKIQAVIDAGVCRRLVELLMHNDYKVVSPALRAVGNIVTGDDIQTQVILNCSALQSLLHLLSSPKESIKKEACWTISNITAGNRAQIQTVIDANIFPALISILQTAEFRTRKEAAWAITNATSGGSAEQIKYLVELGCIKPLCDLLTVMDSKIVQVALNGLENILRLGEQEAKRNGTGINPYCALIEEAYGLDKIEFLQSHENQEIYQKAFDLIEHYFGTEDEDSSIAPQVDLNQQQYIFQQCEAPMEGFQL

>NP_057353.1

MPQLSGGGGGGGGDPELCATDEMIPFKDEGDPQKEKIFAEISHPEEEGDLADIKSSLVNESEIIPASNGHEVARQAQTSQEPYHDKAREHPDDGKHPDGGLYNKGPSYSSYSGYIMMPNMNNDPYMSNGSLSPPIPRTSNKVPVVQPSHAVHPLTPLITYSDEHFSPGSHPSHIPSDVNSKQGMSRHPPAPDIPTFYPLSPGGVGQITPPLGWQGQPVYPITGGFRQPYPSSLSVDTSMSRFSHHMIPGPPGPHTTGIPHPAIVTPQVKQEHPHTDSDLMHVKPQHEQRKEQEPKRPHIKKPLNAFMLYMKEMRANVVAECTLKESAAINQILGRRWHALSREEQAKYYELARKERQLHMQLYPGWSARDNYGKKKKRKREKLQESASGTGPRMTAAYI

473 NP_055830.1 NP_115662.2

>NP_055830.1

MGSEDHGAQNPSCKIMTFRPTMEEFKDFNKYVAYIESQGAHRAGLAKIIPPKEWKPRQTYDDIDDVVIPAPIQQVVTGQSGLFTQYNIQKKAMTVGEYRRLANSEKYCTPRHQDFDDLERKYWKNLTFVSPIYGADISGSLYDDDVAQWNIGSLRTILDMVERECGTIIEGVNTPYLYFGMWKTTFAWHTEDMDLYSINYLHFGEPKSWYAIPPEHGKRLERLAIGFFPGSSQGCDAFLRHKMTLISPIILKKYGIPFSRITQEAGEFMITFPYGYHAGFNHGFNCAESTNFATLRWIDYGKVATQCTCRKDMVKISMDVFVRILQPERYELWKQGKDLTVLDHTRPTALTSPELSSWSASRASLKAKLLRRSHRKRSQPKKPKPEDPKFPGEGTAGAALLEEAGGSVKEEAGPEVDPEEEEEEPQPLPHGREAEGAEEDGRGKLRPTKAKSERKKKSFGLLPPQLPPPPAHFPSEEALWLPSPLEPPVLGPGPAAMEESPLPAPLNVVPPEVPSEELEAKPRPIIPMLYVVPRPGKAAFNQEHVSCQQAFEHFAQKGPTWKEPVSPMELTGPEDGAASSGAGRMETKARAGEGQAPSTFSKLKMEIKKSRRHPLGRPPTRSPLSVVKQEASSDEEASPFSGEEDVSDPDALRPLLSLQWKNRAASFQAERKFNAAAARTEPYCAICTLFYPYCQALQTEKEAPIASLGEGCPATLPSKSRQKTRPLIPEMCFTSGGENTEPLPANSYIGDDGTSPLIACGKCCLQVHASCYGIRPELVNEGWTCSRCAAHAWTAECCLCNLRGGALQMTTDRRWIHVICAIAVPEARFLNVIERHPVDISAIPEQRWKLKCVYCRKRMKKVSGACIQCSYEHCSTSFHVTCAHAAGVLMEPDDWPYVVSITCLKHKSGGHAVQLLRAVSLGQVVITKNRNGLYYRCRVIGAASQTCYEVNFDDGSYSDNLYPESITSRDCVQLGPPSEGELVELRWTDGNLYKAKFISSVTSHIYQVEFEDGSQLTVKRGDIFTLEEELPKRVRSRLSLSTGAPQEPAFSGEEAKAAKRPRVGTPLATEDSGRSQDYVAFVESLLQVQGRPGAPF

>NP_115662.2

MAEKFDHLEEHLEKFVENIRQLGIIVSDFQPSSQAGLNQKLNFIVTGLQDIDKCRQQLHDITVPLEVFEYIDQGRNPQLYTKECLERALAKNEQVKGKIDTMKKFKSLLIQELSKVFPEDMAKYRSIRGEDHPPS

474 NP_005576.3 NP_006107.1

>NP_005576.3

MFRSKRSGLVRRLWRSRVVPDREEGGSGGGGGGDEDGSLGSRAEPAPRAREGGGCGRSEVRPVAPRRPRDAVGQRGAQGAGRRRRAGGPPRPMSEPGAGAGSSLLDVAEPGGPGWLPESDCETVTCCLFSERDAAGAPRDASDPLAGAALEPAGGGRSREARSRLLLLEQELKTVTYSLLKRLKERSLDTLLEAVESRGGVPGGCVLVPRADLRLGGQPAPPQLLLGRLFRWPDLQHAVELKPLCGCHSFAAAADGPTVCCNPYHFSRLCGPESPPPPYSRLSPRDEYKPLDLSDSTLSYTETEATNSLITAPGEFSDASMSPDATKPSHWCSVAYWEHRTRVGRLYAVYDQAVSIFYDLPQGSGFCLGQLNLEQRSESVRRTRSKIGFGILLSKEPDGVWAYNRGEHPIFVNSPTLDAPGGRALVVRKVPPGYSIKVFDFERSGLQHAPEPDAADGPYDPNSVRISFAKGWGPCYSRQFITSCPCWLEILLNNPR

>NP_006107.1

MAAQRRSLLQSEQQPSWTDDLPLCHLSGVGSASNRSYSADGKGTESHPPEDSWLKFRSENNCFLYGVFNGYDGNRVTNFVAQRLSAELLLGQLNAEHAEADVRRVLLQAFDVVERSFLESIDDALAEKASLQSQLPEGVPQHQLPPQYQKILERLKTLEREISGGAMAVVAVLLNNKLYVANVGTNRALLCKSTVDGLQVTQLNVDHTTENEDELFRLSQLGLDAGKIKQVGIICGQESTRRIGDYKVKYGYTDIDLLSAAKSKPIIAEPEIHGAQPLDGVTGFLVLMSEGLYKALEAAHGPGQANQEIAAMIDTEFAKQTSLDAVAQAVVDRVKRIHSDTFASGGERARFCPRHEDMTLLVRNFGYPLGEMSQPTPSPAPAAGGRVYPVSVPYSSAQSTSKTSVTLSLVMPSQGQMVNGAHSASTLDEATPTLTNQSPTLTLQSTNTHTQSSSSSSDGGLFRSRPAHSLPPGEDGRVEPYVDFAEFYRLWSVDHGEQSVVTAP

475 NP_006144.1 NP_002600.1

>NP_006144.1

MAEEVVVVAKFDYVAQQEQELDIKKNERLWLLDDSKSWWRVRNSMNKTGFVPSNYVERKNSARKASIVKNLKDTLGIGKVKRKPSVPDSASPADDSFVDPGERLYDLNMPAYVKFNYMAEREDELSLIKGTKVIVMEKCSDGWWRGSYNGQVGWFPSNYVTEEGDSPLGDHVGSLSEKLAAVVNNLNTGQVLHVVQALYPFSSSNDEELNFEKGDVMDVIEKPENDPEWWKCRKINGMVGLVPKNYVTVMQNNPLTSGLEPSPPQCDYIRPSLTGKFAGNPWYYGKVTRHQAEMALNERGHEGDFLIRDSESSPNDFSVSLKAQGKNKHFKVQLKETVYCIGQRKFSTMEELVEHYKKAPIFTSEQGEKLYLVKHLS

>NP_002600.1

MRLPGAMPALALKGELLLLSLLLLLEPQISQGLVVTPPGPELVLNVSSTFVLTCSGSAPVVWERMSQEPPQEMAKAQDGTFSSVLTLTNLTGLDTGEYFCTHNDSRGLETDERKRLYIFVPDPTVGFLPNDAEELFIFLTEITEITIPCRVTDPQLVVTLHEKKGDVALPVPYDHQRGFSGIFEDRSYICKTTIGDREVDSDAYYVYRLQVSSINVSVNAVQTVVRQGENITLMCIVIGNEVVNFEWTYPRKESGRLVEPVTDFLLDMPYHIRSILHIPSAELEDSGTYTCNVTESVNDHQDEKAINITVVESGYVRLLGEVGTLQFAELHRSRTLQVVFEAYPPPTVLWFKDNRTLGDSSAGEIALSTRNVSETRYVSELTLVRVKVAEAGHYTMRAFHEDAEVQLSFQLQINVPVRVLELSESHPDSGEQTVRCRGRGMPQPNIIWSACRDLKRCPRELPPTLLGNSSEEESQLETNVTYWEEEQEFEVVSTLRLQHVDRPLSVRCTLRNAVGQDTQEVIVVPHSLPFKVVVISAILALVVLTIISLIILIMLWQKKPRYEIRWKVIESVSSDGHEYIYVDPMQLPYDSTWELPRDQLVLGRTLGSGAFGQVVEATAHGLSHSQATMKVAVKMLKSTARSSEKQALMSELKIMSHLGPHLNVVNLLGACTKGGPIYIITEYCRYGDLVDYLHRNKHTFLQHHSDKRRPPSAELYSNALPVGLPLPSHVSLTGESDGGYMDMSKDESVDYVPMLDMKGDVKYADIESSNYMAPYDNYVPSAPERTCRATLINESPVLSYMDLVGFSYQVANGMEFLASKNCVHRDLAARNVLICEGKLVKICDFGLARDIMRDSNYISKGSTFLPLKWMAPESIFNSLYTTLSDVWSFGILLWEIFTLGGTPYPELPMNEQFYNAIKRGYRMAQPAHASDEIYEIMQKCWEEKFEIRPPFSQLVLLLERLLGEGYKKKYQQVDEEFLRSDHPAILRSQARLPGFHGLRSPLDTSSVLYTAVQPNEGDNDYIIPLPDPKPEVADEGPLEGSPSLASSTLNEVNTSSTISCDSPLEPQDEPEPEPQLELQVEPEPELEQLPDSGCPAPRAEAEDSFL

476 NP_002721.1 NP_004956.5

>NP_002721.1

MGNAAAAKKGSEQESVKEFLAKAKEDFLKKWESPAQNTAHLDQFERIKTLGTGSFGRVMLVKHKETGNHYAMKILDKQKVVKLKQIEHTLNEKRILQAVNFPFLVKLEFSFKDNSNLYMVMEYVPGGEMFSHLRRIGRFSEPHARFYAAQIVLTFEYLHSLDLIYRDLKPENLLIDQQGYIQVTDFGFAKRVKGRTWTLCGTPEYLAPEIILSKGYNKAVDWWALGVLIYEMAAGYPPFFADQPIQIYEKIVSGKVRFPSHFSSDLKDLLRNLLQVDLTKRFGNLKNGVNDIKNHKWFATTDWIAIYQRKVEAPFIPKFKGPGDTSNFDDYEEEEIRVSINEKCGKEFSEF

>NP_004956.5

MPKRKVSSAEGAAKEEPKRRSARLSAKPPAKVEAKPKKAAAKDKSSDKKVQTKGKRGAKGKQAEVANQETKEDLPAENGETKTEESPASDEAGEKEAKSD

477 NP_061828.1 BAC87138.1

>NP_061828.1

MQIFVKTLTGKTITLEVEPSDTIENVKAKIQDKEGIPPDQQRLIFAGKQLEDGRTLSDYNIQKESTLHLVLRLRGGMQIFVKTLTGKTITLEVEPSDTIENVKAKIQDKEGIPPDQQRLIFAGKQLEDGRTLSDYNIQKESTLHLVLRLRGGMQIFVKTLTGKTITLEVEPSDTIENVKAKIQDKEGIPPDQQRLIFAGKQLEDGRTLSDYNIQKESTLHLVLRLRGGC

>BAC87138.1

MVVVTGREPDSRRQDGAMSSSDAEDDFLEPATPTATQAGHALPLLPQERCAEFPALRGPPTQGACSSCVQRGPVLCHRAPPGAAGEHAATEGREGAPSVSGTHALLQRPLGADCGDRPAACGPAEGPLCQAQVVSRKKMSLKSERRGIHVDQSDLLCKKGCGYYGNPAWQGFCSKCWREEYHKARQKQIQEDWELAERVLLCCPGWSAMVQFQLTATSASWAQVILLLQPPKWLGLQKLQREEEEAFASSQSSQGAQSLTFSKFEEKKTNEKTRKVTTVKKFFSASSRVGSKKEIQEAKAPSPSINRQTSIETDRVSKEFIEFLKTFHKTGQEIYKQTKLFLEGMHYKRDLSIEEQSECAQDFYHNVAERMQTRGKERRFHHVGQAGLELLTSGDPPASASQSAGNTGVEPPHPAVPPERVEKIMDQIEKYIMTRLYKYVFCPETTDDEKKDLAIQKRIRALRWVTPQMLCVPVNDDIPEVSDMVVKAITDIIEMDSKRVPRDKLACITKCSKHIFNAIKITKNEPASADDFLPTLIYIVLKGNPPRLQSNIQYITRFCNPSRLMTGEDGYYFTNLCCAVAFIEKLDAQSLNLSQEDFDRYMSGQTSPRKQEAESWSPDACLGVKQMYKNLDLLSQLNERQERIMNEAKKLEKDLIDWTDGIAREVQDIVEKYPLEIKPPNQPLAAIDSENVENDKLPPPLQPQVYAG

478 NP_000360.2 NP_006414.1

>NP_000360.2

MRPADLLQLVLLLDLPRDLGGMGCSSPPCECHQEEDFRVTCKDIQRIPSLPPSTQTLKLIETHLRTIPSHAFSNLPNISRIYVSIDVTLQQLESHSFYNLSKVTHIEIRNTRNLTYIDPDALKELPLLKFLGIFNTGLKMFPDLTKVYSTDIFFILEITDNPYMTSIPVNAFQGLCNETLTLKLYNNGFTSVQGYAFNGTKLDAVYLNKNKYLTVIDKDAFGGVYSGPSLLDVSQTSVTALPSKGLEHLKELIARNTWTLKKLPLSLSFLHLTRADLSYPSHCCAFKNQKKIRGILESLMCNESSMQSLRQRKSVNALNSPLHQEYEENLGDSIVGYKEKSKFQDTHNNAHYYVFFEEQEDEIIGFGQELKNPQEETLQAFDSHYDYTICGDSEDMVCTPKSDEFNPCEDIMGYKFLRIVVWFVSLLALLGNVFVLLILLTSHYKLNVPRFLMCNLAFADFCMGMYLLLIASVDLYTHSEYYNHAIDWQTGPGCNTAGFFTVFASELSVYTLTVITLERWYAITFAMRLDRKIRLRHACAIMVGGWVCCFLLALLPLVGISSYAKVSICLPMDTETPLALAYIVFVLTLNIVAFVIVCCCYVKIYITVRNPQYNPGDKDTKIAKRMAVLIFTDFICMAPISFYALSAILNKPLITVSNSKILLVLFYPLNSCANPFLYAIFTKAFQRDVFILLSKFGICKRQAQAYRGQRVPPKNSTDIQVQKVTHEMRQGLHNMEDVYELIENSHLTPKKQGQISEEYMQTVL

>NP_006414.1

MAAQKDQQKDAEAEGLSGTTLLPKLIPSGAGREWLERRRATIRPWSTFVDQQRFSRPRNLGELCQRLVRNVEYYQSNYVFVFLGLILYCVVTSPMLLVALAVFFGACYILYLRTLESKLVLFGREVSPEHQYALAGGISFPFFWLAGAGSAVFWVLGATLVVIGSHAAFHQIEAVDGEELQMEPV

479 NP_000902.3 NP_002060.4

>NP_000902.3

MEPAPSAGAELQPPLFANASDAYPSACPSAGANASGPPGARSASSLALAIAITALYSAVCAVGLLGNVLVMFGIVRYTKMKTATNIYIFNLALADALATSTLPFQSAKYLMETWPFGELLCKAVLSIDYYNMFTSIFTLTMMSVDRYIAVCHPVKALDFRTPAKAKLINICIWVLASGVGVPIMVMAVTRPRDGAVVCMLQFPSPSWYWDTVTKICVFLFAFVVPILIITVCYGLMLLRLRSVRLLSGSKEKDRSLRRITRMVLVVVGAFVVCWAPIHIFVIVWTLVDIDRRDPLVVAALHLCIALGYANSSLNPVLYAFLDENFKRCFRQLCRKPCGRPDPSSFSRAREATARERVTACTPSDGPGGGAAA

>NP_002060.4

MGCTLSAEDKAAVERSKMIDRNLREDGEKAAREVKLLLLGAGESGKSTIVKQMKIIHEAGYSEEECKQYKAVVYSNTIQSIIAIIRAMGRLKIDFGDSARADDARQLFVLAGAAEEGFMTAELAGVIKRLWKDSGVQACFNRSREYQLNDSAAYYLNDLDRIAQPNYIPTQQDVLRTRVKTTGIVETHFTFKDLHFKMFDVGGQRSERKKWIHCFEGVTAIIFCVALSDYDLVLAEDEEMNRMHESMKLFDSICNNKWFTDTSIILFLNKKDLFEEKIKKSPLTICYPEYAGSNTYEEAAAYIQCQFEDLNKRKDTKEIYTHFTCATDTKNVQFVFDAVTDVIIKNNLKDCGLF

480 NP_005617.2 NP_003940.3

>NP_005617.2

MPRVYIGRLSYQARERDVERFFKGYGKILEVDLKNGYGFVEFDDLRDADDAVYELNGKDLCGERVIVEHARGPRRDGSYGSGRSGYGYRRSGRDKYGPPTRTEYRLIVENLSSRCSWQDLKDYMRQAGEVTYADAHKGRKNEGVIEFVSYSDMKRALEKLDGTEVNGRKIRLVEDKPGSRRRRSYSRSRSHSRSRSRSRHSRKSRSRSGSSKSSHSKSRSRSRSGSRSRSKSRSRSQSRSRSKKEKSRSPSKEKSRSRSHSAGKSRSKSKDQAEEKIQNNDNVGKPKSRSPSRHKSKSKSRSRSQERRVEEEKRGSVSRGRSQEKSLRQSRSRSRSKGGSRSRSRSRSKSKDKRKGRKRSREESRSRSRSRSKSERSRKRGSKRDSKAGSSKKKKKEDTDRSQSRSPSRSVSKEREHAKSESSQREGRGESENAGTNQETRSRSRSNSKSKPNLPSESRSRSKSASKTRSRSKSRSRSASRSPSRSRSRSHSRS

>NP_003940.3

MRPKRLGRCCAGSRLGPGDPAALTCAPSPSASPAPEPSAQPQARGTGQRVGSRATSGSQFLSEARTGARPASEAGAKAGARRPSAFSAIQGDVRSMPDNSDAPWTRFVFQGPFGSRATGRGTGKAAGIWKTPAAYVGRRPGVSGPERAAFIRELEEALCPNLPPPVKKITQEDVKVMLYLLEELLPPVWESVTYGMVLQRERDLNTAARIGQSLVKQNSVLMEENSKLEALLGSAKEEILYLRHQVNLRDELLQLYSDSDEEDEDEEEEEEEKEAEEEQEEEEAEEDLQCAHPCDAPKLISQEALLHQHHCPQLEALQEKLRLLEEENHQLREEASQLDTLEDEEQMLILECVEQFSEASQQMAELSEVLVLRLENYERQQQEVARLQAQVLKLQQRCRMYGAETEKLQKQLASEKEIQMQLQEESVWVGSQLQDLREKYMDCGGMLIEMQEEVKTLRQQPPVSTGSATHYPYSVPLETLPGFQETLAEELRTSLRRMISDPVYFMERNYEMPRGDTSSLRYDFRYSEDREQVRGFEAEEGLMLAADIMRGEDFTPAEEFVPQEELGAAKKVPAEEGVMEEAELVSEETEGWEEVELELDEATRMNVVTSALEASGLGPSHLDMNYVLQQLANWQDAHYRRQLRWKMLQKGECPHGALPAASRTSCRSSCR

481 NP_004603.1 NP_001822.2

>NP_004603.1

MEAAVAAPRPRLLLLVLAAAAAAAAALLPGATALQCFCHLCTKDNFTCVTDGLCFVSVTETTDKVIHNSMCIAEIDLIPRDRPFVCAPSSKTGSVTTTYCCNQDHCNKIELPTTVKSSPGLGPVELAAVIAGPVCFVCISLMLMVYICHNRTVIHHRVPNEEDPSLDRPFISEGTTLKDLIYDMTTSGSGSGLPLLVQRTIARTIVLQESIGKGRFGEVWRGKWRGEEVAVKIFSSREERSWFREAEIYQTVMLRHENILGFIAADNKDNGTWTQLWLVSDYHEHGSLFDYLNRYTVTVEGMIKLALSTASGLAHLHMEIVGTQGKPAIAHRDLKSKNILVKKNGTCCIADLGLAVRHDSATDTIDIAPNHRVGTKRYMAPEVLDDSINMKHFESFKRADIYAMGLVFWEIARRCSIGGIHEDYQLPYYDLVPSDPSVEEMRKVVCEQKLRPNIPNRWQSCEALRVMAKIMRECWYANGAARLTALRIKKTLSQLSQQEGIKM

>NP_001822.2

MQVCSQPQRGCVREQSAINTAPPSAHNAASPGGARGHRVPLTEACKDSRIGGMMKTLLLFVGLLLTWESGQVLGDQTVSDNELQEMSNQGSKYVNKEIQNAVNGVKQIKTLIEKTNEERKTLLSNLEEAKKKKEDALNETRESETKLKELPGVCNETMMALWEECKPCLKQTCMKFYARVCRSGSGLVGRQLEEFLNQSSPFYFWMNGDRIDSLLENDRQQTHMLDVMQDHFSRASSIIDELFQDRFFTREPQDTYHYLPFSLPHRRPHFFFPKSRIVRSLMPFSPYEPLNFHAMFQPFLEMIHEAQQAMDIHFHSPAFQHPPTEFIREGDDDRTVCREIRHNSTGCLRMKDQCDKCREILSVDCSTNNPSQAKLRRELDESLQVAERLTRKYNELLKSYQWKMLNTSSLLEQLNEQFNWVSRLANLTQGEDQYYLRVTTVASHTSDSDVPSGVTEVVVKLFDSDPITVTVPVEVSRKNPKFMETVAEKALQEYRKKHREE

482 NP_000524.3 NP_001020272.1

>NP_000524.3

MGLLECCARCLVGAPFASLVATGLCFFGVALFCGCGHEALTGTEKLIETYFSKNYQDYEYLINVIHAFQYVIYGTASFFFLYGALLLAEGFYTTGAVRQIFGDYKTTICGKGLSATVTGGQKGRGSRGQHQAHSLERVCHCLGKWLGHPDKFVGITYALTVVWLLVFACSAVPVYIYFNTWTTCQSIAFPSKTSASIGSLCADARMYGVLPWNAFPGKVCGSNLLSICKTAEFQMTFHLFIAAFVGAAATLVSLLTFMIAATYNFAVLKLMGRGTKF

>NP_001020272.1

MGNHAGKRELNAEKASTNSETNRGESEKKRNLGELSRTTSEDNEVFGEADANQNNGTSSQDTAVTDSKRTADPKNAWQDAHPADPGSRPHLIRLFSRDAPGREDNTFKDRPSESDELQTIQEDSAATSESLDVMASQKRPSQRHGSKYLATASTMDHARHGFLPRHRDTGILDSIGRFFGGDRGAPKRGSGKDSHHPARTAHYGSLPQKSHGRTQDENPVVHFFKNIVTPRTPPPSQGKGRGLSLSRFSWGAEGQRPGFGYGGRASDYKSAHKGFKGVDAQGTLSKIFKLGGRDSRSGSPMARR

483 NP_001070667.1 NP_112738.1

>NP_001070667.1

MNSGGGFGLGLGFGLTPTSVIQVTNLSSAVTSEQMRTLFSFLGEIEELRLYPPDNAPLAFSSKVCYVKFRDPSSVGVAQHLTNTVFIDRALIVVPCAEGKIPEESKALSLLAPAPTMTSLMPGAGLLPIPTPNPLTTLGVSLSSLGAIPAAALDPNIATLGEIPQPPLMGNVDPSKIDEIRRTVYVGNLNSQTTTADQLLEFFKQVGEVKFVRMAGDETQPTRFAFVEFADQNSVPRALAFNGVMFGDRPLKINHSNNAIVKPPEMTPQAAAKELEEVMKRVREAQSFISAAIEPESGKSNERKGGRSRSHTRSKSRSSSKSHSRRKRSQSKHRSRSHNRSRSRQKDRRRSKSPHKKRSKSRERRKSRSRSHSRDKRKDTREKIKEKERVKEKDREKEREREKEREKEKERGKNKDRDKEREKDREKDKEKDREREREKEHEKDRDKEKEKEQDKEKEREKDRSKEIDEKRKKDKKSRTPPRSYNASRRSRSSSRERRRRRSRSSSRSPRTSKTIKRKSSRSPSPRSRNKKDKKREKERDHISERRERERSTSMRKSSNDRDGKEKLEKNSTSLKEKEHNKEPDSSVSKEVDDKDAPRTEENKIQHNGNCQLNEENLSTKTEAV

>NP_112738.1

MSEEQFGGDGAAAAATAAVGGSAGEQEGAMVAATQGAAAAAGSGAGTGGGTASGGTEGGSAESEGAKIDASKNEEDEGHSNSSPRHSEAATAQREEWKMFIGGLSWDTTKKDLKDYFSKFGEVVDCTLKLDPITGRSRGFGFVLFKESESVDKVMDQKEHKLNGKVIDPKRAKAMKTKEPVKKIFVGGLSPDTPEEKIREYFGGFGEVESIELPMDNKTNKRRGFCFITFKEEEPVKKIMEKKYHNVGLSKCEIKVAMSKEQYQQQQQWGSRGGFAGRARGRGGGPSQNWNQGYSNYWNQGYGNYGYNSQGYGGYGGYDYTGYNNYYGYGDYSNQQSGYGKVSRRGGHQNSYKPY

484 NP_002222.1 NP_391987.1

>NP_002222.1

MGSACIKVTKYFLFLFNLIFFILGAVILGFGVWILADKSSFISVLQTSSSSLRMGAYVFIGVGAVTMLMGFLGCIGAVNEVRCLLGLYFAFLLLILIAQVTAGALFYFNMGKLKQEMGGIVTELIRDYNSSREDSLQDAWDYVQAQVKCCGWVSFYNWTDNAELMNRPEVTYPCSCEVKGEEDNSLSVRKGFCEAPGNRTQSGNHPEDWPVYQEGCMEKVQAWLQENLGIILGVGVGVAIIELLGMVLSICLCRHVHSEDYSKVPKY

>NP_391987.1

MNLQPIFWIGLISSVCCVFAQTDENRCLKANAKSCGECIQAGPNCGWCTNSTFLQEGMPTSARCDDLEALKKKGCPPDDIENPRGSKDIKKNKNVTNRSKGTAEKLKPEDITQIQPQQLVLRLRSGEPQTFTLKFKRAEDYPIDLYYLMDLSYSMKDDLENVKSLGTDLMNEMRRITSDFRIGFGSFVEKTVMPYISTTPAKLRNPCTSEQNCTSPFSYKNVLSLTNKGEVFNELVGKQRISGNLDSPEGGFDAIMQVAVCGSLIGWRNVTRLLVFSTDAGFHFAGDGKLGGIVLPNDGQCHLENNMYTMSHYYDYPSIAHLVQKLSENNIQTIFAVTEEFQPVYKELKNLIPKSAVGTLSANSSNVIQLIIDAYNSLSSEVILENGKLSEGVTISYKSYCKNGVNGTGENGRKCSNISIGDEVQFEISITSNKCPKKDSDSFKIRPLGFTEEVEVILQYICECECQSEGIPESPKCHEGNGTFECGACRCNEGRVGRHCECSTDEVNSEDMDAYCRKENSSEICSNNGECVCGQCVCRKRDNTNEIYSGKFCECDNFNCDRSNGLICGGNGVCKCRVCECNPNYTGSACDCSLDTSTCEASNGQICNGRGICECGVCKCTDPKFQGQTCEMCQTCLGVCAEHKECVQCRAFNKGEKKDTCTQECSYFNITKVESRDKLPQPVQPDPVSHCKEKDVDDCWFYFTYSVNGNNEVMVHVVENPECPTGPDIIPIVAGVVAGIVLIGLALLLIWKLLMIIHDRREFAKFEKEKMNAKWDTSLSVAQPGVQWCDISSLQPLTSRFQQFSCLSLPSTWDYRVKILFIRVP

485 NP_001020018.1 NP_114417.1

>NP_001020018.1

MGRGLLRGLWPLHIVLWTRIASTIPPHVQKSDVEMEAQKDEIICPSCNRTAHPLRHINNDMIVTDNNGAVKFPQLCKFCDVRFSTCDNQKSCMSNCSITSICEKPQEVCVAVWRKNDENITLETVCHDPKLPYHDFILEDAASPKCIMKEKKKPGETFFMCSCSSDECNDNIIFSEEYNTSNPDLLLVIFQVTGISLLPPLGVAISVIIIFYCYRVNRQQKLSSTWETGKTRKLMEFSEHCAIILEDDRSDISSTCANNINHNTELLPIELDTLVGKGRFAEVYKAKLKQNTSEQFETVAVKIFPYEEYASWKTEKDIFSDINLKHENILQFLTAEERKTELGKQYWLITAFHAKGNLQEYLTRHVISWEDLRKLGSSLARGIAHLHSDHTPCGRPKMPIVHRDLKSSNILVKNDLTCCLCDFGLSLRLDPTLSVDDLANSGQVGTARYMAPEVLESRMNLENVESFKQTDVYSMALVLWEMTSRCNAVGEVKDYEPPFGSKVREHPCVESMKDNVLRDRGRPEIPSFWLNHQGIQMVCETLTECWDHDPEARLTAQCVAERFSELEHLDRLSGRSCSEEKIPEDGSLNTTK

>NP_114417.1

MDDAAVLKRRGYLLGINLGEGSYAKVKSAYSERLKFNVAIKIIDRKKAPADFLEKFLPREIEILAMLNHCSIIKTYEIFETSHGKVYIVMELAVQGDLLELIKTRGALHEDEARKKFHQLSLAIKYCHDLDVVHRDLKCDNLLLDKDFNIKLSDFSFSKRCLRDDSGRMALSKTFCGSPAYAAPEVLQGIPYQPKVYDIWSLGVILYIMVCGSMPYDDSNIKKMLRIQKEHRVNFPRSKHLTGECKDLIYHMLQPDVNRRLHIDEILSHCWMQPKARGSPSVAINKEGESSRGTEPLWTPEPGSDKKSATKLEPEGEAQPQAQPETKPEGTAMQMSRQSEILGFPSKPSTMETEEGPPQQPPETRAQ

486 NP_057535.1 NP_006792.1

>NP_057535.1

MTAAENVCYTLINVPMDSEPPSEISLKNDLEKGDVKSKTEALKKVIIMILNGEKLPGLLMTIIRFVLPLQDHTIKKLLLVFWEIVPKTTPDGRLLHEMILVCDAYRKDLQHPNEFIRGSTLRFLCKLKEAELLEPLMPAIRACLEHRHSYVRRNAVLAIYTIYRNFEHLIPDAPELIHDFLVNEKDASCKRNAFMMLIHADQDRALDYLSTCIDQVQTFGDILQLVIVELIYKVCHANPSERARFIRCIYNLLQSSSPAVKYEAAGTLVTLSSAPTAIKAAAQCYIDLIIKESDNNVKLIVLDRLIELKEHPAHERVLQDLVMDILRVLSTPDLEVRKKTLQLALDLVSSRNVEELVIVLKKEVIKTNNVSEHEDTDKYRQLLVRTLHSCSVRFPDMAANVIPVLMEFLSDNNEAAAADVLEFVREAIQRFDNLRMLIVEKMLEVFHAIKSVKIYRGALWILGEYCSTKEDIQSVMTEIRRSLGEIPIVESEIKKEAGELKPEEEITVGPVQKLVTEMGTYATQSALSSSRPTKKEEDRPPLRGFLLDGDFFVAASLATTLTKIALRYVALVQEKKKQNSFVAEAMLLMATILHLGKSSLPKKPITDDDVDRISLCLKVLSECSPLMNDIFNKECRQSLSHMLSAKLEEEKLSQKKESEKRNVTVQPDDPISFMQLTAKNEMNCKEDQFQLSLLAAMGNTQRKEAADPLASKLNKVTQLTGFSDPVYAEAYVHVNQYDIVLDVLVVNQTSDTLQNCTLELATLGDLKLVEKPSPLTLAPHDFANIKANVKVASTENGIIFGNIVYDVSGAASDRNCVVLSDIHIDIMDYIQPATCTDAEFRQMWAEFEWENKVTVNTNMVDLNDYLQHILKSTNMKCLTPEKALSGYCGFMAANLYARSIFGEDALANVSIEKPIHQGPDAAVTGHIRIRAKSQGMALSLGDKINLSQKKTSI

>NP_006792.1

MNLFRFLGDLSHLLAIILLLLKIWKSRSCAGISGKSQVLFAVVFTARYLDLFTNYISLYNTCMKVVYIACSFTTVWLIYSKFKATYDGNHDTFRVEFLVVPTAILAFLVNHDFTPLEILWTFSIYLESVAILPQLFMVSKTGEAETITSHYLFALGVYRTLYLFNWIWRYHFEGFFDLIAIVAGLVQTVLYCDFFYLYITKVLKGKKLSLPA

487 NP_001217.2 NP_008878.3

>NP_001217.2

MSSASGLRRGHPAGGEENMTETDAFYKREMFDPAEKYKMDHRRRGIALIFNHERFFWHLTLPERRGTCADRDNLTRRFSDLGFEVKCFNDLKAEELLLKIHEVSTVSHADADCFVCVFLSHGEGNHIYAYDAKIEIQTLTGLFKGDKCHSLVGKPKIFIIQACRGNQHDVPVIPLDVVDNQTEKLDTNITEVDAASVYTLPAGADFLMCYSVAEGYYSHRETVNGSWYIQDLCEMLGKYGSSLEFTELLTLVNRKVSQRRVDFCKDPSAIGKKQVPCFASMLTKKLHFFPKSN

>NP_008878.3

MASGGSGGVSVPALWSEVNRYGQNGDFTRALKTVNKILQINKDDVTALHCKVVCLIQNGSFKEALNVINTHTKVLANNSLSFEKAYCEYRLNRIENALKTIESANQQTDKLKELYGQVLYRLERYDECLAVYRDLVRNSQDDYDEERKTNLSAVVAAQSNWEKVVPENLGLQEGTHELCYNTACALIGQGQLNQAMKILQKAEDLCRRSLSEDTDGTEEDPQAELAIIHGQMAYILQLQGRTEEALQLYNQIIKLKPTDVGLLAVIANNIITINKDQNVFDSKKKVKLTNAEGVEFKLSKKQLQAIEFNKALLAMYTNQAEQCRKISASLQSQSPEHLLPVLIQAAQLCREKQHTKAIELLQEFSDQHPENAAEIKLTMAQLKISQGNISKACLILRSIEELKHKPGMVSALVTMYSHEEDIDSAIEVFTQAIQWYQNHQPKSPAHLSLIREAANFKLKYGRKKEAISDLQQLWKQNPKDIHTLAQLISAYSLVDPEKAKALSKHLPSSDSMSLKVDVEALENSAGATYIRKKGGKVTGDSQPKEQGQGDLKKKKKKKKGKLPKNYDPKVTPDPERWLPMRERSYYRGRKKGKKKDQIGKGTQGATAGASSELDASKTVSSPPTSPRPGSAATVSASTSNIIPPRHQKPAGAPATKKKQQQKKKKGGKGGW

488 NP_004604.2 NP_000203.2

>NP_004604.2

MAEELVLERCDLELETNGRDHHTADLCREKLVVRRGQPFWLTLHFEGRNYEASVDSLTFSVVTGPAPSQEAGTKARFPLRDAVEEGDWTATVVDQQDCTLSLQLTTPANAPIGLYRLSLEASTGYQGSSFVLGHFILLFNAWCPADAVYLDSEEERQEYVLTQQGFIYQGSAKFIKNIPWNFGQFEDGILDICLILLDVNPKFLKNAGRDCSRRSSPVYVGRVVSGMVNCNDDQGVLLGRWDNNYGDGVSPMSWIGSVDILRRWKNHGCQRVKYGQCWVFAAVACTVLRCLGIPTRVVTNYNSAHDQNSNLLIEYFRNEFGEIQGDKSEMIWNFHCWVESWMTRPDLQPGYEGWQALDPTPQEKSEGTYCCGPVPVRAIKEGDLSTKYDAPFVFAEVNADVVDWIQQDDGSVHKSINRSLIVGLKISTKSVGRDEREDITHTYKYPEGSSEEREAFTRANHLNKLAEKEETGMAMRIRVGQSMNMGSDFDVFAHITNNTAEEYVCRLLLCARTVSYNGILGPECGTKYLLNLNLEPFSEKSVPLCILYEKYRDCLTESNLIKVRALLVEPVINSYLLAERDLYLENPEIKIRILGEPKQKRKLVAEVSLQNPLPVALEGCTFTVEGAGLTEEQKTVEIPDPVEAGEEVKVRMDLLPLHMGLHKLVVNFESDKLKAVKGFRNVIIGPA

>NP_000203.2

MRARPRPRPLWATVLALGALAGVGVGGPNICTTRGVSSCQQCLAVSPMCAWCSDEALPLGSPRCDLKENLLKDNCAPESIEFPVSEARVLEDRPLSDKGSGDSSQVTQVSPQRIALRLRPDDSKNFSIQVRQVEDYPVDIYYLMDLSYSMKDDLWSIQNLGTKLATQMRKLTSNLRIGFGAFVDKPVSPYMYISPPEALENPCYDMKTTCLPMFGYKHVLTLTDQVTRFNEEVKKQSVSRNRDAPEGGFDAIMQATVCDEKIGWRNDASHLLVFTTDAKTHIALDGRLAGIVQPNDGQCHVGSDNHYSASTTMDYPSLGLMTEKLSQKNINLIFAVTENVVNLYQNYSELIPGTTVGVLSMDSSNVLQLIVDAYGKIRSKVELEVRDLPEELSLSFNATCLNNEVIPGLKSCMGLKIGDTVSFSIEAKVRGCPQEKEKSFTIKPVGFKDSLIVQVTFDCDCACQAQAEPNSHRCNNGNGTFECGVCRCGPGWLGSQCECSEEDYRPSQQDECSPREGQPVCSQRGECLCGQCVCHSSDFGKITGKYCECDDFSCVRYKGEMCSGHGQCSCGDCLCDSDWTGYYCNCTTRTDTCMSSNGLLCSGRGKCECGSCVCIQPGSYGDTCEKCPTCPDACTFKKECVECKKFDRGALHDENTCNRYCRDEIESVKELKDTGKDAVNCTYKNEDDCVVRFQYYEDSSGKSILYVVEEPECPKGPDILVVLLSVMGAILLIGLAALLIWKLLITIHDRKEFAKFEEERARAKWDTANNPLYKEATSTFTNITYRGT

489 NP_001356.1 NP_004516.2

>NP_001356.1

MSQRPRAPRSALWLLAPPLLRWAPPLLTVLHSDLFQALLDILDYYEASLSESQKYRYQDEDTPPLEHSPAHLPNQANSPPVIVNTDTLEAPGYELQVNGTEGEMEYEEITLERGNSGLGFSIAGGTDNPHIGDDPSIFITKIIPGGAAAQDGRLRVNDSILFVNEVDVREVTHSAAVEALKEAGSIVRLYVMRRKPPAEKVMEIKLIKGPKGLGFSIAGGVGNQHIPGDNSIYVTKIIEGGAAHKDGRLQIGDKILAVNSVGLEDVMHEDAVAALKNTYDVVYLKVAKPSNAYLSDSYAPPDITTSYSQHLDNEISHSSYLGTDYPTAMTPTSPRRYSPVAKDLLGEEDIPREPRRIVIHRGSTGLGFNIVGGEDGEGIFISFILAGGPADLSGELRKGDQILSVNGVDLRNASHEQAAIALKNAGQTVTIIAQYKPEEYSRFEAKIHDLREQLMNSSLGSGTASLRSNPKRGFYIRALFDYDKTKDCGFLSQALSFRFGDVLHVIDASDEEWWQARRVHSDSETDDIGFIPSKRRVERREWSRLKAKDWGSSSGSQGREDSVLSYETVTQMEVHYARPIIILGPTKDRANDDLLSEFPDKFGSCVPHTTRPKREYEIDGRDYHFVSSREKMEKDIQAHKFIEAGQYNSHLYGTSVQSVREVAEQGKHCILDVSANAVRRLQAAHLHPIAIFIRPRSLENVLEINKRITEEQARKAFDRATKLEQEFTECFSAIVEGDSFEEIYHKVKRVIEDLSGPYIWVPARERL

>NP_004516.2

MDRGPAAVACTLLLALVACLAPASGQECDSAHFRCGSGHCIPADWRCDGTKDCSDDADEIGCAVVTCQQGYFKCQSEGQCIPNSWVCDQDQDCDDGSDERQDCSQSTCSSHQITCSNGQCIPSEYRCDHVRDCPDGADENDCQYPTCEQLTCDNGACYNTSQKCDWKVDCRDSSDEINCTEICLHNEFSCGNGECIPRAYVCDHDNDCQDGSDEHACNYPTCGGYQFTCPSGRCIYQNWVCDGEDDCKDNGDEDGCESGPHDVHKCSPREWSCPESGRCISIYKVCDGILDCPGREDENNTSTGKYCSMTLCSALNCQYQCHETPYGGACFCPPGYIINHNDSRTCVEFDDCQIWGICDQKCESRPGRHLCHCEEGYILERGQYCKANDSFGEASIIFSNGRDLLIGDIHGRSFRILVESQNRGVAVGVAFHYHLQRVFWTDTVQNKVFSVDINGLNIQEVLNVSVETPENLAVDWVNNKIYLVETKVNRIDMVNLDGSYRVTLITENLGHPRGIAVDPTVGYLFFSDWESLSGEPKLERAFMDGSNRKDLVKTKLGWPAGVTLDMISKRVYWVDSRFDYIETVTYDGIQRKTVVHGGSLIPHPFGVSLFEGQVFFTDWTKMAVLKANKFTETNPQVYYQASLRPYGVTVYHSLRQPYATNPCKDNNGGCEQVCVLSHRTDNDGLGFRCKCTFGFQLDTDERHCIAVQNFLIFSSQVAIRGIPFTLSTQEDVMVPVSGNPSFFVGIDFDAQDSTIFFSDMSKHMIFKQKIDGTGREILAANRVENVESLAFDWISKNLYWTDSHYKSISVMRLADKTRRTVVQYLNNPRSVVVHPFAGYLFFTDWFRPAKIMRAWSDGSHLLPVINTTLGWPNGLAIDWAASRLYWVDAYFDKIEHSTFDGLDRRRLGHIEQMTHPFGLAIFGEHLFFTDWRLGAIIRVRKADGGEMTVIRSGIAYILHLKSYDVNIQTGSNACNQPTHPNGDCSHFCFPVPNFQRVCGCPYGMRLASNHLTCEGDPTNEPPTEQCGLFSFPCKNGRCVPNYYLCDGVDDCHDNSDEQLCGTLNNTCSSSAFTCGHGECIPAHWRCDKRNDCVDGSDEHNCPTHAPASCLDTQYTCDNHQCISKNWVCDTDNDCGDGSDEKNCNSTETCQPSQFNCPNHRCIDLSFVCDGDKDCVDGSDEVGCVLNCTASQFKCASGDKCIGVTNRCDGVFDCSDNSDEAGCPTRPPGMCHSDEFQCQEDGICIPNFWECDGHPDCLYGSDEHNACVPKTCPSSYFHCDNGNCIHRAWLCDRDNDCGDMSDEKDCPTQPFRCPSWQWQCLGHNICVNLSVVCDGIFDCPNGTDESPLCNGNSCSDFNGGCTHECVQEPFGAKCLCPLGFLLANDSKTCEDIDECDILGSCSQHCYNMRGSFRCSCDTGYMLESDGRTCKVTASESLLLLVASQNKIIADSVTSQVHNIYSLVENGSYIVAVDFDSISGRIFWSDATQGKTWSAFQNGTDRRVVFDSSIILTETIAIDWVGRNLYWTDYALETIEVSKIDGSHRTVLISKNLTNPRGLALDPRMNEHLLFWSDWGHHPRIERASMDGSMRTVIVQDKIFWPCGLTIDYPNRLLYFMDSYLDYMDFCDYNGHHRRQVIASDLIIRHPYALTLFEDSVYWTDRATRRVMRANKWHGGNQSVVMYNIQWPLGIVAVHPSKQPNSVNPCAFSRCSHLCLLSSQGPHFYSCVCPSGWSLSPDLLNCLRDDQPFLITVRQHIIFGISLNPEVKSNDAMVPIAGIQNGLDVEFDDAEQYIYWVENPGEIHRVKTDGTNRTVFASISMVGPSMNLALDWISRNLYSTNPRTQSIEVLTLHGDIRYRKTLIANDGTALGVGFPIGITVDPARGKLYWSDQGTDSGVPAKIASANMDGTSVKTLFTGNLEHLECVTLDIEEQKLYWAVTGRGVIERGNVDGTDRMILVHQLSHPWGIAVHDSFLYYTDEQYEVIERVDKATGANKIVLRDNVPNLRGLQVYHRRNAAESSNGCSNNMNACQQICLPVPGGLFSCACATGFKLNPDNRSCSPYNSFIVVSMLSAIRGFSLELSDHSETMVPVAGQGRNALHVDVDVSSGFIYWCDFSSSVASDNAIRRIKPDGSSLMNIVTHGIGENGVRGIAVDWVAGNLYFTNAFVSETLIEVLRINTTYRRVLLKVTVDMPRHIVVDPKNRYLFWADYGQRPKIERSFLDCTNRTVLVSEGIVTPRGLAVDRSDGYVYWVDDSLDIIARIRINGENSEVIRYGSRYPTPYGITVFENSIIWVDRNLKKIFQASKEPENTEPPTVIRDNINWLRDVTIFDKQVQPRSPAEVNNNPCLENNGGCSHLCFALPGLHTPKCDCAFGTLQSDGKNCAISTENFLIFALSNSLRSLHLDPENHSPPFQTINVERTVMSLDYDSVSDRIYFTQNLASGVGQISYATLSSGIHTPTVIASGIGTADGIAFDWITRRIYYSDYLNQMINSMAEDGSNRTVIARVPKPRAIVLDPCQGYLYWADWDTHAKIERATLGGNFRVPIVNSSLVMPSGLTLDYEEDLLYWVDASLQRIERSTLTGVDREVIVNAAVHAFGLTLYGQYIYWTDLYTQRIYRANKYDGSGQIAMTTNLLSQPRGINTVVKNQKQQCNNPCEQFNGGCSHICAPGPNGAECQCPHEGNWYLANNRKHCIVDNGERCGASSFTCSNGRCISEEWKCDNDNDCGDGSDEMESVCALHTCSPTAFTCANGRCVQYSYRCDYYNDCGDGSDEAGCLFRDCNATTEFMCNNRRCIPREFICNGVDNCHDNNTSDEKNCPDRTCQSGYTKCHNSNICIPRVYLCDGDNDCGDNSDENPTYCTTHTCSSSEFQCASGRCIPQHWYCDQETDCFDASDEPASCGHSERTCLADEFKCDGGRCIPSEWICDGDNDCGDMSDEDKRHQCQNQNCSDSEFLCVNDRPPDRRCIPQSWVCDGDVDCTDGYDENQNCTRRTCSENEFTCGYGLCIPKIFRCDRHNDCGDYSDERGCLYQTCQQNQFTCQNGRCISKTFVCDEDNDCGDGSDELMHLCHTPEPTCPPHEFKCDNGRCIEMMKLCNHLDDCLDNSDEKGCGINECHDPSISGCDHNCTDTLTSFYCSCRPGYKLMSDKRTCVDIDECTEMPFVCSQKCENVIGSYICKCAPGYLREPDGKTCRQNSNIEPYLIFSNRYYLRNLTIDGYFYSLILEGLDNVVALDFDRVEKRLYWIDTQRQVIERMFLNKTNKETIINHRLPAAESLAVDWVSRKLYWLDARLDGLFVSDLNGGHRRMLAQHCVDANNTFCFDNPRGLALHPQYGYLYWADWGHRAYIGRVGMDGTNKSVIISTKLEWPNGITIDYTNDLLYWADAHLGYIEYSDLEGHHRHTVYDGALPHPFAITIFEDTIYWTDWNTRTVEKGNKYDGSNRQTLVNTTHRPFDIHVYHPYRQPIVSNPCGTNNGGCSHLCLIKPGGKGFTCECPDDFRTLQLSGSTYCMPMCSSTQFLCANNEKCIPIWWKCDGQKDCSDGSDELALCPQRFCRLGQFQCSDGNCTSPQTLCNAHQNCPDGSDEDRLLCENHHCDSNEWQCANKRCIPESWQCDTFNDCEDNSDEDSSHCASRTCRPGQFRCANGRCIPQAWKCDVDNDCGDHSDEPIEECMSSAHLCDNFTEFSCKTNYRCIPKWAVCNGVDDCRDNSDEQGCEERTCHPVGDFRCKNHHCIPLRWQCDGQNDCGDNSDEENCAPRECTESEFRCVNQQCIPSRWICDHYNDCGDNSDERDCEMRTCHPEYFQCTSGHCVHSELKCDGSADCLDASDEADCPTRFPDGAYCQATMFECKNHVCIPPYWKCDGDDDCGDGSDEELHLCLDVPCNSPNRFRCDNNRCIYSHEVCNGVDDCGDGTDETEEHCRKPTPKPCTEYEYKCGNGHCIPHDNVCDDADDCGDWSDELGCNKGKERTCAENICEQNCTQLNEGGFICSCTAGFETNVFDRTSCLDINECEQFGTCPQHCRNTKGSYECVCADGFTSMSDRPGKRCAAEGSSPLLLLPDNVRIRKYNLSSERFSEYLQDEEYIQAVDYDWDPKDIGLSVVYYTVRGEGSRFGAIKRAYIPNFESGRNNLVQEVDLKLKYVMQPDGIAVDWVGRHIYWSDVKNKRIEVAKLDGRYRKWLISTDLDQPAAIAVNPKLGLMFWTDWGKEPKIESAWMNGEDRNILVFEDLGWPTGLSIDYLNNDRIYWSDFKEDVIETIKYDGTDRRVIAKEAMNPYSLDIFEDQLYWISKEKGEVWKQNKFGQGKKEKTLVVNPWLTQVRIFHQLRYNKSVPNLCKQICSHLCLLRPGGYSCACPQGSSFIEGSTTECDAAIELPINLPPPCRCMHGGNCYFDETDLPKCKCPSGYTGKYCEMAFSKGISPGTTAVAVLLTILLIVVIGALAIAGFFHYRRTGSLLPALPKLPSLSSLVKPSENGNGVTFRSGADLNMDIGVSGFGPETAIDRSMAMSEDFVMEMGKQPIIFENPMYSARDSAVKVVQPIQVTVSENVDNKNYGSPINPSEIVPETNPTSPAADGTQVTKWNLFKRKSKQTTNFENPIYAQMENEQKESVAATPPPSPSLPAKPKPPSRRDPTPTYSATEDTFKDTANLVKEDSEV

490 NP_000827.1 NP_002729.2

>NP_000827.1

MRGAGGPRGPRGPAKMLLLLALACASPFPEEAPGPGGAGGPGGGLGGARPLNVALVFSGPAYAAEAARLGPAVAAAVRSPGLDVRPVALVLNGSDPRSLVLQLCDLLSGLRVHGVVFEDDSRAPAVAPILDFLSAQTSLPIVAVHGGAALVLTPKEKGSTFLQLGSSTEQQLQVIFEVLEEYDWTSFVAVTTRAPGHRAFLSYIEVLTDGSLVGWEHRGALTLDPGAGEAVLSAQLRSVSAQIRLLFCAREEAEPVFRAAEEAGLTGSGYVWFMVGPQLAGGGGSGAPGEPPLLPGGAPLPAGLFAVRSAGWRDDLARRVAAGVAVVARGAQALLRDYGFLPELGHDCRAQNRTHRGESLHRYFMNITWDNRDYSFNEDGFLVNPSLVVISLTRDRTWEVVGSWEQQTLRLKYPLWSRYGRFLQPVDDTQHLTVATLEERPFVIVEPADPISGTCIRDSVPCRSQLNRTHSPPPDAPRPEKRCCKGFCIDILKRLAHTIGFSYDLYLVTNGKHGKKIDGVWNGMIGEVFYQRADMAIGSLTINEERSEIVDFSVPFVETGISVMVARSNGTVSPSAFLEPYSPAVWVMMFVMCLTVVAVTVFIFEYLSPVGYNRSLATGKRPGGSTFTIGKSIWLLWALVFNNSVPVENPRGTTSKIMVLVWAFFAVIFLASYTANLAAFMIQEEYVDTVSGLSDRKFQRPQEQYPPLKFGTVPNGSTEKNIRSNYPDMHSYMVRYNQPRVEEALTQLKAGKLDAFIYDAAVLNYMARKDEGCKLVTIGSGKVFATTGYGIALHKGSRWKRPIDLALLQFLGDDEIEMLERLWLSGICHNDKIEVMSSKLDIDNMAGVFYMLLVAMGLSLLVFAWEHLVYWRLRHCLGPTHRMDFLLAFSRGMYSCCSAEAAPPPAKPPPPPQPLPSPAYPAPGPAPGPAPFVPRERASVDRWRRTKGAGPPGGAGLADGFHRYYGPIEPQGLGLGLGEARAAPRGAAGRPLSPPAAQPPQKPPASYFAIVRDKEPAEPPAGAFPGFPSPPAPPAAAATAVGPPLCRLAFEDESPPAPARWPRSDPESQPLLGPGAGGAGGTGGAGGGAPAAPPPCCAAPPPCPYLDLEPSPSDSEDSESLGGASLGGLDPWWFADFPYPYAERLGPPPGRYWSVDKLGGWRAGSWDYLPPRSGPAAWHCRHCASLELLPPPRHLSCSHDGLDGGWWAPPPPPWAAGPLPRRRARCGCPRSHPHRPRASHRTPAAAAPHHHRHRRAAGGWDLPPPAPTSRSLEDLSSCPRAAPARRLTGPSRHARRCPHAAHWGPPLPTASHRRHRGGDLGTRRGSAHFSSLESEV

>NP_002729.2

MADPAAGPPPSEGEESTVRFARKGALRQKNVHEVKNHKFTARFFKQPTFCSHCTDFIWGFGKQGFQCQVCCFVVHKRCHEFVTFSCPGADKGPASDDPRSKHKFKIHTYSSPTFCDHCGSLLYGLIHQGMKCDTCMMNVHKRCVMNVPSLCGTDHTERRGRIYIQAHIDRDVLIVLVRDAKNLVPMDPNGLSDPYVKLKLIPDPKSESKQKTKTIKCSLNPEWNETFRFQLKESDKDRRLSVEIWDWDLTSRNDFMGSLSFGISELQKASVDGWFKLLSQEEGEYFNVPVPPEGSEANEELRQKFERAKISQGTKVPEEKTTNTVSKFDNNGNRDRMKLTDFNFLMVLGKGSFGKVMLSERKGTDELYAVKILKKDVVIQDDDVECTMVEKRVLALPGKPPFLTQLHSCFQTMDRLYFVMEYVNGGDLMYHIQQVGRFKEPHAVFYAAEIAIGLFFLQSKGIIYRDLKLDNVMLDSEGHIKIADFGMCKENIWDGVTTKTFCGTPDYIAPEIIAYQPYGKSVDWWAFGVLLYEMLAGQAPFEGEDEDELFQSIMEHNVAYPKSMSKEAVAICKGLMTKHPGKRLGCGPEGERDIKEHAFFRYIDWEKLERKEIQPPYKPKACGRNAENFDRFFTRHPPVLTPPDQEVIRNIDQSEFEGFSFVNSEFLKPEVKS

491 NP_002998.1 NP_064516.2

>NP_002998.1

MKPNIIFVLSLLLILEKQAAVMGQKGGSKGRLPSEFSQFPHGQKGQHYSGQKGKQQTESKGSFSIQYTYHVDANDHDQSRKSQQYDLNALHKTTKSQRHLGGSQQLLHNKQEGRDHDKSKGHFHRVVIHHKGGKAHRGTQNPSQDQGNSPSGKGISSQYSNTEERLWVHGLSKEQTSVSGAQKGRKQGGSQSSYVLQTEELVANKQQRETKNSHQNKGHYQNVVEVREEHSSKVQTSLCPAHQDKLQHGSKDIFSTQDELLVYNKNQHQTKNLNQDQQHGRKANKISYQSSSTEERRLHYGENGVQKDVSQSSIYSQTEEKAQGKSQKQITIPSQEQEHSQKANKISYQSSSTEERRLHYGENGVQKDVSQRSIYSQTEKLVAGKSQIQAPNPKQEPWHGENAKGESGQSTNREQDLLSHEQKGRHQHGSHGGLDIVIIEQEDDSDRHLAQHLNNDRNPLFT

>NP_064516.2

MAEPSGAETRPPIRVTVKTPKDKEEIVICDRASVKEFKEEISRRFKAQQDQLVLIFAGKILKDGDTLNQHGIKDGLTVHLVIKTPQKAQDPAAATASSPSTPDPASAPSTTPASPATPAQPSTSGSASSDAGSGSRRSSGGGPSPGAGEGSPSATASILSGFGGILGLGSLGLGSANFMELQQQMQRQLMSNPEMLSQIMENPLVQDMMSNPDLMRHMIMANPQMQQLMERNPEISHMLNNPELMRQTMELARNPAMMQEMMRNQDRALSNLESIPGGYNALRRMYTDIQEPMFSAAREQFGNNPFSSLAGNSDSSSSQPLRTENREPLPNPWSPSPPTSQAPGSGGEGTGGSGTSQVHPTVSNPFGINAASLGSGMFNSPEMQALLQQISENPQLMQNVISAPYMRSMMQTLAQNPDFAAQMMVNVPLFAGNPQLQEQLRLQLPVFLQQMQNPESLSILTNPRAMQALLQIQQGLQTLQTEAPGLVPSLGSFGISRTPAPSAGSNAGSTPEAPTSSPATPATSSPTGASSAQQQLMQQMIQLLAGSGNSQVQTPEVRFQQQLEQLNSMGFINREANLQALIATGGDINAAIERLLGSQLS

492 NP_000091.1 NP_001903.1

>NP_000091.1

MMCGAPSATQPATAETQHIADQVRSQLEEKENKKFPVFKAVSFKSQVVAGTNYFIKVHVGDEDFVHLRVFQSLPHENKPLTLSNYQTNKAKHDELTYF

>NP_001903.1

MNPTLILAAFCLGIASATLTFDHSLEAQWTKWKAMHNRLYGMNEEGWRRAVWEKNMKMIELHNQEYREGKHSFTMAMNAFGDMTSEEFRQVMNGFQNRKPRKGKVFQEPLFYEAPRSVDWREKGYVTPVKNQGQCGSCWAFSATGALEGQMFRKTGRLISLSEQNLVDCSGPQGNEGCNGGLMDYAFQYVQDNGGLDSEESYPYEATEESCKYNPKYSVANDTGFVDIPKQEKALMKAVATVGPISVAIDAGHESFLFYKEGIYFEPDCSSEDMDHGVLVVGYGFESTESDNNKYWLVKNSWGEEWGMGGYVKMAKDRRNHCGIASAASYPTV

493 NP_006792.1 NP_001649.1

>NP_006792.1

MNLFRFLGDLSHLLAIILLLLKIWKSRSCAGISGKSQVLFAVVFTARYLDLFTNYISLYNTCMKVVYIACSFTTVWLIYSKFKATYDGNHDTFRVEFLVVPTAILAFLVNHDFTPLEILWTFSIYLESVAILPQLFMVSKTGEAETITSHYLFALGVYRTLYLFNWIWRYHFEGFFDLIAIVAGLVQTVLYCDFFYLYITKVLKGKKLSLPA

>NP_001649.1

MGNIFANLFKGLFGKKEMRILMVGLDAAGKTTILYKLKLGEIVTTIPTIGFNVETVEYKNISFTVWDVGGQDKIRPLWRHYFQNTQGLIFVVDSNDRERVNEAREELMRMLAEDELRDAVLLVFANKQDLPNAMNAAEITDKLGLHSLRHRNWYIQATCATSGDGLYEGLDWLSNQLRNQK

494 NP_005558.1 NP_001839.2

>NP_005558.1

MTPPRLFWVWLLVAGTQGVNDGDMRLADGGATNQGRVEIFYRGQWGTVCDNLWDLTDASVVCRALGFENATQALGRAAFGQGSGPIMLDEVQCTGTEASLADCKSLGWLKSNCRHERDAGVVCTNETRSTHTLDLSRELSEALGQIFDSQRGCDLSISVNVQGEDALGFCGHTVILTANLEAQALWKEPGSNVTMSVDAECVPMVRDLLRYFYSRRIDITLSSVKCFHKLASAYGARQLQGYCASLFAILLPQDPSFQMPLDLYAYAVATGDALLEKLCLQFLAWNFEALTQAEAWPSVPTDLLQLLLPRSDLAVPSELALLKAVDTWSWGERASHEEVEGLVEKIRFPMMLPEELFELQFNLSLYWSHEALFQKKTLQALEFHTVPFQLLARYKGLNLTEDTYKPRIYTSPTWSAFVTDSSWSARKSQLVYQSRRGPLVKYSSDYFQAPSDYRYYPYQSFQTPQHPSFLFQDKRVSWSLVYLPTIQSCWNYGFSCSSDELPVLGLTKSGGSDRTIAYENKALMLCEGLFVADVTDFEGWKAAIPSALDTNSSKSTSSFPCPAGHFNGFRTVIRPFYLTNSSGVD

>NP_001839.2

MRAARALLPLLLQACWTAAQDEPETPRAVAFQDCPVDLFFVLDTSESVALRLKPYGALVDKVKSFTKRFIDNLRDRYYRCDRNLVWNAGALHYSDEVEIIQGLTRMPGGRDALKSSVDAVKYFGKGTYTDCAIKKGLEQLLVGGSHLKENKYLIVVTDGHPLEGYKEPCGGLEDAVNEAKHLGVKVFSVAITPDHLEPRLSIIATDHTYRRNFTAADWGQSRDAEEAISQTIDTIVDMIKNNVEQVCCSFECQPARGPPGLRGDPGFEGERGKPGLPGEKGEAGDPGRPGDLGPVGYQGMKGEKGSRGEKGSRGPKGYKGEKGKRGIDGVDGVKGEMGYPGLPGCKGSPGFDGIQGPPGPKGDPGAFGLKGEKGEPGADGEAGRPGSSGPSGDEGQPGEPGPPGEKGEAGDEGNPGPDGAPGERGGPGERGPRGTPGTRGPRGDPGEAGPQGDQGREGPVGVPGDPGEAGPIGPKGYRGDEGPPGSEGARGAPGPAGPPGDPGLMGERGEDGPAGNGTEGFPGFPGYPGNRGAPGINGTKGYPGLKGDEGEAGDPGDDNNDIAPRGVKGAKGYRGPEGPQGPPGHQGPPGPDECEILDIIMKMCSCCECKCGPIDLLFVLDSSESIGLQNFEIAKDFVVKVIDRLSRDELVKFEPGQSYAGVVQYSHSQMQEHVSLRSPSIRNVQELKEAIKSLQWMAGGTFTGEALQYTRDQLLPPSPNNRIALVITDGRSDTQRDTTPLNVLCSPGIQVVSVGIKDVFDFIPGSDQLNVISCQGLAPSQGRPGLSLVKENYAELLEDAFLKNVTAQICIDKKCPDYTCPITFSSPADITILLDGSASVGSHNFDTTKRFAKRLAERFLTAGRTDPAHDVRVAVVQYSGTGQQRPERASLQFLQNYTALASAVDAMDFINDATDVNDALGYVTRFYREASSGAAKKRLLLFSDGNSQGATPAAIEKAVQEAQRAGIEIFVVVVGRQVNEPHIRVLVTGKTAEYDVAYGESHLFRVPSYQALLRGVFHQTVSRKVALG

495 NP_001393.1 NP_004146.1

>NP_001393.1

MGKEKTHINIVVIGHVDSGKSTTTGHLIYKCGGIDKRTIEKFEKEAAEMGKGSFKYAWVLDKLKAERERGITIDISLWKFETSKYYVTIIDAPGHRDFIKNMITGTSQADCAVLIVAAGVGEFEAGISKNGQTREHALLAYTLGVKQLIVGVNKMDSTEPPYSQKRYEEIVKEVSTYIKKIGYNPDTVAFVPISGWNGDNMLEPSANMPWFKGWKVTRKDGNASGTTLLEALDCILPPTRPTDKPLRLPLQDVYKIGGIGTVPVGRVETGVLKPGMVVTFAPVNVTTEVKSVEMHHEALSEALPGDNVGFNVKNVSVKDVRRGNVAGDSKNDPPMEAAGFTAQVIILNHPGQISAGYAPVLDCHTAHIACKFAELKEKIDRRSGKKLEDGPKFLKSGDAAIVDMVPGKPMCVESFSDYPPLGRFAVRDMRQTVAVGVIKAVDKKAAGAGKVTKSAQKAQKAK

>NP_004146.1

METLSNASGTFAIRLLKILCQDNPSHNVFCSPVSISSALAMVLLGAKGNTATQMAQALSLNTEEDIHRAFQSLLTEVNKAGTQYLLRTANRLFGEKTCQFLSTFKESCLQFYHAELKELSFIRAAEESRKHINTWVSKKTEGKIEELLPGSSIDAETRLVLVNAIYFKGKWNEPFDETYTREMPFKINQEEQRPVQMMYQEATFKLAHVGEVRAQLLELPYARKELSLLVLLPDDGVELSTVEKSLTFEKLTAWTKPDCMKSTEVEVLLPKFKLQEDYDMESVLRHLGIVDAFQQGKADLSAMSAERDLCLSKFVHKSFVEVNEEGTEAAAASSCFVVAECCMESGPRFCADHPFLFFIRHNRANSILFCGRFSSP

496 NP_001269.3 NP_006303.3

>NP_001269.3

MERPPGLRPGAGGPWEMRERLGTGGFGNVCLYQHRELDLKIAIKSCRLELSTKNRERWCHEIQIMKKLNHANVVKACDVPEELNILIHDVPLLAMEYCSGGDLRKLLNKPENCCGLKESQILSLLSDIGSGIRYLHENKIIHRDLKPENIVLQDVGGKIIHKIIDLGYAKDVDQGSLCTSFVGTLQYLAPELFENKPYTATVDYWSFGTMVFECIAGYRPFLHHLQPFTWHEKIKKKDPKCIFACEEMSGEVRFSSHLPQPNSLCSLVVEPMENWLQLMLNWDPQQRGGPVDLTLKQPRCFVLMDHILNLKIVHILNMTSAKIISFLLPPDESLHSLQSRIERETGINTGSQELLSETGISLDPRKPASQCVLDGVRGCDSYMVYLFDKSKTVYEGPFASRSLSDCVNYIVQDSKIQLPIIQLRKVWAEAVHYVSGLKEDYSRLFQGQRAAMLSLLRYNANLTKMKNTLISASQQLKAKLEFFHKSIQLDLERYSEQMTYGISSEKMLKAWKEMEEKAIHYAEVGVIGYLEDQIMSLHAEIMELQKSPYGRRQGDLMESLEQRAIDLYKQLKHRPSDHSYSDSTEMVKIIVHTVQSQDRVLKELFGHLSKLLGCKQKIIDLLPKVEVALSNIKEADNTVMFMQGKRQKEIWHLLKIACTQSSARSLVGSSLEGAVTPQTSAWLPPTSAEHDHSLSCVVTPQDGETSAQMIEENLNCLGHLSTIIHEANEEQGNSMMNLDWSWLTE

>NP_006303.3

MSGSTQPVAQTWRATEPRYPPHSLSYPVQIARTHTDVGLLEYQHHSRDYASHLSPGSIIQPQRRRPSLLSEFQPGNERSQELHLRPESHSYLPELGKSEMEFIESKRPRLELLPDPLLRPSPLLATGQPAGSEDLTKDRSLTGKLEPVSPPSPPHTDPELELVPPRLSKEELIQNMDRVDREITMVEQQISKLKKKQQQLEEEAAKPPEPEKPVSPPPIESKHRSLVQIIYDENRKKAEAAHRILEGLGPQVELPLYNQPSDTRQYHENIKINQAMRKKLILYFKRRNHARKQWEQKFCQRYDQLMEAWEKKVERIENNPRRRAKESKVREYYEKQFPEIRKQRELQERMQSRVGQRGSGLSMSAARSEHEVSEIIDGLSEQENLEKQMRQLAVIPPMLYDADQQRIKFINMNGLMADPMKVYKDRQVMNMWSEQEKETFREKFMQHPKNFGLIASFLERKTVAECVLYYYLTKKNENYKSLVRRSYRRRGKSQQQQQQQQQQQQQQQQQPMPRSSQEEKDEKEKEKEAEKEEEKPEVENDKEDLLKEKTDDTSGEDNDEKEAVASKGRKTANSQGRRKGRITRSMANEANSEEAITPQQSAELASMELNESSRWTEEEMETAKKGLLEHGRNWSAIARMVGSKTVSQCKNFYFNYKKRQNLDEILQQHKLKMEKERNARRKKKKAPAAASEEAAFPPVVEDEEMEASGVSGNEEEMVEEAEALHASGNEVPRGECSGPATVNNSSDTESIPSPHTEAAKDTGQNGPKPPATLGADGPPPGPPTPPPEDIPAPTEPTPASEATGAPTPPPAPPSPSAPPPVVPKEEKEEETAAAPPVEEGEEQKPPAAEELAVDTGKAEEPVKSECTEEAEEGPAKGKDAEAAEATAEGALKAEKKEGGSGRATTAKSSGAPQDSDSSATCSADEVDEAEGGDKNRLLSPRPSLLTPTGDPRANASPQKPLDLKQLKQRAAAIPPIVTKVHEPPREDAAPTKPAPPAPPPPQNLQPESDAPQQPGSSPRGKSRSPAPPADKEAEKPVFFPAFAAEAQKLPGDPPCWTSGLPFPVPPREVIKASPHAPDPSAFSYAPPGHPLPLGLHDTARPVLPRPPTISNPPPLISSAKHPSVLERQIGAISQGMSVQLHVPYSEHAKAPVGPVTMGLPLPMDPKKLAPFSGVKQEQLSPRGQAGPPESLGVPTAQEASVLRGTALGSVPGGSITKGIPSTRVPSDSAITYRGSITHGTPADVLYKGTITRIIGEDSPSRLDRGREDSLPKGHVIYEGKKGHVLSYEGGMSVTQCSKEDGRSSSGPPHETAAPKRTYDMMEGRVGRAISSASIEGLMGRAIPPERHSPHHLKEQHHIRGSITQGIPRSYVEAQEDYLRREAKLLKREGTPPPPPPSRDLTEAYKTQALGPLKLKPAHEGLVATVKEAGRSIHEIPREELRHTPELPLAPRPLKEGSITQGTPLKYDTGASTTGSKKHDVRSLIGSPGRTFPPVHPLDVMADARALERACYEESLKSRPGTASSSGGSIARGAPVIVPELGKPRQSPLTYEDHGAPFAGHLPRGSPVTTREPTPRLQEGSLSSSKASQDRKLTSTPREIAKSPHSTVPEHHPHPISPYEHLLRGVSGVDLYRSHIPLAFDPTSIPRGIPLDAAAAYYLPRHLAPNPTYPHLYPPYLIRGYPDTAALENRQTIINDYITSQQMHHNAATAMAQRADMLRGLSPRESSLALNYAAGPRGIIDLSQVPHLPVLVPPTPGTPATAMDRLAYLPTAPQPFSSRHSSSPLSPGGPTHLTKPTTTSSSERERDRDRERDRDREREKSILTSTTTVEHAPIWRPGTEQSSGSSGSSGGGGGSSSRPASHSHAHQHSPISPRTQDALQQRPSVLHNTGMKGIITAVEPSTPTVLRSTSTSSPVRPAATFPPATHCPLGGTLDGVYPTLMEPVLLPKEAPRVARPERPRADTGHAFLAKPPARSGLEPASSPSKGSEPRPLVPPVSGHATIARTPAKNLAPHHASPDPPAPPASASDPHREKTQSKPFSIQELELRSLGYHGSSYSPEGVEPVSPVSSPSLTHDKGLPKHLEELDKSHLEGELRPKQPGPVKLGGEAAHLPHLRPLPESQPSSSPLLQTAPGVKGHQRVVTLAQHISEVITQDYTRHHPQQLSAPLPAPLYSFPGASCPVLDLRRPPSDLYLPPPDHGAPARGSPHSEGGKRSPEPNKTSVLGGGEDGIEPVSPPEGMTEPGHSRSAVYPLLYRDGEQTEPSRMGSKSPGNTSQPPAFFSKLTESNSAMVKSKKQEINKKLNTHNRNEPEYNISQPGTEIFNMPAITGTGLMTYRSQAVQEHASTNMGLEAIIRKALMGKYDQWEESPPLSANAFNPLNASASLPAAMPITAADGRSDHTLTSPGGGGKAKVSGRPSSRKAKSPAPGLASGDRPPSVSSVHSEGDCNRRTPLTNRVWEDRPSSAGSTPFPYNPLIMRLQAGVMASPPPPGLPAGSGPLAGPHHAWDEEPKPLLCSQYETLSDSE

497 NP_001886.1 NP_000442.1

>NP_001886.1

MSGPVPSRARVYTDVNTHRPREYWDYESHVVEWGNQDDYQLVRKLGRGKYSEVFEAINITNNEKVVVKILKPVKKKKIKREIKILENLRGGPNIITLADIVKDPVSRTPALVFEHVNNTDFKQLYQTLTDYDIRFYMYEILKALDYCHSMGIMHRDVKPHNVMIDHEHRKLRLIDWGLAEFYHPGQEYNVRVASRYFKGPELLVDYQMYDYSLDMWSLGCMLASMIFRKEPFFHGHDNYDQLVRIAKVLGTEDLYDYIDKYNIELDPRFNDILGRHSRKRWERFVHSENQHLVSPEALDFLDKLLRYDHQSRLTAREAMEHPYFYTVVKDQARMGSSSMPGGSTPVSSANMMSGISSVPTPSPLGPLAGSPVIAAANPLGMPVPAAAGAQQ

>NP_000442.1

MEELTAFVSKSFDQKSKDGNGGGGGGGGKKDSITYREVLESGLARSRELGTSDSSLQDITEGGGHCPVHLFKDHVDNDKEKLKEFGTARVAEGIYECKEKREDVKSEDEDGQTKLKQRRSRTNFTLEQLNELERLFDETHYPDAFMREELSQRLGLSEARVQVWFQNRRAKCRKQENQMHKGVILGTANHLDACRVAPYVNMGALRMPFQQVQAQLQLEGVAHAHPHLHPHLAAHAPYLMFPPPPFGLPIASLAESASAAAVVAAAAKSNSKNSSIADLRLKARKHAEALGL

498 NP_003178.1 NP_036448.1

>NP_003178.1

MESGKTASPKSMPKDAQMMAQILKDMGITEYEPRVINQMLEFAFRYVTTILDDAKIYSSHAKKATVDADDVRLAIQCRADQSFTSPPPRDFLLDIARQRNQTPLPLIKPYSGPRLPPDRYCLTAPNYRLKSLQKKASTSAGRITVPRLSVGSVTSRPSTPTLGTPTPQTMSVSTKVGTPMSLTGQRFTVQMPTSQSPAVKASIPATSAVQNVLINPSLIGSKNILITTNMMSSQNTANESSNALKRKREDDDDDDDDDDDYDNL

>NP_036448.1

METMASPGKDNYRMKSYKNNALNPEEMRRRREEEGIQLRKQKREQQLFKRRNVELINEEAAMFDSLLMDSYVSSTTGESVITREMVEMLFSDDSDLQLATTQKFRKLLSKEPSPPIDEVINTPRVVDRFVEFLKRNENCTLQFEAAWALTNIASGTSQQTKIVIEAGAVPIFIELLNSDFEDVQEQAVWALGNIAGDSSVCRDYVLNCSILNPLLTLLTKSTRLTMTRNAVWALSNLCRGKNPPPEFAKVSPCLPVLSRLLFSSDSDLLADACWALSYLSDGPNEKIQAVIDSGVCRRLVELLMHNDYKVASPALRAVGNIVTGDDIQTQVILNCSALPCLLHLLSSPKESIRKEACWTISNITAGNRAQIQAVIDANIFPVLIEILQKAEFRTRKEAAWAITNATSGGTPEQIRYLVSLGCIKPLCDLLTVMDSKIVQVALNGLENILRLGEQEGKRSGSGVNPYCGLIEEAYGLDKIEFLQSHENQEIYQKAFDLIEHYFGVEDDDSSLAPQVDETQQQFIFQQPEAPMEGFQL

499 NP_005726.1 NP_001091.1

>NP_005726.1

MESYDIIANQPVVIDNGSGVIKAGFAGDQIPKYCFPNYVGRPKHMRVMAGALEGDLFIGPKAEEHRGLLTIRYPMEHGVVRDWNDMERIWQYVYSKDQLQTFSEEHPVLLTEAPLNPSKNREKAAEVFFETFNVPALFISMQAVLSLYATGRTTGVVLDSGDGVTHAVPIYEGFAMPHSIMRVDIAGRDVSRYLRLLLRKEGVDFHTSAEFEVVRTIKERACYLSINPQKDEALETEKVQYTLPDGSTLDVGPARFRAPELLFQPDLVGDESEGLHEVVAFAIHKSDMDLRRTLFANIVLSGGSTLFKGFGDRLLSEVKKLAPKDIKIKISAPQERLYSTWIGGSILASLDTFKKMWVSKKEYEEDGSRAIHRKTF

>NP_001091.1

MCDEDETTALVCDNGSGLVKAGFAGDDAPRAVFPSIVGRPRHQGVMVGMGQKDSYVGDEAQSKRGILTLKYPIEHGIITNWDDMEKIWHHTFYNELRVAPEEHPTLLTEAPLNPKANREKMTQIMFETFNVPAMYVAIQAVLSLYASGRTTGIVLDSGDGVTHNVPIYEGYALPHAIMRLDLAGRDLTDYLMKILTERGYSFVTTAEREIVRDIKEKLCYVALDFENEMATAASSSSLEKSYELPDGQVITIGNERFRCPETLFQPSFIGMESAGIHETTYNSIMKCDIDIRKDLYANNVMSGGTTMYPGIADRMQKEITALAPSTMKIKIIAPPERKYSVWIGGSILASLSTFQQMWITKQEYDEAGPSIVHRKCF

500 NP_003609.2 NP_003017.1

>NP_003609.2

MNPGFDLSRRNPQEDFELIQRIGSGTYGDVYKARNVNTGELAAIKVIKLEPGEDFAVVQQEIIMMKDCKHPNIVAYFGSYLRRDKLWICMEFCGGGSLQDIYHVTGPLSELQIAYVSRETLQGLYYLHSKGKMHRDIKGANILLTDNGHVKLADFGVSAQITATIAKRKSFIGTPYWMAPEVAAVERKGGYNQLCDLWAVGITAIELAELQPPMFDLHPMRALFLMTKSNFQPPKLKDKMKWSNSFHHFVKMALTKNPKKRPTAEKLLQHPFVTQHLTRSLAIELLDKVNNPDHSTYHDFDDDDPEPLVAVPHRIHSTSRNVREEKTRSEITFGQVKFDPPLRKETEPHHELPDSDGFLDSSEEIYYTARSNLDLQLEYGQGHQGGYFLGANKSLLKSVEEELHQRGHVAHLEDDEGDDDESKHSTLKAKIPPPLPPKPKSIFIPQEMHSTEDENQGTIKRCPMSGSPAKPSQVPPRPPPPRLPPHKPVALGNGMSSFQLNGERDGSLCQQQNEHRGTNLSRKEKKDVPKPISNGLPPTPKVHMGACFSKVFNGCPLKIHCASSWINPDTRDQYLIFGAEEGIYTLNLNELHETSMEQLFPRRCTWLYVMNNCLLSISGKASQLYSHNLPGLFDYARQMQKLPVAIPAHKLPDRILPRKFSVSAKIPETKWCQKCCVVRNPYTGHKYLCGALQTSIVLLEWVEPMQKFMLIKHIDFPIPCPLRMFEMLVVPEQEYPLVCVGVSRGRDFNQVVRFETVNPNSTSSWFTESDTPQTNVTHVTQLERDTILVCLDCCIKIVNLQGRLKSSRKLSSELTFDFQIESIVCLQDSVLAFWKHGMQGRSFRSNEVTQEISDSTRIFRLLGSDRVVVLESRPTDNPTANSNLYILAGHENSY

>NP_003017.1

MSVAGLKKQFHKATQKVSEKVGGAEGTKLDDDFKEMERKVDVTSRAVMEIMTKTIEYLQPNPASRAKLSMINTMSKIRGQEKGPGYPQAEALLAEAMLKFGRELGDDCNFGPALGEVGEAMRELSEVKDSLDIEVKQNFIDPLQNLHDKDLREIQHHLKKLEGRRLDFDYKKKRQGKIPDEELRQALEKFDESKEIAESSMFNLLEMDIEQVSQLSALVQAQLEYHKQAVQILQQVTVRLEERIRQASSQPRREYQPKPRMSLEFPTGDSTQPNGGLSHTGTPKPSGVQMDQPCCRALYDFEPENEGELGFKEGDIITLTNQIDENWYEGMLHGHSGFFPINYVEILVALPH

501 NP_005730.1 NP_005334.1

>NP_005730.1

MGTLGKAREAPRKPSHGCRAASKARLEAKPANSPFPSHPSLAHITQFRMMVSLGHLAKGASLDDLIDSCIQSFDADGNLCRSNQLLQVMLTMHRIVISSAELLQKVITLYKDALAKNSPGLCLKICYFVRYWITEFWVMFKMDASLTDTMEEFQELVKAKGEELHCRLIDTTQINARDWSRKLTQRIKSNTSKKRKVSLLFDHLEPEELSEHLTYLEFKSFRRISFSDYQNYLVNSCVKENPTMERSIALCNGISQWVQLMVLSRPTPQLRAEVFIKFIQVAQKLHQLQNFNTLMAVIGGLCHSSISRLKETSSHVPHEINKVLGEMTELLSSSRNYDNYRRAYGECTDFKIPILGVHLKDLISLYEAMPDYLGDGKVNVHKLLALYNHISELVQLQEVAPPLEANKDLVHLLTLSLDLYYTEDEIYELSYAREPRNHRAPPLTPSKPPVVVDWASGVSPKPDPKTISKHVQRMVDSVFKNYDHDQDGYISQEEFEKIAASFPFSFCVMDKDREGLISRDEITAYFMRASSIYSKLGLGFPHNFQETTYLKPTFCDNCAGFLWGVIKQGYRCKDCGMNCHKQCKDLVVFECKKRAKNPVAPTENNTSVGPVSNLCSLGAKDLLHAPEEGPFTFPNGEAVEHGEESKDRTIMLMGVSSQKISLRLKRAVAHKATQTESQPWIGSEGPSGPFVLSSPRKTAQDTLYVLPSPTSPCPSPVLVRKRAFVKWENKDSLIKSKEELRHLRLPTYQELEQEINTLKADNDALKIQLKYAQKKIESLQLEKSNHVLAQMEQGDCS

>NP_005334.1

MTEYKLVVVGAGGVGKSALTIQLIQNHFVDEYDPTIEDSYRKQVVIDGETCLLDILDTAGQEEYSAMRDQYMRTGEGFLCVFAINNTKSFEDIHQYREQIKRVKDSDDVPMVLVGNKCDLAARTVESRQAQDLARSYGIPYIETSAKTRQGVEDAFYTLVREIRQHKLRKLNPPDESGPGCMSCKCVLS

502 NP_004581.1 NP_001286.1

>NP_004581.1

MKVSEAALSLLVLILIITSASRSQPKVPEWVNTPSTCCLKYYEKVLPRRLVVGYRKALNCHLPAIIFVTKRNREVCTNPNDDWVQEYIKDPNLPLLPTRNLSTVKIITAKNGQPQLLNSQ

>NP_001286.1

METPNTTEDYDTTTEFDYGDATPCQKVNERAFGAQLLPPLYSLVFVIGLVGNILVVLVLVQYKRLKNMTSIYLLNLAISDLLFLFTLPFWIDYKLKDDWVFGDAMCKILSGFYYTGLYSEIFFIILLTIDRYLAIVHAVFALRARTVTFGVITSIIIWALAILASMPGLYFSKTQWEFTHHTCSLHFPHESLREWKLFQALKLNLFGLVLPLLVMIICYTGIIKILLRRPNEKKSKAVRLIFVIMIIFFLFWTPYNLTILISVFQDFLFTHECEQSRHLDLAVQVTEVIAYTHCCVNPVIYAFVGERFRKYLRQLFHRRVAVHLVKWLPFLSVDRLERVSSTSPSTGEHELSAGF

503 NP_073602.2 NP_005598.3

>NP_073602.2

MASPTSTNPAHAHFESFLQAQLCQDVLSSFQELCGALGLEPGGGLPQYHKIKDQLNYWSAKSLWTKLDKRAGQPVYQQGRACTSTKCLVVGAGPCGLRVAVELALLGARVVLVEKRTKFSRHNVLHLWPFTIHDLRALGAKKFYGRFCTGTLDHISIRQLQLLLLKVALLLGVEIHWGVTFTGLQPPPRKGSGWRAQLQPNPPAQLANYEFDVLISAAGGKFVPEGFKVREMRGKLAIGITANFVNGRTVEETQVPEISGVARIYNQSFFQSLLKATGIDLENIVYYKDDTHYFVMTAKKQCLLRLGVLRQDWPDTNRLLGSANVVPEALQRFTRAAADFATHGKLGKLEFAQDAHGQPDVSAFDFTSMMRAESSARVQEKHGARLLLGLVGDCLVEPFWPLGTGVARGFLAAFDAAWMVKRWAEGAESLEVLAERESLYQLLSQTSPENMHRNVAQYGLDPATRYPNLNLRAVTPNQVRDLYDVLAKEPVQRNNDKTDTGMPATGSAGTQEELLRWCQEQTAGYPGVHVSDLSSSWADGLALCALVYRLQPGLLEPSELQGLGALEATAWALKVAENELGITPVVSAQAVVAGSDPLGLIAYLSHFHSAFKSMAHSPGPVSQASPGTSSAVLFLSKLQRTLQRSRAKENAEDAGGKKLRLEMEAETPSTEVPPDPEPGVPLTPPSQHQEAGAGDLCALCGEHLYVLERLCVNGHFFHRSCFRCHTCEATLWPGGYEQHPGDGHFYCLQHLPQTDHKKEGSDRGPESPELPTPSENSMPPGLSTPTASQEGAGPVPDPSQPTRRQIRLSSPERQRLSSLNLTPDPEMEPPPKPPRSCSALARHALESSFVGWGLPVQSPQALVAMEKEEKESPFSSEEEEEDVPLDSDVEQALQTFAKTSGTMNNYPTWRRTLLRRAKEEEMKRFCKAQTIQRRLNEIEAALRELEAEGVKLELALRRQSSSPEQQKKLWVGQLLQLVDKKNSLVAEEAELMITVQELNLEEKQWQLDQELRGYMNREENLKTAADRQAEDQVLRKLVDLVNQRDALIRFQEERRLSELALGTGAQG

>NP_005598.3

MISADCNLCLPEYDRYLASSKIMAAAYLDPNLNHTPNSSTKTHLGTGMERSPGAMERVLKVFHYFESNSEPTTWASIIRHGDATDVRGIIQKIVDSHKVKHVACYGFRLSHLRSEEVHWLHVDMGVSSVREKYELAHPPEEWKYELRIRYLPKGFLNQFTEDKPTLNFFYQQVKSDYMLEIADQVDQEIALKLGCLEIRRSYWEMRGNALEKKSNYEVLEKDVGLKRFFPKSLLDSVKAKTLRKLIQQTFRQFANLNREESILKFFEILSPVYRFDKECFKCALGSSWIISVELAIGPEEGISYLTDKGCNPTHLADFTQVQTIQYSNSEDKDRKGMLQLKIAGAPEPLTVTAPSLTIAENMADLIDGYCRLVNGTSQSFIIRPQKEGERALPSIPKLANSEKQGMRTHAVSVSETDDYAEIIDEEDTYTMPSTRDYEIQRERIELGRCIGEGQFGDVHQGIYMSPENPALAVAIKTCKNCTSDSVREKFLQEALTMRQFDHPHIVKLIGVITENPVWIIMELCTLGELRSFLQVRKYSLDLASLILYAYQLSTALAYLESKRFVHRDIAARNVLVSSNDCVKLGDFGLSRYMEDSTYYKASKGKLPIKWMAPESINFRRFTSASDVWMFGVCMWEILMHGVKPFQGVKNNDVIGRIENGERLPMPPNCPPTLYSLMTKCWAYDPSRRPRFTELKAQLSTILEEEKAQQEERMRMESRRQATVSWDSGGSDEAPPKPSRPGYPSPRSSEGFYPSPQHMVQTNHYQVSGYPGSHGITAMAGSIYPGQASLLDQTDSWNHRPQEIAMWQPNVEDSTVLDLRGIGQVLPTHLMEERLIRQQQEMEEDQRWLEKEERFLKPDVRLSRGSIDREDGSLQGPIGNQHIYQPVGKPDPAAPPKKPPRPGAPGHLGSLASLSSPADSYNEGVKLQPQEISPPPTANLDRSNDKVYENVTGLVKAVIEMSSKIQPAPPEEYVPMVKEVGLALRTLLATVDETIPLLPASTHREIEMAQKLLNSDLGELINKMKLAQQYVMTSLQQEYKKQMLTAAHALAVDAKNLLDVIDQARLKMLGQTRPH

504 NP_002736.3 NP_000112.1

>NP_002736.3

MAAAAAAGAGPEMVRGQVFDVGPRYTNLSYIGEGAYGMVCSAYDNVNKVRVAIKKISPFEHQTYCQRTLREIKILLRFRHENIIGINDIIRAPTIEQMKDVYIVQDLMETDLYKLLKTQHLSNDHICYFLYQILRGLKYIHSANVLHRDLKPSNLLLNTTCDLKICDFGLARVADPDHDHTGFLTEYVATRWYRAPEIMLNSKGYTKSIDIWSVGCILAEMLSNRPIFPGKHYLDQLNHILGILGSPSQEDLNCIINLKARNYLLSLPHKNKVPWNRLFPNADSKALDLLDKMLTFNPHKRIEVEQALAHPYLEQYYDPSDEPIAEAPFKFDMELDDLPKEKLKELIFEETARFQPGYRS

>NP_000112.1

MDHLGASLWPQVGSLCLLLAGAAWAPPPNLPDPKFESKAALLAARGPEELLCFTERLEDLVCFWEEAASAGVGPGNYSFSYQLEDEPWKLCRLHQAPTARGAVRFWCSLPTADTSSFVPLELRVTAASGAPRYHRVIHINEVVLLDAPVGLVARLADESGHVVLRWLPPPETPMTSHIRYEVDVSAGNGAGSVQRVEILEGRTECVLSNLRGRTRYTFAVRARMAEPSFGGFWSAWSEPVSLLTPSDLDPLILTLSLILVVILVLLTVLALLSHRRALKQKIWPGIPSPESEFEGLFTTHKGNFQLWLYQNDGCLWWSPCTPFTEDPPASLEVLSERCWGTMQAVEPGTDDEGPLLEPVGSEHAQDTYLVLDKWLLPRNPPSEDLPGPGGSVDIVAMDEGSEASSCSSALASKPSPEGASAASFEYTILDPSSQLLRPWTLCPELPPTPPHLKYLYLVVSDSGISTDYSSGDSQGAQGGLSDGPYSNPYENSLIPAAEPLPPSYVACS

505 NP_000264.1 NP_002060.4

>NP_000264.1

MTQAGRRGPGTPEPRPRTQPMASPRLGTFCCPTRDAATQLVLSFQPRAFHALCLGSGGLRLALGLLQLLPGRRPAGPGSPATSPPASVRILRAAAACDLLGCLGMVIRSTVWLGFPNFVDSVSDMNHTEIWPAAFCVGSAMWIQLLYSACFWWLFCYAVDAYLVIRRSAGLSTILLYHIMAWGLATLLCVEGAAMLYYPSVSRCERGLDHAIPHYVTMYLPLLLVLVANPILFQKTVTAVASLLKGRQGIYTENERRMGAVIKIRFFKIMLVLIICWLSNIINESLLFYLEMQTDINGGSLKPVRTAAKTTWFIMGILNPAQGFLLSLAFYGWTGCSLGFQSPRKEIQWESLTTSAAEGAHPSPLMPHENPASGKVSQVGGQTSDEALSMLSEGSDASTIEIHTASESCNKNEGDPALPTHGDL

>NP_002060.4

MGCTLSAEDKAAVERSKMIDRNLREDGEKAAREVKLLLLGAGESGKSTIVKQMKIIHEAGYSEEECKQYKAVVYSNTIQSIIAIIRAMGRLKIDFGDSARADDARQLFVLAGAAEEGFMTAELAGVIKRLWKDSGVQACFNRSREYQLNDSAAYYLNDLDRIAQPNYIPTQQDVLRTRVKTTGIVETHFTFKDLHFKMFDVGGQRSERKKWIHCFEGVTAIIFCVALSDYDLVLAEDEEMNRMHESMKLFDSICNNKWFTDTSIILFLNKKDLFEEKIKKSPLTICYPEYAGSNTYEEAAAYIQCQFEDLNKRKDTKEIYTHFTCATDTKNVQFVFDAVTDVIIKNNLKDCGLF

506 NP_001828.1 NP_005239.1

>NP_001828.1

MTTSLDTVETFGTTSYYDDVGLLCEKADTRALMAQFVPPLYSLVFTVGLLGNVVVVMILIKYRRLRIMTNIYLLNLAISDLLFLVTLPFWIHYVRGHNWVFGHGMCKLLSGFYHTGLYSEIFFIILLTIDRYLAIVHAVFALRARTVTFGVITSIVTWGLAVLAALPEFIFYETEELFEETLCSALYPEDTVYSWRHFHTLRMTIFCLVLPLLVMAICYTGIIKTLLRCPSKKKYKAIRLIFVIMAVFFIFWTPYNVAILLSSYQSILFGNDCERSKHLDLVMLVTEVIAYSHCCMNPVIYAFVGERFRKYLRHFFHRHLLMHLGRYIPFLPSEKLERTSSVSPSTAEPELSIVF

>NP_005239.1

MGCVFCKKLEPVATAKEDAGLEGDFRSYGAADHYGPDPTKARPASSFAHIPNYSNFSSQAINPGFLDSGTIRGVSGIGVTLFIALYDYEARTEDDLTFTKGEKFHILNNTEGDWWEARSLSSGKTGCIPSNYVAPVDSIQAEEWYFGKIGRKDAERQLLSPGNPQGAFLIRESETTKGAYSLSIRDWDQTRGDHVKHYKIRKLDMGGYYITTRVQFNSVQELVQHYMEVNDGLCNLLIAPCTIMKPQTLGLAKDAWEISRSSITLERRLGTGCFGDVWLGTWNGSTKVAVKTLKPGTMSPKAFLEEAQVMKLLRHDKLVQLYAVVSEEPIYIVTEFMCHGSLLDFLKNPEGQDLRLPQLVDMAAQVAEGMAYMERMNYIHRDLRAANILVGERLACKIADFGLARLIKDDEYNPCQGSKFPIKWTAPEAALFGRFTIKSDVWSFGILLTELITKGRIPYPGMNKREVLEQVEQGYHMPCPPGCPASLYEAMEQTWRLDPEERPTFEYLQSFLEDYFTSAEPQYQPGDQT

507 NP_056007.1 NP_005604.1

>NP_056007.1

MAGAQPGVHALQLKPVCVSDSLKKGTKFVKWDDDSTIVTPIILRTDPQGFFFYWTDQNKETELLDLSLVKDARCGRHAKAPKDPKLRELLDVGNIGRLEQRMITVVYGPDLVNISHLNLVAFQEEVAKEWTNEVFSLATNLLAQNMSRDAFLEKAYTKLKLQVTPEGRIPLKNIYRLFSADRKRVETALEACSLPSSRNDSIPQEDFTPEVYRVFLNNLCPRPEIDNIFSEFGAKSKPYLTVDQMMDFINLKQRDPRLNEILYPPLKQEQVQVLIEKYEPNNSLARKGQISVDGFMRYLSGEENGVVSPEKLDLNEDMSQPLSHYFINSSHNTYLTAGQLAGNSSVEMYRQVLLSGCRCVELDCWKGRTAEEEPVITHGFTMTTEISFKEVIEAIAECAFKTSPFPILLSFENHVDSPKQQAKMAEYCRLIFGDALLMEPLEKYPLESGVPLPSPMDLMYKILVKNKKKSHKSSEGSGKKKLSEQASNTYSDSSSMFEPSSPGAGEADTESDDDDDDDDCKKSSMDEGTAGSEAMATEEMSNLVNYIQPVKFESFEISKKRNKSFEMSSFVETKGLEQLTKSPVEFVEYNKMQLSRIYPKGTRVDSSNYMPQLFWNAGCQMVALNFQTMDLAMQINMGMYEYNGKSGYRLKPEFMRRPDKHFDPFTEGIVDGIVANTLSVKIISGQFLSDKKVGTYVEVDMFGLPVDTRRKAFKTKTSQGNAVNPVWEEEPIVFKKVVLPTLACLRIAVYEEGGKFIGHRILPVQAIRPGYHYICLRNERNQPLTLPAVFVYIEVKDYVPDTYADVIEALSNPIRYVNLMEQRAKQLAALTLEDEEEVKKEADPGETPSEAPSEARTTPAENGVNHTTTLTPKPPSQALHSQPAPGSVKAPAKTEDLIQSVLTEVEAQTIEELKQQKSFVKLQKKHYKEMKDLVKRHHKKTTDLIKEHTTKYNEIQNDYLRRRAALEKSAKKDSKKKSEPSSPDHGSSTIEQDLAALDAEMTQKLIDLKDKQQQQLLNLRQEQYYSEKYQKREHIKLLIQKLTDVAEECQNNQLKKLKEICEKEKKELKKKMDKKRQEKITEAKSKDKSQMEEEKTEMIRSYIQEVVQYIKRLEEAQSKRQEKLVEKHKEIRQQILDEKPKLQVELEQEYQDKFKRLPLEILEFVQEAMKGKISEDSNHGSAPLSLSSDPGKVNHKTPSSEELGGDIPGKEFDTPL

>NP_005604.1

MCKGLAGLPASCLRSAKDMKHRLGFLLQKSDSCEHNSSHNKKDKVVICQRVSQEEVKKWAESLENLISHECGLAAFKAFLKSEYSEENIDFWISCEEYKKIKSPSKLSPKAKKIYNEFISVQATKEVNLDSCTREETSRNMLEPTITCFDEAQKKIFNLMEKDSYRRFLKSRFYLDLVNPSSCGAEKQKGAKSSADCASLVPQCA

508 NP_005204.1 NP_680090.1

>NP_005204.1

MIPGGLSEAKPATPEIQEIVDKVKPQLEEKTNETYGKLEAVQYKTQVVAGTNYYIKVRAGDNKYMHLKVFKSLPGQNEDLVLTGYQVDKNKDDELTGF

>NP_680090.1

MWQLWASLCCLLVLANARSRPSFHPLSDELVNYVNKRNTTWQAGHNFYNVDMSYLKRLCGTFLGGPKPPQRVMFTEDLKLPASFDAREQWPQCPTIKEIRDQGSCGSCWAFGAVEAISDRICIHTNAHVSVEVSAEDLLTCCGSMCGDGCNGGYPAEAWNFWTRKGLVSGGLYESHVGCRPYSIPPCEHHVNGSRPPCTGEGDTPKCSKICEPGYSPTYKQDKHYGYNSYSVSNSEKDIMAEIYKNGPVEGAFSVYSDFLLYKSGVYQHVTGEMMGGHAIRILGWGVENGTPYWLVANSWNTDWGDNGFFKILRGQDHCGIESEVVAGIPRTDQYWEKI

509 NP_000367.1 NP_005239.1

>NP_000367.1

MEAMAASTSLPDPGDFDRNVPRICGVCGDRATGFHFNAMTCEGCKGFFRRSMKRKALFTCPFNGDCRITKDNRRHCQACRLKRCVDIGMMKEFILTDEEVQRKREMILKRKEEEALKDSLRPKLSEEQQRIIAILLDAHHKTYDPTYSDFCQFRPPVRVNDGGGSHPSRPNSRHTPSFSGDSSSSCSDHCITSSDMMDSSSFSNLDLSEEDSDDPSVTLELSQLSMLPHLADLVSYSIQKVIGFAKMIPGFRDLTSEDQIVLLKSSAIEVIMLRSNESFTMDDMSWTCGNQDYKYRVSDVTKAGHSLELIEPLIKFQVGLKKLNLHEEEHVLLMAICIVSPDRPGVQDAALIEAIQDRLSNTLQTYIRCRHPPPGSHLLYAKMIQKLADLRSLNEEHSKQYRCLSFQPECSMKLTPLVLEVFGNEIS

>NP_005239.1

MGCVFCKKLEPVATAKEDAGLEGDFRSYGAADHYGPDPTKARPASSFAHIPNYSNFSSQAINPGFLDSGTIRGVSGIGVTLFIALYDYEARTEDDLTFTKGEKFHILNNTEGDWWEARSLSSGKTGCIPSNYVAPVDSIQAEEWYFGKIGRKDAERQLLSPGNPQGAFLIRESETTKGAYSLSIRDWDQTRGDHVKHYKIRKLDMGGYYITTRVQFNSVQELVQHYMEVNDGLCNLLIAPCTIMKPQTLGLAKDAWEISRSSITLERRLGTGCFGDVWLGTWNGSTKVAVKTLKPGTMSPKAFLEEAQVMKLLRHDKLVQLYAVVSEEPIYIVTEFMCHGSLLDFLKNPEGQDLRLPQLVDMAAQVAEGMAYMERMNYIHRDLRAANILVGERLACKIADFGLARLIKDDEYNPCQGSKFPIKWTAPEAALFGRFTIKSDVWSFGILLTELITKGRIPYPGMNKREVLEQVEQGYHMPCPPGCPASLYEAMEQTWRLDPEERPTFEYLQSFLEDYFTSAEPQYQPGDQT

510 NP_036558.3 NP_036565.2

>NP_036558.3

MFLYNLTLQRATGISFAIHGNFSGTKQQEIVVSRGKILELLRPDPNTGKVHTLLTVEVFGVIRSLMAFRLTGGTKDYIVVGSDSGRIVILEYQPSKNMFEKIHQETFGKSGCRRIVPGQFLAVDPKGRAVMISAIEKQKLVYILNRDAAARLTISSPLEAHKANTLVYHVVGVDVGFENPMFACLEMDYEEADNDPTGEAAANTQQTLTFYELDLGLNHVVRKYSEPLEEHGNFLITVPGGSDGPSGVLICSENYITYKNFGDQPDIRCPIPRRRNDLDDPERGMIFVCSATHKTKSMFFFLAQTEQGDIFKITLETDEDMVTEIRLKYFDTVPVAAAMCVLKTGFLFVASEFGNHYLYQIAHLGDDDEEPEFSSAMPLEEGDTFFFQPRPLKNLVLVDELDSLSPILFCQIADLANEDTPQLYVACGRGPRSSLRVLRHGLEVSEMAVSELPGNPNAVWTVRRHIEDEFDAYIIVSFVNATLVLSIGETVEEVTDSGFLGTTPTLSCSLLGDDALVQVYPDGIRHIRADKRVNEWKTPGKKTIVKCAVNQRQVVIALTGGELVYFEMDPSGQLNEYTERKEMSADVVCMSLANVPPGEQRSRFLAVGLVDNTVRIISLDPSDCLQPLSMQALPAQPESLCIVEMGGTEKQDELGERGSIGFLYLNIGLQNGVLLRTVLDPVTGDLSDTRTRYLGSRPVKLFRVRMQGQEAVLAMSSRSWLSYSYQSRFHLTPLSYETLEFASGFASEQCPEGIVAISTNTLRILALEKLGAVFNQVAFPLQYTPRKFVIHPESNNLIIIETDHNAYTEATKAQRKQQMAEEMVEAAGEDERELAAEMAAAFLNENLPESIFGAPKAGNGQWASVIRVMNPIQGNTLDLVQLEQNEAAFSVAVCRFSNTGEDWYVLVGVAKDLILNPRSVAGGFVYTYKLVNNGEKLEFLHKTPVEEVPAAIAPFQGRVLIGVGKLLRVYDLGKKKLLRKCENKHIANYISGIQTIGHRVIVSDVQESFIWVRYKRNENQLIIFADDTYPRWVTTASLLDYDTVAGADKFGNICVVRLPPNTNDEVDEDPTGNKALWDRGLLNGASQKAEVIMNYHVGETVLSLQKTTLIPGGSESLVYTTLSGGIGILVPFTSHEDHDFFQHVEMHLRSEHPPLCGRDHLSFRSYYFPVKNVIDGDLCEQFNSMEPNKQKNVSEELDRTPPEVSKKLEDIRTRYAF

>NP_036565.2

MAKIAKTHEDIEAQIREIQGKKAALDEAQGVGLDSTGYYDQEIYGGSDSRFAGYVTSIAATELEDDDDDYSSSTSLLGQKKPGYHAPVALLNDIPQSTEQYDPFAEHRPPKIADREDEYKKHRRTMIISPERLDPFADGGKTPDPKMNARTYMDVMREQHLTKEEREIRQQLAEKAKAGELKVVNGAAASQPPSKRKRRWDQTADQTPGATPKKLSSWDQAETPGHTPSLRWDETPGRAKGSETPGATPGSKIWDPTPSHTPAGAATPGRGDTPGHATPGHGGATSSARKNRWDETPKTERDTPGHGSGWAETPRTDRGGDSIGETPTPGASKRKSRWDETPASQMGGSTPVLTPGKTPIGTPAMNMATPTPGHIMSMTPEQLQAWRWEREIDERNRPLSDEELDAMFPEGYKVLPPPAGYVPIRTPARKLTATPTPLGGMTGFHMQTEDRTMKSVNDQPSGNLPFLKPDDIQYFDKLLVDVDESTLSPEEQKERKIMKLLLKIKNGTPPMRKAALRQITDKAREFGAGPLFNQILPLLMSPTLEDQERHLLVKVIDRILYKLDDLVRPYVHKILVVIEPLLIDEDYYARVEGREIISNLAKAAGLATMISTMRPDIDNMDEYVRNTTARAFAVVASALGIPSLLPFLKAVCKSKKSWQARHTGIKIVQQIAILMGCAILPHLRSLVEIIEHGLVDEQQKVRTISALAIAALAEAATPYGIESFDSVLKPLWKGIRQHRGKGLAAFLKAIGYLIPLMDAEYANYYTREVMLILIREFQSPDEEMKKIVLKVVKQCCGTDGVEANYIKTEILPPFFKHFWQHRMALDRRNYRQLVDTTVELANKVGAAEIISRIVDDLKDEAEQYRKMVMETIEKIMGNLGAADIDHKLEEQLIDGILYAFQEQTTEDSVMLNGFGTVVNALGKRVKPYLPQICGTVLWRLNNKSAKVRQQAADLISRTAVVMKTCQEEKLMGHLGVVLYEYLGEEYPEVLGSILGALKAIVNVIGMHKMTPPIKDLLPRLTPILKNRHEKVQENCIDLVGRIADRGAEYVSAREWMRICFELLELLKAHKKAIRRATVNTFGYIAKAIGPHDVLATLLNNLKVQERQNRVCTTVAIAIVAETCSPFTVLPALMNEYRVPELNVQNGVLKSLSFLFEYIGEMGKDYIYAVTPLLEDALMDRDLVHRQTASAVVQHMSLGVYGFGCEDSLNHLLNYVWPNVFETSPHVIQAVMGALEGLRVAIGPCRMLQYCLQGLFHPARKVRDVYWKIYNSIYIGSQDALIAHYPRIYNDDKNTYIRYELDYIL

511 NP_055731.2 NP_006232.1

>NP_055731.2

MPGPPALRRRLLLLLLVLLIAGSAGAAPLPQTGAGEAPPAAEVSSSFVILCVCSLIILIVLIANCVSCCKDPEIDFKEFEDNFDDEIDFTPPAEDTPSVQSPAEVFTLSVPNISLPAPSQFQPSVEGLKSQVARHSLNYIQEIGNGWFGKVLLGEIYTGTSVARVIVKELKASANPKEQDTFLKNGEPYYILQHPNILQCVGQCVEAIPYLLVFEFCDLGDLKAYLRSEQEHMRGDSQTMLLQRMACEVAAGLAAMHKLHFLHSDLALRNCFLTSDLNVKVGDYGIGFSRYKEDYIETDDKKVFPLRWTAPELVTSFQDRLLTADQTKYSNIWSLGVTLWELFDNAAQPYSNLSNLDVLNQVIRERDTKLPKPQLEQPYSDRWYEVLQFCWLSPEKRPAAEDVHRLLTYLRLQSQRDSEVDFEQQWNALKPNTNSRDSSNNAAFPILDHFARDRLGREMEEVLTVTETSQGLSFEYVWEAAKHDHFDERSRGHLDEGLSYTSIFYPVEVFESSLSDPGPGKQDDSGQDVPLRVPGVVPVFDAHNLSVGSDYYIQLEEKSGSNLELDYPPALLTTDMDNPERTGPELSQLTALRSVELEESSTDEDFFQSSTDPKDSSLPGDLHVTSGPESPFNNIFNDVDKSEDLPSHQKIFDLMELNGVQADFKPATLSSSLDNPKESVITGHFEKEKPRKIFDSEPLCLSDNLMHQDNFDPLNVQELSENFLFLQEKNLLKGSLSSKEHINDLQTELKNAGFTEAMLETSCRNSLDTELQFAENKPGLSLLQENVSTKGDDTDVMLTGDTLSTSLQSSPEVQVPPTSFETEETPRRVPPDSLPTQGETQPTCLDVIVPEDCLHQDISPDAVTVPVEILSTDARTHSLDNRSQDSPGESEETLRLTESDSVLADDILASRVSVGSSLPELGQELHNKPFSEDHHSHRRLEKNLEAVETLNQLNSKDAAKEAGLVSALSSDSTSQDSLLEDSLSAPFPASEPSLETPDSLESVDVHEALLDSLGSHTPQKLVPPDKPADSGYETENLESPEWTLHPAPEGTADSEPATTGDGGHSGLPPNPVIVISDAGDGHRGTEVTPETFTAGSQGSYRDSAYFSDNDSEPEKRSEEVPGTSPSALVLVQEQPLPEPVLPEQSPAAQDSCLEARKSQPDESCLSALHNSSDLELRATPEPAQTGVPQQVHPTEDEASSPWSVLNAELSSGDDFETQDDRPCTLASTGTNTNELLAYTNSALDKSLSSHSEGPKLKEPDIEGKYLGKLGVSGMLDLSEDGMDADEEDENSDDSDEDLRAFNLHSLSSESEDETEHPVPIILSNEDGRHLRSLLKPTAANAPDPLPEDWKKEKKAVTFFDDVTVYLFDQETPTKELGPCGGEACGPDLSGPAPASGSPYLSRCINSESSTDEEGGGFEWDDDFSPDPFMSKTTSNLLSSKPSLQTSKYFSPPPPARSTEQSWPHSAPYSRFSISPANIASFSLTHLTDSDIEQGGSSEDGEKD

>NP_006232.1

MAASTASHRPIKGILKNKTSTTSSMVASAEQPRGNVDEELSKKSQKWDEMNILATYHPADKDYGLMKIDEPSTPYHSMMGDDEDACSDTEATEAMAPDILARKLAAAEGLEPKYRIQEQESSGEEDSDLSPEEREKKRQFEMKRKLHYNEGLNIKLARQLISKDLHDDDEDEEMLETADGESMNTEESNQGSTPSDQQQNKLRSS

512 NP_005329.3 AAH14214.1

>NP_005329.3

MDRMASSMKQVPNPLPKVLSRRGVGAGLEAAERESFERTQTVSINKAINTQEVAVKEKHARTCILGTHHEKGAQTFWSVVNRLPLSSNAVLCWKFCHVFHKLLRDGHPNVLKDSLRYRNELSDMSRMWGHLSEGYGQLCSIYLKLLRTKMEYHTKNPRFPGNLQMSDRQLDEAGESDVNNFFQLTVEMFDYLECELNLFQTVFNSLDMSRSVSVTAAGQCRLAPLIQVILDCSHLYDYTVKLLFKLHSCLPADTLQGHRDRFMEQFTKLKDLFYRSSNLQYFKRLIQIPQLPENPPNFLRASALSEHISPVVVIPAEASSPDSEPVLEKDDLMDMDASQQNLFDNKFDDIFGSSFSSDPFNFNSQNGVNKDEKDHLIERLYREISGLKAQLENMKTESQRVVLQLKGHVSELEADLAEQQHLRQQAADDCEFLRAELDELRRQREDTEKAQRSLSEIERKAQANEQRYSKLKEKYSELVQNHADLLRKNAEVTKQVSMARQAQVDLEREKKELEDSLERISDQGQRKTQEQLEVLESLKQELATSQRELQVLQGSLETSAQSEANWAAEFAELEKERDSLVSGAAHREEELSALRKELQDTQLKLASTEESMCQLAKDQRKMLLVGSRKAAEQVIQDALNQLEEPPLISCAGSADHLLSTVTSISSCIEQLEKSWSQYLACPEDISGLLHSITLLAHLTSDAIAHGATTCLRAPPEPADSLTEACKQYGRETLAYLASLEEEGSLENADSTAMRNCLSKIKAIGEELLPRGLDIKQEELGDLVDKEMAATSAAIETATARIEEMLSKSRAGDTGVKLEVNERILGCCTSLMQAIQVLIVASKDLQREIVESGRGTASPKEFYAKNSRWTEGLISASKAVGWGATVMVDAADLVVQGRGKFEELMVCSHEIAASTAQLVAASKVKADKDSPNLAQLQQASRGVNQATAGVVASTISGKSQIEETDNMDFSSMTLTQIKRQEMDSQVRVLELENELQKERQKLGELRKKHYELAGVAEGWEEGTEASPPTLQEVVTEKE

>AAH14214.1

MPAVSKGDGMRGLAVFISDIRNCKSKEAEIKRINKELANIRSKFKGDKALDGYSKKKYVCKLLFIFLLGHDIDFGHMEAVNLLSSNKYTEKQIGYLFISVLVNSNSELIRLINNAIKNDLASRNPTFMCLALHCIANVGSREMGEAFAADIPRILVAGDSMDSVKQSAALCLLRLYKASPDLVPMGEWTARVVHLLNDQHMGVVTAAVSLITCLCKKNPDDFKTCVSLAVSRLSRIVSSASTDLQDYTYYFVPAPWLSVKLLRLLQCYPLPEDAAVKGRLVECLETVLNKAQEPPKSKKVQHSNAKNAILFETISLIIHYDSEPNLLVRACNQLGQFLQHRETNLRYLALESMCTLASSEFSHEAVKTHIDTVINALKTERDVSVRQRAADLLYAMCDRSNAKQIVSEMLRYLETADYAIREEIVLKVAILAEKYAVDYSWYVDTILNLIRIAGDYVSEEVWYRVLQIVTNRDDVQGYAAKTVFEALQAPACHENMVKVGGYILGEFGNLIAGDPRSSPPVQFSLLHSKFHLCSVATRALLLSTYIKFINLFPETKATIQGVLRAGSQLRNADVELQQRAVEYLTLSSVASTDVLATVLEEMPPFPERESSILAKLKRKKGPGAGSALDDGRRDPSSNDINGGMEPTPSTVSTPSPSADLLGLRAAPPPAAPPASAGAGNLLVDVFDGPAAQPSLGPTPEEAFLSPGPEDIGPPIPEADELLNKFVCKNNGVLFENQLLQIGVKSEFRQNLGRMYLFYGNKTSVQFQNFSPTVVHPGDLQTQLAVQTKRVAAQVDGGAQVQQVLNIECLRDFLTPPLLSVRFRYGGAPQALTLKLPVTINKFFQPTEMAAQDFFQRWKQLSLPQQEAQKIFKANHPMDAEVTKAKLLGFGSALLDNVDPNPGDREDTRVWGMPGTFLRPFVFLFLFICCCLHSGGLGGVPLPPFPPQAQRGEGPGKWMSPPLPPHPVVAPPTPSPSRGCVLL

513 NP_001933.2 NP_009114.1

>NP_001933.2

MDWSFFRVVAMLFIFLVVVEVNSEFRIQVRDYNTKNGTIKWHSIRRQKREWIKFAAACREGEDNSKRNPIAKIHSDCAANQQVTYRISGVGIDQPPYGIFVINQKTGEINITSIVDREVTPFFIIYCRALNSMGQDLERPLELRVRVLDINDNPPVFSMATFAGQIEENSNANTLVMILNATDADEPNNLNSKIAFKIIRQEPSDSPMFIINRNTGEIRTMNNFLDREQYGQYALAVRGSDRDGGADGMSAECECNIKILDVNDNIPYMEQSSYTIEIQENTLNSNLLEIRVIDLDEEFSANWMAVIFFISGNEGNWFEIEMNERTNVGILKVVKPLDYEAMQSLQLSIGVRNKAEFHHSIMSQYKLKASAISVTVLNVIEGPVFRPGSKTYVVTGNMGSNDKVGDFVATDLDTGRPSTTVRYVMGNNPADLLAVDSRTGKLTLKNKVTKEQYNMLGGKYQGTILSIDDNLQRTCTGTININIQSFGNDDRTNTEPNTKITTNTGRQESTSSTNYDTSTTSTDSSQVYSSEPGNGAKDLLSDNVHFGPAGIGLLIMGFLVLGLVPFLMICCDCGGAPRSAAGFEPVPECSDGAIHSWAVEGPQPEPRDITTVIPQIPPDNANIIECIDNSGVYTNEYGGREMQDLGGGERMTGFELTEGVKTSGMPEICQEYSGTLRRNSMRECREGGLNMNFMESYFCQKAYAYADEDEGRPSNDCLLIYDIEGVGSPAGSVGCCSFIGEDLDDSFLDTLGPKFKKLADISLGKESYPDLDPSWPPQSTEPVCLPQETEPVVSGHPPISPHFGTTTVISESTYPSGPGVLHPKPILDPLGYGNVTVTESYTTSDTLKPSVHVHDNRPASNVVVTERVVGPISGADLHGMLEMPDLRDGSNVIVTERVIAPSSSLPTSLTIHHPRESSNVVVTERVIQPTSGMIGSLSMHPELANAHNVIVTERVVSGAGVTGISGTTGISGGIGSSGLVGTSMGAGSGALSGAGISGGGIGLSSLGGTASIGHMRSSSDHHFNQTIGSASPSTARSRITKYSTVQYSK

>NP_009114.1

MQDGNFLLSALQPEAGVCSLALPSDLQLDRRGAEGPEAERLRAARVQEQVRARLLQLGQQPRHNGAAEPEPEAETARGTSRGQYHTLQAGFSSRSQGLSGDKTSGFRPIAKPAYSPASWSSRSAVDLSCSRRLSSAHNGGSAFGAAGYGGAQPTPPMPTRPVSFHERGGVGSRADYDTLSLRSLRLGPGGLDDRYSLVSEQLEPAATSTYRAFAYERQASSSSSRAGGLDWPEATEVSPSRTIRAPAVRTLQRFQSSHRSRGVGGAVPGAVLEPVARAPSVRSLSLSLADSGHLPDVHGFNSYGSHRTLQRLSSGFDDIDLPSAVKYLMASDPNLQVLGAAYIQHKCYSDAAAKKQARSLQAVPRLVKLFNHANQEVQRHATGAMRNLIYDNADNKLALVEENGIFELLRTLREQDDELRKNVTGILWNLSSSDHLKDRLARDTLEQLTDLVLSPLSGAGGPPLIQQNASEAEIFYNATGFLRNLSSASQATRQKMRECHGLVDALVTSINHALDAGKCEDKSVENAVCVLRNLSYRLYDEMPPSALQRLEGRGRRDLAGAPPGEVVGCFTPQSRRLRELPLAADALTFAEVSKDPKGLEWLWSPQIVGLYNRLLQRCELNRHTTEAAAGALQNITAGDRRWAGVLSRLALEQERILNPLLDRVRTADHHQLRSLTGLIRNLSRNARNKDEMSTKVVSHLIEKLPGSVGEKSPPAEVLVNIIAVLNNLVVASPIAARDLLYFDGLRKLIFIKKKRDSPDSEKSSRAASSLLANLWQYNKLHRDFRAKGYRKEDFLGP

514 NP_000048.1 NP_002453.1

>NP_000048.1

MAAVPQNNLQEQLERHSARTLNNKLSLSKPKFSGFTFKKKTSSDNNVSVTNVSVAKTPVLRNKDVNVTEDFSFSEPLPNTTNQQRVKDFFKNAPAGQETQRGGSKSLLPDFLQTPKEVVCTTQNTPTVKKSRDTALKKLEFSSSPDSLSTINDWDDMDDFDTSETSKSFVTPPQSHFVRVSTAQKSKKGKRNFFKAQLYTTNTVKTDLPPPSSESEQIDLTEEQKDDSEWLSSDVICIDDGPIAEVHINEDAQESDSLKTHLEDERDNSEKKKNLEEAELHSTEKVPCIEFDDDDYDTDFVPPSPEEIISASSSSSKCLSTLKDLDTSDRKEDVLSTSKDLLSKPEKMSMQELNPETSTDCDARQISLQQQLIHVMEHICKLIDTIPDDKLKLLDCGNELLQQRNIRRKLLTEVDFNKSDASLLGSLWRYRPDSLDGPMEGDSCPTGNSMKELNFSHLPSNSVSPGDCLLTTTLGKTGFSATRKNLFERPLFNTHLQKSFVSSNWAETPRLGKKNESSYFPGNVLTSTAVKDQNKHTASINDLERETQPSYDIDNFDIDDFDDDDDWEDIMHNLAASKSSTAAYQPIKEGRPIKSVSERLSSAKTDCLPVSSTAQNINFSESIQNYTDKSAQNLASRNLKHERFQSLSFPHTKEMMKIFHKKFGLHNFRTNQLEAINAALLGEDCFILMPTGGGKSLCYQLPACVSPGVTVVISPLRSLIVDQVQKLTSLDIPATYLTGDKTDSEATNIYLQLSKKDPIIKLLYVTPEKICASNRLISTLENLYERKLLARFVIDEAHCVSQWGHDFRQDYKRMNMLRQKFPSVPVMALTATANPRVQKDILTQLKILRPQVFSMSFNRHNLKYYVLPKKPKKVAFDCLEWIRKHHPYDSGIIYCLSRRECDTMADTLQRDGLAALAYHAGLSDSARDEVQQKWINQDGCQVICATIAFGMGIDKPDVRFVIHASLPKSVEGYYQESGRAGRDGEISHCLLFYTYHDVTRLKRLIMMEKDGNHHTRETHFNNLYSMVHYCENITECRRIQLLAYFGENGFNPDFCKKHPDVSCDNCCKTKDYKTRDVTDDVKSIVRFVQEHSSSQGMRNIKHVGPSGRFTMNMLVDIFLGSKSAKIQSGIFGKGSAYSRHNAERLFKKLILDKILDEDLYINANDQAIAYVMLGNKAQTVLNGNLKVDFMETENSSSVKKQKALVAKVSQREEMVKKCLGELTEVCKSLGKVFGVHYFNIFNTVTLKKLAESLSSDPEVLLQIDGVTEDKLEKYGAEVISVLQKYSEWTSPAEDSSPGISLSSSRGPGRSAAEELDEEIPVSSHYFASKTRNERKRKKMPASQRSKRRKTASSGSKAKGGSATCRKISSKTKSSSIIGSSSASHTSQATSGANSKLGIMAPPKPINRPFLKPSYAFS

>NP_002453.1

MVVSEVDIAKADPAAASHPLLLNGDATVAQKNPGSVAENNLCSQYEEKVRPCIDLIDSLRALGVEQDLALPAIAVIGDQSSGKSSVLEALSGVALPRGSGIVTRCPLVLKLKKLVNEDKWRGKVSYQDYEIEISDASEVEKEINKAQNAIAGEGMGISHELITLEISSRDVPDLTLIDLPGITRVAVGNQPADIGYKIKTLIKKYIQRQETISLVVVPSNVDIATTEALSMAQEVDPEGDRTIGILTKPDLVDKGTEDKVVDVVRNLVFHLKKGYMIVKCRGQQEIQDQLSLSEALQREKIFFENHPYFRDLLEEGKATVPCLAEKLTSELITHICKSLPLLENQIKETHQRITEELQKYGVDIPEDENEKMFFLIDKINAFNQDITALMQGEETVGEEDIRLFTRLRHEFHKWSTIIENNFQEGHKILSRKIQKFENQYRGRELPGFVNYRTFETIVKQQIKALEEPAVDMLHTVTDMVRLAFTDVSIKNFEEFFNLHRTAKSKIEDIRAEQEREGEKLIRLHFQMEQIVYCQDQVYRGALQKVREKELEEEKKKKSWDFGAFQSSSATDSSMEEIFQHLMAYHQEASKRISSHIPLIIQFFMLQTYGQQLQKAMLQLLQDKDTYSWLLKERSDTSDKRKFLKERLARLTQARRRLAQFPG

515 NP_055490.2 NP_803136.2

>NP_055490.2

MSLGLAGAQEVELTLETVIQTLESSVLCQEKGLGARDLAQDAQITSLPALIREIVTRNLSQPESPVLLPATEMASLLSLQEENQLLQQELSRVEDLLAQSRAERDELAIKYNAVSERLEQALRLEPGELETQEPRGLVRQSVELRRQLQEEQASYRRKLQAYQEGQQRQAQLVQRLQGKILQYKKRCSELEQQLLERSGELEQQRLRDTEHSQDLESALIRLEEEQQRSASLAQVNAMLREQLDQAGSANQALSEDIRKVTNDWTRCRKELEHREAAWRREEESFNAYFSNEHSRLLLLWRQVVGFRRLVSEVKMFTERDLLQLGGELARTSRAVQEAGLGLSTGLRLAESRAEAALEKQALLQAQLEEQLRDKVLREKDLAQQQMQSDLDKADLSARVTELGLAVKRLEKQNLEKDQVNKDLTEKLEALESLRLQEQAALETEDGEGLQQTLRDLAQAVLSDSESGVQLSGSERTADASNGSLRGLSGQRTPSPPRRSSPGRGRSPRRGPSPACSDSSTLALIHSALHKRQLQVQDMRGRYEASQDLLGTLRKQLSDSESERRALEEQLQRLRDKTDGAMQAHEDAQREVQRLRSANELLSREKSNLAHSLQVAQQQAEELRQEREKLQAAQEELRRQRDRLEEEQEDAVQDGARVRRELERSHRQLEQLEGKRSVLAKELVEVREALSRATLQRDMLQAEKAEVAEALTKAEAGRVELELSMTKLRAEEASLQDSLSKLSALNESLAQDKLDLNRLVAQLEEEKSALQGRQRQAEQEATVAREEQERLEELRLEQEVARQGLEGSLRVAEQAQEALEQQLPTLRHERSQLQEQLAQLSRQLSGREQELEQARREAQRQVEALERAAREKEALAKEHAGLAVQLVAAEREGRTLSEEATRLRLEKEALEGSLFEVQRQLAQLEARREQLEAEGQALLLAKETLTGELAGLRQQIIATQEKASLDKELMAQKLVQAEREAQASLREQRAAHEEDLQRLQREKEAAWRELEAERAQLQSQLQREQEELLARLEAEKEELSEEIAALQQERDEGLLLAESEKQQALSLKESEKTALSEKLMGTRHSLATISLEMERQKRDAQSRQEQDRSTVNALTSELRDLRAQREEAAAAHAQEVRRLQEQARDLGKQRDSCLREAEELRTQLRLLEDARDGLRRELLEAQRKLRESQEGREVQRQEAGELRRSLGEGAKEREALRRSNEELRSAVKKAESERISLKLANEDKEQKLALLEEARTAVGKEAGELRTGLQEVERSRLEARRELQELRRQMKMLDSENTRLGRELAELQGRLALGERAEKESRRETLGLRQRLLKGEASLEVMRQELQVAQRKLQEQEGEFRTRERRLLGSLEEARGTEKQQLDHARGLELKLEAARAEAAELGLRLSAAEGRAQGLEAELARVEVQRRAAEAQLGGLRSALRRGLGLGRAPSPAPRPVPGSPARDAPAEGSGEGLNSPSTLECSPGSQPPSPGPATSPASPDLDPEAVRGALREFLQELRSAQRERDELRTQTSALNRQLAEMEAERDSATSRARQLQKAVAESEEARRSVDGRLSGVQAELALQEESVRRSERERRATLDQVATLERSLQATESELRASQEKISKMKANETKLEGDKRRLKEVLDASESRTVKLELQRRSLEGELQRSRLGLSDREAQAQALQDRVDSLQRQVADSEVKAGTLQLTVERLNGALAKVEESEGALRDKVRGLTEALAQSSASLNSTRDKNLHLQKALTACEHDRQVLQERLDAARQALSEARKQSSSLGEQVQTLRGEVADLELQRVEAEGQLQQLREVLRQRQEGEAAALNTVQKLQDERRLLQERLGSLQRALAQLEAEKREVERSALRLEKDRVALRRTLDKVEREKLRSHEDTVRLSAEKGRLDRTLTGAELELAEAQRQIQQLEAQVVVLEQSHSPAQLEVDAQQQQLELQQEVERLRSAQAQTERTLEARERAHRQRVRGLEEQVSTLKGQLQQELRRSSAPFSPPSGPPEK

>NP_803136.2

MSVQVAAPGSAGLGPERLSPEELVRQTRQVVQGLEALRAEHHGLAGHLAEALAGQGPAAGLEMLEEKQQVVSHSLEAIELGLGEAQVLLALSAHVGALEAEKQRLRSQARRLAQENVWLREELEETQRRLRASEESVAQLEEEKRHLEFLGQLRQYDPPAESQQSESPPRRDSLASLFPSEEEERKGPEAAGAAAAQQGGYEIPARLRTLHNLVIQYAGQGRYEVAVPLCRQALEDLERSSGHCHPDVATMLNILALVYRDQNKYKEATDLLHDALQIREQTLGPEHPAVAATLNNLAVLYGKRGRYREAEPLCQRALEIREKVLGADHPDVAKQLNNLALLCQNQGKFEDVERHYARALSIYEALGGPHDPNVAKTKNNLASAYLKQNKYQQAEELYKEILHKEDLPAPLGAPNTGTAGDAEQALRRSSSLSKIRESIRRGSEKLVSRLRGEAAAGAAGMKRAMSLNTLNVDAPRAPGTQFPSWHLDKAPRTLSASTQDLSPH

516 NP_000651.3 NP_000879.2

>NP_000651.3

MPPSGLRLLPLLLPLLWLLVLTPGRPAAGLSTCKTIDMELVKRKRIEAIRGQILSKLRLASPPSQGEVPPGPLPEAVLALYNSTRDRVAGESAEPEPEPEADYYAKEVTRVLMVETHNEIYDKFKQSTHSIYMFFNTSELREAVPEPVLLSRAELRLLRLKLKVEQHVELYQKYSNNSWRYLSNRLLAPSDSPEWLSFDVTGVVRQWLSRGGEIEGFRLSAHCSCDSRDNTLQVDINGFTTGRRGDLATIHGMNRPFLLLMATPLERAQHLQSSRHRRALDTNYCFSSTEKNCCVRQLYIDFRKDLGWKWIHEPKGYHANFCLGPCPYIWSLDTQYSKVLALYNQHNPGASAAPCCVPQALEPLPIVYYVGRKPKVEQLSNMIVRSCKCS

>NP_000879.2

MGIELLCLFFLFLGRNDHVQGGCALGGAETCEDCLLIGPQCAWCAQENFTHPSGVGERCDTPANLLAKGCQLNFIENPVSQVEILKNKPLSVGRQKNSSDIVQIAPQSLILKLRPGGAQTLQVHVRQTEDYPVDLYYLMDLSASMDDDLNTIKELGSRLSKEMSKLTSNFRLGFGSFVEKPVSPFVKTTPEEIANPCSSIPYFCLPTFGFKHILPLTNDAERFNEIVKNQKISANIDTPEGGFDAIMQAAVCKEKIGWRNDSLHLLVFVSDADSHFGMDSKLAGIVIPNDGLCHLDSKNEYSMSTVLEYPTIGQLIDKLVQNNVLLIFAVTQEQVHLYENYAKLIPGATVGLLQKDSGNILQLIISAYEELRSEVELEVLGDTEGLNLSFTAICNNGTLFQHQKKCSHMKVGDTASFSVTVNIPHCERRSRHIIIKPVGLGDALELLVSPECNCDCQKEVEVNSSKCHHGNGSFQCGVCACHPGHMGPRCECGEDMLSTDSCKEAPDHPSCSGRGDCYCGQCICHLSPYGNIYGPYCQCDNFSCVRHKGLLCGGNGDCDCGECVCRSGWTGEYCNCTTSTDSCVSEDGVLCSGRGDCVCGKCVCTNPGASGPTCERCPTCGDPCNSKRSCIECHLSAAGQAREECVDKCKLAGATISEEEDFSKDGSVSCSLQGENECLITFLITTDNEGKTIIHSINEKDCPKPPNIPMIMLGVSLAILLIGVVLLCIWKLLVSFHDRKEVAKFEAERSKAKWQTGTNPLYRGSTSTFKNVTYKHREKQKVDLSTDC

517 NP_003160.2 NP_006212.1

>NP_003160.2

MSDSEDSNFSEEEDSERSSDGEEAEVDEERRSAAGSEKEEEPEDEEEEEEEEEYDEEEEEEDDDRPPKKPRHGGFILDEADVDDEYEDEDQWEDGAEDILEKEEIEASNIDNVVLDEDRSGARRLQNLWRDQREEELGEYYMKKYAKSSVGETVYGGSDELSDDITQQQLLPGVKDPNLWTVKCKIGEERATAISLMRKFIAYQFTDTPLQIKSVVAPEHVKGYIYVEAYKQTHVKQAIEGVGNLRLGYWNQQMVPIKEMTDVLKVVKEVANLKPKSWVRLKRGIYKDDIAQVDYVEPSQNTISLKMIPRIDYDRIKARMSLKDWFAKRKKFKRPPQRLFDAEKIRSLGGDVASDGDFLIFEGNRYSRKGFLFKSFAMSAVITEGVKPTLSELEKFEDQPEGIDLEVVTESTGKEREHNFQPGDNVEVCEGELINLQGKILSVDGNKITIMPKHEDLKDMLEFPAQELRKYFKMGDHVKVIAGRFEGDTGLIVRVEENFVILFSDLTMHELKVLPRDLQLCSETASGVDVGGQHEWGELVQLDPQTVGVIVRLERETFQVLNMYGKVVTVRHQAVTRKKDNRFAVALDSEQNNIHVKDIVKVIDGPHSGREGEIRHLFRSFAFLHCKKLVENGGMFVCKTRHLVLAGGSKPRDVTNFTVGGFAPMSPRISSPMHPSAGGQRGGFGSPGGGSGGMSRGRGRRDNELIGQTVRISQGPYKGYIGVVKDATESTARVELHSTCQTISVDRQRLTTVGSRRPGGMTSTYGRTPMYGSQTPMYGSGSRTPMYGSQTPLQDGSRTPHYGSQTPLHDGSRTPAQSGAWDPNNPNTPSRAEEEYEYAFDDEPTPSPQAYGGTPNPQTPGYPDPSSPQVNPQYNPQTPGTPAMYNTDQFSPYAAPSPQGSYQPSPSPQSYHQVAPSPAGYQNTHSPASYHPTPSPMAYQASPSPSPVGYSPMTPGAPSPGGYNPHTPGSGIEQNSSDWVTTDIQVKVRDTYLDTQVVGQTGVIRSVTGGMCSVYLKDSEKVVSISSEHLEPITPTKNNKVKVILGEDREATGVLLSIDGEDGIVRMDLDEQLKILNLRFLGKLLEA

>NP_006212.1

MADEEKLPPGWEKRMSRSSGRVYYFNHITNASQWERPSGNSSSGGKNGQGEPARVRCSHLLVKHSQSRRPSSWRQEKITRTKEEALELINGYIQKIKSGEEDFESLASQFSDCSSAKARGDLGAFSRGQMQKPFEDASFALRTGEMSGPVFTDSGIHIILRTE

518 NP_067068.1 NP_009185.2

>NP_067068.1

MPLSGTPAPNKKRKSSKLIMELTGGGQESSGLNLGKKISVPRDVMLEELSLLTNRGSKMFKLRQMRVEKFIYENHPDVFSDSSMDHFQKFLPTVGGQLGTAGQGFSYSKSNGRGGSQAGGSGSAGQYGSDQQHHLGSGSGAGGTGGPAGQAGRGGAAGTAGVGETGSGDQAGGEGKHITVFKTYISPWERAMGVDPQQKMELGIDLLAYGAKAELPKYKSFNRTAMPYGGYEKASKRMTFQMPKFDLGPLLSEPLVLYNQNLSNRPSFNRTPIPWLSSGEPVDYNVDIGIPLDGETEEL

>NP_009185.2

MGEVEAPGRLWLESPPGGAPPIFLPSDGQALVLGRGPLTQVTDRKCSRTQVELVADPETRTVAVKQLGVNPSTTGTQELKPGLEGSLGVGDTLYLVNGLHPLTLRWEETRTPESQPDTPPGTPLVSQDEKRDAELPKKRMRKSNPGWENLEKLLVFTAAGVKPQGKVAGFDLDGTLITTRSGKVFPTGPSDWRILYPEIPRKLRELEAEGYKLVIFTNQMSIGRGKLPAEEFKAKVEAVVEKLGVPFQVLVATHAGLYRKPVTGMWDHLQEQANDGTPISIGDSIFVGDAAGRPANWAPGRKKKDFSCADRLFALNLGLPFATPEEFFLKWPAAGFELPAFDPRTVSRSGPLCLPESRALLSASPEVVVAVGFPGAGKSTFLKKHLVSAGYVHVNRDTLGSWQRCVTTCETALKQGKRVAIDNTNPDAASRARYVQCARAAGVPCRCFLFTATLEQARHNNRFREMTDSSHIPVSDMVMYGYRKQFEAPTLAEGFSAILEIPFRLWVEPRLGRLYCQFSEG

519 NP_036580.2 NP_006303.3

>NP_036580.2

MAVWIQAQQLQGEALHQMQALYGQHFPIEVRHYLSQWIESQAWDSVDLDNPQENIKATQLLEGLVQELQKKAEHQVGEDGFLLKIKLGHYATQLQNTYDRCPMELVRCIRHILYNEQRLVREANNGSSPAGSLADAMSQKHLQINQTFEELRLVTQDTENELKKLQQTQEYFIIQYQESLRIQAQFGPLAQLSPQERLSRETALQQKQVSLEAWLQREAQTLQQYRVELAEKHQKTLQLLRKQQTIILDDELIQWKRRQQLAGNGGPPEGSLDVLQSWCEKLAEIIWQNRQQIRRAEHLCQQLPIPGPVEEMLAEVNATITDIISALVTSTFIIEKQPPQVLKTQTKFAATVRLLVGGKLNVHMNPPQVKATIISEQQAKSLLKNENTRNDYSGEILNNCCVMEYHQATGTLSAHFRNMSLKRIKRSDRRGAESVTEEKFTILFESQFSVGGNELVFQVKTLSLPVVVIVHGSQDNNATATVLWDNAFAEPGRVPFAVPDKVLWPQLCEALNMKFKAEVQSNRGLTKENLVFLAQKLFNNSSSHLEDYSGLSVSWSQFNRENLPGRNYTFWQWFDGVMEVLKKHLKPHWNDGAILGFVNKQQAHDLLINKPDGTFLLRFSDSEIGGITIAWKFDSQERMFWNLMPFTTRDFSIRSLADRLGDLNYLIYVFPDRPKDEVYSKYYTPVPCESATAKAVDGYVKPQIKQVVPEFVNASADAGGGSATYMDQAPSPAVCPQAHYNMYPQNPDSVLDTDGDFDLEDTMDVARRVEELLGRPMDSQWIPHAQS

>NP_006303.3

MSGSTQPVAQTWRATEPRYPPHSLSYPVQIARTHTDVGLLEYQHHSRDYASHLSPGSIIQPQRRRPSLLSEFQPGNERSQELHLRPESHSYLPELGKSEMEFIESKRPRLELLPDPLLRPSPLLATGQPAGSEDLTKDRSLTGKLEPVSPPSPPHTDPELELVPPRLSKEELIQNMDRVDREITMVEQQISKLKKKQQQLEEEAAKPPEPEKPVSPPPIESKHRSLVQIIYDENRKKAEAAHRILEGLGPQVELPLYNQPSDTRQYHENIKINQAMRKKLILYFKRRNHARKQWEQKFCQRYDQLMEAWEKKVERIENNPRRRAKESKVREYYEKQFPEIRKQRELQERMQSRVGQRGSGLSMSAARSEHEVSEIIDGLSEQENLEKQMRQLAVIPPMLYDADQQRIKFINMNGLMADPMKVYKDRQVMNMWSEQEKETFREKFMQHPKNFGLIASFLERKTVAECVLYYYLTKKNENYKSLVRRSYRRRGKSQQQQQQQQQQQQQQQQQPMPRSSQEEKDEKEKEKEAEKEEEKPEVENDKEDLLKEKTDDTSGEDNDEKEAVASKGRKTANSQGRRKGRITRSMANEANSEEAITPQQSAELASMELNESSRWTEEEMETAKKGLLEHGRNWSAIARMVGSKTVSQCKNFYFNYKKRQNLDEILQQHKLKMEKERNARRKKKKAPAAASEEAAFPPVVEDEEMEASGVSGNEEEMVEEAEALHASGNEVPRGECSGPATVNNSSDTESIPSPHTEAAKDTGQNGPKPPATLGADGPPPGPPTPPPEDIPAPTEPTPASEATGAPTPPPAPPSPSAPPPVVPKEEKEEETAAAPPVEEGEEQKPPAAEELAVDTGKAEEPVKSECTEEAEEGPAKGKDAEAAEATAEGALKAEKKEGGSGRATTAKSSGAPQDSDSSATCSADEVDEAEGGDKNRLLSPRPSLLTPTGDPRANASPQKPLDLKQLKQRAAAIPPIVTKVHEPPREDAAPTKPAPPAPPPPQNLQPESDAPQQPGSSPRGKSRSPAPPADKEAEKPVFFPAFAAEAQKLPGDPPCWTSGLPFPVPPREVIKASPHAPDPSAFSYAPPGHPLPLGLHDTARPVLPRPPTISNPPPLISSAKHPSVLERQIGAISQGMSVQLHVPYSEHAKAPVGPVTMGLPLPMDPKKLAPFSGVKQEQLSPRGQAGPPESLGVPTAQEASVLRGTALGSVPGGSITKGIPSTRVPSDSAITYRGSITHGTPADVLYKGTITRIIGEDSPSRLDRGREDSLPKGHVIYEGKKGHVLSYEGGMSVTQCSKEDGRSSSGPPHETAAPKRTYDMMEGRVGRAISSASIEGLMGRAIPPERHSPHHLKEQHHIRGSITQGIPRSYVEAQEDYLRREAKLLKREGTPPPPPPSRDLTEAYKTQALGPLKLKPAHEGLVATVKEAGRSIHEIPREELRHTPELPLAPRPLKEGSITQGTPLKYDTGASTTGSKKHDVRSLIGSPGRTFPPVHPLDVMADARALERACYEESLKSRPGTASSSGGSIARGAPVIVPELGKPRQSPLTYEDHGAPFAGHLPRGSPVTTREPTPRLQEGSLSSSKASQDRKLTSTPREIAKSPHSTVPEHHPHPISPYEHLLRGVSGVDLYRSHIPLAFDPTSIPRGIPLDAAAAYYLPRHLAPNPTYPHLYPPYLIRGYPDTAALENRQTIINDYITSQQMHHNAATAMAQRADMLRGLSPRESSLALNYAAGPRGIIDLSQVPHLPVLVPPTPGTPATAMDRLAYLPTAPQPFSSRHSSSPLSPGGPTHLTKPTTTSSSERERDRDRERDRDREREKSILTSTTTVEHAPIWRPGTEQSSGSSGSSGGGGGSSSRPASHSHAHQHSPISPRTQDALQQRPSVLHNTGMKGIITAVEPSTPTVLRSTSTSSPVRPAATFPPATHCPLGGTLDGVYPTLMEPVLLPKEAPRVARPERPRADTGHAFLAKPPARSGLEPASSPSKGSEPRPLVPPVSGHATIARTPAKNLAPHHASPDPPAPPASASDPHREKTQSKPFSIQELELRSLGYHGSSYSPEGVEPVSPVSSPSLTHDKGLPKHLEELDKSHLEGELRPKQPGPVKLGGEAAHLPHLRPLPESQPSSSPLLQTAPGVKGHQRVVTLAQHISEVITQDYTRHHPQQLSAPLPAPLYSFPGASCPVLDLRRPPSDLYLPPPDHGAPARGSPHSEGGKRSPEPNKTSVLGGGEDGIEPVSPPEGMTEPGHSRSAVYPLLYRDGEQTEPSRMGSKSPGNTSQPPAFFSKLTESNSAMVKSKKQEINKKLNTHNRNEPEYNISQPGTEIFNMPAITGTGLMTYRSQAVQEHASTNMGLEAIIRKALMGKYDQWEESPPLSANAFNPLNASASLPAAMPITAADGRSDHTLTSPGGGGKAKVSGRPSSRKAKSPAPGLASGDRPPSVSSVHSEGDCNRRTPLTNRVWEDRPSSAGSTPFPYNPLIMRLQAGVMASPPPPGLPAGSGPLAGPHHAWDEEPKPLLCSQYETLSDSE

520 NP_003290.1 NP_003226.4

>NP_003290.1

MRALWVLGLCCVLLTFGSVRADDEVDVDGTVEEDLGKSREGSRTDDEVVQREEEAIQLDGLNASQIRELREKSEKFAFQAEVNRMMKLIINSLYKNKEIFLRELISNASDALDKIRLISLTDENALSGNEELTVKIKCDKEKNLLHVTDTGVGMTREELVKNLGTIAKSGTSEFLNKMTEAQEDGQSTSELIGQFGVGFYSAFLVADKVIVTSKHNNDTQHIWESDSNEFSVIADPRGNTLGRGTTITLVLKEEASDYLELDTIKNLVKKYSQFINFPIYVWSSKTETVEEPMEEEEAAKEEKEESDDEAAVEEEEEEKKPKTKKVEKTVWDWELMNDIKPIWQRPSKEVEEDEYKAFYKSFSKESDDPMAYIHFTAEGEVTFKSILFVPTSAPRGLFDEYGSKKSDYIKLYVRRVFITDDFHDMMPKYLNFVKGVVDSDDLPLNVSRETLQQHKLLKVIRKKLVRKTLDMIKKIADDKYNDTFWKEFGTNIKLGVIEDHSNRTRLAKLLRFQSSHHPTDITSLDQYVERMKEKQDKIYFMAGSSRKEAESSPFVERLLKKGYEVIYLTEPVDEYCIQALPEFDGKRFQNVAKEGVKFDESEKTKESREAVEKEFEPLLNWMKDKALKDKIEKAVVSQRLTESPCALVASQYGWSGNMERIMKAQAYQTGKDISTNYYASQKKTFEINPRHPLIRDMLRRIKEDEDDKTVLDLAVVLFETATLRSGYLLPDTKAYGDRIERMLRLSLNIDPDAKVEEEPEEEPEETAEDTTEDTEQDEDEEMDVGTDEEEETAKESTAEKDEL

>NP_003226.4

MALVLEIFTLLASICWVSANIFEYQVDAQPLRPCELQRETAFLKQADYVPQCAEDGSFQTVQCQNDGRSCWCVGANGSEVLGSRQPGRPVACLSFCQLQKQQILLSGYINSTDTSYLPQCQDSGDYAPVQCDVQQVQCWCVDAEGMEVYGTRQLGRPKRCPRSCEIRNRRLLHGVGDKSPPQCSAEGEFMPVQCKFVNTTDMMIFDLVHSYNRFPDAFVTFSSFQRRFPEVSGYCHCADSQGRELAETGLELLLDEIYDTIFAGLDLPSTFTETTLYRILQRRFLAVQSVISGRFRCPTKCEVERFTATSFGHPYVPSCRRNGDYQAVQCQTEGPCWCVDAQGKEMHGTRQQGEPPSCAEGQSCASERQQALSRLYFGTSGYFSQHDLFSSPEKRWASPRVARFATSCPPTIKELFVDSGLLRPMVEGQSQQFSVSENLLKEAIRAIFPSRGLARLALQFTTNPKRLQQNLFGGKFLVNVGQFNLSGALGTRGTFNFSQFFQQLGLASFLNGGRQEDLAKPLSVGLDSNSSTGTPEAAKKDGTMNKPTVGSFGFEINLQENQNALKFLASLLELPEFLLFLQHAISVPEDVARDLGDVMETVLSSQTCEQTPERLFVPSCTTEGSYEDVQCFSGECWCVNSWGKELPGSRVRGGQPRCPTDCEKQRARMQSLMGSQPAGSTLFVPACTSEGHFLPVQCFNSECYCVDAEGQAIPGTRSAIGKPKKCPTPCQLQSEQAFLRTVQALLSNSSMLPTLSDTYIPQCSTDGQWRQVQCNGPPEQVFELYQRWEAQNKGQDLTPAKLLVKIMSYREAASGNFSLFIQSLYEAGQQDVFPVLSQYPSLQDVPLAALEGKRPQPRENILLEPYLFWQILNGQLSQYPGSYSDFSTPLAHFDLRNCWCVDEAGQELEGMRSEPSKLPTCPGSCEEAKLRVLQFIRETEEIVSASNSSRFPLGESFLVAKGIRLRNEDLGLPPLFPPREAFAEQFLRGSDYAIRLAAQSTLSFYQRRRFSPDDSAGASALLRSGPYMPQCDAFGSWEPVQCHAGTGHCWCVDEKGGFIPGSLTARSLQIPQCPTTCEKSRTSGLLSSWKQARSQENPSPKDLFVPACLETGEYARLQASGAGTWCVDPASGEELRPGSSSSAQCPSLCNVLKSGVLSRRVSPGYVPACRAEDGGFSPVQCDQAQGSCWCVMDSGEEVPGTRVTGGQPACESPRCPLPFNASEVVGGTILCETISGPTGSAMQQCQLLCRQGSWSVFPPGPLICSLESGRWESQLPQPRACQRPQLWQTIQTQGHFQLQLPPGKMCSADYADLLQTFQVFILDELTARGFCQIQVKTFGTLVSIPVCNNSSVQVGCLTRERLGVNVTWKSRLEDIPVASLPDLHDIERALVGKDLLGRFTDLIQSGSFQLHLDSKTFPAETIRFLQGDHFGTSPRTWFGCSEGFYQVLTSEASQDGLGCVKCPEGSYSQDEECIPCPVGFYQEQAGSLACVPCPVGRTTISAGAFSQTHCVTDCQRNEAGLQCDQNGQYRASQKDRGSGKAFCVDGEGRRLPWWETEAPLEDSQCLMMQKFEKVPESKVIFDANAPVAVRSKVPDSEFPVMQCLTDCTEDEACSFFTVSTTEPEISCDFYAWTSDNVACMTSDQKRDALGNSKATSFGSLRCQVKVRSHGQDSPAVYLKKGQGSTTTLQKRFEPTGFQNMLSGLYNPIVFSASGANLTDAHLFCLLACDRDLCCDGFVLTQVQGGAIICGLLSSPSVLLCNVKDWMDPSEAWANATCPGVTYDQESHQVILRLGDQEFIKSLTPLEGTQDTFTNFQQVYLWKDSDMGSRPESMGCRKDTVPRPASPTEAGLTTELFSPVDLNQVIVNGNQSLSSQKHWLFKHLFSAQQANLWCLSRCVQEHSFCQLAEITESASLYFTCTLYPEAQVCDDIMESNAQGCRLILPQMPKALFRKKVILEDKVKNFYTRLPFQKLMGISIRNKVPMSEKSISNGFFECERRCDADPCCTGFGFLNVSQLKGGEVTCLTLNSLGIQMCSEENGGAWRILDCGSPDIEVHTYPFGWYQKPIAQNNAPSFCPLVVLPSLTEKVSLDSWQSLALSSVVVDPSIRHFDVAHVSTAATSNFSAVRDLCLSECSQHEACLITTLQTQPGAVRCMFYADTQSCTHSLQGQNCRLLLREEATHIYRKPGISLLSYEASVPSVPISTHGRLLGRSQAIQVGTSWKQVDQFLGVPYAAPPLAERRFQAPEPLNWTGSWDASKPRASCWQPGTRTSTSPGVSEDCLYLNVFIPQNVAPNASVLVFFHNTMDREESEGWPAIDGSFLAAVGNLIVVTASYRVGVFGFLSSGSGEVSGNWGLLDQVAALTWVQTHIRGFGGDPRRVSLAADRGGADVASIHLLTARATNSQLFRRAVLMGGSALSPAAVISHERAQQQAIALAKEVSCPMSSSQEVVSCLRQKPANVLNDAQTKLLAVSGPFHYWGPVIDGHFLREPPARALKRSLWVEVDLLIGSSQDDGLINRAKAVKQFEESRGRTSSKTAFYQALQNSLGGEDSDARVEAAATWYYSLEHSTDDYASFSRALENATRDYFIICPIIDMASAWAKRARGNVFMYHAPENYGHGSLELLADVQFALGLPFYPAYEGQFSLEEKSLSLKIMQYFSHFIRSGNPNYPYEFSRKVPTFATPWPDFVPRAGGENYKEFSELLPNRQGLKKADCSFWSKYISSLKTSADGAKGGQSAESEEEELTAGSGLREDLLSLQEPGSKTYSK

521 NP_009225.1 NP_061828.1

>NP_009225.1

MDLSALRVEEVQNVINAMQKILECPICLELIKEPVSTKCDHIFCKFCMLKLLNQKKGPSQCPLCKNDITKRSLQESTRFSQLVEELLKIICAFQLDTGLEYANSYNFAKKENNSPEHLKDEVSIIQSMGYRNRAKRLLQSEPENPSLQETSLSVQLSNLGTVRTLRTKQRIQPQKTSVYIELGSDSSEDTVNKATYCSVGDQELLQITPQGTRDEISLDSAKKAACEFSETDVTNTEHHQPSNNDLNTTEKRAAERHPEKYQGSSVSNLHVEPCGTNTHASSLQHENSSLLLTKDRMNVEKAEFCNKSKQPGLARSQHNRWAGSKETCNDRRTPSTEKKVDLNADPLCERKEWNKQKLPCSENPRDTEDVPWITLNSSIQKVNEWFSRSDELLGSDDSHDGESESNAKVADVLDVLNEVDEYSGSSEKIDLLASDPHEALICKSERVHSKSVESNIEDKIFGKTYRKKASLPNLSHVTENLIIGAFVTEPQIIQERPLTNKLKRKRRPTSGLHPEDFIKKADLAVQKTPEMINQGTNQTEQNGQVMNITNSGHENKTKGDSIQNEKNPNPIESLEKESAFKTKAEPISSSISNMELELNIHNSKAPKKNRLRRKSSTRHIHALELVVSRNLSPPNCTELQIDSCSSSEEIKKKKYNQMPVRHSRNLQLMEGKEPATGAKKSNKPNEQTSKRHDSDTFPELKLTNAPGSFTKCSNTSELKEFVNPSLPREEKEEKLETVKVSNNAEDPKDLMLSGERVLQTERSVESSSISLVPGTDYGTQESISLLEVSTLGKAKTEPNKCVSQCAAFENPKGLIHGCSKDNRNDTEGFKYPLGHEVNHSRETSIEMEESELDAQYLQNTFKVSKRQSFAPFSNPGNAEEECATFSAHSGSLKKQSPKVTFECEQKEENQGKNESNIKPVQTVNITAGFPVVGQKDKPVDNAKCSIKGGSRFCLSSQFRGNETGLITPNKHGLLQNPYRIPPLFPIKSFVKTKCKKNLLEENFEEHSMSPEREMGNENIPSTVSTISRNNIRENVFKEASSSNINEVGSSTNEVGSSINEIGSSDENIQAELGRNRGPKLNAMLRLGVLQPEVYKQSLPGSNCKHPEIKKQEYEEVVQTVNTDFSPYLISDNLEQPMGSSHASQVCSETPDDLLDDGEIKEDTSFAENDIKESSAVFSKSVQKGELSRSPSPFTHTHLAQGYRRGAKKLESSEENLSSEDEELPCFQHLLFGKVNNIPSQSTRHSTVATECLSKNTEENLLSLKNSLNDCSNQVILAKASQEHHLSEETKCSASLFSSQCSELEDLTANTNTQDPFLIGSSKQMRHQSESQGVGLSDKELVSDDEERGTGLEENNQEEQSMDSNLGEAASGCESETSVSEDCSGLSSQSDILTTQQRDTMQHNLIKLQQEMAELEAVLEQHGSQPSNSYPSIISDSSALEDLRNPEQSTSEKAVLTSQKSSEYPISQNPEGLSADKFEVSADSSTSKNKEPGVERSSPSKCPSLDDRWYMHSCSGSLQNRNYPSQEELIKVVDVEEQQLEESGPHDLTETSYLPRQDLEGTPYLESGISLFSDDPESDPSEDRAPESARVGNIPSSTSALKVPQLKVAESAQSPAAAHTTDTAGYNAMEESVSREKPELTASTERVNKRMSMVVSGLTPEEFMLVYKFARKHHITLTNLITEETTHVVMKTDAEFVCERTLKYFLGIAGGKWVVSYFWVTQSIKERKMLNEHDFEVRGDVVNGRNHQGPKRARESQDRKIFRGLEICCYGPFTNMPTDQLEWMVQLCGASVVKELSSFTLGTGVHPIVVVQPDAWTEDNGFHAIGQMCEAPVVTREWVLDSVALYQCQELDTYLIPQIPHSHY

>NP_061828.1

MQIFVKTLTGKTITLEVEPSDTIENVKAKIQDKEGIPPDQQRLIFAGKQLEDGRTLSDYNIQKESTLHLVLRLRGGMQIFVKTLTGKTITLEVEPSDTIENVKAKIQDKEGIPPDQQRLIFAGKQLEDGRTLSDYNIQKESTLHLVLRLRGGMQIFVKTLTGKTITLEVEPSDTIENVKAKIQDKEGIPPDQQRLIFAGKQLEDGRTLSDYNIQKESTLHLVLRLRGGC

522 NP_775114.1 NP_002735.3

>NP_775114.1

MNAPERQPQPDGGDAPGHEPGGSPQDELDFSILFDYEYLNPNEEEPNAHKVASPPSGPAYPDDVLDYGLKPYSPLASLSGEPPGRFGEPDRVGPQKFLSAAKPAGASGLSPRIEITPSHELIQAVGPLRMRDAGLLVEQPPLAGVAASPRFTLPVPGFEGYREPLCLSPASSGSSASFISDTFSPYTSPCVSPNNGGPDDLCPQFQNIPAHYSPRTSPIMSPRTSLAEDSCLGRHSPVPRPASRSSSPGAKRRHSCAEALVALPPGASPQRSRSPSPQPSSHVAPQDHGSPAGYPPVAGSAVIMDALNSLATDSPCGIPPKMWKTSPDPSPVSAAPSKAGLPRHIYPAVEFLGPCEQGERRNSAPESILLVPPTWPKPLVPAIPICSIPVTASLPPLEWPLSSQSGSYELRIEVQPKPHHRAHYETEGSRGAVKAPTGGHPVVQLHGYMENKPLGLQIFIGTADERILKPHAFYQVHRITGKTVTTTSYEKIVGNTKVLEIPLEPKNNMRATIDCAGILKLRNADIELRKGETDIGRKNTRVRLVFRVHIPESSGRIVSLQTASNPIECSQRSAHELPMVERQDTDSCLVYGGQQMILTGQNFTSESKVVFTEKTTDGQQIWEMEATVDKDKSQPNMLFVEIPEYRNKHIRTPVKVNFYVINGKRKRSQPQHFTYHPVPAIKTEPTDEYDPTLICSPTHGGLGSQPYYPQHPMVAESPSCLVATMAPCQQFRTGLSSPDARYQQQNPAAVLYQRSKSLSPSLLGYQQPALMAAPLSLADAHRSVLVHAGSQGQSSALLHPSPTNQQASPVIHYSPTNQQLRCGSHQEFQHIMYCENFAPGTTRPGPPPVSQGQRLSPGSYPTVIQQQNATSQRAAKNGPPVSDQKEVLPAGVTIKQEQNLDQTYLDDVNEIIRKEFSGPPARNQT

>NP_002735.3

MPSRTGPKMEGSGGRVRLKAHYGGDIFITSVDAATTFEELCEEVRDMCRLHQQHPLTLKWVDSEGDPCTVSSQMELEEAFRLARQCRDEGLIIHVFPSTPEQPGLPCPGEDKSIYRRGARRWRKLYRANGHLFQAKRFNRRAYCGQCSERIWGLARQGYRCINCKLLVHKRCHGLVPLTCRKHMDSVMPSQEPPVDDKNEDADLPSEETDGIAYISSSRKHDSIKDDSEDLKPVIDGMDGIKISQGLGLQDFDLIRVIGRGSYAKVLLVRLKKNDQIYAMKVVKKELVHDDEDIDWVQTEKHVFEQASSNPFLVGLHSCFQTTSRLFLVIEYVNGGDLMFHMQRQRKLPEEHARFYAAEICIALNFLHERGIIYRDLKLDNVLLDADGHIKLTDYGMCKEGLGPGDTTSTFCGTPNYIAPEILRGEEYGFSVDWWALGVLMFEMMAGRSPFDIITDNPDMNTEDYLFQVILEKPIRIPRFLSVKASHVLKGFLNKDPKERLGCRPQTGFSDIKSHAFFRSIDWDLLEKKQALPPFQPQITDDYGLDNFDTQFTSEPVQLTPDDEDAIKRIDQSEFEGFEYINPLLLSTEESV

523 NP_536859.1 NP_004110.2

>NP_536859.1

MVRWFHRDLSGLDAETLLKGRGVHGSFLARPSRKNQGDFSLSVRVGDQVTHIRIQNSGDFYDLYGGEKFATLTELVEYYTQQQGVLQDRDGTIIHLKYPLNCSDPTSERWYHGHMSGGQAETLLQAKGEPWTFLVRESLSQPGDFVLSVLSDQPKAGPGSPLRVTHIKVMCEGGRYTVGGLETFDSLTDLVEHFKKTGIEEASGAFVYLRQPYYATRVNAADIENRVLELNKKQESEDTAKAGFWEEFESLQKQEVKNLHQRLEGQRPENKGKNRYKNILPFDHSRVILQGRDSNIPGSDYINANYIKNQLLGPDENAKTYIASQGCLEATVNDFWQMAWQENSRVIVMTTREVEKGRNKCVPYWPEVGMQRAYGPYSVTNCGEHDTTEYKLRTLQVSPLDNGDLIREIWHYQYLSWPDHGVPSEPGGVLSFLDQINQRQESLPHAGPIIVHCSAGIGRTGTIIVIDMLMENISTKGLDCDIDIQKTIQMVRAQRSGMVQTEAQYKFIYVAIAQFIETTKKKLEVLQSQKGQESEYGNITYPPAMKNAHAKASRTSSKSLESSAGTVAASPVRRGGQRGLPVPGPPVLSPDLHQLPVLAPLHPAADTRRMCMRTCTLRTRGRRK

>NP_004110.2

MPALARDGGQLPLLVVFSAMIFGTITNQDLPVIKCVLINHKNNDSSVGKSSSYPMVSESPEDLGCALRPQSSGTVYEAAAVEVDVSASITLQVLVDAPGNISCLWVFKHSSLNCQPHFDLQNRGVVSMVILKMTETQAGEYLLFIQSEATNYTILFTVSIRNTLLYTLRRPYFRKMENQDALVCISESVPEPIVEWVLCDSQGESCKEESPAVVKKEEKVLHELFGTDIRCCARNELGRECTRLFTIDLNQTPQTTLPQLFLKVGEPLWIRCKAVHVNHGFGLTWELENKALEEGNYFEMSTYSTNRTMIRILFAFVSSVARNDTGYYTCSSSKHPSQSALVTIVEKGFINATNSSEDYEIDQYEEFCFSVRFKAYPQIRCTWTFSRKSFPCEQKGLDNGYSISKFCNHKHQPGEYIFHAENDDAQFTKMFTLNIRRKPQVLAEASASQASCFSDGYPLPSWTWKKCSDKSPNCTEEITEGVWNRKANRKVFGQWVSSSTLNMSEAIKGFLVKCCAYNSLGTSCETILLNSPGPFPFIQDNISFYATIGVCLLFIVVLTLLICHKYKKQFRYESQLQMVQVTGSSDNEYFYVDFREYEYDLKWEFPRENLEFGKVLGSGAFGKVMNATAYGISKTGVSIQVAVKMLKEKADSSEREALMSELKMMTQLGSHENIVNLLGACTLSGPIYLIFEYCCYGDLLNYLRSKREKFHRTWTEIFKEHNFSFYPTFQSHPNSSMPGSREVQIHPDSDQISGLHGNSFHSEDEIEYENQKRLEEEEDLNVLTFEDLLCFAYQVAKGMEFLEFKSCVHRDLAARNVLVTHGKVVKICDFGLARDIMSDSNYVVRGNARLPVKWMAPESLFEGIYTIKSDVWSYGILLWEIFSLGVNPYPGIPVDANFYKLIQNGFKMDQPFYATEEIYIIMQSCWAFDSRKRPSFPNLTSFLGCQLADAEEAMYQNVDGRVSECPHTYQNRRPFSREMDLGLLSPQAQVEDS

524 NP_000537.3 NP_001008709.1

>NP_000537.3

MEEPQSDPSVEPPLSQETFSDLWKLLPENNVLSPLPSQAMDDLMLSPDDIEQWFTEDPGPDEAPRMPEAAPPVAPAPAAPTPAAPAPAPSWPLSSSVPSQKTYQGSYGFRLGFLHSGTAKSVTCTYSPALNKMFCQLAKTCPVQLWVDSTPPPGTRVRAMAIYKQSQHMTEVVRRCPHHERCSDSDGLAPPQHLIRVEGNLRVEYLDDRNTFRHSVVVPYEPPEVGSDCTTIHYNYMCNSSCMGGMNRRPILTIITLEDSSGNLLGRNSFEVRVCACPGRDRRTEEENLRKKGEPHHELPPGSTKRALPNNTSSSPQPKKKPLDGEYFTLQIRGRERFEMFRELNEALELKDAQAGKEPGGSRAHSSHLKSKKGQSTSRHKKLMFKTEGPDSD

>NP_001008709.1

MSDSEKLNLDSIIGRLLEGSRVLTPHCAPVQGSRPGKNVQLTENEIRGLCLKSREIFLSQPILLELEAPLKICGDIHGQYYDLLRLFEYGGFPPESNYLFLGDYVDRGKQSLETICLLLAYKIKYPENFFLLRGNHECASINRIYGFYDECKRRYNIKLWKTFTDCFNCLPIAAIVDEKIFCCHGGLSPDLQSMEQIRRIMRPTDVPDQGLLCDLLWSDPDKDVQGWGENDRGVSFTFGAEVVAKFLHKHDLDLICRAHQVVEDGYEFFAKRQLVTLFSAPNYCGEFDNAGAMMSVDETLMCSFQILKPADKNKGKYGQFSGLNPGGRPITPPRNSAKAKK

525 NP_055717.2 NP_115568.2

>NP_055717.2

MKGLGDSRPRHLSDSLDPPHEPLFAGTDRNPYLLSPTEAFAREARFPGQNTLPGDGLFPLNNQLPPPSSTFPRIHYNSHFEVPEESPFPSHAQATKINRLPANLLDQFEKQLPIHRDGFSTLQFPRGEAKARGESPGRIRHLVHSVQRLFFTKAPSLEGTAGKVGGNGSKKGGMEDGKGRRAKSKERAKAGEPKRRSRSNISGWWSSDDNLDGEAGAFRSSGPASGLMTLGRQAERSQPRYFMHAYNTISGHMLKTTKNNTTELTAPPPPPAPPATCPSLGVGTDTNYVKRGSWSTLTLSHAHEVCQKTSATLDKSLLKSKSCHQGLAYHYLQVPGGGGEWSTTLLSPRETDAAAEGPIPCRRMRSGSYIKAMGDEDSDESGGSPKPSPKTAARRQSYLRATQQSLGEQSNPRRSLDRLDSVDMLLPSKCPSWEEDYTPVSDSLNDSSCISQIFGQASLIPQLFGHEQQVREAELSDQYEAACESACSEAESTAAETLDLPLPSYFRSRSHSYLRAIQAGCSQEEDSVSLQSLSPPPSTGSLSNSRTLPSSSCLVAYKKTPPPVPPRTTSKPFISVTVQSSTESAQDTYLDSQDHKSEVTSQSGLSNSSDSLDSSTRPPSVTRGGVAPAPEAPEPPPKHAALKSEQGTLTSSESHPEAAPKRKLSSIGIQERTRRNGSHLSEDNGPKAIDVMAPSSESSVPSHSMSSRRDTDSDTQDANDSSCKSSERSLPDCTPHPNSISIDAGPRQAPKIAQIKRNLSYGDNSDPALEASSLPPPDPWLETSSSSPAEPAQPGACRRDGYWFLKLLQAETERLEGWCCQMDKETKENNLSEEVLGKVLSAVGSAQLLMSQKFQQFRGLCEQNLNPDANPRPTAQDLAGFWDLLQLSIEDISMKFDELYHLKANSWQLVETPEKRKEEKKPPPPVPKKPAKSKPAVSRDKASDASDKQRQEARKRLLAAKRAASVRQNSATESADSIEIYVPEAQTRL

>NP_115568.2

MDPKDRKKIQFSVPAPPSQLDPRQVEMIRRRRPTPAMLFRLSEHSSPEEEASPHQRASGEGHHLKSKRPNPCAYTPPSLKAVQRIAESHLQSISNLNENQASEEEDELGELRELGYPREEDEEEEEDDEEEEEEEDSQAEVLKVIRQSAGQKTTCGQGLEGPWERPPPLDESERDGGSEDQVEDPALSEPGEEPQRPSPSEPGT

526 AAD02233.1 AAH11837.2

>AAD02233.1

MEELSADEIRRRRLARLAGGQTSQPTTPLTSPQRENPPGPPIAASAPGPSQSLGLNVHNMTPATSPIGASGVAHRSQSSEGVSSLSSSPSNSLETQSQSLSRSQSMDIDGVSCEKSMSQVDVDSGIENMEVDENDRREKRSLSDKEPSSGPEVSEEQALQLVCKIFRVSWKDRDRDVIFLSSLSAQFKQNPKEVFSDFKDLIGQILMEVLMMSTQTRDENPFASLTATSQPIAAAARSPDRNLLLNTGSNPGTSPMFCSVASFGASSLSSLYESSPAPTPSFWSSVPVMGPSLASPSRAASQLAVPSTPLSPHSAASGTAAGSQPSSPRYRPYTVTHPWASSGVSILSSSPSPPALASSPQAVPASSSRQRPSSTGPPLPPASPSATSRRPSSLRISPSLGASGGASNWDSYSDHFTIETCKETDMLNYLIECFDRVGIEEKKAPKMCSQPAVSQLLSNIRSQCISHTALVLQGSLTQPRSLQQPSFLVPYMLCRNLPYGFIQELVRTTHQDEEVFKQIFIPILQGLALAAKECSLDSDYFKYPLMALGELCETKFGKTHPVCNLVASLRLWLPKSLSPGCGRELQRLSYLGAFFSFSVFAEDDVKVVEKYFSGPAITLENTRVVSQSLQHYLELGRQELFKILHSILLNGETREAALSYMAAVVNANMKKAQMQTDDRLVSTDGFMLNFLWVLQQLSTKIKLETVDPTYIFHPRCRITLPNDETRVNATMEDVNDWLTELYGDQPPFSEPKFPTECFFLTLHAHHLSILPSCRRYIRRLRAIRELNRTVEDLKNNESQWKDSPLATRHREMLKRCKTQLKKLVRCKACADAGLLDESFLRRCLNFYGLLIQLLLRILDPAYPDITLPLNSDVPKVFAALPEFYVEDVAEFLFFIVQYSPQALYEPCTQDIVMFLVVMLCNQNYIRNPYLVAKLVEVMFMTNPAVQPRTQKFFEMIENHPLSTKLLVPSLMKFYTDVEHTGATSEFYDKFTIRYHISTIFKSLWQNIAHHGTFMEEFNSGKQFVRYINMLINDTTFLLDESLESLKRIHEVQEEMKNKEQWDQLPRDQQQARQSQLAQDERVSRSYLALATETVDMFHILTKQVQKPFLRPELGPRLAAMLNFNLQQLCGPKCRDLKVENPEKYGFEPKKLLDQLTDIYLQLDCARFAKAIADDQRSYSKELFEEVISKMRKAGIKSTIAIEKFKLLAEKVEEIVAKNARAEIDYSDAPDEFRDPLMDTLMTDPVRLPSGTIMDRSIILRHLLNSPTDPFNRQTLTESMLEPVPELKEQIQAWMREKQNSDH

>AAH11837.2

MAAAAECDVVMAATEPELLDDQEAKREAETFKEQGNAYYAKKDYNEAYNYYTKAIDMCPKNASYYGNRAATLMMLGRFREALGDAQQSVRLDDSFVRGHLREGKCHLSLGNAMAACRSFQRALELDHKNAQAQQEFKNANAVMEYEKIAETDFEKRDFRKVVFCMDRALEFAPACHRFKILKAECLAMLGRYPEAQSVASDILRMDSTNADALYVRGLCLYYEDCIEKAVQFFVQALRMAPDHEKACIACRNAKALKAKKEDGNKAFKEGNYKLAYELYTEALGIDPNNIKTNAKLYCNRGTVNSKLRKLDDAIEDCTNAVKLDDTYIKAYLRRAQCYMDTEQYEEAVRDYEKVYQTEKTKEHKQLLKNAQLELKKSKRKDYYKILGVDKNASEDEIKKAYRKRALMHHPDRHSGASAEVQKEEEKKFKEVGEAFTILSDPKKKTRYDSGQDLDEEGMNMGDFDPNNIFKAFFGGPGGFSFEASGPGNFFFQFG

527 NP_001075032.1 NP_064516.2

>NP_001075032.1

MGGHFWPPEPYTVFMWGSPWEADSPRVKLRGREKGRQTEGGAFPLVSSALSGDPRFFSPTTPPAEPIVVRLKEVRLQRDDFEILKVIGRGAFSEVAVVKMKQTGQVYAMKIMNKWDMLKRGEVSCFREERDVLVNGDRRWITQLHFAFQDENYLYLVMEYYVGGDLLTLLSKFGERIPAEMARFYLAEIVMAIDSVHRLGYVHRDIKPDNILLDRCGHIRLADFGSCLKLRADGTVRSLVAVGTPDYLSPEILQAVGGGPGTGSYGPECDWWALGVFAYEMFYGQTPFYADSTAETYGKIVHYKEHLSLPLVDEGVPEEARDFIQRLLCPPETRLGRGGAGDFRTHPFFFGLDWDGLRDSVPPFTPDFEGATDTCNFDLVEDGLTAMVSGGGETLSDIREGAPLGVHLPFVGYSYSCMALRDSEVPGPTPMELEAEQLLEPHVQAPSLEPSVSPQDETAEVAVPAAVPAAEAEAEVTLRELQEALEEEVLTRQSLSREMEAIRTDNQNFASQLREAEARNRDLEAHVRQLQERMELLQAEGATAVTGVPSPRATDPPSHLDGPPAVAVGQCPLVGPGPMHRRHLLLPARVPRPGLSEALSLLLFAVVLSRAAALGCIGLVAHAGQLTAVWRRPGAARAP

>NP_064516.2

MAEPSGAETRPPIRVTVKTPKDKEEIVICDRASVKEFKEEISRRFKAQQDQLVLIFAGKILKDGDTLNQHGIKDGLTVHLVIKTPQKAQDPAAATASSPSTPDPASAPSTTPASPATPAQPSTSGSASSDAGSGSRRSSGGGPSPGAGEGSPSATASILSGFGGILGLGSLGLGSANFMELQQQMQRQLMSNPEMLSQIMENPLVQDMMSNPDLMRHMIMANPQMQQLMERNPEISHMLNNPELMRQTMELARNPAMMQEMMRNQDRALSNLESIPGGYNALRRMYTDIQEPMFSAAREQFGNNPFSSLAGNSDSSSSQPLRTENREPLPNPWSPSPPTSQAPGSGGEGTGGSGTSQVHPTVSNPFGINAASLGSGMFNSPEMQALLQQISENPQLMQNVISAPYMRSMMQTLAQNPDFAAQMMVNVPLFAGNPQLQEQLRLQLPVFLQQMQNPESLSILTNPRAMQALLQIQQGLQTLQTEAPGLVPSLGSFGISRTPAPSAGSNAGSTPEAPTSSPATPATSSPTGASSAQQQLMQQMIQLLAGSGNSQVQTPEVRFQQQLEQLNSMGFINREANLQALIATGGDINAAIERLLGSQLS

528 NP_003143.2 NP_002736.3

>NP_003143.2

MAGWIQAQQLQGDALRQMQVLYGQHFPIEVRHYLAQWIESQPWDAIDLDNPQDRAQATQLLEGLVQELQKKAEHQVGEDGFLLKIKLGHYATQLQKTYDRCPLELVRCIRHILYNEQRLVREANNCSSPAGILVDAMSQKHLQINQTFEELRLVTQDTENELKKLQQTQEYFIIQYQESLRIQAQFAQLAQLSPQERLSRETALQQKQVSLEAWLQREAQTLQQYRVELAEKHQKTLQLLRKQQTIILDDELIQWKRRQQLAGNGGPPEGSLDVLQSWCEKLAEIIWQNRQQIRRAEHLCQQLPIPGPVEEMLAEVNATITDIISALVTSTFIIEKQPPQVLKTQTKFAATVRLLVGGKLNVHMNPPQVKATIISEQQAKSLLKNENTRNECSGEILNNCCVMEYHQATGTLSAHFRNMSLKRIKRADRRGAESVTEEKFTVLFESQFSVGSNELVFQVKTLSLPVVVIVHGSQDHNATATVLWDNAFAEPGRVPFAVPDKVLWPQLCEALNMKFKAEVQSNRGLTKENLVFLAQKLFNNSSSHLEDYSGLSVSWSQFNRENLPGWNYTFWQWFDGVMEVLKKHHKPHWNDGAILGFVNKQQAHDLLINKPDGTFLLRFSDSEIGGITIAWKFDSPERNLWNLKPFTTRDFSIRSLADRLGDLSYLIYVFPDRPKDEVFSKYYTPVLAKAVDGYVKPQIKQVVPEFVNASADAGGSSATYMDQAPSPAVCPQAPYNMYPQNPDHVLDQDGEFDLDETMDVARHVEELLRRPMDSLDSRLSPPAGLFTSARGSLS

>NP_002736.3

MAAAAAAGAGPEMVRGQVFDVGPRYTNLSYIGEGAYGMVCSAYDNVNKVRVAIKKISPFEHQTYCQRTLREIKILLRFRHENIIGINDIIRAPTIEQMKDVYIVQDLMETDLYKLLKTQHLSNDHICYFLYQILRGLKYIHSANVLHRDLKPSNLLLNTTCDLKICDFGLARVADPDHDHTGFLTEYVATRWYRAPEIMLNSKGYTKSIDIWSVGCILAEMLSNRPIFPGKHYLDQLNHILGILGSPSQEDLNCIINLKARNYLLSLPHKNKVPWNRLFPNADSKALDLLDKMLTFNPHKRIEVEQALAHPYLEQYYDPSDEPIAEAPFKFDMELDDLPKEKLKELIFEETARFQPGYRS

529 NP_001658.2 NP_002592.1

>NP_001658.2

MGLLTILKKMKQKERELRLLMLGLDNAGKTTILKKFNGEDIDTISPTLGFNIKTLEHRGFKLNIWDVGGQKSLRSYWRNYFESTDGLIWVVDSADRQRMQDCQRELQSLLVEERLAGATLLIFANKQDLPGALSSNAIREVLELDSIRSHHWCIQGCSAVTGENLLPGIDWLLDDISSRIFTAD

>NP_002592.1

MSAKDERAREILRGFKLNWMNLRDAETGKILWQGTEDLSVPGVEHEARVPKKILKCKAVSRELNFSSTEQMEKFRLEQKVYFKGQCLEEWFFEFGFVIPNSTNTWQSLIEAAPESQMMPASVLTGNVIIETKFFDDDLLVSTSRVRLFYV

530 NP_065162.1 NP_060729.2

>NP_065162.1

MSNPGTRRNGSSIKIRLTVLCAKNLAKKDFFRLPDPFAKIVVDGSGQCHSTDTVKNTLDPKWNQHYDLYVGKTDSITISVWNHKKIHKKQGAGFLGCVRLLSNAISRLKDTGYQRLDLCKLNPSDTDAVRGQIVVSLQTRDRIGTGGSVVDCRGLLENEGTVYEDSGPGRPLSCFMEEPAPYTDSTGAAAGGGNCRFVESPSQDQRLQAQRLRNPDVRGSLQTPQNRPHGHQSPELPEGYEQRTTVQGQVYFLHTQTGVSTWHDPRIPSPSGTIPGGDAAFLYEFLLQGHTSEPRDLNSVNCDELGPLPPGWEVRSTVSGRIYFVDHNNRTTQFTDPRLHHIMNHQCQLKEPSQPLPLPSEGSLEDEELPAQRYERDLVQKLKVLRHELSLQQPQAGHCRIEVSREEIFEESYRQIMKMRPKDLKKRLMVKFRGEEGLDYGGVAREWLYLLCHEMLNPYYGLFQYSTDNIYMLQINPDSSINPDHLSYFHFVGRIMGLAVFHGHYINGGFTVPFYKQLLGKPIQLSDLESVDPELHKSLVWILENDITPVLDHTFCVEHNAFGRILQHELKPNGRNVPVTEENKKEYVRLYVNWRFMRGIEAQFLALQKGFNELIPQHLLKPFDQKELELIIGGLDKIDLNDWKSNTRLKHCVADSNIVRWFWQAVETFDEERRARLLQFVTGSTRVPLQGFKALQGSTGAAGPRLFTIHLIDANTDNLPKAHTCFNRIDIPPYESYEKLYEKLLTAVEETCGFAVE

>NP_060729.2

MAAAVGVRGRYELPPCSGPGWLLSLSALLSVAARGAFATTHWVVTEDGKIQQQVDSPMNLKHPHDLVILMRQEATVNYLKELEKQLVAQKIHIEENEDRDTGLEQRHNKEDPDCIKAKVPLGDLDLYDGTYITLESKDISPEDYIDTESPVPPDPEQPDCTKILELPYSIHAFQHLRGVQERVNLSAPLLPKEDPIFTYLSKRLGRSIDDIGHLIHEGLQKNTSSWVLYNMASFYWRIKNEPYQVVECAMRALHFSSRHNKDIALVNLANVLHRAHFSADAAVVVHAALDDSDFFTSYYTLGNIYAMLGEYNHSVLCYDHALQARPGFEQAIKRKHAVLCQQKLEQKLEAQHRSLQRTLNELKEYQKQHDHYLRQQEILEKHKLIQEEQILRNIIHETQMAKEAQLGNHQICRLVNQQHSLHCQWDQPVRYHRGDIFENVDYVQFGEDSSTSSMMSVNFDVQSNQSDINDSVKSSPVAHSILWIWGRDSDAYRDKQHILWPKRADCTESYPRVPVGGELPTYFLPPENKGLRIHELSSDDYSTEEEAQTPDCSITDFRKSHTLSYLVKELEVRMDLKAKMPDDHARKILLSRINNYTIPEEEIGSFLFHAINKPNAPIWLILNEAGLYWRAVGNSTFAIACLQRALNLAPLQYQDVPLVNLANLLIHYGLHLDATKLLLQALAINSSEPLTFLSLGNAYLALKNISGALEAFRQALKLTTKCPECENSLKLIRCMQFYPFLYNITSSVCSGTVVEESNGSDEMENSDETKMSEEILALVDEFQQAWPLEGFGGALEMKGRRLDLQGIRVLKKGPQDGVARSSCYGDCRSEDDEATEWITFQVKRVKKPKGDHKKTPGKKVETGQIENGHRYQANLEITGPKVASPGPQGKKRDYQRLGWPSPDECLKLRWVELTAIVSTWLAVSSKNIDITEHIDFATPIQQPAMEPLCNGNLPTSMHTLDHLHGVSNRASLHYTGESQLTEVLQNLGKDQYPQQSLEQIGTRIAKVLEKNQTSWVLSSMAALYWRVKGQGKKAIDCLRQALHYAPHQMKDVPLISLANILHNAKLWNDAVIVATMAVEIAPHFAVNHFTLGNVYVAMEEFEKALVWYESTLKLQPEFVPAKNRIQTIQCHLMLKKGRRSP

531 NP_060719.4 NP_057103.2

>NP_060719.4

MMDLVLEEDVTVPGTLSGCSGLVPSVPDDLDGINPNAGLGNGLLPNVSEETVSPTRARNMKDFENQITELKKENFNLKLRIYFLEERMQQEFHGPTEHIYKTNIELKVEVESLKRELQEREQLLIKASKAVESLAEAGGSEIQRVKEDARKKVQQVEDLLTKRILLLEKDVTAAQAELEKAFAGTETEKALRLRLESKLSEMKKMHEGDLAMALVLDEKDRLIEELKLSLKSKEALIQCLKEEKSQMACPDENVSSGELRGLCAAPREEKERETEAAQMEHQKERNSFEERIQALEEDLREKEREIATEKKNSLKRDKAIQGLTMALKSKEKKVEELNSEIEKLSAAFAKAREALQKAQTQEFQGSEDYETALSGKEALSAALRSQNLTKSTENHRLRRSIKKITQELSDLQQERERLEKDLEEAHREKSKGDCTIRDLRNEVEKLRNEVNEREKAMENRYKSLLSESNKKLHNQEQVIKHLTESTNQKDVLLQKFNEKDLEVIQQNCYLMAAEDLELRSEGLITEKCSSQQPPGSKTIFSKEKKQSSDYEELIQVLKKEQDIYTHLVKSLQESDSINNLQAELNKIFALRKQLEQDVLSYQNLRKTLEEQISEIRRREEESFSLYSDQTSYLSICLEENNRFQVEHFSQEELKKKVSDLIQLVKELYTDNQHLKKTIFDLSCMGFQGNGFPDRLASTEQTELLASKEDEDTIKIGEDDEINFLSDQHLQQSNEIMKDLSKGGCKNGYLRHTESKISDCDGAHAPGCLEEGAFINLLAPLFNEKATLLLESRPDLLKVVRELLLGQLFLTEQEVSGEHLDGKTEKTPKQKGELVHFVQTNSFSKPHDELKLSCEAQLVKAGEVPKVGLKDASVQTVATEGDLLRFKHEATREAWEEKPINTALSAEHRPENLHGVPGWQAALLSLPGITNREAKKSRLPILIKPSRSLGNMYRLPATQEVVTQLQSQILELQGELKEFKTCNKQLHQKLILAEAVMEGRPTPDKTLLNAQPPVGAAYQDSPGEQKGIKTTSSVWRDKEMDSDQQRSYEIDSEICPPDDLASLPSCKENPEDVLSPTSVATYLSSKSQPSAKVSVMGTDQSESINTSNETEYLKQKIHDLETELEGYQNFIFQLQKHSQCSEAIITVLCGTEGAQDGLSKPKNGSDGEEMTFSSLHQVRYVKHVKILGPLAPEMIDSRVLENLKQQLEEQEYKLQKEQNLNMQLFSEIHNLQNKFRDLSPPRYDSLVQSQARELSLQRQQIKDGHGICVISRQHMNTMIKAFEELLQASDVDYCVAEGFQEQLNQCAELLEKLEKLFLNGKSVGVEMNTQNELMERIEEDNLTYQHLLPESPEPSASHALSDYETSEKSFFSRDQKQDNETEKTSVMVNSFSQDLLMEHIQEIRTLRKRLEESIKTNEKLRKQLERQGSEFVQGSTSIFASGSELHSSLTSEIHFLRKQNQALNAMLIKGSRDKQKENDKLRESLSRKTVSLEHLQREYASVKEENERLQKEGSEKERHNQQLIQEVRCSGQELSRVQEEVKLRQQLLSQNDKLLQSLRVELKAYEKLDEEHRRLREASGEGWKGQDPFRDLHSLLMEIQALRLQLERSIETSSTLQSRLKEQLARGAEKAQEGALTLAVQAVSIPEVPLQPDKHDGDKYPMESDNSFDLFDSSQAVTPKSVSETPPLSGNDTDSLSCDSGSSATSTPCVSRLVTGHHLWASKNGRHVLGLIEDYEALLKQISQGQRLLAEMDIQTQEAPSSTSQELGTKGPHPAPLSKFVSSVSTAKLTLEEAYRRLKLLWRVSLPEDGQCPLHCEQIGEMKAEVTKLHKKLFEQEKKLQNTMKLLQLSKRQEKVIFDQLVVTHKILRKARGNLELRPGGAHPGTCSPSRPGS

>NP_057103.2

MSAQAQMRAMLDQLMGTSRDGDTTRQRIKFSDDRVCKSHLLNCCPHDVLSGTRMDLGECLKVHDLALRADYEIASKEQDFFFELDAMDHLQSFIADCDRRTEVAKKRLAETQEEISAEVAAKAERVHELNEEIGKLLAKVEQLGAEGNVEESQKVMDEVEKARAKKREAEEVYRNSMPASSFQQQKLRVCEVCSAYLGLHDNDRRLADHFGGKLHLGFIEIREKLEELKRVVAEKQEKRNQERLKRREEREREEREKLRRSRSHSKNPKRSRSREHRRHRSRSMSRERKRRTRSKSREKRHRHRSRSSSRSRSRSHQRSRHSSRDRSRERSKRRSSKERFRDQDLASCDRDRSSRDRSPRDRDRKDKKRSYESANGRSEDRRSSEEREAGEI

532 NP_002613.2 NP_006830.1

>NP_002613.2

MAAPVDLELKKAFTELQAKVIDTQQKVKLADIQIEQLNRTKKHAHLTDTEIMTLVDETNMYEGVGRMFILQSKEAIHSQLLEKQKIAEEKIKELEQKKSYLERSVKEAEDNIREMLMARRAQ

>NP_006830.1

MLRACQLSGVTAAAQSCLCGKFVLRPLRPCRRYSTSGSSGLTTGKIAGAGLLFVGGGIGGTILYAKWDSHFRESVEKTIPYSDKLFEMVLGPAAYNVPLPKKSIQSGPLKISSVSEVMKESKQSASQLQKQKGDTPASATAPTEAAQIISAAGDTLSVPAPAVQPEESLKTDHPEIGEGKPTPALSEEASSSSIRERPPEEVAARLAQQEKQEQVKIESLAKSLEDALRQTASVTLQAIAAQNAAVQAVNAHSNILKAAMDNSEIAGEKKSAQWRTVEGALKERRKAVDEAADALLKAKEELEKMKSVIENAKKKEVAGAKPHITAAEGKLHNMIVDLDNVVKKVQAAQSEAKVVSQYHELVVQARDDFKRELDSITPEVLPGWKGMSVSDLADKLSTDDLNSLIAHAHRRIDQLNRELAEQKATEKQHITLALEKQKLEEKRAFDSAVAKALEHHRSEIQAEQDRKIEEVRDAMENEMRTQLRRQAAAHTDHLRDVLRVQEQELKSEFEQNLSEKLSEQELQFRRLSQEQVDNFTLDINTAYARLRGIEQAVQSHAVAEEEARKAHQLWLSVEALKYSMKTSSAETPTIPLGSAVEAIKANCSDNEFTQALTAAIPPESLTRGVYSEETLRARFYAVQKLARRVAMIDETRNSLYQYFLSYLQSLLLFPPQQLKPPPELCPEDINTFKLLSYASYCIEHGDLELAAKFVNQLKGESRRVAQDWLKEARMTLETKQIVEILTAYASAVGIGTTQVQPE

533 NP_060436.4 NP_004703.1

>NP_060436.4

METLKDKTLQELEELQNDSEAIDQLALESPEVQDLQLEREMALATNRSLAERNLEFQGPLEISRSNLSDRYQELRKLVERCQEQKAKLEKFSSALQPGTLLDLLQVEGMKIEEESEAMAEKFLEGEVPLETFLENFSSMRMLSHLRRVRVEKLQEVVRKPRASQELAGDAPPPRPPPPVRPVPQGTPPVVEEQPQPPLAMPPYPLPYSPSPSLPVGPTAHGALPPAPFPVVSQPSFYSGPLGPTYPAAQLGPRGAAGYSWSPQRSMPPRPGYPGTPMGASGPGYPLRGGRAPSPGYPQQSPYPATGGKPPYPIQPQLPSFPGQPQPSVPLQPPYPPGPAPPYGFPPPPGPAWPGY

>NP_004703.1

MGRGSGTFERLLDKATSQLLLETDWESILQICDLIRQGDTQAKYAVNSIKKKVNDKNPHVALYALEVMESVVKNCGQTVHDEVANKQTMEELKDLLKRQVEVNVRNKILYLIQAWAHAFRNEPKYKVVQDTYQIMKVEGHVFPEFKESDAMFAAERAPDWVDAEECHRCRVQFGVMTRKHHCRACGQIFCGKCSSKYSTIPKFGIEKEVRVCEPCYEQLNRKAEGKATSTTELPPEYLTSPLSQQSQLPPKRDETALQEEEELQLALALSQSEAEEKERLRQKSTYTSYPKAEPMPSASSAPPASSLYSSPVNSSAPLAEDIDPELARYLNRNYWEKKQEEARKSPTPSAPVPLTEPAAQPGEGHAAPTNVVENPLPETDSQPIPPSGGPFSEPQFHNGESEESHEQFLKALQNAVTTFVNRMKSNHMRGRSITNDSAVLSLFQSINGMHPQLLELLNQLDERRLYYEGLQDKLAQIRDARGALSALREEHREKLRRAAEEAERQRQIQLAQKLEIMRQKKQEYLEVQRQLAIQRLQEQEKERQMRLEQQKQTVQMRAQMPAFPLPYAQLQAMPAAGGVLYQPSGPASFPSTFSPAGSVEGSPMHGVYMSQPAPAAGPYPSMPSTAADPSMVSAYMYPAGATGAQAAPQAQAGPTASPAYSSYQPTPTAGYQNVASQAPQSLPAISQPPQSSTMGYMGSQSVSMGYQPYNMQNLMTTLPSQDASLPPQQPYIAGQQPMYQQMAPSGGPPQQQPPVAQQPQAQGPPAQGSEAQLISFD

534 NP_001966.1 NP_055097.1

>NP_001966.1

MSIEKIWAREILDSRGNPTVEVDLYTAKGLFRAAVPSGASTGIYEALELRDGDKQRYLGKGVLKAVDHINSTIAPALISSGLSVVEQEKLDNLMLELDGTENKSKFGANAILGVSLAVCKAGAAERELPLYRHIAQLAGNSDLILPVPAFNVINGGSHAGNKLAMQEFMILPVGAESFRDAMRLGAEVYHTLKGVIKDKYGKDATNVGDEGGFAPNILENSEALELVKEAIDKAGYTEKIVIGMDVAASEFYRDGKYDLDFKSPTDPSRYITGDQLGALYQDFVRDYPVVSIEDPFDQDDWAAWSKFTANVGIQIVGDDLTVTNPKRIERAVEEKACNCLLLKVNQIGSVTEAIQACKLAQENGWGVMVSHRSGETEDTFIADLVVGLCTGQIKTGAPCRSERLAKYNQLMRIEEELGDEARFAGHNFRNPSVL

>NP_055097.1

MKGALGSPVAAAGAAMQESFGCVVANRFHQLLDDESDPFDILREAERRRQQQLQRKRRDEAAAAAGAGPRGGRSPAGASGHRAGAGGRRESQKERKSLPAPVAHRPDSPGGGLQAPGQKRTPRRGEQQGWNDSRGPEGMLERAERRSYREYRPYETERQADFTAEKFPDEKPGDRFDRDRPLRGRGGPRGGMRGRGRGGPGNRVFDAFDQRGKREFERYGGNDKIAVRTEDNMGGCGVRTWGSGKDTSDVEPTAPMEEPTVVEESQGTPEEESPAKVPELEVEEETQVQEMTLDEWKNLQEQTRPKPEFNIRKPESTVPSKAVVIHKSKYRDDMVKDDYEDDSHVFRKPANDITSQLEINFGNLPRPGRGARGGTRGGRGRIRRAENNYPRAEVVMQDVAPNPDDPEDFPALS

535 NP_057272.1 NP_064708.1

>NP_057272.1

MSGGVYGGDEVGALVFDIGSFSVRAGYAGEDCPKADFPTTVGLLAAEEGGGLELEGDKEKKGKIFHIDTNALHVPRDGAEVMSPLKNGMIEDWECFRAILDHTYSKHVKSEPNLHPVLMSEAPWNTRAKREKLTELMFEQYNIPAFFLCKTAVLTAFANGRSTGLVLDSGATHTTAIPVHDGYVLQQGIVKSPLAGDFISMQCRELFQEMAIDIIPPYMIAAKEPVREGAPPNWKKKEKLPQVSKSWHNYMCNEVIQDFQASVLQVSDSPYDEQVAAQMPTVHYEMPNGYNTDYGAERLRIPEGLFDPSNVKGLSGNTMLGVGHVVTTSIGMCDIDIRPGLYGSVIVTGGNTLLQGFTDRLNRELSQKTPPSMRLKLIASNSTMERKFSPWIGGSILASLGTFQQMWISKQEYEEGGKQCVERKCP

>NP_064708.1

MAAAAVSGALGRAGWRLLQLRCLPVARCRQALVPRAFHASAVGLRSSDEQKQQPPNSFSQQHSETQGAEKPDPESSHSPPRYTDQGGEEEEDYESEEQLQHRILTAALEFVPAHGWTAEAIAEGAQSLGLSSAAASMFGKDGSELILHFVTQCNTRLTRVLEEEQKLVQLGQAEKRKTDQFLRDAVETRLRMLIPYIEHWPRALSILMLPHNIPSSLSLLTSMVDDMWHYAGDQSTDFNWYTRRAMLAAIYNTTELVMMQDSSPDFEDTWRFLENRVNDAMNMGHTAKQVKSTGEALVQGLMGAAVTLKNLTGLNQRR

536 NP_008877.1 NP_001091.1

>NP_008877.1

MSSTLSPTDFDSLEIQGQYSDINNRWDLPDSDWDNDSSSARLFERSRIKALADEREAVQKKTFTKWVNSHLARVTCRVGDLYSDLRDGRNLLRLLEVLSGEILPKPTKGRMRIHCLENVDKALQFLKEQKVHLENMGSHDIVDGNHRLTLGLVWTIILRFQIQDISVETEDNKEKKSAKDALLLWCQMKTAGYPNVNVHNFTTSWRDGLAFNAIVHKHRPDLLDFESLKKCNAHYNLQNAFNLAEKELGLTKLLDPEDVNVDQPDEKSIITYVATYYHYFSKMKALAVEGKRIGKVLDHAMEAERLVEKYESLASELLQWIEQTIVTLNDRQLANSLSGVQNQLQSFNSYRTVEKPPKFTEKGNLEVLLFTIQSKLRANNQKVYTPREGRLISDINKAWERLEKAEHERELALRTELIRQEKLEQLAARFDRKAAMRETWLSENQRLVSQDNFGLELAAVEAAVRKHEAIETDIVAYSGRVQAVDAVAAELAAERYHDIKRIAARQHNVARLWDFLRQMVAARRERLLLNLELQKVFQDLLYLMDWMEEMKGRLQSQDLGRHLAGVEDLLQLHELVEADIAVQAERVRAVSASALRFCNPGKEYRPCDPQLVSERVAKLEQSYEALCELAAARRARLEESRRLWRFLWEVGEAEAWVREQQHLLASADTGRDLTGALRLLNKHTALRGEMSGRLGPLKLTLEQGQQLVAEGHPGASQASARAAELQAQWERLEALAEERAQRLAQAASLYQFQADANDMEAWLVDALRLVSSPELGHDEFSTQALARQHRALEEEIRSHRPTLDALREQAAALPPTLSRTPEVQGRVPTLERHYEELQARAGERARALEAALALYTMLSEAGACGLWVEEKEQWLNGLALPERLEDLEVVQQRFETLEPEMNTLAAQITAVNDIAEQLLKANPPGKDRIVNTQEQLNHRWQQFRRLADGKKAALTSALSIQNYHLECTETQAWMREKTKVIESTQGLGNDLAGVLALQRKLAGTERDLEAIAARVGELTREANALAAGHPAQAVAINARLREVQTGWEDLRATMRRREESLGEARRLQDFLRSLDDFQAWLGRTQTAVASEEGPATLPEAEALLAQHAALRGEVERAQSEYSRLRALGEEVTRDQADPQCLFLRQRLEALGTGWEELGRMWESRQGRLAQAHGFQGFLRDARQAEGVLSSQEYVLSHTEMPGTLQAADAAIKKLEDFMSTMDANGERIHGLLEAGRQLVSEGNIHADKIREKADSIERRHKKNQDAAQQFLGRLRDNREQQHFLQDCHELKLWIDEKMLTAQDVSYDEARNLHTKWQKHQAFMAELAANKDWLDKVDKEGRELTLEKPELKALVSEKLRDLHRRWDELETTTQAKARSLFDANRAELFAQSCCALESWLESLQAQLHSDDYGKDLTSVNILLKKQQMLEWEMAVREKEVEAIQAQAKALAQEDQGAGEVERTSRAVEEKFRALCQPMRERCRRLQASREQHQFHRDVEDEILWVTERLPMASSMEHGKDLPSVQLLMKKNQTLQKEIQGHEPRIADLRERQRALGAAAAGPELAELQEMWKRLGHELELRGKRLEDALRAQQFYRDAAEAEAWMGEQELHMMGQEKAKDELSAQAEVKKHQVLEQALADYAQTIHQLAASSQDMIDHEHPESTRISIRQAQVDKLYAGLKELAGERRERLQEHLRLCQLRRELDDLEQWIQEREVVAASHELGQDYEHVTMLRDKFREFSRDTSTIGQERVDSANALANGLIAGGHAARATVAEWKDSLNEAWADLLELLDTRGQVLAAAYELQRFLHGARQALARVQHKQQQLPDGTGRDLNAAEALQRRHCAYEHDIQALSPQVQQVQDDGHRLQKAYAGDKAEEIGRHMQAVAEAWAQLQGSSAARRQLLLDTTDKFRFFKAVRELMLWMDEVNLQMDAQERPRDVSSADLVIKNQQGIKAEIEARADRFSSCIDMGKELLARSHYAAEEISEKLSQLQARRQETAEKWQEKMDWLQLVLEVLVFGRDAGMAEAWLCSQEPLVRSAELGCTVDEVESLIKRHEAFQKSAVAWEERFCALEKLTALEEREKERKRKREEEERRKQPPAPEPTASVPPGDLVGGQTASDTTWDGTQPRPPPSTQAPSVNGVCTDGEPSQPLLGQQRLEHSSFPEGPGPGSGDEANGPRGERQTRTRGPAPSAMPQSRSTESAHAATLPPRGPEPSAQEQMEGMLCRKQEMEAFGKKAANRSWQNVYCVLRRGSLGFYKDAKAASAGVPYHGEVPVSLARAQGSVAFDYRKRKHVFKLGLQDGKEYLFQAKDEAEMSSWLRVVNAAIATASSASGEPEEPVVPSTTRGMTRAMTMPPVSPVGAEGPVVLRSKDGREREREKRFSFFKKNK

>NP_001091.1

MCDEDETTALVCDNGSGLVKAGFAGDDAPRAVFPSIVGRPRHQGVMVGMGQKDSYVGDEAQSKRGILTLKYPIEHGIITNWDDMEKIWHHTFYNELRVAPEEHPTLLTEAPLNPKANREKMTQIMFETFNVPAMYVAIQAVLSLYASGRTTGIVLDSGDGVTHNVPIYEGYALPHAIMRLDLAGRDLTDYLMKILTERGYSFVTTAEREIVRDIKEKLCYVALDFENEMATAASSSSLEKSYELPDGQVITIGNERFRCPETLFQPSFIGMESAGIHETTYNSIMKCDIDIRKDLYANNVMSGGTTMYPGIADRMQKEITALAPSTMKIKIIAPPERKYSVWIGGSILASLSTFQQMWITKQEYDEAGPSIVHRKCF

537 NP_056279.1 NP_001246.2

>NP_056279.1

MLPPAIHFYLLPLACILMKSCLAFKNDATEILYSHVVKPVPAHPSSNSTLNQARNGGRHFSNTGLDRNTRVQVGCRELRSTKYISDGQCTSISPLKELVCAGECLPLPVLPNWIGGGYGTKYWSRRSSQEWRCVNDKTRTQRIQLQCQDGSTRTYKITVVTACKCKRYTRQHNESSHNFESMSPAKPVQHHRERKRASKSSKHSMS

>NP_001246.2

MAQFAFESDLHSLLQLDAPIPNAPPARWQRKAKEAAGPAPSPMRAANRSHSAGRTPGRTPGKSSSKVQTTPSKPGGDRYIPHRSAAQMEVASFLLSKENQPENSQTPTKKEHQKAWALNLNGFDVEEAKILRLSGKPQNAPEGYQNRLKVLYSQKATPGSSRKTCRYIPSLPDRILDAPEIRNDYYLNLVDWSSGNVLAVALDNSVYLWSASSGDILQLLQMEQPGEYISSVAWIKEGNYLAVGTSSAEVQLWDVQQQKRLRNMTSHSARVGSLSWNSYILSSGSRSGHIHHHDVRVAEHHVATLSGHSQEVCGLRWAPDGRHLASGGNDNLVNVWPSAPGEGGWVPLQTFTQHQGAVKAVAWCPWQSNVLATGGGTSDRHIRIWNVCSGACLSAVDAHSQVCSILWSPHYKELISGHGFAQNQLVIWKYPTMAKVAELKGHTSRVLSLTMSPDGATVASAAADETLRLWRCFELDPARRREREKASAAKSSLIHQGIR

538 NP_003795.2 NP_066961.2

>NP_003795.2

MQPDMSLNVIKMKSSDFLESAELDSGGFGKVSLCFHRTQGLMIMKTVYKGPNCIEHNEALLEEAKMMNRLRHSRVVKLLGVIIEEGKYSLVMEYMEKGNLMHVLKAEMSTPLSVKGRIILEIIEGMCYLHGKGVIHKDLKPENILVDNDFHIKIADLGLASFKMWSKLNNEEHNELREVDGTAKKNGGTLYYMAPEHLNDVNAKPTEKSDVYSFAVVLWAIFANKEPYENAICEQQLIMCIKSGNRPDVDDITEYCPREIISLMKLCWEANPEARPTFPGIEEKFRPFYLSQLEESVEEDVKSLKKEYSNENAVVKRMQSLQLDCVAVPSSRSNSATEQPGSLHSSQGLGMGPVEESWFAPSLEHPQEENEPSLQSKLQDEANYHLYGSRMDRQTKQQPRQNVAYNREEERRRRVSHDPFAQQRPYENFQNTEGKGTAYSSAASHGNAVHQPSGLTSQPQVLYQNNGLYSSHGFGTRPLDPGTAGPRVWYRPIPSHMPSLHNIPVPETNYLGNTPTMPFSSLPPTDESIKYTIYNSTGIQIGAYNYMEIGGTSSSLLDSTNTNFKEEPAAKYQAIFDNTTSLTDKHLDPIRENLGKHWKNCARKLGFTQSQIDEIDHDYERDGLKEKVYQMLQKWVMREGIKGATVGKLAQALHQCSRIDLLSSLIYVSQN

>NP_066961.2

MAAASVTPPGSLELLQPGFSKTLLGTKLEAKYLCSACRNVLRRPFQAQCGHRYCSFCLASILSSGPQNCAACVHEGIYEEGISILESSSAFPDNAARREVESLPAVCPSDGCTWKGTLKEYESCHEGRCPLMLTECPACKGLVRLGEKERHLEHECPERSLSCRHCRAPCCGADVKAHHEVCPKFPLTCDGCGKKKIPREKFQDHVKTCGKCRVPCRFHAIGCLETVEGEKQQEHEVQWLREHLAMLLSSVLEAKPLLGDQSHAGSELLQRCESLEKKTATFENIVCVLNREVERVAMTAEACSRQHRLDQDKIEALSSKVQQLERSIGLKDLAMADLEQKVLEMEASTYDGVFIWKISDFARKRQEAVAGRIPAIFSPAFYTSRYGYKMCLRIYLNGDGTGRGTHLSLFFVVMKGPNDALLRWPFNQKVTLMLLDQNNREHVIDAFRPDVTSSSFQRPVNDMNIASGCPLFCPVSKMEAKNSYVRDDAIFIKAIVDLTGL

539 NP_001560.2 NP_612564.1

>NP_001560.2

MAGGPGPGEPAAPGAQHFLYEVPPWVMCRFYKVMDALEPADWCQFAALIVRDQTELRLCERSGQRTASVLWPWINRNARVADLVHILTHLQLLRARDIITAWHPPAPLPSPGTTAPRPSSIPAPAEAEAWSPRKLPSSASTFLSPAFPGSQTHSGPELGLVPSPASLWPPPPSPAPSSTKPGPESSVSLLQGARPFPFCWPLCEISRGTHNFSEELKIGEGGFGCVYRAVMRNTVYAVKRLKENADLEWTAVKQSFLTEVEQLSRFRHPNIVDFAGYCAQNGFYCLVYGFLPNGSLEDRLHCQTQACPPLSWPQRLDILLGTARAIQFLHQDSPSLIHGDIKSSNVLLDERLTPKLGDFGLARFSRFAGSSPSQSSMVARTQTVRGTLAYLPEEYIKTGRLAVDTDTFSFGVVVLETLAGQRAVKTHGARTKYLKDLVEEEAEEAGVALRSTQSTLQAGLAADAWAAPIAMQIYKKHLDPRPGPCPPELGLGLGQLACCCLHRRAKRRPPMTQVYERLEKLQAVVAGVPGHSEAASCIPPSPQENSYVSSTGRAHSGAAPWQPLAAPSGASAQAAEQLQRGPNQPVESDESLGGLSAALRSWHLTPSCPLDPAPLREAGCPQGDTAGESSWGSGPGSRPTAVEGLALGSSASSSSEPPQIIINPARQKMVQKLALYEDGALDSLQLLSSSSLPGLGLEQDRQGPEESDEFQS

>NP_612564.1

MMSASRLAGTLIPAMAFLSCVRPESWEPCVEVVPNITYQCMELNFYKIPDNLPFSTKNLDLSFNPLRHLGSYSFFSFPELQVLDLSRCEIQTIEDGAYQSLSHLSTLILTGNPIQSLALGAFSGLSSLQKLVAVETNLASLENFPIGHLKTLKELNVAHNLIQSFKLPEYFSNLTNLEHLDLSSNKIQSIYCTDLRVLHQMPLLNLSLDLSLNPMNFIQPGAFKEIRLHKLTLRNNFDSLNVMKTCIQGLAGLEVHRLVLGEFRNEGNLEKFDKSALEGLCNLTIEEFRLAYLDYYLDDIIDLFNCLTNVSSFSLVSVTIERVKDFSYNFGWQHLELVNCKFGQFPTLKLKSLKRLTFTSNKGGNAFSEVDLPSLEFLDLSRNGLSFKGCCSQSDFGTTSLKYLDLSFNGVITMSSNFLGLEQLEHLDFQHSNLKQMSEFSVFLSLRNLIYLDISHTHTRVAFNGIFNGLSSLEVLKMAGNSFQENFLPDIFTELRNLTFLDLSQCQLEQLSPTAFNSLSSLQVLNMSHNNFFSLDTFPYKCLNSLQVLDYSLNHIMTSKKQELQHFPSSLAFLNLTQNDFACTCEHQSFLQWIKDQRQLLVEVERMECATPSDKQGMPVLSLNITCQMNKTIIGVSVLSVLVVSVVAVLVYKFYFHLMLLAGCIKYGRGENIYDAFVIYSSQDEDWVRNELVKNLEEGVPPFQLCLHYRDFIPGVAIAANIIHEGFHKSRKVIVVVSQHFIQSRWCIFEYEIAQTWQFLSSRAGIIFIVLQKVEKTLLRQQVELYRLLSRNTYLEWEDSVLGRHIFWRRLRKALLDGKSWNPEGTVGTGCNWQEATSI

540 NP_055843.1 NP_006144.1

>NP_055843.1

MASDSPARSLDEIDLSALRDPAGIFELVELVGNGTYGQVYKGRHVKTGQLAAIKVMDVTGDEEEEIKQEINMLKKYSHHRNIATYYGAFIKKNPPGMDDQLWLVMEFCGAGSVTDLIKNTKGNTLKEEWIAYICREILRGLSHLHQHKVIHRDIKGQNVLLTENAEVKLVDFGVSAQLDRTVGRRNTFIGTPYWMAPEVIACDENPDATYDFKSDLWSLGITAIEMAEGAPPLCDMHPMRALFLIPRNPAPRLKSKKWSKKFQSFIESCLVKNHSQRPATEQLMKHPFIRDQPNERQVRIQLKDHIDRTKKKRGEKDETEYEYSGSEEEEEENDSGEPSSILNLPGESTLRRDFLRLQLANKERSEALRRQQLEQQQRENEEHKRQLLAERQKRIEEQKEQRRRLEEQQRREKELRKQQEREQRRHYEEQMRREEERRRAEHEQEYIRRQLEEEQRQLEILQQQLLHEQALLLEYKRKQLEEQRQAERLQRQLKQERDYLVSLQHQRQEQRPVEKKPLYHYKEGMSPSEKPAWAKEVEERSRLNRQSSPAMPHKVANRISDPNLPPRSESFSISGVQPARTPPMLRPVDPQIPHLVAVKSQGPALTASQSVHEQPTKGLSGFQEALNVTSHRVEMPRQNSDPTSENPPLPTRIEKFDRSSWLRQEEDIPPKVPQRTTSISPALARKNSPGNGSALGPRLGSQPIRASNPDLRRTEPILESPLQRTSSGSSSSSSTPSSQPSSQGGSQPGSQAGSSERTRVRANSKSEGSPVLPHEPAKVKPEESRDITRPSRPASYKKAIDEDLTALAKELRELRIEETNRPMKKVTDYSSSSEESESSEEEEEDGESETHDGTVAVSDIPRLIPTGAPGSNEQYNVGMVGTHGLETSHADSFSGSISREGTLMIRETSGEKKRSGHSDSNGFAGHINLPDLVQQSHSPAGTPTEGLGRVSTHSQEMDSGTEYGMGSSTKASFTPFVDPRVYQTSPTDEDEEDEESSAAALFTSELLRQEQAKLNEARKISVVNVNPTNIRPHSDTPEIRKYKKRFNSEILCAALWGVNLLVGTENGLMLLDRSGQGKVYNLINRRRFQQMDVLEGLNVLVTISGKKNKLRVYYLSWLRNRILHNDPEVEKKQGWITVGDLEGCIHYKVVKYERIKFLVIALKNAVEIYAWAPKPYHKFMAFKSFADLQHKPLLVDLTVEEGQRLKVIFGSHTGFHVIDVDSGNSYDIYIPSHIQGNITPHAIVILPKTDGMEMLVCYEDEGVYVNTYGRITKDVVLQWGEMPTSVAYIHSNQIMGWGEKAIEIRSVETGHLDGVFMHKRAQRLKFLCERNDKVFFASVRSGGSSQVFFMTLNRNSMMNW

>NP_006144.1

MAEEVVVVAKFDYVAQQEQELDIKKNERLWLLDDSKSWWRVRNSMNKTGFVPSNYVERKNSARKASIVKNLKDTLGIGKVKRKPSVPDSASPADDSFVDPGERLYDLNMPAYVKFNYMAEREDELSLIKGTKVIVMEKCSDGWWRGSYNGQVGWFPSNYVTEEGDSPLGDHVGSLSEKLAAVVNNLNTGQVLHVVQALYPFSSSNDEELNFEKGDVMDVIEKPENDPEWWKCRKINGMVGLVPKNYVTVMQNNPLTSGLEPSPPQCDYIRPSLTGKFAGNPWYYGKVTRHQAEMALNERGHEGDFLIRDSESSPNDFSVSLKAQGKNKHFKVQLKETVYCIGQRKFSTMEELVEHYKKAPIFTSEQGEKLYLVKHLS

541 NP_006023.1 NP_777283.1

>NP_006023.1

MSDPEMGWVPEPPTMTLGASRVELRVSCHGLLDRDTLTKPHPCVLLKLYSDEQWVEVERTEVLRSCSSPVFSRVLALEYFFEEKQPLQFHVFDAEDGATSPRNDTFLGSTECTLGQIVSQTKVTKPLLLKNGKTAGKSTITIVAEEVSGTNDYVQLTFRAYKLDNKDLFSKSDPFMEIYKTNEDQSDQLVWRTEVVKNNLNPSWEPFRLSLHSLCSCDVHRPLKFLVYDYDSSGKHDFIGEFTSTFQEMQEGTANPGQEMQWDCINPKYRDKKKNYKSSGTVVLAQCTVEKVHTFLDYIMGGCQISFTVAIDFTASNGDPRSSQSLHCLSPRQPNHYLQALRAVGGICQDYDSDKRFPAFGFGARIPPNFEVSHDFAINFDPENPECEEISGVIASYRRCLPQIQLYGPTNVAPIINRVAEPAQREQSTGQATKYSVLLVLTDGVVSDMAETRTAIVRASRLPMSIIIVGVGNADFSDMRLLDGDDGPLRCPRGVPAARDIVQFVPFRDFKDAAPSALAKCVLAEVPRQVVEYYASQGISPGAPRPCTLATTPSPSP

>NP_777283.1

MNTNDAKEYLARREIPQLFESLLNGLMCSKPEDPVEYLESCLQKVKELGGCDKVKWDTFVSQEKKTLPPLNGGQSRRSFLRNVMPENSNFPYRRYDRLPPIHQFSIESDTDLSETAELIEEYEVFDPTRPRPKIILVIGGPGSGKGTQSLKIAERYGFQYISVGELLRKKIHSTSSNRKWSLIAKIITTGELAPQETTITEIKQKLMQIPDEEGIVIDGFPRDVAQALSFEDQICTPDLVVFLACANQRLKERLLKRAEQQGRPDDNVKATQRRLMNFKQNAAPLVKYFQEKGLIMTFDADRDEDEVFYDISMAVDNKLFPNKEAAAGSSDLDPSMILDTGEIIDTGSDYEDQGDDQLNVFGEDTMGGFMEDLRKCKIIFIIGGPGSGKGTQCEKLVEKYGFTHLSTGELLREELASESERSKLIRDIMERGDLVPSGIVLELLKEAMVASLGDTRGFLIDGYPREVKQGEEFGRRIGDPQLVICMDCSADTMTNRLLQRSRSSLPVDDTTKTIAKRLEAYYRASIPVIAYYETKTQLHKINAEGTPEDVFLQLCTAIDSIF

542 NP_001306.1 NP_004981.2

>NP_001306.1

MSQERPTFYRQELNKTIWEVPERYQNLSPVGSGAYGSVCAAFDTKTGLRVAVKKLSRPFQSIIHAKRTYRELRLLKHMKHENVIGLLDVFTPARSLEEFNDVYLVTHLMGADLNNIVKCQKLTDDHVQFLIYQILRGLKYIHSADIIHRDLKPSNLAVNEDCELKILDFGLARHTDDEMTGYVATRWYRAPEIMLNWMHYNQTVDIWSVGCIMAELLTGRTLFPGTDHINQLQQIMRLTGTPPAYLINRMPSHEARNYIQSLTQMPKMNFANVFIGANPLAVDLLEKMLVLDSDKRITAAQALAHAYFAQYHDPDDEPVADPYDQSFESRDLLIDEWKSLTYDEVISFVPPPLDQEEMES

>NP_004981.2

MRLFVSDGVPGCLPVLAAAGRARGRAEVLISTVGPEDCVVPFLTRPKVPVLQLDSGNYLFSTSAICRYFFLLSGWEQDDLTNQWLEWEATELQPALSAALYYLVVQGKKGEDVLGSVRRALTHIDHSLSRQNCPFLAGETESLADIVLWGALYPLLQDPAYLPEELSALHSWFQTLSTQEPCQRAAETVLKQQGVLALRPYLQKQPQPSPAEGRAVTNEPEEEELATLSEEEIAMAVTAWEKGLESLPPLRPQQNPVLPVAGERNVLITSALPYVNNVPHLGNIIGCVLSADVFARYSRLRQWNTLYLCGTDEYGTATETKALEEGLTPQEICDKYHIIHADIYRWFNISFDIFGRTTTPQQTKITQDIFQQLLKRGFVLQDTVEQLRCEHCARFLADRFVEGVCPFCGYEEARGDQCDKCGKLINAVELKKPQCKVCRSCPVVQSSQHLFLDLPKLEKRLEEWLGRTLPGSDWTPNAQFITRSWLRDGLKPRCITRDLKWGTPVPLEGFEDKVFYVWFDATIGYLSITANYTDQWERWWKNPEQVDLYQFMAKDNVPFHSLVFPCSALGAEDNYTLVSHLIATEYLNYEDGKFSKSRGVGVFGDMAQDTGIPADIWRFYLLYIRPEGQDSAFSWTDLLLKNNSELLNNLGNFINRAGMFVSKFFGGYVPEMVLTPDDQRLLAHVTLELQHYHQLLEKVRIRDALRSILTISRHGNQYIQVNEPWKRIKGSEADRQRAGTVTGLAVNIAALLSVMLQPYMPTVSATIQAQLQLPPPACSILLTNFLCTLPAGHQIGTVSPLFQKLENDQIESLRQRFGGGQAKTSPKPAVVETVTTAKPQQIQALMDEVTKQGNIVRELKAQKADKNEVAAEVAKLLDLKKQLAVAEGKPPEAPKGKKKK

543 NP_060267.2 AAF19526.1

>NP_060267.2

MAAGGSGGRASCPPGVGVGPGTGGSPGPSANAAATPAPGNAAAAAAAAAAAAAAPGPTPPAPPGPGTDAQAAGAERAEEAAGPGAAALQREAAYNWQASKPTVQERFAFLFNNEVLCDVHFLVGKGLSSQRIPAHRFVLAVGSAVFDAMFNGGMATTSTEIELPDVEPAAFLALLKFLYSDEVQIGPETVMTTLYTAKKYAVPALEAHCVEFLKKNLRADNAFMLLTQARLFDEPQLASLCLENIDKNTADAITAEGFTDIDLDTLVAVLERDTLGIREVRLFNAVVRWSEAECQRQQLQVTPENRRKVLGKALGLIRFPLMTIEEFAAGPAQSGILVDREVVSLFLHFTVNPKPRVEFIDRPRCCLRGKECSINRFQQVESRWGYSGTSDRIRFSVNKRIFVVGFGLYGSIHGPTDYQVNIQIIHTDSNTVLGQNDTGFSCDGSASTFRVMFKEPVEVLPNVNYTACATLKGPDSHYGTKGLRKVTHESPTTGAKTCFTFCYAAGNNNGTSVEDGQIPEVIFYT

>AAF19526.1

MGKKHKKHKSDKHLYEEYVEKPLKLVLKVGGNEVTELSTGSSGHDSSLFEDKNDHDKHKDRKRKKGKKGEKQIPGEEKGRKRTRVKEDKKKRDRDQVENEAEKDLQCHAPVRLDLPPEKPLTSSLAKQEEVEQTPLQEALNQLMRQLQRKDPSAFFSFPVTDFIAPGYSMIIKHPMDFSTMKEKIKNNDYQSIEELKDNFKLMCTNAMIYNKPETIYYKAAKKLLHSGMKILSQERIQSLKQSIDFMADLQKTRKQKDGTDTSQSGEDGGCWQREREDSGDAEAHAFKSPSKENKKKDKDMLEDKFKSNNLEREQEQLDRIVKESGGKLTRRLVNSQCEFERRKPDGTTTLGLLHPVDPIVGEPGYCPVRLGMTTGRLQSGVNTLQGFKEDKRNKVTPVLYLNYGPYSSYAPHYDSTFANISKDDSDLIYSTYGEDSDLPSDFSIHEFLATCQDYPYVMADSLLDVLTKGGHSRTLQEMEMSLPEDEGHTRTLDTAKEMEQITEVEPPGRLDSSTQDRLIALKAVTNFGVPVEVFDSEEAEIFQKKLDETTRLLRELQEAQNERLSTRPPPNMICLLGPSYREMHLAEQVTNNLKELAQQVTPGDIVSTYGVRKAMGISIPSPVMENNFVDLTEDTEEPKKTDVAECGPGGS

544 NP_004442.3 NP_149033.2

>NP_004442.3

MSSQVVGIEPLYIKAEPASPDSPKGSSETETEPPVALAPGPAPTRCLPGHKEEEDGEGAGPGEQGGGKLVLSSLPKRLCLVCGDVASGYHYGVASCEACKAFFKRTIQGSIEYSCPASNECEITKRRRKACQACRFTKCLRVGMLKEGVRLDRVRGGRQKYKRRPEVDPLPFPGPFPAGPLAVAGGPRKTAAPVNALVSHLLVVEPEKLYAMPDPAGPDGHLPAVATLCDLFDREIVVTISWAKSIPGFSSLSLSDQMSVLQSVWMEVLVLGVAQRSLPLQDELAFAEDLVLDEEGARAAGLGELGAALLQLVRRLQALRLEREEYVLLKALALANSDSVHIEDAEAVEQLREALHEALLEYEAGRAGPGGGAERRRAGRLLLTLPLLRQTAGKVLAHFYGVKLEGKVPMHKLFLEMLEAMMD

>NP_149033.2

MQKGLLDQDTGLVLLESQVIMSGLIAPETGENLSLEEGIARNLINPQMYQQLRELQDALALISRLTESRGPLSVVEAIEKRIISETVGLKILEAHLATGGFSLSPSENCINLEEAFHQGLISAWLHSVLESYLRTSKNLIDPNTAEKIGLLDLMQRCIVHQESGFKLLPVKQLAGGMVSLKSGRKVSIFRAVQEGLIDRQVTVRLLEAQLFAGGIVDPRTGHRLTVEEAVRHNLIDQDMACAILIRQLQTGGIIDTVTGQRLTIDEAVSNDLVAAKIALVILESLWSFMGLLWPESGEILPITDALEQGIVSTELAHKILSNRQHIKALFLPATTEILSWKKAIESGILDRDLANNLKSICIPDVMPHMQLADSAEQNINPGAAVLPCSKSHPKATASQSENLLFQLMTHSYINVQNGQRLLLLDKELMETLTSRDEYQTSPPKVVEIGHQRQKTPEGLQESANVKISGTFSSGWTVRLPEFQFSSQNKEYPDREDCTTEKGKKTTVETEDSSVENPEQDLFVEQKERNPNIDALKVINKVKLEVQRQLIGTQREDQTAVSVRENASRGHLLTIPPAEAEGVPLVVDKDVFSVETPKKEHQPLRNTSFTCQNEQAHTLETEYIHDETGGSHIKPQSKKLQVQVKKTLGIKLELKSETDGNVHPLDKKEMLKKTFLAKDDHKESQEAQNIAGGSMMMSEKTDEEDSGREIFLSCSHPLELLEEATLNVLSAQLLDGGIFHEQTGQKLLLNEAISRGIVPSHTAVKLMEKLNMFQGFFDSQTCESLTTEEVINEGLMDEKLLHNVLMADKAISGVLDPRTQTLCSVKDAVTVGLLDKETATRILERQVVTGGIIDLKRGKKVSVTLASTLGLVDVADQPELINLEKASKGRDAEKTVRERLISLQMETTGLIDPDSKAPLTVVQSIDRGLLEREEAVRLLTKQVVDGGIIHHISGMRLSVDNAFRHGLIGEDLAEKLKRVENLNIHQIFNPETKENISLPKAIKLDLITSDLKREIQEVQAFTGNFVDLISGQRLTLAEAKKEGLLTNEAVLSPGMMHGIVDPENCRIVPYSELVKKCKIDIESGQRYLEVIPFSDIKDGVSDKVLTLSQAIQLGKVDFASTLKVLEAQANTGGIIDTATGKRLTLASALEEKLVDENMVRIIASHQVLNGGIVDIFSDQRVTLVEAIEKRLISPELANMIQIDSSEFSDHRAQIEKQEGIEVCALQNEFLGKDMLIACNQTAEMSCNKVEESERLFQVENQSAQEKVKVRVSDGEQAKKSREISLKEFGCKDQRKPRMSSDAKEFISIINPHNLKGKSLGQVSLTHPYSECDFKLKEVARNNMGNDTNEEQEKAVTKIEIISHMKQSTSCLDSEEIRENQGEVILEVQETYCETSGKLPSEQVLQQPMNARVKSKREKREVIVEESIRTCKPAFLSEEKLYQETAIRDEHDSHIKSQPREMTSSEKGKEADTEMGFSITFKIEESSSQVVPQGISVKHLDALTLFSSKQANEGKVNNLSLCLTLKPEENLSREIACGAQSEPFPCMTPRPEGLHYQESDGKAQVTGPSQISKTDKSFQGTTRQETNYQDSWVTSKTKETKHQISSSNECKEKSYQEVSFDPARGLKLEEITVSRPDSKEVRYLEFSDRKDLHHQGSKSDDKLCGTLKSEIATQELTGEKFLEMANPNVAGLEAGSIEDIVTQRGSRVLGSFLPEKLFKGVSQKENTGQQNAIISPTVLETSEEKTVSLTVCSAVKTEKTPQEKLRESPGSEQTPFMTAPEGKGNGGVNPEPFRATQNVFTRQLCLEHDEKLVSYLSLLRNIEMRTKQIQPLELNLAELQDLLCQAKVLERELKDLTTLVSQELECVNQIIISQPQEVPAQLLKALEKDAKNLQKSLSSVSDTWNSRLLHFQNAVEIEKTKVLNQHTQLEGRLQDLRAWVGNKNLILNSKGSNSEIDVDSLNLCLQQYEDLKQPMAERKAQLDALAFDIQFFISEHAQDLSPQQNRQMLRLLNELQRSFQDILEQTAAQVDALQGHLQQMEQEALVKTLQKQQNTCHQQLEDLCSWVGQAERALAGHQGRTTQQDLSALQKNQSDLKDLQDDIQNRATSFATVVKDIEGFMEENQTKLSPRELTALREKLHQAKEQYEALQEETRVAQKELEEAVTSALQQETEKSKAAKELAENKKKIDALLDWVTSVGSSGGQLLTNLPGMEQLSGASLEKGALDTTDGYMGVNQAPEKLDKQCEMMKARHQELLSQQQNFILATQSAQAFLDQHGHNLTPEEQQMLQQKLGELKEQYSTSLAQSEAELKQVQTLQDELQKFLQDHKEFESWLERSEKELENMHKGGSSPETLPSLLKRQGSFSEDVISHKGDLRFVTISGQKVLDMENSFKEGKEPSEIGNLVKDKLKDATERYTALHSKCTRLGSHLNMLLGQYHQFQNSADSLQAWMQACEANVEKLLSDTVASDPGVLQEQLATTKQLQEELAEHQVPVEKLQKVARDIMEIEGEPAPDHRHVQETTDSILSHFQSLSYSLAERSSLLQKAIAQSQSVQESLESLLQSIGEVEQNLEGKQVSSLSSGVIQEALATNMKLKQDIARQKSSLEATREMVTRFMETADSTTAAVLQGKLAEVSQRFEQLCLQQQEKESSLKKLLPQAEMFEHLSGKLQQFMENKSRMLASGNQPDQDITHFFQQIQELNLEMEDQQENLDTLEHLVTELSSCGFALDLCQHQDRVQNLRKDFTELQKTVKEREKDASSCQEQLDEFRKLVRTFQKWLKETEGSIPPTETSMSAKELEKQIEHLKSLLDDWASKGTLVEEINCKGTSLENLIMEITAPDSQGKTGSILPSVGSSVGSVNGYHTCKDLTEIQCDMSDVNLKYEKLGGVLHERQESLQAILNRMEEVHKEANSVLQWLESKEEVLKSMDAMSSPTKTETVKAQAESNKAFLAELEQNSPKIQKVKEALAGLLVTYPNSQEAENWKKIQEELNSRWERATEVTVARQRQLEESASHLACFQAAESQLRPWLMEKELMMGVLGPLSIDPNMLNAQKQQVQFMLKEFEARRQQHEQLNEAAQGILTGPGDVSLSTSQVQKELQSINQKWVELTDKLNSRSSQIDQAIVKSTQYQELLQDLSEKVRAVGQRLSVQSAISTQPEAVKQQLEETSEIRSDLEQLDHEVKEAQTLCDELSVLIGEQYLKDELKKRLETVALPLQGLEDLAADRINRLQAALASTQQFQQMFDELRTWLDDKQSQQAKNCPISAKLERLQSQLQENEEFQKSLNQHSGSYEVIVAEGESLLLSVPPGEEKRTLQNQLVELKNHWEELSKKTADRQSRLKDCMQKAQKYQWHVEDLVPWIEDCKAKMSELRVTLDPVQLESSLLRSKAMLNEVEKRRSLLEILNSAADILINSSEADEDGIRDEKAGINQNMDAVTEELQAKTGSLEEMTQRLREFQESFKNIEKKVEGAKHQLEIFDALGSQACSNKNLEKLRAQQEVLQALEPQVDYLRNFTQGLVEDAPDGSDASQLLHQAEVAQQEFLEVKQRVNSGCVMMENKLEGIGQFHCRVREMFSQLADLDDELDGMGAIGRDTDSLQSQIEDVRLFLNKIHVLKLDIEASEAECRHMLEEEGTLDLLGLKRELEALNKQCGKLTERGKARQEQLELTLGRVEDFYRKLKGLNDATTAAEEAEALQWVVGTEVEIINQQLADFKMFQKEQVDPLQMKLQQVNGLGQGLIQSAGKDCDVQGLEHDMEEINARWNTLNKKVAQRIAQLQEALLHCGKFQDALEPLLSWLADTEELIANQKPPSAEYKVVKAQIQEQKLLQRLLDDRKATVDMLQAEGGRIAQSAELADREKITGQLESLESRWTELLSKAAARQKQLEDILVLAKQFHETAEPISDFLSVTEKKLANSEPVGTQTAKIQQQIIRHKALEEDIENHATDVHQAVKIGQSLSSLTSPAEQGVLSEKIDSLQARYSEIQDRCCRKAALLDQALSNARLFGEDEVEVLNWLAEVEDKLSSVFVKDFKQDVLHRQHADHLALNEEIVNRKKNVDQAIKNGQALLKQTTGEEVLLIQEKLDGIKTRYADITVTSSKALRTLEQARQLATKFQSTYEELTGWLREVEEELATSGGQSPTGEQIPQFQQRQKELKKEVMEHRLVLDTVNEVSRALLELVPWRAREGLDKLVSDANEQYKLVSDTIGQRVDEIDAAIQRSQQYEQAADAELAWVAETKRKLMALGPIRLEQDQTTAQLQVQKAFSIDIIRHKDSMDELFSHRSEIFGTCGEEQKTVLQEKTESLIQQYEAISLLNSERYARLERAQVLVNQFWETYEELSPWIEETRALIAQLPSPAIDHEQLRQQQEEMRQLRESIAEHKPHIDKLLKIGPQLKELNPEEGEMVEEKYQKAENMYAQIKEEVRQRALALDEAVSQSTQITEFHDKIEPMLETLENLSSRLRMPPLIPAEVDKIRECISDNKSATVELEKLQPSFEALKRRGEELIGRSQGADKDLAAKEIQDKLDQMVFFWEDIKARAEEREIKFLDVLELAEKFWYDMAALLTTIKDTQDIVHDLESPGIDPSIIKQQVEAAETIKEETDGLHEELEFIRILGADLIFACGETEKPEVRKSIDEMNNAWENLNKTWKERLEKLEDAMQAAVQYQDTLQAMFDWLDNTVIKLCTMPPVGTDLNTVKDQLNEMKEFKVEVYQQQIEMEKLNHQGELMLKKATDETDRDIIREPLTELKHLWENLGEKIAHRQHKLEGALLALGQFQHALEELMSWLTHTEELLDAQRPISGDPKVIEVELAKHHVLKNDVLAHQATVETVNKAGNELLESSAGDDASSLRSRLEAMNQCWESVLQKTEEREQQLQSTLQQAQGFHSEIEDFLLELTRMESQLSASKPTGGLPETAREQLDTHMELYSQLKAKEETYNQLLDKGRLMLLSRDDSGSGSKTEQSVALLEQKWHVVSSKMEERKSKLEEALNLATEFQNSLQEFINWLTLAEQSLNIASPPSLILNTVLSQIEEHKVFANEVNAHRDQIIELDQTGNQLKFLSQKQDVVLIKNLLVSVQSRWEKVVQRSIERGRSLDDARKRAKQFHEAWKKLIDWLEDAESHLDSELEISNDPDKIKLQLSKHKEFQKTLGGKQPVYDTTIRTGRALKEKTLLPEDSQKLDNFLGEVRDKWDTVCGKSVERQHKLEEALLFSGQFMDALQALVDWLYKVEPQLAEDQPVHGDLDLVMNLMDAHKVFQKELGKRTGTVQVLKRSGRELIENSRDDTTWVKGQLQELSTRWDTVCKLSVSKQSRLEQALKQAEVFRDTVHMLLEWLSEAEQTLRFRGALPDDTEALQSLIDTHKEFMKKVEEKRVDVNSAVAMGEVILAVCHPDCITTIKHWITIIRARFEEVLTWAKQHQQRLETALSELVANAELLEELLAWIQWAETTLIQRDQEPIPQNIDRVKALIAEHQTFMEEMTRKQPDVDRVTKTYKRKNIEPTHAPFIEKSRSGGRKSLSQPTPPPMPILSQSEAKNPRINQLSARWQQVWLLALERQRKLNDALDRLEELKEFANFDFDVWRKKYMRWMNHKKSRVMDFFRRIDKDQDGKITRQEFIDGILASKFPTTKLEMTAVADIFDRDGDGYIDYYEFVAALHPNKDAYRPTTDADKIEDEVTRQVAQCKCAKRFQVEQIGENKYRFFLGNQFGDSQQLRLVRILRSTVMVRVGGGWMALDEFLVKNDPCRARGRTNIELREKFILPEGASQGMTPFRSRGRRSKPSSRAASPTRSSSSASQSNHSCTSMPSSPATPASGTKVIPSSGSKLKRPTPTFHSSRTSLAGDTSNSSSPASTGAKTNRADPKKSASRPGSRAGSRAGSRASSRRGSDASDFDLLETQSACSDTSESSAAGGQGNSRRGLNKPSKIPTMSKKTTTASPRTPGPKR

545 NP_004963.1 NP_005205.2

>NP_004963.1

MGMACLTMTEMEGTSTSSIYQNGDISGNANSMKQIDPVLQVYLYHSLGKSEADYLTFPSGEYVAEEICIAASKACGITPVYHNMFALMSETERIWYPPNHVFHIDESTRHNVLYRIRFYFPRWYCSGSNRAYRHGISRGAEAPLLDDFVMSYLFAQWRHDFVHGWIKVPVTHETQEECLGMAVLDMMRIAKENDQTPLAIYNSISYKTFLPKCIRAKIQDYHILTRKRIRYRFRRFIQQFSQCKATARNLKLKYLINLETLQSAFYTEKFEVKEPGSGPSGEEIFATIIITGNGGIQWSRGKHKESETLTEQDLQLYCDFPNIIDVSIKQANQEGSNESRVVTIHKQDGKNLEIELSSLREALSFVSLIDGYYRLTADAHHYLCKEVAPPAVLENIQSNCHGPISMDFAISKLKKAGNQTGLYVLRCSPKDFNKYFLTFAVERENVIEYKHCLITKNENEEYNLSGTKKNFSSLKDLLNCYQMETVRSDNIIFQFTKCCPPKPKDKSNLLVFRTNGVSDVPTSPTLQRPTHMNQMVFHKIRNEDLIFNESLGQGTFTKIFKGVRREVGDYGQLHETEVLLKVLDKAHRNYSESFFEAASMMSKLSHKHLVLNYGVCVCGDENILVQEFVKFGSLDTYLKKNKNCINILWKLEVAKQLAWAMHFLEENTLIHGNVCAKNILLIREEDRKTGNPPFIKLSDPGISITVLPKDILQERIPWVPPECIENPKNLNLATDKWSFGTTLWEICSGGDKPLSALDSQRKLQFYEDRHQLPAPKWAELANLINNCMDYEPDFRPSFRAIIRDLNSLFTPDYELLTENDMLPNMRIGALGFSGAFEDRDPTQFEERHLKFLQQLGKGNFGSVEMCRYDPLQDNTGEVVAVKKLQHSTEEHLRDFEREIEILKSLQHDNIVKYKGVCYSAGRRNLKLIMEYLPYGSLRDYLQKHKERIDHIKLLQYTSQICKGMEYLGTKRYIHRDLATRNILVENENRVKIGDFGLTKVLPQDKEYYKVKEPGESPIFWYAPESLTESKFSVASDVWSFGVVLYELFTYIEKSKSPPAEFMRMIGNDKQGQMIVFHLIELLKNNGRLPRPDGCPDEIYMIMTECWNNNVNQRPSFRDLALRVDQIRDNMAG

>NP_005205.2

MACLGFQRHKAQLNLATRTWPCTLLFFLLFIPVFCKAMHVAQPAVVLASSRGIASFVCEYASPGKATEVRVTVLRQADSQVTEVCAATYMMGNELTFLDDSICTGTSSGNQVNLTIQGLRAMDTGLYICKVELMYPPPYYLGIGNGTQIYVIDPEPCPDSDFLLWILAAVSSGLFFYSFLLTAVSLSKMLKKRSPLTTGVYVKMPPTEPECEKQFQPYFIPIN

546 NP_003461.1 NP_060620.2

>NP_003461.1

MNHQQQQQQQKAGEQQLSEPEDMEMEAGDTDDPPRITQNPVINGNVALSDGHNTAEEDMEDDTSWRSEATFQFTVERFSRLSESVLSPPCFVRNLPWKIMVMPRFYPDRPHQKSVGFFLQCNAESDSTSWSCHAQAVLKIINYRDDEKSFSRRISHLFFHKENDWGFSNFMAWSEVTDPEKGFIDDDKVTFEVFVQADAPHGVAWDSKKHTGYVGLKNQGATCYMNSLLQTLFFTNQLRKAVYMMPTEGDDSSKSVPLALQRVFYELQHSDKPVGTKKLTKSFGWETLDSFMQHDVQELCRVLLDNVENKMKGTCVEGTIPKLFRGKMVSYIQCKEVDYRSDRREDYYDIQLSIKGKKNIFESFVDYVAVEQLDGDNKYDAGEHGLQEAEKGVKFLTLPPVLHLQLMRFMYDPQTDQNIKINDRFEFPEQLPLDEFLQKTDPKDPANYILHAVLVHSGDNHGGHYVVYLNPKGDGKWCKFDDDVVSRCTKEEAIEHNYGGHDDDLSVRHCTNAYMLVYIRESKLSEVLQAVTDHDIPQQLVERLQEEKRIEAQKRKERQEAHLYMQVQIVAEDQFCGHQGNDMYDEEKVKYTVFKVLKNSSLAEFVQSLSQTMGFPQDQIRLWPMQARSNGTKRPAMLDNEADGNKTMIELSDNENPWTIFLETVDPELAASGATLPKFDKDHDVMLFLKMYDPKTRSLNYCGHIYTPISCKIRDLLPVMCDRAGFIQDTSLILYEEVKPNLTERIQDYDVSLDKALDELMDGDIIVFQKDDPENDNSELPTAKEYFRDLYHRVDVIFCDKTIPNDPGFVVTLSNRMNYFQVAKTVAQRLNTDPMLLQFFKSQGYRDGPGNPLRHNYEGTLRDLLQFFKPRQPKKLYYQQLKMKITDFENRRSFKCIWLNSQFREEEITLYPDKHGCVRDLLEECKKAVELGEKASGKLRLLEIVSYKIIGVHQEDELLECLSPATSRTFRIEEIPLDQVDIDKENEMLVTVAHFHKEVFGTFGIPFLLRIHQGEHFREVMKRIQSLLDIQEKEFEKFKFAIVMTGRHQYINEDEYEVNLKDFEPQPGNMSHPRPWLGLDHFNKAPKRSRYTYLEKAIKIHN

>NP_060620.2

MDLHRAAFKMENSSYLPNPLASPALMVLASTAEASRDASIPCQQPRPFGVPVSVDKDVHIPFTNGSYTFASMYHRQGGVPGTFANRDFPPSLLHLHPQFAPPNLDCTPISMLNHSGVGAFRPFASTEDRESYQSAFTPAKRLKNCHDTESPHLRFSDADGKEYDFGTQLPSSSPGSLKVDDTGKKIFAVSGLISDREASSSPEDRNDRCKKKAAALFDSQAPICPICQVLLRPSELQEHMEQELEQLAQLPSSKNSLLKDAMAPGTPKSLLLSASIKREGESPTASPHSSATDDLHHSDRYQTFLRVRANRQTRLNARIGKMKRRKQDEGQREGSCMAEDDAVDIEHENNNRFEEYEWCGQKRIRATTLLEGGFRGSGFIMCSGKENPDSDADLDVDGDDTLEYGKPQYTEADVIPCTGEEPGEAKEREALRGAVLNGGPPSTRITPEFSKWASDEMPSTSNGESSKQEAMQKTCKNSDIEKITEDSAVTTFEALKARVRELERQLSRGDRYKCLICMDSYSMPLTSIQCWHVHCEECWLRTLGAKKLCPQCNTITAPGDLRRIYL

547 NP_005021.2 NP_004194.3

>NP_005021.2

MSAAVTAGKLARAPADPGKAGVPGVAAPGAPAAAPPAKEIPEVLVDPRSRRRYVRGRFLGKGGFAKCFEISDADTKEVFAGKIVPKSLLLKPHQREKMSMEISIHRSLAHQHVVGFHGFFEDNDFVFVVLELCRRRSLLELHKRRKALTEPEARYYLRQIVLGCQYLHRNRVIHRDLKLGNLFLNEDLEVKIGDFGLATKVEYDGERKKTLCGTPNYIAPEVLSKKGHSFEVDVWSIGCIMYTLLVGKPPFETSCLKETYLRIKKNEYSIPKHINPVAASLIQKMLQTDPTARPTINELLNDEFFTSGYIPARLPITCLTIPPRFSIAPSSLDPSNRKPLTVLNKGLENPLPERPREKEEPVVRETGEVVDCHLSDMLQQLHSVNASKPSERGLVRQEEAEDPACIPIFWVSKWVDYSDKYGLGYQLCDNSVGVLFNDSTRLILYNDGDSLQYIERDGTESYLTVSSHPNSLMKKITLLKYFRNYMSEHLLKAGANITPREGDELARLPYLRTWFRTRSAIILHLSNGSVQINFFQDHTKLILCPLMAAVTYIDEKRDFRTYRLSLLEEYGCCKELASRLRYARTMVDKLLSSRSASNRLKAS

>NP_004194.3

MLERPPALAMPMPTEGTPPPLSGTPIPVPAYFRHAEPGFSLKRPRGLSRSLPPPPPAKGSIPISRLFPPRTPGWHQLQPRRVSFRGEASETLQSPGYDPSRPESFFQQSFQRLSRLGHGSYGEVFKVRSKEDGRLYAVKRSMSPFRGPKDRARKLAEVGSHEKVGQHPCCVRLEQAWEEGGILYLQTELCGPSLQQHCEAWGASLPEAQVWGYLRDTLLALAHLHSQGLVHLDVKPANIFLGPRGRCKLGDFGLLVELGTAGAGEVQEGDPRYMAPELLQGSYGTAADVFSLGLTILEVACNMELPHGGEGWQQLRQGYLPPEFTAGLSSELRSVLVMMLEPDPKLRATAEALLALPVLRQPRAWGVLWCMAAEALSRGWALWQALLALLCWLWHGLAHPASWLQPLGPPATPPGSPPCSLLLDSSLSSNWDDDSLGPSLSPEAVLARTVGSTSTPRSRCTPRDALDLSDINSEPPRGSFPSFEPRNLLSLFEDTLDPT

548 NP_002559.2 NP_002085.1

>NP_002559.2

MNPSAPSYPMASLYVGDLHPDVTEAMLYEKFSPAGPILSIRVCRDMITRRSLGYAYVNFQQPADAERALDTMNFDVIKGKPVRIMWSQRDPSLRKSGVGNIFIKNLDKSIDNKALYDTFSAFGNILSCKVVCDENGSKGYGFVHFETQEAAERAIEKMNGMLLNDRKVFVGRFKSRKEREAELGARAKEFTNVYIKNFGEDMDDERLKDLFGKFGPALSVKVMTDESGKSKGFGFVSFERHEDAQKAVDEMNGKELNGKQIYVGRAQKKVERQTELKRKFEQMKQDRITRYQGVNLYVKNLDDGIDDERLRKEFSPFGTITSAKVMMEGGRSKGFGFVCFSSPEEATKAVTEMNGRIVATKPLYVALAQRKEERQAHLTNQYMQRMASVRAVPNPVINPYQPAPPSGYFMAAIPQTQNRAAYYPPSQIAQLRPSPRWTAQGARPHPFQNMPGAIRPAAPRPPFSTMRPASSQVPRVMSTQRVANTSTQTMGPRPAAAAAAATPAVRTVPQYKYAAGVRNPQQHLNAQPQVTMQQPAVHVQGQEPLTASMLASAPPQEQKQMLGERLFPLIQAMHPTLAGKITGMLLEIDNSELLHMLESPESLRSKVDEAVAVLQAHQAKEAAQKAVNSATGVPTV

>NP_002085.1

MELSEPIVENGETEMSPEESWEHKEEISEAEPGGGSLGDGRPPEESAHEMMEEEEEIPKPKSVVAPPGAPKKEHVNVVFIGHVDAGKSTIGGQIMYLTGMVDKRTLEKYEREAKEKNRETWYLSWALDTNQEERDKGKTVEVGRAYFETEKKHFTILDAPGHKSFVPNMIGGASQADLAVLVISARKGEFETGFEKGGQTREHAMLAKTAGVKHLIVLINKMDDPTVNWSNERYEECKEKLVPFLKKVGFNPKKDIHFMPCSGLTGANLKEQSDFCPWYIGLPFIPYLDNLPNFNRSVDGPIRLPIVDKYKDMGTVVLGKLESGSICKGQQLVMMPNKHNVEVLGILSDDVETDTVAPGENLKIRLKGIEEEEILPGFILCDPNNLCHSGRTFDAQIVIIEHKSIICPGYNAVLHIHTCIEEVEITALICLVDKKSGEKSKTRPRFVKQDQVCIARLRTAGTICLETFKDFPQMGRFTLRDEGKTIAIGKVLKLVPEKD

549 NP_006089.1 NP_112197.1

>NP_006089.1

MTEQMTLRGTLKGHNGWVTQIATTPQFPDMILSASRDKTIIMWKLTRDETNYGIPQRALRGHSHFVSDVVISSDGQFALSGSWDGTLRLWDLTTGTTTRRFVGHTKDVLSVAFSSDNRQIVSGSRDKTIKLWNTLGVCKYTVQDESHSEWVSCVRFSPNSSNPIIVSCGWDKLVKVWNLANCKLKTNHIGHTGYLNTVTVSPDGSLCASGGKDGQAMLWDLNEGKHLYTLDGGDIINALCFSPNRYWLCAATGPSIKIWDLEGKIIVDELKQEVISTSSKAEPPQCTSLAWSADGQTLFAGYTDNLVRVWQVTIGTR

>NP_112197.1

MSGGKKKSSFQITSVTTDYEGPGSPGASDPPTPQPPTGPPPRLPNGEPSPDPGGKGTPRNGSPPPGAPSSRFRVVKLPHGLGEPYRRGRWTCVDVYERDLEPHSFGGLLEGIRGASGGAGGRSLDSRLELASLGLGAPTPPSGLSQGPTSWLRPPPTSPGPQARSFTGGLGQLVVPSKAKAEKPPLSASSPQQRPPEPETGESAGTSRAATPLPSLRVEAEAGGSGARTPPLSRRKAVDMRLRMELGAPEEMGQVPPLDSRPSSPALYFTHDASLVHKSPDPFGAVAAQKFSLAHSMLAISGHLDSDDDSGSGSLVGIDNKIEQAMDLVKSHLMFAVREEVEVLKEQIRELAERNAALEQENGLLRALASPEQLAQLPSSGVPRLGPPAPNGPSV

550 NP_003173.1 NP_997647.1

>NP_003173.1

MKILVALAVFFLVSTQLFAEEIGANDDLNYWSDWYDSDQIKEELPEPFEHLLQRIARRPKPQQFFGLMGKRDADSSIEKQVALLKALYGHGQISHKRHKTDSFVGLMGKRALNSVAYERSAMQNYERRR

>NP_997647.1

MLRGPGPGLLLLAVQCLGTAVPSTGASKSKRQAQQMVQPQSPVAVSQSKPGCYDNGKHYQINQQWERTYLGNALVCTCYGGSRGFNCESKPEAEETCFDKYTGNTYRVGDTYERPKDSMIWDCTCIGAGRGRISCTIANRCHEGGQSYKIGDTWRRPHETGGYMLECVCLGNGKGEWTCKPIAEKCFDHAAGTSYVVGETWEKPYQGWMMVDCTCLGEGSGRITCTSRNRCNDQDTRTSYRIGDTWSKKDNRGNLLQCICTGNGRGEWKCERHTSVQTTSSGSGPFTDVRAAVYQPQPHPQPPPYGHCVTDSGVVYSVGMQWLKTQGNKQMLCTCLGNGVSCQETAVTQTYGGNSNGEPCVLPFTYNGRTFYSCTTEGRQDGHLWCSTTSNYEQDQKYSFCTDHTVLVQTRGGNSNGALCHFPFLYNNHNYTDCTSEGRRDNMKWCGTTQNYDADQKFGFCPMAAHEEICTTNEGVMYRIGDQWDKQHDMGHMMRCTCVGNGRGEWTCIAYSQLRDQCIVDDITYNVNDTFHKRHEEGHMLNCTCFGQGRGRWKCDPVDQCQDSETGTFYQIGDSWEKYVHGVRYQCYCYGRGIGEWHCQPLQTYPSSSGPVEVFITETPSQPNSHPIQWNAPQPSHISKYILRWRPKNSVGRWKEATIPGHLNSYTIKGLKPGVVYEGQLISIQQYGHQEVTRFDFTTTSTSTPVTSNTVTGETTPFSPLVATSESVTEITASSFVVSWVSASDTVSGFRVEYELSEEGDEPQYLDLPSTATSVNIPDLLPGRKYIVNVYQISEDGEQSLILSTSQTTAPDAPPDPTVDQVDDTSIVVRWSRPQAPITGYRIVYSPSVEGSSTELNLPETANSVTLSDLQPGVQYNITIYAVEENQESTPVVIQQETTGTPRSDTVPSPRDLQFVEVTDVKVTIMWTPPESAVTGYRVDVIPVNLPGEHGQRLPISRNTFAEVTGLSPGVTYYFKVFAVSHGRESKPLTAQQTTKLDAPTNLQFVNETDSTVLVRWTPPRAQITGYRLTVGLTRRGQPRQYNVGPSVSKYPLRNLQPASEYTVSLVAIKGNQESPKATGVFTTLQPGSSIPPYNTEVTETTIVITWTPAPRIGFKLGVRPSQGGEAPREVTSDSGSIVVSGLTPGVEYVYTIQVLRDGQERDAPIVNKVVTPLSPPTNLHLEANPDTGVLTVSWERSTTPDITGYRITTTPTNGQQGNSLEEVVHADQSSCTFDNLSPGLEYNVSVYTVKDDKESVPISDTIIPEVPQLTDLSFVDITDSSIGLRWTPLNSSTIIGYRITVVAAGEGIPIFEDFVDSSVGYYTVTGLEPGIDYDISVITLINGGESAPTTLTQQTAVPPPTDLRFTNIGPDTMRVTWAPPPSIDLTNFLVRYSPVKNEEDVAELSISPSDNAVVLTNLLPGTEYVVSVSSVYEQHESTPLRGRQKTGLDSPTGIDFSDITANSFTVHWIAPRATITGYRIRHHPEHFSGRPREDRVPHSRNSITLTNLTPGTEYVVSIVALNGREESPLLIGQQSTVSDVPRDLEVVAATPTSLLISWDAPAVTVRYYRITYGETGGNSPVQEFTVPGSKSTATISGLKPGVDYTITVYAVTGRGDSPASSKPISINYRTEIDKPSQMQVTDVQDNSISVKWLPSSSPVTGYRVTTTPKNGPGPTKTKTAGPDQTEMTIEGLQPTVEYVVSVYAQNPSGESQPLVQTAVTNIDRPKGLAFTDVDVDSIKIAWESPQGQVSRYRVTYSSPEDGIHELFPAPDGEEDTAELQGLRPGSEYTVSVVALHDDMESQPLIGTQSTAIPAPTDLKFTQVTPTSLSAQWTPPNVQLTGYRVRVTPKEKTGPMKEINLAPDSSSVVVSGLMVATKYEVSVYALKDTLTSRPAQGVVTTLENVSPPRRARVTDATETTITISWRTKTETITGFQVDAVPANGQTPIQRTIKPDVRSYTITGLQPGTDYKIYLYTLNDNARSSPVVIDASTAIDAPSNLRFLATTPNSLLVSWQPPRARITGYIIKYEKPGSPPREVVPRPRPGVTEATITGLEPGTEYTIYVIALKNNQKSEPLIGRKKTDELPQLVTLPHPNLHGPEILDVPSTVQKTPFVTHPGYDTGNGIQLPGTSGQQPSVGQQMIFEEHGFRRTTPPTTATPIRHRPRPYPPNVGEEIQIGHIPREDVDYHLYPHGPGLNPNASTGQEALSQTTISWAPFQDTSEYIISCHPVGTDEEPLQFRVPGTSTSATLTGLTRGATYNIIVEALKDQQRHKVREEVVTVGNSVNEGLNQPTDDSCFDPYTVSHYAVGDEWERMSESGFKLLCQCLGFGSGHFRCDSSRWCHDNGVNYKIGEKWDRQGENGQMMSCTCLGNGKGEFKCDPHEATCYDDGKTYHVGEQWQKEYLGAICSCTCFGGQRGWRCDNCRRPGGEPSPEGTTGQSYNQYSQRYHQRTNTNVNCPIECFMPLDVQADREDSRE

551 NP_000042.3 NP_006297.2

>NP_000042.3

MSLVLNDLLICCRQLEHDRATERKKEVEKFKRLIRDPETIKHLDRHSDSKQGKYLNWDAVFRFLQKYIQKETECLRIAKPNVSASTQASRQKKMQEISSLVKYFIKCANRRAPRLKCQELLNYIMDTVKDSSNGAIYGADCSNILLKDILSVRKYWCEISQQQWLELFSVYFRLYLKPSQDVHRVLVARIIHAVTKGCCSQTDGLNSKFLDFFSKAIQCARQEKSSSGLNHILAALTIFLKTLAVNFRIRVCELGDEILPTLLYIWTQHRLNDSLKEVIIELFQLQIYIHHPKGAKTQEKGAYESTKWRSILYNLYDLLVNEISHIGSRGKYSSGFRNIAVKENLIELMADICHQVFNEDTRSLEISQSYTTTQRESSDYSVPCKRKKIELGWEVIKDHLQKSQNDFDLVPWLQIATQLISKYPASLPNCELSPLLMILSQLLPQQRHGERTPYVLRCLTEVALCQDKRSNLESSQKSDLLKLWNKIWCITFRGISSEQIQAENFGLLGAIIQGSLVEVDREFWKLFTGSACRPSCPAVCCLTLALTTSIVPGTVKMGIEQNMCEVNRSFSLKESIMKWLLFYQLEGDLENSTEVPPILHSNFPHLVLEKILVSLTMKNCKAAMNFFQSVPECEHHQKDKEELSFSEVEELFLQTTFDKMDFLTIVRECGIEKHQSSIGFSVHQNLKESLDRCLLGLSEQLLNNYSSEITNSETLVRCSRLLVGVLGCYCYMGVIAEEEAYKSELFQKAKSLMQCAGESITLFKNKTNEEFRIGSLRNMMQLCTRCLSNCTKKSPNKIASGFFLRLLTSKLMNDIADICKSLASFIKKPFDRGEVESMEDDTNGNLMEVEDQSSMNLFNDYPDSSVSDANEPGESQSTIGAINPLAEEYLSKQDLLFLDMLKFLCLCVTTAQTNTVSFRAADIRRKLLMLIDSSTLEPTKSLHLHMYLMLLKELPGEEYPLPMEDVLELLKPLSNVCSLYRRDQDVCKTILNHVLHVVKNLGQSNMDSENTRDAQGQFLTVIGAFWHLTKERKYIFSVRMALVNCLKTLLEADPYSKWAILNVMGKDFPVNEVFTQFLADNHHQVRMLAAESINRLFQDTKGDSSRLLKALPLKLQQTAFENAYLKAQEGMREMSHSAENPETLDEIYNRKSVLLTLIAVVLSCSPICEKQALFALCKSVKENGLEPHLVKKVLEKVSETFGYRRLEDFMASHLDYLVLEWLNLQDTEYNLSSFPFILLNYTNIEDFYRSCYKVLIPHLVIRSHFDEVKSIANQIQEDWKSLLTDCFPKILVNILPYFAYEGTRDSGMAQQRETATKVYDMLKSENLLGKQIDHLFISNLPEIVVELLMTLHEPANSSASQSTDLCDFSGDLDPAPNPPHFPSHVIKATFAYISNCHKTKLKSILEILSKSPDSYQKILLAICEQAAETNNVYKKHRILKIYHLFVSLLLKDIKSGLGGAWAFVLRDVIYTLIHYINQRPSCIMDVSLRSFSLCCDLLSQVCQTAVTYCKDALENHLHVIVGTLIPLVYEQVEVQKQVLDLLKYLVIDNKDNENLYITIKLLDPFPDHVVFKDLRITQQKIKYSRGPFSLLEEINHFLSVSVYDALPLTRLEGLKDLRRQLELHKDQMVDIMRASQDNPQDGIMVKLVVNLLQLSKMAINHTGEKEVLEAVGSCLGEVGPIDFSTIAIQHSKDASYTKALKLFEDKELQWTFIMLTYLNNTLVEDCVKVRSAAVTCLKNILATKTGHSFWEIYKMTTDPMLAYLQPFRTSRKKFLEVPRFDKENPFEGLDDINLWIPLSENHDIWIKTLTCAFLDSGGTKCEILQLLKPMCEVKTDFCQTVLPYLIHDILLQDTNESWRNLLSTHVQGFFTSCLRHFSQTSRSTTPANLDSESEHFFRCCLDKKSQRTMLAVVDYMRRQKRPSSGTIFNDAFWLDLNYLEVAKVAQSCAAHFTALLYAEIYADKKSMDDQEKRSLAFEEGSQSTTISSLSEKSKEETGISLQDLLLEIYRSIGEPDSLYGCGGGKMLQPITRLRTYEHEAMWGKALVTYDLETAIPSSTRQAGIIQALQNLGLCHILSVYLKGLDYENKDWCPELEELHYQAAWRNMQWDHCTSVSKEVEGTSYHESLYNALQSLRDREFSTFYESLKYARVKEVEEMCKRSLESVYSLYPTLSRLQAIGELESIGELFSRSVTHRQLSEVYIKWQKHSQLLKDSDFSFQEPIMALRTVILEILMEKEMDNSQRECIKDILTKHLVELSILARTFKNTQLPERAIFQIKQYNSVSCGVSEWQLEEAQVFWAKKEQSLALSILKQMIKKLDASCAANNPSLKLTYTECLRVCGNWLAETCLENPAVIMQTYLEKAVEVAGNYDGESSDELRNGKMKAFLSLARFSDTQYQRIENYMKSSEFENKQALLKRAKEEVGLLREHKIQTNRYTVKVQRELELDELALRALKEDRKRFLCKAVENYINCLLSGEEHDMWVFRLCSLWLENSGVSEVNGMMKRDGMKIPTYKFLPLMYQLAARMGTKMMGGLGFHEVLNNLISRISMDHPHHTLFIILALANANRDEFLTKPEVARRSRITKNVPKQSSQLDEDRTEAANRIICTIRSRRPQMVRSVEALCDAYIILANLDATQWKTQRKGINIPADQPITKLKNLEDVVVPTMEIKVDHTGEYGNLVTIQSFKAEFRLAGGVNLPKIIDCVGSDGKERRQLVKGRDDLRQDAVMQQVFQMCNTLLQRNTETRKRKLTICTYKVVPLSQRSGVLEWCTGTVPIGEFLVNNEDGAHKRYRPNDFSAFQCQKKMMEVQKKSFEEKYEVFMDVCQNFQPVFRYFCMEKFLDPAIWFEKRLAYTRSVATSSIVGYILGLGDRHVQNILINEQSAELVHIDLGVAFEQGKILPTPETVPFRLTRDIVDGMGITGVEGVFRRCCEKTMEVMRNSQETLLTIVEVLLYDPLFDWTMNPLKALYLQQRPEDETELHPTLNADDQECKRNLSDIDQSFNKVAERVLMRLQEKLKGVEEGTVLSVGGQVNLLIQQAIDPKNLSRLFPGWKAWV

>NP_006297.2

MGFLKLIEIENFKSYKGRQIIGPFQRFTAIIGPNGSGKSNLMDAISFVLGEKTSNLRVKTLRDLIHGAPVGKPAANRAFVSMVYSEEGAEDRTFARVIVGGSSEYKINNKVVQLHEYSEELEKLGILIKARNFLVFQGAVESIAMKNPKERTALFEEISRSGELAQEYDKRKKEMVKAEEDTQFNYHRKKNIAAERKEAKQEKEEADRYQRLKDEVVRAQVQLQLFKLYHNEVEIEKLNKELASKNKEIEKDKKRMDKVEDELKEKKKELGKMMREQQQIEKEIKEKDSELNQKRPQYIKAKENTSHKIKKLEAAKKSLQNAQKHYKKRKGDMDELEKEMLSVEKARQEFEERMEEESQSQGRDLTLEENQVKKYHRLKEEASKRAATLAQELEKFNRDQKADQDRLDLEERKKVETEAKIKQKLREIEENQKRIEKLEEYITTSKQSLEEQKKLEGELTEEVEMAKRRIDEINKELNQVMEQLGDARIDRQESSRQQRKAEIMESIKRLYPGSVYGRLIDLCQPTQKKYQIAVTKVLGKNMDAIIVDSEKTGRDCIQYIKEQRGEPETFLPLDYLEVKPTDEKLRELKGAKLVIDVIRYEPPHIKKALQYACGNALVCDNVEDARRIAFGGHQRHKTVALDGTLFQKSGVISGGASDLKAKARRWDEKAVDKLKEKKERLTEELKEQMKAKRKEAELRQVQSQAHGLQMRLKYSQSDLEQTKTRHLALNLQEKSKLESELANFGPRINDIKRIIQSREREMKDLKEKMNQVEDEVFEEFCREIGVRNIREFEEEKVKRQNEIAKKRLEFENQKTRLGIQLDFEKNQLKEDQDKVHMWEQTVKKDENEIEKLKKEEQRHMKIIDETMAQLQDLKNQHLAKKSEVNDKNHEMEEIRKKLGGANKEMTHLQKEVTAIETKLEQKRSDRHNLLQACKMQDIKLPLSKGTMDDISQEEGSSQGEDSVSGSQRISSIYAREALIEIDYGDLCEDLKDAQAEEEIKQEMNTLQQKLNEQQSVLQRIAAPNMKAMEKLESVRDKFQETSDEFEAARKRAKKAKQAFEQIKKERFDRFNACFESVATNIDEIYKALSRNSSAQAFLGPENPEEPYLDGINYNCVAPGKRFRPMDNLSGGEKTVAALALLFAIHSYKPAPFFVLDEIDAALDNTNIGKVANYIKEQSTCNFQAIVISLKEEFYTKAESLIGVYPEQGDCVISKVLTFDLTKYPDANPNPNEQ

552 NP_002476.2 NP_001229.1

>NP_002476.2

MWKLLPAAGPAGGEPYRLLTGVEYVVGRKNCAILIENDQSISRNHAVLTANFSVTNLSQTDEIPVLTLKDNSKYGTFVNEEKMQNGFSRTLKSGDGITFGVFGSKFRIEYEPLVACSSCLDVSGKTALNQAILQLGGFTVNNWTEECTHLVMVSVKVTIKTICALICGRPIVKPEYFTEFLKAVESKKQPPQIESFYPPLDEPSIGSKNVDLSGRQERKQIFKGKTFIFLNAKQHKKLSSAVVFGGGEARLITEENEEEHNFFLAPGTCVVDTGITNSQTLIPDCQKKWIQSIMDMLQRQGLRPIPEAEIGLAVIFMTTKNYCDPQGHPSTGLKTTTPGPSLSQGVSVDEKLMPSAPVNTTTYVADTESEQADTWDLSERPKEIKVSKMEQKFRMLSQDAPTVKESCKTSSNNNSMVSNTLAKMRIPNYQLSPTKLPSINKSKDRASQQQQTNSIRNYFQPSTKKRERDEENQEMSSCKSARIETSCSLLEQTQPATPSLWKNKEQHLSENEPVDTNSDNNLFTDTDLKSIVKNSASKSHAAEKLRSNKKREMDDVAIEDEVLEQLFKDTKPELEIDVKVQKQEEDVNVRKRPRMDIETNDTFSDEAVPESSKISQENEIGKKRELKEDSLWSAKEISNNDKLQDDSEMLPKKLLLTEFRSLVIKNSTSRNPSGINDDYGQLKNFKKFKKVTYPGAGKLPHIIGGSDLIAHHARKNTELEEWLRQEMEVQNQHAKEESLADDLFRYNPYLKRRR

>NP_001229.1

MPRERRERDAKERDTMKEDGGAEFSARSRKRKANVTVFLQDPDEEMAKIDRTARDQCGSQPWDNNAVCADPCSLIPTPDKEDDDRVYPNSTCKPRIIAPSRGSPLPVLSWANREEVWKIMLNKEKTYLRDQHFLEQHPLLQPKMRAILLDWLMEVCEVYKLHRETFYLAQDFFDRYMATQENVVKTLLQLIGISSLFIAAKLEEIYPPKLHQFAYVTDGACSGDEILTMELMIMKALKWRLSPLTIVSWLNVYMQVAYLNDLHEVLLPQYPQQIFIQIAELLDLCVLDVDCLEFPYGILAASALYHFSSSELMQKVSGYQWCDIENCVKWMVPFAMVIRETGSSKLKHFRGVADEDAHNIQTHRDSLDLLDKARAKKAMLSEQNRASPLPSGLLTPPQSGKKQSSGPEMA

553 NP_009107.1 NP_064527.1

>NP_009107.1

MSRFLNVLRSWLVMVSIIAMGNTLQSFRDHTFLYEKLYTGKPNLVNGLQARTFGIWTLLSSVIRCLCAIDIHNKTLYHITLWTFLLALGHFLSELFVYGTAAPTIGVLAPLMVASFSILGMLVGLRYLEVEPVSRQKKRN

>NP_064527.1

MAGASLGARFYRQIKRHPGIIPMIGLICLGMGSAALYLLRLALRSPDVCWDRKNNPEPWNRLSPNDQYKFLAVSTDYKKLKKDRPDF

554 NP_874369.1 NP_002458.2

>NP_874369.1

MAEVVSPVPGAGRREPGEVGRARGPPVADPGVALSPQGEIIEGCRLPVLRRNQDNEDEWPLAEILSVKDISGRKLFYVHYIDFNKRLDEWVTHERLDLKKIQFPKKEAKTPTKNGLPGSRPGSPEREVPASAQASGKTLPIPVQITLRFNLPKEREAIPGGEPDQPLSSSSCLQPNHRSTKRKVEVVSPATPVPSETAPASVFPQNGAARRAVAAQPGRKRKSNCLGTDEDSQDSSDGIPSAPRMTGSLVSDRSHDDIVTRMKNIECIELGRHRLKPWYFSPYPQELTTLPVLYLCEFCLKYGRSLKCLQRHLTKCDLRHPPGNEIYRKGTISFFEIDGRKNKSYSQNLCLLAKCFLDHKTLYYDTDPFLFYVMTEYDCKGFHIVGYFSKEKESTEDYNVACILTLPPYQRRGYGKLLIEFSYELSKVEGKTGTPEKPLSDLGLLSYRSYWSQTILEILMGLKSESGERPQITINEISEITSIKKEDVISTLQYLNLINYYKGQYILTLSEDIVDGHERAMLKRLLRIDSKCLHFTPKDWSKRGKW

>NP_002458.2

MDFFRVVENQQPPATMPLNVSFTNRNYDLDYDSVQPYFYCDEEENFYQQQQQSELQPPAPSEDIWKKFELLPTPPLSPSRRSGLCSPSYVAVTPFSLRGDNDGGGGSFSTADQLEMVTELLGGDMVNQSFICDPDDETFIKNIIIQDCMWSGFSAAAKLVSEKLASYQAARKDSGSPNPARGHSVCSTSSLYLQDLSAAASECIDPSVVFPYPLNDSSSPKSCASQDSSAFSPSSDSLLSSTESSPQGSPEPLVLHEETPPTTSSDSEEEQEDEEEIDVVSVEKRQAPGKRSESGSPSAGGHSKPPHSPLVLKRCHVSTHQHNYAAPPSTRKDYPAAKRVKLDSVRVLRQISNNRKCTSPRSSDTEENVKRRTHNVLERQRRNELKRSFFALRDQIPELENNEKAPKVVILKKATAYILSVQAEEQKLISEEDLLRKRREQLKHKLEQLRNSCA

555 NP_000928.1 NP_009225.1

>NP_000928.1

MHGGGPPSGDSACPLRTIKRVQFGVLSPDELKRMSVTEGGIKYPETTEGGRPKLGGLMDPRQGVIERTGRCQTCAGNMTECPGHFGHIELAKPVFHVGFLVKTMKVLRCVCFFCSKLLVDSNNPKIKDILAKSKGQPKKRLTHVYDLCKGKNICEGGEEMDNKFGVEQPEGDEDLTKEKGHGGCGRYQPRIRRSGLELYAEWKHVNEDSQEKKILLSPERVHEIFKRISDEECFVLGMEPRYARPEWMIVTVLPVPPLSVRPAVVMQGSARNQDDLTHKLADIVKINNQLRRNEQNGAAAHVIAEDVKLLQFHVATMVDNELPGLPRAMQKSGRPLKSLKQRLKGKEGRVRGNLMGKRVDFSARTVITPDPNLSIDQVGVPRSIAANMTFAEIVTPFNIDRLQELVRRGNSQYPGAKYIIRDNGDRIDLRFHPKPSDLHLQTGYKVERHMCDGDIVIFNRQPTLHKMSMMGHRVRILPWSTFRLNLSVTTPYNADFDGDEMNLHLPQSLETRAEIQELAMVPRMIVTPQSNRPVMGIVQDTLTAVRKFTKRDVFLERGEVMNLLMFLSTWDGKVPQPAILKPRPLWTGKQIFSLIIPGHINCIRTHSTHPDDEDSGPYKHISPGDTKVVVENGELIMGILCKKSLGTSAGSLVHISYLEMGHDITRLFYSNIQTVINNWLLIEGHTIGIGDSIADSKTYQDIQNTIKKAKQDVIEVIEKAHNNELEPTPGNTLRQTFENQVNRILNDARDKTGSSAQKSLSEYNNFKSMVVSGAKGSKINISQVIAVVGQQNVEGKRIPFGFKHRTLPHFIKDDYGPESRGFVENSYLAGLTPTEFFFHAMGGREGLIDTAVKTAETGYIQRRLIKSMESVMVKYDATVRNSINQVVQLRYGEDGLAGESVEFQNLATLKPSNKAFEKKFRFDYTNERALRRTLQEDLVKDVLSNAHIQNELEREFERMREDREVLRVIFPTGDSKVVLPCNLLRMIWNAQKIFHINPRLPSDLHPIKVVEGVKELSKKLVIVNGDDPLSRQAQENATLLFNIHLRSTLCSRRMAEEFRLSGEAFDWLLGEIESKFNQAIAHPGEMVGALAAQSLGEPATQMTLNTFHYAGVSAKNVTLGVPRLKELINISKKPKTPSLTVFLLGQSARDAERAKDILCRLEHTTLRKVTANTAIYYDPNPQSTVVAEDQEWVNVYYEMPDFDVARISPWLLRVELDRKHMTDRKLTMEQIAEKINAGFGDDLNCIFNDDNAEKLVLRIRIMNSDENKMQEEEEVVDKMDDDVFLRCIESNMLTDMTLQGIEQISKVYMHLPQTDNKKKIIITEDGEFKALQEWILETDGVSLMRVLSEKDVDPVRTTSNDIVEIFTVLGIEAVRKALERELYHVISFDGSYVNYRHLALLCDTMTCRGHLMAITRHGVNRQDTGPLMKCSFEETVDVLMEAAAHGESDPMKGVSENIMLGQLAPAGTGCFDLLLDAEKCKYGMEIPTNIPGLGAAGPTGMFFGSAPSPMGGISPAMTPWNQGATPAYGAWSPSVGSGMTPGAAGFSPSAASDASGFSPGYSPAWSPTPGSPGSPGPSSPYIPSPGGAMSPSYSPTSPAYEPRSPGGYTPQSPSYSPTSPSYSPTSPSYSPTSPNYSPTSPSYSPTSPSYSPTSPSYSPTSPSYSPTSPSYSPTSPSYSPTSPSYSPTSPSYSPTSPSYSPTSPSYSPTSPSYSPTSPSYSPTSPSYSPTSPSYSPTSPSYSPTSPNYSPTSPNYTPTSPSYSPTSPSYSPTSPNYTPTSPNYSPTSPSYSPTSPSYSPTSPSYSPSSPRYTPQSPTYTPSSPSYSPSSPSYSPTSPKYTPTSPSYSPSSPEYTPTSPKYSPTSPKYSPTSPKYSPTSPTYSPTTPKYSPTSPTYSPTSPVYTPTSPKYSPTSPTYSPTSPKYSPTSPTYSPTSPKGSTYSPTSPGYSPTSPTYSLTSPAISPDDSDEEN

>NP_009225.1

MDLSALRVEEVQNVINAMQKILECPICLELIKEPVSTKCDHIFCKFCMLKLLNQKKGPSQCPLCKNDITKRSLQESTRFSQLVEELLKIICAFQLDTGLEYANSYNFAKKENNSPEHLKDEVSIIQSMGYRNRAKRLLQSEPENPSLQETSLSVQLSNLGTVRTLRTKQRIQPQKTSVYIELGSDSSEDTVNKATYCSVGDQELLQITPQGTRDEISLDSAKKAACEFSETDVTNTEHHQPSNNDLNTTEKRAAERHPEKYQGSSVSNLHVEPCGTNTHASSLQHENSSLLLTKDRMNVEKAEFCNKSKQPGLARSQHNRWAGSKETCNDRRTPSTEKKVDLNADPLCERKEWNKQKLPCSENPRDTEDVPWITLNSSIQKVNEWFSRSDELLGSDDSHDGESESNAKVADVLDVLNEVDEYSGSSEKIDLLASDPHEALICKSERVHSKSVESNIEDKIFGKTYRKKASLPNLSHVTENLIIGAFVTEPQIIQERPLTNKLKRKRRPTSGLHPEDFIKKADLAVQKTPEMINQGTNQTEQNGQVMNITNSGHENKTKGDSIQNEKNPNPIESLEKESAFKTKAEPISSSISNMELELNIHNSKAPKKNRLRRKSSTRHIHALELVVSRNLSPPNCTELQIDSCSSSEEIKKKKYNQMPVRHSRNLQLMEGKEPATGAKKSNKPNEQTSKRHDSDTFPELKLTNAPGSFTKCSNTSELKEFVNPSLPREEKEEKLETVKVSNNAEDPKDLMLSGERVLQTERSVESSSISLVPGTDYGTQESISLLEVSTLGKAKTEPNKCVSQCAAFENPKGLIHGCSKDNRNDTEGFKYPLGHEVNHSRETSIEMEESELDAQYLQNTFKVSKRQSFAPFSNPGNAEEECATFSAHSGSLKKQSPKVTFECEQKEENQGKNESNIKPVQTVNITAGFPVVGQKDKPVDNAKCSIKGGSRFCLSSQFRGNETGLITPNKHGLLQNPYRIPPLFPIKSFVKTKCKKNLLEENFEEHSMSPEREMGNENIPSTVSTISRNNIRENVFKEASSSNINEVGSSTNEVGSSINEIGSSDENIQAELGRNRGPKLNAMLRLGVLQPEVYKQSLPGSNCKHPEIKKQEYEEVVQTVNTDFSPYLISDNLEQPMGSSHASQVCSETPDDLLDDGEIKEDTSFAENDIKESSAVFSKSVQKGELSRSPSPFTHTHLAQGYRRGAKKLESSEENLSSEDEELPCFQHLLFGKVNNIPSQSTRHSTVATECLSKNTEENLLSLKNSLNDCSNQVILAKASQEHHLSEETKCSASLFSSQCSELEDLTANTNTQDPFLIGSSKQMRHQSESQGVGLSDKELVSDDEERGTGLEENNQEEQSMDSNLGEAASGCESETSVSEDCSGLSSQSDILTTQQRDTMQHNLIKLQQEMAELEAVLEQHGSQPSNSYPSIISDSSALEDLRNPEQSTSEKAVLTSQKSSEYPISQNPEGLSADKFEVSADSSTSKNKEPGVERSSPSKCPSLDDRWYMHSCSGSLQNRNYPSQEELIKVVDVEEQQLEESGPHDLTETSYLPRQDLEGTPYLESGISLFSDDPESDPSEDRAPESARVGNIPSSTSALKVPQLKVAESAQSPAAAHTTDTAGYNAMEESVSREKPELTASTERVNKRMSMVVSGLTPEEFMLVYKFARKHHITLTNLITEETTHVVMKTDAEFVCERTLKYFLGIAGGKWVVSYFWVTQSIKERKMLNEHDFEVRGDVVNGRNHQGPKRARESQDRKIFRGLEICCYGPFTNMPTDQLEWMVQLCGASVVKELSSFTLGTGVHPIVVVQPDAWTEDNGFHAIGQMCEAPVVTREWVLDSVALYQCQELDTYLIPQIPHSHY

556 NP_055368.1 NP_055332.2

>NP_055368.1

MLFWHTQPEHYNQHNSGSYLRDVLALPIFKQEEPQLSPENEARLPPLQYVLCAATSPAVKLHEETLTYLNQGQSYEIRLLENRKLGDFQDLNTKYVKSIIRVVFHDRRLQYTEHQQLEGWRWSRPGDRILDIDIPLSVGILDPRASPTQLNAVEFLWDPAKRASAFIQVHCISTEFTPRKHGGEKGVPFRVQIDTFKQNENGEYTEHLHSASCQIKVFKPKGADRKQKTDREKMEKRTAQEKEKYQPSYETTILTECSPWPDVAYQVNSAPSPSYNGSPNSFGLGEGNASPTHPVEALPVGSDHLLPSASIQDAQQWLHRNRFSQFCRLFASFSGADLLKMSRDDLVQICGPADGIRLFNAIKGRNVRPKMTIYVCQELEQNRVPLQQKRDGSGDSNLSVYHAIFLEELTTLELIEKIANLYSISPQHIHRVYRQGPTGIHVVVSNEMVQNFQDESCFVLSTIKAESNDGYHIILKCGL

>NP_055332.2

MAWVLKMDEVIESGLVHDFDASLSGIGQELGAGAYSMSDVLALPIFKQEDSSLPLDGETEHPPFQYVMCAATSPAVKLHDETLTYLNQGQSYEIRMLDNRKMGDMPEINGKLVKSIIRVVFHDRRLQYTEHQQLEGWKWNRPGDRLLDLDIPMSVGIIDTRTNPSQLNAVEFLWDPAKRTSAFIQVHCISTEFTPRKHGGEKGVPFRIQVDAFKQNENGEYTDHLHSASCQIKVFKPKGADRKQKTDREKMEKRTAHEKEKYQPSYDTTILTEMRLEPIIEDAVEHEQKKSSKRTLPADYGDSLAKRGSCSPWPDAPTAYVNNSPSPAPTFTSPQQSTCSVPDSNSSSPNHQGDGASQTSGEQIQPSATIQETQQWLLKNRFSSYTRLFSNFSGADLLKLTKEDLVQICGAADGIRLYNSLKSRSVRPRLTIYVCREQPSSTVLQGQQQAASSASENGSGAPYVYHAIYLEEMIASEVARKLALVFNIPLHQINQVYRQGPTGIHILVSDQMVQNFQDESCFLFSTVKAESSDGIHIILK

557 NP_003395.1 NP_068660.1

>NP_003395.1

MTMDKSELVQKAKLAEQAERYDDMAAAMKAVTEQGHELSNEERNLLSVAYKNVVGARRSSWRVISSIEQKTERNEKKQQMGKEYREKIEAELQDICNDVLELLDKYLIPNATQPESKVFYLKMKGDYFRYLSEVASGDNKQTTVSNSQQAYQEAFEISKKEMQPTHPIRLGLALNFSVFYYEILNSPEKACSLAKTAFDEAIAELDTLNEESYKDSTLIMQLLRDNLTLWTSENQGDEGDAGEGEN

>NP_068660.1

MEVPQPEPAPGSALSPAGVCGGAQRPGHLPGLLLGSHGLLGSPVRAAASSPVTTLTQTMHDLAGLGSRSRLTHLSLSRRASESSLSSESSESSDAGLCMDSPSPMDPHMAEQTFEQAIQAASRIIRNEQFAIRRFQSMPVRLLGHSPVLRNITNSQAPDGRRKSEAGSGAASSSGEDKENVRFWKAGVGALREEEGACWGGSLACEDPPLPSWLQDGFVFKMPWKPTHPSSTHALAEWASRREAFAQRPSSAPDLMCLSPDRKMEVEELSPLALGRFSLTPAEGDTEEDDGFVDILESDLKDDDAVPPGMESLISAPLVKTLEKEEEKDLVMYSKCQRLFRSPSMPCSVIRPILKRLERPQDRDTPVQNKRRRSVTPPEEQQEAEEPKARVLRSKSLCHDEIENLLDSDHRELIGDYSKAFLLQTVDGKHQDLKYISPETMVALLTGKFSNIVDKFVIVDCRYPYEYEGGHIKTAVNLPLERDAESFLLKSPIAPCSLDKRVILIFHCEFSSERGPRMCRFIRERDRAVNDYPSLYYPEMYILKGGYKEFFPQHPNFCEPQDYRPMNHEAFKDELKTFRLKTRSWAGERSRRELCSRLQDQ

558 NP_031396.1 NP_005139.1

>NP_031396.1

METSALKQQEQPAATKIRNLPWVEKYRPQTLNDLISHQDILSTIQKFINEDRLPHLLLYGPPGTGKTSTILACAKQLYKDKEFGSMVLELNASDDRGIDIIRGPILSFASTRTIFKKGFKLVILDEADAMTQDAQNALRRVIEKFTENTRFCLICNYLSKIIPALQSRCTRFRFGPLTPELMVPRLEHVVEEEKVDISEDGMKALVTLSSGDMRRALNILQSTNMAFGKVTEETVYTCTGHPLKSDIANILDWMLNQDFTTAYRNITELKTLKGLALHDILTEIHLFVHRVDFPSSVRIHLLTKMADIEYRLSVGTNEKIQLSSLIAAFQVTRDLIVAEA

>NP_005139.1

MKVKKGGGGAGTATESAPGPSGQSVAPIPQPPAESESGSESEPDAGPGPRPGPLQRKQPIGPEDVLGLQRITGDYLCSPEENIYKIDFVRFKIRDMDSGTVLFEIKKPPVSERLPINRRDLDPNAGRFVRYQFTPAFLRLRQVGATVEFTVGDKPVNNFRMIERHYFRNQLLKSFDFHFGFCIPSSKNTCEHIYDFPPLSEELISEMIRHPYETQSDSFYFVDDRLVMHNKADYSYSGTP

559 NP_003336.1 NP_002959.2

>NP_003336.1

MSGIALSRLAQERKAWRKDHPFGFVAVPTKNPDGTMNLMNWECAIPGKKGTPWEGGLFKLRMLFKDDYPSSPPKCKFEPPLFHPNVYPSGTVCLSILEEDKDWRPAITIKQILLGIQELLNEPNIQDPAQAEAYTIYCQNRVEYEKRVRAQAKKFAPS

>NP_002959.2

MSRRKQAKPQHFQSDPEVASLPRRDGDTEKGQPSRPTKSKDAHVCGRCCAEFFELSDLLLHKKNCTKNQLVLIVNENPASPPETFSPSPPPDNPDEQMNDTVNKTDQVDCSDLSEHNGLDREESMEVEAPVANKSGSGTSSGSHSSTAPSSSSSSSSSSGGGGSSSTGTSAITTSLPQLGDLTTLGNFSVINSNVIIENLQSTKVAVAQFSQEARCGGASGGKLAVPALMEQLLALQQQQIHQLQLIEQIRHQILLLASQNADLPTSSSPSQGTLRTSANPLSTLSSHLSQQLAAAAGLAQSLASQSASISGVKQLPPIQLPQSSSGNTIIPSNSGSSPNMNILAAAVTTPSSEKVASSAGASHVSNPAVSSSSSPAFAISSLLSPASNPLLPQQASANSVFPSPLPNIGTTAEDLNSLSALAQQRKSKPPNVTAFEAKSTSDEAFFKHKCRFCAKVFGSDSALQIHLRSHTGERPFKCNICGNRFSTKGNLKVHFQRHKEKYPHIQMNPYPVPEHLDNIPTSTGIPYGMSIPPEKPVTSWLDTKPVLPTLTTSVGLPLPPTLPSLIPFIKTEEPAPIPISHSATSPPGSVKSDSGGPESATRNLGGLPEEAEGSTLPPSGGKSEESGMVTNSVPTASSSVLSSPAADCGPAGSATTFTNPLLPLMSEQFKAKFPFGGLLDSAQASETSKLQQLVENIDKKATDPNECIICHRVLSCQSALKMHYRTHTGERPFKCKICGRAFTTKGNLKTHYSVHRAMPPLRVQHSCPICQKKFTNAVVLQQHIRMHMGGQIPNTPVPDSYSESMESDTGSFDEKNFDDLDNFSDENMEDCPEGSIPDTPKSADASQDSLSSSPLPLEMSSIAALENQMKMINAGLAEQLQASLKSVENGSIEGDVLTNDSSSVGGDMESQSAGSPAISESTSSMQALSPSNSTQEFHKSPSIEEKPQRAVPSEFANGLSPTPVNGGALDLTSSHAEKIIKEDSLGILFPFRDRGKFKNTACDICGKTFACQSALDIHYRSHTKERPFICTVCNRGFSTKGNLKQHMLTHQMRDLPSQLFEPSSNLGPNQNSAVIPANSLSSLIKTEVNGFVHVSPQDSKDTPTSHVPSGPLSSSATSPVLLPALPRRTPKQHYCNTCGKTFSSSSALQIHERTHTGEKPFACTICGRAFTTKGNLKVHMGTHMWNSTPARRGRRLSVDGPMTFLGGNPVKFPEMFQKDLAARSGSGDPSSFWNQYAAALSNGLAMKANEISVIQNGGIPPIPGSLGSGNSSPVSGLTGNLERLQNSEPNAPLAGLEKMASSENGTNFRFTRFVEDSKEIVTS

560 NP_005243.1 NP_001871.2

>NP_005243.1

MMFSGFNADYEASSSRCSSASPAGDSLSYYHSPADSFSSMGSPVNAQDFCTDLAVSSANFIPTVTAISTSPDLQWLVQPALVSSVAPSQTRAPHPFGVPAPSAGAYSRAGVVKTMTGGRAQSIGRRGKVEQLSPEEEEKRRIRRERNKMAAAKCRNRRRELTDTLQAETDQLEDEKSALQTEIANLLKEKEKLEFILAAHRPACKIPDDLGFPEEMSVASLDLTGGLPEVATPESEEAFTLPLLNDPEPKPSVEPVKSISSMELKTEPFDDFLFPASSRPSGSETARSVPDMDLSGSFYAADWEPLHSGSLGMGPMATELEPLCTPVVTCTPSCTAYTSSFVFTYPEADSFPSCAAAHRKGSSSNEPSSDSLSSPTLLAL

>NP_001871.2

MKFKLHVNSARQYKDLWNMSDDKPFLCTAPGCGQRFTNEDHLAVHKHKHEMTLKFGPARNDSVIVADQTPTPTRFLKNCEEVGLFNELASPFENEFKKASEDDIKKMPLDLSPLATPIIRSKIEEPSVVETTHQDSPLPHPESTTSDEKEVPLAQTAQPTSAIVRPASLQVPNVLLTSSDSSVIIQQAVPSPTSSTVITQAPSSNRPIVPVPGPFPLLLHLPNGQTMPVAIPASITSSNVHVPAAVPLVRPVTMVPSVPGIPGPSSPQPVQSEAKMRLKAALTQQHPPVTNGDTVKGHGSGLVRTQSEESRPQSLQQPATSTTETPASPAHTTPQTQSTSGRRRRAANEDPDEKRRKFLERNRAAASRCRQKRKVWVQSLEKKAEDLSSLNGQLQSEVTLLRNEVAQLKQLLLAHKDCPVTAMQKKSGYHTADKDDSSEDISVPSSPHTEAIQHSSVSTSNGVSSTSKAEAVATSVLTQMADQSTEPALSQIVMAPSSQSQPSGS

561 NP_002860.2 NP_000381.1

>NP_002860.2

MSTGGDFGNPLRKFKLVFLGEQSVGKTSLITRFMYDSFDNTYQATIGIDFLSKTMYLEDRTIRLQLWDTAGQERFRSLIPSYIRDSAAAVVVYDITNVNSFQQTTKWIDDVRTERGSDVIIMLVGNKTDLADKRQVSIEEGERKAKELNVMFIETSAKAGYNVKQLFRRVAAALPGMESTQDRSREDMIDIKLEKPQEQPVSEGGCSC

>NP_000381.1

MADTLPSEFDVIVIGTGLPESIIAAACSRSGRRVLHVDSRSYYGGNWASFSFSGLLSWLKEYQENSDIVSDSPVWQDQILENEEAIALSRKDKTIQHVEVFCYASQDLHEDVEEAGALQKNHALVTSANSTEAADSAFLPTEDESLSTMSCEMLTEQTPSSDPENALEVNGAEVTGEKENHCDDKTCVPSTSAEDMSENVPIAEDTTEQPKKNRITYSQIIKEGRRFNIDLVSKLLYSRGLLIDLLIKSNVSRYAEFKNITRILAFREGRVEQVPCSRADVFNSKQLTMVEKRMLMKFLTFCMEYEKYPDEYKGYEEITFYEYLKTQKLTPNLQYIVMHSIAMTSETASSTIDGLKATKNFLHCLGRYGNTPFLFPLYGQGELPQCFCRMCAVFGGIYCLRHSVQCLVVDKESRKCKAIIDQFGQRIISEHFLVEDSYFPENMCSRVQYRQISRAVLITDRSVLKTDSDQQISILTVPAEEPGTFAVRVIELCSSTMTCMKGTYLVHLTCTSSKTAREDLESVVQKLFVPYTEMEIENEQVEKPRILWALYFNMRDSSDISRSCYNDLPSNVYVCSGPDCGLGNDNAVKQAETLFQEICPNEDFCPPPPNPEDIILDGDSLQPEASESSAIPEANSETFKESTNLGNLEESSE

562 NP_006471.2 NP_002875.1

>NP_006471.2

MPGKPKHLGVPNGRMVLAVSDGELSSTTGPQGQGEGRGSSLSIHSLPSGPSSPFPTEEQPVASWALSFERLLQDPLGLAYFTEFLKKEFSAENVTFWKACERFQQIPASDTQQLAQEARNIYQEFLSSQALSPVNIDRQAWLGEEVLAEPRPDMFRAQQLQIFNLMKFDSYARFVKSPLYRECLLAEAEGRPLREPGSSRLGSPDATRKKPKLKPGKSLPLGVEELGQLPPVEGPGGRPLRKSFRRELGGTANAALRRESQGSLNSSASLDLGFLAFVSSKSESHRKSLGSTEGESESRPGKYCCVYLPDGTASLALARPGLTIRDMLAGICEKRGLSLPDIKVYLVGNEQALVLDQDCTVLADQEVRLENRITFELELTALERVVRISAKPTKRLQEALQPILEKHGLSPLEVVLHRPGEKQPLDLGKLVSSVAAQRLVLDTLPGVKISKARDKSPCRSQGCPPRTQDKATHPPPASPSSLVKVPSSATGKRQTCDIEGLVELLNRVQSSGAHDQRGLLRKEDLVLPEFLQLPAQGPSSEETPPQTKSAAQPIGGSLNSTTDSAL

>NP_002875.1

MREYKLVVLGSGGVGKSALTVQFVQGIFVEKYDPTIEDSYRKQVEVDCQQCMLEILDTAGTEQFTAMRDLYMKNGQGFALVYSITAQSTFNDLQDLREQILRVKDTEDVPMILVGNKCDLEDERVVGKEQGQNLARQWCNCAFLESSAKSKINVNEIFYDLVRQINRKTPVEKKKPKKKSCLLL

563 NP_852664.1 NP_000737.1

>NP_852664.1

MSAEGYQYRALYDYKKEREEDIDLHLGDILTVNKGSLVALGFSDGQEARPEEIGWLNGYNETTGERGDFPGTYVEYIGRKKISPPTPKPRPPRPLPVAPGSSKTEADVEQQALTLPDLAEQFAPPDIAPPLLIKLVEAIEKKGLECSTLYRTQSSSNLAELRQLLDCDTPSVDLEMIDVHVLADAFKRYLLDLPNPVIPAAVYSEMISLAPEVQSSEEYIQLLKKLIRSPSIPHQYWLTLQYLLKHFFKLSQTSSKNLLNARVLSEIFSPMLFRFSAASSDNTENLIKVIEILISTEWNERQPAPALPPKPPKPTTVANNGMNNNMSLQDAEWYWGDISREEVNEKLRDTADGTFLVRDASTKMHGDYTLTLRKGGNNKLIKIFHRDGKYGFSDPLTFSSVVELINHYRNESLAQYNPKLDVKLLYPVSKYQQDQVVKEDNIEAVGKKLHEYNTQFQEKSREYDRLYEEYTRTSQEIQMKRTAIEAFNETIKIFEEQCQTQERYSKEYIEKFKREGNEKEIQRIMHNYDKLKSRISEIIDSRRRLEEDLKKQAAEYREIDKRMNSIKPDLIQLRKTRDQYLMWLTQKGVRQKKLNEWLGNENTEDQYSLVEDDEDLPHHDEKTWNVGSSNRNKAENLLRGKRDGTFLVRESSKQGCYACSVVVDGEVKHCVINKTATGYGFAEPYNLYSSLKELVLHYQHTSLVQHNDSLNVTLAYPVYAQQRR

>NP_000737.1

MRCSPGGVWLALAASLLHVSLQGEFQRKLYKELVKNYNPLERPVANDSQPLTVYFSLSLLQIMDVDEKNQVLTTNIWLQMSWTDHYLQWNVSEYPGVKTVRFPDGQIWKPDILLYNSADERFDATFHTNVLVNSSGHCQYLPPGIFKSSCYIDVRWFPFDVQHCKLKFGSWSYGGWSLDLQMQEADISGYIPNGEWDLVGIPGKRSERFYECCKEPYPDVTFTVTMRRRTLYYGLNLLIPCVLISALALLVFLLPADSGEKISLGITVLLSLTVFMLLVAEIMPATSDSVPLIAQYFASTMIIVGLSVVVTVIVLQYHHHDPDGGKMPKWTRVILLNWCAWFLRMKRPGEDKVRPACQHKQRRCSLASVEMSAVAPPPASNGNLLYIGFRGLDGVHCVPTPDSGVVCGRMACSPTHDEHLLHGGQPPEGDPDLAKILEEVRYIANRFRCQDESEAVCSEWKFAACVVDRLCLMAFSVFTIICTIGILMSAPNFVEAVSKDFA

564 NP_002460.1 NP_002158.2

>NP_002460.1

MMMDLFETGSYFFYLDGENVTLQPLEVAEGSPLYPGSDGTLSPCQDQMPPEAGSDSSGEEHVLAPPGLQPPHCPGQCLIWACKTCKRKSAPTDRRKAATLRERRRLKKINEAFEALKRRTVANPNQRLPKVEILRSAISYIERLQDLLHRLDQQEKMQELGVDPFSYRPKQENLEGADFLRTCSSQWPSVSDHSRGLVITAKEGGASIDSSASSSLRCLSSIVDSISSEERKLPCVEEVVEK

>NP_002158.2

MKALSPVRGCYEAVCCLSERSLAIARGRGKGPAAEEPLSLLDDMNHCYSRLRELVPGVPRGTQLSQVEILQRVIDYILDLQVVLAEPAPGPPDGPHLPIQTAELAPELVISNDKRSFCH

565 NP_001014809.1 NP_115500.1

>NP_001014809.1

MADRRRAWNTEDDLPVYLARPGSAAQTPRQKYGGMFAAVEGAYENKTIDFDAYSVGRRGSARTPRSAGRPDAVGLPGPGGSEDTASDVSEPSGSAVSSPGERDERPPTLRIRRPAPRDLPLGRDNGQSDRLLIKGGRIINDDQSLYADVYLEDGLIKQIGENLIVPGGVKTIEANGRMVIPGGIDVNTYLQKPSQGMTAADDFFQGTRAALVGGTTMIIDHVVPEPGSSLLTSFEKWHEAADTKSCCDYSLHVDITSWYDGVREELEVLVQDKGVNSFQVYMAYKDVYQMSDSQLYEAFTFLKGLGAVILVHAENGDLIAQEQKRILEMGITGPEGHALSRPEELEAEAVFRAITIAGRINCPVYITKVMSKSAADIIALARKKGPLVFGEPIAASLGTDGTHYWSKNWAKAAAFVTSPPLSPDPTTPDYLTSLLACGDLQVTGSGHCPYSTAQKAVGKDNFTLIPEGVNGIEERMTVVWDKAVATGKMDENQFVAVTSTNAAKIFNLYPRKGRIAVGSDADVVIWDPDKLKTITAKSHKSAVEYNIFEGMECHGSPLVVISQGKIVFEDGNINVNKGMGRFIPRKAFPEHLYQRVKIRNKVFGLQGVSRGMYDGPVYEVPATPKYATPAPSAKSSPSKHQPPPIRNLHQSNFSLSGAQIDDNNPRRTGHRIVAPPGGRSNITSLG

>NP_115500.1

MAACRALKAVLVDLSGTLHIEDAAVPGAQEALKRLRGASVIIRFVTNTTKESKQDLLERLRKLEFDISEDEIFTSLTAARSLLERKQVRPMLLVDDRALPDFKGIQTSDPNAVVMGLAPEHFHYQILNQAFRLLLDGAPLIAIHKARYYKRKDGLALGPGPFVTALEYATDTKATVVGKPEKTFFLEALRGTGCEPEEAVMIGDDCRDDVGGAQDVGMLGILVKTGKYRASDEEKINPPPYLTCESFPHAVDHILQHLL

566 AAQ86961.1 NP_000827.1

>AAQ86961.1

MESHSRAGKSRKSAKFRSISRSLMLCNAKTSDDGSSPDEKYPDPFEISLAQGKEGIFHSSVQLADTSEAGPSSVPDLALASEAAQLQAAGNDRGKTCRRIFFMKESSTASSREKPGKLEAQSSNFLFPKACHQRARSNSTSVNPYCTREIDFPMTKKSAAPTDRQPYSLCSNRKSLSQQLDCPAGKAAGTSRPTRSLSTAQLVQPSGGLQASVISNIVLMKGQAKGLGFSIVGGKDSIYGPIGIYVKTIFAGGAAAADGRLQEGDEILELNGGSMAGLTHQDALQKFKQAKKGLLTLTVRTRLTAPPSLCSHLSPPLCRSLSSSTCITKDSSSFALESPSAPISTAKPNYRIMVEVSLQKEAGVGLGIGLCSVPYFQCISGIFVHTLSPGSVAHLDGRLRCGDEIVEISDSPVHCLTLNEVYTILSHCDPGPVPIIVSRHPDPQVSEQQLKEAVAQAVENTKFGKERHQWSLEGVKRLESSWHGRPTLEKEREKNSAPPHRRAQKVMIRSSSDSSYMSGSPGGSPGSGSAEKPSSDVDISTHSPSLPLAREPVVLSIASSRLPQESPPLPESRDSHPPLRLKKSFEILVRKPMSSKPKPPPRKYFKSDSDPQKSLEERENSSCSSGHTPPTCGQEARELLPLLLPQEDTAGRSPSASAGCPGPGIGPQTKSSTEGEPGWRRASPVTQTSPIKHPLLKRQARMDYSFDTTAEDPWVRISDCIKNLFSPIMSENHGHMPLQPNASLNEEEGTQGHPDGTPPKLDTANGTPKVYKSADSSTVKKGPPVAPKPAWFRQSLKGLRNRASDPRGLPDPALSTQPAPASREHLGSHIRASSSSSSIRQRISSFETFGSSQLPDKGAQRLSLQPSSGEAAKPLGKHEEGRFSGLLGRGAAPTLVPQQPEQVLSSGSPAASEARDPGVSESPPPGRQPNQKTLPPGPDPLLRLLSTQAEESQGPVLKMPSQRARSFPLTRSQSCETKLLDEKTSKLYSISSQVSSAVMKSLLCLPSSISCAQTPCIPKEGASPTSSSNEDSAANGSAETSALDTGFSLNLSELREYTEGLTEAKEDDDGDHSSLQSGQSVISLLSSEELKKLIEEVKVLDEATLKQLDGIHVTILHKEEGAGLGFSLAGGADLENKVITVHRVFPNGLASQEGTIQKGNEVLSINGKSLKGTTHHDALAILRQAREPRQAVIVTRKLTPEAMPDLNSSTDSAASASAASDVSVESTAEATVCTVTLEKMSAGLGFSLEGGKGSLHGDKPLTINRIFKGAASEQSETVQPGDEILQLGGTAMQGLTRFEAWNIIKALPDGPVTIVIRRKSLQSKETTAAGDS

>NP_000827.1

MRGAGGPRGPRGPAKMLLLLALACASPFPEEAPGPGGAGGPGGGLGGARPLNVALVFSGPAYAAEAARLGPAVAAAVRSPGLDVRPVALVLNGSDPRSLVLQLCDLLSGLRVHGVVFEDDSRAPAVAPILDFLSAQTSLPIVAVHGGAALVLTPKEKGSTFLQLGSSTEQQLQVIFEVLEEYDWTSFVAVTTRAPGHRAFLSYIEVLTDGSLVGWEHRGALTLDPGAGEAVLSAQLRSVSAQIRLLFCAREEAEPVFRAAEEAGLTGSGYVWFMVGPQLAGGGGSGAPGEPPLLPGGAPLPAGLFAVRSAGWRDDLARRVAAGVAVVARGAQALLRDYGFLPELGHDCRAQNRTHRGESLHRYFMNITWDNRDYSFNEDGFLVNPSLVVISLTRDRTWEVVGSWEQQTLRLKYPLWSRYGRFLQPVDDTQHLTVATLEERPFVIVEPADPISGTCIRDSVPCRSQLNRTHSPPPDAPRPEKRCCKGFCIDILKRLAHTIGFSYDLYLVTNGKHGKKIDGVWNGMIGEVFYQRADMAIGSLTINEERSEIVDFSVPFVETGISVMVARSNGTVSPSAFLEPYSPAVWVMMFVMCLTVVAVTVFIFEYLSPVGYNRSLATGKRPGGSTFTIGKSIWLLWALVFNNSVPVENPRGTTSKIMVLVWAFFAVIFLASYTANLAAFMIQEEYVDTVSGLSDRKFQRPQEQYPPLKFGTVPNGSTEKNIRSNYPDMHSYMVRYNQPRVEEALTQLKAGKLDAFIYDAAVLNYMARKDEGCKLVTIGSGKVFATTGYGIALHKGSRWKRPIDLALLQFLGDDEIEMLERLWLSGICHNDKIEVMSSKLDIDNMAGVFYMLLVAMGLSLLVFAWEHLVYWRLRHCLGPTHRMDFLLAFSRGMYSCCSAEAAPPPAKPPPPPQPLPSPAYPAPGPAPGPAPFVPRERASVDRWRRTKGAGPPGGAGLADGFHRYYGPIEPQGLGLGLGEARAAPRGAAGRPLSPPAAQPPQKPPASYFAIVRDKEPAEPPAGAFPGFPSPPAPPAAAATAVGPPLCRLAFEDESPPAPARWPRSDPESQPLLGPGAGGAGGTGGAGGGAPAAPPPCCAAPPPCPYLDLEPSPSDSEDSESLGGASLGGLDPWWFADFPYPYAERLGPPPGRYWSVDKLGGWRAGSWDYLPPRSGPAAWHCRHCASLELLPPPRHLSCSHDGLDGGWWAPPPPPWAAGPLPRRRARCGCPRSHPHRPRASHRTPAAAAPHHHRHRRAAGGWDLPPPAPTSRSLEDLSSCPRAAPARRLTGPSRHARRCPHAAHWGPPLPTASHRRHRGGDLGTRRGSAHFSSLESEV

567 NP_004865.1 NP_001056.1

>NP_004865.1

MSALRRSGYGPSDGPSYGRYYGPGGGDVPVHPPPPLYPLRPEPPQPPISWRVRGGGPAETTWLGEGGGGDGYYPSGGAWPEPGRAGGSHQEQPPYPSYNSNYWNSTARSRAPYPSTYPVRPELQGQSLNSYTNGAYGPTYPPGPGANTASYSGAYYAPGYTQTSYSTEVPSTYRSSGNSPTPVSRWIYPQQDCQTEAPPLRGQVPGYPPSQNPGMTLPHYPYGDGNRSVPQSGPTVRPQEDAWASPGAYGMGGRYPWPSSAPSAPPGNLYMTESTSPWPSSGSPQSPPSPPVQQPKDSSYPYSQSDQSMNRHNFPCSVHQYESSGTVNNDDSDLLDSQVQYSAEPQLYGNATSDHPNNQDQSSSLPEECVPSDESTPPSIKKIIHVLEKVQYLEQEVEEFVGKKTDKAYWLLEEMLTKELLELDSVETGGQDSVRQARKEAVCKIQAILEKLEKKGL

>NP_001056.1

MGLSTVPDLLLPLVLLELLVGIYPSGVIGLVPHLGDREKRDSVCPQGKYIHPQNNSICCTKCHKGTYLYNDCPGPGQDTDCRECESGSFTASENHLRHCLSCSKCRKEMGQVEISSCTVDRDTVCGCRKNQYRHYWSENLFQCFNCSLCLNGTVHLSCQEKQNTVCTCHAGFFLRENECVSCSNCKKSLECTKLCLPQIENVKGTEDSGTTVLLPLVIFFGLCLLSLLFIGLMYRYQRWKSKLYSIVCGKSTPEKEGELEGTTTKPLAPNPSFSPTPGFTPTLGFSPVPSSTFTSSSTYTPGDCPNFAAPRREVAPPYQGADPILATALASDPIPNPLQKWEDSAHKPQSLDTDDPATLYAVVENVPPLRWKEFVRRLGLSDHEIDRLELQNGRCLREAQYSMLATWRRRTPRREATLELLGRVLRDMDLLGCLEDIEEALCGPAALPPAPSLLR

568 NP_002884.1 NP_003164.1

>NP_002884.1

MASKEMFEDTVEERVINEEYKIWKKNTPFLYDLVMTHALQWPSLTVQWLPEVTKPEGKDYALHWLVLGTHTSDEQNHLVVARVHIPNDDAQFDASHCDSDKGEFGGFGSVTGKIECEIKINHEGEVNRARYMPQNPHIIATKTPSSDVLVFDYTKHPAKPDPSGECNPDLRLRGHQKEGYGLSWNSNLSGHLLSASDDHTVCLWDINAGPKEGKIVDAKAIFTGHSAVVEDVAWHLLHESLFGSVADDQKLMIWDTRSNTTSKPSHLVDAHTAEVNCLSFNPYSEFILATGSADKTVALWDLRNLKLKLHTFESHKDEIFQVHWSPHNETILASSGTDRRLNVWDLSKIGEEQSAEDAEDGPPELLFIHGGHTAKISDFSWNPNEPWVICSVSEDNIMQIWQMAENIYNDEESDVTTSELEGQGS

>NP_003164.1

MAENLKGCSVCCKSSWNQLQDLCRLAKLSCPALGISKRNLYDFEVEYLCDYKKIREQEYYLVKWRGYPDSESTWEPRQNLKCVRILKQFHKDLERELLRRHHRSKTPRHLDPSLANYLVQKAKQRRALRRWEQELNAKRSHLGRITVENEVDLDGPPRAFVYINEYRVGEGITLNQVAVGCECQDCLWAPTGGCCPGASLHKFAYNDQGQVRLRAGLPIYECNSRCRCGYDCPNRVVQKGIRYDLCIFRTDDGRGWGVRTLEKIRKNSFVMEYVGEIITSEEAERRGQIYDRQGATYLFDLDYVEDVYTVDAAYYGNISHFVNHSCDPNLQVYNVFIDNLDERLPRIAFFATRTIRAGEELTFDYNMQVDPVDMESTRMDSNFGLAGLPGSPKKRVRIECKCGTESCRKYLF

569 NP_004521.1 NP_085059.2

>NP_004521.1

MEALMARGALTGPLRALCLLGCLLSHAAAAPSPIIKFPGDVAPKTDKELAVQYLNTFYGCPKESCNLFVLKDTLKKMQKFFGLPQTGDLDQNTIETMRKPRCGNPDVANYNFFPRKPKWDKNQITYRIIGYTPDLDPETVDDAFARAFQVWSDVTPLRFSRIHDGEADIMINFGRWEHGDGYPFDGKDGLLAHAFAPGTGVGGDSHFDDDELWTLGEGQVVRVKYGNADGEYCKFPFLFNGKEYNSCTDTGRSDGFLWCSTTYNFEKDGKYGFCPHEALFTMGGNAEGQPCKFPFRFQGTSYDSCTTEGRTDGYRWCGTTEDYDRDKKYGFCPETAMSTVGGNSEGAPCVFPFTFLGNKYESCTSAGRSDGKMWCATTANYDDDRKWGFCPDQGYSLFLVAAHEFGHAMGLEHSQDPGALMAPIYTYTKNFRLSQDDIKGIQELYGASPDIDLGTGPTPTLGPVTPEICKQDIVFDGIAQIRGEIFFFKDRFIWRTVTPRDKPMGPLLVATFWPELPEKIDAVYEAPQEEKAVFFAGNEYWIYSASTLERGYPKPLTSLGLPPDVQRVDAAFNWSKNKKTYIFAGDKFWRYNEVKKKMDPGFPKLIADAWNAIPDNLDAVVDLQGGGHSYFFKGAYYLKLENQSLKSVKFGSIKSDWLGC

>NP_085059.2

MAPYPCGCHILLLLFCCLAAARANLLNLNWLWFNNEDTSHAATTIPEPQGPLPVQPTADTTTHVTPRNGSTEPATAPGSPEPPSELLEDGQDTPTSAESPDAPEENIAGVGAEILNVAKGIRSFVQLWNDTVPTESLARAETLVLETPVGPLALAGPSSTPQENGTTLWPSRGIPSSPGAHTTEAGTLPAPTPSPPSLGRPWAPLTGPSVPPPSSERISEEVGLLQLLGDPPPQQVTQTDDPDVGLAYVFGPDANSGQVARYHFPSLFFRDFSLLFHIRPATEGPGVLFAITDSAQAMVLLGVKLSGVQDGHQDISLLYTEPGAGQTHTAASFRLPAFVGQWTHLALSVAGGFVALYVDCEEFQRMPLARSSRGLELEPGAGLFVAQAGGADPDKFQGVIAELKVRRDPQVSPMHCLDEEGDDSDGASGDSGSGLGDARELLREETGAALKPRLPAPPPVTTPPLAGGSSTEDSRSEEVEEQTTVASLGAQTLPGSDSVSTWDGSVRTPGGRVKEGGLKGQKGEPGVPGPPGRAGPPGSPCLPGPPGLPCPVSPLGPAGPALQTVPGPQGPPGPPGRDGTPGRDGEPGDPGEDGKPGDTGPQGFPGTPGDVGPKGDKGDPGVGERGPPGPQGPPGPPGPSFRHDKLTFIDMEGSGFGGDLEALRGPRGFPGPPGPPGVPGLPGEPGRFGVNSSDVPGPAGLPGVPGREGPPGFPGLPGPPGPPGREGPPGRTGQKGSLGEAGAPGHKGSKGAPGPAGARGESGLAGAPGPAGPPGPPGPPGPPGPGLPAGFDDMEGSGGPFWSTARSADGPQGPPGLPGLKGDPGVPGLPGAKGEVGADGVPGFPGLPGREGIAGPQGPKGDRGSRGEKGDPGKDGVGQPGLPGPPGPPGPVVYVSEQDGSVLSVPGPEGRPGFAGFPGPAGPKGNLGSKGERGSPGPKGEKGEPGSIFSPDGGALGPAQKGAKGEPGFRGPPGPYGRPGYKGEIGFPGRPGRPGMNGLKGEKGEPGDASLGFGMRGMPGPPGPPGPPGPPGTPVYDSNVFAESSRPGPPGLPGNQGPPGPKGAKGEVGPPGPPGQFPFDFLQLEAEMKGEKGDRGDAGQKGERGEPGGGGFFGSSLPGPPGPPGPRGYPGIPGPKGESIRGQPGPPGPQGPPGIGYEGRQGPPGPPGPPGPPSFPGPHRQTISVPGPPGPPGPPGPPGTMGASSGVRLWATRQAMLGQVHEVPEGWLIFVAEQEELYVRVQNGFRKVQLEARTPLPRGTDNEVAALQPPVVQLHDSNPYPRREHPHPTARPWRADDILASPPRLPEPQPYPGAPHHSSYVHLRPARPTSPPAHSHRDFQPVLHLVALNSPLSGGMRGIRGADFQCFQQARAVGLAGTFRAFLSSRLQDLYSIVRRADRAAVPIVNLKDELLFPSWEALFSGSEGPLKPGARIFSFDGKDVLRHPTWPQKSVWHGSDPNGRRLTESYCETWRTEAPSATGQASSLLGGRLLGQSAASCHHAYIVLCIENSFMTASK

570 NP_057365.3 NP_001424.2

>NP_057365.3

MRKGVLKDPEIADLFYKDDPEELFIGLHEIGHGSFGAVYFATNAHTSEVVAIKKMSYSGKQTHEKWQDILKEVKFLRQLKHPNTIEYKGCYLKEHTAWLVMEYCLGSASDLLEVHKKPLQEVEIAAITHGALHGLAYLHSHALIHRDIKAGNILLTEPGQVKLADFGSASMASPANSFVGTPYWMAPEVILAMDEGQYDGKVDIWSLGITCIELAERKPPLFNMNAMSALYHIAQNDSPTLQSNEWTDSFRRFVDYCLQKIPQERPTSAELLRHDFVRRDRPLRVLIDLIQRTKDAVRELDNLQYRKMKKILFQETRNGPLNESQEDEEDSEHGTSLNREMDSLGSNHSIPSMSVSTGSQSSSVNSMQEVMDESSSELVMMHDDESTINSSSSVVHKKDHVFIRDEAGHGDPRPEPRPTQSVQSQALHYRNRERFATIKSASLVTRQIHEHEQENELREQMSGYKRMRRQHQKQLIALENKLKAEMDEHRLKLQKEVETHANNSSIELEKLAKKQVAIIEKEAKVAAADEKKFQQQILAQQKKDLTTFLESQKKQYKICKEKIKEEMNEDHSTPKKEKQERISKHKENLQHTQAEEEAHLLTQQRLYYDKNCRFFKRKIMIKRHEVEQQNIREELNKKRTQKEMEHAMLIRHDESTRELEYRQLHTLQKLRMDLIRLQHQTELENQLEYNKRRERELHRKHVMELRQQPKNLKAMEMQIKKQFQDTCKVQTKQYKALKNHQLEVTPKNEHKTILKTLKDEQTRKLAILAEQYEQSINEMMASQALRLDEAQEAECQALRLQLQQEMELLNAYQSKIKMQTEAQHERELQKLEQRVSLRRAHLEQKIEEELAALQKERSERIKNLLERQEREIETFDMESLRMGFGNLVTLDFPKEDYR

>NP_001424.2

MPARRLLLLLTLLLPGLGIFGSTSTVTLPETLLFVSTLDGSLHAVSKRTGSIKWTLKEDPVLQVPTHVEEPAFLPDPNDGSLYTLGSKNNEGLTKLPFTIPELVQASPCRSSDGILYMGKKQDIWYVIDLLTGEKQQTLSSAFADSLCPSTSLLYLGRTEYTITMYDTKTRELRWNATYFDYAASLPEDDVDYKMSHFVSNGDGLVVTVDSESGDVLWIQNYASPVVAFYVWQREGLRKVMHINVAVETLRYLTFMSGEVGRITKWKYPFPKETEAKSKLTPTLYVGKYSTSLYASPSMVHEGVAVVPRGSTLPLLEGPQTDGVTIGDKGECVITPSTDVKFDPGLKSKNKLNYLRNYWLLIGHHETPLSASTKMLERFPNNLPKHRENVIPADSEKKSFEEVINLVDQTSENAPTTVSRDVEEKPAHAPARPEAPVDSMLKDMATIILSTFLLIGWVAFIITYPLSMHQQQQLQHQQFQKELEKIQLLQQQQQQLPFHPPGDTAQDGELLDTSGPYSESSGTSSPSTSPRASNHSLCSGSSASKAGSSPSLEQDDGDEETSVVIVGKISFCPKDVLGHGAEGTIVYRGMFDNRDVAVKRILPECFSFADREVQLLRESDEHPNVIRYFCTEKDRQFQYIAIELCAATLQEYVEQKDFAHLGLEPITLLQQTTSGLAHLHSLNIVHRDLKPHNILISMPNAHGKIKAMISDFGLCKKLAVGRHSFSRRSGVPGTEGWIAPEMLSEDCKENPTYTVDIFSAGCVFYYVISEGSHPFGKSLQRQANILLGACSLDCLHPEKHEDVIARELIEKMIAMDPQKRPSAKHVLKHPFFWSLEKQLQFFQDVSDRIEKESLDGPIVKQLERGGRAVVKMDWRENITDPLQTDLRKFRTYKGGSVRDLLRAMRNKKHHYRELPAEVRETLGTLPDDFVCYFTSRFPHLLAHTYRAMELCSHERLFQPYYFHEPPEPQPPVTPDAL

571 NP_000430.3 NP_000406.1

>NP_000430.3

MERRAWSLQCTAFVLFCAWCALNSAKAKRQFVNEWAAEIPGGPEAASAIAEELGYDLLGQIGSLENHYLFKHKNHPRRSRRSAFHITKRLSDDDRVIWAEQQYEKERSKRSALRDSALNLFNDPMWNQQWYLQDTRMTAALPKLDLHVIPVWQKGITGKGVVITVLDDGLEWNHTDIYANYDPEASYDFNDNDHDPFPRYDPTNENKHGTRCAGEIAMQANNHKCGVGVAYNSKVGGIRMLDGIVTDAIEASSIGFNPGHVDIYSASWGPNDDGKTVEGPGRLAQKAFEYGVKQGRQGKGSIFVWASGNGGRQGDNCDCDGYTDSIYTISISSASQQGLSPWYAEKCSSTLATSYSSGDYTDQRITSADLHNDCTETHTGTSASAPLAAGIFALALEANPNLTWRDMQHLVVWTSEYDPLANNPGWKKNGAGLMVNSRFGFGLLNAKALVDLADPRTWRSVPEKKECVVKDNDFEPRALKANGEVIIEIPTRACEGQENAIKSLEHVQFEATIEYSRRGDLHVTLTSAAGTSTVLLAERERDTSPNGFKNWDFMSVHTWGENPIGTWTLRITDMSGRIQNEGRIVNWKLILHGTSSQPEHMKQPRVYTSYNTVQNDRRGVEKMVDPGEEQPTQENPKENTLVSKSPSSSSVGGRRDELEEGAPSQAMLRLLQSAFSKNSPPKQSPKKSPSAKLNIPYENFYEALEKLNKPSQLKDSEDSLYNDYVDVFYNTKPYKHRDDRLLQALVDILNEEN

>NP_000406.1

MGILKLQVFLIVLSVALNHLKATPIESHQVEKRKCNTATCATQRLANFLVHSSNNFGAILSSTNVGSNTYGKRNAVEVLKREPLNYLPL

572 NP_003874.2 NP_644805.1

>NP_003874.2

MAKTVAYFYDPDVGNFHYGAGHPMKPHRLALTHSLVLHYGLYKKMIVFKPYQASQHDMCRFHSEDYIDFLQRVSPTNMQGFTKSLNAFNVGDDCPVFPGLFEFCSRYTGASLQGATQLNNKICDIAINWAGGLHHAKKFEASGFCYVNDIVIGILELLKYHPRVLYIDIDIHHGDGVQEAFYLTDRVMTVSFHKYGNYFFPGTGDMYEVGAESGRYYCLNVPLRDGIDDQSYKHLFQPVINQVVDFYQPTCIVLQCGADSLGCDRLGCFNLSIRGHGECVEYVKSFNIPLLVLGGGGYTVRNVARCWTYETSLLVEEAISEELPYSEYFEYFAPDFTLHPDVSTRIENQNSRQYLDQIRQTIFENLKMLNHAPSVQIHDVPADLLTYDRTDEADAEERGPEENYSRPEAPNEFYDGDHDNDKESDVEI

>NP_644805.1

MAQWNQLQQLDTRYLEQLHQLYSDSFPMELRQFLAPWIESQDWAYAASKESHATLVFHNLLGEIDQQYSRFLQESNVLYQHNLRRIKQFLQSRYLEKPMEIARIVARCLWEESRLLQTAATAAQQGGQANHPTAAVVTEKQQMLEQHLQDVRKRVQDLEQKMKVVENLQDDFDFNYKTLKSQGDMQDLNGNNQSVTRQKMQQLEQMLTALDQMRRSIVSELAGLLSAMEYVQKTLTDEELADWKRRQQIACIGGPPNICLDRLENWITSLAESQLQTRQQIKKLEELQQKVSYKGDPIVQHRPMLEERIVELFRNLMKSAFVVERQPCMPMHPDRPLVIKTGVQFTTKVRLLVKFPELNYQLKIKVCIDKDSGDVAALRGSRKFNILGTNTKVMNMEESNNGSLSAEFKHLTLREQRCGNGGRANCDASLIVTEELHLITFETEVYHQGLKIDLETHSLPVVVISNICQMPNAWASILWYNMLTNNPKNVNFFTKPPIGTWDQVAEVLSWQFSSTTKRGLSIEQLTTLAEKLLGPGVNYSGCQITWAKFCKENMAGKGFSFWVWLDNIIDLVKKYILALWNEGYIMGFISKERERAILSTKPPGTFLLRFSESSKEGGVTFTWVEKDISGKTQIQSVEPYTKQQLNNMSFAEIIMGYKIMDATNILVSPLVYLYPDIPKEEAFGKYCRPESQEHPEADPGSAAPYLKTKFICVTPTTCSNTIDLPMSPRTLDSLMQFGNNGEGAEPSAGGQFESLTFDMELTSECATSPM

573 NP_874369.1 NP_000316.2

>NP_874369.1

MAEVVSPVPGAGRREPGEVGRARGPPVADPGVALSPQGEIIEGCRLPVLRRNQDNEDEWPLAEILSVKDISGRKLFYVHYIDFNKRLDEWVTHERLDLKKIQFPKKEAKTPTKNGLPGSRPGSPEREVPASAQASGKTLPIPVQITLRFNLPKEREAIPGGEPDQPLSSSSCLQPNHRSTKRKVEVVSPATPVPSETAPASVFPQNGAARRAVAAQPGRKRKSNCLGTDEDSQDSSDGIPSAPRMTGSLVSDRSHDDIVTRMKNIECIELGRHRLKPWYFSPYPQELTTLPVLYLCEFCLKYGRSLKCLQRHLTKCDLRHPPGNEIYRKGTISFFEIDGRKNKSYSQNLCLLAKCFLDHKTLYYDTDPFLFYVMTEYDCKGFHIVGYFSKEKESTEDYNVACILTLPPYQRRGYGKLLIEFSYELSKVEGKTGTPEKPLSDLGLLSYRSYWSQTILEILMGLKSESGERPQITINEISEITSIKKEDVISTLQYLNLINYYKGQYILTLSEDIVDGHERAMLKRLLRIDSKCLHFTPKDWSKRGKW

>NP_000316.2

MNCMKGPLHLEHRAAGTKLSAVSSSSCHHPQPLAMASVLAPGQPRSLDSSKHRLEVHTISDTSSPEAAEKDKSQQGKNEDVGAEDPSKKKRQRRQRTHFTSQQLQELEATFQRNRYPDMSTREEIAVWTNLTEARVRVWFKNRRAKWRKRERNQQAELCKNGFGPQFNGLMQPYDDMYPGYSYNNWAAKGLTSASLSTKSFPFFNSMNVNPLSSQSMFSPPNSISSMSMSSSMVPSAVTGVPGSSLNSLNNLNNLSSPSLNSAVPTPACPYAPPTPPYVYRDTCNSSLASLRLKAKQHSSFGYASVQNPASNLSACQYAVDRPV

574 NP_079463.2 NP_065867.1

>NP_079463.2

MLDLEVVPERSLGNEQWEFTLGMPLAQAVAILQKHCRIIKNVQVLYSEQSPLSHDLILNLTQDGIKLMFDAFNQRLKVIEVCDLTKVKLKYCGVHFNSQAIAPTIEQIDQSFGATHPGVYNSAEQLFHLNFRGLSFSFQLDSWTEAPKYEPNFAHGLASLQIPHGATVKRMYIYSGNSLQDTKAPMMPLSCFLGNVYAESVDVLRDGTGPAGLRLRLLAAGCGPGLLADAKMRVFERSVYFGDSCQDVLSMLGSPHKVFYKSEDKMKIHSPSPHKQVPSKCNDYFFNYFTLGVDILFDANTHKVKKFVLHTNYPGHYNFNIYHRCEFKIPLAIKKENADGQTETCTTYSKWDNIQELLGHPVEKPVVLHRSSSPNNTNPFGSTFCFGLQRMIFEVMQNNHIASVTLYGPPRPGSHLRTAELP

>NP_065867.1

MASEAVKVVVRCRPMNQRERELRCQPVVTVDCARAQCCIQNPGAADEPPKQFTFDGAYHVDHVTEQIYNEIAYPLVEGVTEGYNGTIFAYGQTGSGKSFTMQGLPDPPSQRGIIPRAFEHVFESVQCAENTKFLVRASYLEIYNEDVRDLLGADTKQKLELKEHPEKGVYVKGLSMHTVHSVAQCEHIMETGWKNRSVGYTLMNKDSSRSHSIFTISIEMSAVDERGKDHLRAGKLNLVDLAGSERQSKTGATGERLKEATKINLSLSALGNVISALVDGRCKHVPYRDSKLTRLLQDSLGGNTKTLMVACLSPADNNYDETLSTLRYANRAKNIRNKPRINEDPKDALLREYQEEIKKLKAILTQQMSPSSLSALLSRQVPPDPVQVEEKLLPQPVIQHDMEAEKQLIREEYEERLARLKADYKAEQESRARLEEDITAMRNSYDVRLSTLEENLRKETEAVLQVGVLYKAEVMSRAEFASSAEYPPAFQYETVVKPKVFSTTDTLPSDDVSKTQVSSRFAELPKVEPSKSEISLGSSESSSLEETSVSEAFPGPEEPSNVEVSMPTEESRSRYFLDECLGQEAAGHLLGEQNYLPQEEPQEVPLQGLLGLQDPFAEVEAKLARLSSTVARTDAPQADVPKVPVQVPAPTDLLEPSDARPEAEAADDFPPRPEVDLASEVALEVVRTAEPGVWLEAQAPVALVAQPEPLPATAGVKRESVGMEVAVLTDDPLPVVDQQQVLARLQLLEQQVVGGEQAKNKDLKEKHKRRKRYADERRKQLVAALQNSDEDSGDWVLLNVYDSIQEEVRAKSKLLEKMQRKLRAAEVEIKDLQSEFQLEKIDYLATIRRQERDSMLLQQLLEQVQPLIRRDCNYSNLEKILRESCWDEDNGFWKIPHPVITKTSLPVAVSTGPQNKPARKTSAADNGEPNMEEDRYRLMLSRSNSENIASNYFRSKWASQILSTDARKSLTHHNSPPGLSCPLSNNSAIPPTQAPEMPQPRPFRLESLDIPFTKAKRKKSKSNFGSEPL

575 NP_001420.2 NP_002421.3

>NP_001420.2

MAENVVEPGPPSAKRPKLSSPALSASASDGTDFGSLFDLEHDLPDELINSTELGLTNGGDINQLQTSLGMVQDAASKHKQLSELLRSGSSPNLNMGVGGPGQVMASQAQQSSPGLGLINSMVKSPMTQAGLTSPNMGMGTSGPNQGPTQSTGMMNSPVNQPAMGMNTGMNAGMNPGMLAAGNGQGIMPNQVMNGSIGAGRGRQNMQYPNPGMGSAGNLLTEPLQQGSPQMGGQTGLRGPQPLKMGMMNNPNPYGSPYTQNPGQQIGASGLGLQIQTKTVLSNNLSPFAMDKKAVPGGGMPNMGQQPAPQVQQPGLVTPVAQGMGSGAHTADPEKRKLIQQQLVLLLHAHKCQRREQANGEVRQCNLPHCRTMKNVLNHMTHCQSGKSCQVAHCASSRQIISHWKNCTRHDCPVCLPLKNAGDKRNQQPILTGAPVGLGNPSSLGVGQQSAPNLSTVSQIDPSSIERAYAALGLPYQVNQMPTQPQVQAKNQQNQQPGQSPQGMRPMSNMSASPMGVNGGVGVQTPSLLSDSMLHSAINSQNPMMSENASVPSLGPMPTAAQPSTTGIRKQWHEDITQDLRNHLVHKLVQAIFPTPDPAALKDRRMENLVAYARKVEGDMYESANNRAEYYHLLAEKIYKIQKELEEKRRTRLQKQNMLPNAAGMVPVSMNPGPNMGQPQPGMTSNGPLPDPSMIRGSVPNQMMPRITPQSGLNQFGQMSMAQPPIVPRQTPPLQHHGQLAQPGALNPPMGYGPRMQQPSNQGQFLPQTQFPSQGMNVTNIPLAPSSGQAPVSQAQMSSSSCPVNSPIMPPGSQGSHIHCPQLPQPALHQNSPSPVPSRTPTPHHTPPSIGAQQPPATTIPAPVPTPPAMPPGPQSQALHPPPRQTPTPPTTQLPQQVQPSLPAAPSADQPQQQPRSQQSTAASVPTPTAPLLPPQPATPLSQPAVSIEGQVSNPPSTSSTEVNSQAIAEKQPSQEVKMEAKMEVDQPEPADTQPEDISESKVEDCKMESTETEERSTELKTEIKEEEDQPSTSATQSSPAPGQSKKKIFKPEELRQALMPTLEALYRQDPESLPFRQPVDPQLLGIPDYFDIVKSPMDLSTIKRKLDTGQYQEPWQYVDDIWLMFNNAWLYNRKTSRVYKYCSKLSEVFEQEIDPVMQSLGYCCGRKLEFSPQTLCCYGKQLCTIPRDATYYSYQNRYHFCEKCFNEIQGESVSLGDDPSQPQTTINKEQFSKRKNDTLDPELFVECTECGRKMHQICVLHHEIIWPAGFVCDGCLKKSARTRKENKFSAKRLPSTRLGTFLENRVNDFLRRQNHPESGEVTVRVVHASDKTVEVKPGMKARFVDSGEMAESFPYRTKALFAFEEIDGVDLCFFGMHVQEYGSDCPPPNQRRVYISYLDSVHFFRPKCLRTAVYHEILIGYLEYVKKLGYTTGHIWACPPSEGDDYIFHCHPPDQKIPKPKRLQEWYKKMLDKAVSERIVHDYKDIFKQATEDRLTSAKELPYFEGDFWPNVLEESIKELEQEEEERKREENTSNESTDVTKGDSKNAKKKNNKKTSKNKSSLSRGNKKKPGMPNVSNDLSQKLYATMEKHKEVFFVIRLIAGPAANSLPPIVDPDPLIPCDLMDGRDAFLTLARDKHLEFSSLRRAQWSTMCMLVELHTQSQDRFVYTCNECKHHVETRWHCTVCEDYDLCITCYNTKNHDHKMEKLGLGLDDESNNQQAAATQSPGDSRRLSIQRCIQSLVHACQCRNANCSLPSCQKMKRVVQHTKGCKRKTNGGCPICKQLIALCCYHAKHCQENKCPVPFCLNIKQKLRQQQLQHRLQQAQMLRRRMASMQRTGVVGQQQGLPSPTPATPTTPTGQQPTTPQTPQPTSQPQPTPPNSMPPYLPRTQAAGPVSQGKAAGQVTPPTPPQTAQPPLPGPPPAAVEMAMQIQRAAETQRQMAHVQIFQRPIQHQMPPMTPMAPMGMNPPPMTRGPSGHLEPGMGPTGMQQQPPWSQGGLPQPQQLQSGMPRPAMMSVAQHGQPLNMAPQPGLGQVGISPLKPGTVSQQALQNLLRTLRSPSSPLQQQQVLSILHANPQLLAAFIKQRAAKYANSNPQPIPGQPGMPQGQPGLQPPTMPGQQGVHSNPAMQNMNPMQAGVQRAGLPQQQPQQQLQPPMGGMSPQAQQMNMNHNTMPSQFRDILRRQQMMQQQQQQGAGPGIGPGMANHNQFQQPQGVGYPPQQQQRMQHHMQQMQQGNMGQIGQLPQALGAEAGASLQAYQQRLLQQQMGSPVQPNPMSPQQHMLPNQAQSPHLQGQQIPNSLSNQVRSPQPVPSPRPQSQPPHSSPSPRMQPQPSPHHVSPQTSSPHPGLVAAQANPMEQGHFASPDQNSMLSQLASNPGMANLHGASATDLGLSTDNSDLNSNLSQSTLDIH

>NP_002421.3

MFGLDQFEPQVNSRNAGQGERNFNETGLSMNTHFKAPAFHTGGPPGPVDPAMSALGEPPILGMNMEPYGFHARGHSELHAGGLQAQPVHGFFGGQQPHHGHPGSHHPHQHHPHFGGNFGGPDPGASCLHGGRLLGYGGAAGGLGSQPPFAEGYEHMAESQGPESFGPQRPGNLPDFHSSGASSHAVPAPCLPLDQSPNRAASFHGLPSSSGSDSHSLEPRRVTNQGAVDSLEYNYPGEAPSGHFDMFSPSDSEGQLPHYAAGRQVPGGAFPGASAMPRAAGMVGLSKMHAQPPQQQPQQQQQPQQQQQQHGVFFERFSGARKMPVGLEPSVGSRHPLMQPPQQAPPPPQQQPPQQPPQQQPPPPPGLLVRQNSCPPALPRPQQGEAGTPSGGLQDGGPMLPSQHAQFEYPIHRLENRSMHPYSEPVFSMQHPPPQQAPNQRLQHFDAPPYMNVAKRPRFDFPGSAGVDRCASWNGSMHNGALDNHLSPSAYPGLPGEFTPPVPDSFPSGPPLQHPAPDHQSLQQQQQQQQQQQQQQQQQQQQQQQQQQQQRQNAALMIKQMASRNQQQRLRQPNLAQLGHPGDVGQGGLVHGGPVGGLAQPNFEREGGSTGAGRLGTFEQQAPHLAQESAWFSGPHPPPGDLLPRRMGGSGLPADCGPHDPSLAPPPPPGGSGVLFRGPLQEPMRMPGEGHVPALPSPGLQFGGSLGGLGQLQSPGAGVGLPSAASERRPPPPDFATSALGGQPGFPFGAAGRQSTPHSGPGVNSPPSAGGGGGSSGGGGGGGAYPPQPDFQPSQRTSASKLGALSLGSFNKPSSKDNLFGQSCLAALSTACQNMIASLGAPNLNVTFNKKNPPEGKRKLSQNETDGAAVAGNPGSDYFPGGTAPGAPGPGGPSGTSSSGSKASGPPNPPAQGDGTSLSPNYTLESTSGNDGKPVSGGGGRGRGRRKRDSGHVSPGTFFDKYSAAPDSGGAPGVSPGQQQASGAAVGGSSAGETRGAPTPHEKALTSPSWGKGAELLLGDQPDLIGSLDGGAKSDSSSPNVGEFASDEVSTSYANEDEVSSSSDNPQALVKASRSPLVTGSPKLPPRGVGAGEHGPKAPPPALGLGIMSNSTSTPDSYGGGGGPGHPGTPGLEQVRTPTSSSGAPPPDEIHPLEILQAQIQLQRQQFSISEDQPLGLKGGKKGECAVGASGAQNGDSELGSCCSEAVKSAMSTIDLDSLMAEHSAAWYMPADKALVDSADDDKTLAPWEKAKPQNPNSKEAHDLPANKASASQPGSHLQCLSVHCTDDVGDAKARASVPTWRSLHSDISNRFGTFVAALT

576 NP_060247.2 NP_065147.1

>NP_060247.2

MAETVWSTDTGEAVYRSRDPVRNLRLRVHLQRITSSNFLHYQPAAELGKDLIDLATFRPQPTASGHRPEEDEEEEIVIGWQEKLFSQFEVDLYQNETACQSPLDYQYRQEILKLENSGGKKNRRIFTYTDSDRYTNLEEHCQRMTTAASEVPSFLVERMANVRRRRQDRRGMEGGILKSRIVTWEPSEEFVRNNHVINTPLQTMHIMADLGPYKKLGYKKYEHVLCTLKVDSNGVITVKPDFTGLKGPYRIETEGEKQELWKYTIDNVSPHAQPEEEERERRVFKDLYGRHKEYLSSLVGTDFEMTVPGALRLFVNGEVVSAQGYEYDNLYVHFFVELPTAHWSSPAFQQLSGVTQTCTTKSLAMDKVAHFSYPFTFEAFFLHEDESSDALPEWPVLYCEVLSLDFWQRYRVEGYGAVVLPATPGSHTLTVSTWRPVELGTVAELRRFFIGGSLELEDLSYVRIPGSFKGERLSRFGLRTETTGTVTFRLHCLQQSRAFMESSSLQKRMRSVLDRLEGFSQQSSIHNVLEAFRRARRRMQEARESLPQDLVSPSGTLVS

>NP_065147.1

MKLKDTKSRPKQSSCGKFQTKGIKVVGKWKEVKIDPNMFADGQMDDLVCFEELTDYQLVSPAKNPSSLFSKEAPKRKAQAVSEEEEEEEGKSSSPKKKIKLKKSKNVATEGTSTQKEFEVKDPELEAQGDDMVCDDPEAGEMTSENLVQTAPKKKKNKGKKGLEPSQSTAAKVPKKAKTWIPEVHDQKADVSAWKDLFVPRPVLRALSFLGFSAPTPIQALTLAPAIRDKLDILGAAETGSGKTLAFAIPMIHAVLQWQKRNAAPPPSNTEAPPGETRTEAGAETRSPGKAEAESDALPDDTVIESEALPSDIAAEARAKTGGTVSDQALLFGDDDAGEGPSSLIREKPVPKQNENEEENLDKEQTGNLKQELDDKSATCKAYPKRPLLGLVLTPTRELAVQVKQHIDAVARFTGIKTAILVGGMSTQKQQRMLNRRPEIVVATPGRLWELIKEKHYHLRNLRQLRCLVVDEADRMVEKGHFAELSQLLEMLNDSQYNPKRQTLVFSATLTLVHQAPARILHKKHTKKMDKTAKLDLLMQKIGMRGKPKVIDLTRNEATVETLTETKIHCETDEKDFYLYYFLMQYPGRSLVFANSISCIKRLSGLLKVLDIMPLTLHACMHQKQRLRNLEQFARLEDCVLLATDVAARGLDIPKVQHVIHYQVPRTSEIYVHRSGRTARATNEGLSLMLIGPEDVINFKKIYKTLKKDEDIPLFPVQTKYMDVVKERIRLARQIEKSEYRNFQACLHNSWIEQAAAALEIELEEDMYKGGKADQQEERRRQKQMKVLKKELRHLLSQPLFTESQKTKYPTQSGKPPLLVSAPSKSESALSCLSKQKKKKTKKPKEPQPEQPQPSTSAN

577 P10071 NP_003027.1

>P10071

MEAQSHSSTTTEKKKVENSIVKCSTRTDVSEKAVASSTTSNEDESPGQTYHRERRNAITMQPQNVQGLSKVSEEPSTSSDERASLIKKEIHGSLPHVAEPSVPYRGTVFAMDPRNGYMEPHYHPPHLFPAFHPPVPIDARHHEGRYHYDPSPIPPLHMTSALSSSPTYPDLPFIRISPHRNPAAASESPFSPPHPYINPYMDYIRSLHSSPSLSMISATRGLSPTDAPHAGVSPAEYYHQMALLTGQRSPYADIIPSAATAGTGAIHMEYLHAMDSTRFSSPRLSARPSRKRTLSISPLSDHSFDLQTMIRTSPNSLVTILNNSRSSSSASGSYGHLSASAISPALSFTYSSAPVSLHMHQQILSRQQSLGSAFGHSPPLIHPAPTFPTQRPIPGIPTVLNPVQVSSGPSESSQNKPTSESAVSSTGDPMHNKRSKIKPDEDLPSPGARGQQEQPEGTTLVKEEGDKDESKQEPEVIYETNCHWEGCAREFDTQEQLVHHINNDHIHGEKKEFVCRWLDCSREQKPFKAQYMLVVHMRRHTGEKPHKCTFEGCTKAYSRLENLKTHLRSHTGEKPYVCEHEGCNKAFSNASDRAKHQNRTHSNEKPYVCKIPGCTKRYTDPSSLRKHVKTVHGPEAHVTKKQRGDIHPRPPPPRDSGSHSQSRSPGRPTQGALGEQQDLSNTTSKREECLQVKTVKAEKPMTSQPSPGGQSSCSSQQSPISNYSNSGLELPLTDGGSIGDLSAIDETPIMDSTISTATTALALQARRNPAGTKWMEHVKLERLKQVNGMFPRLNPILPPKAPAVSPLIGNGTQSNNTCSLGGPMTLLPGRSDLSGVDVTMLNMLNRRDSSASTISSAYLSSRRSSGISPCFSSRRSSEASQAEGRPQNVSVADSYDPISTDASRRSSEASQSDGLPSLLSLTPAQQYRLKAKYAAATGGPPPTPLPNMERMSLKTRLALLGDALEPGVALPPVHAPRRCSDGGAHGYGRRHLQPHDALGHGVRRASDPVRTGSEGLALPRVPRFSSLSSCNPPAMATSAEKRSLVLQNYTRPEGGQSRNFHSSPCPPSITENVTLESLTMDADANLNDEDFLPDDVVQYLNSQNQAGYEQHFPSALPDDSKVPHGPGDFDAPGLPDSHAGQQFHALEQPCPEGSKTDLPIQWNEVSSGSADLSSSKLKCGPRPAVPQTRAFGFCNGMVVHPQNPLRSGPAGGYQTLGENSNPYGGPEHLMLHNSPGSGTSGNAFHEQPCKAPQYGNCLNRQPVAPGALDGACGAGIQASKLKSTPMQGSGGQLNFGLPVAPNESAGSMVNGMQNQDPVGQGYLAHQLLGDSMQHPGAGRPGQQMLGQISATSHINIYQGPESCLPGAHGMGSQPSSLAVVRGYQPCASFGGSRRQAMPRDSLALQSGQLSDTSQTCRVNGIKMEMKGQPHPLCSNLQNYSGQFYDQTVGFSQQDTKAGSFSISDASCLLQGTSAKNSELLSPGANQVTSTVDSLDSHDLEGVQIDFDAIIDDGDHSSLMSGALSPSIIQNLSHSSSRLTTPRASLPFPALSMSTTNMAIGDMSSLLTSLAEESKFLAVMQ

>NP_003027.1

MEAAAGGRGCFQPHPGLQKTLEQFHLSSMSSLGGPAAFSARWAQEAYKKESAKEAGAAAVPAPVPAATEPPPVLHLPAIQPPPPVLPGPFFMPSDRSTERCETVLEGETISCFVVGGEKRLCLPQILNSVLRDFSLQQINAVCDELHIYCSRCTADQLEILKVMGILPFSAPSCGLITKTDAERLCNALLYGGAYPPPCKKELAASLALGLELSERSVRVYHECFGKCKGLLVPELYSSPSAACIQCLDCRLMYPPHKFVVHSHKALENRTCHWGFDSANWRAYILLSQDYTGKEEQARLGRCLDDVKEKFDYGNKYKRRVPRVSSEPPASIRPKTDDTSSQSPAPSEKDKPSSWLRTLAGSSNKSLGCVHPRQRLSAFRPWSPAVSASEKELSPHLPALIRDSFYSYKSFETAVAPNVALAPPAQQKVVSSPPCAAAVSRAPEPLATCTQPRKRKLTVDTPGAPETLAPVAAPEEDKDSEAEVEVESREEFTSSLSSLSSPSFTSSSSAKDLGSPGARALPSAVPDAAAPADAPSGLEAELEHLRQALEGGLDTKEAKEKFLHEVVKMRVKQEEKLSAALQAKRSLHQELEFLRVAKKEKLREATEAKRNLRKEIERLRAENEKKMKEANESRLRLKRELEQARQARVCDKGCEAGRLRAKYSAQIEDLQVKLQHAEADREQLRADLLREREAREHLEKVVKELQEQLWPRARPEAAGSEGAAELEP

578 NP_000497.1 NP_004559.4

>NP_000497.1

MAHVRGLQLPGCLALAALCSLVHSQHVFLAPQQARSLLQRVRRANTFLEEVRKGNLERECVEETCSYEEAFEALESSTATDVFWAKYTACETARTPRDKLAACLEGNCAEGLGTNYRGHVNITRSGIECQLWRSRYPHKPEINSTTHPGADLQENFCRNPDSSTTGPWCYTTDPTVRRQECSIPVCGQDQVTVAMTPRSEGSSVNLSPPLEQCVPDRGQQYQGRLAVTTHGLPCLAWASAQAKALSKHQDFNSAVQLVENFCRNPDGDEEGVWCYVAGKPGDFGYCDLNYCEEAVEEETGDGLDEDSDRAIEGRTATSEYQTFFNPRTFGSGEADCGLRPLFEKKSLEDKTERELLESYIDGRIVEGSDAEIGMSPWQVMLFRKSPQELLCGASLISDRWVLTAAHCLLYPPWDKNFTENDLLVRIGKHSRTRYERNIEKISMLEKIYIHPRYNWRENLDRDIALMKLKKPVAFSDYIHPVCLPDRETAASLLQAGYKGRVTGWGNLKETWTANVGKGQPSVLQVVNLPIVERPVCKDSTRIRITDNMFCAGYKPDEGKRGDACEGDSGGPFVMKSPFNNRWYQMGIVSWGEGCDRDGKYGFYTHVFRLKKWIQKVIDQFGE

>NP_004559.4

MDVLAEANGTFALNLLKTLGKDNSKNVFFSPMSMSCALAMVYMGAKGNTAAQMAQILSFNKSGGGGDIHQGFQSLLTEVNKTGTQYLLRVANRLFGEKSCDFLSSFRDSCQKFYQAEMEELDFISAVEKSRKHINTWVAEKTEGKIAELLSPGSVDPLTRLVLVNAVYFRGNWDGQFDKENTEERLFKVSKNEEKPVQMMFKQSTFKKTYIGEIFTQILVLPYVGKELNMIIMLPDETTDLRTVEKELTYEKFVEWTRLDMMDEEEVEVSLPRFKLEESYDMESVLRNLGMTDAFELGKADFSGMSQTDLSLSKVVHKSFVEVNEEGTEAAAATAAIMMMRCARFVPRFCADHPFLFFIQHRKTNGILFCGRFSSP

579 NP_859530.1 NP_003048.1

>NP_859530.1

MLQTLYDYFWWERLWLPVNLTWADLEDRDGRVYAKASDLYITLPLALLFLIVRYFFELYVATPLAALLNIKEKTRLRAPPNATLEHFYLTSGKQPKQVEVELLSRQSGLSGRQVERWFRRRRNQDRPSLLKKFREASWRFTFYLIAFIAGMAVIVDKPWFYDMKKVWEGYPIQSTIPSQYWYYMIELSFYWSLLFSIASDVKRKDFKEQIIHHVATIILISFSWFANYIRAGTLIMALHDSSDYLLESAKMFNYAGWKNTCNNIFIVFAIVFIITRLVILPFWILHCTLVYPLELYPAFFGYYFFNSMMGVLQLLHIFWAYLILRMAHKFITGKLVEDERSDREETESSEGEEAAAGGGAKSRPLANGHPILNNNHRKND

>NP_003048.1

MPTVDDILEQVGESGWFQKQAFLILCLLSAAFAPICVGIVFLGFTPDHHCQSPGVAELSQRCGWSPAEELNYTVPGLGPAGEAFLGQCRRYEVDWNQSALSCVDPLASLATNRSHLPLGPCQDGWVYDTPGSSIVTEFNLVCADSWKLDLFQSCLNAGFLFGSLGVGYFADRFGRKLCLLGTVLVNAVSGVLMAFSPNYMSMLLFRLLQGLVSKGNWMAGYTLITEFVGSGSRRTVAIMYQMAFTVGLVALTGLAYALPHWRWLQLAVSLPTFLFLLYYWCVPESPRWLLSQKRNTEAIKIMDHIAQKNGKLPPADLKMLSLEEDVTEKLSPSFADLFRTPRLRKRTFILMYLWFTDSVLYQGLILHMGATSGNLYLDFLYSALVEIPGAFIALITIDRVGRIYPMAMSNLLAGAACLVMIFISPDLHWLNIIIMCVGRMGITIAIQMICLVNAELYPTFVRNLGVMVCSSLCDIGGIITPFIVFRLREVWQALPLILFAVLGLLAAGVTLLLPETKGVALPETMKDAENLGRKAKPKENTIYLKVQTSEPSGT

580 NP_037398.1 NP_001895.1

>NP_037398.1

MSAETPITLNIDPQDLQVQTFTVEKLLEPLIIQVTTLVNCPQNPSSRKKGRSKRASVLLASVEEATWNLLDKGEKIAQEATVLKDELTASLEEVRKESEALKVSAERFTDDPCFLPKREAVVQAARALLAAVTRLLILADMIDVMCLLQHVSAFQRTFETLKNVANKSDLQKTYQKLGKELENLDYLAFKRQQDLKSPNQRDEIAGARASLKENSPLLHSICSACLEHSDVASLKASKDTVCEEIQNALNVISNASQGIQNMTTPPEPQAATLGSALDELENLIVLNPLTVTEEEIRPSLEKRLEAIISGAALLADSSCTRDLHRERIIAECNAIRQALQDLLSEYMNNAGKKERSNTLNIALDNMCKKTRDLRRQLRKAIIDHVSDSFLDTTVPLLVLIEAAKNGREKEIKEYAAIFHEHTSRLVEVANLACSMSTNEDGIKIVKIAANHLETLCPQIINAALALAARPKSQAVKNTMEMYKRTWENHIHVLTEAVDDITSIDDFLAVSESHILEDVNKCIIALRDQDADNLDRAAGAIRGRAARVAHIVTGEMDSYEPGAYTEGVMRNVNFLTSTVIPEFVTQVNVALEALSKSSLNVLDDNQFVDISKKIYDTIHDIRCSVMMIRTPEELEDVSDLEEEHEVRSHTSIQTEGKTDRAKMTQLPEAEKEKIAEQVADFKKVKSKLDAEIEIWDDTSNDIIVLAKNMCMIMMEMTDFTRGKGPLKHTTDVIYAAKMISESGSRMDVLARQIANQCPDPSCKQDLLAYLEQIKFYSHQLKICSQVKAEIQNLGGELIMSALDSVTSLIQAAKNLMNAVVQTVKMSYIASTKIIRIQSPAGPRHPVVMWRMKAPAKKPLIKREKPEETCAAVRRGSAKKKIHPLQVMSEFRGRQIY

>NP_001895.1

MATQADLMELDMAMEPDRKAAVSHWQQQSYLDSGIHSGATTTAPSLSGKGNPEEEDVDTSQVLYEWEQGFSQSFTQEQVADIDGQYAMTRAQRVRAAMFPETLDEGMQIPSTQFDAAHPTNVQRLAEPSQMLKHAVVNLINYQDDAELATRAIPELTKLLNDEDQVVVNKAAVMVHQLSKKEASRHAIMRSPQMVSAIVRTMQNTNDVETARCTAGTLHNLSHHREGLLAIFKSGGIPALVKMLGSPVDSVLFYAITTLHNLLLHQEGAKMAVRLAGGLQKMVALLNKTNVKFLAITTDCLQILAYGNQESKLIILASGGPQALVNIMRTYTYEKLLWTTSRVLKVLSVCSSNKPAIVEAGGMQALGLHLTDPSQRLVQNCLWTLRNLSDAATKQEGMEGLLGTLVQLLGSDDINVVTCAAGILSNLTCNNYKNKMMVCQVGGIEALVRTVLRAGDREDITEPAICALRHLTSRHQEAEMAQNAVRLHYGLPVVVKLLHPPSHWPLIKATVGLIRNLALCPANHAPLREQGAIPRLVQLLVRAHQDTQRRTSMGGTQQQFVEGVRMEEIVEGCTGALHILARDVHNRIVIRGLNTIPLFVQLLYSPIENIQRVAAGVLCELAQDKEAAEAIEAEGATAPLTELLHSRNEGVATYAAAVLFRMSEDKPQDYKKRLSVELTSSLFRTEPMAWNETADLGLDIGAQGEPLGYRQDDPSYRSFHSGGYGQDALGMDPMMEHEMGGHHPGADYPVDGLPDLGHAQDLMDGLPPGDSNQLAWFDTDL

581 NP_000362.1 NP_036564.2

>NP_000362.1

MASHRLLLLCLAGLVFVSEAGPTGTGESKCPLMVKVLDAVRGSPAINVAVHVFRKAADDTWEPFASGKTSESGELHGLTTEEEFVEGIYKVEIDTKSYWKALGISPFHEHAEVVFTANDSGPRRYTIAALLSPYSYSTTAVVTNPKE

>NP_036564.2

MSSLPGCIGLDAATATVESEEIAELQQAVVEELGISMEELRHFIDEELEKMDCVQQRKKQLAELETWVIQKESEVAHVDQLFDDASRAVTNCESLVKDFYSKLGLQYRDSSSEDESSRPTEIIEIPDEDDDVLSIDSGDAGSRTPKDQKLREAMAALRKSAQDVQKFMDAVNKKSSSQDLHKGTLSQMSGELSKDGDLIVSMRILGKKRTKTWHKGTLIAIQTVGPGKKYKVKFDNKGKSLLSGNHIAYDYHPPADKLYVGSRVVAKYKDGNQVWLYAGIVAETPNVKNKLRFLIFFDDGYASYVTQSELYPICRPLKKTWEDIEDISCRDFIEEYVTAYPNRPMVLLKSGQLIKTEWEGTWWKSRVEEVDGSLVRILFLDDKRCEWIYRGSTRLEPMFSMKTSSASALEKKQGQLRTRPNMGAVRSKGPVVQYTQDLTGTGTQFKPVEPPQPTAPPAPPFPPAPPLSPQAGDSDLESQLAQSRKQVAKKSTSFRPGSVGSGHSSSTSPALSENVSGGKPGINQTYRSPLGSTASAPAPSALPAPPAPPVFHGMLERAPAEPSYRAPMEKLFYLPHVCSYTCLSRVRPMRNEQYRGKNPLLVPLLYDFRRMTARRRVNRKMGFHVIYKTPCGLCLRTMQEIERYLFETGCDFLFLEMFCLDPYVLVDRKFQPYKPFYYILDITYGKEDVPLSCVNEIDTTPPPQVAYSKERIPGKGVFINTGPEFLVGCDCKDGCRDKSKCACHQLTIQATACTPGGQINPNSGYQYKRLEECLPTGVYECNKRCKCDPNMCTNRLVQHGLQVRLQLFKTQNKGWGIRCLDDIAKGSFVCIYAGKILTDDFADKEGLEMGDEYFANLDHIESVENFKEGYESDAPCSSDSSGVDLKDQEDGNSGTEDPEESNDDSSDDNFCKDEDFSTSSVWRSYATRRQTRGQKENGLSETTSKDSHPPDLGPPHIPVPPSIPVGGCNPPSSEETPKNKVASWLSCNSVSEGGFADSDSHSSFKTNEGGEGRAGGSRMEAEKASTSGLGIKDEGDIKQAKKEDTDDRNKMSVVTESSRNYGYNPSPVKPEGLRRPPSKTSMHQSRRLMASAQSNPDDVLTLSSSTESEGESGTSRKPTAGQTSATAVDSDDIQTISSGSEGDDFEDKKNMTGPMKRQVAVKSTRGFALKSTHGIAIKSTNMASVDKGESAPVRKNTRQFYDGEESCYIIDAKLEGNLGRYLNHSCSPNLFVQNVFVDTHDLRFPWVAFFASKIRAGTELTWDYNYEVGSVEGKELLCCCGAIECRGRLL

582 NP_005477.1 NP_056039.1

>NP_005477.1

MAFGKSHRDPYATSVGHLIEKATFAGVQTEDWGQFMHICDIINTTQDAPKDAVKALKKRISKNYNHKEIQLTLSLIDMCVQNCGPSFQSLIVKKEFVKENLVKLLNPRYNLPLDIQNRILNFIKTWSQGFPGGVDVSEVKEVYLDLVKKGVQFPPSEAEAETARQETAQISSNPPTSVPTAPALSSVIAPKNSTVTLVPEQIGKLHSELDMVKMNVRVMSAILMENTPGSENHEDIELLQKLYKTGREMQERIMDLLVVVENEDVTVELIQVNEDLNNAILGYERFTRNQQRILEQNKNQKEATNTTSEPSAPSQDLLDLSPSPRMPRATLGELNTMNNQLSGLNFSLPSSDVTNNLKPSLHPQMNLLALENTEIPPFAQRTSQNLTSSHAYDNFLEHSNSVFLQPVSLQTIAAAPSNQSLPPLPSNHPAMTKSDLQPPNYYEVMEFDPLAPAVTTEAIYEEIDAHQHKGAQNDGD

>NP_056039.1

MSIDHLKQKIPPALFYKETYLGPNEVLKNGMYCSLYEVVEKTRIGSNMESLLQKLDREKLVLVKPLGDRGYLFLLSPYQMVPPYEYQTAKSRVLHALFLFQEPRSIVTSQKGSTNAAPQERHESMPDVLKIAQFLQFSLIQCRKEFKNISAINFHSVVEKYVSEFFKRGFGSGKREFIMFPYDSRLDDKKFLYSAPRNKSHIDTCLHAYIFRPEVYQLPICKLKELFEENRKLQQFSPLSDYEGQEEEMNGTKMKFGKRNNSRGEAIISGKQRSSHSLDYDKDRVKELINLIQCRKKSVGGDSDTEDMRSKTVLKRKLEDLPENMRKLAKTSNLSENCHLYEESPQPIGSLGHDADLRRQQQDTCNSGIADIHRLFNWLSETLANARHSDASLTDTVNKALGLSTDDAYEELRQKHEYELNSTPDKKDYEQPTCAKVENAQFKGTQSLLLEVDATSKYSVAISTSEVGTDHKLHLKEDPNLISVNNFEDCSLCPSVPIEHGFRRQQSKSNNVEETEIHWKLIPITGGNARSPEDQLGKHGEKQTPDTLKGTTEDDVLTGQVEEQCVPAAEAEPPAVSETTERTVLGEYNLFSRKIEEILKQKNVSYVSRVSTPIFSTQEKMKRLSEFIYSKTSKAGVQEFVDGLHEKLNTIIIKASAKGGNLPPVSPNDSGAKIASNPLERHVIPVSSSDFSNKHLLGPLCSDPLKDTNSDEQHSTSALTEVEMNQPQHATELMVTSDHIVPGDMAREPVEETTKSPSDVNISAQPALSNFISQLEPEVFNSLVKIMKDVQKNTVKFYIHEEEESVLCKEIKEYLIKLGNTECHPEQFLERRSKLDKLLIIIQNEDIAGFIHKIPGLVTLKKLPCVSFAGVDSLDDVKNHTYNELFVSGGFIVSDESILNPEVVTVENLKNFLTFLEELSTPEGKWQWKVHCKFQKKLKELGRLNAKALSLLTLLNVYQKKHLVEILSYHNCDSQTRNAPELDCLIRLQAQNIQQRHIVFLTEKNIKMLSSYTDNGIVVATAEDFMQNFKNLVGYHNSITEENLPQLGANENLESQSALLENDEKDEEDMSLDSGDEISHIEVCSNFHSEIWEKETKGSRGTDQKKNTQIELQSSPDVQNSLLEDKTYLDSEERTSIDIVCSEGENSNSTEQDSYSNFQVYHSQLNMSHQFSHFNVLTHQTFLGTPYALSSSQSQENENYFLSAYTESLDRDKSPPPLSWGKSDSSRPYSQEK

583 NP_001519.1 NP_000592.3

>NP_001519.1

MGRWAWVPSPWPPPGLGPFLLLLLLLLLLPRGFQPQPGGNRTESPEPNATATPAIPTILVTSVTSETPATSAPEAEGPQSGGLPPPPRAVPSSSSPQAQALTEDGRPCRFPFRYGGRMLHACTSEGSAHRKWCATTHNYDRDRAWGYCVEATPPPGGPAALDPCASGPCLNGGSCSNTQDPQSYHCSCPRAFTGKDCGTEKCFDETRYEYLEGGDRWARVRQGHVEQCECFGGRTWCEGTRHTACLSSPCLNGGTCHLIVATGTTVCACPPGFAGRLCNIEPDERCFLGNGTGYRGVASTSASGLSCLAWNSDLLYQELHVDSVGAAALLGLGPHAYCRNPDNDERPWCYVVKDSALSWEYCRLEACESLTRVQLSPDLLATLPEPASPGRQACGRRHKKRTFLRPRIIGGSSSLPGSHPWLAAIYIGDSFCAGSLVHTCWVVSAAHCFSHSPPRDSVSVVLGQHFFNRTTDVTQTFGIEKYIPYTLYSVFNPSDHDLVLIRLKKKGDRCATRSQFVQPICLPEPGSTFPAGHKCQIAGWGHLDENVSGYSSSLREALVPLVADHKCSSPEVYGADISPNMLCAGYFDCKSDACQGDSGGPLACEKNGVAYLYGIISWGDGCGRLHKPGVYTRVANYVDWINDRIRPPRRLVAPS

>NP_000592.3

MWVTKLLPALLLQHVLLHLLLLPIAIPYAEGQRKRRNTIHEFKKSAKTTLIKIDPALKIKTKKVNTADQCANRCTRNKGLPFTCKAFVFDKARKQCLWFPFNSMSSGVKKEFGHEFDLYENKDYIRNCIIGKGRSYKGTVSITKSGIKCQPWSSMIPHEHSFLPSSYRGKDLQENYCRNPRGEEGGPWCFTSNPEVRYEVCDIPQCSEVECMTCNGESYRGLMDHTESGKICQRWDHQTPHRHKFLPERYPDKGFDDNYCRNPDGQPRPWCYTLDPHTRWEYCAIKTCADNTMNDTDVPLETTECIQGQGEGYRGTVNTIWNGIPCQRWDSQYPHEHDMTPENFKCKDLRENYCRNPDGSESPWCFTTDPNIRVGYCSQIPNCDMSHGQDCYRGNGKNYMGNLSQTRSGLTCSMWDKNMEDLHRHIFWEPDASKLNENYCRNPDDDAHGPWCYTGNPLIPWDYCPISRCEGDTTPTIVNLDHPVISCAKTKQLRVVNGIPTRTNIGWMVSLRYRNKHICGGSLIKESWVLTARQCFPSRDLKDYEAWLGIHDVHGRGDEKCKQVLNVSQLVYGPEGSDLVLMKLARPAVLDDFVSTIDLPNYGCTIPEKTSCSVYGWGYTGLINYDGLLRVAHLYIMGNEKCSQHHRGKVTLNESEICAGAEKIGSGPCEGDYGGPLVCEQHKMRMVLGVIVPGRGCAIPNRPGIFVRVAYYAKWIHKIILTYKVPQS

584 NP_002156.2 NP_783552.1

>NP_002156.2

MKVASGSTATAAAGPSCALKAGKTASGAGEVVRCLSEQSVAISRCAGGAGARLPALLDEQQVNVLLYDMNGCYSRLKELVPTLPQNRKVSKVEILQHVIDYIRDLQLELNSESEVGTPGGRGLPVRAPLSTLNGEISALTAEAACVPADDRILCR

>NP_783552.1

MISVKRNTWRALSLVIGDCRKKGNFEYCQDRTEKHSTMPDSPVDVKTQSRLTPPTMPPPPTTQGAPRTSSFTPTTLTNGTSHSPTALNGAPSPPNGFSNGPSSSSSSSLANQQLPPACGARQLSKLKRFLTTLQQFGNDISPEIGERVRTLVLGLVNSTLTIEEFHSKLQEATNFPLRPFVIPFLKANLPLLQRELLHCARLAKQNPAQYLAQHEQLLLDASTTSPVDSSELLLDVNENGKRRTPDRTKENGFDREPLHSEHPSKRPCTISPGQRYSPNNGLSYQPNGLPHPTPPPPQHYRLDDMAIAHHYRDSYRHPSHRDLRDRNRPMGLHGTRQEEMIDHRLTDREWAEEWKHLDHLLNCIMDMVEKTRRSLTVLRRCQEADREELNYWIRRYSDAEDLKKGGGSSSSHSRQQSPVNPDPVALDAHREFLHRPASGYVPEEIWKKAEEAVNEVKRQAMTELQKAVSEAERKAHDMITTERAKMERTVAEAKRQAAEDALAVINQQEDSSESCWNCGRKASETCSGCNTARYCGSFCQHKDWEKHHHICGQTLQAQQQGDTPAVSSSVTPNSGAGSPMDTPPAATPRSTTPGTPSTIETTPR

585 NP_207837.2 NP_296373.1

>NP_207837.2

MGSVTGAVLKTLLLLSTQNWNRVEAGNSYDCDDPLVSALPQASFSSSSELSSSHGPGFARLNRRDGAGGWSPLVSNKYQWLQIDLGERMEVTAVATQGGYGSSNWVTSYLLMFSDSGWNWKQYRQEDSIWGFSGNANADSVVYYRLQPSIKARFLRFIPLEWNPKGRIGMRIEVFGCAYRSEVVDLDGKSSLLYRFDQKSLSPIKDIISLKFKTMQSDGILLHREGPNGDHITLQLRRARLFLLINSGEAKLPSTSTLVNLTLGSLLDDQHWHSVLIQRLGKQVNFTVDEHRHHFHARGEFNLMNLDYEISFGGIPAPGKSVSFPHRNFHGCLENLYYNGVDIIDLAKQQKPQIIAMGNVSFSCSQPQSMPVTFLSSRSYLALPDFSGEEEVSATFQFRTWNKAGLLLFSELQLISGGILLFLSDGKLKSNLYQPGKLPSDITAGVELNDGQWHSVSLSAKKNHLSVAVDGQMASAAPLLGPEQIYSGGTYYFGGCPDKSFGSKCKSPLGGFQGCMRLISISGKVVDLISVQQGSLGNFSDLQIDSCGISDRCLPNYCEHGGECSQSWSTFHCNCTNTGYRGATCHNSIYEQSCEAYKHRGNTSGFYYIDSDGSGPLEPFLLYCNMTETAWTIIQHNGSDLTRVRNTNPENPYAGFFEYVASMEQLQATINRAEHCEQEFTYYCKKSRLVNKQDGTPLSWWVGRTNETQTYWGGSSPDLQKCTCGLEGNCIDSQYYCNCDADRNEWTNDTGLLAYKEHLPVTKIVITDTGRLHSEAAYKLGPLLCQGDRSFWNSASFDTEASYLHFPTFHGELSADVSFFFKTTASSGVFLENLGIADFIRIELRSPTVVTFSFDVGNGPFEISVQSPTHFNDNQWHHVRVERNMKEASLQVDQLTPKTQPAPADGHVLLQLNSQLFVGGTATRQRGFLGCIRSLQLNGMTLDLEERAQVTPEVQPGCRGHCSSYGKLCRNGGKCRERPIGFFCDCTFSAYTGPFCSNEISAYFGSGSSVIYNFQENYLLSKNSSSHAASFHGDMKLSREMIKFSFRTTRTPSLLLFVSSFYKEYLSVIIAKNGSLQIRYKLNKYQEPDVVNFDFKNMADGQLHHIMINREEGVVFIEIDDNRRRQVHLSSGTEFSAVKSLVLGRILEHSDVDQDTALAGAQGFTGCLSAVQLSHVAPLKAALHPSHPDPVTVTGHVTESSCMAQPGTDATSRERTHSFADHSGTIDDREPLANAIKSDSAVIGGLIAVVIFILLCITAIAVRIYQQKRLYKRSEAKRSENVDSAEAVLKSELNIQNAVNENQKEYFF

>NP_296373.1

MAAAAVDSAMEVVPALAEEAAPEVAGLSCLVNLPGEVLEYILCCGSLTAADIGRVSSTCRRLRELCQSSGKVWKEQFRVRWPSLMKHYSPTDYVNWLEEYKVRQKAGLEARKIVASFSKRFFSEHVPCNGFSDIENLEGPEIFFEDELVCILNMEGRKALTWKYYAKKILYYLRQQKILNNLKAFLQQPDDYESYLEGAVYIDQYCNPLSDISLKDIQAQIDSIVELVCKTLRGINSRHPSLAFKAGESSMIMEIELQSQVLDAMNYVLYDQLKFKGNRMDYYNALNLYMHQVLIRRTGIPISMSLLYLTIARQLGVPLEPVNFPSHFLLRWCQGAEGATLDIFDYIYIDAFGKGKQLTVKECEYLIGQHVTAALYGVVNVKKVLQRMVGNLLSLGKREGIDQSYQLLRDSLDLYLAMYPDQVQLLLLQARLYFHLGIWPEKSFCLVLKVLDILQHIQTLDPGQHGAVGYLVQHTLEHIERKKEEVGVEVKLRSDEKHRDVCYSIGLIMKHKRYGYNCVIYGWDPTCMMGHEWIRNMNVHSLPHGHHQPFYNVLVEDGSCRYAAQENLEYNVEPQEISHPDVGRYFSEFTGTHYIPNAELEIRYPEDLEFVYETVQNIYSAKKENIDE

586 NP_002651.2 NP_067028.1

>NP_002651.2

MAGAASPCANGCGPGAPSDAEVLHLCRSLEVGTVMTLFYSKKSQRPERKTFQVKLETRQITWSRGADKIEGAIDIREIKEIRPGKTSRDFDRYQEDPAFRPDQSHCFVILYGMEFRLKTLSLQATSEDEVNMWIKGLTWLMEDTLQAPTPLQIERWLRKQFYSVDRNREDRISAKDLKNMLSQVNYRVPNMRFLRERLTDLEQRSGDITYGQFAQLYRSLMYSAQKTMDLPFLEASTLRAGERPELCRVSLPEFQQFLLDYQGELWAVDRLQVQEFMLSFLRDPLREIEEPYFFLDEFVTFLFSKENSVWNSQLDAVCPDTMNNPLSHYWISSSHNTYLTGDQFSSESSLEAYARCLRMGCRCIELDCWDGPDGMPVIYHGHTLTTKIKFSDVLHTIKEHAFVASEYPVILSIEDHCSIAQQRNMAQYFKKVLGDTLLTKPVEISADGLPSPNQLKRKILIKHKKLAEGSAYEEVPTSMMYSENDISNSIKNGILYLEDPVNHEWYPHYFVLTSSKIYYSEETSSDQGNEDEEEPKEVSSSTELHSNEKWFHGKLGAGRDGRHIAERLLTEYCIETGAPDGSFLVRESETFVGDYTLSFWRNGKVQHCRIHSRQDAGTPKFFLTDNLVFDSLYDLITHYQQVPLRCNEFEMRLSEPVPQTNAHESKEWYHASLTRAQAEHMLMRVPRDGAFLVRKRNEPNSYAISFRAEGKIKHCRVQQEGQTVMLGNSEFDSLVDLISYYEKHPLYRKMKLRYPINEEALEKIGTAEPDYGALYEGRNPGFYVEANPMPTFKCAVKALFDYKAQREDELTFIKSAIIQNVEKQEGGWWRGDYGGKKQLWFPSNYVEEMVNPVALEPEREHLDENSPLGDLLRGVLDVPACQIAIRPEGKNNRLFVFSISMASVAHWSLDVAADSQEELQDWVKKIREVAQTADARLTEGKIMERRKKIALELSELVVYCRPVPFDEEKIGTERACYRDMSSFPETKAEKYVNKAKGKKFLQYNRLQLSRIYPKGQRLDSSNYDPLPMWICGSQLVALNFQTPDKPMQMNQALFMTGRHCGYVLQPSTMRDEAFDPFDKSSLRGLEPCAISIEVLGARHLPKNGRGIVCPFVEIEVAGAEYDSTKQKTEFVVDNGLNPVWPAKPFHFQISNPEFAFLRFVVYEEDMFSDQNFLAQATFPVKGLKTGYRAVPLKNNYSEDLELASLLIKIDIFPAKENGDLSPFSGTSLRERGSDASGQLFHGRAREGSFESRYQQPFEDFRISQEHLADHFDSRERRAPRRTRVNGDNRL

>NP_067028.1

MPPQQGDPAFPDRCEAPPVPPRRERGGRGGRGPGEPGGRGRAGGAEGRGVKCVLVGDGAVGKTSLVVSYTTNGYPTEYIPTAFDNFSAVVSVDGRPVRLQLCDTAGQDEFDKLRPLCYTNTDIFLLCFSVVSPSSFQNVSEKWVPEIRCHCPKAPIILVGTQSDLREDVKVLIELDKCKEKPVPEEAAKLCAEEIKAASYIECSALTQKNLKEVFDAAIVAGIQYSDTQQQPKKSKSRTPDKMKNLSKSWWKKYCCFV

587 NP_055774.1 NP_001441.4

>NP_055774.1

MMRQRQSHYCSVLFLSVNYLGGTFPGDICSEENQIVSSYASKVCFEIEEDYKNRQFLGPEGNVDVELIDKSTNRYSVWFPTAGWYLWSATGLGFLVRDEVTVTIAFGSWSQHLALDLQHHEQWLVGGPLFDVTAEPEEAVAEIHLPHFISLQGEVDVSWFLVAHFKNEGMVLEHPARVEPFYAVLESPSFSLMGILLRIASGTRLSIPITSNTLIYYHPHPEDIKFHLYLVPSDALLTKAIDDEEDRFHGVRLQTSPPMEPLNFGSSYIVSNSANLKVMPKELKLSYRSPGEIQHFSKFYAGQMKEPIQLEITEKRHGTLVWDTEVKPVDLQLVAASAPPPFSGAAFVKENHRQLQARMGDLKGVLDDLQDNEVLTENEKELVEQEKTRQSKNEALLSMVEKKGDLALDVLFRSISERDPYLVSYLRQQNL

>NP_001441.4

MTERFDCHHCNESLFGKKYILREESPYCVVCFETLFANTCEECGKPIGCDCKDLSYKDRHWHEACFHCSQCRNSLVDKPFAAKEDQLLCTDCYSNEYSSKCQECKKTIMPGTRKMEYKGSSWHETCFICHRCQQPIGTKSFIPKDNQNFCVPCYEKQHAMQCVQCKKPITTGGVTYREQPWHKECFVCTACRKQLSGQRFTARDDFAYCLNCFCDLYAKKCAGCTNPISGLGGTKYISFEERQWHNDCFNCKKCSLSLVGRGFLTERDDILCPDCGKDI

588 NP_009104.1 NP_056076.1

>NP_009104.1

MAGIPGLLFLLFFLLCAVGQVSPYSAPWKPTWPAYRLPVVLPQSTLNLAKPDFGAEAKLEVSSSCGPQCHKGTPLPTYEEAKQYLSYETLYANGSRTETQVGIYILSSSGDGAQHRDSGSSGKSRRKRQIYGYDSRFSIFGKDFLLNYPFSTSVKLSTGCTGTLVAEKHVLTAAHCIHDGKTYVKGTQKLRVGFLKPKFKDGGRGANDSTSAMPEQMKFQWIRVKRTHVPKGWIKGNANDIGMDYDYALLELKKPHKRKFMKIGVSPPAKQLPGGRIHFSGYDNDRPGNLVYRFCDVKDETYDLLYQQCDAQPGASGSGVYVRMWKRQQQKWERKIIGIFSGHQWVDMNGSPQDFNVAVRITPLKYAQICYWIKGNYLDCREG

>NP_056076.1

MVALRGLGSGLQPWCPLDLRLEWVDTVWELDFTETEPLDPSIEAEIIETGLAAFTKLYESLLPFATGEHGSMESIWTFFIENNVSHSTLVALFYHFVQIVHKKNVSVQYREYGLHAAGLYFLLLEVPGSVANQVFHPVMFDKCIQTLKKSWPQESNLNRKRKKEQPKSSQANPGRHRKRGKPPRREDIEMDEIIEEQEDENICFSARDLSQIRNAIFHLLKNFLRLLPKFSLKEKPQCVQNCIEVFVSLTNFEPVLHECHVTQARALNQAKYIPELAYYGLYLLCSPIHGEGDKVISCVFHQMLSVILMLEVGEGSHRAPLAVTSQVINCRNQAVQFISALVDELKESIFPVVRILLQHICAKVVDKSEYRTFAAQSLVQLLSKLPCGEYAMFIAWLYKYSRSSKIPHRVFTLDVVLALLELPEREVDNTLSLEHQKFLKHKFLVQEIMFDRCLDKAPTVRSKALSSFAHCLELTVTSASESILELLINSPTFSVIESHPGTLLRNSSAFSYQRQTSNRSEPSGEINIDSSGETVGSGERCVMAMLRRRIRDEKTNVRKSALQVLVSILKHCDVSGMKEDLWILQDQCRDPAVSVRKQALQSLTELLMAQPRCVQIQKAWLRGVVPVVMDCESTVQEKALEFLDQLLLQNIRHHSHFHSGDDSQVLAWALLTLLTTESQELSRYLNKAFHIWSKKEKFSPTFINNVISHTGTEHSAPAWMLLSKIAGSSPRLDYSRIIQSWEKISSQQNPNSNTLGHILCVIGHIAKHLPKSTRDKVTDAVKCKLNGFQWSLEVISSAVDALQRLCRASAETPAEEQELLTQVCGDVLSTCEHRLSNIVLKENGTGNMDEDLLVKYIFTLGDIAQLCPARVEKRIFLLIQSVLASSADADHSPSSQGSSEAPASQPPPQVRGSVMPSVIRAHAIITLGKLCLQHEDLAKKSIPALVRELEVCEDVAVRNNVIIVMCDLCIRYTIMVDKYIPNISMCLKDSDPFIRKQTLILLTNLLQEEFVKWKGSLFFRFVSTLIDSHPDIASFGEFCLAHLLLKRNPVMFFQHFIECIFHFNNYEKHEKYNKFPQSEREKRLFSLKGKSNKERRMKIYKFLLEHFTDEQRFNITSKICLSILACFADGILPLDLDASELLSDTFEVLSSKEIKLLAMRSKPDKDLLMEEDDMALANVVMQEAQKKLISQVQKRNFIENIIPIIISLKTVLEKNKIPALRELMHYLREVMQDYRDELKDFFAVDKQLASELEYDMKKYQEQLVQEQELAKHADVAGTAGGAEVAPVAQVALCLETVPVPAGQENPAMSPAVSQPCTPRASAGHVAVSSPTPETGPLQRLLPKARPMSLSTIAILNSVKKAVESKSRHRSRSLGVLPFTLNSGSPEKTCSQVSSYSLEQESNGEIEHVTKRAISTPEKSISDVTFGAGVSYIGTPRTPSSAKEKIEGRSQGNDILCLSLPDKPPPQPQQWNVRSPARNKDTPACSRRSLRKTPLKTAN

589 NP_005761.2 NP_005577.1

>NP_005761.2

MTGFLLPPASRGTRRSCSRSRKRQTRRRRNPSSFVASCPTLLPFACVPGASPTTLAFPPVVLTGPSTDGIPFALSLQRVPFVLPSPQVASLPLGHSRG

>NP_005577.1

MYQVSGQRPSGCDAPYGAPSAAPGPAQTLSLLPGLEVVTGSTHPAEAAPEEGSLEEAATPMPQGNGPGIPQGLDSTDLDVPTEAVTCQPQGNPLGCTPLLPNDSGHPSELGGTRRAGNGALGGPKAHRKLQTHPSLASQGSKKSKSSSKSTTSQIPLQAQEDCCVHCILSCLFCEFLTLCNIVLDCATCGSCSSEDSCLCCCCCGSGECADCDLPCDLDCGILDACCESADCLEICMECCGLCFSS

590 NP_060740.1 NP_996670.1

>NP_060740.1

MGEAEVGGGGAAGDKGPGEAATSPAEETVVWSPEVEVCLFHAMLGHKPVGVNRHFHMICIRDKFSQNIGRQVPSKVIWDHLSTMYDMQALHESEILPFPNPERNFVLPEEIIQEVREGKVMIEEEMKEEMKEDVDPHNGADDVFSSSGSLGKASEKSSKDKEKNSSDLGCKEGADKRKRSRVTDKVLTANSNPSSPSAAKRRRT

>NP_996670.1

MAPKQDPKPKFQEGERVLCFHGPLLYEAKCVKVAIKDKQVKYFIHYSGWNKKSAVRPRRSEKSLKTHEDIVALFPVPEGAPSVHHPLLTSSWDEWVPESRVLKYVDTNLQKQRELQKANQEQYAEGKMRGAAPGKKTSGLQQKNVEVKTKKNKQKTPGNGDGGSTSETPQPPRKKRARVDPTVENEETFMNRVEVKVKIPEELKPWLVDDWDLITRQKQLFYLPAKKNVDSILEDYANYKKSRGNTDNKEYAVNEVVAGIKEYFNVMLGTQLLYKFERPQYAEILADHPDAPMSQVYGAPHLLRLFVRIGAMLAYTPLDEKSLALLLNYLHDFLKYLAKNSATLFSASDYEVAPPEYHRKAV

591 NP_000620.2 NP_997468.1

>NP_000620.2

MMVVLLGATTLVLVAVAPWVLSAAAGGKNLKSPQKVEVDIIDDNFILRWNRSDESVGNVTFSFDYQKTGMDNWIKLSGCQNITSTKCNFSSLKLNVYEEIKLRIRAEKENTSSWYEVDSFTPFRKAQIGPPEVHLEAEDKAIVIHISPGTKDSVMWALDGLSFTYSLVIWKNSSGVEERIENIYSRHKIYKLSPETTYCLKVKAALLTSWKIGVYSPVHCIKTTVENELPPPENIEVSVQNQNYVLKWDYTYANMTFQVQWLHAFLKRNPGNHLYKWKQIPDCENVKTTQCVFPQNVFQKGIYLLRVQASDGNNTSFWSEEIKFDTEIQAFLLPPVFNIRSLSDSFHIYIGAPKQSGNTPVIQDYPLIYEIIFWENTSNAERKIIEKKTDVTVPNLKPLTVYCVKARAHTMDEKLNKSSVFSDAVCEKTKPGNTSKIWLIVGICIALFALPFVIYAAKVFLRCINYVFFPSLKPSSSIDEYFSEQPLKNLLLSTSEEQIEKCFIIENISTIATVEETNQTDEDHKKYSSQTSQDSGNYSNEDESESKTSEELQQDFV

>NP_997468.1

MLLSQNAFIFRSLNLVLMVYISLVFGISYDSPDYTDESCTFKISLRNFRSILSWELKNHSIVPTHYTLLYTIMSKPEDLKVVKNCANTTRSFCDLTDEWRSTHEAYVTVLEGFSGNTTLFSCSHNFWLAIDMSFEPPEFEIVGFTNHINVMVKFPSIVEEELQFDLSLVIEEQSEGIVKKHKPEIKGNMSGNFTYIIDKLIPNTNYCVSVYLEHSDEQAVIKSPLKCTLLPPGQESESAESAKIGGIITVFLIALVLTSTIVTLKWIGYICLRNSLPKVLNFHNFLAWPFPNLPPLEAMDMVEVIYINRKKKVWDYNYDDESDSDTEAAPRTSGGGYTMHGLTVRPLGQASATSTESQLIDPESEEEPDLPEVDVELPTMPKDSPQQLELLSGPCERRKSPLQDPFPEEDYSSTEGSGGRITFNVDLNSVFLRVLDDEDSDDLEAPLMLSSHLEEMVDPEDPDNVQSNHLLASGEGTQPTFPSPSSEGLWSEDAPSDQSDTSESDVDLGDGYIMR

592 NP_473363.1 NP_003818.2

>NP_473363.1

MDPLFQQTHKQVHEIQSCMGRLETADKQSVHIVENEIQASIDQIFSRLERLEILSSKEPPNKRQNARLRVDQLKYDVQHLQTALRNFQHRRHAREQQERQREELLSRTFTTNDSDTTIPMDESLQFNSSLQKVHNGMDDLILDGHNILDGLRTQRLTLKGTQKKILDIANMLGLSNTVMRLIEKRAFQDKYFMIGTQGSCQTAHFGGRSAGSS

>NP_003818.2

MDNSGKEAEAMALLAEAERKVKNSQSFFSGLFGGSSKIEEACEIYARAANMFKMAKNWSAAGNAFCQAAQLHLQLQSKHDAATCFVDAGNAFKKADPQEAINCLMRAIEIYTDMGRFTIAAKHHISIAEIYETELVDIEKAIAHYEQSADYYKGEESNSSANKCLLKVAGYAALLEQYQKAIDIYEQVGTNAMDSPLLKYSAKDYFFKAALCHFCIDMLNAKLAVQKYEELFPAFSDSRECKLMKKLLEAHEEQNVDSYTESVKEYDSISRLDQWLTTMLLRIKKTIQGDEEDLR

593 NP_932170.1 NP_006130.1

>NP_932170.1

MKWKALFTAAILQAQLPITEAQSFGLLDPKLCYLLDGILFIYGVILTALFLRVKFSRSADAPAYQQGQNQLYNELNLGRREEYDVLDKRRGRDPEMGGKPQRRKNPQEGLYNELQKDKMAEAYSEIGMKGERRRGKGHDGLYQGLSTATKDTYDALHMQALPPR

>NP_006130.1

MLRLLLALNLFPSIQVTGNKILVKQSPMLVAYDNAVNLSCKYSYNLFSREFRASLHKGLDSAVEVCVVYGNYSQQLQVYSKTGFNCDGKLGNESVTFYLQNLYVNQTDIYFCKIEVMYPPPYLDNEKSNGTIIHVKGKHLCPSPLFPGPSKPFWVLVVVGGVLACYSLLVTVAFIIFWVRSKRSRLLHSDYMNMTPRRPGPTRKHYQPYAPPRDFAAYRS

594 NP_000955.1 NP_054790.2

>NP_000955.1

MASNSSSCPTPGGGHLNGYPVPPYAFFFPPMLGGLSPPGALTTLQHQLPVSGYSTPSPATIETQSSSSEEIVPSPPSPPPLPRIYKPCFVCQDKSSGYHYGVSACEGCKGFFRRSIQKNMVYTCHRDKNCIINKVTRNRCQYCRLQKCFEVGMSKESVRNDRNKKKKEVPKPECSESYTLTPEVGELIEKVRKAHQETFPALCQLGKYTTNNSSEQRVSLDIDLWDKFSELSTKCIIKTVEFAKQLPGFTTLTIADQITLLKAACLDILILRICTRYTPEQDTMTFSDGLTLNRTQMHNAGFGPLTDLVFAFANQLLPLEMDDAETGLLSAICLICGDRQDLEQPDRVDMLQEPLLEALKVYVRKRRPSRPHMFPKMLMKITDLRSISAKGAERVITLKMEIPGSMPPLIQEMLENSEGLDTLSGQPGGGGRDGGGLAPPPGSCSPSLSPSSNRSSPATHSP

>NP_054790.2

MVLDDLPNLEDIYTSLCSSTMEDSEMDFDSGLEDDDTKSDSILEDSTIFVAFKGNIDDKDFKWKLDAILKNVPNLLHMESSKLKVQKVEPWNSVRVTFNIPREAAERLRILAQSNNQQLRDLGILSVQIEGEGAINLALAQNRSQDVRMNGPMGAGNSVRMEAGFPMASGPGIIRMNNPATVMIPPGGNVSSSMMAPGPNPELQPRTPRPASQSDAMDPLLSGLHIQQQSHPSGSLAPPHHPMQPVSVNRQMNPANFPQLQQQQQQQQQQQQQQQQQQQQQQQQQLQARPPQQHQQQQPQGIRPQFTAPTQVPVPPGWNQLPSGALQPPPAQGSLGTMTANQGWKKAPLPGPMQQQLQARPSLATVQTPSHPPPPYPFGSQQASQAHTNFPQMSNPGQFTAPQMKSLQGGPSRVPTPLQQPHLTNKSPASSPSSFQQGSPASSPTVNQTQQQMGPRPPQNNPLPQGFQQPVSSPGRNPMVQQGNVPPNFMVMQQQPPNQGPQSLHPGLGGMPKRLPPGFSAGQANPNFMQGQVPSTTATTPGNSGAPQLQANQNVQHAGGQGAGPPQNQMQVSHGPPNMMQPSLMGIHGNMNNQQAGTSGVPQVNLSNMQGQPQQGPPSQLMGMHQQIVPSQGQMVQQQGTLNPQNPMILSRAQLMPQGQMMVNPPSQNLGPSPQRMTPPKQMLSQQGPQMMAPHNQMMGPQGQVLLQQNPMIEQIMTNQMQGNKQQFNTQNQSNVMPGPAQIMRGPTPNMQGNMVQFTGQMSGQMLPQQGPVNNSPSQVMGIQGQVLRPPGPSPHMAQQHGDPATTANNDVSLSQMMPDVSIQQTNMVPPHVQAMQGNSASGNHFSGHGMSFNAPFSGAPNGNQMSCGQNPGFPVNKDVTLTSPLLVNLLQSDISAGHFGVNNKQNNTNANKPKKKKPPRKKKNSQQDLNTPDTRPAGLEEADQPPLPGEQGINLDNSGPKLPEFSNRPPGYPSQPVEQRPLQQMPPQLMQHVAPPPQPPQQQPQPQLPQQQQPPPPSQPQSQQQQQQQQQMMMMLMMQQDPKSVRLPVSQNVHPPRGPLNPDSQRMPMQQSGSVPVMVSLQGPASVPPSPDKQRMPMPVNTPLGSNSRKMVYQESPQNPSSSPLAEMASLPEASGSEAPSVPGGPNNMPSHVVLPQNQLMMTGPKPGPSPLSATQGATPQQPPVNSLPSSHGHHFPNVAAPTQTSRPKTPNRASPRPYYPQTPNNRPPSTEPSEISLSPERLNASIAGLFPPQINIPLPPRPNLNRGFDQQGLNPTTLKAIGQAPSNLTMNPSNFATPQTHKLDSVVVNSGKQSNSGATKRASPSNSRRSSPGSSRKTTPSPGRQNSKAPKLTLASQTNAALLQNVELPRNVLVSPTPLANPPVPGSFPNNSGLNPQNSTVSVAAVGGVVEDNKESLNVPQDSDCQNSQSRKEQVNIELKAVPAQEVKMVVPEDQSKKDGQPSDPNKLPSVEENKNLVSPAMREAPTSLSQLLDNSGAPNVTIKPPGLTDLEVTPPVVSGEDLKKASVIPTLQDLSSSKEPSNSLNLPHSNELCSSLVHPELSEVSSNVAPSIPPVMSRPVSSSSISTPLPPNQITVFVTSNPITTSANTSAALPTHLQSALMSTVVTMPNAGSKVMVSEGQSAAQSNARPQFITPVFINSSSIIQVMKGSQPSTIPAAPLTTNSGLMPPSVAVVGPLHIPQNIKFSSAPVPPNALSSSPAPNIQTGRPLVLSSRATPVQLPSPPCTSSPVVPSHPPVQQVKELNPDEASPQVNTSADQNTLPSSQSTTMVSPLLTNSPGSSGNRRSPVSSSKGKGKVDKIGQILLTKACKKVTGSLEKGEEQYGADGETEGQGLDTTAPGLMGTEQLSTELDSKTPTPPAPTLLKMTSSPVGPGTASAGPSLPGGALPTSVRSIVTTLVPSELISAVPTTKSNHGGIASESLAGGLVEEKVGSHPELLPSIAPSQNLVSKETSTTALQASVARPELEVNAAIVSGQSSEPKEIVEKSKIPGRRNSRTEEPTVASESVENGHRKRSSRPASASSSTKDITSAVQSKRRKSK

595 NP_001035.1 NP_000786.1

>NP_001035.1

MSKSKCSVGLMSSVVAPAKEPNAVGPKEVELILVKEQNGVQLTSSTLTNPRQSPVEAQDRETWGKKIDFLLSVIGFAVDLANVWRFPYLCYKNGGGAFLVPYLLFMVIAGMPLFYMELALGQFNREGAAGVWKICPILKGVGFTVILISLYVGFFYNVIIAWALHYLFSSFTTELPWIHCNNSWNSPNCSDAHPGDSSGDSSGLNDTFGTTPAAEYFERGVLHLHQSHGIDDLGPPRWQLTACLVLVIVLLYFSLWKGVKTSGKVVWITATMPYVVLTALLLRGVTLPGAIDGIRAYLSVDFYRLCEASVWIDAATQVCFSLGVGFGVLIAFSSYNKFTNNCYRDAIVTTSINSLTSFSSGFVVFSFLGYMAQKHSVPIGDVAKDGPGLIFIIYPEAIATLPLSSAWAVVFFIMLLTLGIDSAMGGMESVITGLIDEFQLLHRHRELFTLFIVLATFLLSLFCVTNGGIYVFTLLDHFAAGTSILFGVLIEAIGVAWFYGVGQFSDDIQQMTGQRPSLYWRLCWKLVSPCFLLFVVVVSIVTFRPPHYGAYIFPDWANALGWVIATSSMAMVPIYAAYKFCSLPGSFREKLAYAIAPEKDRELVDRGEVRQFTLRHWLKV

>NP_000786.1

MDPLNLSWYDDDLERQNWSRPFNGSDGKADRPHYNYYATLLTLLIAVIVFGNVLVCMAVSREKALQTTTNYLIVSLAVADLLVATLVMPWVVYLEVVGEWKFSRIHCDIFVTLDVMMCTASILNLCAISIDRYTAVAMPMLYNTRYSSKRRVTVMISIVWVLSFTISCPLLFGLNNADQNECIIANPAFVVYSSIVSFYVPFIVTLLVYIKIYIVLRRRRKRVNTKRSSRAFRAHLRAPLKGNCTHPEDMKLCTVIMKSNGSFPVNRRRVEAARRAQELEMEMLSSTSPPERTRYSPIPPSHHQLTLPDPSHHGLHSTPDSPAKPEKNGHAKDHPKIAKIFEIQTMPNGKTRTSLKTMSRRKLSQQKEKKATQMLAIVLGVFIICWLPFFITHILNIHCDCNIPPVLYSAFTWLGYVNSAVNPIIYTTFNIEFRKAFLKILHC

596 NP_001002255.1 NP_003895.1

>NP_001002255.1

MANEKPTEEVKTENNNHINLKVAGQDGSVVQFKIKRQTPLSKLMKAYCEPRGLSVKQIRFRFGGQPISGTDKPAQLEMEDEDTIDVFQQPTGGVY

>NP_003895.1

MAASGAVEPGPPGAAVAPSPAPAPPPAPDHLFRPISAEDEEQQPTEIESLCMNCYCNGMTRLLLTKIPFFREIIVSSFSCEHCGWNNTEIQSAGRIQDQGVRYTLSVRALEDMNREVVKTDSAATRIPELDFEIPAFSQKGALTTVEGLITRAISGLEQDQPARRANKDATAERIDEFIVKLKELKQVASPFTLIIDDPSGNSFVENPHAPQKDDALVITHYNRTRQQEEMLGLQEEAPAEKPEEEDLRNEVLQFSTNCPECNAPAQTNMKLVQIPHFKEVIIMATNCENCGHRTNEVKSGGAVEPLGTRITLHITDASDMTRDLLKSETCSVEIPELEFELGMAVLGGKFTTLEGLLKDIRELVTKNPFTLGDSSNPGQTERLQEFSQKMDQIIEGNMKAHFIMDDPAGNSYLQNVYAPEDDPEMKVERYKRTFDQNEELGLNDMKTEGYEAGLAPQR

597 NP_002721.1 NP_004324.2

>NP_002721.1

MGNAAAAKKGSEQESVKEFLAKAKEDFLKKWESPAQNTAHLDQFERIKTLGTGSFGRVMLVKHKETGNHYAMKILDKQKVVKLKQIEHTLNEKRILQAVNFPFLVKLEFSFKDNSNLYMVMEYVPGGEMFSHLRRIGRFSEPHARFYAAQIVLTFEYLHSLDLIYRDLKPENLLIDQQGYIQVTDFGFAKRVKGRTWTLCGTPEYLAPEIILSKGYNKAVDWWALGVLIYEMAAGYPPFFADQPIQIYEKIVSGKVRFPSHFSSDLKDLLRNLLQVDLTKRFGNLKNGVNDIKNHKWFATTDWIAIYQRKVEAPFIPKFKGPGDTSNFDDYEEEEIRVSINEKCGKEFSEF

>NP_004324.2

MAALSGGGGGGAEPGQALFNGDMEPEAGAGAGAAASSAADPAIPEEVWNIKQMIKLTQEHIEALLDKFGGEHNPPSIYLEAYEEYTSKLDALQQREQQLLESLGNGTDFSVSSSASMDTVTSSSSSSLSVLPSSLSVFQNPTDVARSNPKSPQKPIVRVFLPNKQRTVVPARCGVTVRDSLKKALMMRGLIPECCAVYRIQDGEKKPIGWDTDISWLTGEELHVEVLENVPLTTHNFVRKTFFTLAFCDFCRKLLFQGFRCQTCGYKFHQRCSTEVPLMCVNYDQLDLLFVSKFFEHHPIPQEEASLAETALTSGSSPSAPASDSIGPQILTSPSPSKSIPIPQPFRPADEDHRNQFGQRDRSSSAPNVHINTIEPVNIDDLIRDQGFRGDGGSTTGLSATPPASLPGSLTNVKALQKSPGPQRERKSSSSSEDRNRMKTLGRRDSSDDWEIPDGQITVGQRIGSGSFGTVYKGKWHGDVAVKMLNVTAPTPQQLQAFKNEVGVLRKTRHVNILLFMGYSTKPQLAIVTQWCEGSSLYHHLHIIETKFEMIKLIDIARQTAQGMDYLHAKSIIHRDLKSNNIFLHEDLTVKIGDFGLATVKSRWSGSHQFEQLSGSILWMAPEVIRMQDKNPYSFQSDVYAFGIVLYELMTGQLPYSNINNRDQIIFMVGRGYLSPDLSKVRSNCPKAMKRLMAECLKKKRDERPLFPQILASIELLARSLPKIHRSASEPSLNRAGFQTEDFSLYACASPKTPIQAGGYGAFPVH

598 NP_001427.2 NP_059107.1

>NP_001427.2

MKPGFSPRGGGFGGRGGFGDRGGRGGRGGFGGGRGRGGGFRGRGRGGGGGGGGGGGGGRGGGGFHSGGNRGRGRGGKRGNQSGKNVMVEPHRHEGVFICRGKEDALVTKNLVPGESVYGEKRVSISEGDDKIEYRAWNPFRSKLAAAILGGVDQIHIKPGAKVLYLGAASGTTVSHVSDIVGPDGLVYAVEFSHRSGRDLINLAKKRTNIIPVIEDARHPHKYRMLIAMVDVIFADVAQPDQTRIVALNAHTFLRNGGHFVISIKANCIDSTASAEAVFASEVKKMQQENMKPQEQLTLEPYERDHAVVVGVYRPPPKVKN

>NP_059107.1

MAMSSGGSGGGVPEQEDSVLFRRGTGQSDDSDIWDDTALIKAYDKAVASFKHALKNGDICETSGKPKTTPKRKPAKKNKSQKKNTAASLQQWKVGDKCSAIWSEDGCIYPATIASIDFKRETCVVVYTGYGNREEQNLSDLLSPICEVANNIEQNAQENENESQVSTDESENSRSPGNKSDNIKPKSAPWNSFLPPPPPMPGPRLGPGKPGLKFNGPPPPPPPPPPHLLSCWLPPFPSGPPIIPPPPPICPDSLDDADALGSMLISWYMSGYHTGYYMGFRQNQKEGRCSHSLN

599 NP_000859.1 NP_004304.1

>NP_000859.1

MVNLRNAVHSFLVHLIGLLVWQCDISVSPVAAIVTDIFNTSDGGRFKFPDGVQNWPALSIVIIIIMTIGGNILVIMAVSMEKKLHNATNYFLMSLAIADMLVGLLVMPLSLLAILYDYVWPLPRYLCPVWISLDVLFSTASIMHLCAISLDRYVAIRNPIEHSRFNSRTKAIMKIAIVWAISIGVSVPIPVIGLRDEEKVFVNNTTCVLNDPNFVLIGSFVAFFIPLTIMVITYCLTIYVLRRQALMLLHGHTEEPPGLSLDFLKCCKRNTAEEENSANPNQDQNARRRKKKERRPRGTMQAINNERKASKVLGIVFFVFLIMWCPFFITNILSVLCEKSCNQKLMEKLLNVFVWIGYVCSGINPLVYTLFNKIYRRAFSNYLRCNYKVEKKPPVRQIPRVAATALSGRELNVNIYRHTNEPVIEKASDNEPGIEMQVENLELPVNPSSVVSERISSV

>NP_004304.1

MGEKPGTRVFKKSSPNCKLTVYLGKRDFVDHLDKVDPVDGVVLVDPDYLKDRKVFVTLTCAFRYGREDLDVLGLSFRKDLFIATYQAFPPVPNPPRPPTRLQDRLLRKLGQHAHPFFFTIPQNLPCSVTLQPGPEDTGKACGVDFEIRAFCAKSLEEKSHKRNSVRLVIRKVQFAPEKPGPQPSAETTRHFLMSDRSLHLEASLDKELYYHGEPLNVNVHVTNNSTKTVKKIKVSVRQYADICLFSTAQYKCPVAQLEQDDQVSPSSTFCKVYTITPLLSDNREKRGLALDGKLKHEDTNLASSTIVKEGANKEVLGILVSYRVKVKLVVSRGGDVSVELPFVLMHPKPHDHIPLPRPQSAAPETDVPVDTNLIEFDTNYATDDDIVFEDFARLRLKGMKDDDYDDQLC

600 NP_059345.1 NP_057232.2

>NP_059345.1

MSLSRSEEMHRLTENVYKTIMEQFNPSLRNFIAMGKNYEKALAGVTYAAKGYFDALVKMGELASESQGSKELGDVLFQMAEVHRQIQNQLEEMLKSFHNELLTQLEQKVELDSRYLSAALKKYQTEQRSKGDALDKCQAELKKLRKKSQGSKNPQKYSDKELQYIDAISNKQGELENYVSDGYKTALTEERRRFCFLVEKQCAVAKNSAAYHSKGKELLAQKLPLWQQACADPSKIPERAVQLMQQVASNGATLPSALSASKSNLVISDPIPGAKPLPVPPELAPFVGRMSAQESTPIMNGVTGPDGEDYSPWADRKAAQPKSLSPPQSQSKLSDSYSNTLPVRKSVTPKNSYATTENKTLPRSSSMAAGLERNGRMRVKAIFSHAAGDNSTLLSFKEGDLITLLVPEARDGWHYGESEKTKMRGWFPFSYTRVLDSDGSDRLHMSLQQGKSSSTGNLLDKDDLAIPPPDYGAASRAFPAQTASGFKQRPYSVAVPAFSQGLDDYGARSMSRNPFAHVQLKPTVTNDRCDLSAQGPEGREHGDGSARTLAGR

>NP_057232.2

MTHSPATSEDEERHSASECPEGGSESDSSPDGPGRGPRGTRGQGSGAPGSLASVRGLQGRSMSVPDDAHFSMMVFRIGIPDLHQTKCLRFNPDATIWTAKQQVLCALSESLQDVLNYGLFQPATSGRDANFLEEERLLREYPQSFEKGVPYLEFRYKTRVYKQTNLDEKQLAKLHTKTGLKKFLEYVQLGTSDKVARLLDKGLDPNYHDSDSGETPLTLAAQTEGSVEVIRTLCLGGAHIDFRARDGMTALHKAACARHCLALTALLDLGGSPNYKDRRGLTPLFHTAMVGGDPRCCELLLFNRAQLGIADENGWQEIHQACQRGHSQHLEHLLFYGAEPGAQNASGNTALHICALYNKETCARILLYRGADKDVKNNNGQTPFQVAVIAGNFELGELIRNHREQDVVPFQESPKYAARRRGPPGTGLTVPPALLRANSDTSMALPDWMVFSAPGAASSGAPGPTSGSQGQSQPSAPTTKLSSGTLRSASSPRGARARSPSRGRHPEDAKRQPRGRPSSSGTPREGPAGGTGGSGGPGGSLGSRGRRRKLYSAVPGRSFMAVKSYQAQAEGEISLSKGEKIKVLSIGEGGFWEGQVKGRVGWFPSDCLEEVANRSQESKQESRSDKAKRLFRHYTVGSYDSFDAPSLMDGIGPGSDYIIKEKTVLLQKKDSEGFGFVLRGAKAQTPIEEFTPTPAFPALQYLESVDEGGVAWRAGLRMGDFLIEVNGQNVVKVGHRQVVNMIRQGGNTLMVKVVMVTRHPDMDEAVHKKAPQQAKRLPPPTISLRSKSMTSELEEMEYEQQPAPVPSMEKKRTVYQMALNKLDEILAAAQQTISASESPGPGGLASLGKHRPKGFFATESSFDPHHRAQPSYERPSFLPPGPGLMLRQKSIGAAEDDRPYLAPPAMKFSRSLSVPGSEDIPPPPTTSPPEPPYSTPPVPSSSGRLTPSPRGGPFNPGSGGPLPASSPASFDGPSPPDTRVGSREKSLYHSGPLPPAHHHPPHHHHHHAPPPQPHHHHAHPPHPPEMETGGSPDDPPPRLALGPQPSLRGWRGGGPSPTPGAPSPSHHGSAGGGGGSSQGPALRYFQLPPRAASAAMYVPARSGRGRKGPLVKQTKVEGEPQKGGGLPPAPSPTSPASPQPPPAVAAPSEKNSIPIPTIIIKAPSTSSSGRSSQGSSTEAEPPTQPEPTGGGGGGGSSPSPAPAMSPVPPSPSPVPTPASPSGPATLDFTSQFGAALVGAARREGGWQNEARRRSTLFLSTDAGDEDGGDGGLGTGAAPGPRLRHSKSIDEGMFSAEPYLRLESAGSGAGYGGYGAGSRAYGGGGGSSAFTSFLPPRPLVHPLTGKALDPASPLGLALAARERALKESSEGGGAPQPPPRPPSPRYEAPPPTPHHHSPHAHHEPVLRLWGASPPDPARRELGYRAGLGSQEKSLPASPPAARRSLLHRLPPTAPGVGPLLLQLGTEPPAPHPGVSKPWRSAAPEEPERLPLHVRFLENCQPRAPVTSGRGPPSEDGPGVPPPSPRRSVPPSPTSPRASEENGLPLLVLPPPAPSVDVEDGEFLFVEPLPPPLEFSNSFEKPESPLTPGPPHPLPDTPAPATPLPPVPPPAVAAAPPTLDSTASSLTSYDSEVATLTQGASAAPGDPHPPGPPAPAAPAPAAPQPGPDPPPGTDSGIEEVDSRSSSDHPLETISSASTLSSLSAEGGGSAGGGGGAGAGVASGPELLDTYVAYLDGQAFGGSSTPGPPYPPQLMTPSKLRGRALGASGGLRPGPSGGLRDPVTPTSPTVSVTGAGTDGLLALRACSGPPTAGVAGGPVAVEPEVPPVPLPTASSLPRKLLPWEEGPGPPPPPLPGPLAQPQASALATVKASIISELSSKLQQFGGSSAAGGALPWARGGSGGGGDSHHGGASYVPERTSSLQRQRLSDDSQSSLLSKPVSSLFQNWPKPPLPPLPTGTGVSPTAAAAPGATSPSASSSSTSTRHLQGVEFEMRPPLLRRAPSPSLLPASEHKVSPAPRPSSLPILPSGPLYPGLFDIRGSPTGGAGGSADPFAPVFVPPHPGISGGLGGALSGASRSLSPTRLLSLPPDKPFGAKPLGFWTKFDVADWLEWLGLAEHRAQFLDHEIDGSHLPALTKEDYVDLGVTRVGHRMNIDRALKFFLER

601 NP_203699.1 NP_000475.1

>NP_203699.1

MKLRGVSLAAGLFLLALSLWGQPAEAAACYGCSPGSKCDCSGIKGEKGERGFPGLEGHPGLPGFPGPEGPPGPRGQKGDDGIPGPPGPKGIRGPPGLPGFPGTPGLPGMPGHDGAPGPQGIPGCNGTKGERGFPGSPGFPGLQGPPGPPGIPGMKGEPGSIIMSSLPGPKGNPGYPGPPGIQGLPGPTGIPGPIGPPGPPGLMGPPGPPGLPGPKGNMGLNFQGPKGEKGEQGLQGPPGPPGQISEQKRPIDVEFQKGDQGLPGDRGPPGPPGIRGPPGPPGGEKGEKGEQGEPGKRGKPGKDGENGQPGIPGLPGDPGYPGEPGRDGEKGQKGDTGPPGPPGLVIPRPGTGITIGEKGNIGLPGLPGEKGERGFPGIQGPPGLPGPPGAAVMGPPGPPGFPGERGQKGDEGPPGISIPGPPGLDGQPGAPGLPGPPGPAGPHIPPSDEICEPGPPGPPGSPGDKGLQGEQGVKGDKGDTCFNCIGTGISGPPGQPGLPGLPGPPGSLGFPGQKGEKGQAGATGPKGLPGIPGAPGAPGFPGSKGEPGDILTFPGMKGDKGELGSPGAPGLPGLPGTPGQDGLPGLPGPKGEPGGITFKGERGPPGNPGLPGLPGNIGPMGPPGFGPPGPVGEKGIQGVAGNPGQPGIPGPKGDPGQTITQPGKPGLPGNPGRDGDVGLPGDPGLPGQPGLPGIPGSKGEPGIPGIGLPGPPGPKGFPGIPGPPGAPGTPGRIGLEGPPGPPGFPGPKGEPGFALPGPPGPPGLPGFKGALGPKGDRGFPGPPGPPGRTGLDGLPGPKGDVGPNGQPGPMGPPGLPGIGVQGPPGPPGIPGPIGQPGLHGIPGEKGDPGPPGLDVPGPPGERGSPGIPGAPGPIGPPGSPGLPGKAGASGFPGTKGEMGMMGPPGPPGPLGIPGRSGVPGLKGDDGLQGQPGLPGPTGEKGSKGEPGLPGPPGPMDPNLLGSKGEKGEPGLPGIPGVSGPKGYQGLPGDPGQPGLSGQPGLPGPPGPKGNPGLPGQPGLIGPPGLKGTIGDMGFPGPQGVEGPPGPSGVPGQPGSPGLPGQKGDKGDPGISSIGLPGLPGPKGEPGLPGYPGNPGIKGSVGDPGLPGLPGTPGAKGQPGLPGFPGTPGPPGPKGISGPPGNPGLPGEPGPVGGGGHPGQPGPPGEKGKPGQDGIPGPAGQKGEPGQPGFGNPGPPGLPGLSGQKGDGGLPGIPGNPGLPGPKGEPGFHGFPGVQGPPGPPGSPGPALEGPKGNPGPQGPPGRPGPTGFQGLPGPEGPPGLPGNGGIKGEKGNPGQPGLPGLPGLKGDQGPPGLQGNPGRPGLNGMKGDPGLPGVPGFPGMKGPSGVPGSAGPEGEPGLIGPPGPPGLPGPSGQSIIIKGDAGPPGIPGQPGLKGLPGPQGPQGLPGPTGPPGDPGRNGLPGFDGAGGRKGDPGLPGQPGTRGLDGPPGPDGLQGPPGPPGTSSVAHGFLITRHSQTTDAPQCPQGTLQVYEGFSLLYVQGNKRAHGQDLGTAGSCLRRFSTMPFMFCNINNVCNFASRNDYSYWLSTPEPMPMSMQPLKGQSIQPFISRCAVCEAPAVVIAVHSQTIQIPHCPQGWDSLWIGYSFMMHTSAGAEGSGQALASPGSCLEEFRSAPFIECHGRGTCNYYANSYSFWLATVDVSDMFSKPQSETLKAGDLRTRISRCQVCMKRT

>NP_000475.1

MLPGLALLLLAAWTARALEVPTDGNAGLLAEPQIAMFCGRLNMHMNVQNGKWDSDPSGTKTCIDTKEGILQYCQEVYPELQITNVVEANQPVTIQNWCKRGRKQCKTHPHFVIPYRCLVGEFVSDALLVPDKCKFLHQERMDVCETHLHWHTVAKETCSEKSTNLHDYGMLLPCGIDKFRGVEFVCCPLAEESDNVDSADAEEDDSDVWWGGADTDYADGSEDKVVEVAEEEEVAEVEEEEADDDEDDEDGDEVEEEAEEPYEEATERTTSIATTTTTTTESVEEVVREVCSEQAETGPCRAMISRWYFDVTEGKCAPFFYGGCGGNRNNFDTEEYCMAVCGSAMSQSLLKTTQEPLARDPVKLPTTAASTPDAVDKYLETPGDENEHAHFQKAKERLEAKHRERMSQVMREWEEAERQAKNLPKADKKAVIQHFQEKVESLEQEAANERQQLVETHMARVEAMLNDRRRLALENYITALQAVPPRPRHVFNMLKKYVRAEQKDRQHTLKHFEHVRMVDPKKAAQIRSQVMTHLRVIYERMNQSLSLLYNVPAVAEEIQDEVDELLQKEQNYSDDVLANMISEPRISYGNDALMPSLTETKTTVELLPVNGEFSLDDLQPWHSFGADSVPANTENEVEPVDARPAADRGLTTRPGSGLTNIKTEEISEVKMDAEFRHDSGYEVHHQKLVFFAEDVGSNKGAIIGLMVGGVVIATVIVITLVMLKKKQYTSIHHGVVEVDAAVTPEERHLSKMQQNGYENPTYKFFEQMQN

602 NP_005138.2 NP_002866.2

>NP_005138.2

MAARCSTRWLLVVVGTPRLPAISGRGARPPREGVVGAWLSRKLSVPAFASSLTSCGPRALLTLRPGVSLTGTKHYPFICTASFHTSAPLAKEDYYQILGVPRNASQKEIKKAYYQLAKKYHPDTNKDDPKAKEKFSQLAEAYEVLSDEVKRKQYDAYGSAGFDPGASGSQHSYWKGGPTVDPEELFRKIFGEFSSSSFGDFQTVFDQPQEYFMELTFNQAAKGVNKEFTVNIMDTCERCNGKGNEPGTKVQHCHYCGGSGMETINTGPFVMRSTCRRCGGRGSIIISPCVVCRGAGQAKQKKRVMIPVPAGVEDGQTVRMPVGKREIFITFRVQKSPVFRRDGADIHSDLFISIAQALLGGTARAQGLYETINVTIPPGTQTDQKIRMGGKGIPRINSYGYGDHYIHIKIRVPKRLTSRQQSLILSYAEDETDVEGTVNGVTLTSSGGSTMDSSAGSKARREAGEDEEGFLSKLKKMFTS

>NP_002866.2

MAMQMQLEANADTSVEEESFGPQPISRLEQCGINANDVKKLEEAGFHTVEAVAYAPKKELINIKGISEAKADKILAEAAKLVPMGFTTATEFHQRRSEIIQITTGSKELDKLLQGGIETGSITEMFGEFRTGKTQICHTLAVTCQLPIDRGGGEGKAMYIDTEGTFRPERLLAVAERYGLSGSDVLDNVAYARAFNTDHQTQLLYQASAMMVESRYALLIVDSATALYRTDYSGRGELSARQMHLARFLRMLLRLADEFGVAVVITNQVVAQVDGAAMFAADPKKPIGGNIIAHASTTRLYLRKGRGETRICKIYDSPCLPEAEAMFAINADGVGDAKD

603 NP_003817.1 NP_056285.1

>NP_003817.1

MAAQKINEGLEHLAKAEKYLKTGFLKWKPDYDSAASEYGKAAVAFKNAKQFEQAKDACLREAVAHENNRALFHAAKAYEQAGMMLKEMQKLPEAVQLIEKASMMYLENGTPDTAAMALERAGKLIENVDPEKAVQLYQQTANVFENEERLRQAVELLGKASRLLVRGRRFDEAALSIQKEKNIYKEIENYPTCYKKTIAQVLVHLHRNDYVAAERCVRESYSIPGFNGSEDCAALEQLLEGYDQQDQDQVSDVCNSPLFKYMDNDYAKLGLSLVVPGGGIKKKSPATPQAKPDGVTATAADEEEDEYSGGLC

>NP_056285.1

MALVRGAEPAAGPSRWLPTHVQVTVLRARGLRGKSSGAGSTSDAYTVIQVGREKYSTSVVEKTHGCPEWREECSFELPPGALDGLLRAQEADAGPAPWAASSAAACELVLTTMHRSLIGVDKFLGQATVALDEVFGAGRAQHTQWYKLHSKPGKKEKERGEIEVTIQFTRNNLSASMFDLSMKDKPRSPFSKIRDKMKGKKKYDLESASAILPSSAIEDPDLGSLGKMGKAKGFFLRNKLRKSSLTQSNTSLGSDSTLSSASGSLAYQGPGAELLTRSPSRSSWLSTEGGRDSAQSPKLFTHKRTYSDEANQMRVAPPRALLDLQGHLDAASRSSLCVNGSHIYNEEPQGPVRHRSSISGSLPSSGSLQAVSSRFSEEGPRSTDDTWPRGSRSNSSSEAVLGQEELSAQAKVLAPGASHPGEEEGARLPEGKPVQVATPIVASSEAVAEKEGARKEERKPRMGLFHHHHQGLSRSELGRRSSLGEKGGPILGASPHHSSSGEEKAKSSWFGLREAKDPTQKPSPHPVKPLSAAPVEGSPDRKQSRSSLSIALSSGLEKLKTVTSGSIQPVTQAPQAGQMVDTKRLKDSAVLDQSAKYYHLTHDELISLLLQRERELSQRDEHVQELESYIDRLLVRIMETSPTLLQIPPGPPK

604 NP_006696.1 NP_002835.2

>NP_006696.1

MTLEEVRGQDTVPESTARMQGAGKALHELLLSAQRQGCLTAGVYESAKVLNVDPDNVTFCVLAAGEEDEGDIALQIHFTLIQAFCCENDIDIVRVGDVQRLAAIVGAGEEAGAPGDLHCILISNPNEDAWKDPALEKLSLFCEESRSVNDWVPSITLPE

>NP_002835.2

MDTTAAAALPAFVALLLLSPWPLLGSAQGQFSAGGCTFDDGPGACDYHQDLYDDFEWVHVSAQEPHYLPPEMPQGSYMIVDSSDHDPGEKARLQLPTMKENDTHCIDFSYLLYSQKGLNPGTLNILVRVNKGPLANPIWNVTGFTGRDWLRAELAVSTFWPNEYQVIFEAEVSGGRSGYIAIDDIQVLSYPCDKSPHFLRLGDVEVNAGQNATFQCIATGRDAVHNKLWLQRRNGEDIPVAQTKNINHRRFAASFRLQEVTKTDQDLYRCVTQSERGSGVSNFAQLIVREPPRPIAPPQLLGVGPTYLLIQLNANSIIGDGPIILKEVEYRMTSGSWTETHAVNAPTYKLWHLDPDTEYEIRVLLTRPGEGGTGLPGPPLITRTKCAEPMRTPKTLKIAEIQARRIAVDWESLGYNITRCHTFNVTICYHYFRGHNESKADCLDMDPKAPQHVVNHLPPYTNVSLKMILTNPEGRKESEETIIQTDEDVPGPVPVKSLQGTSFENKIFLNWKEPLDPNGIITQYEISYSSIRSFDPAVPVAGPPQTVSNLWNSTHHVFMHLHPGTTYQFFIRASTVKGFGPATAINVTTNISAPTLPDYEGVDASLNETATTITVLLRPAQAKGAPISAYQIVVEELHPHRTKREAGAMECYQVPVTYQNAMSGGAPYYFAAELPPGNLPEPAPFTVGDNRTYQGFWNPPLAPRKGYNIYFQAMSSVEKETKTQCVRIATKAAATEEPEVIPDPAKQTDRVVKIAGISAGILVFILLLLVVILIVKKSKLAKKRKDAMGNTRQEMTHMVNAMDRSYADQSTLHAEDPLSITFMDQHNFSPRYENHSATAESSRLLDVPRYLCEGTESPYQTGQLHPAIRVADLLQHINLMKTSDSYGFKEEYESFFEGQSASWDVAKKDQNRAKNRYGNIIAYDHSRVILQPVEDDPSSDYINANYIDGYQRPSHYIATQGPVHETVYDFWRMIWQEQSACIVMVTNLVEVGRVKCYKYWPDDTEVYGDFKVTCVEMEPLAEYVVRTFTLERRGYNEIREVKQFHFTGWPDHGVPYHATGLLSFIRRVKLSNPPSAGPIVVHCSAGAGRTGCYIVIDIMLDMAEREGVVDIYNCVKALRSRRINMVQTEEQYIFIHDAILEACLCGETAIPVCEFKAAYFDMIRIDSQTNSSHLKDEFQTLNSVTPRLQAEDCSIACLPRNHDKNRFMDMLPPDRCLPFLITIDGESSNYINAALMDSYRQPAAFIVTQYPLPNTVKDFWRLVYDYGCTSIVMLNEVDLSQGCPQYWPEEGMLRYGPIQVECMSCSMDCDVINRIFRICNLTRPQEGYLMVQQFQYLGWASHREVPGSKRSFLKLILQVEKWQEECEEGEGRTIIHCLNGGGRSGMFCAIGIVVEMVKRQNVVDVFHAVKTLRNSKPNMVEAPEQYRFCYDVALEYLESS

605 NP_003293.2 NP_077721.1

>NP_003293.2

MSGPTWLPPKQPEPARAPQGRAIPRGTPGPPPAHGAALQPHPRVNFCPLPSEQCYQAPGGPEDRGPAWVGSHGVLQHTQGLPADRGGLRPGSLDAEIDLLSSTLAELNGGRGHASRRPDRQAYEPPPPPAYRTGSLKPNPASPLPASPYGGPTPASYTTASTPAGPAFPVQVKVAQPVRGCGPPRRGASQASGPLPGPHFPLPGRGEVWGPGYRSQREPGPGAKEEAAGVSGPAGRGRGGEHGPQVPLSQPPEDELDRLTKKLVHDMNHPPSGEYFGQCGGCGEDVVGDGAGVVALDRVFHVGCFVCSTCRAQLRGQHFYAVERRAYCEGCYVATLEKCATCSQPILDRILRAMGKAYHPGCFTCVVCHRGLDGIPFTVDATSQIHCIEDFHRKFAPRCSVCGGAIMPEPGQEETVRIVALDRSFHIGCYKCEECGLLLSSEGECQGCYPLDGHILCKACSAWRIQELSATVTTDC

>NP_077721.1

MAALSCLLDSVRRDIKKVDRELRQLRCIDEFSTRCLCDLYMHPYCCCDLHPYPYCLCYSKRSRSCGLCDLYPCCLCDYKLYCLRPSLRSLERKAIRAIEDEKRELAKLRRTTNRILASSCCSSNILGSVNVCGFEPDQVKVRVKDGKVCVSAERENRYDCLGSKKYSYMNICKEFSLPPCVDEKDVTYSYGLGSCVKIESPCYPCTSPCSPCSPCNPCNPCSPCNPCSPYDPCNPCYPCGSRFSCRKMIL

606 NP_001339.1 NP_002735.3

>NP_001339.1

MSTFRQEDVEDHYEMGEELGSGQFAIVRKCRQKGTGKEYAAKFIKKRRLSSSRRGVSREEIEREVNILREIRHPNIITLHDIFENKTDVVLILELVSGGELFDFLAEKESLTEDEATQFLKQILDGVHYLHSKRIAHFDLKPENIMLLDKNVPNPRIKLIDFGIAHKIEAGNEFKNIFGTPEFVAPEIVNYEPLGLEADMWSIGVITYILLSGASPFLGETKQETLTNISAVNYDFDEEYFSNTSELAKDFIRRLLVKDPKRRMTIAQSLEHSWIKAIRRRNVRGEDSGRKPERRRLKTTRLKEYTIKSHSSLPPNNSYADFERFSKVLEEAAAAEEGLRELQRSRRLCHEDVEALAAIYEEKEAWYREESDSLGQDLRRLRQELLKTEALKRQAQEEAKGALLGTSGLKRRFSRLENRYEALAKQVASEMRFVQDLVRALEQEKLQGVECGLR

>NP_002735.3

MPSRTGPKMEGSGGRVRLKAHYGGDIFITSVDAATTFEELCEEVRDMCRLHQQHPLTLKWVDSEGDPCTVSSQMELEEAFRLARQCRDEGLIIHVFPSTPEQPGLPCPGEDKSIYRRGARRWRKLYRANGHLFQAKRFNRRAYCGQCSERIWGLARQGYRCINCKLLVHKRCHGLVPLTCRKHMDSVMPSQEPPVDDKNEDADLPSEETDGIAYISSSRKHDSIKDDSEDLKPVIDGMDGIKISQGLGLQDFDLIRVIGRGSYAKVLLVRLKKNDQIYAMKVVKKELVHDDEDIDWVQTEKHVFEQASSNPFLVGLHSCFQTTSRLFLVIEYVNGGDLMFHMQRQRKLPEEHARFYAAEICIALNFLHERGIIYRDLKLDNVLLDADGHIKLTDYGMCKEGLGPGDTTSTFCGTPNYIAPEILRGEEYGFSVDWWALGVLMFEMMAGRSPFDIITDNPDMNTEDYLFQVILEKPIRIPRFLSVKASHVLKGFLNKDPKERLGCRPQTGFSDIKSHAFFRSIDWDLLEKKQALPPFQPQITDDYGLDNFDTQFTSEPVQLTPDDEDAIKRIDQSEFEGFEYINPLLLSTEESV

607 NP_000909.2 NP_001757.1

>NP_000909.2

MLRRALLCLAVAALVRADAPEEEDHVLVLRKSNFAEALAAHKYLLVEFYAPWCGHCKALAPEYAKAAGKLKAEGSEIRLAKVDATEESDLAQQYGVRGYPTIKFFRNGDTASPKEYTAGREADDIVNWLKKRTGPAATTLPDGAAAESLVESSEVAVIGFFKDVESDSAKQFLQAAEAIDDIPFGITSNSDVFSKYQLDKDGVVLFKKFDEGRNNFEGEVTKENLLDFIKHNQLPLVIEFTEQTAPKIFGGEIKTHILLFLPKSVSDYDGKLSNFKTAAESFKGKILFIFIDSDHTDNQRILEFFGLKKEECPAVRLITLEEEMTKYKPESEELTAERITEFCHRFLEGKIKPHLMSQELPEDWDKQPVKVLVGKNFEDVAFDEKKNVFVEFYAPWCGHCKQLAPIWDKLGETYKDHENIVIAKMDSTANEVEAVKVHSFPTLKFFPASADRTVIDYNGERTLDGFKKFLESGGQDGAGDDDDLEDLEEAEEPDMEEDDDQKAVKDEL

>NP_001757.1

MGCLLFLLLWALLQAWGSAEVPQRLFPLRCLQISSFANSSWTRTDGLAWLGELQTHSWSNDSDTVRSLKPWSQGTFSDQQWETLQHIFRVYRSSFTRDVKEFAKMLRLSYPLELQVSAGCEVHPGNASNNFFHVAFQGKDILSFQGTSWEPTQEAPLWVNLAIQVLNQDKWTRETVQWLLNGTCPQFVSGLLESGKSELKKQVKPKAWLSRGPSPGPGRLLLVCHVSGFYPKPVWVKWMRGEQEQQGTQPGDILPNADETWYLRATLDVVAGEAAGLSCRVKHSSLEGQDIVLYWGGSYTSMGLIALAVLACLLFLLIVGFTSRFKRQTSYQGVL

608 NP_127497.1 NP_004338.1

>NP_127497.1

MAGGAWGRLACYLEFLKKEELKEFQLLLANKAHSRSSSGETPAQPEKTSGMEVASYLVAQYGEQRAWDLALHTWEQMGLRSLCAQAQEGAGHSPSFPYSPSEPHLGSPSQPTSTAVLMPWIHELPAGCTQGSERRVLRQLPDTSGRRWREISASLLYQALPSSPDHESPSQESPNAPTSTAVLGSWGSPPQPSLAPREQEAPGTQWPLDETSGIYYTEIREREREKSEKGRPPWAAVVGTPPQAHTSLQPHHHPWEPSVRESLCSTWPWKNEDFNQKFTQLLLLQRPHPRSQDPLVKRSWPDYVEENRGHLIEIRDLFGPGLDTQEPRIVILQGAAGIGKSTLARQVKEAWGRGQLYGDRFQHVFYFSCRELAQSKVVSLAELIGKDGTATPAPIRQILSRPERLLFILDGVDEPGWVLQEPSSELCLHWSQPQPADALLGSLLGKTILPEASFLITARTTALQNLIPSLEQARWVEVLGFSESSRKEYFYRYFTDERQAIRAFRLVKSNKELWALCLVPWVSWLACTCLMQQMKRKEKLTLTSKTTTTLCLHYLAQALQAQPLGPQLRDLCSLAAEGIWQKKTLFSPDDLRKHGLDGAIISTFLKMGILQEHPIPLSYSFIHLCFQEFFAAMSYVLEDEKGRGKHSNCIIDLEKTLEAYGIHGLFGASTTRFLLGLLSDEGEREMENIFHCRLSQGRNLMQWVPSLQLLLQPHSLESLHCLYETRNKTFLTQVMAHFEEMGMCVETDMELLVCTFCIKFSRHVKKLQLIEGRQHRSTWSPTMVVLFRWVPVTDAYWQILFSVLKVTRNLKELDLSGNSLSHSAVKSLCKTLRRPRCLLETLRLAGCGLTAEDCKDLAFGLRANQTLTELDLSFNVLTDAGAKHLCQRLRQPSCKLQRLQLVSCGLTSDCCQDLASVLSASPSLKELDLQQNNLDDVGVRLLCEGLRHPACKLIRLGLDQTTLSDEMRQELRALEQEKPQLLIFSRRKPSVMTPTEGLDTGEMSNSTSSLKRQRLGSERAASHVAQANLKLLDVSKIFPIAEIAEESSPEVVPVELLCVPSPASQGDLHTKPLGTDDDFWGPTGPVATEVVDKEKNLYRVHFPVAGSYRWPNTGLCFVMREAVTVEIEFCVWDQFLGEINPQHSWMVAGPLLDIKAEPGAVEAVHLPHFVALQGGHVDTSLFQMAHFKEEGMLLEKPARVELHHIVLENPSFSPLGVLLKMIHNALRFIPVTSVVLLYHRVHPEEVTFHLYLIPSDCSIRKAIDDLEMKFQFVRIHKPPPLTPLYMGCRYTVSGSGSGMLEILPKELELCYRSPGEDQLFSEFYVGHLGSGIRLQVKDKKDETLVWEALVKPGDLMPATTLIPPARIAVPSPLDAPQLLHFVDQYREQLIARVTSVEVVLDKLHGQVLSQEQYERVLAENTRPSQMRKLFSLSQSWDRKCKDGLYQALKETHPHLIMELWEKGSKKGLLPLSS

>NP_004338.1

MFKGILQSGLDNFVINHMLKNNVAGQTSIQTLVPNTDQKSTSVKKDNHKKKTVKMLEYLGKDVLHGVFNYLAKHDVLTLKEEEKKKYYDAKIEDKALILVDSLRKNRVAHQMFTQTLLNMDQKITSVKPLLQIEAGPPESAESTNILKLCPREEFLRLCKKNHDEIYPIKKREDRRRLALIICNTKFDHLPARNGAHYDIVGMKRLLQGLGYTVVDEKNLTARDMESVLRAFAARPEHKSSDSTFLVLMSHGILEGICGTAHKKKKPDVLLYDTIFQIFNNRNCLSLKDKPKVIIVQACRGEKHGELWVRDSPASLAVISSQSSENLEADSVCKIHEEKDFIAFCSSTPHNVSWRDRTRGSIFITELITCFQKYSCCCHLMEIFRKVQKSFEVPQAKAQMPTIERATLTRDFYLFPGN

609 NP_057250.1 NP_003336.1

>NP_057250.1

MADSAELKQMVMSLRVSELQVLLGYAGRNKHGRKHELLTKALHLLKAGCSPAVQMKIKELYRRRFPQKIMTPADLSIPNVHSSPMPATLSPSTIPQLTYDGHPASSPLLPVSLLGPKHELELPHLTSALHPVHPDIKLQKLPFYDLLDELIKPTSLASDNSQRFRETCFAFALTPQQVQQISSSMDISGTKCDFTVQVQLRFCLSETSCPQEDHFPPNLCVKVNTKPCSLPGYLPPTKNGVEPKRPSRPINITSLVRLSTTVPNTIVVSWTAEIGRNYSMAVYLVKQLSSTVLLQRLRAKGIRNPDHSRALIKEKLTADPDSEIATTSLRVSLLCPLGKMRLTIPCRALTCSHLQCFDATLYIQMNEKKPTWVCPVCDKKAPYEHLIIDGLFMEILKYCTDCDEIQFKEDGTWAPMRSKKEVQEVSASYNGVDGCLSSTLEHQVASHHQSSNKNKKVEVIDLTIDSSSDEEEEEPSAKRTCPSLSPTSPLNNKGILSLPHQASPVSRTPSLPAVDTSYINTSLIQDYRHPFHMTPMPYDLQGLDFFPFLSGDNQHYNTSLLAAAAAAVSDDQDLLHSSRFFPYTSSQMFLDQLSAGGSTSLPTTNGSSSGSNSSLVSSNSLRESHSHTVTNRSSTDTASIFGIIPDIISLD

>NP_003336.1

MSGIALSRLAQERKAWRKDHPFGFVAVPTKNPDGTMNLMNWECAIPGKKGTPWEGGLFKLRMLFKDDYPSSPPKCKFEPPLFHPNVYPSGTVCLSILEEDKDWRPAITIKQILLGIQELLNEPNIQDPAQAEAYTIYCQNRVEYEKRVRAQAKKFAPS

610 NP_066124.1 NP_002736.3

>NP_066124.1

MAKATSGAAGLRLLLLLLLPLLGKVALGLYFSRDAYWEKLYVDQAAGTPLLYVHALRDAPEEVPSFRLGQHLYGTYRTRLHENNWICIQEDTGLLYLNRSLDHSSWEKLSVRNRGFPLLTVYLKVFLSPTSLREGECQWPGCARVYFSFFNTSFPACSSLKPRELCFPETRPSFRIRENRPPGTFHQFRLLPVQFLCPNISVAYRLLEGEGLPFRCAPDSLEVSTRWALDREQREKYELVAVCTVHAGAREEVVMVPFPVTVYDEDDSAPTFPAGVDTASAVVEFKRKEDTVVATLRVFDADVVPASGELVRRYTSTLLPGDTWAQQTFRVEHWPNETSVQANGSFVRATVHDYRLVLNRNLSISENRTMQLAVLVNDSDFQGPGAGVLLLHFNVSVLPVSLHLPSTYSLSVSRRARRFAQIGKVCVENCQAFSGINVQYKLHSSGANCSTLGVVTSAEDTSGILFVNDTKALRRPKCAELHYMVVATDQQTSRQAQAQLLVTVEGSYVAEEAGCPLSCAVSKRRLECEECGGLGSPTGRCEWRQGDGKGITRNFSTCSPSTKTCPDGHCDVVETQDINICPQDCLRGSIVGGHEPGEPRGIKAGYGTCNCFPEEEKCFCEPEDIQDPLCDELCRTVIAAAVLFSFIVSVLLSAFCIHCYHKFAHKPPISSAEMTFRRPAQAFPVSYSSSGARRPSLDSMENQVSVDAFKILEDPKWEFPRKNLVLGKTLGEGEFGKVVKATAFHLKGRAGYTTVAVKMLKENASPSELRDLLSEFNVLKQVNHPHVIKLYGACSQDGPLLLIVEYAKYGSLRGFLRESRKVGPGYLGSGGSRNSSSLDHPDERALTMGDLISFAWQISQGMQYLAEMKLVHRDLAARNILVAEGRKMKISDFGLSRDVYEEDSYVKRSQGRIPVKWMAIESLFDHIYTTQSDVWSFGVLLWEIVTLGGNPYPGIPPERLFNLLKTGHRMERPDNCSEEMYRLMLQCWKQEPDKRPVFADISKDLEKMMVKRRDYLDLAASTPSDSLIYDDGLSEEETPLVDCNNAPLPRALPSTWIENKLYGMSDPNWPGESPVPLTRADGTNTGFPRYPNDSVYANWMLSPSAAKLMDTFDS

>NP_002736.3

MAAAAAAGAGPEMVRGQVFDVGPRYTNLSYIGEGAYGMVCSAYDNVNKVRVAIKKISPFEHQTYCQRTLREIKILLRFRHENIIGINDIIRAPTIEQMKDVYIVQDLMETDLYKLLKTQHLSNDHICYFLYQILRGLKYIHSANVLHRDLKPSNLLLNTTCDLKICDFGLARVADPDHDHTGFLTEYVATRWYRAPEIMLNSKGYTKSIDIWSVGCILAEMLSNRPIFPGKHYLDQLNHILGILGSPSQEDLNCIINLKARNYLLSLPHKNKVPWNRLFPNADSKALDLLDKMLTFNPHKRIEVEQALAHPYLEQYYDPSDEPIAEAPFKFDMELDDLPKEKLKELIFEETARFQPGYRS

611 NP_005405.1 NP_006302.2

>NP_005405.1

MENLQTNFSLVQGSTKKLNGMGDDGSPPAKKMITDIHVNGKTINKVPTVKKEHLDDYGEAPVETDGEHVKRTCTSVPETLHLNPSLKHTLAQFHLSSQSSLGGPAAFSARHSQESMSPTVFLPLPSPQVLPGPLLIPSDSSTELTQTVLEGESISCFQVGGEKRLCLPQVLNSVLREFTLQQINTVCDELYIYCSRCTSDQLHILKVLGILPFNAPSCGLITLTDAQRLCNALLRPRTFPQNGSVLPAKSSLAQLKETGSAFEVEHECLGKCQGLFAPQFYVQPDAPCIQCLECCGMFAPQTFVMHSHRSPDKRTCHWGFESAKWHCYLHVNQKYLGTPEEKKLKIILEEMKEKFSMRSGKRNQSKTDAPSGMELQSWYPVIKQEGDHVSQTHSFLHPSYYLYMCDKVVAPNVSLTSAVSQSKELTKTEASKSISRQSEKAHSSGKLQKTVSYPDVSLEEQEKMDLKTSRELCSRLDASISNNSTSKRKSESATCNLVRDINKVGIGLVAAASSPLLVKDVICEDDKGKIMEEVMRTYLKQQEKLNLILQKKQQLQMEVKMLSSSKSMKELTEEQQNLQKELESLQNEHAQRMEEFYVEQKDLEKKLEQIMKQKCTCDSNLEKDKEAEYAGQLAELRQRLDHAEADRQELQDELRQEREARQKLEMMIKELKLQILKSSKTAKE

>NP_006302.2

MSSSGYPPNQGAFSTEQSRYPPHSVQYTFPNTRHQQEFAVPDYRSSHLEVSQASQLLQQQQQQQLRRRPSLLSEFHPGSDRPQERRTSYEPFHPGPSPVDHDSLESKRPRLEQVSDSHFQRVSAAVLPLVHPLPEGLRASADAKKDPAFGGKHEAPSSPISGQPCGDDQNASPSKLSKEELIQSMDRVDREIAKVEQQILKLKKKQQQLEEEAAKPPEPEKPVSPPPVEQKHRSIVQIIYDENRKKAEEAHKIFEGLGPKVELPLYNQPSDTKVYHENIKTNQVMRKKLILFFKRRNHARKQREQKICQRYDQLMEAWEKKVDRIENNPRRKAKESKTREYYEKQFPEIRKQREQQERFQRVGQRGAGLSATIARSEHEISEIIDGLSEQENNEKQMRQLSVIPPMMFDAEQRRVKFINMNGLMEDPMKVYKDRQFMNVWTDHEKEIFKDKFIQHPKNFGLIASYLERKSVPDCVLYYYLTKKNENYKALVRRNYGKRRGRNQQIARPSQEEKVEEKEEDKAEKTEKKEEEKKDEEEKDEKEDSKENTKEKDKIDGTAEETEEREQATPRGRKTANSQGRRKGRITRSMTNEAAAASAAAAAATEEPPPPLPPPPEPISTEPVETSRWTEEEMEVAKKGLVEHGRNWAAIAKMVGTKSEAQCKNFYFNYKRRHNLDNLLQQHKQKTSRKPREERDVSQCESVASTVSAQEDEDIEASNEEENPEDSEVEAVKPSEDSPENATSRGNTEPAVELEPTTETAPSTSPSLAVPSTKPAEDESVETQVNDSISAETAEQMDVDQQEHSAEEGSVCDPPPATKADSVDVEVRVPENHASKVEGDNTKERDLDRASEKVEPRDEDLVVAQQINAQRPEPQSDNDSSATCSADEDVDGEPERQRMFPMDSKPSLLNPTGSILVSSPLKPNPLDLPQLQHRAAVIPPMVSCTPCNIPIGTPVSGYALYQRHIKAMHESALLEEQRQRQEQIDLECRSSTSPCGTSKSPNREWEVLQPAPHQVITNLPEGVRLPTTRPTRPPPPLIPSSKTTVASEKPSFIMGGSISQGTPGTYLTSHNQASYTQETPKPSVGSISLGLPRQQESAKSATLPYIKQEEFSPRSQNSQPEGLLVRAQHEGVVRGTAGAIQEGSITRGTPTSKISVESIPSLRGSITQGTPALPQTGIPTEALVKGSISRMPIEDSSPEKGREEAASKGHVIYEGKSGHILSYDNIKNAREGTRSPRTAHEISLKRSYESVEGNIKQGMSMRESPVSAPLEGLICRALPRGSPHSDLKERTVLSGSIMQGTPRATTESFEDGLKYPKQIKRESPPIRAFEGAITKGKPYDGITTIKEMGRSIHEIPRQDILTQESRKTPEVVQSTRPIIEGSISQGTPIKFDNNSGQSAIKHNVKSLITGPSKLSRGMPPLEIVPENIKVVERGKYEDVKAGETVRSRHTSVVSSGPSVLRSTLHEAPKAQLSPGIYDDTSARRTPVSYQNTMSRGSPMMNRTSDVTISSNKSTNHERKSTLTPTQRESIPAKSPVPGVDPVVSHSPFDPHHRGSTAGEVYRSHLPTHLDPAMPFHRALDPAAAAYLFQRQLSPTPGYPSQYQLYAMENTRQTILNDYITSQQMQVNLRPDVARGLSPREQPLGLPYPATRGIIDLTNMPPTILVPHPGGTSTPPMDRITYIPGTQITFPPRPYNSASMSPGHPTHLAAAASAEREREREREKERERERIAAASSDLYLRPGSEQPGRPGSHGYVRSPSPSVRTQETMLQQRPSVFQGTNGTSVITPLDPTAQLRIMPLPAGGPSISQGLPASRYNTAADALAALVDAAASAPQMDVSKTKESKHEAARLEENLRSRSAAVSEQQQLEQKTLEVEKRSVQCLYTSSAFPSGKPQPHSSVVYSEAGKDKGPPPKSRYEEELRTRGKTTITAANFIDVIITRQIASDKDARERGSQSSDSSSSLSSHRYETPSDAIEVISPASSPAPPQEKLQTYQPEVVKANQAENDPTRQYEGPLHHYRPQQESPSPQQQLPPSSQAEGMGQVPRTHRLITLADHICQIITQDFARNQVSSQTPQQPPTSTFQNSPSALVSTPVRTKTSNRYSPESQAQSVHHQRPGSRVSPENLVDKSRGSRPGKSPERSHVSSEPYEPISPPQVPVVHEKQDSLLLLSQRGAEPAEQRNDARSPGSISYLPSFFTKLENTSPMVKSKKQEIFRKLNSSGGGDSDMAAAQPGTEIFNLPAVTTSGSVSSRGHSFADPASNLGLEDIIRKALMGSFDDKVEDHGVVMSQPMGVVPGTANTSVVTSGETRREEGDPSPHSGGVCKPKLISKSNSRKSKSPIPGQGYLGTERPSSVSSVHSEGDYHRQTPGWAWEDRPSSTGSTQFPYNPLTMRMLSSTPPTPIACAPSAVNQAAPHQQNRIWEREPAPLLSAQYETLSDSDD

612 NP_031379.2 NP_002728.1

>NP_031379.2

MSGVVRTLSRCLLPAEAGGARERRAGSGARDAEREARRRSRDIDALLARERRAVRRLVKILLLGAGESGKSTFLKQMRIIHGREFDQKALLEFRDTIFDNILKGSRVLVDARDKLGIPWQYSENEKHGMFLMAFENKAGLPVEPATFQLYVPALSALWRDSGIREAFSRRSEFQLGESVKYFLDNLDRIGQLNYFPSKQDILLARKATKGIVEHDFVIKKIPFKMVDVGGQRSQRQKWFQCFDGITSILFMVSSSEYDQVLMEDRRTNRLVESMNIFETIVNNKLFFNVSIILFLNKMDLLVEKVKTVSIKKHFPDFRGDPHRLEDVQRYLVQCFDRKRRNRSKPLFHHFTTAIDTENVRFVFHAVKDTILQENLKDIMLQ

>NP_002728.1

MADVFPGNDSTASQDVANRFARKGALRQKNVHEVKDHKFIARFFKQPTFCSHCTDFIWGFGKQGFQCQVCCFVVHKRCHEFVTFSCPGADKGPDTDDPRSKHKFKIHTYGSPTFCDHCGSLLYGLIHQGMKCDTCDMNVHKQCVINVPSLCGMDHTEKRGRIYLKAEVADEKLHVTVRDAKNLIPMDPNGLSDPYVKLKLIPDPKNESKQKTKTIRSTLNPQWNESFTFKLKPSDKDRRLSVEIWDWDRTTRNDFMGSLSFGVSELMKMPASGWYKLLNQEEGEYYNVPIPEGDEEGNMELRQKFEKAKLGPAGNKVISPSEDRKQPSNNLDRVKLTDFNFLMVLGKGSFGKVMLADRKGTEELYAIKILKKDVVIQDDDVECTMVEKRVLALLDKPPFLTQLHSCFQTVDRLYFVMEYVNGGDLMYHIQQVGKFKEPQAVFYAAEISIGLFFLHKRGIIYRDLKLDNVMLDSEGHIKIADFGMCKEHMMDGVTTRTFCGTPDYIAPEIIAYQPYGKSVDWWAYGVLLYEMLAGQPPFDGEDEDELFQSIMEHNVSYPKSLSKEAVSICKGLMTKHPAKRLGCGPEGERDVREHAFFRRIDWEKLENREIQPPFKPKVCGKGAENFDKFFTRGQPVLTPPDQLVIANIDQSDFEGFSYVNPQFVHPILQSAV

613 NP_005234.1 NP_064585.1

>NP_005234.1

MASTDYSTYSQAAAQQGYSAYTAQPTQGYAQTTQAYGQQSYGTYGQPTDVSYTQAQTTATYGQTAYATSYGQPPTGYTTPTAPQAYSQPVQGYGTGAYDTTTATVTTTQASYAAQSAYGTQPAYPAYGQQPAATAPTRPQDGNKPTETSQPQSSTGGYNQPSLGYGQSNYSYPQVPGSYPMQPVTAPPSYPPTSYSSTQPTSYDQSSYSQQNTYGQPSSYGQQSSYGQQSSYGQQPPTSYPPQTGSYSQAPSQYSQQSSSYGQQSSFRQDHPSSMGVYGQESGGFSGPGENRSMSGPDNRGRGRGGFDRGGMSRGGRGGGRGGMGSAGERGGFNKPGGPMDEGPDLDLGPPVDPDEDSDNSAIYVQGLNDSVTLDDLADFFKQCGVVKMNKRTGQPMIHIYLDKETGKPKGDATVSYEDPPTAKAAVEWFDGKDFQGSKLKVSLARKKPPMNSMRGGLPPREGRGMPPPLRGGPGGPGGPGGPMGRMGGRGGDRGGFPPRGPRGSRGNPSGGGNVQHRAGDWQCPNPGCGNQNFAWRTECNQCKAPKPEGFLPPPFPPPGGDRGRGGPGGMRGGRGGLMDRGGPGGMFRGGRGGDRGGFRGGRGMDRGGFGGGRRGGPGGPPGPLMEQMGGRRGGRGGPGKMDKGEHRQERRDRPY

>NP_064585.1

MAGSSEEAPDYGRGVVIMDDWPGYDLNLFTYPQHYYGDLEYVLIPHGIIVDRIERLAKDIMKDIGYSDIMVLCVLKGGYKFCADLVEHLKNISRNSDRFVSMKVDFIRLKSYRNDQSMGEMQIIGGDDLSTLAGKNVLIVEDVVGTGRTMKALLSNIEKYKPNMIKVASLLVKRTSRSDGFRPDYAGFEIPNLFVVGYALDYNEYFRDLNHICVINEHGKEKYRV

614 NP_542937.1 NP_000494.2

>NP_542937.1

MAVPAALIPPTQLVPPQPPISTSASSSGTTTSTSSATSSPAPSIGPPASSGPTLFRPEPIASAAAAAATVTSTGGGGGGGGSGGGGGSSGNGGGGGGGGGGSNCNPNLAAASNGSGGGGGGISAGGGVASSTPINASTGSSSSSSSSSSSSSSSSSSSSSSSSCGPLPGKPVYSTPSPVENTPQNNECKMVDLRGAKVASFTVEGCELICLPQAFDLFLKHLVGGLHTVYTKLKRLEITPVVCNVEQVRILRGLGAIQPGVNRCKLISRKDFETLYNDCTNASSRPGRPPKRTQSVTSPENSHIMPHSVPGLMSPGIIPPTGLTAAAAAAAAATNAAIAEAMKVKKIKLEAMSNYHASNNQHGADSENGDMNSSVGLELPFMMMPHPLIPVSLPPASVTMAMSQMNHLSTIANMAAAAQVQSPPSRVETSVIKERVPDSPSPAPSLEEGRRPGSHPSSHRSSSVSSSPARTESSSDRIPVHQNGLSMNQMLMGLSPNVLPGPKEGDLAGHDMGHESKRMHIEKDETPLSTPTARDSLDKLSLTGHGQPLPPGFPSPFLFPDGLSSIETLLTNIQGLLKVAIDNARAQEKQVQLEKTELKMDFLRERELRETLEKQLAMEQKNRAIVQKRLKKEKKAKRKLQEALEFETKRREQAEQTLKQAASTDSLRVLNDSLTPEIEADRSGGRTDAERTIQDGRLYLKTTVMY

>NP_000494.2

MEMQDLTSPHSRLSGSSESPSGPKLGNSHINSNSMTPNGTEVKTEPMSSSETASTTADGSLNNFSGSAIGSSSFSPRPTHQFSPPQIYPSNRPYPHILPTPSSQTMAAYGQTQFTTGMQQATAYATYPQPGQPYGISSYGALWAGIKTEGGLSQSQSPGQTGFLSYGTSFSTPQPGQAPYSYQMQGSSFTTSSGIYTGNNSLTNSSGFNSSQQDYPSYPSFGQGQYAQYYNSSPYPAHYMTSSNTSPTTPSTNATYQLQEPPSGITSQAVTDPTAEYSTIHSPSTPIKDSDSDRLRRGSDGKSRGRGRRNNNPSPPPDSDLERVFIWDLDETIIVFHSLLTGSYANRYGRDPPTSVSLGLRMEEMIFNLADTHLFFNDLEECDQVHIDDVSSDDNGQDLSTYNFGTDGFPAAATSANLCLATGVRGGVDWMRKLAFRYRRVKEIYNTYKNNVGGLLGPAKREAWLQLRAEIEALTDSWLTLALKALSLIHSRTNCVNILVTTTQLIPALAKVLLYGLGIVFPIENIYSATKIGKESCFERIIQRFGRKVVYVVIGDGVEEEQGAKKHAMPFWRISSHSDLMALHHALELEYL

615 NP_036611.2 NP_037409.2

>NP_036611.2

MVDREQLVQKARLAEQAERYDDMAAAMKNVTELNEPLSNEERNLLSVAYKNVVGARRSSWRVISSIEQKTSADGNEKKIEMVRAYREKIEKELEAVCQDVLSLLDNYLIKNCSETQYESKVFYLKMKGDYYRYLAEVATGEKRATVVESSEKAYSEAHEISKEHMQPTHPIRLGLALNYSVFYYEIQNAPEQACHLAKTAFDDAIAELDTLNEDSYKDSTLIMQLLRDNLTLWTSDQQDDDGGEGNN

>NP_037409.2

MDTMMLNVRNLFEQLVRRVEILSEGNEVQFIQLAKDFEDFRKKWQRTDHELGKYKDLLMKAETERSALDVKLKHARNQVDVEIKRRQRAEADCEKLERQIQLIREMLMCDTSGSIQLSEEQKSALAFLNRGQPSSSNAGNKRLSTIDESGSILSDISFDKTDESLDWDSSLVKTFKLKKREKRRSTSRQFVDGPPGPVKKTRSIGSAVDQGNESIVAKTTVTVPNDGGPIEAVSTIETVPYWTRSRRKTGTLQPWNSDSTLNSRQLEPRTETDSVGTPQSNGGMRLHDFVSKTVIKPESCVPCGKRIKFGKLSLKCRDCRVVSHPECRDRCPLPCIPTLIGTPVKIGEGMLADFVSQTSPMIPSIVVHCVNEIEQRGLTETGLYRISGCDRTVKELKEKFLRVKTVPLLSKVDDIHAICSLLKDFLRNLKEPLLTFRLNRAFMEAAEITDEDNSIAAMYQAVGELPQANRDTLAFLMIHLQRVAQSPHTKMDVANLAKVFGPTIVAHAVPNPDPVTMLQDIKRQPKVVERLLSLPLEYWSQFMMVEQENIDPLHVIENSNAFSTPQTPDIKVSLLGPVTTPEHQLLKTPSSSSLSQRVRSTLTKNTPRFGSKSKSATNLGRQGNFFASPMLK

616 NP_000714.3 NP_075461.2

>NP_000714.3

MVQKTSMSRGPYPPSQEIPMEVFDPSPQGKYSKRKGRFKRSDGSTSSDTTSNSFVRQGSAESYTSRPSDSDVSLEEDREALRKEAERQALAQLEKAKTKPVAFAVRTNVGYNPSPGDEVPVQGVAITFEPKDFLHIKEKYNNDWWIGRLVKEGCEVGFIPSPVKLDSLRLLQEQKLRQNRLGSSKSGDNSSSSLGDVVTGTRRPTPPASAKQKQKSTEHVPPYDVVPSMRPIILVGPSLKGYEVTDMMQKALFDFLKHRFDGRISITRVTADISLAKRSVLNNPSKHIIIERSNTRSSLAEVQSEIERIFELARTLQLVALDADTINHPAQLSKTSLAPIIVYIKITSPKVLQRLIKSRGKSQSKHLNVQIAASEKLAQCPPEMFDIILDENQLEDACEHLAEYLEAYWKATHPPSSTPPNPLLNRTMATAALAASPAPVSNLQGPYLASGDQPLERATGEHASMHEYPGELGQPPGLYPSSHPPGRAGTLRALSRQDTFDADTPGSRNSAYTELGDSCVDMETDPSEGPGLGDPAGGGTPPARQGSWEDEEEDYEEELTDNRNRGRNKARYCAEGGGPVLGRNKNELEGWGRGVYIR

>NP_075461.2

MARFGDEMPARYGGGGSGAAAGVVVGSGGGRGAGGSRQGGQPGAQRMYKQSMAQRARTMALYNPIPVRQNCLTVNRSLFLFSEDNVVRKYAKKITEWPPFEYMILATIIANCIVLALEQHLPDDDKTPMSERLDDTEPYFIGIFCFEAGIKIIALGFAFHKGSYLRNGWNVMDFVVVLTGILATVGTEFDLRTLRAVRVLRPLKLVSGIPSLQVVLKSIMKAMIPLLQIGLLLFFAILIFAIIGLEFYMGKFHTTCFEEGTDDIQGESPAPCGTEEPARTCPNGTKCQPYWEGPNNGITQFDNILFAVLTVFQCITMEGWTDLLYNSNDASGNTWNWLYFIPLIIIGSFFMLNLVLGVLSGEFAKERERVENRRAFLKLRRQQQIERELNGYMEWISKAEEVILAEDETDGEQRHPFDGALRRTTIKKSKTDLLNPEEAEDQLADIASVGSPFARASIKSAKLENSTFFHKKERRMRFYIRRMVKTQAFYWTVLSLVALNTLCVAIVHYNQPEWLSDFLYYAEFIFLGLFMSEMFIKMYGLGTRPYFHSSFNCFDCGVIIGSIFEVIWAVIKPGTSFGISVLRALRLLRIFKVTKYWASLRNLVVSLLNSMKSIISLLFLLFLFIVVFALLGMQLFGGQFNFDEGTPPTNFDTFPAAIMTVFQILTGEDWNEVMYDGIKSQGGVQGGMVFSIYFIVLTLFGNYTLLNVFLAIAVDNLANAQELTKVEADEQEEEEAANQKLALQKAKEVAEVSPLSAANMSIAVKEQQKNQKPAKSVWEQRTSEMRKQNLLASREALYNEMDPDERWKAAYTRHLRPDMKTHLDRPLVVDPQENRNNNTNKSRAAEPTVDQRLGQQRAEDFLRKQARYHDRARDPSGSAGLDARRPWAGSQEAELSREGPYGRESDHHAREGSLEQPGFWEGEAERGKAGDPHRRHVHRQGGSRESRSGSPRTGADGEHRRHRAHRRPGEEGPEDKAERRARHREGSRPARGGEGEGEGPDGGERRRRHRHGAPATYEGDARREDKERRHRRRKENQGSGVPVSGPNLSTTRPIQQDLGRQDPPLAEDIDNMKNNKLATAESAAPHGSLGHAGLPQSPAKMGNSTDPGPMLAIPAMATNPQNAASRRTPNNPGNPSNPGPPKTPENSLIVTNPSGTQTNSAKTARKPDHTTVDIPPACPPPLNHTVVQVNKNANPDPLPKKEEEKKEEEEDDRGEDGPKPMPPYSSMFILSTTNPLRRLCHYILNLRYFEMCILMVIAMSSIALAAEDPVQPNAPRNNVLRYFDYVFTGVFTFEMVIKMIDLGLVLHQGAYFRDLWNILDFIVVSGALVAFAFTGNSKGKDINTIKSLRVLRVLRPLKTIKRLPKLKAVFDCVVNSLKNVFNILIVYMLFMFIFAVVAVQLFKGKFFHCTDESKEFEKDCRGKYLLYEKNEVKARDREWKKYEFHYDNVLWALLTLFTVSTGEGWPQVLKHSVDATFENQGPSPGYRMEMSIFYVVYFVVFPFFFVNIFVALIIITFQEQGDKMMEEYSLEKNERACIDFAISAKPLTRHMPQNKQSFQYRMWQFVVSPPFEYTIMAMIALNTIVLMMKFYGASVAYENALRVFNIVFTSLFSLECVLKVMAFGILNYFRDAWNIFDFVTVLGSITDILVTEFGNPNNFINLSFLRLFRAARLIKLLRQGYTIRILLWTFVQSFKALPYVCLLIAMLFFIYAIIGMQVFGNIGIDVEDEDSDEDEFQITEHNNFRTFFQALMLLFRSATGEAWHNIMLSCLSGKPCDKNSGILTRECGNEFAYFYFVSFIFLCSFLMLNLFVAVIMDNFEYLTRDSSILGPHHLDEYVRVWAEYDPAAWGRMPYLDMYQMLRHMSPPLGLGKKCPARVAYKRLLRMDLPVADDNTVHFNSTLMALIRTALDIKIAKGGADKQQMDAELRKEMMAIWPNLSQKTLDLLVTPHKSTDLTVGKIYAAMMIMEYYRQSKAKKLQAMREEQDRTPLMFQRMEPPSPTQEGGPGQNALPSTQLDPGGALMAHESGLKESPSWVTQRAQEMFQKTGTWSPEQGPPTDMPNSQPNSQSVEMREMGRDGYSDSEHYLPMEGQGRAASMPRLPAENQRRRGRPRGNNLSTISDTSPMKRSASVLGPKARRLDDYSLERVPPEENQRHHQRRRDRSHRASERSLGRYTDVDTGLGTDLSMTTQSGDLPSKERDQERGRPKDRKHRQHHHHHHHHHHPPPPDKDRYAQERPDHGRARARDQRWSRSPSEGREHMAHRQGSSSVSGSPAPSTSGTSTPRRGRRQLPQTPSTPRPHVSYSPVIRKAGGSGPPQQQQQQQQQQQQQAVARPGRAATSGPRRYPGPTAEPLAGDRPPTGGHSSGRSPRMERRVPGPARSESPRACRHGGARWPASGPHVSEGPPGPRHHGYYRGSDYDEADGPGSGGGEEAMAGAYDAPPPVRHASSGATGRSPRTPRASGPACASPSRHGRRLPNGYYPAHGLARPRGPGSRKGLHEPYSESDDDWC

617 NP_004999.1 NP_002680.2

>NP_004999.1

MTEADVNPKAYPLADAHLTKKLLDLVQQSCNYKQLRKGANEATKTLNRGISEFIVMAADAEPLEIILHLPLLCEDKNVPYVFVRSKQALGRACGVSRPVIACSVTIKEGSQLKQQIQSIQQSIERLLV

>NP_002680.2

MSASAQQLAEELQIFGLDCEEALIEKLVELCVQYGQNEEGMVGELIAFCTSTHKVGLTSEILNSFEHEFLSKRLSKARHSTCKDSGHAGARDIVSIQELIEVEEEEEILLNSYTTPSKGSQKRAISTPETPLTKRSVSTRSPHQLLSPSSFSPSATPSQKYNSRSNRGEVVTSFGLAQGVSWSGRGGAGNISLKVLGCPEALTGSYKSMFQKLPDIREVLTCKIEELGSELKEHYKIEAFTPLLAPAQEPVTLLGQIGCDSNGKLNNKSVILEGDREHSSGAQIPVDLSELKEYSLFPGQVVIMEGINTTGRKLVATKLYEGVPLPFYQPTEEDADFEQSMVLVACGPYTTSDSITYDPLLDLIAVINHDRPDVCILFGPFLDAKHEQVENCLLTSPFEDIFKQCLRTIIEGTRSSGSHLVFVPSLRDVHHEPVYPQPPFSYSDLSREDKKQVQFVSEPCSLSINGVIFGLTSTDLLFHLGAEEISSSSGTSDRFSRILKHILTQRSYYPLYPPQEDMAIDYESFYVYAQLPVTPDVLIIPSELRYFVKDVLGCVCVNPGRLTKGQVGGTFARLYLRRPAADGAERQSPCIAVQVVRI

618 NP_000292.1 NP_004559.4

>NP_000292.1

MEHKEVVLLLLLFLKSGQGEPLDDYVNTQGASLFSVTKKQLGAGSIEECAAKCEEDEEFTCRAFQYHSKEQQCVIMAENRKSSIIIRMRDVVLFEKKVYLSECKTGNGKNYRGTMSKTKNGITCQKWSSTSPHRPRFSPATHPSEGLEENYCRNPDNDPQGPWCYTTDPEKRYDYCDILECEEECMHCSGENYDGKISKTMSGLECQAWDSQSPHAHGYIPSKFPNKNLKKNYCRNPDRELRPWCFTTDPNKRWELCDIPRCTTPPPSSGPTYQCLKGTGENYRGNVAVTVSGHTCQHWSAQTPHTHNRTPENFPCKNLDENYCRNPDGKRAPWCHTTNSQVRWEYCKIPSCDSSPVSTEQLAPTAPPELTPVVQDCYHGDGQSYRGTSSTTTTGKKCQSWSSMTPHRHQKTPENYPNAGLTMNYCRNPDADKGPWCFTTDPSVRWEYCNLKKCSGTEASVVAPPPVVLLPDVETPSEEDCMFGNGKGYRGKRATTVTGTPCQDWAAQEPHRHSIFTPETNPRAGLEKNYCRNPDGDVGGPWCYTTNPRKLYDYCDVPQCAAPSFDCGKPQVEPKKCPGRVVGGCVAHPHSWPWQVSLRTRFGMHFCGGTLISPEWVLTAAHCLEKSPRPSSYKVILGAHQEVNLEPHVQEIEVSRLFLEPTRKDIALLKLSSPAVITDKVIPACLPSPNYVVADRTECFITGWGETQGTFGAGLLKEAQLPVIENKVCNRYEFLNGRVQSTELCAGHLAGGTDSCQGDSGGPLVCFEKDKYILQGVTSWGLGCARPNKPGVYVRVSRFVTWIEGVMRNN

>NP_004559.4

MDVLAEANGTFALNLLKTLGKDNSKNVFFSPMSMSCALAMVYMGAKGNTAAQMAQILSFNKSGGGGDIHQGFQSLLTEVNKTGTQYLLRVANRLFGEKSCDFLSSFRDSCQKFYQAEMEELDFISAVEKSRKHINTWVAEKTEGKIAELLSPGSVDPLTRLVLVNAVYFRGNWDGQFDKENTEERLFKVSKNEEKPVQMMFKQSTFKKTYIGEIFTQILVLPYVGKELNMIIMLPDETTDLRTVEKELTYEKFVEWTRLDMMDEEEVEVSLPRFKLEESYDMESVLRNLGMTDAFELGKADFSGMSQTDLSLSKVVHKSFVEVNEEGTEAAAATAAIMMMRCARFVPRFCADHPFLFFIQHRKTNGILFCGRFSSP

619 NP_004706.3 NP_000929.1

>NP_004706.3

MEVPAAGRVPAEGAPTAAVAEVRCPGPAPLRLLEWRVAAGAAVRIGSVLAVFEAAASAQSSGASQSRVASGGCVRPARPERRLRSERAGVVRELCAQPGQVVAPGAVLVRLEGCSHPVVMKGLCAECGQDLTQLQSKNGKQQVPLSTATVSMVHSVPELMVSSEQAEQLGREDQQRLHRNRKLVLMVDLDQTLIHTTEQHCQQMSNKGIFHFQLGRGEPMLHTRLRPHCKDFLEKIAKLYELHVFTFGSRLYAHTIAGFLDPEKKLFSHRILSRDECIDPFSKTGNLRNLFPCGDSMVCIIDDREDVWKFAPNLITVKKYVYFQGTGDMNAPPGSRESQTRKKVNHSRGTEVSEPSPPVRDPEGVTQAPGVEPSNGLEKPARELNGSEAATPRDSPRPGKPDERDIWPPAQAPTSSQELAGAPEPQGSCAQGGRVAPGQRPAQGATGTDLDFDLSSDSESSSESEGTKSSSSASDGESEGKRGRQKPKAAPEGAGALAQGSSLEPGRPAAPSLPGEAEPGAHAPDKEPELGGQEEGERDGLCGLGNGCADRKEAETESQNSELSGVTAGESLDQSMEEEEEEDTDEDDHLIYLEEILVRVHTDYYAKYDRYLNKEIEEAPDIRKIVPELKSKVLADVAIIFSGLHPTNFPIEKTREHYHATALGAKILTRLVLSPDAPDRATHLIAARAGTEKVLQAQECGHLHVVNPDWLWSCLERWDKVEEQLFPLRDDHTKAQRENSPAAFPDREGVPPTALFHPMPVLPKAQPGPEVRIYDSNTGKLIRTGARGPPAPSSSLPIRQEPSSFRAVPPPQPQMFGEELPDAQDGEQPGPSRRKRQPSMSETMPLYTLCKEDLESMDKEVDDILGEGSDDSDSEKRRPEEQEEEPQPRKPGTRRERTLGAPASSERSAAGGRGPRGHKRKLNEEDAASESSRESSNEDEGSSSEADEMAKALEAELNDLM

>NP_000929.1

MYDADEDMQYDEDDDEITPDLWQEACWIVISSYFDEKGLVRQQLDSFDEFIQMSVQRIVEDAPPIDLQAEAQHASGEVEEPPRYLLKFEQIYLSKPTHWERDGAPSPMMPNEARLRNLTYSAPLYVDITKTVIKEGEEQLQTQHQKTFIGKIPIMLRSTYCLLNGLTDRDLCELNECPLDPGGYFIINGSEKVLIAQEKMATNTVYVFAKKDSKYAYTGECRSCLENSSRPTSTIWVSMLARGGQGAKKSAIGQRIVATLPYIKQEVPIIIVFRALGFVSDRDILEHIIYDFEDPEMMEMVKPSLDEAFVIQEQNVALNFIGSRGAKPGVTKEKRIKYAKEVLQKEMLPHVGVSDFCETKKAYFLGYMVHRLLLAALGRRELDDRDHYGNKRLDLAGPLLAFLFRGMFKNLLKEVRIYAQKFIDRGKDFNLELAIKTRIISDGLKYSLATGNWGDQKKAHQARAGVSQVLNRLTFASTLSHLRRLNSPIGRDGKLAKPRQLHNTLWGMVCPAETPEGHAVGLVKNLALMAYISVGSQPSPILEFLEEWSMENLEEISPAAIADATKIFVNGCWVGIHKDPEQLMNTLRKLRRQMDIIVSEVSMIRDIREREIRIYTDAGRICRPLLIVEKQKLLLKKRHIDQLKEREYNNYSWQDLVASGVVEYIDTLEEETVMLAMTPDDLQEKEVAYCSTYTHCEIHPSMILGVCASIIPFPDHNQSPRNTYQSAMGKQAMGVYITNFHVRMDTLAHVLYYPQKPLVTTRSMEYLRFRELPAGINSIVAIASYTGYNQEDSVIMNRSAVDRGFFRSVFYRSYKEQESKKGFDQEEVFEKPTRETCQGMRHAIYDKLDDDGLIAPGVRVSGDDVIIGKTVTLPENEDELESTNRRYTKRDCSTFLRTSETGIVDQVMVTLNQEGYKFCKIRVRSVRIPQIGDKFASRHGQKGTCGIQYRQEDMPFTCEGITPDIIINPHAIPSRMTIGHLIECLQGKVSANKGEIGDATPFNDAVNVQKISNLLSDYGYHLRGNEVLYNGFTGRKITSQIFIGPTYYQRLKHMVDDKIHSRARGPIQILNRQPMEGRSRDGGLRFGEMERDCQIAHGAAQFLRERLFEASDPYQVHVCNLCGIMAIANTRTHTYECRGCRNKTQISLVRMPYACKLLFQELMSMSIAPRMMSV

620 AAH20590.1 NP_056146.1

>AAH20590.1

MGAAVFFGCTFVAFGPAFALFLITVAGDPLRVIILVAGAFFWLVSLLLASVVWFILVHVTDRSDARLQYGLLIFGAAVSVLLQEVFRFAYYKLLKKADEGLASLSEDGRSPISIRQMAYVSGLSFGIISGVFSVINILADALGPGVVGIHGDSPYYFLTSAFLTAAIILLHTFWGVVFFDACERRRYWALGLVVGSHLLTSGLTFLNPWYEASLLPIYAVTVSMGLWAFITAGGSLRSIQRSLLCRRQEDSRVMVYSALRIPPED

>NP_056146.1

MATAGGGSGADPGSRGLLRLLSFCVLLAGLCRGNSVERKIYIPLNKTAPCVRLLNATHQIGCQSSISGDTGVIHVVEKEEDLQWVLTDGPNPPYMVLLESKHFTRDLMEKLKGRTSRIAGLAVSLTKPSPASGFSPSVQCPNDGFGVYSNSYGPEFAHCREIQWNSLGNGLAYEDFSFPIFLLEDENETKVIKQCYQDHNLSQNGSAPTFPLCAMQLFSHMHAVISTATCMRRSSIQSTFSINPEIVCDPLSDYNVWSMLKPINTTGTLKPDDRVVVAATRLDSRSFFWNVAPGAESAVASFVTQLAAAEALQKAPDVTTLPRNVMFVFFQGETFDYIGSSRMVYDMEKGKFPVQLENVDSFVELGQVALRTSLELWMHTDPVSQKNESVRNQVEDLLATLEKSGAGVPAVILRRPNQSQPLPPSSLQRFLRARNISGVVLADHSGAFHNKYYQSIYDTAENINVSYPEWLSPEEDLNFVTDTAKALADVATVLGRALYELAGGTNFSDTVQADPQTVTRLLYGFLIKANNSWFQSILRQDLRSYLGDGPLQHYIAVSSPTNTTYVVQYALANLTGTVVNLTREQCQDPSKVPSENKDLYEYSWVQGPLHSNETDRLPRCVRSTARLARALSPAFELSQWSSTEYSTWTESRWKDIRARIFLIASKELELITLTVGFGILIFSLIVTYCINAKADVLFIAPREPGAVSY

621 NP_006531.1 NP_002948.1

>NP_006531.1

MSGMGENTSDPSRAETRKRKECPDQLGPSPKRNTEKRNREQENKYIEELAELIFANFNDIDNFNFKPDKCAILKETVKQIRQIKEQEKAAAANIDEVQKSDVSSTGQGVIDKDALGPMMLEALDGFFFVVNLEGNVVFVSENVTQYLRYNQEELMNKSVYSILHVGDHTEFVKNLLPKSIVNGGSWSGEPPRRNSHTFNCRMLVKPLPDSEEEGHDNQEAHQKYETMQCFAVSQPKSIKEEGEDLQSCLICVARRVPMKERPVLPSSESFTTRQDLQGKITSLDTSTMRAAMKPGWEDLVRRCIQKFHAQHEGESVSYAKRHHHEVLRQGLAFSQIYRFSLSDGTLVAAQTKSKLIRSQTTNEPQLVISLHMLHREQNVCVMNPDLTGQTMGKPLNPISSNSPAHQALCSGNPGQDMTLSSNINFPINGPKEQMGMPMGRFGGSGGMNHVSGMQATTPQGSNYALKMNSPSQSSPGMNPGQPTSMLSPRHRMSPGVAGSPRIPPSQFSPAGSLHSPVGVCSSTGNSHSYTNSSLNALQALSEGHGVSLGSSLASPDLKMGNLQNSPVNMNPPPLSKMGSLDSKDCFGLYGEPSEGTTGQAESSCHPGEQKETNDPNLPPAVSSERADGQSRLHDSKGQTKLLQLLTTKSDQMEPSPLASSLSDTNKDSTGSLPGSGSTHGTSLKEKHKILHRLLQDSSSPVDLAKLTAEATGKDLSQESSSTAPGSEVTIKQEPVSPKKKENALLRYLLDKDDTKDIGLPEITPKLERLDSKTDPASNTKLIAMKTEKEEMSFEPGDQPGSELDNLEEILDDLQNSQLPQLFPDTRPGAPAGSVDKQAIINDLMQLTAENSPVTPVGAQKTALRISQSTFNNPRPGQLGRLLPNQNLPLDITLQSPTGAGPFPPIRNSSPYSVIPQPGMMGNQGMIGNQGNLGNSSTGMIGNSASRPTMPSGEWAPQSSAVRVTCAATTSAMNRPVQGGMIRNPAASIPMRPSSQPGQRQTLQSQVMNIGPSELEMNMGGPQYSQQQAPPNQTAPWPESILPIDQASFASQNRQPFGSSPDDLLCPHPAAESPSDEGALLDQLYLALRNFDGLEEIDRALGIPELVSQSQAVDPEQFSSQDSNIMLEQKAPVFPQQYASQAQMAQGSYSPMQDPNFHTMGQRPSYATLRMQPRPGLRPTGLVQNQPNQLRLQLQHRLQAQQNRQPLMNQISNVSNVNLTLRPGVPTQAPINAQMLAQRQREILNQHLRQRQMHQQQQVQQRTLMMRGQGLNMTPSMVAPSGMPATMSNPRIPQANAQQFPFPPNYGISQQPDPGFTGATTPQSPLMSPRMAHTQSPMMQQSQANPAYQAPSDINGWAQGNMGGNSMFSQQSPPHFGQQANTSMYSNNMNINVSMATNTGGMSSMNQMTGQISMTSVTSVPTSGLSSMGPEQVNDPALRGGNLFPNQLPGMDMIKQEGDTTRKYC

>NP_002948.1

MDTKHFLPLDFSTQVNSSLTSPTGRGSMAAPSLHPSLGPGIGSPGQLHSPISTLSSPINGMGPPFSVISSPMGPHSMSVPTTPTLGFSTGSPQLSSPMNPVSSSEDIKPPLGLNGVLKVPAHPSGNMASFTKHICAICGDRSSGKHYGVYSCEGCKGFFKRTVRKDLTYTCRDNKDCLIDKRQRNRCQYCRYQKCLAMGMKREAVQEERQRGKDRNENEVESTSSANEDMPVERILEAELAVEPKTETYVEANMGLNPSSPNDPVTNICQAADKQLFTLVEWAKRIPHFSELPLDDQVILLRAGWNELLIASFSHRSIAVKDGILLATGLHVHRNSAHSAGVGAIFDRVLTELVSKMRDMQMDKTELGCLRAIVLFNPDSKGLSNPAEVEALREKVYASLEAYCKHKYPEQPGRFAKLLLRLPALRSIGLKCLEHLFFFKLIGDTPIDTFLMEMLEAPHQMT

622 BAA89210.1 NP_002583.1

>BAA89210.1

MAPLLGRKPFPLVNPLPGEEPFFTIPHTQEAFRTREEYEARLERYSERIWTCKSTGSSQLTHKEAWEEEQEVAELLKEEFPAWYEKLVLEMVHHNTASLEKLVDTAWLEIMTKYAVGEECDFEVGKEKMLKVKIVKIHPLEKVDEEATEKKSDGACDSPSSDKENSSQIAQDHQKKETVVKEDEGRRESINDRARRSPRKLPTSLKKGERKWAPPKFLPHKYDVKLQNEDKIISNVPADSLIRTERPPNKEIVRYFIRHNALRAGTGENAPWVVEDELVKKYSLPSKFSDFLLDPYKYMTLNPSTKRKNTGSPDRKPSKKSKTDNSSLSSPLNPKLWCHVHLKKSLSGSPLKVKNSKNSKSPEEHLEEMMKMMSPNKLHTNFHIPKKGPPAKKPGKHSDKPLKAKGRSKGILNGQKSTGNSKSPKKGLKTPKTKMKQMTLLDMAKGTQKMTRAPRNSGGTPRTSSKPHKHLPPAALHLIAYYKENKDREDKRSALSCVISKTARLLSSEDRARLPEELRSLVQKRYELLEHKKRWASMSEEQRKEYLKKKREELKKKLKEKAKERREKEMLERLEKQKRYEDQELTGKNLPAFRLVDTPEGLPNTLFGDVAMVVEFLSCYSGLLLPDAQYPITAVSLMEALSADKGGFLYLNRVLVILLQTLLQDEIAEDYGELGMKLSEIPLTLHSVSELVRLCLRRSDVQEESEGSDTDDNKDSAAFEDNEVQDEFLEKLETSEFFELTSEEKLQILTALCHRILMTYSVQDHMETRQQMSAELWKERLAVLKEENDKKRAEKQKRKEMEAKNKENGKVENGLGKTDRKKRIVKFEPQVDTEAEDMISAVKSRRLLAIQAKKEREIQEREMKVKLERQAEEERIRKHKAAAEKAFQEGIAKAKLVMRRTPIGTDRNHNRYWLFSDEVPGLFIEKGWVHDSIDYRFNHHCKDHTVSGDEDYCPRSKKANLGKNASMNTQHGTATEVAVETTTPKQGQNLWFLCDSQKELDELLNCLHPQGIRESQLKERLEKRYQDIIHSIHLARKPNLGLKSCDGNQELLNFLRSDLIEVATRLQKGGLGYVEETSEFEARVISLEKLKDFGECVIALQASVIKKFLQGFMAPKQKRRKLQSEDSAKTEEVDEEKKMVEEAKVASALEKWKTAIREAQTFSRMHVLLGMLDACIKWDMSAENARCKVCPKKGEDDKLILCDECNKAFHLFCLRPALYEVPDGEWQCPACQPATARRNSRGRNYTEESASEDSEDDESDEEEEEEEEEEEEEDYEVAGLRLRPRKTIRGKHSVIPPAARSGRRPGKKPHSTRRSQPKAPPVDDAEVDELVLQTKRSSRRQSLELQKCEEILHKIVKYRFSWPFREPVTRDEAEDYYDVITHPMDFQTVQNKCSCGSYRSVQEFLTDMKQVFTNAEVYNCRGSHVLSCMVKTEQCLVVLLHKHLPGHPYVRRKRKKFPDRLAEDEGDSEPEAVGQSRDEDRRSREAEIQEWLQDTSLYSAKINSKDHNCFMMLVNTQFCMALTDTVT

>NP_002583.1

MFEARLVQGSILKKVLEALKDLINEACWDISSSGVNLQSMDSSHVSLVQLTLRSEGFDTYRCDRNLAMGVNLTSMSKILKCAGNEDIITLRAEDNADTLALVFEAPNQEKVSDYEMKLMDLDVEQLGIPEQEYSCVVKMPSGEFARICRDLSHIGDAVVISCAKDGVKFSASGELGNGNIKLSQTSNVDKEEEAVTIEMNEPVQLTFALRYLNFFTKATPLSSTVTLSMSADVPLVVEYKIADMGHLKYYLAPKIEDEEGS

623 NP_054790.2 NP_001460.1

>NP_054790.2

MVLDDLPNLEDIYTSLCSSTMEDSEMDFDSGLEDDDTKSDSILEDSTIFVAFKGNIDDKDFKWKLDAILKNVPNLLHMESSKLKVQKVEPWNSVRVTFNIPREAAERLRILAQSNNQQLRDLGILSVQIEGEGAINLALAQNRSQDVRMNGPMGAGNSVRMEAGFPMASGPGIIRMNNPATVMIPPGGNVSSSMMAPGPNPELQPRTPRPASQSDAMDPLLSGLHIQQQSHPSGSLAPPHHPMQPVSVNRQMNPANFPQLQQQQQQQQQQQQQQQQQQQQQQQQQLQARPPQQHQQQQPQGIRPQFTAPTQVPVPPGWNQLPSGALQPPPAQGSLGTMTANQGWKKAPLPGPMQQQLQARPSLATVQTPSHPPPPYPFGSQQASQAHTNFPQMSNPGQFTAPQMKSLQGGPSRVPTPLQQPHLTNKSPASSPSSFQQGSPASSPTVNQTQQQMGPRPPQNNPLPQGFQQPVSSPGRNPMVQQGNVPPNFMVMQQQPPNQGPQSLHPGLGGMPKRLPPGFSAGQANPNFMQGQVPSTTATTPGNSGAPQLQANQNVQHAGGQGAGPPQNQMQVSHGPPNMMQPSLMGIHGNMNNQQAGTSGVPQVNLSNMQGQPQQGPPSQLMGMHQQIVPSQGQMVQQQGTLNPQNPMILSRAQLMPQGQMMVNPPSQNLGPSPQRMTPPKQMLSQQGPQMMAPHNQMMGPQGQVLLQQNPMIEQIMTNQMQGNKQQFNTQNQSNVMPGPAQIMRGPTPNMQGNMVQFTGQMSGQMLPQQGPVNNSPSQVMGIQGQVLRPPGPSPHMAQQHGDPATTANNDVSLSQMMPDVSIQQTNMVPPHVQAMQGNSASGNHFSGHGMSFNAPFSGAPNGNQMSCGQNPGFPVNKDVTLTSPLLVNLLQSDISAGHFGVNNKQNNTNANKPKKKKPPRKKKNSQQDLNTPDTRPAGLEEADQPPLPGEQGINLDNSGPKLPEFSNRPPGYPSQPVEQRPLQQMPPQLMQHVAPPPQPPQQQPQPQLPQQQQPPPPSQPQSQQQQQQQQQMMMMLMMQQDPKSVRLPVSQNVHPPRGPLNPDSQRMPMQQSGSVPVMVSLQGPASVPPSPDKQRMPMPVNTPLGSNSRKMVYQESPQNPSSSPLAEMASLPEASGSEAPSVPGGPNNMPSHVVLPQNQLMMTGPKPGPSPLSATQGATPQQPPVNSLPSSHGHHFPNVAAPTQTSRPKTPNRASPRPYYPQTPNNRPPSTEPSEISLSPERLNASIAGLFPPQINIPLPPRPNLNRGFDQQGLNPTTLKAIGQAPSNLTMNPSNFATPQTHKLDSVVVNSGKQSNSGATKRASPSNSRRSSPGSSRKTTPSPGRQNSKAPKLTLASQTNAALLQNVELPRNVLVSPTPLANPPVPGSFPNNSGLNPQNSTVSVAAVGGVVEDNKESLNVPQDSDCQNSQSRKEQVNIELKAVPAQEVKMVVPEDQSKKDGQPSDPNKLPSVEENKNLVSPAMREAPTSLSQLLDNSGAPNVTIKPPGLTDLEVTPPVVSGEDLKKASVIPTLQDLSSSKEPSNSLNLPHSNELCSSLVHPELSEVSSNVAPSIPPVMSRPVSSSSISTPLPPNQITVFVTSNPITTSANTSAALPTHLQSALMSTVVTMPNAGSKVMVSEGQSAAQSNARPQFITPVFINSSSIIQVMKGSQPSTIPAAPLTTNSGLMPPSVAVVGPLHIPQNIKFSSAPVPPNALSSSPAPNIQTGRPLVLSSRATPVQLPSPPCTSSPVVPSHPPVQQVKELNPDEASPQVNTSADQNTLPSSQSTTMVSPLLTNSPGSSGNRRSPVSSSKGKGKVDKIGQILLTKACKKVTGSLEKGEEQYGADGETEGQGLDTTAPGLMGTEQLSTELDSKTPTPPAPTLLKMTSSPVGPGTASAGPSLPGGALPTSVRSIVTTLVPSELISAVPTTKSNHGGIASESLAGGLVEEKVGSHPELLPSIAPSQNLVSKETSTTALQASVARPELEVNAAIVSGQSSEPKEIVEKSKIPGRRNSRTEEPTVASESVENGHRKRSSRPASASSSTKDITSAVQSKRRKSK

>NP_001460.1

MSGWESYYKTEGDEEAEEEQEENLEASGDYKYSGRDSLIFLVDASKAMFESQSEDELTPFDMSIQCIQSVYISKIISSDRDLLAVVFYGTEKDKNSVNFKNIYVLQELDNPGAKRILELDQFKGQQGQKRFQDMMGHGSDYSLSEVLWVCANLFSDVQFKMSHKRIMLFTNEDNPHGNDSAKASRARTKAGDLRDTGIFLDLMHLKKPGGFDISLFYRDIISIAEDEDLRVHFEESSKLEDLLRKVRAKETRKRALSRLKLKLNKDIVISVGIYNLVQKALKPPPIKLYRETNEPVKTKTRTFNTSTGGLLLPSDTKRSQIYGSRQIILEKEETEELKRFDDPGLMLMGFKPLVLLKKHHYLRPSLFVYPEESLVIGSSTLFSALLIKCLEKEVAALCRYTPRRNIPPYFVALVPQEEELDDQKIQVTPPGFQLVFLPFADDKRKMPFTEKIMATPEQVGKMKAIVEKLRFTYRSDSFENPVLQQHFRNLEALALDLMEPEQAVDLTLPKVEAMNKRLGSLVDEFKELVYPPDYNPEGKVTKRKHDNEGSGSKRPKVEYSEEELKTHISKGTLGKFTVPMLKEACRAYGLKSGLKKQELLEALTKHFQD

624 NP_068581.1 AAH30590.1

>NP_068581.1

MSDSDLGEDEGLLSLAGKRKRRGNLPKESVKILRDWLYLHRYNAYPSEQEKLSLSGQTNLSVLQICNWFINARRRLLPDMLRKDGKDPNQFTISRRGGKASDVALPRGSSPSVLAVSVPAPTNVLSLSVCSMPLHSGQGEKPAAPFPRGELESPKPLVTPGSTLTLLTRAEAGSPTGGLFNTPPPTPPEQDKEDFSSFQLLVEVALQRAAEMELQKQQDPSLPLLHTPIPLVSENPQ

>AAH30590.1

MNISGSSCGSPNSADTSSDFKDLWTKLKECHDREVQGLQVKVTKLKQERILDAQRLEEFFTKNQQLREQQKVLHETIKVLEDRLRAGLCDRCAVTEEHMRKKQQEFENIRQQNLKLITELMNERNTLQEENKKLSEQLQQKIENDQQHQAAELECEEDVIPDSPITAFSFSGVNRLRRKENPHVRYIEQTHTKLEHSVCANEMRKVSKSSTHPQHNPNENEILVADTYDQSQSPMAKAHGTSSYTPDKSSFNLATVVAETLGLGVQEESETQGPMSPLGDELYHCLEGNHKKQPFEESTRNTEDSLRFSDSTSKTPPQEELPTRVSSPVFGATSSIKSGLDLNTSLSPSLLQPGKKKHLKTLPFSNTCISRLEKTRSKSEDSALFTHHSLGSEVNKIIIQSSNKQILINKNISESLGEQNRTEYGKDSNTDKHLEPLKSLGGRTSKRKKTEEESEHEVSCPQASFDKENAFPFPMDNQFSMNGDCVMYKPLDLSDRFSAIQRQEKSQGSETSKNKFRQVTLYEALKTIPKGFSSSRKASDGNCTLPKDSPGEPCSQECIILQPLNKCSPDNKPSLQIKEENAVFKIPLRPRESLETENVLDDIKSAGSHEPIKIQTRSDHGGCELASVLQLNPCRTGKIKSLQNNQDVSFENIQWSIDPGADLSQYKMDVTVIDTKDGSQSKLGGETVDMDCTLVSETVLLKMKKQEQKGEKSSMLFYIDEERKMNDSLEDMFDRTTHEEYESCLADSFSQAADEEEELSTATKKLHTHGDKQDKVKQKAFVEPYFKGDERETSLQNFPHIEVVRKKEERRKLLGHTCKECEIYYADMPAEEREKKLASCSRHRFRYIPPNTPENFWEVGFPSTQTCMERGYIKEDLDPCPRPKRRQPYNAIFSPKGKEQKT

625 NP_066961.2 NP_055517.3

>NP_066961.2

MAAASVTPPGSLELLQPGFSKTLLGTKLEAKYLCSACRNVLRRPFQAQCGHRYCSFCLASILSSGPQNCAACVHEGIYEEGISILESSSAFPDNAARREVESLPAVCPSDGCTWKGTLKEYESCHEGRCPLMLTECPACKGLVRLGEKERHLEHECPERSLSCRHCRAPCCGADVKAHHEVCPKFPLTCDGCGKKKIPREKFQDHVKTCGKCRVPCRFHAIGCLETVEGEKQQEHEVQWLREHLAMLLSSVLEAKPLLGDQSHAGSELLQRCESLEKKTATFENIVCVLNREVERVAMTAEACSRQHRLDQDKIEALSSKVQQLERSIGLKDLAMADLEQKVLEMEASTYDGVFIWKISDFARKRQEAVAGRIPAIFSPAFYTSRYGYKMCLRIYLNGDGTGRGTHLSLFFVVMKGPNDALLRWPFNQKVTLMLLDQNNREHVIDAFRPDVTSSSFQRPVNDMNIASGCPLFCPVSKMEAKNSYVRDDAIFIKAIVDLTGL

>NP_055517.3

MDLHKQWENTETNWHKEKMELLDQFDNERKEWESQWKIMQKKIEELCREVKLWRKININESAKIIDLYHEKTIPEKVIESSPNYPDLGQSEFIRTNHKDGLRKENKREQSLVSGGNQMCKEQKATKKSKVGFLDPLATDNQKECEAWPDLRTSEEDSKSCSGALSTALEELAKVSEELCSFQEEIRKRSNHRRMKSDSFLQEMPNVTNIPHGDPMINNDQCILPISLEKEKQKNRKNLSCTNVLQSNSTKKCGIDTIDLKRNETPPVPPPRSTSRNFPSSDSEQAYERWKERLDHNSWVPHEGRSKRNYNPHFPLRQQEMSMLYPNEGKTSKDGIIFSSLVPEVKIDSKPPSNEDVGLSMWSCDIGIGAKRSPSTSWFQKTCSTPSNPKYEMVIPDHPAKSHPDLHVSNDCSSSVAESSSPLRNFSCGFERTTRNEKLAAKTDEFNRTVFRTDRNCQAIQQNHSCSKSSEDLKPCDTSSTHTGSISQSNDVSGIWKTNAHMPVPMENVPDNPTKKSTTGLVRQMQGHLSPRSYRNMLHEHDWRPSNLSGRPRSADPRSNYGVVEKLLKTYETATESALQNSKCFQDNWTKCNSDVSGGATLSQHLEMLQMEQQFQQKTAVWGGQEVKQGIDPKKITEESMSVNASHGKGFSRPARPANRRLPSRWASRSPSAPPALRRTTHNYTISLRSEALMV

626 NP_003965.2 NP_005347.3

>NP_003965.2

MGDGAVKQGFLYLQQQQTFGKKWRRFGASLYGGSDCALARLELQEGPEKPRRCEAARKVIRLSDCLRVAEAGGEASSPRDTSAFFLETKERLYLLAAPAAERGDWVQAICLLAFPGQRKELSGPEGKQSRPCMEENELYSSAVTVGPHKEFAVTMRPTEASERCHLRGSYTLRAGESALELWGGPEPGTQLYDWPYRFLRRFGRDKVTFSFEAGRRCVSGEGNFEFETRQGNEIFLALEEAISAQKNAAPATPQPQPATIPASLPRPDSPYSRPHDSLPPPSPTTPVPAPRPRGQEGEYAVPFDAVARSLGKNFRGILAVPPQLLADPLYDSIEETLPPRPDHIYDEPEGVAALSLYDSPQEPRGEAWRRQATADRDPAGLQHVQPAGQDFSASGWQPGTEYDNVVLKKGPK

>NP_005347.3

MGCGCSSHPEDDWMENIDVCENCHYPIVPLDGKGTLLIRNGSEVRDPLVTYEGSNPPASPLQDNLVIALHSYEPSHDGDLGFEKGEQLRILEQSGEWWKAQSLTTGQEGFIPFNFVAKANSLEPEPWFFKNLSRKDAERQLLAPGNTHGSFLIRESESTAGSFSLSVRDFDQNQGEVVKHYKIRNLDNGGFYISPRITFPGLHELVRHYTNASDGLCTRLSRPCQTQKPQKPWWEDEWEVPRETLKLVERLGAGQFGEVWMGYYNGHTKVAVKSLKQGSMSPDAFLAEANLMKQLQHQRLVRLYAVVTQEPIYIITEYMENGSLVDFLKTPSGIKLTINKLLDMAAQIAEGMAFIEERNYIHRDLRAANILVSDTLSCKIADFGLARLIEDNEYTAREGAKFPIKWTAPEAINYGTFTIKSDVWSFGILLTEIVTHGRIPYPGMTNPEVIQNLERGYRMVRPDNCPEELYQLMRLCWKERPEDRPTFDYLRSVLEDFFTATEGQYQPQP

627 NP_892113.3 NP_066189.2

>NP_892113.3

MNLLPPKPKYNPLRNESLSSMEEGASGSTPPEELPSPPASSLGPILPPLPGDDSPTTLCSFFPRMSNLRLANPAGGRPGSKGEPGRAADDGEGIVGAAMPDSGPLPLLQDMNKLSGGGGRRTRVEGGQLGGEEWTRHGSFVNKPTRGWLHPNDKVMGPGVSYLVRYMGCVEVLQSMRALDFNTRTQVTREAISLVCEAVPGAKGATRRRKPCSRPLSSILGRSNLKFAGMPITLTVSTSSLNLMAADCKQIIANHHMQSISFASGGDPDTAEYVAYVAKDPVNQRACHILECPEGLAQDVISTIGQAFELRFKQYLRNPPKLVTPHDRMAGFDGSAWDEEEEEPPDHQYYNDFPGKEPPLGGVVDMRLREGAAPGAARPTAPNAQTPSHLGATLPVGQPVGGDPEVRKQMPPPPPCPGRELFDDPSYVNVQNLDKARQAVGGAGPPNPAINGSAPRDLFDMKPFEDALRVPPPPQSVSMAEQLRGEPWFHGKLSRREAEALLQLNGDFLVRESTTTPGQYVLTGLQSGQPKHLLLVDPEGVVRTKDHRFESVSHLISYHMDNHLPIISAGSELCLQQPVERKL

>NP_066189.2

MNGAGPGPAAAAPVPVPVPVPDWRQFCELHAQAAAVDFAHKFCRFLRDNPAYDTPDAGASFSRHFAANFLDVFGEEVRRVLVAGPTTRGAAVSAEAMEPELADTSALKAAPYGHSRSSEDVSTHAATKARVRKGFSLRNMSLCVVDGVRDMWHRRASPEPDAAAAPRTAEPRDKWTRRLRLSRTLAAKVELVDIQREGALRFMVADDAAAGSGGSAQWQKCRLLLRRAVAEERFRLEFFVPPKASRPKVSIPLSAIIEVRTTMPLEMPEKDNTFVLKVENGAEYILETIDSLQKHSWVADIQGCVDPGDSEEDTELSCTRGGCLASRVASCSCELLTDAVDLPRPPETTAVGAVVTAPHSRGRDAVRESLIHVPLETFLQTLESPGGSGSDSNNTGEQGAETDPEAEPELELSDYPWFHGTLSRVKAAQLVLAGGPRNHGLFVIRQSETRPGEYVLTFNFQGKAKHLRLSLNGHGQCHVQHLWFQSVLDMLRHFHTHPIPLESGGSADITLRSYVRAQDPPPEPGPTPPAAPASPACWSDSPGQHYFSSLAAAACPPASPSDAAGASSSSASSSSAASGPAPPRPVEGQLSARSRSNSAERLLEAVAATAAEEPPEAAPGRARAVENQYSFY

628 NP_004844.1 NP_003818.2

>NP_004844.1

MAPDPWFSTYDSTCQIAQEIAEKIQQRNQYERKGEKAPKLTVTIRALLQNLKEKIALLKDLLLRAVSTHQITQLEGDRRQNLLDDLVTRERLLLASFKNEGAEPDLIRSSLMSEEAKRGAPNPWLFEEPEETRGLGFDEIRQQQQKIIQEQDAGLDALSSIISRQKQMGQEIGNELDEQNEIIDDLANLVENTDEKLRNETRRVNMVDRKSASCGMIMVILLLLVAIVVVAVWPTN

>NP_003818.2

MDNSGKEAEAMALLAEAERKVKNSQSFFSGLFGGSSKIEEACEIYARAANMFKMAKNWSAAGNAFCQAAQLHLQLQSKHDAATCFVDAGNAFKKADPQEAINCLMRAIEIYTDMGRFTIAAKHHISIAEIYETELVDIEKAIAHYEQSADYYKGEESNSSANKCLLKVAGYAALLEQYQKAIDIYEQVGTNAMDSPLLKYSAKDYFFKAALCHFCIDMLNAKLAVQKYEELFPAFSDSRECKLMKKLLEAHEEQNVDSYTESVKEYDSISRLDQWLTTMLLRIKKTIQGDEEDLR

629 NP_002730.1 NP_001334.2

>NP_002730.1

MAGLGPGVGDSEGGPRPLFCRKGALRQKVVHEVKSHKFTARFFKQPTFCSHCTDFIWGIGKQGLQCQVCSFVVHRRCHEFVTFECPGAGKGPQTDDPRNKHKFRLHSYSSPTFCDHCGSLLYGLVHQGMKCSCCEMNVHRRCVRSVPSLCGVDHTERRGRLQLEIRAPTADEIHVTVGEARNLIPMDPNGLSDPYVKLKLIPDPRNLTKQKTRTVKATLNPVWNETFVFNLKPGDVERRLSVEVWDWDRTSRNDFMGAMSFGVSELLKAPVDGWYKLLNQEEGEYYNVPVADADNCSLLQKFEACNYPLELYERVRMGPSSSPIPSPSPSPTDPKRCFFGASPGRLHISDFSFLMVLGKGSFGKVMLAERRGSDELYAIKILKKDVIVQDDDVDCTLVEKRVLALGGRGPGGRPHFLTQLHSTFQTPDRLYFVMEYVTGGDLMYHIQQLGKFKEPHAAFYAAEIAIGLFFLHNQGIIYRDLKLDNVMLDAEGHIKITDFGMCKENVFPGTTTRTFCGTPDYIAPEIIAYQPYGKSVDWWSFGVLLYEMLAGQPPFDGEDEEELFQAIMEQTVTYPKSLSREAVAICKGFLTKHPGKRLGSGPDGEPTIRAHGFFRWIDWERLERLEIPPPFRPRPCGRSGENFDKFFTRAAPALTPPDRLVLASIDQADFQGFTYVNPDFVHPDARSPTSPVPVPVM

>NP_001334.2

MSNEVETSATNGQPDQQAAPKAPSKKEKKKGPEKTDEYLLARFKGDGVKYKAKLIGIDDVPDARGDKMSQDSMMKLKGMAAAGRSQGQHKQRIWVNISLSGIKIIDEKTGVIEHEHPVNKISFIARDVTDNRAFGYVCGGEGQHQFFAIKTGQQAEPLVVDLKDLFQVIYNVKKKEEEKKKIEEASKAVENGSEALMILDDQTNKLKSGVDQMDLFGDMSTPPDLNSPTESKDILLVDLNSEIDTNQNSLRENPFLTNGITSCSLPRPTPQASFLPENAFSANLNFFPTPNPDPFRDDPFTQPDQSTPSSFDSLKSPDQKKENSSSSSTPLSNGPLNGDVDYFGQQFDQISNRTGKQEAQAGPWPFSSSQTQPAVRTQNGVSEREQNGFSVKSSPNPFVGSPPKGLSIQNGVKQDLESSVQSSPHDSIAIIPPPQSTKPGRGRRTAKSSANDLLASDIFAPPVSEPSGQASPTGQPTALQPNPLDLFKTSAPAPVGPLVGLGGVTVTLPQAGPWNTASLVFNQSPSMAPGAMMGGQPSGFSQPVIFGTSPAVSGWNQPSPFAASTPPPVPVVWGPSASVAPNAWSTTSPLGNPFQSNIFPAPAVSTQPPSMHSSLLVTPPQPPPRAGPPKDISSDAFTALDPLGDKEIKDVKEMFKDFQLRQPPAVPARKGEQTSSGTLSAFASYFNSKVGIPQENADHDDFDANQLLNKINEPPKPAPRQVSLPVTKSTDNAFENPFFKDSFGSSQASVASSQPVSSEMYRDPFGNPFA

630 NP_057065.2 NP_058519.2

>NP_057065.2

MATITCTRFTEEYQLFEELGKGAFSVVRRCVKVLAGQEYAAKIINTKKLSARDHQKLEREARICRLLKHPNIVRLHDSISEEGHHYLIFDLVTGGELFEDIVAREYYSEADASHCIQQILEAVLHCHQMGVVHRDLKPENLLLASKLKGAAVKLADFGLAIEVEGEQQAWFGFAGTPGYLSPEVLRKDPYGKPVDLWACGVILYILLVGYPPFWDEDQHRLYQQIKAGAYDFPSPEWDTVTPEAKDLINKMLTINPSKRITAAEALKHPWISHRSTVASCMHRQETVDCLKKFNARRKLKGAILTTMLATRNFSGGKSGGNKKSDGVKKRKSSSSVQLMESSESTNTTIEDEDTKVRKQEIIKVTEQLIEAISNGDFESYTKMCDPGMTAFEPEALGNLVEGLDFHRFYFENLWSRNSKPVHTTILNPHIHLMGDESACIAYIRITQYLDAGGIPRTAQSEETRVWHRRDGKWQIVHFHRSGAPSVLPH

>NP_058519.2

MAEPRQEFEVMEDHAGTYGLGDRKDQGGYTMHQDQEGDTDAGLKESPLQTPTEDGSEEPGSETSDAKSTPTAEDVTAPLVDEGAPGKQAAAQPHTEIPEGTTAEEAGIGDTPSLEDEAAGHVTQEPESGKVVQEGFLREPGPPGLSHQLMSGMPGAPLLPEGPREATRQPSGTGPEDTEGGRHAPELLKHQLLGDLHQEGPPLKGAGGKERPGSKEEVDEDRDVDESSPQDSPPSKASPAQDGRPPQTAAREATSIPGFPAEGAIPLPVDFLSKVSTEIPASEPDGPSVGRAKGQDAPLEFTFHVEITPNVQKEQAHSEEHLGRAAFPGAPGEGPEARGPSLGEDTKEADLPEPSEKQPAAAPRGKPVSRVPQLKARMVSKSKDGTGSDDKKAKTSTRSSAKTLKNRPCLSPKHPTPGSSDPLIQPSSPAVCPEPPSSPKHVSSVTSRTGSSGAKEMKLKGADGKTKIATPRGAAPPGQKGQANATRIPAKTPPAPKTPPSSGEPPKSGDRSGYSSPGSPGTPGSRSRTPSLPTPPTREPKKVAVVRTPPKSPSSAKSRLQTAPVPMPDLKNVKSKIGSTENLKHQPGGGKVQIINKKLDLSNVQSKCGSKDNIKHVPGGGSVQIVYKPVDLSKVTSKCGSLGNIHHKPGGGQVEVKSEKLDFKDRVQSKIGSLDNITHVPGGGNKKIETHKLTFRENAKAKTDHGAEIVYKSPVVSGDTSPRHLSNVSSTGSIDMVDSPQLATLADEVSASLAKQGL

631 NP_569736.1 NP_036377.1

>NP_569736.1

MMPGQIPDPSVTTGSLPGLGPLTGLPSSALTVEELKYADIRNLGAMIAPLHFLEVKLGKRPQPVKSELDEEEERRKRRREKNKVAAARCRNKKKERTEFLQRESERLELMNAELKTQIEELKQERQQLILMLNRHRPTCIVRTDSVKTPESEGNPLLEQLEKK

>NP_036377.1

MALTSFLPAPTQLSQDQLEAEEKARSQRSRQTSLVSSRREPPPYGYRKGWIPRLLEDFGDGGAFPEIHVAQYPLDMGRKKKMSNALAIQVDSEGKIKYDAIARQGQSKDKVIYSKYTDLVPKEVMNADDPDLQRPDEEAIKEITEKTRVALEKSVSQKVAAAMPVRAADKLAPAQYIRYTPSQQGVAFNSGAKQRVIRMVEMQKDPMEPPRFKINKKIPRGPPSPPAPVMHSPSRKMTVKEQQEWKIPPCISNWKNAKGYTIPLDKRLAADGRGLQTVHINENFAKLAEALYIADRKAREAVEMRAQVERKMAQKEKEKHEEKLREMAQKARERRAGIKTHVEKEDGEARERDEIRHDRRKERQHDRNLSRAAPDKRSKLQRNENRDISEVIALGVPNPRTSNEVQYDQRLFNQSKGMDSGFAGGEDEIYNVYDQAWRGGKDMAQSIYRPSKNLDKDMYGDDLEARIKTNRFVPDKEFSGSDRRQRGREGPVQFEEDPFGLDKFLEEAKQHGGSKRPSDSSRPKEHEHEGKKRRKE

632 NP_036611.2 NP_005879.1

>NP_036611.2

MVDREQLVQKARLAEQAERYDDMAAAMKNVTELNEPLSNEERNLLSVAYKNVVGARRSSWRVISSIEQKTSADGNEKKIEMVRAYREKIEKELEAVCQDVLSLLDNYLIKNCSETQYESKVFYLKMKGDYYRYLAEVATGEKRATVVESSEKAYSEAHEISKEHMQPTHPIRLGLALNYSVFYYEIQNAPEQACHLAKTAFDDAIAELDTLNEDSYKDSTLIMQLLRDNLTLWTSDQQDDDGGEGNN

>NP_005879.1

MFSSVAHLARANPFNTPHLQLVHDGLGDLRSSSPGPTGQPRRPRNLAAAAVEEQYSCDYGSGRFFILCGLGGIISCGTTHTALVPLDLVKCRMQVDPQKYKGIFNGFSVTLKEDGVRGLAKGWAPTFLGYSMQGLCKFGFYEVFKVLYSNMLGEENTYLWRTSLYLAASASAEFFADIALAPMEAAKVRIQTQPGYANTLRDAAPKMYKEEGLKAFYKGVAPLWMRQIPYTMMKFACFERTVEALYKFVVPKPRSECSKPEQLVVTFVAGYIAGVFCAIVSHPADSVVSVLNKEKGSSASLVLKRLGFKGVWKGLFARIIMIGTLTALQWFIYDSVKVYFRLPRPPPPEMPESLKKKLGLTQ

633 NP_002397.1 BAA21128.1

>NP_002397.1

MLKKQSAGLVLWGAILFVAWNALLLLFFWTRPAPGRPPSVSALDGDPASLTREVIRLAQDAEVELERQRGLLQQIGDALSSQRGRVPTAAPPAQPRVPVTPAPAVIPILVIACDRSTVRRCLDKLLHYRPSAELFPIIVSQDCGHEETAQAIASYGSAVTHIRQPDLSSIAVPPDHRKFQGYYKIARHYRWALGQVFRQFRFPAAVVVEDDLEVAPDFFEYFRATYPLLKADPSLWCVSAWNDNGKEQMVDASRPELLYRTDFFPGLGWLLLAELWAELEPKWPKAFWDDWMRRPEQRQGRACIRPEISRTMTFGRKGVSHGQFFDQHLKFIKLNQQFVHFTQLDLSYLQREAYDRDFLARVYGAPQLQVEKVRTNDRKELGEVRVQYTGRDSFKAFAKALGVMDDLKSGVPRAGYRGIVTFQFRGRRVHLAPPPTWEGYDPSWN

>BAA21128.1

MHSTTPISSLFSFTSPAVKRLLGWKQGDEEEKWAEKAVDSLVKKLKKKKGAMDELERALSCPGQPSKCVTIPRSLDGRLQVSHRKGLPHVIYCRVWRWPDLQSHHELKPLECCEFPFGSKQKEVCINPYHYRRVETPVLPPVLVPRHSEYNPQLSLLAKFRSASLHSEPLMPHNATYPDSFQQPPCSALPPSPSHAFSQSPCTASYPHSPGSPSEPESPYQHSVDTPPLPYHATEASETQSGQPVDATADRHVVLSIPNGDFRPVCYEEPQHWCSVAYYELNNRVGETFQASSRSVLIDGFTDPSNNRNRFCLGLLSNVNRNSTIENTRRHIGKGVHLYYVGGEVYAECVSDSSIFVQSRNCNYQHGFHPATVCKIPSGCSLKVFNNQLFAQLLAQSVHHGFEVVYELTKMCTIRMSFVKGWGAEYHRQDVTSTPCWIEIHLHGPLQWLDKVLTQMGSPHNPISSVS

634 NP_004299.1 Q14643

>NP_004299.1

MDPLSELQDDLTLDDTSEALNQLKLASIDEKNWPSDEMPDFPKSDDSKSSSPELVTHLKWDDPYYDIARHQIVEVAGDDKYGRKIIVFSACRMPPSHQLDHSKLLGYLKHTLDQYVESDYTLLYLHHGLTSDNKPSLSWLRDAYREFDRKYKKNIKALYIVHPTMFIKTLLILFKPLISFKFGQKIFYVNYLSELSEHVKLEQLGIPRQVLKYDDFLKSTQKSPATAPKPMPPRPPLPNQQFGVSLQHLQEKNPEQEPIPIVLRETVAYLQAHALTTEGIFRRSANTQVVREVQQKYNMGLPVDFDQYNELHLPAVILKTFLRELPEPLLTFDLYPHVVGFLNIDESQRVPATLQVLQTLPEENYQVLRFLTAFLVQISAHSDQNKMTNTNLAVVFGPNLLWAKDAAITLKAINPINTFTKFLLDHQGELFPSPDPSGL

>Q14643

MSDKMSSFLHIGDICSLYAEGSTNGFISTLGLVDDRCVVQPETGDLNNPPKKFRDCLFKLCPMNRYSAQKQFWKAAKPGANSTTDAVLLNKLHHAADLEKKQNETENRKLLGTVIQYGNVIQLLHLKSNKYLTVNKRLPALLEKNAMRVTLDEAGNEGSWFYIQPFYKLRSIGDSVVIGDKVVLNPVNAGQPLHASSHQLVDNPGCNEVNSVNCNTSWKIVLFMKWSDNKDDILKGGDVVRLFHAEQEKFLTCDEHRKKQHVFLRTTGRQSATSATSSKALWEVEVVQHDPCRGGAGYWNSLFRFKHLATGHYLAAEVDPDFEEECLEFQPSVDPDQDASRSRLRNAQEKMVYSLVSVPEGNDISSIFELDPTTLRGGDSLVPRNSYVRLRHLCTNTWVHSTNIPIDKEEEKPVMLKIGTSPVKEDKEAFAIVPVSPAEVRDLDFANDASKVLGSIAGKLEKGTITQNERRSVTKLLEDLVYFVTGGTNSGQDVLEVVFSKPNRERQKLMREQNILKQIFKLLQAPFTDCGDGPMLRLEELGDQRHAPFRHICRLCYRVLRHSQQDYRKNQEYIAKQFGFMQKQIGYDVLAEDTITALLHNNRKLLEKHITAAEIDTFVSLVRKNREPRFLDYLSDLCVSMNKSIPVTQELICKAVLNPTNADILIETKLVLSRFEFEGVSSTGENALEAGEDEEEVWLFWRDSNKEIRSKSVRELAQDAKEGQKEDRDVLSYYRYQLNLFARMCLDRQYLAINEISGQLDVDLILRCMSDENLPYDLRASFCRLMLHMHVDRDPQEQVTPVKYARLWSEIPSEIAIDDYDSSGASKDEIKERFAQTMEFVEEYLRDVVCQRFPFSDKEKNKLTFEVVNLARNLIYFGFYNFSDLLRLTKILLAILDCVHVTTIFPISKMAKGEENKGNNDVEKLKSSNVMRSIHGVGELMTQVVLRGGGFLPMTPMAAAPEGNVKQAEPEKEDIMVMDTKLKIIEILQFILNVRLDYRISCLLCIFKREFDESNSQTSETSSGNSSQEGPSNVPGALDFEHIEEQAEGIFGGSEENTPLDLDDHGGRTFLRVLLHLTMHDYPPLVSGALQLLFRHFSQRQEVLQAFKQVQLLVTSQDVDNYKQIKQDLDQLRSIVEKSELWVYKGQGPDETMDGASGENEHKKTEEGNNKPQKHESTSSYNYRVVKEILIRLSKLCVQESASVRKSRKQQQRLLRNMGAHAVVLELLQIPYEKAEDTKMQEIMRLAHEFLQNFCAGNQQNQALLHKHINLFLNPGILEAVTMQHIFMNNFQLCSEINERVVQHFVHCIETHGRNVQYIKFLQTIVKAEGKFIKKCQDMVMAELVNSGEDVLVFYNDRASFQTLIQMMRSERDRMDENSPLMYHIHLVELLAVCTEGKNVYTEIKCNSLLPLDDIVRVVTHEDCIPEVKIAYINFLNHCYVDTEVEMKEIYTSNHMWKLFENFLVDICRACNNTSDRKHADSILEKYVTEIVMSIVTTFFSSPFSDQSTTLQTRQPVFVQLLQGVFRVYHCNWLMPSQKASVESCIRVLSDVAKSRAIAIPVDLDSQVNNLFLKSHSIVQKTAMNWRLSARNAARRDSVLAASRDYRNIIERLQDIVSALEDRLRPLVQAELSVLVDVLHRPELLFPENTDARRKCESGGFICKLIKHTKQLLEENEEKLCIKVLQTLREMMTKDRGYGEKLISIDELDNAELPPAPDSENSTEELEPSPPLRQLEDHKRGEALRQVLVNRYYGNVRPSGRRESLTSFGNGPLSAGGPGKPGGGGGGSGSSSMSRGEMSLAEVQCHLDKEGASNLVIDLIMNASSDRVFHESILLAIALLEGGNTTIQHSFFCRLTEDKKSEKFFKVFYDRMKVAQQEIKATVTVNTSDLGNKKKDDEVDRDAPSRKKAKEPTTQITEEVRDQLLEASAATRKAFTTFRREADPDDHYQPGEGTQATADKAKDDLEMSAVITIMQPILRFLQLLCENHNRDLQNFLRCQNNKTNYNLVCETLQFLDCICGSTTGGLGLLGLYINEKNVALINQTLESLTEYCQGPCHENQNCIATHESNGIDIITALILNDINPLGKKRMDLVLELKNNASKLLLAIMESRHDSENAERILYNMRPKELVEVIKKAYMQGEVEFEDGENGEDGAASPRNVGHNIYILAHQLARHNKELQSMLKPGGQVDGDEALEFYAKHTAQIEIVRLDRTMEQIVFPVPSICEFLTKESKLRIYYTTERDEQGSKINDFFLRSEDLFNEMNWQKKLRAQPVLYWCARNMSFWSSISFNLAVLMNLLVAFFYPFKGVRGGTLEPHWSGLLWTAMLISLAIVIALPKPHGIRALIASTILRLIFSVGLQPTLFLLGAFNVCNKIIFLMSFVGNCGTFTRGYRAMVLDVEFLYHLLYLVICAMGLFVHEFFYSLLLFDLVYREETLLNVIKSVTRNGRSIILTAVLALILVYLFSIVGYLFFKDDFILEVDRLPNETAVPETGESLASEFLFSDVCRVESGENCSSPAPREELVPAEETEQDKEHTCETLLMCIVTVLSHGLRSGGGVGDVLRKPSKEEPLFAARVIYDLLFFFMVIIIVLNLIFGVIIDTFADLRSEKQKKEEILKTTCFICGLERDKFDNKTVTFEEHIKEEHNMWHYLCFIVLVKVKDSTEYTGPESYVAEMIKERNLDWFPRMRAMSLVSSDSEGEQNELRNLQEKLESTMKLVTNLSGQLSELKDQMTEQRKQKQRIGLLGHPPHMNVNPQQPA

635 NP_001028687.1 NP_061817.1

>NP_001028687.1

MFPVSSGCFQEQQETNKSLPRSASTPETRTKFTQDNLCHAQRERLDSANLWVLVDCILRDTSEDLGLQCDAVNLAFGCRCEELEDARHKLQHHLHKMLREITDQEHNVVALKEAIKDKEEPLHIAQTRL

>NP_061817.1

MAGVRSLRCSRGCAGGCECGDKGKCSDSSLLGKRLSEDSSRHQLLQKWASMWSSMSEDASVADMERAQLEEEAAAAEERPLVFLCSGCRRPLGDSLSWVASQEDTNCILLRCVSCNVSVDKEQKLSKREKENGCVLETLCCAGCSLNLGYVYRCTPKNLDYKRDLFCLSVEAIESYVLGSSEKQIVSEDKELFNLESRVEIEKSLTQMEDVLKALQMKLWEAESKLSFATCKS

636 NP_001195.2 NP_001060.1

>NP_001195.2

MTSSLQRPWRVPWLPWTILLVSTAAASQNQERLCAFKDPYQQDLGIGESRISHENGTILCSKGSTCYGLWEKSKGDINLVKQGCWSHIGDPQECHYEECVVTTTPPSIQNGTYRFCCCSTDLCNVNFTENFPPPDTTPLSPPHSFNRDETIIIALASVSVLAVLIVALCFGYRMLTGDRKQGLHSMNMMEAAASEPSLDLDNLKLLELIGRGRYGAVYKGSLDERPVAVKVFSFANRQNFINEKNIYRVPLMEHDNIARFIVGDERVTADGRMEYLLVMEYYPNGSLCKYLSLHTSDWVSSCRLAHSVTRGLAYLHTELPRGDHYKPAISHRDLNSRNVLVKNDGTCVISDFGLSMRLTGNRLVRPGEEDNAAISEVGTIRYMAPEVLEGAVNLRDCESALKQVDMYALGLIYWEIFMRCTDLFPGESVPEYQMAFQTEVGNHPTFEDMQVLVSREKQRPKFPEAWKENSLAVRSLKETIEDCWDQDAEARLTAQCAEERMAELMMIWERNKSVSPTVNPMSTAMQNERNLSHNRRVPKIGPYPDYSSSSYIEDSIHHTDSIVKNISSEHSMSSTPLTIGEKNRNSINYERQQAQARIPSPETSVTSLSTNTTTTNTTGLTPSTGMTTISEMPYPDETNLHTTNVAQSIGPTPVCLQLTEEDLETNKLDPKEVDKNLKESSDENLMEHSLKQFSGPDPLSSTSSSLLYPLIKLAVEATGQQDFTQTANGQACLIPDVLPTQIYPLPKQQNLPKRPTSLPLNTKNSTKEPRLKFGSKHKSNLKQVETGVAKMNTINAAEPHVVTVTMNGVAGRNHSVNSHAATTQYANGTVLSGQTTNIVTHRAQEMLQNQFIGEDTRLNINSSPDEHEPLLRREQQAGHDEGVLDRLVDRRERPLEGGRTNSNNNNSNPCSEQDVLAQGVPSTAADPGPSKPRRAQRPNSLDLSATNVLDGSSIQIGESTQDGKSGSGEKIKKRVKTPYSLKRWRPSTWVISTESLDCEVNNNGSNRAVHSKSSTAVYLAEGGTATTMVSKDIGMNCL

>NP_001060.1

MREIVHIQAGQCGNQIGAKFWEVISDEHGIDPTGSYHGDSDLQLERINVYYNEAAGNKYVPRAILVDLEPGTMDSVRSGPFGQIFRPDNFVFGQSGAGNNWAKGHYTEGAELVDSVLDVVRKESESCDCLQGFQLTHSLGGGTGSGMGTLLISKIREEYPDRIMNTFSVMPSPKVSDTVVEPYNATLSVHQLVENTDETYSIDNEALYDICFRTLKLTTPTYGDLNHLVSATMSGVTTCLRFPGQLNADLRKLAVNMVPFPRLHFFMPGFAPLTSRGSQQYRALTVPELTQQMFDSKNMMAACDPRHGRYLTVAAIFRGRMSMKEVDEQMLNVQNKNSSYFVEWIPNNVKTAVCDIPPRGLKMSATFIGNSTAIQELFKRISEQFTAMFRRKAFLHWYTGEGMDEMEFTEAESNMNDLVSEYQQYQDATADEQGEFEEEEGEDEA

637 NP_002871.1 NP_002145.3

>NP_002871.1

MEHIQGAWKTISNGFGFKDAVFDGSSCISPTIVQQFGYQRRASDDGKLTDPSKTSNTIRVFLPNKQRTVVNVRNGMSLHDCLMKALKVRGLQPECCAVFRLLHEHKGKKARLDWNTDAASLIGEELQVDFLDHVPLTTHNFARKTFLKLAFCDICQKFLLNGFRCQTCGYKFHEHCSTKVPTMCVDWSNIRQLLLFPNSTIGDSGVPALPSLTMRRMRESVSRMPVSSQHRYSTPHAFTFNTSSPSSEGSLSQRQRSTSTPNVHMVSTTLPVDSRMIEDAIRSHSESASPSALSSSPNNLSPTGWSQPKTPVPAQRERAPVSGTQEKNKIRPRGQRDSSYYWEIEASEVMLSTRIGSGSFGTVYKGKWHGDVAVKILKVVDPTPEQFQAFRNEVAVLRKTRHVNILLFMGYMTKDNLAIVTQWCEGSSLYKHLHVQETKFQMFQLIDIARQTAQGMDYLHAKNIIHRDMKSNNIFLHEGLTVKIGDFGLATVKSRWSGSQQVEQPTGSVLWMAPEVIRMQDNNPFSFQSDVYSYGIVLYELMTGELPYSHINNRDQIIFMVGRGYASPDLSKLYKNCPKAMKRLVADCVKKVKEERPLFPQILSSIELLQHSLPKINRSASEPSLHRAAHTEDINACTLTTSPRLPVF

>NP_002145.3

MSVVGIDLGFQSCYVAVARAGGIETIANEYSDRCTPACISFGPKNRSIGAAAKSQVISNAKNTVQGFKRFHGRAFSDPFVEAEKSNLAYDIVQLPTGLTGIKVTYMEEERNFTTEQVTAMLLSKLKETAESVLKKPVVDCVVSVPCFYTDAERRSVMDATQIAGLNCLRLMNETTAVALAYGIYKQDLPALEEKPRNVVFVDMGHSAYQVSVCAFNRGKLKVLATAFDTTLGGRKFDEVLVNHFCEEFGKKYKLDIKSKIRALLRLSQECEKLKKLMSANASDLPLSIECFMNDVDVSGTMNRGKFLEMCNDLLARVEPPLRSVLEQTKLKKEDIYAVEIVGGATRIPAVKEKISKFFGKELSTTLNADEAVTRGCALQCAILSPAFKVREFSITDVVPYPISLRWNSPAEEGSSDCEVFSKNHAAPFSKVLTFYRKEPFTLEAYYSSPQDLPYPDPAIAQFSVQKVTPQSDGSSSKVKVKVRVNVHGIFSVSSASLVEVHKSEENEEPMETDQNAKEEEKMQVDQEEPHVEEQQQQTPAENKAESEEMETSQAGSKDKKMDQPPQAKKAKVKTSTVDLPIENQLLWQIDREMLNLYIENEGKMIMQDKLEKERNDAKNAVEEYVYEMRDKLSGEYEKFVSEDDRNSFTLKLEDTENWLYEDGEDQPKQVYVDKLAELKNLGQPIKIRFQESEERPKLFEELGKQIQQYMKIISSFKNKEDQYDHLDAADMTKVEKSTNEAMEWMNNKLNLQNKQSLTMDPVVKSKEIEAKIKELTSTCSPIISKPKPKVEPPKEEQKNAEQNGPVDGQGDNPGPQAAEQGTDTAVPSDSDKKLPEMDID

638 NP_001931.2 NP_003293.2

>NP_001931.2

MKTRQNKDSMSMRSGRKKEAPGPREELRSRGRASPGGVSTSSSDGKAEKSRQTAKKARVEEASTPKVNKQGRSEEISESESEETNAPKKTKTEQELPRPQSPSDLDSLDGRSLNDDGSSDPRDIDQDNRSTSPSIYSPGSVENDSDSSSGLSQGPARPYHPPPLFPPSPQPPDSTPRQPEASFEPHPSVTPTGYHAPMEPPTSRMFQAPPGAPPPHPQLYPGGTGGVLSGPPMGPKGGGAASSVGGPNGGKQHPPPTTPISVSSSGASGAPPTKPPTTPVGGGNLPSAPPPANFPHVTPNLPPPPALRPLNNASASPPGLGAQPLPGHLPSPHAMGQGMGGLPPGPEKGPTLAPSPHSLPPASSSAPAPPMRFPYSSSSSSSAAASSSSSSSSSSASPFPASQALPSYPHSFPPPTSLSVSNQPPKYTQPSLPSQAVWSQGPPPPPPYGRLLANSNAHPGPFPPSTGAQSTAHPPVSTHHHHHQQQQQQQQQQQQQQQQQQQHHGNSGPPPPGAFPHPLEGGSSHHAHPYAMSPSLGSLRPYPPGPAHLPPPHSQVSYSQAGPNGPPVSSSSNSSSSTSQGSYPCSHPSPSQGPQGAPYPFPPVPTVTTSSATLSTVIATVASSPAGYKTASPPGPPPYGKRAPSPGAYKTATPPGYKPGSPPSFRTGTPPGYRGTSPPAGPGTFKPGSPTVGPGPLPPAGPSGLPSLPPPPAAPASGPPLSATQIKQEPAEEYETPESPVPPARSPSPPPKVVDVPSHASQSARFNKHLDRGFNSCARSDLYFVPLEGSKLAKKRADLVEKVRREAEQRAREEKEREREREREKEREREKERELERSVKLAQEGRAPVECPSLGPVPHRPPFEPGSAVATVPPYLGPDTPALRTLSEYARPHVMSPGNRNHPFYVPLGAVDPGLLGYNVPALYSSDPAAREREREARERDLRDRLKPGFEVKPSELEPLHGVPGPGLDPFPRHGGLALQPGPPGLHPFPFHPSLGPLERERLALAAGPALRPDMSYAERLAAERQHAERVAALGNDPLARLQMLNVTPHHHQHSHIHSHLHLHQQDAIHAASASVHPLIDPLASGSHLTRIPYPAGTLPNPLLPHPLHENEVLRHQLFAAPYRDLPASLSAPMSAAHQLQAMHAQSAELQRLALEQQQWLHAHHPLHSVPLPAQEDYYSHLKKESDKPL

>NP_003293.2

MSGPTWLPPKQPEPARAPQGRAIPRGTPGPPPAHGAALQPHPRVNFCPLPSEQCYQAPGGPEDRGPAWVGSHGVLQHTQGLPADRGGLRPGSLDAEIDLLSSTLAELNGGRGHASRRPDRQAYEPPPPPAYRTGSLKPNPASPLPASPYGGPTPASYTTASTPAGPAFPVQVKVAQPVRGCGPPRRGASQASGPLPGPHFPLPGRGEVWGPGYRSQREPGPGAKEEAAGVSGPAGRGRGGEHGPQVPLSQPPEDELDRLTKKLVHDMNHPPSGEYFGQCGGCGEDVVGDGAGVVALDRVFHVGCFVCSTCRAQLRGQHFYAVERRAYCEGCYVATLEKCATCSQPILDRILRAMGKAYHPGCFTCVVCHRGLDGIPFTVDATSQIHCIEDFHRKFAPRCSVCGGAIMPEPGQEETVRIVALDRSFHIGCYKCEECGLLLSSEGECQGCYPLDGHILCKACSAWRIQELSATVTTDC

639 NP_004327.1 NP_006312.1

>NP_004327.1

MDTPENVLQMLEAHMQSYKGNDPLGEWERYIQWVEENFPENKEYLITLLEHLMKEFLDKKKYHNDPRFISYCLKFAEYNSDLHQFFEFLYNHGIGTLSSPLYIAWAGHLEAQGELQHASAVLQRGIQNQAEPREFLQQQYRLFQTRLTETHLPAQARTSEPLHNVQVLNQMITSKSNPGNNMACISKNQGSELSGVISSACDKESNMERRVITISKSEYSVHSSLASKVDVEQVVMYCKEKLIRGESEFSFEELRAQKYNQRRKHEQWVNEDRHYMKRKEANAFEEQLLKQKMDELHKKLHQVVETSHEDLPASQERSEVNPARMGPSVGSQQELRAPCLPVTYQQTPVNMEKNPREAPPVVPPLANAISAALVSPATSQSIAPPVPLKAQTVTDSMFAVASKDAGCVNKSTHEFKPQSGAEIKEGCETHKVANTSSFHTTPNTSLGMVQATPSKVQPSPTVHTKEALGFIMNMFQAPTLPDISDDKDEWQSLDQNEDAFEAQFQKNVRSSGAWGVNKIISSLSSAFHVFEDGNKENYGLPQPKNKPTGARTFGERSVSRLPSKPKEEVPHAEEFLDDSTVWGIRCNKTLAPSPKSPGDFTSAAQLASTPFHKLPVESVHILEDKENVVAKQCTQATLDSCEENMVVPSRDGKFSPIQEKSPKQALSSHMYSASLLRLSQPAAGGVLTCEAELGVEACRLTDTDAAIAEDPPDAIAGLQAEWMQMSSLGTVDAPNFIVGNPWDDKLIFKLLSGLSKPVSSYPNTFEWQCKLPAIKPKTEFQLGSKLVYVHHLLGEGAFAQVYEATQGDLNDAKNKQKFVLKVQKPANPWEFYIGTQLMERLKPSMQHMFMKFYSAHLFQNGSVLVGELYSYGTLLNAINLYKNTPEKVMPQGLVISFAMRMLYMIEQVHDCEIIHGDIKPDNFILGNGFLEQDDEDDLSAGLALIDLGQSIDMKLFPKGTIFTAKCETSGFQCVEMLSNKPWNYQIDYFGVAATVYCMLFGTYMKVKNEGGECKPEGLFRRLPHLDMWNEFFHVMLNIPDCHHLPSLDLLRQKLKKVFQQHYTNKIRALRNRLIVLLLECKRSRK

>NP_006312.1

MSVDMNSQGSDSNEEDYDPNCEEEEEEEEDDPGDIEDYYVGVASDVEQQGADAFDPEEYQFTCLTYKESEGALNEHMTSLASVLKVSHSVAKLILVNFHWQVSEILDRYKSNSAQLLVEARVQPNPSKHVPTSHPPHHCAVCMQFVRKENLLSLACQHQFCRSCWEQHCSVLVKDGVGVGVSCMAQDCPLRTPEDFVFPLLPNEELREKYRRYLFRDYVESHYQLQLCPGADCPMVIRVQEPRARRVQCNRCNEVFCFKCRQMYHAPTDCATIRKWLTKCADDSETANYISAHTKDCPKCNICIEKNGGCNHMQCSKCKHDFCWMCLGDWKTHGSEYYECSRYKENPDIVNQSQQAQAREALKKYLFYFERWENHNKSLQLEAQTYQRIHEKIQERVMNNLGTWIDWQYLQNAAKLLAKCRYTLQYTYPYAYYMESGPRKKLFEYQQAQLEAEIENLSWKVERADSYDRGDLENQMHIAEQRRRTLLKDFHDT

640 NP_001388.1 NP_001946.2

>NP_001388.1

MRGVWPPPVSALLSALGMSTYKRATLDEEDLVDSLSEGDAYPNGLQVNFHSPRSGQRCWAARTQVEKRLVVLVVLLAAGLVACLAALGIQYQTRSPSVCLSEACVSVTSSILSSMDPTVDPCHDFFSYACGGWIKANPVPDGHSRWGTFSNLWEHNQAIIKHLLENSTASVSEAERKAQVYYRACMNETRIEELRAKPLMELIERLGGWNITGPWAKDNFQDTLQVVTAHYRTSPFFSVYVSADSKNSNSNVIQVDQSGLGLPSRDYYLNKTENEKVLTGYLNYMVQLGKLLGGGDEEAIRPQMQQILDFETALANITIPQEKRRDEELIYHKVTAAELQTLAPAINWLPFLNTIFYPVEINESEPIVVYDKEYLEQISTLINTTDRCLLNNYMIWNLVRKTSSFLDQRFQDADEKFMEVMYGTKKTCLPRWKFCVSDTENNLGFALGPMFVKATFAEDSKSIATEIILEIKKAFEESLSTLKWMDEETRKSAKEKADAIYNMIGYPNFIMDPKELDKVFNDYTAVPDLYFENAMRFFNFSWRVTADQLRKAPNRDQWSMTPPMVNAYYSPTKNEIVFPAGILQAPFYTRSSPKALNFGGIGVVVGHELTHAFDDQGREYDKDGNLRPWWKNSSVEAFKRQTECMVEQYSNYSVNGEPVNGRHTLGENIADNGGLKAAYRAYQNWVKKNGAEHSLPTLGLTNNQLFFLGFAQVWCSVRTPESSHEGLITDPHSPSRFRVIGSLSNSKEFSEHFRCPPGSPMNPPHKCEVW

>NP_001946.2

MDYLLMIFSLLFVACQGAPETAVLGAELSAVGENGGEKPTPSPPWRLRRSKRCSCSSLMDKECVYFCHLDIIWVNTPEHVVPYGLGSPRSKRALENLLPTKATDRENRCQCASQKDKKCWNFCQAGKELRAEDIMEKDWNNHKKGKDCSKLGKKCIYQQLVRGRKIRRSSEEHLRQTRSETMRNSVKSSFHDPKLKGNPSRERYVTHNRAHW

641 NP_057563.3 NP_000537.3

>NP_057563.3

MTAPVPAPRILLPLLLLLLLTPPPGARGEVCMASRGLSLFPESCPDFCCGTCDDQYCCSDVLKKFVWSEERCAVPEASVPASVEPVEQLGSALRFRPGYNDPMSGFGATLAVGLTIFVLSVVTIIICFTCSCCCLYKTCRRPRPVVTTTTSTTVVHAPYPQPPSVPPSYPGPSYQGYHTMPPQPGMPAAPYPMQYPPPYPAQPMGPPAYHETLAGGAAAPYPASQPPYNPAYMDAPKAAL

>NP_000537.3

MEEPQSDPSVEPPLSQETFSDLWKLLPENNVLSPLPSQAMDDLMLSPDDIEQWFTEDPGPDEAPRMPEAAPPVAPAPAAPTPAAPAPAPSWPLSSSVPSQKTYQGSYGFRLGFLHSGTAKSVTCTYSPALNKMFCQLAKTCPVQLWVDSTPPPGTRVRAMAIYKQSQHMTEVVRRCPHHERCSDSDGLAPPQHLIRVEGNLRVEYLDDRNTFRHSVVVPYEPPEVGSDCTTIHYNYMCNSSCMGGMNRRPILTIITLEDSSGNLLGRNSFEVRVCACPGRDRRTEEENLRKKGEPHHELPPGSTKRALPNNTSSSPQPKKKPLDGEYFTLQIRGRERFEMFRELNEALELKDAQAGKEPGGSRAHSSHLKSKKGQSTSRHKKLMFKTEGPDSD

642 NP_000917.3 NP_057250.1

>NP_000917.3

MTELKAKGPRAPHVAGGPPSPEVGSPLLCRPAAGPFPGSQTSDTLPEVSAIPISLDGLLFPRPCQGQDPSDEKTQDQQSLSDVEGAYSRAEATRGAGGSSSSPPEKDSGLLDSVLDTLLAPSGPGQSQPSPPACEVTSSWCLFGPELPEDPPAAPATQRVLSPLMSRSGCKVGDSSGTAAAHKVLPRGLSPARQLLLPASESPHWSGAPVKPSPQAAAVEVEEEDGSESEESAGPLLKGKPRALGGAAAGGGAAAVPPGAAAGGVALVPKEDSRFSAPRVALVEQDAPMAPGRSPLATTVMDFIHVPILPLNHALLAARTRQLLEDESYDGGAGAASAFAPPRSSPCASSTPVAVGDFPDCAYPPDAEPKDDAYPLYSDFQPPALKIKEEEEGAEASARSPRSYLVAGANPAAFPDFPLGPPPPLPPRATPSRPGEAAVTAAPASASVSSASSSGSTLECILYKAEGAPPQQGPFAPPPCKAPGASGCLLPRDGLPSTSASAAAAGAAPALYPALGLNGLPQLGYQAAVLKEGLPQVYPPYLNYLRPDSEASQSPQYSFESLPQKICLICGDEASGCHYGVLTCGSCKVFFKRAMEGQHNYLCAGRNDCIVDKIRRKNCPACRLRKCCQAGMVLGGRKFKKFNKVRVVRALDAVALPQPVGVPNESQALSQRFTFSPGQDIQLIPPLINLLMSIEPDVIYAGHDNTKPDTSSSLLTSLNQLGERQLLSVVKWSKSLPGFRNLHIDDQITLIQYSWMSLMVFGLGWRSYKHVSGQMLYFAPDLILNEQRMKESSFYSLCLTMWQIPQEFVKLQVSQEEFLCMKVLLLLNTIPLEGLRSQTQFEEMRSSYIRELIKAIGLRQKGVVSSSQRFYQLTKLLDNLHDLVKQLHLYCLNTFIQSRALSVEFPEMMSEVIAAQLPKILAGMVKPLLFHKK

>NP_057250.1

MADSAELKQMVMSLRVSELQVLLGYAGRNKHGRKHELLTKALHLLKAGCSPAVQMKIKELYRRRFPQKIMTPADLSIPNVHSSPMPATLSPSTIPQLTYDGHPASSPLLPVSLLGPKHELELPHLTSALHPVHPDIKLQKLPFYDLLDELIKPTSLASDNSQRFRETCFAFALTPQQVQQISSSMDISGTKCDFTVQVQLRFCLSETSCPQEDHFPPNLCVKVNTKPCSLPGYLPPTKNGVEPKRPSRPINITSLVRLSTTVPNTIVVSWTAEIGRNYSMAVYLVKQLSSTVLLQRLRAKGIRNPDHSRALIKEKLTADPDSEIATTSLRVSLLCPLGKMRLTIPCRALTCSHLQCFDATLYIQMNEKKPTWVCPVCDKKAPYEHLIIDGLFMEILKYCTDCDEIQFKEDGTWAPMRSKKEVQEVSASYNGVDGCLSSTLEHQVASHHQSSNKNKKVEVIDLTIDSSSDEEEEEPSAKRTCPSLSPTSPLNNKGILSLPHQASPVSRTPSLPAVDTSYINTSLIQDYRHPFHMTPMPYDLQGLDFFPFLSGDNQHYNTSLLAAAAAAVSDDQDLLHSSRFFPYTSSQMFLDQLSAGGSTSLPTTNGSSSGSNSSLVSSNSLRESHSHTVTNRSSTDTASIFGIIPDIISLD

643 NP_005528.3 NP_003397.1

>NP_005528.3

MSFVECPYHSPAERLVAEADEGGPSAITGMGLCFRCLLFSFSGRSGVEGGRVDLNVFGSLGLQPWIGSSRCWGGPCSSALRCGWFSSWPPPSKSAIPIGGGSRGAGRVSRWPPPHWLEAWRVSPRPLSPLSPATFGRGFIAVAVIPGLWARGRGCSSDRLPRPAGPARRQFQAASLLTRGWGRAWPWKQILKELDECYERFSRETDGAQKRRMLHCVQRALIRSQELGDEKIQIVSQMVELVENRTRQVDSHVELFEAQQELGDTAGNSGKAGADRPKGEAAAQADKPNSKRSRRQRNNENRENASSNHDHDDGASGTPKEKKAKTSKKKKRSKAKAEREASPADLPIDPNEPTYCLCNQVSYGEMIGCDNDECPIEWFHFSCVGLNHKPKGKWYCPKCRGENEKTMDKALEKSKKERAYNR

>NP_003397.1

MDKNELVQKAKLAEQAERYDDMAACMKSVTEQGAELSNEERNLLSVAYKNVVGARRSSWRVVSSIEQKTEGAEKKQQMAREYREKIETELRDICNDVLSLLEKFLIPNASQAESKVFYLKMKGDYYRYLAEVAAGDDKKGIVDQSQQAYQEAFEISKKEMQPTHPIRLGLALNFSVFYYEILNSPEKACSLAKTAFDEAIAELDTLSEESYKDSTLIMQLLRDNLTLWTSDTQGDEAEAGEGGEN

644 NP_001902.1 NP_001076.2

>NP_001902.1

MQPLLLLLAFLLPTGAEAGEIIGGRESRPHSRPYMAYLQIQSPAGQSRCGGFLVREDFVLTAAHCWGSNINVTLGAHNIQRRENTQQHITARRAIRHPQYNQRTIQNDIMLLQLSRRVRRNRNVNPVALPRAQEGLRPGTLCTVAGWGRVSMRRGTDTLREVQLRVQRDRQCLRIFGSYDPRRQICVGDRRERKAAFKGDSGGPLLCNNVAHGIVSYGKSSGVPPEVFTRVSSFLPWIRTTMRSFKLLDQMETPL

>NP_001076.2

MERMLPLLALGLLAAGFCPAVLCHPNSPLDEENLTQENQDRGTHVDLGLASANVDFAFSLYKQLVLKAPDKNVIFSPLSISTALAFLSLGAHNTTLTEILKGLKFNLTETSEAEIHQSFQHLLRTLNQSSDELQLSMGNAMFVKEQLSLLDRFTEDAKRLYGSEAFATDFQDSAAAKKLINDYVKNGTRGKITDLIKDLDSQTMMVLVNYIFFKAKWEMPFDPQDTHQSRFYLSKKKWVMVPMMSLHHLTIPYFRDEELSCTVVELKYTGNASALFILPDQDKMEEVEAMLLPETLKRWRDSLEFREIGELYLPKFSISRDYNLNDILLQLGIEEAFTSKADLSGITGARNLAVSQVVHKAVLDVFEEGTEASAATAVKITLLSALVETRTIVRFNRPFLMIIVPTDTQNIFFMSKVTNPKQA

645 NP_005629.1 NP_079495.1

>NP_005629.1

MAILFAVVARGTTILAKHAWCGGNFLEVTEQILAKIPSENNKLTYSHGNYLFHYICQDRIVYLCITDDDFERSRAFNFLNEIKKRFQTTYGSRAQTALPYAMNSEFSSVLAAQLKHHSENKGLDKVMETQAQVDELKGIMVRNIDLVAQRGERLELLIDKTENLVDSSVTFKTTSRNLARAMCMKNLKLTIIIIIVSIVFIYIIVSPLCGGFTWPSCVKK

>NP_079495.1

MADQRQRSLSTSGESLYHVLGLDKNATSDDIKKSYRKLALKYHPDKNPDNPEAADKFKEINNAHAILTDATKRNIYDKYGSLGLYVAEQFGEENVNTYFVLSSWWAKALFVFCGLLTCCYCCCCLCCCFNCCCGKCKPKAPEGEETEFYVSPEDLEAQLQSDEREATDTPIVIQPASATETTQLTADSHPSYHTDGFN

646 NP_055191.2 NP_008921.1

>NP_055191.2

MTTHVTLEDALSNVDLLEELPLPDQQPCIEPPPSSIMYQANFDTNFEDRNAFVTGIARYIEQATVHSSMNEMLEEGHEYAVMLYTWRSCSRAIPQVKCNEQPNRVEIYEKTVEVLEPEVTKLMKFMYFQRKAIERFCSEVKRLCHAERRKDFVSEAYLLTLGKFINMFAVLDELKNMKCSVKNDHSAYKRAAQFLRKMADPQSIQESQNLSMFLANHNRITQCLHQQLEVIPGYEELLADIVNICVDYYENKMYLTPSEKHMLLKVMGFGLYLMDGNVSNIYKLDAKKRINLSKIDKFFKQLQVVPLFGDMQIELARYIKTSAHYEENKSKWTCTQSSISPQYNICEQMVQIRDDHIRFISELARYSNSEVVTGSGLDSQKSDEEYRELFDLALRGLQLLSKWSAHVMEVYSWKLVHPTDKFCNKDCPGTAEEYERATRYNYTSEEKFAFVEVIAMIKGLQVLMGRMESVFNQAIRNTIYAALQDFAQVTLREPLRQAVRKKKNVLISVLQAIRKTICDWEGGREPPNDPCLRGEKDPKGGFDIKVPRRAVGPSSTQLYMVRTMLESLIADKSGSKKTLRSSLDGPIVLAIEDFHKQSFFFTHLLNISEALQQCCDLSQLWFREFFLELTMGRRIQFPIEMSMPWILTDHILETKEPSMMEYVLYPLDLYNDSAYYALTKFKKQFLYDEIEAEVNLCFDQFVYKLADQIFAYYKAMAGSVLLDKRFRAECKNYGVIIPYPPSNRYETLLKQRHVQLLGRSIDLNRLITQRISAAMYKSLDQAISRFESEDLTSIVELEWLLEINRLTHRLLCKHMTLDSFDAMFREANHNVSAPYGRITLHVFWELNFDFLPNYCYNGSTNRFVRTAIPFTQEPQRDKPANVQPYYLYGSKPLNIAYSHIYSSYRNFVGPPHFKTICRLLGYQGIAVVMEELLKIVKSLLQGTILQYVKTLIEVMPKICRLPRHEYGSPGILEFFHHQLKDIIEYAELKTDVFQSLREVGNAILFCLLIEQALSQEEVCDLLHAAPFQNILPRVYIKEGERLEVRMKRLEAKYAPLHLVPLIERLGTPQQIAIAREGDLLTKERLCCGLSMFEVILTRIRSYLQDPIWRGPPPTNGVMHVDECVEFHRLWSAMQFVYCIPVGTNEFTAEQCFGDGLNWAGCSIIVLLGQQRRFDLFDFCYHLLKVQRQDGKDEIIKNVPLKKMADRIRKYQILNNEVFAILNKYMKSVETDSSTVEHVRCFQPPIHQSLATTC

>NP_008921.1

MPLVTRNIEPRHLCRQTLPSVRSELECVTNITLANVIRQLGSLSKYAEDIFGELFTQANTFASRVSSLAERVDRLQVKVTQLDPKEEEVSLQGINTRKAFRSSTIQDQKLFDRNSLPVPVLETYNTCDTPPPLNNLTPYRDDGKEALKFYTDPSYFFDLWKEKMLQDTKDIMKEKRKHRKEKKDNPNRGNVNPRKIKTRKEEWEKMKMGQEFVESKEKLGTSGYPPTLVYQNGSIGCVENVDASSYPPPPQSDSASSPSPSFSEDNLPPPPAEFSYPVDNQRGSGLAGPKRSSVVSPSHPPPAPPLGSPPGPKPGFAPPPAPPPPPPPMIGIPPPPPPVGFGSPGTPPPPSPPSFPPHPDFAAPPPPPPPPAADYPTLPPPPLSQPTGGAPPPPPPPPPPGPPPPPFTGADGQPAIPPPLSDTTKPKSSLPAVSDARSDLLSAIRQGFQLRRVEEQREQEKRDVVGNDVATILSRRIAVEYSDSEDDSSEFDEDDWSD

647 NP_002729.2 NP_000156.1

>NP_002729.2

MADPAAGPPPSEGEESTVRFARKGALRQKNVHEVKNHKFTARFFKQPTFCSHCTDFIWGFGKQGFQCQVCCFVVHKRCHEFVTFSCPGADKGPASDDPRSKHKFKIHTYSSPTFCDHCGSLLYGLIHQGMKCDTCMMNVHKRCVMNVPSLCGTDHTERRGRIYIQAHIDRDVLIVLVRDAKNLVPMDPNGLSDPYVKLKLIPDPKSESKQKTKTIKCSLNPEWNETFRFQLKESDKDRRLSVEIWDWDLTSRNDFMGSLSFGISELQKASVDGWFKLLSQEEGEYFNVPVPPEGSEANEELRQKFERAKISQGTKVPEEKTTNTVSKFDNNGNRDRMKLTDFNFLMVLGKGSFGKVMLSERKGTDELYAVKILKKDVVIQDDDVECTMVEKRVLALPGKPPFLTQLHSCFQTMDRLYFVMEYVNGGDLMYHIQQVGRFKEPHAVFYAAEIAIGLFFLQSKGIIYRDLKLDNVMLDSEGHIKIADFGMCKENIWDGVTTKTFCGTPDYIAPEIIAYQPYGKSVDWWAFGVLLYEMLAGQAPFEGEDEDELFQSIMEHNVAYPKSMSKEAVAICKGLMTKHPGKRLGCGPEGERDIKEHAFFRYIDWEKLERKEIQPPYKPKACGRNAENFDRFFTRHPPVLTPPDQEVIRNIDQSEFEGFSFVNSEFLKPEVKS

>NP_000156.1

MGDWSALGKLLDKVQAYSTAGGKVWLSVLFIFRILLLGTAVESAWGDEQSAFRCNTQQPGCENVCYDKSFPISHVRFWVLQIIFVSVPTLLYLAHVFYVMRKEEKLNKKEEELKVAQTDGVNVDMHLKQIEIKKFKYGIEEHGKVKMRGGLLRTYIISILFKSIFEVAFLLIQWYIYGFSLSAVYTCKRDPCPHQVDCFLSRPTEKTIFIIFMLVVSLVSLALNIIELFYVFFKGVKDRVKGKSDPYHATSGALSPAKDCGSQKYAYFNGCSSPTAPLSPMSPPGYKLVTGDRNNSSCRNYNKQASEQNWANYSAEQNRMGQAGSTISNSHAQPFDFPDDNQNSKKLAAGHELQPLAIVDQRPSSRASSRASSRPRPDDLEI

648 NP_004654.1 NP_000012.1

>NP_004654.1

MGTRDDEYDYLFKVVLIGDSGVGKSNLLSRFTRNEFNLESKSTIGVEFATRSIQVDGKTIKAQIWDTAGQERYRAITSAYYRGAVGALLVYDIAKHLTYENVERWLKELRDHADSNIVIMLVGNKSDLRHLRAVPTDEARAFAEKNGLSFIETSALDSTNVEAAFQTILTEIYRIVSQKQMSDRRENDMSPSNNVVPIHVPPTTENKPKVQCCQNI

>NP_000012.1

MTELPAPLSYFQNAQMSEDNHLSNTVRSQNDNRERQEHNDRRSLGHPEPLSNGRPQGNSRQVVEQDEEEDEELTLKYGAKHVIMLFVPVTLCMVVVVATIKSVSFYTRKDGQLIYTPFTEDTETVGQRALHSILNAAIMISVIVVMTILLVVLYKYRCYKVIHAWLIISSLLLLFFFSFIYLGEVFKTYNVAVDYITVALLIWNFGVVGMISIHWKGPLRLQQAYLIMISALMALVFIKYLPEWTAWLILAVISVYDLVAVLCPKGPLRMLVETAQERNETLFPALIYSSTMVWLVNMAEGDPEAQRRVSKNSKYNAESTERESQDTVAENDDGGFSEEWEAQRDSHLGPHRSTPESRAAVQELSSSILAGEDPEERGVKLGLGDFIFYSVLVGKASATASGDWNTTIACFVAILIGLCLTLLLLAIFKKALPALPISITFGLVFYFATDYLVQPFMDQLAFHQFYI

649 NP_003648.1 NP_003737.1

>NP_003648.1

MGNQMSVPQRVEDQENEPEAETYQDNASALNGVPVVVSTHTVQHLEEVDLGISVKTDNVATSSPETTEISAVADANGKNLGKEAKPEAPAAKSRFFLMLSRPVPGRTGDQAADSSLGSVKLDVSSNKAPANKDPSESWTLPVAAGPGQDTDKTPGHAPAQDKVLSAARDPTLLPPETGGAGGEAPSKPKDSSFFDKFFKLDKGQEKVPGDSQQEAKRAEHQDKVDEVPGLSGQSDDVPAGKDIVDGKEKEGQELGTADCSVPGDPEGLETAKDDSQAAAIAENNNSIMSFFKTLVSPNKAETKKDPEDTGAEKSPTTSADLKSDKANFTSQETQGAGKNSKGCNPSGHTQSVTTPEPAKEGTKEKSGPTSLPLGKLFWKKSVKEDSVPTGAEENVVCESPVEIIKSKEVESALQTVDLNEGDAAPEPTEAKLKREESKPRTSLMAFLRQMSVKGDGGITHSEEINGKDSSCQTSDSTEKTITPPEPEPTGAPQKGKEGSSKDKKSAAEMNKQKSNKQEAKEPAQCTEQATVDTNSLQNGDKLQKRPEKRQQSLGGFFKGLGPKRMLDAQVQTDPVSIGPVGKPK

>NP_003737.1

MCDRKAVIKNADMSEEMQQDSVECATQALEKYNIEKDIAAHIKKEFDKKYNPTWHCIVGRNFGSYVTHETKHFIYFYLGQVAILLFKSG

650 NP_004644.2 NP_001011663.1

>NP_004644.2

MEPGCDEFLPPPECPVFEPSWAEFQDPLGYIAKIRPIAEKSGICKIRPPADWQPPFAVEVDNFRFTPRVQRLNELEAQTRVKLNYLDQIAKFWEIQGSSLKIPNVERKILDLYSLSKIVIEEGGYEAICKDRRWARVAQRLHYPPGKNIGSLLRSHYERIIYPYEMFQSGANHVQCNTHPFDNEVKDKEYKPHSIPLRQSVQPSKFSSYSRRAKRLQPDPEPTEEDIEKHPELKKLQIYGPGPKMMGLGLMAKDKDKTVHKKVTCPPTVTVKDEQSGGGNVSSTLLKQHLSLEPCTKTTMQLRKNHSSAQFIDSYICQVCSRGDEDDKLLFCDGCDDNYHIFCLLPPLPEIPRGIWRCPKCILAECKQPPEAFGFEQATQEYSLQSFGEMADSFKSDYFNMPVHMVPTELVEKEFWRLVSSIEEDVTVEYGADIHSKEFGSGFPVSNSKQNLSPEEKEYATSGWNLNVMPVLDQSVLCHINADISGMKVPWLYVGMVFSAFCWHIEDHWSYSINYLHWGEPKTWYGVPSLAAEHLEEVMKMLTPELFDSQPDLLHQLVTLMNPNTLMSHGVPVVRTNQCAGEFVITFPRAYHSGFNQGYNFAEAVNFCTADWLPAGRQCIEHYRRLRRYCVFSHEELICKMAAFPETLDLNLAVAVHKEMFIMVQEERRLRKALLEKGVTEAEREAFELLPDDERQCIKCKTTCFLSALACYDCPDGLVCLSHINDLCKCSSSRQYLRYRYTLDELPTMLHKLKIRAESFDTWANKVRVALEVEDGRKRSFEELRALESEARERRFPNSELLQRLKNCLSEVEACIAQVLGLVSGQVARMDTPQLTLTELRVLLEQMGSLPCAMHQIGDVKDVLEQVEAYQAEAREALATLPSSPGLLRSLLERGQQLGVEVPEAHQLQQQVEQAQWLDEVKQALAPSAHRGSLVIMQGLLVMGAKIASSPSVDKARAELQELLTIAERWEEKAHFCLEARQKHPPATLEAIIRETENIPVHLPNIQALKEALTKAQAWIADVDEIQNGDHYPCLDDLEGLVAVGRDLPVGLEELRQLELQVLTAHSWREKASKTFLKKNSCYTLLEVLCPCADAGSDSTKRSRWMEKALGLYQCDTELLGLSAQDLRDPGSVIVAFKEGEQKEKEGILQLRRTNSAKPSPLAPSLMASSPTSICVCGQVPAGVGVLQCDLCQDWFHGQCVSVPHLLTSPKPSLTSSPLLAWWEWDTKFLCPLCMRSRRPRLETILALLVALQRLPVRLPEGEALQCLTERAIGWQDRARKALASEDVTALLRQLAELRQQLQAKPRPEEASVYTSATACDPIREGSGNNISKVQGLLENGDSVTSPENMAPGKGSDLELLSSLLPQLTGPVLELPEAIRAPLEELMMEGDLLEVTLDENHSIWQLLQAGQPPDLDRIRTLLELEKFEHQGSRTRSRALERRRRRQKVDQGRNVENLVQQELQSKRARSSGIMSQVGREEEHYQEKADRENMFLTPSTDHSPFLKGNQNSLQHKDSGSSAACPSLMPLLQLSYSDEQQL

>NP_001011663.1

MEGVAVVTAGSVGAAKTEGAAALPPPPPVSPPALTPAPAAGEEGPAPLSETGAPGCSGSRPPELEPERSLGRFRGRFEDEDEELEEEEELEEEEEEEEEDMSHFSLRLEGGRQDSEDEEERLINLSELTPYILCSICKGYLIDATTITECLHTFCKSCIVRHFYYSNRCPKCNIVVHQTQPLYNIRLDRQLQDIVYKLVINLEEREKKQMHDFYKERGLEVPKPAVPQPVPSSKGRSKKVLESVFRIPPELDMSLLLEFIGANEGTGHFKPLEKKFVRVSGEATIGHVEKFLRRKMGLDPACQVDIICGDHLLEQYQTLREIRRAIGDAAMQDGLLVLHYGLVVSPLKIT

651 NP_071356.1 NP_115708.1

>NP_071356.1

MPLGHIMRLDLEKIALEYIVPCLHEVGFCYLDNFLGEVVGDCVLERVKQLHCTGALRDGQLAGPRAGVSKRHLRGDQITWIGGNEEGCEAISFLLSLIDRLVLYCGSRLGKYYVKERSKAMVACYPGNGTGYVRHVDNPNGDGRCITCIYYLNKNWDAKLHGGILRIFPEGKSFIADVEPIFDRLLFFWSDRRNPHEVQPSYATRYAMTVWYFDAEERAEAKKKFRNLTRKTESALTED

>NP_115708.1

MAFPEPKPRPPELPQKRLKTLDCGQGAVRAVRFNVDGNYCLTCGSDKTLKLWNPLRGTLLRTYSGHGYEVLDAAGSFDNSSLCSGGGDKAVVLWDVASGQVVRKFRGHAGKVNTVQFNEEATVILSGSIDSSIRCWDCRSRRPEPVQTLDEARDGVSSVKVSDHEILAGSVDGRVRRYDLRMGQLFSDYVGSPITCTCFSRDGQCTLVSSLDSTLRLLDKDTGELLGEYKGHKNQEYKLDCCLSERDTHVVSCSEDGKVFFWDLVEGALALALPVGSGVVQSLAYHPTEPCLLTAMGGSVQCWREEAYEAEDGAG

652 NP_443096.1 NP_536737.3

>NP_443096.1

MTSFEDADTEETVTCLQMTVYHPGQLQCGIFQSISFNREKLPSSEVVKFGRNSNICHYTFQDKQVSRVQFSLQLFKKFNSSVLSFEIKNMSKKTNLIVDSRELGYLNKMDLPYRCMVRFGEYQFLMEKEDGESLEFFETQFILSPRSLLQENNWPPHRPIPEYGTYSLCSSQSSSPTEMDENES

>NP_536737.3

MSSLYPSLEDLKVDQAIQAQVRASPKMPALPVQATAISPPPVLYPNLAELENYMGLSLSSQEVQESLLQIPEGDSTAVSGPGPGQMVAPVTGYSLGVRRAEIKPGVREIHLCKDERGKTGLRLRKVDQGLFVQLVQANTPASLVGLRFGDQLLQIDGRDCAGWSSHKAHQVVKKASGDKIVVVVRDRPFQRTVTMHKDSMGHVGFVIKKGKIVSLVKGSSAARNGLLTNHYVCEVDGQNVIGLKDKKIMEILATAGNVVTLTIIPSVIYEHMVKKLPPVLLHHTMDHSIPDA

653 NP_068713.2 NP_005484.2

>NP_068713.2

MAWRCPRMGRVPLAWCLALCGWACMAPRGTQAEESPFVGNPGNITGARGLTGTLRCQLQVQGEPPEVHWLRDGQILELADSTQTQVPLGEDEQDDWIVVSQLRITSLQLSDTGQYQCLVFLGHQTFVSQPGYVGLEGLPYFLEEPEDRTVAANTPFNLSCQAQGPPEPVDLLWLQDAVPLATAPGHGPQRSLHVPGLNKTSSFSCEAHNAKGVTTSRTATITVLPQQPRNLHLVSRQPTELEVAWTPGLSGIYPLTHCTLQAVLSDDGMGIQAGEPDPPEEPLTSQASVPPHQLRLGSLHPHTPYHIRVACTSSQGPSSWTHWLPVETPEGVPLGPPENISATRNGSQAFVHWQEPRAPLQGTLLGYRLAYQGQDTPEVLMDIGLRQEVTLELQGDGSVSNLTVCVAAYTAAGDGPWSLPVPLEAWRPGQAQPVHQLVKEPSTPAFSWPWWYVLLGAVVAAACVLILALFLVHRRKKETRYGEVFEPTVERGELVVRYRVRKSYSRRTTEATLNSLGISEELKEKLRDVMVDRHKVALGKTLGEGEFGAVMEGQLNQDDSILKVAVKTMKIAICTRSELEDFLSEAVCMKEFDHPNVMRLIGVCFQGSERESFPAPVVILPFMKHGDLHSFLLYSRLGDQPVYLPTQMLVKFMADIASGMEYLSTKRFIHRDLAARNCMLNENMSVCVADFGLSKKIYNGDYYRQGRIAKMPVKWIAIESLADRVYTSKSDVWSFGVTMWEIATRGQTPYPGVENSEIYDYLRQGNRLKQPADCLDGLYALMSRCWELNPQDRPSFTELREDLENTLKALPPAQEPDEILYVNMDEGGGYPEPPGAAGGADPPTQPDPKDSCSCLTAAEVHPAGRYVLCPSTTPSPAQPADRGSPAAPGQEDGA

>NP_005484.2

MSGQPPPPPPQQQQQQQQLSPPPPAALAPVSGVVLPAPPAVSAGSSPAGSPGGGAGGEGLGAAAAALLLHPPPPPPPATAAPPPPPPPPPPPASAAAPASGPPAPPGLAAGPGPAGGAPTPALVAGSSAAAPFPHGDSALNEQEKELQRRLKRLYPAVDEQETPLPRSWSPKDKFSYIGLSQNNLRVHYKGHGKTPKDAASVRATHPIPAACGIYYFEVKIVSKGRDGYMGIGLSAQGVNMNRLPGWDKHSYGYHGDDGHSFCSSGTGQPYGPTFTTGDVIGCCVNLINNTCFYTKNGHSLGIAFTDLPPNLYPTVGLQTPGEVVDANFGQHPFVFDIEDYMREWRTKIQAQIDRFPIGDREGEWQTMIQKMVSSYLVHHGYCATAEAFARSTDQTVLEELASIKNRQRIQKLVLAGRMGEAIETTQQLYPSLLERNPNLLFTLKVRQFIEMVNGTDSEVRCLGGRSPKSQDSYPVSPRPFSSPSMSPSHGMNIHNLASGKGSTAHFSGFESCSNGVISNKAHQSYCHSNKHQSSNLNVPELNSINMSRSQQVNNFTSNDVDMETDHYSNGVGETSSNGFLNGSSKHDHEMEDCDTEMEVDSSQLRRQLCGGSQAAIERMIHFGRELQAMSEQLRRDCGKNTANKKMLKDAFSLLAYSDPWNSPVGNQLDPIQREPVCSALNSAILETHNLPKQPPLALAMGQATQCLGLMARSGIGSCAFATVEDYLH

654 NP_003844.1 NP_001553.1

>NP_003844.1

MLCLGWIFLWLVAGERIKGFNISGCSTKKLLWTYSTRSEEEFVLFCDLPEPQKSHFCHRNRLSPKQVPEHLPFMGSNDLSDVQWYQQPSNGDPLEDIRKSYPHIIQDKCTLHFLTPGVNNSGSYICRPKMIKSPYDVACCVKMILEVKPQTNASCEYSASHKQDLLLGSTGSISCPSLSCQSDAQSPAVTWYKNGKLLSVERSNRIVVDEVYDYHQGTYVCDYTQSDTVSSWTVRAVVQVRTIVGDTKLKPDILDPVEDTLEVELGKPLTISCKARFGFERVFNPVIKWYIKDSDLEWEVSVPEAKSIKSTLKDEIIERNIILEKVTQRDLRRKFVCFVQNSIGNTTQSVQLKEKRGVVLLYILLGTIGTLVAVLAASALLYRHWIEIVLLYRTYQSKDQTLGDKKDFDAFVSYAKWSSFPSEATSSLSEEHLALSLFPDVLENKYGYSLCLLERDVAPGGVYAEDIVSIIKRSRRGIFILSPNYVNGPSIFELQAAVNLALDDQTLKLILIKFCYFQEPESLPHLVKKALRVLPTVTWRGLKSVPPNSRFWAKMRYHMPVKNSQGFTWNQLRITSRIFQWKGLSRTETTGRSSQPKEW

>NP_001553.1

MAAEPVEDNCINFVAMKFIDNTLYFIAEDDENLESDYFGKLESKLSVIRNLNDQVLFIDQGNRPLFEDMTDSDCRDNAPRTIFIISMYKDSQPRGMAVTISVKCEKISTLSCENKIISFKEMNPPDNIKDTKSDIIFFQRSVPGHDNKMQFESSSYEGYFLACEKERDLFKLILKKEDELGDRSIMFTVQNED

655 NP_002729.2 NP_006167.1

>NP_002729.2

MADPAAGPPPSEGEESTVRFARKGALRQKNVHEVKNHKFTARFFKQPTFCSHCTDFIWGFGKQGFQCQVCCFVVHKRCHEFVTFSCPGADKGPASDDPRSKHKFKIHTYSSPTFCDHCGSLLYGLIHQGMKCDTCMMNVHKRCVMNVPSLCGTDHTERRGRIYIQAHIDRDVLIVLVRDAKNLVPMDPNGLSDPYVKLKLIPDPKSESKQKTKTIKCSLNPEWNETFRFQLKESDKDRRLSVEIWDWDLTSRNDFMGSLSFGISELQKASVDGWFKLLSQEEGEYFNVPVPPEGSEANEELRQKFERAKISQGTKVPEEKTTNTVSKFDNNGNRDRMKLTDFNFLMVLGKGSFGKVMLSERKGTDELYAVKILKKDVVIQDDDVECTMVEKRVLALPGKPPFLTQLHSCFQTMDRLYFVMEYVNGGDLMYHIQQVGRFKEPHAVFYAAEIAIGLFFLQSKGIIYRDLKLDNVMLDSEGHIKIADFGMCKENIWDGVTTKTFCGTPDYIAPEIIAYQPYGKSVDWWAFGVLLYEMLAGQAPFEGEDEDELFQSIMEHNVAYPKSMSKEAVAICKGLMTKHPGKRLGCGPEGERDIKEHAFFRYIDWEKLERKEIQPPYKPKACGRNAENFDRFFTRHPPVLTPPDQEVIRNIDQSEFEGFSFVNSEFLKPEVKS

>NP_006167.1

MDCCTENACSKPDDDILDIPLDDPGANAAAAKIQASFRGHMARKKIKSGERGRKGPGPGGPGGAGVARGGAGGGPSGD

656 NP_006752.1 NP_000539.2

>NP_006752.1

MDDREDLVYQAKLAEQAERYDEMVESMKKVAGMDVELTVEERNLLSVAYKNVIGARRASWRIISSIEQKEENKGGEDKLKMIREYRQMVETELKLICCDILDVLDKHLIPAANTGESKVFYYKMKGDYHRYLAEFATGNDRKEAAENSLVAYKAASDIAMTELPPTHPIRLGLALNFSVFYYEILNSPDRACRLAKAAFDDAIAELDTLSEESYKDSTLIMQLLRDNLTLWTSDMQGDGEEQNKEALQDVEDENQ

>NP_000539.2

MAKPTSKDSGLKEKFKILLGLGTPRPNPRSAEGKQTEFIITAEILRELSMECGLNNRIRMIGQICEVAKTKKFEEHAVEALWKAVADLLQPERPLEARHAVLALLKAIVQGQGERLGVLRALFFKVIKDYPSNEDLHERLEVFKALTDNGRHITYLEEELADFVLQWMDVGLSSEFLLVLVNLVKFNSCYLDEYIARMVQMICLLCVRTASSVDIEVSLQVLDAVVCYNCLPAESLPLFIVTLCRTINVKELCEPCWKLMRNLLGTHLGHSAIYNMCHLMEDRAYMEDAPLLRGAVFFVGMALWGAHRLYSLRNSPTSVLPSFYQAMACPNEVVSYEIVLSITRLIKKYRKELQVVAWDILLNIIERLLQQLQTLDSPELRTIVHDLLTTVEELCDQNEFHGSQERYFELVERCADQRPESSLLNLISYRAQSIHPAKDGWIQNLQALMERFFRSESRGAVRIKVLDVLSFVLLINRQFYEEELINSVVISQLSHIPEDKDHQVRKLATQLLVDLAEGCHTHHFNSLLDIIEKVMARSLSPPPELEERDVAAYSASLEDVKTAVLGLLVILQTKLYTLPASHATRVYEMLVSHIQLHYKHSYTLPIASSIRLQAFDFLLLLRADSLHRLGLPNKDGVVRFSPYCVCDYMEPERGSEKKTSGPLSPPTGPPGPAPAGPAVRLGSVPYSLLFRVLLQCLKQESDWKVLKLVLGRLPESLRYKVLIFTSPCSVDQLCSALCSMLSGPKTLERLRGAPEGFSRTDLHLAVVPVLTALISYHNYLDKTKQREMVYCLEQGLIHRCASQCVVALSICSVEMPDIIIKALPVLVVKLTHISATASMAVPLLEFLSTLARLPHLYRNFAAEQYASVFAISLPYTNPSKFNQYIVCLAHHVIAMWFIRCRLPFRKDFVPFITKGLRSNVLLSFDDTPEKDSFRARSTSLNERPKSLRIARPPKQGLNNSPPVKEFKESSAAEAFRCRSISVSEHVVRSRIQTSLTSASLGSADENSVAQADDSLKNLHLELTETCLDMMARYVFSNFTAVPKRSPVGEFLLAGGRTKTWLVGNKLVTVTTSVGTGTRSLLGLDSGELQSGPESSSSPGVHVRQTKEAPAKLESQAGQQVSRGARDRVRSMSGGHGLRVGALDVPASQFLGSATSPGPRTAPAAKPEKASAGTRVPVQEKTNLAAYVPLLTQGWAEILVRRPTGNTSWLMSLENPLSPFSSDINNMPLQELSNALMAAERFKEHRDTALYKSLSVPAASTAKPPPLPRSNTVASFSSLYQSSCQGQLHRSVSWADSAVVMEEGSPGEVPVLVEPPGLEDVEAALGMDRRTDAYSRSSSVSSQEEKSLHAEELVGRGIPIERVVSSEGGRPSVDLSFQPSQPLSKSSSSPELQTLQDILGDPGDKADVGRLSPEVKARSQSGTLDGESAAWSASGEDSRGQPEGPLPSSSPRSPSGLRPRGYTISDSAPSRRGKRVERDALKSRATASNAEKVPGINPSFVFLQLYHSPFFGDESNKPILLPNESQSFERSVQLLDQIPSYDTHKIAVLYVGEGQSNSELAILSNEHGSYRYTEFLTGLGRLIELKDCQPDKVYLGGLDVCGEDGQFTYCWHDDIMQAVFHIATLMPTKDVDKHRCDKKRHLGNDFVSIVYNDSGEDFKLGTIKGQFNFVHVIVTPLDYECNLVSLQCRKDMEGLVDTSVAKIVSDRNLPFVARQMALHANMASQVHHSRSNPTDIYPSKWIARLRHIKRLRQRICEEAAYSNPSLPLVHPPSHSKAPAQTPAEPTPGYEVGQRKRLISSVEDFTEFV

657 NP_004124.4 NP_006804.1

>NP_004124.4

MDMANYSEVLDPTYTTLEFETMQILYNSSDSSAPETSMNTTDNGVNCLCAICGDRATGKHYGASSCDGCKGFFRRSIRKSHVYSCRFSRQCVVDKDKRNQCRYCRLRKCFRAGMKKEAVQNERDRISTRRSTFDGSNIPSINTLAQAEVRSRQISVSSPGSSTDINVKKIASIGDVCESMKQQLLVLVEWAKYIPAFCELPLDDQVALLRAHAGEHLLLGATKRSMMYKDILLLGNNYVIHRNSCEVEISRVANRVLDELVRPFQEIQIDDNEYACLKAIVFFDPDAKGLSDPVKIKNMRFQVQIGLEDYINDRQYDSRGRFGELLLLLPTLQSITWQMIEQIQFVKLFGMVKIDNLLQEMLLGGASNDGSHLHHPMHPHLSQDPLTGQTILLGPMSTLVHADQISTPETPLPSPPQGSGQEQYKIAANQASVISHQHLSKQKQL

>NP_006804.1

MTVVSVPQREPLVLGGRLAPLGFSSRGYFGALPMVTTAPPPLPRIPDPRALPPTLFLPHFLGGDGPCLTPQPRAPAALPNRSLAVAGGTPRAAPKKRRKKKVRASPAGQLPSRFHQYQQHRPSLEGGRSPATGPSGAQEVPGPAAALAPSPAAAAGTEGASPDLAPLRPAAPGQTPLRKEVLKSKMGKSEKIALPHGQLVHGIHLYEQPKINRQKSKYNLPLTKITSAKRNENNFWQDSVSSDRIQKQEKKPFKNTENIKNSHLKKSAFLTEVSQKENYAGAKFSDPPSPSVLPKPPSHWMGSTVENSNQNRELMAVHLKTLLKVQT

658 NP_004949.1 NP_065812.1

>NP_004949.1

MLGTGPAAATTAATTSSNVSVLQQFASGLKSRNEETRAKAAKELQHYVTMELREMSQEESTRFYDQLNHHIFELVSSSDANERKGGILAIASLIGVEGGNATRIGRFANYLRNLLPSNDPVVMEMASKAIGRLAMAGDTFTAEYVEFEVKRALEWLGADRNEGRRHAAVLVLRELAISVPTFFFQQVQPFFDNIFVAVWDPKQAIREGAVAALRACLILTTQREPKEMQKPQWYRHTFEEAEKGFDETLAKEKGMNRDDRIHGALLILNELVRISSMEGERLREEMEEITQQQLVHDKYCKDLMGFGTKPRHITPFTSFQAVQPQQSNALVGLLGYSSHQGLMGFGTSPSPAKSTLVESRCCRDLMEEKFDQVCQWVLKCRNSKNSLIQMTILNLLPRLAAFRPSAFTDTQYLQDTMNHVLSCVKKEKERTAAFQALGLLSVAVRSEFKVYLPRVLDIIRAALPPKDFAHKRQKAMQVDATVFTCISMLARAMGPGIQQDIKELLEPMLAVGLSPALTAVLYDLSRQIPQLKKDIQDGLLKMLSLVLMHKPLRHPGMPKGLAHQLASPGLTTLPEASDVGSITLALRTLGSFEFEGHSLTQFVRHCADHFLNSEHKEIRMEAARTCSRLLTPSIHLISGHAHVVSQTAVQVVADVLSKLLVVGITDPDPDIRYCVLASLDERFDAHLAQAENLQALFVALNDQVFEIRELAICTVGRLSSMNPAFVMPFLRKMLIQILTELEHSGIGRIKEQSARMLGHLVSNAPRLIRPYMEPILKALILKLKDPDPDPNPGVINNVLATIGELAQVSGLEMRKWVDELFIIIMDMLQDSSLLAKRQVALWTLGQLVASTGYVVEPYRKYPTLLEVLLNFLKTEQNQGTRREAIRVLGLLGALDPYKHKVNIGMIDQSRDASAVSLSESKSSQDSSDYSTSEMLVNMGNLPLDEFYPAVSMVALMRIFRDQSLSHHHTMVVQAITFIFKSLGLKCVQFLPQVMPTFLNVIRVCDGAIREFLFQQLGMLVSFVKSHIRPYMDEIVTLMREFWVMNTSIQSTIILLIEQIVVALGGEFKLYLPQLIPHMLRVFMHDNSPGRIVSIKLLAAIQLFGANLDDYLHLLLPPIVKLFDAPEAPLPSRKAALETVDRLTESLDFTDYASRIIHPIVRTLDQSPELRSTAMDTLSSLVFQLGKKYQIFIPMVNKVLVRHRINHQRYDVLICRIVKGYTLADEEEDPLIYQHRMLRSGQGDALASGPVETGPMKKLHVSTINLQKAWGAARRVSKDDWLEWLRRLSLELLKDSSSPSLRSCWALAQAYNPMARDLFNAAFVSCWSELNEDQQDELIRSIELALTSQDIAEVTQTLLNLAEFMEHSDKGPLPLRDDNGIVLLGERAAKCRAYAKALHYKELEFQKGPTPAILESLISINNKLQQPEAAAGVLEYAMKHFGELEIQATWYEKLHEWEDALVAYDKKMDTNKDDPELMLGRMRCLEALGEWGQLHQQCCEKWTLVNDETQAKMARMAAAAAWGLGQWDSMEEYTCMIPRDTHDGAFYRAVLALHQDLFSLAQQCIDKARDLLDAELTAMAGESYSRAYGAMVSCHMLSELEEVIQYKLVPERREIIRQIWWERLQGCQRIVEDWQKILMVRSLVVSPHEDMRTWLKYASLCGKSGRLALAHKTLVLLLGVDPSRQLDHPLPTVHPQVTYAYMKNMWKSARKIDAFQHMQHFVQTMQQQAQHAIATEDQQHKQELHKLMARCFLKLGEWQLNLQGINESTIPKVLQYYSAATEHDRSWYKAWHAWAVMNFEAVLHYKHQNQARDEKKKLRHASGANITNATTAATTAATATTTASTEGSNSESEAESTENSPTPSPLQKKVTEDLSKTLLMYTVPAVQGFFRSISLSRGNNLQDTLRVLTLWFDYGHWPDVNEALVEGVKAIQIDTWLQVIPQLIARIDTPRPLVGRLIHQLLTDIGRYHPQALIYPLTVASKSTTTARHNAANKILKNMCEHSNTLVQQAMMVSEELIRVAILWHEMWHEGLEEASRLYFGERNVKGMFEVLEPLHAMMERGPQTLKETSFNQAYGRDLMEAQEWCRKYMKSGNVKDLTQAWDLYYHVFRRISKQLPQLTSLELQYVSPKLLMCRDLELAVPGTYDPNQPIIRIQSIAPSLQVITSKQRPRKLTLMGSNGHEFVFLLKGHEDLRQDERVMQLFGLVNTLLANDPTSLRKNLSIQRYAVIPLSTNSGLIGWVPHCDTLHALIRDYREKKKILLNIEHRIMLRMAPDYDHLTLMQKVEVFEHAVNNTAGDDLAKLLWLKSPSSEVWFDRRTNYTRSLAVMSMVGYILGLGDRHPSNLMLDRLSGKILHIDFGDCFEVAMTREKFPEKIPFRLTRMLTNAMEVTGLDGNYRITCHTVMEVLREHKDSVMAVLEAFVYDPLLNWRLMDTNTKGNKRSRTRTDSYSAGQSVEILDGVELGEPAHKKTGTTVPESIHSFIGDGLVKPEALNKKAIQIINRVRDKLTGRDFSHDDTLDVPTQVELLIKQATSHENLCQCYIGWCPFW

>NP_065812.1

MESEMLQSPLLGLGEEDEADLTDWNLPLAFMKKRHCEKIEGSKSLAQSWRMKDRMKTVSVALVLCLNVGVDPPDVVKTTPCARLECWIDPLSMGPQKALETIGANLQKQYENWQPRARYKQSLDPTVDEVKKLCTSLRRNAKEERVLFHYNGHGVPRPTVNGEVWVFNKNYTQYIPLSIYDLQTWMGSPSIFVYDCSNAGLIVKSFKQFALQREQELEVAAINPNHPLAQMPLPPSMKNCIQLAACEATELLPMIPDLPADLFTSCLTTPIKIALRWFCMQKCVSLVPGVTLDLIEKIPGRLNDRRTPLGELNWIFTAITDTIAWNVLPRDLFQKLFRQDLLVASLFRNFLLAERIMRSYNCTPVSSPRLPPTYMHAMWQAWDLAVDICLSQLPTIIEEGTAFRHSPFFAEQLTAFQVWLTMGVENRNPPEQLPIVLQVLLSQVHRLRALDLLGRFLDLGPWAVSLALSVGIFPYVLKLLQSSARELRPLLVFIWAKILAVDSSCQADLVKDNGHKYFLSVLADPYMPAEHRTMTAFILAVIVNSYHTGQEACLQGNLIAICLEQLNDPHPLLRQWVAICLGRIWQNFDSARWCGVRDSAHEKLYSLLSDPIPEVRCAAVFALGTFVGNSAERTDHSTTIDHNVAMMLAQLVSDGSPMVRKELVVALSHLVVQYESNFCTVALQFIEEEKNYALPSPATTEGGSLTPVRDSPCTPRLRSVSSYGNIRAVATARSLNKSLQNLSLTEESGGAVAFSPGNLSTSSSASSTLGSPENEEHILSFETIDKMRRASSYSSLNSLIGVSFNSVYTQIWRVLLHLAADPYPEVSDVAMKVLNSIAYKATVNARPQRVLDTSSLTQSAPASPTNKGVHIHQAGGSPPASSTSSSSLTNDVAKQPVSRDLPSGRPGTTGPAGAQYTPHSHQFPRTRKMFDKGPEQTADDADDAAGHKSFISATVQTGFCDWSARYFAQPVMKIPEEHDLESQIRKEREWRFLRNSRVRRQAQQVIQKGITRLDDQIFLNRNPGVPSVVKFHPFTPCIAVADKDSICFWDWEKGEKLDYFHNGNPRYTRVTAMEYLNGQDCSLLLTATDDGAIRVWKNFADLEKNPEMVTAWQGLSDMLPTTRGAGMVVDWEQETGLLMSSGDVRIVRIWDTDREMKVQDIPTGADSCVTSLSCDSHRSLIVAGLGDGSIRVYDRRMALSECRVMTYREHTAWVVKASLQKRPDGHIVSVSVNGDVRIFDPRMPESVNVLQIVKGLTALDIHPQADLIACGSVNQFTAIYNSSGELINNIKYYDGFMGQRVGAISCLAFHPHWPHLAVGSNDYYISVYSVEKRVR

659 NP_004300.1 NP_006269.1

>NP_004300.1

MAEQEPTAEQLAQIAAENEEDEHSVNYKPPAQKSIQEIQELDKDDESLRKYKEALLGRVAVSADPNVPNVVVTGLTLVCSSAPGPLELDLTGDLESFKKQSFVLKEGVEYRIKISFRVNREIVSGMKYIQHTYRKGVKIDKTDYMVGSYGPRAEEYEFLTPVEEAPKGMLARGSYSIKSRFTDDDKTDHLSWEWNLTIKKDWKD

>NP_006269.1

MVSKSRWKLLAMLALVLVVMVWYSISREDSFYFPIPEKKEPCLQGEAESKASKLFGNYSRDQPIFLRLEDYFWVKTPSAYELPYGTKGSEDLLLRVLAITSSSIPKNIQSLRCRRCVVVGNGHRLRNSSLGDAINKYDVVIRLNNAPVAGYEGDVGSKTTMRLFYPESAHFDPKVENNPDTLLVLVAFKAMDFHWIETILSDKKRVRKGFWKQPPLIWDVNPKQIRILNPFFMEIAADKLLSLPMQQPRKIKQKPTTGLLAITLALHLCDLVHIAGFGYPDAYNKKQTIHYYEQITLKSMAGSGHNVSQEALAIKRMLEMGAIKNLTSF

660 NP_001002255.1 NP_038470.1

>NP_001002255.1

MANEKPTEEVKTENNNHINLKVAGQDGSVVQFKIKRQTPLSKLMKAYCEPRGLSVKQIRFRFGGQPISGTDKPAQLEMEDEDTIDVFQQPTGGVY

>NP_038470.1

MLARAARGTGALLLRGSLLASGRAPRRASSGLPRNTVVLFVPQQEAWVVERMGRFHRILEPGLNILIPVLDRIRYVQSLKEIVINVPEQSAVTLDNVTLQIDGVLYLRIMDPYKASYGVEDPEYAVTQLAQTTMRSELGKLSLDKVFRERESLNASIVDAINQAADCWGIRCLRYEIKDIHVPPRVKESMQMQVEAERRKRATVLESEGTRESAINVAEGKKQAQILASEAEKAEQINQAAGEASAVLAKAKAKAEAIRILAAALTQHNGDAAASLTVAEQYVSAFSKLAKDSNTILLPSNPGDVTSMVAQAMGVYGALTKAPVPGTPDSLSSGSSRDVQGTDASLDEELDRVKMS

661 NP_892113.3 NP_002438.1

>NP_892113.3

MNLLPPKPKYNPLRNESLSSMEEGASGSTPPEELPSPPASSLGPILPPLPGDDSPTTLCSFFPRMSNLRLANPAGGRPGSKGEPGRAADDGEGIVGAAMPDSGPLPLLQDMNKLSGGGGRRTRVEGGQLGGEEWTRHGSFVNKPTRGWLHPNDKVMGPGVSYLVRYMGCVEVLQSMRALDFNTRTQVTREAISLVCEAVPGAKGATRRRKPCSRPLSSILGRSNLKFAGMPITLTVSTSSLNLMAADCKQIIANHHMQSISFASGGDPDTAEYVAYVAKDPVNQRACHILECPEGLAQDVISTIGQAFELRFKQYLRNPPKLVTPHDRMAGFDGSAWDEEEEEPPDHQYYNDFPGKEPPLGGVVDMRLREGAAPGAARPTAPNAQTPSHLGATLPVGQPVGGDPEVRKQMPPPPPCPGRELFDDPSYVNVQNLDKARQAVGGAGPPNPAINGSAPRDLFDMKPFEDALRVPPPPQSVSMAEQLRGEPWFHGKLSRREAEALLQLNGDFLVRESTTTPGQYVLTGLQSGQPKHLLLVDPEGVVRTKDHRFESVSHLISYHMDNHLPIISAGSELCLQQPVERKL

>NP_002438.1

MELLPPLPQSFLLLLLLPAKPAAGEDWQCPRTPYAASRDFDVKYVVPSFSAGGLVQAMVTYEGDRNESAVFVAIRNRLHVLGPDLKSVQSLATGPAGDPGCQTCAACGPGPHGPPGDTDTKVLVLDPALPALVSCGSSLQGRCFLHDLEPQGTAVHLAAPACLFSAHHNRPDDCPDCVASPLGTRVTVVEQGQASYFYVASSLDAAVAGSFSPRSVSIRRLKADASGFAPGFVALSVLPKHLVSYSIEYVHSFHTGAFVYFLTVQPASVTDDPSALHTRLARLSATEPELGDYRELVLDCRFAPKRRRRGAPEGGQPYPVLQVAHSAPVGAQLATELSIAEGQEVLFGVFVTGKDGGPGVGPNSVVCAFPIDLLDTLIDEGVERCCESPVHPGLRRGLDFFQSPSFCPNPPGLEALSPNTSCRHFPLLVSSSFSRVDLFNGLLGPVQVTALYVTRLDNVTVAHMGTMDGRILQVELVRSLNYLLYVSNFSLGDSGQPVQRDVSRLGDHLLFASGDQVFQVPIRGPGCRHFLTCGRCLRAWHFMGCGWCGNMCGQQKECPGSWQQDHCPPKLTEFHPHSGPLRGSTRLTLCGSNFYLHPSGLVPEGTHQVTVGQSPCRPLPKDSSKLRPVPRKDFVEEFECELEPLGTQAVGPTNVSLTVTNMPPGKHFRVDGTSVLRGFSFMEPVLIAVQPLFGPRAGGTCLTLEGQSLSVGTSRAVLVNGTECLLARVSEGQLLCATPPGATVASVPLSLQVGGAQVPGSWTFQYREDPVVLSISPNCGYINSHITICGQHLTSAWHLVLSFHDGLRAVESRCERQLPEQQLCRLPEYVVRDPQGWVAGNLSARGDGAAGFTLPGFRFLPPPHPPSANLVPLKPEEHAIKFEYIGLGAVADCVGINVTVGGESCQHEFRGDMVVCPLPPSLQLGQDGAPLQVCVDGECHILGRVVRPGPDGVPQSTLLGILLPLLLLVAALATALVFSYWWRRKQLVLPPNLNDLASLDQTAGATPLPILYSGSDYRSGLALPAIDGLDSTTCVHGASFSDSEDESCVPLLRKESIQLRDLDSALLAEVKDVLIPHERVVTHSDRVIGKGHFGVVYHGEYIDQAQNRIQCAIKSLSRITEMQQVEAFLREGLLMRGLNHPNVLALIGIMLPPEGLPHVLLPYMCHGDLLQFIRSPQRNPTVKDLISFGLQVARGMEYLAEQKFVHRDLAARNCMLDESFTVKVADFGLARDILDREYYSVQQHRHARLPVKWMALESLQTYRFTTKSDVWSFGVLLWELLTRGAPPYRHIDPFDLTHFLAQGRRLPQPEYCPDSLYQVMQQCWEADPAVRPTFRVLVGEVEQIVSALLGDHYVQLPATYMNLGPSTSHEMNVRPEQPQFSPMPGNVRRPRPLSEPPRPT

662 NP_005671.1 NP_000537.3

>NP_005671.1

MDLEESEEFKERCTQCAAVSWGLTDEGKYYCTSCHNVTERYQEVTNTDLIPNTQIKALNRGLKKKNNTEKGWDWYVCEGFQYILYQQAEALKNLGVGPELKNDVLHNFWKRYLQKSKQAYCKNPVYTTGRKPTVLEDNLSHSDWASEPELLSDVSCPPFLESGAESQSDIHTRKPFPVSKASQSETSVCSGSLDGVEYSQRKEKGIVKMTMPQTLAFCYLSLLWQREAITLSDLLRFVEEDHIPYINAFQHFPEQMKLYGRDRGIFGIESWPDYEDIYKKTIEVGTFLDLPRFPDITEDCYLHPNILCMKYLMEVNLPDEMHSLTCHVVKMTGMGEVDFLTFDPIAKMAKTVKYDVQAVAIIVVVLKLLFLLDDSFEWSLSNLAEKHNEKNKKDKPWFDFRKWYQIMKKAFDEKKQKWEEARAKYLWKSEKPLYYSFVDKPVAYKKREMVVNLQKQFSTLVESTATAGKKSPSSFQFNWTEEDTDRTCFHGHSLQGVLKEKGQSLLTKNSLYWLSTQKFCRCYCTHVTTYEESNYSLSYQFILNLFSFLLRIKTSLLHEEVSLVEKKLFEKKYSVKRKKSRSKKVRRH

>NP_000537.3

MEEPQSDPSVEPPLSQETFSDLWKLLPENNVLSPLPSQAMDDLMLSPDDIEQWFTEDPGPDEAPRMPEAAPPVAPAPAAPTPAAPAPAPSWPLSSSVPSQKTYQGSYGFRLGFLHSGTAKSVTCTYSPALNKMFCQLAKTCPVQLWVDSTPPPGTRVRAMAIYKQSQHMTEVVRRCPHHERCSDSDGLAPPQHLIRVEGNLRVEYLDDRNTFRHSVVVPYEPPEVGSDCTTIHYNYMCNSSCMGGMNRRPILTIITLEDSSGNLLGRNSFEVRVCACPGRDRRTEEENLRKKGEPHHELPPGSTKRALPNNTSSSPQPKKKPLDGEYFTLQIRGRERFEMFRELNEALELKDAQAGKEPGGSRAHSSHLKSKKGQSTSRHKKLMFKTEGPDSD

663 NP_056284.1 NP_057632.2

>NP_056284.1

MLVLRSALTRALASRTLAPQMCSSFATGPRQYDGIFYEFRSYYLKPSKMNEFLENFEKNAHLRTAHSELVGYWSVEFGGRMNTVFHIWKYDNFAHRTEVRKALAKDKEWQEQFLIPNLALIDKQESEITYLVPWCKLEKPPKEGVYELATFQMKPGGPALWGDAFKRAVHAHVNLGYTKLVGVFHTEYGALNRVHVLWWNESADSRAAGRHKSHEDPRVVAAVRESVNYLVSQQNMLLIPTSFSPLK

>NP_057632.2

MMGLGNGRRSMKSPPLVLAALVACIIVLGFNYWIASSRSVDLQTRIMELEGRVRRAAAERGAVELKKNEFQGELEKQREQLDKIQSSHNFQLESVNKLYQDEKAVLVNNITTGERLIRVLQDQLKTLQRNYGRLQQDVLQFQKNQTNLERKFSYDLSQCINQMKEVKEQCEERIEEVTKKGNEAVASRDLSENNDQRQQLQALSEPQPRLQAAGLPHTEVPQGKGNVLGNSKSQTPAPSSEVVLDSKRQVEKEETNEIQVVNEEPQRDRLPQEPGREQVVEDRPVGGRGFGGAGELGQTPQVQAALSVSQENPEMEGPERDQLVIPDGQEEEQEAAGEGRNQQKLRGEDDYNMDENEAESETDKQAALAGNDRNIDVFNVEDQKRDTINLLDQREKRNHTL

664 NP_733765.1 NP_003740.2

>NP_733765.1

MENAHTKTVEEVLGHFGVNESTGLSLEQVKKLKERWGSNELPAEEGKTLLELVIEQFEDLLVRILLLAACISFVLAWFEEGEETITAFVEPFVILLILVANAIVGVWQERNAENAIEALKEYEPEMGKVYRQDRKSVQRIKAKDIVPGDIVEIAVGDKVPADIRLTSIKSTTLRVDQSILTGESVSVIKHTDPVPDPRAVNQDKKNMLFSGTNIAAGKAMGVVVATGVNTEIGKIRDEMVATEQERTPLQQKLDEFGEQLSKVISLICIAVWIINIGHFNDPVHGGSWIRGAIYYFKIAVALAVAAIPEGLPAVITTCLALGTRRMAKKNAIVRSLPSVETLGCTSVICSDKTGTLTTNQMSVCRMFILDRVEGDTCSLNEFTITGSTYAPIGEVHKDDKPVNCHQYDGLVELATICALCNDSALDYNEAKGVYEKVGEATETALTCLVEKMNVFDTELKGLSKIERANACNSVIKQLMKKEFTLEFSRDRKSMSVYCTPNKPSRTSMSKMFVKGAPEGVIDRCTHIRVGSTKVPMTSGVKQKIMSVIREWGSGSDTLRCLALATHDNPLRREEMHLEDSANFIKYETNLTFVGCVGMLDPPRIEVASSVKLCRQAGIRVIMITGDNKGTAVAICRRIGIFGQDEDVTSKAFTGREFDELNPSAQRDACLNARCFARVEPSHKSKIVEFLQSFDEITAMTGDGVNDAPALKKAEIGIAMGSGTAVAKTASEMVLADDNFSTIVAAVEEGRAIYNNMKQFIRYLISSNVGEVVCIFLTAALGFPEALIPVQLLWVNLVTDGLPATALGFNPPDLDIMNKPPRNPKEPLISGWLFFRYLAIGCYVGAATVGAAAWWFIAADGGPRVSFYQLSHFLQCKEDNPDFEGVDCAIFESPYPMTMALSVLVTIEMCNALNSLSENQSLLRMPPWENIWLVGSICLSMSLHFLILYVEPLPLIFQITPLNVTQWLMVLKISLPVILMDETLKFVARNYLEPGKECVQPATKSCSFSACTDGISWPFVLLIMPLVIWVYSTDTNFSDMFWS

>NP_003740.2

MASPPRHGPPGPASGDGPNLNNNNNNNNHSVRKCGYLRKQKHGHKRFFVLRGPGAGGDEATAGGGSAPQPPRLEYYESEKKWRSKAGAPKRVIALDCCLNINKRADAKHKYLIALYTKDEYFAVAAENEQEQEGWYRALTDLVSEGRAAAGDAPPAAAPAASCSASLPGALGGSAGAAGAEDSYGLVAPATAAYREVWQVNLKPKGLGQSKNLTGVYRLCLSARTIGFVKLNCEQPSVTLQLMNIRRCGHSDSFFFIEVGRSAVTGPGELWMQADDSVVAQNIHETILEAMKALKELFEFRPRSKSQSSGSSATHPISVPGARRHHHLVNLPPSQTGLVRRSRTDSLAATPPAAKCSSCRVRTASEGDGGAAAGAAAAGARPVSVAGSPLSPGPVRAPLSRSHTLSGGCGGRGSKVALLPAGGALQHSRSMSMPVAHSPPAATSPGSLSSSSGHGSGSYPPPPGPHPPLPHPLHHGPGQRPSSGSASASGSPSDPGFMSLDEYGSSPGDLRAFCSHRSNTPESIAETPPARDGGGGGEFYGYMTMDRPLSHCGRSYRRVSGDAAQDLDRGLRKRTYSLTTPARQRPVPQPSSASLDEYTLMRATFSGSAGRLCPSCPASSPKVAYHPYPEDYGDIEIGSHRSSSSNLGADDGYMPMTPGAALAGSGSGSCRSDDYMPMSPASVSAPKQILQPRAAAAAAAAVPSAGPAGPAPTSAAGRTFPASGGGYKASSPAESSPEDSGYMRMWCGSKLSMEHADGKLLPNGDYLNVSPSDAVTTGTPPDFFSAALHPGGEPLRGVPGCCYSSLPRSYKAPYTCGGDSDQYVLMSSPVGRILEEERLEPQATPGPSQAASAFGAGPTQPPHPVVPSPVRPSGGRPEGFLGQRGRAVRPTRLSLEGLPSLPSMHEYPLPPEPKSPGEYINIDFGEPGARLSPPAPPLLASAASSSSLLSASSPASSLGSGTPGTSSDSRQRSPLSDYMNLDFSSPKSPKPGAPSGHPVGSLDGLLSPEASSPYPPLPPRPSASPSSSLQPPPPPPAPGELYRLPPASAVATAQGPGAASSLSSDTGDNGDYTEMAFGVAATPPQPIAAPPKPEAARVASPTSGVKRLSLMEQVSGVEAFLQASQPPDPHRGAKVIRADPQGGRRRHSSETFSSTTTVTPVSPSFAHNPKRHNSASVENVSLRKSSEGGVGVGPGGGDEPPTSPRQLQPAPPLAPQGRPWTPGQPGGLVGCPGSGGSPMRRETSAGFQNGLNYIAIDVREEPGLPPQPQPPPPPLPQPGDKSSWGRTRSLGGLISAVGVGSTGGGCGGPGPGALPPANTYASIDFLSHHLKEATIVKE

665 NP_006604.1 NP_055202.1

>NP_006604.1

MESVALYSFQATESDELAFNKGDTLKILNMEDDQNWYKAELRGVEGFIPKNYIRVKPHPWYSGRISRQLAEEILMKRNHLGAFLIRESESSPGEFSVSVNYGDQVQHFKVLREASGKYFLWEEKFNSLNELVDFYRTTTIAKKRQIFLRDEEPLLKSPGACFAQAQFDFSAQDPSQLSFRRGDIIEVLERPDPHWWRGRSCGRVGFFPRSYVQPVHL

>NP_055202.1

MEEAILVPCVLGLLLLPILAMLMALCVHCHRLPGSYDSTSSDSLYPRGIQFKRPHTVAPWPPAYPPVTSYPPLSQPDLLPIPRSPQPLGGSHRTPSSRRDSDGANSVASYENEGASGIRGAQAGWGVWGPSWTRLTPVSLPPEPACEDADEDEDDYHNPGYLVVLPDSTPATSTAAPSAPALSTPGIRDSAFSMESIDDYVNVPESGESAEASLDGSREYVNVSQELHPGAAKTEPAALSSQEAEEVEEEGAPDYENLQELN

666 NP_003244.2 NP_002730.1

>NP_003244.2

MGNAESQHVEHEFYGEKHASLGRKHTSRSLRLSHKTRRTRHASSGKVIHRNSEVSTRSSSTPSIPQSLAENGLEPFSQDGTLEDFGSPIWVDRVDMGLRPVSYTDSSVTPSVDSSIVLTAASVQSMPDTEESRLYGDDATYLAEGGRRQHSYTSNGPTFMETASFKKKRSKSADIWREDSLEFSLSDLSQEHLTSNEEILGSAEEKDCEEARGMETRASPRQLSTCQRANSLGDLYAQKNSGVTANGGPGSKFAGYCRNLVSDIPNLANHKMPPAAAEETPPYSNYNTLPCRKSHCLSEGATNPQISHSNSMQGRRAKTTQDVNAGEGSEFADSGIEGATTDTDLLSRRSNATNSSYSPTTGRAFVGSDSGSSSTGDAARQGVYENFRRELEMSTTNSESLEEAGSAHSDEQSSGTLSSPGQSDILLTAAQGTVRKAGALAVKNFLVHKKNKKVESATRRKWKHYWVSLKGCTLFFYESDGRSGIDHNSIPKHAVWVENSIVQAVPEHPKKDFVFCLSNSLGDAFLFQTTSQTELENWITAIHSACATAVARHHHKEDTLRLLKSEIKKLEQKIDMDEKMKKMGEMQLSSVTDSKKKKTILDQIFVWEQNLEQFQMDLFRFRCYLASLQGGELPNPKRLLAFASRPTKVAMGRLGIFSVSSFHALVAARTGETGVRRRTQAMSRSASKRRSRFSSLWGLDTTSKKKQGRPSINQVFGEGTEAVKKSLEGIFDDIVPDGKREKEVVLPNVHQHNPDCDIWVHEYFTPSWFCLPNNQPALTVVRPGDTARDTLELICKTHQLDHSAHYLRLKFLIENKMQLYVPQPEEDIYELLYKEIEICPKVTQSIHIEKSDTAADTYGFSLSSVEEDGIRRLYVNSVKETGLASKKGLKAGDEILEINNRAADALNSSMLKDFLSQPSLGLLVRTYPELEEGVELLESPPHRVDGPADLGESPLAFLTSNPGHSLCSEQGSSAETAPEETEGPDLESSDETDHSSKSTEQVAAFCRSLHEMNPSDQSPSPQDSTGPQLATMRQLSDADKLRKVICELLETERTYVKDLNCLMERYLKPLQKETFLTQDELDVLFGNLTEMVEFQVEFLKTLEDGVRLVPDLEKLEKVDQFKKVLFSLGGSFLYYADRFKLYSAFCASHTKVPKVLVKAKTDTAFKAFLDAQNPKQQHSSTLESYLIKPIQRILKYPLLLRELFALTDAESEEHYHLDVAIKTMNKVASHINEMQKIHEEFGAVFDQLIAEQTGEKKEVADLSMGDLLLHTTVIWLNPPASLGKWKKEPELAAFVFKTAVVLVYKDGSKQKKKLVGSHRLSIYEDWDPFRFRHMIPTEALQVRALASADAEANAVCEIVHVKSESEGRPERVFHLCCSSPESRKDFLKAVHSILRDKHRRQLLKTESLPSSQQYVPFGGKRLCALKGARPAMSRAVSAPSKSLGRRRRRLARNRFTIDSDAVSASSPEKESQQPPGGGDTDRWVEEQFDLAQYEEQDDIKETDILSDDDEFCESVKGASVDRDLQERLQATSISQRERGRKTLDSHASRMAQLKKQAALSGINGGLESASEEVIWVRREDFAPSRKLNTEI

>NP_002730.1

MAGLGPGVGDSEGGPRPLFCRKGALRQKVVHEVKSHKFTARFFKQPTFCSHCTDFIWGIGKQGLQCQVCSFVVHRRCHEFVTFECPGAGKGPQTDDPRNKHKFRLHSYSSPTFCDHCGSLLYGLVHQGMKCSCCEMNVHRRCVRSVPSLCGVDHTERRGRLQLEIRAPTADEIHVTVGEARNLIPMDPNGLSDPYVKLKLIPDPRNLTKQKTRTVKATLNPVWNETFVFNLKPGDVERRLSVEVWDWDRTSRNDFMGAMSFGVSELLKAPVDGWYKLLNQEEGEYYNVPVADADNCSLLQKFEACNYPLELYERVRMGPSSSPIPSPSPSPTDPKRCFFGASPGRLHISDFSFLMVLGKGSFGKVMLAERRGSDELYAIKILKKDVIVQDDDVDCTLVEKRVLALGGRGPGGRPHFLTQLHSTFQTPDRLYFVMEYVTGGDLMYHIQQLGKFKEPHAAFYAAEIAIGLFFLHNQGIIYRDLKLDNVMLDAEGHIKITDFGMCKENVFPGTTTRTFCGTPDYIAPEIIAYQPYGKSVDWWSFGVLLYEMLAGQPPFDGEDEEELFQAIMEQTVTYPKSLSREAVAICKGFLTKHPGKRLGSGPDGEPTIRAHGFFRWIDWERLERLEIPPPFRPRPCGRSGENFDKFFTRAAPALTPPDRLVLASIDQADFQGFTYVNPDFVHPDARSPTSPVPVPVM

667 NP_006069.1 NP_000818.1

>NP_006069.1

MGLFDRGVQMLLTTVGAFAAFSLMTIAVGTDYWLYSRGVCKTKSVSENETSKKNEEVMTHSGLWRTCCLEGNFKGLCKQIDHFPEDADYEADTAEYFLRAVRASSIFPILSVILLFMGGLCIAASEFYKTRHNIILSAGIFFVSAGLSNIIGIIVYISANAGDPSKSDSKKNSYSYGWSFYFGALSFIIAEMVGVLAVHMFIDRHKQLRATARATDYLQASAITRIPSYRYRYQRRSRSSSRSTEPSHSRDASPVGIKGFNTLPSTEISMYTLSRDPLKAATTPTATYNSDRDNSFLQVHNCIQKENKDSLHSNTANRRTTPV

>NP_000818.1

MQHIFAFFCTGFLGAVVGANFPNNIQIGGLFPNQQSQEHAAFRFALSQLTEPPKLLPQIDIVNISDSFEMTYRFCSQFSKGVYAIFGFYERRTVNMLTSFCGALHVCFITPSFPVDTSNQFVLQLRPELQDALISIIDHYKWQKFVYIYDADRGLSVLQKVLDTAAEKNWQVTAVNILTTTEEGYRMLFQDLEKKKERLVVVDCESERLNAILGQIIKLEKNGIGYHYILANLGFMDIDLNKFKESGANVTGFQLVNYTDTIPAKIMQQWKNSDARDHTRVDWKRPKYTSALTYDGVKVMAEAFQSLRRQRIDISRRGNAGDCLANPAVPWGQGIDIQRALQQVRFEGLTGNVQFNEKGRRTNYTLHVIEMKHDSIRKIGYWNEDDKFVPAATDAQAGGDNSSVQNRTYIVTTILEDPYVMLKKNANQFEGNDRYEGYCVELAAEIAKHVGYSYRLEIVSDGKYGARDPDTKAWNGMVGELVYGRADVAVAPLTITLVREEVIDFSKPFMSLGISIMIKKPQKSKPGVFSFLDPLAYEIWMCIVFAYIGVSVVLFLVSRFSPYEWHSEEFEEGRDQTTSDQSNEFGIFNSLWFSLGAFMQQGCDISPRSLSGRIVGGVWWFFTLIIISSYTANLAAFLTVERMVSPIESAEDLAKQTEIAYGTLEAGSTKEFFRRSKIAVFEKMWTYMKSAEPSVFVRTTEEGMIRVRKSKGKYAYLLESTMNEYIEQRKPCDTMKVGGNLDSKGYGIATPKGSALRNPVNLAVLKLNEQGLLDKLKNKWWYDKGECGSGGGDSKDKTSALSLSNVAGVFYILIGGLGLAMLVALIEFCYKSRSESKRMKGFCLIPQQSINEAIRTSTLPRNSGAGASSGGSGENGRVVSHDFPKSMQSIPCMSHSSGMPLGATGL

668 NP_001019801.2 NP_005672.1

>NP_001019801.2

MLHSPHKQPQNHKCGANFLQEDSKKSLVFKWLISAGHYQPPRPTESFKAASSIYNRGYKFYLKKKGGTMASNSLFSTVTPCQQNFFWDPSTSRRFSPPSSSLQPGKMSDVSPVVAAQQQQQQQQQQQQQQQQQQQQQQQEAAAAAAAAAAAAAAAAAVPRLRPPHDNRTMVEIIADHPAELVRTDSPNFLCSVLPSHWRCNKTLPVAFKVVALGEVPDGTVVTVMAGNDENYSAELRNASAVMKNQVARFNDLRFVGRSGRGKSFTLTITVFTNPPQVATYHRAIKVTVDGPREPRRHRQKLDDSKPSLFSDRLSDLGRIPHPSMRVGVPPQNPRPSLNSAPSPFNPQGQSQITDPRQAQSSPPWSYDQSYPSYLSQMTSPSIHSTTPLSSTRGTGLPAITDVPRRISDDDTATSDFCLWPSTLSKKSQAGASELGPFSDPRQFPSISSLTESRFSNPRMHYPATFTYTPPVTSGMSLGMSATTHYHTYLPPPYPGSSQSQSGPFQTSSTPYLYYGTSSGSYQFPMVPGGDRSPSRMLPPCTTTSNGSTLLNPNLPNQNDGVDADGSHSSSPTVLNSSGRMDESVWRPY

>NP_005672.1

MSDFSEELKGPVTDDEEVETSVLSGAGMHFPWLQTYVETVAIGGKRRKDFAQTTSACLSFIQEALLKHQWQQAAEYMYSYFQTLEDSDSYKRQAAPEIIWKLGSEILFYHPKSNMESFNTFANRMKNIGVMNYLKISLQHALYLLHHGMLKDAKRNLSEAETWRHGENTSSREILINLIQAYKGLLQYYTWSEKKMELSKLDKDDYAYNAVAQDVFNHSWKTSANISALIKIPGVWDPFVKSYVEMLEFYGDRDGAQEVLTNYAYDEKFPSNPNAHIYLYNFLKRQKAPRSKLISVLKILYQIVPSHKLMLEFHTLLRKSEKEEHRKLGLEVLFGVLDFAGCTKNITAWKYLAKYLKNILMGNHLAWVQEEWNSRKNWWPGFHFSYFWAKSDWKEDTALACEKAFVAGLLLGKGCRYFRYILKQDHQILGKKIKRMKRSVKKYSIVNPRL

669 NP_006087.2 NP_001014.1

>NP_006087.2

MSREMQDVDLAEVKPLVEKGETITGLLQEFDVQEQDIETLHGSVHVTLCGTPKGNRPVILTYHDIGMNHKTCYNPLFNYEDMQEITQHFAVCHVDAPGQQDGAASFPAGYMYPSMDQLAEMLPGVLQQFGLKSIIGMGTGAGAYILTRFALNNPEMVEGLVLINVNPCAEGWMDWAASKISGWTQALPDMVVSHLFGKEEMQSNVEVVHTYRQHIVNDMNPGNLHLFINAYNSRRDLEIERPMPGTHTVTLQCPALLVVGDSSPAVDAVVECNSKLDPTKTTLLKMADCGGLPQISQPAKLAEAFKYFVQGMGYMPSASMTRLMRSRTASGSSVTSLDGTRSRSHTSEGTRSRSHTSEGTRSRSHTSEGAHLDITPNSGAAGNSAGPKSMEVSC

>NP_001014.1

MAFKDTGKTPVEPEVAIHRIRITLTSRNVKSLEKVCADLIRGAKEKNLKVKGPVRMPTKTLRITTRKTPCGEGSKTWDRFQMRIHKRLIDLHSPSEIVKQITSISIEPGVEVEVTIADA

670 NP_659480.1 NP_001420.2

>NP_659480.1

MPEGPLVRKFHHLVSPFVGQQVVKTGGSSKKLQPASLQSLWLQDTQVHGKKLFLRFDLDEEMGPPGSSPTPEPPQKEVQKEGAADPKQVGEPSGQKTLDGSSRSAELVPQGEDDSEYLERDAPAGDAGRWLRVSFGLFGSVWVNDFSRAKKANKRGDWRDPSPRLVLHFGGGGFLAFYNCQLSWSSSPVVTPTCDILSEKFHRGQALEALGQAQPVCYTLLDQRYFSGLGNIIKNEALYRAGIHPLSLGSVLSASRREVLVDHVVEFSTAWLQGKFQGRPQHTQVYQKEQCPAGHQVMKEAFGPEDGLQRLTWWCPQCQPQLSEEPEQCQFS

>NP_001420.2

MAENVVEPGPPSAKRPKLSSPALSASASDGTDFGSLFDLEHDLPDELINSTELGLTNGGDINQLQTSLGMVQDAASKHKQLSELLRSGSSPNLNMGVGGPGQVMASQAQQSSPGLGLINSMVKSPMTQAGLTSPNMGMGTSGPNQGPTQSTGMMNSPVNQPAMGMNTGMNAGMNPGMLAAGNGQGIMPNQVMNGSIGAGRGRQNMQYPNPGMGSAGNLLTEPLQQGSPQMGGQTGLRGPQPLKMGMMNNPNPYGSPYTQNPGQQIGASGLGLQIQTKTVLSNNLSPFAMDKKAVPGGGMPNMGQQPAPQVQQPGLVTPVAQGMGSGAHTADPEKRKLIQQQLVLLLHAHKCQRREQANGEVRQCNLPHCRTMKNVLNHMTHCQSGKSCQVAHCASSRQIISHWKNCTRHDCPVCLPLKNAGDKRNQQPILTGAPVGLGNPSSLGVGQQSAPNLSTVSQIDPSSIERAYAALGLPYQVNQMPTQPQVQAKNQQNQQPGQSPQGMRPMSNMSASPMGVNGGVGVQTPSLLSDSMLHSAINSQNPMMSENASVPSLGPMPTAAQPSTTGIRKQWHEDITQDLRNHLVHKLVQAIFPTPDPAALKDRRMENLVAYARKVEGDMYESANNRAEYYHLLAEKIYKIQKELEEKRRTRLQKQNMLPNAAGMVPVSMNPGPNMGQPQPGMTSNGPLPDPSMIRGSVPNQMMPRITPQSGLNQFGQMSMAQPPIVPRQTPPLQHHGQLAQPGALNPPMGYGPRMQQPSNQGQFLPQTQFPSQGMNVTNIPLAPSSGQAPVSQAQMSSSSCPVNSPIMPPGSQGSHIHCPQLPQPALHQNSPSPVPSRTPTPHHTPPSIGAQQPPATTIPAPVPTPPAMPPGPQSQALHPPPRQTPTPPTTQLPQQVQPSLPAAPSADQPQQQPRSQQSTAASVPTPTAPLLPPQPATPLSQPAVSIEGQVSNPPSTSSTEVNSQAIAEKQPSQEVKMEAKMEVDQPEPADTQPEDISESKVEDCKMESTETEERSTELKTEIKEEEDQPSTSATQSSPAPGQSKKKIFKPEELRQALMPTLEALYRQDPESLPFRQPVDPQLLGIPDYFDIVKSPMDLSTIKRKLDTGQYQEPWQYVDDIWLMFNNAWLYNRKTSRVYKYCSKLSEVFEQEIDPVMQSLGYCCGRKLEFSPQTLCCYGKQLCTIPRDATYYSYQNRYHFCEKCFNEIQGESVSLGDDPSQPQTTINKEQFSKRKNDTLDPELFVECTECGRKMHQICVLHHEIIWPAGFVCDGCLKKSARTRKENKFSAKRLPSTRLGTFLENRVNDFLRRQNHPESGEVTVRVVHASDKTVEVKPGMKARFVDSGEMAESFPYRTKALFAFEEIDGVDLCFFGMHVQEYGSDCPPPNQRRVYISYLDSVHFFRPKCLRTAVYHEILIGYLEYVKKLGYTTGHIWACPPSEGDDYIFHCHPPDQKIPKPKRLQEWYKKMLDKAVSERIVHDYKDIFKQATEDRLTSAKELPYFEGDFWPNVLEESIKELEQEEEERKREENTSNESTDVTKGDSKNAKKKNNKKTSKNKSSLSRGNKKKPGMPNVSNDLSQKLYATMEKHKEVFFVIRLIAGPAANSLPPIVDPDPLIPCDLMDGRDAFLTLARDKHLEFSSLRRAQWSTMCMLVELHTQSQDRFVYTCNECKHHVETRWHCTVCEDYDLCITCYNTKNHDHKMEKLGLGLDDESNNQQAAATQSPGDSRRLSIQRCIQSLVHACQCRNANCSLPSCQKMKRVVQHTKGCKRKTNGGCPICKQLIALCCYHAKHCQENKCPVPFCLNIKQKLRQQQLQHRLQQAQMLRRRMASMQRTGVVGQQQGLPSPTPATPTTPTGQQPTTPQTPQPTSQPQPTPPNSMPPYLPRTQAAGPVSQGKAAGQVTPPTPPQTAQPPLPGPPPAAVEMAMQIQRAAETQRQMAHVQIFQRPIQHQMPPMTPMAPMGMNPPPMTRGPSGHLEPGMGPTGMQQQPPWSQGGLPQPQQLQSGMPRPAMMSVAQHGQPLNMAPQPGLGQVGISPLKPGTVSQQALQNLLRTLRSPSSPLQQQQVLSILHANPQLLAAFIKQRAAKYANSNPQPIPGQPGMPQGQPGLQPPTMPGQQGVHSNPAMQNMNPMQAGVQRAGLPQQQPQQQLQPPMGGMSPQAQQMNMNHNTMPSQFRDILRRQQMMQQQQQQGAGPGIGPGMANHNQFQQPQGVGYPPQQQQRMQHHMQQMQQGNMGQIGQLPQALGAEAGASLQAYQQRLLQQQMGSPVQPNPMSPQQHMLPNQAQSPHLQGQQIPNSLSNQVRSPQPVPSPRPQSQPPHSSPSPRMQPQPSPHHVSPQTSSPHPGLVAAQANPMEQGHFASPDQNSMLSQLASNPGMANLHGASATDLGLSTDNSDLNSNLSQSTLDIH

671 NP_004146.1 AAF19526.1

>NP_004146.1

METLSNASGTFAIRLLKILCQDNPSHNVFCSPVSISSALAMVLLGAKGNTATQMAQALSLNTEEDIHRAFQSLLTEVNKAGTQYLLRTANRLFGEKTCQFLSTFKESCLQFYHAELKELSFIRAAEESRKHINTWVSKKTEGKIEELLPGSSIDAETRLVLVNAIYFKGKWNEPFDETYTREMPFKINQEEQRPVQMMYQEATFKLAHVGEVRAQLLELPYARKELSLLVLLPDDGVELSTVEKSLTFEKLTAWTKPDCMKSTEVEVLLPKFKLQEDYDMESVLRHLGIVDAFQQGKADLSAMSAERDLCLSKFVHKSFVEVNEEGTEAAAASSCFVVAECCMESGPRFCADHPFLFFIRHNRANSILFCGRFSSP

>AAF19526.1

MGKKHKKHKSDKHLYEEYVEKPLKLVLKVGGNEVTELSTGSSGHDSSLFEDKNDHDKHKDRKRKKGKKGEKQIPGEEKGRKRTRVKEDKKKRDRDQVENEAEKDLQCHAPVRLDLPPEKPLTSSLAKQEEVEQTPLQEALNQLMRQLQRKDPSAFFSFPVTDFIAPGYSMIIKHPMDFSTMKEKIKNNDYQSIEELKDNFKLMCTNAMIYNKPETIYYKAAKKLLHSGMKILSQERIQSLKQSIDFMADLQKTRKQKDGTDTSQSGEDGGCWQREREDSGDAEAHAFKSPSKENKKKDKDMLEDKFKSNNLEREQEQLDRIVKESGGKLTRRLVNSQCEFERRKPDGTTTLGLLHPVDPIVGEPGYCPVRLGMTTGRLQSGVNTLQGFKEDKRNKVTPVLYLNYGPYSSYAPHYDSTFANISKDDSDLIYSTYGEDSDLPSDFSIHEFLATCQDYPYVMADSLLDVLTKGGHSRTLQEMEMSLPEDEGHTRTLDTAKEMEQITEVEPPGRLDSSTQDRLIALKAVTNFGVPVEVFDSEEAEIFQKKLDETTRLLRELQEAQNERLSTRPPPNMICLLGPSYREMHLAEQVTNNLKELAQQVTPGDIVSTYGVRKAMGISIPSPVMENNFVDLTEDTEEPKKTDVAECGPGGS

672 NP_002241.1 NP_002724.1

>NP_002241.1

MGGDLVLGLGALRRRKRLLEQEKSLAGWALVLAGTGIGLMVLHAEMLWFGGCSWALYLFLVKCTISISTFLLLCLIVAFHAKEVQLFMTDNGLRDWRVALTGRQAAQIVLELVVCGLHPAPVRGPPCVQDLGAPLTSPQPWPGFLGQGEALLSLAMLLRLYLVPRAVLLRSGVLLNASYRSIGALNQVRFRHWFVAKLYMNTHPGRLLLGLTLGLWLTTAWVLSVAERQAVNATGHLSDTLWLIPITFLTIGYGDVVPGTMWGKIVCLCTGVMGVCCTALLVAVVARKLEFNKAEKHVHNFMMDIQYTKEMKESAARVLQEAWMFYKHTRRKESHAARRHQRKLLAAINAFRQVRLKHRKLREQVNSMVDISKMHMILYDLQQNLSSSHRALEKQIDTLAGKLDALTELLSTALGPRQLPEPSQQSK

>NP_002724.1

METVISSDSSPAVENEHPQETPESNNSVYTSFMKSHRCYDLIPTSSKLVVFDTSLQVKKAFFALVTNGVRAAPLWDSKKQSFVGMLTITDFINILHRYYKSALVQIYELEEHKIETWREVYLQDSFKPLVCISPNASLFDAVSSLIRNKIHRLPVIDPESGNTLYILTHKRILKFLKLFITEFPKPEFMSKSLEELQIGTYANIAMVRTTTPVYVALGIFVQHRVSALPVVDEKGRVVDIYSKFDVINLAAEKTYNNLDVSVTKALQHRSHYFEGVLKCYLHETLETIINRLVEAEVHRLVVVDENDVVKGIVSLSDILQALVLTGGEKKP

673 NP_001559.1 NP_003940.3

>NP_001559.1

MAEYDLTTRIAHFLDRHLVFPLLEFLSVKEIYNEKELLQGKLDLLSDTNMVDFAMDVYKNLYSDDIPHALREKRTTVVAQLKQLQAETEPIVKMFEDPETTRQMQSTRDGRMLFDYLADKHGFRQEYLDTLYRYAKFQYECGNYSGAAEYLYFFRVLVPATDRNALSSLWGKLASEILMQNWDAAMEDLTRLKETIDNNSVSSPLQSLQQRTWLIHWSLFVFFNHPKGRDNIIDLFLYQPQYLNAIQTMCPHILRYLTTAVITNKDVRKRRQVLKDLVKVIQQESYTYKDPITEFVECLYVNFDFDGAQKKLRECESVLVNDFFLVACLEDFIENARLFIFETFCRIHQCISINMLADKLNMTPEEAERWIVNLIRNARLDAKIDSKLGHVVMGNNAVSPYQQVIEKTKSLSFRSQMLAMNIEKKLNQNSRSEAPNWATQDSGFY

>NP_003940.3

MRPKRLGRCCAGSRLGPGDPAALTCAPSPSASPAPEPSAQPQARGTGQRVGSRATSGSQFLSEARTGARPASEAGAKAGARRPSAFSAIQGDVRSMPDNSDAPWTRFVFQGPFGSRATGRGTGKAAGIWKTPAAYVGRRPGVSGPERAAFIRELEEALCPNLPPPVKKITQEDVKVMLYLLEELLPPVWESVTYGMVLQRERDLNTAARIGQSLVKQNSVLMEENSKLEALLGSAKEEILYLRHQVNLRDELLQLYSDSDEEDEDEEEEEEEKEAEEEQEEEEAEEDLQCAHPCDAPKLISQEALLHQHHCPQLEALQEKLRLLEEENHQLREEASQLDTLEDEEQMLILECVEQFSEASQQMAELSEVLVLRLENYERQQQEVARLQAQVLKLQQRCRMYGAETEKLQKQLASEKEIQMQLQEESVWVGSQLQDLREKYMDCGGMLIEMQEEVKTLRQQPPVSTGSATHYPYSVPLETLPGFQETLAEELRTSLRRMISDPVYFMERNYEMPRGDTSSLRYDFRYSEDREQVRGFEAEEGLMLAADIMRGEDFTPAEEFVPQEELGAAKKVPAEEGVMEEAELVSEETEGWEEVELELDEATRMNVVTSALEASGLGPSHLDMNYVLQQLANWQDAHYRRQLRWKMLQKGECPHGALPAASRTSCRSSCR

674 NP_005834.3 NP_000206.2

>NP_005834.3

MPLFTANPFEQDVEKATNEYNTTEDWSLIMDICDKVGSTPNGAKDCLKAIMKRVNHKVPHVALQALTLLGACVANCGKIFHLEVCSRDFATEVRAVIKNKAHPKVCEKLKSLMVEWSEEFQKDPQFSLISATIKSMKEEGITFPPAGSQTVSAAAKNGTSSNKNKEDEDIAKAIELSLQEQKQQHTETKSLYPSSEIQLNNKVARKVRALYDFEAVEDNELTFKHGEIIIVLDDSDANWWKGENHRGIGLFPSDFVTTNLNIETEVAAVDKLNVIDDDVEEIKKSEPEPVYIDEDKMDRALQVLQSIDPTDSKPDSQDLLDLEDICQQMGPMIDEKLEEIDRKHSELSELNVKVLEALELYNKLVNEAPVYSVYSKLHPPAHYPPASSGVPMQTYPVQSHGGNYMGQSIHQVTVAQSYSLGPDQIGPLRSLPPNVNSSVTAQPAQTSYLSTGQDTVSNPTYMNQNSNLQSATGTTAYTQQMGMSVDMSSYQNTTSNLPQLAGFPVTVPAHPVAQQHTNYHQQPLL

>NP_000206.2

MAPPSEETPLIPQRSCSLLSTEAGALHVLLPARGPGPPQRLSFSFGDHLAEDLCVQAAKASGILPVYHSLFALATEDLSCWFPPSHIFSVEDASTQVLLYRIRFYFPNWFGLEKCHRFGLRKDLASAILDLPVLEHLFAQHRSDLVSGRLPVGLSLKEQGECLSLAVLDLARMAREQAQRPGELLKTVSYKACLPPSLRDLIQGLSFVTRRRIRRTVRRALRRVAACQADRHSLMAKYIMDLERLDPAGAAETFHVGLPGALGGHDGLGLLRVAGDGGIAWTQGEQEVLQPFCDFPEIVDISIKQAPRVGPAGEHRLVTVTRTDNQILEAEFPGLPEALSFVALVDGYFRLTTDSQHFFCKEVAPPRLLEEVAEQCHGPITLDFAINKLKTGGSRPGSYVLRRSPQDFDSFLLTVCVQNPLGPDYKGCLIRRSPTGTFLLVGLSRPHSSLRELLATCWDGGLHVDGVAVTLTSCCIPRPKEKSNLIVVQRGHSPPTSSLVQPQSQYQLSQMTFHKIPADSLEWHENLGHGSFTKIYRGCRHEVVDGEARKTEVLLKVMDAKHKNCMESFLEAASLMSQVSYRHLVLLHGVCMAGDSTMVQEFVHLGAIDMYLRKRGHLVPASWKLQVVKQLAYALNYLEDKGLPHGNVSARKVLLAREGADGSPPFIKLSDPGVSPAVLSLEMLTDRIPWVAPECLREAQTLSLEADKWGFGATVWEVFSGVTMPISALDPAKKLQFYEDRQQLPAPKWTELALLIQQCMAYEPVQRPSFRAVIRDLNSLISSDYELLSDPTPGALAPRDGLWNGAQLYACQDPTIFEERHLKYISQLGKGNFGSVELCRYDPLGDNTGALVAVKQLQHSGPDQQRDFQREIQILKALHSDFIVKYRGVSYGPGRQSLRLVMEYLPSGCLRDFLQRHRARLDASRLLLYSSQICKGMEYLGSRRCVHRDLAARNILVESEAHVKIADFGLAKLLPLDKDYYVVREPGQSPIFWYAPESLSDNIFSRQSDVWSFGVVLYELFTYCDKSCSPSAEFLRMMGCERDVPALCRLLELLEEGQRLPAPPACPAEVHELMKLCWAPSPQDRPSFSALGPQLDMLWSGSRGCETHAFTAHPEGKHHSLSFS

675 NP_004570.2 NP_066961.2

>NP_004570.2

MALLRDVSLQDPRDRFELLQRVGAGTYGDVYKARDTVTSELAAVKIVKLDPGDDISSLQQEITILRECRHPNVVAYIGSYLRNDRLWICMEFCGGGSLQEIYHATGPLEERQIAYVCREALKGLHHLHSQGKIHRDIKGANLLLTLQGDVKLADFGVSGELTASVAKRRSFIGTPYWMAPEVAAVERKGGYNELCDVWALGITAIELGELQPPLFHLHPMRALMLMSKSSFQPPKLRDKTRWTQNFHHFLKLALTKNPKKRPTAEKLLQHPFTTQQLPRALLTQLLDKASDPHLGTPSPEDCELETYDMFPDTIHSRGQHGPAERTPSEIQFHQVKFGAPRRKETDPLNEPWEEEWTLLGKEELSGSLLQSVQEALEERSLTIRSASEFQELDSPDDTMGTIKRAPFLGPLPTDPPAEEPLSSPPGTLPPPPSGPNSSPLLPTAWATMKQREDPERSSCHGLPPTPKVHMGACFSKVFNGCPLRIHAAVTWIHPVTRDQFLVVGAEEGIYTLNLHELHEDTLEKLISHRCSWLYCVNNVLLSLSGKSTHIWAHDLPGLFEQRRLQQQVPLSIPTNRLTQRIIPRRFALSTKIPDTKGCLQCRVVRNPYTGATFLLAALPTSLLLLQWYEPLQKFLLLKNFSSPLPSPAGMLEPLVLDGKELPQVCVGAEGPEGPGCRVLFHVLPLEAGLTPDILIPPEGIPGSAQQVIQVDRDTILVSFERCVRIVNMQGEPTATLAPELTFDFPIETVVCLQDSVLAFWSHGMQGRSLDTNEVTQEITDETRIFRVLGAHRDIILESIPTDNPEAHSNLYILTGHQSTY

>NP_066961.2

MAAASVTPPGSLELLQPGFSKTLLGTKLEAKYLCSACRNVLRRPFQAQCGHRYCSFCLASILSSGPQNCAACVHEGIYEEGISILESSSAFPDNAARREVESLPAVCPSDGCTWKGTLKEYESCHEGRCPLMLTECPACKGLVRLGEKERHLEHECPERSLSCRHCRAPCCGADVKAHHEVCPKFPLTCDGCGKKKIPREKFQDHVKTCGKCRVPCRFHAIGCLETVEGEKQQEHEVQWLREHLAMLLSSVLEAKPLLGDQSHAGSELLQRCESLEKKTATFENIVCVLNREVERVAMTAEACSRQHRLDQDKIEALSSKVQQLERSIGLKDLAMADLEQKVLEMEASTYDGVFIWKISDFARKRQEAVAGRIPAIFSPAFYTSRYGYKMCLRIYLNGDGTGRGTHLSLFFVVMKGPNDALLRWPFNQKVTLMLLDQNNREHVIDAFRPDVTSSSFQRPVNDMNIASGCPLFCPVSKMEAKNSYVRDDAIFIKAIVDLTGL

676 NP_001471.1 NP_057057.2

>NP_001471.1

MELAVGNLSEGNASWPEPPAPEPGPLFGIGVENFVTLVVFGLIFALGVLGNSLVITVLARSKPGKPRSTTNLFILNLSIADLAYLLFCIPFQATVYALPTWVLGAFICKFIHYFFTVSMLVSIFTLAAMSVDRYVAIVHSRRSSSLRVSRNALLGVGCIWALSIAMASPVAYHQGLFHPRASNQTFCWEQWPDPRHKKAYVVCTFVFGYLLPLLLICFCYAKVLNHLHKKLKNMSKKSEASKKKTAQTVLVVVVVFGISWLPHHIIHLWAEFGVFPLTPASFLFRITAHCLAYSNSSVNPIIYAFLSENFRKAYKQVFKCHIRKDSHLSDTKENKSRIDTPPSTNCTHV

>NP_057057.2

MARGSALLLASLLLAAALSASAGLWSPAKEKRGWTLNSAGYLLGPHAVGNHRSFSDKNGLTSKRELRPEDDMKPGSFDRSIPENNIMRTIIEFLSFLHLKEAGALDRLLDLPAAASSEDIERS

677 NP_003891.1 NP_003627.1

>NP_003891.1

MASLTVKAYLLGKEDAAREIRRFSFCCSPEPEAEAEAAAGPGPCERLLSRVAALFPALRPGGFQAHYRDEDGDLVAFSSDEELTMAMSYVKDDIFRIYIKEKKECRRDHRPPCAQEAPRNMVHPNVICDGCNGPVVGTRYKCSVCPDYDLCSVCEGKGLHRGHTKLAFPSPFGHLSEGFSHSRWLRKVKHGHFGWPGWEMGPPGNWSPRPPRAGEARPGPTAESASGPSEDPSVNFLKNVGESVAAALSPLGIEVDIDVEHGGKRSRLTPVSPESSSTEEKSSSQPSSCCSDPSKPGGNVEGATQSLAEQMRKIALESEGRPEEQMESDNCSGGDDDWTHLSSKEVDPSTGELQSLQMPESEGPSSLDPSQEGPTGLKEAALYPHLPPEADPRLIESLSQMLSMGFSDEGGWLTRLLQTKNYDIGAALDTIQYSKHPPPL

>NP_003627.1

MYPESTTGSPARLSLRQTGSPGMIYSTRYGSPKRQLQFYRNLGKSGLRVSCLGLGTWVTFGGQITDEMAEQLMTLAYDNGINLFDTAEVYAAGKAEVVLGNIIKKKGWRRSSLVITTKIFWGGKAETERGLSRKHIIEGLKASLERLQLEYVDVVFANRPDPNTPMEETVRAMTHVINQGMAMYWGTSRWSSMEIMEAYSVARQFNLTPPICEQAEYHMFQREKVEVQLPELFHKIGVGAMTWSPLACGIVSGKYDSGIPPYSRASLKGYQWLKDKILSEEGRRQQAKLKELQAIAERLGCTLPQLAIAWCLRNEGVSSVLLGASNADQLMENIGAIQVLPKLSSSIIHEIDSILGNKPYSKKDYRS

678 NP_067637.2 NP_002060.4

>NP_067637.2

MPDTNSTINLSLSTRVTLAFFMSLVAFAIMLGNALVILAFVVDKNLRHRSSYFFLNLAISDFFVGVISIPLYIPHTLFEWDFGKEICVFWLTTDYLLCTASVYNIVLISYDRYLSVSNAVSYRTQHTGVLKIVTLMVAVWVLAFLVNGPMILVSESWKDEGSECEPGFFSEWYILAITSFLEFVIPVILVAYFNMNIYWSLWKRDHLSRCQSHPGLTAVSSNICGHSFRGRLSSRRSLSASTEVPASFHSERQRRKSSLMFSSRTKMNSNTIASKMGSFSQSDSVALHQREHVELLRARRLAKSLAILLGVFAVCWAPYSLFTIVLSFYSSATGPKSVWYRIAFWLQWFNSFVNPLLYPLCHKRFQKAFLKIFCIKKQPLPSQHSRSVSS

>NP_002060.4

MGCTLSAEDKAAVERSKMIDRNLREDGEKAAREVKLLLLGAGESGKSTIVKQMKIIHEAGYSEEECKQYKAVVYSNTIQSIIAIIRAMGRLKIDFGDSARADDARQLFVLAGAAEEGFMTAELAGVIKRLWKDSGVQACFNRSREYQLNDSAAYYLNDLDRIAQPNYIPTQQDVLRTRVKTTGIVETHFTFKDLHFKMFDVGGQRSERKKWIHCFEGVTAIIFCVALSDYDLVLAEDEEMNRMHESMKLFDSICNNKWFTDTSIILFLNKKDLFEEKIKKSPLTICYPEYAGSNTYEEAAAYIQCQFEDLNKRKDTKEIYTHFTCATDTKNVQFVFDAVTDVIIKNNLKDCGLF

679 NP_000323.2 NP_001025053.1

>NP_000323.2

MKSNQERSNECLPPKKREIPATSRSSEEKAPTLPSDNHRVEGTAWLPGNPGGRGHGGGRHGPAGTSVELGLQQGIGLHKALSTGLDYSPPSAPRSVPVATTLPAAYATPQPGTPVSPVQYAHLPHTFQFIGSSQYSGTYASFIPSQLIPPTANPVTSAVASAAGATTPSQRSQLEAYSTLLANMGSLSQTPGHKAEQQQQQQQQQQQQHQHQQQQQQQQQQQQQQHLSRAPGLITPGSPPPAQQNQYVHISSSPQNTGRTASPPAIPVHLHPHQTMIPHTLTLGPPSQVVMQYADSGSHFVPREATKKAESSRLQQAIQAKEVLNGEMEKSRRYGAPSSADLGLGKAGGKSVPHPYESRHVVVHPSPSDYSSRDPSGVRASVMVLPNSNTPAADLEVQQATHREASPSTLNDKSGLHLGKPGHRSYALSPHTVIQTTHSASEPLPVGLPATAFYAGTQPPVIGYLSGQQQAITYAGSLPQHLVIPGTQPLLIPVGSTDMEASGAAPAIVTSSPQFAAVPHTFVTTALPKSENFNPEALVTQAAYPAMVQAQIHLPVVQSVASPAAAPPTLPPYFMKGSIIQLANGELKKVEDLKTEDFIQSAEISNDLKIDSSTVERIEDSHSPGVAVIQFAVGEHRAQVSVEVLVEYPFFVFGQGWSSCCPERTSQLFDLPCSKLSVGDVCISLTLKNLKNGSVKKGQPVDPASVLLKHSKADGLAGSRHRYAEQENGINQGSAQMLSENGELKFPEKMGLPAAPFLTKIEPSKPAATRKRRWSAPESRKLEKSEDEPPLTLPKPSLIPQEVKICIEGRSNVGK

>NP_001025053.1

MRVKPQGLVVTSSAVCSSPDYLREPKYYPGGPPTPRPLLPTRPPASPPDKAFSTHAFSENPRPPPRRDPSTRRPPVLAKGDDPLPPRAARPVSQARCPTPVGDGSSSRRCWDNGRVNLRPVVQLIDIMKDLTRLSQDLQHSGVHLDCGGLRLSRPPAPPPGDLQYSFFSSPSLANSIRSPEERATPHAKSERPSHPLYEPEPEPRDSPQPGQGHSPGATAAATGLPPEPEPDSTDYSELADADILSELASLTCPEAQLLEAQALEPPSPEPEPQLLDPQPRFLDPQALEPLGEALELPPLQPLADPLGLPGLALQALDTLPDSLESQLLDPQALDPLPKLLDVPGRRLEPQQPLGHCPLAEPLRLDLCSPHGPPGPEGHPKYALRRTDRPKILCRRRKAGRGRKADAGPEGRLLPLPMPTGLVAALAEPPPPPPPPPPALPGPGPVSVPELKPESSQTPVVSTRKGKCRGVRRMVVKMAKIPVSLGRRNKTTYKVSSLSSSLSVEGKELGLRVSAEPTPLLKMKNNGRNVVVVFPPGEMPIILKRKRGRPPKNLLLGPGKPKEPAVVAAEAATVAAATMAMPEVKKRRRRKQKLASPQPSYAADANDSKAEYSDVLAKLAFLNRQSQCAGRCSPPRCWTPSEPESVHQAPDTQSISHFLHRVQGFRRRGGKAGGFGGRGGGHAAKSARCSFSDFFEGIGKKKKVVAVAAAGVGGPGLTELGHPRKRGRGEVDAVTGKPKRKRRSRKNGTLFPEQVPSGPGFGEAGAEWAGDKGGGWAPHHGHPGGQAGRNCGFQGTEARAFASTGLESGASGRGSYYSTGAPSGQTELSQERQNLFTGYFRSLLDSDDSSDLLDFALSASRPESRKASGTYAGPPTSALPAQRGLATFPSRGAKASPVAVGSSGAGADPSFQPVLSARQTFPPGRAASYGLTPAASDCRAAETFPKLVPPPSAMARSPTTHPPANTYLPQYGGYGAGQSVFAPTKPFTGQDCANSKDCSFAYGSGNSLPASPSSAHSAGYAPPPTGGPCLPPSKASFFSSSEGAPFSGSAPTPLRCDSRASTVSPGGYMVPKGTTASATSAASAASSSSSSFQPSPENCRQFAGASQWPFRQGYGGLDWASEAFSQLYNPSFDCHVSEPNVILDISNYTPQKVKQQTAVSETFSESSSDSTQFNQPVGGGGFRRANSEASSSEGQSSLSSLEKLMMDWNEASSAPGYNWNQSVLFQSSSKPGRGRRKKVDLFEASHLGFPTSASAAASGYPSKRSTGPRQPRGGRGGGACSAKKERGGAAAKAKFIPKPQPVNPLFQDSPDLGLDYYSGDSSMSPLPSQSRAFGVGERDPCDFIGPYSMNPSTPSDGTFGQGFHCDSPSLGAPELDGKHFPPLAHPPTVFDAGLQKAYSPTCSPTLGFKEELRPPPTKLAACEPLKHGLQGASLGHAAAAQAHLSCRDLPLGQPHYDSPSCKGTAYWYPPGSAARSPPYEGKVGTGLLADFLGRTEAACLSAPHLASPPATPKADKEPLEMARPPGPPRGPAAAAAGYGCPLLSDLTLSPVPRDSLLPLQDTAYRYPGFMPQAHPGLGGGPKSGFLGPMAEPHPEDTFTVTSL

680 NP_891548.1 NP_002219.1

>NP_891548.1

MASTIKEALSVVSEDQSLFECAYGTPHLAKTEMTASSSSDYGQTSKMSPRVPQQDWLSQPPARVTIKMECNPSQVNGSRNSPDECSVAKGGKMVGSPDTVGMNYGSYMEEKHMPPPNMTTNERRVIVPADPTLWSTDHVRQWLEWAVKEYGLPDVNILLFQNIDGKELCKMTKDDFQRLTPSYNADILLSHLHYLRETPLPHLTSDDVDKALQNSPRLMHARNTGGAAFIFPNTSVYPEATQRITTRPDLPYEPPRRSAWTGHGHPTPQSKAAQPSPSTVPKTEDQRPQLDPYQILGPTSSRLANPGSGQIQLWQFLLELLSDSSNSSCITWEGTNGEFKMTDPDEVARRWGERKSKPNMNYDKLSRALRYYYDKNIMTKVHGKRYAYKFDFHGIAQALQPHPPESSLYKYPSDLPYMGSYHAHPQKMNFVAPHPPALPVTSSSFFAAPNPYWNSPTGGIYPNTRLPTSHMPSHLGTYY

>NP_002219.1

MTAKMETTFYDDALNASFLPSESGPYGYSNPKILKQSMTLNLADPVGSLKPHLRAKNSDLLTSPDVGLLKLASPELERLIIQSSNGHITTTPTPTQFLCPKNVTDEQEGFAEGFVRALAELHSQNTLPSVTSAAQPVNGAGMVAPAVASVAGGSGSGGFSASLHSEPPVYANLSNFNPGALSSGGGAPSYGAAGLAFPAQPQQQQQPPHHLPQQMPVQHPRLQALKEEPQTVPEMPGETPPLSPIDMESQERIKAERKRMRNRIAASKCRKRKLERIARLEEKVKTLKAQNSELASTANMLREQVAQLKQKVMNHVNSGCQLMLTQQLQTF

681 NP_038474.1 NP_852480.1

>NP_038474.1

MAEAATPGTTATTSGAGAAAATAAAASPTPIPTVTAPSLGAGGGGGGSDGSGGGWTKQVTCRYFMHGVCKEGDNCRYSHDLSDSPYSVVCKYFQRGYCIYGDRCRYEHSKPLKQEEATATELTTKSSLAASSSLSSIVGPLVEMNTGEAESRNSNFATVGAGSEDWVNAIEFVPGQPYCGRTAPSCTEAPLQGSVTKEESEKEQTAVETKKQLCPYAAVGECRYGENCVYLHGDSCDMCGLQLLHPMDAAQRSQHIKSCIEAHEKDMELSFAVQRSKDMVCGICMEVVYEKANPSERRFGILSNCNHTYCLKCIRKWRSAKQFESKIIKSCPECRITSNFVIPSEYWVEEKEEKQKLILKYKEAMSNKACRYFDEGRGSCPFGGNCFYKHAYPDGRREEPQRQKVGTSSRYRAQRRNHFWELIEERENSNPFDNDEEEVVTFELGEMLLMLLAAGGDDELTDSEDEWDLFHDELEDFYDLDL

>NP_852480.1

MAAGFKTVEPLEYYRRFLKENCRPDGRELGEFRTTTVNIGSISTADGSALVKLGNTTVICGVKAEFAAPSTDAPDKGYVVPNVDLPPLCSSRFRSGPPGEEAQVASQFIADVIENSQIIQKEDLCISPGKLVWVLYCDLICLDYDGNILDACTFALLAALKNVQLPEVTINEETALAEVNLKKKSYLNIRTHPVATSFAVFDDTLLIVDPTGEEEHLATGTLTIVMDEEGKLCCLHKPGGSGLTGAKLQDCMSRAVTRHKEVKKLMDEVIKSMKPK

682 NP_004417.2 NP_006588.1

>NP_004417.2

METESEQNSNSTNGSSSSGGSSRPQIAQMSLYERQAVQALQALQRQPNAAQYFHQFMLQQQLSNAQLHSLAAVQQATIAASRQASSPNTSTTQQQTTTTQASINLATTSAAQLISRSQSVSSPSATTLTQSVLLGNTTSPPLNQSQAQMYLRPQLGNLLQVNRTLGRNVPLASQLILMPNGAVAAVQQEVPSAQSPGVHADADQVQNLAVRNQQASAQGPQMQGSTQKAIPPGASPVSSLSQASSQALAVAQASSGATNQSLNLSQAGGGSGNSIPGSMGPGGGGQAHGGLGQLPSSGMGGGSCPRKGTGVVQPLPAAQTVTVSQGSQTEAESAAAKKAEADGSGQQNVGMNLTRTATPAPSQTLISSATYTQIQPHSLIQQQQQIHLQQKQVVIQQQIAIHHQQQFQHRQSQLLHTATHLQLAQQQQQQQQQQQQQQQPQATTLTAPQPPQVPPTQQVPPSQSQQQAQTLVVQPMLQSSPLSLPPDAAPKPPIPIQSKPPVAPIKPPQLGAAKMSAAQQPPPHIPVQVVGTRQPGTAQAQALGLAQLAAAVPTSRGMPGTVQSGQAHLASSPPSSQAPGALQECPPTLAPGMTLAPVQGTAHVVKGGATTSSPVVAQVPAAFYMQSVHLPGKPQTLAVKRKADSEEERDDVSTLGSMLPAKASPVAESPKVMDEKSSLGEKAESVANVNANTPSSELVALTPAPSVPPPTLAMVSRQMGDSKPPQAIVKPQILTHIIEGFVIQEGAEPFPVGCSQLLKESEKPLQTGLPTGLTENQSGGPLGVDSPSAELDKKANLLKCEYCGKYAPAEQFRGSKRFCSMTCAKRYNVSCSHQFRLKRKKMKEFQEANYARVRRRGPRRSSSDIARAKIQGKCHRGQEDSSRGSDNSSYDEALSPTSPGPLSVRAGHGERDLGNPNTAPPTPELHGINPVFLSSNPSRWSVEEVYEFIASLQGCQEIAEEFRSQEIDGQALLLLKEEHLMSAMNIKLGPALKICAKINVLKET

>NP_006588.1

MSKGPAVGIDLGTTYSCVGVFQHGKVEIIANDQGNRTTPSYVAFTDTERLIGDAAKNQVAMNPTNTVFDAKRLIGRRFDDAVVQSDMKHWPFMVVNDAGRPKVQVEYKGETKSFYPEEVSSMVLTKMKEIAEAYLGKTVTNAVVTVPAYFNDSQRQATKDAGTIAGLNVLRIINEPTAAAIAYGLDKKVGAERNVLIFDLGGGTFDVSILTIEDGIFEVKSTAGDTHLGGEDFDNRMVNHFIAEFKRKHKKDISENKRAVRRLRTACERAKRTLSSSTQASIEIDSLYEGIDFYTSITRARFEELNADLFRGTLDPVEKALRDAKLDKSQIHDIVLVGGSTRIPKIQKLLQDFFNGKELNKSINPDEAVAYGAAVQAAILSGDKSENVQDLLLLDVTPLSLGIETAGGVMTVLIKRNTTIPTKQTQTFTTYSDNQPGVLIQVYEGERAMTKDNNLLGKFELTGIPPAPRGVPQIEVTFDIDANGILNVSAVDKSTGKENKITITNDKGRLSKEDIERMVQEAEKYKAEDEKQRDKVSSKNSLESYAFNMKATVEDEKLQGKINDEDKQKILDKCNEIINWLDKNQTAEKEEFEHQQKELEKVCNPIITKLYQSAGGMPGGMPGGFPGGGAPPSGGASSGPTIEEVD

683 NP_570720.1 NP_037364.1

>NP_570720.1

MKKMSNIYESAANTLGIFNSPCLTKVELRVACKGISDRDALSKPDPCVILKMQSHGQWFEVDRTEVIRTCINPVYSKLFTVDFYFEEVQRLRFEVHDISSNHNGLKEADFLGGMECTLGQIVSQRKLSKSLLKHGNTAGKSSITVIAEELSGNDDYVELAFNARKLDDKDFFSKSDPFLEIFRMNDDATQQLVHRTEVVMNNLSPAWKSFKVSVNSLCSGDPDRRLKCIVWDWDSNGKHDFIGEFTSTFKEMRGAMEGKQVQWECINPKYKAKKKNYKNSGTVILNLCKIHKMHSFLDYIMGGCQIQFTVAIDFTASNGDPRNSCSLHYIHPYQPNEYLKALVAVGEICQDYDSDKMFPAFGFGARIPPEYTVSHDFAINFNEDNPECAGIQGVVEAYQSCLPKLQLYGPTNIAPIIQKVAKSASEETNTKEASQYFILLILTDGVITDMADTREAIVHASHLPMSVIIVGVGNADFSDMQMLDGDDGILRSPKGEPVLRDIVQFVPFRNFKHASPAALAKSVLAEVPNQVVDYYNGKGIKPKCSSEMYESSRTLAP

>NP_037364.1

MAAYSYRPGPGAGPGPAAGAALPDQSFLWNVFQRVDKDRSGVISDTELQQALSNGTWTPFNPVTVRSIISMFDRENKAGVNFSEFTGVWKYITDWQNVFRTYDRDNSGMIDKNELKQALSGFGYRLSDQFHDILIRKFDRQGRGQIAFDDFIQGCIVLQRLTDIFRRYDTDQDGWIQVSYEQYLSMVFSIV

684 NP_002119.1 NP_009107.1

>NP_002119.1

MGKGDPKKPRGKMSSYAFFVQTCREEHKKKHPDASVNFSEFSKKCSERWKTMSAKEKGKFEDMAKADKARYEREMKTYIPPKGETKKKFKDPNAPKRPPSAFFLFCSEYRPKIKGEHPGLSIGDVAKKLGEMWNNTAADDKQPYEKKAAKLKEKYEKDIAAYRAKGKPDAAKKGVVKAEKSKKKKEEEEDEEDEEDEEEEEDEEDEDEEEDDDDE

>NP_009107.1

MSRFLNVLRSWLVMVSIIAMGNTLQSFRDHTFLYEKLYTGKPNLVNGLQARTFGIWTLLSSVIRCLCAIDIHNKTLYHITLWTFLLALGHFLSELFVYGTAAPTIGVLAPLMVASFSILGMLVGLRYLEVEPVSRQKKRN

685 NP_036611.2 NP_004563.2

>NP_036611.2

MVDREQLVQKARLAEQAERYDDMAAAMKNVTELNEPLSNEERNLLSVAYKNVVGARRSSWRVISSIEQKTSADGNEKKIEMVRAYREKIEKELEAVCQDVLSLLDNYLIKNCSETQYESKVFYLKMKGDYYRYLAEVATGEKRATVVESSEKAYSEAHEISKEHMQPTHPIRLGLALNYSVFYYEIQNAPEQACHLAKTAFDDAIAELDTLNEDSYKDSTLIMQLLRDNLTLWTSDQQDDDGGEGNN

>NP_004563.2

MAAPGAPAEYGYIRTVLGQQILGQLDSSSLALPSEAKLKLAGSSGRGGQTVKSLRIQEQVQQTLARKGRSSVGNGNLHRTSSVPEYVYNLHLVENDFVGGRSPVPKTYDMLKAGTTATYEGRWGRGTAQYSSQKSVEERSLRHPLRRLEISPDSSPERAHYTHSDYQYSQRSQAGHTLHHQESRRAALLVPPRYARSEIVGVSRAGTTSRQRHFDTYHRQYQHGSVSDTVFDSIPANPALLTYPRPGTSRSMGNLLEKENYLTAGLTVGQVRPLVPLQPVTQNRASRSSWHQSSFHSTRTLREAGPSVAVDSSGRRAHLTVGQAAAGGSGNLLTERSTFTDSQLGNADMEMTLERAVSMLEADHMLPSRISAAATFIQHECFQKSEARKRVNQLRGILKLLQLLKVQNEDVQRAVCGALRNLVFEDNDNKLEVAELNGVPRLLQVLKQTRDLETKKQITDHTVNLRSRNGWPGAVAHACNPSTLGGQGGRITRSGVRDQPDQHGLLWNLSSNDKLKNLMITEALLTLTENIIIPFSGWPEGDYPKANGLLDFDIFYNVTGCLRNMSSAGADGRKAMRRCDGLIDSLVHYVRGTIADYQPDDKATENCVCILHNLSYQLEAELPEKYSQNIYIQNRNIQTDNNKSIGCFGSRSRKVKEQYQDVPMPEEKSNPKGVEWLWHSIVIRMYLSLIAKSVRNYTQEASLGALQNLTAGSGPMPTSVAQTVVQKESGLQHTRKMLHVGDPSVKKTAISLLRNLSRNLSLQNEIAKETLPDLVSIIPDTVPSTDLLIETTASACYTLNNIIQNSYQNARDLLNTGGIQKIMAISAGDAYASNKASKAASVLLYSLWAHTELHHAYKKAQFKKTDFVNSRTAKAYHSLKD

686 NP_003121.1 NP_001026.2

>NP_003121.1

MAYPGHPGAGGGYYPGGYGGAPGGPAFPGQTQDPLYGYFAAVAGQDGQIDADELQRCLTQSGIAGGYKPFNLETCRLMVSMLDRDMSGTMGFNEFKELWAVLNGWRQHFISFDTDRSGTVDPQELQKALTTMGFRLSPQAVNSIAKRYSTNGKITFDDYIACCVKLRALTDSFRRRDTAQQGVVNFPYDDFIQCVMSV

>NP_001026.2

MADGGEGEDEIQFLRTDDEVVLQCTATIHKEQQKLCLAAEGFGNRLCFLESTSNSKNVPPDLSICTFVLEQSLSVRALQEMLANTVEKSEGQVDVEKWKFMMKTAQGGGHRTLLYGHAILLRHSYSGMYLCCLSTSRSSTDKLAFDVGLQEDTTGEACWWTIHPASKQRSEGEKVRVGDDLILVSVSSERYLHLSYGNGSLHVDAAFQQTLWSVAPISSGSEAAQGYLIGGDVLRLLHGHMDECLTVPSGEHGEEQRRTVHYEGGAVSVHARSLWRLETLRVAWSGSHIRWGQPFRLRHVTTGKYLSLMEDKNLLLMDKEKADVKSTAFTFRSSKEKLDVGVRKEVDGMGTSEIKYGDSVCYIQHVDTGLWLTYQSVDVKSVRMGSIQRKAIMHHEGHMDDGISLSRSQHEESRTARVIRSTVFLFNRFIRGLDALSKKAKASTVDLPIESVSLSLQDLIGYFHPPDEHLEHEDKQNRLRALKNRQNLFQEEGMINLVLECIDRLHVYSSAAHFADVAGREAGESWKSILNSLYELLAALIRGNRKNCAQFSGSLDWLISRLERLEASSGILEVLHCVLVESPEALNIIKEGHIKSIISLLDKHGRNHKVLDVLCSLCVCHGVAVRSNQHLICDNLLPGRDLLLQTRLVNHVSSMRPNIFLGVSEGSAQYKKWYYELMVDHTEPFVTAEATHLRVGWASTEGYSPYPGGGEEWGGNGVGDDLFSYGFDGLHLWSGCIARTVSSPNQHLLRTDDVISCCLDLSAPSISFRINGQPVQGMFENFNIDGLFFPVVSFSAGIKVRFLLGGRHGEFKFLPPPGYAPCYEAVLPKEKLKVEHSREYKQERTYTRDLLGPTVSLTQAAFTPIPVDTSQIVLPPHLERIREKLAENIHELWVMNKIELGWQYGPVRDDNKRQHPCLVEFSKLPEQERNYNLQMSLETLKTLLALGCHVGISDEHAEDKVKKMKLPKNYQLTSGYKPAPMDLSFIKLTPSQEAMVDKLAENAHNVWARDRIRQGWTYGIQQDVKNRRNPRLVPYTLLDDRTKKSNKDSLREAVRTLLGYGYNLEAPDQDHAARAEVCSGTGERFRIFRAEKTYAVKAGRWYFEFETVTAGDMRVGWSRPGCQPDQELGSDERAFAFDGFKAQRWHQGNEHYGRSWQAGDVVGCMVDMNEHTMMFTLNGEILLDDSGSELAFKDFDVGDGFIPVCSLGVAQVGRMNFGKDVSTLKYFTICGLQEGYEPFAVNTNRDITMWLSKRLPQFLQVPSNHEHIEVTRIDGTIDSSPCLKVTQKSFGSQNSNTDIMFYRLSMPIECAEVFSKTVAGGLPGAGLFGPKNDLEDYDADSDFEVLMKTAHGHLVPDRVDKDKEATKPEFNNHKDYAQEKPSRLKQRFLLRRTKPDYSTSHSARLTEDVLADDRDDYDFLMQTSTYYYSVRIFPGQEPANVWVGWITSDFHQYDTGFDLDRVRTVTVTLGDEKGKVHESIKRSNCYMVCAGESMSPGQGRNNNGLEIGCVVDAASGLLTFIANGKELSTYYQVEPSTKLFPAVFAQATSPNVFQFELGRIKNVMPLSAGLFKSEHKNPVPQCPPRLHVQFLSHVLWSRMPNQFLKVDVSRISERQGWLVQCLDPLQFMSLHIPEENRSVDILELTEQEELLKFHYHTLRLYSAVCALGNHRVAHALCSHVDEPQLLYAIENKYMPGLLRAGYYDLLIDIHLSSYATARLMMNNEYIVPMTEETKSITLFPDENKKHGLPGIGLSTSLRPRMQFSSPSFVSISNECYQYSPEFPLDILKSKTIQMLTEAVKEGSLHARDPVGGTTEFLFVPLIKLFYTLLIMGIFHNEDLKHILQLIEPSVFKEAATPEEESDTLEKELSVDDAKLQGAGEEEAKGGKRPKEGLLQMKLPEPVKLQMCLLLQYLCDCQVRHRIEAIVAFSDDFVAKLQDNQRFRYNEVMQALNMSAALTARKTKEFRSPPQEQINMLLNFKDDKSECPCPEEIRDQLLDFHEDLMTHCGIELDEDGSLDGNSDLTIRGRLLSLVEKVTYLKKKQAEKPVESDSKKSSTLQQLISETMVRWAQESVIEDPELVRAMFVLLHRQYDGIGGLVRALPKTYTINGVSVEDTINLLASLGQIRSLLSVRMGKEEEKLMIRGLGDIMNNKVFYQHPNLMRALGMHETVMEVMVNVLGGGESKEITFPKMVANCCRFLCYFCRISRQNQKAMFDHLSYLLENSSVGLASPAMRGSTPLDVAAASVMDNNELALALREPDLEKVVRYLAGCGLQSCQMLVSKGYPDIGWNPVEGERYLDFLRFAVFCNGESVEENANVVVRLLIRRPECFGPALRGEGGNGLLAAMEEAIKIAEDPSRDGPSPNSGSSKTLDTEEEEDDTIHMGNAIMTFYSALIDLLGRCAPEMHLIHAGKGEAIRIRSILRSLIPLGDLVGVISIAFQMPTIAKDGNVVEPDMSAGFCPDHKAAMVLFLDRVYGIEVQDFLLHLLEVGFLPDLRAAASLDTAALSATDMALALNRYLCTAVLPLLTRCAPLFAGTEHHASLIDSLLHTVYRLSKGCSLTKAQRDSIEVCLLSICGQLRPSMMQHLLRRLVFDVPLLNEHAKMPLKLLTNHYERCWKYYCLPGGWGNFGAASEEELHLSRKLFWGIFDALSQKKYEQELFKLALPCLSAVAGALPPDYMESNYVSMMEKQSSMDSEGNFNPQPVDTSNITIPEKLEYFINKYAEHSHDKWSMDKLANGWIYGEIYSDSSKVQPLMKPYKLLSEKEKEIYRWPIKESLKTMLAWGWRIERTREGDSMALYNRTRRISQTSQVSVDAAHGYSPRAIDMSNVTLSRDLHAMAEMMAENYHNIWAKKKKMELESKGGGNHPLLVPYDTLTAKEKAKDREKAQDILKFLQINGYAVSRGFKDLELDTPSIEKRFAYSFLQQLIRYVDEAHQYILEFDGGSRGKGEHFPYEQEIKFFAKVVLPLIDQYFKNHRLYFLSAASRPLCSGGHASNKEKEMVTSLFCKLGVLVRHRISLFGNDATSIVNCLHILGQTLDARTVMKTGLESVKSALRAFLDNAAEDLEKTMENLKQGQFTHTRNQPKGVTQIINYTTVALLPMLSSLFEHIGQHQFGEDLILEDVQVSCYRILTSLYALGTSKSIYVERQRSALGECLAAFAGAFPVAFLETHLDKHNIYSIYNTKSSRERAALSLPTNVEDVCPNIPSLEKLMEEIVELAESGIRYTQMPHVMEVILPMLCSYMSRWWEHGPENNPERAEMCCTALNSEHMNTLLGNILKIIYNNLGIDEGAWMKRLAVFSQPIINKVKPQLLKTHFLPLMEKLKKKAATVVSEEDHLKAEARGDMSEAELLILDEFTTLARDLYAFYPLLIRFVDYNRAKWLKEPNPEAEELFRMVAEVFIYWSKSHNFKREEQNFVVQNEINNMSFLITDTKSKMSKAAVSDQERKKMKRKGDRYSMQTSLIVAALKRLLPIGLNICAPGDQELIALAKNRFSLKDTEDEVRDIIRSNIHLQGKLEDPAIRWQMALYKDLPNRTDDTSDPEKTVERVLDIANVLFHLEQKSKRVGRRHYCLVEHPQRSKKAVWHKLLSKQRKRAVVACFRMAPLYNLPRHRAVNLFLQGYEKSWIETEEHYFEDKLIEDLAKPGAEPPEEDEGTKRVDPLHQLILLFSRTALTEKCKLEEDFLYMAYADIMAKSCHDEEDDDGEEEVKSFEEKEMEKQKLLYQQARLHDRGAAEMVLQTISASKGETGPMVAATLKLGIAILNGGNSTVQQKMLDYLKEKKDVGFFQSLAGLMQSCSVLDLNAFERQNKAEGLGMVTEEGSGEKVLQDDEFTCDLFRFLQLLCEGHNSDFQNYLRTQTGNNTTVNIIISTVDYLLRVQESISDFYWYYSGKDVIDEQGQRNFSKAIQVAKQVFNTLTEYIQGPCTGNQQSLAHSRLWDAVVGFLHVFAHMQMKLSQDSSQIELLKELMDLQKDMVVMLLSMLEGNVVNGTIGKQMVDMLVESSNNVEMILKFFDMFLKLKDLTSSDTFKEYDPDGKGVISKRDFHKAMESHKHYTQSETEFLLSCAETDENETLDYEEFVKRFHEPAKDIGFNVAVLLTNLSEHMPNDTRLQTFLELAESVLNYFQPFLGRIEIMGSAKRIERVYFEISESSRTQWEKPQVKESKRQFIFDVVNEGGEKEKMELFVNFCEDTIFEMQLAAQISESDLNERSANKEESEKERPEEQGPRMAFFSILTVRSALFALRYNILTLMRMLSLKSLKKQMKKVKKMTVKDMVTAFFSSYWSIFMTLLHFVASVFRGFFRIICSLLLGGSLVEGAKKIKVAELLANMPDPTQDEVRGDGEEGERKPLEAALPSEDLTDLKELTEESDLLSDIFGLDLKREGGQYKLIPHNPNAGLSDLMSNPVPMPEVQEKFQEQKAKEEEKEEKEETKSEPEKAEGEDGEKEEKAKEDKGKQKLRQLHTHRYGEPEVPESAFWKKIIAYQQKLLNYFARNFYNMRMLALFVAFAINFILLFYKVSTSSVVEGKELPTRSSSENAKVTSLDSSSHRIIAVHYVLEESSGYMEPTLRILAILHTVISFFCIIGYYCLKVPLVIFKREKEVARKLEFDGLYITEQPSEDDIKGQWDRLVINTQSFPNNYWDKFVKRKVMDKYGEFYGRDRISELLGMDKAALDFSDAREKKKPKKDSSLSAVLNSIDVKYQMWKLGVVFTDNSFLYLAWYMTMSVLGHYNNFFFAAHLLDIAMGFKTLRTILSSVTHNGKQLVLTVGLLAVVVYLYTVVAFNFFRKFYNKSEDGDTPDMKCDDMLTCYMFHMYVGVRAGGGIGDEIEDPAGDEYEIYRIIFDITFFFFVIVILLAIIQGLIIDAFGELRDQQEQVKEDMETKCFICGIGNDYFDTVPHGFETHTLQEHNLANYLFFLMYLINKDETEHTGQESYVWKMYQERCWEFFPAGDCFRKQYEDQLN

687 NP_001019978.1 NP_003197.2

>NP_001019978.1

MGPASPAARGLSRRPGQPPLPLLLPLLLLLLRAQPAIGSLAGGSPGAAEAPGSAQVAGLCGRLTLHRDLRTGRWEPDPQRSRRCLRDPQRVLEYCRQMYPELQIARVEQATQAIPMERWCGGSRSGSCAHPHHQVVPFRCLPGEFVSEALLVPEGCRFLHQERMDQCESSTRRHQEAQEACSSQGLILHGSGMLLPCGSDRFRGVEYVCCPPPGTPDPSGTAVGDPSTRSWPPGSRVEGAEDEEEEESFPQPVDDYFVEPPQAEEEEETVPPPSSHTLAVVGKVTPTPRPTDGVDIYFGMPGEISEHEGFLRAKMDLEERRMRQINEVMREWAMADNQSKNLPKADRQALNEHFQSILQTLEEQVSGERQRLVETHATRVIALINDQRRAALEGFLAALQADPPQAERVLLALRRYLRAEQKEQRHTLRHYQHVAAVDPEKAQQMRFQVHTHLQVIEERVNQSLGLLDQNPHLAQELRPQIQELLHSEHLGPSELEAPAPGGSSEDKGGLQPPDSKDADTPMTLPKGSTEQDAASPEKEKMNPLEQYERKVNASVPRGFPFHSSEIQRDELAPAGTGVSREAVSGLLIMGAGGGSLIVLSMLLLRRKKPYGAISHGVVEVDPMLTLEEQQLRELQRHGYENPTYRFLEERP

>NP_003197.2

MSTGSLSDVEDLQEVEMLECDGLKMDSNKEFVTSNESTEESSNCENGSPQKGRGGLGKRRKAPTKKSPLSGVSQEGKQVQRNAANARERARMRVLSKAFSRLKTTLPWVPPDTKLSKLDTLRLASSYIAHLRQILANDKYENGYIHPVNLTWPFMVAGKPESDLKEVVTASRLCGTTAS

688 NP_001460.1 NP_002102.4

>NP_001460.1

MSGWESYYKTEGDEEAEEEQEENLEASGDYKYSGRDSLIFLVDASKAMFESQSEDELTPFDMSIQCIQSVYISKIISSDRDLLAVVFYGTEKDKNSVNFKNIYVLQELDNPGAKRILELDQFKGQQGQKRFQDMMGHGSDYSLSEVLWVCANLFSDVQFKMSHKRIMLFTNEDNPHGNDSAKASRARTKAGDLRDTGIFLDLMHLKKPGGFDISLFYRDIISIAEDEDLRVHFEESSKLEDLLRKVRAKETRKRALSRLKLKLNKDIVISVGIYNLVQKALKPPPIKLYRETNEPVKTKTRTFNTSTGGLLLPSDTKRSQIYGSRQIILEKEETEELKRFDDPGLMLMGFKPLVLLKKHHYLRPSLFVYPEESLVIGSSTLFSALLIKCLEKEVAALCRYTPRRNIPPYFVALVPQEEELDDQKIQVTPPGFQLVFLPFADDKRKMPFTEKIMATPEQVGKMKAIVEKLRFTYRSDSFENPVLQQHFRNLEALALDLMEPEQAVDLTLPKVEAMNKRLGSLVDEFKELVYPPDYNPEGKVTKRKHDNEGSGSKRPKVEYSEEELKTHISKGTLGKFTVPMLKEACRAYGLKSGLKKQELLEALTKHFQD

>NP_002102.4

MATLEKLMKAFESLKSFQQQQQQQQQQQQQQQQQQQQQQQPPPPPPPPPPPQLPQPPPQAQPLLPQPQPPPPPPPPPPGPAVAEEPLHRPKKELSATKKDRVNHCLTICENIVAQSVRNSPEFQKLLGIAMELFLLCSDDAESDVRMVADECLNKVIKALMDSNLPRLQLELYKEIKKNGAPRSLRAALWRFAELAHLVRPQKCRPYLVNLLPCLTRTSKRPEESVQETLAAAVPKIMASFGNFANDNEIKVLLKAFIANLKSSSPTIRRTAAGSAVSICQHSRRTQYFYSWLLNVLLGLLVPVEDEHSTLLILGVLLTLRYLVPLLQQQVKDTSLKGSFGVTRKEMEVSPSAEQLVQVYELTLHHTQHQDHNVVTGALELLQQLFRTPPPELLQTLTAVGGIGQLTAAKEESGGRSRSGSIVELIAGGGSSCSPVLSRKQKGKVLLGEEEALEDDSESRSDVSSSALTASVKDEISGELAASSGVSTPGSAGHDIITEQPRSQHTLQADSVDLASCDLTSSATDGDEEDILSHSSSQVSAVPSDPAMDLNDGTQASSPISDSSQTTTEGPDSAVTPSDSSEIVLDGTDNQYLGLQIGQPQDEDEEATGILPDEASEAFRNSSMALQQAHLLKNMSHCRQPSDSSVDKFVLRDEATEPGDQENKPCRIKGDIGQSTDDDSAPLVHCVRLLSASFLLTGGKNVLVPDRDVRVSVKALALSCVGAAVALHPESFFSKLYKVPLDTTEYPEEQYVSDILNYIDHGDPQVRGATAILCGTLICSILSRSRFHVGDWMGTIRTLTGNTFSLADCIPLLRKTLKDESSVTCKLACTAVRNCVMSLCSSSYSELGLQLIIDVLTLRNSSYWLVRTELLETLAEIDFRLVSFLEAKAENLHRGAHHYTGLLKLQERVLNNVVIHLLGDEDPRVRHVAAASLIRLVPKLFYKCDQGQADPVVAVARDQSSVYLKLLMHETQPPSHFSVSTITRIYRGYNLLPSITDVTMENNLSRVIAAVSHELITSTTRALTFGCCEALCLLSTAFPVCIWSLGWHCGVPPLSASDESRKSCTVGMATMILTLLSSAWFPLDLSAHQDALILAGNLLAASAPKSLRSSWASEEEANPAATKQEEVWPALGDRALVPMVEQLFSHLLKVINICAHVLDDVAPGPAIKAALPSLTNPPSLSPIRRKGKEKEPGEQASVPLSPKKGSEASAASRQSDTSGPVTTSKSSSLGSFYHLPSYLKLHDVLKATHANYKVTLDLQNSTEKFGGFLRSALDVLSQILELATLQDIGKCVEEILGYLKSCFSREPMMATVCVQQLLKTLFGTNLASQFDGLSSNPSKSQGRAQRLGSSSVRPGLYHYCFMAPYTHFTQALADASLRNMVQAEQENDTSGWFDVLQKVSTQLKTNLTSVTKNRADKNAIHNHIRLFEPLVIKALKQYTTTTCVQLQKQVLDLLAQLVQLRVNYCLLDSDQVFIGFVLKQFEYIEVGQFRESEAIIPNIFFFLVLLSYERYHSKQIIGIPKIIQLCDGIMASGRKAVTHAIPALQPIVHDLFVLRGTNKADAGKELETQKEVVVSMLLRLIQYHQVLEMFILVLQQCHKENEDKWKRLSRQIADIILPMLAKQQMHIDSHEALGVLNTLFEILAPSSLRPVDMLLRSMFVTPNTMASVSTVQLWISGILAILRVLISQSTEDIVLSRIQELSFSPYLISCTVINRLRDGDSTSTLEEHSEGKQIKNLPEETFSRFLLQLVGILLEDIVTKQLKVEMSEQQHTFYCQELGTLLMCLIHIFKSGMFRRITAAATRLFRSDGCGGSFYTLDSLNLRARSMITTHPALVLLWCQILLLVNHTDYRWWAEVQQTPKRHSLSSTKLLSPQMSGEEEDSDLAAKLGMCNREIVRRGALILFCDYVCQNLHDSEHLTWLIVNHIQDLISLSHEPPVQDFISAVHRNSAASGLFIQAIQSRCENLSTPTMLKKTLQCLEGIHLSQSGAVLTLYVDRLLCTPFRVLARMVDILACRRVEMLLAANLQSSMAQLPMEELNRIQEYLQSSGLAQRHQRLYSLLDRFRLSTMQDSLSPSPPVSSHPLDGDGHVSLETVSPDKDWYVHLVKSQCWTRSDSALLEGAELVNRIPAEDMNAFMMNSEFNLSLLAPCLSLGMSEISGGQKSALFEAAREVTLARVSGTVQQLPAVHHVFQPELPAEPAAYWSKLNDLFGDAALYQSLPTLARALAQYLVVVSKLPSHLHLPPEKEKDIVKFVVATLEALSWHLIHEQIPLSLDLQAGLDCCCLALQLPGLWSVVSSTEFVTHACSLIYCVHFILEAVAVQPGEQLLSPERRTNTPKAISEEEEEVDPNTQNPKYITAACEMVAEMVESLQSVLALGHKRNSGVPAFLTPLLRNIIISLARLPLVNSYTRVPPLVWKLGWSPKPGGDFGTAFPEIPVEFLQEKEVFKEFIYRINTLGWTSRTQFEETWATLLGVLVTQPLVMEQEESPPEEDTERTQINVLAVQAITSLVLSAMTVPVAGNPAVSCLEQQPRNKPLKALDTRFGRKLSIIRGIVEQEIQAMVSKRENIATHHLYQAWDPVPSLSPATTGALISHEKLLLQINPERELGSMSYKLGQVSIHSVWLGNSITPLREEEWDEEEEEEADAPAPSSPPTSPVNSRKHRAGVDIHSCSQFLLELYSRWILPSSSARRTPAILISEVVRSLLVVSDLFTERNQFELMYVTLTELRRVHPSEDEILAQYLVPATCKAAAVLGMDKAVAEPVSRLLESTLRSSHLPSRVGALHGVLYVLECDLLDDTAKQLIPVISDYLLSNLKGIAHCVNIHSQQHVLVMCATAFYLIENYPLDVGPEFSASIIQMCGVMLSGSEESTPSIIYHCALRGLERLLLSEQLSRLDAESLVKLSVDRVNVHSPHRAMAALGLMLTCMYTGKEKVSPGRTSDPNPAAPDSESVIVAMERVSVLFDRIRKGFPCEARVVARILPQFLDDFFPPQDIMNKVIGEFLSNQQPYPQFMATVVYKVFQTLHSTGQSSMVRDWVMLSLSNFTQRAPVAMATWSLSCFFVSASTSPWVAAILPHVISRMGKLEQVDVNLFCLVATDFYRHQIEEELDRRAFQSVLEVVAAPGSPYHRLLTCLRNVHKVTTC

689 NP_078799.2 NP_057285.3

>NP_078799.2

MEKFGMNFGGGPSKKDLLETIETQKKQLLQYQARLKDVVRAYKSLLKEKEALEASIKVLSVSHEADVGLAGVQLPGLTFPDSVDDRCSTHSEDSTGTATSLDTAASLTSTKGEFGVEDDRPARGPPPPKSEEASWSESGVSSSSGDGPFAGGEVDKRLHQLKTQLATLTSSLATVTQEKSRMEASYLADKKKMKQDLEDASNKAEEERARLEGELKGLQEQIAETKARLITQQHDRAQEQSDHALMLRELQKLLQEERTQRQDLELRLEETREALAGRAYAAEQMEGFELQTKQLTREVEELKSELQAIRDEKNQPDPRLQELQEEAARLKSHFQAQLQQEMRKTALAEDQLRQQSQVEEQRVAALENQISEVSELLGTYEKAKQKDQLAIQKLKERILQLDLENKTLALAASSRSPLDSHGEESSLDVNVLKDKMEKLKRLLQVAARKSQVTLDVEKLCDLEIMPSSEAADGEKATALYYQQELKQLKEEFERYKMRAQVVLKSKNTKDGNLGKELEAAQEQLAELKEKYISLRLSCEELEHQHQQEADDWKQELARLQQLHRQELERCQLDFRDRTLKLEEELHKQRDRALAVLTEKDLELEQLRSVALASGLPGRRSPVGGGGPGDPADTSSSDSLTQALQLAAANEPTFFLYAEQLARKEVEITSLRKQKHRLEVEVHQLQDRLLEEGERHREEVAALQSHIEKNIRDQSREGANLEYLKNIIYRFLTLPDSLGRQQTLTAILTILHFSPEEKQVIMRLPTSASWWPSGKR

>NP_057285.3

MRTLEDSSGTVLHRLIQEQLRYGNLTETRTLLAIQQQALRGGAGTGGTGSPQASLEILAPEDSQVLQQATRQEPQGQEHQGGENHLAENTLYRLCPQPSKGEELPTYEEAKAHSQYYAAQQAGTRPHAGDRDPRGAPGGSRRQDEALRELRHGHVRSLSERLLQLSLERNGARAPSHMSSSHSFPQLARNQQGPPLRGPPAEGPESRGPPPQYPHVVLAHETTTAVTDPRYRARGSPHFQHAEVRILQAQVPPVFLQQQQQYQYLQQSQEHPPPPHPAALGHGPLSSLSPPAVEGPVSAQASSATSGSAHLAQMEAVLRENARLQRDNERLQRELESSAEKAGRIEKLESEIQRLSEAHESLTRASSKREALEKTMRNKMDSEMRRLQDFNRDLRERLESANRRLASKTQEAQAGSQDMVAKLLAQSYEQQQEQEKLEREMALLRGAIEDQRRRAELLEQALGNAQGRAARAEEELRKKQAYVEKVERLQQALGQLQAACEKREQLELRLRTRLEQELKALRAQQRQAGAPGGSSGSGGSPELSALRLSEQLREKEEQILALEADMTKWEQKYLEERAMRQFAMDAAATAAAQRDTTLIRHSPQPSPSSSFNEGLLTGGHRHQEMESRLKVLHAQILEKDAVIKVLQQRSRRDPGKAIQGSLRPAKSVPSVFAAAAAGTQGWQGLSSSERQTADAPARLTTADRAPTEEPVVTAPPAAHAKHGSRDGSTQTEGPPDSTSTCLPPEPDSLLGCSSSQRAASLDSVATSRVQDLSDMVEILI

690 NP_000130.1 NP_002077.1

>NP_000130.1

MDTESNRRANLALPQEPSSVPAFEVLEISPQEVSSGRLLKSASSPPLHTWLTVLKKEQEFLGVTQILTAMICLCFGTVVCSVLDISHIEGDIFSSFKAGYPFWGAIFFSISGMLSIISERRNATYLVRGSLGANTASSIAGGTGITILIINLKKSLAYIHIHSCQKFFETKCFMASFSTEIVVMMLFLTILGLGSAVSLTICGAGEELKGNKVPEDRVYEELNIYSATYSELEDPGEMSPPIDL

>NP_002077.1

MEAIAKYDFKATADDELSFKRGDILKVLNEECDQNWYKAELNGKDGFIPKNYIEMKPHPWFFGKIPRAKAEEMLSKQRHDGAFLIRESESAPGDFSLSVKFGNDVQHFKVLRDGAGKYFLWVVKFNSLNELVDYHRSTSVSRNQQIFLRDIEQVPQQPTYVQALFDFDPQEDGELGFRRGDFIHVMDNSDPNWWKGACHGQTGMFPRNYVTPVNRNV

691 NP_003346.2 NP_003397.1

>NP_003346.2

MVGFKATDVPPTATVKFLGAGTAACIADLITFPLDTAKVRLQIQGESQGPVRATASAQYRGVMGTILTMVRTEGPRSLYNGLVAGLQRQMSFASVRIGLYDSVKQFYTKGSEHASIGSRLLAGSTTGALAVAVAQPTDVVKVRFQAQARAGGGRRYQSTVNAYKTIAREEGFRGLWKGTSPNVARNAIVNCAELVTYDLIKDALLKANLMTDDLPCHFTSAFGAGFCTTVIASPVDVVKTRYMNSALGQYSSAGHCALTMLQKEGPRAFYKGFMPSFLRLGSWNVVMFVTYEQLKRALMAACTSREAPF

>NP_003397.1

MDKNELVQKAKLAEQAERYDDMAACMKSVTEQGAELSNEERNLLSVAYKNVVGARRSSWRVVSSIEQKTEGAEKKQQMAREYREKIETELRDICNDVLSLLEKFLIPNASQAESKVFYLKMKGDYYRYLAEVAAGDDKKGIVDQSQQAYQEAFEISKKEMQPTHPIRLGLALNFSVFYYEILNSPEKACSLAKTAFDEAIAELDTLSEESYKDSTLIMQLLRDNLTLWTSDTQGDEAEAGEGGEN

692 NP_003397.1 NP_005535.1

>NP_003397.1

MDKNELVQKAKLAEQAERYDDMAACMKSVTEQGAELSNEERNLLSVAYKNVVGARRSSWRVVSSIEQKTEGAEKKQQMAREYREKIETELRDICNDVLSLLEKFLIPNASQAESKVFYLKMKGDYYRYLAEVAAGDDKKGIVDQSQQAYQEAFEISKKEMQPTHPIRLGLALNFSVFYYEILNSPEKACSLAKTAFDEAIAELDTLSEESYKDSTLIMQLLRDNLTLWTSDTQGDEAEAGEGGEN

>NP_005535.1

MASPPESDGFSDVRKVGYLRKPKSMHKRFFVLRAASEAGGPARLEYYENEKKWRHKSSAPKRSIPLESCFNINKRADSKNKHLVALYTRDEHFAIAADSEAEQDSWYQALLQLHNRAKGHHDGAAALGAGGGGGSCSGSSGLGEAGEDLSYGDVPPGPAFKEVWQVILKPKGLGQTKNLIGIYRLCLTSKTISFVKLNSEAAAVVLQLMNIRRCGHSENFFFIEVGRSAVTGPGEFWMQVDDSVVAQNMHETILEAMRAMSDEFRPRSKSQSSSNCSNPISVPLRRHHLNNPPPSQVGLTRRSRTESITATSPASMVGGKPGSFRVRASSDGEGTMSRPASVDGSPVSPSTNRTHAHRHRGSARLHPPLNHSRSIPMPASRCSPSATSPVSLSSSSTSGHGSTSDCLFPRRSSASVSGSPSDGGFISSDEYGSSPCDFRSSFRSVTPDSLGHTPPARGEEELSNYICMGGKGPSTLTAPNGHYILSRGGNGHRCTPGTGLGTSPALAGDEAASAADLDNRFRKRTHSAGTSPTITHQKTPSQSSVASIEEYTEMMPAYPPGGGSGGRLPGHRHSAFVPTRSYPEEGLEMHPLERRGGHHRPDSSTLHTDDGYMPMSPGVAPVPSGRKGSGDYMPMSPKSVSAPQQIINPIRRHPQRVDPNGYMMMSPSGGCSPDIGGGPSSSSSSSNAVPSGTSYGKLWTNGVGGHHSHVLPHPKPPVESSGGKLLPCTGDYMNMSPVGDSNTSSPSDCYYGPEDPQHKPVLSYYSLPRSFKHTQRPGEPEEGARHQHLRLSTSSGRLLYAATADDSSSSTSSDSLGGGYCGARLEPSLPHPHHQVLQPHLPRKVDTAAQTNSRLARPTRLSLGDPKASTLPRAREQQQQQQPLLHPPEPKSPGEYVNIEFGSDQSGYLSGPVAFHSSPSVRCPSQLQPAPREEETGTEEYMKMDLGPGRRAAWQESTGVEMGRLGPAPPGAASICRPTRAVPSSRGDYMTMQMSCPRQSYVDTSPAAPVSYADMRTGIAAEEVSLPRATMAAASSSSAASASPTGPQGAAELAAHSSLLGGPQGPGGMSAFTRVNLSPNRNQSAKVIRADPQGCRRRHSSETFSSTPSATRVGNTVPFGAGAAVGGGGGSSSSSEDVKRHSSASFENVWLRPGELGGAPKEPAKLCGAAGGLENGLNYIDLDLVKDFKQCPQECTPEPQPPPPPPPHQPLGSGESSSTRRSSEDLSAYASISFQKQPEDRQ

693 NP_065145.2 NP_150628.2

>NP_065145.2

MSNMEKHLFNLKFAAKELSRSAKKCDKEEKAEKAKIKKAIQKGNMEVARIHAENAIRQKNQAVNFLRMSARVDAVAARVQTAVTMGKVTKSMAGVVKSMDATLKTMNLEKISALMDKFEHQFETLDVQTQQMEDTMSSTTTLTTPQNQVDMLLQEMADEAGLDLNMELPQGQTGSVGTSVASAEQDELSQRLARLRDQV

>NP_150628.2

MAAPEAPPLDRVFRTTWLSTECDSHPLPPSYRKFLFETQEADLAGGTTVAAGNLLNESEKDCGQDRRAPGVQPCLLVTMTSVVKTVYSLQPSSALSGGQPADTQTRATSKSLLPVRSKEVDVSKQLHSGGPENDVTKITKLRRENGQMKATDTATRRNVRKGYKPLSKQKSEEELKDKNQLLEAVNKQLHQKLTETQGELKDLTQKVELLEKFRDNCLAILESKGLDPALGGETLASRQESTTDHMDSMLLLETLQEELKLFNETAKKQMEELQALKVKLEMKEERVRFLEQQTLCNNQVNDLTTALKEMEQLLEM

694 NP_733832.1 NP_060644.4

>NP_733832.1

MSGEPELIELRELAPAGRAGKGRTRLERANALRIARGTACNPTRQLVPGRGHRFQPAGPATHTWCDLCGDFIWGVVRKGLQCARLSADCKFTCHYRCRALVCLDCCGPRDLGWEPAVERDTNVDEPVEWETPDLSQAEIEQKIKEYNAQINSNLFMSLNKDGSYTGFIKVQLKLVRPVSVPSSKKPPSLQDARRGPGRGTSVRRRTSFYLPKDAVKHLHVLSRTRAREVIEALLRKFLVVDDPRKFALFERAERHGQVYLRKLLDDEQPLRLRLLAGPSDKALSFVLKENDSGEVNWDAFSMPELHNFLRILQREEEEHLRQILQKYSYCRQKIQEALHACPLG

>NP_060644.4

MAAVAGSGAAAAPSSLLLVVGSEFGSPGLLTYVLEELERGIRSWDVDPGVCNLDEQLKVFVSRHSATFSSIVKGQRSLHHRGDNLETLVLLNPSDKSLYDELRNLLLDPASHKLLVLAGPCLEETGELLLQTGGFSPHHFLQVLKDREIRDILATTPPPVQPPILTITCPTFGDWAQLAPAVPGLQGALRLQLRLNPPAQLPNSEGLCEFLEYVAESLEPPSPFELLEPPTSGGFLRLGRPCCYIFPGGLGDAAFFAVNGFTVLVNGGSNPKSSFWKLVRHLDRVDAVLVTHPGADSLPGLNSLLRRKLAERSEVAAGGGSWDDRLRRLISPNLGVVFFNACEAASRLARGEDEAELALSLLAQLGITPLPLSRGPVPAKPTVLFEKMGVGRLDMYVLHPPSAGAERTLASVCALLVWHPAGPGEKVVRVLFPGCTPPACLLDGLVRLQHLRFLREPVVTPQDLEGPGRAESKESVGSRDSSKREGLLATHPRPGQERPGVARKEPARAEAPRKTEKEAKTPRELKKDPKPSVSRTQPREVRRAASSVPNLKKTNAQAAPKPRKAPSTSHSGFPPVANGPRSPPSLRCGEASPPSAACGSPASQLVATPSLELGPIPAGEEKALELPLAASSIPRPRTPSPESHRSPAEGSERLSLSPLRGGEAGPDASPTVTTPTVTTPSLPAEVGSPHSTEVDESLSVSFEQVLPPSAPTSEAGLSLPLRGPRARRSASPHDVDLCLVSPCEFEHRKAVPMAPAPASPGSSNDSSARSQERAGGLGAEETPPTSVSESLPTLSDSDPVPLAPGAADSDEDTEGFGVPRHDPLPDPLKVPPPLPDPSSICMVDPEMLPPKTARQTENVSRTRKPLARPNSRAAAPKATPVAAAKTKGLAGGDRASRPLSARSEPSEKGGRAPLSRKSSTPKTATRGPSGSASSRPGVSATPPKSPVYLDLAYLPSGSSAHLVDEEFFQRVRALCYVISGQDQRKEEGMRAVLDALLASKQHWDRDLQVTLIPTFDSVAMHTWYAETHARHQALGITVLGSNSMVSMQDDAFPACKVEF

695 NP_006368.3 NP_003577.2

>NP_006368.3

MSLLCVRVKRAKFQGSPDKFNTYVTLKVQNVKSTTVAVRGDQPSWEQDFMFEISRLDLGLSVEVWNKGLIWDTMVGTVWIALKTIRQSDEEGPGEWSTLEAETLMKDDEICGTRNPTPHKILLDTRFELPFDIPEEEARYWTYKWEQINALGADNEYSSQEESQRKPLPTAAAQCSFEDPDSAVDDRDSDYRSETSNSFPPPYHTASQPNASVHQFPVPVRSPQQLLLQGSSRDSCNDSMQSYDLDYPERRAISPTSSSRYGSSCNVSQGSSQLSELDQYHEQDDDHRETDSIHSCHSSHSLSRDGQAGFGEQEKPLEVTGQAEKEAACEPKEMKEDATTHPPPDLVLQKDHFLGPQESFPEENASSPFTQARAHWIRAVTKVRLQLQEIPDDGDPSLPQWLPEGPAGGLYGIDSMPDLRRKKPLPLVSDLSLVQSRKAGITSAMATRTSLKDEELKSHVYKKTLQALIYPISCTTPHNFEVWTATTPTYCYECEGLLWGIARQGMRCSECGVKCHEKCQDLLNADCLQRAAEKSCKHGAEDRTQNIIMAMKDRMKIRERNKPEIFEVIRDVFTVNKAAHVQQMKTVKQSVLDGTSKWSAKITITVVCAQGLQAKDKTGSSDPYVTVQVSKTKKRTKTIFGNLNPVWEEKFHFECHNSSDRIKVRVWDEDDDIKSRVKQRLKRESDDFLGQTIIEVRTLSGEMDVWYNLEKRTDKSAVSGAIRLQISVEIKGEEKVAPYHVQYTCLHENLFHYLTDIQGSGGVRIPEARGDDAWKVYFDETAQEIVDEFAMRYGIESIYQAMTHFACLSSKYMCPGVPAVMSTLLANINAYYAHTTASTNVSASDRFAASNFGKERFVKLLDQLHNSLRIDLSTYRNNFPAGSPERLQDLKSTVDLLTSITFFRMKVQELQSPPRASQVVKDCVKACLNSTYEYIFNNCHDLYSRQYQLKQELPPEEQGPSIRNLDFWPKLITLIVSIIEEDKNSYTPVLNQFPQELNVGKVSAEVMWHLFAQDMKYALEEHEKDHLCKSADYMNLHFKVKWLHNEYVRDLPVLQGQVPEYPAWFEQFVLQWLDENEDVSLEFLRGALERDKKDGFQQTSEHALFSCSVVDVFTQLNQSFEIIRKLECPDPSILAHYMRRFAKTIGKVLMQYADILSKDFPAYCTKEKLPCILMNNVQQLRVQLEKMFEAMGGKELDLEAADSLKELQVKLNTVLDELSMVFGNSFQVRIDECVRQMADILGQVRGTGNASPDARASAAQDADSVLRPLMDFLDGNLTLFATVCEKTVLKRVLKELWRVVMNTMERMIVLPPLTDQTGTQLIFTAAKELSHLSKLKDHMVREETRNLTPKQCAVLDLALDTIKQYFHAGGNGLKKTFLEKSPDLQSLRYALSLYTQTTDTLIKTFVRSQTTQGSGVDDPVGEVSIQVDLFTHPGTGEHKVTVKVVAANDLKWQTAGMFRPFVEVTMVGPHQSDKKRKFTTKSKSNNWAPKYNETFHFLLGNEEGPESYELQICVKDYCFAREDRVLGLAVMPLRDVTAKGSCACWCPLGRKIHMDETGLTILRILSQRSNDEVAREFVKLKSESRSTEEGS

>NP_003577.2

MRGRRGDRMTINIQEHMAINVCPGPIRPIRQISDYFPRGPGPEGGGGGGGEAPAHLVPLALAPPAALLGATTPEDGAEVDSYDSDDATALGTLEFDLLYDRASCTLHCSILRAKGLKPMDFNGLADPYVKLHLLPGACKANKLKTKTQRNTLNPVWNEDLTYSGITDDDITHKVLRIAVCDEDKLSHNEFIGEIRVPLRRLKPSQKKHFNICLERQVPLASPSSMSAALRGISCYLKELEQAEQGQGLLEERGRILLSLSYSSRRRGLLVGILRCAHLAAMDVNGYSDPYVKTYLRPDVDKKSKHKTCVKKKTLNPEFNEEFFYEIELSTLATKTLEVTVWDYDIGKSNDFIGGVSLGPGARGEARKHWSDCLQQPDAALERWHTLTSELPPAAGALSSA

696 NP_005347.3 NP_852664.1

>NP_005347.3

MGCGCSSHPEDDWMENIDVCENCHYPIVPLDGKGTLLIRNGSEVRDPLVTYEGSNPPASPLQDNLVIALHSYEPSHDGDLGFEKGEQLRILEQSGEWWKAQSLTTGQEGFIPFNFVAKANSLEPEPWFFKNLSRKDAERQLLAPGNTHGSFLIRESESTAGSFSLSVRDFDQNQGEVVKHYKIRNLDNGGFYISPRITFPGLHELVRHYTNASDGLCTRLSRPCQTQKPQKPWWEDEWEVPRETLKLVERLGAGQFGEVWMGYYNGHTKVAVKSLKQGSMSPDAFLAEANLMKQLQHQRLVRLYAVVTQEPIYIITEYMENGSLVDFLKTPSGIKLTINKLLDMAAQIAEGMAFIEERNYIHRDLRAANILVSDTLSCKIADFGLARLIEDNEYTAREGAKFPIKWTAPEAINYGTFTIKSDVWSFGILLTEIVTHGRIPYPGMTNPEVIQNLERGYRMVRPDNCPEELYQLMRLCWKERPEDRPTFDYLRSVLEDFFTATEGQYQPQP

>NP_852664.1

MSAEGYQYRALYDYKKEREEDIDLHLGDILTVNKGSLVALGFSDGQEARPEEIGWLNGYNETTGERGDFPGTYVEYIGRKKISPPTPKPRPPRPLPVAPGSSKTEADVEQQALTLPDLAEQFAPPDIAPPLLIKLVEAIEKKGLECSTLYRTQSSSNLAELRQLLDCDTPSVDLEMIDVHVLADAFKRYLLDLPNPVIPAAVYSEMISLAPEVQSSEEYIQLLKKLIRSPSIPHQYWLTLQYLLKHFFKLSQTSSKNLLNARVLSEIFSPMLFRFSAASSDNTENLIKVIEILISTEWNERQPAPALPPKPPKPTTVANNGMNNNMSLQDAEWYWGDISREEVNEKLRDTADGTFLVRDASTKMHGDYTLTLRKGGNNKLIKIFHRDGKYGFSDPLTFSSVVELINHYRNESLAQYNPKLDVKLLYPVSKYQQDQVVKEDNIEAVGKKLHEYNTQFQEKSREYDRLYEEYTRTSQEIQMKRTAIEAFNETIKIFEEQCQTQERYSKEYIEKFKREGNEKEIQRIMHNYDKLKSRISEIIDSRRRLEEDLKKQAAEYREIDKRMNSIKPDLIQLRKTRDQYLMWLTQKGVRQKKLNEWLGNENTEDQYSLVEDDEDLPHHDEKTWNVGSSNRNKAENLLRGKRDGTFLVRESSKQGCYACSVVVDGEVKHCVINKTATGYGFAEPYNLYSSLKELVLHYQHTSLVQHNDSLNVTLAYPVYAQQRR

697 NP_004508.1 NP_066932.1

>NP_004508.1

MDDIFTQCREGNAVAVRLWLDNTENDLNQGDDHGFSPLHWACREGRSAVVEMLIMRGARINVMNRGDDTPLHLAASHGHRDIVQKLLQYKADINAVNEHGNVPLHYACFWGQDQVAEDLVANGALVSICNKYGEMPVDKAKAPLRELLRERAEKMGQNLNRIPYKDTFWKGTTRTRPRNGTLNKHSGIDFKQLNFLTKLNENHSGELWKGRWQGNDIVVKVLKVRDWSTRKSRDFNEECPRLRIFSHPNVLPVLGACQSPPAPHPTLITHWMPYGSLYNVLHEGTNFVVDQSQAVKFALDMARGMAFLHTLEPLIPRHALNSRSVMIDEDMTARISMADVKFSFQCPGRMYAPAWVAPEALQKKPEDTNRRSADMWSFAVLLWELVTREVPFADLSNMEIGMKVALEGLRPTIPPGISPHVCKLMKICMNEDPAKRPKFDMIVPILEKMQDK

>NP_066932.1

MSDKPDMAEIEKFDKSKLKKTETQEKNPLPSKETIEQEKQAGES

698 NP_002196.2 NP_075261.1

>NP_002196.2

MGSRTPESPLHAVQLRWGPRRRPPLLPLLLLLLPPPPRVGGFNLDAEAPAVLSGPPGSFFGFSVEFYRPGTDGVSVLVGAPKANTSQPGVLQGGAVYLCPWGASPTQCTPIEFDSKGSRLLESSLSSSEGEEPVEYKSLQWFGATVRAHGSSILACAPLYSWRTEKEPLSDPVGTCYLSTDNFTRILEYAPCRSDFSWAAGQGYCQGGFSAEFTKTGRVVLGGPGSYFWQGQILSATQEQIAESYYPEYLINLVQGQLQTRQASSIYDDSYLGYSVAVGEFSGDDTEDFVAGVPKGNLTYGYVTILNGSDIRSLYNFSGEQMASYFGYAVAATDVNGDGLDDLLVGAPLLMDRTPDGRPQEVGRVYVYLQHPAGIEPTPTLTLTGHDEFGRFGSSLTPLGDLDQDGYNDVAIGAPFGGETQQGVVFVFPGGPGGLGSKPSQVLQPLWAASHTPDFFGSALRGGRDLDGNGYPDLIVGSFGVDKAVVYRGRPIVSASASLTIFPAMFNPEERSCSLEGNPVACINLSFCLNASGKHVADSIGFTVELQLDWQKQKGGVRRALFLASRQATLTQTLLIQNGAREDCREMKIYLRNESEFRDKLSPIHIALNFSLDPQAPVDSHGLRPALHYQSKSRIEDKAQILLDCGEDNICVPDLQLEVFGEQNHVYLGDKNALNLTFHAQNVGEGGAYEAELRVTAPPEAEYSGLVRHPGNFSSLSCDYFAVNQSRLLVCDLGNPMKAGASLWGGLRFTVPHLRDTKKTIQFDFQILSKNLNNSQSDVVSFRLSVEAQAQVTLNGVSKPEAVLFPVSDWHPRDQPQKEEDLGPAVHHVYELINQGPSSISQGVLELSCPQALEGQQLLYVTRVTGLNCTTNHPINPKGLELDPEGSLHHQQKREAPSRSSASSGPQILKCPEAECFRLRCELGPLHQQESQSLQLHFRVWAKTFLQREHQPFSLQCEAVYKALKMPYRILPRQLPQKERQVATAVQWTKAEGSYGVPLWIIILAILFGLLLLGLLIYILYKLGFFKRSLPYGTAMEKAQLKPPATSDA

>NP_075261.1

MVSWGRFICLVVVTMATLSLARPSFSLVEDTTLEPEEPPTKYQISQPEVYVAAPGESLEVRCLLKDAAVISWTKDGVHLGPNNRTVLIGEYLQIKGATPRDSGLYACTASRTVDSETWYFMVNVTDAISSGDDEDDTDGAEDFVSENSNNKRAPYWTNTEKMEKRLHAVPAANTVKFRCPAGGNPMPTMRWLKNGKEFKQEHRIGGYKVRNQHWSLIMESVVPSDKGNYTCVVENEYGSINHTYHLDVVERSPHRPILQAGLPANASTVVGGDVEFVCKVYSDAQPHIQWIKHVEKNGSKYGPDGLPYLKVLKVLKAAGVNTTDKEIEVLYIRNVTFEDAGEYTCLAGNSIGISFHSAWLTVLPAPGREKEITASPDYLEIAIYCIGVFLIACMVVTVILCRMKNTTKKPDFSSQPAVHKLTKRIPLRRQVSAESSSSMNSNTPLVRITTRLSSTADTPMLAGVSEYELPEDPKWEFPRDKLTLGKPLGEGCFGQVVMAEAVGIDKDKPKEAVTVAVKMLKDDATEKDLSDLVSEMEMMKMIGKHKNIINLLGACTQDGPLYVIVEYASKGNLREYLRARRPPGMEYSYDINRVPEEQMTFKDLVSCTYQLARGMEYLASQKCIHRDLAARNVLVTENNVMKIADFGLARDINNIDYYKKTTNGRLPVKWMAPEALFDRVYTHQSDVWSFGVLMWEIFTLGGSPYPGIPVEELFKLLKEGHRMDKPANCTNELYMMMRDCWHAVPSQRPTFKQLVEDLDRILTLTTNEEYLDLSQPLEQYSPSYPDTRSSCSSGDDSVFSPDPMPYEPCLPQYPHINGSVKT

699 NP_006793.1 NP_006860.1

>NP_006793.1

METILEQQRRYHEEKERLMDVMAKEMLTKKSTLRDQINSDHRTRAMQDRYMEVSGNLRDLYDDKDGLRKEELNAISGPNEFAEFYNRLKQIKEFHRKHPNEICVPMSVEFEELLKARENPSEEAQNLVEFTDEEGYGRYLDLHDCYLKYINLKASEKLDYITYLSIFDQLFDIPKERKNAEYKRYLEMLLEYLQDYTDRVKPLQDQNELFGKIQAEFEKKWENGTFPGWPKETSSALTHAGAHLDLSAFSSWEELASLGLDRLKSALLALGLKCGGTLEERAQRLFSTKGKSLESLDTSLFAKNPKSKGTKRDTERNKDIAFLEAQIYEYVEILGEQRHLTHENVQRKQARTGEEREEEEEEQISESESEDEENEIIYNPKNLPLGWDGKPIPYWLYKLHGLNINYNCEICGNYTYRGPKAFQRHFAEWRHAHGMRCLGIPNTAHFANVTQIEDAVSLWAKLKLQKASERWQPDTEEEYEDSSGNVVNKKTYEDLKRQGLL

>NP_006860.1

MAKERRRAVLELLQRPGNARCADCGAPDPDWASYTLGVFICLSCSGIHRNIPQVSKVKSVRLDAWEEAQVEFMASHGNDAARARFESKVPSFYYRPTPSDCQLLREQWIRAKYERQEFIYPEKQEPYSAGYREGFLWKRGRDNGQFLSRKFVLTEREGALKYFNRNDAKEPKAVMKIEHLNATFQPAKIGHPHGLQVTYLKDNSTRNIFIYHEDGKEIVDWFNALRAARFHYLQVAFPGASDADLVPKLSRNYLKEGYMEKTGPKQTEGFRKRWFTMDDRRLMYFKDPLDAFARGEVFIGSKESGYTVLHGFPPSTQGHHWPHGITIVTPDRKFLFACETESDQREWVAAFQKAVDRPMLPQEYAVEAHFKHKP

700 NP_612482.2 NP_002688.2

>NP_612482.2

MSDQDHSMDEMTAVVKIEKGVGGNNGGNGNGGGAFSQARSSSTGSSSSTGGGGQESQPSPLALLAATCSRIESPNENSNNSQGPSQSGGTGELDLTATQLSQGANGWQIISSSSGATPTSKEQSGSSTNGSNGSESSKNRTVSGGQYVVAAAPNLQNQQVLTGLPGVMPNIQYQVIPQFQTVDGQQLQFAATGAQVQQDGSGQIQIIPGANQQIITNRGSGGNIIAAMPNLLQQAVPLQGLANNVLSGQTQYVTNVPVALNGNITLLPVNSVSAATLTPSSQAVTISSSGSQESGSQPVTSGTTISSASLVSSQASSSSFFTNANSYSTTTTTSNMGIMNFTTSGSSGTNSQGQTPQRVSGLQGSDALNIQQNQTSGGSLQAGQQKEGEQNQQTQQQQILIQPQLVQGGQALQALQAAPLSGQTFTTQAISQETLQNLQLQAVPNSGPIIIRTPTVGPNGQVSWQTLQLQNLQVQNPQAQTITLAPMQGVSLGQTSSSNTTLTPIASAASIPAGTVTVNAAQLSSMPGLQTINLSALGTSGIQVHPIQGLPLAIANAPGDHGAQLGLHGAGGDGIHDDTAGGEEGENSPDAQPQAGRRTRREACTCPYCKDSEGRGSGDPGKKKQHICHIQGCGKVYGKTSHLRAHLRWHTGERPFMCTWSYCGKRFTRSDELQRHKRTHTGEKKFACPECPKRFMRSDHLSKHIKTHQNKKGGPGVALSVGTLPLDSGAGSEGSGTATPSALITTNMVAMEAICPEGIARLANSGINVMQVADLQSINISGNGF

>NP_002688.2

MNNPSETSKPSMESGDGNTGTQTNGLDFQKQPVPVGGAISTAQAQAFLGHLHQVQLAGTSLQAAAQSLNVQSKSNEESGDSQQPSQPSQQPSVQAAIPQTQLMLAGGQITGLTLTPAQQQLLLQQAQAQAQLLAAAVQQHSASQQHSAAGATISASAATPMTQIPLSQPIQIAQDLQQLQQLQQQNLNLQQFVLVHPTTNLQPAQFIISQTPQGQQGLLQAQNLLTQLPQQSQANLLQSQPSITLTSQPATPTRTIAATPIQTLPQSQSTPKRIDTPSLEEPSDLEELEQFAKTFKQRRIKLGFTQGDVGLAMGKLYGNDFSQTTISRFEALNLSFKNMCKLKPLLEKWLNDAENLSSDSSLSSPSALNSPGIEGLSRRRKKRTSIETNIRVALEKSFLENQKPTSEEITMIADQLNMEKEVIRVWFCNRRQKEKRINPPSSGGTSSSPIKAIFPSPTSLVATTPSLVTSSAATTLTVSPVLPLTSAAVTNLSVTGTSDTTSNNTATVISTAPPASSAVTSPSLSPSPSASASTSEASSASETSTTQTTSTPLSSPLGTSQVMVTASGLQTAAAAALQGAAQLPANASLAAMAAAAGLNPSLMAPSQFAAGGALLSLNPGTLSGALSPALMSNSTLATIQALASGGSLPITSLDATGNLVFANAGGAPNIVTAPLFLNPQNLSLLTSNPVSLVSAAAASAGNSAPVASLHATSTSAESIQNSLFTVASASGAASTTTTASKAQ

701 NP_004318.3 NP_003396.1

>NP_004318.3

MVDPVGFAEAWKAQFPDSEPPRMELRSVGDIEQELERCKASIRRLEQEVNQERFRMIYLQTLLAKEKKSYDRQRWGFRRAAQAPDGASEPRASASRPQPAPADGADPPPAEEPEARPDGEGSPGKARPGTARRPGAAASGERDDRGPPASVAALRSNFERIRKGHGQPGADAEKPFYVNVEFHHERGLVKVNDKEVSDRISSLGSQAMQMERKKSQHGAGSSVGDASRPPYRGRSSESSCGVDGDYEDAELNPRFLKDNLIDANGGSRPPWPPLEYQPYQSIYVGGMMEGEGKGPLLRSQSTSEQEKRLTWPRRSYSPRSFEDCGGGYTPDCSSNENLTSSEEDFSSGQSSRVSPSPTTYRMFRDKSRSPSQNSQQSFDSSSPPTPQCHKRHRHCPVVVSEATIVGVRKTGQIWPNDGEGAFHGDADGSFGTPPGYGCAADRAEEQRRHQDGLPYIDDSPSSSPHLSSKGRGSRDALVSGALESTKASELDLEKGLEMRKWVLSGILASEETYLSHLEALLLPMKPLKAAATTSQPVLTSQQIETIFFKVPELYEIHKEFYDGLFPRVQQWSHQQRVGDLFQKLASQLGVYRAFVDNYGVAMEMAEKCCQANAQFAEISENLRARSNKDAKDPTTKNSLETLLYKPVDRVTRSTLVLHDLLKHTPASHPDHPLLQDALRISQNFLSSINEEITPRRQSMTVKKGEHRQLLKDSFMVELVEGARKLRHVFLFTDLLLCTKLKKQSGGKTQQYDCKWYIPLTDLSFQMVDELEAVPNIPLVPDEELDALKIKISQIKNDIQREKRANKGSKATERLKKKLSEQESLLLLMSPSMAFRVHSRNGKSYTFLISSDYERAEWRENIREQQKKCFRSFSLTSVELQMLTNSCVKLQTVHSIPLTINKEDDESPGLYGFLNVIVHSATGFKQSSNLYCTLEVDSFGYFVNKAKTRVYRDTAEPNWNEEFEIELEGSQTLRILCYEKCYNKTKIPKEDGESTDRLMGKGQVQLDPQALQDRDWQRTVIAMNGIEVKLSVKFNSREFSLKRMPSRKQTGVFGVKIAVVTKRERSKVPYIVRQCVEEIERRGMEEVGIYRVSGVATDIQALKAAFDVNNKDVSVMMSEMDVNAIAGTLKLYFRELPEPLFTDEFYPNFAEGIALSDPVAKESCMLNLLLSLPEANLLTFLFLLDHLKRVAEKEAVNKMSLHNLATVFGPTLLRPSEKESKLPANPSQPITMTDSWSLEVMSQVQVLLYFLQLEAIPAPDSKRQSILFSTEV

>NP_003396.1

MGDREQLLQRARLAEQAERYDDMASAMKAVTELNEPLSNEDRNLLSVAYKNVVGARRSSWRVISSIEQKTMADGNEKKLEKVKAYREKIEKELETVCNDVLSLLDKFLIKNCNDFQYESKVFYLKMKGDYYRYLAEVASGEKKNSVVEASEAAYKEAFEISKEQMQPTHPIRLGLALNFSVFYYEIQNAPEQACLLAKQAFDDAIAELDTLNEDSYKDSTLIMQLLRDNLTLWTSDQQDEEAGEGN

702 NP_001020330.1 NP_002113.2

>NP_001020330.1

MHRRRSRSCREDQKPVMDDQRDLISNNEQLPMLGRRPGAPESKCSRGALYTGFSILVTLLLAGQATTAYFLYQQQGRLDKLTVTSQNLQLENLRMKLPKPPKPVSKMRMATPLLMQALPMGALPQGPMQNATKYGNMTEDHVMHLLQNADPLKVYPPLKGSFPENLRHLKNTMETIDWKVFESWMHHWLLFEMSRHSLEQKPTDAPPKVLTKCQEEVSHIPAVHPGSFRPKCDENGNYLPLQCYGSIGYCWCVFPNGTEVPNTRSRGHHNCSESLELEDPSSGLGVTKQDLGPVPM

>NP_002113.2

MILNKALLLGALALTTVMSPCGGEDIVADHVASCGVNLYQFYGPSGQYTHEFDGDEQFYVDLERKETAWRWPEFSKFGGFDPQGALRNMAVAKHNLNIMIKRYNSTAATNEVPEVTVFSKSPVTLGQPNTLICLVDNIFPPVVNITWLSNGQSVTEGVSETSFLSKSDHSFFKISYLTFLPSADEIYDCKVEHWGLDQPLLKHWEPEIPAPMSELTETVVCALGLSVGLMGIVVGTVFIIQGLRSVGASRHQGPL

703 NP_002712.1 NP_001542.1

>NP_002712.1

MAPLDLDKYVEIARLCKYLPENDLKRLCDYVCDLLLEESNVQPVSTPVTVCGDIHGQFYDLCELFRTGGQVPDTNYIFMGDFVDRGYYSLETFTYLLALKAKWPDRITLLRGNHESRQITQVYGFYDECQTKYGNANAWRYCTKVFDMLTVAALIDEQILCVHGGLSPDIKTLDQIRTIERNQEIPHKGAFCDLVWSDPEDVDTWAISPRGAGWLFGAKVTNEFVHINNLKLICRAHQLVHEGYKFMFDEKLVTVWSAPNYCYRCGNIASIMVFKDVNTREPKLFRAVPDSERVIPPRTTTPYFL

>NP_001542.1

MAAEDELQLPRLPELFETGRQLLDEVEVATEPAGSRIVQEKVFKGLDLLEKAAEMLSQLDLFSRNEDLEEIASTDLKYLLVPAFQGALTMKQVNPSKRLDHLQRAREHFINYLTQCHCYHVAEFELPKTMNNSAENHTANSSMAYPSLVAMASQRQAKIQRYKQKKELEHRLSAMKSAVESGQADDERVREYYLLHLQRWIDISLEEIESIDQEIKILRERDSSREASTSNSSRQERPPVKPFILTRNMAQAKVFGAGYPSLPTMTVSDWYEQHRKYGALPDQGIAKAAPEEFRKAAQQQEEQEEKEEEDDEQTLHRAREWDDWKDTHPRGYGNRQNMG

704 NP_001561.3 NP_001560.2

>NP_001561.3

MACYIYQLPSWVLDDLCRNMDALSEWDWMEFASYVITDLTQLRKIKSMERVQGVSITRELLWWWGMRQATVQQLVDLLCRLELYRAAQIILNWKPAPEIRCPIPAFPDSVKPEKPLAASVRKAEDEQEEGQPVRMATFPGPGSSPARAHQPAFLQPPEEDAPHSLRSDLPTSSDSKDFSTSIPKQEKLLSLAGDSLFWSEADVVQATDDFNQNRKISQGTFADVYRGHRHGKPFVFKKLRETACSSPGSIERFFQAELQICLRCCHPNVLPVLGFCAARQFHSFIYPYMANGSLQDRLQGQGGSDPLPWPQRVSICSGLLCAVEYLHGLEIIHSNVKSSNVLLDQNLTPKLAHPMAHLCPVNKRSKYTMMKTHLLRTSAAYLPEDFIRVGQLTKRVDIFSCGIVLAEVLTGIPAMDNNRSPVYLKDLLLSDIPSSTASLCSRKTGVENVMAKEICQKYLEKGAGRLPEDCAEALATAACLCLRRRNTSLQEVCGSVAAVEERLRGRETLLPWSGLSEGTGSSSNTPEETDDVDNSSLDASSSMSVAPWAGAATPLLPTENGEGRLRVIVGREADSSSEACVGLEPPQDVTETSWQIEINEAKRKLMENILLYKEEKVDSIELFGP

>NP_001560.2

MAGGPGPGEPAAPGAQHFLYEVPPWVMCRFYKVMDALEPADWCQFAALIVRDQTELRLCERSGQRTASVLWPWINRNARVADLVHILTHLQLLRARDIITAWHPPAPLPSPGTTAPRPSSIPAPAEAEAWSPRKLPSSASTFLSPAFPGSQTHSGPELGLVPSPASLWPPPPSPAPSSTKPGPESSVSLLQGARPFPFCWPLCEISRGTHNFSEELKIGEGGFGCVYRAVMRNTVYAVKRLKENADLEWTAVKQSFLTEVEQLSRFRHPNIVDFAGYCAQNGFYCLVYGFLPNGSLEDRLHCQTQACPPLSWPQRLDILLGTARAIQFLHQDSPSLIHGDIKSSNVLLDERLTPKLGDFGLARFSRFAGSSPSQSSMVARTQTVRGTLAYLPEEYIKTGRLAVDTDTFSFGVVVLETLAGQRAVKTHGARTKYLKDLVEEEAEEAGVALRSTQSTLQAGLAADAWAAPIAMQIYKKHLDPRPGPCPPELGLGLGQLACCCLHRRAKRRPPMTQVYERLEKLQAVVAGVPGHSEAASCIPPSPQENSYVSSTGRAHSGAAPWQPLAAPSGASAQAAEQLQRGPNQPVESDESLGGLSAALRSWHLTPSCPLDPAPLREAGCPQGDTAGESSWGSGPGSRPTAVEGLALGSSASSSSEPPQIIINPARQKMVQKLALYEDGALDSLQLLSSSSLPGLGLEQDRQGPEESDEFQS

705 NP_000452.2 NP_068811.1

>NP_000452.2

MTPNSMTENGLTAWDKPKHCPDREHDWKLVGMSEACLHRKSHSERRSTLKNEQSSPHLIQTTWTSSIFHLDHDDVNDQSVSSAQTFQTEEKKCKGYIPSYLDKDELCVVCGDKATGYHYRCITCEGCKGFFRRTIQKNLHPSYSCKYEGKCVIDKVTRNQCQECRFKKCIYVGMATDLVLDDSKRLAKRKLIEENREKRRREELQKSIGHKPEPTDEEWELIKTVTEAHVATNAQGSHWKQKRKFLPEDIGQAPIVNAPEGGKVDLEAFSHFTKIITPAITRVVDFAKKLPMFCELPCEDQIILLKGCCMEIMSLRAAVRYDPESETLTLNGEMAVTRGQLKNGGLGVVSDAIFDLGMSLSSFNLDDTEVALLQAVLLMSSDRPGLACVERIEKYQDSFLLAFEHYINYRKHHVTHFWPKLLMKVTDLRMIGACHASRFLHMKVECPTELFPPLFLEVFED

>NP_068811.1

MSWAARPPFLPQRHAAGQCGPVGVRKEMHCGVASRWRRRRPWLDPAAAAAAAVAGGEQQTPEPEPGEAGRDGMGDSGRDSRSPDSSSPNPLPQGVPPPSPPGPPLPPSTAPSLGGSGAPPPPPMPPPPLGSPFPVISSSMGSPGLPPPAPPGFSGPVSSPQINSTVSLPGGGSGPPEDVKPPVLGVRGLHCPPPPGGPGAGKRLCAICGDRSSGKHYGVYSCEGCKGFFKRTIRKDLTYSCRDNKDCTVDKRQRNRCQYCRYQKCLATGMKREAVQEERQRGKDKDGDGEGAGGAPEEMPVDRILEAELAVEQKSDQGVEGPGGTGGSGSSPNDPVTNICQAADKQLFTLVEWAKRIPHFSSLPLDDQVILLRAGWNELLIASFSHRSIDVRDGILLATGLHVHRNSAHSAGVGAIFDRVLTELVSKMRDMRMDKTELGCLRAIILFNPDAKGLSNPSEVEVLREKVYASLETYCKQKYPEQQGRFAKLLLRLPALRSIGLKCLEHLFFFKLIGDTPIDTFLMEMLEAPHQLA

706 NP_775736.2 NP_057284.1

>NP_775736.2

MGPGERAGGGGDAGKGNAAGGGGGGRSATTAGSRAVSALCLLLSVGSAAACLLLGVQAAALQGRVAALEEERELLRRAGPPGALDAWAEPHLERLLREKLDGLAKIRTAREAPSECVCPPGPPGRRGKPGRRGDPGPPGQSGRDGYPGPLGLDGKPGLPGPKGEKGAPGDFGPRGDQGQDGAAGPPGPPGPPGARGPPGDTGKDGPRGAQGPAGPKGEPGQDGEMGPKGPPGPKGEPGVPGKKGDDGTPSQPGPPGPKGEPGSMGPRGENGVDGAPGPKGEPGHRGTDGAAGPRGAPGLKGEQGDTVVIDYDGRILDALKGPPGPQGPPGPPGIPGAKGELGLPGAPGIDGEKGPKGQKGDPGEPGPAGLKGEAGEMGLSGLPGADGLKGEKGESASDSLQESLAQLIVEPGPPGPPGPPGPMGLQGIQGPKGLDGAKGEKGASGERGPSGLPGPVGPPGLIGLPGTKGEKGRPGEPGLDGFPGPRGEKGDRSERGEKGERGVPGRKGVKGQKGEPGPPGLDQPCPVGPDGLPVPGCWHK

>NP_057284.1

MTSALENYINRTVAVITSDGRMIVGTLKGFDQTINLILDESHERVFSSSQGVEQVVLGLYIVRGDNVAVIGEIDEETDSALDLGNIRAEPLNSVAH

707 NP_057424.2 NP_066361.1

>NP_057424.2

MNSPVDPGARQALRKKPPERTPEDLNTIYSYLHGMEILSNLREHQLRLMSARARYERYSGNQVLFCSETIARCWYILLSGSVLVKGSMVLPPCSFGKQFGGKRGCDCLVLEPSEMIVVENAKDNEDSILQREIPARQSRRRFRKINYKGERQTITDDVEVNSYLSLPADLTKMHLTENPHPQVTHVSSSQSGCSIASDSGSSSLSDIYQATESEVGDVDLTRLPEGPVDSEDDEEEDEEIDRTDPLQGRDLVRECLEKEPADKTDDDIEQLLEFMHQLPAFANMTMSVRRELCSVMIFEVVEQAGAIILEDGQELDSWYVILNGTVEISHPDGKVENLFMGNSFGITPTLDKQYMHGIVRTKVDDCQFVCIAQQDYWRILNHVEKNTHKVEEEGEIVMVHEHRELDRSGTRKGHIVIKATPERLIMHLIEEHSIVDPTYIEDFLLTYRTFLESPLDVGIKLLEWFKIDSLRDKVTRIVLLWVNNHFNDFEGDPAMTRFLEEFEKNLEDTKMNGHLRLLNIACAAKAKWRQVVLQKASRESPLQFSLNGGSEKGFGIFVEGVEPGSKAADSGLKRGDQIMEVNGQNFENITFMKAVEILRNNTHLALTVKTNIFVFKELLFRTEQEKSGVPHIPKIAEKKSNRHSIQHVPGDIEQTSQEKGSKKVKANTVSGGRNKIRKILDKTRFSILPPKLFSDGGLSQSQDDSIVGTRHCRHSLAIMPIPGTLSSSSPDLLQPTTSMLDFSNPSDIPDQVIRVFKVDQQSCYIIISKDTTAKEVVFHAVHEFGLTGASDTYSLCEVSVTPEGVIKQRRLPDQFSKLADRIQLNGRYYLKNNMETETLCSDEDAQELVKESQLSMLQLSTIEVATQLSMRDFDLFRNIEPTEYIDDLFKLNSKTGNTHLKRFEDIVNQETFWVASEILTEANQLKRMKIIKHFIKIALHCRECKNFNSMFAIISGLNLASVARLRGTWEKLPSKYEKHLQDLQDIFDPSRNMAKYRNILSSQSMQPPIIPLFPVVKKDMTFLHEGNDSKVDGLVNFEKLRMISKEIRQVVRMTSANMDPAMMFRQRSLSQGSTNSNMLDVQGGAHKKRARRSSLLNAKKLYEDAQMARKVKQYLSSLDVETDEEKFQMMSLQWEPAYGTLTKNLSEKRSAKSSEMSPVPMRSAGQTTKAHLHQPHRVSQVLQVPAVNLHPIRKKGQTKDPALNTSLPQKVLGTTEEISGKKHTEDTISVASSLHSSPPASPQGSPHKGYTLIPSAKSDNLSDSSHSEISSRSSIVSNCSVDSMSAALQDERCSSQALAVPESTGALEKTEHASGIGDHSQHGPGWTLLKPSLIKCLAVSSSVSNEEISQEHIIIEAADSGRGSWTSCSSSSHDNFQSLPNPKSWDFLNSYRHTHLDDPIAEVEPTDSEPYSCSKSCSRTCGQCKGSLERKSWTSSSSLSDTYEPNYGTVKRRVLESTPAESSEGLDPKDATDPVYKTVTSSTEKGLIVYCVTSPKKDDRYREPPPTPPGYLGISLADLKEGPHTHLKPPDYSVAVQRSKMMHNSLSRLPPASLSSNLVACVPSKIVTQPQRHNLQPFHPKLGDVTDADSEADENEQ

>NP_066361.1

MREYKVVVLGSGGVGKSALTVQFVTGTFIEKYDPTIEDFYRKEIEVDSSPSVLEILDTAGTEQFASMRDLYIKNGQGFILVYSLVNQQSFQDIKPMRDQIIRVKRYEKVPVILVGNKVDLESEREVSSSEGRALAEEWGCPFMETSAKSKTMVDELFAEIVRQMNYAAQPDKDDPCCSACNIQ

708 NP_003216.1 NP_872342.2

>NP_003216.1

MATMENKVICALVLVSMLALGTLAEAQTETCTVAPRERQNCGFPGVTPSQCANKGCCFDDTVRGVPWCFYPNTIDVPPEEECEF

>NP_872342.2

MKILVAFLVVLTIFGIQSHGYEVFNIISPSNNGGNVQETVTIDNEKNTAIINIHAGSCSSTTIFDYKHGYIASRVLSRRACFILKMDHQNIPPLNNLQWYIYEKQALDNMFSSKYTWVKYNPLESLIKDVDWFLLGSPIEKLCKHIPLYKGEVVENTHNVGAGGCAKAGLLGILGISICADIHV

709 NP_001005355.1 NP_065393.1

>NP_001005355.1

MWNPNAGQPGPNPYPPNIGCPGGSNPAHPPPINPPFPPGPCPPPPGAPHGNPAFPPGGPPHPVPQPGYPGCQPLGPYPPPYPPPAPGIPPVNPLAPGMVGPAVIVDKKMQKKMKKAHKKMHKHQKHHKYHKHGKHSSSSSSSSSSDSD

>NP_065393.1

MEDLDQSPLVSSSDSPPRPQPAFKYQFVREPEDEEEEEEEEEEDEDEDLEELEVLERKPAAGLSAAPVPTAPAAGAPLMDFGNDFVPPAPRGPLPAAPPVAPERQPSWDPSPVSSTVPAPSPLSAAAVSPSKLPEDDEPPARPPPPPPASVSPQAEPVWTPPAPAPAAPPSTPAAPKRRGSSGSVDETLFALPAASEPVIRSSAENMDLKEQPGNTISAGQEDFPSVLLETAASLPSLSPLSAASFKEHEYLGNLSTVLPTEGTLQENVSEASKEVSEKAKTLLIDRDLTEFSELEYSEMGSSFSVSPKAESAVIVANPREEIIVKNKDEEEKLVSNNILHNQQELPTALTKLVKEDEVVSSEKAKDSFNEKRVAVEAPMREEYADFKPFERVWEVKDSKEDSDMLAAGGKIESNLESKVDKKCFADSLEQTNHEKDSESSNDDTSFPSTPEGIKDRSGAYITCAPFNPAATESIATNIFPLLGDPTSENKTDEKKIEEKKAQIVTEKNTSTKTSNPFLVAAQDSETDYVTTDNLTKVTEEVVANMPEGLTPDLVQEACESELNEVTGTKIAYETKMDLVQTSEVMQESLYPAAQLCPSFEESEATPSPVLPDIVMEAPLNSAVPSAGASVIQPSSSPLEASSVNYESIKHEPENPPPYEEAMSVSLKKVSGIKEEIKEPENINAALQETEAPYISIACDLIKETKLSAEPAPDFSDYSEMAKVEQPVPDHSELVEDSSPDSEPVDLFSDDSIPDVPQKQDETVMLVKESLTETSFESMIEYENKEKLSALPPEGGKPYLESFKLSLDNTKDTLLPDEVSTLSKKEKIPLQMEELSTAVYSNDDLFISKEAQIRETETFSDSSPIEIIDEFPTLISSKTDSFSKLAREYTDLEVSHKSEIANAPDGAGSLPCTELPHDLSLKNIQPKVEEKISFSDDFSKNGSATSKVLLLPPDVSALATQAEIESIVKPKVLVKEAEKKLPSDTEKEDRSPSAIFSAELSKTSVVDLLYWRDIKKTGVVFGASLFLLLSLTVFSIVSVTAYIALALLSVTISFRIYKGVIQAIQKSDEGHPFRAYLESEVAISEELVQKYSNSALGHVNCTIKELRRLFLVDDLVDSLKFAVLMWVFTYVGALFNGLTLLILALISLFSVPVIYERHQAQIDHYLGLANKNVKDAMAKIQAKIPGLKRKAE

710 NP_071935.1 NP_061887.2

>NP_071935.1

MAAEVLPSARWQYCGAPDGSQRAVLVQFSNGKLQSPGNMRFTLYENKDSTNPRKRNQRILAAETDRLSYVGNNFGTGALKCNTLCRHFVGILNKTSGQMEVYDAELFNMQPLFSDVSVESELALESQTKTYREKMDSCIEAFGTTKQKRALNTRRMNRVGNESLNRAVAKAAETIIDTKGVTALVSDAIHNDLQDDSLYLPPCYDDAAKPEDVYKFEDLLSPAEYEALQSPSEAFRNVTSEEILKMIEENSHCTFVIEALKSLPSDVESRDRQARCIWFLDTLIKFRAHRVVKRKSALGPGVPHIINTKLLKHFTCLTYNNGRLRNLISDSMKAKITAYVIILALHIHDFQIDLTVLQRDLKLSEKRMMEIAKAMRLKISKRRVSVAAGSEEDHKLGTLSLPLPPAQTSDRLAKRRKIT

>NP_061887.2

MDPGSRWRNLPSGPSLKHLTDPSYGIPREQQKAALQELTRAHVESFNYAVHEGLGLAVQAIPPFEFAFKDERISFTILDAVISPPTVPKGTICKEANVYPAECRGRRSTYRGKLTADINWAVNGISKGIIKQFLGYVPIMVKSKLCNLRNLPPQALIEHHEEAEEMGGYFIINGIEKVIRMLIMPRRNFPIAMIRPKWKTRGPGYTQYGVSMHCVREEHSAVNMNLHYLENGTVMLNFIYRKELFFLPLGFALKALVSFSDYQIFQELIKGKEDDSFLRNSVSQMLRIVMEEGCSTQKQVLNYLGECFRVKLNVPDWYPNEQAAEFLFNQCICIHLKSNTEKFYMLCLMTRKLFALAKGECMEDNPDSLVNQEVLTPGQLFLMFLKEKLEGWLVSIKIAFDKKAQKTSVSMNTDNLMRIFTMGIDLTKPFEYLFATGNLRSKTGLGLLQDSGLCVVADKLNFIRYLSHFRCVHRGADFAKMRTTTVRRLLPESWGFLCPVHTPDGEPCGLMNHLTAVCEVVTQFVYTASIPALLCNLGVTPIDGAPHRSYSECYPVLLDGVMVGWVDKDLAPGIADSLRHFKVLREKRIPPWMEVVLIPMTGKPSLYPGLFLFTTPCRLVRPVQNLALGKEELIGTMEQIFMNVAIFEDEVFAGVTTHQELFPHSLLSVIANFIPFSDHNQSPRNMYQCQMGKQTMGFPLLTYQDRSDNKLYRLQTPQSPLVRPSMYDYYDMDNYPIGTNAIVAVISYTGYDMEDAMIVNKASWERGFAHGSVYKSEFIDLSEKIKQGDSSLVFGIKPGDPRVLQKLDDDGLPFIGAKLQYGDPYYSYLNLNTGESFVMYYKSKENCVVDNIKVCSNDTGSGKFKCVCITMRVPRNPTIGDKFASRHGQKGILSRLWPAEDMPFTESGMVPDILFNPHGFPSRMTIGMLIESMAGKSAALHGLCHDATPFIFSEENSALEYFGEMLKAAGYNFYGTERLYSGISGLELEADIFIGVVYYQRLRHMVSDKFQVRTTGARDRVTNQPIGGRNVQGGIRFGEMERDALLAHGTSFLLHDRLFNCSDRSVAHVCVKCGSLLSPLLEKPPPSWSAMRNRKYNCTLCSRSDTIDTVSVPYVFRYFVAELAAMNIKVKLDVV

711 NP_005299.1 NP_000697.1

>NP_005299.1

MELENIVANTVLLKAREGGGGKRKGKSKKWKEILKFPHISQCEDLRRTIDRDYCSLCDKQPIGRLLFRQFCETRPGLECYIQFLDSVAEYEVTPDEKLGEKGKEIMTKYLTPKSPVFIAQVGQDLVSQTEEKLLQKPCKELFSACAQSVHEYLRGEPFHEYLDSMFFDRFLQWKWLERQPVTKNTFRQYRVLGKGGFGEVCACQVRATGKMYACKRLEKKRIKKRKGESMALNEKQILEKVNSQFVVNLAYAYETKDALCLVLTIMNGGDLKFHIYNMGNPGFEEERALFYAAEILCGLEDLHRENTVYRDLKPENILLDDYGHIRISDLGLAVKIPEGDLIRGRVGTVGYMAPEVLNNQRYGLSPDYWGLGCLIYEMIEGQSPFRGRKEKVKREEVDRRVLETEEVYSHKFSEEAKSICKMLLTKDAKQRLGCQEEGAAEVKRHPFFRNMNFKRLEAGMLDPPFVPDPRAVYCKDVLDIEQFSTVKGVNLDHTDDDFYSKFSTGSVSIPWQNEMIETECFKELNVFGPNGTLPPDLNRNHPPEPPKKGLLQRLFKRQHQNNSKSSPSSKTSFNHHINSNHVSSNSTGSS

>NP_000697.1

MRLSAGPDAGPSGNSSPWWPLATGAGNTSREAEALGEGNGPPRDVRNEELAKLEIAVLAVTFAVAVLGNSSVLLALHRTPRKTSRMHLFIRHLSLADLAVAFFQVLPQMCWDITYRFRGPDWLCRVVKHLQVFGMFASAYMLVVMTADRYIAVCHPLKTLQQPARRSRLMIAAAWVLSFVLSTPQYFVFSMIEVNNVTKARDCWATFIQPWGSRAYVTWMTGGIFVAPVVILGTCYGFICYNIWCNVRGKTASRQSKGAEQAGVAFQKGFLLAPCVSSVKSISRAKIRTVKMTFVIVTAYIVCWAPFFIIQMWSVWDPMSVWTESENPTITITALLGSLNSCCNPWIYMFFSGHLLQDCVQSFPCCQNMKEKFNKEDTDSMSRRQTFYSNNRSPTNSTGMWKDSPKSSKSIKFIPVST

712 NP_000975.2 NP_006382.1

>NP_000975.2

MAPKAKKEAPAPPKAEAKAKALKAKKAVLKGVHSHKKKKIRTSPTFRRPKTLRLRRQPKYPRKSAPRRNKLDHYAIIKFPLTTESAMKKIEDNNTLVFIVDVKANKHQIKQAVKKLYDIDVAKVNTLIRPDGEKKAYVRLAPDYDALDVANKIGII

>NP_006382.1

MDPNTIIEALRGTMDPALREAAERQLNEAHKSLNFVSTLLQITMSEQLDLPVRQAGVIYLKNMITQYWPDRETAPGDISPYTIPEEDRHCIRENIVEAIIHSPELIRVQLTTCIHHIIKHDYPSRWTAIVDKIGFYLQSDNSACWLGILLCLYQLVKNYEYKKPEERSPLVAAMQHFLPVLKDRFIQLLSDQSDQSVLIQKQIFKIFYALVQYTLPLELINQQNLTEWIEILKTVVNRDVPNETLQVEEDDRPELPWWKCKKWALHILARLFERYGSPGNVSKEYNEFAEVFLKAFAVGVQQVLLKVLYQYKEKQYMAPRVLQQTLNYINQGVSHALTWKNLKPHIQGIIQDVIFPLMCYTDADEELWQEDPYEYIRMKFDVFEDFISPTTAAQTLLFTACSKRKEVLQKTMGFCYQILTEPNADPRKKDGALHMIGSLAEILLKKKIYKDQMEYMLQNHVFPLFSSELGYMRARACWVLHYFCEVKFKSDQNLQTALELTRRCLIDDREMPVKVEAAIALQVLISNQEKAKEYITPFIRPVMQALLHIIRETENDDLTNVIQKMICEYSEEVTPIAVEMTQHLAMTFNQVIQTGPDEEGSDDKAVTAMGILNTIDTLLSVVEDHKEITQQLEGICLQVIGTVLQQHVLEFYEEIFSLAHSLTCQQVSPQMWQLLPLVFEVFQQDGFDYFTDMMPLLHNYVTVDTDTLLSDTKYLEMIYSMCKKVLTGVAGEDAECHAAKLLEVIILQCKGRGIDQCIPLFVEAALERLTREVKTSELRTMCLQVAIAALYYNPHLLLNTLENLRFPNNVEPVTNHFITQWLNDVDCFLGLHDRKMCVLGLCALIDMEQIPQVLNQVSGQILPAFILLFNGLKRAYACHAEHENDSDDDDEAEDDDETEELGSDEDDIDEDGQEYLEILAKQAGEDGDDEDWEEDDAEETALEGYSTIIDDEDNPVDEYQIFKAIFQTIQNRNPVWYQALTHGLNEEQRKQLQDIATLADQRRAAHESKMIEKHGGYKFSAPVVPSSFNFGGPAPGMN

713 NP_003336.1 NP_789782.1

>NP_003336.1

MSGIALSRLAQERKAWRKDHPFGFVAVPTKNPDGTMNLMNWECAIPGKKGTPWEGGLFKLRMLFKDDYPSSPPKCKFEPPLFHPNVYPSGTVCLSILEEDKDWRPAITIKQILLGIQELLNEPNIQDPAQAEAYTIYCQNRVEYEKRVRAQAKKFAPS

>NP_789782.1

MSVFGKLFGAGGGKAGKGGPTPQEAIQRLRDTEEMLSKKQEFLEKKIEQELTAAKKHGTKNKRAALQALKRKKRYEKQLAQIDGTLSTIEFQREALENANTNTEVLKNMGYAAKAMKAAHDNMDIDKVDELMQDIADQQELAEEISTAISKPVGFGEEFDEDELMAELEELEQEELDKNLLEISGPETVPLPNVPSIALPSKPAKKKEEEDDDMKELENWAGSM

714 NP_003874.2 NP_006028.1

>NP_003874.2

MAKTVAYFYDPDVGNFHYGAGHPMKPHRLALTHSLVLHYGLYKKMIVFKPYQASQHDMCRFHSEDYIDFLQRVSPTNMQGFTKSLNAFNVGDDCPVFPGLFEFCSRYTGASLQGATQLNNKICDIAINWAGGLHHAKKFEASGFCYVNDIVIGILELLKYHPRVLYIDIDIHHGDGVQEAFYLTDRVMTVSFHKYGNYFFPGTGDMYEVGAESGRYYCLNVPLRDGIDDQSYKHLFQPVINQVVDFYQPTCIVLQCGADSLGCDRLGCFNLSIRGHGECVEYVKSFNIPLLVLGGGGYTVRNVARCWTYETSLLVEEAISEELPYSEYFEYFAPDFTLHPDVSTRIENQNSRQYLDQIRQTIFENLKMLNHAPSVQIHDVPADLLTYDRTDEADAEERGPEENYSRPEAPNEFYDGDHDNDKESDVEI

>NP_006028.1

MSSQSHPDGLSGRDQPVELLNPARVNHMPSTVDVATALPLQVAPSAVPMDLRLDHQFSLPVAEPALREQQLQQELLALKQKQQIQRQILIAEFQRQHEQLSRQHEAQLHEHIKQQQEMLAMKHQQELLEHQRKLERHRQEQELEKQHREQKLQQLKNKEKGKESAVASTEVKMKLQEFVLNKKKALAHRNLNHCISSDPRYWYGKTQHSSLDQSSPPQSGVSTSYNHPVLGMYDAKDDFPLRKTASEPNLKLRSRLKQKVAERRSSPLLRRKDGPVVTALKKRPLDVTDSACSSAPGSGPSSPNNSSGSVSAENGIAPAVPSIPAETSLAHRLVAREGSAAPLPLYTSPSLPNITLGLPATGPSAGTAGQQDTERLTLPALQQRLSLFPGTHLTPYLSTSPLERDGGAAHSPLLQHMVLLEQPPAQAPLVTGLGALPLHAQSLVGADRVSPSIHKLRQHRPLGRTQSAPLPQNAQALQHLVIQQQHQQFLEKHKQQFQQQQLQMNKIIPKPSEPARQPESHPEETEEELREHQALLDEPYLDRLPGQKEAHAQAGVQVKQEPIESDEEEAEPPREVEPGQRQPSEQELLFRQQALLLEQQRIHQLRNYQASMEAAGIPVSFGGHRPLSRAQSSPASATFPVSVQEPPTKPRFTTGLVYDTLMLKHQCTCGSSSSHPEHAGRIQSIWSRLQETGLRGKCECIRGRKATLEELQTVHSEAHTLLYGTNPLNRQKLDSKKLLGSLASVFVRLPCGGVGVDSDTIWNEVHSAGAARLAVGCVVELVFKVATGELKNGFAVVRPPGHHAEESTPMGFCYFNSVAVAAKLLQQRLSVSKILIVDWDVHHGNGTQQAFYSDPSVLYMSLHRYDDGNFFPGSGAPDEVGTGPGVGFNVNMAFTGGLDPPMGDAEYLAAFRTVVMPIASEFAPDVVLVSSGFDAVEGHPTPLGGYNLSARCFGYLTKQLMGLAGGRIVLALEGGHDLTAICDASEACVSALLGNELDPLPEKVLQQRPNANAVRSMEKVMEIHSKYWRCLQRTTSTAGRSLIEAQTCENEEAETVTAMASLSVGVKPAEKRPDEEPMEEEPPL

715 NP_036611.2 NP_001265.1

>NP_036611.2

MVDREQLVQKARLAEQAERYDDMAAAMKNVTELNEPLSNEERNLLSVAYKNVVGARRSSWRVISSIEQKTSADGNEKKIEMVRAYREKIEKELEAVCQDVLSLLDNYLIKNCSETQYESKVFYLKMKGDYYRYLAEVATGEKRATVVESSEKAYSEAHEISKEHMQPTHPIRLGLALNYSVFYYEIQNAPEQACHLAKTAFDDAIAELDTLNEDSYKDSTLIMQLLRDNLTLWTSDQQDDDGGEGNN

>NP_001265.1

MAVPFVEDWDLVQTLGEGAYGEVQLAVNRVTEEAVAVKIVDMKRAVDCPENIKKEICINKMLNHENVVKFYGHRREGNIQYLFLEYCSGGELFDRIEPDIGMPEPDAQRFFHQLMAGVVYLHGIGITHRDIKPENLLLDERDNLKISDFGLATVFRYNNRERLLNKMCGTLPYVAPELLKRREFHAEPVDVWSCGIVLTAMLAGELPWDQPSDSCQEYSDWKEKKTYLNPWKKIDSAPLALLHKILVENPSARITIPDIKKDRWYNKPLKKGAKRPRVTSGGVSESPSGFSKHIQSNLDFSPVNSASSEENVKYSSSQPEPRTGLSLWDTSPSYIDKLVQGISFSQPTCPDHMLLNSQLLGTPGSSQNPWQRLVKRMTRFFTKLDADKSYQCLKETCEKLGYQWKKSCMNQVTISTTDRRNNKLIFKVNLLEMDDKILVDFRLSKGDGLEFKRHFLKIKGKLIDIVSSQKVWLPAT

716 NP_116259.1 AAB48304.1

>NP_116259.1

MWDQGGQPWQQWPLNQQQWMQSFQHQQDPSQIDWAALAQAWIAQREASGQQSMVEQPPGMMPNGQDMSTMESGPNNHGNFQGDSNFNRMWQPEWGMHQQPPHPPPDQPWMPPTPGPMDIVPPSEDSNSQDSGEFAPDNRHIFNQNNHNFGGPPDNFAVGPVNQFDYQHGAAFGPPQGGFHPPYWQPGPPGPPAPPQNRRERPSSFRDRQRSPIALPVKQEPPQIDAVKRRTLPAWIREGLEKMEREKQKKLEKERMEQQRSQLSKKEKKATEDAEGGDGPRLPQRSKFDSDEEEEDTENVEAASSGKVTRSPSPVPQEEHSDPEMTEEEKEYQMMLLTKMLLTEILLDVTDEEIYYVAKDAHRKATKAPAKQLAQSSALASLTGLGGLGGYGSGDSEDERSDRGSESSDTDDEELRHRIRQKQEAFWRKEKEQQLLHDKQMEEEKQQTERVTKEMNEFIHKEQNSLSLLEAREADGDVVNEKKRTPNETTSVLEPKKEHKEKEKQGRSRSGSSSSGSPSSNSRTSSTSSTVSSSSYSSSSGSSRTSSRSSSPKRKKRHSRSRSPTIKARRSRSRSYSRRIKIESNRARVKIRDRRRSNRNSIERERRRNRSPSRERRRSRSRSRDRRTNRASRSRSRDRRKIDDQRGNLSGNSHKHKGEAKEQERKKERSRSIDKDRKKKDKEREREQDKRKEKQKREEKDFKFSSQDDRLKRKRESERTFSRSGSISVKIIRHDSRQDSKKSTTKDSKKHSGSDSSGRSSSESPGSSKEKKAKKPKHSRSRSAEKSQRSGKKASRKHKSKSRSR

>AAB48304.1

MAVAVRTLQEQLEKAKESLKNVDENIRKLTGRDPNDVRPIQARLLALSGPGGGRGRGSLLLRRGFSDSGGPPAKQRDLEGAVSRLGGERRTRRESRQESDPEDDDVKKPALQSSVVATSKERTRRDLIQDQNMDEKGKQRNRRIFGLLMGTLQKFKQESTVATERQNRRQEIEQKLEVQAEEERKQVENERRELFEERRAKQTELRLLEQKVELAQLQEEWNEHNAKIIKYIRTKTKPHLFYIPGRMCPATQKLIEESQRKMNALFDGRRIEFAEQINKMEARPRRQSMKEKEHQVVRNEEHKAEQEEGKVAQREEELVETGNQHNDVEIEEAGEEEEKEIGIVHSDAEKEQEEEEQKQEMEVKMEEETEVRESEKQQDSQPEEVMDVLEMVENVKHVIADQEVMETNRVESVEPSENEASKELEPEMEFEIEPDKECKSLSPGKENVSALDMEKESDEKEEKESEPQPEPVAQPQAQSQPQLQLQSQSEPQPQLQPEPAQPQLQSQPQLQLQSQCHAVLQSHPPSQPEDLSLAVLQPTPQVTQEHGHFLPERKDFPVESVKLTEVPVDPVLTVHPESESETNTRSRSRGRTRNRTTKSRSRSSSSSSSSSSSTSSSSGSSSSSGSSSSRSSSSSSSSTSGSSSRDSSSSTSSSSESRSRSRGRGHNRDRKHRRSVDRKRRDTSGLERSHKSSKGGSSRDTKGSKDKNSRSDRKRSISESSRSGKRSSRSERDRKSDRKDKRR

717 NP_006592.3 NP_006810.1

>NP_006592.3

MQPASAKWYDRRDYVFIEFCVEDSKDVNVNFEKSKLTFSCLGGSDNFKHLNEIDLFHCIDPNDSKHKRTDRSILCCLRKGESGQSWPRLTKERAKLNWLSVDFNNWKDWEDDSDEDMSNFDRFSEMMNNMGGDEDVDLPEVDGADDDSQDSDDEKMPDLE

>NP_006810.1

MEQVNELKEKGNKALSVGNIDDALQCYSEAIKLDPHNHVLYSNRSAAYAKKGDYQKAYEDGCKTVDLKPDWGKGYSRKAAALEFLNRFEEAKRTYEEGLKHEANNPQLKEGLQNMEARLAERKFMNPFNMPNLYQKLESDPRTRTLLSDPTYRELIEQLRNKPSDLGTKLQDPRIMTTLSVLLGVDLGSMDEEEEIATPPPPPPPKKETKPEPMEEDLPENKKQALKEKELGNDAYKKKDFDTALKHYDKAKELDPTNMTYITNQAAVYFEKGDYNKCRELCEKAIEVGRENREDYRQIAKAYARIGNSYFKEEKYKDAIHFYNKSLAEHRTPDVLKKCQQAEKILKEQERLAYINPDLALEEKNKGNECFQKGDYPQAMKHYTEAIKRNPKDAKLYSNRAACYTKLLEFQLALKDCEECIQLEPTFIKGYTRKAAALEAMKDYTKAMDVYQKALDLDSSCKEAADGYQRCMMAQYNRHDSPEDVKRRAMADPEVQQIMSDPAMRLILEQMQKDPQALSEHLKNPVIAQKIQKLMDVGLIAIR

718 NP_005892.1 NP_003274.2

>NP_005892.1

MSSILPFTPPVVKRLLGWKKSAGGSGGAGGGEQNGQEEKWCEKAVKSLVKKLKKTGRLDELEKAITTQNCNTKCVTIPSTCSEIWGLSTPNTIDQWDTTGLYSFSEQTRSLDGRLQVSHRKGLPHVIYCRLWRWPDLHSHHELKAIENCEYAFNLKKDEVCVNPYHYQRVETPVLPPVLVPRHTEILTELPPLDDYTHSIPENTNFPAGIEPQSNYIPETPPPGYISEDGETSDQQLNQSMDTGSPAELSPTTLSPVNHSLDLQPVTYSEPAFWCSIAYYELNQRVGETFHASQPSLTVDGFTDPSNSERFCLGLLSNVNRNATVEMTRRHIGRGVRLYYIGGEVFAECLSDSAIFVQSPNCNQRYGWHPATVCKIPPGCNLKIFNNQEFAALLAQSVNQGFEAVYQLTRMCTIRMSFVKGWGAEYRRQTVTSTPCWIELHLNGPLQWLDKVLTQMGSPSVRCSSMS

>NP_003274.2

MSDTEEQEYEEEQPEEEAADEEEEAPEEPEPVAEPEEERPKPSRPVVPPLIPPKIPEGERVDFDDIHRKRMEKDLLELQTLIDVHFEQRKKEEEELVALKERIERRRSERAEQQRFRTEKERERQAKLAEEKMRKEEEEAKKRAEDDAKKKKVLSNMGAHFGGYLVKAEQKRGKRQTGREMKVRILSERKKPLDIDYMGEEQLRARSAWLPPSQPSCPAREKAQELSDWIHQLESEKFDLMAKLKQQKYEINVLYNRISHAQKFRKGAGKGRVGGRWK

719 NP_001973.2 P15941

>NP_001973.2

MRANDALQVLGLLFSLARGSEVGNSQAVCPGTLNGLSVTGDAENQYQTLYKLYERCEVVMGNLEIVLTGHNADLSFLQWIREVTGYVLVAMNEFSTLPLPNLRVVRGTQVYDGKFAIFVMLNYNTNSSHALRQLRLTQLTEILSGGVYIEKNDKLCHMDTIDWRDIVRDRDAEIVVKDNGRSCPPCHEVCKGRCWGPGSEDCQTLTKTICAPQCNGHCFGPNPNQCCHDECAGGCSGPQDTDCFACRHFNDSGACVPRCPQPLVYNKLTFQLEPNPHTKYQYGGVCVASCPHNFVVDQTSCVRACPPDKMEVDKNGLKMCEPCGGLCPKACEGTGSGSRFQTVDSSNIDGFVNCTKILGNLDFLITGLNGDPWHKIPALDPEKLNVFRTVREITGYLNIQSWPPHMHNFSVFSNLTTIGGRSLYNRGFSLLIMKNLNVTSLGFRSLKEISAGRIYISANRQLCYHHSLNWTKVLRGPTEERLDIKHNRPRRDCVAEGKVCDPLCSSGGCWGPGPGQCLSCRNYSRGGVCVTHCNFLNGEPREFAHEAECFSCHPECQPMEGTATCNGSGSDTCAQCAHFRDGPHCVSSCPHGVLGAKGPIYKYPDVQNECRPCHENCTQGCKGPELQDCLGQTLVLIGKTHLTMALTVIAGLVVIFMMLGGTFLYWRGRRIQNKRAMRRYLERGESIEPLDPSEKANKVLARIFKETELRKLKVLGSGVFGTVHKGVWIPEGESIKIPVCIKVIEDKSGRQSFQAVTDHMLAIGSLDHAHIVRLLGLCPGSSLQLVTQYLPLGSLLDHVRQHRGALGPQLLLNWGVQIAKGMYYLEEHGMVHRNLAARNVLLKSPSQVQVADFGVADLLPPDDKQLLYSEAKTPIKWMALESIHFGKYTHQSDVWSYGVTVWELMTFGAEPYAGLRLAEVPDLLEKGERLAQPQICTIDVYMVMVKCWMIDENIRPTFKELANEFTRMARDPPRYLVIKRESGPGIAPGPEPHGLTNKKLEEVELEPELDLDLDLEAEEDNLATTTLGSALSLPVGTLNRPRGSQSLLSPSSGYMPMNQGNLGESCQESAVSGSSERCPRPVSLHPMPRGCLASESSEGHVTGSEAELQEKVSMCRSRSRSRSPRPRGDSAYHSQRHSLLTPVTPLSPPGLEEEDVNGYVMPDTHLKGTPSSREGTLSSVGLSSVLGTEEEDEDEEYEYMNRRRRHSPPHPPRPSSLEELGYEYMDVGSDLSASLGSTQSCPLHPVPIMPTAGTTPDEDYEYMNRQRDGGGPGGDYAAMGACPASEQGYEEMRAFQGPGHQAPHVHYARLKTLRSLEATDSAFDNPDYWHSRLFPKANAQRT

>P15941

MTPGTQSPFFLLLLLTVLTVVTGSGHASSTPGGEKETSATQRSSVPSSTEKNAVSMTSSVLSSHSPGSGSSTTQGQDVTLAPATEPASGSAATWGQDVTSVPVTRPALGSTTPPAHDVTSAPDNKPAPGSTAPPAHGVTSAPDTRPAPGSTAPPAHGVTSAPDTRPAPGSTAPPAHGVTSAPDTRPAPGSTAPPAHGVTSAPDTRPAPGSTAPPAHGVTSAPDTRPAPGSTAPPAHGVTSAPDTRPAPGSTAPPAHGVTSAPDTRPAPGSTAPPAHGVTSAPDTRPAPGSTAPPAHGVTSAPDTRPAPGSTAPPAHGVTSAPDTRPAPGSTAPPAHGVTSAPDTRPAPGSTAPPAHGVTSAPDTRPAPGSTAPPAHGVTSAPDTRPAPGSTAPPAHGVTSAPDTRPAPGSTAPPAHGVTSAPDTRPAPGSTAPPAHGVTSAPDTRPAPGSTAPPAHGVTSAPDTRPAPGSTAPPAHGVTSAPDTRPAPGSTAPPAHGVTSAPDTRPAPGSTAPPAHGVTSAPDTRPAPGSTAPPAHGVTSAPDTRPAPGSTAPPAHGVTSAPDTRPAPGSTAPPAHGVTSAPDTRPAPGSTAPPAHGVTSAPDTRPAPGSTAPPAHGVTSAPDTRPAPGSTAPPAHGVTSAPDTRPAPGSTAPPAHGVTSAPDTRPAPGSTAPPAHGVTSAPDTRPAPGSTAPPAHGVTSAPDTRPAPGSTAPPAHGVTSAPDTRPAPGSTAPPAHGVTSAPDTRPAPGSTAPPAHGVTSAPDTRPAPGSTAPPAHGVTSAPDTRPAPGSTAPPAHGVTSAPDTRPAPGSTAPPAHGVTSAPDTRPAPGSTAPPAHGVTSAPDTRPAPGSTAPPAHGVTSAPDTRPAPGSTAPPAHGVTSAPDTRPAPGSTAPPAHGVTSAPDTRPAPGSTAPPAHGVTSAPDTRPAPGSTAPPAHGVTSAPDNRPALGSTAPPVHNVTSASGSASGSASTLVHNGTSARATTTPASKSTPFSIPSHHSDTPTTLASHSTKTDASSTHHSSVPPLTSSNHSTSPQLSTGVSFFFLSFHISNLQFNSSLEDPSTDYYQELQRDISEMFLQIYKQGGFLGLSNIKFRPGSVVVQLTLAFREGTINVHDVETQFNQYKTEAASRYNLTISDVSVSDVPFPFSAQSGAGVPGWGIALLVLVCVLVALAIVYLIALAVCQCRRKNYGQLDIFPARDTYHPMSEYPTYHTHGRYVPPSSTDRSPYEKVSAGNGGSSLSYTNPAVAAASANL

720 NP_003835.2 NP_001073594.1

>NP_003835.2

MAPPPARVHLGAFLAVTPNPGSAASGTEAAAATPSKVWGSSAGRIEPRGGGRGALPTSMGQHGPSARARAGRAPGPRPAREASPRLRVHKTFKFVVVGVLLQVVPSSAATIKLHDQSIGTQQWEHSPLGELCPPGSHRSEHPGACNRCTEGVGYTNASNNLFACLPCTACKSDEEERSPCTTTRNTACQCKPGTFRNDNSAEMCRKCSRGCPRGMVKVKDCTPWSDIECVHKESGNGHNIWVILVVTLVVPLLLVAVLIVCCCIGSGCGGDPKCMDRVCFWRLGLLRGPGAEDNAHNEILSNADSLSTFVSEQQMESQEPADLTGVTVQSPGEAQCLLGPAEAEGSQRRRLLVPANGADPTETLMLFFDKFANIVPFDSWDQLMRQLDLTKNEIDVVRAGTAGPGDALYAMLMKWVNKTGRNASIHTLLDALERMEERHAKEKIQDLLVDSGKFIYLEDGTGSAVSLE

>NP_001073594.1

MEGGRRARVVIESKRNFFLGAFPTPFPAEHVELGRLGDSETAMVPGKGGADYILLPFKKMDFSRNLYDIGEQLDSEDLASLKFLSLDYIPQRKQEPIKDALMLFQRLQEKRMLEESNLSFLKELLFRINRLDLLITYLNTRKEEMERELQTPGRAQISAYRVMLYQISEEVSRSELRSFKFLLQEEISKCKLDDDMNLLDIFIEMEKRVILGEGKLDILKRVCAQINKSLLKIINDYEEFSKERSSSLEGSPDEFSNGEELCGVMTISDSPREQDSESQTLDKVYQMKSKPRGYCLIINNHNFAKAREKVPKLHSIRDRNGTHLDAGALTTTFEELHFEIKPHDDCTVEQIYEILKIYQLMDHSNMDCFICCILSHGDKGIIYGTDGQEAPIYELTSQFTGLKCPSLAGKPKVFFIQACQGDNYQKGIPVETDSEEQPYLEMDLSSPQTRYIPDEADFLLGMATVNNCVSYRNPAEGTWYIQSLCQSLRERCPRGDDILTILTEVNYEVSNKDDKKNMGKQMPQPTFTLRKKLVFPSD

721 NP_004337.2 BAA18998.1

>NP_004337.2

MENTENSVDSKSIKNLEPKIIHGSESMDSGISLDNSYKMDYPEMGLCIIINNKNFHKSTGMTSRSGTDVDAANLRETFRNLKYEVRNKNDLTREEIVELMRDVSKEDHSKRSSFVCVLLSHGEEGIIFGTNGPVDLKKITNFFRGDRCRSLTGKPKLFIIQACRGTELDCGIETDSGVDDDMACHKIPVEADFLYAYSTAPGYYSWRNSKDGSWFIQSLCAMLKQYADKLEFMHILTRVNRKVATEFESFSFDATFHAKKQIPCIVSMLTKELYFYH

>BAA18998.1

MDDLDALLADLESTTSHISKRPVFLSEETPYSYPTGNHTYQEIAVPPPVPPPPSSEALNGTILDPLDQWQPSGSRFIHQQPQSSSPVYGSSAKTSSVSNPQDSVGSPCSRVGEEEHVYSFPNKQKSAEPSPTVMSTSLGSNLSELDRLLLELNAVQHNPPGFPADEANSSPPLPGALSPLYGVPETNSPLGGKAGPLTKEKPKRNGGRGLEDVRPSVESLLDELESSVPSPVPAITVNQGEMSSPQRVTSTQQQTRISASSATRELDELMASLSDFKGSWPLEEVVLLVSISSSVQEGEKYPHPCAARHRTPSLRSPDQPPPCPQFMAQGKTGSSSPPGGPPKPGSQLDSMLGSLQSDLNKLGVATVAKGVCGACKKPIAGQVVTAMGKTWHPEHFVCTHCQEEIGSRNFFERDGQPYCEKDYHNLFSPRCYYCNGPILDKVVTALDRTWHPEHFFCAQCGAFFGPEGFHEKDGKAYCRKDYFDMFAPKCGGCARAILENYISALNTLWHPECFVCRECFTPFVNGSFFEHDGQPYCEVHYHERRGSLCSGCQKPITGRCITAMAKKFHPEHFVCAFCLKQLNKGTFKEQNDKPYCQNCFLKLFC

722 NP_005021.2 NP_001005333.1

>NP_005021.2

MSAAVTAGKLARAPADPGKAGVPGVAAPGAPAAAPPAKEIPEVLVDPRSRRRYVRGRFLGKGGFAKCFEISDADTKEVFAGKIVPKSLLLKPHQREKMSMEISIHRSLAHQHVVGFHGFFEDNDFVFVVLELCRRRSLLELHKRRKALTEPEARYYLRQIVLGCQYLHRNRVIHRDLKLGNLFLNEDLEVKIGDFGLATKVEYDGERKKTLCGTPNYIAPEVLSKKGHSFEVDVWSIGCIMYTLLVGKPPFETSCLKETYLRIKKNEYSIPKHINPVAASLIQKMLQTDPTARPTINELLNDEFFTSGYIPARLPITCLTIPPRFSIAPSSLDPSNRKPLTVLNKGLENPLPERPREKEEPVVRETGEVVDCHLSDMLQQLHSVNASKPSERGLVRQEEAEDPACIPIFWVSKWVDYSDKYGLGYQLCDNSVGVLFNDSTRLILYNDGDSLQYIERDGTESYLTVSSHPNSLMKKITLLKYFRNYMSEHLLKAGANITPREGDELARLPYLRTWFRTRSAIILHLSNGSVQINFFQDHTKLILCPLMAAVTYIDEKRDFRTYRLSLLEEYGCCKELASRLRYARTMVDKLLSSRSASNRLKAS

>NP_001005333.1

MAQKMDCGAGLLGFQNPDACRAVCHPLPQPPASTLPLSAFPTLCDPPYSQLRDPPAVLSCYCTPLGASPAPAEASVEDSALLMQTLMEAIQISEAPPTNQATAAASPQSSQPPTANEMADIQVSAAAARPKSAFKVQNATTKGPNGVYDFSQAHNAKDVPNTQPKAAFKSQNATPKGPNAAYDFSQAATTGELAANKSEMAFKAQNATTKVGPNATYNFSQSLNANDLANSRPKTPFKAWNDTTKAPTADTQTQNVNQAKMATSQADIETDPGISEPDGATAQTSADGSQAQNLESRTIIRGKRTRKINNLNVEENSSGDQRRAPLAAGTWRSAPVPVTTQNPPGAPPNVLWQTPLAWQNPSGWQNQTARQTPPARQSPPARQTPPAWQNPVAWQNPVIWPNPVIWQNPVIWPNPIVWPGPVVWPNPLAWQNPPGWQTPPGWQTPPGWQGPPDWQGPPDWPLPPDWPLPPDWPLPTDWPLPPDWIPADWPIPPDWQNLRPSPNLRPSPNSRASQNPGAAQPRDVALLQERANKLVKYLMLKDYTKVPIKRSEMLRDIIREYTDVYPEIIERACFVLEKKFGIQLKEIDKEEHLYILISTPESLAGILGTTKDTPKLGLLLVILGVIFMNGNRASEAVLWEALRKMGLRPGVRHPLLGDLRKLLTYEFVKQKYLDYRRVPNSNPPEYEFLWGLRSYHETSKMKVLRFIAEVQKRDPRDWTAQFMEAADEALDALDAAAAEAEARAEARTRMGIGDEAVSGPWSWDDIEFELLTWDEEGDFGDPWSRIPFTFWARYHQNARSRFPQTFAGPIIGPGGTASANFAANFGAIGFFWVE

723 NP_057645.1 NP_001073594.1

>NP_057645.1

MEEPQKSYVNTMDLERDEPLKSTGPQISVSEFSCHCCYDILVNPTTLNCGHSFCRHCLALWWASSKKTECPECREKWEGFPKVSILLRDAIEKLFPDAIRLRFEDIQQNNDIVQSLAAFQKYGNDQIPLAPNTGRANQQMGGGFFSGVLTALTGVAVVLLVYHWSSRESEHDLLVHKAVAKWTAEEVVLWLEQLGPWASLYRERFLSERVNGRLLLTLTEEEFSKTPYTIENSSHRRAILMELERVKALGVKPPQNLWEYKAVNPGRSLFLLYALKSSPRLSLLYLYLFDYTDTFLPFIHTICPLQEDSSGEDIVTKLLDLKEPTWKQWREFLVKYSFLPYQLIAEFAWDWLEVHYWTSRFLIINAMLLSVLELFSFWRIWSRSELKTVPQRMWSHFWKVSTQGLFVAMFWPLIPQFVCNCLFYWALYFNPIINIDLVVKELRRLETQVL

>NP_001073594.1

MEGGRRARVVIESKRNFFLGAFPTPFPAEHVELGRLGDSETAMVPGKGGADYILLPFKKMDFSRNLYDIGEQLDSEDLASLKFLSLDYIPQRKQEPIKDALMLFQRLQEKRMLEESNLSFLKELLFRINRLDLLITYLNTRKEEMERELQTPGRAQISAYRVMLYQISEEVSRSELRSFKFLLQEEISKCKLDDDMNLLDIFIEMEKRVILGEGKLDILKRVCAQINKSLLKIINDYEEFSKERSSSLEGSPDEFSNGEELCGVMTISDSPREQDSESQTLDKVYQMKSKPRGYCLIINNHNFAKAREKVPKLHSIRDRNGTHLDAGALTTTFEELHFEIKPHDDCTVEQIYEILKIYQLMDHSNMDCFICCILSHGDKGIIYGTDGQEAPIYELTSQFTGLKCPSLAGKPKVFFIQACQGDNYQKGIPVETDSEEQPYLEMDLSSPQTRYIPDEADFLLGMATVNNCVSYRNPAEGTWYIQSLCQSLRERCPRGDDILTILTEVNYEVSNKDDKKNMGKQMPQPTFTLRKKLVFPSD

724 NP_055901.1 NP_056053.1

>NP_055901.1

MDFQASHWARGFQNRTCGPRPGSPQPPPRRPWASRVLQEATNWRAGPLAEVRAREQEKRKAASQEREAKETERKRRKAGGARRSPPGRPRPEPRNAPRVAQLAGLPAPLRPERLAPVGRAPRPSAQPQSDPGSAWAGPWGGRRPGPPSYEAHLLLRGSAGTAPRRRWDRPPPYVAPPSYEGPHRTLGTKRGPGNSQVPTSSAPAATPARTDGGRTKKRLDPRIYRDVLGAWGLRQGQGLLGGSPGCGAARARPEPGKGVVEKSLGLAAADLNSGSDSHPQAKATGSAGTEIAPAGSATAAPCAPHPAPRSRHHLKGSREGKEGEQIWFPKCWIPSPKKQPPRHSQTLPRPWAPGGTGWRESLGLGEGAGPETLEGWKATRRAHTLPRSSQGLSRGEGVFVIDATCVVIRSQYVPTPRTQQVQLLPSGVTRVVGDSPSQSKPGKEEGEGATVFPSPCQKRLSSSRLLHQPGGGRGGEAEGGRPGDSTLEERTFRILGLPAPEVNLRDAPTQPGSPEHQALGPAASGAQGRAEGSEVAVVQRRAGRGWARTPGPYAGALREAVSRIRRHTAPDSDTDEAEELSVHSGSSDGSDTEAPGASWRNERTLPEVGNSSPEEDGKTAELSDSVGEILDVISQTEEVLFGVRDIRGTQQGNRKRQ

>NP_056053.1

MPRPELPLPEGWEEARDFDGKVYYIDHTNRTTSWIDPRDRYTKPLTFADCISDELPLGWEEAYDPQVGDYFIDHNTKTTQIEDPRVQWRREQEHMLKDYLVVAQEALSAQKEIYQVKQQRLELAQQEYQQLHAVWEHKLGSQVSLVSGSSSSSKYDPEILKAEIATAKSRVNKLKREMVHLQHELQFKERGFQTLKKIDKKMSDAQGSYKLDEAQAVLRETKAIKKAITCGEKEKQDLIKSLAMLKDGFRTDRGSHSDLWSSSSSLESSSFPLPKQYLDVSSQTDISGSFGINSNNQLAEKVRLRLRYEEAKRRIANLKIQLAKLDSEAWPGVLDSERDRLILINEKEELLKEMRFISPRKWTQGEVEQLEMARKRLEKDLQAARDTQSKALTERLKLNSKRNQLVRELEEATRQVATLHSQLKSLSSSMQSLSSGSSPGSLTSSRGSLVASSLDSSTSASFTDLYYDPFEQLDSELQSKVEFLLLEGATGFRPSGCITTIHEDEVAKTQKAEGGGRLQALRSLSGTPKSMTSLSPRSSLSSPSPPCSPLMADPLLAGDAFLNSLEFEDPELSATLCELSLGNSAQERYRLEEPGTEGKQLGQAVNTAQGCGLKVACVSAAVSDESVAGDSGVYEASVQRLGASEAAAFDSDESEAVGATRIQIALKYDEKNKQFAILIIQLSNLSALLQQQDQKVNIRVAVLPCSESTTCLFRTRPLDASDTLVFNEVFWVSMSYPALHQKTLRVDVCTTDRSHLEECLGGAQISLAEVCRSGERSTRWYNLLSYKYLKKQSRELKPVGVMAPASGPASTDAVSALLEQTAVELEKRQEGRSSTQTLEDSWRYEETSENEAVAEEEEEEVEEEEGEEDVFTEKASPDMDGYPALKVDKETNTETPAPSPTVVRPKDRRVGTPSQGPFLRGSTIIRSKTFSPGPQSQYVCRLNRSDSDSSTLSKKPPFVRNSLERRSVRMKRPSSVKSLRSERLIRTSLDLELDLQATRTWHSQLTQEISVLKELKEQLEQAKSHGEKELPQWLREDERFRLLLRMLEKRQMDRAEHKGELQTDKMMRAAAKDVHRLRGQSCKEPPEVQSFREKMAFFTRPRMNIPALSADDV

725 NP_005461.2 NP_005624.2

>NP_005461.2

MAELQMLLEEEIPSGKRALIESYQNLTRVADYCENNYIQATDKRKALEETKAYTTQSLASVAYQINALANNVLQLLDIQASQLRRMESSINHISQTVDIHKEKVARREIGILTTNKNTSRTHKIIAPANMERPVRYIRKPIDYTVLDDVGHGVKWLKAKHGNNQPARTGTLSRTNPPTQKPPSPPMSGRGTLGRNTPYKTLEPVKPPTVPNDYMTSPARLGSQHSPGRTASLNQRPRTHSGSSGGSGSRENSGSSSIGIPIAVPTPSPPTIGPENISVPPPSGAPPAPPLAPLLPVSTVIAAPGSAPGSQYGTMTRQISRHNSTTSSTSSGGYRRTPSVTAQFSAQPHVNGGPLYSQNSISIAPPPPPMPQLTPQIPLTGFVARVQENIADSPTPPPPPPPDDIPMFDDSPPPPPPPPVDYEDEEAAVVQYNDPYADGDPAWAPKNYIEKVVAIYDYTKDKDDELSFMEGAIIYVIKKNDDGWYEGVCNRVTGLFPGNYVESIMHYTD

>NP_005624.2

MQAQQLPYEFFSEENAPKWRGLLVPALKKVQGQVHPTLESNDDALQYVEELILQLLNMLCQAQPRSASDVEERVQKSFPHPIDKWAIADAQSAIEKRKRRNPLSLPVEKIHPLLKEVLGYKIDHQVSVYIVAVLEYISADILKLVGNYVRNIRHYEITKQDIKVAMCADKVLMDMFHQDVEDINILSLTDEEPSTSGEQTYYDLVKAFMAEIRQYIRELNLIIKVFREPFVSNSKLFSANDVENIFSRIVDIHELSVKLLGHIEDTVEMTDEGSPHPLVGSCFEDLAEELAFDPYESYARDILRPGFHDRFLSQLSKPGAALYLQSIGEGFKEAVQYVLPRLLLAPVYHCLHYFELLKQLEEKSEDQEDKECLKQAITALLNVQSGMEKICSKSLAKRRLSESACRFYSQQMKGKQLAIKKMNEIQKNIDGWEGKDIGQCCNEFIMEGTLTRVGAKHERHIFLFDGLMICCKSNHGQPRLPGASNAEYRLKEKFFMRKVQINDKDDTNEYKHAFEIILKDENSVIFSAKSAEEKNNWMAALISLQYRSTLERMLDVTMLQEEKEEQMRLPSADVYRFAEPDSEENIIFEENMQPKAGIPIIKAGTVIKLIERLTYHMYADPNFVRTFLTTYRSFCKPQELLSLIIERFEIPEPEPTEADRIAIENGDQPLSAELKRFRKEYIQPVQLRVLNVCRHWVEHHFYDFERDAYLLQRMEEFIGTVRGKAMKKWVESITKIIQRKKIARDNGPGHNITFQSSPPTVEWHISRPGHIETFDLLTLHPIEIARQLTLLESDLYRAVQPSELVGSVWTKEDKEINSPNLLKMIRHTTNLTLWFEKCIVETENLEERVAVVSRIIEILQVFQELNNFNGVLEVVSAMNSSPVYRLDHTFEQIPSRQKKILEEAHELSEDHYKKYLAKLRSINPPCVPFFGIYLTNILKTEEGNPEVLKRHGKELINFSKRRKVAEITGEIQQYQNQPYCLRVESDIKRFFENLNPMGNSMEKEFTDYLFNKSLEIEPRNPKPLPRFPKKYSYPLKSPGVRPSNPRPGTMRHPTPLQQEPRKISYSRIPESETESTASAPNSPRTPLTPPPASGASSTTDVCSVFDSDHSSPFHSSNDTVFIQVTLPHGPRSASVSSISLTKGTDEVPVPPPVPPRRRPESAPAESSPSKIMSKHLDSPPAIPPRQPTSKAYSPRYSISDRTSISDPPESPPLLPPREPVRTPDVFSSSPLHLQPPPLGKKSDHGNAFFPNSPSPFTPPPPQTPSPHGTRRHLPSPPLTQEVDLHSIAGPPVPPRQSTSQHIPKLPPKTYKREHTHPSMHRDGPPLLENAHSS

726 NP_002829.2 NP_001762.1

>NP_002829.2

MYLWLKLLAFGFAFLDTEVFVTGQSPTPSPTGLTTAKMPSVPLSSDPLPTHTTAFSPASTFERENDFSETTTSLSPDNTSTQVSPDSLDNASAFNTTGVSSVQTPHLPTHADSQTPSAGTDTQTFSGSAANAKLNPTPGSNAISDVPGERSTASTFPTDPVSPLTTTLSLAHHSSAALPARTSNTTITANTSDAYLNASETTTLSPSGSAVISTTTIATTPSKPTCDEKYANITVDYLYNKETKLFTAKLNVNENVECGNNTCTNNEVHNLTECKNASVSISHNSCTAPDKTLILDVPPGVEKFQLHDCTQVEKADTTICLKWKNIETFTCDTQNITYRFQCGNMIFDNKEIKLENLEPEHEYKCDSEILYNNHKFTNASKIIKTDFGSPGEPQIIFCRSEAAHQGVITWNPPQRSFHNFTLCYIKETEKDCLNLDKNLIKYDLQNLKPYTKYVLSLHAYIIAKVQRNGSAAMCHFTTKSAPPSQVWNMTVSMTSDNSMHVKCRPPRDRNGPHERYHLEVEAGNTLVRNESHKNCDFRVKDLQYSTDYTFKAYFHNGDYPGEPFILHHSTSYNSKALIAFLAFLIIVTSIALLVVLYKIYDLHKKRSCNLDEQQELVERDDEKQLMNVEPIHADILLETYKRKIADEGRLFLAEFQSIPRVFSKFPIKEARKPFNQNKNRYVDILPYDYNRVELSEINGDAGSNYINASYIDGFKEPRKYIAAQGPRDETVDDFWRMIWEQKATVIVMVTRCEEGNRNKCAEYWPSMEEGTRAFGDVVVKINQHKRCPDYIIQKLNIVNKKEKATGREVTHIQFTSWPDHGVPEDPHLLLKLRRRVNAFSNFFSGPIVVHCSAGVGRTGTYIGIDAMLEGLEAENKVDVYGYVVKLRRQRCLMVQVEAQYILIHQALVEYNQFGETEVNLSELHPYLHNMKKRDPPSEPSPLEAEFQRLPSYRSWRTQHIGNQEENKSKNRNSNVIPYDYNRVPLKHELEMSKESEHDSDESSDDDSDSEEPSKYINASFIMSYWKPEVMIAAQGPLKETIGDFWQMIFQRKVKVIVMLTELKHGDQEICAQYWGEGKQTYGDIEVDLKDTDKSSTYTLRVFELRHSKRKDSRTVYQYQYTNWSVEQLPAEPKELISMIQVVKQKLPQKNSSEGNKHHKSTPLLIHCRDGSQQTGIFCALLNLLESAETEEVVDIFQVVKALRKARPGMVSTFEQYQFLYDVIASTYPAQNGQVKKNNHQEDKIEFDNEVDKVKQDANCVNPLGAPEKLPEAKEQAEGSEPTSGTEGPEHSVNGPASPALNQGS

>NP_001762.1

MHLLGPWLLLLVLEYLAFSDSSKWVFEHPETLYAWEGACVWIPCTYRALDGDLESFILFHNPEYNKNTSKFDGTRLYESTKDGKVPSEQKRVQFLGDKNKNCTLSIHPVHLNDSGQLGLRMESKTEKWMERIHLNVSERPFPPHIQLPPEIQESQEVTLTCLLNFSCYGYPIQLQWLLEGVPMRQAAVTSTSLTIKSVFTRSELKFSPQWSHHGKIVTCQLQDADGKFLSNDTVQLNVKHTPKLEIKVTPSDAIVREGDSVTMTCEVSSSNPEYTTVSWLKDGTSLKKQNTFTLNLREVTKDQSGKYCCQVSNDVGPGRSEEVFLQVQYAPEPSTVQILHSPAVEGSQVEFLCMSLANPLPTNYTWYHNGKEMQGRTEEKVHIPKILPWHAGTYSCVAENILGTGQRGPGAELDVQYPPKKVTTVIQNPMPIREGDTVTLSCNYNSSNPSVTRYEWKPHGAWEEPSLGVLKIQNVGWDNTTIACARCNSWCSWASPVALNVQYAPRDVRVRKIKPLSEIHSGNSVSLQCDFSSSHPKEVQFFWEKNGRLLGKESQLNFDSISPEDAGSYSCWVNNSIGQTASKAWTLEVLYAPRRLRVSMSPGDQVMEGKSATLTCESDANPPVSHYTWFDWNNQSLPHHSQKLRLEPVKVQHSGAYWCQGTNSVGKGRSPLSTLTVYYSPETIGRRVAVGLGSCLAILILAICGLKLQRRWKRTQSQQGLQENSSGQSFFVRNKKVRRAPLSEGPHSLGCYNPMMEDGISYTTLRFPEMNIPRTGDAESSEMQRPPRTCDDTVTYSALHKRQVGDYENVIPDFPEDEGIHYSELIQFGVGERPQAQENVDYVILKH

727 NP_006410.1 NP_001707.1

>NP_006410.1

MKFISTSLLLMLLVSSLSPVQGVLEVYYTSLRCRCVQESSVFIPRRFIDRIQILPRGNGCPRKEIIVWKKNKSIVCVDPQAEWIQRMMEVLRKRSSSTLPVPVFKRKIP

>NP_001707.1

MNYPLTLEMDLENLEDLFWELDRLDNYNDTSLVENHLCPATEGPLMASFKAVFVPVAYSLIFLLGVIGNVLVLVILERHRQTRSSTETFLFHLAVADLLLVFILPFAVAEGSVGWVLGTFLCKTVIALHKVNFYCSSLLLACIAVDRYLAIVHAVHAYRHRRLLSIHITCGTIWLVGFLLALPEILFAKVSQGHHNNSLPRCTFSQENQAETHAWFTSRFLYHVAGFLLPMLVMGWCYVGVVHRLRQAQRRPQRQKAVRVAILVTSIFFLCWSPYHIVIFLDTLARLKAVDNTCKLNGSLPVAITMCEFLGLAHCCLNPMLYTFAGVKFRSDLSRLLTKLGCTGPASLCQLFPSWRRSSLSESENATSLTTF

728 NP_001008493.1 NP_061928.4

>NP_001008493.1

MSEQSICQARAAVMVYDDANKKWVPAGGSTGFSRVHIYHHTGNNTFRVVGRKIQDHQVVINCAIPKGLKYNQATQTFHQWRDARQVYGLNFGSKEDANVFASAMMHALEVLNSQETGPTLPRQNSQLPAQVQNGPSQEELEIQRRQLQEQQRQKELERERLERERMERERLERERLERERLERERLEQEQLERERQERERQERLERQERLERQERLERQERLDRERQERQERERLERLERERQERERQEQLEREQLEWERERRISSAAAPASVETPLNSVLGDSSASEPGLQAASQPAETPSQQGIVLGPLAPPPPPPLPPGPAQASVALPPPPGPPPPPPLPSTGPPPPPPPPPLPNQVPPPPPPPPAPPLPASGFFLASMSEDNRPLTGLAAAIAGAKLRKVSRMEDTSFPSGGNAIGVNSASSKTDTGRGNGPLPLGGSGLMEEMSALLARRRRIAEKGSTIETEQKEDKGEDSEPVTSKASSTSTPEPTRKPWERTNTMNGSKSPVISRRDSPRKNQIVFDNRSYDSLHRPKSTPLSQPSANGVQTEGLDYDRLKQDILDEMRKELTKLKEELIDAIRQELSKSNTA

>NP_061928.4

MGSGGDSLLGGRGSLPLLLLLIMGGMAQDSPPQILVHPQDQLFQGPGPARMSCQASGQPPPTIRWLLNGQPLSMVPPDPHHLLPDGTLLLLQPPARGHAHDGQALSTDLGVYTCEASNRLGTAVSRGARLSVAVLREDFQIQPRDMVAVVGEQFTLECGPPWGHPEPTVSWWKDGKPLALQPGRHTVSGGSLLMARAEKSDEGTYMCVATNSAGHRESRAARVSIQEPQDYTEPVELLAVRIQLENVTLLNPDPAEGPKPRPAVWLSWKVSGPAAPAQSYTALFRTQTAPGGQGAPWAEELLAGWQSAELGGLHWGQDYEFKVRPSSGRARGPDSNVLLLRLPEKVPSAPPQEVTLKPGNGTVFVSWVPPPAENHNGIIRGYQVWSLGNTSLPPANWTVVGEQTQLEIATHMPGSYCVQVAAVTGAGAGEPSRPVCLLLEQAMERATQEPSEHGPWTLEQLRATLKRPEVIATCGVALWLLLLGTAVCIHRRRRARVHLGPGLYRYTSEDAILKHRMDHSDSQWLADTWRSTSGSRDLSSSSSLSSRLGADARDPLDCRRSLLSWDSRSPGVPLLPDTSTFYGSLIAELPSSTPARPSPQVPAVRRLPPQLAQLSSPCSSSDSLCSRRGLSSPRLSLAPAEAWKAKKKQELQHANSSPLLRGSHSLELRACELGNRGSKNLSQSPGAVPQALVAWRALGPKLLSSSNELVTRHLPPAPLFPHETPPTQSQQTQPPVAPQAPSSILLPAAPIPILSPCSPPSPQASSLSGPSPASSRLSSSSLSSLGEDQDSVLTPEEVALCLELSEGEETPRNSVSPMPRAPSPPTTYGYISVPTASEFTDMGRTGGGVGPKGGVLLCPPRPCLTPTPSEGSLANGWGSASEDNAASARASLVSSSDGSFLADAHFARALAVAVDSFGFGLEPREADCVFIDASSPPSPRDEIFLTPNLSLPLWEWRPDWLEDMEVSHTQRLGRGMPPWPPDSQISSQRSQLHCRMPKAGASPVDYS

729 NP_005329.3 NP_001005.1

>NP_005329.3

MDRMASSMKQVPNPLPKVLSRRGVGAGLEAAERESFERTQTVSINKAINTQEVAVKEKHARTCILGTHHEKGAQTFWSVVNRLPLSSNAVLCWKFCHVFHKLLRDGHPNVLKDSLRYRNELSDMSRMWGHLSEGYGQLCSIYLKLLRTKMEYHTKNPRFPGNLQMSDRQLDEAGESDVNNFFQLTVEMFDYLECELNLFQTVFNSLDMSRSVSVTAAGQCRLAPLIQVILDCSHLYDYTVKLLFKLHSCLPADTLQGHRDRFMEQFTKLKDLFYRSSNLQYFKRLIQIPQLPENPPNFLRASALSEHISPVVVIPAEASSPDSEPVLEKDDLMDMDASQQNLFDNKFDDIFGSSFSSDPFNFNSQNGVNKDEKDHLIERLYREISGLKAQLENMKTESQRVVLQLKGHVSELEADLAEQQHLRQQAADDCEFLRAELDELRRQREDTEKAQRSLSEIERKAQANEQRYSKLKEKYSELVQNHADLLRKNAEVTKQVSMARQAQVDLEREKKELEDSLERISDQGQRKTQEQLEVLESLKQELATSQRELQVLQGSLETSAQSEANWAAEFAELEKERDSLVSGAAHREEELSALRKELQDTQLKLASTEESMCQLAKDQRKMLLVGSRKAAEQVIQDALNQLEEPPLISCAGSADHLLSTVTSISSCIEQLEKSWSQYLACPEDISGLLHSITLLAHLTSDAIAHGATTCLRAPPEPADSLTEACKQYGRETLAYLASLEEEGSLENADSTAMRNCLSKIKAIGEELLPRGLDIKQEELGDLVDKEMAATSAAIETATARIEEMLSKSRAGDTGVKLEVNERILGCCTSLMQAIQVLIVASKDLQREIVESGRGTASPKEFYAKNSRWTEGLISASKAVGWGATVMVDAADLVVQGRGKFEELMVCSHEIAASTAQLVAASKVKADKDSPNLAQLQQASRGVNQATAGVVASTISGKSQIEETDNMDFSSMTLTQIKRQEMDSQVRVLELENELQKERQKLGELRKKHYELAGVAEGWEEGTEASPPTLQEVVTEKE

>NP_001005.1

MLMPKKNRIAIYELLFKEGVMVAKKDVHMPKHPELADKNVPNLHVMKAMQSLKSRGYVKEQFAWRHFYWYLTNEGIQYLRDYLHLPPEIVPATLRRSRPETGRPRPKGLEGERPARLTRGEADRDTYRRSAVPPGADKKAEAGAGSATEFQFRGGFGRGRGQPPQ

730 NP_061329.3 NP_005264.2

>NP_061329.3

MSSKTASTNNIAQARRTVQQLRLEASIERIKVSKASADLMSYCEEHARSDPLLIGIPTSENPFKDKKTCIIL

>NP_005264.2

MSELEQLRQEAEQLRNQIRDARKACGDSTLTQITAGLDPVGRIQMRTRRTLRGHLAKIYAMHWGTDSRLLVSASQDGKLIIWDSYTTNKVHAIPLRSSWVMTCAYAPSGNFVACGGLDNICSIYSLKTREGNVRVSRELPGHTGYLSCCRFLDDNQIITSSGDTTCALWDIETGQQTVGFAGHSGDVMSLSLAPDGRTFVSGACDASIKLWDVRDSMCRQTFIGHESDINAVAFFPNGYAFTTGSDDATCRLFDLRADQELLMYSHDNIICGITSVAFSRSGRLLLAGYDDFNCNIWDAMKGDRAGVLAGHDNRVSCLGVTDDGMAVATGSWDSFLKIWN

731 NP_072166.1 NP_005350.1

>NP_072166.1

MAPGSRTSLLLAFALLCLPWLQEAGAVQTVPLSRLFDHAMLQAHRAHQLAIDTYQEFEETYIPKDQKYSFLHDSQTSFCFSDSIPTPSNMEETQQKSNLELLRISLLLIESWLEPVRFLRSMFANNLVYDTSDSDDYHLLKDLEEGIQTLMGVRVAPGVTNPGTPLASRAGGEKYCCPLFSSKALTQENSPYSSFRLVNPPGLSLHPEGEGGKWINERGREQCPSAWPLLLFLHFAEAGRRQPPDWADPQADLQQV

>NP_005350.1

MDNMSITNTPTSNDACLSIVHSLMCHRQGGESETFAKRAIESLVKKLKEKKDELDSLITAITTNGAHPSKCVTIQRTLDGRLQVAGRKGFPHVIYARLWRWPDLHKNELKHVKYCQYAFDLKCDSVCVNPYHYERVVSPGIDLSGLTLQSNAPSSMMVKDEYVHDFEGQPSLSTEGHSIQTIQHPPSNRASTETYSTPALLAPSESNATSTANFPNIPVASTSQPASILGGSHSEGLLQIASGPQPGQQQNGFTGQPATYHHNSTTTWTGSRTAPYTPNLPHHQNGHLQHHPPMPPHPGHYWPVHNELAFQPPISNHPAPEYWCSIAYFEMDVQVGETFKVPSSCPIVTVDGYVDPSGGDRFCLGQLSNVHRTEAIERARLHIGKGVQLECKGEGDVWVRCLSDHAVFVQSYYLDREAGRAPGDAVHKIYPSAYIKVFDLRQCHRQMQQQAATAQAAAAAQAAAVAGNIPGPGSVGGIAPAISLSAAAGIGVDDLRRLCILRMSFVKGWGPDYPRQSIKETPCWIEIHLHRALQLLDEVLHTMPIADPQPLD

732 NP_001073286.1 NP_002077.1

>NP_001073286.1

MAAAGQLCLLYLSAGLLSRLGAAFNLDTREDNVIRKYGDPGSLFGFSLAMHWQLQPEDKRLLLVGAPRAEALPLQRANRTGGLYSCDITARGPCTRIEFDNDADPTSESKEDQWMGVTVQSQGPGGKVVTCAHRYEKRQHVNTKQESRDIFGRCYVLSQNLRIEDDMDGGDWSFCDGRLRGHEKFGSCQQGVAATFTKDFHYIVFGAPGTYNWKGIVRVEQKNNTFFDMNIFEDGPYEVGGETEHDESLVPVPANSYLGFSLDSGKGIVSKDEITFVSGAPRANHSGAVVLLKRDMKSAHLLPEHIFDGEGLASSFGYDVAVVDLNKDGWQDIVIGAPQYFDRDGEVGGAVYVYMNQQGRWNNVKPIRLNGTKDSMFGIAVKNIGDINQDGYPDIAVGAPYDDLGKVFIYHGSANGINTKPTQVLKGISPYFGYSIAGNMDLDRNSYPDVAVGSLSDSVTIFRSRPVINIQKTITVTPNRIDLRQKTACGAPSGICLQVKSCFEYTANPAGYNPSISIVGTLEAEKERRKSGLSSRVQFRNQGSEPKYTQELTLKRQKQKVCMEETLWLQDNIRDKLRPIPITASVEIQEPSSRRRVNSLPEVLPILNSDEPKTAHIDVHFLKEGCGDDNVCNSNLKLEYKFCTREGNQDKFSYLPIQKGVPELVLKDQKDIALEITVTNSPSNPRNPTKDGDDAHEAKLIATFPDTLTYSAYRELRAFPEKQLSCVANQNGSQADCELGNPFKRNSNVTFYLVLSTTEVTFDTPDLDINLKLETTSNQDNLAPITAKAKVVIELLLSVSGVAKPSQVYFGGTVVGEQAMKSEDEVGSLIEYEFRVINLGKPLTNLGTATLNIQWPKEISNGKWLLYLVKVESKGLEKVTCEPQKEINSLNLTESHNSRKKREITEKQIDDNRKFSLFAERKYQTLNCSVNVNCVNIRCPLRGLDSKASLILRSRLWNSTFLEEYSKLNYLDILMRAFIDVTAAAENIRLPNAGTQVRVTVFPSKTVAQYSGVPWWIILVAILAGILMLALLVFILWKCGFFKRSRYDDSVPRYHAVRIRKEEREIKDEKYIDNLEKKQWITKWNENESYS

>NP_002077.1

MEAIAKYDFKATADDELSFKRGDILKVLNEECDQNWYKAELNGKDGFIPKNYIEMKPHPWFFGKIPRAKAEEMLSKQRHDGAFLIRESESAPGDFSLSVKFGNDVQHFKVLRDGAGKYFLWVVKFNSLNELVDYHRSTSVSRNQQIFLRDIEQVPQQPTYVQALFDFDPQEDGELGFRRGDFIHVMDNSDPNWWKGACHGQTGMFPRNYVTPVNRNV

733 NP_006140.1 NP_005710.1

>NP_006140.1

MAYVPAPGYQPTYNPTLPYYQPIPGGLNVGMSVYIQGVASEHMKRFFVNFVVGQDPGSDVAFHFNPRFDGWDKVVFNTLQGGKWGSEERKRSMPFKKGAAFELVFIVLAEHYKVVVNGNPFYEYGHRLPLQMVTHLQVDGDLQLQSINFIGGQPLRPQGPPMMPPYPGPGHCHQQLNSLPTMEGPPTFNPPVPYFGRLQGGLTARRTIIIKGYVPPTGKSFAINFKVGSSGDIALHINPRMGNGTVVRNSLLNGSWGSEEKKITHNPFGPGQFFDLSIRCGLDRFKVYANGQHLFDFAHRLSAFQRVDTLEIQGDVTLSYVQI

>NP_005710.1

MPAYHSSLMDPDTKLIGNMALLPIRSQFKGPAPRETKDTDIVDEAIYYFKANVFFKNYEIKNEADRTLIYITLYISECLKKLQKCNSKSQGEKEMYTLGITNFPIPGEPGFPLNAIYAKPANKQEDEVMRAYLQQLRQETGLRLCEKVFDPQNDKPSKWWTCFVKRQFMNKSLSGPGQ

734 NP_006501.1 NP_004179.2

>NP_006501.1

MASGSVAECLQQETTCPVCLQYFAEPMMLDCGHNICCACLARCWGTAETNVSCPQCRETFPQRHMRPNRHLANVTQLVKQLRTERPSGPGGEMGVCEKHREPLKLYCEEDQMPICVVCDRSREHRGHSVLPLEEAVEGFKEQIQNQLDHLKRVKDLKKRRRAQGEQARAELLSLTQMEREKIVWEFEQLYHSLKEHEYRLLARLEELDLAIYNSINGAITQFSCNISHLSSLIAQLEEKQQQPTRELLQDIGDTLSRAERIRIPEPWITPPDLQEKIHIFAQKCLFLTESLKQFTEKMQSDMEKIQELREAQLYSVDVTLDPDTAYPSLILSDNLRQVRYSYLQQDLPDNPERFNLFPCVLGSPCFIAGRHYWEVEVGDKAKWTIGVCEDSVCRKGGVTSAPQNGFWAVSLWYGKEYWALTSPMTALPLRTPLQRVGIFLDYDAGEVSFYNVTERCHTFTFSHATFCGPVRPYFSLSYSGGKSAAPLIICPMSGIDGFSGHVGNHGHSMETSP

>NP_004179.2

MPRSFLVKSKMAHTYHQPRVQEDEPLWPPALTPVPRDQAPSNSPVLSTLFPNQCLDWTNLKREPELEQDQNLARMAPAPEGPIVLSRPQDGDSPLSDSPPFYKPSFSWDTLATTYGHSYRQAPSTMQSAFLEHSVSLYGSPLVPSTEPALDFSLRYSPGMDAYHCVKCNKVFSTPHGLEVHVRRSHSGTWPFACDICGKTFGHAVSLEQHTHVHSQERSFECRMCGKAFKRSSTLSTHLLIHSDTRPYPCQFCGKRFHQKSDMKKHTYIHTGEKPHKCQVCGKAFSQSSNLITHSRKHTGFKPFSCELCTKGFQRKVDLRRHRESQHNLK

735 NP_001542.1 NP_000372.1

>NP_001542.1

MAAEDELQLPRLPELFETGRQLLDEVEVATEPAGSRIVQEKVFKGLDLLEKAAEMLSQLDLFSRNEDLEEIASTDLKYLLVPAFQGALTMKQVNPSKRLDHLQRAREHFINYLTQCHCYHVAEFELPKTMNNSAENHTANSSMAYPSLVAMASQRQAKIQRYKQKKELEHRLSAMKSAVESGQADDERVREYYLLHLQRWIDISLEEIESIDQEIKILRERDSSREASTSNSSRQERPPVKPFILTRNMAQAKVFGAGYPSLPTMTVSDWYEQHRKYGALPDQGIAKAAPEEFRKAAQQQEEQEEKEEEDDEQTLHRAREWDDWKDTHPRGYGNRQNMG

>NP_000372.1

METLESELTCPICLELFEDPLLLPCAHSLCFNCAHRILVSHCATNESVESITAFQCPTCRHVITLSQRGLDGLKRNVTLQNIIDRFQKASVSGPNSPSETRRERAFDANTMTSAEKVLCQFCDQDPAQDAVKTCVTCEVSYCDECLKATHPNKKPFTGHRLIEPIPDSHIRGLMCLEHEDEKVNMYCVTDDQLICALCKLVGRHRDHQVAALSERYDKLKQNLESNLTNLIKRNTELETLLAKLIQTCQHVEVNASRQEAKLTEECDLLIEIIQQRRQIIGTKIKEGKVMRLRKLAQQIANCKQCIERSASLISQAEHSLKENDHARFLQTAKNITERVSMATASSQVLIPEINLNDTFDTFALDFSREKKLLECLDYLTAPNPPTIREELCTASYDTITVHWTSDDEFSVVSYELQYTIFTGQANVVSLCNSADSWMIVPNIKQNHYTVHGLQSGTKYIFMVKAINQAGSRSSEPGKLKTNSQPFKLDPKSAHRKLKVSHDNLTVERDESSSKKSHTPERFTSQGSYGVAGNVFIDSGRHYWEVVISGSTWYAIGLAYKSAPKHEWIGKNSASWALCRCNNNWVVRHNSKEIPIEPAPHLRRVGILLDYDNGSIAFYDALNSIHLYTFDVAFAQPVCPTFTVWNKCLTIITGLPIPDHLDCTEQLP

736 NP_945327.1 NP_001645.1

>NP_945327.1

MAGAVPGAIMDEDYYGSAAEWGDEADGGQQEDDSGEGEDDAEVQQECLHKFSTRDYIMEPSIFNTLKRYFQAGGSPENVIQLLSENYTAVAQTVNLLAEWLIQTGVEPVQVQETVENHLKSLLIKHFDPRKADSIFTEEGETPAWLEQMIAHTTWRDLFYKLAEAHPDCLMLNFTVKLISDAGYQGEITSVSTACQQLEVFSRVLRTSLATILDGGEENLEKNLPEFAKMVCHGEHTYLFAQAMMSVLAQEEQGGSAVRRIAQEVQRFAQEKGHDASQITLALGTAASYPRACQALGAMLSKGALNPADITVLFKMFTSMDPPPVELIRVPAFLDLFMQSLFKPGARINQDHKHKYIHILAYAASVVETWKKNKRVSINKDELKSTSKAVETVHNLCCNENKGASELVAELSTLYQCIRFPVVAMGVLKWVDWTVSEPRYFQLQTDHTPVHLALLDEISTCHQLLHPQVLQLLVKLFETEHSQLDVMEQLELKKTLLDRMVHLLSRGYVLPVVSYIRKCLEKLDTDISLIRYFVTEVLDVIAPPYTSDFVQLFLPILENDSIAGTIKTEGEHDPVTEFIAHCKSNFIMVN

>NP_001645.1

MEPPRGPPANGAEPSRAVGTVKVYLPNKQRTVVTVRDGMSVYDSLDKALKVRGLNQDCCVVYRLIKGRKTVTAWDTAIAPLDGEELIVEVLEDVPLTMHNFVRKTFFSLAFCDFCLKFLFHGFRCQTCGYKFHQHCSSKVPTVCVDMSTNRQQFYHSVQDLSGGSRQHEAPSNRPLNELLTPQGPSPRTQHCDPEHFPFPAPANAPLQRIRSTSTPNVHMVSTTAPMDSNLIQLTGQSFSTDAAGSRGGSDGTPRGSPSPASVSSGRKSPHSKSPAEQRERKSLADDKKKVKNLGYRDSGYYWEVPPSEVQLLKRIGTGSFGTVFRGRWHGDVAVKVLKVSQPTAEQAQAFKNEMQVLRKTRHVNILLFMGFMTRPGFAIITQWCEGSSLYHHLHVADTRFDMVQLIDVARQTAQGMDYLHAKNIIHRDLKSNNIFLHEGLTVKIGDFGLATVKTRWSGAQPLEQPSGSVLWMAAEVIRMQDPNPYSFQSDVYAYGVVLYELMTGSLPYSHIGCRDQIIFMVGRGYLSPDLSKISSNCPKAMRRLLSDCLKFQREERPLFPQILATIELLQRSLPKIERSASEPSLHRTQADELPACLLSAARLVP

737 NP_061154.1 NP_076974.1

>NP_061154.1

MADEQEIMCKLESIKEIRNKTLQMEKIKARLKAEFEALESEERHLKEYKQEMDLLLQEKMAHVEELRLIHADINVMENTIKQSENDLNKLLESTRRLHDEYKPLKEHVDALRMTLGLQRLPDLCEEEEKLSLDYFEKQKAEWQTEPQEPPIPESLAAAAAAAQQLQVARKQDTRQTATFRQQPPPMKACLSCHQQIHRNAPICPLCKAKSRSRNPKKPKRKQDE

>NP_076974.1

MDLPDSASRVFCGRILSMVNTDDVNAIILAQKNMLDRFEKTNEMLLNFNNLSSARLQQMSERFLHHTRTLVEMKRDLDSIFRRIRTLKGKLARQHPEAFSHIPEASFLEEEDEDPIPPSTTTTIATSEQSTGSCDTSPDTVSPSLSPGFEDLSHVQAGSPAINGRSQTDDEEMTGE

738 NP_004675.2 NP_000302.1

>NP_004675.2

MKTGPFFLCLLGTAAAIPTNARLLSDHSKPTAETVAPDNTAIPSLWAEAEENEKETAVSTEDDSHHKAEKSSVLKSKEESHEQSAEQGKSSSQELGLKDQEDSDGHLSVNLEYAPTEGTLDIKEDMIEPQEKKLSENTDFLAPGVSSFTDSNQQESITKREENQEQPRNYSHHQLNRSSKHSQGLRDQGNQEQDPNISNGEEEEEKEPGEVGTHNDNQERKTELPREHANSKQEEDNTQSDDILEESDQPTQVSKMQEDEFDQGNQEQEDNSNAEMEEENASNVNKHIQETEWQSQEGKTGLEAISNHKETEEKTVSEALLMEPTDDGNTTPRNHGVDDDGDDDGDDGGTDGPRHSASDDYFIPSQAFLEAERAQSIAYHLKIEEQREKVHENENIGTTEPGEHQEAKKAENSSNEEETSSEGNMRVHAVDSCMSFQCKRGHICKADQQGKPHCVCQDPVTCPPTKPLDQVCGTDNQTYASSCHLFATKCRLEGTKKGHQLQLDYFGACKSIPTCTDFEVIQFPLRMRDWLKNILMQLYEANSEHAGYLNEKQRNKVKKIYLDEKRLLAGDHPIDLLLRDFKKNYHMYVYPVHWQFSELDQHPMDRVLTHSELAPLRASLVPMEHCITRFFEECDPNKDKHITLKEWGHCFGIKEEDIDENLLF

>NP_000302.1

MANLGCWMLVLFVATWSDLGLCKKRPKPGGWNTGGSRYPGQGSPGGNRYPPQGGGGWGQPHGGGWGQPHGGGWGQPHGGGWGQPHGGGWGQGGGTHSQWNKPSKPKTNMKHMAGAAAAGAVVGGLGGYMLGSAMSRPIIHFGSDYEDRYYRENMHRYPNQVYYRPMDEYSNQNNFVHDCVNITIKQHTVTTTTKGENFTETDVKMMERVVEQMCITQYERESQAYYQRGSSMVLFSSPPVILLISFLIFLIVG

739 NP_002860.2 NP_002667.1

>NP_002860.2

MSTGGDFGNPLRKFKLVFLGEQSVGKTSLITRFMYDSFDNTYQATIGIDFLSKTMYLEDRTIRLQLWDTAGQERFRSLIPSYIRDSAAAVVVYDITNVNSFQQTTKWIDDVRTERGSDVIIMLVGNKTDLADKRQVSIEEGERKAKELNVMFIETSAKAGYNVKQLFRRVAAALPGMESTQDRSREDMIDIKLEKPQEQPVSEGGCSC

>NP_002667.1

MAVTAQAARRRERVLCLFDVDGTLTPARQKIDPEVAAFLQKLRSRVQIGVVGGSDYCKIAEQLGDGDEVIEKFDYVFAENGTVQYKHGRLLSKQTIQNHLGEELLQDLINFCLSYMALLRLPKKRGTFIEFRNGMLNISPIGRSCTLEERIEFSELDKKEKIREKFVEDLKTEFAGKGLRFSRGGMISFDVFPEGWDKRYCLDSLDQDSFDTIHFFGNETSPGGNDFEIFADPRTVGHSVVSPQDTVQRCREIFFPETAHEA

740 NP_071330.3 NP_061485.1

>NP_071330.3

MALRKELLKSIWYAFTALDVEKSGKVSKSQLKVLSHNLYTVLHIPHDPVALEEHFRDDDDGPVSSQGYMPYLNKYILDKVEEGAFVKEHFDELCWTLTAKKNYRADSNGNSMLSNQDAFRLWCLFNFLSEDKYPLIMVPDEVEYLLKKVLSSMSLEVSLGELEELLAQEAQVAQTTGGLSVWQFLELFNSGRCLRGVGRDTLSMAIHEVYQELIQDVLKQGYLWKRGHLRRNWAERWFQLQPSCLCYFGSEECKEKRGIIPLDAHCCVEVLPDRDGKRCMFCVKTANRTYEMSASDTRQRQEWTAAIQMAIRLQAEGKTSLHKDLKQKRREQREQRERRRAAKEEELLRLQQLQEEKERKLQELELLQEAQRQAERLLQEEEERRRSQHRELQQALEGQLREAEQARASMQAEMELKEEEAARQRQRIKELEEMQQRLQEALQLEVKARRDEESVRIAQTRLLEEEEEKLKQLMQLKEEQERYIERAQQEKEELQQEMAQQSRSLQQAQQQLEEVRQNRQRADEDVEAAQRKLRQASTNVKHWNVQMNRLMHPIEPGDKRPVTSSSFSGFQPPLLAHRDSSLKRLTRWGSQGNRTPSPNSNEQQKSLNGGDEAPAPASTPQEDKLDPAPEN

>NP_061485.1

MQAIKCVVVGDGAVGKTCLLISYTTNAFPGEYIPTVFDNYSANVMVDGKPVNLGLWDTAGQEDYDRLRPLSYPQTVGETYGKDITSRGKDKPIADVFLICFSLVSPASFENVRAKWYPEVRHHCPNTPIILVGTKLDLRDDKDTIEKLKEKKLTPITYPQGLAMAKEIGAVKYLECSALTQRGLKTVFDEAIRAVLCPPPVKKRKRKCLLL

741 NP_000240.1 NP_689543.1

>NP_000240.1

MSFVAGVIRRLDETVVNRIAAGEVIQRPANAIKEMIENCLDAKSTSIQVIVKEGGLKLIQIQDNGTGIRKEDLDIVCERFTTSKLQSFEDLASISTYGFRGEALASISHVAHVTITTKTADGKCAYRASYSDGKLKAPPKPCAGNQGTQITVEDLFYNIATRRKALKNPSEEYGKILEVVGRYSVHNAGISFSVKKQGETVADVRTLPNASTVDNIRSIFGNAVSRELIEIGCEDKTLAFKMNGYISNANYSVKKCIFLLFINHRLVESTSLRKAIETVYAAYLPKNTHPFLYLSLEISPQNVDVNVHPTKHEVHFLHEESILERVQQHIESKLLGSNSSRMYFTQTLLPGLAGPSGEMVKSTTSLTSSSTSGSSDKVYAHQMVRTDSREQKLDAFLQPLSKPLSSQPQAIVTEDKTDISSGRARQQDEEMLELPAPAEVAAKNQSLEGDTTKGTSEMSEKRGPTSSNPRKRHREDSDVEMVEDDSRKEMTAACTPRRRIINLTSVLSLQEEINEQGHEVLREMLHNHSFVGCVNPQWALAQHQTKLYLLNTTKLSEELFYQILIYDFANFGVLRLSEPAPLFDLAMLALDSPESGWTEEDGPKEGLAEYIVEFLKKKAEMLADYFSLEIDEEGNLIGLPLLIDNYVPPLEGLPIFILRLATEVNWDEEKECFESLSKECAMFYSIRKQYISEESTLSGQQSEVPGSIPNSWKWTVEHIVYKALRSHILPPKHFTEDGNILQLANLPDLYKVFERC

>NP_689543.1

MNKLNFHNNRVMQDRRSVCIFLPNDESLNIIINVKILCHQLLVQVCDLLRLKDCHLFGLSVIQNNEHVYMELSQKLYKYCPKEWKKEASKGIDQFGPPMIIHFRVQYYVENGRLISDRAARYYYYWHLRKQVLHSQCVLREEAYFLLAAFALQADLGNFKRNKHYGKYFEPEAYFPSWVVSKRGKDYILKHIPNMHKDQFALTASEAHLKYIKEAVRLDDVAVHYYRLYKDKREIEASLTLGLTMRGIQIFQNLDEEKQLLYDFPWTNVGKLVFVGKKFEILPDGLPSARKLIYYTGCPMRSRHLLQLLSNSHRLYMNLQPVLRHIRKLEENEEKKQYRESYISDNLDLDMDQLEKRSRASGSSAGSMKHKRLSRHSTASHSSSHTSGIEADTKPRDTGPEDSYSSSAIHRKLKTCSSMTSHGSSHTSGVESGGKDRLEEDLQDDEIEMLVDDPRDLEQMNEESLEVSPDMCIYITEDMLMSRKLNGHSGLIVKEIGSSTSSSSETVVKLRGQSTDSLPQTICRKPKTSTDRHSLSLDDIRLYQKDFLRIAGLCQDTAQSYTFGCGHELDEEGLYCNSCLAQQCINIQDAFPVKRTSKYFSLDLTHDEVPEFVV

742 NP_002736.3 NP_115984.3

>NP_002736.3

MAAAAAAGAGPEMVRGQVFDVGPRYTNLSYIGEGAYGMVCSAYDNVNKVRVAIKKISPFEHQTYCQRTLREIKILLRFRHENIIGINDIIRAPTIEQMKDVYIVQDLMETDLYKLLKTQHLSNDHICYFLYQILRGLKYIHSANVLHRDLKPSNLLLNTTCDLKICDFGLARVADPDHDHTGFLTEYVATRWYRAPEIMLNSKGYTKSIDIWSVGCILAEMLSNRPIFPGKHYLDQLNHILGILGSPSQEDLNCIINLKARNYLLSLPHKNKVPWNRLFPNADSKALDLLDKMLTFNPHKRIEVEQALAHPYLEQYYDPSDEPIAEAPFKFDMELDDLPKEKLKELIFEETARFQPGYRS

>NP_115984.3

MMKTEPRGPGGPLRSASPHRSAYEAGIQALKPPDAPGPDEAPKGAHHKKYGSNVHRIKSMFLQMGTTAGPSGEAGGGAGLAEAPRASERGVRLSLPRASSLNENVDHSALLKLGTSVSERVSRFDSKPAPSAQPAPPPHPPSRLQETRKLFERSAPAAAGGDKEAAARRLLRQERAGLQDRKLDVVVRFNGSTEALDKLDADAVSPTVSQLSAVFEKADSRTGLHRGPGLPRAAGVPQVNSKLVSKRSRVFQPPPPPPPAPSGDAPAEKERCPAGQQPPQHRVAPARPPPKPREVRKIKPVEVEESGESEAESAPGEVIQAEVTVHAALENGSTVATAASPAPEEPKAQAAPEKEAAAVAPPERGVGNGRAPDVAPEEVDESKKEDFSEADLVDVSAYSGLGEDSAGSALEEDDEDDEEDGEPPYEPESGCVEIPGLSEEEDPAPSRKIHFSTAPIQVFSTYSNEDYDRRNEDVDPMAASAEYELEKRVERLELFPVELEKDSEGLGISIIGMGAGADMGLEKLGIFVKTVTEGGAAHRDGRIQVNDLLVEVDGTSLVGVTQSFAASVLRNTKGRVRFMIGRERPGEQSEVAQLIQQTLEQERWQREMMEQRYAQYGEDDEETGEYATDEDEELSPTFPGGEMAIEVFELAENEDALSPVDMEPEKLVHKFKELQIKHAVTEAEIQQLKRKLQSLEQEKGRWRVEKAQLEQSVEENKERMEKLEGYWGEAQSLCQAVDEHLRETQAQYQALERKYSKAKRLIKDYQQKEIEFLKKETAQRRVLEESELARKEEMDKLLDKISELEGNLQTLRNSNST

743 AAH06201.1 NP_060560.1

>AAH06201.1

MTDSKYFTTNKKGEIFELKAELNNEKKEKRKEAVKKVIAAMTVGKDVSSLFPDVVNCMQTDNLELKKLVYLYLMNYAKSQPDMAIMAVNSFVKDCEDPNPLIRALAVRTMGCIRVDKITEYLCEPLRKCLKDEDPYVRKTAAVCVAKLHDINAQMVEDQGFLDSLRDLIADSNPMVVANAVAALSEISESHPNSNLLDLNPQNINKLLTALNECTEWGQIFILDCLSNYNPKDDREAQSICERVTPRLSHANSAVVLSAVKVLMKFLELLPKDSDYYNMLLKKLAPPLVTLLSGEPEVQYVALRNINLIVQKRPEILKQEIKVFFVKYNDPIYVKLEKLDIMIRLASQANIAQVLAELKEYATEVDVDFVRKAVRAIGRCAIKVEQSAERCVSTLLDLIQTKVNYVVQEAIVVIRDIFRKYPNKYESIIATLCENLDSLDEPDARAAMIWIVGEYAERIDNADELLESFLEGFHDESTQVQLTLLTAIVKLFLKKPSETQELVQQVLSLATQDSDNPDLRDRGYIYWRLLSTDPVTAKEVVLSEKPLISEETDLIEPTLLDELICHIGSLASVYHKPPNAFVEGSHGIHRKHLPIHHGSTDAGDSPVGTTTATNLEQPQVIPSQGDLLGDLLNLDLGPPVNVPQVSSMQMGAVDLLGGGLDSLLGSDLGGGIGGSPAVGQSFIPSSVPATFAPSPTPAVVSSGLNDLFELSTGIGMAPGGYVAPKAVWLPAVKAKGLEISGTFTHRQGHIYMEMNFTNKALQHMTDFAIQFNKNSFGVIPSTPLAIHTPLMPNQSIDVSLPLNTLGPVMKMEPLNNLQVAVKNNIDVFYFSCLIPLNVLFVEDGKMERQVFLATWKDIPNENELQFQIKECHLNADTVSSKLQNNNVYTIAKRNVEGQDMLYQSLKLTNGIWILAELRIQPGNPNYTLSLKCRAPEVSQYIYQVYDSILKN

>NP_060560.1

MEESGYESVLCVKPDVHVYRIPPRATNRGYRAAEWQLDQPSWSGRLRITAKGQMAYIKLEDRTSGELFAQAPVDQFPGTAVESVTDSSRYFVIRIEDGNGRRAFIGIGFGDRGDAFDFNVALQDHFKWVKQQCEFAKQAQNPDQGPKLDLGFKEGQTIKLNIANMKKKEGAAGNPRVRPASTGGLSLLPPPPGGKTSTLIPPPGEQLAVGGSLVQPAVAPSSGGAPVPWPQPNPATADIWGDFTKSTGSTSSQTQPGTGWVQF

744 NP_444252.1 NP_076422.1

>NP_444252.1

MAGWQSYVDNLMCDGCCQEAAIVGYCDAKYVWAATAGGVFQSITPIEIDMIVGKDREGFFTNGLTLGAKKCSVIRDSLYVDGDCTMDIRTKSQGGEPTYNVAVGRAGRVLVFVMGKEGVHGGGLNKKAYSMAKYLRDSGF

>NP_076422.1

MEQDDPVEALTELRERRLGALELLQAAAGSGLAAYAVWALLLQPGFRRVPLRLQVPYVGASARQVEHVLSLLRGRPGKTVDLGSGDGRIVLAAHRCGLRPAVGYELNPWLVALARLHAWRAGCAGSVCYRRKDLWKVSLRDCRNVSVFLAPSVLPLLEDKLRTELPAGARVVSGRFPLPTWQPVTAVGEGLDRVWAYDVPEGGQAGEAASSRIPIQAAPGPSSAPIPGGLISQAS

745 NP_004975.2 NP_068707.1

>NP_004975.2

MAETNNECSIKVLCRFRPLNQAEILRGDKFIPIFQGDDSVVIGGKPYVFDRVFPPNTTQEQVYHACAMQIVKDVLAGYNGTIFAYGQTSSGKTHTMEGKLHDPQLMGIIPRIARDIFNHIYSMDENLEFHIKVSYFEIYLDKIRDLLDVTKTNLSVHEDKNRVPFVKGCTERFVSSPEEILDVIDEGKSNRHVAVTNMNEHSSRSHSIFLINIKQENMETEQKLSGKLYLVDLAGSEKVSKTGAEGAVLDEAKNINKSLSALGNVISALAEGTKSYVPYRDSKMTRILQDSLGGNCRTTMFICCSPSSYNDAETKSTLMFGQRAKTIKNTASVNLELTAEQWKKKYEKEKEKTKAQKETIAKLEAELSRWRNGENVPETERLAGEEAALGAELCEETPVNDNSSIVVRIAPEERQKYEEEIRRLYKQLDDKDDEINQQSQLIEKLKQQMLDQEELLVSTRGDNEKVQRELSHLQSENDAAKDEVKEVLQALEELAVNYDQKSQEVEEKSQQNQLLVDELSQKVATMLSLESELQRLQEVSGHQRKRIAEVLNGLMKDLSEFSVIVGNGEIKLPVEISGAIEEEFTVARLYISKIKSEVKSVVKRCRQLENLQVECHRKMEVTGRELSSCQLLISQHEAKIRSLTEYMQSVELKKRHLEESYDSLSDELAKLQAQETVHEVALKDKEPDTQDADEVKKALELQMESHREAHHRQLARLRDEINEKQKTIDELKDLNQKLQLELEKLQADYEKLKSEEHEKSTKLQELTFLYERHEQSKQDLKGLEETVARELQTLHNLRKLFVQDVTTRVKKSAEMEPEDSGGIHSQKQKISFLENNLEQLTKVHKQLVRDNADLRCELPKLEKRLRATAERVKALEGALKEAKEGAMKDKRRYQQEVDRIKEAVRYKSSGKRGHSAQIAKPVRPGHYPASSPTNPYGTRSPECISYTNSLFQNYQNLYLQATPSSTSDMYFANSCTSSGATSSGGPLASYQKANMDNGNATDINDNRSDLPCGYEAEDQAKLFPLHQETAAS

>NP_068707.1

MIEESGNKRKTMAEKRQLFIEMRAQNFDVIRLSTYRTACKLRFVQKRCNLHLVDIWNMIEAFRDNGLNTLDHTTEISVSRLETVISSIYYQLNKRLPSTHQISVEQSISLLLNFMIAAYDSEGRGKLTVFSVKAMLATMCGGKMLDKLRYVFSQMSDSNGLMIFSKFDQFLKEVLKLPTAVFEGPSFGYTEHSVRTCFPQQRKIMLNMFLDTMMADPPPQCLVWLPLMHRLAHVENVFHPVECSYCRCESMMGFRYRCQQCHNYQLCQNCFWRGHAGGPHSNQHQMKEHSSWKSPAKKLSHAISKSLGCVPTREPPHPVFPEQPEKPLDLAHIVPPRPLTNMNDTMVSHMSSGVPTPTKRLQYSQDIPSHLADEHALIASYVARLQHCARVLDSPSRLDEEHRLIARYAARLAAEAGNVTRPPTDLSFNFDANKQQRQLIAELENKNREILQEIQRLRLEHEQASQPTPEKAQQNPTLLAELRLLRQRKDELEQRMSALQESRRELMVQLEELMKLLKEEEQKQAAQATGSPHTSPTHGGGRPMPMPVRSTSAGSTPTHCPQDSLSGVGGDVQEAFAQGTRRNLRNDLLVAADSITNTMSSLVKELHSAEEGAEEEEEKMQNGKDRG

746 BAA92038.1 NP_115689.1

>BAA92038.1

MLVHLFRVGIRGGPFPGRLLPPLRFQTFSAVRNTWRNGKTGQLHKAEGEYSDGYRSSSLLRAVAHLRSQLWAHLPRAPLAPRWSPSAWCWVGGALLGPMVLSKHPHLCLVALCEAEEAPPASSTPHVVGSRFNWKLFWQFLHPHLLVLGVAVVLALGAALVNVQIPLLLGQLVEVVAKYTRDHVGSFMTESQNLSTHLLILYGVQGLLTFGYLVLLSHVGERMAVDMRRALFSSLLRQDITFFDANKTGQLVSRLTTDVQEFKSSFKLVISQGLRSCTQVAGCLVSLSMLSTRLTLLLMVATPALMGVGTLMGSGLRKLSRQCQEQIARAMGVADEALGNVRTVRAFAMEQREEERYGAELEACHCRAEELGRGIALFQGLSNIAFNCMVLGTLFIGGSLVAGQQLTGGDLMSFLVASQTVQRSMANLSVLFGQVVRGLSAGARVFEYMALNPCIPLSGGCCVPKEQLRGSVTFQNVCFSYPCRPGFEVLKDFTLTLPPGKIVALVGQSGGGKTTVASLLERFYDPTAGVVMLDGRDLRTLDPSWLRGQVVGFISQEPVLFGTTIMENIRFGKLEASDEEVYTAAREANAHEFITSFPEGYNTVVGERGTTLSGGQKQRLAIARALIKQPTVLILDEATSALDAESERVVQEALDRASAGRTVLVIAHRLSTVRGAHCIVVMADGRVWEAGTHEELLKKGGLYAELIRRQALDAPRTAAPPPKKPEGPRSHQHKS

>NP_115689.1

MLPARLPFRLLSLFLRGSAPTAARHGLREPLLERRCAAASSFQHSSSLGRELPYDPVDTEGFGEGGDMQERFLFPEYILDPEPQPTREKQLQELQQQQEEEERQRQQRREERRQQNLRARSREHPVVGHPDPALPPSGVNCSGCGAELHCQDAGVPGYLPREKFLRTAEADGGLARTVCQRCWLLSHHRRALRLQVSREQYLELVSAALRRPGPSLVLYMVDLLDLPDALLPDLPALVGPKQLIVLGNKVDLLPQDAPGYRQRLRERLWEDCARAGLLLAPGHQGPQRPVKDEPQDGENPNPPNWSRTVVRDVRLISAKTGYGVEELISALQRSWRYRGDVYLVGATNAGKSTLFNTLLESDYCTAKGSEAIDRATISPWPGTTLNLLKFPICNPTPYRMFKRHQRLKKDSTQAEEDLSEQEQNQLNVLKKHGYVVGRVGRTFLYSEEQKDNIPFEFDADSLAFDMENDPVMGTHKSTKQVELTAQDVKDAHWFYDTPGITKENCILNLLTEKEVNIVLPTQSIVPRTFVLKPGMVLFLGAIGRIDFLQGNQSAWFTVVASNILPVHITSLDRADALYQKHAGHTLLQIPMGGKERMAGFPPLVAEDIMLKEGLGASEAVADIKFSSAGWVSVTPNFKDRLHLRGYTPEGTVLTVRPPLLPYIVNIKGQRIKKSVAYKTKKPPSLMYNVRKKKGKINV

747 NP_001013861.1 NP_005393.2

>NP_001013861.1

MIPPQEASARRREIEDKLKQEEETLSFIRDSLEKSDQLTKNMVSILSSFESRLMKLENSIIPVHKQTENLQRLQENVEKTLSCLDHVISYYHVASDTEKIIREGPTGRLEEYLGSMAKIQKAVEYFQDNSPDSPELNKVKLLFERGKEALESEFRSLMTRHSKVVSPVLILDLISGDDDLEAQEDVTLEHLPESVLQDVIRISRWLVEYGRNQDFMNVYYQIRSSQLDRSIKGLKEHFHKSSSSSGVPYSPAIPNKRKDTPTKKPVKRPGTIRKAQNLLKQYSQHGLDGKKGGSNLIPLEGRDDMLDVETDAYIHCVSAFVKLAQSEYQLLADIIPEHHQKKTFDSLIQDALDGLMLEGENIVSAARKAIVRHDFSTVLTVFPILRHLKQTKPEFDQVLQGTAASTKNKLPGLITSMETIGAKALEDFADNIKNDPDKEYNMPKDGTVHELTSNAILFLQQLLDFQETAGAMLASQETSSSATSYSSEFSKRLLSTYICKVLGNLQLNLLSKSKVYEDPALSAIFLHNNYNYILKSLEKSELIQLVAVTQKTAERSYREHIEQQIQTYQRSWLKVTDYIAEKNLPVFQPGVKLRDKERQIIKERFKGFNDGLEELCKIQKAWAIPDTEQRDRIRQAQKTIVKETYGAFLQKFGSVPFTKNPEKYIKYGVEQVGDMIDRLFDTSA

>NP_005393.2

MAANKPKGQNSLALHKVIMVGSGGVGKSALTLQFMYDEFVEDYEPTKADSYRKKVVLDGEEVQIDILDTAGQEDYAAIRDNYFRSGEGFLCVFSITEMESFAATADFREQILRVKEDENVPFLLVGNKSDLEDKRQVSVEEAKNRAEQWNVNYVETSAKTRANVDKVFFDLMREIRARKMEDSKEKNGKKKRKSLAKRIRERCCIL

748 NP_066964.1 NP_005517.1

>NP_066964.1

MVRSGNKAAVVLCMDVGFTMSNSIPGIESPFEQAKKVITMFVQRQVFAENKDEIALVLFGTDGTDNPLSGGDQYQNITVHRHLMLPDFDLLEDIESKIQPGSQQADFLDALIVSMDVIQHETIGKKFEKRHIEIFTDLSSRFSKSQLDIIIHSLKKCDISLQFFLPFSLGKEDGSGDRGDGPFRLGGHGPSFPLKGITEQQKEGLEIVKMVMISLEGEDGLDEIYSFSESLRKLCVFKKIERHSIHWPCRLTIGSNLSIRIAAYKSILQERVKKTWTVVDAKTLKKEDIQKETVYCLNDDDETEVLKEDIIQGFRYGSDIVPFSKVDEEQMKYKSEGKCFSVLGFCKSSQVQRRFFMGNQVLKVFAARDDEAAAVALSSLIHALDDLDMVAIVRYAYDKRANPQVGVAFPHIKHNYECLVYVQLPFMEDLRQYMFSSLKNSKKYAPTEAQLNAVDALIDSMSLAKKDEKTDTLEDLFPTTKIPNPRFQRLFQCLLHRALHPREPLPPIQQHIWNMLNPPAEVTTKSQIPLSKIKTLFPLIEAKKKDQVTAQEIFQDNHEDGPTAKKLKTEQGGAHFSVSSLAEGSVTSVGSVNPAENFRVLVKQKKASFEEASNQLINHIEQFLDTNETPYFMKSIDCIRAFREEAIKFSEEQRFNNFLKALQEKVEIKQLNHFWEIVVQDGITLITKEEASGSSVTAEEAKKFLAPKDKPSGDTAAVFEEGGDVDDLLDMI

>NP_005517.1

MDLPVGPGAAGPSNVPAFLTKLWTLVSDPDTDALICWSPSGNSFHVFDQGQFAKEVLPKYFKHNNMASFVRQLNMYGFRKVVHIEQGGLVKPERDDTEFQHPCFLRGQEQLLENIKRKVTSVSTLKSEDIKIRQDSVTKLLTDVQLMKGKQECMDSKLLAMKHENEALWREVASLRQKHAQQQKVVNKLIQFLISLVQSNRILGVKRKIPLMLNDSGSAHSMPKYSRQFSLEHVHGSGPYSAPSPAYSSSSLYAPDAVASSGPIISDITELAPASPMASPGGSIDERPLSSSPLVRVKEEPPSPPQSPRVEEASPGRPSSVDTLLSPTALIDSILRESEPAPASVTALTDARGHTDTEGRPPSPPPTSTPEKCLSVACLDKNELSDHLDAMDSNLDNLQTMLSSHGFSVDTSALLDLFSPSVTVPDMSLPDLDSSLASIQELLSPQEPPRPPEAENSSPDSGKQLVHYTAQPLFLLDPGSVDTGSNDLPVLFELGEGSYFSEGDGFAEDPTISLLTGSEPPKAKDPTVS

749 NP_004374.1 NP_005347.3

>NP_004374.1

MSAIQAAWPSGTECIAKYNFHGTAEQDLPFCKGDVLTIVAVTKDPNWYKAKNKVGREGIIPANYVQKREGVKAGTKLSLMPWFHGKITREQAERLLYPPETGLFLVRESTNYPGDYTLCVSCDGKVEHYRIMYHASKLSIDEEVYFENLMQLVEHYTSDADGLCTRLIKPKVMEGTVAAQDEFYRSGWALNMKELKLLQTIGKGEFGDVMLGDYRGNKVAVKCIKNDATAQAFLAEASVMTQLRHSNLVQLLGVIVEEKGGLYIVTEYMAKGSLVDYLRSRGRSVLGGDCLLKFSLDVCEAMEYLEGNNFVHRDLAARNVLVSEDNVAKVSDFGLTKEASSTQDTGKLPVKWTAPEALREKKFSTKSDVWSFGILLWEIYSFGRVPYPRIPLKDVVPRVEKGYKMDAPDGCPPAVYEVMKNCWHLDAAMRPSFLQLREQLEHIKTHELHL

>NP_005347.3

MGCGCSSHPEDDWMENIDVCENCHYPIVPLDGKGTLLIRNGSEVRDPLVTYEGSNPPASPLQDNLVIALHSYEPSHDGDLGFEKGEQLRILEQSGEWWKAQSLTTGQEGFIPFNFVAKANSLEPEPWFFKNLSRKDAERQLLAPGNTHGSFLIRESESTAGSFSLSVRDFDQNQGEVVKHYKIRNLDNGGFYISPRITFPGLHELVRHYTNASDGLCTRLSRPCQTQKPQKPWWEDEWEVPRETLKLVERLGAGQFGEVWMGYYNGHTKVAVKSLKQGSMSPDAFLAEANLMKQLQHQRLVRLYAVVTQEPIYIITEYMENGSLVDFLKTPSGIKLTINKLLDMAAQIAEGMAFIEERNYIHRDLRAANILVSDTLSCKIADFGLARLIEDNEYTAREGAKFPIKWTAPEAINYGTFTIKSDVWSFGILLTEIVTHGRIPYPGMTNPEVIQNLERGYRMVRPDNCPEELYQLMRLCWKERPEDRPTFDYLRSVLEDFFTATEGQYQPQP

750 NP_861445.3 NP_536859.1

>NP_861445.3

MKTLPAMLGTGKLFWVFFLIPYLDIWNIHGKESCDVQLYIKRQSEHSILAGDPFELECPVKYCANRPHVTWCKLNGTTCVKLEDRQTSWKEEKNISFFILHFEPVLPNDNGSYRCSANFQSNLIESHSTTLYVTDVKSASERPSKDEMASRPWLLYSLLPLGGLPLLITTCFCLFCCLRRHQGKQNELSDTAGREINLVDAHLKSEQTEASTRQNSQVLLSETGIYDNDPDLCFRMQEGSEVYSNPCLEENKPGIVYASLNHSVIGPNSRLARNVKEAPTEYASICVRS

>NP_536859.1

MVRWFHRDLSGLDAETLLKGRGVHGSFLARPSRKNQGDFSLSVRVGDQVTHIRIQNSGDFYDLYGGEKFATLTELVEYYTQQQGVLQDRDGTIIHLKYPLNCSDPTSERWYHGHMSGGQAETLLQAKGEPWTFLVRESLSQPGDFVLSVLSDQPKAGPGSPLRVTHIKVMCEGGRYTVGGLETFDSLTDLVEHFKKTGIEEASGAFVYLRQPYYATRVNAADIENRVLELNKKQESEDTAKAGFWEEFESLQKQEVKNLHQRLEGQRPENKGKNRYKNILPFDHSRVILQGRDSNIPGSDYINANYIKNQLLGPDENAKTYIASQGCLEATVNDFWQMAWQENSRVIVMTTREVEKGRNKCVPYWPEVGMQRAYGPYSVTNCGEHDTTEYKLRTLQVSPLDNGDLIREIWHYQYLSWPDHGVPSEPGGVLSFLDQINQRQESLPHAGPIIVHCSAGIGRTGTIIVIDMLMENISTKGLDCDIDIQKTIQMVRAQRSGMVQTEAQYKFIYVAIAQFIETTKKKLEVLQSQKGQESEYGNITYPPAMKNAHAKASRTSSKSLESSAGTVAASPVRRGGQRGLPVPGPPVLSPDLHQLPVLAPLHPAADTRRMCMRTCTLRTRGRRK

751 NP_065746.3 NP_003189.1

>NP_065746.3

MLRSTGFFRAIDCPYWSGAPGGPCRRPYCHFRHRGARGSGAPGDGGEAPPAAGLGYDPYNPELPKPPAQRENGTLGLGEEPRPDVLELELVNQAIEAVRSEVELEQRRYRELLETTREHRSAEAPALAPRGPNASPTVGPDEDAFPLAFDYSPGSHGLLSPDAGYQPTPLAAPAEPGSKYSLASLDRGQGRGGGGGGALEYVPKAVSQPRRHSRPVPSGKYVVDNSRPPTDLEYDPLSNYSARHLSRASSRDERAAKRPRGSRGSEPYTPAPKKLCDPFGSCDARFSDSEDEAATVPGNEPTTASTPKARADPEIKATGQPPSKEGLEAEGGGLRETKETAVQCDVGDLQPPPAKPASPAQVQSSQDGGCPKEGKPKKKKTGAPPAPSCKDGAQGKDKTKDKGRGRPVEKPRADKKGPQASSPRRKAERPEGTKKKPSSATPVATSGKGRPDRPARRPSPTSGDSRPAAGRGPPRPLQLPDRKSTKAPSGKLVERKARSLDEGASQDAPKLKKRALSHADLFGDESEDEAAGPGVPSVWPSALPSLSSDSDSDSDSSLGFPEAQGPPKRLKASPPPSPAPSSSSSSSSSTSSAGADVDYSALEKEVDFDSDPMEECLRIFNESTSVKTEDRGRLARQPPKEEKSEEKGLSGLTTLFPGQKRRISHLSKQGQEVEPPRRGPAVPPARPPTAQEVCYLRAQQAQRASASLLQAPARLAEKSPSVHISAPGEKRRIAHIPNPRLAAAPTGAKRTLAASGSQSSNGPEPGGQQLKTRTLSGMASKTTTTIIPKRIAHSPSLQSLKKPIIPKEFGGKVPTVIRQRYLNLFIEECLKFCTSNQEAIEKALNEEKVAYDRSPSKNIYLNVAVNTLKKLRGLAPSAVPGLSKTSGRRVVSHEVVLGGRLAAKTSFSLSRPSSPRVEDLKGAALYSRLREYLLTQDQLKENGYPFPHPERPGGAIIFTAEEKRPKDSSCRTCCRCGTEYLVSSSGRCIRDEECYYHWGRLRRNRVAGGWETQYMCCSAAAGSVGCQVAKQHVQDGRKERLEGFVKTFEKELSGDTHPGIYALDCEMSYTTYGLELTRVTVVDTDVHVVYDTFVKPDNEIVDYNTRFSGVTEADLADTSVTLRDVQAVLLSMFSADTILIGHSLESDLLALKVIHSTVVDTSVLFPHRLGLPYKRSLRNLMADYLRQIIQDNVDGHSSSEDAGACMHLVIWKVREDAKTKR

>NP_003189.1

MAAESALQVVEKLQARLAANPDPKKLLKYLKKLSTLPITVDILAETGVGKTVNSLRKHEHVGSFARDLVAQWKKLVPVERNAEPDEQDFEKSNSRKRPRDALQKEEEMEGDYQETWKATGSRSYSPDHRQKKHRKLSELERPHKVSHGHERRDERKRCHRMSPTYSSDPESSDYGHVQSPPSCTSPHQMYVDHYRSLEEDQEPIVSHQKPGKGHSNAFQDRLGASQERHLGEPHGKGVVSQNKEHKSSHKDKRPVDAKSDEKASVVSREKSHKALSKEENRRPPSGDNAREKPPSSGVKKEKDREGSSLKKKCLPPSEAASDNHLKKPKHRDPEKAKLDKSKQGLDSFDTGKGAGDLLPKVKEKGSNNLKTPEGKVKTNLDRKSLGSLPKVEETDMEDEFEQPTMSFESYLSYDQPRKKKKKIVKTSATALGDKGLKKNDSKSTGKNLDSVQKLPKVNKTKSEKPAGADLAKLRKVPDVLPVLPDLPLPAIQANYRPLPSLELISSFQPKRKAFSSPQEEEEAGFTGRRMNSKMQVYSGSKCAYLPKMMTLHQQCIRVLKNNIDSIFEVGGVPYSVLEPVLERCTPDQLYRIEEYNHVLIEETDQLWKVHCHRDFKEERPEEYESWREMYLRLQDAREQRLRVLTKNIQFAHANKPKGRQAKMAFVNSVAKPPRDVRRRQEKFGTGGAAVPEKIKIKPAPYPMGSSHASASSISFNPSPEEPAYDGPSTSSAHLAPVVSSTVSYDPRKPTVKKIAPMMAKTIKAFKNRFSRR

752 NP_003833.3 NP_001073594.1

>NP_003833.3

MEQRGQNAPAASGARKRHGPGPREARGARPGLRVPKTLVLVVAAVLLLVSAESALITQQDLAPQQRAAPQQKRSSPSEGLCPPGHHISEDGRDCISCKYGQDYSTHWNDLLFCLRCTRCDSGEVELSPCTTTRNTVCQCEEGTFREEDSPEMCRKCRTGCPRGMVKVGDCTPWSDIECVHKESGTKHSGEAPAVEETVTSSPGTPASPCSLSGIIIGVTVAAVVLIVAVFVCKSLLWKKVLPYLKGICSGGGGDPERVDRSSQRPGAEDNVLNEIVSILQPTQVPEQEMEVQEPAEPTGVNMLSPGESEHLLEPAEAERSQRRRLLVPANEGDPTETLRQCFDDFADLVPFDSWEPLMRKLGLMDNEIKVAKAEAAGHRDTLYTMLIKWVNKTGRDASVHTLLDALETLGERLAKQKIEDHLLSSGKFMYLEGNADSAMS

>NP_001073594.1

MEGGRRARVVIESKRNFFLGAFPTPFPAEHVELGRLGDSETAMVPGKGGADYILLPFKKMDFSRNLYDIGEQLDSEDLASLKFLSLDYIPQRKQEPIKDALMLFQRLQEKRMLEESNLSFLKELLFRINRLDLLITYLNTRKEEMERELQTPGRAQISAYRVMLYQISEEVSRSELRSFKFLLQEEISKCKLDDDMNLLDIFIEMEKRVILGEGKLDILKRVCAQINKSLLKIINDYEEFSKERSSSLEGSPDEFSNGEELCGVMTISDSPREQDSESQTLDKVYQMKSKPRGYCLIINNHNFAKAREKVPKLHSIRDRNGTHLDAGALTTTFEELHFEIKPHDDCTVEQIYEILKIYQLMDHSNMDCFICCILSHGDKGIIYGTDGQEAPIYELTSQFTGLKCPSLAGKPKVFFIQACQGDNYQKGIPVETDSEEQPYLEMDLSSPQTRYIPDEADFLLGMATVNNCVSYRNPAEGTWYIQSLCQSLRERCPRGDDILTILTEVNYEVSNKDDKKNMGKQMPQPTFTLRKKLVFPSD

753 AAH07560.1 NP_004459.2

>AAH07560.1

MNPNCARCGKIVYPTEKVNCLDKFWHKACFHCETCKMTLNMKNYKGYEKKPYCNAHYPKQSFTMVADTPENLRLKQQSELQSQVRYKEEFEKNKGKGFSVVADTPELQRIKKTQDQISNIKYHEEFEKSRMGPSGGEGMEPERRDSQDGSSYRRPLEQQQPHHIPTSAPVYQQPQQQPVAQSYGGYKEPAAPVSIQRSAPICLQHIPRHRIRPGRDPSILQCLCFLKPATACDSYPSSSFFCQLKPSSATSAGSLLWQASPLIDFLVFSLDGTGMGLSGGGRGPWGRAGMGDLLACGPHLPLCSLPSHPPAQLLTYPHIPGLG

>NP_004459.2

MSESFDCAKCNESLYGRKYIQTDSGPYCVPCYDNTFANTCAECQQLIGHDSRELFYEDRHFHEGCFRCCRCQRSLADEPFTCQDSELLCNDCYCSAFSSQCSACGETVMPGSRKLEYGGQTWHEHCFLCSGCEQPLGSRSFVPDKGAHYCVPCYENKFAPRCARCSKTLTQGGVTYRDQPWHRECLVCTGCQTPLAGQQFTSRDEDPYCVACFGELFAPKCSSCKRPIVGLGGGKYVSFEDRHWHHNCFSCARCSTSLVGQGFVPDGDQVLCQGCSQAGP

754 NP_975010.1 NP_001057.1

>NP_975010.1

MDTKSILEELLLKRSQQKKKMSPNNYKERLFVLTKTNLSYYEYDKMKRGSRKGSIEIKKIRCVEKVNLEEQTPVERQYPFQIVYKDGLLYVYASNEESRSQWLKALQKEIRGNPHLLVKYHSGFFVDGKFLCCQQSCKAAPGCTLWEAYANLHTAVNEEKHRVPTFPDRVLKIPRAVPVLKMDAPSSSTTLAQYDNESKKNYGSQPPSSSTSLAQYDSNSKKIYGSQPNFNMQYIPREDFPDWWQVRKLKSSSSSEDVASSNQKERNVNHTTSKISWEFPESSSSEEEENLDDYDWFAGNISRSQSEQLLRQKGKEGAFMVRNSSQVGMYTVSLFSKAVNDKKGTVKHYHVHTNAENKLYLAENYCFDSIPKLIHYHQHNSAGMITRLRHPVSTKANKVPDSVSLGNGIWELKREEITLLKELGSGQFGVVQLGKWKGQYDVAVKMIKEGSMSEDEFFQEAQTMMKLSHPKLVKFYGVCSKEYPIYIVTEYISNGCLLNYLRSHGKGLEPSQLLEMCYDVCEGMAFLESHQFIHRDLAARNCLVDRDLCVKVSDFGMTRYVLDDQYVSSVGTKFPVKWSAPEVFHYFKYSSKSDVWAFGILMWEVFSLGKQPYDLYDNSQVVLKVSQGHRLYRPHLASDTIYQIMYSCWHELPEKRPTFQQLLSSIEPLREKDKH

>NP_001057.1

MAPVAVWAALAVGLELWAAAHALPAQVAFTPYAPEPGSTCRLREYYDQTAQMCCSKCSPGQHAKVFCTKTSDTVCDSCEDSTYTQLWNWVPECLSCGSRCSSDQVETQACTREQNRICTCRPGWYCALSKQEGCRLCAPLRKCRPGFGVARPGTETSDVVCKPCAPGTFSNTTSSTDICRPHQICNVVAIPGNASMDAVCTSTSPTRSMAPGAVHLPQPVSTRSQHTQPTPEPSTAPSTSFLLPMGPSPPAEGSTGDFALPVGLIVGVTALGLLIIGVVNCVIMTQVKKKPLCLQREAKVPHLPADKARGTQGPEQQHLLITAPSSSSSSLESSASALDRRAPTRNQPQAPGVEASGAGEARASTGSSDSSPGGHGTQVNVTCIVNVCSSSDHSSQCSSQASSTMGDTDSSPSESPKDEQVPFSKEECAFRSQLETPETLLGSTEEKPLPLGVPDAGMKPS

755 NP_000261.1 NP_068597.2

>NP_000261.1

MENGYTYEDYKNTAEWLLSHTKHRPQVAIICGSGLGGLTDKLTQAQIFDYSEIPNFPRSTVPGHAGRLVFGFLNGRACVMMQGRFHMYEGYPLWKVTFPVRVFHLLGVDTLVVTNAAGGLNPKFEVGDIMLIRDHINLPGFSGQNPLRGPNDERFGDRFPAMSDAYDRTMRQRALSTWKQMGEQRELQEGTYVMVAGPSFETVAECRVLQKLGADAVGMSTVPEVIVARHCGLRVFGFSLITNKVIMDYESLEKANHEEVLAAGKQAAQKLEQFVSILMASIPLPDKAS

>NP_068597.2

MNSRQAWRLFLSQGRGDRWVSRPRGHFSPALRREFFTTTTKEGYDRRPVDITPLEQRKLTFDTHALVQDLETHGFDKTQAETIVSALTALSNVSLDTIYKEMVTQAQQEITVQQLMAHLDAIRKDMVILEKSEFANLRAENEKMKIELDQVKQQLMHETSRIRADNKLDINLERSRVTDMFTDQEKQLMETTTEFTKKDTQTKSIISETSNKIDAEIASLKTLMESNKLETIRYLAASVFTCLAIALGFYRFWK

756 NP_001075106.1 NP_002107.3

>NP_001075106.1

MTPILTVLICLGLSLGPRTHVQAGHLPKPTLWAEPGSVITQGSPVTLRCQGGQETQEYRLYREKKTALWITRIPQELVKKGQFPIPSITWEHAGRYRCYYGSDTAGRSESSDPLELVVTGAYIKPTLSAQPSPVVNSGGNVILQCDSQVAFDGFSLCKEGEDEHPQCLNSQPHARGSSRAIFSVGPVSPSRRWWYRCYAYDSNSPYEWSLPSDLLELLVLGVSKKPSLSVQPGPIVAPEETLTLQCGSDAGYNRFVLYKDGERDFLQLAGAQPQAGLSQANFTLGPVSRSYGGQYRCYGAHNLSSEWSAPSDPLDILIAGQFYDRVSLSVQPGPTVASGENVTLLCQSQGWMQTFLLTKEGAADDPWRLRSTYQSQKYQAEFPMGPVTSAHAGTYRCYGSQSSKPYLLTHPSDPLELVVSGPSGGPSSPTTGPTSTSAGPEDQPLTPTGSDPQSGLGRHLGVVIGILVAVILLLLLLLLLFLILRHRRQGKHWTSTQRKADFQHPAGAVGPEPTDRGLQWRSSPAADAQEENLYAAVKHTQPEDGVEMDTRQSPHDEDPQAVTYAEVKHSRPRREMASPPSPLSGEFLDTKDRQAEEDRQMDTEAAASEAPQDVTYAQLHSLTLRREATEPPPSQEGPSPAVPSIYATLAIH

>NP_002107.3

MAVMAPRTLLLLLSGALALTQTWAGSHSMRYFFTSVSRPGRGEPRFIAVGYVDDTQFVRFDSDAASQRMEPRAPWIEQEGPEYWDQETRNVKAQSQTDRVDLGTLRGYYNQSEAGSHTIQIMYGCDVGSDGRFLRGYRQDAYDGKDYIALNEDLRSWTAADMAAQITKRKWEAAHEAEQLRAYLDGTCVEWLRRYLENGKETLQRTDPPKTHMTHHPISDHEATLRCWALGFYPAEITLTWQRDGEDQTQDTELVETRPAGDGTFQKWAAVVVPSGEEQRYTCHVQHEGLPKPLTLRWELSSQPTIPIVGIIAGLVLLGAVITGAVVAAVMWRRKSSDRKGGSYTQAASSDSAQGSDVSLTACKV

757 NP_653086.1 NP_001663.2

>NP_653086.1

MGTKAQVERKLLCLFILAILLCSLALGSVTVHSSEPEVRIPENNPVKLSCAYSGFSSPRVEWKFDQGDTTRLVCYNNKITASYEDRVTFLPTGITFKSVTREDTGTYTCMVSEEGGNSYGEVKVKLIVLVPPSKPTVNIPSSATIGNRAVLTCSEQDGSPPSEYTWFKDGIVMPTNPKSTRAFSNSSYVLNPTTGELVFDPLSASDTGEYSCEARNGYGTPMTSNAVRMEAVERNVGVIVAAVLVTLILLGILVFGIWFAYSRGHFDRTKKGTSSKKVIYSQPSARSEGEFKQTSSFLV

>NP_001663.2

MDVTRLLLATLLVFLCFFTANSHLPPEEKLRDDRSLRSNSSVNLLDVPSVSIVALNKKSKQIGRKAAEKKRSSKKEASMKKVVRPRTPLSAPCVATRNSCKPPAPACCDPCASCQCRFFRSACSCRVLSLNC

758 NP_056085.1 NP_000323.2

>NP_056085.1

MSWFSGLLVPKVDERKTAWGERNGQKRSRRRGTRAGGFCTPRYMSCLRDAEPPSPTPAGPPRCPWQDDAFIRRGGPGKGKELGLRAVALGFEDTEVTTTAGGTAEVAPDAVPRSGRSCWRRLVQVFQSKQFRSAKLERLYQRYFFQMNQSSLTLLMAVLVLLTAVLLAFHAAPARPQPAYVALLACAAALFVGLMVVCNRHSFRQDSMWVVSYVVLGILAAVQVGGALAADPRSPSAGLWCPVFFVYIAYTLLPIRMRAAVLSGLGLSTLHLILAWQLNRGDAFLWKQLGANVLLFLCTNVIGICTHYPAEVSQRQAFQETRGYIQARLHLQHENRQQERLLLSVLPQHVAMEMKEDINTKKEDMMFHKIYIQKHDNVSILFADIEGFTSLASQCTAQELVMTLNELFARFDKLAAENHCLRIKILGDCYYCVSGLPEARADHAHCCVEMGVDMIEAISLVREVTGVNVNMRVGIHSGRVHCGVLGLRKWQFDVWSNDVTLANHMEAGGRAGRIHITRATLQYLNGDYEVEPGRGGERNAYLKEQHIETFLILGASQKRKEEKAMLAKLQRTRANSMEGLMPRWVPDRAFSRTKDSKAFRQMGIDDSSKDNRGTQDALNPEDEVDEFLSRAIDARSIDQLRKDHVRRFLLTFQREDLEKKYSRKVDPRFGAYVACALLVFCFICFIQLLIFPHSTLMLGIYASIFLLLLITVLICAVYSCGSLFPKALQRLSRSIVRSRAHSTAVGIFSVLLVFTSAIANMFTCNHTPIRSCAARMLNLTPADITACHLQQLNYSLGLDAPLCEGTMPTCSFPEYFIGNMLLSLLASSVFLHISSIGKLAMIFVLGLIYLVLLLLGPPATIFDNYDLLLGVHGLASSNETFDGLDCPAAGRVALKYMTPVILLVFALALYLHAQQVESTARLDFLWKLQATGEKEEMEELQAYNRRLLHNILPKDVAAHFLARERRNDELYYQSCECVAVMFASIANFSEFYVELEANNEGVECLRLLNEIIADFDEIISEERFRQLEKIKTIGSTYMAASGLNASTYDQVGRSHITALADYAMRLMEQMKHINEHSFNNFQMKIGLNMGPVVAGVIGARKPQYDIWGNTVNVSSRMDSTGVPDRIQVTTDLYQVLAAKGYQLECRGVVKVKGKGEMTTYFLNGGPSS

>NP_000323.2

MKSNQERSNECLPPKKREIPATSRSSEEKAPTLPSDNHRVEGTAWLPGNPGGRGHGGGRHGPAGTSVELGLQQGIGLHKALSTGLDYSPPSAPRSVPVATTLPAAYATPQPGTPVSPVQYAHLPHTFQFIGSSQYSGTYASFIPSQLIPPTANPVTSAVASAAGATTPSQRSQLEAYSTLLANMGSLSQTPGHKAEQQQQQQQQQQQQHQHQQQQQQQQQQQQQQHLSRAPGLITPGSPPPAQQNQYVHISSSPQNTGRTASPPAIPVHLHPHQTMIPHTLTLGPPSQVVMQYADSGSHFVPREATKKAESSRLQQAIQAKEVLNGEMEKSRRYGAPSSADLGLGKAGGKSVPHPYESRHVVVHPSPSDYSSRDPSGVRASVMVLPNSNTPAADLEVQQATHREASPSTLNDKSGLHLGKPGHRSYALSPHTVIQTTHSASEPLPVGLPATAFYAGTQPPVIGYLSGQQQAITYAGSLPQHLVIPGTQPLLIPVGSTDMEASGAAPAIVTSSPQFAAVPHTFVTTALPKSENFNPEALVTQAAYPAMVQAQIHLPVVQSVASPAAAPPTLPPYFMKGSIIQLANGELKKVEDLKTEDFIQSAEISNDLKIDSSTVERIEDSHSPGVAVIQFAVGEHRAQVSVEVLVEYPFFVFGQGWSSCCPERTSQLFDLPCSKLSVGDVCISLTLKNLKNGSVKKGQPVDPASVLLKHSKADGLAGSRHRYAEQENGINQGSAQMLSENGELKFPEKMGLPAAPFLTKIEPSKPAATRKRRWSAPESRKLEKSEDEPPLTLPKPSLIPQEVKICIEGRSNVGK

759 NP_001137.2 NP_002196.2

>NP_001137.2

MTVFLSFAFLAAILTHIGCSNQRRSPENSGRRYNRIQHGQCAYTFILPEHDGNCRESTTDQYNTNALQRDAPHVEPDFSSQKLQHLEHVMENYTQWLQKLENYIVENMKSEMAQIQQNAVQNHTATMLEIGTSLLSQTAEQTRKLTDVETQVLNQTSRLEIQLLENSLSTYKLEKQLLQQTNEILKIHEKNSLLEHKILEMEGKHKEELDTLKEEKENLQGLVTRQTYIIQELEKQLNRATTNNSVLQKQQLELMDTVHNLVNLCTKEGVLLKGGKREEEKPFRDCADVYQAGFNKSGIYTIYINNMPEPKKVFCNMDVNGGGWTVIQHREDGSLDFQRGWKEYKMGFGNPSGEYWLGNEFIFAITSQRQYMLRIELMDWEGNRAYSQYDRFHIGNEKQNYRLYLKGHTGTAGKQSSLILHGADFSTKDADNDNCMCKCALMLTGGWWFDACGPSNLNGMFYTAGQNHGKLNGIKWHYFKGPSYSLRSTTMMIRPLDF

>NP_002196.2

MGSRTPESPLHAVQLRWGPRRRPPLLPLLLLLLPPPPRVGGFNLDAEAPAVLSGPPGSFFGFSVEFYRPGTDGVSVLVGAPKANTSQPGVLQGGAVYLCPWGASPTQCTPIEFDSKGSRLLESSLSSSEGEEPVEYKSLQWFGATVRAHGSSILACAPLYSWRTEKEPLSDPVGTCYLSTDNFTRILEYAPCRSDFSWAAGQGYCQGGFSAEFTKTGRVVLGGPGSYFWQGQILSATQEQIAESYYPEYLINLVQGQLQTRQASSIYDDSYLGYSVAVGEFSGDDTEDFVAGVPKGNLTYGYVTILNGSDIRSLYNFSGEQMASYFGYAVAATDVNGDGLDDLLVGAPLLMDRTPDGRPQEVGRVYVYLQHPAGIEPTPTLTLTGHDEFGRFGSSLTPLGDLDQDGYNDVAIGAPFGGETQQGVVFVFPGGPGGLGSKPSQVLQPLWAASHTPDFFGSALRGGRDLDGNGYPDLIVGSFGVDKAVVYRGRPIVSASASLTIFPAMFNPEERSCSLEGNPVACINLSFCLNASGKHVADSIGFTVELQLDWQKQKGGVRRALFLASRQATLTQTLLIQNGAREDCREMKIYLRNESEFRDKLSPIHIALNFSLDPQAPVDSHGLRPALHYQSKSRIEDKAQILLDCGEDNICVPDLQLEVFGEQNHVYLGDKNALNLTFHAQNVGEGGAYEAELRVTAPPEAEYSGLVRHPGNFSSLSCDYFAVNQSRLLVCDLGNPMKAGASLWGGLRFTVPHLRDTKKTIQFDFQILSKNLNNSQSDVVSFRLSVEAQAQVTLNGVSKPEAVLFPVSDWHPRDQPQKEEDLGPAVHHVYELINQGPSSISQGVLELSCPQALEGQQLLYVTRVTGLNCTTNHPINPKGLELDPEGSLHHQQKREAPSRSSASSGPQILKCPEAECFRLRCELGPLHQQESQSLQLHFRVWAKTFLQREHQPFSLQCEAVYKALKMPYRILPRQLPQKERQVATAVQWTKAEGSYGVPLWIIILAILFGLLLLGLLIYILYKLGFFKRSLPYGTAMEKAQLKPPATSDA

760 NP_954986.2 NP_000336.1

>NP_954986.2

MPTPDATTPQAKGFRRAVSELDAKQAEAIMVRGQGAPGPSLTGSPWPGTAAPAASYTPTPRSPRFIGRRQSLIEDARKEREAAVAAAAAAVPSEPGDPLEAVAFEEKEGKAVLNLLFSPRATKPSALSRAVKVFETFEAKIHHLETRPAQRPRAGGPHLEYFVRLEVRRGDLAALLSGVRQVSEDVRSPAGPKVPWFPRKVSELDKCHHLVTKFDPDLDLDHPGFSDQVYRQRRKLIAEIAFQYRHGDPIPRVEYTAEEIATWKEVYTTLKGLYATHACGEHLEAFALLERFSGYREDNIPQLEDVSRFLKERTGFQLRPVAGLLSARDFLASLAFRVFQCTQYIRHASSPMHSPEPDCCHELLGHVPMLADRTFAQFSQDIGLASLGASDEEIEKLSTLYWFTVEFGLCKQNGEVKAYGAGLLSSYGELLHCLSEEPEIRAFDPEAAAVQPYQDQTYQSVYFVSESFSDAKDKLRSYASRIQRPFSVKFDPYTLAIDVLDSPQAVRRSLEGVQDELDTLAHALSAIG

>NP_000336.1

MDVFMDVMKGLSKAKEGVVAAAEKTKQGVAEAAGKTKEGVLYVGSKTKEGVVHGVATVAEKTKEQVTNVGGAVVTGVTAVAQKTVEGAGSIAAATGFVKKDQLGKNEEGAPQEGILEDMPVDPDNEAYEMPSEEGYQDYEPEA

761 NP_003815.1 NP_005585.1

>NP_003815.1

MDPFLVLLHSVSSSLSSSELTELKFLCLGRVGKRKLERVQSGLDLFSMLLEQNDLEPGHTELLRELLASLRRHDLLRRVDDFEAGAAAGAAPGEEDLCAAFNVICDNVGKDWRRLARQLKVSDTKIDSIEDRYPRNLTERVRESLRIWKNTEKENATVAHLVGALRSCQMNLVADLVQEVQQARDLQNRSGAMSPMSWNSDASTSEAS

>NP_005585.1

MPGEATETVPATEQELPQPQAETGSGTESDSDESVPELEEQDSTQATTQQAQLAAAAEIDEEPVSKAKQSRSEKKARKAMSKLGLRQVTGVTRVTIRKSKNILFVITKPDVYKSPASDTYIVFGEAKIEDLSQQAQLAAAEKFKVQGEAVSNIQENTQTPTVQEESEEEEVDETGVEVKDIELVMSQANVSRAKAVRALKNNSNDIVNAIMELTM

762 NP_001918.3 NP_004406.2

>NP_001918.3

MSQAYSSSQRVSSYRRTFGGAPGFPLGSPLSSPVFPRAGFGSKGSSSSVTSRVYQVSRTSGGAGGLGSLRASRLGTTRTPSSYGAGELLDFSLADAVNQEFLTTRTNEKVELQELNDRFANYIEKVRFLEQQNAALAAEVNRLKGREPTRVAELYEEELRELRRQVEVLTNQRARVDVERDNLLDDLQRLKAKLQEEIQLKEEAENNLAAFRADVDAATLARIDLERRIESLNEEIAFLKKVHEEEIRELQAQLQEQQVQVEMDMSKPDLTAALRDIRAQYETIAAKNISEAEEWYKSKVSDLTQAANKNNDALRQAKQEMMEYRHQIQSYTCEIDALKGTNDSLMRQMRELEDRFASEASGYQDNIARLEEEIRHLKDEMARHLREYQDLLNVKMALDVEIATYRKLLEGEESRINLPIQTYSALNFRETSPEQRGSEVHTKKTVMIKTIETRDGEVVSEATQQQHEVL

>NP_004406.2

MSCNGGSHPRINTLGRMIRAESGPDLRYEVTSGGGGTSRMYYSRRGVITDQNSDGYCQTGTMSRHQNQNTIQELLQNCSDCLMRAELIVQPELKYGDGIQLTRSRELDECFAQANDQMEILDSLIREMRQMGQPCDAYQKRLLQLQEQMRALYKAISVPRVRRASSKGGGGYTCQSGSGWDEFTKHVTSECLGWMRQQRAEMDMVAWGVDLASVEQHINSHRGIHNSIGDYRWQLDKIKADLREKSAIYQLEEEYENLLKASFERMDHLRQLQNIIQATSREIMWINDCEEEELLYDWSDKNTNIAQKQEAFSIRMSQLEVKEKELNKLKQESDQLVLNQHPASDKIEAYMDTLQTQWSWILQITKCIDVHLKENAAYFQFFEEAQSTEAYLKGLQDSIRKKYPCDKNMPLQHLLEQIKELEKEREKILEYKRQVQNLVNKSKKIVQLKPRNPDYRSNKPIILRALCDYKQDQKIVHKGDECILKDNNERSKWYVTGPGGVDMLVPSVGLIIPPPNPLAVDLSCKIEQYYEAILALWNQLYINMKSLVSWHYCMIDIEKIRAMTIAKLKTMRQEDYMKTIADLELHYQEFIRNSQGSEMFGDDDKRKIQSQFTDAQKHYQTLVIQLPGYPQHQTVTTTEITHHGTCQDVNHNKVIETNRENDKQETWMLMELQKIRRQIEHCEGRMTLKNLPLADQGSSHHITVKINELKSVQNDSQAIAEVLNQLKDMLANFRGSEKYCYLQNEVFGLFQKLENINGVTDGYLNSLCTVRALLQAILQTEDMLKVYEARLTEEETVCLDLDKVEAYRCGLKKIKNDLNLKKSLLATMKTELQKAQQIHSQTSQQYPLYDLDLGKFGEKVTQLTDRWQRIDKQIDFRLWDLEKQIKQLRNYRDNYQAFCKWLYDAKRRQDSLESMKFGDSNTVMRFLNEQKNLHSEISGKRDKSEEVQKIAELCANSIKDYELQLASYTSGLETLLNIPIKRTMIQSPSGVILQEAADVHARYIELLTRSGDYYRFLSEMLKSLEDLKLKNTKIEVLEEELRLARDANSENCNKNKFLDQNLQKYQAECSQFKAKLASLEELKRQAELDGKSAKQNLDKCYGQIKELNEKITRLTYEIEDEKRRRKSVEDRFDQQKNDYDQLQKARQCEKENLGWQKLESEKAIKEKEYEIERLRVLLQEEGTRKREYENELAKVRNHYNEEMSNLRNKYETEINITKTTIKEISMQKEDDSKNLRNQLDRLSRENRDLKDEIVRLNDSILQATEQRRRAEENALQQKACGSEIMQKKQHLEIELKQVMQQRSEDNARHKQSLEEAAKTIQDKNKEIERLKAEFQEEAKRRWEYENELSKVRNNYDEEIISLKNQFETEINITKTTIHQLTMQKEEDTSGYRAQIDNLTRENRSLSEEIKRLKNTLTQTTENLRRVEEDIQQQKATGSEVSQRKQQLEVELRQVTQMRTEESVRYKQSLDDAAKTIQDKNKEIERLKQLIDKETNDRKCLEDENARLQRVQYDLQKANSSATETINKLKVQEQELTRLRIDYERVSQERTVKDQDITRFQNSLKELQLQKQKVEEELNRLKRTASEDSCKRKKLEEELEGMRRSLKEQAIKITNLTQQLEQASIVKKRSEDDLRQQRDVLDGHLREKQRTQEELRRLSSEVEALRRQLLQEQESVKQAHLRNEHFQKAIEDKSRSLNESKIEIERLQSLTENLTKEHLMLEEELRNLRLEYDDLRRGRSEADSDKNATILELRSQLQISNNRTLELQGLINDLQRERENLRQEIEKFQKQALEASNRIQESKNQCTQVVQERESLLVKIKVLEQDKARLQRLEDELNRAKSTLEAETRVKQRLECEKQQIQNDLNQWKTQYSRKEEAIRKIESEREKSEREKNSLRSEIERLQAEIKRIEERCRRKLEDSTRETQSQLETERSRYQREIDKLRQRPYGSHRETQTECEWTVDTSKLVFDGLRKKVTAMQLYECQLIDKTTLDKLLKGKKSVEEVASEIQPFLRGAGSIAGASASPKEKYSLVEAKRKKLISPESTVMLLEAQAATGGIIDPHRNEKLTVDSAIARDLIDFDDRQQIYAAEKAITGFDDPFSGKTVSVSEAIKKNLIDRETGMRLLEAQIASGGVVDPVNSVFLPKDVALARGLIDRDLYRSLNDPRDSQKNFVDPVTKKKVSYVQLKERCRIEPHTGLLLLSVQKRSMSFQGIRQPVTVTELVDSGILRPSTVNELESGQISYDEVGERIKDFLQGSSCIAGIYNETTKQKLGIYEAMKIGLVRPGTALELLEAQAATGFIVDPVSNLRLPVEEAYKRGLVGIEFKEKLLSAERAVTGYNDPETGNIISLFQAMNKELIEKGHGIRLLEAQIATGGIIDPKESHRLPVDIAYKRGYFNEELSEILSDPSDDTKGFFDPNTEENLTYLQLKERCIKDEETGLCLLPLKEKKKQVQTSQKNTLRKRRVVIVDPETNKEMSVQEAYKKGLIDYETFKELCEQECEWEEITITGSDGSTRVVLVDRKTGSQYDIQDAIDKGLVDRKFFDQYRSGSLSLTQFADMISLKNGVGTSSSMGSGVSDDVFSSSRHESVSKISTISSVRNLTIRSSSFSDTLEESSPIAAIFDTENLEKISITEGIERGIVDSITGQRLLEAQACTGGIIHPTTGQKLSLQDAVSQGVIDQDMATRLKPAQKAFIGFEGVKGKKKMSAAEAVKEKWLPYEAGQRFLEFQYLTGGLVDPEVHGRISTEEAIRKGFIDGRAAQRLQDTSSYAKILTCPKTKLKISYKDAINRSMVEDITGLRLLEAASVSSKGLPSPYNMSSAPGSRSGSRSGSRSGSRSGSRSGSRRGSFDATGNSSYSYSYSFSSSSIGH

763 NP_003749.2 NP_003741.1

>NP_003749.2

MAAAAAAAGDSDSWDADAFSVEDPVRKVGGGGTAGGDRWEGEDEDEDVKDNWDDDDDEKKEEAEVKPEVKISEKKKIAEKIKEKERQQKKRQEEIKKRLEEPEEPKVLTPEEQLADKLRLKKLQEESDLELAKETFGVNNAVYGIDAMNPSSRDDFTEFGKLLKDKITQYEKSLYYASFLEVLVRDVCISLEIDDLKKITNSLTVLCSEKQKQEKQSKAKKKKKGVVPGGGLKATMKDDLADYGGYDGGYVQDYEDFM

>NP_003741.1

MPAYFQRPENALKRANEFLEVGKKQPALDVLYDVMKSKKHRTWQKIHEPIMLKYLELCVDLRKSHLAKEGLYQYKNICQQVNIKSLEDVVRAYLKMAEEKTEAAKEESQQMVLDIEDLDNIQTPESVLLSAVSGEDTQDRTDRLLLTPWVKFLWESYRQCLDLLRNNSRVERLYHDIAQQAFKFCLQYTRKAEFRKLCDNLRMHLSQIQRHHNQSTAINLNNPESQSMHLETRLVQLDSAISMELWQEAFKAVEDIHGLFSLSKKPPKPQLMANYYNKVSTVFWKSGNALFHASTLHRLYHLSREMRKNLTQDEMQRMSTRVLLATLSIPITPERTDIARLLDMDGIIVEKQRRLATLLGLQAPPTRIGLINDMVRFNVLQYVVPEVKDLYNWLEVEFNPLKLCERVTKVLNWVREQPEKEPELQQYVPQLQNNTILRLLQQVSQIYQSIEFSRLTSLVPFVDAFQLERAIVDAARHCDLQVRIDHTSRTLSFGSDLNYATREDAPIGPHLQSMPSEQIRNQLTAMSSVLAKALEVIKPAHILQEKEEQHQLAVTAYLKNSRKEHQRILARRQTIEERKERLESLNIQREKEELEQREAELQKVRKAEEERLRQEAKEREKERILQEHEQIKKKTVRERLEQIKKTELGAKAFKDIDIEDLEELDPDFIMAKQVEQLEKEKKELQERLKNQEKKIDYFERAKRLEEIPLIKSAYEEQRIKDMDLWEQQEEERITTMQLEREKALEHKNRMSRMLEDRDLFVMRLKAARQSVYEEKLKQFEERLAEERHNRLEERKRQRKEERRITYYREKEEEEQRRAEEQMLKEREERERAERAKREEELREYQERVKKLEEVERKKRQRELEIEERERRREEERRLGDSSLSRKDSRWGDRDSEGTWRKGPEADSEWRRGPPEKEWRRGEGRDEDRSHRRDEERPRRLGDDEDREPSLRPDDDRVPRRGMDDDRGPRRGPEEDRFSRRGADDDRPSWRNTDDDRPPRRIADEDRGNWRHADDDRPPRRGLDEDRGSWRTADEDRGPRRGMDDDRGPRRGGADDERSSWRNADDDRGPRRGLDDDRGPRRGMDDDRGPRRGMDDDRGPRRGMDDDRGPRRGLDDDRGPWRNADDDRIPRRGAEDDRGPWRNMDDDRLSRRADDDRFPRRGDDSRPGPWRPLVKPGGWREKEKAREESWGPPRESRPSEEREWDREKERDRDNQDREENDKDPERERDRERDVDREDRFRRPRDEGGWRRGPAEESSSWRDSSRRDDRDRDDRRRERDDRRDLRERRDLRDDRDRRGPPLRSEREEVSSWRRADDRKDDRVEERDPPRRVPPPALSRDRERDRDREREGEKEKASWRAEKDRESLRRTKNETDEDGWTTVRR

764 NP_001078923.1 NP_001035919.1

>NP_001078923.1

MSRKGPRAEVCADCSAPDPGWASISRGVLVCDECCSVHRSLGRHISIVKHLRHSAWPPTLLQMVHTLASNGANSIWEHSLLDPAQVQSGRRKANPQDKVHPIKSEFIRAKYQMLAFVHKLPCRDDDGVTAKDLSKQLHSSVRTGNLETCLRLLSLGAQANFFHPEKGTTPLHVAAKAGQTLQAELLVVYGADPGSPDVNGRTPIDYARQAGHHELAERLVECQYELTDRLAFYLCGRKPDHKNGHYIIPQMADRSRQKCMSQSLDLSELAKAAKKKLQALSNRLFEELAMDVYDEVDRRENDAVWLATQNHSTLVTERSAVPFLPVNPEYSATRNQGRQKLARFNAREFATLIIDILSEAKRRQQGKSLSSPTDNLELSLRSQSDLDDQHDYDSVASDEDTDQEPLRSTGATRSNRARSMDSSDLSDGAVTLQEYLELKKALATSEAKVQQLMKVNSSLSDELRRLQREIHKLQAENLQLRQPPGPVPTPPLPSERAEHTPMAPGGSTHRRDRQAFSMYEPGSALKPFGGPPGDELTTRLQPFHSTELEDDAIYSVHVPAGLYRIRKGVSASAVPFTPSSPLLSCSQEGSRHTSKLSRHGSGADSDYENTQSGDPLLGLEGKRFLELGKEEDFHPELESLDGDLDPGLPSTEDVILKTEQVTKNIQELLRAAQEFKHDSFVPCSEKIHLAVTEMASLFPKRPALEPVRSSLRLLNASAYRLQSECRKTVPPEPGAPVDFQLLTQQVIQCAYDIAKAAKQLVTITTREKKQ

>NP_001035919.1

MEDLDALLSDLETTTSHMPRSGAPKERPAEPLTPPPSYGHQPQTGSGESSGASGDKDHLYSTVCKPRSPKPAAPAAPPFSSSSGVLGTGLCELDRLLQELNATQFNITDEIMSQFPSSKVASGEQKEDQSEDKKRPSLPSSPSPGLPKASATSATLELDRLMASLSDFRVQNHLPASGPTQPPVVSSTNEGSPSPPEPTGKGSLDTMLGLLQSDLSRRGVPTQAKGLCGSCNKPIAGQVVTALGRAWHPEHFVCGGCSTALGGSSFFEKDGAPFCPECYFERFSPRCGFCNQPIRHKMVTALGTHWHPEHFCCVSCGEPFGDEGFHEREGRPYCRRDFLQLFAPRCQGCQGPILDNYISALSALWHPDCFVCRECFAPFSGGSFFEHEGRPLCENHFHARRGSLCATCGLPVTGRCVSALGRRFHPDHFTCTFCLRPLTKGSFQERAGKPYCQPCFLKLFG

765 NP_006624.2 NP_001005386.1

>NP_006624.2

MPHEELPSLQRPRYGSIVDDERLSAEEMDERRRQNIAYEYLCHLEEAKRWMEVCLVEELPPTTELEEGLRNGVYLAKLAKFFAPKMVSEKKIYDVEQTRYKKSGLHFRHTDNTVQWLRAMESIGLPKIFYPETTDVYDRKNIPRMIYCIHALSLYLFKLGIAPQIQDLLGKVDFTEEEISNMRKELEKYGIQMPSFSKIGGILANELSVDEAALHAAVIAINEAVEKGIAEQTVVTLRNPNAVLTLVDDNLAPEYQKELWDAKKKKEENARLKNSCISEEERDAYEELLTQAEIQGNINKVNRQAAVDHINAVIPEGDPENTLLALKKPEAQLPAVYPFAAAMYQNELFNLQKQNTMNYLAHEELLIAVEMLSAVALLNQALESNDLVSVQNQLRSPAIGLNNLDKAYVERYANTLLSVKLEVLSQGQDNLSWNEIQNCIDMVNAQIQEENDRVVAVGYINEAIDEGNPLRTLETLLLPTANISDVDPAHAQHYQDVLYHAKSQKLGDSESVSKVLWLDEIQQAVDDANVDEDRAKQWVTLVVDVNQCLEGKKSSDILSVLKSSTSNANDIIPECADKYYDALVKAKELKSERVSSDGSWLKLNLHKKYDYYYNTDSKESSWVTPESCLYKESWLTGKEIEDIIEEVTVGYIRENIWSASEELLLRFQATSSGPILREEFEARKSFLHEQEENVVKIQAFWKGYKQRKEYMHRRQTFIDNTDSIVKIQSWFRMATARKSYLSRLQYFRDHNNEIVKIQSLLRANKARDDYKTLVGSENPPLTVIRKFVYLLDQSDLDFQEELEVARLREEVVTKIRANQQLEKDLNLMDIKIGLLVKNRITLEDVISHSKKLNKKKGGEMEILNNTDNQGIKSLSKERRKTLETYQQLFYLLQTNPLYLAKLIFQMPQNKSTKFMDTVIFTLYNYASNQREEYLLLKLFKTALEEEIKSKVDQVQDIVTGNPTVIKMVVSFNRGARGQNTLRQLLAPVVKEIIDDKSLIINTNPVEVYKAWVNQLETQTGEASKLPYDVTTEQALTYPEVKNKLEASIENLRRVTDKVLNSIISSLDLLPYGLRYIAKVLKNSIHEKFPDATEDELLKIVGNLLYYRYMNPAIVAPDGFDIIDMTAGGQINSDQRRNLGSVAKVLQHAASNKLFEGENEHLSSMNNYLSETYQEFRKYFKEACNVPEPEEKFNMDKYTDLVTVSKPVIYISIEEIISTHSLLLEHQDAIAPEKNDLLSELLGSLGEVPTVESFLGEGAVDPNDPNKANTLSQLSKTEISLVLTSKYDIEDGEAIDSRSLMIKTKKLIIDVIRNQPGNTLTEILETPATAQQEVDHATDMVSRAMIDSRTPEEMKHSQSMIEDAQLPLEQKKRKIQRNLRTLEQTGHVSSENKYQDILNEIAKDIRNQRIYRKLRKAELAKLQQTLNALNKKAAFYEEQINYYDTYIKTCLDNLKRKNTRRSIKLDGKGEPKGAKRAKPVKYTAAKLHEKGVLLDIDDLQTNQFKNVTFDIIATEDVGIFDVRSKFLGVEMEKVQLNIQDLLQMQYEGVAVMKMFDKVKVNVNLLIYLLNKKFYGK

>NP_001005386.1

MDSQGRKVVVCDNGTGFVKCGYAGSNFPEHIFPALVGRPIIRSTTKVGNIEIKNNKKMDLMVGDEASELRSMLEVNYPMENGIVRNWDDMKHLWDYTFGPEKLNIDTRNCKILLTEPPMNPTKNREKIVEVMFETYQFSGVYVAIQAVLTLYAQGLLTGVVVDSGDGVTHICPVYEGFSLPHLTRRLDIAGRDITRYLIKLLLLRGYAFNHSADFETVRMIKEKLCYVGYNIEQEQKLALETTVLVESYTLPDGRIIKVGGERFEAPEALFQPHLINVEGVGVAELLFNTIQAADIDTRSEFYKHIVLSGGSTMYPGLPSRLERELKQLYLERVLKGDVEKLSKFKIRIEDPPRRKHMVFLGGAVLADIMKDKDNFWMTRQEYQEKGVRVLEKLGVTVR

766 NP_006262.1 NP_001918.3

>NP_006262.1

MGSELETAMETLINVFHAHSGKEGDKYKLSKKELKELLQTELSGFLDAQKDVDAVDKVMKELDENGDGEVDFQEYVVLVAALTVACNNFFWENS

>NP_001918.3

MSQAYSSSQRVSSYRRTFGGAPGFPLGSPLSSPVFPRAGFGSKGSSSSVTSRVYQVSRTSGGAGGLGSLRASRLGTTRTPSSYGAGELLDFSLADAVNQEFLTTRTNEKVELQELNDRFANYIEKVRFLEQQNAALAAEVNRLKGREPTRVAELYEEELRELRRQVEVLTNQRARVDVERDNLLDDLQRLKAKLQEEIQLKEEAENNLAAFRADVDAATLARIDLERRIESLNEEIAFLKKVHEEEIRELQAQLQEQQVQVEMDMSKPDLTAALRDIRAQYETIAAKNISEAEEWYKSKVSDLTQAANKNNDALRQAKQEMMEYRHQIQSYTCEIDALKGTNDSLMRQMRELEDRFASEASGYQDNIARLEEEIRHLKDEMARHLREYQDLLNVKMALDVEIATYRKLLEGEESRINLPIQTYSALNFRETSPEQRGSEVHTKKTVMIKTIETRDGEVVSEATQQQHEVL

767 NP_009133.2 NP_002725.1

>NP_009133.2

MRGAGPSPRQSPRTLRPDPGPAMSFFRRKVKGKEQEKTSDVKSIKASISVHSPQKSTKNHALLEAAGPSHVAINAISANMDSFSSSRTATLKKQPSHMEAAHFGDLGRSCLDYQTQETKSSLSKTLEQVLHDTIVLPYFIQFMELRRMEHLVKFWLEAESFHSTTWSRIRAHSLNTVKQSSLAEPVSPSKKHETTASFLTDSLDKRLEDSGSAQLFMTHSEGIDLNNRTNSTQNHLLLSQECDSAHSLRLEMARAGTHQVSMETQESSSTLTVASRNSPASPLKELSGKLMKSIEQDAVNTFTKYISPDAAKPIPITEAMRNDIIARICGEDGQVDPNCFVLAQSIVFSAMEQEHFSEFLRSHHFCKYQIEVLTSGTVYLADILFCESALFYFSEYMEKEDAVNILQFWLAADNFQSQLAAKKGQYDGQEAQNDAMILYDKYFSLQATHPLGFDDVVRLEIESNICREGGPLPNCFTTPLRQAWTTMEKVFLPGFLSSNLYYKYLNDLIHSVRGDEFLGGNVSLTAPGSVGPPDESHPGSSDSSASQSSVKKASIKILKNFDEAIIVDAASLDPESLYQRTYAGKMTFGRVSDLGQFIRESEPEPDVRKSKGSMFSQAMKKWVQGNTDEAQEELAWKIAKMIVSDIMQQAQYDQPLEKSTKL

>NP_002725.1

MESGSTAASEEARSLRECELYVQKHNIQALLKDSIVQLCTARPERPMAFLREYFERLEKEEAKQIQNLQKAGTRTDSREDEISPPPPNPVVKGRRRRGAISAEVYTEEDAASYVRKVIPKDYKTMAALAKAIEKNVLFSHLDDNERSDIFDAMFSVSFIAGETVIQQGDEGDNFYVIDQGETDVYVNNEWATSVGEGGSFGELALIYGTPRAATVKAKTNVKLWGIDRDSYRRILMGSTLRKRKMYEEFLSKVSILESLDKWERLTVADALEPVQFEDGQKIVVQGEPGDEFFIILEGSAAVLQRRSENEEFVEVGRLGPSDYFGEIALLMNRPRAATVVARGPLKCVKLDRPRFERVLGPCSDILKRNIQQYNSFVSLSV

768 NP_002084.2 NP_068779.1

>NP_002084.2

MSGRPRTTSFAESCKPVQQPSAFGSMKVSRDKDGSKVTTVVATPGQGPDRPQEVSYTDTKVIGNGSFGVVYQAKLCDSGELVAIKKVLQDKRFKNRELQIMRKLDHCNIVRLRYFFYSSGEKKDEVYLNLVLDYVPETVYRVARHYSRAKQTLPVIYVKLYMYQLFRSLAYIHSFGICHRDIKPQNLLLDPDTAVLKLCDFGSAKQLVRGEPNVSYICSRYYRAPELIFGATDYTSSIDVWSAGCVLAELLLGQPIFPGDSGVDQLVEIIKVLGTPTREQIREMNPNYTEFKFPQIKAHPWTKDSSGTGHFTSGVRVFRPRTPPEAIALCSRLLEYTPTARLTPLEACAHSFFDELRDPNVKLPNGRDTPALFNFTTQELSSNPPLATILIPPHARIQAAASTPTNATAASDANTGDRGQTNNAASASASNST

>NP_068779.1

MFGLKRNAVIGLNLYCGGAGLGAGSGGATRPGGRLLATEKEASARREIGGGEAGAVIGGSAGASPPSTLTPDSRRVARPPPIGAEVPDVTATPARLLFFAPTRRAAPLEEMEAPAADAIMSPEEELDGYEPEPLGKRPAVLPLLELVGESGNNTSTDGSLPSTPPPAEEEEDELYRQSLEIISRYLREQATGAKDTKPMGRSGATSRKALETLRRVGDGVQRNHETAFQGMLRKLDIKNEDDVKSLSRVMIHVFSDGVTNWGRIVTLISFGAFVAKHLKTINQESCIEPLAESITDVLVRTKRDWLVKQRGWDGFVEFFHVEDLEGGIRNVLLAFAGVAGVGAGLAYLIR

769 NP_001194.1 NP_003274.2

>NP_001194.1

MLLRSAGKLNVGTKKEDGESTAPTPRPKVLRCKCHHHCPEDSVNNICSTDGYCFTMIEEDDSGLPVVTSGCLGLEGSDFQCRDTPIPHQRRSIECCTERNECNKDLHPTLPPLKNRDFVDGPIHHRALLISVTVCSLLLVLIILFCYFRYKRQETRPRYSIGLEQDETYIPPGESLRDLIEQSQSSGSGSGLPLLVQRTIAKQIQMVKQIGKGRYGEVWMGKWRGEKVAVKVFFTTEEASWFRETEIYQTVLMRHENILGFIAADIKGTGSWTQLYLITDYHENGSLYDYLKSTTLDAKSMLKLAYSSVSGLCHLHTEIFSTQGKPAIAHRDLKSKNILVKKNGTCCIADLGLAVKFISDTNEVDIPPNTRVGTKRYMPPEVLDESLNRNHFQSYIMADMYSFGLILWEVARRCVSGGIVEEYQLPYHDLVPSDPSYEDMREIVCIKKLRPSFPNRWSSDECLRQMGKLMTECWAHNPASRLTALRVKKTLAKMSESQDIKL

>NP_003274.2

MSDTEEQEYEEEQPEEEAADEEEEAPEEPEPVAEPEEERPKPSRPVVPPLIPPKIPEGERVDFDDIHRKRMEKDLLELQTLIDVHFEQRKKEEEELVALKERIERRRSERAEQQRFRTEKERERQAKLAEEKMRKEEEEAKKRAEDDAKKKKVLSNMGAHFGGYLVKAEQKRGKRQTGREMKVRILSERKKPLDIDYMGEEQLRARSAWLPPSQPSCPAREKAQELSDWIHQLESEKFDLMAKLKQQKYEINVLYNRISHAQKFRKGAGKGRVGGRWK

770 NP_005850.1 NP_000312.2

>NP_005850.1

MADRDSGSEQGGAALGSGGSLGHPGSGSGSGGGGGGGGGGGGSGGGGGGAPGGLQHETQELASKRVDIQNKRFYLDVKQNAKGRFLKIAEVGAGGNKSRLTLSMSVAVEFRDYLGDFIEHYAQLGPSQPPDLAQAQDEPRRALKSEFLVRENRKYYMDLKENQRGRFLRIRQTVNRGPGLGSTQGQTIALPAQGLIEFRDALAKLIDDYGVEEEPAELPEGTSLTVDNKRFFFDVGSNKYGVFMRVSEVKPTYRNSITVPYKVWAKFGHTFCKYSEEMKKIQEKQREKRAACEQLHQQQQQQQEETAAATLLLQGEEEGEED

>NP_000312.2

MPPKTPRKTAATAAAAAAEPPAPPPPPPPEEDPEQDSGPEDLPLVRLEFEETEEPDFTALCQKLKIPDHVRERAWLTWEKVSSVDGVLGGYIQKKKELWGICIFIAAVDLDEMSFTFTELQKNIEISVHKFFNLLKEIDTSTKVDNAMSRLLKKYDVLFALFSKLERTCELIYLTQPSSSISTEINSALVLKVSWITFLLAKGEVLQMEDDLVISFQLMLCVLDYFIKLSPPMLLKEPYKTAVIPINGSPRTPRRGQNRSARIAKQLENDTRIIEVLCKEHECNIDEVKNVYFKNFIPFMNSLGLVTSNGLPEVENLSKRYEEIYLKNKDLDARLFLDHDKTLQTDSIDSFETQRTPRKSNLDEEVNVIPPHTPVRTVMNTIQQLMMILNSASDQPSENLISYFNNCTVNPKESILKRVKDIGYIFKEKFAKAVGQGCVEIGSQRYKLGVRLYYRVMESMLKSEEERLSIQNFSKLLNDNIFHMSLLACALEVVMATYSRSTSQNLDSGTDLSFPWILNVLNLKAFDFYKVIESFIKAEGNLTREMIKHLERCEHRIMESLAWLSDSPLFDLIKQSKDREGPTDHLESACPLNLPLQNNHTAADMYLSPVRSPKKKGSTTRVNSTANAETQATSAFQTQKPLKSTSLSLFYKKVYRLAYLRLNTLCERLLSEHPELEHIIWTLFQHTLQNEYELMRDRHLDQIMMCSMYGICKVKNIDLKFKIIVTAYKDLPHAVQETFKRVLIKEEEYDSIIVFYNSVFMQRLKTNILQYASTRPPTLSPIPHIPRSPYKFPSSPLRIPGGNIYISPLKSPYKISEGLPTPTKMTPRSRILVSIGESFGTSEKFQKINQMVCNSDRVLKRSAEGSNPPKPLKKLRFDIEGSDEADGSKHLPGESKFQQKLAEMTSTRTRMQKQKMNDSMDTSNKEEK

771 NP_002568.2 NP_001092759.1

>NP_002568.2

MSDNGELEDKPPAPPVRMSSTIFSTGGKDPLSANHSLKPLPSVPEEKKPRHKIISIFSGTEKGSKKKEKERPEISPPSDFEHTIHVGFDAVTGEFTGMPEQWARLLQTSNITKLEQKKNPQAVLDVLKFYDSNTVKQKYLSFTPPEKDGFPSGTPALNAKGTEAPAVVTEEEDDDEETAPPVIAPRPDHTKSIYTRSVIDPVPAPVGDSHVDGAAKSLDKQKKKTKMTDEEIMEKLRTIVSIGDPKKKYTRYEKIGQGASGTVFTATDVALGQEVAIKQINLQKQPKKELIINEILVMKELKNPNIVNFLDSYLVGDELFVVMEYLAGGSLTDVVTETCMDEAQIAAVCRECLQALEFLHANQVIHRDIKSDNVLLGMEGSVKLTDFGFCAQITPEQSKRSTMVGTPYWMAPEVVTRKAYGPKVDIWSLGIMAIEMVEGEPPYLNENPLRALYLIATNGTPELQNPEKLSPIFRDFLNRCLEMDVEKRGSAKELLQHPFLKLAKPLSSLTPLIMAAKEAMKSNR

>NP_001092759.1

MLLGASWLCASKAAAAAAQSEGDEDRPGERRRRRAAATAAGAGEDMDESSLLDLLECSVCLERLDTTAKVLPCQHTFCRRCLESIVCSRHELRCPECRILVGCGVDELPANILLVRLLDGIRQRPRAGTSPGGSPPARPIPGQSAAPTLAGGGGGAAGSTPGSPVFLSAAAGSTAGSLRELATSRTAPAAKNPCLLPYGKALYSYEGKEPGDLKFNKGDIIVLRRKVDEQWYHGELHGTQGFLPASYIQCIQPLPHAPPQGKALYDFEMKDKDQDKDCLTFTKDEILTVLRRVDENWAEGMLGDKIGIFPLLYVELNDSAKQLIEMDKPCPAAASSCNASLPSDSGAVASVAPSPTLSSSGAVSAFQRRVDGKKNTKKRHSFTALSVTHRSSQAASHRHSMEISAPVLISSSDPRAAARIGDLAHLSCAAPTQDVSSSAGSTPTAVPRAASVSGEQGTPPKVQLPLNVYLALYAYKPQKSDELELHKGEMYRVLEKCQDGWFKGASLRTGVSGVFPGNYVTPVSRVPAGGAGPPRNNVVGGSPLAKGITTTMHPGSGSLSSLATATRPALPITTPQAHAQHPTASPPTGSCLRHSAQPTASQARSTISTAAHSAAQAQDRPTATVSPLRTQNSPSRLPATSLRPHSVVSPQHSHQPPVQMCPRPAIPLTSAASAITPPNVSAANLNGEAGGGPIGVLSTSSPTNTGCKLDEKKSEKKEKKSGLLKLLAGASTKKKSRSPPSVSPTHDPQVAVDALLQGAVGPEVSSLSIHGRAGSCPIESEMQGAMGMEPLHRKAGSLDLNFTSPSRQAPLSMAAIRPEPKLLPRERYRVVVSYPPQSEAEIELKEGDIVFVHKKREDGWYKGTLQRNGRTGLFPGSFVESF

772 NP_054762.2 NP_057163.1

>NP_054762.2

MASLFKKKTVDDVIKEQNRELRGTQRAIIRDRAALEKQEKQLELEIKKMAKIGNKEACKVLAKQLVHLRKQKTRTFAVSSKVTSMSTQTKVMNSQMKMAGAMSTTAKTMQAVNKKMDPQKTLQTMQNFQKENMKMEMTEEMINDTLDDIFDGSDDEEESQDIVNQVLDEIGIEISGKMAKAPSAARSLPSASTSKATISDEEIERQLKALGVD

>NP_057163.1

MGLFGKTQEKPPKELVNEWSLKIRKEMRVVDRQIRDIQREEEKVKRSVKDAAKKGQKDVCIVLAKEMIRSRKAVSKLYASKAHMNSVLMGMKNQLAVLRVAGSLQKSTEVMKAMQSLVKIPEIQATMRELSKEMMKAGIIEEMLEDTFESMDDQEEMEEEAEMEIDRILFEITAGALGKAPSKVTDALPEPEPPGAMAASEDEEEEEEALEAMQSRLATLRS

773 NP_037390.2 NP_150634.1

>NP_037390.2

MGRARDAILDALENLTAEELKKFKLKLLSVPLREGYGRIPRGALLSMDALDLTDKLVSFYLETYGAELTANVLRDMGLQEMAGQLQAATHQGSGAAPAGIQAPPQSAAKPGLHFIDQHRAALIARVTNVEWLLDALYGKVLTDEQYQAVRAEPTNPSKMRKLFSFTPAWNWTCKDLLLQALRESQSYLVEDLERS

>NP_150634.1

MADKVLKEKRKLFIRSMGEGTINGLLDELLQTRVLNKEEMEKVKRENATVMDKTRALIDSVIPKGAQACQICITYICEEDSYLAGTLGLSADQTSGNYLNMQDSQGVLSSFPAPQAVQDNPAMPTSSGSEGNVKLCSLEEAQRIWKQKSAEIYPIMDKSSRTRLALIICNEEFDSIPRRTGAEVDITGMTMLLQNLGYSVDVKKNLTASDMTTELEAFAHRPEHKTSDSTFLVFMSHGIREGICGKKHSEQVPDILQLNAIFNMLNTKNCPSLKDKPKVIIIQACRGDSPGVVWFKDSVGVSGNLSLPTTEEFEDDAIKKAHIEKDFIAFCSSTPDNVSWRHPTMGSVFIGRLIEHMQEYACSCDVEEIFRKVRFSFEQPDGRAQMPTTERVTLTRCFYLFPGH

774 NP_002721.1 NP_848928.1

>NP_002721.1

MGNAAAAKKGSEQESVKEFLAKAKEDFLKKWESPAQNTAHLDQFERIKTLGTGSFGRVMLVKHKETGNHYAMKILDKQKVVKLKQIEHTLNEKRILQAVNFPFLVKLEFSFKDNSNLYMVMEYVPGGEMFSHLRRIGRFSEPHARFYAAQIVLTFEYLHSLDLIYRDLKPENLLIDQQGYIQVTDFGFAKRVKGRTWTLCGTPEYLAPEIILSKGYNKAVDWWALGVLIYEMAAGYPPFFADQPIQIYEKIVSGKVRFPSHFSSDLKDLLRNLLQVDLTKRFGNLKNGVNDIKNHKWFATTDWIAIYQRKVEAPFIPKFKGPGDTSNFDDYEEEEIRVSINEKCGKEFSEF

>NP_848928.1

MSETQNSTSQKAMDEDNKAASQTMPNTQDKNYEDELTQVALALVEDVINYAVKIVEEERNPLKNIKWMTHGEFTVEKGLKQIDEYFSKCVSKKCWAHGVEFVERKDLIHSFLYIYYVHWSISTADLPVARISAGTYFTMKVSKTKPPDAPIVVSYVGDHQALVHRPGMVRFRENWQKNLTDAKYSFMESFPFLFNRV

775 NP_003395.1 NP_004808.2

>NP_003395.1

MTMDKSELVQKAKLAEQAERYDDMAAAMKAVTEQGHELSNEERNLLSVAYKNVVGARRSSWRVISSIEQKTERNEKKQQMGKEYREKIEAELQDICNDVLELLDKYLIPNATQPESKVFYLKMKGDYFRYLSEVASGDNKQTTVSNSQQAYQEAFEISKKEMQPTHPIRLGLALNFSVFYYEILNSPEKACSLAKTAFDEAIAELDTLNEESYKDSTLIMQLLRDNLTLWTSENQGDEGDAGEGEN

>NP_004808.2

MPVRGDRGFPPRRELSGWLRAPGMEELIWEQYTVTLQKDSKRGFGIAVSGGRDNPHFENGETSIVISDVLPGGPADGLLQENDRVVMVNGTPMEDVLHSFAVQQLRKSGKVAAIVVKRPRKVQVAALQASPPLDQDDRAFEVMDEFDGRSFRSGYSERSRLNSHGGRSRSWEDSPERGRPHERARSRERDLSRDRSRGRSLERGLDQDHARTRDRSRGRSLERGLDHDFGPSRDRDRDRSRGRSIDQDYERAYHRAYDPDYERAYSPEYRRGARHDARSRGPRSRSREHPHSRSPSPEPRGRPGPIGVLLMKSRANEEYGLRLGSQIFVKEMTRTGLATKDGNLHEGDIILKINGTVTENMSLTDARKLIEKSRGKLQLVVLRDSQQTLINIPSLNDSDSEIEDISEIESNRSFSPEERRHQYSDYDYHSSSEKLKERPSSREDTPSRLSRMGATPTPFKSTGDIAGTVVPETNKEPRYQEDPPAPQPKAAPRTFLRPSPEDEAIYGPNTKMVRFKKGDSVGLRLAGGNDVGIFVAGIQEGTSAEQEGLQEGDQILKVNTQDFRGLVREDAVLYLLEIPKGEMVTILAQSRADVYRDILACGRGDSFFIRSHFECEKETPQSLAFTRGEVFRVVDTLYDGKLGNWLAVRIGNELEKGLIPNKSRAEQMASVQNAQRDNAGDRADFWRMRGQRSGVKKNLRKSREDLTAVVSVSTKFPAYERVLLREAGFKRPVVLFGPIADIAMEKLANELPDWFQTAKTEPKDAGSEKSTGVVRLNTVRQIIEQDKHALLDVTPKAVDLLNYTQWFPIVIFFNPDSRQGVKTMRQRLNPTSNKSSRKLFDQANKLKKTCAHLFTATINLNSANDSWFGSLKDTIQHQQGEAVWVSEGKMEGMDDDPEDRMSYLTAMGADYLSCDSRLISDFEDTDGEGGAYTDNELDEPAEEPLVSSITRSSEPVQHEESIRKPSPEPRAQMRRAASSDQLRDNSPPPAFKPEPPKAKTQNKEESYDFSKSYEYKSNPSAVAGNETPGASTKGYPPPVAAKPTFGRSILKPSTPIPPQEGEEVGESSEEQDNAPKSVLGKVKIFEKMDHKARLQRMQELQEAQNARIEIAQKHPDIYAVPIKTHKPDPGTPQHTSSRPPEPQKAPSRPYQDTRGSYGSDAEEEEYRQQLSEHSKRGYYGQSARYRDTEL

776 NP_003395.1 NP_005535.1

>NP_003395.1

MTMDKSELVQKAKLAEQAERYDDMAAAMKAVTEQGHELSNEERNLLSVAYKNVVGARRSSWRVISSIEQKTERNEKKQQMGKEYREKIEAELQDICNDVLELLDKYLIPNATQPESKVFYLKMKGDYFRYLSEVASGDNKQTTVSNSQQAYQEAFEISKKEMQPTHPIRLGLALNFSVFYYEILNSPEKACSLAKTAFDEAIAELDTLNEESYKDSTLIMQLLRDNLTLWTSENQGDEGDAGEGEN

>NP_005535.1

MASPPESDGFSDVRKVGYLRKPKSMHKRFFVLRAASEAGGPARLEYYENEKKWRHKSSAPKRSIPLESCFNINKRADSKNKHLVALYTRDEHFAIAADSEAEQDSWYQALLQLHNRAKGHHDGAAALGAGGGGGSCSGSSGLGEAGEDLSYGDVPPGPAFKEVWQVILKPKGLGQTKNLIGIYRLCLTSKTISFVKLNSEAAAVVLQLMNIRRCGHSENFFFIEVGRSAVTGPGEFWMQVDDSVVAQNMHETILEAMRAMSDEFRPRSKSQSSSNCSNPISVPLRRHHLNNPPPSQVGLTRRSRTESITATSPASMVGGKPGSFRVRASSDGEGTMSRPASVDGSPVSPSTNRTHAHRHRGSARLHPPLNHSRSIPMPASRCSPSATSPVSLSSSSTSGHGSTSDCLFPRRSSASVSGSPSDGGFISSDEYGSSPCDFRSSFRSVTPDSLGHTPPARGEEELSNYICMGGKGPSTLTAPNGHYILSRGGNGHRCTPGTGLGTSPALAGDEAASAADLDNRFRKRTHSAGTSPTITHQKTPSQSSVASIEEYTEMMPAYPPGGGSGGRLPGHRHSAFVPTRSYPEEGLEMHPLERRGGHHRPDSSTLHTDDGYMPMSPGVAPVPSGRKGSGDYMPMSPKSVSAPQQIINPIRRHPQRVDPNGYMMMSPSGGCSPDIGGGPSSSSSSSNAVPSGTSYGKLWTNGVGGHHSHVLPHPKPPVESSGGKLLPCTGDYMNMSPVGDSNTSSPSDCYYGPEDPQHKPVLSYYSLPRSFKHTQRPGEPEEGARHQHLRLSTSSGRLLYAATADDSSSSTSSDSLGGGYCGARLEPSLPHPHHQVLQPHLPRKVDTAAQTNSRLARPTRLSLGDPKASTLPRAREQQQQQQPLLHPPEPKSPGEYVNIEFGSDQSGYLSGPVAFHSSPSVRCPSQLQPAPREEETGTEEYMKMDLGPGRRAAWQESTGVEMGRLGPAPPGAASICRPTRAVPSSRGDYMTMQMSCPRQSYVDTSPAAPVSYADMRTGIAAEEVSLPRATMAAASSSSAASASPTGPQGAAELAAHSSLLGGPQGPGGMSAFTRVNLSPNRNQSAKVIRADPQGCRRRHSSETFSSTPSATRVGNTVPFGAGAAVGGGGGSSSSSEDVKRHSSASFENVWLRPGELGGAPKEPAKLCGAAGGLENGLNYIDLDLVKDFKQCPQECTPEPQPPPPPPPHQPLGSGESSSTRRSSEDLSAYASISFQKQPEDRQ

777 NP_001005271.1 NP_079351.1

>NP_001005271.1

MVVSEEEEEEEEEGDEEEEEEVEAADEDDEEDDDEGVLGRGPGHDRGRDRHSPPGCHLFPPPPPPPPPLPPPPPPPPPDKDDIRLLPSALGVKKRKRGPKKQKENKPGKPRKRKKRDSEEEFGSERDEYREKSESGGSEYGTGPGRKRRRKHREKKEKKTKRRKKGEGDGGQKQVEQKSSATLLLTWGLEDVEHVFSEEDYHTLTNYKAFSQFMRPLIAKKNPKIPMSKMMTILGAKWREFSANNPFKGSAAAVAAAAAAAAAAVAEQVSAAVSSATPIAPSGPPALPPPPAADIQPPPIRRAKTKEGKGPGHKRRSKSPRVPDGRKKLRGKKMAPLKIKLGLLGGKRKKGGSYVFQSDEGPEPEAEESDLDSGSVHSASGRPDGPVRTKKLKRGRPGRKKKKVLGCPAVAGEEEVDGYETDHQDYCEVCQQGGEIILCDTCPRAYHLVCLDPELDRAPEGKWSCPHCEKEGVQWEAKEEEEEYEEEGEEEGEKEEEDDHMEYCRVCKDGGELLCCDACISSYHIHCLNPPLPDIPNGEWLCPRCTCPVLKGRVQKILHWRWGEPPVAVPAPQQADGNPDVPPPRPLQGRSEREFFVKWVGLSYWHCSWAKELQLEIFHLVMYRNYQRKNDMDEPPPLDYGSGEDDGKSDKRKVKDPHYAEMEEKYYRFGIKPEWMTVHRIINHSVDKKGNYHYLVKWRDLPYDQSTWEEDEMNIPEYEEHKQSYWRHRELIMGEDPAQPRKYKKKKKELQGDGPPSSPTNDPTVKYETQPRFITATGGTLHMYQLEGLNWLRFSWAQGTDTILADEMGLGKTIQTIVFLYSLYKEGHTKGPFLVSAPLSTIINWEREFQMWAPKFYVVTYTGDKDSRAIIRENEFSFEDNAIKGGKKAFKMKREAQVKFHVLLTSYELITIDQAALGSIRWACLVVDEAHRLKNNQSKFFRVLNGYKIDHKLLLTGTPLQNNLEELFHLLNFLTPERFNNLEGFLEEFADISKEDQIKKLHDLLGPHMLRRLKADVFKNMPAKTELIVRVELSPMQKKYYKYILTRNFEALNSRGGGNQVSLLNIMMDLKKCCNHPYLFPVAAMESPKLPSGAYEGGALIKSSGKLMLLQKMLRKLKEQGHRVLIFSQMTKMLDLLEDFLDYEGYKYERIDGGITGALRQEAIDRFNAPGAQQFCFLLSTRAGGLGINLATADTVIIFDSDWNPHNDIQAFSRAHRIGQANKVMIYRFVTRASVEERITQVAKRKMMLTHLVVRPGLGSKAGSMSKQELDDILKFGTEELFKDENEGENKEEDSSVIHYDNEAIARLLDRNQDATEDTDVQNMNEYLSSFKVAQYVVREEDKIEEIEREIIKQEENVDPDYWEKLLRHHYEQQQEDLARNLGKGKRVRKQVNYNDAAQEDQDNQSEYSVGSEEEDEDFDERPEGRRQSKRQLRNEKDKPLPPLLARVGGNIEVLGFNTRQRKAFLNAVMRWGMPPQDAFTTQWLVRDLRGKTEKEFKAYVSLFMRHLCEPGADGSETFADGVPREGLSRQQVLTRIGVMSLVKKKVQEFEHINGRWSMPELMPDPSADSKRSSRASSPTKTSPTTPEASATNSPCTSKPATPAPSEKGEGIRTPLEKEEAENQEEKPEKNSRIGEKMETEADAPSPAPSLGERLEPRKIPLEDEVPGVPGEMEPEPGYRGDREKSATESTPGERGEEKPLDGQEHRERPEGETGDLGKREDVKGDRELRPGPRDEPRSNGRREEKTEKPRFMFNIADGGFTELHTLWQNEERAAISSGKLNEIWHRRHDYWLLAGIVLHGYARWQDIQNDAQFAIINEPFKTEANKGNFLEMKNKFLARRFKLLEQALVIEEQLRRAAYLNLSQEPAHPAMALHARFAEAECLAESHQHLSKESLAGNKPANAVLHKVLNQLEELLSDMKADVTRLPATLSRIPPIAARLQMSERSILSRLASKGTEPHPTPAYPPGPYATPPGYGAAFSAAPVGALAAAGANYSQMPAGSFITAATNGPPVLVKKEKEMVGALVSDGLDRKEPRAGEVICIDD

>NP_079351.1

MGAVTDDEVIRKRLLIDGDGAGGDRRINLLVKSFIKWCNSGSQEEGYSQYQRMLSTLSQCEFSMGKTLLVYDMNLREMENYEKIYKEIECSIAGAHEKIAECKKQILQAKRIRKNRQEYDALAKVIQHHPDRHETLKELEALGKELEHLSHIKESVEDKLELRRKQFHVLLSTIHELQQTLENDEKLSEVEEAQEASMESDPKP

778 NP_002640.2 NP_065387.1

>NP_002640.2

MELENYKQPVVLREDNCRRRRRMKPRSAAASLSSMELIPIEFVLPTSQRKCKSPETALLHVAGHGNVEQMKAQVWLRALETSVAADFYHRLGPHHFLLLYQKKGQWYEIYDKYQVVQTLDCLRYWKATHRSPGQIHLVQRHPPSEESQAFQRQLTALIGYDVTDVSNVHDDELEFTRRGLVTPRMAEVASRDPKLYAMHPWVTSKPLPEYLWKKIANNCIFIVIHRSTTSQTIKVSPDDTPGAILQSFFTKMAKKKSLMDIPESQSEQDFVLRVCGRDEYLVGETPIKNFQWVRHCLKNGEEIHVVLDTPPDPALDEVRKEEWPLVDDCTGVTGYHEQLTIHGKDHESVFTVSLWDCDRKFRVKIRGIDIPVLPRNTDLTVFVEANIQHGQQVLCQRRTSPKPFTEEVLWNVWLEFSIKIKDLPKGALLNLQIYCGKAPALSSKASAESPSSESKGKVQLLYYVNLLLIDHRFLLRRGEYVLHMWQISGKGEDQGSFNADKLTSATNPDKENSMSISILLDNYCHPIALPKHQPTPDPEGDRVRAEMPNQLRKQLEAIIATDPLNPLTAEDKELLWHFRYESLKHPKAYPKLFSSVKWGQQEIVAKTYQLLARREVWDQSALDVGLTMQLLDCNFSDENVRAIAVQKLESLEDDDVLHYLLQLVQAVKFEPYHDSALARFLLKRGLRNKRIGHFLFWFLRSEIAQSRHYQQRFAVILEAYLRGCGTAMLHDFTQQVQVIEMLQKVTLDIKSLSAEKYDVSSQVISQLKQKLENLQNSQLPESFRVPYDPGLKAGALAIEKCKVMASKKKPLWLEFKCADPTALSNETIGIIFKHGDDLRQDMLILQILRIMESIWETESLDLCLLPYGCISTGDKIGMIEIVKDATTIAKIQQSTVGNTGAFKDEVLNHWLKEKSPTEEKFQAAVERFVYSCAGYCVATFVLGIGDRHNDNIMITETGNLFHIDFGHILGNYKSFLGINKERVPFVLTPDFLFVMGTSGKKTSPHFQKFQDICVKAYLALRHHTNLLIILFSMMLMTGMPQLTSKEDIEYIRDALTVGKNEEDAKKYFLDQIEVCRDKGWTVQFNWFLHLVLGIKQGEKHSA

>NP_065387.1

MAPARGRLPPALWVVTAAAAAATCVSAARGEVNLLDTSTIHGDWGWLTYPAHGWDSINEVDESFQPIHTYQVCNVMSPNQNNWLRTSWVPRDGARRVYAEIKFTLRDCNSMPGVLGTCKETFNLYYLESDRDLGASTQESQFLKIDTIAADESFTGADLGVRRLKLNTEVRSVGPLSKRGFYLAFQDIGACLAILSLRIYYKKCPAMVRNLAAFSEAVTGADSSSLVEVRGQCVRHSEERDTPKMYCSAEGEWLVPIGKCVCSAGYEERRDACVACELGFYKSAPGDQLCARCPPHSHSAAPAAQACHCDLSYYRAALDPPSSACTRPPSAPVNLISSVNGTSVTLEWAPPLDPGGRSDITYNAVCRRCPWALSRCEACGSGTRFVPQQTSLVQASLLVANLLAHMNYSFWIEAVNGVSDLSPEPRRAAVVNITTNQAAPSQVVVIRQERAGQTSVSLLWQEPEQPNGIILEYEIKYYEKDKEMQSYSTLKAVTTRATVSGLKPGTRYVFQVRARTSAGCGRFSQAMEVETGKPRPRYDTRTIVWICLTLITGLVVLLLLLICKKRHCGYSKAFQDSDEEKMHYQNGQAPPPVFLPLHHPPGKLPEPQFYAEPHTYEEPGRAGRSFTREIEASRIHIEKIIGSGDSGEVCYGRLRVPGQRDVPVAIKALKAGYTERQRRDFLSEASIMGQFDHPNIIRLEGVVTRGRLAMIVTEYMENGSLDTFLRTHDGQFTIMQLVGMLRGVGAGMRYLSDLGYVHRDLAARNVLVDSNLVCKVSDFGLSRVLEDDPDAAYTTTGGKIPIRWTAPEAIAFRTFSSASDVWSFGVVMWEVLAYGERPYWNMTNRDVISSVEEGYRLPAPMGCPHALHQLMLDCWHKDRAQRPRFSQIVSVLDALIRSPESLRATATVSRCPPPAFVRSCFDLRGGSGGGGGLTVGDWLDSIRMGRYRDHFAAGGYSSLGMVLRMNAQDVRALGITLMGHQKKILGSIQTMRAQLTSTQGPRRHL

779 NP_000973.2 NP_006866.2

>NP_000973.2

MTNTKGKRRGTRYMFSRPFRKHGVVPLATYMRIYKKGDIVDIKGMGTVQKGMPHKCYHGKTGRVYNVTQHAVGIVVNKQVKGKILAKRINVRIEHIKHSKSRDSFLKRVKENDQKKKEAKEKGTWVQLKRQPAPPREAHFVRTNGKEPELLEPIPYEFMA

>NP_006866.2

MLTKPLQGPPAPPGTPTPPPGGKDREAFEAEYRLGPLLGKGGFGTVFAGHRLTDRLQVAIKVIPRNRVLGWSPLSDSVTCPLEVALLWKVGAGGGHPGVIRLLDWFETQEGFMLVLERPLPAQDLFDYITEKGPLGEGPSRCFFGQVVAAIQHCHSRGVVHRDIKDENILIDLRRGCAKLIDFGSGALLHDEPYTDFDGTRVYSPPEWISRHQYHALPATVWSLGILLYDMVCGDIPFERDQEILEAELHFPAHVSPDCCALIRRCLAPKPSSRPSLEEILLDPWMQTPAEDVPLNPSKGGPAPLAWSLLP

780 NP_001735.1 NP_004371.2

>NP_001735.1

MLKVTVPSCSASSCSSVTASAAPGTASLVPDYWIDGSNRDALSDFFEVESELGRGATSIVYRCKQKGTQKPYALKVLKKTVDKKIVRTEIGVLLRLSHPNIIKLKEIFETPTEISLVLELVTGGELFDRIVEKGYYSERDAADAVKQILEAVAYLHENGIVHRDLKPENLLYATPAPDAPLKIADFGLSKIVEHQVLMKTVCGTPGYCAPEILRGCAYGPEVDMWSVGIITYILLCGFEPFYDERGDQFMFRRILNCEYYFISPWWDEVSLNAKDLVRKLIVLDPKKRLTTFQALQHPWVTGKAANFVHMDTAQKKLQEFNARRKLKAAVKAVVASSRLGSASSSHGSIQESHKASRDPSPIQDGNEDMKAIPEGEKIQGDGAQAAVKGAQAELMKVQALEKVKGADINAEEAPKMVPKAVEDGIKVADLELEEGLAEEKLKTVEEAAAPREGQGSSAVGFEVPQQDVILPEY

>NP_004371.2

MAENLLDGPPNPKRAKLSSPGFSANDSTDFGSLFDLENDLPDELIPNGGELGLLNSGNLVPDAASKHKQLSELLRGGSGSSINPGIGNVSASSPVQQGLGGQAQGQPNSANMASLSAMGKSPLSQGDSSAPSLPKQAASTSGPTPAASQALNPQAQKQVGLATSSPATSQTGPGICMNANFNQTHPGLLNSNSGHSLINQASQGQAQVMNGSLGAAGRGRGAGMPYPTPAMQGASSSVLAETLTQVSPQMTGHAGLNTAQAGGMAKMGITGNTSPFGQPFSQAGGQPMGATGVNPQLASKQSMVNSLPTFPTDIKNTSVTNVPNMSQMQTSVGIVPTQAIATGPTADPEKRKLIQQQLVLLLHAHKCQRREQANGEVRACSLPHCRTMKNVLNHMTHCQAGKACQVAHCASSRQIISHWKNCTRHDCPVCLPLKNASDKRNQQTILGSPASGIQNTIGSVGTGQQNATSLSNPNPIDPSSMQRAYAALGLPYMNQPQTQLQPQVPGQQPAQPQTHQQMRTLNPLGNNPMNIPAGGITTDQQPPNLISESALPTSLGATNPLMNDGSNSGNIGTLSTIPTAAPPSSTGVRKGWHEHVTQDLRSHLVHKLVQAIFPTPDPAALKDRRMENLVAYAKKVEGDMYESANSRDEYYHLLAEKIYKIQKELEEKRRSRLHKQGILGNQPALPAPGAQPPVIPQAQPVRPPNGPLSLPVNRMQVSQGMNSFNPMSLGNVQLPQAPMGPRAASPMNHSVQMNSMGSVPGMAISPSRMPQPPNMMGAHTNNMMAQAPAQSQFLPQNQFPSSSGAMSVGMGQPPAQTGVSQGQVPGAALPNPLNMLGPQASQLPCPPVTQSPLHPTPPPASTAAGMPSLQHTTPPGMTPPQPAAPTQPSTPVSSSGQTPTPTPGSVPSATQTQSTPTVQAAAQAQVTPQPQTPVQPPSVATPQSSQQQPTPVHAQPPGTPLSQAAASIDNRVPTPSSVASAETNSQQPGPDVPVLEMKTETQAEDTEPDPGESKGEPRSEMMEEDLQGASQVKEETDIAEQKSEPMEVDEKKPEVKVEVKEEEESSSNGTASQSTSPSQPRKKIFKPEELRQALMPTLEALYRQDPESLPFRQPVDPQLLGIPDYFDIVKNPMDLSTIKRKLDTGQYQEPWQYVDDVWLMFNNAWLYNRKTSRVYKFCSKLAEVFEQEIDPVMQSLGYCCGRKYEFSPQTLCCYGKQLCTIPRDAAYYSYQNRYHFCEKCFTEIQGENVTLGDDPSQPQTTISKDQFEKKKNDTLDPEPFVDCKECGRKMHQICVLHYDIIWPSGFVCDNCLKKTGRPRKENKFSAKRLQTTRLGNHLEDRVNKFLRRQNHPEAGEVFVRVVASSDKTVEVKPGMKSRFVDSGEMSESFPYRTKALFAFEEIDGVDVCFFGMHVQEYGSDCPPPNTRRVYISYLDSIHFFRPRCLRTAVYHEILIGYLEYVKKLGYVTGHIWACPPSEGDDYIFHCHPPDQKIPKPKRLQEWYKKMLDKAFAERIIHDYKDIFKQATEDRLTSAKELPYFEGDFWPNVLEESIKELEQEEEERKKEESTAASETTEGSQGDSKNAKKKNNKKTNKNKSSISRANKKKPSMPNVSNDLSQKLYATMEKHKEVFFVIHLHAGPVINTLPPIVDPDPLLSCDLMDGRDAFLTLARDKHWEFSSLRRSKWSTLCMLVELHTQGQDRFVYTCNECKHHVETRWHCTVCEDYDLCINCYNTKSHAHKMVKWGLGLDDEGSSQGEPQSKSPQESRRLSIQRCIQSLVHACQCRNANCSLPSCQKMKRVVQHTKGCKRKTNGGCPVCKQLIALCCYHAKHCQENKCPVPFCLNIKHKLRQQQIQHRLQQAQLMRRRMATMNTRNVPQQSLPSPTSAPPGTPTQQPSTPQTPQPPAQPQPSPVSMSPAGFPSVARTQPPTTVSTGKPTSQVPAPPPPAQPPPAAVEAARQIEREAQQQQHLYRVNINNSMPPGRTGMGTPGSQMAPVSLNVPRPNQVSGPVMPSMPPGQWQQAPLPQQQPMPGLPRPVISMQAQAAVAGPRMPSVQPPRSISPSALQDLLRTLKSPSSPQQQQQVLNILKSNPQLMAAFIKQRTAKYVANQPGMQPQPGLQSQPGMQPQPGMHQQPSLQNLNAMQAGVPRPGVPPQQQAMGGLNPQGQALNIMNPGHNPNMASMNPQYREMLRRQLLQQQQQQQQQQQQQQQQQQGSAGMAGGMAGHGQFQQPQGPGGYPPAMQQQQRMQQHLPLQGSSMGQMAAQMGQLGQMGQPGLGADSTPNIQQALQQRILQQQQMKQQIGSPGQPNPMSPQQHMLSGQPQASHLPGQQIATSLSNQVRSPAPVQSPRPQSQPPHSSPSPRIQPQPSPHHVSPQTGSPHPGLAVTMASSIDQGHLGNPEQSAMLPQLNTPSRSALSSELSLVGDTTGDTLEKFVEGL

781 NP_003241.2 NP_006302.2

>NP_003241.2

MEQKPSKVECGSDPEENSARSPDGKRKRKNGQCSLKTSMSGYIPSYLDKDEQCVVCGDKATGYHYRCITCEGCKGFFRRTIQKNLHPTYSCKYDSCCVIDKITRNQCQLCRFKKCIAVGMAMDLVLDDSKRVAKRKLIEQNRERRRKEEMIRSLQQRPEPTPEEWDLIHIATEAHRSTNAQGSHWKQRRKFLPDDIGQSPIVSMPDGDKVDLEAFSEFTKIITPAITRVVDFAKKLPMFSELPCEDQIILLKGCCMEIMSLRAAVRYDPESDTLTLSGEMAVKREQLKNGGLGVVSDAIFELGKSLSAFNLDDTEVALLQAVLLMSTDRSGLLCVDKIEKSQEAYLLAFEHYVNHRKHNIPHFWPKLLMKEREVQSSILYKGAAAEGRPGGSLGVHPEGQQLLGMHVVQGPQVRQLEQQLGEAGSLQGPVLQHQSPKSPQQRLLELLHRSGILHARAVCGEDDSSEADSPSSSEEEPEVCEDLAGNAASP

>NP_006302.2

MSSSGYPPNQGAFSTEQSRYPPHSVQYTFPNTRHQQEFAVPDYRSSHLEVSQASQLLQQQQQQQLRRRPSLLSEFHPGSDRPQERRTSYEPFHPGPSPVDHDSLESKRPRLEQVSDSHFQRVSAAVLPLVHPLPEGLRASADAKKDPAFGGKHEAPSSPISGQPCGDDQNASPSKLSKEELIQSMDRVDREIAKVEQQILKLKKKQQQLEEEAAKPPEPEKPVSPPPVEQKHRSIVQIIYDENRKKAEEAHKIFEGLGPKVELPLYNQPSDTKVYHENIKTNQVMRKKLILFFKRRNHARKQREQKICQRYDQLMEAWEKKVDRIENNPRRKAKESKTREYYEKQFPEIRKQREQQERFQRVGQRGAGLSATIARSEHEISEIIDGLSEQENNEKQMRQLSVIPPMMFDAEQRRVKFINMNGLMEDPMKVYKDRQFMNVWTDHEKEIFKDKFIQHPKNFGLIASYLERKSVPDCVLYYYLTKKNENYKALVRRNYGKRRGRNQQIARPSQEEKVEEKEEDKAEKTEKKEEEKKDEEEKDEKEDSKENTKEKDKIDGTAEETEEREQATPRGRKTANSQGRRKGRITRSMTNEAAAASAAAAAATEEPPPPLPPPPEPISTEPVETSRWTEEEMEVAKKGLVEHGRNWAAIAKMVGTKSEAQCKNFYFNYKRRHNLDNLLQQHKQKTSRKPREERDVSQCESVASTVSAQEDEDIEASNEEENPEDSEVEAVKPSEDSPENATSRGNTEPAVELEPTTETAPSTSPSLAVPSTKPAEDESVETQVNDSISAETAEQMDVDQQEHSAEEGSVCDPPPATKADSVDVEVRVPENHASKVEGDNTKERDLDRASEKVEPRDEDLVVAQQINAQRPEPQSDNDSSATCSADEDVDGEPERQRMFPMDSKPSLLNPTGSILVSSPLKPNPLDLPQLQHRAAVIPPMVSCTPCNIPIGTPVSGYALYQRHIKAMHESALLEEQRQRQEQIDLECRSSTSPCGTSKSPNREWEVLQPAPHQVITNLPEGVRLPTTRPTRPPPPLIPSSKTTVASEKPSFIMGGSISQGTPGTYLTSHNQASYTQETPKPSVGSISLGLPRQQESAKSATLPYIKQEEFSPRSQNSQPEGLLVRAQHEGVVRGTAGAIQEGSITRGTPTSKISVESIPSLRGSITQGTPALPQTGIPTEALVKGSISRMPIEDSSPEKGREEAASKGHVIYEGKSGHILSYDNIKNAREGTRSPRTAHEISLKRSYESVEGNIKQGMSMRESPVSAPLEGLICRALPRGSPHSDLKERTVLSGSIMQGTPRATTESFEDGLKYPKQIKRESPPIRAFEGAITKGKPYDGITTIKEMGRSIHEIPRQDILTQESRKTPEVVQSTRPIIEGSISQGTPIKFDNNSGQSAIKHNVKSLITGPSKLSRGMPPLEIVPENIKVVERGKYEDVKAGETVRSRHTSVVSSGPSVLRSTLHEAPKAQLSPGIYDDTSARRTPVSYQNTMSRGSPMMNRTSDVTISSNKSTNHERKSTLTPTQRESIPAKSPVPGVDPVVSHSPFDPHHRGSTAGEVYRSHLPTHLDPAMPFHRALDPAAAAYLFQRQLSPTPGYPSQYQLYAMENTRQTILNDYITSQQMQVNLRPDVARGLSPREQPLGLPYPATRGIIDLTNMPPTILVPHPGGTSTPPMDRITYIPGTQITFPPRPYNSASMSPGHPTHLAAAASAEREREREREKERERERIAAASSDLYLRPGSEQPGRPGSHGYVRSPSPSVRTQETMLQQRPSVFQGTNGTSVITPLDPTAQLRIMPLPAGGPSISQGLPASRYNTAADALAALVDAAASAPQMDVSKTKESKHEAARLEENLRSRSAAVSEQQQLEQKTLEVEKRSVQCLYTSSAFPSGKPQPHSSVVYSEAGKDKGPPPKSRYEEELRTRGKTTITAANFIDVIITRQIASDKDARERGSQSSDSSSSLSSHRYETPSDAIEVISPASSPAPPQEKLQTYQPEVVKANQAENDPTRQYEGPLHHYRPQQESPSPQQQLPPSSQAEGMGQVPRTHRLITLADHICQIITQDFARNQVSSQTPQQPPTSTFQNSPSALVSTPVRTKTSNRYSPESQAQSVHHQRPGSRVSPENLVDKSRGSRPGKSPERSHVSSEPYEPISPPQVPVVHEKQDSLLLLSQRGAEPAEQRNDARSPGSISYLPSFFTKLENTSPMVKSKKQEIFRKLNSSGGGDSDMAAAQPGTEIFNLPAVTTSGSVSSRGHSFADPASNLGLEDIIRKALMGSFDDKVEDHGVVMSQPMGVVPGTANTSVVTSGETRREEGDPSPHSGGVCKPKLISKSNSRKSKSPIPGQGYLGTERPSSVSSVHSEGDYHRQTPGWAWEDRPSSTGSTQFPYNPLTMRMLSSTPPTPIACAPSAVNQAAPHQQNRIWEREPAPLLSAQYETLSDSDD

782 NP_003109.1 NP_000080.2

>NP_003109.1

MRAWIFFLLCLAGRALAAPQQEALPDETEVVEETVAEVTEVSVGANPVQVEVGEFDDGAEETEEEVVAENPCQNHHCKHGKVCELDENNTPMCVCQDPTSCPAPIGEFEKVCSNDNKTFDSSCHFFATKCTLEGTKKGHKLHLDYIGPCKYIPPCLDSELTEFPLRMRDWLKNVLVTLYERDEDNNLLTEKQKLRVKKIHENEKRLEAGDHPVELLARDFEKNYNMYIFPVHWQFGQLDQHPIDGYLSHTELAPLRAPLIPMEHCTTRFFETCDLDNDKYIALDEWAGCFGIKQKDIDKDLVI

>NP_000080.2

MLSFVDTRTLLLLAVTLCLATCQSLQEETVRKGPAGDRGPRGERGPPGPPGRDGEDGPTGPPGPPGPPGPPGLGGNFAAQYDGKGVGLGPGPMGLMGPRGPPGAAGAPGPQGFQGPAGEPGEPGQTGPAGARGPAGPPGKAGEDGHPGKPGRPGERGVVGPQGARGFPGTPGLPGFKGIRGHNGLDGLKGQPGAPGVKGEPGAPGENGTPGQTGARGLPGERGRVGAPGPAGARGSDGSVGPVGPAGPIGSAGPPGFPGAPGPKGEIGAVGNAGPAGPAGPRGEVGLPGLSGPVGPPGNPGANGLTGAKGAAGLPGVAGAPGLPGPRGIPGPVGAAGATGARGLVGEPGPAGSKGESGNKGEPGSAGPQGPPGPSGEEGKRGPNGEAGSAGPPGPPGLRGSPGSRGLPGADGRAGVMGPPGSRGASGPAGVRGPNGDAGRPGEPGLMGPRGLPGSPGNIGPAGKEGPVGLPGIDGRPGPIGPAGARGEPGNIGFPGPKGPTGDPGKNGDKGHAGLAGARGAPGPDGNNGAQGPPGPQGVQGGKGEQGPPGPPGFQGLPGPSGPAGEVGKPGERGLHGEFGLPGPAGPRGERGPPGESGAAGPTGPIGSRGPSGPPGPDGNKGEPGVVGAVGTAGPSGPSGLPGERGAAGIPGGKGEKGEPGLRGEIGNPGRDGARGAPGAVGAPGPAGATGDRGEAGAAGPAGPAGPRGSPGERGEVGPAGPNGFAGPAGAAGQPGAKGERGAKGPKGENGVVGPTGPVGAAGPAGPNGPPGPAGSRGDGGPPGMTGFPGAAGRTGPPGPSGISGPPGPPGPAGKEGLRGPRGDQGPVGRTGEVGAVGPPGFAGEKGPSGEAGTAGPPGTPGPQGLLGAPGILGLPGSRGERGLPGVAGAVGEPGPLGIAGPPGARGPPGAVGSPGVNGAPGEAGRDGNPGNDGPPGRDGQPGHKGERGYPGNIGPVGAAGAPGPHGPVGPAGKHGNRGETGPSGPVGPAGAVGPRGPSGPQGIRGDKGEPGEKGPRGLPGLKGHNGLQGLPGIAGHHGDQGAPGSVGPAGPRGPAGPSGPAGKDGRTGHPGTVGPAGIRGPQGHQGPAGPPGPPGPPGPPGVSGGGYDFGYDGDFYRADQPRSAPSLRPKDYEVDATLKSLNNQIETLLTPEGSRKNPARTCRDLRLSHPEWSSGYYWIDPNQGCTMDAIKVYCDFSTGETCIRAQPENIPAKNWYRSSKDKKHVWLGETINAGSQFEYNVEGVTSKEMATQLAFMRLLANYASQNITYHCKNSIAYMDEETGNLKKAVILQGSNDVELVAEGNSRFTYTVLVDGCSKKTNEWGKTIIEYKTNKPSRLPFLDIAPLDIGGADQEFFVDIGPVCFK

783 NP_056980.2 NP_112197.1

>NP_056980.2

MGDLELLLPGEAEVLVRGLRSFPLREMGSEGWNQQHENLEKLNMQAILDATVSQGEPIQELLVTHGKVPTLVEELIAVEMWKQKVFPVFCRVEDFKPQNTFPIYMVVHHEASIINLLETVFFHKEVCESAEDTVLDLVDYCHRKLTLLVAQSGCGGPPEGEGSQDSNPMQELQKQAELMEFEIALKALSVLRYITDCVDSLSLSTLSRMLSTHNLPCLLVELLEHSPWSRREGGKLQQFEGSRWHTVAPSEQQKLSKLDGQVWIALYNLLLSPEAQARYCLTSFAKGRLLKLRAFLTDTLLDQLPNLAHLQSFLAHLTLTETQPPKKDLVLEQIPEIWERLERENRGKWQAIAKHQLQHVFSPSEQDLRLQARRWAETYRLDVLEAVAPERPRCAYCSAEASKRCSRCQNEWYCCRECQVKHWEKHGKTCVLAAQGDRAK

>NP_112197.1

MSGGKKKSSFQITSVTTDYEGPGSPGASDPPTPQPPTGPPPRLPNGEPSPDPGGKGTPRNGSPPPGAPSSRFRVVKLPHGLGEPYRRGRWTCVDVYERDLEPHSFGGLLEGIRGASGGAGGRSLDSRLELASLGLGAPTPPSGLSQGPTSWLRPPPTSPGPQARSFTGGLGQLVVPSKAKAEKPPLSASSPQQRPPEPETGESAGTSRAATPLPSLRVEAEAGGSGARTPPLSRRKAVDMRLRMELGAPEEMGQVPPLDSRPSSPALYFTHDASLVHKSPDPFGAVAAQKFSLAHSMLAISGHLDSDDDSGSGSLVGIDNKIEQAMDLVKSHLMFAVREEVEVLKEQIRELAERNAALEQENGLLRALASPEQLAQLPSSGVPRLGPPAPNGPSV

784 NP_004260.2 NP_542382.1

>NP_004260.2

MADVINVSVNLEAFSQAISAIQALRSSVSRVFDCLKDGMRNKETLEGREKAFIAHFQDNLHSVNRDLNELERLSNLVGKPSENHPLHNSGLLSLDPVQDKTPLYSQLLQAYKWSNKLQYHAGLASGLLNQQSLKRSANQMGVSAKRRPKAQPTTLVLPPQYVDDVISRIDRMFPEMSIHLSRPNGTSAMLLVTLGKVLKVIVVMRSLFIDRTIVKGYNENVYTEDGKLDIWSKSNYQVFQKVTDHATTALLHYQLPQMPDVVVRSFMTWLRSYIKLFQAPCQRCGKFLQDGLPPTWRDFRTLEAFHDTCRQ

>NP_542382.1

MSTPPLAASGMAPGPFAGPQAQQAAREVNTASLCRIGQETVQDIVYRTMEIFQLLRNMQLPNGVTYHTGTYQDRLTKLQDNLRQLSVLFRKLRLVYDKCNENCGGMDPIPVEQLIPYVEEDGSKNDDRAGPPRFASEERREIAEVNKKLKQKNQQLKQIMDQLRNLIWDINAMLAMRN

785 NP_596868.1 NP_002681.1

>NP_596868.1

MPLLTQQIQDEDDQYSLVASLDNVRNLSTILKAIHFREHATCFATKNGIKVTVENAKCVQANAFIQAGIFQEFKVQEESVTFRINLTVLLDCLSIFGSSPMPGTLTALRMCYQGYGYPLMLFLEEGGVVTVCKINTQEPEETLDFDFCSTNVINKIILQSEGLREAFSELDMTSEVLQITMSPDKPYFRLSTFGNAGSSHLDYPKDSDLMEAFHCNQTQVNRYKISLLKPSTKALVLSCKVSIRTDNRGFLSLQYMIRNEDGQICFVEYYCCPDEEVPESES

>NP_002681.1

MSKRKAPQETLNGGITDMLTELANFEKNVSQAIHKYNAYRKAASVIAKYPHKIKSGAEAKKLPGVGTKIAEKIDEFLATGKLRKLEKIRQDDTSSSINFLTRVSGIGPSAARKFVDEGIKTLEDLRKNEDKLNHHQRIGLKYFGDFEKRIPREEMLQMQDIVLNEVKKVDSEYIATVCGSFRRGAESSGDMDVLLTHPSFTSESTKQPKLLHQVVEQLQKVHFITDTLSKGETKFMGVCQLPSKNDEKEYPHRRIDIRLIPKDQYYCGVLYFTGSDIFNKNMRAHALEKGFTINEYTIRPLGVTGVAGEPLPVDSEKDIFDYIQWKYREPKDRSE

786 NP_848928.1 NP_004148.1

>NP_848928.1

MSETQNSTSQKAMDEDNKAASQTMPNTQDKNYEDELTQVALALVEDVINYAVKIVEEERNPLKNIKWMTHGEFTVEKGLKQIDEYFSKCVSKKCWAHGVEFVERKDLIHSFLYIYYVHWSISTADLPVARISAGTYFTMKVSKTKPPDAPIVVSYVGDHQALVHRPGMVRFRENWQKNLTDAKYSFMESFPFLFNRV

>NP_004148.1

MSHIQIPPGLTELLQGYTVEVLRQQPPDLVEFAVEYFTRLREARAPASVLPAATPRQSLGHPPPEPGPDRVADAKGDSESEEDEDLEVPVPSRFNRRVSVCAETYNPDEEEEDTDPRVIHPKTDEQRCRLQEACKDILLFKNLDQEQLSQVLDAMFERIVKADEHVIDQGDDGDNFYVIERGTYDILVTKDNQTRSVGQYDNRGSFGELALMYNTPRAATIVATSEGSLWGLDRVTFRRIIVKNNAKKRKMFESFIESVPLLKSLEVSERMKIVDVIGEKIYKDGERIITQGEKADSFYIIESGEVSILIRSRTKSNKDGGNQEVEIARCHKGQYFGELALVTNKPRAASAYAVGDVKCLVMDVQAFERLLGPCMDIMKRNISHYEEQLVKMFGSSVDLGNLGQ

787 O15049 NP_940682.2

>O15049

MATAPGPAGIAMGSVGSLLERQDFSPEELRAALAGSRGSRQPDGLLRKGLGQREFLSYLHLPKKDSKSTKNTKRAPRNEPADYATLYYREHSRAGDFSKTSLPERGRFDKCRIRPSVFKPTAGNGKGFLSMQSLASHKGQKLWRSNGSLHTLACHPPLSPGPQASQARAQLLHALSLDEGGPEPEPSLSDSSSGGSFGRSPGTGPSPFSSSLGHLNHLGGSLDRASQGPKEAGPPAVLSCLPEPPPPYEFSCSSAEEMGAVLPETCEELKRGLGDEDGSNPFTQVLEERQRLWLAELKRLYVERLHEVTQKAERSERNLQLQLFMAQQEQRRLRKELRAQQGLAPEPRAPGTLPEADPSARPEEEARWEVCQKTAEISLLKQQLREAQAELAQKLAEIFSLKTQLRGSRAQAQAQDAELVRLREAVRSLQEQAPREEAPGSCETDDCKSRGLLGEAGGSEARDSAEQLRAELLQERLRGQEQALRFEQERRTWQEEKERVLRYQREIQGGYMDMYRRNQALEQELRALREPPTPWSPRLESSKI

>NP_940682.2

MAQSLRLHFAARRSNTYPLSETSGDDLDSHVHMCFKRPTRISTSNVVQMKLTPRQTALAPLIKENVQSQERSSVPSSENVNKKSSCLQISLQPTRYSGYLQSSNVLADSDDASFTCILKDGIYSSAVVDNELNAVNDGHLVSSPAICSGSLSNFSTSDNGSYSSNGSDFGSCASITSGGSYTNSVISDSSSYTFPPSDDTFLGGNLPSDSTSNRSVPNRNTTPCEIFSRSTSTDPFVQDDLEHGLEIMKLPVSRNTKIPLKRYSSLVIFPRSPSTTRPTSPTSLCTLLSKGSYQTSHQFIISPSEIAHNEDGTSAKGFLSTAVNGLRLSKTICTPGEVRDIRPLHRKGSLQKKIVLSNNTPRQTVCEKSSEGYSCVSVHFTQRKAATLDCETTNGDCKPEMSEIKLNSDSEYIKLMHRTSACLPSSQNVDCQININGELERPHSQMNKNHGILRRSISLGGAYPNISCLSSLKHNCSKGGPSQLLIKFASGNEGKVDNLSRDSNRDCTNELSNSCKPGWVVLDQPDAACHLQQQQEPSPLPPGWEERQDILGRTYYVNHESRRTQWKRPTPQDNLTDAENGNIQLQAQRAFTTRRQISEETESVDNRESSENWEIIREDEATMYSNQAFPSPPPSSNLDVPTHLAEELNARLTIFGNSAVSQPASSSNHSSRRGSLQAYTFEEQPTLPVLLPTSSGLPPGWEEKQDERGRSYYVDHNSRTTTWTKPTVQATVETSQLTSSQSSAGPQSQASTSDSGQQVTQPSEIEQGFLPKGWEVRHAPNGRPFFIDHNTKTTTWEDPRLKIPAHLRGKTSLDTSNDLGPLPPGWEERTHTDGRIFYINHNIKRTQWEDPRLENVAITGPAVPYSRDYKRKYEFFRRKLKKQNDIPNKFEMKLRRATVLEDSYRRIMGVKRADFLKARLWIEFDGEKGLDYGGVAREWFFLISKEMFNPYYGLFEYSATDNYTLQINPNSGLCNEDHLSYFKFIGRVAGMAVYHGKLLDGFFIRPFYKMMLHKPITLHDMESVDSEYYNSLRWILENDPTELDLRFIIDEELFGQTHQHELKNGGSEIVVTNKNKKEYIYLVIQWRFVNRIQKQMAAFKEGFFELIPQDLIKIFDENELELLMCGLGDVDVNDWREHTKYKNGYSANHQVIQWFWKAVLMMDSEKRIRLLQFVTGTSRVPMNGFAELYGSNGPQSFTVEQWGTPEKLPRAHTCFNRLDLPPYESFEELWDKLQMAIENTQGFDGVD

788 NP_057442.1 NP_000955.1

>NP_057442.1

MSYPQFGYPYSSAPQFLMATNSLSTCCESGGRTLADSGPAASAQAPVYCPVYESRLLATARHELNSAAALGVYGGPYGGSQGYGNYVTYGSEASAFYSLNSFDSKDGSGSAHGGLAPAAAAYYPYEPALGQYPYDRYGTMDSGTRRKNATRETTSTLKAWLQEHRKNPYPTKGEKIMLAIITKMTLTQVSTWFANARRRLKKENKMTWPPRNKCADEKRPYAEGEEEEGGEEEAREEPLKSSKNAEPVGKEEKELELSDLDDFDPLEAEPPACELKPPFHSLDGGLERVPAAPDGPVKEASGALRMSLAAGGGAALDEDLERARSCLRSAAAGPEPLPGAEGGPQVCEAKLGFVPAGASAGLEAKPRIWSLAHTATAAAAAATSLSQTEFPSCMLKRQGPAAPAAVSSAPATSPSVALPHSGALDRHQDSPVTSLRNWVDGVFHDPILRHSTLNQAWATAKGALLDPGPLGRSLGAGANVLTAPLARAFPPAVPQDAPAAGAARELLALPKAGGKPFCA

>NP_000955.1

MASNSSSCPTPGGGHLNGYPVPPYAFFFPPMLGGLSPPGALTTLQHQLPVSGYSTPSPATIETQSSSSEEIVPSPPSPPPLPRIYKPCFVCQDKSSGYHYGVSACEGCKGFFRRSIQKNMVYTCHRDKNCIINKVTRNRCQYCRLQKCFEVGMSKESVRNDRNKKKKEVPKPECSESYTLTPEVGELIEKVRKAHQETFPALCQLGKYTTNNSSEQRVSLDIDLWDKFSELSTKCIIKTVEFAKQLPGFTTLTIADQITLLKAACLDILILRICTRYTPEQDTMTFSDGLTLNRTQMHNAGFGPLTDLVFAFANQLLPLEMDDAETGLLSAICLICGDRQDLEQPDRVDMLQEPLLEALKVYVRKRRPSRPHMFPKMLMKITDLRSISAKGAERVITLKMEIPGSMPPLIQEMLENSEGLDTLSGQPGGGGRDGGGLAPPPGSCSPSLSPSSNRSSPATHSP

789 NP_690862.1 NP_003191.1

>NP_690862.1

MAAYPESCVDTTVLDFVADLSLASPRRPLLCDFAPGVSLGDPALALREGRPRRMARFEEGDPEEEECEVDQGDGEEEEEEERGRGVSLLGRPKRKRVITYAQRQAANIRERKRMFNLNEAFDQLRRKVPTFAYEKRLSRIETLRLAIVYISFMTELLESCEKKESG

>NP_003191.1

MNQPQRMAPVGTDKELSDLLDFSMMFPLPVTNGKGRPASLAGAQFGGSGLEDRPSSGSWGSGDQSSSSFDPSRTFSEGTHFTESHSSLSSSTFLGPGLGGKSGERGAYASFGRDAGVGGLTQAGFLSGELALNSPGPLSPSGMKGTSQYYPSYSGSSRRRAADGSLDTQPKKVRKVPPGLPSSVYPPSSGEDYGRDATAYPSAKTPSSTYPAPFYVADGSLHPSAELWSPPGQAGFGPMLGGGSSPLPLPPGSGPVGSSGSSSTFGGLHQHERMGYQLHGAEVNGGLPSASSFSSAPGATYGGVSSHTPPVSGADSLLGSRGTTAGSSGDALGKALASIYSPDHSSNNFSSSPSTPVGSPQGLAGTSQWPRAGAPGALSPSYDGGLHGLQSKIEDHLDEAIHVLRSHAVGTAGDMHTLLPGHGALASGFTGPMSLGGRHAGLVGGSHPEDGLAGSTSLMHNHAALPSQPGTLPDLSRPPDSYSGLGRAGATAAASEIKREEKEDEENTSAADHSEEEKKELKAPRARTSPDEDEDDLLPPEQKAEREKERRVANNARERLRVRDINEAFKELGRMCQLHLNSEKPQTKLLILHQAVSVILNLEQQVRERNLNPKAACLKRREEEKVSGVVGDPQMVLSAPHPGLSEAHNPAGHM

790 NP_078976.2 NP_068810.2

>NP_078976.2

MKAVKSERERGSRRRHRDGDVVLPAGVVVKQERLSPEVAPPAHRRPDHSGGSPSPPTSEPARSGHRGNRARGVSRSPPKKKNKASGRRSKSPRSKRNRSPHHSTVKVKQEREDHPRRGREDRQHREPSEQEHRRARNSDRDRHRGHSHQRRTSNERPGSGQGQGRDRDTQNLQAQEEEREFYNARRREHRQRNDVGGGGSESQELVPRPGGNNKEKEVPAKEKPSFELSGALLEDTNTFRGVVIKYSEPPEARIPKKRWRLYPFKNDEVLPVMYIHRQSAYLLGRHRRIADIPIDHPSCSKQHAVFQYRLVEYTRADGTVGRRVKPYIIDLGSGNGTFLNNKRIEPQRYYELKEKDVLKFGFSSREYVLLHESSDTSEIDRKDDEDEEEEEEVSDS

>NP_068810.2

MDELFPLIFPAEPAQASGPYVEIIEQPKQRGMRFRYKCEGRSAGSIPGERSTDTTKTHPTIKINGYTGPGTVRISLVTKDPPHRPHPHELVGKDCRDGFYEAELCPDRCIHSFQNLGIQCVKKRDLEQAISQRIQTNNNPFQVPIEEQRGDYDLNAVRLCFQVTVRDPSGRPLRLPPVLPHPIFDNRAPNTAELKICRVNRNSGSCLGGDEIFLLCDKVQKEDIEVYFTGPGWEARGSFSQADVHRQVAIVFRTPPYADPSLQAPVRVSMQLRRPSDRELSEPMEFQYLPDTDDRHRIEEKRKRTYETFKSIMKKSPFSGPTDPRPPPRRIAVPSRSSASVPKPAPQPYPFTSSLSTINYDEFPTMVFPSGQISQASALAPAPPQVLPQAPAPAPAPAMVSALAQAPAPVPVLAPGPPQAVAPPAPKPTQAGEGTLSEALLQLQFDDEDLGALLGNSTDPAVFTDLASVDNSEFQQLLNQGIPVAPHTTEPMLMEYPEAITRLVTGAQRPPDPAPAPLGAPGLPNGLLSGDEDFSSIADMDFSALLSQISS

791 NP_473364.1 NP_006761.1

>NP_473364.1

MKLVTIFLLVTISLCSYSATAFLINKVPLPVDKLAPLPLDNILPFMDPLKLLLKTLGISVEHLVEGLRKCVNELGPEASEAVKKLLEALSHLV

>NP_006761.1

MRNKKILKEDELLSETQQAAFHQIAMEPFEINVPKPKRRNGVNFSLAVVVIYLILLTAGAGLLVVQVLNLQARLRVLEMYFLNDTLAAEDSPSFSLLQSAHPGEHLAQGASRLQVLQAQLTWVRVSHEHLLQRVDNFTQNPGMFRIKGEQGAPGLQGHKGAMGMPGAPGPPGPPAEKGAKGAMGRDGATGPSGPQGPPGVKGEAGLQGPQGAPGKQGATGTPGPQGEKGSKGDGGLIGPKGETGTKGEKGDLGLPGSKGDRGMKGDAGVMGPPGAQGSKGDFGRPGPPGLAGFPGAKGDQGQPGLQGVPGPPGAVGHPGAKGEPGSAGSPGRAGLPGSPGSPGATGLKGSKGDTGLQGQQGRKGESGVPGPAGVKGEQGSPGLAGPKGAPGQAGQKGDQGVKGSSGEQGVKGEKGERGENSVSVRIVGSSNRGRAEVYYSGTWGTICDDEWQNSDAIVFCRMLGYSKGRALYKVGAGTGQIWLDNVQCRGTESTLWSCTKNSWGHHDCSHEEDAGVECSV

792 NP_006197.1 NP_000203.2

>NP_006197.1

MGTSHPAFLVLGCLLTGLSLILCQLSLPSILPNENEKVVQLNSSFSLRCFGESEVSWQYPMSEEESSDVEIRNEENNSGLFVTVLEVSSASAAHTGLYTCYYNHTQTEENELEGRHIYIYVPDPDVAFVPLGMTDYLVIVEDDDSAIIPCRTTDPETPVTLHNSEGVVPASYDSRQGFNGTFTVGPYICEATVKGKKFQTIPFNVYALKATSELDLEMEALKTVYKSGETIVVTCAVFNNEVVDLQWTYPGEVKGKGITMLEEIKVPSIKLVYTLTVPEATVKDSGDYECAARQATREVKEMKKVTISVHEKGFIEIKPTFSQLEAVNLHEVKHFVVEVRAYPPPRISWLKNNLTLIENLTEITTDVEKIQEIRYRSKLKLIRAKEEDSGHYTIVAQNEDAVKSYTFELLTQVPSSILDLVDDHHGSTGGQTVRCTAEGTPLPDIEWMICKDIKKCNNETSWTILANNVSNIITEIHSRDRSTVEGRVTFAKVEETIAVRCLAKNLLGAENRELKLVAPTLRSELTVAAAVLVLLVIVIISLIVLVVIWKQKPRYEIRWRVIESISPDGHEYIYVDPMQLPYDSRWEFPRDGLVLGRVLGSGAFGKVVEGTAYGLSRSQPVMKVAVKMLKPTARSSEKQALMSELKIMTHLGPHLNIVNLLGACTKSGPIYIITEYCFYGDLVNYLHKNRDSFLSHHPEKPKKELDIFGLNPADESTRSYVILSFENNGDYMDMKQADTTQYVPMLERKEVSKYSDIQRSLYDRPASYKKKSMLDSEVKNLLSDDNSEGLTLLDLLSFTYQVARGMEFLASKNCVHRDLAARNVLLAQGKIVKICDFGLARDIMHDSNYVSKGSTFLPVKWMAPESIFDNLYTTLSDVWSYGILLWEIFSLGGTPYPGMMVDSTFYNKIKSGYRMAKPDHATSEVYEIMVKCWNSEPEKRPSFYHLSEIVENLLPGQYKKSYEKIHLDFLKSDHPAVARMRVDSDNAYIGVTYKNEEDKLKDWEGGLDEQRLSADSGYIIPLPDIDPVPEEEDLGKRNRHSSQTSEESAIETGSSSSTFIKREDETIEDIDMMDDIGIDSSDLVEDSFL

>NP_000203.2

MRARPRPRPLWATVLALGALAGVGVGGPNICTTRGVSSCQQCLAVSPMCAWCSDEALPLGSPRCDLKENLLKDNCAPESIEFPVSEARVLEDRPLSDKGSGDSSQVTQVSPQRIALRLRPDDSKNFSIQVRQVEDYPVDIYYLMDLSYSMKDDLWSIQNLGTKLATQMRKLTSNLRIGFGAFVDKPVSPYMYISPPEALENPCYDMKTTCLPMFGYKHVLTLTDQVTRFNEEVKKQSVSRNRDAPEGGFDAIMQATVCDEKIGWRNDASHLLVFTTDAKTHIALDGRLAGIVQPNDGQCHVGSDNHYSASTTMDYPSLGLMTEKLSQKNINLIFAVTENVVNLYQNYSELIPGTTVGVLSMDSSNVLQLIVDAYGKIRSKVELEVRDLPEELSLSFNATCLNNEVIPGLKSCMGLKIGDTVSFSIEAKVRGCPQEKEKSFTIKPVGFKDSLIVQVTFDCDCACQAQAEPNSHRCNNGNGTFECGVCRCGPGWLGSQCECSEEDYRPSQQDECSPREGQPVCSQRGECLCGQCVCHSSDFGKITGKYCECDDFSCVRYKGEMCSGHGQCSCGDCLCDSDWTGYYCNCTTRTDTCMSSNGLLCSGRGKCECGSCVCIQPGSYGDTCEKCPTCPDACTFKKECVECKKFDRGALHDENTCNRYCRDEIESVKELKDTGKDAVNCTYKNEDDCVVRFQYYEDSSGKSILYVVEEPECPKGPDILVVLLSVMGAILLIGLAALLIWKLLITIHDRKEFAKFEEERARAKWDTANNPLYKEATSTFTNITYRGT

793 NP_004963.1 NP_000638.1

>NP_004963.1

MGMACLTMTEMEGTSTSSIYQNGDISGNANSMKQIDPVLQVYLYHSLGKSEADYLTFPSGEYVAEEICIAASKACGITPVYHNMFALMSETERIWYPPNHVFHIDESTRHNVLYRIRFYFPRWYCSGSNRAYRHGISRGAEAPLLDDFVMSYLFAQWRHDFVHGWIKVPVTHETQEECLGMAVLDMMRIAKENDQTPLAIYNSISYKTFLPKCIRAKIQDYHILTRKRIRYRFRRFIQQFSQCKATARNLKLKYLINLETLQSAFYTEKFEVKEPGSGPSGEEIFATIIITGNGGIQWSRGKHKESETLTEQDLQLYCDFPNIIDVSIKQANQEGSNESRVVTIHKQDGKNLEIELSSLREALSFVSLIDGYYRLTADAHHYLCKEVAPPAVLENIQSNCHGPISMDFAISKLKKAGNQTGLYVLRCSPKDFNKYFLTFAVERENVIEYKHCLITKNENEEYNLSGTKKNFSSLKDLLNCYQMETVRSDNIIFQFTKCCPPKPKDKSNLLVFRTNGVSDVPTSPTLQRPTHMNQMVFHKIRNEDLIFNESLGQGTFTKIFKGVRREVGDYGQLHETEVLLKVLDKAHRNYSESFFEAASMMSKLSHKHLVLNYGVCVCGDENILVQEFVKFGSLDTYLKKNKNCINILWKLEVAKQLAWAMHFLEENTLIHGNVCAKNILLIREEDRKTGNPPFIKLSDPGISITVLPKDILQERIPWVPPECIENPKNLNLATDKWSFGTTLWEICSGGDKPLSALDSQRKLQFYEDRHQLPAPKWAELANLINNCMDYEPDFRPSFRAIIRDLNSLFTPDYELLTENDMLPNMRIGALGFSGAFEDRDPTQFEERHLKFLQQLGKGNFGSVEMCRYDPLQDNTGEVVAVKKLQHSTEEHLRDFEREIEILKSLQHDNIVKYKGVCYSAGRRNLKLIMEYLPYGSLRDYLQKHKERIDHIKLLQYTSQICKGMEYLGTKRYIHRDLATRNILVENENRVKIGDFGLTKVLPQDKEYYKVKEPGESPIFWYAPESLTESKFSVASDVWSFGVVLYELFTYIEKSKSPPAEFMRMIGNDKQGQMIVFHLIELLKNNGRLPRPDGCPDEIYMIMTECWNNNVNQRPSFRDLALRVDQIRDNMAG

>NP_000638.1

MLSTSRSRFIRNTNESGEEVTTFFDYDYGAPCHKFDVKQIGAQLLPPLYSLVFIFGFVGNMLVVLILINCKKLKCLTDIYLLNLAISDLLFLITLPLWAHSAANEWVFGNAMCKLFTGLYHIGYFGGIFFIILLTIDRYLAIVHAVFALKARTVTFGVVTSVITWLVAVFASVPGIIFTKCQKEDSVYVCGPYFPRGWNNFHTIMRNILGLVLPLLIMVICYSGILKTLLRCRNEKKRHRAVRVIFTIMIVYFLFWTPYNIVILLNTFQEFFGLSNCESTSQLDQATQVTETLGMTHCCINPIIYAFVGEKFRSLFHIALGCRIAPLQKPVCGGPGVRPGKNVKVTTQGLLDGRGKGKSIGRAPEASLQDKEGA

794 NP_001256.1 NP_001306.1

>NP_001256.1

MYVSYLLDKDVSMYPSSVRHSGGLNLAPQNFVSPPQYPDYGGYHVAAAAAAAANLDSAQSPGPSWPAAYGAPLREDWNGYAPGGAAAAANAVAHGLNGGSPAAAMGYSSPADYHPHHHPHHHPHHPAAAPSCASGLLQTLNPGPPGPAATAAAEQLSPGGQRRNLCEWMRKPAQQSLGSQVKTRTKDKYRVVYTDHQRLELEKEFHYSRYITIRRKAELAATLGLSERQVKIWFQNRRAKERKINKKKLQQQQQQQPPQPPPPPPQPPQPQPGPLRSVPEPLSPVSSLQASVSGSVPGVLGPTGGVLNPTVTQ

>NP_001306.1

MSQERPTFYRQELNKTIWEVPERYQNLSPVGSGAYGSVCAAFDTKTGLRVAVKKLSRPFQSIIHAKRTYRELRLLKHMKHENVIGLLDVFTPARSLEEFNDVYLVTHLMGADLNNIVKCQKLTDDHVQFLIYQILRGLKYIHSADIIHRDLKPSNLAVNEDCELKILDFGLARHTDDEMTGYVATRWYRAPEIMLNWMHYNQTVDIWSVGCIMAELLTGRTLFPGTDHINQLQQIMRLTGTPPAYLINRMPSHEARNYIQSLTQMPKMNFANVFIGANPLAVDLLEKMLVLDSDKRITAAQALAHAYFAQYHDPDDEPVADPYDQSFESRDLLIDEWKSLTYDEVISFVPPPLDQEEMES

795 NP_056953.2 NP_004312.2

>NP_056953.2

MGETLGDSPIDPESDSFTDTLSANISQEMTMVDTEMPFWPTNFGISSVDLSVMEDHSHSFDIKPFTTVDFSSISTPHYEDIPFTRTDPVVADYKYDLKLQEYQSAIKVEPASPPYYSEKTQLYNKPHEEPSNSLMAIECRVCGDKASGFHYGVHACEGCKGFFRRTIRLKLIYDRCDLNCRIHKKSRNKCQYCRFQKCLAVGMSHNAIRFGRMPQAEKEKLLAEISSDIDQLNPESADLRALAKHLYDSYIKSFPLTKAKARAILTGKTTDKSPFVIYDMNSLMMGEDKIKFKHITPLQEQSKEVAIRIFQGCQFRSVEAVQEITEYAKSIPGFVNLDLNDQVTLLKYGVHEIIYTMLASLMNKDGVLISEGQGFMTREFLKSLRKPFGDFMEPKFEFAVKFNALELDDSDLAIFIAVIILSGDRPGLLNVKPIEDIQDNLLQALELQLKLNHPESSQLFAKLLQKMTDLRQIVTEHVQLLQVIKKTETDMSLHPLLQEIYKDLY

>NP_004312.2

MAGASVKVAVRVRPFNSREMSRDSKCIIQMSGSTTTIVNPKQPKETPKSFSFDYSYWSHTSPEDINYASQKQVYRDIGEEMLQHAFEGYNVCIFAYGQTGAGKSYTMMGKQEKDQQGIIPQLCEDLFSRINDTTNDNMSYSVEVSYMEIYCERVRDLLNPKNKGNLRVREHPLLGPYVEDLSKLAVTSYNDIQDLMDSGNKARTVAATNMNETSSRSHAVFNIIFTQKRHDAETNITTEKVSKISLVDLAGSERADSTGAKGTRLKEGANINKSLTTLGKVISALAEMDSGPNKNKKKKKTDFIPYRDSVLTWLLRENLGGNSRTAMVAALSPADINYDETLSTLRYADRAKQIRCNAVINEDPNNKLIRELKDEVTRLRDLLYAQGLGDITDMTNALVGMSPSSSLSALSSRAASVSSLHERILFAPGSEEAIERLKETEKIIAELNETWEEKLRRTEAIRMEREALLAEMGVAMREDGGTLGVFSPKKTPHLVNLNEDPLMSECLLYYIKDGITRVGREDGERRQDIVLSGHFIKEEHCVFRSDSRGGSEAVVTLEPCEGADTYVNGKKVTEPSILRSGNRIIMGKSHVFRFNHPEQARQERERTPCAETPAEPVDWAFAQRELLEKQGIDMKQEMEQRLQELEDQYRREREEATYLLEQQRLDYESKLEALQKQMDSRYYPEVNEEEEEPEDEVQWTERECELALWAFRKWKWYQFTSLRDLLWGNAIFLKEANAISVELKKKVQFQFVLLTDTLYSPLPPDLLPPEAAKDRETRPFPRTIVAVEVQDQKNGATHYWTLEKLRQRLDLMREMYDRAAEVPSSVIEDCDNVVTGGDPFYDRFPWFRLVGRAFVYLSNLLYPVPLVHRVAIVSEKGEVKGFLRVAVQAISADEEAPDYGSGVRQSGTAKISFDDQHFEKFQSESCPVVGMSRSGTSQEELRIVEGQGQGADVGPSADEVNNNTCSAVPPEGLLLDSSEKAALDGPLDAALDHLRLGNTFTFRVTVLQASSISAEYADIFCQFNFIHRHDEAFSTEPLKNTGRGPPLGFYHVQNIAVEVTKSFIEYIKSQPIVFEVFGHYQQHPFPPLCKDVLSPLRPSRRHFPRVMPLSKPVPATKLSTLTRPCPGPCHCKYDLLVYFEICELEANGDYIPAVVDHRGGMPCMGTFLLHQGIQRRITVTLLHETGSHIRWKEVRELVVGRIRNTPETDESLIDPNILSLNILSSGYIHPAQDDRTFYQFEAAWDSSMHNSLLLNRVTPYREKIYMTLSAYIEMENCTQPAVVTKDFCMVFYSRDAKLPASRSIRNLFGSGSLRASESNRVTGVYELSLCHVADAGSPGMQRRRRRVLDTSVAYVRGEENLAGWRPRSDSLILDHQWELEKLSLLQEVEKTRHYLLLREKLETAQRPVPEALSPAFSEDSESHGSSSASSPLSAEGRPSPLEAPNERQRELAVKCLRLLTHTFNREYTHSHVCVSASESKLSEMSVTLLRDPSMSPLGVATLTPSSTCPSLVEGRYGATDLRTPQPCSRPASPEPELLPEADSKKLPSPARATETDKEPQRLLVPDIQEIRVSPIVSKKGYLHFLEPHTSGWARRFVVVRRPYAYMYNSDKDTVERFVLNLATAQVEYSEDQQAMLKTPNTFAVCTEHRGILLQAASDKDMHDWLYAFNPLLAGTIRSKLSRRRSAQMRV

796 NP_036376.2 NP_001012679.1

>NP_036376.2

MEEGARHRNNTEKKHPGGGESDASPEAGSGGGGVALKKEIGLVSACGIIVGNIIGSGIFVSPKGVLENAGSVGLALIVWIVTGFITVVGALCYAELGVTIPKSGGDYSYVKDIFGGLAGFLRLWIAVLVIYPTNQAVIALTFSNYVLQPLFPTCFPPESGLRLLAAICLLLLTWVNCSSVRWATRVQDIFTAGKLLALALIIIMGIVQICKGEYFWLEPKNAFENFQEPDIGLVALAFLQGSFAYGGWNFLNYVTEELVDPYKNLPRAIFISIPLVTFVYVFANVAYVTAMSPQELLASNAVAVTFGEKLLGVMAWIMPISVALSTFGGVNGSLFTSSRLFFAGAREGHLPSVLAMIHVKRCTPIPALLFTCISTLLMLVTSDMYTLINYVGFINYLFYGVTVAGQIVLRWKKPDIPRPIKINLLFPIIYLLFWAFLLVFSLWSEPVVCGIGLAIMLTGVPVYFLGVYWQHKPKCFSDFIELLTLVSQKMCVVVYPEVERGSGTEEANEDMEEQQQPMYQPTPTKDKDVAGQPQP

>NP_001012679.1

MELQPPEASIAVVSIPRQLPGSHSEAGVQGLSAGDDSELGSHCVAQTGLELLASGDPLPSASQNAEMIETGSDCVTQAGLQLLASSDPPALASKNAEVTETGFHHVSQADIEFLTSIDPTASASGSAGITGTMSQDTEVDMKEVELNELEPEKQPMNAASGAAMSLAGAEKNGLVKIKVAEDEAEAAAAAKFTGLSKEELLKVAGSPGWVRTRWALLLLFWLGWLGMLAGAVVIIVRAPRCRELPAQKWWHTGALYRIGDLQAFQGHGAGNLAGLKGRLDYLSSLKVKGLVLGPIHKNQKDDVAQTDLLQIDPNFGSKEDFDSLLQSAKKKSIRVILDLTPNYRGENSWFSTQVDTVATKVKDALEFWLQAGVDGFQVRDIENLKDASSFLAEWQNITKGFSEDRLLIAGTNSSDLQQILSLLESNKDLLLTSSYLSDSGSTGEHTKSLVTQYLNATGNRWCSWSLSQARLLTSFLPAQLLRLYQLMLFTLPGTPVFSYGDEIGLDAAALPGQPMEAPVMLWDESSFPDIPGAVSANMTVKGQSEDPGSLLSLFRRLSDQRSKERSLLHGDFHAFSAGPGLFSYIRHWDQNERFLVVLNFGDVGLSAGLQASDLPASASLPAKADLLLSTQPGREEGSPLELERLKLEPHEGLLLRFPYAA

797 NP_004447.2 NP_061109.1

>NP_004447.2

MGQTGKKSEKGPVCWRKRVKSEYMRLRQLKRFRRADEVKSMFSSNRQKILERTEILNQEWKQRRIQPVHILTSVSSLRGTRECSVTSDLDFPTQVIPLKTLNAVASVPIMYSWSPLQQNFMVEDETVLHNIPYMGDEVLDQDGTFIEELIKNYDGKVHGDRECGFINDEIFVELVNALGQYNDDDDDDDGDDPEEREEKQKDLEDHRDDKESRPPRKFPSDKIFEAISSMFPDKGTAEELKEKYKELTEQQLPGALPPECTPNIDGPNAKSVQREQSLHSFHTLFCRRCFKYDCFLHRKCNYSFHATPNTYKRKNTETALDNKPCGPQCYQHLEGAKEFAAALTAERIKTPPKRPGGRRRGRLPNNSSRPSTPTINVLESKDTDSDREAGTETGGENNDKEEEEKKDETSSSSEANSRCQTPIKMKPNIEPPENVEWSGAEASMFRVLIGTYYDNFCAIARLIGTKTCRQVYEFRVKESSIIAPAPAEDVDTPPRKKKRKHRLWAAHCRKIQLKKDGSSNHVYNYQPCDHPRQPCDSSCPCVIAQNFCEKFCQCSSECQNRFPGCRCKAQCNTKQCPCYLAVRECDPDLCLTCGAADHWDSKNVSCKNCSIQRGSKKHLLLAPSDVAGWGIFIKDPVQKNEFISEYCGEIISQDEADRRGKVYDKYMCSFLFNLNNDFVVDATRKGNKIRFANHSVNPNCYAKVMMVNGDHRIGIFAKRAIQTGEELFFDYRYSQADALKYVGIEREMEIP

>NP_061109.1

MEAGEEPLLLAELKPGRPHQFDWKSSCETWSVAFSPDGSWFAWSQGHCIVKLIPWPLEEQFIPKGFEAKSRSSKNETKGRGSPKEKTLDCGQIVWGLAFSPWPSPPSRKLWARHHPQVPDVSCLVLATGLNDGQIKIWEVQTGLLLLNLSGHQDVVRDLSFTPSGSLILVSASRDKTLRIWDLNKHGKQIQVLSGHLQWVYCCSISPDCSMLCSAAGEKSVFLWSMRSYTLIRKLEGHQSSVVSCDFSPDSALLVTASYDTNVIMWDPYTGERLRSLHHTQVDPAMDDSDVHISSLRSVCFSPEGLYLATVADDRLLRIWALELKTPIAFAPMTNGLCCTFFPHGGVIATGTRDGHVQFWTAPRVLSSLKHLCRKALRSFLTTYQVLALPIPKKMKEFLTYRTF

798 NP_002023.2 NP_001014809.1

>NP_002023.2

MTTASTSQVRQNYHQDSEAAINRQINLELYASYVYLSMSYYFDRDDVALKNFAKYFLHQSHEEREHAEKLMKLQNQRGGRIFLQDIKKPDCDDWESGLNAMECALHLEKNVNQSLLELHKLATDKNDPHLCDFIETHYLNEQVKAIKELGDHVTNLRKMGAPESGLAEYLFDKHTLGDSDNES

>NP_001014809.1

MADRRRAWNTEDDLPVYLARPGSAAQTPRQKYGGMFAAVEGAYENKTIDFDAYSVGRRGSARTPRSAGRPDAVGLPGPGGSEDTASDVSEPSGSAVSSPGERDERPPTLRIRRPAPRDLPLGRDNGQSDRLLIKGGRIINDDQSLYADVYLEDGLIKQIGENLIVPGGVKTIEANGRMVIPGGIDVNTYLQKPSQGMTAADDFFQGTRAALVGGTTMIIDHVVPEPGSSLLTSFEKWHEAADTKSCCDYSLHVDITSWYDGVREELEVLVQDKGVNSFQVYMAYKDVYQMSDSQLYEAFTFLKGLGAVILVHAENGDLIAQEQKRILEMGITGPEGHALSRPEELEAEAVFRAITIAGRINCPVYITKVMSKSAADIIALARKKGPLVFGEPIAASLGTDGTHYWSKNWAKAAAFVTSPPLSPDPTTPDYLTSLLACGDLQVTGSGHCPYSTAQKAVGKDNFTLIPEGVNGIEERMTVVWDKAVATGKMDENQFVAVTSTNAAKIFNLYPRKGRIAVGSDADVVIWDPDKLKTITAKSHKSAVEYNIFEGMECHGSPLVVISQGKIVFEDGNINVNKGMGRFIPRKAFPEHLYQRVKIRNKVFGLQGVSRGMYDGPVYEVPATPKYATPAPSAKSSPSKHQPPPIRNLHQSNFSLSGAQIDDNNPRRTGHRIVAPPGGRSNITSLG

799 NP_005822.1 NP_006490.3

>NP_005822.1

MEETIKDPPTSAVLLDHCHFSQVIFNSVEKFYIPGGDVTCHYTFTQHFIPRRKDWIGIFRVGWKTTREYYTFMWVTLPIDLNNKSAKQQEVQFKAYYLPKDDEYYQFCYVDEDGVVRGASIPFQFRPENEEDILVVTTQGEVEEIEQHNKELCKENQELKDSCISLQKQNSDMQAELQKKQEELETLQSINKKLELKVKEQKDYWETELLQLKEQNQKMSSENEKMGIRVDQLQAQLSTQEKEMEKLVQGDQDKTEQLEQLKKENDHLFLSLTEQRKDQKKLEQTVEQMKQNETTAMKKQQELMDENFDLSKRLSENEIICNALQRQKERLEGENDLLKRENSRLLSYMGLDFNSLPYQVPTSDEGGARQNPGLAYGNPYSGIQESSSPSPLSIKKCPICKADDICDHTLEQQQMQPLCFNCPICDKIFPATEKQIFEDHVFCHSL

>NP_006490.3

MMLSLNNLQNIIYNPVIPFVGTIPDQLDPGTLIVIRGHVPSDADRFQVDLQNGSSMKPRADVAFHFNPRFKRAGCIVCNTLINEKWGREEITYDTPFKREKSFEIVIMVLKDKFQVAVNGKHTLLYGHRIGPEKIDTLGIYGKVNIHSIGFSFSSDLQSTQASSLELTEISRENVPKSGTPQLPSNRGGDISKIAPRTVYTKSKDSTVNHTLTCTKIPPMNYVSKRLPFAARLNTPMGPGRTVVVKGEVNANAKSFNVDLLAGKSKDIALHLNPRLNIKAFVRNSFLQESWGEEERNITSFPFSPGMYFEMIIYCDVREFKVAVNGVHSLEYKHRFKELSSIDTLEINGDIHLLEVRSW

800 NP_490647.1 NP_000215.1

>NP_490647.1

MVDYYEVLGVQRHASPEDIKKAYRKLALKWHPDKNPENKEEAERKFKQVAEAYEVLSDAKKRDIYDKYGKEGLNGGGGGGSHFDSPFEFGFTFRNPDDVFREFFGGRDPFSFDFFEDPFEDFFGNRRGPRGSRSRGTGSFFSAFSGFPSFGSGFSSFDTGFTSFGSLGHGGLTSFSSTSFGGSGMGNFKSISTSTKMVNGRKITTKRIVENGQERVEVEEDGQLKSLTINGVADDDALAEERMRRGQNALPAQPAGLRPPKPPRPASLLRHAPHCLSEEEGEQDRPRAPGPWDPLASAAGLKEGGKRKKQKQREESKKKKSTKGNH

>NP_000215.1

MSFTTRSTFSTNYRSLGSVQAPSYGARPVSSAASVYAGAGGSGSRISVSRSTSFRGGMGSGGLATGIAGGLAGMGGIQNEKETMQSLNDRLASYLDRVRSLETENRRLESKIREHLEKKGPQVRDWSHYFKIIEDLRAQIFANTVDNARIVLQIDNARLAADDFRVKYETELAMRQSVENDIHGLRKVIDDTNITRLQLETEIEALKEELLFMKKNHEEEVKGLQAQIASSGLTVEVDAPKSQDLAKIMADIRAQYDELARKNREELDKYWSQQIEESTTVVTTQSAEVGAAETTLTELRRTVQSLEIDLDSMRNLKASLENSLREVEARYALQMEQLNGILLHLESELAQTRAEGQRQAQEYEALLNIKVKLEAEIATYRRLLEDGEDFNLGDALDSSNSMQTIQKTTTRRIVDGKVVSETNDTKVLRH

801 NP_619527.1 NP_004041.1

>NP_619527.1

MAKQPSDVSSECDREGRQLQPAERPPQLRPGAPTSLQTEPQGNPEGNHGGEGDSCPHGSPQGPLAPPASPGPFATRSPLFIFMRRSSLLSRSSSGYFSFDTDRSPAPMSCDKSTQTPSPPCQAFNHYLSAMASMRQAEPADMRPEIWIAQELRRIGDEFNAYYARRVFLNNYQAAEDHPRMVILRLLRYIVRLVWRMH

>NP_004041.1

MATPASAPDTRALVADFVGYKLRQKGYVCGAGPGEGPAADPLHQAMRAAGDEFETRFRRTFSDLAAQLHVTPGSAQQRFTQVSDELFQGGPNWGRLVAFFVFGAALCAESVNKEMEPLVGQVQEWMVAYLETRLADWIHSSGGWAEFTALYGDGALEEARRLREGNWASVRTVLTGAVALGALVTVGAFFASK

802 NP_001728.1 NP_000602.1

>NP_001728.1

MSACRSFAVAICILEISILTAQYTTSYDPELTESSGSASHIDCRMSPWSEWSQCDPCLRQMFRSRSIEVFGQFNGKRCTDAVGDRRQCVPTEPCEDAEDDCGNDFQCSTGRCIKMRLRCNGDNDCGDFSDEDDCESEPRPPCRDRVVEESELARTAGYGINILGMDPLSTPFDNEFYNGLCNRDRDGNTLTYYRRPWNVASLIYETKGEKNFRTEHYEEQIEAFKSIIQEKTSNFNAAISLKFTPTETNKAEQCCEETASSISLHGKGSFRFSYSKNETYQLFLSYSSKKEKMFLHVKGEIHLGRFVMRNRDVVLTTTFVDDIKALPTTYEKGEYFAFLETYGTHYSSSGSLGGLYELIYVLDKASMKRKGVELKDIKRCLGYHLDVSLAFSEISVGAEFNKDDCVKRGEGRAVNITSENLIDDVVSLIRGGTRKYAFELKEKLLRGTVIDVTDFVNWASSINDAPVLISQKLSPIYNLVPVKMKNAHLKKQNLERAIEDYINEFSVRKCHTCQNGGTVILMDGKCLCACPFKFEGIACEISKQKISEGLPALEFPNEK

>NP_000602.1

MGIQGGSVLFGLLLVLAVFCHSGHSLQCYNCPNPTADCKTAVNCSSDFDACLITKAGLQVYNKCWKFEHCNFNDVTTRLRENELTYYCCKKDLCNFNEQLENGGTSLSEKTVLLLVTPFLAAAWSLHP

803 NP_003395.1 NP_077007.1

>NP_003395.1

MTMDKSELVQKAKLAEQAERYDDMAAAMKAVTEQGHELSNEERNLLSVAYKNVVGARRSSWRVISSIEQKTERNEKKQQMGKEYREKIEAELQDICNDVLELLDKYLIPNATQPESKVFYLKMKGDYFRYLSEVASGDNKQTTVSNSQQAYQEAFEISKKEMQPTHPIRLGLALNFSVFYYEILNSPEKACSLAKTAFDEAIAELDTLNEESYKDSTLIMQLLRDNLTLWTSENQGDEGDAGEGEN

>NP_077007.1

MRKETPPPLVPPAAREWNLPPNAPACMERQLEAARYRSDGALLLGASSLSGRCWAGSLWLFKDPCAAPNEGFCSAGVQTEAGVADLTWVGERGILVASDSGAVELWELDENETLIVSKFCKYEHDDIVSTVSVLSSGTQAVSGSKDICIKVWDLAQQVVLSSYRAHAAQVTCVAASPHKDSVFLSCSEDNRILLWDTRCPKPASQIGCSAPGYLPTSLAWHPQQSEVFVFGDENGTVSLVDTKSTSCVLSSAVHSQCVTGLVFSPHSVPFLASLSEDCSLAVLDSSLSELFRSQAHRDFVRDATWSPLNHSLLTTVGWDHQVVHHVVPTEPLPAPGPASVTE

804 NP_006779.1 NP_005393.2

>NP_006779.1

MTECFLPPTSSPSEHRRVEHGSGLTRTPSSEEISPTKFPGLYRTGEPSPPHDILHEPPDVVSDDEKDHGKKKGKFKKKEKRTEGYAAFQEDSSGDEAESPSKMKRSKGIHVFKKPSFSKKKEKDFKIKEKPKEEKHKEEKHKEEKHKEKKSKDLTAADVVKQWKEKKKKKKPIQEPEVPQIDVPNLKPIFGIPLADAVERTMMYDGIRLPAVFRECIDYVEKYGMKCEGIYRVSGIKSKVDELKAAYDREESTNLEDYEPNTVASLLKQYLRDLPENLLTKELMPRFEEACGRTTETEKVQEFQRLLKELPECNYLLISWLIVHMDHVIAKELETKMNIQNISIVLSPTVQISNRVLYVFFTHVQELFGNVVLKQVMKPLRWSNMATMPTLPETQAGIKEEIRRQEFLLNCLHRDLQGGIKDLSKEERLWEVQRILTALKRKLREAKRQECETKIAQEIASLSKEDVSKEEMNENEEVINILLAQENEILTEQEELLAMEQFLRRQIASEKEEIERLRAEIAEIQSRQQHGRSETEEYSSESESESEDEEELQIILEDLQRQNEELEIKNNHLNQAIHEEREAIIELRVQLRLLQMQRAKAEQQAQEDEEPEWRGGAVQPPRDGVLEPKAAKEQPKAGKEPAKPSPSRDRKETSI

>NP_005393.2

MAANKPKGQNSLALHKVIMVGSGGVGKSALTLQFMYDEFVEDYEPTKADSYRKKVVLDGEEVQIDILDTAGQEDYAAIRDNYFRSGEGFLCVFSITEMESFAATADFREQILRVKEDENVPFLLVGNKSDLEDKRQVSVEEAKNRAEQWNVNYVETSAKTRANVDKVFFDLMREIRARKMEDSKEKNGKKKRKSLAKRIRERCCIL

805 NP_003251.2 NP_009056.3

>NP_003251.2

MYPQGRHPTPLQSGQPFKFSILEICDRIKEEFQFLQAQYHSLKLECEKLASEKTEMQRHYVMYYEMSYGLNIEMHKQAEIVKRLSGICAQIIPFLTQEHQQQVLQAVERAKQVTVGELNSLIGQQLQPLSHHAPPVPLTPRPAGLVGGSATGLLALSGALAAQAQLAAAVKEDRAGVEAEGSRVERAPSRSASPSPPESLVEEERPSGPGGGGKQRADEKEPSGPYESDEDKSDYNLVVDEDQPSEPPSPATTPCGKVPICIPARRDLVDSPASLASSLGSPLPRAKELILNDLPASTPASKSCDSSPPQDASTPGPSSASHLCQLAAKPAPSTDSVALRSPLTLSSPFTTSFSLGSHSTLNGDLSVPSSYVSLHLSPQVSSSVVYGRSPVMAFESHPHLRGSSVSSSLPSIPGGKPAYSFHVSADGQMQPVPFPSDALVGAGIPRHARQLHTLAHGEVVCAVTISGSTQHVYTGGKGCVKVWDVGQPGAKTPVAQLDCLNRDNYIRSCKLLPDGRSLIVGGEASTLSIWDLAAPTPRIKAELTSSAPACYALAVSPDAKVCFSCCSDGNIVVWDLQNQTMVRQFQGHTDGASCIDISDYGTRLWTGGLDNTVRCWDLREGRQLQQHDFSSQIFSLGHCPNQDWLAVGMESSNVEILHVRKPEKYQLHLHESCVLSLKFASCGRWFVSTGKDNLLNAWRTPYGASIFQSKESSSVLSCDISRNNKYIVTGSGDKKATVYEVVY

>NP_009056.3

MKSCAVSLTTAAVAFGDEAKKMAEGKASRESEEESVSLTVEEREALGGMDSRLFGFVRLHEDGARTKTLLGKAVRCYESLILKAEGKVESDFFCQLGHFNLLLEDYSKALSAYQRYYSLQADYWKNAAFLYGLGLVYFYYNAFHWAIKAFQDVLYVDPSFCRAKEIHLRLGLMFKVNTDYKSSLKHFQLALIDCNPCTLSNAEIQFHIAHLYETQRKYHSAKEAYEQLLQTENLPAQVKATVLQQLGWMHHNMDLVGDKATKESYAIQYLQKSLEADPNSGQSWYFLGRCYSSIGKVQDAFISYRQSIDKSEASADTWCSIGVLYQQQNQPMDALQAYICAVQLDHGHAAAWMDLGTLYESCNQPQDAIKCYLNAARSKRCSNTSTLAARIKFLQNGSDNWNGGQSLSHHPVQQVYSLCLTPQKLQHLEQLRANRDNLNPAQKHQLEQLESQFVLMQQMRHKEVAQVRTTGIHNGAITDSSLPTNSVSNRQPHGALTRVSSVSQPGVRPACVEKLLSSGAFSAGCIPCGTSKILGSTDTILLGSNCIAGSESNGNVPYLQQNTHTLPHNHTDLNSSTEEPWRKQLSNSAQGLHKSQSSCLSGPNEEQPLFSTGSAQYHQATSTGIKKANEHLTLPSNSVPQGDADSHLSCHTATSGGQQGIMFTKESKPSKNRSLVPETSRHTGDTSNGCADVKGLSNHVHQLIADAVSSPNHGDSPNLLIADNPQLSALLIGKANGNVGTGTCDKVNNIHPAVHTKTDHSVASSPSSAISTATPSPKSTEQRSINSVTSLNSPHSGLHTVNGEGLGKSQSSTKVDLPLASHRSTSQILPSMSVSICPSSTEVLKACRNPGKNGLSNSCILLDKCPPPRPPTSPYPPLPKDKLNPPTPSIYLENKRDAFFPPLHQFCTNPKNPVTVIRGLAGALKLDLGLFSTKTLVEANNEHMVEVRTQLLQPADENWDPTGTKKIWRCESNRSHTTIAKYAQYQASSFQESLREENEKRTQHKDHSDNESTSSENSGRRRKGPFKTIKFGTNIDLSDNKKWKLQLHELTKLPAFARVVSAGNLLTHVGHTILGMNTVQLYMKVPGSRTPGHQENNNFCSVNINIGPGDCEWFVVPEDYWGVLNDFCEKNNLNFLMSSWWPNLEDLYEANVPVYRFIQRPGDLVWINAGTVHWVQAVGWCNNIAWNVGPLTACQYKLAVERYEWNKLKSVKSPVPMVHLSWNMARNIKVSDPKLFEMIKYCLLKILKQYQTLREALVAAGKEVIWHGRTNDEPAHYCSICEVEVFNLLFVTNESNTQKTYIVHCHDCARKTSKSLENFVVLEQYKMEDLIQVYDQFTLALSLSSSS

806 NP_003698.1 NP_065853.2

>NP_003698.1

MKIEEVKSTTKTQRIASHSHVKGLGLDESGLAKQAASGLVGQENAREACGVIVELIKSKKMAGRAVLLAGPPGTGKTALALAIAQELGSKVPFCPMVGSEVYSTEIKKTEVLMENFRRAIGLRIKETKEVYEGEVTELTPCETENPMGGYGKTISHVIIGLKTAKGTKQLKLDPSIFESLQKERVEAGDVIYIEANSGAVKRQGRCDTYATEFDLEAEEYVPLPKGDVHKKKEIIQDVTLHDLDVANARPQGGQDILSMMGQLMKPKKTEITDKLRGEINKVVNKYIDQGIAELVPGVLFVDEVHMLDIECFTYLHRALESSIAPIVIFASNRGNCVIRGTEDITSPHGIPLDLLDRVMIIRTMLYTPQEMKQIIKIRAQTEGINISEEALNHLGEIGTKTTLRYSVQLLTPANLLAKINGKDSIEKEHVEEISELFYDAKSSAKILADQQDKYMK

>NP_065853.2

MLAGRPGTRSAVGELGTESSDNLDRAPLGPRESGGHHRPGSYLDMKIHLEKNLEEERQILLQQQKICRNRARKYFVESNRRKKAFEEKRKEQEEKEHQIREQILQQRKQKFEEVTEKFQRAHVPLSQRRKAVSRKPVPPLEEALKQIQESNLKSEVNLPFSRRPTINWRAIDSALPSALSKNDHKHQKQLLSKINCEKEMNENMRATLATSKNVFQLKLEETQKLLEDQHLSNLQKFGDEVNQITNSETLSSIDSLEATEHEEIYLTLNKEHSTSIQRNTISLKPANMQSTNLSCFDEDKLAFSKTQHINNWLTNLDASNTQNVTAFSDILSKSNVLPSWEYFNSKEQNPSPLNGTVERATNTANNSVPFVSSPPMFVLDKKCEKTSETSTMRTTDSTSGAFKRERPLVTESPTFKFSKSQSTSDSLTQEVATFPDQEKYSELNQENGTTSIPTSCVPVATPLVLPSNIQSARPSAKNSIHIKEIDAVQCSDKLDELKDGKEEEIKYFNCNKEELPLFSDSFQDAYIPHNPDSKDEKQKLAETSSLSNVTSNYDFVGQHKKMKYNIHERNGVRFLKSILKKESKYEHGYLKALIINQSFKFGNQKAAAIRDSIELTKEKGAEIPKTIKKLRWFDETSNIENNAENSHSLKNKTGTTQQHSQQFHIQSGAGSNIISVSTCAVNSADTKKSREDSISENVTTLGGSGADHMPLNCFIPSGYNFAKHAWPASKKEESKIPVHDDSKTKQGKPQRGRAKIIRKPGSAKVQSGFICTNRKGAVIQPQSASKVNIFTQAQGKLIIPCPPPQSTSNIRSGKNIQVSQCQPVTPENPQNIITHNSFNSKHVLPTEHSLNQWNQESSSPLSNACSDLVTVIPSLPSYCSSECQTFAKINHSNGTQAVARQDATLYCTQRSPVCEESYPSVTLRTAEEESVPLWKRGPNVLHQNKRATGSTVMRRKRIAETKRRNILEQKRQNPGSVGQKYSEQINNFGQSVLLSSSEPKQTTRGTSYIEEVSDSTSEFLMAENLVKASVPEDEILTVLNSKQIQKSNLPLNKTQQFNICTLSAEEQKILESLNDLSERLHYIQESICKNPSIKNTLQIIPLLEKREDRTSSCRDKR

807 NP_001093.1 NP_005357.2

>NP_001093.1

MDHYDSQQTNDYMQPEEDWDRDLLLDPAWEKQQRKTFTAWCNSHLRKAGTQIENIEEDFRDGLKLMLLLEVISGERLAKPERGKMRVHKISNVNKALDFIASKGVKLVSIGAEEIVDGNVKMTLGMIWTIILRFAIQDISVEETSAKEGLLLWCQRKTAPYKNVNIQNFHISWKDGLGFCALIHRHRPELIDYGKLRKDDPLTNLNTAFDVAEKYLDIPKMLDAEDIVGTARPDEKAIMTYVSSFYHAFSGAQKAETAANRICKVLAVNQENEQLMEDYEKLASDLLEWIRRTIPWLENRVPENTMHAMQQKLEDFRDYRRLHKPPKVQEKCQLEINFNTLQTKLRLSNRPAFMPSEGRMVSDINNAWGCLEQVEKGYEEWLLNEIRRLERLDHLAEKFRQKASIHEAWTDGKEAMLRQKDYETATLSEIKALLKKHEAFESDLAAHQDRVEQIAAIAQELNELDYYDSPSVNARCQKICDQWDNLGALTQKRREALERTEKLLETIDQLYLEYAKRAAPFNNWMEGAMEDLQDTFIVHTIEEIQGLTTAHEQFKATLPDADKERLAILGIHNEVSKIVQTYHVNMAGTNPYTTITPQEINGKWDHVRQLVPRRDQALTEEHARQQHNERLRKQFGAQANVIGPWIQTKMEEIGRISIEMHGTLEDQLSHLRQYEKSIVNYKPKIDQLEGDHQLIQEALIFDNKHTNYTMEHIRVGWEQLLTTIARTINEVENQILTRDAKGISQEQMNEFRASFNHFDRDHSGTLGPEEFKACLISLGYDIGNDPQGEAEFARIMSIVDPNRLGVVTFQAFIDFMSRETADTDTADQVMASFKILAGDKNYITMDELRRELPPDQAEYCIARMAPYTGPDSVPGALDYMSFSTALYGESDL

>NP_005357.2

METQFRRGGLGCSPASIKRKKKREDSGDFGLQVSTMFSEDDFQSTERAPYGPQLQWSQDLPRVQVFREQANLEDRSPRRTQRITGGEQVLWGPITQIFPTVRPADLTRVIMPLEQRSQHCKPEEGLQAQEEDLGLVGAQALQAEEQEAAFFSSTLNVGTLEELPAAESPSPPQSPQEESFSPTAMDAIFGSLSDEGSGSQEKEGPSTSPDLIDPESFSQDILHDKIIDLVHLLLRKYRVKGLITKAEMLGSVIKNYEDYFPEIFREASVCMQLLFGIDVKEVDPTSHSYVLVTSLNLSYDGIQCNEQSMPKSGLLIIVLGVIFMEGNCIPEEVMWEVLSIMGVYAGREHFLFGEPKRLLTQNWVQEKYLVYRQVPGTDPACYEFLWGPRAHAETSKMKVLEYIANANGRDPTSYPSLYEDALREEGEGV

808 NP_001888.2 NP_066973.1

>NP_001888.2

MQSGPRPPLPAPGLALALTLTMLARLASAASFFGENHLEVPVATALTDIDLQLQFSTSQPEALLLLAAGPADHLLLQLYSGRLQVRLVLGQEELRLQTPAETLLSDSIPHTVVLTVVEGWATLSVDGFLNASSAVPGAPLEVPYGLFVGGTGTLGLPYLRGTSRPLRGCLHAATLNGRSLLRPLTPDVHEGCAEEFSASDDVALGFSGPHSLAAFPAWGTQDEGTLEFTLTTQSRQAPLAFQAGGRRGDFIYVDIFEGHLRAVVEKGQGTVLLHNSVPVADGQPHEVSVHINAHRLEISVDQYPTHTSNRGVLSYLEPRGSLLLGGLDAEASRHLQEHRLGLTPEATNASLLGCMEDLSVNGQRRGLREALLTRNMAAGCRLEEEEYEDDAYGHYEAFSTLAPEAWPAMELPEPCVPEPGLPPVFANFTQLLTISPLVVAEGGTAWLEWRHVQPTLDLMEAELRKSQVLFSVTRGARHGELELDIPGAQARKMFTLLDVVNRKARFIHDGSEDTSDQLVLEVSVTARVPMPSCLRRGQTYLLPIQVNPVNDPPHIIFPHGSLMVILEHTQKPLGPEVFQAYDPDSACEGLTFQVLGTSSGLPVERRDQPGEPATEFSCRELEAGSLVYVHRGGPAQDLTFRVSDGLQASPPATLKVVAIRPAIQIHRSTGLRLAQGSAMPILPANLSVETNAVGQDVSVLFRVTGALQFGELQKQGAGGVEGAEWWATQAFHQRDVEQGRVRYLSTDPQHHAYDTVENLALEVQVGQEILSNLSFPVTIQRATVWMLRLEPLHTQNTQQETLTTAHLEATLEEAGPSPPTFHYEVVQAPRKGNLQLQGTRLSDGQGFTQDDIQAGRVTYGATARASEAVEDTFRFRVTAPPYFSPLYTFPIHIGGDPDAPVLTNVLLVVPEGGEGVLSADHLFVKSLNSASYLYEVMERPRHGRLAWRGTQDKTTMVTSFTNEDLLRGRLVYQHDDSETTEDDIPFVATRQGESSGDMAWEEVRGVFRVAIQPVNDHAPVQTISRIFHVARGGRRLLTTDDVAFSDADSGFADAQLVLTRKDLLFGSIVAVDEPTRPIYRFTQEDLRKRRVLFVHSGADRGWIQLQVSDGQHQATALLEVQASEPYLRVANGSSLVVPQGGQGTIDTAVLHLDTNLDIRSGDEVHYHVTAGPRWGQLVRAGQPATAFSQQDLLDGAVLYSHNGSLSPRDTMAFSVEAGPVHTDATLQVTIALEGPLAPLKLVRHKKIYVFQGEAAEIRRDQLEAAQEAVPPADIVFSVKSPPSAGYLVMVSRGALADEPPSLDPVQSFSQEAVDTGRVLYLHSRPEAWSDAFSLDVASGLGAPLEGVLVELEVLPAAIPLEAQNFSVPEGGSLTLAPPLLRVSGPYFPTLLGLSLQVLEPPQHGALQKEDGPQARTLSAFSWRMVEEQLIRYVHDGSETLTDSFVLMANASEMDRQSHPVAFTVTVLPVNDQPPILTTNTGLQMWEGATAPIPAEALRSTDGDSGSEDLVYTIEQPSNGRVVLRGAPGTEVRSFTQAQLDGGLVLFSHRGTLDGGFRFRLSDGEHTSPGHFFRVTAQKQVLLSLKGSQTLTVCPGSVQPLSSQTLRASSSAGTDPQLLLYRVVRGPQLGRLFHAQQDSTGEALVNFTQAEVYAGNILYEHEMPPEPFWEAHDTLELQLSSPPARDVAATLAVAVSFEAACPQRPSHLWKNKGLWVPEGQRARITVAALDASNLLASVPSPQRSEHDVLFQVTQFPSRGQLLVSEEPLHAGQPHFLQSQLAAGQLVYAHGGGGTQQDGFHFRAHLQGPAGASVAGPQTSEAFAITVRDVNERPPQPQASVPLRLTRGSRAPISRAQLSVVDPDSAPGEIEYEVQRAPHNGFLSLVGGGLGPVTRFTQADVDSGRLAFVANGSSVAGIFQLSMSDGASPPLPMSLAVDILPSAIEVQLRAPLEVPQALGRSSLSQQQLRVVSDREEPEAAYRLIQGPQYGHLLVGGRPTSAFSQFQIDQGEVVFAFTNFSSSHDHFRVLALARGVNASAVVNVTVRALLHVWAGGPWPQGATLRLDPTVLDAGELANRTGSVPRFRLLEGPRHGRVVRVPRARTEPGGSQLVEQFTQQDLEDGRLGLEVGRPEGRAPGPAGDSLTLELWAQGVPPAVASLDFATEPYNAARPYSVALLSVPEAARTEAGKPESSTPTGEPGPMASSPEPAVAKGGFLSFLEANMFSVIIPMCLVLLLLALILPLLFYLRKRNKTGKHDVQVLTAKPRNGLAGDTETFRKVEPGQAIPLTAVPGQGPPPGGQPDPELLQFCRTPNPALKNGQYWV

>NP_066973.1

MIAVSFKCRCQILRRLTKDESPYTKSASQTKPPDGALAVRRQSIPEEFKGSTVVELMKKEGTTLGLTVSGGIDKDGKPRVSNLRQGGIAARSDQLDVGDYIKAVNGINLAKFRHDEIISLLKNVGERVVLEVEYELPPVSVQGSSVIFRTVEVTLHKEGNTFGFVIRGGAHDDRNKSRPVVITCVRPGGPADREGTIKPGDRLLSVDGIRLLGTTHAEAMSILKQCGQEAALLIEYDVSVMDSVATASGPLLVEVAKTPGASLGVALTTSMCCNKQVIVIDKIKSASIADRCGALHVGDHILSIDGTSMEYCTLAEATQFLANTTDQVKLEILPHHQTRLALKGPDHAALVSSSFSPTSMSAYSLSSLNMGTLPRSLYSTSPRGTMMRRRLKKKDFKSSLSLASSTVGLAGQVVHTETTEVVLTADPVTGFGIQLQGSVFATETLSSPPLISYIEADSPAERCGVLQIGDRVMAINGIPTEDSTFEEASQLLRDSSITSKVTLEIEFDVAESVIPSSGTFHVKLPKKHNVELGITISSPSSRKPGDPLVISDIKKGSVAHRTGTLELGDKLLAIDNIRLDNCSMEDAVQILQQCEDLVKLKIRKDEDNSDEQESSGAIIYTVELKRYGGPLGITISGTEEPFDPIIISSLTKGGLAERTGAIHIGDRILAINSSSLKGKPLSEAIHLLQMAGETVTLKIKKQTDAQSASSPKKFPISSHLSDLGDVEEDSSPAQKPGKLSDMYPSTVPSVDSAVDSWDGSAIDTSYGTEGTSFQASGYNFNTYDWRSPKQRGSLSPVTKPRSQTYPDVGLSYEDWDRSTASGFAGAADSAETEQEENFWSQALEDLETCGQSGILRELEEKADRRVSLRNMTLLATIMSGSTMSLNHEAPTPRSQLGRQASFQERSSSRPHYSQTTRSNTLPSDVGRKSVTLRKMKQEIKEIMSPTPVELHKVTLYKDSDMEDFGFSVADGLLEKGVYVKNIRPAGPGDLGGLKPYDRLLQVNHVRTRDFDCCLVVPLIAESGNKLDLVISRNPLASQKSIDQQSLPGDWSEQNSAFFQQPSHGGNLETREPTNTL

809 NP_057671.2 NP_056989.2

>NP_057671.2

MASNKTTLQKMGKKQNGKSKKVEEAEPEEFVVEKVLDRRVVNGKVEYFLKWKGFTDADNTWEPEENLDCPELIEAFLNSQKAGKEKDGTKRKSLSDSESDDSKSKKKRDAADKPRGFARGLDPERIIGATDSSGELMFLMKWKDSDEADLVLAKEANMKCPQIVIAFYEERLTWHSCPEDEAQ

>NP_056989.2

MEVAVEKAVAAAAAASAAASGGPSAAPSGENEAESRQGPDSERGGEAARLNLLDTCAVCHQNIQSRAPKLLPCLHSFCQRCLPAPQRYLMLPAPMLGSAETPPPVPAPGSPVSGSSPFATQVGVIRCPVCSQECAERHIIDNFFVKDTTEVPSSTVEKSNQVCTSCEDNAEANGFCVECVEWLCKTCIRAHQRVKFTKDHTVRQKEEVSPEAVGVTSQRPVFCPFHKKEQLKLYCETCDKLTCRDCQLLEHKEHRYQFIEEAFQNQKVIIDTLITKLMEKTKYIKFTGNQIQNRIIEVNQNQKQVEQDIKVAIFTLMVEINKKGKALLHQLESLAKDHRMKLMQQQQEVAGLSKQLEHVMHFSKWAVSSGSSTALLYSKRLITYRLRHLLRARCDASPVTNNTIQFHCDPSFWAQNIINLGSLVIEDKESQPQMPKQNPVVEQNSQPPSGLSSNQLSKFPTQISLAQLRLQHMQQQVMAQRQQVQRRPAPVGLPNPRMQGPIQQPSISHQQPPPRLINFQNHSPKPNGPVLPPHPQQLRYPPNQNIPRQAIKPNPLQMAFLAQQAIKQWQISSGQGTPSTTNSTSSTPSSPTITSAAGYDGKAFGSPMIDLSSPVGGSYNLPSLPDIDCSSTIMLDNIVRKDTNIDHGQPRPPSNRTVQSPNSSVPSPGLAGPVTMTSVHPPIRSPSASSVGSRGSSGSSSKPAGADSTHKVPVVMLEPIRIKQENSGPPENYDFPVVIVKQESDEESRPQNANYPRSILTSLLLNSSQSSTSEETVLRSDAPDSTGDQPGLHQDNSSNGKSEWLDPSQKSPLHVGETRKEDDPNEDWCAVCQNGGELLCCEKCPKVFHLSCHVPTLTNFPSGEWICTFCRDLSKPEVEYDCDAPSHNSEKKKTEGLVKLTPIDKRKCERLLLFLYCHEMSLAFQDPVPLTVPDYYKIIKNPMDLSTIKKRLQEDYSMYSKPEDFVADFRLIFQNCAEFNEPDSEVANAGIKLENYFEELLKNLYPEKRFPKPEFRNESEDNKFSDDSDDDFVQPRKKRLKSIEERQLLK

810 NP_002736.3 NP_005766.2

>NP_002736.3

MAAAAAAGAGPEMVRGQVFDVGPRYTNLSYIGEGAYGMVCSAYDNVNKVRVAIKKISPFEHQTYCQRTLREIKILLRFRHENIIGINDIIRAPTIEQMKDVYIVQDLMETDLYKLLKTQHLSNDHICYFLYQILRGLKYIHSANVLHRDLKPSNLLLNTTCDLKICDFGLARVADPDHDHTGFLTEYVATRWYRAPEIMLNSKGYTKSIDIWSVGCILAEMLSNRPIFPGKHYLDQLNHILGILGSPSQEDLNCIINLKARNYLLSLPHKNKVPWNRLFPNADSKALDLLDKMLTFNPHKRIEVEQALAHPYLEQYYDPSDEPIAEAPFKFDMELDDLPKEKLKELIFEETARFQPGYRS

>NP_005766.2

MQGPPRSLRAGLSLDDFIPGHLQSHIGSSSRGTRVPVIRNGGSNTLNFQFHDPAPRTVCNGGYTPRRDASQHPDPAWYQTWPGPGSKPSASTKIPASQHTQNWSATWTKDSKRRDKRWVKYEGIGPVDESGMPIAPRSSVDRPRDWYRRMFQQIHRKMPDLQLDWTFEEPPRDPRHLGAQQRPAHRPGPATSSSGRSWDHSEELPRSTFNYRPGAFSTVLQPSNQVLRRREKVDNVWTEESWNQFLQELETGQRPKKPLVDDPGEKPSQPIEVLLERELAELSAELDKDLRAIETRLPSPKSSPAPRRAPEQRPPAGPASAWSSSYPHAPYLGSARSLSPHKMADGGSPFLGRRDFVYPSSTRDPSASNGGGSPARREEKKRKAARLKFDFQAQSPKELTLQKGDIVYIHKEVDKNWLEGEHHGRLGIFPANYVEVLPADEIPKPIKPPTYQVLEYGEAVAQYTFKGDLEVELSFRKGEHICLIRKVNENWYEGRITGTGRQGIFPASYVQVSREPRLRLCDDGPQLPTSPRLTAAARSARHPSSPSALRSPADPTDLGGQTSPRRTGFSFPTQEPRPQTQNLGTPGPALSHSRGPSHPLDLGTSSPNTSQIHWTPYRAMYQYRPQNEDELELREGDRVDVMQQCDDGWFVGVSRRTQKFGTFPGNYVAPV

811 NP_003338.1 NP_000453.2

>NP_003338.1

MAASRRLMKELEEIRKCGMKNFRNIQVDEANLLTWQGLIVPDNPPYDKGAFRIEINFPAEYPFKPPKITFKTKIYHPNIDEKGQVCLPVISAENWKPATKTDQVIQSLIALVNDPQPEHPLRADLAEEYSKDRKKFCKNAEEFTKKYGEKRPVD

>NP_000453.2

MEKLHQCYWKSGEPQSDDIEASRMKRAAAKHLIERYYHQLTEGCGNEACTNEFCASCPTFLRMDNNAAAIKALELYKINAKLCDPHPSKKGASSAYLENSKGAPNNSCSEIKMNKKGARIDFKDVTYLTEEKVYEILELCREREDYSPLIRVIGRVFSSAEALVQSFRKVKQHTKEELKSLQAKDEDKDEDEKEKAACSAAAMEEDSEASSSRIGDSSQGDNNLQKLGPDDVSVDIDAIRRVYTRLLSNEKIETAFLNALVYLSPNVECDLTYHNVYSRDPNYLNLFIIVMENRNLHSPEYLEMALPLFCKAMSKLPLAAQGKLIRLWSKYNADQIRRMMETFQQLITYKVISNEFNSRNLVNDDDAIVAASKCLKMVYYANVVGGEVDTNHNEEDDEEPIPESSELTLQELLGEERRNKKGPRVDPLETELGVKTLDCRKPLIPFEEFINEPLNEVLEMDKDYTFFKVETENKFSFMTCPFILNAVTKNLGLYYDNRIRMYSERRITVLYSLVQGQQLNPYLRLKVRRDHIIDDALVRLEMIAMENPADLKKQLYVEFEGEQGVDEGGVSKEFFQLVVEEIFNPDIGMFTYDESTKLFWFNPSSFETEGQFTLIGIVLGLAIYNNCILDVHFPMVVYRKLMGKKGTFRDLGDSHPVLYQSLKDLLEYEGNVEDDMMITFQISQTDLFGNPMMYDLKENGDKIPITNENRKEFVNLYSDYILNKSVEKQFKAFRRGFHMVTNESPLKYLFRPEEIELLICGSRNLDFQALEETTEYDGGYTRDSVLIREFWEIVHSFTDEQKRLFLQFTTGTDRAPVGGLGKLKMIIAKNGPDTERLPTSHTCFNVLLLPEYSSKEKLKERLLKAITYAKGFGML

812 NP_057250.1 NP_000035.2

>NP_057250.1

MADSAELKQMVMSLRVSELQVLLGYAGRNKHGRKHELLTKALHLLKAGCSPAVQMKIKELYRRRFPQKIMTPADLSIPNVHSSPMPATLSPSTIPQLTYDGHPASSPLLPVSLLGPKHELELPHLTSALHPVHPDIKLQKLPFYDLLDELIKPTSLASDNSQRFRETCFAFALTPQQVQQISSSMDISGTKCDFTVQVQLRFCLSETSCPQEDHFPPNLCVKVNTKPCSLPGYLPPTKNGVEPKRPSRPINITSLVRLSTTVPNTIVVSWTAEIGRNYSMAVYLVKQLSSTVLLQRLRAKGIRNPDHSRALIKEKLTADPDSEIATTSLRVSLLCPLGKMRLTIPCRALTCSHLQCFDATLYIQMNEKKPTWVCPVCDKKAPYEHLIIDGLFMEILKYCTDCDEIQFKEDGTWAPMRSKKEVQEVSASYNGVDGCLSSTLEHQVASHHQSSNKNKKVEVIDLTIDSSSDEEEEEPSAKRTCPSLSPTSPLNNKGILSLPHQASPVSRTPSLPAVDTSYINTSLIQDYRHPFHMTPMPYDLQGLDFFPFLSGDNQHYNTSLLAAAAAAVSDDQDLLHSSRFFPYTSSQMFLDQLSAGGSTSLPTTNGSSSGSNSSLVSSNSLRESHSHTVTNRSSTDTASIFGIIPDIISLD

>NP_000035.2

MEVQLGLGRVYPRPPSKTYRGAFQNLFQSVREVIQNPGPRHPEAASAAPPGASLLLLQQQQQQQQQQQQQQQQQQQQQQQETSPRQQQQQQGEDGSPQAHRRGPTGYLVLDEEQQPSQPQSALECHPERGCVPEPGAAVAASKGLPQQLPAPPDEDDSAAPSTLSLLGPTFPGLSSCSADLKDILSEASTMQLLQQQQQEAVSEGSSSGRAREASGAPTSSKDNYLGGTSTISDNAKELCKAVSVSMGLGVEALEHLSPGEQLRGDCMYAPLLGVPPAVRPTPCAPLAECKGSLLDDSAGKSTEDTAEYSPFKGGYTKGLEGESLGCSGSAAAGSSGTLELPSTLSLYKSGALDEAAAYQSRDYYNFPLALAGPPPPPPPPHPHARIKLENPLDYGSAWAAAAAQCRYGDLASLHGAGAAGPGSGSPSAAASSSWHTLFTAEEGQLYGPCGGGGGGGGGGGGGGGGGGGGGGGEAGAVAPYGYTRPPQGLAGQESDFTAPDVWYPGGMVSRVPYPSPTCVKSEMGPWMDSYSGPYGDMRLETARDHVLPIDYYFPPQKTCLICGDEASGCHYGALTCGSCKVFFKRAAEGKQKYLCASRNDCTIDKFRRKNCPSCRLRKCYEAGMTLGARKLKKLGNLKLQEEGEASSTTSPTEETTQKLTVSHIEGYECQPIFLNVLEAIEPGVVCAGHDNNQPDSFAALLSSLNELGERQLVHVVKWAKALPGFRNLHVDDQMAVIQYSWMGLMVFAMGWRSFTNVNSRMLYFAPDLVFNEYRMHKSRMYSQCVRMRHLSQEFGWLQITPQEFLCMKALLLFSIIPVDGLKNQKFFDELRMNYIKELDRIIACKRKNPTSCSRRFYQLTKLLDSVQPIARELHQFTFDLLIKSHMVSVDFPEMMAEIISVQVPKILSGKVKPIYFHTQ

813 NP_001019801.2 NP_074036.1

>NP_001019801.2

MLHSPHKQPQNHKCGANFLQEDSKKSLVFKWLISAGHYQPPRPTESFKAASSIYNRGYKFYLKKKGGTMASNSLFSTVTPCQQNFFWDPSTSRRFSPPSSSLQPGKMSDVSPVVAAQQQQQQQQQQQQQQQQQQQQQQQEAAAAAAAAAAAAAAAAAVPRLRPPHDNRTMVEIIADHPAELVRTDSPNFLCSVLPSHWRCNKTLPVAFKVVALGEVPDGTVVTVMAGNDENYSAELRNASAVMKNQVARFNDLRFVGRSGRGKSFTLTITVFTNPPQVATYHRAIKVTVDGPREPRRHRQKLDDSKPSLFSDRLSDLGRIPHPSMRVGVPPQNPRPSLNSAPSPFNPQGQSQITDPRQAQSSPPWSYDQSYPSYLSQMTSPSIHSTTPLSSTRGTGLPAITDVPRRISDDDTATSDFCLWPSTLSKKSQAGASELGPFSDPRQFPSISSLTESRFSNPRMHYPATFTYTPPVTSGMSLGMSATTHYHTYLPPPYPGSSQSQSGPFQTSSTPYLYYGTSSGSYQFPMVPGGDRSPSRMLPPCTTTSNGSTLLNPNLPNQNDGVDADGSHSSSPTVLNSSGRMDESVWRPY

>NP_074036.1

MPRVVPDQRSKFENEEFFRKLSRECEIKYTGFRDRPHEERQARFQNACRDGRSEIAFVATGTNLSLQFFPASWQGEQRQTPSREYVDLEREAGKVYLKAPMILNGVCVIWKGWIDLQRLDGMGCLEFDEERAQQEDALAQQAFEEARRRTREFEDRDRSHREEMEARRQQDPSPGSNLGGGDDLKLR

814 NP_001341.1 NP_036377.1

>NP_001341.1

MATANSIIVLDDDDEDEAAAQPGPSHPLPNAASPGAEAPSSSEPHGARGSSSSGGKKCYKLENEKLFEEFLELCKMQTADHPEVVPFLYNRQQRAHSLFLASAEFCNILSRVLSRARSRPAKLYVYINELCTVLKAHSAKKKLNLAPAATTSNEPSGNNPPTHLSLDPTNAENTASQSPRTRGSRRQIQRLEQLLALYVAEIRRLQEKELDLSELDDPDSAYLQEARLKRKLIRLFGRLCELKDCSSLTGRVIEQRIPYRGTRYPEVNRRIERLINKPGPDTFPDYGDVLRAVEKAAARHSLGLPRQQLQLMAQDAFRDVGIRLQERRHLDLIYNFGCHLTDDYRPGVDPALSDPVLARRLRENRSLAMSRLDEVISKYAMLQDKSEEGERKKRRARLQGTSSHSADTPEASLDSGEGPSGMASQGCPSASRAETDDEDDEESDEEEEEEEEEEEEEATDSEEEEDLEQMQEGQEDDEEEDEEEEAAAGKDGDKSPMSSLQISNEKNLEPGKQISRSSGEQQNKGRIVSPSLLSEEPLAPSSIDAESNGEQPEELTLEEESPVSQLFELEIEALPLDTPSSVETDISSSRKQSEEPFTTVLENGAGMVSSTSFNGGVSPHNWGDSGPPCKKSRKEKKQTGSGPLGNSYVERQRSVHEKNGKKICTLPSPPSPLASLAPVADSSTRVDSPSHGLVTSSLCIPSPARLSQTPHSQPPRPGTCKTSVATQCDPEEIIVLSDSD

>NP_036377.1

MALTSFLPAPTQLSQDQLEAEEKARSQRSRQTSLVSSRREPPPYGYRKGWIPRLLEDFGDGGAFPEIHVAQYPLDMGRKKKMSNALAIQVDSEGKIKYDAIARQGQSKDKVIYSKYTDLVPKEVMNADDPDLQRPDEEAIKEITEKTRVALEKSVSQKVAAAMPVRAADKLAPAQYIRYTPSQQGVAFNSGAKQRVIRMVEMQKDPMEPPRFKINKKIPRGPPSPPAPVMHSPSRKMTVKEQQEWKIPPCISNWKNAKGYTIPLDKRLAADGRGLQTVHINENFAKLAEALYIADRKAREAVEMRAQVERKMAQKEKEKHEEKLREMAQKARERRAGIKTHVEKEDGEARERDEIRHDRRKERQHDRNLSRAAPDKRSKLQRNENRDISEVIALGVPNPRTSNEVQYDQRLFNQSKGMDSGFAGGEDEIYNVYDQAWRGGKDMAQSIYRPSKNLDKDMYGDDLEARIKTNRFVPDKEFSGSDRRQRGREGPVQFEEDPFGLDKFLEEAKQHGGSKRPSDSSRPKEHEHEGKKRRKE

815 NP_002077.1 NP_060276.1

>NP_002077.1

MEAIAKYDFKATADDELSFKRGDILKVLNEECDQNWYKAELNGKDGFIPKNYIEMKPHPWFFGKIPRAKAEEMLSKQRHDGAFLIRESESAPGDFSLSVKFGNDVQHFKVLRDGAGKYFLWVVKFNSLNELVDYHRSTSVSRNQQIFLRDIEQVPQQPTYVQALFDFDPQEDGELGFRRGDFIHVMDNSDPNWWKGACHGQTGMFPRNYVTPVNRNV

>NP_060276.1

MGLPVSWAPPALWVLGCCALLLSLWALCTACRRPEDAVAPRKRARRQRARLQGSATAAEASLLRRTHLCSLSKSDTRLHELHRGPRSSRALRPASMDLLRPHWLEVSRDITGPQAAPSAFPHQELPRALPAAAATAGCAGLEATYSNVGLAALPGVSLAASPVVAEYARVQKRKGTHRSPQEPQQGKTEVTPAAQVDVLYSRVCKPKRRDPGPTTDPLDPKGQGAILALAGDLAYQTLPLRALDVGSGPLENVYESIRELGDPAGRSSTCGAGTPPASSCPSLGRGWRPLPASLP

816 NP_008835.5 NP_002303.2

>NP_008835.5

MAGSGAGVRCSLLRLQETLSAADRCGAALAGHQLIRGLGQECVLSSSPAVLALQTSLVFSRDFGLLVFVRKSLNSIEFRECREEILKFLCIFLEKMGQKIAPYSVEIKNTCTSVYTKDRAAKCKIPALDLLIKLLQTFRSSRLMDEFKIGELFSKFYGELALKKKIPDTVLEKVYELLGLLGEVHPSEMINNAENLFRAFLGELKTQMTSAVREPKLPVLAGCLKGLSSLLCNFTKSMEEDPQTSREIFNFVLKAIRPQIDLKRYAVPSAGLRLFALHASQFSTCLLDNYVSLFEVLLKWCAHTNVELKKAALSALESFLKQVSNMVAKNAEMHKNKLQYFMEQFYGIIRNVDSNNKELSIAIRGYGLFAGPCKVINAKDVDFMYVELIQRCKQMFLTQTDTGDDRVYQMPSFLQSVASVLLYLDTVPEVYTPVLEHLVVMQIDSFPQYSPKMQLVCCRAIVKVFLALAAKGPVLRNCISTVVHQGLIRICSKPVVLPKGPESESEDHRASGEVRTGKWKVPTYKDYVDLFRHLLSSDQMMDSILADEAFFSVNSSSESLNHLLYDEFVKSVLKIVEKLDLTLEIQTVGEQENGDEAPGVWMIPTSDPAANLHPAKPKDFSAFINLVEFCREILPEKQAEFFEPWVYSFSYELILQSTRLPLISGFYKLLSITVRNAKKIKYFEGVSPKSLKHSPEDPEKYSCFALFVKFGKEVAVKMKQYKDELLASCLTFLLSLPHNIIELDVRAYVPALQMAFKLGLSYTPLAEVGLNALEEWSIYIDRHVMQPYYKDILPCLDGYLKTSALSDETKNNWEVSALSRAAQKGFNKVVLKHLKKTKNLSSNEAISLEEIRIRVVQMLGSLGGQINKNLLTVTSSDEMMKSYVAWDREKRLSFAVPFREMKPVIFLDVFLPRVTELALTASDRQTKVAACELLHSMVMFMLGKATQMPEGGQGAPPMYQLYKRTFPVLLRLACDVDQVTRQLYEPLVMQLIHWFTNNKKFESQDTVALLEAILDGIVDPVDSTLRDFCGRCIREFLKWSIKQITPQQQEKSPVNTKSLFKRLYSLALHPNAFKRLGASLAFNNIYREFREEESLVEQFVFEALVIYMESLALAHADEKSLGTIQQCCDAIDHLCRIIEKKHVSLNKAKKRRLPRGFPPSASLCLLDLVKWLLAHCGRPQTECRHKSIELFYKFVPLLPGNRSPNLWLKDVLKEEGVSFLINTFEGGGCGQPSGILAQPTLLYLRGPFSLQATLCWLDLLLAALECYNTFIGERTVGALQVLGTEAQSSLLKAVAFFLESIAMHDIIAAEKCFGTGAAGNRTSPQEGERYNYSKCTVVVRIMEFTTTLLNTSPEGWKLLKKDLCNTHLMRVLVQTLCEPASIGFNIGDVQVMAHLPDVCVNLMKALKMSPYKDILETHLREKITAQSIEELCAVNLYGPDAQVDRSRLAAVVSACKQLHRAGLLHNILPSQSTDLHHSVGTELLSLVYKGIAPGDERQCLPSLDLSCKQLASGLLELAFAFGGLCERLVSLLLNPAVLSTASLGSSQGSVIHFSHGEYFYSLFSETINTELLKNLDLAVLELMQSSVDNTKMVSAVLNGMLDQSFRERANQKHQGLKLATTILQHWKKCDSWWAKDSPLETKMAVLALLAKILQIDSSVSFNTSHGSFPEVFTTYISLLADTKLDLHLKGQAVTLLPFFTSLTGGSLEELRRVLEQLIVAHFPMQSREFPPGTPRFNNYVDCMKKFLDALELSQSPMLLELMTEVLCREQQHVMEELFQSSFRRIARRGSCVTQVGLLESVYEMFRKDDPRLSFTRQSFVDRSLLTLLWHCSLDALREFFSTIVVDAIDVLKSRFTKLNESTFDTQITKKMGYYKILDVMYSRLPKDDVHAKESKINQVFHGSCITEGNELTKTLIKLCYDAFTENMAGENQLLERRRLYHCAAYNCAISVICCVFNELKFYQGFLFSEKPEKNLLIFENLIDLKRRYNFPVEVEVPMERKKKYIEIRKEAREAANGDSDGPSYMSSLSYLADSTLSEEMSQFDFSTGVQSYSYSSQDPRPATGRFRRREQRDPTVHDDVLELEMDELNRHECMAPLTALVKHMHRSLGPPQGEEDSVPRDLPSWMKFLHGKLGNPIVPLNIRLFLAKLVINTEEVFRPYAKHWLSPLLQLAASENNGGEGIHYMVVEIVATILSWTGLATPTGVPKDEVLANRLLNFLMKHVFHPKRAVFRHNLEIIKTLVECWKDCLSIPYRLIFEKFSGKDPNSKDNSVGIQLLGIVMANDLPPYDPQCGIQSSEYFQALVNNMSFVRYKEVYAAAAEVLGLILRYVMERKNILEESLCELVAKQLKQHQNTMEDKFIVCLNKVTKSFPPLADRFMNAVFFLLPKFHGVLKTLCLEVVLCRVEGMTELYFQLKSKDFVQVMRHRDDERQKVCLDIIYKMMPKLKPVELRELLNPVVEFVSHPSTTCREQMYNILMWIHDNYRDPESETDNDSQEIFKLAKDVLIQGLIDENPGLQLIIRNFWSHETRLPSNTLDRLLALNSLYSPKIEVHFLSLATNFLLEMTSMSPDYPNPMFEHPLSECEFQEYTIDSDWRFRSTVLTPMFVETQASQGTLQTRTQEGSLSARWPVAGQIRATQQQHDFTLTQTADGRSSFDWLTGSSTDPLVDHTSPSSDSLLFAHKRSERLQRAPLKSVGPDFGKKRLGLPGDEVDNKVKGAAGRTDLLRLRRRFMRDQEKLSLMYARKGVAEQKREKEIKSELKMKQDAQVVLYRSYRHGDLPDIQIKHSSLITPLQAVAQRDPIIAKQLFSSLFSGILKEMDKFKTLSEKNNITQKLLQDFNRFLNTTFSFFPPFVSCIQDISCQHAALLSLDPAAVSAGCLASLQQPVGIRLLEEALLRLLPAELPAKRVRGKARLPPDVLRWVELAKLYRSIGEYDVLRGIFTSEIGTKQITQSALLAEARSDYSEAAKQYDEALNKQDWVDGEPTEAEKDFWELASLDCYNHLAEWKSLEYCSTASIDSENPPDLNKIWSEPFYQETYLPYMIRSKLKLLLQGEADQSLLTFIDKAMHGELQKAILELHYSQELSLLYLLQDDVDRAKYYIQNGIQSFMQNYSSIDVLLHQSRLTKLQSVQALTEIQEFISFISKQGNLSSQVPLKRLLNTWTNRYPDAKMDPMNIWDDIITNRCFFLSKIEEKLTPLPEDNSMNVDQDGDPSDRMEVQEQEEDISSLIRSCKFSMKMKMIDSARKQNNFSLAMKLLKELHKESKTRDDWLVSWVQSYCRLSHCRSRSQGCSEQVLTVLKTVSLLDENNVSSYLSKNILAFRDQNILLGTTYRIIANALSSEPACLAEIEEDKARRILELSGSSSEDSEKVIAGLYQRAFQHLSEAVQAAEEEAQPPSWSCGPAAGVIDAYMTLADFCDQQLRKEEENASVIDSAELQAYPALVVEKMLKALKLNSNEARLKFPRLLQIIERYPEETLSLMTKEISSVPCWQFISWISHMVALLDKDQAVAVQHSVEEITDNYPQAIVYPFIISSESYSFKDTSTGHKNKEFVARIKSKLDQGGVIQDFINALDQLSNPELLFKDWSNDVRAELAKTPVNKKNIEKMYERMYAALGDPKAPGLGAFRRKFIQTFGKEFDKHFGKGGSKLLRMKLSDFNDITNMLLLKMNKDSKPPGNLKECSPWMSDFKVEFLRNELEIPGQYDGRGKPLPEYHVRIAGFDERVTVMASLRRPKRIIIRGHDEREHPFLVKGGEDLRQDQRVEQLFQVMNGILAQDSACSQRALQLRTYSVVPMTSRLGLIEWLENTVTLKDLLLNTMSQEEKAAYLSDPRAPPCEYKDWLTKMSGKHDVGAYMLMYKGANRTETVTSFRKRESKVPADLLKRAFVRMSTSPEAFLALRSHFASSHALICISHWILGIGDRHLNNFMVAMETGGVIGIDFGHAFGSATQFLPVPELMPFRLTRQFINLMLPMKETGLMYSIMVHALRAFRSDPGLLTNTMDVFVKEPSFDWKNFEQKMLKKGGSWIQEINVAEKNWYPRQKICYAKRKLAGANPAVITCDELLLGHEKAPAFRDYVAVARGSKDHNIRAQEPESGLSEETQVKCLMDQATDPNILGRTWEGWEPWM

>NP_002303.2

MAASQTSQTVASHVPFADLCSTLERIQKSKGRAEKIRHFREFLDSWRKFHDALHKNHKDVTDSFYPAMRLILPQLERERMAYGIKETMLAKLYIELLNLPRDGKDALKLLNYRTPTGTHGDAGDFAMIAYFVLKPRCLQKGSLTIQQVNDLLDSIASNNSAKRKDLIKKSLLQLITQSSALEQKWLIRMIIKDLKLGVSQQTIFSVFHNDAAELHNVTTDLEKVCRQLHDPSVGLSDISITLFSAFKPMLAAIADIEHIEKDMKHQSFYIETKLDGERMQMHKDGDVYKYFSRNGYNYTDQFGASPTEGSLTPFIHNAFKADIQICILDGEMMAYNPNTQTFMQKGTKFDIKRMVEDSDLQTCYCVFDVLMVNNKKLGHETLRKRYEILSSIFTPIPGRIEIVQKTQAHTKNEVIDALNEAIDKREEGIMVKQPLSIYKPDKRGEGWLKIKPEYVSGLMDELDILIVGGYWGKGSRGGMMSHFLCAVAEKPPPGEKPSVFHTLSRVGSGCTMKELYDLGLKLAKYWKPFHRKAPPSSILCGTEKPEVYIEPCNSVIVQIKAAEIVPSDMYKTGCTLRFPRIEKIRDDKEWHECMTLDDLEQLRGKASGKLASKHLYIGGDDEPQEKKRKAAPKMKKVIGIIEHLKAPNLTNVNKISNIFEDVEFCVMSGTDSQPKPDLENRIAEFGGYIVQNPGPDTYCVIAGSENIRVKNIILSNKHDVVKPAWLLECFKTKSFVPWQPRFMIHMCPSTKEHFAREYDCYGDSYFIDTDLNQLKEVFSGIKNSNEQTPEEMASLIADLEYRYSWDCSPLSMFRRHTVYLDSYAVINDLSTKNEGTRLAIKALELRFHGAKVVSCLAEGVSHVIIGEDHSRVADFKAFRRTFKRKFKILKESWVTDSIDKCELQEENQYLI

817 NP_009117.2 NP_060649.3

>NP_009117.2

METRSPGLNNMKPQSLQLVLEEQVLALQQQMAENQAASWRKLKNSQEAQQRQATLVRKLQAKVLQYRSWCQELEKRLEATGGPIPQRWENVEEPNLDELLVRLEEEQQRCESLAEVNTQLRLHMEKADVVNKALREDVEKLTVDWSRARDELMRKESQWQMEQEFFKGYLKGEHGRLLSLWREVVTFRRHFLEMKSATDRDLMELKAEHVRLSGSLLTCCLRLTVGAQSREPNGSGRMDGREPAQLLLLLAKTQELEKEAHERSQELIQLKSQGDLEKAELQDRVTELSALLTQSQKQNEDYEKMIKALRETVEILETNHTELMEHEASLSRNAQEEKLSLQQVIKDITQVMVEEGDNIAQGSGHENSLELDSSIFSQFDYQDADKALTLVRSVLTRRRQAVQDLRQQLAGCQEAVNLLQQQHDQWEEEGKALRQRLQKLTGERDTLAGQTVDLQGEVDSLSKERELLQKAREELRQQLEVLEQEAWRLRRVNVELQLQGDSAQGQKEEQQEELHLAVRERERLQEMLMGLEAKQSESLSELITLREALESSHLEGELLRQEQTEVTAALARAEQSIAELSSSENTLKTEVADLRAAAVKLSALNEALALDKVGLNQQLLQLEEENQSVCSRMEAAEQARNALQVDLAEAEKRREALWEKNTHLEAQLQKAEEAGAELQADLRDIQEEKEEIQKKLSESRHQQEAATTQLEQLHQEAKRQEEVLARAVQEKEALVREKAALEVRLQAVERDRQDLAEQLQGLSSAKELLESSLFEAQQQNSVIEVTKGQLEVQIQTVTQAKEVIQGEVRCLKLELDTERSQAEQERDAAARQLAQAEQEGKTALEQQKAAHEKEVNQLREKWEKERSWHQQELAKALESLEREKMELEMRLKEQQTEMEAIQAQREEERTQAESALCQMQLETEKERVSLLETLLQTQKELADASQQLERLRQDMKVQKLKEQETTGILQTQLQEAQRELKEAARQHRDDLAALQEESSSLLQDKMDLQKQVEDLKSQLVAQDDSQRLVEQEVQEKLRETQEYNRIQKELEREKASLTLSLMEKEQRLLVLQEADSIRQQELSALRQDMQEAQGEQKELSAQMELLRQEVKEKEADFLAQEAQLLEELEASHITEQQLRASLWAQEAKAAQLQLRLRSTESQLEALAAEQQPGNQAQAQAQLASLYSALQQALGSVCESRPELSGGGDSAPSVWGLEPDQNGARSLFKRGPLLTALSAEAVASALHKLHQDLWKTQQTRDVLRDQVQKLEERLTDTEAEKSQVHTELQDLQRQLSQNQEEKSKWEGKQNSLESELMELHETMASLQSRLRRAELQRMEAQGERELLQAAKENLTAQVEHLQAAVVEARAQASAAGILEEDLRTARSALKLKNEEVESERERAQALQEQGELKVAQGKALQENLALLTQTLAEREEEVETLRGQIQELEKQREMQKAALELLSLDLKKRNQEVDLQQEQIQELEKCRSVLEHLPMAVQEREQKLTVQREQIRELEKDRETQRNVLEHQLLELEKKDQMIESQRGQVQDLKKQLVTLECLALELEENHHKMECQQKLIKELEGQRETQRVALTHLTLDLEERSQELQAQSSQIHDLESHSTVLARELQERDQEVKSQREQIEELQRQKEHLTQDLERRDQELMLQKERIQVLEDQRTRQTKILEEDLEQIKLSLRERGRELTTQRQLMQERAEEGKGPSKAQRGSLEHMKLILRDKEKEVECQQEHIHELQELKDQLEQQLQGLHRKVGETSLLLSQREQEIVVLQQQLQEAREQGELKEQSLQSQLDEAQRALAQRDQELEALQQEQQQAQGQEERVKEKADALQGALEQAHMTLKERHGELQDHKEQARRLEEELAVEGRRVQALEEVLGDLRAESREQEKALLALQQQCAEQAQEHEVETRALQDSWLQAQAVLKERDQELEALRAESQSSRHQEEAARARAEALQEALGKAHAALQGKEQHLLEQAELSRSLEASTATLQASLDACQAHSRQLEEALRIQEGEIQDQDLRYQEDVQQLQQALAQRDEELRHQQEREQLLEKSLAQRVQENMIQEKQNLGQEREEEEIRGLHQSVRELQLTLAQKEQEILELRETQQRNNLEALPHSHKTSPMEEQSLKLDSLEPRLQRELERLQAALRQTEAREIEWREKAQDLALSLAQTKASVSSLQEVAMFLQASVLERDSEQQRLQDELELTRRALEKERLHSPGATSTAELGSRGEQGVQLGEVSGVEAEPSPDGMEKQSWRQRLEHLQQAVARLEIDRSRLQRHNVQLRSTLEQVERERRKLKREAMRAAQAGSLEISKATASSPTQQDGRGQKNSDAKCVAELQKEVVLLQAQLTLERKQKQDYITRSAQTSRELAGLHHSLSHSLLAVAQAPEATVLEAETRRLDESLTQSLTSPGPVLLHPSPSTTQAASR

>NP_060649.3

MDSLEEPQKKVFKARKTMRVSDRQQLEAVYKVKEELLKTDVKLLNGNHENGDLDPTSPLENMDYIKDKEEVNGIEEICFDPEGSKAEWKETPCILSVNVKNKQDDDLNCEPLSPHNITPEPVSKLPAEPVSGDPAPGDLDAGDPASGVLASGDSTSGDPTSSEPSSSDAASGDATSGDAPSGDVSPGDATSGDATADDLSSGDPTSSDPIPGEPVPVEPISGDCAADDIASSEITSVDLASGAPASTDPASDDLASGDLSSSELASDDLATGELASDELTSESTFDRTFEPKSVPVCEPVPEIDNIEPSSNKDDDFLEKNGADEKLEQIQSKDSLDEKNKADNNIDANEETLETDDTTICSDRPPENEKKVEEDIITELALGEDAISSSMEIDQGEKNEDETSADLVETINENVIEDNKSENILENTDSMETDEIIPILEKLAPSEDELTCFSKTSLLPIDETNPDLEEKMESSFGSPSKQESSESLPKEAFLVLSDEEDISGEKDESEVISQNETCSPAEVESNEKDNKPEEEEQVIHEDDERPSEKNEFSRRKRSKSEDMDNVQSKRRRYMEEEYEAEFQVKITAKGDINQKLQKVIQWLLEEKLCALQCAVFDKTLAELKTRVEKIECNKRHKTVLTELQAKIARLTKRFEAAKEDLKKRHEHPPNPPVSPGKTVNDVNSNNNMSYRNAGTVRQMLESKRNVSESAPPSFQTPVNTVSSTNLVTPPAVVSSQPKLQTPVTSGSLTATSVLPAPNTATVVATTQVPSGNPQPTISLQPLPVILHVPVAVSSQPQLLQSHPGTLVTNQPSGNVEFISVQSPPTVSGLTKNPVSLPSLPNPTKPNNVPSVPSPSIQRNPTASAAPLGTTLAVQAVPTAHSIVQATRTSLPTVGPSGLYSPSTNRGPIQMKIPISAFSTSSAAEQNSNTTPRIENQTNKTIDASVSKKAADSTSQCGKATGSDSSGVIDLTMDDEESGASQDPKKLNHTPVSTMSSSQPVSRPLQPIQPAPPLQPSGVPTSGPSQTTIHLLPTAPTTVNVTHRPVTQVTTRLPVPRAPANHQVVYTTLPAPPAQAPLRGTVMQAPAVRQVNPQNSVTVRVPQTTTYVVNNGLTLGSTGPQLTVHHRPPQVHTEPPRPVHPAPLPEAPQPQRLPPEAASTSLPQKPHLKLARVQSQNGIVLSWSVLEVDRSCATVDSYHLYAYHEEPSATVPSQWKKIGEVKALPLPMACTLTQFVSGSKYYFAVRAKDIYGRFGPFCDPQSTDVISSTQSS

818 NP_003444.1 NP_005057.1

>NP_003444.1

MDTSSVGGLELTDQTPVLLGSTAMATSLTNVGNSFSGPANPLVSRSNKFQNSSVEDDDDVVFIEPVQPPPPSVPVVADQRTITFTSSKNEELQGNDSKITPSSKELASQKGSVSETIVIDDEEDMETNQGQEKNSSNFIERRPPETKNRTNDVDFSTSSFSRSKVNAGMGNSGITTEPDSEIQIANVTTLETGVSSVNDGQLENTDGRDMNLMITHVTSLQNTNLGDVSNGLQSSNFGVNIQTYTPSLTSQTKTGVGPFNPGRMNVAGDVFQNGESATHHNPDSWISQSASFPRNQKQPGVDSLSPVASLPKQIFQPSVQQQPTKPVKVTCANCKKPLQKGQTAYQRKGSAHLFCSTTCLSSFSHKPAPKKLCVMCKKDITTMKGTIVAQVDSSESFQEFCSTSCLSLYEDKQNPTKGALNKSRCTICGKLTEIRHEVSFKNMTHKLCSDHCFNRYRMANGLIMNCCEQCGEYLPSKGAGNNVLVIDGQQKRFCCQSCVSEYKQVGSHPSFLKEVRDHMQDSFLMQPEKYGKLTTCTGCRTQCRFFDMTQCIGPNGYMEPYCSTACMNSHKTKYAKSQSLGIICHFCKRNSLPQYQATMPDGKLYNFCNSSCVAKFQALSMQSSPNGQFVAPSDIQLKCNYCKNSFCSKPEILEWENKVHQFCSKTCSDDYKKLHCIVTYCEYCQEEKTLHETVNFSGVKRPFCSEGCKLLYKQDFARRLGLRCVTCNYCSQLCKKGATKELDGVVRDFCSEDCCKKFQDWYYKAARCDCCKSQGTLKERVQWRGEMKHFCDQHCLLRFYCQQNEPNMTTQKGPENLHYDQGCQTSRTKMTGSAPPPSPTPNKEMKNKAVLCKPLTMTKATYCKPHMQTKSCQTDDTWRTEYVPVPIPVPVYIPVPMHMYSQNIPVPTTVPVPVPVPVFLPAPLDSSEKIPAAIEELKSKVSSDALDTELLTMTDMMSEDEGKTETTNINSVIIETDIIGSDLLKNSDPETQSSMPDVPYEPDLDIEIDFPRAAEELDMENEFLLPPVFGEEYEEQPRPRSKKKGAKRKAVSGYQSHDDSSDNSECSFPFKYTYGVNAWKHWVKTRQLDEDLLVLDELKSSKSVKLKEDLLSHTTAELNYGLAHFVNEIRRPNGENYAPDSIYYLCLGIQEYLCGSNRKDNIFIDPGYQTFEQELNKILRSWQPSILPDGSIFSRVEEDYLWRIKQLGSHSPVALLNTLFYFNTKYFGLKTVEQHLRLSFGTVFRHWKKNPLTMENKACLRYQVSSLCGTDNEDKITTGKRKHEDDEPVFEQIENTANPSRCPVKMFECYLSKSPQNLNQRMDVFYLQPECSSSTDSPVWYTSTSLDRNTLENMLVRVLLVKDIYDKDNYELDEDTD

>NP_005057.1

MSRDRFRSRGGGGGGFHRRGGGGGRGGLHDFRSPPPGMGLNQNRGPMGPGPGQSGPKPPIPPPPPHQQQQQPPPQQPPPQQPPPHQPPPHPQPHQQQQPPPPPQDSSKPVVAQGPGPAPGVGSAPPASSSAPPATPPTSGAPPGSGPGPTPTPPPAVTSAPPGAPPPTPPSSGVPTTPPQAGGPPPPPAAVPGPGPGPKQGPGPGGPKGGKMPGGPKPGGGPGLSTPGGHPKPPHRGGGEPRGGRQHHPPYHQQHHQGPPPGGPGGRSEEKISDSEGFKANLSLLRRPGEKTYTQRCRLFVGNLPADITEDEFKRLFAKYGEPGEVFINKGKGFGFIKLESRALAEIAKAELDDTPMRGRQLRVRFATHAAALSVRNLSPYVSNELLEEAFSQFGPIERAVVIVDDRGRSTGKGIVEFASKPAARKAFERCSEGVFLLTTTPRPVIVEPLEQLDDEDGLPEKLAQKNPMYQKERETPPRFAQHGTFEYEYSQRWKSLDEMEKQQREQVEKNMKDAKDKLESEMEDAYHEHQANLLRQDLMRRQEELRRMEELHNQEMQKRKEMQLRQEEERRRREEEMMIRQREMEEQMRRQREESYSRMGYMDPRERDMRMGGGGAMNMGDPYGSGGQKFPPLGGGGGIGYEANPGVPPATMSGSMMGSDMRTERFGQGGAGPVGGQGPRGMGPGTPAGYGRGREEYEGPNKKPRF

819 NP_003063.2 NP_005410.1

>NP_003063.2

MSTPDPPLGGTPRPGPSPGPGPSPGAMLGPSPGPSPGSAHSMMGPSPGPPSAGHPIPTQGPGGYPQDNMHQMHKPMESMHEKGMSDDPRYNQMKGMGMRSGGHAGMGPPPSPMDQHSQGYPSPLGGSEHASSPVPASGPSSGPQMSSGPGGAPLDGADPQALGQQNRGPTPFNQNQLHQLRAQIMAYKMLARGQPLPDHLQMAVQGKRPMPGMQQQMPTLPPPSVSATGPGPGPGPGPGPGPGPAPPNYSRPHGMGGPNMPPPGPSGVPPGMPGQPPGGPPKPWPEGPMANAAAPTSTPQKLIPPQPTGRPSPAPPAVPPAASPVMPPQTQSPGQPAQPAPMVPLHQKQSRITPIQKPRGLDPVEILQEREYRLQARIAHRIQELENLPGSLAGDLRTKATIELKALRLLNFQRQLRQEVVVCMRRDTALETALNAKAYKRSKRQSLREARITEKLEKQQKIEQERKRRQKHQEYLNSILQHAKDFKEYHRSVTGKIQKLTKAVATYHANTEREQKKENERIEKERMRRLMAEDEEGYRKLIDQKKDKRLAYLLQQTDEYVANLTELVRQHKAAQVAKEKKKKKKKKKAENAEGQTPAIGPDGEPLDETSQMSDLPVKVIHVESGKILTGTDAPKAGQLEAWLEMNPGYEVAPRSDSEESGSEEEEEEEEEEQPQAAQPPTLPVEEKKKIPDPDSDDVSEVDARHIIENAKQDVDDEYGVSQALARGLQSYYAVAHAVTERVDKQSALMVNGVLKQYQIKGLEWLVSLYNNNLNGILADEMGLGKTIQTIALITYLMEHKRINGPFLIIVPLSTLSNWAYEFDKWAPSVVKVSYKGSPAARRAFVPQLRSGKFNVLLTTYEYIIKDKHILAKIRWKYMIVDEGHRMKNHHCKLTQVLNTHYVAPRRLLLTGTPLQNKLPELWALLNFLLPTIFKSCSTFEQWFNAPFAMTGEKVDLNEEETILIIRRLHKVLRPFLLRRLKKEVEAQLPEKVEYVIKCDMSALQRVLYRHMQAKGVLLTDGSEKDKKGKGGTKTLMNTIMQLRKICNHPYMFQHIEESFSEHLGFTGGIVQGLDLYRASGKFELLDRILPKLRATNHKVLLFCQMTSLMTIMEDYFAYRGFKYLRLDGTTKAEDRGMLLKTFNEPGSEYFIFLLSTRAGGLGLNLQSADTVIIFDSDWNPHQDLQAQDRAHRIGQQNEVRVLRLCTVNSVEEKILAAAKYKLNVDQKVIQAGMFDQKSSSHERRAFLQAILEHEEQDESRHCSTGSGSASFAHTAPPPAGVNPDLEEPPLKEEDEVPDDETVNQMIARHEEEFDLFMRMDLDRRREEARNPKRKPRLMEEDELPSWIIKDDAEVERLTCEEEEEKMFGRGSRHRKEVDYSDSLTEKQWLKAIEEGTLEEIEEEVRQKKSSRKRKRDSDAGSSTPTTSTRSRDKDDESKKQKKRGRPPAEKLSPNPPNLTKKMKKIVDAVIKYKDSSSGRQLSEVFIQLPSRKELPEYYELIRKPVDFKKIKERIRNHKYRSLNDLEKDVMLLCQNAQTFNLEGSLIYEDSIVLQSVFTSVRQKIEKEDDSEGEESEEEEEGEEEGSESESRSVKVKIKLGRKEKAQDRLKGGRRRPSRGSRAKPVVSDDDSEEEQEEDRSGSGSEED

>NP_005410.1

MAQWEMLQNLDSPFQDQLHQLYSHSLLPVDIRQYLAVWIEDQNWQEAALGSDDSKATMLFFHFLDQLNYECGRCSQDPESLLLQHNLRKFCRDIQPFSQDPTQLAEMIFNLLLEEKRILIQAQRAQLEQGEPVLETPVESQQHEIESRILDLRAMMEKLVKSISQLKDQQDVFCFRYKIQAKGKTPSLDPHQTKEQKILQETLNELDKRRKEVLDASKALLGRLTTLIELLLPKLEEWKAQQQKACIRAPIDHGLEQLETWFTAGAKLLFHLRQLLKELKGLSCLVSYQDDPLTKGVDLRNAQVTELLQRLLHRAFVVETQPCMPQTPHRPLILKTGSKFTVRTRLLVRLQEGNESLTVEVSIDRNPPQLQGFRKFNILTSNQKTLTPEKGQSQGLIWDFGYLTLVEQRSGGSGKGSNKGPLGVTEELHIISFTVKYTYQGLKQELKTDTLPVVIISNMNQLSIAWASVLWFNLLSPNLQNQQFFSNPPKAPWSLLGPALSWQFSSYVGRGLNSDQLSMLRNKLFGQNCRTEDPLLSWADFTKRESPPGKLPFWTWLDKILELVHDHLKDLWNDGRIMGFVSRSQERRLLKKTMSGTFLLRFSESSEGGITCSWVEHQDDDKVLIYSVQPYTKEVLQSLPLTEIIRHYQLLTEENIPENPLRFLYPRIPRDEAFGCYYQEKVNLQERRKYLKHRLIVVSNRQVDELQQPLELKPEPELESLELELGLVPEPELSLDLEPLLKAGLDLGPELESVLESTLEPVIEPTLCMVSQTVPEPDQGPVSQPVPEPDLPCDLRHLNTEPMEIFRNCVKIEEIMPNGDPLLAGQNTVDEVYVSRPSHFYTDGPLMPSDF

820 NP_003572.2 NP_005598.3

>NP_003572.2

MTEEVIVIAKWDYTAQQDQELDIKKNERLWLLDDSKTWWRVRNAANRTGYVPSNYVERKNSLKKGSLVKNLKDTLGLGKTRRKTSARDASPTPSTDAEYPANGSGADRIYDLNIPAFVKFAYVAEREDELSLVKGSRVTVMEKCSDGWWRGSYNGQIGWFPSNYVLEEVDEAAAESPSFLSLRKGASLSNGQGSRVLHVVQTLYPFSSVTEEELNFEKGETMEVIEKPENDPEWWKCKNARGQVGLVPKNYVVVLSDGPALHPAHAPQISYTGPSSSGRFAGREWYYGNVTRHQAECALNERGVEGDFLIRDSESSPSDFSVSLKASGKNKHFKVQLVDNVYCIGQRRFHTMDELVEHYKKAPIFTSEHGEKLYLVRALQ

>NP_005598.3

MISADCNLCLPEYDRYLASSKIMAAAYLDPNLNHTPNSSTKTHLGTGMERSPGAMERVLKVFHYFESNSEPTTWASIIRHGDATDVRGIIQKIVDSHKVKHVACYGFRLSHLRSEEVHWLHVDMGVSSVREKYELAHPPEEWKYELRIRYLPKGFLNQFTEDKPTLNFFYQQVKSDYMLEIADQVDQEIALKLGCLEIRRSYWEMRGNALEKKSNYEVLEKDVGLKRFFPKSLLDSVKAKTLRKLIQQTFRQFANLNREESILKFFEILSPVYRFDKECFKCALGSSWIISVELAIGPEEGISYLTDKGCNPTHLADFTQVQTIQYSNSEDKDRKGMLQLKIAGAPEPLTVTAPSLTIAENMADLIDGYCRLVNGTSQSFIIRPQKEGERALPSIPKLANSEKQGMRTHAVSVSETDDYAEIIDEEDTYTMPSTRDYEIQRERIELGRCIGEGQFGDVHQGIYMSPENPALAVAIKTCKNCTSDSVREKFLQEALTMRQFDHPHIVKLIGVITENPVWIIMELCTLGELRSFLQVRKYSLDLASLILYAYQLSTALAYLESKRFVHRDIAARNVLVSSNDCVKLGDFGLSRYMEDSTYYKASKGKLPIKWMAPESINFRRFTSASDVWMFGVCMWEILMHGVKPFQGVKNNDVIGRIENGERLPMPPNCPPTLYSLMTKCWAYDPSRRPRFTELKAQLSTILEEEKAQQEERMRMESRRQATVSWDSGGSDEAPPKPSRPGYPSPRSSEGFYPSPQHMVQTNHYQVSGYPGSHGITAMAGSIYPGQASLLDQTDSWNHRPQEIAMWQPNVEDSTVLDLRGIGQVLPTHLMEERLIRQQQEMEEDQRWLEKEERFLKPDVRLSRGSIDREDGSLQGPIGNQHIYQPVGKPDPAAPPKKPPRPGAPGHLGSLASLSSPADSYNEGVKLQPQEISPPPTANLDRSNDKVYENVTGLVKAVIEMSSKIQPAPPEEYVPMVKEVGLALRTLLATVDETIPLLPASTHREIEMAQKLLNSDLGELINKMKLAQQYVMTSLQQEYKKQMLTAAHALAVDAKNLLDVIDQARLKMLGQTRPH

821 NP_003391.1 NP_000237.1

>NP_003391.1

MPAIMTMLADHAARQLLDFSQKLDINLLDNVVNCLYHGEGAQQRMAQEVLTHLKEHPDAWTRVDTILEFSQNMNTKYYGLQILENVIKTRWKILPRNQCEGIKKYVVGLIIKTSSDPTCVEKEKVYIGKLNMILVQILKQEWPKHWPTFISDIVGASRTSESLCQNNMVILKLLSEEVFDFSSGQITQVKSKHLKDSMCNEFSQIFQLCQFVMENSQNAPLVHATLETLLRFLNWIPLGYIFETKLISTLIYKFLNVPMFRNVSLKCLTEIAGVSVSQYEEQFVTLFTLTMMQLKQMLPLNTNIRLAYSNGKDDEQNFIQNLSLFLCTFLKEHDQLIEKRLNLRETLMEALHYMLLVSEVEETEIFKICLEYWNHLAAELYRESPFSTSASPLLSGSQHFDVPPRRQLYLPMLFKVRLLMVSRMAKPEEVLVVENDQGEVVREFMKDTDSINLYKNMRETLVYLTHLDYVDTERIMTEKLHNQVNGTEWSWKNLNTLCWAIGSISGAMHEEDEKRFLVTVIKDLLGLCEQKRGKDNKAIIASNIMYIVGQYPRFLRAHWKFLKTVVNKLFEFMHETHDGVQDMACDTFIKIAQKCRRHFVQVQVGEVMPFIDEILNNINTIICDLQPQQVHTFYEAVGYMIGAQTDQTVQEHLIEKYMLLPNQVWDSIIQQATKNVDILKDPETVKQLGSILKTNVRACKAVGHPFVIQLGRIYLDMLNVYKCLSENISAAIQANGEMVTKQPLIRSMRTVKRETLKLISGWVSRSNDPQMVAENFVPPLLDAVLIDYQRNVPAAREPEVLSTMAIIVNKLGGHITAEIPQIFDAVFECTLNMINKDFEEYPEHRTNFFLLLQAVNSHCFPAFLAIPPTQFKLVLDSIIWAFKHTMRNVADTGLQILFTLLQNVAQEEAAAQSFYQTYFCDILQHIFSVVTDTSHTAGLTMHASILAYMFNLVEEGKISTSLNPGNPVNNQIFLQEYVANLLKSAFPHLQDAQVKLFVTGLFSLNQDIPAFKEHLRDFLVQIKEFAGEDTSDLFLEEREIALRQADEEKHKRQMSVPGIFNPHEIPEEMCD

>NP_000237.1

MRCLAPRPAGSYLSEPQGSSQCATMELGPLEGGYLELLNSDADPLCLYHFYDQMDLAGEEEIELYSEPDTDTINCDQFSRLLCDMEGDEETREAYANIAELDQYVFQDSQLEGLSKDIFKHIGPDEVIGESMEMPAEVGQKSQKRPFPEELPADLKHWKPAEPPTVVTGSLLVGPVSDCSTLPCLPLPALFNQEPASGQMRLEKTDQIPMPFSSSSLSCLNLPEGPIQFVPTISTLPHGLWQISEAGTGVSSIFIYHGEVPQASQVPPPSGFTVHGLPTSPDRPGSTSPFAPSATDLPSMPEPALTSRANMTEHKTSPTQCPAAGEVSNKLPKWPEPVEQFYRSLQDTYGAEPAGPDGILVEVDLVQARLERSSSKSLERELATPDWAERQLAQGGLAEVLLAAKEHRRPRETRVIAVLGKAGQGKSYWAGAVSRAWACGRLPQYDFVFSVPCHCLNRPGDAYGLQDLLFSLGPQPLVAADEVFSHILKRPDRVLLILDAFEELEAQDGFLHSTCGPAPAEPCSLRGLLAGLFQKKLLRGCTLLLTARPRGRLVQSLSKADALFELSGFSMEQAQAYVMRYFESSGMTEHQDRALTLLRDRPLLLSHSHSPTLCRAVCQLSEALLELGEDAKLPSTLTGLYVGLLGRAALDSPPGALAELAKLAWELGRRHQSTLQEDQFPSADVRTWAMAKGLVQHPPRAAESELAFPSFLLQCFLGALWLALSGEIKDKELPQYLALTPRKKRPYDNWLEGVPRFLAGLIFQPPARCLGALLGPSAAASVDRKQKVLARYLKRLQPGTLRARQLLELLHCAHEAEEAGIWQHVVQELPGRLSFLGTRLTPPDAHVLGKALEAAGQDFSLDLRSTGICPSGLGSLVGLSCVTRFRAALSDTVALWESLRQHGETKLLQAAEEKFTIEPFKAKSLKDVEDLGKLVQTQRTRSSSEDTAGELPAVRDLKKLEFALGPVSGPQAFPKLVRILTAFSSLQHLDLDALSENKIGDEGVSQLSATFPQLKSLETLNLSQNNITDLGAYKLAEALPSLAASLLRLSLYNNCICDVGAESLARVLPDMVSLRVMDVQYNKFTAAGAQQLAASLRRCPHVETLAMWTPTIPFSVQEHLQQQDSRISLR

822 NP_056293.4 NP_057219.1

>NP_056293.4

MRRREGHGTDSEMGQGPVRESQSSDPPALQFRISEYKPLNMAGVEQPPSPELRQEGVTEYEDGGAPAGDGEAGPQQAEDHPQNPPEDPNQDPPEDDSTCQCQACGPHQAAGPDLGSSNDGCPQLFQERSVIVENSSGSTSASELLKPMKKRKRREYQSPSEEESEPEAMEKQEEGKDPEGQPTASTPESEEWSSSQPATGEKKECWSWESYLEEQKAITAPVSLFQDSQAVTHNKNGFKLGMKLEGIDPQHPSMYFILTVAEVCGYRLRLHFDGYSECHDFWVNANSPDIHPAGWFEKTGHKLQPPKGYKEEEFSWSQYLRSTRAQAAPKHLFVSQSHSPPPLGFQVGMKLEAVDRMNPSLVCVASVTDVVDSRFLVHFDNWDDTYDYWCDPSSPYIHPVGWCQKQGKPLTPPQDYPDPDNFCWEKYLEETGASAVPTWAFKVRPPHSFLVNMKLEAVDRRNPALIRVASVEDVEDHRIKIHFDGWSHGYDFWIDADHPDIHPAGWCSKTGHPLQPPLGPREPSSASPGGCPPLSYRSLPHTRTSKYSFHHRKCPTPGCDGSGHVTGKFTAHHCLSGCPLAERNQSRLKAELSDSEASARKKNLSGFSPRKKPRHHGRIGRPPKYRKIPQEDFQTLTPDVVHQSLFMSALSAHPDRSLSVCWEQHCKLLPGVAGISASTVAKWTIDEVFGFVQTLTGCEDQARLFKDEARIVRVTHVSGKTLVWTVAQLGDLVCSDHLQEGKGILETGVHSLLCSLPTHLLAKLSFASDSQY

>NP_057219.1

MQEGELAISPISPVAAMPPLGTHVQARCEAQINLLGEGGICKLPGRLRIQPALWSREDVLHWLRWAEQEYSLPCTAEHGFEMNGRALCILTKDDFRHRAPSSGDVLYELLQYIKTQRRALVCGPFFGGIFRLKTPTQHSPVPPEEVTGPSQMDTRRGHLLQPPDPGLTSNFGHLDDPGLARWTPGKEESLNLCHCAELGCRTQGVCSFPAMPQAPIDGRIADCRLLWDYVYQLLLDTRYEPYIKWEDKDAKIFRVVDPNGLARLWGNHKNRVNMTYEKMSRALRHYYKLNIIKKEPGQKLLFRFLKTPGKMVQDKHSHLEPLESQEQDRIEFKDKRPEISP

823 NP_001015055.1 NP_004603.1

>NP_001015055.1

MFSRNHRSRVTVARGSALEMEFKRGRFRLSLFSDLPEDTELQRKLDHEIRMREGACKLLAACSQREQALEATKSLLVCNSRILSYMGELQRRKEAQVLGKTSRRPSDSGPPAERSPCRGRVCISDLRIPLMWKDTEYFKNKGDLHRWAVFLLLQLGEHIQDTEMILVDRTLTDISFQSNVLFAEAGPDFELRLELYGACVEEEGALTGGPKRLATKLSSSLGRSSGRRVRASLDSAGGSGSSPILLPTPVVGGPRYHLLAHTTLTLAAVQDGFRTHDLTLASHEENPAWLPLYGSVCCRLAAQPLCMTQPTASGTLRVQQAGEMQNWAQVHGVLKGTNLFCYRQPEDADTGEEPLLTIAVNKETRVRAGELDQALGRPFTLSISNQYGDDEVTHTLQTESREALQSWMEALWQLFFDMSQWKQCCDEIMKIETPAPRKPPQALAKQGSLYHEMAIEPLDDIAAVTDILTQREGARLETPPPWLAMFTDQPALPNPCSPASVAPAPDWTHPLPWGRPRTFSLDAVPPDHSPRARSVAPLPPQRSPRTRGLCSKGQPRTWLQSPV

>NP_004603.1

MEAAVAAPRPRLLLLVLAAAAAAAAALLPGATALQCFCHLCTKDNFTCVTDGLCFVSVTETTDKVIHNSMCIAEIDLIPRDRPFVCAPSSKTGSVTTTYCCNQDHCNKIELPTTVKSSPGLGPVELAAVIAGPVCFVCISLMLMVYICHNRTVIHHRVPNEEDPSLDRPFISEGTTLKDLIYDMTTSGSGSGLPLLVQRTIARTIVLQESIGKGRFGEVWRGKWRGEEVAVKIFSSREERSWFREAEIYQTVMLRHENILGFIAADNKDNGTWTQLWLVSDYHEHGSLFDYLNRYTVTVEGMIKLALSTASGLAHLHMEIVGTQGKPAIAHRDLKSKNILVKKNGTCCIADLGLAVRHDSATDTIDIAPNHRVGTKRYMAPEVLDDSINMKHFESFKRADIYAMGLVFWEIARRCSIGGIHEDYQLPYYDLVPSDPSVEEMRKVVCEQKLRPNIPNRWQSCEALRVMAKIMRECWYANGAARLTALRIKKTLSQLSQQEGIKM

824 NP_079452.3 NP_057608.2

>NP_079452.3

MDEEENHYVSQLREVYSSCDTTGTGFLDRQELTQLCLKLHLEQQLPVLLQTLLGNDHFARVNFEEFKEGFVAVLSSNAGVRPSDEDSSSLESAASSAIPPKYVNGSKWYGRRSRPELCDAATEARRVPEQQTQASLKSHLWRSASLESVESPKSDEEAESTKEAQNELFEAQGQLQTWDSEDFGSPQKSCSPSFDTPESQIRGVWEELGVGSSGHLSEQELAVVCQSVGLQGLEKEELEDLFNKLDQDGDGKVSLEEFQLGLFSHEPALLLESSTRVKPSKAWSHYQVPEESGCHTTTTSSLVSLCSSLRLFSSIDDGSGFAFPDQVLAMWTQEGIQNGREILQSLDFSVDEKVNLLELTWALDNELMTVDSAVQQAALACYHQELSYQQGQVEQLARERDKARQDLERAEKRNLEFVKEMDDCHSTLEQLTEKKIKHLEQGYRERLSLLRSEVEAERELFWEQAHRQRAALEWDVGRLQAEEAGLREKLTLALKENSRLQKEIVEVVEKLSDSERLALKLQKDLEFVLKDKLEPQSAELLAQEERFAAVLKEYELKCRDLQDRNDELQAELEGLWARLPKNRHSPSWSPDGRRRQLPGLGPAGISFLGNSAPVSIETELMMEQVKEHYQDLRTQLETKVNYYEREIAALKRNFEKERKDMEQARRREVSVLEGQKADLEELHEKSQEVIWGLQEQLQDTARGPEPEQMGLAPCCTQALCGLALRHHSHLQQIRREAEAELSGELSGLGALPARRDLTLELEEPPQGPLPRGSQRSEQLELERALKLQPCASEKRAQMCVSLALEEEELELARGKRVDGPSLEAEMQALPKDGLVAGSGQEGTRGLLPLRPGCGERPLAWLAPGDGRESEEAAGAGPRRRQAQDTEATQSPAPAPAPASHGPSERWSRMQPCGVDGDIVPKEPEPFGASAAGLEQPGARELPLLGTERDASQTQPRMWEPPLRPAASCRGQAERLQAIQEERARSWSRGTQEQASEQQARAEGALEPGCHKHSVEVARRGSLPSHLQLADPQGSWQEQLAAPEEGETKIALEREKDDMETKLLHLEDVVRALEKHVDLRENDRLEFHRLSEENTLLKNDLGRVRQELEAAESTHDAQRKEIEVLKKDKEKACSEMEVLNRQNQNYKDQLSQLNVRVLQLGQEASTHQAQNEEHRVTIQMLTQSLEEVVRSGQQQSDQIQKLRVELECLNQEHQSLQLPWSELTQTLEESQDQVQGAHLRLRQAQAQHLQEVRLVPQDRVAELHRLLSLQGEQARRRLDAQREEHEKQLKATEERVEEAEMILKNMEMLLQEKVDKLKEQFEKNTKSDLLLKELYVENAHLVRALQATEEKQRGAEKQSRLLEEKVRALNKLVSRIAPAALSV

>NP_057608.2

MAYIQLEPLNEGFLSRISGLLLCRWTCRHCCQKCYESSCCQSSEDEVEILGPFPAQTPPWLMASRSSDKDGDSVHTASEVPLTPRTNSPDGRRSSSDTSKSTYSLTRRISSLESRRPSSPLIDIKPIEFGVLSAKKEPIQPSVLRRTYNPDDYFRKFEPHLYSLDSNSDDVDSLTDEEILSKYQLGMLHFSTQYDLLHNHLTVRVIEARDLPPPISHDGSRQDMAHSNPYVKICLLPDQKNSKQTGVKRKTQKPVFEERYTFEIPFLEAQRRTLLLTVVDFDKFSRHCVIGKVSVPLCEVDLVKGGHWWKALIPSSQNEVELGELLLSLNYLPSAGRLNVDVIRAKQLLQTDVSQGSDPFVKIQLVHGLKLVKTKKTSFLRGTIDPFYNESFSFKVPQEELENASLVFTVFGHNMKSSNDFIGRIVIGQYSSGPSETNHWRRMLNTHRTAVEQWHSLRSRAECDRVSPASLEVT

825 NP_002537.3 NP_997647.1

>NP_002537.3

MNNLLCCALVFLDISIKWTTQETFPPKYLHYDEETSHQLLCDKCPPGTYLKQHCTAKWKTVCAPCPDHYYTDSWHTSDECLYCSPVCKELQYVKQECNRTHNRVCECKEGRYLEIEFCLKHRSCPPGFGVVQAGTPERNTVCKRCPDGFFSNETSSKAPCRKHTNCSVFGLLLTQKGNATHDNICSGNSESTQKCGIDVTLCEEAFFRFAVPTKFTPNWLSVLVDNLPGTKVNAESVERIKRQHSSQEQTFQLLKLWKHQNKDQDIVKKIIQDIDLCENSVQRHIGHANLTFEQLRSLMESLPGKKVGAEDIEKTIKACKPSDQILKLLSLWRIKNGDQDTLKGLMHALKHSKTYHFPKTVTQSLKKTIRFLHSFTMYKLYQKLFLEMIGNQVQSVKISCL

>NP_997647.1

MLRGPGPGLLLLAVQCLGTAVPSTGASKSKRQAQQMVQPQSPVAVSQSKPGCYDNGKHYQINQQWERTYLGNALVCTCYGGSRGFNCESKPEAEETCFDKYTGNTYRVGDTYERPKDSMIWDCTCIGAGRGRISCTIANRCHEGGQSYKIGDTWRRPHETGGYMLECVCLGNGKGEWTCKPIAEKCFDHAAGTSYVVGETWEKPYQGWMMVDCTCLGEGSGRITCTSRNRCNDQDTRTSYRIGDTWSKKDNRGNLLQCICTGNGRGEWKCERHTSVQTTSSGSGPFTDVRAAVYQPQPHPQPPPYGHCVTDSGVVYSVGMQWLKTQGNKQMLCTCLGNGVSCQETAVTQTYGGNSNGEPCVLPFTYNGRTFYSCTTEGRQDGHLWCSTTSNYEQDQKYSFCTDHTVLVQTRGGNSNGALCHFPFLYNNHNYTDCTSEGRRDNMKWCGTTQNYDADQKFGFCPMAAHEEICTTNEGVMYRIGDQWDKQHDMGHMMRCTCVGNGRGEWTCIAYSQLRDQCIVDDITYNVNDTFHKRHEEGHMLNCTCFGQGRGRWKCDPVDQCQDSETGTFYQIGDSWEKYVHGVRYQCYCYGRGIGEWHCQPLQTYPSSSGPVEVFITETPSQPNSHPIQWNAPQPSHISKYILRWRPKNSVGRWKEATIPGHLNSYTIKGLKPGVVYEGQLISIQQYGHQEVTRFDFTTTSTSTPVTSNTVTGETTPFSPLVATSESVTEITASSFVVSWVSASDTVSGFRVEYELSEEGDEPQYLDLPSTATSVNIPDLLPGRKYIVNVYQISEDGEQSLILSTSQTTAPDAPPDPTVDQVDDTSIVVRWSRPQAPITGYRIVYSPSVEGSSTELNLPETANSVTLSDLQPGVQYNITIYAVEENQESTPVVIQQETTGTPRSDTVPSPRDLQFVEVTDVKVTIMWTPPESAVTGYRVDVIPVNLPGEHGQRLPISRNTFAEVTGLSPGVTYYFKVFAVSHGRESKPLTAQQTTKLDAPTNLQFVNETDSTVLVRWTPPRAQITGYRLTVGLTRRGQPRQYNVGPSVSKYPLRNLQPASEYTVSLVAIKGNQESPKATGVFTTLQPGSSIPPYNTEVTETTIVITWTPAPRIGFKLGVRPSQGGEAPREVTSDSGSIVVSGLTPGVEYVYTIQVLRDGQERDAPIVNKVVTPLSPPTNLHLEANPDTGVLTVSWERSTTPDITGYRITTTPTNGQQGNSLEEVVHADQSSCTFDNLSPGLEYNVSVYTVKDDKESVPISDTIIPEVPQLTDLSFVDITDSSIGLRWTPLNSSTIIGYRITVVAAGEGIPIFEDFVDSSVGYYTVTGLEPGIDYDISVITLINGGESAPTTLTQQTAVPPPTDLRFTNIGPDTMRVTWAPPPSIDLTNFLVRYSPVKNEEDVAELSISPSDNAVVLTNLLPGTEYVVSVSSVYEQHESTPLRGRQKTGLDSPTGIDFSDITANSFTVHWIAPRATITGYRIRHHPEHFSGRPREDRVPHSRNSITLTNLTPGTEYVVSIVALNGREESPLLIGQQSTVSDVPRDLEVVAATPTSLLISWDAPAVTVRYYRITYGETGGNSPVQEFTVPGSKSTATISGLKPGVDYTITVYAVTGRGDSPASSKPISINYRTEIDKPSQMQVTDVQDNSISVKWLPSSSPVTGYRVTTTPKNGPGPTKTKTAGPDQTEMTIEGLQPTVEYVVSVYAQNPSGESQPLVQTAVTNIDRPKGLAFTDVDVDSIKIAWESPQGQVSRYRVTYSSPEDGIHELFPAPDGEEDTAELQGLRPGSEYTVSVVALHDDMESQPLIGTQSTAIPAPTDLKFTQVTPTSLSAQWTPPNVQLTGYRVRVTPKEKTGPMKEINLAPDSSSVVVSGLMVATKYEVSVYALKDTLTSRPAQGVVTTLENVSPPRRARVTDATETTITISWRTKTETITGFQVDAVPANGQTPIQRTIKPDVRSYTITGLQPGTDYKIYLYTLNDNARSSPVVIDASTAIDAPSNLRFLATTPNSLLVSWQPPRARITGYIIKYEKPGSPPREVVPRPRPGVTEATITGLEPGTEYTIYVIALKNNQKSEPLIGRKKTDELPQLVTLPHPNLHGPEILDVPSTVQKTPFVTHPGYDTGNGIQLPGTSGQQPSVGQQMIFEEHGFRRTTPPTTATPIRHRPRPYPPNVGEEIQIGHIPREDVDYHLYPHGPGLNPNASTGQEALSQTTISWAPFQDTSEYIISCHPVGTDEEPLQFRVPGTSTSATLTGLTRGATYNIIVEALKDQQRHKVREEVVTVGNSVNEGLNQPTDDSCFDPYTVSHYAVGDEWERMSESGFKLLCQCLGFGSGHFRCDSSRWCHDNGVNYKIGEKWDRQGENGQMMSCTCLGNGKGEFKCDPHEATCYDDGKTYHVGEQWQKEYLGAICSCTCFGGQRGWRCDNCRRPGGEPSPEGTTGQSYNQYSQRYHQRTNTNVNCPIECFMPLDVQADREDSRE

826 NP_000233.1 NP_001733.1

>NP_000233.1

MSLFPSLPLLLLSMVAASYSETVTCEDAQKTCPAVIACSSPGINGFPGKDGRDGTKGEKGEPGQGLRGLQGPPGKLGPPGNPGPSGSPGPKGQKGDPGKSPDGDSSLAASERKALQTEMARIKKWLTFSLGKQVGNKFFLTNGEIMTFEKVKALCVKFQASVATPRNAAENGAIQNLIKEEAFLGITDEKTEGQFVDLTGNRLTYTNWNEGEPNNAGSDEDCVLLLKNGQWNDVPCSTSHLAVCEFPI

>NP_001733.1

MRFTFTSRCLALFLLLNHPTPILPAFSNQTYPTIEPKPFLYVVGRKKMMDAQYKCYDRMQQLPAYQGEGPYCNRTWDGWLCWDDTPAGVLSYQFCPDYFPDFDPSEKVTKYCDEKGVWFKHPENNRTWSNYTMCNAFTPEKLKNAYVLYYLAIVGHSLSIFTLVISLGIFVFFRSLGCQRVTLHKNMFLTYILNSMIIIIHLVEVVPNGELVRRDPVSCKILHFFHQYMMACNYFWMLCEGIYLHTLIVVAVFTEKQRLRWYYLLGWGFPLVPTTIHAITRAVYFNDNCWLSVETHLLYIIHGPVMAALVVNFFFLLNIVRVLVTKMRETHEAESHMYLKAVKATMILVPLLGIQFVVFPWRPSNKMLGKIYDYVMHSLIHFQGFFVATIYCFCNNEVQTTVKRQWAQFKIQWNQRWGRRPSNRSARAAAAAAEAGDIPIYICHQELRNEPANNQGEESAEIIPLNIIEQESSA

827 NP_001012300.1 NP_057137.1

>NP_001012300.1

MTRGTGGTAQRGRSGPGLSPDGIWMAKELYLKTSSVKEAGEGPRGLAGEGGWGGVPFAEALRILGGPNPTISLLARSQGLLDSSLMASGTASRSEDEESLAGQKRASSQALGTIPKRRSSSRFIKRKKFDDELVESSLAKSSTRAKGASGVEPGRCSGSEPSSSEKKKVSKAPSTPVPPSPAPAPGLTKRVKKSKQPLQVTKDLGRWKPADDLLLINAVLQTNDLTSVHLGVKFSCRFTLREVQERWYALLYDPVISKLACQAMRQLHPEAIAAIQSKALFSKAEEQLLSKVGSTSQPTLETFQDLLHRHPDAFYLARTAKALQAHWQLMKQYYLLEDQTVQPLPKGDQVLNFSDAEDLIDDSKLKDMRDEVLEHELMVADRRQKREIRQLEQELHKWQVLVDSITGMSSPDFDNQTLAVLRGRMVRYLMRSREITLGRATKDNQIDVDLSLEGPAWKISRKQGVIKLKNNGDFFIANEGRRPIYIDGRPVLCGSKWRLSNNSVVEIASLRFVFLINQDLIALIRAEAAKITPQ

>NP_057137.1

MDEDGLPLMGSGIDLTKVPAIQQKRTVAFLNQFVVHTVQFLNRFSTVCEEKLADLSLRIQQIETTLNILDAKLSSIPGLDDVTVEVSPLNVTSVTNGAHPEATSEQPQQNSTQDSGLQESEVSAENILTVAKDPRYARYLKMVQVGVPVMAIRNKMISEGLDPDLLERPDAPVPDGESEKTVEESSDSESSFSD

828 NP_055859.1 NP_001650.1

>NP_055859.1

MAATAVAAAVAGTESAQGPPGPAASLELWLNKATDPSMSEQDWSAIQNFCEQVNTDPNGPTHAPWLLAHKIQSPQEKEALYALTVLEMCMNHCGEKFHSEVAKFRFLNELIKVLSPKYLGSWATGKVKGRVIEILFSWTVWFPEDIKIRDAYQMLKKQGIIKQDPKLPVDKILPPPSPWPKSSIFDADEEKSKLLTRLLKSNHPEDLQAANRLIKNLVKEEQEKSEKVSKRVSAVEEVRSHVKVLQEMLSMYRRPGQAPPDQEALQVVYERCEKLRPTLFRLASDTTDDDDALAEILQANDLLTQGVLLYKQVMEGRVTFGNRVTSSLGDIPVSRVFQNPAGCMKTCPLIDLEVDNGPAQMGTVVPSLLHQDLAALGISDAPVTGMVSGQNCCEEKRNPSSSTLPGGGVQNPSADRNLLDLLSAQPAPCPLNYVSQKSVPKEVPPGTKSSPGWSWEAGPLAPSPSSQNTPLAQVFVPLESVKPSSLPPLIVYDRNGFRILLHFSQTGAPGHPEVQVLLLTMMSTAPQPVWDIMFQVAVPKSMRVKLQPASSSKLPAFSPLMPPAVISQMLLLDNPHKEPIRLRYKLTFNQGGQPFSEVGEVKDFPDLAVLGAA

>NP_001650.1

MGNIFGNLLKSLIGKKEMRILMVGLDAAGKTTILYKLKLGEIVTTIPTIGFNVETVEYKNISFTVWDVGGQDKIRPLWRHYFQNTQGLIFVVDSNDRERVNEAREELMRMLAEDELRDAVLLVFANKQDLPNAMNAAEITDKLGLHSLRHRNWYIQATCATSGDGLYEGLDWLANQLKNKK

829 NP_003591.2 NP_663735.2

>NP_003591.2

MDRSKENCISGPVKATAPVGGPKRVLVTQQFPCQNPLPVNSGQAQRVLCPSNSSQRIPLQAQKLVSSHKPVQNQKQKQLQATSVPHPVSRPLNNTQKSKQPLPSAPENNPEEELASKQKNEESKKRQWALEDFEIGRPLGKGKFGNVYLAREKQSKFILALKVLFKAQLEKAGVEHQLRREVEIQSHLRHPNILRLYGYFHDATRVYLILEYAPLGTVYRELQKLSKFDEQRTATYITELANALSYCHSKRVIHRDIKPENLLLGSAGELKIADFGWSVHAPSSRRTTLCGTLDYLPPEMIEGRMHDEKVDLWSLGVLCYEFLVGKPPFEANTYQETYKRISRVEFTFPDFVTEGARDLISRLLKHNPSQRPMLREVLEHPWITANSSKPSNCQNKESASKQS

>NP_663735.2

METLSFPRYNVAEIVIHIRNKILTGADGKNLTKNDLYPNPKPEVLHMIYMRALQIVYGIRLEHFYMMPVNSEVMYPHLMEGFLPFSNLVTHLDSFLPICRVNDFETADILCPKAKRTSRFLSGIINFIHFREACRETYMEFLWQYKSSADKMQQLNAAHQEALMKLERLDSVPVEEQEEFKQLSDGIQELQQSLNQDFHQKTIVLQEGNSQKKSNISEKTKRLNELKLSVVSLKEIQESLKTKIVDSPEKLKNYKEKMKDTVQKLKNARQEVVEKYEIYGDSVDCLPSCQLEVQLYQKKIQDLSDNREKLASILKESLNLEDQIESDESELKKLKTEENSFKRLMIVKKEKLATAQFKINKKHEDVKQYKRTVIEDCNKVQEKRGAVYERVTTINQEIQKIKLGIQQLKDAAEREKLKSQEIFLNLKTALEKYHDGIEKAAEDSYAKIDEKTAELKRKMFKMST

830 NP_003295.1 NP_000818.1

>NP_003295.1

MMAALYPSTDLSGASSSSLPSSPSSSSPNEVMALKDVREVKEENTLNEKLFLLACDKGDYYMVKKILEENSSGDLNINCVDVLGRNAVTITIENENLDILQLLLDYGCQKLMERIQNPEYSTTMDVAPVILAAHRNNYEILTMLLKQDVSLPKPHAVGCECTLCSAKNKKDSLRHSRFRLDIYRCLASPALIMLTEEDPILRAFELSADLKELSLVEVEFRNDYEELARQCKMFAKDLLAQARNSRELEVILNHTSSDEPLDKRGLLEERMNLSRLKLAIKYNQKEFVSQSNCQQFLNTVWFGQMSGYRRKPTCKKIMTVLTVGIFWPVLSLCYLIAPKSQFGRIIHTPFMKFIIHGASYFTFLLLLNLYSLVYNEDKKNTMGPALERIDYLLILWIIGMIWSDIKRLWYEGLEDFLEESRNQLSFVMNSLYLATFALKVVAHNKFHDFADRKDWDAFHPTLVAEGLFAFANVLSYLRLFFMYTTSSILGPLQISMGQMLQDFGKFLGMFLLVLFSFTIGLTQLYDKGYTSKEQKDCVGIFCEQQSNDTFHSFIGTCFALFWYIFSLAHVAIFVTRFSYGEELQSFVGAVIVGTYNVVVVIVLTKLLVAMLHKSFQLIANHEDKEWKFARAKLWLSYFDDKCTLPPPFNIIPSPKTICYMISSLSKWICSHTSKGKVKRQNSLKEWRNLKQKRDENYQKVMCCLVHRYLTSMRQKMQSTDQATVENLNELRQDLSKFRNEIRDLLGFRTSKYAMFYPRN

>NP_000818.1

MQHIFAFFCTGFLGAVVGANFPNNIQIGGLFPNQQSQEHAAFRFALSQLTEPPKLLPQIDIVNISDSFEMTYRFCSQFSKGVYAIFGFYERRTVNMLTSFCGALHVCFITPSFPVDTSNQFVLQLRPELQDALISIIDHYKWQKFVYIYDADRGLSVLQKVLDTAAEKNWQVTAVNILTTTEEGYRMLFQDLEKKKERLVVVDCESERLNAILGQIIKLEKNGIGYHYILANLGFMDIDLNKFKESGANVTGFQLVNYTDTIPAKIMQQWKNSDARDHTRVDWKRPKYTSALTYDGVKVMAEAFQSLRRQRIDISRRGNAGDCLANPAVPWGQGIDIQRALQQVRFEGLTGNVQFNEKGRRTNYTLHVIEMKHDSIRKIGYWNEDDKFVPAATDAQAGGDNSSVQNRTYIVTTILEDPYVMLKKNANQFEGNDRYEGYCVELAAEIAKHVGYSYRLEIVSDGKYGARDPDTKAWNGMVGELVYGRADVAVAPLTITLVREEVIDFSKPFMSLGISIMIKKPQKSKPGVFSFLDPLAYEIWMCIVFAYIGVSVVLFLVSRFSPYEWHSEEFEEGRDQTTSDQSNEFGIFNSLWFSLGAFMQQGCDISPRSLSGRIVGGVWWFFTLIIISSYTANLAAFLTVERMVSPIESAEDLAKQTEIAYGTLEAGSTKEFFRRSKIAVFEKMWTYMKSAEPSVFVRTTEEGMIRVRKSKGKYAYLLESTMNEYIEQRKPCDTMKVGGNLDSKGYGIATPKGSALRNPVNLAVLKLNEQGLLDKLKNKWWYDKGECGSGGGDSKDKTSALSLSNVAGVFYILIGGLGLAMLVALIEFCYKSRSESKRMKGFCLIPQQSINEAIRTSTLPRNSGAGASSGGSGENGRVVSHDFPKSMQSIPCMSHSSGMPLGATGL

831 NP_003884.1 NP_067021.1

>NP_003884.1

MLDRDVGPTPMYPPTYLEPGIGRHTPYGNQTDYRIFELNKRLQNWTEECDNLWWDAFTTEFFEDDAMLTITFCLEDGPKRYTIGRTLIPRYFRSIFEGGATELYYVLKHPKEAFHSNFVSLDCDQGSMVTQHGKPMFTQVCVEGRLYLEFMFDDMMRIKTWHFSIRQHRELIPRSILAMHAQDPQMLDQLSKNITRCGLSNSTLNYLRLCVILEPMQELMSRHKTYSLSPRDCLKTCLFQKWQRMVAPPAEPTRQQPSKRRKRKMSGGSTMSSGGGNTNNSNSKKKSPASTFALSSQVPDVMVVGEPTLMGGEFGDEDERLITRLENTQFDAANGIDDEDSFNNSPALGANSPWNSKPPSSQESKSENPTSQASQ

>NP_067021.1

MDSSAVITQISKEEARGPLRGKGDQKSAASQKPRSRGILHSLFCCVCRDDGEALPAHSGAPLLVEENGAIPKQTPVQYLLPEAKAQDSDKICVVIDLDETLVHSSFKPVNNADFIIPVEIDGVVHQVYVLKRPHVDEFLQRMGELFECVLFTASLAKYADPVADLLDKWGAFRARLFRESCVFHRGNYVKDLSRLGRDLRRVLILDNSPASYVFHPDNAVPVASWFDNMSDTELHDLLPFFEQLSRVDDVYSVLRQPRPGS

832 NP_002960.2 NP_066301.1

>NP_002960.2

MSSPPPARSGFYRQEVTKTAWEVRAVYRDLQPVGSGAYGAVCSAVDGRTGAKVAIKKLYRPFQSELFAKRAYRELRLLKHMRHENVIGLLDVFTPDETLDDFTDFYLVMPFMGTDLGKLMKHEKLGEDRIQFLVYQMLKGLRYIHAAGIIHRDLKPGNLAVNEDCELKILDFGLARQADSEMTGYVVTRWYRAPEVILNWMRYTQTVDIWSVGCIMAEMITGKTLFKGSDHLDQLKEIMKVTGTPPAEFVQRLQSDEAKNYMKGLPELEKKDFASILTNASPLAVNLLEKMLVLDAEQRVTAGEALAHPYFESLHDTEDEPQVQKYDDSFDDVDRTLDEWKRVTYKEVLSFKPPRQLGARVSKETPL

>NP_066301.1

MAVAAAAAAAGPAGAGGGRAQRSGLLEVLVRDRWHKVLVNLSEDALVLSSEEGAAAYNGIGTATNGSFCRGAGAGHPGAGGAQPPDSPAGVRTAFTDLPEQVPESISNQKRGVKVLKQELGGLGISIKGGKENKMPILISKIFKGLAADQTQALYVGDAILSVNGADLRDATHDEAVQALKRAGKEVLLEVKYMREATPYVKKGSPVSEIGWETPPPESPRLGGSTSDPPSSQSFSFHRDRKSIPLKMCYVTRSMALADPENRQLEIHSPDAKHTVILRSKDSATAQAWFSAIHSNVNDLLTRVIAEVREQLGKTGIAGSREIRHLGWLAEKVPGESKKQWKPALVVLTEKDLLIYDSMPRRKEAWFSPVHTYPLLATRLVHSGPGKGSPQAGVDLSFATRTGTRQGIETHLFRAETSRDLSHWTRSIVQGCHNSAELIAEISTACTYKNQECRLTIHYENGFSITTEPQEGAFPKTIIQSPYEKLKMSSDDGIRMLYLDFGGKDGEIQLDLHSCPKPIVFIIHSFLSAKITRLGLVA

833 BAA18998.1 NP_005198.1

>BAA18998.1

MDDLDALLADLESTTSHISKRPVFLSEETPYSYPTGNHTYQEIAVPPPVPPPPSSEALNGTILDPLDQWQPSGSRFIHQQPQSSSPVYGSSAKTSSVSNPQDSVGSPCSRVGEEEHVYSFPNKQKSAEPSPTVMSTSLGSNLSELDRLLLELNAVQHNPPGFPADEANSSPPLPGALSPLYGVPETNSPLGGKAGPLTKEKPKRNGGRGLEDVRPSVESLLDELESSVPSPVPAITVNQGEMSSPQRVTSTQQQTRISASSATRELDELMASLSDFKGSWPLEEVVLLVSISSSVQEGEKYPHPCAARHRTPSLRSPDQPPPCPQFMAQGKTGSSSPPGGPPKPGSQLDSMLGSLQSDLNKLGVATVAKGVCGACKKPIAGQVVTAMGKTWHPEHFVCTHCQEEIGSRNFFERDGQPYCEKDYHNLFSPRCYYCNGPILDKVVTALDRTWHPEHFFCAQCGAFFGPEGFHEKDGKAYCRKDYFDMFAPKCGGCARAILENYISALNTLWHPECFVCRECFTPFVNGSFFEHDGQPYCEVHYHERRGSLCSGCQKPITGRCITAMAKKFHPEHFVCAFCLKQLNKGTFKEQNDKPYCQNCFLKLFC

>NP_005198.1

MSSARFDSSDRSAWYMGPVSRQEAQTRLQGQRHGMFLVRDSSTCPGDYVLSVSENSRVSHYIINSLPNRRFKIGDQEFDHLPALLEFYKIHYLDTTTLIEPAPRYPSPPMGSVSAPNLPTAEDNLEYVRTLYDFPGNDAEDLPFKKGEILVIIEKPEEQWWSARNKDGRVGMIPVPYVEKLVRSSPHGKHGNRNSNSYGIPEPAHAYAQPQTTTPLPAVSGSPGAAITPLPSTQNGPVFAKAIQKRVPCAYDKTALALEVGDIVKVTRMNINGQWEGEVNGRKGLFPFTHVKIFDPQNPDENE

834 NP_004992.2 NP_006244.2

>NP_004992.2

MASATRLIQRLRNWASGHDLQGKLQLRYQEISKRTQPPPKLPVGPSHKLSNNYYCTRDGRRESVPPSIIMSSQKALVSGKPAESSAVAATEKKAVTPAPPIKRWELSSDQPYL

>NP_006244.2

MGNTSSERAALERHGGHKTPRRDSSGGTKDGDRPKILMDSPEDADLFHSEEIKAPEKEEFLAWQHDLEVNDKAPAQARPTVFRWTGGGKEVYLSGSFNNWSKLPLTRSHNNFVAILDLPEGEHQYKFFVDGQWTHDPSEPIVTSQLGTVNNIIQVKKTDFEVFDALMVDSQKCSDVSELSSSPPGPYHQEPYVCKPEERFRAPPILPPHLLQVILNKDTGISCDPALLPEPNHVMLNHLYALSIKDGVMVLSATHRYKKKYVTTLLYKPI

835 NP_000114.2 NP_001508.1

>NP_000114.2

MGVQGLWKLLECSGRQVSPEALEGKILAVDISIWLNQALKGVRDRHGNSIENPHLLTLFHRLCKLLFFRIRPIFVFDGDAPLLKKQTLVKRRQRKDLASSDSRKTTEKLLKTFLKRQAIKTAFRSKRDEALPSLTQVRRENDLYVLPPLQEEEKHSSEEEDEKEWQERMNQKQALQEEFFHNPQAIDIESEDFSSLPPEVKHEILTDMKEFTKRRRTLFEAMPEESDDFSQYQLKGLLKKNYLNQHIEHVQKEMNQQHSGHIRRQYEDEGGFLKEVESRRVVSEDTSHYILIKGIQAKTVAEVDSESLPSSSKMHGMSFDVKSSPCEKLKTEKEPDATPPSPRTLLAMQAALLGSSSEEELESENRRQARGRNAPAAVDEGSISPRTLSAIKRALDDDEDVKVCAGDDVQTGGPGAEEMRINSSTENSDEGLKVRDGKGIPFTATLASSSVNSAEEHVASTNEGREPTDSVPKEQMSLVHVGTEAFPISDESMIKDRKDRLPLESAVVRHSDAPGLPNGRELTPASPTCTNSVSKNETHAEVLEQQNELCPYESKFDSSLLSSDDETKCKPNSASEVIGPVSLQETSSIVSVPSEAVDNVENVVSFNAKEHENFLETIQEQQTTESAGQDLISIPKAVEPMEIDSEESESDGSFIEVQSVISDEELQAEFPETSKPPSEQGEEELVGTREGEAPAESESLLRDNSERDDVDGEPQEAEKDAEDSLHEWQDINLEELETLESNLLAQQNSLKAQKQQQERIAATVTGQMFLESQELLRLFGIPYIQAPMEAEAQCAILDLTDQTSGTITDDSDIWLFGARHVYRNFFNKNKFVEYYQYVDFHNQLGLDRNKLINLAYLLGSDYTEGIPTVGCVTAMEILNEFPGHGLEPLLKFSEWWHEAQKNPKIRPNPHDTKVKKKLRTLQLTPGFPNPAVAEAYLKPVVDDSKGSFLWGKPDLDKIREFCQRYFGWNRTKTDESLFPVLKQLDAQQTQLRIDSFFRLAQQEKEDAKRIKSQRLNRAVTCMLRKEKEAAASEIEAVSVAMEKEFELLDKAKRKTQKRGITNTLEESSSLKRKRLSDSKRKNTCGGFLGETCLSESSDGSSSEDAESSSLMNVQRRTAAKEPKTSASDSQNSVKEAPVKNGGATTSSSSDSDDDGGKEKMVLVTARSVFGKKRRKLRRARGRKRKT

>NP_001508.1

MESTPSRGLNRVHLQCRNLQEFLGGLSPGVLDRLYGHPATCLAVFRELPSLAKNWVMRMLFLEQPLPQAAVALWVKKEFSKAQEESTGLLSGLRIWHTQLLPGGLQGLILNPIFRQNLRIALLGGGKAWSDDTSQLGPDKHARDVPSLDKYAEERWEVVLHFMVGSPSAAVSQDLAQLLSQAGLMKSTEPGEPPCITSAGFQFLLLDTPAQLWYFMLQYLQTAQSRGMDLVEILSFLFQLSFSTLGKDYSVEGMSDSLLNFLQHLREFGLVFQRKRKSRRYYPTRLAINLSSGVSGAGGTVHQPGFIVVETNYRLYAYTESELQIALIALFSEMLYRFPNMVVAQVTRESVQQAIASGITAQQIIHFLRTRAHPVMLKQTPVLPPTITDQIRLWELERDRLRFTEGVLYNQFLSQVDFELLLAHARELGVLVFENSAKRLMVVTPAGHSDVKRFWKRQKHSS

836 NP_005441.1 NP_066551.1

>NP_005441.1

MERCPSLGVTLYALVVVLGLRATPAGGQHYLHIRPAPSDNLPLVDLIEHPDPIFDPKEKDLNETLLRSLLGGHYDPGFMATSPPEDRPGGGGGAAGGAEDLAELDQLLRQRPSGAMPSEIKGLEFSEGLAQGKKQRLSKKLRRKLQMWLWSQTFCPVLYAWNDLGSRFWPRYVKVGSCFSKRSCSVPEGMVCKPSKSVHLTVLRWRCQRRGGQRCGWIPIQYPIISECKCSC

>NP_066551.1

MHLTVFLLKGIVGFLWSCWVLVGYAKGGLGDNHVHSSFIYRRLRNHERREIQREILSILGLPHRPRPFSPGKQASSAPLFMLDLYNAMTNEENPEESEYSVRASLAEETRGARKGYPASPNGYPRRIQLSRTTPLTTQSPPLASLHDTNFLNDADMVMSFVNLVERDKDFSHQRRHYKEFRFDLTQIPHGEAVTAAEFRIYKDRSNNRFENETIKISIYQIIKEYTNRDADLFLLDTRKAQALDVGWLVFDITVTSNHWVINPQNNLGLQLCAETGDGRSINVKSAGLVGRQGPQSKQPFMVAFFKASEVLLRSVRAANKRKNQNRNKSSSHQDSSRMSSVGDYNTSEQKQACKKHELYVSFRDLGWQDWIIAPEGYAAFYCDGECSFPLNAHMNATNHAIVQTLVHLMFPDHVPKPCCAPTKLNAISVLYFDDSSNVILKKYRNMVVRSCGCH

837 NP_777584.1 NP_055399.1

>NP_777584.1

MDLLWMPLLLVAACVSAVHSSPEVNAGVSSIHITKPVHILEERSLLVLTPAGLTQMLNQTRFLMVLFHNPSSKQSRNLAEELGKAVEIMGKGKNGIGFGKVDITIEKELQQEFGITKAPELKLFFEGNRSEPISCKGVVESAALVVWLRRQISQKAFLFNSSEQVAEFVISRPLVIVGFFQDLEEEVAELFYDVIKDFPELTFGVITIGNVIGRFHVTLDSVLVFKKGKIVNRQKLINDSTNKQELNRVIKQHLTDFVIEYNTENKDLISELHIMSHMLLFVSKSSESYGIIIQHYKLASKEFQNKILFILVDADEPRNGRVFKYFRVTEVDIPSVQILNLSSDARYKMPSDDITYESLKKFGRSFLSKNATKHQSSEEIPKYWDQGLVKQLVGKNFNVVVFDKEKDVFVMFYAPWSKKCKMLFPLLEELGRKYQNHSTIIIAKIDVTANDIQLMYLDRYPFFRLFPSGSQQAVLYKGEHTLKGFSDFLESHIKTKIEDEDELLSVEQNEVIEEEVLAEEKEVPMMRKGLPEQQSPELENMTKYVSKLEEPAGKKKTSEEVVVVVAKPKGPPVQKKKPKVKEEL

>NP_055399.1

MGRGWGFLFGLLGAVWLLSSGHGEEQPPETAAQRCFCQVSGYLDDCTCDVETIDRFNNYRLFPRLQKLLESDYFRYYKVNLKRPCPFWNDISQCGRRDCAVKPCQSDEVPDGIKSASYKYSEEANNLIEECEQAERLGAVDESLSEETQKAVLQWTKHDDSSDNFCEADDIQSPEAEYVDLLLNPERYTGYKGPDAWKIWNVIYEENCFKPQTIKRPLNPLASGQGTSEENTFYSWLEGLCVEKRAFYRLISGLHASINVHLSARYLLQETWLEKKWGHNITEFQQRFDGILTEGEGPRRLKNLYFLYLIELRALSKVLPFFERPDFQLFTGNKIQDEENKMLLLEILHEIKSFPLHFDENSFFAGDKKEAHKLKEDFRLHFRNISRIMDCVGCFKCRLWGKLQTQGLGTALKILFSEKLIANMPESGPSYEFHLTRQEIVSLFNAFGRISTSVKELENFRNLLQNIH

838 NP_055561.2 NP_003338.1

>NP_055561.2

MTTTRYRPTWDLALDPLVSCKLCLGEYPVEQMTTIAQCQCIFCTLCLKQYVELLIKEGLETAISCPDAACPKQGHLQENEIECMVAAEIMQRYKKLQFEREVLFDPCRTWCPASTCQAVCQLQDVGLQTPQPVQCKACRMEFCSTCKASWHPGQGCPETMPITFLPGETSAAFKMEEDDAPIKRCPKCKVYIERDEGCAQMMCKNCKHAFCWYCLESLDDDFLLIHYDKGPCRNKLGHSRASVIWHRTQVVGIFAGFGLLLLVASPFLLLATPFVLCCKCKCSKGDDDPLPT

>NP_003338.1

MAASRRLMKELEEIRKCGMKNFRNIQVDEANLLTWQGLIVPDNPPYDKGAFRIEINFPAEYPFKPPKITFKTKIYHPNIDEKGQVCLPVISAENWKPATKTDQVIQSLIALVNDPQPEHPLRADLAEEYSKDRKKFCKNAEEFTKKYGEKRPVD

839 NP_004417.2 NP_002922.2

>NP_004417.2

METESEQNSNSTNGSSSSGGSSRPQIAQMSLYERQAVQALQALQRQPNAAQYFHQFMLQQQLSNAQLHSLAAVQQATIAASRQASSPNTSTTQQQTTTTQASINLATTSAAQLISRSQSVSSPSATTLTQSVLLGNTTSPPLNQSQAQMYLRPQLGNLLQVNRTLGRNVPLASQLILMPNGAVAAVQQEVPSAQSPGVHADADQVQNLAVRNQQASAQGPQMQGSTQKAIPPGASPVSSLSQASSQALAVAQASSGATNQSLNLSQAGGGSGNSIPGSMGPGGGGQAHGGLGQLPSSGMGGGSCPRKGTGVVQPLPAAQTVTVSQGSQTEAESAAAKKAEADGSGQQNVGMNLTRTATPAPSQTLISSATYTQIQPHSLIQQQQQIHLQQKQVVIQQQIAIHHQQQFQHRQSQLLHTATHLQLAQQQQQQQQQQQQQQQPQATTLTAPQPPQVPPTQQVPPSQSQQQAQTLVVQPMLQSSPLSLPPDAAPKPPIPIQSKPPVAPIKPPQLGAAKMSAAQQPPPHIPVQVVGTRQPGTAQAQALGLAQLAAAVPTSRGMPGTVQSGQAHLASSPPSSQAPGALQECPPTLAPGMTLAPVQGTAHVVKGGATTSSPVVAQVPAAFYMQSVHLPGKPQTLAVKRKADSEEERDDVSTLGSMLPAKASPVAESPKVMDEKSSLGEKAESVANVNANTPSSELVALTPAPSVPPPTLAMVSRQMGDSKPPQAIVKPQILTHIIEGFVIQEGAEPFPVGCSQLLKESEKPLQTGLPTGLTENQSGGPLGVDSPSAELDKKANLLKCEYCGKYAPAEQFRGSKRFCSMTCAKRYNVSCSHQFRLKRKKMKEFQEANYARVRRRGPRRSSSDIARAKIQGKCHRGQEDSSRGSDNSSYDEALSPTSPGPLSVRAGHGERDLGNPNTAPPTPELHGINPVFLSSNPSRWSVEEVYEFIASLQGCQEIAEEFRSQEIDGQALLLLKEEHLMSAMNIKLGPALKICAKINVLKET

>NP_002922.2

MTTPANAQNASKTWELSLYELHRTPQEAIMDGTEIAVSPRSLHSELMCPICLDMLKNTMTTKECLHRFCSDCIVTALRSGNKECPTCRKKLVSKRSLRPDPNFDALISKIYPSREEYEAHQDRVLIRLSRLHNQQALSSSIEEGLRMQAMHRAQRVRRPIPGSDQTTTMSGGEGEPGEGEGDGEDVSSDSAPDSAPGPAPKRPRGGGAGGSSVGTGGGGTGGVGGGAGSEDSGDRGGTLGGGTLGPPSPPGAPSPPEPGGEIELVFRPHPLLVEKGEYCQTRYVKTTGNATVDHLSKYLALRIALERRQQQEAGEPGGPGGGASDTGGPDGCGGEGGGAGGGDGPEEPALPSLEGVSEKQYTIYIAPGGGAFTTLNGSLTLELVNEKFWKVSRPLELCYAPTKDPK

840 NP_004443.2 NP_858045.1

>NP_004443.2

MSSDDRHLGSSCGSFIKTEPSSPSSGIDALSHHSPSGSSDASGGFGLALGTHANGLDSPPMFAGAGLGGTPCRKSYEDCASGIMEDSAIKCEYMLNAIPKRLCLVCGDIASGYHYGVASCEACKAFFKRTIQGNIEYSCPATNECEITKRRRKSCQACRFMKCLKVGMLKEGVRLDRVRGGRQKYKRRLDSESSPYLSLQISPPAKKPLTKIVSYLLVAEPDKLYAMPPPGMPEGDIKALTTLCDLADRELVVIIGWAKHIPGFSSLSLGDQMSLLQSAWMEILILGIVYRSLPYDDKLVYAEDYIMDEEHSRLAGLLELYRAILQLVRRYKKLKVEKEEFVTLKALALANSDSMYIEDLEAVQKLQDLLHEALQDYELSQRHEEPWRTGKLLLTLPLLRQTAAKAVQHFYSVKLQGKVPMHKLFLEMLEAKAWARADSLQEWRPLEQVPSPLHRATKRQHVHFLTPLPPPPSVAWVGTAQAGYHLEVFLPQRAGWPRAA

>NP_858045.1

MSGLGENLDPLASDSRKRKLPCDTPGQGLTCSGEKRRREQESKYIEELAELISANLSDIDNFNVKPDKCAILKETVRQIRQIKEQGKTISNDDDVQKADVSSTGQGVIDKDSLGPLLLQALDGFLFVVNRDGNIVFVSENVTQYLQYKQEDLVNTSVYNILHEEDRKDFLKNLPKSTVNGVSWTNETQRQKSHTFNCRMLMKTPHDILEDINASPEMRQRYETMQCFALSQPRAMMEEGEDLQSCMICVARRITTGERTFPSNPESFITRHDLSGKVVNIDTNSLRSSMRPGFEDIIRRCIQRFFSLNDGQSWSQKRHYQEAYLNGHAETPVYRFSLADGTIVTAQTKSKLFRNPVTNDRHGFVSTHFLQREQNGYRPNPNPVGQGIRPPMAGCNSSVGGMSMSPNQGLQMPSSRAYGLADPSTTGQMSGARYGGSSNIASLTPGPGMQSPSSYQNNNYGLNMSSPPHGSPGLAPNQQNIMISPRNRGSPKIASHQFSPVAGVHSPMASSGNTGNHSFSSSSLSALQAISEGVGTSLLSTLSSPGPKLDNSPNMNITQPSKVSNQDSKSPLGFYCDQNPVESSMCQSNSRDHLSDKESKESSVEGAENQRGPLESKGHKKLLQLLTCSSDDRGHSSLTNSPLDSSCKESSVSVTSPSGVSSSTSGGVSSTSNMHGSLLQEKHRILHKLLQNGNSPAEVAKITAEATGKDTSSITSCGDGNVVKQEQLSPKKKENNALLRYLLDRDDPSDALSKELQPQVEGVDNKMSQCTSSTIPSSSQEKDPKIKTETSEEGSGDLDNLDAILGDLTSSDFYNNSISSNGSHLGTKQQVFQGTNSLGLKSSQSVQSIRPPYNRAVSLDSPVSVGSSPPVKNISAFPMLPKQPMLGGNPRMMDSQENYGSSMGGPNRNVTVTQTPSSGDWGLPNSKAGRMEPMNSNSMGRPGGDYNTSLPRPALGGSIPTLPLRSNSIPGARPVLQQQQQMLQMRPGEIPMGMGANPYGQAAASNQLGSWPDGMLSMEQVSHGTQNRPLLRNSLDDLVGPPSNLEGQSDERALLDQLHTLLSNTDATGLEEIDRALGIPELVNQGQALEPKQDAFQGQEAAVMMDQKAGLYGQTYPAQGPPMQGGFHLQGQSPSFNSMMNQMNQQGNFPLQGMHPRANIMRPRTNTPKQLRMQLQQRLQGQQFLNQSRQALELKMENPTAGGAAVMRPMMQPQVSSQQGFLNAQMVAQRSRELLSHHFRQQRVAMMMQQQQQQQQQQQQQQQQQQQQQQQQQQQQQTQAFSPPPNVTASPSMDGLLAGPTMPQAPPQQFPYQPNYGMGQQPDPAFGRVSSPPNAMMSSRMGPSQNPMMQHPQAASIYQSSEMKGWPSGNLARNSSFSQQQFAHQGNPAVYSMVHMNGSSGHMGQMNMNPMPMSGMPMGPDQKYC

841 NP_001025178.1 NP_001020029.1

>NP_001025178.1

MPAPIRLRELIRTIRTARTQAEEREMIQKECAAIRSSFREEDNTYRCRNVAKLLYMHMLGYPAHFGQLECLKLIASQKFTDKRIGYLGAMLLLDERQDVHLLMTNCIKNDLNHSTQFVQGLALCTLGCMGSSEMCRDLAGEVEKLLKTSNSYLRKKAALCAVHVIRKVPELMEMFLPATKNLLNEKNHGVLHTSVVLLTEMCERSPDMLAHFRKNEKLVPQLVRILKNLIMSGYSPEHDVSGISDPFLQVRILRLLRILGRNDDDSSEAMNDILAQVATNTETSKNVGNAILYETVLTIMDIKSESGLRVLAINILGRFLLNNDKNIRYVALTSLLKTVQTDHNAVQRHRSTIVDCLKDLDVSIKRRAMELSFALVNGNNIRGMMKELLYFLDSCEPEFKADCASGIFLAAEKYAPSKRWHIDTIMRVLTTAGSYVRDDAVPNLIQLITNSVEMHAYTVQRLYKAILGDYSQQPLVQVAAWCIGEYGDLLVSGQCEEEEPIQVTEDEVLDILESVLISNMSTSVTRGYALTAIMKLSTRFTCTVNRIKKVVSIYGSSIDVELQQRAVEYNALFKKYDHMRSALLERMPVMEKVTTNGPTEIVQTNGETEPAPLETKPPPSGPQPTSQANDLLDLLGGNDITPVIPTAPTSKPSSAGGELLDLLGDINLTGAPAAAPAPASVPQISQPPFLLDGLSSQPLFNDIAAGIPSITAYSKNGLKIEFTFERSNTNPSVTVITIQASNSTELDMTDFVFQAAVPKTFQLQLLSPSSSIVPAFNTGTITQVIKVLNPQKQQLRMRIKLTYNHKGSAMQDLAEVNNFPPQSWQ

>NP_001020029.1

MTSATEFENVGNQPPYSRINARWDAPDDELDNDNSSARLFERSRIKALADEREVVQKKTFTKWVNSHLARVSCRITDLYKDLRDGRMLIKLLEVLSGEMLPKPTKGKMRIHCLENVDKALQFLKEQRVHLENMGSHDIVDGNHRLVLGLIWTIILRFQIQDIVVQTQEGRETRSAKDALLLWCQMKTAGYPHVNVTNFTSSWKDGLAFNALIHKHRPDLIDFDKLKDSNARHNLEHAFNVAERQLGIIPLLDPEDVFTENPDEKSIITYVVAFYHYFSKMKVLAVEGKRVGKVIDHAIETEKMIEKYSGLASDLLTWIEQTITVLNSRKFANSLTGVQQQLQAFSTYRTVEKPPKFQEKGNLEVLLFTIQSRMRANNQKVYTPHDGKLVSDINRAWESLEEAEYRRELALRNELIRQEKLEQLARRFDRKAAMRETWLSENQRLVAQDNFGYDLAAVEAAKKKHEAIETDTAAYEERVRALEDLAQELEKENYHDQKRITARKDNILRLWSYLQELLQSRRQRLETTLALQKLFQDMLHSIDWMDEIKAHLLSAEFGKHLLEVEDLLQKHKLMEADIAIQGDKVKAITAATLKFTEGKGYQPCDPQVIQDRISHLEQCFEELSNMAAGRKAQLEQSKRLWKFFWEMDEAESWIKEKEQIYSSLDYGKDLTSVLILQRKHKAFEDELRGLDAHLEQIFQEAHGMVARKQFGHPQIEARIKEVSAQWDQLKDLAAFCKKNLQDAENFFQFQGDADDLKAWLQDAHRLLSGEDVGQDEGATRALGKKHKDFLEELEESRGVMEHLEQQAQGFPEEFRDSPDVTHRLQALRELYQQVVAQADLRQQRLQEALDLYTVFGETDACELWMGEKEKWLAEMEMPDTLEDLEVVQHRFDILDQEMKTLMTQIDGVNLAANSLVESGHPRSREVKQYQDHLNTRWQAFQTLVSERREAVDSALRVHNYCVDCEETSKWITDKTKVVESTKDLGRDLAGIIAIQRKLSGLERDVAAIQARVDALERESQQLMDSHPEQKEDIGQRQKHLEELWQGLQQSLQGQEDLLGEVSQLQAFLQDLDDFQAWLSITQKAVASEDMPESLPEAEQLLQQHAGIKDEIDGHQDSYQRVKESGEKVIQGQTDPEYLLLGQRLEGLDTGWNALGRMWESRSHTLAQCLGFQEFQKDAKQAEAILSNQEYTLAHLEPPDSLEAAEAGIRKFEDFLGSMENNRDKVLSPVDSGNKLVAEGNLYSDKIKEKVQLIEDRHRKNNEKAQEASVLLRDNLELQNFLQNCQELTLWINDKLLTSQDVSYDEARNLHNKWLKHQAFVAELASHEGWLENIDAEGKQLMDEKPQFTALVSQKLEALHRLWDELQATTKEKTQHLSAARSSDLRLQTHADLNKWISAMEDQLRSDDPGKDLTSVNRMLAKLKRVEDQVNVRKEELGELFAQVPSMGEEGGDADLSIEKRFLDLLEPLGRRKKQLESSRAKLQISRDLEDETLWVEERLPLAQSADYGTNLQTVQLFMKKNQTLQNEILGHTPRVEDVLQRGQQLVEAAEIDCQDLEERLGHLQSSWDRLREAAAGRLQRLRDANEAQQYYLDADEAEAWIGEQELYVISDEIPKDEEGAIVMLKRHLRQQRAVEDYGRNIKQLASRAQGLLSAGHPEGEQIIRLQGQVDKHYAGLKDVAEERKRKLENMYHLFQLKRETDDLEQWISEKELVASSPEMGQDFDHVTLLRDKFRDFARETGAIGQERVDNVNAFIERLIDAGHSEAATIAEWKDGLNEMWADLLELIDTRMQLLAASYDLHRYFYTGAEILGLIDEKHRELPEDVGLDASTAESFHRVHTAFERELHLLGVQVQQFQDVATRLQTAYAGEKAEAIQNKEQEVSAAWQALLDACAGRRTQLVDTADKFRFFSMARDLLSWMESIIRQIETQERPRDVSSVELLMKYHQGINAEIETRSKNFSACLELGESLLQRQHQASEEIREKLQQVMSRRKEMNEKWEARWERLRMLLEVCQFSRDASVAEAWLIAQEPYLASGDFGHTVDSVEKLIKRHEAFEKSTASWAERFAALEKPTTLELKERQIAERPAEETGPQEEEGETAGEAPVSHHAATERTSPGEEEGTWPQNLQQPPPPGQHKDGQKSTGDERPTTEPLFKVLDTPLSEGDEPATLPAPRDHGQSVQMEGYLGRKHDLEGPNKKASNRSWNNLYCVLRNSELTFYKDAKNLALGMPYHGEEPLALRHAICEIAANYKKKKHVFKLRLSNGSEWLFHGKDEEEMLSWLQGVSTAINESQSIRVKAQSLPLPSLSGPDASLGKKDKEKRFSFFPKKK

842 NP_008872.1 NP_002219.1

>NP_008872.1

MAEEQDLSEVELSPVGSEEPRCLSPGSAPSLGPDGGGGGSGLRASPGPGELGKVKKEQQDGEADDDKFPVCIREAVSQVLSGYDWTLVPMPVRVNGASKSKPHVKRPMNAFMVWAQAARRKLADQYPHLHNAELSKTLGKLWRLLNESDKRPFIEEAERLRMQHKKDHPDYKYQPRRRKNGKAAQGEAECPGGEAEQGGTAAIQAHYKSAHLDHRHPGEGSPMSDGNPEHPSGQSHGPPTPPTTPKTELQSGKADPKRDGRSMGEGGKPHIDFGNVDIGEISHEVMSNMETFDVAELDQYLPPNGHPGHVSSYSAAGYGLGSALAVASGHSAWISKPPGVALPTVSPPGVDAKAQVKTETAGPQGPPHYTDQPSTSQIAYTSLSLPHYGSAFPSISRPQFDYSDHQPSGPYYGHSGQASGLYSAFSYMGPSQRPLYTAISDPSPSGPQSHSPTHWEQPVYTTLSRP

>NP_002219.1

MTAKMETTFYDDALNASFLPSESGPYGYSNPKILKQSMTLNLADPVGSLKPHLRAKNSDLLTSPDVGLLKLASPELERLIIQSSNGHITTTPTPTQFLCPKNVTDEQEGFAEGFVRALAELHSQNTLPSVTSAAQPVNGAGMVAPAVASVAGGSGSGGFSASLHSEPPVYANLSNFNPGALSSGGGAPSYGAAGLAFPAQPQQQQQPPHHLPQQMPVQHPRLQALKEEPQTVPEMPGETPPLSPIDMESQERIKAERKRMRNRIAASKCRKRKLERIARLEEKVKTLKAQNSELASTANMLREQVAQLKQKVMNHVNSGCQLMLTQQLQTF

843 NP_071396.3 NP_005307.1

>NP_071396.3

MSDTKVKVAVRVRPMNRRELELNTKCVVEMEGNQTVLHPPPSNTKQGERKPPKVFAFDYCFWSMDESNTTKYAGQEVVFKCLGEGILEKAFQGYNACIFAYGQTGSGKSFSMMGHAEQLGLIPRLCCALFKRISLEQNESQTFKVEVSYMEIYNEKVRDLLDPKGSRQSLKVREHKVLGPYVDGLSQLAVTSFEDIESLMSEGNKSRTVAATNMNEESSRSHAVFNIIITQTLYDLQSGNSGEKVSKVSLVDLAGSERVSKTGAAGERLKEGSNINKSLTTLGLVISSLADQAAGKGKSKFVPYRDSVLTWLLKDNLGGNSQTSMIATISPAADNYEETLSTLRYADRAKRIVNHAVVNEDPNAKVIRELREEVEKLREQLSQAEAMKAPELKEKLEESEKLIKELTVTWEEKLRKTEEIAQERQRQLESMGISLEMSGIKVGDDKCYLVNLNADPALNELLVYYLKDHTRVGADTSQDIQLFGIGIQPQHCEIDIASDGDVTLTPKENARSCVNGTLVCSTTQLWHGDRILWGNNHFFRINLPKRKRRDWLKDFEKETGPPEHDLDAASEASSEPDYNYEFAQMEVIMKTLNSNDPVQNVVQVLEKQYLEEKRSALEEQRLMYERELEQLRQQLSPDRQPQSSGPDRLAYSSQTAQQKVTQWAEERDELFRQGLAKLREQLVKANTLVREANFLAEEMSKLTDYQVTLQIPAANLSANRKRGAIVSEPAIQVRRKGKSTQVWTIEKLENKLIDMRDLYQEWKEKVPEAKRLYGKRGDPFYEAQENHNLIGVANVFLECLFCDVKLQYAVPIISQQGEVAGRLHVEVMRVTGAVPERVVEDDSSENSSESGSLEVVDSSGEIIHRVKKLTCRVKIKEATGLPLNLSNFVFCQYTFWDQCESTVAAPVVDPEVPSPQSKDAQYTVAFSHCKDYVVNVTEEFLEFISDGALAIEVWGHRCAGNGSSIWEVDSLHAKTRTLHDRWNEVTRRIEMWISILELNELGEYAAVELHQAKDVNTGGIFQLRQGHSRRVQVTVKPVQHSGTLPLMVEAILSVSIGCVTARSTKLQRGLDSYQRDDEDGDDMDSYQEEDLNCVRERWSDALIKRREYLDEQIKKVSNKTEKTEDDVEREAQLVEQWVGLTEERNAVLVPAPGSGIPGAPADWIPPPGMETHIPVLFLDLNADDPSANEQLVGPHASGVNSILPKEHGSQFFYLPIIKHSDDEVSATASWDSSVHDSVHLNRVTPQNERIYLIVKTTVQLSHPAAMELVLRKRIAANIYNKQSFTQSLKRRISLKNIFYSCGVTYEIVSNIPKATEEIEDRETLALLAARSENEGTSDGETYIEKYTRGVLQVENILSLERLRQAVTVKEALSTKARHIRRSLSTPNVHNVSSSRPDLSGFDEDDKGWPENQLDMSDYSSSYQDVACYGTLPRDSPRRNKEGCTSETPHALTVSPFKAFSPQPPKFFKPLMPVKEEHKKRIALEARPLLSQESMPPPQAHNPGCIVPSGSNGSSMPVEHNNKREKKIDSEEEENELEAINRKLISSQPYVPVEFADFSVYNASLENREWFSSKVDLSNSRVLEKEVSRSPTTSSITSGYFSHSASNATLSDMVVPSSDSSDQLAIQTKDADSTEHSTPSLVHDFRPSSNKELTEVEKGLVKDKIIVVPLKENSALAKGSPSSQSIPEKNSKSLCRTGSCSELDACPSKISQPARGFCPREVTVEHTTNILEDHSFTEFMGVSEGKDFDGLTDSSAGELSSRRSLPNKTGGKTVSDGLHHPSQLHSKLENDQVIIPEAAFWVLCCQ

>NP_005307.1

MATSSEEVLLIVKKVRQKKQDGALYLMAERIAWAPEGKDRFTISHMYADIKCQKISPEGKAKIQLQLVLHAGDTTNFHFSNESTAVKERDAVKDLLQQLLPKFKRKANKELEEKNRMLQEDPVLFQLYKDLVVSQVISAEEFWANRLNVNATDSSSTSNHKQDVGISAAFLADVRPQTDGCNGLRYNLTSDIIESIFRTYPAVKMKYAENVPHNMTEKEFWTRFFQSHYFHRDRLNTGSKDLFAECAKIDEKGLKTMVSLGVKNPLLDLTALEDKPLDEGYGISSVPSASNSKSIKENSNAAIIKRFNHHSAMVLAAGLRKQEAQNEQTSEPSNMDGNSGDADCFQPAVKRAKLQESIEYEDLGKNNSVKTIALNLKKSDRYYHGPTPIQSLQYATSQDIINSFQSIRQEMEAYTPKLTQVLSSSAASSTITALSPGGALMQGGTQQAINQMVPNDIQSELKHLYVAVGELLRHFWSCFPVNTPFLEEKVVKMKSNLERFQVTKLCPFQEKIRRQYLSTNLVSHIEEMLQTAYNKLHTWQSRRLMKKT

844 NP_150593.1 NP_002988.3

>NP_150593.1

MKFTTLLFLAAVAGALVYAEDASSDSTGADPAQEAGTSKPNEEISGPAEPASPPETTTTAQETSAAAVQGTAKVTSSRQELNPLKSIVEKSILLTEQALAKAGKGMHGGVPGGKQFIENGSEFAQKLLKKFSLLKPWA

>NP_002988.3

MRRAALWLWLCALALSLQPALPQIVATNLPPEDQDGSGDDSDNFSGSGAGALQDITLSQQTPSTWKDTQLLTAIPTSPEPTGLEATAASTSTLPAGEGPKEGEAVVLPEVEPGLTAREQEATPRPRETTQLPTTHQASTTTATTAQEPATSHPHRDMQPGHHETSTPAGPSQADLHTPHTEDGGPSATERAAEDGASSQLPAAEGSGEQDFTFETSGENTAVVAVEPDRRNQSPVDQGATGASQGLLDRKEVLGGVIAGGLVGLIFAVCLVGFMLYRMKKKDEGSYSLEEPKQANGGAYQKPTKQEEFYA

845 NP_003089.1 NP_008819.1

>NP_003089.1

MASGRRAPRTGLLELRAGAGSGAGGERWQRVLLSLAEDVLTVSPADGDPGPEPGAPREQEPAQLNGAAEPGAGPPQLPEALLLQRRRVTVRKADAGGLGISIKGGRENKMPILISKIFKGLAADQTEALFVGDAILSVNGEDLSSATHDEAVQVLKKTGKEVVLEVKYMKDVSPYFKNSTGGTSVGWDSPPASPLQRQPSSPGPTPRNFSEAKHMSLKMAYVSKRCTPNDPEPRYLEICSADGQDTLFLRAKDEASARSWATAIQAQVNTLTPRVKDELQALLAATSTAGSQDIKQIGWLTEQLPSGGTAPTLALLTEKELLLYLSLPETREALSRPARTAPLIATRLVHSGPSKGSVPYDAELSFALRTGTRHGVDTHLFSVESPQELAAWTRQLVDGCHRAAEGVQEVSTACTWNGRPCSLSVHIDKGFTLWAAEPGAARAVLLRQPFEKLQMSSDDGASLLFLDFGGAEGEIQLDLHSCPKTIVFIIHSFLSAKVTRLGLLA

>NP_008819.1

MADQLTEEQIAEFKEAFSLFDKDGDGTITTKELGTVMRSLGQNPTEAELQDMINEVDADGNGTIDFPEFLTMMARKMKDTDSEEEIREAFRVFDKDGNGYISAAELRHVMTNLGEKLTDEEVDEMIREADIDGDGQVNYEEFVQMMTAK

846 NP_002384.2 NP_005216.1

>NP_002384.2

MTSFSTSAQCSTSDSACRISPGQINQVRPKLPLLKILHAAGAQGEMFTVKEVMHYLGQYIMVKQLYDQQEQHMVYCGGDLLGELLGRQSFSVKDPSPLYDMLRKNLVTLATATTDAAQTLALAQDHSMDIPSQDQLKQSAEESSTSRKRTTEDDIPTLPTSEHKCIHSREDEDLIENLAQDETSRLDLGFEEWDVAGLPWWFLGNLRSNYTPRSNGSTDLQTNQDVGTAIVSDTTDDLWFLNESVSEQLGVGIKVEAADTEQTSEEVGKVSDKKVIEVGKNDDLEDSKSLSDDTDVEVTSEDEWQCTECKKFNSPSKRYCFRCWALRKDWYSDCSKLTHSLSTSDITAIPEKENEGNDVPDCRRTISAPVVRPKDAYIKKENSKLFDPCNSVEFLDLAHSSESQETISSMGEQLDNLSEQRTDTENMEDCQNLLKPCSLCEKRPRDGNIIHGRTGHLVTCFHCARRLKKAGASCPICKKEIQLVIKVFIA

>NP_005216.1

MALAGAPAGGPCAPALEALLGAGALRLLDSSQIVIISAAQDASAPPAPTGPAAPAAGPCDPDLLLFATPQAPRPTPSAPRPALGRPPVKRRLDLETDHQYLAESSGPARGRGRHPGKGVKSPGEKSRYETSLNLTTKRFLELLSHSADGVVDLNWAAEVLKVQKRRIYDITNVLEGIQLIAKKSKNHIQWLGSHTTVGVGGRLEGLTQDLRQLQESEQQLDHLMNICTTQLRLLSEDTDSQRLAYVTCQDLRSIADPAEQMVMVIKAPPETQLQAVDSSENFQISLKSKQGPIDVFLCPEETVGGISPGKTPSQEVTSEEENRATDSATIVSPPPSSPPSSLTTDPSQSLLSLEQEPLLSRMGSLRAPVDEDRLSPLVAADSLLEHVREDFSGLLPEEFISLSPPHEALDYHFGLEEGEGIRDLFDCDFGDLTPLDF

847 NP_000726.1 NP_002816.1

>NP_000726.1

MDYYRKYAAIFLVTLSVFLHVLHSAPDVQDCPECTLQENPFFSQPGAPILQCMGCCFSRAYPTPLRSKKTMLVQKNVTSESTCCVAKSYNRVTVMGGFKVENHTACHCSTCYYHKS

>NP_002816.1

MQAQQYQQQRRKFAAAFLAFIFILAAVDTAEAGKKEKPEKKVKKSDCGEWQWSVCVPTSGDCGLGTREGTRTGAECKQTMKTQRCKIPCNWKKQFGAECKYQFQAWGECDLNTALKTRTGSLKRALHNAECQKTVTISKPCGKLTKPKPQAESKKKKKEGKKQEKMLD

848 NP_001012410.1 NP_000994.1

>NP_001012410.1

MAKERCLKKSFQDSLEDIKKRMKEKRNKNLAEIGKRRSFIAAPCQIITNTSTLLKNYQDNNKMLVLALENEKSKVKEAQDIILQLRKECYYLTCQLYALKGKLTSQQTVEPAQNQEICSSGMDPNSDDSSRNLFVKDLPQIPLEETELPGQGESFQIEDQIPTIPQDTLGVDFDSGEAKSTDNVLPRTVSVRSSLKKHCNSICQFDSLDDFETSHLAGKSFEFERVGFLDPLVNMHIPENVQHNACQWSKDQVNLSPKLIQPGTFTKTKEDILESKSEQTKSKQRDTQERKREEKRKANRRKSKRMSKYKENKSENKKTVPQKKMHKSVSSNDAYNFNLEEGVHLTPFRQKVSNDSNREENNESEVSLCESSGSGDDSDDLYLPTCKYIQNPTSNSDRPVTRPLAKRALKYTDEKETEGSKPTKTPTTTPPETQQSPHLSLKDITNVSLYPVVKIRRLSLSPKKNKASPAVALPKRRCTASVNYKEPTLASKLRRGDPFTDLCFLNSPIFKQKKDLRRSKKRALEVSPAKEAIFILYYVREFVSRFPDCRKCKLETHICLR

>NP_000994.1

MASVSELACIYSALILHDDEVTVTEDKINALIKAAGVNVEPFWPGLFAKALANVNIGSLICNVGAGGPAPAAGAAPAGGPAPSTAAAPAEEKKVEAKKEESEESDDDMGFGLFD

849 NP_003755.2 NP_076974.1

>NP_003755.2

MKDRLAELLDLSKQYDQQFPDGDDEFDSPHEDIVFETDHILESLYRDIRDIQDENQLLVADVKRLGKQNARFLTSMRRLSSIKRDTNSIAKAIKARGEVIHCKLRAMKELSEAAEAQHGPHSAVARISRAQYNALTLTFQRAMHDYNQAEMKQRDNCKIRIQRQLEIMGKEVSGDQIEDMFEQGKWDVFSENLLADVKGARAALNEIESRHRELLRLESRIRDVHELFLQMAVLVEKQADTLNVIELNVQKTVDYTGQAKAQVRKAVQYEEKNPCRTLCCFCCPCLK

>NP_076974.1

MDLPDSASRVFCGRILSMVNTDDVNAIILAQKNMLDRFEKTNEMLLNFNNLSSARLQQMSERFLHHTRTLVEMKRDLDSIFRRIRTLKGKLARQHPEAFSHIPEASFLEEEDEDPIPPSTTTTIATSEQSTGSCDTSPDTVSPSLSPGFEDLSHVQAGSPAINGRSQTDDEEMTGE

850 NP_001922.2 NP_002602.2

>NP_001922.2

MWRVCARRAQNVAPWAGLEARWTALQEVPGTPRVTSRSGPAPARRNSVTTGYGGVRALCGWTPSSGATPRNRLLLQLLGSPGRRYYSLPPHQKVPLPSLSPTMQAGTIARWEKKEGDKINEGDLIAEVETDKATVGFESLEECYMAKILVAEGTRDVPIGAIICITVGKPEDIEAFKNYTLDSSAAPTPQAAPAPTPAATASPPTPSAQAPGSSYPPHMQVLLPALSPTMTMGTVQRWEKKVGEKLSEGDLLAEIETDKATIGFEVQEEGYLAKILVPEGTRDVPLGTPLCIIVEKEADISAFADYRPTEVTDLKPQVPPPTPPPVAAVPPTPQPLAPTPSAPCPATPAGPKGRVFVSPLAKKLAVEKGIDLTQVKGTGPDGRITKKDIDSFVPSKVAPAPAAVVPPTGPGMAPVPTGVFTDIPISNIRRVIAQRLMQSKQTIPHYYLSIDVNMGEVLLVRKELNKILEGRSKISVNDFIIKASALACLKVPEANSSWMDTVIRQNHVVDVSVAVSTPAGLITPIVFNAHIKGVETIANDVVSLATKAREGKLQPHEFQGGTFTISNLGMFGIKNFSAIINPPQACILAIGASEDKLVPADNEKGFDVASMMSVTLSCDHRVVDGAVGAQWLAEFRKYLEKPITMLL

>NP_002602.2

MRWVWALLKNASLAGAPKYIEHFSKFSPSPLSMKQFLDFGSSNACEKTSFTFLRQELPVRLANIMKEINLLPDRVLSTPSVQLVQSWYVQSLLDIMEFLDKDPEDHRTLSQFTDALVTIRNRHNDVVPTMAQGVLEYKDTYGDDPVSNQNIQYFLDRFYLSRISIRMLINQHTLIFDGSTNPAHPKHIGSIDPNCNVSEVVKDAYDMAKLLCDKYYMASPDLEIQEINAANSKQPIHMVYVPSHLYHMLFELFKNAMRATVESHESSLILPPIKVMVALGEEDLSIKMSDRGGGVPLRKIERLFSYMYSTAPTPQPGTGGTPLAGFGYGLPISRLYAKYFQGDLQLFSMEGFGTDAVIYLKALSTDSVERLPVYNKSAWRHYQTIQEAGDWCVPSTEPKNTSTYRVS

851 NP_001447.2 NP_001655.1

>NP_001447.2

MSSSHSRAGQSAAGAAPGGGVDTRDAEMPATEKDLAEDAPWKKIQQNTFTRWCNEHLKCVSKRIANLQTDLSDGLRLIALLEVLSQKKMHRKHNQRPTFRQMQLENVSVALEFLDRESIKLVSIDSKAIVDGNLKLILGLIWTLILHYSISMPMWDEEEDEEAKKQTPKQRLLGWIQNKLPQLPITNFSRDWQSGRALGALVDSCAPGLCPDWDSWDASKPVTNAREAMQQADDWLGIPQVITPEEIVDPNVDEHSVMTYLSQFPKAKLKPGAPLRPKLNPKKARAYGPGIEPTGNMVKKRAEFTVETRSAGQGEVLVYVEDPAGHQEEAKVTANNDKNRTFSVWYVPEVTGTHKVTVLFAGQHIAKSPFEVYVDKSQGDASKVTAQGPGLEPSGNIANKTTYFEIFTAGAGTGEVEVVIQDPMGQKGTVEPQLEARGDSTYRCSYQPTMEGVHTVHVTFAGVPIPRSPYTVTVGQACNPSACRAVGRGLQPKGVRVKETADFKVYTKGAGSGELKVTVKGPKGEERVKQKDLGDGVYGFEYYPMVPGTYIVTITWGGQNIGRSPFEVKVGTECGNQKVRAWGPGLEGGVVGKSADFVVEAIGDDVGTLGFSVEGPSQAKIECDDKGDGSCDVRYWPQEAGEYAVHVLCNSEDIRLSPFMADIRDAPQDFHPDRVKARGPGLEKTGVAVNKPAEFTVDAKHGGKAPLRVQVQDNEGCPVEALVKDNGNGTYSCSYVPRKPVKHTAMVSWGGVSIPNSPFRVNVGAGSHPNKVKVYGPGVAKTGLKAHEPTYFTVDCAEAGQGDVSIGIKCAPGVVGPAEADIDFDIIRNDNDTFTVKYTPRGAGSYTIMVLFADQATPTSPIRVKVEPSHDASKVKAEGPGLSRTGVELGKPTHFTVNAKAAGKGKLDVQFSGLTKGDAVRDVDIIDHHDNTYTVKYTPVQQGPVGVNVTYGGDPIPKSPFSVAVSPSLDLSKIKVSGLGEKVDVGKDQEFTVKSKGAGGQGKVASKIVGPSGAAVPCKVEPGLGADNSVVRFLPREEGPYEVEVTYDGVPVPGSPFPLEAVAPTKPSKVKAFGPGLQGGSAGSPARFTIDTKGAGTGGLGLTVEGPCEAQLECLDNGDGTCSVSYVPTEPGDYNINILFADTHIPGSPFKAHVVPCFDASKVKCSGPGLERATAGEVGQFQVDCSSAGSAELTIEICSEAGLPAEVYIQDHGDGTHTITYIPLCPGAYTVTIKYGGQPVPNFPSKLQVEPAVDTSGVQCYGPGIEGQGVFREATTEFSVDARALTQTGGPHVKARVANPSGNLTETYVQDRGDGMYKVEYTPYEEGLHSVDVTYDGSPVPSSPFQVPVTEGCDPSRVRVHGPGIQSGTTNKPNKFTVETRGAGTGGLGLAVEGPSEAKMSCMDNKDGSCSVEYIPYEAGTYSLNVTYGGHQVPGSPFKVPVHDVTDASKVKCSGPGLSPGMVRANLPQSFQVDTSKAGVAPLQVKVQGPKGLVEPVDVVDNADGTQTVNYVPSREGPYSISVLYGDEEVPRSPFKVKVLPTHDASKVKASGPGLNTTGVPASLPVEFTIDAKDAGEGLLAVQITDPEGKPKKTHIQDNHDGTYTVAYVPDVTGRYTILIKYGGDEIPFSPYRVRAVPTGDASKCTVTGAGIGPTIQIGEETVITVDTKAAGKGKVTCTVCTPDGSEVDVDVVENEDGTFDIFYTAPQPGKYVICVRFGGEHVPNSPFQVTALAGDQPSVQPPLRSQQLAPQYTYAQGGQQTWAPERPLVGVNGLDVTSLRPFDLVIPFTIKKGEITGEVRMPSGKVAQPTITDNKDGTVTVRYAPSEAGLHEMDIRYDNMHIPGSPLQFYVDYVNCGHVTAYGPGLTHGVVNKPATFTVNTKDAGEGGLSLAIEGPSKAEISCTDNQDGTCSVSYLPVLPGDYSILVKYNEQHVPGSPFTARVTGDDSMRMSHLKVGSAADIPINISETDLSLLTATVVPPSGREEPCLLKRLRNGHVGISFVPKETGEHLVHVKKNGQHVASSPIPVVISQSEIGDASRVRVSGQGLHEGHTFEPAEFIIDTRDAGYGGLSLSIEGPSKVDINTEDLEDGTCRVTYCPTEPGNYIINIKFADQHVPGSPFSVKVTGEGRVKESITRRRRAPSVANVGSHCDLSLKIPEISIQDMTAQVTSPSGKTHEAEIVEGENHTYCIRFVPAEMGTHTVSVKYKGQHVPGSPFQFTVGPLGEGGAHKVRAGGPGLERAEAGVPAEFSIWTREAGAGGLAIAVEGPSKAEISFEDRKDGSCGVAYVVQEPGDYEVSVKFNEEHIPDSPFVVPVASPSGDARRLTVSSLQESGLKVNQPASFAVSLNGAKGAIDAKVHSPSGALEECYVTEIDQDKYAVRFIPRENGVYLIDVKFNGTHIPGSPFKIRVGEPGHGGDPGLVSAYGAGLEGGVTGNPAEFVVNTSNAGAGALSVTIDGPSKVKMDCQECPEGYRVTYTPMAPGSYLISIKYGGPYHIGGSPFKAKVTGPRLVSNHSLHETSSVFVDSLTKATCAPQHGAPGPGPADASKVVAKGLGLSKAYVGQKSSFTVDCSKAGNNMLLVGVHGPRTPCEEILVKHVGSRLYSVSYLLKDKGEYTLVVKWGDEHIPGSPYRVVVP

>NP_001655.1

MAAIRKKLVIVGDGACGKTCLLIVFSKDQFPEVYVPTVFENYVADIEVDGKQVELALWDTAGQEDYDRLRPLSYPDTDVILMCFSIDSPDSLENIPEKWTPEVKHFCPNVPIILVGNKKDLRNDEHTRRELAKMKQEPVKPEEGRDMANRIGAFGYMECSAKTKDGVREVFEMATRAALQARRGKKKSGCLVL

852 NP_003130.2 NP_055621.1

>NP_003130.2

MLDFFTIFSKGGLVLWCFQGVSDSCTGPVNALIRSVLLQERGGNNSFTHEALTLKYKLDNQFELVFVVGFQKILTLTYVDKLIDDVHRLFRDKYRTEIQQQSALSLLNGTFDFQNDFLRLLREAEESSKIRAPTTMKKFEDSEKAKKPVRSMIETRGEKPKEKAKNSKKKGAKKEGSDGPLATSKPVPAEKSGLPVGPENGVELSKEELIRRKREEFIQKHGRGMEKSNKSTKSDAPKEKGKKAPRVWELGGCANKEVLDYSTPTTNGTPEAALSEDINLIRGTGSGGQLQDLDCSSSDDEGAAQNSTKPSATKGTLGGMFGMLKGLVGSKSLSREDMESVLDKMRDHLIAKNVAADIAVQLCESVANKLEGKVMGTFSTVTSTVKQALQESLVQILQPQRRVDMLRDIMDAQRRQRPYVVTFCGVNGVGKSTNLAKISFWLLENGFSVLIAACDTFRAGAVEQLRTHTRRLSALHPPEKHGGRTMVQLFEKGYGKDAAGIAMEAIAFARNQGFDVVLVDTAGRMQDNAPLMTALAKLITVNTPDLVLFVGEALVGNEAVDQLVKFNRALADHSMAQTPRLIDGIVLTKFDTIDDKVGAAISMTYITSKPIVFVGTGQTYCDLRSLNAKAVVAALMKA

>NP_055621.1

MDSPPKLTGETLIVHHIPLVHCQVPDRQCCGGAGGGGGSTRPNPFCPPELGITQPDQDLGQADSLLFSSLHSAPGGTARSIDSTKSRSRDGRGPGAPKRHNPFLLQEGVGEPGLGDLYDDSIGDSATQQSFHLHGTGQPNFHLSSFQLPPSGPRVGRPWGTTRSRAGVVEGQEQEPVMTLDTQQCGTSHCCRPELEAETMELDECGGPGGSGSGGGASDTSGFSFDQEWKLSSDESPRNPGCSGSGDQHCRCSSTSSQSEAADQSMGYVSDSSCNSSDGVLVTFSTLYNKMHGTPRANLNSAPQSCSDSSFCSHSDPGAFYLDLQPSPFESKMSYESHHPESGGREGGYGCPHASSPELDANCNSYRPHCEPCPAVADLTACFQSQARLVVATQNYYKLVTCDLSSQSSPSPAGSSITSCSEEHTKISPPPGPGPDPGPSQPSEYYLFQKPEVQPEEQEAVSSSTQAAAAVGPTVLEGQVYTNTSPPNLSTGRQRSRSYDRSLQRSPPVRLGSLERMLSCPVRLSEGPAAMAGPGSPPRRVTSFAELAKGRKKTGGSGSPPLRVSVGDSSQEFSPIQEAQQDRGAPLDEGTCCSHSLPPMPLGPGMDLLGPDPSPPWSTQVCQGPHSSEMPPAGLRATGQGPLAQLMDPGPALPGSPANSHTQRDARARADGGGTESRPVLRYSKEQRPTTLPIQPFVFQHHFPKQLAKARALHSLSQLYSLSGCSRTQQPAPLAAPAAQVSVPAPSGEPQASTPRATGRGARKAGSEPETSRPSPLGSYSPIRSVGPFGPSTDSSASTSCSPPPEQPTATESLPPWSHSCPSAVRPATSQQPQKEDQKILTLTEYRLHGTGSLPPLGSWRSGLSRAESLARGGGEGSMATRPSNANHLSPQALKWREYRRKNPLGPPGLSGSLDRRSQEARLARRNPIFEFPGSLSAASHLNCRLNGQAVKPLPLTCPDFQDPFSLTEKPPAEFCLSPDGSSEAISIDLLQKKGLVKAVNIAVDLIVAHFGTSRDPGVKAKLGNSSVSPNVGHLVLKYLCPAVRAVLEDGLKAFVLDVIIGQRKNMPWSVVEASTQLGPSTKVLHGLYNKVSQFPELTSHTMRFNAFILGLLNIRSLEFWFNHLYNHEDIIQTHYQPWGFLSAAHTVCPGLFEELLLLLQPLALLPFSLDLLFQHRLLQSGQQQRQHKELLRVSQDLLLSAHSTLQLARARGQEGPGDVDRAAQGERVKGVGASEGGEEEEEEEETEEVAEAAGGSGRARWARGGQAGWWYQLMQSSQVYIDGSIEGSRFPRGSSNSSSEKKKGAGGGGPPQAPPPREGVVEGAEACPASEEALGRERGWPFWMGSPPDSVLAELRRSREREGPAASPAENEEGASEPSPGGIKWGHLFGSRKAQREARPTNRLPSDWLSLDKSMFQLVAQTVGSRREPEPKESLQEPHSPALPSSPPCEVQALCHHLATGPGQLSFHKGDILRVLGRAGGDWLRCSRGPDSGLVPLAYVTLTPTPSPTPGSSQN

853 NP_000825.1 NP_057065.2

>NP_000825.1

MKPRAECCSPKFWLVLAVLAVSGSRARSQKSPPSIGIAVILVGTSDEVAIKDAHEKDDFHHLSVVPRVELVAMNETDPKSIITRICDLMSDRKIQGVVFADDTDQEAIAQILDFISAQTLTPILGIHGGSSMIMADKDESSMFFQFGPSIEQQASVMLNIMEEYDWYIFSIVTTYFPGYQDFVNKIRSTIENSFVGWELEEVLLLDMSLDDGDSKIQNQLKKLQSPIILLYCTKEEATYIFEVANSVGLTGYGYTWIVPSLVAGDTDTVPAEFPTGLISVSYDEWDYGLPARVRDGIAIITTAASDMLSEHSFIPEPKSSCYNTHEKRIYQSNMLNRYLINVTFEGRNLSFSEDGYQMHPKLVIILLNKERKWERVGKWKDKSLQMKYYVWPRMCPETEEQEDDHLSIVTLEEAPFVIVESVDPLSGTCMRNTVPCQKRIVTENKTDEEPGYIKKCCKGFCIDILKKISKSVKFTYDLYLVTNGKHGKKINGTWNGMIGEVVMKRAYMAVGSLTINEERSEVVDFSVPFIETGISVMVSRSNGTVSPSAFLEPFSADVWVMMFVMLLIVSAVAVFVFEYFSPVGYNRCLADGREPGGPSFTIGKAIWLLWGLVFNNSVPVQNPKGTTSKIMVSVWAFFAVIFLASYTANLAAFMIQEEYVDQVSGLSDKKFQRPNDFSPPFRFGTVPNGSTERNIRNNYAEMHAYMGKFNQRGVDDALLSLKTGKLDAFIYDAAVLNYMAGRDEGCKLVTIGSGKVFASTGYGIAIQKDSGWKRQVDLAILQLFGDGEMEELEALWLTGICHNEKNEVMSSQLDIDNMAGVFYMLGAAMALSLITFICEHLFYWQFRHCFMGVCSGKPGMVFSISRGIYSCIHGVAIEERQSVMNSPTATMNNTHSNILRLLRTAKNMANLSGVNGSPQSALDFIRRESSVYDISEHRRSFTHSDCKSYNNPPCEENLFSDYISEVERTFGNLQLKDSNVYQDHYHHHHRPHSIGSASSIDGLYDCDNPPFTTQSRSISKKPLDIGLPSSKHSQLSDLYGKFSFKSDRYSGHDDLIRSDVSDISTHTVTYGNIEGNAAKRRKQQYKDSLKKRPASAKSRREFDEIELAYRRRPPRSPDHKRYFRDKEGLRDFYLDQFRTKENSPHWEHVDLTDIYKERSDDFKRDSISGGGPCTNRSHIKHGTGDKHGVVSGVPAPWEKNLTNVEWEDRSGGNFCRSCPSKLHNYSTTVTGQNSGRQACIRCEACKKAGNLYDISEDNSLQELDQPAAPVAVTSNASTTKYPQSPTNSKAQKKNRNKLRRQHSYDTFVDLQKEEAALAPRSVSLKDKGRFMDGSPYAHMFEMSAGESTFANNKSSVPTAGHHHHNNPGGGYMLSKSLYPDRVTQNPFIPTFGDDQCLLHGSKSYFFRQPTVAGASKARPDFRALVTNKPVVSALHGAVPARFQKDICIGNQSNPCVPNNKNPRAFNGSSNGHVYEKLSSIESDV

>NP_057065.2

MATITCTRFTEEYQLFEELGKGAFSVVRRCVKVLAGQEYAAKIINTKKLSARDHQKLEREARICRLLKHPNIVRLHDSISEEGHHYLIFDLVTGGELFEDIVAREYYSEADASHCIQQILEAVLHCHQMGVVHRDLKPENLLLASKLKGAAVKLADFGLAIEVEGEQQAWFGFAGTPGYLSPEVLRKDPYGKPVDLWACGVILYILLVGYPPFWDEDQHRLYQQIKAGAYDFPSPEWDTVTPEAKDLINKMLTINPSKRITAAEALKHPWISHRSTVASCMHRQETVDCLKKFNARRKLKGAILTTMLATRNFSGGKSGGNKKSDGVKKRKSSSSVQLMESSESTNTTIEDEDTKVRKQEIIKVTEQLIEAISNGDFESYTKMCDPGMTAFEPEALGNLVEGLDFHRFYFENLWSRNSKPVHTTILNPHIHLMGDESACIAYIRITQYLDAGGIPRTAQSEETRVWHRRDGKWQIVHFHRSGAPSVLPH

854 NP_008855.1 NP_002496.1

>NP_008855.1

MSGGGVIRGPAGNNDCRIYVGNLPPDIRTKDIEDVFYKYGAIRDIDLKNRRGGPPFAFVEFEDPRDAEDAVYGRDGYDYDGYRLRVEFPRSGRGTGRGGGGGGGGGAPRGRYGPPSRRSENRVVVSGLPPSGSWQDLKDHMREAGDVCYADVYRDGTGVVEFVRKEDMTYAVRKLDNTKFRSHEGETAYIRVKVDGPRSPSYGRSRSRSRSRSRSRSRSNSRSRSYSPRRSRGSPRYSPRHSRSRSRT

>NP_002496.1

MEQYTANSNSSTEQIVVQAGQIQQQQQGGVTAVQLQTEAQVASASGQQVQTLQVVQGQPLMVQVSGGQLITSTGQPIMVQAVPGGQGQTIMQVPVSGTQGLQQIQLVPPGQIQIQGGQAVQVQGQQGQTQQIIIQQPQTAVTAGQTQTQQQIAVQGQQVAQTAEGQTIVYQPVNADGTILQQVTVPVSGMITIPAASLAGAQIVQTGANTNTTSSGQGTVTVTLPVAGNVVNSGGMVMMVPGAGSVPAIQRIPLPGAEMLEEEPLYVNAKQYHRILKRRQARAKLEAEGKIPKERRKYLHESRHRHAMARKRGEGGRFFSPKEKDSPHMQDPNQADEEAMTQIIRVS

855 NP_002231.1 NP_000786.1

>NP_002231.1

MAKLTESMTNVLEGDSMDQDVESPVAIHQPKLPKQARDDLPRHISRDRTKRKIQRYVRKDGKCNVHHGNVRETYRYLTDIFTTLVDLKWRFNLLIFVMVYTVTWLFFGMIWWLIAYIRGDMDHIEDPSWTPCVTNLNGFVSAFLFSIETETTIGYGYRVITDKCPEGIILLLIQSVLGSIVNAFMVGCMFVKISQPKKRAETLVFSTHAVISMRDGKLCLMFRVGDLRNSHIVEASIRAKLIKSKQTSEGEFIPLNQTDINVGYYTGDDRLFLVSPLIISHEINQQSPFWEISKAQLPKEELEIVVILEGMVEATGMTCQARSSYITSEILWGYRFTPVLTLEDGFYEVDYNSFHETYETSTPSLSAKELAELASRAELPLSWSVSSKLNQHAELETEEEEKNLEEQTERNGDVANLENESKV

>NP_000786.1

MDPLNLSWYDDDLERQNWSRPFNGSDGKADRPHYNYYATLLTLLIAVIVFGNVLVCMAVSREKALQTTTNYLIVSLAVADLLVATLVMPWVVYLEVVGEWKFSRIHCDIFVTLDVMMCTASILNLCAISIDRYTAVAMPMLYNTRYSSKRRVTVMISIVWVLSFTISCPLLFGLNNADQNECIIANPAFVVYSSIVSFYVPFIVTLLVYIKIYIVLRRRRKRVNTKRSSRAFRAHLRAPLKGNCTHPEDMKLCTVIMKSNGSFPVNRRRVEAARRAQELEMEMLSSTSPPERTRYSPIPPSHHQLTLPDPSHHGLHSTPDSPAKPEKNGHAKDHPKIAKIFEIQTMPNGKTRTSLKTMSRRKLSQQKEKKATQMLAIVLGVFIICWLPFFITHILNIHCDCNIPPVLYSAFTWLGYVNSAVNPIIYTTFNIEFRKAFLKILHC

856 NP_000278.2 NP_005893.1

>NP_000278.2

MALAVLRVLEPFPTETPPLAVLLPPGGPWPAAELGLVLALRPAGESPAGPALLVAALEGPDAGTEEQGPGPPQLLVSRALLRLLALGSGAWVRARAVRRPPALGWALLGTSLGPGLGPRVGPLLVRRGETLPVPGPRVLETRPALQGLLGPGTRLAVTELRGRARLCPESGDSSRPPPPPVVSSFAVSGTVRRLQGVLGGTGDSLGVSRSCLRGLGLFQGEWVWVAQARESSNTSQPHLARVQVLEPRWDLSDRLGPGSGPLGEPLADGLALVPATLAFNLGCDPLEMGELRIQRYLEGSIAPEDKGSCSLLPGPPFARELHIEIVSSPHYSTNGNYDGVLYRHFQIPRVVQEGDVLCVPTIGQVEILEGSPEKLPRWREMFFKVKKTVGEAPDGPASAYLADTTHTSLYMVGSTLSPVPWLPSEESTLWSSLSPPGLEALVSELCAVLKPRLQPGGALLTGTSSVLLRGPPGCGKTTVVAAACSHLGLHLLKVPCSSLCAESSGAVETKLQAIFSRARRCRPAVLLLTAVDLLGRDRDGLGEDARVMAVLRHLLLNEDPLNSCPPLMVVATTSRAQDLPADVQTAFPHELEVPALSEGQRLSILRALTAHLPLGQEVNLAQLARRCAGFVVGDLYALLTHSSRAACTRIKNSGLAGGLTEEDEGELCAAGFPLLAEDFGQALEQLQTAHSQAVGAPKIPSVSWHDVGGLQEVKKEILETIQLPLEHPELLSLGLRRSGLLLHGPPGTGKTLLAKAVATECSLTFLSVKGPELINMYVGQSEENVREVFARARAAAPCIIFFDELDSLAPSRGRSGDSGGVMDRVVSQLLAELDGLHSTQDVFVIGATNRPDLLDPALLRPGRFDKLVFVGANEDRASQLRVLSAITRKFKLEPSVSLVNVLDCCPPQLTGADLYSLCSDAMTAALKRRVHDLEEGLEQGSSALMLTMEDLLQAAARLQPSVSEQELLRYKRIQRKFAAC

>NP_005893.1

MSSILPFTPPIVKRLLGWKKGEQNGQEEKWCEKAVKSLVKKLKKTGQLDELEKAITTQNVNTKCITIPRSLDGRLQVSHRKGLPHVIYCRLWRWPDLHSHHELRAMELCEFAFNMKKDEVCVNPYHYQRVETPVLPPVLVPRHTEIPAEFPPLDDYSHSIPENTNFPAGIEPQSNIPETPPPGYLSEDGETSDHQMNHSMDAGSPNLSPNPMSPAHNNLDLQPVTYCEPAFWCSISYYELNQRVGETFHASQPSMTVDGFTDPSNSERFCLGLLSNVNRNAAVELTRRHIGRGVRLYYIGGEVFAECLSDSAIFVQSPNCNQRYGWHPATVCKIPPGCNLKIFNNQEFAALLAQSVNQGFEAVYQLTRMCTIRMSFVKGWGAEYRRQTVTSTPCWIELHLNGPLQWLDKVLTQMGSPSIRCSSVS

857 NP_005632.1 NP_004597.2

>NP_005632.1

MAEEKKLKLSNTVLPSESMKVVAESMGIAQIQEETCQLLTDEVSYRIKEIAQDALKFMHMGKRQKLTTSDIDYALKLKNVEPLYGFHAQEFIPFRFASGGGRELYFYEEKEVDLSDIINTPLPRVPLDVCLKAHWLSIEGCQPAIPENPPPAPKEQQKAEATEPLKSAKPGQEEDGPLKGKGQGATTADGKGKEKKAPPLLEGAPLRLKPRSIHELSVEQQLYYKEITEACVGSCEAKRAEALQSIATDPGLYQMLPRFSTFISEGVRVNVVQNNLALLIYLMRMVKALMDNPTLYLEKYVHELIPAVMTCIVSRQLCLRPDVDNHWALRDFAARLVAQICKHFSTTTNNIQSRITKTFTKSWVDEKTPWTTRYGSIAGLAELGHDVIKTLILPRLQQEGERIRSVLDGPVLSNIDRIGADHVQSLLLKHCAPVLAKLRPPPDNQDAYRAEFGSLGPLLCSQVVKARAQAALQAQQVNRTTLTITQPRPTLTLSQAPQPGPRTPGLLKVPGSIALPVQTLVSARAAAPPQPSPPPTKFIVMSSSSSAPSTQQVLSLSTSAPGSGSTTTSPVTTTVPSVQPIVKLVSTATTAPPSTAPSGPGSVQKYIVVSLPPTGEGKGGPTSHPSPVPPPASSPSPLSGSALCGGKQEAGDSPPPAPGTPKANGSQPNSGSPQPAP

>NP_004597.2

MGPGCDLLLRTAATITAAAIMSDTDSDEDSAGGGPFSLAGFLFGNINGAGQLEGESVLDDECKKHLAGLGALGLGSLITELTANEELTGTDGALVNDEGWVRSTEDAVDYSDINEVAEDESRRYQQTMGSLQPLCHSDYDEDDYDADCEDIDCKLMPPPPPPPGPMKKDKDQDSITGVSENGEGIILPSIIAPSSLASEKVDFSSSSDSESEMGPQEATQAESEDGKLTLPLAGIMQHDATKLLPSVTELFPEFRPGKVLRFLRLFGPGKNVPSVWRSARRKRKKKHRELIQEEQIQEVECSVESEVSQKSLWNYDYAPPPPPEQCLSDDEITMMAPVESKFSQSTGDIDKVTDTKPRVAEWRYGPARLWYDMLGVPEDGSGFDYGFKLRKTEHEPVIKSRMIEEFRKLEENNGTDLLADENFLMVTQLHWEDDIIWDGEDVKHKGTKPQRASLAGWLPSSMTRNAMAYNVQQGFAATLDDDKPWYSIFPIDNEDLVYGRWEDNIIWDAQAMPRLLEPPVLTLDPNDENLILEIPDEKEEATSNSPSKESKKESSLKKSRILLGKTGVIKEEPQQNMSQPEVKDPWNLSNDEYYYPKQQGLRGTFGGNIIQHSIPAVELRQPFFPTHMGPIKLRQFHRPPLKKYSFGALSQPGPHSVQPLLKHIKKKAKMREQERQASGGGEMFFMRTPQDLTGKDGDLILAEYSEENGPLMMQVGMATKIKNYYKRKPGKDPGAPDCKYGETVYCHTSPFLGSLHPGQLLQAFENNLFRAPIYLHKMPETDFLIIRTRQGYYIRELVDIFVVGQQCPLFEVPGPNSKRANTHIRDFLQVFIYRLFWKSKDRPRRIRMEDIKKAFPSHSESSIRKRLKLCADFKRTGMDSNWWVLKSDFRLPTEEEIRAMVSPEQCCAYYSMIAAEQRLKDAGYGEKSFFAPEEENEEDFQMKIDDEVRTAPWNTTRAFIAAMKGKCLLEVTGVADPTGCGEGFSYVKIPNKPTQQKDDKEPQPVKKTVTGTDADLRRLSLKNAKQLLRKFGVPEEEIKKLSRWEVIDVVRTMSTEQARSGEGPMSKFARGSRFSVAEHQERYKEECQRIFDLQNKVLSSTEVLSTDTDSSSAEDSDFEEMGKNIENMLQNKKTSSQLSREREEQERKELQRMLLAAGSAASGNNHRDDDTASVTSLNSSATGRCLKIYRTFRDEEGKEYVRCETVRKPAVIDAYVRIRTTKDEEFIRKFALFDEQHREEMRKERRRIQEQLRRLKRNQEKEKLKGPPEKKPKKMKERPDLKLKCGACGAIGHMRTNKFCPLYYQTNAPPSNPVAMTEEQEEELEKTVIHNDNEELIKVEGTKIVLGKQLIESADEVRRKSLVLKFPKQQLPPKKKRRVGTTVHCDYLNRPHKSIHRRRTDPMVTLSSILESIINDMRDLPNTYPFHTPVNAKVVKDYYKIITRPMDLQTLRENVRKRLYPSREEFREHLELIVKNSATYNGPKHSLTQISQSMLDLCDEKLKEKEDKLARLEKAINPLLDDDDQVAFSFILDNIVTQKMMAVPDSWPFHHPVNKKFVPDYYKVIVNPMDLETIRKNISKHKYQSRESFLDDVNLILANSVKYNGPESQYTKTAQEIVNVCYQTLTEYDEHLTQLEKDICTAKEAALEEAELESLDPMTPGPYTPQPPDLYDTNTSLSMSRDASVFQDESNMSVLDIPSATPEKQVTQEGEDGDGDLADEEEGTVQQPQASVLYEDLLMSEGEDDEEDAGSDEEGDNPFSAIQLSESGSDSDVGSGGIRPKQPRMLQENTRMDMENEESMMSYEGDGGEASHGLEDSNISYGSYEEPDPKSNTQDTSFSSIGGYEVSEEEEDEEEEEQRSGPSVLSQVHLSEDEEDSEDFHSIAGDSDLDSDE

858 NP_005707.1 NP_000224.2

>NP_005707.1

MPLGLGRRKKAPPLVENEEAEPGRGGLGVGEPGPLGGGGSGGPQMGLPPPPPALRPRLVFHTQLAHGSPTGRIEGFTNVKELYGKIAEAFRLPTAEVMFCTLNTHKVDMDKLLGGQIGLEDFIFAHVKGQRKEVEVFKSEDALGLTITDNGAGYAFIKRIKEGSVIDHIHLISVGDMIEAINGQSLLGCRHYEVARLLKELPRGRTFTLKLTEPRKAFDMISQRSAGGRPGSGPQLGTGRGTLRLRSRGPATVEDLPSAFEEKAIEKVDDLLESYMGIRDTELAATMVELGKDKRNPDELAEALDERLGDFAFPDEFVFDVWGAIGDAKVGRY

>NP_000224.2

MKQRFSALQLLKLLLLLQPPLPRALREALCPEPCNCVPDGALRCPGPTAGLTRLSLAYLPVKVIPSQAFRGLNEVIKIEISQIDSLERIEANAFDNLLNLSEILIQNTKNLRYIEPGAFINLPRLKYLSICNTGIRKFPDVTKVFSSESNFILEICDNLHITTIPGNAFQGMNNESVTLKLYGNGFEEVQSHAFNGTTLTSLELKENVHLEKMHNGAFRGATGPKTLDISSTKLQALPSYGLESIQRLIATSSYSLKKLPSRETFVNLLEATLTYPSHCCAFRNLPTKEQNFSHSISENFSKQCESTVRKVNNKTLYSSMLAESELSGWDYEYGFCLPKTPRCAPEPDAFNPCEDIMGYDFLRVLIWLINILAIMGNMTVLFVLLTSRYKLTVPRFLMCNLSFADFCMGLYLLLIASVDSQTKGQYYNHAIDWQTGSGCSTAGFFTVFASELSVYTLTVITLERWHTITYAIHLDQKLRLRHAILIMLGGWLFSSLIAMLPLVGVSNYMKVSICFPMDVETTLSQVYILTILILNVVAFFIICACYIKIYFAVRNPELMATNKDTKIAKKMAILIFTDFTCMAPISFFAISAAFKVPLITVTNSKVLLVLFYPINSCANPFLYAIFTKTFQRDFFLLLSKFGCCKRRAELYRRKDFSAYTSNCKNGFTGSNKPSQSTLKLSTLHCQGTALLDKTRYTEC

859 NP_002382.1 NP_004295.2

>NP_002382.1

MQHRGFLLLTLLALLALTSAVAKKKDKVKKGGPGSECAEWAWGPCTPSSKDCGVGFREGTCGAQTQRIRCRVPCNWKKEFGADCKYKFENWGACDGGTGTKVRQGTLKKARYNAQCQETIRVTKPCTPKTKAKAKAKKGKGKD

>NP_004295.2

MGAIGLLWLLPLLLSTAAVGSGMGTGQRAGSPAAGPPLQPREPLSYSRLQRKSLAVDFVVPSLFRVYARDLLLPPSSSELKAGRPEARGSLALDCAPLLRLLGPAPGVSWTAGSPAPAEARTLSRVLKGGSVRKLRRAKQLVLELGEEAILEGCVGPPGEAAVGLLQFNLSELFSWWIRQGEGRLRIRLMPEKKASEVGREGRLSAAIRASQPRLLFQIFGTGHSSLESPTNMPSPSPDYFTWNLTWIMKDSFPFLSHRSRYGLECSFDFPCELEYSPPLHDLRNQSWSWRRIPSEEASQMDLLDGPGAERSKEMPRGSFLLLNTSADSKHTILSPWMRSSSEHCTLAVSVHRHLQPSGRYIAQLLPHNEAAREILLMPTPGKHGWTVLQGRIGRPDNPFRVALEYISSGNRSLSAVDFFALKNCSEGTSPGSKMALQSSFTCWNGTVLQLGQACDFHQDCAQGEDESQMCRKLPVGFYCNFEDGFCGWTQGTLSPHTPQWQVRTLKDARFQDHQDHALLLSTTDVPASESATVTSATFPAPIKSSPCELRMSWLIRGVLRGNVSLVLVENKTGKEQGRMVWHVAAYEGLSLWQWMVLPLLDVSDRFWLQMVAWWGQGSRAIVAFDNISISLDCYLTISGEDKILQNTAPKSRNLFERNPNKELKPGENSPRQTPIFDPTVHWLFTTCGASGPHGPTQAQCNNAYQNSNLSVEVGSEGPLKGIQIWKVPATDTYSISGYGAAGGKGGKNTMMRSHGVSVLGIFNLEKDDMLYILVGQQGEDACPSTNQLIQKVCIGENNVIEEEIRVNRSVHEWAGGGGGGGGATYVFKMKDGVPVPLIIAAGGGGRAYGAKTDTFHPERLENNSSVLGLNGNSGAAGGGGGWNDNTSLLWAGKSLQEGATGGHSCPQAMKKWGWETRGGFGGGGGGCSSGGGGGGYIGGNAASNNDPEMDGEDGVSFISPLGILYTPALKVMEGHGEVNIKHYLNCSHCEVDECHMDPESHKVICFCDHGTVLAEDGVSCIVSPTPEPHLPLSLILSVVTSALVAALVLAFSGIMIVYRRKHQELQAMQMELQSPEYKLSKLRTSTIMTDYNPNYCFAGKTSSISDLKEVPRKNITLIRGLGHGAFGEVYEGQVSGMPNDPSPLQVAVKTLPEVCSEQDELDFLMEALIISKFNHQNIVRCIGVSLQSLPRFILLELMAGGDLKSFLRETRPRPSQPSSLAMLDLLHVARDIACGCQYLEENHFIHRDIAARNCLLTCPGPGRVAKIGDFGMARDIYRASYYRKGGCAMLPVKWMPPEAFMEGIFTSKTDTWSFGVLLWEIFSLGYMPYPSKSNQEVLEFVTSGGRMDPPKNCPGPVYRIMTQCWQHQPEDRPNFAIILERIEYCTQDPDVINTALPIEYGPLVEEEEKVPVRPKDPEGVPPLLVSQQAKREEERSPAAPPPLPTTSSGKAAKKPTAAEISVRVPRGPAVEGGHVNMAFSQSNPPSELHKVHGSRNKPTSLWNPTYGSWFTEKPTKKNNPIAKKEPHDRGNLGLEGSCTVPPNVATGRLPGASLLLEPSSLTANMKEVPLFRLRHFPCGNVNYGYQQQGLPLEAATAPGAGHYEDTILKSKNSMNQPGP

860 NP_612144.1 NP_004407.2

>NP_612144.1

MASHLRPPSPLLVRVYKSGPRVRRKLESYFQSSKSSGGGECTVSTQEHEAPGTFRVEFSERAAKERVLKKGEHQILVDEKPVPIFLVPTENSIKKNTRPQISSLTQSQAETPSGDMHQHEGHIPNAVDSCLQKIFLTVTADLNCNLFSKEQRAYITTLCPSIRKMEGHDGIEKVCGDFQDIERIHQFLSEQFLESEQKQQFSPSMTERKPLSQQERDSCISPSEPETKAEQKSNYFEVPLPYFEYFKYICPDKINSIEKRFGVNIEIQESSPNMVCLDFTSSRSGDLEAARESFASEFQKNTEPLKQECVSLADSKQANKFKQELNHQFTKLLIKEKGGELTLLGTQDDISAAKQKISEAFVKIPVKLFAANYMMNVIEVDSAHYKLLETELLQEISEIEKRYDICSKVSEKGQKTCILFESKDRQVDLSVHAYASFIDAFQHASCQLMREVLLLKSLGKERKHLHQTKFADDFRKRHPNVHFVLNQESMTLTGLPNHLAKAKQYVLKGGGMSSLAGKKLKEGHETPMDIDSDDSKAASPPLKGSVSSEASELDKKEKGICVICMDTISNKKVLPKCKHEFCAPCINKAMSYKPICPTCQTSYGIQKGNQPEGSMVFTVSRDSLPGYESFGTIVITYSMKAGIQTEEHPNPGKRYPGIQRTAYLPDNKEGRKVLKLLYRAFDQKLIFTVGYSRVLGVSDVITWNDIHHKTSRFGGPEMYGYPDPSYLKRVKEELKAKGIE

>NP_004407.2

MSRPGHGGLMPVNGLGFPPQNVARVVVWEWLNEHSRWRPYTATVCHHIENVLKEDARGSVVLGQVDAQLVPYIIDLQSMHQFRQDTGTMRPVRRNFYDPSSAPGKGIVWEWENDGGAWTAYDMDICITIQNAYEKQHPWLDLSSLGFCYLIYFNSMSQMNRQTRRRRRLRRRLDLAYPLTVGSIPKSQSWPVGASSGQPCSCQQCLLVNSTRAASNAILASQRRKAPPAPPLPPPPPPGGPPGALAVRPSATFTGAALWAAPAAGPAEPAPPPGAPPRSPGAPGGARTPGQNNLNRPGPQRTTSVSARASIPPGVPALPVKNLNGTGPVHPALAGMTGILLCAAGLPVCLTRAPKPILHPPPVSKSDVKPVPGVPGVCRKTKKKHLKKSKNPEDVVRRYMQKVKNPPDEDCTICMERLVTASGYEGVLRHKGVRPELVGRLGRCGHMYHLLCLVAMYSNGNKDGSLQCPTCKAIYGEKTGTQPPGKMEFHLIPHSLPGFPDTQTIRIVYDIPTGIQGPEHPNPGKKFTARGFPRHCYLPNNEKGRKVLRLLITAWERRLIFTIGTSNTTGESDTVVWNEIHHKTEFGSNLTGHGYPDASYLDNVLAELTAQGVSEAAAKA

861 NP_006020.4 NP_077292.1

>NP_006020.4

MAMTLLEDWCRGMDVNSQRALLVWGIPVNCDEAEIEETLQAAMPQVSYRMLGRMFWREENAKAALLELTGAVDYAAIPREMPGKGGVWKVLFKPPTSDAEFLERLHLFLAREGWTVQDVARVLGFQNPTPTPGPEMPAEMLNYILDNVIQPLVESIWYKRLTLFSGRDIPGPGEETFDPWLEHTNEVLEEWQVSDVEKRRRLMESLRGPAADVIRILKSNNPAITTAECLKALEQVFGSVESSRDAQIKFLNTYQNPGEKLSAYVIRLEPLLQKVVEKGAIDKDNVNQARLEQVIAGANHSGAIRRQLWLTGAGEGPAPNLFQLLVQIREEEAKEEEEEAEATLLQLGLEGHF

>NP_077292.1

MNILPKKSWHVRNKDNVARVRRDEAQAREEEKERERRVLLAQQEARTEFLRKKARHQNSLPELEAAEAGAPGSGPVDLFRELLEEGKGVIRGNKEYEEEKRQEKERQEKALGILTYLGQSAAEAQTQPPWYQLPPGRGGPPPGPAPDEKIKSRLDPLREMQKHLGKKRQHGGDEGSRSRKEKEGSEKQRPKEPPSLDQLRAERLRREAAERSRAEALLARVQGRALQEGQPEEDETDDRRRRYNSQFNPQLARRPRQQDPHLTH

862 NP_005782.1 NP_219484.1

>NP_005782.1

MAPQVWRRRTLERCLTEVGKATGRPECFLTIQEGLASKFTSLTKVLYDFNKILENGRIHGSPLQKLVIENFDDEQIWQQLELQNEPILQYFQNAVSETINDEDISLLPESEEQEREEDGSEIEADDKEDLEDLEEEEVSDMGNDDPEMGERAENSSKSDLRKSPVFSDEDSDLDFDISKLEQQSKVQNKGQGKPREKSIVDDKFFKLSEMEAYLENIEKEEERKDDNDEEEEDIDFFEDIDSDEDEGGLFGSKKLKSGKSSRNLKYKDFFDPVESDEDITNVHDDELDSNKEDDEIAEEEAEELSISETDEDDDLQENEDNKQHKESLKRVTFALPDDAETEDTGVLNVKKNSDEVKSSFEKRQEKMNEKIASLEKELLEKKPWQLQGEVTAQKRPENSLLEETLHFDHAVRMAPVITEETTLQLEDIIKQRIRDQAWDDVVRKEKPKEDAYEYKKRLTLDHEKSKLSLAEIYEQEYIKLNQQKTAEEENPEHVEIQKMMDSLFLKLDALSNFHFIPKPPVPEIKVVSNLPAITMEEVAPVSVSDAALLAPEEIKEKNKAGDIKTAAEKTATDKKRERRKKKYQKRMKIKEKEKRRKLLEKSSVDQAGKYSKTVASEKLKQLTKTGKASFIKDEGKDKALKSSQAFFSKLQDQVKMQINDAKKTEKKKKKRQDISVHKLKL

>NP_219484.1

MLRREARLRREYLYRKAREEAQRSAQERKERLRRALEENRLIPTELRREALALQGSLEFDDAGGEGVTSHVDDEYRWAGVEDPKVMITTSRDPSSRLKMFAKELKLVFPGAQRMNRGRHEVGALVRACKANGVTDLLVVHEHRGTPVGLIVSHLPFGPTAYFTLCNVVMRHDIPDLGTMSEAKPHLITHGFSSRLGKRVSDILRYLFPVPKDDSHRVITFANQDDYISFRHHVYKKTDHRNVELTEVGPRFELKLYMIRLGTLEQEATADVEWRWHPYTNTARKRVFLSTE

863 NP_001931.2 NP_003572.2

>NP_001931.2

MKTRQNKDSMSMRSGRKKEAPGPREELRSRGRASPGGVSTSSSDGKAEKSRQTAKKARVEEASTPKVNKQGRSEEISESESEETNAPKKTKTEQELPRPQSPSDLDSLDGRSLNDDGSSDPRDIDQDNRSTSPSIYSPGSVENDSDSSSGLSQGPARPYHPPPLFPPSPQPPDSTPRQPEASFEPHPSVTPTGYHAPMEPPTSRMFQAPPGAPPPHPQLYPGGTGGVLSGPPMGPKGGGAASSVGGPNGGKQHPPPTTPISVSSSGASGAPPTKPPTTPVGGGNLPSAPPPANFPHVTPNLPPPPALRPLNNASASPPGLGAQPLPGHLPSPHAMGQGMGGLPPGPEKGPTLAPSPHSLPPASSSAPAPPMRFPYSSSSSSSAAASSSSSSSSSSASPFPASQALPSYPHSFPPPTSLSVSNQPPKYTQPSLPSQAVWSQGPPPPPPYGRLLANSNAHPGPFPPSTGAQSTAHPPVSTHHHHHQQQQQQQQQQQQQQQQQQQHHGNSGPPPPGAFPHPLEGGSSHHAHPYAMSPSLGSLRPYPPGPAHLPPPHSQVSYSQAGPNGPPVSSSSNSSSSTSQGSYPCSHPSPSQGPQGAPYPFPPVPTVTTSSATLSTVIATVASSPAGYKTASPPGPPPYGKRAPSPGAYKTATPPGYKPGSPPSFRTGTPPGYRGTSPPAGPGTFKPGSPTVGPGPLPPAGPSGLPSLPPPPAAPASGPPLSATQIKQEPAEEYETPESPVPPARSPSPPPKVVDVPSHASQSARFNKHLDRGFNSCARSDLYFVPLEGSKLAKKRADLVEKVRREAEQRAREEKEREREREREKEREREKERELERSVKLAQEGRAPVECPSLGPVPHRPPFEPGSAVATVPPYLGPDTPALRTLSEYARPHVMSPGNRNHPFYVPLGAVDPGLLGYNVPALYSSDPAAREREREARERDLRDRLKPGFEVKPSELEPLHGVPGPGLDPFPRHGGLALQPGPPGLHPFPFHPSLGPLERERLALAAGPALRPDMSYAERLAAERQHAERVAALGNDPLARLQMLNVTPHHHQHSHIHSHLHLHQQDAIHAASASVHPLIDPLASGSHLTRIPYPAGTLPNPLLPHPLHENEVLRHQLFAAPYRDLPASLSAPMSAAHQLQAMHAQSAELQRLALEQQQWLHAHHPLHSVPLPAQEDYYSHLKKESDKPL

>NP_003572.2

MTEEVIVIAKWDYTAQQDQELDIKKNERLWLLDDSKTWWRVRNAANRTGYVPSNYVERKNSLKKGSLVKNLKDTLGLGKTRRKTSARDASPTPSTDAEYPANGSGADRIYDLNIPAFVKFAYVAEREDELSLVKGSRVTVMEKCSDGWWRGSYNGQIGWFPSNYVLEEVDEAAAESPSFLSLRKGASLSNGQGSRVLHVVQTLYPFSSVTEEELNFEKGETMEVIEKPENDPEWWKCKNARGQVGLVPKNYVVVLSDGPALHPAHAPQISYTGPSSSGRFAGREWYYGNVTRHQAECALNERGVEGDFLIRDSESSPSDFSVSLKASGKNKHFKVQLVDNVYCIGQRRFHTMDELVEHYKKAPIFTSEHGEKLYLVRALQ

864 NP_006387.1 NP_079331.3

>NP_006387.1

MALNGAEVDDFSWEPPTEAETKVLQARRERQDRISRLMGDYLLRGYRMLGETCADCGTILLQDKQRKIYCVACQELDSDVDKDNPALNAQAALSQAREHQLASASELPLGSRPAPQPPVPRPEHCEGAAAGLKAAQGPPAPAVPPNTDVMACTQTALLQKLTWASAELGSSTSLETSIQLCGLIRACAEALRSLQQLQH

>NP_079331.3

MGLKNKKNTEDPEEPLIASQSTEPEIGHLSPSKKETIMVTLHGATNLPACKDGSEPWPYVVVKSTSEEKNNQSSKAVTSVTSEPTRAPIWGDTVNVEIQAEDAGQEDVILKVVDNRKKQELLSYKIPIKYLRVFHPYHFELVKPTESGKADEATAKTQLYATVVRKSSFIPRYIGCNHMALEIFLRGVNEPLANNPNPIVVIARVVPNYKEFKVSQANRDLASVGLPITPLSFPIPSMMNFDVPRVSQNGCPQLSKPGGPPEQPLWNQSFLFQGRDGATSFSEDTALVLEYYSSTSMKGSQPWTLNQPLGISVLPLKSRLYQKMLTGKGLDGLHVERLPIMDTSLKTINDEAPTVALSFQLLSSERPENFLTPNNSKALPTLDPKILDKKLRTIQESWSKDTVSSTMDLSTSTPREAEEEPLVPEMSHDTEMNNYRRAMQKMAEDILSLRRQASILEGENRILRSRLAQQEEEEGQGKASEAQNTVSMKQKLLLSELDMKKLRDRVQHLQNELIRKNDREKELLLLYQAQQPQAALLKQYQGKLQKMKALEETVRHQEKVIEKMERVLEDRLQDRSKPPPLNRQQGKPYTGFPMLSASGLPLGSMGENLPVELYSVLLAENAKLRTELDKNRHQQAPIILQQQALPDLLSGTSDKFNLLAKLEHAQSRILSLESQLEDSARRWGREKQDLATRLQEQEKGFRHPSNSIIIEQPSALTHSMDLKQPSELEPLLPSSDSKLNKPLSPQKETANSQQT

865 NP_003921.2 NP_001456.3

>NP_003921.2

MPNPSSTSSPYPLPEEIRNLLADVETFVADILKGENLSKKAKEKRESLIKKIKDVKSIYLQEFQDKGDAEDGEEYDDPFAGPPDTISLASERYDKDDEAPSDGAQFPPIAAQDLPFVLKAGYLEKRRKDHSFLGFEWQKRWCALSKTVFYYYGSDKDKQQKGEFAIDGYSVRMNNTLRKDGKKDCCFEISAPDKRIYQFTAASPKDAEEWVQQLKFVLQDMESDIIPEDYDERGELYDDVDHPLPISNPLTSSQPIDDEIYEELPEEEEDSAPVKVEEQRKMSQDSVHHTSGDKSTDYANFYQGLWDCTGAFSDELSFKRGDVIYILSKEYNRYGWWVGEMKGAIGLVPKAYIMEMYDI

>NP_001456.3

MAKYNTGGNPTEDVSVNSRPFRVTGPNSSSGIQARKNLFNNQGNASPPAGPSNVPKFGSPKPPVAVKPSSEEKPDKEPKPPFLKPTGAGQRFGTPASLTTRDPEAKVGFLKPVGPKPINLPKEDSKPTFPWPPGNKPSLHSVNQDHDLKPLGPKSGPTPPTSENEQKQAFPKLTGVKGKFMSASQDLEPKPLFPKPAFGQKPPLSTENSHEDESPMKNVSSSKGSPAPLGVRSKSGPLKPAREDSENKDHAGEISSLPFPGVVLKPAASRGGPGLSKNGEEKKEDRKIDAAKNTFQSKINQEELASGTPPARFPKAPSKLTVGGPWGQSQEKEKGDKNSATPKQKPLPPLFTLGPPPPKPNRPPNVDLTKFHKTSSGNSTSKGQTSYSTTSLPPPPPSHPASQPPLPASHPSQPPVPSLPPRNIKPPFDLKSPVNEDNQDGVTHSDGAGNLDEEQDSEGETYEDIEASKEREKKREKEEKKRLELEKKEQKEKEKKEQEIKKKFKLTGPIQVIHLAKACCDVKGGKNELSFKQGEQIEIIRITDNPEGKWLGRTARGSYGYIKTTAVEIDYDSLKLKKDSLGAPSRPIEDDQEVYDDVAEQDDISSHSQSGSGGIFPPPPDDDIYDGIEEEDADDGSTLQVQEKSNTWSWGILKMLKGKDDRKKSIREKPKVSDSDNNEGSSFPAPPKQLDMGDEVYDDVDTSDFPVSSAEMSQGTNVGKAKTEEKDLKKLKKQEKEEKDFRKKFKYDGEIRVLYSTKVTTSITSKKWGTRDLQVKPGESLEVIQTTDDTKVLCRNEEGKYGYVLRSYLADNDGEIYDDIADGCIYDND

866 NP_976225.1 NP_003394.1

>NP_976225.1

MSTKNFRVSDGDWICPDKKCGNVNFARRTSCNRCGREKTTEAKMMKAGGTEIGKTLAEKSRGLFSANDWQCKTCSNVNWARRSECNMCNTPKYAKLEERTGYGGGFNERENVEYIEREESDGEYDEFGRKKKKYRGKAVGPASILKEVEDKESEGEEEDEDEDLSKYKLDEDEDEDDADLSKYNLDASEEEDSNKKKSNRRSRSKSRSSHSRSSSRSSSPSSSRSRSRSRSRSSSSSQSRSRSSSRERSRSRGSKSRSSSRSHRGSSSPRKRSYSSSSSSPERNRKRSRSRSSSSGDRKKRRTRSRSPERRHRSSSGSSHSGSRSSSKKK

>NP_003394.1

MASGDTLYIATDGSEMPAEIVELHEIEVETIPVETIETTVVGEEEEEDDDDEDGGGGDHGGGGGHGHAGHHHHHHHHHHHPPMIALQPLVTDDPTQVHHHQEVILVQTREEVVGGDDSDGLRAEDGFEDQILIPVPAPAGGDDDYIEQTLVTVAAAGKSGGGGSSSSGGGRVKKGGGKKSGKKSYLSGGAGAAGGGGADPGNKKWEQKQVQIKTLEGEFSVTMWSSDEKKDIDHETVVEEQIIGENSPPDYSEYMTGKKLPPGGIPGIDLSDPKQLAEFARMKPRKIKEDDAPRTIACPHKGCTKMFRDNSAMRKHLHTHGPRVHVCAECGKAFVESSKLKRHQLVHTGEKPFQCTFEGCGKRFSLDFNLRTHVRIHTGDRPYVCPFDGCNKKFAQSTNLKSHILTHAKAKNNQ

867 NP_003397.1 NP_004584.1

>NP_003397.1

MDKNELVQKAKLAEQAERYDDMAACMKSVTEQGAELSNEERNLLSVAYKNVVGARRSSWRVVSSIEQKTEGAEKKQQMAREYREKIETELRDICNDVLSLLEKFLIPNASQAESKVFYLKMKGDYYRYLAEVAAGDDKKGIVDQSQQAYQEAFEISKKEMQPTHPIRLGLALNFSVFYYEILNSPEKACSLAKTAFDEAIAELDTLSEESYKDSTLIMQLLRDNLTLWTSDTQGDEAEAGEGGEN

>NP_004584.1

MSDSGEQNYGERESRSASRSGSAHGSGKSARHTPARSRSKEDSRRSRSKSRSRSESRSRSRRSSRRHYTRSRSRSRSHRRSRSRSYSRDYRRRHSHSHSPMSTRRRHVGNRANPDPNCCLGVFGLSLYTTERDLREVFSKYGPIADVSIVYDQQSRRSRGFAFVYFENVDDAKEAKERANGMELDGRRIRVDFSITKRPHTPTPGIYMGRPTYGSSRRRDYYDRGYDRGYDDRDYYSRSYRGGGGGGGGWRAAQDRDQIYRRRSPSPYYSRGGYRSRSRSRSYSPRRY

868 NP_620637.1 NP_008835.5

>NP_620637.1

MSRSKRDNNFYSVEIGDSTFTVLKRYQNLKPIGSGAQGIVCAAYDAILERNVAIKKLSRPFQNQTHAKRAYRELVLMKCVNHKNIIGLLNVFTPQKSLEEFQDVYIVMELMDANLCQVIQMELDHERMSYLLYQMLCGIKHLHSAGIIHRDLKPSNIVVKSDCTLKILDFGLARTAGTSFMMTPYVVTRYYRAPEVILGMGYKENVDLWSVGCIMGEMVCHKILFPGRDYIDQWNKVIEQLGTPCPEFMKKLQPTVRTYVENRPKYAGYSFEKLFPDVLFPADSEHNKLKASQARDLLSKMLVIDASKRISVDEALQHPYINVWYDPSEAEAPPPKIPDKQLDEREHTIEEWKELIYKEVMDLEERTKNGVIRGQPSPLGAAVINGSQHPSSSSSVNDVSSMSTDPTLASDTDSSLEAAAGPLGCCR

>NP_008835.5

MAGSGAGVRCSLLRLQETLSAADRCGAALAGHQLIRGLGQECVLSSSPAVLALQTSLVFSRDFGLLVFVRKSLNSIEFRECREEILKFLCIFLEKMGQKIAPYSVEIKNTCTSVYTKDRAAKCKIPALDLLIKLLQTFRSSRLMDEFKIGELFSKFYGELALKKKIPDTVLEKVYELLGLLGEVHPSEMINNAENLFRAFLGELKTQMTSAVREPKLPVLAGCLKGLSSLLCNFTKSMEEDPQTSREIFNFVLKAIRPQIDLKRYAVPSAGLRLFALHASQFSTCLLDNYVSLFEVLLKWCAHTNVELKKAALSALESFLKQVSNMVAKNAEMHKNKLQYFMEQFYGIIRNVDSNNKELSIAIRGYGLFAGPCKVINAKDVDFMYVELIQRCKQMFLTQTDTGDDRVYQMPSFLQSVASVLLYLDTVPEVYTPVLEHLVVMQIDSFPQYSPKMQLVCCRAIVKVFLALAAKGPVLRNCISTVVHQGLIRICSKPVVLPKGPESESEDHRASGEVRTGKWKVPTYKDYVDLFRHLLSSDQMMDSILADEAFFSVNSSSESLNHLLYDEFVKSVLKIVEKLDLTLEIQTVGEQENGDEAPGVWMIPTSDPAANLHPAKPKDFSAFINLVEFCREILPEKQAEFFEPWVYSFSYELILQSTRLPLISGFYKLLSITVRNAKKIKYFEGVSPKSLKHSPEDPEKYSCFALFVKFGKEVAVKMKQYKDELLASCLTFLLSLPHNIIELDVRAYVPALQMAFKLGLSYTPLAEVGLNALEEWSIYIDRHVMQPYYKDILPCLDGYLKTSALSDETKNNWEVSALSRAAQKGFNKVVLKHLKKTKNLSSNEAISLEEIRIRVVQMLGSLGGQINKNLLTVTSSDEMMKSYVAWDREKRLSFAVPFREMKPVIFLDVFLPRVTELALTASDRQTKVAACELLHSMVMFMLGKATQMPEGGQGAPPMYQLYKRTFPVLLRLACDVDQVTRQLYEPLVMQLIHWFTNNKKFESQDTVALLEAILDGIVDPVDSTLRDFCGRCIREFLKWSIKQITPQQQEKSPVNTKSLFKRLYSLALHPNAFKRLGASLAFNNIYREFREEESLVEQFVFEALVIYMESLALAHADEKSLGTIQQCCDAIDHLCRIIEKKHVSLNKAKKRRLPRGFPPSASLCLLDLVKWLLAHCGRPQTECRHKSIELFYKFVPLLPGNRSPNLWLKDVLKEEGVSFLINTFEGGGCGQPSGILAQPTLLYLRGPFSLQATLCWLDLLLAALECYNTFIGERTVGALQVLGTEAQSSLLKAVAFFLESIAMHDIIAAEKCFGTGAAGNRTSPQEGERYNYSKCTVVVRIMEFTTTLLNTSPEGWKLLKKDLCNTHLMRVLVQTLCEPASIGFNIGDVQVMAHLPDVCVNLMKALKMSPYKDILETHLREKITAQSIEELCAVNLYGPDAQVDRSRLAAVVSACKQLHRAGLLHNILPSQSTDLHHSVGTELLSLVYKGIAPGDERQCLPSLDLSCKQLASGLLELAFAFGGLCERLVSLLLNPAVLSTASLGSSQGSVIHFSHGEYFYSLFSETINTELLKNLDLAVLELMQSSVDNTKMVSAVLNGMLDQSFRERANQKHQGLKLATTILQHWKKCDSWWAKDSPLETKMAVLALLAKILQIDSSVSFNTSHGSFPEVFTTYISLLADTKLDLHLKGQAVTLLPFFTSLTGGSLEELRRVLEQLIVAHFPMQSREFPPGTPRFNNYVDCMKKFLDALELSQSPMLLELMTEVLCREQQHVMEELFQSSFRRIARRGSCVTQVGLLESVYEMFRKDDPRLSFTRQSFVDRSLLTLLWHCSLDALREFFSTIVVDAIDVLKSRFTKLNESTFDTQITKKMGYYKILDVMYSRLPKDDVHAKESKINQVFHGSCITEGNELTKTLIKLCYDAFTENMAGENQLLERRRLYHCAAYNCAISVICCVFNELKFYQGFLFSEKPEKNLLIFENLIDLKRRYNFPVEVEVPMERKKKYIEIRKEAREAANGDSDGPSYMSSLSYLADSTLSEEMSQFDFSTGVQSYSYSSQDPRPATGRFRRREQRDPTVHDDVLELEMDELNRHECMAPLTALVKHMHRSLGPPQGEEDSVPRDLPSWMKFLHGKLGNPIVPLNIRLFLAKLVINTEEVFRPYAKHWLSPLLQLAASENNGGEGIHYMVVEIVATILSWTGLATPTGVPKDEVLANRLLNFLMKHVFHPKRAVFRHNLEIIKTLVECWKDCLSIPYRLIFEKFSGKDPNSKDNSVGIQLLGIVMANDLPPYDPQCGIQSSEYFQALVNNMSFVRYKEVYAAAAEVLGLILRYVMERKNILEESLCELVAKQLKQHQNTMEDKFIVCLNKVTKSFPPLADRFMNAVFFLLPKFHGVLKTLCLEVVLCRVEGMTELYFQLKSKDFVQVMRHRDDERQKVCLDIIYKMMPKLKPVELRELLNPVVEFVSHPSTTCREQMYNILMWIHDNYRDPESETDNDSQEIFKLAKDVLIQGLIDENPGLQLIIRNFWSHETRLPSNTLDRLLALNSLYSPKIEVHFLSLATNFLLEMTSMSPDYPNPMFEHPLSECEFQEYTIDSDWRFRSTVLTPMFVETQASQGTLQTRTQEGSLSARWPVAGQIRATQQQHDFTLTQTADGRSSFDWLTGSSTDPLVDHTSPSSDSLLFAHKRSERLQRAPLKSVGPDFGKKRLGLPGDEVDNKVKGAAGRTDLLRLRRRFMRDQEKLSLMYARKGVAEQKREKEIKSELKMKQDAQVVLYRSYRHGDLPDIQIKHSSLITPLQAVAQRDPIIAKQLFSSLFSGILKEMDKFKTLSEKNNITQKLLQDFNRFLNTTFSFFPPFVSCIQDISCQHAALLSLDPAAVSAGCLASLQQPVGIRLLEEALLRLLPAELPAKRVRGKARLPPDVLRWVELAKLYRSIGEYDVLRGIFTSEIGTKQITQSALLAEARSDYSEAAKQYDEALNKQDWVDGEPTEAEKDFWELASLDCYNHLAEWKSLEYCSTASIDSENPPDLNKIWSEPFYQETYLPYMIRSKLKLLLQGEADQSLLTFIDKAMHGELQKAILELHYSQELSLLYLLQDDVDRAKYYIQNGIQSFMQNYSSIDVLLHQSRLTKLQSVQALTEIQEFISFISKQGNLSSQVPLKRLLNTWTNRYPDAKMDPMNIWDDIITNRCFFLSKIEEKLTPLPEDNSMNVDQDGDPSDRMEVQEQEEDISSLIRSCKFSMKMKMIDSARKQNNFSLAMKLLKELHKESKTRDDWLVSWVQSYCRLSHCRSRSQGCSEQVLTVLKTVSLLDENNVSSYLSKNILAFRDQNILLGTTYRIIANALSSEPACLAEIEEDKARRILELSGSSSEDSEKVIAGLYQRAFQHLSEAVQAAEEEAQPPSWSCGPAAGVIDAYMTLADFCDQQLRKEEENASVIDSAELQAYPALVVEKMLKALKLNSNEARLKFPRLLQIIERYPEETLSLMTKEISSVPCWQFISWISHMVALLDKDQAVAVQHSVEEITDNYPQAIVYPFIISSESYSFKDTSTGHKNKEFVARIKSKLDQGGVIQDFINALDQLSNPELLFKDWSNDVRAELAKTPVNKKNIEKMYERMYAALGDPKAPGLGAFRRKFIQTFGKEFDKHFGKGGSKLLRMKLSDFNDITNMLLLKMNKDSKPPGNLKECSPWMSDFKVEFLRNELEIPGQYDGRGKPLPEYHVRIAGFDERVTVMASLRRPKRIIIRGHDEREHPFLVKGGEDLRQDQRVEQLFQVMNGILAQDSACSQRALQLRTYSVVPMTSRLGLIEWLENTVTLKDLLLNTMSQEEKAAYLSDPRAPPCEYKDWLTKMSGKHDVGAYMLMYKGANRTETVTSFRKRESKVPADLLKRAFVRMSTSPEAFLALRSHFASSHALICISHWILGIGDRHLNNFMVAMETGGVIGIDFGHAFGSATQFLPVPELMPFRLTRQFINLMLPMKETGLMYSIMVHALRAFRSDPGLLTNTMDVFVKEPSFDWKNFEQKMLKKGGSWIQEINVAEKNWYPRQKICYAKRKLAGANPAVITCDELLLGHEKAPAFRDYVAVARGSKDHNIRAQEPESGLSEETQVKCLMDQATDPNILGRTWEGWEPWM

869 NP_063949.2 NP_006222.2

>NP_063949.2

MAAAAAAGSGTPREEEGPAGEAAASQPQAPTSVPGARLSRLPLARVKALVKADPDVTLAGQEAIFILARAAELFVETIAKDAYCCAQQGKRKTLQRRDLDNAIEAVDEFAFLEGTLD

>NP_006222.2

MSLRSGGRRRADPGADGEASRDDGATSSVSALKRLERSQWTDKMDLRFGFERLKEPGEKTGWLINMHPTEILDEDKRLGSAVDYYFIQDDGSRFKVALPYKPYFYIATRKGCEREVSSFLSKKFQGKIAKVETVPKEDLDLPNHLVGLKRNYIRLSFHTVEDLVKVRKEISPAVKKNREQDHASDAYTALLSSVLQRGGVITDEEETSKKIADQLDNIVDMREYDVPYHIRLSIDLKIHVAHWYNVRYRGNAFPVEITRRDDLVERPDPVVLAFDIETTKLPLKFPDAETDQIMMISYMIDGQGYLITNREIVSEDIEDFEFTPKPEYEGPFCVFNEPDEAHLIQRWFEHVQETKPTIMVTYNGDFFDWPFVEARAAVHGLSMQQEIGFQKDSQGEYKAPQCIHMDCLRWVKRDSYLPVGSHNLKAAAKAKLGYDPVELDPEDMCRMATEQPQTLATYSVSDAVATYYLYMKYVHPFIFALCTIIPMEPDEVLRKGSGTLCEALLMVQAFHANIIFPNKQEQEFNKLTDDGHVLDSETYVGGHVEALESGVFRSDIPCRFRMNPAAFDFLLQRVEKTLRHALEEEEKVPVEQVTNFEEVCDEIKSKLASLKDVPSRIECPLIYHLDVGAMYPNIILTNRLQPSAMVDEATCAACDFNKPGANCQRKMAWQWRGEFMPASRSEYHRIQHQLESEKFPPLFPEGPARAFHELSREEQAKYEKRRLADYCRKAYKKIHITKVEERLTTICQRENSFYVDTVRAFRDRRYEFKGLHKVWKKKLSAAVEVGDAAEVKRCKNMEVLYDSLQLAHKCILNSFYGYVMRKGARWYSMEMAGIVCFTGANIITQARELIEQIGRPLELDTDGIWCVLPNSFPENFVFKTTNVKKPKVTISYPGAMLNIMVKEGFTNDQYQELAEPSSLTYVTRSENSIFFEVDGPYLAMILPASKEEGKKLKKRYAVFNEDGSLAELKGFEVKRRGELQLIKIFQSSVFEAFLKGSTLEEVYGSVAKVADYWLDVLYSKAANMPDSELFELISENRSMSRKLEDYGEQKSTSISTAKRLAEFLGDQMVKDAGLSCRYIISRKPEGSPVTERAIPLAIFQAEPTVRKHFLRKWLKSSSLQDFDIRAILDWDYYIERLGSAIQKIITIPAALQQVKNPVPRVKHPDWLHKKLLEKNDVYKQKKISELFTLEGRRQVTMAEASEDSPRPSAPDMEDFGLVKLPHPAAPVTVKRKRVLWESQEESQDLTPTVPWQEILGQPPALGTSQEEWLVWLRFHKKKWQLQARQRLARRKRQRLESAEGVLRPGAIRDGPATGLGSFLRRTARSILDLPWQIVQISETSQAGLFRLWALVGSDLHCIRLSIPRVFYVNQRVAKAEEGASYRKVNRVLPRSNMVYNLYEYSVPEDMYQEHINEINAELSAPDIEGVYETQVPLLFRALVHLGCVCVVNKQLVRHLSGWEAETFALEHLEMRSLAQFSYLEPGSIRHIYLYHHAQAHKALFGIFIPSQRRASVFVLDTVRSNQMPSLGALYSAEHGLLLEKVGPELLPPPKHTFEVRAETDLKTICRAIQRFLLAYKEERRGPTLIAVQSSWELKRLASEIPVLEEFPLVPICVADKINYGVLDWQRHGARRMIRHYLNLDTCLSQAFEMSRYFHIPIGNLPEDISTFGSDLFFARHLQRHNHLLWLSPTARPDLGGKEADDNCLVMEFDDQATVEINSSGCYSTVCVELDLQNLAVNTILQSHHVNDMEGADSMGISFDVIQQASLEDMITGGQAASAPASYDETALCSNTFRILKSMVVGWVKEITQYHNIYADNQVMHFYRWLRSPSSLLHDPALHRTLHNMMKKLFLQLIAEFKRLGSSVIYANFNRIILCTKKRRVEDAIAYVEYITSSIHSKETFHSLTISFSRCWEFLLWMDPSNYGGIKGKVSSRIHCGLQDSQKAGGAEDEQENEDDEEERDGEEEEEAEESNVEDLLENNWNILQFLPQAASCQNYFLMIVSAYIVAVYHCMKDGLRRSAPGSTPVRRRGASQLSQEAEGAVGALPGMITFSQDYVANELTQSFFTITQKIQKKVTGSRNSTELSEMFPVLPGSHLLLNNPALEFIKYVCKVLSLDTNITNQVNKLNRDLLRLVDVGEFSEEAQFRDPCRSYVLPEVICRSCNFCRDLDLCKDSSFSEDGAVLPQWLCSNCQAPYDSSAIEMTLVEVLQKKLMAFTLQDLVCLKCRGVKETSMPVYCSCAGDFALTIHTQVFMEQIGIFRNIAQHYGMSYLLETLEWLLQKNPQLGH

870 NP_009148.2 NP_003567.2

>NP_009148.2

MRMTMEEMKNEAETTSMVSMPLYAVMYPVFNELERVNLSAAQTLRAAFIKAEKENPGLTQDIIMKILEKKSVEVNFTESLLRMAADDVEEYMIERPEPEFQDLNEKARALKQILSKIPDEINDRVRFLQTIKDIASAIKELLDTVNNVFKKYQYQNRRALEHQKKEFVKYSKSFSDTLKTYFKDGKAINVFVSANRLIHQTNLILQTFKTVA

>NP_003567.2

MDSRAQLWGLALNKRRATLPHPGGSTNLKADPEELFTKLEKIGKGSFGEVFKGIDNRTQKVVAIKIIDLEEAEDEIEDIQQEITVLSQCDSPYVTKYYGSYLKDTKLWIIMEYLGGGSALDLLEPGPLDETQIATILREILKGLDYLHSEKKIHRDIKAANVLLSEHGEVKLADFGVAGQLTDTQIKRNTFVGTPFWMAPEVIKQSAYDSKADIWSLGITAIELARGEPPHSELHPMKVLFLIPKNNPPTLEGNYSKPLKEFVEACLNKEPSFRPTAKELLKHKFILRNAKKTSYLTELIDRYKRWKAEQSHDDSSSEDSDAETDGQASGGSDSGDWIFTIREKDPKNLENGALQPSDLDRNKMKDIPKRPFSQCLSTIISPLFAELKEKSQACGGNLGSIEELRGAIYLAEEACPGISDTMVAQLVQRLQRYSLSGGGTSSH

871 NP_002721.1 NP_004313.1

>NP_002721.1

MGNAAAAKKGSEQESVKEFLAKAKEDFLKKWESPAQNTAHLDQFERIKTLGTGSFGRVMLVKHKETGNHYAMKILDKQKVVKLKQIEHTLNEKRILQAVNFPFLVKLEFSFKDNSNLYMVMEYVPGGEMFSHLRRIGRFSEPHARFYAAQIVLTFEYLHSLDLIYRDLKPENLLIDQQGYIQVTDFGFAKRVKGRTWTLCGTPEYLAPEIILSKGYNKAVDWWALGVLIYEMAAGYPPFFADQPIQIYEKIVSGKVRFPSHFSSDLKDLLRNLLQVDLTKRFGNLKNGVNDIKNHKWFATTDWIAIYQRKVEAPFIPKFKGPGDTSNFDDYEEEEIRVSINEKCGKEFSEF

>NP_004313.1

MFQIPEFEPSEQEDSSSAERGLGPSPAGDGPSGSGKHHRQAPGLLWDASHQQEQPTSSSHHGGAGAVEIRSRHSSYPAGTEDDEGMGEEPSPFRGRSRSAPPNLWAAQRYGRELRRMSDEFVDSFKKGLPRPKSAGTATQMRQSSSWTRVFQSWWDRNLGRGSSAPSQ

872 NP_000607.1 NP_001900.1

>NP_000607.1

MNRGVPFRHLLLVLQLALLPAATQGKKVVLGKKGDTVELTCTASQKKSIQFHWKNSNQIKILGNQGSFLTKGPSKLNDRADSRRSLWDQGNFPLIIKNLKIEDSDTYICEVEDQKEEVQLLVFGLTANSDTHLLQGQSLTLTLESPPGSSPSVQCRSPRGKNIQGGKTLSVSQLELQDSGTWTCTVLQNQKKVEFKIDIVVLAFQKASSIVYKKEGEQVEFSFPLAFTVEKLTGSGELWWQAERASSSKSWITFDLKNKEVSVKRVTQDPKLQMGKKLPLHLTLPQALPQYAGSGNLTLALEAKTGKLHQEVNLVVMRATQLQKNLTCEVWGPTSPKLMLSLKLENKEAKVSKREKAVWVLNPEAGMWQCLLSDSGQVLLESNIKVLPTWSTPVQPMALIVLGGVAGLLLFIGLGIFFCVRCRHRRRQAERMSQIKRLLSEKKTCQCPHRFQKTCSPI

>NP_001900.1

MQPSSLLPLALCLLAAPASALVRIPLHKFTSIRRTMSEVGGSVEDLIAKGPVSKYSQAVPAVTEGPIPEVLKNYMDAQYYGEIGIGTPPQCFTVVFDTGSSNLWVPSIHCKLLDIACWIHHKYNSDKSSTYVKNGTSFDIHYGSGSLSGYLSQDTVSVPCQSASSASALGGVKVERQVFGEATKQPGITFIAAKFDGILGMAYPRISVNNVLPVFDNLMQQKLVDQNIFSFYLSRDPDAQPGGELMLGGTDSKYYKGSLSYLNVTRKAYWQVHLDQVEVASGLTLCKEGCEAIVDTGTSLMVGPVDEVRELQKAIGAVPLIQGEYMIPCEKVSTLPAITLKLGGKGYKLSPEDYTLKVSQAGKTLCLSGFMGMDIPPPSGPLWILGDVFIGRYYTVFDRDNNRVGFAEAARL

873 NP_689729.1 NP_060298.2

>NP_689729.1

MAAGELEGGKPLSGLLNALAQDTFHGYPGITEELLRSQLYPEVPPEEFRPFLAKMRGILKSIASADMDFNQLEAFLTAQTKKQGGITSDQAAVISKFWKSHKTKIRESLMNQSRWNSGLRGLSWRVDGKSQSRHSAQIHTPVAIIELELGKYGQESEFLCLEFDEVKVNQILKTLSEVEESISTLISQPN

>NP_060298.2

MRFRFCGDLDCPDWVLAEISTLAKMSSVKLRLLCSQVLKELLGQGIDYEKILKLTADAKFESGDVKATVAVLSFILSSAAKHSVDGESLSSELQQLGLPKEHAASLCRCYEEKQSPLQKHLRVCSLRMNRLAGVGWRVDYTLSSSLLQSVEEPMVHLRLEVAAAPGTPAQPVAMSLSADKFQVLLAELKQAQTLMSSLG

874 NP_115687.2 NP_006221.1

>NP_115687.2

MADISLDELIRKRGAAAKGRLNARPGVGGVRSRVGIQQGLLSQSTRTATFQQRFDARQKIGLSDARLKLGVKDAREKLLQKDARFRIKGKVQDAREMLNSRKQQTTVPQKPRQVADAREKISLKRSSPAAFINPPIGTVTPALKLTKTIQNLYDLDEDDDGIASVPTKQMKFAASGGFLHHMAGLSSSKLSMSKALPLTKVVQNDAYTAPALPSSIRTKALTNMSRTLVNKEEPPKELPAAEPVLSPLEGTKMTVNNLHPRVTEEDIVELFCVCGALKRARLVHPGVAEVVFVKKDDAITAYKKYNNRCLDGQPMKCNLHMNGNVITSDQPILLRLSDSPSMKKESELPRRVNSASSSNPPAEVDPDTILKALFKSSGASVTTQPTEFKIKL

>NP_006221.1

MFSEQAAQRAHTLLSPPSANNATFARVPVATYTNSSQPFRLGERSFSRQYAHIYATRLIQMRPFLENRAQQHWGSGVGVKKLCELQPEEKCCVVGTLFKAMPLQPSILREVSEEHNLLPQPPRSKYIHPDDELVLEDELQRIKLKGTIDVSKLVTGTVLAVFGSVRDDGKFLVEDYCFADLAPQKPAPPLDTDRFVLLVSGLGLGGGGGESLLGTQLLVDVVTGQLGDEGEQCSAAHVSRVILAGNLLSHSTQSRDSINKAKYLTKKTQAASVEAVKMLDEILLQLSASVPVDVMPGEFDPTNYTLPQQPLHPCMFPLATAYSTLQLVTNPYQATIDGVRFLGTSGQNVSDIFRYSSMEDHLEILEWTLRVRHISPTAPDTLGCYPFYKTDPFIFPECPHVYFCGNTPSFGSKIIRGPEDQTVLLVTVPDFSATQTACLVNLRSLACQPISFSGFGAEDDDLGGLGLGP

875 NP_002361.1 NP_006817.1

>NP_002361.1

MESDFYLRYYVGHKGKFGHEFLEFEFRPDGKLRYANNSNYKNDVMIRKEAYVHKSVMEELKRIIDDSEITKEDDALWPPPDRVGRQELEIVIGDEHISFTTSKIGSLIDVNQSKDPEGLRVFYYLVQDLKCLVFSLIGLHFKIKPI

>NP_006817.1

MEKTELIQKAKLAEQAERYDDMATCMKAVTEQGAELSNEERNLLSVAYKNVVGGRRSAWRVISSIEQKTDTSDKKLQLIKDYREKVESELRSICTTVLELLDKYLIANATNPESKVFYLKMKGDYFRYLAEVACGDDRKQTIDNSQGAYQEAFDISKKEMQPTHPIRLGLALNFSVFYYEILNNPELACTLAKTAFDEAIAELDTLNEDSYKDSTLIMQLLRDNLTLWTSDSAGEECDAAEGAEN

876 NP_001524.2 NP_006330.2

>NP_001524.2

MSRRLLPRAEKRRRRLEQRQQPDEQRRRSGAMVKMAAAGGGGGGGRYYGGGSEGGRAPKRLKTDNAGDQHGGGGGGGGGAGAAGGGGGGENYDDPHKTPASPVVHIRGLIDGVVEADLVEALQEFGPISYVVVMPKKRQALVEFEDVLGACNAVNYAADNQIYIAGHPAFVNYSTSQKISRPGDSDDSRSVNSVLLFTILNPIYSITTDVLYTICNPCGPVQRIVIFRKNGVQAMVEFDSVQSAQRAKASLNGADIYSGCCTLKIEYAKPTRLNVFKNDQDTWDYTNPNLSGQGDPGSNPNKRQRQPPLLGDHPAEYGGPHGGYHSHYHDEGYGPPPPHYEGRRMGPPVGGHRRGPSRYGPQYGHPPPPPPPPEYGPHADSPVLMVYGLDQSKMNCDRVFNVFCLYGNVEKVKFMKSKPGAAMVEMADGYAVDRAITHLNNNFMFGQKLNVCVSKQPAIMPGQSYGLEDGSCSYKDFSESRNNRFSTPEQAAKNRIQHPSNVLHFFNAPLEVTEENFFEICDELGVKRPSSVKVFSGKSERSSSGLLEWESKSDALETLGFLNHYQMKNPNGPYPYTLKLCFSTAQHAS

>NP_006330.2

MSHGPKQPGAAAAPAGGKAPGQHGGFVVTVKQERGEGPRAGEKGSHEEEPVKKRGWPKGKKRKKILPNGPKAPVTGYVRFLNERREQIRTRHPDLPFPEITKMLGAEWSKLQPTEKQRYLDEAEREKQQYMKELRAYQQSEAYKMCTEKIQEKKIKKEDSSSGLMNTLLNGHKGGDCDGFSTFDVPIFTEEFLDQNKAREAELRRLRKMNVAFEEQNAVLQRHTQSMSSARERLEQELALEERRTLALQQQLQAVRQALTASFASLPVPGTGETPTLGTLDFYMARLHGAIERDPAQHEKLIVRIKEILAQVASEHL

877 NP_001306.1 NP_620777.1

>NP_001306.1

MSQERPTFYRQELNKTIWEVPERYQNLSPVGSGAYGSVCAAFDTKTGLRVAVKKLSRPFQSIIHAKRTYRELRLLKHMKHENVIGLLDVFTPARSLEEFNDVYLVTHLMGADLNNIVKCQKLTDDHVQFLIYQILRGLKYIHSADIIHRDLKPSNLAVNEDCELKILDFGLARHTDDEMTGYVATRWYRAPEIMLNWMHYNQTVDIWSVGCIMAELLTGRTLFPGTDHINQLQQIMRLTGTPPAYLINRMPSHEARNYIQSLTQMPKMNFANVFIGANPLAVDLLEKMLVLDSDKRITAAQALAHAYFAQYHDPDDEPVADPYDQSFESRDLLIDEWKSLTYDEVISFVPPPLDQEEMES

>NP_620777.1

MSEESDMDKAIKETSILEEYSINWTQKLGAGISGPVRVCVKKSTQERFALKILLDRPKARNEVRLHMMCATHPNIVQIIEVFANSVQFPHESSPRARLLIVMEMMEGGELFHRISQHRHFTEKQASQVTKQIALALRHCHLLNIAHRDLKPENLLFKDNSLDAPVKLCDFGFAKIDQGDLMTPQFTPYYVAPQVLEAQRRHQKEKSGIIPTSPTPYTYNKSCDLWSLGVIIYVMLCGYPPFYSKHHSRTIPKDMRRKIMTGSFEFPEEEWSQISEMAKDVVRKLLKVKPEERLTIEGVLDHPWLNSTEALDNVLPSAQLMMDKAVVAGIQQAHAEQLANMRIQDLKVSLKPLHSVNNPILRKRKLLGTKPKDSVYIHDHENGAEDSNVALEKLRDVIAQCILPQAGKGENEDEKLNEVMQEAWKYNRECKLLRDTLQSFSWNGRGFTDKVDRLKLAEIVKQVIEEQTTSHESQ

878 NP_006716.2 NP_001618.2

>NP_006716.2

MWLFFGITGLLTAALSGHPSPAPPDQLNTSSAESELWEPGERLPVRLTNGSSSCSGTVEVRLEASWEPACGALWDSRAAEAVCRALGCGGAEAASQLAPPTPELPPPPAAGNTSVAANATLAGAPALLCSGAEWRLCEVVEHACRSDGRRARVTCAENRALRLVDGGGACAGRVEMLEHGEWGSVCDDTWDLEDAHVVCRQLGCGWAVQALPGLHFMPGRGPIHRDQVNCSGAEAYLWDCPGLPGQHYCGHKEDAGVVCSEHQSWRLTGGADRCEGQVEVHFRGVWNTVCDSEWYPSEAKVLCQSLGCGTAVERPKGLPHSLSGRMYYSCNGEELTLSNCSWRFNNSNLCSQSLAARVLCSASRSLHNLSTPEVPASVQTVTIESSVTVKIENKESRELMLLIPSIVLGILLLGSLIFIAFILLRIKGKYALPVMVNHQHLPTTIPAGSNSYQPVPITIPKEVFMLPIQVQAPPPEDSDSGSDSDYEHYDFSAQPPVALTTFYNSQRHRVTDEEVQQSRFQMPPLEEGLEELHASHIPTANPGHCITDPPSLGPQYHPRSNSESSTSSGEDYCNSPKSKLPPWNPQVFSSERSSFLEQPPNLELASTQPAFSAGPPADDSSSTSSGEWYQNFQPPPQPPSEEQFGCPGSPSPQPDSTDNDDYDDISAA

>NP_001618.2

MESKGASSCRLLFCLLISATVFRPGLGWYTVNSAYGDTIIIPCRLDVPQNLMFGKWKYEKPDGSPVFIAFRSSTKKSVQYDDVPEYKDRLNLSENYTLSISNARISDEKRFVCMLVTEDNVFEAPTIVKVFKQPSKPEIVSKALFLETEQLKKLGDCISEDSYPDGNITWYRNGKVLHPLEGAVVIIFKKEMDPVTQLYTMTSTLEYKTTKADIQMPFTCSVTYYGPSGQKTIHSEQAVFDIYYPTEQVTIQVLPPKNAIKEGDNITLKCLGNGNPPPEEFLFYLPGQPEGIRSSNTYTLTDVRRNATGDYKCSLIDKKSMIASTAITVHYLDLSLNPSGEVTRQIGDALPVSCTISASRNATVVWMKDNIRLRSSPSFSSLHYQDAGNYVCETALQEVEGLKKRESLTLIVEGKPQIKMTKKTDPSGLSKTIICHVEGFPKPAIQWTITGSGSVINQTEESPYINGRYYSKIIISPEENVTLTCTAENQLERTVNSLNVSAISIPEHDEADEISDENREKVNDQAKLIVGIVVGLLLAALVAGVVYWLYMKKSKTASKHVNKDLGNMEENKKLEENNHKTEA

879 NP_000866.1 NP_003595.1

>NP_000866.1

MKSGSGGGSPTSLWGLLFLSAALSLWPTSGEICGPGIDIRNDYQQLKRLENCTVIEGYLHILLISKAEDYRSYRFPKLTVITEYLLLFRVAGLESLGDLFPNLTVIRGWKLFYNYALVIFEMTNLKDIGLYNLRNITRGAIRIEKNADLCYLSTVDWSLILDAVSNNYIVGNKPPKECGDLCPGTMEEKPMCEKTTINNEYNYRCWTTNRCQKMCPSTCGKRACTENNECCHPECLGSCSAPDNDTACVACRHYYYAGVCVPACPPNTYRFEGWRCVDRDFCANILSAESSDSEGFVIHDGECMQECPSGFIRNGSQSMYCIPCEGPCPKVCEEEKKTKTIDSVTSAQMLQGCTIFKGNLLINIRRGNNIASELENFMGLIEVVTGYVKIRHSHALVSLSFLKNLRLILGEEQLEGNYSFYVLDNQNLQQLWDWDHRNLTIKAGKMYFAFNPKLCVSEIYRMEEVTGTKGRQSKGDINTRNNGERASCESDVLHFTSTTTSKNRIIITWHRYRPPDYRDLISFTVYYKEAPFKNVTEYDGQDACGSNSWNMVDVDLPPNKDVEPGILLHGLKPWTQYAVYVKAVTLTMVENDHIRGAKSEILYIRTNASVPSIPLDVLSASNSSSQLIVKWNPPSLPNGNLSYYIVRWQRQPQDGYLYRHNYCSKDKIPIRKYADGTIDIEEVTENPKTEVCGGEKGPCCACPKTEAEKQAEKEEAEYRKVFENFLHNSIFVPRPERKRRDVMQVANTTMSSRSRNTTAADTYNITDPEELETEYPFFESRVDNKERTVISNLRPFTLYRIDIHSCNHEAEKLGCSASNFVFARTMPAEGADDIPGPVTWEPRPENSIFLKWPEPENPNGLILMYEIKYGSQVEDQRECVSRQEYRKYGGAKLNRLNPGNYTARIQATSLSGNGSWTDPVFFYVQAKTGYENFIHLIIALPVAVLLIVGGLVIMLYVFHRKRNNSRLGNGVLYASVNPEYFSAADVYVPDEWEVAREKITMSRELGQGSFGMVYEGVAKGVVKDEPETRVAIKTVNEAASMRERIEFLNEASVMKEFNCHHVVRLLGVVSQGQPTLVIMELMTRGDLKSYLRSLRPEMENNPVLAPPSLSKMIQMAGEIADGMAYLNANKFVHRDLAARNCMVAEDFTVKIGDFGMTRDIYETDYYRKGGKGLLPVRWMSPESLKDGVFTTYSDVWSFGVVLWEIATLAEQPYQGLSNEQVLRFVMEGGLLDKPDNCPDMLFELMRMCWQYNPKMRPSFLEIISSIKEEMEPGFREVSFYYSEENKLPEPEELDLEPENMESVPLDPSASSSSLPLPDRHSGHKAENGPGPGVLVLRASFDERQPYAHMNGGRKNERALPLPQSSTC

>NP_003595.1

MASCSFTRDQATRRLRGAAAAAAAALAAVVTTPLLSSGTPTALIGTGSSCPGAMWLSTATGSRSDSESEEEDLPVGEEVCKRGYLRKQKHGHRRYFVLKLETADAPARLEYYENARKFRHSVRAAAAAAAAAASGAAIPPLIPPRRVITLYQCFSVSQRADARYRHLIALFTQDEYFAMVAENESEQESWYLLLSRLILESKRRRCGTLGAQPDGEPAALAAAAAAEPPFYKDVWQVIVKPRGLGHRKELSGVFRLCLTDEEVVFVRLNTEVASVVVQLLSIRRCGHSEQYFFLEVGRSTVIGPGELWMQVDDCVVAQNMHELFLEKMRALCADEYRARCRSYSISIGAHLLTLLSARRHLGLVPLEPGGWLRRSRFEQFCHLRAIGDGEDEMLFTRRFVTPSEPVAHSRRGRLHLPRGRRSRRAVSVPASFFRRLAPSPARPRHPAEAPNNGARLSSEVSGSGSGNFGEEGNPQGKEDQEGSGGDYMPMNNWGSGNGRGSGGGQGSNGQGSSSHSSGGNQCSGEGQGSRGGQGSNGQGSGGNQCSRDGQGTAGGHGSGGGQRPGGGHGSGGGQGPGDGHGSGGGKNSGGGKGSGSGKGSDGDGERGKSLKKRSYFGKLTQSKQQQMPPPPPPPPPPPPAGGTGGKGKSGGRFRLYFCVDRGATKECKEAKEVKDAEIPEGAARGPHRARAFDEDEDDPYVPMRPGVATPLVSSSDYMPMAPQNVSASKKRHSRSPFEDSRGYMMMFPRVSPPPAPSPPKAPDTNKEDDSKDNDSESDYMFMAPGAGAIPKNPRNPQGGSSSKSWSSYFSLPNPFRSSPLGQNDNSEYVPMLPGKFLGRGLDKEVSYNWDPKDAASKPSGEGSFSKPGDGGSPSKPSDHEPPKNKAKRPNRLSFITKGYKIKPKPQKPTHEQREADSSSDYVNMDFTKRESNTPAPSTQGLPDSWGIIAEPRQSAFSNYVNVEFGVPFPNPANDLSDLLRAIPRANPLSLDSARWPLPPLPLSATGSNAIEEEGDYIEVIFNSAMTPAMALADSAIRYDAETGRIYVVDPFSECCMDISLSPSRCSEPPPVARLLQEEEQERRRPQSRSQSFFAAARAAVSAFPTDSLERDLSPSSAPAVASAAEPTLALSQVVAAASALAAAPGIGAAAAAAGFDSASARWFQPVANAADAEAVRGAQDVAGGSNPGAHNPSANLARGDNQAGGAAAAAAAPEPPPRSRRVPRPPEREDSDNDDDTHVRMDFARRDNQFDSPKRGR

880 NP_003110.1 NP_060842.3

>NP_003110.1

MAVLLLLLRALRRGPGPGPRPLWGPGPAWSPGFPARPGRGRPYMASRPPGDLAEAGGRALQSLQLRLLTPTFEGINGLLLKQHLVQNPVRLWQLLGGTFYFNTSRLKQKNKEKDKSKGKAPEEDEEERRRRERDDQMYRERLRTLLVIAVVMSLLNALSTSGGSISWNDFVHEMLAKGEVQRVQVVPESDVVEVYLHPGAVVFGRPRLALMYRMQVANIDKFEEKLRAAEDELNIEAKDRIPVSYKRTGFFGNALYSVGMTAVGLAILWYVFRLAGMTGREGGFSAFNQLKMARFTIVDGKMGKGVSFKDVAGMHEAKLEVREFVDYLKSPERFLQLGAKVPKGALLLGPPGCGKTLLAKAVATEAQVPFLAMAGPEFVEVIGGLGAARVRSLFKEARARAPCIVYIDEIDAVGKKRSTTMSGFSNTEEEQTLNQLLVEMDGMGTTDHVIVLASTNRADILDGALMRPGRLDRHVFIDLPTLQERREIFEQHLKSLKLTQSSTFYSQRLAELTPGFSGADIANICNEAALHAAREGHTSVHTLNFEYAVERVLAGTAKKSKILSKEEQKVVAFHESGHALVGWMLEHTEAVMKVSITPRTNAALGFAQMLPRDQHLFTKEQLFERMCMALGGRASEALSFNEVTSGAQDDLRKVTRIAYSMVKQFGMAPGIGPISFPEAQEGLMGIGRRPFSQGLQQMMDHEARLLVAKAYRHTEKVLQDNLDKLQALANALLEKEVINYEDIEALIGPPPHGPKKMIAPQRWIDAQREKQDLGEEETEETQQPPLGGEEPTWPK

>NP_060842.3

MSNNLRRVFLKPAEENSGNASRCVSGCMYQVVQTIGSDGKNLLQLLPIPKSSGNLIPLVQSSVMSDALKGNTGKPVQVTFQTQISSSSTSASVQLPIFQPASSSNYFLTRTVDTSEKGRVTSVGTGNFSSSVSKVQSHGVKIDGLTMQTFAVPPSTQKDSSFIVVNTQSLPVTVKSPVLPSGHHLQIPAHAEVKSVPASSLPPSVQQKILATATTSTSGMVEASQMPTVIYVSPVNTVKNVVTKNFQNIYPKPVTEIAKPVILNTTQIPKNVATETQLKGGQHSQAAPVKWIFQDNLQPFTPSLVPVKSSNNVASKILKTFVDRKNLGDNTINMPPLSTIDPSGTRSKNMPIKDNALVMFNGKVYLLAKKGTDVLPSQIDQQNSVSPDTPVRKDTLQTVSSSPVTEISREVVNIVLAKSKSSQMETKSLSNTQLASMANLRAEKNKVEKPSPSTTNPHMNQSSNYLKQSKTLFTNPIFPVGFSTGHNAPRKVTAVIYARKGSVLQSIEKISSSVDATTVTSQQCVFRDQEPKIHNEMASTSDKGAQGRNDKKDSQGRSNKALHLKSDAEFKKIFGLTKDLRVCLTRIPDHLTSGEGFDSFSSLVKSGTYKETEFMVKEGERKQQNFDKKRKAKTNKKMDHIKKRKTENAYNAIINGEANVTGSQLLSSILPTSDVSQHNILTSHSKTRQEKRTEMEYYTHEKQEKGTLNSNAAYEQSHFFNKNYTEDIFPVTPPELEETIRDEKIRRLKQVLREKEAALEEMRKKMHQK

881 NP_000451.1 NP_005364.1

>NP_000451.1

MELTELLLVVMLLLTARLTLSSPAPPACDLRVLSKLLRDSHVLHSRLSQCPEVHPLPTPVLLPAVDFSLGEWKTQMEETKAQDILGAVTLLLEGVMAARGQLGPTCLSSLLGQLSGQVRLLLGALQSLLGTQLPPQGRTTAHKDPNAIFLSFQHLLRGKVRFLMLVGGSTLCVRRAPPTTAVPSRTSLVLTLNELPNRTSGLLETNFTASARTTGSGLLKWQQGFRAKIPGLLNQTSRSLDQIPGYLNRIHELLNGTRGLFPGPSRRTLGAPDISSGTSDTGSLPPNLQPGYSPSPTHPPTGQYTLFPLPPTLPTPVVQLHPLLPDPSAPTPTPTSPLLNTSYTHSQNLSQEG

>NP_005364.1

MPSWALFMVTSCLLLAPQNLAQVSSQDVSLLASDSEPLKCFSRTFEDLTCFWDEEEAAPSGTYQLLYAYPREKPRACPLSSQSMPHFGTRYVCQFPDQEEVRLFFPLHLWVKNVFLNQTRTQRVLFVDSVGLPAPPSIIKAMGGSQPGELQISWEEPAPEISDFLRYELRYGPRDPKNSTGPTVIQLIATETCCPALQRPHSASALDQSPCAQPTMPWQDGPKQTSPSREASALTAEGGSCLISGLQPGNSYWLQLRSEPDGISLGGSWGSWSLPVTVDLPGDAVALGLQCFTLDLKNVTCQWQQQDHASSQGFFYHSRARCCPRDRYPIWENCEEEEKTNPGLQTPQFSRCHFKSRNDSIIHILVEVTTAPGTVHSYLGSPFWIHQAVRLPTPNLHWREISSGHLELEWQHPSSWAAQETCYQLRYTGEGHQDWKVLEPPLGARGGTLELRPRSRYRLQLRARLNGPTYQGPWSSWSDPTRVETATETAWISLVTALHLVLGLSAVLGLLLLRWQFPAHYRRLRHALWPSLPDLHRVLGQYLRDTAALSPPKATVSDTCEEVEPSLLEILPKSSERTPLPLCSSQAQMDYRRLQPSCLGTMPLSVCPPMAESGSCCTTHIANHSYLPLSYWQQP

882 NP_001008712.1 NP_113640.1

>NP_001008712.1

MNNGGKAEKENTPSEANLQEEEVRTLFVSGLPLDIKPRELYLLFRPFKGYEGSLIKLTSKQPVGFVSFDSRSEAEAAKNALNGIRFDPEIPQTLRLEFAKANTKMAKNKLVGTPNPSTPLPNTVPQFIAREPYELTVPALYPSSPEVWAPYPLYPAELAPALPPPAFTYPASLHAQCFSPEAKPNTPVFCPLLQQIRFVSGNVFVTYQPTADQQRELPC

>NP_113640.1

MTDTAEAVPKFEEMFASRFTENDKEYQEYLKRPPESPPIVEEWNSRAGGNQRNRGNRLQDNRQFRGRDNRWGWPSDNRSNQWHGRSWGNNYPQHRQEPYYPQQYGHYGYNQRPPYGYY

883 NP_057254.1 NP_003396.1

>NP_057254.1

MEPGMLGPHNLPHHEPISFGIDQILSGPETPGGGLGLGRGGQGHGENGAFSGGYHGASGYGPAGSLAPLPGSSGVGPGGVIRVPAHRPLPVPPPAGGAPAVPGPSGLGGAGGLAGLTFPWMDSGRRFAKDRLTAALSPFSGTRRIGHPYQNRTPPKRKKPRTSFSRSQVLELERRFLRQKYLASAERAALAKALRMTDAQVKTWFQNRRTKWRRQTAEEREAERHRAGRLLLHLQQDALPRPLRPPLPPDPLCLHNSSLFALQNLQPWAEDNKVASVSGLASVV

>NP_003396.1

MGDREQLLQRARLAEQAERYDDMASAMKAVTELNEPLSNEDRNLLSVAYKNVVGARRSSWRVISSIEQKTMADGNEKKLEKVKAYREKIEKELETVCNDVLSLLDKFLIKNCNDFQYESKVFYLKMKGDYYRYLAEVASGEKKNSVVEASEAAYKEAFEISKEQMQPTHPIRLGLALNFSVFYYEIQNAPEQACLLAKQAFDDAIAELDTLNEDSYKDSTLIMQLLRDNLTLWTSDQQDEEAGEGN

884 NP_002651.2 NP_002244.1

>NP_002651.2

MAGAASPCANGCGPGAPSDAEVLHLCRSLEVGTVMTLFYSKKSQRPERKTFQVKLETRQITWSRGADKIEGAIDIREIKEIRPGKTSRDFDRYQEDPAFRPDQSHCFVILYGMEFRLKTLSLQATSEDEVNMWIKGLTWLMEDTLQAPTPLQIERWLRKQFYSVDRNREDRISAKDLKNMLSQVNYRVPNMRFLRERLTDLEQRSGDITYGQFAQLYRSLMYSAQKTMDLPFLEASTLRAGERPELCRVSLPEFQQFLLDYQGELWAVDRLQVQEFMLSFLRDPLREIEEPYFFLDEFVTFLFSKENSVWNSQLDAVCPDTMNNPLSHYWISSSHNTYLTGDQFSSESSLEAYARCLRMGCRCIELDCWDGPDGMPVIYHGHTLTTKIKFSDVLHTIKEHAFVASEYPVILSIEDHCSIAQQRNMAQYFKKVLGDTLLTKPVEISADGLPSPNQLKRKILIKHKKLAEGSAYEEVPTSMMYSENDISNSIKNGILYLEDPVNHEWYPHYFVLTSSKIYYSEETSSDQGNEDEEEPKEVSSSTELHSNEKWFHGKLGAGRDGRHIAERLLTEYCIETGAPDGSFLVRESETFVGDYTLSFWRNGKVQHCRIHSRQDAGTPKFFLTDNLVFDSLYDLITHYQQVPLRCNEFEMRLSEPVPQTNAHESKEWYHASLTRAQAEHMLMRVPRDGAFLVRKRNEPNSYAISFRAEGKIKHCRVQQEGQTVMLGNSEFDSLVDLISYYEKHPLYRKMKLRYPINEEALEKIGTAEPDYGALYEGRNPGFYVEANPMPTFKCAVKALFDYKAQREDELTFIKSAIIQNVEKQEGGWWRGDYGGKKQLWFPSNYVEEMVNPVALEPEREHLDENSPLGDLLRGVLDVPACQIAIRPEGKNNRLFVFSISMASVAHWSLDVAADSQEELQDWVKKIREVAQTADARLTEGKIMERRKKIALELSELVVYCRPVPFDEEKIGTERACYRDMSSFPETKAEKYVNKAKGKKFLQYNRLQLSRIYPKGQRLDSSNYDPLPMWICGSQLVALNFQTPDKPMQMNQALFMTGRHCGYVLQPSTMRDEAFDPFDKSSLRGLEPCAISIEVLGARHLPKNGRGIVCPFVEIEVAGAEYDSTKQKTEFVVDNGLNPVWPAKPFHFQISNPEFAFLRFVVYEEDMFSDQNFLAQATFPVKGLKTGYRAVPLKNNYSEDLELASLLIKIDIFPAKENGDLSPFSGTSLRERGSDASGQLFHGRAREGSFESRYQQPFEDFRISQEHLADHFDSRERRAPRRTRVNGDNRL

>NP_002244.1

MQSKVLLAVALWLCVETRAASVGLPSVSLDLPRLSIQKDILTIKANTTLQITCRGQRDLDWLWPNNQSGSEQRVEVTECSDGLFCKTLTIPKVIGNDTGAYKCFYRETDLASVIYVYVQDYRSPFIASVSDQHGVVYITENKNKTVVIPCLGSISNLNVSLCARYPEKRFVPDGNRISWDSKKGFTIPSYMISYAGMVFCEAKINDESYQSIMYIVVVVGYRIYDVVLSPSHGIELSVGEKLVLNCTARTELNVGIDFNWEYPSSKHQHKKLVNRDLKTQSGSEMKKFLSTLTIDGVTRSDQGLYTCAASSGLMTKKNSTFVRVHEKPFVAFGSGMESLVEATVGERVRIPAKYLGYPPPEIKWYKNGIPLESNHTIKAGHVLTIMEVSERDTGNYTVILTNPISKEKQSHVVSLVVYVPPQIGEKSLISPVDSYQYGTTQTLTCTVYAIPPPHHIHWYWQLEEECANEPSQAVSVTNPYPCEEWRSVEDFQGGNKIEVNKNQFALIEGKNKTVSTLVIQAANVSALYKCEAVNKVGRGERVISFHVTRGPEITLQPDMQPTEQESVSLWCTADRSTFENLTWYKLGPQPLPIHVGELPTPVCKNLDTLWKLNATMFSNSTNDILIMELKNASLQDQGDYVCLAQDRKTKKRHCVVRQLTVLERVAPTITGNLENQTTSIGESIEVSCTASGNPPPQIMWFKDNETLVEDSGIVLKDGNRNLTIRRVRKEDEGLYTCQACSVLGCAKVEAFFIIEGAQEKTNLEIIILVGTAVIAMFFWLLLVIILRTVKRANGGELKTGYLSIVMDPDELPLDEHCERLPYDASKWEFPRDRLKLGKPLGRGAFGQVIEADAFGIDKTATCRTVAVKMLKEGATHSEHRALMSELKILIHIGHHLNVVNLLGACTKPGGPLMVIVEFCKFGNLSTYLRSKRNEFVPYKTKGARFRQGKDYVGAIPVDLKRRLDSITSSQSSASSGFVEEKSLSDVEEEEAPEDLYKDFLTLEHLICYSFQVAKGMEFLASRKCIHRDLAARNILLSEKNVVKICDFGLARDIYKDPDYVRKGDARLPLKWMAPETIFDRVYTIQSDVWSFGVLLWEIFSLGASPYPGVKIDEEFCRRLKEGTRMRAPDYTTPEMYQTMLDCWHGEPSQRPTFSELVEHLGNLLQANAQQDGKDYIVLPISETLSMEEDSGLSLPTSPVSCMEEEEVCDPKFHYDNTAGISQYLQNSKRKSRPVSVKTFEDIPLEEPEVKVIPDDNQTDSGMVLASEELKTLEDRTKLSPSFGGMVPSKSRESVASEGSNQTSGYQSGYHSDDTDTTVYSSEEAELLKLIEIGVQTGSTAQILQPDSGTTLSSPPV

885 NP_005602.2 NP_116090.2

>NP_005602.2

MPSGGDQSPPPPPPPPAAAASDEEEEDDGEAEDAAPSAESPTPQIQQRFDELCSRLNMDEAARPEAWDSYRSMSESYTLEGNDLHWLACALYVACRKSVPTVSKGTVEGNYVSLTRILKCSEQSLIEFFNKMKKWEDMANLPPHFRERTERLERNFTVSAVIFKKYEPIFQDIFKYPQEEQPRQQRGRKQRRQPCTVSEIFHFCWVLFIYAKGNFPMISDDLVNSYHLLLCALDLVYGNALQCSNRKELVNPNFKGLSEDFHAKDSKPSSDPPCIIEKLCSLHDGLVLEAKGIKEHFWKPYIRKLYEKKLLKGKEENLTGFLEPGNFGESFKAINKAYEEYVLSVGNLDERIFLGEDAEEEIGTLSRCLNAGSGTETAERVQMKNILQQHFDKSKALRISTPLTGVRYIKENSPCVTPVSTATHSLSRLHTMLTGLRNAPSEKLEQILRTCSRDPTQAIANRLKEMFEIYSQHFQPDEDFSNCAKEIASKHFRFAEMLYYKVLESVIEQEQKRLGDMDLSGILEQDAFHRSLLACCLEVVTFSYKPPGNFPFITEIFDVPLYHFYKVIEVFIRAEDGLCREVVKHLNQIEEQILDHLAWKPESPLWEKIRDNENRVPTCEEVMPPQNLERADEICIAGSPLTPRRVTEVRADTGGLGRSITSPTTLYDRYSSPPASTTRRRLFVENDSPSDGGTPGRMPPQPLVNAVPVQNVSGETVSVTPVPGQTLVTMATATVTANNGQTVTIPVQGIANENGGITFFPVQVNVGGQAQAVTGSIQPLSAQALAGSLSSQQVTGTTLQVPGQVAIQQISPGGQQQKQGQSVTSSSNRPRKTSSLSLFFRKVYHLAAVRLRDLCAKLDISDELRKKIWTCFEFSIIQCPELMMDRHLDQLLMCAIYVMAKVTKEDKSFQNIMRCYRTQPQARSQVYRSVLIKGKRKRRNSGSSDSRSHQNSPTELNKDRTSRDSSPVMRSSSTLPVPQPSSAPPTPTRLTGANSDMEEEERGDLIQFYNNIYIKQIKTFAMKYSQANMDAPPLSPYPFVRTGSPRRIQLSQNHPVYISPHKNETMLSPREKIFYYFSNSPSKRLREINSMIRTGETPTKKRGILLEDGSESPAKRICPENHSALLRRLQDVANDRGSH

>NP_116090.2

MGPDRVTARELCENDDLATSLVLDPYLGFRTHKMNVSPVPPLRRQQHLRSALETFLRQRDLEAAYRALTLGGWTARYFQSRGPRQEAALKTHVYRYLRAFLPESGFTILPCTRYSMETNGAKIVSTRAWKKNEKLELLVGCIAELREADEGLLRAGENDFSIMYSTRKRSAQLWLGPAAFINHDCKPNCKFVPADGNAACVKVLRDIEPGDEVTCFYGEGFFGEKNEHCECHTCERKGEGAFRTRPREPALPPRPLDKYQLRETKRRLQQGLDSGSRQGLLGPRACVHPSPLRRDPFCAACQPLRLPACSARPDTSPLWLQWLPQPQPRVRPRKRRRPRPRRAPVLSTHHAARVSLHRWGGCGPHCRLRGEALVALGQPPHARWAPQQDWHWARRYGLPYVVRVDLRRLAPAPPATPAPAGTPGPILIPKQALAFAPFSPPKRLRLVVSHGSIDLDVGGEEL

886 NP_055807.1 NP_001655.1

>NP_055807.1

MAPRKRGGRGISFIFCCFRNNDHPEITYRLRNDSNFALQTMEPALPMPPVEELDVMFSELVDELDLTDKHREAMFALPAEKKWQIYCSKKKDQEENKGATSWPEFYIDQLNSMAARKSLLALEKEEEEERSKTIESLKTALRTKPMRFVTRFIDLDGLSCILNFLKTMDYETSESRIHTSLIGCIKALMNNSQGRAHVLAHSESINVIAQSLSTENIKTKVAVLEILGAVCLVPGGHKKVLQAMLHYQKYASERTRFQTLINDLDKSTGRYRDEVSLKTAIMSFINAVLSQGAGVESLDFRLHLRYEFLMLGIQPVIDKLREHENSTLDRHLDFFEMLRNEDELEFAKRFELVHIDTKSATQMFELTRKRLTHSEAYPHFMSILHHCLQMPYKRSGNTVQYWLLLDRIIQQIVIQNDKGQDPDSTPLENFNIKNVVRMLVNENEVKQWKEQAEKMRKEHNELQQKLEKKERECDAKTQEKEEMMQTLNKMKEKLEKETTEHKQVKQQVADLTAQLHELSRRAVCASIPGGPSPGAPGGPFPSSVPGSLLPPPPPPPLPGGMLPPPPPPLPPGGPPPPPGPPPLGAIMPPPGAPMGLALKKKSIPQPTNALKSFNWSKLPENKLEGTVWTEIDDTKVFKILDLEDLERTFSAYQRQQDFFVNSNSKQKEADAIDDTLSSKLKVKELSVIDGRRAQNCNILLSRLKLSNDEIKRAILTMDEQEDLPKDMLEQLLKFVPEKSDIDLLEEHKHELDRMAKADRFLFEMSRINHYQQRLQSLYFKKKFAERVAEVKPKVEAIRSGSEEVFRSGALKQLLEVVLAFGNYMNKGQRGNAYGFKISSLNKIADTKSSIDKNITLLHYLITIVENKYPSVLNLNEELRDIPQAAKVNMTELDKEISTLRSGLKAVETELEYQKSQPPQPGDKFVSVVSQFITVASFSFSDVEDLLAEAKDLFTKAVKHFGEEAGKIQPDEFFGIFDQFLQAVSEAKQENENMRKKKEEEERRARMEAQLKEQRERERKMRKAKENSEESGEFDDLVSALRSGEVFDKDLSKLKRNRKRITNQMTDSSRERPITKLNF

>NP_001655.1

MAAIRKKLVIVGDGACGKTCLLIVFSKDQFPEVYVPTVFENYVADIEVDGKQVELALWDTAGQEDYDRLRPLSYPDTDVILMCFSIDSPDSLENIPEKWTPEVKHFCPNVPIILVGNKKDLRNDEHTRRELAKMKQEPVKPEEGRDMANRIGAFGYMECSAKTKDGVREVFEMATRAALQARRGKKKSGCLVL

887 NP_065921.2 NP_002410.1

>NP_065921.2

MDESALLDLLECPVCLERLDASAKVLPCQHTFCKRCLLGIVGSRNELRCPECRTLVGSGVEELPSNILLVRLLDGIKQRPWKPGPGGGSGTNCTNALRSQSSTVANCSSKDLQSSQGGQQPRVQSWSPPVRGIPQLPCAKALYNYEGKEPGDLKFSKGDIIILRRQVDENWYHGEVNGIHGFFPTNFVQIIKPLPQPPPQCKALYDFEVKDKEADKDCLPFAKDDVLTVIRRVDENWAEGMLADKIGIFPISYVEFNSAAKQLIEWDKPPVPGVDAGECSSAAAQSSTAPKHSDTKKNTKKRHSFTSLTMANKSSQASQNRHSMEISPPVLISSSNPTAAARISELSGLSCSAPSQVHISTTGLIVTPPPSSPVTTGPSFTFPSDVPYQAALGTLNPPLPPPPLLAATVLASTPPGATAAAAAAGMGPRPMAGSTDQIAHLRPQTRPSVYVAIYPYTPRKEDELELRKGEMFLVFERCQDGWFKGTSMHTSKIGVFPGNYVAPVTRAVTNASQAKVPMSTAGQTSRGVTMVSPSTAGGPAQKLQGNGVAGSPSVVPAAVVSAAHIQTSPQAKVLLHMTGQMTVNQARNAVRTVAAHNQERPTAAVTPIQVQNAAGLSPASVGLSHHSLASPQPAPLMPGSATHTAAISISRASAPLACAAAAPLTSPSITSASLEAEPSGRIVTVLPGLPTSPDSASSACGNSSATKPDKDSKKEKKGLLKLLSGASTKRKPRVSPPASPTLEVELGSAELPLQGAVGPELPPGGGHGRAGSCPVDGDGPVTTAVAGAALAQDAFHRKASSLDSAVPIAPPPRQACSSLGPVLNESRPVVCERHRVVVSYPPQSEAELELKEGDIVFVHKKREDGWFKGTLQRNGKTGLFPGSFVENI

>NP_002410.1

MEPLKSLFLKSPLGSWNGSGSGGGGGGGGGRPEGSPKAAGYANPVWTALFDYEPSGQDELALRKGDRVEVLSRDAAISGDEGWWAGQVGGQVGIFPSNYVSRGGGPPPCEVASFQELRLEEVIGIGGFGKVYRGSWRGELVAVKAARQDPDEDISVTAESVRQEARLFAMLAHPNIIALKAVCLEEPNLCLVMEYAAGGPLSRALAGRRVPPHVLVNWAVQIARGMHYLHCEALVPVIHRDLKSNNILLLQPIESDDMEHKTLKITDFGLAREWHKTTQMSAAGTYAWMAPEVIKASTFSKGSDVWSFGVLLWELLTGEVPYRGIDCLAVAYGVAVNKLTLPIPSTCPEPFAQLMADCWAQDPHRRPDFASILQQLEALEAQVLREMPRDSFHSMQEGWKREIQGLFDELRAKEKELLSREEELTRAAREQRSQAEQLRRREHLLAQWELEVFERELTLLLQQVDRERPHVRRRRGTFKRSKLRARDGGERISMPLDFKHRITVQASPGLDRRRNVFEVGPGDSPTFPRFRAIQLEPAEPGQAWGRQSPRRLEDSSNGERRACWAWGPSSPKPGEAQNGRRRSRMDEATWYLDSDDSSPLGSPSTPPALNGNPPRPSLEPEEPKRPVPAERGSSSGTPKLIQRALLRGTALLASLGLGRDLQPPGGPGRERGESPTTPPTPTPAPCPTEPPPSPLICFSLKTPDSPPTPAPLLLDLGIPVGQRSAKSPRREEEPRGGTVSPPPGTSRSAPGTPGTPRSPPLGLISRPRPSPLRSRIDPWSFVSAGPRPSPLPSPQPAPRRAPWTLFPDSDPFWDSPPANPFQGGPQDCRAQTKDMGAQAPWVPEAGP

888 NP_937818.1 NP_059523.1

>NP_937818.1

MVLLSTLGIVFQGEGPPISSCDTGTMANCERTFIAIKPDGVQRGLVGEIIKRFEQKGFRLVGLKFMQASEDLLKEHYVDLKDRPFFAGLVKYMHSGPVVAMVWEGLNVVKTGRVMLGETNPADSKPGTIRGDFCIQVGRNIIHGSDSVESAEKEIGLWFHPEELVDYTSCAQNWIYE

>NP_059523.1

MAEDVSSAAPSPRRCADGRDADPTEEQMAETERNDEEQFECQELLECQVQVGAPEEEEEEEEDAGLVAEAEAVAAGWMLDFLCLSLCRAFRDGRSEDFRRTRNSAEAIIHGLSSLTACQLRTIYICQFLTRIAAGKTLDAQFENDERITPLESALMIWGSIEKEHDKLHEEIQNLIKIQAIAVCMENGNFKEAEEVFERIFGDPNSHMPFKSKLLMIISQKDTFHSFFQHFSYNHMMEKIKSYVNYVLSEKSSTFLMKAAAKVVESKRTRTITSQDKPSGNDVEMETEANLDTRKSVSDKQSAVTESSEGTVSLLRSHKNLFLSKLQHGTQQQDLNKKERRVGTPQSTKKKKESRRATESRIPVSKSQPVTPEKHRARKRQAWLWEEDKNLRSGVRKYGEGNWSKILLHYKFNNRTSVMLKDRWRTMKKLKLISSDSED

889 NP_003018.2 NP_112486.1

>NP_003018.2

MDGIFAGIICNQANRCLTWTSQQLFSEKISGAEGTKLDDEFLDMERKIDVTNKVVAEILSKTTEYLQPNPAYRAKLGMLNTVSKIRGQVKTTGYPQTEGLLGDCMLKYGKELGEDSTFGNALIEVGESMKLMAEVKDSLDINVKQTFIDPLQLLQDKDLKEIGHHLKKLEGRRLDYDYKKKRVGKIPDEEVRQAVEKFEESKELAERSMFNFLENDVEQVSQLAVFIEAALDYHRQSTEILQELQSKLQMRISAASSVPRREYKPRPVKRSSSELNGVSTTSVVKTTGSNIPMDQPCCRGLYDFEPENQGELGFKEGDIITLTNQIDENWYEGMIHGESGFFPINYVEVIVPLPQ

>NP_112486.1

MAGAATQASLESAPRIMRLVAECSRSRARAGELWLPHGTVATPVFMPVGTQATMKGITTEQLDALGCRICLGNTYHLGLRPGPELIQKANGLHGFMNWPHNLLTDSGGFQMVSLVSLSEVTEEGVRFRSPYDGNETLLSPEKSVQIQNALGSDIIMQLDDVVSSTVTGPRVEEAMYRSIRWLDRCIAAHQRPDKQNLFAIIQGGLDADLRATCLEEMTKRDVPGFAIGGLSGGESKSQFWRMVALSTSRLPKDKPRYLMGVGYATDLVVCVALGCDMFDCVFPTRTARFGSALVPTGNLQLRKKVFEKDFGPIDPECTCPTCQKHSRAFLHALLHSDNTAALHHLTVHNIAYQLQLMSAVRTSIVEKRFPDFVRDFMGAMYGDPTLCPTWATDALASVGITLG

890 NP_006205.1 NP_005477.1

>NP_006205.1

MEQLRAAARLQIVLGHLGRPSAGAVVAHPTSGTISSASFHPQQFQYTLDNNVLTLEQRKFYEENGFLVIKNLVPDADIQRFRNEFEKICRKEVKPLGLTVMRDVTISKSEYAPSEKMITKVQDFQEDKELFRYCTLPEILKYVECFTGPNIMAMHTMLINKPPDSGKKTSRHPLHQDLHYFPFRPSDLIVCAWTAMEHISRNNGCLVVLPGTHKGSLKPHDYPKWEGGVNKMFHGIQDYEENKARVHLVMEKGDTVFFHPLLIHGSGQNKTQGFRKAISCHFASADCHYIDVKGTSQENIEKEVVGIAHKFFGAENSVNLKDIWMFRARLVKGERTNL

>NP_005477.1

MAFGKSHRDPYATSVGHLIEKATFAGVQTEDWGQFMHICDIINTTQDAPKDAVKALKKRISKNYNHKEIQLTLSLIDMCVQNCGPSFQSLIVKKEFVKENLVKLLNPRYNLPLDIQNRILNFIKTWSQGFPGGVDVSEVKEVYLDLVKKGVQFPPSEAEAETARQETAQISSNPPTSVPTAPALSSVIAPKNSTVTLVPEQIGKLHSELDMVKMNVRVMSAILMENTPGSENHEDIELLQKLYKTGREMQERIMDLLVVVENEDVTVELIQVNEDLNNAILGYERFTRNQQRILEQNKNQKEATNTTSEPSAPSQDLLDLSPSPRMPRATLGELNTMNNQLSGLNFSLPSSDVTNNLKPSLHPQMNLLALENTEIPPFAQRTSQNLTSSHAYDNFLEHSNSVFLQPVSLQTIAAAPSNQSLPPLPSNHPAMTKSDLQPPNYYEVMEFDPLAPAVTTEAIYEEIDAHQHKGAQNDGD

891 NP_938150.1 NP_036367.2

>NP_938150.1

MPRLHDHFWSCSCAHSARRRGPPRASAAGLAAKVGEMINVSVSGPSLLAAHGAPDADPAPRGRSAAMSGPEPGSPYPNTWHHRLLQRSLVLFSVGVVLALVLNLLQIQRNVTLFPEEVIATIFSSAWWVPPCCGTAAAVVGLLYPCIDSHLGEPHKFKREWASVMRCIAVFVGINHASAKLDFANNVQLSLTLAALSLGLWWTFDRSRSGLGLGITIAFLATLITQFLVYNGVYQYTSPDFLYIRSWLPCIFFSGGVTVGNIGRQLAMLIPFCEELNLKTTWLFHKTRSNYRVFLKSPIVIESSKPPILRARKILEENLTVDYDKDYLFS

>NP_036367.2

MTLTERLREKISRAFYNHGLLCASYPIPIILFTGFCILACCYPLLKLPLPGTGPVEFTTPVKDYSPPPVDSDRKQGEPTEQPEWYVGAPVAYVQQIFVKSSVFPWHKNLLAVDVFRSPLSRAFQLVEEIRNHVLRDSSGIRSLEELCLQVTDLLPGLRKLRNLLPEHGCLLLSPGNFWQNDWERFHADPDIIGTIHQHEPKTLQTSATLKDLLFGVPGKYSGVSLYTRKRMVSYTITLVFQHYHAKFLGSLRARLMLLHPSPNCSLRAESLVHVHFKEEIGVAELIPLVTTYIILFAYIYFSTRKIDMVKSKWGLALAAVVTVLSSLLMSVGLCTLFGLTPTLNGGEIFPYLVVVIGLENVLVLTKSVVSTPVDLEVKLRIAQGLSSESWSIMKNMATELGIILIGYFTLVPAIQEFCLFAVVGLVSDFFLQMLFFTTVLSIDIRRMELADLNKRLPPEACLPSAKPVGQPTRYERQLAVRPSTPHTITLQPSSFRNLRLPKRLRVVYFLARTRLAQRLIMAGTVVWIGILVYTDPAGLRNYLAAQVTEQSPLGEGALAPMPVPSGMLPPSHPDPAFSIFPPDAPKLPENQTSPGESPERGGPAEVVHDSPVPEVTWGPEDEELWRKLSFRHWPTLFSYYNITLAKRYISLLPVIPVTLRLNPREALEGRHPQDGRSAWPPPGPIPAGHWEAGPKGPGGVQAHGDVTLYKVAALGLATGIVLVLLLLCLYRVLCPRNYGQLGGGPGRRRRGELPCDDYGYAPPETEIVPLVLRGHLMDIECLASDGMLLVSCCLAGHVCVWDAQTGDCLTRIPRPGRQRRDSGVGSGLEAQESWERLSDGGKAGPEEPGDSPPLRHRPRGPPPPSLFGDQPDLTCLIDTNFSAQPRSSQPTQPEPRHRAVCGRSRDSPGYDFSCLVQRVYQEEGLAAVCTPALRPPSPGPVLSQAPEDEGGSPEKGSPSLAWAPSAEGSIWSLELQGNLIVVGRSSGRLEVWDAIEGVLCCSSEEVSSGITALVFLDKRIVAARLNGSLDFFSLETHTALSPLQFRGTPGRGSSPASPVYSSSDTVACHLTHTVPCAHQKPITALKAAAGRLVTGSQDHTLRVFRLEDSCCLFTLQGHSGAITTVYIDQTMVLASGGQDGAICLWDVLTGSRVSHVFAHRGDVTSLTCTTSCVISSGLDDLISIWDRSTGIKFYSIQQDLGCGASLGVISDNLLVTGGQGCVSFWDLNYGDLLQTVYLGKNSEAQPARQILVLDNAAIVCNFGSELSLVYVPSVLEKLD

892 NP_001027024.3 NP_689739.4

>NP_001027024.3

MRRPPGNGEAASEGPGGWGLWGVQESRRLCCAGHDRCKQALLQIGINMMALPGGRHLDSVTLPGQRLHLMQVDSVQRWMEDLKLMTECECMCVLQAKPISLEEDAQGDLILAGGPGPGDPLQLLLKRGWVISTELRRIGQKLAQDRWARVHSMSVRLTCHARSMVSEYSAVSRNSLKEMGEIEKLLMEKCSELSAVTERCLQVENEHVLKSMKACVSETLSMLGQHFGQLLELALTREVQALVRKIDASDNIYTTESTTGNLFSLTQEGAPLCRIIAKEGGVVALFKVCRQDSFRCLYPQALRTLASICCVEEGVHQLEKVDGVLCLADILTDNSHSEATRAEAAAVVAQVTSPHLPVTQHLSSFLESMEEIVTALVKLCQEASSGEVFLLASAALANITFFDTMACEMLLQLNAIRVLLEACSDKQRVDTPYTRDQIVTILANMSVLEQCASDIIQENGVQLIMGMLSEKPRSGTPAEVAACERVQQKAAVTLARLSRDPDVAREAVRLSCMSRLIELCRSPSERNSSDAVLVACLAALRRLAGVCPEGLQDSDFQQLVQPRLVDSFLLCSNMEESFV

>NP_689739.4

MKVTVCFGRTGIVVPCKEGQLRVGELTQQALQRYLKTREKGPGYWVKIHHLEYTDGGILDPDDVLADVVEDKDKLIAVFEEQEPLHKIESPSGNPADRQSPDAFETEVAAQLAAFKPIGGEIEVTPSALKLGTPLLVRRSSDPVPGPPADTQPSASHPGGQSLKLVVPDSTQNLEDREVLNGVQTELLTSPRTKDTLSDMTRTVEISGEGGPLGIHVVPFFSSLSGRILGLFIRGIEDNSRSKREGLFHENECIVKINNVDLVDKTFAQAQDVFRQAMKSPSVLLHVLPPQNREQYEKSVIGSLNIFGNNDGVLKTKVPPPVHGKSGLKTANLTGTDSPETDASASLQQNKSPRVPRLGGKPSSPSLSPLMGFGSNKNAKKIKIDLKKGPEGLGFTVVTRDSSIHGPGPIFVKNILPKGAAIKDGRLQSGDRILEVNGRDVTGRTQEELVAMLRSTKQGETASLVIARQEGHFLPRELDGRLRMNDQLIAVNGESLLGKSNHEAMETLRRSMSMEGNIRGMIQLVILRRPERPMEDPAECGAFSKPCFENCQNAVTTSRRNDNSILHPLGTCSPQDKQKGLLLPNDGWAESEVPPSPTPHSALGLGLEDYSHSSGVDSAVYFPDQHINFRSVTPARQPESINLKASKSMDLVPDESKVHSLAGQKSESPSKDFGPTLGLKKSSSLESLQTAVAEVRKNDLPFHRPRPHMVRGRGCNESFRAAIDKSYDGPEEIEADGLSDKSSHSGQGALNCESAPQGNSELEDMENKARKVKKTKEKEKKKEKGKLKVKEKKRKEENEDPERKIKKKGFGAMLRFGKKKEDKGGKAEQKGTLKHGGLREEELEKMKEERERIGAKHQELREKQARGLLDYATGAIGSVYDMDDDEMDPNYARVNHFREPCTSANVFRSPSPPRAGPFGYPRDGHPLSPERDHLEGLYAKVNKPYHPLVPADSGRPTGGSTDRIQKLRKEYYQARREGFPLYEDDEGRARPSEYDLLWVPGRGPDGNAHNLRFEGMERQYASLPRGGPADPVDYLPAAPRGLYKERELPYYPGAHPMHPPKGSYPRPTELRVADLRYPQHYPPPPAPQHKGPFRQDVPPSPPQHQRMPAYQETGRPGPRGGSPDQYPYRTQDSRQKNPMTAAV

893 NP_004655.1 NP_003035.2

>NP_004655.1

MLKPSVTSAPTADMATLTVVQPLTLDRDVARAIELLEKLQESGEVPVHKLQSLKKVLQSEFCTAIREVYQYMHETITVNGCPEFRARATAKATVAAFAASEGHSHPRVVELPKTDEGLGFNVMGGKEQNSPIYISRIIPGGVAERHGGLKRGDQLLSVNGVSVEGEHHEKAVELLKAAKDSVKLVVRYTPKVLEEMEARFEKLRTARRRQQQQLLIQQQQQQQQQQTQQNHMS

>NP_003035.2

MDGKVAVQERGPPAVSWVPEEGEKLDQEDEDQVKDRGQWTNKMEFVLSVAGEIIGLGNVWRFPYLCYKNGGGAFFIPYFIFFFVCGIPVFFLEVALGQYTSQGSVTAWRKICPLFQGIGLASVVIESYLNVYYIIILAWALFYLFSSFTSELPWTTCNNFWNTEHCTDFLNHSGAGTVTPFENFTSPVMEFWERRVLGITSGIHDLGSLRWELALCLLLAWVICYFCIWKGVKSTGKVVYFTATFPYLMLVILLIRGVTLPGAYQGIIYYLKPDLFRLKDPQVWMDAGTQIFFSFAICQGCLTALGSYNKYHNNCYKDCIALCFLNSATSFVAGFVVFSILGFMSQEQGVPISEVAESGPGLAFIAFPKAVTMMPLSQLWSCLFFIMLIFLGLDSQFVCVECLVTASIDMFPRQLRKSGRRELLILTIAVMCYLIGLFLVTEGGMYIFQLFDYYASSGICLLFLSLFEVVCISWVYGADRFYDNIEDMIGYRPWPLVKISWLFLTPGLCLATFLFSLSKYTPLKYNNVYVYPPWGYSIGWFLALSSMVCVPLFVVITLLKTRGPFRKRLRHVITPDSSLPQPKQHPCLDGSAGRNFGPSPTREGLIAGEKETHL

894 NP_006399.1 NP_068597.2

>NP_006399.1

MEKIPVSAFLLLVALSYTLARDTTVKPGAKKDTKDSRPKLPQTLSRGWGDQLIWTQTYEEALYKSKTSNKPLMIIHHLDECPHSQALKKVFAENKEIQKLAEQFVLLNLVYETTDKHLSPDGQYVPRIMFVDPSLTVRADITGRYSNRLYAYEPADTALLLDNMKKALKLLKTEL

>NP_068597.2

MNSRQAWRLFLSQGRGDRWVSRPRGHFSPALRREFFTTTTKEGYDRRPVDITPLEQRKLTFDTHALVQDLETHGFDKTQAETIVSALTALSNVSLDTIYKEMVTQAQQEITVQQLMAHLDAIRKDMVILEKSEFANLRAENEKMKIELDQVKQQLMHETSRIRADNKLDINLERSRVTDMFTDQEKQLMETTTEFTKKDTQTKSIISETSNKIDAEIASLKTLMESNKLETIRYLAASVFTCLAIALGFYRFWK

895 NP_005399.1 NP_000638.1

>NP_005399.1

MKVSAVLLCLLLMTAAFNPQGLAQPDALNVPSTCCFTFSSKKISLQRLKSYVITTSRCPQKAVIFRTKLGKEICADPKEKWVQNYMKHLGRKAHTLKT

>NP_000638.1

MLSTSRSRFIRNTNESGEEVTTFFDYDYGAPCHKFDVKQIGAQLLPPLYSLVFIFGFVGNMLVVLILINCKKLKCLTDIYLLNLAISDLLFLITLPLWAHSAANEWVFGNAMCKLFTGLYHIGYFGGIFFIILLTIDRYLAIVHAVFALKARTVTFGVVTSVITWLVAVFASVPGIIFTKCQKEDSVYVCGPYFPRGWNNFHTIMRNILGLVLPLLIMVICYSGILKTLLRCRNEKKRHRAVRVIFTIMIVYFLFWTPYNIVILLNTFQEFFGLSNCESTSQLDQATQVTETLGMTHCCINPIIYAFVGEKFRSLFHIALGCRIAPLQKPVCGGPGVRPGKNVKVTTQGLLDGRGKGKSIGRAPEASLQDKEGA

896 NP_002736.3 NP_005185.2

>NP_002736.3

MAAAAAAGAGPEMVRGQVFDVGPRYTNLSYIGEGAYGMVCSAYDNVNKVRVAIKKISPFEHQTYCQRTLREIKILLRFRHENIIGINDIIRAPTIEQMKDVYIVQDLMETDLYKLLKTQHLSNDHICYFLYQILRGLKYIHSANVLHRDLKPSNLLLNTTCDLKICDFGLARVADPDHDHTGFLTEYVATRWYRAPEIMLNSKGYTKSIDIWSVGCILAEMLSNRPIFPGKHYLDQLNHILGILGSPSQEDLNCIINLKARNYLLSLPHKNKVPWNRLFPNADSKALDLLDKMLTFNPHKRIEVEQALAHPYLEQYYDPSDEPIAEAPFKFDMELDDLPKEKLKELIFEETARFQPGYRS

>NP_005185.2

MQRLVAWDPACLPLPPPPPAFKSMEVANFYYEADCLAAAYGGKAAPAAPPAARPGPRPPAGELGSIGDHERAIDFSPYLEPLGAPQAPAPATATDTFEAAPPAPAPAPASSGQHHDFLSDLFSDDYGGKNCKKPAEYGYVSLGRLGAAKGALHPGCFAPLHPPPPPPPPPAELKAEPGFEPADCKRKEEAGAPGGGAGMAAGFPYALRAYLGYQAVPSGSSGSLSTSSSSSPPGTPSPADAKAPPTACYAGAAPAPSQVKSKAKKTVDKHSDEYKIRRERNNIAVRKSRDKAKMRNLETQHKVLELTAENERLQKKVEQLSRELSTLRNLFKQLPEPLLASSGHC

897 NP_005177.2 NP_003842.1

>NP_005177.2

MSEEIITPVYCTGVSAQVQKQRARELGLGRHENAIKYLGQDYEQLRVRCLQSGTLFRDEAFPPVPQSLGYKDLGPNSSKTYGIKWKRPTELLSNPQFIVDGATRTDICQGALGDCWLLAAIASLTLNDTLLHRVVPHGQSFQNGYAGIFHFQLWQFGEWVDVVVDDLLPIKDGKLVFVHSAEGNEFWSALLEKAYAKVNGSYEALSGGSTSEGFEDFTGGVTEWYELRKAPSDLYQIILKALERGSLLGCSIDISSVLDMEAITFKKLVKGHAYSVTGAKQVNYRGQVVSLIRMRNPWGEVEWTGAWSDSSSEWNNVDPYERDQLRVKMEDGEFWMSFRDFMREFTRLEICNLTPDALKSRTIRKWNTTLYEGTWRRGSTAGGCRNYPATFWVNPQFKIRLDETDDPDDYGDRESGCSFVLALMQKHRRRERRFGRDMETIGFAVYEVPPELVGQPAVHLKRDFFLANASRARSEQFINLREVSTRFRLPPGEYVVVPSTFEPNKEGDFVLRFFSEKSAGTVELDDQIQANLPDEQVLSEEEIDENFKALFRQLAGEDMEISVKELRTILNRIISKHKDLRTKGFSLESCRSMVNLMDRDGNGKLGLVEFNILWNRIRNYLSIFRKFDLDKSGSMSAYEMRMAIESAGFKLNKKLYELIITRYSEPDLAVDFDNFVCCLVRLETMFRFFKTLDTDLDGVVTFDLFKWLQLTMFA

>NP_003842.1

MAGLSRGSARALLAALLASTLLALLVSPARGRGGRDHGDWDEASRLPPLPPREDAARVARFVTHVSDWGALATISTLEAVRGRPFADVLSLSDGPPGAGSGVPYFYLSPLQLSVSNLQENPYATLTMTLAQTNFCKKHGFDPQSPLCVHIMLSGTVTKVNETEMDIAKHSLFIRHPEMKTWPSSHNWFFAKLNITNIWVLDYFGGPKIVTPEEYYNVTVQ

898 NP_002341.1 NP_000112.1

>NP_002341.1

MGCIKSKGKDSLSDDGVDLKTQPVRNTERTIYVRDPTSNKQQRPVPESQLLPGQRFQTKDPEEQGDIVVALYPYDGIHPDDLSFKKGEKMKVLEEHGEWWKAKSLLTKKEGFIPSNYVAKLNTLETEEWFFKDITRKDAERQLLAPGNSAGAFLIRESETLKGSFSLSVRDFDPVHGDVIKHYKIRSLDNGGYYISPRITFPCISDMIKHYQKQADGLCRRLEKACISPKPQKPWDKDAWEIPRESIKLVKRLGAGQFGEVWMGYYNNSTKVAVKTLKPGTMSVQAFLEEANLMKTLQHDKLVRLYAVVTREEPIYIITEYMAKGSLLDFLKSDEGGKVLLPKLIDFSAQIAEGMAYIERKNYIHRDLRAANVLVSESLMCKIADFGLARVIEDNEYTAREGAKFPIKWTAPEAINFGCFTIKSDVWSFGILLYEIVTYGKIPYPGRTNADVMTALSQGYRMPRVENCPDELYDIMKMCWKEKAEERPTFDYLQSVLDDFYTATEGQYQQQP

>NP_000112.1

MDHLGASLWPQVGSLCLLLAGAAWAPPPNLPDPKFESKAALLAARGPEELLCFTERLEDLVCFWEEAASAGVGPGNYSFSYQLEDEPWKLCRLHQAPTARGAVRFWCSLPTADTSSFVPLELRVTAASGAPRYHRVIHINEVVLLDAPVGLVARLADESGHVVLRWLPPPETPMTSHIRYEVDVSAGNGAGSVQRVEILEGRTECVLSNLRGRTRYTFAVRARMAEPSFGGFWSAWSEPVSLLTPSDLDPLILTLSLILVVILVLLTVLALLSHRRALKQKIWPGIPSPESEFEGLFTTHKGNFQLWLYQNDGCLWWSPCTPFTEDPPASLEVLSERCWGTMQAVEPGTDDEGPLLEPVGSEHAQDTYLVLDKWLLPRNPPSEDLPGPGGSVDIVAMDEGSEASSCSSALASKPSPEGASAASFEYTILDPSSQLLRPWTLCPELPPTPPHLKYLYLVVSDSGISTDYSSGDSQGAQGGLSDGPYSNPYENSLIPAAEPLPPSYVACS

899 NP_001030126.1 NP_005179.2

>NP_001030126.1

MSSECDGGSKAVMNGLAPGSNGQDKATADPLRARSISAVKIIPVKTVKNASGLVLPTDMDPTKICTGKGAVTLRASSSYRETPSSSPASPQETRQHESKPGLEPEPSSADEWRLSSSADANGNAQPSSLAAKGYRSVHPNLPSDKSQDATSSSAAQPEVIVVPLYLVNTDRGQEGTARPPTPLGPLGCVPTIPATASAASPLTFPTLDDFIPPHLQRWPHHSQPARASGSFAPISQTPPSFSPPPPLVPPAPEDLRRVSEPDLTGAVSSTDSSPLLNEVSSSLIGTDSQAFPSVSKPSSAYPSTTIVNPTIVLLQHNREQQKRLSSLSDPVSERRVGEQDSAPTQEKPTSPGKAIEKRAKDDSRRVVKSTQDLSDVSMDEVGIPLRNTERSKDWYKTMFKQIHKLNRDTPEENPYFPTYKFPELPEIQQTSEEDNPYTPTYQFPASTPSPKSEDDDSDLYSPRYSFSEDTKSPLSVPRSKSEMSYIDGEKVVKRSATLPLPARSSSLKSSSERNDWEPPDKKVDTRKYRAEPKSIYEYQPGKSSVLTNEKMSRDISPEEIDLKNEPWYKFFSELEFGKPPPKKIWDYTPGDCSILPREDRKTNLDKDLSLCQTELEADLEKMETLNKAPSANVPQSSAISPTPEISSETPGYIYSSNFHAVKRESDGAPGDLTSLENERQIYKSVLEGGDIPLQGLSGLKRPSSSASTKDSESPRHFIPADYLESTEEFIRRRHDDKEKLLADQRRLKREQEEADIAARRHTGVIPTHHQFITNERFGDLLNIDDTAKRKSGSEMRPARAKFDFKAQTLKELPLQKGDIVYIYKQIDQNWYEGEHHGRVGIFPRTYIELLPPAEKAQPKKLTPVQVLEYGEAIAKFNFNGDTQVEMSFRKGERITLLRQVDENWYEGRIPGTSRQGIFPITYVDVIKRPLVKNPVDYMDLPFSSSPSRSATASPQFSSHSKLITPAPSSLPHSRRALSPEMHAVTSEWISLTVGVPGRRSLALTPPLPPLPEASIYNTDHLALSPRASPSLSLSLPHLSWSDRPTPRSVASPLALPSPHKTYSLAPTSQASLHMNGDGGVHTPSSGIHQDSFLQLPLGSSDSVISQLSDAFSSQSKRQPWREESGQYERKAERGAGERGPGGPKISKKSCLKPSDVVRCLSTEQRLSDLNTPEESRPGKPLGSAFPGSEAEQTERHRGGEQAGRKAARRGGSQQPQAQQRRVTPDRSQTSQDLFSYQALYSYIPQNDDELELRDGDIVDVMEKCDDGWFVGTSRRTKQFGTFPGNYVKPLYL

>NP_005179.2

MAGNVKKSSGAGGGSGSGGSGSGGLIGLMKDAFQPHHHHHHHLSPHPPGTVDKKMVEKCWKLMDKVVRLCQNPKLALKNSPPYILDLLPDTYQHLRTILSRYEGKMETLGENEYFRVFMENLMKKTKQTISLFKEGKERMYEENSQPRRNLTKLSLIFSHMLAELKGIFPSGLFQGDTFRITKADAAEFWRKAFGEKTIVPWKSFRQALHEVHPISSGLEAMALKSTIDLTCNDYISVFEFDIFTRLFQPWSSLLRNWNSLAVTHPGYMAFLTYDEVKARLQKFIHKPGSYIFRLSCTRLGQWAIGYVTADGNILQTIPHNKPLFQALIDGFREGFYLFPDGRNQNPDLTGLCEPTPQDHIKVTQEQYELYCEMGSTFQLCKICAENDKDVKIEPCGHLMCTSCLTSWQESEGQGCPFCRCEIKGTEPIVVDPFDPRGSGSLLRQGAEGAPSPNYDDDDDERADDTLFMMKELAGAKVERPPSPFSMAPQASLPPVPPRLDLLPQRVCVPSSASALGTASKAASGSLHKDKPLPVPPTLRDLPPPPPPDRPYSVGAESRPQRRPLPCTPGDCPSRDKLPPVPSSRLGDSWLPRPIPKVPVSAPSSSDPWTGRELTNRHSLPFSLPSQMEPRPDVPRLGSTFSLDTSMSMNSSPLVGPECDHPKIKPSSSANAIYSLAARPLPVPKLPPGEQCEGEEDTEYMTPSSRPLRPLDTSQSSRACDCDQQIDSCTYEAMYNIQSQAPSITESSTFGEGNLAAAHANTGPEESENEDDGYDVPKPPVPAVLARRTLSDISNASSSFGWLSLDGDPTTNVTEGSQVPERPPKPFPRRINSERKAGSCQQGSGPAASAATASPQLSSEIENLMSQGYSYQDIQKALVIAQNNIEMAKNILREFVSISSPAHVAT

900 NP_002729.2 BAA05892.1

>NP_002729.2

MADPAAGPPPSEGEESTVRFARKGALRQKNVHEVKNHKFTARFFKQPTFCSHCTDFIWGFGKQGFQCQVCCFVVHKRCHEFVTFSCPGADKGPASDDPRSKHKFKIHTYSSPTFCDHCGSLLYGLIHQGMKCDTCMMNVHKRCVMNVPSLCGTDHTERRGRIYIQAHIDRDVLIVLVRDAKNLVPMDPNGLSDPYVKLKLIPDPKSESKQKTKTIKCSLNPEWNETFRFQLKESDKDRRLSVEIWDWDLTSRNDFMGSLSFGISELQKASVDGWFKLLSQEEGEYFNVPVPPEGSEANEELRQKFERAKISQGTKVPEEKTTNTVSKFDNNGNRDRMKLTDFNFLMVLGKGSFGKVMLSERKGTDELYAVKILKKDVVIQDDDVECTMVEKRVLALPGKPPFLTQLHSCFQTMDRLYFVMEYVNGGDLMYHIQQVGRFKEPHAVFYAAEIAIGLFFLQSKGIIYRDLKLDNVMLDSEGHIKIADFGMCKENIWDGVTTKTFCGTPDYIAPEIIAYQPYGKSVDWWAFGVLLYEMLAGQAPFEGEDEDELFQSIMEHNVAYPKSMSKEAVAICKGLMTKHPGKRLGCGPEGERDIKEHAFFRYIDWEKLERKEIQPPYKPKACGRNAENFDRFFTRHPPVLTPPDQEVIRNIDQSEFEGFSFVNSEFLKPEVKS

>BAA05892.1

MVLLLILSVLLLKEDVRGSAQSSERRVVAHMPGDIIIGALFSVHHQPTVDKVHERKCGAVREQYGIQRVEAMLHTLERINSDPTLLPNITLGCEIRDSCWHSAVALEQSIEFIRDSLISSEEEEGLVRCVDGSSSSFRSKKPIVGVIGPGSSSVAIQVQNLLQLFNIPQIAYSATSMDLSDKTLFKYFMRVVPSDAQQARAMVDIVKRYNWTYVSAVHTEGNYGESGMEAFKDMSAKEGICIAHSYKIYSNAGEQSFDKLLKKLTSHLPKARVVACFCEGMTVRGLLMAMRRLGLAGEFLLLGSDGWADRYDVTDGYQREAVGGITIKLQSPDVKWFDDYYLKLRPETNHRNPWFQEFWQHRFQCRLEGFPQENSKYNKTCNSSLTLKTHHVQDSKMGFVINAIYSMAYGLHNMQMSLCPGYAGLCDAMKPIDGRKLLESLMKTNFTGVSGDTILFDENGDSPGRYEIMNFKEMGKDYFDYINVGSWDNGELKMDDDEVWSKKSNIIRSVCSEPCEKGQIKVIRKGEVSCCWTCTPCKENEYVFDEYTCKACQLGSWPTDDLTGCDLIPVQYLRWGDPEPIAAVVFACLGLLATLFVTVVFIIYRDTPVVKSSSRELCYIILAGICLGYLCTFCLIAKPKQIYCYLQRIGIGLSPAMSYSALVTKTNRIARILAGSKKKICTKKPRFMSACAQLVIAFILICIQLGIIVALFIMEPPDIMHDYPSIREVYLICNTTNLGVVTPLGYNGLLILSCTFYAFKTRNVPANFNEAKYIAFTMYTTCIIWLAFVPIYFGSNYKIITMCFSVSLSATVALGCMFVPKVYIILAKPERNVRSAFTTSTVVRMHVGDGKSSSAASRSSSLVNLWKRRGSSGETLRYKDRRLAQHKSEIECFTPKGSMGNGGRATMSSSNGKSVTWAQNEKSSRGQHLWQRLSIHINKKENPNQTAVIKPFPKSTESRGLGAGAGAGGSAGGVGATGGAGCAGAGPGGPESPDAGPKALYDVAEAEEHFPAPARPRSPSPISTLSHRAGSASRTDDDVPSLHSEPVARSSSSQGSLMEQISSVVTRFTANISELNSMMLSTAAPSPGVGAPLCSSYLIPKEIQLPTTMTTFAEIQPLPAIEVTGGAQPAAGAQAAGDAARESPAAGPEAAAAKPDLEELVALTPPSPFRDSVDSGSTTPNSPVSESALCIPSSPKYDTLIIRDYTQSSSSL

901 NP_003780.1 NP_000034.1

>NP_003780.1

MAAGQNGHEEWVGSAYLFVESSLDKVVLSDAYAHPQQKVAVYRALQAALAESGGSPDVLQMLKIHRSDPQLIVQLRFCGRQPCGRFLRAYREGALRAALQRSLAAALAQHSVPLQLELRAGAERLDALLADEERCLSCILAQQPDRLRDEELAELEDALRNLKCGSGARGGDGEVASAPLQPPVPSLSEVKPPPPPPPAQTFLFQGQPVVNRPLSLKDQQTFARSVGLKWRKVGRSLQRGCRALRDPALDSLAYEYEREGLYEQAFQLLRRFVQAEGRRATLQRLVEALEENELTSLAEDLLGLTDPNGGLA

>NP_000034.1

MLGIWTLLPLVLTSVARLSSKSVNAQVTDINSKGLELRKTVTTVETQNLEGLHHDGQFCHKPCPPGERKARDCTVNGDEPDCVPCQEGKEYTDKAHFSSKCRRCRLCDEGHGLEVEINCTRTQNTKCRCKPNFFCNSTVCEHCDPCTKCEHGIIKECTLTSNTKCKEEGSRSNLGWLCLLLLPIPLIVWVKRKEVQKTCRKHRKENQGSHESPTLNPETVAINLSDVDLSKYITTIAGVMTLSQVKGFVRKNGVNEAKIDEIKNDNVQDTAEQKVQLLRNWHQLHGKKEAYDTLIKDLKKANLCTLAEKIQTIILKDITSDSENSNFRNEIQSLV

902 NP_115516.1 NP_077817.2

>NP_115516.1

MCEGPSRISGPIPPDPTLCPDNYRRPTSAQGRLEGNALKLDLLTSDRALDTTAPRGPCIGPGAGEILERGQRGVGDVLLQLEGISLGPGASLKRKDPKDHEKENLRRIREIQKRFREQERSREQGQPRPLKALWRSPKYDKVESRVKAQLQEPGPASGTESAHFLRAHSRCGPGLPPPHVSSPQPTPPGPEAKEPGLGVDFIRHNARAAKRAPRRHSCSLQVLAQVLEQQRQAQEHYNATQKGHVPHYLLERRDLWRREAEARKQSQPDPAMPPGHTRMPENQRLETLTKLLQSQSQLLRELVLLPAGADSLRAQSHRAELDRKLVQVEEAIKIFSRPKVFVKMDD

>NP_077817.2

MFPVAPKPQDSSQPSDRLMTEKQQEEAEWESINVLLMMHGLKPLSLVKRTDLKDLIIFDKQSSQRMRQNLKLLVEETSCQQNMIQELIETNQQLRNELQLEQSRAANQEQRANDLEQIMESVKSKIGELEDESLSRACHQQNKIKDLQKEQKTLQVKCQHYKKKRTEQEETIASLQMEVCRLKKEEEDRIVTQNRVFAYLCKRVPHTVLDRQLLCLIDYYESKIRKIHTQRQYKEDESQSEEENDYRNLDASPTYKGLLMSLQNQLKESKSKIDALSSEKLNLQKDLETRPTQHELRLYKQQVKKLEKALKKNVKLQELINHKKAEDTEKKDEPSKYNQQQALIDQRYFQVLCSINSIIHNPRAPVIIYKQTKGGVQNFNKDLVQDCGFEHLVPVIEMWADQLTSLKDLYKSLKTLSAELVPWLNLKKQDENEGIKVEDLLFIVDTMLEEVENKEKDSNMPHFQTLQAIVSHFQKLFDVPSLNGVYPRMNEVYTRLGEMNNAVRNLQELLELDSSSSLCVLVSTVGKLCRLINEDVNEQVMQVLGPEDLQSIIYKLEEHEEFFPAFQAFTNDLLEILEIDDLDAIVPAVKKLKVLSY

903 NP_006147.1 NP_004350.1

>NP_006147.1

MLIKVKTLTGKEIEIDIEPTDKVERIKERVEEKEGIPPQQQRLIYSGKQMNDEKTAADYKILGGSVLHLVLALRGGGGLRQ

>NP_004350.1

MARPLVPSSQKALLLELKGLQEEPVEGFRVTLVDEGDLYNWEVAIFGPPNTYYEGGYFKARLKFPIDYPYSPPAFRFLTKMWHPNIYETGDVCISILHPPVDDPQSGELPSERWNPTQNVRTILLSVISLLNEPNTFSPANVDASVMYRKWKESKGKDREYTDIIRKQVLGTKVDAERDGVKVPTTLAEYCVKTKAPAPDEGSDLFYDDYYEDGEVEEEADSCFGDDEDDSGTEES

904 NP_005214.2 NP_001091.1

>NP_005214.2

MRGMKLLGALLALAALLQGAVSLKIAAFNIQTFGETKMSNATLVSYIVQILSRYDIALVQEVRDSHLTAVGKLLDNLNQDAPDTYHYVVSEPLGRNSYKERYLFVYRPDQVSAVDSYYYDDGCEPCGNDTFNREPAIVRFFSRFTEVREFAIVPLHAAPGDAVAEIDALYDVYLDVQEKWGLEDVMLMGDFNAGCSYVRPSQWSSIRLWTSPTFQWLIPDSADTTATPTHCAYDRIVVAGMLLRGAVVPDSALPFNFQAAYGLSDQLAQAISDHYPVEVMLK

>NP_001091.1

MCDEDETTALVCDNGSGLVKAGFAGDDAPRAVFPSIVGRPRHQGVMVGMGQKDSYVGDEAQSKRGILTLKYPIEHGIITNWDDMEKIWHHTFYNELRVAPEEHPTLLTEAPLNPKANREKMTQIMFETFNVPAMYVAIQAVLSLYASGRTTGIVLDSGDGVTHNVPIYEGYALPHAIMRLDLAGRDLTDYLMKILTERGYSFVTTAEREIVRDIKEKLCYVALDFENEMATAASSSSLEKSYELPDGQVITIGNERFRCPETLFQPSFIGMESAGIHETTYNSIMKCDIDIRKDLYANNVMSGGTTMYPGIADRMQKEITALAPSTMKIKIIAPPERKYSVWIGGSILASLSTFQQMWITKQEYDEAGPSIVHRKCF

905 NP_002728.1 NP_000910.2

>NP_002728.1

MADVFPGNDSTASQDVANRFARKGALRQKNVHEVKDHKFIARFFKQPTFCSHCTDFIWGFGKQGFQCQVCCFVVHKRCHEFVTFSCPGADKGPDTDDPRSKHKFKIHTYGSPTFCDHCGSLLYGLIHQGMKCDTCDMNVHKQCVINVPSLCGMDHTEKRGRIYLKAEVADEKLHVTVRDAKNLIPMDPNGLSDPYVKLKLIPDPKNESKQKTKTIRSTLNPQWNESFTFKLKPSDKDRRLSVEIWDWDRTTRNDFMGSLSFGVSELMKMPASGWYKLLNQEEGEYYNVPIPEGDEEGNMELRQKFEKAKLGPAGNKVISPSEDRKQPSNNLDRVKLTDFNFLMVLGKGSFGKVMLADRKGTEELYAIKILKKDVVIQDDDVECTMVEKRVLALLDKPPFLTQLHSCFQTVDRLYFVMEYVNGGDLMYHIQQVGKFKEPQAVFYAAEISIGLFFLHKRGIIYRDLKLDNVMLDSEGHIKIADFGMCKEHMMDGVTTRTFCGTPDYIAPEIIAYQPYGKSVDWWAYGVLLYEMLAGQPPFDGEDEDELFQSIMEHNVSYPKSLSKEAVSICKGLMTKHPAKRLGCGPEGERDVREHAFFRRIDWEKLENREIQPPFKPKVCGKGAENFDKFFTRGQPVLTPPDQLVIANIDQSDFEGFSYVNPQFVHPILQSAV

>NP_000910.2

MAGRVPSLLVLLVFPSSCLAFRSPLSVFKRFKETTRPFSNECLGTTRPVVPIDSSDFALDIRMPGVTPKQSDTYFCMSMRIPVDEEAFVIDFKPRASMDTVHHMLLFGCNMPSSTGSYWFCDEGTCTDKANILYAWARNAPPTRLPKGVGFRVGGETGSKYFVLQVHYGDISAFRDNNKDCSGVSLHLTRLPQPLIAGMYLMMSVDTVIPAGEKVVNSDISCHYKNYPMHVFAYRVHTHHLGKVVSGYRVRNGQWTLIGRQSPQLPQAFYPVGHPVDVSFGDLLAARCVFTGEGRTEATHIGGTSSDEMCNLYIMYYMEAKHAVSFMTCTQNVAPDMFRTIPPEANIPIPVKSDMVMMHEHHKETEYKDKIPLLQQPKREEEEVLDQGDFYSLLSKLLGEREDVVHVHKYNPTEKAESESDLVAEIANVVQKKDLGRSDAREGAEHERGNAILVRDRIHKFHRLVSTLRPPESRVFSLQQPPPGEGTWEPEHTGDFHMEEALDWPGVYLLPGQVSGVALDPKNNLVIFHRGDHVWDGNSFDSKFVYQQIGLGPIEEDTILVIDPNNAAVLQSSGKNLFYLPHGLSIDKDGNYWVTDVALHQVFKLDPNNKEGPVLILGRSMQPGSDQNHFCQPTDVAVDPGTGAIYVSDGYCNSRIVQFSPSGKFITQWGEESSGSSPLPGQFTVPHSLALVPLLGQLCVADRENGRIQCFKTDTKEFVREIKHSSFGRNVFAISYIPGLLFAVNGKPHFGDQEPVQGFVMNFSNGEIIDIFKPVRKHFDMPHDIVASEDGTVYIGDAHTNTVWKFTLTEKLEHRSVKKAGIEVQEIKEAEAVVETKMENKPTSSELQKMQEKQKLIKEPGSGVPVVLITTLLVIPVVVLLAIAIFIRWKKSRAFGADSEHKLETSSGRVLGRFRGKGSGGLNLGNFFASRKGYSRKGFDRLSTEGSDQEKEDDGSESEEEYSAPLPALAPSSS

906 NP_005234.1 NP_005089.2

>NP_005234.1

MASTDYSTYSQAAAQQGYSAYTAQPTQGYAQTTQAYGQQSYGTYGQPTDVSYTQAQTTATYGQTAYATSYGQPPTGYTTPTAPQAYSQPVQGYGTGAYDTTTATVTTTQASYAAQSAYGTQPAYPAYGQQPAATAPTRPQDGNKPTETSQPQSSTGGYNQPSLGYGQSNYSYPQVPGSYPMQPVTAPPSYPPTSYSSTQPTSYDQSSYSQQNTYGQPSSYGQQSSYGQQSSYGQQPPTSYPPQTGSYSQAPSQYSQQSSSYGQQSSFRQDHPSSMGVYGQESGGFSGPGENRSMSGPDNRGRGRGGFDRGGMSRGGRGGGRGGMGSAGERGGFNKPGGPMDEGPDLDLGPPVDPDEDSDNSAIYVQGLNDSVTLDDLADFFKQCGVVKMNKRTGQPMIHIYLDKETGKPKGDATVSYEDPPTAKAAVEWFDGKDFQGSKLKVSLARKKPPMNSMRGGLPPREGRGMPPPLRGGPGGPGGPGGPMGRMGGRGGDRGGFPPRGPRGSRGNPSGGGNVQHRAGDWQCPNPGCGNQNFAWRTECNQCKAPKPEGFLPPPFPPPGGDRGRGGPGGMRGGRGGLMDRGGPGGMFRGGRGGDRGGFRGGRGMDRGGFGGGRRGGPGGPPGPLMEQMGGRRGGRGGPGKMDKGEHRQERRDRPY

>NP_005089.2

MSTGSVSDPEEMELRGLQREYPVPASKRPPLRGVERSYASPSDNSSAEEEDPDGEEERCALGTAGSAEGCKRKRPRVAGGGGAGGSAGGGGKKPLPAKGSAAECKQSQRNAANARERARMRVLSKAFSRLKTSLPWVPPDTKLSKLDTLRLASSYIAHLRQLLQEDRYENGYVHPVNLTWPFVVSGRPDSDTKEVSAANRLCGTTA

907 NP_003905.1 NP_000312.2

>NP_003905.1

METGFPAIMYPGSFIGGWGEEYLSWEGPGLPDFVFQQQPVESEAMHCSNPKSGVVLATVARGPDACQILTRAPLGQDPPQRTVLGLLTANGQYRRTCGQGITRIRCYSGSENAFPPAGKKALPDCGVQEPPKQGFDIYMDELEQGDRDSCSVREGMAFEDVYEVDTGTLKSDLHFLLDFNTVSPMLVDSSLLSQSEDISSLGTDVINVTEYAEEIYQYLREAEIRHRPKAHYMKKQPDITEGMRTILVDWLVEVGEEYKLRAETLYLAVNFLDRFLSCMSVLRGKLQLVGTAAMLLASKYEEIYPPEVDEFVYITDDTYTKRQLLKMEHLLLKVLAFDLTVPTTNQFLLQYLRRQGVCVRTENLAKYVAELSLLEADPFLKYLPSLIAAAAFCLANYTVNKHFWPETLAAFTGYSLSEIVPCLSELHKAYLDIPHRPQQAIREKYKASKYLCVSLMEPPAVLLLQ

>NP_000312.2

MPPKTPRKTAATAAAAAAEPPAPPPPPPPEEDPEQDSGPEDLPLVRLEFEETEEPDFTALCQKLKIPDHVRERAWLTWEKVSSVDGVLGGYIQKKKELWGICIFIAAVDLDEMSFTFTELQKNIEISVHKFFNLLKEIDTSTKVDNAMSRLLKKYDVLFALFSKLERTCELIYLTQPSSSISTEINSALVLKVSWITFLLAKGEVLQMEDDLVISFQLMLCVLDYFIKLSPPMLLKEPYKTAVIPINGSPRTPRRGQNRSARIAKQLENDTRIIEVLCKEHECNIDEVKNVYFKNFIPFMNSLGLVTSNGLPEVENLSKRYEEIYLKNKDLDARLFLDHDKTLQTDSIDSFETQRTPRKSNLDEEVNVIPPHTPVRTVMNTIQQLMMILNSASDQPSENLISYFNNCTVNPKESILKRVKDIGYIFKEKFAKAVGQGCVEIGSQRYKLGVRLYYRVMESMLKSEEERLSIQNFSKLLNDNIFHMSLLACALEVVMATYSRSTSQNLDSGTDLSFPWILNVLNLKAFDFYKVIESFIKAEGNLTREMIKHLERCEHRIMESLAWLSDSPLFDLIKQSKDREGPTDHLESACPLNLPLQNNHTAADMYLSPVRSPKKKGSTTRVNSTANAETQATSAFQTQKPLKSTSLSLFYKKVYRLAYLRLNTLCERLLSEHPELEHIIWTLFQHTLQNEYELMRDRHLDQIMMCSMYGICKVKNIDLKFKIIVTAYKDLPHAVQETFKRVLIKEEEYDSIIVFYNSVFMQRLKTNILQYASTRPPTLSPIPHIPRSPYKFPSSPLRIPGGNIYISPLKSPYKISEGLPTPTKMTPRSRILVSIGESFGTSEKFQKINQMVCNSDRVLKRSAEGSNPPKPLKKLRFDIEGSDEADGSKHLPGESKFQQKLAEMTSTRTRMQKQKMNDSMDTSNKEEK

908 NP_006245.2 NP_065574.2

>NP_006245.2

MAPFLRIAFNSYELGSLQAEDEANQPFCAVKMKEALSTERGKTLVQKKPTMYPEWKSTFDAHIYEGRVIQIVLMRAAEEPVSEVTVGVSVLAERCKKNNGKAEFWLDLQPQAKVLMSVQYFLEDVDCKQSMRSEDEAKFPTMNRRGAIKQAKIHYIKNHEFIATFFGQPTFCSVCKDFVWGLNKQGYKCRQCNAAIHKKCIDKIIGRCTGTAANSRDTIFQKERFNIDMPHRFKVHNYMSPTFCDHCGSLLWGLVKQGLKCEDCGMNVHHKCREKVANLCGINQKLLAEALNQVTQRASRRSDSASSEPVGIYQGFEKKTGVAGEDMQDNSGTYGKIWEGSSKCNINNFIFHKVLGKGSFGKVLLGELKGRGEYFAIKALKKDVVLIDDDVECTMVEKRVLTLAAENPFLTHLICTFQTKDHLFFVMEFLNGGDLMYHIQDKGRFELYRATFYAAEIMCGLQFLHSKGIIYRDLKLDNVLLDRDGHIKIADFGMCKENIFGESRASTFCGTPDYIAPEILQGLKYTFSVDWWSFGVLLYEMLIGQSPFHGDDEDELFESIRVDTPHYPRWITKESKDILEKLFEREPTKRLGVTGNIKIHPFFKTINWTLLEKRRLEPPFRPKVKSPRDYSNFDQEFLNEKARLSYSDKNLIDSMDQSAFAGFSFVNPKFEHLLED

>NP_065574.2

MGLRTAKKRGLGGGGKWKREEGGGTRGRREVRPACFLQSGGRGDPGDVGGPAGNPGCSPHPRAATRPPPLPAHTPAHTPEWCGAASAEAAEPRRAGPHLCIPAPGLTKTPILEKVPRKMAAKTPSSEESGLPKLPVPPLQQTLATYLQCMRHLVSEEQFRKSQAIVQQFGAPGGLGETLQQKLLERQEKTANWVSEYWLNDMYLNNRLALPVNSSPAVIFARQHFPGTDDQLRFAASLISGVLSYKALLDSHSIPTDCAKGQLSGQPLCMKQYYGLFSSYRLPGHTQDTLVAQNSSIMPEPEHVIVACCNQFFVLDVVINFRRLSEGDLFTQLRKIVKMASNEDERLPPIGLLTSDGRSEWAEARTVLVKDSTNRDSLDMIERCICLVCLDAPGGVELSDTHRALQLLHGGGYSKNGANRWYDKSLQFVVGRDGTCGVVCEHSPFDGIVLVQCTEHLLKHVTQSSRKLIRADSVSELPAPRRLRWKCSPEIQGHLASSAEKLQRIVKNLDFIVYKFDNYGKTFIKKQKCSPDAFIQVALQLAFYRLHRRLVPTYESASIRRFQEGRVDNIRSATPEALAFVRAVTDHKAAVPASEKLLLLKDAIRAQTAYTVMAITGMAIDNHLLALRELARAMCKELPEMFMDETYLMSNRFVLSTSQVPTTTEMFCCYGPVVPNGYGACYNPQPETILFCISSFHSCKETSSSKFAKAVEESLIDMRDLCSLLPPTESKPLATKEKATRPSQGHQP

909 NP_085153.1 NP_001076783.1

>NP_085153.1

MGGLFSRWRTKPSTVEVLESIDKEIQALEEFREKNQRLQKLWVGRLILYSSVLYLFTCLIVYLWYLPDEFTARLAMTLPFFAFPLIIWSIRTVIIFFFSKRTERNNEALDDLKSQRKKILEEVMEKETYKTAKLILERFDPDSKKAKECEPPSAGAAVTARPGQEIRQRTAAQRNLSPTPASPNQGPPPQVPVSPGPPKDSSAPGGPPERTVTPALSSNVLPRHLGSPATSVPGMGLHPPGPPLARPILPRERGALDRIVEYLVGDGPQNRYALICQQCFSHNGMALKEEFEYIAFRCAYCFFLNPARKTRPQAPRLPEFSFEKRQVVEGSSSVGPLPSGSVLSSDNQFNEESLEHDVLDDNTEQTDDKIPATEQTNQVIEKASDSEEPEEKQETENEEASVIETNSTVPGADSIPDPELSGESLTAE

>NP_001076783.1

MDVHGEAAGEAGQEGGEGLQGGAGQSEEGPSAEKCRVCPCVCRERHPQEERRCELASDGVPRRRSGLQGADSCDYEGGDQEYGPGDQSPGQGPEHHGPAEGLLSDGQVRAAGAEPGRPYIGDGGLHELGHHPDHAAGAGGQPHHADRRGEWPGGAGPAQPAARGRLCRGRELCAQPGGPAVTEVGRLEELAVPRRCAPPLPRDVLEGSCPLPTASCLCADPAGLRPAATLRLSPARPAWP

910 BAB62909.1 NP_003395.1

>BAB62909.1

MDRSKRNSIAGFPPRVERLEEFEGGGGGEGNVSQVGRVWPSSYRALISAFSRLTRLDDFTCEKIGSGFFSEVFKVRHRASGQVMALKMNTLSSNRANMLKEVQLMNRLSHPNILRFMGVCVHQGQLHALTEYINSGNLEQLLDSNLHLPWTVRVKLAYDIAVGLSYLHFKGIFHRDLTSKNCLIKRDENGYSAVVADFGLAEKIPDVSMGSEKLAVVGSPFWMAPEVLRDEPYNEKADVFSYGIILCEIIARIQADPDYLPRTENFGLDYDAFQHMVGDCPPDFLQLTFNCCNMDPKLRPSFVEIGKTLEEILSRLQEEEQERDRKLQPTARGLLEKAPGVKRLSSLDDKIPHKSPCPRRTIWLSRSQSDIFSRKPPRTVSVLDPYYRPRDGAARTPKVNPFSARQDLMGGKIKFFDLPSKSVISLVFDLDAPGPGTMPLADWQEPLAPPIRRWRSLPGSPEFLHQEACPFVGREESLSDGPPPRLSSLKYRVKEIPPFRASALPAAQAHEAMDCSILQEENGFGSRPQGTSPCPAGASEEMEVEERPAGSTPATFSTSGIGLQTQGKQDG

>NP_003395.1

MTMDKSELVQKAKLAEQAERYDDMAAAMKAVTEQGHELSNEERNLLSVAYKNVVGARRSSWRVISSIEQKTERNEKKQQMGKEYREKIEAELQDICNDVLELLDKYLIPNATQPESKVFYLKMKGDYFRYLSEVASGDNKQTTVSNSQQAYQEAFEISKKEMQPTHPIRLGLALNFSVFYYEILNSPEKACSLAKTAFDEAIAELDTLNEESYKDSTLIMQLLRDNLTLWTSENQGDEGDAGEGEN

911 NP_004111.2 NP_036379.2

>NP_004111.2

MAPEINLPGPMSLIDNTKGQLVVNPEALKILSAITQPVVVVAIVGLYRTGKSYLMNKLAGKKNGFSLGSTVKSHTKGIWMWCVPHPKKPEHTLVLLDTEGLGDIEKGDNENDSWIFALAILLSSTFVYNSMGTINQQAMDQLHYVTELTDRIKANSSPGNNSVDDSADFVSFFPAFVWTLRDFTLELEVDGEPITADDYLELSLKLRKGTDKKSKSFNDPRLCIRKFFPKRKCFVFDWPAPKKYLAHLEQLKEEELNPDFIEQVAEFCSYILSHSNVKTLSGGIPVNGPRLESLVLTYVNAISSGDLPCMENAVLALAQIENSAAVEKAIAHYEQQMGQKVQLPTETLQELLDLHRDSEREAIEVFMKNSFKDVDQMFQRKLGAQLEARRDDFCKQNSKASSDCCMALLQDIFGPLEEDVKQGTFSKPGGYRLFTQKLQELKNKYYQVPRKGIQAKEVLKKYLESKEDVADALLQTDQSLSEKEKAIEVERIKAESAEAAKKMLEEIQKKNEEMMEQKEKSYQEHVKQLTEKMERDRAQLMAEQEKTLALKLQEQERLLKEGFENESKRLQKDIWDIQMRSKSLEPICNIL

>NP_036379.2

MSTRESFNPESYELDKSFRLTRFTELKGTGCKVPQDVLQKLLESLQENHFQEDEQFLGAVMPRLGIGMDTCVIPLRHGGLSLVQTTDYIYPIVDDPYMMGRIACANVLSDLYAMGVTECDNMLMLLGVSNKMTDRERDKVMPLIIQGFKDAAEEAGTSVTGGQTVLNPWIVLGGVATTVCQPNEFIMPDNAVPGDVLVLTKPLGTQVAVAVHQWLDIPEKWNKIKLVVTQEDVELAYQEAMMNMARLNRTAAGLMHTFNAHAATDITGFGILGHAQNLAKQQRNEVSFVIHNLPVLAKMAAVSKACGNMFGLMHGTCPETSGGLLICLPREQAARFCAEIKSPKYGEGHQAWIIGIVEKGNRTARIIDKPRIIEVAPQVATQNVNPTPGATS

912 NP_002728.1 NP_001002878.1

>NP_002728.1

MADVFPGNDSTASQDVANRFARKGALRQKNVHEVKDHKFIARFFKQPTFCSHCTDFIWGFGKQGFQCQVCCFVVHKRCHEFVTFSCPGADKGPDTDDPRSKHKFKIHTYGSPTFCDHCGSLLYGLIHQGMKCDTCDMNVHKQCVINVPSLCGMDHTEKRGRIYLKAEVADEKLHVTVRDAKNLIPMDPNGLSDPYVKLKLIPDPKNESKQKTKTIRSTLNPQWNESFTFKLKPSDKDRRLSVEIWDWDRTTRNDFMGSLSFGVSELMKMPASGWYKLLNQEEGEYYNVPIPEGDEEGNMELRQKFEKAKLGPAGNKVISPSEDRKQPSNNLDRVKLTDFNFLMVLGKGSFGKVMLADRKGTEELYAIKILKKDVVIQDDDVECTMVEKRVLALLDKPPFLTQLHSCFQTVDRLYFVMEYVNGGDLMYHIQQVGKFKEPQAVFYAAEISIGLFFLHKRGIIYRDLKLDNVMLDSEGHIKIADFGMCKEHMMDGVTTRTFCGTPDYIAPEIIAYQPYGKSVDWWAYGVLLYEMLAGQPPFDGEDEDELFQSIMEHNVSYPKSLSKEAVSICKGLMTKHPAKRLGCGPEGERDVREHAFFRRIDWEKLENREIQPPFKPKVCGKGAENFDKFFTRGQPVLTPPDQLVIANIDQSDFEGFSYVNPQFVHPILQSAV

>NP_001002878.1

MSSESSKKRKPKVIRSDGAPAEGKRNRSDTEQEGKYYSEEAEVDLRDPGRDYELYKYTCQELQRLMAEIQDLKSRGGKDVAIEIEERRIQSCVHFMTLKKLNRLAHIRLKKGRDQTHEAKQKVDAYHLQLQNLLYEVMHLQKEITKCLEFKSKHEEIDLVSLEEFYKEAPPDISKAEVTMGDPHQQTLARLDWELEQRKRLAEKYRECLSNKEKILKEIEVKKEYLSSLQPRLNSIMQASLPVQEYLFMPFDQAHKQYETARHLPPPLYVLFVQATAYGQACDKTLSVAIEGSVDEAKALFKPPEDSQDDESDSDAEEEQTTKRRRPTLGVQLDDKRKEMLKRHPLSVMLDLKCKDDSVLHLTFYYLMNLNIMTVKAKVTTAMELITPISAGDLLSPDSVLSCLYPGDHGKKTPNPANQYQFDKVGILTLSDYVLELGHPYLWVQKLGGLHFPKEQPQQTVIADHSLSASHMETTMKLLKTRVQSRLALHKQFASLEHGIVPVTSDCQYLFPAKVVSRLVKWVTVAHEDYMELHFTKDIVDAGLAGDTNLYYMALIERGTAKLQAAVVLNPGYSSIPPVFQLCLNWKGEKTNSNDDNIRAMEGEVNVCYKELCGPWPSHQLLTNQLQRLCVLLDVYLETESHDDSVEGPKEFPQEKMCLRLFRGPSRMKPFKYNHPQGFFSHR

913 NP_000624.2 NP_000012.1

>NP_000624.2

MAHAGRTGYDNREIVMKYIHYKLSQRGYEWDAGDVGAAPPGAAPAPGIFSSQPGHTPHPAASRDPVARTSPLQTPAAPGAAAGPALSPVPPVVHLTLRQAGDDFSRRYRRDFAEMSSQLHLTPFTARGRFATVVEELFRDGVNWGRIVAFFEFGGVMCVESVNREMSPLVDNIALWMTEYLNRHLHTWIQDNGGWDAFVELYGPSMRPLFDFSWLSLKTLLSLALVGACITLGAYLGHK

>NP_000012.1

MTELPAPLSYFQNAQMSEDNHLSNTVRSQNDNRERQEHNDRRSLGHPEPLSNGRPQGNSRQVVEQDEEEDEELTLKYGAKHVIMLFVPVTLCMVVVVATIKSVSFYTRKDGQLIYTPFTEDTETVGQRALHSILNAAIMISVIVVMTILLVVLYKYRCYKVIHAWLIISSLLLLFFFSFIYLGEVFKTYNVAVDYITVALLIWNFGVVGMISIHWKGPLRLQQAYLIMISALMALVFIKYLPEWTAWLILAVISVYDLVAVLCPKGPLRMLVETAQERNETLFPALIYSSTMVWLVNMAEGDPEAQRRVSKNSKYNAESTERESQDTVAENDDGGFSEEWEAQRDSHLGPHRSTPESRAAVQELSSSILAGEDPEERGVKLGLGDFIFYSVLVGKASATASGDWNTTIACFVAILIGLCLTLLLLAIFKKALPALPISITFGLVFYFATDYLVQPFMDQLAFHQFYI

914 NP_036204.2 NP_000082.2

>NP_036204.2

MATSMGLLLLLLLLLTQPGAGTGADTEAVVCVGTACYTAHSGKLSAAEAQNHCNQNGGNLATVKSKEEAQHVQRVLAQLLRREAALTARMSKFWIGLQREKGKCLDPSLPLKGFSWVGGGEDTPYSNWHKELRNSCISKRCVSLLLDLSQPLLPSRLPKWSEGPCGSPGSPGSNIEGFVCKFSFKGMCRPLALGGPGQVTYTTPFQTTSSSLEAVPFASAANVACGEGDKDETQSHYFLCKEKAPDVFDWGSSGPLCVSPKYGCNFNNGGCHQDCFEGGDGSFLCGCRPGFRLLDDLVTCASRNPCSSSPCRGGATCVLGPHGKNYTCRCPQGYQLDSSQLDCVDVDECQDSPCAQECVNTPGGFRCECWVGYEPGGPGEGACQDVDECALGRSPCAQGCTNTDGSFHCSCEEGYVLAGEDGTQCQDVDECVGPGGPLCDSLCFNTQGSFHCGCLPGWVLAPNGVSCTMGPVSLGPPSGPPDEEDKGEKEGSTVPRAATASPTRGPEGTPKATPTTSRPSLSSDAPITSAPLKMLAPSGSPGVWREPSIHHATAASGPQEPAGGDSSVATQNNDGTDGQKLLLFYILGTVVAILLLLALALGLLVYRKRRAKREEKKEKKPQNAADSYSWVPERAESRAMENQYSPTPGTDC

>NP_000082.2

MSARTAPRPQVLLLPLLLVLLAAAPAASKGCVCKDKGQCFCDGAKGEKGEKGFPGPPGSPGQKGFTGPEGLPGPQGPKGFPGLPGLTGSKGVRGISGLPGFSGSPGLPGTPGNTGPYGLVGVPGCSGSKGEQGFPGLPGTLGYPGIPGAAGLKGQKGAPAKEEDIELDAKGDPGLPGAPGPQGLPGPPGFPGPVGPPGPPGFFGFPGAMGPRGPKGHMGERVIGHKGERGVKGLTGPPGPPGTVIVTLTGPDNRTDLKGEKGDKGAMGEPGPPGPSGLPGESYGSEKGAPGDPGLQGKPGKDGVPGFPGSEGVKGNRGFPGLMGEDGIKGQKGDIGPPGFRGPTEYYDTYQEKGDEGTPGPPGPRGARGPQGPSGPPGVPGSPGSSRPGLRGAPGWPGLKGSKGERGRPGKDAMGTPGSPGCAGSPGLPGSPGPPGPPGDIVFRKGPPGDHGLPGYLGSPGIPGVDGPKGEPGLLCTQCPYIPGPPGLPGLPGLHGVKGIPGRQGAAGLKGSPGSPGNTGLPGFPGFPGAQGDPGLKGEKGETLQPEGQVGVPGDPGLRGQPGRKGLDGIPGTPGVKGLPGPKGELALSGEKGDQGPPGDPGSPGSPGPAGPAGPPGYGPQGEPGLQGTQGVPGAPGPPGEAGPRGELSVSTPVPGPPGPPGPPGHPGPQGPPGIPGSLGKCGDPGLPGPDGEPGIPGIGFPGPPGPKGDQGFPGTKGSLGCPGKMGEPGLPGKPGLPGAKGEPAVAMPGGPGTPGFPGERGNSGEHGEIGLPGLPGLPGTPGNEGLDGPRGDPGQPGPPGEQGPPGRCIEGPRGAQGLPGLNGLKGQQGRRGKTGPKGDPGIPGLDRSGFPGETGSPGIPGHQGEMGPLGQRGYPGNPGILGPPGEDGVIGMMGFPGAIGPPGPPGNPGTPGQRGSPGIPGVKGQRGTPGAKGEQGDKGNPGPSEISHVIGDKGEPGLKGFAGNPGEKGNRGVPGMPGLKGLKGLPGPAGPPGPRGDLGSTGNPGEPGLRGIPGSMGNMGMPGSKGKRGTLGFPGRAGRPGLPGIHGLQGDKGEPGYSEGTRPGPPGPTGDPGLPGDMGKKGEMGQPGPPGHLGPAGPEGAPGSPGSPGLPGKPGPHGDLGFKGIKGLLGPPGIRGPPGLPGFPGSPGPMGIRGDQGRDGIPGPAGEKGETGLLRAPPGPRGNPGAQGAKGDRGAPGFPGLPGRKGAMGDAGPRGPTGIEGFPGPPGLPGAIIPGQTGNRGPPGSRGSPGAPGPPGPPGSHVIGIKGDKGSMGHPGPKGPPGTAGDMGPPGRLGAPGTPGLPGPRGDPGFQGFPGVKGEKGNPGFLGSIGPPGPIGPKGPPGVRGDPGTLKIISLPGSPGPPGTPGEPGMQGEPGPPGPPGNLGPCGPRGKPGKDGKPGTPGPAGEKGNKGSKGEPGPAGSDGLPGLKGKRGDSGSPATWTTRGFVFTRHSQTTAIPSCPEGTVPLYSGFSFLFVQGNQRAHGQDLGTLGSCLQRFTTMPFLFCNVNDVCNFASRNDYSYWLSTPALMPMNMAPITGRALEPYISRCTVCEGPAIAIAVHSQTTDIPPCPHGWISLWKGFSFIMFTSAGSEGTGQALASPGSCLEEFRASPFLECHGRGTCNYYSNSYSFWLASLNPERMFRKPIPSTVKAGELEKIISRCQVCMKKRH

915 NP_444252.1 NP_002650.1

>NP_444252.1

MAGWQSYVDNLMCDGCCQEAAIVGYCDAKYVWAATAGGVFQSITPIEIDMIVGKDREGFFTNGLTLGAKKCSVIRDSLYVDGDCTMDIRTKSQGGEPTYNVAVGRAGRVLVFVMGKEGVHGGGLNKKAYSMAKYLRDSGF

>NP_002650.1

MGHPPLLPLLLLLHTCVPASWGLRCMQCKTNGDCRVEECALGQDLCRTTIVRLWEEGEELELVEKSCTHSEKTNRTLSYRTGLKITSLTEVVCGLDLCNQGNSGRAVTYSRSRYLECISCGSSDMSCERGRHQSLQCRSPEEQCLDVVTHWIQEGEEGRPKDDRHLRGCGYLPGCPGSNGFHNNDTFHFLKCCNTTKCNEGPILELENLPQNGRQCYSCKGNSTHGCSSEETFLIDCRGPMNQCLVATGTHEPKNQSYMVRGCATASMCQHAHLGDAFSMNHIDVSCCTKSGCNHPDLDVQYRSGAAPQPGPAHLSLTITLLMTARLWGGTLLWT

916 NP_874369.1 NP_008896.1

>NP_874369.1

MAEVVSPVPGAGRREPGEVGRARGPPVADPGVALSPQGEIIEGCRLPVLRRNQDNEDEWPLAEILSVKDISGRKLFYVHYIDFNKRLDEWVTHERLDLKKIQFPKKEAKTPTKNGLPGSRPGSPEREVPASAQASGKTLPIPVQITLRFNLPKEREAIPGGEPDQPLSSSSCLQPNHRSTKRKVEVVSPATPVPSETAPASVFPQNGAARRAVAAQPGRKRKSNCLGTDEDSQDSSDGIPSAPRMTGSLVSDRSHDDIVTRMKNIECIELGRHRLKPWYFSPYPQELTTLPVLYLCEFCLKYGRSLKCLQRHLTKCDLRHPPGNEIYRKGTISFFEIDGRKNKSYSQNLCLLAKCFLDHKTLYYDTDPFLFYVMTEYDCKGFHIVGYFSKEKESTEDYNVACILTLPPYQRRGYGKLLIEFSYELSKVEGKTGTPEKPLSDLGLLSYRSYWSQTILEILMGLKSESGERPQITINEISEITSIKKEDVISTLQYLNLINYYKGQYILTLSEDIVDGHERAMLKRLLRIDSKCLHFTPKDWSKRGKW

>NP_008896.1

MSAQSVEEDSILIIPTPDEEEKILRVKLEEDPDGEEGSSIPWNHLPDPEIFRQRFRQFGYQDSPGPREAVSQLRELCRLWLRPETHTKEQILELVVLEQFVAILPKELQTWVRDHHPENGEEAVTVLEDLESELDDPGQPVSLRRRKREVLVEDMVSQEEAQGLPSSELDAVENQLKWASWELHSLRHCDDDGRTENGALAPKQELPSALESHEVPGTLSMGVPQIFKYGETCFPKGRFERKRNPSRKKQHICDECGKHFSQGSALILHQRIHSGEKPYGCVECGKAFSRSSILVQHQRVHTGEKPYKCLECGKAFSQNSGLINHQRIHTGEKPYECVQCGKSYSQSSNLFRHQRRHNAEKLLNVVEV

917 NP_982260.1 NP_996919.1

>NP_982260.1

METRKPAERLALPYSLRTAPLGVPGTLPGLPRRDPLRVALRLDAACWEWARSGCARGWQYLPVPLDSAFEPAFLRKRNERERQRVRCVNEGYARLRDHLPRELADKRLSKVETLRAAIDYIKHLQELLERQAWGLEGAAGAVPQRRAECNSDGESKASSAPSPSSEPEEGGS

>NP_996919.1

MNPQQQRMAAIGTDKELSDLLDFSAMFSPPVNSGKTRPTTLGSSQFSGSGIDERGGTTSWGTSGQPSPSYDSSRGFTDSPHYSDHLNDSRLGAHEGLSPTPFMNSNLMGKTSERGSFSLYSRDTGLPGCQSSLLRQDLGLGSPAQLSSSGKPGTAYYSFSATSSRRRPLHDSAALDPLQAKKVRKVPPGLPSSVYAPSPNSDDFNRESPSYPSPKPPTSMFASTFFMQDGTHNSSDLWSSSNGMSQPGFGGILGTSTSHMSQSSSYGNLHSHDRLSYPPHSVSPTDINTSLPPMSSFHRGSTSSSPYVAASHTPPINGSDSILGTRGNAAGSSQTGDALGKALASIYSPDHTSSSFPSNPSTPVGSPSPLTGTSQWPRPGGQAPSSPSYENSLHSLKNRVEQQLHEHLQDAMSFLKDVCEQSRMEDRLDRLDDAIHVLRNHAVGPSTSLPAGHSDIHSLLGPSHNAPIGSLNSNYGGSSLVASSRSASMVGTHREDSVSLNGNHSVLSSTVTTSSTDLNHKTQENYRGGLQSQSGTVVTTEIKTENKEKDENLHEPPSSDDMKSDDESSQKDIKVSSRGRTSSTNEDEDLNPEQKIEREKERRMANNARERLRVRDINEAFKELGRMCQLHLKSEKPQTKLLILHQAVAVILSLEQQVRERNLNPKAACLKRREEEKVSAVSAEPPTTLPGTHPGLSETTNPMGHM

918 NP_057336.2 NP_004337.2

>NP_057336.2

MAAAAAAASGPGCSSAAGAGAAGVSEWLVLRDGCMHCDADGLHSLSYHPALNAILAVTSRGTIKVIDGTSGATLQASALSAKPGGQVKCQYISAVDKVIFVDDYAVGCRKDLNGILLLDTALQTPVSKQDDVVQLELPVTEAQQLLSACLEKVDISSTEGYDLFITQLKDGLKNTSHETAANHKVAKWATVTFHLPHHVLKSIASAIVNELKKINQNVAALPVASSVMDRLSYLLPSARPELGVGPGRSVDRSLMYSEANRRETFTSWPHVGYRWAQPDPMAQAGFYHQPASSGDDRAMCFTCSVCLVCWEPTDEPWSEHERHSPNCPFVKGEHTQNVPLSVTLATSPAQFPCTDGTDRISCFGSGSCPHFLAAATKRGKICIWDVSKLMKVHLKFEINAYDPAIVQQLILSGDPSSGVDSRRPTLAWLEDSSSCSDIPKLEGDSDDLLEDSDSEEHSRSDSVTGHTSQKEAMEVSLDITALSILQQPEKLQWEIVANVLEDTVKDLEELGANPCLTNSKSEKTKEKHQEQHNIPFPCLLAGGLLTYKSPATSPISSNSHRSLDGLSRTQGESISEQGSTDNESCTNSELNSPLVRRTLPVLLLYSIKESDEKAGKIFSQMNNIMSKSLHDDGFTVPQIIEMELDSQEQLLLQDPPVTYIQQFADAAANLTSPDSEKWNSVFPKPGTLVQCLRLPKFAEEENLCIDSITPCADGIHLLVGLRTCPVESLSAINQVEALNNLNKLNSALCNRRKGELESNLAVVNGANISVIQHESPADVQTPLIIQPEQRNVSGGYLVLYKMNYATRIVTLEEEPIKIQHIKDPQDTITSLILLPPDILDNREDDCEEPIEDMQLTSKNGFEREKTSDISTLGHLVITTQGGYVKILDLSNFEILAKVEPPKKEGTEEQDTFVSVIYCSGTDRLCACTKGGELHFLQIGGTCDDIDEADILVDGSLSKGIEPSSEGSKPLSNPSSPGISGVDLLVDQPFTLEILTSLVELTRFETLTPRFSATVPPCWVEVQQEQQQRRHPQHLHQQHHGDAAQHTRTWKLQTDSNSWDEHVFELVLPKACMVGHVDFKFVLNSNITNIPQIQVTLLKNKAPGLGKVNALNIEVEQNGKPSLVDLNEEMQHMDVEESQCLRLCPFLEDHKEDILCGPVWLASGLDLSGHAGMLTLTSPKLVKGMAGGKYRSFLIHVKAVNERGTEEICNGGMRPVVRLPSLKHQSNKGYSLASLLAKVAAGKEKSSNVKNENTSGTRKSENLRGCDLLQEVSVTIRRFKKTSISKERVQRCAMLQFSEFHEKLLNTLCRKTDDGQITEHAQSLVLDTLCWLAGVHSNGPGSSKEGNENLLSKTRKFLSDIVRVCFFEAGRSIAHKCARFLALCISNGKCDPCQPAFGPVLLKALLDNMSFLPAATTGGSVYWYFVLLNYVKDEDLAGCSTACASLLTAVSRQLQDRLTPMEALLQTRYGLYSSPFDPVLFDLEMSGSSCKNVYNSSIGVQSDEIDLSDVLSGNGKVSSCTAAEGSFTSLTGLLEVEPLHFTCVSTSDGTRIERDDAMSSFGVTPAVGGLSSGTVGEASTALSSAAQVALQSLSHAMASAEQQLQVLQEKQQQLLKLQQQKAKLEAKLHQTTAAAAAAASAVGPVHNSVPSNPVAAPGFFIHPSDVIPPTPKTTPLFMTPPLTPPNEAVSVVINAELAQLFPGSVIDPPAVNLAAHNKNSNKSRMNPLGSGLALAISHASHFLQPPPHQSIIIERMHSGARRFVTLDFGRPILLTDVLIPTCGDLASLSIDIWTLGEEVDGRRLVVATDISTHSLILHDLIPPPVCRFMKITVIGRYGSTNARAKIPLGFYYGHTYILPWESELKLMHDPLKGEGESANQPEIDQHLAMMVALQEDIQCRYNLACHRLETLLQSIDLPPLNSANNAQYFLRKPDKAVEEDSRVFSAYQDCIQLQLQLNLAHNAVQRLKVALGASRKMLSETSNPEDLIQTSSTEQLRTIIRYLLDTLLSLLHASNGHSVPAVLQSTFHAQACEELFKHLCISGTPKIRLHTGLLLVQLCGGERWWGQFLSNVLQELYNSEQLLIFPQDRVFMLLSCIGQRSLSNSGVLESLLNLLDNLLSPLQPQLPMHRRTEGVLDIPMISWVVMLVSRLLDYVATVEDEAAAAKKPLNGNQWSFINNNLHTQSLNRSSKGSSSLDRLYSRKIRKQLVHHKQQLNLLKAKQKALVEQMEKEKIQSNKGSSYKLLVEQAKLKQATSKHFKDLIRLRRTAEWSRSNLDTEVTTAKESPEIEPLPFTLAHERCISVVQKLVLFLLSMDFTCHADLLLFVCKVLARIANATRPTIHLCEIVNEPQLERLLLLLVGTDFNRGDISWGGAWAQYSLTCMLQDILAGELLAPVAAEAMEEGTVGDDVGATAGDSDDSLQQSSVQLLETIDEPLTHDITGAPPLSSLEKDKEIDLELLQDLMEVDIDPLDIDLEKDPLAAKVFKPISSTWYDYWGADYGTYNYNPYIGGLGIPVAKPPANTEKNGSQTVSVSVSQALDARLEVGLEQQAELMLKMMSTLEADSILQALTNTSPTLSQSPTGTDDSLLGGLQAANQTSQLIIQLSSVPMLNVCFNKLFSMLQVHHVQLESLLQLWLTLSLNSSSTGNKENGADIFLYNANRIPVISLNQASITSFLTVLAWYPNTLLRTWCLVLHSLTLMTNMQLNSGSSSAIGTQESTAHLLVSDPNLIHVLVKFLSGTSPHGTNQHSPQVGPTATQAMQEFLTRLQVHLSSTCPQIFSEFLLKLIHILSTERGAFQTGQGPLDAQVKLLEFTLEQNFEVVSVSTISAVIESVTFLVHHYITCSDKVMSRSGSDSSVGARACFGGLFANLIRPGDAKAVCGEMTRDQLMFDLLKLVNILVQLPLSGNREYSARVSVTTNTTDSVSDEEKVSGGKDGNGSSTSVQGSPAYVADLVLANQQIMSQILSALGLCNSSAMAMIIGASGLHLTKHENFHGGLDAISVGDGLFTILTTLSKKASTVHMMLQPILTYMACGYMGRQGSLATCQLSEPLLWFILRVLDTSDALKAFHDMGGVQLICNNMVTSTRAIVNTARSMVSTIMKFLDSGPNKAVDSTLKTRILASEPDNAEGIHNFAPLGTITSSSPTAQPAEVLLQATPPHRRARSAAWSYIFLPEEAWCDLTIHLPAAVLLKEIHIQPHLASLATCPSSVSVEVSADGVNMLPLSTPVVTSGLTYIKIQLVKAEVASAVCLRLHRPRDASTLGLSQIKLLGLTAFGTTSSATVNNPFLPSEDQVSKTSIGWLRLLHHCLTHISDLEGMMASAAAPTANLLQTCAALLMSPYCGMHSPNIEVVLVKIGLQSTRIGLKLIDILLRNCAASGSDPTDLNSPLLFGRLNGLSSDSTIDILYQLGTTQDPGTKDRIQALLKWVSDSARVAAMKRSGRMNYMCPNSSTVEYGLLMPSPSHLHCVAAILWHSYELLVEYDLPALLDQELFELLFNWSMSLPCNMVLKKAVDSLLCSMCHVHPNYFSLLMGWMGITPPPVQCHHRLSMTDDSKKQDLSSSLTDDSKNAQAPLALTESHLATLASSSQSPEAIKQLLDSGLPSLLVRSLASFCFSHISSSESIAQSIDISQDKLRRHHVPQQCNKMPITADLVAPILRFLTEVGNSHIMKDWLGGSEVNPLWTALLFLLCHSGSTSGSHNLGAQQTSARSASLSSAATTGLTTQQRTAIENATVAFFLQCISCHPNNQKLMAQVLCELFQTSPQRGNLPTSGNISGFIRRLFLQLMLEDEKVTMFLQSPCPLYKGRINATSHVIQHPMYGAGHKFRTLHLPVSTTLSDVLDRVSDTPSITAKLISEQKDDKEKKNHEEKEKVKAENGFQDNYSVVVASGLKSQSKRAVSATPPRPPSRRGRTIPDKIGSTSGAEAANKIITVPVFHLFHKLLAGQPLPAEMTLAQLLTLLYDRKLPQGYRSIDLTVKLGSRVITDPSLSKTDSYKRLHPEKDHGDLLASCPEDEALTPGDECMDGILDESLLETCPIQSPLQVFAGMGGLALIAERLPMLYPEVIQQVSAPVVTSTTQEKPKDSDQFEWVTIEQSGELVYEAPETVAAEPPPIKSAVQTMSPIPAHSLAAFGLFLRLPGYAEVLLKERKHAQCLLRLVLGVTDDGEGSHILQSPSANVLPTLPFHVLRSLFSTTPLTTDDGVLLRRMALEIGALHLILVCLSALSHHSPRVPNSSVNQTEPQVSSSHNPTSTEEQQLYWAKGTGFGTGSTASGWDVEQALTKQRLEEEHVTCLLQVLASYINPVSSAVNGEAQSSHETRGQNSNALPSVLLELLSQSCLIPAMSSYLRNDSVLDMARHVPLYRALLELLRAIASCAAMVPLLLPLSTENGEEEEEQSECQTSVGTLLAKMKTCVDTYTNRLRSKRENVKTGVKPDASDQEPEGLTLLVPDIQKTAEIVYAATTSLRQANQEKKLGEYSKKAAMKPKPLSVLKSLEEKYVAVMKKLQFDTFEMVSEDEDGKLGFKVNYHYMSQVKNANDANSAARARRLAQEAVTLSTSLPLSSSSSVFVRCDEERLDIMKVLITGPADTPYANGCFEFDVYFPQDYPSSPPLVNLETTGGHSVRFNPNLYNDGKVCLSILNTWHGRPEEKWNPQTSSFLQVLVSVQSLILVAEPYFNEPGYERSRGTPSGTQSSREYDGNIRQATVKWAMLEQIRNPSPCFKEVIHKHFYLKRVEIMAQCEEWIADIQQYSSDKRVGRTMSHHAAALKRHTAQLREELLKLPCPEGLDPDTDDAPEVCRATTGAEETLMHDQVKPSSSKELPSDFQL

>NP_004337.2

MENTENSVDSKSIKNLEPKIIHGSESMDSGISLDNSYKMDYPEMGLCIIINNKNFHKSTGMTSRSGTDVDAANLRETFRNLKYEVRNKNDLTREEIVELMRDVSKEDHSKRSSFVCVLLSHGEEGIIFGTNGPVDLKKITNFFRGDRCRSLTGKPKLFIIQACRGTELDCGIETDSGVDDDMACHKIPVEADFLYAYSTAPGYYSWRNSKDGSWFIQSLCAMLKQYADKLEFMHILTRVNRKVATEFESFSFDATFHAKKQIPCIVSMLTKELYFYH

919 NP_002728.1 NP_004235.3

>NP_002728.1

MADVFPGNDSTASQDVANRFARKGALRQKNVHEVKDHKFIARFFKQPTFCSHCTDFIWGFGKQGFQCQVCCFVVHKRCHEFVTFSCPGADKGPDTDDPRSKHKFKIHTYGSPTFCDHCGSLLYGLIHQGMKCDTCDMNVHKQCVINVPSLCGMDHTEKRGRIYLKAEVADEKLHVTVRDAKNLIPMDPNGLSDPYVKLKLIPDPKNESKQKTKTIRSTLNPQWNESFTFKLKPSDKDRRLSVEIWDWDRTTRNDFMGSLSFGVSELMKMPASGWYKLLNQEEGEYYNVPIPEGDEEGNMELRQKFEKAKLGPAGNKVISPSEDRKQPSNNLDRVKLTDFNFLMVLGKGSFGKVMLADRKGTEELYAIKILKKDVVIQDDDVECTMVEKRVLALLDKPPFLTQLHSCFQTVDRLYFVMEYVNGGDLMYHIQQVGKFKEPQAVFYAAEISIGLFFLHKRGIIYRDLKLDNVMLDSEGHIKIADFGMCKEHMMDGVTTRTFCGTPDYIAPEIIAYQPYGKSVDWWAYGVLLYEMLAGQPPFDGEDEDELFQSIMEHNVSYPKSLSKEAVSICKGLMTKHPAKRLGCGPEGERDVREHAFFRRIDWEKLENREIQPPFKPKVCGKGAENFDKFFTRGQPVLTPPDQLVIANIDQSDFEGFSYVNPQFVHPILQSAV

>NP_004235.3

MSKLRMVLLEDSGSADFRRHFVNLSPFTITVVLLLSACFVTSSLGGTDKELRLVDGENKCSGRVEVKVQEEWGTVCNNGWSMEAVSVICNQLGCPTAIKAPGWANSSAGSGRIWMDHVSCRGNESALWDCKHDGWGKHSNCTHQQDAGVTCSDGSNLEMRLTRGGNMCSGRIEIKFQGRWGTVCDDNFNIDHASVICRQLECGSAVSFSGSSNFGEGSGPIWFDDLICNGNESALWNCKHQGWGKHNCDHAEDAGVICSKGADLSLRLVDGVTECSGRLEVRFQGEWGTICDDGWDSYDAAVACKQLGCPTAVTAIGRVNASKGFGHIWLDSVSCQGHEPAVWQCKHHEWGKHYCNHNEDAGVTCSDGSDLELRLRGGGSRCAGTVEVEIQRLLGKVCDRGWGLKEADVVCRQLGCGSALKTSYQVYSKIQATNTWLFLSSCNGNETSLWDCKNWQWGGLTCDHYEEAKITCSAHREPRLVGGDIPCSGRVEVKHGDTWGSICDSDFSLEAASVLCRELQCGTVVSILGGAHFGEGNGQIWAEEFQCEGHESHLSLCPVAPRPEGTCSHSRDVGVVCSRYTEIRLVNGKTPCEGRVELKTLGAWGSLCNSHWDIEDAHVLCQQLKCGVALSTPGGARFGKGNGQIWRHMFHCTGTEQHMGDCPVTALGASLCPSEQVASVICSGNQSQTLSSCNSSSLGPTRPTIPEESAVACIESGQLRLVNGGGRCAGRVEIYHEGSWGTICDDSWDLSDAHVVCRQLGCGEAINATGSAHFGEGTGPIWLDEMKCNGKESRIWQCHSHGWGQQNCRHKEDAGVICSEFMSLRLTSEASREACAGRLEVFYNGAWGTVGKSSMSETTVGVVCRQLGCADKGKINPASLDKAMSIPMWVDNVQCPKGPDTLWQCPSSPWEKRLASPSEETWITCDNKIRLQEGPTSCSGRVEIWHGGSWGTVCDDSWDLDDAQVVCQQLGCGPALKAFKEAEFGQGTGPIWLNEVKCKGNESSLWDCPARRWGHSECGHKEDAAVNCTDISVQKTPQKATTGRSSRQSSFIAVGILGVVLLAIFVALFFLTKKRRQRQRLAVSSRGENLVHQIQYREMNSCLNADDLDLMNSSENSHESADFSAAELISVSKFLPISGMEKEAILSHTEKENGNL

920 NP_003675.2 NP_003751.2

>NP_003675.2

MVSSQKLEKPIEMGSSEPLPIADGDRRRKKKRRGRATDSLPGKFEDMYKLTSELLGEGAYAKVQGAVSLQNGKEYAVKIIEKQAGHSRSRVFREVETLYQCQGNKNILELIEFFEDDTRFYLVFEKLQGGSILAHIQKQKHFNEREASRVVRDVAAALDFLHTKDKVSLCHLGWSAMAPSGLTAAPTSLGSSDPPTSASQVAGTTGIAHRDLKPENILCESPEKVSPVKICDFDLGSGMKLNNSCTPITTPELTTPCGSAEYMAPEVVEVFTDQATFYDKRCDLWSLGVVLYIMLSGYPPFVGHCGADCGWDRGEVCRVCQNKLFESIQEGKYEFPDKDWAHISSEAKDLISKLLVRDAKQRLSAAQVLQHPWVQGQAPEKGLPTPQVLQRNSSTMDLTLFAAEAIALNRQLSQHEENELAEEPEALADGLCSMKLSPPCKSRLARRRALAQAGRGEDRSPPTAL

>NP_003751.2

MNSQPQTRSPFFQRPQIQPPRATIPNSSPSIRPGAQTPTAVYQANQHIMMVNHLPMPYPVPQGPQYCIPQYRHSGPPYVGPPQQYPVQPPGPGPFYPGPGPGDFPNAYGTPFYPSQPVYQSAPIIVPTQQQPPPAKREKKTIRIRDPNQGGKDITEEIMSGGGSRNPTPPIGRPTSTPTPPQQLPSQVPEHSPVVYGTVESAHLAASTPVTAASDQKQEEKPKPDPVLKSPSPVLRLVLSGEKKEQEGQTSETTAIVSIAELPLPPSPTTVSSVARSTIAAPTSSALSSQPIFTTAIDDRCELSSPREDTIPIPSLTSCTETSDPLPTNENDDDICKKPCSVAPNDIPLVSSTNLINEINGVSEKLSATESIVEIVKQEVLPLTLELEILENPPEEMKLECIPAPITPSTVPSFPPTPPTPPASPPHTPVIVPAAATTVSSPSAAITVQRVLEEDESIRTCLSEDAKEIQNKIEVEADGQTEEILDSQNLNSRRSPVPAQIAITVPKTWKKPKDRTRTTEEMLEAELELKAEEELSIDKVLESEQDKMSQGFHPERDPSDLKKVKAVEENGEEAEPVRNGAESVSEGEGIDANSGSTDSSGDGVTFPFKPESWKPTDTEGKKQYDREFLLDFQFMPACIQKPEGLPPISDVVLDKINQPKLPMRTLDPRILPRGPDFTPAFADFGRQTPGGRGVPLLNVGSRRSQPGQRREPRKIITVSVKEDVHLKKAENAWKPSQKRDSQADDPENIKTQELFRKVRSILNKLTPQMFNQLMKQVSGLTVDTEERLKGVIDLVFEKAIDEPSFSVAYANMCRCLVTLKVPMADKPGNTVNFRKLLLNRCQKEFEKDKADDDVFEKKQKELEAASAPEERTRLHDELEEAKDKARRRSIGNIKFIGELFKLKMLTEAIMHDCVVKLLKNHDEESLECLCRLLTTIGKDLDFEKAKPRMDQYFNQMEKIVKERKTSSRIRFMLQDVIDLRLCNWVSRRADQGPKTIEQIHKEAKIEEQEEQRKVQQLMTKEKRRPGVQRVDEGGWNTVQGAKNSRVLDPSKFLKITKPTIDEKIQLVPKAQLGSWGKGSSGGAKASETDALRSSASSLNRFSALQPPAPSGSTPSTPVEFDSRRTLTSRGSMGREKNDKPLPSATARPNTFMRGGSSKDLLDNQSQEEQRREMLETVKQLTGGVDVERNSTEAERNKTRESAKPEISAMSAHDKAALSEEELERKSKSIIDEFLHINDFKEAMQCVEELNAQGLLHVFVRVGVESTLERSQITRDHMGQLLYQLVQSEKLSKQDFFKGFSETLELADDMAIDIPHIWLYLAELVTPMLKEGGISMRELTIEFSKPLLPVGRAGVLLSEILHLLCKQMSHKKVGALWREADLSWKDFLPEGEDVHNFLLEQKLDFIESDSPCSSEALSKKELSAEELYKRLEKLIIEDKANDEQIFDWVEANLDEIQMSSPTFLRALMTAVCKAAIIADSSTFRVDTAVIKQRVPILLKYLDSDTEKELQALYALQASIVKLDQPANLLRMFFDCLYDEEVISEDAFYKWESSKDPAEQNGKGVALKSVTAFFTWLREAEEESEDN

921 NP_055889.2 NP_001356.1

>NP_055889.2

MSGASVKVAVRVRPFNSRETSKESKCIIQMQGNSTSIINPKNPKEAPKSFSFDYSYWSHTSPEDPCFASQNRVYNDIGKEMLLHAFEGYNVCIFAYGQTGAGKSYTMMGKQEESQAGIIPQLCEELFEKINDNCNEEMSYSVEVSYMEIYCERVRDLLNPKNKGNLRVREHPLLGPYVEDLSKLAVTSYTDIADLMDAGNKARTVAATNMNETSSRSHAVFTIVFTQKKHDNETNLSTEKVSKISLVDLAGSERADSTGAKGTRLKEGANINKSLTTLGKVISALAEVSKKKKKTDFIPYRDSVLTWLLRENLGGNSRTAMVAALSPADINYDETLSTLRYADRAKQIKCNAVINEDPNAKLVRELKEEVTRLKDLLRAQGLGDIIDTSMGSLTSSPSSCSLSSQVGLTSVTSIQERIMSTPGGEEAIERLKESEKIIAELNETWEEKLRKTEAIRMEREALLAEMGVAIREDGGTLGVFSPKKTPHLVNLNEDPLMSECLLYYIKDGITRVGQADAERRQDIVLSGAHIKEEHCIFRSERSNSGEVIVTLEPCERSETYVNGKRVSQPVQLRSGNRIIMGKNHVFRFNHPEQARAEREKTPSAETPSEPVDWTFAQRELLEKQGIDMKQEMEKRLQEMEILYKKEKEEADLLLEQQRLDYESKLQALQKQVETRSLAAETTEEEEEEEEVPWTQHEFELAQWAFRKWKSHQFTSLRDLLWGNAVYLKEANAISVELKKKVQFQFVLLTDTLYSPLPPELLPTEMEKTHEDRPFPRTVVAVEVQDLKNGATHYWSLEKLKQRLDLMREMYDRAGEMASSAQDESETTVTGSDPFYDRFHWFKLVGSSPIFHGCVNERLADRTPSPTFSTADSDITELADEQQDEMEDFDDEAFVDDAGSDAGTEEGSDLFSDGHDPFYDRSPWFILVGRAFVYLSNLLYPVPLIHRVAIVSEKGEVRGFLRVAVQAIAADEEAPDYGSGIRQSGTAKISFDNEYFNQSDFSSVAMTRSGLSLEELRIVEGQGQSSEVITPPEEISRINDLDLKSSTLLDGKMVMEGFSEEIGNHLKLGSAFTFRVTVLQASGILPEYADIFCQFNFLHRHDEAFSTEPLKNNGRGSPLAFYHVQNIAVEITESFVDYIKTKPIVFEVFGHYQQHPLHLQGQELNSPPQPCRRFFPPPMPLSKPVPATKLNTMSKTSLGQSMSKYDLLVWFEISELEPTGEYIPAVVDHTAGLPCQGTFLLHQGIQRRITVTIIHEKGSELHWKDVRELVVGRIRNKPEVDEAAVDAILSLNIISAKYLKSSHNSSRTFYRFEAVWDSSLHNSLLLNRVTPYGEKIYMTLSAYLELDHCIQPAVITKDVCMVFYSRDAKISPPRSLRSLFGSGYSKSPDSNRVTGIYELSLCKMSDTGSPGMQRRRRKILDTSVAYVRGEENLAGWRPRGDSLILEHQWELEKLELLHEVEKTRHFLLLRERLGDSIPKSLSDSLSPSLSSGTLSTSTSISSQISTTTFESAITPSESSGYDSGDIESLVDREKELATKCLQLLTHTFNREFSQVHGSVSDCKLSDISPIGRDPSESSFSSATLTPSSTCPSLVDSRSNSLDQKTPEANSRASSPCPEFEQFQIVPAVETPYLARAGKNEFLNLVPDIEEIRPSSVVSKKGYLHFKEPLYSNWAKHFVVVRRPYVFIYNSDKDPVERGIINLSTAQVEYSEDQQAMVKTPNTFAVCTKHRGVLLQALNDKDMNDWLYAFNPLLAGTIRSKLSRRCPSQSKY

>NP_001356.1

MSQRPRAPRSALWLLAPPLLRWAPPLLTVLHSDLFQALLDILDYYEASLSESQKYRYQDEDTPPLEHSPAHLPNQANSPPVIVNTDTLEAPGYELQVNGTEGEMEYEEITLERGNSGLGFSIAGGTDNPHIGDDPSIFITKIIPGGAAAQDGRLRVNDSILFVNEVDVREVTHSAAVEALKEAGSIVRLYVMRRKPPAEKVMEIKLIKGPKGLGFSIAGGVGNQHIPGDNSIYVTKIIEGGAAHKDGRLQIGDKILAVNSVGLEDVMHEDAVAALKNTYDVVYLKVAKPSNAYLSDSYAPPDITTSYSQHLDNEISHSSYLGTDYPTAMTPTSPRRYSPVAKDLLGEEDIPREPRRIVIHRGSTGLGFNIVGGEDGEGIFISFILAGGPADLSGELRKGDQILSVNGVDLRNASHEQAAIALKNAGQTVTIIAQYKPEEYSRFEAKIHDLREQLMNSSLGSGTASLRSNPKRGFYIRALFDYDKTKDCGFLSQALSFRFGDVLHVIDASDEEWWQARRVHSDSETDDIGFIPSKRRVERREWSRLKAKDWGSSSGSQGREDSVLSYETVTQMEVHYARPIIILGPTKDRANDDLLSEFPDKFGSCVPHTTRPKREYEIDGRDYHFVSSREKMEKDIQAHKFIEAGQYNSHLYGTSVQSVREVAEQGKHCILDVSANAVRRLQAAHLHPIAIFIRPRSLENVLEINKRITEEQARKAFDRATKLEQEFTECFSAIVEGDSFEEIYHKVKRVIEDLSGPYIWVPARERL

922 NP_004577.1 NP_058519.2

>NP_004577.1

MPLAQLADPWQKMAVESPSDSAENGQQIMDEPMGEEEINPQTEEVSIKEIAITHHVKEGHEKADPSQFELLKVLGQGSFGKVFLVKKISGSDARQLYAMKVLKKATLKVRDRVRTKMERDILVEVNHPFIVKLHYAFQTEGKLYLILDFLRGGDLFTRLSKEVMFTEEDVKFYLAELALALDHLHSLGIIYRDLKPENILLDEEGHIKLTDFGLSKESIDHEKKAYSFCGTVEYMAPEVVNRRGHTQSADWWSFGVLMFEMLTGTLPFQGKDRKETMTMILKAKLGMPQFLSPEAQSLLRMLFKRNPANRLGAGPDGVEEIKRHSFFSTIDWNKLYRREIHPPFKPATGRPEDTFYFDPEFTAKTPKDSPGIPPSANAHQLFRGFSFVAITSDDESQAMQTVGVHSIVQQLHRNSIQFTDGYEVKEDIGVGSYSVCKRCIHKATNMEFAVKIIDKSKRDPTEEIEILLRYGQHPNIITLKDVYDDGKYVYVVTELMKGGELLDKILRQKFFSEREASAVLFTITKTVEYLHAQGVVHRDLKPSNILYVDESGNPESIRICDFGFAKQLRAENGLLMTPCYTANFVAPEVLKRQGYDAACDIWSLGVLLYTMLTGYTPFANGPDDTPEEILARIGSGKFSLSGGYWNSVSDTAKDLVSKMLHVDPHQRLTAALVLRHPWIVHWDQLPQYQLNRQDAPHLVKGAMAATYSALNRNQSPVLEPVGRSTLAQRRGIKKITSTAL

>NP_058519.2

MAEPRQEFEVMEDHAGTYGLGDRKDQGGYTMHQDQEGDTDAGLKESPLQTPTEDGSEEPGSETSDAKSTPTAEDVTAPLVDEGAPGKQAAAQPHTEIPEGTTAEEAGIGDTPSLEDEAAGHVTQEPESGKVVQEGFLREPGPPGLSHQLMSGMPGAPLLPEGPREATRQPSGTGPEDTEGGRHAPELLKHQLLGDLHQEGPPLKGAGGKERPGSKEEVDEDRDVDESSPQDSPPSKASPAQDGRPPQTAAREATSIPGFPAEGAIPLPVDFLSKVSTEIPASEPDGPSVGRAKGQDAPLEFTFHVEITPNVQKEQAHSEEHLGRAAFPGAPGEGPEARGPSLGEDTKEADLPEPSEKQPAAAPRGKPVSRVPQLKARMVSKSKDGTGSDDKKAKTSTRSSAKTLKNRPCLSPKHPTPGSSDPLIQPSSPAVCPEPPSSPKHVSSVTSRTGSSGAKEMKLKGADGKTKIATPRGAAPPGQKGQANATRIPAKTPPAPKTPPSSGEPPKSGDRSGYSSPGSPGTPGSRSRTPSLPTPPTREPKKVAVVRTPPKSPSSAKSRLQTAPVPMPDLKNVKSKIGSTENLKHQPGGGKVQIINKKLDLSNVQSKCGSKDNIKHVPGGGSVQIVYKPVDLSKVTSKCGSLGNIHHKPGGGQVEVKSEKLDFKDRVQSKIGSLDNITHVPGGGNKKIETHKLTFRENAKAKTDHGAEIVYKSPVVSGDTSPRHLSNVSSTGSIDMVDSPQLATLADEVSASLAKQGL

923 NP_116584.2 NP_003397.1

>NP_116584.2

MLSNSQGQSPPVPFPAPAPPPQPPTPALPHPPAQPPPPPPQQFPQFHVKSGLQIKKNAIIDDYKVTSQVLGLGINGKVLQIFNKRTQEKFALKMLQDCPKARREVELHWRASQCPHIVRIVDVYENLYAGRKCLLIVMECLDGGELFSRIQDRGDQAFTEREASEIMKSIGEAIQYLHSINIAHRDVKPENLLYTSKRPNAILKLTDFGFAKETTSHNSLTTPCYTPYYVAPEVLGPEKYDKSCDMWSLGVIMYILLCGYPPFYSNHGLAISPGMKTRIRMGQYEFPNPEWSEVSEEVKMLIRNLLKTEPTQRMTITEFMNHPWIMQSTKVPQTPLHTSRVLKEDKERWEDVKEEMTSALATMRVDYEQIKIKKIEDASNPLLLKRRKKARALEAAALAH

>NP_003397.1

MDKNELVQKAKLAEQAERYDDMAACMKSVTEQGAELSNEERNLLSVAYKNVVGARRSSWRVVSSIEQKTEGAEKKQQMAREYREKIETELRDICNDVLSLLEKFLIPNASQAESKVFYLKMKGDYYRYLAEVAAGDDKKGIVDQSQQAYQEAFEISKKEMQPTHPIRLGLALNFSVFYYEILNSPEKACSLAKTAFDEAIAELDTLSEESYKDSTLIMQLLRDNLTLWTSDTQGDEAEAGEGGEN

924 NP_057457.1 NP_005418.1

>NP_057457.1

MAALRYAGLDDTDSEDELPPGWEERTTKDGWVYYANHTEEKTQWEHPKTGKRKRVAGDLPYGWEQETDENGQVFFVDHINKRTTYLDPRLAFTVDDNPTKPTTRQRYDGSTTAMEILQGRDFTGKVVVVTGANSGIGFETAKSFALHGAHVILACRNMARASEAVSRILEEWHKAKVEAMTLDLALLRSVQHFAEAFKAKNVPLHVLVCNAATFALPWSLTKDGLETTFQVNHLGHFYLVQLLQDVLCRSAPARVIVVSSESHRFTDINDSLGKLDFSRLSPTKNDYWAMLAYNRSKLCNILFSNELHRRLSPRGVTSNAVHPGNMMYSNIHRSWWVYTLLFTLARPFTKSMQQGAATTVYCAAVPELEGLGGMYFNNCCRCMPSPEAQSEETARTLWALSERLIQERLGSQSG

>NP_005418.1

MAQSTATSPDGGTTFEHLWSSLEPDSTYFDLPQSSRGNNEVVGGTDSSMDVFHLEGMTTSVMAQFNLLSSTMDQMSSRAASASPYTPEHAASVPTHSPYAQPSSTFDTMSPAPVIPSNTDYPGPHHFEVTFQQSSTAKSATWTYSPLLKKLYCQIAKTCPIQIKVSTPPPPGTAIRAMPVYKKAEHVTDVVKRCPNHELGRDFNEGQSAPASHLIRVEGNNLSQYVDDPVTGRQSVVVPYEPPQVGTEFTTILYNFMCNSSCVGGMNRRPILIIITLEMRDGQVLGRRSFEGRICACPGRDRKADEDHYREQQALNESSAKNGAASKRAFKQSPPAVPALGAGVKKRRHGDEDTYYLQVRGRENFEILMKLKESLELMELVPQPLVDSYRQQQQLLQRPSHLQPPSYGPVLSPMNKVHGGMNKLPSVNQLVGQPPPHSSAATPNLGPVGPGMLNNHGHAVPANGEMSSSHSAQSMVSGSHCTPPPPYHADPSLVSFLTGLGCPNCIEYFTSQGLQSIYHLQNLTIEDLGALKIPEQYRMTIWRGLQDLKQGHDYSTAQQLLRSSNAATISIGGSGELQRQRVMEAVHFRVRHTITIPNRGGPGGGPDEWADFGFDLPDCKARKQPIKEEFTEAEIH

925 NP_000629.3 NP_000930.1

>NP_000629.3

MAPLRPLLILALLAWVALADQESCKGRCTEGFNVDKKCQCDELCSYYQSCCTDYTAECKPQVTRGDVFTMPEDEYTVYDDGEEKNNATVHEQVGGPSLTSDLQAQSKGNPEQTPVLKPEEEAPAPEVGASKPEGIDSRPETLHPGRPQPPAEEELCSGKPFDAFTDLKNGSLFAFRGQYCYELDEKAVRPGYPKLIRDVWGIEGPIDAAFTRINCQGKTYLFKGSQYWRFEDGVLDPDYPRNISDGFDGIPDNVDAALALPAHSYSGRERVYFFKGKQYWEYQFQHQPSQEECEGSSLSAVFEHFAMMQRDSWEDIFELLFWGRTSAGTRQPQFISRDWHGVPGQVDAAMAGRIYISGMAPRPSLAKKQRFRHRNRKGYRSQRGHSRGRNQNSRRPSRATWLSLFSSEESNLGANNYDDYRMDWLVPATCEPIQSVFFFSGDKYYRVNLRTRRVDTVDPPYPRSIAQYWLGCPAPGHL

>NP_000930.1

MPRSCCSRSGALLLALLLQASMEVRGWCLESSQCQDLTTESNLLECIRACKPDLSAETPMFPGNGDEQPLTENPRKYVMGHFRWDRFGRRNSSSSGSSGAGQKREDVSAGEDCGPLPEGGPEPRSDGAKPGPREGKRSYSMEHFRWGKPVGKKRRPVKVYPNGAEDESAEAFPLEFKRELTGQRLREGDGPDGPADDGAGAQADLEHSLLVAAEKKDEGPYRMEHFRWGSPPKDKRYGGFMTSEKSQTPLVTLFKNAIIKNAYKKGE

926 NP_000592.3 NP_892028.1

>NP_000592.3

MWVTKLLPALLLQHVLLHLLLLPIAIPYAEGQRKRRNTIHEFKKSAKTTLIKIDPALKIKTKKVNTADQCANRCTRNKGLPFTCKAFVFDKARKQCLWFPFNSMSSGVKKEFGHEFDLYENKDYIRNCIIGKGRSYKGTVSITKSGIKCQPWSSMIPHEHSFLPSSYRGKDLQENYCRNPRGEEGGPWCFTSNPEVRYEVCDIPQCSEVECMTCNGESYRGLMDHTESGKICQRWDHQTPHRHKFLPERYPDKGFDDNYCRNPDGQPRPWCYTLDPHTRWEYCAIKTCADNTMNDTDVPLETTECIQGQGEGYRGTVNTIWNGIPCQRWDSQYPHEHDMTPENFKCKDLRENYCRNPDGSESPWCFTTDPNIRVGYCSQIPNCDMSHGQDCYRGNGKNYMGNLSQTRSGLTCSMWDKNMEDLHRHIFWEPDASKLNENYCRNPDDDAHGPWCYTGNPLIPWDYCPISRCEGDTTPTIVNLDHPVISCAKTKQLRVVNGIPTRTNIGWMVSLRYRNKHICGGSLIKESWVLTARQCFPSRDLKDYEAWLGIHDVHGRGDEKCKQVLNVSQLVYGPEGSDLVLMKLARPAVLDDFVSTIDLPNYGCTIPEKTSCSVYGWGYTGLINYDGLLRVAHLYIMGNEKCSQHHRGKVTLNESEICAGAEKIGSGPCEGDYGGPLVCEQHKMRMVLGVIVPGRGCAIPNRPGIFVRVAYYAKWIHKIILTYKVPQS

>NP_892028.1

MAQKEGGRTVPCCSRPKVAALTAGTLLLLTAIGAASWAIVAVLLRSDQEPLYPVQVSSADARLMVFDKTEGTWRLLCSSRSNARVAGLSCEEMGFLRALTHSELDVRTAGANGTSGFFCVDEGRLPHTQRLLEVISVCDCPRGRFLAAICQDCGRRKLPVDRIVGGRDTSLGRWPWQVSLRYDGAHLCGGSLLSGDWVLTAAHCFPERNRVLSRWRVFAGAVAQASPHGLQLGVQAVVYHGGYLPFRDPNSEENSNDIALVHLSSPLPLTEYIQPVCLPAAGQALVDGKICTVTGWGNTQYYGQQAGVLQEARVPIISNDVCNGADFYGNQIKPKMFCAGYPEGGIDACQGDSGGPFVCEDSISRTPRWRLCGIVSWGTGCALAQKPGVYTKVSDFREWIFQAIKTHSEASGMVTQL

927 NP_005808.2 NP_004242.1

>NP_005808.2

MSADGAEADGSTQVTVEEPVQQPSVVDRVASMPLISSTCDMVSAAYASTKESYPHIKTVCDAAEKGVRTLTAAAVSGAQPILSKLEPQIASASEYAHRGLDKLEENLPILQQPTEKVLADTKELVSSKVSGAQEMVSSAKDTVATQLSEAVDATRGAVQSGVDKTKSVVTGGVQSVMGSRLGQMVLSGVDTVLGKSEEWADNHLPLTDAELARIATSLDGFDVASVQQQRQEQSYFVRLGSLSERLRQHAYEHSLGKLRATKQRAQEALLQLSQALSLMETVKQGVDQKLVEGQEKLHQMWLSWNQKQLQGPEKEPPKPEQVESRALTMFRDIAQQLQATCTSLGSSIQGLPTNVKDQVQQARRQVEDLQATFSSIHSFQDLSSSILAQSRERVASAREALDHMVEYVAQNTPVTWLVGPFAPGITEKAPEEKK

>NP_004242.1

MAGKSSLFKVILLGDGGVGKSSLMNRYVTNKFDTQLFHTIGVEFLNKDLEVDGHFVTMQIWDTAGQERFRSLRTPFYRGSDCCLLTFSVDDSQSFQNLSNWKKEFIYYADVKEPESFPFVILGNKIDISERQVSTEEAQAWCRDNGDYPYFETSAKDATNVAAAFEEAVRRVLATEDRSDHLIQTDTVNLHRKPKPSSSCC

928 NP_055809.1 NP_620637.1

>NP_055809.1

MAVEGSTITSRIKNLLRSPSIKLRRSKAGNRREDLSSKVTLEKVLGITVSGGRGLACDPRSGLVAYPAGCVVVLFNPRKHKQHHILNSSRKTITALAFSPDGKYLVTGESGHMPAVRVWDVAEHSQVAELQEHKYGVACVAFSPSAKYIVSVGYQHDMIVNVWAWKKNIVVASNKVSSRVTAVSFSEDCSYFVTAGNRHIKFWYLDDSKTSKVNATVPLLGRSGLLGELRNNLFTDVACGRGKKADSTFCITSSGLLCEFSDRRLLDKWVELRTTVAHCISVSQDYIFCGCADGTVRLFNPSNLHFLSTLPRPHALGTDIASVTEASRLFSGVANARYPDTIALTFDPTNQWLSCVYNDHSIYVWDVRDPKKVGKVYSALYHSSCVWSVEVYPEVKDSNQACLPPSSFITCSSDNTIRLWNTESSGVHGSTLHRNILSSDLIKIIYVDGNTQALLDTELPGGDKADASLLDPRVGIRSVCVSPNGQHLASGDRMGTLRVHELQSLSEMLKVEAHDSEILCLEYSKPDTGLKLLASASRDRLIHVLDAGREYSLQQTLDEHSSSITAVKFAASDGQVRMISCGADKSIYFRTAQKSGDGVQFTRTHHVVRKTTLYDMDVEPSWKYTAIGCQDRNIRIFNISSGKQKKLFKGSQGEDGTLIKVQTDPSGIYIATSCSDKNLSIFDFSSGECVATMFGHSEIVTGMKFSNDCKHLISVSGDSCIFVWRLSSEMTISMRQRLAELRQRQRGGKQQGPSSPQRASGPNRHQAPSMLSPGPALSSDSDKEGEDEGTEEELPALPVLAKSTKKALASVPSPALPRSLSHWEMSRAQESVGFLDPAPAANPGPRRRGRWVQPGVELSVRSMLDLRQLETLAPSLQDPSQDSLAIIPSGPRKHGQEALETSLTSQNEKPPRPQASQPCSYPHIIRLLSQEEGVFAQDLEPAPIEDGIVYPEPSDNPTMDTSEFQVQAPARGTLGRVYPGSRSSEKHSPDSACSVDYSSSCLSSPEHPTEDSESTEPLSVDGISSDLEEPAEGDEEEEEEEGGMGPYGLQEGSPQTPDQEQFLKQHFETLASGAAPGAPVQVPERSESRSISSRFLLQVQTRPLREPSPSSSSLALMSRPAQVPQASGEQPRGNGANPPGAPPEVEPSSGNPSPQQAASVLLPRCRLNPDSSWAPKRVATASPFSGLQKAQSVHSLVPQERHEASLQAPSPGALLSREIEAQDGLGSLPPADGPPSRPHSYQNPTTSSMAKISRSISVGENLGLVAEPQAHAPIRVSPLSKLALPSRAHLVLDIPKPLPDRPTLAAFSPVTKGRAPGEAEKPGFPVGLGKAHSTTERWACLGEGTTPKPRTECQAHPGPSSPCAQQLPVSSLFQGPENLQPPPPEKTPNPMECTKPGAALSQDSEPAVSLEQCEQLVAELRGSVRQAVRLYHSVAGCRMPSAEQSRIAQLLRDTFSSVRQELEAVAGAVLSSPGSSPGAVGAEQTQALLEQYSELLLRAVERRMERKL

>NP_620637.1

MSRSKRDNNFYSVEIGDSTFTVLKRYQNLKPIGSGAQGIVCAAYDAILERNVAIKKLSRPFQNQTHAKRAYRELVLMKCVNHKNIIGLLNVFTPQKSLEEFQDVYIVMELMDANLCQVIQMELDHERMSYLLYQMLCGIKHLHSAGIIHRDLKPSNIVVKSDCTLKILDFGLARTAGTSFMMTPYVVTRYYRAPEVILGMGYKENVDLWSVGCIMGEMVCHKILFPGRDYIDQWNKVIEQLGTPCPEFMKKLQPTVRTYVENRPKYAGYSFEKLFPDVLFPADSEHNKLKASQARDLLSKMLVIDASKRISVDEALQHPYINVWYDPSEAEAPPPKIPDKQLDEREHTIEEWKELIYKEVMDLEERTKNGVIRGQPSPLGAAVINGSQHPSSSSSVNDVSSMSTDPTLASDTDSSLEAAAGPLGCCR

929 NP_536859.1 NP_000620.2

>NP_536859.1

MVRWFHRDLSGLDAETLLKGRGVHGSFLARPSRKNQGDFSLSVRVGDQVTHIRIQNSGDFYDLYGGEKFATLTELVEYYTQQQGVLQDRDGTIIHLKYPLNCSDPTSERWYHGHMSGGQAETLLQAKGEPWTFLVRESLSQPGDFVLSVLSDQPKAGPGSPLRVTHIKVMCEGGRYTVGGLETFDSLTDLVEHFKKTGIEEASGAFVYLRQPYYATRVNAADIENRVLELNKKQESEDTAKAGFWEEFESLQKQEVKNLHQRLEGQRPENKGKNRYKNILPFDHSRVILQGRDSNIPGSDYINANYIKNQLLGPDENAKTYIASQGCLEATVNDFWQMAWQENSRVIVMTTREVEKGRNKCVPYWPEVGMQRAYGPYSVTNCGEHDTTEYKLRTLQVSPLDNGDLIREIWHYQYLSWPDHGVPSEPGGVLSFLDQINQRQESLPHAGPIIVHCSAGIGRTGTIIVIDMLMENISTKGLDCDIDIQKTIQMVRAQRSGMVQTEAQYKFIYVAIAQFIETTKKKLEVLQSQKGQESEYGNITYPPAMKNAHAKASRTSSKSLESSAGTVAASPVRRGGQRGLPVPGPPVLSPDLHQLPVLAPLHPAADTRRMCMRTCTLRTRGRRK

>NP_000620.2

MMVVLLGATTLVLVAVAPWVLSAAAGGKNLKSPQKVEVDIIDDNFILRWNRSDESVGNVTFSFDYQKTGMDNWIKLSGCQNITSTKCNFSSLKLNVYEEIKLRIRAEKENTSSWYEVDSFTPFRKAQIGPPEVHLEAEDKAIVIHISPGTKDSVMWALDGLSFTYSLVIWKNSSGVEERIENIYSRHKIYKLSPETTYCLKVKAALLTSWKIGVYSPVHCIKTTVENELPPPENIEVSVQNQNYVLKWDYTYANMTFQVQWLHAFLKRNPGNHLYKWKQIPDCENVKTTQCVFPQNVFQKGIYLLRVQASDGNNTSFWSEEIKFDTEIQAFLLPPVFNIRSLSDSFHIYIGAPKQSGNTPVIQDYPLIYEIIFWENTSNAERKIIEKKTDVTVPNLKPLTVYCVKARAHTMDEKLNKSSVFSDAVCEKTKPGNTSKIWLIVGICIALFALPFVIYAAKVFLRCINYVFFPSLKPSSSIDEYFSEQPLKNLLLSTSEEQIEKCFIIENISTIATVEETNQTDEDHKKYSSQTSQDSGNYSNEDESESKTSEELQQDFV

930 NP_361014.1 NP_004553.1

>NP_361014.1

MNQELLSVGSKRRRTGGSLRGNPSSSQVDEEQMNRVVEEEQQQQLRQQEEEHTARNGEVVGVEPRPGGQNDSQQGQLEENNNRFISVDEDSSGNQEEQEEDEEHAGEQDEEDEEEEEMDQESDDFDQSDDSSREDEHTHTNSVTNSSSIVDLPVHQLSSPFYTKTTKMKRKLDHGSEVRSFSLGKKPCKVSEYTSTTGLVPCSATPTTFGDLRAANGQGQQRRRITSVQPPTGLQEWLKMFQSWSGPEKLLALDELIDSCEPTQVKHMMQVIEPQFQRDFISLLPKELALYVLSFLEPKDLLQAAQTCRYWRILAEDNLLWREKCKEEGIDEPLHIKRRKVIKPGFIHSPWKSAYIRQHRIDTNWRRGELKSPKVLKGHDDHVITCLQFCGNRIVSGSDDNTLKVWSAVTGKCLRTLVGHTGGVWSSQMRDNIIISGSTDRTLKVWNAETGECIHTLYGHTSTVRCMHLHEKRVVSGSRDATLRVWDIETGQCLHVLMGHVAAVRCVQYDGRRVVSGAYDFMVKVWDPETETCLHTLQGHTNRVYSLQFDGIHVVSGSLDTSIRVWDVETGNCIHTLTGHQSLTSGMELKDNILVSGNADSTVKIWDIKTGQCLQTLQGPNKHQSAVTCLQFNKNFVITSSDDGTVKLWDLKTGEFIRNLVTLESGGSGGVVWRIRASNTKLVCAVGSRNGTEETKLLVLDFDVDMK

>NP_004553.1

MIVFVRFNSSHGFPVEVDSDTSIFQLKEVVAKRQGVPADQLRVIFAGKELRNDWTVQNCDLDQQSIVHIVQRPWRKGQEMNATGGDDPRNAAGGCEREPQSLTRVDLSSSVLPGDSVGLAVILHTDSRKDSPPAGSPAGRSIYNSFYVYCKGPCQRVQPGKLRVQCSTCRQATLTLTQGPSCWDDVLIPNRMSGECQSPHCPGTSAEFFFKCGAHPTSDKETPVALHLIATNSRNITCITCTDVRSPVLVFQCNSRHVICLDCFHLYCVTRLNDRQFVHDPQLGYSLPCVAGCPNSLIKELHHFRILGEEQYNRYQQYGAEECVLQMGGVLCPRPGCGAGLLPEPDQRKVTCEGGNGLGCGFAFCRECKEAYHEGECSAVFEASGTTTQAYRVDERAAEQARWEAASKETIKKTTKPCPRCHVPVEKNGGCMHMKCPQPQCRLEWCWNCGCEWNRVCMGDHWFDV

931 NP_003391.1 NP_009330.1

>NP_003391.1

MPAIMTMLADHAARQLLDFSQKLDINLLDNVVNCLYHGEGAQQRMAQEVLTHLKEHPDAWTRVDTILEFSQNMNTKYYGLQILENVIKTRWKILPRNQCEGIKKYVVGLIIKTSSDPTCVEKEKVYIGKLNMILVQILKQEWPKHWPTFISDIVGASRTSESLCQNNMVILKLLSEEVFDFSSGQITQVKSKHLKDSMCNEFSQIFQLCQFVMENSQNAPLVHATLETLLRFLNWIPLGYIFETKLISTLIYKFLNVPMFRNVSLKCLTEIAGVSVSQYEEQFVTLFTLTMMQLKQMLPLNTNIRLAYSNGKDDEQNFIQNLSLFLCTFLKEHDQLIEKRLNLRETLMEALHYMLLVSEVEETEIFKICLEYWNHLAAELYRESPFSTSASPLLSGSQHFDVPPRRQLYLPMLFKVRLLMVSRMAKPEEVLVVENDQGEVVREFMKDTDSINLYKNMRETLVYLTHLDYVDTERIMTEKLHNQVNGTEWSWKNLNTLCWAIGSISGAMHEEDEKRFLVTVIKDLLGLCEQKRGKDNKAIIASNIMYIVGQYPRFLRAHWKFLKTVVNKLFEFMHETHDGVQDMACDTFIKIAQKCRRHFVQVQVGEVMPFIDEILNNINTIICDLQPQQVHTFYEAVGYMIGAQTDQTVQEHLIEKYMLLPNQVWDSIIQQATKNVDILKDPETVKQLGSILKTNVRACKAVGHPFVIQLGRIYLDMLNVYKCLSENISAAIQANGEMVTKQPLIRSMRTVKRETLKLISGWVSRSNDPQMVAENFVPPLLDAVLIDYQRNVPAAREPEVLSTMAIIVNKLGGHITAEIPQIFDAVFECTLNMINKDFEEYPEHRTNFFLLLQAVNSHCFPAFLAIPPTQFKLVLDSIIWAFKHTMRNVADTGLQILFTLLQNVAQEEAAAQSFYQTYFCDILQHIFSVVTDTSHTAGLTMHASILAYMFNLVEEGKISTSLNPGNPVNNQIFLQEYVANLLKSAFPHLQDAQVKLFVTGLFSLNQDIPAFKEHLRDFLVQIKEFAGEDTSDLFLEEREIALRQADEEKHKRQMSVPGIFNPHEIPEEMCD

>NP_009330.1

MSQWYELQQLDSKFLEQVHQLYDDSFPMEIRQYLAQWLEKQDWEHAANDVSFATIRFHDLLSQLDDQYSRFSLENNFLLQHNIRKSKRNLQDNFQEDPIQMSMIIYSCLKEERKILENAQRFNQAQSGNIQSTVMLDKQKELDSKVRNVKDKVMCIEHEIKSLEDLQDEYDFKCKTLQNREHETNGVAKSDQKQEQLLLKKMYLMLDNKRKEVVHKIIELLNVTELTQNALINDELVEWKRRQQSACIGGPPNACLDQLQNWFTIVAESLQQVRQQLKKLEELEQKYTYEHDPITKNKQVLWDRTFSLFQQLIQSSFVVERQPCMPTHPQRPLVLKTGVQFTVKLRLLVKLQELNYNLKVKVLFDKDVNERNTVKGFRKFNILGTHTKVMNMEESTNGSLAAEFRHLQLKEQKNAGTRTNEGPLIVTEELHSLSFETQLCQPGLVIDLETTSLPVVVISNVSQLPSGWASILWYNMLVAEPRNLSFFLTPPCARWAQLSEVLSWQFSSVTKRGLNVDQLNMLGEKLLGPNASPDGLIPWTRFCKENINDKNFPFWLWIESILELIKKHLLPLWNDGCIMGFISKERERALLKDQQPGTFLLRFSESSREGAITFTWVERSQNGGEPDFHAVEPYTKKELSAVTFPDIIRNYKVMAAENIPENPLKYLYPNIDKDHAFGKYYSRPKEAPEPMELDGPKGTGYIKTELISVSEVHPSRLQTTDNLLPMSPEEFDEVSRIVGSVEFDSMMNTV

932 NP_005822.1 NP_009108.1

>NP_005822.1

MEETIKDPPTSAVLLDHCHFSQVIFNSVEKFYIPGGDVTCHYTFTQHFIPRRKDWIGIFRVGWKTTREYYTFMWVTLPIDLNNKSAKQQEVQFKAYYLPKDDEYYQFCYVDEDGVVRGASIPFQFRPENEEDILVVTTQGEVEEIEQHNKELCKENQELKDSCISLQKQNSDMQAELQKKQEELETLQSINKKLELKVKEQKDYWETELLQLKEQNQKMSSENEKMGIRVDQLQAQLSTQEKEMEKLVQGDQDKTEQLEQLKKENDHLFLSLTEQRKDQKKLEQTVEQMKQNETTAMKKQQELMDENFDLSKRLSENEIICNALQRQKERLEGENDLLKRENSRLLSYMGLDFNSLPYQVPTSDEGGARQNPGLAYGNPYSGIQESSSPSPLSIKKCPICKADDICDHTLEQQQMQPLCFNCPICDKIFPATEKQIFEDHVFCHSL

>NP_009108.1

MYSEIQRERADIGGLMARPEYREWNPELIKPKKLLNPVKASRSHQELHRELLMNHRRGLGVDSKPELQRVLEHRRRNQLIKKKKEELEAKRLQCPFEQELLRRQQRLNQLEKPPEKEEDHAPEFIKVRENLRRIATLTSEEREL

933 NP_066402.2 NP_002308.2

>NP_066402.2

MPEPAKSAPAPKKGSKKAVTKAQKKDGKKRKRSRKESYSIYVYKVLKQVHPDTGISSKAMGIMNSFVNDIFERIAGEASRLAHYNKRSTITSREIQTAVRLLLPGELAKHAVSEGTKAVTKYTSAK

>NP_002308.2

MRFAWTVLLLGPLQLCALVHCAPPAAGQQQPPREPPAAPGAWRQQIQWENNGQVFSLLSLGSQYQPQRRRDPGAAVPGAANASAQQPRTPILLIRDNRTAAARTRTAGSSGVTAGRPRPTARHWFQAGYSTSRAREAGASRAENQTAPGEVPALSNLRPPSRVDGMVGDDPYNPYKYSDDNPYYNYYDTYERPRPGGRYRPGYGTGYFQYGLPDLVADPYYIQASTYVQKMSMYNLRCAAEENCLASTAYRADVRDYDHRVLLRFPQRVKNQGTSDFLPSRPRYSWEWHSCHQHYHSMDEFSHYDLLDANTQRRVAEGHKASFCLEDTSCDYGYHRRFACTAHTQGLSPGCYDTYGADIDCQWIDITDVKPGNYILKVSVNPSYLVPESDYTNNVVRCDIRYTGHHAYASGCTISPY

934 NP_612808.1 NP_066285.1

>NP_612808.1

MSRRKQGNPQHLSQRELITPEADHVEAAILEEDEGLEIEEPSGLGLMVGGPDPDLLTCGQCQMNFPLGDILVFIEHKRKQCGGSLGACYDKALDKDSPPPSSRSELRKVSEPVEIGIQVTPDEDDHLLSPTKGICPKQENIAGPCRPAQLPAVAPIAASSHPHSSVITSPLRALGALPPCLPLPCCSARPVSGDGTQGEGQTEAPFGCQCQLSGKDEPSSYICTTCKQPFNSAWFLLQHAQNTHGFRIYLEPGPASSSLTPRLTIPPPLGPEAVAQSPLMNFLGDSNPFNLLRMTGPILRDHPGFGEGRLPGTPPLFSPPPRHHLDPHRLSAEEMGLVAQHPSAFDRVMRLNPMAIDSPAMDFSRRLRELAGNSSTPPPVSPGRGNPMHRLLNPFQPSPKSPFLSTPPLPPMPPGGTPPPQPPAKSKSCEFCGKTFKFQSNLIVHRRSHTGEKPYKCQLCDHACSQASKLKRHMKTHMHKAGSLAGRSDDGLSAASSPEPGTSELAGEGLKAADGDFRHHESDPSLGHEPEEEDEEEEEEEEELLLENESRPESSFSMDSELSRNRENGGGGVPGVPGAGGGAAKALADEKALVLGKVMENVGLGALPQYGELLADKQKRGAFLKRAAGGGDAGDDDDAGGCGDAGAGGAVNGRGGGFAPGTEPFPGLFPRKPAPLPSPGLNSAAKRIKVEKDLELPPAALIPSENVYSQWLVGYAASRHFMKDPFLGFTDARQSPFATSSEHSSENGSLRFSTPPGDLLDGGLSGRSGTASGGSTPHLGGPGPGRPSSKEGRRSDTCEYCGKVFKNCSNLTVHRRSHTGERPYKCELCNYACAQSSKLTRHMKTHGQIGKEVYRCDICQMPFSVYSTLEKHMKKWHGEHLLTNDVKIEQAERS

>NP_066285.1

MAMVVSTWRDPQDEVPGSQGSQASQAPPVPGPPPGAPHTPQTPGQGGPASTPAQTAAGGQGGPGGPGSDKQQQQQHIECVVCGDKSSGKHYGQFTCEGCKSFFKRSVRRNLSYTCRANRNCPIDQHHRNQCQYCRLKKCLKVGMRREAVQRGRMPPTQPTHGQFALTNGDPLNCHSYLSGYISLLLRAEPYPTSRFGSQCMQPNNIMGIENICELAARMLFSAVEWARNIPFFPDLQITDQVALLRLTWSELFVLNAAQCSMPLHVAPLLAAAGLHASPMSADRVVAFMDHIRIFQEQVEKLKALHVDSAEYSCLKAIVLFTSDACGLSDVAHVESLQEKSQCALEEYVRSQYPNQPTRFGKLLLRLPSLRTVSSSVIEQLFFVRLVGKTPIETLIRDMLLSGSSFNWPYMAIQ

935 AAH11604.1 NP_006273.1

>AAH11604.1

MEPVETWTPGKVATWLRGLDDSLQDYPFEDWQLPGKNLLQLCPQSLEALAVRSLGHQELILGGVEQLQALSSRLQTENLQSLTEGLLGATHDFQSIVQGCLGDCAKTPIDVLCAAVELLHEADALLFWLSRYLFSHLNDFSACQEIRDLLEELSQVLHEDGPAAEKEGTVLRICSHVAGICHNILVCCPKELLEQKAVLEQVQLDSPLGLEIHTTSNCQHFVSQVDTQVPTDSRLQIQPGDEVVQINEQVVVREERDMVGWPRKNMVRELLREPAGLSLVLKKIPIPETPPQTPPQVLDSPHQRSPSLSLAPLSPRAPSEDVFAFDLSSNPSPGPSPAWTDSASLGPEPLPIPPEPPAILPAGVAGTPGLPESPDKSPVGRKKSKGLATRLSRRRVSCRELGRPDCDGWLLLRKAPGGFMGPRWRRRWFVLKGHTLYWYRQPQDEKAEGLINVSNYSLESGHDQKKKYVFQLTHDVYKPFIFAADTLTDLSMWVRHLITCISKYQSPGRAPPPREEDCYSETEAEDPDDEAGSHSASPSPAQAGSPLHGDTSPAATPTQRSPRTSFGSLTDSSEEALEGMVRGLRQGGVSLLGQPQPLTQEQWRSSFMRRNRDPQLNERVHRVRALQSTLKAKLQELQVLEEVLGDPELTGEKFRQWKEQNRELYSEGLGAWGVAQAEGSSHILTSDSTEQSPHSLPSDPEEHSHLCPLTSESSLRPPDL

>NP_006273.1

METVQLRNPPRRQLKKLDEDSLTKQPEEVFDVLEKLGEGSYGSVYKAIHKETGQIVAIKQVPVESDLQEIIKEISIMQQCDSPHVVKYYGSYFKNTDLWIVMEYCGAGSVSDIIRLRNKTLTEDEIATILQSTLKGLEYLHFMRKIHRDIKAGNILLNTEGHAKLADFGVAGQLTDTMAKRNTVIGTPFWMAPEVIQEIGYNCVADIWSLGITAIEMAEGKPPYADIHPMRAIFMIPTNPPPTFRKPELWSDNFTDFVKQCLVKSPEQRATATQLLQHPFVRSAKGVSILRDLINEAMDVKLKRQESQQREVDQDDEENSEEDEMDSGTMVRAVGDEMGTVRVASTMTDGANTMIEHDDTLPSQLGTMVINAEDEEEEGTMKRRDETMQPAKPSFLEYFEQKEKENQINSFGKSVPGPLKNSSDWKIPQDGDYEFLKSWTVEDLQKRLLALDPMMEQEIEEIRQKYQSKRQPILDAIEAKKRRQQNF

936 NP_055382.2 NP_068375.3

>NP_055382.2

MNHLNVLAKALYDNVAESPDELSFRKGDIMTVLEQDTQGLDGWWLCSLHGRQGIVPGNRLKILVGMYDKKPAGPGPGPPATPAQPQPGLHAPAPPASQYTPMLPNTYQPQPDSVYLVPTPSKAQQGLYQVPGPSPQFQSPPAKQTSTFSKQTPHHPFPSPATDLYQVPPGPGGPAQDIYQVPPSAGMGHDIYQVPPSMDTRSWEGTKPPAKVVVPTRVGQGYVYEAAQPEQDEYDIPRHLLAPGPQDIYDVPPVRGLLPSQYGQEVYDTPPMAVKGPNGRDPLLEVYDVPPSVEKGLPPSNHHAVYDVPPSVSKDVPDGPLLREETYDVPPAFAKAKPFDPARTPLVLAAPPPDSPPAEDVYDVPPPAPDLYDVPPGLRRPGPGTLYDVPRERVLPPEVADGGVVDSGVYAVPPPAEREAPAEGKRLSASSTGSTRSSQSASSLEVAGPGREPLELEVAVEALARLQQGVSATVAHLLDLAGSAGATGSWRSPSEPQEPLVQDLQAAVAAVQSAVHELLEFARSAVGNAAHTSDRALHAKLSRQLQKMEDVHQTLVAHGQALDAGRGGSGATLEDLDRLVACSRAVPEDAKQLASFLHGNASLLFRRTKATAPGPEGGGTLHPNPTDKTSSIQSRPLPSPPKFTSQDSPDGQYENSEGGWMEDYDYVHLQGKEEFEKTQKELLEKGSITRQGKSQLELQQLKQFERLEQEVSRPIDHDLANWTPAQPLAPGRTGGLGPSDRQLLLFYLEQCEANLTTLTNAVDAFFTAVATNQPPKIFVAHSKFVILSAHKLVFIGDTLSRQAKAADVRSQVTHYSNLLCDLLRGIVATTKAAALQYPSPSAAQDMVERVKELGHSTQQFRRVLGQLAAA

>NP_068375.3

MAAYLQWRRFVFFDKELVKEPLSNDGAAPGATPASGSAASKFLCLPPGITVCDSGRGSLVFGDMEGQIWFLPRSLQLTGFQAYKLRVTHLYQLKQHNILASVGEDEEGINPLVKIWNLEKRDGGNPLCTRIFPAIPGTEPTVVSCLTVHENLNFMAIGFTDGSVTLNKGDITRDRHSKTQILHKGNYPVTGLAFRQAGKTTHLFVVTTENVQSYIVSGKDYPRVELDTHGCGLRCSALSDPSQDLQFIVAGDECVYLYQPDERGPCFAFEGHKLIAHWFRGYLIIVSRDRKVSPKSEFTSRDSQSSDKQILNIYDLCNKFIAYSTVFEDVVDVLAEWGSLYVLTRDGRVHALQEKDTQTKLEMLFKKNLFEMAINLAKSQHLDSDGLAQIFMQYGDHLYSKGNHDGAVQQYIRTIGKLEPSYVIRKFLDAQRIHNLTAYLQTLHRQSLANADHTTLLLNCYTKLKDSSKLEEFIKKKSESEVHFDVETAIKVLRQAGYYSHALYLAENHAHHEWYLKIQLEDIKNYQEALRYIGKLPFEQAESNMKRYGKILMHHIPEQTTQLLKGLCTDYRPSLEGRSDREAPGCRANSEEFIPIFANNPRELKAFLEHMSEVQPDSPQGIYDTLLELRLQNWAHEKDPQVKEKLHAEAISLLKSGRFCDVFDKALVLCQMHDFQDGVLYLYEQGKLFQQIMHYHMQHEQYRQVISVCERHGEQDPSLWEQALSYFARKEEDCKEYVAAVLKHIENKNLMPPLLVVQTLAHNSTATLSVIRDYLVQKLQKQSQQIAQDELRVRRYREETTRIRQEIQELKASPKIFQKTKCSICNSALELPSVHFLCGHSFHQHCFESYSESDADCPTCLPENRKVMDMIRAQEQKRDLHDQFQHQLKCSNDSFSVIADYFGRGVFNKLTLLTDPPTARLTSSLEAGLQRDLLMHSRRGT

937 NP_065701.2 NP_612193.1

>NP_065701.2

MMWRPSVLLLLLLLRHGAQGKPSPDAGPHGQGRVHQAAPLSDAPHDDAHGNFQYDHEAFLGREVAKEFDQLTPEESQARLGRIVDRMDRAGDGDGWVSLAELRAWIAHTQQRHIRDSVSAAWDTYDTDRDGRVGWEELRNATYGHYAPGEEFHDVEDAETYKKMLARDERRFRVADQDGDSMATREELTAFLHPEEFPHMRDIVIAETLEDLDRNKDGYVQVEEYIADLYSAEPGEEEPAWVQTERQQFRDFRDLNKDGHLDGSEVGHWVLPPAQDQPLVEANHLLHESDTDKDGRLSKAEILGNWNMFVGSQATNYGEDLTRHHDEL

>NP_612193.1

MPPRAPPAPGPRPPPRAAAATDTAAGAGGAGGAGGAGGPGFRPLAPRPWRWLLLLALPAACSAPPPRPVYTNHWAVQVLGGPAEADRVAAAHGYLNLGQIGNLEDYYHFYHSKTFKRSTLSSRGPHTFLRMDPQVKWLQQQEVKRRVKRQVRSDPQALYFNDPIWSNMWYLHCGDKNSRCRSEMNVQAAWKRGYTGKNVVVTILDDGIERNHPDLAPNYDSYASYDVNGNDYDPSPRYDASNENKHGTRCAGEVAASANNSYCIVGIAYNAKIGGIRMLDGDVTDVVEAKSLGIRPNYIDIYSASWGPDDDGKTVDGPGRLAKQAFEYGIKKGRQGLGSIFVWASGNGGREGDYCSCDGYTNSIYTISVSSATENGYKPWYLEECASTLATTYSSGAFYERKIVTTDLRQRCTDGHTGTSVSAPMVAGIIALALEANSQLTWRDVQHLLVKTSRPAHLKASDWKVNGAGHKVSHFYGFGLVDAEALVVEAKKWTAVPSQHMCVAASDKRPRSIPLVQVLRTTALTSACAEHSDQRVVYLEHVVVRTSISHPRRGDLQIYLVSPSGTKSQLLAKRLLDLSNEGFTNWEFMTVHCWGEKAEGQWTLEIQDLPSQVRNPEKQGKLKEWSLILYGTAEHPYHTFSAHQSRSRMLELSAPELEPPKAALSPSQVEVPEDEEDYTAQSTPGSANILQTSVCHPECGDKGCDGPNADQCLNCVHFSLGSVKTSRKCVSVCPLGYFGDTAARRCRRCHKGCETCSSRAATQCLSCRRGFYHHQEMNTCVTLCPAGFYADESQKNCLKCHPSCKKCVDEPEKCTVCKEGFSLARGSCIPDCEPGTYFDSELIRCGECHHTCGTCVGPGREECIHCAKNFHFHDWKCVPACGEGFYPEEMPGLPHKVCRRYGPPGGERQAAVSSKGVPGGQSLAASSPGAGEGMLHHPTVDRSPFTELLRGLRPFVHWMHICWVPAVGRHRAAAG

938 NP_005234.1 NP_077299.3

>NP_005234.1

MASTDYSTYSQAAAQQGYSAYTAQPTQGYAQTTQAYGQQSYGTYGQPTDVSYTQAQTTATYGQTAYATSYGQPPTGYTTPTAPQAYSQPVQGYGTGAYDTTTATVTTTQASYAAQSAYGTQPAYPAYGQQPAATAPTRPQDGNKPTETSQPQSSTGGYNQPSLGYGQSNYSYPQVPGSYPMQPVTAPPSYPPTSYSSTQPTSYDQSSYSQQNTYGQPSSYGQQSSYGQQSSYGQQPPTSYPPQTGSYSQAPSQYSQQSSSYGQQSSFRQDHPSSMGVYGQESGGFSGPGENRSMSGPDNRGRGRGGFDRGGMSRGGRGGGRGGMGSAGERGGFNKPGGPMDEGPDLDLGPPVDPDEDSDNSAIYVQGLNDSVTLDDLADFFKQCGVVKMNKRTGQPMIHIYLDKETGKPKGDATVSYEDPPTAKAAVEWFDGKDFQGSKLKVSLARKKPPMNSMRGGLPPREGRGMPPPLRGGPGGPGGPGGPMGRMGGRGGDRGGFPPRGPRGSRGNPSGGGNVQHRAGDWQCPNPGCGNQNFAWRTECNQCKAPKPEGFLPPPFPPPGGDRGRGGPGGMRGGRGGLMDRGGPGGMFRGGRGGDRGGFRGGRGMDRGGFGGGRRGGPGGPPGPLMEQMGGRRGGRGGPGKMDKGEHRQERRDRPY

>NP_077299.3

MTKRKKLRTSGEGLCPPKPLKNPRLGDFYGDPQSSMLGCLHHPEEPEGKLGPVPSTQQHGEEPGKAVSSSPDEETGSPCRLLRQPEKEPAPLPPSQNSFGRFVPQFAKSRKTVTRKEEMKDEDRGSGAFSLETIAESSAQSPGCQLLVETLGVPLQEATELGDPTQADSARPEQSSQSPVQAVPGSGDSQPDDPPDRGTGLSASQRASQDHLSEQGADDSKPETDRVPGDGGQKEHLPSIDSEGEKPDRGAPQEGGAQRTAGAGLPGGPQEEGDGVPCTPASAPTSGPAPGLGPASWCLEPGSVAQGSPDPQQTPSRMGREGEGTHSSLGCSSLGMVVIADLSTDPTELEERALEVAGPDGQASAISPASPRRKAADGGHRRALPGCTSLTGETTGESGEAGQDGKPPGDVLVGPTASLALAPGSGESMMGAGDSGHASPDTGPCVNQKQEPGPAQEEAELGGQNLERDLEGFRVSPQASVVLEHREIADDPLQEPGAQQGIPDTTSELAGQRDHLPHSADQGTWADSLAVELDFLLDSQIQDALDASDFEAPPEQLFPSGNKPGPCWPGPSSHANGDPVAVAKAQPSRLIMGTHRDLEAFKRLNYRKTKLGGKAPLPYPSKGPGNIPRGDPPWREL

939 NP_000292.1 NP_002119.1

>NP_000292.1

MEHKEVVLLLLLFLKSGQGEPLDDYVNTQGASLFSVTKKQLGAGSIEECAAKCEEDEEFTCRAFQYHSKEQQCVIMAENRKSSIIIRMRDVVLFEKKVYLSECKTGNGKNYRGTMSKTKNGITCQKWSSTSPHRPRFSPATHPSEGLEENYCRNPDNDPQGPWCYTTDPEKRYDYCDILECEEECMHCSGENYDGKISKTMSGLECQAWDSQSPHAHGYIPSKFPNKNLKKNYCRNPDRELRPWCFTTDPNKRWELCDIPRCTTPPPSSGPTYQCLKGTGENYRGNVAVTVSGHTCQHWSAQTPHTHNRTPENFPCKNLDENYCRNPDGKRAPWCHTTNSQVRWEYCKIPSCDSSPVSTEQLAPTAPPELTPVVQDCYHGDGQSYRGTSSTTTTGKKCQSWSSMTPHRHQKTPENYPNAGLTMNYCRNPDADKGPWCFTTDPSVRWEYCNLKKCSGTEASVVAPPPVVLLPDVETPSEEDCMFGNGKGYRGKRATTVTGTPCQDWAAQEPHRHSIFTPETNPRAGLEKNYCRNPDGDVGGPWCYTTNPRKLYDYCDVPQCAAPSFDCGKPQVEPKKCPGRVVGGCVAHPHSWPWQVSLRTRFGMHFCGGTLISPEWVLTAAHCLEKSPRPSSYKVILGAHQEVNLEPHVQEIEVSRLFLEPTRKDIALLKLSSPAVITDKVIPACLPSPNYVVADRTECFITGWGETQGTFGAGLLKEAQLPVIENKVCNRYEFLNGRVQSTELCAGHLAGGTDSCQGDSGGPLVCFEKDKYILQGVTSWGLGCARPNKPGVYVRVSRFVTWIEGVMRNN

>NP_002119.1

MGKGDPKKPRGKMSSYAFFVQTCREEHKKKHPDASVNFSEFSKKCSERWKTMSAKEKGKFEDMAKADKARYEREMKTYIPPKGETKKKFKDPNAPKRPPSAFFLFCSEYRPKIKGEHPGLSIGDVAKKLGEMWNNTAADDKQPYEKKAAKLKEKYEKDIAAYRAKGKPDAAKKGVVKAEKSKKKKEEEEDEEDEEDEEEEEDEEDEDEEEDDDDE

940 NP_005893.1 NP_001895.1

>NP_005893.1

MSSILPFTPPIVKRLLGWKKGEQNGQEEKWCEKAVKSLVKKLKKTGQLDELEKAITTQNVNTKCITIPRSLDGRLQVSHRKGLPHVIYCRLWRWPDLHSHHELRAMELCEFAFNMKKDEVCVNPYHYQRVETPVLPPVLVPRHTEIPAEFPPLDDYSHSIPENTNFPAGIEPQSNIPETPPPGYLSEDGETSDHQMNHSMDAGSPNLSPNPMSPAHNNLDLQPVTYCEPAFWCSISYYELNQRVGETFHASQPSMTVDGFTDPSNSERFCLGLLSNVNRNAAVELTRRHIGRGVRLYYIGGEVFAECLSDSAIFVQSPNCNQRYGWHPATVCKIPPGCNLKIFNNQEFAALLAQSVNQGFEAVYQLTRMCTIRMSFVKGWGAEYRRQTVTSTPCWIELHLNGPLQWLDKVLTQMGSPSIRCSSVS

>NP_001895.1

MATQADLMELDMAMEPDRKAAVSHWQQQSYLDSGIHSGATTTAPSLSGKGNPEEEDVDTSQVLYEWEQGFSQSFTQEQVADIDGQYAMTRAQRVRAAMFPETLDEGMQIPSTQFDAAHPTNVQRLAEPSQMLKHAVVNLINYQDDAELATRAIPELTKLLNDEDQVVVNKAAVMVHQLSKKEASRHAIMRSPQMVSAIVRTMQNTNDVETARCTAGTLHNLSHHREGLLAIFKSGGIPALVKMLGSPVDSVLFYAITTLHNLLLHQEGAKMAVRLAGGLQKMVALLNKTNVKFLAITTDCLQILAYGNQESKLIILASGGPQALVNIMRTYTYEKLLWTTSRVLKVLSVCSSNKPAIVEAGGMQALGLHLTDPSQRLVQNCLWTLRNLSDAATKQEGMEGLLGTLVQLLGSDDINVVTCAAGILSNLTCNNYKNKMMVCQVGGIEALVRTVLRAGDREDITEPAICALRHLTSRHQEAEMAQNAVRLHYGLPVVVKLLHPPSHWPLIKATVGLIRNLALCPANHAPLREQGAIPRLVQLLVRAHQDTQRRTSMGGTQQQFVEGVRMEEIVEGCTGALHILARDVHNRIVIRGLNTIPLFVQLLYSPIENIQRVAAGVLCELAQDKEAAEAIEAEGATAPLTELLHSRNEGVATYAAAVLFRMSEDKPQDYKKRLSVELTSSLFRTEPMAWNETADLGLDIGAQGEPLGYRQDDPSYRSFHSGGYGQDALGMDPMMEHEMGGHHPGADYPVDGLPDLGHAQDLMDGLPPGDSNQLAWFDTDL

941 NP_065949.1 NP_005888.1

>NP_065949.1

MEESPLSRAPSRGGVNFLNVARTYIPNTKVECHYTLPPGTMPSASDWIGIFKVEAACVRDYHTFVWSSVPESTTDGSPIHTSVQFQASYLPKPGAQLYQFRYVNRQGQVCGQSPPFQFREPRPMDELVTLEEADGGSDILLVVPKATVLQNQLDESQQERNDLMQLKLQLEGQVTELRSRVQELERALATARQEHTELMEQYKGISRSHGEITEERDILSRQQGDHVARILELEDDIQTISEKVLTKEVELDRLRDTVKALTREQEKLLGQLKEVQADKEQSEAELQVAQQENHHLNLDLKEAKSWQEEQSAQAQRLKDKVAQMKDTLGQAQQRVAELEPLKEQLRGAQELAASSQQKATLLGEELASAAAARDRTIAELHRSRLEVAEVNGRLAELGLHLKEEKCQWSKERAGLLQSVEAEKDKILKLSAEILRLEKAVQEERTQNQVFKTELAREKDSSLVQLSESKRELTELRSALRVLQKEKEQLQEEKQELLEYMRKLEARLEKVADEKWNEDATTEDEEAAVGLSCPAALTDSEDESPEDMRLPPYGLCERGDPGSSPAGPREASPLVVISQPAPISPHLSGPAEDSSSDSEAEDEKSVLMAAVQSGGEEANLLLPELGSAFYDMASGFTVGTLSETSTGGPATPTWKECPICKERFPAESDKDALEDHMDGHFFFSTQDPFTFE

>NP_005888.1

MANEDCPKAADSPFSSDKHAQLILAQINKMRNGQHFCDVQLQVGQESFKAHRLVLAASSPYFAALFTGGMKESSKDVVPILGIEAGIFQILLDFIYTGIVNIGVNNVQELIIAADMLQLTEVVHLCCEFLKGQIDPLNCIGIFQFSEQIACHDLLEFSENYIHVHFLEVHSGEEFLALTKDQLIKILRSEELSIEDEYQVFLAAMQWILKDLGKRRKHVVEVLDPIRFPLLPPQRLLKYIEGVSDFNLRVALQTLLKEYCEVCKSPKENKFCSFLQTSKVRPRKKARKYLYAVGGYTRLQGGRWSDSRALSCVERFDTFSQYWTTVSSLHQARSGLGVTVLGGMVYAIGGEKDSMIFDCTECYDPVTKQWTTVASMNHPRCGLGVCVCYGAIYALGGWVGAEIGNTIERFDPDENKWEVVGNMAVSRYYFGCCEMQGLIYVIGGISNEGIELRSFEVYDPLSKRWSPLPPMGTRRAYLGVAALNDCIYSVGGWNETQDALHTVEKYSFEEEKWVEVASMKVPRAGMCVVAVNGLLYVSGGRSSSHDFLAPGTLDSVEVYNPHSDTWTEIGNMITSRCEGGVAVL

942 NP_057363.3 NP_004352.2

>NP_057363.3

MRTYHYIPLFIWTYMFHTVDTILLQEKPNSYLSSKKIAGLTKDDGKMLRRTKRGWMWNQFFLLEEYTGTDTQYVGKLHTDQDKGDGNLKYILTGDGAGSLFVIDENTGDIHAAKKLDREEKSLYILRAKAIDRKTGRQVEPESEFIIKIHDINDNEPKFTKDLYTASVPEMSGVGTSVIQVTATDADDANYGNSAKVVYSILQGQPYFSVDPESGIIKTALPDMSRENREQYQVVIQAKDMGGQMGGLSGTTTVNITLTDVNNNPPRFPQSTYQFNSPESVPLGTHLGRIKANDPDVGENAEMEYSIAEGDGADMFDVITDKDTQEGIITVKQNLDFENQMLYTLRVDASNTHPDPRFLHLGPFKDTAVVKISVEDIDEPPVFTKVSYLIEVDEDVKEGSIIGQVTAYDPDARNNLIKYSVDRHTDMDRIFGIHSENGSIFTLKALDRESSPWHNITVTATEINNPKQSSHIPVFIRILDINDHAPEFAMYYETFVCENAKPGQLIQTVSVMDKDDPPRGHKFFFEPVPEFTLNPNFTIVDNKDNTAGIMTRKDGYSRNKMSTYLLPILIFDNDYPIQSSTGTLTIRVCACDNQGNMQSCTAEALILSAGLSTGALVAILLCVLILLILVVLFAALKRQRKKEPLIISKDDVRDNIVTYNDEGGGEEDTQAFDIGTLRNPEAREDSKLRRDVMPETIFQIRRTVPLWENIDVQDFIHRRLKENDADPSAPPYDSLATYAYEGNDSIADSLSSLESLTADCNQDYDYLSDWGPRFKKLADMYGGDDSDRD

>NP_004352.2

MKLGKVEFCHFLQLIALFLCFSGMSQAELSRSRSKPYFQSGRSRTKRSWVWNQFFVLEEYMGSDPLYVGKLHSDVDKGDGSIKYILSGEGASSIFIIDENTGDIHATKRLDREEQAYYTLRAQALDRLTNKPVEPESEFVIKIQDINDNEPKFLDGPYTAGVPEMSPVGTSVVQVTATDADDPTYGNSARVVYSILQGQPYFSVEPKTGVIKTALPNMDREAKDQYLLVIQAKDMVGQNGGLSGTTSVTVTLTDVNDNPPRFPRRSYQYNVPESLPVASVVARIKAADADIGANAEMEYKIVDGDGLGIFKISVDKETQEGIITIQKELDFEAKTSYTLRIEAANKDADPRFLSLGPFSDTTTVKIIVEDVDEPPVFSSPLYPMEVSEATQVGNIIGTVAAHDPDSSNSPVRYSIDRNTDLERYFNIDANSGVITTAKSLDRETNAIHNITVLAMESQNPSQVGRGYVAITILDINDNAPEFAMDYETTVCENAQPGQVIQKISAVDKDEPSNGHQFYFSLTTDATNNHNFSLKDNKDNTASILTRRNGFRRQEQSVYYLPIFIVDSGSPSLSSTNTLTIRVCDCDADGVAQTCNAEAYVLPAGLSTGALIAILACVLTLLVLILLIVTMRRRKKEPLIFDEERDIRENIVRYDDEGGGEEDTEAFDMAALRNLNVIRDTKTRRDVTPEIQFLSRPAFKSIPDNVIFREFIWERLKEADVDPGAPPYDSLQTYAFEGNGSVAESLSSLDSISSNSDQNYDYLSDWGPRFKRLADMYGTGQESLYS

943 NP_002349.1 NP_000386.1

>NP_002349.1

MALQLSREQGITLRGSAEIVAEFFSFGINSILYQRGIYPSETFTRVQKYGLTLLVTTDLELIKYLNNVVEQLKDWLYKCSVQKLVVVISNIESGEVLERWQFDIECDKTAKDDSAPREKSQKAIQDEIRSVIRQITATVTFLPLLEVSCSFDLLIYTDKDLVVPEKWEESGPQFITNSEEVRLRSFTTTIHKVNSMVAYKIPVND

>NP_000386.1

MVLAQGLLSMALLALCWERSLAGAEETIPLQTLRCYNDYTSHITCRWADTQDAQRLVNVTLIRRVNEDLLEPVSCDLSDDMPWSACPHPRCVPRRCVIPCQSFVVTDVDYFSFQPDRPLGTRLTVTLTQHVQPPEPRDLQISTDQDHFLLTWSVALGSPQSHWLSPGDLEFEVVYKRLQDSWEDAAILLSNTSQATLGPEHLMPSSTYVARVRTRLAPGSRLSGRPSKWSPEVCWDSQPGDEAQPQNLECFFDGAAVLSCSWEVRKEVASSVSFGLFYKPSPDAGEEECSPVLREGLGSLHTRHHCQIPVPDPATHGQYIVSVQPRRAEKHIKSSVNIQMAPPSLNVTKDGDSYSLRWETMKMRYEHIDHTFEIQYRKDTATWKDSKTETLQNAHSMALPALEPSTRYWARVRVRTSRTGYNGIWSEWSEARSWDTESVLPMWVLALIVIFLTIAVLLALRFCGIYGYRLRRKWEEKIPNPSKSHLFQNGSAELWPPGSMSAFTSGSPPHQGPWGSRFPELEGVFPVGFGDSEVSPLTIEDPKHVCDPPSGPDTTPAASDLPTEQPPSPQPGPPAASHTPEKQASSFDFNGPYLGPPHSRSLPDILGQPEPPQEGGSQKSPPPGSLEYLCLPAGGQVQLVPLAQAMGPGQAVEVERRPSQGAAGSPSLESGGGPAPPALGPRVGGQDQKDSPVAIPMSSGDTEDPGVASGYVSSADLVFTPNSGASSVSLVPSLGLPSDQTPSLCPGLASGPPGAPGPVKSGFEGYVELPPIEGRSPRSPRNNPVPPEAKSPVLNPGERPADVSPTSPQPEGLLVLQQVGDYCFLPGLGPGPLSLRSKPSSPGPGPEIKNLDQAFQVKKPPGQAVPQVPVIQLFKALKQQDYLSLPPWEVNKPGEVC

944 NP_001002255.1 NP_001524.2

>NP_001002255.1

MANEKPTEEVKTENNNHINLKVAGQDGSVVQFKIKRQTPLSKLMKAYCEPRGLSVKQIRFRFGGQPISGTDKPAQLEMEDEDTIDVFQQPTGGVY

>NP_001524.2

MSRRLLPRAEKRRRRLEQRQQPDEQRRRSGAMVKMAAAGGGGGGGRYYGGGSEGGRAPKRLKTDNAGDQHGGGGGGGGGAGAAGGGGGGENYDDPHKTPASPVVHIRGLIDGVVEADLVEALQEFGPISYVVVMPKKRQALVEFEDVLGACNAVNYAADNQIYIAGHPAFVNYSTSQKISRPGDSDDSRSVNSVLLFTILNPIYSITTDVLYTICNPCGPVQRIVIFRKNGVQAMVEFDSVQSAQRAKASLNGADIYSGCCTLKIEYAKPTRLNVFKNDQDTWDYTNPNLSGQGDPGSNPNKRQRQPPLLGDHPAEYGGPHGGYHSHYHDEGYGPPPPHYEGRRMGPPVGGHRRGPSRYGPQYGHPPPPPPPPEYGPHADSPVLMVYGLDQSKMNCDRVFNVFCLYGNVEKVKFMKSKPGAAMVEMADGYAVDRAITHLNNNFMFGQKLNVCVSKQPAIMPGQSYGLEDGSCSYKDFSESRNNRFSTPEQAAKNRIQHPSNVLHFFNAPLEVTEENFFEICDELGVKRPSSVKVFSGKSERSSSGLLEWESKSDALETLGFLNHYQMKNPNGPYPYTLKLCFSTAQHAS

945 NP_005822.1 NP_149981.2

>NP_005822.1

MEETIKDPPTSAVLLDHCHFSQVIFNSVEKFYIPGGDVTCHYTFTQHFIPRRKDWIGIFRVGWKTTREYYTFMWVTLPIDLNNKSAKQQEVQFKAYYLPKDDEYYQFCYVDEDGVVRGASIPFQFRPENEEDILVVTTQGEVEEIEQHNKELCKENQELKDSCISLQKQNSDMQAELQKKQEELETLQSINKKLELKVKEQKDYWETELLQLKEQNQKMSSENEKMGIRVDQLQAQLSTQEKEMEKLVQGDQDKTEQLEQLKKENDHLFLSLTEQRKDQKKLEQTVEQMKQNETTAMKKQQELMDENFDLSKRLSENEIICNALQRQKERLEGENDLLKRENSRLLSYMGLDFNSLPYQVPTSDEGGARQNPGLAYGNPYSGIQESSSPSPLSIKKCPICKADDICDHTLEQQQMQPLCFNCPICDKIFPATEKQIFEDHVFCHSL

>NP_149981.2

MDSVAFEDVAVNFTQEEWALLSPSQKNLYRDVTLETFRNLASVGIQWKDQDIENLYQNLGIKLRSLVERLCGRKEGNEHRETFSQIPDCHLNKKSQTGVKPCKCSVCGKVFLRHSFLDRHMRAHAGHKRSECGGEWRETPRKQKQHGKASISPSSGARRTVTPTRKRPYECKVCGKAFNSPNLFQIHQRTHTGKRSYKCREIVRAFTVSSFFRKHGKMHTGEKRYECKYCGKPIDYPSLFQIHVRTHTGEKPYKCKQCGKAFISAGYLRTHEIRSHALEKSHQCQECGKKLSCSSSLHRHERTHSGGKLYECQKCAKVFRCPTSLQAHERAHTGERPYECNKCGKTFNYPSCFRRHKKTHSGEKPYECTRCGKAFGWCSSLRRHEMTHTGEKPFDCKQCGKVFTFSNYLRLHERTHLAGRSQCFGRRQGDHLSPGV

946 NP_683725.1 NP_061820.1

>NP_683725.1

MAEQSDEAVKYYTLEEIQKHNHSKSTWLILHHKVYDLTKFLEEHPGGEEVLREQAGGDATENFEDVGHSTDAREMSKTFIIGELHPDDRPKLNKPPETLITTIDSSSSWWTNWVIPAISAVAVALMYRLYMAED

>NP_061820.1

MGDVEKGKKIFIMKCSQCHTVEKGGKHKTGPNLHGLFGRKTGQAPGYSYTAANKNKGIIWGEDTLMEYLENPKKYIPGTKMIFVGIKKKEERADLIAYLKKATNE

947 AAM77350.1 NP_004508.1

>AAM77350.1

MSDALANAVCQRCQARFSPAERIVNSNGELYHEHCFVCAQCFRPFPEGLFYEFEGRKYCEHDFQMLFAPCCGSCGEFIIGRVIKAMNNNWHPGCFRCELCDVELADLGFVKNAGRHLCRPCHNREKAKGLGKYICQRCHLVIDEQPLMFRSDAYHPDHFNCTHCGKELTAEARELKGELYCLPCHDKMGVPICGACRRPIEGRVVNALGKQWHVEHFVCAKCEKPFLGHRHYEKKGLAYCETHYNQLFGDVCYNCSHVIEGDVVSALNKAWCVSCFSCSTCNSKLTLKNKFVEFDMKPVCKRCYEKFPLELKKRLKKLSELTSRKPSPRPQTSTLPEGPLAQCLSAPPPSPLLLSMLGPLVPIHLCPPHLTLPFSFLIAFSLPVPSSLPSPCLSSPWPWLLSVRRQELGSGSL

>NP_004508.1

MDDIFTQCREGNAVAVRLWLDNTENDLNQGDDHGFSPLHWACREGRSAVVEMLIMRGARINVMNRGDDTPLHLAASHGHRDIVQKLLQYKADINAVNEHGNVPLHYACFWGQDQVAEDLVANGALVSICNKYGEMPVDKAKAPLRELLRERAEKMGQNLNRIPYKDTFWKGTTRTRPRNGTLNKHSGIDFKQLNFLTKLNENHSGELWKGRWQGNDIVVKVLKVRDWSTRKSRDFNEECPRLRIFSHPNVLPVLGACQSPPAPHPTLITHWMPYGSLYNVLHEGTNFVVDQSQAVKFALDMARGMAFLHTLEPLIPRHALNSRSVMIDEDMTARISMADVKFSFQCPGRMYAPAWVAPEALQKKPEDTNRRSADMWSFAVLLWELVTREVPFADLSNMEIGMKVALEGLRPTIPPGISPHVCKLMKICMNEDPAKRPKFDMIVPILEKMQDK

948 NP_001744.2 NP_005334.1

>NP_001744.2

MSGGKYVDSEGHLYTVPIREQGNIYKPNNKAMADELSEKQVYDAHTKEIDLVNRDPKHLNDDVVKIDFEDVIAEPEGTHSFDGIWKASFTTFTVTKYWFYRLLSALFGIPMALIWGIYFAILSFLHIWAVVPCIKSFLIEIQCISRVYSIYVHTVCDPLFEAVGKIFSNVRINLQKEI

>NP_005334.1

MTEYKLVVVGAGGVGKSALTIQLIQNHFVDEYDPTIEDSYRKQVVIDGETCLLDILDTAGQEEYSAMRDQYMRTGEGFLCVFAINNTKSFEDIHQYREQIKRVKDSDDVPMVLVGNKCDLAARTVESRQAQDLARSYGIPYIETSAKTRQGVEDAFYTLVREIRQHKLRKLNPPDESGPGCMSCKCVLS

949 NP_001777.1 NP_004569.2

>NP_001777.1

MEDYTKIEKIGEGTYGVVYKGRHKTTGQVVAMKKIRLESEEEGVPSTAIREISLLKELRHPNIVSLQDVLMQDSRLYLIFEFLSMDLKKYLDSIPPGQYMDSSLVKSYLYQILQGIVFCHSRRVLHRDLKPQNLLIDDKGTIKLADFGLARAFGIPIRVYTHEVVTLWYRSPEVLLGSARYSTPVDIWSIGTIFAELATKKPLFHGDSEIDQLFRIFRALGTPNNEVWPEVESLQDYKNTFPKWKPGSLASHVKNLDENGLDLLSKMLIYDPAKRISGKMALNHPYFNDLDNQIKKM

>NP_004569.2

MSQTAMSETYDFLFKFLVIGNAGTGKSCLLHQFIEKKFKDDSNHTIGVEFGSKIINVGGKYVKLQIWDTAGQERFRSVTRSYYRGAAGALLVYDITSRETYNALTNWLTDARMLASQNIVIILCGNKKDLDADREVTFLEASRFAQENELMFLETSALTGENVEEAFVQCARKILNKIESGELDPERMGSGIQYGDAALRQLRSPRRAQAPNAQECGC

950 NP_037394.2 NP_055070.1

>NP_037394.2

MLCYVTRPDAVLMEVEVEAKANGEDCLNQVCRRLGIIEVDYFGLQFTGSKGESLWLNLRNRISQQMDGLAPYRLKLRVKFFVEPHLILQEQTRHIFFLHIKEALLAGHLLCSPEQAVELSALLAQTKFGDYNQNTAKYNYEELCAKELSSATLNSIVAKHKELEGTSQASAEYQVLQIVSAMENYGIEWHSVRDSEGQKLLIGVGPEGISICKDDFSPINRIAYPVVQMATQSGKNVYLTVTKESGNSIVLLFKMISTRAASGLYRAITETHAFYRCDTVTSAVMMQYSRDLKGHLASLFLNENINLGKKYVFDIKRTSKEVYDHARRALYNAGVVDLVSRNNQSPSHSPLKSSESSMNCSSCEGLSCQQTRVLQEKLRKLKEAMLCMVCCEEEINSTFCPCGHTVCCESCAAQLQSCPVCRSRVEHVQHVYLPTHTSLLNLTVI

>NP_055070.1

MKGWGWLALLLGALLGTAWARRSQDLHCGACRALVDELEWEIAQVDPKKTIQMGSFRINPDGSQSVVEVPYARSEAHLTELLEEICDRMKEYGEQIDPSTHRKNYVRVVGRNGESSELDLQGIRIDSDISGTLKFACESIVEEYEDELIEFFSREADNVKDKLCSKRTDLCDHALHISHDEL

951 NP_002730.1 NP_001782.1

>NP_002730.1

MAGLGPGVGDSEGGPRPLFCRKGALRQKVVHEVKSHKFTARFFKQPTFCSHCTDFIWGIGKQGLQCQVCSFVVHRRCHEFVTFECPGAGKGPQTDDPRNKHKFRLHSYSSPTFCDHCGSLLYGLVHQGMKCSCCEMNVHRRCVRSVPSLCGVDHTERRGRLQLEIRAPTADEIHVTVGEARNLIPMDPNGLSDPYVKLKLIPDPRNLTKQKTRTVKATLNPVWNETFVFNLKPGDVERRLSVEVWDWDRTSRNDFMGAMSFGVSELLKAPVDGWYKLLNQEEGEYYNVPVADADNCSLLQKFEACNYPLELYERVRMGPSSSPIPSPSPSPTDPKRCFFGASPGRLHISDFSFLMVLGKGSFGKVMLAERRGSDELYAIKILKKDVIVQDDDVDCTLVEKRVLALGGRGPGGRPHFLTQLHSTFQTPDRLYFVMEYVTGGDLMYHIQQLGKFKEPHAAFYAAEIAIGLFFLHNQGIIYRDLKLDNVMLDAEGHIKITDFGMCKENVFPGTTTRTFCGTPDYIAPEIIAYQPYGKSVDWWSFGVLLYEMLAGQPPFDGEDEEELFQAIMEQTVTYPKSLSREAVAICKGFLTKHPGKRLGSGPDGEPTIRAHGFFRWIDWERLERLEIPPPFRPRPCGRSGENFDKFFTRAAPALTPPDRLVLASIDQADFQGFTYVNPDFVHPDARSPTSPVPVPVM

>NP_001782.1

MQTIKCVVVGDGAVGKTCLLISYTTNKFPSEYVPTVFDNYAVTVMIGGEPYTLGLFDTAGQEDYDRLRPLSYPQTDVFLVCFSVVSPSSFENVKEKWVPEITHHCPKTPFLLVGTQIDLRDDPSTIEKLAKNKQKPITPETAEKLARDLKAVKYVECSALTQKGLKNVFDEAILAALEPPEPKKSRRCVLL

952 NP_005220.1 NP_659732.1

>NP_005220.1

MDPSVTLWQFLLQLLREQGNGHIISWTSRDGGEFKLVDAEEVARLWGLRKNKTNMNYDKLSRALRYYYDKNIIRKVSGQKFVYKFVSYPEVAGCSTEDCPPQPEVSVTSTMPNVAPAAIHAAPGDTVSGKPGTPKGAGMAGPGGLARSSRNEYMRSGLYSTFTIQSLQPQPPPHPRPAVVLPNAAPAGAAAPPSGSRSTSPSPLEACLEAEEAGLPLQVILTPPEAPNLKSEELNVEPGLGRALPPEVKVEGPKEELEVAGERGFVPETTKAEPEVPPQEGVPARLPAVVMDTAGQAGGHAASSPEISQPQKGRKPRDLELPLSPSLLGGPGPERTPGSGSGSGLQAPGPALTPSLLPTHTLTPVLLTPSSLPPSIHFWSTLSPIAPRSPAKLSFQFPSSGSAQVHIPSISVDGLSTPVVLSPGPQKP

>NP_659732.1

MGVQGTLMSRDSQTPHLLSILGKSKRKKDLRISCMSKPPAPNPTPPRNLDSRTFITIGDRNFEVEADDLVTISELGRGAYGVVEKVRHAQSGTIMAVKRIRATVNSQEQKRLLMDLDINMRTVDCFYTVTFYGALFREGDVWICMELMDTSLDKFYRKVLDKNMTIPEDILGEIAVSIVRALEHLHSKLSVIHRDVKPSNVLINKEGHVKMCDFGISGYLVDSVAKTMDAGCKPYMAPERINPELNQKGYNVKSDVWSLGITMIEMAILRFPYESWGTPFQQLKQVVEEPSPQLPADRFSPEFVDFTAQCLRKNPAERMSYLELMEHPFFTLHKTKKTDIAAFVKEILGEDS

953 NP_068810.2 NP_002189.1

>NP_068810.2

MDELFPLIFPAEPAQASGPYVEIIEQPKQRGMRFRYKCEGRSAGSIPGERSTDTTKTHPTIKINGYTGPGTVRISLVTKDPPHRPHPHELVGKDCRDGFYEAELCPDRCIHSFQNLGIQCVKKRDLEQAISQRIQTNNNPFQVPIEEQRGDYDLNAVRLCFQVTVRDPSGRPLRLPPVLPHPIFDNRAPNTAELKICRVNRNSGSCLGGDEIFLLCDKVQKEDIEVYFTGPGWEARGSFSQADVHRQVAIVFRTPPYADPSLQAPVRVSMQLRRPSDRELSEPMEFQYLPDTDDRHRIEEKRKRTYETFKSIMKKSPFSGPTDPRPPPRRIAVPSRSSASVPKPAPQPYPFTSSLSTINYDEFPTMVFPSGQISQASALAPAPPQVLPQAPAPAPAPAMVSALAQAPAPVPVLAPGPPQAVAPPAPKPTQAGEGTLSEALLQLQFDDEDLGALLGNSTDPAVFTDLASVDNSEFQQLLNQGIPVAPHTTEPMLMEYPEAITRLVTGAQRPPDPAPAPLGAPGLPNGLLSGDEDFSSIADMDFSALLSQISS

>NP_002189.1

MPITRMRMRPWLEMQINSNQIPGLIWINKEEMIFQIPWKHAAKHGWDINKDACLFRSWAIHTGRYKAGEKEPDPKTWKANFRCAMNSLPDIEEVKDQSRNKGSSAVRVYRMLPPLTKNQRKERKSKSSRDAKSKAKRKSCGDSSPDTFSDGLSSSTLPDDHSSYTVPGYMQDLEVEQALTPALSPCAVSSTLPDWHIPVEVVPDSTSDLYNFQVSPMPSTSEATTDEDEEGKLPEDIMKLLEQSEWQPTNVDGKGYLLNEPGVQPTSVYGDFSCKEEPEIDSPGGDIGLSLQRVFTDLKNMDATWLDSLLTPVRLPSIQAIPCAP

954 NP_004283.2 NP_006752.1

>NP_004283.2

MESPGESGAGSPGAPSPSSFTTGHLAREKPAQDPLYDVPNASGGQAGGPQRPGRVVSLRERLLLTRPVWLQLQANAAAALHMLRTEPPGTFLVRKSNTRQCQALCMRLPEASGPSFVSSHYILESPGGVSLEGSELMFPDLVQLICAYCHTRDILLLPLQLPRAIHHAATHKELEAISHLGIEFWSSSLNIKAQRGPAGGPVLPQLKARSPQELDQGTGAALCFFNPLFPGDLGPTKREKFKRSFKVRVSTETSSPLSPPAVPPPPVPVLPGAVPSQTERLPPCQLLRRESSVGYRVPAGSGPSLPPMPSLQEVDCGSPSSSEEEGVPGSRGSPATSPHLGRRRPLLRSMSAAFCSLLAPERQVGRAAAALMQDRHTAAGQLVQDLLTQVRAGPEPQELQGIRQALSRARAMLSAELGPEKLLSPKRLEHVLEKSLHCSVLKPLRPILAARLRRRLAADGSLGRLAEGLRLARAQGPGAFGSHLSLPSPVELEQVRQKLLQLLRTYSPSAQVKRLLQACKLLYMALRTQEGEGAGADEFLPLLSLVLAHCDLPELLLEAEYMSELLEPSLLTGEGGYYLTSLSASLALLSGLGQAHTLPLSPVQELRRSLSLWEQRRLPATHCFQHLLRVAYQDPSSGCTSKTLAVPPEASIATLNQLCATKFRVTQPNTFGLFLYKEQGYHRLPPGALAHRLPTTGYLVYRRAEWPETQGAVTEEEGSGQSEARSRGEEQGCQGDGDAGVKASPRDIREQSETTAEGGQGQAQEGPAQPGEPEAEGSRAAEE

>NP_006752.1

MDDREDLVYQAKLAEQAERYDEMVESMKKVAGMDVELTVEERNLLSVAYKNVIGARRASWRIISSIEQKEENKGGEDKLKMIREYRQMVETELKLICCDILDVLDKHLIPAANTGESKVFYYKMKGDYHRYLAEFATGNDRKEAAENSLVAYKAASDIAMTELPPTHPIRLGLALNFSVFYYEILNSPDRACRLAKAAFDDAIAELDTLSEESYKDSTLIMQLLRDNLTLWTSDMQGDGEEQNKEALQDVEDENQ

955 NP_005442.2 NP_005252.1

>NP_005442.2

MDSFKVVLEGPAPWGFRLQGGKDFNVPLSISRLTPGGKAAQAGVAVGDWVLSIDGENAGSLTHIEAQNKIRACGERLSLGLSRAQPVQSKPQKASAPAADPPRYTFAPSVSLNKTARPFGAPPPADSAPQQNGQPLRPLVPDASKQRLMENTEDWRPRPGTGQSRSFRILAHLTGTEFMQDPDEEHLKKSSQVPRTEAPAPASSTPQEPWPGPTAPSPTSRPPWAVDPAFAERYAPDKTSTVLTRHSQPATPTPLQSRTSIVQAAAGGVPGGGSNNGKTPVCHQCHKVIRGRYLVALGHAYHPEEFVCSQCGKVLEEGGFFEEKGAIFCPPCYDVRYAPSCAKCKKKITGEIMHALKMTWHVHCFTCAACKTPIRNRAFYMEEGVPYCERDYEKMFGTKCHGCDFKIDAGDRFLEALGFSWHDTCFVCAICQINLEGKTFYSKKDRPLCKSHAFSHV

>NP_005252.1

MTLNNVTMRQGTVGMQPQQQRWSIPADGRHLMVQKEPHQYSHRNRHSATPEDHCRRSWSSDSTDSVISSESGNTYYRVVLIGEQGVGKSTLANIFAGVHDSMDSDCEVLGEDTYERTLMVDGESATIILLDMWENKGENEWLHDHCMQVGDAYLIVYSITDRASFEKASELRIQLRRARQTEDIPIILVGNKSDLVRCREVSVSEGRACAVVFDCKFIETSAAVQHNVKELFEGIVRQVRLRRDSKEKNERRLAYQKRKESMPRKARRFWGKIVAKNNKNMAFKLKSKSCHDLSVL

956 NP_005891.1 NP_057682.1

>NP_005891.1

MNVTSLFSFTSPAVKRLLGWKQGDEEEKWAEKAVDALVKKLKKKKGAMEELEKALSCPGQPSNCVTIPRSLDGRLQVSHRKGLPHVIYCRVWRWPDLQSHHELKPLECCEFPFGSKQKEVCINPYHYKRVESPVLPPVLVPRHSEYNPQHSLLAQFRNLGQNEPHMPLNATFPDSFQQPNSHPFPHSPNSSYPNSPGSSSSTYPHSPTSSDPGSPFQMPADTPPPAYLPPEDPMTQDGSQPMDTNMMAPPLPSEINRGDVQAVAYEEPKHWCSIVYYELNNRVGEAFHASSTSVLVDGFTDPSNNKNRFCLGLLSNVNRNSTIENTRRHIGKGVHLYYVGGEVYAECLSDSSIFVQSRNCNYHHGFHPTTVCKIPSGCSLKIFNNQEFAQLLAQSVNHGFETVYELTKMCTIRMSFVKGWGAEYHRQDVTSTPCWIEIHLHGPLQWLDKVLTQMGSPHNPISSVS

>NP_057682.1

MMLIPTHHFRNIERKPEYLQPEKCVPPPYPGPVGTMWFIRDGCGIACAIVTWFLVLYAEFVVLFVMLIPSRDYVYSIINGIVFNLLAFLALASHCRAMLTDPGAVPKGNATKEFIESLQLKPGQVVYKCPKCCSIKPDRAHHCSVCKRCIRKMDHHCPWVNNCVGENNQKYFVLFTMYIALISLHALIMVGFHFLHCFEEDWTTYGLNREEMAETGISLHEKMQPLNFSSTECSSFSPPTTVILLILLCFEGLLFLIFTSVMFGTQVHSICTDETGIEQLKKEERRWAKKTKWMNMKAVFGHPFSLGWASPFATPDQGKADPYQYVV

957 NP_003083.1 NP_001348.2

>NP_003083.1

MDIRPNHTIYINNMNDKIKKEELKRSLYALFSQFGHVVDIVALKTMKMRGQAFVIFKELGSSTNALRQLQGFPFYGKPMRIQYAKTDSDIISKMRGTFADKEKKKEKKKAKTVEQTATTTNKKPGQGTPNSANTQGNSTPNPQVPDYPPNYILFLNNLPEETNEMMLSMLFNQFPGFKEVRLVPGRHDIAFVEFENDGQAGAARDALQGFKITPSHAMKITYAKK

>NP_001348.2

MGDVKNFLYAWCGKRKMTPSYEIRAVGNKNRQKFMCEVQVEGYNYTGMGNSTNKKDAQSNAARDFVNYLVRINEIKSEEVPAFGVASPPPLTDTPDTTANAEGDLPTTMGGPLPPHLALKAENNSEVGASGYGVPGPTWDRGANLKDYYSRKEEQEVQATLESEEVDLNAGLHGNWTLENAKARLNQYFQKEKIQGEYKYTQVGPDHNRSFIAEMTIYIKQLGRRIFAREHGSNKKLAAQSCALSLVRQLYHLGVVEAYSGLTKKKEGETVEPYKVNLSQDLEHQLQNIIQELNLEILPPPEDPSVPVALNIGKLAQFEPSQRQNQVGVVPWSPPQSNWNPWTSSNIDEGPLAFATPEQISMDLKNELMYQLEQDHDLQAILQERELLPVKKFESEILEAISQNSVVIIRGATGCGKTTQVPQFILDDFIQNDRAAECNIVVTQPRRISAVSVAERVAFERGEEPGKSCGYSVRFESILPRPHASIMFCTVGVLLRKLEAGIRGISHVIVDEIHERDINTDFLLVVLRDVVQAYPEVRIVLMSATIDTSMFCEYFFNCPIIEVYGRTYPVQEYFLEDCIQMTHFVPPPKDKKKKDKDDDGGEDDDANCNLICGDEYGPETRLSMSQLNEKETPFELIEALLKYIETLNVPGAVLVFLPGWNLIYTMQKHLEMNPHFGSHRYQILPLHSQIPREEQRKVFDPVPVGVTKVILSTNIAETSITINDVVYVIDSCKQKVKLFTAHNNMTNYATVWASKTNLEQRKGRAGRVRPGFCFHLCSRARFERLETHMTPEMFRTPLHEIALSIKLLRLGGIGQFLAKAIEPPPLDAVIEAEHTLRELDALDANDELTPLGRILAKLPIEPRFGKMMIMGCIFYVGDAICTIAAATCFPEPFINEGKRLGYIHRNFAGNRFSDHVALLSVFQAWDDARMGGEEAEIRFCEHKRLNMATLRMTWEAKVQLKEILINSGFPEDCLLTQVFTNTGPDNNLDVVISLLAFGVYPNVCYHKEKRKILTTEGRNALIHKSSVNCPFSSQDMKYPSPFFVFGEKIRTRAISAKGMTLVTPLQLLLFASKKVQSDGQIVLVDDWIKLQISHEAAACITGLRAAMEALVVEVTKQPAIISQLDPVNERMLNMIRQISRPSAAGINLMIGSTRYGDGPRPPKMARYDNGSGYRRGGSSYSGGGYGGGYSSGGYGSGGYGGSANSFRAGYGAGVGGGYRGVSRGGFRGNSGGDYRGPSGGYRGSGGFQRGGGRGAYGTGYFGQGRGGGGY

958 NP_000389.1 NP_683725.1

>NP_000389.1

MGAQLSTLGHMVLFPVWFLYSLLMKLFQRSTPAITLESPDIKYPLRLIDREIISHDTRRFRFALPSPQHILGLPVGQHIYLSARIDGNLVVRPYTPISSDDDKGFVDLVIKVYFKDTHPKFPAGGKMSQYLESMQIGDTIEFRGPSGLLVYQGKGKFAIRPDKKSNPIIRTVKSVGMIAGGTGITPMLQVIRAIMKDPDDHTVCHLLFANQTEKDILLRPELEELRNKHSARFKLWYTLDRAPEAWDYGQGFVNEEMIRDHLPPPEEEPLVLMCGPPPMIQYACLPNLDHVGHPTERCFVF

>NP_683725.1

MAEQSDEAVKYYTLEEIQKHNHSKSTWLILHHKVYDLTKFLEEHPGGEEVLREQAGGDATENFEDVGHSTDAREMSKTFIIGELHPDDRPKLNKPPETLITTIDSSSSWWTNWVIPAISAVAVALMYRLYMAED

959 NP_055102.3 NP_064555.2

>NP_055102.3

MLVGQGAGPLGPAVVTAAVVLLLSGVGPAHGSEDIVVGCGGFVKSDVEINYSLIEIKLYTKHGTLKYQTDCAPNNGYFMIPLYDKGDFILKIEPPLGWSFEPTTVELHVDGVSDICTKGGDINFVFTGFSVNGKVLSKGQPLGPAGVQVSLRNTGTEAKIQSTVTQPGGKFAFFKVLPGDYEILATHPTWALKEASTTVRVTNSNANAASPLIVAGYNVSGSVRSDGEPMKGVKFLLFSSLVTKEDVLGCNVSPVPGFQPQDESLVYLCYTVSREDGSFSFYSLPSGGYTVIPFYRGERITFDVAPSRLDFTVEHDSLKIEPVFHVMGFSVTGRVLNGPEGDGVPEAVVTLNNQIKVKTKADGSFRLENITTGTYTIHAQKEHLYFETVTIKIAPNTPQLADIIATGFSVCGQISIIRFPDTVKQMNKYKVVLSSQDKDKSLVTVETDAHGSFCFKAKPGTYKVQVMVPEAETRAGLTLKPQTFPLTVTNRPMMDVAFVQFLASVSGKVSCLDTCGDLLVTLQSLSRQGEKRSLQLSGKVNAMTFTFDNVLPGKYKISIMHEDWCWKNKSLEVEVLEDDMSAVEFRQTGYMLRCSLSHAITLEFYQDGNGRENVGIYNLSKGVNRFCLSKPGVYKVTPRSCHRFEQAFYTYDTSSPSILTLTAIRHHVLGTITTDKMMDVTVTIKSSIDSEPALVLGPLKSVQELRREQQLAEIEARRQEREKNGNEEGEERMTKPPVQEMVDELQGPFSYDFSYWARSGEKITVTPSSKELLFYPPSMEAVVSGESCPGKLIEIHGKAGLFLEGQIHPELEGVEIVISEKGASSPLITVFTDDKGAYSVGPLHSDLEYTVTSQKEGYVLTAVEGTIGDFKAYALAGVSFEIKAEDDQPLPGVLLSLSGGLFRSNLLTQDNGILTFSNLSPGQYYFKPMMKEFRFEPSSQMIEVQEGQNLKITITGYRTAYSCYGTVSSLNGEPEQGVAMEAVGQNDCSIYGEDTVTDEEGKFRLRGLLPGCVYHVQLKAEGNDHIERALPHHRVIEVGNNDIDDVNIIVFRQINQFDLSGNVITSSEYLPTLWVKLYKSENLDNPIQTVSLGQSLFFHFPPLLRDGENYVVLLDSTLPRSQYDYILPQVSFTAVGYHKHITLIFNPTRKLPEQDIAQGSYIALPLTLLVLLAGYNHDKLIPLLLQLTSRLQGVRALGQAASDNSGPEDAKRQAKKQKTRRT

>NP_064555.2

MLEEAGEVLENMLKASCLPLGFIVFLPAVLLLVAPPLPAADAAHEFTVYRMQQYDLQGQPYGTRNAVLNTEARTMAAEVLSRRCVLMRLLDFSYEQYQKALRQSAGAVVIILPRAMAAVPQDVVRQFMEIEPEMLAMETAVPVYFAVEDEALLSIYKQTQAASASQGSASAAEVLLRTATANGFQMVTSGVQSKAVSDWLIASVEGRLTGLGGEDLPTIVIVAHYDAFGVAPWLSLGADSNGSGVSVLLELARLFSRLYTYKRTHAAYNLLFFASGGGKFNYQGTKRWLEDNLDHTDSSLLQDNVAFVLCLDTVGRGSSLHLHVSKPPREGTLQHAFLRELETVAAHQFPEVRFSMVHKRINLAEDVLAWEHERFAIRRLPAFTLSHLESHRDGQRSSIMDVRSRVDSKTLTRNTRIIAEALTRVIYNLTEKGTPPDMPVFTEQMQIQQEQLDSVMDWLTNQPRAAQLVDKDSTFLSTLEHHLSRYLKDVKQHHVKADKRDPEFVFYDQLKQVMNAYRVKPAVFDLLLAVGIAAYLGMAYVAVQHFSLLYKTVQRLLVKAKTQ

960 NP_002266.2 NP_004196.3

>NP_002266.2

MTTTFLQTSSSTFGGGSTRGGSLLAGGGGFGGGSLSGGGGSRSISASSARFVSSGSGGGYGGGMRVCGFGGGAGSVFGGGFGGGVGGGFGGGFGGGDGGLLSGNEKITMQNLNDRLASYLDKVRALEEANADLEVKIHDWYQKQTPASPECDYSQYFKTIEELRDKIMATTIDNSRVILEIDNARLAADDFRLKYENELALRQGVEADINGLRRVLDELTLARTDLEMQIEGLNEELAYLKKNHEEEMKEFSSQLAGQVNVEMDAAPGVDLTRVLAEMREQYEAMAEKNRRDVEAWFFSKTEELNKEVASNTEMIQTSKTEITDLRRTMQELEIELQSQLSMKAGLENSLAETECRYATQLQQIQGLIGGLEAQLSELRCEMEAQNQEYKMLLDIKTRLEQEIATYRSLLEGQDAKMAGIGIREASSGGGGSSSNFHINVEESVDGQVVSSHKREI

>NP_004196.3

MSQLSSTLKRYTESARYTDAHYAKSGYGAYTPSSYGANLAASLLEKEKLGFKPVPTSSFLTRPRTYGPSSLLDYDRGRPLLRPDITGGGKRAESQTRGTERPLGSGLSGGSGFPYGVTNNCLSYLPINAYDQGVTLTQKLDSQSDLARDFSSLRTSDSYRIDPRNLGRSPMLARTRKELCTLQGLYQTASCPEYLVDYLENYGRKGSASQVPSQAPPSRVPEIISPTYRPIGRYTLWETGKGQAPGPSRSSSPGRDGMNSKSAQGLAGLRNLGNTCFMNSILQCLSNTRELRDYCLQRLYMRDLHHGSNAHTALVEEFAKLIQTIWTSSPNDVVSPSEFKTQIQRYAPRFVGYNQQDAQEFLRFLLDGLHNEVNRVTLRPKSNPENLDHLPDDEKGRQMWRKYLEREDSRIGDLFVGQLKGSLTCTDCGYCSTVFDPFWDLSLPIAKRGYPEVTLMDCMRLFTKEDVLDGDEKPTCCRCRGRKRCIKKFSIQRFPKILVLRLKRFSESRIRTSKLTTFVNFPLRDLDLREFASENTNHAVYNLYAVSNHSGTTMGGHYTAYCRSPGTGEWHTFNDSSVTPMSSSQVRTSDAYLLFYELASPPSRM

961 NP_055498.2 NP_057103.2

>NP_055498.2

MEVVGDFEYSKRDLVGHGAFAVVFRGRHRQKTDWEVAIKSINKKNLSKSQILLGKEIKILKELQHENIVALYDVQELPNSVFLVMEYCNGGDLADYLQAKGTLSEDTIRVFLHQIAAAMRILHSKGIIHRDLKPQNILLSYANRRKSSVSGIRIKIADFGFARYLHSNMMAATLCGSPMYMAPEVIMSQHYDAKADLWSIGTVIYQCLVGKPPFQANSPQDLRMFYEKNRSLMPSIPRETSPYLANLLLGLLQRNQKDRMDFEAFFSHPFLEQGPVKKSCPVPVPMYSGSVSGSSCGSSPSCRFASPPSLPDMQHIQEENLSSPPLGPPNYLQVSKDSASTSSKNSSCDTDDFVLVPHNISSDHSCDMPMGTAGRRASNEFLVCGGQCQPTVSPHSETAPIPVPTQIRNYQRIEQNLTSTASSGTNVHGSPRSAVVRRSNTSPMGFLRPGSCSPVPADTAQTVGRRLSTGSSRPYSPSPLVGTIPEQFSQCCCGHPQGHDSRSRNSSGSPVPQAQSPQSLLSGARLQSAPTLTDIYQNKQKLRKQHSDPVCPSHTGAGYSYSPQPSRPGSLGTSPTKHLGSSPRSSDWFFKTPLPTIIGSPTKTTAPFKIPKTQASSNLLALVTRHGPAEEQSKDGNEPRECAHCLLVQGSERQRAEQQSKAVFGRSVSTGKLSDQQGKTPICRHQGSTDSLNTERPMDIAPAGACGGVLAPPAGTAASSKAVLFTVGSPPHSAAAPTCTHMFLRTRTTSVGPSNSGGSLCAMSGRVCVGSPPGPGFGSSPPGAEAAPSLRYVPYGASPPSLEGLITFEAPELPEETLMEREHTDTLRHLNVMLMFTECVLDLTAMRGGNPELCTSAVSLYQIQESVVVDQISQLSKDWGWVEQLVLYMKAAQLLAASLHLAKAQIKSGKLSPSTAVKQVVKNLNERYKFCITMCKKLTEKLNRFFSDKQRFIDEINSVTAEKLIYNCAVEMVQSAALDEMFQQTEDIVYRYHKAALLLEGLSRILQDPADIENVHKYKCSIERRLSALCHSTATV

>NP_057103.2

MSAQAQMRAMLDQLMGTSRDGDTTRQRIKFSDDRVCKSHLLNCCPHDVLSGTRMDLGECLKVHDLALRADYEIASKEQDFFFELDAMDHLQSFIADCDRRTEVAKKRLAETQEEISAEVAAKAERVHELNEEIGKLLAKVEQLGAEGNVEESQKVMDEVEKARAKKREAEEVYRNSMPASSFQQQKLRVCEVCSAYLGLHDNDRRLADHFGGKLHLGFIEIREKLEELKRVVAEKQEKRNQERLKRREEREREEREKLRRSRSHSKNPKRSRSREHRRHRSRSMSRERKRRTRSKSREKRHRHRSRSSSRSRSRSHQRSRHSSRDRSRERSKRRSSKERFRDQDLASCDRDRSSRDRSPRDRDRKDKKRSYESANGRSEDRRSSEEREAGEI

962 NP_006702.1 NP_003127.1

>NP_006702.1

MDLSGVKKKSLLGVKENNKKSSTRAPSPTKRKDRSDEKSKDRSKDKGATKESSEKDRGRDKTRKRRSASSGSSSTRSRSSSTSSSGSSTSTGSSSGSSSSSASSRSGSSSTSRSSSSSSSSGSPSPSRRRHDNRRRSRSKSKPPKRDEKERKRRSPSPKPTKVHIGRLTRNVTKDHIMEIFSTYGKIKMIDMPVERMHPHLSKGYAYVEFENPDEAEKALKHMDGGQIDGQEITATAVLAPWPRPPPRRFSPPRRMLPPPPMWRRSPPRMRRRSRSPRRRSPVRRRSRSPGRRRHRSRSSSNSSR

>NP_003127.1

MVLADLGRKITSALRSLSNATIINEEVLNAMLKEVCTALLEADVNIKLVKQLRENVKSAIDLEEMASGLNKRKMIQHAVFKELVKLVDPGVKAWTPTKGKQNVIMFVGLQGSGKTTTCSKLAYYYQRKGWKTCLICADTFRAGAFDQLKQNATKARIPFYGSYTEMDPVIIASEGVEKFKNENFEIIIVDTSGRHKQEDSLFEEMLQVANAIQPDNIVYVMDASIGQACEAQAKAFKDKVDVASVIVTKLDGHAKGGGALSAVAATKSPIIFIGTGEHIDDFEPFKTQPFISKLLGMGDIEGLIDKVNELKLDDNEALIEKLKHGQFTLRDMYEQFQNIMKMGPFSQILGMIPGFGTDFMSKGNEQESMARLKKLMTIMDSMNDQELDSTDGAKVFSKQPGRIQRVARGSGVSTRDVQELLTQYTKFAQMVKKMGGIKGLFKGGDMSKNVSQSQMAKLNQQMAKMMDPRVLHHMGGMAGLQSMMRQFQQGAAGNMKGMMGFNNM

963 NP_149033.2 NP_054860.1

>NP_149033.2

MQKGLLDQDTGLVLLESQVIMSGLIAPETGENLSLEEGIARNLINPQMYQQLRELQDALALISRLTESRGPLSVVEAIEKRIISETVGLKILEAHLATGGFSLSPSENCINLEEAFHQGLISAWLHSVLESYLRTSKNLIDPNTAEKIGLLDLMQRCIVHQESGFKLLPVKQLAGGMVSLKSGRKVSIFRAVQEGLIDRQVTVRLLEAQLFAGGIVDPRTGHRLTVEEAVRHNLIDQDMACAILIRQLQTGGIIDTVTGQRLTIDEAVSNDLVAAKIALVILESLWSFMGLLWPESGEILPITDALEQGIVSTELAHKILSNRQHIKALFLPATTEILSWKKAIESGILDRDLANNLKSICIPDVMPHMQLADSAEQNINPGAAVLPCSKSHPKATASQSENLLFQLMTHSYINVQNGQRLLLLDKELMETLTSRDEYQTSPPKVVEIGHQRQKTPEGLQESANVKISGTFSSGWTVRLPEFQFSSQNKEYPDREDCTTEKGKKTTVETEDSSVENPEQDLFVEQKERNPNIDALKVINKVKLEVQRQLIGTQREDQTAVSVRENASRGHLLTIPPAEAEGVPLVVDKDVFSVETPKKEHQPLRNTSFTCQNEQAHTLETEYIHDETGGSHIKPQSKKLQVQVKKTLGIKLELKSETDGNVHPLDKKEMLKKTFLAKDDHKESQEAQNIAGGSMMMSEKTDEEDSGREIFLSCSHPLELLEEATLNVLSAQLLDGGIFHEQTGQKLLLNEAISRGIVPSHTAVKLMEKLNMFQGFFDSQTCESLTTEEVINEGLMDEKLLHNVLMADKAISGVLDPRTQTLCSVKDAVTVGLLDKETATRILERQVVTGGIIDLKRGKKVSVTLASTLGLVDVADQPELINLEKASKGRDAEKTVRERLISLQMETTGLIDPDSKAPLTVVQSIDRGLLEREEAVRLLTKQVVDGGIIHHISGMRLSVDNAFRHGLIGEDLAEKLKRVENLNIHQIFNPETKENISLPKAIKLDLITSDLKREIQEVQAFTGNFVDLISGQRLTLAEAKKEGLLTNEAVLSPGMMHGIVDPENCRIVPYSELVKKCKIDIESGQRYLEVIPFSDIKDGVSDKVLTLSQAIQLGKVDFASTLKVLEAQANTGGIIDTATGKRLTLASALEEKLVDENMVRIIASHQVLNGGIVDIFSDQRVTLVEAIEKRLISPELANMIQIDSSEFSDHRAQIEKQEGIEVCALQNEFLGKDMLIACNQTAEMSCNKVEESERLFQVENQSAQEKVKVRVSDGEQAKKSREISLKEFGCKDQRKPRMSSDAKEFISIINPHNLKGKSLGQVSLTHPYSECDFKLKEVARNNMGNDTNEEQEKAVTKIEIISHMKQSTSCLDSEEIRENQGEVILEVQETYCETSGKLPSEQVLQQPMNARVKSKREKREVIVEESIRTCKPAFLSEEKLYQETAIRDEHDSHIKSQPREMTSSEKGKEADTEMGFSITFKIEESSSQVVPQGISVKHLDALTLFSSKQANEGKVNNLSLCLTLKPEENLSREIACGAQSEPFPCMTPRPEGLHYQESDGKAQVTGPSQISKTDKSFQGTTRQETNYQDSWVTSKTKETKHQISSSNECKEKSYQEVSFDPARGLKLEEITVSRPDSKEVRYLEFSDRKDLHHQGSKSDDKLCGTLKSEIATQELTGEKFLEMANPNVAGLEAGSIEDIVTQRGSRVLGSFLPEKLFKGVSQKENTGQQNAIISPTVLETSEEKTVSLTVCSAVKTEKTPQEKLRESPGSEQTPFMTAPEGKGNGGVNPEPFRATQNVFTRQLCLEHDEKLVSYLSLLRNIEMRTKQIQPLELNLAELQDLLCQAKVLERELKDLTTLVSQELECVNQIIISQPQEVPAQLLKALEKDAKNLQKSLSSVSDTWNSRLLHFQNAVEIEKTKVLNQHTQLEGRLQDLRAWVGNKNLILNSKGSNSEIDVDSLNLCLQQYEDLKQPMAERKAQLDALAFDIQFFISEHAQDLSPQQNRQMLRLLNELQRSFQDILEQTAAQVDALQGHLQQMEQEALVKTLQKQQNTCHQQLEDLCSWVGQAERALAGHQGRTTQQDLSALQKNQSDLKDLQDDIQNRATSFATVVKDIEGFMEENQTKLSPRELTALREKLHQAKEQYEALQEETRVAQKELEEAVTSALQQETEKSKAAKELAENKKKIDALLDWVTSVGSSGGQLLTNLPGMEQLSGASLEKGALDTTDGYMGVNQAPEKLDKQCEMMKARHQELLSQQQNFILATQSAQAFLDQHGHNLTPEEQQMLQQKLGELKEQYSTSLAQSEAELKQVQTLQDELQKFLQDHKEFESWLERSEKELENMHKGGSSPETLPSLLKRQGSFSEDVISHKGDLRFVTISGQKVLDMENSFKEGKEPSEIGNLVKDKLKDATERYTALHSKCTRLGSHLNMLLGQYHQFQNSADSLQAWMQACEANVEKLLSDTVASDPGVLQEQLATTKQLQEELAEHQVPVEKLQKVARDIMEIEGEPAPDHRHVQETTDSILSHFQSLSYSLAERSSLLQKAIAQSQSVQESLESLLQSIGEVEQNLEGKQVSSLSSGVIQEALATNMKLKQDIARQKSSLEATREMVTRFMETADSTTAAVLQGKLAEVSQRFEQLCLQQQEKESSLKKLLPQAEMFEHLSGKLQQFMENKSRMLASGNQPDQDITHFFQQIQELNLEMEDQQENLDTLEHLVTELSSCGFALDLCQHQDRVQNLRKDFTELQKTVKEREKDASSCQEQLDEFRKLVRTFQKWLKETEGSIPPTETSMSAKELEKQIEHLKSLLDDWASKGTLVEEINCKGTSLENLIMEITAPDSQGKTGSILPSVGSSVGSVNGYHTCKDLTEIQCDMSDVNLKYEKLGGVLHERQESLQAILNRMEEVHKEANSVLQWLESKEEVLKSMDAMSSPTKTETVKAQAESNKAFLAELEQNSPKIQKVKEALAGLLVTYPNSQEAENWKKIQEELNSRWERATEVTVARQRQLEESASHLACFQAAESQLRPWLMEKELMMGVLGPLSIDPNMLNAQKQQVQFMLKEFEARRQQHEQLNEAAQGILTGPGDVSLSTSQVQKELQSINQKWVELTDKLNSRSSQIDQAIVKSTQYQELLQDLSEKVRAVGQRLSVQSAISTQPEAVKQQLEETSEIRSDLEQLDHEVKEAQTLCDELSVLIGEQYLKDELKKRLETVALPLQGLEDLAADRINRLQAALASTQQFQQMFDELRTWLDDKQSQQAKNCPISAKLERLQSQLQENEEFQKSLNQHSGSYEVIVAEGESLLLSVPPGEEKRTLQNQLVELKNHWEELSKKTADRQSRLKDCMQKAQKYQWHVEDLVPWIEDCKAKMSELRVTLDPVQLESSLLRSKAMLNEVEKRRSLLEILNSAADILINSSEADEDGIRDEKAGINQNMDAVTEELQAKTGSLEEMTQRLREFQESFKNIEKKVEGAKHQLEIFDALGSQACSNKNLEKLRAQQEVLQALEPQVDYLRNFTQGLVEDAPDGSDASQLLHQAEVAQQEFLEVKQRVNSGCVMMENKLEGIGQFHCRVREMFSQLADLDDELDGMGAIGRDTDSLQSQIEDVRLFLNKIHVLKLDIEASEAECRHMLEEEGTLDLLGLKRELEALNKQCGKLTERGKARQEQLELTLGRVEDFYRKLKGLNDATTAAEEAEALQWVVGTEVEIINQQLADFKMFQKEQVDPLQMKLQQVNGLGQGLIQSAGKDCDVQGLEHDMEEINARWNTLNKKVAQRIAQLQEALLHCGKFQDALEPLLSWLADTEELIANQKPPSAEYKVVKAQIQEQKLLQRLLDDRKATVDMLQAEGGRIAQSAELADREKITGQLESLESRWTELLSKAAARQKQLEDILVLAKQFHETAEPISDFLSVTEKKLANSEPVGTQTAKIQQQIIRHKALEEDIENHATDVHQAVKIGQSLSSLTSPAEQGVLSEKIDSLQARYSEIQDRCCRKAALLDQALSNARLFGEDEVEVLNWLAEVEDKLSSVFVKDFKQDVLHRQHADHLALNEEIVNRKKNVDQAIKNGQALLKQTTGEEVLLIQEKLDGIKTRYADITVTSSKALRTLEQARQLATKFQSTYEELTGWLREVEEELATSGGQSPTGEQIPQFQQRQKELKKEVMEHRLVLDTVNEVSRALLELVPWRAREGLDKLVSDANEQYKLVSDTIGQRVDEIDAAIQRSQQYEQAADAELAWVAETKRKLMALGPIRLEQDQTTAQLQVQKAFSIDIIRHKDSMDELFSHRSEIFGTCGEEQKTVLQEKTESLIQQYEAISLLNSERYARLERAQVLVNQFWETYEELSPWIEETRALIAQLPSPAIDHEQLRQQQEEMRQLRESIAEHKPHIDKLLKIGPQLKELNPEEGEMVEEKYQKAENMYAQIKEEVRQRALALDEAVSQSTQITEFHDKIEPMLETLENLSSRLRMPPLIPAEVDKIRECISDNKSATVELEKLQPSFEALKRRGEELIGRSQGADKDLAAKEIQDKLDQMVFFWEDIKARAEEREIKFLDVLELAEKFWYDMAALLTTIKDTQDIVHDLESPGIDPSIIKQQVEAAETIKEETDGLHEELEFIRILGADLIFACGETEKPEVRKSIDEMNNAWENLNKTWKERLEKLEDAMQAAVQYQDTLQAMFDWLDNTVIKLCTMPPVGTDLNTVKDQLNEMKEFKVEVYQQQIEMEKLNHQGELMLKKATDETDRDIIREPLTELKHLWENLGEKIAHRQHKLEGALLALGQFQHALEELMSWLTHTEELLDAQRPISGDPKVIEVELAKHHVLKNDVLAHQATVETVNKAGNELLESSAGDDASSLRSRLEAMNQCWESVLQKTEEREQQLQSTLQQAQGFHSEIEDFLLELTRMESQLSASKPTGGLPETAREQLDTHMELYSQLKAKEETYNQLLDKGRLMLLSRDDSGSGSKTEQSVALLEQKWHVVSSKMEERKSKLEEALNLATEFQNSLQEFINWLTLAEQSLNIASPPSLILNTVLSQIEEHKVFANEVNAHRDQIIELDQTGNQLKFLSQKQDVVLIKNLLVSVQSRWEKVVQRSIERGRSLDDARKRAKQFHEAWKKLIDWLEDAESHLDSELEISNDPDKIKLQLSKHKEFQKTLGGKQPVYDTTIRTGRALKEKTLLPEDSQKLDNFLGEVRDKWDTVCGKSVERQHKLEEALLFSGQFMDALQALVDWLYKVEPQLAEDQPVHGDLDLVMNLMDAHKVFQKELGKRTGTVQVLKRSGRELIENSRDDTTWVKGQLQELSTRWDTVCKLSVSKQSRLEQALKQAEVFRDTVHMLLEWLSEAEQTLRFRGALPDDTEALQSLIDTHKEFMKKVEEKRVDVNSAVAMGEVILAVCHPDCITTIKHWITIIRARFEEVLTWAKQHQQRLETALSELVANAELLEELLAWIQWAETTLIQRDQEPIPQNIDRVKALIAEHQTFMEEMTRKQPDVDRVTKTYKRKNIEPTHAPFIEKSRSGGRKSLSQPTPPPMPILSQSEAKNPRINQLSARWQQVWLLALERQRKLNDALDRLEELKEFANFDFDVWRKKYMRWMNHKKSRVMDFFRRIDKDQDGKITRQEFIDGILASKFPTTKLEMTAVADIFDRDGDGYIDYYEFVAALHPNKDAYRPTTDADKIEDEVTRQVAQCKCAKRFQVEQIGENKYRFFLGNQFGDSQQLRLVRILRSTVMVRVGGGWMALDEFLVKNDPCRARGRTNIELREKFILPEGASQGMTPFRSRGRRSKPSSRAASPTRSSSSASQSNHSCTSMPSSPATPASGTKVIPSSGSKLKRPTPTFHSSRTSLAGDTSNSSSPASTGAKTNRADPKKSASRPGSRAGSRAGSRASSRRGSDASDFDLLETQSACSDTSESSAAGGQGNSRRGLNKPSKIPTMSKKTTTASPRTPGPKR

>NP_054860.1

MQAAPRAGCGAALLLWIVSSCLCRAWTAPSTSQKCDEPLVSGLPHVAFSSSSSISGSYSPGYAKINKRGGAGGWSPSDSDHYQWLQVDFGNRKQISAIATQGRYSSSDWVTQYRMLYSDTGRNWKPYHQDGNIWAFPGNINSDGVVRHELQHPIIARYVRIVPLDWNGEGRIGLRIEVYGCSYWADVINFDGHVVLPYRFRNKKMKTLKDVIALNFKTSESEGVILHGEGQQGDYITLELKKAKLVLSLNLGSNQLGPIYGHTSVMTGSLLDDHHWHSVVIERQGRSINLTLDRSMQHFRTNGEFDYLDLDYEITFGGIPFSGKPSSSSRKNFKGCMESINYNGVNITDLARRKKLEPSNVGNLSFSCVEPYTVPVFFNATSYLEVPGRLNQDLFSVSFQFRTWNPNGLLVFSHFADNLGNVEIDLTESKVGVHINITQTKMSQIDISSGSGLNDGQWHEVRFLAKENFAILTIDGDEASAVRTNSPLQVKTGEKYFFGGFLNQMNNSSHSVLQPSFQGCMQLIQVDDQLVNLYEVAQRKPGSFANVSIDMCAIIDRCVPNHCEHGGKCSQTWDSFKCTCDETGYSGATCHNSIYEPSCEAYKHLGQTSNYYWIDPDGSGPLGPLKVYCNMTEDKVWTIVSHDLQMQTPVVGYNPEKYSVTQLVYSASMDQISAITDSAEYCEQYVSYFCKMSRLLNTPDGSPYTWWVGKANEKHYYWGGSGPGIQKCACGIERNCTDPKYYCNCDADYKQWRKDAGFLSYKDHLPVSQVVVGDTDRQGSEAKLSVGPLRCQGDRNYWNAASFPNPSSYLHFSTFQGETSADISFYFKTLTPWGVFLENMGKEDFIKLELKSATEVSFSFDVGNGPVEIVVRSPTPLNDDQWHRVTAERNVKQASLQVDRLPQQIRKAPTEGHTRLELYSQLFVGGAGGQQGFLGCIRSLRMNGVTLDLEERAKVTSGFISGCSGHCTSYGTNCENGGKCLERYHGYSCDCSNTAYDGTFCNKDVGAFFEEGMWLRYNFQAPATNARDSSSRVDNAPDQQNSHPDLAQEEIRFSFSTTKAPCILLYISSFTTDFLAVLVKPTGSLQIRYNLGGTREPYNIDVDHRNMANGQPHSVNITRHEKTIFLKLDHYPSVSYHLPSSSDTLFNSPKSLFLGKVIETGKIDQEIHKYNTPGFTGCLSRVQFNQIAPLKAALRQTNASAHVHIQGELVESNCGASPLTLSPMSSATDPWHLDHLDSASADFPYNPGQGQAIRNGVNRNSAIIGGVIAVVIFTILCTLVFLIRYMFRHKGTYHTNEAKGAESAESADAAIMNNDPNFTETIDESKKEWLI

964 NP_005585.1 NP_002219.1

>NP_005585.1

MPGEATETVPATEQELPQPQAETGSGTESDSDESVPELEEQDSTQATTQQAQLAAAAEIDEEPVSKAKQSRSEKKARKAMSKLGLRQVTGVTRVTIRKSKNILFVITKPDVYKSPASDTYIVFGEAKIEDLSQQAQLAAAEKFKVQGEAVSNIQENTQTPTVQEESEEEEVDETGVEVKDIELVMSQANVSRAKAVRALKNNSNDIVNAIMELTM

>NP_002219.1

MTAKMETTFYDDALNASFLPSESGPYGYSNPKILKQSMTLNLADPVGSLKPHLRAKNSDLLTSPDVGLLKLASPELERLIIQSSNGHITTTPTPTQFLCPKNVTDEQEGFAEGFVRALAELHSQNTLPSVTSAAQPVNGAGMVAPAVASVAGGSGSGGFSASLHSEPPVYANLSNFNPGALSSGGGAPSYGAAGLAFPAQPQQQQQPPHHLPQQMPVQHPRLQALKEEPQTVPEMPGETPPLSPIDMESQERIKAERKRMRNRIAASKCRKRKLERIARLEEKVKTLKAQNSELASTANMLREQVAQLKQKVMNHVNSGCQLMLTQQLQTF

965 P33316 NP_001001930.1

>P33316

MTPLCPRPALCYHFLTSLLRSAMQNARGTAEGRSRGTLRARPAPRPPAAQHGIPRPLSSAGRLSQGCRGASTVGAAGWKGELPKAGGSPAPGPETPAISPSKRARPAEVGGMQLRFARLSEHATAPTRGSARAAGYDLYSAYDYTIPPMEKAVVKTDIQIALPSGCYGRVAPRSGLAAKHFIDVGAGVIDEDYRGNVGVVLFNFGKEKFEVKKGDRIAQLICERIFYPEIEEVQALDDTERGSGGFGSTGKN

>NP_001001930.1

MVDTESPLCPLSPLEAGDLESPLSEEFLQEMGNIQEISQSIGEDSSGSFGFTEYQYLGSCPGSDGSVITDTLSPASSPSSVTYPVVPGSVDESPSGALNIECRICGDKASGYHYGVHACEGCKGFFRRTIRLKLVYDKCDRSCKIQKKNRNKCQYCRFHKCLSVGMSHNAIRFGRMPRSEKAKLKAEILTCEHDIEDSETADLKSLAKRIYEAYLKNFNMNKVKARVILSGKASNNPPFVIHDMETLCMAEKTLVAKLVANGIQNKEAEVRIFHCCQCTSVETVTELTEFAKAIPGFANLDLNDQVTLLKYGVYEAIFAMLSSVMNKDGMLVAYGNGFITREFLKSLRKPFCDIMEPKFDFAMKFNALELDDSDISLFVAAIICCGDRPGLLNVGHIEKMQEGIVHVLRLHLQSNHPDDIFLFPKLLQKMADLRQLVTEHAQLVQIIKKTESDAALHPLLQEIYRDMY

966 NP_000575.1 NP_001548.1

>NP_000575.1

MTSKLAVALLAAFLISAALCEGAVLPRSAKELRCQCIKTYSKPFHPKFIKELRVIESGPHCANTEIIVKLSDGRELCLDPKENWVQRVVEKFLKRAENS

>NP_001548.1

MEDFNMESDSFEDFWKGEDLSNYSYSSTLPPFLLDAAPCEPESLEINKYFVVIIYALVFLLSLLGNSLVMLVILYSRVGRSVTDVYLLNLALADLLFALTLPIWAASKVNGWIFGTFLCKVVSLLKEVNFYSGILLLACISVDRYLAIVHATRTLTQKRYLVKFICLSIWGLSLLLALPVLLFRRTVYSSNVSPACYEDMGNNTANWRMLLRILPQSFGFIVPLLIMLFCYGFTLRTLFKAHMGQKHRAMRVIFAVVLIFLLCWLPYNLVLLADTLMRTQVIQETCERRNHIDRALDATEILGILHSCLNPLIYAFIGQKFRHGLLKILAIHGLISKDSLPKDSRPSFVGSSSGHTSTTL

967 NP_000612.1 NP_690001.3

>NP_000612.1

MDILCEENTSLSSTTNSLMQLNDDTRLYSNDFNSGEANTSDAFNWTVDSENRTNLSCEGCLSPSCLSLLHLQEKNWSALLTAVVIILTIAGNILVIMAVSLEKKLQNATNYFLMSLAIADMLLGFLVMPVSMLTILYGYRWPLPSKLCAVWIYLDVLFSTASIMHLCAISLDRYVAIQNPIHHSRFNSRTKAFLKIIAVWTISVGISMPIPVFGLQDDSKVFKEGSCLLADDNFVLIGSFVSFFIPLTIMVITYFLTIKSLQKEATLCVSDLGTRAKLASFSFLPQSSLSSEKLFQRSIHREPGSYTGRRTMQSISNEQKACKVLGIVFFLFVVMWCPFFITNIMAVICKESCNEDVIGALLNVFVWIGYLSSAVNPLVYTLFNKTYRSAFSRYIQCQYKENKKPLQLILVNTIPALAYKSSQLQMGQKKNSKQDAKTTDNDCSMVALGKQHSEEASKDNSDGVNEKVSCV

>NP_690001.3

MGKDQELLEAARTGNVALVEKLLSGRKGGILGGGSGPLPLSNLLSIWRGPNVNCTDSSGYTALHHAALNGHKDIVLKLLQYEASTNVADNKGYFPIHLAAWKGDVEIVKILIHHGPSHSRVNEQNNENETALHCAAQYGHSEVVAVLLEELTDPTIRNSKLETPLDLAALYGRLRVVKMIISAHPNLMSCNTRKHTPLHLAARNGHKAVVQVLLEAGMDVSCQTEKGSALHEAALFGKVDVVRVLLETGIDANIKDSLGRTVLDILKEHPSQKSLQIATLLQEYLEGVGRSTVLEEPVQEDATQETHISSPVESPSQKTKSETVTGELSKLLDEIKLCQEKDYSFEDLCHTISDHYLDNLSKISEEELGKNGSQSVRTSSTINLSPGEVEEEDDDENTCGPSGLWEALTPCNGCRNLGFPMLAQESYPKKRNYTMEIVPSASLDTFPSENENFLCDLMDTAVTKKPCSLEIARAPSPRTDNASEVAVTTPGTSNHRNSSTGPTPDCSPPSPDTALKNIVKVIRPQPKQRTSIVSSLDFHRMNHNQEYFEINTSTGCTSFTASPPASPPTSSVGTTEVKNEGTNHTDDLSRQDDNDPPKEYDPGQFAGLLHGSSPACESPENPFHLYGKREQCEKGQDEVSLANSPLPFKQSPIENNSEPLVKKIKPKVVSRTIFHKKSNQLENHTIVGTRSTRSGSRNGDQWVMNAGGFVERACTLGRIRSLPKALIDMHLSKSVSKSDSDLIAYPSNEKTSRVNWSESSTAEHSSKGNSERTPSFTSEWEEIDKIMSSIDVGINNELKEMNGETTRPRCPVQTVGQWLESIGLPQYENHLMANGFDNVQFMGSNVMEDQDLLEIGILNSGHRQRILQAIQLLPKMRPIGHDGYHPTSVAEWLDSIELGDYTKAFLINGYTSMDLLKKIWEVELINVLKINLIGHRKRILASLGDRLHDDPPQKPPRSITLREPSGNHTPPQLSPSLSQSTYTTGGSLDVPHIIMQGDARRRRNENYFDDIPRSKLERQMAQTGDWGEPSITLRPPNEATASTPVQYWQHHPEKLIFQSCDYKAFYLGSMLIKELRGTESTQDACAKMRANCQKSTEQMKKVPTIILSVSYKGVKFIDATNKNIIAEHEIRNISCAAQDPEDLSTFAYITKDLKSNHHYCHVFTAFDVNLAYEIILTLGQAFEVAYQLALQARKGGHSSTLPESFENKPSKPIPKPRVSIRKSVDLLHASHTGQEPSERHTEEALRKF

968 NP_665802.1 NP_005408.1

>NP_665802.1

MSLLNCENSCGSSQSESDCCVAMASSCSAVTKDDSVGGTASTGNLSSSFMEEIQGYDVEFDPPLESKYECPICLMALREAVQTPCGHRFCKACIIKSIRDAGHKCPVDNEILLENQLFPDNFAKREILSLMVKCPNEGCLHKMELRHLEDHQAHCEFALMDCPQCQRPFQKFHINIHILKDCPRRQVSCDNCAASMAFEDKEIHDQNCPLANVICEYCNTILIREQMPNHYDLDCPTAPIPCTFSTFGCHEKMQRNHLARHLQENTQSHMRMLAQAVHSLSVIPDSGYISEVRNFQETIHQLEGRLVRQDHQIRELTAKMETQSMYVSELKRTIRTLEDKVAEIEAQQCNGIYIWKIGNFGMHLKCQEEEKPVVIHSPGFYTGKPGYKLCMRLHLQLPTAQRCANYISLFVHTMQGEYDSHLPWPFQGTIRLTILDQSEAPVRQNHEEIMDAKPELLAFQRPTIPRNPKGFGYVTFMHLEALRQRTFIKDDTLLVRCEVSTRFDMGSLRREGFQPRSTDAGV

>NP_005408.1

MGSNKSKPKDASQRRRSLEPAENVHGAGGGAFPASQTPSKPASADGHRGPSAAFAPAAAEPKLFGGFNSSDTVTSPQRAGPLAGGVTTFVALYDYESRTETDLSFKKGERLQIVNNTEGDWWLAHSLSTGQTGYIPSNYVAPSDSIQAEEWYFGKITRRESERLLLNAENPRGTFLVRESETTKGAYCLSVSDFDNAKGLNVKHYKIRKLDSGGFYITSRTQFNSLQQLVAYYSKHADGLCHRLTTVCPTSKPQTQGLAKDAWEIPRESLRLEVKLGQGCFGEVWMGTWNGTTRVAIKTLKPGTMSPEAFLQEAQVMKKLRHEKLVQLYAVVSEEPIYIVTEYMSKGSLLDFLKGETGKYLRLPQLVDMAAQIASGMAYVERMNYVHRDLRAANILVGENLVCKVADFGLARLIEDNEYTARQGAKFPIKWTAPEAALYGRFTIKSDVWSFGILLTELTTKGRVPYPGMVNREVLDQVERGYRMPCPPECPESLHDLMCQCWRKEPEERPTFEYLQAFLEDYFTSTEPQYQPGENL

969 NP_060947.1 NP_001091.1

>NP_060947.1

MPLYEGLGSGGEKTAVVIDLGEAFTKCGFAGETGPRCIIPSVIKRAGMPKPVRVVQYNINTEELYSYLKEFIHILYFRHLLVNPRDRRVVIIESVLCPSHFRETLTRVLFKYFEVPSVLLAPSHLMALLTLGINSAMVLDCGYRESLVLPIYEGIPVLNCWGALPLGGKALHKELETQLLEQCTVDTSVAKEQSLPSVMGSVPEGVLEDIKARTCFVSDLKRGLKIQAAKFNIDGNNERPSPPPNVDYPLDGEKILHILGSIRDSVVEILFEQDNEEQSVATLILDSLIQCPIDTRKQLAENLVVIGGTSMLPGFLHRLLAEIRYLVEKPKYKKALGTKTFRIHTPPAKANCVAWLGGAIFGALQDILGSRSVSKEYYNQTGRIPDWCSLNNPPLEMMFDVGKTQPPLMKRAFSTEK

>NP_001091.1

MCDEDETTALVCDNGSGLVKAGFAGDDAPRAVFPSIVGRPRHQGVMVGMGQKDSYVGDEAQSKRGILTLKYPIEHGIITNWDDMEKIWHHTFYNELRVAPEEHPTLLTEAPLNPKANREKMTQIMFETFNVPAMYVAIQAVLSLYASGRTTGIVLDSGDGVTHNVPIYEGYALPHAIMRLDLAGRDLTDYLMKILTERGYSFVTTAEREIVRDIKEKLCYVALDFENEMATAASSSSLEKSYELPDGQVITIGNERFRCPETLFQPSFIGMESAGIHETTYNSIMKCDIDIRKDLYANNVMSGGTTMYPGIADRMQKEITALAPSTMKIKIIAPPERKYSVWIGGSILASLSTFQQMWITKQEYDEAGPSIVHRKCF

970 NP_000480.2 NP_065853.2

>NP_000480.2

MTAEPMSESKLNTLVQKLHDFLAHSSEESEETSSPPRLAMNQNTDKISGSGSNSDMMENSKEEGTSSSEKSKSSGSSRSKRKPSIVTKYVESDDEKPLDDETVNEDASNENSENDITMQSLPKGTVIVQPEPVLNEDKDDFKGPEFRSRSKMKTENLKKRGEDGLHGIVSCTACGQQVNHFQKDSIYRHPSLQVLICKNCFKYYMSDDISRDSDGMDEQCRWCAEGGNLICCDFCHNAFCKKCILRNLGRKELSTIMDENNQWYCYICHPEPLLDLVTACNSVFENLEQLLQQNKKKIKVDSEKSNKVYEHTSRFSPKKTSSNCNGEEKKLDDSCSGSVTYSYSALIVPKEMIKKAKKLIETTANMNSSYVKFLKQATDNSEISSATKLRQLKAFKSVLADIKKAHLALEEDLNSEFRAMDAVNKEKNTKEHKVIDAKFETKARKGEKPCALEKKDISKSEAKLSRKQVDSEHMHQNVPTEEQRTNKSTGGEHKKSDRKEEPQYEPANTSEDLDMDIVSVPSSVPEDIFENLETAMEVQSSVDHQGDGSSGTEQEVESSSVKLNISSKDNRGGIKSKTTAKVTKELYVKLTPVSLSNSPIKGADCQEVPQDKDGYKSCGLNPKLEKCGLGQENSDNEHLVENEVSLLLEESDLRRSPRVKTTPLRRPTETNPVTSNSDEECNETVKEKQKLSVPVRKKDKRNSSDSAIDNPKPNKLPKSKQSETVDQNSDSDEMLAILKEVSRMSHSSSSDTDINEIHTNHKTLYDLKTQAGKDDKGKRKRKSSTSGSDFDTKKGKSAKSSIISKKKRQTQSESSNYDSELEKEIKSMSKIGAARTTKKRIPNTKDFDSSEDEKHSKKGMDNQGHKNLKTSQEGSSDDAERKQERETFSSAEGTVDKDTTIMELRDRLPKKQQASASTDGVDKLSGKEQSFTSLEVRKVAETKEKSKHLKTKTCKKVQDGLSDIAEKFLKKDQSDETSEDDKKQSKKGTEEKKKPSDFKKKVIKMEQQYESSSDGTEKLPEREEICHFPKGIKQIKNGTTDGEKKSKKIRDKTSKKKDELSDYAEKSTGKGDSCDSSEDKKSKNGAYGREKKRCKLLGKSSRKRQDCSSSDTEKYSMKEDGCNSSDKRLKRIELRERRNLSSKRNTKEIQSGSSSSDAEESSEDNKKKKQRTSSKKKAVIVKEKKRNSLRTSTKRKQADITSSSSSDIEDDDQNSIGEGSSDEQKIKPVTENLVLSSHTGFCQSSGDEALSKSVPVTVDDDDDDNDPENRIAKKMLLEEIKANLSSDEDGSSDDEPEEGKKRTGKQNEENPGDEEAKNQVNSESDSDSEESKKPRYRHRLLRHKLTVSDGESGEEKKTKPKEHKEVKGRNRRKVSSEDSEDSDFQESGVSEEVSESEDEQRPRTRSAKKAELEENQRSYKQKKKRRRIKVQEDSSSENKSNSEEEEEEKEEEEEEEEEEEEEEEDENDDSKSPGKGRKKIRKILKDDKLRTETQNALKEEEERRKRIAEREREREKLREVIEIEDASPTKCPITTKLVLDEDEETKEPLVQVHRNMVIKLKPHQVDGVQFMWDCCCESVKKTKKSPGSGCILAHCMGLGKTLQVVSFLHTVLLCDKLDFSTALVVCPLNTALNWMNEFEKWQEGLKDDEKLEVSELATVKRPQERSYMLQRWQEDGGVMIIGYEMYRNLAQGRNVKSRKLKEIFNKALVDPGPDFVVCDEGHILKNEASAVSKAMNSIRSRRRIILTGTPLQNNLIEYHCMVNFIKENLLGSIKEFRNRFINPIQNGQCADSTMVDVRVMKKRAHILYEMLAGCVQRKDYTALTKFLPPKHEYVLAVRMTSIQCKLYQYYLDHLTGVGNNSEGGRGKAGAKLFQDFQMLSRIWTHPWCLQLDYISKENKGYFDEDSMDEFIASDSDETSMSLSSDDYTKKKKKGKKGKKDSSSSGSGSDNDVEVIKVWNSRSRGGGEGNVDETGNNPSVSLKLEESKATSSSNPSSPAPDWYKDFVTDADAEVLEHSGKMVLLFEILRMAEEIGDKVLVFSQSLISLDLIEDFLELASREKTEDKDKPLIYKGEGKWLRNIDYYRLDGSTTAQSRKKWAEEFNDETNVRGRLFIISTKAGSLGINLVAANRVIIFDASWNPSYDIQSIFRVYRFGQTKPVYVYRFLAQGTMEDKIYDRQVTKQSLSFRVVDQQQVERHFTMNELTELYTFEPDLLDDPNSEKKKKRDTPMLPKDTILAELLQIHKEHIVGYHEHDSLLDHKEEEELTEEERKAAWAEYEAEKKGLTMRFNIPTGTNLPPVSFNSQTPYIPFNLGALSAMSNQQLEDLINQGREKVVEATNSVTAVRIQPLEDIISAVWKENMNLSEAQVQALALSRQASQELDVKRREAIYNDVLTKQQMLISCVQRILMNRRLQQQYNQQQQQQMTYQQATLGHLMMPKPPNLIMNPSNYQQIDMRGMYQPVAGGMQPPPLQRAPPPMRSKNPGPSQGKSM

>NP_065853.2

MLAGRPGTRSAVGELGTESSDNLDRAPLGPRESGGHHRPGSYLDMKIHLEKNLEEERQILLQQQKICRNRARKYFVESNRRKKAFEEKRKEQEEKEHQIREQILQQRKQKFEEVTEKFQRAHVPLSQRRKAVSRKPVPPLEEALKQIQESNLKSEVNLPFSRRPTINWRAIDSALPSALSKNDHKHQKQLLSKINCEKEMNENMRATLATSKNVFQLKLEETQKLLEDQHLSNLQKFGDEVNQITNSETLSSIDSLEATEHEEIYLTLNKEHSTSIQRNTISLKPANMQSTNLSCFDEDKLAFSKTQHINNWLTNLDASNTQNVTAFSDILSKSNVLPSWEYFNSKEQNPSPLNGTVERATNTANNSVPFVSSPPMFVLDKKCEKTSETSTMRTTDSTSGAFKRERPLVTESPTFKFSKSQSTSDSLTQEVATFPDQEKYSELNQENGTTSIPTSCVPVATPLVLPSNIQSARPSAKNSIHIKEIDAVQCSDKLDELKDGKEEEIKYFNCNKEELPLFSDSFQDAYIPHNPDSKDEKQKLAETSSLSNVTSNYDFVGQHKKMKYNIHERNGVRFLKSILKKESKYEHGYLKALIINQSFKFGNQKAAAIRDSIELTKEKGAEIPKTIKKLRWFDETSNIENNAENSHSLKNKTGTTQQHSQQFHIQSGAGSNIISVSTCAVNSADTKKSREDSISENVTTLGGSGADHMPLNCFIPSGYNFAKHAWPASKKEESKIPVHDDSKTKQGKPQRGRAKIIRKPGSAKVQSGFICTNRKGAVIQPQSASKVNIFTQAQGKLIIPCPPPQSTSNIRSGKNIQVSQCQPVTPENPQNIITHNSFNSKHVLPTEHSLNQWNQESSSPLSNACSDLVTVIPSLPSYCSSECQTFAKINHSNGTQAVARQDATLYCTQRSPVCEESYPSVTLRTAEEESVPLWKRGPNVLHQNKRATGSTVMRRKRIAETKRRNILEQKRQNPGSVGQKYSEQINNFGQSVLLSSSEPKQTTRGTSYIEEVSDSTSEFLMAENLVKASVPEDEILTVLNSKQIQKSNLPLNKTQQFNICTLSAEEQKILESLNDLSERLHYIQESICKNPSIKNTLQIIPLLEKREDRTSSCRDKR

971 NP_115634.1 NP_061485.1

>NP_115634.1

MDVVEVAGSWWAQEREDIIMKYEKGHRAGLPEDKGPKPFRSYNNNVDHLGIVHETELPPLTAREAKQIRREISRKSKWVDMLGDWEKYKSSRKLIDRAYKGMPMNIRGPMWSVLLNTEEMKLKNPGRYQIMKEKGKKSSEHIQRIDRDVSGTLRKHIFFRDRYGTKQRELLHILLAYEEYNPEVGYCRDLSHIAALFLLYLPEEDAFWALVQLLASERHSLQGFHSPNGGTVQGLQDQQEHVVATSQPKTMGHQDKKDLCGQCSPLGCLIRILIDGISLGLTLRLWDVYLVEGEQALMPITRIAFKVQQKRLTKTSRCGPWARFCNRFVDTWARDEDTVLKHLRASMKKLTRKKGDLPPPAKPEQGSSASRPVPASRGGKTLCKGDRQAPPGPPARFPRPIWSASPPRAPRSSTPCPGGAVREDTYPVGTQGVPSPALAQGGPQGSWRFLQWNSMPRLPTDLDVEGPWFRHYDFRQSCWVRAISQEDQLAPCWQAEHPAERVRSAFAAPSTDSDQGTPFRARDEQQCAPTSGPCLCGLHLESSQFPPGF

>NP_061485.1

MQAIKCVVVGDGAVGKTCLLISYTTNAFPGEYIPTVFDNYSANVMVDGKPVNLGLWDTAGQEDYDRLRPLSYPQTVGETYGKDITSRGKDKPIADVFLICFSLVSPASFENVRAKWYPEVRHHCPNTPIILVGTKLDLRDDKDTIEKLKEKKLTPITYPQGLAMAKEIGAVKYLECSALTQRGLKTVFDEAIRAVLCPPPVKKRKRKCLLL

972 NP_001008656.1 NP_006383.2

>NP_001008656.1

MAEARKRRELLPLIYHHLLRAGYVRAAREVKEQSGQKCFLAQPVTLLDIYTHWQQTSELGRKRKAEEDAALQAKKTRVSDPISTSESSEEEEEAEAETAKATPRLASTNSSVLGADLPSSMKEKAKAETEKAGKTGNSMPHPATGKTVANLLSGKSPRKSAEPSANTTLVSETEEEGSVPAFGAAAKPGMVSAGQADSSSEDTSSSSDETDVEGKPSVKPAQVKASSVSTKESPARKAAPAPGKVGDVTPQVKGGALPPAKRAKKPEEESESSEEGSESEEEAPAGTRSQVKASEKILQVRAASAPAKGTPGKGATPAPPGKAGAVASQTKAGKPEEDSESSSEESSDSEEETPAAKALLQAKASGKTSQVGAASAPAKESPRKGAAPAPPGKTGPAVAKAQAGKREEDSQSSSEESDSEEEAPAQAKPSGKAPQVRAASAPAKESPRKGAAPAPPRKTGPAAAQVQVGKQEEDSRSSSEESDSDREALAAMNAAQVKPLGKSPQVKPASTMGMGPLGKGAGPVPPGKVGPATPSAQVGKWEEDSESSSEESSDSSDGEVPTAVAPAQEKSLGNILQAKPTSSPAKGPPQKAGPVAVQVKAEKPMDNSESSEESSDSADSEEAPAAMTAAQAKPALKIPQTKACPKKTNTTASAKVAPVRVGTQAPRKAGTATSPAGSSPAVAGGTQRPAEDSSSSEESDSEEEKTGLAVTVGQAKSVGKGLQVKAASVPVKGSLGQGTAPVLPGKTGPTVTQVKAEKQEDSESSEEESDSEEAAASPAQVKTSVKKTQAKANPAAARAPSAKGTISAPGKVVTAAAQAKQRSPSKVKPPVRNPQNSTVLARGPASVPSVGKAVATAAQAQTGPEEDSGSSEEESDSEEEAETLAQVKPSGKTHQIRAALAPAKESPRKGAAPTPPGKTGPSAAQAGKQDDSGSSSEESDSDGEAPAAVTSAQVIKPPLIFVDPNRSPAGPAATPAQAQAASTPRKARASESTARSSSSESEDEDVIPATQCLTPGIRTNVVTMPTAHPRIAPKASMAGASSSKESSRISDGKKQEGPATQVDSAVGTLPATSPQSTSVQAKGTNKLRKPKLPEVQQATKAPESSDDSEDSSDSSSGSEEDGEGPQGAKSAHTLGPTPSRTETLVEETAAESSEDDVVAPSQSLLSGYMTPGLTPANSQASKATPKLDSSPSVSSTLAAKDDPDGKQEAKPQQAAGMLSPKTGGKEAASGTTPQKSRKPKKGAGNPQASTLALQSNITQCLLGQPWPLNEAQVQASVVKVLTELLEQERKKVVDTTKESSRKGWESRKRKLSGDQPAARTPRSKKKKKLGAGEGGEASVSPEKTSTTSKGKAKRDKASGDVKEKKGKGSLGSQGAKDEPEEELQKGMGTVEGGDQSNPKSKKEKKKSDKRKKDKEKKEKKKKAKKASTKDSESPSQKKKKKKKKTAEQTV

>NP_006383.2

MVLLHVLFEHAVGYALLALKEVEEISLLQPQVEESVLNLGKFHSIVRLVAFCPFASSQVALENANAVSEGVVHEDLRLLLETHLPSKKKKVLLGVGDPKIGAAIQEELGYNCQTGGVIAEILRGVRLHFHNLVKGLTDLSACKAQLGLGHSYSRAKVKFNVNRVDNMIIQSISLLDQLDKDINTFSMRVREWYGYHFPELVKIINDNATYCRLAQFIGNRRELNEDKLEKLEELTMDGAKAKAILDASRSSMGMDISAIDLINIESFSSRVVSLSEYRQSLHTYLRSKMSQVAPSLSALIGEAVGARLIAHAGSLTNLAKYPASTVQILGAEKALFRALKTRGNTPKYGLIFHSTFIGRAAAKNKGRISRYLANKCSIASRIDCFSEVPTSVFGEKLREQVEERLSFYETGEIPRKNLDVMKEAMVQAEEAAAEITRKLEKQEKKRLKKEKKRLAALALASSENSSSTPEECEEMSEKPKKKKKQKPQEVPQENGMEDPSISFSKPKKKKSFSKEELMSSDLEETAGSTSIPKRKKSTPKEETVNDPEEAGHRSGSKKKRKFSKEEPVSSGPEEAVGKSSSKKKKKFHKASQED

973 NP_000161.2 NP_112218.2

>NP_000161.2

MQSCARAWGLRLGRGVGGGRRLAGGSGPCWAPRSRDSSSGGGDSAAAGASRLLERLLPRHDDFARRHIGPGDKDQREMLQTLGLASIDELIEKTVPANIRLKRPLKMEDPVCENEILATLHAISSKNQIWRSYIGMGYYNCSVPQTILRNLLENSGWITQYTPYQPEVSQGRLESLLNYQTMVCDITGLDMANASLLDEGTAAAEALQLCYRHNKRRKFLVDPRCHPQTIAVVQTRAKYTGVLTELKLPCEMDFSGKDVSGVLFQYPDTEGKVEDFTELVERAHQSGSLACCATDLLALCILRPPGEFGVDIALGSSQRFGVPLGYGGPHAAFFAVRESLVRMMPGRMVGVTRDATGKEVYRLALQTREQHIRRDKATSNICTAQALLANMAAMFAIYHGSHGLEHIARRVHNATLILSEGLKRAGHQLQHDLFFDTLKIQCGCSVKEVLGRAAQRQINFRLFEDGTLGISLDETVNEKDLDDLLWIFGCESSAELVAESMGEECRGIPGSVFKRTSPFLTHQVFNSYHSETNIVRYMKKLENKDISLVHSMIPLGSCTMKLNSSSELAPITWKEFANIHPFVPLDQAQGYQQLFRELEKDLCELTGYDQVCFQPNSGAQGEYAGLATIRAYLNQKGEGHRTVCLIPKSAHGTNPASAHMAGMKIQPVEVDKYGNIDAVHLKAMVDKHKENLAAIMITYPSTNGVFEENISDVCDLIHQHGGQVYLDGANMNAQVGICRPGDFGSDVSHLNLHKTFCIPHGGGGPGMGPIGVKKHLAPFLPNHPVISLKRNEDACPVGTVSAAPWGSSSILPISWAYIKMMGGKGLKQATETAILNANYMAKRLETHYRILFRGARGYVGHEFILDTRPFKKSANIEAVDVAKRLQDYGFHAPTMSWPVAGTLMVEPTESEDKAELDRFCDAMISIRQEIADIEEGRIDPRVNPLKMSPHSLTCVTSSHWDRPYSREVAAFPLPFVKPENKFWPTIARIDDIYGDQHLVCTCPPMEVYESPFSEQKRASS

>NP_112218.2

MRLIRNIYIFCSIVMTAEGDAPELPEERELMTNCSNMSLRKVPADLTPATTTLDLSYNLLFQLQSSDFHSVSKLRVLILCHNRIQQLDLKTFEFNKELRYLDLSNNRLKSVTWYLLAGLRYLDLSFNDFDTMPICEEAGNMSHLEILGLSGAKIQKSDFQKIAHLHLNTVFLGFRTLPHYEEGSLPILNTTKLHIVLPMDTNFWVLLRDGIKTSKILEMTNIDGKSQFVSYEMQRNLSLENAKTSVLLLNKVDLLWDDLFLILQFVWHTSVEHFQIRNVTFGGKAYLDHNSFDYSNTVMRTIKLEHVHFRVFYIQQDKIYLLLTKMDIENLTISNAQMPHMLFPNYPTKFQYLNFANNILTDELFKRTIQLPHLKTLILNGNKLETLSLVSCFANNTPLEHLDLSQNLLQHKNDENCSWPETVVNMNLSYNKLSDSVFRCLPKSIQILDLNNNQIQTVPKETIHLMALRELNIAFNFLTDLPGCSHFSRLSVLNIEMNFILSPSLDFVQSCQEVKTLNAGRNPFRCTCELKNFIQLETYSEVMMVGWSDSYTCEYPLNLRGTRLKDVHLHELSCNTALLIVTIVVIMLVLGLAVAFCCLHFDLPWYLRMLGQCTQTWHRVRKTTQEQLKRNVRFHAFISYSEHDSLWVKNELIPNLEKEDGSILICLYESYFDPGKSISENIVSFIEKSYKSIFVLSPNFVQNEWCHYEFYFAHHNLFHENSDHIILILLEPIPFYCIPTRYHKLKALLEKKAYLEWPKDRRKCGLFWANLRAAINVNVLATREMYELQTFTELNEESRGSTISLMRTDCL

974 AAH02810.1 NP_064516.2

>AAH02810.1

MSADGGGIQDTQDKETPPEVPDRGHPHQEMPSKLGEAVPSGDAQESLHIKMEPEEPHSEGASQEDGAQGAWGWAPLSHGSKEKALFLPGGALPSPRIPVLSREGRTRDRQMAAALLTAWSQMPVTFEDVALYLSREEWGRLDHTQQNFYRDVLQKKNGLSLGFPFSRPFWAPQAHGKGEASGSSRQAGDEKEWRGACTGAVEVGQRVQTSSVAALGNVKPFRTRAGRVQWGVPQCAQEAACGRSSGPAKDSGQPAEPDRTPDAAPPDPSPTEPQEYRVPEKPNEEEKGAPESGEEGLAPDSEVGRKSYRCEQCGKGFSWHSHLVTHRRTHTGEKPYACTDCGKRFGRSSHLIQHQIIHTGEKPYTCPACRKSFSHHSTLIQHQRIHTGEKPYVCDRCAKRFTRRSDLVTHQGTHTGAKPHKCPICAKCFTQSSALVTHQRTHTGVKPYPCPECGKCFSQRSNLIAHNRTHTGEKPYHCLDCGKSFSHSSHLTAHQRTHRGVRPYACPLCGKSFSRRSNLHRHEKIHTTGPKALAMLMLGAAAAGALATPPPAPT

>NP_064516.2

MAEPSGAETRPPIRVTVKTPKDKEEIVICDRASVKEFKEEISRRFKAQQDQLVLIFAGKILKDGDTLNQHGIKDGLTVHLVIKTPQKAQDPAAATASSPSTPDPASAPSTTPASPATPAQPSTSGSASSDAGSGSRRSSGGGPSPGAGEGSPSATASILSGFGGILGLGSLGLGSANFMELQQQMQRQLMSNPEMLSQIMENPLVQDMMSNPDLMRHMIMANPQMQQLMERNPEISHMLNNPELMRQTMELARNPAMMQEMMRNQDRALSNLESIPGGYNALRRMYTDIQEPMFSAAREQFGNNPFSSLAGNSDSSSSQPLRTENREPLPNPWSPSPPTSQAPGSGGEGTGGSGTSQVHPTVSNPFGINAASLGSGMFNSPEMQALLQQISENPQLMQNVISAPYMRSMMQTLAQNPDFAAQMMVNVPLFAGNPQLQEQLRLQLPVFLQQMQNPESLSILTNPRAMQALLQIQQGLQTLQTEAPGLVPSLGSFGISRTPAPSAGSNAGSTPEAPTSSPATPATSSPTGASSAQQQLMQQMIQLLAGSGNSQVQTPEVRFQQQLEQLNSMGFINREANLQALIATGGDINAAIERLLGSQLS

975 NP_006177.1 NP_068811.1

>NP_006177.1

MPCVQAQYGSSPQGASPASQSYSYHSSGEYSSDFLTPEFVKFSMDLTNTEITATTSLPSFSTFMDNYSTGYDVKPPCLYQMPLSGQQSSIKVEDIQMHNYQQHSHLPPQSEEMMPHSGSVYYKPSSPPTPTTPGFQVQHSPMWDDPGSLHNFHQNYVATTHMIEQRKTPVSRLSLFSFKQSPPGTPVSSCQMRFDGPLHVPMNPEPAGSHHVVDGQTFAVPNPIRKPASMGFPGLQIGHASQLLDTQVPSPPSRGSPSNEGLCAVCGDNAACQHYGVRTCEGCKGFFKRTVQKNAKYVCLANKNCPVDKRRRNRCQYCRFQKCLAVGMVKEVVRTDSLKGRRGRLPSKPKSPQEPSPPSPPVSLISALVRAHVDSNPAMTSLDYSRFQANPDYQMSGDDTQHIQQFYDLLTGSMEIIRGWAEKIPGFADLPKADQDLLFESAFLELFVLRLAYRSNPVEGKLIFCNGVVLHRLQCVRGFGEWIDSIVEFSSNLQNMNIDISAFSCIAALAMVTERHGLKEPKRVEELQNKIVNCLKDHVTFNNGGLNRPNYLSKLLGKLPELRTLCTQGLQRIFYLKLEDLVPPPAIIDKLFLDTLPF

>NP_068811.1

MSWAARPPFLPQRHAAGQCGPVGVRKEMHCGVASRWRRRRPWLDPAAAAAAAVAGGEQQTPEPEPGEAGRDGMGDSGRDSRSPDSSSPNPLPQGVPPPSPPGPPLPPSTAPSLGGSGAPPPPPMPPPPLGSPFPVISSSMGSPGLPPPAPPGFSGPVSSPQINSTVSLPGGGSGPPEDVKPPVLGVRGLHCPPPPGGPGAGKRLCAICGDRSSGKHYGVYSCEGCKGFFKRTIRKDLTYSCRDNKDCTVDKRQRNRCQYCRYQKCLATGMKREAVQEERQRGKDKDGDGEGAGGAPEEMPVDRILEAELAVEQKSDQGVEGPGGTGGSGSSPNDPVTNICQAADKQLFTLVEWAKRIPHFSSLPLDDQVILLRAGWNELLIASFSHRSIDVRDGILLATGLHVHRNSAHSAGVGAIFDRVLTELVSKMRDMRMDKTELGCLRAIILFNPDAKGLSNPSEVEVLREKVYASLETYCKQKYPEQQGRFAKLLLRLPALRSIGLKCLEHLFFFKLIGDTPIDTFLMEMLEAPHQLA

976 NP_071285.1 NP_001094.1

>NP_071285.1

MTVTRTHHFKEGSLRAPAIPLHSAAAELASNHPRGPEANLEVRPKESWNHADFVHCEDTESVPGKPSVNADEEVGGPQICRVCGDKATGYHFNVMTCEGCKGFFRRAMKRNARLRCPFRKGACEITRKTRRQCQACRLRKCLESGMKKEMIMSDEAVEERRALIKRKKSERTGTQPLGVQGLTEEQRMMIRELMDAQMKTFDTTFSHFKNFRLPGVLSSGCELPESLQAPSREEAAKWSQVRKDLCSLKVSLQLRGEDGSVWNYKPPADSGGKEIFSLLPHMADMSTYMFKGIISFAKVISYFRDLPIEDQISLLKGAAFELCQLRFNTVFNAETGTWECGRLSYCLEDTAGGFQQLLLEPMLKFHYMLKKLQLHEEEYVLMQAISLFSPDRPGVLQHRVVDQLQEQFAITLKSYIECNRPQPAHRFLFLKIMAMLTELRSINAQHTQRLLRIQDIHPFATPLMQELFGITGS

>NP_001094.1

MNQIEPGVQYNYVYDEDEYMIQEEEWDRDLLLDPAWEKQQRKTFTAWCNSHLRKAGTQIENIEEDFRNGLKLMLLLEVISGERLPKPDRGKMRFHKIANVNKALDYIASKGVKLVSIGAEEIVDGNVKMTLGMIWTIILRFAIQDISVEETSAKEGLLLWCQRKTAPYRNVNIQNFHTSWKDGLGLCALIHRHRPDLIDYSKLNKDDPIGNINLAMEIAEKHLDIPKMLDAEDIVNTPKPDERAIMTYVSCFYHAFAGAEQAETAANRICKVLAVNQENERLMEEYERLASELLEWIRRTIPWLENRTPEKTMQAMQKKLEDFRDYRRKHKPPKVQEKCQLEINFNTLQTKLRISNRPAFMPSEGKMVSDIAGAWQRLEQAEKGYEEWLLNEIRRLERLEHLAEKFRQKASTHETWAYGKEQILLQKDYESASLTEVRALLRKHEAFESDLAAHQDRVEQIAAIAQELNELDYHDAVNVNDRCQKICDQWDRLGTLTQKRREALERMEKLLETIDQLHLEFAKRAAPFNNWMEGAMEDLQDMFIVHSIEEIQSLITAHEQFKATLPEADGERQSIMAIQNEVEKVIQSYNIRISSSNPYSTVTMDELRTKWDKVKQLVPIRDQSLQEELARQHANERLRRQFAAQANAIGPWIQNKMEEIARSSIQITGALEDQMNQLKQYEHNIINYKNNIDKLEGDHQLIQEALVFDNKHTNYTMEHIRVGWELLLTTIARTINEVETQILTRDAKGITQEQMNEFRASFNHFDRRKNGLMDHEDFRACLISMGYDLGEAEFARIMTLVDPNGQGTVTFQSFIDFMTRETADTDTAEQVIASFRILASDKPYILAEELRRELPPDQAQYCIKRMPAYSGPGSVPGALDYAAFSSALYGESDL

977 NP_005736.3 NP_002107.3

>NP_005736.3

MSLQWTAVATFLYAEVFVVLLLCIPFISPKRWQKIFKSRLVELLVSYGNTFFVVLIVILVLLVIDAVREIRKYDDVTEKVNLQNNPGAMEHFHMKLFRAQRNLYIAGFSLLLSFLLRRLVTLISQQATLLASNEAFKKQAESASEAAKKYMEENDQLKKGAAVDGGKLDVGNAEVKLEEENRSLKADLQKLKDELASTKQKLEKAENQVLAMRKQSEGLTKEYDRLLEEHAKLQAAVDGPMDKKEE

>NP_002107.3

MAVMAPRTLLLLLSGALALTQTWAGSHSMRYFFTSVSRPGRGEPRFIAVGYVDDTQFVRFDSDAASQRMEPRAPWIEQEGPEYWDQETRNVKAQSQTDRVDLGTLRGYYNQSEAGSHTIQIMYGCDVGSDGRFLRGYRQDAYDGKDYIALNEDLRSWTAADMAAQITKRKWEAAHEAEQLRAYLDGTCVEWLRRYLENGKETLQRTDPPKTHMTHHPISDHEATLRCWALGFYPAEITLTWQRDGEDQTQDTELVETRPAGDGTFQKWAAVVVPSGEEQRYTCHVQHEGLPKPLTLRWELSSQPTIPIVGIIAGLVLLGAVITGAVVAAVMWRRKSSDRKGGSYTQAASSDSAQGSDVSLTACKV

978 NP_071910.3 NP_071911.3

>NP_071910.3

MHTPPALPRRFQGGGRVRTPGSHRQGKDNLERDPSGGCVPDFLPQAQDSNHFIMESLFCESSGDSSLEKEFLGAPVGPSVSTPNSQHSSPSRSLSANSIKVEMYSDEESSRLLGPDERLLEKDDSVIVEDSLSEPLGYCDGSGPEPHSPGGIRLPNGKLKCDVCGMVCIGPNVLMVHKRSHTGERPFHCNQCGASFTQKGNLLRHIKLHSGEKPFKCPFCNYACRRRDALTGHLRTHSVSSPTVGKPYKCNYCGRSYKQQSTLEEHKERCHNYLQSLSTEAQALAGQPGDEIRDLEMVPDSMLHSSSERPTFIDRLANSLTKRKRSTPQKFVGEKQMRFSLSDLPYDVNSGGYEKDVELVAHHSLEPGFGSSLAFVGAEHLRPLRLPPTNCISELTPVISSVYTQMQPLPGRLELPGSREAGEGPEDLADGGPLLYRPRGPLTDPGASPSNGCQDSTDTESNHEDRVAGVVSLPQGPPPQPPPTIVVGRHSPAYAKEDPKPQEGLLRGTPGPSKEVLRVVGESGEPVKAFKCEHCRILFLDHVMFTIHMGCHGFRDPFECNICGYHSQDRYEFSSHIVRGEHKVG

>NP_071911.3

MGEKKPEPLDFVKDFQEYLTQQTHHVNMISGSVSGDKEAEALQGAGTDGDQNGLDHPSVEVSLDENSGMLVDGFERTFDGKLKCRYCNYASKGTARLIEHIRIHTGEKPHRCHLCPFASAYERHLEAHMRSHTGEKPYKCELCSFRCSDRSNLSHHRRRKHKMVPIKGTRSSLSSKKMWGVLQKKTSNLGYSRRALINLSPPSMVVQKPDYLNDFTHEIPNIQTDSYESMAKTTPTGGLPRDPQELMVDNPLNQLSTLAGQLSSLPPENQNPASPDVVPCPDEKPFMIQQPSTQAVVSAVSASIPQSSSPTSPEPRPSHSQRNYSPVAGPSSEPSAHTSTPSIGNSQPSTPAPALPVQDPQLLHHCQHCDMYFADNILYTIHMGCHGYENPFQCNICGCKCKNKYDFACHFARGQHNQH

979 NP_115496.2 NP_006696.1

>NP_115496.2

MASSGGELGSLFDHHVQRAVCDTRAKYREGRRPRAVKVYTINLESQYLLIQGVPAVGVMKELVERFALYGAIEQYNALDEYPAEDFTEVYLIKFMNLQSARTAKRKMDEQSFFGGLLHVCYAPEFETVEETRKKLQMRKAYVVKTTENKDHYVTKKKLVTEHKDTEDFRQDFHSEMSGFCKAALNTSAGNSNPYLPYSCELPLCYFSSKCMCSSGGPVDRAPDSSKDGRNHHKTMGHYNHNDSLRKTQINSLKNSVACPGAQKAITSSEAVDRFMPRTTQLQERKRRREDDRKLGTFLQTNPTGNEIMIGPLLPDISKVDMHDDSLNTTANLIRHKLKEVISSVPKPPEDKPEDVHTSHPLKQRRRI

>NP_006696.1

MTLEEVRGQDTVPESTARMQGAGKALHELLLSAQRQGCLTAGVYESAKVLNVDPDNVTFCVLAAGEEDEGDIALQIHFTLIQAFCCENDIDIVRVGDVQRLAAIVGAGEEAGAPGDLHCILISNPNEDAWKDPALEKLSLFCEESRSVNDWVPSITLPE

980 NP_060290.3 NP_055579.1

>NP_060290.3

MDPGDPAGDPAAGERHRMGRDPLLLLQALQTLWSTRERKQLREEAWRGFAALDDPLAGLLDMLESCRGQRGEGPSLAAWISHQLQCWLQAQPCPSLAQHSLRLKQLQARAVKVLTESPPSLAAPLASIFQLQDADRSCLLAHVHRLHHEGRFREAATLGATLKLQSELGVEKMSIPLLLQDKVALVERYVAGFPDLQRRLLVLMDSWCQPGFDIKDVARRYPEVTSLSLEKLSPKALSRQVLRLQERYGVAPALCPNAAIQQRLAALRHLCHKRFVEKSLSQENWTDHVQGLVGQSPWLQEQLSQLLVSHSDPVTAAQCAMELLLPEERLPAAVAVELRRFRLQGRATEADSRLEVKDMKDRYYQLPIPRENVHLLASWEDLTRHEGALLQCHQVVGVDVEWTPVFVAGGRPRPSLLQVAVEGHVFLLDVLALSQPPTGQGAQAFSRLVAQLLSDPSITKLGYGMVGDLQKLGTSCPALAHVEKQILGGMDLLLVHRQMRVASVPAPAVDRARELRGLSLLVQQVLGTALDKTQQLSNWDRRPLCEEQVIYAAADAYCLLEVHQALCREPARFHLSEDLAGSRRPRHRERPGARKPPGLQKASAPAAPRQVPVAVAVSEGAAPQIPARAFRVVCDNMLQGLARSLRCLGVDARMLGNGEDHRRAAEVARQEGRIILTSGQPFHKLRAQVGAGRCLSVDCSLKAQQQAKAVLKHFNVRVTHADIFSRCQACNCDQYLKVSRDMMKQLMWLSSHQEGPRSSGDEATQSQAVQEPGPAPDAAPEGCTYDRPCRWLQMADLRAETPDMLADGTRLQLAGVPVGVLRTPGLRCFYCCTGCGKVFWDGSHLGRVATHFRDMLESAPSPCEPSPAPSPASSPF

>NP_055579.1

MNSKGQYPTQPTYPVQPPGNPVYPQTLHLPQAPPYTDAPPAYSELYRPSFVHPGAATVPTMSAAFPGASLYLPMAQSVAVGPLGSTIPMAYYPVGPIYPPGSTVLVEGGYDAGARFGAGATAGNIPPPPPGCPPNAAQLAVMQGANVLVTQRKGNFFMGGSDGGYTIW

981 NP_001702.1 NP_000919.1

>NP_001702.1

MWPLWRLVSLLALSQALPFEQRGFWDFTLDDGPFMMNDEEASGADTSGVLDPDSVTPTYSAMCPFGCHCHLRVVQCSDLGLKSVPKEISPDTTLLDLQNNDISELRKDDFKGLQHLYALVLVNNKISKIHEKAFSPLRKLQKLYISKNHLVEIPPNLPSSLVELRIHDNRIRKVPKGVFSGLRNMNCIEMGGNPLENSGFEPGAFDGLKLNYLRISEAKLTGIPKDLPETLNELHLDHNKIQAIELEDLLRYSKLYRLGLGHNQIRMIENGSLSFLPTLRELHLDNNKLARVPSGLPDLKLLQVVYLHSNNITKVGVNDFCPMGFGVKRAYYNGISLFNNPVPYWEVQPATFRCVTDRLAIQFGNYKK

>NP_000919.1

MKLLVLAVLLTVAAADSGISPRAVWQFRKMIKCVIPGSDPFLEYNNYGCYCGLGGSGTPVDELDKCCQTHDNCYDQAKKLDSCKFLLDNPYTHTYSYSCSGSAITCSSKNKECEAFICNCDRNAAICFSKAPYNKAHKNLDTKKYCQS

982 NP_000312.2 NP_002871.1

>NP_000312.2

MPPKTPRKTAATAAAAAAEPPAPPPPPPPEEDPEQDSGPEDLPLVRLEFEETEEPDFTALCQKLKIPDHVRERAWLTWEKVSSVDGVLGGYIQKKKELWGICIFIAAVDLDEMSFTFTELQKNIEISVHKFFNLLKEIDTSTKVDNAMSRLLKKYDVLFALFSKLERTCELIYLTQPSSSISTEINSALVLKVSWITFLLAKGEVLQMEDDLVISFQLMLCVLDYFIKLSPPMLLKEPYKTAVIPINGSPRTPRRGQNRSARIAKQLENDTRIIEVLCKEHECNIDEVKNVYFKNFIPFMNSLGLVTSNGLPEVENLSKRYEEIYLKNKDLDARLFLDHDKTLQTDSIDSFETQRTPRKSNLDEEVNVIPPHTPVRTVMNTIQQLMMILNSASDQPSENLISYFNNCTVNPKESILKRVKDIGYIFKEKFAKAVGQGCVEIGSQRYKLGVRLYYRVMESMLKSEEERLSIQNFSKLLNDNIFHMSLLACALEVVMATYSRSTSQNLDSGTDLSFPWILNVLNLKAFDFYKVIESFIKAEGNLTREMIKHLERCEHRIMESLAWLSDSPLFDLIKQSKDREGPTDHLESACPLNLPLQNNHTAADMYLSPVRSPKKKGSTTRVNSTANAETQATSAFQTQKPLKSTSLSLFYKKVYRLAYLRLNTLCERLLSEHPELEHIIWTLFQHTLQNEYELMRDRHLDQIMMCSMYGICKVKNIDLKFKIIVTAYKDLPHAVQETFKRVLIKEEEYDSIIVFYNSVFMQRLKTNILQYASTRPPTLSPIPHIPRSPYKFPSSPLRIPGGNIYISPLKSPYKISEGLPTPTKMTPRSRILVSIGESFGTSEKFQKINQMVCNSDRVLKRSAEGSNPPKPLKKLRFDIEGSDEADGSKHLPGESKFQQKLAEMTSTRTRMQKQKMNDSMDTSNKEEK

>NP_002871.1

MEHIQGAWKTISNGFGFKDAVFDGSSCISPTIVQQFGYQRRASDDGKLTDPSKTSNTIRVFLPNKQRTVVNVRNGMSLHDCLMKALKVRGLQPECCAVFRLLHEHKGKKARLDWNTDAASLIGEELQVDFLDHVPLTTHNFARKTFLKLAFCDICQKFLLNGFRCQTCGYKFHEHCSTKVPTMCVDWSNIRQLLLFPNSTIGDSGVPALPSLTMRRMRESVSRMPVSSQHRYSTPHAFTFNTSSPSSEGSLSQRQRSTSTPNVHMVSTTLPVDSRMIEDAIRSHSESASPSALSSSPNNLSPTGWSQPKTPVPAQRERAPVSGTQEKNKIRPRGQRDSSYYWEIEASEVMLSTRIGSGSFGTVYKGKWHGDVAVKILKVVDPTPEQFQAFRNEVAVLRKTRHVNILLFMGYMTKDNLAIVTQWCEGSSLYKHLHVQETKFQMFQLIDIARQTAQGMDYLHAKNIIHRDMKSNNIFLHEGLTVKIGDFGLATVKSRWSGSQQVEQPTGSVLWMAPEVIRMQDNNPFSFQSDVYSYGIVLYELMTGELPYSHINNRDQIIFMVGRGYASPDLSKLYKNCPKAMKRLVADCVKKVKEERPLFPQILSSIELLQHSLPKINRSASEPSLHRAAHTEDINACTLTTSPRLPVF

983 NP_004604.2 NP_001092.1

>NP_004604.2

MAEELVLERCDLELETNGRDHHTADLCREKLVVRRGQPFWLTLHFEGRNYEASVDSLTFSVVTGPAPSQEAGTKARFPLRDAVEEGDWTATVVDQQDCTLSLQLTTPANAPIGLYRLSLEASTGYQGSSFVLGHFILLFNAWCPADAVYLDSEEERQEYVLTQQGFIYQGSAKFIKNIPWNFGQFEDGILDICLILLDVNPKFLKNAGRDCSRRSSPVYVGRVVSGMVNCNDDQGVLLGRWDNNYGDGVSPMSWIGSVDILRRWKNHGCQRVKYGQCWVFAAVACTVLRCLGIPTRVVTNYNSAHDQNSNLLIEYFRNEFGEIQGDKSEMIWNFHCWVESWMTRPDLQPGYEGWQALDPTPQEKSEGTYCCGPVPVRAIKEGDLSTKYDAPFVFAEVNADVVDWIQQDDGSVHKSINRSLIVGLKISTKSVGRDEREDITHTYKYPEGSSEEREAFTRANHLNKLAEKEETGMAMRIRVGQSMNMGSDFDVFAHITNNTAEEYVCRLLLCARTVSYNGILGPECGTKYLLNLNLEPFSEKSVPLCILYEKYRDCLTESNLIKVRALLVEPVINSYLLAERDLYLENPEIKIRILGEPKQKRKLVAEVSLQNPLPVALEGCTFTVEGAGLTEEQKTVEIPDPVEAGEEVKVRMDLLPLHMGLHKLVVNFESDKLKAVKGFRNVIIGPA

>NP_001092.1

MDDDIAALVVDNGSGMCKAGFAGDDAPRAVFPSIVGRPRHQGVMVGMGQKDSYVGDEAQSKRGILTLKYPIEHGIVTNWDDMEKIWHHTFYNELRVAPEEHPVLLTEAPLNPKANREKMTQIMFETFNTPAMYVAIQAVLSLYASGRTTGIVMDSGDGVTHTVPIYEGYALPHAILRLDLAGRDLTDYLMKILTERGYSFTTTAEREIVRDIKEKLCYVALDFEQEMATAASSSSLEKSYELPDGQVITIGNERFRCPEALFQPSFLGMESCGIHETTFNSIMKCDVDIRKDLYANTVLSGGTTMYPGIADRMQKEITALAPSTMKIKIIAPPERKYSVWIGGSILASLSTFQQMWISKQEYDESGPSIVHRKCF

984 NP_036433.2 NP_005384.2

>NP_036433.2

MSKSLKKKSHWTSKVHESVIGRNPEGQLGFELKGGAENGQFPYLGEVKPGKVAYESGSKLVSEELLLEVNETPVAGLTIRDVLAVIKHCKDPLRLKCVKQGGIVDKDLRHYLNLRFQKGSVDHELQQIIRDNLYLRTVPCTTRPHKEGEVPGVDYIFITVEDFMELEKSGALLESGTYEDNYYGTPKPPAEPAPLLLNVTDQILPGATPSAEGKRKRNKSVSNMEKASIEPPEEEEEERPVVNGNGVVVTPESSEHEDKSAGASGEMPSQPYPAPVYSQPEELKEQMDDTKPTKPEDNEEPDPLPDNWEMAYTEKGEVYFIDHNTKTTSWLDPRLAKKAKPPEECKENELPYGWEKIDDPIYGTYYVDHINRRTQFENPVLEAKRKLQQHNMPHTELGTKPLQAPGFREKPLFTRDASQLKGTFLSTTLKKSNMGFGFTIIGGDEPDEFLQVKSVIPDGPAAQDGKMETGDVIVYINEVCVLGHTHADVVKLFQSVPIGQSVNLVLCRGYPLPFDPEDPANSMVPPLAIMERPPPVMVNGRHNYETYLEYISRTSQSVPDITDRPPHSLHSMPTDGQLDGTYPPPVHDDNVSMASSGATQAELMTLTIVKGAQGFGFTIADSPTGQRVKQILDIQGCPGLCEGDLIVEINQQNVQNLSHTEVVDILKDCPIGSETSLIIHRGGFFSPWKTPKPIMDRWENQGSPQTSLSAPAIPQNLPFPPALHRSSFPDSTEAFDPRKPDPYELYEKSRAIYESRQQVPPRTSFRMDSSGPDYKELDVHLRRMESGFGFRILGGDEPGQPILIGAVIAMGSADRDGRLHPGDELVYVDGIPVAGKTHRYVIDLMHHAARNGQVNLTVRRKVLCGGEPCPENGRSPGSVSTHHSSPRSDYATYTNSNHAAPSSNASPPEGFASHSLQTSDVVIHRKENEGFGFVIISSLNRPESGSTITVPHKIGRIIDGSPADRCAKLKVGDRILAVNGQSIINMPHADIVKLIKDAGLSVTLRIIPQEELNSPTSAPSSEKQSPMAQQSPLAQQSPLAQPSPATPNSPIAQPAPPQPLQLQGHENSYRSEVKARQDVKPDIRQPPFTDYRQPPLDYRQPPGGDYQQPPPLDYRQPPLLDYRQHSPDTRQYPLSDYRQPQDFDYFTVDMEKGAKGFGFSIRGGREYKMDLYVLRLAEDGPAIRNGRMRVGDQIIEINGESTRDMTHARAIELIKSGGRRVRLLLKRGTGQVPEYDEPAPWSSPAAAAPGLPEVGVSLDDGLAPFSPSHPAPPSDPSHQISPGPTWDIKREHDVRKPKELSACGQKKQRLGEQRERSASPQRAARPRLEEAPGGQGRPEAGRPASEARAPGLAAADAADAARAGGKEAPRAAAGSELCRREGPGAAPAFAGPGGGGSGALEAEGRAGARAGPRPGPRPPGGAPARKAAVAPGPWKVPGSDKLPSVLKPGASAASR

>NP_005384.2

MARWPPFGLCLLLLLLSPPPLPLTGAHRFSAPNTTLNHLALAPGRGTLYVGAVNRLFQLSPELQLEAVAVTGPVIDSPDCVPFRDPAECPQAQLTDNANQLLLVSSRAQELVACGQVRQGVCETRRLGDVAEVLYQAEDPGDGQFVAANTPGVATVGLVVPLPGRDLLLVARGLAGKLSAGVPPLAIRQLAGSQPFSSEGLGRLVVGDFSDYNNSYVGAFADARSAYFVFRRRGARAQAEYRSYVARVCLGDTNLYSYVEVPLACQGQGLIQAAFLAPGTLLGVFAAGPRGTQAALCAFPMVELGASMEQARRLCYTAGGRGPSGAEEATVEYGVTSRCVTLPLDSPESYPCGDEHTPSPIAGRQPLEVQPLLKLGQPVSAVAALQADGHMIAFLGDTQGQLYKVFLHGSQGQVYHSQQVGPPGSAISPDLLLDSSGSHLYVLTAHQVDRIPVAACPQFPDCASCLQAQDPLCGWCVLQGRCTRKGQCGRAGQLNQWLWSYEEDSHCLHIQSLLPGHHPRQEQGQVTLSVPRLPILDADEYFHCAFGDYDSLAHVEGPHVACVTPPQDQVPLNPPGTDHVTVPLALMFEDVTVAATNFSFYDCSAVQALEAAAPCRACVGSIWRCHWCPQSSHCVYGEHCPEGERTIYSAQEVDIQVRGPGACPQVEGLAGPHLVPVGWESHLALRVRNLQHFRGLPASFHCWLELPGELRGLPATLEETAGDSGLIHCQAHQFYPSMSQRELPVPIYVTQGEAQRLDNTHALYVILYDCAMGHPDCSHCQAANRSLGCLWCADGQPACRYGPLCPPGAVELLCPAPSIDAVEPLTGPPEGGLALTILGSNLGRAFADVQYAVSVASRPCNPEPSLYRTSARIVCVTSPAPNGTTGPVRVAIKSQPPGISSQHFTYQDPVLLSLSPRWGPQAGGTQLTIRGQHLQTGGNTSAFVGGQPCPILEPVCPEAIVCRTRPQAAPGEAAVLVVFGHAQRTLLASPFRYTANPQLVAAEPSASFRGGGRLIRVRGTGLDVVQRPLLSVWLEADAEVQASRAQPQDPQPRRSCGAPAADPQACIQLGGGLLQCSTVCSVNSSSLLLCRSPAVPDRAHPQRVFFTLDNVQVDFASASGGQGFLYQPNPRLAPLSREGPARPYRLKPGHVLDVEGEGLNLGISKEEVRVHIGRGECLVKTLTRTHLYCEPPAHAPQPANGSGLPQFVVQMGNVQLALGPVQYEAEPPLSAFPVEAQAGVGMGAAVLIAAVLLLTLMYRHKSKQALRDYQKVLVQLESLETGVGDQCRKEFTDLMTEMTDLSSDLEGSGIPFLDYRTYAERAFFPGHGGCPLQPKPEGPGEDGHCATVRQGLTQLSNLLNSKLFLLTLIHTLEEQPSFSQRDRCHVASLLSLALHGKLEYLTDIMRTLLGDLAAHYVHRNPKLMLRRTETMVEKLLTNWLSICLYAFLREVAGEPLYMLFRAIQYQVDKGPVDAVTGKAKRTLNDSRLLREDVEFQPLTLMVLVGPGAGGAAGSSEMQRVPARVLDTDTITQVKEKVLDQVYKGTPFSQRPSVHALDLEWRSGLAGHLTLSDEDLTSVTQNHWKRLNTLQHYKVPDGATVGLVPQLHRGSTISQSLAQRCPLGENIPTLEDGEEGGVCLWHLVKATEEPEGAKVRCSSLREREPARAKAIPEIYLTRLLSMKGTLQKFVDDTFQAILSVNRPIPIAVKYLFDLLDELAEKHGIEDPGTLHIWKTNSLLLRFWVNALKNPQLIFDVRVSDNVDAILAVIAQTFIDSCTTSEHKVGRDSPVNKLLYAREIPRYKQMVERYYADIRQSSPASYQEMNSALAELSGNYTSAPHCLEALQELYNHIHRYYDQIISALEEDPVGQKLQLACRLQQVAALVENKVTDL

985 NP_005185.2 NP_612482.2

>NP_005185.2

MQRLVAWDPACLPLPPPPPAFKSMEVANFYYEADCLAAAYGGKAAPAAPPAARPGPRPPAGELGSIGDHERAIDFSPYLEPLGAPQAPAPATATDTFEAAPPAPAPAPASSGQHHDFLSDLFSDDYGGKNCKKPAEYGYVSLGRLGAAKGALHPGCFAPLHPPPPPPPPPAELKAEPGFEPADCKRKEEAGAPGGGAGMAAGFPYALRAYLGYQAVPSGSSGSLSTSSSSSPPGTPSPADAKAPPTACYAGAAPAPSQVKSKAKKTVDKHSDEYKIRRERNNIAVRKSRDKAKMRNLETQHKVLELTAENERLQKKVEQLSRELSTLRNLFKQLPEPLLASSGHC

>NP_612482.2

MSDQDHSMDEMTAVVKIEKGVGGNNGGNGNGGGAFSQARSSSTGSSSSTGGGGQESQPSPLALLAATCSRIESPNENSNNSQGPSQSGGTGELDLTATQLSQGANGWQIISSSSGATPTSKEQSGSSTNGSNGSESSKNRTVSGGQYVVAAAPNLQNQQVLTGLPGVMPNIQYQVIPQFQTVDGQQLQFAATGAQVQQDGSGQIQIIPGANQQIITNRGSGGNIIAAMPNLLQQAVPLQGLANNVLSGQTQYVTNVPVALNGNITLLPVNSVSAATLTPSSQAVTISSSGSQESGSQPVTSGTTISSASLVSSQASSSSFFTNANSYSTTTTTSNMGIMNFTTSGSSGTNSQGQTPQRVSGLQGSDALNIQQNQTSGGSLQAGQQKEGEQNQQTQQQQILIQPQLVQGGQALQALQAAPLSGQTFTTQAISQETLQNLQLQAVPNSGPIIIRTPTVGPNGQVSWQTLQLQNLQVQNPQAQTITLAPMQGVSLGQTSSSNTTLTPIASAASIPAGTVTVNAAQLSSMPGLQTINLSALGTSGIQVHPIQGLPLAIANAPGDHGAQLGLHGAGGDGIHDDTAGGEEGENSPDAQPQAGRRTRREACTCPYCKDSEGRGSGDPGKKKQHICHIQGCGKVYGKTSHLRAHLRWHTGERPFMCTWSYCGKRFTRSDELQRHKRTHTGEKKFACPECPKRFMRSDHLSKHIKTHQNKKGGPGVALSVGTLPLDSGAGSEGSGTATPSALITTNMVAMEAICPEGIARLANSGINVMQVADLQSINISGNGF

986 NP_006303.3 NP_620309.1

>NP_006303.3

MSGSTQPVAQTWRATEPRYPPHSLSYPVQIARTHTDVGLLEYQHHSRDYASHLSPGSIIQPQRRRPSLLSEFQPGNERSQELHLRPESHSYLPELGKSEMEFIESKRPRLELLPDPLLRPSPLLATGQPAGSEDLTKDRSLTGKLEPVSPPSPPHTDPELELVPPRLSKEELIQNMDRVDREITMVEQQISKLKKKQQQLEEEAAKPPEPEKPVSPPPIESKHRSLVQIIYDENRKKAEAAHRILEGLGPQVELPLYNQPSDTRQYHENIKINQAMRKKLILYFKRRNHARKQWEQKFCQRYDQLMEAWEKKVERIENNPRRRAKESKVREYYEKQFPEIRKQRELQERMQSRVGQRGSGLSMSAARSEHEVSEIIDGLSEQENLEKQMRQLAVIPPMLYDADQQRIKFINMNGLMADPMKVYKDRQVMNMWSEQEKETFREKFMQHPKNFGLIASFLERKTVAECVLYYYLTKKNENYKSLVRRSYRRRGKSQQQQQQQQQQQQQQQQQPMPRSSQEEKDEKEKEKEAEKEEEKPEVENDKEDLLKEKTDDTSGEDNDEKEAVASKGRKTANSQGRRKGRITRSMANEANSEEAITPQQSAELASMELNESSRWTEEEMETAKKGLLEHGRNWSAIARMVGSKTVSQCKNFYFNYKKRQNLDEILQQHKLKMEKERNARRKKKKAPAAASEEAAFPPVVEDEEMEASGVSGNEEEMVEEAEALHASGNEVPRGECSGPATVNNSSDTESIPSPHTEAAKDTGQNGPKPPATLGADGPPPGPPTPPPEDIPAPTEPTPASEATGAPTPPPAPPSPSAPPPVVPKEEKEEETAAAPPVEEGEEQKPPAAEELAVDTGKAEEPVKSECTEEAEEGPAKGKDAEAAEATAEGALKAEKKEGGSGRATTAKSSGAPQDSDSSATCSADEVDEAEGGDKNRLLSPRPSLLTPTGDPRANASPQKPLDLKQLKQRAAAIPPIVTKVHEPPREDAAPTKPAPPAPPPPQNLQPESDAPQQPGSSPRGKSRSPAPPADKEAEKPVFFPAFAAEAQKLPGDPPCWTSGLPFPVPPREVIKASPHAPDPSAFSYAPPGHPLPLGLHDTARPVLPRPPTISNPPPLISSAKHPSVLERQIGAISQGMSVQLHVPYSEHAKAPVGPVTMGLPLPMDPKKLAPFSGVKQEQLSPRGQAGPPESLGVPTAQEASVLRGTALGSVPGGSITKGIPSTRVPSDSAITYRGSITHGTPADVLYKGTITRIIGEDSPSRLDRGREDSLPKGHVIYEGKKGHVLSYEGGMSVTQCSKEDGRSSSGPPHETAAPKRTYDMMEGRVGRAISSASIEGLMGRAIPPERHSPHHLKEQHHIRGSITQGIPRSYVEAQEDYLRREAKLLKREGTPPPPPPSRDLTEAYKTQALGPLKLKPAHEGLVATVKEAGRSIHEIPREELRHTPELPLAPRPLKEGSITQGTPLKYDTGASTTGSKKHDVRSLIGSPGRTFPPVHPLDVMADARALERACYEESLKSRPGTASSSGGSIARGAPVIVPELGKPRQSPLTYEDHGAPFAGHLPRGSPVTTREPTPRLQEGSLSSSKASQDRKLTSTPREIAKSPHSTVPEHHPHPISPYEHLLRGVSGVDLYRSHIPLAFDPTSIPRGIPLDAAAAYYLPRHLAPNPTYPHLYPPYLIRGYPDTAALENRQTIINDYITSQQMHHNAATAMAQRADMLRGLSPRESSLALNYAAGPRGIIDLSQVPHLPVLVPPTPGTPATAMDRLAYLPTAPQPFSSRHSSSPLSPGGPTHLTKPTTTSSSERERDRDRERDRDREREKSILTSTTTVEHAPIWRPGTEQSSGSSGSSGGGGGSSSRPASHSHAHQHSPISPRTQDALQQRPSVLHNTGMKGIITAVEPSTPTVLRSTSTSSPVRPAATFPPATHCPLGGTLDGVYPTLMEPVLLPKEAPRVARPERPRADTGHAFLAKPPARSGLEPASSPSKGSEPRPLVPPVSGHATIARTPAKNLAPHHASPDPPAPPASASDPHREKTQSKPFSIQELELRSLGYHGSSYSPEGVEPVSPVSSPSLTHDKGLPKHLEELDKSHLEGELRPKQPGPVKLGGEAAHLPHLRPLPESQPSSSPLLQTAPGVKGHQRVVTLAQHISEVITQDYTRHHPQQLSAPLPAPLYSFPGASCPVLDLRRPPSDLYLPPPDHGAPARGSPHSEGGKRSPEPNKTSVLGGGEDGIEPVSPPEGMTEPGHSRSAVYPLLYRDGEQTEPSRMGSKSPGNTSQPPAFFSKLTESNSAMVKSKKQEINKKLNTHNRNEPEYNISQPGTEIFNMPAITGTGLMTYRSQAVQEHASTNMGLEAIIRKALMGKYDQWEESPPLSANAFNPLNASASLPAAMPITAADGRSDHTLTSPGGGGKAKVSGRPSSRKAKSPAPGLASGDRPPSVSSVHSEGDCNRRTPLTNRVWEDRPSSAGSTPFPYNPLIMRLQAGVMASPPPPGLPAGSGPLAGPHHAWDEEPKPLLCSQYETLSDSE

>NP_620309.1

MASPADSCIQFTRHASDVLLNLNRLRSRDILTDVVIVVSREQFRAHKTVLMACSGLFYSIFTDQLKCNLSVINLDPEINPEGFCILLDFMYTSRLNLREGNIMAVMATAMYLQMEHVVDTCRKFIKASEAEMVSAIKPPREEFLNSRMLMPQDIMAYRGREVVENNLPLRSAPGCESRAFAPSLYSGLSTPPASYSMYSHLPVSSLLFSDEEFRDVRMPVANPFPKERALPCDSARPVPGEYSRPTLEVSPNVCHSNIYSPKETIPEEARSDMHYSVAEGLKPAAPSARNAPYFPCDKASKEEERPSSEDEIALHFEPPNAPLNRKGLVSPQSPQKSDCQPNSPTESCSSKNACILQASGSPPAKSPTDPKACNWKKYKFIVLNSLNQNAKPEGPEQAELGRLSPRAYTAPPACQPPMEPENLDLQSPTKLSASGEDSTIPQASRLNNIVNRSMTGSPRSSSESHSPLYMHPPKCTSCGSQSPQHAEMCLHTAGPTFPEEMGETQSEYSDSSCENGAFFCNECDCRFSEEASLKRHTLQTHSDKPYKCDRCQASFRYKGNLASHKTVHTGEKPYRCNICGAQFNRPANLKTHTRIHSGEKPYKCETCGARFVQVAHLRAHVLIHTGEKPYPCEICGTRFRHLQTLKSHLRIHTGEKPYHCEKCNLHFRHKSQLRLHLRQKHGAITNTKVQYRVSATDLPPELPKAC

987 AAK69623.1 NP_004078.2

>AAK69623.1

MFHCIPLWRCNRHVESIDKRHCSLVYVPEEIYRYARSLEELLLDANQLRELPEQFFQLVKLRKLGLSDNEIQRLPPEIANFMQLVELDVSRNEIPEIPESISFCKALQVADFSGNPLTRLPESFPELQNLTCLSVNDISLQSLPENIGNLYNLASLELRENLLTYLPDSLTQLRRLEELDLGNNEIYNLPESIGALLHLKDLWLDGNQLSELPQEIGNLKNLLCLDVSENRLERLPEEISGLTSLTDLVISQNLLETIPDGIGKLKKLSILKVDQNRLTQLPEAVGECESLTELVLTENQLLTLPKSIGKLKKLSNLNADRNKLVSLPKEIGGCCSLTVFCVRDNRLTRIPAEVSQATELHVLDVAGNRLLHLPLSLTALKLKALWLSDNQSQPLLTFQTDTDYTTGEKILTCVLLPQLPSEPTCQENLPRCGALENLVNDVSDEAWNERAVNRVSAIRFVEDEKDEEDNETRTLLRRATPHPGELKHMKKTVENLRNDMNAAKGLDSNKNEVNHAIDRVTTSV

>NP_004078.2

MPVRKQDTQRALHLLEEYRSKLSQTEDRQLRSSIERVINIFQSNLFQALIDIQEFYEVTLLDNPKCIDRSKPSEPIQPVNTWEISSLPSSTVTSETLPSSLSPSVEKYRYQDEDTPPQEHISPQITNEVIGPELVHVSEKNLSEIENVHGFVSHSHISPIKPTEAVLPSPPTVPVIPVLPVPAENTVILPTIPQANPPPVLVNTDSLETPTYVNGTDADYEYEEITLERGNSGLGFSIAGGTDNPHIGDDSSIFITKIITGGAAAQDGRLRVNDCILRVNEVDVRDVTHSKAVEALKEAGSIVRLYVKRRKPVSEKIMEIKLIKGPKGLGFSIAGGVGNQHIPGDNSIYVTKIIEGGAAHKDGKLQIGDKLLAVNNVCLEEVTHEEAVTALKNTSDFVYLKVAKPTSMYMNDGYAPPDITNSSSQPVDNHVSPSSFLGQTPASPARYSPVSKAVLGDDEITREPRKVVLHRGSTGLGFNIVGGEDGEGIFISFILAGGPADLSGELRKGDRIISVNSVDLRAASHEQAAAALKNAGQAVTIVAQYRPEEYSRFEAKIHDLREQMMNSSISSGSGSLRTSQKRSLYVRALFDYDKTKDSGLPSQGLNFKFGDILHVINASDDEWWQARQVTPDGESDEVGVIPSKRRVEKKERARLKTVKFNSKTRDKGQSFNDKRKKNLFSRKFPFYKNKDQSEQETSDADQHVTSNASDSESSYRGQEEYVLSYEPVNQQEVNYTRPVIILGPMKDRINDDLISEFPDKFGSCVPHTTRPKRDYEVDGRDYHFVTSREQMEKDIQEHKFIEAGQYNNHLYGTSVQSVREVAEKGKHCILDVSGNAIKRLQIAQLYPISIFIKPKSMENIMEMNKRLTEEQARKTFERAMKLEQEFTEHFTAIVQGDTLEDIYNQVKQIIEEQSGSYIWVPAKEKL

988 NP_000545.1 NP_620159.2

>NP_000545.1

MMAYMNPGPHYSVNALALSGPSVDLMHQAVPYPSAPRKQRRERTTFTRSQLEELEALFAKTQYPDVYAREEVALKINLPESRVQVWFKNRRAKCRQQRQQQKQQQQPPGGQAKARPAKRKAGTSPRPSTDVCPDPLGISDSYSPPLPGPSGSPTTAVATVSIWSPASESPLPEAQRAGLVASGPSLTSAPYAMTYAPASAFCSSPSAYGSPSSYFSGLDPYLSPMVPQLGGPALSPLSGPSVGPSLAQSPTSLSGQSYGAYSPVDSLEFKDPTGTWKFTYNPMDPLDYKDQSAWKFQIL

>NP_620159.2

MHPGRTTGKGPSTHTQIDQQPPRLLIVHIALPSWADICTNLCEALQNFFSLACSLMGPSRMSLFSLYMVQDQHECILPFVQVKGNFARLQTCISELRMLQREGCFRSQGASLRLAVEDGLQQFKQYSRHVTTRAALTYTSLEITILTSQPGKEVVKQLEEGLKDTDLARVRRFQVVEVTKGILEHVDSASPVEDTSNDESSILGTDIDLQTIDNDIVSMEIFFKAWLHNSGTDQEQIHLLLSSQCFSNISRPRDNPMCLKCDLQERLLCPSLLAGTADGSLRMDDPKGDFITLYQMASQSSASHYKLQVIKALKSSGLCESLTYGLPFILRPTSCWQLDWDELETNQQHFHALCHSLLKREWLLLAKGEPPGPGHSQRIPASTFYVIMPSHSLTLLVKAVATRELMLPSTFPLLPEDPHDDSLKNVESMLDSLELEPTYNPLHVQSHLYSHLSSIYAKPQGRLHPHWESRAPRKHPCKTGQLQTNRARATVAPLPMTPVPGRASKMPAASKSSSDAFFLPSEWEKDPSRP

989 CAA71622.1 NP_000217.2

>CAA71622.1

CGKIDHWTHSETVSMTDFDIQYTYLGGHVCLSAHARNGSKQWGTYAEAISACGQHRLCRFQGCKYLSDINITRYNINCRENRACNELFHCQCDPGYAPPDCEPPISSPGGVFNDGFRLTVEKSVPLFPKRRAAPKNNGLLISFYISFSPLLILIAIVSLKWNKMKRFWSKGGTISSISISEDSSRNNNESQS

>NP_000217.2

MSCRQFSSSYLSRSGGGGGGGLGSGGSIRSSYSRFSSSGGGGGGGRFSSSSGYGGGSSRVCGRGGGGSFGYSYGGGSGGGFSASSLGGGFGGGSRGFGGASGGGYSSSGGFGGGFGGGSGGGFGGGYGSGFGGFGGFGGGAGGGDGGILTANEKSTMQELNSRLASYLDKVQALEEANNDLENKIQDWYDKKGPAAIQKNYSPYYNTIDDLKDQIVDLTVGNNKTLLDIDNTRMTLDDFRIKFEMEQNLRQGVDADINGLRQVLDNLTMEKSDLEMQYETLQEELMALKKNHKEEMSQLTGQNSGDVNVEINVAPGKDLTKTLNDMRQEYEQLIAKNRKDIENQYETQITQIEHEVSSSGQEVQSSAKEVTQLRHGVQELEIELQSQLSKKAALEKSLEDTKNRYCGQLQMIQEQISNLEAQITDVRQEIECQNQEYSLLLSIKMRLEKEIETYHNLLEGGQEDFESSGAGKIGLGGRGGSGGSYGRGSRGGSGGSYGGGGSGGGYGGGSGSRGGSGGSYGGGSGSGGGSGGGYGGGSGGGHSGGSGGGHSGGSGGNYGGGSGSGGGSGGGYGGGSGSRGGSGGSHGGGSGFGGESGGSYGGGEEASGSGGGYGGGSGKSSHS

990 NP_003842.1 NP_000867.2

>NP_003842.1

MAGLSRGSARALLAALLASTLLALLVSPARGRGGRDHGDWDEASRLPPLPPREDAARVARFVTHVSDWGALATISTLEAVRGRPFADVLSLSDGPPGAGSGVPYFYLSPLQLSVSNLQENPYATLTMTLAQTNFCKKHGFDPQSPLCVHIMLSGTVTKVNETEMDIAKHSLFIRHPEMKTWPSSHNWFFAKLNITNIWVLDYFGGPKIVTPEEYYNVTVQ

>NP_000867.2

MGAAAGRSPHLGPAPARRPQRSLLLLQLLLLVAAPGSTQAQAAPFPELCSYTWEAVDTKNNVLYKINICGSVDIVQCGPSSAVCMHDLKTRTYHSVGDSVLRSATRSLLEFNTTVSCDQQGTNHRVQSSIAFLCGKTLGTPEFVTATECVHYFEWRTTAACKKDIFKANKEVPCYVFDEELRKHDLNPLIKLSGAYLVDDSDPDTSLFINVCRDIDTLRDPGSQLRACPPGTAACLVRGHQAFDVGQPRDGLKLVRKDRLVLSYVREEAGKLDFCDGHSPAVTITFVCPSERREGTIPKLTAKSNCRYEIEWITEYACHRDYLESKTCSLSGEQQDVSIDLTPLAQSGGSSYISDGKEYLFYLNVCGETEIQFCNKKQAAVCQVKKSDTSQVKAAGRYHNQTLRYSDGDLTLIYFGGDECSSGFQRMSVINFECNKTAGNDGKGTPVFTGEVDCTYFFTWDTEYACVKEKEDLLCGATDGKKRYDLSALVRHAEPEQNWEAVDGSQTETEKKHFFINICHRVLQEGKARGCPEDAAVCAVDKNGSKNLGKFISSPMKEKGNIQLSYSDGDDCGHGKKIKTNITLVCKPGDLESAPVLRTSGEGGCFYEFEWHTAAACVLSKTEGENCTVFDSQAGFSFDLSPLTKKNGAYKVETKKYDFYINVCGPVSVSPCQPDSGACQVAKSDEKTWNLGLSNAKLSYYDGMIQLNYRGGTPYNNERHTPRATLITFLCDRDAGVGFPEYQEEDNSTYNFRWYTSYACPEEPLECVVTDPSTLEQYDLSSLAKSEGGLGGNWYAMDNSGEHVTWRKYYINVCRPLNPVPGCNRYASACQMKYEKDQGSFTEVVSISNLGMAKTGPVVEDSGSLLLEYVNGSACTTSDGRQTTYTTRIHLVCSRGRLNSHPIFSLNWECVVSFLWNTEAACPIQTTTDTDQACSIRDPNSGFVFNLNPLNSSQGYNVSGIGKIFMFNVCGTMPVCGTILGKPASGCEAETQTEELKNWKPARPVGIEKSLQLSTEGFITLTYKGPLSAKGTADAFIVRFVCNDDVYSGPLKFLHQDIDSGQGIRNTYFEFETALACVPSPVDCQVTDLAGNEYDLTGLSTVRKPWTAVDTSVDGRKRTFYLSVCNPLPYIPGCQGSAVGSCLVSEGNSWNLGVVQMSPQAAANGSLSIMYVNGDKCGNQRFSTRITFECAQISGSPAFQLQDGCEYVFIWRTVEACPVVRVEGDNCEVKDPRHGNLYDLKPLGLNDTIVSAGEYTYYFRVCGKLSSDVCPTSDKSKVVSSCQEKREPQGFHKVAGLLTQKLTYENGLLKMNFTGGDTCHKVYQRSTAIFFYCDRGTQRPVFLKETSDCSYLFEWRTQYACPPFDLTECSFKDGAGNSFDLSSLSRYSDNWEAITGTGDPEHYLINVCKSLAPQAGTEPCPPEAAACLLGGSKPVNLGRVRDGPQWRDGIIVLKYVDGDLCPDGIRKKSTTIRFTCSESQVNSRPMFISAVEDCEYTFAWPTATACPMKSNEHDDCQVTNPSTGHLFDLSSLSGRAGFTAAYSEKGLVYMSICGENENCPPGVGACFGQTRISVGKANKRLRYVDQVLQLVYKDGSPCPSKSGLSYKSVISFVCRPEAGPTNRPMLISLDKQTCTLFFSWHTPLACEQATECSVRNGSSIVDLSPLIHRTGGYEAYDESEDDASDTNPDFYINICQPLNPMHGVPCPAGAAVCKVPIDGPPIDIGRVAGPPILNPIANEIYLNFESSTPCLADKHFNYTSLIAFHCKRGVSMGTPKLLRTSECDFVFEWETPVVCPDEVRMDGCTLTDEQLLYSFNLSSLSTSTFKVTRDSRTYSVGVCTFAVGPEQGGCKDGGVCLLSGTKGASFGRLQSMKLDYRHQDEAVVLSYVNGDRCPPETDDGVPCVFPFIFNGKSYEECIIESRAKLWCSTTADYDRDHEWGFCRHSNSYRTSSIIFKCDEDEDIGRPQVFSEVRGCDVTFEWKTKVVCPPKKLECKFVQKHKTYDLRLLSSLTGSWSLVHNGVSYYINLCQKIYKGPLGCSERASICRRTTTGDVQVLGLVHTQKLGVIGDKVVVTYSKGYPCGGNKTASSVIELTCTKTVGRPAFKRFDIDSCTYYFSWDSRAACAVKPQEVQMVNGTITNPINGKSFSLGDIYFKLFRASGDMRTNGDNYLYEIQLSSITSSRNPACSGANICQVKPNDQHFSRKVGTSDKTKYYLQDGDLDVVFASSSKCGKDKTKSVSSTIFFHCDPLVEDGIPEFSHETADCQYLFSWYTSAVCPLGVGFDSENPGDDGQMHKGLSERSQAVGAVLSLLLVALTCCLLALLLYKKERRETVISKLTTCCRRSSNVSYKYSKVNKEEETDENETEWLMEEIQLPPPRQGKEGQENGHITTKSVKALSSLHGDDQDSEDEVLTIPEVKVHSGRGAGAESSHPVRNAQSNALQEREDDRVGLVRGEKARKGKSSSAQQKTVSSTKLVSFHDDSDEDLLHI

991 NP_006243.2 NP_006203.2

>NP_006243.2

MAEKQKHDGRVKIGHYVLGDTLGVGTFGKVKIGEHQLTGHKVAVKILNRQKIRSLDVVGKIKREIQNLKLFRHPHIIKLYQVISTPTDFFMVMEYVSGGELFDYICKHGRVEEMEARRLFQQILSAVDYCHRHMVVHRDLKPENVLLDAHMNAKIADFGLSNMMSDGEFLRTSCGSPNYAAPEVISGRLYAGPEVDIWSCGVILYALLCGTLPFDDEHVPTLFKKIRGGVFYIPEYLNRSVATLLMHMLQVDPLKRATIKDIREHEWFKQDLPSYLFPEDPSYDANVIDDEAVKEVCEKFECTESEVMNSLYSGDPQDQLAVAYHLIIDNRRIMNQASEFYLASSPPSGSFMDDSAMHIPPGLKPHPERMPPLIADSPKARCPLDALNTTKPKSLAVKKAKWHLGIRSQSKPYDIMAEVYRAMKQLDFEWKVVNAYHLRVRRKNPVTGNYVKMSLQLYLVDNRSYLLDFKSIDDEVVEQRSGSSTPQRSCSAAGLHRPRSSFDSTTAESHSLSGSLTGSLTGSTLSSVSPRLGSHTMDFFEMCASLITTLAR

>NP_006203.2

MSGASSSEQNNNSYETKTPNLRMSEKKCSWASYMTNSPTLIVMIGLPARGKTYVSKKLTRYLNWIGVPTKVFNLGVYRREAVKSYKSYDFFRHDNEEAMKIRKQCALVALEDVKAYLTEENGQIAVFDATNTTRERRDMILNFAEQNSFKVFFVESVCDDPDVIAANILEVKVSSPDYPERNRENVMEDFLKRIECYKVTYRPLDPDNYDKDLSFIKVINVGQRFLVNRVQDYIQSKIVYYLMNIHVQPRTIYLCRHGESEFNLLGKIGGDSGLSVRGKQFAQALRKFLEEQEITDLKVWTSQLKRTIQTAESLGVPYEQWKILNEIDAGVCEEMTYAEIEKRYPEEFALRDQEKYLYRYPGGESYQDLVQRLEPVIMELERQGNVLVISHQAVMRCLLAYFLDKGADELPYLRCPLHTIFKLTPVAYGCKVETIKLNVEAVNTHRDKPTNNFPKNQTPVRMRRNSFTPLSSSNTIRRPRNYSVGSRPLKPLSPLRAQDMQEGAD

992 NP_003467.1 NP_002470.2

>NP_003467.1

MPNWGGGAKCGACEKTVYHAEEIQCNGRSFHKTCFHCMACRKALDSTTVAAHESEIYCKVCYGRRYGPKGIGYGQGAGCLSTDTGEHLGLQFQQSPKPARSVTTSNPSKFTAKFGESEKCPRCGKSVYAAEKVMGGGKPWHKTCFRCAICGKSLESTNVTDKDGELYCKVCYAKNFGPTGIGFGGLTQQVEKKE

>NP_002470.2

MELYETSPYFYQEPRFYDGENYLPVHLQGFEPPGYERTELTLSPEAPGPLEDKGLGTPEHCPGQCLPWACKVCKRKSVSVDRRRAATLREKRRLKKVNEAFEALKRSTLLNPNQRLPKVEILRSAIQYIERLQALLSSLNQEERDLRYRGGGGPQPGVPSECSSHSASCSPEWGSALEFSANPGDHLLTADPTDAHNLHSLTSIVDSITVEDVSVAFPDETMPN

993 NP_000691.1 NP_002020.1

>NP_000691.1

MAMVSEFLKQAWFIENEEQEYVQTVKSSKGGPGSAVSPYPTFNPSSDVAALHKAIMVKGVDEATIIDILTKRNNAQRQQIKAAYLQETGKPLDETLKKALTGHLEEVVLALLKTPAQFDADELRAAMKGLGTDEDTLIEILASRTNKEIRDINRVYREELKRDLAKDITSDTSGDFRNALLSLAKGDRSEDFGVNEDLADSDARALYEAGERRKGTDVNVFNTILTTRSYPQLRRVFQKYTKYSKHDMNKVLDLELKGDIEKCLTAIVKCATSKPAFFAEKLHQAMKGVGTRHKALIRIMVSRSEIDMNDIKAFYQKMYGISLCQAILDETKGDYEKILVALCGGN

>NP_002020.1

METNSSLPTNISGGTPAVSAGYLFLDIITYLVFAVTFVLGVLGNGLVIWVAGFRMTHTVTTISYLNLAVADFCFTSTLPFFMVRKAMGGHWPFGWFLCKFVFTIVDINLFGSVFLIALIALDRCVCVLHPVWTQNHRTVSLAKKVIIGPWVMALLLTLPVIIRVTTVPGKTGTVACTFNFSPWTNDPKERINVAVAMLTVRGIIRFIIGFSAPMSIVAVSYGLIATKIHKQGLIKSSRPLRVLSFVAAAFFLCWSPYQVVALIATVRIRELLQGMYKEIGIAVDVTSALAFFNSCLNPMLYVFMGQDFRERLIHALPASLERALTEDSTQTSDTATNSTLPSAEVELQAK

994 NP_001395.1 NP_004419.2

>NP_001395.1

MAAGTLYTYPENWRAFKALIAAQYSGAQVRVLSAPPHFHFGQTNRTPEFLRKFPAGKVPAFEGDDGFCVFESNAIAYYVSNEELRGSTPEAAAQVVQWVSFADSDIVPPASTWVFPTLGIMHHNKQATENAKEEVRRILGLLDAYLKTRTFLVGERVTLADITVVCTLLWLYKQVLEPSFRQAFPNTNRWFLTCINQPQFRAVLGEVKLCEKMAQFDAKKFAETQPKKDTPRKEKGSREEKQKPQAERKEEKKAAAPAPEEEMDECEQALAAEPKAKDPFAHLPKSTFVLDEFKRKYSNEDTLSVALPYFWEHFDKDGWSLWYSEYRFPEELTQTFMSCNLITGMFQRLDKLRKNAFASVILFGTNNSSSISGVWVFRGQELAFPLSPDWQVDYESYTWRKLDPGSEETQTLVREYFSWEGAFQHVGKAFNQGKIFK

>NP_004419.2

MEFLWAPLLGLCCSLAAADRHTVFWNSSNPKFRNEDYTIHVQLNDYVDIICPHYEDHSVADAAMEQYILYLVEHEEYQLCQPQSKDQVRWQCNRPSAKHGPEKLSEKFQRFTPFTLGKEFKEGHSYYYISKPIHQHEDRCLRLKVTVSGKITHSPQAHDNPQEKRLAADDPEVRVLHSIGHSAAPRLFPLAWTVLLLPLLLLQTP

995 AAA36585.1 NP_004274.1

>AAA36585.1

MNEVSVIKEGWLHKRGEYIKTWRPRYFLLKSDGSFIGYKERPEAPDQTLPPLNNFSVAECQLMKTERPRPNTFVIRCLQWTTVIERTFHVDSPDEREEWMRAIQMVANSLKQRAPGEDPMDYKCGSPSDSSTTEEMEVAVSKARAKVTMNDFDYLKLLGKGTFGKVILVREKATGRYYAMKILRKEVIIAKDEVAHTVTESRVLQNTRHPFLTALKYAFQTHDRLCFVMEYANGGELFFHLSRERVFTEERARFYGAEIVSALEYLHSRDVVYRDIKLENLMLDKDGHIKITDFGLCKEGISDGATMKTFCGTPEYLAPEVLEDNDYGRAVDWWGLGVVMYEMMCGRLPFYNQDHERLFELILMEEIRFPRTLSPEAKSLLAGLLKKDPKQRLGGGPSDAKEVMEHRFFLSINWQDVVQKKLLPPFKPQVTSEVDTRYFDDEFTAQSITITPPDRYDSLGLLELDQRTHFPQFSYSAFREEKDLLMSLFVSLILFSDFSSLKSHSFSSNFILLSFSSLKK

>NP_004274.1

MASAGDTQAGPRDAADQNFDYMFKLLLIGNSSVGKTSFLFRYADDSFTPAFVSTVGIDFKVKTVYRHDKRIKLQIWDTAGQERYRTITTAYYRGAMGFLLMYDIANQESFAAVQDWATQIKTYSWDNAQVILVGNKCDLEDERVVPAEDGRRLADDLGFEFFEASAKENINVKQVFERLVDVICEKMNESLEPSSSSGSNGKGPAVGDAPAPQPSSCSC

996 NP_444252.1 NP_002841.3

>NP_444252.1

MAGWQSYVDNLMCDGCCQEAAIVGYCDAKYVWAATAGGVFQSITPIEIDMIVGKDREGFFTNGLTLGAKKCSVIRDSLYVDGDCTMDIRTKSQGGEPTYNVAVGRAGRVLVFVMGKEGVHGGGLNKKAYSMAKYLRDSGF

>NP_002841.3

MAPTWGPGMVSVVGPMGLLVVLLVGGCAAEEPPRFIKEPKDQIGVSGGVASFVCQATGDPKPRVTWNKKGKKVNSQRFETIEFDESAGAVLRIQPLRTPRDENVYECVAQNSVGEITVHAKLTVLREDQLPSGFPNIDMGPQLKVVERTRTATMLCAASGNPDPEITWFKDFLPVDPSASNGRIKQLRSETFESTPIRGALQIESSEETDQGKYECVATNSAGVRYSSPANLYVRELREVRRVAPRFSILPMSHEIMPGGNVNITCVAVGSPMPYVKWMQGAEDLTPEDDMPVGRNVLELTDVKDSANYTCVAMSSLGVIEAVAQITVKSLPKAPGTPMVTENTATSITITWDSGNPDPVSYYVIEYKSKSQDGPYQIKEDITTTRYSIGGLSPNSEYEIWVSAVNSIGQGPPSESVVTRTGEQAPASAPRNVQARMLSATTMIVQWEEPVEPNGLIRGYRVYYTMEPEHPVGNWQKHNVDDSLLTTVGSLLEDETYTVRVLAFTSVGDGPLSDPIQVKTQQGVPGQPMNLRAEARSETSITLSWSPPRQESIIKYELLFREGDHGREVGRTFDPTTSYVVEDLKPNTEYAFRLAARSPQGLGAFTPVVRQRTLQSKPSAPPQDVKCVSVRSTAILVSWRPPPPETHNGALVGYSVRYRPLGSEDPEPKEVNGIPPTTTQILLEALEKWTQYRITTVAHTEVGPGPESSPVVVRTDEDVPSAPPRKVEAEALNATAIRVLWRSPAPGRQHGQIRGYQVHYVRMEGAEARGPPRIKDVMLADAQWETDDTAEYEMVITNLQPETAYSITVAAYTMKGDGARSKPKVVVTKGAVLGRPTLSVQQTPEGSLLARWEPPAGTAEDQVLGYRLQFGREDSTPLATLEFPPSEDRYTASGVHKGATYVFRLAARSRGGLGEEAAEVLSIPEDTPRGHPQILEAAGNASAGTVLLRWLPPVPAERNGAIVKYTVAVREAGALGPARETELPAAAEPGAENALTLQGLKPDTAYDLQVRAHTRRGPGPFSPPVRYRTFLRDQVSPKNFKVKMIMKTSVLLSWEFPDNYNSPTPYKIQYNGLTLDVDGRTTKKLITHLKPHTFYNFVLTNRGSSLGGLQQTVTAWTAFNLLNGKPSVAPKPDADGFIMVYLPDGQSPVPVQSYFIVMVPLRKSRGGQFLTPLGSPEDMDLEELIQDISRLQRRSLRHSRQLEVPRPYIAARFSVLPPTFHPGDQKQYGGFDNRGLEPGHRYVLFVLAVLQKSEPTFAASPFSDPFQLDNPDPQPIVDGEEGLIWVIGPVLAVVFIICIVIAILLYKNKPDSKRKDSEPRTKCLLNNADLAPHHPKDPVEMRRINFQTPDSGLRSPLREPGFHFESMLSHPPIPIADMAEHTERLKANDSLKLSQEYESIDPGQQFTWEHSNLEVNKPKNRYANVIAYDHSRVILQPIEGIMGSDYINANYVDGYRCQNAYIATQGPLPETFGDFWRMVWEQRSATIVMMTRLEEKSRIKCDQYWPNRGTETYGFIQVTLLDTIELATFCVRTFSLHKNGSSEKREVRQFQFTAWPDHGVPEYPTPFLAFLRRVKTCNPPDAGPIVVHCSAGVGRTGCFIVIDAMLERIKPEKTVDVYGHVTLMRSQRNYMVQTEDQYSFIHEALLEAVGCGNTEVPARSLYAYIQKLAQVEPGEHVTGMELEFKRLANSKAHTSRFISANLPCNKFKNRLVNIMPYESTRVCLQPIRGVEGSDYINASFIDGYRQQKAYIATQGPLAETTEDFWRMLWENNSTIVVMLTKLREMGREKCHQYWPAERSARYQYFVVDPMAEYNMPQYILREFKVTDARDGQSRTVRQFQFTDWPEQGVPKSGEGFIDFIGQVHKTKEQFGQDGPISVHCSAGVGRTGVFITLSIVLERMRYEGVVDIFQTVKMLRTQRPAMVQTEDEYQFCYQAALEYLGSFDHYAT

997 NP_003706.1 NP_004477.2

>NP_003706.1

MNFLRGVMGGQSAGPQHTEAETIQKLCDRVASSTLLDDRRNAVRALKSLSKKYRLEVGIQAMEHLIHVLQTDRSDSEIIGYALDILYNIISNEEEEEVEENSTRQSEDLGSQFTEIFIKQQENVTLLLSLLEEFDFHVRWPGVKLLTSLLKQLGPQVQQIILVSPMGVSRLMDLLADSREVIRNDGVLLLQALTRSNGAIQKIVAFENAFERLLDIISEEGNSDGGIVVEDCLILLQNLLKNNNSNQNFFKEGSYIQRMKPWFEVGDENSGWSAQKVTNLHLMLQLVRVLVSPTNPPGATSSCQKAMFQCGLLQQLCTILMATGVPADILTETINTVSEVIRGCQVNQDYFASVNAPSNPPRPAIVVLLMSMVNERQPFVLRCAVLYCFQCFLYKNQKGQGEIVSTLLPSTIDATGNSVSAGQLLCGGLFSTDSLSNWCAAVALAHALQENATQKEQLLRVQLATSIGNPPVSLLQQCTNILSQGSKIQTRVGLLMLLCTWLSNCPIAVTHFLHNSANVPFLTGQIAENLGEEEQLVQGLCALLLGISIYFNDNSLESYMKEKLKQLIEKRIGKENFIEKLGFISKHELYSRASQKPQPNFPSPEYMIFDHEFTKLVKELEGVITKAIYKSSEEDKKEEEVKKTLEQHDNIVTHYKNMIREQDLQLEELRQQVSTLKCQNEQLQTAVTQQVSQIQQHKDQYNLLKIQLGKDNQHQGSYSEGAQMNGIQPEEIGRLREEIEELKRNQELLQSQLTEKDSMIENMKSSQTSGTNEQSSAIVSARDSEQVAELKQELATLKSQLNSQSVEITKLQTEKQELLQKTEAFAKSVEVQGETETIIATKTTDVEGRLSALLQETKELKNEIKALSEERTAIKEQLDSSNSTIAILQTEKDKLELEITDSKKEQDDLLVLLADQDQKILSLKNKLKDLGHPVEEEDELESGDQEDEDDESEDPGKDLDHI

>NP_004477.2

MSEETRQSKLAAAKKKLREYQQRNSPGVPTGAKKKKKIKNGSNPETTTSGGCHSPEDTPKDNAATLQPSDDTVLPGGVPSPGASLTSMAASQNHDADNVPNLMDETKTFSSTESLRQLSQQLNGLVCESATCVNGEGPASSANLKDLESRYQQLAVALDSSYVTNKQLNITIEKLKQQNQEITDQLEEEKKECHQKQGALREQLQVHIQTIGILVSEKAELQTALAHTQHAARQKEGESEDLASRLQYSRRRVGELERALSAVSTQQKKADRYNKELTKERDALRLELYKNTQSNEDLKQEKSELEEKLRVLVTEKAGMQLNLEELQKKLEMTELLLQQFSSRCEAPDANQQLQQAMEERAQLEAHLGQVMESVRQLQMERDKYAENLKGESAMWRQRMQQMSEQVHTLREEKECSMSRVQELETSLAELRNQMAEPPPPEPPAGPSEVEQQLQAEAEHLRKELEGLAGQLQAQVQDNEGLSRLNREQEERLLELERAAELWGEQAEARRQILETMQNDRTTISRALSQNRELKEQLAELQSGFVKLTNENMEITSALQSEQHVKRELGKKLGELQEKLSELKETVELKSQEAQSLQQQRDQYLGHLQQYVAAYQQLTSEKEVLHNQLLLQTQLVDQLQQQEAQGKAVAEMARQELQETQERLEAATQQNQQLRAQLSLMAHPGEGDGLDREEEEDEEEEEEEAVAVPQPMPSIPEDLESREAMVAFFNSAVASAEEEQARLRGQLKEQRVRCRRLAHLLASAQKEPEAAAPAPGTGGDSVCGETHRALQGAMEKLQSRFMELMQEKADLKERVEELEHRCIQLSGETDTIGEYIALYQSQRAVLKERHREKEEYISRLAQDKEEMKVKLLELQELVLRLVGDRNEWHGRFLAAAQNPADEPTSGAPAPQELGAANQQGDLCEVSLAGSVEPAQGEAREGSPRDNPTAQQIMQLLREMQNPRERPGLGSNPCIPFFYRADENDEVKITVI

998 NP_005307.1 NP_002651.2

>NP_005307.1

MATSSEEVLLIVKKVRQKKQDGALYLMAERIAWAPEGKDRFTISHMYADIKCQKISPEGKAKIQLQLVLHAGDTTNFHFSNESTAVKERDAVKDLLQQLLPKFKRKANKELEEKNRMLQEDPVLFQLYKDLVVSQVISAEEFWANRLNVNATDSSSTSNHKQDVGISAAFLADVRPQTDGCNGLRYNLTSDIIESIFRTYPAVKMKYAENVPHNMTEKEFWTRFFQSHYFHRDRLNTGSKDLFAECAKIDEKGLKTMVSLGVKNPLLDLTALEDKPLDEGYGISSVPSASNSKSIKENSNAAIIKRFNHHSAMVLAAGLRKQEAQNEQTSEPSNMDGNSGDADCFQPAVKRAKLQESIEYEDLGKNNSVKTIALNLKKSDRYYHGPTPIQSLQYATSQDIINSFQSIRQEMEAYTPKLTQVLSSSAASSTITALSPGGALMQGGTQQAINQMVPNDIQSELKHLYVAVGELLRHFWSCFPVNTPFLEEKVVKMKSNLERFQVTKLCPFQEKIRRQYLSTNLVSHIEEMLQTAYNKLHTWQSRRLMKKT

>NP_002651.2

MAGAASPCANGCGPGAPSDAEVLHLCRSLEVGTVMTLFYSKKSQRPERKTFQVKLETRQITWSRGADKIEGAIDIREIKEIRPGKTSRDFDRYQEDPAFRPDQSHCFVILYGMEFRLKTLSLQATSEDEVNMWIKGLTWLMEDTLQAPTPLQIERWLRKQFYSVDRNREDRISAKDLKNMLSQVNYRVPNMRFLRERLTDLEQRSGDITYGQFAQLYRSLMYSAQKTMDLPFLEASTLRAGERPELCRVSLPEFQQFLLDYQGELWAVDRLQVQEFMLSFLRDPLREIEEPYFFLDEFVTFLFSKENSVWNSQLDAVCPDTMNNPLSHYWISSSHNTYLTGDQFSSESSLEAYARCLRMGCRCIELDCWDGPDGMPVIYHGHTLTTKIKFSDVLHTIKEHAFVASEYPVILSIEDHCSIAQQRNMAQYFKKVLGDTLLTKPVEISADGLPSPNQLKRKILIKHKKLAEGSAYEEVPTSMMYSENDISNSIKNGILYLEDPVNHEWYPHYFVLTSSKIYYSEETSSDQGNEDEEEPKEVSSSTELHSNEKWFHGKLGAGRDGRHIAERLLTEYCIETGAPDGSFLVRESETFVGDYTLSFWRNGKVQHCRIHSRQDAGTPKFFLTDNLVFDSLYDLITHYQQVPLRCNEFEMRLSEPVPQTNAHESKEWYHASLTRAQAEHMLMRVPRDGAFLVRKRNEPNSYAISFRAEGKIKHCRVQQEGQTVMLGNSEFDSLVDLISYYEKHPLYRKMKLRYPINEEALEKIGTAEPDYGALYEGRNPGFYVEANPMPTFKCAVKALFDYKAQREDELTFIKSAIIQNVEKQEGGWWRGDYGGKKQLWFPSNYVEEMVNPVALEPEREHLDENSPLGDLLRGVLDVPACQIAIRPEGKNNRLFVFSISMASVAHWSLDVAADSQEELQDWVKKIREVAQTADARLTEGKIMERRKKIALELSELVVYCRPVPFDEEKIGTERACYRDMSSFPETKAEKYVNKAKGKKFLQYNRLQLSRIYPKGQRLDSSNYDPLPMWICGSQLVALNFQTPDKPMQMNQALFMTGRHCGYVLQPSTMRDEAFDPFDKSSLRGLEPCAISIEVLGARHLPKNGRGIVCPFVEIEVAGAEYDSTKQKTEFVVDNGLNPVWPAKPFHFQISNPEFAFLRFVVYEEDMFSDQNFLAQATFPVKGLKTGYRAVPLKNNYSEDLELASLLIKIDIFPAKENGDLSPFSGTSLRERGSDASGQLFHGRAREGSFESRYQQPFEDFRISQEHLADHFDSRERRAPRRTRVNGDNRL

999 NP_005949.1 NP_001737.1

>NP_005949.1

MQGNGSALPNASQPVLRGDGARPSWLASALACVLIFTIVVDILGNLLVILSVYRNKKLRNAGNIFVVSLAVADLVVAIYPYPLVLMSIFNNGWNLGYLHCQVSGFLMGLSVIGSIFNITGIAINRYCYICHSLKYDKLYSSKNSLCYVLLIWLLTLAAVLPNLRAGTLQYDPRIYSCTFAQSVSSAYTIAVVVFHFLVPMIIVIFCYLRIWILVLQVRQRVKPDRKPKLKPQDFRNFVTMFVVFVLFAICWAPLNFIGLAVASDPASMVPRIPEWLFVASYYMAYFNSCLNAIIYGLLNQNFRKEYRRIIVSLCTARVFFVDSSNDVADRVKWKPSPLMTNNNVVKVDSV

>NP_001737.1

MEGKWLLCMLLVLGTAIVEAHDGHDDDVIDIEDDLDDVIEEVEDSKPDTTAPPSSPKVTYKAPVPTGEVYFADSFDRGTLSGWILSKAKKDDTDDEIAKYDGKWEVEEMKESKLPGDKGLVLMSRAKHHAISAKLNKPFLFDTKPLIVQYEVNFQNGIECGGAYVKLLSKTPELNLDQFHDKTPYTIMFGPDKCGEDYKLHFIFRHKNPKTGIYEEKHAKRPDADLKTYFTDKKTHLYTLILNPDNSFEILVDQSVVNSGNLLNDMTPPVNPSREIEDPEDRKPEDWDERPKIPDPEAVKPDDWDEDAPAKIPDEEATKPEGWLDDEPEYVPDPDAEKPEDWDEDMDGEWEAPQIANPRCESAPGCGVWQRPVIDNPNYKGKWKPPMIDNPSYQGIWKPRKIPNPDFFEDLEPFRMTPFSAIGLELWSMTSDIFFDNFIICADRRIVDDWANDGWGLKKAADGAAEPGVVGQMIEAAEERPWLWVVYILTVALPVFLVILFCCSGKKQTSGMEYKKTDAPQPDVKEEEEEKEEEKDKGDEEEEGEEKLEEKQKSDAEEDGGTVSQEEEDRKPKAEEDEILNRSPRNRKPRRE

1000 NP_057327.2 NP_064516.2

>NP_057327.2

MGIQTSPVLLASLGVGLVTLLGLAVGSYLVRRSRRPQVTLLDPNEKYLLRLLDKTTVSHNTKRFRFALPTAHHTLGLPVGKHIYLSTRIDGSLVIRPYTPVTSDEDQGYVDLVIKVYLKGVHPKFPEGGKMSQYLDSLKVGDVVEFRGPSGLLTYTGKGHFNIQPNKKSPPEPRVAKKLGMIAGGTGITPMLQLIRAILKVPEDPTQCFLLFANQTEKDIILREDLEELQARYPNRFKLWFTLDHPPKDWAYSKGFVTADMIREHLPAPGDDVLVLLCGPPPMVQLACHPNLDKLGYSQKMRFTY

>NP_064516.2

MAEPSGAETRPPIRVTVKTPKDKEEIVICDRASVKEFKEEISRRFKAQQDQLVLIFAGKILKDGDTLNQHGIKDGLTVHLVIKTPQKAQDPAAATASSPSTPDPASAPSTTPASPATPAQPSTSGSASSDAGSGSRRSSGGGPSPGAGEGSPSATASILSGFGGILGLGSLGLGSANFMELQQQMQRQLMSNPEMLSQIMENPLVQDMMSNPDLMRHMIMANPQMQQLMERNPEISHMLNNPELMRQTMELARNPAMMQEMMRNQDRALSNLESIPGGYNALRRMYTDIQEPMFSAAREQFGNNPFSSLAGNSDSSSSQPLRTENREPLPNPWSPSPPTSQAPGSGGEGTGGSGTSQVHPTVSNPFGINAASLGSGMFNSPEMQALLQQISENPQLMQNVISAPYMRSMMQTLAQNPDFAAQMMVNVPLFAGNPQLQEQLRLQLPVFLQQMQNPESLSILTNPRAMQALLQIQQGLQTLQTEAPGLVPSLGSFGISRTPAPSAGSNAGSTPEAPTSSPATPATSSPTGASSAQQQLMQQMIQLLAGSGNSQVQTPEVRFQQQLEQLNSMGFINREANLQALIATGGDINAAIERLLGSQLS

1001 NP_004991.1 NP_055579.1

>NP_004991.1

MAGVLKKTTGLVGLAVCNTPHERLRILYTKILDVLEEIPKNAAYRKYTEQITNEKLAMVKAEPDVKKLEDQLQGGQLEEVILQAEHELNLARKMREWKLWEPLVEEPPADQWKWPI

>NP_055579.1

MNSKGQYPTQPTYPVQPPGNPVYPQTLHLPQAPPYTDAPPAYSELYRPSFVHPGAATVPTMSAAFPGASLYLPMAQSVAVGPLGSTIPMAYYPVGPIYPPGSTVLVEGGYDAGARFGAGATAGNIPPPPPGCPPNAAQLAVMQGANVLVTQRKGNFFMGGSDGGYTIW

1002 NP_000035.2 NP_002383.2

>NP_000035.2

MEVQLGLGRVYPRPPSKTYRGAFQNLFQSVREVIQNPGPRHPEAASAAPPGASLLLLQQQQQQQQQQQQQQQQQQQQQQQETSPRQQQQQQGEDGSPQAHRRGPTGYLVLDEEQQPSQPQSALECHPERGCVPEPGAAVAASKGLPQQLPAPPDEDDSAAPSTLSLLGPTFPGLSSCSADLKDILSEASTMQLLQQQQQEAVSEGSSSGRAREASGAPTSSKDNYLGGTSTISDNAKELCKAVSVSMGLGVEALEHLSPGEQLRGDCMYAPLLGVPPAVRPTPCAPLAECKGSLLDDSAGKSTEDTAEYSPFKGGYTKGLEGESLGCSGSAAAGSSGTLELPSTLSLYKSGALDEAAAYQSRDYYNFPLALAGPPPPPPPPHPHARIKLENPLDYGSAWAAAAAQCRYGDLASLHGAGAAGPGSGSPSAAASSSWHTLFTAEEGQLYGPCGGGGGGGGGGGGGGGGGGGGGGGEAGAVAPYGYTRPPQGLAGQESDFTAPDVWYPGGMVSRVPYPSPTCVKSEMGPWMDSYSGPYGDMRLETARDHVLPIDYYFPPQKTCLICGDEASGCHYGALTCGSCKVFFKRAAEGKQKYLCASRNDCTIDKFRRKNCPSCRLRKCYEAGMTLGARKLKKLGNLKLQEEGEASSTTSPTEETTQKLTVSHIEGYECQPIFLNVLEAIEPGVVCAGHDNNQPDSFAALLSSLNELGERQLVHVVKWAKALPGFRNLHVDDQMAVIQYSWMGLMVFAMGWRSFTNVNSRMLYFAPDLVFNEYRMHKSRMYSQCVRMRHLSQEFGWLQITPQEFLCMKALLLFSIIPVDGLKNQKFFDELRMNYIKELDRIIACKRKNPTSCSRRFYQLTKLLDSVQPIARELHQFTFDLLIKSHMVSVDFPEMMAEIISVQVPKILSGKVKPIYFHTQ

>NP_002383.2

MVRSRQMCNTNMSVPTDGAVTTSQIPASEQETLVRPKPLLLKLLKSVGAQKDTYTMKEVLFYLGQYIMTKRLYDEKQQHIVYCSNDLLGDLFGVPSFSVKEHRKIYTMIYRNLVVVNQQESSDSGTSVSENRCHLEGGSDQKDLVQELQEEKPSSSHLVSRPSTSSRRRAISETEENSDELSGERQRKRHKSDSISLSFDESLALCVIREICCERSSSSESTGTPSNPDLDAGVSEHSGDWLDQDSVSDQFSVEFEVESLDSEDYSLSEEGQELSDEDDEVYQVTVYQAGESDTDSFEEDPEISLADYWKCTSCNEMNPPLPSHCNRCWALRENWLPEDKGKDKGEISEKAKLENSTQAEEGFDVPDCKKTIVNDSRESCVEENDDKITQASQSQESEDYSQPSTSSSIIYSSQEDVKEFEREETQDKEESVESSLPLNAIEPCVICQGRPKNGCIVHGKTGHLMACFTCAKKLKKRNKPCPVCRQPIQMIVLTYFP

1003 NP_003835.2 NP_001835.3

>NP_003835.2

MAPPPARVHLGAFLAVTPNPGSAASGTEAAAATPSKVWGSSAGRIEPRGGGRGALPTSMGQHGPSARARAGRAPGPRPAREASPRLRVHKTFKFVVVGVLLQVVPSSAATIKLHDQSIGTQQWEHSPLGELCPPGSHRSEHPGACNRCTEGVGYTNASNNLFACLPCTACKSDEEERSPCTTTRNTACQCKPGTFRNDNSAEMCRKCSRGCPRGMVKVKDCTPWSDIECVHKESGNGHNIWVILVVTLVVPLLLVAVLIVCCCIGSGCGGDPKCMDRVCFWRLGLLRGPGAEDNAHNEILSNADSLSTFVSEQQMESQEPADLTGVTVQSPGEAQCLLGPAEAEGSQRRRLLVPANGADPTETLMLFFDKFANIVPFDSWDQLMRQLDLTKNEIDVVRAGTAGPGDALYAMLMKWVNKTGRNASIHTLLDALERMEERHAKEKIQDLLVDSGKFIYLEDGTGSAVSLE

>NP_001835.3

MIRLGAPQTLVLLTLLVAAVLRCQGQDVQEAGSCVQDGQRYNDKDVWKPEPCRICVCDTGTVLCDDIICEDVKDCLSPEIPFGECCPICPTDLATASGQPGPKGQKGEPGDIKDIVGPKGPPGPQGPAGEQGPRGDRGDKGEKGAPGPRGRDGEPGTPGNPGPPGPPGPPGPPGLGGNFAAQMAGGFDEKAGGAQLGVMQGPMGPMGPRGPPGPAGAPGPQGFQGNPGEPGEPGVSGPMGPRGPPGPPGKPGDDGEAGKPGKAGERGPPGPQGARGFPGTPGLPGVKGHRGYPGLDGAKGEAGAPGVKGESGSPGENGSPGPMGPRGLPGERGRTGPAGAAGARGNDGQPGPAGPPGPVGPAGGPGFPGAPGAKGEAGPTGARGPEGAQGPRGEPGTPGSPGPAGASGNPGTDGIPGAKGSAGAPGIAGAPGFPGPRGPPGPQGATGPLGPKGQTGEPGIAGFKGEQGPKGEPGPAGPQGAPGPAGEEGKRGARGEPGGVGPIGPPGERGAPGNRGFPGQDGLAGPKGAPGERGPSGLAGPKGANGDPGRPGEPGLPGARGLTGRPGDAGPQGKVGPSGAPGEDGRPGPPGPQGARGQPGVMGFPGPKGANGEPGKAGEKGLPGAPGLRGLPGKDGETGAAGPPGPAGPAGERGEQGAPGPSGFQGLPGPPGPPGEGGKPGDQGVPGEAGAPGLVGPRGERGFPGERGSPGAQGLQGPRGLPGTPGTDGPKGASGPAGPPGAQGPPGLQGMPGERGAAGIAGPKGDRGDVGEKGPEGAPGKDGGRGLTGPIGPPGPAGANGEKGEVGPPGPAGSAGARGAPGERGETGPPGPAGFAGPPGADGQPGAKGEQGEAGQKGDAGAPGPQGPSGAPGPQGPTGVTGPKGARGAQGPPGATGFPGAAGRVGPPGSNGNPGPPGPPGPSGKDGPKGARGDSGPPGRAGEPGLQGPAGPPGEKGEPGDDGPSGAEGPPGPQGLAGQRGIVGLPGQRGERGFPGLPGPSGEPGKQGAPGASGDRGPPGPVGPPGLTGPAGEPGREGSPGADGPPGRDGAAGVKGDRGETGAVGAPGAPGPPGSPGPAGPTGKQGDRGEAGAQGPMGPSGPAGARGIQGPQGPRGDKGEAGEPGERGLKGHRGFTGLQGLPGPPGPSGDQGASGPAGPSGPRGPPGPVGPSGKDGANGIPGPIGPPGPRGRSGETGPAGPPGNPGPPGPPGPPGPGIDMSAFAGLGPREKGPDPLQYMRADQAAGGLRQHDAEVDATLKSLNNQIESIRSPEGSRKNPARTCRDLKLCHPEWKSGDYWIDPNQGCTLDAMKVFCNMETGETCVYPNPANVPKKNWWSSKSKEKKHIWFGETINGGFHFSYGDDNLAPNTANVQMTFLRLLSTEGSQNITYHCKNSIAYLDEAAGNLKKALLIQGSNDVEIRAEGNSRFTYTALKDGCTKHTGKWGKTVIEYRSQKTSRLPIIDIAPMDIGGPEQEFGVDIGPVCFL

1004 NP_001096.1 NP_001813.1

>NP_001096.1

MVDGVMILPVLIMIALPSPSMEDEKPKVNPKLYMCVCEGLSCGNEDHCEGQQCFSSLSINDGFHVYQKGCFQVYEQGKMTCKTPPSPGQAVECCQGDWCNRNITAQLPTKGKSFPGTQNFHLEVGLIILSVVFAVCLLACLLGVALRKFKRRNQERLNPRDVEYGTIEGLITTNVGDSTLADLLDHSCTSGSGSGLPFLVQRTVARQITLLECVGKGRYGEVWRGSWQGENVAVKIFSSRDEKSWFRETELYNTVMLRHENILGFIASDMTSRHSSTQLWLITHYHEMGSLYDYLQLTTLDTVSCLRIVLSIASGLAHLHIEIFGTQGKPAIAHRDLKSKNILVKKNGQCCIADLGLAVMHSQSTNQLDVGNNPRVGTKRYMAPEVLDETIQVDCFDSYKRVDIWAFGLVLWEVARRMVSNGIVEDYKPPFYDVVPNDPSFEDMRKVVCVDQQRPNIPNRWFSDPTLTSLAKLMKECWYQNPSARLTALRIKKTLTKIDNSLDKLKTDC

>NP_001813.1

MALTLFDTDEYRPPVWKSYLYQLQQEAPHPRRITCTCEVENRPKYYGREFHGMISREAADQLLIVAEGSYLIRESQRQPGTYTLALRFGSQTRNFRLYYDGKHFVGEKRFESIHDLVTDGLITLYIETKAAEYIAKMTINPIYEHVGYTTLNREPAYKKHMPVLKETHDERDSTGQDGVSEKRLTSLVRRATLKENEQIPKYEKIHNFKVHTFRGPHWCEYCANFMWGLIAQGVKCADCGLNVHKQCSKMVPNDCKPDLKHVKKVYSCDLTTLVKAHTTKRPMVVDMCIREIESRGLNSEGLYRVSGFSDLIEDVKMAFDRDGEKADISVNMYEDINIITGALKLYFRDLPIPLITYDAYPKFIESAKIMDPDEQLETLHEALKLLPPAHCETLRYLMAHLKRVTLHEKENLMNAENLGIVFGPTLMRSPELDAMAALNDIRYQRLVVELLIKNEDILF

1005 NP_775832.1 NP_003989.2

>NP_775832.1

MCPQESSFQPSQFLLLVGVPVASVLLLAQCLRWHCPRRLLGACWTLNGQEEPVSQPTPQLENEVSRQHLPATLPEMVAFYQELHTPTQGQTMVRQLMHKLLVFSAREVDHRGGCLMLQDTGISLLIPPGAVAVGRQERVSLILVWDLSDAPSLSQAQGLVSPVVACGPHGASFLKPCTLTFKHCAEQPSHARTYSSNTTLLDAKVWRPLGRPGAHASRDECRIHLSHFSLYTCVLEAPVGREARKWLQLAVFCSPLVPGQSHLQLRIYFLNNTPCALQWALTNEQPHGGRLRGPCQLFDFNGARGDQCLKLTYISEGWENVDDSSCQLVPHLHIWHGKCPFRSFCFRRKAADENEDCSALTNEIIVTMHTFQDGLETKYMEILRFQASEEESWAAPPPVSQPPPCNRLPPELFEQLRMLLEPNSITGNDWRGLASHLGLCGMKIRFLSCQRSPAAAILELFEEQNGSLQELHYLMTVMERLDCASAIQNYLSGTHGGSPGPERGGARDNQGLELDEKL

>NP_003989.2

MAEDDPYLGRPEQMFHLDPSLTHTIFNPEVFQPQMALPTADGPYLQILEQPKQRGFRFRYVCEGPSHGGLPGASSEKNKKSYPQVKICNYVGPAKVIVQLVTNGKNIHLHAHSLVGKHCEDGICTVTAGPKDMVVGFANLGILHVTKKKVFETLEARMTEACIRGYNPGLLVHPDLAYLQAEGGGDRQLGDREKELIRQAALQQTKEMDLSVVRLMFTAFLPDSTGSFTRRLEPVVSDAIYDSKAPNASNLKIVRMDRTAGCVTGGEEIYLLCDKVQKDDIQIRFYEEEENGGVWEGFGDFSPTDVHRQFAIVFKTPKYKDINITKPASVFVQLRRKSDLETSEPKPFLYYPEIKDKEEVQRKRQKLMPNFSDSFGGGSGAGAGGGGMFGSGGGGGGTGSTGPGYSFPHYGFPTYGGITFHPGTTKSNAGMKHGTMDTESKKDPEGCDKSDDKNTVNLFGKVIETTEQDQEPSEATVGNGEVTLTYATGTKEESAGVQDNLFLEKAMQLAKRHANALFDYAVTGDVKMLLAVQRHLTAVQDENGDSVLHLAIIHLHSQLVRDLLEVTSGLISDDIINMRNDLYQTPLHLAVITKQEDVVEDLLRAGADLSLLDRLGNSVLHLAAKEGHDKVLSILLKHKKAALLLDHPNGDGLNAIHLAMMSNSLPCLLLLVAAGADVNAQEQKSGRTALHLAVEHDNISLAGCLLLEGDAHVDSTTYDGTTPLHIAAGRGSTRLAALLKAAGADPLVENFEPLYDLDDSWENAGEDEGVVPGTTPLDMATSWQVFDILNGKPYEPEFTSDDLLAQGDMKQLAEDVKLQLYKLLEIPDPDKNWATLAQKLGLGILNNAFRLSPAPSKTLMDNYEVSGGTVRELVEALRQMGYTEAIEVIQAASSPVKTTSQAHSLPLSPASTRQQIDELRDSDSVCDSGVETSFRKLSFTESLTSGASLLTLNKMPHDYGQEGPLEGKI

1006 NP_066973.1 NP_001019265.1

>NP_066973.1

MIAVSFKCRCQILRRLTKDESPYTKSASQTKPPDGALAVRRQSIPEEFKGSTVVELMKKEGTTLGLTVSGGIDKDGKPRVSNLRQGGIAARSDQLDVGDYIKAVNGINLAKFRHDEIISLLKNVGERVVLEVEYELPPVSVQGSSVIFRTVEVTLHKEGNTFGFVIRGGAHDDRNKSRPVVITCVRPGGPADREGTIKPGDRLLSVDGIRLLGTTHAEAMSILKQCGQEAALLIEYDVSVMDSVATASGPLLVEVAKTPGASLGVALTTSMCCNKQVIVIDKIKSASIADRCGALHVGDHILSIDGTSMEYCTLAEATQFLANTTDQVKLEILPHHQTRLALKGPDHAALVSSSFSPTSMSAYSLSSLNMGTLPRSLYSTSPRGTMMRRRLKKKDFKSSLSLASSTVGLAGQVVHTETTEVVLTADPVTGFGIQLQGSVFATETLSSPPLISYIEADSPAERCGVLQIGDRVMAINGIPTEDSTFEEASQLLRDSSITSKVTLEIEFDVAESVIPSSGTFHVKLPKKHNVELGITISSPSSRKPGDPLVISDIKKGSVAHRTGTLELGDKLLAIDNIRLDNCSMEDAVQILQQCEDLVKLKIRKDEDNSDEQESSGAIIYTVELKRYGGPLGITISGTEEPFDPIIISSLTKGGLAERTGAIHIGDRILAINSSSLKGKPLSEAIHLLQMAGETVTLKIKKQTDAQSASSPKKFPISSHLSDLGDVEEDSSPAQKPGKLSDMYPSTVPSVDSAVDSWDGSAIDTSYGTEGTSFQASGYNFNTYDWRSPKQRGSLSPVTKPRSQTYPDVGLSYEDWDRSTASGFAGAADSAETEQEENFWSQALEDLETCGQSGILRELEEKADRRVSLRNMTLLATIMSGSTMSLNHEAPTPRSQLGRQASFQERSSSRPHYSQTTRSNTLPSDVGRKSVTLRKMKQEIKEIMSPTPVELHKVTLYKDSDMEDFGFSVADGLLEKGVYVKNIRPAGPGDLGGLKPYDRLLQVNHVRTRDFDCCLVVPLIAESGNKLDLVISRNPLASQKSIDQQSLPGDWSEQNSAFFQQPSHGGNLETREPTNTL

>NP_001019265.1

MDSKESLTPGREENPSSVLAQERGDVMDFYKTLRGGATVKVSASSPSLAVASQSDSKQRRLLVDFPKGSVSNAQQPDLSKAVSLSMGLYMGETETKVMGNDLGFPQQGQISLSSGETDLKLLEESIANLNRSTSVPENPKSSASTAVSAAPTEKEFPKTHSDVSSEQQHLKGQTGTNGGNVKLYTTDQSTFDILQDLEFSSGSPGKETNESPWRSDLLIDENCLLSPLAGEDDSFLLEGNSNEDCKPLILPDTKPKIKDNGDLVLSSPSNVTLPQVKTEKEDFIELCTPGVIKQEKLGTVYCQASFPGANIIGNKMSAISVHGVSTSGGQMYHYDMNTASLSQQQDQKPIFNVIPPIPVGSENWNRCQGSGDDNLTSLGTLNFPGRTVFSNGYSSPSMRPDVSSPPSSSSTATTGPPPKLCLVCSDEASGCHYGVLTCGSCKVFFKRAVEGRQHNYLCAGRNDCIIDKIRRKNCPACRYRKCLQAGMNLEARKTKKKIKGIQQATTGVSQETSENPGNKTIVPATLPQLTPTLVSLLEVIEPEVLYAGYDSSVPDSTWRIMTTLNMLGGRQVIAAVKWAKAIPGFRNLHLDDQMTLLQYSWMFLMAFALGWRSYRQSSANLLCFAPDLIINEQRMTLPCMYDQCKHMLYVSSELHRLQVSYEEYLCMKTLLLLSSVPKDGLKSQELFDEIRMTYIKELGKAIVKREGNSSQNWQRFYQLTKLLDSMHEVVENLLNYCFQTFLDKTMSIEFPEMLAEIITNQIPKYSNGNIKKLLFHQK

1007 NP_006863.2 NP_002158.2

>NP_006863.2

MACLNPVPKLYRSVIEDVIEGVRNLFAEEGIEEQVLKDLKQLWETKVLQSKATEDFFRNSIQSPLFTLQLPHSLHQTLQSSTASLVIPAGRTLPSFTTAELGTSNSSANFTFPGYPIHVPAGVTLQTVSGHLYKVNVPIMVTETSGRAGILQHPIQQVFQQLGQPSVIQTSVPQLNPWSLQATTEKSQRIETVLQQPAILPSGPVDRKHLENATSDILVSPGNEHKIVPEALLCHQESSHYISLPGVVFSPQVSQTNSNVESVLSGSASMAQNLHDESLSTSPHGALHQHVTDIQLHILKNRMYGCDSVKQPRNIEEPSNIPVSEKDSNSQVDLSIRVTDDDIGEIIQVDGSGDTSSNEEIGSTRDADENEFLGNIDGGDLKVPEEEADSISNEDSATNSSDNEDPQVNIVEEDPLNSGDDVSEQDVPDLFDTDNVIVCQYDKIHRSKNKWKFYLKDGVMCFGGRDYVFAKAIGDAEW

>NP_002158.2

MKALSPVRGCYEAVCCLSERSLAIARGRGKGPAAEEPLSLLDDMNHCYSRLRELVPGVPRGTQLSQVEILQRVIDYILDLQVVLAEPAPGPPDGPHLPIQTAELAPELVISNDKRSFCH

1008 NP_997080.1 NP_002077.1

>NP_997080.1

MRLALLWALGLLGAGSPLPSWPLPNIGGTEEQQAESEKAPREPLEPQVLQDDLPISLKKVLQTSLPEPLRIKLELDGDSHILELLQNRELVPGRPTLVWYQPDGTRVVSEGHTLENCCYQGRVRGYAGSWVSICTCSGLRGLVVLTPERSYTLEQGPGDLQGPPIISRIQDLHLPGHTCALSWRESVHTQKPPEHPLGQRHIRRRRDVVTETKTVELVIVADHSEAQKYRDFQHLLNRTLEVALLLDTFFRPLNVRVALVGLEAWTQRDLVEISPNPAVTLENFLHWRRAHLLPRLPHDSAQLVTGTSFSGPTVGMAIQNSICSPDFSGGVNMDHSTSILGVASSIAHELGHSLGLDHDLPGNSCPCPGPAPAKTCIMEASTDFLPGLNFSNCSRRALEKALLDGMGSCLFERLPSLPPMAAFCGNMFVEPGEQCDCGFLDDCVDPCCDSLTCQLRPGAQCASDGPCCQNCQLRPSGWQCRPTRGDCDLPEFCPGDSSQCPPDVSLGDGEPCAGGQAVCMHGRCASYAQQCQSLWGPGAQPAAPLCLQTANTRGNAFGSCGRNPSGSYVSCTPRDAICGQLQCQTGRTQPLLGSIRDLLWETIDVNGTELNCSWVHLDLGSDVAQPLLTLPGTACGPGLVCIDHRCQRVDLLGAQECRSKCHGHGVCDSNRHCYCEEGWAPPDCTTQLKATSSLTTGLLLSLLVLLVLVMLGASYWYRARLHQRLCQLKGPTCQYRAAQSGPSERPGPPQRALLARGTKQASALSFPAPPSRPLPPDPVSKRLQAELADRPNPPTRPLPADPVVRSPKSQGPAKPPPPRKPLPADPQGRCPSGDLPGPGAGIPPLVVPSRPAPPPPTVSSLYL

>NP_002077.1

MEAIAKYDFKATADDELSFKRGDILKVLNEECDQNWYKAELNGKDGFIPKNYIEMKPHPWFFGKIPRAKAEEMLSKQRHDGAFLIRESESAPGDFSLSVKFGNDVQHFKVLRDGAGKYFLWVVKFNSLNELVDYHRSTSVSRNQQIFLRDIEQVPQQPTYVQALFDFDPQEDGELGFRRGDFIHVMDNSDPNWWKGACHGQTGMFPRNYVTPVNRNV

1009 NP_004844.1 NP_068375.3

>NP_004844.1

MAPDPWFSTYDSTCQIAQEIAEKIQQRNQYERKGEKAPKLTVTIRALLQNLKEKIALLKDLLLRAVSTHQITQLEGDRRQNLLDDLVTRERLLLASFKNEGAEPDLIRSSLMSEEAKRGAPNPWLFEEPEETRGLGFDEIRQQQQKIIQEQDAGLDALSSIISRQKQMGQEIGNELDEQNEIIDDLANLVENTDEKLRNETRRVNMVDRKSASCGMIMVILLLLVAIVVVAVWPTN

>NP_068375.3

MAAYLQWRRFVFFDKELVKEPLSNDGAAPGATPASGSAASKFLCLPPGITVCDSGRGSLVFGDMEGQIWFLPRSLQLTGFQAYKLRVTHLYQLKQHNILASVGEDEEGINPLVKIWNLEKRDGGNPLCTRIFPAIPGTEPTVVSCLTVHENLNFMAIGFTDGSVTLNKGDITRDRHSKTQILHKGNYPVTGLAFRQAGKTTHLFVVTTENVQSYIVSGKDYPRVELDTHGCGLRCSALSDPSQDLQFIVAGDECVYLYQPDERGPCFAFEGHKLIAHWFRGYLIIVSRDRKVSPKSEFTSRDSQSSDKQILNIYDLCNKFIAYSTVFEDVVDVLAEWGSLYVLTRDGRVHALQEKDTQTKLEMLFKKNLFEMAINLAKSQHLDSDGLAQIFMQYGDHLYSKGNHDGAVQQYIRTIGKLEPSYVIRKFLDAQRIHNLTAYLQTLHRQSLANADHTTLLLNCYTKLKDSSKLEEFIKKKSESEVHFDVETAIKVLRQAGYYSHALYLAENHAHHEWYLKIQLEDIKNYQEALRYIGKLPFEQAESNMKRYGKILMHHIPEQTTQLLKGLCTDYRPSLEGRSDREAPGCRANSEEFIPIFANNPRELKAFLEHMSEVQPDSPQGIYDTLLELRLQNWAHEKDPQVKEKLHAEAISLLKSGRFCDVFDKALVLCQMHDFQDGVLYLYEQGKLFQQIMHYHMQHEQYRQVISVCERHGEQDPSLWEQALSYFARKEEDCKEYVAAVLKHIENKNLMPPLLVVQTLAHNSTATLSVIRDYLVQKLQKQSQQIAQDELRVRRYREETTRIRQEIQELKASPKIFQKTKCSICNSALELPSVHFLCGHSFHQHCFESYSESDADCPTCLPENRKVMDMIRAQEQKRDLHDQFQHQLKCSNDSFSVIADYFGRGVFNKLTLLTDPPTARLTSSLEAGLQRDLLMHSRRGT

1010 NP_001155.1 NP_001008493.1

>NP_001155.1

MSVPSSLSQSAINANSHGGPALSLPLPLHAAHNQLLNAKLQATAVGPKDLRSAMGEGGGPEPGPANAKWLKEGQNQLRRAATAHRDQNRNVTLTLAEEASQEPEMAPLGPKGLIHLYSELELSAHNAANRGLRGPGLIISTQEQGPDEGEEKAAGEAEEEEEDDDDEEEEEDLSSPPGLPEPLESVEAPPRPQALTDGPREHSKSASLLFGMRNSAASDEDSSWATLSQGSPSYGSPEDTDSFWNPNAFETDSDLPAGWMRVQDTSGTYYWHIPTGTTQWEPPGRASPSQGSSPQEESQLTWTGFAHGEGFEDGEFWKDEPSDEAPMELGLKEPEEGTLTFPAQSLSPEPLPQEEEKLPPRNTNPGIKCFAVRSLGWVEMTEEELAPGRSSVAVNNCIRQLSYHKNNLHDPMSGGWGEGKDLLLQLEDETLKLVEPQSQALLHAQPIISIRVWGVGRDSGRERDFAYVARDKLTQMLKCHVFRCEAPAKNIATSLHEICSKIMAERRNARCLVNGLSLDHSKLVDVPFQVEFPAPKNELVQKFQVYYLGNVPVAKPVGVDVINGALESVLSSSSREQWTPSHVSVAPATLTILHQQTEAVLGECRVRFLSFLAVGRDVHTFAFIMAAGPASFCCHMFWCEPNAASLSEAVQAACMLRYQKCLDARSQASTSCLPAPPAESVARRVGWTVRRGVQSLWGSLKPKRLGAHTP

>NP_001008493.1

MSEQSICQARAAVMVYDDANKKWVPAGGSTGFSRVHIYHHTGNNTFRVVGRKIQDHQVVINCAIPKGLKYNQATQTFHQWRDARQVYGLNFGSKEDANVFASAMMHALEVLNSQETGPTLPRQNSQLPAQVQNGPSQEELEIQRRQLQEQQRQKELERERLERERMERERLERERLERERLERERLEQEQLERERQERERQERLERQERLERQERLERQERLDRERQERQERERLERLERERQERERQEQLEREQLEWERERRISSAAAPASVETPLNSVLGDSSASEPGLQAASQPAETPSQQGIVLGPLAPPPPPPLPPGPAQASVALPPPPGPPPPPPLPSTGPPPPPPPPPLPNQVPPPPPPPPAPPLPASGFFLASMSEDNRPLTGLAAAIAGAKLRKVSRMEDTSFPSGGNAIGVNSASSKTDTGRGNGPLPLGGSGLMEEMSALLARRRRIAEKGSTIETEQKEDKGEDSEPVTSKASSTSTPEPTRKPWERTNTMNGSKSPVISRRDSPRKNQIVFDNRSYDSLHRPKSTPLSQPSANGVQTEGLDYDRLKQDILDEMRKELTKLKEELIDAIRQELSKSNTA

1011 NP_005893.1 NP_065162.1

>NP_005893.1

MSSILPFTPPIVKRLLGWKKGEQNGQEEKWCEKAVKSLVKKLKKTGQLDELEKAITTQNVNTKCITIPRSLDGRLQVSHRKGLPHVIYCRLWRWPDLHSHHELRAMELCEFAFNMKKDEVCVNPYHYQRVETPVLPPVLVPRHTEIPAEFPPLDDYSHSIPENTNFPAGIEPQSNIPETPPPGYLSEDGETSDHQMNHSMDAGSPNLSPNPMSPAHNNLDLQPVTYCEPAFWCSISYYELNQRVGETFHASQPSMTVDGFTDPSNSERFCLGLLSNVNRNAAVELTRRHIGRGVRLYYIGGEVFAECLSDSAIFVQSPNCNQRYGWHPATVCKIPPGCNLKIFNNQEFAALLAQSVNQGFEAVYQLTRMCTIRMSFVKGWGAEYRRQTVTSTPCWIELHLNGPLQWLDKVLTQMGSPSIRCSSVS

>NP_065162.1

MSNPGTRRNGSSIKIRLTVLCAKNLAKKDFFRLPDPFAKIVVDGSGQCHSTDTVKNTLDPKWNQHYDLYVGKTDSITISVWNHKKIHKKQGAGFLGCVRLLSNAISRLKDTGYQRLDLCKLNPSDTDAVRGQIVVSLQTRDRIGTGGSVVDCRGLLENEGTVYEDSGPGRPLSCFMEEPAPYTDSTGAAAGGGNCRFVESPSQDQRLQAQRLRNPDVRGSLQTPQNRPHGHQSPELPEGYEQRTTVQGQVYFLHTQTGVSTWHDPRIPSPSGTIPGGDAAFLYEFLLQGHTSEPRDLNSVNCDELGPLPPGWEVRSTVSGRIYFVDHNNRTTQFTDPRLHHIMNHQCQLKEPSQPLPLPSEGSLEDEELPAQRYERDLVQKLKVLRHELSLQQPQAGHCRIEVSREEIFEESYRQIMKMRPKDLKKRLMVKFRGEEGLDYGGVAREWLYLLCHEMLNPYYGLFQYSTDNIYMLQINPDSSINPDHLSYFHFVGRIMGLAVFHGHYINGGFTVPFYKQLLGKPIQLSDLESVDPELHKSLVWILENDITPVLDHTFCVEHNAFGRILQHELKPNGRNVPVTEENKKEYVRLYVNWRFMRGIEAQFLALQKGFNELIPQHLLKPFDQKELELIIGGLDKIDLNDWKSNTRLKHCVADSNIVRWFWQAVETFDEERRARLLQFVTGSTRVPLQGFKALQGSTGAAGPRLFTIHLIDANTDNLPKAHTCFNRIDIPPYESYEKLYEKLLTAVEETCGFAVE

1012 NP_001252.1 NP_002175.2

>NP_001252.1

MAKQYDSVECPFCDEVSKYEKLAKIGQGTFGEVFKARHRKTGQKVALKKVLMENEKEGFPITALREIKILQLLKHENVVNLIEICRTKASPYNRCKGSIYLVFDFCEHDLAGLLSNVLVKFTLSEIKRVMQMLLNGLYYIHRNKILHRDMKAANVLITRDGVLKLADFGLARAFSLAKNSQPNRYTNRVVTLWYRPPELLLGERDYGPPIDLWGAGCIMAEMWTRSPIMQGNTEQHQLALISQLCGSITPEVWPNVDNYELYEKLELVKGQKRKVKDRLKAYVRDPYALDLIDKLLVLDPAQRIDSDDALNHDFFWSDPMPSDLKGMLSTHLTSMFEYLAPPRRKGSQITQQSTNQSRNPATTNQTEFERVF

>NP_002175.2

MLTLQTWLVQALFIFLTTESTGELLDPCGYISPESPVVQLHSNFTAVCVLKEKCMDYFHVNANYIVWKTNHFTIPKEQYTIINRTASSVTFTDIASLNIQLTCNILTFGQLEQNVYGITIISGLPPEKPKNLSCIVNEGKKMRCEWDGGRETHLETNFTLKSEWATHKFADCKAKRDTPTSCTVDYSTVYFVNIEVWVEAENALGKVTSDHINFDPVYKVKPNPPHNLSVINSEELSSILKLTWTNPSIKSVIILKYNIQYRTKDASTWSQIPPEDTASTRSSFTVQDLKPFTEYVFRIRCMKEDGKGYWSDWSEEASGITYEDRPSKAPSFWYKIDPSHTQGYRTVQLVWKTLPPFEANGKILDYEVTLTRWKSHLQNYTVNATKLTVNLTNDRYLATLTVRNLVGKSDAAVLTIPACDFQATHPVMDLKAFPKDNMLWVEWTTPRESVKKYILEWCVLSDKAPCITDWQQEDGTVHRTYLRGNLAESKCYLITVTPVYADGPGSPESIKAYLKQAPPSKGPTVRTKKVGKNEAVLEWDQLPVDVQNGFIRNYTIFYRTIIGNETAVNVDSSHTEYTLSSLTSDTLYMVRMAAYTDEGGKDGPEFTFTTPKFAQGEIEAIVVPVCLAFLLTTLLGVLFCFNKRDLIKKHIWPNVPDPSKSHIAQWSPHTPPRHNFNSKDQMYSDGNFTDVSVVEIEANDKKPFPEDLKSLDLFKKEKINTEGHSSGIGGSSCMSSSRPSISSSDENESSQNTSSTVQYSTVVHSGYRHQVPSVQVFSRSESTQPLLDSEERPEDLQLVDHVDGGDGILPRQQYFKQNCSQHESSPDISHFERSKQVSSVNEEDFVRLKQQISDHISQSCGSGQMKMFQEVSAADAFGPGTEGQVERFETVGMEAATDEGMPKSYLPQTVRQGGYMPQ

1013 NP_055128.1 NP_060745.3

>NP_055128.1

MSGFLEGLRCSECIDWGEKRNTIASIAAGVLFFTGWWIIIDAAVIYPTMKDFNHSYHACGVIATIAFLMINAVSNGQVRGDSYSEGCLGQTGARIWLFVGFMLAFGSLIASMWILFGGYVAKEKDIVYPGIAVFFQNAFIFFGGLVFKFGRTEDLWQ

>NP_060745.3

MESQCDYSMYFPAVPLPPRAELAGDPGRYRALPRRNHLYLGETVRFLLVLRCRGGAGSGTGGGPGLGSRGAWAELATALAALASVSAGGGMPGGGGAGDQDSEPPGGGDPGGGGLFRGCSPLLTHGPGPATSGGATTLPVEEPIVSTDEVIFPLTVSLDRLPPGTPKAKIVVTVWKREIEAPEVRDQGYLRLLQTRSPGETFRGEQSAFKAQVSTLLTLLPPPVLRCRQFTVAGKHLTVLKVLNSSSQEEISIWDIRILPNFNASYLPVMPDGSVLLVDNVCHQSGEVSMGSFCRLPGTSGCFPCPLNALEEHNFLFQLRGGEQPPPGAKEGLEVPLIAVVQWSTPKLPFTQSIYTHYRLPSVRLDRPCFVMTASCKSPVRTYERFTVTYTLLNNLQDFLAVRLVWTPEHAQAGKQLCEEERRAMQAALDSVVCHTPLNNLGFSRKGSALTFSVAFQALRTGLFELSQHMKLKLQFTASVSHPPPEARPLSRKSSPSSPAVRDLVERHQASLGRSQSFSHQQPSRSHLMRSGSVMERRAITPPVASPVGRPLYLPPDKAVLSLDKIAKRECKVLVVEPVK

1014 NP_001194.1 NP_075567.1

>NP_001194.1

MLLRSAGKLNVGTKKEDGESTAPTPRPKVLRCKCHHHCPEDSVNNICSTDGYCFTMIEEDDSGLPVVTSGCLGLEGSDFQCRDTPIPHQRRSIECCTERNECNKDLHPTLPPLKNRDFVDGPIHHRALLISVTVCSLLLVLIILFCYFRYKRQETRPRYSIGLEQDETYIPPGESLRDLIEQSQSSGSGSGLPLLVQRTIAKQIQMVKQIGKGRYGEVWMGKWRGEKVAVKVFFTTEEASWFRETEIYQTVLMRHENILGFIAADIKGTGSWTQLYLITDYHENGSLYDYLKSTTLDAKSMLKLAYSSVSGLCHLHTEIFSTQGKPAIAHRDLKSKNILVKKNGTCCIADLGLAVKFISDTNEVDIPPNTRVGTKRYMPPEVLDESLNRNHFQSYIMADMYSFGLILWEVARRCVSGGIVEEYQLPYHDLVPSDPSYEDMREIVCIKKLRPSFPNRWSSDECLRQMGKLMTECWAHNPASRLTALRVKKTLAKMSESQDIKL

>NP_075567.1

MSIYKEPPPGMFVVPDTVDMTKIHALITGPFDTPYEGGFFLFVFRCPPDYPIHPPRVKLMTTGNNTVRFNPNFYRNGKVCLSILGTWTGPAWSPAQSISSVLISIQSLMTENPYHNEPGFEQERHPGDSKNYNECIRHETIRVAVCDMMEGKCPCPEPLRGVMEKSFLEYYDFYEVACKDRLHLQGQTMQDPFGEKRGHFDYQSLLMRLGLIRQKVLERLHNENAEMDSDSSSSGTETDLHGSLRV

1015 NP_006487.1 NP_001769.2

>NP_006487.1

MGCTLSAEDKAAVERSKMIDRNLREDGEKAAKEVKLLLLGAGESGKSTIVKQMKIIHEDGYSEDECKQYKVVVYSNTIQSIIAIIRAMGRLKIDFGEAARADDARQLFVLAGSAEEGVMTPELAGVIKRLWRDGGVQACFSRSREYQLNDSASYYLNDLDRISQSNYIPTQQDVLRTRVKTTGIVETHFTFKDLYFKMFDVGGQRSERKKWIHCFEGVTAIIFCVALSDYDLVLAEDEEMNRMHESMKLFDSICNNKWFTETSIILFLNKKDLFEEKIKRSPLTICYPEYTGSNTYEEAAAYIQCQFEDLNRRKDTKEIYTHFTCATDTKNVQFVFDAVTDVIIKNNLKECGLY

>NP_001769.2

MCSRGWDSCLALELLLLPLSLLVTSIQGHLVHMTVVSGSNVTLNISESLPENYKQLTWFYTFDQKIVEWDSRKSKYFESKFKGRVRLDPQSGALYISKVQKEDNSTYIMRVLKKTGNEQEWKIKLQVLDPVPKPVIKIEKIEDMDDNCYLKLSCVIPGESVNYTWYGDKRPFPKELQNSVLETTLMPHNYSRCYTCQVSNSVSSKNGTVCLSPPCTLARSFGVEWIASWLVVTVPTILGLLLT

1016 NP_005517.1 NP_006752.1

>NP_005517.1

MDLPVGPGAAGPSNVPAFLTKLWTLVSDPDTDALICWSPSGNSFHVFDQGQFAKEVLPKYFKHNNMASFVRQLNMYGFRKVVHIEQGGLVKPERDDTEFQHPCFLRGQEQLLENIKRKVTSVSTLKSEDIKIRQDSVTKLLTDVQLMKGKQECMDSKLLAMKHENEALWREVASLRQKHAQQQKVVNKLIQFLISLVQSNRILGVKRKIPLMLNDSGSAHSMPKYSRQFSLEHVHGSGPYSAPSPAYSSSSLYAPDAVASSGPIISDITELAPASPMASPGGSIDERPLSSSPLVRVKEEPPSPPQSPRVEEASPGRPSSVDTLLSPTALIDSILRESEPAPASVTALTDARGHTDTEGRPPSPPPTSTPEKCLSVACLDKNELSDHLDAMDSNLDNLQTMLSSHGFSVDTSALLDLFSPSVTVPDMSLPDLDSSLASIQELLSPQEPPRPPEAENSSPDSGKQLVHYTAQPLFLLDPGSVDTGSNDLPVLFELGEGSYFSEGDGFAEDPTISLLTGSEPPKAKDPTVS

>NP_006752.1

MDDREDLVYQAKLAEQAERYDEMVESMKKVAGMDVELTVEERNLLSVAYKNVIGARRASWRIISSIEQKEENKGGEDKLKMIREYRQMVETELKLICCDILDVLDKHLIPAANTGESKVFYYKMKGDYHRYLAEFATGNDRKEAAENSLVAYKAASDIAMTELPPTHPIRLGLALNFSVFYYEILNSPDRACRLAKAAFDDAIAELDTLSEESYKDSTLIMQLLRDNLTLWTSDMQGDGEEQNKEALQDVEDENQ

1017 NP_891549.1 NP_004022.2

>NP_891549.1

MACTGPSLPSAFDILGAAGQDKLLYLKHKLKTPRPGCQGQDLLHAMVLLKLGQETEARISLEALKADAVARLVARQWAGVDSTEDPEEPPDVSWAVARLYHLLAEEKLCPASLRDVAYQEAVRTLSSRDDHRLGELQDEARNRCGWDIAGDPGSIRTLQSNLGCLPPSSALPSGTRSLPRPIDGVSDWSQGCSLRSTGSPASLASNLEISQSPTMPFLSLHRSPHGPSKLCDDPQASLVPEPVPGGCQEPEEMSWPPSGEIASPPELPSSPPPGLPEVAPDATSTGLPDTPAAPETSTNYPVECTEGSAGPQSLPLPILEPVKNPCSVKDQTPLQLSVEDTTSPNTKPCPPTPTTPETSPPPPPPPPSSTPCSAHLTPSSLFPSSLESSSEQKFYNFVILHARADEHIALRVREKLEALGVPDGATFCEDFQVPGRGELSCLQDAIDHSAFIILLLTSNFDCRLSLHQVNQAMMSNLTRQGSPDCVIPFLPLESSPAQLSSDTASLLSGLVRLDEHSQIFARKVANTFKPHRLQARKAMWRKEQDTRALREQSQHLDGERMQAAALNAAYSAYLQSYLSYQAQMEQLQVAFGSHMSFGTGAPYGARMPFGGQVPLGAPPPFPTWPGCPQPPPLHAWQAGTPPPPSPQPAAFPQSLPFPQSPAFPTASPAPPQSPGLQPLIIHHAQMVQLGLNNHMWNQRGSQAPEDKTQEAE

>NP_004022.2

MPVPERPAAGPDSPRPGTRRAAPRVLFGEWLLGEISSGCYEGLQWLDEARTCFRVPWKHFARKDLSEADARIFKAWAVARGRWPPSSRGGGPPPEAETAERAGWKTNFRCALRSTRRFVMLRDNSGDPADPHKVYALSRELCWREGPGTDQTEAEAPAAVPPPQGGPPGPFLAHTHAGLQAPGPLPAPAGDKGDLLLQAVQQSCLADHLLTASWGADPVPTKAPGEGQEGLPLTGACAGGPGLPAGELYGWAVETTPSPGPQPAALTTGEAAAPESPHQAEPYLSPSPSACTAVQEPSPGALDVTIMYKGRTVLQKVVGHPSCTFLYGPPDPAVRATDPQQVAFPSPAELPDQKQLRYTEELLRHVAPGLHLELRGPQLWARRMGKCKVYWEVGGPPGSASPSTPACLLPRNCDTPIFDFRVFFQELVEFRARQRRGSPRYTIYLGFGQDLSAGRPKEKSLVLVKLEPWLCRVHLEGTQREGVSSLDSSSLSLCLSSANSLYDDIECFLMELEQPA

1018 NP_006793.1 NP_006749.1

>NP_006793.1

METILEQQRRYHEEKERLMDVMAKEMLTKKSTLRDQINSDHRTRAMQDRYMEVSGNLRDLYDDKDGLRKEELNAISGPNEFAEFYNRLKQIKEFHRKHPNEICVPMSVEFEELLKARENPSEEAQNLVEFTDEEGYGRYLDLHDCYLKYINLKASEKLDYITYLSIFDQLFDIPKERKNAEYKRYLEMLLEYLQDYTDRVKPLQDQNELFGKIQAEFEKKWENGTFPGWPKETSSALTHAGAHLDLSAFSSWEELASLGLDRLKSALLALGLKCGGTLEERAQRLFSTKGKSLESLDTSLFAKNPKSKGTKRDTERNKDIAFLEAQIYEYVEILGEQRHLTHENVQRKQARTGEEREEEEEEQISESESEDEENEIIYNPKNLPLGWDGKPIPYWLYKLHGLNINYNCEICGNYTYRGPKAFQRHFAEWRHAHGMRCLGIPNTAHFANVTQIEDAVSLWAKLKLQKASERWQPDTEEEYEDSSGNVVNKKTYEDLKRQGLL

>NP_006749.1

MAEYLASIFGTEKDKVNCSFYFKIGACRHGDRCSRLHNKPTFSQTIALLNIYRNPQNSSQSADGLRCAVSDVEMQEHYDEFFEEVFTEMEEKYGEVEEMNVCDNLGDHLVGNVYVKFRREEDAEKAVIDLNNRWFNGQPIHAELSPVTDFREACCRQYEMGECTRGGFCNFMHLKPISRELRRELYGRRRKKHRSRSRSRERRSRSRDRGRGGGGGGGGGGGGRERDRRRSRDRERSGRF

1019 NP_005985.2 NP_004378.1

>NP_005985.2

MAYHPFHAPRPADFPMSAFLAAAQPSFFPALALPPGALAKPLPDPGLAGAAAAAAAAAAAAEAGLHVSALGPHPPAAHLRSLKSLEPEDEVEDDPKVTLEAKELWDQFHKLGTEMVITKSGRRMFPPFKVRVSGLDKKAKYILLMDIVAADDCRYKFHNSRWMVAGKADPEMPKRMYIHPDSPATGEQWMAKPVAFHKLKLTNNISDKHGFTILNSMHKYQPRFHIVRANDILKLPYSTFRTYVFPETDFIAVTAYQNDKITQLKIDNNPFAKGFRDTGNGRREKRKQLTLPSLRLYEEHCKPERDGAESDASSCDPPPAREPPTSPGAAPSPLRLHRARAEEKSCAADSDPEPERLSEERAGAPLGRSPAPDSASPTRLTEPERARERRSPERGKEPAESGGDGPFGLRSLEKERAEARRKDEGRKEAAEGKEQGLAPLVVQTDSASPLGAGHLPGLAFSSHLHGQQFFGPLGAGQPLFLHPGQFTMGPGAFSAMGMGHLLASVAGGGNGGGGGPGTAAGLDAGGLGPAASAASTAAPFPFHLSQHMLASQGIPMPTFGGLFPYPYTYMAAAAAAASALPATSAAAAAAAAAGSLSRSPFLGSARPRLRFSPYQIPVTIPPSTSLLTTGLASEGSKAAGGNSREPSPLPELALRKVGAPSRGALSPSGSAKEAANELQSIQRLVSGLESQRALSPGRESPK

>NP_004378.1

MFPSPALTPTPFSVKDILNLEQQQRSLAAAGELSARLEATLAPSSCMLAAFKPEAYAGPEAAAPGLPELRAELGRAPSPAKCASAFPAAPAFYPRAYSDPDPAKDPRAEKKELCALQKAVELEKTEADNAERPRARRRRKPRVLFSQAQVYELERRFKQQRYLSAPERDQLASVLKLTSTQVKIWFQNRRYKCKRQRQDQTLELVGLPPPPPPPARRIAVPVLVRDGKPCLGDSAPYAPAYGVGLNPYGYNAYPAYPGYGGAACSPGYSCTAAYPAGPSPAQPATAAANNNFVNFGVGDLNAVQSPGIPQSNSGVSTLHGIRAW

1020 NP_006101.1 AAA51946.1

>NP_006101.1

MPKRKVTFQGVGDEEDEDEIIVPKKKLVDPVAGSGGPGSRFKGKHSLDSDEEEDDDDGGSSKYDILASEDVEGQEAATLPSEGGVRITPFNLQEEMEEGHFDADGNYFLNRDAQIRDSWLDNIDWVKIRERPPGQRQASDSEEEDSLGQTSMSAQALLEGLLELLLPRETVAGALRRLGARGGGKGRKGPGQPSSPQRLDRLSGLADQMVARGNLGVYQETRERLAMRLKGLGCQTLGPHNPTPPPSLDMFAEELAEEELETPTPTQRGEAESRGDGLVDVMWEYKWENTGDAELYGPFTSAQMQTWVSEGYFPDGVYCRKLDPPGGQFYNSKRIDFDLYT

>AAA51946.1

MSFPCKFVASFLLIFNVSSKGAVSKEITNALETWGALGQDINLDIPSFQMSDDIDDIKWEKTSDKKKIAQFRKEKETFKEKDTYKLFKNGTLKIKHLKTDDQDIYKVSIYDTKGKNVLEKIFDLKIQERVSKPKISWTCINTTLTCEVMNGTDPELNLYQDGKHLKLSQRVITHKWTTSLSAKFKCTAGNKVSKESSVEPVSCPEKGLDIYLIIGICGGGSLLMVFVALLVFYITKRKKQRSRRNDEELETRAHRVATEERGRKPHQIPASTPQNPATSQHPPPPPGHRSQAPSHRPPPPGHRVQHQPQKRPPAPSGTQVHQQKGPPLPRPRVQPKPPMGQQKTHCPLPLIKKDRNCLFQ

1021 NP_002788.1 NP_002786.2

>NP_002788.1

MALASVLERPLPVNQRGFFGLGGRADLLDLGPGSLSDGLSLAAPGWGVPEEPGIEMLHGTTTLAFKFRHGVIVAADSRATAGAYIASQTVKKVIEINPYLLGTMAGGAADCSFWERLLARQCRIYELRNKERISVAAASKLLANMVYQYKGMGLSMGTMICGWDKRGPGLYYVDSEGNRISGATFSVGSGSVYAYGVMDRGYSYDLEVEQAYDLARRAIYQATYRDAYSGGAVNLYHVREDGWIRVSSDNVADLHEKYSGSTP

>NP_002786.2

MSIMSYNGGAVMAMKGKNCVAIAADRRFGIQAQMVTTDFQKIFPMGDRLYIGLAGLATDVQTVAQRLKFRLNLYELKEGRQIKPYTLMSMVANLLYEKRFGPYYTEPVIAGLDPKTFKPFICSLDLIGCPMVTDDFVVSGTCAEQMYGMCESLWEPNMDPDHLFETISQAMLNAVDRDAVSGMGVIVHIIEKDKITTRTLKARMD

1022 NP_004442.3 NP_006245.2

>NP_004442.3

MSSQVVGIEPLYIKAEPASPDSPKGSSETETEPPVALAPGPAPTRCLPGHKEEEDGEGAGPGEQGGGKLVLSSLPKRLCLVCGDVASGYHYGVASCEACKAFFKRTIQGSIEYSCPASNECEITKRRRKACQACRFTKCLRVGMLKEGVRLDRVRGGRQKYKRRPEVDPLPFPGPFPAGPLAVAGGPRKTAAPVNALVSHLLVVEPEKLYAMPDPAGPDGHLPAVATLCDLFDREIVVTISWAKSIPGFSSLSLSDQMSVLQSVWMEVLVLGVAQRSLPLQDELAFAEDLVLDEEGARAAGLGELGAALLQLVRRLQALRLEREEYVLLKALALANSDSVHIEDAEAVEQLREALHEALLEYEAGRAGPGGGAERRRAGRLLLTLPLLRQTAGKVLAHFYGVKLEGKVPMHKLFLEMLEAMMD

>NP_006245.2

MAPFLRIAFNSYELGSLQAEDEANQPFCAVKMKEALSTERGKTLVQKKPTMYPEWKSTFDAHIYEGRVIQIVLMRAAEEPVSEVTVGVSVLAERCKKNNGKAEFWLDLQPQAKVLMSVQYFLEDVDCKQSMRSEDEAKFPTMNRRGAIKQAKIHYIKNHEFIATFFGQPTFCSVCKDFVWGLNKQGYKCRQCNAAIHKKCIDKIIGRCTGTAANSRDTIFQKERFNIDMPHRFKVHNYMSPTFCDHCGSLLWGLVKQGLKCEDCGMNVHHKCREKVANLCGINQKLLAEALNQVTQRASRRSDSASSEPVGIYQGFEKKTGVAGEDMQDNSGTYGKIWEGSSKCNINNFIFHKVLGKGSFGKVLLGELKGRGEYFAIKALKKDVVLIDDDVECTMVEKRVLTLAAENPFLTHLICTFQTKDHLFFVMEFLNGGDLMYHIQDKGRFELYRATFYAAEIMCGLQFLHSKGIIYRDLKLDNVLLDRDGHIKIADFGMCKENIFGESRASTFCGTPDYIAPEILQGLKYTFSVDWWSFGVLLYEMLIGQSPFHGDDEDELFESIRVDTPHYPRWITKESKDILEKLFEREPTKRLGVTGNIKIHPFFKTINWTLLEKRRLEPPFRPKVKSPRDYSNFDQEFLNEKARLSYSDKNLIDSMDQSAFAGFSFVNPKFEHLLED

1023 NP_005553.1 NP_006120.1

>NP_005553.1

MPALWLGCCLCFSLLLPAARATSRREVCDCNGKSRQCIFDRELHRQTGNGFRCLNCNDNTDGIHCEKCKNGFYRHRERDRCLPCNCNSKGSLSARCDNSGRCSCKPGVTGARCDRCLPGFHMLTDAGCTQDQRLLDSKCDCDPAGIAGPCDAGRCVCKPAVTGERCDRCRSGYYNLDGGNPEGCTQCFCYGHSASCRSSAEYSVHKITSTFHQDVDGWKAVQRNGSPAKLQWSQRHQDVFSSAQRLDPVYFVAPAKFLGNQQVSYGQSLSFDYRVDRGGRHPSAHDVILEGAGLRITAPLMPLGKTLPCGLTKTYTFRLNEHPSNNWSPQLSYFEYRRLLRNLTALRIRATYGEYSTGYIDNVTLISARPVSGAPAPWVEQCICPVGYKGQFCQDCASGYKRDSARLGPFGTCIPCNCQGGGACDPDTGDCYSGDENPDIECADCPIGFYNDPHDPRSCKPCPCHNGFSCSVMPETEEVVCNNCPPGVTGARCELCADGYFGDPFGEHGPVRPCQPCQCNNNVDPSASGNCDRLTGRCLKCIHNTAGIYCDQCKAGYFGDPLAPNPADKCRACNCNPMGSEPVGCRSDGTCVCKPGFGGPNCEHGAFSCPACYNQVKIQMDQFMQQLQRMEALISKAQGGDGVVPDTELEGRMQQAEQALQDILRDAQISEGASRSLGLQLAKVRSQENSYQSRLDDLKMTVERVRALGSQYQNRVRDTHRLITQMQLSLAESEASLGNTNIPASDHYVGPNGFKSLAQEATRLAESHVESASNMEQLTRETEDYSKQALSLVRKALHEGVGSGSGSPDGAVVQGLVEKLEKTKSLAQQLTREATQAEIEADRSYQHSLRLLDSVSRLQGVSDQSFQVEEAKRIKQKADSLSTLVTRHMDEFKRTQKNLGNWKEEAQQLLQNGKSGREKSDQLLSRANLAKSRAQEALSMGNATFYEVESILKNLREFDLQVDNRKAEAEEAMKRLSYISQKVSDASDKTQQAERALGSAAADAQRAKNGAGEALEISSEIEQEIGSLNLEANVTADGALAMEKGLASLKSEMREVEGELERKELEFDTNMDAVQMVITEAQKVDTRAKNAGVTIQDTLNTLDGLLHLMDQPLSVDEEGLVLLEQKLSRAKTQINSQLRPMMSELEERARQQRGHLHLLETSIDGILADVKNLENIRDNLPPGCYNTQALEQQ

>NP_006120.1

MPGVARLPLLLGLLLLPRPGRPLDLADYTYDLAEEDDSEPLNYKDPCKAAAFLGDIALDEEDLRAFQVQQAVDLRRHTARKSSIKAAVPGNTSTPSCQSTNGQPQRGACGRWRGRSRSRRAATSRPERVWPDGVIPFVIGGNFTGSQRAVFRQAMRHWEKHTCVTFLERTDEDSYIVFTYRPCGCCSYVGRRGGGPQAISIGKNCDKFGIVVHELGHVVGFWHEHTRPDRDRHVSIVRENIQPGQEYNFLKMEPQEVESLGETYDFDSIMHYARNTFSRGIFLDTIVPKYEVNGVKPPIGQRTRLSKGDIAQARKLYKCPACGETLQDSTGNFSSPEYPNGYSAHMHCVWRISVTPGEKIILNFTSLDLYRSRLCWYDYVEVRDGFWRKAPLRGRFCGSKLPEPIVSTDSRLWVEFRSSSNWVGKGFFAVYEAICGGDVKKDYGHIQSPNYPDDYRPSKVCIWRIQVSEGFHVGLTFQSFEIERHDSCAYDYLEVRDGHSESSTLIGRYCGYEKPDDIKSTSSRLWLKFVSDGSINKAGFAVNFFKEVDECSRPNRGGCEQRCLNTLGSYKCSCDPGYELAPDKRRCEAACGGFLTKLNGSITSPGWPKEYPPNKNCIWQLVAPTQYRISLQFDFFETEGNDVCKYDFVEVRSGLTADSKLHGKFCGSEKPEVITSQYNNMRVEFKSDNTVSKKGFKAHFFSDKDECSKDNGGCQQDCVNTFGSYECQCRSGFVLHDNKHDCKEAGCDHKVTSTSGTITSPNWPDKYPSKKECTWAISSTPGHRVKLTFMEMDIESQPECAYDHLEVFDGRDAKAPVLGRFCGSKKPEPVLATGSRMFLRFYSDNSVQRKGFQASHATECGGQVRADVKTKDLYSHAQFGDNNYPGGVDCEWVIVAEEGYGVELVFQTFEVEEETDCGYDYMELFDGYDSTAPRLGRYCGSGPPEEVYSAGDSVLVKFHSDDTITKKGFHLRYTSTKFQDTLHSRK

1024 P07910 NP_057619.1

>P07910

MASNVTNKTDPRSMNSRVFIGNLNTLVVKKSDVEAIFSKYGKIVGCSVHKGFAFVQYVNERNARAAVAGEDGRMIAGQVLDINLAAEPKVNRGKAGVKRSAAEMYGSVTEHPSPSPLLSSSFDLDYDFQRDYYDRMYSYPARVPPPPPIARAVVPSKRQRVSGNTSRRGKSGFNSKSGQRGSSKSGKLKGDDLQAIKKELTQIKQKVDSLLENLEKMEKEQSKQAVEMKNDKSEEEQSSSSVKKDETNVKMESEGGADDSAEEGDLLDDDDNEDRGDDQLELIKDDEKEAEEGEDDRDSANGEDDS

>NP_057619.1

MLVLPSPCPQPLAFSSVETMEGPPRRTCRSPEPGPSSSIGSPQASSPPRPNHYLLIDTQGVPYTVLVDEESQREPGASGAPGQKKCYSCPVCSRVFEYMSYLQRHSITHSEVKPFECDICGKAFKRASHLARHHSIHLAGGGRPHGCPLCPRRFRDAGELAQHSRVHSGERPFQCPHCPRRFMEQNTLQKHTRWKHP

1025 NP_001886.1 NP_000629.3

>NP_001886.1

MSGPVPSRARVYTDVNTHRPREYWDYESHVVEWGNQDDYQLVRKLGRGKYSEVFEAINITNNEKVVVKILKPVKKKKIKREIKILENLRGGPNIITLADIVKDPVSRTPALVFEHVNNTDFKQLYQTLTDYDIRFYMYEILKALDYCHSMGIMHRDVKPHNVMIDHEHRKLRLIDWGLAEFYHPGQEYNVRVASRYFKGPELLVDYQMYDYSLDMWSLGCMLASMIFRKEPFFHGHDNYDQLVRIAKVLGTEDLYDYIDKYNIELDPRFNDILGRHSRKRWERFVHSENQHLVSPEALDFLDKLLRYDHQSRLTAREAMEHPYFYTVVKDQARMGSSSMPGGSTPVSSANMMSGISSVPTPSPLGPLAGSPVIAAANPLGMPVPAAAGAQQ

>NP_000629.3

MAPLRPLLILALLAWVALADQESCKGRCTEGFNVDKKCQCDELCSYYQSCCTDYTAECKPQVTRGDVFTMPEDEYTVYDDGEEKNNATVHEQVGGPSLTSDLQAQSKGNPEQTPVLKPEEEAPAPEVGASKPEGIDSRPETLHPGRPQPPAEEELCSGKPFDAFTDLKNGSLFAFRGQYCYELDEKAVRPGYPKLIRDVWGIEGPIDAAFTRINCQGKTYLFKGSQYWRFEDGVLDPDYPRNISDGFDGIPDNVDAALALPAHSYSGRERVYFFKGKQYWEYQFQHQPSQEECEGSSLSAVFEHFAMMQRDSWEDIFELLFWGRTSAGTRQPQFISRDWHGVPGQVDAAMAGRIYISGMAPRPSLAKKQRFRHRNRKGYRSQRGHSRGRNQNSRRPSRATWLSLFSSEESNLGANNYDDYRMDWLVPATCEPIQSVFFFSGDKYYRVNLRTRRVDTVDPPYPRSIAQYWLGCPAPGHL

1026 NP_001035948.1 NP_031375.3

>NP_001035948.1

MATFPPATSAPQQPPGPEDEDSSLDESDLYSLAHSYLGPLIMPMPTSPLTPALVTGGGGRKGRTKREAAANTNRPSPGGHERKLVTKLQNSERKKRGARR

>NP_031375.3

MSDQAPKVPEEMFREVKYYAVGDIDPQVIQLLKAGKAKEVSYNALASHIISEDGDNPEVGEAREVFDLPVVKPSWVILSVQCGTLLPVNGFSPESCQIFFGITACLSQVSSEDRSALWALVTFYGGDCQLTLNKKCTHLIVPEPKGEKYECALKRASIKIVTPDWVLDCVSEKTKKDEAFYHPRLIIYEEEEEEEEEEEEVENEEQDSQNEGSTDEKSSPASSQEGSPSGDQQFSPKSNTEKSKGELMFDDSSDSSPEKQERNLNWTPAEVPQLAAAKRRLPQGKEPGLINLCANVPPVPGNILPPEVRGNLMAAGQNLQSSERSEMIATWSPAVRTLRNITNNADIQQMNRPSNVAHILQTLSAPTKNLEQQVNHSQQGHTNANAVLFSQVKVTPETHMLQQQQQAQQQQQQHPVLHLQPQQIMQLQQQQQQQISQQPYPQQPPHPFSQQQQQQQQAHPHQFSQQQLQFPQQQLHPPQQLHRPQQQLQPFQQQHALQQQFHQLQQHQLQQQQLAQLQQQHSLLQQQQQQQIQQQQLQRMHQQQQQQQMQSQTAPHLSQTSQALQHQVPPQQPPQQQQQQQPPPSPQQHQLFGHDPAVEIPEEGFLLGCVFAIADYPEQMSDKQLLATWKRIIQAHGGTVDPTFTSRCTHLLCESQVSSAYAQAIRERKRCVTAHWLNTVLKKKKMVPPHRALHFPVAFPPGGKPCSQHIISVTGFVDSDRDDLKLMAYLAGAKYTGYLCRSNTVLICKEPTGLKYEKAKEWRIPCVNAQWLGDILLGNFEALRQIQYSRYTAFSLQDPFAPTQHLVLNLLDAWRVPLKVSAELLMSIRLPPKLKQNEVANVQPSSKRARIEDVPPPTKKLTPELTPFVLFTGFEPVQVQQYIKKLYILGGEVAESAQKCTHLIASKVTRTVKFLTAISVVKHIVTPEWLEECFRCQKFIDEQNYILRDAEAEVLFSFSLEESLKRAHVSPLFKAKYFYITPGICPSLSTMKAIVECAGGKVLSKQPSFRKLMEHKQNSSLSEIILISCENDLHLCREYFARGIDVHNAEFVLTGVLTQTLDYESYKFN

1027 NP_004210.1 NP_001246.2

>NP_004210.1

MATLIYVDKENGEPGTRVVAKDGLKLGSGPSIKALDGRSQVSTPRFGKTFDAPPALPKATRKALGTVNRATEKSVKTKGPLKQKQPSFSAKKMTEKTVKAKSSVPASDDAYPEIEKFFPFNPLDFESFDLPEEHQIAHLPLSGVPLMILDEERELEKLFQLGPPSPVKMPSPPWESNLLQSPSSILSTLDVELPPVCCDIDI

>NP_001246.2

MAQFAFESDLHSLLQLDAPIPNAPPARWQRKAKEAAGPAPSPMRAANRSHSAGRTPGRTPGKSSSKVQTTPSKPGGDRYIPHRSAAQMEVASFLLSKENQPENSQTPTKKEHQKAWALNLNGFDVEEAKILRLSGKPQNAPEGYQNRLKVLYSQKATPGSSRKTCRYIPSLPDRILDAPEIRNDYYLNLVDWSSGNVLAVALDNSVYLWSASSGDILQLLQMEQPGEYISSVAWIKEGNYLAVGTSSAEVQLWDVQQQKRLRNMTSHSARVGSLSWNSYILSSGSRSGHIHHHDVRVAEHHVATLSGHSQEVCGLRWAPDGRHLASGGNDNLVNVWPSAPGEGGWVPLQTFTQHQGAVKAVAWCPWQSNVLATGGGTSDRHIRIWNVCSGACLSAVDAHSQVCSILWSPHYKELISGHGFAQNQLVIWKYPTMAKVAELKGHTSRVLSLTMSPDGATVASAAADETLRLWRCFELDPARRREREKASAAKSSLIHQGIR

1028 NP_000044.2 NP_004036.1

>NP_000044.2

MPEQERQITAREGASRKILSKLSLPTRAWEPAMKKSFAFDNVGYEGGLDGLGPSSQVATSTVRILGMTCQSCVKSIEDRISNLKGIISMKVSLEQGSATVKYVPSVVCLQQVCHQIGDMGFEASIAEGKAASWPSRSLPAQEAVVKLRVEGMTCQSCVSSIEGKVRKLQGVVRVKVSLSNQEAVITYQPYLIQPEDLRDHVNDMGFEAAIKSKVAPLSLGPIDIERLQSTNPKRPLSSANQNFNNSETLGHQGSHVVTLQLRIDGMHCKSCVLNIEENIGQLLGVQSIQVSLENKTAQVKYDPSCTSPVALQRAIEALPPGNFKVSLPDGAEGSGTDHRSSSSHSPGSPPRNQVQGTCSTTLIAIAGMTCASCVHSIEGMISQLEGVQQISVSLAEGTATVLYNPSVISPEELRAAIEDMGFEASVVSESCSTNPLGNHSAGNSMVQTTDGTPTSVQEVAPHTGRLPANHAPDILAKSPQSTRAVAPQKCFLQIKGMTCASCVSNIERNLQKEAGVLSVLVALMAGKAEIKYDPEVIQPLEIAQFIQDLGFEAAVMEDYAGSDGNIELTITGMTCASCVHNIESKLTRTNGITYASVALATSKALVKFDPEIIGPRDIIKIIEEIGFHASLAQRNPNAHHLDHKMEIKQWKKSFLCSLVFGIPVMALMIYMLIPSNEPHQSMVLDHNIIPGLSILNLIFFILCTFVQLLGGWYFYVQAYKSLRHRSANMDVLIVLATSIAYVYSLVILVVAVAEKAERSPVTFFDTPPMLFVFIALGRWLEHLAKSKTSEALAKLMSLQATEATVVTLGEDNLIIREEQVPMELVQRGDIVKVVPGGKFPVDGKVLEGNTMADESLITGEAMPVTKKPGSTVIAGSINAHGSVLIKATHVGNDTTLAQIVKLVEEAQMSKAPIQQLADRFSGYFVPFIIIMSTLTLVVWIVIGFIDFGVVQRYFPNPNKHISQTEVIIRFAFQTSITVLCIACPCSLGLATPTAVMVGTGVAAQNGILIKGGKPLEMAHKIKTVMFDKTGTITHGVPRVMRVLLLGDVATLPLRKVLAVVGTAEASSEHPLGVAVTKYCKEELGTETLGYCTDFQAVPGCGIGCKVSNVEGILAHSERPLSAPASHLNEAGSLPAEKDAVPQTFSVLIGNREWLRRNGLTISSDVSDAMTDHEMKGQTAILVAIDGVLCGMIAIADAVKQEAALAVHTLQSMGVDVVLITGDNRKTARAIATQVGINKVFAEVLPSHKVAKVQELQNKGKKVAMVGDGVNDSPALAQADMGVAIGTGTDVAIEAADVVLIRNDLLDVVASIHLSKRTVRRIRINLVLALIYNLVGIPIAAGVFMPIGIVLQPWMGSAAMAASSVSVVLSSLQLKCYKKPDLERYEAQAHGHMKPLTASQVSVHIGMDDRWRDSPRATPWDQVSYVSQVSLSSLTSDKPSRHSAAADDDGDKWSLLLNGRDEEQYI

>NP_004036.1

MPKHEFSVDMTCGGCAEAVSRVLNKLGGVKYDIDLPNKKVCIESEHSMDTLLATLKKTGKTVSYLGLE

1029 AAH07560.1 NP_005577.1

>AAH07560.1

MNPNCARCGKIVYPTEKVNCLDKFWHKACFHCETCKMTLNMKNYKGYEKKPYCNAHYPKQSFTMVADTPENLRLKQQSELQSQVRYKEEFEKNKGKGFSVVADTPELQRIKKTQDQISNIKYHEEFEKSRMGPSGGEGMEPERRDSQDGSSYRRPLEQQQPHHIPTSAPVYQQPQQQPVAQSYGGYKEPAAPVSIQRSAPICLQHIPRHRIRPGRDPSILQCLCFLKPATACDSYPSSSFFCQLKPSSATSAGSLLWQASPLIDFLVFSLDGTGMGLSGGGRGPWGRAGMGDLLACGPHLPLCSLPSHPPAQLLTYPHIPGLG

>NP_005577.1

MYQVSGQRPSGCDAPYGAPSAAPGPAQTLSLLPGLEVVTGSTHPAEAAPEEGSLEEAATPMPQGNGPGIPQGLDSTDLDVPTEAVTCQPQGNPLGCTPLLPNDSGHPSELGGTRRAGNGALGGPKAHRKLQTHPSLASQGSKKSKSSSKSTTSQIPLQAQEDCCVHCILSCLFCEFLTLCNIVLDCATCGSCSSEDSCLCCCCCGSGECADCDLPCDLDCGILDACCESADCLEICMECCGLCFSS

1030 NP_005376.2 NP_006702.1

>NP_005376.2

MGAQDRPQCHFDIEINREPVGRIMFQLFSDICPKTCKNFLCLCSGEKGLGKTTGKKLCYKGSTFHRVVKNFMIQGGDFSEGNGKGGESIYGGYFKDENFILKHDRAFLLSMANRGKHTNGSQFFITTKPAPHLDGVHVVFGLVISGFEVIEQIENLKTDAASRPYADVRVIDCGVLATKSIKDVFEKKRKKPTHSEGSDSSSNSSSSSESSSESELEHERSRRRKHKRRPKVKRSKKRRKEASSSEEPRNKHAMNPKGHSERSDTNEKRSVDSSAKREKPVVRPEEIPPVPENRFLLRRDMPVVTAEPEPKIPDVAPIVSDQKPSVSKSGRKIKGRGTIRYHTPPRSRSCSESDDDDSSETPPHWKEEMQRLRAYRPPSGEKWSKGDKLSDPCSSRWDERSLSQRSRSWSYNGYYSDLSTARHSGHHKKRRKEKKVKHKKKGKKQKHCRRHKQTKKRRILIPSDIESSKSSTRRMKSSCDRERSSRSSSLSSHHSSKRDWSKSDKDVQSSLTHSSRDSYRSKSHSQSYSRGSSRSRTASKSSSHSRSRSKSRSSSKSGHRKRASKSPRKTASQLSENKPVKTEPLRATMAQNENVVVQPVVAENIPVIPLSDSPPPSRWKPGQKPWKPSYERIQEMKAKTTHLLPIQSTYSLANIKETGSSSSYHKREKNSESDQSTYSKYSDRSSESSPRSRSRSSRSRSYSRSYTRSRSLASSHSRSRSPSSRSHSRNKYSDHSQCSRSSSYTSISSDDGRRAKRRLRSSGKKNSVSHKKHSSSSEKTLHSKYVKGRDRSSCVRKYSESRSSLDYSSDSEQSSVQATQSAQEKEKQGQMERTHNKQEKNRGEEKSKSERECPHSKKRTLKENLSDHLRNGSKPKRKNYAGSKWDSESNSERDVTKNSKNDSHPSSDKEEGEATSDSESEVSEIHIKVKPTTKSSTNTSLPDDNGAWKSSKQRTSTSDSEGSCSNSENNRGKPQKHKHGSKENLKREHTKKVKEKLKGKKDKKHKAPKRKQAFHWQPPLEFGEEEEEEIDDKQVTQESKEKKVSENNETIKDNILKTEKSSEEDLSGKHDTVTVSSDLDQFTKDDSKLSISPTALNTEENVACLQNIQHVEESVPNGVEDVLQTDDNMEICTPDRSSPAKVEETSPLGNARLDTPDINIVLKQDMATEHPQAEVVKQESSMSESKVLGEVGKQDSSSASLASAGESTGKKEVAEKSQINLIDKKWKPLQGVGNLAAPNAATSSAVEVKVLTTVPEMKPQGLRIEIKSKNKVRPGSLFDEVRKTARLNRRPRNQESSSDEQTPSRDDDSQSRSPSRSRSKSETKSRHRTRSVSYSHSRSRSRSSTSSYRSRSYSRSRSRGWYSRGRTRSRSSSYRSYKSHRTSSRSRSRSSSYDPHSRSRSYTYDSYYSRSRSRSRSQRSDSYHRGRSYNRRSRSCRSYGSDSESDRSYSHHRSPSESSRYS

>NP_006702.1

MDLSGVKKKSLLGVKENNKKSSTRAPSPTKRKDRSDEKSKDRSKDKGATKESSEKDRGRDKTRKRRSASSGSSSTRSRSSSTSSSGSSTSTGSSSGSSSSSASSRSGSSSTSRSSSSSSSSGSPSPSRRRHDNRRRSRSKSKPPKRDEKERKRRSPSPKPTKVHIGRLTRNVTKDHIMEIFSTYGKIKMIDMPVERMHPHLSKGYAYVEFENPDEAEKALKHMDGGQIDGQEITATAVLAPWPRPPPRRFSPPRRMLPPPPMWRRSPPRMRRRSRSPRRRSPVRRRSRSPGRRRHRSRSSSNSSR

1031 NP_000307.1 NP_003396.1

>NP_000307.1

MGTARIAPGLALLLCCPVLSSAYALVDADDVMTKEEQIFLLHRAQAQCEKRLKEVLQRPASIMESDKGWTSASTSGKPRKDKASGKLYPESEEDKEAPTGSRYRGRPCLPEWDHILCWPLGAPGEVVAVPCPDYIYDFNHKGHAYRRCDRNGSWELVPGHNRTWANYSECVKFLTNETREREVFDRLGMIYTVGYSVSLASLTVAVLILAYFRRLHCTRNYIHMHLFLSFMLRAVSIFVKDAVLYSGATLDEAERLTEEELRAIAQAPPPPATAAAGYAGCRVAVTFFLYFLATNYYWILVEGLYLHSLIFMAFFSEKKYLWGFTVFGWGLPAVFVAVWVSVRATLANTGCWDLSSGNKKWIIQVPILASIVLNFILFINIVRVLATKLRETNAGRCDTRQQYRKLLKSTLVLMPLFGVHYIVFMATPYTEVSGTLWQVQMHYEMLFNSFQGFFVAIIYCFCNGEVQAEIKKSWSRWTLALDFKRKARSGSSSYSYGPMVSHTSVTNVGPRVGLGLPLSPRLLPTATTNGHPQLPGHAKPGTPALETLETTPPAMAAPKDDGFLNGSCSGLDEEASGPERPPALLQEEWETVM

>NP_003396.1

MGDREQLLQRARLAEQAERYDDMASAMKAVTELNEPLSNEDRNLLSVAYKNVVGARRSSWRVISSIEQKTMADGNEKKLEKVKAYREKIEKELETVCNDVLSLLDKFLIKNCNDFQYESKVFYLKMKGDYYRYLAEVASGEKKNSVVEASEAAYKEAFEISKEQMQPTHPIRLGLALNFSVFYYEIQNAPEQACLLAKQAFDDAIAELDTLNEDSYKDSTLIMQLLRDNLTLWTSDQQDEEAGEGN

1032 NP_002512.1 NP_000899.1

>NP_002512.1

MDPQTAPSRALLLLLFLHLAFLGGRSHPLGSPGSASDLETSGLQEQRNHLQGKLSELQVEQTSLEPLQESPRPTGVWKSREVATEGIRGHRKMVLYTLRAPRSPKMVQGSGCFGRKMDRISSSSGLGCKVLRRH

>NP_000899.1

MPSLLVLTFSPCVLLGWALLAGGTGGGGVGGGGGGAGIGGGRQEREALPPQKIEVLVLLPQDDSYLFSLTRVRPAIEYALRSVEGNGTGRRLLPPGTRFQVAYEDSDCGNRALFSLVDRVAAARGAKPDLILGPVCEYAAAPVARLASHWDLPMLSAGALAAGFQHKDSEYSHLTRVAPAYAKMGEMMLALFRHHHWSRAALVYSDDKLERNCYFTLEGVHEVFQEEGLHTSIYSFDETKDLDLEDIVRNIQASERVVIMCASSDTIRSIMLVAHRHGMTSGDYAFFNIELFNSSSYGDGSWKRGDKHDFEAKQAYSSLQTVTLLRTVKPEFEKFSMEVKSSVEKQGLNMEDYVNMFVEGFHDAILLYVLALHEVLRAGYSKKDGGKIIQQTWNRTFEGIAGQVSIDANGDRYGDFSVIAMTDVEAGTQEVIGDYFGKEGRFEMRPNVKYPWGPLKLRIDENRIVEHTNSSPCKSCGLEESAVTGIVVGALLGAGLLMAFYFFRKKYRITIERRTQQEESNLGKHRELREDSIRSHFSVA

1033 NP_066983.1 NP_112420.1

>NP_066983.1

MAKLLSCVLGPRLYKIYRERDSERAPASVPETPTAVTAPHSSSWDTYYQPRALEKHADSILALASVFWSISYYSSPFAFFYLYRKGYLSLSKVVPFSHYAGTLLLLLAGVACLRGIGRWTNPQYRQFITILEATHRNQSSENKRQLANYNFDFRSWPVDFHWEEPSSRKESRGGPSRRGVALLRPEPLHRGTADTLLNRVKKLPCQITSYLVAHTLGRRMLYPGSVYLLQKALMPVLLQGQARLVEECNGRRAKLLACDGNEIDTMFVDRRGTAEPQGQKLVICCEGNAGFYEVGCVSTPLEAGYSVLGWNHPGFAGSTGVPFPQNEANAMDVVVQFAIHRLGFQPQDIIIYAWSIGGFTATWAAMSYPDVSAMILDASFDDLVPLALKVMPDSWRGLVTRTVRQHLNLNNAEQLCRYQGPVLLIRRTKDEIITTTVPEDIMSNRGNDLLLKLLQHRYPRVMAEEGLRVVRQWLEASSQLEEASIYSRWEVEEDWCLSVLRSYQAEHGPDFPWSVGEDMSADGRRQLALFLARKHLHNFEATHCTPLPAQNFQMPWHL

>NP_112420.1

MSKSESPKEPEQLRKLFIGGLSFETTDESLRSHFEQWGTLTDCVVMRDPNTKRSRGFGFVTYATVEEVDAAMNARPHKVDGRVVEPKRAVSREDSQRPGAHLTVKKIFVGGIKEDTEEHHLRDYFEQYGKIEVIEIMTDRGSGKKRGFAFVTFDDHDSVDKIVIQKYHTVNGHNCEVRKALSKQEMASASSSQRGRSGSGNFGGGRGGGFGGNDNFGRGGNFSGRGGFGGSRGGGGYGGSGDGYNGFGNDGGYGGGGPGYSGGSRGYGSGGQGYGNQGSGYGGSGSYDSYNNGGGGGFGGGSGSNFGGGGSYNDFGNYNNQSSNFGPMKGGNFGGRSSGPYGGGGQYFAKPRNQGGYGGSSSSSSYGSGRRF

1034 NP_001895.1 NP_002948.1

>NP_001895.1

MATQADLMELDMAMEPDRKAAVSHWQQQSYLDSGIHSGATTTAPSLSGKGNPEEEDVDTSQVLYEWEQGFSQSFTQEQVADIDGQYAMTRAQRVRAAMFPETLDEGMQIPSTQFDAAHPTNVQRLAEPSQMLKHAVVNLINYQDDAELATRAIPELTKLLNDEDQVVVNKAAVMVHQLSKKEASRHAIMRSPQMVSAIVRTMQNTNDVETARCTAGTLHNLSHHREGLLAIFKSGGIPALVKMLGSPVDSVLFYAITTLHNLLLHQEGAKMAVRLAGGLQKMVALLNKTNVKFLAITTDCLQILAYGNQESKLIILASGGPQALVNIMRTYTYEKLLWTTSRVLKVLSVCSSNKPAIVEAGGMQALGLHLTDPSQRLVQNCLWTLRNLSDAATKQEGMEGLLGTLVQLLGSDDINVVTCAAGILSNLTCNNYKNKMMVCQVGGIEALVRTVLRAGDREDITEPAICALRHLTSRHQEAEMAQNAVRLHYGLPVVVKLLHPPSHWPLIKATVGLIRNLALCPANHAPLREQGAIPRLVQLLVRAHQDTQRRTSMGGTQQQFVEGVRMEEIVEGCTGALHILARDVHNRIVIRGLNTIPLFVQLLYSPIENIQRVAAGVLCELAQDKEAAEAIEAEGATAPLTELLHSRNEGVATYAAAVLFRMSEDKPQDYKKRLSVELTSSLFRTEPMAWNETADLGLDIGAQGEPLGYRQDDPSYRSFHSGGYGQDALGMDPMMEHEMGGHHPGADYPVDGLPDLGHAQDLMDGLPPGDSNQLAWFDTDL

>NP_002948.1

MDTKHFLPLDFSTQVNSSLTSPTGRGSMAAPSLHPSLGPGIGSPGQLHSPISTLSSPINGMGPPFSVISSPMGPHSMSVPTTPTLGFSTGSPQLSSPMNPVSSSEDIKPPLGLNGVLKVPAHPSGNMASFTKHICAICGDRSSGKHYGVYSCEGCKGFFKRTVRKDLTYTCRDNKDCLIDKRQRNRCQYCRYQKCLAMGMKREAVQEERQRGKDRNENEVESTSSANEDMPVERILEAELAVEPKTETYVEANMGLNPSSPNDPVTNICQAADKQLFTLVEWAKRIPHFSELPLDDQVILLRAGWNELLIASFSHRSIAVKDGILLATGLHVHRNSAHSAGVGAIFDRVLTELVSKMRDMQMDKTELGCLRAIVLFNPDSKGLSNPAEVEALREKVYASLEAYCKHKYPEQPGRFAKLLLRLPALRSIGLKCLEHLFFFKLIGDTPIDTFLMEMLEAPHQMT

1035 NP_000570.1 NP_002976.2

>NP_000570.1

MDYQVSSPIYDINYYTSEPCQKINVKQIAARLLPPLYSLVFIFGFVGNMLVILILINCKRLKSMTDIYLLNLAISDLFFLLTVPFWAHYAAAQWDFGNTMCQLLTGLYFIGFFSGIFFIILLTIDRYLAVVHAVFALKARTVTFGVVTSVITWVVAVFASLPGIIFTRSQKEGLHYTCSSHFPYSQYQFWKNFQTLKIVILGLVLPLLVMVICYSGILKTLLRCRNEKKRHRAVRLIFTIMIVYFLFWAPYNIVLLLNTFQEFFGLNNCSSSNRLDQAMQVTETLGMTHCCINPIIYAFVGEKFRNYLLVFFQKHIAKRFCKCCSIFQQEAPERASSVYTRSTGEQEISVGL

>NP_002976.2

MKVSAAALAVILIATALCAPASASPYSSDTTPCCFAYIARPLPRAHIKEYFYTSGKCSNPAVVFVTRKNRQVCANPEKKWVREYINSLEMS

1036 NP_037380.1 NP_003391.1

>NP_037380.1

MASVDFKTYVDQACRAAEEFVNVYYTTMDKRRRLLSRLYMGTATLVWNGNAVSGQESLSEFFEMLPSSEFQISVVDCQPVHDEATPSQTTVLVVICGSVKFEGNKQRDFNQNFILTAQASPSNTVWKIASDCFRFQDWAS

>NP_003391.1

MPAIMTMLADHAARQLLDFSQKLDINLLDNVVNCLYHGEGAQQRMAQEVLTHLKEHPDAWTRVDTILEFSQNMNTKYYGLQILENVIKTRWKILPRNQCEGIKKYVVGLIIKTSSDPTCVEKEKVYIGKLNMILVQILKQEWPKHWPTFISDIVGASRTSESLCQNNMVILKLLSEEVFDFSSGQITQVKSKHLKDSMCNEFSQIFQLCQFVMENSQNAPLVHATLETLLRFLNWIPLGYIFETKLISTLIYKFLNVPMFRNVSLKCLTEIAGVSVSQYEEQFVTLFTLTMMQLKQMLPLNTNIRLAYSNGKDDEQNFIQNLSLFLCTFLKEHDQLIEKRLNLRETLMEALHYMLLVSEVEETEIFKICLEYWNHLAAELYRESPFSTSASPLLSGSQHFDVPPRRQLYLPMLFKVRLLMVSRMAKPEEVLVVENDQGEVVREFMKDTDSINLYKNMRETLVYLTHLDYVDTERIMTEKLHNQVNGTEWSWKNLNTLCWAIGSISGAMHEEDEKRFLVTVIKDLLGLCEQKRGKDNKAIIASNIMYIVGQYPRFLRAHWKFLKTVVNKLFEFMHETHDGVQDMACDTFIKIAQKCRRHFVQVQVGEVMPFIDEILNNINTIICDLQPQQVHTFYEAVGYMIGAQTDQTVQEHLIEKYMLLPNQVWDSIIQQATKNVDILKDPETVKQLGSILKTNVRACKAVGHPFVIQLGRIYLDMLNVYKCLSENISAAIQANGEMVTKQPLIRSMRTVKRETLKLISGWVSRSNDPQMVAENFVPPLLDAVLIDYQRNVPAAREPEVLSTMAIIVNKLGGHITAEIPQIFDAVFECTLNMINKDFEEYPEHRTNFFLLLQAVNSHCFPAFLAIPPTQFKLVLDSIIWAFKHTMRNVADTGLQILFTLLQNVAQEEAAAQSFYQTYFCDILQHIFSVVTDTSHTAGLTMHASILAYMFNLVEEGKISTSLNPGNPVNNQIFLQEYVANLLKSAFPHLQDAQVKLFVTGLFSLNQDIPAFKEHLRDFLVQIKEFAGEDTSDLFLEEREIALRQADEEKHKRQMSVPGIFNPHEIPEEMCD

1037 NP_005584.1 NP_005175.2

>NP_005584.1

MDVMDGCQFSPSEYFYDGSCIPSPEGEFGDEFVPRVAAFGAHKAELQGSDEDEHVRAPTGHHQAGHCLMWACKACKRKSTTMDRRKAATMRERRRLKKVNQAFETLKRCTTTNPNQRLPKVEILRNAIRYIESLQELLREQVENYYSLPGQSCSEPTSPTSNCSDGMPECNSPVWSRKSSTFDSIYCPDVSNVYATDKNSLSSLDCLSNIVDRITSSEQPGLPLQDLASLSPVASTDSQPRTPGASSSRLIYHVL

>NP_005175.2

MADQLTEEQIAEFKEAFSLFDKDGDGTITTKELGTVMRSLGQNPTEAELQDMINEVDADGNGTIDFPEFLTMMARKMKDTDSEEEIREAFRVFDKDGNGYISAAELRHVMTNLGEKLTDEEVDEMIREADIDGDGQVNYEEFVQMMTAK

1038 NP_002145.3 NP_002040.1

>NP_002145.3

MSVVGIDLGFQSCYVAVARAGGIETIANEYSDRCTPACISFGPKNRSIGAAAKSQVISNAKNTVQGFKRFHGRAFSDPFVEAEKSNLAYDIVQLPTGLTGIKVTYMEEERNFTTEQVTAMLLSKLKETAESVLKKPVVDCVVSVPCFYTDAERRSVMDATQIAGLNCLRLMNETTAVALAYGIYKQDLPALEEKPRNVVFVDMGHSAYQVSVCAFNRGKLKVLATAFDTTLGGRKFDEVLVNHFCEEFGKKYKLDIKSKIRALLRLSQECEKLKKLMSANASDLPLSIECFMNDVDVSGTMNRGKFLEMCNDLLARVEPPLRSVLEQTKLKKEDIYAVEIVGGATRIPAVKEKISKFFGKELSTTLNADEAVTRGCALQCAILSPAFKVREFSITDVVPYPISLRWNSPAEEGSSDCEVFSKNHAAPFSKVLTFYRKEPFTLEAYYSSPQDLPYPDPAIAQFSVQKVTPQSDGSSSKVKVKVRVNVHGIFSVSSASLVEVHKSEENEEPMETDQNAKEEEKMQVDQEEPHVEEQQQQTPAENKAESEEMETSQAGSKDKKMDQPPQAKKAKVKTSTVDLPIENQLLWQIDREMLNLYIENEGKMIMQDKLEKERNDAKNAVEEYVYEMRDKLSGEYEKFVSEDDRNSFTLKLEDTENWLYEDGEDQPKQVYVDKLAELKNLGQPIKIRFQESEERPKLFEELGKQIQQYMKIISSFKNKEDQYDHLDAADMTKVEKSTNEAMEWMNNKLNLQNKQSLTMDPVVKSKEIEAKIKELTSTCSPIISKPKPKVEPPKEEQKNAEQNGPVDGQGDNPGPQAAEQGTDTAVPSDSDKKLPEMDID

>NP_002040.1

MEFPGLGSLGTSEPLPQFVDPALVSSTPESGVFFPSGPEGLDAAASSTAPSTATAAAAALAYYRDAEAYRHSPVFQVYPLLNCMEGIPGGSPYAGWAYGKTGLYPASTVCPTREDSPPQAVEDLDGKGSTSFLETLKTERLSPDLLTLGPALPSSLPVPNSAYGGPDFSSTFFSPTGSPLNSAAYSSPKLRGTLPLPPCEARECVNCGATATPLWRRDRTGHYLCNACGLYHKMNGQNRPLIRPKKRLIVSKRAGTQCTNCQTTTTTLWRRNASGDPVCNACGLYYKLHQVNRPLTMRKDGIQTRNRKASGKGKKKRGSSLGGTGAAEGPAGGFMVVAGGSGSGNCGEVASGLTLGPPGTAHLYQGLGPVVLSGPVSHLMPFPGPLLGSPTGSFPTGPMPPTTSTTVVAPLSS

1039 NP_036555.1 NP_116196.3

>NP_036555.1

MAEVQVLVLDGRGHLLGRLAAIVAKQVLLGRKVVVVRCEGINISGNFYRNKLKYLAFLRKRMNTNPSRGPYHFRAPSRIFWRTVRGMLPHKTKRGQAALDRLKVFDGIPPPYDKKKRMVVPAALKVVRLKPTRKFAYLGRLAHEVGWKYQAVTATLEEKRKEKAKIHYRKKKQLMRLRKQAEKNVEKKIDKYTEVLKTHGLLV

>NP_116196.3

MSYEVTSGCHWTCQVPESCDNGLHCAGPLGHLHRRCQRTSAHLLVFTEHAEMRRFKRKHLTAIDCQHLARSHLAVTQPFGQRWTNRDPNHGLYPKPRTKRGSRGQGSQRCIPEFFLAGKQPCTNDMAKSNSVGQDSCQDSEGDMIFPAESSCALPQEGSAGPGSPGSAPPSRKRSWSSEEESNQATGTSRWDGVSKKAPRHHLSVPCTRPREARQEAEDSTSRLSAESGETDQDAGDVGPDPIPDSYYGLLGTLPCQEALSHICSLPSEVLRHVFAFLPVEDLYWNLSLVCHLWREIISDPLFIPWKKLYHRYLMNEEQAVSKVDGILSNCGIEKESDLCVLNLIRYTATTKCSPSVDPERVLWSLRDHPLLPEAEACVRQHLPDLYAAAGGVNIWALVAAVVLLSSSVNDIQRLLFCLRRPSSTVTMPDVTETLYCIAVLLYAMREKGINISNRIHYNIFYCLYLQENSCTQATKVKEEPSVWPGKKTIQLTHEQQLILNHKMEPLQVVKIMAFAGTGKTSTLVKYAEKWSQSRFLYVTFNKSIAKQAERVFPSNVICKTFHSMAYGHIGRKYQSKKKLNLFKLTPFMVNSVLAEGKGGFIRAKLVCKTLENFFASADEELTIDHVPIWCKNSQGQRVMVEQSEKLNGVLEASRLWDNMRKLGECTEEAHQMTHDGYLKLWQLSKPSLASFDAIFVDEAQDCTPAIMNIVLSQPCGKIFVGDPHQQIYTFRGAVNALFTVPHTHVFYLTQSFRFGVEIAYVGATILDVCKRVRKKTLVGGNHQSGIRGDAKGQVALLSRTNANVFDEAVRVTEGEFPSRIHLIGGIKSFGLDRIIDIWILLQPEEERRKQNLVIKDKFIRRWVHKEGFSGFKRYVTAAEDKELEAKIAVVEKYNIRIPELVQRIEKCHIEDLDFAEYILGTVHKAKGLEFDTVHVLDDFVKVPCARHNLPQLPHFRVESFSEDEWNLLYVAVTRAKKRLIMTKSLENILTLAGEYFLQAELTSNVLKTGVVRCCVGQCNNAIPVDTVLTMKKLPITYSNRKENKGGYLCHSCAEQRIGPLAFLTASPEQVRAMERTVENIVLPRHEALLFLVF

1040 NP_004094.3 NP_006507.1

>NP_004094.3

MSGVSEPLSRVKLGTLRRPEGPAEPMVVVPVDVEKEDVRILKVCFYSNSFNPGKNFKLVKCTVQTEIREIITSILLSGRIGPNIRLAECYGLRLKHMKSDEIHWLHPQMTVGEVQDKYECLHVEAEWRYDLQIRYLPEDFMESLKEDRTTLLYFYQQLRNDYMQRYASKVSEGMALQLGCLELRRFFKDMPHNALDKKSNFELLEKEVGLDLFFPKQMQENLKPKQFRKMIQQTFQQYASLREEECVMKFFNTLAGFANIDQETYRCELIQGWNITVDLVIGPKGIRQLTSQDAKPTCLAEFKQIRSIRCLPLEEGQAVLQLGIEGAPQALSIKTSSLAEAENMADLIDGYCRLQGEHQGSLIIHPRKDGEKRNSLPQIPMLNLEARRSHLSESCSIESDIYAEIPDETLRRPGGPQYGIAREDVVLNRILGEGFFGEVYEGVYTNHKGEKINVAVKTCKKDCTLDNKEKFMSEAVIMKNLDHPHIVKLIGIIEEEPTWIIMELYPYGELGHYLERNKNSLKVLTLVLYSLQICKAMAYLESINCVHRDIAVRNILVASPECVKLGDFGLSRYIEDEDYYKASVTRLPIKWMSPESINFRRFTTASDVWMFAVCMWEILSFGKQPFFWLENKDVIGVLEKGDRLPKPDLCPPVLYTLMTRCWDYDPSDRPRFTELVCSLSDVYQMEKDIAMEQERNARYRTPKILEPTAFQEPPPKPSRPKYRPPPQTNLLAPKLQFQVPEGLCASSPTLTSPMEYPSPVNSLHTPPLHRHNVFKRHSMREEDFIQPSSREEAQQLWEAEKVKMRQILDKQQKQMVEDYQWLRQEEKSLDPMVYMNDKSPLTPEKEVGYLEFTGPPQKPPRLGAQSIQPTANLDRTDDLVYLNVMELVRAVLELKNELCQLPPEGYVVVVKNVGLTLRKLIGSVDDLLPSLPSSSRTEIEGTQKLLNKDLAELINKMRLAQQNAVTSLSEECKRQMLTASHTLAVDAKNLLDAVDQAKVLANLAHPPAE

>NP_006507.1

MEPSSKKLTGRLMLAVGGAVLGSLQFGYNTGVINAPQKVIEEFYNQTWVHRYGESILPTTLTTLWSLSVAIFSVGGMIGSFSVGLFVNRFGRRNSMLMMNLLAFVSAVLMGFSKLGKSFEMLILGRFIIGVYCGLTTGFVPMYVGEVSPTAFRGALGTLHQLGIVVGILIAQVFGLDSIMGNKDLWPLLLSIIFIPALLQCIVLPFCPESPRFLLINRNEENRAKSVLKKLRGTADVTHDLQEMKEESRQMMREKKVTILELFRSPAYRQPILIAVVLQLSQQLSGINAVFYYSTSIFEKAGVQQPVYATIGSGIVNTAFTVVSLFVVERAGRRTLHLIGLAGMAGCAILMTIALALLEQLPWMSYLSIVAIFGFVAFFEVGPGPIPWFIVAELFSQGPRPAAIAVAGFSNWTSNFIVGMCFQYVEQLCGPYVFIIFTVLLVLFFIFTYFKVPETKGRTFDEIASGFRQGGASQSDKTPEELFHPLGADSQV

1041 NP_006087.2 NP_004059.2

>NP_006087.2

MSREMQDVDLAEVKPLVEKGETITGLLQEFDVQEQDIETLHGSVHVTLCGTPKGNRPVILTYHDIGMNHKTCYNPLFNYEDMQEITQHFAVCHVDAPGQQDGAASFPAGYMYPSMDQLAEMLPGVLQQFGLKSIIGMGTGAGAYILTRFALNNPEMVEGLVLINVNPCAEGWMDWAASKISGWTQALPDMVVSHLFGKEEMQSNVEVVHTYRQHIVNDMNPGNLHLFINAYNSRRDLEIERPMPGTHTVTLQCPALLVVGDSSPAVDAVVECNSKLDPTKTTLLKMADCGGLPQISQPAKLAEAFKYFVQGMGYMPSASMTRLMRSRTASGSSVTSLDGTRSRSHTSEGTRSRSHTSEGTRSRSHTSEGAHLDITPNSGAAGNSAGPKSMEVSC

>NP_004059.2

MIGGLFIYNHKGEVLISRVYRDDIGRNAVDAFRVNVIHARQQVRSPVTNIARTSFFHVKRSNIWLAAVTKQNVNAAMVFEFLYKMCDVMAAYFGKISEENIKNNFVLIYELLDEILDFGYPQNSETGALKTFITQQGIKSQHQTKEEQSQITSQVTGQIGWRREGIKYRRNELFLDVLESVNLLMSPQGQVLSAHVSGRVVMKSYLSGMPECKFGMNDKIVIEKQGKGTADETSKSGKQSIAIDDCTFHQCVRLSKFDSERSISFIPPDGEFELMRYRTTKDIILPFRVIPLVREVGRTKLEVKVVIKSNFKPSLLAQKIEVRIPTPLNTSGVQVICMKGKAKYKASENAIVWKIKRMAGMKESQISAEIELLPTNDKKKWARPPISMNFEVPFAPSGLKVRYLKVFEPKLNYSDHDVIKWVRYIGRSGIYETRC

1042 NP_002720.1 NP_002779.1

>NP_002720.1

MQYPHPGPAAGAVGVPLYAPTPLLQPAHPTPFYIEDILGRGPAAPTPAPTLPSPNSSFTSLVSPYRTPVYEPTPIHPAFSHHSAAALAAAYGPGGFGGPLYPFPRTVNDYTHALLRHDPLGKPLLWSPFLQRPLHKRKGGQVRFSNDQTIELEKKFETQKYLSPPERKRLAKMLQLSERQVKTWFQNRRAKWRRLKQENPQSNKKEELESLDSSCDQRQDLPSEQNKGASLDSSQCSPSPASQEDLESEISEDSDQEVDIEGDKSYFNAG

>NP_002779.1

MSSIGTGYDLSASTFSPDGRVFQVEYAMKAVENSSTAIGIRCKDGVVFGVEKLVLSKLYEEGSNKRLFNVDRHVGMAVAGLLADARSLADIAREEASNFRSNFGYNIPLKHLADRVAMYVHAYTLYSAVRPFGCSFMLGSYSVNDGAQLYMIDPSGVSYGYWGCAIGKARQAAKTEIEKLQMKEMTCRDIVKEVAKIIYIVHDEVKDKAFELELSWVGELTNGRHEIVPKDIREEAEKYAKESLKEEDESDDDNM

1043 NP_115705.2 NP_000456.1

>NP_115705.2

MATAMYLEHYLDSIENLPCELQRNFQLMRELDQRTEDKKAEIDILAAEYISTVKTLSPDQRVERLQKIQNAYSKCKEYSDDKVQLAMQTYEMVDKHIRRLDADLARFEADLKDKMEGSDFESSGGRGLKKGRGQKEKRGSRGRGRRTSEEDTPKKKKHKGGSEFTDTILSVHPSDVLDMPVDPNEPTYCLCHQVSYGEMIGCDNPDCPIEWFHFACVDLTTKPKGKWFCPRCVQEKRKKK

>NP_000456.1

MPDNRQPRNRQPRIRSGNEPRSAPAMEPDGRGAWAHSRAALDRLEKLLRCSRCTNILREPVCLGGCEHIFCSNCVSDCIGTGCPVCYTPAWIQDLKINRQLDSMIQLCSKLRNLLHDNELSDLKEDKPRKSLFNDAGNKKNSIKMWFSPRSKKVRYVVSKASVQTQPAIKKDASAQQDSYEFVSPSPPADVSERAKKASARSGKKQKKKTLAEINQKWNLEAEKEDGEFDSKEESKQKLVSFCSQPSVISSPQINGEIDLLASGSLTESECFGSLTEVSLPLAEQIESPDTKSRNEVVTPEKVCKNYLTSKKSLPLENNGKRGHHNRLSSPISKRCRTSILSTSGDFVKQTVPSENIPLPECSSPPSCKRKVGGTSGRKNSNMSDEFISLSPGTPPSTLSSSSYRQVMSSPSAMKLLPNMAVKRNHRGETLLHIASIKGDIPSVEYLLQNGSDPNVKDHAGWTPLHEACNHGHLKVVELLLQHKALVNTTGYQNDSPLHDAAKNGHVDIVKLLLSYGASRNAVNIFGLRPVDYTDDESMKSLLLLPEKNESSSASHCSVMNTGQRRDGPLVLIGSGLSSEQQKMLSELAVILKAKKYTEFDSTVTHVVVPGDAVQSTLKCMLGILNGCWILKFEWVKACLRRKVCEQEEKYEIPEGPRRSRLNREQLLPKLFDGCYFYLWGTFKHHPKDNLIKLVTAGGGQILSRKPKPDSDVTQTINTVAYHARPDSDQRFCTQYIIYEDLCNYHPERVRQGKVWKAPSSWFIDCVMSFELLPLDS

1044 NP_002222.1 NP_005219.2

>NP_002222.1

MGSACIKVTKYFLFLFNLIFFILGAVILGFGVWILADKSSFISVLQTSSSSLRMGAYVFIGVGAVTMLMGFLGCIGAVNEVRCLLGLYFAFLLLILIAQVTAGALFYFNMGKLKQEMGGIVTELIRDYNSSREDSLQDAWDYVQAQVKCCGWVSFYNWTDNAELMNRPEVTYPCSCEVKGEEDNSLSVRKGFCEAPGNRTQSGNHPEDWPVYQEGCMEKVQAWLQENLGIILGVGVGVAIIELLGMVLSICLCRHVHSEDYSKVPKY

>NP_005219.2

MRPSGTAGAALLALLAALCPASRALEEKKVCQGTSNKLTQLGTFEDHFLSLQRMFNNCEVVLGNLEITYVQRNYDLSFLKTIQEVAGYVLIALNTVERIPLENLQIIRGNMYYENSYALAVLSNYDANKTGLKELPMRNLQEILHGAVRFSNNPALCNVESIQWRDIVSSDFLSNMSMDFQNHLGSCQKCDPSCPNGSCWGAGEENCQKLTKIICAQQCSGRCRGKSPSDCCHNQCAAGCTGPRESDCLVCRKFRDEATCKDTCPPLMLYNPTTYQMDVNPEGKYSFGATCVKKCPRNYVVTDHGSCVRACGADSYEMEEDGVRKCKKCEGPCRKVCNGIGIGEFKDSLSINATNIKHFKNCTSISGDLHILPVAFRGDSFTHTPPLDPQELDILKTVKEITGFLLIQAWPENRTDLHAFENLEIIRGRTKQHGQFSLAVVSLNITSLGLRSLKEISDGDVIISGNKNLCYANTINWKKLFGTSGQKTKIISNRGENSCKATGQVCHALCSPEGCWGPEPRDCVSCRNVSRGRECVDKCNLLEGEPREFVENSECIQCHPECLPQAMNITCTGRGPDNCIQCAHYIDGPHCVKTCPAGVMGENNTLVWKYADAGHVCHLCHPNCTYGCTGPGLEGCPTNGPKIPSIATGMVGALLLLLVVALGIGLFMRRRHIVRKRTLRRLLQERELVEPLTPSGEAPNQALLRILKETEFKKIKVLGSGAFGTVYKGLWIPEGEKVKIPVAIKELREATSPKANKEILDEAYVMASVDNPHVCRLLGICLTSTVQLITQLMPFGCLLDYVREHKDNIGSQYLLNWCVQIAKGMNYLEDRRLVHRDLAARNVLVKTPQHVKITDFGLAKLLGAEEKEYHAEGGKVPIKWMALESILHRIYTHQSDVWSYGVTVWELMTFGSKPYDGIPASEISSILEKGERLPQPPICTIDVYMIMVKCWMIDADSRPKFRELIIEFSKMARDPQRYLVIQGDERMHLPSPTDSNFYRALMDEEDMDDVVDADEYLIPQQGFFSSPSTSRTPLLSSLSATSNNSTVACIDRNGLQSCPIKEDSFLQRYSSDPTGALTEDSIDDTFLPVPEYINQSVPKRPAGSVQNPVYHNQPLNPAPSRDPHYQDPHSTAVGNPEYLNTVQPTCVNSTFDSPAHWAQKGSHQISLDNPDYQQDFFPKEAKPNGIFKGSTAENAEYLRVAPQSSEFIGA

1045 NP_001702.1 NP_000080.2

>NP_001702.1

MWPLWRLVSLLALSQALPFEQRGFWDFTLDDGPFMMNDEEASGADTSGVLDPDSVTPTYSAMCPFGCHCHLRVVQCSDLGLKSVPKEISPDTTLLDLQNNDISELRKDDFKGLQHLYALVLVNNKISKIHEKAFSPLRKLQKLYISKNHLVEIPPNLPSSLVELRIHDNRIRKVPKGVFSGLRNMNCIEMGGNPLENSGFEPGAFDGLKLNYLRISEAKLTGIPKDLPETLNELHLDHNKIQAIELEDLLRYSKLYRLGLGHNQIRMIENGSLSFLPTLRELHLDNNKLARVPSGLPDLKLLQVVYLHSNNITKVGVNDFCPMGFGVKRAYYNGISLFNNPVPYWEVQPATFRCVTDRLAIQFGNYKK

>NP_000080.2

MLSFVDTRTLLLLAVTLCLATCQSLQEETVRKGPAGDRGPRGERGPPGPPGRDGEDGPTGPPGPPGPPGPPGLGGNFAAQYDGKGVGLGPGPMGLMGPRGPPGAAGAPGPQGFQGPAGEPGEPGQTGPAGARGPAGPPGKAGEDGHPGKPGRPGERGVVGPQGARGFPGTPGLPGFKGIRGHNGLDGLKGQPGAPGVKGEPGAPGENGTPGQTGARGLPGERGRVGAPGPAGARGSDGSVGPVGPAGPIGSAGPPGFPGAPGPKGEIGAVGNAGPAGPAGPRGEVGLPGLSGPVGPPGNPGANGLTGAKGAAGLPGVAGAPGLPGPRGIPGPVGAAGATGARGLVGEPGPAGSKGESGNKGEPGSAGPQGPPGPSGEEGKRGPNGEAGSAGPPGPPGLRGSPGSRGLPGADGRAGVMGPPGSRGASGPAGVRGPNGDAGRPGEPGLMGPRGLPGSPGNIGPAGKEGPVGLPGIDGRPGPIGPAGARGEPGNIGFPGPKGPTGDPGKNGDKGHAGLAGARGAPGPDGNNGAQGPPGPQGVQGGKGEQGPPGPPGFQGLPGPSGPAGEVGKPGERGLHGEFGLPGPAGPRGERGPPGESGAAGPTGPIGSRGPSGPPGPDGNKGEPGVVGAVGTAGPSGPSGLPGERGAAGIPGGKGEKGEPGLRGEIGNPGRDGARGAPGAVGAPGPAGATGDRGEAGAAGPAGPAGPRGSPGERGEVGPAGPNGFAGPAGAAGQPGAKGERGAKGPKGENGVVGPTGPVGAAGPAGPNGPPGPAGSRGDGGPPGMTGFPGAAGRTGPPGPSGISGPPGPPGPAGKEGLRGPRGDQGPVGRTGEVGAVGPPGFAGEKGPSGEAGTAGPPGTPGPQGLLGAPGILGLPGSRGERGLPGVAGAVGEPGPLGIAGPPGARGPPGAVGSPGVNGAPGEAGRDGNPGNDGPPGRDGQPGHKGERGYPGNIGPVGAAGAPGPHGPVGPAGKHGNRGETGPSGPVGPAGAVGPRGPSGPQGIRGDKGEPGEKGPRGLPGLKGHNGLQGLPGIAGHHGDQGAPGSVGPAGPRGPAGPSGPAGKDGRTGHPGTVGPAGIRGPQGHQGPAGPPGPPGPPGPPGVSGGGYDFGYDGDFYRADQPRSAPSLRPKDYEVDATLKSLNNQIETLLTPEGSRKNPARTCRDLRLSHPEWSSGYYWIDPNQGCTMDAIKVYCDFSTGETCIRAQPENIPAKNWYRSSKDKKHVWLGETINAGSQFEYNVEGVTSKEMATQLAFMRLLANYASQNITYHCKNSIAYMDEETGNLKKAVILQGSNDVELVAEGNSRFTYTVLVDGCSKKTNEWGKTIIEYKTNKPSRLPFLDIAPLDIGGADQEFFVDIGPVCFK

1046 NP_003168.2 NP_000052.1

>NP_003168.2

MASSGMADSANHLPFFFGNITREEAEDYLVQGGMSDGLYLLRQSRNYLGGFALSVAHGRKAHHYTIERELNGTYAIAGGRTHASPADLCHYHSQESDGLVCLLKKPFNRPQGVQPKTGPFEDLKENLIREYVKQTWNLQGQALEQAIISQKPQLEKLIATTAHEKMPWFHGKISREESEQIVLIGSKTNGKFLIRARDNNGSYALCLLHEGKVLHYRIDKDKTGKLSIPEGKKFDTLWQLVEHYSYKADGLLRVLTVPCQKIGTQGNVNFGGRPQLPGSHPATWSAGGIISRIKSYSFPKPGHRKSSPAQGNRQESTVSFNPYEPELAPWAADKGPQREALPMDTEVYESPYADPEEIRPKEVYLDRKLLTLEDKELGSGNFGTVKKGYYQMKKVVKTVAVKILKNEANDPALKDELLAEANVMQQLDNPYIVRMIGICEAESWMLVMEMAELGPLNKYLQQNRHVKDKNIIELVHQVSMGMKYLEESNFVHRDLAARNVLLVTQHYAKISDFGLSKALRADENYYKAQTHGKWPVKWYAPECINYYKFSSKSDVWSFGVLMWEAFSYGQKPYRGMKGSEVTAMLEKGERMGCPAGCPREMYDLMNLCWTYDVENRPGFAAVELRLRNYYYDVVN

>NP_000052.1

MAAVILESIFLKRSQQKKKTSPLNFKKRLFLLTVHKLSYYEYDFERGRRGSKKGSIDVEKITCVETVVPEKNPPPERQIPRRGEESSEMEQISIIERFPYPFQVVYDEGPLYVFSPTEELRKRWIHQLKNVIRYNSDLVQKYHPCFWIDGQYLCCSQTAKNAMGCQILENRNGSLKPGSSHRKTKKPLPPTPEEDQILKKPLPPEPAAAPVSTSELKKVVALYDYMPMNANDLQLRKGDEYFILEESNLPWWRARDKNGQEGYIPSNYVTEAEDSIEMYEWYSKHMTRSQAEQLLKQEGKEGGFIVRDSSKAGKYTVSVFAKSTGDPQGVIRHYVVCSTPQSQYYLAEKHLFSTIPELINYHQHNSAGLISRLKYPVSQQNKNAPSTAGLGYGSWEIDPKDLTFLKELGTGQFGVVKYGKWRGQYDVAIKMIKEGSMSEDEFIEEAKVMMNLSHEKLVQLYGVCTKQRPIFIITEYMANGCLLNYLREMRHRFQTQQLLEMCKDVCEAMEYLESKQFLHRDLAARNCLVNDQGVVKVSDFGLSRYVLDDEYTSSVGSKFPVRWSPPEVLMYSKFSSKSDIWAFGVLMWEIYSLGKMPYERFTNSETAEHIAQGLRLYRPHLASEKVYTIMYSCWHEKADERPTFKILLSNILDVMDEES

1047 NP_002028.1 NP_002221.1

>NP_002028.1

MGCVQCKDKEATKLTEERDGSLNQSSGYRYGTDPTPQHYPSFGVTSIPNYNNFHAAGGQGLTVFGGVNSSSHTGTLRTRGGTGVTLFVALYDYEARTEDDLSFHKGEKFQILNSSEGDWWEARSLTTGETGYIPSNYVAPVDSIQAEEWYFGKLGRKDAERQLLSFGNPRGTFLIRESETTKGAYSLSIRDWDDMKGDHVKHYKIRKLDNGGYYITTRAQFETLQQLVQHYSERAAGLCCRLVVPCHKGMPRLTDLSVKTKDVWEIPRESLQLIKRLGNGQFGEVWMGTWNGNTKVAIKTLKPGTMSPESFLEEAQIMKKLKHDKLVQLYAVVSEEPIYIVTEYMNKGSLLDFLKDGEGRALKLPNLVDMAAQVAAGMAYIERMNYIHRDLRSANILVGNGLICKIADFGLARLIEDNEYTARQGAKFPIKWTAPEAALYGRFTIKSDVWSFGILLTELVTKGRVPYPGMNNREVLEQVERGYRMPCPQDCPISLHELMIHCWKKDPEERPTFEYLQSFLEDYFTATEPQYQPGENL

>NP_002221.1

MEVMNLMEQPIKVTEWQQTYTYDSGIHSGANTCVPSVSSKGIMEEDEACGRQYTLKKTTTYTQGVPPSQGDLEYQMSTTARAKRVREAMCPGVSGEDSSLLLATQVEGQATNLQRLAEPSQLLKSAIVHLINYQDDAELATRALPELTKLLNDEDPVVVTKAAMIVNQLSKKEASRRALMGSPQLVAAVVRTMQNTSDLDTARCTTSILHNLSHHREGLLAIFKSGGIPALVRMLSSPVESVLFYAITTLHNLLLYQEGAKMAVRLADGLQKMVPLLNKNNPKFLAITTDCLQLLAYGNQESKLIILANGGPQALVQIMRNYSYEKLLWTTSRVLKVLSVCPSNKPAIVEAGGMQALGKHLTSNSPRLVQNCLWTLRNLSDVATKQEGLESVLKILVNQLSVDDVNVLTCATGTLSNLTCNNSKNKTLVTQNSGVEALIHAILRAGDKDDITEPAVCALRHLTSRHPEAEMAQNSVRLNYGIPAIVKLLNQPNQWPLVKATIGLIRNLALCPANHAPLQEAAVIPRLVQLLVKAHQDAQRHVAAGTQQPYTDGVRMEEIVEGCTGALHILARDPMNRMEIFRLNTIPLFVQLLYSSVENIQRVAAGVLCELAQDKEAADAIDAEGASAPLMELLHSRNEGTATYAAAVLFRISEDKNPDYRKRVSVELTNSLFKHDPAAWEAAQSMIPINEPYGDDMDATYRPMYSSDVPLDPLEMHMDMDGDYPIDTYSDGLRPPYPTADHMLA

1048 NP_775841.2 NP_000611.1

>NP_775841.2

MTQKGSMKPVKKKKTEEPELEPLCCCEYIDRNGEKNHVATCLCDCQDLDEGCDRWITCKSLQPETCERIMDTISDRLRIPWLRGAKKVNISIIPPLVLLPVFLHVASWHFLLGVVVLTSLPVLALWYYYLTHRRKEQTLFFLSLGLFSLGYMYYVFLQEVVPKGRVGPVQLAVLTCGLFLILLALHRAKKNPGYLSNPASGDRSLSSSQLECLSRKGQEKTKGFPGADMSGSLNNRTTKDDPKGSSKMPAGSPTKAKEDWCAKCQLVRPARAWHCRICGICVRRMDHHCVWINSCVGESNHQAFILALLIFLLTSVYGITLTLDTICRDRSVFTALFYCPGVYANYSSALSFTCVWYSVIITAGMAYIFLIQLINISYNVTEREVQQALRQKTGRRLLCGLIVDTGLLG

>NP_000611.1

MEDHMFGVQQIQPNVISVRLFKRKVGGLGFLVKERVSKPPVIISDLIRGGAAEQSGLIQAGDIILAVNGRPLVDLSYDSALEVLRGIASETHVVLILRGPEGFTTHLETTFTGDGTPKTIRVTQPLGPPTKAVDLSHQPPAGKEQPLAVDGASGPGNGPQHAYDDGQEAGSLPHANGLAPRPPGQDPAKKATRVSLQGRGENNELLKEIEPVLSLLTSGSRGVKGGAPAKAEMKDMGIQVDRDLDGKSHKPLPLGVENDRVFNDLWGKGNVPVVLNNPYSEKEQPPTSGKQSPTKNGSPSKCPRFLKVKNWETEVVLTDTLHLKSTLETGCTEYICMGSIMHPSQHARRPEDVRTKGQLFPLAKEFIDQYYSSIKRFGSKAHMERLEEVNKEIDTTSTYQLKDTELIYGAKHAWRNASRCVGRIQWSKLQVFDARDCTTAHGMFNYICNHVKYATNKGNLRSAITIFPQRTDGKHDFRVWNSQLIRYAGYKQPDGSTLGDPANVQFTEICIQQGWKPPRGRFDVLPLLLQANGNDPELFQIPPELVLEVPIRHPKFEWFKDLGLKWYGLPAVSNMLLEIGGLEFSACPFSGWYMGTEIGVRDYCDNSRYNILEEVAKKMNLDMRKTSSLWKDQALVEINIAVLYSFQSDKVTIVDHHSATESFIKHMENEYRCRGGCPADWVWIVPPMSGSITPVFHQEMLNYRLTPSFEYQPDPWNTHVWKGTNGTPTKRRAIGFKKLAEAVKFSAKLMGQAMAKRVKATILYATETGKSQAYAKTLCEIFKHAFDAKVMSMEEYDIVHLEHETLVLVVTSTFGNGDPPENGEKFGCALMEMRHPNSVQEERKSYKVRFNSVSSYSDSQKSSGDGPDLRDNFESAGPLANVRFSVFGLGSRAYPHFCAFGHAVDTLLEELGGERILKMREGDELCGQEEAFRTWAKKVFKAACDVFCVGDDVNIEKANNSLISNDRSWKRNKFRLTFVAEAPELTQGLSNVHKKRVSAARLLSRQNLQSPKSSRSTIFVRLHTNGSQELQYQPGDHLGVFPGNHEDLVNALIERLEDAPPVNQMVKVELLEERNTALGVISNWTDELRLPPCTIFQAFKYYLDITTPPTPLQLQQFASLATSEKEKQRLLVLSKGLQEYEEWKWGKNPTIVEVLEEFPSIQMPATLLLTQLSLLQPRYYSISSSPDMYPDEVHLTVAIVSYRTRDGEGPIHHGVCSSWLNRIQADELVPCFVRGAPSFHLPRNPQVPCILVGPGTGIAPFRSFWQQRQFDIQHKGMNPCPMVLVFGCRQSKIDHIYREETLQAKNKGVFRELYTAYSREPDKPKKYVQDILQEQLAESVYRALKEQGGHIYVCGDVTMAADVLKAIQRIMTQQGKLSAEDAGVFISRMRDDNRYHEDIFGVTLRTYEVTNRLRSESIAFIEESKKDTDEVFSS

1049 NP_699202.1 NP_001005743.1

>NP_699202.1

MGTTSDEMVSVEQTSSSSLNPLCFECGQQHWTRENHLYNYQNEVDDDLVCHICLQPLLQPLDTPCGHTFCYKCLRNFLQEKDFCPLDRKRLHFKLCKKSSILVHKLLDKLLVLCPFSSVCKDVMQRCDLEAHLKNRCPGASHRRVALERRKTSRTQAEIENENGPTLLDPAGTLSPEADCLGTGAVPVERHLTSASLSTWSEEPGLDNPAFEESAGADTTQQPLSLPEGEITTIEIHRSNPYIQLGISIVGGNETPLINIVIQEVYRDGVIARDGRLLAGDQILQVNNYNISNVSHNYARAVLSQPCNTLHLTVLRERRFGNRAHNHSDSNSPREEIFQVALHKRDSGEQLGIKLVRRTDEPGVFILDLLEGGLAAQDGRLSSNDRVLAINGHDLKYGTPELAAQIIQASGERVNLTIARPGKPQPGNTIREAGNHSSSSQHHTPPPYYSRPSSHKDLTQCVTCQEKHITVKKEPHESLGMTVAGGRGSKSGELPIFVTSVPPHGCLARDGRIKRGDVLLNINGIDLTNLSHSEAVAMLKASAASPAVALKALEVQIVEEATQNAEEQPSTFSENEYDASWSPSWVMWLGLPSTLHSCHDIVLRRSYLGSWGFSIVGGYEENHTNQPFFIKTIVLGTPAYYDGRLKCGDMIVAVNGLSTVGMSHSALVPMLKEQRNKVTLTVICWPGSLV

>NP_001005743.1

MNKLRQSFRRKKDVYVPEASRPHQWQTDEEGVRTGKCSFPVKYLGHVEVDESRGMHICEDAVKRLKAERKFFKGFFGKTGKKAVKAVLWVSADGLRVVDEKTKDLIVDQTIEKVSFCAPDRNFDRAFSYICRDGTTRRWICHCFMAVKDTGERLSHAVGCAFAACLERKQKREKECGVTATFDASRTTFTREGSFRVTTATEQAEREEIMKQMQDAKKAETDKIVVGSSVAPGNTAPSPSSPTSPTSDATTSLEMNNPHAIPRRHAPIEQLARQGSFRGFPALSQKMSPFKRQLSLRINELPSTMQRKTDFPIKNAVPEVEGEAESISSLCSQITNAFSTPEDPFSSAPMTKPVTVVAPQSPTFQANGTDSAFHVLAKPAHTALAPVAMPVRETNPWAHAPDAANKEIAATCSGTEWGQSSGAASPGLFQAGHRRTPSEADRWLEEVSKSVRAQQPQASAAPLQPVLQPPPPTAISQPASPFQGNAFLTSQPVPVGVVPALQPAFVPAQSYPVANGMPYPAPNVPVVGITPSQMVANVFGTAGHPQAAHPHQSPSLVRQQTFPHYEASSATTSPFFKPPAQHLNGSAAFNGVDDGRLASADRHTEVPTGTCPVDPFEAQWAALENKSKQRTNPSPTNPFSSDLQKTFEIEL

1050 NP_000452.2 NP_000537.3

>NP_000452.2

MTPNSMTENGLTAWDKPKHCPDREHDWKLVGMSEACLHRKSHSERRSTLKNEQSSPHLIQTTWTSSIFHLDHDDVNDQSVSSAQTFQTEEKKCKGYIPSYLDKDELCVVCGDKATGYHYRCITCEGCKGFFRRTIQKNLHPSYSCKYEGKCVIDKVTRNQCQECRFKKCIYVGMATDLVLDDSKRLAKRKLIEENREKRRREELQKSIGHKPEPTDEEWELIKTVTEAHVATNAQGSHWKQKRKFLPEDIGQAPIVNAPEGGKVDLEAFSHFTKIITPAITRVVDFAKKLPMFCELPCEDQIILLKGCCMEIMSLRAAVRYDPESETLTLNGEMAVTRGQLKNGGLGVVSDAIFDLGMSLSSFNLDDTEVALLQAVLLMSSDRPGLACVERIEKYQDSFLLAFEHYINYRKHHVTHFWPKLLMKVTDLRMIGACHASRFLHMKVECPTELFPPLFLEVFED

>NP_000537.3

MEEPQSDPSVEPPLSQETFSDLWKLLPENNVLSPLPSQAMDDLMLSPDDIEQWFTEDPGPDEAPRMPEAAPPVAPAPAAPTPAAPAPAPSWPLSSSVPSQKTYQGSYGFRLGFLHSGTAKSVTCTYSPALNKMFCQLAKTCPVQLWVDSTPPPGTRVRAMAIYKQSQHMTEVVRRCPHHERCSDSDGLAPPQHLIRVEGNLRVEYLDDRNTFRHSVVVPYEPPEVGSDCTTIHYNYMCNSSCMGGMNRRPILTIITLEDSSGNLLGRNSFEVRVCACPGRDRRTEEENLRKKGEPHHELPPGSTKRALPNNTSSSPQPKKKPLDGEYFTLQIRGRERFEMFRELNEALELKDAQAGKEPGGSRAHSSHLKSKKGQSTSRHKKLMFKTEGPDSD

1051 NP_003927.1 NP_004926.1

>NP_003927.1

MGTVLSLSPASSAKGRRPGGLPEEKKKAPPAGDEALGGYGAPPVGKGGKGESRLKRPSVLISALTWKRLVAASAKKKKGSKKVTPKPASTGPDPLVQQRNRENLLRKGRDPPDGGGTAKPLAVPVPTVPAAAATCEPPSGGSAAAQPPGSGGGKPPPPPPPAPQVAPPVPGGSPRRVIVQASTGELLRCLGDFVCRRCYRLKELSPGELVGWFRGVDRSLLLQGWQDQAFITPANLVFVYLLCRESLRGDELASAAELQAAFLTCLYLAYSYMGNEISYPLKPFLVEPDKERFWQRCLRLIQRLSPQMLRLNADPHFFTQVFQDLKNEGEAAASGGGPPSGGAPAASSAARDSCAAGTKHWTMNLDR

>NP_004926.1

MQKYEKLEKIGEGTYGTVFKAKNRETHEIVALKRVRLDDDDEGVPSSALREICLLKELKHKNIVRLHDVLHSDKKLTLVFEFCDQDLKKYFDSCNGDLDPEIVKSFLFQLLKGLGFCHSRNVLHRDLKPQNLLINRNGELKLADFGLARAFGIPVRCYSAEVVTLWYRPPDVLFGAKLYSTSIDMWSAGCIFAELANAGRPLFPGNDVDDQLKRIFRLLGTPTEEQWPSMTKLPDYKPYPMYPATTSLVNVVPKLNATGRDLLQNLLKCNPVQRISAEEALQHPYFSDFCPP

1052 NP_009145.1 NP_003253.1

>NP_009145.1

MAGQQFQYDDSGNTFFYFLTSFVGLIVIPATYYLWPRDQNAEQIRLKNIRKVYGRCMWYRLRLLKPQPNIIPTVKKIVLLAGWALFLFLAYKVSKTDREYQEYNPYEVLNLDPGATVAEIKKQYRLLSLKYHPDKGGDEVMFMRIAKAYAALTDEESRKNWEEFGNPDGPQATSFGIALPAWIVDQKNSILVLLVYGLAFMVILPVVVGSWWYRSIRYSGDQILIRTTQIYTYFVYKTRNMDMKRLIMVLAGASEFDPQYNKDATSRPTDNILIPQLIREIGSINLKKNEPPLTCPYSLKARVLLLSHLARMKIPETLEEDQQFMLKKCPALLQEMVNVICQLIVMARNREEREFRAPTLASLENCMKLSQMAVQGLQQFKSPLLQLPHIEEDNLRRVSNHKKYKIKTIQDLVSLKESDRHTLLHFLEDEKYEEVMAVLGSFPYVTMDIKSQVLDDEDSNNITVGSLVTVLVKLTRQTMAEVFEKEQSICAAEEQPAEDGQGETNKNRTKGGWQQKSKGPKKTAKSKKKKPLKKKPTPVLLPQSKQQKQKQANGVVGNEAAVKEDEEEVSDKGSDSEEEETNRDSQSEKDDGSDRDSDREQDEKQNKDDEAEWQELQQSIQRKERALLETKSKITHPVYSLYFPEEKQEWWWLYIADRKEQTLISMPYHVCTLKDTEEVELKFPAPGKPGNYQYTVFLRSDSYMGLDQIKPLKLEVHEAKPVPENHPQWDTAIEGDEDQEDSEGFEDSFEEEEEEEEDDD

>NP_003253.1

MAERRRHKKRIQEVGEPSKEEKAVAKYLRFNCPTKSTNMMGHRVDYFIASKAVDCLLDSKWAKAKKGEEALFTTRESVVDYCNRLLKKQFFHRALKVMKMKYDKDIKKEKDKGKAESGKEEDKKSKKENIKDEKTKKEKEKKKDGEKEESKKEETPGTPKKKETKKKFKLEPHDDQVFLDGNEVYVWIYDPVHFKTFVMGLILVIAVIAATLFPLWPAEMRVGVYYLSVGAGCFVASILLLAVARCILFLIIWLITGGRHHFWFLPNLTADVGFIDSFRPLYTHEYKGPKADLKKDEKSETKKQQKSDSEEKSDSEKKEDEEGKVGPGNHGTEGSGGERHSDTDSDRREDDRSQHSSGNGNDFEMITKEELEQQTDGDCEEDEEEENDGETPKSSHEKS

1053 NP_000203.2 NP_068656.2

>NP_000203.2

MRARPRPRPLWATVLALGALAGVGVGGPNICTTRGVSSCQQCLAVSPMCAWCSDEALPLGSPRCDLKENLLKDNCAPESIEFPVSEARVLEDRPLSDKGSGDSSQVTQVSPQRIALRLRPDDSKNFSIQVRQVEDYPVDIYYLMDLSYSMKDDLWSIQNLGTKLATQMRKLTSNLRIGFGAFVDKPVSPYMYISPPEALENPCYDMKTTCLPMFGYKHVLTLTDQVTRFNEEVKKQSVSRNRDAPEGGFDAIMQATVCDEKIGWRNDASHLLVFTTDAKTHIALDGRLAGIVQPNDGQCHVGSDNHYSASTTMDYPSLGLMTEKLSQKNINLIFAVTENVVNLYQNYSELIPGTTVGVLSMDSSNVLQLIVDAYGKIRSKVELEVRDLPEELSLSFNATCLNNEVIPGLKSCMGLKIGDTVSFSIEAKVRGCPQEKEKSFTIKPVGFKDSLIVQVTFDCDCACQAQAEPNSHRCNNGNGTFECGVCRCGPGWLGSQCECSEEDYRPSQQDECSPREGQPVCSQRGECLCGQCVCHSSDFGKITGKYCECDDFSCVRYKGEMCSGHGQCSCGDCLCDSDWTGYYCNCTTRTDTCMSSNGLLCSGRGKCECGSCVCIQPGSYGDTCEKCPTCPDACTFKKECVECKKFDRGALHDENTCNRYCRDEIESVKELKDTGKDAVNCTYKNEDDCVVRFQYYEDSSGKSILYVVEEPECPKGPDILVVLLSVMGAILLIGLAALLIWKLLITIHDRKEFAKFEEERARAKWDTANNPLYKEATSTFTNITYRGT

>NP_068656.2

MSWSLHPRNLILYFYALLFLSSTCVAYVATRDNCCILDERFGSYCPTTCGIADFLSTYQTKVDKDLQSLEDILHQVENKTSEVKQLIKAIQLTYNPDESSKPNMIDAATLKSRKMLEEIMKYEASILTHDSSIRYLQEIYNSNNQKIVNLKEKVAQLEAQCQEPCKDTVQIHDITGKDCQDIANKGAKQSGLYFIKPLKANQQFLVYCEIDGSGNGWTVFQKRLDGSVDFKKNWIQYKEGFGHLSPTGTTEFWLGNEKIHLISTQSAIPYALRVELEDWNGRTSTADYAMFKVGPEADKYRLTYAYFAGGDAGDAFDGFDFGDDPSDKFFTSHNGMQFSTWDNDNDKFEGNCAEQDGSGWWMNKCHAGHLNGVYYQGGTYSKASTPNGYDNGIIWATWKTRWYSMKKTTMKIIPFNRLTIGEGQQHHLGGAKQVRPEHPAETEYDSLYPEDDL

1054 NP_003371.2 NP_054750.1

>NP_003371.2

MSTRSVSSSSYRRMFGGPGTASRPSSSRSYVTTSTRTYSLGSALRPSTSRSLYASSPGGVYATRSSAVRLRSSVPGVRLLQDSVDFSLADAINTEFKNTRTNEKVELQELNDRFANYIDKVRFLEQQNKILLAELEQLKGQGKSRLGDLYEEEMRELRRQVDQLTNDKARVEVERDNLAEDIMRLREKLQEEMLQREEAENTLQSFRQDVDNASLARLDLERKVESLQEEIAFLKKLHEEEIQELQAQIQEQHVQIDVDVSKPDLTAALRDVRQQYESVAAKNLQEAEEWYKSKFADLSEAANRNNDALRQAKQESTEYRRQVQSLTCEVDALKGTNESLERQMREMEENFAVEAANYQDTIGRLQDEIQNMKEEMARHLREYQDLLNVKMALDIEIATYRKLLEGEESRISLPLPNFSSLNLRETNLDSLPLVDTHSKRTLLIKTVETRDGQVINETSQHHDDLE

>NP_054750.1

MLLSWLTVLGAGMVVLHFLQKLLFPYFWDDFWFVLKVVLIIIRLKKYEKRGELVTVLDKFLSHAKRQPRKPFIIYEGDIYTYQDVDKRSSRVAHVFLNHSSLKKGDTVALLMSNEPDFVHVWFGLAKLGCVVAFLNTNIRSNSLLNCIRACGPRALVVGADLLGTVEEILPSLSENISVWGMKDSVPQGVISLKEKLSTSPDEPVPRSHHVVSLLKSTCLYIFTSGTTGLPKAAVISQLQVLRGSAVLWAFGCTAHDIVYITLPLYHSSAAILGISGCVELGATCVLKKKFSASQFWSDCKKYDVTVFQYIGELCRYLCKQSKREGEKDHKVRLAIGNGIRSDVWREFLDRFGNIKVCELYAATESSISFMNYTGRIGAIGRTNLFYKLLSTFDLIKYDFQKDEPMRNEQGWCIHVKKGEPGLLISRVNAKNPFFGYAGPYKHTKDKLLCDVFKKGDVYLNTGDLIVQDQDNFLYFWDRTGDTFRWKGENVATTEVADVIGMLDFIQEANVYGVAISGYEGRAGMASIILKPNTSLDLEKVYEQVVTFLPAYACPRFLRIQEKMEATGTFKLLKHQLVEDGFNPLKISEPLYFMDNLKKSYVLLTRELYDQIMLGEIKL

1055 NP_056981.2 NP_005350.1

>NP_056981.2

MAAELVEAKNMVMSFRVSDLQMLLGFVGRSKSGLKHELVTRALQLVQFDCSPELFKKIKELYETRYAKKNSEPAPQPHRPLDPLTMHSTYDRAGAVPRTPLAGPNIDYPVLYGKYLNGLGRLPAKTLKPEVRLVKLPFFNMLDELLKPTELVPQNNEKLQESPCIFALTPRQVELIRNSRELQPGVKAVQVVLRICYSDTSCPQEDQYPPNIAVKVNHSYCSVPGYYPSNKPGVEPKRPCRPINLTHLMYLSSATNRITVTWGNYGKSYSVALYLVRQLTSSELLQRLKTIGVKHPELCKALVKEKLRLDPDSEIATTGVRVSLICPLVKMRLSVPCRAETCAHLQCFDAVFYLQMNEKKPTWMCPVCDKPAPYDQLIIDGLLSKILSECEDADEIEYLVDGSWCPIRAEKERSCSPQGAILVLGPSDANGLLPAPSVNGSGALGSTGGGGPVGSMENGKPGADVVDLTLDSSSSSEDEEEEEEEEEDEDEEGPRPKRRCPFQKGLVPAC

>NP_005350.1

MDNMSITNTPTSNDACLSIVHSLMCHRQGGESETFAKRAIESLVKKLKEKKDELDSLITAITTNGAHPSKCVTIQRTLDGRLQVAGRKGFPHVIYARLWRWPDLHKNELKHVKYCQYAFDLKCDSVCVNPYHYERVVSPGIDLSGLTLQSNAPSSMMVKDEYVHDFEGQPSLSTEGHSIQTIQHPPSNRASTETYSTPALLAPSESNATSTANFPNIPVASTSQPASILGGSHSEGLLQIASGPQPGQQQNGFTGQPATYHHNSTTTWTGSRTAPYTPNLPHHQNGHLQHHPPMPPHPGHYWPVHNELAFQPPISNHPAPEYWCSIAYFEMDVQVGETFKVPSSCPIVTVDGYVDPSGGDRFCLGQLSNVHRTEAIERARLHIGKGVQLECKGEGDVWVRCLSDHAVFVQSYYLDREAGRAPGDAVHKIYPSAYIKVFDLRQCHRQMQQQAATAQAAAAAQAAAVAGNIPGPGSVGGIAPAISLSAAAGIGVDDLRRLCILRMSFVKGWGPDYPRQSIKETPCWIEIHLHRALQLLDEVLHTMPIADPQPLD

1056 NP_004025.1 NP_003121.1

>NP_004025.1

MSYPGYPPTGYPPFPGYPPAGQESSFPPSGQYPYPSGFPPMGGGAYPQVPSSGYPGAGGYPAPGGYPAPGGYPGAPQPGGAPSYPGVPPGQGFGVPPGGAGFSGYPQPPSQSYGGGPAQVPLPGGFPGGQMPSQYPGGQPTYPSQINTDSFSSYPVFSPVSLDYSSEPATVTQVTQGTIRPAANFDAIRDAEILRKAMKGFGTDEQAIVDVVANRSNDQRQKIKAAFKTSYGKDLIKDLKSELSGNMEELILALFMPPTYYDAWSLRKAMQGAGTQERVLIEILCTRTNQEIREIVRCYQSEFGRDLEKDIRSDTSGHFERLLVSMCQGNRDENQSINHQMAQEDAQRLYQAGEGRLGTDESCFNMILATRSFPQLRATMEAYSRMANRDLLSSVSREFSGYVESGLKTILQCALNRPAFFAERLYYAMKGAGTDDSTLVRIVVTRSEIDLVQIKQMFAQMYQKTLGTMIAGDTSGDYRRLLLAIVGQ

>NP_003121.1

MAYPGHPGAGGGYYPGGYGGAPGGPAFPGQTQDPLYGYFAAVAGQDGQIDADELQRCLTQSGIAGGYKPFNLETCRLMVSMLDRDMSGTMGFNEFKELWAVLNGWRQHFISFDTDRSGTVDPQELQKALTTMGFRLSPQAVNSIAKRYSTNGKITFDDYIACCVKLRALTDSFRRRDTAQQGVVNFPYDDFIQCVMSV

1057 NP_055912.1 NP_003379.3

>NP_055912.1

MEPRSMEYFCAQVQQKDVGGRLQVGQELLLYLGAPGAISDLEEDLGRLGKTVDALTGWVGSSNYRVSLMGLEILSAFVDRLSTRFKSYVAMVIVALIDRMGDAKDKVRDEAQTLILKLMDQVAPPMYIWEQLASGFKHKNFRSREGVCLCLIETLNIFGAQPLVISKLIPHLCILFGDSNSQVRDAAILAIVEIYRHVGEKVRMDLYKRGIPPARLEMIFAKFDEVQSSGGMILSVCKDKSFDDEESVDGNRPSSAASAFKVPAPKTSGNPANSARKPGSAGGPKVGGASKEGGAGAVDEDDFIKAFTDVPSIQIYSSRELEETLNKIREILSDDKHDWDQRANALKKIRSLLVAGAAQYDCFFQHLRLLDGALKLSAKDLRSQVVREACITVAHLSTVLGNKFDHGAEAIVPTLFNLVPNSAKVMATSGCAAIRFIIRHTHVPRLIPLITSNCTSKSVPVRRRSFEFLDLLLQEWQTHSLERHAAVLVETIKKGIHDADAEARVEARKTYMGLRNHFPGEAETLYNSLEPSYQKSLQTYLKSSGSVASLPQSDRSSSSSQESLNRPFSSKWSTANPSTVAGRVSAGSSKASSLPGSLQRSRSDIDVNAAAGAKAHHAAGQSVRSGRLGAGALNAGSYASLEDTSDKLDGTASEDGRVRAKLSAPLAGMGNAKADSRGRSRTKMVSQSQPGSRSGSPGRVLTTTALSTVSSGVQRVLVNSASAQKRSKIPRSQGCSREASPSRLSVARSSRIPRPSVSQGCSREASRESSRDTSPVRSFQPLGPGYGISQSSRLSSSVSAMRVLNTGSDVEEAVADALKKPARRRYESYGMHSDDDANSDASSACSERSYSSRNGSIPTYMRQTEDVAEVLNRCASSNWSERKEGLLGLQNLLKNQRTLSRVELKRLCEIFTRMFADPHGKRVFSMFLETLVDFIQVHKDDLQDWLFVLLTQLLKKMGADLLGSVQAKVQKALDVTRESFPNDLQFNILMRFTVDQTQTPSLKVKVAILKYIETLAKQMDPGDFINSSETRLAVSRVITWTTEPKSSDVRKAAQSVLISLFELNTPEFTMLLGALPKTFQDGATKLLHNHLRNTGNGTQSSMGSPLTRPTPRSPANWSSPLTSPTNTSQNTLSPSAFDYDTENMNSEDIYSSLRGVTEAIQNFSFRSQEDMNEPLKRDSKKDDGDSMCGGPGMSDPRAGGDATDSSQTALDNKASLLHSMPTHSSPRSRDYNPYNYSDSISPFNKSALKEAMFDDDADQFPDDLSLDHSDLVAELLKELSNHNERVEERKIALYELMKLTQEESFSVWDEHFKTILLLLLETLGDKEPTIRALALKVLREILRHQPARFKNYAELTVMKTLEAHKDPHKEVVRSAEEAASVLATSISPEQCIKVLCPIIQTADYPINLAAIKMQTKVIERVSKETLNLLLPEIMPGLIQGYDNSESSVRKACVFCLVAVHAVIGDELKPHLSQLTGSKMKLLNLYIKRAQTGSGGADPTTDVSGQS

>NP_003379.3

MQKPSGLKPPGRGGKHSSPMGRTSTGSASSSAAVAASSKEGSPLHKQSSGPSSSPAAAAAPEKPGPKAAEVGDDFLGDFVVGERVWVNGVKPGVVQYLGETQFAPGQWAGVVLDDPVGKNDGAVGGVRYFECPALQGIFTRPSKLTRQPTAEGSGSDAHSVESLTAQNLSLHSGTATPPLTSRVIPLRESVLNSSVKTGNESGSNLSDSGSVKRGEKDLRLGDRVLVGGTKTGVVRYVGETDFAKGEWCGVELDEPLGKNDGAVAGTRYFQCPPKFGLFAPIHKVIRIGFPSTSPAKAKKTKRMAMGVSALTHSPSSSSISSVSSVASSVGGRPSRSGLLTETSSRYARKISGTTALQEALKEKQQHIEQLLAERDLERAEVAKATSHICEVEKEIALLKAQHEQYVAEAEEKLQRARLLVESVRKEKVDLSNQLEEERRKVEDLQFRVEEESITKGDLETQTQLEHARIGELEQSLLLEKAQAERLLRELADNRLTTVAEKSRVLQLEEELTLRRGEIEELQQCLLHSGPPPPDHPDAAEILRLRERLLSASKEHQRESGVLRDKYEKALKAYQAEVDKLRAANEKYAQEVAGLKDKVQQATSENMGLMDNWKSKLDSLASDHQKSLEDLKATLNSGPGAQQKEIGELKAVMEGIKMEHQLELGNLQAKHDLETAMHVKEKEALREKLQEAQEELAGLQRHWRAQLEVQASQHRLELQEAQDQRRDAELRVHELEKLDVEYRGQAQAIEFLKEQISLAEKKMLDYERLQRAEAQGKQEVESLREKLLVAENRLQAVEALCSSQHTHMIESNDISEETIRTKETVEGLQDKLNKRDKEVTALTSQTEMLRAQVSALESKCKSGEKKVDALLKEKRRLEAELETVSRKTHDASGQLVLISQELLRKERSLNELRVLLLEANRHSPGPERDLSREVHKAEWRIKEQKLKDDIRGLREKLTGLDKEKSLSDQRRYSLIDPSSAPELLRLQHQLMSTEDALRDALDQAQQVEKLMEAMRSCPDKAQTIGNSGSANGIHQQDKAQKQEDKH

1058 NP_006087.2 NP_001952.1

>NP_006087.2

MSREMQDVDLAEVKPLVEKGETITGLLQEFDVQEQDIETLHGSVHVTLCGTPKGNRPVILTYHDIGMNHKTCYNPLFNYEDMQEITQHFAVCHVDAPGQQDGAASFPAGYMYPSMDQLAEMLPGVLQQFGLKSIIGMGTGAGAYILTRFALNNPEMVEGLVLINVNPCAEGWMDWAASKISGWTQALPDMVVSHLFGKEEMQSNVEVVHTYRQHIVNDMNPGNLHLFINAYNSRRDLEIERPMPGTHTVTLQCPALLVVGDSSPAVDAVVECNSKLDPTKTTLLKMADCGGLPQISQPAKLAEAFKYFVQGMGYMPSASMTRLMRSRTASGSSVTSLDGTRSRSHTSEGTRSRSHTSEGTRSRSHTSEGAHLDITPNSGAAGNSAGPKSMEVSC

>NP_001952.1

MVNFTVDQIRAIMDKKANIRNMSVIAHVDHGKSTLTDSLVCKAGIIASARAGETRFTDTRKDEQERCITIKSTAISLFYELSENDLNFIKQSKDGAGFLINLIDSPGHVDFSSEVTAALRVTDGALVVVDCVSGVCVQTETVLRQAIAERIKPVLMMNKMDRALLELQLEPEELYQTFQRIVENVNVIISTYGEGESGPMGNIMIDPVLGTVGFGSGLHGWAFTLKQFAEMYVAKFAAKGEGQLGPAERAKKVEDMMKKLWGDRYFDPANGKFSKSATSPEGKKLPRTFCQLILDPIFKVFDAIMNFKKEETAKLIEKLDIKLDSEDKDKEGKPLLKAVMRRWLPAGDALLQMITIHLPSPVTAQKYRCELLYEGPPDDEAAMGIKSCDPKGPLMMYISKMVPTSDKGRFYAFGRVFSGLVSTGLKVRIMGPNYTPGKKEDLYLKPIQRTILMMGRYVEPIEDVPCGNIVGLVGVDQFLVKTGTITTFEHAHNMRVMKFSVSPVVRVAVEAKNPADLPKLVEGLKRLAKSDPMVQCIIEESGEHIIAGAGELHLEICLKDLEEDHACIPIKKSDPVVSYRETVSEESNVLCLSKSPNKHNRLYMKARPFPDGLAEDIDKGEVSARQELKQRARYLAEKYEWDVAEARKIWCFGPDGTGPNILTDITKGVQYLNEIKDSVVAGFQWATKEGALCEENMRGVRFDVHDVTLHADAIHRGGGQIIPTARRCLYASVLTAQPRLMEPIYLVEIQCPEQVVGGIYGVLNRKRGHVFEESQVAGTPMFVVKAYLPVNESFGFTADLRSNTGGQAFPQCVFDHWQILPGDPFDNSSRPSQVVAETRKRKGLKEGIPALDNFLDKL

1059 NP_001026855.2 NP_000624.2

>NP_001026855.2

MRFGSKMMPMFLTVYLSNNEQHFTEVPVTPETICRDVVDLCKEPGESDCHLAEVWCGSERPVADNERMFDVLQRFGSQRNEVRFFLRHERPPGRDIVSGPRSQDPSLKRNGVKVPGEYRRKENGVNSPRMDLTLAELQEMASRQQQQIEAQQQLLATKEQRLKFLKQQDQRQQQQVAEQEKLKRLKEIAENQEAKLKKVRALKGHVEQKRLSNGKLVEEIEQMNNLFQQKQRELVLAVSKVEELTRQLEMLKNGRIDSHHDNQSAVAELDRLYKELQLRNKLNQEQNAKLQQQRECLNKRNSEVAVMDKRVNELRDRLWKKKAALQQKENLPVSSDGNLPQQAASAPSRVAAVGPYIQSSTMPRMPSRPELLVKPALPDGSLVIQASEGPMKIQTLPNMRSGAASQTKGSKIHPVGPDWSPSNADLFPSQGSASVPQSTGNALDQVDDGEVPLREKEKKVRPFSMFDAVDQSNAPPSFGTLRKNQSSEDILRDAQVANKNVAKVPPPVPTKPKQINLPYFGQTNQPPSDIKPDGSSQQLSTVVPSMGTKPKPAGQQPRVLLSPSIPSVGQDQTLSPGSKQESPPAAAVRPFTPQPSKDTLLPPFRKPQTVAASSIYSMYTQQQAPGKNFQQAVQSALTKTHTRGPHFSSVYGKPVIAAAQNQQQHPENIYSNSQGKPGSPEPETEPVSSVQENHENERIPRPLSPTKLLPFLSNPYRNQSDADLEALRKKLSNAPRPLKKRSSITEPEGPNGPNIQKLLYQRTTIAAMETISVPSYPSKSASVTASSESPVEIQNPYLHVEPEKEVVSLVPESLSPEDVGNASTENSDMPAPSPGLDYEPEGVPDNSPNLQNNPEEPNPEAPHVLDVYLEEYPPYPPPPYPSGEPEGPGEDSVSMRPPEITGQVSLPPGKRTNLRKTGSERIAHGMRVKFNPLALLLDSSLEGEFDLVQRIIYEVDDPSLPNDEGITALHNAVCAGHTEIVKFLVQFGVNVNAADSDGWTPLHCAASCNNVQVCKFLVESGAAVFAMTYSDMQTAADKCEEMEEGYTQCSQFLYGVQEKMGIMNKGVIYALWDYEPQNDDELPMKEGDCMTIIHREDEDEIEWWWARLNDKEGYVPRNLLGLYPRIKPRQRSLA

>NP_000624.2

MAHAGRTGYDNREIVMKYIHYKLSQRGYEWDAGDVGAAPPGAAPAPGIFSSQPGHTPHPAASRDPVARTSPLQTPAAPGAAAGPALSPVPPVVHLTLRQAGDDFSRRYRRDFAEMSSQLHLTPFTARGRFATVVEELFRDGVNWGRIVAFFEFGGVMCVESVNREMSPLVDNIALWMTEYLNRHLHTWIQDNGGWDAFVELYGPSMRPLFDFSWLSLKTLLSLALVGACITLGAYLGHK

1060 NP_006477.2 NP_002499.2

>NP_006477.2

MERAAPSRRVPLPLLLLGGLALLAAGVDADVLLEACCADGHRMATHQKDCSLPYATESKECRMVQEQCCHSQLEELHCATGISLANEQDRCATPHGDNASLEATFVKRCCHCCLLGRAAQAQGQSCEYSLMVGYQCGQVFRACCVKSQETGDLDVGGLQETDKIIEVEEEQEDPYLNDRCRGGGPCKQQCRDTGDEVVCSCFVGYQLLSDGVSCEDVNECITGSHSCRLGESCINTVGSFRCQRDSSCGTGYELTEDNSCKDIDECESGIHNCLPDFICQNTLGSFRCRPKLQCKSGFIQDALGNCIDINECLSISAPCPIGHTCINTEGSYTCQKNVPNCGRGYHLNEEGTRCVDVDECAPPAEPCGKGHRCVNSPGSFRCECKTGYYFDGISRMCVDVNECQRYPGRLCGHKCENTLGSYLCSCSVGFRLSVDGRSCEDINECSSSPCSQECANVYGSYQCYCRRGYQLSDVDGVTCEDIDECALPTGGHICSYRCINIPGSFQCSCPSSGYRLAPNGRNCQDIDECVTGIHNCSINETCFNIQGGFRCLAFECPENYRRSAATLQQEKTDTVRCIKSCRPNDVTCVFDPVHTISHTVISLPTFREFTRPEEIIFLRAITPPHPASQANIIFDITEGNLRDSFDIIKRYMDGMTVGVVRQVRPIVGPFHAVLKLEMNYVVGGVVSHRNVVNVHIFVSEYWF

>NP_002499.2

MLASSSRIRAAWTRALLLPLLLAGPVGCLSRQELFPFGPGQGDLELEDGDDFVSPALELSGALRFYDRSDIDAVYVTTNGIIATSEPPAKESHPGLFPPTFGAVAPFLADLDTTDGLGKVYYREDLSPSITQRAAECVHRGFPEISFQPSSAVVVTWESVAPYQGPSRDPDQKGKRNTFQAVLASSDSSSYAIFLYPEDGLQFHTTFSKKENNQVPAVVAFSQGSVGFLWKSNGAYNIFANDRESVENLAKSSNSGQQGVWVFEIGSPATTNGVVPADVILGTEDGAEYDDEDEDYDLATTRLGLEDVGTTPFSYKALRRGGADTYSVPSVLSPRRAATERPLGPPTERTRSFQLAVETFHQQHPQVIDVDEVEETGVVFSYNTDSRQTCANNRHQCSVHAECRDYATGFCCSCVAGYTGNGRQCVAEGSPQRVNGKVKGRIFVGSSQVPIVFENTDLHSYVVMNHGRSYTAISTIPETVGYSLLPLAPVGGIIGWMFAVEQDGFKNGFSITGGEFTRQAEVTFVGHPGNLVIKQRFSGIDEHGHLTIDTELEGRVPQIPFGSSVHIEPYTELYHYSTSVITSSSTREYTVTEPERDGASPSRIYTYQWRQTITFQECVHDDSRPALPSTQQLSVDSVFVLYNQEEKILRYALSNSIGPVREGSPDALQNPCYIGTHGCDTNAACRPGPRTQFTCECSIGFRGDGRTCYDIDECSEQPSVCGSHTICNNHPGTFRCECVEGYQFSDEGTCVAVVDQRPINYCETGLHNCDIPQRAQCIYTGGSSYTCSCLPGFSGDGQACQDVDECQPSRCHPDAFCYNTPGSFTCQCKPGYQGDGFRCVPGEVEKTRCQHEREHILGAAGATDPQRPIPPGLFVPECDAHGHYAPTQCHGSTGYCWCVDRDGREVEGTRTRPGMTPPCLSTVAPPIHQGPAVPTAVIPLPPGTHLLFAQTGKIERLPLEGNTMRKTEAKAFLHVPAKVIIGLAFDCVDKMVYWTDITEPSIGRASLHGGEPTTIIRQDLGSPEGIAVDHLGRNIFWTDSNLDRIEVAKLDGTQRRVLFETDLVNPRGIVTDSVRGNLYWTDWNRDNPKIETSYMDGTNRRILVQDDLGLPNGLTFDAFSSQLCWVDAGTNRAECLNPSQPSRRKALEGLQYPFAVTSYGKNLYFTDWKMNSVVALDLAISKETDAFQPHKQTRLYGITTALSQCPQGHNYCSVNNGGCTHLCLATPGSRTCRCPDNTLGVDCIEQK

1061 NP_002643.1 NP_000607.1

>NP_002643.1

MRLLQLLFRASPATLLLVLCLQLGANKAQDNTRKIIIKNFDIPKSVRPNDEVTAVLAVQTELKECMVVKTYLISSIPLQGAFNYKYTACLCDDNPKTFYWDFYTNRTVQIAAVVDVIRELGICPDDAAVIPIKNNRFYTIEILKVE

>NP_000607.1

MNRGVPFRHLLLVLQLALLPAATQGKKVVLGKKGDTVELTCTASQKKSIQFHWKNSNQIKILGNQGSFLTKGPSKLNDRADSRRSLWDQGNFPLIIKNLKIEDSDTYICEVEDQKEEVQLLVFGLTANSDTHLLQGQSLTLTLESPPGSSPSVQCRSPRGKNIQGGKTLSVSQLELQDSGTWTCTVLQNQKKVEFKIDIVVLAFQKASSIVYKKEGEQVEFSFPLAFTVEKLTGSGELWWQAERASSSKSWITFDLKNKEVSVKRVTQDPKLQMGKKLPLHLTLPQALPQYAGSGNLTLALEAKTGKLHQEVNLVVMRATQLQKNLTCEVWGPTSPKLMLSLKLENKEAKVSKREKAVWVLNPEAGMWQCLLSDSGQVLLESNIKVLPTWSTPVQPMALIVLGGVAGLLLFIGLGIFFCVRCRHRRRQAERMSQIKRLLSEKKTCQCPHRFQKTCSPI

1062 NP_003168.2 NP_005991.1

>NP_003168.2

MASSGMADSANHLPFFFGNITREEAEDYLVQGGMSDGLYLLRQSRNYLGGFALSVAHGRKAHHYTIERELNGTYAIAGGRTHASPADLCHYHSQESDGLVCLLKKPFNRPQGVQPKTGPFEDLKENLIREYVKQTWNLQGQALEQAIISQKPQLEKLIATTAHEKMPWFHGKISREESEQIVLIGSKTNGKFLIRARDNNGSYALCLLHEGKVLHYRIDKDKTGKLSIPEGKKFDTLWQLVEHYSYKADGLLRVLTVPCQKIGTQGNVNFGGRPQLPGSHPATWSAGGIISRIKSYSFPKPGHRKSSPAQGNRQESTVSFNPYEPELAPWAADKGPQREALPMDTEVYESPYADPEEIRPKEVYLDRKLLTLEDKELGSGNFGTVKKGYYQMKKVVKTVAVKILKNEANDPALKDELLAEANVMQQLDNPYIVRMIGICEAESWMLVMEMAELGPLNKYLQQNRHVKDKNIIELVHQVSMGMKYLEESNFVHRDLAARNVLLVTQHYAKISDFGLSKALRADENYYKAQTHGKWPVKWYAPECINYYKFSSKSDVWSFGVLMWEAFSYGQKPYRGMKGSEVTAMLEKGERMGCPAGCPREMYDLMNLCWTYDVENRPGFAAVELRLRNYYYDVVN

>NP_005991.1

MRECISVHVGQAGVQMGNACWELYCLEHGIQPDGQMPSDKTIGGGDDSFTTFFCETGAGKHVPRAVFVDLEPTVIDEIRNGPYRQLFHPEQLITGKEDAANNYARGHYTIGKEIIDPVLDRIRKLSDQCTGLQGFLVFHSFGGGTGSGFTSLLMERLSVDYGKKSKLEFSIYPAPQVSTAVVEPYNSILTTHTTLEHSDCAFMVDNEAIYDICRRNLDIERPTYTNLNRLISQIVSSITASLRFDGALNVDLTEFQTNLVPYPRIHFPLATYAPVISAEKAYHEQLSVAEITNACFEPANQMVKCDPRHGKYMACCLLYRGDVVPKDVNAAIAAIKTKRSIQFVDWCPTGFKVGINYQPPTVVPGGDLAKVQRAVCMLSNTTAIAEAWARLDHKFDLMYAKRAFVHWYVGEGMEEGEFSEAREDMAALEKDYEEVGIDSYEDEDEGEE

1063 NP_005408.1 NP_005649.1

>NP_005408.1

MGSNKSKPKDASQRRRSLEPAENVHGAGGGAFPASQTPSKPASADGHRGPSAAFAPAAAEPKLFGGFNSSDTVTSPQRAGPLAGGVTTFVALYDYESRTETDLSFKKGERLQIVNNTEGDWWLAHSLSTGQTGYIPSNYVAPSDSIQAEEWYFGKITRRESERLLLNAENPRGTFLVRESETTKGAYCLSVSDFDNAKGLNVKHYKIRKLDSGGFYITSRTQFNSLQQLVAYYSKHADGLCHRLTTVCPTSKPQTQGLAKDAWEIPRESLRLEVKLGQGCFGEVWMGTWNGTTRVAIKTLKPGTMSPEAFLQEAQVMKKLRHEKLVQLYAVVSEEPIYIVTEYMSKGSLLDFLKGETGKYLRLPQLVDMAAQIASGMAYVERMNYVHRDLRAANILVGENLVCKVADFGLARLIEDNEYTARQGAKFPIKWTAPEAALYGRFTIKSDVWSFGILLTELTTKGRVPYPGMVNREVLDQVERGYRMPCPPECPESLHDLMCQCWRKEPEERPTFEYLQAFLEDYFTSTEPQYQPGENL

>NP_005649.1

MASSSGSSPRPAPDENEFPFGCPPTVCQDPKEPRALCCAGCLSENPRNGEDQICPKCRGEDLQSISPGSRLRTQEKAHPEVAEAGIGCPFAGVGCSFKGSPQSVQEHEVTSQTSHLNLLLGFMKQWKARLGCGLESGPMALEQNLSDLQLQAAVEVAGDLEVDCYRAPCSESQEELALQHFMKEKLLAELEGKLRVFENIVAVLNKEVEASHLALATSIHQSQLDRERILSLEQRVVELQQTLAQKDQALGKLEQSLRLMEEASFDGTFLWKITNVTRRCHESACGRTVSLFSPAFYTAKYGYKLCLRLYLNGDGTGKRTHLSLFIVIMRGEYDALLPWPFRNKVTFMLLDQNNREHAIDAFRPDLSSASFQRPQSETNVASGCPLFFPLSKLQSPKHAYVKDDTMFLKCIVETST

1064 NP_060918.2 NP_003579.3

>NP_060918.2

MASASYHISNLLEKMTSSDKDFRFMATNDLMTELQKDSIKLDDDSERKVVKMILKLLEDKNGEVQNLAVKCLGPLVSKVKEYQVETIVDTLCTNMLSDKEQLRDISSIGLKTVIGELPPASSGSALAANVCKKITGRLTSAIAKQEDVSVQLEALDIMADMLSRQGGLLVNFHPSILTCLLPQLTSPRLAVRKRTIIALGHLVMSCGNIVFVDLIEHLLSELSKNDSMSTTRTYIQCIAAISRQAGHRIGEYLEKIIPLVVKFCNVDDDELREYCIQAFESFVRRCPKEVYPHVSTIINICLKYLTYDPNYNYDDEDEDENAMDADGGDDDDQGSDDEYSDDDDMSWKVRRAAAKCLDAVVSTRHEMLPEFYKTVSPALISRFKEREENVKADVFHAYLSLLKQTRPVQSWLCDPDAMEQGETPLTMLQSQVPNIVKALHKQMKEKSVKTRQCCFNMLTELVNVLPGALTQHIPVLVPGIIFSLNDKSSSSNLKIDALSCLYVILCNHSPQVFHPHVQALVPPVVACVGDPFYKITSEALLVTQQLVKVIRPLDQPSSFDATPYIKDLFTCTIKRLKAADIDQEVKERAISCMGQIICNLGDNLGSDLPNTLQIFLERLKNEITRLTTVKALTLIAGSPLKIDLRPVLGEGVPILASFLRKNQRALKLGTLSALDILIKNYSDSLTAAMIDAVLDELPPLISESDMHVSQMAISFLTTLAKVYPSSLSKISGSILNELIGLVRSPLLQGGALSAMLDFFQALVVTGTNNLGYMDLLRMLTGPVYSQSTALTHKQSYYSIAKCVAALTRACPKEGPAVVGQFIQDVKNSRSTDSIRLLALLSLGEVGHHIDLSGQLELKSVILEAFSSPSEEVKSAASYALGSISVGNLPEYLPFVLQEITSQPKRQYLLLHSLKEIISSASVVGLKPYVENIWALLLKHCECAEEGTRNVVAECLGKLTLIDPETLLPRLKGYLISGSSYARSSVVTAVKFTISDHPQPIDPLLKNCIGDFLKTLEDPDLNVRRVALVTFNSAAHNKPSLIRDLLDTVLPHLYNETKVRKELIREVEMGPFKHTVDDGLDIRKAAFECMYTLLDSCLDRLDIFEFLNHVEDGLKDHYDIKMLTFLMLVRLSTLCPSAVLQRLDRLVEPLRATCTTKVKANSVKQEFEKQDELKRSAMRAVAALLTIPEAEKSPLMSEFQSQISSNPELAAIFESIQKDSSSTNLESMDTS

>NP_003579.3

MMSQSSGSGDGNDDEATTSKDGGFSSPSPSAAAAAQEVRSATDGNTSTTPPTSAKKRKLNSSSSSSSNSSNEREDFDSTSSSSSTPPLQPRDSASPSTSSFCLGVSVAASSHVPIQKKLRFEDTLEFVGFDAKMAEESSSSSSSSSPTAATSQQQQLKNKSILISSVASVHHANGLAKSSTTVSSFANSKPGSAKKLVIKNFKDKPKLPENYTDETWQKLKEAVEAIQNSTSIKYNLEELYQAVENLCSYKISANLYKQLRQICEDHIKAQIHQFREDSLDSVLFLKKIDRCWQNHCRQMIMIRSIFLFLDRTYVLQNSMLPSIWDMGLELFRAHIISDQKVQNKTIDGILLLIERERNGEAIDRSLLRSLLSMLSDLQIYQDSFEQRFLEETNRLYAAEGQKLMQEREVPEYLHHVNKRLEEEADRLITYLDQTTQKSLIATVEKQLLGEHLTAILQKGLNNLLDENRIQDLSLLYQLFSRVRGGVQVLLQQWIEYIKAFGSTIVINPEKDKTMVQELLDFKDKVDHIIDICFLKNEKFINAMKEAFETFINKRPNKPAELIAKYVDSKLRAGNKEATDEELEKMLDKIMIIFRFIYGKDVFEAFYKKDLAKRLLVGKSASVDAEKSMLSKLKHECGAAFTSKLEGMFKDMELSKDIMIQFKQYMQNQNVPGNIELTVNILTMGYWPTYVPMEVHLPPEMVKLQEIFKTFYLGKHSGRKLQWQSTLGHCVLKAEFKEGKKELQVSLFQTLVLLMFNEGEEFSLEEIKQATGIEDGELRRTLQSLACGKARVLAKNPKGKDIEDGDKFICNDDFKHKLFRIKINQIQMKETVEEQASTTERVFQDRQYQIDAAIVRIMKMRKTLSHNLLVSEVYNQLKFPVKPADLKKRIESLIDRDYMERDKENPNQYNYIA

1065 NP_001887.1 NP_001028849.1

>NP_001887.1

MPGPAAGSRARVYAEVNSLRSREYWDYEAHVPSWGNQDDYQLVRKLGRGKYSEVFEAINITNNERVVVKILKPVKKKKIKREVKILENLRGGTNIIKLIDTVKDPVSKTPALVFEYINNTDFKQLYQILTDFDIRFYMYELLKALDYCHSKGIMHRDVKPHNVMIDHQQKKLRLIDWGLAEFYHPAQEYNVRVASRYFKGPELLVDYQMYDYSLDMWSLGCMLASMIFRREPFFHGQDNYDQLVRIAKVLGTEELYGYLKKYHIDLDPHFNDILGQHSRKRWENFIHSENRHLVSPEALDLLDKLLRYDHQQRLTAKEAMEHPYFYPVVKEQSQPCADNAVLSSGLTAAR

>NP_001028849.1

MGGGDGAAFKRPGDGARLQRVLGLGSRREPRSLPAGGPAPRRTAPPPPGHASAGPAAMSSHIAKSESKTSLLKAAAAAASGGSRAPRHGPARDPGLPSRRLPGSCPATPQSSGDPSSRRPLCRPAPREEGARGSQRVLPQAHCRPREALPAAASRPSPSSPLPPARGRDGEERGLSPALGLRGSLRARGRGDSVPAAASEADPFLHRLRPMLSSAFGQDRSLRPEEIEELREAFREFDKDKDGYINCRDLGNCMRTMGYMPTEMELIELSQQINMNLGGHVDFDDFVELMGPKLLAETADMIGVKELRDAFREFDTNGDGEISTSELREAMRKLLGHQVGHRDIEEIIRDVDLNGDGRVDFEEFVRMMSR

1066 NP_001060.1 NP_058519.2

>NP_001060.1

MREIVHIQAGQCGNQIGAKFWEVISDEHGIDPTGSYHGDSDLQLERINVYYNEAAGNKYVPRAILVDLEPGTMDSVRSGPFGQIFRPDNFVFGQSGAGNNWAKGHYTEGAELVDSVLDVVRKESESCDCLQGFQLTHSLGGGTGSGMGTLLISKIREEYPDRIMNTFSVMPSPKVSDTVVEPYNATLSVHQLVENTDETYSIDNEALYDICFRTLKLTTPTYGDLNHLVSATMSGVTTCLRFPGQLNADLRKLAVNMVPFPRLHFFMPGFAPLTSRGSQQYRALTVPELTQQMFDSKNMMAACDPRHGRYLTVAAIFRGRMSMKEVDEQMLNVQNKNSSYFVEWIPNNVKTAVCDIPPRGLKMSATFIGNSTAIQELFKRISEQFTAMFRRKAFLHWYTGEGMDEMEFTEAESNMNDLVSEYQQYQDATADEQGEFEEEEGEDEA

>NP_058519.2

MAEPRQEFEVMEDHAGTYGLGDRKDQGGYTMHQDQEGDTDAGLKESPLQTPTEDGSEEPGSETSDAKSTPTAEDVTAPLVDEGAPGKQAAAQPHTEIPEGTTAEEAGIGDTPSLEDEAAGHVTQEPESGKVVQEGFLREPGPPGLSHQLMSGMPGAPLLPEGPREATRQPSGTGPEDTEGGRHAPELLKHQLLGDLHQEGPPLKGAGGKERPGSKEEVDEDRDVDESSPQDSPPSKASPAQDGRPPQTAAREATSIPGFPAEGAIPLPVDFLSKVSTEIPASEPDGPSVGRAKGQDAPLEFTFHVEITPNVQKEQAHSEEHLGRAAFPGAPGEGPEARGPSLGEDTKEADLPEPSEKQPAAAPRGKPVSRVPQLKARMVSKSKDGTGSDDKKAKTSTRSSAKTLKNRPCLSPKHPTPGSSDPLIQPSSPAVCPEPPSSPKHVSSVTSRTGSSGAKEMKLKGADGKTKIATPRGAAPPGQKGQANATRIPAKTPPAPKTPPSSGEPPKSGDRSGYSSPGSPGTPGSRSRTPSLPTPPTREPKKVAVVRTPPKSPSSAKSRLQTAPVPMPDLKNVKSKIGSTENLKHQPGGGKVQIINKKLDLSNVQSKCGSKDNIKHVPGGGSVQIVYKPVDLSKVTSKCGSLGNIHHKPGGGQVEVKSEKLDFKDRVQSKIGSLDNITHVPGGGNKKIETHKLTFRENAKAKTDHGAEIVYKSPVVSGDTSPRHLSNVSSTGSIDMVDSPQLATLADEVSASLAKQGL

1067 NP_150241.2 NP_001886.1

>NP_150241.2

MEPAPARSPRPQQDPARPQEPTMPPPETPSEGRQPSPSPSPTERAPASEEEFQFLRCQQCQAEAKCPKLLPCLHTLCSGCLEASGMQCPICQAPWPLGADTPALDNVFFESLQRRLSVYRQIVDAQAVCTRCKESADFWCFECEQLLCAKCFEAHQWFLKHEARPLAELRNQSVREFLDGTRKTNNIFCSNPNHRTPTLTSIYCRGCSKPLCCSCALLDSSHSELKCDISAEIQQRQEELDAMTQALQEQDSAFGAVHAQMHAAVGQLGRARAETEELIRERVRQVVAHVRAQERELLEAVDARYQRDYEEMASRLGRLDAVLQRIRTGSALVQRMKCYASDQEVLDMHGFLRQALCRLRQEEPQSLQAAVRTDGFDEFKVRLQDLSSCITQGKDAAVSKKASPEAASTPRDPIDVDLPEEAERVKAQVQALGLAEAQPMAVVQSVPGAHPVPVYAFSIKGPSYGEDVSNTTTAQKRKCSQTQCPRKVIKMESEEGKEARLARSSPEQPRPSTSKAVSPPHLDGPPSPRSPVIGSEVFLPNSNHVASGAGEAEERVVVISSSEDSDAENSSSRELDDSSSESSDLQLEGPSTLRVLDENLADPQAEDRPLVFFDLKIDNETQKISQLAAVNRESKFRVVIQPEAFFSIYSKAVSLEVGLQHFLSFLSSMRRPILACYKLWGPGLPNFFRALEDINRLWEFQEAISGFLAALPLIRERVPGASSFKLKNLAQTYLARNMSERSAMAAVLAMRDLCRLLEVSPGPQLAQHVYPFSSLQCFASLQPLVQAAVLPRAEARLLALHNVSFMELLSAHRRDRQGGLKKYSRYLSLQTTTLPPAQPAFNLQALGTYFEGLLEGPALARAEGVSTPLAGRGLAERASQQS

>NP_001886.1

MSGPVPSRARVYTDVNTHRPREYWDYESHVVEWGNQDDYQLVRKLGRGKYSEVFEAINITNNEKVVVKILKPVKKKKIKREIKILENLRGGPNIITLADIVKDPVSRTPALVFEHVNNTDFKQLYQTLTDYDIRFYMYEILKALDYCHSMGIMHRDVKPHNVMIDHEHRKLRLIDWGLAEFYHPGQEYNVRVASRYFKGPELLVDYQMYDYSLDMWSLGCMLASMIFRKEPFFHGHDNYDQLVRIAKVLGTEDLYDYIDKYNIELDPRFNDILGRHSRKRWERFVHSENQHLVSPEALDFLDKLLRYDHQSRLTAREAMEHPYFYTVVKDQARMGSSSMPGGSTPVSSANMMSGISSVPTPSPLGPLAGSPVIAAANPLGMPVPAAAGAQQ

1068 NP_004802.1 NP_742017.1

>NP_004802.1

MEELDALLEELERSTLQDSDEYSNPAPLPLDQHSRKETNLDETSEILSIQDNTSPLPAQLVYTTNIQELNVYSEAQEPKESPPPSKTSAAAQLDELMAHLTEMQAKVAVRADAGKKHLPDKQDHKASLDSMLGGLEQELQDLGIATVPKGHCASCQKPIAGKVIHALGQSWHPEHFVCTHCKEEIGSSPFFERSGLAYCPNDYHQLFSPRCAYCAAPILDKVLTAMNQTWHPEHFFCSHCGEVFGAEGFHEKDKKPYCRKDFLAMFSPKCGGCNRPVLENYLSAMDTVWHPECFVCGDCFTSFSTGSFFELDGRPFCELHYHHRRGTLCHGCGQPITGRCISAMGYKFHPEHFVCAFCLTQLSKGIFREQNDKTYCQPCFNKLFPL

>NP_742017.1

MVCSPVTVRIAPPDRRFSRSAIPEQIISSTLSSPSSNAPDPCAKETVLSALKEKEKKRTVEEEDQIFLDGQENKRRRHDSSGSGHSAFEPLVANGVPASFVPKPGSLKRGLNSQSSDDHLNKRSRSSSMSSLTGAYASGIPSSSRNAITSSYSSTRGISQLWKRNGPSSSPFSSPASSRSQTPERPAKKIREEELCHHSSSSTPLAADRESQGEKAADTTPRKKQNSNSQSTPGSSGQRKRKVQLLPSRRGEQLTLPPPPQLGYSITAEDLDLEKKASLQWFNQALEDKSDAASNSVTETPPITQPSFTFTLPAAAPASPPTSLLAPSTNPLLESLKKMQTPPSLPPCPESAGAATTEALSPPKTPSLLPPLGLSQSGPPGLLPSPSFDSKPPTTLLGLIPAPSMVPATDTKAPPTLQAETATKPQATSAPSPAPKQSFLFGTQNTSPSSPAAPAASSAPPMFKPIFTAPPKSEKEGPTPPGPSVTATAPSSSSLPTTTSTTAPTFQPVFSSMGPPASVPLPAPFFKQTTTPATAPTTTAPLFTGLASATSAVAPITSASPSTDSASKPAFGFGINSVSSSSVSTTTSTATAASQPFLFGAPQASAASFTPAMGSIFQFGKPPALPTTTTVTTFSQSLHTAVPTATSSSAADFSGFGSTLATSAPATSSQPTLTFSNTSTPTFNIPFGSSAKSPLPSYPGANPQPAFGAAEGQPPGAAKPALAPSFGSSFTFGNSAAPAAAPTPAPPSMIKVVPAYVPTPIHPIFGGATHSAFGLKATASAFGAPASSQPAFGGSTAVFFGAATSSGFGATTQTASSGSSSSVFGSTTPSPFTFGGSAAPAGSGSFGINVATPGSSTTTGAFSFGAGQSGSTATSTPFAGGLGQNALGTTGQSTPFAFNVSSTTESKPVFGGTATPTFGLNTPAPGVGTSGSSLSFGASSAPAQGFVGVAPFGSAALSFSIGAGSKTPGARQRLQARRQHTRKK

1069 NP_001027026.1 NP_065162.1

>NP_001027026.1

MESVRIEQMLSLPAEVSSDNLESAERGASAAQVDMGPHPKVAAEGPAPLPTREPEQEQSPGTSTPESKVLLTQADALASRGRIREALEVYRQLSERQQLVAEQLEQLVRCLAEKVPQGEALAPAPPDEGSTASGTVAAEETGAAAAAAATEVWDGFKCRKCHGFLSDPVSLSCGHTFCKLCLERGRAADRRCALCGVKLSALMVATGRARGARRAGQQPPPPLRVNVVLSGLLGKLFPGPARASQLRHEGNRLYRERQVEAALLKYNEAVKLAPNDHLLYSNRSQIYFTLESHENALHDAEIACKLRPMGFKAHFRKAQALATLGKVEEALREFLYCVSLDGKNKRARCEAQRDNLELPHCSSQEEAAARGDGSSLMDPAKVKGDGQQHHMKDQEEEEEKWDATSPKAASSKTGKCQEKKRKHCQIESQEETGMPNKASKQDPPTDQGDKPALSLPLASFDASDLECALCMRLFYEPVTTPCGHTFCLKCLERCLDHNAKCPLCKDGLSQCLASRKYSKNVIMEELIAKFLPEELKERRKLYEEEMEELSNLNKNVPIFVCTMAYPTVPCPLHIFEPCYRLMIRRCIETGTRQFGMCLGDPVKGFAEYGCILEIRNVQFFADGRSVVDSIGKRRFRVLHQSQRDGYNTADIEYIEDQKVQGEDCAELMGLHNCVYQQASLWFHSLKLSLKNRILNHFGPMPEKDADPQMNPNGPAWCWWMLAVLPLESRAQLPFLAMRSLKDRLNGIRRVLAFISRNQN

>NP_065162.1

MSNPGTRRNGSSIKIRLTVLCAKNLAKKDFFRLPDPFAKIVVDGSGQCHSTDTVKNTLDPKWNQHYDLYVGKTDSITISVWNHKKIHKKQGAGFLGCVRLLSNAISRLKDTGYQRLDLCKLNPSDTDAVRGQIVVSLQTRDRIGTGGSVVDCRGLLENEGTVYEDSGPGRPLSCFMEEPAPYTDSTGAAAGGGNCRFVESPSQDQRLQAQRLRNPDVRGSLQTPQNRPHGHQSPELPEGYEQRTTVQGQVYFLHTQTGVSTWHDPRIPSPSGTIPGGDAAFLYEFLLQGHTSEPRDLNSVNCDELGPLPPGWEVRSTVSGRIYFVDHNNRTTQFTDPRLHHIMNHQCQLKEPSQPLPLPSEGSLEDEELPAQRYERDLVQKLKVLRHELSLQQPQAGHCRIEVSREEIFEESYRQIMKMRPKDLKKRLMVKFRGEEGLDYGGVAREWLYLLCHEMLNPYYGLFQYSTDNIYMLQINPDSSINPDHLSYFHFVGRIMGLAVFHGHYINGGFTVPFYKQLLGKPIQLSDLESVDPELHKSLVWILENDITPVLDHTFCVEHNAFGRILQHELKPNGRNVPVTEENKKEYVRLYVNWRFMRGIEAQFLALQKGFNELIPQHLLKPFDQKELELIIGGLDKIDLNDWKSNTRLKHCVADSNIVRWFWQAVETFDEERRARLLQFVTGSTRVPLQGFKALQGSTGAAGPRLFTIHLIDANTDNLPKAHTCFNRIDIPPYESYEKLYEKLLTAVEETCGFAVE

1070 NP_056468.1 NP_006839.2

>NP_056468.1

MRQNDKIMCILENRKKRDRKNLCRAINDFQQSFQKPETRREFDLSDPLALKKDLPARQSDNDVRNTISGMQKFMGEDLNFHERKKFQEEQNREWSLQQQREWKNARAEQKCAEALYTETRLQFDETAKHLQKLESTTRKAVCASVKDFNKSQAIESVERKKQEKKQEQEDNLAEITNLLRGDLLSENPQQAASSFGPHRVVPDRWKGMTQEQLEQIRLVQKQQIQEKLRLQEEKRQRDLDWDRRRIQGARATLLFERQQWRRQRDLRRALDSSNLSLAKEQHLQKKYMNEVYTNQPTGDYFTQFNTGSR

>NP_006839.2

MEAEAGGLEELTDEEMAALGKEELVRRLRREEAARLAALVQRGRLMQEVNRQLQGHLGEIRELKQLNRRLQAENRELRDLCCFLDSERQRGRRAARQWQLFGTQASRAVREDLGGCWQKLAELEGRQEELLRENLALKELCLALGEEWGPRGGPSGAGGSGAGPAPELALPPCGPRDLGDGSSSTGSVGSPDQLPLACSPDD

1071 NP_000386.1 NP_002101.2

>NP_000386.1

MVLAQGLLSMALLALCWERSLAGAEETIPLQTLRCYNDYTSHITCRWADTQDAQRLVNVTLIRRVNEDLLEPVSCDLSDDMPWSACPHPRCVPRRCVIPCQSFVVTDVDYFSFQPDRPLGTRLTVTLTQHVQPPEPRDLQISTDQDHFLLTWSVALGSPQSHWLSPGDLEFEVVYKRLQDSWEDAAILLSNTSQATLGPEHLMPSSTYVARVRTRLAPGSRLSGRPSKWSPEVCWDSQPGDEAQPQNLECFFDGAAVLSCSWEVRKEVASSVSFGLFYKPSPDAGEEECSPVLREGLGSLHTRHHCQIPVPDPATHGQYIVSVQPRRAEKHIKSSVNIQMAPPSLNVTKDGDSYSLRWETMKMRYEHIDHTFEIQYRKDTATWKDSKTETLQNAHSMALPALEPSTRYWARVRVRTSRTGYNGIWSEWSEARSWDTESVLPMWVLALIVIFLTIAVLLALRFCGIYGYRLRRKWEEKIPNPSKSHLFQNGSAELWPPGSMSAFTSGSPPHQGPWGSRFPELEGVFPVGFGDSEVSPLTIEDPKHVCDPPSGPDTTPAASDLPTEQPPSPQPGPPAASHTPEKQASSFDFNGPYLGPPHSRSLPDILGQPEPPQEGGSQKSPPPGSLEYLCLPAGGQVQLVPLAQAMGPGQAVEVERRPSQGAAGSPSLESGGGPAPPALGPRVGGQDQKDSPVAIPMSSGDTEDPGVASGYVSSADLVFTPNSGASSVSLVPSLGLPSDQTPSLCPGLASGPPGAPGPVKSGFEGYVELPPIEGRSPRSPRNNPVPPEAKSPVLNPGERPADVSPTSPQPEGLLVLQQVGDYCFLPGLGPGPLSLRSKPSSPGPGPEIKNLDQAFQVKKPPGQAVPQVPVIQLFKALKQQDYLSLPPWEVNKPGEVC

>NP_002101.2

MGGRSSCEDPGCPRDEERAPRMGCMKSKFLQVGGNTFSKTETSASPHCPVYVPDPTSTIKPGPNSHNSNTPGIREAGSEDIIVVALYDYEAIHHEDLSFQKGDQMVVLEESGEWWKARSLATRKEGYIPSNYVARVDSLETEEWFFKGISRKDAERQLLAPGNMLGSFMIRDSETTKGSYSLSVRDYDPRQGDTVKHYKIRTLDNGGFYISPRSTFSTLQELVDHYKKGNDGLCQKLSVPCMSSKPQKPWEKDAWEIPRESLKLEKKLGAGQFGEVWMATYNKHTKVAVKTMKPGSMSVEAFLAEANVMKTLQHDKLVKLHAVVTKEPIYIITEFMAKGSLLDFLKSDEGSKQPLPKLIDFSAQIAEGMAFIEQRNYIHRDLRAANILVSASLVCKIADFGLARVIEDNEYTAREGAKFPIKWTAPEAINFGSFTIKSDVWSFGILLMEIVTYGRIPYPGMSNPEVIRALERGYRMPRPENCPEELYNIMMRCWKNRPEERPTFEYIQSVLDDFYTATESQYQQQP

1072 NP_003912.1 NP_003339.1

>NP_003912.1

MEPTAPSLTEEDLTEVKKDALENLRVYLCEKIIAERHFDHLRAKKILSREDTEEISCRTSSRKRAGKLLDYLQENPKGLDTLVESIRREKTQNFLIQKITDEVLKLRNIKLEHLKGLKCSSCEPFPDGATNNLSRSNSDESNFSEKLRASTVMYHPEGESSTTPFFSTNSSLNLPVLEVGRTENTIFSSTTLPRPGDPGAPPLPPDLQLEEEGTCANSSEMFLPLRSRTVSRQ

>NP_003339.1

MAGLPRRIIKETQRLLAEPVPGIKAEPDESNARYFHVVIAGPQDSPFEGGTFKLELFLPEEYPMAAPKVRFMTKIYHPNVDKLGRICLDILKDKWSPALQIRTVLLSIQALLSAPNPDDPLANDVAEQWKTNEAQAIETARAWTRLYAMNNI

1073 NP_002415.1 NP_002973.1

>NP_002415.1

MFSLKTLPFLLLLHVQISKAFPVSSKEKNTKTVQDYLEKFYQLPSNQYQSTRKNGTNVIVEKLKEMQRFFGLNVTGKPNEETLDMMKKPRCGVPDSGGFMLTPGNPKWERTNLTYRIRNYTPQLSEAEVERAIKDAFELWSVASPLIFTRISQGEADINIAFYQRDHGDNSPFDGPNGILAHAFQPGQGIGGDAHFDAEETWTNTSANYNLFLVAAHEFGHSLGLAHSSDPGALMYPNYAFRETSNYSLPQDDIDGIQAIYGLSSNPIQPTGPSTPKPCDPSLTFDAITTLRGEILFFKDRYFWRRHPQLQRVEMNFISLFWPSLPTGIQAAYEDFDRDLIFLFKGNQYWALSGYDILQGYPKDISNYGFPSSVQAIDAAVFYRSKTYFFVNDQFWRYDNQRQFMEPGYPKSISGAFPGIESKVDAVFQQEHFFHVFSGPRYYAFDLIAQRVTRVARGNKWLNCRYG

>NP_002973.1

MKVSAALLCLLLIAATFIPQGLAQPDAINAPVTCCYNFTNRKISVQRLASYRRITSSKCPKEAVIFKTIVAKEICADPKQKWVQDSMDHLDKQTQTPKT

1074 NP_005139.1 NP_060125.4

>NP_005139.1

MKVKKGGGGAGTATESAPGPSGQSVAPIPQPPAESESGSESEPDAGPGPRPGPLQRKQPIGPEDVLGLQRITGDYLCSPEENIYKIDFVRFKIRDMDSGTVLFEIKKPPVSERLPINRRDLDPNAGRFVRYQFTPAFLRLRQVGATVEFTVGDKPVNNFRMIERHYFRNQLLKSFDFHFGFCIPSSKNTCEHIYDFPPLSEELISEMIRHPYETQSDSFYFVDDRLVMHNKADYSYSGTP

>NP_060125.4

MPLKLRGKKKAKSKETAGLVEGEPTGAGGGSLSASRAPARRLVFHAQLAHGSATGRVEGFSSIQELYAQIAGAFEISPSEILYCTLNTPKIDMERLLGGQLGLEDFIFAHVKGIEKEVNVYKSEDSLGLTITDNGVGYAFIKRIKDGGVIDSVKTICVGDHIESINGENIVGWRHYDVAKKLKELKKEELFTMKLIEPKKAFEIELRSKAGKSSGEKIGCGRATLRLRSKGPATVEEMPSETKAKAIEKIDDVLELYMGIRDIDLATTMFEAGKDKVNPDEFAVALDETLGDFAFPDEFVFDVWGVIGDAKRRGL

1075 NP_478126.1 NP_055299.1

>NP_478126.1

MPGDHRRIRGPEESQPPQLYAADEEEAPGTRDPTRLRPVYARAGLLSQAKGSAYLEAGGTKVLCAVSGPRQAEGGERGGGPAGAGGEAPAALRGRLLCDFRRAPFAGRRRRAPPGGCEERELALALQEALEPAVRLGRYPRAQLEVSALLLEDGGSALAAALTAAALALADAGVEMYDLVVGCGLSLAPGPAPTWLLDPTRLEEERAAAGLTVALMPVLNQVAGLLGSGEGGLTESWAEAVRLGLEGCQRLYPVLQQSLVRAARRRGAAAQP

>NP_055299.1

MASREEVLALQAEVAQREEELNSLKQKLASALLAEQEPQPERLVPVSPLPPKAALSRDEILRYSRQLVLPELGVHGQLRLGTACVLIVGCGGLGCPLAQYLAAAGVGRLGLVDYDVVEMSNLARQVLHGEALAGQAKAFSAAASLRRLNSAVECVPYTQALTPATALDLVRRYDVVADCSDNVPTRYLVNDACVLAGRPLVSASALRFEGQITVYHYDGGPCYRCIFPQPPPAETVTNCADGGVLGVVTGVLGCLQALEVLKIAAGLGPSYSGSLLLFDALRGHFRSIRLRSRRLDCAACGERPTVTDLLDYEAFCGSSATDKCRSLQLLSPEERVSVTDYKRLLDSGAFHLLLDVRPQVEVDICRLPHALHIPLKHLERRDAESLKLLKEAIWEEKQGTQEGAAVPIYVICKLGNDSQKAVKILQSLSAAQELDPLTVRDVVGGLMAWAAKIDGTFPQY

1076 NP_061848.2 NP_005723.2

>NP_061848.2

MAEAMDLGKDPNGPTHSSTLFVRDDGSSMSFYVRPSPAKRRLSTLILHGGGTVCRVQEPGAVLLAQPGEALAEASGDFISTQYILDCVERNERLELEAYRLGPASAADTGSEAKPGALAEGAAEPEPQRHAGRIAFTDADDVAILTYVKENARSPSSVTGNALWKAMEKSSLTQHSWQSLKDRYLKHLRGQEHKYLLGDAPVSPSSQKLKRKAEEDPEAADSGEPQNKRTPDLPEEEYVKEEIQENEEAVKKMLVEATREFEEVVVDESPPDFEIHITMCDDDPPTPEEDSETQPDEEEEEEEEKVSQPEVGAAIKIIRQLMEKFNLDLSTVTQAFLKNSGELEATSAFLASGQRADGYPIWSRQDDIDLQKDDEDTREALVKKFGAQNVARRIEFRKK

>NP_005723.2

MSRIEKMSILGVRSFGIEDKDKQIITFFSPLTILVGPNGAGKTTIIECLKYICTGDFPPGTKGNTFVHDPKVAQETDVRAQIRLQFRDVNGELIAVQRSMVCTQKSKKTEFKTLEGVITRTKHGEKVSLSSKCAEIDREMISSLGVSKAVLNNVIFCHQEDSNWPLSEGKALKQKFDEIFSATRYIKALETLRQVRQTQGQKVKEYQMELKYLKQYKEKACEIRDQITSKEAQLTSSKEIVKSYENELDPLKNRLKEIEHNLSKIMKLDNEIKALDSRKKQMEKDNSELEEKMEKVFQGTDEQLNDLYHNHQRTVREKERKLVDCHRELEKLNKESRLLNQEKSELLVEQGRLQLQADRHQEHIRARDSLIQSLATQLELDGFERGPFSERQIKNFHKLVRERQEGEAKTANQLMNDFAEKETLKQKQIDEIRDKKTGLGRIIELKSEILSKKQNELKNVKYELQQLEGSSDRILELDQELIKAERELSKAEKNSNVETLKMEVISLQNEKADLDRTLRKLDQEMEQLNHHTTTRTQMEMLTKDKADKDEQIRKIKSRHSDELTSLLGYFPNKKQLEDWLHSKSKEINQTRDRLAKLNKELASSEQNKNHINNELKRKEEQLSSYEDKLFDVCGSQDFESDLDRLKEEIEKSSKQRAMLAGATAVYSQFITQLTDENQSCCPVCQRVFQTEAELQEVISDLQSKLRLAPDKLKSTESELKKKEKRRDEMLGLVPMRQSIIDLKEKEIPELRNKLQNVNRDIQRLKNDIEEQETLLGTIMPEEESAKVCLTDVTIMERFQMELKDVERKIAQQAAKLQGIDLDRTVQQVNQEKQEKQHKLDTVSSKIELNRKLIQDQQEQIQHLKSTTNELKSEKLQISTNLQRRQQLEEQTVELSTEVQSLYREIKDAKEQVSPLETTLEKFQQEKEELINKKNTSNKIAQDKLNDIKEKVKNIHGYMKDIENYIQDGKDDYKKQKETELNKVIAQLSECEKHKEKINEDMRLMRQDIDTQKIQERWLQDNLTLRKRNEELKEVEEERKQHLKEMGQMQVLQMKSEHQKLEENIDNIKRNHNLALGRQKGYEEEIIHFKKELREPQFRDAEEKYREMMIVMRTTELVNKDLDIYYKTLDQAIMKFHSMKMEEINKIIRDLWRSTYRGQDIEYIEIRSDADENVSASDKRRNYNYRVVMLKGDTALDMRGRCSAGQKVLASLIIRLALAETFCLNCGIIALDEPTTNLDRENIESLAHALVEIIKSRSQQRNFQLLVITHDEDFVELLGRSEYVEKFYRIKKNIDQCSEIVKCSVSSLGFNVH

1077 NP_005239.1 NP_001372.1

>NP_005239.1

MGCVFCKKLEPVATAKEDAGLEGDFRSYGAADHYGPDPTKARPASSFAHIPNYSNFSSQAINPGFLDSGTIRGVSGIGVTLFIALYDYEARTEDDLTFTKGEKFHILNNTEGDWWEARSLSSGKTGCIPSNYVAPVDSIQAEEWYFGKIGRKDAERQLLSPGNPQGAFLIRESETTKGAYSLSIRDWDQTRGDHVKHYKIRKLDMGGYYITTRVQFNSVQELVQHYMEVNDGLCNLLIAPCTIMKPQTLGLAKDAWEISRSSITLERRLGTGCFGDVWLGTWNGSTKVAVKTLKPGTMSPKAFLEEAQVMKLLRHDKLVQLYAVVSEEPIYIVTEFMCHGSLLDFLKNPEGQDLRLPQLVDMAAQVAEGMAYMERMNYIHRDLRAANILVGERLACKIADFGLARLIKDDEYNPCQGSKFPIKWTAPEAALFGRFTIKSDVWSFGILLTELITKGRIPYPGMNKREVLEQVEQGYHMPCPPGCPASLYEAMEQTWRLDPEERPTFEYLQSFLEDYFTSAEPQYQPGDQT

>NP_001372.1

MDGAVMEGPLFLQSQRFGTKRWRKTWAVLYPASPHGVARLEFFDHKGSSSGGGRGSSRRLDCKVIRLAECVSVAPVTVETPPEPGATAFRLDTAQRSHLLAADAPSSAAWVQTLCRNAFPKGSWTLAPTDNPPKLSALEMLENSLYSPTWEGSQFWVTVQRTEAAERCGLHGSYVLRVEAERLTLLTVGAQSQILEPLLSWPYTLLRRYGRDKVMFSFEAGRRCPSGPGTFTFQTAQGNDIFQAVETAIHRQKAQGKAGQGHDVLRADSHEGEVAEGKLPSPPGPQELLDSPPALYAEPLDSLRIAPCPSQDSLYSDPLDSTSAQAGEGVQRKKPLYWDLYEHAQQQLLKAKLTDPKEDPIYDEPEGLAPVPPQGLYDLPREPKDAWWCQARVKEEGYELPYNPATDDYAVPPPRSTKPLLAPKPQGPAFPEPGTATGSGIKSHNSALYSQVQKSGASGSWDCGLSRVGTDKTGVKSEGST

1078 NP_036428.1 NP_003168.2

>NP_036428.1

MSGGGDVVCTGWLRKSPPEKKLRRYAWKKRWFILRSGRMSGDPDVLEYYKNDHSKKPLRIINLNFCEQVDAGLTFNKKELQDSFVFDIKTSERTFYLVAETEEDMNKWVQSICQICGFNQAEESTDSLRNVSSAGHGPRSSPAELSSSSQHLLRERKSSAPSHSSQPTLFTFEPPVSNHMQPTLSTSAPQEYLYLHQCISRRAENARSASFSQGTRASFLMRSDTAVQKLAQGNGHCVNGISGQVHGFYSLPKPSRHNTEFRDSTYDLPRSLASHGHTKGSLTGSETDNEDVYTFKTPSNTLCREFGDLLVDNMDVPATPLSAYQIPRTFTLDKNHNAMTVATPGDSAIAPPPRPPKPSQAETPRWGSPQQRPPISENSRSVAATIPRRNTLPAMDNSRLHRASSCETYEYPQRGGESAGRSAESMSDGVGSFLPGKMIVGRSDSTNSEDNYVPMNPGSSTLLAMERAGDNSQSVYIPMSPGAHHFDSLGYPSTTLPVHRGPSRGSEIQPPPVNRNLKPDRKAKPTPLDLRNNTVIDELPFKSPITKSWSRANHTFNSSSSQYCRPISTQSITSTDSGDSEENYVPMQNPVSASPVPSGTNSPAPKKSTGSVDYLALDFQPSSPSPHRKPSTSSVTSDEKVDYVQVDKEKTQALQNTMQEWTDVRQSSEPSKGAKL

>NP_003168.2

MASSGMADSANHLPFFFGNITREEAEDYLVQGGMSDGLYLLRQSRNYLGGFALSVAHGRKAHHYTIERELNGTYAIAGGRTHASPADLCHYHSQESDGLVCLLKKPFNRPQGVQPKTGPFEDLKENLIREYVKQTWNLQGQALEQAIISQKPQLEKLIATTAHEKMPWFHGKISREESEQIVLIGSKTNGKFLIRARDNNGSYALCLLHEGKVLHYRIDKDKTGKLSIPEGKKFDTLWQLVEHYSYKADGLLRVLTVPCQKIGTQGNVNFGGRPQLPGSHPATWSAGGIISRIKSYSFPKPGHRKSSPAQGNRQESTVSFNPYEPELAPWAADKGPQREALPMDTEVYESPYADPEEIRPKEVYLDRKLLTLEDKELGSGNFGTVKKGYYQMKKVVKTVAVKILKNEANDPALKDELLAEANVMQQLDNPYIVRMIGICEAESWMLVMEMAELGPLNKYLQQNRHVKDKNIIELVHQVSMGMKYLEESNFVHRDLAARNVLLVTQHYAKISDFGLSKALRADENYYKAQTHGKWPVKWYAPECINYYKFSSKSDVWSFGVLMWEAFSYGQKPYRGMKGSEVTAMLEKGERMGCPAGCPREMYDLMNLCWTYDVENRPGFAAVELRLRNYYYDVVN

1079 NP_001015053.1 NP_060215.4

>NP_001015053.1

MNSPNESADGMSGREPSLEILPRTSLHSIPVTVEVKPVLPRAMPSSMGGGGGGSPSPVELRGALVGSVDPTLREQQLQQELLALKQQQQLQKQLLFAEFQKQHDHLTRQHEVQLQKHLKQQQEMLAAKQQQEMLAAKRQQELEQQRQREQQRQEELEKQRLEQQLLILRNKEKSKESAIASTEVKLRLQEFLLSKSKEPTPGGLNHSLPQHPKCWGAHHASLDQSSPPQSGPPGTPPSYKLPLPGPYDSRDDFPLRKTASEPNLKVRSRLKQKVAERRSSPLLRRKDGTVISTFKKRAVEITGAGPGASSVCNSAPGSGPSSPNSSHSTIAENGFTGSVPNIPTEMLPQHRALPLDSSPNQFSLYTSPSLPNISLGLQATVTVTNSHLTASPKLSTQQEAERQALQSLRQGGTLTGKFMSTSSIPGCLLGVALEGDGSPHGHASLLQHVLLLEQARQQSTLIAVPLHGQSPLVTGERVATSMRTVGKLPRHRPLSRTQSSPLPQSPQALQQLVMQQQHQQFLEKQKQQQLQLGKILTKTGELPRQPTTHPEETEEELTEQQEVLLGEGALTMPREGSTESESTQEDLEEEDEEDDGEEEEDCIQVKDEEGESGAEEGPDLEEPGAGYKKLFSDAQPLQPLQVYQAPLSLATVPHQALGRTQSSPAAPGGMKSPPDQPVKHLFTTGVVYDTFMLKHQCMCGNTHVHPEHAGRIQSIWSRLQETGLLSKCERIRGRKATLDEIQTVHSEYHTLLYGTSPLNRQKLDSKKLLGPISQKMYAVLPCGGIGVDSDTVWNEMHSSSAVRMAVGCLLELAFKVAAGELKNGFAIIRPPGHHAEESTAMGFCFFNSVAITAKLLQQKLNVGKVLIVDWDIHHGNGTQQAFYNDPSVLYISLHRYDNGNFFPGSGAPEEVGGGPGVGYNVNVAWTGGVDPPIGDVEYLTAFRTVVMPIAHEFSPDVVLVSAGFDAVEGHLSPLGGYSVTARCFGHLTRQLMTLAGGRVVLALEGGHDLTAICDASEACVSALLSVELQPLDEAVLQQKPNINAVATLEKVIEIQSKHWSCVQKFAAGLGRSLREAQAGETEEAETVSAMALLSVGAEQAQAAAAREHSPRPAEEPMEQEPAL

>NP_060215.4

MLSATPLYGNVHSWMNSERVRMCGASEDRKILVNDGDASKARLELREENPLNHNVVDASTAHRIDGLAALSMDRTGLIREGLRVPGNIVYSSLCGLGSEKGREAATSTLGGLGFSSERNPEMQFKPNTPETVEASAVSGKPPNGFSAIYKTPPGIQKSAVATAEALGLDRPASDKQSPLNINGASYLRLPWVNPYMEGATPAIYPFLDSPNKYSLNMYKALLPQQSYSLAQPLYSPVCTNGERFLYLPPPHYVGPHIPSSLASPMRLSTPSASPAIPPLVHCADKSLPWKMGVSPGNPVDSHAYPHIQNSKQPRVPSAKAVTSGLPGDTALLLPPSPRPSPRVHLPTQPAADTYSEFHKHYARISTSPSVALSKPYMTVSSEFPAARLSNGKYPKAPEGGEGAQPVPGHARKTAVQDRKDGSSPPLLEKQTVTKDVTDKPLDLSSKVVDVDASKADHMKKMAPTVLVHSRAGSGLVLSGSEIPKETLSPPGNGCAIYRSEIISTAPSSWVVPGPSPNEENNGKSMSLKNKALDWAIPQQRSSSCPRMGGTDAVITNVSGSVSSAGRPASASPAPNANADGTKTSRSSVETTPSVIQHVGQPPATPAKHSSSTSSKGAKASNPEPSFKANENGLPPSSIFLSPNEAFRSPPIPYPRSYLPYPAPEGIAVSPLSLHGKGPVYPHPVLLPNGSLFPGHLAPKPGLPYGLPTGRPEFVTYQDALGLGMVHPMLIPHTPIEITKEEKPERRSRSHERARYEDPTLRNRFSEILETSSTKLHPDVPTDKNLKPNPNWNQGKTVVKSDKLVYVDLLREEPDAKTDTNVSKPSFAAESVGQSAEPPKPSVEPALQQHRDFIALREELGRISDFHETYTFKQPVFTVSKDSVLAGTNKENLGLPVSTPFLEPPLGSDGPAVTFGKTQEDPKPFCVGSAPPSVDVTPTYTKDGADEAESNDGKVLKPKPSKLAKRIANSAGYVGDRFKCVTTELYADSSQLSREQRALQMEGLQEDSILCLPAAYCERAMMRFSELEMKEREGGHPATKDSEMCKFSPADWERLKGNQDKKPKSVTLEEAIAEQNESERCEYSVGNKHRDPFEAPEDKDLPVEKYFVERQPVSEPPADQVASDMPHSPTLRVDRKRKVSGDSSHTETTAEEVPEDPLLKAKRRRVSKGLHPKKQRHLLHLRERWEQQVSAADGKPGRQSRKEVTQATQPEAIPQGTNITEEKPGRKRAEAKGNRSWSEESLKPSDNEQGLPVFSGSPPMKSLSSTSAGGKKQAQPSCAPASRPPAKQQKIKENQKTDVLCADEEEDCQAASLLQKYTDNSEKPSGKRLCKTKHLIPQESRRGLPLTGEYYVENADGKVTVRRFRKRPEPSSDYDLSPAKQEPKPFDRLQQLLPASQSTQLPCSSSPQETTQSRPMPPEARRLIVNKNAGETLLQRAARLGYEEVVLYCLENKICDVNHRDNAGYCALHEACARGWLNIVRHLLEYGADVNCSAQDGTRPLHDAVENDHLEIVRLLLSYGADPTLATYSGRTIMKMTHSELMEKFLTDYLNDLQGRNDDDASGTWDFYGSSVCEPDDESGYDVLANPPGPEDQDDDDDAYSDVFEFEFSETPLLPCYNIQVSVAQGPRNWLLLSDVLKKLKMSSRIFRCNFPNVEIVTIAEAEFYRQVSASLLFSCSKDLEAFNPESKELLDLVEFTNEIQTLLGSSVEWLHPSDLASDNYW

1080 NP_060346.2 NP_057067.1

>NP_060346.2

MAEASPHPGRYFCHCCSVEIVPRLPDYICPRCESGFIEELPEETRSTENGSAPSTAPTDQSRPPLEHVDQHLFTLPQGYGQFAFGIFDDSFEIPTFPPGAQADDGRDPESRRERDHPSRHRYGARQPRARLTTRRATGRHEGVPTLEGIIQQLVNGIITPATIPSLGPWGVLHSNPMDYAWGANGLDAIITQLLNQFENTGPPPADKEKIQALPTVPVTEEHVGSGLECPVCKDDYALGERVRQLPCNHLFHDGCIVPWLEQHDSCPVCRKSLTGQNTATNPPGLTGVSFSSSSSSSSSSSPSNENATWSPLGRPQPPRPLSNLTL

>NP_057067.1

MALKRIQKELTDLQRDPPAQCSAGPVGDDLFHWQATIMGPNDSPYQGGVFFLTIHFPTDYPFKPPKVAFTTKIYHPNINSNGSICLDILRSQWSPALTVSKVLLSICSLLCDPNPDDPLVPEIAHTYKADREKYNRLAREWTQKYAM

1081 NP_061039.2 NP_037497.1

>NP_061039.2

MGNCCWTQCFGLLRKEAGRLQRVGGGGGSKYFRTCSRGEHLTIEFENLVESDEGESPGSSHRPLTEEEIVDLRERHYDSIAEKQKDLDEKIQKELALQEEKLRLEEEALYAAQREAARAAKQRKLLEQERQRIVQQYHPSNNGEYQSSGPEDDFESCLRNMKSQYEVFRSSRLSSDATVLTPNTESSCDLMTKTKSTSGNDDSTSLDLEWEDEEGMNRMLPMRERSKTEEDILRAALKYSNKKTGSNPTSASDDSNGLEWENDFVSAEMDDNGNSEYSGFVNPVLELSDSGIRHSDTDQQTR

>NP_037497.1

MEPAMEPETLEARINRATNPLNKELDWASINGFCEQLNEDFEGPPLATRLLAHKIQSPQEWEAIQALTVLETCMKSCGKRFHDEVGKFRFLNELIKVVSPKYLGSRTSEKVKNKILELLYSWTVGLPEEVKIAEAYQMLKKQGIVKSDPKLPDDTTFPLPPPRPKNVIFEDEEKSKMLARLLKSSHPEDLRAANKLIKEMVQEDQKRMEKISKRVNAIEEVNNNVKLLTEMVMSHSQGGAAAGSSEDLMKELYQRCERMRPTLFRLASDTEDNDEALAEILQANDNLTQVINLYKQLVRGEEVNGDATAGSIPGSTSALLDLSGLDLPPAGTTYPAMPTRPGEQASPEQPSASVSLLDDELMSLGLSDPTPPSGPSLDGTGWNSFQSSDATEPPAPALAQAPSMESRPPAQTSLPASSGLDDLDLLGKTLLQQSLPPESQQVRWEKQQPTPRLTLRDLQNKSSSCSSPSSSATSLLHTVSPEPPRPPQQPVPTELSLASITVPLESIKPSNILPVTVYDQHGFRILFHFARDPLPGRSDVLVVVVSMLSTAPQPIRNIVFQSAVPKVMKVKLQPPSGTELPAFNPIVHPSAITQVLLLANPQKEKVRLRYKLTFTMGDQTYNEMGDVDQFPPPETWGSL

1082 NP_000116.2 NP_005345.3

>NP_000116.2

MTMTLHTKASGMALLHQIQGNELEPLNRPQLKIPLERPLGEVYLDSSKPAVYNYPEGAAYEFNAAAAANAQVYGQTGLPYGPGSEAAAFGSNGLGGFPPLNSVSPSPLMLLHPPPQLSPFLQPHGQQVPYYLENEPSGYTVREAGPPAFYRPNSDNRRQGGRERLASTNDKGSMAMESAKETRYCAVCNDYASGYHYGVWSCEGCKAFFKRSIQGHNDYMCPATNQCTIDKNRRKSCQACRLRKCYEVGMMKGGIRKDRRGGRMLKHKRQRDDGEGRGEVGSAGDMRAANLWPSPLMIKRSKKNSLALSLTADQMVSALLDAEPPILYSEYDPTRPFSEASMMGLLTNLADRELVHMINWAKRVPGFVDLTLHDQVHLLECAWLEILMIGLVWRSMEHPGKLLFAPNLLLDRNQGKCVEGMVEIFDMLLATSSRFRMMNLQGEEFVCLKSIILLNSGVYTFLSSTLKSLEEKDHIHRVLDKITDTLIHLMAKAGLTLQQQHQRLAQLLLILSHIRHMSNKGMEHLYSMKCKNVVPLYDLLLEMLDAHRLHAPTSRGGASVEETDQSHLATAGSTSSHSLQKYYITGEAEGFPATV

>NP_005345.3

METPFYGDEALSGLGGGASGSGGSFASPGRLFPGAPPTAAAGSMMKKDALTLSLSEQVAAALKPAAAPPPTPLRADGAPSAAPPDGLLASPDLGLLKLASPELERLIIQSNGLVTTTPTSSQFLYPKVAASEEQEFAEGFVKALEDLHKQNQLGAGAAAAAAAAAAGGPSGTATGSAPPGELAPAAAAPEAPVYANLSSYAGGAGGAGGAATVAFAAEPVPFPPPPPPGALGPPRLAALKDEPQTVPDVPSFGESPPLSPIDMDTQERIKAERKRLRNRIAASKCRKRKLERISRLEEKVKTLKSQNTELASTASLLREQVAQLKQKVLSHVNSGCQLLPQHQVPAY

1083 NP_000323.2 NP_998820.1

>NP_000323.2

MKSNQERSNECLPPKKREIPATSRSSEEKAPTLPSDNHRVEGTAWLPGNPGGRGHGGGRHGPAGTSVELGLQQGIGLHKALSTGLDYSPPSAPRSVPVATTLPAAYATPQPGTPVSPVQYAHLPHTFQFIGSSQYSGTYASFIPSQLIPPTANPVTSAVASAAGATTPSQRSQLEAYSTLLANMGSLSQTPGHKAEQQQQQQQQQQQQHQHQQQQQQQQQQQQQQHLSRAPGLITPGSPPPAQQNQYVHISSSPQNTGRTASPPAIPVHLHPHQTMIPHTLTLGPPSQVVMQYADSGSHFVPREATKKAESSRLQQAIQAKEVLNGEMEKSRRYGAPSSADLGLGKAGGKSVPHPYESRHVVVHPSPSDYSSRDPSGVRASVMVLPNSNTPAADLEVQQATHREASPSTLNDKSGLHLGKPGHRSYALSPHTVIQTTHSASEPLPVGLPATAFYAGTQPPVIGYLSGQQQAITYAGSLPQHLVIPGTQPLLIPVGSTDMEASGAAPAIVTSSPQFAAVPHTFVTTALPKSENFNPEALVTQAAYPAMVQAQIHLPVVQSVASPAAAPPTLPPYFMKGSIIQLANGELKKVEDLKTEDFIQSAEISNDLKIDSSTVERIEDSHSPGVAVIQFAVGEHRAQVSVEVLVEYPFFVFGQGWSSCCPERTSQLFDLPCSKLSVGDVCISLTLKNLKNGSVKKGQPVDPASVLLKHSKADGLAGSRHRYAEQENGINQGSAQMLSENGELKFPEKMGLPAAPFLTKIEPSKPAATRKRRWSAPESRKLEKSEDEPPLTLPKPSLIPQEVKICIEGRSNVGK

>NP_998820.1

MYELLVLFMLIQPQSMAHPCGGTPTYPESQIFFPTIHERPVSFSPPPTCPPKVAISQRRKSTSFLEAQTHHFQPLLRTVGQSLLPPGGSPTNWTPEAVVMLGTTASRVTGESCEIQVHPMFEPSQVYSDYRPGLVLPEEAHYFIPQEAVYVAGVHYQARVAEQYEGIPYNSSVLSSPMKQIPEQKPVQGGPTSSSVFEFPSGQAFLVGHLQNLRLDSGLGPGSPLSSISAPISTDATRLKFHPVFVPHSAPAVLTHNNESRSNCVFEFHVHTPSSSSGEGGGILPQRVYRNRQVAVDLNQEELPPQSVGLHGYLQPVTEEKHNYHAPELTVSVVEPIGQNWPIGSPEYSSDSSQITSSDPSDFQSPPPTGGAAAPFGSDVSMPFIHLPQTVLQESPLFFCFPQGTTSQQVLTASFSSGGSALHPQVIGKLPQLF

1084 NP_060080.6 NP_005576.3

>NP_060080.6

MSQWTPEYNELYTLKVDMKSEIPSDAPKTQESLKGILLHPEPIGAAKSFPAGVEMINSKVGNEFSHLCDDSQKQEKEMNGNQQEQEKSLVVRKKRKSQQAGPSYVQNCVKENQGILGLRQHLGTPSDEDNDSSFSDCLSSPSSSLHFGDSDTVTSDEDKEVSVRHSQTILNAKSRSHSARSHKWPRTETESVSGLLMKRPCLHGSSLRRLPCRKRFVKNNSSQRTQKQKERILMQRKKREVLARRKYALLPSSSSSSENDLSSESSSSSSTEGEEDLFVSASENHQNNPAVPSGSIDEDVVVIEASSTPQVTANEEINVTSTDSEVEIVTVGESYRSRSTLGHSRSHWSQGSSSHASRPQEPRNRSRISTVIQPLRQNAAEVVDLTVDEDEPTVVPTTSARMESQATSASINNSNPSTSEQASDTASAVTSSQPSTVSETSATLTSNSTTGTSIGDDSRRTTSSAVTETGPPAMPRLPSCCPQHSPCGGSSQNHHALGHPHTSCFQQHGHHFQHHHHHHHTPHPAVPVSPSFSDPACPVERPPQVQAPCGANSSSGTSYHEQQALPVDLSNSGIRSHGSGSFHGASAFDPCCPVSSSRAAIFGHQAAAAAPSQPLSSIDGYGSSMVAQPQPQPPPQPSLSSCRHYMPPPYASLTRPLHHQASACPHSHGNPPPQTQPPPQVDYVIPHPVHAFHSQISSHATSHPVAPPPPTHLASTAAPIPQHLPPTHQPISHHIPATAPPAQRLHPHEVMQRMEVQRRRMMQHPTRAHERPPPHPHRMHPNYGHGHHIHVPQTMSSHPRQAPERSAWELGIEAGVTAATYTPGALHPHLAHYHAPPRLHHLQLGALPLMVPDMAGYPHIRYISSGLDGTSFRGPFRGNFEELIHLEERLGNVNRGASQGTIERCTYPHKYKKRKLHCKQDGEEGTEEDTEEKCTICLSILEEGEDVRRLPCMHLFHQVCVDQWLITNKKCPICRVDIEAQLPSES

>NP_005576.3

MFRSKRSGLVRRLWRSRVVPDREEGGSGGGGGGDEDGSLGSRAEPAPRAREGGGCGRSEVRPVAPRRPRDAVGQRGAQGAGRRRRAGGPPRPMSEPGAGAGSSLLDVAEPGGPGWLPESDCETVTCCLFSERDAAGAPRDASDPLAGAALEPAGGGRSREARSRLLLLEQELKTVTYSLLKRLKERSLDTLLEAVESRGGVPGGCVLVPRADLRLGGQPAPPQLLLGRLFRWPDLQHAVELKPLCGCHSFAAAADGPTVCCNPYHFSRLCGPESPPPPYSRLSPRDEYKPLDLSDSTLSYTETEATNSLITAPGEFSDASMSPDATKPSHWCSVAYWEHRTRVGRLYAVYDQAVSIFYDLPQGSGFCLGQLNLEQRSESVRRTRSKIGFGILLSKEPDGVWAYNRGEHPIFVNSPTLDAPGGRALVVRKVPPGYSIKVFDFERSGLQHAPEPDAADGPYDPNSVRISFAKGWGPCYSRQFITSCPCWLEILLNNPR

1085 NP_663788.1 BAA18998.1

>NP_663788.1

MNSAEQTVTWLITLGVLESPKKTISDPEGFLQASLKDGVVLCRLLERLLPGTIEKVYPEPRSESECLSNIREFLRGCGASLRLETFDANDLYQGQNFNKVLSSLVTLNKVTADIGLGSDSVCARPSSHRIKSFDSLGSQSLHTRTSKLFQGQYRSLDMTDNSNNQLVVRAKFNFQQTNEDELSFSKGDVIHVTRVEEGGWWEGTLNGRTGWFPSNYVREVKASEKPVSPKSGTLKSPPKGFDTTAINKSYYNVVLQNILETENEYSKELQTVLSTYLRPLQTSEKLSSANISYLMGNLEEICSFQQMLVQSLEECTKLPEAQQRVGGCFLNLMPQMKTLYLTYCANHPSAVNVLTEHSEELGEFMETKGASSPGILVLTTGLSKPFMRLDKYPTLLKELERHMEDYHTDRQDIQKSMAAFKNLSAQCQEVRKRKELELQILTEAIRNWEGDDIKTLGNVTYMSQVLIQCAGSEEKNERYLLLFPNVLLMLSASPRMSGFIYQGKLPTTGMTITKLEDSENHRNAFEISGSMIERILVSCNNQQDLQEWVEHLQKQTKVTSVGNPTIKPHSVPSHTLPSHPVTPSSKHADSKPAPLTPAYHTLPHPSHHGTPHTTINWGPLEPPKTPKPWSLSCLRPAPPLRPSAALCYKEDLSKSPKTMKKLLPKRKPERKPSDEEFASRKSTAALEEDAQILKVIEAYCTSAKTRQTLNSTWQGTDLMHNHVLADDDQPSLDSLGRRSSLSRLEPSDLSEDSDYDSIWTAHSYRMGSTSRKSCCSYISHQN

>BAA18998.1

MDDLDALLADLESTTSHISKRPVFLSEETPYSYPTGNHTYQEIAVPPPVPPPPSSEALNGTILDPLDQWQPSGSRFIHQQPQSSSPVYGSSAKTSSVSNPQDSVGSPCSRVGEEEHVYSFPNKQKSAEPSPTVMSTSLGSNLSELDRLLLELNAVQHNPPGFPADEANSSPPLPGALSPLYGVPETNSPLGGKAGPLTKEKPKRNGGRGLEDVRPSVESLLDELESSVPSPVPAITVNQGEMSSPQRVTSTQQQTRISASSATRELDELMASLSDFKGSWPLEEVVLLVSISSSVQEGEKYPHPCAARHRTPSLRSPDQPPPCPQFMAQGKTGSSSPPGGPPKPGSQLDSMLGSLQSDLNKLGVATVAKGVCGACKKPIAGQVVTAMGKTWHPEHFVCTHCQEEIGSRNFFERDGQPYCEKDYHNLFSPRCYYCNGPILDKVVTALDRTWHPEHFFCAQCGAFFGPEGFHEKDGKAYCRKDYFDMFAPKCGGCARAILENYISALNTLWHPECFVCRECFTPFVNGSFFEHDGQPYCEVHYHERRGSLCSGCQKPITGRCITAMAKKFHPEHFVCAFCLKQLNKGTFKEQNDKPYCQNCFLKLFC

1086 NP_004964.2 NP_004378.1

>NP_004964.2

MSKERPKRNIIQKKYDDSDGIPWSEERVVRKVLYLSLKEFKNSQKRQHAEGIAGSLKTVNGLLGNDQSKGLGPASEQSENEKDDASQVSSTSNDVSSSDFEEGPSRKRPRLQAQRKFAQSQPNSPSTTPVKIVEPLLPPPATQISDLSKRKPKTEDFLTFLCLRGSPALPNSMVYFGSSQDEEEVEEEDDETEDVKTATNNASSSCQSTPRKGKTHKHVHNGHVFNGSSRSTREKEPVQKHKSKEATPAKEKHSDHRADSRREQASANHPAAAPSTGSSAKGLAATHHHPPLHRSAQDLRKQVSKVNGVTRMSSLGAGVTSAKKMREVRPSPSKTVKYTATVTKGAVTYTKAKRELVKDTKPNHHKPSSAVNHTISGKTESSNAKTRKQVLSLGGASKSTGPAVNGLKVSGRLNPKSCTKEVGGRQLREGLQLREGLRNSKRRLEEAHQAEKPQSPPKKMKGAAGPAEGPGKKAPAERGLLNGHVKKEVPERSLERNRPKRATAGKSTPGRQAHGKADSASCENRSTSQPESVHKPQDSGKAEKGGGKAGWAAMDEIPVLRPSAKEFHDPLIYIESVRAQVEKFGMCRVIPPPDWRPECKLNDEMRFVTQIQHIHKLGRRWGPNVQRLACIKKHLKSQGITMDELPLIGGCELDLACFFRLINEMGGMQQVTDLKKWNKLADMLRIPRTAQDRLAKLQEAYCQYLLSYDSLSPEEHRRLEKEVLMEKEILEKRKGPLEGHTENDHHKFHPLPRFEPKNGLIHGVAPRNGFRSKLKEVGQAQLKTGRRRLFAQEKEVVKEEEEDKGVLNDFHKCIYKGRSVSLTTFYRTARNIMSMCFSKEPAPAEIEQEYWRLVEEKDCHVAVHCGKVDTNTHGSGFPVGKSEPFSRHGWNLTVLPNNTGSILRHLGAVPGVTIPWLNIGMVFSTSCWSRDQNHLPYIDYLHTGADCIWYCIPAEEENKLEDVVHTLLQANGTPGLQMLESNVMISPEVLCKEGIKVHRTVQQSGQFVVCFPGSFVSKVCCGYSVSETVHFATTQWTSMGFETAKEMKRRHIAKPFSMEKLLYQIAQAEAKKENGPTLSTISALLDELRDTELRQRRQLFEAGLHSSARYGSHDGSSTVADGKKKPRKWLQLETSERRCQICQHLCYLSMVVQENENVVFCLECALRHVEKQKSCRGLKLMYRYDEEQIISLVNQICGKVSGKNGSIENCLSKPTPKRGPRKRATVDVPPSRLSASSSSKSASSSS

>NP_004378.1

MFPSPALTPTPFSVKDILNLEQQQRSLAAAGELSARLEATLAPSSCMLAAFKPEAYAGPEAAAPGLPELRAELGRAPSPAKCASAFPAAPAFYPRAYSDPDPAKDPRAEKKELCALQKAVELEKTEADNAERPRARRRRKPRVLFSQAQVYELERRFKQQRYLSAPERDQLASVLKLTSTQVKIWFQNRRYKCKRQRQDQTLELVGLPPPPPPPARRIAVPVLVRDGKPCLGDSAPYAPAYGVGLNPYGYNAYPAYPGYGGAACSPGYSCTAAYPAGPSPAQPATAAANNNFVNFGVGDLNAVQSPGIPQSNSGVSTLHGIRAW

1087 NP_005234.1 NP_542408.1

>NP_005234.1

MASTDYSTYSQAAAQQGYSAYTAQPTQGYAQTTQAYGQQSYGTYGQPTDVSYTQAQTTATYGQTAYATSYGQPPTGYTTPTAPQAYSQPVQGYGTGAYDTTTATVTTTQASYAAQSAYGTQPAYPAYGQQPAATAPTRPQDGNKPTETSQPQSSTGGYNQPSLGYGQSNYSYPQVPGSYPMQPVTAPPSYPPTSYSSTQPTSYDQSSYSQQNTYGQPSSYGQQSSYGQQSSYGQQPPTSYPPQTGSYSQAPSQYSQQSSSYGQQSSFRQDHPSSMGVYGQESGGFSGPGENRSMSGPDNRGRGRGGFDRGGMSRGGRGGGRGGMGSAGERGGFNKPGGPMDEGPDLDLGPPVDPDEDSDNSAIYVQGLNDSVTLDDLADFFKQCGVVKMNKRTGQPMIHIYLDKETGKPKGDATVSYEDPPTAKAAVEWFDGKDFQGSKLKVSLARKKPPMNSMRGGLPPREGRGMPPPLRGGPGGPGGPGGPMGRMGGRGGDRGGFPPRGPRGSRGNPSGGGNVQHRAGDWQCPNPGCGNQNFAWRTECNQCKAPKPEGFLPPPFPPPGGDRGRGGPGGMRGGRGGLMDRGGPGGMFRGGRGGDRGGFRGGRGMDRGGFGGGRRGGPGGPPGPLMEQMGGRRGGRGGPGKMDKGEHRQERRDRPY

>NP_542408.1

MSDRKAVIKNADMSEDMQQDAVDCATQAMEKYNIEKDIAAYIKKEFDKKYNPTWHCIVGRNFGSYVTHETKHFIYFYLGQVAILLFKSG

1088 NP_004984.2 NP_009057.1

>NP_004984.2

MESIFHEKQEGSLCAQHCLNNLLQGEYFSPVELSSIAHQLDEEERMRMAEGGVTSEDYRTFLQQPSGNMDDSGFFSIQVISNALKVWGLELILFNSPEYQRLRIDPINERSFICNYKEHWFTVRKLGKQWFNLNSLLTGPELISDTYLALFLAQLQQEGYSIFVVKGDLPDCEADQLLQMIRVQQMHRPKLIGEELAQLKEQRVHKTDLERVLEANDGSGMLDEDEEDLQRALALSRQEIDMEDEEADLRRAIQLSMQGSSRNISQDMTQTSGTNLTSEELRKRREAYFEKQQQKQQQQQQQQQQGDLSGQSSHPCERPATSSGALGSDLGDAMSEEDMLQAAVTMSLETVRNDLKTEGKK

>NP_009057.1

MASGADSKGDDLSTAILKQKNRPNRLIVDEAINEDNSVVSLSQPKMDELQLFRGDTVLLKGKKRREAVCIVLSDDTCSDEKIRMNRVVRNNLRVRLGDVISIQPCPDVKYGKRIHVLPIDDTVEGITGNLFEVYLKPYFLEAYRPIRKGDIFLVRGGMRAVEFKVVETDPSPYCIVAPDTVIHCEGEPIKREDEEESLNEVGYDDIGGCRKQLAQIKEMVELPLRHPALFKAIGVKPPRGILLYGPPGTGKTLIARAVANETGAFFFLINGPEIMSKLAGESESNLRKAFEEAEKNAPAIIFIDELDAIAPKREKTHGEVERRIVSQLLTLMDGLKQRAHVIVMAATNRPNSIDPALRRFGRFDREVDIGIPDATGRLEILQIHTKNMKLADDVDLEQVANETHGHVGADLAALCSEAALQAIRKKMDLIDLEDETIDAEVMNSLAVTMDDFRWALSQSNPSALRETVVEVPQVTWEDIGGLEDVKRELQELVQYPVEHPDKFLKFGMTPSKGVLFYGPPGCGKTLLAKAIANECQANFISIKGPELLTMWFGESEANVREIFDKARQAAPCVLFFDELDSIAKARGGNIGDGGGAADRVINQILTEMDGMSTKKNVFIIGATNRPDIIDPAILRPGRLDQLIYIPLPDEKSRVAILKANLRKSPVAKDVDLEFLAKMTNGFSGADLTEICQRACKLAIRESIESEIRRERERQTNPSAMEVEEDDPVPEIRRDHFEEAMRFARRSVSDNDIRKYEMFAQTLQQSRGFGSFRFPSGNQGGAGPSQGSGGGTGGSVYTEDNDDDLYG

1089 NP_115791.2 NP_059830.1

>NP_115791.2

MDDYMETLKDEEDALWENVECNRHMLSRYINPAKLTPYLRQCKVIDEQDEDEVLNAPMLPSKINRAGRLLDILHTKGQRGYVVFLESLEFYYPELYKLVTGKEPTRRFSTIVVEEGHEGLTHFLMNEVIKLQQQMKAKDLQRCELLARLRQLEDEKKQMTLTRVELLTFQERYYKMKEERDSYNDELVKVKDDNYNLAMRYAQLSEEKNMAVMRSRDLQLEIDQLKHRLNKMEEECKLERNQSLKLKNDIENRPKKEQVLELERENEMLKTKNQELQSIIQAGKRSLPDSDKAILDILEHDRKEALEDRQELVNRIYNLQEEARQAEELRDKYLEEKEDLELKCSTLGKDCEMYKHRMNTVMLQLEEVERERDQAFHSRDEAQTQYSQCLIEKDKYRKQIRELEEKNDEMRIEMVRREACIVNLESKLRRLSKDSNNLDQSLPRNLPVTIISQDFGDASPRTNGQEADDSSTSEESPEDSKYFLPYHPPQRRMNLKGIQLQRAKSPISLKRTSDFQAKGHEEEGTDASPSSCGSLPITNSFTKMQPPRSRSSIMSITAEPPGNDSIVRRYKEDAPHRSTVEEDNDSGGFDALDLDDDSHERYSFGPSSIHSSSSSHQSEGLDAYDLEQVNLMFRKFSLERPFRPSVTSVGHVRGPGPSVQHTTLNGDSLTSQLTLLGGNARGSFVHSVKPGSLAEKAGLREGHQLLLLEGCIRGERQSVPLDTCTKEEAHWTIQRCSGPVTLHYKVNHEGYRKLVKDMEDGLITSGDSFYIRLNLNISSQLDACTMSLKCDDVVHVRDTMYQDRHEWLCARVDPFTDHDLDMGTIPSYSRAQQLLLVKLQRLMHRGSREEVDGTHHTLRALRNTLQPEEALSTSDPRVSPRLSRASFLFGQLLQFVSRSENKYKRMNSNERVRIISGSPLGSLARSSLDATKLLTEKQEELDPESELGKNLSLIPYSLVRAFYCERRRPVLFTPTVLAKTLVQRLLNSGGAMEFTICKSDIVTRDEFLRRQKTETIIYSREKNPNAFECIAPANIEAVAAKNKHCLLEAGIGCTRDLIKSNIYPIVLFIRVCEKNIKRFRKLLPRPETEEEFLRVCRLKEKELEALPCLYATVEPDMWGSVEELLRVVKDKIGEEQRKTIWVDEDQL

>NP_059830.1

MPYEIKKVFASLPQVERGVSKIIGGDPKGNNFLYTNGKCVILRNIDNPALADIYTEHAHQVVVAKYAPSGFYIASGDVSGKLRIWDTTQKEHLLKYEYQPFAGKIKDIAWTEDSKRIAVVGEGREKFGAVFLWDSGSSVGEITGHNKVINSVDIKQSRPYRLATGSDDNCAAFFEGPPFKFKFTIGDHSRFVNCVRFSPDGNRFATASADGQIYIYDGKTGEKVCALGGSKAHDGGIYAISWSPDSTHLLSASGDKTSKIWDVSVNSVVSTFPMGSTVLDQQLGCLWQKDHLLSVSLSGYINYLDRNNPSKPLHVIKGHSKSIQCLTVHKNGGKSYIYSGSHDGHINYWDSETGENDSFAGKGHTNQVSRMTVDESGQLISCSMDDTVRYTSLMLRDYSGQGVVKLDVQPKCVAVGPGGYAVVVCIGQIVLLKDQRKCFSIDNPGYEPEVVAVHPGGDTVAIGGVDGNVRLYSILGTTLKDEGKLLEAKGPVTDVAYSHDGAFLAVCDASKVVTVFSVADGYSENNVFYGHHAKIVCLAWSPDNEHFASGGMDMMVYVWTLSDPETRVKIQDAHRLHHVSSLAWLDEHTLVTTSHDASVKEWTITY

1090 NP_057403.1 NP_000537.3

>NP_057403.1

MSAEVKVTGQNQEQFLLLAKSAKGAALATLIHQVLEAPGVYVFGELLDMPNVRELAESDFASTFRLLTVFAYGTYADYLAEARNLPPLTEAQKNKLRHLSVVTLAAKVKCIPYAVLLEALALRNVRQLEDLVIEAVYADVLRGSLDQRNQRLEVDYSIGRDIQRQDLSAIARTLQEWCVGCEVVLSGIEEQVSRANQHKEQQLGLKQQIESEVANLKKTIKVTTAAAAAATSQDPEQHLTELREPAPGTNQRQPSKKASKGKGLRGSAKIWSKSN

>NP_000537.3

MEEPQSDPSVEPPLSQETFSDLWKLLPENNVLSPLPSQAMDDLMLSPDDIEQWFTEDPGPDEAPRMPEAAPPVAPAPAAPTPAAPAPAPSWPLSSSVPSQKTYQGSYGFRLGFLHSGTAKSVTCTYSPALNKMFCQLAKTCPVQLWVDSTPPPGTRVRAMAIYKQSQHMTEVVRRCPHHERCSDSDGLAPPQHLIRVEGNLRVEYLDDRNTFRHSVVVPYEPPEVGSDCTTIHYNYMCNSSCMGGMNRRPILTIITLEDSSGNLLGRNSFEVRVCACPGRDRRTEEENLRKKGEPHHELPPGSTKRALPNNTSSSPQPKKKPLDGEYFTLQIRGRERFEMFRELNEALELKDAQAGKEPGGSRAHSSHLKSKKGQSTSRHKKLMFKTEGPDSD

1091 NP_002261.3 NP_003126.1

>NP_002261.3

MVWDRQTKMEYEWKPDEQGLQQILQLLKESQSPDTTIQRTVQQKLEQLNQYPDFNNYLIFVLTKLKSEDEPTRSLSGLILKNNVKAHFQNFPNGVTDFIKSECLNNIGDSSPLIRATVGILITTIASKGELQNWPDLLPKLCSLLDSEDYNTCEGAFGALQKICEDSAEILDSDVLDRPLNIMIPKFLQFFKHSSPKIRSHAVACVNQFIISRTQALMLHIDSFIENLFALAGDEEPEVRKNVCRALVMLLEVRMDRLLPHMHNIVEYMLQRTQDQDENVALEACEFWLTLAEQPICKDVLVRHLPKLIPVLVNGMKYSDIDIILLKGDVEEDETIPDSEQDIRPRFHRSRTVAQQHDEDGIEEEDDDDDEIDDDDTISDWNLRKCSAAALDVLANVYRDELLPHILPLLKELLFHHEWVVKESGILVLGAIAEGCMQGMIPYLPELIPHLIQCLSDKKALVRSITCWTLSRYAHWVVSQPPDTYLKPLMTELLKRILDSNKRVQEAACSAFATLEEEACTELVPYLAYILDTLVFAFSKYQHKNLLILYDAIGTLADSVGHHLNKPEYIQMLMPPLIQKWNMLKDEDKDLFPLLECLSSVATALQSGFLPYCEPVYQRCVNLVQKTLAQAMLNNAQPDQYEAPDKDFMIVALDLLSGLAEGLGGNIEQLVARSNILTLMYQCMQDKMPEVRQSSFALLGDLTKACFQHVKPCIADFMPILGTNLNPEFISVCNNATWAIGEISIQMGIEMQPYIPMVLHQLVEIINRPNTPKTLLENTAITIGRLGYVCPQEVAPMLQQFIRPWCTSLRNIRDNEEKDSAFRGICTMISVNPSGVIQDFIFFCDAVASWINPKDDLRDMFCKILHGFKNQVGDENWRRFSDQFPLPLKERLAAFYGV

>NP_003126.1

MACAAARSPADQDRFICIYPAYLNNKKTIAEGRRIPISKAVENPTATEIQDVCSAVGLNVFLEKNKMYSREWNRDVQYRGRVRVQLKQEDGSLCLVQFPSRKSVMLYAAEMIPKLKTRTQKTGGADQSLQQGEGSKKGKGKKKK

1092 NP_060740.1 NP_631938.1

>NP_060740.1

MGEAEVGGGGAAGDKGPGEAATSPAEETVVWSPEVEVCLFHAMLGHKPVGVNRHFHMICIRDKFSQNIGRQVPSKVIWDHLSTMYDMQALHESEILPFPNPERNFVLPEEIIQEVREGKVMIEEEMKEEMKEDVDPHNGADDVFSSSGSLGKASEKSSKDKEKNSSDLGCKEGADKRKRSRVTDKVLTANSNPSSPSAAKRRRT

>NP_631938.1

MATGTGKHKLLSTGPTEPWSIREKLCLASSVMRSGDQNWVSVSRAIKPFAEPGRPPDWFSQKHCASQYSELLETTETPKRKRGEKGEVVETVEDVIVRKLTAERVEELKKVIKETQERYRRLKRDAELIQAGHMDSRLDELCNDIATKKKLEEEEAEVKRKATDAAYQARQAVKTPPRRLPTVMVRSPIDSASPGGDYPLGDLTPTTMEEATSGVNESEMAVASGHLNSTGVLLEVGGVLPMIHGGEIQQTPNTVAASPAASGAPTLSRLLEAGPTQFTTPLASFTTVASEPPVKLVPPPVESVSQATIVMMPALPAPSSAPAVSTTESVAPVSQPDNCVPMEAVGDPHTVTVSMDSSEISMIINSIKEECFRSGVAEAPVGSKAPSIDGKEELDLAEKMDIAVSYTGEELDFETVGDIIAIIEDKVDDHPEVLDVAAVEAALSFCEENDDPQSLPGPWEHPIQQERDKPVPLPAPEMTVKQERLDFEETENKGIHELVDIREPSAEIKVEPAEPEPVISGAEIVAGVVPATSMEPPELRSQDLDEELGSTAAGEIVEADVAIGKGDETPLTNVKTEASPESMLSPSHGSNPIEDPLEAETQHKFEMSDSLKEESGTIFGSQIKDAPGEDEEEDGVSEAASLEEPKEEDQGEGYLSEMDNEPPVSESDDGFSIHNATLQSHTLADSIPSSPASSQFSVCSEDQEAIQAQKIWKKAIMLVWRAAANHRYANVFLQPVTDDIAPGYHSIVQRPMDLSTIKKNIENGLIRSTAEFQRDIMLMFQNAVMYNSSDHDVYHMAVEMQRDVLEQIQQFLATQLIMQTSESGISAKSLRGRDSTRKQDASEKDSVPMGSPAFLLSLFMGHEWVWLDSEQDHPNDSELSNDCRSLFSSWDSSLDLDVGNWRETEDPEAEELEESSPEREPSELLVGDGGSEESQEAARKASHQNLLHFLSEVAYLMEPLCISSNESSEGCCPPSGTRQEGREIKASEGERELCRETEELSAKGDPLVAEKPLGENGKPEVASAPSVICTVQGLLTESEEGEAQQESKGEDQGEVYVSEMEDQPPSGECDDAFNIKETPLVDTLFSHATSSKLTDLSQDDPVQDHLLFKKTLLPVWKMIASHRFSSPFLKPVSERQAPGYKDVVKRPMDLTSLKRNLSKGRIRTMAQFLRDLMLMFQNAVMYNDSDHHVYHMAVEMRREVLEQIQVLNIWLDKRKGSSSLEGEPANPVDDGKPVF

1093 NP_619525.1 NP_004850.1

>NP_619525.1

MAEAEGESLESWLNKATNPSNRQEDWEYIIGFCDQINKELEGPQIAVRLLAHKIQSPQEWEALQALTVLEACMKNCGRRFHNEVGKFRFLNELIKVVSPKYLGDRVSEKVKTKVIELLYSWTMALPEEAKIKDAYHMLKRQGIVQSDPPIPVDRTLIPSPPPRPKNPVFDDEEKSKLLAKLLKSKNPDDLQEANKLIKSMVKEDEARIQKVTKRLHTLEEVNNNVRLLSEMLLHYSQEDSSDGDRELMKELFDQCENKRRTLFKLASETEDNDNSLGDILQASDNLSRVINSYKTIIEGQVINGEVATLTLPDSEGNSQCSNQGTLIDLAELDTTNSLSSVLAPAPTPPSSGIPILPPPPQASGPPRSRSSSQAEATLGPSSTSNALSWLDEELLCLGLADPAPNVPPKESAGNSQWHLLQREQSDLDFFSPRPGTAACGASDAPLLQPSAPSSSSSQAPLPPPFPAPVVPASVPAPSAGSSLFSTGVAPALAPKVEPAVPGHHGLALGNSALHHLDALDQLLEEAKVTSGLVKPTTSPLIPTTTPARPLLPFSTGPGSPLFQPLSFQSQGSPPKGPELSLASIHVPLESIKPSSALPVTAYDKNGFRILFHFAKECPPGRPDVLVVVVSMLNTAPLPVKSIVLQAAVPKSMKVKLQPPSGTELSPFSPIQPPAAITQVMLLANPLKEKVRLRYKLTFALGEQLSTEVGEVDQFPPVEQWGNL

>NP_004850.1

MAQILPIRFQEHLQLQNLGINPANIGFSTLTMESDKFICIREKVGEQAQVVIIDMNDPSNPIRRPISADSAIMNPASKVIALKAGKTLQIFNIEMKSKMKAHTMTDDVTFWKWISLNTVALVTDNAVYHWSMEGESQPVKMFDRHSSLAGCQIINYRTDAKQKWLLLTGISAQQNRVVGAMQLYSVDRKVSQPIEGHAASFAQFKMEGNAEESTLFCFAVRGQAGGKLHIIEVGTPPTGNQPFPKKAVDVFFPPEAQNDFPVAMQISEKHDVVFLITKYGYIHLYDLETGTCIYMNRISGETIFVTAPHEATAGIIGVNRKGQVLSVCVEEENIIPYITNVLQNPDLALRMAVRNNLAGAEELFARKFNALFAQGNYSEAAKVAANAPKGILRTPDTIRRFQSVPAQPGQTSPLLQYFGILLDQGQLNKYESLELCRPVLQQGRKQLLEKWLKEDKLECSEELGDLVKSVDPTLALSVYLRANVPNKVIQCFAETGQVQKIVLYAKKVGYTPDWIFLLRNVMRISPDQGQQFAQMLVQDEEPLADITQIVDVFMEYNLIQQCTAFLLDALKNNRPSEGPLQTRLLEMNLMHAPQVADAILGNQMFTHYDRAHIAQLCEKAGLLQRALEHFTDLYDIKRAVVHTHLLNPEWLVNYFGSLSVEDSLECLRAMLSANIRQNLQICVQVASKYHEQLSTQSLIELFESFKSFEGLFYFLGSIVNFSQDPDVHFKYIQAACKTGQIKEVERICRESNCYDPERVKNFLKEAKLTDQLPLIIVCDRFDFVHDLVLYLYRNNLQKYIEIYVQKVNPSRLPVVIGGLLDVDCSEDVIKNLILVVRGQFSTDELVAEVEKRNRLKLLLPWLEARIHEGCEEPATHNALAKIYIDSNNNPERFLRENPYYDSRVVGKYCEKRDPHLACVAYERGQCDLELINVCNENSLFKSLSRYLVRRKDPELWGSVLLESNPYRRPLIDQVVQTALSETQDPEEVSVTVKAFMTADLPNELIELLEKIVLDNSVFSEHRNLQNLLILTAIKADRTRVMEYINRLDNYDAPDIANIAISNELFEEAFAIFRKFDVNTSAVQVLIEHIGNLDRAYEFAERCNEPAVWSQLAKAQLQKGMVKEAIDSYIKADDPSSYMEVVQAANTSGNWEELVKYLQMARKKARESYVETELIFALAKTNRLAELEEFINGPNNAHIQQVGDRCYDEKMYDAAKLLYNNVSNFGRLASTLVHLGEYQAAVDGARKANSTRTWKEVCFACVDGKEFRLAQMCGLHIVVHADELEELINYYQDRGYFEELITMLEAALGLERAHMGMFTELAILYSKFKPQKMREHLELFWSRVNIPKVLRAAEQAHLWAELVFLYDKYEEYDNAIITMMNHPTDAWKEGQFKDIITKVANVELYYRAIQFYLEFKPLLLNDLLMVLSPRLDHTRAVNYFSKVKQLPLVKPYLRSVQNHNNKSVNESLNNLFITEEDYQALRTSIDAYDNFDNISLAQRLEKHELIEFRRIAAYLFKGNNRWKQSVELCKKDSLYKDAMQYASESKDTELAEELLQWFLQEEKRECFGACLFTCYDLLRPDVVLETAWRHNIMDFAMPYFIQVMKEYLTKVDKLDASESLRKEEEQATETQPIVYGQPQLMLTAGPSVAVPPQAPFGYGYTAPPYGQPQPGFGYSM

1094 NP_002605.2 NP_001044.1

>NP_002605.2

MTSTFNPRECKLSKQEGQNYGFFLRIEKDTEGHLVRVVEKCSPAEKAGLQDGDRVLRINGVFVDKEEHMQVVDLVRKSGNSVTLLVLDGDSYEKAVKTRVDLKELGQSQKEQGLSDNILSPVMNGGVQTWTQPRLCYLVKEGGSYGFSLKTVQGKKGVYMTDITPQGVAMRAGVLADDHLIEVNGENVEDASHEEVVEKVKKSGSRVMFLLVDKETDKRHVEQKIQFKRETASLKLLPHQPRIVEMKKGSNGYGFYLRAGSEQKGQIIKDIDSGSPAEEAGLKNNDLVVAVNGESVETLDHDSVVEMIRKGGDQTSLLVVDKETDNMYRLAHFSPFLYYQSQELPNGSVKEAPAPTPTSLEVSSPPDTTEEVDHKPKLCRLAKGENGYGFHLNAIRGLPGSFIKEVQKGGPADLAGLEDEDVIIEVNGVNVLDEPYEKVVDRIQSSGKNVTLLVCGKKAYDYFQAKKIPIVSSLADPLDTPPDSKEGIVVESNHDSHMAKERAHSTASHSSSNSEDTEM

>NP_001044.1

MEPLFPASTPSWNASSPGAASGGGDNRTLVGPAPSAGARAVLVPVLYLLVCAAGLGGNTLVIYVVLRFAKMKTVTNIYILNLAVADVLYMLGLPFLATQNAASFWPFGPVLCRLVMTLDGVNQFTSVFCLTVMSVDRYLAVVHPLSSARWRRPRVAKLASAAAWVLSLCMSLPLLVFADVQEGGTCNASWPEPVGLWGAVFIIYTAVLGFFAPLLVICLCYLLIVVKVRAAGVRVGCVRRRSERKVTRMVLVVVLVFAGCWLPFFTVNIVNLAVALPQEPASAGLYFFVVILSYANSCANPVLYGFLSDNFRQSFQKVLCLRKGSGAKDADATEPRPDRIRQQQEATPPAHRAAANGLMQTSKL

1095 NP_005816.2 NP_078832.1

>NP_005816.2

MGTQRLCGRGTQGWPGSSEQHVQEATSSAGLHSGVDELGVRSEPGGRLPERSLGPAHPAPAAMAGTLDLDKGCTVEELLRGCIEAFDDSGKVRDPQLVRMFLMMHPWYIPSSQLAAKLLHIYQQSRKDNSNSLQVKTCHLVRYWISAFPAEFDLNPELAEQIKELKALLDQEGNRRHSSLIDIDSVPTYKWKRQVTQRNPVGQKKRKMSLLFDHLEPMELAEHLTYLEYRSFCKILFQDYHSFVTHGCTVDNPVLERFISLFNSVSQWVQLMILSKPTAPQRALVITHFVHVAEKLLQLQNFNTLMAVVGGLSHSSISRLKETHSHVSPETIKLWEGLTELVTATGNYGNYRRRLAACVGFRFPILGVHLKDLVALQLALPDWLDPARTRLNGAKMKQLFSILEELAMVTSLRPPVQANPDLLSLLTVSLDQYQTEDELYQLSLQREPRSKSSPTSPTSCTPPPRPPVLEEWTSAAKPKLDQALVVEHIEKMVESVFRNFDVDGDGHISQEEFQIIRGNFPYLSAFGDLDQNQDGCISREEMVSYFLRSSSVLGGRMGFVHNFQESNSLRPVACRHCKALILGIYKQGLKCRACGVNCHKQCKDRLSVECRRRAQSVSLEGSAPSPSPMHSHHHRAFSFSLPRPGRRGSRPPEIREEEVQTVEDGVFDIHL

>NP_078832.1

MASTGSQASDIDEIFGFFNDGEPPTKKPRKLLPSLKTKKPRELVLVIGTGISAAVAPQVPALKSWKGLIQALLDAAIDFDLLEDEESKKFQKCLHEDKNLVHVAHDLIQKLSPRTSNVRSTFFKDCLYEVFDDLESKMEDSGKQLLQSVLHLMENGALVLTTNFDNLLELYAADQGKQLESLDLTDEKKVLEWAQEKRKLSVLHIHGVYTNPSGIVLHPAGYQNVLRNTEVMREIQKLYENKSFLFLGCGWTVDDTTFQALFLEAVKHKSDLEHFMLVRRGDVDEFKKLRENMLDKGIKVISYGDDYADLPEYFKRLTCEISTRGTSAGMVREGQLNGSSAAHSEIRGCST

1096 NP_079404.2 NP_001005735.1

>NP_079404.2

MAAPVRLGRKRPLPACPNPLFVRWLTEWRDEATRSRHRTRFVFQKALRSLRRYPLPLRSGKEAKILQHFGDGLCRMLDERLQRHRTSGGDHAPDSPSGENSPAPQGRLAEVQDSSMPVPAQPKAGGSGSYWPARHSGARVILLVLYREHLNPNGHHFLTKEELLQRCAQKSPRVAPGSAPPWPALRSLLHRNLVLRTHQPARYSLTPEGLELAQKLAESEGLSLLNVGIGPKEPPGEETAVPGAASAELASEAGVQQQPLELRPGEYRVLLCVDIGETRGGGHRPELLRELQRLHVTHTVRKLHVGDFVWVAQETNPRDPANPGELVLDHIVERKRLDDLCSSIIDGRFREQKFRLKRCGLERRVYLVEEHGSVHNLSLPESTLLQAVTNTQVIDGFFVKRTADIKESAAYLALLTRGLQRLYQGHTLRSRPWGTPGNPESGAMTSPNPLCSLLTFSDFNAGAIKNKAQSVREVFARQLMQVRGVSGEKAAALVDRYSTPASLLAAYDACATPKEQETLLSTIKCGRLQRNLGPALSRTLSQLYCSYGPLT

>NP_001005735.1

MSRESDVEAQQSHGSSACSQPHGSVTQSQGSSSQSQGISSSSTSTMPNSSQSSHSSSGTLSSLETVSTQELYSIPEDQEPEDQEPEEPTPAPWARLWALQDGFANLETESGHVTQSDLELLLSSDPPASASQSAGIRGVRHHPRPVCSLKCVNDNYWFGRDKSCEYCFDEPLLKRTDKYRTYSKKHFRIFREVGPKNSYIAYIEDHSGNGTFVNTELVGKGKRRPLNNNSEIALSLSRNKVFVFFDLTVDDQSVYPKALRDEYIMSKTLGSGACGEVKLAFERKTCKKVAIKIISKRKFAIGSAREADPALNVETEIEILKKLNHPCIIKIKNFFDAEDYYIVLELMEGGELFDKVVGNKRLKEATCKLYFYQMLLAVQYLHENGIIHRDLKPENVLLSSQEEDCLIKITDFGHSKILGETSLMRTLCGTPTYLAPEVLVSVGTAGYNRAVDCWSLGVILFICLSGYPPFSEHRTQVSLKDQITSGKYNFIPEVWAEVSEKALDLVKKLLVVDPKARFTTEEALRHPWLQDEDMKRKFQDLLSEENESTALPQVLAQPSTSRKRPREGEAEGAETTKRPAVCAAVL

1097 NP_000561.3 NP_001630.1

>NP_000561.3

MWQLLLPTALLLLVSAGMRTEDLPKAVVFLEPQWYSVLEKDSVTLKCQGAYSPEDNSTQWFHNENLISSQASSYFIDAATVNDSGEYRCQTNLSTLSDPVQLEVHIGWLLLQAPRWVFKEEDPIHLRCHSWKNTALHKVTYLQNGKDRKYFHHNSDFHIPKATLKDSGSYFCRGLVGSKNVSSETVNITITQGLAVSTISSFSPPGYQVSFCLVMVLLFAVDTGLYFSVKTNI

>NP_001630.1

MNKPLLWISVLTSLLEAFAHTDLSGKVFVFPRESVTDHVNLITPLEKPLQNFTLCFRAYSDLSRAYSLFSYNTQGRDNELLVYKERVGEYSLYIGRHKVTSKVIEKFPAPVHICVSWESSSGIAEFWINGTPLVKKGLRQGYFVEAQPKIVLGQEQDSYGGKFDRSQSFVGEIGDLYMWDSVLPPENILSAYQGTPLPANILDWQALNYEIRGYVIIKPLVWV

1098 NP_003498.1 NP_066016.1

>NP_003498.1

MRDPGAAAPLSSLGLCALVLALLGALSAGAGAQPYHGEKGISVPDHGFCQPISIPLCTDIAYNQTILPNLLGHTNQEDAGLEVHQFYPLVKVQCSPELRFFLCSMYAPVCTVLDQAIPPCRSLCERARQGCEALMNKFGFQWPERLRCENFPVHGAGEICVGQNTSDGSGGPGGGPTAYPTAPYLPDLPFTALPPGASDGRGRPAFPFSCPRQLKVPPYLGYRFLGERDCGAPCEPGRANGLMYFKEEERRFARLWVGVWSVLCCASTLFTVLTYLVDMRRFSYPERPIIFLSGCYFMVAVAHVAGFLLEDRAVCVERFSDDGYRTVAQGTKKEGCTILFMVLYFFGMASSIWWVILSLTWFLAAGMKWGHEAIEANSQYFHLAAWAVPAVKTITILAMGQVDGDLLSGVCYVGLSSVDALRGFVLAPLFVYLFIGTSFLLAGFVSLFRIRTIMKHDGTKTEKLEKLMVRIGVFSVLYTVPATIVLACYFYEQAFREHWERTWLLQTCKSYAVPCPPGHFPPMSPDFTVFMIKYLMTMIVGITTGFWIWSGKTLQSWRRFYHRLSHSSKGETAV

>NP_066016.1

MSKTLKKKKHWLSKVQECAVSWAGPPGDFGAEIRGGAERGEFPYLGRLREEPGGGTCCIVSGKAPNPSDVLLEVNGTPVSGLTNRDTLAVIRHFREPIRLKTVKPGKVINKDLRHYLSLQFQKGSIDHKLQQVIRDNLYLRTIPCTTRAPRDGEVPGVDYNFISVEQFKALEESGALLESGTYDGNFYGTPKPPAEPSPFQPDPVDQVLFDNEFDAESQRKRTTSVSKMERMDSSLPEEEEDEDKGAINGSGNAENRERHSESSDWMKTVPSYNQTNSSMDFRNYMMRDETLEPLPKNWEMAYTDTGMIYFIDHNTKTTTWLDPRLCKKAKAPEDCEDGELPYGWEKIEDPQYGTYYVDFTLVAQAGVQWHDLGSLQPPPPGFNHLNQKTQFENPVEEAKRKKQLGQVEIGSSKPDMEKSHFTRDPSQLKGVLVRASLKKSTMGFGFTIIGGDRPDEFLQVKNVLKDGPAAQDGKIAPGDVIVDINGNCVFGHTHADVVQMFQLVPVNQYVNLTLCRGYPLPDDSEDPVVDIVAATPVINGQSLTKGETCMNPQDFKPGAMVLEQNGKSGHTSTGDGLNGPSDASEQRVSMASSGSSQPELVTIPLIKGPKGFGFAIADSPTGQKVKMILDSQWCQGLQKEDIIKEIYHQNVQNLTHLQVVEVLKQFPVGADVPLLILRGGPPSTTKTAKMKTDKKENAGSLEAINEPIPQPMPFPPSIIRSGSPKLDPSEVYLKSKTLYEDKPPNTKDLDVFLRKQESGFGFRVLGGDGPDQSIYIGAIIPLGAAEKDGRLRAADELMCIDGIPVKGKSHKQVLDLMTTAARNGHVLLTVRRKIFYGEKQPEDDSSQAFISTQNGSPRLNRAEVPARPAPQEPYDVVLQRKENEGFGFVILTSKNKPPPGVIPHKIGRVIEGSPADRCGKLKVGDHISAVNGQSIVELSHANIVQLIKDAGVTVTLTVIAEEEHHGPPSGTNSARQSPALQHRPMGQSQANHIPGDRSALEGEIGKDVSTSYRHSWSDHKHLAQPDTAVISVVGSRHNQNLGCYPVELERGPRGFGFSLRGGKEYNMGLFILRLAEDGPAIKDGRIHVGDQIVEINGEPTQGITHTRAIELIQAGGNKVLLLLRPGTGLIPDHGLAPSGLCSYVKPEQH

1099 BAA88957.1 NP_002836.2

>BAA88957.1

MGPWSRSLSALLLLLQVSSWLCQEPEPCHPGFDAESYTFTVPRRHLERGRVLGRVNFEDCTGRQRTAYFSLDTRFKVGTDGVITVKRPLRFHNPQIHFLVYAWDSTYRKFSTKVTLNTVGHHHRPPPHQASVSGIQAELLTFPNSSPGLRRQKRDWVIPPISCPENEKGPFPKNLVQIKSNKDKEGKVFYSITGQGADTPPVGVFIIERETGWLKVTEPLDRERIATYTLFSHAVSSNGNAVEDPMEILITVTDQNDNKPEFTQEVFKGSVMEGALPGTSVMEVTATDADDDVNTYNAAIAYTILSQDPELPDKNMFTINRNTGVISVVTTGLDRESFPTYTLVVQAADLQGEGLSTTATAVITVTDTNDNPPIFNPTTYKGQVPENEANVVITTLKVTDADAPNTPAWEAVYTILNDDGGQFVVTTNPVNNDGILKTAKDLARCRNWHPHSCSYPCPLGLDFEAKQQYILHVAVTNVVPFEVSLTTSTATVTVDVLDVNEAPIFVPPEKRVEVSEDFGVGQEITSYTAQEPDTFMEQKITYRIWRDTANWLEINPDTGAISTRAELDREDFEHVKNSTYTALIIATDNGSPVATGTGTLLLILSDVNDNAPIPEPRTIFFCERNPKPQVINIIDADLPPNTSPFTAELTHGASANWTIQYNDPTQESIILKPKMALEVGDYKINLKLMDNQNKDQVTTLEVSVCDCEGAAGVCRKAQPVEAGLQIPAILGILGGILALLILILLLLLFLRRRAVVKEPLLPPEDDTRDNVYYYDEEGGGEEDQDFDLSQLHRGLDARPEVTRNDVAPTLMSVPRYLPRPANPDEIGNFIDENLKAADTDPTAPPYDSLLVFDYEGSGSEAASLSSLNSSESDKDQDYDYLNEWGNRFKKLADMYGGGEDD

>NP_002836.2

MRGLGTCLATLAGLLLTAAGETFSGGCLFDEPYSTCGYSQSEGDDFNWEQVNTLTKPTSDPWMPSGSLMLVNASGRPEGQRAHLLLPQLKENDTHCIDFHYFVSSKSNSPPGLLNVYVKVNNGPLGNPIWNISGDPTRTWNRAELAISTFWPNFYQVIFEVITSGHQGYLAIDEVKVLGHPCTRTPHFLRIQNVEVNAGQFATFQCSAIGRTVAGDRLWLQGIDVRDAPLKEIKVTSSRRFIASFNVVNTTKRDAGKYRCMIRTEGGVGISNYAELVVKEPPVPIAPPQLASVGATYLWIQLNANSINGDGPIVAREVEYCTASGSWNDRQPVDSTSYKIGHLDPDTEYEISVLLTRPGEGGTGSPGPALRTRTKCADPMRGPRKLEVVEVKSRQITIRWEPFGYNVTRCHSYNLTVHYCYQVGGQEQVREEVSWDTENSHPQHTITNLSPYTNVSVKLILMNPEGRKESQELIVQTDEDLPGAVPTESIQGSTFEEKIFLQWREPTQTYGVITLYEITYKAVSSFDPEIDLSNQSGRVSKLGNETHFLFFGLYPGTTYSFTIRASTAKGFGPPATNQFTTKISAPSMPAYELETPLNQTDNTVTVMLKPAHSRGAPVSVYQIVVEEERPRRTKKTTEILKCYPVPIHFQNASLLNSQYYFAAEFPADSLQAAQPFTIGDNKTYNGYWNTPLLPYKSYRIYFQAASRANGETKIDCVQVATKGAATPKPVPEPEKQTDHTVKIAGVIAGILLFVIIFLGVVLVMKKRKLAKKRKETMSSTRQEMTVMVNSMDKSYAEQGTNCDEAFSFMDTHNLNGRSVSSPSSFTMKTNTLSTSVPNSYYPDETHTMASDTSSLVQSHTYKKREPADVPYQTGQLHPAIRVADLLQHITQMKCAEGYGFKEEYESFFEGQSAPWDSAKKDENRMKNRYGNIIAYDHSRVRLQTIEGDTNSDYINGNYIDGYHRPNHYIATQGPMQETIYDFWRMVWHENTASIIMVTNLVEVGRVKCCKYWPDDTEIYKDIKVTLIETELLAEYVIRTFAVEKRGVHEIREIRQFHFTGWPDHGVPYHATGLLGFVRQVKSKSPPSAGPLVVHCSAGAGRTGCFIVIDIMLDMAEREGVVDIYNCVRELRSRRVNMVQTEEQYVFIHDAILEACLCGDTSVPASQVRSLYYDMNKLDPQTNSSQIKEEFRTLNMVTPTLRVEDCSIALLPRNHEKNRCMDILPPDRCLPFLITIDGESSNYINAALMDSYKQPSAFIVTQHPLPNTVKDFWRLVLDYHCTSVVMLNDVDPAQLCPQYWPENGVHRHGPIQVEFVSADLEEDIISRIFRIYNAARPQDGYRMVQQFQFLGWPMYRDTPVSKRSFLKLIRQVDKWQEEYNGGEGPTVVHCLNGGGRSGTFCAISIVCEMLRHQRTVDVFHAVKTLRNNKPNMVDLLDQYKFCYEVALEYLNSG

1100 NP_060369.2 NP_060788.3

>NP_060369.2

MRPRSGPTRNPRLRAFAGVPTRGRTRGQSRRCAAEASAGPERDARPGAPAAGTMGAAHSASEEVRELEGKTGFSSDQIEQLHRRFKQLSGDQPTIRKENFNNVPDLELNPIRSKIVRAFFDNRNLRKGPSGLADEINFEDFLTIMSYFRPIDTTMDEEQVELSRKEKLRFLFHMYDSDSDGRITLEEYRNVVEELLSGNPHIEKESARSIADGAMMEAASVCMGQMEPDQVYEGITFEDFLKIWQGIDIETKMHVRFLNMETMALCH

>NP_060788.3

MDDDDFGGFEAAETFDGGSGETQTTSPAIPWAAFPAVSGVHLSPSSPEIVLDRDHSSSIGCLSSDAIISSPENTHAANSIVSQTIPKAQIQQSTHTHLDISLFPLGLTDEKSNGTIALVDDSEDPGANVSNIQLQQKISSLEIKLKVSEEEKQRIKQDVESLMEKHNVLEKGFLKEKEQEAISFQDRYKELQEKHKQELEDMRKAGHEALSIIVDEYKALLQSSVKQQVEAIEKQYISAIEKQAHKCEELLNAQHQRLLEMLDTEKELLKEKIKEALIQQSQEQKEILEKCLEEERQRNKEALVSAAKLEKEAVKDAVLKVVEEERKNLEKAHAEERELWKTEHAKDQEKVSQEIQKAIQEQRKISQETVKAAIIEEQKRSEKAVEEAVKRTRDELIEYIKEQKRLDQVIRQRSLSSLELFLSCAQKQLSALIATEPVDIE

1101 NP_002056.2 NP_078889.1

>NP_002056.2

MTTSASSHLNKGIKQVYMSLPQGEKVQAMYIWIDGTGEGLRCKTRTLDSEPKCVEELPEWNFDGSSTLQSEGSNSDMYLVPAAMFRDPFRKDPNKLVLCEVFKYNRRPAETNLRHTCKRIMDMVSNQHPWFGMEQEYTLMGTDGHPFGWPSNGFPGPQGPYYCGVGADRAYGRDIVEAHYRACLYAGVKIAGTNAEVMPAQWEFQIGPCEGISMGDHLWVARFILHRVCEDFGVIATFDPKPIPGNWNGAGCHTNFSTKAMREENGLKYIEEAIEKLSKRHQYHIRAYDPKGGLDNARRLTGFHETSNINDFSAGVANRSASIRIPRTVGQEKKGYFEDRRPSANCDPFSVTEALIRTCLLNETGDEPFQYKN

>NP_078889.1

MVDRLANSEANTRRISIVENCFGAAGQPLTIPGRVLIGEGVLTKLCRKKPKARQFFLFNDILVYGNIVIQKKKYNKQHIIPLENVTIDSIKDEGDLRNGWLIKTPTKSFAVYAATATEKSEWMNHINKCVTDLLSKSGKTPSNEHAAVWVPDSEATVCMRCQKAKFTPVNRRHHCRKCGFVVCGPCSEKRFLLPSQSSKPVRICDFCYDLLSAGDMATCQPARSDSYSQSLKSPLNDMSDDDDDDDSSD

1102 NP_056453.1 NP_003795.2

>NP_056453.1

MAAAPVAAGSGAGRGRRSAATVAAWGGWGGRPRPGNILLQLRQGQLTGRGLVRAVQFTETFLTERDKQSKWSGIPQLLLKLHTTSHLHSDFVECQNILKEISPLLSMEAMAFVTEERKLTQETTYPNTYIFDLFGGVDLLVEILMRPTISIRGQKLKISDEMSKDCLSILYNTCVCTEGVTKRLAEKNDFVIFLFTLMTSKKTFLQTATLIEDILGVKKEMIRLDEVPNLSSLVSNFDQQQLANFCRILAVTISEMDTGNDDKHTLLAKNAQQKKSLSLGPSAAEINQAALLSIPGFVERLCKLATRKVSESTGTASFLQELEEWYTWLDNALVLDALMRVANEESEHNQASIVFPPPGASEENGLPHTSARTQLPQSMKIMHEIMYKLEVLYVLCVLLMGRQRNQVHRMIAEFKLIPGLNNLFDKLIWRKHSASALVLHGHNQNCDCSPDITLKIQFLRLLQSFSDHHENKYLLLNNQELNELSAISLKANIPEVEAVLNTDRSLVCDGKRGLLTRLLQVMKKEPAESSFRFWQARAVESFLRGTTSYADQMFLLKRGLLEHILYCIVDSECKSRDVLQSYFDLLGELMKFNVDAFKRFNKYINTDAKFQVFLKQINSSLVDSNMLVRCVTLSLDRFENQVDMKVAEVLSECRLLAYISQVPTQMSFLFRLINIIHVQTLTQENVSCLNTSLVILMLARRKERLPLYLRLLQRMEHSKKYPGFLLNNFHNLLRFWQQHYLHKDKDSTCLENSSCISFSYWKETVSILLNPDRQSPSALVSYIEEPYMDIDRDFTEE

>NP_003795.2

MQPDMSLNVIKMKSSDFLESAELDSGGFGKVSLCFHRTQGLMIMKTVYKGPNCIEHNEALLEEAKMMNRLRHSRVVKLLGVIIEEGKYSLVMEYMEKGNLMHVLKAEMSTPLSVKGRIILEIIEGMCYLHGKGVIHKDLKPENILVDNDFHIKIADLGLASFKMWSKLNNEEHNELREVDGTAKKNGGTLYYMAPEHLNDVNAKPTEKSDVYSFAVVLWAIFANKEPYENAICEQQLIMCIKSGNRPDVDDITEYCPREIISLMKLCWEANPEARPTFPGIEEKFRPFYLSQLEESVEEDVKSLKKEYSNENAVVKRMQSLQLDCVAVPSSRSNSATEQPGSLHSSQGLGMGPVEESWFAPSLEHPQEENEPSLQSKLQDEANYHLYGSRMDRQTKQQPRQNVAYNREEERRRRVSHDPFAQQRPYENFQNTEGKGTAYSSAASHGNAVHQPSGLTSQPQVLYQNNGLYSSHGFGTRPLDPGTAGPRVWYRPIPSHMPSLHNIPVPETNYLGNTPTMPFSSLPPTDESIKYTIYNSTGIQIGAYNYMEIGGTSSSLLDSTNTNFKEEPAAKYQAIFDNTTSLTDKHLDPIRENLGKHWKNCARKLGFTQSQIDEIDHDYERDGLKEKVYQMLQKWVMREGIKGATVGKLAQALHQCSRIDLLSSLIYVSQN

1103 NP_002509.2 NP_000955.1

>NP_002509.2

MDEDEKDRAKRASRNKSEKKRRDQFNVLIKELSSMLPGNTRKMDKTTVLEKVIGFLQKHNEVSAQTEICDIQQDWKPSFLSNEEFTQLMLEALDGFIIAVTTDGSIIYVSDSITPLLGHLPSDVMDQNLLNFLPEQEHSEVYKILSSHMLVTDSPSPEYLKSDSDLEFYCHLLRGSLNPKEFPTYEYIKFVGNFRSYNNVPSPSCNGFDNTLSRPCRVPLGKEVCFIATVRLATPQFLKEMCIVDEPLEEFTSRHSLEWKFLFLDHRAPPIIGYLPFEVLGTSGYDYYHIDDLELLARCHQHLMQFGKGKSCCYRFLTKGQQWIWLQTHYYITYHQWNSKPEFIVCTHSVVSYADVRVERRQELALEDPPSEALHSSALKDKGSSLEPRQHFNTLDVGASGLNTSHSPSASSRSSHKSSHTAMSEPTSTPTKLMAEASTPALPRSATLPQELPVPGLSQAATMPAPLPSPSSCDLTQQLLPQTVLQSTPAPMAQFSAQFSMFQTIKDQLEQRTRILQANIRWQQEELHKIQEQLCLVQDSNVQMFLQQPAVSLSFSSTQRPEAQQQLQQRSAAVTQPQLGAGPQLPGQISSAQVTSQHLLRESSVISTQGPKPMRSSQLMQSSGRSGSSLVSPFSSATAALPPSLNLTTPASTSQDASQCQPSPDFSHDRQLRLLLSQPIQPMMPGSCDARQPSEVSRTGRQVKYAQSQTVFQNPDAHPANSSSAPMPVLLMGQAVLHPSFPASQPSPLQPAQARQQPPQHYLQVQAPTSLHSEQQDSLLLSTYSQQPGTLGYPQPPPAQPQPLRPPRRVSSLSESSGLQQPPR

>NP_000955.1

MASNSSSCPTPGGGHLNGYPVPPYAFFFPPMLGGLSPPGALTTLQHQLPVSGYSTPSPATIETQSSSSEEIVPSPPSPPPLPRIYKPCFVCQDKSSGYHYGVSACEGCKGFFRRSIQKNMVYTCHRDKNCIINKVTRNRCQYCRLQKCFEVGMSKESVRNDRNKKKKEVPKPECSESYTLTPEVGELIEKVRKAHQETFPALCQLGKYTTNNSSEQRVSLDIDLWDKFSELSTKCIIKTVEFAKQLPGFTTLTIADQITLLKAACLDILILRICTRYTPEQDTMTFSDGLTLNRTQMHNAGFGPLTDLVFAFANQLLPLEMDDAETGLLSAICLICGDRQDLEQPDRVDMLQEPLLEALKVYVRKRRPSRPHMFPKMLMKITDLRSISAKGAERVITLKMEIPGSMPPLIQEMLENSEGLDTLSGQPGGGGRDGGGLAPPPGSCSPSLSPSSNRSSPATHSP

1104 NP_003102.1 NP_005216.1

>NP_003102.1

MTAPEKPVKQEEMAALDVDSGGGGGGGGGHGEYLQQQQQHGNGAVAAAAAAQDTQPSPLALLAATCSKIGPPSPGDDEEEAAAAAGAPAAAGATGDLASAQLGGAPNRWEVLSATPTTIKDEAGNLVQIPSAATSSGQYVLPLQNLQNQQIFSVAPGSDSSNGTVSSVQYQVIPQIQSADGQQVQIGFTGSSDNGGINQESSQIQIIPGSNQTLLASGTPSANIQNLIPQTGQVQVQGVAIGGSSFPGQTQVVANVPLGLPGNITFVPINSVDLDSLGLSGSSQTMTAGINADGHLINTGQAMDSSDNSERTGERVSPDINETNTDTDLFVPTSSSSQLPVTIDSTGILQQNTNSLTTSSGQVHSSDLQGNYIQSPVSEETQAQNIQVSTAQPVVQHLQLQESQQPTSQAQIVQGITPQTIHGVQASGQNISQQALQNLQLQLNPGTFLIQAQTVTPSGQVTWQTFQVQGVQNLQNLQIQNTAAQQITLTPVQTLTLGQVAAGGAFTSTPVSLSTGQLPNLQTVTVNSIDSAGIQLHPGENADSPADIRIKEEEPDPEEWQLSGDSTLNTNDLTHLRVQVVDEEGDQQHQEGKRLRRVACTCPNCKEGGGRGTNLGKKKQHICHIPGCGKVYGKTSHLRAHLRWHSGERPFVCNWMYCGKRFTRSDELQRHRRTHTGEKKFVCPECSKRFMRSDHLAKHIKTHQNKKGIHSSSTVLASVEAARDDTLITAGGTTLILANIQQGSVSGIGTVNTSATSNQDILTNTEIPLQLVTVSGNETME

>NP_005216.1

MALAGAPAGGPCAPALEALLGAGALRLLDSSQIVIISAAQDASAPPAPTGPAAPAAGPCDPDLLLFATPQAPRPTPSAPRPALGRPPVKRRLDLETDHQYLAESSGPARGRGRHPGKGVKSPGEKSRYETSLNLTTKRFLELLSHSADGVVDLNWAAEVLKVQKRRIYDITNVLEGIQLIAKKSKNHIQWLGSHTTVGVGGRLEGLTQDLRQLQESEQQLDHLMNICTTQLRLLSEDTDSQRLAYVTCQDLRSIADPAEQMVMVIKAPPETQLQAVDSSENFQISLKSKQGPIDVFLCPEETVGGISPGKTPSQEVTSEEENRATDSATIVSPPPSSPPSSLTTDPSQSLLSLEQEPLLSRMGSLRAPVDEDRLSPLVAADSLLEHVREDFSGLLPEEFISLSPPHEALDYHFGLEEGEGIRDLFDCDFGDLTPLDF

1105 NP_116738.1 NP_000570.1

>NP_116738.1

MKISVAAIPFFLLITIALGTKTESSSQTGGKPKVVKIQLKLVGGPYHPSECCFTYTTYKIPRQRIMDYYETNSQCSKPGIVFITKRGHSVCTNPSDKWVQDYIKDMKEN

>NP_000570.1

MDYQVSSPIYDINYYTSEPCQKINVKQIAARLLPPLYSLVFIFGFVGNMLVILILINCKRLKSMTDIYLLNLAISDLFFLLTVPFWAHYAAAQWDFGNTMCQLLTGLYFIGFFSGIFFIILLTIDRYLAVVHAVFALKARTVTFGVVTSVITWVVAVFASLPGIIFTRSQKEGLHYTCSSHFPYSQYQFWKNFQTLKIVILGLVLPLLVMVICYSGILKTLLRCRNEKKRHRAVRLIFTIMIVYFLFWAPYNIVLLLNTFQEFFGLNNCSSSNRLDQAMQVTETLGMTHCCINPIIYAFVGEKFRNYLLVFFQKHIAKRFCKCCSIFQQEAPERASSVYTRSTGEQEISVGL

1106 NP_002806.2 NP_068597.2

>NP_002806.2

MAAAAVVEFQRAQSLLSTDREASIDILHSIVKRDIQENDEEAVQVKEQSILELGSLLAKTGQAAELGGLLKYVRPFLNSISKAKAARLVRSLLDLFLDMEAATGQEVELCLECIEWAKSEKRTFLRQALEARLVSLYFDTKRYQEALHLGSQLLRELKKMDDKALLVEVQLLESKTYHALSNLPKARAALTSARTTANAIYCPPKLQATLDMQSGIIHAAEEKDWKTAYSYFYEAFEGYDSIDSPKAITSLKYMLLCKIMLNTPEDVQALVSGKLALRYAGRQTEALKCVAQASKNRSLADFEKALTDYRAELRDDPIISTHLAKLYDNLLEQNLIRVIEPFSRVQIEHISSLIKLSKADVERKLSQMILDKKFHGILDQGEGVLIIFDEPPVDKTYEAALETIQNMSKVVDSLYNKAKKLT

>NP_068597.2

MNSRQAWRLFLSQGRGDRWVSRPRGHFSPALRREFFTTTTKEGYDRRPVDITPLEQRKLTFDTHALVQDLETHGFDKTQAETIVSALTALSNVSLDTIYKEMVTQAQQEITVQQLMAHLDAIRKDMVILEKSEFANLRAENEKMKIELDQVKQQLMHETSRIRADNKLDINLERSRVTDMFTDQEKQLMETTTEFTKKDTQTKSIISETSNKIDAEIASLKTLMESNKLETIRYLAASVFTCLAIALGFYRFWK

1107 NP_004929.2 NP_734465.2

>NP_004929.2

MTVFRQENVDDYYDTGEELGSGQFAVVKKCREKSTGLQYAAKFIKKRRTKSSRRGVSREDIEREVSILKEIQHPNVITLHEVYENKTDVILILELVAGGELFDFLAEKESLTEEEATEFLKQILNGVYYLHSLQIAHFDLKPENIMLLDRNVPKPRIKIIDFGLAHKIDFGNEFKNIFGTPEFVAPEIVNYEPLGLEADMWSIGVITYILLSGASPFLGDTKQETLANVSAVNYEFEDEYFSNTSALAKDFIRRLLVKDPKKRMTIQDSLQHPWIKPKDTQQALSRKASAVNMEKFKKFAARKKWKQSVRLISLCQRLSRSFLSRSNMSVARSDDTLDEEDSFVMKAIIHAINDDNVPGLQHLLGSLSNYDVNQPNKHGTPPLLIAAGCGNIQILQLLIKRGSRIDVQDKGGSNAVYWAARHGHVDTLKFLSENKCPLDVKDKSGEMALHVAARYGHADVAQLLCSFGSNPNIQDKEEETPLHCAAWHGYYSVAKALCEAGCNVNIKNREGETPLLTASARGYHDIVECLAEHGADLNACDKDGHIALHLAVRRCQMEVIKTLLSQGCFVDYQDRHGNTPLHVACKDGNMPIVVALCEANCNLDISNKYGRTPLHLAANNGILDVVRYLCLMGASVEALTTDGKTAEDLARSEQHEHVAGLLARLRKDTHRGLFIQQLRPTQNLQPRIKLKLFGHSGSGKTTLVESLKCGLLRSFFRRRRPRLSSTNSSRFPPSPLASKPTVSVSINNLYPGCENVSVRSRSMMFEPGLTKGMLEVFVAPTHHPHCSADDQSTKAIDIQNAYLNGVGDFSVWEFSGNPVYFCCYDYFAANDPTSIHVVVFSLEEPYEIQLNQVIFWLSFLKSLVPVEEPIAFGGKLKNPLQVVLVATHADIMNVPRPAGGEFGYDKDTSLLKEIRNRFGNDLHISNKLFVLDAGASGSKDMKVLRNHLQEIRSQIVSVCPPMTHLCEKIISTLPSWRKLNGPNQLMSLQQFVYDVQDQLNPLASEEDLRRIAQQLHSTGEINIMQSETVQDVLLLDPRWLCTNVLGKLLSVETPRALHHYRGRYTVEDIQRLVPDSDVEELLQILDAMDICARDLSSGTMVDVPALIKTDNLHRSWADEEDEVMVYGGVRIVPVEHLTPFPCGIFHKVQVNLCRWIHQQSTEGDADIRLWVNGCKLANRGAELLVLLVNHGQGIEVQVRGLETEKIKCCLLLDSVCSTIENVMATTLPGLLTVKHYLSPQQLREHHEPVMIYQPRDFFRAQTLKETSLTNTMGGYKESFSSIMCFGCHDVYSQASLGMDIHASDLNLLTRRKLSRLLDPPDPLGKDWCLLAMNLGLPDLVAKYNTSNGAPKDFLPSPLHALLREWTTYPESTVGTLMSKLRELGRRDAADFLLKASSVFKINLDGNGQEAYASSCNSGTSYNSISSVVSR

>NP_734465.2

MGARSGARGALLLALLLCWDPRLSQAGTDSGSEVLPDSFPSAPAEPLPYFLQEPQDAYIVKNKPVELRCRAFPATQIYFKCNGEWVSQNDHVTQEGLDEATGLRVREVQIEVSRQQVEELFGLEDYWCQCVAWSSAGTTKSRRAYVRIAYLRKNFDQEPLGKEVPLDHEVLLQCRPPEGVPVAEVEWLKNEDVIDPTQDTNFLLTIDHNLIIRQARLSDTANYTCVAKNIVAKRRSTTATVIVYVNGGWSSWAEWSPCSNRCGRGWQKRTRTCTNPAPLNGGAFCEGQAFQKTACTTICPVDGAWTEWSKWSACSTECAHWRSRECMAPPPQNGGRDCSGTLLDSKNCTDGLCMQNKKTLSDPNSHLLEASGDAALYAGLVVAIFVVVAILMAVGVVVYRRNCRDFDTDITDSSAALTGGFHPVNFKTARPSNPQLLHPSVPPDLTASAGIYRGPVYALQDSTDKIPMTNSPLLDPLPSLKVKVYSSSTTGSGPGLADGADLLGVLPPGTYPSDFARDTHFLHLRSASLGSQQLLGLPRDPGSSVSGTFGCLGGRLSIPGTGVSLLVPNGAIPQGKFYEMYLLINKAESTLPLSEGTQTVLSPSVTCGPTGLLLCRPVILTMPHCAEVSARDWIFQLKTQAHQGHWEEVVTLDEETLNTPCYCQLEPRACHILLDQLGTYVFTGESYSRSAVKRLQLAVFAPALCTSLEYSLRVYCLEDTPVALKEVLELERTLGGYLVEEPKPLMFKDSYHNLRLSLHDLPHAHWRSKLLAKYQEIPFYHIWSGSQKALHCTFTLERHSLASTELTCKICVRQVEGEGQIFQLHTTLAETPAGSLDTLCSAPGSTVTTQLGPYAFKIPLSIRQKICNSLDAPNSRGNDWRMLAQKLSMDRYLNYFATKASPTGVILDLWEALQQDDGDLNSLASALEEMGKSEMLVAVATDGDC

1108 NP_001009566.1 NP_073733.1

>NP_001009566.1

MLRRPAPALAPAARLLLAGLLCGGGVWAARVNKHKPWLEPTYHGIVTENDNTVLLDPPLIALDKDAPLRFAESFEVTVTKEGEICGFKIHGQNVPFDAVVVDKSTGEGVIRSKEKLDCELQKDYSFTIQAYDCGKGPDGTNVKKSHKATVHIQVNDVNEYAPVFKEKSYKATVIEGKQYDSILRVEAVDADCSPQFSQICSYEIITPDVPFTVDKDGYIKNTEKLNYGKEHQYKLTVTAYDCGKKRATEDVLVKISIKPTCTPGWQGWNNRIEYEPGTGALAVFPNIHLETCDEPVASVQATVELETSHIGKGCDRDTYSEKSLHRLCGAAAGTAELLPSPSGSLNWTMGLPTDNGHDSDQVFEFNGTQAVRIPDGVVSVSPKEPFTISVWMRHGPFGRKKETILCSSDKTDMNRHHYSLYVHGCRLIFLFRQDPSEEKKYRPAEFHWKLNQVCDEEWHHYVLNVEFPSVTLYVDGTSHEPFSVTEDYPLHPSKIETQLVVGACWQEFSGVENDNETEPVTVASAGGDLHMTQFFRGNLAGLTLRSGKLADKKVIDCLYTCKEGLDLQVLEDSGRGVQIQAHPSQLVLTLEGEDLGELDKAMQHISYLNSRQFPTPGIRRLKITSTIKCFNEATCISVPPVDGYVMVLQPEEPKISLSGVHHFARAASEFESSEGVFLFPELRIISTITREVEPEGDGAEDPTVQESLVSEEIVHDLDTCEVTVEGEELNHEQESLEVDMARLQQKGIEVSSSELGMTFTGVDTMASYEEVLHLLRYRNWHARSLLDRKFKLICSELNGRYISNEFKVEVNVIHTANPMEHANHMAAQPQFVHPEHRSFVDLSGHNLANPHPFAVVPSTATVVIVVCVSFLVFMIILGVFRIRAAHRRTMRDQDTGKENEMDWDDSALTITVNPMETYEDQHSSEEEEEEEEEEESEDGEEEDDITSAESESSEEEEGEQGDPQNATRQQQLEWDDSTLSY

>NP_073733.1

MAMMVFPREEKLSQDEIVLGTKAVIQGLETLRGEHRALLAPLVAPEAGEAEPGSQERCILLRRSLEAIELGLGEAQVILALSSHLGAVESEKQKLRAQVRRLVQENQWLREELAGTQQKLQRSEQAVAQLEEEKQHLLFMSQIRKLDEDASPNEEKGDVPKDTLDDLFPNEDEQSPAPSPGGGDVSGQHGGYEIPARLRTLHNLVIQYASQGRYEVAVPLCKQALEDLEKTSGHDHPDVATMLNILALVYRDQNKYKEAAHLLNDALAIREKTLGKDHPAVAATLNNLAVLYGKRGKYKEAEPLCKRALEIREKVLGKFHPDVAKQLSNLALLCQNQGKAEEVEYYYRRALEIYATRLGPDDPNVAKTKNNLASCYLKQGKYQDAETLYKEILTRAHEKEFGSVNGDNKPIWMHAEEREESKDKRRDSAPYGEYGSWYKACKVDSPTVNTTLRSLGALYRRQGKLEAAHTLEDCASRNRKQGLDPASQTKVVELLKDGSGRRGDRRSSRDMAGGAGPRSESDLEDVGPTAEWNGDGSGSLRRSGSFGKLRDALRRSSEMLVKKLQGGTPQEPPNPRMKRASSLNFLNKSVEEPTQPGGTGLSDSRTLSSSSMDLSRRSSLVG

1109 NP_002568.2 NP_061485.1

>NP_002568.2

MSDNGELEDKPPAPPVRMSSTIFSTGGKDPLSANHSLKPLPSVPEEKKPRHKIISIFSGTEKGSKKKEKERPEISPPSDFEHTIHVGFDAVTGEFTGMPEQWARLLQTSNITKLEQKKNPQAVLDVLKFYDSNTVKQKYLSFTPPEKDGFPSGTPALNAKGTEAPAVVTEEEDDDEETAPPVIAPRPDHTKSIYTRSVIDPVPAPVGDSHVDGAAKSLDKQKKKTKMTDEEIMEKLRTIVSIGDPKKKYTRYEKIGQGASGTVFTATDVALGQEVAIKQINLQKQPKKELIINEILVMKELKNPNIVNFLDSYLVGDELFVVMEYLAGGSLTDVVTETCMDEAQIAAVCRECLQALEFLHANQVIHRDIKSDNVLLGMEGSVKLTDFGFCAQITPEQSKRSTMVGTPYWMAPEVVTRKAYGPKVDIWSLGIMAIEMVEGEPPYLNENPLRALYLIATNGTPELQNPEKLSPIFRDFLNRCLEMDVEKRGSAKELLQHPFLKLAKPLSSLTPLIMAAKEAMKSNR

>NP_061485.1

MQAIKCVVVGDGAVGKTCLLISYTTNAFPGEYIPTVFDNYSANVMVDGKPVNLGLWDTAGQEDYDRLRPLSYPQTVGETYGKDITSRGKDKPIADVFLICFSLVSPASFENVRAKWYPEVRHHCPNTPIILVGTKLDLRDDKDTIEKLKEKKLTPITYPQGLAMAKEIGAVKYLECSALTQRGLKTVFDEAIRAVLCPPPVKKRKRKCLLL

1110 NP_008843.1 NP_060842.3

>NP_008843.1

MDSGTRPVGSCCSSPAGLSREYKLVMLGAGGVGKSAMTMQFISHRFPEDHDPTIEDAYKIRIRIDDEPANLDILDTAGQAEFTAMRDQYMRAGEGFIICYSITDRRSFHEVREFKQLIYRVRRTDDTPVVLVGNKSDLKQLRQVTKEEGLALAREFSCPFFETSAAYRYYIDDVFHALVREIRRKEKEAVLAMEKKSKPKNSVWKRLKSPFRKKKDSVT

>NP_060842.3

MSNNLRRVFLKPAEENSGNASRCVSGCMYQVVQTIGSDGKNLLQLLPIPKSSGNLIPLVQSSVMSDALKGNTGKPVQVTFQTQISSSSTSASVQLPIFQPASSSNYFLTRTVDTSEKGRVTSVGTGNFSSSVSKVQSHGVKIDGLTMQTFAVPPSTQKDSSFIVVNTQSLPVTVKSPVLPSGHHLQIPAHAEVKSVPASSLPPSVQQKILATATTSTSGMVEASQMPTVIYVSPVNTVKNVVTKNFQNIYPKPVTEIAKPVILNTTQIPKNVATETQLKGGQHSQAAPVKWIFQDNLQPFTPSLVPVKSSNNVASKILKTFVDRKNLGDNTINMPPLSTIDPSGTRSKNMPIKDNALVMFNGKVYLLAKKGTDVLPSQIDQQNSVSPDTPVRKDTLQTVSSSPVTEISREVVNIVLAKSKSSQMETKSLSNTQLASMANLRAEKNKVEKPSPSTTNPHMNQSSNYLKQSKTLFTNPIFPVGFSTGHNAPRKVTAVIYARKGSVLQSIEKISSSVDATTVTSQQCVFRDQEPKIHNEMASTSDKGAQGRNDKKDSQGRSNKALHLKSDAEFKKIFGLTKDLRVCLTRIPDHLTSGEGFDSFSSLVKSGTYKETEFMVKEGERKQQNFDKKRKAKTNKKMDHIKKRKTENAYNAIINGEANVTGSQLLSSILPTSDVSQHNILTSHSKTRQEKRTEMEYYTHEKQEKGTLNSNAAYEQSHFFNKNYTEDIFPVTPPELEETIRDEKIRRLKQVLREKEAALEEMRKKMHQK

1111 NP_973728.1 NP_005565.1

>NP_973728.1

MTSAVVDSGGTILELSSNGVENQEESEKVSEYPAVIVEPVPSARLEQGYAAQVLVYDDETYMMQDVAEEQEVETENVETVEASVHSSNAHCTDKTIEAAEALLHMESPTCLRDSRSPEFIHAAMRPDVITETVVEVSTEESEPMDTSPIPTSPDSHEPMKKKKVGRKPKTQQSPISNGSPELGIKKKPREGKGNTTYLWEFLLDLLQDKNTCPRYIKWTQREKGIFKLVDSKAVSKLWGKHKNKPDMNYETMGRALRYYYQRGILAKVEGQRLVYQFKDMPKNIVVIDDDKSETCNEDLAGTTDEKSLERVSLSAESLLKAASSVRSGKNSSPINCSRAEKGVARVVNITSPGHDASSRSPTTTASVSATAAPRTVRVAMQVPVVMTSLGQKISTVAVQSVNAGAPLITSTSPTTATSPKVVIQTIPTVMPASTENGDKITMQPAKIITIPATQLAQCQLQTKSNLTGSGSINIVGTPLAVRALTPVSIAHGTPVMRLSMPTQQASGQTPPRVISAVIKGPEVKSEAVAKKQEHDVKTLQLVEEKPADGNKTVTHVVVVSAPSAIALPVTMKTEGLVTCEK

>NP_005565.1

MSSAIERKSLDPSEEPVDEVLQIPPSLLTCGGCQQNIGDRYFLKAIDQYWHEDCLSCDLCGCRLGEVGRRLYYKLGRKLCRRDYLRLFGQDGLCASCDKRIRAYEMTMRVKDKVYHLECFKCAACQKHFCVGDRYLLINSDIVCEQDIYEWTKINGMI

1112 NP_003156.1 Q15531

>NP_003156.1

MAPIGLKAVVGEKIMHDVIKKVKKKGEWKVLVVDQLSMRMLSSCCKMTDIMTEGITIVEDINKRREPLPSLEAVYLITPSEKSVHSLISDFKDPPTAKYRAAHVFFTDSCPDALFNELVKSRAAKVIKTLTEINIAFLPYESQVYSLDSADSFQSFYSPHKAQMKNPILERLAEQIATLCATLKEYPAVRYRGEYKDNALLAQLIQDKLDAYKADDPTMGEGPDKARSQLLILDRGFDPSSPVLHELTFQAMSYDLLPIENDVYKYETSGIGEARVKEVLLDEDDDLWIALRHKHIAEVSQEVTRSLKDFSSSKRMNTGEKTTMRDLSQMLKKMPQYQKELSKYSTHLHLAEDCMKHYQGTVDKLCRVEQDLAMGTDAEGEKIKDPMRAIVPILLDANVSTYDKIRIILLYIFLKNGITEENLNKLIQHAQIPPEDSEIITNMAHLGVPIVTDSTLRRRSKPERKERISEQTYQLSRWTPIIKDIMEDTIEDKLDTKHYPYISTRSSASFSTTAVSARYGHWHKNKAPGEYRSGPRLIIFILGGVSLNEMRCAYEVTQANGKWEVLIGSTHILTPTKFLMDLRHPDFRESSRVSFEDQAPTME

>Q15531

MKDRTQVLRTRRNSDDKEEVVHVDRDHFMDEFFEQEEEIRGCIEKLSEDVEQVKKQHSAILAAPNPDERTKQELEDLTADIKKTANKVRSKLKAIEQSIEQEEGSTAPRPILRIRKTQHSTLSRKFVEVMTEYNATQSKYRDRCKDRIQRQLEITGRTTTNEELEDMLESGKLPIFTDDIKMDSQMTKQALNEIETRHNEIIKLETSIRELHDMFVDMAMLVESQGEMIDRIEYNVEHSVDYVERAVSDTKKAVKYQSKARRKKIIIIICCVVLGVVLASSIGCTLGL

1113 NP_057559.2 NP_620637.1

>NP_057559.2

MAVDIQPACLGLYCGKTLLFKNGSTEIYGECGVCPRGQRTNAQKYCQPCTESPELYDWLYLGFMAMLPLVLHWFFIEWYSGKKSSSALFQHITALFECSMAAIITLLVSDPVGVLYIRSCRVLMLSDWYTMLYNPSPDYVTTVHCTHEAVYPLYTIVFIYYAFCLVLMMLLRPLLVKKIACGLGKSDRFKSIYAALYFFPILTVLQAVGGGLLYYAFPYIILVLSLVTLAVYMSASEIENCYDLLVRKKRLIVLFSHWLLHAYGIISISRVDKLEQDLPLLALVPTPALFYLFTAKFTEPSRILSEGANGH

>NP_620637.1

MSRSKRDNNFYSVEIGDSTFTVLKRYQNLKPIGSGAQGIVCAAYDAILERNVAIKKLSRPFQNQTHAKRAYRELVLMKCVNHKNIIGLLNVFTPQKSLEEFQDVYIVMELMDANLCQVIQMELDHERMSYLLYQMLCGIKHLHSAGIIHRDLKPSNIVVKSDCTLKILDFGLARTAGTSFMMTPYVVTRYYRAPEVILGMGYKENVDLWSVGCIMGEMVCHKILFPGRDYIDQWNKVIEQLGTPCPEFMKKLQPTVRTYVENRPKYAGYSFEKLFPDVLFPADSEHNKLKASQARDLLSKMLVIDASKRISVDEALQHPYINVWYDPSEAEAPPPKIPDKQLDEREHTIEEWKELIYKEVMDLEERTKNGVIRGQPSPLGAAVINGSQHPSSSSSVNDVSSMSTDPTLASDTDSSLEAAAGPLGCCR

1114 NP_000090.1 NP_009224.2

>NP_000090.1

MAGPLRAPLLLLAILAVALAVSPAAGSSPGKPPRLVGGPMDASVEEEGVRRALDFAVGEYNKASNDMYHSRALQVVRARKQIVAGVNYFLDVELGRTTCTKTQPNLDNCPFHDQPHLKRKAFCSFQIYAVPWQGTMTLSKSTCQDA

>NP_009224.2

MRLLWGLIWASSFFTLSLQKPRLLLFSPSVVHLGVPLSVGVQLQDVPRGQVVKGSVFLRNPSRNNVPCSPKVDFTLSSERDFALLSLQVPLKDAKSCGLHQLLRGPEVQLVAHSPWLKDSLSRTTNIQGINLLFSSRRGHLFLQTDQPIYNPGQRVRYRVFALDQKMRPSTDTITVMVENSHGLRVRKKEVYMPSSIFQDDFVIPDISEPGTWKISARFSDGLESNSSTQFEVKKYVLPNFEVKITPGKPYILTVPGHLDEMQLDIQARYIYGKPVQGVAYVRFGLLDEDGKKTFFRGLESQTKLVNGQSHISLSKAEFQDALEKLNMGITDLQGLRLYVAAAIIESPGGEMEEAELTSWYFVSSPFSLDLSKTKRHLVPGAPFLLQALVREMSGSPASGIPVKVSATVSSPGSVPEVQDIQQNTDGSGQVSIPIIIPQTISELQLSVSAGSPHPAIARLTVAAPPSGGPGFLSIERPDSRPPRVGDTLNLNLRAVGSGATFSHYYYMILSRGQIVFMNREPKRTLTSVSVFVDHHLAPSFYFVAFYYHGDHPVANSLRVDVQAGACEGKLELSVDGAKQYRNGESVKLHLETDSLALVALGALDTALYAAGSKSHKPLNMGKVFEAMNSYDLGCGPGGGDSALQVFQAAGLAFSDGDQWTLSRKRLSCPKEKTTRKKRNVNFQKAINEKLGQYASPTAKRCCQDGVTRLPMMRSCEQRAARVQQPDCREPFLSCCQFAESLRKKSRDKGQAGLQRALEILQEEDLIDEDDIPVRSFFPENWLWRVETVDRFQILTLWLPDSLTTWEIHGLSLSKTKGLCVATPVQLRVFREFHLHLRLPMSVRRFEQLELRPVLYNYLDKNLTVSVHVSPVEGLCLAGGGGLAQQVLVPAGSARPVAFSVVPTAAAAVSLKVVARGSFEFPVGDAVSKVLQIEKEGAIHREELVYELNPLDHRGRTLEIPGNSDPNMIPDGDFNSYVRVTASDPLDTLGSEGALSPGGVASLLRLPRGCGEQTMIYLAPTLAASRYLDKTEQWSTLPPETKDHAVDLIQKGYMRIQQFRKADGSYAAWLSRDSSTWLTAFVLKVLSLAQEQVGGSPEKLQETSNWLLSQQQADGSFQDPCPVLDRSMQGGLVGNDETVALTAFVTIALHHGLAVFQDEGAEPLKQRVEASISKANSFLGEKASAGLLGAHAAAITAYALTLTKAPVDLLGVAHNNLMAMAQETGDNLYWGSVTGSQSNAVSPTPAPRNPSDPMPQAPALWIETTAYALLHLLLHEGKAEMADQASAWLTRQGSFQGGFRSTQDTVIALDALSAYWIASHTTEERGLNVTLSSTGRNGFKSHALQLNNRQIRGLEEELQFSLGSKINVKVGGNSKGTLKVLRTYNVLDMKNTTCQDLQIEVTVKGHVEYTMEANEDYEDYEYDELPAKDDPDAPLQPVTPLQLFEGRRNRRRREAPKVVEEQESRVHYTVCIWRNGKVGLSGMAIADVTLLSGFHALRADLEKLTSLSDRYVSHFETEGPHVLLYFDSVPTSRECVGFEAVQEVPVGLVQPASATLYDYYNPERRCSVFYGAPSKSRLLATLCSAEVCQCAEGKCPRQRRALERGLQDEDGYRMKFACYYPRVEYGFQVKVLREDSRAAFRLFETKITQVLHFTKDVKAAANQMRNFLVRASCRLRLEPGKEYLIMGLDGATYDLEGHPQYLLDSNSWIEEMPSERLCRSTRQRAACAQLNDFLQEYGTQGCQV

1115 NP_002833.2 BAA18998.1

>NP_002833.2

MAGAGGGLGVWGNLVLLGLCSWTGARAPAPNPGRNLTVETQTTSSISLSWEVPDGLDSQNSNYWVQCTGDGGTTETRNTTATNVTVDGLGPGSLYTCSVWVEKDGVNSSVGTVTTATAPNPVRNLRVEAQTNSSIALTWEVPDGPDPQNSTYGVEYTGDGGRAGTRSTAHTNITVDGLEPGCLYAFSMWVGKNGINSSRETRNATTAHNPVRNLRVEAQTTSSISLSWEVPDGTDPQNSTYCVQCTGDGGRTETRNTTDTRVTVDGLGPGSLYTCSVWVEKDGVNSSVEIVTSATAPNPVRNLTVEAQTNSSIALTWEVPDGPDPQNSTYGVEYTGDGGRAGTRSTAHTNITVDRLEPGCLYVFSVWVGKNGINSSRETRNATTAPNPVRNLHMETQTNSSIALCWEVPDGPYPQDYTYWVEYTGDGGGTETRNTTNTSVTAERLEPGTLYTFSVWAEKNGARGSRQNVSISTVPNAVTSLSKQDWTNSTIALRWTAPQGPGQSSYSYWVSWVREGMTDPRTQSTSGTDITLKELEAGSLYHLTVWAERNEVRGYNSTLTAATAPNEVTDLQNETQTKNSVMLWWKAPGDPHSQLYVYWVQWASKGHPRRGQDPQANWVNQTSRTNETWYKVEALEPGTLYNFTVWAERNDVASSTQSLCASTYPDTVTITSCVSTSAGYGVNLIWSCPQGGYEAFELEVGGQRGSQDRSSCGEAVSVLGLGPARSYPATITTIWDGMKVVSHSVVCHTESAGVIAGAFVGILLFLILVGLLIFFLKRRNKKKQQKPELRDLVFSSPGDIPAEDFADHVRKNERDSNCGFADEYQQLSLVGHSQSQMVASASENNAKNRYRNVLPYDWSRVPLKPIHEEPGSDYINASFMPGLWSPQEFIATQGPLPQTVGDFWRLVWEQQSHTLVMLTNCMEAGRVKCEHYWPLDSQPCTHGHLRVTLVGEEVMENWTVRELLLLQVEEQKTLSVRQFHYQAWPDHGVPSSPDTLLAFWRMLRQWLDQTMEGGPPIVHCSAGVGRTGTLIALDVLLRQLQSEGLLGPFSFVRKMRESRPLMVQTEAQYVFLHQCILRFLQQSAQAPAEKEVPYEDVENLIYENVAAIQAHKLEV

>BAA18998.1

MDDLDALLADLESTTSHISKRPVFLSEETPYSYPTGNHTYQEIAVPPPVPPPPSSEALNGTILDPLDQWQPSGSRFIHQQPQSSSPVYGSSAKTSSVSNPQDSVGSPCSRVGEEEHVYSFPNKQKSAEPSPTVMSTSLGSNLSELDRLLLELNAVQHNPPGFPADEANSSPPLPGALSPLYGVPETNSPLGGKAGPLTKEKPKRNGGRGLEDVRPSVESLLDELESSVPSPVPAITVNQGEMSSPQRVTSTQQQTRISASSATRELDELMASLSDFKGSWPLEEVVLLVSISSSVQEGEKYPHPCAARHRTPSLRSPDQPPPCPQFMAQGKTGSSSPPGGPPKPGSQLDSMLGSLQSDLNKLGVATVAKGVCGACKKPIAGQVVTAMGKTWHPEHFVCTHCQEEIGSRNFFERDGQPYCEKDYHNLFSPRCYYCNGPILDKVVTALDRTWHPEHFFCAQCGAFFGPEGFHEKDGKAYCRKDYFDMFAPKCGGCARAILENYISALNTLWHPECFVCRECFTPFVNGSFFEHDGQPYCEVHYHERRGSLCSGCQKPITGRCITAMAKKFHPEHFVCAFCLKQLNKGTFKEQNDKPYCQNCFLKLFC

1116 NP_036547.1 NP_056345.3

>NP_036547.1

MRRSAAPSQLQGNSFKKPKFIPPGRSNPGLNEEITKLNPDIKLFEGVAINNTFLPSQNDLRICSLNLPSEESTREINNRDNCSGKYCFEAPTLATLDPPHTVHSAPKEVAVSKEQEEKSDSLVKYFSVVWCKPSKKKHKKWEGDAVLIVKGKSFILKNLEGKDIGRGIGYKFKELEKIEEGQTLMICGKEIEVMGVISPDDFSSGRCFQLGGGSTAISHSSQVARKCFSNPFKSVCKPSSKENRQNDFQNCKPRHDPYTPNSLVMPRPDKNHQWVFNKNCFPLVDVVIDPYLVYHLRPHQKEGIIFLYECVMGMRMNGRCGAILADEMGLGKTLQCISLIWTLQCQGPYGGKPVIKKTLIVTPGSLVNNWKKEFQKWLGSERIKIFTVDQDHKVEEFIKSIFYSVLIISYEMLLRSLDQIKNIKFDLLICDEGHRLKNSAIKTTTALISLSCEKRIILTGTPIQNDLQEFFALIDFVNPGILGSLSSYRKIYEEPIILSREPSASEEEKELGERRAAELTCLTGLFILRRTQEIINKYLPPKIENVVFCRPGALQIELYRKLLNSQVVRFCLQGLLENSPHLICIGALKKLCNHPCLLFNSIKEKECSSTCDKNEEKSLYKGLLSVFPADYNPLLFTEKESGKLQVLSKLLAVIHELRPTEKVVLVSNYTQTLNILQEVCKRHGYAYTRLDGQTPISQRQQIVDGFNSQHSSFFIFLLSSKAGGVGLNLIGGSHLILYDIDWNPATDIQAMSRVWRDGQKYPVHIYRLLTTGTIEEKIYQRQISKQGLCGAVVDLTKTSEHIQFSVEELKNLFTLHESSDCVTHDLLDCECTGEEVHTGDSLEKFIVSRDCQLGPHHQKSNSLKPLSMSQLKQWKHFSGDHLNLTDPFLERITENVSFIFQNITTQATGT

>NP_056345.3

MGSSQSVEIPGGGTEGYHVLRVQENSPGHRAGLEPFFDFIVSINGSRLNKDNDTLKDLLKANVEKPVKMLIYSSKTLELRETSVTPSNLWGGQGLLGVSIRFCSFDGANENVWHVLEVESNSPAALAGLRPHSDYIIGADTVMNESEDLFSLIETHEAKPLKLYVYNTDTDNCREVIITPNSAWGGEGSLGCGIGYGYLHRIPTRPFEEGKKISLPGQMAGTPITPLKDGFTEVQLSSVNPPSLSPPGTTGIEQSLTGLSISSTPPAVSSVLSTGVPTVPLLPPQVNQSLTSVPPMNPATTLPGLMPLPAGLPNLPNLNLNLPAPHIMPGVGLPELVNPGLPPLPSMPPRNLPGIAPLPLPSEFLPSFPLVPESSSAASSGELLSSLPPTSNAPSDPATTTAKADAASSLTVDVTPPTAKAPTTVEDRVGDSTPVSEKPVSAAVDANASESP

1117 NP_004773.1 NP_003560.1

>NP_004773.1

MSAYPKSYNPFDDDGEDEGARPAPWRDARDLPDGPDAPADRQQYLRQEVLRRAEATAASTSRSLALMYESEKVGVASSEELARQRGVLERTEKMVDKMDQDLKISQKHINSIKSVFGGLVNYFKSKPVETPPEQNGTLTSQPNNRLKEAISTSKEQEAKYQASHPNLRKLDDTDPVPRGAGSAMSTDAYPKNPHLRAYHQKIDSNLDELSMGLGRLKDIALGMQTEIEEQDDILDRLTTKVDKLDVNIKSTERKVRQL

>NP_003560.1

MSYTPGVGGDPTQLAQRISSNIQKITQCSVEIQRTLNQLGTPQDSPELRQQLQQKQQYTNQLAKETDKYIKEFGSLPTTPSEQRQRKIQKDRLVAEFTTSLTNFQKVQRQAAEREKEFVARVRASSRVSGSFPEDSSKERNLVSWESQTQPQVQVQDEEITEDDLRLIHERESSIRQLEADIMDINEIFKDLGMMIHEQGDVIDSIEANVENAEVHVQQANQQLSRAADYQRKSRKTLCIIILILVIGVAIISLIIWGLNH

1118 NP_060926.2 NP_037474.1

>NP_060926.2

MNSAAGFSHLDRRERVLKLGESFEKQPRCAFHTVRYDFKPASIDTSSEGYLEVGEGEQVTITLPNIEGSTPPVTVFKGSKKPYLKECILIINHDTGECRLEKLSSNITVKKTRVEGSSKIQYRKEQQQQQMWNSARTPNLVKHSPSEDKMSPASPIDDIERELKAEASLMDQMSSCDSSSDSKSSSSSSSEDSSSDSEDEDCKSSTSDTGNCVSGHPTMTQYRIPDIDASHNRFRDNSGLLMNTLRNDLQLSESGSDSDD

>NP_037474.1

MELEQREGTMAAVGFEEFSAPPGSELALPPLFGGHILESELETEVEFVSGGLGGSGLRERDEEEEAARGRRRRQRELNRRKYQALGRRCREIEQVNERVLNRLHQVQRITRRLQQERRFLMRVLDSYGDDYRASQFTIVLEDEGSQGTDAPTPGNAENEPPEKETLSPPRRTPAPPEPGSPAPGEGPSGRKRRRVPRDGRRAGNALTPELAPVQIKVEEDFGFEADEALDSSWVSRGPDKLLPYPTLASPASD

1119 NP_003072.2 NP_004703.1

>NP_003072.2

MAEDADMRNELEEMQRRADQLADESLESTRRMLQLVEESKDAGIRTLVMLDEQGEQLDRVEEGMNHINQDMKEAEKNLKDLGKCCGLFICPCNKLKSSDAYKKAWGNNQDGVVASQPARVVDEREQMAISGGFIRRVTNDARENEMDENLEQVSGIIGNLRHMALDMGNEIDTQNRQIDRIMEKADSNKTRIDEANQRATKMLGSG

>NP_004703.1

MGRGSGTFERLLDKATSQLLLETDWESILQICDLIRQGDTQAKYAVNSIKKKVNDKNPHVALYALEVMESVVKNCGQTVHDEVANKQTMEELKDLLKRQVEVNVRNKILYLIQAWAHAFRNEPKYKVVQDTYQIMKVEGHVFPEFKESDAMFAAERAPDWVDAEECHRCRVQFGVMTRKHHCRACGQIFCGKCSSKYSTIPKFGIEKEVRVCEPCYEQLNRKAEGKATSTTELPPEYLTSPLSQQSQLPPKRDETALQEEEELQLALALSQSEAEEKERLRQKSTYTSYPKAEPMPSASSAPPASSLYSSPVNSSAPLAEDIDPELARYLNRNYWEKKQEEARKSPTPSAPVPLTEPAAQPGEGHAAPTNVVENPLPETDSQPIPPSGGPFSEPQFHNGESEESHEQFLKALQNAVTTFVNRMKSNHMRGRSITNDSAVLSLFQSINGMHPQLLELLNQLDERRLYYEGLQDKLAQIRDARGALSALREEHREKLRRAAEEAERQRQIQLAQKLEIMRQKKQEYLEVQRQLAIQRLQEQEKERQMRLEQQKQTVQMRAQMPAFPLPYAQLQAMPAAGGVLYQPSGPASFPSTFSPAGSVEGSPMHGVYMSQPAPAAGPYPSMPSTAADPSMVSAYMYPAGATGAQAAPQAQAGPTASPAYSSYQPTPTAGYQNVASQAPQSLPAISQPPQSSTMGYMGSQSVSMGYQPYNMQNLMTTLPSQDASLPPQQPYIAGQQPMYQQMAPSGGPPQQQPPVAQQPQAQGPPAQGSEAQLISFD

1120 NP_001217.2 NP_005564.1

>NP_001217.2

MSSASGLRRGHPAGGEENMTETDAFYKREMFDPAEKYKMDHRRRGIALIFNHERFFWHLTLPERRGTCADRDNLTRRFSDLGFEVKCFNDLKAEELLLKIHEVSTVSHADADCFVCVFLSHGEGNHIYAYDAKIEIQTLTGLFKGDKCHSLVGKPKIFIIQACRGNQHDVPVIPLDVVDNQTEKLDTNITEVDAASVYTLPAGADFLMCYSVAEGYYSHRETVNGSWYIQDLCEMLGKYGSSLEFTELLTLVNRKVSQRRVDFCKDPSAIGKKQVPCFASMLTKKLHFFPKSN

>NP_005564.1

MATATPVPPRMGSRAGGPTTPLSPTRLSRLQEKEELRELNDRLAVYIDKVRSLETENSALQLQVTEREEVRGRELTGLKALYETELADARRALDDTARERAKLQIELGKCKAEHDQLLLNYAKKESDLNGAQIKLREYEAALNSKDAALATALGDKKSLEGDLEDLKDQIAQLEASLAAAKKQLADETLLKVDLENRCQSLTEDLEFRKSMYEEEINETRRKHETRLVEVDSGRQIEYEYKLAQALHEMREQHDAQVRLYKEELEQTYHAKLENARLSSEMNTSTVNSAREELMESRMRIESLSSQLSNLQKESRACLERIQELEDLLAKEKDNSRRMLTDKEREMAEIRDQMQQQLNDYEQLLDVKLALDMEISAYRKLLEGEEERLKLSPSPSSRVTVSRASSSRSVRTTRGKRKRVDVEESEASSSVSISHSASATGNVCIEEIDVDGKFIRLKNTSEQDQPMGGWEMIRKIGDTSVSYKYTSRYVLKAGQTVTIWAANAGVTASPPTDLIWKNQNSWGTGEDVKVILKNSQGEEVAQRSTVFKTTIPEEEEEEEEAAGVVVEEELFHQQGTPRASNRSCAIM

1121 NP_002153.1 NP_005997.2

>NP_002153.1

MATMVPSVLWPRACWTLLVCCLLTPGVQGQEFLLRVEPQNPVLSAGGSLFVNCSTDCPSSEKIALETSLSKELVASGMGWAAFNLSNVTGNSRILCSVYCNGSQITGSSNITVYGLPERVELAPLPPWQPVGQNFTLRCQVEGGSPRTSLTVVLLRWEEELSRQPAVEEPAEVTATVLASRDDHGAPFSCRTELDMQPQGLGLFVNTSAPRQLRTFVLPVTPPRLVAPRFLEVETSWPVDCTLDGLFPASEAQVYLALGDQMLNATVMNHGDTLTATATATARADQEGAREIVCNVTLGGERREARENLTVFSFLGPIVNLSEPTAHEGSTVTVSCMAGARVQVTLDGVPAAAPGQPAQLQLNATESDDGRSFFCSATLEVDGEFLHRNSSVQLRVLYGPKIDRATCPQHLKWKDKTRHVLQCQARGNPYPELRCLKEGSSREVPVGIPFFVNVTHNGTYQCQASSSRGKYTLVVVMDIEAGSSHFVPVFVAVLLTLGVVTIVLALMYVFREHQRSGSYHVREESTYLPLTSMQPTEAMGEEPSRAE

>NP_005997.2

MDLTKMGMIQLQNPSHPTGLLCKANQMRLAGTLCDVVIMVDSQEFHAHRTVLACTSKMFEILFHRNSQHYTLDFLSPKTFQQILEYAYTATLQAKAEDLDDLLYAAEILEIEYLEEQCLKMLETIQASDDNDTEATMADGGAEEEEDRKARYLKNIFISKHSSEESGYASVAGQSLPGPMVDQSPSVSTSFGLSAMSPTKAAVDSLMTIGQSLLQGTLQPPAGPEEPTLAGGGRHPGVAEVKTEMMQVDEVPSQDSPGAAESSISGGMGDKVEERGKEGPGTPTRSSVITSARELHYGREESAEQVPPPAEAGQAPTGRPEHPAPPPEKHLGIYSVLPNHKADAVLSMPSSVTSGLHVQPALAVSMDFSTYGGLLPQGFIQRELFSKLGELAVGMKSESRTIGEQCSVCGVELPDNEAVEQHRKLHSGMKTYGCELCGKRFLDSLRLRMHLLAHSAGAKAFVCDQCGAQFSKEDALETHRQTHTGTDMAVFCLLCGKRFQAQSALQQHMEVHAGVRSYICSECNRTFPSHTALKRHLRSHTGDHPYECEFCGSCFRDESTLKSHKRIHTGEKPYECNGCGKKFSLKHQLETHYRVHTGEKPFECKLCHQRSRDYSAMIKHLRTHNGASPYQCTICTEYCPSLSSMQKHMKGHKPEEIPPDWRIEKTYLYLCYV

1122 NP_006814.1 NP_004495.2

>NP_006814.1

MTDVETTYADFIASGRTGRRNAIHDILVSSASGNSNELALKLAGLDINKTEGEEDAQRSSTEQSGEAQGEAAKSES

>NP_004495.2

MAASAKRKQEEKHLKMLRDMTGLPHNRKCFDCDQRGPTYVNMTVGSFVCTSCSGSLRGLNPPHRVKSISMTTFTQQEIEFLQKHGNEVCKQIWLGLFDDRSSAIPDFRDPQKVKEFLQEKYEKKRWYVPPEQAKVVASVHASISGSSASSTSSTPEVKPLKSLLGDSAPTLHLNKGTPSQSPVVGRSQGQQQEKKQFDLLSDLGSDIFAAPAPQSTATANFANFAHFNSHAAQNSANADFANFDAFGQSSGSSNFGGFPTASHSPFQPQTTGGSAASVNANFAHFDNFPKSSSADFGTFNTSQSHQTASAVSKVSTNKAGLQTADKYAALANLDNIFSAGQGGDQGSGFGTTGKAPVGSVVSVPSQSSASSDKYAALAELDSVFSSAATSSNAYTSTSNASSNVFGTVPVVASAQTQPASSSVPAPFGATPSTNPFVAAAGPSVASSTNPFQTNARGATAATFGTASMSMPTGFGTPAPYSLPTSFSGSFQQPAFPAQAAFPQQTAFSQQPNGAGFAAFGQTKPVVTPFGQVAAAGVSSNPFMTGAPTGQFPTGSSSTNPFL

1123 NP_066285.1 NP_060103.2

>NP_066285.1

MAMVVSTWRDPQDEVPGSQGSQASQAPPVPGPPPGAPHTPQTPGQGGPASTPAQTAAGGQGGPGGPGSDKQQQQQHIECVVCGDKSSGKHYGQFTCEGCKSFFKRSVRRNLSYTCRANRNCPIDQHHRNQCQYCRLKKCLKVGMRREAVQRGRMPPTQPTHGQFALTNGDPLNCHSYLSGYISLLLRAEPYPTSRFGSQCMQPNNIMGIENICELAARMLFSAVEWARNIPFFPDLQITDQVALLRLTWSELFVLNAAQCSMPLHVAPLLAAAGLHASPMSADRVVAFMDHIRIFQEQVEKLKALHVDSAEYSCLKAIVLFTSDACGLSDVAHVESLQEKSQCALEEYVRSQYPNQPTRFGKLLLRLPSLRTVSSSVIEQLFFVRLVGKTPIETLIRDMLLSGSSFNWPYMAIQ

>NP_060103.2

MAEGEGYFAMSEDELACSPYIPLGGDFGGGDFGGGDFGGGDFGGGGSFGGHCLDYCESPTAHCNVLNWEQVQRLDGILSETIPIHGRGNFPTLELQPSLIVKVVRRRLAEKRIGVRDVRLNGSAASHVLHQDSGLGYKDLDLIFCADLRGEGEFQTVKDVVLDCLLDFLPEGVNKEKITPLTLKEAYVQKMVKVCNDSDRWSLISLSNNSGKNVELKFVDSLRRQFEFSVDSFQIKLDSLLLFYECSENPMTETFHPTIIGESVYGDFQEAFDHLCNKIIATRNPEEIRGGGLLKYCNLLVRGFRPASDEIKTLQRYMCSRFFIDFSDIGEQQRKLESYLQNHFVGLEDRKYEYLMTLHGVVNESTVCLMGHERRQTLNLITMLAIRVLADQNVIPNVANVTCYYQPAPYVADANFSNYYIAQVQPVFTCQQQTYSTWLPCN

1124 NP_006597.2 NP_005602.2

>NP_006597.2

MASPSKAVIVPGNGGGDVTTHGWYGWVKKELEKIPGFQCLAKNMPDPITARESIWLPFMETELHCDEKTIIIGHSSGAIAAMRYAETHRVYAIVLVSAYTSDLGDENERASGYFTRPWQWEKIKANCPYIVQFGSTDDPFLPWKEQQEVADRLETKLHKFTDCGHFQNTEFHELITVVKSLLKVPA

>NP_005602.2

MPSGGDQSPPPPPPPPAAAASDEEEEDDGEAEDAAPSAESPTPQIQQRFDELCSRLNMDEAARPEAWDSYRSMSESYTLEGNDLHWLACALYVACRKSVPTVSKGTVEGNYVSLTRILKCSEQSLIEFFNKMKKWEDMANLPPHFRERTERLERNFTVSAVIFKKYEPIFQDIFKYPQEEQPRQQRGRKQRRQPCTVSEIFHFCWVLFIYAKGNFPMISDDLVNSYHLLLCALDLVYGNALQCSNRKELVNPNFKGLSEDFHAKDSKPSSDPPCIIEKLCSLHDGLVLEAKGIKEHFWKPYIRKLYEKKLLKGKEENLTGFLEPGNFGESFKAINKAYEEYVLSVGNLDERIFLGEDAEEEIGTLSRCLNAGSGTETAERVQMKNILQQHFDKSKALRISTPLTGVRYIKENSPCVTPVSTATHSLSRLHTMLTGLRNAPSEKLEQILRTCSRDPTQAIANRLKEMFEIYSQHFQPDEDFSNCAKEIASKHFRFAEMLYYKVLESVIEQEQKRLGDMDLSGILEQDAFHRSLLACCLEVVTFSYKPPGNFPFITEIFDVPLYHFYKVIEVFIRAEDGLCREVVKHLNQIEEQILDHLAWKPESPLWEKIRDNENRVPTCEEVMPPQNLERADEICIAGSPLTPRRVTEVRADTGGLGRSITSPTTLYDRYSSPPASTTRRRLFVENDSPSDGGTPGRMPPQPLVNAVPVQNVSGETVSVTPVPGQTLVTMATATVTANNGQTVTIPVQGIANENGGITFFPVQVNVGGQAQAVTGSIQPLSAQALAGSLSSQQVTGTTLQVPGQVAIQQISPGGQQQKQGQSVTSSSNRPRKTSSLSLFFRKVYHLAAVRLRDLCAKLDISDELRKKIWTCFEFSIIQCPELMMDRHLDQLLMCAIYVMAKVTKEDKSFQNIMRCYRTQPQARSQVYRSVLIKGKRKRRNSGSSDSRSHQNSPTELNKDRTSRDSSPVMRSSSTLPVPQPSSAPPTPTRLTGANSDMEEEERGDLIQFYNNIYIKQIKTFAMKYSQANMDAPPLSPYPFVRTGSPRRIQLSQNHPVYISPHKNETMLSPREKIFYYFSNSPSKRLREINSMIRTGETPTKKRGILLEDGSESPAKRICPENHSALLRRLQDVANDRGSH

1125 NP_057250.1 NP_005598.3

>NP_057250.1

MADSAELKQMVMSLRVSELQVLLGYAGRNKHGRKHELLTKALHLLKAGCSPAVQMKIKELYRRRFPQKIMTPADLSIPNVHSSPMPATLSPSTIPQLTYDGHPASSPLLPVSLLGPKHELELPHLTSALHPVHPDIKLQKLPFYDLLDELIKPTSLASDNSQRFRETCFAFALTPQQVQQISSSMDISGTKCDFTVQVQLRFCLSETSCPQEDHFPPNLCVKVNTKPCSLPGYLPPTKNGVEPKRPSRPINITSLVRLSTTVPNTIVVSWTAEIGRNYSMAVYLVKQLSSTVLLQRLRAKGIRNPDHSRALIKEKLTADPDSEIATTSLRVSLLCPLGKMRLTIPCRALTCSHLQCFDATLYIQMNEKKPTWVCPVCDKKAPYEHLIIDGLFMEILKYCTDCDEIQFKEDGTWAPMRSKKEVQEVSASYNGVDGCLSSTLEHQVASHHQSSNKNKKVEVIDLTIDSSSDEEEEEPSAKRTCPSLSPTSPLNNKGILSLPHQASPVSRTPSLPAVDTSYINTSLIQDYRHPFHMTPMPYDLQGLDFFPFLSGDNQHYNTSLLAAAAAAVSDDQDLLHSSRFFPYTSSQMFLDQLSAGGSTSLPTTNGSSSGSNSSLVSSNSLRESHSHTVTNRSSTDTASIFGIIPDIISLD

>NP_005598.3

MISADCNLCLPEYDRYLASSKIMAAAYLDPNLNHTPNSSTKTHLGTGMERSPGAMERVLKVFHYFESNSEPTTWASIIRHGDATDVRGIIQKIVDSHKVKHVACYGFRLSHLRSEEVHWLHVDMGVSSVREKYELAHPPEEWKYELRIRYLPKGFLNQFTEDKPTLNFFYQQVKSDYMLEIADQVDQEIALKLGCLEIRRSYWEMRGNALEKKSNYEVLEKDVGLKRFFPKSLLDSVKAKTLRKLIQQTFRQFANLNREESILKFFEILSPVYRFDKECFKCALGSSWIISVELAIGPEEGISYLTDKGCNPTHLADFTQVQTIQYSNSEDKDRKGMLQLKIAGAPEPLTVTAPSLTIAENMADLIDGYCRLVNGTSQSFIIRPQKEGERALPSIPKLANSEKQGMRTHAVSVSETDDYAEIIDEEDTYTMPSTRDYEIQRERIELGRCIGEGQFGDVHQGIYMSPENPALAVAIKTCKNCTSDSVREKFLQEALTMRQFDHPHIVKLIGVITENPVWIIMELCTLGELRSFLQVRKYSLDLASLILYAYQLSTALAYLESKRFVHRDIAARNVLVSSNDCVKLGDFGLSRYMEDSTYYKASKGKLPIKWMAPESINFRRFTSASDVWMFGVCMWEILMHGVKPFQGVKNNDVIGRIENGERLPMPPNCPPTLYSLMTKCWAYDPSRRPRFTELKAQLSTILEEEKAQQEERMRMESRRQATVSWDSGGSDEAPPKPSRPGYPSPRSSEGFYPSPQHMVQTNHYQVSGYPGSHGITAMAGSIYPGQASLLDQTDSWNHRPQEIAMWQPNVEDSTVLDLRGIGQVLPTHLMEERLIRQQQEMEEDQRWLEKEERFLKPDVRLSRGSIDREDGSLQGPIGNQHIYQPVGKPDPAAPPKKPPRPGAPGHLGSLASLSSPADSYNEGVKLQPQEISPPPTANLDRSNDKVYENVTGLVKAVIEMSSKIQPAPPEEYVPMVKEVGLALRTLLATVDETIPLLPASTHREIEMAQKLLNSDLGELINKMKLAQQYVMTSLQQEYKKQMLTAAHALAVDAKNLLDVIDQARLKMLGQTRPH

1126 NP_006381.2 NP_003126.1

>NP_006381.2

MDLNRIIQALKGTIDPKLRIAAENELNQSYKIINFAPSLLRIIVSDHVEFPVRQAAAIYLKNMVTQYWPDREPPPGEAIFPFNIHENDRQQIRDNIVEGIIRSPDLVRVQLTMCLRAIIKHDFPGHWPGVVDKIDYYLQSQSSASWLGSLLCLYQLVKTYEYKKAEEREPLIIAMQIFLPRIQQQIVQLLPDSSYYSVLLQKQILKIFYALVQYALPLQLVNNQTMTTWMEIFRTIIDRTVPPETLHIDEDDRPELVWWKCKKWALHIVARLFERYGSPGNVTKEYFEFSEFFLKTYAVGIQQVLLKILDQYRQKEYVAPRVLQQAFNYLNQGVVHSITWKQMKPHIQNISEDVIFSVMCYKDEDEELWQEDPYEYIRMKFDIFEDYASPTTAAQTLLYTAAKKRKEVLPKMMAFCYQILTDPNFDPRKKDGALHVIGSLAEILLKKSLFKDQMELFLQNHVFPLLLSNLGYLRARSCWVLHAFSSLKFHNELNLRNAVELAKKSLIEDKEMPVKVEAALALQSLISNQIQAKEYMKPHVRPIMQELLHIVRETENDDVTNVIQKMICEYSQEVASIAVDMTQHLAEIFGKVLQSDEYEEVEDKTVMAMGILHTIDTILTVVEDHKEITQQLENICLRIIDLVLQKHVIEFYEEILSLAYSLTCHSISPQMWQLLGILYEVFQQDCFEYFTDMMPLLHNYVTIDTDTLLSNAKHLEILFTMCRKVLCGDAGEDAECHAAKLLEVIILQCKGRGIDQCIPLFVQLVLERLTRGVKTSELRTMCLQVAIAALYYNPDLLLHTLERIQLPHNPGPITVQFINQWMNDTDCFLGHHDRKMCIIGLSILLELQNRPPAVDAVVGQIVPSILFLFLGLKQVCATRQLVNREDRSKAEKADMEENEEISSDEEETNVTAQAMQSNNGRGEDEEEEDDDWDEEVLEETALEGFSTPLDLDNSVDEYQFFTQALITVQSRDAAWYQLLMAPLSEDQRTALQEVYTLAEHRRTVAEAKKKIEQQGGFTFENKGVLSAFNFGTVPSNN

>NP_003126.1

MACAAARSPADQDRFICIYPAYLNNKKTIAEGRRIPISKAVENPTATEIQDVCSAVGLNVFLEKNKMYSREWNRDVQYRGRVRVQLKQEDGSLCLVQFPSRKSVMLYAAEMIPKLKTRTQKTGGADQSLQQGEGSKKGKGKKKK

1127 NP_002022.1 NP_000312.2

>NP_002022.1

MSNICQRLWEYLEPYLPCLSTEADKSTVIENPGALCSPQSQRHGHYFVALFDYQARTAEDLSFRAGDKLQVLDTLHEGWWFARHLEKRRDGSSQQLQGYIPSNYVAEDRSLQAEPWFFGAIGRSDAEKQLLYSENKTGSFLIRESESQKGEFSLSVLDGAVVKHYRIKRLDEGGFFLTRRRIFSTLNEFVSHYTKTSDGLCVKLGKPCLKIQVPAPFDLSYKTVDQWEIDRNSIQLLKRLGSGQFGEVWEGLWNNTTPVAVKTLKPGSMDPNDFLREAQIMKNLRHPKLIQLYAVCTLEDPIYIITELMRHGSLQEYLQNDTGSKIHLTQQVDMAAQVASGMAYLESRNYIHRDLAARNVLVGEHNIYKVADFGLARVFKVDNEDIYESRHEIKLPVKWTAPEAIRSNKFSIKSDVWSFGILLYEIITYGKMPYSGMTGAQVIQMLAQNYRLPQPSNCPQQFYNIMLECWNAEPKERPTFETLRWKLEDYFETDSSYSDANNFIR

>NP_000312.2

MPPKTPRKTAATAAAAAAEPPAPPPPPPPEEDPEQDSGPEDLPLVRLEFEETEEPDFTALCQKLKIPDHVRERAWLTWEKVSSVDGVLGGYIQKKKELWGICIFIAAVDLDEMSFTFTELQKNIEISVHKFFNLLKEIDTSTKVDNAMSRLLKKYDVLFALFSKLERTCELIYLTQPSSSISTEINSALVLKVSWITFLLAKGEVLQMEDDLVISFQLMLCVLDYFIKLSPPMLLKEPYKTAVIPINGSPRTPRRGQNRSARIAKQLENDTRIIEVLCKEHECNIDEVKNVYFKNFIPFMNSLGLVTSNGLPEVENLSKRYEEIYLKNKDLDARLFLDHDKTLQTDSIDSFETQRTPRKSNLDEEVNVIPPHTPVRTVMNTIQQLMMILNSASDQPSENLISYFNNCTVNPKESILKRVKDIGYIFKEKFAKAVGQGCVEIGSQRYKLGVRLYYRVMESMLKSEEERLSIQNFSKLLNDNIFHMSLLACALEVVMATYSRSTSQNLDSGTDLSFPWILNVLNLKAFDFYKVIESFIKAEGNLTREMIKHLERCEHRIMESLAWLSDSPLFDLIKQSKDREGPTDHLESACPLNLPLQNNHTAADMYLSPVRSPKKKGSTTRVNSTANAETQATSAFQTQKPLKSTSLSLFYKKVYRLAYLRLNTLCERLLSEHPELEHIIWTLFQHTLQNEYELMRDRHLDQIMMCSMYGICKVKNIDLKFKIIVTAYKDLPHAVQETFKRVLIKEEEYDSIIVFYNSVFMQRLKTNILQYASTRPPTLSPIPHIPRSPYKFPSSPLRIPGGNIYISPLKSPYKISEGLPTPTKMTPRSRILVSIGESFGTSEKFQKINQMVCNSDRVLKRSAEGSNPPKPLKKLRFDIEGSDEADGSKHLPGESKFQQKLAEMTSTRTRMQKQKMNDSMDTSNKEEK

1128 NP_115676.1 NP_002264.1

>NP_115676.1

MALPTLPSYWCSQQRLNQQLARQREQEARLRQQWEQNSRYFRMSDICSSKQAEWSSKTSYQRSMHAYQREKMKEEKRRSLEARREKLRQLMQEEQDLLARELEELRLSMNLQERRIREQHGKLKSAKEEQRKLIAEQLLYEHWKKNNPKLREMELDLHQKHVVNSWEMQKEEKKQQEATAEQENKRYENEYERARREALERMKAEEERRQLEDKLQAEALLQQMEELKLKEVEATKLKKEQENLLKQRWELERLEEERKQMEAFRQKAELGRFLRHQYNAQLSRRTQQIQEELEADRRILQALLEKEDESQRLHLARREQVMADVAWMKQAIEEQLQLERAREAELQMLLREEAKEMWEKREAEWARERSARDRLMSEVLTGRQQQIQEKIEQNRRAQEESLKHREQLIRNLEEVRELARREKEESEKLKSARKQELEAQVAERRLQAWEADQQEEEEEEEARRVEQLSDALLQQEAETMAEQGYRPKPYGHPKIAWN

>NP_002264.1

MSIRVTQKSYKVSTSGPRAFSSRSYTSGPGSRISSSSFSRVGSSNFRGGLGGGYGGASGMGGITAVTVNQSLLSPLVLEVDPNIQAVRTQEKEQIKTLNNKFASFIDKVRFLEQQNKMLETKWSLLQQQKTARSNMDNMFESYINNLRRQLETLGQEKLKLEAELGNMQGLVEDFKNKYEDEINKRTEMENEFVLIKKDVDEAYMNKVELESRLEGLTDEINFLRQLYEEEIRELQSQISDTSVVLSMDNSRSLDMDSIIAEVKAQYEDIANRSRAEAESMYQIKYEELQSLAGKHGDDLRRTKTEISEMNRNISRLQAEIEGLKGQRASLEAAIADAEQRGELAIKDANAKLSELEAALQRAKQDMARQLREYQELMNVKLALDIEIATYRKLLEGEESRLESGMQNMSIHTKTTSGYAGGLSSAYGGLTSPGLSYSLGSSFGSGAGSSSFSRTSSSRAVVVKKIETRDGKLVSESSDVLPK

1129 NP_055948.2 NP_002410.1

>NP_055948.2

MMEIQMDEGGGVVVYQDDYCSGSVMSERVSGLAGSIYREFERLIHCYDEEVVKELMPLVVNVLENLDSVLSENQEHEVELELLREDNEQLLTQYEREKALRRQAEEKFIEFEDALEQEKKELQIQVEHYEFQTRQLELKAKNYADQISRLEERESEMKKEYNALHQRHTEMIQTYVEHIERSKMQQVGGNSQTESSLPGRRKERPTSLNVFPLADGTVRAQIGGKLVPAGDHWHLSDLGQLQSSSSYQCPQDEMSESGQSSAAATPSTTGTKSNTPTSSVPSAAVTPLNESLQPLGDYGVGSKNSKRAREKRDSRNMEVQVTQEMRNVSIGMGSSDEWSDVQDIIDSTPELDMCPETRLDRTGSSPTQGIVNKAFGINTDSLYHELSTAGSEVIGDVDEGADLLGEFSVRDDFFGMGKEVGNLLLENSQLLETKNALNVVKNDLIAKVDQLSGEQEVLRGELEAAKQAKVKLENRIKELEEELKRVKSEAIIARREPKEEAEDVSSYLCTESDKIPMAQRRRFTRVEMARVLMERNQYKERLMELQEAVRWTEMIRASREHPSVQEKKKSTIWQFFSRLFSSSSSPPPAKRPYPSVNIHYKSPTTAGFSQRRNHAMCPISAGSRPLEFFPDDDCTSSARREQKREQYRQVREHVRNDDGRLQACGWSLPAKYKQLSPNGGQEDTRMKNVPVPVYCRPLVEKDPTMKLWCAAGVNLSGWRPNEDDAGNGVKPAPGRDPLTCDREGDGEPKSAHTSPEKKKAKELPEMDATSSRVWILTSTLTTSKVVIIDANQPGTVVDQFTVCNAHVLCISSIPAASDSDYPPGEMFLDSDVNPEDPGADGVLAGITLVGCATRCNVPRSNCSSRGDTPVLDKGQGEVATIANGKVNPSQSTEEATEATEVPDPGPSEPETATLRPGPLTEHVFTDPAPTPSSGPQPGSENGPEPDSSSTRPEPEPSGDPTGAGSSAAPTMWLGAQNGWLYVHSAVANWKKCLHSIKLKDSVLSLVHVKGRVLVALADGTLAIFHRGEDGQWDLSNYHLMDLGHPHHSIRCMAVVYDRVWCGYKNKVHVIQPKTMQIEKSFDAHPRRESQVRQLAWIGDGVWVSIRLDSTLRLYHAHTHQHLQDVDIEPYVSKMLGTGKLGFSFVRITALLVAGSRLWVGTGNGVVISIPLTETVVLHRGQLLGLRANKTSPTSGEGARPGGIIHVYGDDSSDRAASSFIPYCSMAQAQLCFHGHRDAVKFFVSVPGNVLATLNGSVLDSPAEGPGPAAPASEVEGQKLRNVLVLSGGEGYIDFRIGDGEDDETEEGAGDMSQVKPVLSKAERSHIIVWQVSYTPE

>NP_002410.1

MEPLKSLFLKSPLGSWNGSGSGGGGGGGGGRPEGSPKAAGYANPVWTALFDYEPSGQDELALRKGDRVEVLSRDAAISGDEGWWAGQVGGQVGIFPSNYVSRGGGPPPCEVASFQELRLEEVIGIGGFGKVYRGSWRGELVAVKAARQDPDEDISVTAESVRQEARLFAMLAHPNIIALKAVCLEEPNLCLVMEYAAGGPLSRALAGRRVPPHVLVNWAVQIARGMHYLHCEALVPVIHRDLKSNNILLLQPIESDDMEHKTLKITDFGLAREWHKTTQMSAAGTYAWMAPEVIKASTFSKGSDVWSFGVLLWELLTGEVPYRGIDCLAVAYGVAVNKLTLPIPSTCPEPFAQLMADCWAQDPHRRPDFASILQQLEALEAQVLREMPRDSFHSMQEGWKREIQGLFDELRAKEKELLSREEELTRAAREQRSQAEQLRRREHLLAQWELEVFERELTLLLQQVDRERPHVRRRRGTFKRSKLRARDGGERISMPLDFKHRITVQASPGLDRRRNVFEVGPGDSPTFPRFRAIQLEPAEPGQAWGRQSPRRLEDSSNGERRACWAWGPSSPKPGEAQNGRRRSRMDEATWYLDSDDSSPLGSPSTPPALNGNPPRPSLEPEEPKRPVPAERGSSSGTPKLIQRALLRGTALLASLGLGRDLQPPGGPGRERGESPTTPPTPTPAPCPTEPPPSPLICFSLKTPDSPPTPAPLLLDLGIPVGQRSAKSPRREEEPRGGTVSPPPGTSRSAPGTPGTPRSPPLGLISRPRPSPLRSRIDPWSFVSAGPRPSPLPSPQPAPRRAPWTLFPDSDPFWDSPPANPFQGGPQDCRAQTKDMGAQAPWVPEAGP

1130 NP_060624.1 NP_003522.1

>NP_060624.1

MAKVSVLNVAVLENPSPFHSPFRFEISFECSEALADDLEWKIIYVGSAESEEFDQILDSVLVGPVPAGRHMFVFQADAPNPSLIPETDAVGVTVVLITCTYHGQEFIRVGYYVNNEYLNPELRENPPMKPDFSQLQRNILASNPRVTRFHINWDNNMDRLEAIETQDPSLGCGLPLNCTPIKGLGLPGCIPGLLPENSMDCI

>NP_003522.1

MARTKQTARKSTGGKAPRKQLATKAARKSAPATGGVKKPHRYRPGTVALREIRRYQKSTELLIRKLPFQRLVREIAQDFKTDLRFQSSAVMALQEACEAYLVGLFEDTNLCAIHAKRVTIMPKDIQLARRIRGERA

1131 NP_056953.2 NP_001434.1

>NP_056953.2

MGETLGDSPIDPESDSFTDTLSANISQEMTMVDTEMPFWPTNFGISSVDLSVMEDHSHSFDIKPFTTVDFSSISTPHYEDIPFTRTDPVVADYKYDLKLQEYQSAIKVEPASPPYYSEKTQLYNKPHEEPSNSLMAIECRVCGDKASGFHYGVHACEGCKGFFRRTIRLKLIYDRCDLNCRIHKKSRNKCQYCRFQKCLAVGMSHNAIRFGRMPQAEKEKLLAEISSDIDQLNPESADLRALAKHLYDSYIKSFPLTKAKARAILTGKTTDKSPFVIYDMNSLMMGEDKIKFKHITPLQEQSKEVAIRIFQGCQFRSVEAVQEITEYAKSIPGFVNLDLNDQVTLLKYGVHEIIYTMLASLMNKDGVLISEGQGFMTREFLKSLRKPFGDFMEPKFEFAVKFNALELDDSDLAIFIAVIILSGDRPGLLNVKPIEDIQDNLLQALELQLKLNHPESSQLFAKLLQKMTDLRQIVTEHVQLLQVIKKTETDMSLHPLLQEIYKDLY

>NP_001434.1

MSFSGKYQLQSQENFEAFMKAIGLPEELIQKGKDIKGVSEIVQNGKHFKFTITAGSKVIQNEFTVGEECELETMTGEKVKTVVQLEGDNKLVTTFKNIKSVTELNGDIITNTMTLGDIVFKRISKRI

1132 NP_004299.1 NP_002881.1

>NP_004299.1

MDPLSELQDDLTLDDTSEALNQLKLASIDEKNWPSDEMPDFPKSDDSKSSSPELVTHLKWDDPYYDIARHQIVEVAGDDKYGRKIIVFSACRMPPSHQLDHSKLLGYLKHTLDQYVESDYTLLYLHHGLTSDNKPSLSWLRDAYREFDRKYKKNIKALYIVHPTMFIKTLLILFKPLISFKFGQKIFYVNYLSELSEHVKLEQLGIPRQVLKYDDFLKSTQKSPATAPKPMPPRPPLPNQQFGVSLQHLQEKNPEQEPIPIVLRETVAYLQAHALTTEGIFRRSANTQVVREVQQKYNMGLPVDFDQYNELHLPAVILKTFLRELPEPLLTFDLYPHVVGFLNIDESQRVPATLQVLQTLPEENYQVLRFLTAFLVQISAHSDQNKMTNTNLAVVFGPNLLWAKDAAITLKAINPINTFTKFLLDHQGELFPSPDPSGL

>NP_002881.1

MMAAEAGSEEGGPVTAGAGGGGAAAGSSAYPAVCRVKIPAALPVAAAPYPGLVETGVAGTLGGGAALGSEFLGAGSVAGALGGAGLTGGGTAAGVAGAAAGVAGAAVAGPSGDMALTKLPTSLLAETLGPGGGFPPLPPPPYLPPLGAGLGTVDEGDSLDGPEYEEEEVAIPLTAPPTNQWYHGKLDRTIAEERLRQAGKSGSYLIRESDRRPGSFVLSFLSQMNVVNHFRIIAMCGDYYIGGRRFSSLSDLIGYYSHVSCLLKGEKLLYPVAPPEPVEDRRRVRAILPYTKVPDTDEISFLKGDMFIVHNELEDGWMWVTNLRTDEQGLIVEDLVEEVGREEDPHEGKIWFHGKISKQEAYNLLMTVGQVCSFLVRPSDNTPGDYSLYFRTNENIQRFKICPTPNNQFMMGGRYYNSIGDIIDHYRKEQIVEGYYLKEPVPMQDQEQVLNDTVDGKEIYNTIRRKTKDAFYKNIVKKGYLLKKGKGKRWKNLYFILEGSDAQLIYFESEKRATKPKGLIDLSVCSVYVVHDSLFGRPNCFQIVVQHFSEEHYIFYFAGETPEQAEDWMKGLQAFCNLRKSSPGTSNKRLRQVSSLVLHIEEAHKLPVKHFTNPYCNIYLNSVQVAKTHAREGQNPVWSEEFVFDDLPPDINRFEITLSNKTKKSKDPDILFMRCQLSRLQKGHATDEWFLLSSHIPLKGIEPGSLRVRARYSMEKIMPEEEYSEFKELILQKELHVVYALSHVCGQDRTLLASILLRIFLHEKLESLLLCTLNDREISMEDEATTLFRATTLASTLMEQYMKATATQFVHHALKDSILKIMESKQSCELSPSKLEKNEDVNTNLTHLLNILSELVEKIFMASEILPPTLRYIYGCLQKSVQHKWPTNTTMRTRVVSGFVFLRLICPAILNPRMFNIISDSPSPIAARTLILVAKSVQNLANLVEFGAKEPYMEGVNPFIKSNKHRMIMFLDELGNVPELPDTTEHSRTDLSRDLAALHEICVAHSDELRTLSNERGAQQHVLKKLLAITELLQQKQNQYTKTNDVR

1133 NP_001035147.1 NP_060828.2

>NP_001035147.1

MRIAVICFCLLGITCAIPVKQADSGSSEEKQLYNKYPDAVATWLNPDPSQKQNLLAPQNAVSSEETNDFKQETLPSKSNESHDHMDDMDDEDDDDHVDSQDSIDSNDSDDVDDTDDSHQSDESHHSDESDELVTDFPTDLPATEVFTPVVPTVDTYDGRGDSVVYGLRSKSKKFRRPDIQYPDATDEDITSHMESEELNGAYKAIPVAQDLNAPSDWDSRGKDSYETSQLDDQSAETHSHKQSRLYKRKANDESNEHSDVIDSQELSKVSREFHSHEFHSHEDMLVVDPKSKEEDKHLKFRISHELDSASSEVN

>NP_060828.2

MATCAEILRSEFPEIDGQVFDYVTGVLHSGSADFESVDDLVEAVGELLQEVSGDSKDDAGIRAVCQRMYNTLRLAEPQSQGNSQVLLDAPIQLSKITENYDCGTKLPGLLKREQSSTVNAKKLEKAEARLKAKQEKRSEKDTLKTSNPLVLEEASASQAGSRKESRLESSGKNKSYDVRIENFDVSFGDRVLLAGADVNLAWGRRYGLVGRNGLGKTTLLKMLATRSLRVPAHISLLHVEQEVAGDDTPALQSVLESDSVREDLLRRERELTAQIAAGRAEGSEAAELAEIYAKLEEIEADKAPARASVILAGLGFTPKMQQQPTREFSGGWRMRLALARALFARPDLLLLDEPTNMLDVRAILWLENYLQTWPSTILVVSHDRNFLNAIATDIIHLHSQRLDGYRGDFETFIKSKQERLLNQQREYEAQQQYRQHIQVFIDRFRYNANRASQVQSKLKMLEKLPELKPVDKESEVVMKFPDGFEKFSPPILQLDEVDFYYDPKHVIFSRLSVSADLESRICVVGENGAGKSTMLKLLLGDLAPVRGIRHAHRNLKIGYFSQHHVEQLDLNVSAVELLARKFPGRPEEEYRHQLGRYGISGELAMRPLASLSGGQKSRVAFAQMTMPCPNFYILDEPTNHLDMETIEALGRALNNFRGGVILVSHDERFIRLVCRELWVCEGGGVTRVEGGFDQYRALLQEQFRREGFL

1134 NP_079331.3 NP_006839.2

>NP_079331.3

MGLKNKKNTEDPEEPLIASQSTEPEIGHLSPSKKETIMVTLHGATNLPACKDGSEPWPYVVVKSTSEEKNNQSSKAVTSVTSEPTRAPIWGDTVNVEIQAEDAGQEDVILKVVDNRKKQELLSYKIPIKYLRVFHPYHFELVKPTESGKADEATAKTQLYATVVRKSSFIPRYIGCNHMALEIFLRGVNEPLANNPNPIVVIARVVPNYKEFKVSQANRDLASVGLPITPLSFPIPSMMNFDVPRVSQNGCPQLSKPGGPPEQPLWNQSFLFQGRDGATSFSEDTALVLEYYSSTSMKGSQPWTLNQPLGISVLPLKSRLYQKMLTGKGLDGLHVERLPIMDTSLKTINDEAPTVALSFQLLSSERPENFLTPNNSKALPTLDPKILDKKLRTIQESWSKDTVSSTMDLSTSTPREAEEEPLVPEMSHDTEMNNYRRAMQKMAEDILSLRRQASILEGENRILRSRLAQQEEEEGQGKASEAQNTVSMKQKLLLSELDMKKLRDRVQHLQNELIRKNDREKELLLLYQAQQPQAALLKQYQGKLQKMKALEETVRHQEKVIEKMERVLEDRLQDRSKPPPLNRQQGKPYTGFPMLSASGLPLGSMGENLPVELYSVLLAENAKLRTELDKNRHQQAPIILQQQALPDLLSGTSDKFNLLAKLEHAQSRILSLESQLEDSARRWGREKQDLATRLQEQEKGFRHPSNSIIIEQPSALTHSMDLKQPSELEPLLPSSDSKLNKPLSPQKETANSQQT

>NP_006839.2

MEAEAGGLEELTDEEMAALGKEELVRRLRREEAARLAALVQRGRLMQEVNRQLQGHLGEIRELKQLNRRLQAENRELRDLCCFLDSERQRGRRAARQWQLFGTQASRAVREDLGGCWQKLAELEGRQEELLRENLALKELCLALGEEWGPRGGPSGAGGSGAGPAPELALPPCGPRDLGDGSSSTGSVGSPDQLPLACSPDD

1135 NP_002458.2 NP_002373.3

>NP_002458.2

MDFFRVVENQQPPATMPLNVSFTNRNYDLDYDSVQPYFYCDEEENFYQQQQQSELQPPAPSEDIWKKFELLPTPPLSPSRRSGLCSPSYVAVTPFSLRGDNDGGGGSFSTADQLEMVTELLGGDMVNQSFICDPDDETFIKNIIIQDCMWSGFSAAAKLVSEKLASYQAARKDSGSPNPARGHSVCSTSSLYLQDLSAAASECIDPSVVFPYPLNDSSSPKSCASQDSSAFSPSSDSLLSSTESSPQGSPEPLVLHEETPPTTSSDSEEEQEDEEEIDVVSVEKRQAPGKRSESGSPSAGGHSKPPHSPLVLKRCHVSTHQHNYAAPPSTRKDYPAAKRVKLDSVRVLRQISNNRKCTSPRSSDTEENVKRRTHNVLERQRRNELKRSFFALRDQIPELENNEKAPKVVILKKATAYILSVQAEEQKLISEEDLLRKRREQLKHKLEQLRNSCA

>NP_002373.3

MSDNDDIEVESDEEQPRFQSAADKRAHHNALERKRRDHIKDSFHSLRDSVPSLQGEKASRAQILDKATEYIQYMRRKNHTHQQDIDDLKRQNALLEQQVRALEKARSSAQLQTNYPSSDNSLYTNAKGSTISAFDGGSDSSSESEPEEPQSRKKLRMEAS

1136 NP_000066.1 NP_510867.1

>NP_000066.1

MATSRYEPVAEIGVGAYGTVYKARDPHSGHFVALKSVRVPNGGGGGGGLPISTVREVALLRRLEAFEHPNVVRLMDVCATSRTDREIKVTLVFEHVDQDLRTYLDKAPPPGLPAETIKDLMRQFLRGLDFLHANCIVHRDLKPENILVTSGGTVKLADFGLARIYSYQMALTPVVVTLWYRAPEVLLQSTYATPVDMWSVGCIFAEMFRRKPLFCGNSEADQLGKIFDLIGLPPEDDWPRDVSLPRGAFPPRGPRPVQSVVPEMEESGAQLLLEMLTFNPHKRISAFRALQHSYLHKDEGNPE

>NP_510867.1

MSEPAGDVRQNPCGSKACRRLFGPVDSEQLSRDCDALMAGCIQEARERWNFDFVTETPLEGDFAWERVRGLGLPKLYLPTGPRRGRDELGGGRRPGTSPALLQGTAEEDHVDLSLSCTLVPRSGEQAEGSPGGPGDSQGRKRRQTSMTDFYHSKRRLIFSKRKP

1137 NP_056399.1 NP_002583.1

>NP_056399.1

MAACTARRALAVGSRWWSRSLTGARWPRPLCAAAGAGAFSPASTTTTRRHLSSRNRPEGKVLETVGVFEVPKQNGKYETGQLFLHSIFGYRGVVLFPWQARLYDRDVASAAPEKAENPAGHGSKEVKGKTHTYYQVLIDARDCPHISQRSQTEAVTFLANHDDSRALYAIPGLDYVSHEDILPYTSTDQVPIQHELFERFLLYDQTKAPPFVARETLRAWQEKNHPWLELSDVHRETTENIRVTVIPFYMGMREAQNSHVYWWRYCIRLENLDSDVVQLRERHWRIFSLSGTLETVRGRGVVGREPVLSKEQPAFQYSSHVSLQASSGHMWGTFRFERPDGSHFDVRIPPFSLESNKDEKTPPSGLHW

>NP_002583.1

MFEARLVQGSILKKVLEALKDLINEACWDISSSGVNLQSMDSSHVSLVQLTLRSEGFDTYRCDRNLAMGVNLTSMSKILKCAGNEDIITLRAEDNADTLALVFEAPNQEKVSDYEMKLMDLDVEQLGIPEQEYSCVVKMPSGEFARICRDLSHIGDAVVISCAKDGVKFSASGELGNGNIKLSQTSNVDKEEEAVTIEMNEPVQLTFALRYLNFFTKATPLSSTVTLSMSADVPLVVEYKIADMGHLKYYLAPKIEDEEGS

1138 NP_000593.1 NP_002323.2

>NP_000593.1

MQMSPALTCLVLGLALVFGEGSAVHHPPSYVAHLASDFGVRVFQQVAQASKDRNVVFSPYGVASVLAMLQLTTGGETQQQIQAAMGFKIDDKGMAPALRHLYKELMGPWNKDEISTTDAIFVQRDLKLVQGFMPHFFRLFRSTVKQVDFSEVERARFIINDWVKTHTKGMISNLLGKGAVDQLTRLVLVNALYFNGQWKTPFPDSSTHRRLFHKSDGSTVSVPMMAQTNKFNYTEFTTPDGHYYDILELPYHGDTLSMFIAAPYEKEVPLSALTNILSAQLISHWKGNMTRLPRLLVLPKFSLETEVDLRKPLENLGMTDMFRQFQADFTSLSDQEPLHVAQALQKVKIEVNESGTVASSSTAVIVSARMAPEEIIMDRPFLFVVRHNPTGTVLFMGQVMEP

>NP_002323.2

MLTPPLLLLLPLLSALVAAAIDAPKTCSPKQFACRDQITCISKGWRCDGERDCPDGSDEAPEICPQSKAQRCQPNEHNCLGTELCVPMSRLCNGVQDCMDGSDEGPHCRELQGNCSRLGCQHHCVPTLDGPTCYCNSSFQLQADGKTCKDFDECSVYGTCSQLCTNTDGSFICGCVEGYLLQPDNRSCKAKNEPVDRPPVLLIANSQNILATYLSGAQVSTITPTSTRQTTAMDFSYANETVCWVHVGDSAAQTQLKCARMPGLKGFVDEHTINISLSLHHVEQMAIDWLTGNFYFVDDIDDRIFVCNRNGDTCVTLLDLELYNPKGIALDPAMGKVFFTDYGQIPKVERCDMDGQNRTKLVDSKIVFPHGITLDLVSRLVYWADAYLDYIEVVDYEGKGRQTIIQGILIEHLYGLTVFENYLYATNSDNANAQQKTSVIRVNRFNSTEYQVVTRVDKGGALHIYHQRRQPRVRSHACENDQYGKPGGCSDICLLANSHKARTCRCRSGFSLGSDGKSCKKPEHELFLVYGKGRPGIIRGMDMGAKVPDEHMIPIENLMNPRALDFHAETGFIYFADTTSYLIGRQKIDGTERETILKDGIHNVEGVAVDWMGDNLYWTDDGPKKTISVARLEKAAQTRKTLIEGKMTHPRAIVVDPLNGWMYWTDWEEDPKDSRRGRLERAWMDGSHRDIFVTSKTVLWPNGLSLDIPAGRLYWVDAFYDRIETILLNGTDRKIVYEGPELNHAFGLCHHGNYLFWTEYRSGSVYRLERGVGGAPPTVTLLRSERPPIFEIRMYDAQQQQVGTNKCRVNNGGCSSLCLATPGSRQCACAEDQVLDADGVTCLANPSYVPPPQCQPGEFACANSRCIQERWKCDGDNDCLDNSDEAPALCHQHTCPSDRFKCENNRCIPNRWLCDGDNDCGNSEDESNATCSARTCPPNQFSCASGRCIPISWTCDLDDDCGDRSDESASCAYPTCFPLTQFTCNNGRCININWRCDNDNDCGDNSDEAGCSHSCSSTQFKCNSGRCIPEHWTCDGDNDCGDYSDETHANCTNQATRPPGGCHTDEFQCRLDGLCIPLRWRCDGDTDCMDSSDEKSCEGVTHVCDPSVKFGCKDSARCISKAWVCDGDNDCEDNSDEENCESLACRPPSHPCANNTSVCLPPDKLCDGNDDCGDGSDEGELCDQCSLNNGGCSHNCSVAPGEGIVCSCPLGMELGPDNHTCQIQSYCAKHLKCSQKCDQNKFSVKCSCYEGWVLEPDGESCRSLDPFKPFIIFSNRHEIRRIDLHKGDYSVLVPGLRNTIALDFHLSQSALYWTDVVEDKIYRGKLLDNGALTSFEVVIQYGLATPEGLAVDWIAGNIYWVESNLDQIEVAKLDGTLRTTLLAGDIEHPRAIALDPRDGILFWTDWDASLPRIEAASMSGAGRRTVHRETGSGGWPNGLTVDYLEKRILWIDARSDAIYSARYDGSGHMEVLRGHEFLSHPFAVTLYGGEVYWTDWRTNTLAKANKWTGHNVTVVQRTNTQPFDLQVYHPSRQPMAPNPCEANGGQGPCSHLCLINYNRTVSCACPHLMKLHKDNTTCYEFKKFLLYARQMEIRGVDLDAPYYNYIISFTVPDIDNVTVLDYDAREQRVYWSDVRTQAIKRAFINGTGVETVVSADLPNAHGLAVDWVSRNLFWTSYDTNKKQINVARLDGSFKNAVVQGLEQPHGLVVHPLRGKLYWTDGDNISMANMDGSNRTLLFSGQKGPVGLAIDFPESKLYWISSGNHTINRCNLDGSGLEVIDAMRSQLGKATALAIMGDKLWWADQVSEKMGTCSKADGSGSVVLRNSTTLVMHMKVYDESIQLDHKGTNPCSVNNGDCSQLCLPTSETTRSCMCTAGYSLRSGQQACEGVGSFLLYSVHEGIRGIPLDPNDKSDALVPVSGTSLAVGIDFHAENDTIYWVDMGLSTISRAKRDQTWREDVVTNGIGRVEGIAVDWIAGNIYWTDQGFDVIEVARLNGSFRYVVISQGLDKPRAITVHPEKGYLFWTEWGQYPRIERSRLDGTERVVLVNVSISWPNGISVDYQDGKLYWCDARTDKIERIDLETGENREVVLSSNNMDMFSVSVFEDFIYWSDRTHANGSIKRGSKDNATDSVPLRTGIGVQLKDIKVFNRDRQKGTNVCAVANGGCQQLCLYRGRGQRACACAHGMLAEDGASCREYAGYLLYSERTILKSIHLSDERNLNAPVQPFEDPEHMKNVIALAFDYRAGTSPGTPNRIFFSDIHFGNIQQINDDGSRRITIVENVGSVEGLAYHRGWDTLYWTSYTTSTITRHTVDQTRPGAFERETVITMSGDDHPRAFVLDECQNLMFWTNWNEQHPSIMRAALSGANVLTLIEKDIRTPNGLAIDHRAEKLYFSDATLDKIERCEYDGSHRYVILKSEPVHPFGLAVYGEHIFWTDWVRRAVQRANKHVGSNMKLLRVDIPQQPMGIIAVANDTNSCELSPCRINNGGCQDLCLLTHQGHVNCSCRGGRILQDDLTCRAVNSSCRAQDEFECANGECINFSLTCDGVPHCKDKSDEKPSYCNSRRCKKTFRQCSNGRCVSNMLWCNGADDCGDGSDEIPCNKTACGVGEFRCRDGTCIGNSSRCNQFVDCEDASDEMNCSATDCSSYFRLGVKGVLFQPCERTSLCYAPSWVCDGANDCGDYSDERDCPGVKRPRCPLNYFACPSGRCIPMSWTCDKEDDCEHGEDETHCNKFCSEAQFECQNHRCISKQWLCDGSDDCGDGSDEAAHCEGKTCGPSSFSCPGTHVCVPERWLCDGDKDCADGADESIAAGCLYNSTCDDREFMCQNRQCIPKHFVCDHDRDCADGSDESPECEYPTCGPSEFRCANGRCLSSRQWECDGENDCHDQSDEAPKNPHCTSQEHKCNASSQFLCSSGRCVAEALLCNGQDDCGDSSDERGCHINECLSRKLSGCSQDCEDLKIGFKCRCRPGFRLKDDGRTCADVDECSTTFPCSQRCINTHGSYKCLCVEGYAPRGGDPHSCKAVTDEEPFLIFANRYYLRKLNLDGSNYTLLKQGLNNAVALDFDYREQMIYWTDVTTQGSMIRRMHLNGSNVQVLHRTGLSNPDGLAVDWVGGNLYWCDKGRDTIEVSKLNGAYRTVLVSSGLREPRALVVDVQNGYLYWTDWGDHSLIGRIGMDGSSRSVIVDTKITWPNGLTLDYVTERIYWADAREDYIEFASLDGSNRHVVLSQDIPHIFALTLFEDYVYWTDWETKSINRAHKTTGTNKTLLISTLHRPMDLHVFHALRQPDVPNHPCKVNNGGCSNLCLLSPGGGHKCACPTNFYLGSDGRTCVSNCTASQFVCKNDKCIPFWWKCDTEDDCGDHSDEPPDCPEFKCRPGQFQCSTGICTNPAFICDGDNDCQDNSDEANCDIHVCLPSQFKCTNTNRCIPGIFRCNGQDNCGDGEDERDCPEVTCAPNQFQCSITKRCIPRVWVCDRDNDCVDGSDEPANCTQMTCGVDEFRCKDSGRCIPARWKCDGEDDCGDGSDEPKEECDERTCEPYQFRCKNNRCVPGRWQCDYDNDCGDNSDEESCTPRPCSESEFSCANGRCIAGRWKCDGDHDCADGSDEKDCTPRCDMDQFQCKSGHCIPLRWRCDADADCMDGSDEEACGTGVRTCPLDEFQCNNTLCKPLAWKCDGEDDCGDNSDENPEECARFVCPPNRPFRCKNDRVCLWIGRQCDGTDNCGDGTDEEDCEPPTAHTTHCKDKKEFLCRNQRCLSSSLRCNMFDDCGDGSDEEDCSIDPKLTSCATNASICGDEARCVRTEKAAYCACRSGFHTVPGQPGCQDINECLRFGTCSQLCNNTKGGHLCSCARNFMKTHNTCKAEGSEYQVLYIADDNEIRSLFPGHPHSAYEQAFQGDESVRIDAMDVHVKAGRVYWTNWHTGTISYRSLPPAAPPTTSNRHRRQIDRGVTHLNISGLKMPRGIAIDWVAGNVYWTDSGRDVIEVAQMKGENRKTLISGMIDEPHAIVVDPLRGTMYWSDWGNHPKIETAAMDGTLRETLVQDNIQWPTGLAVDYHNERLYWADAKLSVIGSIRLNGTDPIVAADSKRGLSHPFSIDVFEDYIYGVTYINNRVFKIHKFGHSPLVNLTGGLSHASDVVLYHQHKQPEVTNPCDRKKCEWLCLLSPSGPVCTCPNGKRLDNGTCVPVPSPTPPPDAPRPGTCNLQCFNGGSCFLNARRQPKCRCQPRYTGDKCELDQCWEHCRNGGTCAASPSGMPTCRCPTGFTGPKCTQQVCAGYCANNSTCTVNQGNQPQCRCLPGFLGDRCQYRQCSGYCENFGTCQMAADGSRQCRCTAYFEGSRCEVNKCSRCLEGACVVNKQSGDVTCNCTDGRVAPSCLTCVGHCSNGGSCTMNSKMMPECQCPPHMTGPRCEEHVFSQQQPGHIASILIPLLLLLLLVLVAGVVFWYKRRVQGAKGFQHQRMTNGAMNVEIGNPTYKMYEGGEPDDVGGLLDADFALDPDKPTNFTNPVYATLYMGGHGSRHSLASTDEKRELLGRGPEDEIGDPLA

1139 NP_006092.1 NP_036520.1

>NP_006092.1

MKRSSVSSGGAGRLSMQELRSQDVNKQGLYTPQTKEKPTFGKLSINKPTSERKVSLFGKRTSGHGSRNSQLGIFSSSEKIKDPRPLNDKAFIQQCIRQLCEFLTENGYAHNVSMKSLQAPSVKDFLKIFTFLYGFLCPSYELPDTKFEEEVPRIFKDLGYPFALSKSSMYTVGAPHTWPHIVAALVWLIDCIKIHTAMKESSPLFDDGQPWGEETEDGIMHNKLFLDYTIKCYESFMSGADSFDEMNAELQSKLKDLFNVDAFKLESLEAKNRALNEQIARLEQEREKEPNRLESLRKLKASLQGDVQKYQAYMSNLESHSAILDQKLNGLNEEIARVELECETIKQENTRLQNIIDNQKYSVADIERINHERNELQQTINKLTKDLEAEQQKLWNEELKYARGKEAIETQLAEYHKLARKLKLIPKGAENSKGYDFEIKFNPEAGANCLVKYRAQVYVPLKELLNETEEEINKALNKKMGLEDTLEQLNAMITESKRSVRTLKEEVQKLDDLYQQKIKEAEEEDEKCASELESLEKHKHLLESTVNQGLSEAMNELDAVQREYQLVVQTTTEERRKVGNNLQRLLEMVATHVGSVEKHLEEQIAKVDREYEECMSEDLSENIKEIRDKYEKKATLIKSSEE

>NP_036520.1

MSVPGPSSPDGALTRPPYCLEAGEPTPGLSDTSPDEGLIEDLTIEDKAVEQLAEGLLSHYLPDLQRSKQALQELTQNQVVLLDTLEQEISKFKECHSMLDINALFAEAKHYHAKLVNIRKEMLMLHEKTSKLKKRALKLQQKRQKEELEREQQREKEFEREKQLTARPAKRM

1140 NP_002334.2 NP_000087.1

>NP_002334.2

MKLVFLVLLFLGALGLCLAGRRRSVQWCAVSQPEATKCFQWQRNMRKVRGPPVSCIKRDSPIQCIQAIAENRADAVTLDGGFIYEAGLAPYKLRPVAAEVYGTERQPRTHYYAVAVVKKGGSFQLNELQGLKSCHTGLRRTAGWNVPIGTLRPFLNWTGPPEPIEAAVARFFSASCVPGADKGQFPNLCRLCAGTGENKCAFSSQEPYFSYSGAFKCLRDGAGDVAFIRESTVFEDLSDEAERDEYELLCPDNTRKPVDKFKDCHLARVPSHAVVARSVNGKEDAIWNLLRQAQEKFGKDKSPKFQLFGSPSGQKDLLFKDSAIGFSRVPPRIDSGLYLGSGYFTAIQNLRKSEEEVAARRARVVWCAVGEQELRKCNQWSGLSEGSVTCSSASTTEDCIALVLKGEADAMSLDGGYVYTAGKCGLVPVLAENYKSQQSSDPDPNCVDRPVEGYLAVAVVRRSDTSLTWNSVKGKKSCHTAVDRTAGWNIPMGLLFNQTGSCKFDEYFSQSCAPGSDPRSNLCALCIGDEQGENKCVPNSNERYYGYTGAFRCLAENAGDVAFVKDVTVLQNTDGNNNEAWAKDLKLADFALLCLDGKRKPVTEARSCHLAMAPNHAVVSRMDKVERLKQVLLHQQAKFGRNGSDCPDKFCLFQSETKNLLFNDNTECLARLHGKTTYEKYLGPQYVAGITNLKKCSTSPLLEACEFLRK

>NP_000087.1

MKILILGIFLFLCSTPAWAKEKHYYIGIIETTWDYASDHGEKKLISVDTEHSNIYLQNGPDRIGRLYKKALYLQYTDETFRTTIEKPVWLGFLGPIIKAETGDKVYVHLKNLASRPYTFHSHGITYYKEHEGAIYPDNTTDFQRADDKVYPGEQYTYMLLATEEQSPGEGDGNCVTRIYHSHIDAPKDIASGLIGPLIICKKDSLDKEKEKHIDREFVVMFSVVDENFSWYLEDNIKTYCSEPEKVDKDNEDFQESNRMYSVNGYTFGSLPGLSMCAEDRVKWYLFGMGNEVDVHAAFFHGQALTNKNYRIDTINLFPATLFDAYMVAQNPGEWMLSCQNLNHLKAGLQAFFQVQECNKSSSKDNIRGKHVRHYYIAAEEIIWNYAPSGIDIFTKENLTAPGSDSAVFFEQGTTRIGGSYKKLVYREYTDASFTNRKERGPEEEHLGILGPVIWAEVGDTIRVTFHNKGAYPLSIEPIGVRFNKNNEGTYYSPNYNPQSRSVPPSASHVAPTETFTYEWTVPKEVGPTNADPVCLAKMYYSAVDPTKDIFTGLIGPMKICKKGSLHANGRQKDVDKEFYLFPTVFDENESLLLEDNIRMFTTAPDQVDKEDEDFQESNKMHSMNGFMYGNQPGLTMCKGDSVVWYLFSAGNEADVHGIYFSGNTYLWRGERRDTANLFPQTSLTLHMWPDTEGTFNVECLTTDHYTGGMKQKYTVNQCRRQSEDSTFYLGERTYYIAAVEVEWDYSPQREWEKELHHLQEQNVSNAFLDKGEFYIGSKYKKVVYRQYTDSTFRVPVERKAEEEHLGILGPQLHADVGDKVKIIFKNMATRPYSIHAHGVQTESSTVTPTLPGETLTYVWKIPERSGAGTEDSACIPWAYYSTVDQVKDLYSGLIGPLIVCRRPYLKVFNPRRKLEFALLFLVFDENESWYLDDNIKTYSDHPEKVNKDDEEFIESNKMHAINGRMFGNLQGLTMHVGDEVNWYLMGMGNEIDLHTVHFHGHSFQYKHRGVYSSDVFDIFPGTYQTLEMFPRTPGIWLLHCHVTDHIHAGMETTYTVLQNEDTKSG

1141 NP_003395.1 NP_003381.1

>NP_003395.1

MTMDKSELVQKAKLAEQAERYDDMAAAMKAVTEQGHELSNEERNLLSVAYKNVVGARRSSWRVISSIEQKTERNEKKQQMGKEYREKIEAELQDICNDVLELLDKYLIPNATQPESKVFYLKMKGDYFRYLSEVASGDNKQTTVSNSQQAYQEAFEISKKEMQPTHPIRLGLALNFSVFYYEILNSPEKACSLAKTAFDEAIAELDTLNEESYKDSTLIMQLLRDNLTLWTSENQGDEGDAGEGEN

>NP_003381.1

MSFLSRQQPPPPRRAGAACTLRQKLIFSPCSDCEEEEEEEEEEGSGHSTGEDSAFQEPDSPLPPARSPTEPGPERRRSPGPAPGSPGELEEDLLLPGACPGADEAGGGAEGDSWEEEGFGSSSPVKSPAAPYFLGSSFSPVRCGGPGDASPRGCGARRAGEGRRSPRPDHPGTPPHKTFRKLRLFDTPHTPKSLLSKARGIDSSSVKLRGSSLFMDTEKSGKREFDVRQTPQVNINPFTPDSLLLHSSGQCRRRKRTYWNDSCGEDMEASDYELEDETRPAKRITITESNMKSRYTTEFHELEKIGSGEFGSVFKCVKRLDGCIYAIKRSKKPLAGSVDEQNALREVYAHAVLGQHSHVVRYFSAWAEDDHMLIQNEYCNGGSLADAISENYRIMSYFKEAELKDLLLQVGRGLRYIHSMSLVHMDIKPSNIFISRTSIPNAASEEGDEDDWASNKVMFKIGDLGHVTRISSPQVEEGDSRFLANEVLQENYTHLPKADIFALALTVVCAAGAEPLPRNGDQWHEIRQGRLPRIPQVLSQEFTELLKVMIHPDPERRPSAMALVKHSVLLSASRKSAEQLRIELNAEKFKNSLLQKELKKAQMAKAAAEERALFTDRMATRSTTQSNRTSRLIGKKMNRSVSLTIY

1142 NP_056250.3 NP_004214.1

>NP_056250.3

MQEQEIGFISKYNEGLCVNTDPVSILTSILDMSLHRQMGSDRDLQSSASSVSLPSVKKAPKKRRISIGSLFRRKKDNKRKSRELNGGVDGIASIESIHSEMCTDKNSIFSTNTSSDNGLTSISKQIGDFIECPLCLLRHSKDRFPDIMTCHHRSCVDCLRQYLRIEISESRVNISCPECTERFNPHDIRLILSDDVLMEKYEEFMLRRWLVADPDCRWCPAPDCGYAVIAFGCASCPKLTCGREGCGTEFCYHCKQIWHPNQTCDAARQERAQSLRLRTIRSSSISYSQESGAAADDIKPCPRCAAYIIKMNDGSCNHMTCAVCGCEFCWLCMKEISDLHYLSPSGCTFWGKKPWSRKKKILWQLGTLVGAPVGIALIAGIAIPAMIIGIPVYVGRKIHNRYEGKDVSKHKRNLAIAGGVTLSVIVSPVVAAVTVGIGVPIMLAYVYGVVPISLCRSGGCGVSAGNGKGVRIEFDDENDINVGGTNTAVDTTSVAEARHNPSIGEGSVGGLTGSLSASGSHMDRIGAIRDNLSETASTMALAGASITGSLSGSAMVNCFNRLEVQADVQKERYSLSGESGTVSLGTVSDNASTKAMAGSILNSYIPLDKEGNSMEVQVDIESKPSKFRHNSGSSSVDDGSATRSYAGGSSSGLPEGKSSATKWSKEATAGKKSKSGKLRKKGNMKINETREDMDAQLLEQQSTNSSEFEAPSLSDSMPSVADSHSSHFSEFSCSDLESMKTSCSHGSSDYHTRFATVNILPEVENDRLENSPHQCSISVVTQTASCSEVSQLNHIAEEHGNNGIKPNVDLYFGDALKETNNNHSHQTMELKVAIQTEI

>NP_004214.1

MMASMRVVKELEDLQKKPPPYLRNLSSDDANVLVWHALLLPDQPPYHLKAFNLRISFPPEYPFKPPMIKFTTKIYHPNVDENGQICLPIISSENWKPCTKTCQVLEALNVLVNRPNIREPLRMDLADLLTQNPELFRKNAEEFTLRFGVDRPS

1143 NP_056219.2 NP_065980.1

>NP_056219.2

MNAPLDGLSVSSSSTGSLGSAAGAGGGGGAGLRLLSANVRQLHQALTALLSEAEREQFTHCLNAYHARRNVFDLVRTLRVLLDSPVKRRLLPMLRLVIPRSDQLLFDQYTAEGLYLPATTPYRQPAWGGPDSAGPGEVRLVSLRRAKAHEGLGFSIRGGSEHGVGIYVSLVEPGSLAEKEGLRVGDQILRVNDKSLARVTHAEAVKALKGSKKLVLSVYSAGRIPGGYVTNHIYTWVDPQGRSISPPSGLPQPHGGALRQQEGDRRSTLHLLQGGDEKKVNLVLGDGRSLGLTIRGGAEYGLGIYITGVDPGSEAEGSGLKVGDQILEVNGRSFLNILHDEAVRLLKSSRHLILTVKDVGRLPHARTTVDETKWIASSRIRETMANSAGFLGDLTTEGINKPGFYKGPAGSQVTLSSLGNQTRVLLEEQARHLLNEQEHATMAYYLDEYRGGSVSVEALVMALFKLLNTHAKFSLLSEVRGTISPQDLERFDHLVLRREIESMKARQPPGPGAGDTYSMVSYSDTGSSTGSHGTSTTVSSARNTLDLEETGEAVQGNINALPDVSVDDVRSTSQGLSSFKPLPRPPPLAQGNDLPLGQPRKLGREDLQPPSSMPSCSGTVFSAPQNRSPPAGTAPTPGTSSAQDLPSSPIYASVSPANPSSKRPLDAHLALVNQHPIGPFPRVQSPPHLKSPSAEATVAGGCLLPPSPSGHPDQTGTNQHFVMVEVHRPDSEPDVNEVRALPQTRTASTLSHLSDSGQTLSEDSGVDAGEAEASAPGRGRQSVSTKSRSSKELPRNERPTDGANKPPGLLEPTSTLVRVKKSAATLGIAIEGGANTRQPLPRIVTIQRGGSAHNCGQLKVGHVILEVNGLTLRGKEHREAARIIAEAFKTKDRDYIDFLVTEFNVML

>NP_065980.1

MLNKMTLHPQQIMIGPRFNRALFDPLLVVLLALQLLVVAGLVRAQTCPSVCSCSNQFSKVICVRKNLREVPDGISTNTRLLNLHENQIQIIKVNSFKHLRHLEILQLSRNHIRTIEIGAFNGLANLNTLELFDNRLTTIPNGAFVYLSKLKELWLRNNPIESIPSYAFNRIPSLRRLDLGELKRLSYISEGAFEGLSNLRYLNLAMCNLREIPNLTPLIKLDELDLSGNHLSAIRPGSFQGLMHLQKLWMIQSQIQVIERNAFDNLQSLVEINLAHNNLTLLPHDLFTPLHHLERIHLHHNPWNCNCDILWLSWWIKDMAPSNTACCARCNTPPNLKGRYIGELDQNYFTCYAPVIVEPPADLNVTEGMAAELKCRASTSLTSVSWITPNGTVMTHGAYKVRIAVLSDGTLNFTNVTVQDTGMYTCMVSNSVGNTTASATLNVTAATTTPFSYFSTVTVETMEPSQDEARTTDNNVGPTPVVDWETTNVTTSLTPQSTRSTEKTFTIPVTDINSGIPGIDEVMKTTKIIIGCFVAITLMAAVMLVIFYKMRKQHHRQNHHAPTRTVEIINVDDEITGDTPMESHLPMPAIEHEHLNHYNSYKSPFNHTTTVNTINSIHSSVHEPLLIRMNSKDNVQETQI

1144 NP_573566.2 NP_705582.1

>NP_573566.2

MAALLRSARWLLRAGAAPRLPLSLRLLPGGPGRLHAASYLPAARAGPVAGGLLSPARLYAIAAKEKDIQEESTFSSRKISNQFDWALMRLDLSVRRTGRIPKKLLQKVFNDTCRSGGLGGSHALLLLRSCGSLLPELKLEERTEFAHRIWDTLQKLGAVYDVSHYNALLKVYLQNEYKFSPTDFLAKMEEANIQPNRVTYQRLIASYCNVGDIEGASKILGFMKTKDLPVTEAVFSALVTGHARAGDMENAENILTVMRDAGIEPGPDTYLALLNAYAEKGDIDHVKQTLEKVEKSELHLMDRDLLQIIFSFSKAGYPQYVSEILEKVTCERRYIPDAMNLILLLVTEKLEDVALQILLACPVSKEDGPSVFGSFFLQHCVTMNTPVEKLTDYCKKLKEVQMHSFPLQFTLHCALLANKTDLAKALMKAVKEEGFPIRPHYFWPLLVGRRKEKNVQGIIEILKGMQELGVHPDQETYTDYVIPCFDSVNSARAILQENGCLSDSDMFSQAGLRSEAANGNLDFVLSFLKSNTLPISLQSIRSSLLLGFRRSMNINLWSEITELLYKDGRYCQEPRGPTEAVGYFLYNLIDSMSDSEVQAKEEHLRQYFHQLEKMNVKIPENIYRGIRNLLESYHVPELIKDAHLLVESKNLDFQKTVQLTSSELESTLETLKAENQPIRDVLKQLILVLCSEENMQKALELKAKYESDMVTGGYAALINLCCRHDKVEDALNLKEEFDRLDSSAVLDTGKYVGLVRVLAKHGKLQDAINILKEMKEKDVLIKDTTALSFFHMLNGAALRGEIETVKQLHEAIVTLGLAEPSTNISFPLVTVHLEKGDLSTALEVAIDCYEKYKVLPRIHDVLCKLVEKGETDLIQKAMDFVSQEQGEMVMLYDLFFAFLQTGNYKEAKKIIETPGIRARSARLQWFCDRCVANNQVETLEKLVELTQKLFECDRDQMYYNLLKLYKINGDWQRADAVWNKIQEENVIPREKTLRLLAEILREGNQEVPFDVPELWYEDEKHSLNSSSASTTEPDFQKDILIACRLNQKKGAYDIFLNAKEQNIVFNAETYSNLIKLLMSEDYFTQAMEVKAFAETHIKGFTLNDAANSRLIITQVRRDYLKEAVTTLKTVLDQQQTPSRLAVTRVIQALAMKGDVENIEVVQKMLNGLEDSIGLSKMVFINNIALAQIKNNNIDAAIENIENMLTSENKVIEPQYFGLAYLFRKVIEEQLEPAVEKISIMAERLANQFAIYKPVTDFFLQLVDAGKVDDARALLQRCGAIAEQTPILLLFLLRNSRKQGKASTVKSVLELIPELNEKEEAYNSLMKSYVSEKDVTSAKALYEHLTAKNTKLDDLFLKRYASLLKYAGEPVPFIEPPESFEFYAQQLRKLRENSS

>NP_705582.1

MVFPLPTPQEPIMATPPKRRAVEATGEKVLRYETFISDVLQRDLRKVLDHRDKVYEQLAKYLQLRNVIERLQEAKHSELYMQVDLGCNFFVDTVVPDTSRIYVALGYGFFLELTLAEALKFIDRKSSLLTELSNSLTKDSMNIKAHIHMLLEGLRELQGLQNFPEKPHH

1145 NP_004737.2 NP_004078.2

>NP_004737.2

MKGLSGSRSHHHGVTCDSACDSLSHHSDRKPYLLSPVEHHPADHPYYTQRNSFQAECVGPFSDPLASSTFPRRHYTSQQELKDECALVPRTLATKANRIPANLLDQFERQLPLSRDGYHTLQYKRTAVEHRSDSPGRIRHLVHSVQKLFTKSHSLEGPSKGSVNGGKASPDEAQAARYGKRSKSKERRAEPKARPSTSPGWWSSDDNLDGDMCIYHAPSGVMTMGRCPDRSASQYFLEAYNTISEQAVKASRSNNDVKCSTCANLPVSLDTPLLKKSAWSSTLTVSRAREVYQKASVNMDQAMVKSESCQQERSCQYLQVPQDEWTGYTPRGKDDEIPCRRMRSGSYIKAMGDEDSGDSDTSPKPSPKVAARRESYLKATQPSLTELTTLKISNEHSPKLQIRSHSYLRAVSEVSINRSLDSLDPAGLLTSPKFRSRNESYMRAMSTISQVSEMEVNGQFESVCESVFSELESQAVEALDLPMPGCFRMRSHSYVRAIEKGCSQDDECVSLRSSSPPRTTTTVRTIQSSTVSSCITTYKKTPPPVPPRTTTKPFISITAQSSTESAQDAYMDGQGQRGDIISQSGLSNSTESLDSMKALTAAIEAANAQIHGPASQHMGNNTATVTTTTTIATVTTEDRKKDHFKKNRCLSIGIQVDDAEEPDKTGENKAPSKFQSVGVQVEEEKCFRRFTRSNSVTTAVQADLDFHDNLENSLESIEDNSCPGPMARQFSRDASTSTVSIQGSGNHYHACAADDDFDTDFDPSILPPPDPWIDSITEDPLEAVQRSVCHRDGHWFLKLLQAERDRMEGWCQQMEREERENNLPEDILGKIRTAVGSAQLLMAQKFYQFRELCEENLNPNAHPRPTSQDLAGFWDMLQLSIENISMKFDELHQLKANNWKQMDPLDKKERRAPPPVPKKPAKGPAPLIRERSLESSQRQEARKRLMAAKRAASVRQNSATESAESIEIYIPEAQTRL

>NP_004078.2

MPVRKQDTQRALHLLEEYRSKLSQTEDRQLRSSIERVINIFQSNLFQALIDIQEFYEVTLLDNPKCIDRSKPSEPIQPVNTWEISSLPSSTVTSETLPSSLSPSVEKYRYQDEDTPPQEHISPQITNEVIGPELVHVSEKNLSEIENVHGFVSHSHISPIKPTEAVLPSPPTVPVIPVLPVPAENTVILPTIPQANPPPVLVNTDSLETPTYVNGTDADYEYEEITLERGNSGLGFSIAGGTDNPHIGDDSSIFITKIITGGAAAQDGRLRVNDCILRVNEVDVRDVTHSKAVEALKEAGSIVRLYVKRRKPVSEKIMEIKLIKGPKGLGFSIAGGVGNQHIPGDNSIYVTKIIEGGAAHKDGKLQIGDKLLAVNNVCLEEVTHEEAVTALKNTSDFVYLKVAKPTSMYMNDGYAPPDITNSSSQPVDNHVSPSSFLGQTPASPARYSPVSKAVLGDDEITREPRKVVLHRGSTGLGFNIVGGEDGEGIFISFILAGGPADLSGELRKGDRIISVNSVDLRAASHEQAAAALKNAGQAVTIVAQYRPEEYSRFEAKIHDLREQMMNSSISSGSGSLRTSQKRSLYVRALFDYDKTKDSGLPSQGLNFKFGDILHVINASDDEWWQARQVTPDGESDEVGVIPSKRRVEKKERARLKTVKFNSKTRDKGQSFNDKRKKNLFSRKFPFYKNKDQSEQETSDADQHVTSNASDSESSYRGQEEYVLSYEPVNQQEVNYTRPVIILGPMKDRINDDLISEFPDKFGSCVPHTTRPKRDYEVDGRDYHFVTSREQMEKDIQEHKFIEAGQYNNHLYGTSVQSVREVAEKGKHCILDVSGNAIKRLQIAQLYPISIFIKPKSMENIMEMNKRLTEEQARKTFERAMKLEQEFTEHFTAIVQGDTLEDIYNQVKQIIEEQSGSYIWVPAKEKL

1146 NP_003940.3 NP_060621.3

>NP_003940.3

MRPKRLGRCCAGSRLGPGDPAALTCAPSPSASPAPEPSAQPQARGTGQRVGSRATSGSQFLSEARTGARPASEAGAKAGARRPSAFSAIQGDVRSMPDNSDAPWTRFVFQGPFGSRATGRGTGKAAGIWKTPAAYVGRRPGVSGPERAAFIRELEEALCPNLPPPVKKITQEDVKVMLYLLEELLPPVWESVTYGMVLQRERDLNTAARIGQSLVKQNSVLMEENSKLEALLGSAKEEILYLRHQVNLRDELLQLYSDSDEEDEDEEEEEEEKEAEEEQEEEEAEEDLQCAHPCDAPKLISQEALLHQHHCPQLEALQEKLRLLEEENHQLREEASQLDTLEDEEQMLILECVEQFSEASQQMAELSEVLVLRLENYERQQQEVARLQAQVLKLQQRCRMYGAETEKLQKQLASEKEIQMQLQEESVWVGSQLQDLREKYMDCGGMLIEMQEEVKTLRQQPPVSTGSATHYPYSVPLETLPGFQETLAEELRTSLRRMISDPVYFMERNYEMPRGDTSSLRYDFRYSEDREQVRGFEAEEGLMLAADIMRGEDFTPAEEFVPQEELGAAKKVPAEEGVMEEAELVSEETEGWEEVELELDEATRMNVVTSALEASGLGPSHLDMNYVLQQLANWQDAHYRRQLRWKMLQKGECPHGALPAASRTSCRSSCR

>NP_060621.3

MTARGQSPLAPLLETLEDPSASHGGQTDAYLTLTSRMTGEEGKEVITEIEKKLPRLYKVLKTHISSQNSELSSAALQALGFCLYNPKITSELSEANALELLSKLNDTIKNSDKNVRTRALWVISKQTFPSEVVGKMVSSIIDSLEILFNKGETHSAVVDFEALNVIVRLIEQAPIQMGEEAVRWAKLVIPLVVHSAQKVHLRGATALEMGMPLLLQKQQEIASITEQLMTTKLISELQKLFMSKNETYVLKLWPLFVKLLGRTLHRSGSFINSLLQLEELGFRSGAPMIKKIAFIAWKSLIDNFALNPDILCSAKRLKLLMQPLSSIHVRTETLALTKLEVWWYLLMRLGPHLPANFEQVCVPLIQSTISIDSNASPQGNSCHVATSPGLNPMTPVHKGASSPYGAPGTPRMNLSSNLGGMATIPSIQLLGLEMLLHFLLGPEALSFAKQNKLVLSLEPLEHPLISSPSFFSKHANTLITAVHDSFVAVGKDAPDVVVSAIWKELISLVKSVTESGNKKEKPGSEVLTLLLKSLESIVKSEVFPVSKTLVLMEITIKGLPQKVLGSPAYQVANMDILNGTPALFLIQLIFNNFLECGVSDERFFLSLESLVGCVLSGPTSPLAFSDSVLNVINQNAKQLENKEHLWKMWSVIVTPLTELINQTNEVNQGDALEHNFSAIYGALTLPVNHIFSEQRFPVATMKTLLRTWSELYRAFARCAALVATAEENLCCEELSSKIMSSLEDEGFSNLLFVDRIIYIITVMVDCIDFSPYNIKYQPKVKSPQRPSDWSKKKNEPLGKLTSLFKLIVKVIYSFHTLSFKEAHSDTLFTIGNSITGIISSVLGHISLPSMIRKIFATLTRPLALFYENSKLDEVPKVYSCLNNKLEKLLGEIIACLQFSYTGTYDSELLEQLSPLLCIIFLHKNKQIRKQSAQFWNATFAKVMMLVYPEELKPVLTQAKQKFLLLLPGLETVEMMEESSGPYSDGTENSQLNVKISGMERKSNGKRDSFLAQTKNKKENMKPAAKLKLESSSLKVKGEILLEEEKSTDFVFIPPEGKDAKERILTDHQKEVLKTKRCDIPAMYNNLDVSQDTLFTQYSQEEPMEIPTLTRKPKEDSKMMITEEQMDSDIVIPQDVTEDCGMAEHLEKSSLSNNECGSLDKTSPEMSNSNNDERKKALISSRKTSTECASSTENSFVVSSSSVSNTTVAGTPPYPTSRRQTFITLEKFDGSENRPFSPSPLNNISSTVTVKNNQETMIKTDFLPKAKQREGTFSKSDSEKIVNGTKRSSRRAGKAEQTGNKRSKPLMRSEPEKNTEESVEGIVVLENNPPGLLNQTECVSDNQVHLSESTMEHDNTKLKAATVENAVLLETNTVEEKNVEINLESKENTPPVVISADQMVNEDSQVQITPNQKTLRRSSRRRSEVVESTTESQDKENSHQKKERRKEEEKPLQKSPLHIKDDVLPKQKLIAEQTLQENLIEKGSNLHEKTLGETSANAETEQNKKKADPENIKSEGDGTQDIVDKSSEKLVRGRTRYQTRRASQGLLSSIENSESDSSEAKEEGSRKKRSGKWKNKSNESVDIQDQEEKVVKQECIKAENQSHDYKATSEEDVSIKSPICEKQDESNTVICQDSTVTSDLLQVPDDLPNVCEEKNETSKYAEYSFTSLPVPESNLRTRNAIKRLHKRDSFDNCSLGESSKIGISDISSLSEKTFQTLECQHKRSRRVRRSKGCDCCGEKSQPQEKSLIGLKNTENNDVEISETKKADVQAPVSPSETSQANPYSEGQFLDEHHSVNFHLGLKEDNDTINDSLIVSETKSKENTMQESLPSGIVNFREEICDMDSSEAMSLESQESPNENFKTVGPCLGDSKNVSQESLETKEEKPEETPKMELSLENVTVEGNACKVTESNLEKAKTMELNVGNEASFHGQERTKTGISEEAAIEENKRNDDSEADTAKLNAKEVATEEFNSDISLSDNTTPVKLNAQTEISEQTAAGELDGGNDVSDLHSSEETNTKMKNNEEMMIGEAMAETGHDGETENEGITTKTSKPDEAETNMLTAEMDNFVCDTVEMSTEEGIIDANKTETNTEYSKSEEKLDNNQMVMESDILQEDHHTSQKVEEPSQCLASGTAISELIIEDNNASPQKLRELDPSLVSANDSPSGMQTRCVWSPLASPSTSILKRGLKRSQEDEISSPVNKVRRVSFADPIYQAGLADDIDRRCSIVRSHSSNSSPIGKSVKTSPTTQSKHNTTSAKGFLSPGSRSPKFKSSKKCLISEMAKESIPCPTESVYPPLVNCVAPVDIILPQITSNMWARGLGQLIRAKNIKTIGDLSTLTASEIKTLPIRSPKVSNVKKALRIYHEQQVKTRGLEEIPVFDISEKTVNGIENKSLSPDEERLVSDIIDPVALEIPLSKNLLAQISALALQLDSEDLHNYSGSQLFEMHEKLSCMANSVIKNLQSRWRSPSHENSI

1147 NP_075380.1 NP_065393.1

>NP_075380.1

MKRASAGGSRLLAWVLWLQAWQVAAPCPGACVCYNEPKVTTSCPQQGLQAVPVGIPAASQRIFLHGNRISHVPAASFRACRNLTILWLHSNVLARIDAAAFTGLALLEQLDLSDNAQLRSVDPATFHGLGRLHTLHLDRCGLQELGPGLFRGLAALQYLYLQDNALQALPDDTFRDLGNLTHLFLHGNRISSVPERAFRGLHSLDRLLLHQNRVAHVHPHAFRDLGRLMTLYLFANNLSALPTEALAPLRALQYLRLNDNPWVCDCRARPLWAWLQKFRGSSSEVPCSLPQRLAGRDLKRLAANDLQGCAVATGPYHPIWTGRATDEEPLGLPKCCQPDAADKASVLEPGRPASAGNALKGRVPPGDSPPGNGSGPRHINDSPFGTLPGSAEPPLTAVRPEGSEPPGFPTSGPRRRPGCSRKNRTRSHCRLGQAGSGGGGTGDSEGSGALPSLTCSLTPLGLALVLWTVLGPC

>NP_065393.1

MEDLDQSPLVSSSDSPPRPQPAFKYQFVREPEDEEEEEEEEEEDEDEDLEELEVLERKPAAGLSAAPVPTAPAAGAPLMDFGNDFVPPAPRGPLPAAPPVAPERQPSWDPSPVSSTVPAPSPLSAAAVSPSKLPEDDEPPARPPPPPPASVSPQAEPVWTPPAPAPAAPPSTPAAPKRRGSSGSVDETLFALPAASEPVIRSSAENMDLKEQPGNTISAGQEDFPSVLLETAASLPSLSPLSAASFKEHEYLGNLSTVLPTEGTLQENVSEASKEVSEKAKTLLIDRDLTEFSELEYSEMGSSFSVSPKAESAVIVANPREEIIVKNKDEEEKLVSNNILHNQQELPTALTKLVKEDEVVSSEKAKDSFNEKRVAVEAPMREEYADFKPFERVWEVKDSKEDSDMLAAGGKIESNLESKVDKKCFADSLEQTNHEKDSESSNDDTSFPSTPEGIKDRSGAYITCAPFNPAATESIATNIFPLLGDPTSENKTDEKKIEEKKAQIVTEKNTSTKTSNPFLVAAQDSETDYVTTDNLTKVTEEVVANMPEGLTPDLVQEACESELNEVTGTKIAYETKMDLVQTSEVMQESLYPAAQLCPSFEESEATPSPVLPDIVMEAPLNSAVPSAGASVIQPSSSPLEASSVNYESIKHEPENPPPYEEAMSVSLKKVSGIKEEIKEPENINAALQETEAPYISIACDLIKETKLSAEPAPDFSDYSEMAKVEQPVPDHSELVEDSSPDSEPVDLFSDDSIPDVPQKQDETVMLVKESLTETSFESMIEYENKEKLSALPPEGGKPYLESFKLSLDNTKDTLLPDEVSTLSKKEKIPLQMEELSTAVYSNDDLFISKEAQIRETETFSDSSPIEIIDEFPTLISSKTDSFSKLAREYTDLEVSHKSEIANAPDGAGSLPCTELPHDLSLKNIQPKVEEKISFSDDFSKNGSATSKVLLLPPDVSALATQAEIESIVKPKVLVKEAEKKLPSDTEKEDRSPSAIFSAELSKTSVVDLLYWRDIKKTGVVFGASLFLLLSLTVFSIVSVTAYIALALLSVTISFRIYKGVIQAIQKSDEGHPFRAYLESEVAISEELVQKYSNSALGHVNCTIKELRRLFLVDDLVDSLKFAVLMWVFTYVGALFNGLTLLILALISLFSVPVIYERHQAQIDHYLGLANKNVKDAMAKIQAKIPGLKRKAE

1148 NP_002919.2 NP_006487.1

>NP_002919.2

MCRTLAAFPTTCLERAKEFKTRLGIFLHKSELGCDTGSTGKFEWGSKHSKENRNFSEDVLGWRESFDLLLSSKNGVAAFHAFLKTEFSEENLEFWLACEEFKKIRSATKLASRAHQIFEEFICSEAPKEVNIDHETRELTRMNLQTATATCFDAAQGKTRTLMEKDSYPRFLKSPAYRDLAAQASAASATLSSCSLDEPSHT

>NP_006487.1

MGCTLSAEDKAAVERSKMIDRNLREDGEKAAKEVKLLLLGAGESGKSTIVKQMKIIHEDGYSEDECKQYKVVVYSNTIQSIIAIIRAMGRLKIDFGEAARADDARQLFVLAGSAEEGVMTPELAGVIKRLWRDGGVQACFSRSREYQLNDSASYYLNDLDRISQSNYIPTQQDVLRTRVKTTGIVETHFTFKDLYFKMFDVGGQRSERKKWIHCFEGVTAIIFCVALSDYDLVLAEDEEMNRMHESMKLFDSICNNKWFTETSIILFLNKKDLFEEKIKRSPLTICYPEYTGSNTYEEAAAYIQCQFEDLNRRKDTKEIYTHFTCATDTKNVQFVFDAVTDVIIKNNLKECGLY

1149 NP_009115.2 NP_003595.1

>NP_009115.2

MATARTFGPEREAEPAKEARVVGSELVDTYTVYIIQVTDGSHEWTVKHRYSDFHDLHEKLVAERKIDKNLLPPKKIIGKNSRSLVEKREKDLEVYLQKLLAAFPGVTPRVLAHFLHFHFYEINGITAALAEELFEKGEQLLGAGEVFAIGPLQLYAVTEQLQQGKPTCASGDAKTDLGHILDFTCRLKYLKVSGTEGPFGTSNIQEQLLPFDLSIFKSLHQVEISHCDAKHIRGLVASKPTLATLSVRFSATSMKEVLVPEASEFDEWEPEGTTLEGPVTAVIPTWQALTTLDLSHNSISEIDESVKLIPKIEFLDLSHNGLLVVDNLQHLYNLVHLDLSYNKLSSLEGLHTKLGNIKTLNLAGNLLESLSGLHKLYSLVNLDLRDNRIEQMEEVRSIGSLPCLEHVSLLNNPLSIIPDYRTKVLAQFGERASEVCLDDTVTTEKELDTVEVLKAIQKAKEVKSKLSNPEKKGGEDSRLSAAPCIRPSSSPPTVAPASASLPQPILSNQGIMFVQEEALASSLSSTDSLTPEHQPIAQGCSDSLESIPAGQAASDDLRDVPGAVGGASPEHAEPEVQVVPGSGQIIFLPFTCIGYTATNQDFIQRLSTLIRQAIERQLPAWIEAANQREEGQGEQGEEEDEEEEEEEDVAENRYFEMGPPDVEEEEGGGQGEEEEEEEEDEEAEEERLALEWALGADEDFLLEHIRILKVLWCFLIHVQGSIRQFAACLVLTDFGIAVFEIPHQESRGSSQHILSSLRFVFCFPHGDLTEFGFLMPELCLVLKVRHSENTLFIISDAANLHEFHADLRSCFAPQHMAMLCSPILYGSHTSLQEFLRQLLTFYKVAGGCQERSQGCFPVYLVYSDKRMVQTAAGDYSGNIEWASCTLCSAVRRSCCAPSEAVKSAAIPYWLLLTPQHLNVIKADFNPMPNRGTHNCRNRNSFKLSRVPLSTVLLDPTRSCTQPRGAFADGHVLELLVGYRFVTAIFVLPHEKFHFLRVYNQLRASLQDLKTVVIAKTPGTGGSPQGSFADGQPAERRASNDQRPQEVPAEALAPAPAEVPAPAPAAASASGPAKTPAPAEASTSALVPEETPVEAPAPPPAEAPAQYPSEHLIQATSEENQIPSHLPACPSLRHVASLRGSAIIELFHSSIAEVENEELRHLMWSSVVFYQTPGLEVTACVLLSTKAVYFVLHDGLRRYFSEPLQDFWHQKNTDYNNSPFHISQCFVLKLSDLQSVNVGLFDQHFRLTGSTPMQVVTCLTRDSYLTHCFLQHLMVVLSSLERTPSPEPVDKDFYSEFGNKTTGKMENYELIHSSRVKFTYPSEEEIGDLTFTVAQKMAEPEKAPALSILLYVQAFQVGMPPPGCCRGPLRPKTLLLTSSEIFLLDEDCVHYPLPEFAKEPPQRDRYRLDDGRRVRDLDRVLMGYQTYPQALTLVFDDVQGHDLMGSVTLDHFGEVPGGPARASQGREVQWQVFVPSAESREKLISLLARQWEALCGRELPVELTG

>NP_003595.1

MASCSFTRDQATRRLRGAAAAAAAALAAVVTTPLLSSGTPTALIGTGSSCPGAMWLSTATGSRSDSESEEEDLPVGEEVCKRGYLRKQKHGHRRYFVLKLETADAPARLEYYENARKFRHSVRAAAAAAAAAASGAAIPPLIPPRRVITLYQCFSVSQRADARYRHLIALFTQDEYFAMVAENESEQESWYLLLSRLILESKRRRCGTLGAQPDGEPAALAAAAAAEPPFYKDVWQVIVKPRGLGHRKELSGVFRLCLTDEEVVFVRLNTEVASVVVQLLSIRRCGHSEQYFFLEVGRSTVIGPGELWMQVDDCVVAQNMHELFLEKMRALCADEYRARCRSYSISIGAHLLTLLSARRHLGLVPLEPGGWLRRSRFEQFCHLRAIGDGEDEMLFTRRFVTPSEPVAHSRRGRLHLPRGRRSRRAVSVPASFFRRLAPSPARPRHPAEAPNNGARLSSEVSGSGSGNFGEEGNPQGKEDQEGSGGDYMPMNNWGSGNGRGSGGGQGSNGQGSSSHSSGGNQCSGEGQGSRGGQGSNGQGSGGNQCSRDGQGTAGGHGSGGGQRPGGGHGSGGGQGPGDGHGSGGGKNSGGGKGSGSGKGSDGDGERGKSLKKRSYFGKLTQSKQQQMPPPPPPPPPPPPAGGTGGKGKSGGRFRLYFCVDRGATKECKEAKEVKDAEIPEGAARGPHRARAFDEDEDDPYVPMRPGVATPLVSSSDYMPMAPQNVSASKKRHSRSPFEDSRGYMMMFPRVSPPPAPSPPKAPDTNKEDDSKDNDSESDYMFMAPGAGAIPKNPRNPQGGSSSKSWSSYFSLPNPFRSSPLGQNDNSEYVPMLPGKFLGRGLDKEVSYNWDPKDAASKPSGEGSFSKPGDGGSPSKPSDHEPPKNKAKRPNRLSFITKGYKIKPKPQKPTHEQREADSSSDYVNMDFTKRESNTPAPSTQGLPDSWGIIAEPRQSAFSNYVNVEFGVPFPNPANDLSDLLRAIPRANPLSLDSARWPLPPLPLSATGSNAIEEEGDYIEVIFNSAMTPAMALADSAIRYDAETGRIYVVDPFSECCMDISLSPSRCSEPPPVARLLQEEEQERRRPQSRSQSFFAAARAAVSAFPTDSLERDLSPSSAPAVASAAEPTLALSQVVAAASALAAAPGIGAAAAAAGFDSASARWFQPVANAADAEAVRGAQDVAGGSNPGAHNPSANLARGDNQAGGAAAAAAAPEPPPRSRRVPRPPEREDSDNDDDTHVRMDFARRDNQFDSPKRGR

1150 NP_001207.1 BAA88957.1

>NP_001207.1

MAPLCPSPWLPLLIPAPAPGLTVQLLLSLLLLMPVHPQRLPRMQEDSPLGGGSSGEDDPLGEEDLPSEEDSPREEDPPGEEDLPGEEDLPGEEDLPEVKPKSEEEGSLKLEDLPTVEAPGDPQEPQNNAHRDKEGDDQSHWRYGGDPPWPRVSPACAGRFQSPVDIRPQLAAFCPALRPLELLGFQLPPLPELRLRNNGHSVQLTLPPGLEMALGPGREYRALQLHLHWGAAGRPGSEHTVEGHRFPAEIHVVHLSTAFARVDEALGRPGGLAVLAAFLEEGPEENSAYEQLLSRLEEIAEEGSETQVPGLDISALLPSDFSRYFQYEGSLTTPPCAQGVIWTVFNQTVMLSAKQLHTLSDTLWGPGDSRLQLNFRATQPLNGRVIEASFPAGVDSSPRAAEPVQLNSCLAAGDILALVFGLLFAVTSVAFLVQMRRQHRRGTKGGVSYRPAEVAETGA

>BAA88957.1

MGPWSRSLSALLLLLQVSSWLCQEPEPCHPGFDAESYTFTVPRRHLERGRVLGRVNFEDCTGRQRTAYFSLDTRFKVGTDGVITVKRPLRFHNPQIHFLVYAWDSTYRKFSTKVTLNTVGHHHRPPPHQASVSGIQAELLTFPNSSPGLRRQKRDWVIPPISCPENEKGPFPKNLVQIKSNKDKEGKVFYSITGQGADTPPVGVFIIERETGWLKVTEPLDRERIATYTLFSHAVSSNGNAVEDPMEILITVTDQNDNKPEFTQEVFKGSVMEGALPGTSVMEVTATDADDDVNTYNAAIAYTILSQDPELPDKNMFTINRNTGVISVVTTGLDRESFPTYTLVVQAADLQGEGLSTTATAVITVTDTNDNPPIFNPTTYKGQVPENEANVVITTLKVTDADAPNTPAWEAVYTILNDDGGQFVVTTNPVNNDGILKTAKDLARCRNWHPHSCSYPCPLGLDFEAKQQYILHVAVTNVVPFEVSLTTSTATVTVDVLDVNEAPIFVPPEKRVEVSEDFGVGQEITSYTAQEPDTFMEQKITYRIWRDTANWLEINPDTGAISTRAELDREDFEHVKNSTYTALIIATDNGSPVATGTGTLLLILSDVNDNAPIPEPRTIFFCERNPKPQVINIIDADLPPNTSPFTAELTHGASANWTIQYNDPTQESIILKPKMALEVGDYKINLKLMDNQNKDQVTTLEVSVCDCEGAAGVCRKAQPVEAGLQIPAILGILGGILALLILILLLLLFLRRRAVVKEPLLPPEDDTRDNVYYYDEEGGGEEDQDFDLSQLHRGLDARPEVTRNDVAPTLMSVPRYLPRPANPDEIGNFIDENLKAADTDPTAPPYDSLLVFDYEGSGSEAASLSSLNSSESDKDQDYDYLNEWGNRFKKLADMYGGGEDD

1151 NP_660095.1 NP_001005333.1

>NP_660095.1

MGQESSKPVWPNPTGGYQSNTGRRYGRRHAYVSFRPPTSQRERIASQRKTNSEVPMHRSAPSQTTKRSRSPFSTTRRSWDDSESSGTNLNIDNEDYSRYPPREYRASGSRRGMAYGHIDSYGADDSEEEGAGPVERPPVRGKTGKFKDDKLYDPEKGARSLAGPPPHFSSFSRDVREERDKLDPVPAARCSASRADFLPQSSVASQSSSEGKLATKGDSSERERREQNLPARPSRAPVSICGGGENTSKSAEEPVVRPKIRNLASPNCVKPKIFFDTDDDDDMPHSTSRWRDTANDNEGHSDGLARRGRGESSSGYPEPKYPEDKREARSDQVKPEKVPRRRRTMADPDFWTHSDDYYKYCDEDSDSDKEWIAALRRKYRSREQTLSSSGESWETLPGKEEREPPQAKVSASTGTSPGPGASASAGAGAGASAGSNGSNYLEEVREPSLQEEQASLEEGEIPWLQYHENDSSSEGDNDSGHELMQPGVFMLDGNNNLEDDSSVSEDLEVDWSLFDGFADGLGVAEAISYVDPQFLTYMALEERLAQAMETALAHLESLAVDVEVANPPASKESIDALPEILVTEDHGAVGQEMCCPICCSEYVKGEVATELPCHHYFHKPCVSIWLQKSGTCPVCRCMFPPPL

>NP_001005333.1

MAQKMDCGAGLLGFQNPDACRAVCHPLPQPPASTLPLSAFPTLCDPPYSQLRDPPAVLSCYCTPLGASPAPAEASVEDSALLMQTLMEAIQISEAPPTNQATAAASPQSSQPPTANEMADIQVSAAAARPKSAFKVQNATTKGPNGVYDFSQAHNAKDVPNTQPKAAFKSQNATPKGPNAAYDFSQAATTGELAANKSEMAFKAQNATTKVGPNATYNFSQSLNANDLANSRPKTPFKAWNDTTKAPTADTQTQNVNQAKMATSQADIETDPGISEPDGATAQTSADGSQAQNLESRTIIRGKRTRKINNLNVEENSSGDQRRAPLAAGTWRSAPVPVTTQNPPGAPPNVLWQTPLAWQNPSGWQNQTARQTPPARQSPPARQTPPAWQNPVAWQNPVIWPNPVIWQNPVIWPNPIVWPGPVVWPNPLAWQNPPGWQTPPGWQTPPGWQGPPDWQGPPDWPLPPDWPLPPDWPLPTDWPLPPDWIPADWPIPPDWQNLRPSPNLRPSPNSRASQNPGAAQPRDVALLQERANKLVKYLMLKDYTKVPIKRSEMLRDIIREYTDVYPEIIERACFVLEKKFGIQLKEIDKEEHLYILISTPESLAGILGTTKDTPKLGLLLVILGVIFMNGNRASEAVLWEALRKMGLRPGVRHPLLGDLRKLLTYEFVKQKYLDYRRVPNSNPPEYEFLWGLRSYHETSKMKVLRFIAEVQKRDPRDWTAQFMEAADEALDALDAAAAEAEARAEARTRMGIGDEAVSGPWSWDDIEFELLTWDEEGDFGDPWSRIPFTFWARYHQNARSRFPQTFAGPIIGPGGTASANFAANFGAIGFFWVE

1152 NP_002520.2 NP_003891.1

>NP_002520.2

MLRGGRRGQLGWHSWAAGPGSLLAWLILASAGAAPCPDACCPHGSSGLRCTRDGALDSLHHLPGAENLTELYIENQQHLQHLELRDLRGLGELRNLTIVKSGLRFVAPDAFHFTPRLSRLNLSFNALESLSWKTVQGLSLQELVLSGNPLHCSCALRWLQRWEEEGLGGVPEQKLQCHGQGPLAHMPNASCGVPTLKVQVPNASVDVGDDVLLRCQVEGRGLEQAGWILTELEQSATVMKSGGLPSLGLTLANVTSDLNRKNVTCWAENDVGRAEVSVQVNVSFPASVQLHTAVEMHHWCIPFSVDGQPAPSLRWLFNGSVLNETSFIFTEFLEPAANETVRHGCLRLNQPTHVNNGNYTLLAANPFGQASASIMAAFMDNPFEFNPEDPIPVSFSPVDTNSTSGDPVEKKDETPFGVSVAVGLAVFACLFLSTLLLVLNKCGRRNKFGINRPAVLAPEDGLAMSLHFMTLGGSSLSPTEGKGSGLQGHIIENPQYFSDACVHHIKRRDIVLKWELGEGAFGKVFLAECHNLLPEQDKMLVAVKALKEASESARQDFQREAELLTMLQHQHIVRFFGVCTEGRPLLMVFEYMRHGDLNRFLRSHGPDAKLLAGGEDVAPGPLGLGQLLAVASQVAAGMVYLAGLHFVHRDLATRNCLVGQGLVVKIGDFGMSRDIYSTDYYRVGGRTMLPIRWMPPESILYRKFTTESDVWSFGVVLWEIFTYGKQPWYQLSNTEAIDCITQGRELERPRACPPEVYAIMRGCWQREPQQRHSIKDVHARLQALAQAPPVYLDVLG

>NP_003891.1

MASLTVKAYLLGKEDAAREIRRFSFCCSPEPEAEAEAAAGPGPCERLLSRVAALFPALRPGGFQAHYRDEDGDLVAFSSDEELTMAMSYVKDDIFRIYIKEKKECRRDHRPPCAQEAPRNMVHPNVICDGCNGPVVGTRYKCSVCPDYDLCSVCEGKGLHRGHTKLAFPSPFGHLSEGFSHSRWLRKVKHGHFGWPGWEMGPPGNWSPRPPRAGEARPGPTAESASGPSEDPSVNFLKNVGESVAAALSPLGIEVDIDVEHGGKRSRLTPVSPESSSTEEKSSSQPSSCCSDPSKPGGNVEGATQSLAEQMRKIALESEGRPEEQMESDNCSGGDDDWTHLSSKEVDPSTGELQSLQMPESEGPSSLDPSQEGPTGLKEAALYPHLPPEADPRLIESLSQMLSMGFSDEGGWLTRLLQTKNYDIGAALDTIQYSKHPPPL

1153 NP_001250.1 NP_510867.1

>NP_001250.1

MEKDGLCRADQQYECVAEIGEGAYGKVFKARDLKNGGRFVALKRVRVQTGEEGMPLSTIREVAVLRHLETFEHPNVVRLFDVCTVSRTDRETKLTLVFEHVDQDLTTYLDKVPEPGVPTETIKDMMFQLLRGLDFLHSHRVVHRDLKPQNILVTSSGQIKLADFGLARIYSFQMALTSVVVTLWYRAPEVLLQSSYATPVDLWSVGCIFAEMFRRKPLFRGSSDVDQLGKILDVIGLPGEEDWPRDVALPRQAFHSKSAQPIEKFVTDIDELGKDLLLKCLTFNPAKRISAYSALSHPYFQDLERCKENLDSHLPPSQNTSELNTA

>NP_510867.1

MSEPAGDVRQNPCGSKACRRLFGPVDSEQLSRDCDALMAGCIQEARERWNFDFVTETPLEGDFAWERVRGLGLPKLYLPTGPRRGRDELGGGRRPGTSPALLQGTAEEDHVDLSLSCTLVPRSGEQAEGSPGGPGDSQGRKRRQTSMTDFYHSKRRLIFSKRKP

1154 NP_004356.2 NP_060842.3

>NP_004356.2

MSLALRSELVVDKTKRKKRRELSEEQKQEIKDAFELFDTDKDEAIDYHELKVAMRALGFDVKKADVLKILKDYDREATGKITFEDFNEVVTDWILERDPHEEILKAFKLFDDDDSGKISLRNLRRVARELGENMSDEELRAMIEEFDKDGDGEINQEEFIAIMTGDI

>NP_060842.3

MSNNLRRVFLKPAEENSGNASRCVSGCMYQVVQTIGSDGKNLLQLLPIPKSSGNLIPLVQSSVMSDALKGNTGKPVQVTFQTQISSSSTSASVQLPIFQPASSSNYFLTRTVDTSEKGRVTSVGTGNFSSSVSKVQSHGVKIDGLTMQTFAVPPSTQKDSSFIVVNTQSLPVTVKSPVLPSGHHLQIPAHAEVKSVPASSLPPSVQQKILATATTSTSGMVEASQMPTVIYVSPVNTVKNVVTKNFQNIYPKPVTEIAKPVILNTTQIPKNVATETQLKGGQHSQAAPVKWIFQDNLQPFTPSLVPVKSSNNVASKILKTFVDRKNLGDNTINMPPLSTIDPSGTRSKNMPIKDNALVMFNGKVYLLAKKGTDVLPSQIDQQNSVSPDTPVRKDTLQTVSSSPVTEISREVVNIVLAKSKSSQMETKSLSNTQLASMANLRAEKNKVEKPSPSTTNPHMNQSSNYLKQSKTLFTNPIFPVGFSTGHNAPRKVTAVIYARKGSVLQSIEKISSSVDATTVTSQQCVFRDQEPKIHNEMASTSDKGAQGRNDKKDSQGRSNKALHLKSDAEFKKIFGLTKDLRVCLTRIPDHLTSGEGFDSFSSLVKSGTYKETEFMVKEGERKQQNFDKKRKAKTNKKMDHIKKRKTENAYNAIINGEANVTGSQLLSSILPTSDVSQHNILTSHSKTRQEKRTEMEYYTHEKQEKGTLNSNAAYEQSHFFNKNYTEDIFPVTPPELEETIRDEKIRRLKQVLREKEAALEEMRKKMHQK

1155 NP_277050.2 NP_057531.2

>NP_277050.2

MSWLSSSQGVVLTAYHPSGKDQTVGNSHAKAGEEATSSRRYGQYTMNQESTTIKVMEKPPFDRSISQDSLDELSMEDYWIELENIKKSSENSQEDQEVVVVKEPDEGELEEEWLKEAGLSNLFGESAGDPQESIVFLSTLTRTQAAAVQKRVETVSQTLRKKNKQYQIPDVRDIFAQQRESKETAPGGTESQSLRTNENKYQGRDDEASNLVGEEKLIPPEETPAPETDINLEVSFAEQALNQKESSKEKIQKSKGDDATLPSFRLPKDKTGTTRIGDLAPQDMKKVCHLALIELTALYDVLGIELKQQKAVKIKTKDSGLFCVPLTALLEQDQRKVPGMRIPLIFQKLISRIEERGLETEGLLRIPGAAIRIKNLCQELEAKFYEGTFNWESVKQHDAASLLKLFIRELPQPLLSVEYLKAFQAVQNLPTKKQQLQALNLLVILLPDANRDTLKALLEFLQRVIDNKEKNKMTVMNVAMVMAPNLFMCHALGLKSSEQREFVMAAGTANTMHLLIKYQKLLWTIPKFIVNQVRKQNTENHKKDKRAMKKLLKKMAYDREKYEKQDKSTNDADVPQGVIRVQAPHLSKVSMAIQLTEELKASDVLARFLSQESGVAQTLKKGEVFLYEIGGNIGERCLDDDTYMKDLYQLNPNAEWVIKSKPL

>NP_057531.2

MQQVLENLTELPSSTGAEEIDLIFLKGIMENPIVKSLAKAHERLEDSKLEAVSDNNLELVNEILEDITPLINVDENVAELVGILKEPHFQSLLEAHDIVASKCYDSPPSSPEMNNSSINNQLLPVDAIRILGIHKRAGEPLGVTFRVENNDLVIARILHGGMIDRQGLLHVGDIIKEVNGHEVGNNPKELQELLKNISGSVTLKILPSYRDTITPQQVFVKCHFDYNPYNDNLIPCKEAGLKFSKGEILQIVNREDPNWWQASHVKEGGSAGLIPSQFLEEKRKAFVRRDWDNSGPFCGTISSKKKKKMMYLTTRNAEFDRHEIQIYEEVAKMPPFQRKTLVLIGAQGVGRRSLKNRFIVLNPTRFGTTVPFTSRKPREDEKDGQAYKFVSRSEMEADIKAGKYLEHGEYEGNLYGTKIDSILEVVQTGRTCILDVNPQALKVLRTSEFMPYVVFIAAPELETLRAMHKAVVDAGITTKLLTDSDLKKTVDESARIQRAYNHYFDLIIINDNLDKAFEKLQTAIEKLRMEPQWVPISWVY

1156 NP_006697.2 NP_008855.1

>NP_006697.2

MAERGGDGGESERFNPGELRMAQQQALRFRGPAPPPNAVMRGPPPLMRPPPPFGMMRGPPPPPRPPFGRPPFDPNMPPMPPPGGIPPPMGPPHLQRPPFMPPPMSSMPPPPGMMFPPGMPPVTAPGTPALPPTEEIWVENKTPDGKVYYYNARTRESAWTKPDGVKVIQQSELTPMLAAQAQVQAQAQAQAQAQAQAQAQAQAQAQAQAQAQAQAQAQAQAQAQAQAQAQAQAQAQAQAQAQVQAQVQAQVQAQAVGASTPTTSSPAPAVSTSTSSSTPSSTTSTTTTATSVAQTVSTPTTQDQTPSSAVSVATPTVSVSTPAPTATPVQTVPQPHPQTLPPAVPHSVPQPTTAIPAFPPVMVPPFRVPLPGMPIPLPGVAMMQIVSCPYVKTVATTKTGVLPGMAPPIVPMIHPQVAIAASPATLAGATAVSEWTEYKTADGKTYYYNNRTLESTWEKPQELKEKEKLEEKIKEPIKEPSEEPLPMETEEEDPKEEPIKEIKEEPKEEEMTEEEKAAQKAKPVATAPIPGTPWCVVWTGDERVFFYNPTTRLSMWDRPDDLIGRADVDKIIQEPPHKKGMEELKKLRHPTPTMLSIQKWQFSMSAIKEEQELMEEINEDEPVKAKKRKRDDNKDIDSEKEAAMEAEIKAARERAIVPLEARMKQFKDMLLERGVSAFSTWEKELHKIVFDPRYLLLNPKERKQVFDQYVKTRAEEERREKKNKIMQAKEDFKKMMEEAKFNPRATFSEFAAKHAKDSRFKAIEKMKDREALFNEFVAAARKKEKEDSKTRGEKIKSDFFELLSNHHLDSQSRWSKVKDKVESDPRYKAVDSSSMREDLFKQYIEKIAKNLDSEKEKELERQARIEASLREREREVQKARSEQTKEIDREREQHKREEAIQNFKALLSDMVRSSDVSWSDTRRTLRKDHRWESGSLLEREEKEKLFNEHIEALTKKKREHFRQLLDETSAITLTSTWKEVKKIIKEDPRCIKFSSSDRKKQREFEEYIRDKYITAKADFRTLLKETKFITYRSKKLIQESDQHLKDVEKILQNDKRYLVLDCVPEERRKLIVAYVDDLDRRGPPPPPTASEPTRRSTK

>NP_008855.1

MSGGGVIRGPAGNNDCRIYVGNLPPDIRTKDIEDVFYKYGAIRDIDLKNRRGGPPFAFVEFEDPRDAEDAVYGRDGYDYDGYRLRVEFPRSGRGTGRGGGGGGGGGAPRGRYGPPSRRSENRVVVSGLPPSGSWQDLKDHMREAGDVCYADVYRDGTGVVEFVRKEDMTYAVRKLDNTKFRSHEGETAYIRVKVDGPRSPSYGRSRSRSRSRSRSRSRSNSRSRSYSPRRSRGSPRYSPRHSRSRSRT

1157 NP_570138.1 NP_002063.2

>NP_570138.1

METTLLFFSQINMCESKEKTFFKLIHGSGKEETSKEAKIRAKEKRNRLSLLVQKPEFHEDTRSSRSGHLAKETRVSPEEAVKWGESFDKLLSHRDGLEAFTRFLKTEFSEENIEFWIACEDFKKSKGPQQIHLKAKAIYEKFIQTDAPKEVNLDFHTKEVITNSITQPTLHSFDAAQSRVYQLMEQDSYTRFLKSDIYLDLMEGRPQRPTNLRRRSRSFTCNEFQDVQSDVAIWL

>NP_002063.2

MTLESIMACCLSEEAKEARRINDEIERQLRRDKRDARRELKLLLLGTGESGKSTFIKQMRIIHGSGYSDEDKRGFTKLVYQNIFTAMQAMIRAMDTLKIPYKYEHNKAHAQLVREVDVEKVSAFENPYVDAIKSLWNDPGIQECYDRRREYQLSDSTKYYLNDLDRVADPAYLPTQQDVLRVRVPTTGIIEYPFDLQSVIFRMVDVGGQRSERRKWIHCFENVTSIMFLVALSEYDQVLVESDNENRMEESKALFRTIITYPWFQNSSVILFLNKKDLLEEKIMYSHLVDYFPEYDGPQRDAQAAREFILKMFVDLNPDSDKIIYSHFTCATDTENIRFVFAAVKDTILQLNLKEYNLV

1158 NP_004259.3 NP_000537.3

>NP_004259.3

MSGVRAVRISIESACEKQVHEVGLDGTETYLPPLSMSQNLARLAQRIDFSQGSGSEEEEAAGTEGDAQEWPGAGSSADQDDEEGVVKFQPSLWPWDSVRNNLRSALTEMCVLYDVLSIVRDKKFMTLDPVSQDALPPKQNPQTLQLISKKKSLAGAAQILLKGAERLTKSVTENQENKLQRDFNSELLRLRQHWKLRKVGDKILGDLSYRSAGSLFPHHGTFEVIKNTDLDLDKKIPEDYCPLDVQIPSDLEGSAYIKVSIQKQAPDIGDLGTVNLFKRPLPKSKPGSPHWQTKLEAAQNVLLCKEIFAQLSREAVQIKSQVPHIVVKNQIISQPFPSLQLSISLCHSSNDKKSQKFATEKQCPEDHLYVLEHNLHLLIREFHKQTLSSIMMPHPASAPFGHKRMRLSGPQAFDKNEINSLQSSEGLLEKIIKQAKHIFLRSRAAATIDSLASRIEDPQIQAHWSNINDVYESSVKVLITSQGYEQICKSIQLQLNIGVEQIRVVHRDGRVITLSYQEQELQDFLLSQMSQHQVHAVQQLAKVMGWQVLSFSNHVGLGPIESIGNASAITVASPSGDYAISVRNGPESGSKIMVQFPRNQCKDLPKSDVLQDNKWSHLRGPFKEVQWNKMEGRNFVYKMELLMSALSPCLL

>NP_000537.3

MEEPQSDPSVEPPLSQETFSDLWKLLPENNVLSPLPSQAMDDLMLSPDDIEQWFTEDPGPDEAPRMPEAAPPVAPAPAAPTPAAPAPAPSWPLSSSVPSQKTYQGSYGFRLGFLHSGTAKSVTCTYSPALNKMFCQLAKTCPVQLWVDSTPPPGTRVRAMAIYKQSQHMTEVVRRCPHHERCSDSDGLAPPQHLIRVEGNLRVEYLDDRNTFRHSVVVPYEPPEVGSDCTTIHYNYMCNSSCMGGMNRRPILTIITLEDSSGNLLGRNSFEVRVCACPGRDRRTEEENLRKKGEPHHELPPGSTKRALPNNTSSSPQPKKKPLDGEYFTLQIRGRERFEMFRELNEALELKDAQAGKEPGGSRAHSSHLKSKKGQSTSRHKKLMFKTEGPDSD

1159 NP_005234.1 NP_062815.2

>NP_005234.1

MASTDYSTYSQAAAQQGYSAYTAQPTQGYAQTTQAYGQQSYGTYGQPTDVSYTQAQTTATYGQTAYATSYGQPPTGYTTPTAPQAYSQPVQGYGTGAYDTTTATVTTTQASYAAQSAYGTQPAYPAYGQQPAATAPTRPQDGNKPTETSQPQSSTGGYNQPSLGYGQSNYSYPQVPGSYPMQPVTAPPSYPPTSYSSTQPTSYDQSSYSQQNTYGQPSSYGQQSSYGQQSSYGQQPPTSYPPQTGSYSQAPSQYSQQSSSYGQQSSFRQDHPSSMGVYGQESGGFSGPGENRSMSGPDNRGRGRGGFDRGGMSRGGRGGGRGGMGSAGERGGFNKPGGPMDEGPDLDLGPPVDPDEDSDNSAIYVQGLNDSVTLDDLADFFKQCGVVKMNKRTGQPMIHIYLDKETGKPKGDATVSYEDPPTAKAAVEWFDGKDFQGSKLKVSLARKKPPMNSMRGGLPPREGRGMPPPLRGGPGGPGGPGGPMGRMGGRGGDRGGFPPRGPRGSRGNPSGGGNVQHRAGDWQCPNPGCGNQNFAWRTECNQCKAPKPEGFLPPPFPPPGGDRGRGGPGGMRGGRGGLMDRGGPGGMFRGGRGGDRGGFRGGRGMDRGGFGGGRRGGPGGPPGPLMEQMGGRRGGRGGPGKMDKGEHRQERRDRPY

>NP_062815.2

MGGFLPKAEGPGSQLQKLLPSFLVREQDWDQHLDKLHMLQQKRILESPLLRASKENDLSVLRQLLLDCTCDVRQRGALGETALHIAALYDNLEAALVLMEAAPELVFEPTTCEAFAGQTALHIAVVNQNVNLVRALLTRRASVSARATGTAFRRSPRNLIYFGEHPLSFAACVNSEEIVRLLIEHGADIRAQDSLGNTVLHILILQPNKTFACQMYNLLLSYDGHGDHLQPLDLVPNHQGLTPFKLAGVEGNTVMFQHLMQKRRHIQWTYGPLTSILYDLTEIDSWGEELSFLELVVSSDKREARQILEQTPVKELVSFKWNKYGRPYFCILAALYLLYMICFTTCCVYRPLKFRGGNRTHSRDITILQQKLLQEAYETREDIIRLVGELVSIVGAVIILLLEIPDIFRVGASRYFGKTILGGPFHVIIITYASLVLVTMVMRLTNTNGEVVPMSFALVLGWCSVMYFTRGFQMLGPFTIMIQKMIFGDLMRFCWLMAVVILGFASAFYIIFQTEDPTSLGQFYDYPMALFTTFELFLTVIDAPANYDVDLPFMFSIVNFAFAIIATLLMLNLFIAMMGDTHWRVAQERDELWRAQVVATTVMLERKLPRCLWPRSGICGCEFGLGDRWFLRVENHNDQNPLRVLRYVEVFKNSDKEDDQEHPSEKQPSGAESGTLARASLALPTSSLSRTASQSSSHRGWEILRQNTLGHLNLGLNLSEGDGEEVYHF

1160 NP_003248.3 NP_001661.1

>NP_003248.3

MSARAAAAKSTAMEETAIWEQHTVTLHRAPGFGFGIAISGGRDNPHFQSGETSIVISDVLKGGPAEGQLQENDRVAMVNGVSMDNVEHAFAVQQLRKSGKNAKITIRRKKKVQIPVSRPDPEPVSDNEEDSYDEEIHDPRSGRSGVVNRRSEKIWPRDRSASRERSLSPRSDRRSVASSQPAKPTKVTLVKSRKNEEYGLRLASHIFVKEISQDSLAARDGNIQEGDVVLKINGTVTENMSLTDAKTLIERSKGKLKMVVQRDERATLLNVPDLSDSIHSANASERDDISEIQSLASDHSGRSHDRPPRRSRSRSPDQRSEPSDHSRHSPQQPSNGSLRSRDEERISKPGAVSTPVKHADDHTPKTVEEVTVERNEKQTPSLPEPKPVYAQVGQPDVDLPVSPSDGVLPNSTHEDGILRPSMKLVKFRKGDSVGLRLAGGNDVGIFVAGVLEDSPAAKEGLEEGDQILRVNNVDFTNIIREEAVLFLLDLPKGEEVTILAQKKKDVYRRIVESDVGDSFYIRTHFEYEKESPYGLSFNKGEVFRVVDTLYNGKLGSWLAIRIGKNHKEVERGIIPNKNRAEQLASVQYTLPKTAGGDRADFWRFRGLRSSKRNLRKSREDLSAQPVQTKFPAYERVVLREAGFLRPVTIFGPIADVAREKLAREEPDIYQIAKSEPRDAGTDQRSSGIIRLHTIKQIIDQDKHALLDVTPNAVDRLNYAQWYPIVVFLNPDSKQGVKTMRMRLCPESRKSARKLYERSHKLRKNNHHLFTTTINLNSMNDGWYGALKEAIQQQQNQLVWVSEGKADGATSDDLDLHDDRLSYLSAPGSEYSMYSTDSRHTSDYEDTDTEGGAYTDQELDETLNDEVGTPPESAITRSSEPVREDSSGMHHENQTYPPYSPQAQPQPIHRIDSPGFKPASQQKAEASSPVPYLSPETNPASSTSAVNHNVNLTNVRLEEPTPAPSTSYSPQADSLRTPSTEAAHIMLRDQEPSLSSHVDPTKVYRKDPYPEEMMRQNHVLKQPAVSHPGHRPDKEPNLTYEPQLPYVEKQASRDLEQPTYRYESSSYTDQFSRNYEHRLRYEDRVPMYEEQWSYYDDKQPYPSRPPFDNQHSQDLDSRQHPEESSERGYFPRFEEPAPLSYDSRPRYEQAPRASALRHEEQPAPGYDTHGRLRPEAQPHPSAGPKPAESKQYFEQYSRSYEQVPPQGFTSRAGHFEPLHGAAAVPPLIPSSQHKPEALPSNTKPLPPPPTQTEEEEDPAMKPQSVLTRVKMFENKRSASLETKKDVNDTGSFKPPEVASKPSGAPIIGPKPTSQNQFSEHDKTLYRIPEPQKPQLKPPEDIVRSNHYDPEEDEEYYRKQLSYFDRRSFENKPPAHIAASHLSEPAKPAHSQNQSNFSSYSSKGKPPEADGVDRSFGEKRYEPIQATPPPPPLPSQYAQPSQPVTSASLHIHSKGAHGEGNSVSLDFQNSLVSKPDPPPSQNKPATFRPPNREDTAQAAFYPQKSFPDKAPVNGTEQTQKTVTPAYNRFTPKPYTSSARPFERKFESPKFNHNLLPSETAHKPDLSSKTPTSPKTLVKSHSLAQPPEFDSGVETFSIHAEKPKYQINNISTVPKAIPVSPSAVEEDEDEDGHTVVATARGIFNSNGGVLSSIETGVSIIIPQGAIPEGVEQEIYFKVCRDNSILPPLDKEKGETLLSPLVMCGPHGLKFLKPVELRLPHCDPKTWQNKCLPGDPNYLVGANCVSVLIDHF

>NP_001661.1

MEDCNVHSAASILASVKEQEARFERLTRALEQERRHVALQLERAQQPGMVSGGMGSGQPLPMAWQQLVLQEQSPGSQASLATMPEAPDVLEETVTVEEDPGTPTSHVSIVTSEDGTTRRTETKVTKTVKTVTTRTVRQVPVGPDGLPLLDGGPPLGPFADGALDRHFLLRGGGPVATLSRAYLSSGGGFPEGPEPRDSPSYGSLSRGLGMRPPRAGPLGPGPGDGCFTLPGHREAFPVGPEPGPPGGRSLPERFQAEPYGLEDDTRSLAADDEGGPELEPDYGTATRRRPECGRGLHTRAYEDTADDGGELADERPAFPMVTAPLAQPERGSMGSLDRLVRRSPSVDSARKEPRWRDPELPEVLAMLRHPVDPVKANAAAYLQHLCFENEGVKRRVRQLRGLPLLVALLDHPRAEVRRRACGALRNLSYGRDTDNKAAIRDCGGVPALVRLLRAARDNEVRELVTGTLWNLSSYEPLKMVIIDHGLQTLTHEVIVPHSGWEREPNEDSKPRDAEWTTVFKNTSGCLRNVSSDGAEARRRLRECEGLVDALLHALQSAVGRKDTDNKSVENCVCIMRNLSYHVHKEVPGADRYQEAEPGPLGSAVGSQRRRRDDASCFGGKKAKEEWFHQGKKDGEMDRNFDTLDLPKRTEAAKGFELLYQPEVVRLYLSLLTESRNFNTLEAAAGALQNLSAGNWMWATYIRATVRKERGLPVLVELLQSETDKVVRAVAIALRNLSLDRRNKDLIGSYAMAELVRNVRNAQAPPRPGACLEEDTVVAVLNTIHEIVSDSLDNARSLLQARGVPALVALVASSQSVREAKAASHVLQTVWSYKELRGTLQKDGWTKARFQSAAATAKGPKGALSPGGFDDSTLPLVDKSLEGEKTGSRDVIPMDALGPDGYSTVDRRERRPRGASSAGEASEKEPLKLDPSRKAPPPGPSRPAVRLVDAVGDAKPQPVDSWV

1161 AAK69431.1 NP_899236.1

>AAK69431.1

MTTKRSLFVRLVPCRCLRGEEETVTTLDYSHCSLEQVPKEIFTFEKTLEELYLDANQIEELPKQLFNCQSLHKLSLPDNDLTTLPASIANLINLRELDVSKNGIQEFPENIKNCKVLTIVEASVNPISKLPDGFSQLLNLTQLYLNDAFLEFLPANFGRLTKLQILELRENQLKMLPKTMNRLTQLERLDLGSNEFTEVPEVLEQLSGLKEFWMDANRLTFIPGFIGSLKQLTYLDVSKNNIEMVEEGISTCENLQDLLLSSNSLQQLPETIGSLKNITTLKIDENQLMYLPDSIGGLISVEELDCSFNEVEALPSSIGQLTNLRTFAADHNYLQQLPPEIGSWKNITVLFLHSNKLETLPEEMGDMQKLKVINLSDNRLKNLPFSFTKLQQLTAMWLSDNQSKPLIPLQKETDSETQKMVLTNYMFPQQPRTEDVMFISDNESFNPSLWEEQRKQRAQVAFECDEDKDEREAPPREGNLKRYPTPYPDELKNMVKTVQTIVHRLKDEETNEDSGRDLKPHEDQQDINKDVGVKTSESTTTVKSKVDEREKYMIGNSVQKISEPEAEISPGSLPVTANMKASENLKHIVNHDDVFEESEELSSDEEMKMAEMRPPLIETSINQPKVVALSNNKKDDTKETDSLSDEVTHNSNQNNSNCSSPSRMSDSVSLNTDSSQDTSLCSPVKQTHIDINSKIRQEDENFNSLLQNGDILNSSTEEKFKAHDKKDFNLPEYDLNVEERLVLIEKSVDSTATADDTHKLDHINMNLNKLITNDTFQPEIMERSKTQDIVLGTSFLSINSKEETEHLENGNKCPNLESVNKVNGHSEETSQSPNRTEPHDSDCSVDLGISKSTEDLSPQKSGPVGSVVKSHSITNMEIGGLKIYDILSDNGPQQPSTTVKITSAVDGKNIVRSKSATLLYDQPLQVFTGSSSSSDLISGTKAIFKFDSNHNPEEPNIIRGPTSGPQSAPQIYGPPQYNIQYSSSAAVKDTLWHSKQNPQIDHASFPPQLLPRSESTENQSYAKHSANMNFSNHNNVRANTAYHLHQRLGPARHGEMWAISPNDRLIPAVTRSTIQRQSSVSSTASVNLGDPGSTRRAQIPEGDYLSYREFHSAGRTPPMMPGSQRPLSARTYSIDGPNASRPQSARPSINEIPERTMSVSDFNYSRTSPSKRPNARVGSEHSLLDPPGKSKVPRDWREQVLRHIEAKKLEKKHPQTSSSGDPCQDGIFISGQQNYSSATLSHKDVPPDSLMKMPLSNGQMGQPLRPQANYSQIHHPPQASVARHPSREQLIDYLMLKVAHQPPYTQPHCSPRQGHELAKQEIRVRVEKDPELGFSISGGVGGRGNPFRPDDDGIFVTRVQPEGPASKLLQPGDKIIQANGYSFINIEHGQAVSLLKTFQNTVELIIVREVSS

>NP_899236.1

MAGYLSPAAYLYVEEQEYLQAYEDVLERYKDERDKVQKKTFTKWINQHLMKVRKHVNDLYEDLRDGHNLISLLEVLSGDTLPREKGRMRFHRLQNVQIALDYLKRRQVKLVNIRNDDITDGNPKLTLGLIWTIILHFQISDIHVTGESEDMSAKERLLLWTQQATEGYAGIRCENFTTCWRDGKLFNAIIHKYRPDLIDMNTVAVQSNLANLEHAFYVAEKIGVIRLLDPEDVDVSSPDEKSVITYVSSLYDAFPKVPEGGEGIGANDVEVKWIEYQNMVNYLIQWIRHHVTTMSERTFPNNPVELKALYNQYLQFKETEIPPKETEKSKIKRLYKLLEIWIEFGRIKLLQGYHPNDIEKEWGKLIIAMLEREKALRPEVERLEMLQQIANRVQRDSVICEDKLILAGNALQSDSKRLESGVQFQNEAEIAGYILECENLLRQHVIDVQILIDGKYYQADQLVQRVAKLRDEIMALRNECSSVYSKGRILTTEQTKLMISGITQSLNSGFAQTLHPSLTSGLTQSLTPSLTSSSMTSGLSSGMTSRLTPSVTPAYTPGFPSGLVPNFSSGVEPNSLQTLKLMQIRKPLLKSSLLDQNLTEEEINMKFVQDLLNWVDEMQVQLDRTEWGSDLPSVESHLENHKNVHRAIEEFESSLKEAKISEIQMTAPLKLTYAEKLHRLESQYAKLLNTSRNQERHLDTLHNFVSRATNELIWLNEKEEEEVAYDWSERNTNIARKKDYHAELMRELDQKEENIKSVQEIAEQLLLENHPARLTIEAYRAAMQTQWSWILQLCQCVEQHIKENTAYFEFFNDAKEATDYLRNLKDAIQRKYSCDRSSSIHKLEDLVQESMEEKEELLQYKSTIANLMGKAKTIIQLKPRNSDCPLKTSIPIKAICDYRQIEITIYKDDECVLANNSHRAKWKVISPTGNEAMVPSVCFTVPPPNKEAVDLANRIEQQYQNVLTLWHESHINMKSVVSWHYLINEIDRIRASNVASIKTMLPGEHQQVLSNLQSRFEDFLEDSQESQVFSGSDITQLEKEVNVCKQYYQELLKSAEREEQEESVYNLYISEVRNIRLRLENCEDRLIRQIRTPLERDDLHESVFRITEQEKLKKELERLKDDLGTITNKCEEFFSQAAASSSVPTLRSELNVVLQNMNQVYSMSSTYIDKLKTVNLVLKNTQAAEALVKLYETKLCEEEAVIADKNNIENLISTLKQWRSEVDEKRQVFHALEDELQKAKAISDEMFKTYKERDLDFDWHKEKADQLVERWQNVHVQIDNRLRDLEGIGKSLKYYRDTYHPLDDWIQQVETTQRKIQENQPENSKTLATQLNQQKMLVSEIEMKQSKMDECQKYAEQYSATVKDYELQTMTYRAMVDSQQKSPVKRRRMQSSADLIIQEFMDLRTRYTALVTLMTQYIKFAGDSLKRLEEEEKSLEEEKKEHVEKAKELQKWVSNISKTLKDAEKAGKPPFSKQKISSEEISTKKEQLSEALQTIQLFLAKHGDKMTDEERNELEKQVKTLQESYNLLFSESLKQLQESQTSGDVKVEEKIVAERQQEYKEKLQGICDLLTQTENRLIGHQEAFMIGDGTVELKKYQSKQEELQKDMQGSAQALAEVVKNTENFLKENGEKLSQEDKALIEQKLNEAKIKCEQLNLKAEQSKKELDKVVTTAIKEETEKVAAVKQLEESKTKIENLLDWLSNVDKDSERAGTKHKQVIEQNGTHFQEGDGKSAIGEEDEVNGNLLETDVDGQVGTTQENLNQQYQKVKAQHEKIISQHQAVIIATQSAQVLLEKQGQYLSPEEKEKLQKNMKELKVHYETALAESEKKMKLTHSLQEELEKFDADYTEFEHWLQQSEQELENLEAGADDINGLMTKLKRQKSFSEDVISHKGDLRYITISGNRVLEAAKSCSKRDGGKVDTSATHREVQRKLDHATDRFRSLYSKCNVLGNNLKDLVDKYQHYEDASCGLLAGLQACEATASKHLSEPIAVDPKNLQRQLEETKALQGQISSQQVAVEKLKKTAEVLLDARGSLLPAKNDIQKTLDDIVGRYEDLSKSVNERNEKLQITLTRSLSVQDGLDEMLDWMGNVESSLKEQGQVPLNSTALQDIISKNIMLEQDIAGRQSSINAMNEKVKKFMETTDPSTASSLQAKMKDLSARFSEASHKHKETLAKMEELKTKVELFENLSEKLQTFLETKTQALTEVDVPGKDVTELSQYMQESTSEFLEHKKHLEVLHSLLKEISSHGLPSDKALVLEKTNNLSKKFKEMEDTIKEKKEAVTSCQEQLDAFQVLVKSLKSWIKETTKKVPIVQPSFGAEDLGKSLEDTKKLQEKWSLKTPEIQKVNNSGISLCNLISAVTTPAKAIAAVKSGGAVLNGEGTATNTEEFWANKGLTSIKKDMTDISHGYEDLGLLLKDKIAELNTKLSKLQKAQEESSAMMQWLQKMNKTATKWQQTPAPTDTEAVKTQVEQNKSFEAELKQNVNKVQELKDKLTELLEENPDTPEAPRWKQMLTEIDSKWQELNQLTIDRQQKLEESSNNLTQFQTVEAQLKQWLVEKELMVSVLGPLSIDPNMLNTQRQQVQILLQEFATRKPQYEQLTAAGQGILSRPGEDPSLRGIVKEQLAAVTQKWDSLTGQLSDRCDWIDQAIVKSTQYQSLLRSLSDKLSDLDNKLSSSLAVSTHPDAMNQQLETAQKMKQEIQQEKKQIKVAQALCEDLSALVKEEYLKAELSRQLEGILKSFKDVEQKAENHVQHLQSACASSHQFQQMSRDFQAWLDTKKEEQNKSHPISAKLDVLESLIKDHKDFSKTLTAQSHMYEKTIAEGENLLLKTQGSEKAALQLQLNTIKTNWDTFNKQVKERENKLKESLEKALKYKEQVETLWPWIDKCQNNLEEIKFCLDPAEGENSIAKLKSLQKEMDQHFGMVELLNNTANSLLSVCEIDKEVVTDENKSLIQKVDMVTEQLHSKKFCLENMTQKFKEFQEVSKESKRQLQCAKEQLDIHDSLGSQAYSNKYLTMLQTQQKSLQALKHQVDLAKRLAQDLVVEASDSKGTSDVLLQVETIAQEHSTLSQQVDEKCSFLETKLQGIGHFQNTIREMFSQFAEFDDELDSMAPVGRDAETLQKQKETIKAFLKKLEALMASNDNANKTCKMMLATEETSPDLVGIKRDLEALSKQCNKLLDRAQAREEQVEGTIKRLEEFYSKLKEFSILLQKAEEHEESQGPVGMETETINQQLNMFKVFQKEEIEPLQGKQQDVNWLGQGLIQSAAKSTSTQGLEHDLDDVNARWKTLNKKVAQRAAQLQEALLHCGRFQDALESLLSWMVDTEELVANQKPPSAEFKVVKAQIQEQKLLQRLLDDRKSTVEVIKREGEKIATTAEPADKVKILKQLSLLDSRWEALLNKAETRNRQLEGISVVAQQFHETLEPLNEWLTTIEKRLVNCEPIGTQASKLEEQIAQHKALEDDIINHNKHLHQAVSIGQSLKVLSSREDKDMVQSKLDFSQVWYIEIQEKSHSRSELLQQALCNAKIFGEDEVELMNWLNEVHDKLSKLSVQDYSTEGLWKQQSELRVLQEDILLRKQNVDQALLNGLELLKQTTGDEVLIIQDKLEAIKARYKDITKLSTDVAKTLEQALQLARRLHSTHEELCTWLDKVEVELLSYETQVLKGEEASQAQMRPKELKKEAKNNKALLDSLNEVSSALLELVPWRAREGLEKMVAEDNERYRLVSDTITQKVEEIDAAILRSQQFDQAADAELSWITETEKKLMSLGDIRLEQDQTSAQLQVQKTFTMEILRHKDIIDDLVKSGHKIMTACSEEEKQSMKKKLDKVLKNYDTICQINSERYLQLERAQSLVNQFWETYEELWPWLTETQSIISQLPAPALEYETLRQQQEEHRQLRELIAEHKPHIDKMNKTGPQLLELSPGEGFSIQEKYVAADTLYSQIKEDVKKRAVALDEAISQSTQFHDKIDQILESLERIVERLRQPPSISAEVEKIKEQISENKNVSVDMEKLQPLYETLKQRGEEMIARSGGTDKDISAKAVQDKLDQMVFIWENIHTLVEEREAKLLDVMELAEKFWCDHMSLIVTIKDTQDFIRDLEDPGIDPSVVKQQQEAAETIREEIDGLQEELDIVINLGSELIAACGEPDKPIVKKSIDELNSAWDSLNKAWKDRIDKLEEAMQAAVQYQDGLQAVFDWVDIAGGKLASMSPIGTDLETVKQQIEELKQFKSEAYQQQIEMERLNHQAELLLKKVTEESDKHTVQDPLMELKLIWDSLEERIINRQHKLEGALLALGQFQHALDELLAWLTHTEGLLSEQKPVGGDPKAIEIELAKHHVLQNDVLAHQSTVEAVNKAGNDLIESSAGEEASNLQNKLEVLNQRWQNVLEKTEQRKQQLDGALRQAKGFHGEIEDLQQWLTDTERHLLASKPLGGLPETAKEQLNVHMEVCAAFEAKEETYKSLMQKGQQMLARCPKSAETNIDQDINNLKEKWESVETKLNERKTKLEEALNLAMEFHNSLQDFINWLTQAEQTLNVASRPSLILDTVLFQIDEHKVFANEVNSHREQIIELDKTGTHLKYFSQKQDVVLIKNLLISVQSRWEKVVQRLVERGRSLDDARKRAKQFHEAWSKLMEWLEESEKSLDSELEIANDPDKIKTQLAQHKEFQKSLGAKHSVYDTTNRTGRSLKEKTSLADDNLKLDDMLSELRDKWDTICGKSVERQNKLEEALLFSGQFTDALQALIDWLYRVEPQLAEDQPVHGDIDLVMNLIDNHKAFQKELGKRTSSVQALKRSARELIEGSRDDSSWVKVQMQELSTRWETVCALSISKQTRLEAALRQAEEFHSVVHALLEWLAEAEQTLRFHGVLPDDEDALRTLIDQHKEFMKKLEEKRAELNKATTMGDTVLAICHPDSITTIKHWITIIRARFEEVLAWAKQHQQRLASALAGLIAKQELLEALLAWLQWAETTLTDKDKEVIPQEIEEVKALIAEHQTFMEEMTRKQPDVDKVTKTYKRRAADPSSLQSHIPVLDKGRAGRKRFPASSLYPSGSQTQIETKNPRVNLLVSKWQQVWLLALERRRKLNDALDRLEELREFANFDFDIWRKKYMRWMNHKKSRVMDFFRRIDKDQDGKITRQEFIDGILSSKFPTSRLEMSAVADIFDRDGDGYIDYYEFVAALHPNKDAYKPITDADKIEDEVTRQVAKCKCAKRFQVEQIGDNKYRFFLGNQFGDSQQLRLVRILRSTVMVRVGGGWMALDEFLVKNDPCRAKGRTNMELREKFILADGASQGMAAFRPRGRRSRPSSRGASPNRSTSVSSQAAQAASPQVPATTTPKILHPLTRNYGKPWLTNSKMSTPCKAAECSDFPVPSAEGTPIQGSKLRLPGYLSGKGFHSGEDSGLITTAAARVRTQFADSKKTPSRPGSRAGSKAGSRASSRRGSDASDFDISEIQSVCSDVETVPQTHRPTPRAGSRPSTAKPSKIPTPQRKSPASKLDKSSKR

1162 NP_076944.1 NP_006092.1

>NP_076944.1

MSVDPMTYEAQFFGFTPQTCMLRIYIAFQDYLFEVMQAVEQVILKKLDGIPDCDISPVQIRKCTEKFLCFMKGHFDNLFSKMEQLFLQLILRIPSNILLPEDKCKETPYSEEDFQHLQKEIEQLQEKYKTELCTKQALLAELEEQKIVQAKLKQTLTFFDELHNVGRDHGTSDFRESLVSLVQNSRKLQNIRDNVEKESKRLKIS

>NP_006092.1

MKRSSVSSGGAGRLSMQELRSQDVNKQGLYTPQTKEKPTFGKLSINKPTSERKVSLFGKRTSGHGSRNSQLGIFSSSEKIKDPRPLNDKAFIQQCIRQLCEFLTENGYAHNVSMKSLQAPSVKDFLKIFTFLYGFLCPSYELPDTKFEEEVPRIFKDLGYPFALSKSSMYTVGAPHTWPHIVAALVWLIDCIKIHTAMKESSPLFDDGQPWGEETEDGIMHNKLFLDYTIKCYESFMSGADSFDEMNAELQSKLKDLFNVDAFKLESLEAKNRALNEQIARLEQEREKEPNRLESLRKLKASLQGDVQKYQAYMSNLESHSAILDQKLNGLNEEIARVELECETIKQENTRLQNIIDNQKYSVADIERINHERNELQQTINKLTKDLEAEQQKLWNEELKYARGKEAIETQLAEYHKLARKLKLIPKGAENSKGYDFEIKFNPEAGANCLVKYRAQVYVPLKELLNETEEEINKALNKKMGLEDTLEQLNAMITESKRSVRTLKEEVQKLDDLYQQKIKEAEEEDEKCASELESLEKHKHLLESTVNQGLSEAMNELDAVQREYQLVVQTTTEERRKVGNNLQRLLEMVATHVGSVEKHLEEQIAKVDREYEECMSEDLSENIKEIRDKYEKKATLIKSSEE

1163 NP_004776.3 NP_003051.1

>NP_004776.3

MAAPEPLRPRLCRLVRGEQGYGFHLHGEKGRRGQFIRRVEPGSPAEAAALRAGDRLVEVNGVNVEGETHHQVVQRIKAVEGQTRLLVVDQETDEELRRRQLTCTEEMAQRGLPPAHDPWEPKPDWAHTGSHSSEAGKKDVSGPLRELRPRLCHLRKGPQGYGFNLHSDKSRPGQYIRSVDPGSPAARSGLRAQDRLIEVNGQNVEGLRHAEVVASIKAREDEARLLVVDPETDEHFKRLRVTPTEEHVEGPLPSPVTNGTSPAQLNGGSACSSRSDLPGSDKDTEESGLHLSPTAAEAKEKARAMRVNKRAPQMDWNRKREIFSNF

>NP_003051.1

MRDYDEVTAFLGEWGPFQRLIFFLLSASIIPNGFTGLSSVFLIATPEHRCRVPDAANLSSAWRNHTVPLRLRDGREVPHSCRRYRLATIANFSALGLEPGRDVDLGQLEQESCLDGWEFSQDVYLSTIVTEWNLVCEDDWKAPLTISLFFVGVLLGSFISGQLSDRFGRKNVLFVTMGMQTGFSFLQIFSKNFEMFVVLFVLVGMGQISNYVAAFVLGTEILGKSVRIIFSTLGVCIFYAFGYMVLPLFAYFIRDWRMLLVALTMPGVLCVALWWFIPESPRWLISQGRFEEAEVIIRKAAKANGIVVPSTIFDPSELQDLSSKKQQSHNILDLLRTWNIRMVTIMSIMLWMTISVGYFGLSLDTPNLHGDIFVNCFLSAMVEVPAYVLAWLLLQYLPRRYSMATALFLGGSVLLFMQLVPPDLYYLATVLVMVGKFGVTAAFSMVYVYTAELYPTVVRNMGVGVSSTASRLGSILSPYFVYLGAYDRFLPYILMGSLTILTAILTLFLPESFGTPLPDTIDQMLRVKGMKHRKTPSHTRMLKDGQERPTILKSTAF

1164 NP_004371.2 NP_004022.2

>NP_004371.2

MAENLLDGPPNPKRAKLSSPGFSANDSTDFGSLFDLENDLPDELIPNGGELGLLNSGNLVPDAASKHKQLSELLRGGSGSSINPGIGNVSASSPVQQGLGGQAQGQPNSANMASLSAMGKSPLSQGDSSAPSLPKQAASTSGPTPAASQALNPQAQKQVGLATSSPATSQTGPGICMNANFNQTHPGLLNSNSGHSLINQASQGQAQVMNGSLGAAGRGRGAGMPYPTPAMQGASSSVLAETLTQVSPQMTGHAGLNTAQAGGMAKMGITGNTSPFGQPFSQAGGQPMGATGVNPQLASKQSMVNSLPTFPTDIKNTSVTNVPNMSQMQTSVGIVPTQAIATGPTADPEKRKLIQQQLVLLLHAHKCQRREQANGEVRACSLPHCRTMKNVLNHMTHCQAGKACQVAHCASSRQIISHWKNCTRHDCPVCLPLKNASDKRNQQTILGSPASGIQNTIGSVGTGQQNATSLSNPNPIDPSSMQRAYAALGLPYMNQPQTQLQPQVPGQQPAQPQTHQQMRTLNPLGNNPMNIPAGGITTDQQPPNLISESALPTSLGATNPLMNDGSNSGNIGTLSTIPTAAPPSSTGVRKGWHEHVTQDLRSHLVHKLVQAIFPTPDPAALKDRRMENLVAYAKKVEGDMYESANSRDEYYHLLAEKIYKIQKELEEKRRSRLHKQGILGNQPALPAPGAQPPVIPQAQPVRPPNGPLSLPVNRMQVSQGMNSFNPMSLGNVQLPQAPMGPRAASPMNHSVQMNSMGSVPGMAISPSRMPQPPNMMGAHTNNMMAQAPAQSQFLPQNQFPSSSGAMSVGMGQPPAQTGVSQGQVPGAALPNPLNMLGPQASQLPCPPVTQSPLHPTPPPASTAAGMPSLQHTTPPGMTPPQPAAPTQPSTPVSSSGQTPTPTPGSVPSATQTQSTPTVQAAAQAQVTPQPQTPVQPPSVATPQSSQQQPTPVHAQPPGTPLSQAAASIDNRVPTPSSVASAETNSQQPGPDVPVLEMKTETQAEDTEPDPGESKGEPRSEMMEEDLQGASQVKEETDIAEQKSEPMEVDEKKPEVKVEVKEEEESSSNGTASQSTSPSQPRKKIFKPEELRQALMPTLEALYRQDPESLPFRQPVDPQLLGIPDYFDIVKNPMDLSTIKRKLDTGQYQEPWQYVDDVWLMFNNAWLYNRKTSRVYKFCSKLAEVFEQEIDPVMQSLGYCCGRKYEFSPQTLCCYGKQLCTIPRDAAYYSYQNRYHFCEKCFTEIQGENVTLGDDPSQPQTTISKDQFEKKKNDTLDPEPFVDCKECGRKMHQICVLHYDIIWPSGFVCDNCLKKTGRPRKENKFSAKRLQTTRLGNHLEDRVNKFLRRQNHPEAGEVFVRVVASSDKTVEVKPGMKSRFVDSGEMSESFPYRTKALFAFEEIDGVDVCFFGMHVQEYGSDCPPPNTRRVYISYLDSIHFFRPRCLRTAVYHEILIGYLEYVKKLGYVTGHIWACPPSEGDDYIFHCHPPDQKIPKPKRLQEWYKKMLDKAFAERIIHDYKDIFKQATEDRLTSAKELPYFEGDFWPNVLEESIKELEQEEEERKKEESTAASETTEGSQGDSKNAKKKNNKKTNKNKSSISRANKKKPSMPNVSNDLSQKLYATMEKHKEVFFVIHLHAGPVINTLPPIVDPDPLLSCDLMDGRDAFLTLARDKHWEFSSLRRSKWSTLCMLVELHTQGQDRFVYTCNECKHHVETRWHCTVCEDYDLCINCYNTKSHAHKMVKWGLGLDDEGSSQGEPQSKSPQESRRLSIQRCIQSLVHACQCRNANCSLPSCQKMKRVVQHTKGCKRKTNGGCPVCKQLIALCCYHAKHCQENKCPVPFCLNIKHKLRQQQIQHRLQQAQLMRRRMATMNTRNVPQQSLPSPTSAPPGTPTQQPSTPQTPQPPAQPQPSPVSMSPAGFPSVARTQPPTTVSTGKPTSQVPAPPPPAQPPPAAVEAARQIEREAQQQQHLYRVNINNSMPPGRTGMGTPGSQMAPVSLNVPRPNQVSGPVMPSMPPGQWQQAPLPQQQPMPGLPRPVISMQAQAAVAGPRMPSVQPPRSISPSALQDLLRTLKSPSSPQQQQQVLNILKSNPQLMAAFIKQRTAKYVANQPGMQPQPGLQSQPGMQPQPGMHQQPSLQNLNAMQAGVPRPGVPPQQQAMGGLNPQGQALNIMNPGHNPNMASMNPQYREMLRRQLLQQQQQQQQQQQQQQQQQQGSAGMAGGMAGHGQFQQPQGPGGYPPAMQQQQRMQQHLPLQGSSMGQMAAQMGQLGQMGQPGLGADSTPNIQQALQQRILQQQQMKQQIGSPGQPNPMSPQQHMLSGQPQASHLPGQQIATSLSNQVRSPAPVQSPRPQSQPPHSSPSPRIQPQPSPHHVSPQTGSPHPGLAVTMASSIDQGHLGNPEQSAMLPQLNTPSRSALSSELSLVGDTTGDTLEKFVEGL

>NP_004022.2

MPVPERPAAGPDSPRPGTRRAAPRVLFGEWLLGEISSGCYEGLQWLDEARTCFRVPWKHFARKDLSEADARIFKAWAVARGRWPPSSRGGGPPPEAETAERAGWKTNFRCALRSTRRFVMLRDNSGDPADPHKVYALSRELCWREGPGTDQTEAEAPAAVPPPQGGPPGPFLAHTHAGLQAPGPLPAPAGDKGDLLLQAVQQSCLADHLLTASWGADPVPTKAPGEGQEGLPLTGACAGGPGLPAGELYGWAVETTPSPGPQPAALTTGEAAAPESPHQAEPYLSPSPSACTAVQEPSPGALDVTIMYKGRTVLQKVVGHPSCTFLYGPPDPAVRATDPQQVAFPSPAELPDQKQLRYTEELLRHVAPGLHLELRGPQLWARRMGKCKVYWEVGGPPGSASPSTPACLLPRNCDTPIFDFRVFFQELVEFRARQRRGSPRYTIYLGFGQDLSAGRPKEKSLVLVKLEPWLCRVHLEGTQREGVSSLDSSSLSLCLSSANSLYDDIECFLMELEQPA

1165 NP_002084.2 NP_077719.2

>NP_002084.2

MSGRPRTTSFAESCKPVQQPSAFGSMKVSRDKDGSKVTTVVATPGQGPDRPQEVSYTDTKVIGNGSFGVVYQAKLCDSGELVAIKKVLQDKRFKNRELQIMRKLDHCNIVRLRYFFYSSGEKKDEVYLNLVLDYVPETVYRVARHYSRAKQTLPVIYVKLYMYQLFRSLAYIHSFGICHRDIKPQNLLLDPDTAVLKLCDFGSAKQLVRGEPNVSYICSRYYRAPELIFGATDYTSSIDVWSAGCVLAELLLGQPIFPGDSGVDQLVEIIKVLGTPTREQIREMNPNYTEFKFPQIKAHPWTKDSSGTGHFTSGVRVFRPRTPPEAIALCSRLLEYTPTARLTPLEACAHSFFDELRDPNVKLPNGRDTPALFNFTTQELSSNPPLATILIPPHARIQAAASTPTNATAASDANTGDRGQTNNAASASASNST

>NP_077719.2

MPALRPALLWALLALWLCCAAPAHALQCRDGYEPCVNEGMCVTYHNGTGYCKCPEGFLGEYCQHRDPCEKNRCQNGGTCVAQAMLGKATCRCASGFTGEDCQYSTSHPCFVSRPCLNGGTCHMLSRDTYECTCQVGFTGKECQWTDACLSHPCANGSTCTTVANQFSCKCLTGFTGQKCETDVNECDIPGHCQHGGTCLNLPGSYQCQCPQGFTGQYCDSLYVPCAPSPCVNGGTCRQTGDFTFECNCLPGFEGSTCERNIDDCPNHRCQNGGVCVDGVNTYNCRCPPQWTGQFCTEDVDECLLQPNACQNGGTCANRNGGYGCVCVNGWSGDDCSENIDDCAFASCTPGSTCIDRVASFSCMCPEGKAGLLCHLDDACISNPCHKGALCDTNPLNGQYICTCPQGYKGADCTEDVDECAMANSNPCEHAGKCVNTDGAFHCECLKGYAGPRCEMDINECHSDPCQNDATCLDKIGGFTCLCMPGFKGVHCELEINECQSNPCVNNGQCVDKVNRFQCLCPPGFTGPVCQIDIDDCSSTPCLNGAKCIDHPNGYECQCATGFTGVLCEENIDNCDPDPCHHGQCQDGIDSYTCICNPGYMGAICSDQIDECYSSPCLNDGRCIDLVNGYQCNCQPGTSGVNCEINFDDCASNPCIHGICMDGINRYSCVCSPGFTGQRCNIDIDECASNPCRKGATCINGVNGFRCICPEGPHHPSCYSQVNECLSNPCIHGNCTGGLSGYKCLCDAGWVGINCEVDKNECLSNPCQNGGTCDNLVNGYRCTCKKGFKGYNCQVNIDECASNPCLNQGTCFDDISGYTCHCVLPYTGKNCQTVLAPCSPNPCENAAVCKESPNFESYTCLCAPGWQGQRCTIDIDECISKPCMNHGLCHNTQGSYMCECPPGFSGMDCEEDIDDCLANPCQNGGSCMDGVNTFSCLCLPGFTGDKCQTDMNECLSEPCKNGGTCSDYVNSYTCKCQAGFDGVHCENNINECTESSCFNGGTCVDGINSFSCLCPVGFTGSFCLHEINECSSHPCLNEGTCVDGLGTYRCSCPLGYTGKNCQTLVNLCSRSPCKNKGTCVQKKAESQCLCPSGWAGAYCDVPNVSCDIAASRRGVLVEHLCQHSGVCINAGNTHYCQCPLGYTGSYCEEQLDECASNPCQHGATCSDFIGGYRCECVPGYQGVNCEYEVDECQNQPCQNGGTCIDLVNHFKCSCPPGTRGLLCEENIDDCARGPHCLNGGQCMDRIGGYSCRCLPGFAGERCEGDINECLSNPCSSEGSLDCIQLTNDYLCVCRSAFTGRHCETFVDVCPQMPCLNGGTCAVASNMPDGFICRCPPGFSGARCQSSCGQVKCRKGEQCVHTASGPRCFCPSPRDCESGCASSPCQHGGSCHPQRQPPYYSCQCAPPFSGSRCELYTAPPSTPPATCLSQYCADKARDGVCDEACNSHACQWDGGDCSLTMENPWANCSSPLPCWDYINNQCDELCNTVECLFDNFECQGNSKTCKYDKYCADHFKDNHCDQGCNSEECGWDGLDCAADQPENLAEGTLVIVVLMPPEQLLQDARSFLRALGTLLHTNLRIKRDSQGELMVYPYYGEKSAAMKKQRMTRRSLPGEQEQEVAGSKVFLEIDNRQCVQDSDHCFKNTDAAAALLASHAIQGTLSYPLVSVVSESLTPERTQLLYLLAVAVVIILFIILLGVIMAKRKRKHGSLWLPEGFTLRRDASNHKRREPVGQDAVGLKNLSVQVSEANLIGTGTSEHWVDDEGPQPKKVKAEDEALLSEEDDPIDRRPWTQQHLEAADIRRTPSLALTPPQAEQEVDVLDVNVRGPDGCTPLMLASLRGGSSDLSDEDEDAEDSSANIITDLVYQGASLQAQTDRTGEMALHLAARYSRADAAKRLLDAGADANAQDNMGRCPLHAAVAADAQGVFQILIRNRVTDLDARMNDGTTPLILAARLAVEGMVAELINCQADVNAVDDHGKSALHWAAAVNNVEATLLLLKNGANRDMQDNKEETPLFLAAREGSYEAAKILLDHFANRDITDHMDRLPRDVARDRMHHDIVRLLDEYNVTPSPPGTVLTSALSPVICGPNRSFLSLKHTPMGKKSRRPSAKSTMPTSLPNLAKEAKDAKGSRRKKSLSEKVQLSESSVTLSPVDSLESPHTYVSDTTSSPMITSPGILQASPNPMLATAAPPAPVHAQHALSFSNLHEMQPLAHGASTVLPSVSQLLSHHHIVSPGSGSAGSLSRLHPVPVPADWMNRMEVNETQYNEMFGMVLAPAEGTHPGIAPQSRPPEGKHITTPREPLPPIVTFQLIPKGSIAQPAGAPQPQSTCPPAVAGPLPTMYQIPEMARLPSVAFPTAMMPQQDGQVAQTILPAYHPFPASVGKYPTPPSQHSYASSNAAERTPSHSGHLQGEHPYLTPSPESPDQWSSSSPHSASDWSDVTTSPTPGGAGGGQRGPGTHMSEPPHNNMQVYA

1166 NP_036520.1 NP_065209.2

>NP_036520.1

MSVPGPSSPDGALTRPPYCLEAGEPTPGLSDTSPDEGLIEDLTIEDKAVEQLAEGLLSHYLPDLQRSKQALQELTQNQVVLLDTLEQEISKFKECHSMLDINALFAEAKHYHAKLVNIRKEMLMLHEKTSKLKKRALKLQQKRQKEELEREQQREKEFEREKQLTARPAKRM

>NP_065209.2

MPYSVGFREADAATSFLRAARSGNLDKALDHLRNGVDINTCNQNGLNGLHLASKEGHVKMVVELLHKEIILETTTKKGNTALHIAALAGQDEVVRELVNYGANVNAQSQKGFTPLYMAAQENHLEVVKFLLENGANQNVATEDGFTPLAVALQQGHENVVAHLINYGTKGKVRLPALHIAARNDDTRTAAVLLQNDPNPDVLSKTGFTPLHIAAHYENLNVAQLLLNRGASVNFTPQNGITPLHIASRRGNVIMVRLLLDRGAQIETKTKDELTPLHCAARNGHVRISEILLDHGAPIQAKTKNGLSPIHMAAQGDHLDCVRLLLQYDAEIDDITLDHLTPLHVAAHCGHHRVAKVLLDKGAKPNSRALNGFTPLHIACKKNHVRVMELLLKTGASIDAVTESGLTPLHVASFMGHLPIVKNLLQRGASPNVSNVKVETPLHMAARAGHTEVAKYLLQNKAKVNAKAKDDQTPLHCAARIGHTNMVKLLLENNANPNLATTAGHTPLHIAAREGHVETVLALLEKEASQACMTKKGFTPLHVAAKYGKVRVAELLLERDAHPNAAGKNGLTPLHVAVHHNNLDIVKLLLPRGGSPHSPAWNGYTPLHIAAKQNQVEVARSLLQYGGSANAESVQGVTPLHLAAQEGHAEMVALLLSKQANGNLGNKSGLTPLHLVAQEGHVPVADVLIKHGVMVDATTRMGYTPLHVASHYGNIKLVKFLLQHQADVNAKTKLGYSPLHQAAQQGHTDIVTLLLKNGASPNEVSSDGTTPLAIAKRLGYISVTDVLKVVTDETSFVLVSDKHRMSFPETVDEILDVSEDEGEELISFKAERRDSRDVDEEKELLDFVPKLDQVVESPAIPRIPCAMPETVVIRSEEQEQASKEYDEDSLIPSSPATETSDNISPVASPVHTGFLVSFMVDARGGSMRGSRHNGLRVVIPPRTCAAPTRITCRLVKPQKLSTPPPLAEEEGLASRIIALGPTGAQFLSPVIVEIPHFASHGRGDRELVVLRSENGSVWKEHRSRYGESYLDQILNGMDEELGSLEELEKKRVCRIITTDFPLYFVIMSRLCQDYDTIGPEGGSLKSKLVPLVQATFPENAVTKRVKLALQAQPVPDELVTKLLGNQATFSPIVTVEPRRRKFHRPIGLRIPLPPSWTDNPRDSGEGDTTSLRLLCSVIGGTDQAQWEDITGTTKLVYANECANFTTNVSARFWLSDCPRTAEAVNFATLLYKELTAVPYMAKFVIFAKMNDPREGRLRCYCMTDDKVDKTLEQHENFVEVARSRDIEVLEGMSLFAELSGNLVPVKKAAQQRSFHFQSFRENRLAMPVKVRDSSREPGGSLSFLRKAMKYEDTQHILCHLNITMPPCAKGSGAEDRRRTPTPLALRYSILSESTPGSLSGTEQAEMKMAVISEHLGLSWAELARELQFSVEDINRIRVENPNSLLEQSVALLNLWVIREGQNANMENLYTALQSIDRGEIVNMLEGSGRQSRNLKPDRRHTDRDYSLSPSQMNGYSSLQDELLSPASLGCALSSPLRADQYWNEVAVLDAIPLAATEHDTMLEMSDMQVWSAGLTPSLVTAEDSSLECSKAEDSDATGHEWKLEGALSEEPRGPELGSLELVEDDTVDSDATNGLIDLLEQEEGQRSEEKLPGSKRQDDATGAGQDSENEVSLVSGHQRGQARITHSPTVSQVTERSQDRLQDWDADGSIVSYLQDAAQGSWQEEVTQGPHSFQGTSTMTEGLEPGGSQEYEKVLVSVSEHTWTEQPEAESSQADRDRRQQGQEEQVQEAKNTFTQVVQGNEFQNIPGEQVTEEQFTDEQGNIVTKKIIRKVVRQIDLSSADAAQEHEEVTVEGPLEDPSELEVDIDYFMKHSKDHTSTPNP

1167 NP_003072.2 NP_055047.1

>NP_003072.2

MAEDADMRNELEEMQRRADQLADESLESTRRMLQLVEESKDAGIRTLVMLDEQGEQLDRVEEGMNHINQDMKEAEKNLKDLGKCCGLFICPCNKLKSSDAYKKAWGNNQDGVVASQPARVVDEREQMAISGGFIRRVTNDARENEMDENLEQVSGIIGNLRHMALDMGNEIDTQNRQIDRIMEKADSNKTRIDEANQRATKMLGSG

>NP_055047.1

MSATAATAPPAAPAGEGGPPAPPPNLTSNRRLQQTQAQVDEVVDIMRVNVDKVLERDQKLSELDDRADALQAGASQFETSAAKLKRKYWWKNLKMMIILGVICAIILIIIIVYFSS

1168 NP_001420.2 NP_940851.1

>NP_001420.2

MAENVVEPGPPSAKRPKLSSPALSASASDGTDFGSLFDLEHDLPDELINSTELGLTNGGDINQLQTSLGMVQDAASKHKQLSELLRSGSSPNLNMGVGGPGQVMASQAQQSSPGLGLINSMVKSPMTQAGLTSPNMGMGTSGPNQGPTQSTGMMNSPVNQPAMGMNTGMNAGMNPGMLAAGNGQGIMPNQVMNGSIGAGRGRQNMQYPNPGMGSAGNLLTEPLQQGSPQMGGQTGLRGPQPLKMGMMNNPNPYGSPYTQNPGQQIGASGLGLQIQTKTVLSNNLSPFAMDKKAVPGGGMPNMGQQPAPQVQQPGLVTPVAQGMGSGAHTADPEKRKLIQQQLVLLLHAHKCQRREQANGEVRQCNLPHCRTMKNVLNHMTHCQSGKSCQVAHCASSRQIISHWKNCTRHDCPVCLPLKNAGDKRNQQPILTGAPVGLGNPSSLGVGQQSAPNLSTVSQIDPSSIERAYAALGLPYQVNQMPTQPQVQAKNQQNQQPGQSPQGMRPMSNMSASPMGVNGGVGVQTPSLLSDSMLHSAINSQNPMMSENASVPSLGPMPTAAQPSTTGIRKQWHEDITQDLRNHLVHKLVQAIFPTPDPAALKDRRMENLVAYARKVEGDMYESANNRAEYYHLLAEKIYKIQKELEEKRRTRLQKQNMLPNAAGMVPVSMNPGPNMGQPQPGMTSNGPLPDPSMIRGSVPNQMMPRITPQSGLNQFGQMSMAQPPIVPRQTPPLQHHGQLAQPGALNPPMGYGPRMQQPSNQGQFLPQTQFPSQGMNVTNIPLAPSSGQAPVSQAQMSSSSCPVNSPIMPPGSQGSHIHCPQLPQPALHQNSPSPVPSRTPTPHHTPPSIGAQQPPATTIPAPVPTPPAMPPGPQSQALHPPPRQTPTPPTTQLPQQVQPSLPAAPSADQPQQQPRSQQSTAASVPTPTAPLLPPQPATPLSQPAVSIEGQVSNPPSTSSTEVNSQAIAEKQPSQEVKMEAKMEVDQPEPADTQPEDISESKVEDCKMESTETEERSTELKTEIKEEEDQPSTSATQSSPAPGQSKKKIFKPEELRQALMPTLEALYRQDPESLPFRQPVDPQLLGIPDYFDIVKSPMDLSTIKRKLDTGQYQEPWQYVDDIWLMFNNAWLYNRKTSRVYKYCSKLSEVFEQEIDPVMQSLGYCCGRKLEFSPQTLCCYGKQLCTIPRDATYYSYQNRYHFCEKCFNEIQGESVSLGDDPSQPQTTINKEQFSKRKNDTLDPELFVECTECGRKMHQICVLHHEIIWPAGFVCDGCLKKSARTRKENKFSAKRLPSTRLGTFLENRVNDFLRRQNHPESGEVTVRVVHASDKTVEVKPGMKARFVDSGEMAESFPYRTKALFAFEEIDGVDLCFFGMHVQEYGSDCPPPNQRRVYISYLDSVHFFRPKCLRTAVYHEILIGYLEYVKKLGYTTGHIWACPPSEGDDYIFHCHPPDQKIPKPKRLQEWYKKMLDKAVSERIVHDYKDIFKQATEDRLTSAKELPYFEGDFWPNVLEESIKELEQEEEERKREENTSNESTDVTKGDSKNAKKKNNKKTSKNKSSLSRGNKKKPGMPNVSNDLSQKLYATMEKHKEVFFVIRLIAGPAANSLPPIVDPDPLIPCDLMDGRDAFLTLARDKHLEFSSLRRAQWSTMCMLVELHTQSQDRFVYTCNECKHHVETRWHCTVCEDYDLCITCYNTKNHDHKMEKLGLGLDDESNNQQAAATQSPGDSRRLSIQRCIQSLVHACQCRNANCSLPSCQKMKRVVQHTKGCKRKTNGGCPICKQLIALCCYHAKHCQENKCPVPFCLNIKQKLRQQQLQHRLQQAQMLRRRMASMQRTGVVGQQQGLPSPTPATPTTPTGQQPTTPQTPQPTSQPQPTPPNSMPPYLPRTQAAGPVSQGKAAGQVTPPTPPQTAQPPLPGPPPAAVEMAMQIQRAAETQRQMAHVQIFQRPIQHQMPPMTPMAPMGMNPPPMTRGPSGHLEPGMGPTGMQQQPPWSQGGLPQPQQLQSGMPRPAMMSVAQHGQPLNMAPQPGLGQVGISPLKPGTVSQQALQNLLRTLRSPSSPLQQQQVLSILHANPQLLAAFIKQRAAKYANSNPQPIPGQPGMPQGQPGLQPPTMPGQQGVHSNPAMQNMNPMQAGVQRAGLPQQQPQQQLQPPMGGMSPQAQQMNMNHNTMPSQFRDILRRQQMMQQQQQQGAGPGIGPGMANHNQFQQPQGVGYPPQQQQRMQHHMQQMQQGNMGQIGQLPQALGAEAGASLQAYQQRLLQQQMGSPVQPNPMSPQQHMLPNQAQSPHLQGQQIPNSLSNQVRSPQPVPSPRPQSQPPHSSPSPRMQPQPSPHHVSPQTSSPHPGLVAAQANPMEQGHFASPDQNSMLSQLASNPGMANLHGASATDLGLSTDNSDLNSNLSQSTLDIH

>NP_940851.1

MRALPGLLEARARTPRLLLLQCLLAAARPSSADGSAPDSPFTSPPLREEIMANNFSLESHNISLTEHSSMPVEKNITLERPSNVNLTCQFTTSGDLNAVNVTWKKDGEQLENNYLVSATGSTLYTQYRFTIINSKQMGSYSCFFREEKEQRGTFNFKVPELHGKNKPLISYVGDSTVLTCKCQNCFPLNWTWYSSNGSVKVPVGVQMNKYVINGTYANETKLKITQLLEEDGESYWCRALFQLGESEEHIELVVLSYLVPLKPFLVIVAEVILLVATILLCEKYTQKKKKHSDEGKEFEQIEQLKSDDSNGIENNVPRHRKNESLGQ

1169 NP_001420.2 NP_006229.1

>NP_001420.2

MAENVVEPGPPSAKRPKLSSPALSASASDGTDFGSLFDLEHDLPDELINSTELGLTNGGDINQLQTSLGMVQDAASKHKQLSELLRSGSSPNLNMGVGGPGQVMASQAQQSSPGLGLINSMVKSPMTQAGLTSPNMGMGTSGPNQGPTQSTGMMNSPVNQPAMGMNTGMNAGMNPGMLAAGNGQGIMPNQVMNGSIGAGRGRQNMQYPNPGMGSAGNLLTEPLQQGSPQMGGQTGLRGPQPLKMGMMNNPNPYGSPYTQNPGQQIGASGLGLQIQTKTVLSNNLSPFAMDKKAVPGGGMPNMGQQPAPQVQQPGLVTPVAQGMGSGAHTADPEKRKLIQQQLVLLLHAHKCQRREQANGEVRQCNLPHCRTMKNVLNHMTHCQSGKSCQVAHCASSRQIISHWKNCTRHDCPVCLPLKNAGDKRNQQPILTGAPVGLGNPSSLGVGQQSAPNLSTVSQIDPSSIERAYAALGLPYQVNQMPTQPQVQAKNQQNQQPGQSPQGMRPMSNMSASPMGVNGGVGVQTPSLLSDSMLHSAINSQNPMMSENASVPSLGPMPTAAQPSTTGIRKQWHEDITQDLRNHLVHKLVQAIFPTPDPAALKDRRMENLVAYARKVEGDMYESANNRAEYYHLLAEKIYKIQKELEEKRRTRLQKQNMLPNAAGMVPVSMNPGPNMGQPQPGMTSNGPLPDPSMIRGSVPNQMMPRITPQSGLNQFGQMSMAQPPIVPRQTPPLQHHGQLAQPGALNPPMGYGPRMQQPSNQGQFLPQTQFPSQGMNVTNIPLAPSSGQAPVSQAQMSSSSCPVNSPIMPPGSQGSHIHCPQLPQPALHQNSPSPVPSRTPTPHHTPPSIGAQQPPATTIPAPVPTPPAMPPGPQSQALHPPPRQTPTPPTTQLPQQVQPSLPAAPSADQPQQQPRSQQSTAASVPTPTAPLLPPQPATPLSQPAVSIEGQVSNPPSTSSTEVNSQAIAEKQPSQEVKMEAKMEVDQPEPADTQPEDISESKVEDCKMESTETEERSTELKTEIKEEEDQPSTSATQSSPAPGQSKKKIFKPEELRQALMPTLEALYRQDPESLPFRQPVDPQLLGIPDYFDIVKSPMDLSTIKRKLDTGQYQEPWQYVDDIWLMFNNAWLYNRKTSRVYKYCSKLSEVFEQEIDPVMQSLGYCCGRKLEFSPQTLCCYGKQLCTIPRDATYYSYQNRYHFCEKCFNEIQGESVSLGDDPSQPQTTINKEQFSKRKNDTLDPELFVECTECGRKMHQICVLHHEIIWPAGFVCDGCLKKSARTRKENKFSAKRLPSTRLGTFLENRVNDFLRRQNHPESGEVTVRVVHASDKTVEVKPGMKARFVDSGEMAESFPYRTKALFAFEEIDGVDLCFFGMHVQEYGSDCPPPNQRRVYISYLDSVHFFRPKCLRTAVYHEILIGYLEYVKKLGYTTGHIWACPPSEGDDYIFHCHPPDQKIPKPKRLQEWYKKMLDKAVSERIVHDYKDIFKQATEDRLTSAKELPYFEGDFWPNVLEESIKELEQEEEERKREENTSNESTDVTKGDSKNAKKKNNKKTSKNKSSLSRGNKKKPGMPNVSNDLSQKLYATMEKHKEVFFVIRLIAGPAANSLPPIVDPDPLIPCDLMDGRDAFLTLARDKHLEFSSLRRAQWSTMCMLVELHTQSQDRFVYTCNECKHHVETRWHCTVCEDYDLCITCYNTKNHDHKMEKLGLGLDDESNNQQAAATQSPGDSRRLSIQRCIQSLVHACQCRNANCSLPSCQKMKRVVQHTKGCKRKTNGGCPICKQLIALCCYHAKHCQENKCPVPFCLNIKQKLRQQQLQHRLQQAQMLRRRMASMQRTGVVGQQQGLPSPTPATPTTPTGQQPTTPQTPQPTSQPQPTPPNSMPPYLPRTQAAGPVSQGKAAGQVTPPTPPQTAQPPLPGPPPAAVEMAMQIQRAAETQRQMAHVQIFQRPIQHQMPPMTPMAPMGMNPPPMTRGPSGHLEPGMGPTGMQQQPPWSQGGLPQPQQLQSGMPRPAMMSVAQHGQPLNMAPQPGLGQVGISPLKPGTVSQQALQNLLRTLRSPSSPLQQQQVLSILHANPQLLAAFIKQRAAKYANSNPQPIPGQPGMPQGQPGLQPPTMPGQQGVHSNPAMQNMNPMQAGVQRAGLPQQQPQQQLQPPMGGMSPQAQQMNMNHNTMPSQFRDILRRQQMMQQQQQQGAGPGIGPGMANHNQFQQPQGVGYPPQQQQRMQHHMQQMQQGNMGQIGQLPQALGAEAGASLQAYQQRLLQQQMGSPVQPNPMSPQQHMLPNQAQSPHLQGQQIPNSLSNQVRSPQPVPSPRPQSQPPHSSPSPRMQPQPSPHHVSPQTSSPHPGLVAAQANPMEQGHFASPDQNSMLSQLASNPGMANLHGASATDLGLSTDNSDLNSNLSQSTLDIH

>NP_006229.1

MEQPQEEAPEVREEEEKEEVAEAEGAPELNGGPQHALPSSSYTDLSRSSSPPSLLDQLQMGCDGASCGSLNMECRVCGDKASGFHYGVHACEGCKGFFRRTIRMKLEYEKCERSCKIQKKNRNKCQYCRFQKCLALGMSHNAIRFGRMPEAEKRKLVAGLTANEGSQYNPQVADLKAFSKHIYNAYLKNFNMTKKKARSILTGKASHTAPFVIHDIETLWQAEKGLVWKQLVNGLPPYKEISVHVFYRCQCTTVETVRELTEFAKSIPSFSSLFLNDQVTLLKYGVHEAIFAMLASIVNKDGLLVANGSGFVTREFLRSLRKPFSDIIEPKFEFAVKFNALELDDSDLALFIAAIILCGDRPGLMNVPRVEAIQDTILRALEFHLQANHPDAQYLFPKLLQKMADLRQLVTEHAQMMQRIKKTETETSLHPLLQEIYKDMY

1170 AAH28049.1 NP_055878.1

>AAH28049.1

MAAPEPARAAPPPPPPPPPPPGADRVVKAVPFPPTHRLTSEEVFDLDGIPRVDVLKNHLVKEGRVDEEIALRIINEGAAILRREKTMIEVEAPITVCGDIHGQFFDLMKLFEVGGSPANTRYLFLGDYVDRGYFSIECVLYLWVLKILYPSTLFLLRGNHECRHLTEYFTFKQECKIKYSERVYEACMEAFDSLPLAALLNQQFLCVHGGLSPEIHTLDDIRRLDRFKEPPAFGPMCDLLWSDPSEDFGNEKSQEHFSHNTVRGCSYFYNYPAVCEFLQNNNLLSIIRAHEAQDAGYRMYRKSQTTGFPSLITIFSAPNYLDVYNNKAAVLKYENNVMNIRQFNCSPHPYWLPNFMDVFTWSLPFVGEKVTEMLVNVLSICSDDELMTEGEDQFDVGSAAARKEIIRNKIRAIGKMARVFSVLREESESVLTLKGLTPTGMLPSGVLAGGRQTLQSATVEAIEAEKAIRGFSPPHRICSFEEAKGLDRINERMPPRKDAVQQDGFNSLNTAHATENHGTGNHTAQ

>NP_055878.1

MAPLALVGVTLLLAAPPCSGAATPTPSLPPPPANDSDTSTGGCQGSYRCQPGVLLPVWEPDDPSLGDKAARAVVYFVAMVYMFLGVSIIADRFMAAIEVITSKEKEITITKANGETSVGTVRIWNETVSNLTLMALGSSAPEILLSVIEVCGHNFQAGELGPGTIVGSAAFNMFVVIAVCIYVIPAGESRKIKHLRVFFVTASWSIFAYVWLYLILAVFSPGVVQVWEALLTLVFFPVCVVFAWMADKRLLFYKYVYKRYRTDPRSGIIIGAEGDPPKSIELDGTFVGAEAPGELGGLGPGPAEARELDASRREVIQILKDLKQKHPDKDLEQLVGIANYYALLHQQKSRAFYRIQATRLMTGAGNVLRRHAADASRRAAPAEGAGEDEDDGASRIFFEPSLYHCLENCGSVLLSVTCQGGEGNSTFYVDYRTEDGSAKAGSDYEYSEGTLVFKPGETQKELRIGIIDDDIFEEDEHFFVRLLNLRVGDAQGMFEPDGGGRPKGRLVAPLLATVTILDDDHAGIFSFQDRLLHVSECMGTVDVRVVRSSGARGTVRLPYRTVDGTARGGGVHYEDACGELEFGDDETMKTLQVKIVDDEEYEKKDNFFIELGQPQWLKRGISALLLNQGDGDRKLTAEEEEARRIAEMGKPVLGENCRLEVIIEESYDFKNTVDKLIKKTNLALVIGTHSWREQFLEAITVSAGDEEEEEDGSREERLPSCFDYVMHFLTVFWKVLFACVPPTEYCHGWACFGVSILVIGLLTALIGDLASHFGCTVGLKDSVNAVVFVALGTSIPDTFASKVAALQDQCADASIGNVTGSNAVNVFLGLGVAWSVAAVYWAVQGRPFEVRTGTLAFSVTLFTVFAFVGIAVLLYRRRPHIGGELGGPRGPKLATTALFLGLWLLYILFASLEAYCHIRGF

1171 NP_060564.2 NP_002085.1

>NP_060564.2

MDSGSSSSDSAPDCWDQVDMESPGSAPSGDGVSSAVAEAQREPLSSAFSRKLNVNAKPFVPNVHAAEFVPSFLRGPTQPPTLPAGSGSNDETCTGAGYPQGKRMGRGAPVEPSREEPLVSLEGSNSAVTMELSEPVVENGEVEMALEESWEHSKEVSEAEPGGGSSGDSGPPEESGQEMMEEKEEIRKSKSVIVPSGAPKKEHVNVVFIGHVDAGKSTIGGQIMFLTGMVDKRTLEKYEREAKEKNRETWYLSWALDTNQEERDKGKTVEVGRAYFETERKHFTILDAPGHKSFVPNMIGGASQADLAVLVISARKGEFETGFEKGGQTREHAMLAKTAGVKHLIVLINKMDDPTVNWSIERYEECKEKLVPFLKKVGFSPKKDIHFMPCSGLTGANIKEQSDFCPWYTGLPFIPYLDNLPNFNRSIDGPIRLPIVDKYKDMGTVVLGKLESGSIFKGQQLVMMPNKHNVEVLGILSDDTETDFVAPGENLKIRLKGIEEEEILPGFILCDPSNLCHSGRTFDVQIVIIEHKSIICPGYNAVLHIHTCIEEVEITALISLVDKKSGEKSKTRPRFVKQDQVCIARLRTAGTICLETFKDFPQMGRFTLRDEGKTIAIGKVLKLVPEKD

>NP_002085.1

MELSEPIVENGETEMSPEESWEHKEEISEAEPGGGSLGDGRPPEESAHEMMEEEEEIPKPKSVVAPPGAPKKEHVNVVFIGHVDAGKSTIGGQIMYLTGMVDKRTLEKYEREAKEKNRETWYLSWALDTNQEERDKGKTVEVGRAYFETEKKHFTILDAPGHKSFVPNMIGGASQADLAVLVISARKGEFETGFEKGGQTREHAMLAKTAGVKHLIVLINKMDDPTVNWSNERYEECKEKLVPFLKKVGFNPKKDIHFMPCSGLTGANLKEQSDFCPWYIGLPFIPYLDNLPNFNRSVDGPIRLPIVDKYKDMGTVVLGKLESGSICKGQQLVMMPNKHNVEVLGILSDDVETDTVAPGENLKIRLKGIEEEEILPGFILCDPNNLCHSGRTFDAQIVIIEHKSIICPGYNAVLHIHTCIEEVEITALICLVDKKSGEKSKTRPRFVKQDQVCIARLRTAGTICLETFKDFPQMGRFTLRDEGKTIAIGKVLKLVPEKD

1172 NP_001395.1 NP_005878.1

>NP_001395.1

MAAGTLYTYPENWRAFKALIAAQYSGAQVRVLSAPPHFHFGQTNRTPEFLRKFPAGKVPAFEGDDGFCVFESNAIAYYVSNEELRGSTPEAAAQVVQWVSFADSDIVPPASTWVFPTLGIMHHNKQATENAKEEVRRILGLLDAYLKTRTFLVGERVTLADITVVCTLLWLYKQVLEPSFRQAFPNTNRWFLTCINQPQFRAVLGEVKLCEKMAQFDAKKFAETQPKKDTPRKEKGSREEKQKPQAERKEEKKAAAPAPEEEMDECEQALAAEPKAKDPFAHLPKSTFVLDEFKRKYSNEDTLSVALPYFWEHFDKDGWSLWYSEYRFPEELTQTFMSCNLITGMFQRLDKLRKNAFASVILFGTNNSSSISGVWVFRGQELAFPLSPDWQVDYESYTWRKLDPGSEETQTLVREYFSWEGAFQHVGKAFNQGKIFK

>NP_005878.1

MRPCIWIHVHLKPPCRLVELLPFSSALQGLSHLSLGTTLPVILPERNEEQNLQELSHNADKYQMGDCCKEEI

1173 NP_000052.1 NP_079055.2

>NP_000052.1

MAAVILESIFLKRSQQKKKTSPLNFKKRLFLLTVHKLSYYEYDFERGRRGSKKGSIDVEKITCVETVVPEKNPPPERQIPRRGEESSEMEQISIIERFPYPFQVVYDEGPLYVFSPTEELRKRWIHQLKNVIRYNSDLVQKYHPCFWIDGQYLCCSQTAKNAMGCQILENRNGSLKPGSSHRKTKKPLPPTPEEDQILKKPLPPEPAAAPVSTSELKKVVALYDYMPMNANDLQLRKGDEYFILEESNLPWWRARDKNGQEGYIPSNYVTEAEDSIEMYEWYSKHMTRSQAEQLLKQEGKEGGFIVRDSSKAGKYTVSVFAKSTGDPQGVIRHYVVCSTPQSQYYLAEKHLFSTIPELINYHQHNSAGLISRLKYPVSQQNKNAPSTAGLGYGSWEIDPKDLTFLKELGTGQFGVVKYGKWRGQYDVAIKMIKEGSMSEDEFIEEAKVMMNLSHEKLVQLYGVCTKQRPIFIITEYMANGCLLNYLREMRHRFQTQQLLEMCKDVCEAMEYLESKQFLHRDLAARNCLVNDQGVVKVSDFGLSRYVLDDEYTSSVGSKFPVRWSPPEVLMYSKFSSKSDIWAFGVLMWEIYSLGKMPYERFTNSETAEHIAQGLRLYRPHLASEKVYTIMYSCWHEKADERPTFKILLSNILDVMDEES

>NP_079055.2

MASSSVPPATVSAATAGPGPGFGFASKTKKKHFVQQKVKVFRAADPLVGVFLWGVAHSINELSQVPPPVMLLPDDFKASSKIKVNNHLFHRENLPSHFKFKEYCPQVFRNLRDRFGIDDQDYLVSLTRNPPSESEGSDGRFLISYDRTLVIKEVSSEDIADMHSNLSNYHQYIVKCHGNTLLPQFLGMYRVSVDNEDSYMLVMRNMFSHRLPVHRKYDLKGSLVSREASDKEKVKELPTLRDMDFLNKNQKVYIGEEEKKIFLEKLKRDVEFLVQLKIMDYSLLLGIHDIIRGSEPEEEAPVREDESEVDGDCSLTGPPALVGSYGTSPEGIGGYIHSHRPLGPGEFESFIDVYAIRSAEGAPQKEVYFMGLIDILTQYDAKKKAAHAAKTVKHGAGAEISTVHPEQYAKRFLDFITNIFA

1174 NP_002825.3 NP_004318.3

>NP_002825.3

MTSRRWFHPNITGVEAENLLLTRGVDGSFLARPSKSNPGDFTLSVRRNGAVTHIKIQNTGDYYDLYGGEKFATLAELVQYYMEHHGQLKEKNGDVIELKYPLNCADPTSERWFHGHLSGKEAEKLLTEKGKHGSFLVRESQSHPGDFVLSVRTGDDKGESNDGKSKVTHVMIRCQELKYDVGGGERFDSLTDLVEHYKKNPMVETLGTVLQLKQPLNTTRINAAEIESRVRELSKLAETTDKVKQGFWEEFETLQQQECKLLYSRKEGQRQENKNKNRYKNILPFDHTRVVLHDGDPNEPVSDYINANIIMPEFETKCNNSKPKKSYIATQGCLQNTVNDFWRMVFQENSRVIVMTTKEVERGKSKCVKYWPDEYALKEYGVMRVRNVKESAAHDYTLRELKLSKVGQGNTERTVWQYHFRTWPDHGVPSDPGGVLDFLEEVHHKQESIMDAGPVVVHCSAGIGRTGTFIVIDILIDIIREKGVDCDIDVPKTIQMVRSQRSGMVQTEAQYRFIYMAVQHYIETLQRRIEEEQKSKRKGHEYTNIKYSLADQTSGDQSPLPPCTPTPPCAEMREDSARVYENVGLMQQQKSFR

>NP_004318.3

MVDPVGFAEAWKAQFPDSEPPRMELRSVGDIEQELERCKASIRRLEQEVNQERFRMIYLQTLLAKEKKSYDRQRWGFRRAAQAPDGASEPRASASRPQPAPADGADPPPAEEPEARPDGEGSPGKARPGTARRPGAAASGERDDRGPPASVAALRSNFERIRKGHGQPGADAEKPFYVNVEFHHERGLVKVNDKEVSDRISSLGSQAMQMERKKSQHGAGSSVGDASRPPYRGRSSESSCGVDGDYEDAELNPRFLKDNLIDANGGSRPPWPPLEYQPYQSIYVGGMMEGEGKGPLLRSQSTSEQEKRLTWPRRSYSPRSFEDCGGGYTPDCSSNENLTSSEEDFSSGQSSRVSPSPTTYRMFRDKSRSPSQNSQQSFDSSSPPTPQCHKRHRHCPVVVSEATIVGVRKTGQIWPNDGEGAFHGDADGSFGTPPGYGCAADRAEEQRRHQDGLPYIDDSPSSSPHLSSKGRGSRDALVSGALESTKASELDLEKGLEMRKWVLSGILASEETYLSHLEALLLPMKPLKAAATTSQPVLTSQQIETIFFKVPELYEIHKEFYDGLFPRVQQWSHQQRVGDLFQKLASQLGVYRAFVDNYGVAMEMAEKCCQANAQFAEISENLRARSNKDAKDPTTKNSLETLLYKPVDRVTRSTLVLHDLLKHTPASHPDHPLLQDALRISQNFLSSINEEITPRRQSMTVKKGEHRQLLKDSFMVELVEGARKLRHVFLFTDLLLCTKLKKQSGGKTQQYDCKWYIPLTDLSFQMVDELEAVPNIPLVPDEELDALKIKISQIKNDIQREKRANKGSKATERLKKKLSEQESLLLLMSPSMAFRVHSRNGKSYTFLISSDYERAEWRENIREQQKKCFRSFSLTSVELQMLTNSCVKLQTVHSIPLTINKEDDESPGLYGFLNVIVHSATGFKQSSNLYCTLEVDSFGYFVNKAKTRVYRDTAEPNWNEEFEIELEGSQTLRILCYEKCYNKTKIPKEDGESTDRLMGKGQVQLDPQALQDRDWQRTVIAMNGIEVKLSVKFNSREFSLKRMPSRKQTGVFGVKIAVVTKRERSKVPYIVRQCVEEIERRGMEEVGIYRVSGVATDIQALKAAFDVNNKDVSVMMSEMDVNAIAGTLKLYFRELPEPLFTDEFYPNFAEGIALSDPVAKESCMLNLLLSLPEANLLTFLFLLDHLKRVAEKEAVNKMSLHNLATVFGPTLLRPSEKESKLPANPSQPITMTDSWSLEVMSQVQVLLYFLQLEAIPAPDSKRQSILFSTEV

1175 CAA53046.1 NP_536350.2

>CAA53046.1

MRPPSPLPARWLCVLAGALAWALGPAGGQAARLQEECDYVQMIEVQHKQCLEEAQLENETIGCSKMWDNLTCWPATPRGQVVVLACPLIFKLFSSIQGRNVSRSCTDEGWTHLEPGPYPIACGLDDKAASLDEQQTMFYGSVKTGYTIGYGLSLATLLVATAILSLFRKLHCTRNYIHMHLFISFILRAAAVFIKDLALFDSGESDQCSEGSVGCKAAMVFFQYCVMANFFWLLVEGLYLYTLLAVSFFSERKYFWGYILIGWGVPSTFTMVWTIARIHFEDYGLLRCWDTINSSLWWIIKGPILTSILVNFILFICIIRILLQKLRPPDIRKSDSSPYSRLARSTLLLIPLFGVHYIMFAFFPDNFKPEVKMVFELVVGSFQGFVVAILYCFLNGEVQAELRRKWRRWHLQGVLGWNPKYRHPSGGSNGATCSTQVSMLTRVSPGARRSSSFQAEVSLV

>NP_536350.2

MGVRNCLYGNNMSGQRDIPPEIGEQPEQPPLEAPGAAAPGAGPSPAEEMETEPPHNEPIPVENDGEACGPPEVSRPNFQVLNPAFREAGAHGSYSPPPEEAMPFEAEQPSLGGFWPTLEQPGFPSGVHAGLEAFGPALMEPGAFSGARPGLGGYSPPPEEAMPFEFDQPAQRGCSQLLLQVPDLAPGGPGAAGVPGAPPEEPQALRPAKAGSRGGYSPPPEETMPFELDGEGFGDDSPPPGLSRVIAQVDGSSQFAAVAASSAVRLTPAANAPPLWVPGAIGSPSQEAVRPPSNFTGSSPWMEISGPPFEIGSAPAGVDDTPVNMDSPPIALDGPPIKVSGAPDKRERAERPPVEEEAAEMEGAADAAEGGKVPSPGYGSPAAGAASADTAARAAPAAPADPDSGATPEDPDSGTAPADPDSGAFAADPDSGAAPAAPADPDSGAAPDAPADPDSGAAPDAPADPDAGAAPEAPAAPAAAETRAAHVAPAAPDAGAPTAPAASATRAAQVRRAASAAPASGARRKIHLRPPSPEIQAADPPTPRPTRASAWRGKSESSRGRRVYYDEGVASSDDDSSGDESDDGTSGCLRWFQHRRNRRRRKPQRNLLRNFLVQAFGGCFGRSESPQPKASRSLKVKKVPLAEKRRQMRKEALEKRAQKRAEKKRSKLIDKQLQDEKMGYMCTHRLLLLGAGESGKSTIVKQMRILHVNGFNGEGGEEDPQAARSNSDGEKATKVQDIKNNLKEAIETIVAAMSNLVPPVELANPENQFRVDYILSVMNVPDFDFPPEFYEHAKALWEDEGVRACYERSNEYQLIDCAQYFLDKIDVIKQADYVPSDQDLLRCRVLTSGIFETKFQVDKVNFHMFDVGGQRDERRKWIQCFNDVTAIIFVVASSSYNMVIREDNQTNRLQEALNLFKSIWNNRWLRTISVILFLNKQDLLAEKVLAGKSKIEDYFPEFARYTTPEDATPEPGEDPRVTRAKYFIRDEFLRISTASGDGRHYCYPHFTCAVDTENIRRVFNDCRDIIQRMHLRQYELL

1176 NP_060649.3 NP_004578.2

>NP_060649.3

MDSLEEPQKKVFKARKTMRVSDRQQLEAVYKVKEELLKTDVKLLNGNHENGDLDPTSPLENMDYIKDKEEVNGIEEICFDPEGSKAEWKETPCILSVNVKNKQDDDLNCEPLSPHNITPEPVSKLPAEPVSGDPAPGDLDAGDPASGVLASGDSTSGDPTSSEPSSSDAASGDATSGDAPSGDVSPGDATSGDATADDLSSGDPTSSDPIPGEPVPVEPISGDCAADDIASSEITSVDLASGAPASTDPASDDLASGDLSSSELASDDLATGELASDELTSESTFDRTFEPKSVPVCEPVPEIDNIEPSSNKDDDFLEKNGADEKLEQIQSKDSLDEKNKADNNIDANEETLETDDTTICSDRPPENEKKVEEDIITELALGEDAISSSMEIDQGEKNEDETSADLVETINENVIEDNKSENILENTDSMETDEIIPILEKLAPSEDELTCFSKTSLLPIDETNPDLEEKMESSFGSPSKQESSESLPKEAFLVLSDEEDISGEKDESEVISQNETCSPAEVESNEKDNKPEEEEQVIHEDDERPSEKNEFSRRKRSKSEDMDNVQSKRRRYMEEEYEAEFQVKITAKGDINQKLQKVIQWLLEEKLCALQCAVFDKTLAELKTRVEKIECNKRHKTVLTELQAKIARLTKRFEAAKEDLKKRHEHPPNPPVSPGKTVNDVNSNNNMSYRNAGTVRQMLESKRNVSESAPPSFQTPVNTVSSTNLVTPPAVVSSQPKLQTPVTSGSLTATSVLPAPNTATVVATTQVPSGNPQPTISLQPLPVILHVPVAVSSQPQLLQSHPGTLVTNQPSGNVEFISVQSPPTVSGLTKNPVSLPSLPNPTKPNNVPSVPSPSIQRNPTASAAPLGTTLAVQAVPTAHSIVQATRTSLPTVGPSGLYSPSTNRGPIQMKIPISAFSTSSAAEQNSNTTPRIENQTNKTIDASVSKKAADSTSQCGKATGSDSSGVIDLTMDDEESGASQDPKKLNHTPVSTMSSSQPVSRPLQPIQPAPPLQPSGVPTSGPSQTTIHLLPTAPTTVNVTHRPVTQVTTRLPVPRAPANHQVVYTTLPAPPAQAPLRGTVMQAPAVRQVNPQNSVTVRVPQTTTYVVNNGLTLGSTGPQLTVHHRPPQVHTEPPRPVHPAPLPEAPQPQRLPPEAASTSLPQKPHLKLARVQSQNGIVLSWSVLEVDRSCATVDSYHLYAYHEEPSATVPSQWKKIGEVKALPLPMACTLTQFVSGSKYYFAVRAKDIYGRFGPFCDPQSTDVISSTQSS

>NP_004578.2

MDIYDTQTLGVVVFGGFMVVSAIGIFLVSTFSMKETSYEEALANQRKEMAKTHHQKVEKKKKEKTVEKKGKTKKKEEKPNGKIPDHDPAPNVTVLLREPVRAPAVAVAPTPVQPPIIVAPVATVPAMPQEKLASSPKDKKKKEKKVAKVEPAVSSVVNSIQVLTSKAAILETAPKEGRNTDVAQSPEAPKQEAPAKKKSGSKKKGPPDADGPLYLPYKTLVSTVGSMVFNEGEAQRLIEILSEKAGIIQDTWHKATQKGDPVAILKRQLEEKEKLLATEQEDAAVAKSKLRELNKEMAAEKAKAAAGEAKVKKQLVAREQEITAVQARMQASYREHVKEVQQLQGKIRTLQEQLENGPNTQLARLQQENSILRDALNQATSQVESKQNAELAKLRQELSKVSKELVEKSEAVRQDEQQRKALEAKAAAFEKQVLQLQASHRESEEALQKRLDEVSRELCHTQSSHASLRADAEKAQEQQQQMAELHSKLQSSEAEVRSKCEELSGLHGQLQEARAENSQLTERIRSIEALLEAGQARDAQDVQASQAEADQQQTRLKELESQVSGLEKEAIELREAVEQQKVKNNDLREKNWKAMEALATAEQACKEKLHSLTQAKEESEKQLCLIEAQTMEALLALLPELSVLAQQNYTEWLQDLKEKGPTLLKHPPAPAEPSSDLASKLREAEETQSTLQAECDQYRSILAETEGMLRDLQKSVEEEEQVWRAKVGAAEEELQKSRVTVKHLEEIVEKLKGELESSDQVREHTSHLEAELEKHMAAASAECQNYAKEVAGLRQLLLESQSQLDAAKSEAQKQSDELALVRQQLSEMKSHVEDGDIAGAPASSPEAPPAEQDPVQLKTQLEWTEAILEDEQTQRQKLTAEFEEAQTSACRLQEELEKLRTAGPLESSETEEASQLKERLEKEKKLTSDLGRAATRLQELLKTTQEQLAREKDTVKKLQEQLEKAEDGSSSKEGTSV

1177 NP_003783.1 NP_001871.2

>NP_003783.1

MAESDWDTVTVLRKKGPTAAQAKSKQAILAAQRRGEDVETSKKWAAGQNKQHSITKNTAKLDRETEELHHDRVTLEVGKVIQQGRQSKGLTQKDLATKINEKPQVIADYESGRAIPNNQVLGKIERAIGLKLRGKDIGKPIEKGPRAK

>NP_001871.2

MKFKLHVNSARQYKDLWNMSDDKPFLCTAPGCGQRFTNEDHLAVHKHKHEMTLKFGPARNDSVIVADQTPTPTRFLKNCEEVGLFNELASPFENEFKKASEDDIKKMPLDLSPLATPIIRSKIEEPSVVETTHQDSPLPHPESTTSDEKEVPLAQTAQPTSAIVRPASLQVPNVLLTSSDSSVIIQQAVPSPTSSTVITQAPSSNRPIVPVPGPFPLLLHLPNGQTMPVAIPASITSSNVHVPAAVPLVRPVTMVPSVPGIPGPSSPQPVQSEAKMRLKAALTQQHPPVTNGDTVKGHGSGLVRTQSEESRPQSLQQPATSTTETPASPAHTTPQTQSTSGRRRRAANEDPDEKRRKFLERNRAAASRCRQKRKVWVQSLEKKAEDLSSLNGQLQSEVTLLRNEVAQLKQLLLAHKDCPVTAMQKKSGYHTADKDDSSEDISVPSSPHTEAIQHSSVSTSNGVSSTSKAEAVATSVLTQMADQSTEPALSQIVMAPSSQSQPSGS

1178 NP_006131.2 NP_000749.2

>NP_006131.2

MLLLVTSLLLCELPHPAFLLIPEKSDLRTVAPASSLNVRFDSRTMNLSWDCQENTTFSKCFLTDKKNRVVEPRLSNNECSCTFREICLHEGVTFEVHVNTSQRGFQQKLLYPNSGREGTAAQNFSCFIYNADLMNCTWARGPTAPRDVQYFLYIRNSKRRREIRCPYYIQDSGTHVGCHLDNLSGLTSRNYFLVNGTSREIGIQFFDSLLDTKKIERFNPPSNVTVRCNTTHCLVRWKQPRTYQKLSYLDFQYQLDVHRKNTQPGTENLLINVSGDLENRYNFPSSEPRAKHSVKIRAADVRILNWSSWSEAIEFGSDDGNLGSVYIYVLLIVGTLVCGIVLGFLFKRFLRIQRLFPPVPQIKDKLNDNHEVEDEIIWEEFTPEEGKGYREEVLTVKEIT

>NP_000749.2

MWLQSLLLLGTVACSISAPARSPSPSTQPWEHVNAIQEARRLLNLSRDTAAEMNETVEVISEMFDLQEPTCLQTRLELYKQGLRGSLTKLKGPLTMMASHYKQHCPPTPETSCATQIITFESFKENLKDFLLVIPFDCWEPVQE

1179 NP_000357.3 AAD42222.1

>NP_000357.3

MDAIKKKMQMLKLDKENALDRAEQAEADKKAAEDRSKQLEDELVSLQKKLKGTEDELDKYSEALKDAQEKLELAEKKATDAEADVASLNRRIQLVEEELDRAQERLATALQKLEEAEKAADESERGMKVIESRAQKDEEKMEIQEIQLKEAKHIAEDADRKYEEVARKLVIIESDLERAEERAELSEGQVRQLEEQLRIMDQTLKALMAAEDKYSQKEDRYEEEIKVLSDKLKEAETRAEFAERSVTKLEKSIDDLEDELYAQKLKYKAISEELDHALNDMTSM

>AAD42222.1

MTTEKSLVTEAENSQHQQKEEGEEAINSGQQEPQQEESCQTAAEGDNWCEHKLKASNGDTPTHEDLTKNKERTSENRGLSRLFSSFLKRPKSQVSEEEGKEVESDKEKGEGGQKEIEFGTSLDEEIILKAPIAAPEPELKTDPSLDLHSLSSAETQPAQEELREDPDFEIKEGEGLEECSKIEVKEESPQSKAETELKASQKPIRKHRNMHCKVSLLDDTVYECVVEKHAKGQDLLKRVCEHLNLLEEDYFGLAIWDNTTSKTWLDSAKEIKKQVRGVPWNFTFNVKFYPPDPAQLTEDITRYYLCLQLRQDIVAGRLPCSFATLALLGSYTIQSELGDYDPELHGVDYVSDFKLAPNQTKELEEKVMELHKSYRSMTPAQADLEFLENAKKLSMYGVDLHKAKDLEGVDIILGVCSSGLLVYKDKLRINRFPWPKVLKISYKRSSFFIKIRPGEQEQYESTIGFKLPSYRAAKKLWKVCVEHHTFFRLTSTDTIPKSKFLALGSKFRYSGRTQAQTRQASALIDRPAPHFERTASKRASRSLDGAAAVDSADRSPRPTSAPAITQGQVAEGGVLDASAKKTVVPKAQKETVKAEVKKEDEPPEQAEPEPTEAWKVEKTHIEVTVPTSNGDQTQKLAEKTEDLIRMRKKKRERLDGENIYIRHSSLMLEDLDKSQEEIEKHHASISELKKNFMESVPEPRPSEWDKRLSTHSPFRTLNINGQIPTGEGPPLVKTQTVTISDNANAVKSEIPTKDVPIVHTETKTITYEAAQTDDNSGDLDPGVLLTAQTITSETPSSTTTTQITKTVKGGISETRIEKRIVITGDADIDHDQVLVQAIKEAKEQHPDMSVTKVVVHQETEIADE

1180 NP_004152.1 NP_056955.1

>NP_004152.1

MSSMNPEYDYLFKLLLIGDSGVGKSCLLLRFADDTYTESYISTIGVDFKIRTIELDGKTIKLQIWDTAGQERFRTITSSYYRGAHGIIVVYDVTDQESFNNVKQWLQEIDRYASENVNKLLVGNKCDLTTKKVVDYTTAKEFADSLGIPFLETSAKNATNVEQSFMTMAAEIKKRMGPGATAGGAEKSNVKIQSTPVKQSGGGCC

>NP_056955.1

MKAKRRRPDLDEIHRELRPQGSARPQPDPNAEFDPDLPGGGLHRCLACARYFIDSTNLKTHFRSKDHKKRLKQLSVEPYSQEEAERAAGMGSYVPPRRLAVPTEVSTEVPEMDTST

1181 NP_008990.2 NP_005150.1

>NP_008990.2

MMGEAAVAAGPCPLREDSFTRFSSQSNVYGLAGGAGGRGELLAATLKGKVLGFRYQDLRQKIRPVAKELQFNYIPVDAEIVSIDTFNKSPPKRGLVVGITFIKDSGDKGSPFLNIYCDYEPGSEYNLDSIAQSCLNLELQFTPFQLCHAEVQVGDQLETVFLLSGNDPAIHLYKENEGLHQFEEQPVENLFPELTNLTSSVLWLDVHNFPGTSRRLSALGCQSGYVRVAHVDQRSREVLQMWSVLQDGPISRVIVFSLSAAKETKDRPLQDEYSVLVASMLEPAVVYRDLLNRGLEDQLLLPGSDQFDSVLCSLVTDVDLDGRPEVLVATYGQELLCYKYRGPESGLPEAQHGFHLLWQRSFSSPLLAMAHVDLTGDGLQELAVVSLKGVHILQHSLIQASELVLTRLRHQVEQRRRRLQGLEDGAGAGPAENAAS

>NP_005150.1

MCDDEETTALVCDNGSGLVKAGFAGDDAPRAVFPSIVGRPRHQGVMVGMGQKDSYVGDEAQSKRGILTLKYPIEHGIITNWDDMEKIWHHTFYNELRVAPEEHPTLLTEAPLNPKANREKMTQIMFETFNVPAMYVAIQAVLSLYASGRTTGIVLDSGDGVTHNVPIYEGYALPHAIMRLDLAGRDLTDYLMKILTERGYSFVTTAEREIVRDIKEKLCYVALDFENEMATAASSSSLEKSYELPDGQVITIGNERFRCPETLFQPSFIGMESAGIHETTYNSIMKCDIDIRKDLYANNVLSGGTTMYPGIADRMQKEITALAPSTMKIKIIAPPERKYSVWIGGSILASLSTFQQMWISKQEYDEAGPSIVHRKCF

1182 NP_003322.2 NP_002028.1

>NP_003322.2

MPLRHWGMARGSKPVGDGAQPMAAMGGLKVLLHWAGPGGGEPWVTFSESSLTAEEVCIHIAHKVGITPPCFNLFALFDAQAQVWLPPNHILEIPRDASLMLYFRIRFYFRNWHGMNPREPAVYRCGPPGTEASSDQTAQGMQLLDPASFEYLFEQGKHEFVNDVASLWELSTEEEIHHFKNESLGMAFLHLCHLALRHGIPLEEVAKKTSFKDCIPRSFRRHIRQHSALTRLRLRNVFRRFLRDFQPGRLSQQMVMVKYLATLERLAPRFGTERVPVCHLRLLAQAEGEPCYIRDSGVAPTDPGPESAAGPPTHEVLVTGTGGIQWWPVEEEVNKEEGSSGSSGRNPQASLFGKKAKAHKAVGQPADRPREPLWAYFCDFRDITHVVLKEHCVSIHRQDNKCLELSLPSRAAALSFVSLVDGYFRLTADSSHYLCHEVAPPRLVMSIRDGIHGPLLEPFVQAKLRPEDGLYLIHWSTSHPYRLILTVAQRSQAPDGMQSLRLRKFPIEQQDGAFVLEGWGRSFPSVRELGAALQGCLLRAGDDCFSLRRCCLPQPGETSNLIIMRGARASPRTLNLSQLSFHRVDQKEITQLSHLGQGTRTNVYEGRLRVEGSGDPEEGKMDDEDPLVPGRDRGQELRVVLKVLDPSHHDIALAFYETASLMSQVSHTHLAFVHGVCVRGPENIMVTEYVEHGPLDVWLRRERGHVPMAWKMVVAQQLASALSYLENKNLVHGNVCGRNILLARLGLAEGTSPFIKLSDPGVGLGALSREERVERIPWLAPECLPGGANSLSTAMDKWGFGATLLEICFDGEAPLQSRSPSEKEHFYQRQHRLPEPSCPQLATLTSQCLTYEPTQRPSFRTILRDLTRLQPHNLADVLTVNPDSPASDPTVFHKRYLKKIRDLGEGHFGKVSLYCYDPTNDGTGEMVAVKALKADCGPQHRSGWKQEIDILRTLYHEHIIKYKGCCEDQGEKSLQLVMEYVPLGSLRDYLPRHSIGLAQLLLFAQQICEGMAYLHSQHYIHRDLAARNVLLDNDRLVKIGDFGLAKAVPEGHEYYRVREDGDSPVFWYAPECLKEYKFYYASDVWSFGVTLYELLTHCDSSQSPPTKFLELIGIAQGQMTVLRLTELLERGERLPRPDKCPCEVYHLMKNCWETEASFRPTFENLIPILKTVHEKYQGQAPSVFSVC

>NP_002028.1

MGCVQCKDKEATKLTEERDGSLNQSSGYRYGTDPTPQHYPSFGVTSIPNYNNFHAAGGQGLTVFGGVNSSSHTGTLRTRGGTGVTLFVALYDYEARTEDDLSFHKGEKFQILNSSEGDWWEARSLTTGETGYIPSNYVAPVDSIQAEEWYFGKLGRKDAERQLLSFGNPRGTFLIRESETTKGAYSLSIRDWDDMKGDHVKHYKIRKLDNGGYYITTRAQFETLQQLVQHYSERAAGLCCRLVVPCHKGMPRLTDLSVKTKDVWEIPRESLQLIKRLGNGQFGEVWMGTWNGNTKVAIKTLKPGTMSPESFLEEAQIMKKLKHDKLVQLYAVVSEEPIYIVTEYMNKGSLLDFLKDGEGRALKLPNLVDMAAQVAAGMAYIERMNYIHRDLRSANILVGNGLICKIADFGLARLIEDNEYTARQGAKFPIKWTAPEAALYGRFTIKSDVWSFGILLTELVTKGRVPYPGMNNREVLEQVERGYRMPCPQDCPISLHELMIHCWKKDPEERPTFEYLQSFLEDYFTATEPQYQPGENL

1183 NP_003191.1 NP_001895.1

>NP_003191.1

MNQPQRMAPVGTDKELSDLLDFSMMFPLPVTNGKGRPASLAGAQFGGSGLEDRPSSGSWGSGDQSSSSFDPSRTFSEGTHFTESHSSLSSSTFLGPGLGGKSGERGAYASFGRDAGVGGLTQAGFLSGELALNSPGPLSPSGMKGTSQYYPSYSGSSRRRAADGSLDTQPKKVRKVPPGLPSSVYPPSSGEDYGRDATAYPSAKTPSSTYPAPFYVADGSLHPSAELWSPPGQAGFGPMLGGGSSPLPLPPGSGPVGSSGSSSTFGGLHQHERMGYQLHGAEVNGGLPSASSFSSAPGATYGGVSSHTPPVSGADSLLGSRGTTAGSSGDALGKALASIYSPDHSSNNFSSSPSTPVGSPQGLAGTSQWPRAGAPGALSPSYDGGLHGLQSKIEDHLDEAIHVLRSHAVGTAGDMHTLLPGHGALASGFTGPMSLGGRHAGLVGGSHPEDGLAGSTSLMHNHAALPSQPGTLPDLSRPPDSYSGLGRAGATAAASEIKREEKEDEENTSAADHSEEEKKELKAPRARTSPDEDEDDLLPPEQKAEREKERRVANNARERLRVRDINEAFKELGRMCQLHLNSEKPQTKLLILHQAVSVILNLEQQVRERNLNPKAACLKRREEEKVSGVVGDPQMVLSAPHPGLSEAHNPAGHM

>NP_001895.1

MATQADLMELDMAMEPDRKAAVSHWQQQSYLDSGIHSGATTTAPSLSGKGNPEEEDVDTSQVLYEWEQGFSQSFTQEQVADIDGQYAMTRAQRVRAAMFPETLDEGMQIPSTQFDAAHPTNVQRLAEPSQMLKHAVVNLINYQDDAELATRAIPELTKLLNDEDQVVVNKAAVMVHQLSKKEASRHAIMRSPQMVSAIVRTMQNTNDVETARCTAGTLHNLSHHREGLLAIFKSGGIPALVKMLGSPVDSVLFYAITTLHNLLLHQEGAKMAVRLAGGLQKMVALLNKTNVKFLAITTDCLQILAYGNQESKLIILASGGPQALVNIMRTYTYEKLLWTTSRVLKVLSVCSSNKPAIVEAGGMQALGLHLTDPSQRLVQNCLWTLRNLSDAATKQEGMEGLLGTLVQLLGSDDINVVTCAAGILSNLTCNNYKNKMMVCQVGGIEALVRTVLRAGDREDITEPAICALRHLTSRHQEAEMAQNAVRLHYGLPVVVKLLHPPSHWPLIKATVGLIRNLALCPANHAPLREQGAIPRLVQLLVRAHQDTQRRTSMGGTQQQFVEGVRMEEIVEGCTGALHILARDVHNRIVIRGLNTIPLFVQLLYSPIENIQRVAAGVLCELAQDKEAAEAIEAEGATAPLTELLHSRNEGVATYAAAVLFRMSEDKPQDYKKRLSVELTSSLFRTEPMAWNETADLGLDIGAQGEPLGYRQDDPSYRSFHSGGYGQDALGMDPMMEHEMGGHHPGADYPVDGLPDLGHAQDLMDGLPPGDSNQLAWFDTDL

1184 NP_001518.2 NP_061973.1

>NP_001518.2

MRSPPCGLLRWFGGPLLASWCRCHLRFRAFGTSAGWYRAFPAPPPLLPPACPSPRDYRPHVSLSPFLSRPSRGGSSSSSSSRRRSPVAAVAGEPMAYSQGGGKKKVCYYYDGDIGNYYYGQGHPMKPHRIRMTHNLLLNYGLYRKMEIYRPHKATAEEMTKYHSDEYIKFLRSIRPDNMSEYSKQMQRFNVGEDCPVFDGLFEFCQLSTGGSVAGAVKLNRQQTDMAVNWAGGLHHAKKSEASGFCYVNDIVLAILELLKYHQRVLYIDIDIHHGDGVEEAFYTTDRVMTVSFHKYGEYFPGTGDLRDIGAGKGKYYAVNFPMRDGIDDESYGQIFKPIISKVMEMYQPSAVVLQCGADSLSGDRLGCFNLTVKGHAKCVEVVKTFNLPLLMLGGGGYTIRNVARCWTYETAVALDCEIPNELPYNDYFEYFGPDFKLHISPSNMTNQNTPEYMEKIKQRLFENLRMLPHAPGVQMQAIPEDAVHEDSGDEDGEDPDKRISIRASDKRIACDEEFSDSEDEGEGGRRNVADHKKGAKKARIEEDKKETEDKKTDVKEEDKSKDNSGEKTDTKGTKSEQLSNP

>NP_061973.1

MATGADVRDILELGGPEGDAASGTISKKDIINPDKKKSKKSSETLTFKRPEGMHREVYALLYSDKKDAPPLLPSDTGQGYRTVKAKLGSKKVRPWKWMPFTNPARKDGAMFFHWRRAAEEGKDYPFARFNKTVQVPVYSEQEYQLYLHDDAWTKAETDHLFDLSRRFDLRFVVIHDRYDHQQFKKRSVEDLKERYYHICAKLANVRAVPGTDLKIPVFDAGHERRRKEQLERLYNRTPEQVAEEEYLLQELRKIEARKKEREKRSQDLQKLITAADTTAEQRRTERKAPKKKLPQKKEAEKPAVPETAGIKFPDFKSAGVTLRSQRMKLPSSVGQKKIKALEQMLLELGVELSPTPTEELVHMFNELRSDLVLLYELKQACANCEYELQMLRHRHEALARAGVLGGPATPASGPGPASAEPAVTEPGLGPDPKDTIIDVVGAPLTPNSRKRRESASSSSSVKKAKKP

1185 NP_068808.1 NP_002511.1

>NP_068808.1

MSLVGGFPHHPVVHHEGYPFAAAAAAAAAAAASRCSHEENPYFHGWLIGHPEMSPPDYSMALSYSPEYASGAAGLDHSHYGGVPPGAGPPGLGGPRPVKRRGTANRKERRRTQSINSAFAELRECIPNVPADTKLSKIKTLRLATSYIAYLMDLLAKDDQNGEAEAFKAEIKKTDVKEEKRKKELNEILKSTVSSNDKKTKGRTGWPQHVWALELKQ

>NP_002511.1

MEDSMDMDMSPLRPQNYLFGCELKADKDYHFKVDNDENEHQLSLRTVSLGAGAKDELHIVEAEAMNYEGSPIKVTLATLKMSVQPTVSLGGFEITPPVVLRLKCGSGPVHISGQHLVAVEEDAESEDEEEEDVKLLSISGKRSAPGGGSKVPQKKVKLAADEDDDDDDEEDDDEDDDDDDFDDEEAEEKAPVKKSIRDTPAKNAQKSNQNGKDSKPSSTPRSKGQESFKKQEKTPKTPKGPSSVEDIKAKMQASIEKGGSLPKVEAKFINYVKNCFRMTDQEAIQDLWQWRKSL

1186 NP_003746.2 NP_003741.1

>NP_003746.2

MPTGDFDSKPSWADQVEEEGEDDKCVTSELLKGIPLATGDTSPEPELLPGAPLPPPKEVINGNIKTVTEYKIDEDGKKFKIVRTFRIETRKASKAVARRKNWKKFGNSEFDPPGPNVATTTVSDDVSMTFITSKEDLNCQEEEDPMNKLKGQKIVSCRICKGDHWTTRCPYKDTLGPMQKELAEQLGLSTGEKEKLPGELEPVQATQNKTGKYVPPSLRDGASRRGESMQPNRRADDNATIRVTNLSEDTRETDLQELFRPFGSISRIYLAKDKTTGQSKGFAFISFHRREDAARAIAGVSGFGYDHLILNVEWAKPSTN

>NP_003741.1

MPAYFQRPENALKRANEFLEVGKKQPALDVLYDVMKSKKHRTWQKIHEPIMLKYLELCVDLRKSHLAKEGLYQYKNICQQVNIKSLEDVVRAYLKMAEEKTEAAKEESQQMVLDIEDLDNIQTPESVLLSAVSGEDTQDRTDRLLLTPWVKFLWESYRQCLDLLRNNSRVERLYHDIAQQAFKFCLQYTRKAEFRKLCDNLRMHLSQIQRHHNQSTAINLNNPESQSMHLETRLVQLDSAISMELWQEAFKAVEDIHGLFSLSKKPPKPQLMANYYNKVSTVFWKSGNALFHASTLHRLYHLSREMRKNLTQDEMQRMSTRVLLATLSIPITPERTDIARLLDMDGIIVEKQRRLATLLGLQAPPTRIGLINDMVRFNVLQYVVPEVKDLYNWLEVEFNPLKLCERVTKVLNWVREQPEKEPELQQYVPQLQNNTILRLLQQVSQIYQSIEFSRLTSLVPFVDAFQLERAIVDAARHCDLQVRIDHTSRTLSFGSDLNYATREDAPIGPHLQSMPSEQIRNQLTAMSSVLAKALEVIKPAHILQEKEEQHQLAVTAYLKNSRKEHQRILARRQTIEERKERLESLNIQREKEELEQREAELQKVRKAEEERLRQEAKEREKERILQEHEQIKKKTVRERLEQIKKTELGAKAFKDIDIEDLEELDPDFIMAKQVEQLEKEKKELQERLKNQEKKIDYFERAKRLEEIPLIKSAYEEQRIKDMDLWEQQEEERITTMQLEREKALEHKNRMSRMLEDRDLFVMRLKAARQSVYEEKLKQFEERLAEERHNRLEERKRQRKEERRITYYREKEEEEQRRAEEQMLKEREERERAERAKREEELREYQERVKKLEEVERKKRQRELEIEERERRREEERRLGDSSLSRKDSRWGDRDSEGTWRKGPEADSEWRRGPPEKEWRRGEGRDEDRSHRRDEERPRRLGDDEDREPSLRPDDDRVPRRGMDDDRGPRRGPEEDRFSRRGADDDRPSWRNTDDDRPPRRIADEDRGNWRHADDDRPPRRGLDEDRGSWRTADEDRGPRRGMDDDRGPRRGGADDERSSWRNADDDRGPRRGLDDDRGPRRGMDDDRGPRRGMDDDRGPRRGMDDDRGPRRGLDDDRGPWRNADDDRIPRRGAEDDRGPWRNMDDDRLSRRADDDRFPRRGDDSRPGPWRPLVKPGGWREKEKAREESWGPPRESRPSEEREWDREKERDRDNQDREENDKDPERERDRERDVDREDRFRRPRDEGGWRRGPAEESSSWRDSSRRDDRDRDDRRRERDDRRDLRERRDLRDDRDRRGPPLRSEREEVSSWRRADDRKDDRVEERDPPRRVPPPALSRDRERDRDREREGEKEKASWRAEKDRESLRRTKNETDEDGWTTVRR

1187 NP_859530.1 NP_550434.1

>NP_859530.1

MLQTLYDYFWWERLWLPVNLTWADLEDRDGRVYAKASDLYITLPLALLFLIVRYFFELYVATPLAALLNIKEKTRLRAPPNATLEHFYLTSGKQPKQVEVELLSRQSGLSGRQVERWFRRRRNQDRPSLLKKFREASWRFTFYLIAFIAGMAVIVDKPWFYDMKKVWEGYPIQSTIPSQYWYYMIELSFYWSLLFSIASDVKRKDFKEQIIHHVATIILISFSWFANYIRAGTLIMALHDSSDYLLESAKMFNYAGWKNTCNNIFIVFAIVFIITRLVILPFWILHCTLVYPLELYPAFFGYYFFNSMMGVLQLLHIFWAYLILRMAHKFITGKLVEDERSDREETESSEGEEAAAGGGAKSRPLANGHPILNNNHRKND

>NP_550434.1

MAKDFQDIQQLSSEENDHPFHQGEGPGTRRLNPRRGNPFLKGPPPAQPLAQRLCSMVCFSLLALSFNILLLVVICVTGSQSEGHRGAQLQAELRSLKEAFSNFSSSTLTEVQAISTHGGSVGDKITSLGAKLEKQQQDLKADHDALLFHLKHFPVDLRFVACQMELLHSNGSQRTCCPVNWVEHQGSCYWFSHSGKAWAEAEKYCQLENAHLVVINSWEEQKFIVQHTNPFNTWIGLTDSDGSWKWVDGTDYRHNYKNWAVTQPDNWHGHELGGSEDCVEVQPDGRWNDDFCLQVYRWVCEKRRNATGEVA

1188 NP_150634.1 NP_932070.1

>NP_150634.1

MADKVLKEKRKLFIRSMGEGTINGLLDELLQTRVLNKEEMEKVKRENATVMDKTRALIDSVIPKGAQACQICITYICEEDSYLAGTLGLSADQTSGNYLNMQDSQGVLSSFPAPQAVQDNPAMPTSSGSEGNVKLCSLEEAQRIWKQKSAEIYPIMDKSSRTRLALIICNEEFDSIPRRTGAEVDITGMTMLLQNLGYSVDVKKNLTASDMTTELEAFAHRPEHKTSDSTFLVFMSHGIREGICGKKHSEQVPDILQLNAIFNMLNTKNCPSLKDKPKVIIIQACRGDSPGVVWFKDSVGVSGNLSLPTTEEFEDDAIKKAHIEKDFIAFCSSTPDNVSWRHPTMGSVFIGRLIEHMQEYACSCDVEEIFRKVRFSFEQPDGRAQMPTTERVTLTRCFYLFPGH

>NP_932070.1

MCSGAGVMMARWAARGRAGWRSTVRILSPLGHCEPGVSRSCRAAQAMDCEVNNGSSLRDECITNLLVFGFLQSCSDNSFRRELDALGHELPVLAPQWEGYDELQTDGNRSSHSRLGRIEADSESQEDIIRNIARHLAQVGDSMDRSIPPGLVNGLALQLRNTSRSEEDRNRDLATALEQLLQAYPRDMEKEKTMLVLALLLAKKVASHTPSLLRDVFHTTVNFINQNLRTYVRSLARNGMD

1189 NP_002881.1 NP_004374.1

>NP_002881.1

MMAAEAGSEEGGPVTAGAGGGGAAAGSSAYPAVCRVKIPAALPVAAAPYPGLVETGVAGTLGGGAALGSEFLGAGSVAGALGGAGLTGGGTAAGVAGAAAGVAGAAVAGPSGDMALTKLPTSLLAETLGPGGGFPPLPPPPYLPPLGAGLGTVDEGDSLDGPEYEEEEVAIPLTAPPTNQWYHGKLDRTIAEERLRQAGKSGSYLIRESDRRPGSFVLSFLSQMNVVNHFRIIAMCGDYYIGGRRFSSLSDLIGYYSHVSCLLKGEKLLYPVAPPEPVEDRRRVRAILPYTKVPDTDEISFLKGDMFIVHNELEDGWMWVTNLRTDEQGLIVEDLVEEVGREEDPHEGKIWFHGKISKQEAYNLLMTVGQVCSFLVRPSDNTPGDYSLYFRTNENIQRFKICPTPNNQFMMGGRYYNSIGDIIDHYRKEQIVEGYYLKEPVPMQDQEQVLNDTVDGKEIYNTIRRKTKDAFYKNIVKKGYLLKKGKGKRWKNLYFILEGSDAQLIYFESEKRATKPKGLIDLSVCSVYVVHDSLFGRPNCFQIVVQHFSEEHYIFYFAGETPEQAEDWMKGLQAFCNLRKSSPGTSNKRLRQVSSLVLHIEEAHKLPVKHFTNPYCNIYLNSVQVAKTHAREGQNPVWSEEFVFDDLPPDINRFEITLSNKTKKSKDPDILFMRCQLSRLQKGHATDEWFLLSSHIPLKGIEPGSLRVRARYSMEKIMPEEEYSEFKELILQKELHVVYALSHVCGQDRTLLASILLRIFLHEKLESLLLCTLNDREISMEDEATTLFRATTLASTLMEQYMKATATQFVHHALKDSILKIMESKQSCELSPSKLEKNEDVNTNLTHLLNILSELVEKIFMASEILPPTLRYIYGCLQKSVQHKWPTNTTMRTRVVSGFVFLRLICPAILNPRMFNIISDSPSPIAARTLILVAKSVQNLANLVEFGAKEPYMEGVNPFIKSNKHRMIMFLDELGNVPELPDTTEHSRTDLSRDLAALHEICVAHSDELRTLSNERGAQQHVLKKLLAITELLQQKQNQYTKTNDVR

>NP_004374.1

MSAIQAAWPSGTECIAKYNFHGTAEQDLPFCKGDVLTIVAVTKDPNWYKAKNKVGREGIIPANYVQKREGVKAGTKLSLMPWFHGKITREQAERLLYPPETGLFLVRESTNYPGDYTLCVSCDGKVEHYRIMYHASKLSIDEEVYFENLMQLVEHYTSDADGLCTRLIKPKVMEGTVAAQDEFYRSGWALNMKELKLLQTIGKGEFGDVMLGDYRGNKVAVKCIKNDATAQAFLAEASVMTQLRHSNLVQLLGVIVEEKGGLYIVTEYMAKGSLVDYLRSRGRSVLGGDCLLKFSLDVCEAMEYLEGNNFVHRDLAARNVLVSEDNVAKVSDFGLTKEASSTQDTGKLPVKWTAPEALREKKFSTKSDVWSFGILLWEIYSFGRVPYPRIPLKDVVPRVEKGYKMDAPDGCPPAVYEVMKNCWHLDAAMRPSFLQLREQLEHIKTHELHL

1190 NP_065120.2 NP_004603.1

>NP_065120.2

MGNGTEEDYNFVFKVVLIGESGVGKTNLLSRFTRNEFSHDSRTTIGVEFSTRTVMLGTAAVKAQIWDTAGLERYRAITSAYYRGAVGALLVFDLTKHQTYAVVERWLKELYDHAEATIVVMLVGNKSDLSQAREVPTEEARMFAENNGLLFLETSALDSTNVELAFETVLKEIFAKVSKQRQNSIRTNAITLGSAQAGQEPGPGEKRACCISL

>NP_004603.1

MEAAVAAPRPRLLLLVLAAAAAAAAALLPGATALQCFCHLCTKDNFTCVTDGLCFVSVTETTDKVIHNSMCIAEIDLIPRDRPFVCAPSSKTGSVTTTYCCNQDHCNKIELPTTVKSSPGLGPVELAAVIAGPVCFVCISLMLMVYICHNRTVIHHRVPNEEDPSLDRPFISEGTTLKDLIYDMTTSGSGSGLPLLVQRTIARTIVLQESIGKGRFGEVWRGKWRGEEVAVKIFSSREERSWFREAEIYQTVMLRHENILGFIAADNKDNGTWTQLWLVSDYHEHGSLFDYLNRYTVTVEGMIKLALSTASGLAHLHMEIVGTQGKPAIAHRDLKSKNILVKKNGTCCIADLGLAVRHDSATDTIDIAPNHRVGTKRYMAPEVLDDSINMKHFESFKRADIYAMGLVFWEIARRCSIGGIHEDYQLPYYDLVPSDPSVEEMRKVVCEQKLRPNIPNRWQSCEALRVMAKIMRECWYANGAARLTALRIKKTLSQLSQQEGIKM

1191 NP_005616.2 NP_004420.1

>NP_005616.2

MSLYPSLEDLKVDKVIQAQTAFSANPANPAILSEASAPIPHDGNLYPRLYPELSQYMGLSLNEEEIRANVAVVSGAPLQGQLVARPSSINYMVAPVTGNDVGIRRAEIKQGIREVILCKDQDGKIGLRLKSIDNGIFVQLVQANSPASLVGLRFGDQVLQINGENCAGWSSDKAHKVLKQAFGEKITMTIRDRPFERTITMHKDSTGHVGFIFKNGKITSIVKDSSAARNGLLTEHNICEINGQNVIGLKDSQIADILSTSGTVVTITIMPAFIFEHIIKRMAPSIMKSLMDHTIPEV

>NP_004420.1

MARPGQRWLGKWLVAMVVWALCRLATPLAKNLEPVSWSSLNPKFLSGKGLVIYPKIGDKLDIICPRAEAGRPYEYYKLYLVRPEQAAACSTVLDPNVLVTCNRPEQEIRFTIKFQEFSPNYMGLEFKKHHDYYITSTSNGSLEGLENREGGVCRTRTMKIIMKVGQDPNAVTPEQLTTSRPSKEADNTVKMATQAPGSRGSLGDSDGKHETVNQEEKSGPGASGGSSGDPDGFFNSKVALFAAVGAGCVIFLLIIIFLTVLLLKLRKRHRKHTQQRAAALSLSTLASPKGGSGTAGTEPSDIIIPLRTTENNYCPHYEKVSGDYGHPVYIVQEMPPQSPANIYYKV

1192 NP_003216.1 NP_038466.2

>NP_003216.1

MATMENKVICALVLVSMLALGTLAEAQTETCTVAPRERQNCGFPGVTPSQCANKGCCFDDTVRGVPWCFYPNTIDVPPEEECEF

>NP_038466.2

MAESGESGGPPGSQDSAAGAEGAGAPAAAASAEPKIMKVTVKTPKEKEEFAVPENSSVQQFKEEISKRFKSHTDQLVLIFAGKILKDQDTLSQHGIHDGLTVHLVIKTQNRPQDHSAQQTNTAGSNVTTSSTPNSNSTSGSATSNPFGLGGLGGLAGLSSLGLNTTNFSELQSQMQRQLLSNPEMMVQIMENPFVQSMLSNPDLMRQLIMANPQMQQLIQRNPEISHMLNNPDIMRQTLELARNPAMMQEMMRNQDRALSNLESIPGGYNALRRMYTDIQEPMLSAAQEQFGGNPFASLVSNTSSGEGSQPSRTENRDPLPNPWAPQTSQSSSASSGTASTVGGTTGSTASGTSGQSTTAPNLVPGVGASMFNTPGMQSLLQQITENPQLMQNMLSAPYMRSMMQSLSQNPDLAAQMMLNNPLFAGNPQLQEQMRQQLPTFLQQMQNPDTLSAMSNPRAMQALLQIQQGLQTLATEAPGLIPGFTPGLGALGSTGGSSGTNGSNATPSENTSPTAGTTEPGHQQFIQQMLQALAGVNPQLQNPEVRFQQQLEQLSAMGFLNREANLQALIATGGDINAAIERLLGSQPS

1193 NP_000315.1 NP_001034221.1

>NP_000315.1

MRFTFPLMAIVLEIAMIVLFGLFVEYETDQTVLEQLNITKPTDMGIFFELYPLFQDVHVMIFVGFGFLMTFLKKYGFSSVGINLLVAALGLQWGTIVQGILQSQGQKFNIGIKNMINADFSAATVLISFGAVLGKTSPTQMLIMTILEIVFFAHNEYLVSEIFKASDIGASMTIHAFGAYFGLAVAGILYRSGLRKGHENEESAYYSDLFAMIGTLFLWMFWPSFNSAIAEPGDKQCRAIVDTYFSLAACVLTAFAFSSLVEHRGKLNMVHIQNATLAGGVAVGTCADMAIHPFGSMIIGSIAGMVSVLGYKFLTPLFTTKLRIHDTCGVHNLHGLPGVVGGLAGIVAVAMGASNTSMAMQAAALGSSIGTAVVGGLMTGLILKLPLWGQPSDQNCYDDSVYWKVPKTR

>NP_001034221.1

MGSLFPLSLLFFLAAAYPGVGSALGRRTKRAQSPKGSPLAPSGTSVPFWVRMSPEFVAVQPGKSVQLNCSNSCPQPQNSSLRTPLRQGKTLRGPGWVSYQLLDVRAWSSLAHCLVTCAGKTRWATSRITAYSVPGGLLGGDPEAWKPGHLFRKPGALHRPGSGQRDLDLRVCCWTPRLLAARDLPRAPQSRRPGGPQQLGTHYTDARLEPRAHSFGLRFHRCPCRDPPHCGRCVPMQVPSYEVPGVKGDVLCRLSEKKRNMKQSGEMAIHGG

1194 NP_001155.1 NP_004516.2

>NP_001155.1

MSVPSSLSQSAINANSHGGPALSLPLPLHAAHNQLLNAKLQATAVGPKDLRSAMGEGGGPEPGPANAKWLKEGQNQLRRAATAHRDQNRNVTLTLAEEASQEPEMAPLGPKGLIHLYSELELSAHNAANRGLRGPGLIISTQEQGPDEGEEKAAGEAEEEEEDDDDEEEEEDLSSPPGLPEPLESVEAPPRPQALTDGPREHSKSASLLFGMRNSAASDEDSSWATLSQGSPSYGSPEDTDSFWNPNAFETDSDLPAGWMRVQDTSGTYYWHIPTGTTQWEPPGRASPSQGSSPQEESQLTWTGFAHGEGFEDGEFWKDEPSDEAPMELGLKEPEEGTLTFPAQSLSPEPLPQEEEKLPPRNTNPGIKCFAVRSLGWVEMTEEELAPGRSSVAVNNCIRQLSYHKNNLHDPMSGGWGEGKDLLLQLEDETLKLVEPQSQALLHAQPIISIRVWGVGRDSGRERDFAYVARDKLTQMLKCHVFRCEAPAKNIATSLHEICSKIMAERRNARCLVNGLSLDHSKLVDVPFQVEFPAPKNELVQKFQVYYLGNVPVAKPVGVDVINGALESVLSSSSREQWTPSHVSVAPATLTILHQQTEAVLGECRVRFLSFLAVGRDVHTFAFIMAAGPASFCCHMFWCEPNAASLSEAVQAACMLRYQKCLDARSQASTSCLPAPPAESVARRVGWTVRRGVQSLWGSLKPKRLGAHTP

>NP_004516.2

MDRGPAAVACTLLLALVACLAPASGQECDSAHFRCGSGHCIPADWRCDGTKDCSDDADEIGCAVVTCQQGYFKCQSEGQCIPNSWVCDQDQDCDDGSDERQDCSQSTCSSHQITCSNGQCIPSEYRCDHVRDCPDGADENDCQYPTCEQLTCDNGACYNTSQKCDWKVDCRDSSDEINCTEICLHNEFSCGNGECIPRAYVCDHDNDCQDGSDEHACNYPTCGGYQFTCPSGRCIYQNWVCDGEDDCKDNGDEDGCESGPHDVHKCSPREWSCPESGRCISIYKVCDGILDCPGREDENNTSTGKYCSMTLCSALNCQYQCHETPYGGACFCPPGYIINHNDSRTCVEFDDCQIWGICDQKCESRPGRHLCHCEEGYILERGQYCKANDSFGEASIIFSNGRDLLIGDIHGRSFRILVESQNRGVAVGVAFHYHLQRVFWTDTVQNKVFSVDINGLNIQEVLNVSVETPENLAVDWVNNKIYLVETKVNRIDMVNLDGSYRVTLITENLGHPRGIAVDPTVGYLFFSDWESLSGEPKLERAFMDGSNRKDLVKTKLGWPAGVTLDMISKRVYWVDSRFDYIETVTYDGIQRKTVVHGGSLIPHPFGVSLFEGQVFFTDWTKMAVLKANKFTETNPQVYYQASLRPYGVTVYHSLRQPYATNPCKDNNGGCEQVCVLSHRTDNDGLGFRCKCTFGFQLDTDERHCIAVQNFLIFSSQVAIRGIPFTLSTQEDVMVPVSGNPSFFVGIDFDAQDSTIFFSDMSKHMIFKQKIDGTGREILAANRVENVESLAFDWISKNLYWTDSHYKSISVMRLADKTRRTVVQYLNNPRSVVVHPFAGYLFFTDWFRPAKIMRAWSDGSHLLPVINTTLGWPNGLAIDWAASRLYWVDAYFDKIEHSTFDGLDRRRLGHIEQMTHPFGLAIFGEHLFFTDWRLGAIIRVRKADGGEMTVIRSGIAYILHLKSYDVNIQTGSNACNQPTHPNGDCSHFCFPVPNFQRVCGCPYGMRLASNHLTCEGDPTNEPPTEQCGLFSFPCKNGRCVPNYYLCDGVDDCHDNSDEQLCGTLNNTCSSSAFTCGHGECIPAHWRCDKRNDCVDGSDEHNCPTHAPASCLDTQYTCDNHQCISKNWVCDTDNDCGDGSDEKNCNSTETCQPSQFNCPNHRCIDLSFVCDGDKDCVDGSDEVGCVLNCTASQFKCASGDKCIGVTNRCDGVFDCSDNSDEAGCPTRPPGMCHSDEFQCQEDGICIPNFWECDGHPDCLYGSDEHNACVPKTCPSSYFHCDNGNCIHRAWLCDRDNDCGDMSDEKDCPTQPFRCPSWQWQCLGHNICVNLSVVCDGIFDCPNGTDESPLCNGNSCSDFNGGCTHECVQEPFGAKCLCPLGFLLANDSKTCEDIDECDILGSCSQHCYNMRGSFRCSCDTGYMLESDGRTCKVTASESLLLLVASQNKIIADSVTSQVHNIYSLVENGSYIVAVDFDSISGRIFWSDATQGKTWSAFQNGTDRRVVFDSSIILTETIAIDWVGRNLYWTDYALETIEVSKIDGSHRTVLISKNLTNPRGLALDPRMNEHLLFWSDWGHHPRIERASMDGSMRTVIVQDKIFWPCGLTIDYPNRLLYFMDSYLDYMDFCDYNGHHRRQVIASDLIIRHPYALTLFEDSVYWTDRATRRVMRANKWHGGNQSVVMYNIQWPLGIVAVHPSKQPNSVNPCAFSRCSHLCLLSSQGPHFYSCVCPSGWSLSPDLLNCLRDDQPFLITVRQHIIFGISLNPEVKSNDAMVPIAGIQNGLDVEFDDAEQYIYWVENPGEIHRVKTDGTNRTVFASISMVGPSMNLALDWISRNLYSTNPRTQSIEVLTLHGDIRYRKTLIANDGTALGVGFPIGITVDPARGKLYWSDQGTDSGVPAKIASANMDGTSVKTLFTGNLEHLECVTLDIEEQKLYWAVTGRGVIERGNVDGTDRMILVHQLSHPWGIAVHDSFLYYTDEQYEVIERVDKATGANKIVLRDNVPNLRGLQVYHRRNAAESSNGCSNNMNACQQICLPVPGGLFSCACATGFKLNPDNRSCSPYNSFIVVSMLSAIRGFSLELSDHSETMVPVAGQGRNALHVDVDVSSGFIYWCDFSSSVASDNAIRRIKPDGSSLMNIVTHGIGENGVRGIAVDWVAGNLYFTNAFVSETLIEVLRINTTYRRVLLKVTVDMPRHIVVDPKNRYLFWADYGQRPKIERSFLDCTNRTVLVSEGIVTPRGLAVDRSDGYVYWVDDSLDIIARIRINGENSEVIRYGSRYPTPYGITVFENSIIWVDRNLKKIFQASKEPENTEPPTVIRDNINWLRDVTIFDKQVQPRSPAEVNNNPCLENNGGCSHLCFALPGLHTPKCDCAFGTLQSDGKNCAISTENFLIFALSNSLRSLHLDPENHSPPFQTINVERTVMSLDYDSVSDRIYFTQNLASGVGQISYATLSSGIHTPTVIASGIGTADGIAFDWITRRIYYSDYLNQMINSMAEDGSNRTVIARVPKPRAIVLDPCQGYLYWADWDTHAKIERATLGGNFRVPIVNSSLVMPSGLTLDYEEDLLYWVDASLQRIERSTLTGVDREVIVNAAVHAFGLTLYGQYIYWTDLYTQRIYRANKYDGSGQIAMTTNLLSQPRGINTVVKNQKQQCNNPCEQFNGGCSHICAPGPNGAECQCPHEGNWYLANNRKHCIVDNGERCGASSFTCSNGRCISEEWKCDNDNDCGDGSDEMESVCALHTCSPTAFTCANGRCVQYSYRCDYYNDCGDGSDEAGCLFRDCNATTEFMCNNRRCIPREFICNGVDNCHDNNTSDEKNCPDRTCQSGYTKCHNSNICIPRVYLCDGDNDCGDNSDENPTYCTTHTCSSSEFQCASGRCIPQHWYCDQETDCFDASDEPASCGHSERTCLADEFKCDGGRCIPSEWICDGDNDCGDMSDEDKRHQCQNQNCSDSEFLCVNDRPPDRRCIPQSWVCDGDVDCTDGYDENQNCTRRTCSENEFTCGYGLCIPKIFRCDRHNDCGDYSDERGCLYQTCQQNQFTCQNGRCISKTFVCDEDNDCGDGSDELMHLCHTPEPTCPPHEFKCDNGRCIEMMKLCNHLDDCLDNSDEKGCGINECHDPSISGCDHNCTDTLTSFYCSCRPGYKLMSDKRTCVDIDECTEMPFVCSQKCENVIGSYICKCAPGYLREPDGKTCRQNSNIEPYLIFSNRYYLRNLTIDGYFYSLILEGLDNVVALDFDRVEKRLYWIDTQRQVIERMFLNKTNKETIINHRLPAAESLAVDWVSRKLYWLDARLDGLFVSDLNGGHRRMLAQHCVDANNTFCFDNPRGLALHPQYGYLYWADWGHRAYIGRVGMDGTNKSVIISTKLEWPNGITIDYTNDLLYWADAHLGYIEYSDLEGHHRHTVYDGALPHPFAITIFEDTIYWTDWNTRTVEKGNKYDGSNRQTLVNTTHRPFDIHVYHPYRQPIVSNPCGTNNGGCSHLCLIKPGGKGFTCECPDDFRTLQLSGSTYCMPMCSSTQFLCANNEKCIPIWWKCDGQKDCSDGSDELALCPQRFCRLGQFQCSDGNCTSPQTLCNAHQNCPDGSDEDRLLCENHHCDSNEWQCANKRCIPESWQCDTFNDCEDNSDEDSSHCASRTCRPGQFRCANGRCIPQAWKCDVDNDCGDHSDEPIEECMSSAHLCDNFTEFSCKTNYRCIPKWAVCNGVDDCRDNSDEQGCEERTCHPVGDFRCKNHHCIPLRWQCDGQNDCGDNSDEENCAPRECTESEFRCVNQQCIPSRWICDHYNDCGDNSDERDCEMRTCHPEYFQCTSGHCVHSELKCDGSADCLDASDEADCPTRFPDGAYCQATMFECKNHVCIPPYWKCDGDDDCGDGSDEELHLCLDVPCNSPNRFRCDNNRCIYSHEVCNGVDDCGDGTDETEEHCRKPTPKPCTEYEYKCGNGHCIPHDNVCDDADDCGDWSDELGCNKGKERTCAENICEQNCTQLNEGGFICSCTAGFETNVFDRTSCLDINECEQFGTCPQHCRNTKGSYECVCADGFTSMSDRPGKRCAAEGSSPLLLLPDNVRIRKYNLSSERFSEYLQDEEYIQAVDYDWDPKDIGLSVVYYTVRGEGSRFGAIKRAYIPNFESGRNNLVQEVDLKLKYVMQPDGIAVDWVGRHIYWSDVKNKRIEVAKLDGRYRKWLISTDLDQPAAIAVNPKLGLMFWTDWGKEPKIESAWMNGEDRNILVFEDLGWPTGLSIDYLNNDRIYWSDFKEDVIETIKYDGTDRRVIAKEAMNPYSLDIFEDQLYWISKEKGEVWKQNKFGQGKKEKTLVVNPWLTQVRIFHQLRYNKSVPNLCKQICSHLCLLRPGGYSCACPQGSSFIEGSTTECDAAIELPINLPPPCRCMHGGNCYFDETDLPKCKCPSGYTGKYCEMAFSKGISPGTTAVAVLLTILLIVVIGALAIAGFFHYRRTGSLLPALPKLPSLSSLVKPSENGNGVTFRSGADLNMDIGVSGFGPETAIDRSMAMSEDFVMEMGKQPIIFENPMYSARDSAVKVVQPIQVTVSENVDNKNYGSPINPSEIVPETNPTSPAADGTQVTKWNLFKRKSKQTTNFENPIYAQMENEQKESVAATPPPSPSLPAKPKPPSRRDPTPTYSATEDTFKDTANLVKEDSEV

1195 NP_003620.2 NP_000866.1

>NP_003620.2

MYNTVWSMDRDDADWREVMMPYSTELIFYIEMDPPALPPKPPKPMTSAVPNGMKDSSVSLQDAEWYWGDISREEVNDKLRDMPDGTFLVRDASTKMQGDYTLTLRKGGNNKLIKIYHRDGKYGFSDPLTFNSVVELINHYHHESLAQYNPKLDVKLMYPVSRYQQDQLVKEDNIDAVGKKLQEYHSQYQEKSKEYDRLYEEYTRTSQEIQMKRTAIEAFNETIKIFEEQCHTQEQHSKEYIERFRREGNEKEIERIMMNYDKLKSRLGEIHDSKMRLEQDLKNQALDNREIDKKMNSIKPDLIQLRKIRDQHLVWLNHKGVRQKRLNVWLGIKNEDAAENYFINEEDENLPHYDEKTWFVEDINRVQAEDLLYGKPDGAFLIRESSKKGCYACSVVADGEVKHCVIYSTARGYGFAEPYNLYSSLKELVLHYQQTSLVQHNDSLNVRLAYPVHAQMPSLCR

>NP_000866.1

MKSGSGGGSPTSLWGLLFLSAALSLWPTSGEICGPGIDIRNDYQQLKRLENCTVIEGYLHILLISKAEDYRSYRFPKLTVITEYLLLFRVAGLESLGDLFPNLTVIRGWKLFYNYALVIFEMTNLKDIGLYNLRNITRGAIRIEKNADLCYLSTVDWSLILDAVSNNYIVGNKPPKECGDLCPGTMEEKPMCEKTTINNEYNYRCWTTNRCQKMCPSTCGKRACTENNECCHPECLGSCSAPDNDTACVACRHYYYAGVCVPACPPNTYRFEGWRCVDRDFCANILSAESSDSEGFVIHDGECMQECPSGFIRNGSQSMYCIPCEGPCPKVCEEEKKTKTIDSVTSAQMLQGCTIFKGNLLINIRRGNNIASELENFMGLIEVVTGYVKIRHSHALVSLSFLKNLRLILGEEQLEGNYSFYVLDNQNLQQLWDWDHRNLTIKAGKMYFAFNPKLCVSEIYRMEEVTGTKGRQSKGDINTRNNGERASCESDVLHFTSTTTSKNRIIITWHRYRPPDYRDLISFTVYYKEAPFKNVTEYDGQDACGSNSWNMVDVDLPPNKDVEPGILLHGLKPWTQYAVYVKAVTLTMVENDHIRGAKSEILYIRTNASVPSIPLDVLSASNSSSQLIVKWNPPSLPNGNLSYYIVRWQRQPQDGYLYRHNYCSKDKIPIRKYADGTIDIEEVTENPKTEVCGGEKGPCCACPKTEAEKQAEKEEAEYRKVFENFLHNSIFVPRPERKRRDVMQVANTTMSSRSRNTTAADTYNITDPEELETEYPFFESRVDNKERTVISNLRPFTLYRIDIHSCNHEAEKLGCSASNFVFARTMPAEGADDIPGPVTWEPRPENSIFLKWPEPENPNGLILMYEIKYGSQVEDQRECVSRQEYRKYGGAKLNRLNPGNYTARIQATSLSGNGSWTDPVFFYVQAKTGYENFIHLIIALPVAVLLIVGGLVIMLYVFHRKRNNSRLGNGVLYASVNPEYFSAADVYVPDEWEVAREKITMSRELGQGSFGMVYEGVAKGVVKDEPETRVAIKTVNEAASMRERIEFLNEASVMKEFNCHHVVRLLGVVSQGQPTLVIMELMTRGDLKSYLRSLRPEMENNPVLAPPSLSKMIQMAGEIADGMAYLNANKFVHRDLAARNCMVAEDFTVKIGDFGMTRDIYETDYYRKGGKGLLPVRWMSPESLKDGVFTTYSDVWSFGVVLWEIATLAEQPYQGLSNEQVLRFVMEGGLLDKPDNCPDMLFELMRMCWQYNPKMRPSFLEIISSIKEEMEPGFREVSFYYSEENKLPEPEELDLEPENMESVPLDPSASSSSLPLPDRHSGHKAENGPGPGVLVLRASFDERQPYAHMNGGRKNERALPLPQSSTC

1196 NP_000359.1 NP_003395.1

>NP_000359.1

MAQQANVGELLAMLDSPMLGVRDDVTAVFKENLNSDRGPMLVNTLVDYYLETSSQPALHILTTLQEPHDKHLLDRINEYVGKAATRLSILSLLGHVIRLQPSWKHKLSQAPLLPSLLKCLKMDTDVVVLTTGVLVLITMLPMIPQSGKQHLLDFFDIFGRLSSWCLKKPGHVAEVYLVHLHASVYALFHRLYGMYPCNFVSFLRSHYSMKENLETFEEVVKPMMEHVRIHPELVTGSKDHELDPRRWKRLETHDVVIECAKISLDPTEASYEDGYSVSHQISARFPHRSADVTTSPYADTQNSYGCATSTPYSTSRLMLLNMPGQLPQTLSSPSTRLITEPPQATLWSPSMVCGMTTPPTSPGNVPPDLSHPYSKVFGTTAGGKGTPLGTPATSPPPAPLCHSDDYVHISLPQATVTPPRKEERMDSARPCLHRQHHLLNDRGSEEPPGSKGSVTLSDLPGFLGDLASEEDSIEKDKEEAAISRELSEITTAEAEPVVPRGGFDSPFYRDSLPGSQRKTHSAASSSQGASVNPEPLHSSLDKLGPDTPKQAFTPIDLPCGSADESPAGDRECQTSLETSIFTPSPCKIPPPTRVGFGSGQPPPYDHLFEVALPKTAHHFVIRKTEELLKKAKGNTEEDGVPSTSPMEVLDRLIQQGADAHSKELNKLPLPSKSVDWTHFGGSPPSDEIRTLRDQLLLLHNQLLYERFKRQQHALRNRRLLRKVIKAAALEEHNAAMKDQLKLQEKDIQMWKVSLQKEQARYNQLQEQRDTMVTKLHSQIRQLQHDREEFYNQSQELQTKLEDCRNMIAELRIELKKANNKVCHTELLLSQVSQKLSNSESVQQQMEFLNRQLLVLGEVNELYLEQLQNKHSDTTKEVEMMKAAYRKELEKNRSHVLQQTQRLDTSQKRILELESHLAKKDHLLLEQKKYLEDVKLQARGQLQAAESRYEAQKRITQVFELEILDLYGRLEKDGLLKKLEEEKAEAAEAAEERLDCCNDGCSDSMVGHNEEASGHNGETKTPRPSSARGSSGSRGGGGSSSSSSELSTPEKPPHQRAGPFSSRWETTMGEASASIPTTVGSLPSSKSFLGMKARELFRNKSESQCDEDGMTSSLSESLKTELGKDLGVEAKIPLNLDGPHPSPPTPDSVGQLHIMDYNETHHEHS

>NP_003395.1

MTMDKSELVQKAKLAEQAERYDDMAAAMKAVTEQGHELSNEERNLLSVAYKNVVGARRSSWRVISSIEQKTERNEKKQQMGKEYREKIEAELQDICNDVLELLDKYLIPNATQPESKVFYLKMKGDYFRYLSEVASGDNKQTTVSNSQQAYQEAFEISKKEMQPTHPIRLGLALNFSVFYYEILNSPEKACSLAKTAFDEAIAELDTLNEESYKDSTLIMQLLRDNLTLWTSENQGDEGDAGEGEN

1197 NP_001645.1 NP_872270.1

>NP_001645.1

MEPPRGPPANGAEPSRAVGTVKVYLPNKQRTVVTVRDGMSVYDSLDKALKVRGLNQDCCVVYRLIKGRKTVTAWDTAIAPLDGEELIVEVLEDVPLTMHNFVRKTFFSLAFCDFCLKFLFHGFRCQTCGYKFHQHCSSKVPTVCVDMSTNRQQFYHSVQDLSGGSRQHEAPSNRPLNELLTPQGPSPRTQHCDPEHFPFPAPANAPLQRIRSTSTPNVHMVSTTAPMDSNLIQLTGQSFSTDAAGSRGGSDGTPRGSPSPASVSSGRKSPHSKSPAEQRERKSLADDKKKVKNLGYRDSGYYWEVPPSEVQLLKRIGTGSFGTVFRGRWHGDVAVKVLKVSQPTAEQAQAFKNEMQVLRKTRHVNILLFMGFMTRPGFAIITQWCEGSSLYHHLHVADTRFDMVQLIDVARQTAQGMDYLHAKNIIHRDLKSNNIFLHEGLTVKIGDFGLATVKTRWSGAQPLEQPSGSVLWMAAEVIRMQDPNPYSFQSDVYAYGVVLYELMTGSLPYSHIGCRDQIIFMVGRGYLSPDLSKISSNCPKAMRRLLSDCLKFQREERPLFPQILATIELLQRSLPKIERSASEPSLHRTQADELPACLLSAARLVP

>NP_872270.1

MSKPHSEAGTAFIQTQQLHAAMADTFLEHMCRLDIDSPPITARNTGIICTIGPASRSVETLKEMIKSGMNVARLNFSHGTHEYHAETIKNVRTATESFASDPILYRPVAVALDTKGPEIRTGLIKGSGTAEVELKKGATLKITLDNAYMEKCDENILWLDYKNICKVVEVGSKIYVDDGLISLQVKQKGADFLVTEVENGGSLGSKKGVNLPGAAVDLPAVSEKDIQDLKFGVEQDVDMVFASFIRKASDVHEVRKVLGEKGKNIKIISKIENHEGVRRFDEILEASDGIMVARGDLGIEIPAEKVFLAQKMMIGRCNRAGKPVICATQMLESMIKKPRPTRAEGSDVANAVLDGADCIMLSGETAKGDYPLEAVRMQHLIAREAEAAMFHRKLFEELVRASSHSTDLMEAMAMGSVEASYKCLAAALIVLTESGRSAHQVARYRPRAPIIAVTRNPQTARQAHLYRGIFPVLCKDPVQEAWAEDVDLRVNFAMNVGKARGFFKKGDVVIVLTGWRPGSGFTNTMRVVPVP

1198 NP_000035.2 NP_004337.2

>NP_000035.2

MEVQLGLGRVYPRPPSKTYRGAFQNLFQSVREVIQNPGPRHPEAASAAPPGASLLLLQQQQQQQQQQQQQQQQQQQQQQQETSPRQQQQQQGEDGSPQAHRRGPTGYLVLDEEQQPSQPQSALECHPERGCVPEPGAAVAASKGLPQQLPAPPDEDDSAAPSTLSLLGPTFPGLSSCSADLKDILSEASTMQLLQQQQQEAVSEGSSSGRAREASGAPTSSKDNYLGGTSTISDNAKELCKAVSVSMGLGVEALEHLSPGEQLRGDCMYAPLLGVPPAVRPTPCAPLAECKGSLLDDSAGKSTEDTAEYSPFKGGYTKGLEGESLGCSGSAAAGSSGTLELPSTLSLYKSGALDEAAAYQSRDYYNFPLALAGPPPPPPPPHPHARIKLENPLDYGSAWAAAAAQCRYGDLASLHGAGAAGPGSGSPSAAASSSWHTLFTAEEGQLYGPCGGGGGGGGGGGGGGGGGGGGGGGEAGAVAPYGYTRPPQGLAGQESDFTAPDVWYPGGMVSRVPYPSPTCVKSEMGPWMDSYSGPYGDMRLETARDHVLPIDYYFPPQKTCLICGDEASGCHYGALTCGSCKVFFKRAAEGKQKYLCASRNDCTIDKFRRKNCPSCRLRKCYEAGMTLGARKLKKLGNLKLQEEGEASSTTSPTEETTQKLTVSHIEGYECQPIFLNVLEAIEPGVVCAGHDNNQPDSFAALLSSLNELGERQLVHVVKWAKALPGFRNLHVDDQMAVIQYSWMGLMVFAMGWRSFTNVNSRMLYFAPDLVFNEYRMHKSRMYSQCVRMRHLSQEFGWLQITPQEFLCMKALLLFSIIPVDGLKNQKFFDELRMNYIKELDRIIACKRKNPTSCSRRFYQLTKLLDSVQPIARELHQFTFDLLIKSHMVSVDFPEMMAEIISVQVPKILSGKVKPIYFHTQ

>NP_004337.2

MENTENSVDSKSIKNLEPKIIHGSESMDSGISLDNSYKMDYPEMGLCIIINNKNFHKSTGMTSRSGTDVDAANLRETFRNLKYEVRNKNDLTREEIVELMRDVSKEDHSKRSSFVCVLLSHGEEGIIFGTNGPVDLKKITNFFRGDRCRSLTGKPKLFIIQACRGTELDCGIETDSGVDDDMACHKIPVEADFLYAYSTAPGYYSWRNSKDGSWFIQSLCAMLKQYADKLEFMHILTRVNRKVATEFESFSFDATFHAKKQIPCIVSMLTKELYFYH

1199 NP_114060.1 NP_064532.1

>NP_114060.1

MVNSCCGSVCSDQGCGLENCCRPSCCQTTCCRTTCCRPSCCVSSCCRPQCCQSVCCQPTCCRPSCCQTTCCRTTCCRPSCCVSSCCRPQCCQSVCCQPTCCRPSCCQTTCCRTTCCRPSCCVSSCCRPQCCQSVCCQPTCCRPSCCISSSCCPSCCESSCCRPCCCLRPVCGRVSCHTTCYRPTCVISTCPRPLCCASSCC

>NP_064532.1

MPARCVAAHCGNTTKSGKSLFRFPKDRAVRLLWDRFVRGCRADWYGGNDRSVICSDHFAPACFDVSSVIQKNLRFSQRLRLVAGAVPTLHRVPAPAPKRGEEGDQAGRLDTRGELQAARHSEAAPGPVSCTRPRAGKQAAASQITCENELVQTQPHADNPSNTVTSVPTHCEEGPVHKSTQISLKRPRHRSVGIQAKVKAFGKRLCNATTQTEELWSRTSSLFDIYSSDSETDTDWDIKSEQSDLSYMAVQVKEETC

1200 NP_005949.1 NP_002061.1

>NP_005949.1

MQGNGSALPNASQPVLRGDGARPSWLASALACVLIFTIVVDILGNLLVILSVYRNKKLRNAGNIFVVSLAVADLVVAIYPYPLVLMSIFNNGWNLGYLHCQVSGFLMGLSVIGSIFNITGIAINRYCYICHSLKYDKLYSSKNSLCYVLLIWLLTLAAVLPNLRAGTLQYDPRIYSCTFAQSVSSAYTIAVVVFHFLVPMIIVIFCYLRIWILVLQVRQRVKPDRKPKLKPQDFRNFVTMFVVFVLFAICWAPLNFIGLAVASDPASMVPRIPEWLFVASYYMAYFNSCLNAIIYGLLNQNFRKEYRRIIVSLCTARVFFVDSSNDVADRVKWKPSPLMTNNNVVKVDSV

>NP_002061.1

MGCTVSAEDKAAAERSKMIDKNLREDGEKAAREVKLLLLGAGESGKSTIVKQMKIIHEDGYSEEECRQYRAVVYSNTIQSIMAIVKAMGNLQIDFADPSRADDARQLFALSCTAEEQGVLPDDLSGVIRRLWADHGVQACFGRSREYQLNDSAAYYLNDLERIAQSDYIPTQQDVLRTRVKTTGIVETHFTFKDLHFKMFDVGGQRSERKKWIHCFEGVTAIIFCVALSAYDLVLAEDEEMNRMHESMKLFDSICNNKWFTDTSIILFLNKKDLFEEKITHSPLTICFPEYTGANKYDEAASYIQSKFEDLNKRKDTKEIYTHFTCATDTKNVQFVFDAVTDVIIKNNLKDCGLF

1201 NP_031374.2 NP_003122.1

>NP_031374.2

MGEPAGVAGTMESPFSPGLFHRLDEDWDSALFAELGYFTDTDELQLEAANETYENNFDNLDFDLDLMPWESDIWDINNQICTVKDIKAEPQPLSPASSSYSVSSPRSVDSYSSTQHVPEELDLSSSSQMSPLSLYGENSNSLSSAEPLKEDKPVTGPRNKTENGLTPKKKIQVNSKPSIQPKPLLLPAAPKTQTNSSVPAKTIIIQTVPTLMPLAKQQPIISLQPAPTKGQTVLLSQPTVVQLQAPGVLPSAQPVLAVAGGVTQLPNHVVNVVPAPSANSPVNGKLSVTKPVLQSTMRNVGSDIAVLRRQQRMIKNRESACQSRKKKKEYMLGLEARLKAALSENEQLKKENGTLKRQLDEVVSENQRLKVPSPKRRVVCVMIVLAFIILNYGPMSMLEQDSRRMNPSVSPANQRRHLLGFSAKEAQDTSDGIIQKNSYRYDHSVSNDKALMVLTEEPLLYIPPPPCQPLINTTESLRLNHELRGWVHRHEVERTKSRRMTNNQQKTRILQGALEQGSNSQLMAVQYTETTSSISRNSGSELQVYYASPRSYQDFFEAIRRRGDTFYVVSFRRDHLLLPATTHNKTTRPKMSIVLPAININENVINGQDYEVMMQIDCQVMDTRILHIKSSSVPPYLRDQQRNQTNTFFGSPPAATEATHVVSTIPESLQ

>NP_003122.1

MLPTQAGAAAALGRGSALGGSLNRTPTGRPGGGGGTRGANGGRVPGNGAGLGPGRLEREAAAAAATTPAPTAGALYSGSEGDSESGEEEELGAERRGLKRSLSEMEIGMVVGGPEASAAATGGYGPVSGAVSGAKPGKKTRGRVKIKMEFIDNKLRRYTTFSKRKTGIMKKAYELSTLTGTQVLLLVASETGHVYTFATRKLQPMITSETGKALIQTCLNSPDSPPRSDPTTDQRMSATGFEETDLTYQVSESDSSGETKDTLKPAFTVTNLPGTTSTIQTAPSTSTTMQVSSGPSFPITNYLAPVSASVSPSAVSSANGTVLKSTGSGPVSSGGLMQLPTSFTLMPGGAVAQQVPVQAIQVHQAPQQASPSRDSSTDLTQTSSSGTVTLPATIMTSSVPTTVGGHMMYPSPHAVMYAPTSGLGDGSLTVLNAFSQAPSTMQVSHSQVQEPGGVPQVFLTASSGTVQIPVSAVQLHQMAVIGQQAGSSSNLTELQVVNLDTAHSTKSE

1202 NP_055826.1 NP_002701.1

>NP_055826.1

MEIDQCLLESLPLGQRQRLVKRMRCEQIKAYYEREKAFQKQEGFLKRLKHAKNPKVHFNLTDMLQDAIIHHNDKEVLRLLKEGADPHTLVSSGGSLLHLCARYDNAFIAEILIDRGVNVNHQDEDFWTPMHIACACDNPDIVLLLVLAGANVLLQDVNGNIPLDYAVEGTESSSILLTYLDENGVDLTSLRQMKLQRPMSMLTDVKHFLSSGGNVNEKNDEGVTLLHMACASGYKEVVSLILEHGGDLNIVDDQYWTPLHLAAKYGQTNLVKLLLMHQANPHLVNCNEEKASDIAASEFIEEMLLKAEIAWEEKMKEPLSASTLAQEEPYEEIIHDLPVLSSKLSPLVLPIAKQDSLLEKDIMFKDATKGLCKQQSQDSIPENPMMSGSTKPEQVKLMPPAPNDDLATLSELNDGSLLYEIQKRFGNNQIYTFIGDILLLVNPYKELPIYSSMVSQLYFSSSGKLCSSLPPHLFSCVERAFHQLFREQRPQCFILSGERGSGKSEASKQIIRHLTCRAGASRATLDSRFKHVVCILEAFGHAKTTLNDLSSCFIKYFELQFCERKQQLTGARIYTYLLEKSRLVSQPLGQSNFLIFYLLMDGLSAEEKYGLHLNNLCAHRYLNQTIQDDASTGERSLNREKLAVLKRALNVVGFSSLEVENLFVILAAILHLGDIRFTALNEGNSAFVSDLQLLEQVAGMLQVSTDELASALTTDIQYFKGDMIIRRHTIQIAEFFRDLLAKSLYSRLFSFLVNTMNSCLHSQDEQKSMQTLDIGILDIFGFEEFQKNEFEQLCVNMTNEKMHHYINEVLFLHEQVECVQEGVTMETAYSPGNQNGVLDFFFQKPSGFLTLLDEESQMIWSVESNFPKKLQSLLESSNTNAVYSPMKDGNGNVALKDHGTAFTIMHYAGRVMYDVVGAIEKNKDSLSQNLLFVMKTSENVVINHLFQSKLSQTGSLVSAYPSFKFRGHKSALLSKKMTASSIIGENKNYLELSKLLKKKGTSTFLQRLERGDPVTIASQLRKSLMDIIGKLQKCTPHFIHCIRPNNSKLPDTFDNFYVSAQLQYIGVLEMVKIFRYGYPVRLSFSDFLSRYKPLADTFLREKKEQSAAERCRLVLQQCKLQGWQMGVRKVFLKYWHADQLNDLCLQLQRKIITCQKVIRGFLARQHLLQRISIRQQEVTSINSFLQNTEDMGLKTYDALVIQNASDIARENDRLRSEMNAPYHKEKLEVRNMQEEGSKRTDDKSGPRHFHPSSMSVCAAVDGLGQCLVGPSIWSPSLHSVFSMDDSSSLPSPRKQPPPKPKRDPNTRLSASYEAVSACLSAAREAANEALARPRPHSDDYSTMKKIPPRKPKRSPNTKLSGSYEEISGSRPGDARPAGAPGAAARVLTPGTPQCALPPAAPPGDEDDSEPVYIEMLGHAARPDSPDPGESVYEEMKCCLPDDGGPGAGSFLLHGASPPLLHRAPEDEAAGPPGDACDIPPPFPNLLPHRPPLLVFPPTPVTCSPASDESPLTPLEVKKLPVLETNLKYPVQPEGSSPLSPQYSKSQKGDGDRPASPGLALFNGSGRASPPSTPPPPPPPPGPPPAPYRPCAHLAFPPEPAPVNAGKAGPSAEAPKVHPKPNSAPVAGPCSSFPKIPYSPVKATRADARKAGSSASPPAPYSPPSSRPLSSPLDELASLFNSGRSVLRKSAAGRKIREAEGFETNMNISSRDDPSTSEITSETQDRNANNHGIQLSNSLSSAITAENGNSISNGLPEEDGYSRLSISGTGTSTFQRHRDSHTTQVIHQLRLSENESVALQELLDWRRKLCEEGQDWQQILHHAEPRVPPPPPCKKPSLLKKPEGASCNRLPSELWDTTI

>NP_002701.1

MADLDKLNIDSIIQRLLEVRGSKPGKNVQLQENEIRGLCLKSREIFLSQPILLELEAPLKICGDIHGQYYDLLRLFEYGGFPPESNYLFLGDYVDRGKQSLETICLLLAYKIKYPENFFLLRGNHECASINRIYGFYDECKRRYNIKLWKTFTDCFNCLPIAAIVDEKIFCCHGGLSPDLQSMEQIRRIMRPTDVPDQGLLCDLLWSDPDKDVLGWGENDRGVSFTFGAEVVAKFLHKHDLDLICRAHQVVEDGYEFFAKRQLVTLFSAPNYCGEFDNAGAMMSVDETLMCSFQILKPAEKKKPNATRPVTPPRGMITKQAKK

1203 NP_054790.2 NP_009052.3

>NP_054790.2

MVLDDLPNLEDIYTSLCSSTMEDSEMDFDSGLEDDDTKSDSILEDSTIFVAFKGNIDDKDFKWKLDAILKNVPNLLHMESSKLKVQKVEPWNSVRVTFNIPREAAERLRILAQSNNQQLRDLGILSVQIEGEGAINLALAQNRSQDVRMNGPMGAGNSVRMEAGFPMASGPGIIRMNNPATVMIPPGGNVSSSMMAPGPNPELQPRTPRPASQSDAMDPLLSGLHIQQQSHPSGSLAPPHHPMQPVSVNRQMNPANFPQLQQQQQQQQQQQQQQQQQQQQQQQQQLQARPPQQHQQQQPQGIRPQFTAPTQVPVPPGWNQLPSGALQPPPAQGSLGTMTANQGWKKAPLPGPMQQQLQARPSLATVQTPSHPPPPYPFGSQQASQAHTNFPQMSNPGQFTAPQMKSLQGGPSRVPTPLQQPHLTNKSPASSPSSFQQGSPASSPTVNQTQQQMGPRPPQNNPLPQGFQQPVSSPGRNPMVQQGNVPPNFMVMQQQPPNQGPQSLHPGLGGMPKRLPPGFSAGQANPNFMQGQVPSTTATTPGNSGAPQLQANQNVQHAGGQGAGPPQNQMQVSHGPPNMMQPSLMGIHGNMNNQQAGTSGVPQVNLSNMQGQPQQGPPSQLMGMHQQIVPSQGQMVQQQGTLNPQNPMILSRAQLMPQGQMMVNPPSQNLGPSPQRMTPPKQMLSQQGPQMMAPHNQMMGPQGQVLLQQNPMIEQIMTNQMQGNKQQFNTQNQSNVMPGPAQIMRGPTPNMQGNMVQFTGQMSGQMLPQQGPVNNSPSQVMGIQGQVLRPPGPSPHMAQQHGDPATTANNDVSLSQMMPDVSIQQTNMVPPHVQAMQGNSASGNHFSGHGMSFNAPFSGAPNGNQMSCGQNPGFPVNKDVTLTSPLLVNLLQSDISAGHFGVNNKQNNTNANKPKKKKPPRKKKNSQQDLNTPDTRPAGLEEADQPPLPGEQGINLDNSGPKLPEFSNRPPGYPSQPVEQRPLQQMPPQLMQHVAPPPQPPQQQPQPQLPQQQQPPPPSQPQSQQQQQQQQQMMMMLMMQQDPKSVRLPVSQNVHPPRGPLNPDSQRMPMQQSGSVPVMVSLQGPASVPPSPDKQRMPMPVNTPLGSNSRKMVYQESPQNPSSSPLAEMASLPEASGSEAPSVPGGPNNMPSHVVLPQNQLMMTGPKPGPSPLSATQGATPQQPPVNSLPSSHGHHFPNVAAPTQTSRPKTPNRASPRPYYPQTPNNRPPSTEPSEISLSPERLNASIAGLFPPQINIPLPPRPNLNRGFDQQGLNPTTLKAIGQAPSNLTMNPSNFATPQTHKLDSVVVNSGKQSNSGATKRASPSNSRRSSPGSSRKTTPSPGRQNSKAPKLTLASQTNAALLQNVELPRNVLVSPTPLANPPVPGSFPNNSGLNPQNSTVSVAAVGGVVEDNKESLNVPQDSDCQNSQSRKEQVNIELKAVPAQEVKMVVPEDQSKKDGQPSDPNKLPSVEENKNLVSPAMREAPTSLSQLLDNSGAPNVTIKPPGLTDLEVTPPVVSGEDLKKASVIPTLQDLSSSKEPSNSLNLPHSNELCSSLVHPELSEVSSNVAPSIPPVMSRPVSSSSISTPLPPNQITVFVTSNPITTSANTSAALPTHLQSALMSTVVTMPNAGSKVMVSEGQSAAQSNARPQFITPVFINSSSIIQVMKGSQPSTIPAAPLTTNSGLMPPSVAVVGPLHIPQNIKFSSAPVPPNALSSSPAPNIQTGRPLVLSSRATPVQLPSPPCTSSPVVPSHPPVQQVKELNPDEASPQVNTSADQNTLPSSQSTTMVSPLLTNSPGSSGNRRSPVSSSKGKGKVDKIGQILLTKACKKVTGSLEKGEEQYGADGETEGQGLDTTAPGLMGTEQLSTELDSKTPTPPAPTLLKMTSSPVGPGTASAGPSLPGGALPTSVRSIVTTLVPSELISAVPTTKSNHGGIASESLAGGLVEEKVGSHPELLPSIAPSQNLVSKETSTTALQASVARPELEVNAAIVSGQSSEPKEIVEKSKIPGRRNSRTEEPTVASESVENGHRKRSSRPASASSSTKDITSAVQSKRRKSK

>NP_009052.3

MSSPTTSSLDTPLPGNGPPQPGAPSSSPTVKEEGPEPWPGGPDPDVPGTDEASSACSTDWVIPDPEEEPERKRKKGPAPKMLGHELCRVCGDKASGFHYNVLSCEGCKGFFRRSVVRGGARRYACRGGGTCQMDAFMRRKCQQCRLRKCKEAGMREQCVLSEEQIRKKKIRKQQQQESQSQSQSPVGPQGSSSSASGPGASPGGSEAGSQGSGEGEGVQLTAAQELMIQQLVAAQLQCNKRSFSDQPKVTPWPLGADPQSRDARQQRFAHFTELAIISVQEIVDFAKQVPGFLQLGREDQIALLKASTIEIMLLETARRYNHETECITFLKDFTYSKDDFHRAGLQVEFINPIFEFSRAMRRLGLDDAEYALLIAINIFSADRPNVQEPGRVEALQQPYVEALLSYTRIKRPQDQLRFPRMLMKLVSLRTLSSVHSEQVFALRLQDKKLPPLLSEIWDVHE

1204 NP_003785.1 NP_647593.1

>NP_003785.1

MEQAPPDPERQLQPAPLEPLGSPDAGLGAAVGKEAEGAGEESSGVDTMTHNNFWLKKIEISVSEAEKRTGRNAMNMQETYTAYLIETRSVEHTDGQSVLTDSLWRRYSEFELLRSYLLVYYPHIVVPPLPEKRAEFVWHKLSADNMDPDFVERRRIGLENFLLRIASHPILCRDKIFYLFLTQEGNWKETVNETGFQLKADSRLKALNATFRVKNPDKRFTDLKHYSDELQSVISHLLRVRARVADRLYGVYKVHGNYGRVFSEWSAIEKEMGDGLQSAGHHMDVYASSIDDILEDEEHYADQLKEYLFYAEALRAVCRKHELMQYDLEMAAQDLASKKQQCEELVTGTVRTFSLKGMTTKLFGQETPEQREARIKVLEEQINEGEQQLKSKNLEGREFVKNAWADIERFKEQKNRDLKEALISYAVMQISMCKKGIQVWTNAKECFSKM

>NP_647593.1

MAEMGSKGVTAGKIASNVQKKLTRAQEKVLQKLGKADETKDEQFEQCVQNFNKQLTEGTRLQKDLRTYLASVKAMHEASKKLNECLQEVYEPDWPGRDEANKIAENNDLLWMDYHQKLVDQALLTMDTYLGQFPDIKSRIAKRGRKLVDYDSARHHYESLQTAKKKDEAKIAKPVSLLEKAAPQWCQGKLQAHLVAQTNLLRNQAEEELIKAQKVFEEMNVDLQEELPSLWNSRVGFYVNTFQSIAGLEENFHKEMSKLNQNLNDVLVGLEKQHGSNTFTVKAQPSDNAPAKGNKSPSPPDGSPAATPEIRVNHEPEPAGGATPGATLPKSPSQLRKGPPVPPPPKHTPSKEVKQEQILSLFEDTFVPEISVTTPSQFEAPGPFSEQASLLDLDFDPLPPVTSPVKAPTPSGQSIPWDLWEPTESPAGSLPSGEPSAAEGTFAVSWPSQTAEPGPAQPAEASEVAGGTQPAAGAQEPGETAASEAASSSLPAVVVETFPATVNGTVEGGSGAGRLDLPPGFMFKVQAQHDYTATDTDELQLKAGDVVLVIPFQNPEEQDEGWLMGVKESDWNQHKELEKCRGVFPENFTERVP

1205 NP_001015053.1 NP_006303.3

>NP_001015053.1

MNSPNESADGMSGREPSLEILPRTSLHSIPVTVEVKPVLPRAMPSSMGGGGGGSPSPVELRGALVGSVDPTLREQQLQQELLALKQQQQLQKQLLFAEFQKQHDHLTRQHEVQLQKHLKQQQEMLAAKQQQEMLAAKRQQELEQQRQREQQRQEELEKQRLEQQLLILRNKEKSKESAIASTEVKLRLQEFLLSKSKEPTPGGLNHSLPQHPKCWGAHHASLDQSSPPQSGPPGTPPSYKLPLPGPYDSRDDFPLRKTASEPNLKVRSRLKQKVAERRSSPLLRRKDGTVISTFKKRAVEITGAGPGASSVCNSAPGSGPSSPNSSHSTIAENGFTGSVPNIPTEMLPQHRALPLDSSPNQFSLYTSPSLPNISLGLQATVTVTNSHLTASPKLSTQQEAERQALQSLRQGGTLTGKFMSTSSIPGCLLGVALEGDGSPHGHASLLQHVLLLEQARQQSTLIAVPLHGQSPLVTGERVATSMRTVGKLPRHRPLSRTQSSPLPQSPQALQQLVMQQQHQQFLEKQKQQQLQLGKILTKTGELPRQPTTHPEETEEELTEQQEVLLGEGALTMPREGSTESESTQEDLEEEDEEDDGEEEEDCIQVKDEEGESGAEEGPDLEEPGAGYKKLFSDAQPLQPLQVYQAPLSLATVPHQALGRTQSSPAAPGGMKSPPDQPVKHLFTTGVVYDTFMLKHQCMCGNTHVHPEHAGRIQSIWSRLQETGLLSKCERIRGRKATLDEIQTVHSEYHTLLYGTSPLNRQKLDSKKLLGPISQKMYAVLPCGGIGVDSDTVWNEMHSSSAVRMAVGCLLELAFKVAAGELKNGFAIIRPPGHHAEESTAMGFCFFNSVAITAKLLQQKLNVGKVLIVDWDIHHGNGTQQAFYNDPSVLYISLHRYDNGNFFPGSGAPEEVGGGPGVGYNVNVAWTGGVDPPIGDVEYLTAFRTVVMPIAHEFSPDVVLVSAGFDAVEGHLSPLGGYSVTARCFGHLTRQLMTLAGGRVVLALEGGHDLTAICDASEACVSALLSVELQPLDEAVLQQKPNINAVATLEKVIEIQSKHWSCVQKFAAGLGRSLREAQAGETEEAETVSAMALLSVGAEQAQAAAAREHSPRPAEEPMEQEPAL

>NP_006303.3

MSGSTQPVAQTWRATEPRYPPHSLSYPVQIARTHTDVGLLEYQHHSRDYASHLSPGSIIQPQRRRPSLLSEFQPGNERSQELHLRPESHSYLPELGKSEMEFIESKRPRLELLPDPLLRPSPLLATGQPAGSEDLTKDRSLTGKLEPVSPPSPPHTDPELELVPPRLSKEELIQNMDRVDREITMVEQQISKLKKKQQQLEEEAAKPPEPEKPVSPPPIESKHRSLVQIIYDENRKKAEAAHRILEGLGPQVELPLYNQPSDTRQYHENIKINQAMRKKLILYFKRRNHARKQWEQKFCQRYDQLMEAWEKKVERIENNPRRRAKESKVREYYEKQFPEIRKQRELQERMQSRVGQRGSGLSMSAARSEHEVSEIIDGLSEQENLEKQMRQLAVIPPMLYDADQQRIKFINMNGLMADPMKVYKDRQVMNMWSEQEKETFREKFMQHPKNFGLIASFLERKTVAECVLYYYLTKKNENYKSLVRRSYRRRGKSQQQQQQQQQQQQQQQQQPMPRSSQEEKDEKEKEKEAEKEEEKPEVENDKEDLLKEKTDDTSGEDNDEKEAVASKGRKTANSQGRRKGRITRSMANEANSEEAITPQQSAELASMELNESSRWTEEEMETAKKGLLEHGRNWSAIARMVGSKTVSQCKNFYFNYKKRQNLDEILQQHKLKMEKERNARRKKKKAPAAASEEAAFPPVVEDEEMEASGVSGNEEEMVEEAEALHASGNEVPRGECSGPATVNNSSDTESIPSPHTEAAKDTGQNGPKPPATLGADGPPPGPPTPPPEDIPAPTEPTPASEATGAPTPPPAPPSPSAPPPVVPKEEKEEETAAAPPVEEGEEQKPPAAEELAVDTGKAEEPVKSECTEEAEEGPAKGKDAEAAEATAEGALKAEKKEGGSGRATTAKSSGAPQDSDSSATCSADEVDEAEGGDKNRLLSPRPSLLTPTGDPRANASPQKPLDLKQLKQRAAAIPPIVTKVHEPPREDAAPTKPAPPAPPPPQNLQPESDAPQQPGSSPRGKSRSPAPPADKEAEKPVFFPAFAAEAQKLPGDPPCWTSGLPFPVPPREVIKASPHAPDPSAFSYAPPGHPLPLGLHDTARPVLPRPPTISNPPPLISSAKHPSVLERQIGAISQGMSVQLHVPYSEHAKAPVGPVTMGLPLPMDPKKLAPFSGVKQEQLSPRGQAGPPESLGVPTAQEASVLRGTALGSVPGGSITKGIPSTRVPSDSAITYRGSITHGTPADVLYKGTITRIIGEDSPSRLDRGREDSLPKGHVIYEGKKGHVLSYEGGMSVTQCSKEDGRSSSGPPHETAAPKRTYDMMEGRVGRAISSASIEGLMGRAIPPERHSPHHLKEQHHIRGSITQGIPRSYVEAQEDYLRREAKLLKREGTPPPPPPSRDLTEAYKTQALGPLKLKPAHEGLVATVKEAGRSIHEIPREELRHTPELPLAPRPLKEGSITQGTPLKYDTGASTTGSKKHDVRSLIGSPGRTFPPVHPLDVMADARALERACYEESLKSRPGTASSSGGSIARGAPVIVPELGKPRQSPLTYEDHGAPFAGHLPRGSPVTTREPTPRLQEGSLSSSKASQDRKLTSTPREIAKSPHSTVPEHHPHPISPYEHLLRGVSGVDLYRSHIPLAFDPTSIPRGIPLDAAAAYYLPRHLAPNPTYPHLYPPYLIRGYPDTAALENRQTIINDYITSQQMHHNAATAMAQRADMLRGLSPRESSLALNYAAGPRGIIDLSQVPHLPVLVPPTPGTPATAMDRLAYLPTAPQPFSSRHSSSPLSPGGPTHLTKPTTTSSSERERDRDRERDRDREREKSILTSTTTVEHAPIWRPGTEQSSGSSGSSGGGGGSSSRPASHSHAHQHSPISPRTQDALQQRPSVLHNTGMKGIITAVEPSTPTVLRSTSTSSPVRPAATFPPATHCPLGGTLDGVYPTLMEPVLLPKEAPRVARPERPRADTGHAFLAKPPARSGLEPASSPSKGSEPRPLVPPVSGHATIARTPAKNLAPHHASPDPPAPPASASDPHREKTQSKPFSIQELELRSLGYHGSSYSPEGVEPVSPVSSPSLTHDKGLPKHLEELDKSHLEGELRPKQPGPVKLGGEAAHLPHLRPLPESQPSSSPLLQTAPGVKGHQRVVTLAQHISEVITQDYTRHHPQQLSAPLPAPLYSFPGASCPVLDLRRPPSDLYLPPPDHGAPARGSPHSEGGKRSPEPNKTSVLGGGEDGIEPVSPPEGMTEPGHSRSAVYPLLYRDGEQTEPSRMGSKSPGNTSQPPAFFSKLTESNSAMVKSKKQEINKKLNTHNRNEPEYNISQPGTEIFNMPAITGTGLMTYRSQAVQEHASTNMGLEAIIRKALMGKYDQWEESPPLSANAFNPLNASASLPAAMPITAADGRSDHTLTSPGGGGKAKVSGRPSSRKAKSPAPGLASGDRPPSVSSVHSEGDCNRRTPLTNRVWEDRPSSAGSTPFPYNPLIMRLQAGVMASPPPPGLPAGSGPLAGPHHAWDEEPKPLLCSQYETLSDSE

1206 NP_005139.1 NP_004441.1

>NP_005139.1

MKVKKGGGGAGTATESAPGPSGQSVAPIPQPPAESESGSESEPDAGPGPRPGPLQRKQPIGPEDVLGLQRITGDYLCSPEENIYKIDFVRFKIRDMDSGTVLFEIKKPPVSERLPINRRDLDPNAGRFVRYQFTPAFLRLRQVGATVEFTVGDKPVNNFRMIERHYFRNQLLKSFDFHFGFCIPSSKNTCEHIYDFPPLSEELISEMIRHPYETQSDSFYFVDDRLVMHNKADYSYSGTP

>NP_004441.1

MSHTILLVQPTKRPEGRTYADYESVNECMEGVCKMYEEHLKRMNPNSPSITYDISQLFDFIDDLADLSCLVYRADTQTYQPYNKDWIKEKIYVLLRRQAQQAGK

1207 NP_127491.1 NP_004337.2

>NP_127491.1

MAGLKRRASQVWPEEHGEQEHGLYSLHRMFDIVGTHLTHRDVRVLSFLFVDVIDDHERGLIRNGRDFLLALERQGRCDESNFRQVLQLLRIITRHDLLPYVTLKRRRAVCPDLVDKYLEETSIRYVTPRALSDPEPRPPQPSKTVPPHYPVVCCPTSGPQMCSKRPARGRATLGSQRKRRKSVTPDPKEKQTCDIRLRVRAEYCQHETALQGNVFSNKQDPLERQFERFNQANTILKSRDLGSIICDIKFSELTYLDAFWRDYINGSLLEALKGVFITDSLKQAVGHEAIKLLVNVDEEDYELGRQKLLRNLMLQALP

>NP_004337.2

MENTENSVDSKSIKNLEPKIIHGSESMDSGISLDNSYKMDYPEMGLCIIINNKNFHKSTGMTSRSGTDVDAANLRETFRNLKYEVRNKNDLTREEIVELMRDVSKEDHSKRSSFVCVLLSHGEEGIIFGTNGPVDLKKITNFFRGDRCRSLTGKPKLFIIQACRGTELDCGIETDSGVDDDMACHKIPVEADFLYAYSTAPGYYSWRNSKDGSWFIQSLCAMLKQYADKLEFMHILTRVNRKVATEFESFSFDATFHAKKQIPCIVSMLTKELYFYH

1208 NP_733832.1 NP_001061.2

>NP_733832.1

MSGEPELIELRELAPAGRAGKGRTRLERANALRIARGTACNPTRQLVPGRGHRFQPAGPATHTWCDLCGDFIWGVVRKGLQCARLSADCKFTCHYRCRALVCLDCCGPRDLGWEPAVERDTNVDEPVEWETPDLSQAEIEQKIKEYNAQINSNLFMSLNKDGSYTGFIKVQLKLVRPVSVPSSKKPPSLQDARRGPGRGTSVRRRTSFYLPKDAVKHLHVLSRTRAREVIEALLRKFLVVDDPRKFALFERAERHGQVYLRKLLDDEQPLRLRLLAGPSDKALSFVLKENDSGEVNWDAFSMPELHNFLRILQREEEEHLRQILQKYSYCRQKIQEALHACPLG

>NP_001061.2

MPREIITLQLGQCGNQIGFEFWKQLCAEHGISPEGIVEEFATEGTDRKDVFFYQADDEHYIPRAVLLDLEPRVIHSILNSPYAKLYNPENIYLSEHGGGAGNNWASGFSQGEKIHEDIFDIIDREADGSDSLEGFVLCHSIAGGTGSGLGSYLLERLNDRYPKKLVQTYSVFPNQDEMSDVVVQPYNSLLTLKRLTQNADCVVVLDNTALNRIATDRLHIQNPSFSQINQLVSTIMSASTTTLRYPGYMNNDLIGLIASLIPTPRLHFLMTGYTPLTTDQSVASVRKTTVLDVMRRLLQPKNVMVSTGRDRQTNHCYIAILNIIQGEVDPTQVHKSLQRIRERKLANFIPWGPASIQVALSRKSPYLPSAHRVSGLMMANHTSISSLFERTCRQYDKLRKREAFLEQFRKEDMFKDNFDEMDTSREIVQQLIDEYHAATRPDYISWGTQEQ

1209 NP_004422.2 NP_002077.1

>NP_004422.2

MELQAARACFALLWGCALAAAAAAQGKEVVLLDFAAAGGELGWLTHPYGKGWDLMQNIMNDMPIYMYSVCNVMSGDQDNWLRTNWVYRGEAERIFIELKFTVRDCNSFPGGASSCKETFNLYYAESDLDYGTNFQKRLFTKIDTIAPDEITVSSDFEARHVKLNVEERSVGPLTRKGFYLAFQDIGACVALLSVRVYYKKCPELLQGLAHFPETIAGSDAPSLATVAGTCVDHAVVPPGGEEPRMHCAVDGEWLVPIGQCLCQAGYEKVEDACQACSPGFFKFEASESPCLECPEHTLPSPEGATSCECEEGFFRAPQDPASMPCTRPPSAPHYLTAVGMGAKVELRWTPPQDSGGREDIVYSVTCEQCWPESGECGPCEASVRYSEPPHGLTRTSVTVSDLEPHMNYTFTVEARNGVSGLVTSRSFRTASVSINQTEPPKVRLEGRSTTSLSVSWSIPPPQQSRVWKYEVTYRKKGDSNSYNVRRTEGFSVTLDDLAPDTTYLVQVQALTQEGQGAGSKVHEFQTLSPEGSGNLAVIGGVAVGVVLLLVLAGVGFFIHRRRKNQRARQSPEDVYFSKSEQLKPLKTYVDPHTYEDPNQAVLKFTTEIHPSCVTRQKVIGAGEFGEVYKGMLKTSSGKKEVPVAIKTLKAGYTEKQRVDFLGEAGIMGQFSHHNIIRLEGVISKYKPMMIITEYMENGALDKFLREKDGEFSVLQLVGMLRGIAAGMKYLANMNYVHRDLAARNILVNSNLVCKVSDFGLSRVLEDDPEATYTTSGGKIPIRWTAPEAISYRKFTSASDVWSFGIVMWEVMTYGERPYWELSNHEVMKAINDGFRLPTPMDCPSAIYQLMMQCWQQERARRPKFADIVSILDKLIRAPDSLKTLADFDPRVSIRLPSTSGSEGVPFRTVSEWLESIKMQQYTEHFMAAGYTAIEKVVQMTNDDIKRIGVRLPGHQKRIAYSLLGLKDQVNTVGIPI

>NP_002077.1

MEAIAKYDFKATADDELSFKRGDILKVLNEECDQNWYKAELNGKDGFIPKNYIEMKPHPWFFGKIPRAKAEEMLSKQRHDGAFLIRESESAPGDFSLSVKFGNDVQHFKVLRDGAGKYFLWVVKFNSLNELVDYHRSTSVSRNQQIFLRDIEQVPQQPTYVQALFDFDPQEDGELGFRRGDFIHVMDNSDPNWWKGACHGQTGMFPRNYVTPVNRNV

1210 NP_612639.2 NP_006275.1

>NP_612639.2

MADAAATAGAGGSGTRSGSKQSTNPADNYHLARRRTLQVVVSSLLTEAGFESAEKASVETLTEMLQSYISEIGRSAKSYCEHTARTQPTLSDIVVTLVEMGFNVDTLPAYAKRSQRMVITAPPVTNQPVTPKALTAGQNRPHPPHIPSHFPEFPDPHTYIKTPTYREPVSDYQVLREKAASQRRDVERALTRFMAKTGETQSLFKDDVSTFPLIAARPFTIPYLTALLPSELEMQQMEETDSSEQDEQTDTENLALHISMEDSGAEKENTSVLQQNPSLSGSRNGEENIIDNPYLRPVKKPKIRRKKSLS

>NP_006275.1

MSCSGSGADPEAAPASAASAPGPAPPVSAPAALPSSTAAENKASPAGTAGGPGAGAAAGGTGPLAARAGEPAERRGAAPVSAGGAAPPEGAISNGVYVLPSAANGDVKPVVSSTPLVDFLMQLEDYTPTIPDAVTGYYLNRAGFEASDPRIIRLISLAAQKFISDIANDALQHCKMKGTASGSSRSKSKDRKYTLTMEDLTPALSEYGINVKKPHYFT

1211 NP_005556.1 AAH11772.1

>NP_005556.1

MALRNVPFRSEVLGWDPDSLADYFKKLNYKDCEKAVKKYHIDGARFLNLTENDIQKFPKLRVPILSKLSQEINKNEERRSIFTRKPQVPRFPEETESHEEDNGGWSSFEEDDYESPNDDQDGEDDGDYESPNEEEEAPVEDDADYEPPPSNDEEALQNSILPAKPFPNSNSMYIDRPPSGKTPQQPPVPPQRPMAALPPPPAGRNHSPLPPPQTNHEEPSRSRNHKTAKLPAPSIDRSTKPPLDRSLAPFDREPFTLGKKPPFSDKPSIPAGRSLGEHLPKIQKPPLPPTTERHERSSPLPGKKPPVPKHGWGPDRRENDEDDVHQRPLPQPALLPMSSNTFPSRSTKPSPMNPLPSSHMPGAFSESNSSFPQSASLPPYFSQGPSNRPPIRAEGRNFPLPLPNKPRPPSPAEEENSLNEEWYVSYITRPEAEAALRKINQDGTFLVRDSSKKTTTNPYVLMVLYKDKVYNIQIRYQKESQVYLLGTGLRGKEDFLSVSDIIDYFRKMPLLLIDGKNRGSRYQCTLTHAAGYP

>AAH11772.1

MSTTVNVDSLAEYEKSQIKRALELGTVMTVFSFRKSTPERRTVQVIMETRQVAWSKTADKIEGFLDIMEIKEIRPGKNSKDFERAKAVRQKEDCCFTILYGTQFVLSTLSLAADSKEDAVNWLSGLKILHQEAMNASTPTIIESWLRKQIYSVDQTRRNSISLRELKTILPLINFKVSSAKFLKDKFVEIGAHKDELSFEQFHLFYKKLMFEQQKSILDEFKKDSSVFILGNTDRPDASAVYLRDFQRFLIHEQQEHWAQDLNKVRERMTKFIDDTMRETAEPFLFVDEFLTYLFSRENSIWDEKYDAVDMQDMNNPLSHYWISSSHNTYLTGDQLRSESSPEAYIRCLRMGCRCIELDCWDGPDGKPVIYHGWTRTTKIKFDDVVQAIKDHAFVTSSFPVILSIEEHCSVEQQRHMAKAFKEVFGDLLLTKPTEASADQLPSPSQLREKIIIKHKKLGPRGDVDVNMEDKKDEHKQQGELYMWDSIDQKWTRHYCAIADAKLSFSDDIEQTMEEEVPQDIPPTELHFGEKWFHKKVEKRTSAEKLLQEYCMETGGKDGTFLVRESETFPNDYTLSFWRSGRVQHCRIRSTMEGGTLKYYLTDNLTFSSIYALIQHYRETHLRCAEFELRLTDPVPNPNPHESKPWYYDSLSRGEAEDMLMRIPRDGAFLIRKREGSDSYAITFRARGKVKHCRINRDGRHFVLGTSAYFESLVELVSYYEKHSLYRKMRLRYPVTPELLERYNMERDINSLYDVSRMYVDPSEINPSMPQRTVKALYDYKAKRSDELSFCRGALIHNVSKEPGGWWKGDYGTRIQQYFPSNYVEDISTADFEELEKQIIEDNPLGSLCRGILDLNTYNVVKAPQGKNQKSFVFILEPKQQGYPPVEFATDRVEELFEWFQSIREITWKIDTKENNMKYWEKNQSIAIELSDLVVYCKPTSKTKDNLENPDFREIRSFVETKADSIIRQKPVDLLKYNQKGLTRVYPKGQRVDSSNYDPFRLWLCGSQMVALNFQTADKYMQMNHALFSLNGRTGYVLQPESMRTEKYDPMPPESQRKILMTLTVKVLGARHLPKLGRSIACPFVEVEICGAEYDNNKFKTTVVNDNGLSPIWAPTQEKVTFEIYDPNLAFLRFVVYEEDMFSDPNFLAHATYPIKAVKSGFRSVPLKNGYSEDIELASLLVFCEMRPVLESEEELYSSCRQLRRRQEELNNQLFLYDTHQNLRNANRDALVKEFSVNENQLQLYQEKCNKRLREKRVSNSKFYS

1212 NP_004577.1 NP_000215.1

>NP_004577.1

MPLAQLADPWQKMAVESPSDSAENGQQIMDEPMGEEEINPQTEEVSIKEIAITHHVKEGHEKADPSQFELLKVLGQGSFGKVFLVKKISGSDARQLYAMKVLKKATLKVRDRVRTKMERDILVEVNHPFIVKLHYAFQTEGKLYLILDFLRGGDLFTRLSKEVMFTEEDVKFYLAELALALDHLHSLGIIYRDLKPENILLDEEGHIKLTDFGLSKESIDHEKKAYSFCGTVEYMAPEVVNRRGHTQSADWWSFGVLMFEMLTGTLPFQGKDRKETMTMILKAKLGMPQFLSPEAQSLLRMLFKRNPANRLGAGPDGVEEIKRHSFFSTIDWNKLYRREIHPPFKPATGRPEDTFYFDPEFTAKTPKDSPGIPPSANAHQLFRGFSFVAITSDDESQAMQTVGVHSIVQQLHRNSIQFTDGYEVKEDIGVGSYSVCKRCIHKATNMEFAVKIIDKSKRDPTEEIEILLRYGQHPNIITLKDVYDDGKYVYVVTELMKGGELLDKILRQKFFSEREASAVLFTITKTVEYLHAQGVVHRDLKPSNILYVDESGNPESIRICDFGFAKQLRAENGLLMTPCYTANFVAPEVLKRQGYDAACDIWSLGVLLYTMLTGYTPFANGPDDTPEEILARIGSGKFSLSGGYWNSVSDTAKDLVSKMLHVDPHQRLTAALVLRHPWIVHWDQLPQYQLNRQDAPHLVKGAMAATYSALNRNQSPVLEPVGRSTLAQRRGIKKITSTAL

>NP_000215.1

MSFTTRSTFSTNYRSLGSVQAPSYGARPVSSAASVYAGAGGSGSRISVSRSTSFRGGMGSGGLATGIAGGLAGMGGIQNEKETMQSLNDRLASYLDRVRSLETENRRLESKIREHLEKKGPQVRDWSHYFKIIEDLRAQIFANTVDNARIVLQIDNARLAADDFRVKYETELAMRQSVENDIHGLRKVIDDTNITRLQLETEIEALKEELLFMKKNHEEEVKGLQAQIASSGLTVEVDAPKSQDLAKIMADIRAQYDELARKNREELDKYWSQQIEESTTVVTTQSAEVGAAETTLTELRRTVQSLEIDLDSMRNLKASLENSLREVEARYALQMEQLNGILLHLESELAQTRAEGQRQAQEYEALLNIKVKLEAEIATYRRLLEDGEDFNLGDALDSSNSMQTIQKTTTRRIVDGKVVSETNDTKVLRH

1213 NP_055538.2 NP_004594.1

>NP_055538.2

MAMSLPGSRRTSAGSRRRTSPPVSVRDAYGTSSLSSSSNSGSYKGSDSSPTPRRSMKYTLCSDNHGIKPPTPEQYLTPLQQKEVCIRHLKARLKDTQDRLQDRDTEIDDLKTQLSRMQEDWIEEECHRVEAQLALKEARKEIKQLKQVIDTVKNNLIDKDKGLQKYFVDINIQNKKLETLLHSMEVAQNGMAKEDGTGESAGGSPARSLTRSSTYTKLSDPAVCGDRQPGDPSSGSAEDGADSGFAAADDTLSRTDALEASSLLSSGVDCGTEETSLHSSFGLGPRFPASNTYEKLLCGMEAGVQASCMQERAIQTDFVQYQPDLDTILEKVTQAQVCGTDPESGDRCPELDAHPSGPRDPNSAVVVTVGDELEAPEPITRGPTPQRPGANPNPGQSVSVVCPMEEEEEAAVAEKEPKSYWSRHYIVDLLAVVVPAVPTVAWLCRSQRRQGQPIYNISSLLRGCCTVALHSIRRISCRSLSQPSPSPAGGGSQL

>NP_004594.1

MKDRTQELRTAKDSDDDDDVAVTVDRDRFMDEFFEQVEEIRGFIDKIAENVEEVKRKHSAILASPNPDEKTKEELEELMSDIKKTANKVRSKLKSIEQSIEQEEGLNRSSADLRIRKTQHSTLSRKFVEVMSEYNATQSDYRERCKGRIQRQLEITGRTTTSEELEDMLESGNPAIFASGIIMDSSISKQALSEIETRHSEIIKLENSIRELHDMFMDMAMLVESQGEMIDRIEYNVEHAVDYVERAVSDTKKAVKYQSKARRKKIMIIICCVILGIVIASTVGGIFA

1214 NP_003206.2 NP_000940.1

>NP_003206.2

MNFNTILEEILIKRSQQKKKTSPLNYKERLFVLTKSMLTYYEGRAEKKYRKGFIDVSKIKCVEIVKNDDGVIPCQNKYPFQVVHDANTLYIFAPSPQSRDLWVKKLKEEIKNNNNIMIKYHPKFWTDGSYQCCRQTEKLAPGCEKYNLFESSIRKALPPAPETKKRRPPPPIPLEEEDNSEEIVVAMYDFQAAEGHDLRLERGQEYLILEKNDVHWWRARDKYGNEGYIPSNYVTGKKSNNLDQYEWYCRNMNRSKAEQLLRSEDKEGGFMVRDSSQPGLYTVSLYTKFGGEGSSGFRHYHIKETTTSPKKYYLAEKHAFGSIPEIIEYHKHNAAGLVTRLRYPVSVKGKNAPTTAGFSYEKWEINPSELTFMRELGSGLFGVVRLGKWRAQYKVAIKAIREGAMCEEDFIEEAKVMMKLTHPKLVQLYGVCTQQKPIYIVTEFMERGCLLNFLRQRQGHFSRDVLLSMCQDVCEGMEYLERNSFIHRDLAARNCLVSEAGVVKVSDFGMARYVLDDQYTSSSGAKFPVKWCPPEVFNYSRFSSKSDVWSFGVLMWEVFTEGRMPFEKYTNYEVVTMVTRGHRLYQPKLASNYVYEVMLRCWQEKPEGRPSFEDLLRTIDELVECEETFGR

>NP_000940.1

MKENVASATVFTLLLFLNTCLLNGQLPPGKPEIFKCRSPNKETFTCWWRPGTDGGLPTNYSLTYHREGETLMHECPDYITGGPNSCHFGKQYTSMWRTYIMMVNATNQMGSSFSDELYVDVTYIVQPDPPLELAVEVKQPEDRKPYLWIKWSPPTLIDLKTGWFTLLYEIRLKPEKAAEWEIHFAGQQTEFKILSLHPGQKYLVQVRCKPDHGYWSAWSPATFIQIPSDFTMNDTTVWISVAVLSAVICLIIVWAVALKGYSMVTCIFPPVPGPKIKGFDAHLLEKGKSEELLSALGCQDFPPTSDYEDLLVEYLEVDDSEDQHLMSVHSKEHPSQGMKPTYLDPDTDSGRGSCDSPSLLSEKCEEPQANPSTFYDPEVIEKPENPETTHTWDPQCISMEGKIPYFHAGGSKCSTWPLPQPSQHNPRSSYHNITDVCELAVGPAGAPATLLNEAGKDALKSSQTIKSREEGKATQQREVESFHSETDQDTPWLLPQEKTPFGSAKPLDYVEIHKVNKDGALSLLPKQRENSGKPKKPGTPENNKEYAKVSGVMDNNILVLVPDPHAKNVACFEESAKEAPPSLEQNQAEKALANFTATSSKCRLQLGGLDYLDPACFTHSFH

1215 NP_059107.1 NP_008869.1

>NP_059107.1

MAMSSGGSGGGVPEQEDSVLFRRGTGQSDDSDIWDDTALIKAYDKAVASFKHALKNGDICETSGKPKTTPKRKPAKKNKSQKKNTAASLQQWKVGDKCSAIWSEDGCIYPATIASIDFKRETCVVVYTGYGNREEQNLSDLLSPICEVANNIEQNAQENENESQVSTDESENSRSPGNKSDNIKPKSAPWNSFLPPPPPMPGPRLGPGKPGLKFNGPPPPPPPPPPHLLSCWLPPFPSGPPIIPPPPPICPDSLDDADALGSMLISWYMSGYHTGYYMGFRQNQKEGRCSHSLN

>NP_008869.1

MKLVRFLMKLSHETVTIELKNGTQVHGTITGVDVSMNTHLKAVKMTLKNREPVQLETLSIRGNNIRYFILPDSLPLDTLLVDVEPKVKSKKREAVAGRGRGRGRGRGRGRGRGRGGPRR

1216 NP_056281.1 NP_065147.1

>NP_056281.1

MEAVPRMPMIWLDLKEAGDFHFQPAVKKFVLKNYGENPEAYNEELKKLELLRQNAVRVPRDFEGCSVLRKYLGQLHYLQSRVPMGSGQEAAVPVTWTEIFSGKSVAHEDIKYEQACILYNLGALHSMLGAMDKRVSEEGMKVSCTHFQCAAGAFAYLREHFPQAYSVDMSRQILTLNVNLMLGQAQECLLEKSMLDNRKSFLVARISAQVVDYYKEACRALENPDTASLLGRIQKDWKKLVQMKIYYFAAVAHLHMGKQAEEQQKFGERVAYFQSALDKLNEAIKLAKGQPDTVQDALRFTMDVIGGKYNSAKKDNDFIYHEAVPALDTLQPVKGAPLVKPLPVNPTDPAVTGPDIFAKLVPMAAHEASSLYSEEKAKLLREMMAKIEDKNEVLDQFMDSMQLDPETVDNLDAYSHIPPQLMEKCAALSVRPDTVRNLVQSMQVLSGVFTDVEASLKDIRDLLEEDELLEQKFQEAVGQAGAISITSKAELAEVRREWAKYMEVHEKASFTNSELHRAMNLHVGNLRLLSGPLDQVRAALPTPALSPEDKAVLQNLKRILAKVQEMRDQRVSLEQQLRELIQKDDITASLVTTDHSEMKKLFEEQLKKYDQLKVYLEQNLAAQDRVLCALTEANVQYAAVRRVLSDLDQKWNSTLQTLVASYEAYEDLMKKSQEGRDFYADLESKVAALLERTQSTCQAREAARQQLLDRELKKKPPPRPTAPKPLLPRREESEAVEAGDPPEELRSLPPDMVAGPRLPDTFLGSATPLHFPPSPFPSSTGPGPHYLSGPLPPGTYSGPTQLIQPRAPGPHAMPVAPGPALYPAPAYTPELGLVPRSSPQHGVVSSPYVGVGPAPPVAGLPSAPPPQFSGPELAMAVRPATTTVDSIQAPIPSHTAPRPNPTPAPPPPCFPVPPPQPLPTPYTYPAGAKQPIPAQHHFSSGIPAGFPAPRIGPQPQPHPQPHPSQAFGPQPPQQPLPLQHPHLFPPQAPGLLPPQSPYPYAPQPGVLGQPPPPLHTQLYPGPAQDPLPAHSGALPFPSPGPPQPPHPPLAYGPAPSTRPMGPQAAPLTIRGPSSAGQSTPSPHLVPSPAPSPGPGPVPPRPPAAEPPPCLRRGAAAADLLSSSPESQHGGTQSPGGGQPLLQPTKVDAAEGRRPQALRLIERDPYEHPERLRQLQQELEAFRGQLGDVGALDTVWRELQDAQEHDARGRSIAIARCYSLKNRHQDVMPYDSNRVVLRSGKDDYINASCVEGLSPYCPPLVATQAPLPGTAADFWLMVHEQKVSVIVMLVSEAEMEKQKVARYFPTERGQPMVHGALSLALSSVRSTETHVERVLSLQFRDQSLKRSLVHLHFPTWPELGLPDSPSNLLRFIQEVHAHYLHQRPLHTPIIVHCSSGVGRTGAFALLYAAVQEVEAGNGIPELPQLVRRMRQQRKHMLQEKLHLRFCYEAVVRHVEQVLQRHGVPPPCKPLASASISQKNHLPQDSQDLVLGGDVPISSIQATIAKLSIRPPGGLESPVASLPGPAEPPGLPPASLPESTPIPSSSPPPLSSPLPEAPQPKEEPPVPEAPSSGPPSSSLELLASLTPEAFSLDSSLRGKQRMSKHNFLQAHNGQGLRATRPSDDPLSLLDPLWTLNKT

>NP_065147.1

MKLKDTKSRPKQSSCGKFQTKGIKVVGKWKEVKIDPNMFADGQMDDLVCFEELTDYQLVSPAKNPSSLFSKEAPKRKAQAVSEEEEEEEGKSSSPKKKIKLKKSKNVATEGTSTQKEFEVKDPELEAQGDDMVCDDPEAGEMTSENLVQTAPKKKKNKGKKGLEPSQSTAAKVPKKAKTWIPEVHDQKADVSAWKDLFVPRPVLRALSFLGFSAPTPIQALTLAPAIRDKLDILGAAETGSGKTLAFAIPMIHAVLQWQKRNAAPPPSNTEAPPGETRTEAGAETRSPGKAEAESDALPDDTVIESEALPSDIAAEARAKTGGTVSDQALLFGDDDAGEGPSSLIREKPVPKQNENEEENLDKEQTGNLKQELDDKSATCKAYPKRPLLGLVLTPTRELAVQVKQHIDAVARFTGIKTAILVGGMSTQKQQRMLNRRPEIVVATPGRLWELIKEKHYHLRNLRQLRCLVVDEADRMVEKGHFAELSQLLEMLNDSQYNPKRQTLVFSATLTLVHQAPARILHKKHTKKMDKTAKLDLLMQKIGMRGKPKVIDLTRNEATVETLTETKIHCETDEKDFYLYYFLMQYPGRSLVFANSISCIKRLSGLLKVLDIMPLTLHACMHQKQRLRNLEQFARLEDCVLLATDVAARGLDIPKVQHVIHYQVPRTSEIYVHRSGRTARATNEGLSLMLIGPEDVINFKKIYKTLKKDEDIPLFPVQTKYMDVVKERIRLARQIEKSEYRNFQACLHNSWIEQAAAALEIELEEDMYKGGKADQQEERRRQKQMKVLKKELRHLLSQPLFTESQKTKYPTQSGKPPLLVSAPSKSESALSCLSKQKKKKTKKPKEPQPEQPQPSTSAN

1217 NP_061027.2 NP_002328.1

>NP_061027.2

MSEFLLALLTLSGLLPIARVLTVGADRDQQLCDPGEFLCHDHVTCVSQSWLCDGDPDCPDDSDESLDTCPEEVEIKCPLNHIACLGTNKCVHLSQLCNGVLDCPDGYDEGVHCQELLSNCQQLNCQYKCTMVRNSTRCYCEDGFEITEDGRSCKDQDECAVYGTCSQTCRNTHGSYTCSCVEGYLMQPDNRSCKAKIEPTDRPPILLIANFETIEVFYLNGSKMATLSSVNGNEIHTLDFIYNEDMICWIESRESSNQLKCIQITKAGGLTDEWTINILQSFHNVQQMAIDWLTRNLYFVDHVGDRIFVCNSNGSVCVTLIDLELHNPKAIAVDPIAGKLFFTDYGNVAKVERCDMDGMNRTRIIDSKTEQPAALALDLVNKLVYWVDLYLDYVGVVDYQGKNRHTVIQGRQVRHLYGITVFEDYLYATNSDNYNIVRINRFNGTDIHSLIKIENAWGIRIYQKRTQPTVRSHACEVDPYGMPGGCSHICLLSSSYKTRTCRCRTGFNLGSDGRSCKRPKNELFLFYGKGRPGIVRGMDLNTKIADEYMIPIENLVNPRALDFHAETNYIYFADTTSFLIGRQKIDGTERETILKDDLDNVEGIAVDWIGNNLYWTNDGHRKTINVARLEKASQSRKTLLEGEMSHPRGIVVDPVNGWMYWTDWEEDEIDDSVGRIEKAWMDGFNRQIFVTSKMLWPNGLTLDFHTNTLYWCDAYYDHIEKVFLNGTHRKIVYSGRELNHPFGLSHHGNYVFWTDYMNGSIFQLDLITSEVTLLRHERPPLFGLQIYDPRKQQGDNMCRVNNGGCSTLCLAIPGGRVCACADNQLLDENGTTCTFNPGEALPHICKAGEFRCKNRHCIQARWKCDGDDDCLDGSDEDSVNCFNHSCPDDQFKCQNNRCIPKRWLCDGANDCGSNEDESNQTCTARTCQVDQFSCGNGRCIPRAWLCDREDDCGDQTDEMASCEFPTCEPLTQFVCKSGRCISSKWHCDSDDDCGDGSDEVGCVHSCFDNQFRCSSGRCIPGHWACDGDNDCGDFSDEAQINCTKEEIHSPAGCNGNEFQCHPDGNCVPDLWRCDGEKDCEDGSDEKGCNGTIRLCDHKTKFSCWSTGRCINKAWVCDGDIDCEDQSDEDDCDSFLCGPPKHPCANDTSVCLQPEKLCNGKKDCPDGSDEGYLCDECSLNNGGCSNHCSVVPGRGIVCSCPEGLQLNKDNKTCEIVDYCSNHLKCSQVCEQHKHTVKCSCYEGWKLDVDGESCTSVDPFEAFIIFSIRHEIRRIDLHKRDYSLLVPGLRNTIALDFHFNQSLLYWTDVVEDRIYRGKLSESGGVSAIEVVVEHGLATPEGLTVDWIAGNIYWIDSNLDQIEVAKLDGSLRTTLIAGAMEHPRAIALDPRYGILFWTDWDANFPRIESASMSGAGRKTIYKDMKTGAWPNGLTVDHFEKRIVWTDARSDAIYSALYDGTNMIEIIRGHEYLSHPFAVSLYGSEVYWTDWRTNTLSKANKWTGQNVSVIQKTSAQPFDLQIYHPSRQPQAPNPCAANDGKGPCSHMCLINHNRSAACACPHLMKLSSDKKTCYEMKKFLLYARRSEIRGVDIDNPYFNFITAFTVPDIDDVTVIDFDASEERLYWTDIKTQTIKRAFINGTGLETVISRDIQSIRGLAVDWVSRNLYWISSEFDETQINVARLDGSLKTSIIHGIDKPQCLAAHPVRGKLYWTDGNTINMANMDGSNSKILFQNQKEPVGLSIDYVENKLYWISSGNGTINRCNLDGGNLEVIESMKEELTKATALTIMDKKLWWADQNLAQLGTCSKRDGRNPTILRNKTSGVVHMKVYDKEAQQGSNSCQLNNGGCSQLCLPTSETTRTCMCTVGYYLQKNRMSCQGIESFLMYSVHEGIRGIPLEPSDKMDALMPISGTSFAVGIDFHAENDTIYWTDMGFNKISRAKRDQTWKEDIITNGLGRVEGIAVDWIAGNIYWTDHGFNLIEVARLNGSFRYVIISQGLDQPRSIAVHPEKGLLFWTEWGQMPCIGKARLDGSEKVVLVSMGIAWPNGISIDYEENKLYWCDARTDKIERIDLETGGNREMVLSGSNVDMFSVAVFGAYIYWSDRAHANGSVRRGHKNDATETITMRTGLGVNLKEVKIFNRVREKGTNVCARDNGGCKQLCLYRGNSRRTCACAHGYLAEDGVTCLRHEGYLLYSGRTILKSIHLSDETNLNSPIRPYENPRYFKNVIALAFDYNQRRKGTNRIFYSDAHFGNIQLIKDNWEDRQVIVENVGSVEGLAYHRAWDTLYWTSSTTSSITRHTVDQTRPGAFDREAVITMSEDDHPHVLALDECQNLMFWTNWNEQHPSIMRSTLTGKNAQVVVSTDILTPNGLTIDYRAEKLYFSDGSLGKIERCEYDGSQRHVIVKSGPGTFLSLAVYDNYIFWSDWGRRAILRSNKYTGGDTKILRSDIPHQPMGIIAVANDTNSCELSPCALLNGGCHDLCLLTPNGRVNCSCRGDRILLEDNRCVTKNSSCNAYSEFECGNGECIDYQLTCDGIPHCKDKSDEKLLYCENRSCRRGFKPCYNRRCIPHGKLCDGENDCGDNSDELDCKVSTCATVEFRCADGTCIPRSARCNQNIDCADASDEKNCNNTDCTHFYKLGVKTTGFIRCNSTSLCVLPTWICDGSNDCGDYSDELKCPVQNKHKCEENYFSCPSGRCILNTWICDGQKDCEDGRDEFHCDSSCSWNQFACSAQKCISKHWICDGEDDCGDGLDESDSICGAITCAADMFSCQGSRACVPRHWLCDGERDCPDGSDELSTAGCAPNNTCDENAFMCHNKVCIPKQFVCDHDDDCGDGSDESPQCGYRQCGTEEFSCADGRCLLNTQWQCDGDFDCPDHSDEAPLNPKCKSAEQSCNSSFFMCKNGRCIPSGGLCDNKDDCGDGSDERNCHINECLSKKVSGCSQDCQDLPVSYKCKCWPGFQLKDDGKTCVDIDECSSGFPCSQQCINTYGTYKCLCTDGYEIQPDNPNGCKSLSDEEPFLILADHHEIRKISTDGSNYTLLKQGLNNVIAIDFDYREEFIYWIDSSRPNGSRINRMCLNGSDIKVVHNTAVPNALAVDWIGKNLYWSDTEKRIIEVSKLNGLYPTILVSKRLKFPRDLSLDPQAGYLYWIDCCEYPHIGRVGMDGTNQSVVIETKISRPMALTIDYVNRRLYWADENHIEFSNMDGSHRHKVPNQDIPGVIALTLFEDYIYWTDGKTKSLSRAHKTSGADRLSLIYSWHAITDIQVYHSYRQPDVSKHLCMINNGGCSHLCLLAPGKTHTCACPTNFYLAADNRTCLSNCTASQFRCKTDKCIPFWWKCDTVDDCGDGSDEPDDCPEFRCQPGRFQCGTGLCALPAFICDGENDCGDNSDELNCDTHVCLSGQFKCTKNQKCIPVNLRCNGQDDCGDEEDERDCPENSCSPDYFQCKTTKHCISKLWVCDEDPDCADASDEANCDKKTCGPHEFQCKNNNCIPDHWRCDSQNDCSDNSDEENCKPQTCTLKDFLCANGDCVSSRFWCDGDFDCADGSDERNCETSCSKDQFRCSNGQCIPAKWKCDGHEDCKYGEDEKSCEPASPTCSSREYICASDGCISASLKCNGEYDCADGSDEMDCVTECKEDQFRCKNKAHCIPIRWLCDGIHDCVDGSDEENCERGGNICRADEFLCNNSLCKLHFWVCDGEDDCGDNSDEAPDMCVKFLCPSTRPHRCRNNRICLQSEQMCNGIDECGDNSDEDHCGGKLTYKARPCKKDEFACSNKKCIPMDLQCDRLDDCGDGSDEQGCRIAPTEYTCEDNVNPCGDDAYCNQIKTSVFCRCKPGFQRNMKNRQCEDLNECLVFGTCSHQCINVEGSYKCVCDQNFQERNNTCIAEGSEDQVLYIANDTDILGFIYPFNYSGDHQQISHIEHNSRITGMDVYYQRDMIIWSTQFNPGGIFYKRIHGREKRQANSGLICPEFKRPRDIAVDWVAGNIYWTDHSRMHWFSYYTTHWTSLRYSINVGQLNGPNCTRLLTNMAGEPYAIAVNPKRGMMYWTVVGDHSHIEEAAMDGTLRRILVQKNLQRPTGLAVDYFSERIYWADFELSIIGSVLYDGSNSVVSVSSKQGLLHPHRIDIFEDYIYGAGPKNGVFRVQKFGHGSVEYLALNIDKTKGVLISHRYKQLDLPNPCLDLACEFLCLLNPSGATCVCPEGKYLINGTCNDDSLLDDSCKLTCENGGRCILNEKGDLRCHCWPSYSGERCEVNHCSNYCQNGGTCVPSVLGRPTCSCALGFTGPNCGKTVCEDFCQNGGTCIVTAGNQPYCHCQPEYTGDRCQYYVCHHYCVNSESCTIGDDGSVECVCPTRYEGPKCEVDKCVRCHGGHCIINKDSEDIFCNCTNGKIASSCQLCDGYCYNGGTCQLDPETNVPVCLCSTNWSGTQCERPAPKSSKSDHISTRSIAIIVPLVLLVTLITTLVIGLVLCKRKRRTKTIRRQPIINGGINVEIGNPSYNMYEVDHDHNDGGLLDPGFMIDPTKARYIGGGPSAFKLPHTAPPIYLNSDLKGPLTAGPTNYSNPVYAKLYMDGQNCRNSLGSVDERKELLPKKIEIGIRETVA

>NP_002328.1

MAPRRVRSFLRGLPALLLLLLFLGPWPAASHGGKYSREKNQPKPSPKRESGEEFRMEKLNQLWEKAQRLHLPPVRLAELHADLKIQERDELAWKKLKLDGLDEDGEKEARLIRNLNVILAKYGLDGKKDARQVTSNSLSGTQEDGLDDPRLEKLWHKAKTSGKFSGEELDKLWREFLHHKEKVHEYNVLLETLSRTEEIHENVISPSDLSDIKGSVLHSRHTELKEKLRSINQGLDRLRRVSHQGYSTEAEFEEPRVIDLWDLAQSANLTDKELEAFREELKHFEAKIEKHNHYQKQLEIAHEKLRHAESVGDGERVSRSREKHALLEGRTKELGYTVKKHLQDLSGRISRARHNEL

1218 NP_612384.1 NP_004926.1

>NP_612384.1

MLSKRGCHARIYADFPIRRLISQRSSLETLEDIEENAPLRRCRTLSGSPRPKNFKKIHFIKNMRQHDTRNGRIVLISGRRSFCSIFSVLPYRDSTQVGDLKLDGGRQSTGAVSLKEIIGLEGVELGADGKTVSYTQFLLPTNAFGARRNTIDSTSSFSQFRNLSHRSLSIGRASGTQGSLDTGSDLGDFMDYDPNLLDDPQWPCGKHKRVLIFPSYMTTVIDYVKPSDLKKDMNETFKEKFPHIKLTLSKIRSLKREMRKLAQEDCGLEEPTVAMAFVYFEKLALKGKLNKQNRKLCAGACVLLAAKIGSDLKKHEVKHLIDKLEEKFRLNRRELIAFEFPVLVALEFALHLPEHEVMPHYRRLVQSS

>NP_004926.1

MQKYEKLEKIGEGTYGTVFKAKNRETHEIVALKRVRLDDDDEGVPSSALREICLLKELKHKNIVRLHDVLHSDKKLTLVFEFCDQDLKKYFDSCNGDLDPEIVKSFLFQLLKGLGFCHSRNVLHRDLKPQNLLINRNGELKLADFGLARAFGIPVRCYSAEVVTLWYRPPDVLFGAKLYSTSIDMWSAGCIFAELANAGRPLFPGNDVDDQLKRIFRLLGTPTEEQWPSMTKLPDYKPYPMYPATTSLVNVVPKLNATGRDLLQNLLKCNPVQRISAEEALQHPYFSDFCPP

1219 NP_000072.2 NP_001311.3

>NP_000072.2

MSTDSNSLAREFLTDVNRLCNAVVQRVEAREEEEEETHMATLGQYLVHGRGFLLLTKLNSIIDQALTCREELLTLLLSLLPLVWKIPVQEEKATDFNLPLSADIILTKEKNSSSQRSTQEKLHLEGSALSSQVSAKVNVFRKSRRQRKITHRYSVRDARKTQLSTSDSEANSDEKGIAMNKHRRPHLLHHFLTSFPKQDHPKAKLDRLATKEQTPPDAMALENSREIIPRQGSNTDILSEPAALSVISNMNNSPFDLCHVLLSLLEKVCKFDVTLNHNSPLAASVVPTLTEFLAGFGDCCSLSDNLESRVVSAGWTEEPVALIQRMLFRTVLHLLSVDVSTAEMMPENLRKNLTELLRAALKIRICLEKQPDPFAPRQKKTLQEVQEDFVFSKYRHRALLLPELLEGVLQILICCLQSAASNPFYFSQAMDLVQEFIQHHGFNLFETAVLQMEWLVLRDGVPPEASEHLKALINSVMKIMSTVKKVKSEQLHHSMCTRKRHRRCEYSHFMHHHRDLSGLLVSAFKNQVSKNPFEETADGDVYYPERCCCIAVCAHQCLRLLQQASLSSTCVQILSGVHNIGICCCMDPKSVIIPLLHAFKLPALKNFQQHILNILNKLILDQLGGAEISPKIKKAACNICTVDSDQLAQLEETLQGNLCDAELSSSLSSPSYRFQGILPSSGSEDLLWKWDALKAYQNFVFEEDRLHSIQIANHICNLIQKGNIVVQWKLYNYIFNPVLQRGVELAHHCQHLSVTSAQSHVCSHHNQCLPQDVLQIYVKTLPILLKSRVIRDLFLSCNGVSQIIELNCLNGIRSHSLKAFETLIISLGEQQKDASVPDIDGIDIEQKELSSVHVGTSFHHQQAYSDSPQSLSKFYAGLKEAYPKRRKTVNQDVHINTINLFLCVAFLCVSKEAESDRESANDSEDTSGYDSTASEPLSHMLPCISLESLVLPSPEHMHQAADIWSMCRWIYMLSSVFQKQFYRLGGFRVCHKLIFMIIQKLFRSHKEEQGKKEGDTSVNENQDLNRISQPKRTMKEDLLSLAIKSDPIPSELGSLKKSADSLGKLELQHISSINVEEVSATEAAPEEAKLFTSQESETSLQSIRLLEALLAICLHGARTSQQKMELELPNQNLSVESILFEMRDHLSQSKVIETQLAKPLFDALLRVALGNYSADFEHNDAMTEKSHQSAEELSSQPGDFSEEAEDSQCCSFKLLVEEEGYEADSESNPEDGETQDDGVDLKSETEGFSASSSPNDLLENLTQGEIIYPEICMLELNLLSASKAKLDVLAHVFESFLKIIRQKEKNVFLLMQQGTVKNLLGGFLSILTQDDSDFQACQRVLVDLLVSLMSSRTCSEELTLLLRIFLEKSPCTKILLLGILKIIESDTTMSPSQYLTFPLLHAPNLSNGVSSQKYPGILNSKAMGLLRRARVSRSKKEADRESFPHRLLSSWHIAPVHLPLLGQNCWPHLSEGFSVSLWFNVECIHEAESTTEKGKKIKKRNKSLILPDSSFDGTESDRPEGAEYINPGERLIEEGCIHIISLGSKALMIQVWADPHNATLIFRVCMDSNDDMKAVLLAQVESQENIFLPSKWQHLVLTYLQQPQGKRRIHGKISIWVSGQRKPDVTLDFMLPRKTSLSSDSNKTFCMIGHCLSSQEEFLQLAGKWDLGNLLLFNGAKVGSQEAFYLYACGPNHTSVMPCKYGKPVNDYSKYINKEILRCEQIRELFMTKKDVDIGLLIESLSVVYTTYCPAQYTIYEPVIRLKGQMKTQLSQRPFSSKEVQSILLEPHHLKNLQPTEYKTIQGILHEIGGTGIFVFLFARVVELSSCEETQALALRVILSLIKYNQQRVHELENCNGLSMIHQVLIKQKCIVGFYILKTLLEGCCGEDIIYMNENGEFKLDVDSNAIIQDVKLLEELLLDWKIWSKAEQGVWETLLAALEVLIRADHHQQMFNIKQLLKAQVVHHFLLTCQVLQEYKEGQLTPMPREVCRSFVKIIAEVLGSPPDLELLTIIFNFLLAVHPPTNTYVCHNPTNFYFSLHIDGKIFQEKVRSIMYLRHSSSGGRSLMSPGFMVISPSGFTASPYEGENSSNIIPQQMAAHMLRSRSLPAFPTSSLLTQSQKLTGSLGCSIDRLQNIADTYVATQSKKQNSLGSSDTLKKGKEDAFISSCESAKTVCEMEAVLSAQVSVSDVPKGVLGFPVVKADHKQLGAEPRSEDDSPGDESCPRRPDYLKGLASFQRSHSTIASLGLAFPSQNGSAAVGRWPSLVDRNTDDWENFAYSLGYEPNYNRTASAHSVTEDCLVPICCGLYELLSGVLLILPDVLLEDVMDKLIQADTLLVLVNHPSPAIQQGVIKLLDAYFARASKEQKDKFLKNRGFSLLANQLYLHRGTQELLECFIEMFFGRHIGLDEEFDLEDVRNMGLFQKWSVIPILGLIETSLYDNILLHNALLLLLQILNSCSKVADMLLDNGLLYVLCNTVAALNGLEKNIPMSEYKLLACDIQQLFIAVTIHACSSSGSQYFRVIEDLIVMLGYLQNSKNKRTQNMAVALQLRVLQAAMEFIRTTANHDSENLTDSLQSPSAPHHAVVQKRKSIAGPRKFPLAQTESLLMKMRSVANDELHVMMQRRMSQENPSQATETELAQRLQRLTVLAVNRIIYQEFNSDIIDILRTPENVTQSKTSVFQTEISEENIHHEQSSVFNPFQKEIFTYLVEGFKVSIGSSKASGSKQQWTKILWSCKETFRMQLGRLLVHILSPAHAAQERKQIFEIVHEPNHQEILRDCLSPSLQHGAKLVLYLSELIHNHQGELTEEELGTAELLMNALKLCGHKCIPPSASTKADLIKMIKEEQKKYETEEGVNKAAWQKTVNNNQQSLFQRLDSKSKDISKIAADITQAVSLSQGNERKKVIQHIRGMYKVDLSASRHWQELIQQLTHDRAVWYDPIYYPTSWQLDPTEGPNRERRRLQRCYLTIPNKYLLRDRQKSEDVVKPPLSYLFEDKTHSSFSSTVKDKAASESIRVNRRCISVAPSRETAGELLLGKCGMYFVEDNASDTVESSSLQGELEPASFSWTYEEIKEVHKRWWQLRDNAVEIFLTNGRTLLLAFDNTKVRDDVYHNILTNNLPNLLEYGNITALTNLWYTGQITNFEYLTHLNKHAGRSFNDLMQYPVFPFILADYVSETLDLNDLLIYRNLSKPIAVQYKEKEDRYVDTYKYLEEEYRKGAREDDPMPPVQPYHYGSHYSNSGTVLHFLVRMPPFTKMFLAYQDQSFDIPDRTFHSTNTTWRLSSFESMTDVKELIPEFFYLPEFLVNREGFDFGVRQNGERVNHVNLPPWARNDPRLFILIHRQALESDYVSQNICQWIDLVFGYKQKGKASVQAINVFHPATYFGMDVSAVEDPVQRRALETMIKTYGQTPRQLFHMAHVSRPGAKLNIEGELPAAVGLLVQFAFRETREQVKEITYPSPLSWIKGLKWGEYVGSPSAPVPVVCFSQPHGERFGSLQALPTRAICGLSRNFCLLMTYSKEQGVRSMNSTDIQWSAILSWGYADNILRLKSKQSEPPVNFIQSSQQYQVTSCAWVPDSCQLFTGSKCGVITAYTNRFTSSTPSEIEMETQIHLYGHTEEITSLFVCKPYSILISVSRDGTCIIWDLNRLCYVQSLAGHKSPVTAVSASETSGDIATVCDSAGGGSDLRLWTVNGDLVGHVHCREIICSVAFSNQPEGVSINVIAGGLENGIVRLWSTWDLKPVREITFPKSNKPIISLTFSCDGHHLYTANSDGTVIAWCRKDQQRLKQPMFYSFLSSYAAG

>NP_001311.3

MSSSEEVSWISWFCGLRGNEFFCEVDEDYIQDKFNLTGLNEQVPHYRQALDMILDLEPDEELEDNPNQSDLIEQAAEMLYGLIHARYILTNRGIAQMLEKYQQGDFGYCPRVYCENQPMLPIGLSDIPGEAMVKLYCPKCMDVYTPKSSRHHHTDGAYFGTGFPHMLFMVHPEYRPKRPANQFVPRLYGFKIHPMAYQLQLQAASNFKSPVKTIR

1220 NP_997647.1 NP_000085.1

>NP_997647.1

MLRGPGPGLLLLAVQCLGTAVPSTGASKSKRQAQQMVQPQSPVAVSQSKPGCYDNGKHYQINQQWERTYLGNALVCTCYGGSRGFNCESKPEAEETCFDKYTGNTYRVGDTYERPKDSMIWDCTCIGAGRGRISCTIANRCHEGGQSYKIGDTWRRPHETGGYMLECVCLGNGKGEWTCKPIAEKCFDHAAGTSYVVGETWEKPYQGWMMVDCTCLGEGSGRITCTSRNRCNDQDTRTSYRIGDTWSKKDNRGNLLQCICTGNGRGEWKCERHTSVQTTSSGSGPFTDVRAAVYQPQPHPQPPPYGHCVTDSGVVYSVGMQWLKTQGNKQMLCTCLGNGVSCQETAVTQTYGGNSNGEPCVLPFTYNGRTFYSCTTEGRQDGHLWCSTTSNYEQDQKYSFCTDHTVLVQTRGGNSNGALCHFPFLYNNHNYTDCTSEGRRDNMKWCGTTQNYDADQKFGFCPMAAHEEICTTNEGVMYRIGDQWDKQHDMGHMMRCTCVGNGRGEWTCIAYSQLRDQCIVDDITYNVNDTFHKRHEEGHMLNCTCFGQGRGRWKCDPVDQCQDSETGTFYQIGDSWEKYVHGVRYQCYCYGRGIGEWHCQPLQTYPSSSGPVEVFITETPSQPNSHPIQWNAPQPSHISKYILRWRPKNSVGRWKEATIPGHLNSYTIKGLKPGVVYEGQLISIQQYGHQEVTRFDFTTTSTSTPVTSNTVTGETTPFSPLVATSESVTEITASSFVVSWVSASDTVSGFRVEYELSEEGDEPQYLDLPSTATSVNIPDLLPGRKYIVNVYQISEDGEQSLILSTSQTTAPDAPPDPTVDQVDDTSIVVRWSRPQAPITGYRIVYSPSVEGSSTELNLPETANSVTLSDLQPGVQYNITIYAVEENQESTPVVIQQETTGTPRSDTVPSPRDLQFVEVTDVKVTIMWTPPESAVTGYRVDVIPVNLPGEHGQRLPISRNTFAEVTGLSPGVTYYFKVFAVSHGRESKPLTAQQTTKLDAPTNLQFVNETDSTVLVRWTPPRAQITGYRLTVGLTRRGQPRQYNVGPSVSKYPLRNLQPASEYTVSLVAIKGNQESPKATGVFTTLQPGSSIPPYNTEVTETTIVITWTPAPRIGFKLGVRPSQGGEAPREVTSDSGSIVVSGLTPGVEYVYTIQVLRDGQERDAPIVNKVVTPLSPPTNLHLEANPDTGVLTVSWERSTTPDITGYRITTTPTNGQQGNSLEEVVHADQSSCTFDNLSPGLEYNVSVYTVKDDKESVPISDTIIPEVPQLTDLSFVDITDSSIGLRWTPLNSSTIIGYRITVVAAGEGIPIFEDFVDSSVGYYTVTGLEPGIDYDISVITLINGGESAPTTLTQQTAVPPPTDLRFTNIGPDTMRVTWAPPPSIDLTNFLVRYSPVKNEEDVAELSISPSDNAVVLTNLLPGTEYVVSVSSVYEQHESTPLRGRQKTGLDSPTGIDFSDITANSFTVHWIAPRATITGYRIRHHPEHFSGRPREDRVPHSRNSITLTNLTPGTEYVVSIVALNGREESPLLIGQQSTVSDVPRDLEVVAATPTSLLISWDAPAVTVRYYRITYGETGGNSPVQEFTVPGSKSTATISGLKPGVDYTITVYAVTGRGDSPASSKPISINYRTEIDKPSQMQVTDVQDNSISVKWLPSSSPVTGYRVTTTPKNGPGPTKTKTAGPDQTEMTIEGLQPTVEYVVSVYAQNPSGESQPLVQTAVTNIDRPKGLAFTDVDVDSIKIAWESPQGQVSRYRVTYSSPEDGIHELFPAPDGEEDTAELQGLRPGSEYTVSVVALHDDMESQPLIGTQSTAIPAPTDLKFTQVTPTSLSAQWTPPNVQLTGYRVRVTPKEKTGPMKEINLAPDSSSVVVSGLMVATKYEVSVYALKDTLTSRPAQGVVTTLENVSPPRRARVTDATETTITISWRTKTETITGFQVDAVPANGQTPIQRTIKPDVRSYTITGLQPGTDYKIYLYTLNDNARSSPVVIDASTAIDAPSNLRFLATTPNSLLVSWQPPRARITGYIIKYEKPGSPPREVVPRPRPGVTEATITGLEPGTEYTIYVIALKNNQKSEPLIGRKKTDELPQLVTLPHPNLHGPEILDVPSTVQKTPFVTHPGYDTGNGIQLPGTSGQQPSVGQQMIFEEHGFRRTTPPTTATPIRHRPRPYPPNVGEEIQIGHIPREDVDYHLYPHGPGLNPNASTGQEALSQTTISWAPFQDTSEYIISCHPVGTDEEPLQFRVPGTSTSATLTGLTRGATYNIIVEALKDQQRHKVREEVVTVGNSVNEGLNQPTDDSCFDPYTVSHYAVGDEWERMSESGFKLLCQCLGFGSGHFRCDSSRWCHDNGVNYKIGEKWDRQGENGQMMSCTCLGNGKGEFKCDPHEATCYDDGKTYHVGEQWQKEYLGAICSCTCFGGQRGWRCDNCRRPGGEPSPEGTTGQSYNQYSQRYHQRTNTNVNCPIECFMPLDVQADREDSRE

>NP_000085.1

MTLRLLVAALCAGILAEAPRVRAQHRERVTCTRLYAADIVFLLDGSSSIGRSNFREVRSFLEGLVLPFSGAASAQGVRFATVQYSDDPRTEFGLDALGSGGDVIRAIRELSYKGGNTRTGAAILHVADHVFLPQLARPGVPKVCILITDGKSQDLVDTAAQRLKGQGVKLFAVGIKNADPEELKRVASQPTSDFFFFVNDFSILRTLLPLVSRRVCTTAGGVPVTRPPDDSTSAPRDLVLSEPSSQSLRVQWTAASGPVTGYKVQYTPLTGLGQPLPSERQEVNVPAGETSVRLRGLRPLTEYQVTVIALYANSIGEAVSGTARTTALEGPELTIQNTTAHSLLVAWRSVPGATGYRVTWRVLSGGPTQQQELGPGQGSVLLRDLEPGTDYEVTVSTLFGRSVGPATSLMARTDASVEQTLRPVILGPTSILLSWNLVPEARGYRLEWRRETGLEPPQKVVLPSDVTRYQLDGLQPGTEYRLTLYTLLEGHEVATPATVVPTGPELPVSPVTDLQATELPGQRVRVSWSPVPGATQYRIIVRSTQGVERTLVLPGSQTAFDLDDVQAGLSYTVRVSARVGPREGSASVLTVRREPETPLAVPGLRVVVSDATRVRVAWGPVPGASGFRISWSTGSGPESSQTLPPDSTATDITGLQPGTTYQVAVSVLRGREEGPAAVIVARTDPLGPVRTVHVTQASSSSVTITWTRVPGATGYRVSWHSAHGPEKSQLVSGEATVAELDGLEPDTEYTVHVRAHVAGVDGPPASVVVRTAPEPVGRVSRLQILNASSDVLRITWVGVTGATAYRLAWGRSEGGPMRHQILPGNTDSAEIRGLEGGVSYSVRVTALVGDREGTPVSIVVTTPPEAPPALGTLHVVQRGEHSLRLRWEPVPRAQGFLLHWQPEGGQEQSRVLGPELSSYHLDGLEPATQYRVRLSVLGPAGEGPSAEVTARTESPRVPSIELRVVDTSIDSVTLAWTPVSRASSYILSWRPLRGPGQEVPGSPQTLPGISSSQRVTGLEPGVSYIFSLTPVLDGVRGPEASVTQTPVCPRGLADVVFLPHATQDNAHRAEATRRVLERLVLALGPLGPQAVQVGLLSYSHRPSPLFPLNGSHDLGIILQRIRDMPYMDPSGNNLGTAVVTAHRYMLAPDAPGRRQHVPGVMVLLVDEPLRGDIFSPIREAQASGLNVVMLGMAGADPEQLRRLAPGMDSVQTFFAVDDGPSLDQAVSGLATALCQASFTTQPRPEPCPVYCPKGQKGEPGEMGLRGQVGPPGDPGLPGRTGAPGPQGPPGSATAKGERGFPGADGRPGSPGRAGNPGTPGAPGLKGSPGLPGPRGDPGERGPRGPKGEPGAPGQVIGGEGPGLPGRKGDPGPSGPPGPRGPLGDPGPRGPPGLPGTAMKGDKGDRGERGPPGPGEGGIAPGEPGLPGLPGSPGPQGPVGPPGKKGEKGDSEDGAPGLPGQPGSPGEQGPRGPPGAIGPKGDRGFPGPLGEAGEKGERGPPGPAGSRGLPGVAGRPGAKGPEGPPGPTGRQGEKGEPGRPGDPAVVGPAVAGPKGEKGDVGPAGPRGATGVQGERGPPGLVLPGDPGPKGDPGDRGPIGLTGRAGPPGDSGPPGEKGDPGRPGPPGPVGPRGRDGEVGEKGDEGPPGDPGLPGKAGERGLRGAPGVRGPVGEKGDQGDPGEDGRNGSPGSSGPKGDRGEPGPPGPPGRLVDTGPGAREKGEPGDRGQEGPRGPKGDPGLPGAPGERGIEGFRGPPGPQGDPGVRGPAGEKGDRGPPGLDGRSGLDGKPGAAGPSGPNGAAGKAGDPGRDGLPGLRGEQGLPGPSGPPGLPGKPGEDGKPGLNGKNGEPGDPGEDGRKGEKGDSGASGREGRDGPKGERGAPGILGPQGPPGLPGPVGPPGQGFPGVPGGTGPKGDRGETGSKGEQGLPGERGLRGEPGSVPNVDRLLETAGIKASALREIVETWDESSGSFLPVPERRRGPKGDSGEQGPPGKEGPIGFPGERGLKGDRGDPGPQGPPGLALGERGPPGPSGLAGEPGKPGIPGLPGRAGGVGEAGRPGERGERGEKGERGEQGRDGPPGLPGTPGPPGPPGPKVSVDEPGPGLSGEQGPPGLKGAKGEPGSNGDQGPKGDRGVPGIKGDRGEPGPRGQDGNPGLPGERGMAGPEGKPGLQGPRGPPGPVGGHGDPGPPGAPGLAGPAGPQGPSGLKGEPGETGPPGRGLTGPTGAVGLPGPPGPSGLVGPQGSPGLPGQVGETGKPGAPGRDGASGKDGDRGSPGVPGSPGLPGPVGPKGEPGPTGAPGQAVVGLPGAKGEKGAPGGLAGDLVGEPGAKGDRGLPGPRGEKGEAGRAGEPGDPGEDGQKGAPGPKGFKGDPGVGVPGSPGPPGPPGVKGDLGLPGLPGAPGVVGFPGQTGPRGEMGQPGPSGERGLAGPPGREGIPGPLGPPGPPGSVGPPGASGLKGDKGDPGVGLPGPRGERGEPGIRGEDGRPGQEGPRGLTGPPGSRGERGEKGDVGSAGLKGDKGDSAVILGPPGPRGAKGDMGERGPRGLDGDKGPRGDNGDPGDKGSKGEPGDKGSAGLPGLRGLLGPQGQPGAAGIPGDPGSPGKDGVPGIRGEKGDVGFMGPRGLKGERGVKGACGLDGEKGDKGEAGPPGRPGLAGHKGEMGEPGVPGQSGAPGKEGLIGPKGDRGFDGQPGPKGDQGEKGERGTPGIGGFPGPSGNDGSAGPPGPPGSVGPRGPEGLQGQKGERGPPGERVVGAPGVPGAPGERGEQGRPGPAGPRGEKGEAALTEDDIRGFVRQEMSQHCACQGQFIASGSRPLPSYAADTAGSQLHAVPVLRVSHAEEEERVPPEDDEYSEYSEYSVEEYQDPEAPWDSDDPCSLPLDEGSCTAYTLRWYHRAVTGSTEACHPFVYGGCGGNANRFGTREACERRCPPRVVQSQGTGTAQD

1221 NP_001652.2 NP_061936.2

>NP_001652.2

MGNHLTEMAPTASSFLPHFQALHVVVIGLDSAGKTSLLYRLKFKEFVQSVPTKGFNTEKIRVPLGGSRGITFQVWDVGGQEKLRPLWRSYTRRTDGLVFVVDAAEAERLEEAKVELHRISRASDNQGVPVLVLANKQDQPGALSAAEVEKRLAVRELAAATLTHVQGCSAVDGLGLQQGLERLYEMILKRKKAARGGKKRR

>NP_061936.2

MDGFAGSLDDSISAASTSDVQDRLSALESRVQQQEDEITVLKAALADVLRRLAISEDHVASVKKSVSSKGQPSPRAVIPMSCITNGSGANRKPSHTSAVSIAGKETLSSAAKSGTEKKKEKPQGQREKKEESHSNDQSPQIRASPSPQPSSQPLQIHRQTPESKNATPTKSIKRPSPAEKSHNSWENSDDSRNKLSKIPSTPKLIPKVTKTADKHKDVIINQEGEYIKMFMRGRPITMFIPSDVDNYDDIRTELPPEKLKLEWAYGYRGKDCRANVYLLPTGEIVYFIASVVVLFNYEERTQRHYLGHTDCVKCLAIHPDKIRIATGQIAGVDKDGRPLQPHVRVWDSVTLSTLQIIGLGTFERGVGCLDFSKADSGVHLCVIDDSNEHMLTVWDWQKKAKGAEIKTTNEVVLAVEFHPTDANTIITCGKSHIFFWTWSGNSLTRKQGIFGKYEKPKFVQCLAFLGNGDVLTGDSGGVMLIWSKTTVEPTPGKGPKGVYQISKQIKAHDGSVFTLCQMRNGMLLTGGGKDRKIILWDHDLNPEREIEVPDQYGTIRAVAEGKADQFLVGTSRNFILRGTFNDGFQIEVQGHTDELWGLATHPFKDLLLTCAQDRQVCLWNSMEHRLEWTRLVDEPGHCADFHPSGTVVAIGTHSGRWFVLDAETRDLVSIHTDGNEQLSVMRYSIDGTFLAVGSHDNFIYLYVVSENGRKYSRYGRCTGHSSYITHLDWSPDNKYIMSNSGDYEILYWDIPNGCKLIRNRSDCKDIDWTTYTCVLGFQVFGVWPEGSDGTDINALVRSHNRKVIAVADDFCKVHLFQYPCSKAKAPSHKYSAHSSHVTNVSFTHNDSHLISTGGKDMSIIQWKLVEKLSLPQNETVADTTLTKAPVSSTESVIQSNTPTPPPSQPLNETAEEESRISSSPTLLENSLEQTVEPSEDHSEEESEEGSGDLGEPLYEEPCNEISKEQAKATLLEDQQDPSPSS

1222 NP_060910.3 NP_536859.1

>NP_060910.3

MGPAGSLLGSGQMQITLWGSLAAVAIFFVITFLIFLCSSCDREKKPRQHSGDHENLMNVPSDKEMFSRSVTSLATDAPASSEQNGALTNGDILSEDSTLTCMQHYEEVQTSASDLLDSQDSTGKPKCHQSRELPRIPPESAVDTMLTARSVDGDQGLGMEGPYEVLKDSSSQENMVEDCLYETVKEIKEVAAAAHLEKGHSGKAKSTSASKELPGPQTEGKAEFAEYASVDRNKKCRQSVNVESILGNSCDPEEEAPPPVPVKLLDENENLQEKEGGEAEESATDTTSETNKRFSSLSYKSREEDPTLTEEEISAMYSSVNKPGQLVNKSGQSLTVPESTYTSIQGDPQRSPSSCNDLYATVKDFEKTPNSTLPPAGRPSEEPEPDYEAIQTLNREEEKATLGTNGHHGLVPKENDYESISDLQQGRDITRL

>NP_536859.1

MVRWFHRDLSGLDAETLLKGRGVHGSFLARPSRKNQGDFSLSVRVGDQVTHIRIQNSGDFYDLYGGEKFATLTELVEYYTQQQGVLQDRDGTIIHLKYPLNCSDPTSERWYHGHMSGGQAETLLQAKGEPWTFLVRESLSQPGDFVLSVLSDQPKAGPGSPLRVTHIKVMCEGGRYTVGGLETFDSLTDLVEHFKKTGIEEASGAFVYLRQPYYATRVNAADIENRVLELNKKQESEDTAKAGFWEEFESLQKQEVKNLHQRLEGQRPENKGKNRYKNILPFDHSRVILQGRDSNIPGSDYINANYIKNQLLGPDENAKTYIASQGCLEATVNDFWQMAWQENSRVIVMTTREVEKGRNKCVPYWPEVGMQRAYGPYSVTNCGEHDTTEYKLRTLQVSPLDNGDLIREIWHYQYLSWPDHGVPSEPGGVLSFLDQINQRQESLPHAGPIIVHCSAGIGRTGTIIVIDMLMENISTKGLDCDIDIQKTIQMVRAQRSGMVQTEAQYKFIYVAIAQFIETTKKKLEVLQSQKGQESEYGNITYPPAMKNAHAKASRTSSKSLESSAGTVAASPVRRGGQRGLPVPGPPVLSPDLHQLPVLAPLHPAADTRRMCMRTCTLRTRGRRK

1223 NP_055150.1 NP_001420.2

>NP_055150.1

MSEMAELSELYEESSDLQMDVMPGEGDLPQMEVGSGSRELSLRPSRSGAQQLEEEGPMEEEEAQPMAAPEGKRSLANGPNAGEQPGQVAGADFESEDEGEEFDDWEDDYDYPEEEQLSGAGYRVSAALEEADKMFLRTREPALDGGFQMHYEKTPFDQLAFIEELFSLMVVNRLTEELGCDEIIDRE

>NP_001420.2

MAENVVEPGPPSAKRPKLSSPALSASASDGTDFGSLFDLEHDLPDELINSTELGLTNGGDINQLQTSLGMVQDAASKHKQLSELLRSGSSPNLNMGVGGPGQVMASQAQQSSPGLGLINSMVKSPMTQAGLTSPNMGMGTSGPNQGPTQSTGMMNSPVNQPAMGMNTGMNAGMNPGMLAAGNGQGIMPNQVMNGSIGAGRGRQNMQYPNPGMGSAGNLLTEPLQQGSPQMGGQTGLRGPQPLKMGMMNNPNPYGSPYTQNPGQQIGASGLGLQIQTKTVLSNNLSPFAMDKKAVPGGGMPNMGQQPAPQVQQPGLVTPVAQGMGSGAHTADPEKRKLIQQQLVLLLHAHKCQRREQANGEVRQCNLPHCRTMKNVLNHMTHCQSGKSCQVAHCASSRQIISHWKNCTRHDCPVCLPLKNAGDKRNQQPILTGAPVGLGNPSSLGVGQQSAPNLSTVSQIDPSSIERAYAALGLPYQVNQMPTQPQVQAKNQQNQQPGQSPQGMRPMSNMSASPMGVNGGVGVQTPSLLSDSMLHSAINSQNPMMSENASVPSLGPMPTAAQPSTTGIRKQWHEDITQDLRNHLVHKLVQAIFPTPDPAALKDRRMENLVAYARKVEGDMYESANNRAEYYHLLAEKIYKIQKELEEKRRTRLQKQNMLPNAAGMVPVSMNPGPNMGQPQPGMTSNGPLPDPSMIRGSVPNQMMPRITPQSGLNQFGQMSMAQPPIVPRQTPPLQHHGQLAQPGALNPPMGYGPRMQQPSNQGQFLPQTQFPSQGMNVTNIPLAPSSGQAPVSQAQMSSSSCPVNSPIMPPGSQGSHIHCPQLPQPALHQNSPSPVPSRTPTPHHTPPSIGAQQPPATTIPAPVPTPPAMPPGPQSQALHPPPRQTPTPPTTQLPQQVQPSLPAAPSADQPQQQPRSQQSTAASVPTPTAPLLPPQPATPLSQPAVSIEGQVSNPPSTSSTEVNSQAIAEKQPSQEVKMEAKMEVDQPEPADTQPEDISESKVEDCKMESTETEERSTELKTEIKEEEDQPSTSATQSSPAPGQSKKKIFKPEELRQALMPTLEALYRQDPESLPFRQPVDPQLLGIPDYFDIVKSPMDLSTIKRKLDTGQYQEPWQYVDDIWLMFNNAWLYNRKTSRVYKYCSKLSEVFEQEIDPVMQSLGYCCGRKLEFSPQTLCCYGKQLCTIPRDATYYSYQNRYHFCEKCFNEIQGESVSLGDDPSQPQTTINKEQFSKRKNDTLDPELFVECTECGRKMHQICVLHHEIIWPAGFVCDGCLKKSARTRKENKFSAKRLPSTRLGTFLENRVNDFLRRQNHPESGEVTVRVVHASDKTVEVKPGMKARFVDSGEMAESFPYRTKALFAFEEIDGVDLCFFGMHVQEYGSDCPPPNQRRVYISYLDSVHFFRPKCLRTAVYHEILIGYLEYVKKLGYTTGHIWACPPSEGDDYIFHCHPPDQKIPKPKRLQEWYKKMLDKAVSERIVHDYKDIFKQATEDRLTSAKELPYFEGDFWPNVLEESIKELEQEEEERKREENTSNESTDVTKGDSKNAKKKNNKKTSKNKSSLSRGNKKKPGMPNVSNDLSQKLYATMEKHKEVFFVIRLIAGPAANSLPPIVDPDPLIPCDLMDGRDAFLTLARDKHLEFSSLRRAQWSTMCMLVELHTQSQDRFVYTCNECKHHVETRWHCTVCEDYDLCITCYNTKNHDHKMEKLGLGLDDESNNQQAAATQSPGDSRRLSIQRCIQSLVHACQCRNANCSLPSCQKMKRVVQHTKGCKRKTNGGCPICKQLIALCCYHAKHCQENKCPVPFCLNIKQKLRQQQLQHRLQQAQMLRRRMASMQRTGVVGQQQGLPSPTPATPTTPTGQQPTTPQTPQPTSQPQPTPPNSMPPYLPRTQAAGPVSQGKAAGQVTPPTPPQTAQPPLPGPPPAAVEMAMQIQRAAETQRQMAHVQIFQRPIQHQMPPMTPMAPMGMNPPPMTRGPSGHLEPGMGPTGMQQQPPWSQGGLPQPQQLQSGMPRPAMMSVAQHGQPLNMAPQPGLGQVGISPLKPGTVSQQALQNLLRTLRSPSSPLQQQQVLSILHANPQLLAAFIKQRAAKYANSNPQPIPGQPGMPQGQPGLQPPTMPGQQGVHSNPAMQNMNPMQAGVQRAGLPQQQPQQQLQPPMGGMSPQAQQMNMNHNTMPSQFRDILRRQQMMQQQQQQGAGPGIGPGMANHNQFQQPQGVGYPPQQQQRMQHHMQQMQQGNMGQIGQLPQALGAEAGASLQAYQQRLLQQQMGSPVQPNPMSPQQHMLPNQAQSPHLQGQQIPNSLSNQVRSPQPVPSPRPQSQPPHSSPSPRMQPQPSPHHVSPQTSSPHPGLVAAQANPMEQGHFASPDQNSMLSQLASNPGMANLHGASATDLGLSTDNSDLNSNLSQSTLDIH

1224 NP_036413.1 NP_055101.2

>NP_036413.1

MAAGVAAWLPFARAAAIGWMPVASGPMPAPPRQERKRTQDALIVLNVSGTRFQTWQDTLERYPDTLLGSSERDFFYHPETQQYFFDRDPDIFRHILNFYRTGKLHYPRHECISAYDEELAFFGLIPEIIGDCCYEEYKDRRRENAERLQDDADTDTAGESALPTMTARQRVWRAFENPHTSTMALVFYYVTGFFIAVSVIANVVETVPCGSSPGHIKELPCGERYAVAFFCLDTACVMIFTVEYLLRLAAAPSRYRFVRSVMSIIDVVAILPYYIGLVMTDNEDVSGAFVTLRVFRVFRIFKFSRHSQGLRILGYTLKSCASELGFLLFSLTMAIIIFATVMFYAEKGSSASKFTSIPAAFWYTIVTMTTLGYGDMVPKTIAGKIFGSICSLSGVLVIALPVPVIVSNFSRIYHQNQRADKRRAQKKARLARIRAAKSGSANAYMQSKRNGLLSNQLQSSEDEQAFVSKSGSSFETQHHHLLHCLEKTTNHEFVDEQVFEESCMEVATVNRPSSHSPSLSSQQGVTSTCCSRRHKKTFRIPNANVSGSHQGSIQELSTIQIRCVERTPLSNSRSSLNAKMEECVKLNCEQPYVTTAIISIPTPPVTTPEGDDRPESPEYSGGNIVRVSAL

>NP_055101.2

MGKSNSKLKPEVVEELTRKTYFTEKEVQQWYKGFIKDCPSGQLDAAGFQKIYKQFFPFGDPTKFATFVFNVFDENKDGRIEFSEFIQALSVTSRGTLDEKLRWAFKLYDLDNDGYITRNEMLDIVDAIYQMVGNTVELPEEENTPEKRVDRIFAMMDKNADGKLTLQEFQEGSKADPSIVQALSLYDGLV

1225 NP_001238.1 NP_000323.2

>NP_001238.1

MKKGIRYETSRKTSYIFQQPQHGPWQTRMRKISNHGSLRVAKVAYPLGLCVGVFIYVAYIKWHRATATQAFFSITRAAPGARWGQQAHSPLGTAADGHEVFYGIMFDAGSTGTRVHVFQFTRPPRETPTLTHETFKAVKPGLSAYADDVEKSAQGIRELLDVAKQDIPFDFWKATPLVLKATAGLRLLPGEKAQKLLQKVKEVFKASPFLVGDDCVSIMNGTDEGVSAWITINFLTGSLKTPGGSSVGMLDLGGGSTQIAFLPRVEGTLQASPPGYLTALRMFNRTYKLYSYSYLGLGLMSARLAILGGVEGQPAKDGKELVSPCLSPSFKGEWEHAEVTYRVSGQKAAASLHELCAARVSEVLQNRVHRTEEVKHVDFYAFSYYYDLAAGVGLIDAEKGGSLVVGDFEIAAKYVCRTLETQPQSSPFSCMDLTYVSLLLQEFGFPRSKVLKLTRKIDNVETSWALGAIFHYIDSLNRQKSPAS

>NP_000323.2

MKSNQERSNECLPPKKREIPATSRSSEEKAPTLPSDNHRVEGTAWLPGNPGGRGHGGGRHGPAGTSVELGLQQGIGLHKALSTGLDYSPPSAPRSVPVATTLPAAYATPQPGTPVSPVQYAHLPHTFQFIGSSQYSGTYASFIPSQLIPPTANPVTSAVASAAGATTPSQRSQLEAYSTLLANMGSLSQTPGHKAEQQQQQQQQQQQQHQHQQQQQQQQQQQQQQHLSRAPGLITPGSPPPAQQNQYVHISSSPQNTGRTASPPAIPVHLHPHQTMIPHTLTLGPPSQVVMQYADSGSHFVPREATKKAESSRLQQAIQAKEVLNGEMEKSRRYGAPSSADLGLGKAGGKSVPHPYESRHVVVHPSPSDYSSRDPSGVRASVMVLPNSNTPAADLEVQQATHREASPSTLNDKSGLHLGKPGHRSYALSPHTVIQTTHSASEPLPVGLPATAFYAGTQPPVIGYLSGQQQAITYAGSLPQHLVIPGTQPLLIPVGSTDMEASGAAPAIVTSSPQFAAVPHTFVTTALPKSENFNPEALVTQAAYPAMVQAQIHLPVVQSVASPAAAPPTLPPYFMKGSIIQLANGELKKVEDLKTEDFIQSAEISNDLKIDSSTVERIEDSHSPGVAVIQFAVGEHRAQVSVEVLVEYPFFVFGQGWSSCCPERTSQLFDLPCSKLSVGDVCISLTLKNLKNGSVKKGQPVDPASVLLKHSKADGLAGSRHRYAEQENGINQGSAQMLSENGELKFPEKMGLPAAPFLTKIEPSKPAATRKRRWSAPESRKLEKSEDEPPLTLPKPSLIPQEVKICIEGRSNVGK

1226 NP_005234.1 NP_001411.2

>NP_005234.1

MASTDYSTYSQAAAQQGYSAYTAQPTQGYAQTTQAYGQQSYGTYGQPTDVSYTQAQTTATYGQTAYATSYGQPPTGYTTPTAPQAYSQPVQGYGTGAYDTTTATVTTTQASYAAQSAYGTQPAYPAYGQQPAATAPTRPQDGNKPTETSQPQSSTGGYNQPSLGYGQSNYSYPQVPGSYPMQPVTAPPSYPPTSYSSTQPTSYDQSSYSQQNTYGQPSSYGQQSSYGQQSSYGQQPPTSYPPQTGSYSQAPSQYSQQSSSYGQQSSFRQDHPSSMGVYGQESGGFSGPGENRSMSGPDNRGRGRGGFDRGGMSRGGRGGGRGGMGSAGERGGFNKPGGPMDEGPDLDLGPPVDPDEDSDNSAIYVQGLNDSVTLDDLADFFKQCGVVKMNKRTGQPMIHIYLDKETGKPKGDATVSYEDPPTAKAAVEWFDGKDFQGSKLKVSLARKKPPMNSMRGGLPPREGRGMPPPLRGGPGGPGGPGGPMGRMGGRGGDRGGFPPRGPRGSRGNPSGGGNVQHRAGDWQCPNPGCGNQNFAWRTECNQCKAPKPEGFLPPPFPPPGGDRGRGGPGGMRGGRGGLMDRGGPGGMFRGGRGGDRGGFRGGRGMDRGGFGGGRRGGPGGPPGPLMEQMGGRRGGRGGPGKMDKGEHRQERRDRPY

>NP_001411.2

MVTQILGAMESQVGGGPAGPALPNGPLLGTNGATDDSKTNLIVNYLPQNMTQDEFKSLFGSIGDIESCKLVRDKITGQSLGYGFVNYSDPNDADKAINTLNGLKLQTKTIKVSYARPSSASIRDANLYVSGLPKTMSQKEMEQLFSQYGRIITSRILVDQVTGVSRGVGFIRFDKRIEAEEAIKGLNGQKPLGAAEPITVKFANNPSQKTGQALLTHLYQSSARRYAGPLHHQTQRFRLDNLLNMAYGVKSPLSLIARFSPIAIDGMSGLAGVGLSGGAAGAGWCIFVYNLSPEADESVLWQLFGPFGAVTNVKVIRDFTTNKCKGFGFVTMTNYDEAAMAIASLNGYRLGERVLQVSFKTSKQHKA

1227 NP_001014809.1 NP_003899.2

>NP_001014809.1

MADRRRAWNTEDDLPVYLARPGSAAQTPRQKYGGMFAAVEGAYENKTIDFDAYSVGRRGSARTPRSAGRPDAVGLPGPGGSEDTASDVSEPSGSAVSSPGERDERPPTLRIRRPAPRDLPLGRDNGQSDRLLIKGGRIINDDQSLYADVYLEDGLIKQIGENLIVPGGVKTIEANGRMVIPGGIDVNTYLQKPSQGMTAADDFFQGTRAALVGGTTMIIDHVVPEPGSSLLTSFEKWHEAADTKSCCDYSLHVDITSWYDGVREELEVLVQDKGVNSFQVYMAYKDVYQMSDSQLYEAFTFLKGLGAVILVHAENGDLIAQEQKRILEMGITGPEGHALSRPEELEAEAVFRAITIAGRINCPVYITKVMSKSAADIIALARKKGPLVFGEPIAASLGTDGTHYWSKNWAKAAAFVTSPPLSPDPTTPDYLTSLLACGDLQVTGSGHCPYSTAQKAVGKDNFTLIPEGVNGIEERMTVVWDKAVATGKMDENQFVAVTSTNAAKIFNLYPRKGRIAVGSDADVVIWDPDKLKTITAKSHKSAVEYNIFEGMECHGSPLVVISQGKIVFEDGNINVNKGMGRFIPRKAFPEHLYQRVKIRNKVFGLQGVSRGMYDGPVYEVPATPKYATPAPSAKSSPSKHQPPPIRNLHQSNFSLSGAQIDDNNPRRTGHRIVAPPGGRSNITSLG

>NP_003899.2

MSGDEMIFDPTMSKKKKKKKKPFMLDEEGDTQTEETQPSETKEVEPEPTEDKDLEADEEDTRKKDASDDLDDLNFFNQKKKKKKTKKIFDIDEAEEGVKDLKIESDVQEPTEPEDDLDIMLGNKKKKKKNVKFPDEDEILEKDEALEDEDNKKDDGISFSNQTGPAWAGSERDYTYEELLNRVFNIMREKNPDMVAGEKRKFVMKPPQVVRVGTKKTSFVNFTDICKLLHRQPKHLLAFLLAELGTSGSIDGNNQLVIKGRFQQKQIENVLRRYIKEYVTCHTCRSPDTILQKDTRLYFLQCETCHSRCSVASIKTGFQAVTGKRAQLRAKAN

1228 NP_852664.1 NP_004439.2

>NP_852664.1

MSAEGYQYRALYDYKKEREEDIDLHLGDILTVNKGSLVALGFSDGQEARPEEIGWLNGYNETTGERGDFPGTYVEYIGRKKISPPTPKPRPPRPLPVAPGSSKTEADVEQQALTLPDLAEQFAPPDIAPPLLIKLVEAIEKKGLECSTLYRTQSSSNLAELRQLLDCDTPSVDLEMIDVHVLADAFKRYLLDLPNPVIPAAVYSEMISLAPEVQSSEEYIQLLKKLIRSPSIPHQYWLTLQYLLKHFFKLSQTSSKNLLNARVLSEIFSPMLFRFSAASSDNTENLIKVIEILISTEWNERQPAPALPPKPPKPTTVANNGMNNNMSLQDAEWYWGDISREEVNEKLRDTADGTFLVRDASTKMHGDYTLTLRKGGNNKLIKIFHRDGKYGFSDPLTFSSVVELINHYRNESLAQYNPKLDVKLLYPVSKYQQDQVVKEDNIEAVGKKLHEYNTQFQEKSREYDRLYEEYTRTSQEIQMKRTAIEAFNETIKIFEEQCQTQERYSKEYIEKFKREGNEKEIQRIMHNYDKLKSRISEIIDSRRRLEEDLKKQAAEYREIDKRMNSIKPDLIQLRKTRDQYLMWLTQKGVRQKKLNEWLGNENTEDQYSLVEDDEDLPHHDEKTWNVGSSNRNKAENLLRGKRDGTFLVRESSKQGCYACSVVVDGEVKHCVINKTATGYGFAEPYNLYSSLKELVLHYQHTSLVQHNDSLNVTLAYPVYAQQRR

>NP_004439.2

MELAALCRWGLLLALLPPGAASTQVCTGTDMKLRLPASPETHLDMLRHLYQGCQVVQGNLELTYLPTNASLSFLQDIQEVQGYVLIAHNQVRQVPLQRLRIVRGTQLFEDNYALAVLDNGDPLNNTTPVTGASPGGLRELQLRSLTEILKGGVLIQRNPQLCYQDTILWKDIFHKNNQLALTLIDTNRSRACHPCSPMCKGSRCWGESSEDCQSLTRTVCAGGCARCKGPLPTDCCHEQCAAGCTGPKHSDCLACLHFNHSGICELHCPALVTYNTDTFESMPNPEGRYTFGASCVTACPYNYLSTDVGSCTLVCPLHNQEVTAEDGTQRCEKCSKPCARVCYGLGMEHLREVRAVTSANIQEFAGCKKIFGSLAFLPESFDGDPASNTAPLQPEQLQVFETLEEITGYLYISAWPDSLPDLSVFQNLQVIRGRILHNGAYSLTLQGLGISWLGLRSLRELGSGLALIHHNTHLCFVHTVPWDQLFRNPHQALLHTANRPEDECVGEGLACHQLCARGHCWGPGPTQCVNCSQFLRGQECVEECRVLQGLPREYVNARHCLPCHPECQPQNGSVTCFGPEADQCVACAHYKDPPFCVARCPSGVKPDLSYMPIWKFPDEEGACQPCPINCTHSCVDLDDKGCPAEQRASPLTSIISAVVGILLVVVLGVVFGILIKRRQQKIRKYTMRRLLQETELVEPLTPSGAMPNQAQMRILKETELRKVKVLGSGAFGTVYKGIWIPDGENVKIPVAIKVLRENTSPKANKEILDEAYVMAGVGSPYVSRLLGICLTSTVQLVTQLMPYGCLLDHVRENRGRLGSQDLLNWCMQIAKGMSYLEDVRLVHRDLAARNVLVKSPNHVKITDFGLARLLDIDETEYHADGGKVPIKWMALESILRRRFTHQSDVWSYGVTVWELMTFGAKPYDGIPAREIPDLLEKGERLPQPPICTIDVYMIMVKCWMIDSECRPRFRELVSEFSRMARDPQRFVVIQNEDLGPASPLDSTFYRSLLEDDDMGDLVDAEEYLVPQQGFFCPDPAPGAGGMVHHRHRSSSTRSGGGDLTLGLEPSEEEAPRSPLAPSEGAGSDVFDGDLGMGAAKGLQSLPTHDPSPLQRYSEDPTVPLPSETDGYVAPLTCSPQPEYVNQPDVRPQPPSPREGPLPAARPAGATLERPKTLSPGKNGVVKDVFAFGGAVENPEYLTPQGGAAPQPHPPPAFSPAFDNLYYWDQDPPERGAPPSTFKGTPTAENPEYLGLDVPV

1229 NP_009123.1 NP_002379.2

>NP_009123.1

MAVTLDKDAYYRRVKRLYSNWRKGEDEYANVDAIVVSVGVDEEIVYAKSTALQTWLFGYELTDTIMVFCDDKIIFMASKKKVEFLKQIANTKGNENANGAPAITLLIREKNESNKSSFDKMIEAIKESKNGKKIGVFSKDKFPGEFMKSWNDCLNKEGFDKIDISAVVAYTIAVKEDGELNLMKKAASITSEVFNKFFKERVMEIVDADEKVRHSKLAESVEKAIEEKKYLAGADPSTVEMCYPPIIQSGGNYNLKFSVVSDKNHMHFGAITCAMGIRFKSYCSNLVRTLMVDPSQEVQENYNFLLQLQEELLKELRHGVKICDVYNAVMDVVKKQKPELLNKITKNLGFGMGIEFREGSLVINSKNQYKLKKGMVFSINLGFSDLTNKEGKKPEEKTYALFIGDTVLVDEDGPATVLTSVKKKVKNVGIFLKNEDEEEEEEEKDEAEDLLGRGSRAALLTERTRNEMTAEEKRRAHQKELAAQLNEEAKRRLTEQKGEQQIQKARKSNVSYKNPSLMPKEPHIREMKIYIDKKYETVIMPVFGIATPFHIATIKNISMSVEGDYTYLRINFYCPGSALGRNEGNIFPNPEATFVKEITYRASNIKAPGEQTVPALNLQNAFRIIKEVQKRYKTREAEEKEKEGIVKQDSLVINLNRSNPKLKDLYIRPNIAQKRMQGSLEAHVNGFRFTSVRGDKVDILYNNIKHALFQPCDGEMIIVLHFHLKNAIMFGKKRHTDVQFYTEVGEITTDLGKHQHMHDRDDLYAEQMEREMRHKLKTAFKNFIEKVEALTKEELEFEVPFRDLGFNGAPYRSTCLLQPTSSALVNATEWPPFVVTLDEVELIHFERVQFHLKNFDMVIVYKDYSKKVTMINAIPVASLDPIKEWLNSCDLKYTEGVQSLNWTKIMKTIVDDPEGFFEQGGWSFLEPEGEGSDAEEGDSESEIEDETFNPSEDDYEEEEEDSDEDYSSEAEESDYSKESLGSEEESGKDWDELEEEARKADRESRYEEEEEQSRSMSRKRKASVHSSGRGSNRGSRHSSAPPKKKRK

>NP_002379.2

MAGTVVLDDVELREAQRDYLDFLDDEEDQGIYQSKVRELISDNQYRLIVNVNDLRRKNEKRANRLLNNAFEELVAFQRALKDFVASIDATYAKQYEEFYVGLEGSFGSKHVSPRTLTSCFLSCVVCVEGIVTKCSLVRPKVVRSVHYCPATKKTIERRYSDLTTLVAFPSSSVYPTKDEENNPLETEYGLSVYKDHQTITIQEMPEKAPAGQLPRSVDVILDDDLVDKAKPGDRVQVVGTYRCLPGKKGGYTSGTFRTVLIACNVKQMSKDAQPSFSAEDIAKIKKFSKTRSKDIFDQLAKSLAPSIHGHDYVKKAILCLLLGGVERDLENGSHIRGDINILLIGDPSVAKSQLLRYVLCTAPRAIPTTGRGSSGVGLTAAVTTDQETGERRLEAGAMVLADRGVVCIDEFDKMSDMDRTAIHEVMEQGRVTIAKAGIHARLNARCSVLAAANPVYGRYDQYKTPMENIGLQDSLLSRFDLLFIMLDQMDPEQDREISDHVLRMHRYRAPGEQDGDAMPLGSAVDILATDDPNFSQEDQQDTQIYEKHDNLLHGTKKKKEKMVSAAFMKKYIHVAKIIKPVLTQESATYIAEEYSRLRSQDSMSSDTARTSPVTARTLETLIRLATAHAKARMSKTVDLQDAEEAVELVQYAYFKKVLEKEKKRKKRSEDESETEDEEEKSQEDQEQKRKRRKTRQPDAKDGDSYDPYDFSDTEEEMPQVHTPKTADSQETKESQKVELSESRLKAFKVALLDVFREAHAQSIGMNRLTESINRDSEEPFSSVEIQAALSKMQDDNQVMVSEGIIFLI

1230 NP_003557.2 NP_004153.2

>NP_003557.2

MLRRILQRTPGRVGSQGSDLDSSATPINTVDVNNESSSEGFICPQCMKSLGSADELFKHYEAVHDAGNDSGHGGESNLALKRDDVTLLRQEVQDLQASLKEEKWYSEELKKELEKYQGLQQQEAKPDGLVTDSSAELQSLEQQLEEAQTENFNIKQMKDLFEQKAAQLATEIADIKSKYDEERSLREAAEQKVTRLTEELNKEATVIQDLKTELLQRPGIEDVAVLKKELVQVQTLMDNMTLERERESEKLKDECKKLQSQYASSEATISQLRSELAKGPQEVAVYVQELQKLKSSVNELTQKNQTLTENLLKKEQDYTKLEEKHNEESVSKKNIQATLHQKDLDCQQLQSRLSASETSLHRIHVELSEKGEATQKLKEELSEVETKYQHLKAEFKQLQQQREEKEQHGLQLQSEINQLHSKLLETERQLGEAHGRLKEQRQLSSEKLMDKEQQVADLQLKLSRLEEQLKEKVTNSTELQHQLDKTKQQHQEQQALQQSTTAKLREAQNDLEQVLRQIGDKDQKIQNLEALLQKSKENISLLEKEREDLYAKIQAGEGETAVLNQLQEKNHTLQEQVTQLTEKLKNQSESHKQAQENLHDQVQEQKAHLRAAQDRVLSLETSVNELNSQLNESKEKVSQLDIQIKAKTELLLSAEAAKTAQRADLQNHLDTAQNALQDKQQELNKITTQLDQVTAKLQDKQEHCSQLESHLKEYKEKYLSLEQKTEELEGQIKKLEADSLEVKASKEQALQDLQQQRQLNTDLELRATELSKQLEMEKEIVSSTRLDLQKKSEALESIKQKLTKQEEEKQILKQDFETLSQETKIQHEELNNRIQTTVTELQKVKMEKEALMTELSTVKDKLSKVSDSLKNSKSEFEKENQKGKAAILDLEKTCKELKHQLQVQMENTLKEQKELKKSLEKEKEASHQLKLELNSMQEQLIQAQNTLKQNEKEEQQLQGNINELKQSSEQKKKQIEALQGELKIAVLQKTELENKLQQQLTQAAQELAAEKEKISVLQNNYEKSQETFKQLQSDFYGRESELLATRQDLKSVEEKLSLAQEDLISNRNQIGNQNKLIQELKTAKATLEQDSAKKEQQLQERCKALQDIQKEKSLKEKELVNEKSKLAEIEEIKCRQEKEITKLNEELKSHKLESIKEITNLKDAKQLLIQQKLELQGKADSLKAAVEQEKRNQQILKDQVKKEEEELKKEFIEKEAKLHSEIKEKEVGMKKHEENEAKLTMQITALNENLGTVKKEWQSSQRRVSELEKQTDDLRGEIAVLEATVQNNQDERRALLERCLKGEGEIEKLQTKVLELQRKLDNTTAAVQELGRENQSLQIKHTQALNRKWAEDNEVQNCMACGKGFSVTVRRHHCRQCGNIFCAECSAKNALTPSSKKPVRVCDACFNDLQG

>NP_004153.2

MASRGATRPNGPNTGNKICQFKLVLLGESAVGKSSLVLRFVKGQFHEFQESTIGAAFLTQTVCLDDTTVKFEIWDTAGQERYHSLAPMYYRGAQAAIVVYDITNEESFARAKNWVKELQRQASPNIVIALSGNKADLANKRAVDFQEAQSYADDNSLLFMETSAKTSMNVNEIFMAIAKKLPKNEPQNPGANSARGRGVDLTEPTQPTRNQCCSN

1231 NP_001001433.1 NP_002826.2

>NP_001001433.1

MATRRLTDAFLLLRNNSIQNRQLLAEQVSSHITSSPLHSRSIAAELDELADDRMALVSGISLDPEAAIGVTKRPPPKWVDGVDEIQYDVGRIKQKMKELASLHDKHLNRPTLDDSSEEEHAIEITTQEITQLFHRCQRAVQALPSRARACSEQEGRLLGNVVASLAQALQELSTSFRHAQSGYLKRMKNREERSQHFFDTSVPLMDDGDDNTLYHRGFTEDQLVLVEQNTLMVEEREREIRQIVQSISDLNEIFRDLGAMIVEQGTVLDRIDYNVEQSCIKTEDGLKQLHKAEQYQKKNRKMLVILILFVIIIVLIVVLVGVKSR

>NP_002826.2

MEQVEILRKFIQRVQAMKSPDHNGEDNFARDFMRLRRLSTKYRTEKIYPTATGEKEENVKKNRYKDILPFDHSRVKLTLKTPSQDSDYINANFIKGVYGPKAYVATQGPLANTVIDFWRMIWEYNVVIIVMACREFEMGRKKCERYWPLYGEDPITFAPFKISCEDEQARTDYFIRTLLLEFQNESRRLYQFHYVNWPDHDVPSSFDSILDMISLMRKYQEHEDVPICIHCSAGCGRTGAICAIDYTWNLLKAGKIPEEFNVFNLIQEMRTQRHSAVQTKEQYELVHRAIAQLFEKQLQLYEIHGAQKIADGVNEINTENMISSIEPEKQDSPPPKPPRTRSCLVEGDAKEEILQPPEPHPVPPILTPSPPSAFPTVTTVWQDNDRYHPKPVLHMVSSEQHSADLNRNYSKSTELPGKNESTIEQIDKKLERNLSFEIKKVPLQEGPKSFDGNTLLNRGHAIKIKSASPCIADKISKPQELSSDLNVGDTSQNSCVDCSVTQSNKVSVTPPEESQNSDTPPRPDRLPLDEKGHVTWSFHGPENAIPIPDLSEGNSSDINYQTRKTVSLTPSPTTQVETPDLVDHDNTSPLFRTPLSFTNPLHSDDSDSDERNSDGAVTQNKTNISTASATVSAATSTESISTRKVLPMSIARHNIAGTTHSGAEKDVDVSEDSPPPLPERTPESFVLASEHNTPVRSEWSELQSQERSEQKKSEGLITSENEKCDHPAGGIHYEMCIECPPTFSDKREQISENPTEATDIGFGNRCGKPKGPRDPPSEWT

1232 NP_005609.3 NP_000426.2

>NP_005609.3

MGSRCALALAVLSALLCQVWSSGVFELKLQEFVNKKGLLGNRNCCRGGAGPPPCACRTFFRVCLKHYQASVSPEPPCTYGSAVTPVLGVDSFSLPDGGGADSAFSNPIRFPFGFTWPGTFSLIIEALHTDSPDDLATENPERLISRLATQRHLTVGEEWSQDLHSSGRTDLKYSYRFVCDEHYYGEGCSVFCRPRDDAFGHFTCGERGEKVCNPGWKGPYCTEPICLPGCDEQHGFCDKPGECKCRVGWQGRYCDECIRYPGCLHGTCQQPWQCNCQEGWGGLFCNQDLNYCTHHKPCKNGATCTNTGQGSYTCSCRPGYTGATCELGIDECDPSPCKNGGSCTDLENSYSCTCPPGFYGKICELSAMTCADGPCFNGGRCSDSPDGGYSCRCPVGYSGFNCEKKIDYCSSSPCSNGAKCVDLGDAYLCRCQAGFSGRHCDDNVDDCASSPCANGGTCRDGVNDFSCTCPPGYTGRNCSAPVSRCEHAPCHNGATCHERGHRYVCECARGYGGPNCQFLLPELPPGPAVVDLTEKLEGQGGPFPWVAVCAGVILVLMLLLGCAAVVVCVRLRLQKHRPPADPCRGETETMNNLANCQREKDISVSIIGATQIKNTNKKADFHGDHSADKNGFKARYPAVDYNLVQDLKGDDTAVRDAHSKRDTKCQPQGSSGEEKGTPTTLRGGEASERKRPDSGCSTSKDTKYQSVYVISEEKDECVIATEV

>NP_000426.2

MGPGARGRRRRRRPMSPPPPPPPVRALPLLLLLAGPGAAAPPCLDGSPCANGGRCTQLPSREAACLCPPGWVGERCQLEDPCHSGPCAGRGVCQSSVVAGTARFSCRCPRGFRGPDCSLPDPCLSSPCAHGARCSVGPDGRFLCSCPPGYQGRSCRSDVDECRVGEPCRHGGTCLNTPGSFRCQCPAGYTGPLCENPAVPCAPSPCRNGGTCRQSGDLTYDCACLPGFEGQNCEVNVDDCPGHRCLNGGTCVDGVNTYNCQCPPEWTGQFCTEDVDECQLQPNACHNGGTCFNTLGGHSCVCVNGWTGESCSQNIDDCATAVCFHGATCHDRVASFYCACPMGKTGLLCHLDDACVSNPCHEDAICDTNPVNGRAICTCPPGFTGGACDQDVDECSIGANPCEHLGRCVNTQGSFLCQCGRGYTGPRCETDVNECLSGPCRNQATCLDRIGQFTCICMAGFTGTYCEVDIDECQSSPCVNGGVCKDRVNGFSCTCPSGFSGSTCQLDVDECASTPCRNGAKCVDQPDGYECRCAEGFEGTLCDRNVDDCSPDPCHHGRCVDGIASFSCACAPGYTGTRCESQVDECRSQPCRHGGKCLDLVDKYLCRCPSGTTGVNCEVNIDDCASNPCTFGVCRDGINRYDCVCQPGFTGPLCNVEINECASSPCGEGGSCVDGENGFRCLCPPGSLPPLCLPPSHPCAHEPCSHGICYDAPGGFRCVCEPGWSGPRCSQSLARDACESQPCRAGGTCSSDGMGFHCTCPPGVQGRQCELLSPCTPNPCEHGGRCESAPGQLPVCSCPQGWQGPRCQQDVDECAGPAPCGPHGICTNLAGSFSCTCHGGYTGPSCDQDINDCDPNPCLNGGSCQDGVGSFSCSCLPGFAGPRCARDVDECLSNPCGPGTCTDHVASFTCTCPPGYGGFHCEQDLPDCSPSSCFNGGTCVDGVNSFSCLCRPGYTGAHCQHEADPCLSRPCLHGGVCSAAHPGFRCTCLESFTGPQCQTLVDWCSRQPCQNGGRCVQTGAYCLCPPGWSGRLCDIRSLPCREAAAQIGVRLEQLCQAGGQCVDEDSSHYCVCPEGRTGSHCEQEVDPCLAQPCQHGGTCRGYMGGYMCECLPGYNGDNCEDDVDECASQPCQHGGSCIDLVARYLCSCPPGTLGVLCEINEDDCGPGPPLDSGPRCLHNGTCVDLVGGFRCTCPPGYTGLRCEADINECRSGACHAAHTRDCLQDPGGGFRCLCHAGFSGPRCQTVLSPCESQPCQHGGQCRPSPGPGGGLTFTCHCAQPFWGPRCERVARSCRELQCPVGVPCQQTPRGPRCACPPGLSGPSCRSFPGSPPGASNASCAAAPCLHGGSCRPAPLAPFFRCACAQGWTGPRCEAPAAAPEVSEEPRCPRAACQAKRGDQRCDRECNSPGCGWDGGDCSLSVGDPWRQCEALQCWRLFNNSRCDPACSSPACLYDNFDCHAGGRERTCNPVYEKYCADHFADGRCDQGCNTEECGWDGLDCASEVPALLARGVLVLTVLLPPEELLRSSADFLQRLSAILRTSLRFRLDAHGQAMVFPYHRPSPGSEPRARRELAPEVIGSVVMLEIDNRLCLQSPENDHCFPDAQSAADYLGALSAVERLDFPYPLRDVRGEPLEPPEPSVPLLPLLVAGAVLLLVILVLGVMVARRKREHSTLWFPEGFSLHKDVASGHKGRREPVGQDALGMKNMAKGESLMGEVATDWMDTECPEAKRLKVEEPGMGAEEAVDCRQWTQHHLVAADIRVAPAMALTPPQGDADADGMDVNVRGPDGFTPLMLASFCGGALEPMPTEEDEADDTSASIISDLICQGAQLGARTDRTGETALHLAARYARADAAKRLLDAGADTNAQDHSGRTPLHTAVTADAQGVFQILIRNRSTDLDARMADGSTALILAARLAVEGMVEELIASHADVNAVDELGKSALHWAAAVNNVEATLALLKNGANKDMQDSKEETPLFLAAREGSYEAAKLLLDHFANREITDHLDRLPRDVAQERLHQDIVRLLDQPSGPRSPPGPHGLGPLLCPPGAFLPGLKAAQSGSKKSRRPPGKAGLGPQGPRGRGKKLTLACPGPLADSSVTLSPVDSLDSPRPFGGPPASPGGFPLEGPYAAATATAVSLAQLGGPGRAGLGRQPPGGCVLSLGLLNPVAVPLDWARLPPPAPPGPSFLLPLAPGPQLLNPGTPVSPQERPPPYLAVPGHGEEYPAAGAHSSPPKARFLRVPSEHPYLTPSPESPEHWASPSPPSLSDWSESTPSPATATGAMATTTGALPAQPLPLSVPSSLAQAQTQLGPQPEVTPKRQVLA

1233 NP_001216.1 NP_116759.2

>NP_001216.1

MAEGNHRKKPLKVLESLGKDFLTGVLDNLVEQNVLNWKEEEKKKYYDAKTEDKVRVMADSMQEKQRMAGQMLLQTFFNIDQISPNKKAHPNMEAGPPESGESTDALKLCPHEEFLRLCKERAEEIYPIKERNNRTRLALIICNTEFDHLPPRNGADFDITGMKELLEGLDYSVDVEENLTARDMESALRAFATRPEHKSSDSTFLVLMSHGILEGICGTVHDEKKPDVLLYDTIFQIFNNRNCLSLKDKPKVIIVQACRGANRGELWVRDSPASLEVASSQSSENLEEDAVYKTHVEKDFIAFCSSTPHNVSWRDSTMGSIFITQLITCFQKYSWCCHLEEVFRKVQQSFETPRAKAQMPTIERLSMTRYFYLFPGN

>NP_116759.2

MKSQGQHWYSSSDKNCKVSFREKLLIIDSNLGVQDVENLKFLCIGLVPNKKLEKSSSASDVFEHLLAEDLLSEEDPFFLAELLYIIRQKKLLQHLNCTKEEVERLLPTRQRVSLFRNLLYELSEGIDSENLKDMIFLLKDSLPKTEMTSLSFLAFLEKQGKIDEDNLTCLEDLCKTVVPKLLRNIEKYKREKAIQIVTPPVDKEAESYQGEEELVSQTDVKTFLEALPQESWQNKHAGSNGNRATNGAPSLVSRGMQGASANTLNSETSTKRAAVYRMNRNHRGLCVIVNNHSFTSLKDRQGTHKDAEILSHVFQWLGFTVHIHNNVTKVEMEMVLQKQKCNPAHADGDCFVFCILTHGRFGAVYSSDEALIPIREIMSHFTALQCPRLAEKPKLFFIQACQGEEIQPSVSIEADALNPEQAPTSLQDSIPAEADFLLGLATVPGYVSFRHVEEGSWYIQSLCNHLKKLVPRHEDILSILTAVNDDVSRRVDKQGTKKQMPQPAFTLRKKLVFPVPLDALSL

1234 NP_000723.1 NP_001737.1

>NP_000723.1

MEHSTFLSGLVLATLLSQVSPFKIPIEELEDRVFVNCNTSITWVEGTVGTLLSDITRLDLGKRILDPRGIYRCNGTDIYKDKESTVQVHYRMCQSCVELDPATVAGIIVTDVIATLLLALGVFCFAGHETGRLSGAADTQALLRNDQVYQPLRDRDDAQYSHLGGNWARNK

>NP_001737.1

MEGKWLLCMLLVLGTAIVEAHDGHDDDVIDIEDDLDDVIEEVEDSKPDTTAPPSSPKVTYKAPVPTGEVYFADSFDRGTLSGWILSKAKKDDTDDEIAKYDGKWEVEEMKESKLPGDKGLVLMSRAKHHAISAKLNKPFLFDTKPLIVQYEVNFQNGIECGGAYVKLLSKTPELNLDQFHDKTPYTIMFGPDKCGEDYKLHFIFRHKNPKTGIYEEKHAKRPDADLKTYFTDKKTHLYTLILNPDNSFEILVDQSVVNSGNLLNDMTPPVNPSREIEDPEDRKPEDWDERPKIPDPEAVKPDDWDEDAPAKIPDEEATKPEGWLDDEPEYVPDPDAEKPEDWDEDMDGEWEAPQIANPRCESAPGCGVWQRPVIDNPNYKGKWKPPMIDNPSYQGIWKPRKIPNPDFFEDLEPFRMTPFSAIGLELWSMTSDIFFDNFIICADRRIVDDWANDGWGLKKAADGAAEPGVVGQMIEAAEERPWLWVVYILTVALPVFLVILFCCSGKKQTSGMEYKKTDAPQPDVKEEEEEKEEEKDKGDEEEEGEEKLEEKQKSDAEEDGGTVSQEEEDRKPKAEEDEILNRSPRNRKPRRE

1235 NP_003049.1 NP_006226.1

>NP_003049.1

MPTTVDDVLEHGGEFHFFQKQMFFLLALLSATFAPIYVGIVFLGFTPDHRCRSPGVAELSLRCGWSPAEELNYTVPGPGPAGEASPRQCRRYEVDWNQSTFDCVDPLASLDTNRSRLPLGPCRDGWVYETPGSSIVTEFNLVCANSWMLDLFQSSVNVGFFIGSMSIGYIADRFGRKLCLLTTVLINAAAGVLMAISPTYTWMLIFRLIQGLVSKAGWLIGYILITEFVGRRYRRTVGIFYQVAYTVGLLVLAGVAYALPHWRWLQFTVALPNFFFLLYYWCIPESPRWLISQNKNAEAMRIIKHIAKKNGKSLPASLQRLRLEEETGKKLNPSFLDLVRTPQIRKHTMILMYNWFTSSVLYQGLIMHMGLAGDNIYLDFFYSALVEFPAAFMIILTIDRIGRRYPWAASNMVAGAACLASVFIPGDLQWLKIIISCLGRMGITMAYEIVCLVNAELYPTFIRNLGVHICSSMCDIGGIITPFLVYRLTNIWLELPLMVFGVLGLVAGGLVLLLPETKGKALPETIEEAENMQRPRKNKEKMIYLQVQKLDIPLN

>NP_006226.1

MLWQKPTAPEQAPAPARPYQGVRVKEPVKELLRRKRGHASSGAAPAPTAVVLPHQPLATYTTVGPSCLDMEGSVSAVTEEAALCAGWLSQPTPATLQPLAPWTPYTEYVPHEAVSCPYSADMYVQPVCPSYTVVGPSSVLAYASPPLITNVTTRSSATPAVGPPLEGPEHQAPLTYFPWPQPLSTLPTSTLQYRPPAPALPGPQFVQLPISIPEPVLQDMEDPRRAASSLTIDKLLLEEEDSDAYALNHTLSVEGF

1236 NP_000410.2 NP_002077.1

>NP_000410.2

MARALCPLQALWLLEWVLLLLGPCAAPPAWALNLDPVQLTFYAGPNGSQFGFSLDFHKDSHGRVAIVVGAPRTLGPSQEETGGVFLCPWRAEGGQCPSLLFDLRDETRNVGSQTLQTFKARQGLGASVVSWSDVIVACAPWQHWNVLEKTEEAEKTPVGSCFLAQPESGRRAEYSPCRGNTLSRIYVENDFSWDKRYCEAGFSSVVTQAGELVLGAPGGYYFLGLLAQAPVADIFSSYRPGILLWHVSSQSLSFDSSNPEYFDGYWGYSVAVGEFDGDLNTTEYVVGAPTWSWTLGAVEILDSYYQRLHRLRGEQMASYFGHSVAVTDVNGDGRHDLLVGAPLYMESRADRKLAEVGRVYLFLQPRGPHALGAPSLLLTGTQLYGRFGSAIAPLGDLDRDGYNDIAVAAPYGGPSGRGQVLVFLGQSEGLRSRPSQVLDSPFPTGSAFGFSLRGAVDIDDNGYPDLIVGAYGANQVAVYRAQPVVKASVQLLVQDSLNPAVKSCVLPQTKTPVSCFNIQMCVGATGHNIPQKLSLNAELQLDRQKPRQGRRVLLLGSQQAGTTLNLDLGGKHSPICHTTMAFLRDEADFRDKLSPIVLSLNVSLPPTEAGMAPAVVLHGDTHVQEQTRIVLDCGEDDVCVPQLQLTASVTGSPLLVGADNVLELQMDAANEGEGAYEAELAVHLPQGAHYMRALSNVEGFERLICNQKKENETRVVLCELGNPMKKNAQIGIAMLVSVGNLEEAGESVSFQLQIRSKNSQNPNSKIVLLDVPVRAEAQVELRGNSFPASLVVAAEEGEREQNSLDSWGPKVEHTYELHNNGPGTVNGLHLSIHLPGQSQPSDLLYILDIQPQGGLQCFPQPPVNPLKVDWGLPIPSPSPIHPAHHKRDRRQIFLPEPEQPSRLQDPVLVSCDSAPCTVVQCDLQEMARGQRAMVTVLAFLWLPSLYQRPLDQFVLQSHAWFNVSSLPYAVPPLSLPRGEAQVWTQLLRALEERAIPIWWVLVGVLGGLLLLTILVLAMWKVGFFKRNRPPLEEDDEEGE

>NP_002077.1

MEAIAKYDFKATADDELSFKRGDILKVLNEECDQNWYKAELNGKDGFIPKNYIEMKPHPWFFGKIPRAKAEEMLSKQRHDGAFLIRESESAPGDFSLSVKFGNDVQHFKVLRDGAGKYFLWVVKFNSLNELVDYHRSTSVSRNQQIFLRDIEQVPQQPTYVQALFDFDPQEDGELGFRRGDFIHVMDNSDPNWWKGACHGQTGMFPRNYVTPVNRNV

1237 NP_663788.1 NP_057466.1

>NP_663788.1

MNSAEQTVTWLITLGVLESPKKTISDPEGFLQASLKDGVVLCRLLERLLPGTIEKVYPEPRSESECLSNIREFLRGCGASLRLETFDANDLYQGQNFNKVLSSLVTLNKVTADIGLGSDSVCARPSSHRIKSFDSLGSQSLHTRTSKLFQGQYRSLDMTDNSNNQLVVRAKFNFQQTNEDELSFSKGDVIHVTRVEEGGWWEGTLNGRTGWFPSNYVREVKASEKPVSPKSGTLKSPPKGFDTTAINKSYYNVVLQNILETENEYSKELQTVLSTYLRPLQTSEKLSSANISYLMGNLEEICSFQQMLVQSLEECTKLPEAQQRVGGCFLNLMPQMKTLYLTYCANHPSAVNVLTEHSEELGEFMETKGASSPGILVLTTGLSKPFMRLDKYPTLLKELERHMEDYHTDRQDIQKSMAAFKNLSAQCQEVRKRKELELQILTEAIRNWEGDDIKTLGNVTYMSQVLIQCAGSEEKNERYLLLFPNVLLMLSASPRMSGFIYQGKLPTTGMTITKLEDSENHRNAFEISGSMIERILVSCNNQQDLQEWVEHLQKQTKVTSVGNPTIKPHSVPSHTLPSHPVTPSSKHADSKPAPLTPAYHTLPHPSHHGTPHTTINWGPLEPPKTPKPWSLSCLRPAPPLRPSAALCYKEDLSKSPKTMKKLLPKRKPERKPSDEEFASRKSTAALEEDAQILKVIEAYCTSAKTRQTLNSTWQGTDLMHNHVLADDDQPSLDSLGRRSSLSRLEPSDLSEDSDYDSIWTAHSYRMGSTSRKSCCSYISHQN

>NP_057466.1

MLGQVVTLILLLLLKVYQGKGCQGSADHVVSISGVPLQLQPNSIQTKVDSIAWKKLLPSQNGFHHILKWENGSLPSNTSNDRFSFIVKNLSLLIKAAQQQDSGLYCLEVTSISGKVQTATFQVFVFDKVEKPRLQGQGKILDRGRCQVALSCLVSRDGNVSYAWYRGSKLIQTAGNLTYLDEEVDINGTHTYTCNVSNPVSWESHTLNLTQDCQNAHQEFRFWPFLVIIVILSALFLGTLACFCVWRRKRKEKQSETSPKEFLTIYEDVKDLKTRRNHEQEQTFPGGGSTIYSMIQSQSSAPTSQEPAYTLYSLIQPSRKSGSRKRNHSPSFNSTIYEVIGKSQPKAQNPARLSRKELENFDVYS

1238 NP_892113.3 NP_004295.2

>NP_892113.3

MNLLPPKPKYNPLRNESLSSMEEGASGSTPPEELPSPPASSLGPILPPLPGDDSPTTLCSFFPRMSNLRLANPAGGRPGSKGEPGRAADDGEGIVGAAMPDSGPLPLLQDMNKLSGGGGRRTRVEGGQLGGEEWTRHGSFVNKPTRGWLHPNDKVMGPGVSYLVRYMGCVEVLQSMRALDFNTRTQVTREAISLVCEAVPGAKGATRRRKPCSRPLSSILGRSNLKFAGMPITLTVSTSSLNLMAADCKQIIANHHMQSISFASGGDPDTAEYVAYVAKDPVNQRACHILECPEGLAQDVISTIGQAFELRFKQYLRNPPKLVTPHDRMAGFDGSAWDEEEEEPPDHQYYNDFPGKEPPLGGVVDMRLREGAAPGAARPTAPNAQTPSHLGATLPVGQPVGGDPEVRKQMPPPPPCPGRELFDDPSYVNVQNLDKARQAVGGAGPPNPAINGSAPRDLFDMKPFEDALRVPPPPQSVSMAEQLRGEPWFHGKLSRREAEALLQLNGDFLVRESTTTPGQYVLTGLQSGQPKHLLLVDPEGVVRTKDHRFESVSHLISYHMDNHLPIISAGSELCLQQPVERKL

>NP_004295.2

MGAIGLLWLLPLLLSTAAVGSGMGTGQRAGSPAAGPPLQPREPLSYSRLQRKSLAVDFVVPSLFRVYARDLLLPPSSSELKAGRPEARGSLALDCAPLLRLLGPAPGVSWTAGSPAPAEARTLSRVLKGGSVRKLRRAKQLVLELGEEAILEGCVGPPGEAAVGLLQFNLSELFSWWIRQGEGRLRIRLMPEKKASEVGREGRLSAAIRASQPRLLFQIFGTGHSSLESPTNMPSPSPDYFTWNLTWIMKDSFPFLSHRSRYGLECSFDFPCELEYSPPLHDLRNQSWSWRRIPSEEASQMDLLDGPGAERSKEMPRGSFLLLNTSADSKHTILSPWMRSSSEHCTLAVSVHRHLQPSGRYIAQLLPHNEAAREILLMPTPGKHGWTVLQGRIGRPDNPFRVALEYISSGNRSLSAVDFFALKNCSEGTSPGSKMALQSSFTCWNGTVLQLGQACDFHQDCAQGEDESQMCRKLPVGFYCNFEDGFCGWTQGTLSPHTPQWQVRTLKDARFQDHQDHALLLSTTDVPASESATVTSATFPAPIKSSPCELRMSWLIRGVLRGNVSLVLVENKTGKEQGRMVWHVAAYEGLSLWQWMVLPLLDVSDRFWLQMVAWWGQGSRAIVAFDNISISLDCYLTISGEDKILQNTAPKSRNLFERNPNKELKPGENSPRQTPIFDPTVHWLFTTCGASGPHGPTQAQCNNAYQNSNLSVEVGSEGPLKGIQIWKVPATDTYSISGYGAAGGKGGKNTMMRSHGVSVLGIFNLEKDDMLYILVGQQGEDACPSTNQLIQKVCIGENNVIEEEIRVNRSVHEWAGGGGGGGGATYVFKMKDGVPVPLIIAAGGGGRAYGAKTDTFHPERLENNSSVLGLNGNSGAAGGGGGWNDNTSLLWAGKSLQEGATGGHSCPQAMKKWGWETRGGFGGGGGGCSSGGGGGGYIGGNAASNNDPEMDGEDGVSFISPLGILYTPALKVMEGHGEVNIKHYLNCSHCEVDECHMDPESHKVICFCDHGTVLAEDGVSCIVSPTPEPHLPLSLILSVVTSALVAALVLAFSGIMIVYRRKHQELQAMQMELQSPEYKLSKLRTSTIMTDYNPNYCFAGKTSSISDLKEVPRKNITLIRGLGHGAFGEVYEGQVSGMPNDPSPLQVAVKTLPEVCSEQDELDFLMEALIISKFNHQNIVRCIGVSLQSLPRFILLELMAGGDLKSFLRETRPRPSQPSSLAMLDLLHVARDIACGCQYLEENHFIHRDIAARNCLLTCPGPGRVAKIGDFGMARDIYRASYYRKGGCAMLPVKWMPPEAFMEGIFTSKTDTWSFGVLLWEIFSLGYMPYPSKSNQEVLEFVTSGGRMDPPKNCPGPVYRIMTQCWQHQPEDRPNFAIILERIEYCTQDPDVINTALPIEYGPLVEEEEKVPVRPKDPEGVPPLLVSQQAKREEERSPAAPPPLPTTSSGKAAKKPTAAEISVRVPRGPAVEGGHVNMAFSQSNPPSELHKVHGSRNKPTSLWNPTYGSWFTEKPTKKNNPIAKKEPHDRGNLGLEGSCTVPPNVATGRLPGASLLLEPSSLTANMKEVPLFRLRHFPCGNVNYGYQQQGLPLEAATAPGAGHYEDTILKSKNSMNQPGP

1239 NP_003480.2 NP_001015053.1

>NP_003480.2

MTHGEELGSDVHQDSIVLTYLEGLLMHQAAGGSGTAVDKKSAGHNEEDQNFNISGSAFPTCQSNGPVLNTHTYQGSGMLHLKKARLLQSSEDWNAAKRKRLSDSIMNLNVKKEALLAGMVDSVPKGKQDSTLLASLLQSFSSRLQTVALSQQIRQSLKEQGYALSHDSLKVEKDLRCYGVASSHLKTLLKKSKVKDQKPDTNLPDVTKNLIRDRFAESPHHVGQSGTKVMSEPLSCAARLQAVASMVEKRASPATSPKPSVACSQLALLLSSEAHLQQYSREHALKTQNANQAASERLAAMARLQENGQKDVGSYQLPKGMSSHLNGQARTSSSKLMASKSSATVFQNPMGIIPSSPKNAGYKNSLERNNIKQAANNSLLLHLLKSQTIPKPMNGHSHSERGSIFEESSTPTTIDEYSDNNPSFTDDSSGDESSYSNCVPIDLSCKHRTEKSESDQPVSLDNFTQSLLNTWDPKVPDVDIKEDQDTSKNSKLNSHQKVTLLQLLLGHKNEENVEKNTSPQGVHNDVSKFNTQNYARTSVIESPSTNRTTPVSTPPLLTSSKAGSPINLSQHSLVIKWNSPPYVCSTQSEKLTNTASNHSMDLTKSKDPPGEKPAQNEGAQNSATFSASKLLQNLAQCGMQSSMSVEEQRPSKQLLTGNTDKPIGMIDRLNSPLLSNKTNAVEENKAFSSQPTGPEPGLSGSEIENLLERRTVLQLLLGNPNKGKSEKKEKTPLRDESTQEHSERALSEQILMVKIKSEPCDDLQIPNTNVHLSHDAKSAPFLGMAPAVQRSAPALPVSEDFKSEPVSPQDFSFSKNGLLSRLLRQNQDSYLADDSDRSHRNNEMALLESKNLCMVPKKRKLYTEPLENPFKKMKNNIVDAANNHSAPEVLYGSLLNQEELKFSRNDLEFKYPAGHGSASESEHRSWARESKSFNVLKQLLLSENCVRDLSPHRSNSVADSKKKGHKNNVTNSKPEFSISSLNGLMYSSTQPSSCMDNRTFSYPGVVKTPVSPTFPEHLGCAGSRPESGLLNGCSMPSEKGPIKWVITDAEKNEYEKDSPRLTKTNPILYYMLQKGGNSVTSRETQDKDIWREASSAESVSQVTAKEELLPTAETKASFFNLRSPYNSHMGNNASRPHSANGEVYGLLGSVLTIKKESE

>NP_001015053.1

MNSPNESADGMSGREPSLEILPRTSLHSIPVTVEVKPVLPRAMPSSMGGGGGGSPSPVELRGALVGSVDPTLREQQLQQELLALKQQQQLQKQLLFAEFQKQHDHLTRQHEVQLQKHLKQQQEMLAAKQQQEMLAAKRQQELEQQRQREQQRQEELEKQRLEQQLLILRNKEKSKESAIASTEVKLRLQEFLLSKSKEPTPGGLNHSLPQHPKCWGAHHASLDQSSPPQSGPPGTPPSYKLPLPGPYDSRDDFPLRKTASEPNLKVRSRLKQKVAERRSSPLLRRKDGTVISTFKKRAVEITGAGPGASSVCNSAPGSGPSSPNSSHSTIAENGFTGSVPNIPTEMLPQHRALPLDSSPNQFSLYTSPSLPNISLGLQATVTVTNSHLTASPKLSTQQEAERQALQSLRQGGTLTGKFMSTSSIPGCLLGVALEGDGSPHGHASLLQHVLLLEQARQQSTLIAVPLHGQSPLVTGERVATSMRTVGKLPRHRPLSRTQSSPLPQSPQALQQLVMQQQHQQFLEKQKQQQLQLGKILTKTGELPRQPTTHPEETEEELTEQQEVLLGEGALTMPREGSTESESTQEDLEEEDEEDDGEEEEDCIQVKDEEGESGAEEGPDLEEPGAGYKKLFSDAQPLQPLQVYQAPLSLATVPHQALGRTQSSPAAPGGMKSPPDQPVKHLFTTGVVYDTFMLKHQCMCGNTHVHPEHAGRIQSIWSRLQETGLLSKCERIRGRKATLDEIQTVHSEYHTLLYGTSPLNRQKLDSKKLLGPISQKMYAVLPCGGIGVDSDTVWNEMHSSSAVRMAVGCLLELAFKVAAGELKNGFAIIRPPGHHAEESTAMGFCFFNSVAITAKLLQQKLNVGKVLIVDWDIHHGNGTQQAFYNDPSVLYISLHRYDNGNFFPGSGAPEEVGGGPGVGYNVNVAWTGGVDPPIGDVEYLTAFRTVVMPIAHEFSPDVVLVSAGFDAVEGHLSPLGGYSVTARCFGHLTRQLMTLAGGRVVLALEGGHDLTAICDASEACVSALLSVELQPLDEAVLQQKPNINAVATLEKVIEIQSKHWSCVQKFAAGLGRSLREAQAGETEEAETVSAMALLSVGAEQAQAAAAREHSPRPAEEPMEQEPAL

1240 NP_004243.1 NP_001895.1

>NP_004243.1

MSADAAAGAPLPRLCCLEKGPNGYGFHLHGEKGKLGQYIRLVEPGSPAEKAGLLAGDRLVEVNGENVEKETHQQVVSRIRAALNAVRLLVVDPETDEQLQKLGVQVREELLRAQEAPGQAEPPAAAEVQGAGNENEPREADKSHPEQRELRPRLCTMKKGPSGYGFNLHSDKSKPGQFIRSVDPDSPAEASGLRAQDRIVEVNGVCMEGKQHGDVVSAIRAGGDETKLLVVDRETDEFFKKCRVIPSQEHLNGPLPVPFTNGEIQKENSREALAEAALESPRPALVRSASSDTSEELNSQDSPPKQDSTAPSSTSSSDPILDFNISLAMAKERAHQKRSSKRAPQMDWSKKNELFSNL

>NP_001895.1

MATQADLMELDMAMEPDRKAAVSHWQQQSYLDSGIHSGATTTAPSLSGKGNPEEEDVDTSQVLYEWEQGFSQSFTQEQVADIDGQYAMTRAQRVRAAMFPETLDEGMQIPSTQFDAAHPTNVQRLAEPSQMLKHAVVNLINYQDDAELATRAIPELTKLLNDEDQVVVNKAAVMVHQLSKKEASRHAIMRSPQMVSAIVRTMQNTNDVETARCTAGTLHNLSHHREGLLAIFKSGGIPALVKMLGSPVDSVLFYAITTLHNLLLHQEGAKMAVRLAGGLQKMVALLNKTNVKFLAITTDCLQILAYGNQESKLIILASGGPQALVNIMRTYTYEKLLWTTSRVLKVLSVCSSNKPAIVEAGGMQALGLHLTDPSQRLVQNCLWTLRNLSDAATKQEGMEGLLGTLVQLLGSDDINVVTCAAGILSNLTCNNYKNKMMVCQVGGIEALVRTVLRAGDREDITEPAICALRHLTSRHQEAEMAQNAVRLHYGLPVVVKLLHPPSHWPLIKATVGLIRNLALCPANHAPLREQGAIPRLVQLLVRAHQDTQRRTSMGGTQQQFVEGVRMEEIVEGCTGALHILARDVHNRIVIRGLNTIPLFVQLLYSPIENIQRVAAGVLCELAQDKEAAEAIEAEGATAPLTELLHSRNEGVATYAAAVLFRMSEDKPQDYKKRLSVELTSSLFRTEPMAWNETADLGLDIGAQGEPLGYRQDDPSYRSFHSGGYGQDALGMDPMMEHEMGGHHPGADYPVDGLPDLGHAQDLMDGLPPGDSNQLAWFDTDL

1241 NP_001420.2 NP_005229.1

>NP_001420.2

MAENVVEPGPPSAKRPKLSSPALSASASDGTDFGSLFDLEHDLPDELINSTELGLTNGGDINQLQTSLGMVQDAASKHKQLSELLRSGSSPNLNMGVGGPGQVMASQAQQSSPGLGLINSMVKSPMTQAGLTSPNMGMGTSGPNQGPTQSTGMMNSPVNQPAMGMNTGMNAGMNPGMLAAGNGQGIMPNQVMNGSIGAGRGRQNMQYPNPGMGSAGNLLTEPLQQGSPQMGGQTGLRGPQPLKMGMMNNPNPYGSPYTQNPGQQIGASGLGLQIQTKTVLSNNLSPFAMDKKAVPGGGMPNMGQQPAPQVQQPGLVTPVAQGMGSGAHTADPEKRKLIQQQLVLLLHAHKCQRREQANGEVRQCNLPHCRTMKNVLNHMTHCQSGKSCQVAHCASSRQIISHWKNCTRHDCPVCLPLKNAGDKRNQQPILTGAPVGLGNPSSLGVGQQSAPNLSTVSQIDPSSIERAYAALGLPYQVNQMPTQPQVQAKNQQNQQPGQSPQGMRPMSNMSASPMGVNGGVGVQTPSLLSDSMLHSAINSQNPMMSENASVPSLGPMPTAAQPSTTGIRKQWHEDITQDLRNHLVHKLVQAIFPTPDPAALKDRRMENLVAYARKVEGDMYESANNRAEYYHLLAEKIYKIQKELEEKRRTRLQKQNMLPNAAGMVPVSMNPGPNMGQPQPGMTSNGPLPDPSMIRGSVPNQMMPRITPQSGLNQFGQMSMAQPPIVPRQTPPLQHHGQLAQPGALNPPMGYGPRMQQPSNQGQFLPQTQFPSQGMNVTNIPLAPSSGQAPVSQAQMSSSSCPVNSPIMPPGSQGSHIHCPQLPQPALHQNSPSPVPSRTPTPHHTPPSIGAQQPPATTIPAPVPTPPAMPPGPQSQALHPPPRQTPTPPTTQLPQQVQPSLPAAPSADQPQQQPRSQQSTAASVPTPTAPLLPPQPATPLSQPAVSIEGQVSNPPSTSSTEVNSQAIAEKQPSQEVKMEAKMEVDQPEPADTQPEDISESKVEDCKMESTETEERSTELKTEIKEEEDQPSTSATQSSPAPGQSKKKIFKPEELRQALMPTLEALYRQDPESLPFRQPVDPQLLGIPDYFDIVKSPMDLSTIKRKLDTGQYQEPWQYVDDIWLMFNNAWLYNRKTSRVYKYCSKLSEVFEQEIDPVMQSLGYCCGRKLEFSPQTLCCYGKQLCTIPRDATYYSYQNRYHFCEKCFNEIQGESVSLGDDPSQPQTTINKEQFSKRKNDTLDPELFVECTECGRKMHQICVLHHEIIWPAGFVCDGCLKKSARTRKENKFSAKRLPSTRLGTFLENRVNDFLRRQNHPESGEVTVRVVHASDKTVEVKPGMKARFVDSGEMAESFPYRTKALFAFEEIDGVDLCFFGMHVQEYGSDCPPPNQRRVYISYLDSVHFFRPKCLRTAVYHEILIGYLEYVKKLGYTTGHIWACPPSEGDDYIFHCHPPDQKIPKPKRLQEWYKKMLDKAVSERIVHDYKDIFKQATEDRLTSAKELPYFEGDFWPNVLEESIKELEQEEEERKREENTSNESTDVTKGDSKNAKKKNNKKTSKNKSSLSRGNKKKPGMPNVSNDLSQKLYATMEKHKEVFFVIRLIAGPAANSLPPIVDPDPLIPCDLMDGRDAFLTLARDKHLEFSSLRRAQWSTMCMLVELHTQSQDRFVYTCNECKHHVETRWHCTVCEDYDLCITCYNTKNHDHKMEKLGLGLDDESNNQQAAATQSPGDSRRLSIQRCIQSLVHACQCRNANCSLPSCQKMKRVVQHTKGCKRKTNGGCPICKQLIALCCYHAKHCQENKCPVPFCLNIKQKLRQQQLQHRLQQAQMLRRRMASMQRTGVVGQQQGLPSPTPATPTTPTGQQPTTPQTPQPTSQPQPTPPNSMPPYLPRTQAAGPVSQGKAAGQVTPPTPPQTAQPPLPGPPPAAVEMAMQIQRAAETQRQMAHVQIFQRPIQHQMPPMTPMAPMGMNPPPMTRGPSGHLEPGMGPTGMQQQPPWSQGGLPQPQQLQSGMPRPAMMSVAQHGQPLNMAPQPGLGQVGISPLKPGTVSQQALQNLLRTLRSPSSPLQQQQVLSILHANPQLLAAFIKQRAAKYANSNPQPIPGQPGMPQGQPGLQPPTMPGQQGVHSNPAMQNMNPMQAGVQRAGLPQQQPQQQLQPPMGGMSPQAQQMNMNHNTMPSQFRDILRRQQMMQQQQQQGAGPGIGPGMANHNQFQQPQGVGYPPQQQQRMQHHMQQMQQGNMGQIGQLPQALGAEAGASLQAYQQRLLQQQMGSPVQPNPMSPQQHMLPNQAQSPHLQGQQIPNSLSNQVRSPQPVPSPRPQSQPPHSSPSPRMQPQPSPHHVSPQTSSPHPGLVAAQANPMEQGHFASPDQNSMLSQLASNPGMANLHGASATDLGLSTDNSDLNSNLSQSTLDIH

>NP_005229.1

MKAAVDLKPTLTIIKTEKVDLELFPSPDMECADVPLLTPSSKEMMSQALKATFSGFTKEQQRLGIPKDPRQWTETHVRDWVMWAVNEFSLKGVDFQKFCMNGAALCALGKDCFLELAPDFVGDILWEHLEILQKEDVKPYQVNGVNPAYPESRYTSDYFISYGIEHAQCVPPSEFSEPSFITESYQTLHPISSEELLSLKYENDYPSVILRDPLQTDTLQNDYFAIKQEVVTPDNMCMGRTSRGKLGGQDSFESIESYDSCDRLTQSWSSQSSFNSLQRVPSYDSFDSEDYPAALPNHKPKGTFKDYVRDRADLNKDKPVIPAAALAGYTGSGPIQLWQFLLELLTDKSCQSFISWTGDGWEFKLSDPDEVARRWGKRKNKPKMNYEKLSRGLRYYYDKNIIHKTAGKRYVYRFVCDLQSLLGYTPEELHAMLDVKPDADE

1242 NP_055548.2 NP_003557.2

>NP_055548.2

MDSYFKAAVSDLDKLLDDFEQNPDEQDYLQDVQNAYDSNHCSVSSELASSQRTSLLPKDQECVNSCASSETSYGTNESSLNEKTLKGLTSIQNEKNVTGLDLLSSVDGGTSDEIQPLYMGRCSKPICDLISDMGNLVHATNSEEDIKKLLPDDFKSNADSLIGLDLSSVSDTPCVSSTDHDSDTVREQQNDTSSELQNREIGGIKELGIKVDTTLSDSYNYSGTENLKDKKIFNQLESIVDFNMSSALTRQSSKMFHAKDKLQHKSQPCGLLKDVGLVKEEVDVAVITAAECLKEEGKTSALTCSLPKNEDLCLNDSNSRDENFKLPDFSFQEDKTVIKQSAQEDSKSLDLKDNDVIQDSSSALHVSSKDVPSSLSCLPASGSMSGSLIESKARGDFLPQHEHKDNIQDAVTIHEEIQNSVVLGGEPFKENDLLKQEKCKSILLQSLIEGMEDRKIDPDQTVIRAESLDGGDTSSTVVESQEGLSGTHVPESSDCCEGFINTFSSNDMDGQDLDYFNIDEGAKSGPLISDAELDAFLTEQYLQTTNIKSFEENVNDSKSQMNQIDMKGLDDGNINNIYFNAEAGAIGESHGINIICETVDKQNTIENGLSLGEKSTIPVQQGLPTSKSEITNQLSVSDINSQSVGGARPKQLFSLPSRTRSSKDLNKPDVPDTIESEPSTADTVVPITCAIDSTADPQVSFNSNYIDIESNSEGGSSFVTANEDSVPENTCKEGLVLGQKQPTWVPDSEAPNCMNCQVKFTFTKRRHHCRACGKVFCGVCCNRKCKLQYLEKEARVCVVCYETISKAQAFERMMSPTGSNLKSNHSDECTTVQPPQENQTSSIPSPATLPVSALKQPGVEGLCSKEQKRVWFADGILPNGEVADTTKLSSGSKRCSEDFSPLSPDVPMTVNTVDHSHSTTVEKPNNETGDITRNEIIQSPISQVPSVEKLSMNTGNEGLPTSGSFTLDDDVFAETEEPSSPTGVLVNSNLPIASISDYRLLCDINKYVCNKISLLPNDEDSLPPLLVASGEKGSVPVVEEHPSHEQIILLLEGEGFHPVTFVLNANLLVNVKFIFYSSDKYWYFSTNGLHGLGQAEIIILLLCLPNEDTIPKDIFRLFITIYKDALKGKYIENLDNITFTESFLSSKDHGGFLFITPTFQKLDDLSLPSNPFLCGILIQKLEIPWAKVFPMRLMLRLGAEYKAYPAPLTSIRGRKPLFGEIGHTIMNLLVDLRNYQYTLHNIDQLLIHMEMGKSCIKIPRKKYSDVMKVLNSSNEHVISIGASFSTEADSHLVCIQNDGIYETQANSTTGHPRKVTGASFVVFNGALKTSSGFLAKSSIVEDGLMVQITPETMNGLRLALREQKDFKITCGKVDAVDLREYVDICWVDAEEKGNKGVISSVDGISLQGFPSEKIKLEADFETDEKIVKCTEVFYFLKDQDLSILSTSYQFAKEIAMACSAALCPHLKTLKSNGMNKIGLRVSIDTDMVEFQAGSEGQLLPQHYLNDLDSALIPVIHGGTSNSSLPLEIELVFFIIEHLF

>NP_003557.2

MLRRILQRTPGRVGSQGSDLDSSATPINTVDVNNESSSEGFICPQCMKSLGSADELFKHYEAVHDAGNDSGHGGESNLALKRDDVTLLRQEVQDLQASLKEEKWYSEELKKELEKYQGLQQQEAKPDGLVTDSSAELQSLEQQLEEAQTENFNIKQMKDLFEQKAAQLATEIADIKSKYDEERSLREAAEQKVTRLTEELNKEATVIQDLKTELLQRPGIEDVAVLKKELVQVQTLMDNMTLERERESEKLKDECKKLQSQYASSEATISQLRSELAKGPQEVAVYVQELQKLKSSVNELTQKNQTLTENLLKKEQDYTKLEEKHNEESVSKKNIQATLHQKDLDCQQLQSRLSASETSLHRIHVELSEKGEATQKLKEELSEVETKYQHLKAEFKQLQQQREEKEQHGLQLQSEINQLHSKLLETERQLGEAHGRLKEQRQLSSEKLMDKEQQVADLQLKLSRLEEQLKEKVTNSTELQHQLDKTKQQHQEQQALQQSTTAKLREAQNDLEQVLRQIGDKDQKIQNLEALLQKSKENISLLEKEREDLYAKIQAGEGETAVLNQLQEKNHTLQEQVTQLTEKLKNQSESHKQAQENLHDQVQEQKAHLRAAQDRVLSLETSVNELNSQLNESKEKVSQLDIQIKAKTELLLSAEAAKTAQRADLQNHLDTAQNALQDKQQELNKITTQLDQVTAKLQDKQEHCSQLESHLKEYKEKYLSLEQKTEELEGQIKKLEADSLEVKASKEQALQDLQQQRQLNTDLELRATELSKQLEMEKEIVSSTRLDLQKKSEALESIKQKLTKQEEEKQILKQDFETLSQETKIQHEELNNRIQTTVTELQKVKMEKEALMTELSTVKDKLSKVSDSLKNSKSEFEKENQKGKAAILDLEKTCKELKHQLQVQMENTLKEQKELKKSLEKEKEASHQLKLELNSMQEQLIQAQNTLKQNEKEEQQLQGNINELKQSSEQKKKQIEALQGELKIAVLQKTELENKLQQQLTQAAQELAAEKEKISVLQNNYEKSQETFKQLQSDFYGRESELLATRQDLKSVEEKLSLAQEDLISNRNQIGNQNKLIQELKTAKATLEQDSAKKEQQLQERCKALQDIQKEKSLKEKELVNEKSKLAEIEEIKCRQEKEITKLNEELKSHKLESIKEITNLKDAKQLLIQQKLELQGKADSLKAAVEQEKRNQQILKDQVKKEEEELKKEFIEKEAKLHSEIKEKEVGMKKHEENEAKLTMQITALNENLGTVKKEWQSSQRRVSELEKQTDDLRGEIAVLEATVQNNQDERRALLERCLKGEGEIEKLQTKVLELQRKLDNTTAAVQELGRENQSLQIKHTQALNRKWAEDNEVQNCMACGKGFSVTVRRHHCRQCGNIFCAECSAKNALTPSSKKPVRVCDACFNDLQG

1243 NP_001518.2 NP_004955.2

>NP_001518.2

MRSPPCGLLRWFGGPLLASWCRCHLRFRAFGTSAGWYRAFPAPPPLLPPACPSPRDYRPHVSLSPFLSRPSRGGSSSSSSSRRRSPVAAVAGEPMAYSQGGGKKKVCYYYDGDIGNYYYGQGHPMKPHRIRMTHNLLLNYGLYRKMEIYRPHKATAEEMTKYHSDEYIKFLRSIRPDNMSEYSKQMQRFNVGEDCPVFDGLFEFCQLSTGGSVAGAVKLNRQQTDMAVNWAGGLHHAKKSEASGFCYVNDIVLAILELLKYHQRVLYIDIDIHHGDGVEEAFYTTDRVMTVSFHKYGEYFPGTGDLRDIGAGKGKYYAVNFPMRDGIDDESYGQIFKPIISKVMEMYQPSAVVLQCGADSLSGDRLGCFNLTVKGHAKCVEVVKTFNLPLLMLGGGGYTIRNVARCWTYETAVALDCEIPNELPYNDYFEYFGPDFKLHISPSNMTNQNTPEYMEKIKQRLFENLRMLPHAPGVQMQAIPEDAVHEDSGDEDGEDPDKRISIRASDKRIACDEEFSDSEDEGEGGRRNVADHKKGAKKARIEEDKKETEDKKTDVKEEDKSKDNSGEKTDTKGTKSEQLSNP

>NP_004955.2

MAQTQGTRRKVCYYYDGDVGNYYYGQGHPMKPHRIRMTHNLLLNYGLYRKMEIYRPHKANAEEMTKYHSDDYIKFLRSIRPDNMSEYSKQMQRFNVGEDCPVFDGLFEFCQLSTGGSVASAVKLNKQQTDIAVNWAGGLHHAKKSEASGFCYVNDIVLAILELLKYHQRVLYIDIDIHHGDGVEEAFYTTDRVMTVSFHKYGEYFPGTGDLRDIGAGKGKYYAVNYPLRDGIDDESYEAIFKPVMSKVMEMFQPSAVVLQCGSDSLSGDRLGCFNLTIKGHAKCVEFVKSFNLPMLMLGGGGYTIRNVARCWTYETAVALDTEIPNELPYNDYFEYFGPDFKLHISPSNMTNQNTNEYLEKIKQRLFENLRMLPHAPGVQMQAIPEDAIPEESGDEDEDDPDKRISICSSDKRIACEEEFSDSEEEGEGGRKNSSNFKKAKRVKTEDEKEKDPEEKKEVTEEEKTKEEKPEAKGVKEEVKLA

1244 NP_002568.2 NP_001782.1

>NP_002568.2

MSDNGELEDKPPAPPVRMSSTIFSTGGKDPLSANHSLKPLPSVPEEKKPRHKIISIFSGTEKGSKKKEKERPEISPPSDFEHTIHVGFDAVTGEFTGMPEQWARLLQTSNITKLEQKKNPQAVLDVLKFYDSNTVKQKYLSFTPPEKDGFPSGTPALNAKGTEAPAVVTEEEDDDEETAPPVIAPRPDHTKSIYTRSVIDPVPAPVGDSHVDGAAKSLDKQKKKTKMTDEEIMEKLRTIVSIGDPKKKYTRYEKIGQGASGTVFTATDVALGQEVAIKQINLQKQPKKELIINEILVMKELKNPNIVNFLDSYLVGDELFVVMEYLAGGSLTDVVTETCMDEAQIAAVCRECLQALEFLHANQVIHRDIKSDNVLLGMEGSVKLTDFGFCAQITPEQSKRSTMVGTPYWMAPEVVTRKAYGPKVDIWSLGIMAIEMVEGEPPYLNENPLRALYLIATNGTPELQNPEKLSPIFRDFLNRCLEMDVEKRGSAKELLQHPFLKLAKPLSSLTPLIMAAKEAMKSNR

>NP_001782.1

MQTIKCVVVGDGAVGKTCLLISYTTNKFPSEYVPTVFDNYAVTVMIGGEPYTLGLFDTAGQEDYDRLRPLSYPQTDVFLVCFSVVSPSSFENVKEKWVPEITHHCPKTPFLLVGTQIDLRDDPSTIEKLAKNKQKPITPETAEKLARDLKAVKYVECSALTQKGLKNVFDEAILAALEPPEPKKSRRCVLL

1245 NP_000252.1 NP_006358.1

>NP_000252.1

MRFFCARCCSFGPEMPAVQLLLLACLVWDVGARTAQLRKANDQSGRCQYTFSVASPNESSCPEQSQAMSVIHNLQRDSSTQRLDLEATKARLSSLESLLHQLTLDQAARPQETQEGLQRELGTLRRERDQLETQTRELETAYSNLLRDKSVLEEEKKRLRQENENLARRLESSSQEVARLRRGQCPQTRDTARAVPPGSREVSTWNLDTLAFQELKSELTEVPASRILKESPSGYLRSGEGDTGCGELVWVGEPLTLRTAETITGKYGVWMRDPKPTYPYTQETTWRIDTVGTDVRQVFEYDLISQFMQGYPSKVHILPRPLESTGAVVYSGSLYFQGAESRTVIRYELNTETVKAEKEIPGAGYHGQFPYSWGGYTDIDLAVDEAGLWVIYSTDEAKGAIVLSKLNPENLELEQTWETNIRKQSVANAFIICGTLYTVSSYTSADATVNFAYDTGTGISKTLTIPFKNRYKYSSMIDYNPLEKKLFAWDNLNMVTYDIKLSKM

>NP_006358.1

MADMQNLVERLERAVGRLEAVSHTSDMHRGYADSPSKAGAAPYVQAFDSLLAGPVAEYLKISKEIGGDVQKHAEMVHTGLKLERALLVTASQCQQPAENKLSDLLAPISEQIKEVITFREKNRGSKLFNHLSAVSESIQALGWVAMAPKPGPYVKEMNDAAMFYTNRVLKEYKDVDKKHVDWVKAYLSIWTELQAYIKEFHTTGLAWSKTGPVAKELSGLPSGPSAGSGPPPPPPGPPPPPVSTSSGSDESASRSALFAQINQGESITHALKHVSDDMKTHKNPALKAQSGPVRSGPKPFSAPKPQTSPSPKRATKKEPAVLELEGKKWRVENQENVSNLVIEDTELKQVAYIYKCVNTTLQIKGKINSITVDNCKKLGLVFDDVVGIVEIINSKDVKVQVMGKVPTISINKTDGCHAYLSKNSLDCEIVSAKSSEMNVLIPTEGGDFNEFPVPEQFKTLWNGQKLVTTVTEIAG

1246 NP_127492.1 NP_073207.1

>NP_127492.1

MAQVAMSTLPVEDEESSESRMVVTFLMSALESMCKELAKSKAEVACIAVYETDVFVVGTERGRAFVNTRKDFQKDFVKYCVEEEEKAAEMHKMKSTTQANRMSVDAVEIETLRKTVEDYFCFCYGKALGKSTVVPVPYEKMLRDQSAVVVQGLPEGVAFKHPENYDLATLKWILENKAGISFIIKRPFLEPKKHVGGRVMVTDADRSILSPGGSCGPIKVKTEPTEDSGISLEMAAVTVKEESEDPDYYQYNIQAGPSETDDVDEKQPLSKPLQGSHHSSEGNEGTEMEVPAEDSTQHVPSETSEDPEVEVTIEDDDYSPPSKRPKANELPQPPVPEPANAGKRKVREFNFEKWNARITDLRKQVEELFERKYAQAIKAKGPVTIPYPLFQSHVEDLYVEGLPEGIPFRRPSTYGIPRLERILLAKERIRFVIKKHELLNSTREDLQLDKPASGVKEEWYARITKLRKMVDQLFCKKFAEALGSTEAKAVPYQKFEAHPNDLYVEGLPENIPFRSPSWYGIPRLEKIIQVGNRIKFVIKRPELLTHSTTEVTQPRTNTPVKEDWNVRITKLRKQVEEIFNLKFAQALGLTEAVKVPYPVFESNPEFLYVEGLPEGIPFRSPTWFGIPRLERIVRGSNKIKFVVKKPELVISYLPPGMASKINTKALQSPKRPRSPGSNSKVPEIEVTVEGPNNNNPQTSAVRTPTQTNGSNVPFKPRGREFSFEAWNAKITDLKQKVENLFNEKCGEALGLKQAVKVPFALFESFPEDFYVEGLPEGVPFRRPSTFGIPRLEKILRNKAKIKFIIKKPEMFETAIKESTSSKSPPRKINSSPNVNTTASGVEDLNIIQVTIPDDDNERLSKVEKARQLREQVNDLFSRKFGEAIGMGFPVKVPYRKITINPGCVVVDGMPPGVSFKAPSYLEISSMRRILDSAEFIKFTVIRPFPGLVINNQLVDQSESEGPVIQESAEPSQLEVPATEEIKETDGSSQIKQEPDPTW

>NP_073207.1

MTSSYGHVLERQPALGGRLDSPGNLDTLQAKKNFSVSHLLDLEEAGDMVAAQADENVGEAGRSLLESPGLTSGSDTPQQDNDQLNSEEKKKRKQRRNRTTFNSSQLQALERVFERTHYPDAFVREDLARRVNLTEARVQVWFQNRRAKFRRNERAMLANKNASLLKSYSGDVTAVEQPIVPRPAPRPTDYLSWGTASPYSAMATYSATCANNSPAQGINMANSIANLRLKAKEYSLQRNQVPTVN

1247 NP_000123.1 NP_000303.1

>NP_000123.1

MQIELSTCFFLCLLRFCFSATRRYYLGAVELSWDYMQSDLGELPVDARFPPRVPKSFPFNTSVVYKKTLFVEFTDHLFNIAKPRPPWMGLLGPTIQAEVYDTVVITLKNMASHPVSLHAVGVSYWKASEGAEYDDQTSQREKEDDKVFPGGSHTYVWQVLKENGPMASDPLCLTYSYLSHVDLVKDLNSGLIGALLVCREGSLAKEKTQTLHKFILLFAVFDEGKSWHSETKNSLMQDRDAASARAWPKMHTVNGYVNRSLPGLIGCHRKSVYWHVIGMGTTPEVHSIFLEGHTFLVRNHRQASLEISPITFLTAQTLLMDLGQFLLFCHISSHQHDGMEAYVKVDSCPEEPQLRMKNNEEAEDYDDDLTDSEMDVVRFDDDNSPSFIQIRSVAKKHPKTWVHYIAAEEEDWDYAPLVLAPDDRSYKSQYLNNGPQRIGRKYKKVRFMAYTDETFKTREAIQHESGILGPLLYGEVGDTLLIIFKNQASRPYNIYPHGITDVRPLYSRRLPKGVKHLKDFPILPGEIFKYKWTVTVEDGPTKSDPRCLTRYYSSFVNMERDLASGLIGPLLICYKESVDQRGNQIMSDKRNVILFSVFDENRSWYLTENIQRFLPNPAGVQLEDPEFQASNIMHSINGYVFDSLQLSVCLHEVAYWYILSIGAQTDFLSVFFSGYTFKHKMVYEDTLTLFPFSGETVFMSMENPGLWILGCHNSDFRNRGMTALLKVSSCDKNTGDYYEDSYEDISAYLLSKNNAIEPRSFSQNSRHPSTRQKQFNATTIPENDIEKTDPWFAHRTPMPKIQNVSSSDLLMLLRQSPTPHGLSLSDLQEAKYETFSDDPSPGAIDSNNSLSEMTHFRPQLHHSGDMVFTPESGLQLRLNEKLGTTAATELKKLDFKVSSTSNNLISTIPSDNLAAGTDNTSSLGPPSMPVHYDSQLDTTLFGKKSSPLTESGGPLSLSEENNDSKLLESGLMNSQESSWGKNVSSTESGRLFKGKRAHGPALLTKDNALFKVSISLLKTNKTSNNSATNRKTHIDGPSLLIENSPSVWQNILESDTEFKKVTPLIHDRMLMDKNATALRLNHMSNKTTSSKNMEMVQQKKEGPIPPDAQNPDMSFFKMLFLPESARWIQRTHGKNSLNSGQGPSPKQLVSLGPEKSVEGQNFLSEKNKVVVGKGEFTKDVGLKEMVFPSSRNLFLTNLDNLHENNTHNQEKKIQEEIEKKETLIQENVVLPQIHTVTGTKNFMKNLFLLSTRQNVEGSYDGAYAPVLQDFRSLNDSTNRTKKHTAHFSKKGEEENLEGLGNQTKQIVEKYACTTRISPNTSQQNFVTQRSKRALKQFRLPLEETELEKRIIVDDTSTQWSKNMKHLTPSTLTQIDYNEKEKGAITQSPLSDCLTRSHSIPQANRSPLPIAKVSSFPSIRPIYLTRVLFQDNSSHLPAASYRKKDSGVQESSHFLQGAKKNNLSLAILTLEMTGDQREVGSLGTSATNSVTYKKVENTVLPKPDLPKTSGKVELLPKVHIYQKDLFPTETSNGSPGHLDLVEGSLLQGTEGAIKWNEANRPGKVPFLRVATESSAKTPSKLLDPLAWDNHYGTQIPKEEWKSQEKSPEKTAFKKKDTILSLNACESNHAIAAINEGQNKPEIEVTWAKQGRTERLCSQNPPVLKRHQREITRTTLQSDQEEIDYDDTISVEMKKEDFDIYDEDENQSPRSFQKKTRHYFIAAVERLWDYGMSSSPHVLRNRAQSGSVPQFKKVVFQEFTDGSFTQPLYRGELNEHLGLLGPYIRAEVEDNIMVTFRNQASRPYSFYSSLISYEEDQRQGAEPRKNFVKPNETKTYFWKVQHHMAPTKDEFDCKAWAYFSDVDLEKDVHSGLIGPLLVCHTNTLNPAHGRQVTVQEFALFFTIFDETKSWYFTENMERNCRAPCNIQMEDPTFKENYRFHAINGYIMDTLPGLVMAQDQRIRWYLLSMGSNENIHSIHFSGHVFTVRKKEEYKMALYNLYPGVFETVEMLPSKAGIWRVECLIGEHLHAGMSTLFLVYSNKCQTPLGMASGHIRDFQITASGQYGQWAPKLARLHYSGSINAWSTKEPFSWIKVDLLAPMIIHGIKTQGARQKFSSLYISQFIIMYSLDGKKWQTYRGNSTGTLMVFFGNVDSSGIKHNIFNPPIIARYIRLHPTHYSIRSTLRMELMGCDLNSCSMPLGMESKAISDAQITASSYFTNMFATWSPSKARLHLQGRSNAWRPQVNNPKEWLQVDFQKTMKVTGVTTQGVKSLLTSMYVKEFLISSSQDGHQWTLFFQNGKVKVFQGNQDSFTPVVNSLDPPLLTRYLRIHPQSWVHQIALRMEVLGCEAQDLY

>NP_000303.1

MWQLTSLLLFVATWGISGTPAPLDSVFSSSERAHQVLRIRKRANSFLEELRHSSLERECIEEICDFEEAKEIFQNVDDTLAFWSKHVDGDQCLVLPLEHPCASLCCGHGTCIDGIGSFSCDCRSGWEGRFCQREVSFLNCSLDNGGCTHYCLEEVGWRRCSCAPGYKLGDDLLQCHPAVKFPCGRPWKRMEKKRSHLKRDTEDQEDQVDPRLIDGKMTRRGDSPWQVVLLDSKKKLACGAVLIHPSWVLTAAHCMDESKKLLVRLGEYDLRRWEKWELDLDIKEVFVHPNYSKSTTDNDIALLHLAQPATLSQTIVPICLPDSGLAERELNQAGQETLVTGWGYHSSREKEAKRNRTFVLNFIKIPVVPHNECSEVMSNMVSENMLCAGILGDRQDACEGDSGGPMVASFHGTWFLVGLVSWGEGCGLLHNYGVYTKVSRYLDWIHGHIRDKEAPQKSWAP

1248 NP_057064.1 NP_060347.2

>NP_057064.1

MVKLNSNPSEKGTKPPSVEDGFQTVPLITPLEVNHLQLPAPEKVIVKTRTEYQPEQKNKGKFRVPKIAEFTVTILVSLALAFLACIVFLVVYKAFTYDHSCPEGFVYKHKRCIPASLDAYYSSQDPNSRSRFYTVISHYSVAKQSTARAIGPWLSAAAVIHEPKPPKTQGH

>NP_060347.2

MAWTKYQLFLAGLMLVTGSINTLSAKWADNFMAEGCGGSKEHSFQHPFLQAVGMFLGEFSCLAAFYLLRCRAAGQSDSSVDPQQPFNPLLFLPPALCDMTGTSLMYVALNMTSASSFQMLRGAVIIFTGLFSVAFLGRRLVLSQWLGILATIAGLVVVGLADLLSKHDSQHKLSEVITGDLLIIMAQIIVAIQMVLEEKFVYKHNVHPLRAVGTEGLFGFVILSLLLVPMYYIPAGSFSGNPRGTLEDALDAFCQVGQQPLIAVALLGNISSIAFFNFAGISVTKELSATTRMVLDSLRTVVIWALSLALGWEAFHALQILGFLILLIGTALYNGLHRPLLGRLSRGRPLAEESEQERLLGGTRTPINDAS

1249 NP_005139.1 NP_002799.3

>NP_005139.1

MKVKKGGGGAGTATESAPGPSGQSVAPIPQPPAESESGSESEPDAGPGPRPGPLQRKQPIGPEDVLGLQRITGDYLCSPEENIYKIDFVRFKIRDMDSGTVLFEIKKPPVSERLPINRRDLDPNAGRFVRYQFTPAFLRLRQVGATVEFTVGDKPVNNFRMIERHYFRNQLLKSFDFHFGFCIPSSKNTCEHIYDFPPLSEELISEMIRHPYETQSDSFYFVDDRLVMHNKADYSYSGTP

>NP_002799.3

MEEGGRDKAPVQPQQSPAAAPGGTDEKPSGKERRDAGDKDKEQELSEEDKQLQDELEMLVERLGEKDTSLYRPALEELRRQIRSSTTSMTSVPKPLKFLRPHYGKLKEIYENMAPGENKRFAADIISVLAMTMSGERECLKYRLVGSQEELASWGHEYVRHLAGEVAKEWQELDDAEKVQREPLLTLVKEIVPYNMAHNAEHEACDLLMEIEQVDMLEKDIDENAYAKVCLYLTSCVNYVPEPENSALLRCALGVFRKFSRFPEALRLALMLNDMELVEDIFTSCKDVVVQKQMAFMLGRHGVFLELSEDVEEYEDLTEIMSNVQLNSNFLALARELDIMEPKVPDDIYKTHLENNRFGGSGSQVDSARMNLASSFVNGFVNAAFGQDKLLTDDGNKWLYKNKDHGMLSAAASLGMILLWDVDGGLTQIDKYLYSSEDYIKSGALLACGIVNSGVRNECDPALALLSDYVLHNSNTMRLGSIFGLGLAYAGSNREDVLTLLLPVMGDSKSSMEVAGVTALACGMIAVGSCNGDVTSTILQTIMEKSETELKDTYARWLPLGLGLNHLGKGEAIEAILAALEVVSEPFRSFANTLVDVCAYAGSGNVLKVQQLLHICSEHFDSKEKEEDKDKKEKKDKDKKEAPADMGAHQGVAVLGIALIAMGEEIGAEMALRTFGHLLRYGEPTLRRAVPLALALISVSNPRLNILDTLSKFSHDADPEVSYYSIFAMGMVGSGTNNARLAAMLRQLAQYHAKDPNNLFMVRLAQGLTHLGKGTLTLCPYHSDRQLMSQVAVAGLLTVLVSFLDVRNIILGKSHYVLYGLVAAMQPRMLVTFDEELRPLPVSVRVGQAVDVVGQAGKPKTITGFQTHTTPVLLAHGERAELATEEFLPVTPILEGFVILRKNPNYDL

1250 NP_078957.1 NP_055259.2

>NP_078957.1

MRMEAGEAAPPAGAGGRAAGGWGKWVRLNVGGTVFLTTRQTLCREQKSFLSRLCQGEELQSDRDETGAYLIDRDPTYFGPILNFLRHGKLVLDKDMAEEGVLEEAEFYNIGPLIRIIKDRMEEKDYTVTQVPPKHVYRVLQCQEEELTQMVSTMSDGWRFEQLVNIGSSYNYGSEDQAEFLCVVSKELHSTPNGLSSESSRKTKSTEEQLEEQQQQEEEVEEVEVEQVQVEADAQEKGSRPHPLRPEAELAVRASPRPLARPQSCHPCCYKPEAPGCEAPDHLQGLGVPI

>NP_055259.2

MIHELLLALSGYPGSIFTWNKRSGLQVSQDFPFLHPSETSVLNRLCRLGTDYIRFTEFIEQYTGHVQQQDHHPSQQGQGGLHGIYLRAFCTGLDSVLQPYRQALLDLEQEFLGDPHLSISHVNYFLDQFQLLFPSVMVVVEQIKSQKIHGCQILETVYKHSCGGLPPVRSALEKILAVCHGVMYKQLSAWMLHGLLLDQHEEFFIKQGPSSGNVSAQPEEDEEDLGIGGLTGKQLRELQDLRLIEEENMLAPSLKQFSLRVEILPSYIPVRVAEKILFVGESVQMFENQNVNLTRKGSILKNQEDTFAAELHRLKQQPLFSLVDFEQVVDRIRSTVAEHLWKLMVEESDLLGQLKIIKDFYLLGRGELFQAFIDTAQHMLKTPPTAVTEHDVNVAFQQSAHKVLLDDDNLLPLLHLTIEYHGKEHKDATQAREGPSRETSPREAPASGWAALGLSYKVQWPLHILFTPAVLEKYNVVFKYLLSVRRVQAELQHCWALQMQRKHLKSNQTDAIKWRLRNHMAFLVDNLQYYLQVDVLESQFSQLLHQINSTRDFESIRLAHDHFLSNLLAQSFILLKPVFHCLNEILDLCHSFCSLVSQNLGPLDERGAAQLSILVKGFSRQSSLLFKILSSVRNHQINSDLAQLLLRLDYNKYYTQAGGTLGSFGM

1251 NP_006839.2 NP_060901.2

>NP_006839.2

MEAEAGGLEELTDEEMAALGKEELVRRLRREEAARLAALVQRGRLMQEVNRQLQGHLGEIRELKQLNRRLQAENRELRDLCCFLDSERQRGRRAARQWQLFGTQASRAVREDLGGCWQKLAELEGRQEELLRENLALKELCLALGEEWGPRGGPSGAGGSGAGPAPELALPPCGPRDLGDGSSSTGSVGSPDQLPLACSPDD

>NP_060901.2

MASNFNDIVKQGYVRIRSRRLGIYQRCWLVFKKASSKGPKRLEKFSDERAAYFRCYHKVTELNNVKNVARLPKSTKKHAIGIYFNDDTSKTFACESDLEADEWCKVLQMECVGTRINDISLGEPDLLATGVEREQSERFNVYLMPSPNLDVHGECALQITYEYICLWDVQNPRVKLISWPLSALRRYGRDTTWFTFEAGRMCETGEGLFIFQTRDGEAIYQKVHSAALAIAEQHERLLQSVKNSMLQMKMSERAASLSTMVPLPRSAYWQHITRQHSTGQLYRLQDVSSPLKLHRTETFPAYRSEH

1252 NP_005297.1 NP_066268.1

>NP_005297.1

MLPDWKSSLILMAYIIIFLTGLPANLLALRAFVGRIRQPQPAPVHILLLSLTLADLLLLLLLPFKIIEAASNFRWYLPKVVCALTSFGFYSSIYCSTWLLAGISIERYLGVAFPVQYKLSRRPLYGVIAALVAWVMSFGHCTIVIIVQYLNTTEQVRSGNEITCYENFTDNQLDVVLPVRLELCLVLFFIPMAVTIFCYWRFVWIMLSQPLVGAQRRRRAVGLAVVTLLNFLVCFGPYNVSHLVGYHQRKSPWWRSIAVVFSSLNASLDPLLFYFSSSVVRRAFGRGLQVLRNQGSSLLGRRGKDTAEGTNEDRGVGQGEGMPSSDFTTE

>NP_066268.1

MGCTLSAEERAALERSKAIEKNLKEDGISAAKDVKLLLLGAGESGKSTIVKQMKIIHEDGFSGEDVKQYKPVVYSNTIQSLAAIVRAMDTLGIEYGDKERKADAKMVCDVVSRMEDTEPFSAELLSAMMRLWGDSGIQECFNRSREYQLNDSAKYYLDSLDRIGAADYQPTEQDILRTRVKTTGIVETHFTFKNLHFRLFDVGGQRSERKKWIHCFEDVTAIIFCVALSGYDQVLHEDETTNRMHESLMLFDSICNNKFFIDTSIILFLNKKDLFGEKIKKSPLTICFPEYTGPNTYEDAAAYIQAQFESKNRSPNKEIYCHMTCATDTNNIQVVFDAVTDIIIANNLRGCGLY

1253 NP_001217.2 NP_000012.1

>NP_001217.2

MSSASGLRRGHPAGGEENMTETDAFYKREMFDPAEKYKMDHRRRGIALIFNHERFFWHLTLPERRGTCADRDNLTRRFSDLGFEVKCFNDLKAEELLLKIHEVSTVSHADADCFVCVFLSHGEGNHIYAYDAKIEIQTLTGLFKGDKCHSLVGKPKIFIIQACRGNQHDVPVIPLDVVDNQTEKLDTNITEVDAASVYTLPAGADFLMCYSVAEGYYSHRETVNGSWYIQDLCEMLGKYGSSLEFTELLTLVNRKVSQRRVDFCKDPSAIGKKQVPCFASMLTKKLHFFPKSN

>NP_000012.1

MTELPAPLSYFQNAQMSEDNHLSNTVRSQNDNRERQEHNDRRSLGHPEPLSNGRPQGNSRQVVEQDEEEDEELTLKYGAKHVIMLFVPVTLCMVVVVATIKSVSFYTRKDGQLIYTPFTEDTETVGQRALHSILNAAIMISVIVVMTILLVVLYKYRCYKVIHAWLIISSLLLLFFFSFIYLGEVFKTYNVAVDYITVALLIWNFGVVGMISIHWKGPLRLQQAYLIMISALMALVFIKYLPEWTAWLILAVISVYDLVAVLCPKGPLRMLVETAQERNETLFPALIYSSTMVWLVNMAEGDPEAQRRVSKNSKYNAESTERESQDTVAENDDGGFSEEWEAQRDSHLGPHRSTPESRAAVQELSSSILAGEDPEERGVKLGLGDFIFYSVLVGKASATASGDWNTTIACFVAILIGLCLTLLLLAIFKKALPALPISITFGLVFYFATDYLVQPFMDQLAFHQFYI

1254 NP_647479.2 NP_758872.1

>NP_647479.2

MPGGKRGLVAPQNTFLENIVRRSSESSFLLGNAQIVDWPVVYSNDGFCKLSGYHRADVMQKSSTCSFMYGELTDKKTIEKVRQTFDNYESNCFEVLLYKKNRTPVWFYMQIAPIRNEHEKVVLFLCTFKDITLFKQPIEDDSTKGWTKFARLTRALTNSRSVLQQLTPMNKTEVVHKHSRLAEVLQLGSDILPQYKQEAPKTPPHIILHYCAFKTTWDWVILILTFYTAIMVPYNVSFKTKQNNIAWLVLDSVVDVIFLVDIVLNFHTTFVGPGGEVISDPKLIRMNYLKTWFVIDLLSCLPYDIINAFENVDEGISSLFSSLKVVRLLRLGRVARKLDHYLEYGAAVLVLLVCVFGLVAHWLACIWYSIGDYEVIDEVTNTIQIDSWLYQLALSIGTPYRYNTSAGIWEGGPSKDSLYVSSLYFTMTSLTTIGFGNIAPTTDVEKMFSVAMMMVGSLLYATIFGNVTTIFQQMYANTNRYHEMLNNVRDFLKLYQVPKGLSERVMDYIVSTWSMSKGIDTEKVLSICPKDMRADICVHLNRKVFNEHPAFRLASDGCLRALAVEFQTIHCAPGDLIYHAGESVDALCFVVSGSLEVIQDDEVVAILGKGDVFGDIFWKETTLAHACANVRALTYCDLHIIKREALLKVLDFYTAFANSFSRNLTLTCNLRKRIIFRKISDVKKEEEERLRQKNEVTLSIPVDHPVRKLFQKFKQQKELRNQGSTQGDPERNQLQVESRSLQNGASITGTSVVTVSQITPIQTSLAYVKTSESLKQNNRDAMELKPNGGADQKCLKVNSPIRMKNGNGKGWLRLKNNMGAHEEKKEDWNNVTKAESMGLLSEDPKSSDSENSVTKNPLRKTDSCDSGITKSDLRLDKAGEARSPLEHSPIQADAKHPFYPIPEQALQTTLQEVKHELKEDIQLLSCRMTALEKQVAEILKILSEKSVPQASSPKSQMPLQVPPQIPCQDIFSVSRPESPESDKDEIHF

>NP_758872.1

MTMAGGRRGLVAPQNTFLENIVRRSNDTNFVLGNAQIVDWPIVYSNDGFCKLSGYHRAEVMQKSSTCSFMYGELTDKDTIEKVRQTFENYEMNSFEILMYKKNRTPVWFFVKIAPIRNEQDKVVLFLCTFSDITAFKQPIEDDSCKGWGKFARLTRALTSSRGVLQQLAPSVQKGENVHKHSRLAEVLQLGSDILPQYKQEAPKTPPHIILHYCVFKTTWDWIILILTFYTAILVPYNVSFKTRQNNVAWLVVDSIVDVIFLVDIVLNFHTTFVGPAGEVISDPKLIRMNYLKTWFVIDLLSCLPYDVINAFENVDEVSAFMGDPGKIGFADQIPPPLEGRESQGISSLFSSLKVVRLLRLGRVARKLDHYIEYGAAVLVLLVCVFGLAAHWMACIWYSIGDYEIFDEDTKTIRNNSWLYQLAMDIGTPYQFNGSGSGKWEGGPSKNSVYISSLYFTMTSLTSVGFGNIAPSTDIEKIFAVAIMMIGSLLYATIFGNVTTIFQQMYANTNRYHEMLNSVRDFLKLYQVPKGLSERVMDYIVSTWSMSRGIDTEKVLQICPKDMRADICVHLNRKVFKEHPAFRLASDGCLRALAMEFQTVHCAPGDLIYHAGESVDSLCFVVSGSLEVIQDDEVVAILGKGDVFGDVFWKEATLAQSCANVRALTYCDLHVIKRDALQKVLEFYTAFSHSFSRNLILTYNLRKRIVFRKISDVKREEEERMKRKNEAPLILPPDHPVRRLFQRFRQQKEARLAAERGGRDLDDLDVEKGNVLTEHASANHSLVKASVVTVRESPATPVSFQAASTSGVPDHAKLQAPGSECLGPKGGGGDCAKRKSWARFKDACGKSEDWNKVSKAESMETLPERTKASGEATLKKTDSCDSGITKSDLRLDNVGEARSPQDRSPILAEVKHSFYPIPEQTLQATVLEVRHELKEDIKALNAKMTNIEKQLSEILRILTSRRSSQSPQELFEISRPQSPESERDIFGAS

1255 NP_001887.1 NP_000368.1

>NP_001887.1

MPGPAAGSRARVYAEVNSLRSREYWDYEAHVPSWGNQDDYQLVRKLGRGKYSEVFEAINITNNERVVVKILKPVKKKKIKREVKILENLRGGTNIIKLIDTVKDPVSKTPALVFEYINNTDFKQLYQILTDFDIRFYMYELLKALDYCHSKGIMHRDVKPHNVMIDHQQKKLRLIDWGLAEFYHPAQEYNVRVASRYFKGPELLVDYQMYDYSLDMWSLGCMLASMIFRREPFFHGQDNYDQLVRIAKVLGTEELYGYLKKYHIDLDPHFNDILGQHSRKRWENFIHSENRHLVSPEALDLLDKLLRYDHQQRLTAKEAMEHPYFYPVVKEQSQPCADNAVLSSGLTAAR

>NP_000368.1

MSGGPMGGRPGGRGAPAVQQNIPSTLLQDHENQRLFEMLGRKCLTLATAVVQLYLALPPGAEHWTKEHCGAVCFVKDNPQKSYFIRLYGLQAGRLLWEQELYSQLVYSTPTPFFHTFAGDDCQAGLNFADEDEAQAFRALVQEKIQKRNQRQSGDRRQLPPPPTPANEERRGGLPPLPLHPGGDQGGPPVGPLSLGLATVDIQNPDITSSRYRGLPAPGPSPADKKRSGKKKISKADIGAPSGFKHVSHVGWDPQNGFDVNNLDPDLRSLFSRAGISEAQLTDAETSKLIYDFIEDQGGLEAVRQEMRRQEPLPPPPPPSRGGNQLPRPPIVGGNKGRSGPLPPVPLGIAPPPPTPRGPPPPGRGGPPPPPPPATGRSGPLPPPPPGAGGPPMPPPPPPPPPPPSSGNGPAPPPLPPALVPAGGLAPGGGRGALLDQIRQGIQLNKTPGAPESSALQPPPQSSEGLVGALMHVMQKRSRAIHSSDEGEDQAGDEDEDDEWDD

1256 NP_004433.2 NP_002881.1

>NP_004433.2

MALRRLGAALLLLPLLAAVEETLMDSTTATAELGWMVHPPSGWEEVSGYDENMNTIRTYQVCNVFESSQNNWLRTKFIRRRGAHRIHVEMKFSVRDCSSIPSVPGSCKETFNLYYYEADFDSATKTFPNWMENPWVKVDTIAADESFSQVDLGGRVMKINTEVRSFGPVSRSGFYLAFQDYGGCMSLIAVRVFYRKCPRIIQNGAIFQETLSGAESTSLVAARGSCIANAEEVDVPIKLYCNGDGEWLVPIGRCMCKAGFEAVENGTVCRGCPSGTFKANQGDEACTHCPINSRTTSEGATNCVCRNGYYRADLDPLDMPCTTIPSAPQAVISSVNETSLMLEWTPPRDSGGREDLVYNIICKSCGSGRGACTRCGDNVQYAPRQLGLTEPRIYISDLLAHTQYTFEIQAVNGVTDQSPFSPQFASVNITTNQAAPSAVSIMHQVSRTVDSITLSWSQPDQPNGVILDYELQYYEKELSEYNATAIKSPTNTVTVQGLKAGAIYVFQVRARTVAGYGRYSGKMYFQTMTEAEYQTSIQEKLPLIIGSSAAGLVFLIAVVVIAIVCNRRRGFERADSEYTDKLQHYTSGHMTPGMKIYIDPFTYEDPNEAVREFAKEIDISCVKIEQVIGAGEFGEVCSGHLKLPGKREIFVAIKTLKSGYTEKQRRDFLSEASIMGQFDHPNVIHLEGVVTKSTPVMIITEFMENGSLDSFLRQNDGQFTVIQLVGMLRGIAAGMKYLADMNYVHRDLAARNILVNSNLVCKVSDFGLSRFLEDDTSDPTYTSALGGKIPIRWTAPEAIQYRKFTSASDVWSYGIVMWEVMSYGERPYWDMTNQDVINAIEQDYRLPPPMDCPSALHQLMLDCWQKDRNHRPKFGQIVNTLDKMIRNPNSLKAMAPLSSGINLPLLDRTIPDYTSFNTVDEWLEAIKMGQYKESFANAGFTSFDVVSQMMMEDILRVGVTLAGHQKKILNSIQVMRAQMNQIQSVEV

>NP_002881.1

MMAAEAGSEEGGPVTAGAGGGGAAAGSSAYPAVCRVKIPAALPVAAAPYPGLVETGVAGTLGGGAALGSEFLGAGSVAGALGGAGLTGGGTAAGVAGAAAGVAGAAVAGPSGDMALTKLPTSLLAETLGPGGGFPPLPPPPYLPPLGAGLGTVDEGDSLDGPEYEEEEVAIPLTAPPTNQWYHGKLDRTIAEERLRQAGKSGSYLIRESDRRPGSFVLSFLSQMNVVNHFRIIAMCGDYYIGGRRFSSLSDLIGYYSHVSCLLKGEKLLYPVAPPEPVEDRRRVRAILPYTKVPDTDEISFLKGDMFIVHNELEDGWMWVTNLRTDEQGLIVEDLVEEVGREEDPHEGKIWFHGKISKQEAYNLLMTVGQVCSFLVRPSDNTPGDYSLYFRTNENIQRFKICPTPNNQFMMGGRYYNSIGDIIDHYRKEQIVEGYYLKEPVPMQDQEQVLNDTVDGKEIYNTIRRKTKDAFYKNIVKKGYLLKKGKGKRWKNLYFILEGSDAQLIYFESEKRATKPKGLIDLSVCSVYVVHDSLFGRPNCFQIVVQHFSEEHYIFYFAGETPEQAEDWMKGLQAFCNLRKSSPGTSNKRLRQVSSLVLHIEEAHKLPVKHFTNPYCNIYLNSVQVAKTHAREGQNPVWSEEFVFDDLPPDINRFEITLSNKTKKSKDPDILFMRCQLSRLQKGHATDEWFLLSSHIPLKGIEPGSLRVRARYSMEKIMPEEEYSEFKELILQKELHVVYALSHVCGQDRTLLASILLRIFLHEKLESLLLCTLNDREISMEDEATTLFRATTLASTLMEQYMKATATQFVHHALKDSILKIMESKQSCELSPSKLEKNEDVNTNLTHLLNILSELVEKIFMASEILPPTLRYIYGCLQKSVQHKWPTNTTMRTRVVSGFVFLRLICPAILNPRMFNIISDSPSPIAARTLILVAKSVQNLANLVEFGAKEPYMEGVNPFIKSNKHRMIMFLDELGNVPELPDTTEHSRTDLSRDLAALHEICVAHSDELRTLSNERGAQQHVLKKLLAITELLQQKQNQYTKTNDVR

1257 NP_001096.1 NP_057647.1

>NP_001096.1

MVDGVMILPVLIMIALPSPSMEDEKPKVNPKLYMCVCEGLSCGNEDHCEGQQCFSSLSINDGFHVYQKGCFQVYEQGKMTCKTPPSPGQAVECCQGDWCNRNITAQLPTKGKSFPGTQNFHLEVGLIILSVVFAVCLLACLLGVALRKFKRRNQERLNPRDVEYGTIEGLITTNVGDSTLADLLDHSCTSGSGSGLPFLVQRTVARQITLLECVGKGRYGEVWRGSWQGENVAVKIFSSRDEKSWFRETELYNTVMLRHENILGFIASDMTSRHSSTQLWLITHYHEMGSLYDYLQLTTLDTVSCLRIVLSIASGLAHLHIEIFGTQGKPAIAHRDLKSKNILVKKNGQCCIADLGLAVMHSQSTNQLDVGNNPRVGTKRYMAPEVLDETIQVDCFDSYKRVDIWAFGLVLWEVARRMVSNGIVEDYKPPFYDVVPNDPSFEDMRKVVCVDQQRPNIPNRWFSDPTLTSLAKLMKECWYQNPSARLTALRIKKTLTKIDNSLDKLKTDC

>NP_057647.1

MSSVFGKPRAGSGPQSAPLEVNLAILGRRGAGKSALTVKFLTKRFISEYDPNLEDTYSSEETVDHQPVHLRVMDTADLDTPRNCERYLNWAHAFLVVYSVDSRQSFDSSSSYLELLALHAKETQRSIPALLLGNKLDMAQYRQVTKAEGVALAGRFGCLFFEVSACLDFEHVQHVFHEAVREARRELEKSPLTRPLFISEERALPHQAPLTARHGLASCTFNTLSTINLKEMPTVAQAKLVTVKSSRAQSKRKAPTLTLLKGFKIF

1258 NP_055467.2 NP_002361.1

>NP_055467.2

MERREEQPGAAGAGAAPALDFTVENVEKALHQLYYDPNIENKNLAQKWLMQAQVSPQAWHFSWQLLQPDKVPEIQYFGASALHIKISRYWSDIPTDQYESLKAQLFTQITRFASGSKIVLTRLCVALASLALSMMPDAWPCAVADMVRLFQAEDSPVDGQGRCLALLELLTVLPEEFQTSRLPQYRKGLVRTSLAVECGAVFPLLEQLLQQPSSPSCVRQKVLKCFSSWVQLEVPLQDCEALIQAAFAALQDSELFDSSVEAIVNAISQPDAQRYVNTLLKLIPLVLGLQEQLRQAVQNGDMETSHGICRIAVALGENHSRALLDQVEHWQSFLALVNMIMFCTGIPGHYPVNETTSSLTLTFWYTLQDDILSFEAEKQAVYQQVYRPVYFQLVDVLLHKAQFPSDEEYGFWSSDEKEQFRIYRVDISDTLMYVYEMLGAELLSNLYDKLGRLLTSSEEPYSWQHTEALLYGFQSIAETIDVNYSDVVPGLIGLIPRISISNVQLADTVMFTIGALSEWLADHPVMINSVLPLVLHALGNPELSVSSVSTLKKICRECKYDLPPYAANIVAVSQDVLMKQIHKTSQCMWLMQALGFLLSALQVEEILKNLHSLISPYIQQLEKLAEEIPNPSNKLAIVHILGLLSNLFTTLDISHHEDDHEGPELRKLPVPQGPNPVVVVLQQVFQLIQKVLSKWLNDAQVVEAVCAIFEKSVKTLLDDFAPMVPQLCEMLGRMYSTIPQASALDLTRQLVHIFAHEPAHFPPIEALFLLVTSVTLTLFQQGPRDHPDIVDSFMQLLAQALKRKPDLFLCERLDVKSVFQCAVLALKFPEAPTVKASCGFFTELLPRCGEVESVGKVVQEDGRMLLIAVLEAIGGQASRSLMDCFADILFALNKHCFSLLSMWIKEALQPPGFPSARLSPEQKDTFSQQILRERVNKRRVKEMVKEFTLLCRGLHGTDYTADY

>NP_002361.1

MESDFYLRYYVGHKGKFGHEFLEFEFRPDGKLRYANNSNYKNDVMIRKEAYVHKSVMEELKRIIDDSEITKEDDALWPPPDRVGRQELEIVIGDEHISFTTSKIGSLIDVNQSKDPEGLRVFYYLVQDLKCLVFSLIGLHFKIKPI

1259 NP_003912.1 NP_066961.2

>NP_003912.1

MEPTAPSLTEEDLTEVKKDALENLRVYLCEKIIAERHFDHLRAKKILSREDTEEISCRTSSRKRAGKLLDYLQENPKGLDTLVESIRREKTQNFLIQKITDEVLKLRNIKLEHLKGLKCSSCEPFPDGATNNLSRSNSDESNFSEKLRASTVMYHPEGESSTTPFFSTNSSLNLPVLEVGRTENTIFSSTTLPRPGDPGAPPLPPDLQLEEEGTCANSSEMFLPLRSRTVSRQ

>NP_066961.2

MAAASVTPPGSLELLQPGFSKTLLGTKLEAKYLCSACRNVLRRPFQAQCGHRYCSFCLASILSSGPQNCAACVHEGIYEEGISILESSSAFPDNAARREVESLPAVCPSDGCTWKGTLKEYESCHEGRCPLMLTECPACKGLVRLGEKERHLEHECPERSLSCRHCRAPCCGADVKAHHEVCPKFPLTCDGCGKKKIPREKFQDHVKTCGKCRVPCRFHAIGCLETVEGEKQQEHEVQWLREHLAMLLSSVLEAKPLLGDQSHAGSELLQRCESLEKKTATFENIVCVLNREVERVAMTAEACSRQHRLDQDKIEALSSKVQQLERSIGLKDLAMADLEQKVLEMEASTYDGVFIWKISDFARKRQEAVAGRIPAIFSPAFYTSRYGYKMCLRIYLNGDGTGRGTHLSLFFVVMKGPNDALLRWPFNQKVTLMLLDQNNREHVIDAFRPDVTSSSFQRPVNDMNIASGCPLFCPVSKMEAKNSYVRDDAIFIKAIVDLTGL

1260 NP_006089.1 NP_002218.2

>NP_006089.1

MTEQMTLRGTLKGHNGWVTQIATTPQFPDMILSASRDKTIIMWKLTRDETNYGIPQRALRGHSHFVSDVVISSDGQFALSGSWDGTLRLWDLTTGTTTRRFVGHTKDVLSVAFSSDNRQIVSGSRDKTIKLWNTLGVCKYTVQDESHSEWVSCVRFSPNSSNPIIVSCGWDKLVKVWNLANCKLKTNHIGHTGYLNTVTVSPDGSLCASGGKDGQAMLWDLNEGKHLYTLDGGDIINALCFSPNRYWLCAATGPSIKIWDLEGKIIVDELKQEVISTSSKAEPPQCTSLAWSADGQTLFAGYTDNLVRVWQVTIGTR

>NP_002218.2

MQYLNIKEDCNAMAFCAKMRSSKKTEVNLEAPEPGVEVIFYLSDREPLRLGSGEYTAEELCIRAAQACRISPLCHNLFALYDENTKLWYAPNRTITVDDKMSLRLHYRMRFYFTNWHGTNDNEQSVWRHSPKKQKNGYEKKKIPDATPLLDASSLEYLFAQGQYDLVKCLAPIRDPKTEQDGHDIENECLGMAVLAISHYAMMKKMQLPELPKDISYKRYIPETLNKSIRQRNLLTRMRINNVFKDFLKEFNNKTICDSSVSTHDLKVKYLATLETLTKHYGAEIFETSMLLISSENEMNWFHSNDGGNVLYYEVMVTGNLGIQWRHKPNVVSVEKEKNKLKRKKLENKHKKDEEKNKIREEWNNFSYFPEITHIVIKESVVSINKQDNKKMELKLSSHEEALSFVSLVDGYFRLTADAHHYLCTDVAPPLIVHNIQNGCHGPICTEYAINKLRQEGSEEGMYVLRWSCTDFDNILMTVTCFEKSEQVQGAQKQFKNFQIEVQKGRYSLHGSDRSFPSLGDLMSHLKKQILRTDNISFMLKRCCQPKPREISNLLVATKKAQEWQPVYPMSQLSFDRILKKDLVQGEHLGRGTRTHIYSGTLMDYKDDEGTSEEKKIKVILKVLDPSHRDISLAFFEAASMMRQVSHKHIVYLYGVCVRDVENIMVEEFVEGGPLDLFMHRKSDVLTTPWKFKVAKQLASALSYLEDKDLVHGNVCTKNLLLAREGIDSECGPFIKLSDPGIPITVLSRQECIERIPWIAPECVEDSKNLSVAADKWSFGTTLWEICYNGEIPLKDKTLIEKERFYESRCRPVTPSCKELADLMTRCMNYDPNQRPFFRAIMRDINKLEEQNPDIVSEKKPATEVDPTHFEKRFLKRIRDLGEGHFGKVELCRYDPEGDNTGEQVAVKSLKPESGGNHIADLKKEIEILRNLYHENIVKYKGICTEDGGNGIKLIMEFLPSGSLKEYLPKNKNKINLKQQLKYAVQICKGMDYLGSRQYVHRDLAARNVLVESEHQVKIGDFGLTKAIETDKEYYTVKDDRDSPVFWYAPECLMQSKFYIASDVWSFGVTLHELLTYCDSDSSPMALFLKMIGPTHGQMTVTRLVNTLKEGKRLPCPPNCPDEVYQLMRKCWEFQPSNRTSFQNLIEGFEALLK

1261 NP_002820.2 NP_001070.2

>NP_002820.2

MTSRLRALGGRINNIRTSELPKEKTRSEVICSIHFLDGVVQTFKVTKQDTGQVLLDMVHNHLGVTEKEYFGLQHDDDSVDSPRWLEASKAIRKQLKGGFPCTLHFRVRFFIPDPNTLQQEQTRHLYFLQLKMDICEGRLTCPLNSAVVLASYAVQSHFGDYNSSIHHPGYLSDSHFIPDQNEDFLTKVESLHEQHSGLKQSEAESCYINIARTLDFYGVELHSGRDLHNLDLMIGIASAGVAVYRKYICTSFYPWVNILKISFKRKKFFIHQRQKQAESREHIVAFNMLNYRSCKNLWKSCVEHHTFFQAKKLLPQEKNVLSQYWTMGSRNTKKSVNNQYCKKVIGGMVWNPAMRRSLSVEHLETKSLPSRSPPITPNWRSPRLRHEIRKPRHSSADNLANEMTYITETEDVFYTYKGSLAPQDSDSEVSQNRSPHQESLSENNPAQSYLTQKSSSSVSPSSNAPGSCSPDGVDQQLLDDFHRVTKGGSTEDASQYYCDKNDNGDSYLVLIRITPDEDGKFGFNLKGGVDQKMPLVVSRINPESPADTCIPKLNEGDQIVLINGRDISEHTHDQVVMFIKASRESHSRELALVIRRRAVRSFADFKSEDELNQLFPEAIFPMCPEGGDTLEGSMAQLKKGLESGTVLIQFEQLYRKKPGLAITFAKLPQNLDKNRYKDVLPYDTTRVLLQGNEDYINASYVNMEIPAANLVNKYIATQGPLPHTCAQFWQVVWDQKLSLIVMLTTLTERGRTKCHQYWPDPPDVMNHGGFHIQCQSEDCTIAYVSREMLVTNTQTGEEHTVTHLQYVAWPDHGVPDDSSDFLEFVNYVRSLRVDSEPLLVHCSAGIGRTGVLVTMETAMCLTERNLPIYPLDIVRKMRDQRAMMVQTSSQYKFVCEAILRVYEEGLVQMLDPS

>NP_001070.2

MPDPAAHLPFFYGSISRAEAEEHLKLAGMADGLFLLRQCLRSLGGYVLSLVHDVRFHHFPIERQLNGTYAIAGGKAHCGPAELCEFYSRDPDGLPCNLRKPCNRPSGLEPQPGVFDCLRDAMVRDYVRQTWKLEGEALEQAIISQAPQVEKLIATTAHERMPWYHSSLTREEAERKLYSGAQTDGKFLLRPRKEQGTYALSLIYGKTVYHYLISQDKAGKYCIPEGTKFDTLWQLVEYLKLKADGLIYCLKEACPNSSASNASGAAAPTLPAHPSTLTHPQRRIDTLNSDGYTPEPARITSPDKPRPMPMDTSVYESPYSDPEELKDKKLFLKRDNLLIADIELGCGNFGSVRQGVYRMRKKQIDVAIKVLKQGTEKADTEEMMREAQIMHQLDNPYIVRLIGVCQAEALMLVMEMAGGGPLHKFLVGKREEIPVSNVAELLHQVSMGMKYLEEKNFVHRDLAARNVLLVNRHYAKISDFGLSKALGADDSYYTARSAGKWPLKWYAPECINFRKFSSRSDVWSYGVTMWEALSYGQKPYKKMKGPEVMAFIEQGKRMECPPECPPELYALMSDCWIYKWEDRPDFLTVEQRMRACYYSLASKVEGPPGSTQKAEAACA

1262 NP_055543.1 NP_056505.1

>NP_055543.1

MDVFSFVKIAKLSSHRTKSSGWPPPSGTWGLSQVPPYGWEMTANRDGRDYFINHMTQAIPFDDPRLESCQIIPPAPRKVEMRRDPVLGFGFVAGSEKPVVVRSVTPGGPSEGKLIPGDQIVMINDEPVSTAPRERVIDLVRSCKESILLTVIQPYPSPKSAFISAAKKARLKSNPVKVRFSEEVIINGQVSETVKDNSLLFMPNVLKVYLENGQTKSFRFDCSTSIKDVILTLQEKLSIKGIEHFSLMLEQRTEGAGTKLLLLHEQETLTQVTQRPSSHKMRCLFRISFVPKDPIDLLRRDPVAFEYLYVQSCNDVVQERFGPELKYDIALRLAALQMYIATVTTKQTQKISLKYIEKEWGLETFLPSAVLQSMKEKNIKKALSHLVKANQNLVPPGKKLSALQAKVHYLKFLSDLRLYGGRVFKATLVQAEKRSEVTLLVGPRYGISHVINTKTNLVALLADFSHVNRIEMFSEEESLVRVELHVLDVKPITLLMESSDAMNLACLTAGYYRLLVDSRRSIFNMANKKNTATQETGPENKGKHNLLGPDWNCIPQMTTFIGEGEQEAQITYIDSKQKTVEITDSTMCPKEHRHLYIDNAYSSDGLNQQLSQPGEAPCEADYRSLAQRSLLTLSGPETLKKAQESPRGAKVSFIFGDFALDDGISPPTLGYETLLDEGPEMLEKQRNLYIGSANDMKGLDLTPEAEGIQFVENSVYANIGDVKSFQAAEGIEEPLLHDICYAENTDDAEDEDEVSCEEDLVVGEMNQPAILNLSGSSDDIIDLTSLPPPEGDDNEDDFLLRSLNMAIAAPPPGFRDSSDEEDSQSQAASFPEDKEKGSSLQNDEIPVSLIDAVPTSAEGKCEKGLDNAVVSTLGALEALSVSEEQQTSDNSGVAILRAYSPESSSDSGNETNSSEMTESSELATAQKQSENLSRMFLATHEGYHPLAEEQTEFPASKTPAGGLPPKSSHALAARPATDLPPKVVPSKQLLHSDHMEMEPETMETKSVTDYFSKLHMGSVAYSCTSKRKSKLADGEGKAPPNGNTTGKKQQGTKTAEMEEEASGKFGTVSSRDSQHLSTFNLERTAFRKDSQRWYVATEGGMAEKSGLEAATGKTFPRASGLGAREAEGKEEGAPDGETSDGSGLGQGDRFLTDVTCASSAKDLDNPEDADSSTCDHPSKLPEADESVARLCDYHLAKRMSSLQSEGHFSLQSSQGSSVDAGCGTGSSGSACATPVESPLCPSLGKHLIPDASGKGVNYIPSEERAPGLPNHGATFKELHPQTEGMCPRMTVPALHTAINTEPLFGTLRDGCHRLPKIKETTV

>NP_056505.1

MEKYHVLEMIGEGSFGRVYKGRRKYSAQVVALKFIPKLGRSEKELRNLQREIEIMRGLRHPNIVHMLDSFETDKEVVVVTDYAEGELFQILEDDGKLPEDQVQAIAAQLVSALYYLHSHRILHRDMKPQNILLAKGGGIKLCDFGFARAMSTNTMVLTSIKGTPLYMSPELVEERPYDHTADLWSVGCILYELAVGTPPFYATSIFQLVSLILKDPVRWPSTISPCFKDFLQGLLTKDPRQRLSWPDLLYHPFIAGHVTIITEPAGPDLGTPFTSRLPPELQVLKDEQAHRLAPKGNQSRILTQAYKRMAEEAMQKKHQNTGPALEQEDKTSKVAPGTAPLPRLGATPQESSLLAGILASELKSSWAKSGTGEVPSAPRENRTTPDCERAFPEERPEVLGQRSTDVVDLENEEPDSDNEWQHLLETTEPVPIQLKAPLTLLCNPDFCQRIQSQLHEAGGQILNGILEGASHILPAFRVLSSLLSSCSDSVALYSFCREAGLPGLLLSLLRHSQESNSLQQQSWYGTFLQDLMAVIQAYFACTFNLERSQTSDSLQVFQEAANLFLDLLGKLLAQPDDSEQTLRRDSLMCFTVLCEAMDGNSRAISKAFYSSLLTTQQVVLDGLLHGLTVPQLPVHTPQGAPQVSQPLREQSEDIPGAISSALAAICTAPVGLPDCWDAKEQVCWHLANQLTEDSSQLRPSLISGLQHPILCLHLLKVLYSCCLVSEGLCRLLGQEPLALESLFMLIQGKVKVVDWEESTEVTLYFLTLLVFRLQNLPCGMEKLGSDVATLFTHSHVVSLVSAAACLLGQLGQQGVTFDLQPMEWMAAATHALSAPAEVRLTPPGSCGFYDGLLILLLQLLTEQGKASLIRDMSSSEMWTVLWHRFSMVLRLPEEASAQEGELSLSSPPSPEPDWTLISPQGMAALLSLAMATFTQEPQLCLSCLSQHGSILMSILKHLLCPSFLNQLRQAPHGSEFLPVVVLSVCQLLCFPFALDMDADLLIGVLADLRDSEVAAHLLQVCCYHLPLMQVELPISLLTRLALMDPTSLNQFVNTVSASPRTIVSFLSVALLSDQPLLTSDLLSLLAHTARVLSPSHLSFIQELLAGSDESYRPLRSLLGHPENSVRAHTYRLLGHLLQHSMALRGALQSQSGLLSLLLLGLGDKDPVVRCSASFAVGNAAYQAGPLGPALAAAVPSMTQLLGDPQAGIRRNVASALGNLGPEGLGEELLQCEVPQRLLEMACGDPQPNVKEAALIALRSLQQEPGIHQVLVSLGASEKLSLLSLGNQSLPHSSPRPASAKHCRKLIHLLRPAHSM

1263 NP_002739.1 NP_002729.2

>NP_002739.1

MAEKFESLMNIHGFDLGSRYMDLKPLGCGGNGLVFSAVDNDCDKRVAIKKIVLTDPQSVKHALREIKIIRRLDHDNIVKVFEILGPSGSQLTDDVGSLTELNSVYIVQEYMETDLANVLEQGPLLEEHARLFMYQLLRGLKYIHSANVLHRDLKPANLFINTEDLVLKIGDFGLARIMDPHYSHKGHLSEGLVTKWYRSPRLLLSPNNYTKAIDMWAAGCIFAEMLTGKTLFAGAHELEQMQLILESIPVVHEEDRQELLSVIPVYIRNDMTEPHKPLTQLLPGISREALDFLEQILTFSPMDRLTAEEALSHPYMSIYSFPMDEPISSHPFHIEDEVDDILLMDETHSHIYNWERYHDCQFSEHDWPVHNNFDIDEVQLDPRALSDVTDEEEVQVDPRKYLDGDREKYLEDPAFDTNYSTEPCWQYSDHHENKYCDLECSHTCNYKTRSSSYLDNLVWRESEVNHYYEPKLIIDLSNWKEQSKEKSDKKGKSKCERNGLVKAQIALEEASQQLAGKEREKNQGFDFDSFIAGTIQLSSQHEPTDVVDKLNDLNSSVSQLELKSLISKSVSQEKQEKGMANLAQLEALYQSSWDSQFVSGGEDCFFINQFCEVRKDEQVEKENTYTSYLDKFFSRKEDTEMLETEPVEDGKLGERGHEEGFLNNSGEFLFNKQLESIGIPQFHSPVGSPLKSIQATLTPSAMKSSPQIPHQTYSSILKHLN

>NP_002729.2

MADPAAGPPPSEGEESTVRFARKGALRQKNVHEVKNHKFTARFFKQPTFCSHCTDFIWGFGKQGFQCQVCCFVVHKRCHEFVTFSCPGADKGPASDDPRSKHKFKIHTYSSPTFCDHCGSLLYGLIHQGMKCDTCMMNVHKRCVMNVPSLCGTDHTERRGRIYIQAHIDRDVLIVLVRDAKNLVPMDPNGLSDPYVKLKLIPDPKSESKQKTKTIKCSLNPEWNETFRFQLKESDKDRRLSVEIWDWDLTSRNDFMGSLSFGISELQKASVDGWFKLLSQEEGEYFNVPVPPEGSEANEELRQKFERAKISQGTKVPEEKTTNTVSKFDNNGNRDRMKLTDFNFLMVLGKGSFGKVMLSERKGTDELYAVKILKKDVVIQDDDVECTMVEKRVLALPGKPPFLTQLHSCFQTMDRLYFVMEYVNGGDLMYHIQQVGRFKEPHAVFYAAEIAIGLFFLQSKGIIYRDLKLDNVMLDSEGHIKIADFGMCKENIWDGVTTKTFCGTPDYIAPEIIAYQPYGKSVDWWAFGVLLYEMLAGQAPFEGEDEDELFQSIMEHNVAYPKSMSKEAVAICKGLMTKHPGKRLGCGPEGERDIKEHAFFRYIDWEKLERKEIQPPYKPKACGRNAENFDRFFTRHPPVLTPPDQEVIRNIDQSEFEGFSFVNSEFLKPEVKS

1264 NP_055525.3 NP_114038.1

>NP_055525.3

MTGAEIESGAQVKPEKKPGEEVVGGAEIENDVPLVVRPKVRTQAQIMPGARPKNKSKVMPGASTKVETSAVGGARPKSKAKAIPVSRFKEEAQMWAQPRFGAERLSKTERNSQTNIIASPLVSTDSVLVAKTKYLSEDRELVNTDTESFPRRKAHYQAGFQPSFRSKEETNMGSWCCPRPTSKQEASPNSDFKWVDKSVSSLFWSGDEVTAKFHPGNRVKDSNRSMHMANQEANTMSRSQTNQELYIASSSGSEDESVKTPWFWARDKTNTWSGPREDPNSRSRFRSKKEVYVESSSGSEHEDHLESWFGAGKEAKFRSKMRAGKEANNRARHRAKREACIDFMPGSIDVIKKESCFWPEENANTFSRPMIKKEARARAMTKEEAKTKARARAKQEARSEEEALIGTWFWATDESSMADEASIESSLQVEDESIIGSWFWTEEEASMGTGASSKSRPRTDGERIGDSLFGAREKTSMKTGAEATSESILAADDEQVIIGSWFWAGEEVNQEAEEETIFGSWFWVIDAASVESGVGVSCESRTRSEEEEVIGPWFWSGEQVDIEAGIGEEARPGAEEETIFGSWFWAENQTYMDCRAETSCDTMQGAEEEEPIIGSWFWTRVEACVEGDVNSKSSLEDKEEAMIPCFGAKEEVSMKHGTGVRCRFMAGAEETNNKSCFWAEKEPCMYPAGGGSWKSRPEEEEDIVNSWFWSRKYTKPEAIIGSWLWATEESNIDGTGEKAKLLTEEETIINSWFWKEDEAISEATDREESRPEAEEGDIIGSWFWAGEEDRLEPAAETREEDRLAAEKEGIVGSWFGAREETIRREAGSCSKSSPKAEEEEVIIGSWFWEEEASPEAVAGVGFESKPGTEEEEITVGSWFWPEEEASIQAGSQAVEEMESETEEETIFGSWFWDGKEVSEEAGPCCVSKPEDDEEMIVESWFWSRDKAIKETGTVATCESKPENEEGAIVGSWFEAEDEVDNRTDNGSNCGSRTLADEDEAIVGSWFWAGDEAHFESNPSPVFRAICRSTCSVEQEPDPSRRPQSWEEVTVQFKPGPWGRVGFPSISPFRFPKEAASLFCEMFGGKPRNMVLSPEGEDQESLLQPDQPSPEFPFQYDPSYRSVQEIREHLRAKESTEPESSSCNCIQCELKIGSEEFEELLLLMEKIRDPFIHEISKIAMGMRSASQFTRDFIRDSGVVSLIETLLNYPSSRVRTSFLENMIRMAPPYPNLNIIQTYICKVCEETLAYSVDSPEQLSGIRMIRHLTTTTDYHTLVANYMSGFLSLLATGNAKTRFHVLKMLLNLSENLFMTKELLSAEAVSEFIGLFNREETNDNIQIVLAIFENIGNNIKKETVFSDDDFNIEPLISAFHKVEKFAKELQGKTDNQNDPEGDQEN

>NP_114038.1

MILNSSTEDGIKRIQDDCPKAGRHNYIFVMIPTLYSIIFVVGIFGNSLVVIVIYFYMKLKTVASVFLLNLALADLCFLLTLPLWAVYTAMEYRWPFGNYLCKIASASVSFNLYASVFLLTCLSIDRYLAIVHPMKSRLRRTMLVAKVTCIIIWLLAGLASLPAIIHRNVFFIENTNITVCAFHYESQNSTLPIGLGLTKNILGFLFPFLIILTSYTLIWKALKKAYEIQKNKPRNDDIFKIIMAIVLFFFFSWIPHQIFTFLDVLIQLGIIRDCRIADIVDTAMPITICIAYFNNCLNPLFYGFLGKKFKRYFLQLLKYIPPKAKSHSNLSTKMSTLSYRPSDNVSSSTKKPAPCFEVE

1265 AAH15714.1 NP_006330.2

>AAH15714.1

MELQTLQEALKVEIQVHQKLVAQMKQDPQNADLKKQLHELQAKITALSEKQKRVVEQLRKNLIVKQEQPDKFQIQPLPQSENKLQTAQQQPLQQLQQQQQYHHHHAQQSAAASPNLTASQKTVTTASMITTKTLPLVLKAATATMPASVVGQRPTIAMVTAINSQKAVLSTDVQNTPVNLQTSSKVTGPGAEAVQIVAKNTVTLVQATPPQPIKVPQFIPPPRLTPRPNFLPQVRPKPVAQNNIPIAPAPPPMLAAPQLIQRPVMLTKFTPTTLPTSQNSIHPVRVVNGQTATIAKTFPMAQLTSIVIATPGTRLAGPQTVQLSKPSLEKQTVKSHTETDEKQTESRTITPPAAPKPKREENPQKLAFMVSLGLVTHDHLEEIQSKRQERKRRTTANPVYSGAVFEPERKKSAVTYLNSTMHPGTRKRGRPPKYNAVLGFGALTPTSPQSSHPDSPENEKTETTFTFPAPVQPVSLPSPTSTDGDIHEDFCSVCRKSGQLLMCDTCSRVYHLDCLDPPLKTIPKGMWICPRCQDQMLKKEEAIPWPGTLAIVHSYIAYKAAKEEEKQKLLKWSSDLKQEREQLEQKVKQLSNSISKCMEMKNTILARQKEMHSSLEKVKQLIRLIHGIDLSKPVDSEATVGAISNGPDCTPPANAATSTPAPSPSSQSCTANCNQGEETK

>NP_006330.2

MSHGPKQPGAAAAPAGGKAPGQHGGFVVTVKQERGEGPRAGEKGSHEEEPVKKRGWPKGKKRKKILPNGPKAPVTGYVRFLNERREQIRTRHPDLPFPEITKMLGAEWSKLQPTEKQRYLDEAEREKQQYMKELRAYQQSEAYKMCTEKIQEKKIKKEDSSSGLMNTLLNGHKGGDCDGFSTFDVPIFTEEFLDQNKAREAELRRLRKMNVAFEEQNAVLQRHTQSMSSARERLEQELALEERRTLALQQQLQAVRQALTASFASLPVPGTGETPTLGTLDFYMARLHGAIERDPAQHEKLIVRIKEILAQVASEHL

1266 NP_001077431.1 NP_001460.1

>NP_001077431.1

MHHQQRMAALGTDKELSDLLDFSAMFSPPVSSGKNGPTSLASGHFTGSNVEDRSSSGSWGNGGHPSPSRNYGDGTPYDHMTSRDLGSHDNLSPPFVNSRIQSKTERGSYSSYGRESNLQGCHQQSLLGGDMDMGNPGTLSPTKPGSQYYQYSSNNPRRRPLHSSAMEVQTKKVRKVPPGLPSSVYAPSASTADYNRDSPGYPSSKPATSTFPSSFFMQDGHHSSDPWSSSSGMNQPGYAGMLGNSSHIPQSSSYCSLHPHERLSYPSHSSADINSSLPPMSTFHRSGTNHYSTSSCTPPANGTDSIMANRGSGAAGSSQTGDALGKALASIYSPDHTNNSFSSNPSTPVGSPPSLSAGTAVWSRNGGQASSSPNYEGPLHSLQSRIEDRLERLDDAIHVLRNHAVGPSTAMPGGHGDMHGIIGPSHNGAMGGLGSGYGTGLLSANRHSLMVGTHREDGVALRGSHSLLPNQVPVPQLPVQSATSPDLNPPQDPYRGMPPGLQGQSVSSGSSEIKSDDEGDENLQDTKSSEDKKLDDDKKDIKSITRSRSSNNDDEDLTPEQKAEREKERRMANNARERLRVRDINEAFKELGRMVQLHLKSDKPQTKLLILHQAVAVILSLEQQVRERNLNPKAACLKRREEEKVSSEPPPLSLAGPHPGMGDASNHMGQM

>NP_001460.1

MSGWESYYKTEGDEEAEEEQEENLEASGDYKYSGRDSLIFLVDASKAMFESQSEDELTPFDMSIQCIQSVYISKIISSDRDLLAVVFYGTEKDKNSVNFKNIYVLQELDNPGAKRILELDQFKGQQGQKRFQDMMGHGSDYSLSEVLWVCANLFSDVQFKMSHKRIMLFTNEDNPHGNDSAKASRARTKAGDLRDTGIFLDLMHLKKPGGFDISLFYRDIISIAEDEDLRVHFEESSKLEDLLRKVRAKETRKRALSRLKLKLNKDIVISVGIYNLVQKALKPPPIKLYRETNEPVKTKTRTFNTSTGGLLLPSDTKRSQIYGSRQIILEKEETEELKRFDDPGLMLMGFKPLVLLKKHHYLRPSLFVYPEESLVIGSSTLFSALLIKCLEKEVAALCRYTPRRNIPPYFVALVPQEEELDDQKIQVTPPGFQLVFLPFADDKRKMPFTEKIMATPEQVGKMKAIVEKLRFTYRSDSFENPVLQQHFRNLEALALDLMEPEQAVDLTLPKVEAMNKRLGSLVDEFKELVYPPDYNPEGKVTKRKHDNEGSGSKRPKVEYSEEELKTHISKGTLGKFTVPMLKEACRAYGLKSGLKKQELLEALTKHFQD

1267 NP_061916.3 NP_057421.1

>NP_061916.3

MGESSEDIDQMFSTLLGEMDLLTQSLGVDTLPPPDPNPPRAEFNYSVGFKDLNESLNALEDQDLDALMADLVADISEAEQRTIQAQKESLQNQHHSASLQASIFSGAASLGYGTNVAATGISQYEDDLPPPPADPVLDLPLPPPPPEPLSQEEEEAQAKADKIKLALEKLKEAKVKKLVVKVHMNDNSTKSLMVDERQLARDVLDNLFEKTHCDCNVDWCLYEIYPELQIERFFEDHENVVEVLSDWTRDTENKILFLEKEEKYAVFKNPQNFYLDNRGKKESKETNEKMNAKNKESLLEESFCGTSIIVPELEGALYLKEDGKKSWKRRYFLLRASGIYYVPKGKTKTSRDLACFIQFENVNIYYGTQHKMKYKAPTDYCFVLKHPQIQKESQYIKYLCCDDTRTLNQWVMGIRIAKYGKTLYDNYQRAVAKAGLASRWTNLGTVNAAAPAQPSTGPKTGTTQPNGQIPQATHSVSAVLQEAQRHAETSKDKKPALGNHHDPAVPRAPHAPKSSLPPPPPVRRSSDTSGSPATPLKAKGTGGGGLPAPPDDFLPPPPPPPPLDDPELPPPPPDFMEPPPDFVPPPPPSYAGIAGSELPPPPPPPPAPAPAPVPDSARPPPAVAKRPPVPPKRQENPGHPGGAGGGEQDFMSDLMKALQKKRGNVS

>NP_057421.1

MATSEQSICQARASVMVYDDTSKKWVPIKPGQQGFSRINIYHNTASNTFRVVGVKLQDQQVVINYSIVKGLKYNQATPTFHQWRDARQVYGLNFASKEEATTFSNAMLFALNIMNSQEGGPSSQRQVQNGPSPDEMDIQRRQVMEQHQQQRQESLERRTSATGPILPPGHPSSAASAPVSCSGPPPPPPPPVPPPPTGATPPPPPPLPAGGAQGSSHDESSMSGLAAAIAGAKLRRVQRPEDASGGSSPSGTSKSDANRASSGGGGGGLMEEMNKLLAKRRKAASQSDKPAEKKEDESQMEDPSTSPSPGTRAASQPPNSSEAGRKPWERSNSVEKPVSSILSRTPSVAKSPEAKSPLQSQPHSRMKPAGSVNDMALDAFDLDRMKQEILEEVVRELHKVKEEIIDAIRQELSGISTT

1268 NP_000928.1 NP_006212.1

>NP_000928.1

MHGGGPPSGDSACPLRTIKRVQFGVLSPDELKRMSVTEGGIKYPETTEGGRPKLGGLMDPRQGVIERTGRCQTCAGNMTECPGHFGHIELAKPVFHVGFLVKTMKVLRCVCFFCSKLLVDSNNPKIKDILAKSKGQPKKRLTHVYDLCKGKNICEGGEEMDNKFGVEQPEGDEDLTKEKGHGGCGRYQPRIRRSGLELYAEWKHVNEDSQEKKILLSPERVHEIFKRISDEECFVLGMEPRYARPEWMIVTVLPVPPLSVRPAVVMQGSARNQDDLTHKLADIVKINNQLRRNEQNGAAAHVIAEDVKLLQFHVATMVDNELPGLPRAMQKSGRPLKSLKQRLKGKEGRVRGNLMGKRVDFSARTVITPDPNLSIDQVGVPRSIAANMTFAEIVTPFNIDRLQELVRRGNSQYPGAKYIIRDNGDRIDLRFHPKPSDLHLQTGYKVERHMCDGDIVIFNRQPTLHKMSMMGHRVRILPWSTFRLNLSVTTPYNADFDGDEMNLHLPQSLETRAEIQELAMVPRMIVTPQSNRPVMGIVQDTLTAVRKFTKRDVFLERGEVMNLLMFLSTWDGKVPQPAILKPRPLWTGKQIFSLIIPGHINCIRTHSTHPDDEDSGPYKHISPGDTKVVVENGELIMGILCKKSLGTSAGSLVHISYLEMGHDITRLFYSNIQTVINNWLLIEGHTIGIGDSIADSKTYQDIQNTIKKAKQDVIEVIEKAHNNELEPTPGNTLRQTFENQVNRILNDARDKTGSSAQKSLSEYNNFKSMVVSGAKGSKINISQVIAVVGQQNVEGKRIPFGFKHRTLPHFIKDDYGPESRGFVENSYLAGLTPTEFFFHAMGGREGLIDTAVKTAETGYIQRRLIKSMESVMVKYDATVRNSINQVVQLRYGEDGLAGESVEFQNLATLKPSNKAFEKKFRFDYTNERALRRTLQEDLVKDVLSNAHIQNELEREFERMREDREVLRVIFPTGDSKVVLPCNLLRMIWNAQKIFHINPRLPSDLHPIKVVEGVKELSKKLVIVNGDDPLSRQAQENATLLFNIHLRSTLCSRRMAEEFRLSGEAFDWLLGEIESKFNQAIAHPGEMVGALAAQSLGEPATQMTLNTFHYAGVSAKNVTLGVPRLKELINISKKPKTPSLTVFLLGQSARDAERAKDILCRLEHTTLRKVTANTAIYYDPNPQSTVVAEDQEWVNVYYEMPDFDVARISPWLLRVELDRKHMTDRKLTMEQIAEKINAGFGDDLNCIFNDDNAEKLVLRIRIMNSDENKMQEEEEVVDKMDDDVFLRCIESNMLTDMTLQGIEQISKVYMHLPQTDNKKKIIITEDGEFKALQEWILETDGVSLMRVLSEKDVDPVRTTSNDIVEIFTVLGIEAVRKALERELYHVISFDGSYVNYRHLALLCDTMTCRGHLMAITRHGVNRQDTGPLMKCSFEETVDVLMEAAAHGESDPMKGVSENIMLGQLAPAGTGCFDLLLDAEKCKYGMEIPTNIPGLGAAGPTGMFFGSAPSPMGGISPAMTPWNQGATPAYGAWSPSVGSGMTPGAAGFSPSAASDASGFSPGYSPAWSPTPGSPGSPGPSSPYIPSPGGAMSPSYSPTSPAYEPRSPGGYTPQSPSYSPTSPSYSPTSPSYSPTSPNYSPTSPSYSPTSPSYSPTSPSYSPTSPSYSPTSPSYSPTSPSYSPTSPSYSPTSPSYSPTSPSYSPTSPSYSPTSPSYSPTSPSYSPTSPSYSPTSPSYSPTSPSYSPTSPNYSPTSPNYTPTSPSYSPTSPSYSPTSPNYTPTSPNYSPTSPSYSPTSPSYSPTSPSYSPSSPRYTPQSPTYTPSSPSYSPSSPSYSPTSPKYTPTSPSYSPSSPEYTPTSPKYSPTSPKYSPTSPKYSPTSPTYSPTTPKYSPTSPTYSPTSPVYTPTSPKYSPTSPTYSPTSPKYSPTSPTYSPTSPKGSTYSPTSPGYSPTSPTYSLTSPAISPDDSDEEN

>NP_006212.1

MADEEKLPPGWEKRMSRSSGRVYYFNHITNASQWERPSGNSSSGGKNGQGEPARVRCSHLLVKHSQSRRPSSWRQEKITRTKEEALELINGYIQKIKSGEEDFESLASQFSDCSSAKARGDLGAFSRGQMQKPFEDASFALRTGEMSGPVFTDSGIHIILRTE

1269 NP_000928.1 AAP88787.1

>NP_000928.1

MHGGGPPSGDSACPLRTIKRVQFGVLSPDELKRMSVTEGGIKYPETTEGGRPKLGGLMDPRQGVIERTGRCQTCAGNMTECPGHFGHIELAKPVFHVGFLVKTMKVLRCVCFFCSKLLVDSNNPKIKDILAKSKGQPKKRLTHVYDLCKGKNICEGGEEMDNKFGVEQPEGDEDLTKEKGHGGCGRYQPRIRRSGLELYAEWKHVNEDSQEKKILLSPERVHEIFKRISDEECFVLGMEPRYARPEWMIVTVLPVPPLSVRPAVVMQGSARNQDDLTHKLADIVKINNQLRRNEQNGAAAHVIAEDVKLLQFHVATMVDNELPGLPRAMQKSGRPLKSLKQRLKGKEGRVRGNLMGKRVDFSARTVITPDPNLSIDQVGVPRSIAANMTFAEIVTPFNIDRLQELVRRGNSQYPGAKYIIRDNGDRIDLRFHPKPSDLHLQTGYKVERHMCDGDIVIFNRQPTLHKMSMMGHRVRILPWSTFRLNLSVTTPYNADFDGDEMNLHLPQSLETRAEIQELAMVPRMIVTPQSNRPVMGIVQDTLTAVRKFTKRDVFLERGEVMNLLMFLSTWDGKVPQPAILKPRPLWTGKQIFSLIIPGHINCIRTHSTHPDDEDSGPYKHISPGDTKVVVENGELIMGILCKKSLGTSAGSLVHISYLEMGHDITRLFYSNIQTVINNWLLIEGHTIGIGDSIADSKTYQDIQNTIKKAKQDVIEVIEKAHNNELEPTPGNTLRQTFENQVNRILNDARDKTGSSAQKSLSEYNNFKSMVVSGAKGSKINISQVIAVVGQQNVEGKRIPFGFKHRTLPHFIKDDYGPESRGFVENSYLAGLTPTEFFFHAMGGREGLIDTAVKTAETGYIQRRLIKSMESVMVKYDATVRNSINQVVQLRYGEDGLAGESVEFQNLATLKPSNKAFEKKFRFDYTNERALRRTLQEDLVKDVLSNAHIQNELEREFERMREDREVLRVIFPTGDSKVVLPCNLLRMIWNAQKIFHINPRLPSDLHPIKVVEGVKELSKKLVIVNGDDPLSRQAQENATLLFNIHLRSTLCSRRMAEEFRLSGEAFDWLLGEIESKFNQAIAHPGEMVGALAAQSLGEPATQMTLNTFHYAGVSAKNVTLGVPRLKELINISKKPKTPSLTVFLLGQSARDAERAKDILCRLEHTTLRKVTANTAIYYDPNPQSTVVAEDQEWVNVYYEMPDFDVARISPWLLRVELDRKHMTDRKLTMEQIAEKINAGFGDDLNCIFNDDNAEKLVLRIRIMNSDENKMQEEEEVVDKMDDDVFLRCIESNMLTDMTLQGIEQISKVYMHLPQTDNKKKIIITEDGEFKALQEWILETDGVSLMRVLSEKDVDPVRTTSNDIVEIFTVLGIEAVRKALERELYHVISFDGSYVNYRHLALLCDTMTCRGHLMAITRHGVNRQDTGPLMKCSFEETVDVLMEAAAHGESDPMKGVSENIMLGQLAPAGTGCFDLLLDAEKCKYGMEIPTNIPGLGAAGPTGMFFGSAPSPMGGISPAMTPWNQGATPAYGAWSPSVGSGMTPGAAGFSPSAASDASGFSPGYSPAWSPTPGSPGSPGPSSPYIPSPGGAMSPSYSPTSPAYEPRSPGGYTPQSPSYSPTSPSYSPTSPSYSPTSPNYSPTSPSYSPTSPSYSPTSPSYSPTSPSYSPTSPSYSPTSPSYSPTSPSYSPTSPSYSPTSPSYSPTSPSYSPTSPSYSPTSPSYSPTSPSYSPTSPSYSPTSPSYSPTSPNYSPTSPNYTPTSPSYSPTSPSYSPTSPNYTPTSPNYSPTSPSYSPTSPSYSPTSPSYSPSSPRYTPQSPTYTPSSPSYSPSSPSYSPTSPKYTPTSPSYSPSSPEYTPTSPKYSPTSPKYSPTSPKYSPTSPTYSPTTPKYSPTSPTYSPTSPVYTPTSPKYSPTSPTYSPTSPKYSPTSPTYSPTSPKGSTYSPTSPGYSPTSPTYSLTSPAISPDDSDEEN

>AAP88787.1

MASSAQSGGSSGGPAVPTVQRGIIKMVLSGCAIIVRGQPRGGPPPERQINLSNIRAGNLARRAAATQPDAKDTPDEPWAFPAREFLRKKLIGKEVCFTIENKTPQGREYGMIYLGKDTNGENIAESLVAEGLATRREGMRANNPEQNRLSECEEQAKAAKKGMWSEGNGSHTIRDLKYTIENPRHFVDSHHQKPVNAIIEHVRDGSVVRALLLPDYYLVTVMLSGIKCPTFRREADGSETPEPFAAEAKFFTESRLLQRDVQIILESCHNQNILGTILHPNGNITELLLKEGFARCVDWSIAVYTRGAEKLRAAERFAKERRLRIWRDYVAPTANLDQKDKQFVAKVMQVLNADAIVVKLNSGDYKTIHLSSIRPPRLEGENTQDKNKKLRPLYDIPYMFEAREFLRKKLIGKKVNVTVDYIRPASPATETVPAFSERTCATVTIGGINIAEALVSKGLATVIRYRQDDDQRSSHYDELLAAEARAIKNGKGLHSKKEVPIHRVADISGDTQKAKQFLPFLQRAGRSEAVVEYVFSGSRLKLYLPKETCLITFLLAGIECPRGARNLPGLVQEGEPFSEEATLFTKELVLQREVEVEVESMDKAGNFIGWLHIDGANLSVLLVEHALSKVHFTAERSSYYKSLLSAEEAAKQKKEKVWAHYEEQPVEEVMPVLEEKERSASYKPVFVTEITDDLHFYVQDVETGTQLEKLMENMRNDIASHPPVEGSYAPRRGEFCIAKFVDGEWYRARVEKVESPAKIHVFYIDYGNREVLPSTRLGTLSPAFSTRVLPAQATEYAFAFIQVPQDDDARTDAVDSVVRDIQNTQCLLNVEHLSAGCPHVTLQFADSKGDVGLGLVKEGLVMVEVRKEKQFQKVITEYLNAQESAKSARLNLWRYGDFRADDADEFGYSR

1270 NP_061185.1 NP_061485.1

>NP_061185.1

MPRKKAAAAAWEEPSSGNGTARAGPRKRGGPAGRKRERPERCSSSSGGGSSGDEDGLELDGAPGGGKRAARPATAGKAGGAAVVITEPEHTKERVKLEGSKCKGQLLIFGATNWDLIGRKEVPKQQAAYRNLGQNLWGPHRYGCLAGVRVRTVVSGSCAAHSLLITTEGKLWSWGRNEKGQLGHGDTKRVEAPRLIEGLSHEVIVSAACGRNHTLALTETGSVFAFGENKMGQLGLGNQTDAVPSPAQIMYNGQPITKMACGAEFSMIMDCKGNLYSFGCPEYGQLGHNSDGKFIARAQRIEYDCELVPRRVAIFIEKTKDGQILPVPNVVVRDVACGANHTLVLDSQKRVFSWGFGGYGRLGHAEQKDEMVPRLVKLFDFPGRGASQIYAGYTCSFAVSEVGGLFFWGATNTSRESTMYPKAVQDLCGWRIRSLACGKSSIIVAADESTISWGPSPTFGELGYGDHKPKSSTAAQEVKTLDGIFSEQVAMGYSHSLVIARDESETEKEKIKKLPEYNPRTL

>NP_061485.1

MQAIKCVVVGDGAVGKTCLLISYTTNAFPGEYIPTVFDNYSANVMVDGKPVNLGLWDTAGQEDYDRLRPLSYPQTVGETYGKDITSRGKDKPIADVFLICFSLVSPASFENVRAKWYPEVRHHCPNTPIILVGTKLDLRDDKDTIEKLKEKKLTPITYPQGLAMAKEIGAVKYLECSALTQRGLKTVFDEAIRAVLCPPPVKKRKRKCLLL

1271 NP_002784.1 NP_002786.2

>NP_002784.1

MLSSTAMYSAPGRDLGMEPHRAAGPLQLRFSPYVFNGGTILAIAGEDFAIVASDTRLSEGFSIHTRDSPKCYKLTDKTVIGCSGFHGDCLTLTKIIEARLKMYKHSNNKAMTTGAIAAMLSTILYSRRFFPYYVYNIIGGLDEEGKGAVYSFDPVGSYQRDSFKAGGSASAMLQPLLDNQVGFKNMQNVEHVPLSLDRAMRLVKDVFISAAERDVYTGDALRICIVTKEGIREETVSLRKD

>NP_002786.2

MSIMSYNGGAVMAMKGKNCVAIAADRRFGIQAQMVTTDFQKIFPMGDRLYIGLAGLATDVQTVAQRLKFRLNLYELKEGRQIKPYTLMSMVANLLYEKRFGPYYTEPVIAGLDPKTFKPFICSLDLIGCPMVTDDFVVSGTCAEQMYGMCESLWEPNMDPDHLFETISQAMLNAVDRDAVSGMGVIVHIIEKDKITTRTLKARMD

1272 NP_008835.5 NP_006375.1

>NP_008835.5

MAGSGAGVRCSLLRLQETLSAADRCGAALAGHQLIRGLGQECVLSSSPAVLALQTSLVFSRDFGLLVFVRKSLNSIEFRECREEILKFLCIFLEKMGQKIAPYSVEIKNTCTSVYTKDRAAKCKIPALDLLIKLLQTFRSSRLMDEFKIGELFSKFYGELALKKKIPDTVLEKVYELLGLLGEVHPSEMINNAENLFRAFLGELKTQMTSAVREPKLPVLAGCLKGLSSLLCNFTKSMEEDPQTSREIFNFVLKAIRPQIDLKRYAVPSAGLRLFALHASQFSTCLLDNYVSLFEVLLKWCAHTNVELKKAALSALESFLKQVSNMVAKNAEMHKNKLQYFMEQFYGIIRNVDSNNKELSIAIRGYGLFAGPCKVINAKDVDFMYVELIQRCKQMFLTQTDTGDDRVYQMPSFLQSVASVLLYLDTVPEVYTPVLEHLVVMQIDSFPQYSPKMQLVCCRAIVKVFLALAAKGPVLRNCISTVVHQGLIRICSKPVVLPKGPESESEDHRASGEVRTGKWKVPTYKDYVDLFRHLLSSDQMMDSILADEAFFSVNSSSESLNHLLYDEFVKSVLKIVEKLDLTLEIQTVGEQENGDEAPGVWMIPTSDPAANLHPAKPKDFSAFINLVEFCREILPEKQAEFFEPWVYSFSYELILQSTRLPLISGFYKLLSITVRNAKKIKYFEGVSPKSLKHSPEDPEKYSCFALFVKFGKEVAVKMKQYKDELLASCLTFLLSLPHNIIELDVRAYVPALQMAFKLGLSYTPLAEVGLNALEEWSIYIDRHVMQPYYKDILPCLDGYLKTSALSDETKNNWEVSALSRAAQKGFNKVVLKHLKKTKNLSSNEAISLEEIRIRVVQMLGSLGGQINKNLLTVTSSDEMMKSYVAWDREKRLSFAVPFREMKPVIFLDVFLPRVTELALTASDRQTKVAACELLHSMVMFMLGKATQMPEGGQGAPPMYQLYKRTFPVLLRLACDVDQVTRQLYEPLVMQLIHWFTNNKKFESQDTVALLEAILDGIVDPVDSTLRDFCGRCIREFLKWSIKQITPQQQEKSPVNTKSLFKRLYSLALHPNAFKRLGASLAFNNIYREFREEESLVEQFVFEALVIYMESLALAHADEKSLGTIQQCCDAIDHLCRIIEKKHVSLNKAKKRRLPRGFPPSASLCLLDLVKWLLAHCGRPQTECRHKSIELFYKFVPLLPGNRSPNLWLKDVLKEEGVSFLINTFEGGGCGQPSGILAQPTLLYLRGPFSLQATLCWLDLLLAALECYNTFIGERTVGALQVLGTEAQSSLLKAVAFFLESIAMHDIIAAEKCFGTGAAGNRTSPQEGERYNYSKCTVVVRIMEFTTTLLNTSPEGWKLLKKDLCNTHLMRVLVQTLCEPASIGFNIGDVQVMAHLPDVCVNLMKALKMSPYKDILETHLREKITAQSIEELCAVNLYGPDAQVDRSRLAAVVSACKQLHRAGLLHNILPSQSTDLHHSVGTELLSLVYKGIAPGDERQCLPSLDLSCKQLASGLLELAFAFGGLCERLVSLLLNPAVLSTASLGSSQGSVIHFSHGEYFYSLFSETINTELLKNLDLAVLELMQSSVDNTKMVSAVLNGMLDQSFRERANQKHQGLKLATTILQHWKKCDSWWAKDSPLETKMAVLALLAKILQIDSSVSFNTSHGSFPEVFTTYISLLADTKLDLHLKGQAVTLLPFFTSLTGGSLEELRRVLEQLIVAHFPMQSREFPPGTPRFNNYVDCMKKFLDALELSQSPMLLELMTEVLCREQQHVMEELFQSSFRRIARRGSCVTQVGLLESVYEMFRKDDPRLSFTRQSFVDRSLLTLLWHCSLDALREFFSTIVVDAIDVLKSRFTKLNESTFDTQITKKMGYYKILDVMYSRLPKDDVHAKESKINQVFHGSCITEGNELTKTLIKLCYDAFTENMAGENQLLERRRLYHCAAYNCAISVICCVFNELKFYQGFLFSEKPEKNLLIFENLIDLKRRYNFPVEVEVPMERKKKYIEIRKEAREAANGDSDGPSYMSSLSYLADSTLSEEMSQFDFSTGVQSYSYSSQDPRPATGRFRRREQRDPTVHDDVLELEMDELNRHECMAPLTALVKHMHRSLGPPQGEEDSVPRDLPSWMKFLHGKLGNPIVPLNIRLFLAKLVINTEEVFRPYAKHWLSPLLQLAASENNGGEGIHYMVVEIVATILSWTGLATPTGVPKDEVLANRLLNFLMKHVFHPKRAVFRHNLEIIKTLVECWKDCLSIPYRLIFEKFSGKDPNSKDNSVGIQLLGIVMANDLPPYDPQCGIQSSEYFQALVNNMSFVRYKEVYAAAAEVLGLILRYVMERKNILEESLCELVAKQLKQHQNTMEDKFIVCLNKVTKSFPPLADRFMNAVFFLLPKFHGVLKTLCLEVVLCRVEGMTELYFQLKSKDFVQVMRHRDDERQKVCLDIIYKMMPKLKPVELRELLNPVVEFVSHPSTTCREQMYNILMWIHDNYRDPESETDNDSQEIFKLAKDVLIQGLIDENPGLQLIIRNFWSHETRLPSNTLDRLLALNSLYSPKIEVHFLSLATNFLLEMTSMSPDYPNPMFEHPLSECEFQEYTIDSDWRFRSTVLTPMFVETQASQGTLQTRTQEGSLSARWPVAGQIRATQQQHDFTLTQTADGRSSFDWLTGSSTDPLVDHTSPSSDSLLFAHKRSERLQRAPLKSVGPDFGKKRLGLPGDEVDNKVKGAAGRTDLLRLRRRFMRDQEKLSLMYARKGVAEQKREKEIKSELKMKQDAQVVLYRSYRHGDLPDIQIKHSSLITPLQAVAQRDPIIAKQLFSSLFSGILKEMDKFKTLSEKNNITQKLLQDFNRFLNTTFSFFPPFVSCIQDISCQHAALLSLDPAAVSAGCLASLQQPVGIRLLEEALLRLLPAELPAKRVRGKARLPPDVLRWVELAKLYRSIGEYDVLRGIFTSEIGTKQITQSALLAEARSDYSEAAKQYDEALNKQDWVDGEPTEAEKDFWELASLDCYNHLAEWKSLEYCSTASIDSENPPDLNKIWSEPFYQETYLPYMIRSKLKLLLQGEADQSLLTFIDKAMHGELQKAILELHYSQELSLLYLLQDDVDRAKYYIQNGIQSFMQNYSSIDVLLHQSRLTKLQSVQALTEIQEFISFISKQGNLSSQVPLKRLLNTWTNRYPDAKMDPMNIWDDIITNRCFFLSKIEEKLTPLPEDNSMNVDQDGDPSDRMEVQEQEEDISSLIRSCKFSMKMKMIDSARKQNNFSLAMKLLKELHKESKTRDDWLVSWVQSYCRLSHCRSRSQGCSEQVLTVLKTVSLLDENNVSSYLSKNILAFRDQNILLGTTYRIIANALSSEPACLAEIEEDKARRILELSGSSSEDSEKVIAGLYQRAFQHLSEAVQAAEEEAQPPSWSCGPAAGVIDAYMTLADFCDQQLRKEEENASVIDSAELQAYPALVVEKMLKALKLNSNEARLKFPRLLQIIERYPEETLSLMTKEISSVPCWQFISWISHMVALLDKDQAVAVQHSVEEITDNYPQAIVYPFIISSESYSFKDTSTGHKNKEFVARIKSKLDQGGVIQDFINALDQLSNPELLFKDWSNDVRAELAKTPVNKKNIEKMYERMYAALGDPKAPGLGAFRRKFIQTFGKEFDKHFGKGGSKLLRMKLSDFNDITNMLLLKMNKDSKPPGNLKECSPWMSDFKVEFLRNELEIPGQYDGRGKPLPEYHVRIAGFDERVTVMASLRRPKRIIIRGHDEREHPFLVKGGEDLRQDQRVEQLFQVMNGILAQDSACSQRALQLRTYSVVPMTSRLGLIEWLENTVTLKDLLLNTMSQEEKAAYLSDPRAPPCEYKDWLTKMSGKHDVGAYMLMYKGANRTETVTSFRKRESKVPADLLKRAFVRMSTSPEAFLALRSHFASSHALICISHWILGIGDRHLNNFMVAMETGGVIGIDFGHAFGSATQFLPVPELMPFRLTRQFINLMLPMKETGLMYSIMVHALRAFRSDPGLLTNTMDVFVKEPSFDWKNFEQKMLKKGGSWIQEINVAEKNWYPRQKICYAKRKLAGANPAVITCDELLLGHEKAPAFRDYVAVARGSKDHNIRAQEPESGLSEETQVKCLMDQATDPNILGRTWEGWEPWM

>NP_006375.1

MGGSGSRLSKELLAEYQDLTFLTKQEILLAHRRFCELLPQEQRTVESSLRAQVPFEQILSLPELKANPFKERICRVFSTSPAKDSLSFEDFLDLLSVFSDTATPDIKSHYAFRIFDFDDDGTLNREDLSRLVNCLTGEGEDTRLSASEMKQLIDNILEESDIDRDGTINLSEFQHVISRSPDFASSFKIVL

1273 AAH11604.1 NP_733832.1

>AAH11604.1

MEPVETWTPGKVATWLRGLDDSLQDYPFEDWQLPGKNLLQLCPQSLEALAVRSLGHQELILGGVEQLQALSSRLQTENLQSLTEGLLGATHDFQSIVQGCLGDCAKTPIDVLCAAVELLHEADALLFWLSRYLFSHLNDFSACQEIRDLLEELSQVLHEDGPAAEKEGTVLRICSHVAGICHNILVCCPKELLEQKAVLEQVQLDSPLGLEIHTTSNCQHFVSQVDTQVPTDSRLQIQPGDEVVQINEQVVVREERDMVGWPRKNMVRELLREPAGLSLVLKKIPIPETPPQTPPQVLDSPHQRSPSLSLAPLSPRAPSEDVFAFDLSSNPSPGPSPAWTDSASLGPEPLPIPPEPPAILPAGVAGTPGLPESPDKSPVGRKKSKGLATRLSRRRVSCRELGRPDCDGWLLLRKAPGGFMGPRWRRRWFVLKGHTLYWYRQPQDEKAEGLINVSNYSLESGHDQKKKYVFQLTHDVYKPFIFAADTLTDLSMWVRHLITCISKYQSPGRAPPPREEDCYSETEAEDPDDEAGSHSASPSPAQAGSPLHGDTSPAATPTQRSPRTSFGSLTDSSEEALEGMVRGLRQGGVSLLGQPQPLTQEQWRSSFMRRNRDPQLNERVHRVRALQSTLKAKLQELQVLEEVLGDPELTGEKFRQWKEQNRELYSEGLGAWGVAQAEGSSHILTSDSTEQSPHSLPSDPEEHSHLCPLTSESSLRPPDL

>NP_733832.1

MSGEPELIELRELAPAGRAGKGRTRLERANALRIARGTACNPTRQLVPGRGHRFQPAGPATHTWCDLCGDFIWGVVRKGLQCARLSADCKFTCHYRCRALVCLDCCGPRDLGWEPAVERDTNVDEPVEWETPDLSQAEIEQKIKEYNAQINSNLFMSLNKDGSYTGFIKVQLKLVRPVSVPSSKKPPSLQDARRGPGRGTSVRRRTSFYLPKDAVKHLHVLSRTRAREVIEALLRKFLVVDDPRKFALFERAERHGQVYLRKLLDDEQPLRLRLLAGPSDKALSFVLKENDSGEVNWDAFSMPELHNFLRILQREEEEHLRQILQKYSYCRQKIQEALHACPLG

1274 NP_005185.2 NP_002119.1

>NP_005185.2

MQRLVAWDPACLPLPPPPPAFKSMEVANFYYEADCLAAAYGGKAAPAAPPAARPGPRPPAGELGSIGDHERAIDFSPYLEPLGAPQAPAPATATDTFEAAPPAPAPAPASSGQHHDFLSDLFSDDYGGKNCKKPAEYGYVSLGRLGAAKGALHPGCFAPLHPPPPPPPPPAELKAEPGFEPADCKRKEEAGAPGGGAGMAAGFPYALRAYLGYQAVPSGSSGSLSTSSSSSPPGTPSPADAKAPPTACYAGAAPAPSQVKSKAKKTVDKHSDEYKIRRERNNIAVRKSRDKAKMRNLETQHKVLELTAENERLQKKVEQLSRELSTLRNLFKQLPEPLLASSGHC

>NP_002119.1

MGKGDPKKPRGKMSSYAFFVQTCREEHKKKHPDASVNFSEFSKKCSERWKTMSAKEKGKFEDMAKADKARYEREMKTYIPPKGETKKKFKDPNAPKRPPSAFFLFCSEYRPKIKGEHPGLSIGDVAKKLGEMWNNTAADDKQPYEKKAAKLKEKYEKDIAAYRAKGKPDAAKKGVVKAEKSKKKKEEEEDEEDEEDEEEEEDEEDEDEEEDDDDE

1275 NP_005326.1 NP_001005386.1

>NP_005326.1

MWKSVVGHDVSVSVETQGDDWDTDPDFVNDISEKEQRWGAKTIEGSGRTEHINIHQLRNKVSEEHDVLRKKEMESGPKASHGYGGRFGVERDRMDKSAVGHEYVAEVEKHSSQTDAAKGFGGKYGVERDRADKSAVGFDYKGEVEKHTSQKDYSRGFGGRYGVEKDKWDKAALGYDYKGETEKHESQRDYAKGFGGQYGIQKDRVDKSAVGFNEMEAPTTAYKKTTPIEAASSGARGLKAKFESMAEEKRKREEEEKAQQVARRQQERKAVTKRSPEAPQPVIAMEEPAVPAPLPKKISSEAWPPVGTPPSSESEPVRTSREHPVPLLPIRQTLPEDNEEPPALPPRTLEGLQVEEEPVYEAEPEPEPEPEPEPENDYEDVEEMDRHEQEDEPEGDYEEVLEPEDSSFSSALAGSSGCPAGAGAGAVALGISAVALYDYQGEGSDELSFDPDDVITDIEMVDEGWWRGRCHGHFGLFPANYVKLLE

>NP_001005386.1

MDSQGRKVVVCDNGTGFVKCGYAGSNFPEHIFPALVGRPIIRSTTKVGNIEIKNNKKMDLMVGDEASELRSMLEVNYPMENGIVRNWDDMKHLWDYTFGPEKLNIDTRNCKILLTEPPMNPTKNREKIVEVMFETYQFSGVYVAIQAVLTLYAQGLLTGVVVDSGDGVTHICPVYEGFSLPHLTRRLDIAGRDITRYLIKLLLLRGYAFNHSADFETVRMIKEKLCYVGYNIEQEQKLALETTVLVESYTLPDGRIIKVGGERFEAPEALFQPHLINVEGVGVAELLFNTIQAADIDTRSEFYKHIVLSGGSTMYPGLPSRLERELKQLYLERVLKGDVEKLSKFKIRIEDPPRRKHMVFLGGAVLADIMKDKDNFWMTRQEYQEKGVRVLEKLGVTVR

1276 NP_877428.1 NP_003494.1

>NP_877428.1

MDEEEDNLSLLTALLEENESALDCNSEENNFLTRENGEPDAFDELFDADGDGESYTEEADDGETGETRDEKENLATLFGDMEDLTDEEEVPASQSTENRVLPAPAPRREKTNEELQEELRNLQEQMKALQEQLKVTTIKQTASPARLQKSPVEKSPRPPLKERRVQRIQESTCFSAELDVPALPRTKRVARTPKASPPDPKSSSSRMTSAPSQPLQTISRNKPSGITRGQIVGTPGSSGETTQPICVEAFSGLRLRRPRVSSTEMNKKMTGRKLIRLSQIKEKMAREKLEEIDWVTFGVILKKVTPQSVNSGKTFSIWKLNDLRDLTQCVSLFLFGEVHKALWKTEQGTVVGILNANPMKPKDGSEEVCLSIDHPQKVLIMGEALDLGTCKAKKKNGEPCTQTVNLRDCEYCQYHVQAQYKKLSAKRADLQSTFSGGRIPKKFARRGTSLKERLCQDGFYYGGVSSASYAASIAAAVAPKKKIQTTLSNLVVKGTNLIIQETRQKLGIPQKSLSCSEEFKELMDLPTCGARNLKQHLAKATASGIMGSPKPAIKSISASALLKQQKQRMLEMRRRKSEEIQKRFLQSSSEVESPAVPSSSRQPPAQPPRTGSEFPRLEGAPATMTPKLGRGVLEGDDVLFYDESPPPRPKLSALAEAKKLAAITKLRAKGQVLTKTNPNSIKKKQKDPQDILEVKERVEKNTMFSSQAEDELEPARKKRREQLAYLESEEFQKILKAKSKHTGILKEAEAEMQERYFEPLVKKEQMEEKMRNIREVKCRVVTCKTCAYTHFKLLETCVSEQHEYHWHDGVKRFFKCPCGNRSISLDRLPNKHCSNCGLYKWERDGMLKEKTGPKIGGETLLPRGEEHAKFLNSLK

>NP_003494.1

MEASLGIQMDEPMAFSPQRDRFQAEGSLKKNEQNFKLAGVKKDIEKLYEAVPQLSNVFKIEDKIGEGTFSSVYLATAQLQVGPEEKIALKHLIPTSHPIRIAAELQCLTVAGGQDNVMGVKYCFRKNDHVVIAMPYLEHESFLDILNSLSFQEVREYMLNLFKALKRIHQFGIVHRDVKPSNFLYNRRLKKYALVDFGLAQGTHDTKIELLKFVQSEAQQERCSQNKSHIITGNKIPLSGPVPKELDQQSTTKASVKRPYTNAQIQIKQGKDGKEGSVGLSVQRSVFGERNFNIHSSISHESPAVKLMKQSKTVDVLSRKLATKKKAISTKVMNSAVMRKTASSCPASLTCDCYATDKVCSICLSRRQQVAPRAGTPGFRAPEVLTKCPNQTTAIDMWSAGVIFLSLLSGRYPFYKASDDLTALAQIMTIRGSRETIQAAKTFGKSILCSKEVPAQDLRKLCERLRGMDSSTPKLTSDIQGHASHQPAISEKTDHKASCLVQTPPGQYSGNSFKKGDSNSCEHCFDEYNTNLEGWNEVPDEAYDLLDKLLDLNPASRITAEEALLHPFFKDMSL

1277 NP_001914.3 NP_006760.1

>NP_001914.3

MSYNYVVTAQKPTAVNGCVTGHFTSAEDLNLLIAKNTRLEIYVVTAEGLRPVKEVGMYGKIAVMELFRPKGESKDLLFILTAKYNACILEYKQSGESIDIITRAHGNVQDRIGRPSETGIIGIIDPECRMIGLRLYDGLFKVIPLDRDNKELKAFNIRLEELHVIDVKFLYGCQAPTICFVYQDPQGRHVKTYEVSLREKEFNKGPWKQENVEAEASMVIAVPEPFGGAIIIGQESITYHNGDKYLAIAPPIIKQSTIVCHNRVDPNGSRYLLGDMEGRLFMLLLEKEEQMDGTVTLKDLRVELLGETSIAECLTYLDNGVVFVGSRLGDSQLVKLNVDSNEQGSYVVAMETFTNLGPIVDMCVVDLERQGQGQLVTCSGAFKEGSLRIIRNGIGIHEHASIDLPGIKGLWPLRSDPNRETDDTLVLSFVGQTRVLMLNGEEVEETELMGFVDDQQTFFCGNVAHQQLIQITSASVRLVSQEPKALVSEWKEPQAKNISVASCNSSQVVVAVGRALYYLQIHPQELRQISHTEMEHEVACLDITPLGDSNGLSPLCAIGLWTDISARILKLPSFELLHKEMLGGEIIPRSILMTTFESSHYLLCALGDGALFYFGLNIETGLLSDRKKVTLGTQPTVLRTFRSLSTTNVFACSDRPTVIYSSNHKLVFSNVNLKEVNYMCPLNSDGYPDSLALANNSTLTIGTIDEIQKLHIRTVPLYESPRKICYQEVSQCFGVLSSRIEVQDTSGGTTALRPSASTQALSSSVSSSKLFSSSTAPHETSFGEEVEVHNLLIIDQHTFEVLHAHQFLQNEYALSLVSCKLGKDPNTYFIVGTAMVYPEEAEPKQGRIVVFQYSDGKLQTVAEKEVKGAVYSMVEFNGKLLASINSTVRLYEWTTEKELRTECNHYNNIMALYLKTKGDFILVGDLMRSVLLLAYKPMEGNFEEIARDFNPNWMSAVEILDDDNFLGAENAFNLFVCQKDSAATTDEERQHLQEVGLFHLGEFVNVFCHGSLVMQNLGETSTPTQGSVLFGTVNGMIGLVTSLSESWYNLLLDMQNRLNKVIKSVGKIEHSFWRSFHTERKTEPATGFIDGDLIESFLDISRPKMQEVVANLQYDDGSGMKREATADDLIKVVEELTRIH

>NP_006760.1

MVNPGSSSQPPPVTAGSLSWKRCAGCGGKIADRFLLYAMDSYWHSRCLKCSCCQAQLGDIGTSCYTKSGMILCRNDYIRLFGNSGACSACGQSIPASELVMRAQGNVYHLKCFTCSTCRNRLVPGDRFHYINGSLFCEHDRPTALINGHLNSLQSNPLLPDQKVC

1278 NP_060952.2 NP_004094.3

>NP_060952.2

MRSSASRLSSFSSRDSLWNRMPDQISVSEFIAETTEDYNSPTTSSFTTRLHNCRNTVTLLEEALDQDRTALQKVKKSVKAIYNSGQDHVQNEENYAQVLDKFGSNFLSRDNPDLGTAFVKFSTLTKELSTLLKNLLQGLSHNVIFTLDSLLKGDLKGVKGDLKKPFDKAWKDYETKFTKIEKEKREHAKQHGMIRTEITGAEIAEEMEKERRLFQLQMCEYLIKVNEIKTKKGVDLLQNLIKYYHAQCNFFQDGLKTADKLKQYIEKLAADLYNIKQTQDEEKKQLTALRDLIKSSLQLDQKEDSQSRQGGYSMHQLQGNKEYGSEKKGYLLKKSDGIRKVWQRRKCSVKNGILTISHATSNRQPAKLNLLTCQVKPNAEDKKSFDLISHNRTYHFQAEDEQDYVAWISVLTNSKEEALTMAFRGEQSAGENSLEDLTKAIIEDVQRLPGNDICCDCGSSEPTWLSTNLGILTCIECSGIHREMGVHISRIQSLELDKLGTSELLLAKNVGNNSFNDIMEANLPSPSPKPTPSSDMTVRKEYITAKYVDHRFSRKTCSTSSAKLNELLEAIKSRDLLALIQVYAEGVELMEPLLEPGQELGETALHLAVRTADQTSLHLVDFLVQNCGNLDKQTALGNTVLHYCSMYSKPECLKLLLRSKPTVDIVNQAGETALDIAKRLKATQCEDLLSQAKSGKFNPHVHVEYEWNLRQEEIDESDDDLDDKPSPIKKERSPRPQSFCHSSSISPQDKLALPGFSTPRDKQRLSYGAFTNQIFVSTSTDSPTSPTTEAPPLPPRNAGKGPTGPPSTLPLSTQTSSGSSTLSKKRPPPPPPGHKRTLSDPPSPLPHGPPNKGAVPWGNDGGPSSSSKTTNKFEGLSQQSSTSSAKTALGPRVLPKLPQKVALRKTDHLSLDKATIPPEIFQKSSQLAELPQKPPPGDLPPKPTELAPKPQIGDLPPKPGELPPKPQLGDLPPKPQLSDLPPKPQMKDLPPKPQLGDLLAKSQTGDVSPKAQQPSEVTLKSHPLDLSPNVQSRDAIQKQASEDSNDLTPTLPETPVPLPRKINTGKNKVRRVKTIYDCQADNDDELTFIEGEVIIVTGEEDQEWWIGHIEGQPERKGVFPVSFVHILSD

>NP_004094.3

MSGVSEPLSRVKLGTLRRPEGPAEPMVVVPVDVEKEDVRILKVCFYSNSFNPGKNFKLVKCTVQTEIREIITSILLSGRIGPNIRLAECYGLRLKHMKSDEIHWLHPQMTVGEVQDKYECLHVEAEWRYDLQIRYLPEDFMESLKEDRTTLLYFYQQLRNDYMQRYASKVSEGMALQLGCLELRRFFKDMPHNALDKKSNFELLEKEVGLDLFFPKQMQENLKPKQFRKMIQQTFQQYASLREEECVMKFFNTLAGFANIDQETYRCELIQGWNITVDLVIGPKGIRQLTSQDAKPTCLAEFKQIRSIRCLPLEEGQAVLQLGIEGAPQALSIKTSSLAEAENMADLIDGYCRLQGEHQGSLIIHPRKDGEKRNSLPQIPMLNLEARRSHLSESCSIESDIYAEIPDETLRRPGGPQYGIAREDVVLNRILGEGFFGEVYEGVYTNHKGEKINVAVKTCKKDCTLDNKEKFMSEAVIMKNLDHPHIVKLIGIIEEEPTWIIMELYPYGELGHYLERNKNSLKVLTLVLYSLQICKAMAYLESINCVHRDIAVRNILVASPECVKLGDFGLSRYIEDEDYYKASVTRLPIKWMSPESINFRRFTTASDVWMFAVCMWEILSFGKQPFFWLENKDVIGVLEKGDRLPKPDLCPPVLYTLMTRCWDYDPSDRPRFTELVCSLSDVYQMEKDIAMEQERNARYRTPKILEPTAFQEPPPKPSRPKYRPPPQTNLLAPKLQFQVPEGLCASSPTLTSPMEYPSPVNSLHTPPLHRHNVFKRHSMREEDFIQPSSREEAQQLWEAEKVKMRQILDKQQKQMVEDYQWLRQEEKSLDPMVYMNDKSPLTPEKEVGYLEFTGPPQKPPRLGAQSIQPTANLDRTDDLVYLNVMELVRAVLELKNELCQLPPEGYVVVVKNVGLTLRKLIGSVDDLLPSLPSSSRTEIEGTQKLLNKDLAELINKMRLAQQNAVTSLSEECKRQMLTASHTLAVDAKNLLDAVDQAKVLANLAHPPAE

1279 NP_006550.1 NP_002651.2

>NP_006550.1

MQRRDDPAARMSRSSGRSGSMDPSGAHPSVRQTPSRQPPLPHRSRGGGGGSRGGARASPATQPPPLLPPSATGPDATVGGPAPTPLLPPSATASVKMEPENKYLPELMAEKDSLDPSFTHAMQLLTAEIEKIQKGDSKKDDEENYLDLFSHKNMKLKERVLIPVKQYPKFNFVGKILGPQGNTIKRLQEETGAKISVLGKGSMRDKAKEEELRKGGDPKYAHLNMDLHVFIEVFGPPCEAYALMAHAMEEVKKFLVPDMMDDICQEQFLELSYLNGVPEPSRGRGVPVRGRGAAPPPPPVPRGRGVGPPRGALVRGTPVRGAITRGATVTRGVPPPPTVRGAPAPRARTAGIQRIPLPPPPAPETYEEYGYDDTYAEQSYEGYEGYYSQSQGDSEYYDYGHGEVQDSYEAYGQDDWNGTRPSLKAPPARPVKGAYREHPYGRY

>NP_002651.2

MAGAASPCANGCGPGAPSDAEVLHLCRSLEVGTVMTLFYSKKSQRPERKTFQVKLETRQITWSRGADKIEGAIDIREIKEIRPGKTSRDFDRYQEDPAFRPDQSHCFVILYGMEFRLKTLSLQATSEDEVNMWIKGLTWLMEDTLQAPTPLQIERWLRKQFYSVDRNREDRISAKDLKNMLSQVNYRVPNMRFLRERLTDLEQRSGDITYGQFAQLYRSLMYSAQKTMDLPFLEASTLRAGERPELCRVSLPEFQQFLLDYQGELWAVDRLQVQEFMLSFLRDPLREIEEPYFFLDEFVTFLFSKENSVWNSQLDAVCPDTMNNPLSHYWISSSHNTYLTGDQFSSESSLEAYARCLRMGCRCIELDCWDGPDGMPVIYHGHTLTTKIKFSDVLHTIKEHAFVASEYPVILSIEDHCSIAQQRNMAQYFKKVLGDTLLTKPVEISADGLPSPNQLKRKILIKHKKLAEGSAYEEVPTSMMYSENDISNSIKNGILYLEDPVNHEWYPHYFVLTSSKIYYSEETSSDQGNEDEEEPKEVSSSTELHSNEKWFHGKLGAGRDGRHIAERLLTEYCIETGAPDGSFLVRESETFVGDYTLSFWRNGKVQHCRIHSRQDAGTPKFFLTDNLVFDSLYDLITHYQQVPLRCNEFEMRLSEPVPQTNAHESKEWYHASLTRAQAEHMLMRVPRDGAFLVRKRNEPNSYAISFRAEGKIKHCRVQQEGQTVMLGNSEFDSLVDLISYYEKHPLYRKMKLRYPINEEALEKIGTAEPDYGALYEGRNPGFYVEANPMPTFKCAVKALFDYKAQREDELTFIKSAIIQNVEKQEGGWWRGDYGGKKQLWFPSNYVEEMVNPVALEPEREHLDENSPLGDLLRGVLDVPACQIAIRPEGKNNRLFVFSISMASVAHWSLDVAADSQEELQDWVKKIREVAQTADARLTEGKIMERRKKIALELSELVVYCRPVPFDEEKIGTERACYRDMSSFPETKAEKYVNKAKGKKFLQYNRLQLSRIYPKGQRLDSSNYDPLPMWICGSQLVALNFQTPDKPMQMNQALFMTGRHCGYVLQPSTMRDEAFDPFDKSSLRGLEPCAISIEVLGARHLPKNGRGIVCPFVEIEVAGAEYDSTKQKTEFVVDNGLNPVWPAKPFHFQISNPEFAFLRFVVYEEDMFSDQNFLAQATFPVKGLKTGYRAVPLKNNYSEDLELASLLIKIDIFPAKENGDLSPFSGTSLRERGSDASGQLFHGRAREGSFESRYQQPFEDFRISQEHLADHFDSRERRAPRRTRVNGDNRL

1280 BAA05892.1 NP_004829.2

>BAA05892.1

MVLLLILSVLLLKEDVRGSAQSSERRVVAHMPGDIIIGALFSVHHQPTVDKVHERKCGAVREQYGIQRVEAMLHTLERINSDPTLLPNITLGCEIRDSCWHSAVALEQSIEFIRDSLISSEEEEGLVRCVDGSSSSFRSKKPIVGVIGPGSSSVAIQVQNLLQLFNIPQIAYSATSMDLSDKTLFKYFMRVVPSDAQQARAMVDIVKRYNWTYVSAVHTEGNYGESGMEAFKDMSAKEGICIAHSYKIYSNAGEQSFDKLLKKLTSHLPKARVVACFCEGMTVRGLLMAMRRLGLAGEFLLLGSDGWADRYDVTDGYQREAVGGITIKLQSPDVKWFDDYYLKLRPETNHRNPWFQEFWQHRFQCRLEGFPQENSKYNKTCNSSLTLKTHHVQDSKMGFVINAIYSMAYGLHNMQMSLCPGYAGLCDAMKPIDGRKLLESLMKTNFTGVSGDTILFDENGDSPGRYEIMNFKEMGKDYFDYINVGSWDNGELKMDDDEVWSKKSNIIRSVCSEPCEKGQIKVIRKGEVSCCWTCTPCKENEYVFDEYTCKACQLGSWPTDDLTGCDLIPVQYLRWGDPEPIAAVVFACLGLLATLFVTVVFIIYRDTPVVKSSSRELCYIILAGICLGYLCTFCLIAKPKQIYCYLQRIGIGLSPAMSYSALVTKTNRIARILAGSKKKICTKKPRFMSACAQLVIAFILICIQLGIIVALFIMEPPDIMHDYPSIREVYLICNTTNLGVVTPLGYNGLLILSCTFYAFKTRNVPANFNEAKYIAFTMYTTCIIWLAFVPIYFGSNYKIITMCFSVSLSATVALGCMFVPKVYIILAKPERNVRSAFTTSTVVRMHVGDGKSSSAASRSSSLVNLWKRRGSSGETLRYKDRRLAQHKSEIECFTPKGSMGNGGRATMSSSNGKSVTWAQNEKSSRGQHLWQRLSIHINKKENPNQTAVIKPFPKSTESRGLGAGAGAGGSAGGVGATGGAGCAGAGPGGPESPDAGPKALYDVAEAEEHFPAPARPRSPSPISTLSHRAGSASRTDDDVPSLHSEPVARSSSSQGSLMEQISSVVTRFTANISELNSMMLSTAAPSPGVGAPLCSSYLIPKEIQLPTTMTTFAEIQPLPAIEVTGGAQPAAGAQAAGDAARESPAAGPEAAAAKPDLEELVALTPPSPFRDSVDSGSTTPNSPVSESALCIPSSPKYDTLIIRDYTQSSSSL

>NP_004829.2

MSTAREQPIFSTRAHVFQIDPATKRNWIPAGKHALTVSYFYDATRNVYRIISIGGAKAIINSTVTPNMTFTKTSQKFGQWADSRANTVYGLGFASEQHLTQFAEKFQEVKEAARLAREKSQDGGELTSPALGLASHQVPPSPLVSANGPGEEKLFRSQSADAPGPTERERLKKMLSEGSVGEVQWEAEFFALQDSNNKLAGALREANAAAAQWRQQLEAQRAEAERLRQRVAELEAQAASEVTPTGEKEGLGQGQSLEQLEALVQTKDQEIQTLKSQTGGPREALEAAEREETQQKVQDLETRNAELEHQLRAMERSLEEARAERERARAEVGRAAQLLDVRLFELSELREGLARLAEAAP

1281 NP_036449.1 NP_003480.2

>NP_036449.1

MVDELVLLLHALLMRHRALSIENSQLMEQLRLLVCERASLLRQVRPPSCPVPFPETFNGESSRLPEFIVQTASYMLVNENRFCNDAMKVAFLISLLTGEAEEWVVPYIEMDSPILGDYRAFLDEMKQCFGWDDDEDDDDEEEEDDY

>NP_003480.2

MTHGEELGSDVHQDSIVLTYLEGLLMHQAAGGSGTAVDKKSAGHNEEDQNFNISGSAFPTCQSNGPVLNTHTYQGSGMLHLKKARLLQSSEDWNAAKRKRLSDSIMNLNVKKEALLAGMVDSVPKGKQDSTLLASLLQSFSSRLQTVALSQQIRQSLKEQGYALSHDSLKVEKDLRCYGVASSHLKTLLKKSKVKDQKPDTNLPDVTKNLIRDRFAESPHHVGQSGTKVMSEPLSCAARLQAVASMVEKRASPATSPKPSVACSQLALLLSSEAHLQQYSREHALKTQNANQAASERLAAMARLQENGQKDVGSYQLPKGMSSHLNGQARTSSSKLMASKSSATVFQNPMGIIPSSPKNAGYKNSLERNNIKQAANNSLLLHLLKSQTIPKPMNGHSHSERGSIFEESSTPTTIDEYSDNNPSFTDDSSGDESSYSNCVPIDLSCKHRTEKSESDQPVSLDNFTQSLLNTWDPKVPDVDIKEDQDTSKNSKLNSHQKVTLLQLLLGHKNEENVEKNTSPQGVHNDVSKFNTQNYARTSVIESPSTNRTTPVSTPPLLTSSKAGSPINLSQHSLVIKWNSPPYVCSTQSEKLTNTASNHSMDLTKSKDPPGEKPAQNEGAQNSATFSASKLLQNLAQCGMQSSMSVEEQRPSKQLLTGNTDKPIGMIDRLNSPLLSNKTNAVEENKAFSSQPTGPEPGLSGSEIENLLERRTVLQLLLGNPNKGKSEKKEKTPLRDESTQEHSERALSEQILMVKIKSEPCDDLQIPNTNVHLSHDAKSAPFLGMAPAVQRSAPALPVSEDFKSEPVSPQDFSFSKNGLLSRLLRQNQDSYLADDSDRSHRNNEMALLESKNLCMVPKKRKLYTEPLENPFKKMKNNIVDAANNHSAPEVLYGSLLNQEELKFSRNDLEFKYPAGHGSASESEHRSWARESKSFNVLKQLLLSENCVRDLSPHRSNSVADSKKKGHKNNVTNSKPEFSISSLNGLMYSSTQPSSCMDNRTFSYPGVVKTPVSPTFPEHLGCAGSRPESGLLNGCSMPSEKGPIKWVITDAEKNEYEKDSPRLTKTNPILYYMLQKGGNSVTSRETQDKDIWREASSAESVSQVTAKEELLPTAETKASFFNLRSPYNSHMGNNASRPHSANGEVYGLLGSVLTIKKESE

1282 NP_055416.2 NP_009097.2

>NP_055416.2

MFSWLKRGGARGQQPEAIRTVTSALKELYRTKLLPLEEHYRFGAFHSPALEDADFDGKPMVLVAGQYSTGKTSFIQYLLEQEVPGSRVGPEPTTDCFVAVMHGDTEGTVPGNALVVDPDKPFRKLNPFGNTFLNRFMCAQLPNQVLESISIIDTPGILSGAKQRVSRGYDFPAVLRWFAERVDLIILLFDAHKLEISDEFSEAIGALRGHEDKIRVVLNKADMVETQQLMRVYGALMWALGKVVGTPEVLRVYIGSFWSQPLLVPDNRRLFELEEQDLFRDIQGLPRHAALRKLNDLVKRARLVRVHAYIISYLKKEMPSVFGKENKKKQLILKLPVIFAKIQLEHHISPGDFPDCQKMQELLMAHDFTKFHSLKPKLLEALDEMLTHDIAKLMPLLRQEELESTEVGVQGGAFEGTHMGPFVERGPDEAMEDGEEGSDDEAEWVVTKDKSKYDEIFYNLAPADGKLSGSKAKTWMVGTKLPNSVLGRIWKLSDVDRDGMLDDEEFALASHLIEAKLEGHGLPANLPRRLVPPSKRRHKGSAE

>NP_009097.2

MSGQSLTDRITAAQHSVTGSAVSKTVCKATTHEIMGPKKKHLDYLIQCTNEMNVNIPQLADSLFERTTNSSWVVVFKSLITTHHLMVYGNERFIQYLASRNTLFNLSNFLDKSGLQGYDMSTFIRRYSRYLNEKAVSYRQVAFDFTKVKRGADGVMRTMNTEKLLKTVPIIQNQMDALLDFNVNSNELTNGVINAAFMLLFKDAIRLFAAYNEGIINLLEKYFDMKKNQCKEGLDIYKKFLTRMTRISEFLKVAEQVGIDRGDIPDLSQAPSSLLDALEQHLASLEGKKIKDSTAASRATTLSNAVSSLASTGLSLTKVDEREKQAALEEEQARLKALKEQRLKELAKKPHTSLTTAASPVSTSAGGIMTAPAIDIFSTPSSSNSTSKLPNDLLDLQQPTFHPSVHPMSTASQVASTWGDPFSATVDAVDDAIPSLNPFLTKSSGDVHLSISSDVSTFTTRTPTHEMFVGFTPSPVAQPHPSAGLNVDFESVFGNKSTNVIVDSGGFDELGGLLKPTVASQNQNLPVAKLPPSKLVSDDLDSSLANLVGNLGIGNGTTKNDVNWSQPGEKKLTGGSNWQPKVAPTTAWNAATMAPPVMAYPATTPTGMIGYGIPPQMGSVPVMTQPTLIYSQPVMRPPNPFGPVSGAQIQFM

1283 NP_005350.1 NP_002219.1

>NP_005350.1

MDNMSITNTPTSNDACLSIVHSLMCHRQGGESETFAKRAIESLVKKLKEKKDELDSLITAITTNGAHPSKCVTIQRTLDGRLQVAGRKGFPHVIYARLWRWPDLHKNELKHVKYCQYAFDLKCDSVCVNPYHYERVVSPGIDLSGLTLQSNAPSSMMVKDEYVHDFEGQPSLSTEGHSIQTIQHPPSNRASTETYSTPALLAPSESNATSTANFPNIPVASTSQPASILGGSHSEGLLQIASGPQPGQQQNGFTGQPATYHHNSTTTWTGSRTAPYTPNLPHHQNGHLQHHPPMPPHPGHYWPVHNELAFQPPISNHPAPEYWCSIAYFEMDVQVGETFKVPSSCPIVTVDGYVDPSGGDRFCLGQLSNVHRTEAIERARLHIGKGVQLECKGEGDVWVRCLSDHAVFVQSYYLDREAGRAPGDAVHKIYPSAYIKVFDLRQCHRQMQQQAATAQAAAAAQAAAVAGNIPGPGSVGGIAPAISLSAAAGIGVDDLRRLCILRMSFVKGWGPDYPRQSIKETPCWIEIHLHRALQLLDEVLHTMPIADPQPLD

>NP_002219.1

MTAKMETTFYDDALNASFLPSESGPYGYSNPKILKQSMTLNLADPVGSLKPHLRAKNSDLLTSPDVGLLKLASPELERLIIQSSNGHITTTPTPTQFLCPKNVTDEQEGFAEGFVRALAELHSQNTLPSVTSAAQPVNGAGMVAPAVASVAGGSGSGGFSASLHSEPPVYANLSNFNPGALSSGGGAPSYGAAGLAFPAQPQQQQQPPHHLPQQMPVQHPRLQALKEEPQTVPEMPGETPPLSPIDMESQERIKAERKRMRNRIAASKCRKRKLERIARLEEKVKTLKAQNSELASTANMLREQVAQLKQKVMNHVNSGCQLMLTQQLQTF

1284 NP_004888.2 NP_003122.1

>NP_004888.2

MLRAPGCLLRTSVAPAAALAAALLSSLARCSLLEPRDPVASSLSPYFGTKTRYEDVNPVLLSGPEAPWRDPELLEGTCTPVQLVALIRHGTRYPTVKQIRKLRQLHGLLQARGSRDGGASSTGSRDLGAALADWPLWYADWMDGQLVEKGRQDMRQLALRLASLFPALFSRENYGRLRLITSSKHRCMDSSAAFLQGLWQHYHPGLPPPDVADMEFGPPTVNDKLMRFFDHCEKFLTEVEKNATALYHVEAFKTGPEMQNILKKVAATLQVPVNDLNADLIQVAFFTCSFDLAIKGVKSPWCDVFDIDDAKVLEYLNDLKQYWKRGYGYTINSRSSCTLFQDIFQHLDKAVEQKQRSQPISSPVILQFGHAETLLPLLSLMGYFKDKEPLTAYNYKKQMHRKFRSGLIVPYASNLIFVLYHCENAKTPKEQFRVQMLLNEKVLPLAYSQETVSFYEDLKNHYKDILQSCQTSEECELARANSTSDEL

>NP_003122.1

MLPTQAGAAAALGRGSALGGSLNRTPTGRPGGGGGTRGANGGRVPGNGAGLGPGRLEREAAAAAATTPAPTAGALYSGSEGDSESGEEEELGAERRGLKRSLSEMEIGMVVGGPEASAAATGGYGPVSGAVSGAKPGKKTRGRVKIKMEFIDNKLRRYTTFSKRKTGIMKKAYELSTLTGTQVLLLVASETGHVYTFATRKLQPMITSETGKALIQTCLNSPDSPPRSDPTTDQRMSATGFEETDLTYQVSESDSSGETKDTLKPAFTVTNLPGTTSTIQTAPSTSTTMQVSSGPSFPITNYLAPVSASVSPSAVSSANGTVLKSTGSGPVSSGGLMQLPTSFTLMPGGAVAQQVPVQAIQVHQAPQQASPSRDSSTDLTQTSSSGTVTLPATIMTSSVPTTVGGHMMYPSPHAVMYAPTSGLGDGSLTVLNAFSQAPSTMQVSHSQVQEPGGVPQVFLTASSGTVQIPVSAVQLHQMAVIGQQAGSSSNLTELQVVNLDTAHSTKSE

1285 NP_002604.1 NP_005391.1

>NP_002604.1

MARTTSQLYDAVPIQSSVVLCSCPSPSMVRTQTESSTPPGIPGGSRQGPAMDGTAAEPRPGAGSLQHAQPPPQPRKKRPEDFKFGKILGEGSFSTVVLARELATSREYAIKILEKRHIIKENKVPYVTRERDVMSRLDHPFFVKLYFTFQDDEKLYFGLSYAKNGELLKYIRKIGSFDETCTRFYTAEIVSALEYLHGKGIIHRDLKPENILLNEDMHIQITDFGTAKVLSPESKQARANSFVGTAQYVSPELLTEKSACKSSDLWALGCIIYQLVAGLPPFRAGNEYLIFQKIIKLEYDFPEKFFPKARDLVEKLLVLDATKRLGCEEMEGYGPLKAHPFFESVTWENLHQQTPPKLTAYLPAMSEDDEDCYGNYDNLLSQFGCMQVSSSSSSHSLSASDTGLPQRSGSNIEQYIHDLDSNSFELDLQFSEDEKRLLLEKQAGGNPWHQFVENNLILKMGPVDKRKGLFARRRQLLLTEGPHLYYVDPVNKVLKGEIPWSQELRPEAKNFKTFFVHTPNRTYYLMDPSGNAHKWCRKIQEVWRQRYQSHPDAAVQ

>NP_005391.1

MVVFNGLLKIKICEAVSLKPTAWSLRHAVGPRPQTFLLDPYIALNVDDSRIGQTATKQKTNSPAWHDEFVTDVCNGRKIELAVFHDAPIGYDDFVANCTIQFEELLQNGSRHFEDWIDLEPEGRVYVIIDLSGSSGEAPKDNEERVFRERMRPRKRQGAVRRRVHQVNGHKFMATYLRQPTYCSHCRDFIWGVIGKQGYQCQVCTCVVHKRCHELIITKCAGLKKQETPDQVGSQRFSVNMPHKFGIHNYKVPTFCDHCGSLLWGLLRQGLQCKVCKMNVHRRCETNVAPNCGVDARGIAKVLADLGVTPDKITNSGQRRKKLIAGAESPQPASGSSPSEEDRSKSAPTSPCDQEIKELENNIRKALSFDNRGEEHRAASSPDGQLMSPGENGEVRQGQAKRLGLDEFNFIKVLGKGSFGKVMLAELKGKDEVYAVKVLKKDVILQDDDVDCTMTEKRILALARKHPYLTQLYCCFQTKDRLFFVMEYVNGGDLMFQIQRSRKFDEPRSRFYAAEVTSALMFLHQHGVIYRDLKLDNILLDAEGHCKLADFGMCKEGILNGVTTTTFCGTPDYIAPEILQELEYGPSVDWWALGVLMYEMMAGQPPFEADNEDDLFESILHDDVLYPVWLSKEAVSILKAFMTKNPHKRLGCVASQNGEDAIKQHPFFKEIDWVLLEQKKIKPPFKPRIKTKRDVNNFDQDFTREEPVLTLVDEAIVKQINQEEFKGFSYFGEDLMP

1286 NP_001886.1 NP_002961.1

>NP_001886.1

MSGPVPSRARVYTDVNTHRPREYWDYESHVVEWGNQDDYQLVRKLGRGKYSEVFEAINITNNEKVVVKILKPVKKKKIKREIKILENLRGGPNIITLADIVKDPVSRTPALVFEHVNNTDFKQLYQTLTDYDIRFYMYEILKALDYCHSMGIMHRDVKPHNVMIDHEHRKLRLIDWGLAEFYHPGQEYNVRVASRYFKGPELLVDYQMYDYSLDMWSLGCMLASMIFRKEPFFHGHDNYDQLVRIAKVLGTEDLYDYIDKYNIELDPRFNDILGRHSRKRWERFVHSENQHLVSPEALDFLDKLLRYDHQSRLTAREAMEHPYFYTVVKDQARMGSSSMPGGSTPVSSANMMSGISSVPTPSPLGPLAGSPVIAAANPLGMPVPAAAGAQQ

>NP_002961.1

MAKFVIRPATAADCSDILRLIKELAKYEYMEEQVILTEKDLLEDGFGEHPFYHCLVAEVPKEHWTPEGHSIVGFAMYYFTYDPWIGKLLYLEDFFVMSDYRGFGIGSEILKNLSQVAMRCRCSSMHFLVAEWNEPSINFYKRRGASDLSSEEGWRLFKIDKEYLLKMATEE

1287 NP_005520.4 NP_005194.3

>NP_005520.4

MGWRAAGALLLALLLHGRLLAVTHGLRAYDGLSLPEDIETVTASQMRWTHSYLSDDEDMLADSISGDDLGSGDLGSGDFQMVYFRALVNFTRSIEYSPQLEDAGSREFREVSEAVVDTLESEYLKIPGDQVVSVVFIKELDGWVFVELDVGSEGNADGAQIQEMLLRVISSGSVASYVTSPQGFQFRRLGTVPQFPRACTEAEFACHSYNECVALEYRCDRRPDCRDMSDELNCEEPVLGISPTFSLLVETTSLPPRPETTIMRQPPVTHAPQPLLPGSVRPLPCGPQEAACRNGHCIPRDYLCDGQEDCEDGSDELDCGPPPPCEPNEFPCGNGHCALKLWRCDGDFDCEDRTDEANCPTKRPEEVCGPTQFRCVSTNMCIPASFHCDEESDCPDRSDEFGCMPPQVVTPPRESIQASRGQTVTFTCVAIGVPTPIINWRLNWGHIPSHPRVTVTSEGGRGTLIIRDVKESDQGAYTCEAMNARGMVFGIPDGVLELVPQRGPCPDGHFYLEHSAACLPCFCFGITSVCQSTRRFRDQIRLRFDQPDDFKGVNVTMPAQPGTPPLSSTQLQIDPSLHEFQLVDLSRRFLVHDSFWALPEQFLGNKVDSYGGSLRYNVRYELARGMLEPVQRPDVVLMGAGYRLLSRGHTPTQPGALNQRQVQFSEEHWVHESGRPVQRAELLQVLQSLEAVLIQTVYNTKMASVGLSDIAMDTTVTHATSHGRAHSVEECRCPIGYSGLSCESCDAHFTRVPGGPYLGTCSGCNCNGHASSCDPVYGHCLNCQHNTEGPQCNKCKAGFFGDAMKATATSCRPCPCPYIDASRRFSDTCFLDTDGQATCDACAPGYTGRRCESCAPGYEGNPIQPGGKCRPVNQEIVRCDERGSMGTSGEACRCKNNVVGRLCNECADGSFHLSTRNPDGCLKCFCMGVSRHCTSSSWSRAQLHGASEEPGHFSLTNAASTHTTNEGIFSPTPGELGFSSFHRLLSGPYFWSLPSRFLGDKVTSYGGELRFTVTQRSQPGSTPLHGQPLVVLQGNNIILEHHVAQEPSPGQPSTFIVPFREQAWQRPDGQPATREHLLMALAGIDTLLIRASYAQQPAESRVSGISMDVAVPEETGQDPALEVEQCSCPPGYRGPSCQDCDTGYTRTPSGLYLGTCERCSCHGHSEACEPETGACQGCQHHTEGPRCEQCQPGYYGDAQRGTPQDCQLCPCYGDPAAGQAAHTCFLDTDGHPTCDACSPGHSGRHCERCAPGYYGNPSQGQPCQRDSQVPGPIGCNCDPQGSVSSQCDAAGQCQCKAQVEGLTCSHCRPHHFHLSASNPDGCLPCFCMGITQQCASSAYTRHLISTHFAPGDFQGFALVNPQRNSRLTGEFTVEPVPEGAQLSFGNFAQLGHESFYWQLPETYQGDKVAAYGGKLRYTLSYTAGPQGSPLSDPDVQITGNNIMLVASQPALQGPERRSYEIMFREEFWRRPDGQPATREHLLMALADLDELLIRATFSSVPLAASISAVSLEVAQPGPSNRPRALEVEECRCPPGYIGLSCQDCAPGYTRTGSGLYLGHCELCECNGHSDLCHPETGACSQCQHNAAGEFCELCAPGYYGDATAGTPEDCQPCACPLTNPENMFSRTCESLGAGGYRCTACEPGYTGQYCEQCGPGYVGNPSVQGGQCLPETNQAPLVVEVHPARSIVPQGGSHSLRCQVSGSPPHYFYWSREDGRPVPSGTQQRHQGSELHFPSVQPSDAGVYICTCRNLHQSNTSRAELLVTEAPSKPITVTVEEQRSQSVRPGADVTFICTAKSKSPAYTLVWTRLHNGKLPTRAMDFNGILTIRNVQLSDAGTYVCTGSNMFAMDQGTATLHVQASGTLSAPVVSIHPPQLTVQPGQLAEFRCSATGSPTPTLEWTGGPGGQLPAKAQIHGGILRLPAVEPTDQAQYLCRAHSSAGQQVARAVLHVHGGGGPRVQVSPERTQVHAGRTVRLYCRAAGVPSATITWRKEGGSLPPQARSERTDIATLLIPAITTADAGFYLCVATSPAGTAQARIQVVVLSASDASPPPVKIESSSPSVTEGQTLDLNCVVAGSAHAQVTWYRRGGSLPPHTQVHGSRLRLPQVSPADSGEYVCRVENGSGPKEASITVSVLHGTHSGPSYTPVPGSTRPIRIEPSSSHVAEGQTLDLNCVVPGQAHAQVTWHKRGGSLPARHQTHGSLLRLHQVTPADSGEYVCHVVGTSGPLEASVLVTIEASVIPGPIPPVRIESSSSTVAEGQTLDLSCVVAGQAHAQVTWYKRGGSLPARHQVRGSRLYIFQASPADAGQYVCRASNGMEASITVTVTGTQGANLAYPAGSTQPIRIEPSSSQVAEGQTLDLNCVVPGQSHAQVTWHKRGGSLPVRHQTHGSLLRLYQASPADSGEYVCRVLGSSVPLEASVLVTIEPAGSVPALGVTPTVRIESSSSQVAEGQTLDLNCLVAGQAHAQVTWHKRGGSLPARHQVHGSRLRLLQVTPADSGEYVCRVVGSSGTQEASVLVTIQQRLSGSHSQGVAYPVRIESSSASLANGHTLDLNCLVASQAPHTITWYKRGGSLPSRHQIVGSRLRIPQVTPADSGEYVCHVSNGAGSRETSLIVTIQGSGSSHVPSVSPPIRIESSSPTVVEGQTLDLNCVVARQPQAIITWYKRGGSLPSRHQTHGSHLRLHQMSVADSGEYVCRANNNIDALEASIVISVSPSAGSPSAPGSSMPIRIESSSSHVAEGETLDLNCVVPGQAHAQVTWHKRGGSLPSHHQTRGSRLRLHHVSPADSGEYVCRVMGSSGPLEASVLVTIEASGSSAVHVPAPGGAPPIRIEPSSSRVAEGQTLDLKCVVPGQAHAQVTWHKRGGNLPARHQVHGPLLRLNQVSPADSGEYSCQVTGSSGTLEASVLVTIEPSSPGPIPAPGLAQPIYIEASSSHVTEGQTLDLNCVVPGQAHAQVTWYKRGGSLPARHQTHGSQLRLHLVSPADSGEYVCRAASGPGPEQEASFTVTVPPSEGSSYRLRSPVISIDPPSSTVQQGQDASFKCLIHDGAAPISLEWKTRNQELEDNVHISPNGSIITIVGTRPSNHGTYRCVASNAYGVAQSVVNLSVHGPPTVSVLPEGPVWVKVGKAVTLECVSAGEPRSSARWTRISSTPAKLEQRTYGLMDSHAVLQISSAKPSDAGTYVCLAQNALGTAQKQVEVIVDTGAMAPGAPQVQAEEAELTVEAGHTATLRCSATGSPAPTIHWSKLRSPLPWQHRLEGDTLIIPRVAQQDSGQYICNATSPAGHAEATIILHVESPPYATTVPEHASVQAGETVQLQCLAHGTPPLTFQWSRVGSSLPGRATARNELLHFERAAPEDSGRYRCRVTNKVGSAEAFAQLLVQGPPGSLPATSIPAGSTPTVQVTPQLETKSIGASVEFHCAVPSDRGTQLRWFKEGGQLPPGHSVQDGVLRIQNLDQSCQGTYICQAHGPWGKAQASAQLVIQALPSVLINIRTSVQTVVVGHAVEFECLALGDPKPQVTWSKVGGHLRPGIVQSGGVVRIAHVELADAGQYRCTATNAAGTTQSHVLLLVQALPQISMPQEVRVPAGSAAVFPCIASGYPTPDISWSKLDGSLPPDSRLENNMLMLPSVRPQDAGTYVCTATNRQGKVKAFAHLQVPERVVPYFTQTPYSFLPLPTIKDAYRKFEIKITFRPDSADGMLLYNGQKRVPGSPTNLANRQPDFISFGLVGGRPEFRFDAGSGMATIRHPTPLALGHFHTVTLLRSLTQGSLIVGDLAPVNGTSQGKFQGLDLNEELYLGGYPDYGAIPKAGLSSGFIGCVRELRIQGEEIVFHDLNLTAHGISHCPTCRDRPCQNGGQCHDSESSSYVCVCPAGFTGSRCEHSQALHCHPEACGPDATCVNRPDGRGYTCRCHLGRSGLRCEEGVTVTTPSLSGAGSYLALPALTNTHHELRLDVEFKPLAPDGVLLFSGGKSGPVEDFVSLAMVGGHLEFRYELGSGLAVLRSAEPLALGRWHRVSAERLNKDGSLRVNGGRPVLRSSPGKSQGLNLHTLLYLGGVEPSVPLSPATNMSAHFRGCVGEVSVNGKRLDLTYSFLGSQGIGQCYDSSPCERQPCQHGATCMPAGEYEFQCLCRDGFKGDLCEHEENPCQLREPCLHGGTCQGTRCLCLPGFSGPRCQQGSGHGIAESDWHLEGSGGNDAPGQYGAYFHDDGFLAFPGHVFSRSLPEVPETIELEVRTSTASGLLLWQGVEVGEAGQGKDFISLGLQDGHLVFRYQLGSGEARLVSEDPINDGEWHRVTALREGRRGSIQVDGEELVSGRSPGPNVAVNAKGSVYIGGAPDVATLTGGRFSSGITGCVKNLVLHSARPGAPPPQPLDLQHRAQAGANTRPCPS

>NP_005194.3

MVAERTHKAAATGARGPGELGAPGTVALVAARAERGARLPSPGSCGLLTLALCSLALSLLAHFRTAELQARVLRLEAERGEQQMETAILGRVNQLLDEKWKLHSRRRREAPKTSPGCNCPPAFQGPTGRPGLPGDKGAIGMPGRVGSPGDAGLSIIGPRGPPGQPGTRGFPGFPGPIGLDGKPGHPGPKGDMGLTGPPGQPGPQGQKGEKGQCGEYPHRECLSSMPAALRSSQIIALKLLPLLNSVRLAPPPVIKRRTFQGEQSQASIQGPPGPPGPPGPSGPLGHPGLPGPMGPPGLPGPPGPKGDPGIQGYHGRKGERGMPGMPGKHGAKGAPGIAVAGMKGEPGIPGTKGEKGAEGSPGLPGLLGQKGEKGDAGNSIGGGRGEPGPPGLPGPPGPKGEAGVDGQVGPPGQPGDKGERGAAGEQGPDGPKGSKGEPGKGEMVDYNGNINEALQEIRTLALMGPPGLPGQIGPPGAPGIPGQKGEIGLPGPPGHDGEKGPRGKPGDMGPPGPQGPPGKDGPPGVKGENGHPGSPGEKGEKGETGQAGSPGEKGEAGEKGNPGAEVPGLPGPEGPPGPPGLQGVPGPKGEAGLDGAKGEKGFQGEKGDRGPLGLPGASGLDGRPGPPGTPGPIGVPGPAGPKGERGSKGDPGMTGPTGAAGLPGLHGPPGDKGNRGERGKKGSRGPKGDKGDQGAPGLDAPCPLGEDGLPVQGCWNK

1288 NP_077295.1 NP_536737.3

>NP_077295.1

MFGSSRGGVRGGQDQFNWEDVKTDKQRENYLGNSLMAPVGRWQKGRDLTWYAKGRAPCAGPSREEELAAVREAEREALLAALGYKNVKKQPTGLSKEDFAEVCKREGGDPEEKGVDRLLGLGSASGSVGRVAMSREDKEAAKLGLSVFTHHRVESGGPGTSAASARRKPRAEDQTESSCESHRKSKKEKKKKKKRKHKKEKKKKDKEHRRPAEATSSPTSPERPRHHHHDSDSNSPCCKRRKRGHSGDRRSPSRRWHDRGSEA

>NP_536737.3

MSSLYPSLEDLKVDQAIQAQVRASPKMPALPVQATAISPPPVLYPNLAELENYMGLSLSSQEVQESLLQIPEGDSTAVSGPGPGQMVAPVTGYSLGVRRAEIKPGVREIHLCKDERGKTGLRLRKVDQGLFVQLVQANTPASLVGLRFGDQLLQIDGRDCAGWSSHKAHQVVKKASGDKIVVVVRDRPFQRTVTMHKDSMGHVGFVIKKGKIVSLVKGSSAARNGLLTNHYVCEVDGQNVIGLKDKKIMEILATAGNVVTLTIIPSVIYEHMVKKLPPVLLHHTMDHSIPDA

1289 NP_002341.1 NP_006245.2

>NP_002341.1

MGCIKSKGKDSLSDDGVDLKTQPVRNTERTIYVRDPTSNKQQRPVPESQLLPGQRFQTKDPEEQGDIVVALYPYDGIHPDDLSFKKGEKMKVLEEHGEWWKAKSLLTKKEGFIPSNYVAKLNTLETEEWFFKDITRKDAERQLLAPGNSAGAFLIRESETLKGSFSLSVRDFDPVHGDVIKHYKIRSLDNGGYYISPRITFPCISDMIKHYQKQADGLCRRLEKACISPKPQKPWDKDAWEIPRESIKLVKRLGAGQFGEVWMGYYNNSTKVAVKTLKPGTMSVQAFLEEANLMKTLQHDKLVRLYAVVTREEPIYIITEYMAKGSLLDFLKSDEGGKVLLPKLIDFSAQIAEGMAYIERKNYIHRDLRAANVLVSESLMCKIADFGLARVIEDNEYTAREGAKFPIKWTAPEAINFGCFTIKSDVWSFGILLYEIVTYGKIPYPGRTNADVMTALSQGYRMPRVENCPDELYDIMKMCWKEKAEERPTFDYLQSVLDDFYTATEGQYQQQP

>NP_006245.2

MAPFLRIAFNSYELGSLQAEDEANQPFCAVKMKEALSTERGKTLVQKKPTMYPEWKSTFDAHIYEGRVIQIVLMRAAEEPVSEVTVGVSVLAERCKKNNGKAEFWLDLQPQAKVLMSVQYFLEDVDCKQSMRSEDEAKFPTMNRRGAIKQAKIHYIKNHEFIATFFGQPTFCSVCKDFVWGLNKQGYKCRQCNAAIHKKCIDKIIGRCTGTAANSRDTIFQKERFNIDMPHRFKVHNYMSPTFCDHCGSLLWGLVKQGLKCEDCGMNVHHKCREKVANLCGINQKLLAEALNQVTQRASRRSDSASSEPVGIYQGFEKKTGVAGEDMQDNSGTYGKIWEGSSKCNINNFIFHKVLGKGSFGKVLLGELKGRGEYFAIKALKKDVVLIDDDVECTMVEKRVLTLAAENPFLTHLICTFQTKDHLFFVMEFLNGGDLMYHIQDKGRFELYRATFYAAEIMCGLQFLHSKGIIYRDLKLDNVLLDRDGHIKIADFGMCKENIFGESRASTFCGTPDYIAPEILQGLKYTFSVDWWSFGVLLYEMLIGQSPFHGDDEDELFESIRVDTPHYPRWITKESKDILEKLFEREPTKRLGVTGNIKIHPFFKTINWTLLEKRRLEPPFRPKVKSPRDYSNFDQEFLNEKARLSYSDKNLIDSMDQSAFAGFSFVNPKFEHLLED

1290 NP_005400.1 NP_001828.1

>NP_005400.1

MSVKGMAIALAVILCATVVQGFPMFKRGRCLCIGPGVKAVKVADIEKASIMYPSNNCDKIEVIITLKENKGQRCLNPKSKQARLIIKKVERKNF

>NP_001828.1

MTTSLDTVETFGTTSYYDDVGLLCEKADTRALMAQFVPPLYSLVFTVGLLGNVVVVMILIKYRRLRIMTNIYLLNLAISDLLFLVTLPFWIHYVRGHNWVFGHGMCKLLSGFYHTGLYSEIFFIILLTIDRYLAIVHAVFALRARTVTFGVITSIVTWGLAVLAALPEFIFYETEELFEETLCSALYPEDTVYSWRHFHTLRMTIFCLVLPLLVMAICYTGIIKTLLRCPSKKKYKAIRLIFVIMAVFFIFWTPYNVAILLSSYQSILFGNDCERSKHLDLVMLVTEVIAYSHCCMNPVIYAFVGERFRKYLRHFFHRHLLMHLGRYIPFLPSEKLERTSSVSPSTAEPELSIVF

1291 NP_002406.1 NP_001020330.1

>NP_002406.1

MPMFIVNTNVPRASVPDGFLSELTQQLAQATGKPPQYIAVHVVPDQLMAFGGSSEPCALCSLHSIGKIGGAQNRSYSKLLCGLLAERLRISPDRVYINYYDMNAANVGWNNSTFA

>NP_001020330.1

MHRRRSRSCREDQKPVMDDQRDLISNNEQLPMLGRRPGAPESKCSRGALYTGFSILVTLLLAGQATTAYFLYQQQGRLDKLTVTSQNLQLENLRMKLPKPPKPVSKMRMATPLLMQALPMGALPQGPMQNATKYGNMTEDHVMHLLQNADPLKVYPPLKGSFPENLRHLKNTMETIDWKVFESWMHHWLLFEMSRHSLEQKPTDAPPKVLTKCQEEVSHIPAVHPGSFRPKCDENGNYLPLQCYGSIGYCWCVFPNGTEVPNTRSRGHHNCSESLELEDPSSGLGVTKQDLGPVPM

1292 NP_005810.1 NP_003816.2

>NP_005810.1

MSMEDPFFVVKGEVQKAVNTAQGLFQRWTELLQDPSTATREEIDWTTNELRNNLRSIEWDLEDLDETISIVEANPRKFNLDATELSIRKAFITSTRQVVRDMKDQMSTSSVQALAERKNRQALLGDSGSQNWSTGTTDKYGRLDRELQRANSHFIEEQQAQQQLIVEQQDEQLELVSGSIGVLKNMSQRIGGELEEQAVMLEDFSHELESTQSRLDNVMKKLAKVSHMTSDRRQWCAIAILFAVLLVVLILFLVL

>NP_003816.2

MDNLSSEEIQQRAHQITDESLESTRRILGLAIESQDAGIKTITMLDEQKEQLNRIEEGLDQINKDMRETEKTLTELNKCCGLCVCPCNRTKNFESGKAYKTTWGDGGENSPCNVVSKQPGPVTNGQLQQPTTGAASGGYIKRITNDAREDEMEENLTQVGSILGNLKDMALNIGNEIDAQNPQIKRITDKADTNRDRIDIANARAKKLIDS

1293 NP_000525.1 NP_000240.1

>NP_000525.1

MKQLPAATVRLLSSSQIITSVVSVVKELIENSLDAGATSVDVKLENYGFDKIEVRDNGEGIKAVDAPVMAMKYYTSKINSHEDLENLTTYGFRGEALGSICCIAEVLITTRTAADNFSTQYVLDGSGHILSQKPSHLGQGTTVTALRLFKNLPVRKQFYSTAKKCKDEIKKIQDLLMSFGILKPDLRIVFVHNKAVIWQKSRVSDHKMALMSVLGTAVMNNMESFQYHSEESQIYLSGFLPKCDADHSFTSLSTPERSFIFINSRPVHQKDILKLIRHHYNLKCLKESTRLYPVFFLKIDVPTADVDVNLTPDKSQVLLQNKESVLIALENLMTTCYGPLPSTNSYENNKTDVSAADIVLSKTAETDVLFNKVESSGKNYSNVDTSVIPFQNDMHNDESGKNTDDCLNHQISIGDFGYGHCSSEISNIDKNTKNAFQDISMSNVSWENSQTEYSKTCFISSVKHTQSENGNKDHIDESGENEEEAGLENSSEISADEWSRGNILKNSVGENIEPVKILVPEKSLPCKVSNNNYPIPEQMNLNEDSCNKKSNVIDNKSGKVTAYDLLSNRVIKKPMSASALFVQDHRPQFLIENPKTSLEDATLQIEELWKTLSEEEKLKYEEKATKDLERYNSQMKRAIEQESQMSLKDGRKKIKPTSAWNLAQKHKLKTSLSNQPKLDELLQSQIEKRRSQNIKMVQIPFSMKNLKINFKKQNKVDLEEKDEPCLIHNLRFPDAWLMTSKTEVMLLNPYRVEEALLFKRLLENHKLPAEPLEKPIMLTESLFNGSHYLDVLYKMTADDQRYSGSTYLSDPRLTANGFKIKLIPGVSITENYLEIEGMANCLPFYGVADLKEILNAILNRNAKEVYECRPRKVISYLEGEAVRLSRQLPMYLSKEDIQDIIYRMKHQFGNEIKECVHGRPFFHHLTYLPETT

>NP_000240.1

MSFVAGVIRRLDETVVNRIAAGEVIQRPANAIKEMIENCLDAKSTSIQVIVKEGGLKLIQIQDNGTGIRKEDLDIVCERFTTSKLQSFEDLASISTYGFRGEALASISHVAHVTITTKTADGKCAYRASYSDGKLKAPPKPCAGNQGTQITVEDLFYNIATRRKALKNPSEEYGKILEVVGRYSVHNAGISFSVKKQGETVADVRTLPNASTVDNIRSIFGNAVSRELIEIGCEDKTLAFKMNGYISNANYSVKKCIFLLFINHRLVESTSLRKAIETVYAAYLPKNTHPFLYLSLEISPQNVDVNVHPTKHEVHFLHEESILERVQQHIESKLLGSNSSRMYFTQTLLPGLAGPSGEMVKSTTSLTSSSTSGSSDKVYAHQMVRTDSREQKLDAFLQPLSKPLSSQPQAIVTEDKTDISSGRARQQDEEMLELPAPAEVAAKNQSLEGDTTKGTSEMSEKRGPTSSNPRKRHREDSDVEMVEDDSRKEMTAACTPRRRIINLTSVLSLQEEINEQGHEVLREMLHNHSFVGCVNPQWALAQHQTKLYLLNTTKLSEELFYQILIYDFANFGVLRLSEPAPLFDLAMLALDSPESGWTEEDGPKEGLAEYIVEFLKKKAEMLADYFSLEIDEEGNLIGLPLLIDNYVPPLEGLPIFILRLATEVNWDEEKECFESLSKECAMFYSIRKQYISEESTLSGQQSEVPGSIPNSWKWTVEHIVYKALRSHILPPKHFTEDGNILQLANLPDLYKVFERC

1294 NP_005875.1 NP_002204.2

>NP_005875.1

MFGKRKKRVEISAPSNFEHRVHTGFDQHEQKFTGLPRQWQSLIEESARRPKPLVDPACITSIQPGAPKTIVRGSKGAKDGALTLLLDEFENMSVTRSNSLRRDSPPPPARARQENGMPEEPATTARGGPGKAGSRGRFAGHSEAGGGSGDRRRAGPEKRPKSSREGSGGPQESSRDKRPLSGPDVGTPQPAGLASGAKLAAGRPFNTYPRADTDHPSRGAQGEPHDVAPNGPSAGGLAIPQSSSSSSRPPTRARGAPSPGVLGPHASEPQLAPPACTPAAPAVPGPPGPRSPQREPQRVSHEQFRAALQLVVDPGDPRSYLDNFIKIGEGSTGIVCIATVRSSGKLVAVKKMDLRKQQRRELLFNEVVIMRDYQHENVVEMYNSYLVGDELWVVMEFLEGGALTDIVTHTRMNEEQIAAVCLAVLQALSVLHAQGVIHRDIKSDSILLTHDGRVKLSDFGFCAQVSKEVPRRKSLVGTPYWMAPELISRLPYGPEVDIWSLGIMVIEMVDGEPPYFNEPPLKAMKMIRDNLPPRLKNLHKVSPSLKGFLDRLLVRDPAQRATAAELLKHPFLAKAGPPASIVPLMRQNRTR

>NP_002204.2

MPRAPAPLYACLLGLCALLPRLAGLNICTSGSATSCEECLLIHPKCAWCSKEDFGSPRSITSRCDLRANLVKNGCGGEIESPASSFHVLRSLPLSSKGSGSAGWDVIQMTPQEIAVNLRPGDKTTFQLQVRQVEDYPVDLYYLMDLSLSMKDDLDNIRSLGTKLAEEMRKLTSNFRLGFGSFVDKDISPFSYTAPRYQTNPCIGYKLFPNCVPSFGFRHLLPLTDRVDSFNEEVRKQRVSRNRDAPEGGFDAVLQAAVCKEKIGWRKDALHLLVFTTDDVPHIALDGKLGGLVQPHDGQCHLNEANEYTASNQMDYPSLALLGEKLAENNINLIFAVTKNHYMLYKNFTALIPGTTVEILDGDSKNIIQLIINAYNSIRSKVELSVWDQPEDLNLFFTATCQDGVSYPGQRKCEGLKIGDTASFEVSLEARSCPSRHTEHVFALRPVGFRDSLEVGVTYNCTCGCSVGLEPNSARCNGSGTYVCGLCECSPGYLGTRCECQDGENQSVYQNLCREAEGKPLCSGRGDCSCNQCSCFESEFGKIYGPFCECDNFSCARNKGVLCSGHGECHCGECKCHAGYIGDNCNCSTDISTCRGRDGQICSERGHCLCGQCQCTEPGAFGEMCEKCPTCPDACSTKRDCVECLLLHSGKPDNQTCHSLCRDEVITWVDTIVKDDQEAVLCFYKTAKDCVMMFTYVELPSGKSNLTVLREPECGNTPNAMTILLAVVGSILLVGLALLAIWKLLVTIHDRREFAKFQSERSRARYEMASNPLYRKPISTHTVDFTFNKFNKSYNGTVD

1295 NP_006097.1 NP_003205.2

>NP_006097.1

MDPGQQPPPQPAPQGQGQPPSQPPQGQGPPSGPGQPAPAATQAAPQAPPAGHQIVHVRGDSETDLEALFNAVMNPKTANVPQTVPMRLRKLPDSFFKPPEPKSHSRQASTDAGTAGALTPQHVRAHSSPASLQLGAVSPGTLTPTGVVSGPAATPTAQHLRQSSFEIPDDVPLPAGWEMAKTSSGQRYFLNHIDQTTTWQDPRKAMLSQMNVTAPTSPPVQQNMMNSASAMNQRISQSAPVKQPPPLAPQSPQGGVMGGSNSNQQQQMRLQQLQMEKERLRLKQQELLRQVRPQELALRSQLPTLEQDGGTQNPVSSPGMSQELRTMTTNSSDPFLNSGTYHSRDESTDSGLSMSSYSVPRTPDDFLNSVDEMDTGDTINQSTLPSQQNRFPDYLEAIPGTNVDLGTLEGDGMNIEGEELMPSLQEALSSDILNDMESVLAATKLDKESFLTWL

>NP_003205.2

MASNSWNASSSPGEAREDGPEGLDKGLDNDAEGVWSPDIEQSFQEALAIYPPCGRRKIILSDEGKMYGRNELIARYIKLRTGKTRTRKQVSSHIQVLARKKVREYQVGIKAMNLDQVSKDKALQSMASMSSAQIVSASVLQNKFSPPSPLPQAVFSTSSRFWSSPPLLGQQPGPSQDIKPFAQPAYPIQPPLPPTLSSYEPLAPLPSAAASVPVWQDRTIASSRLRLLEYSAFMEVQRDPDTYSKHLFVHIGQTNPAFSDPPLEAVDVRQIYDKFPEKKGGLKELYEKGPPNAFFLVKFWADLNSTIQEGPGAFYGVSSQYSSADSMTISVSTKVCSFGKQVVEKVETEYARLENGRFVYRIHRSPMCEYMINFIHKLKHLPEKYMMNSVLENFTILQVVTSRDSQETLLVIAFVFEVSTSEHGAQHHVYKLVKD

1296 NP_057698.2 NP_001241.1

>NP_057698.2

MELGSCLEGGREAAEEEGEPEVKKRRLLCVEFASVASCDAAVAQCFLAENDWEMERALNSYFEPPVEESALERRPETISEPKTYVDLTNEETTDSTTSKISPSEDTQQENGSMFSLITWNIDGLDLNNLSERARGVCSYLALYSPDVIFLQEVIPPYYSYLKKRSSNYEIITGHEEGYFTAIMLKKSRVKLKSQEIIPFPSTKMMRNLLCVHVNVSGNELCLMTSHLESTRGHAAERMNQLKMVLKKMQEAPESATVIFAGDTNLRDREVTRCGGLPNNIVDVWEFLGKPKHCQYTWDTQMNSNLGITAACKLRFDRIFFRAAAEEGHIIPRSLDLLGLEKLDCGRFPSDHWGLLCNLDIIL

>NP_001241.1

MVRLPLQCVLWGCLLTAVHPEPPTACREKQYLINSQCCSLCQPGQKLVSDCTEFTETECLPCGESEFLDTWNRETHCHQHKYCDPNLGLRVQQKGTSETDTICTCEEGWHCTSEACESCVLHRSCSPGFGVKQIATGVSDTICEPCPVGFFSNVSSAFEKCHPWTSCETKDLVVQQAGTNKTDVVCGPQDRLRALVVIPIIFGILFAILLVLVFIKKVAKKPTNKAPHPKQEPQEINFPDDLPGSNTAAPVQETLHGCQPVTQEDGKESRISVQERQ

1297 NP_005234.1 NP_008944.1

>NP_005234.1

MASTDYSTYSQAAAQQGYSAYTAQPTQGYAQTTQAYGQQSYGTYGQPTDVSYTQAQTTATYGQTAYATSYGQPPTGYTTPTAPQAYSQPVQGYGTGAYDTTTATVTTTQASYAAQSAYGTQPAYPAYGQQPAATAPTRPQDGNKPTETSQPQSSTGGYNQPSLGYGQSNYSYPQVPGSYPMQPVTAPPSYPPTSYSSTQPTSYDQSSYSQQNTYGQPSSYGQQSSYGQQSSYGQQPPTSYPPQTGSYSQAPSQYSQQSSSYGQQSSFRQDHPSSMGVYGQESGGFSGPGENRSMSGPDNRGRGRGGFDRGGMSRGGRGGGRGGMGSAGERGGFNKPGGPMDEGPDLDLGPPVDPDEDSDNSAIYVQGLNDSVTLDDLADFFKQCGVVKMNKRTGQPMIHIYLDKETGKPKGDATVSYEDPPTAKAAVEWFDGKDFQGSKLKVSLARKKPPMNSMRGGLPPREGRGMPPPLRGGPGGPGGPGGPMGRMGGRGGDRGGFPPRGPRGSRGNPSGGGNVQHRAGDWQCPNPGCGNQNFAWRTECNQCKAPKPEGFLPPPFPPPGGDRGRGGPGGMRGGRGGLMDRGGPGGMFRGGRGGDRGGFRGGRGMDRGGFGGGRRGGPGGPPGPLMEQMGGRRGGRGGPGKMDKGEHRQERRDRPY

>NP_008944.1

MATASPRSDTSNNHSGRLQLQVTVSSAKLKRKKNWFGTAIYTEVVVDGEITKTAKSSSSSNPKWDEQLTVNVTPQTTLEFQVWSHRTLKADALLGKATIDLKQALLIHNRKLERVKEQLKLSLENKNGIAQTGELTVVLDGLVIEQENITNCSSSPTIEIQENGDALHENGEPSARTTARLAVEGTNGIDNHVPTSTLVQNSCCSYVVNGDNTPSSPSQVAARPKNTPAPKPLASEPADDTVNGESSSFAPTDNASVTGTPVVSEENALSPNCTSTTVEDPPVQEILTSSENNECIPSTSAELESEARSILEPDTSNSRSSSAFEAAKSRQPDGCMDPVRQQSGNANTETLPSGWEQRKDPHGRTYYVDHNTRTTTWERPQPLPPGWERRVDDRRRVYYVDHNTRTTTWQRPTMESVRNFEQWQSQRNQLQGAMQQFNQRYLYSASMLAAENDPYGPLPPGWEKRVDSTDRVYFVNHNTKTTQWEDPRTQGLQNEEPLPEGWEIRYTREGVRYFVDHNTRTTTFKDPRNGKSSVTKGGPQIAYERGFRWKLAHFRYLCQSNALPSHVKINVSRQTLFEDSFQQIMALKPYDLRRRLYVIFRGEEGLDYGGLAREWFFLLSHEVLNPMYCLFEYAGKNNYCLQINPASTINPDHLSYFCFIGRFIAMALFHGKFIDTGFSLPFYKRMLSKKLTIKDLESIDTEFYNSLIWIRDNNIEECGLEMYFSVDMEILGKVTSHDLKLGGSNILVTEENKDEYIGLMTEWRFSRGVQEQTKAFLDGFNEVVPLQWLQYFDEKELEVMLCGMQEVDLADWQRNTVYRHYTRNSKQIIWFWQFVKETDNEVRMRLLQFVTGTCRLPLGGFAELMGSNGPQKFCIEKVGKDTWLPRSHTCFNRLDLPPYKSYEQLKEKLLFAIEETEGFGQE

1298 NP_510867.1 NP_001750.1

>NP_510867.1

MSEPAGDVRQNPCGSKACRRLFGPVDSEQLSRDCDALMAGCIQEARERWNFDFVTETPLEGDFAWERVRGLGLPKLYLPTGPRRGRDELGGGRRPGTSPALLQGTAEEDHVDLSLSCTLVPRSGEQAEGSPGGPGDSQGRKRRQTSMTDFYHSKRRLIFSKRKP

>NP_001750.1

MELLCHEVDPVRRAVRDRNLLRDDRVLQNLLTIEERYLPQCSYFKCVQKDIQPYMRRMVATWMLEVCEEQKCEEEVFPLAMNYLDRFLAGVPTPKSHLQLLGAVCMFLASKLKETSPLTAEKLCIYTDNSIKPQELLEWELVVLGKLKWNLAAVTPHDFIEHILRKLPQQREKLSLIRKHAQTFIALCATDFKFAMYPPSMIATGSVGAAICGLQQDEEVSSLTCDALTELLAKITNTDVDCLKACQEQIEAVLLNSLQQYRQDQRDGSKSEDELDQASTPTDVRDIDL

1299 NP_002266.2 NP_001003690.1

>NP_002266.2

MTTTFLQTSSSTFGGGSTRGGSLLAGGGGFGGGSLSGGGGSRSISASSARFVSSGSGGGYGGGMRVCGFGGGAGSVFGGGFGGGVGGGFGGGFGGGDGGLLSGNEKITMQNLNDRLASYLDKVRALEEANADLEVKIHDWYQKQTPASPECDYSQYFKTIEELRDKIMATTIDNSRVILEIDNARLAADDFRLKYENELALRQGVEADINGLRRVLDELTLARTDLEMQIEGLNEELAYLKKNHEEEMKEFSSQLAGQVNVEMDAAPGVDLTRVLAEMREQYEAMAEKNRRDVEAWFFSKTEELNKEVASNTEMIQTSKTEITDLRRTMQELEIELQSQLSMKAGLENSLAETECRYATQLQQIQGLIGGLEAQLSELRCEMEAQNQEYKMLLDIKTRLEQEIATYRSLLEGQDAKMAGIGIREASSGGGGSSSNFHINVEESVDGQVVSSHKREI

>NP_001003690.1

MARVPLGRSLTLSPRLEHNGMTSAHHNFRLPGSRDSPASASQVAEIIDLEWYEKSEETHASQIELLETSSTQEPLNASEAFCPRDCMVPVVFPGPVSQEGCCQFTCELLKHIMYQRQQLPLPYEQLKHFYRKPSPQAEEMLKKKPRATTEVSSRKCQQALAELESVLSHLEDFFARTLVPRVLILLGGNALSPKEFYELDLSLLAPYSVDQSLSTAACLRRLFRAIFMADAFSELQAPPLMGTVVMAQGHRNCGEDWFRPKLNYRVPSRGHKLTVTLSCGRPSIRTTAWEDYIWFQAPVTFKGFRE

1300 NP_006501.1 NP_115528.3

>NP_006501.1

MASGSVAECLQQETTCPVCLQYFAEPMMLDCGHNICCACLARCWGTAETNVSCPQCRETFPQRHMRPNRHLANVTQLVKQLRTERPSGPGGEMGVCEKHREPLKLYCEEDQMPICVVCDRSREHRGHSVLPLEEAVEGFKEQIQNQLDHLKRVKDLKKRRRAQGEQARAELLSLTQMEREKIVWEFEQLYHSLKEHEYRLLARLEELDLAIYNSINGAITQFSCNISHLSSLIAQLEEKQQQPTRELLQDIGDTLSRAERIRIPEPWITPPDLQEKIHIFAQKCLFLTESLKQFTEKMQSDMEKIQELREAQLYSVDVTLDPDTAYPSLILSDNLRQVRYSYLQQDLPDNPERFNLFPCVLGSPCFIAGRHYWEVEVGDKAKWTIGVCEDSVCRKGGVTSAPQNGFWAVSLWYGKEYWALTSPMTALPLRTPLQRVGIFLDYDAGEVSFYNVTERCHTFTFSHATFCGPVRPYFSLSYSGGKSAAPLIICPMSGIDGFSGHVGNHGHSMETSP

>NP_115528.3

MAHHLPAAMESHQDFRSIKAKFQASQPEPSDLPKKPPKPEFGKLKKFSQPELSEHPKKAPLPEFGAVSLKPPPPEVTDLPKKPPPPEVTDLPKKPPPPEVTDLPKKPPPPEVTDLPKKPSKLELSDLSKKFPQLGATPFPRKPLQPEVGEAPLKASLPEPGAPARKPLQPDELSHPARPPSEPKSGAFPRKLWQPEAGEATPRSPQPELSTFPKKPAQPEFNVYPKKPPQPQVGGLPKKSVPQPEFSEAAQTPLWKPQSSEPKRDSSAFPKKASQPPLSDFPKKPPQPELGDLTRTSSEPEVSVLPKRPRPAEFKALSKKPPQPELGGLPRTSSEPEFNSLPRKLLQPERRGPPRKFSQPEPSAVLKRHPQPEFFGDLPRKPPLPSSASESSLPAAVAGFSSRHPLSPGFGAAGTPRWRSGGLVHSGGARPGLRPSHPPRRRPLPPASSLGHPPAKPPLPPGPVDMQSFRRPSAASIDLRRTRSAAGLHFQDRQPEDIPVPDEIYELYDDVEPRDDSSPSPKGRDEAPSVQQAARRPPQDPALRKEKDPQPQQLPPMDPKLLKQLRKAEKAEREFRKKFKFEGEIVVHTKMMIDPNAKTRRGGGKHLGIRRGEILEVIEFTSNEEMLCRDPKGKYGYVPRTALLPLETEVYDDVDFCDPLENQPLPLGR

1301 NP_001895.1 NP_000438.1

>NP_001895.1

MATQADLMELDMAMEPDRKAAVSHWQQQSYLDSGIHSGATTTAPSLSGKGNPEEEDVDTSQVLYEWEQGFSQSFTQEQVADIDGQYAMTRAQRVRAAMFPETLDEGMQIPSTQFDAAHPTNVQRLAEPSQMLKHAVVNLINYQDDAELATRAIPELTKLLNDEDQVVVNKAAVMVHQLSKKEASRHAIMRSPQMVSAIVRTMQNTNDVETARCTAGTLHNLSHHREGLLAIFKSGGIPALVKMLGSPVDSVLFYAITTLHNLLLHQEGAKMAVRLAGGLQKMVALLNKTNVKFLAITTDCLQILAYGNQESKLIILASGGPQALVNIMRTYTYEKLLWTTSRVLKVLSVCSSNKPAIVEAGGMQALGLHLTDPSQRLVQNCLWTLRNLSDAATKQEGMEGLLGTLVQLLGSDDINVVTCAAGILSNLTCNNYKNKMMVCQVGGIEALVRTVLRAGDREDITEPAICALRHLTSRHQEAEMAQNAVRLHYGLPVVVKLLHPPSHWPLIKATVGLIRNLALCPANHAPLREQGAIPRLVQLLVRAHQDTQRRTSMGGTQQQFVEGVRMEEIVEGCTGALHILARDVHNRIVIRGLNTIPLFVQLLYSPIENIQRVAAGVLCELAQDKEAAEAIEAEGATAPLTELLHSRNEGVATYAAAVLFRMSEDKPQDYKKRLSVELTSSLFRTEPMAWNETADLGLDIGAQGEPLGYRQDDPSYRSFHSGGYGQDALGMDPMMEHEMGGHHPGADYPVDGLPDLGHAQDLMDGLPPGDSNQLAWFDTDL

>NP_000438.1

MLTFMASDSEEEVCDERTSLMSAESPTPRSCQEGRQGPEDGENTAQWRSQENEEDGEEDPDRYVCSGVPGRPPGLEEELTLKYGAKHVIMLFVPVTLCMIVVVATIKSVRFYTEKNGQLIYTTFTEDTPSVGQRLLNSVLNTLIMISVIVVMTIFLVVLYKYRCYKFIHGWLIMSSLMLLFLFTYIYLGEVLKTYNVAMDYPTLLLTVWNFGAVGMVCIHWKGPLVLQQAYLIMISALMALVFIKYLPEWSAWVILGAISVYDLVAVLCPKGPLRMLVETAQERNEPIFPALIYSSAMVWTVGMAKLDPSSQGALQLPYDPEMEEDSYDSFGEPSYPEVFEPPLTGYPGEELEEEEERGVKLGLGDFIFYSVLVGKAAATGSGDWNTTLACFVAILIGLCLTLLLLAVFKKALPALPISITFGLIFYFSTDNLVRPFMDTLASHQLYI

1302 NP_002782.1 NP_002779.1

>NP_002782.1

MSRGSSAGFDRHITIFSPEGRLYQVEYAFKAINQGGLTSVAVRGKDCAVIVTQKKVPDKLLDSSTVTHLFKITENIGCVMTGMTADSRSQVQRARYEAANWKYKYGYEIPVDMLCKRIADISQVYTQNAEMRPLGCCMILIGIDEEQGPQVYKCDPAGYYCGFKATAAGVKQTESTSFLEKKVKKKFDWTFEQTVETAITCLSTVLSIDFKPSEIEVGVVTVENPKFRILTEAEIDAHLVALAERD

>NP_002779.1

MSSIGTGYDLSASTFSPDGRVFQVEYAMKAVENSSTAIGIRCKDGVVFGVEKLVLSKLYEEGSNKRLFNVDRHVGMAVAGLLADARSLADIAREEASNFRSNFGYNIPLKHLADRVAMYVHAYTLYSAVRPFGCSFMLGSYSVNDGAQLYMIDPSGVSYGYWGCAIGKARQAAKTEIEKLQMKEMTCRDIVKEVAKIIYIVHDEVKDKAFELELSWVGELTNGRHEIVPKDIREEAEKYAKESLKEEDESDDDNM

1303 NP_002458.2 NP_002886.2

>NP_002458.2

MDFFRVVENQQPPATMPLNVSFTNRNYDLDYDSVQPYFYCDEEENFYQQQQQSELQPPAPSEDIWKKFELLPTPPLSPSRRSGLCSPSYVAVTPFSLRGDNDGGGGSFSTADQLEMVTELLGGDMVNQSFICDPDDETFIKNIIIQDCMWSGFSAAAKLVSEKLASYQAARKDSGSPNPARGHSVCSTSSLYLQDLSAAASECIDPSVVFPYPLNDSSSPKSCASQDSSAFSPSSDSLLSSTESSPQGSPEPLVLHEETPPTTSSDSEEEQEDEEEIDVVSVEKRQAPGKRSESGSPSAGGHSKPPHSPLVLKRCHVSTHQHNYAAPPSTRKDYPAAKRVKLDSVRVLRQISNNRKCTSPRSSDTEENVKRRTHNVLERQRRNELKRSFFALRDQIPELENNEKAPKVVILKKATAYILSVQAEEQKLISEEDLLRKRREQLKHKLEQLRNSCA

>NP_002886.2

MFEDKPHAEGAAVVAAAGEALQALCQELNLDEGSAAEALDDFTAIRGNYSLEGEVTHWLACSLYVACRKSIIPTVGKGIMEGNCVSLTRILRSAKLSLIQFFSKMKKWMDMSNLPQEFRERIERLERNFEVSTVIFKKYEPIFLDIFQNPYEEPPKLPRSRKQRRIPCSVKDLFNFCWTLFVYTKGNFRMIGDDLVNSYHLLLCCLDLIFANAIMCPNRQDLLNPSFKGLPSDFHTADFTASEEPPCIIAVLCELHDGLLVEAKGIKEHYFKPYISKLFDRKILKGECLLDLSSFTDNSKAVNKEYEEYVLTVGDFDERIFLGADAEEEIGTPRKFTRDTPLGKLTAQANVEYNLQQHFEKKRSFAPSTPLTGRRYLREKEAVITPVASATQSVSRLQSIVAGLKNAPSDQLINIFESCVRNPVENIMKILKGIGETFCQHYTQSTDEQPGSHIDFAVNRLKLAEILYYKILETVMVQETRRLHGMDMSVLLEQDIFHRSLMACCLEIVLFAYSSPRTFPWIIEVLNLQPFYFYKVIEVVIRSEEGLSRDMVKHLNSIEEQILESLAWSHDSALWEALQVSANKVPTCEEVIFPNNFETGNGGNVQGHLPLMPMSPLMHPRVKEVRTDSGSLRRDMQPLSPISVHERYSSPTAGSAKRRLFGEDPPKEMLMDKIITEGTKLKIAPSSSITAENVSILPGQTLLTMATAPVTGTTGHKVTIPLHGVANDAGEITLIPLSMNTNQESKVKSPVSLTAHSLIGASPKQTNLTKAQEVHSTGINRPKRTGSLALFYRKVYHLASVRLRDLCLKLDVSNELRRKIWTCFEFTLVHCPDLMKDRHLDQLLLCAFYIMAKVTKEERTFQEIMKSYRNQPQANSHVYRSVLLKSIPREVVAYNKNINDDFEMIDCDLEDATKTPDCSSGPVKEERGDLIKFYNTIYVGRVKSFALKYDLANQDHMMDAPPLSPFPHIKQQPGSPRRISQQHSIYISPHKNGSGLTPRSALLYKFNGSPSKSLKDINNMIRQGEQRTKKRVIAIDSDAESPAKRVCQENDDVLLKRLQDVVSERANH

1304 NP_001789.2 NP_001230.1

>NP_001789.2

MENFQKVEKIGEGTYGVVYKARNKLTGEVVALKKIRLDTETEGVPSTAIREISLLKELNHPNIVKLLDVIHTENKLYLVFEFLHQDLKKFMDASALTGIPLPLIKSYLFQLLQGLAFCHSHRVLHRDLKPQNLLINTEGAIKLADFGLARAFGVPVRTYTHEVVTLWYRAPEILLGCKYYSTAVDIWSLGCIFAEMVTRRALFPGDSEIDQLFRIFRTLGTPDEVVWPGVTSMPDYKPSFPKWARQDFSKVVPPLDEDGRSLLSQMLHYDPNKRISAKAALAHPFFQDVTKPVPHLRL

>NP_001230.1

MYHNSSQKRHWTFSSEEQLARLRADANRKFRCKAVANGKVLPNDPVFLEPHEEMTLCKYYEKRLLEFCSVFKPAMPRSVVGTACMYFKRFYLNNSVMEYHPRIIMLTCAFLACKVDEFNVSSPQFVGNLRESPLGQEKALEQILEYELLLIQQLNFHLIVHNPYRPFEGFLIDLKTRYPILENPEILRKTADDFLNRIALTDAYLLYTPSQIALTAILSSASRAGITMESYLSESLMLKENRTCLSQLLDIMKSMRNLVKKYEPPRSEEVAVLKQKLERCHSAELALNVITKKRKGYEDDDYVSKKSKHEEEEWTDDDLVESL

1305 NP_055908.1 NP_001560.2

>NP_055908.1

MAQGSHQIDFQVLHDLRQKFPEVPEVVVSRCMLQNNNNLDACCAVLSQESTRYLYGEGDLNFSDDSGISGLRNHMTSLNLDLQSQNIYHHGREGSRMNGSRTLTHSISDGQLQGGQSNSELFQQEPQTAPAQVPQGFNVFGMSSSSGASNSAPHLGFHLGSKGTSSLSQQTPRFNPIMVTLAPNIQTGRNTPTSLHIHGVPPPVLNSPQGNSIYIRPYITTPGGTTRQTQQHSGWVSQFNPMNPQQVYQPSQPGPWTTCPASNPLSHTSSQQPNQQGHQTSHVYMPISSPTTSQPPTIHSSGSSQSSAHSQYNIQNISTGPRKNQIEIKLEPPQRNNSSKLRSSGPRTSSTSSSVNSQTLNRNQPTVYIAASPPNTDELMSRSQPKVYISANAATGDEQVMRNQPTLFISTNSGASAASRNMSGQVSMGPAFIHHHPPKSRAIGNNSATSPRVVVTQPNTKYTFKITVSPNKPPAVSPGVVSPTFELTNLLNHPDHYVETENIQHLTDPTLAHVDRISETRKLSMGSDDAAYTQALLVHQKARMERLQRELEIQKKKLDKLKSEVNEMENNLTRRRLKRSNSISQIPSLEEMQQLRSCNRQLQIDIDCLTKEIDLFQARGPHFNPSAIHNFYDNIGFVGPVPPKPKDQRSIIKTPKTQDTEDDEGAQWNCTACTFLNHPALIRCEQCEMPRHF

>NP_001560.2

MAGGPGPGEPAAPGAQHFLYEVPPWVMCRFYKVMDALEPADWCQFAALIVRDQTELRLCERSGQRTASVLWPWINRNARVADLVHILTHLQLLRARDIITAWHPPAPLPSPGTTAPRPSSIPAPAEAEAWSPRKLPSSASTFLSPAFPGSQTHSGPELGLVPSPASLWPPPPSPAPSSTKPGPESSVSLLQGARPFPFCWPLCEISRGTHNFSEELKIGEGGFGCVYRAVMRNTVYAVKRLKENADLEWTAVKQSFLTEVEQLSRFRHPNIVDFAGYCAQNGFYCLVYGFLPNGSLEDRLHCQTQACPPLSWPQRLDILLGTARAIQFLHQDSPSLIHGDIKSSNVLLDERLTPKLGDFGLARFSRFAGSSPSQSSMVARTQTVRGTLAYLPEEYIKTGRLAVDTDTFSFGVVVLETLAGQRAVKTHGARTKYLKDLVEEEAEEAGVALRSTQSTLQAGLAADAWAAPIAMQIYKKHLDPRPGPCPPELGLGLGQLACCCLHRRAKRRPPMTQVYERLEKLQAVVAGVPGHSEAASCIPPSPQENSYVSSTGRAHSGAAPWQPLAAPSGASAQAAEQLQRGPNQPVESDESLGGLSAALRSWHLTPSCPLDPAPLREAGCPQGDTAGESSWGSGPGSRPTAVEGLALGSSASSSSEPPQIIINPARQKMVQKLALYEDGALDSLQLLSSSSLPGLGLEQDRQGPEESDEFQS

1306 NP_003143.2 NP_932170.1

>NP_003143.2

MAGWIQAQQLQGDALRQMQVLYGQHFPIEVRHYLAQWIESQPWDAIDLDNPQDRAQATQLLEGLVQELQKKAEHQVGEDGFLLKIKLGHYATQLQKTYDRCPLELVRCIRHILYNEQRLVREANNCSSPAGILVDAMSQKHLQINQTFEELRLVTQDTENELKKLQQTQEYFIIQYQESLRIQAQFAQLAQLSPQERLSRETALQQKQVSLEAWLQREAQTLQQYRVELAEKHQKTLQLLRKQQTIILDDELIQWKRRQQLAGNGGPPEGSLDVLQSWCEKLAEIIWQNRQQIRRAEHLCQQLPIPGPVEEMLAEVNATITDIISALVTSTFIIEKQPPQVLKTQTKFAATVRLLVGGKLNVHMNPPQVKATIISEQQAKSLLKNENTRNECSGEILNNCCVMEYHQATGTLSAHFRNMSLKRIKRADRRGAESVTEEKFTVLFESQFSVGSNELVFQVKTLSLPVVVIVHGSQDHNATATVLWDNAFAEPGRVPFAVPDKVLWPQLCEALNMKFKAEVQSNRGLTKENLVFLAQKLFNNSSSHLEDYSGLSVSWSQFNRENLPGWNYTFWQWFDGVMEVLKKHHKPHWNDGAILGFVNKQQAHDLLINKPDGTFLLRFSDSEIGGITIAWKFDSPERNLWNLKPFTTRDFSIRSLADRLGDLSYLIYVFPDRPKDEVFSKYYTPVLAKAVDGYVKPQIKQVVPEFVNASADAGGSSATYMDQAPSPAVCPQAPYNMYPQNPDHVLDQDGEFDLDETMDVARHVEELLRRPMDSLDSRLSPPAGLFTSARGSLS

>NP_932170.1

MKWKALFTAAILQAQLPITEAQSFGLLDPKLCYLLDGILFIYGVILTALFLRVKFSRSADAPAYQQGQNQLYNELNLGRREEYDVLDKRRGRDPEMGGKPQRRKNPQEGLYNELQKDKMAEAYSEIGMKGERRRGKGHDGLYQGLSTATKDTYDALHMQALPPR

1307 NP_005618.2 NP_004163.2

>NP_005618.2

MTVKTEAAKGTLTYSRMRGMVAILIAFMKQRRMGLNDFIQKIANNSYACKHPEVQSILKISQPQEPELMNANPSPPPSPSQQINLGPSSNPHAKPSDFHFLKVIGKGSFGKVLLARHKAEEVFYAVKVLQKKAILKKKEEKHIMSERNVLLKNVKHPFLVGLHFSFQTADKLYFVLDYINGGELFYHLQRERCFLEPRARFYAAEIASALGYLHSLNIVYRDLKPENILLDSQGHIVLTDFGLCKENIEHNSTTSTFCGTPEYLAPEVLHKQPYDRTVDWWCLGAVLYEMLYGLPPFYSRNTAEMYDNILNKPLQLKPNITNSARHLLEGLLQKDRTKRLGAKDDFMEIKSHVFFSLINWDDLINKKITPPFNPNVSGPNDLRHFDPEFTEEPVPNSIGKSPDSVLVTASVKEAAEAFLGFSYAPPTDSFL

>NP_004163.2

MTKSNGEEPKMGGRMERFQQGVSKRTLLAKKKVQNITKEDVKSYLFRNAFVLLTVTAVIVGTILGFTLRPYRMSYREVKYFSFPGELLMRMLQMLVLPLIISSLVTGMAALDSKASGKMGMRAVVYYMTTTIIAVVIGIIIVIIIHPGKGTKENMHREGKIVRVTAADAFLDLIRNMFPPNLVEACFKQFKTNYEKRSFKVPIQANETLVGAVINNVSEAMETLTRITEELVPVPGSVNGVNALGLVVFSMCFGFVIGNMKEQGQALREFFDSLNEAIMRLVAVIMWYAPVGILFLIAGKIVEMEDMGVIGGQLAMYTVTVIVGLLIHAVIVLPLLYFLVTRKNPWVFIGGLLQALITALGTSSSSATLPITFKCLEENNGVDKRVTRFVLPVGATINMDGTALYEALAAIFIAQVNNFELNFGQIITISITATAASIGAAGIPQAGLVTMVIVLTSVGLPTDDITLIIAVDWFLDRLRTTTNVLGDSLGAGIVEHLSRHELKNRDVEMGNSVIEENEMKKPYQLIAQDNETEKPIDSETKM

1308 NP_056292.1 NP_001319.1

>NP_056292.1

MKRRLDDQESPVYAAQQRRIPGSTEAFPHQHRVLAPAPPVYEAVSETMQSATGIQYSVTPSYQVSAMPQSSGSHGPAIAAVHSSHHHPTAVQPHGGQVVQSHAHPAPPVAPVQGQQQFQRLKVEDALSYLDQVKLQFGSQPQVYNDFLDIMKEFKSQSIDTPGVISRVSQLFKGHPDLIMGFNTFLPPGYKIEVQTNDMVNVTTPGQVHQIPTHGIQPQPQPPPQHPSQPSAQSAPAPAQPAPQPPPAKVSKPSQLQAHTPASQQTPPLPPYASPRSPPVQPHTPVTISLGTAPSLQNNQPVEFNHAINYVNKIKNRFQGQPDIYKAFLEILHTYQKEQRNAKEAGGNYTPALTEQEVYAQVARLFKNQEDLLSEFGQFLPDANSSVLLSKTTAEKVDSVRNDHGGTVKKPQLNNKPQRPSQNGCQIRRHPTGTTPPVKKKPKLLNLKDSSMADASKHGGGTESLFFDKVRKALRSAEAYENFLRCLVIFNQEVISRAELVQLVSPFLGKFPELFNWFKNFLGYKESVHLETYPKERATEGIAMEIDYASCKRLGSSYRALPKSYQQPKCTGRTPLCKEVLNDTWVSFPSWSEDSTFVSSKKTQYEEHIYRCEDERFELDVVLETNLATIRVLEAIQKKLSRLSAEEQAKFRLDNTLGGTSEVIHRKALQRIYADKAADIIDGLRKNPSIAVPIVLKRLKMKEEEWREAQRGFNKVWREQNEKYYLKSLDHQGINFKQNDTKVLRSKSLLNEIESIYDERQEQATEENAGVPVGPHLSLAYEDKQILEDAAALIIHHVKRQTGIQKEDKYKIKQIMHHFIPDLLFAQRGDLSDVEEEEEEEMDVDEATGAVKKHNGVGGSPPKSKLLFSNTAAQKLRGMDEVYNLFYVNNNWYIFMRLHQILCLRLLRICSQAERQIEEENREREWEREVLGIKRDKSDSPAIQLRLKEPMDVDVEDYYPAFLDMVRSLLDGNIDSSQYEDSLREMFTIHAYIAFTMDKLIQSIVRQLQHIVSDEICVQVTDLYLAENNNGATGGQLNTQNSRSLLESTYQRKAEQLMSDENCFKLMFIQSQGQVQLTIELLDTEEENSDDPVEAERWSDYVERYMNSDTTSPELREHLAQKPVFLPRNLRRIRKCQRGREQQEKEGKEGNSKKTMENVDSLDKLECRFKLNSYKMVYVIKSEDYMYRRTALLRAHQSHERVSKRLHQRFQAWVDKWTKEHVPREMAAETSKWLMGEGLEGLVPCTTTCDTETLHFVSINKYRVKYGTVFKAP

>NP_001319.1

MGSSHLLNKGLPLGVRPPIMNGPLHPRPLVALLDGRDCTVEMPILKDVATVAFCDAQSTQEIHEKVLNEAVGALMYHTITLTREDLEKFKALRIIVRIGSGFDNIDIKSAGDLGIAVCNVPAASVEETADSTLCHILNLYRRATWLHQALREGTRVQSVEQIREVASGAARIRGETLGIIGLGRVGQAVALRAKAFGFNVLFYDPYLSDGVERALGLQRVSTLQDLLFHSDCVTLHCGLNEHNHHLINDFTVKQMRQGAFLVNTARGGLVDEKALAQALKEGRIRGAALDVHESEPFSFSQGPLKDAPNLICTPHAAWYSEQASIEMREEAAREIRRAITGRIPDSLKNCVNKDHLTAATHWASMDPAVVHPELNGAAYRYPPGVVGVAPTGIPAAVEGIVPSAMSLSHGLPPVAHPPHAPSPGQTVKPEADRDHASDQL

1309 NP_009330.1 NP_005408.1

>NP_009330.1

MSQWYELQQLDSKFLEQVHQLYDDSFPMEIRQYLAQWLEKQDWEHAANDVSFATIRFHDLLSQLDDQYSRFSLENNFLLQHNIRKSKRNLQDNFQEDPIQMSMIIYSCLKEERKILENAQRFNQAQSGNIQSTVMLDKQKELDSKVRNVKDKVMCIEHEIKSLEDLQDEYDFKCKTLQNREHETNGVAKSDQKQEQLLLKKMYLMLDNKRKEVVHKIIELLNVTELTQNALINDELVEWKRRQQSACIGGPPNACLDQLQNWFTIVAESLQQVRQQLKKLEELEQKYTYEHDPITKNKQVLWDRTFSLFQQLIQSSFVVERQPCMPTHPQRPLVLKTGVQFTVKLRLLVKLQELNYNLKVKVLFDKDVNERNTVKGFRKFNILGTHTKVMNMEESTNGSLAAEFRHLQLKEQKNAGTRTNEGPLIVTEELHSLSFETQLCQPGLVIDLETTSLPVVVISNVSQLPSGWASILWYNMLVAEPRNLSFFLTPPCARWAQLSEVLSWQFSSVTKRGLNVDQLNMLGEKLLGPNASPDGLIPWTRFCKENINDKNFPFWLWIESILELIKKHLLPLWNDGCIMGFISKERERALLKDQQPGTFLLRFSESSREGAITFTWVERSQNGGEPDFHAVEPYTKKELSAVTFPDIIRNYKVMAAENIPENPLKYLYPNIDKDHAFGKYYSRPKEAPEPMELDGPKGTGYIKTELISVSEVHPSRLQTTDNLLPMSPEEFDEVSRIVGSVEFDSMMNTV

>NP_005408.1

MGSNKSKPKDASQRRRSLEPAENVHGAGGGAFPASQTPSKPASADGHRGPSAAFAPAAAEPKLFGGFNSSDTVTSPQRAGPLAGGVTTFVALYDYESRTETDLSFKKGERLQIVNNTEGDWWLAHSLSTGQTGYIPSNYVAPSDSIQAEEWYFGKITRRESERLLLNAENPRGTFLVRESETTKGAYCLSVSDFDNAKGLNVKHYKIRKLDSGGFYITSRTQFNSLQQLVAYYSKHADGLCHRLTTVCPTSKPQTQGLAKDAWEIPRESLRLEVKLGQGCFGEVWMGTWNGTTRVAIKTLKPGTMSPEAFLQEAQVMKKLRHEKLVQLYAVVSEEPIYIVTEYMSKGSLLDFLKGETGKYLRLPQLVDMAAQIASGMAYVERMNYVHRDLRAANILVGENLVCKVADFGLARLIEDNEYTARQGAKFPIKWTAPEAALYGRFTIKSDVWSFGILLTELTTKGRVPYPGMVNREVLDQVERGYRMPCPPECPESLHDLMCQCWRKEPEERPTFEYLQAFLEDYFTSTEPQYQPGENL

1310 NP_004475.1 NP_001997.4

>NP_004475.1

MAGTVRTACLVVAMLLSLDFPGQAQPPPPPPDATCHQVRSFFQRLQPGLKWVPETPVPGSDLQVCLPKGPTCCSRKMEEKYQLTARLNMEQLLQSASMELKFLIIQNAAVFQEAFEIVVRHAKNYTNAMFKNNYPSLTPQAFEFVGEFFTDVSLYILGSDINVDDMVNELFDSLFPVIYTQLMNPGLPDSALDINECLRGARRDLKVFGNFPKLIMTQVSKSLQVTRIFLQALNLGIEVINTTDHLKFSKDCGRMLTRMWYCSYCQGLMMVKPCGGYCNVVMQGCMAGVVEIDKYWREYILSLEELVNGMYRIYDMENVLLGLFSTIHDSIQYVQKNAGKLTTTIGKLCAHSQQRQYRSAYYPEDLFIDKKVLKVAHVEHEETLSSRRRELIQKLKSFISFYSALPGYICSHSPVAENDTLCWNGQELVERYSQKAARNGMKNQFNLHELKMKGPEPVVSQIIDKLKHINQLLRTMSMPKGRVLDKNLDEEGFESGDCGDDEDECIGGSGDGMIKVKNQLRFLAELAYDLDVDDAPGNSQQATPKDNEISTFHNLGNVHSPLKLLTSMAISVVCFFFLVH

>NP_001997.4

MVGVGGGDVEDVTPRPGGCQISGRAARGCNGIPGAAAWEAALPRRRPRRHPSVNPRSRAAGSPRTRGRRTEERPSGSRLGDRGRGRALPGGRLGGRGRGRAPERVGGRGRGRGTAAPRAAPAARGSRPGPAGTMAAGSITTLPALPEDGGSGAFPPGHFKDPKRLYCKNGGFFLRIHPDGRVDGVREKSDPHIKLQLQAEERGVVSIKGVCANRYLAMKEDGRLLASKCVTDECFFFERLESNNYNTYRSRKYTSWYVALKRTGQYKLGSKTGPGQKAILFLPMSAKS

1311 NP_009166.2 NP_005327.1

>NP_009166.2

MDEQALLGLNPNADSDFRQRALAYFEQLKISPDAWQVCAEALAQRTYSDDHVKFFCFQVLEHQVKYKYSELTTVQQQLIRETLISWLQAQMLNPQPEKTFIRNKAAQVFALLFVTEYLTKWPKFFFDILSVVDLNPRGVDLYLRILMAIDSELVDRDVVHTSEEARRNTLIKDTMREQCIPNLVESWYQILQNYQFTNSEVTCQCLEVVGAYVSWIDLSLIANDRFINMLLGHMSIEVLREEACDCLFEVVNKGMDPVDKMKLVESLCQVLQSAGFFSIDQEEDVDFLARFSKLVNGMGQSLIVSWSKLIKNGDIKNAQEALQAIETKVALMLQLLIHEDDDISSNIIGFCYDYLHILKQLTVLSDQQKANVEAIMLAVMKKLTYDEEYNFENEGEDEAMFVEYRKQLKLLLDRLAQVSPELLLASVRRVFSSTLQNWQTTRFMEVEVAIRLLYMLAEALPVSHGAHFSGDVSKASALQDMMRTLVTSGVSSYQHTSVTLEFFETVVRYEKFFTVEPQHIPCVLMAFLDHRGLRHSSAKVRSRTAYLFSRFVKSLNKQMNPFIEDILNRIQDLLELSPPENGHQSLLSSDDQLFIYETAGVLIVNSEYPAERKQALMRNLLTPLMEKFKILLEKLMLAQDEERQASLADCLNHAVGFASRTSKAFSNKQTVKQCGCSEVYLDCLQTFLPALSCPLQKDILRSGVRTFLHRMIICLEEEVLPFIPSASEHMLKDCEAKDLQEFIPLINQITAKFKIQVSPFLQQMFMPLLHAIFEVLLRPAEENDQSAALEKQMLRRSYFAFLQTVTGSGMSEVIANQGAENVERVLVTVIQGAVEYPDPIAQKTCFIILSKLVELWGGKDGPVGFADFVYKHIVPACFLAPLKQTFDLADAQTVLALSECAVTLKTIHLKRGPECVQYLQQEYLPSLQVAPEIIQEFCQALQQPDAKVFKNYLKVFFQRAKP

>NP_005327.1

MSSVAVLTQESFAEHRSGLVPQQIKVATLNSEEESDPPTYKDAFPPLPEKAACLESAQEPAGAWGNKIRPIKASVITQVFHVPLEERKYKDMNQFGEGEQAKICLEIMQRTGAHLELSLAKDQGLSIMVSGKLDAVMKARKDIVARLQTQASATVAIPKEHHRFVIGKNGEKLQDLELKTATKIQIPRPDDPSNQIKITGTKEGIEKARHEVLLISAEQDKRAVERLEVEKAFHPFIAGPYNRLVGEIMQETGTRINIPPPSVNRTEIVFTGEKEQLAQAVARIKKIYEEKKKKTTTIAVEVKKSQHKYVIGPKGNSLQEILERTGVSVEIPPSDSISETVILRGEPEKLGQALTEVYAKANSFTVSSVAAPSWLHRFIIGKKGQNLAKITQQMPKVHIEFTEGEDKITLEGPTEDVNVAQEQIEGMVKDLINRMDYVEINIDHKFHRHLIGKSGANINRIKDQYKVSVRIPPDSEKSNLIRIEGDPQGVQQAKRELLELASRMENERTKDLIIEQRFHRTIIGQKGERIREIRDKFPEVIINFPDPAQKSDIVQLRGPKNEVEKCTKYMQKMVADLVENSYSISVPIFKQFHKNIIGKGGANIKKIREESNTKIDLPAENSNSETIIITGKRANCEAARSRILSIQKDLANIAEVEVSIPAKLHNSLIGTKGRLIRSIMEECGGVHIHFPVEGSGSDTVVIRGPSSDVEKAKKQLLHLAEEKQTKSFTVDIRAKPEYHKFLIGKGGGKIRKVRDSTGARVIFPAAEDKDQDLITIIGKEDAVREAQKELEALIQNLDNVVEDSMLVDPKHHRHFVIRRGQVLREIAEEYGGVMVSFPRSGTQSDKVTLKGAKDCVEAAKKRIQEIIEDLEAQVTLECAIPQKFHRSVMGPKGSRIQQITRDFSVQIKFPDREENAVHSTEPVVQENGDEAGEGREAKDCDPGSPRRCDIIIISGRKEKCEAAKEALEALVPVTIEVEVPFDLHRYVIGQKGSGIRKMMDEFEVNIHVPAPELQSDIIAITGLAANLDRAKAGLLERVKELQAEQEDRALRSFKLSVTVDPKYHPKIIGRKGAVITQIRLEHDVNIQFPDKDDGNQPQDQITITGYEKNTEAARDAILRIVGELEQMVSEDVPLDHRVHARIIGARGKAIRKIMDEFKVDIRFPQSGAPDPNCVTVTGLPENVEEAIDHILNLEEEYLADVVDSEALQVYMKPPAHEEAKAPSRGFVVRDAPWTASSSEKAPDMSSSEEFPSFGAQVAPKTLPWGPKR

1312 NP_787062.1 NP_005554.1

>NP_787062.1

MAGSGCAWGAEPPRFLEAFGRLWQVQSRLGSGSSASVYRVRCCGNPGSPPGALKQFLPPGTTGAAASAAEYGFRKERAALEQLQGHRNIVTLYGVFTIHFSPNVPSRCLLLELLDVSVSELLLYSSHQGCSMWMIQHCARDVLEALAFLHHEGYVHADLKPRNILWSAENECFKLIDFGLSFKEGNQDVKYIQTDGYRAPEAELQNCLAQAGLQSDTECTSAVDLWSLGIILLEMFSGMKLKHTVRSQEWKANSSAIIDHIFASKAVVNAAIPAYHLRDLIKSMLHDDPSRRIPAEMALCSPFFSIPFAPHIEDLVMLPTPVLRLLNVLDDDYLENEEEYEDVVEDVKEECQKYGPVVSLLVPKENPGRGQVFVEYANAGDSKAAQKLLTGRMFDGKFVVATFYPLSAYKRGYLYQTLL

>NP_005554.1

MASSDIQVKELEKRASGQAFELILSPRSKESVPEFPLSPPKKKDLSLEEIQKKLEAAEERRKSHEAEVLKQLAEKREHEKEVLQKAIEENNNFSKMAEEKLTHKMEANKENREAQMAAKLERLREKDKHIEEVRKNKESKDPADETEAD

1313 NP_056979.1 NP_008827.2

>NP_056979.1

MNPSMKQKQEEIKENIKNSSVPRRTLKMIQPSASGSLVGRENELSAGLSKRKHRNDHLTSTTSSPGVIVPESSENKNLGGVTQESFDLMIKENPSSQYWKEVAEKRRKALYEALKENEKLHKEIEQKDNEIARLKKENKELAEVAEHVQYMAELIERLNGEPLDNFESLDNQEFDSEEETVEDSLVEDSEIGTCAEGTVSSSTDAKPCI

>NP_008827.2

MSSSYYVNALFSKYTAGASLFQNAEPTSCSFAPNSQRSGYGAGAGAFASTVPGLYNVNSPLYQSPFASGYGLGADAYGNLPCASYDQNIPGLCSDLAKGACDKTDEGALHGAAEANFRIYPWMRSSGPDRKRGRQTYTRYQTLELEKEFHFNRYLTRRRRIEIAHALCLTERQIKIWFQNRRMKWKKEHKDEGPTAAAAPEGAVPSAAATAAADKADEEDDDEEEEDEEE

1314 NP_005185.2 NP_005219.2

>NP_005185.2

MQRLVAWDPACLPLPPPPPAFKSMEVANFYYEADCLAAAYGGKAAPAAPPAARPGPRPPAGELGSIGDHERAIDFSPYLEPLGAPQAPAPATATDTFEAAPPAPAPAPASSGQHHDFLSDLFSDDYGGKNCKKPAEYGYVSLGRLGAAKGALHPGCFAPLHPPPPPPPPPAELKAEPGFEPADCKRKEEAGAPGGGAGMAAGFPYALRAYLGYQAVPSGSSGSLSTSSSSSPPGTPSPADAKAPPTACYAGAAPAPSQVKSKAKKTVDKHSDEYKIRRERNNIAVRKSRDKAKMRNLETQHKVLELTAENERLQKKVEQLSRELSTLRNLFKQLPEPLLASSGHC

>NP_005219.2

MRPSGTAGAALLALLAALCPASRALEEKKVCQGTSNKLTQLGTFEDHFLSLQRMFNNCEVVLGNLEITYVQRNYDLSFLKTIQEVAGYVLIALNTVERIPLENLQIIRGNMYYENSYALAVLSNYDANKTGLKELPMRNLQEILHGAVRFSNNPALCNVESIQWRDIVSSDFLSNMSMDFQNHLGSCQKCDPSCPNGSCWGAGEENCQKLTKIICAQQCSGRCRGKSPSDCCHNQCAAGCTGPRESDCLVCRKFRDEATCKDTCPPLMLYNPTTYQMDVNPEGKYSFGATCVKKCPRNYVVTDHGSCVRACGADSYEMEEDGVRKCKKCEGPCRKVCNGIGIGEFKDSLSINATNIKHFKNCTSISGDLHILPVAFRGDSFTHTPPLDPQELDILKTVKEITGFLLIQAWPENRTDLHAFENLEIIRGRTKQHGQFSLAVVSLNITSLGLRSLKEISDGDVIISGNKNLCYANTINWKKLFGTSGQKTKIISNRGENSCKATGQVCHALCSPEGCWGPEPRDCVSCRNVSRGRECVDKCNLLEGEPREFVENSECIQCHPECLPQAMNITCTGRGPDNCIQCAHYIDGPHCVKTCPAGVMGENNTLVWKYADAGHVCHLCHPNCTYGCTGPGLEGCPTNGPKIPSIATGMVGALLLLLVVALGIGLFMRRRHIVRKRTLRRLLQERELVEPLTPSGEAPNQALLRILKETEFKKIKVLGSGAFGTVYKGLWIPEGEKVKIPVAIKELREATSPKANKEILDEAYVMASVDNPHVCRLLGICLTSTVQLITQLMPFGCLLDYVREHKDNIGSQYLLNWCVQIAKGMNYLEDRRLVHRDLAARNVLVKTPQHVKITDFGLAKLLGAEEKEYHAEGGKVPIKWMALESILHRIYTHQSDVWSYGVTVWELMTFGSKPYDGIPASEISSILEKGERLPQPPICTIDVYMIMVKCWMIDADSRPKFRELIIEFSKMARDPQRYLVIQGDERMHLPSPTDSNFYRALMDEEDMDDVVDADEYLIPQQGFFSSPSTSRTPLLSSLSATSNNSTVACIDRNGLQSCPIKEDSFLQRYSSDPTGALTEDSIDDTFLPVPEYINQSVPKRPAGSVQNPVYHNQPLNPAPSRDPHYQDPHSTAVGNPEYLNTVQPTCVNSTFDSPAHWAQKGSHQISLDNPDYQQDFFPKEAKPNGIFKGSTAENAEYLRVAPQSSEFIGA

1315 NP_002996.1 NP_002024.1

>NP_002996.1

MANCQIAILYQRFQRVVFGISQLLCFSALISELTNQKEVAAWTYHYSTKAYSWNISRKYCQNRYTDLVAIQNKNEIDYLNKVLPYYSSYYWIGIRKNNKTWTWVGTKKALTNEAENWADNEPNNKRNNEDCVEIYIKSPSAPGKWNDEHCLKKKHALCYTASCQDMSCSKQGECLETIGNYTCSCYPGFYGPECEYVRECGELELPQHVLMNCSHPLGNFSFNSQCSFHCTDGYQVNGPSKLECLASGIWTNKPPQCLAAQCPPLKIPERGNMICLHSAKAFQHQSSCSFSCEEGFALVGPEVVQCTASGVWTAPAPVCKAVQCQHLEAPSEGTMDCVHPLTAFAYGSSCKFECQPGYRVRGLDMLRCIDSGHWSAPLPTCEAISCEPLESPVHGSMDCSPSLRAFQYDTNCSFRCAEGFMLRGADIVRCDNLGQWTAPAPVCQALQCQDLPVPNEARVNCSHPFGAFRYQSVCSFTCNEGLLLVGASVLQCLATGNWNSVPPECQAIPCTPLLSPQNGTMTCVQPLGSSSYKSTCQFICDEGYSLSGPERLDCTRSGRWTDSPPMCEAIKCPELFAPEQGSLDCSDTRGEFNVGSTCHFSCNNGFKLEGPNNVECTTSGRWSATPPTCKGIASLPTPGLQCPALTTPGQGTMYCRHHPGTFGFNTTCYFGCNAGFTLIGDSTLSCRPSGQWTAVTPACRAVKCSELHVNKPIAMNCSNLWGNFSYGSICSFHCLEGQLLNGSAQTACQENGHWSTTVPTCQAGPLTIQEALTYFGGAVASTIGLIMGGTLLALLRKRFRQKDDGKCPLNPHSHLGTYGVFTNAAFDPSP

>NP_002024.1

MRRLWGAARKPSGAGWEKEWAEAPQEAPGAWSGRLGPGRSGRKGRAVPGWASWPAHLALAARPARHLGGAGQGPRPLHSGTAPFHSRASGERQRRLEPQLQHESRCRSSTPADAWRAEAALPVRAMGAPWGSPTAAAGGRRGWRRGRGLPWTVCVLAAAGLTCTALITYACWGQLPPLPWASPTPSRPVGVLLWWEPFGGRDSAPRPPPDCRLRFNISGCRLLTDRASYGEAQAVLFHHRDLVKGPPDWPPPWGIQAHTAEEVDLRVLDYEEAAAAAEALATSSPRPPGQRWVWMNFESPSHSPGLRSLASNLFNWTLSYRADSDVFVPYGYLYPRSHPGDPPSGLAPPLSRKQGLVAWVVSHWDERQARVRYYHQLSQHVTVDVFGRGGPGQPVPEIGLLHTVARYKFYLAFENSQHLDYITEKLWRNALLAGAVPVVLGPDRANYERFVPRGAFIHVDDFPSASSLASYLLFLDRNPAVYRRYFHWRRSYAVHITSFWDEPWCRVCQAVQRAGDRPKSIRNLASWFER

1316 NP_036580.2 NP_001008540.1

>NP_036580.2

MAVWIQAQQLQGEALHQMQALYGQHFPIEVRHYLSQWIESQAWDSVDLDNPQENIKATQLLEGLVQELQKKAEHQVGEDGFLLKIKLGHYATQLQNTYDRCPMELVRCIRHILYNEQRLVREANNGSSPAGSLADAMSQKHLQINQTFEELRLVTQDTENELKKLQQTQEYFIIQYQESLRIQAQFGPLAQLSPQERLSRETALQQKQVSLEAWLQREAQTLQQYRVELAEKHQKTLQLLRKQQTIILDDELIQWKRRQQLAGNGGPPEGSLDVLQSWCEKLAEIIWQNRQQIRRAEHLCQQLPIPGPVEEMLAEVNATITDIISALVTSTFIIEKQPPQVLKTQTKFAATVRLLVGGKLNVHMNPPQVKATIISEQQAKSLLKNENTRNDYSGEILNNCCVMEYHQATGTLSAHFRNMSLKRIKRSDRRGAESVTEEKFTILFESQFSVGGNELVFQVKTLSLPVVVIVHGSQDNNATATVLWDNAFAEPGRVPFAVPDKVLWPQLCEALNMKFKAEVQSNRGLTKENLVFLAQKLFNNSSSHLEDYSGLSVSWSQFNRENLPGRNYTFWQWFDGVMEVLKKHLKPHWNDGAILGFVNKQQAHDLLINKPDGTFLLRFSDSEIGGITIAWKFDSQERMFWNLMPFTTRDFSIRSLADRLGDLNYLIYVFPDRPKDEVYSKYYTPVPCESATAKAVDGYVKPQIKQVVPEFVNASADAGGGSATYMDQAPSPAVCPQAHYNMYPQNPDSVLDTDGDFDLEDTMDVARRVEELLGRPMDSQWIPHAQS

>NP_001008540.1

MSIPLPLLQIYTSDNYTEEMGSGDYDSMKEPCFREENANFNKIFLPTIYSIIFLTGIVGNGLVILVMGYQKKLRSMTDKYRLHLSVADLLFVITLPFWAVDAVANWYFGNFLCKAVHVIYTVNLYSSVLILAFISLDRYLAIVHATNSQRPRKLLAEKVVYVGVWIPALLLTIPDFIFANVSEADDRYICDRFYPNDLWVVVFQFQHIMVGLILPGIVILSCYCIIISKLSHSKGHQKRKALKTTVILILAFFACWLPYYIGISIDSFILLEIIKQGCEFENTVHKWISITEALAFFHCCLNPILYAFLGAKFKTSAQHALTSVSRGSSLKILSKGKRGGHSSVSTESESSSFHSS

1317 NP_004837.1 NP_004086.1

>NP_004837.1

MNNKFDALKDDDSGDHDQNEENSTQKDGEKEKTERDKNQSSSKRKAVVPGPAEHPLQYNYTFWYSRRTPGRPTSSQSYEQNIKQIGTFASVEQFWRFYSHMVRPGDLTGHSDFHLFKEGIKPMWEDDANKNGGKWIIRLRKGLASRCWENLILAMLGEQFMVGEEICGAVVSVRFQEDIISIWNKTASDQATTARIRDTLRRVLNLPPNTIMEYKTHTDSIKMPGRLGPQRLLFQNLWKPRLNVP

>NP_004086.1

MSGGSSCSQTPSRAIPATRRVVLGDGVQLPPGDYSTTPGGTLFSTTPGGTRIIYDRKFLMECRNSPVTKTPPRDLPTIPGVTSPSSDEPPMEASQSHLRNSPEDKRAGGEESQFEMDI

1318 NP_000033.1 NP_005568.2

>NP_000033.1

MISPVLILFSSFLCHVAIAGRTCPKPDDLPFSTVVPLKTFYEPGEEITYSCKPGYVSRGGMRKFICPLTGLWPINTLKCTPRVCPFAGILENGAVRYTTFEYPNTISFSCNTGFYLNGADSAKCTEEGKWSPELPVCAPIICPPPSIPTFATLRVYKPSAGNNSLYRDTAVFECLPQHAMFGNDTITCTTHGNWTKLPECREVKCPFPSRPDNGFVNYPAKPTLYYKDKATFGCHDGYSLDGPEEIECTKLGNWSAMPSCKASCKLPVKKATVVYQGERVKIQEKFKNGMLHGDKVSFFCKNKEKKCSYTEDAQCIDGTIEVPKCFKEHSSLAFWKTDASDVKPC

>NP_005568.2

MEHKEVVLLLLLFLKSAAPEQSHVVQDCYHGDGQSYRGTYSTTVTGRTCQAWSSMTPHQHNRTTENYPNAGLIMNYCRNPDAVAAPYCYTRDPGVRWEYCNLTQCSDAEGTAVAPPTVTPVPSLEAPSEQAPTEQRPGVQECYHGNGQSYRGTYSTTVTGRTCQAWSSMTPHSHSRTPEYYPNAGLIMNYCRNPDAVAAPYCYTRDPGVRWEYCNLTQCSDAEGTAVAPPTVTPVPSLEAPSEQAPTEQRPGVQECYHGNGQSYRGTYSTTVTGRTCQAWSSMTPHSHSRTPEYYPNAGLIMNYCRNPDAVAAPYCYTRDPGVRWEYCNLTQCSDAEGTAVAPPTVTPVPSLEAPSEQAPTEQRPGVQECYHGNGQSYRGTYSTTVTGRTCQAWSSMTPHSHSRTPEYYPNAGLIMNYCRNPDAVAAPYCYTRDPGVRWEYCNLTQCSDAEGTAVAPPTVTPVPSLEAPSEQAPTEQRPGVQECYHGNGQSYRGTYSTTVTGRTCQAWSSMTPHSHSRTPEYYPNAGLIMNYCRNPDAVAAPYCYTRDPGVRWEYCNLTQCSDAEGTAVAPPTVTPVPSLEAPSEQAPTEQRPGVQECYHGNGQSYRGTYSTTVTGRTCQAWSSMTPHSHSRTPEYYPNAGLIMNYCRNPDAVAAPYCYTRDPGVRWEYCNLTQCSDAEGTAVAPPTVTPVPSLEAPSEQAPTEQRPGVQECYHGNGQSYRGTYSTTVTGRTCQAWSSMTPHSHSRTPEYYPNAGLIMNYCRNPDAVAAPYCYTRDPGVRWEYCNLTQCSDAEGTAVAPPTVTPVPSLEAPSEQAPTEQRPGVQECYHGNGQSYRGTYSTTVTGRTCQAWSSMTPHSHSRTPEYYPNAGLIMNYCRNPDPVAAPYCYTRDPSVRWEYCNLTQCSDAEGTAVAPPTITPIPSLEAPSEQAPTEQRPGVQECYHGNGQSYQGTYFITVTGRTCQAWSSMTPHSHSRTPAYYPNAGLIKNYCRNPDPVAAPWCYTTDPSVRWEYCNLTRCSDAEWTAFVPPNVILAPSLEAFFEQALTEETPGVQDCYYHYGQSYRGTYSTTVTGRTCQAWSSMTPHQHSRTPENYPNAGLTRNYCRNPDAEIRPWCYTMDPSVRWEYCNLTQCLVTESSVLATLTVVPDPSTEASSEEAPTEQSPGVQDCYHGDGQSYRGSFSTTVTGRTCQSWSSMTPHWHQRTTEYYPNGGLTRNYCRNPDAEISPWCYTMDPNVRWEYCNLTQCPVTESSVLATSTAVSEQAPTEQSPTVQDCYHGDGQSYRGSFSTTVTGRTCQSWSSMTPHWHQRTTEYYPNGGLTRNYCRNPDAEIRPWCYTMDPSVRWEYCNLTQCPVMESTLLTTPTVVPVPSTELPSEEAPTENSTGVQDCYRGDGQSYRGTLSTTITGRTCQSWSSMTPHWHRRIPLYYPNAGLTRNYCRNPDAEIRPWCYTMDPSVRWEYCNLTRCPVTESSVLTTPTVAPVPSTEAPSEQAPPEKSPVVQDCYHGDGRSYRGISSTTVTGRTCQSWSSMIPHWHQRTPENYPNAGLTENYCRNPDSGKQPWCYTTDPCVRWEYCNLTQCSETESGVLETPTVVPVPSMEAHSEAAPTEQTPVVRQCYHGNGQSYRGTFSTTVTGRTCQSWSSMTPHRHQRTPENYPNDGLTMNYCRNPDADTGPWCFTMDPSIRWEYCNLTRCSDTEGTVVAPPTVIQVPSLGPPSEQDCMFGNGKGYRGKKATTVTGTPCQEWAAQEPHRHSTFIPGTNKWAGLEKNYCRNPDGDINGPWCYTMNPRKLFDYCDIPLCASSSFDCGKPQVEPKKCPGSIVGGCVAHPHSWPWQVSLRTRFGKHFCGGTLISPEWVLTAAHCLKKSSRPSSYKVILGAHQEVNLESHVQEIEVSRLFLEPTQADIALLKLSRPAVITDKVMPACLPSPDYMVTARTECYITGWGETQGTFGTGLLKEAQLLVIENEVCNHYKYICAEHLARGTDSCQGDSGGPLVCFEKDKYILQGVTSWGLGCARPNKPGVYARVSRFVTWIEGMMRNN

1319 NP_002531.3 NP_077721.1

>NP_002531.3

MKDRSSTPPLHVHVDENTPVHVHIKKLPKPSATSSQKSHKRGMKGDTVNVRRSVRVKTKNPPHCLEITPPSSEKLVSVMRLSDLSTEDDDSGHCKMNRYDKKIDSLMNAVGCLKSEVKMQKGERQMAKRFLEERKEELEEVAHELAETEHENTVLRHNIERMKEEKDFTILQKKHLQQEKECLMSKLVEAEMDGAAAAKQVMALKDTIGKLKTEKQMTCTDINTLTRQKELLLQKLSTFEETNRTLRDLLREQHCKEDSERLMEQQGALLKRLAEADSEKARLLLLLQDKDKEVEELLQEIQCEKAQAKTASELSKSMESMRGHLQAQLRSKEAENSRLCMQIKNLERSGNQHKAEVEAIMEQLKELKQKGDRDKESLKKAIRAQKERAEKSEEYAEQLHVQLADKDLYVAEALSTLESWRSRYNQVVKEKGDLELEIIVLNDRVTDLVNQQQTLEEKMREDRDSLVERLHRQTAEYSAFKLENERLKASFAPMEDKLNQAHLEVQQLKASVKNYEGMIDNYKSQVMKTRLEADEVAAQLERCDKENKILKDEMNKEIEAARRQFQSQLADLQQLPDILKITEAKLAECQDQLQGYERKNIDLTAIISDLRSRIEHQGDKLEMAREKHQASQKENKQLSLKVDELERKLEATSAQNIEFLQVIAKREEAIHQSQLRLEEKTRECGTLARQLESAIEDARRQVEQTKEHALSKERAAQNKILDLETQLSRTKTELSQLRRSRDDADRRYQSRLQDLKDRLEQSESTNRSMQNYVQFLKSSYANVFGDGPYSTFLTSSPIRSRSPPA

>NP_077721.1

MAALSCLLDSVRRDIKKVDRELRQLRCIDEFSTRCLCDLYMHPYCCCDLHPYPYCLCYSKRSRSCGLCDLYPCCLCDYKLYCLRPSLRSLERKAIRAIEDEKRELAKLRRTTNRILASSCCSSNILGSVNVCGFEPDQVKVRVKDGKVCVSAERENRYDCLGSKKYSYMNICKEFSLPPCVDEKDVTYSYGLGSCVKIESPCYPCTSPCSPCSPCNPCNPCSPCNPCSPYDPCNPCYPCGSRFSCRKMIL

1320 NP_036252.1 NP_055382.2

>NP_036252.1

MVDYIVEYDYDAVHDDELTIRVGEIIRNVKKLQEEGWLEGELNGRRGMFPDNFVKEIKRETEFKDDSLPIKRERHGNVASLVQRISTYGLPAGGIQPHPQTKNIKKKTKKRQCKVLFEYIPQNEDELELKVGDIIDINEEVEEGWWSGTLNNKLGLFPSNFVKELEVTDDGETHEAQDDSETVLAGPTSPIPSLGNVSETASGSVTQPKKIRGIGFGDIFKEGSVKLRTRTSSSETEEKKPEKPLILQSLGPKTQSVEITKTDTEGKIKAKEYCRTLFAYEGTNEDELTFKEGEIIHLISKETGEAGWWRGELNGKEGVFPDNFAVQINELDKDFPKPKKPPPPAKAPAPKPELIAAEKKYFSLKPEEKDEKSTLEQKPSKPAAPQVPPKKPTPPTKASNLLRSSGTVYPKRPEKPVPPPPPIAKINGEVSSISSKFETEPVSKLKLDSEQLPLRPKSVDFDSLTVRTSKETDVVNFDDIASSENLLHLTANRPKMPGRRLPGRFNGGHSPTHSPEKILKLPKEEDSANLKPSELKKDTCYSPKPSVYLSTPSSASKANTTAFLTPLEIKAKVETDDVKKNSLDELRAQIIELLCIVEALKKDHGKELEKLRKDLEEEKTMRSNLEMEIEKLKKAVLSS

>NP_055382.2

MNHLNVLAKALYDNVAESPDELSFRKGDIMTVLEQDTQGLDGWWLCSLHGRQGIVPGNRLKILVGMYDKKPAGPGPGPPATPAQPQPGLHAPAPPASQYTPMLPNTYQPQPDSVYLVPTPSKAQQGLYQVPGPSPQFQSPPAKQTSTFSKQTPHHPFPSPATDLYQVPPGPGGPAQDIYQVPPSAGMGHDIYQVPPSMDTRSWEGTKPPAKVVVPTRVGQGYVYEAAQPEQDEYDIPRHLLAPGPQDIYDVPPVRGLLPSQYGQEVYDTPPMAVKGPNGRDPLLEVYDVPPSVEKGLPPSNHHAVYDVPPSVSKDVPDGPLLREETYDVPPAFAKAKPFDPARTPLVLAAPPPDSPPAEDVYDVPPPAPDLYDVPPGLRRPGPGTLYDVPRERVLPPEVADGGVVDSGVYAVPPPAEREAPAEGKRLSASSTGSTRSSQSASSLEVAGPGREPLELEVAVEALARLQQGVSATVAHLLDLAGSAGATGSWRSPSEPQEPLVQDLQAAVAAVQSAVHELLEFARSAVGNAAHTSDRALHAKLSRQLQKMEDVHQTLVAHGQALDAGRGGSGATLEDLDRLVACSRAVPEDAKQLASFLHGNASLLFRRTKATAPGPEGGGTLHPNPTDKTSSIQSRPLPSPPKFTSQDSPDGQYENSEGGWMEDYDYVHLQGKEEFEKTQKELLEKGSITRQGKSQLELQQLKQFERLEQEVSRPIDHDLANWTPAQPLAPGRTGGLGPSDRQLLLFYLEQCEANLTTLTNAVDAFFTAVATNQPPKIFVAHSKFVILSAHKLVFIGDTLSRQAKAADVRSQVTHYSNLLCDLLRGIVATTKAAALQYPSPSAAQDMVERVKELGHSTQQFRRVLGQLAAA

1321 NP_005892.1 NP_003891.1

>NP_005892.1

MSSILPFTPPVVKRLLGWKKSAGGSGGAGGGEQNGQEEKWCEKAVKSLVKKLKKTGRLDELEKAITTQNCNTKCVTIPSTCSEIWGLSTPNTIDQWDTTGLYSFSEQTRSLDGRLQVSHRKGLPHVIYCRLWRWPDLHSHHELKAIENCEYAFNLKKDEVCVNPYHYQRVETPVLPPVLVPRHTEILTELPPLDDYTHSIPENTNFPAGIEPQSNYIPETPPPGYISEDGETSDQQLNQSMDTGSPAELSPTTLSPVNHSLDLQPVTYSEPAFWCSIAYYELNQRVGETFHASQPSLTVDGFTDPSNSERFCLGLLSNVNRNATVEMTRRHIGRGVRLYYIGGEVFAECLSDSAIFVQSPNCNQRYGWHPATVCKIPPGCNLKIFNNQEFAALLAQSVNQGFEAVYQLTRMCTIRMSFVKGWGAEYRRQTVTSTPCWIELHLNGPLQWLDKVLTQMGSPSVRCSSMS

>NP_003891.1

MASLTVKAYLLGKEDAAREIRRFSFCCSPEPEAEAEAAAGPGPCERLLSRVAALFPALRPGGFQAHYRDEDGDLVAFSSDEELTMAMSYVKDDIFRIYIKEKKECRRDHRPPCAQEAPRNMVHPNVICDGCNGPVVGTRYKCSVCPDYDLCSVCEGKGLHRGHTKLAFPSPFGHLSEGFSHSRWLRKVKHGHFGWPGWEMGPPGNWSPRPPRAGEARPGPTAESASGPSEDPSVNFLKNVGESVAAALSPLGIEVDIDVEHGGKRSRLTPVSPESSSTEEKSSSQPSSCCSDPSKPGGNVEGATQSLAEQMRKIALESEGRPEEQMESDNCSGGDDDWTHLSSKEVDPSTGELQSLQMPESEGPSSLDPSQEGPTGLKEAALYPHLPPEADPRLIESLSQMLSMGFSDEGGWLTRLLQTKNYDIGAALDTIQYSKHPPPL

1322 NP_004716.1 NP_004327.1

>NP_004716.1

MTGSNEFKLNQPPEDGISSVKFSPNTSQFLLVSSWDTSVRLYDVPANSMRLKYQHTGAVLDCAFYDPTHAWSGGLDHQLKMHDLNTDQENLVGTHDAPIRCVEYCPEVNVMVTGSWDQTVKLWDPRTPCNAGTFSQPEKVYTLSVSGDRLIVGTAGRRVLVWDLRNMGYVQQRRESSLKYQTRCIRAFPNKQGYVLSSIEGRVAVEYLDPSPEVQKKKYAFKCHRLKENNIEQIYPVNAISFHNIHNTFATGGSDGFVNIWDPFNKKRLCQFHRYPTSIASLAFSNDGTTLAIASSYMYEMDDTEHPEDGIFIRQVTDAETKPKSPCT

>NP_004327.1

MDTPENVLQMLEAHMQSYKGNDPLGEWERYIQWVEENFPENKEYLITLLEHLMKEFLDKKKYHNDPRFISYCLKFAEYNSDLHQFFEFLYNHGIGTLSSPLYIAWAGHLEAQGELQHASAVLQRGIQNQAEPREFLQQQYRLFQTRLTETHLPAQARTSEPLHNVQVLNQMITSKSNPGNNMACISKNQGSELSGVISSACDKESNMERRVITISKSEYSVHSSLASKVDVEQVVMYCKEKLIRGESEFSFEELRAQKYNQRRKHEQWVNEDRHYMKRKEANAFEEQLLKQKMDELHKKLHQVVETSHEDLPASQERSEVNPARMGPSVGSQQELRAPCLPVTYQQTPVNMEKNPREAPPVVPPLANAISAALVSPATSQSIAPPVPLKAQTVTDSMFAVASKDAGCVNKSTHEFKPQSGAEIKEGCETHKVANTSSFHTTPNTSLGMVQATPSKVQPSPTVHTKEALGFIMNMFQAPTLPDISDDKDEWQSLDQNEDAFEAQFQKNVRSSGAWGVNKIISSLSSAFHVFEDGNKENYGLPQPKNKPTGARTFGERSVSRLPSKPKEEVPHAEEFLDDSTVWGIRCNKTLAPSPKSPGDFTSAAQLASTPFHKLPVESVHILEDKENVVAKQCTQATLDSCEENMVVPSRDGKFSPIQEKSPKQALSSHMYSASLLRLSQPAAGGVLTCEAELGVEACRLTDTDAAIAEDPPDAIAGLQAEWMQMSSLGTVDAPNFIVGNPWDDKLIFKLLSGLSKPVSSYPNTFEWQCKLPAIKPKTEFQLGSKLVYVHHLLGEGAFAQVYEATQGDLNDAKNKQKFVLKVQKPANPWEFYIGTQLMERLKPSMQHMFMKFYSAHLFQNGSVLVGELYSYGTLLNAINLYKNTPEKVMPQGLVISFAMRMLYMIEQVHDCEIIHGDIKPDNFILGNGFLEQDDEDDLSAGLALIDLGQSIDMKLFPKGTIFTAKCETSGFQCVEMLSNKPWNYQIDYFGVAATVYCMLFGTYMKVKNEGGECKPEGLFRRLPHLDMWNEFFHVMLNIPDCHHLPSLDLLRQKLKKVFQQHYTNKIRALRNRLIVLLLECKRSRK

1323 NP_003897.2 NP_002379.2

>NP_003897.2

MNPTNPFSGQQPSAFSASSSNVGTLPSKPPFRFGQPSLFGQNSTLSGKSSGFSQVSSFPASSGVSHSSSVQTLGFTQTSSVGPFSGLEHTSTFVATSGPSSSSVLGNTGFSFKSPTSVGAFPSTSAFGQEAGEIVNSGFGKTEFSFKPLENAVFKPILGAESEPEKTQSQIASGFFTFSHPISSAPGGLAPFSFPQVTSSSATTSNFTFSKPVSSNNSLSAFTPALSNQNVEEEKRGPKSIFGSSNNSFSSFPVSSAVLGEPFQASKAGVRQGCEEAVSQVEPLPSLMKGLKRKEDQDRSPRRHGHEPAEDSDPLSRGDHPPDKRPVRLNRPRGGTLFGRTIQDVFKSNKEVGRLGNKEAKKETGFVESAESDHMAIPGGNQSVLAPSRIPGVNKEEETESREKKEDSLRGTPARQSNRSESTDSLGGLSPSEVTAIQCKNIPDYLNDRTILENHFGKIAKVQRIFTRRSKKLAVVHFFDHASAALARKKGKSLHKDMAIFWHRKKISPNKKPFSLKEKKPGDGEVSPSTEDAPFQHSPLGKAAGRTGASSLLNKSSPVKKPSLLKAHQFEGDSFDSASEGSEGLGPCVLSLSTLIGTVAETSKEKYRLLDQRDRIMRQARVKRTDLDKARTFVGTCLDMCPEKERYMRETRSQLSVFEVVPGTDQVDHAAAVKEYSRSSADQEEPLPHELRPLPVLSRTMDYLVTQIMDQKEGSLRDWYDFVWNRTRGIRKDITQQHLCDPLTVSLIEKCTRFHIHCAHFMCEEPMSSFDAKINNENMTKCLQSLKEMYQDLRNKGVFCASEAEFQGYNVLLSLNKGDILREVQQFHPAVRNSSEVKFAVQAFAALNSNNFVRFFKLVQSASYLNACLLHCYFSQIRKDALRALNFAYTVSTQRSTIFPLDGVVRMLLFRDCEEATDFLTCHGLTVSDGCVELNRSAFLEPEGLSKTRKSVFITRKLTVSVGEIVNGGPLPPVPRHTPVCSFNSQNKYIGESLAAELPVSTQRPGSDTVGGGRGEECGVEPDAPLSSLPQSLPAPAPSPVPLPPVLALTPSVAPSLFQLSVQPEPPPPEPVPMYSDEDLAQVVDELIQEALQRDCEEVGSAGAAYAAAALGVSNAAMEDLLTAATTGILRHIAAEEVSKERERREQERQRAEEERLKQERELVLSELSQGLAVELMERVMMEFVRETCSQELKNAVETDQRVRVARCCEDVCAHLVDLFLVEEIFQTAKETLQELQCFCKYLQRWREAVTARKKLRRQMRAFPAAPCCVDVSDRLRALAPSAECPIAEENLARGLLDLGHAGRLGISCTRLRRLRNKTAHQMKVQHFYQQLLSDVAWASLDLPSLVAEHLPGRQEHVFWKLVLVLPDVEEQSPESCGRILANWLKVKFMGDEGSVDDTSSDAGGIQTLSLFNSLSSKGDQMISVNVCIKVAHGALSDGAIDAVETQKDLLGASGLMLLLPPKMKSEDMAEEDVYWLSALLQLKQLLQAKPFQPALPLVVLVPSPGGDAVEKEVEDGLMLQDLVSAKLISDYTVTEIPDTINDLQGSTKVLQAVQWLVSHCPHSLDLCCQTLIQYVEDGIGHEFSGRFFHDRRERRLGGLASQEPGAIIELFNSVLQFLASVVSSEQLCDLSWPVTEFAEAGGSRLLPHLHWNAPEHLAWLKQAVLGFQLPQMDLPPLGAPWLPVCSMVVQYASQIPSSRQTQPVLQSQVENLLHRTYCRWKSKSPSPVHGAGPSVMEIPWDDLIALCINHKLRDWTPPRLPVTSEALSEDGQICVYFFKNDLKKYDVPLSWEQARLQTQKELQLREGRLAIKPFHPSANNFPIPLLHMHRNWKRSTECAQEGRIPSTEDLMRGASAEELLAQCLSSSLLLEKEENKRFEDQLQQWLSEDSGAFTDLTSLPLYLPQTLVSLSHTIEPVMKTSVTTSPQSDMMREQLQLSEATGTCLGERLKHLERLIRSSREEEVASELHLSALLDMVDI

>NP_002379.2

MAGTVVLDDVELREAQRDYLDFLDDEEDQGIYQSKVRELISDNQYRLIVNVNDLRRKNEKRANRLLNNAFEELVAFQRALKDFVASIDATYAKQYEEFYVGLEGSFGSKHVSPRTLTSCFLSCVVCVEGIVTKCSLVRPKVVRSVHYCPATKKTIERRYSDLTTLVAFPSSSVYPTKDEENNPLETEYGLSVYKDHQTITIQEMPEKAPAGQLPRSVDVILDDDLVDKAKPGDRVQVVGTYRCLPGKKGGYTSGTFRTVLIACNVKQMSKDAQPSFSAEDIAKIKKFSKTRSKDIFDQLAKSLAPSIHGHDYVKKAILCLLLGGVERDLENGSHIRGDINILLIGDPSVAKSQLLRYVLCTAPRAIPTTGRGSSGVGLTAAVTTDQETGERRLEAGAMVLADRGVVCIDEFDKMSDMDRTAIHEVMEQGRVTIAKAGIHARLNARCSVLAAANPVYGRYDQYKTPMENIGLQDSLLSRFDLLFIMLDQMDPEQDREISDHVLRMHRYRAPGEQDGDAMPLGSAVDILATDDPNFSQEDQQDTQIYEKHDNLLHGTKKKKEKMVSAAFMKKYIHVAKIIKPVLTQESATYIAEEYSRLRSQDSMSSDTARTSPVTARTLETLIRLATAHAKARMSKTVDLQDAEEAVELVQYAYFKKVLEKEKKRKKRSEDESETEDEEEKSQEDQEQKRKRRKTRQPDAKDGDSYDPYDFSDTEEEMPQVHTPKTADSQETKESQKVELSESRLKAFKVALLDVFREAHAQSIGMNRLTESINRDSEEPFSSVEIQAALSKMQDDNQVMVSEGIIFLI

1324 NP_002543.2 NP_006181.1

>NP_002543.2

MSSRKSKSNSLIHTECLSQVQRILRERFCRQSPHSNLFGVQVQYKHLSELLKRTALHGESNSVLIIGPRGSGKTMLINHALKELMEIEEVSENVLQVHLNGLLQINDKIALKEITRQLNLENVVGDKVFGSFAENLSFLLEALKKGDRTSSCPVIFILDEFDLFAHHKNQTLLYNLFDISQSAQTPIAVIGLTCRLDILELLEKRVKSRFSHRQIHLMNSFGFPQYVKIFKEQLSLPAEFPDKVFAEKWNENVQYLSEDRSVQEVLQKHFNISKNLRSLHMLLMLALNRVTASHPFMTAVDLMEASQLCSMDSKANIVHGLSVLEICLIIAMKHLNDIYEEEPFNFQMVYNEFQKFVQRKAHSVYNFEKPVVMKAFEHLQQLELIKPMERTSGNSQREYQLMKLLLDNTQIMNALQKYPNCPTDVRQWATSSLSWL

>NP_006181.1

MSKPELKEDKMLEVHFVGDDDVLNHILDREGGAKLKKERAQLLVNPKKIIKKPEYDLEEDDQEVLKDQNYVEIMGRDVQESLKNGSATGGGNKVYSFQNRKHSEKMAKLASELAKTPQKSVSFSLKNDPEITINVPQSSKGHSASDKVQPKNNDKSEFLSTAPRSLRKRLIVPRSHSDSESEYSASNSEDDEGVAQEHEEDTNAVIFSQKIQAQNRVVSAPVGKETPSKRMKRDKTSDLVEEYFEAHSSSKVLTSDRTLQKLKRAKLDQQTLRNLLSKVSPSFSAELKQLNQQYEKLFHKWMLQLHLGFNIVLYGLGSKRDLLERFRTTMLQDSIHVVINGFFPGISVKSVLNSITEEVLDHMGTFRSILDQLDWIVNKFKEDSSLELFLLIHNLDSQMLRGEKSQQIIGQLSSLHNIYLIASIDHLNAPLMWDHAKQSLFNWLWYETTTYSPYTEETSYENSLLVKQSGSLPLSSLTHVLRSLTPNARGIFRLLIKYQLDNQDNPSYIGLSFQDFYQQCREAFLVNSDLTLRAQLTEFRDHKLIRTKKGTDGVEYLLIPVDNGTLTDFLEKEEEEA

1325 NP_002328.1 NP_002680.2

>NP_002328.1

MAPRRVRSFLRGLPALLLLLLFLGPWPAASHGGKYSREKNQPKPSPKRESGEEFRMEKLNQLWEKAQRLHLPPVRLAELHADLKIQERDELAWKKLKLDGLDEDGEKEARLIRNLNVILAKYGLDGKKDARQVTSNSLSGTQEDGLDDPRLEKLWHKAKTSGKFSGEELDKLWREFLHHKEKVHEYNVLLETLSRTEEIHENVISPSDLSDIKGSVLHSRHTELKEKLRSINQGLDRLRRVSHQGYSTEAEFEEPRVIDLWDLAQSANLTDKELEAFREELKHFEAKIEKHNHYQKQLEIAHEKLRHAESVGDGERVSRSREKHALLEGRTKELGYTVKKHLQDLSGRISRARHNEL

>NP_002680.2

MSASAQQLAEELQIFGLDCEEALIEKLVELCVQYGQNEEGMVGELIAFCTSTHKVGLTSEILNSFEHEFLSKRLSKARHSTCKDSGHAGARDIVSIQELIEVEEEEEILLNSYTTPSKGSQKRAISTPETPLTKRSVSTRSPHQLLSPSSFSPSATPSQKYNSRSNRGEVVTSFGLAQGVSWSGRGGAGNISLKVLGCPEALTGSYKSMFQKLPDIREVLTCKIEELGSELKEHYKIEAFTPLLAPAQEPVTLLGQIGCDSNGKLNNKSVILEGDREHSSGAQIPVDLSELKEYSLFPGQVVIMEGINTTGRKLVATKLYEGVPLPFYQPTEEDADFEQSMVLVACGPYTTSDSITYDPLLDLIAVINHDRPDVCILFGPFLDAKHEQVENCLLTSPFEDIFKQCLRTIIEGTRSSGSHLVFVPSLRDVHHEPVYPQPPFSYSDLSREDKKQVQFVSEPCSLSINGVIFGLTSTDLLFHLGAEEISSSSGTSDRFSRILKHILTQRSYYPLYPPQEDMAIDYESFYVYAQLPVTPDVLIIPSELRYFVKDVLGCVCVNPGRLTKGQVGGTFARLYLRRPAADGAERQSPCIAVQVVRI

1326 NP_002728.1 NP_004156.1

>NP_002728.1

MADVFPGNDSTASQDVANRFARKGALRQKNVHEVKDHKFIARFFKQPTFCSHCTDFIWGFGKQGFQCQVCCFVVHKRCHEFVTFSCPGADKGPDTDDPRSKHKFKIHTYGSPTFCDHCGSLLYGLIHQGMKCDTCDMNVHKQCVINVPSLCGMDHTEKRGRIYLKAEVADEKLHVTVRDAKNLIPMDPNGLSDPYVKLKLIPDPKNESKQKTKTIRSTLNPQWNESFTFKLKPSDKDRRLSVEIWDWDRTTRNDFMGSLSFGVSELMKMPASGWYKLLNQEEGEYYNVPIPEGDEEGNMELRQKFEKAKLGPAGNKVISPSEDRKQPSNNLDRVKLTDFNFLMVLGKGSFGKVMLADRKGTEELYAIKILKKDVVIQDDDVECTMVEKRVLALLDKPPFLTQLHSCFQTVDRLYFVMEYVNGGDLMYHIQQVGKFKEPQAVFYAAEISIGLFFLHKRGIIYRDLKLDNVMLDSEGHIKIADFGMCKEHMMDGVTTRTFCGTPDYIAPEIIAYQPYGKSVDWWAYGVLLYEMLAGQPPFDGEDEDELFQSIMEHNVSYPKSLSKEAVSICKGLMTKHPAKRLGCGPEGERDVREHAFFRRIDWEKLENREIQPPFKPKVCGKGAENFDKFFTRGQPVLTPPDQLVIANIDQSDFEGFSYVNPQFVHPILQSAV

>NP_004156.1

MTLNGGGSGAGGSRGGGQERERRRGSTPWGPAPPLHRRSMPVDERDLQAALTPGALTAAAAGTGTQGPRLDWPEDSEDSLSSGGSDSDESVYKVLLLGAPGVGKSALARIFGGVEDGPEAEAAGHTYDRSIVVDGEEASLMVYDIWEQDGGRWLPGHCMAMGDAYVIVYSVTDKGSFEKASELRVQLRRARQTDDVPIILVGNKSDLVRSREVSVDEGRACAVVFDCKFIETSAALHHNVQALFEGVVRQIRLRRDSKEANARRQAGTRRRESLGKKAKRFLGRIVARNSRKMAFRAKSKSCHDLSVL

1327 NP_599022.1 NP_060231.1

>NP_599022.1

MNEGAPGDSDLETEARVPWSIMGHCLRTGQARMSATPTPAGEGARRDELFGILQILHQCILSSGDAFVLTGVCCSWRQNGKPPYSQKEDKEVQTGYMNAQIEIIPCKICGDKSSGIHYGVITCEGCKGFFRRSQQSNATYSCPRQKNCLIDRTSRNRCQHCRLQKCLAVGMSRDAVKFGRMSKKQRDSLYAEVQKHRMQQQQRDHQQQPGEAEPLTPTYNISANGLTELHDDLSNYIDGHTPEGSKADSAVSSFYLDIQPSPDQSGLDINGIKPEPICDYTPASGFFPYCSFTNGETSPTVSMAELEHLAQNISKSHLETCQYLREELQQITWQTFLQEEIENYQNKQREVMWQLCAIKITEAIQYVVEFAKRIDGFMELCQNDQIVLLKAGSLEVVFIRMCRAFDSQNNTVYFDGKYASPDVFKSLGCEDFISFVFEFGKSLCSMHLTEDEIALFSAFVLMSADRSWLQEKVKIEKLQQKIQLALQHVLQKNHREDGILTKLICKVSTLRALCGRHTEKLMAFKAIYPDIVRLHFPPLYKELFTSEFEPAMQIDG

>NP_060231.1

MGGGERYNIPAPQSRNVSKNQQQLNRQKTKEQNSQMKIVHKKKERGHGYNSSAAAWQAMQNGGKNKNFPNNQSWNSSLSGPRLLFKSQANQNYAGAKFSEPPSPSVLPKPPSHWVPVSFNPSDKEIMTFQLKTLLKVQV

1328 NP_057421.1 NP_009297.2

>NP_057421.1

MATSEQSICQARASVMVYDDTSKKWVPIKPGQQGFSRINIYHNTASNTFRVVGVKLQDQQVVINYSIVKGLKYNQATPTFHQWRDARQVYGLNFASKEEATTFSNAMLFALNIMNSQEGGPSSQRQVQNGPSPDEMDIQRRQVMEQHQQQRQESLERRTSATGPILPPGHPSSAASAPVSCSGPPPPPPPPVPPPPTGATPPPPPPLPAGGAQGSSHDESSMSGLAAAIAGAKLRRVQRPEDASGGSSPSGTSKSDANRASSGGGGGGLMEEMNKLLAKRRKAASQSDKPAEKKEDESQMEDPSTSPSPGTRAASQPPNSSEAGRKPWERSNSVEKPVSSILSRTPSVAKSPEAKSPLQSQPHSRMKPAGSVNDMALDAFDLDRMKQEILEEVVRELHKVKEEIIDAIRQELSGISTT

>NP_009297.2

MGQQPGKVLGDQRRPSLPALHFIKGAGKKESSRHGGPHCNVFVEHEALQRPVASDFEPQGLSEAARWNSKENLLAGPSENDPNLFVALYDFVASGDNTLSITKGEKLRVLGYNHNGEWCEAQTKNGQGWVPSNYITPVNSLEKHSWYHGPVSRNAAEYLLSSGINGSFLVRESESSPGQRSISLRYEGRVYHYRINTASDGKLYVSSESRFNTLAELVHHHSTVADGLITTLHYPAPKRNKPTVYGVSPNYDKWEMERTDITMKHKLGGGQYGEVYEGVWKKYSLTVAVKTLKEDTMEVEEFLKEAAVMKEIKHPNLVQLLGVCTREPPFYIITEFMTYGNLLDYLRECNRQEVNAVVLLYMATQISSAMEYLEKKNFIHRDLAARNCLVGENHLVKVADFGLSRLMTGDTYTAHAGAKFPIKWTAPESLAYNKFSIKSDVWAFGVLLWEIATYGMSPYPGIDLSQVYELLEKDYRMERPEGCPEKVYELMRACWQWNPSDRPSFAEIHQAFETMFQESSISDEVEKELGKQGVRGAVSTLLQAPELPTKTRTSRRAAEHRDTTDVPEMPHSKGQGESDPLDHEPAVSPLLPRKERGPPEGGLNEDERLLPKDKKTNLFSALIKKKKKTAPTPPKRSSSFREMDGQPERRGAGEEEGRDISNGALAFTPLDTADPAKSPKPSNGAGVPNGALRESGGSGFRSPHLWKKSSTLTSSRLATGEEEGGGSSSKRFLRSCSASCVPHGAKDTEWRSVTLPRDLQSTGRQFDSSTFGGHKSEKPALPRKRAGENRSDQVTRGTVTPPPRLVKKNEEAADEVFKDIMESSPGSSPPNLTPKPLRRQVTVAPASGLPHKEEAGKGSALGTPAAAEPVTPTSKAGSGAPGGTSKGPAEESRVRRHKHSSESPGRDKGKLSRLKPAPPPPPAASAGKAGGKPSQSPSQEAAGEAVLGAKTKATSLVDAVNSDAAKPSQPGEGLKKPVLPATPKPQSAKPSGTPISPAPVPSTLPSASSALAGDQPSSTAFIPLISTRVSLRKTRQPPERIASGAITKGVVLDSTEALCLAISRNSEQMASHSAVLEAGKNLYTFCVSYVDSIQQMRNKFAFREAINKLENNLRELQICPATAGSGPAATQDFSKLLSSVKEISDIVQR

1329 NP_002583.1 NP_000225.1

>NP_002583.1

MFEARLVQGSILKKVLEALKDLINEACWDISSSGVNLQSMDSSHVSLVQLTLRSEGFDTYRCDRNLAMGVNLTSMSKILKCAGNEDIITLRAEDNADTLALVFEAPNQEKVSDYEMKLMDLDVEQLGIPEQEYSCVVKMPSGEFARICRDLSHIGDAVVISCAKDGVKFSASGELGNGNIKLSQTSNVDKEEEAVTIEMNEPVQLTFALRYLNFFTKATPLSSTVTLSMSADVPLVVEYKIADMGHLKYYLAPKIEDEEGS

>NP_000225.1

MQRSIMSFFHPKKEGKAKKPEKEASNSSRETEPPPKAALKEWNGVVSESDSPVKRPGRKAARVLGSEGEEEDEALSPAKGQKPALDCSQVSPPRPATSPENNASLSDTSPMDSSPSGIPKRRTARKQLPKRTIQEVLEEQSEDEDREAKRKKEEEEEETPKESLTEAEVATEKEGEDGDQPTTPPKPLKTSKAETPTESVSEPEVATKQELQEEEEQTKPPRRAPKTLSSFFTPRKPAVKKEVKEEEPGAPGKEGAAEGPLDPSGYNPAKNNYHPVEDACWKPGQKVPYLAVARTFEKIEEVSARLRMVETLSNLLRSVVALSPPDLLPVLYLSLNHLGPPQQGLELGVGDGVLLKAVAQATGRQLESVRAEAAEKGDVGLVAENSRSTQRLMLPPPPLTASGVFSKFRDIARLTGSASTAKKIDIIKGLFVACRHSEARFIARSLSGRLRLGLAEQSVLAALSQAVSLTPPGQEFPPAMVDAGKGKTAEARKTWLEEQGMILKQTFCEVPDLDRIIPVLLEHGLERLPEHCKLSPGIPLKPMLAHPTRGISEVLKRFEEAAFTCEYKYDGQRAQIHALEGGEVKIFSRNQEDNTGKYPDIISRIPKIKLPSVTSFILDTEAVAWDREKKQIQPFQVLTTRKRKEVDASEIQVQVCLYAFDLIYLNGESLVREPLSRRRQLLRENFVETEGEFVFATSLDTKDIEQIAEFLEQSVKDSCEGLMVKTLDVDATYEIAKRSHNWLKLKKDYLDGVGDTLDLVVIGAYLGRGKRAGRYGGFLLASYDEDSEELQAICKLGTGFSDEELEEHHQSLKALVLPSPRPYVRIDGAVIPDHWLDPSAVWEVKCADLSLSPIYPAARGLVDSDKGISLRFPRFIRVREDKQPEQATTSAQVACLYRKQSQIQNQQGEDSGSDPEDTY

1330 NP_002728.1 NP_061116.2

>NP_002728.1

MADVFPGNDSTASQDVANRFARKGALRQKNVHEVKDHKFIARFFKQPTFCSHCTDFIWGFGKQGFQCQVCCFVVHKRCHEFVTFSCPGADKGPDTDDPRSKHKFKIHTYGSPTFCDHCGSLLYGLIHQGMKCDTCDMNVHKQCVINVPSLCGMDHTEKRGRIYLKAEVADEKLHVTVRDAKNLIPMDPNGLSDPYVKLKLIPDPKNESKQKTKTIRSTLNPQWNESFTFKLKPSDKDRRLSVEIWDWDRTTRNDFMGSLSFGVSELMKMPASGWYKLLNQEEGEYYNVPIPEGDEEGNMELRQKFEKAKLGPAGNKVISPSEDRKQPSNNLDRVKLTDFNFLMVLGKGSFGKVMLADRKGTEELYAIKILKKDVVIQDDDVECTMVEKRVLALLDKPPFLTQLHSCFQTVDRLYFVMEYVNGGDLMYHIQQVGKFKEPQAVFYAAEISIGLFFLHKRGIIYRDLKLDNVMLDSEGHIKIADFGMCKEHMMDGVTTRTFCGTPDYIAPEIIAYQPYGKSVDWWAYGVLLYEMLAGQPPFDGEDEDELFQSIMEHNVSYPKSLSKEAVSICKGLMTKHPAKRLGCGPEGERDVREHAFFRRIDWEKLENREIQPPFKPKVCGKGAENFDKFFTRGQPVLTPPDQLVIANIDQSDFEGFSYVNPQFVHPILQSAV

>NP_061116.2

MGLSLPKEKGLILCLWSKFCRWFQRRESWAQSRDEQNLLQQKRIWESPLLLAAKDNDVQALNKLLKYEDCKVHQRGAMGETALHIAALYDNLEAAMVLMEAAPELVFEPMTSELYEGQTALHIAVVNQNMNLVRALLARRASVSARATGTAFRRSPCNLIYFGEHPLSFAACVNSEEIVRLLIEHGADIRAQDSLGNTVLHILILQPNKTFACQMYNLLLSYDRHGDHLQPLDLVPNHQGLTPFKLAGVEGNTVMFQHLMQKRKHTQWTYGPLTSTLYDLTEIDSSGDEQSLLELIITTKKREARQILDQTPVKELVSLKWKRYGRPYFCMLGAIYLLYIICFTMCCIYRPLKPRTNNRTSPRDNTLLQQKLLQEAYMTPKDDIRLVGELVTVIGAIIILLVEVPDIFRMGVTRFFGQTILGGPFHVLIITYAFMVLVTMVMRLISASGEVVPMSFALVLGWCNVMYFARGFQMLGPFTIMIQKMIFGDLMRFCWLMAVVILGFASAFYIIFQTEDPEELGHFYDYPMALFSTFELFLTIIDGPANYNVDLPFMYSITYAAFAIIATLLMLNLLIAMMGDTHWRVAHERDELWRAQIVATTVMLERKLPRCLWPRSGICGREYGLGDRWFLRVEDRQDLNRQRIQRYAQAFHTRGSEDLDKDSVEKLELGCPFSPHLSLPMPSVSRSTSRSSANWERLRQGTLRRDLRGIINRGLEDGESWEYQI

1331 NP_997274.2 O75916

>NP_997274.2

MAREECKALLDGLNKTTACYHHLVLTVGGSADSQNLRQELQKTRQKAQELAVSTCARLTAVLRDRGLAADERAEFERLWVAFSGCLDLLEADMRRALELGAAFPLHAPRRPLVRTGVAGASSGVAARALSTRSLRLEAEGDFDVADLRELEREVLQVGEMIDNMEMKVNVPRWTVQARQAAGAELLSTVSAGPSSVVSLQERGGGCDPRKALAAILFGAVLLAAVALAVCVAKLS

>O75916

MTIRHQGQQYRPRMAFLQKIEALVKDMQNPETGVRMQNQRVLVTSVPHAMTGSDVLQWIVQRLWISSLEAQNLGNFIVRYGYIYPLQDPKNLILKPDGSLYRFQTPYFWPTQQWPAEDTDYAIYLAKRNIKKKGILEEYEKENYNFLNQKMNYKWDFVIMQAKEQYRAGKERNKADRYALDCQEKAYWLVHRCPPGMDNVLDYGLDRVTNPNEVKVNQKQTVVAVKKEIMYYQQALMRSTVKSSVSLGGIVKYSEQFSSNDAIMSGCLPSNPWITDDTQFWDLNAKLVEIPTKMRVERWAFNFSELIRDPKGRQSFQYFLKKEFSGENLGFWEACEDLKYGDQSKVKEKAEEIYKLFLAPGARRWINIDGKTMDITVKGLKHPHRYVLDAAQTHIYMLMKKDSYARYLKSPIYKDMLAKAIEPQETTKKSSTLPFMRRHLRSSPSPVILRQLEEEAKAREAANTVDITQPGQHMAPSPHLTVYTGTCMPPSPSSPFSSSCRSPRKPFASPSRFIRRPSTTICPSPIRVALESSSGLEQKGECSGSMAPRGPSVTESSEASLDTSWPRSRPRAPPKARMALSFSRFLRRGCLASPVFARLSPKCPAVSHGRVQPLGDVGQQLPRLKSKRVANFFQIKMDVPTGSGTCLMDSEDAGTGESGDRATEKEVICPWESL

1332 NP_005131.1 NP_008819.1

>NP_005131.1

MTEKTNGVKSSPANNHNHHAPPAIKANGKDDHRTSSRPHSAADDDTSSELQRLADVDAPQQGRSGFRRIVRLVGIIREWANKNFREEEPRPDSFLERFRGPELQTVTTQEGDGKGDKDGEDKGTKKKFELFVLDPAGDWYYCWLFVIAMPVLYNWCLLVARACFSDLQKGYYLVWLVLDYVSDVVYIADLFIRLRTGFLEQGLLVKDTKKLRDNYIHTLQFKLDVASIIPTDLIYFAVDIHSPEVRFNRLLHFARMFEFFDRTETRTNYPNIFRISNLVLYILVIIHWNACIYYAISKSIGFGVDTWVYPNITDPEYGYLAREYIYCLYWSTLTLTTIGETPPPVKDEEYLFVIFDFLIGVLIFATIVGNVGSMISNMNATRAEFQAKIDAVKHYMQFRKVSKGMEAKVIRWFDYLWTNKKTVDEREILKNLPAKLRAEIAINVHLSTLKKVRIFHDCEAGLLVELVLKLRPQVFSPGDYICRKGDIGKEMYIIKEGKLAVVADDGVTQYALLSAGSCFGEISILNIKGSKMGNRRTANIRSLGYSDLFCLSKDDLMEAVTEYPDAKKVLEERGREILMKEGLLDENEVATSMEVDVQEKLGQLETNMETLYTRFGRLLAEYTGAQQKLKQRITVLETKMKQNNEDDYLSDGMNSPELAAADEP

>NP_008819.1

MADQLTEEQIAEFKEAFSLFDKDGDGTITTKELGTVMRSLGQNPTEAELQDMINEVDADGNGTIDFPEFLTMMARKMKDTDSEEEIREAFRVFDKDGNGYISAAELRHVMTNLGEKLTDEEVDEMIREADIDGDGQVNYEEFVQMMTAK

1333 NP_006387.1 NP_006420.1

>NP_006387.1

MALNGAEVDDFSWEPPTEAETKVLQARRERQDRISRLMGDYLLRGYRMLGETCADCGTILLQDKQRKIYCVACQELDSDVDKDNPALNAQAALSQAREHQLASASELPLGSRPAPQPPVPRPEHCEGAAAGLKAAQGPPAPAVPPNTDVMACTQTALLQKLTWASAELGSSTSLETSIQLCGLIRACAEALRSLQQLQH

>NP_006420.1

MMPTPVILLKEGTDSSQGIPQLVSNISACQVIAEAVRTTLGPRGMDKLIVDGRGKATISNDGATILKLLDVVHPAAKTLVDIAKSQDAEVGDGTTSVTLLAAEFLKQVKPYVEEGLHPQIIIRAFRTATQLAVNKIKEIAVTVKKADKVEQRKLLEKCAMTALSSKLISQQKAFFAKMVVDAVMMLDDLLQLKMIGIKKVQGGALEDSQLVAGVAFKKTFSYAGFEMQPKKYHNPKIALLNVELELKAEKDNAEIRVHTVEDYQAIVDAEWNILYDKLEKIHHSGAKVVLSKLPIGDVATQYFADRDMFCAGRVPEEDLKRTMMACGGSIQTSVNALSADVLGRCQVFEETQIGGERYNFFTGCPKAKTCTFILRGGAEQFMEETERSLHDAIMIVRRAIKNDSVVAGGGAIEMELSKYLRDYSRTIPGKQQLLIGAYAKALEIIPRQLCDNAGFDATNILNKLRARHAQGGTWYGVDINNEDIADNFEAFVWEPAMVRINALTAASEAACLIVSVDETIKNPRSTVDAPTAAGRGRGRGRPH

1334 NP_002761.1 NP_000286.3

>NP_002761.1

MNLLLILTFVAAAVAAPFDDDDKIVGGYICEENSVPYQVSLNSGYHFCGGSLISEQWVVSAGHCYKSRIQVRLGEHNIEVLEGNEQFINAAKIIRHPKYNSRTLDNDILLIKLSSPAVINSRVSAISLPTAPPAAGTESLISGWGNTLSSGADYPDELQCLDAPVLSQAECEASYPGKITNNMFCVGFLEGGKDSCQGDSGGPVVSNGELQGIVSWGYGCAQKNRPGVYTKVYNYVDWIKDTIAANS

>NP_000286.3

MPSSVSWGILLLAGLCCLVPVSLAEDPQGDAAQKTDTSHHDQDHPTFNKITPNLAEFAFSLYRQLAHQSNSTNIFFSPVSIATAFAMLSLGTKADTHDEILEGLNFNLTEIPEAQIHEGFQELLRTLNQPDSQLQLTTGNGLFLSEGLKLVDKFLEDVKKLYHSEAFTVNFGDTEEAKKQINDYVEKGTQGKIVDLVKELDRDTVFALVNYIFFKGKWERPFEVKDTEEEDFHVDQVTTVKVPMMKRLGMFNIQHCKKLSSWVLLMKYLGNATAIFFLPDEGKLQHLENELTHDIITKFLENEDRRSASLHLPKLSITGTYDLKSVLGQLGITKVFSNGADLSGVTEEAPLKLSKAVHKAVLTIDEKGTEAAGAMFLEAIPMSIPPEVKFNKPFVFLMIEQNTKSPLFMGKVVNPTQK

1335 NP_056399.1 NP_115580.2

>NP_056399.1

MAACTARRALAVGSRWWSRSLTGARWPRPLCAAAGAGAFSPASTTTTRRHLSSRNRPEGKVLETVGVFEVPKQNGKYETGQLFLHSIFGYRGVVLFPWQARLYDRDVASAAPEKAENPAGHGSKEVKGKTHTYYQVLIDARDCPHISQRSQTEAVTFLANHDDSRALYAIPGLDYVSHEDILPYTSTDQVPIQHELFERFLLYDQTKAPPFVARETLRAWQEKNHPWLELSDVHRETTENIRVTVIPFYMGMREAQNSHVYWWRYCIRLENLDSDVVQLRERHWRIFSLSGTLETVRGRGVVGREPVLSKEQPAFQYSSHVSLQASSGHMWGTFRFERPDGSHFDVRIPPFSLESNKDEKTPPSGLHW

>NP_115580.2

MPALPLDQLQITHKDPKTGKLRTSPALHPEQKADRYFVLYKPPPKDNIPALVEEYLERATFVANDLDWLLALPHDKFWCQVIFDETLQKCLDSYLRYVPRKFDEGVASAPEVVDMQKRLHRSVFLTFLRMSTHKESKDHFISPSAFGEILYNNFLFDIPKILDLCVLFGKGNSPLLQKMIGNIFTQQPSYYSDLDETLPTILQVFSNILQHCGLQGDGANTTPQKLEERGRLTPSDMPLLELKDIVLYLCDTCTTLWAFLDIFPLACQTFQKHDFCYRLASFYEAAIPEMESAIKKRRLEDSKLLGDLWQRLSHSRKKLMEIFHIILNQICLLPILESSCDNIQGFIEEFLQIFSSLLQEKRFLRDYDALFPVAEDISLLQQASSVLDETRTAYILQAVESAWEGVDRRKATDAKDPSVIEEPNGEPNGVTVTAEAVSQASSHPENSEEEECMGAAAAVGPAMCGVELDSLISQVKDLLPDLGEGFILACLEYYHYDPEQVINNILEERLAPTLSQLDRNLDREMKPDPTPLLTSRHNVFQNDEFDVFSRDSVDLSRVHKGKSTRKEENTRSLLNDKRAVAAQRQRYEQYSVVVEEVPLQPGESLPYHSVYYEDEYDDTYDGNQVGANDADSDDELISRRPFTIPQVLRTKVPREGQEEDDDDEEDDADEEAPKPDHFVQDPAVLREKAEARRMAFLAKKGYRHDSSTAVAGSPRGHGQSRETTQERRKKEANKATRANHNRRTMADRKRSKGMIPS

1336 NP_001341.1 NP_003874.2

>NP_001341.1

MATANSIIVLDDDDEDEAAAQPGPSHPLPNAASPGAEAPSSSEPHGARGSSSSGGKKCYKLENEKLFEEFLELCKMQTADHPEVVPFLYNRQQRAHSLFLASAEFCNILSRVLSRARSRPAKLYVYINELCTVLKAHSAKKKLNLAPAATTSNEPSGNNPPTHLSLDPTNAENTASQSPRTRGSRRQIQRLEQLLALYVAEIRRLQEKELDLSELDDPDSAYLQEARLKRKLIRLFGRLCELKDCSSLTGRVIEQRIPYRGTRYPEVNRRIERLINKPGPDTFPDYGDVLRAVEKAAARHSLGLPRQQLQLMAQDAFRDVGIRLQERRHLDLIYNFGCHLTDDYRPGVDPALSDPVLARRLRENRSLAMSRLDEVISKYAMLQDKSEEGERKKRRARLQGTSSHSADTPEASLDSGEGPSGMASQGCPSASRAETDDEDDEESDEEEEEEEEEEEEEATDSEEEEDLEQMQEGQEDDEEEDEEEEAAAGKDGDKSPMSSLQISNEKNLEPGKQISRSSGEQQNKGRIVSPSLLSEEPLAPSSIDAESNGEQPEELTLEEESPVSQLFELEIEALPLDTPSSVETDISSSRKQSEEPFTTVLENGAGMVSSTSFNGGVSPHNWGDSGPPCKKSRKEKKQTGSGPLGNSYVERQRSVHEKNGKKICTLPSPPSPLASLAPVADSSTRVDSPSHGLVTSSLCIPSPARLSQTPHSQPPRPGTCKTSVATQCDPEEIIVLSDSD

>NP_003874.2

MAKTVAYFYDPDVGNFHYGAGHPMKPHRLALTHSLVLHYGLYKKMIVFKPYQASQHDMCRFHSEDYIDFLQRVSPTNMQGFTKSLNAFNVGDDCPVFPGLFEFCSRYTGASLQGATQLNNKICDIAINWAGGLHHAKKFEASGFCYVNDIVIGILELLKYHPRVLYIDIDIHHGDGVQEAFYLTDRVMTVSFHKYGNYFFPGTGDMYEVGAESGRYYCLNVPLRDGIDDQSYKHLFQPVINQVVDFYQPTCIVLQCGADSLGCDRLGCFNLSIRGHGECVEYVKSFNIPLLVLGGGGYTVRNVARCWTYETSLLVEEAISEELPYSEYFEYFAPDFTLHPDVSTRIENQNSRQYLDQIRQTIFENLKMLNHAPSVQIHDVPADLLTYDRTDEADAEERGPEENYSRPEAPNEFYDGDHDNDKESDVEI

1337 NP_066961.2 NP_112589.1

>NP_066961.2

MAAASVTPPGSLELLQPGFSKTLLGTKLEAKYLCSACRNVLRRPFQAQCGHRYCSFCLASILSSGPQNCAACVHEGIYEEGISILESSSAFPDNAARREVESLPAVCPSDGCTWKGTLKEYESCHEGRCPLMLTECPACKGLVRLGEKERHLEHECPERSLSCRHCRAPCCGADVKAHHEVCPKFPLTCDGCGKKKIPREKFQDHVKTCGKCRVPCRFHAIGCLETVEGEKQQEHEVQWLREHLAMLLSSVLEAKPLLGDQSHAGSELLQRCESLEKKTATFENIVCVLNREVERVAMTAEACSRQHRLDQDKIEALSSKVQQLERSIGLKDLAMADLEQKVLEMEASTYDGVFIWKISDFARKRQEAVAGRIPAIFSPAFYTSRYGYKMCLRIYLNGDGTGRGTHLSLFFVVMKGPNDALLRWPFNQKVTLMLLDQNNREHVIDAFRPDVTSSSFQRPVNDMNIASGCPLFCPVSKMEAKNSYVRDDAIFIKAIVDLTGL

>NP_112589.1

MGSAKSVPVTPARPPPHNKHLARVADPRSPSAGILRTPIQVESSPQPGLPAGEQLEGLKHAQDSDPRSPTLGIARTPMKTSSGDPPSPLVKQLSEVFETEDSKSNLPPEPVLPPEAPLSSELDLPLGTQLSVEEQMPPWNQTEFPSKQVFSKEEARQPTETPVASQSSDKPSRDPETPRSSGSMRNRWKPNSSKVLGRSPLTILQDDNSPGTLTLRQGKRPSPLSENVSELKEGAILGTGRLLKTGGRAWEQGQDHDKENQHFPLVES

1338 NP_005234.1 NP_115887.1

>NP_005234.1

MASTDYSTYSQAAAQQGYSAYTAQPTQGYAQTTQAYGQQSYGTYGQPTDVSYTQAQTTATYGQTAYATSYGQPPTGYTTPTAPQAYSQPVQGYGTGAYDTTTATVTTTQASYAAQSAYGTQPAYPAYGQQPAATAPTRPQDGNKPTETSQPQSSTGGYNQPSLGYGQSNYSYPQVPGSYPMQPVTAPPSYPPTSYSSTQPTSYDQSSYSQQNTYGQPSSYGQQSSYGQQSSYGQQPPTSYPPQTGSYSQAPSQYSQQSSSYGQQSSFRQDHPSSMGVYGQESGGFSGPGENRSMSGPDNRGRGRGGFDRGGMSRGGRGGGRGGMGSAGERGGFNKPGGPMDEGPDLDLGPPVDPDEDSDNSAIYVQGLNDSVTLDDLADFFKQCGVVKMNKRTGQPMIHIYLDKETGKPKGDATVSYEDPPTAKAAVEWFDGKDFQGSKLKVSLARKKPPMNSMRGGLPPREGRGMPPPLRGGPGGPGGPGGPMGRMGGRGGDRGGFPPRGPRGSRGNPSGGGNVQHRAGDWQCPNPGCGNQNFAWRTECNQCKAPKPEGFLPPPFPPPGGDRGRGGPGGMRGGRGGLMDRGGPGGMFRGGRGGDRGGFRGGRGMDRGGFGGGRRGGPGGPPGPLMEQMGGRRGGRGGPGKMDKGEHRQERRDRPY

>NP_115887.1

MEPPDQCSQYMTSLLSPAVDDEKELQDMNAMVLSLTEEVKEEEEDAQPEPEQGTAAGEKLKSAGAQGGEEKDGGGEEKDGGGAGVPGHLWEGDLEGTSGSDGNVEDSDQSEKEPGQQYSRPQGAVGGLEPGNAQQPNVHAFTPLQLQELERIFQREQFPSEFLRRRLARSMNVTELAVQIWFENRRAKWRRHQRALMARNMLPFMAVGQPVMVTAAEAITAPLFISGMRDDYFWDHSHSSSLCFPMPPFPPPSLPLPLMLLPPMPPAGQAEFGPFPFVIVPSFTFPNV

1339 NP_008985.3 NP_002245.4

>NP_008985.3

MPINKSEKPESCDNVKVVVRCRPLNEREKSMCYKQAVSVDEMRGTITVHKTDSSNEPPKTFTFDTVFGPESKQLDVYNLTARPIIDSVLEGYNGTIFAYGQTGTGKTFTMEGVRAIPELRGIIPNSFAHIFGHIAKAEGDTRFLVRVSYLEIYNEEVRDLLGKDQTQRLEVKERPDVGVYIKDLSAYVVNNADDMDRIMTLGHKNRSVGATNMNEHSSRSHAIFTITIECSEKGIDGNMHVRMGKLHLVDLAGSERQAKTGATGQRLKEATKINLSLSTLGNVISALVDGKSTHVPYRNSKLTRLLQDSLGGNSKTMMCANIGPADYNYDETISTLRYANRAKNIKNKARINEDPKDALLRQFQKEIEELKKKLEEGEEISGSDISGSEEDDDEEGEVGEDGEKRKKRRGKKKVSPDKMIEMQAKIDEERKALETKLDMEEEERNKARAELEKREKDLLKAQQEHQSLLEKLSALEKKVIVGGVDLLAKAEEQEKLLEESNMELEERRKRAEQLRRELEEKEQERLDIEEKYTSLQEEAQGKTKKLKKVWTMLMAAKSEMADLQQEHQREIEGLLENIRQLSRELRLQMLIIDNFIPRDYQEMIENYVHWNEDIGEWQLKCVAYTGNNMRKQTPVPDKKEKDPFEVDLSHVYLAYTEESLRQSLMKLERPRTSKGKARPKTGRRKRSAKPETVIDSLLQ

>NP_002245.4

MASKTKASEALKVVARCRPLSRKEEAAGHEQILTMDVKLGQVTLRNPRAAPGELPKTFTFDAVYDASSKQADLYDETVRPLIDSVLQGFNGTVFAYGQTGTGKTYTMQGTWVEPELRGVIPNAFEHIFTHISRSQNQQYLVRASYLEIYQEEIRDLLSKEPGKRLELKENPETGVYIKDLSSFVTKNVKEIEHVMNLGNQTRAVGSTHMNEVSSRSHAIFIITVECSERGSDGQDHIRVGKLNLVDLAGSERQNKAGPNTAGGAATPSSGGGGGGGGSGGGAGGERPKEASKINLSLSALGNVIAALAGNRSTHIPYRDSKLTRLLQDSLGGNAKTIMVATLGPASHSYDESLSTLRFANRAKNIKNKPRVNEDPKDTLLREFQEEIARLKAQLEKRGMLGKRPRRKSSRRKKAVSAPPGYPEGPVIEAWVAEEEDDNNNNHRPPQPILESALEKNMENYLQEQKERLEEEKAAIQDDRSLVSEEKQKLLEEKEKMLEDLRREQQATELLAAKYKAMESKLLIGGRNIMDHTNEQQKMLELKRQEIAEQKRREREMQQEMMLRDEETMELRGTYTSLQQEVEVKTKKLKKLYAKLQAVKAEIQDQHDEYIRVRQDLEEAQNEQTRELKLKYLIIENFIPPEEKNKIMNRLFLDCEEEQWKFQPLVPAGVSSSQMKKRPTSAVGYKRPISQYARVAMAMGSHPRYRAENIMFLELDVSPPAVFEMEFSHDQEQDPRALHMERLMRLDSFLERPSTSKVRKSRSWCQSPQRPPPSTTHASLASASLRPATVADHE

1340 NP_060561.3 NP_065162.1

>NP_060561.3

MRQKRKGDLSPAELMMLTIGDVIKQLIEAHEQGKDIDLNKVKTKTAAKYGLSAQPRLVDIIAAVPPQYRKVLMPKLKAKPIRTASGIAVVAVMCKPHRCPHISFTGNICVYCPGGPDSDFEYSTQSYTGYEPTSMRAIRARYDPFLQTRHRIEQLKQLGHSVDKVEFIVMGGTFMALPEEYRDYFIRNLHDALSGHTSNNIYEAVKYSERSLTKCIGITIETRPDYCMKRHLSDMLTYGCTRLEIGVQSVYEDVARDTNRGHTVKAVCESFHLAKDSGFKVVAHMMPDLPNVGLERDIEQFTEFFENPAFRPDGLKLYPTLVIRGTGLYELWKSGRYKSYSPSDLVELVARILALVPPWTRVYRVQRDIPMPLVSSGVEHGNLRELALARMKDLGIQCRDVRTREVGIQEIHHKVRPYQVELVRRDYVANGGWETFLSYEDPDQDILIGLLRLRKCSEETFRFELGGGVSIVRELHVYGSVVPVSSRDPTKFQHQGFGMLLMEEAERIAREEHGSGKIAVISGVGTRNYYRKIGYRLQGPYMVKMLK

>NP_065162.1

MSNPGTRRNGSSIKIRLTVLCAKNLAKKDFFRLPDPFAKIVVDGSGQCHSTDTVKNTLDPKWNQHYDLYVGKTDSITISVWNHKKIHKKQGAGFLGCVRLLSNAISRLKDTGYQRLDLCKLNPSDTDAVRGQIVVSLQTRDRIGTGGSVVDCRGLLENEGTVYEDSGPGRPLSCFMEEPAPYTDSTGAAAGGGNCRFVESPSQDQRLQAQRLRNPDVRGSLQTPQNRPHGHQSPELPEGYEQRTTVQGQVYFLHTQTGVSTWHDPRIPSPSGTIPGGDAAFLYEFLLQGHTSEPRDLNSVNCDELGPLPPGWEVRSTVSGRIYFVDHNNRTTQFTDPRLHHIMNHQCQLKEPSQPLPLPSEGSLEDEELPAQRYERDLVQKLKVLRHELSLQQPQAGHCRIEVSREEIFEESYRQIMKMRPKDLKKRLMVKFRGEEGLDYGGVAREWLYLLCHEMLNPYYGLFQYSTDNIYMLQINPDSSINPDHLSYFHFVGRIMGLAVFHGHYINGGFTVPFYKQLLGKPIQLSDLESVDPELHKSLVWILENDITPVLDHTFCVEHNAFGRILQHELKPNGRNVPVTEENKKEYVRLYVNWRFMRGIEAQFLALQKGFNELIPQHLLKPFDQKELELIIGGLDKIDLNDWKSNTRLKHCVADSNIVRWFWQAVETFDEERRARLLQFVTGSTRVPLQGFKALQGSTGAAGPRLFTIHLIDANTDNLPKAHTCFNRIDIPPYESYEKLYEKLLTAVEETCGFAVE

1341 NP_004303.1 NP_005606.1

>NP_004303.1

MSKVFKKTSSNGKLSIYLGKRDFVDHVDTVEPIDGVVLVDPEYLKCRKLFVMLTCAFRYGRDDLEVIGLTFRKDLYVQTLQVVPAESSSPQGALTVLQERLLHKLGDNAYPFTLQMVTNLPCSVTLQPGPEDAGKPCGIDFEVKSFCAENPEETVSKRDYVRLVVRKVQFAPPEAGPGPSAQTIRRFLLSAQPLQLQAWMDREVHYHGEPISVNVSINNCTNKVIKKIKISVDQITDVVLYSLDKYTKTVFIQEFTETVAANSSFSQSFAVTPILAASCQKRGLALDGKLKHEDTNLASSTIIRPGMDKELLGILVSYKVRVNLMVSCGGILGDLTASDVGVELPLVLIHPKPSHEAASSEDIVIEEFTRKGEEESQKAVEAEGDEGS

>NP_005606.1

MVLCFPLLLLLLVLWGPVCPLHAWPKRLTKAHWFEIQHIQPSPLQCNRAMSGINNYTQHCKHQNTFLHDSFQNVAAVCDLLSIVCKNRRHNCHQSSKPVNMTDCRLTSGKYPQCRYSAAAQYKFFIVACDPPQKSDPPYKLVPVHLDSIL

1342 NP_002816.1 NP_077021.1

>NP_002816.1

MQAQQYQQQRRKFAAAFLAFIFILAAVDTAEAGKKEKPEKKVKKSDCGEWQWSVCVPTSGDCGLGTREGTRTGAECKQTMKTQRCKIPCNWKKQFGAECKYQFQAWGECDLNTALKTRTGSLKRALHNAECQKTVTISKPCGKLTKPKPQAESKKKKKEGKKQEKMLD

>NP_077021.1

MDKSGIDSLDHVTSDAVELANRSDNSSDSSLFKTQCIPYSPKGEKRNPIRKFVRTPESVHASDSSSDSSFEPIPLTIKAIFERFKNRKKRYKKKKKRRYQPTGRPRGRPEGRRNPIYSLIDKKKQFRSRGSGFPFLESENEKNAPWRKILTFEQAVARGFFNYIEKLKYEHHLKESLKQMNVGEDLENEDFDSRRYKFLDDDGSISPIEESTAEDEDATHLEDNECDIKLAGDSFIVSSEFPVRLSVYLEEEDITEEAALSKKRATKAKNTGQRGLKM

1343 NP_006245.2 NP_066928.1

>NP_006245.2

MAPFLRIAFNSYELGSLQAEDEANQPFCAVKMKEALSTERGKTLVQKKPTMYPEWKSTFDAHIYEGRVIQIVLMRAAEEPVSEVTVGVSVLAERCKKNNGKAEFWLDLQPQAKVLMSVQYFLEDVDCKQSMRSEDEAKFPTMNRRGAIKQAKIHYIKNHEFIATFFGQPTFCSVCKDFVWGLNKQGYKCRQCNAAIHKKCIDKIIGRCTGTAANSRDTIFQKERFNIDMPHRFKVHNYMSPTFCDHCGSLLWGLVKQGLKCEDCGMNVHHKCREKVANLCGINQKLLAEALNQVTQRASRRSDSASSEPVGIYQGFEKKTGVAGEDMQDNSGTYGKIWEGSSKCNINNFIFHKVLGKGSFGKVLLGELKGRGEYFAIKALKKDVVLIDDDVECTMVEKRVLTLAAENPFLTHLICTFQTKDHLFFVMEFLNGGDLMYHIQDKGRFELYRATFYAAEIMCGLQFLHSKGIIYRDLKLDNVLLDRDGHIKIADFGMCKENIFGESRASTFCGTPDYIAPEILQGLKYTFSVDWWSFGVLLYEMLIGQSPFHGDDEDELFESIRVDTPHYPRWITKESKDILEKLFEREPTKRLGVTGNIKIHPFFKTINWTLLEKRRLEPPFRPKVKSPRDYSNFDQEFLNEKARLSYSDKNLIDSMDQSAFAGFSFVNPKFEHLLED

>NP_066928.1

MDKQNSQMNASHPETNLPVGYPPQYPPTAFQGPPGYSGYPGPQVSYPPPPAGHSGPGPAGFPVPNQPVYNQPVYNQPVGAAGVPWMPAPQPPLNCPPGLEYLSQIDQILIHQQIELLEVLTGFETNNKYEIKNSFGQRVYFAAEDTDCCTRNCCGPSRPFTLRIIDNMGQEVITLERPLRCSSCCCPCCLQEIEIQAPPGVPIGYVIQTWHPCLPKFTIQNEKREDVLKISGPCVVCSCCGDVDFEIKSLDEQCVVGKISKHWTGILREAFTDADNFGIQFPLDLDVKMKAVMIGACFLIDFMFFESTGSQEQKSGVW

1344 NP_055602.1 NP_004850.1

>NP_055602.1

MKDSENKGASSPDMEPSYGGGLFDMVKGGAGRLFSNLKDNLKDTLKDTSSRVIQSVTSYTKGDLDFTYVTSRIIVMSFPLDNVDIGFRNQVDDIRSFLDSRHLDHYTVYNLSPKSYRTAKFHSRVSECSWPIRQAPSLHNLFAVCRNMYNWLLQNPKNVCVVHCLDGRAASSILVGAMFIFCNLYSTPGPAIRLLYAKRPGIGLSPSHRRYLGYMCDLLADKPYRPHFKPLTIKSITVSPIPFFNKQRNGCRPYCDVLIGETKIYSTCTDFERMKEYRVQDGKIFIPLNITVQGDVVVSMYHLRSTIGSRLQAKVTNTQIFQLQFHTGFIPLDTTVLKFTKPELDACDVPEKYPQLFQVTLDVELQPHDKVIDLTPPWEHYCTKDVNPSILFSSHQEHQDTLALGGQAPIDIPPDNPRHYGQSGFFASLCWQDQKSEKSFCEEDHAALVNQESEQSDDELLTLSSPHGNANGDKPHGVKKPSKKQQEPAAPPPPEDVDLLGLEGSAMSNSFSPPAAPPTNSELLSDLFGGGGAAGPTQAGQSGVEDVFHPSGPASTQSTPRRSATSTSASPTLRVGEGATFDPFGAPSKPSGQDLLGSFLNTSSASSDPFLQPTRSPSPTVHASSTPAVNIQPDVSGGWDWHAKPGGFGMGSKSAATSPTGSSHGTPTHQSKPQTLDPFADLGTLGSSSFASKPTTPTGLGGGFPPLSSPQKASPQPMGGGWQQGGAYNWQQPQPKPQPSMPHSSPQNRPNYNVSFSAMPGGQNERGKGSSNLEGKQKAADFEDLLSGQGFNAHKDKKGPRTIAEMRKEEMAKEMDPEKLKILEWIEGKERNIRALLSTMHTVLWAGETKWKPVGMADLVTPEQVKKVYRKAVLVVHPDKATGQPYEQYAKMIFMELNDAWSEFENQGQKPLY

>NP_004850.1

MAQILPIRFQEHLQLQNLGINPANIGFSTLTMESDKFICIREKVGEQAQVVIIDMNDPSNPIRRPISADSAIMNPASKVIALKAGKTLQIFNIEMKSKMKAHTMTDDVTFWKWISLNTVALVTDNAVYHWSMEGESQPVKMFDRHSSLAGCQIINYRTDAKQKWLLLTGISAQQNRVVGAMQLYSVDRKVSQPIEGHAASFAQFKMEGNAEESTLFCFAVRGQAGGKLHIIEVGTPPTGNQPFPKKAVDVFFPPEAQNDFPVAMQISEKHDVVFLITKYGYIHLYDLETGTCIYMNRISGETIFVTAPHEATAGIIGVNRKGQVLSVCVEEENIIPYITNVLQNPDLALRMAVRNNLAGAEELFARKFNALFAQGNYSEAAKVAANAPKGILRTPDTIRRFQSVPAQPGQTSPLLQYFGILLDQGQLNKYESLELCRPVLQQGRKQLLEKWLKEDKLECSEELGDLVKSVDPTLALSVYLRANVPNKVIQCFAETGQVQKIVLYAKKVGYTPDWIFLLRNVMRISPDQGQQFAQMLVQDEEPLADITQIVDVFMEYNLIQQCTAFLLDALKNNRPSEGPLQTRLLEMNLMHAPQVADAILGNQMFTHYDRAHIAQLCEKAGLLQRALEHFTDLYDIKRAVVHTHLLNPEWLVNYFGSLSVEDSLECLRAMLSANIRQNLQICVQVASKYHEQLSTQSLIELFESFKSFEGLFYFLGSIVNFSQDPDVHFKYIQAACKTGQIKEVERICRESNCYDPERVKNFLKEAKLTDQLPLIIVCDRFDFVHDLVLYLYRNNLQKYIEIYVQKVNPSRLPVVIGGLLDVDCSEDVIKNLILVVRGQFSTDELVAEVEKRNRLKLLLPWLEARIHEGCEEPATHNALAKIYIDSNNNPERFLRENPYYDSRVVGKYCEKRDPHLACVAYERGQCDLELINVCNENSLFKSLSRYLVRRKDPELWGSVLLESNPYRRPLIDQVVQTALSETQDPEEVSVTVKAFMTADLPNELIELLEKIVLDNSVFSEHRNLQNLLILTAIKADRTRVMEYINRLDNYDAPDIANIAISNELFEEAFAIFRKFDVNTSAVQVLIEHIGNLDRAYEFAERCNEPAVWSQLAKAQLQKGMVKEAIDSYIKADDPSSYMEVVQAANTSGNWEELVKYLQMARKKARESYVETELIFALAKTNRLAELEEFINGPNNAHIQQVGDRCYDEKMYDAAKLLYNNVSNFGRLASTLVHLGEYQAAVDGARKANSTRTWKEVCFACVDGKEFRLAQMCGLHIVVHADELEELINYYQDRGYFEELITMLEAALGLERAHMGMFTELAILYSKFKPQKMREHLELFWSRVNIPKVLRAAEQAHLWAELVFLYDKYEEYDNAIITMMNHPTDAWKEGQFKDIITKVANVELYYRAIQFYLEFKPLLLNDLLMVLSPRLDHTRAVNYFSKVKQLPLVKPYLRSVQNHNNKSVNESLNNLFITEEDYQALRTSIDAYDNFDNISLAQRLEKHELIEFRRIAAYLFKGNNRWKQSVELCKKDSLYKDAMQYASESKDTELAEELLQWFLQEEKRECFGACLFTCYDLLRPDVVLETAWRHNIMDFAMPYFIQVMKEYLTKVDKLDASESLRKEEEQATETQPIVYGQPQLMLTAGPSVAVPPQAPFGYGYTAPPYGQPQPGFGYSM

1345 NP_004635.2 NP_001275.1

>NP_004635.2

MSAAPAYSEDKGGSAGPGEPEYGHDPASGGIFSSDYKRHDDLKEMLDTNKDSLKLEAMKRIVAMIARGKNASDLFPAVVKNVACKNIEVKKLVYVYLVRYAEEQQDLALLSISTFQRGLKDPNQLIRASALRVLSSIRVPIIVPIMMLAIKEAASDMSPYVRKTAAHAIPKLYSLDSDQKDQLIEVIEKLLADKTTLVAGSVVMAFEEVCPERIDLIHKNYRKLCNLLIDVEEWGQVVIISMLTRYARTQFLSPTQNESLLEENAEKAFYGSEEDEAKGAGSEETAAAAAPSRKPYVMDPDHRLLLRNTKPLLQSRSAAVVMAVAQLYFHLAPKAEVGVIAKALVRLLRSHSEVQYVVLQNVATMSIKRRGMFEPYLKSFYIRSTDPTQIKILKLEVLTNLANETNIPTVLREFQTYIRSMDKDFVAATIQAIGRCATNIGRVRDTCLNGLVQLLSNRDELVVAESVVVIKKLLQMQPAQHGEIIKHLAKLTDNIQVPMARASILWLIGEYCEHVPRIAPDVLRKMAKSFTAEEDIVKLQVINLAAKLYLTNSKQTKLLTQYVLSLAKYDQNYDIRDRARFTRQLIVPSEQGGALSRHAKKLFLAPKPAPVLESSFKDRDHFQLGSLSHLLNAKATGYQELPDWPEEAPDPSVRNVEVPEWTKCSNREKRKEKEKPFYSDSEGESGPTESADSDPESESESDSKSSSESGSGESSSESDNEDQDEDEEKGRGSESEQSEEDGKRKTKKKVPERKGEASSSDEGSDSSSSSSESEMTSESEEEQLEPASWSRKTPPSSKSAPATKEISLLDLEDFTPPSVQPVSPPAIVSTSLAADLEGLTLTDSTLVPSLLSPVSGVGRQELLHRVAGEGLAVDYTFSRQPFSGDPHMVSVHIHFSNSSDTPIKGLHVGTPKLPAGISIQEFPEIESLAPGESATAVMGINFCDSTQAANFQLCTQTRQFYVSIQPPVGELMAPVFMSENEFKKEQGKLMGMNEITEKLMLPDTCRSDHIVVQKVTATANLGRVPCGTSDEYRFAGRTLTGGSLVLLTLDARPAGAAQLTVNSEKMVIGTMLVKDVIQALTQ

>NP_001275.1

MIKAILIFNNHGKPRLSKFYQPYSEDTQQQIIRETFHLVSKRDENVCNFLEGGLLIGGSDNKLIYRHYATLYFVFCVDSSESELGILDLIQVFVETLDKCFENVCELDLIFHVDKVHNILAEMVMGGMVLETNMNEIVTQIDAQNKLEKSEAGLAGAPARAVSAVKNMNLPEIPRNINIGDISIKVPNLPSFK

1346 NP_006275.1 NP_003175.1

>NP_006275.1

MSCSGSGADPEAAPASAASAPGPAPPVSAPAALPSSTAAENKASPAGTAGGPGAGAAAGGTGPLAARAGEPAERRGAAPVSAGGAAPPEGAISNGVYVLPSAANGDVKPVVSSTPLVDFLMQLEDYTPTIPDAVTGYYLNRAGFEASDPRIIRLISLAAQKFISDIANDALQHCKMKGTASGSSRSKSKDRKYTLTMEDLTPALSEYGINVKKPHYFT

>NP_003175.1

MPLTGVEPARMNRKKGDKGFESPRPYKLTHQVVCINNINFQRKSVVGFVELTIFPTVANLNRIKLNSKQCRIYRVRINDLEAAFIYNDPTLEVCHSESKQRNLNYFSNAYAAAVSAVDPDAGNGELCIKVPSELWKHVDELKVLKIHINFSLDQPKGGLHFVVPSVEGSMAERGAHVFSCGYQNSTRFWFPCVDSYSELCTWKLEFTVDAAMVAVSNGDLVETVYTHDMRKKTFHYMLTIPTAASNISLAIGPFEILVDPYMHEVTHFCLPQLLPLLKHTTSYLHEVFEFYEEILTCRYPYSCFKTVFIDEAYVEVAAYASMSIFSTNLLHSAMIIDETPLTRRCLAQSLAQQFFGCFISRMSWSDEWVLKGISGYIYGLWMKKTFGVNEYRHWIKEELDKIVAYELKTGGVLLHPIFGGGKEKDNPASHLHFSIKHPHTLSWEYYTMFQCKAHLVMRLIENRISMEFMLQVFNKLLSLASTASSQKFQSHMWSQMLVSTSGFLKSISNVSGKDIQPLIKQWVDQSGVVKFYGSFAFNRKRNVLELEIKQDYTSPGTQKYVGPLKVTVQELDGSFNHTLQIEENSLKHDIPCHSKSRRNKKKKIPLMNGEEVDMDLSAMDADSPLLWIRIDPDMSVLRKVEFEQADFMWQYQLRYERDVVAQQESILALEKFPTPASRLALTDILEQEQCFYRVRMSACFCLAKIANSMVSTWTGPPAMKSLFTRMFCCKSCPNIVKTNNFMSFQSYFLQKTMPVAMALLRDVHNLCPKEVLTFILDLIKYNDNRKNKFSDNYYRAEMIDALANSVTPAVSVNNEVRTLDNLNPDVRLILEEITRFLNMEKLLPSYRHTITVSCLRAIRVLQKNGHVPSDPALFKSYAEYGHFVDIRIAALEAVVDYTKVDRSYEELQWLLNMIQNDPVPYVRHKILNMLTKNPPFTKNMESPLCNEALVDQLWKLMNSGTSHDWRLRCGAVDLYFTLFGLSRPSCLPLPELGLVLNLKEKKAVLNPTIIPESVAGNQEAANNPSSHPQLVGFQNPFSSSQDEEEIDMDTVHDSQAFISHHLNMLERPSTPGLSKYRPASSRSALIPQHSAGCDSTPTTKPQWSLELARKGTGKEQAPLEMSMHPAASAPLSVFTKESTASKHSDHHHHHHHEHKKKKKKHKHKHKHKHKHDSKEKDKEPFTFSSPASGRSIRSPSLSD

1347 NP_002654.2 NP_000396.2

>NP_002654.2

MTATPESLFPTGDELDSSQLQMESDEVDTLKEGEDPADRMHPFLAIYELQSLKVHPLVFAPGVPVTAQVVGTERYTSGSKVGTCTLYSVRLTHGDFSWTTKKKYRHFQELHRDLLRHKVLMSLLPLARFAVAYSPARDAGNREMPSLPRAGPEGSTRHAASKQKYLENYLNCLLTMSFYRNYHAMTEFLEVSQLSFIPDLGRKGLEGMIRKRSGGHRVPGLTCCGRDQVCYRWSKRWLVVKDSFLLYMCLETGAISFVQLFDPGFEVQVGKRSTEARHGVRIDTSHRSLILKCSSYRQARWWAQEITELAQGPGRDFLQLHRHDSYAPPRPGTLARWFVNGAGYFAAVADAILRAQEEIFITDWWLSPEVYLKRPAHSDDWRLDIMLKRKAEEGVRVSILLFKEVELALGINSGYSKRALMLLHPNIKVMRHPDQVTLWAHHEKLLVVDQVVAFLGGLDLAYGRWDDLHYRLTDLGDSSESAASQPPTPRPDSPATPDLSHNQFFWLGKDYSNLITKDWVQLDRPFEDFIDRETTPRMPWRDVGVVVHGLPARDLARHFIQRWNFTKTTKAKYKTPIYPYLLPKSTSTANQLPFTLPGGQCTTVQVLRSVDRWSAGTLENSILNAYLHTIRESQHFLYIENQFFISCSDGRTVLNKVGDEIVDRILKAHKQGWCYRVYVLLPLLPGFEGDISTGGGNSIQAILHFTYRTLCRGEYSILHRLKAAMGTAWRDYISICGLRTHGELGGHPVSELIYIHSKVLIADDRTVIIGSANINDRSLLGKRDSELAVLIEDTETEPSLMNGAEYQAGRFALSLRKHCFGVILGANTRPDLDLRDPICDDFFQLWQDMAESNANIYEQIFRCLPSNATRSLRTLREYVAVEPLATVSPPLARSELTQVQGHLVHFPLKFLEDESLLPPLGSKEGMIPLEVWT

>NP_000396.2

MQSLMQAPLLIALGLLLAAPAQAHLKKPSQLSSFSWDNCDEGKDPAVIRSLTLEPDPIIVPGNVTLSVMGSTSVPLSSPLKVDLVLEKEVAGLWIKIPCTDYIGSCTFEHFCDVLDMLIPTGEPCPEPLRTYGLPCHCPFKEGTYSLPKSEFVVPDLELPSWLTTGNYRIESVLSSSGKRLGCIKIAASLKGI

1348 NP_005598.3 NP_002010.1

>NP_005598.3

MISADCNLCLPEYDRYLASSKIMAAAYLDPNLNHTPNSSTKTHLGTGMERSPGAMERVLKVFHYFESNSEPTTWASIIRHGDATDVRGIIQKIVDSHKVKHVACYGFRLSHLRSEEVHWLHVDMGVSSVREKYELAHPPEEWKYELRIRYLPKGFLNQFTEDKPTLNFFYQQVKSDYMLEIADQVDQEIALKLGCLEIRRSYWEMRGNALEKKSNYEVLEKDVGLKRFFPKSLLDSVKAKTLRKLIQQTFRQFANLNREESILKFFEILSPVYRFDKECFKCALGSSWIISVELAIGPEEGISYLTDKGCNPTHLADFTQVQTIQYSNSEDKDRKGMLQLKIAGAPEPLTVTAPSLTIAENMADLIDGYCRLVNGTSQSFIIRPQKEGERALPSIPKLANSEKQGMRTHAVSVSETDDYAEIIDEEDTYTMPSTRDYEIQRERIELGRCIGEGQFGDVHQGIYMSPENPALAVAIKTCKNCTSDSVREKFLQEALTMRQFDHPHIVKLIGVITENPVWIIMELCTLGELRSFLQVRKYSLDLASLILYAYQLSTALAYLESKRFVHRDIAARNVLVSSNDCVKLGDFGLSRYMEDSTYYKASKGKLPIKWMAPESINFRRFTSASDVWMFGVCMWEILMHGVKPFQGVKNNDVIGRIENGERLPMPPNCPPTLYSLMTKCWAYDPSRRPRFTELKAQLSTILEEEKAQQEERMRMESRRQATVSWDSGGSDEAPPKPSRPGYPSPRSSEGFYPSPQHMVQTNHYQVSGYPGSHGITAMAGSIYPGQASLLDQTDSWNHRPQEIAMWQPNVEDSTVLDLRGIGQVLPTHLMEERLIRQQQEMEEDQRWLEKEERFLKPDVRLSRGSIDREDGSLQGPIGNQHIYQPVGKPDPAAPPKKPPRPGAPGHLGSLASLSSPADSYNEGVKLQPQEISPPPTANLDRSNDKVYENVTGLVKAVIEMSSKIQPAPPEEYVPMVKEVGLALRTLLATVDETIPLLPASTHREIEMAQKLLNSDLGELINKMKLAQQYVMTSLQQEYKKQMLTAAHALAVDAKNLLDVIDQARLKMLGQTRPH

>NP_002010.1

MVSYWDTGVLLCALLSCLLLTGSSSGSKLKDPELSLKGTQHIMQAGQTLHLQCRGEAAHKWSLPEMVSKESERLSITKSACGRNGKQFCSTLTLNTAQANHTGFYSCKYLAVPTSKKKETESAIYIFISDTGRPFVEMYSEIPEIIHMTEGRELVIPCRVTSPNITVTLKKFPLDTLIPDGKRIIWDSRKGFIISNATYKEIGLLTCEATVNGHLYKTNYLTHRQTNTIIDVQISTPRPVKLLRGHTLVLNCTATTPLNTRVQMTWSYPDEKNKRASVRRRIDQSNSHANIFYSVLTIDKMQNKDKGLYTCRVRSGPSFKSVNTSVHIYDKAFITVKHRKQQVLETVAGKRSYRLSMKVKAFPSPEVVWLKDGLPATEKSARYLTRGYSLIIKDVTEEDAGNYTILLSIKQSNVFKNLTATLIVNVKPQIYEKAVSSFPDPALYPLGSRQILTCTAYGIPQPTIKWFWHPCNHNHSEARCDFCSNNEESFILDADSNMGNRIESITQRMAIIEGKNKMASTLVVADSRISGIYICIASNKVGTVGRNISFYITDVPNGFHVNLEKMPTEGEDLKLSCTVNKFLYRDVTWILLRTVNNRTMHYSISKQKMAITKEHSITLNLTIMNVSLQDSGTYACRARNVYTGEEILQKKEITIRDQEAPYLLRNLSDHTVAISSSTTLDCHANGVPEPQITWFKNNHKIQQEPGIILGPGSSTLFIERVTEEDEGVYHCKATNQKGSVESSAYLTVQGTSDKSNLELITLTCTCVAATLFWLLLTLLIRKMKRSSSEIKTDYLSIIMDPDEVPLDEQCERLPYDASKWEFARERLKLGKSLGRGAFGKVVQASAFGIKKSPTCRTVAVKMLKEGATASEYKALMTELKILTHIGHHLNVVNLLGACTKQGGPLMVIVEYCKYGNLSNYLKSKRDLFFLNKDAALHMEPKKEKMEPGLEQGKKPRLDSVTSSESFASSGFQEDKSLSDVEEEEDSDGFYKEPITMEDLISYSFQVARGMEFLSSRKCIHRDLAARNILLSENNVVKICDFGLARDIYKNPDYVRKGDTRLPLKWMAPESIFDKIYSTKSDVWSYGVLLWEIFSLGGSPYPGVQMDEDFCSRLREGMRMRAPEYSTPEIYQIMLDCWHRDPKERPRFAELVEKLGDLLQANVQQDGKDYIPINAILTGNSGFTYSTPAFSEDFFKESISAPKFNSGSSDDVRYVNAFKFMSLERIKTFEELLPNATSMFDDYQGDSSTLLASPMLKRFTWTDSKPKASLKIDLRVTSKSKESGLSDVSRPSFCHSSCGHVSEGKRRFTYDHAELERKIACCSPPPDYNSVVLYSTPPI

1349 NP_004078.2 NP_066943.2

>NP_004078.2

MPVRKQDTQRALHLLEEYRSKLSQTEDRQLRSSIERVINIFQSNLFQALIDIQEFYEVTLLDNPKCIDRSKPSEPIQPVNTWEISSLPSSTVTSETLPSSLSPSVEKYRYQDEDTPPQEHISPQITNEVIGPELVHVSEKNLSEIENVHGFVSHSHISPIKPTEAVLPSPPTVPVIPVLPVPAENTVILPTIPQANPPPVLVNTDSLETPTYVNGTDADYEYEEITLERGNSGLGFSIAGGTDNPHIGDDSSIFITKIITGGAAAQDGRLRVNDCILRVNEVDVRDVTHSKAVEALKEAGSIVRLYVKRRKPVSEKIMEIKLIKGPKGLGFSIAGGVGNQHIPGDNSIYVTKIIEGGAAHKDGKLQIGDKLLAVNNVCLEEVTHEEAVTALKNTSDFVYLKVAKPTSMYMNDGYAPPDITNSSSQPVDNHVSPSSFLGQTPASPARYSPVSKAVLGDDEITREPRKVVLHRGSTGLGFNIVGGEDGEGIFISFILAGGPADLSGELRKGDRIISVNSVDLRAASHEQAAAALKNAGQAVTIVAQYRPEEYSRFEAKIHDLREQMMNSSISSGSGSLRTSQKRSLYVRALFDYDKTKDSGLPSQGLNFKFGDILHVINASDDEWWQARQVTPDGESDEVGVIPSKRRVEKKERARLKTVKFNSKTRDKGQSFNDKRKKNLFSRKFPFYKNKDQSEQETSDADQHVTSNASDSESSYRGQEEYVLSYEPVNQQEVNYTRPVIILGPMKDRINDDLISEFPDKFGSCVPHTTRPKRDYEVDGRDYHFVTSREQMEKDIQEHKFIEAGQYNNHLYGTSVQSVREVAEKGKHCILDVSGNAIKRLQIAQLYPISIFIKPKSMENIMEMNKRLTEEQARKTFERAMKLEQEFTEHFTAIVQGDTLEDIYNQVKQIIEEQSGSYIWVPAKEKL

>NP_066943.2

MHKHQHCCKCPECYEVTRLAALRRLEPPGYGDWQVPDPYGPGGGNGASAGYGGYSSQTLPSQAGATPTPRTKAKLIPTGRDVGPVPPKPVPGKSTPKLNGSGPSWWPECTCTNRDWYEQVNGSDGMFKYEEIVLERGNSGLGFSIAGGIDNPHVPDDPGIFITKIIPGGAAAMDGRLGVNDCVLRVNEVDVSEVVHSRAVEALKEAGPVVRLVVRRRQPPPETIMEVNLLKGPKGLGFSIAGGIGNQHIPGDNSIYITKIIEGGAAQKDGRLQIGDRLLAVNNTNLQDVRHEEAVASLKNTSDMVYLKVAKPGSLHLNDMYAPPDYASTFTALADNHISHNSSLGYLGAVESKVSYPAPPQVPPTRYSPIPRHMLAEEDFTREPRKIILHKGSTGLGFNIVGGEDGEGIFVSFILAGGPADLSGELRRGDRILSVNGVNLRNATHEQAAAALKRAGQSVTIVAQYRPEEYSRFESKIHDLREQMMNSSMSSGSGSLRTSEKRSLYVRALFDYDRTRDSCLPSQGLSFSYGDILHVINASDDEWWQARLVTPHGESEQIGVIPSKKRVEKKERARLKTVKFHARTGMIESNRDFPGLSDDYYGAKNLKGQEDAILSYEPVTRQEIHYARPVIILGPMKDRVNDDLISEFPHKFGSCVPHTTRPRRDNEVDGQDYHFVVSREQMEKDIQDNKFIEAGQFNDNLYGTSIQSVRAVAERGKHCILDVSGNAIKRLQQAQLYPIAIFIKPKSIEALMEMNRRQTYEQANKIYDKAMKLEQEFGEYFTAIVQGDSLEEIYNKIKQIIEDQSGHYIWVPSPEKL

1350 NP_115976.2 NP_003812.1

>NP_115976.2

MATESTPSEIIERERKKLLEILQHDPDSILDTLTSRRLISEEEYETLENVTDLLKKSRKLLILVQKKGEATCQHFLKCLFSTFPQSAAICGLRHEVLKHENTVPPQSMGASSNSEDAFSPGIKQPEAPEITVFFSEKEHLDLETSEFFRDKKTSYRETALSARKNEKEYDTPEVTLSYSVEKVGCEVPATITYIKDGQRYEELDDSLYLGKEEYLGSVDTPEDAEATVEEEVYDDPEHVGYDGEEDFENSETTEFSGEEPSYEGSETSLSLEEEQEKSIEERKKVFKDVLLCLNMDRSRKVLPDFVKQFSLDRGCKWTPESPGDLAWNFLMKVQARDVTARDSILSHKVLDEDSKEDLLAGVENLEIRDIQTINPLDVLCATMLCSDSSLQRQVMSNMYQCQFALPLLLPDAENNKSILMLGAMKDIVKKQSTQFSGGPTEDTEKFLTLMKMPVISFVRLGYCSFSKSRILNTLLSPAQLKLHKIFLHQDLPLLVLPRQISDGLVEITWCFPDSDDRKENPFFQKPVALANLRGNLESFWTQFGFLMEVSSAVFFFTDCLGEKEWDLLMFLGEAAIERCYFVLSSQARESEEAQIFQRILNLKPAQLLFWERGDAGDRRKNMEGLQAALQEVMFSSCLRCVSVEDMAALARELGIQVDEDFENTQRIQVSSGENMAGTAEGEGQQRHSQLKSSSKSQALMPIQEPGTQCELSQNLQNLYGTPVFRPVLENSWLFPTRIGGNFNHVSLKASWVMGRPFGSEQRPKWFHPLPFQNAGAQGRGKSFGIQSFHPQIFYSGERFMKFSRVARGCHSNGTFGRLPRPICQHVQACPERPQMMGTLERSRAVASKIGHSYSLDSQPARAVGKPWPQQACTRVTELTEATGKLIRTSHIGKPHPQSFQPAAATQKLRPASQQGVQMKTQGGASNPALQIGSHPMCKSSQFKSDQSNPSTVKHSQPKPFHSVPSQPKSSQTKSCQSQPSQTKPSPCKSTQPKPSQPWPPQSKPSQPRPPQPKSSSTNPSQAKAHHSKAGQKRGGKH

>NP_003812.1

MNGEAICSALPTIPYHKLADLRYLSRGASGTVSSARHADWRVQVAVKHLHIHTPLLDSERKDVLREAEILHKARFSYILPILGICNEPEFLGIVTEYMPNGSLNELLHRKTEYPDVAWPLRFRILHEIALGVNYLHNMTPPLLHHDLKTQNILLDNEFHVKIADFGLSKWRMMSLSQSRSSKSAPEGGTIIYMPPENYEPGQKSRASIKHDIYSYAVITWEVLSRKQPFEDVTNPLQIMYSVSQGHRPVINEESLPYDIPHRARMISLIESGWAQNPDERPSFLKCLIELEPVLRTFEEITFLEAVIQLKKTKLQSVSSAIHLCDKKKMELSLNIPVNHGPQEESCGSSQLHENSGSPETSRSLPAPQDNDFLSRKAQDCYFMKLHHCPGNHSWDSTISGSQRAAFCDHKTTPCSSAIINPLSTAGNSERLQPGIAQQWIQSKREDIVNQMTEACLNQSLDALLSRDLIMKEDYELVSTKPTRTSKVRQLLDTTDIQGEEFAKVIVQKLKDNKQMGLQPYPEILVVSRSPSLNLLQNKSM

1351 NP_001217.2 NP_000438.1

>NP_001217.2

MSSASGLRRGHPAGGEENMTETDAFYKREMFDPAEKYKMDHRRRGIALIFNHERFFWHLTLPERRGTCADRDNLTRRFSDLGFEVKCFNDLKAEELLLKIHEVSTVSHADADCFVCVFLSHGEGNHIYAYDAKIEIQTLTGLFKGDKCHSLVGKPKIFIIQACRGNQHDVPVIPLDVVDNQTEKLDTNITEVDAASVYTLPAGADFLMCYSVAEGYYSHRETVNGSWYIQDLCEMLGKYGSSLEFTELLTLVNRKVSQRRVDFCKDPSAIGKKQVPCFASMLTKKLHFFPKSN

>NP_000438.1

MLTFMASDSEEEVCDERTSLMSAESPTPRSCQEGRQGPEDGENTAQWRSQENEEDGEEDPDRYVCSGVPGRPPGLEEELTLKYGAKHVIMLFVPVTLCMIVVVATIKSVRFYTEKNGQLIYTTFTEDTPSVGQRLLNSVLNTLIMISVIVVMTIFLVVLYKYRCYKFIHGWLIMSSLMLLFLFTYIYLGEVLKTYNVAMDYPTLLLTVWNFGAVGMVCIHWKGPLVLQQAYLIMISALMALVFIKYLPEWSAWVILGAISVYDLVAVLCPKGPLRMLVETAQERNEPIFPALIYSSAMVWTVGMAKLDPSSQGALQLPYDPEMEEDSYDSFGEPSYPEVFEPPLTGYPGEELEEEEERGVKLGLGDFIFYSVLVGKAAATGSGDWNTTLACFVAILIGLCLTLLLLAVFKKALPALPISITFGLIFYFSTDNLVRPFMDTLASHQLYI

1352 NP_008830.2 NP_004126.1

>NP_008830.2

MAALSGVRWLTRALVSAGNPGAWRGLSTSAAAHAASRSQAEDVRVEGSFPVTMLPGDGVGPELMHAVKEVFKAAAVPVEFQEHHLSEVQNMASEEKLEQVLSSMKENKVAIIGKIHTPMEYKGELASYDMRLRRKLDLFANVVHVKSLPGYMTRHNNLDLVIIREQTEGEYSSLEHESARGVIECLKIVTRAKSQRIAKFAFDYATKKGRGKVTAVHKANIMKLGDGLFLQCCEEVAELYPKIKFETMIIDNCCMQLVQNPYQFDVLVMPNLYGNIIDNLAAGLVGGAGVVPGESYSAEYAVFETGARHPFAQAVGRNIANPTAMLLSASNMLRHLNLEYHSSMIADAVKKVIKVGKVRTRDMGGYSTTTDFIKSVIGHLQTKGS

>NP_004126.1

MALKVATVAGSAAKAVLGPALLCRPWEVLGAHEVPSRNIFSEQTIPPSAKYGGRHTVTMIPGDGIGPELMLHVKSVFRHACVPVDFEEVHVSSNADEEDIRNAIMAIRRNRVALKGNIETNHNLPPSHKSRNNILRTSLDLYANVIHCKSLPGVVTRHKDIDILIVRENTEGEYSSLEHESVAGVVESLKIITKAKSLRIAEYAFKLAQESGRKKVTAVHKANIMKLGDGLFLQCCREVAARYPQITFENMIVDNTTMQLVSRPQQFDVMVMPNLYGNIVNNVCAGLVGGPGLVAGANYGHVYAVFETATRNTGKSIANKNIANPTATLLASCMMLDHLKLHSYATSIRKAVLASMDNENMHTPDIGGQGTTSEAIQDVIRHIRVINGRAVEA

1353 NP_001828.1 NP_002976.2

>NP_001828.1

MTTSLDTVETFGTTSYYDDVGLLCEKADTRALMAQFVPPLYSLVFTVGLLGNVVVVMILIKYRRLRIMTNIYLLNLAISDLLFLVTLPFWIHYVRGHNWVFGHGMCKLLSGFYHTGLYSEIFFIILLTIDRYLAIVHAVFALRARTVTFGVITSIVTWGLAVLAALPEFIFYETEELFEETLCSALYPEDTVYSWRHFHTLRMTIFCLVLPLLVMAICYTGIIKTLLRCPSKKKYKAIRLIFVIMAVFFIFWTPYNVAILLSSYQSILFGNDCERSKHLDLVMLVTEVIAYSHCCMNPVIYAFVGERFRKYLRHFFHRHLLMHLGRYIPFLPSEKLERTSSVSPSTAEPELSIVF

>NP_002976.2

MKVSAAALAVILIATALCAPASASPYSSDTTPCCFAYIARPLPRAHIKEYFYTSGKCSNPAVVFVTRKNRQVCANPEKKWVREYINSLEMS

1354 NP_060879.2 NP_003644.2

>NP_060879.2

MKLTSEKLPKNPFYASVSQYAAKNQKFFQWKKEKTDYTHANLVDKALQLLKERILKGDTLAYFLRGQLYFEEGWYEEALEQFEEIKEKDHQATYQLGVMYYDGLGTTLDAEKGVDYMKKILDSPCPKARHLKFAAAYNLGRAYYEGKGVKRSNEEAERLWLIAADNGNPKASVKAQSMLGLYYSTKEPKELEKAFYWHSEACGNGNLESQGALGLMYLYGQGIRQDTEAALQCLREAAERGNVYAQGNLVEYYYKMKFFTKCVAFSKRIADYDEVHDIPMIAQVTDCLPEFIGRGMAMASFYHARCLQLGLGITRDETTAKHYYSKACRLNPALADELHSLLIRQRI

>NP_003644.2

MASALEQFVNSVRQLSAQGQMTQLCELINKSGELLAKNLSHLDTVLGALDVQEHSLGVLAVLFVKFSMPSVPDFETLFSQVQLFISTCNGEHIRYATDTFAGLCHQLTNALVERKQPLRGIGILKQAIDKMQMNTNQLTSIHADLCQLCLLAKCFKPALPYLDVDMMDICKENGAYDAKHFLCYYYYGGMIYTGLKNFERALYFYEQAITTPAMAVSHIMLESYKKYILVSLILLGKVQQLPKYTSQIVGRFIKPLSNAYHELAQVYSTNNPSELRNLVNKHSETFTRDNNMGLVKQCLSSLYKKNIQRLTKTFLTLSLQDMASRVQLSGPQEAEKYVLHMIEDGEIFASINQKDGMVSFHDNPEKYNNPAMLHNIDQEMLKCIELDERLKAMDQEITVNPQFVQKSMGSQEDDSGNKPSSYS

1355 NP_976226.1 NP_036526.2

>NP_976226.1

MDEQEALNSIMNDLVALQMNRRHRMPGYETMKNKDTGHSNRQKKHNSSSSALLNSPTVTTSSCAGASEKKKFLSDVRIKFEHNGERRIIAFSRPVKYEDVEHKVTTVFGQPLDLHYMNNELSILLKNQDDLDKAIDILDRSSSMKSLRILLLSQDRNHNSSSPHSGVSRQVRIKASQSAGDINTIYQPPEPRSRHLSVSSQNPGRSSPPPGYVPERQQHIARQGSYTSINSEGEFIPETSEQCMLDPLSSAENSLSGSCQSLDRSADSPSFRKSRMSRAQSFPDNRQEYSDRETQLYDKGVKGGTYPRRYHVSVHHKDYSDGRRTFPRIRRHQGNLFTLVPSSRSLSTNGENMGLAVQYLDPRGRLRSADSENALSVQERNVPTKSPSAPINWRRGKLLGQGAFGRVYLCYDVDTGRELASKQVQFDPDSPETSKEVSALECEIQLLKNLQHERIVQYYGCLRDRAEKTLTIFMEYMPGGSVKDQLKAYGALTESVTRKYTRQILEGMSYLHSNMIVHRDIKGANILRDSAGNVKLGDFGASKRLQTICMSGTGMRSVTGTPYWMSPEVISGEGYGRKADVWSLGCTVVEMLTEKPPWAEYEAMAAIFKIATQPTNPQLPSHISEHGRDFLRRIFVEARQRPSAEELLTHHFAQLMY

>NP_036526.2

MAENSGRAGKSSGSGAGKGAVSAEQVIAGFNRLRQEQRGLASKAAELEMELNEHSLVIDTLKEVDETRKCYRMVGGVLVERTVKEVLPALENNKEQIQKIIETLTQQLQAKGKELNEFREKHNIRLMGEDEKPAAKENSEGAGAKASSAGVLVS

1356 NP_031375.3 NP_003981.2

>NP_031375.3

MSDQAPKVPEEMFREVKYYAVGDIDPQVIQLLKAGKAKEVSYNALASHIISEDGDNPEVGEAREVFDLPVVKPSWVILSVQCGTLLPVNGFSPESCQIFFGITACLSQVSSEDRSALWALVTFYGGDCQLTLNKKCTHLIVPEPKGEKYECALKRASIKIVTPDWVLDCVSEKTKKDEAFYHPRLIIYEEEEEEEEEEEEVENEEQDSQNEGSTDEKSSPASSQEGSPSGDQQFSPKSNTEKSKGELMFDDSSDSSPEKQERNLNWTPAEVPQLAAAKRRLPQGKEPGLINLCANVPPVPGNILPPEVRGNLMAAGQNLQSSERSEMIATWSPAVRTLRNITNNADIQQMNRPSNVAHILQTLSAPTKNLEQQVNHSQQGHTNANAVLFSQVKVTPETHMLQQQQQAQQQQQQHPVLHLQPQQIMQLQQQQQQQISQQPYPQQPPHPFSQQQQQQQQAHPHQFSQQQLQFPQQQLHPPQQLHRPQQQLQPFQQQHALQQQFHQLQQHQLQQQQLAQLQQQHSLLQQQQQQQIQQQQLQRMHQQQQQQQMQSQTAPHLSQTSQALQHQVPPQQPPQQQQQQQPPPSPQQHQLFGHDPAVEIPEEGFLLGCVFAIADYPEQMSDKQLLATWKRIIQAHGGTVDPTFTSRCTHLLCESQVSSAYAQAIRERKRCVTAHWLNTVLKKKKMVPPHRALHFPVAFPPGGKPCSQHIISVTGFVDSDRDDLKLMAYLAGAKYTGYLCRSNTVLICKEPTGLKYEKAKEWRIPCVNAQWLGDILLGNFEALRQIQYSRYTAFSLQDPFAPTQHLVLNLLDAWRVPLKVSAELLMSIRLPPKLKQNEVANVQPSSKRARIEDVPPPTKKLTPELTPFVLFTGFEPVQVQQYIKKLYILGGEVAESAQKCTHLIASKVTRTVKFLTAISVVKHIVTPEWLEECFRCQKFIDEQNYILRDAEAEVLFSFSLEESLKRAHVSPLFKAKYFYITPGICPSLSTMKAIVECAGGKVLSKQPSFRKLMEHKQNSSLSEIILISCENDLHLCREYFARGIDVHNAEFVLTGVLTQTLDYESYKFN

>NP_003981.2

MDMHCKADPFSAMHPGHGGVNQLGGVFVNGRPLPDVVRQRIVELAHQGVRPCDISRQLRVSHGCVSKILGRYYETGSIKPGVIGGSKPKVATPKVVDKIAEYKRQNPTMFAWEIRDRLLAEGICDNDTVPSVSSINRIIRTKVQQPFHPTPDGAGTGVTAPGHTIVPSTASPPVSSASNDPVGSYSINGILGIPRSNGEKRKRDEVEVYTDPAHIRGGGGLHLVWTLRDVSEGSVPNGDSQSGVDSLRKHLRADTFTQQQLEALDRVFERPSYPDVFQASEHIKSEQGNEYSLPALTPGLDEVKSSLSASTNPELGSNVSGTQTYPVVTGRDMASTTLPGYPPHVPPTGQGSYPTSTLAGMVPGSEFSGNPYSHPQYTAYNEAWRFSNPALLMPPPGPPLPLLPLPMTATSYRGDHIKLQADSFGLHIVPV

1357 NP_002255.2 NP_783552.1

>NP_002255.2

MTTPGKENFRLKSYKNKSLNPDEMRRRREEEGLQLRKQKREEQLFKRRNVATAEEETEEEVMSDGGFHEAQINNMEMAPGGVITSDMIEMIFSKSPEQQLSATQKFRKLLSKEPNPPIDEVISTPGVVARFVEFLKRKENCTLQFESAWVLTNIASGNSLQTRIVIQAGAVPIFIELLSSEFEDVQEQAVWALGNIAGDSTMCRDYVLDCNILPPLLQLFSKQNRLTMTRNAVWALSNLCRGKSPPPEFAKVSPCLNVLSWLLFVSDTDVLADACWALSYLSDGPNDKIQAVIDAGVCRRLVELLMHNDYKVVSPALRAVGNIVTGDDIQTQVILNCSALQSLLHLLSSPKESIKKEACWTISNITAGNRAQIQTVIDANIFPALISILQTAEFRTRKEAAWAITNATSGGSAEQIKYLVELGCIKPLCDLLTVMDSKIVQVALNGLENILRLGEQEAKRNGTGINPYCALIEEAYGLDKIEFLQSHENQEIYQKAFDLIEHYFGTEDEDSSIAPQVDLNQQQYIFQQCEAPMEGFQL

>NP_783552.1

MISVKRNTWRALSLVIGDCRKKGNFEYCQDRTEKHSTMPDSPVDVKTQSRLTPPTMPPPPTTQGAPRTSSFTPTTLTNGTSHSPTALNGAPSPPNGFSNGPSSSSSSSLANQQLPPACGARQLSKLKRFLTTLQQFGNDISPEIGERVRTLVLGLVNSTLTIEEFHSKLQEATNFPLRPFVIPFLKANLPLLQRELLHCARLAKQNPAQYLAQHEQLLLDASTTSPVDSSELLLDVNENGKRRTPDRTKENGFDREPLHSEHPSKRPCTISPGQRYSPNNGLSYQPNGLPHPTPPPPQHYRLDDMAIAHHYRDSYRHPSHRDLRDRNRPMGLHGTRQEEMIDHRLTDREWAEEWKHLDHLLNCIMDMVEKTRRSLTVLRRCQEADREELNYWIRRYSDAEDLKKGGGSSSSHSRQQSPVNPDPVALDAHREFLHRPASGYVPEEIWKKAEEAVNEVKRQAMTELQKAVSEAERKAHDMITTERAKMERTVAEAKRQAAEDALAVINQQEDSSESCWNCGRKASETCSGCNTARYCGSFCQHKDWEKHHHICGQTLQAQQQGDTPAVSSSVTPNSGAGSPMDTPPAATPRSTTPGTPSTIETTPR

1358 NP_000465.1 P10071

>NP_000465.1

MMQDVSSSPVSPADDSLSNSEEEPDRQQPPSGKRGGRKRRSSRRSAGGGAGPGGAAGGGVGGGDEPGSPAQGKRGKKSAGCGGGGGAGGGGGSSSGGGSPQSYEELQTQRVMANVRERQRTQSLNEAFAALRKIIPTLPSDKLSKIQTLKLAARYIDFLYQVLQSDELDSKMASCSYVAHERLSYAFSVWRMEGAWSMSASH

>P10071

MEAQSHSSTTTEKKKVENSIVKCSTRTDVSEKAVASSTTSNEDESPGQTYHRERRNAITMQPQNVQGLSKVSEEPSTSSDERASLIKKEIHGSLPHVAEPSVPYRGTVFAMDPRNGYMEPHYHPPHLFPAFHPPVPIDARHHEGRYHYDPSPIPPLHMTSALSSSPTYPDLPFIRISPHRNPAAASESPFSPPHPYINPYMDYIRSLHSSPSLSMISATRGLSPTDAPHAGVSPAEYYHQMALLTGQRSPYADIIPSAATAGTGAIHMEYLHAMDSTRFSSPRLSARPSRKRTLSISPLSDHSFDLQTMIRTSPNSLVTILNNSRSSSSASGSYGHLSASAISPALSFTYSSAPVSLHMHQQILSRQQSLGSAFGHSPPLIHPAPTFPTQRPIPGIPTVLNPVQVSSGPSESSQNKPTSESAVSSTGDPMHNKRSKIKPDEDLPSPGARGQQEQPEGTTLVKEEGDKDESKQEPEVIYETNCHWEGCAREFDTQEQLVHHINNDHIHGEKKEFVCRWLDCSREQKPFKAQYMLVVHMRRHTGEKPHKCTFEGCTKAYSRLENLKTHLRSHTGEKPYVCEHEGCNKAFSNASDRAKHQNRTHSNEKPYVCKIPGCTKRYTDPSSLRKHVKTVHGPEAHVTKKQRGDIHPRPPPPRDSGSHSQSRSPGRPTQGALGEQQDLSNTTSKREECLQVKTVKAEKPMTSQPSPGGQSSCSSQQSPISNYSNSGLELPLTDGGSIGDLSAIDETPIMDSTISTATTALALQARRNPAGTKWMEHVKLERLKQVNGMFPRLNPILPPKAPAVSPLIGNGTQSNNTCSLGGPMTLLPGRSDLSGVDVTMLNMLNRRDSSASTISSAYLSSRRSSGISPCFSSRRSSEASQAEGRPQNVSVADSYDPISTDASRRSSEASQSDGLPSLLSLTPAQQYRLKAKYAAATGGPPPTPLPNMERMSLKTRLALLGDALEPGVALPPVHAPRRCSDGGAHGYGRRHLQPHDALGHGVRRASDPVRTGSEGLALPRVPRFSSLSSCNPPAMATSAEKRSLVLQNYTRPEGGQSRNFHSSPCPPSITENVTLESLTMDADANLNDEDFLPDDVVQYLNSQNQAGYEQHFPSALPDDSKVPHGPGDFDAPGLPDSHAGQQFHALEQPCPEGSKTDLPIQWNEVSSGSADLSSSKLKCGPRPAVPQTRAFGFCNGMVVHPQNPLRSGPAGGYQTLGENSNPYGGPEHLMLHNSPGSGTSGNAFHEQPCKAPQYGNCLNRQPVAPGALDGACGAGIQASKLKSTPMQGSGGQLNFGLPVAPNESAGSMVNGMQNQDPVGQGYLAHQLLGDSMQHPGAGRPGQQMLGQISATSHINIYQGPESCLPGAHGMGSQPSSLAVVRGYQPCASFGGSRRQAMPRDSLALQSGQLSDTSQTCRVNGIKMEMKGQPHPLCSNLQNYSGQFYDQTVGFSQQDTKAGSFSISDASCLLQGTSAKNSELLSPGANQVTSTVDSLDSHDLEGVQIDFDAIIDDGDHSSLMSGALSPSIIQNLSHSSSRLTTPRASLPFPALSMSTTNMAIGDMSSLLTSLAEESKFLAVMQ

1359 NP_932157.1 NP_061110.2

>NP_932157.1

MENELPVPHTSSSACATSSTSGASSSSGCNNSSSGGSGRPTGPQISVYSGIPDRQTVQVIQQALHRQPSTAAQYLQQMYAAQQQHLMLQTAALQQQHLSSAQLQSLAAVQQASLVSNRQGSTSGSNVSAQAPAQSSSINLAASPAAAQLLNRAQSVNSAAASGIAQQAVLLGNTSSPALTASQAQMYLRAQMLIFTPTATVATVQPELGTGSPARPPTPAQVQNLTLRTQQTPAAAASGPTPTQPVLPSLALKPTPGGSQPLPTPAQSRNTAQASPAGAKPGIADSVMEPHKKGDGNSSVPGSMEGRAGLSRTVPAVAAHPLIAPAYAQLQPHQLLPQPSSKHLQPQFVIQQQPQPQQQQPPPQQSRPVLQAEPHPQLASVSPSVALQPSSEAHAMPLGPVTPALPLQCPTANLHKPGGSQQCHPPTPDTGPQNGHPEGVPHTPQRRFQHTSAVILQLQPASPPQQCVPDDWKEVAPGEKSVPETRSGPSPHQQAIVTAMPGGLPVPTSPNIQPSPAHETGQGIVHALTDLSSPGMTSGNGNSASSIAGTAPQNGENKPPQAIVKPQILTHVIEGFVIQEGAEPFPVGRSSLLVGNLKKKYAQGFLPEKLPQQDHTTTTDSEMEEPYLQESKEEGAPLKLKCELCGRVDFAYKFKRSKRFCSMACAKRYNVGCTKRVGLFHSDRSKLQKAGAATHNRRRASKASLPPLTKDTKKQPTGTVPLSVTAALQLTHSQEDSSRCSDNSSYEEPLSPISASSSTSRRRQGQRDLELPDMHMRDLVGMGHHFLPSEPTKWNVEDVYEFIRSLPGCQEIAEEFRAQEIDGQALLLLKEDHLMSAMNIKLGPALKIYARISMLKDS

>NP_061110.2

MLSVQPDTKPKGCAGCNRKIKDRYLLKALDKYWHEDCLKCACCDCRLGEVGSTLYTKANLILCRRDYLRLFGVTGNCAACSKLIPAFEMVMRAKDNVYHLDCFACQLCNQRFCVGDKFFLKNNMILCQTDYEEGLMKEGYAPQVR

1360 AAH26916.1 NP_001341.1

>AAH26916.1

MPGHLQEGFGCVVTNRFDQLFDDESDPFEVLKAAENKKKEAGGGGVGGPGAKSAAQAAAQTNSNAAGKQLRKESQKDRKNPLPPSVGVVDKKEETQPPVALKKEGIRRVGRRPDQQLQGEGKIIDRRPERRPPRERRFEKPLEEKGEGGEFSVDRPIIDRPIRGRGGLGRGRGGRGRGMGRGDGFDSRGKREFDRHSGSDRSSFSHYSGLKHEDKRGGSGSHNWGTVKDELTESPKYIQKQISYNYSDLDQSNVTEETPEGEEHHPVADTENKENEVEEVKEEGPKEMTLDEWKAIQNKDRAKVEFNIRKPNEGADGQWKKGFVLHKSKSEEAHAEDSVMDHHFRKPANDITSQLEINFGDLGRPGRGGRGGRGGRGRGGRPNRGSRTDKSSASAPDVDDPEAFPALA

>NP_001341.1

MATANSIIVLDDDDEDEAAAQPGPSHPLPNAASPGAEAPSSSEPHGARGSSSSGGKKCYKLENEKLFEEFLELCKMQTADHPEVVPFLYNRQQRAHSLFLASAEFCNILSRVLSRARSRPAKLYVYINELCTVLKAHSAKKKLNLAPAATTSNEPSGNNPPTHLSLDPTNAENTASQSPRTRGSRRQIQRLEQLLALYVAEIRRLQEKELDLSELDDPDSAYLQEARLKRKLIRLFGRLCELKDCSSLTGRVIEQRIPYRGTRYPEVNRRIERLINKPGPDTFPDYGDVLRAVEKAAARHSLGLPRQQLQLMAQDAFRDVGIRLQERRHLDLIYNFGCHLTDDYRPGVDPALSDPVLARRLRENRSLAMSRLDEVISKYAMLQDKSEEGERKKRRARLQGTSSHSADTPEASLDSGEGPSGMASQGCPSASRAETDDEDDEESDEEEEEEEEEEEEEATDSEEEEDLEQMQEGQEDDEEEDEEEEAAAGKDGDKSPMSSLQISNEKNLEPGKQISRSSGEQQNKGRIVSPSLLSEEPLAPSSIDAESNGEQPEELTLEEESPVSQLFELEIEALPLDTPSSVETDISSSRKQSEEPFTTVLENGAGMVSSTSFNGGVSPHNWGDSGPPCKKSRKEKKQTGSGPLGNSYVERQRSVHEKNGKKICTLPSPPSPLASLAPVADSSTRVDSPSHGLVTSSLCIPSPARLSQTPHSQPPRPGTCKTSVATQCDPEEIIVLSDSD

1361 NP_005442.2 NP_076964.1

>NP_005442.2

MDSFKVVLEGPAPWGFRLQGGKDFNVPLSISRLTPGGKAAQAGVAVGDWVLSIDGENAGSLTHIEAQNKIRACGERLSLGLSRAQPVQSKPQKASAPAADPPRYTFAPSVSLNKTARPFGAPPPADSAPQQNGQPLRPLVPDASKQRLMENTEDWRPRPGTGQSRSFRILAHLTGTEFMQDPDEEHLKKSSQVPRTEAPAPASSTPQEPWPGPTAPSPTSRPPWAVDPAFAERYAPDKTSTVLTRHSQPATPTPLQSRTSIVQAAAGGVPGGGSNNGKTPVCHQCHKVIRGRYLVALGHAYHPEEFVCSQCGKVLEEGGFFEEKGAIFCPPCYDVRYAPSCAKCKKKITGEIMHALKMTWHVHCFTCAACKTPIRNRAFYMEEGVPYCERDYEKMFGTKCHGCDFKIDAGDRFLEALGFSWHDTCFVCAICQINLEGKTFYSKKDRPLCKSHAFSHV

>NP_076964.1

MSTHVAGLGLDKMKLGNPQSFLDQEEADDQQLLEPEAWKTYTERRNALREFLTSDLSPHLLKRHHARMQLLRKCSYYIEVLPKHLALGDQNPLVLPSALFQLIDPWKFQRMKKVGTAQTKIQLLLLGDLLEQLDHGRAELDALLRSPDPRPFLADWALVERRLADVSAVMDSFLTMMVPGRLHVKHRLVSDVSATKIPHIWLMLSTKMPVVFDRKASAAHQDWARLRWFVTIQPATSEQYELRFRLLDPRTQQECAQCGVIPVAACTFDVRNLLPNRSYKFTIKRAETSTLVYEPWRDSLTLHTKPEPLEGPALSHSV

1362 NP_055202.1 NP_000607.1

>NP_055202.1

MEEAILVPCVLGLLLLPILAMLMALCVHCHRLPGSYDSTSSDSLYPRGIQFKRPHTVAPWPPAYPPVTSYPPLSQPDLLPIPRSPQPLGGSHRTPSSRRDSDGANSVASYENEGASGIRGAQAGWGVWGPSWTRLTPVSLPPEPACEDADEDEDDYHNPGYLVVLPDSTPATSTAAPSAPALSTPGIRDSAFSMESIDDYVNVPESGESAEASLDGSREYVNVSQELHPGAAKTEPAALSSQEAEEVEEEGAPDYENLQELN

>NP_000607.1

MNRGVPFRHLLLVLQLALLPAATQGKKVVLGKKGDTVELTCTASQKKSIQFHWKNSNQIKILGNQGSFLTKGPSKLNDRADSRRSLWDQGNFPLIIKNLKIEDSDTYICEVEDQKEEVQLLVFGLTANSDTHLLQGQSLTLTLESPPGSSPSVQCRSPRGKNIQGGKTLSVSQLELQDSGTWTCTVLQNQKKVEFKIDIVVLAFQKASSIVYKKEGEQVEFSFPLAFTVEKLTGSGELWWQAERASSSKSWITFDLKNKEVSVKRVTQDPKLQMGKKLPLHLTLPQALPQYAGSGNLTLALEAKTGKLHQEVNLVVMRATQLQKNLTCEVWGPTSPKLMLSLKLENKEAKVSKREKAVWVLNPEAGMWQCLLSDSGQVLLESNIKVLPTWSTPVQPMALIVLGGVAGLLLFIGLGIFFCVRCRHRRRQAERMSQIKRLLSEKKTCQCPHRFQKTCSPI

1363 NP_005350.1 NP_061828.1

>NP_005350.1

MDNMSITNTPTSNDACLSIVHSLMCHRQGGESETFAKRAIESLVKKLKEKKDELDSLITAITTNGAHPSKCVTIQRTLDGRLQVAGRKGFPHVIYARLWRWPDLHKNELKHVKYCQYAFDLKCDSVCVNPYHYERVVSPGIDLSGLTLQSNAPSSMMVKDEYVHDFEGQPSLSTEGHSIQTIQHPPSNRASTETYSTPALLAPSESNATSTANFPNIPVASTSQPASILGGSHSEGLLQIASGPQPGQQQNGFTGQPATYHHNSTTTWTGSRTAPYTPNLPHHQNGHLQHHPPMPPHPGHYWPVHNELAFQPPISNHPAPEYWCSIAYFEMDVQVGETFKVPSSCPIVTVDGYVDPSGGDRFCLGQLSNVHRTEAIERARLHIGKGVQLECKGEGDVWVRCLSDHAVFVQSYYLDREAGRAPGDAVHKIYPSAYIKVFDLRQCHRQMQQQAATAQAAAAAQAAAVAGNIPGPGSVGGIAPAISLSAAAGIGVDDLRRLCILRMSFVKGWGPDYPRQSIKETPCWIEIHLHRALQLLDEVLHTMPIADPQPLD

>NP_061828.1

MQIFVKTLTGKTITLEVEPSDTIENVKAKIQDKEGIPPDQQRLIFAGKQLEDGRTLSDYNIQKESTLHLVLRLRGGMQIFVKTLTGKTITLEVEPSDTIENVKAKIQDKEGIPPDQQRLIFAGKQLEDGRTLSDYNIQKESTLHLVLRLRGGMQIFVKTLTGKTITLEVEPSDTIENVKAKIQDKEGIPPDQQRLIFAGKQLEDGRTLSDYNIQKESTLHLVLRLRGGC

1364 NP_000545.1 NP_001420.2

>NP_000545.1

MMAYMNPGPHYSVNALALSGPSVDLMHQAVPYPSAPRKQRRERTTFTRSQLEELEALFAKTQYPDVYAREEVALKINLPESRVQVWFKNRRAKCRQQRQQQKQQQQPPGGQAKARPAKRKAGTSPRPSTDVCPDPLGISDSYSPPLPGPSGSPTTAVATVSIWSPASESPLPEAQRAGLVASGPSLTSAPYAMTYAPASAFCSSPSAYGSPSSYFSGLDPYLSPMVPQLGGPALSPLSGPSVGPSLAQSPTSLSGQSYGAYSPVDSLEFKDPTGTWKFTYNPMDPLDYKDQSAWKFQIL

>NP_001420.2

MAENVVEPGPPSAKRPKLSSPALSASASDGTDFGSLFDLEHDLPDELINSTELGLTNGGDINQLQTSLGMVQDAASKHKQLSELLRSGSSPNLNMGVGGPGQVMASQAQQSSPGLGLINSMVKSPMTQAGLTSPNMGMGTSGPNQGPTQSTGMMNSPVNQPAMGMNTGMNAGMNPGMLAAGNGQGIMPNQVMNGSIGAGRGRQNMQYPNPGMGSAGNLLTEPLQQGSPQMGGQTGLRGPQPLKMGMMNNPNPYGSPYTQNPGQQIGASGLGLQIQTKTVLSNNLSPFAMDKKAVPGGGMPNMGQQPAPQVQQPGLVTPVAQGMGSGAHTADPEKRKLIQQQLVLLLHAHKCQRREQANGEVRQCNLPHCRTMKNVLNHMTHCQSGKSCQVAHCASSRQIISHWKNCTRHDCPVCLPLKNAGDKRNQQPILTGAPVGLGNPSSLGVGQQSAPNLSTVSQIDPSSIERAYAALGLPYQVNQMPTQPQVQAKNQQNQQPGQSPQGMRPMSNMSASPMGVNGGVGVQTPSLLSDSMLHSAINSQNPMMSENASVPSLGPMPTAAQPSTTGIRKQWHEDITQDLRNHLVHKLVQAIFPTPDPAALKDRRMENLVAYARKVEGDMYESANNRAEYYHLLAEKIYKIQKELEEKRRTRLQKQNMLPNAAGMVPVSMNPGPNMGQPQPGMTSNGPLPDPSMIRGSVPNQMMPRITPQSGLNQFGQMSMAQPPIVPRQTPPLQHHGQLAQPGALNPPMGYGPRMQQPSNQGQFLPQTQFPSQGMNVTNIPLAPSSGQAPVSQAQMSSSSCPVNSPIMPPGSQGSHIHCPQLPQPALHQNSPSPVPSRTPTPHHTPPSIGAQQPPATTIPAPVPTPPAMPPGPQSQALHPPPRQTPTPPTTQLPQQVQPSLPAAPSADQPQQQPRSQQSTAASVPTPTAPLLPPQPATPLSQPAVSIEGQVSNPPSTSSTEVNSQAIAEKQPSQEVKMEAKMEVDQPEPADTQPEDISESKVEDCKMESTETEERSTELKTEIKEEEDQPSTSATQSSPAPGQSKKKIFKPEELRQALMPTLEALYRQDPESLPFRQPVDPQLLGIPDYFDIVKSPMDLSTIKRKLDTGQYQEPWQYVDDIWLMFNNAWLYNRKTSRVYKYCSKLSEVFEQEIDPVMQSLGYCCGRKLEFSPQTLCCYGKQLCTIPRDATYYSYQNRYHFCEKCFNEIQGESVSLGDDPSQPQTTINKEQFSKRKNDTLDPELFVECTECGRKMHQICVLHHEIIWPAGFVCDGCLKKSARTRKENKFSAKRLPSTRLGTFLENRVNDFLRRQNHPESGEVTVRVVHASDKTVEVKPGMKARFVDSGEMAESFPYRTKALFAFEEIDGVDLCFFGMHVQEYGSDCPPPNQRRVYISYLDSVHFFRPKCLRTAVYHEILIGYLEYVKKLGYTTGHIWACPPSEGDDYIFHCHPPDQKIPKPKRLQEWYKKMLDKAVSERIVHDYKDIFKQATEDRLTSAKELPYFEGDFWPNVLEESIKELEQEEEERKREENTSNESTDVTKGDSKNAKKKNNKKTSKNKSSLSRGNKKKPGMPNVSNDLSQKLYATMEKHKEVFFVIRLIAGPAANSLPPIVDPDPLIPCDLMDGRDAFLTLARDKHLEFSSLRRAQWSTMCMLVELHTQSQDRFVYTCNECKHHVETRWHCTVCEDYDLCITCYNTKNHDHKMEKLGLGLDDESNNQQAAATQSPGDSRRLSIQRCIQSLVHACQCRNANCSLPSCQKMKRVVQHTKGCKRKTNGGCPICKQLIALCCYHAKHCQENKCPVPFCLNIKQKLRQQQLQHRLQQAQMLRRRMASMQRTGVVGQQQGLPSPTPATPTTPTGQQPTTPQTPQPTSQPQPTPPNSMPPYLPRTQAAGPVSQGKAAGQVTPPTPPQTAQPPLPGPPPAAVEMAMQIQRAAETQRQMAHVQIFQRPIQHQMPPMTPMAPMGMNPPPMTRGPSGHLEPGMGPTGMQQQPPWSQGGLPQPQQLQSGMPRPAMMSVAQHGQPLNMAPQPGLGQVGISPLKPGTVSQQALQNLLRTLRSPSSPLQQQQVLSILHANPQLLAAFIKQRAAKYANSNPQPIPGQPGMPQGQPGLQPPTMPGQQGVHSNPAMQNMNPMQAGVQRAGLPQQQPQQQLQPPMGGMSPQAQQMNMNHNTMPSQFRDILRRQQMMQQQQQQGAGPGIGPGMANHNQFQQPQGVGYPPQQQQRMQHHMQQMQQGNMGQIGQLPQALGAEAGASLQAYQQRLLQQQMGSPVQPNPMSPQQHMLPNQAQSPHLQGQQIPNSLSNQVRSPQPVPSPRPQSQPPHSSPSPRMQPQPSPHHVSPQTSSPHPGLVAAQANPMEQGHFASPDQNSMLSQLASNPGMANLHGASATDLGLSTDNSDLNSNLSQSTLDIH

1365 NP_663777.1 NP_891549.1

>NP_663777.1

MESSKKMDSPGALQTNPPLKLHTDRSAGTPVFVPEQGGYKEKFVKTVEDKYKCEKCHLVLCSPKQTECGHRFCESCMAALLSSSSPKCTACQESIVKDKVFKDNCCKREILALQIYCRNESRGCAEQLMLGHLLVHLKNDCHFEELPCVRPDCKEKVLRKDLRDHVEKACKYREATCSHCKSQVPMIALQKHEDTDCPCVVVSCPHKCSVQTLLRSELSAHLSECVNAPSTCSFKRYGCVFQGTNQQIKAHEASSAVQHVNLLKEWSNSLEKKVSLLQNESVEKNKSIQSLHNQICSFEIEIERQKEMLRNNESKILHLQRVIDSQAEKLKELDKEIRPFRQNWEEADSMKSSVESLQNRVTELESVDKSAGQVARNTGLLESQLSRHDQMLSVHDIRLADMDLRFQVLETASYNGVLIWKIRDYKRRKQEAVMGKTLSLYSQPFYTGYFGYKMCARVYLNGDGMGKGTHLSLFFVIMRGEYDALLPWPFKQKVTLMLMDQGSSRRHLGDAFKPDPNSSSFKKPTGEMNIASGCPVFVAQTVLENGTYIKDDTIFIKVIVDTSDLPDP

>NP_891549.1

MACTGPSLPSAFDILGAAGQDKLLYLKHKLKTPRPGCQGQDLLHAMVLLKLGQETEARISLEALKADAVARLVARQWAGVDSTEDPEEPPDVSWAVARLYHLLAEEKLCPASLRDVAYQEAVRTLSSRDDHRLGELQDEARNRCGWDIAGDPGSIRTLQSNLGCLPPSSALPSGTRSLPRPIDGVSDWSQGCSLRSTGSPASLASNLEISQSPTMPFLSLHRSPHGPSKLCDDPQASLVPEPVPGGCQEPEEMSWPPSGEIASPPELPSSPPPGLPEVAPDATSTGLPDTPAAPETSTNYPVECTEGSAGPQSLPLPILEPVKNPCSVKDQTPLQLSVEDTTSPNTKPCPPTPTTPETSPPPPPPPPSSTPCSAHLTPSSLFPSSLESSSEQKFYNFVILHARADEHIALRVREKLEALGVPDGATFCEDFQVPGRGELSCLQDAIDHSAFIILLLTSNFDCRLSLHQVNQAMMSNLTRQGSPDCVIPFLPLESSPAQLSSDTASLLSGLVRLDEHSQIFARKVANTFKPHRLQARKAMWRKEQDTRALREQSQHLDGERMQAAALNAAYSAYLQSYLSYQAQMEQLQVAFGSHMSFGTGAPYGARMPFGGQVPLGAPPPFPTWPGCPQPPPLHAWQAGTPPPPSPQPAAFPQSLPFPQSPAFPTASPAPPQSPGLQPLIIHHAQMVQLGLNNHMWNQRGSQAPEDKTQEAE

1366 NP_036611.2 NP_004324.2

>NP_036611.2

MVDREQLVQKARLAEQAERYDDMAAAMKNVTELNEPLSNEERNLLSVAYKNVVGARRSSWRVISSIEQKTSADGNEKKIEMVRAYREKIEKELEAVCQDVLSLLDNYLIKNCSETQYESKVFYLKMKGDYYRYLAEVATGEKRATVVESSEKAYSEAHEISKEHMQPTHPIRLGLALNYSVFYYEIQNAPEQACHLAKTAFDDAIAELDTLNEDSYKDSTLIMQLLRDNLTLWTSDQQDDDGGEGNN

>NP_004324.2

MAALSGGGGGGAEPGQALFNGDMEPEAGAGAGAAASSAADPAIPEEVWNIKQMIKLTQEHIEALLDKFGGEHNPPSIYLEAYEEYTSKLDALQQREQQLLESLGNGTDFSVSSSASMDTVTSSSSSSLSVLPSSLSVFQNPTDVARSNPKSPQKPIVRVFLPNKQRTVVPARCGVTVRDSLKKALMMRGLIPECCAVYRIQDGEKKPIGWDTDISWLTGEELHVEVLENVPLTTHNFVRKTFFTLAFCDFCRKLLFQGFRCQTCGYKFHQRCSTEVPLMCVNYDQLDLLFVSKFFEHHPIPQEEASLAETALTSGSSPSAPASDSIGPQILTSPSPSKSIPIPQPFRPADEDHRNQFGQRDRSSSAPNVHINTIEPVNIDDLIRDQGFRGDGGSTTGLSATPPASLPGSLTNVKALQKSPGPQRERKSSSSSEDRNRMKTLGRRDSSDDWEIPDGQITVGQRIGSGSFGTVYKGKWHGDVAVKMLNVTAPTPQQLQAFKNEVGVLRKTRHVNILLFMGYSTKPQLAIVTQWCEGSSLYHHLHIIETKFEMIKLIDIARQTAQGMDYLHAKSIIHRDLKSNNIFLHEDLTVKIGDFGLATVKSRWSGSHQFEQLSGSILWMAPEVIRMQDKNPYSFQSDVYAFGIVLYELMTGQLPYSNINNRDQIIFMVGRGYLSPDLSKVRSNCPKAMKRLMAECLKKKRDERPLFPQILASIELLARSLPKIHRSASEPSLNRAGFQTEDFSLYACASPKTPIQAGGYGAFPVH

1367 NP_003118.1 NP_056456.1

>NP_003118.1

MDPSGVKVLETAEDIQERRQQVLDRYHRFKELSTLRRQKLEDSYRFQFFQRDAEELEKWIQEKLQIASDENYKDPTNLQGKLQKHQAFEAEVQANSGAIVKLDETGNLMISEGHFASETIRTRLMELHRQWELLLEKMREKGIKLLQAQNLVQYLRECEDVMDWINDKEAIVTSEELGQDLEHVEVLQKKFEEFQTDMAAHEERVNEVNQFAAKLIQEQHPEEELIKTKQDEVNAAWQRLKGLALQRQGKLFGAAEVQRFNRDVDETISWIKEKEQLMASDDFGRDLASVQALLRKHEGLERDLAALEDKVKALCAEADRLQQSHPLSATQIQVKREELITNWEQIRTLAAERHARLNDSYRLQRFLADFRDLTSWVTEMKALINADELASDVAGAEALLDRHQEHKGEIDAHEDSFKSADESGQALLAAGHYASDEVREKLTVLSEERAALLELWELRRQQYEQCMDLQLFYRDTEQVDNWMSKQEAFLLNEDLGDFLDSVEALLKKHEDFEKSLSAQEEKITALDEFATKLIQNNHYAMEDVATRRDALLSRRNALHERAMRRRAQLADSFHLQQFFRDSDELKSWVNEKMKTATDEAYKDPSNLQGKVQKHQAFEAELSANQSRIDALEKAGQKLIDVNHYAKDEVAARMNEVISLWKKLLEATELKGIKLREANQQQQFNRNVEDIELWLYEVEGHLASDDYGKDLTNVQNLQKKHALLEADVAAHQDRIDGVTIQARQFQDAGHFDAENIKKKQEALVARYEALKEPMVARKQKLADSLRLQQLFRDVEDEETWIREKEPIAASTNRGKDLIGVQNLLKKHQALQAEIAGHEPRIKAVTQKGNAMVEEGHFAAEDVKAKLHELNQKWEALKAKASQRRQDLEDSLQAQQYFADANEAESWMREKEPIVGSTDYGKDEDSAEALLKKHEALMSDLSAYGSSIQALREQAQSCRQQVAPTDDETGKELVLALYDYQEKSPREVTMKKGDILTLLNSTNKDWWKVEVNDRQGFVPAAYVKKLDPAQSASRENLLEEQGSIALRQEQIDNQTRITKEAGSVSLRMKQVEELYHSLLELGEKRKGMLEKSCKKFMLFREANELQQWINEKEAALTSEEVGADLEQVEVLQKKFDDFQKDLKANESRLKDINKVAEDLESEGLMAEEVQAVQQQEVYGMMPRDETDSKTASPWKSARLMVHTVATFNSIKELNERWRSLQQLAEERSQLLGSAHEVQRFHRDADETKEWIEEKNQALNTDNYGHDLASVQALQRKHEGFERDLAALGDKVNSLGETAERLTQSHPESAEDLQEKCTELNQAWSSLGKRADQRKAKLGDSHDLQRFLSDFRDLMSWINGIRGLVSSDELAKDVTGAEALLERHQEHRTEIDARAGTFQAFEQFGQQLLAHGHYASPEIKQKLDILDQERADLEKAWVQRRMMLDQCLELQLFHRDCEQAENWMAAREAFLNTEDKGDSLDSVEALIKKHEDFDKAINVQEEKIAALQAFADQLIAAGHYAKGDISSRRNEVLDRWRRLKAQMIEKRSKLGESQTLQQFSRDVDEIEAWISEKLQTASDESYKDPTNIQSKHQKHQAFEAELHANADRIRGVIDMGNSLIERGACAGNEDAVKARLAALADQWQFLVQKSAEKSQKLKEANKQQNFNTGIKDIAFWLSEVEALLASEDYGKDLASVNNLLKKHQLLEADISAHEDRLKDLNSQADSLMTSSAFDTSQVKDKRDTINGRFQKIKSMAASRRAKLNESHRLHQFFRDMDDEESWIKEKKLLVGSEDYGRDLTGVQNLRKKHKRLEAELAAHEPAIQGVLDTGKKLSDDNTIGKEEIQQRLAQFVEHWKELKQLAAARGQRLEESLEYQQFVANVEEEEAWINEKMTLVASEDYGDTLAAIQGLLKKHEAFETAFTVHKDRVNDVCTNGQDLIKKNNHHEENISSKMKGLNGKVSDLEKAAAQRKANVDENSAFLQFNWKADVVESWIGEKENSLKTDDYGRDLSSVQTLLTKQETFDAGLQAFQQEGIANITALKDQLLAAKHVQSKAIEARHASLMKRWSQLLANSAARKKKLLEAQSHFRKVEDLFLTFAKKASAFNSWFENAEEDLTDPVRCNSLEEIKALREAHDAFRSSLSSAQADFNQLAELDRQIKSFRVASNPYTWFTMEALEETWRNLQKIIKERELELQKEQRRQEENDKLRQEFAQHANAFHQWIQETRTYLLDGSCMVEESGTLESQLEATKRKHQEIRAMRSQLKKIEDLGAAMEEALILDNKYTEHSTVGLAQQWDQLDQLGMRMQHNLEQQIQARNTTGVTEEALKEFSMMFKHFDKDKSGRLNHQDGKSCLRSLGYDLPMVEEGEPDPEFEAILDTVDPNRDGHVSLQEYMAFMISRETENVKSSEEIESAFRALSSEGKPYVTKEELYQNLTREQADYCVSHMKPIVDGKGRELPTAFDYVEFTRSLFVN

>NP_056456.1

MDLENKVKKMGLGHEQGFGAPCLKCKEKCEGFELHFWRKICRNCKCGQEEHDVLLSNEEDRKVGKLFEDTKYTTLIAKLKSDGIPMYKRNVMILTNPVAAKKNVSINTVTYEWAPPVQNQALARQYMQMLPKEKQPVAGSEGAQYRKKQLAKQLPAHDQDPSKCHELSPREVKEMEQFVKKYKSEALGVGDVKLPCEMDAQGPKQMNIPGGDRSTPAAVGAMEDKSAEHKRTQYSCYCCKLSMKEGDPAIYAERAGYDKLWHPACFVCSTCHELLVDMIYFWKNEKLYCGRHYCDSEKPRCAGCDELIFSNEYTQAENQNWHLKHFCCFDCDSILAGEIYVMVNDKPVCKPCYVKNHAVVCQGCHNAIDPEVQRVTYNNFSWHASTECFLCSCCSKCLIGQKFMPVEGMVFCSVECKKRMS

1368 NP_874369.1 NP_006457.2

>NP_874369.1

MAEVVSPVPGAGRREPGEVGRARGPPVADPGVALSPQGEIIEGCRLPVLRRNQDNEDEWPLAEILSVKDISGRKLFYVHYIDFNKRLDEWVTHERLDLKKIQFPKKEAKTPTKNGLPGSRPGSPEREVPASAQASGKTLPIPVQITLRFNLPKEREAIPGGEPDQPLSSSSCLQPNHRSTKRKVEVVSPATPVPSETAPASVFPQNGAARRAVAAQPGRKRKSNCLGTDEDSQDSSDGIPSAPRMTGSLVSDRSHDDIVTRMKNIECIELGRHRLKPWYFSPYPQELTTLPVLYLCEFCLKYGRSLKCLQRHLTKCDLRHPPGNEIYRKGTISFFEIDGRKNKSYSQNLCLLAKCFLDHKTLYYDTDPFLFYVMTEYDCKGFHIVGYFSKEKESTEDYNVACILTLPPYQRRGYGKLLIEFSYELSKVEGKTGTPEKPLSDLGLLSYRSYWSQTILEILMGLKSESGERPQITINEISEITSIKKEDVISTLQYLNLINYYKGQYILTLSEDIVDGHERAMLKRLLRIDSKCLHFTPKDWSKRGKW

>NP_006457.2

MAEVKVKVQPPDADPVEIENRIIELCHQFPHGITDQVIQNEMPHIEAQQRAVAINRLLSMGQLDLLRSNTGLLYRIKDSQNAGKMKGSDNQEKLVYQIIEDAGNKGIWSRDIRYKSNLPLTEINKILKNLESKKLIKAVKSVAASKKKVYMLYNLQPDRSVTGGAWYSDQDFESEFVEVLNQQCFKFLQSKAETARESKQNPMIQRNSSFASSHEVWKYICELGISKVELSMEDIETILNTLIYDGKVEMTIIAAKEGTVGSVDGHMKLYRAVNPIIPPTGLVRAPCGLCPVFDDCHEGGEISPSNCIYMTEWLEF

1369 NP_002933.1 NP_004778.1

>NP_002933.1

MSLLMFTQLLLCGFLYVRVDGSRLRQEDFPPRIVEHPSDVIVSKGEPTTLNCKAEGRPTPTIEWYKDGERVETDKDDPRSHRMLLPSGSLFFLRIVHGRRSKPDEGSYVCVARNYLGEAVSRNASLEVALLRDDFRQNPTDVVVAAGEPAILECQPPRGHPEPTIYWKKDKVRIDDKEERISIRGGKLMISNTRKSDAGMYTCVGTNMVGERDSDPAELTVFERPTFLRRPINQVVLEEEAVEFRCQVQGDPQPTVRWKKDDADLPRGRYDIKDDYTLRIKKTMSTDEGTYMCIAENRVGKMEASATLTVRAPPQFVVRPRDQIVAQGRTVTFPCETKGNPQPAVFWQKEGSQNLLFPNQPQQPNSRCSVSPTGDLTITNIQRSDAGYYICQALTVAGSILAKAQLEVTDVLTDRPPPIILQGPANQTLAVDGTALLKCKATGDPLPVISWLKEGFTFPGRDPRATIQEQGTLQIKNLRISDTGTYTCVATSSSGETSWSAVLDVTESGATISKNYDLSDLPGPPSKPQVTDVTKNSVTLSWQPGTPGTLPASAYIIEAFSQSVSNSWQTVANHVKTTLYTVRGLRPNTIYLFMVRAINPQGLSDPSPMSDPVRTQDISPPAQGVDHRQVQKELGDVLVRLHNPVVLTPTTVQVTWTVDRQPQFIQGYRVMYRQTSGLQATSSWQNLDAKVPTERSAVLVNLKKGVTYEIKVRPYFNEFQGMDSESKTVRTTEEAPSAPPQSVTVLTVGSYNSTSISVSWDPPPPDHQNGIIQEYKIWCLGNETRFHINKTVDAAIRSVIIGGLFPGIQYRVEVAASTSAGVGVKSEPQPIIIGRRNEVVITENNNSITEQITDVVKQPAFIAGIGGACWVILMGFSIWLYWRRKKRKGLSNYAVTFQRGDGGLMSNGSRPGLLNAGDPSYPWLADSWPATSLPVNNSNSGPNEIGNFGRGDVLPPVPGQGDKTATMLSDGAIYSSIDFTTKTSYNSSSQITQATPYATTQILHSNSIHELAVDLPDPQWKSSIQQKTDLMGFGYSLPDQNKGNNGGKGGKKKKNKNSSKPQKNNGSTWANVPLPPPPVQPLPGTELEHYAVEQQENGYDSDSWCPPLPVQTYLHQGLEDELEEDDDRVPTPPVRGVASSPAISFGQQSTATLTPSPREEMQPMLQAHLDELTRAYQFDIAKQTWHIQSNNQPPQPPVPPLGYVSGALISDLETDVADDDADDEEEALEIPRPLRALDQTPGSSMDNLDSSVTGKAFTSSQRPRPTSPFSTDSNTSAALSQSQRPRPTKKHKGGRMDQQPALPHRREGMTDEEALVPYSKPSFPSPGGHSSSGTASSKGSTGPRKTEVLRAGHQRNASDLLDIGYMGSNSQGQFTGEL

>NP_004778.1

MRGVGWQMLSLSLGLVLAILNKVAPQACPAQCSCSGSTVDCHGLALRSVPRNIPRNTERLDLNGNNITRITKTDFAGLRHLRVLQLMENKISTIERGAFQDLKELERLRLNRNHLQLFPELLFLGTAKLYRLDLSENQIQAIPRKAFRGAVDIKNLQLDYNQISCIEDGAFRALRDLEVLTLNNNNITRLSVASFNHMPKLRTFRLHSNNLYCDCHLAWLSDWLRQRPRVGLYTQCMGPSHLRGHNVAEVQKREFVCSGHQSFMAPSCSVLHCPAACTCSNNIVDCRGKGLTEIPTNLPETITEIRLEQNTIKVIPPGAFSPYKKLRRIDLSNNQISELAPDAFQGLRSLNSLVLYGNKITELPKSLFEGLFSLQLLLLNANKINCLRVDAFQDLHNLNLLSLYDNKLQTIAKGTFSPLRAIQTMHLAQNPFICDCHLKWLADYLHTNPIETSGARCTSPRRLANKRIGQIKSKKFRCSAKEQYFIPGTEDYRSKLSGDCFADLACPEKCRCEGTTVDCSNQKLNKIPEHIPQYTAELRLNNNEFTVLEATGIFKKLPQLRKINFSNNKITDIEEGAFEGASGVNEILLTSNRLENVQHKMFKGLESLKTLMLRSNRITCVGNDSFIGLSSVRLLSLYDNQITTVAPGAFDTLHSLSTLNLLANPFNCNCYLAWLGEWLRKKRIVTGNPRCQKPYFLKEIPIQDVAIQDFTCDDGNDDNSCSPLSRCPTECTCLDTVVRCSNKGLKVLPKGIPRDVTELYLDGNQFTLVPKELSNYKHLTLIDLSNNRISTLSNQSFSNMTQLLTLILSYNRLRCIPPRTFDGLKSLRLLSLHGNDISVVPEGAFNDLSALSHLAIGANPLYCDCNMQWLSDWVKSEYKEPGIARCAGPGEMADKLLLTTPSKKFTCQGPVDVNILAKCNPCLSNPCKNDGTCNSDPVDFYRCTCPYGFKGQDCDVPIHACISNPCKHGGTCHLKEGEEDGFWCICADGFEGENCEVNVDDCEDNDCENNSTCVDGINNYTCLCPPEYTGELCEEKLDFCAQDLNPCQHDSKCILTPKGFKCDCTPGYVGEHCDIDFDDCQDNKCKNGAHCTDAVNGYTCICPEGYSGLFCEFSPPMVLPRTSPCDNFDCQNGAQCIVRINEPICQCLPGYQGEKCEKLVSVNFINKESYLQIPSAKVRPQTNITLQIATDEDSGILLYKGDKDHIAVELYRGRVRASYDTGSHPASAIYSVETINDGNFHIVELLALDQSLSLSVDGGNPKIITNLSKQSTLNFDSPLYVGGMPGKSNVASLRQAPGQNGTSFHGCIRNLYINSELQDFQKVPMQTGILPGCEPCHKKVCAHGTCQPSSQAGFTCECQEGWMGPLCDQRTNDPCLGNKCVHGTCLPINAFSYSCKCLEGHGGVLCDEEEDLFNPCQAIKCKHGKCRLSGLGQPYCECSSGYTGDSCDREISCRGERIRDYYQKQQGYAACQTTKKVSRLECRGGCAGGQCCGPLRSKRRKYSFECTDGSSFVDEVEKVVKCGCTRCVS

1370 NP_004290.2 NP_001012533.1

>NP_004290.2

MALLAMHSWRWAAAAAAFEKRRHSAILIRPLVSVSGSGPQWRPHQLGALGTARAYQQIPESLKSITWQRLGKGNSGQFLDAAKALQVWPLIEKRTCWHGHAGGGLHTDPKEGLKDVDTRKIIKAMLSYVWPKDRPDLRARVAISLGFLGGAKAMNIVVPFMFKYAVDSLNQMSGNMLNLSDAPNTVATMATAVLIGYGVSRAGAAFFNEVRNAVFGKVAQNSIRRIAKNVFLHLHNLDLGFHLSRQTGALSKAIDRGTRGISFVLSALVFNLLPIMFEVMLVSGVLYYKCGAQFALVTLGTLGTYTAFTVAVTRWRTRFRIEMNKADNDAGNAAIDSLLNYETVKYFNNERYEAQRYDGFLKTYETASLKSTSTLAMLNFGQSAIFSVGLTAIMVLASQGIVAGTLTVGDLVMVNGLLFQLSLPLNFLGTVYRETRQALIDMNTLFTLLKVDTQIKDKVMASPLQITPQTATVAFDNVHFEYIEGQKVLSGISFEVPAGKKVAIVGGSGSGKSTIVRLLFRFYEPQKGSIYLAGQNIQDVSLESLRRAVGVVPQDAVLFHNTIYYNLLYGNISASPEEVYAVAKLAGLHDAILRMPHGYDTQVGERGLKLSGGEKQRVAIARAILKDPPVILYDEATSSLDSITEETILGAMKDVVKHRTSIFIAHRLSTVVDADEIIVLDQGKVAERGTHHGLLANPHSIYSEMWHTQSSRVQNHDNPKWEAKKENISKEEERKKLQEEIVNSVKGCGNCSC

>NP_001012533.1

MRSLGANMAAALRAAGVLLRDPLASSSWRVCQPWRWKSGAAAAAVTTETAQHAQGAKPQVQPQKRYESNIRKPKTGILMLNMGGPETLGDVHDFLLRLFLDRDLMTLPIQNKLAPFIAKRRTPKIQEQYRRIGGGSPIKIWTSKQGEGMVKLLDELSPNTAPHKYYIGFRYVHPLTEEAIEEMERDGLERAIAFTQYPQYSCSTTGSSLNAIYRYYNQVGRKPTMKWSTIDRWPTHHLLIQCFADHILKELDHFPLEKRSEVVILFSAHSLPMSVVNRGDPYPQEVSATVQKVMERLEYCNPYRLVWQSKVGPMPWLGPQTDESIKGLCERGRKNILLVPIAFTSDHIETLYELDIEYSQVLAKECGVENIRRAESLNGNPLFSKALADLVHSHIQSNELCSKQLTLSCPLCVNPVCRETKSFFTSQQL

1371 NP_002559.2 NP_001408.2

>NP_002559.2

MNPSAPSYPMASLYVGDLHPDVTEAMLYEKFSPAGPILSIRVCRDMITRRSLGYAYVNFQQPADAERALDTMNFDVIKGKPVRIMWSQRDPSLRKSGVGNIFIKNLDKSIDNKALYDTFSAFGNILSCKVVCDENGSKGYGFVHFETQEAAERAIEKMNGMLLNDRKVFVGRFKSRKEREAELGARAKEFTNVYIKNFGEDMDDERLKDLFGKFGPALSVKVMTDESGKSKGFGFVSFERHEDAQKAVDEMNGKELNGKQIYVGRAQKKVERQTELKRKFEQMKQDRITRYQGVNLYVKNLDDGIDDERLRKEFSPFGTITSAKVMMEGGRSKGFGFVCFSSPEEATKAVTEMNGRIVATKPLYVALAQRKEERQAHLTNQYMQRMASVRAVPNPVINPYQPAPPSGYFMAAIPQTQNRAAYYPPSQIAQLRPSPRWTAQGARPHPFQNMPGAIRPAAPRPPFSTMRPASSQVPRVMSTQRVANTSTQTMGPRPAAAAAAATPAVRTVPQYKYAAGVRNPQQHLNAQPQVTMQQPAVHVQGQEPLTASMLASAPPQEQKQMLGERLFPLIQAMHPTLAGKITGMLLEIDNSELLHMLESPESLRSKVDEAVAVLQAHQAKEAAQKAVNSATGVPTV

>NP_001408.2

MAASAKKKNKKGKTISLTDFLAEDGGTGGGSTYVSKPVSWADETDDLEGDVSTTWHSNDDDVYRAPPIDRSILPTAPRAAREPNIDRSRLPKSPPYTAFLGNLPYDVTEESIKEFFRGLNISAVRLPREPSNPERLKGFGYAEFEDLDSLLSALSLNEESLGNRRIRVDVADQAQDKDRDDRSFGRDRNRDSDKTDTDWRARPATDSFDDYPPRRGDDSFGDKYRDRYDSDRYRDGYRDGYRDGPRRDMDRYGGRDRYDDRGSRDYDRGYDSRIGSGRRAFGSGYRRDDDYRGGGDRYEDRYDRRDDRSWSSRDDYSRDDYRRDDRGPPQRPKLNLKPRSTPKEDDSSASTSQSTRAASIFGGAKPVDTAAREREVEERLQKEQEKLQRQLDEPKLERRPRERHPSWRSEETQERERSRTGSESSQTGTSTTSSRNARRRESEKSLENETLNKEEDCHSPTSKPPKPDQPLKVMPAPPPKENAWVKRSSNPPARSQSSDTEQQSPTSGGGKVAPAQPSEEGPGRKDENKVDGMNAPKGQTGNSSRGPGDGGNRDHWKESDRKDGKKDQDSRSAPEPKKPEENPASKFSSASKYAALSVDGEDENEGEDYAE

1372 NP_002266.2 NP_055517.3

>NP_002266.2

MTTTFLQTSSSTFGGGSTRGGSLLAGGGGFGGGSLSGGGGSRSISASSARFVSSGSGGGYGGGMRVCGFGGGAGSVFGGGFGGGVGGGFGGGFGGGDGGLLSGNEKITMQNLNDRLASYLDKVRALEEANADLEVKIHDWYQKQTPASPECDYSQYFKTIEELRDKIMATTIDNSRVILEIDNARLAADDFRLKYENELALRQGVEADINGLRRVLDELTLARTDLEMQIEGLNEELAYLKKNHEEEMKEFSSQLAGQVNVEMDAAPGVDLTRVLAEMREQYEAMAEKNRRDVEAWFFSKTEELNKEVASNTEMIQTSKTEITDLRRTMQELEIELQSQLSMKAGLENSLAETECRYATQLQQIQGLIGGLEAQLSELRCEMEAQNQEYKMLLDIKTRLEQEIATYRSLLEGQDAKMAGIGIREASSGGGGSSSNFHINVEESVDGQVVSSHKREI

>NP_055517.3

MDLHKQWENTETNWHKEKMELLDQFDNERKEWESQWKIMQKKIEELCREVKLWRKININESAKIIDLYHEKTIPEKVIESSPNYPDLGQSEFIRTNHKDGLRKENKREQSLVSGGNQMCKEQKATKKSKVGFLDPLATDNQKECEAWPDLRTSEEDSKSCSGALSTALEELAKVSEELCSFQEEIRKRSNHRRMKSDSFLQEMPNVTNIPHGDPMINNDQCILPISLEKEKQKNRKNLSCTNVLQSNSTKKCGIDTIDLKRNETPPVPPPRSTSRNFPSSDSEQAYERWKERLDHNSWVPHEGRSKRNYNPHFPLRQQEMSMLYPNEGKTSKDGIIFSSLVPEVKIDSKPPSNEDVGLSMWSCDIGIGAKRSPSTSWFQKTCSTPSNPKYEMVIPDHPAKSHPDLHVSNDCSSSVAESSSPLRNFSCGFERTTRNEKLAAKTDEFNRTVFRTDRNCQAIQQNHSCSKSSEDLKPCDTSSTHTGSISQSNDVSGIWKTNAHMPVPMENVPDNPTKKSTTGLVRQMQGHLSPRSYRNMLHEHDWRPSNLSGRPRSADPRSNYGVVEKLLKTYETATESALQNSKCFQDNWTKCNSDVSGGATLSQHLEMLQMEQQFQQKTAVWGGQEVKQGIDPKKITEESMSVNASHGKGFSRPARPANRRLPSRWASRSPSAPPALRRTTHNYTISLRSEALMV

1373 NP_002728.1 NP_002558.1

>NP_002728.1

MADVFPGNDSTASQDVANRFARKGALRQKNVHEVKDHKFIARFFKQPTFCSHCTDFIWGFGKQGFQCQVCCFVVHKRCHEFVTFSCPGADKGPDTDDPRSKHKFKIHTYGSPTFCDHCGSLLYGLIHQGMKCDTCDMNVHKQCVINVPSLCGMDHTEKRGRIYLKAEVADEKLHVTVRDAKNLIPMDPNGLSDPYVKLKLIPDPKNESKQKTKTIRSTLNPQWNESFTFKLKPSDKDRRLSVEIWDWDRTTRNDFMGSLSFGVSELMKMPASGWYKLLNQEEGEYYNVPIPEGDEEGNMELRQKFEKAKLGPAGNKVISPSEDRKQPSNNLDRVKLTDFNFLMVLGKGSFGKVMLADRKGTEELYAIKILKKDVVIQDDDVECTMVEKRVLALLDKPPFLTQLHSCFQTVDRLYFVMEYVNGGDLMYHIQQVGKFKEPQAVFYAAEISIGLFFLHKRGIIYRDLKLDNVMLDSEGHIKIADFGMCKEHMMDGVTTRTFCGTPDYIAPEIIAYQPYGKSVDWWAYGVLLYEMLAGQPPFDGEDEDELFQSIMEHNVSYPKSLSKEAVSICKGLMTKHPAKRLGCGPEGERDVREHAFFRRIDWEKLENREIQPPFKPKVCGKGAENFDKFFTRGQPVLTPPDQLVIANIDQSDFEGFSYVNPQFVHPILQSAV

>NP_002558.1

MPVDLSKWSGPLSLQEVDEQPQHPLHVTYAGAAVDELGKVLTPTQVKNRPTSISWDGLDSGKLYTLVLTDPDAPSRKDPKYREWHHFLVVNMKGNDISSGTVLSDYVGSGPPKGTGLHRYVWLVYEQDRPLKCDEPILSNRSGDHRGKFKVASFRKKYELRAPVAGTCYQAEWDDYVPKLYEQLSGK

1374 NP_001195.2 NP_002729.2

>NP_001195.2

MTSSLQRPWRVPWLPWTILLVSTAAASQNQERLCAFKDPYQQDLGIGESRISHENGTILCSKGSTCYGLWEKSKGDINLVKQGCWSHIGDPQECHYEECVVTTTPPSIQNGTYRFCCCSTDLCNVNFTENFPPPDTTPLSPPHSFNRDETIIIALASVSVLAVLIVALCFGYRMLTGDRKQGLHSMNMMEAAASEPSLDLDNLKLLELIGRGRYGAVYKGSLDERPVAVKVFSFANRQNFINEKNIYRVPLMEHDNIARFIVGDERVTADGRMEYLLVMEYYPNGSLCKYLSLHTSDWVSSCRLAHSVTRGLAYLHTELPRGDHYKPAISHRDLNSRNVLVKNDGTCVISDFGLSMRLTGNRLVRPGEEDNAAISEVGTIRYMAPEVLEGAVNLRDCESALKQVDMYALGLIYWEIFMRCTDLFPGESVPEYQMAFQTEVGNHPTFEDMQVLVSREKQRPKFPEAWKENSLAVRSLKETIEDCWDQDAEARLTAQCAEERMAELMMIWERNKSVSPTVNPMSTAMQNERNLSHNRRVPKIGPYPDYSSSSYIEDSIHHTDSIVKNISSEHSMSSTPLTIGEKNRNSINYERQQAQARIPSPETSVTSLSTNTTTTNTTGLTPSTGMTTISEMPYPDETNLHTTNVAQSIGPTPVCLQLTEEDLETNKLDPKEVDKNLKESSDENLMEHSLKQFSGPDPLSSTSSSLLYPLIKLAVEATGQQDFTQTANGQACLIPDVLPTQIYPLPKQQNLPKRPTSLPLNTKNSTKEPRLKFGSKHKSNLKQVETGVAKMNTINAAEPHVVTVTMNGVAGRNHSVNSHAATTQYANGTVLSGQTTNIVTHRAQEMLQNQFIGEDTRLNINSSPDEHEPLLRREQQAGHDEGVLDRLVDRRERPLEGGRTNSNNNNSNPCSEQDVLAQGVPSTAADPGPSKPRRAQRPNSLDLSATNVLDGSSIQIGESTQDGKSGSGEKIKKRVKTPYSLKRWRPSTWVISTESLDCEVNNNGSNRAVHSKSSTAVYLAEGGTATTMVSKDIGMNCL

>NP_002729.2

MADPAAGPPPSEGEESTVRFARKGALRQKNVHEVKNHKFTARFFKQPTFCSHCTDFIWGFGKQGFQCQVCCFVVHKRCHEFVTFSCPGADKGPASDDPRSKHKFKIHTYSSPTFCDHCGSLLYGLIHQGMKCDTCMMNVHKRCVMNVPSLCGTDHTERRGRIYIQAHIDRDVLIVLVRDAKNLVPMDPNGLSDPYVKLKLIPDPKSESKQKTKTIKCSLNPEWNETFRFQLKESDKDRRLSVEIWDWDLTSRNDFMGSLSFGISELQKASVDGWFKLLSQEEGEYFNVPVPPEGSEANEELRQKFERAKISQGTKVPEEKTTNTVSKFDNNGNRDRMKLTDFNFLMVLGKGSFGKVMLSERKGTDELYAVKILKKDVVIQDDDVECTMVEKRVLALPGKPPFLTQLHSCFQTMDRLYFVMEYVNGGDLMYHIQQVGRFKEPHAVFYAAEIAIGLFFLQSKGIIYRDLKLDNVMLDSEGHIKIADFGMCKENIWDGVTTKTFCGTPDYIAPEIIAYQPYGKSVDWWAFGVLLYEMLAGQAPFEGEDEDELFQSIMEHNVAYPKSMSKEAVAICKGLMTKHPGKRLGCGPEGERDIKEHAFFRYIDWEKLERKEIQPPYKPKACGRNAENFDRFFTRHPPVLTPPDQEVIRNIDQSEFEGFSFVNSEFLKPEVKS

1375 NP_003795.2 NP_000034.1

>NP_003795.2

MQPDMSLNVIKMKSSDFLESAELDSGGFGKVSLCFHRTQGLMIMKTVYKGPNCIEHNEALLEEAKMMNRLRHSRVVKLLGVIIEEGKYSLVMEYMEKGNLMHVLKAEMSTPLSVKGRIILEIIEGMCYLHGKGVIHKDLKPENILVDNDFHIKIADLGLASFKMWSKLNNEEHNELREVDGTAKKNGGTLYYMAPEHLNDVNAKPTEKSDVYSFAVVLWAIFANKEPYENAICEQQLIMCIKSGNRPDVDDITEYCPREIISLMKLCWEANPEARPTFPGIEEKFRPFYLSQLEESVEEDVKSLKKEYSNENAVVKRMQSLQLDCVAVPSSRSNSATEQPGSLHSSQGLGMGPVEESWFAPSLEHPQEENEPSLQSKLQDEANYHLYGSRMDRQTKQQPRQNVAYNREEERRRRVSHDPFAQQRPYENFQNTEGKGTAYSSAASHGNAVHQPSGLTSQPQVLYQNNGLYSSHGFGTRPLDPGTAGPRVWYRPIPSHMPSLHNIPVPETNYLGNTPTMPFSSLPPTDESIKYTIYNSTGIQIGAYNYMEIGGTSSSLLDSTNTNFKEEPAAKYQAIFDNTTSLTDKHLDPIRENLGKHWKNCARKLGFTQSQIDEIDHDYERDGLKEKVYQMLQKWVMREGIKGATVGKLAQALHQCSRIDLLSSLIYVSQN

>NP_000034.1

MLGIWTLLPLVLTSVARLSSKSVNAQVTDINSKGLELRKTVTTVETQNLEGLHHDGQFCHKPCPPGERKARDCTVNGDEPDCVPCQEGKEYTDKAHFSSKCRRCRLCDEGHGLEVEINCTRTQNTKCRCKPNFFCNSTVCEHCDPCTKCEHGIIKECTLTSNTKCKEEGSRSNLGWLCLLLLPIPLIVWVKRKEVQKTCRKHRKENQGSHESPTLNPETVAINLSDVDLSKYITTIAGVMTLSQVKGFVRKNGVNEAKIDEIKNDNVQDTAEQKVQLLRNWHQLHGKKEAYDTLIKDLKKANLCTLAEKIQTIILKDITSDSENSNFRNEIQSLV

1376 NP_002040.1 NP_002737.2

>NP_002040.1

MEFPGLGSLGTSEPLPQFVDPALVSSTPESGVFFPSGPEGLDAAASSTAPSTATAAAAALAYYRDAEAYRHSPVFQVYPLLNCMEGIPGGSPYAGWAYGKTGLYPASTVCPTREDSPPQAVEDLDGKGSTSFLETLKTERLSPDLLTLGPALPSSLPVPNSAYGGPDFSSTFFSPTGSPLNSAAYSSPKLRGTLPLPPCEARECVNCGATATPLWRRDRTGHYLCNACGLYHKMNGQNRPLIRPKKRLIVSKRAGTQCTNCQTTTTTLWRRNASGDPVCNACGLYYKLHQVNRPLTMRKDGIQTRNRKASGKGKKKRGSSLGGTGAAEGPAGGFMVVAGGSGSGNCGEVASGLTLGPPGTAHLYQGLGPVVLSGPVSHLMPFPGPLLGSPTGSFPTGPMPPTTSTTVVAPLSS

>NP_002737.2

MAAAAAQGGGGGEPRRTEGVGPGVPGEVEMVKGQPFDVGPRYTQLQYIGEGAYGMVSSAYDHVRKTRVAIKKISPFEHQTYCQRTLREIQILLRFRHENVIGIRDILRASTLEAMRDVYIVQDLMETDLYKLLKSQQLSNDHICYFLYQILRGLKYIHSANVLHRDLKPSNLLINTTCDLKICDFGLARIADPEHDHTGFLTEYVATRWYRAPEIMLNSKGYTKSIDIWSVGCILAEMLSNRPIFPGKHYLDQLNHILGILGSPSQEDLNCIINMKARNYLQSLPSKTKVAWAKLFPKSDSKALDLLDRMLTFNPNKRITVEEALAHPYLEQYYDPTDEPVAEEPFTFAMELDDLPKERLKELIFQETARFQPGVLEAP

1377 NP_000456.1 NP_001460.1

>NP_000456.1

MPDNRQPRNRQPRIRSGNEPRSAPAMEPDGRGAWAHSRAALDRLEKLLRCSRCTNILREPVCLGGCEHIFCSNCVSDCIGTGCPVCYTPAWIQDLKINRQLDSMIQLCSKLRNLLHDNELSDLKEDKPRKSLFNDAGNKKNSIKMWFSPRSKKVRYVVSKASVQTQPAIKKDASAQQDSYEFVSPSPPADVSERAKKASARSGKKQKKKTLAEINQKWNLEAEKEDGEFDSKEESKQKLVSFCSQPSVISSPQINGEIDLLASGSLTESECFGSLTEVSLPLAEQIESPDTKSRNEVVTPEKVCKNYLTSKKSLPLENNGKRGHHNRLSSPISKRCRTSILSTSGDFVKQTVPSENIPLPECSSPPSCKRKVGGTSGRKNSNMSDEFISLSPGTPPSTLSSSSYRQVMSSPSAMKLLPNMAVKRNHRGETLLHIASIKGDIPSVEYLLQNGSDPNVKDHAGWTPLHEACNHGHLKVVELLLQHKALVNTTGYQNDSPLHDAAKNGHVDIVKLLLSYGASRNAVNIFGLRPVDYTDDESMKSLLLLPEKNESSSASHCSVMNTGQRRDGPLVLIGSGLSSEQQKMLSELAVILKAKKYTEFDSTVTHVVVPGDAVQSTLKCMLGILNGCWILKFEWVKACLRRKVCEQEEKYEIPEGPRRSRLNREQLLPKLFDGCYFYLWGTFKHHPKDNLIKLVTAGGGQILSRKPKPDSDVTQTINTVAYHARPDSDQRFCTQYIIYEDLCNYHPERVRQGKVWKAPSSWFIDCVMSFELLPLDS

>NP_001460.1

MSGWESYYKTEGDEEAEEEQEENLEASGDYKYSGRDSLIFLVDASKAMFESQSEDELTPFDMSIQCIQSVYISKIISSDRDLLAVVFYGTEKDKNSVNFKNIYVLQELDNPGAKRILELDQFKGQQGQKRFQDMMGHGSDYSLSEVLWVCANLFSDVQFKMSHKRIMLFTNEDNPHGNDSAKASRARTKAGDLRDTGIFLDLMHLKKPGGFDISLFYRDIISIAEDEDLRVHFEESSKLEDLLRKVRAKETRKRALSRLKLKLNKDIVISVGIYNLVQKALKPPPIKLYRETNEPVKTKTRTFNTSTGGLLLPSDTKRSQIYGSRQIILEKEETEELKRFDDPGLMLMGFKPLVLLKKHHYLRPSLFVYPEESLVIGSSTLFSALLIKCLEKEVAALCRYTPRRNIPPYFVALVPQEEELDDQKIQVTPPGFQLVFLPFADDKRKMPFTEKIMATPEQVGKMKAIVEKLRFTYRSDSFENPVLQQHFRNLEALALDLMEPEQAVDLTLPKVEAMNKRLGSLVDEFKELVYPPDYNPEGKVTKRKHDNEGSGSKRPKVEYSEEELKTHISKGTLGKFTVPMLKEACRAYGLKSGLKKQELLEALTKHFQD

1378 NP_004553.1 NP_652759.3

>NP_004553.1

MIVFVRFNSSHGFPVEVDSDTSIFQLKEVVAKRQGVPADQLRVIFAGKELRNDWTVQNCDLDQQSIVHIVQRPWRKGQEMNATGGDDPRNAAGGCEREPQSLTRVDLSSSVLPGDSVGLAVILHTDSRKDSPPAGSPAGRSIYNSFYVYCKGPCQRVQPGKLRVQCSTCRQATLTLTQGPSCWDDVLIPNRMSGECQSPHCPGTSAEFFFKCGAHPTSDKETPVALHLIATNSRNITCITCTDVRSPVLVFQCNSRHVICLDCFHLYCVTRLNDRQFVHDPQLGYSLPCVAGCPNSLIKELHHFRILGEEQYNRYQQYGAEECVLQMGGVLCPRPGCGAGLLPEPDQRKVTCEGGNGLGCGFAFCRECKEAYHEGECSAVFEASGTTTQAYRVDERAAEQARWEAASKETIKKTTKPCPRCHVPVEKNGGCMHMKCPQPQCRLEWCWNCGCEWNRVCMGDHWFDV

>NP_652759.3

MPVIPALWEVEMGRSQGQEIETILANRSHSDSTPLPNFLSGSHRPECCTCRLLTASGAQDSLPFGRRLYSGPWRSCEEVCHVSVLSVLSTSCGLSLSLPIFPGWMEWLSPDIALPRRDEWTQTSPARKRITHAKVQGAGQLRLSIDAQDRVLLLHIIEGKGLISKQPGTCDPYVKISLIPEDSRLRHQKTQTVPDCRDPAFHEHFFFPVQEEDDQKRLLVTVWNRASQSRQSGLIGCMSFGVKSLLTPDKEISGWYYLLGEHLGRTKHLKVARRRLRPLRDPLLRMPGGGDTENGKKLKITIPRGKDGFGFTICCDSPVRVQAVDSGGPAERAGLQQLDTVLQLNERPVEHWKCVELAHEIRSCPSEIILLVWRMVPQVKPGPDGGVLRRASCKSTHDLQSPPNKREKNCTHGVQARPEQRHSCHLVCDSSDGLLLGGWERYTEVAKRGGQHTLPALSRATAPTDPNYIILAPLNPGSQLLRPVYQEDTIPEESGSPSKGKSYTGLGKKSRLMKTVQTMKGHGNYQNCPVVRPHATHSSYGTYVTLAPKVLVFPVFVQPLDLCNPARTLLLSEELLLYEGRNKAAEVTLFAYSDLLLFTKEDEPGRCDVLRNPLYLQSVKLQEGSSEDLKFCVLYLAEKAECLFTLEAHSQEQKKRVCWCLSENIAKQQQLAASPPDSKMFETEADEKREMALEEGKGPGAEDSPPSKEPSPGQELPPGQDLPPNKDSPSGQEPAPSQEPLSSKDSATSEGSPPGPDAPPSKDVPPCQEPPPAQDLSPCQDLPAGQEPLPHQDPLLTKDLPAIQESPTRDLPPCQDLPPSQVSLPAKALTEDTMSSGDLLAATGDPPAAPRPAFVIPEVRLDSTYSQKAGAEQGCSGDEEDAEEAEEVEEGEEGEEDEDEDTSDDNYGERSEAKRSSMIETGQGAEGGLSLRVQNSLRRRTHSEGSLLQEPRGPCFASDTTLHCSDGEGAASTWGMPSPSTLKKELGRNGGSMHHLSLFFTGHRKMSGADTVGDDDEASRKRKSKNLAKDMKNKLGIFRRRNESPGAPPAGKADKMMKSFKPTSEEALKWGESLEKLLVHKYGLAVFQAFLRTEFSEENLEFWLACEDFKKVKSQSKMASKAKKIFAEYIAIQACKEVNLDSYTREHTKDNLQSVTRGCFDLAQKRIFGLMEKDSYPRFLRSDLYLDLINQKKMSPPL

1379 NP_005535.1 NP_001269.3

>NP_005535.1

MASPPESDGFSDVRKVGYLRKPKSMHKRFFVLRAASEAGGPARLEYYENEKKWRHKSSAPKRSIPLESCFNINKRADSKNKHLVALYTRDEHFAIAADSEAEQDSWYQALLQLHNRAKGHHDGAAALGAGGGGGSCSGSSGLGEAGEDLSYGDVPPGPAFKEVWQVILKPKGLGQTKNLIGIYRLCLTSKTISFVKLNSEAAAVVLQLMNIRRCGHSENFFFIEVGRSAVTGPGEFWMQVDDSVVAQNMHETILEAMRAMSDEFRPRSKSQSSSNCSNPISVPLRRHHLNNPPPSQVGLTRRSRTESITATSPASMVGGKPGSFRVRASSDGEGTMSRPASVDGSPVSPSTNRTHAHRHRGSARLHPPLNHSRSIPMPASRCSPSATSPVSLSSSSTSGHGSTSDCLFPRRSSASVSGSPSDGGFISSDEYGSSPCDFRSSFRSVTPDSLGHTPPARGEEELSNYICMGGKGPSTLTAPNGHYILSRGGNGHRCTPGTGLGTSPALAGDEAASAADLDNRFRKRTHSAGTSPTITHQKTPSQSSVASIEEYTEMMPAYPPGGGSGGRLPGHRHSAFVPTRSYPEEGLEMHPLERRGGHHRPDSSTLHTDDGYMPMSPGVAPVPSGRKGSGDYMPMSPKSVSAPQQIINPIRRHPQRVDPNGYMMMSPSGGCSPDIGGGPSSSSSSSNAVPSGTSYGKLWTNGVGGHHSHVLPHPKPPVESSGGKLLPCTGDYMNMSPVGDSNTSSPSDCYYGPEDPQHKPVLSYYSLPRSFKHTQRPGEPEEGARHQHLRLSTSSGRLLYAATADDSSSSTSSDSLGGGYCGARLEPSLPHPHHQVLQPHLPRKVDTAAQTNSRLARPTRLSLGDPKASTLPRAREQQQQQQPLLHPPEPKSPGEYVNIEFGSDQSGYLSGPVAFHSSPSVRCPSQLQPAPREEETGTEEYMKMDLGPGRRAAWQESTGVEMGRLGPAPPGAASICRPTRAVPSSRGDYMTMQMSCPRQSYVDTSPAAPVSYADMRTGIAAEEVSLPRATMAAASSSSAASASPTGPQGAAELAAHSSLLGGPQGPGGMSAFTRVNLSPNRNQSAKVIRADPQGCRRRHSSETFSSTPSATRVGNTVPFGAGAAVGGGGGSSSSSEDVKRHSSASFENVWLRPGELGGAPKEPAKLCGAAGGLENGLNYIDLDLVKDFKQCPQECTPEPQPPPPPPPHQPLGSGESSSTRRSSEDLSAYASISFQKQPEDRQ

>NP_001269.3

MERPPGLRPGAGGPWEMRERLGTGGFGNVCLYQHRELDLKIAIKSCRLELSTKNRERWCHEIQIMKKLNHANVVKACDVPEELNILIHDVPLLAMEYCSGGDLRKLLNKPENCCGLKESQILSLLSDIGSGIRYLHENKIIHRDLKPENIVLQDVGGKIIHKIIDLGYAKDVDQGSLCTSFVGTLQYLAPELFENKPYTATVDYWSFGTMVFECIAGYRPFLHHLQPFTWHEKIKKKDPKCIFACEEMSGEVRFSSHLPQPNSLCSLVVEPMENWLQLMLNWDPQQRGGPVDLTLKQPRCFVLMDHILNLKIVHILNMTSAKIISFLLPPDESLHSLQSRIERETGINTGSQELLSETGISLDPRKPASQCVLDGVRGCDSYMVYLFDKSKTVYEGPFASRSLSDCVNYIVQDSKIQLPIIQLRKVWAEAVHYVSGLKEDYSRLFQGQRAAMLSLLRYNANLTKMKNTLISASQQLKAKLEFFHKSIQLDLERYSEQMTYGISSEKMLKAWKEMEEKAIHYAEVGVIGYLEDQIMSLHAEIMELQKSPYGRRQGDLMESLEQRAIDLYKQLKHRPSDHSYSDSTEMVKIIVHTVQSQDRVLKELFGHLSKLLGCKQKIIDLLPKVEVALSNIKEADNTVMFMQGKRQKEIWHLLKIACTQSSARSLVGSSLEGAVTPQTSAWLPPTSAEHDHSLSCVVTPQDGETSAQMIEENLNCLGHLSTIIHEANEEQGNSMMNLDWSWLTE

1380 NP_001030.2 NP_004594.1

>NP_001030.2

MAPGEKIKAKIKKNLPVTGPQAPTIKELMRWYCLNTNTHGCRRIVVSRGRLRRLLWIGFTLTAVALILWQCALLVFSFYTVSVSIKVHFRKLDFPAVTICNINPYKYSTVRHLLADLEQETREALKSLYGFPESRKRREAESWNSVSEGKQPRFSHRIPLLIFDQDEKGKARDFFTGRKRKVGGSIIHKASNVMHIESKQVVGFQLCSNDTSDCATYTFSSGINAIQEWYKLHYMNIMAQVPLEKKINMSYSAEELLVTCFFDGVSCDARNFTLFHHPMHGNCYTFNNRENETILSTSMGGSEYGLQVILYINEEEYNPFLVSSTGAKVIIHRQDEYPFVEDVGTEIETAMVTSIGMHLTESFKLSEPYSQCTEDGSDVPIRNIYNAAYSLQICLHSCFQTKMVEKCGCAQYSQPLPPAANYCNYQQHPNWMYCYYQLHRAFVQEELGCQSVCKEACSFKEWTLTTSLAQWPSVVSEKWLLPVLTWDQGRQVNKKLNKTDLAKLLIFYKDLNQRSIMESPANSIEMLLSNFGGQLGLWMSCSVVCVIEIIEVFFIDFFSIIARRQWQKAKEWWAWKQAPPCPEAPRSPQGQDNPALDIDDDLPTFNSALHLPPALGTQVPGTPPPKYNTLRLERAFSNQLTDTQMLDEL

>NP_004594.1

MKDRTQELRTAKDSDDDDDVAVTVDRDRFMDEFFEQVEEIRGFIDKIAENVEEVKRKHSAILASPNPDEKTKEELEELMSDIKKTANKVRSKLKSIEQSIEQEEGLNRSSADLRIRKTQHSTLSRKFVEVMSEYNATQSDYRERCKGRIQRQLEITGRTTTSEELEDMLESGNPAIFASGIIMDSSISKQALSEIETRHSEIIKLENSIRELHDMFMDMAMLVESQGEMIDRIEYNVEHAVDYVERAVSDTKKAVKYQSKARRKKIMIIICCVILGIVIASTVGGIFA

1381 NP_001931.2 NP_006452.3

>NP_001931.2

MKTRQNKDSMSMRSGRKKEAPGPREELRSRGRASPGGVSTSSSDGKAEKSRQTAKKARVEEASTPKVNKQGRSEEISESESEETNAPKKTKTEQELPRPQSPSDLDSLDGRSLNDDGSSDPRDIDQDNRSTSPSIYSPGSVENDSDSSSGLSQGPARPYHPPPLFPPSPQPPDSTPRQPEASFEPHPSVTPTGYHAPMEPPTSRMFQAPPGAPPPHPQLYPGGTGGVLSGPPMGPKGGGAASSVGGPNGGKQHPPPTTPISVSSSGASGAPPTKPPTTPVGGGNLPSAPPPANFPHVTPNLPPPPALRPLNNASASPPGLGAQPLPGHLPSPHAMGQGMGGLPPGPEKGPTLAPSPHSLPPASSSAPAPPMRFPYSSSSSSSAAASSSSSSSSSSASPFPASQALPSYPHSFPPPTSLSVSNQPPKYTQPSLPSQAVWSQGPPPPPPYGRLLANSNAHPGPFPPSTGAQSTAHPPVSTHHHHHQQQQQQQQQQQQQQQQQQQHHGNSGPPPPGAFPHPLEGGSSHHAHPYAMSPSLGSLRPYPPGPAHLPPPHSQVSYSQAGPNGPPVSSSSNSSSSTSQGSYPCSHPSPSQGPQGAPYPFPPVPTVTTSSATLSTVIATVASSPAGYKTASPPGPPPYGKRAPSPGAYKTATPPGYKPGSPPSFRTGTPPGYRGTSPPAGPGTFKPGSPTVGPGPLPPAGPSGLPSLPPPPAAPASGPPLSATQIKQEPAEEYETPESPVPPARSPSPPPKVVDVPSHASQSARFNKHLDRGFNSCARSDLYFVPLEGSKLAKKRADLVEKVRREAEQRAREEKEREREREREKEREREKERELERSVKLAQEGRAPVECPSLGPVPHRPPFEPGSAVATVPPYLGPDTPALRTLSEYARPHVMSPGNRNHPFYVPLGAVDPGLLGYNVPALYSSDPAAREREREARERDLRDRLKPGFEVKPSELEPLHGVPGPGLDPFPRHGGLALQPGPPGLHPFPFHPSLGPLERERLALAAGPALRPDMSYAERLAAERQHAERVAALGNDPLARLQMLNVTPHHHQHSHIHSHLHLHQQDAIHAASASVHPLIDPLASGSHLTRIPYPAGTLPNPLLPHPLHENEVLRHQLFAAPYRDLPASLSAPMSAAHQLQAMHAQSAELQRLALEQQQWLHAHHPLHSVPLPAQEDYYSHLKKESDKPL

>NP_006452.3

MWRVKKLSLSLSPSPQTGKPSMRTPLRELTLQPGALTNSGKRSPACSSLTPSLCKLGLQEGSNNSSPVDFVNNKRTDLSSEHFSHSSKWLETCQHESDEQPLDPIPQISSTPKTSEEAVDPLGNYMVKTIVLVPSPLGQQQDMIFEARLDTMAETNSISLNGPLRTDDLVREEVAPCMGDRFSEVAAVSEKPIFQESPSHLLEESPPNPCSEQLHCSKESLSSRTEAVREDLVPSESNAFLPSSVLWLSPSTALAADFRVNHVDPEEEIVEHGAMEEREMRFPTHPKESETEDQALVSSVEDILSTCLTPNLVEMESQEAPGPAVEDVGRILGSDTESWMSPLAWLEKGVNTSVMLENLRQSLSLPSMLRDAAIGTTPFSTCSVGTWFTPSAPQEKSTNTSQTGLVGTKHSTSETEQLLCGRPPDLTALSRHDLEDNLLSSLVILEVLSRQLRDWKSQLAVPHPETQDSSTQTDTSHSGITNKLQHLKESHEMGQALQQARNVMQSWVLISKELISLLHLSLLHLEEDKTTVSQESRRAETLVCCCFDLLKKLRAKLQSLKAEREEARHREEMALRGKDAAEIVLEAFCAHASQRISQLEQDLASMREFRGLLKDAQTQLVGLHAKQEELVQQTVSLTSTLQQDWRSMQLDYTTWTALLSRSRQLTEKLTVKSQQALQERDVAIEEKQEVSRVLEQVSAQLEECKGQTEQLELENSRLATDLRAQLQILANMDSQLKELQSQHTHCAQDLAMKDELLCQLTQSNEEQAAQWQKEEMALKHMQAELQQQQAVLAKEVRDLKETLEFADQENQVAHLELGQVECQLKTTLEVLRERSLQCENLKDTVENLTAKLASTIADNQEQDLEKTRQYSQKLGLLTEQLQSLTLFLQTKLKEKTEQETLLLSTACPPTQEHPLPNDRTFLGSILTAVADEEPESTPVPLLGSDKSAFTRVASMVSLQPAETPGMEESLAEMSIMTTELQSLCSLLQESKEEAIRTLQRKICELQARLQAQEEQHQEVQKAKEADIEKLNQALCLRYKNEKELQEVIQQQNEKILEQIDKSGELISLREEVTHLTRSLRRAETETKVLQEALAGQLDSNCQPMATNWIQEKVWLSQEVDKLRVMFLEMKNEKEKLMIKFQSHRNILEENLRRSDKELEKLDDIVQHIYKTLLSIPEVVRGCKELQGLLEFLS

1382 NP_002244.1 NP_000386.1

>NP_002244.1

MQSKVLLAVALWLCVETRAASVGLPSVSLDLPRLSIQKDILTIKANTTLQITCRGQRDLDWLWPNNQSGSEQRVEVTECSDGLFCKTLTIPKVIGNDTGAYKCFYRETDLASVIYVYVQDYRSPFIASVSDQHGVVYITENKNKTVVIPCLGSISNLNVSLCARYPEKRFVPDGNRISWDSKKGFTIPSYMISYAGMVFCEAKINDESYQSIMYIVVVVGYRIYDVVLSPSHGIELSVGEKLVLNCTARTELNVGIDFNWEYPSSKHQHKKLVNRDLKTQSGSEMKKFLSTLTIDGVTRSDQGLYTCAASSGLMTKKNSTFVRVHEKPFVAFGSGMESLVEATVGERVRIPAKYLGYPPPEIKWYKNGIPLESNHTIKAGHVLTIMEVSERDTGNYTVILTNPISKEKQSHVVSLVVYVPPQIGEKSLISPVDSYQYGTTQTLTCTVYAIPPPHHIHWYWQLEEECANEPSQAVSVTNPYPCEEWRSVEDFQGGNKIEVNKNQFALIEGKNKTVSTLVIQAANVSALYKCEAVNKVGRGERVISFHVTRGPEITLQPDMQPTEQESVSLWCTADRSTFENLTWYKLGPQPLPIHVGELPTPVCKNLDTLWKLNATMFSNSTNDILIMELKNASLQDQGDYVCLAQDRKTKKRHCVVRQLTVLERVAPTITGNLENQTTSIGESIEVSCTASGNPPPQIMWFKDNETLVEDSGIVLKDGNRNLTIRRVRKEDEGLYTCQACSVLGCAKVEAFFIIEGAQEKTNLEIIILVGTAVIAMFFWLLLVIILRTVKRANGGELKTGYLSIVMDPDELPLDEHCERLPYDASKWEFPRDRLKLGKPLGRGAFGQVIEADAFGIDKTATCRTVAVKMLKEGATHSEHRALMSELKILIHIGHHLNVVNLLGACTKPGGPLMVIVEFCKFGNLSTYLRSKRNEFVPYKTKGARFRQGKDYVGAIPVDLKRRLDSITSSQSSASSGFVEEKSLSDVEEEEAPEDLYKDFLTLEHLICYSFQVAKGMEFLASRKCIHRDLAARNILLSEKNVVKICDFGLARDIYKDPDYVRKGDARLPLKWMAPETIFDRVYTIQSDVWSFGVLLWEIFSLGASPYPGVKIDEEFCRRLKEGTRMRAPDYTTPEMYQTMLDCWHGEPSQRPTFSELVEHLGNLLQANAQQDGKDYIVLPISETLSMEEDSGLSLPTSPVSCMEEEEVCDPKFHYDNTAGISQYLQNSKRKSRPVSVKTFEDIPLEEPEVKVIPDDNQTDSGMVLASEELKTLEDRTKLSPSFGGMVPSKSRESVASEGSNQTSGYQSGYHSDDTDTTVYSSEEAELLKLIEIGVQTGSTAQILQPDSGTTLSSPPV

>NP_000386.1

MVLAQGLLSMALLALCWERSLAGAEETIPLQTLRCYNDYTSHITCRWADTQDAQRLVNVTLIRRVNEDLLEPVSCDLSDDMPWSACPHPRCVPRRCVIPCQSFVVTDVDYFSFQPDRPLGTRLTVTLTQHVQPPEPRDLQISTDQDHFLLTWSVALGSPQSHWLSPGDLEFEVVYKRLQDSWEDAAILLSNTSQATLGPEHLMPSSTYVARVRTRLAPGSRLSGRPSKWSPEVCWDSQPGDEAQPQNLECFFDGAAVLSCSWEVRKEVASSVSFGLFYKPSPDAGEEECSPVLREGLGSLHTRHHCQIPVPDPATHGQYIVSVQPRRAEKHIKSSVNIQMAPPSLNVTKDGDSYSLRWETMKMRYEHIDHTFEIQYRKDTATWKDSKTETLQNAHSMALPALEPSTRYWARVRVRTSRTGYNGIWSEWSEARSWDTESVLPMWVLALIVIFLTIAVLLALRFCGIYGYRLRRKWEEKIPNPSKSHLFQNGSAELWPPGSMSAFTSGSPPHQGPWGSRFPELEGVFPVGFGDSEVSPLTIEDPKHVCDPPSGPDTTPAASDLPTEQPPSPQPGPPAASHTPEKQASSFDFNGPYLGPPHSRSLPDILGQPEPPQEGGSQKSPPPGSLEYLCLPAGGQVQLVPLAQAMGPGQAVEVERRPSQGAAGSPSLESGGGPAPPALGPRVGGQDQKDSPVAIPMSSGDTEDPGVASGYVSSADLVFTPNSGASSVSLVPSLGLPSDQTPSLCPGLASGPPGAPGPVKSGFEGYVELPPIEGRSPRSPRNNPVPPEAKSPVLNPGERPADVSPTSPQPEGLLVLQQVGDYCFLPGLGPGPLSLRSKPSSPGPGPEIKNLDQAFQVKKPPGQAVPQVPVIQLFKALKQQDYLSLPPWEVNKPGEVC

1383 NP_003874.2 NP_002711.1

>NP_003874.2

MAKTVAYFYDPDVGNFHYGAGHPMKPHRLALTHSLVLHYGLYKKMIVFKPYQASQHDMCRFHSEDYIDFLQRVSPTNMQGFTKSLNAFNVGDDCPVFPGLFEFCSRYTGASLQGATQLNNKICDIAINWAGGLHHAKKFEASGFCYVNDIVIGILELLKYHPRVLYIDIDIHHGDGVQEAFYLTDRVMTVSFHKYGNYFFPGTGDMYEVGAESGRYYCLNVPLRDGIDDQSYKHLFQPVINQVVDFYQPTCIVLQCGADSLGCDRLGCFNLSIRGHGECVEYVKSFNIPLLVLGGGGYTVRNVARCWTYETSLLVEEAISEELPYSEYFEYFAPDFTLHPDVSTRIENQNSRQYLDQIRQTIFENLKMLNHAPSVQIHDVPADLLTYDRTDEADAEERGPEENYSRPEAPNEFYDGDHDNDKESDVEI

>NP_002711.1

MAEISDLDRQIEQLRRCELIKESEVKALCAKAREILVEESNVQRVDSPVTVCGDIHGQFYDLKELFRVGGDVPETNYLFMGDFVDRGFYSVETFLLLLALKVRYPDRITLIRGNHESRQITQVYGFYDECLRKYGSVTVWRYCTEIFDYLSLSAIIDGKIFCVHGGLSPSIQTLDQIRTIDRKQEVPHDGPMCDLLWSDPEDTTGWGVSPRGAGYLFGSDVVAQFNAANDIDMICRAHQLVMEGYKWHFNETVLTVWSAPNYCYRCGNVAAILELDEHLQKDFIIFEAAPQETRGIPSKKPVADYFL

1384 NP_150593.1 NP_000629.3

>NP_150593.1

MKFTTLLFLAAVAGALVYAEDASSDSTGADPAQEAGTSKPNEEISGPAEPASPPETTTTAQETSAAAVQGTAKVTSSRQELNPLKSIVEKSILLTEQALAKAGKGMHGGVPGGKQFIENGSEFAQKLLKKFSLLKPWA

>NP_000629.3

MAPLRPLLILALLAWVALADQESCKGRCTEGFNVDKKCQCDELCSYYQSCCTDYTAECKPQVTRGDVFTMPEDEYTVYDDGEEKNNATVHEQVGGPSLTSDLQAQSKGNPEQTPVLKPEEEAPAPEVGASKPEGIDSRPETLHPGRPQPPAEEELCSGKPFDAFTDLKNGSLFAFRGQYCYELDEKAVRPGYPKLIRDVWGIEGPIDAAFTRINCQGKTYLFKGSQYWRFEDGVLDPDYPRNISDGFDGIPDNVDAALALPAHSYSGRERVYFFKGKQYWEYQFQHQPSQEECEGSSLSAVFEHFAMMQRDSWEDIFELLFWGRTSAGTRQPQFISRDWHGVPGQVDAAMAGRIYISGMAPRPSLAKKQRFRHRNRKGYRSQRGHSRGRNQNSRRPSRATWLSLFSSEESNLGANNYDDYRMDWLVPATCEPIQSVFFFSGDKYYRVNLRTRRVDTVDPPYPRSIAQYWLGCPAPGHL

1385 NP_002389.1 NP_055436.2

>NP_002389.1

MAQRYDDLPHYGGMDGVGIPSTMYGDPHAARSMQPVHHLNHGPPLHSHQYPHTAHTNAMAPSMGSSVNDALKRDKDAIYGHPLFPLLALIFEKCELATCTPREPGVAGGDVCSSESFNEDIAVFAKQIRAEKPLFSSNPELDNLMIQAIQVLRFHLLELEKVHELCDNFCHRYISCLKGKMPIDLVIDDREGGSKSDSEDITRSANLTDQPSWNRDHDDTASTRSGGTPGPSSGGHTSHSGDNSSEQGDGLDNSVASPSTGDDDDPDKDKKRHKKRGIFPKVATNIMRAWLFQHLTHPYPSEEQKKQLAQDTGLTILQVNNWFINARRRIVQPMIDQSNRAVSQGTPYNPDGQPMGGFVMDGQQHMGIRAPGPMSGMGMNMGMEGQWHYM

>NP_055436.2

MVMSSYMVNSKYVDPKFPPCEEYLQGGYLGEQGADYYGGGAQGADFQPPGLYPRPDFGEQPFGGSGPGPGSALPARGHGQEPGGPGGHYAAPGEPCPAPPAPPPAPLPGARAYSQSDPKQPPSGTALKQPAVVYPWMKKVHVNSVNPNYTGGEPKRSRTAYTRQQVLELEKEFHFNRYLTRRRRIEIAHTLCLSERQIKIWFQNRRMKWKKDHKLPNTKGRSSSSSSSSSCSSSVAPSQHLQPMAKDHHTDLTTL

1386 NP_002010.1 NP_001895.1

>NP_002010.1

MVSYWDTGVLLCALLSCLLLTGSSSGSKLKDPELSLKGTQHIMQAGQTLHLQCRGEAAHKWSLPEMVSKESERLSITKSACGRNGKQFCSTLTLNTAQANHTGFYSCKYLAVPTSKKKETESAIYIFISDTGRPFVEMYSEIPEIIHMTEGRELVIPCRVTSPNITVTLKKFPLDTLIPDGKRIIWDSRKGFIISNATYKEIGLLTCEATVNGHLYKTNYLTHRQTNTIIDVQISTPRPVKLLRGHTLVLNCTATTPLNTRVQMTWSYPDEKNKRASVRRRIDQSNSHANIFYSVLTIDKMQNKDKGLYTCRVRSGPSFKSVNTSVHIYDKAFITVKHRKQQVLETVAGKRSYRLSMKVKAFPSPEVVWLKDGLPATEKSARYLTRGYSLIIKDVTEEDAGNYTILLSIKQSNVFKNLTATLIVNVKPQIYEKAVSSFPDPALYPLGSRQILTCTAYGIPQPTIKWFWHPCNHNHSEARCDFCSNNEESFILDADSNMGNRIESITQRMAIIEGKNKMASTLVVADSRISGIYICIASNKVGTVGRNISFYITDVPNGFHVNLEKMPTEGEDLKLSCTVNKFLYRDVTWILLRTVNNRTMHYSISKQKMAITKEHSITLNLTIMNVSLQDSGTYACRARNVYTGEEILQKKEITIRDQEAPYLLRNLSDHTVAISSSTTLDCHANGVPEPQITWFKNNHKIQQEPGIILGPGSSTLFIERVTEEDEGVYHCKATNQKGSVESSAYLTVQGTSDKSNLELITLTCTCVAATLFWLLLTLLIRKMKRSSSEIKTDYLSIIMDPDEVPLDEQCERLPYDASKWEFARERLKLGKSLGRGAFGKVVQASAFGIKKSPTCRTVAVKMLKEGATASEYKALMTELKILTHIGHHLNVVNLLGACTKQGGPLMVIVEYCKYGNLSNYLKSKRDLFFLNKDAALHMEPKKEKMEPGLEQGKKPRLDSVTSSESFASSGFQEDKSLSDVEEEEDSDGFYKEPITMEDLISYSFQVARGMEFLSSRKCIHRDLAARNILLSENNVVKICDFGLARDIYKNPDYVRKGDTRLPLKWMAPESIFDKIYSTKSDVWSYGVLLWEIFSLGGSPYPGVQMDEDFCSRLREGMRMRAPEYSTPEIYQIMLDCWHRDPKERPRFAELVEKLGDLLQANVQQDGKDYIPINAILTGNSGFTYSTPAFSEDFFKESISAPKFNSGSSDDVRYVNAFKFMSLERIKTFEELLPNATSMFDDYQGDSSTLLASPMLKRFTWTDSKPKASLKIDLRVTSKSKESGLSDVSRPSFCHSSCGHVSEGKRRFTYDHAELERKIACCSPPPDYNSVVLYSTPPI

>NP_001895.1

MATQADLMELDMAMEPDRKAAVSHWQQQSYLDSGIHSGATTTAPSLSGKGNPEEEDVDTSQVLYEWEQGFSQSFTQEQVADIDGQYAMTRAQRVRAAMFPETLDEGMQIPSTQFDAAHPTNVQRLAEPSQMLKHAVVNLINYQDDAELATRAIPELTKLLNDEDQVVVNKAAVMVHQLSKKEASRHAIMRSPQMVSAIVRTMQNTNDVETARCTAGTLHNLSHHREGLLAIFKSGGIPALVKMLGSPVDSVLFYAITTLHNLLLHQEGAKMAVRLAGGLQKMVALLNKTNVKFLAITTDCLQILAYGNQESKLIILASGGPQALVNIMRTYTYEKLLWTTSRVLKVLSVCSSNKPAIVEAGGMQALGLHLTDPSQRLVQNCLWTLRNLSDAATKQEGMEGLLGTLVQLLGSDDINVVTCAAGILSNLTCNNYKNKMMVCQVGGIEALVRTVLRAGDREDITEPAICALRHLTSRHQEAEMAQNAVRLHYGLPVVVKLLHPPSHWPLIKATVGLIRNLALCPANHAPLREQGAIPRLVQLLVRAHQDTQRRTSMGGTQQQFVEGVRMEEIVEGCTGALHILARDVHNRIVIRGLNTIPLFVQLLYSPIENIQRVAAGVLCELAQDKEAAEAIEAEGATAPLTELLHSRNEGVATYAAAVLFRMSEDKPQDYKKRLSVELTSSLFRTEPMAWNETADLGLDIGAQGEPLGYRQDDPSYRSFHSGGYGQDALGMDPMMEHEMGGHHPGADYPVDGLPDLGHAQDLMDGLPPGDSNQLAWFDTDL

1387 NP_006752.1 NP_055511.2

>NP_006752.1

MDDREDLVYQAKLAEQAERYDEMVESMKKVAGMDVELTVEERNLLSVAYKNVIGARRASWRIISSIEQKEENKGGEDKLKMIREYRQMVETELKLICCDILDVLDKHLIPAANTGESKVFYYKMKGDYHRYLAEFATGNDRKEAAENSLVAYKAASDIAMTELPPTHPIRLGLALNFSVFYYEILNSPDRACRLAKAAFDDAIAELDTLSEESYKDSTLIMQLLRDNLTLWTSDMQGDGEEQNKEALQDVEDENQ

>NP_055511.2

MSSSRPEPGPWAPLSPRLQPLSQSSSSLLGEGREQRPELRKTASSTVWQAQLGEASTRPQAPEEEGNPPESMKPARASGPKARPSAGGHWWSSTVGNVSTMGGSDLCRLRAPSAAAMQRSHSDLVRSTQMRGHSGARKASLSCSALGSSPVHRAQLQPGGTSGQGGQAPAGLERDLAPEDETSNSAWMLGASQLSVPPLDLGDTTAHSSSAQAEPKAAEQLATTTCHALPPAALLCGMREVRAGGCCHALPATGILAFPKLVASVSESGLQAQHGVKIHCRLSGGLPGHSHCCAHLWGPAGLVPEPGSRTKDVWTMTSANDLAPAEASPLSAQDAGVQAAPVAACKAVATSPSLEAPAALHVFPEVTLGSSLEEVPSPVRDVRWDAEGMTWEVYGAAVDLEVLGVAIQKHLEMQFEQLQRAPASEDSLSVEGRRGPLRAVMQSLRRPSCCGCSGAAPE

1388 NP_003019.2 NP_002077.1

>NP_003019.2

MAKWLNKYFSLGNSKTKSPPQPPRPDYREQRRRGERPSQPPQAVPQASSAASASCGPATASCFSASSGSLPDDSGSTSDLIRAYRAQKERDFEDPYNGPGSSLRKLRAMCRLDYCGGSGEPGGVQRAFSASSASGAAGCCCASSGAGAAASSSSSSGSPHLYRSSSERRPATPAEVRYISPKHRLIKVESAAGGGAGDPLGGACAGGRTWSPTACGGKKLLNKCAASAAEESGAGKKDKVTIADDYSDPFDAKNDLKSKAGKGESAGYMEPYEAQRIMTEFQRQESVRSQHKGIQLYDTPYEPEGQSVDSDSESTVSPRLRESKLPQDDDRPADEYDQPWEWNRVTIPALAAQFNGNEKRQSSPSPSRDRRRQLRAPGGGFKPIKHGSPEFCGILGERVDPAVPLEKQIWYHGAISRGDAENLLRLCKECSYLVRNSQTSKHDYSLSLRSNQGFMHMKLAKTKEKYVLGQNSPPFDSVPEVIHYYTTRKLPIKGAEHLSLLYPVAVRTL

>NP_002077.1

MEAIAKYDFKATADDELSFKRGDILKVLNEECDQNWYKAELNGKDGFIPKNYIEMKPHPWFFGKIPRAKAEEMLSKQRHDGAFLIRESESAPGDFSLSVKFGNDVQHFKVLRDGAGKYFLWVVKFNSLNELVDYHRSTSVSRNQQIFLRDIEQVPQQPTYVQALFDFDPQEDGELGFRRGDFIHVMDNSDPNWWKGACHGQTGMFPRNYVTPVNRNV

1389 NP_004413.1 NP_036325.2

>NP_004413.1

MAGSSTGGGGVGETKVIYHLDEEETPYLVKIPVPAERITLGDFKSVLQRPAGAKYFFKSMDQDFGVVKEEISDDNARLPCFNGRVVSWLVSSDNPQPEMAPPVHEPRAELAPPAPPLPPLPPERTSGIGDSRPPSFHPNVSSSHENLEPETETESVVSLRRERPRRRDSSEHGAGGHRTGGPSRLERHLAGYESSSTLMTSELESTSLGDSDEEDTMSRFSSSTEQSSASRLLKRHRRRRKQRPPRLERTSSFSSVTDSTMSLNIITVTLNMEKYNFLGISIVGQSNERGDGGIYIGSIMKGGAVAADGRIEPGDMLLQVNDMNFENMSNDDAVRVLRDIVHKPGPIVLTVAKCWDPSPQAYFTLPRNEPIQPIDPAAWVSHSAALTGTFPAYPGSSSMSTITSGSSLPDGCEGRGLSVHTDMASVTKAMAAPESGLEVRDRMWLKITIPNAFLGSDVVDWLYHHVEGFPERREARKYASGLLKAGLIRHTVNKITFSEQCYYVFGDLSGGCESYLVNLSLNDNDGSSGASDQDTLAPLPGATPWPLLPTFSYQYPAPHPYSPQPPPYHELSSYTYGGGSASSQHSEGSRSSGSTRSDGGAGRTGRPEERAPESKSGSGSESEPSSRGGSLRRGGEASGTSDGGPPPSRGSTGGAPNLRAHPGLHPYGPPPGMALPYNPMMVVMMPPPPPPVPPAVQPPGAPPVRDLGSVPPELTASRQSFHMAMGNPSEFFVDVM

>NP_036325.2

MAWRGAGPSVPGAPGGVGLSLGLLLQLLLLLGPARGFGDEEERRCDPIRISMCQNLGYNVTKMPNLVGHELQTDAELQLTTFTPLIQYGCSSQLQFFLCSVYVPMCTEKINIPIGPCGGMCLSVKRRCEPVLKEFGFAWPESLNCSKFPPQNDHNHMCMEGPGDEEVPLPHKTPIQPGEECHSVGTNSDQYIWVKRSLNCVLKCGYDAGLYSRSAKEFTDIWMAVWASLCFISTAFTVLTFLIDSSRFSYPERPIIFLSMCYNIYSIAYIVRLTVGRERISCDFEEAAEPVLIQEGLKNTGCAIIFLLMYFFGMASSIWWVILTLTWFLAAGLKWGHEAIEMHSSYFHIAAWAIPAVKTIVILIMRLVDADELTGLCYVGNQNLDALTGFVVAPLFTYLVIGTLFIAAGLVALFKIRSNLQKDGTKTDKLERLMVKIGVFSVLYTVPATCVIACYFYEISNWALFRYSADDSNMAVEMLKTFMSLLVGITSGMWIWSAKSLHTWQKCSNRLVNSGKVKREKRGNGWVKPGKGSETVV

1390 NP_003343.1 NP_057698.2

>NP_003343.1

MSDQEAKPSTEDLGDKKEGEYIKLKVIGQDSSEIHFKVKMTTHLKKLKESYCQRQGVPMNSLRFLFEGQRIADNHTPKELGMEEEDVIEVYQEQTGGHSTV

>NP_057698.2

MELGSCLEGGREAAEEEGEPEVKKRRLLCVEFASVASCDAAVAQCFLAENDWEMERALNSYFEPPVEESALERRPETISEPKTYVDLTNEETTDSTTSKISPSEDTQQENGSMFSLITWNIDGLDLNNLSERARGVCSYLALYSPDVIFLQEVIPPYYSYLKKRSSNYEIITGHEEGYFTAIMLKKSRVKLKSQEIIPFPSTKMMRNLLCVHVNVSGNELCLMTSHLESTRGHAAERMNQLKMVLKKMQEAPESATVIFAGDTNLRDREVTRCGGLPNNIVDVWEFLGKPKHCQYTWDTQMNSNLGITAACKLRFDRIFFRAAAEEGHIIPRSLDLLGLEKLDCGRFPSDHWGLLCNLDIIL

1391 NP_001521.1 NP_612482.2

>NP_001521.1

MEGAGGANDKKKISSERRKEKSRDAARSRRSKESEVFYELAHQLPLPHNVSSHLDKASVMRLTISYLRVRKLLDAGDLDIEDDMKAQMNCFYLKALDGFVMVLTDDGDMIYISDNVNKYMGLTQFELTGHSVFDFTHPCDHEEMREMLTHRNGLVKKGKEQNTQRSFFLRMKCTLTSRGRTMNIKSATWKVLHCTGHIHVYDTNSNQPQCGYKKPPMTCLVLICEPIPHPSNIEIPLDSKTFLSRHSLDMKFSYCDERITELMGYEPEELLGRSIYEYYHALDSDHLTKTHHDMFTKGQVTTGQYRMLAKRGGYVWVETQATVIYNTKNSQPQCIVCVNYVVSGIIQHDLIFSLQQTECVLKPVESSDMKMTQLFTKVESEDTSSLFDKLKKEPDALTLLAPAAGDTIISLDFGSNDTETDDQQLEEVPLYNDVMLPSPNEKLQNINLAMSPLPTAETPKPLRSSADPALNQEVALKLEPNPESLELSFTMPQIQDQTPSPSDGSTRQSSPEPNSPSEYCFYVDSDMVNEFKLELVEKLFAEDTEAKNPFSTQDTDLDLEMLAPYIPMDDDFQLRSFDQLSPLESSSASPESASPQSTVTVFQQTQIQEPTANATTTTATTDELKTVTKDRMEDIKILIASPSPTHIHKETTSATSSPYRDTQSRTASPNRAGKGVIEQTEKSHPRSPNVLSVALSQRTTVPEEELNPKILALQNAQRKRKMEHDGSLFQAVGIGTLLQQPDDHAATTSLSWKRVKGCKSSEQNGMEQKTIILIPSDLACRLLGQSMDESGLPQLTSYDCEVNAPIQGSRNLLQGEELLRALDQVN

>NP_612482.2

MSDQDHSMDEMTAVVKIEKGVGGNNGGNGNGGGAFSQARSSSTGSSSSTGGGGQESQPSPLALLAATCSRIESPNENSNNSQGPSQSGGTGELDLTATQLSQGANGWQIISSSSGATPTSKEQSGSSTNGSNGSESSKNRTVSGGQYVVAAAPNLQNQQVLTGLPGVMPNIQYQVIPQFQTVDGQQLQFAATGAQVQQDGSGQIQIIPGANQQIITNRGSGGNIIAAMPNLLQQAVPLQGLANNVLSGQTQYVTNVPVALNGNITLLPVNSVSAATLTPSSQAVTISSSGSQESGSQPVTSGTTISSASLVSSQASSSSFFTNANSYSTTTTTSNMGIMNFTTSGSSGTNSQGQTPQRVSGLQGSDALNIQQNQTSGGSLQAGQQKEGEQNQQTQQQQILIQPQLVQGGQALQALQAAPLSGQTFTTQAISQETLQNLQLQAVPNSGPIIIRTPTVGPNGQVSWQTLQLQNLQVQNPQAQTITLAPMQGVSLGQTSSSNTTLTPIASAASIPAGTVTVNAAQLSSMPGLQTINLSALGTSGIQVHPIQGLPLAIANAPGDHGAQLGLHGAGGDGIHDDTAGGEEGENSPDAQPQAGRRTRREACTCPYCKDSEGRGSGDPGKKKQHICHIQGCGKVYGKTSHLRAHLRWHTGERPFMCTWSYCGKRFTRSDELQRHKRTHTGEKKFACPECPKRFMRSDHLSKHIKTHQNKKGGPGVALSVGTLPLDSGAGSEGSGTATPSALITTNMVAMEAICPEGIARLANSGINVMQVADLQSINISGNGF

1392 NP_003494.1 NP_055136.1

>NP_003494.1

MEASLGIQMDEPMAFSPQRDRFQAEGSLKKNEQNFKLAGVKKDIEKLYEAVPQLSNVFKIEDKIGEGTFSSVYLATAQLQVGPEEKIALKHLIPTSHPIRIAAELQCLTVAGGQDNVMGVKYCFRKNDHVVIAMPYLEHESFLDILNSLSFQEVREYMLNLFKALKRIHQFGIVHRDVKPSNFLYNRRLKKYALVDFGLAQGTHDTKIELLKFVQSEAQQERCSQNKSHIITGNKIPLSGPVPKELDQQSTTKASVKRPYTNAQIQIKQGKDGKEGSVGLSVQRSVFGERNFNIHSSISHESPAVKLMKQSKTVDVLSRKLATKKKAISTKVMNSAVMRKTASSCPASLTCDCYATDKVCSICLSRRQQVAPRAGTPGFRAPEVLTKCPNQTTAIDMWSAGVIFLSLLSGRYPFYKASDDLTALAQIMTIRGSRETIQAAKTFGKSILCSKEVPAQDLRKLCERLRGMDSSTPKLTSDIQGHASHQPAISEKTDHKASCLVQTPPGQYSGNSFKKGDSNSCEHCFDEYNTNLEGWNEVPDEAYDLLDKLLDLNPASRITAEEALLHPFFKDMSL

>NP_055136.1

MGSELIGRLAPRLGLAEPDMLRKAEEYLRLSRVKCVGLSARTTETSSAVMCLDLAASWMKCPLDRAYLIKLSGLNKETYQSCLKSFECLLGLNSNIGIRDLAVQFSCIEAVNMASKILKSYESSLPQTQQVDLDLSRPLFTSAALLSACKILKLKVDKNKMVATSGVKKAIFDRLCKQLEKIGQQVDREPGDVATPPRKRKKIVVEAPAKEMEKVEEMPHKPQKDEDLTQDYEEWKRKILENAASAQKATAE

1393 NP_002736.3 NP_001001930.1

>NP_002736.3

MAAAAAAGAGPEMVRGQVFDVGPRYTNLSYIGEGAYGMVCSAYDNVNKVRVAIKKISPFEHQTYCQRTLREIKILLRFRHENIIGINDIIRAPTIEQMKDVYIVQDLMETDLYKLLKTQHLSNDHICYFLYQILRGLKYIHSANVLHRDLKPSNLLLNTTCDLKICDFGLARVADPDHDHTGFLTEYVATRWYRAPEIMLNSKGYTKSIDIWSVGCILAEMLSNRPIFPGKHYLDQLNHILGILGSPSQEDLNCIINLKARNYLLSLPHKNKVPWNRLFPNADSKALDLLDKMLTFNPHKRIEVEQALAHPYLEQYYDPSDEPIAEAPFKFDMELDDLPKEKLKELIFEETARFQPGYRS

>NP_001001930.1

MVDTESPLCPLSPLEAGDLESPLSEEFLQEMGNIQEISQSIGEDSSGSFGFTEYQYLGSCPGSDGSVITDTLSPASSPSSVTYPVVPGSVDESPSGALNIECRICGDKASGYHYGVHACEGCKGFFRRTIRLKLVYDKCDRSCKIQKKNRNKCQYCRFHKCLSVGMSHNAIRFGRMPRSEKAKLKAEILTCEHDIEDSETADLKSLAKRIYEAYLKNFNMNKVKARVILSGKASNNPPFVIHDMETLCMAEKTLVAKLVANGIQNKEAEVRIFHCCQCTSVETVTELTEFAKAIPGFANLDLNDQVTLLKYGVYEAIFAMLSSVMNKDGMLVAYGNGFITREFLKSLRKPFCDIMEPKFDFAMKFNALELDDSDISLFVAAIICCGDRPGLLNVGHIEKMQEGIVHVLRLHLQSNHPDDIFLFPKLLQKMADLRQLVTEHAQLVQIIKKTESDAALHPLLQEIYRDMY

1394 NP_005822.1 NP_055579.1

>NP_005822.1

MEETIKDPPTSAVLLDHCHFSQVIFNSVEKFYIPGGDVTCHYTFTQHFIPRRKDWIGIFRVGWKTTREYYTFMWVTLPIDLNNKSAKQQEVQFKAYYLPKDDEYYQFCYVDEDGVVRGASIPFQFRPENEEDILVVTTQGEVEEIEQHNKELCKENQELKDSCISLQKQNSDMQAELQKKQEELETLQSINKKLELKVKEQKDYWETELLQLKEQNQKMSSENEKMGIRVDQLQAQLSTQEKEMEKLVQGDQDKTEQLEQLKKENDHLFLSLTEQRKDQKKLEQTVEQMKQNETTAMKKQQELMDENFDLSKRLSENEIICNALQRQKERLEGENDLLKRENSRLLSYMGLDFNSLPYQVPTSDEGGARQNPGLAYGNPYSGIQESSSPSPLSIKKCPICKADDICDHTLEQQQMQPLCFNCPICDKIFPATEKQIFEDHVFCHSL

>NP_055579.1

MNSKGQYPTQPTYPVQPPGNPVYPQTLHLPQAPPYTDAPPAYSELYRPSFVHPGAATVPTMSAAFPGASLYLPMAQSVAVGPLGSTIPMAYYPVGPIYPPGSTVLVEGGYDAGARFGAGATAGNIPPPPPGCPPNAAQLAVMQGANVLVTQRKGNFFMGGSDGGYTIW

1395 NP_115977.2 NP_596869.3

>NP_115977.2

MDYKSSLIQDGNPMENLEKQLICPICLEMFTKPVVILPCQHNLCRKCANDIFQAANPYWTSRGSSVSMSGGRFRCPTCRHEVIMDRHGVYGLQRNLLVENIIDIYKQECSSRPLQKGSHPMCKEHEDEKINIYCLTCEVPTCSMCKVFGIHKACEVAPLQSVFQGQKTELNNCISMLVAGNDRVQTIITQLEDSRRVTKENSHQVKEELSQKFDTLYAILDEKKSELLQRITQEQEKKLSFIEALIQQYQEQLDKSTKLVETAIQSLDEPGGATFLLTAKQLIKSIVEASKGCQLGKTEQGFENMDFFTLDLEHIADALRAIDFGTDEEEEEFIEEEDQEEEESTEGKEEGHQ

>NP_596869.3

MTTQAPTFTQPLQSVVVLEGSTATFEAHISGFPVPEVSWFRDGQVISTSTLPGVQISFSDGRAKLTIPAVTKANSGRYSLKATNGSGQATSTAELLVKAETAPPNFVQRLQSMTVRQGSQVRLQVRVTGIPTPVVKFYRDGAEIQSSLDFQISQEGDLYSLLIAEAYPEDSGTYSVNATNSVGRATSTAELLVQGEEEVPAKKTKTIVSTAQISESRQTRIEKKIEAHFDARSIATVEMVIDGAAGQQLPHKTPPRIPPKPKSRSPTPPSIAAKAQLARQQSPSPIRHSPSPVRHVRAPTPSPVRSVSPAARISTSPIRSVRSPLLMRKTQASTVATGPEVPPPWKQEGYVASSSEAEMRETTLTTSTQIRTEERWEGRYGVQEQVTISGAAGAAASVSASASYAAEAVATGAKEVKQDADKSAAVATVVAAVDMARVREPVISAVEQTAQRTTTTAVHIQPAQEQVRKEAEKTAVTKVVVAADKAKEQELKSRTKEVITTKQEQMHVTHEQIRKETEKTFVPKVVISAAKAKEQETRISEEITKKQKQVTQEAIRQETEITAASMVVVATAKSTKLETVPGAQEETTTQQDQMHLSYEKIMKETRKTVVPKVIVATPKVKEQDLVSRGREGITTKREQVQITQEKMRKEAEKTALSTIAVATAKAKEQETILRTRETMATRQEQIQVTHGKVDVGKKAEAVATVVAAVDQARVREPREPGHLEESYAQQTTLEYGYKERISAAKVAEPPQRPASEPHVVPKAVKPRVIQAPSETHIKTTDQKGMHISSQIKKTTDLTTERLVHVDKRPRTASPHFTVSKISVPKTEHGYEASIAGSAIATLQKELSATSSAQKITKSVKAPTVKPSETRVRAEPTPLPQFPFADTPDTYKSEAGVEVKKEVGVSITGTTVREERFEVLHGREAKVTETARVPAPVEIPVTPPTLVSGLKNVTVIEGESVTLECHISGYPSPTVTWYREDYQIESSIDFQITFQSGIARLMIREAFAEDSGRFTCSAVNEAGTVSTSCYLAVQVSEEFEKETTAVTEKFTTEEKRFVESRDVVMTDTSLTEEQAGPGEPAAPYFITKPVVQKLVEGGSVVFGCQVGGNPKPHVYWKKSGVPLTTGYRYKVSYNKQTGECKLVISMTFADDAGEYTIVVRNKHGETSASASLLEEADYELLMKSQQEMLYQTQVTAFVQEPKVGETAPGFVYSEYEKEYEKEQALIRKKMAKDTVVVRTYVEDQEFHISSFEERLIKEIEYRIIKTTLEELLEEDGEEKMAVDISESEAVESGFDSRIKNYRILEGMGVTFHCKMSGYPLPKIAWYKDGKRIKHGERYQMDFLQDGRASLRIPVVLPEDEGIYTAFASNIKGNAICSGKLYVEPAAPLGAPTYIPTLEPVSRIRSLSPRSVSRSPIRMSPARMSPARMSPARMSPARMSPGRRLEETDESQLERLYKPVFVLKPVSFKCLEGQTARFDLKVVGRPMPETFWFHDGQQIVNDYTHKVVIKEDGTQSLIIVPATPSDSGEWTVVAQNRAGRSSISVILTVEAVEHQVKPMFVEKLKNVNIKEGSRLEMKVRATGNPNPDIVWLKNSDIIVPHKYPKIRIEGTKGEAALKIDSTVSQDSAWYTATAINKAGRDTTRCKVNVEVEFAEPEPERKLIIPRGTYRAKEIAAPELEPLHLRYGQEQWEEGDLYDKEKQQKPFFKKKLTSLRLKRFGPAHFECRLTPIGDPTMVVEWLHDGKPLEAANRLRMINEFGYCSLDYGVAYSRDSGIITCRATNKYGTDHTSATLIVKDEKSLVEESQLPEGRKGLQRIEELERMAHEGALTGVTTDQKEKQKPDIVLYPEPVRVLEGETARFRCRVTGYPQPKVNWYLNGQLIRKSKRFRVRYDGIHYLDIVDCKSYDTGEVKVTAENPEGVIEHKVKLEIQQREDFRSVLRRAPEPRPEFHVHEPGKLQFEVQKVDRPVDTTETKEVVKLKRAERITHEKVPEESEELRSKFKRRTEEGYYEAITAVELKSRKKDESYEELLRKTKDELLHWTKELTEEEKKALAEEGKITIPTFKPDKIELSPSMEAPKIFERIQSQTVGQGSDAHFRVRVVGKPDPECEWYKNGVKIERSDRIYWYWPEDNVCELVIRDVTAEDSASIMVKAINIAGETSSHAFLLVQAKQLITFTQELQDVVAKEKDTMATFECETSEPFVKVKWYKDGMEVHEGDKYRMHSDRKVHFLSILTIDTSDAEDYSCVLVEDENVKTTAKLIVEGAVVEFVKELQDIEVPESYSGELECIVSPENIEGKWYHNDVELKSNGKYTITSRRGRQNLTVKDVTKEDQGEYSFVIDGKKTTCKLKMKPRPIAILQGLSDQKVCEGDIVQLEVKVSLESVEGVWMKDGQEVQPSDRVHIVIDKQSHMLLIEDMTKEDAGNYSFTIPALGLSTSGRVSVYSVDVITPLKDVNVIEGTKAVLECKVSVPDVTSVKWYLNDEQIKPDDRVQAIVKGTKQRLVINRTHASDEGPYKLIVGRVETNCNLSVEKIKIIRGLRDLTCTETQNVVFEVELSHSGIDVLWNFKDKEIKPSSKYKIEAHGKIYKLTVLNMMKDDEGKYTFYAGENMTSGKLTVAGGAISKPLTDQTVAESQEAVFECEVANPDSKGEWLRDGKHLPLTNNIRSESDGHKRRLIIAATKLDDIGEYTYKVATSKTSAKLKVEAVKIKKTLKNLTVTETQDAVFTVELTHPNVKGVQWIKNGVVLESNEKYAISVKGTIYSLRIKNCAIVDESVYGFRLGRLGASARLHVETVKIIKKPKDVTALENATVAFEVSVSHDTVPVKWFHKSVEIKPSDKHRLVSERKVHKLMLQNISPSDAGEYTAVVGQLECKAKLFVETLHITKTMKNIEVPETKTASFECEVSHFNVPSMWLKNGVEIEMSEKFKIVVQGKLHQLIIMNTSTEDSAEYTFVCGNDQVSATLTVTPIMITSMLKDINAEEKDTITFEVTVNYEGISYKWLKNGVEIKSTDKCQMRTKKLTHSLNIRNVHFGDAADYTFVAGKATSTATLYVEARHIEFRKHIKDIKVLEKKRAMFECEVSEPDITVQWMKDDQELQITDRIKIQKEKYVHRLLIPSTRMSDAGKYTVVAGGNVSTAKLFVEGRDVRIRSIKKEVQVIEKQRAVVEFEVNEDDVDAHWYKDGIEINFQVQERHKYVVERRIHRMFISETRQSDAGEYTFVAGRNRSSVTLYVNAPEPPQVLQELQPVTVQSGKPARFCAVISGRPQPKISWYKEEQLLSTGFKCKFLHDGQEYTLLLIEAFPEDAAVYTCEAKNDYGVATTSASLSVEVPEVVSPDQEMPVYPPAIITPLQDTVTSEGQPARFQCRVSGTDLKVSWYSKDKKIKPSRFFRMTQFEDTYQLEIAEAYPEDEGTYTFVASNAVGQVSSTANLSLEAPESILHERIEQEIEMEMKAAPVIKRKIEPLEVALGHLAKFTCEIQSAPNVRFQWFKAGREIYESDKCSIRSSKYISSLEILRTQVVDCGEYTCKASNEYGSVSCTATLTVTEAYPPTFLSRPKSLTTFVGKAAKFICTVTGTPVIETIWQKDGAALSPSPNWRISDAENKHILELSNLTIQDRGVYSCKASNKFGADICQAELIIIDKPHFIKELEPVQSAINKKVHLECQVDEDRKVTVTWSKDGQKLPPGKDYKICFEDKIATLEIPLAKLKDSGTYVCTASNEAGSSSCSATVTVREPPSFVKKVDPSYLMLPGESARLHCKLKGSPVIQVTWFKNNKELSESNTVRMYFVNSEAILDITDVKVEDSGSYSCEAVNDVGSDSCSTEIVIKEPPSFIKTLEPADIVRGTNALLQCEVSGTGPFEISWFKDKKQIRSSKKYRLFSQKSLVCLEIFSFNSADVGEYECVVANEVGKCGCMATHLLKEPPTFVKKVDDLIALGGQTVTLQAAVRGSEPISVTWMKGQEVIREDGKIKMSFSNGVAVLIIPDVQISFGGKYTCLAENEAGSQTSVGELIVKEPAKIIERAELIQVTAGDPATLEYTVAGTPELKPKWYKDGRPLVASKKYRISFKNNVAQLKFYSAELHDSGQYTFEISNEVGSSSCETTFTVLDRDIAPFFTKPLRNVDSVVNGTCRLDCKIAGSLPMRVSWFKDGKEIAASDRYRIAFVEGTASLEIIRVDMNDAGNFTCRATNSVGSKDSSGALIVQEPPSFVTKPGSKDVLPGSAVCLKSTFQGSTPLTIRWFKGNKELVSGGSCYITKEALESSLELYLVKTSDSGTYTCKVSNVAGGVECSANLFVKEPATFVEKLEPSQLLKKGDATQLACKVTGTPPIKITWFANDREIKESSKHRMSFVESTAVLRLTDVGIEDSGEYMCEAQNEAGSDHCSSIVIVKESPYFTKEFKPIEVLKEYDVMLLAEVAGTPPFEITWFKDNTILRSGRKYKTFIQDHLVSLQILKFVAADAGEYQCRVTNEVGSSICSARVTLREPPSFIKKIESTSSLRGGTAAFQATLKGSLPITVTWLKDSDEITEDDNIRMTFENNVASLYLSGIEVKHDGKYVCQAKNDAGIQRCSALLSVKEPATITEEAVSIDVTQGDPATLQVKFSGTKEITAKWFKDGQELTLGSKYKISVTDTVSILKIISTEKKDSGEYTFEVQNDVGRSSCKARINVLDLIIPPSFTKKLKKMDSIKGSFIDLECIVAGSHPISIQWFKDDQEISASEKYKFSFHDNTAFLEISQLEGTDSGTYTCSATNKAGHNQCSGHLTVKEPPYFVEKPQSQDVNPNTRVQLKALVGGTAPMTIKWFKDNKELHSGAARSVWKDDTSTSLELFAAKATDSGTYICQLSNDVGTATSKATLFVKEPPQFIKKPSPVLVLRNGQSTTFECQITGTPKIRVSWYLDGNEITAIQKHGISFIDGLATFQISGARVENSGTYVCEARNDAGTASCSIELKVKEPPTFIRELKPVEVVKYSDVELECEVTGTPPFEVTWLKNNREIRSSKKYTLTDRVSVFNLHITKCDPSDTGEYQCIVSNEGGSCSCSTRVALKEPPSFIKKIENTTTVLKSSATFQSTVAGSPPISITWLKDDQILDEDDNVYISFVDSVATLQIRSVDNGHSGRYTCQAKNESGVERCYAFLLVQEPAQIVEKAKSVDVTEKDPMTLECVVAGTPELKVKWLKDGKQIVPSRYFSMSFENNVASFRIQSVMKQDSGQYTFKVENDFGSSSCDAYLRVLDQNIPPSFTKKLTKMDKVLGSSIHMECKVSGSLPISAQWFKDGKEISTSAKYRLVCHERSVSLEVNNLELEDTANYTCKVSNVAGDDACSGILTVKEPPSFLVKPGRQQAIPDSTVEFKAILKGTPPFKIKWFKDDVELVSGPKCFIGLEGSTSFLNLYSVDASKTGQYTCHVTNDVGSDSCTTMLLVTEPPKFVKKLEASKIVKAGDSSRLECKIAGSPEIRVVWFRNEHELPASDKYRMTFIDSVAVIQMNNLSTEDSGDFICEAQNPAGSTSCSTKVIVKEPPVFSSFPPIVETLKNAEVSLECELSGTPPFEVVWYKDKRQLRSSKKYKIASKNFHTSIHILNVDTSDIGEYHCKAQNEVGSDTCVCTVKLKEPPRFVSKLNSLTVVAGEPAELQASIEGAQPIFVQWLKEKEEVIRESENIRITFVENVATLQFAKAEPANAGKYICQIKNDGGMRENMATLMVLEPAVIVEKAGPMTVTVGETCTLECKVAGTPELSVEWYKDGKLLTSSQKHKFSFYNKISSLRILSVERQDAGTYTFQVQNNVGKSSCTAVVDVSDRAVPPSFTRRLKNTGGVLGASCILECKVAGSSPISVAWFHEKTKIVSGAKYQTTFSDNVCTLQLNSLDSSDMGNYTCVAANVAGSDECRAVLTVQEPPSFVKEPEPLEVLPGKNVTFTSVIRGTPPFKVNWFRGARELVKGDRCNIYFEDTVAELELFNIDISQSGEYTCVVSNNAGQASCTTRLFVKEPAAFLKRLSDHSVEPGKSIILESTYTGTLPISVTWKKDGFNITTSEKCNIVTTEKTCILEILNSTKRDAGQYSCEIENEAGRDVCGALVSTLEPPYFVTELEPLEAAVGDSVSLQCQVAGTPEITVSWYKGDTKLRPTPEYRTYFTNNVATLVFNKVNINDSGEYTCKAENSIGTASSKTVFRIQERQLPPSFARQLKDIEQTVGLPVTLTCRLNGSAPIQVCWYRDGVLLRDDENLQTSFVDNVATLKILQTDLSHSGQYSCSASNPLGTASSSARLTAREPKKSPFFDIKPVSIDVIAGESADFECHVTGAQPMRITWSKDNKEIRPGGNYTITCVGNTPHLRILKVGKGDSGQYTCQATNDVGKDMCSAQLSVKEPPKFVKKLEASKVAKQGESIQLECKISGSPEIKVSWFRNDSELHESWKYNMSFINSVALLTINEASAEDSGDYICEAHNGVGDASCSTALTVKAPPVFTQKPSPVGALKGSDVILQCEISGTPPFEVVWVKDRKQVRNSKKFKITSKHFDTSLHILNLEASDVGEYHCKATNEVGSDTCSCSVKFKEPPRFVKKLSDTSTLIGDAVELRAIVEGFQPISVVWLKDRGEVIRESENTRISFIDNIATLQLGSPEASNSGKYICQIKNDAGMRECSAVLTVLEPARIIEKPEPMTVTTGNPFALECVVTGTPELSAKWFKDGRELSADSKHHITFINKVASLKIPCAEMSDKGLYSFEVKNSVGKSNCTVSVHVSDRIVPPSFIRKLKDVNAILGASVVLECRVSGSAPISVGWFQDGNEIVSGPKCQSSFSENVCTLNLSLLEPSDTGIYTCVAANVAGSDECSAVLTVQEPPSFEQTPDSVEVLPGMSLTFTSVIRGTPPFKVKWFKGSRELVPGESCNISLEDFVTELELFEVQPLESGDYSCLVTNDAGSASCTTHLFVKEPATFVKRLADFSVETGSPIVLEATYTGTPPISVSWIKDEYLISQSERCSITMTEKSTILEILESTIEDYAQYSCLIENEAGQDICEALVSVLEPPYFIEPLEHVEAVIGEPATLQCKVDGTPEIRISWYKEHTKLRSAPAYKMQFKNNVASLVINKVDHSDVGEYSCKADNSVGAVASSAVLVIKARKLPPFFARKLKDVHETLGFPVAFECRINGSEPLQVSWYKDGVLLKDDANLQTSFVHNVATLQILQTDQSHIGQYNCSASNPLGTASSSAKLILSEHEVPPFFDLKPVSVDLALGESGTFKCHVTGTAPIKITWAKDNREIRPGGNYKMTLVENTATLTVLKVGKGDAGQYTCYASNIAGKDSCSAQLGVQEPPRFIKKLEPSRIVKQDEFTRYECKIGGSPEIKVLWYKDETEIQESSKFRMSFVDSVAVLEMHNLSVEDSGDYTCEAHNAAGSASSSTSLKVKEPPIFRKKPHPIETLKGADVHLECELQGTPPFHVSWYKDKRELRSGKKYKIMSENFLTSIHILNVDAADIGEYQCKATNDVGSDTCVGSIALKAPPRFVKKLSDISTVVGKEVQLQTTIEGAEPISVVWFKDKGEIVRESDNIWISYSENIATLQFSRVEPANAGKYTCQIKNDAGMQECFATLSVLEPATIVEKPESIKVTTGDTCTLECTVAGTPELSTKWFKDGKELTSDNKYKISFFNKVSGLKIINVAPSDSGVYSFEVQNPVGKDSCTASLQVSDRTVPPSFTRKLKETNGLSGSSVVMECKVYGSPPISVSWFHEGNEISSGRKYQTTLTDNTCALTVNMLEESDSGDYTCIATNMAGSDECSAPLTVREPPSFVQKPDPMDVLTGTNVTFTSIVKGTPPFSVSWFKGSSELVPGDRCNVSLEDSVAELELFDVDTSQSGEYTCIVSNEAGKASCTTHLYIKAPAKFVKRLNDYSIEKGKPLILEGTFTGTPPISVTWKKNGINVTPSQRCNITTTEKSAILEIPSSTVEDAGQYNCYIENASGKDSCSAQILILEPPYFVKQLEPVKVSVGDSASLQCQLAGTPEIGVSWYKGDTKLRPTTTYKMHFRNNVATLVFNQVDINDSGEYICKAENSVGEVSASTFLTVQEQKLPPSFSRQLRDVQETVGLPVVFDCAISGSEPISVSWYKDGKPLKDSPNVQTSFLDNTATLNIFKTDRSLAGQYSCTATNPIGSASSSARLILTEGKNPPFFDIRLAPVDAVVGESADFECHVTGTQPIKVSWAKDSREIRSGGKYQISYLENSAHLTVLKVDKGDSGQYTCYAVNEVGKDSCTAQLNIKERLIPPSFTKRLSETVEETEGNSFKLEGRVAGSQPITVAWYKNNIEIQPTSNCEITFKNNTLVLQVRKAGMNDAGLYTCKVSNDAGSALCTSSIVIKEPKKPPVFDQHLTPVTVSEGEYVQLSCHVQGSEPIRIQWLKAGREIKPSDRCSFSFASGTAVLELRDVAKADSGDYVCKASNVAGSDTTKSKVTIKDKPAVAPATKKAAVDGRLFFVSEPQSIRVVEKTTATFIAKVGGDPIPNVKWTKGKWRQLNQGGRVFIHQKGDEAKLEIRDTTKTDSGLYRCVAFNEHGEIESNVNLQVDERKKQEKIEGDLRAMLKKTPILKKGAGEEEEIDIMELLKNVDPKEYEKYARMYGITDFRGLLQAFELLKQSQEEETHRLEIEEIERSERDEKEFEELVSFIQQRLSQTEPVTLIKDIENQTVLKDNDAVFEIDIKINYPEIKLSWYKGTEKLEPSDKFEISIDGDRHTLRVKNCQLKDQGNYRLVCGPHIASAKLTVIEPAWERHLQDVTLKEGQTCTMTCQFSVPNVKSEWFRNGRILKPQGRHKTEVEHKVHKLTIADVRAEDQGQYTCKYEDLETSAELRIEAEPIQFTKRIQNIVVSEHQSATFECEVSFDDAIVTWYKGPTELTESQKYNFRNDGRCHYMTIHNVTPDDEGVYSVIARLEPRGEARSTAELYLTTKEIKLELKPPDIPDSRVPIPTMPIRAVPPEEIPPVVAPPIPLLLPTPEEKKPPPKRIEVTKKAVKKDAKKVVAKPKEMTPREEIVKKPPPPTTLIPAKAPEIIDVSSKAEEVKIMTITRKKEVQKEKEAVYEKKQAVHKEKRVFIESFEEPYDELEVEPYTEPFEQPYYEEPDEDYEEIKVEAKKEVHEEWEEDFEEGQEYYEREEGYDEGEEEWEEAYQEREVIQVQKEVYEESHERKVPAKVPEKKAPPPPKVIKKPVIEKIEKTSRRMEEEKVQVTKVPEVSKKIVPQKPSRTPVQEEVIEVKVPAVHTKKMVISEEKMFFASHTEEEVSVTVPEVQKEIVTEEKIHVAISKRVEPPPKVPELPEKPAPEEVAPVPIPKKVEPPAPKVPEVPKKPVPEEKKPVPVPKKEPAAPPKVPEVPKKPVPEEKIPVPVAKKKEAPPAKVPEVQKGVVTEEKITIVTQREESPPPAVPEIPKKKVPEERKPVPRKEEEVPPPPKVPALPKKPVPEEKVAVPVPVAKKAPPPRAEVSKKTVVEEKRFVAEEKLSFAVPQRVEVTRHEVSAEEEWSYSEEEEGVSISVYREEEREEEEEAEVTEYEVMEEPEEYVVEEKLHIISKRVEAEPAEVTERQEKKIVLKPKIPAKIEEPPPAKVPEAPKKIVPEKKVPAPVPKKEKVPPPKVPEEPKKPVPEKKVPPKVIKMEEPLPAKVTERHMQITQEEKVLVAVTKKEAPPKARVPEEPKRAVPEEKVLKLKPKREEEPPAKVTEFRKRVVKEEKVSIEAPKREPQPIKEVTIMEEKERAYTLEEEAVSVQREEEYEEYEEYDYKEFEEYEPTEEYDQYEEYEEREYERYEEHEEYITEPEKPIPVKPVPEEPVPTKPKAPPAKVLKKAVPEEKVPVPIPKKLKPPPPKVPEEPKKVFEEKIRISITKREKEQVTEPAAKVPMKPKRVVAEEKVPVPRKEVAPPVRVPEVPKELEPEEVAFEEEVVTHVEEYLVEEEEEYIHEEEEFITEEEVVPVIPVKVPEVPRKPVPEEKKPVPVPKKKEAPPAKVPEVPKKPEEKVPVLIPKKEKPPPAKVPEVPKKPVPEEKVPVPVPKKVEAPPAKVPEVPKKPVPEKKVPVPAPKKVEAPPAKVPEVPKKLIPEEKKPTPVPKKVEAPPPKVPKKREPVPVPVALPQEEEVLFEEEIVPEEEVLPEEEEVLPEEEEVLPEEEEVLPEEEEIPPEEEEVPPEEEYVPEEEEFVPEEEVLPEVKPKVPVPAPVPEIKKKVTEKKVVIPKKEEAPPAKVPEVPKKVEEKRIILPKEEEVLPVEVTEEPEEEPISEEEIPEEPPSIEEVEEVAPPRVPEVIKKAVPEAPTPVPKKVEAPPAKVSKKIPEEKVPVPVQKKEAPPAKVPEVPKKVPEKKVLVPKKEAVPPAKGRTVLEEKVSVAFRQEVVVKERLELEVVEAEVEEIPEEEEFHEVEEYFEEGEFHEVEEFIKLEQHRVEEEHRVEKVHRVIEVFEAEEVEVFEKPKAPPKGPEISEKIIPPKKPPTKVVPRKEPPAKVPEVPKKIVVEEKVRVPEEPRVPPTKVPEVLPPKEVVPEKKVPVPPAKKPEAPPPKVPEAPKEVVPEKKVPVPPPKKPEVPPTKVPEVPKAAVPEKKVPEAIPPKPESPPPEVPEAPKEVVPEKKVPAAPPKKPEVTPVKVPEAPKEVVPEKKVPVPPPKKPEVPPTKVPEVPKVAVPEKKVPEAIPPKPESPPPEVFEEPEEVALEEPPAEVVEEPEPAAPPQVTVPPKKPVPEKKAPAVVAKKPELPPVKVPEVPKEVVPEKKVPLVVPKKPEAPPAKVPEVPKEVVPEKKVAVPKKPEVPPAKVPEVPKKPVLEEKPAVPVPERAESPPPEVYEEPEEIAPEEEIAPEEEKPVPVAEEEEPEVPPPAVPEEPKKIIPEKKVPVIKKPEAPPPKEPEPEKVIEKPKLKPRPPPPPPAPPKEDVKEKIFQLKAIPKKKVPEKPQVPEKVELTPLKVPGGEKKVRKLLPERKPEPKEEVVLKSVLRKRPEEEEPKVEPKKLEKVKKPAVPEPPPPKPVEEVEVPTVTKRERKIPEPTKVPEIKPAIPLPAPEPKPKPEAEVKTIKPPPVEPEPTPIAAPVTVPVVGKKAEAKAPKEEAAKPKGPIKGVPKKTPSPIEAERRKLRPGSGGEKPPDEAPFTYQLKAVPLKFVKEIKDIILTESEFVGSSAIFECLVSPSTAITTWMKDGSNIRESPKHRFIADGKDRKLHIIDVQLSDAGEYTCVLRLGNKEKTSTAKLVVEELPVRFVKTLEEEVTVVKGQPLYLSCELNKERDVVWRKDGKIVVEKPGRIVPGVIGLMRALTINDADDTDAGTYTVTVENANNLECSSCVKVVEVIRDWLVKPIRDQHVKPKGTAIFACDIAKDTPNIKWFKGYDEIPAEPNDKTEILRDGNHLYLKIKNAMPEDIAEYAVEIEGKRYPAKLTLGEREVELLKPIEDVTIYEKESASFDAEISEADIPGQWKLKGELLRPSPTCEIKAEGGKRFLTLHKVKLDQAGEVLYQALNAITTAILTVKEIELDFAVPLKDVTVPERRQARFECVLTREANVIWSKGPDIIKSSDKFDIIADGKKHILVINDSQFDDEGVYTAEVEGKKTSARLFVTGIRLKFMSPLEDQTVKEGETATFVCELSHEKMHVVWFKNDAKLHTSRTVLISSEGKTHKLEMKEVTLDDISQIKAQVKELSSTAQLKVLEADPYFTVKLHDKTAVEKDEITLKCEVSKDVPVKWFKDGEEIVPSPKYSIKADGLRRILKIKKADLKDKGEYVCDCGTDKTKANVTVEARLIKVEKPLYGVEVFVGETAHFEIELSEPDVHGQWKLKGQPLTASPDCEIIEDGKKHILILHNCQLGMTGEVSFQAANAKSAANLKVKELPLIFITPLSDVKVFEKDEAKFECEVSREPKTFRWLKGTQEITGDDRFELIKDGTKHSMVIKSAAFEDEAKYMFEAEDKHTSGKLIIEGIRLKFLTPLKDVTAKEKESAVFTVELSHDNIRVKWFKNDQRLHTTRSVSMQDEGKTHSITFKDLSIDDTSQIRVEAMGMSSEAKLTVLEGDPYFTGKLQDYTGVEKDEVILQCEISKADAPVKWFKDGKEIKPSKNAVIKADGKKRMLILKKALKSDIGQYTCDCGTDKTSGKLDIEDREIKLVRPLHSVEVMETETARFETEISEDDIHANWKLKGEALLQTPDCEIKEEGKIHSLVLHNCRLDQTGGVDFQAANVKSSAHLRVKPRVIGLLRPLKDVTVTAGETATFDCELSYEDIPVEWYLKGKKLEPSDKVVPRSEGKVHTLTLRDVKLEDAGEVQLTAKDFKTHANLFVKEPPVEFTKPLEDQTVEEGATAVLECEVSRENAKVKWFKNGTEILKSKKYEIVADGRVRKLVIHDCTPEDIKTYTCDAKDFKTSCNLNVVPPHVEFLRPLTDLQVREKEMARFECELSRENAKVKWFKDGAEIKKGKKYDIISKGAVRILVINKCLLDDEAEYSCEVRTARTSGMLTVLEEEAVFTKNLANIEVSETDTIKLVCEVSKPGAEVIWYKGDEEIIETGRYEILTEGRKRILVIQNAHLEDAGNYNCRLPSSRTDGKVKVHELAAEFISKPQNLEILEGEKAEFVCSISKESFPVQWKRDDKTLESGDKYDVIADGKKRVLVVKDATLQDMGTYVVMVGAARAAAHLTVIEKLRIVVPLKDTRVKEQQEVVFNCEVNTEGAKAKWFRNEEAIFDSSKYIILQKDLVYTLRIRDAHLDDQANYNVSLTNHRGENVKSAANLIVEEEDLRIVEPLKDIETMEKKSVTFWCKVNRLNVTLKWTKNGEEVPFDNRVSYRVDKYKHMLTIKDCGFPDEGEYIVTAGQDKSVAELLIIEAPTEFVEHLEDQTVTEFDDAVFSCQLSREKANVKWYRNGREIKEGKKYKFEKDGSIHRLIIKDCRLDDECEYACGVEDRKSRARLFVEEIPVEIIRPPQDILEAPGADVVFLAELNKDKVEVQWLRNNMVVVQGDKHQMMSEGKIHRLQICDIKPRDQGEYRFIAKDKEARAKLELAAAPKIKTADQDLVVDVGKPLTMVVPYDAYPKAEAEWFKENEPLSTKTIDTTAEQTSFRILEAKKGDKGRYKIVLQNKHGKAEGFINLKVIDVPGPVRNLEVTETFDGEVSLAWEEPLTDGGSKIIGYVVERRDIKRKTWVLATDRAESCEFTVTGLQKGGVEYLFRVSARNRVGTGEPVETDNPVEARSKYDVPGPPLNVTITDVNRFGVSLTWEPPEYDGGAEITNYVIELRDKTSIRWDTAMTVRAEDLSATVTDVVEGQEYSFRVRAQNRIGVGKPSAATPFVKVADPIERPSPPVNLTSSDQTQSSVQLKWEPPLKDGGSPILGYIIERCEEGKDNWIRCNMKLVPELTYKVTGLEKGNKYLYRVSAENKAGVSDPSEILGPLTADDAFVEPTMDLSAFKDGLEVIVPNPITILVPSTGYPRPTATWCFGDKVLETGDRVKMKTLSAYAELVISPSERSDKGIYTLKLENRVKTISGEIDVNVIARPSAPKELKFGDITKDSVHLTWEPPDDDGGSPLTGYVVEKREVSRKTWTKVMDFVTDLEFTVPDLVQGKEYLFKVCARNKCGPGEPAYVDEPVNMSTPATVPDPPENVKWRDRTANSIFLTWDPPKNDGGSRIKGYIVERCPRGSDKWVACGEPVAETKMEVTGLEEGKWYAYRVKALNRQGASKPSRPTEEIQAVDTQEAPEIFLDVKLLAGLTVKAGTKIELPATVTGKPEPKITWTKADMILKQDKRITIENVPKKSTVTIVDSKRSDTGTYIIEAVNVCGRATAVVEVNVLDKPGPPAAFDITDVTNESCLLTWNPPRDDGGSKITNYVVERRATDSEVWHKLSSTVKDTNFKATKLIPNKEYIFRVAAENMYGVGEPVQASPITAKYQFDPPGPPTRLEPSDITKDAVTLTWCEPDDDGGSPITGYWVERLDPDTDKWVRCNKMPVKDTTYRVKGLTNKKKYRFRVLAENLAGPGKPSKSTEPILIKDPIDPPWPPGKPTVKDVGKTSVRLNWTKPEHDGGAKIESYVIEMLKTGTDEWVRVAEGVPTTQHLLPGLMEGQEYSFRVRAVNKAGESEPSEPSDPVLCREKLYPPSPPRWLEVINITKNTADLKWTVPEKDGGSPITNYIVEKRDVRRKGWQTVDTTVKDTKCTVTPLTEGSLYVFRVAAENAIGQSDYTEIEDSVLAKDTFTTPGPPYALAVVDVTKRHVDLKWEPPKNDGGRPIQRYVIEKKERLGTRWVKAGKTAGPDCNFRVTDVIEGTEVQFQVRAENEAGVGHPSEPTEILSIEDPTSPPSPPLDLHVTDAGRKHIAIAWKPPEKNGGSPIIGYHVEMCPVGTEKWMRVNSRPIKDLKFKVEEGVVPDKEYVLRVRAVNAIGVSEPSEISENVVAKDPDCKPTIDLETHDIIVIEGEKLSIPVPFRAVPVPTVSWHKDGKEVKASDRLTMKNDHISAHLEVPKSVRADAGIYTITLENKLGSATASINVKVIGLPGPCKDIKASDITKSSCKLTWEPPEFDGGTPILHYVLERREAGRRTYIPVMSGENKLSWTVKDLIPNGEYFFRVKAVNKVGGGEYIELKNPVIAQDPKQPPDPPVDVEVHNPTAEAMTITWKPPLYDGGSKIMGYIIEKIAKGEERWKRCNEHLVPILTYTAKGLEEGKEYQFRVRAENAAGISEPSRATPPTKAVDPIDAPKVILRTSLEVKRGDEIALDASISGSPYPTITWIKDENVIVPEEIKKRAAPLVRRRKGEVQEEEPFVLPLTQRLSIDNSKKGESQLRVRDSLRPDHGLYMIKVENDHGIAKAPCTVSVLDTPGPPINFVFEDIRKTSVLCKWEPPLDDGGSEIINYTLEKKDKTKPDSEWIVVTSTLRHCKYSVTKLIEGKEYLFRVRAENRFGPGPPCVSKPLVAKDPFGPPDAPDKPIVEDVTSNSMLVKWNEPKDNGSPILGYWLEKREVNSTHWSRVNKSLLNALKANVDGLLEGLTYVFRVCAENAAGPGKFSPPSDPKTAHDPISPPGPPIPRVTDTSSTTIELEWEPPAFNGGGEIVGYFVDKQLVGTNEWSRCTEKMIKVRQYTVKEIREGADYKLRVSAVNAAGEGPPGETQPVTVAEPQEPPAVELDVSVKGGIQIMAGKTLRIPAVVTGRPVPTKVWTKEEGELDKDRVVIDNVGTKSELIIKDALRKDHGRYVITATNSCGSKFAAARVEVFDVPGPVLDLKPVVTNRKMCLLNWSDPEDDGGSEITGFIIERKDAKMHTWRQPIETERSKCDITGLLEGQEYKFRVIAKNKFGCGPPVEIGPILAVDPLGPPTSPERLTYTERTKSTITLDWKEPRSNGGSPIQGYIIEKRRHDKPDFERVNKRLCPTTSFLVENLDEHQMYEFRVKAVNEIGESEPSLPLNVVIQDDEVPPTIKLRLSVRGDTIKVKAGEPVHIPADVTGLPMPKIEWSKNETVIEKPTDALQITKEEVSRSEAKTELSIPKAVREDKGTYTVTASNRLGSVFRNVHVEVYDRPSPPRNLAVTDIKAESCYLTWDAPLDNGGSEITHYVIDKRDASRKKAEWEEVTNTAVEKRYGIWKLIPNGQYEFRVRAVNKYGISDECKSDKVVIQDPYRLPGPPGKPKVLARTKGSMLVSWTPPLDNGGSPITGYWLEKREEGSPYWSRVSRAPITKVGLKGVEFNVPRLLEGVKYQFRAMAINAAGIGPPSEPSDPEVAGDPIFPPGPPSCPEVKDKTKSSISLGWKPPAKDGGSPIKGYIVEMQEEGTTDWKRVNEPDKLITTCECVVPNLKELRKYRFRVKAVNEAGESEPSDTTGEIPATDIQEEPEVFIDIGAQDCLVCKAGSQIRIPAVIKGRPTPKSSWEFDGKAKKAMKDGVHDIPEDAQLETAENSSVIIIPECKRSHTGKYSITAKNKAGQKTANCRVKVMDVPGPPKDLKVSDITRGSCRLSWKMPDDDGGDRIKGYVIEKRTIDGKAWTKVNPDCGSTTFVVPDLLSEQQYFFRVRAENRFGIGPPVETIQRTTARDPIYPPDPPIKLKIGLITKNTVHLSWKPPKNDGGSPVTHYIVECLAWDPTGTKKEAWRQCNKRDVEELQFTVEDLVEGGEYEFRVKAVNAAGVSKPSATVGPCDCQRPDMPPSIDLKEFMEVEEGTNVNIVAKIKGVPFPTLTWFKAPPKKPDNKEPVLYDTHVNKLVVDDTCTLVIPQSRRSDTGLYTITAVNNLGTASKEMRLNVLGRPGPPVGPIKFESVSADQMTLSWFPPKDDGGSKITNYVIEKREANRKTWVHVSSEPKECTYTIPKLLEGHEYVFRIMAQNKYGIGEPLDSEPETARNLFSVPGAPDKPTVSSVTRNSMTVNWEEPEYDGGSPVTGYWLEMKDTTSKRWKRVNRDPIKAMTLGVSYKVTGLIEGSDYQFRVYAINAAGVGPASLPSDPATARDPIAPPGPPFPKVTDWTKSSADLEWSPPLKDGGSKVTGYIVEYKEEGKEEWEKGKDKEVRGTKLVVTGLKEGAFYKFRVRAVNIAGIGEPGEVTDVIEMKDRLVSPDLQLDASVRDRIVVHAGGVIRIIAYVSGKPPPTVTWNMNERTLPQEATIETTAISSSMVIKNCQRSHQGVYSLLAKNEAGERKKTIIVDVLDVPGPVGTPFLAHNLTNESCKLTWFSPEDDGGSPITNYVIEKRESDRRAWTPVTYTVTRQNATVQGLIQGKAYFFRIAAENSIGMGPFVETSEALVIREPITVPERPEDLEVKEVTKNTVTLTWNPPKYDGGSEIINYVLESRLIGTEKFHKVTNDNLLSRKYTVKGLKEGDTYEYRVSAVNIVGQGKPSFCTKPITCKDELAPPTLHLDFRDKLTIRVGEAFALTGRYSGKPKPKVSWFKDEADVLEDDRTHIKTTPATLALEKIKAKRSDSGKYCVVVENSTGSRKGFCQVNVVDRPGPPVGPVSFDEVTKDYMVISWKPPLDDGGSKITNYIIEKKEVGKDVWMPVTSASAKTTCKVSKLLEGKDYIFRIHAENLYGISDPLVSDSMKAKDRFRVPDAPDQPIVTEVTKDSALVTWNKPHDGGKPITNYILEKRETMSKRWARVTKDPIHPYTKFRVPDLLEGCQYEFRVSAENEIGIGDPSPPSKPVFAKDPIAKPSPPVNPEAIDTTCNSVDLTWQPPRHDGGSKILGYIVEYQKVGDEEWRRANHTPESCPETKYKVTGLRDGQTYKFRVLAVNAAGESDPAHVPEPVLVKDRLEPPELILDANMAREQHIKVGDTLRLSAIIKGVPFPKVTWKKEDRDAPTKARIDVTPVGSKLEIRNAAHEDGGIYSLTVENPAGSKTVSVKVLVLDKPGPPRDLEVSEIRKDSCYLTWKEPLDDGGSVITNYVVERRDVASAQWSPLSATSKKKSHFAKHLNEGNQYLFRVAAENQYGRGPFVETPKPIKALDPLHPPGPPKDLHHVDVDKTEVSLVWNKPDRDGGSPITGYLVEYQEEGTQDWIKFKTVTNLECVVTGLQQGKTYRFRVKAENIVGLGLPDTTIPIECQEKLVPPSVELDVKLIEGLVVKAGTTVRFPAIIRGVPVPTAKWTTDGSEIKTDEHYTVETDNFSSVLTIKNCLRRDTGEYQITVSNAAGSKTVAVHLTVLDVPGPPTGPINILDVTPEHMTISWQPPKDDGGSPVINYIVEKQDTRKDTWGVVSSGSSKTKLKIPHLQKGCEYVFRVRAENKIGVGPPLDSTPTVAKHKFSPPSPPGKPVVTDITENAATVSWTLPKSDGGSPITGYYMERREVTGKWVRVNKTPIADLKFRVTGLYEGNTYEFRVFAENLAGLSKPSPSSDPIKACRPIKPPGPPINPKLKDKSRETADLVWTKPLSDGGSPILGYVVECQKPGTAQWNRINKDELIRQCAFRVPGLIEGNEYRFRIKAANIVGEGEPRELAESVIAKDILHPPEVELDVTCRDVITVRVGQTIRILARVKGRPEPDITWTKEGKVLVREKRVDLIQDLPRVELQIKEAVRADHGKYIISAKNSSGHAQGSAIVNVLDRPGPCQNLKVTNVTKENCTISWENPLDNGGSEITNFIVEYRKPNQKGWSIVASDVTKRLIKANLLANNEYYFRVCAENKVGVGPTIETKTPILAINPIDRPGEPENLHIADKGKTFVYLKWRRPDYDGGSPNLSYHVERRLKGSDDWERVHKGSIKETHYMVDRCVENQIYEFRVQTKNEGGESDWVKTEEVVVKEDLQKPVLDLKLSGVLTVKAGDTIRLEAGVRGKPFPEVAWTKDKDATDLTRSPRVKIDTRADSSKFSLTKAKRSDGGKYVVTATNTAGSFVAYATVNVLDKPGPVRNLKIVDVSSDRCTVCWDPPEDDGGCEIQNYILEKCETKRMVWSTYSATVLTPGTTVTRLIEGNEYIFRVRAENKIGTGPPTESKPVIAKTKYDKPGRPDPPEVTKVSKEEMTVVWNPPEYDGGKSITGYFLEKKEKHSTRWVPVNKSAIPERRMKVQNLLPDHEYQFRVKAENEIGIGEPSLPSRPVVAKDPIEPPGPPTNFRVVDTTKHSITLGWGKPVYDGGAPIIGYVVEMRPKIADASPDEGWKRCNAAAQLVRKEFTVTSLDENQEYEFRVCAQNQVGIGRPAELKEAIKPKEILEPPEIDLDASMRKLVIVRAGCPIRLFAIVRGRPAPKVTWRKVGIDNVVRKGQVDLVDTMAFLVIPNSTRDDSGKYSLTLVNPAGEKAVFVNVRVLDTPGPVSDLKVSDVTKTSCHVSWAPPENDGGSQVTHYIVEKREADRKTWSTVTPEVKKTSFHVTNLVPGNEYYFRVTAVNEYGPGVPTDVPKPVLASDPLSEPDPPRKLEVTEMTKNSATLAWLPPLRDGGAKIDGYITSYREEEQPADRWTEYSVVKDLSLVVTGLKEGKKYKFRVAARNAVGVSLPREAEGVYEAKEQLLPPKILMPEQITIKAGKKLRIEAHVYGKPHPTCKWKKGEDEVVTSSHLAVHKADSSSILIIKDVTRKDSGYYSLTAENSSGTDTQKIKVVVMDAPGPPQPPFDISDIDADACSLSWHIPLEDGGSNITNYIVEKCDVSRGDWVTALASVTKTSCRVGKLIPGQEYIFRVRAENRFGISEPLTSPKMVAQFPFGVPSEPKNARVTKVNKDCIFVAWDRPDSDGGSPIIGYLIERKERNSLLWVKANDTLVRSTEYPCAGLVEGLEYSFRIYALNKAGSSPPSKPTEYVTARMPVDPPGKPEVIDVTKSTVSLIWARPKHDGGSKIIGYFVEACKLPGDKWVRCNTAPHQIPQEEYTATGLEEKAQYQFRAIARTAVNISPPSEPSDPVTILAENVPPRIDLSVAMKSLLTVKAGTNVCLDATVFGKPMPTVSWKKDGTLLKPAEGIKMAMQRNLCTLELFSVNRKDSGDYTITAENSSGSKSATIKLKVLDKPGPPASVKINKMYSDRAMLSWEPPLEDGGSEITNYIVDKRETSRPNWAQVSATVPITSCSVEKLIEGHEYQFRICAENKYGVGDPVFTEPAIAKNPYDPPGRCDPPVISNITKDHMTVSWKPPADDGGSPITGYLLEKRETQAVNWTKVNRKPIIERTLKATGLQEGTEYEFRVTAINKAGPGKPSDASKAAYARDPQYPPGPPAFPKVYDTTRSSVSLSWGKPAYDGGSPIIGYLVEVKRADSDNWVRCNLPQNLQKTRFEVTGLMEDTQYQFRVYAVNKIGYSDPSDVPDKHYPKDILIPPEGELDADLRKTLILRAGVTMRLYVPVKGRPPPKITWSKPNVNLRDRIGLDIKSTDFDTFLRCENVNKYDAGKYILTLENSCGKKEYTIVVKVLDTPGPPVNVTVKEISKDSAYVTWEPPIIDGGSPIINYVVQKRDAERKSWSTVTTECSKTSFRVANLEEGKSYFFRVFAENEYGIGDPGETRDAVKASQTPGPVVDLKVRSVSKSSCSIGWKKPHSDGGSRIIGYVVDFLTEENKWQRVMKSLSLQYSAKDLTEGKEYTFRVSAENENGEGTPSEITVVARDDVVAPDLDLKGLPDLCYLAKENSNFRLKIPIKGKPAPSVSWKKGEDPLATDTRVSVESSAVNTTLIVYDCQKSDAGKYTITLKNVAGTKEGTISIKVVGKPGIPTGPIKFDEVTAEAMTLKWAPPKDDGGSEITNYILEKRDSVNNKWVTCASAVQKTTFRVTRLHEGMEYTFRVSAENKYGVGEGLKSEPIVARHPFDVPDAPPPPNIVDVRHDSVSLTWTDPKKTGGSPITGYHLEFKERNSLLWKRANKTPIRMRDFKVTGLTEGLEYEFRVMAINLAGVGKPSLPSEPVVALDPIDPPGKPEVINITRNSVTLIWTEPKYDGGHKLTGYIVEKRDLPSKSWMKANHVNVPECAFTVTDLVEGGKYEFRIRAKNTAGAISAPSESTETIICKDEYEAPTIVLDPTIKDGLTIKAGDTIVLNAISILGKPLPKSSWSKAGKDIRPSDITQITSTPTSSMLTIKYATRKDAGEYTITATNPFGTKVEHVKVTVLDVPGPPGPVEISNVSAEKATLTWTPPLEDGGSPIKSYILEKRETSRLLWTVVSEDIQSCRHVATKLIQGNEYIFRVSAVNHYGKGEPVQSEPVKMVDRFGPPGPPEKPEVSNVTKNTATVSWKRPVDDGGSEITGYHVERREKKSLRWVRAIKTPVSDLRCKVTGLQEGSTYEFRVSAENRAGIGPPSEASDSVLMKDAAYPPGPPSNPHVTDTTKKSASLAWGKPHYDGGLEITGYVVEHQKVGDEAWIKDTTGTALRITQFVVPDLQTKEKYNFRISAINDAGVGEPAVIPDVEIVEREMAPDFELDAELRRTLVVRAGLSIRIFVPIKGRPAPEVTWTKDNINLKNRANIENTESFTLLIIPECNRYDTGKFVMTIENPAGKKSGFVNVRVLDTPGPVLNLRPTDITKDSVTLHWDLPLIDGGSRITNYIVEKREATRKSYSTATTKCHKCTYKVTGLSEGCEYFFRVMAENEYGIGEPTETTEPVKASEAPSPPDSLNIMDITKSTVSLAWPKPKHDGGSKITGYVIEAQRKGSDQWTHITTVKGLECVVRNLTEGEEYTFQVMAVNSAGRSAPRESRPVIVKEQTMLPELDLRGIYQKLVIAKAGDNIKVEIPVLGRPKPTVTWKKGDQILKQTQRVNFETTATSTILNINECVRSDSGPYPLTARNIVGEVGDVITIQVHDIPGPPTGPIKFDEVSSDFVTFSWDPPENDGGVPISNYVVEMRQTDSTTWVELATTVIRTTYKATRLTTGLEYQFRVKAQNRYGVGPGITSACIVANYPFKVPGPPGTPQVTAVTKDSMTISWHEPLSDGGSPILGYHVERKERNGILWQTVSKALVPGNIFKSSGLTDGIAYEFRVIAENMAGKSKPSKPSEPMLALDPIDPPGKPVPLNITRHTVTLKWAKPEYTGGFKITSYIVEKRDLPNGRWLKANFSNILENEFTVSGLTEDAAYEFRVIAKNAAGAISPPSEPSDAITCRDDVEAPKIKVDVKFKDTVILKAGEAFRLEADVSGRPPPTMEWSKDGKELEGTAKLEIKIADFSTNLVNKDSTRRDSGAYTLTATNPGGFAKHIFNVKVLDRPGPPEGPLAVTEVTSEKCVLSWFPPLDDGGAKIDHYIVQKRETSRLAWTNVASEVQVTKLKVTKLLKGNEYIFRVMAVNKYGVGEPLESEPVLAVNPYGPPDPPKNPEVTTITKDSMVVCWGHPDSDGGSEIINYIVERRDKAGQRWIKCNKKTLTDLRYKVSGLTEGHEYEFRIMAENAAGISAPSPTSPFYKACDTVFKPGPPGNPRVLDTSRSSISIAWNKPIYDGGSEITGYMVEIALPEEDEWQIVTPPAGLKATSYTITGLTENQEYKIRIYAMNSEGLGEPALVPGTPKAEDRMLPPEIELDADLRKVVTIRACCTLRLFVPIKGRPAPEVKWARDHGESLDKASIESTSSYTLLIVGNVNRFDSGKYILTVENSSGSKSAFVNVRVLDTPGPPQDLKVKEVTKTSVTLTWDPPLLDGGSKIKNYIVEKRESTRKAYSTVATNCHKTSWKVDQLQEGCSYYFRVLAENEYGIGLPAETAESVKASERPLPPGKITLMDVTRNSVSLSWEKPEHDGGSRILGYIVEMQTKGSDKWATCATVKVTEATITGLIQGEEYSFRVSAQNEKGISDPRQLSVPVIAKDLVIPPAFKLLFNTFTVLAGEDLKVDVPFIGRPTPAVTWHKDNVPLKQTTRVNAESTENNSLLTIKDACREDVGHYVVKLTNSAGEAIETLNVIVLDKPGPPTGPVKMDEVTADSITLSWGPPKYDGGSSINNYIVEKRDTSTTTWQIVSATVARTTIKACRLKTGCEYQFRIAAENRYGKSTYLNSEPTVAQYPFKVPGPPGTPVVTLSSRDSMEVQWNEPISDGGSRVIGYHLERKERNSILWVKLNKTPIPQTKFKTTGLEEGVEYEFRVSAENIVGIGKPSKVSECYVARDPCDPPGRPEAIIVTRNSVTLQWKKPTYDGGSKITGYIVEKKELPEGRWMKASFTNIIDTHFEVTGLVEDHRYEFRVIARNAAGVFSEPSESTGAITARDEVDPPRISMDPKYKDTIVVHAGESFKVDADIYGKPIPTIQWIKGDQELSNTARLEIKSTDFATSLSVKDAVRVDSGNYILKAKNVAGERSVTVNVKVLDRPGPPEGPVVISGVTAEKCTLAWKPPLQDGGSDIINYIVERRETSRLVWTVVDANVQTLSCKVTKLLEGNEYTFRIMAVNKYGVGEPLESEPVVAKNPFVVPDAPKAPEVTTVTKDSMIVVWERPASDGGSEILGYVLEKRDKEGIRWTRCHKRLIGELRLRVTGLIENHDYEFRVSAENAAGLSEPSPPSAYQKACDPIYKPGPPNNPKVIDITRSSVFLSWSKPIYDGGCEIQGYIVEKCDVSVGEWTMCTPPTGINKTNIEVEKLLEKHEYNFRICAINKAGVGEHADVPGPIIVEEKLEAPDIDLDLELRKIINIRAGGSLRLFVPIKGRPTPEVKWGKVDGEIRDAAIIDVTSSFTSLVLDNVNRYDSGKYTLTLENSSGTKSAFVTVRVLDTPSPPVNLKVTEITKDSVSITWEPPLLDGGSKIKNYIVEKREATRKSYAAVVTNCHKNSWKIDQLQEGCSYYFRVTAENEYGIGLPAQTADPIKVAEVPQPPGKITVDDVTRNSVSLSWTKPEHDGGSKIIQYIVEMQAKHSEKWSECARVKSLQAVITNLTQGEEYLFRVVAVNEKGRSDPRSLAVPIVAKDLVIEPDVKPAFSSYSVQVGQDLKIEVPISGRPKPTITWTKDGLPLKQTTRINVTDSLDLTTLSIKETHKDDGGQYGITVANVVGQKTASIEIVTLDKPDPPKGPVKFDDVSAESITLSWNPPLYTGGCQITNYIVQKRDTTTTVWDVVSATVARTTLKVTKLKTGTEYQFRIFAENRYGQSFALESDPIVAQYPYKEPGPPGTPFATAISKDSMVIQWHEPVNNGGSPVIGYHLERKERNSILWTKVNKTIIHDTQFKAQNLEEGIEYEFRVYAENIVGVGKASKNSECYVARDPCDPPGTPEPIMVKRNEITLQWTKPVYDGGSMITGYIVEKRDLPDGRWMKASFTNVIETQFTVSGLTEDQRYEFRVIAKNAAGAISKPSDSTGPITAKDEVELPRISMDPKFRDTIVVNAGETFRLEADVHGKPLPTIEWLRGDKEIEESARCEIKNTDFKALLIVKDAIRIDGGQYILRASNVAGSKSFPVNVKVLDRPGPPEGPVQVTGVTSEKCSLTWSPPLQDGGSDISHYVVEKRETSRLAWTVVASEVVTNSLKVTKLLEGNEYVFRIMAVNKYGVGEPLESAPVLMKNPFVLPGPPKSLEVTNIAKDSMTVCWNRPDSDGGSEIIGYIVEKRDRSGIRWIKCNKRRITDLRLRVTGLTEDHEYEFRVSAENAAGVGEPSPATVYYKACDPVFKPGPPTNAHIVDTTKNSITLAWGKPIYDGGSEILGYVVEICKADEEEWQIVTPQTGLRVTRFEISKLTEHQEYKIRVCALNKVGLGEATSVPGTVKPEDKLEAPELDLDSELRKGIVVRAGGSARIHIPFKGRPTPEITWSREEGEFTDKVQIEKGVNYTQLSIDNCDRNDAGKYILKLENSSGSKSAFVTVKVLDTPGPPQNLAVKEVRKDSAFLVWEPPIIDGGAKVKNYVIDKRESTRKAYANVSSKCSKTSFKVENLTEGAIYYFRVMAENEFGVGVPVETVDAVKAAEPPSPPGKVTLTDVSQTSASLMWEKPEHDGGSRVLGYVVEMQPKGTEKWSIVAESKVCNAVVTGLSSGQEYQFRVKAYNEKGKSDPRVLGVPVIAKDLTIQPSLKLPFNTYSIQAGEDLKIEIPVIGRPRPNISWVKDGEPLKQTTRVNVEETATSTVLHIKEGNKDDFGKYTVTATNSAGTATENLSVIVLEKPGPPVGPVRFDEVSADFVVISWEPPAYTGGCQISNYIVEKRDTTTTTWHMVSATVARTTIKITKLKTGTEYQFRIFAENRYGKSAPLDSKAVIVQYPFKEPGPPGTPFVTSISKDQMLVQWHEPVNDGGTKIIGYHLEQKEKNSILWVKLNKTPIQDTKFKTTGLDEGLEYEFKVSAENIVGIGKPSKVSECFVARDPCDPPGRPEAIVITRNNVTLKWKKPAYDGGSKITGYIVEKKDLPDGRWMKASFTNVLETEFTVSGLVEDQRYEFRVIARNAAGNFSEPSDSSGAITARDEIDAPNASLDPKYKDVIVVHAGETFVLEADIRGKPIPDVVWSKDGKELEETAARMEIKSTIQKTTLVVKDCIRTDGGQYILKLSNVGGTKSIPITVKVLDRPGPPEGPLKVTGVTAEKCYLAWNPPLQDGGANISHYIIEKRETSRLSWTQVSTEVQALNYKVTKLLPGNEYIFRVMAVNKYGIGEPLESGPVTACNPYKPPGPPSTPEVSAITKDSMVVTWARPVDDGGTEIEGYILEKRDKEGVRWTKCNKKTLTDLRLRVTGLTEGHSYEFRVAAENAAGVGEPSEPSVFYRACDALYPPGPPSNPKVTDTSRSSVSLAWSKPIYDGGAPVKGYVVEVKEAAADEWTTCTPPTGLQGKQFTVTKLKENTEYNFRICAINSEGVGEPATLPGSVVAQERIEPPEIELDADLRKVVVLRASATLRLFVTIKGRPEPEVKWEKAEGILTDRAQIEVTSSFTMLVIDNVTRFDSGRYNLTLENNSGSKTAFVNVRVLDSPSAPVNLTIREVKKDSVTLSWEPPLIDGGAKITNYIVEKRETTRKAYATITNNCTKTTFRIENLQEGCSYYFRVLASNEYGIGLPAETTEPVKVSEPPLPPGRVTLVDVTRNTATIKWEKPESDGGSKITGYVVEMQTKGSEKWSTCTQVKTLEATISGLTAGEEYVFRVAAVNEKGRSDPRQLGVPVIARDIEIKPSVELPFHTFNVKAREQLKIDVPFKGRPQATVNWRKDGQTLKETTRVNVSSSKTVTSLSIKEASKEDVGTYELCVSNSAGSITVPITIIVLDRPGPPGPIRIDEVSCDSITISWNPPEYDGGCQISNYIVEKKETTSTTWHIVSQAVARTSIKIVRLTTGSEYQFRVCAENRYGKSSYSESSAVVAEYPFSPPGPPGTPKVVHATKSTMLVTWQVPVNDGGSRVIGYHLEYKERSSILWSKANKILIADTQMKVSGLDEGLMYEYRVYAENIAGIGKCSKSCEPVPARDPCDPPGQPEVTNITRKSVSLKWSKPHYDGGAKITGYIVERRELPDGRWLKCNYTNIQETYFEVTELTEDQRYEFRVFARNAADSVSEPSESTGPIIVKDDVEPPRVMMDVKFRDVIVVKAGEVLKINADIAGRPLPVISWAKDGIEIEERARTEIISTDNHTLLTVKDCIRRDTGQYVLTLKNVAGTRSVAVNCKVLDKPGPPAGPLEINGLTAEKCSLSWGRPQEDGGADIDYYIVEKRETSHLAWTICEGELQMTSCKVTKLLKGNEYIFRVTGVNKYGVGEPLESVAIKALDPFTVPSPPTSLEITSVTKESMTLCWSRPESDGGSEISGYIIERREKNSLRWVRVNKKPVYDLRVKSTGLREGCEYEYRVYAENAAGLSLPSETSPLIRAEDPVFLPSPPSKPKIVDSGKTTITIAWVKPLFDGGAPITGYTVEYKKSDDTDWKTSIQSLRGTEYTISGLTTGAEYVFRVKSVNKVGASDPSDSSDPQIAKEREEEPLFDIDSEMRKTLIVKAGASFTMTVPFRGRPVPNVLWSKPDTDLRTRAYVDTTDSRTSLTIENANRNDSGKYTLTIQNVLSAASLTLVVKVLDTPGPPTNITVQDVTKESAVLSWDVPENDGGAPVKNYHIEKREASKKAWVSVTNNCNRLSYKVTNLQEGAIYYFRVSGENEFGVGIPAETKEGVKITEKPSPPEKLGVTSISKDSVSLTWLKPEHDGGSRIVHYVVEALEKGQKNWVKCAVAKSTHHVVSGLRENSEYFFRVFAENQAGLSDPRELLLPVLIKEQLEPPEIDMKNFPSHTVYVRAGSNLKVDIPISGKPLPKVTLSRDGVPLKATMRFNTEITAENLTINLKESVTADAGRYEITAANSSGTTKAFINIVVLDRPGPPTGPVVISDITEESVTLKWEPPKYDGGSQVTNYILLKRETSTAVWTEVSATVARTMMKVMKLTTGEEYQFRIKAENRFGISDHIDSACVTVKLPYTTPGPPSTPWVTNVTRESITVGWHEPVSNGGSAVVGYHLEMKDRNSILWQKANKLVIRTTHFKVTTISAGLIYEFRVYAENAAGVGKPSHPSEPVLAIDACEPPRNVRITDISKNSVSLSWQQPAFDGGSKITGYIVERRDLPDGRWTKASFTNVTETQFIISGLTQNSQYEFRVFARNAVGSISNPSEVVGPITCIDSYGGPVIDLPLEYTEVVKYRAGTSVKLRAGISGKPAPTIEWYKDDKELQTNALVCVENTTDLASILIKDADRLNSGCYELKLRNAMGSASATIRVQILDKPGPPGGPIEFKTVTAEKITLLWRPPADDGGAKITHYIVEKRETSRVVWSMVSEHLEECIITTTKIIKGNEYIFRVRAVNKYGIGEPLESDSVVAKNAFVTPGPPGIPEVTKITKNSMTVVWSRPIADGGSDISGYFLEKRDKKSLGWFKVLKETIRDTRQKVTGLTENSDYQYRVCAVNAAGQGPFSEPSEFYKAADPIDPPGPPAKIRIADSTKSSITLGWSKPVYDGGSAVTGYVVEIRQGEEEEWTTVSTKGEVRTTEYVVSNLKPGVNYYFRVSAVNCAGQGEPIEMNEPVQAKDILEAPEIDLDVALRTSVIAKAGEDVQVLIPFKGRPPPTVTWRKDEKNLGSDARYSIENTDSSSLLTIPQVTRNDTGKYILTIENGVGEPKSSTVSVKVLDTPAACQKLQVKHVSRGTVTLLWDPPLIDGGSPIINYVIEKRDATKRTWSVVSHKCSSTSFKLIDLSEKTPFFFRVLAENEIGIGEPCETTEPVKAAEVPAPIRDLSMKDSTKTSVILSWTKPDFDGGSVITEYVVERKGKGEQTWSHAGISKTCEIEVSQLKEQSVLEFRVFAKNEKGLSDPVTIGPITVKELIITPEVDLSDIPGAQVTVRIGHNVHLELPYKGKPKPSISWLKDGLPLKESEFVRFSKTENKITLSIKNAKKEHGGKYTVILDNAVCRIAVPITVITLGPPSKPKGPIRFDEIKADSVILSWDVPEDNGGGEITCYSIEKRETSQTNWKMVCSSVARTTFKVPNLVKDAEYQFRVRAENRYGVSQPLVSSIIVAKHQFRIPGPPGKPVIYNVTSDGMSLTWDAPVYDGGSEVTGFHVEKKERNSILWQKVNTSPISGREYRATGLVEGLDYQFRVYAENSAGLSSPSDPSKFTLAVSPVDPPGTPDYIDVTRETITLKWNPPLRDGGSKIVGYSIEKRQGNERWVRCNFTDVSECQYTVTGLSPGDRYEFRIIARNAVGTISPPSQSSGIIMTRDENVPPIVEFGPEYFDGLIIKSGESLRIKALVQGRPVPRVTWFKDGVEIEKRMNMEITDVLGSTSLFVRDATRDHRGVYTVEAKNASGSAKAEIKVKVQDTPGKVVGPIRFTNITGEKMTLWWDAPLNDGCAPITHYIIEKRETSRLAWALIEDKCEAQSYTAIKLINGNEYQFRVSAVNKFGVGRPLDSDPVVAQIQYTVPDAPGIPEPSNITGNSITLTWARPESDGGSEIQQYILERREKKSTRWVKVISKRPISETRFKVTGLTEGNEYEFHVMAENAAGVGPASGISRLIKCREPVNPPGPPTVVKVTDTSKTTVSLEWSKPVFDGGMEIIGYIIEMCKADLGDWHKVNAEACVKTRYTVTDLQAGEEYKFRVSAINGAGKGDSCEVTGTIKAVDRLTAPELDIDANFKQTHVVRAGASIRLFIAYQGRPTPTAVWSKPDSNLSLRADIHTTDSFSTLTVENCNRNDAGKYTLTVENNSGSKSITFTVKVLDTPGPPGPITFKDVTRGSATLMWDAPLLDGGARIHHYVVEKREASRRSWQVISEKCTRQIFKVNDLAEGVPYYFRVSAVNEYGVGEPYEMPEPIVATEQPAPPRRLDVVDTSKSSAVLAWLKPDHDGGSRITGYLLEMRQKGSDFWVEAGHTKQLTFTVERLVEKTEYEFRVKAKNDAGYSEPREAFSSVIIKEPQIEPTADLTGITNQLITCKAGSPFTIDVPISGRPAPKVTWKLEEMRLKETDRVSITTTKDRTTLTVKDSMRGDSGRYFLTLENTAGVKTFSVTVVVIGRPGPVTGPIEVSSVSAESCVLSWGEPKDGGGTEITNYIVEKRESGTTAWQLVNSSVKRTQIKVTHLTKYMEYSFRVSSENRFGVSKPLESAPIIAEHPFVPPSAPTRPEVYHVSANAMSIRWEEPYHDGGSKIIGYWVEKKERNTILWVKENKVPCLECNYKVTGLVEGLEYQFRTYALNAAGVSKASEASRPIMAQNPVDAPGRPEVTDVTRSTVSLIWSAPAYDGGSKVVGYIIERKPVSEVGDGRWLKCNYTIVSDNFFTVTALSEGDTYEFRVLAKNAAGVISKGSESTGPVTCRDEYAPPKAELDARLHGDLVTIRAGSDLVLDAAVGGKPEPKIIWTKGDKELDLCEKVSLQYTGKRATAVIKFCDRSDSGKYTLTVKNASGTKAVSVMVKVLDSPGPCGKLTVSRVTQEKCTLAWSLPQEDGGAEITHYIVERRETSRLNWVIVEGECPTLSYVVTRLIKNNEYIFRVRAVNKYGPGVPVESEPIVARNSFTIPSPPGIPEEVGTGKEHIIIQWTKPESDGGNEISNYLVDKREKKSLRWTRVNKDYVVYDTRLKVTSLMEGCDYQFRVTAVNAAGNSEPSEASNFISCREPSYTPGPPSAPRVVDTTKHSISLAWTKPMYDGGTDIVGYVLEMQEKDTDQWYRVHTNATIRNTEFTVPDLKMGQKYSFRVAAVNVKGMSEYSESIAEIEPVERIEIPDLELADDLKKTVTIRAGASLRLMVSVSGRPPPVITWSKQGIDLASRAIIDTTESYSLLIVDKVNRYDAGKYTIEAENQSGKKSATVLVKVYDTPGPCPSVKVKEVSRDSVTITWEIPTIDGGAPVNNYIVEKREAAMRAFKTVTTKCSKTLYRISGLVEGTMYYFRVLPENIYGIGEPCETSDAVLVSEVPLVPAKLEVVDVTKSTVTLAWEKPLYDGGSRLTGYVLEACKAGTERWMKVVTLKPTVLEHTVTSLNEGEQYLFRIRAQNEKGVSEPRETVTAVTVQDLRVLPTIDLSTMPQKTIHVPAGRPVELVIPIAGRPPPAASWFFAGSKLRESERVTVETHTKVAKLTIRETTIRDTGEYTLELKNVTGTTSETIKVIILDKPGPPTGPIKIDEIDATSITISWEPPELDGGAPLSGYVVEQRDAHRPGWLPVSESVTRSTFKFTRLTEGNEYVFRVAATNRFGIGSYLQSEVIECRSSIRIPGPPETLQIFDVSRDGMTLTWYPPEDDGGSQVTGYIVERKEVRADRWVRVNKVPVTMTRYRSTGLTEGLEYEHRVTAINARGSGKPSRPSKPIVAMDPIAPPGKPQNPRVTDTTRTSVSLAWSVPEDEGGSKVTGYLIEMQKVDQHEWTKCNTTPTKIREYTLTHLPQGAEYRFRVLACNAGGPGEPAEVPGTVKVTEMLEYPDYELDERYQEGIFVRQGGVIRLTIPIKGKPFPICKWTKEGQDISKRAMIATSETHTELVIKEADRGDSGTYDLVLENKCGKKAVYIKVRVIGSPNSPEGPLEYDDIQVRSVRVSWRPPADDGGADILGYILERREVPKAAWYTIDSRVRGTSLVVKGLKENVEYHFRVSAENQFGISKPLKSEEPVTPKTPLNPPEPPSNPPEVLDVTKSSVSLSWSRPKDDGGSRVTGYYIERKETSTDKWVRHNKTQITTTMYTVTGLVPDAEYQFRIIAQNDVGLSETSPASEPVVCKDPFDKPSQPGELEILSISKDSVTLQWEKPECDGGKEILGYWVEYRQSGDSAWKKSNKERIKDKQFTIGGLLEATEYEFRVFAENETGLSRPRRTAMSIKTKLTSGEAPGIRKEMKDVTTKLGEAAQLSCQIVGRPLPDIKWYRFGKELIQSRKYKMSSDGRTHTLTVMTEEQEDEGVYTCIATNEVGEVETSSKLLLQATPQFHPGYPLKEKYYGAVGSTLRLHVMYIGRPVPAMTWFHGQKLLQNSENITIENTEHYTHLVMKNVQRKTHAGKYKVQLSNVFGTVDAILDVEIQDKPDKPTGPIVIEALLKNSAVISWKPPADDGGSWITNYVVEKCEAKEGAEWQLVSSAISVTTCRIVNLTENAGYYFRVSAQNTFGISDPLEVSSVVIIKSPFEKPGAPGKPTITAVTKDSCVVAWKPPASDGGAKIRNYYLEKREKKQNKWISVTTEEIRETVFSVKNLIEGLEYEFRVKCENLGGESEWSEISEPITPKSDVPIQAPHFKEELRNLNVRYQSNATLVCKVTGHPKPIVKWYRQGKEIIADGLKYRIQEFKGGYHQLIIASVTDDDATVYQVRATNQGGSVSGTASLEVEVPAKIHLPKTLEGMGAVHALRGEVVSIKIPFSGKPDPVITWQKGQDLIDNNGHYQVIVTRSFTSLVFPNGVERKDAGFYVVCAKNRFGIDQKTVELDVADVPDPPRGVKVSDVSRDSVNLTWTEPASDGGSKITNYIVEKCATTAERWLRVGQARETRYTVINLFGKTSYQFRVIAENKFGLSKPSEPSEPTITKEDKTRAMNYDEEVDETREVSMTKASHSSTKELYEKYMIAEDLGRGEFGIVHRCVETSSKKTYMAKFVKVKGTDQVLVKKEISILNIARHRNILHLHESFESMEELVMIFEFISGLDIFERINTSAFELNEREIVSYVHQVCEALQFLHSHNIGHFDIRPENIIYQTRRSSTIKIIEFGQARQLKPGDNFRLLFTAPEYYAPEVHQHDVVSTATDMWSLGTLVYVLLSGINPFLAETNQQIIENIMNAEYTFDEEAFKEISIEAMDFVDRLLVKERKSRMTASEALQHPWLKQKIERVSTKVIRTLKHRRYYHTLIKKDLNMVVSAARISCGGAIRSQKGVSVAKVKVASIEIGPVSGQIMHAVGEEGGHVKYVCKIENYDQSTQVTWYFGVRQLENSEKYEITYEDGVAILYVKDITKLDDGTYRCKVVNDYGEDSSYAELFVKGVREVYDYYCRRTMKKIKRRTDTMRLLERPPEFTLPLYNKTAYVGENVRFGVTITVHPEPHVTWYKSGQKIKPGDNDKKYTFESDKGLYQLTINSVTTDDDAEYTVVARNKYGEDSCKAKLTVTLHPPPTDSTLRPMFKRLLANAECQEGQSVCFEIRVSGIPPPTLKWEKDGQPLSLGPNIEIIHEGLDYYALHIRDTLPEDTGYYRVTATNTAGSTSCQAHLQVERLRYKKQEFKSKEEHERHVQKQIDKTLRMAEILSGTESVPLTQVAKEALREAAVLYKPAVSTKTVKGEFRLEIEEKKEERKLRMPYDVPEPRKYKQTTIEEDQRIKQFVPMSDMKWYKKIRDQYEMPGKLDRVVQKRPKRIRLSRWEQFYVMPLPRITDQYRPKWRIPKLSQDDLEIVRPARRRTPSPDYDFYYRPRRRSLGDISDEELLLPIDDYLAMKRTEEERLRLEEELELGFSASPPSRSPPHFELSSLRYSSPQAHVKVEETRKDFRYSTYHIPTKAEASTSYAELRERHAQAAYRQPKQRQRIMAEREDEELLRPVTTTQHLSEYKSELDFMSKEEKSRKKSRRQREVTEITEIEEEYEISKHAQRESSSSASRLLRRRRSLSPTYIELMRPVSELIRSRPQPAEEYEDDTERRSPTPERTRPRSPSPVSSERSLSRFERSARFDIFSRYESMKAALKTQKTSERKYEVLSQQPFTLDHAPRITLRMRSHRVPCGQNTRFILNVQSKPTAEVKWYHNGVELQESSKIHYTNTSGVLTLEILDCHTDDSGTYRAVCTNYKGEASDYATLDVTGGDYTTYASQRRDEEVPRSVFPELTRTEAYAVSSFKKTSEMEASSSVREVKSQMTETRESLSSYEHSASAEMKSAALEEKSLEEKSTTRKIKTTLAARILTKPRSMTVYEGESARFSCDTDGEPVPTVTWLRKGQVLSTSARHQVTTTKYKSTFEISSVQASDEGNYSVVVENSEGKQEAEFTLTIQKARVTEKAVTSPPRVKSPEPRVKSPEAVKSPKRVKSPEPSHPKAVSPTETKPTPTEKVQHLPVSAPPKITQFLKAEASKEIAKLTCVVESSVLRAKEVTWYKDGKKLKENGHFQFHYSADGTYELKINNLTESDQGEYVCEISGEGGTSKTNLQFMGQAFKSIHEKVSKISETKKSDQKTTESTVTRKTEPKAPEPISSKPVIVTGLQDTTVSSDSVAKFAVKATGEPRPTAIWTKDGKAITQGGKYKLSEDKGGFFLEIHKTDTSDSGLYTCTVKNSAGSVSSSCKLTIKAIKDTEAQKVSTQKTSEITPQKKAVVQEEISQKALRSEEIKMSEAKSQEKLALKEEASKVLISEEVKKSAATSLEKSIVHEEITKTSQASEEVRTHAEIKAFSTQMSINEGQRLVLKANIAGATDVKWVLNGVELTNSEEYRYGVSGSDQTLTIKQASHRDEGILTCISKTKEGIVKCQYDLTLSKELSDAPAFISQPRSQNINEGQNVLFTCEISGEPSPEIEWFKNNLPISISSNVSISRSRNVYSLEIRNASVSDSGKYTIKAKNFRGQCSATASLMVLPLVEEPSREVVLRTSGDTSLQGSFSSQSVQMSASKQEASFSSFSSSSASSMTEMKFASMSAQSMSSMQESFVEMSSSSFMGISNMTQLESSTSKMLKAGIRGIPPKIEALPSDISIDEGKVLTVACAFTGEPTPEVTWSCGGRKIHSQEQGRFHIENTDDLTTLIIMDVQKQDGGLYTLSLGNEFGSDSATVNIHIRSI

1396 NP_055720.2 NP_058644.1

>NP_055720.2

MMRLRGSGMLRDLLLRSPAGVSATLRRAQPLVTLCRRPRGGGRPAAGPAAAARLHPWWGGGGWPAEPLARGLSSSPSEILQELGKGSTHPQPGVSPPAAPAAPGPKDGPGETDAFGNSEGKELVASGENKIKQGLLPSLEDLLFYTIAEGQEKIPVHKFITALKSTGLRTSDPRLKECMDMLRLTLQTTSDGVMLDKDLFKKCVQSNIVLLTQAFRRKLVIPDFMSFTSHIDELYESAKKQSGGKVADYIPQLAKFSPDLWGVSVCTVDGQRHSTGDTKVPFCLQSCVKPLKYAIAVNDLGTEYVHRYVGKEPSGLRFNKLFLNEDDKPHNPMVNAGAIVVTSLIKQGVNNAEKFDYVMQFLNKMAGNEYVGFSNATFQSERESGDRNFAIGYYLKEKKCFPEGTDMVGILDFYFQLCSIEVTCESASVMAATLANGGFCPITGERVLSPEAVRNTLSLMHSCGMYDFSGQFAFHVGLPAKSGVAGGILLVVPNVMGMMCWSPPLDKMGNSVKGIHFCHDLVSLCNFHNYDNLRHFAKKLDPRREGGDQRVKSVINLLFAAYTGDVSALRRFALSAMDMEQRDYDSRTALHVAAAEGHVEVVKFLLEACKVNPFPKDRWNNTPMDEALHFGHHDVFKILQEYQVQYTPQGDSDNGKENQTVHKNLDGLL

>NP_058644.1

MARPQRTPARSPDSIVEVKSKFDAEFRRFALPRASVSGFQEFSRLLRAVHQIPGLDVLLGYTDAHGDLLPLTNDDSLHRALASGPPPLRLLVQKRAEADSSGLAFASNSLQRRKKGLLLRPVAPLRTRPPLLISLPQDFRQVSSVIDVDLLPETHRRVRLHKHGSDRPLGFYIRDGMSVRVAPQGLERVPGIFISRLVRGGLAESTGLLAVSDEILEVNGIEVAGKTLDQVTDMMVANSHNLIVTVKPANQRNNVVRGASGRLTGPPSAGPGPAEPDSDDDSSDLVIENRQPPSSNGLSQGPPCWDLHPGCRHPGTRSSLPSLDDQEQASSGWGSRIRGDGSGFSL

1397 NP_005387.2 NP_001823.1

>NP_005387.2

MMLPPWTLGLLLLATVRGKEVCYGQLGCFSDEKPWAGTLQRPVKLLPWSPEDIDTRFLLYTNENPNNFQLITGTEPDTIEASNFQLDRKTRFIIHGFLDKAEDSWPSDMCKKMFEVEKVNCICVDWRHGSRAMYTQAVQNIRVVGAETAFLIQALSTQLGYSLEDVHVIGHSLGAHTAAEAGRRLGGRVGRITGLDPAGPCFQDEPEEVRLDPSDAVFVDVIHTDSSPIVPSLGFGMSQKVGHLDFFPNGGKEMPGCKKNVLSTITDIDGIWEGIGGFVSCNHLRSFEYYSSSVLNPDGFLGYPCASYDEFQESKCFPCPAEGCPKMGHYADQFKGKTSAVEQTFFLNTGESGNFTSWRYKVSVTLSGKEKVNGYIRIALYGSNENSKQYEIFKGSLKPDASHTCAIDVDFNVGKIQKVKFLWNKRGINLSEPKLGASQITVQSGEDGTEYNFCSSDTVEENVLQSLYPC

>NP_001823.1

MEKILILLLVALSVAYAAPGPRGIIINLENGELCMNSAQCKSNCCQHSSALGLARCTSMASENSECSVKTLYGIYYKCPCERGLTCEGDKTIVGSITNTNFGICHDAGRSKQ

1398 NP_005949.1 NP_003850.1

>NP_005949.1

MQGNGSALPNASQPVLRGDGARPSWLASALACVLIFTIVVDILGNLLVILSVYRNKKLRNAGNIFVVSLAVADLVVAIYPYPLVLMSIFNNGWNLGYLHCQVSGFLMGLSVIGSIFNITGIAINRYCYICHSLKYDKLYSSKNSLCYVLLIWLLTLAAVLPNLRAGTLQYDPRIYSCTFAQSVSSAYTIAVVVFHFLVPMIIVIFCYLRIWILVLQVRQRVKPDRKPKLKPQDFRNFVTMFVVFVLFAICWAPLNFIGLAVASDPASMVPRIPEWLFVASYYMAYFNSCLNAIIYGLLNQNFRKEYRRIIVSLCTARVFFVDSSNDVADRVKWKPSPLMTNNNVVKVDSV

>NP_003850.1

MASLEVSRSPRRSRRELEVRSPRQNKYSVLLPTYNERENLPLIVWLLVKSFSESGINYEIIIIDDGSPDGTRDVAEQLEKIYGSDRILLRPREKKLGLGTAYIHGMKHATGNYIIIMDADLSHHPKFIPEFIRKQKEGNFDIVSGTRYKGNGGVYGWDLKRKIISRGANFLTQILLRPGASDLTGSFRLYRKEVLEKLIEKCVSKGYVFQMEMIVRARQLNYTIGEVPISFVDRVYGESKLGGNEIVSFLKGLLTLFATT

1399 NP_059107.1 NP_001029097.1

>NP_059107.1

MAMSSGGSGGGVPEQEDSVLFRRGTGQSDDSDIWDDTALIKAYDKAVASFKHALKNGDICETSGKPKTTPKRKPAKKNKSQKKNTAASLQQWKVGDKCSAIWSEDGCIYPATIASIDFKRETCVVVYTGYGNREEQNLSDLLSPICEVANNIEQNAQENENESQVSTDESENSRSPGNKSDNIKPKSAPWNSFLPPPPPMPGPRLGPGKPGLKFNGPPPPPPPPPPHLLSCWLPPFPSGPPIIPPPPPICPDSLDDADALGSMLISWYMSGYHTGYYMGFRQNQKEGRCSHSLN

>NP_001029097.1

MMEDDGQPRTLYVGNLSRDVTEVLILQLFSQIGPCKSCKMITEQPDSRRVNSSVGFSVLQHTSNDPYCFVEFYEHRDAAAALAAMNGRKILGKEVKVNWATTPSSQKKDTSNHFHVFVGDLSPEITTEDIKSAFAPFGKISDARVVKDMATGKSKGYGFVSFYNKLDAENAIVHMGGQWLGGRQIRTNWATRKPPAPKSTQENNTKQLRFEDVVNQSSPKNCTVYCGGIASGLTDQLMRQTFSPFGQIMEIRVFPEKGYSFVRFSTHESAAHAIVSVNGTTIEGHVVKCYWGKESPDMTKNFQQVDYSQWGQWSQVYGNPQQYGQYMANGWQVPPYGVYGQPWNQQGFGVDQSPSAAWMGGFGAQPPQGQAPPPVIPPPNQAGYGMASYQTQ

1400 NP_001008701.1 NP_001356.1

>NP_001008701.1

MARLAAVLWNLCVTAVLVTSATQGLSRAGLPFGLMRRELACEGYPIELRCPGSDVIMVENANYGRTDDKICDADPFQMENVQCYLPDAFKIMSQRCNNRTQCVVVAGSDAFPDPCPGTYKYLEVQYDCVPYKVEQKVFVCPGTLQKVLEPTSTHESEHQSGAWCKDPLQAGDRIYVMPWIPYRTDTLTEYASWEDYVAARHTTTYRLPNRVDGTGFVVYDGAVFYNKERTRNIVKYDLRTRIKSGETVINTANYHDTSPYRWGGKTDIDLAVDENGLWVIYATEGNNGRLVVSQLNPYTLRFEGTWETGYDKRSASNAFMVCGVLYVLRSVYVDDDSEAAGNRVDYAFNTNANREEPVSLTFPNPYQFISSVDYNPRDNQLYVWNNYFVVRYSLEFGPPDPSAGPATSPPLSTTTTARPTPLTSTASPAATTPLRRAPLTTHPVGAINQLGPDLPPATAPVPSTRRPPAPNLHVSPELFCEPREVRRVQWPATQQGMLVERPCPKGTRGIASFQCLPALGLWNPRGPDLSNCTSPWVNQVAQKIKSGENAANIASELARHTRGSIYAGDVSSSVKLMEQLLDILDAQLQALRPIERESAGKNYNKMHKRERTCKDYIKAVVETVDNLLRPEALESWKDMNATEQVHTATMLLDVLEEGAFLLADNVREPARFLAAKENVVLEVTVLNTEGQVQELVFPQEEYPRKNSIQLSAKTIKQNSRNGVVKVVFILYNNLGLFLSTENATVKLAGEAGPGGPGGASLVVNSQVIAASINKESSRVFLMDPVIFTVAHLEDKNHFNANCSFWNYSERSMLGYWSTQGCRLVESNKTHTTCACSHLTNFAVLMAHREIYQGRINELLLSVITWVGIVISLVCLAICISTFCFLRGLQTDRNTIHKNLCINLFLAELLFLVGIDKTQYEIACPIFAGLLHYFFLAAFSWLCLEGVHLYLLLVEVFESEYSRTKYYYLGGYCFPALVVGIAAAIDYRSYGTEKACWLRVDNYFIWSFIGPVSFVIVVNLVFLMVTLHKMIRSSSVLKPDSSRLDNIKSWALGAIALLFLLGLTWAFGLLFINKESVVMAYLFTTFNAFQGVFIFVFHCALQKKVHKEYSKCLRHSYCCIRSPPGGTHGSLKTSAMRSNTRYYTGTQSRIRRMWNDTVRKQTESSFMAGDINSTPTLNRGTMGNHLLTNPVLQPRGGTSPYNTLIAESVGFNPSSPPVFNSPGSYREPKHPLGGREACGMDTLPLNGNFNNSYSLRSGDFPPGDGGPEPPRGRNLADAAAFEKMIISELVHNNLRGSSSAAKGPPPPEPPVPPVPGGGGEEEAGGPGGADRAEIELLYKALEEPLLLPRAQSVLYQSDLDESESCTAEDGATSRPLSSPPGRDSLYASGANLRDSPSYPDSSPEGPSEALPPPPPAPPGPPEIYYTSRPPALVARNPLQGYYQVRRPSHEGYLAAPGLEGPGPDGDGQMQLVTSL

>NP_001356.1

MSQRPRAPRSALWLLAPPLLRWAPPLLTVLHSDLFQALLDILDYYEASLSESQKYRYQDEDTPPLEHSPAHLPNQANSPPVIVNTDTLEAPGYELQVNGTEGEMEYEEITLERGNSGLGFSIAGGTDNPHIGDDPSIFITKIIPGGAAAQDGRLRVNDSILFVNEVDVREVTHSAAVEALKEAGSIVRLYVMRRKPPAEKVMEIKLIKGPKGLGFSIAGGVGNQHIPGDNSIYVTKIIEGGAAHKDGRLQIGDKILAVNSVGLEDVMHEDAVAALKNTYDVVYLKVAKPSNAYLSDSYAPPDITTSYSQHLDNEISHSSYLGTDYPTAMTPTSPRRYSPVAKDLLGEEDIPREPRRIVIHRGSTGLGFNIVGGEDGEGIFISFILAGGPADLSGELRKGDQILSVNGVDLRNASHEQAAIALKNAGQTVTIIAQYKPEEYSRFEAKIHDLREQLMNSSLGSGTASLRSNPKRGFYIRALFDYDKTKDCGFLSQALSFRFGDVLHVIDASDEEWWQARRVHSDSETDDIGFIPSKRRVERREWSRLKAKDWGSSSGSQGREDSVLSYETVTQMEVHYARPIIILGPTKDRANDDLLSEFPDKFGSCVPHTTRPKREYEIDGRDYHFVSSREKMEKDIQAHKFIEAGQYNSHLYGTSVQSVREVAEQGKHCILDVSANAVRRLQAAHLHPIAIFIRPRSLENVLEINKRITEEQARKAFDRATKLEQEFTECFSAIVEGDSFEEIYHKVKRVIEDLSGPYIWVPARERL

1401 NP_002914.1 NP_002728.1

>NP_002914.1

MQSAMFLAVQHDCRPMDKSAGSGHKSEEKREKMKRTLLKDWKTRLSYFLQNSSTPGKPKTGKKSKQQAFIKPSPEEAQLWSEAFDELLASKYGLAAFRAFLKSEFCEENIEFWLACEDFKKTKSPQKLSSKARKIYTDFIEKEAPKEINIDFQTKTLIAQNIQEATSGCFTTAQKRVYSLMENNSYPRFLESEFYQDLCKKPQITTEPHAT

>NP_002728.1

MADVFPGNDSTASQDVANRFARKGALRQKNVHEVKDHKFIARFFKQPTFCSHCTDFIWGFGKQGFQCQVCCFVVHKRCHEFVTFSCPGADKGPDTDDPRSKHKFKIHTYGSPTFCDHCGSLLYGLIHQGMKCDTCDMNVHKQCVINVPSLCGMDHTEKRGRIYLKAEVADEKLHVTVRDAKNLIPMDPNGLSDPYVKLKLIPDPKNESKQKTKTIRSTLNPQWNESFTFKLKPSDKDRRLSVEIWDWDRTTRNDFMGSLSFGVSELMKMPASGWYKLLNQEEGEYYNVPIPEGDEEGNMELRQKFEKAKLGPAGNKVISPSEDRKQPSNNLDRVKLTDFNFLMVLGKGSFGKVMLADRKGTEELYAIKILKKDVVIQDDDVECTMVEKRVLALLDKPPFLTQLHSCFQTVDRLYFVMEYVNGGDLMYHIQQVGKFKEPQAVFYAAEISIGLFFLHKRGIIYRDLKLDNVMLDSEGHIKIADFGMCKEHMMDGVTTRTFCGTPDYIAPEIIAYQPYGKSVDWWAYGVLLYEMLAGQPPFDGEDEDELFQSIMEHNVSYPKSLSKEAVSICKGLMTKHPAKRLGCGPEGERDVREHAFFRRIDWEKLENREIQPPFKPKVCGKGAENFDKFFTRGQPVLTPPDQLVIANIDQSDFEGFSYVNPQFVHPILQSAV

1402 NP_002789.1 NP_002790.1

>NP_002789.1

MAATLLAARGAGPAPAWGPEAFTPDWESREVSTGTTIMAVQFDGGVVLGADSRTTTGSYIANRVTDKLTPIHDRIFCCRSGSAADTQAVADAVTYQLGFHSIELNEPPLVHTAASLFKEMCYRYREDLMAGIIIAGWDPQEGGQVYSVPMGGMMVRQSFAIGGSGSSYIYGYVDATYREGMTKEECLQFTANALALAMERDGSSGGVIRLAAIAESGVERQVLLGDQIPKFAVATLPPA

>NP_002790.1

MAAVSVYAPPVGGFSFDNCRRNAVLEADFAKRGYKLPKVRKTGTTIAGVVYKDGIVLGADTRATEGMVVADKNCSKIHFISPNIYCCGAGTAADTDMTTQLISSNLELHSLSTGRLPRVVTANRMLKQMLFRYQGYIGAALVLGGVDVTGPHLYSIYPHGSTDKLPYVTMGSGSLAAMAVFEDKFRPDMEEEEAKNLVSEAIAAGIFNDLGSGSNIDLCVISKNKLDFLRPYTVPNKKGTRLGRYRCEKGTTAVLTEKITPLEIEVLEETVQTMDTS

1403 NP_000787.2 NP_001950.1

>NP_000787.2

MASLSQLSGHLNYTCGAENSTGASQARPHAYYALSYCALILAIVFGNGLVCMAVLKERALQTTTNYLVVSLAVADLLVATLVMPWVVYLEVTGGVWNFSRICCDVFVTLDVMMCTASILNLCAISIDRYTAVVMPVHYQHGTGQSSCRRVALMITAVWVLAFAVSCPLLFGFNTTGDPTVCSISNPDFVIYSSVVSFYLPFGVTVLVYARIYVVLKQRRRKRILTRQNSQCNSVRPGFPQQTLSPDPAHLELKRYYSICQDTALGGPGFQERGGELKREEKTRNSLSPTIAPKLSLEVRKLSNGRLSTSLKLGPLQPRGVPLREKKATQMVAIVLGAFIVCWLPFFLTHVLNTHCQTCHVSPELYSATTWLGYVNSALNPVIYTTFNIEFRKAFLKILSC

>NP_001950.1

MGFGDLKSPAGLQVLNDYLADKSYIEGYVPSQADVAVFEAVSSPPPADLCHALRWYNHIKSYEKEKASLPGVKKALGKYGPADVEDTTGSGATDSKDDDDIDLFGSDDEEESEEAKRLREERLAQYESKKAKKPALVAKSSILLDVKPWDDETDMAKLEECVRSIQADGLVWGSSKLVPVGYGIKKLQIQCVVEDDKVGTDMLEEQITAFEDYVQSMDVAAFNKI

1404 NP_005251.1 NP_015556.1

>NP_005251.1

MARPNKFLLWFCCFAWLCFPISLGSQASGGEAQIAASAELESGAMPWSLLQHIDERDRAGLLPALFKVLSVGRGGSPRLQPDSRALHYMKKLYKTYATKEGIPKSNRSHLYNTVRLFTPCTRHKQAPGDQVTGILPSVELLFNLDRITTVEHLLKSVLLYNINNSVSFSSAVKCVCNLMIKEPKSSSRTLGRAPYSFTFNSQFEFGKKHKWIQIDVTSLLQPLVASNKRSIHMSINFTCMKDQLEHPSAQNGLFNMTLVSPSLILYLNDTSAQAYHSWYSLHYKRRPSQGPDQERSLSAYPVGEEAAEDGRSSHHRHRRGQETVSSELKKPLGPASFNLSEYFRQFLLPQNECELHDFRLSFSQLKWDNWIVAPHRYNPRYCKGDCPRAVGHRYGSPVHTMVQNIIYEKLDSSVPRPSCVPAKYSPLSVLTIEPDGSIAYKEYEDMIATKCTCR

>NP_015556.1

MAAGGSTQQRRREMAAASAAAISGAGRCRLSKIGATRRPPPARVRVAVRLRPFVDGTAGASDPPCVRGMDSCSLEIANWRNHQETLKYQFDAFYGERSTQQDIYAGSVQPILRHLLEGQNASVLAYGPTGAGKTHTMLGSPEQPGVIPRALMDLLQLTREEGAEGRPWALSVTMSYLEIYQEKVLDLLDPASGDLVIREDCRGNILIPGLSQKPISSFADFERHFLPASRNRTVGATRLNQRSSRSHAVLLVKVDQRERLAPFRQREGKLYLIDLAGSEDNRRTGNKGLRLKESGAINTSLFVLGKVVDALNQGLPRVPYRDSKLTRLLQDSLGGSAHSILIANIAPERRFYLDTVSALNFAARSKEVINRPFTNESLQPHALGPVKLSQKELLGPPEAKRARGPEEEEIGSPEPMAAPASASQKLSPLQKLSSMDPAMLERLLSLDRLLASQGSQGAPLLSTPKRERMVLMKTVEEKDLEIERLKTKQKELEAKMLAQKAEEKENHCPTMLRPLSHRTVTGAKPLKKAVVMPLQLIQEQAASPNAEIHILKNKGRKRKLESLDALEPEEKAEDCWELQISPELLAHGRQKILDLLNEGSARDLRSLQRIGPKKAQLIVGWRELHGPFSQVEDLERVEGITGKQMESFLKANILGLAAGQRCGAS

1405 NP_006061.2 NP_065853.2

>NP_006061.2

MNGQLDLSGKLIIKAQLGEDIRRIPIHNEDITYDELVLMMQRVFRGKLLSNDEVTIKYKDEDGDLITIFDSSDLSFAIQCSRILKLTLFVNGQPRPLESSQVKYLRRELIELRNKVNRLLDSLEPPGEPGPSTNIPENDTVDGREEKSASDSSGKQSTQVMAASMSAFDPLKNQDEINKNVMSAFGLTDDQVSGPPSAPAEDRSGTPDSIASSSSAAHPPGVQPQQPPYTGAQTQAGQIEGQMYQQYQQQAGYGAQQPQAPPQQPQQYGIQYSASYSQQTGPQQPQQFQGYGQQPTSQAPAPAFSGQPQQLPAQPPQQYQASNYPAQTYTAQTSQPTNYTVAPASQPGMAPSQPGAYQPRPGFTSLPGSTMTPPPSGPNPYARNRPPFGQGYTQPGPGYR

>NP_065853.2

MLAGRPGTRSAVGELGTESSDNLDRAPLGPRESGGHHRPGSYLDMKIHLEKNLEEERQILLQQQKICRNRARKYFVESNRRKKAFEEKRKEQEEKEHQIREQILQQRKQKFEEVTEKFQRAHVPLSQRRKAVSRKPVPPLEEALKQIQESNLKSEVNLPFSRRPTINWRAIDSALPSALSKNDHKHQKQLLSKINCEKEMNENMRATLATSKNVFQLKLEETQKLLEDQHLSNLQKFGDEVNQITNSETLSSIDSLEATEHEEIYLTLNKEHSTSIQRNTISLKPANMQSTNLSCFDEDKLAFSKTQHINNWLTNLDASNTQNVTAFSDILSKSNVLPSWEYFNSKEQNPSPLNGTVERATNTANNSVPFVSSPPMFVLDKKCEKTSETSTMRTTDSTSGAFKRERPLVTESPTFKFSKSQSTSDSLTQEVATFPDQEKYSELNQENGTTSIPTSCVPVATPLVLPSNIQSARPSAKNSIHIKEIDAVQCSDKLDELKDGKEEEIKYFNCNKEELPLFSDSFQDAYIPHNPDSKDEKQKLAETSSLSNVTSNYDFVGQHKKMKYNIHERNGVRFLKSILKKESKYEHGYLKALIINQSFKFGNQKAAAIRDSIELTKEKGAEIPKTIKKLRWFDETSNIENNAENSHSLKNKTGTTQQHSQQFHIQSGAGSNIISVSTCAVNSADTKKSREDSISENVTTLGGSGADHMPLNCFIPSGYNFAKHAWPASKKEESKIPVHDDSKTKQGKPQRGRAKIIRKPGSAKVQSGFICTNRKGAVIQPQSASKVNIFTQAQGKLIIPCPPPQSTSNIRSGKNIQVSQCQPVTPENPQNIITHNSFNSKHVLPTEHSLNQWNQESSSPLSNACSDLVTVIPSLPSYCSSECQTFAKINHSNGTQAVARQDATLYCTQRSPVCEESYPSVTLRTAEEESVPLWKRGPNVLHQNKRATGSTVMRRKRIAETKRRNILEQKRQNPGSVGQKYSEQINNFGQSVLLSSSEPKQTTRGTSYIEEVSDSTSEFLMAENLVKASVPEDEILTVLNSKQIQKSNLPLNKTQQFNICTLSAEEQKILESLNDLSERLHYIQESICKNPSIKNTLQIIPLLEKREDRTSSCRDKR

1406 NP_114437.2 NP_008977.1

>NP_114437.2

MWQPRRPWPRVPWRWALALLALVGAGLCHAGPQPGYPARPSARNKNWCAYIVNKNVSCSVLEGSESFIQAQYNCAWNQMPCPSALVYRVNFRPRYVTRYKTVTQLEWRCCPGFRGGDCQEGPKDPVKTLRPTPARPRNSLKKATDNEPSQFSEPRKTLSPTGTAQPSWGVDPKEGPQELQEKKIQVLEEKVLRLTRTVLDLQSSLAGVSENLKHATQDDASRTRAPGLSSQHPKPDTTVSGDTETGQSPGVFNTKESGMKDIKSELAEVKDTLKNKSDKLEELDGKVKGYEGQLRQLQEAAQGPTVTMTTNELYQAYVDSKIDALREELMEGMDRKLADLKNSCEYKLTGLQQQCDDYGSSYLGVIELIGEKETSLRKEINNLRARLQEPSAQANCCDSEKNGDIGQQIKTLDQKIERVAEATRMLNGRLDNEFDRLIVPEPDVDFDAKWNELDARINVTEKNAEEHCFYIEETLRGAINGEVGDLKQLVDQKIQSLEDRLGSVLLQMTNNTGAELSPPGAAALPGVSGSGDERVMMELNHLKDKVQVVEDICLLNIQGKPHGMEGALPNREDRAVRDSLHLLKSLNDTMHRKFQETEQTIQKLQQDFSFLYSQLNHTENDVTHLQKEMSNCRAGENAGMGRFTKVGEQERTVDTLPSPQHPVAHCCSQLEERWQRLQSQVISELDACKECTQGVQREVSMVEGRVSHMEKTCSKLDSISGNLQRIKEGLNKHVSSLWNCVRQMNGTLRSHSRDISGLKNSVQQFYSHVFQISTDLQDLVKFQPSAKAPSPPPPAEAPKEPLQPEPAPPRPSGPATAEDPGRRPVLPQRPPEERPPQPPGSTGVIAETGQAGPPAGAGVSGRGLPRGVDGQTGSGTVPGAEGFAGAPGYPKSPPVASPGAPVPSLVSFSAGLTQKPFPSDGGVVLFNKVLVNDGDVYNPSTGVFTAPYDGRYLITATLTPERDAYVEAVLSVSNASVAQLHTAGYRREFLEYHRPPGALHTCGGPGAFHLIVHLKAGDAVNVVVTGGKLAHTDFDEMYSTFSGVFLYPFLSHL

>NP_008977.1

MAPRTLWSCYLCCLLTAAAGAASYPPRGFSLYTGSSGALSPGGPQAQIAPRPASRHRNWCAYVVTRTVSCVLEDGVETYVKYQPCAWGQPQCPQSIMYRRFLRPRYRVAYKTVTDMEWRCCQGYGGDDCAESPAPALGPASSTPRPLARPARPNLSGSSAGSPLSGLGGEGPGESEKVQQLEEQVQSLTKELQGLRGVLQGLSGRLAEDVQRAVETAFNGRQQPADAAARPGVHETLNEIQHQLQLLDTRVSTHDQELGHLNNHHGGSSSSGGSRAPAPASAPPGPSEELLRQLEQRLQESCSVCLAGLDGFRRQQQEDRERLRAMEKLLASVEERQRHLAGLAVGRRPPQECCSPELGRRLAELERRLDVVAGSVTVLSGRRGTELGGAAGQGGHPPGYTSLASRLSRLEDRFNSTLGPSEEQEESWPGAPGGLSHWLPAARGRLEQLGGLLANVSGELGGRLDLLEEQVAGAMQACGQLCSGAPGEQDSQVSEILSALERRVLDSEGQLRLVGSGLHTVEAAGEARQATLEGLQEVVGRLQDRVDAQDETAAEFTLRLNLTAARLGQLEGLLQAHGDEGCGACGGVQEELGRLRDGVERCSCPLLPPRGPGAGPGVGGPSRGPLDGFSVFGGSSGSALQALQGELSEVILSFSSLNDSLNELQTTVEGQGADLADLGATKDRIISEINRLQQEATEHATESEERFRGLEEGQAQAGQCPSLEGRLGRLEGVCERLDTVAGGLQGLREGLSRHVAGLWAGLRETNTTSQMQAALLEKLVGGQAGLGRRLGALNSSLQLLEDRLHQLSLKDLTGPAGEAGPPGPPGLQGPPGPAGPPGSPGKDGQEGPIGPPGPQGEQGVEGAPAAPVPQVAFSAALSLPRSEPGTVPFDRVLLNDGGYYDPETGVFTAPLAGRYLLSAVLTGHRHEKVEAVLSRSNQGVARVDSGGYEPEGLENKPVAESQPSPGTLGVFSLILPLQAGDTVCVDLVMGQLAHSEEPLTIFSGALLYGDPELEHA

1407 NP_000495.1 NP_006278.1

>NP_000495.1

MGRPLHLVLLSASLAGLLLLGESLFIRREQANNILARVTRANSFLEEMKKGHLERECMEETCSYEEAREVFEDSDKTNEFWNKYKDGDQCETSPCQNQGKCKDGLGEYTCTCLEGFEGKNCELFTRKLCSLDNGDCDQFCHEEQNSVVCSCARGYTLADNGKACIPTGPYPCGKQTLERRKRSVAQATSSSGEAPDSITWKPYDAADLDPTENPFDLLDFNQTQPERGDNNLTRIVGGQECKDGECPWQALLINEENEGFCGGTILSEFYILTAAHCLYQAKRFKVRVGDRNTEQEEGGEAVHEVEVVIKHNRFTKETYDFDIAVLRLKTPITFRMNVAPACLPERDWAESTLMTQKTGIVSGFGRTHEKGRQSTRLKMLEVPYVDRNSCKLSSSFIITQNMFCAGYDTKQEDACQGDSGGPHVTRFKDTYFVTGIVSWGEGCARKGKYGIYTKVTAFLKWIDRSMKTRGLPKAKSHAPEVITSSPLK

>NP_006278.1

MIYTMKKVHALWASVCLLLNLAPAPLNADSEEDEEHTIITDTELPPLKLMHSFCAFKADDGPCKAIMKRFFFNIFTRQCEEFIYGGCEGNQNRFESLEECKKMCTRDNANRIIKTTLQQEKPDFCFLEEDPGICRGYITRYFYNNQTKQCERFKYGGCLGNMNNFETLEECKNICEDGPNGFQVDNYGTQLNAVNNSLTPQSTKVPSLFEFHGPSWCLTPADRGLCRANENRFYYNSVIGKCRPFKYSGCGGNENNFTSKQECLRACKKGFIQRISKGGLIKTKRKRKKQRVKIAYEEIFVKNM

1408 NP_858045.1 NP_004442.3

>NP_858045.1

MSGLGENLDPLASDSRKRKLPCDTPGQGLTCSGEKRRREQESKYIEELAELISANLSDIDNFNVKPDKCAILKETVRQIRQIKEQGKTISNDDDVQKADVSSTGQGVIDKDSLGPLLLQALDGFLFVVNRDGNIVFVSENVTQYLQYKQEDLVNTSVYNILHEEDRKDFLKNLPKSTVNGVSWTNETQRQKSHTFNCRMLMKTPHDILEDINASPEMRQRYETMQCFALSQPRAMMEEGEDLQSCMICVARRITTGERTFPSNPESFITRHDLSGKVVNIDTNSLRSSMRPGFEDIIRRCIQRFFSLNDGQSWSQKRHYQEAYLNGHAETPVYRFSLADGTIVTAQTKSKLFRNPVTNDRHGFVSTHFLQREQNGYRPNPNPVGQGIRPPMAGCNSSVGGMSMSPNQGLQMPSSRAYGLADPSTTGQMSGARYGGSSNIASLTPGPGMQSPSSYQNNNYGLNMSSPPHGSPGLAPNQQNIMISPRNRGSPKIASHQFSPVAGVHSPMASSGNTGNHSFSSSSLSALQAISEGVGTSLLSTLSSPGPKLDNSPNMNITQPSKVSNQDSKSPLGFYCDQNPVESSMCQSNSRDHLSDKESKESSVEGAENQRGPLESKGHKKLLQLLTCSSDDRGHSSLTNSPLDSSCKESSVSVTSPSGVSSSTSGGVSSTSNMHGSLLQEKHRILHKLLQNGNSPAEVAKITAEATGKDTSSITSCGDGNVVKQEQLSPKKKENNALLRYLLDRDDPSDALSKELQPQVEGVDNKMSQCTSSTIPSSSQEKDPKIKTETSEEGSGDLDNLDAILGDLTSSDFYNNSISSNGSHLGTKQQVFQGTNSLGLKSSQSVQSIRPPYNRAVSLDSPVSVGSSPPVKNISAFPMLPKQPMLGGNPRMMDSQENYGSSMGGPNRNVTVTQTPSSGDWGLPNSKAGRMEPMNSNSMGRPGGDYNTSLPRPALGGSIPTLPLRSNSIPGARPVLQQQQQMLQMRPGEIPMGMGANPYGQAAASNQLGSWPDGMLSMEQVSHGTQNRPLLRNSLDDLVGPPSNLEGQSDERALLDQLHTLLSNTDATGLEEIDRALGIPELVNQGQALEPKQDAFQGQEAAVMMDQKAGLYGQTYPAQGPPMQGGFHLQGQSPSFNSMMNQMNQQGNFPLQGMHPRANIMRPRTNTPKQLRMQLQQRLQGQQFLNQSRQALELKMENPTAGGAAVMRPMMQPQVSSQQGFLNAQMVAQRSRELLSHHFRQQRVAMMMQQQQQQQQQQQQQQQQQQQQQQQQQQQQQTQAFSPPPNVTASPSMDGLLAGPTMPQAPPQQFPYQPNYGMGQQPDPAFGRVSSPPNAMMSSRMGPSQNPMMQHPQAASIYQSSEMKGWPSGNLARNSSFSQQQFAHQGNPAVYSMVHMNGSSGHMGQMNMNPMPMSGMPMGPDQKYC

>NP_004442.3

MSSQVVGIEPLYIKAEPASPDSPKGSSETETEPPVALAPGPAPTRCLPGHKEEEDGEGAGPGEQGGGKLVLSSLPKRLCLVCGDVASGYHYGVASCEACKAFFKRTIQGSIEYSCPASNECEITKRRRKACQACRFTKCLRVGMLKEGVRLDRVRGGRQKYKRRPEVDPLPFPGPFPAGPLAVAGGPRKTAAPVNALVSHLLVVEPEKLYAMPDPAGPDGHLPAVATLCDLFDREIVVTISWAKSIPGFSSLSLSDQMSVLQSVWMEVLVLGVAQRSLPLQDELAFAEDLVLDEEGARAAGLGELGAALLQLVRRLQALRLEREEYVLLKALALANSDSVHIEDAEAVEQLREALHEALLEYEAGRAGPGGGAERRRAGRLLLTLPLLRQTAGKVLAHFYGVKLEGKVPMHKLFLEMLEAMMD

1409 NP_061828.1 NP_002520.2

>NP_061828.1

MQIFVKTLTGKTITLEVEPSDTIENVKAKIQDKEGIPPDQQRLIFAGKQLEDGRTLSDYNIQKESTLHLVLRLRGGMQIFVKTLTGKTITLEVEPSDTIENVKAKIQDKEGIPPDQQRLIFAGKQLEDGRTLSDYNIQKESTLHLVLRLRGGMQIFVKTLTGKTITLEVEPSDTIENVKAKIQDKEGIPPDQQRLIFAGKQLEDGRTLSDYNIQKESTLHLVLRLRGGC

>NP_002520.2

MLRGGRRGQLGWHSWAAGPGSLLAWLILASAGAAPCPDACCPHGSSGLRCTRDGALDSLHHLPGAENLTELYIENQQHLQHLELRDLRGLGELRNLTIVKSGLRFVAPDAFHFTPRLSRLNLSFNALESLSWKTVQGLSLQELVLSGNPLHCSCALRWLQRWEEEGLGGVPEQKLQCHGQGPLAHMPNASCGVPTLKVQVPNASVDVGDDVLLRCQVEGRGLEQAGWILTELEQSATVMKSGGLPSLGLTLANVTSDLNRKNVTCWAENDVGRAEVSVQVNVSFPASVQLHTAVEMHHWCIPFSVDGQPAPSLRWLFNGSVLNETSFIFTEFLEPAANETVRHGCLRLNQPTHVNNGNYTLLAANPFGQASASIMAAFMDNPFEFNPEDPIPVSFSPVDTNSTSGDPVEKKDETPFGVSVAVGLAVFACLFLSTLLLVLNKCGRRNKFGINRPAVLAPEDGLAMSLHFMTLGGSSLSPTEGKGSGLQGHIIENPQYFSDACVHHIKRRDIVLKWELGEGAFGKVFLAECHNLLPEQDKMLVAVKALKEASESARQDFQREAELLTMLQHQHIVRFFGVCTEGRPLLMVFEYMRHGDLNRFLRSHGPDAKLLAGGEDVAPGPLGLGQLLAVASQVAAGMVYLAGLHFVHRDLATRNCLVGQGLVVKIGDFGMSRDIYSTDYYRVGGRTMLPIRWMPPESILYRKFTTESDVWSFGVVLWEIFTYGKQPWYQLSNTEAIDCITQGRELERPRACPPEVYAIMRGCWQREPQQRHSIKDVHARLQALAQAPPVYLDVLG

1410 NP_005617.2 NP_004951.1

>NP_005617.2

MPRVYIGRLSYQARERDVERFFKGYGKILEVDLKNGYGFVEFDDLRDADDAVYELNGKDLCGERVIVEHARGPRRDGSYGSGRSGYGYRRSGRDKYGPPTRTEYRLIVENLSSRCSWQDLKDYMRQAGEVTYADAHKGRKNEGVIEFVSYSDMKRALEKLDGTEVNGRKIRLVEDKPGSRRRRSYSRSRSHSRSRSRSRHSRKSRSRSGSSKSSHSKSRSRSRSGSRSRSKSRSRSQSRSRSKKEKSRSPSKEKSRSRSHSAGKSRSKSKDQAEEKIQNNDNVGKPKSRSPSRHKSKSKSRSRSQERRVEEEKRGSVSRGRSQEKSLRQSRSRSRSKGGSRSRSRSRSKSKDKRKGRKRSREESRSRSRSRSKSERSRKRGSKRDSKAGSSKKKKKEDTDRSQSRSPSRSVSKEREHAKSESSQREGRGESENAGTNQETRSRSRSNSKSKPNLPSESRSRSKSASKTRSRSKSRSRSASRSPSRSRSRSHSRS

>NP_004951.1

MASNDYTQQATQSYGAYPTQPGQGYSQQSSQPYGQQSYSGYSQSTDTSGYGQSSYSSYGQSQNTGYGTQSTPQGYGSTGGYGSSQSSQSSYGQQSSYPGYGQQPAPSSTSGSYGSSSQSSSYGQPQSGSYSQQPSYGGQQQSYGQQQSYNPPQGYGQQNQYNSSSGGGGGGGGGGNYGQDQSSMSSGGGSGGGYGNQDQSGGGGSGGYGQQDRGGRGRGGSGGGGGGGGGGYNRSSGGYEPRGRGGGRGGRGGMGGSDRGGFNKFGGPRDQGSRHDSEQDNSDNNTIFVQGLGENVTIESVADYFKQIGIIKTNKKTGQPMINLYTDRETGKLKGEATVSFDDPPSAKAAIDWFDGKEFSGNPIKVSFATRRADFNRGGGNGRGGRGRGGPMGRGGYGGGGSGGGGRGGFPSGGGGGGGQQRAGDWKCPNPTCENMNFSWRNECNQCKAPKPDGPGGGPGGSHMGGNYGDDRRGGRGGYDRGGYRGRGGDRGGFRGGRGGGDRGGFGPGKMDSRGEHRQDRRERPY

1411 NP_001816.1 NP_064575.1

>NP_001816.1

MASIFSKLLTGRNASLLFATMGTSVLTTGYLLNRQKVCAEVREQPRLFPPSADYPDLRKHNNCMAECLTPAIYSKLRNKVTPNGYTLDQCIQTGVDNPGHPFIKTVGMVAGDEESYEVFADLFDPVIKLRHNGYDPRVMKHTTDLDASKITQGQFDEHYVLSSRVRTGRSIRGLSLPPACTRAERREVENVAITALEGLKGDLAGRYYKLSEMTEQDQQRLIDDHFLFDKPVSPLLTCAGMARDWPDARGIWHNYDKTFLIWINEEDHTRVISMEKGGNMKRVFERFCRGLKEVERLIQERGWEFMWNERLGYILTCPSNLGTGLRAGVHVRIPKLSKDPRFSKILENLRLQKRGTGGVDTAAVADVYDISNIDRIGRSEVELVQIVIDGVNYLVDCEKKLERGQDIKVPPPLPQFGKK

>NP_064575.1

MGPSTPLLILFLLSWSGPLQGQQHHLVEYMERRLAALEERLAQCQDQSSRHAAELRDFKNKMLPLLEVAEKEREALRTEADTISGRVDRLEREVDYLETQNPALPCVEFDEKVTGGPGTKGKGRRNEKYDMVTDCGYTISQVRSMKILKRFGGPAGLWTKDPLGQTEKIYVLDGTQNDTAFVFPRLRDFTLAMAARKASRVRVPFPWVGTGQLVYGGFLYFARRPPGRPGGGGEMENTLQLIKFHLANRTVVDSSVFPAEGLIPPYGLTADTYIDLAADEEGLWAVYATREDDRHLCLAKLDPQTLDTEQQWDTPCPRENAEAAFVICGTLYVVYNTRPASRARIQCSFDASGTLTPERAALPYFPRRYGAHASLRYNPRERQLYAWDDGYQIVYKLEMRKKEEEV

1412 NP_001019801.2 NP_005229.1

>NP_001019801.2

MLHSPHKQPQNHKCGANFLQEDSKKSLVFKWLISAGHYQPPRPTESFKAASSIYNRGYKFYLKKKGGTMASNSLFSTVTPCQQNFFWDPSTSRRFSPPSSSLQPGKMSDVSPVVAAQQQQQQQQQQQQQQQQQQQQQQQEAAAAAAAAAAAAAAAAAVPRLRPPHDNRTMVEIIADHPAELVRTDSPNFLCSVLPSHWRCNKTLPVAFKVVALGEVPDGTVVTVMAGNDENYSAELRNASAVMKNQVARFNDLRFVGRSGRGKSFTLTITVFTNPPQVATYHRAIKVTVDGPREPRRHRQKLDDSKPSLFSDRLSDLGRIPHPSMRVGVPPQNPRPSLNSAPSPFNPQGQSQITDPRQAQSSPPWSYDQSYPSYLSQMTSPSIHSTTPLSSTRGTGLPAITDVPRRISDDDTATSDFCLWPSTLSKKSQAGASELGPFSDPRQFPSISSLTESRFSNPRMHYPATFTYTPPVTSGMSLGMSATTHYHTYLPPPYPGSSQSQSGPFQTSSTPYLYYGTSSGSYQFPMVPGGDRSPSRMLPPCTTTSNGSTLLNPNLPNQNDGVDADGSHSSSPTVLNSSGRMDESVWRPY

>NP_005229.1

MKAAVDLKPTLTIIKTEKVDLELFPSPDMECADVPLLTPSSKEMMSQALKATFSGFTKEQQRLGIPKDPRQWTETHVRDWVMWAVNEFSLKGVDFQKFCMNGAALCALGKDCFLELAPDFVGDILWEHLEILQKEDVKPYQVNGVNPAYPESRYTSDYFISYGIEHAQCVPPSEFSEPSFITESYQTLHPISSEELLSLKYENDYPSVILRDPLQTDTLQNDYFAIKQEVVTPDNMCMGRTSRGKLGGQDSFESIESYDSCDRLTQSWSSQSSFNSLQRVPSYDSFDSEDYPAALPNHKPKGTFKDYVRDRADLNKDKPVIPAAALAGYTGSGPIQLWQFLLELLTDKSCQSFISWTGDGWEFKLSDPDEVARRWGKRKNKPKMNYEKLSRGLRYYYDKNIIHKTAGKRYVYRFVCDLQSLLGYTPEELHAMLDVKPDADE

1413 NP_658985.2 NP_000030.1

>NP_658985.2

MSRLRALLGLGLLVAGSRVPRIKSQTIACRSGPTWWGPQRLNSGGRWDSEVMASTVVKYLSQEEAQAVDQELFNEYQFSVDQLMELAGLSCATAIAKAYPPTSMSRSPPTVLVICGPGNNGGDGLVCARHLKLFGYEPTIYYPKRPNKPLFTALVTQCQKMDIPFLGEMPAEPMTIDELYELVVDAIFGFSFKGDVREPFHSILSVLKGLTVPIASIDIPSGWDVEKGNAGGIQPDLLISLTAPKKSATQFTGRYHYLGGRFVPPALEKKYQLNLPPYPDTECVYRLQ

>NP_000030.1

MKAAVLTLAVLFLTGSQARHFWQQDEPPQSPWDRVKDLATVYVDVLKDSGRDYVSQFEGSALGKQLNLKLLDNWDSVTSTFSKLREQLGPVTQEFWDNLEKETEGLRQEMSKDLEEVKAKVQPYLDDFQKKWQEEMELYRQKVEPLRAELQEGARQKLHELQEKLSPLGEEMRDRARAHVDALRTHLAPYSDELRQRLAARLEALKENGGARLAEYHAKATEHLSTLSEKAKPALEDLRQGLLPVLESFKVSFLSALEEYTKKLNTQ

1414 NP_000772.1 BAA21128.1

>NP_000772.1

MLAKGLPPRSVLVKGYQTFLSAPREGLGRLRVPTGEGAGISTRSPRPFNEIPSPGDNGWLNLYHFWRETGTHKVHLHHVQNFQKYGPIYREKLGNVESVYVIDPEDVALLFKSEGPNPERFLIPPWVAYHQYYQRPIGVLLKKSAAWKKDRVALNQEVMAPEATKNFLPLLDAVSRDFVSVLHRRIKKAGSGNYSGDISDDLFRFAFESITNVIFGERQGMLEEVVNPEAQRFIDAIYQMFHTSVPMLNLPPDLFRLFRTKTWKDHVAAWDVIFSKADIYTQNFYWELRQKGSVHHDYRGMLYRLLGDSKMSFEDIKANVTEMLAGGVDTTSMTLQWHLYEMARNLKVQDMLRAEVLAARHQAQGDMATMLQLVPLLKASIKETLRLHPISVTLQRYLVNDLVLRDYMIPAKTLVQVAIYALGREPTFFFDPENFDPTRWLSKDKNITYFRNLGFGWGVRQCLGRRIAELEMTIFLINMLENFRVEIQHLSDVGTTFNLILMPEKPISFTFWPFNQEATQQ

>BAA21128.1

MHSTTPISSLFSFTSPAVKRLLGWKQGDEEEKWAEKAVDSLVKKLKKKKGAMDELERALSCPGQPSKCVTIPRSLDGRLQVSHRKGLPHVIYCRVWRWPDLQSHHELKPLECCEFPFGSKQKEVCINPYHYRRVETPVLPPVLVPRHSEYNPQLSLLAKFRSASLHSEPLMPHNATYPDSFQQPPCSALPPSPSHAFSQSPCTASYPHSPGSPSEPESPYQHSVDTPPLPYHATEASETQSGQPVDATADRHVVLSIPNGDFRPVCYEEPQHWCSVAYYELNNRVGETFQASSRSVLIDGFTDPSNNRNRFCLGLLSNVNRNSTIENTRRHIGKGVHLYYVGGEVYAECVSDSSIFVQSRNCNYQHGFHPATVCKIPSGCSLKVFNNQLFAQLLAQSVHHGFEVVYELTKMCTIRMSFVKGWGAEYHRQDVTSTPCWIEIHLHGPLQWLDKVLTQMGSPHNPISSVS

1415 NP_006414.1 NP_004574.2

>NP_006414.1

MAAQKDQQKDAEAEGLSGTTLLPKLIPSGAGREWLERRRATIRPWSTFVDQQRFSRPRNLGELCQRLVRNVEYYQSNYVFVFLGLILYCVVTSPMLLVALAVFFGACYILYLRTLESKLVLFGREVSPEHQYALAGGISFPFFWLAGAGSAVFWVLGATLVVIGSHAAFHQIEAVDGEELQMEPV

>NP_004574.2

MAGRGGAARPNGPAAGNKICQFKLVLLGESAVGKSSLVLRFVKGQFHEYQESTIGAAFLTQTVCLDDTTVKFEIWDTAGQERYHSLAPMYYRGAQAAIVVYDITNTDTFARAKNWVKELQRQASPNIVIALAGNKADLASKRAVEFQEAQAYADDNSLLFMETSAKTAMNVNEIFMAIAKKLPKNEPQNATGAPGRNRGVDLQENNPASRSQCCSN

1416 NP_015566.1 NP_003248.3

>NP_015566.1

MSTMRLLTLALLFSCSVARAACDPKIVNIGAVLSTRKHEQMFREAVNQANKRHGSWKIQLNATSVTHKPNAIQMALSVCEDLISSQVYAILVSHPPTPNDHFTPTPVSYTAGFYRIPVLGLTTRMSIYSDKSIHLSFLRTVPPYSHQSSVWFEMMRVYSWNHIILLVSDDHEGRAAQKRLETLLEERESKAEKVLQFDPGTKNVTALLMEAKELEARVIILSASEDDAATVYRAAAMLNMTGSGYVWLVGEREISGNALRYAPDGILGLQLINGKNESAHISDAVGVVAQAVHELLEKENITDPPRGCVGNTNIWKTGPLFKRVLMSSKYADGVTGRVEFNEDGDRKFANYSIMNLQNRKLVQVGIYNGTHVIPNDRKIIWPGGETEKPRGYQMSTRLKIVTIHQEPFVYVKPTLSDGTCKEEFTVNGDPVKKVICTGPNDTSPGSPRHTVPQCCYGFCIDLLIKLARTMNFTYEVHLVADGKFGTQERVNNSNKKEWNGMMGELLSGQADMIVAPLTINNERAQYIEFSKPFKYQGLTILVKKEIPRSTLDSFMQPFQSTLWLLVGLSVHVVAVMLYLLDRFSPFGRFKVNSEEEEEDALTLSSAMWFSWGVLLNSGIGEGAPRSFSARILGMVWAGFAMIIVASYTANLAAFLVLDRPEERITGINDPRLRNPSDKFIYATVKQSSVDIYFRRQVELSTMYRHMEKHNYESAAEAIQAVRDNKLHAFIWDSAVLEFEASQKCDLVTTGELFFRSGFGIGMRKDSPWKQNVSLSILKSHENGFMEDLDKTWVRYQECDSRSNAPATLTFENMAGVFMLVAGGIVAGIFLIFIEIAYKRHKDARRKQMQLAFAAVNVWRKNLQDRKSGRAEPDPKKKATFRAITSTLASSFKRRRSSKDTSTGGGRGALQNQKDTVLPRRAIEREEGQLQLCSRHRES

>NP_003248.3

MSARAAAAKSTAMEETAIWEQHTVTLHRAPGFGFGIAISGGRDNPHFQSGETSIVISDVLKGGPAEGQLQENDRVAMVNGVSMDNVEHAFAVQQLRKSGKNAKITIRRKKKVQIPVSRPDPEPVSDNEEDSYDEEIHDPRSGRSGVVNRRSEKIWPRDRSASRERSLSPRSDRRSVASSQPAKPTKVTLVKSRKNEEYGLRLASHIFVKEISQDSLAARDGNIQEGDVVLKINGTVTENMSLTDAKTLIERSKGKLKMVVQRDERATLLNVPDLSDSIHSANASERDDISEIQSLASDHSGRSHDRPPRRSRSRSPDQRSEPSDHSRHSPQQPSNGSLRSRDEERISKPGAVSTPVKHADDHTPKTVEEVTVERNEKQTPSLPEPKPVYAQVGQPDVDLPVSPSDGVLPNSTHEDGILRPSMKLVKFRKGDSVGLRLAGGNDVGIFVAGVLEDSPAAKEGLEEGDQILRVNNVDFTNIIREEAVLFLLDLPKGEEVTILAQKKKDVYRRIVESDVGDSFYIRTHFEYEKESPYGLSFNKGEVFRVVDTLYNGKLGSWLAIRIGKNHKEVERGIIPNKNRAEQLASVQYTLPKTAGGDRADFWRFRGLRSSKRNLRKSREDLSAQPVQTKFPAYERVVLREAGFLRPVTIFGPIADVAREKLAREEPDIYQIAKSEPRDAGTDQRSSGIIRLHTIKQIIDQDKHALLDVTPNAVDRLNYAQWYPIVVFLNPDSKQGVKTMRMRLCPESRKSARKLYERSHKLRKNNHHLFTTTINLNSMNDGWYGALKEAIQQQQNQLVWVSEGKADGATSDDLDLHDDRLSYLSAPGSEYSMYSTDSRHTSDYEDTDTEGGAYTDQELDETLNDEVGTPPESAITRSSEPVREDSSGMHHENQTYPPYSPQAQPQPIHRIDSPGFKPASQQKAEASSPVPYLSPETNPASSTSAVNHNVNLTNVRLEEPTPAPSTSYSPQADSLRTPSTEAAHIMLRDQEPSLSSHVDPTKVYRKDPYPEEMMRQNHVLKQPAVSHPGHRPDKEPNLTYEPQLPYVEKQASRDLEQPTYRYESSSYTDQFSRNYEHRLRYEDRVPMYEEQWSYYDDKQPYPSRPPFDNQHSQDLDSRQHPEESSERGYFPRFEEPAPLSYDSRPRYEQAPRASALRHEEQPAPGYDTHGRLRPEAQPHPSAGPKPAESKQYFEQYSRSYEQVPPQGFTSRAGHFEPLHGAAAVPPLIPSSQHKPEALPSNTKPLPPPPTQTEEEEDPAMKPQSVLTRVKMFENKRSASLETKKDVNDTGSFKPPEVASKPSGAPIIGPKPTSQNQFSEHDKTLYRIPEPQKPQLKPPEDIVRSNHYDPEEDEEYYRKQLSYFDRRSFENKPPAHIAASHLSEPAKPAHSQNQSNFSSYSSKGKPPEADGVDRSFGEKRYEPIQATPPPPPLPSQYAQPSQPVTSASLHIHSKGAHGEGNSVSLDFQNSLVSKPDPPPSQNKPATFRPPNREDTAQAAFYPQKSFPDKAPVNGTEQTQKTVTPAYNRFTPKPYTSSARPFERKFESPKFNHNLLPSETAHKPDLSSKTPTSPKTLVKSHSLAQPPEFDSGVETFSIHAEKPKYQINNISTVPKAIPVSPSAVEEDEDEDGHTVVATARGIFNSNGGVLSSIETGVSIIIPQGAIPEGVEQEIYFKVCRDNSILPPLDKEKGETLLSPLVMCGPHGLKFLKPVELRLPHCDPKTWQNKCLPGDPNYLVGANCVSVLIDHF

1417 NP_002205.1 NP_000651.3

>NP_002205.1

MCGSALAFFTAAFVCLQNDRRGPASFLWAAWVFSLVLGLGQGEDNRCASSNAASCARCLALGPECGWCVQEDFISGGSRSERCDIVSNLISKGCSVDSIEYPSVHVIIPTENEINTQVTPGEVSIQLRPGAEANFMLKVHPLKKYPVDLYYLVDVSASMHNNIEKLNSVGNDLSRKMAFFSRDFRLGFGSYVDKTVSPYISIHPERIHNQCSDYNLDCMPPHGYIHVLSLTENITEFEKAVHRQKISGNIDTPEGGFDAMLQAAVCESHIGWRKEAKRLLLVMTDQTSHLALDSKLAGIVVPNDGNCHLKNNVYVKSTTMEHPSLGQLSEKLIDNNINVIFAVQGKQFHWYKDLLPLLPGTIAGEIESKAANLNNLVVEAYQKLISEVKVQVENQVQGIYFNITAICPDGSRKPGMEGCRNVTSNDEVLFNVTVTMKKCDVTGGKNYAIIKPIGFNETAKIHIHRNCSCQCEDNRGPKGKCVDETFLDSKCFQCDENKCHFDEDQFSSESCKSHKDQPVCSGRGVCVCGKCSCHKIKLGKVYGKYCEKDDFSCPYHHGNLCAGHGECEAGRCQCFSGWEGDRCQCPSAAAQHCVNSKGQVCSGRGTCVCGRCECTDPRSIGRFCEHCPTCYTACKENWNCMQCLHPHNLSQAILDQCKTSCALMEQQHYVDQTSECFSSPSYLRIFFIIFIVTFLIGLLKVLIIRQVILQWNSNKIKSSSDYRVSASKKDKLILQSVCTRAVTYRREKPEEIKMDISKLNAHETFRCNF

>NP_000651.3

MPPSGLRLLPLLLPLLWLLVLTPGRPAAGLSTCKTIDMELVKRKRIEAIRGQILSKLRLASPPSQGEVPPGPLPEAVLALYNSTRDRVAGESAEPEPEPEADYYAKEVTRVLMVETHNEIYDKFKQSTHSIYMFFNTSELREAVPEPVLLSRAELRLLRLKLKVEQHVELYQKYSNNSWRYLSNRLLAPSDSPEWLSFDVTGVVRQWLSRGGEIEGFRLSAHCSCDSRDNTLQVDINGFTTGRRGDLATIHGMNRPFLLLMATPLERAQHLQSSRHRRALDTNYCFSSTEKNCCVRQLYIDFRKDLGWKWIHEPKGYHANFCLGPCPYIWSLDTQYSKVLALYNQHNPGASAAPCCVPQALEPLPIVYYVGRKPKVEQLSNMIVRSCKCS

1418 NP_006248.1 NP_733772.1

>NP_006248.1

MSPFLRIGLSNFDCGSCQSCQGEAVNPYCAVLVKEYVESENGQMYIQKKPTMYPPWDSTFDAHINKGRVMQIIVKGKNVDLISETTVELYSLAERCRKNNGKTEIWLELKPQGRMLMNARYFLEMSDTKDMNEFETEGFFALHQRRGAIKQAKVHHVKCHEFTATFFPQPTFCSVCHEFVWGLNKQGYQCRQCNAAIHKKCIDKVIAKCTGSAINSRETMFHKERFKIDMPHRFKVYNYKSPTFCEHCGTLLWGLARQGLKCDACGMNVHHRCQTKVANLCGINQKLMAEALAMIESTQQARCLRDTEQIFREGPVEIGLPCSIKNEARPPCLPTPGKREPQGISWESPLDEVDKMCHLPEPELNKERPSLQIKLKIEDFILHKMLGKGSFGKVFLAEFKKTNQFFAIKALKKDVVLMDDDVECTMVEKRVLSLAWEHPFLTHMFCTFQTKENLFFVMEYLNGGDLMYHIQSCHKFDLSRATFYAAEIILGLQFLHSKGIVYRDLKLDNILLDKDGHIKIADFGMCKENMLGDAKTNTFCGTPDYIAPEILLGQKYNHSVDWWSFGVLLYEMLIGQSPFHGQDEEELFHSIRMDNPFYPRWLEKEAKDLLVKLFVREPEKRLGVRGDIRQHPLFREINWEELERKEIDPPFRPKVKSPFDCSNFDKEFLNEKPRLSFADRALINSMDQNMFRNFSFMNPGMERLIS

>NP_733772.1

MGSSGLGKAATLDELLCTCIEMFDDNGELDNSYLPRIVLLMHRWYLSSTELAEKLLCMYRNATGESCNEFRLKICYFMRYWILKFPAEFNLDLGLIRMTEEFREVASQLGYEKHVSLIDISSIPSYDWMRRVTQRKKVSKKGKACLLFDHLEPIELAEHLTFLEHKSFRRISFTDYQSYVIHGCLENNPTLERSIALFNGISKWVQLMVLSKPTPQQRAEVITKFINVAKKLLQLKNFNTLMAVVGGLSHSSISRLKETHSHLSSEVTKNWNEMTELVSSNGNYCNYRKAFADCDGFKIPILGVHLKDLIAVHVIFPDWTEENKVNIVKMHQLSVTLSELVSLQNASHHLEPNMDLINLLTLSLDLYHTEDDIYKLSLVLEPRNSKSQPTSPTTPNKPVVPLEWALGVMPKPDPTVINKHIRKLVESVFRNYDHDHDGYISQEDFESIAANFPFLDSFCVLDKDQDGLISKDEMMAYFLRAKSQLHCKMGPGFIHNFQEMTYLKPTFCEHCAGFLWGIIKQGYKCKDCGANCHKQCKDLLVLACRRFARAPSLSSGHGSLPGSPSLPPAQDEVFEFPGVTAGHRDLDSRAITLVTGSSRKISVRLQRATTSQATQTEPVWSEAGWGDSGSHTFPKMKSKFHDKAAKDKGFAKWENEKPRVHAGVDVVDRGTEFELDQDEGEETRQDGEDG

1419 NP_036233.2 NP_002728.1

>NP_036233.2

MEAADASRSNGSSPEARDARSPSGPSGSLENGTKADGKDAKTTNGHGGEAAEGKSLGSALKPGEGRSALFAGNEWRRPIIQFVESGDDKNSNYFSMDSMEGKRSPYAGLQLGAAKKPPVTFAEKGELRKSIFSESRKPTVSIMEPGETRRNSYPRADTGLFSRSKSGSEEVLCDSCIGNKQKAVKSCLVCQASFCELHLKPHLEGAAFRDHQLLEPIRDFEARKCPVHGKTMELFCQTDQTCICYLCMFQEHKNHSTVTVEEAKAEKETELSLQKEQLQLKIIEIEDEAEKWQKEKDRIKSFTTNEKAILEQNFRDLVRDLEKQKEEVRAALEQREQDAVDQVKVIMDALDERAKVLHEDKQTREQLHSISDSVLFLQEFGALMSNYSLPPPLPTYHVLLEGEGLGQSLGNFKDDLLNVCMRHVEKMCKADLSRNFIERNHMENGGDHRYVNNYTNSFGGEWSAPDTMKRYSMYLTPKGGVRTSYQPSSPGRFTKETTQKNFNNLYGTKGNYTSRVWEYSSSIQNSDNDLPVVQGSSSFSLKGYPSLMRSQSPKAQPQTWKSGKQTMLSHYRPFYVNKGNGIGSNEAP

>NP_002728.1

MADVFPGNDSTASQDVANRFARKGALRQKNVHEVKDHKFIARFFKQPTFCSHCTDFIWGFGKQGFQCQVCCFVVHKRCHEFVTFSCPGADKGPDTDDPRSKHKFKIHTYGSPTFCDHCGSLLYGLIHQGMKCDTCDMNVHKQCVINVPSLCGMDHTEKRGRIYLKAEVADEKLHVTVRDAKNLIPMDPNGLSDPYVKLKLIPDPKNESKQKTKTIRSTLNPQWNESFTFKLKPSDKDRRLSVEIWDWDRTTRNDFMGSLSFGVSELMKMPASGWYKLLNQEEGEYYNVPIPEGDEEGNMELRQKFEKAKLGPAGNKVISPSEDRKQPSNNLDRVKLTDFNFLMVLGKGSFGKVMLADRKGTEELYAIKILKKDVVIQDDDVECTMVEKRVLALLDKPPFLTQLHSCFQTVDRLYFVMEYVNGGDLMYHIQQVGKFKEPQAVFYAAEISIGLFFLHKRGIIYRDLKLDNVMLDSEGHIKIADFGMCKEHMMDGVTTRTFCGTPDYIAPEIIAYQPYGKSVDWWAYGVLLYEMLAGQPPFDGEDEDELFQSIMEHNVSYPKSLSKEAVSICKGLMTKHPAKRLGCGPEGERDVREHAFFRRIDWEKLENREIQPPFKPKVCGKGAENFDKFFTRGQPVLTPPDQLVIANIDQSDFEGFSYVNPQFVHPILQSAV

1420 NP_002721.1 NP_002033.1

>NP_002721.1

MGNAAAAKKGSEQESVKEFLAKAKEDFLKKWESPAQNTAHLDQFERIKTLGTGSFGRVMLVKHKETGNHYAMKILDKQKVVKLKQIEHTLNEKRILQAVNFPFLVKLEFSFKDNSNLYMVMEYVPGGEMFSHLRRIGRFSEPHARFYAAQIVLTFEYLHSLDLIYRDLKPENLLIDQQGYIQVTDFGFAKRVKGRTWTLCGTPEYLAPEIILSKGYNKAVDWWALGVLIYEMAAGYPPFFADQPIQIYEKIVSGKVRFPSHFSSDLKDLLRNLLQVDLTKRFGNLKNGVNDIKNHKWFATTDWIAIYQRKVEAPFIPKFKGPGDTSNFDDYEEEEIRVSINEKCGKEFSEF

>NP_002033.1

MRFGIFLLWWGWVLATESRMHWPGREVHEMSKKGRPQRQRREVHEDAHKQVSPILRRSPDITKSPLTKSEQLLRIDDHDFSMRPGFGGPAIPVGVDVQVESLDSISEVDMDFTMTLYLRHYWKDERLSFPSTNNLSMTFDGRLVKKIWVPDMFFVHSKRSFIHDTTTDNVMLRVQPDGKVLYSLRVTVTAMCNMDFSRFPLDTQTCSLEIESYAYTEDDLMLYWKKGNDSLKTDERISLSQFLIQEFHTTTKLAFYSSTGWYNRLYINFTLRRHIFFFLLQTYFPATLMVMLSWVSFWIDRRAVPARVPLGITTVLTMSTIITGVNASMPRVSYIKAVDIYLWVSFVFVFLSVLEYAAVNYLTTVQERKEQKLREKLPCTSGLPPPRTAMLDGNYSDGEVNDLDNYMPENGEKPDRMMVQLTLASERSSPQRKSQRSSYVSMRIDTHAIDKYSRIIFPAAYILFNLIYWSIFS

1421 NP_001008493.1 NP_056036.1

>NP_001008493.1

MSEQSICQARAAVMVYDDANKKWVPAGGSTGFSRVHIYHHTGNNTFRVVGRKIQDHQVVINCAIPKGLKYNQATQTFHQWRDARQVYGLNFGSKEDANVFASAMMHALEVLNSQETGPTLPRQNSQLPAQVQNGPSQEELEIQRRQLQEQQRQKELERERLERERMERERLERERLERERLERERLEQEQLERERQERERQERLERQERLERQERLERQERLDRERQERQERERLERLERERQERERQEQLEREQLEWERERRISSAAAPASVETPLNSVLGDSSASEPGLQAASQPAETPSQQGIVLGPLAPPPPPPLPPGPAQASVALPPPPGPPPPPPLPSTGPPPPPPPPPLPNQVPPPPPPPPAPPLPASGFFLASMSEDNRPLTGLAAAIAGAKLRKVSRMEDTSFPSGGNAIGVNSASSKTDTGRGNGPLPLGGSGLMEEMSALLARRRRIAEKGSTIETEQKEDKGEDSEPVTSKASSTSTPEPTRKPWERTNTMNGSKSPVISRRDSPRKNQIVFDNRSYDSLHRPKSTPLSQPSANGVQTEGLDYDRLKQDILDEMRKELTKLKEELIDAIRQELSKSNTA

>NP_056036.1

MEAGSVVRAIFDFCPSVSEELPLFVGDIIEVLAVVDEFWLLGKKEDVTGQFPSSFVEIVTIPSLKEGERLFVCICEFTSQELDNLPLHRGDLVILDGIPTAGWLQGRSCWGARGFFPSSCVRELCLSSQSRQWHSQSALFQIPEYSMGQARALMGLSAQLDEELDFREGDVITIIGVPEPGWFEGELEGRRGIFPEGFVELLGPLRTVDESVSSGNQDDCIVNGEVDTPVGEEEIGPDEDEEEPGTYGVALYRFQALEPNELDFEVGDKIRILATLEDGWLEGSLKGRTGIFPYRFVKLCPDTRVEETMALPQEGSLARIPETSLDCLENTLGVEEQRHETSDHEAEEPDCIISEAPTSPLGHLTSEYDTDRNSYQDEDTAGGPPRSPGVEWEMPLATDSPTSDPTEVVNGISSQPQVPFHPNLQKSQYYSTVGGSHPHSEQYPDLLPLEARTRDYASLPPKRMYSQLKTLQKPVLPLYRGSSVSASRVVKPRQSSPQLHNLASYTKKHHTSSVYSISERLEMKPGPQAQGLVMEAATHSQGDGSTDLDSKLTQQLIEFEKSLAGPGTEPDKILRHFSIMDFNSEKDIVRGSSKLITEQELPERRKALRPPPPRPCTPVSTSPHLLVDQNLKPAPPLVVRPSRPAPLPPSAQQRTNAVSPKLLSRHRPTCETLEKEGPGHMGRSLDQTSPCPLVLVRIEEMERDLDMYSRAQEELNLMLEEKQDESSRAETLEDLKFCESNIESLNMELQQLREMTLLSSQSSSLVAPSGSVSAENPEQRMLEKRAKVIEELLQTERDYIRDLEMCIERIMVPMQQAQVPNIDFEGLFGNMQMVIKVSKQLLAALEISDAVGPVFLGHRDELEGTYKIYCQNHDEAIALLEIYEKDEKIQKHLQDSLADLKSLYNEWGCTNYINLGSFLIKPVQRVMRYPLLLMELLNSTPESHPDKVPLTNAVLAVKEINVNINEYKRRKDLVLKYRKGDEDSLMEKISKLNIHSIIKKSNRVSSHLKHLTGFAPQIKDEVFEETEKNFRMQERLIKSFIRDLSLYLQHIRESACVKVVAAVSMWDVCMERGHRDLEQFERVHRYISDQLFTNFKERTERLVISPLNQLLSMFTGPHKLVQKRFDKLLDFYNCTERAEKLKDKKTLEELQSARNNYEALNAQLLDELPKFHQYAQGLFTNCVHGYAEAHCDFVHQALEQLKPLLSLLKVAGREGNLIAIFHEEHSRVLQQLQVFTFFPESLPATKKPFERKTIDRQSARKPLLGLPSYMLQSEELRASLLARYPPEKLFQAERNFNAAQDLDVSLLEGDLVGVIKKKDPMGSQNRWLIDNGVTKGFVYSSFLKPYNPRRSHSDASVGSHSSTESEHGSSSPRFPRQNSGSTLTFNPSSMAVSFTSGSCQKQPQDASPPPKECDQGTLSASLNPSNSESSPSRCPSDPDSTSQPRSGDSADVARDVKQPTATPRSYRNFRHPEIVGYSVPGRNGQSQDLVKGCARTAQAPEDRSTEPDGSEAEGNQVYFAVYTFKARNPNELSVSANQKLKILEFKDVTGNTEWWLAEVNGKKGYVPSNYIRKTEYT

1422 NP_060654.1 NP_001060.1

>NP_060654.1

MLALISRLLDWFRSLFWKEEMELTLVGLQYSGKTTFVNVIASGQFSEDMIPTVGFNMRKVTKGNVTIKIWDIGGQPRFRSMWERYCRGVNAIVYMIDAADREKIEASRNELHNLLDKPQLQGIPVLVLGNKRDLPNALDEKQLIEKMNLSAIQDREICCYSISCKEKDNIDITLQWLIQHSKSRRS

>NP_001060.1

MREIVHIQAGQCGNQIGAKFWEVISDEHGIDPTGSYHGDSDLQLERINVYYNEAAGNKYVPRAILVDLEPGTMDSVRSGPFGQIFRPDNFVFGQSGAGNNWAKGHYTEGAELVDSVLDVVRKESESCDCLQGFQLTHSLGGGTGSGMGTLLISKIREEYPDRIMNTFSVMPSPKVSDTVVEPYNATLSVHQLVENTDETYSIDNEALYDICFRTLKLTTPTYGDLNHLVSATMSGVTTCLRFPGQLNADLRKLAVNMVPFPRLHFFMPGFAPLTSRGSQQYRALTVPELTQQMFDSKNMMAACDPRHGRYLTVAAIFRGRMSMKEVDEQMLNVQNKNSSYFVEWIPNNVKTAVCDIPPRGLKMSATFIGNSTAIQELFKRISEQFTAMFRRKAFLHWYTGEGMDEMEFTEAESNMNDLVSEYQQYQDATADEQGEFEEEEGEDEA

1423 NP_006303.3 NP_068810.2

>NP_006303.3

MSGSTQPVAQTWRATEPRYPPHSLSYPVQIARTHTDVGLLEYQHHSRDYASHLSPGSIIQPQRRRPSLLSEFQPGNERSQELHLRPESHSYLPELGKSEMEFIESKRPRLELLPDPLLRPSPLLATGQPAGSEDLTKDRSLTGKLEPVSPPSPPHTDPELELVPPRLSKEELIQNMDRVDREITMVEQQISKLKKKQQQLEEEAAKPPEPEKPVSPPPIESKHRSLVQIIYDENRKKAEAAHRILEGLGPQVELPLYNQPSDTRQYHENIKINQAMRKKLILYFKRRNHARKQWEQKFCQRYDQLMEAWEKKVERIENNPRRRAKESKVREYYEKQFPEIRKQRELQERMQSRVGQRGSGLSMSAARSEHEVSEIIDGLSEQENLEKQMRQLAVIPPMLYDADQQRIKFINMNGLMADPMKVYKDRQVMNMWSEQEKETFREKFMQHPKNFGLIASFLERKTVAECVLYYYLTKKNENYKSLVRRSYRRRGKSQQQQQQQQQQQQQQQQQPMPRSSQEEKDEKEKEKEAEKEEEKPEVENDKEDLLKEKTDDTSGEDNDEKEAVASKGRKTANSQGRRKGRITRSMANEANSEEAITPQQSAELASMELNESSRWTEEEMETAKKGLLEHGRNWSAIARMVGSKTVSQCKNFYFNYKKRQNLDEILQQHKLKMEKERNARRKKKKAPAAASEEAAFPPVVEDEEMEASGVSGNEEEMVEEAEALHASGNEVPRGECSGPATVNNSSDTESIPSPHTEAAKDTGQNGPKPPATLGADGPPPGPPTPPPEDIPAPTEPTPASEATGAPTPPPAPPSPSAPPPVVPKEEKEEETAAAPPVEEGEEQKPPAAEELAVDTGKAEEPVKSECTEEAEEGPAKGKDAEAAEATAEGALKAEKKEGGSGRATTAKSSGAPQDSDSSATCSADEVDEAEGGDKNRLLSPRPSLLTPTGDPRANASPQKPLDLKQLKQRAAAIPPIVTKVHEPPREDAAPTKPAPPAPPPPQNLQPESDAPQQPGSSPRGKSRSPAPPADKEAEKPVFFPAFAAEAQKLPGDPPCWTSGLPFPVPPREVIKASPHAPDPSAFSYAPPGHPLPLGLHDTARPVLPRPPTISNPPPLISSAKHPSVLERQIGAISQGMSVQLHVPYSEHAKAPVGPVTMGLPLPMDPKKLAPFSGVKQEQLSPRGQAGPPESLGVPTAQEASVLRGTALGSVPGGSITKGIPSTRVPSDSAITYRGSITHGTPADVLYKGTITRIIGEDSPSRLDRGREDSLPKGHVIYEGKKGHVLSYEGGMSVTQCSKEDGRSSSGPPHETAAPKRTYDMMEGRVGRAISSASIEGLMGRAIPPERHSPHHLKEQHHIRGSITQGIPRSYVEAQEDYLRREAKLLKREGTPPPPPPSRDLTEAYKTQALGPLKLKPAHEGLVATVKEAGRSIHEIPREELRHTPELPLAPRPLKEGSITQGTPLKYDTGASTTGSKKHDVRSLIGSPGRTFPPVHPLDVMADARALERACYEESLKSRPGTASSSGGSIARGAPVIVPELGKPRQSPLTYEDHGAPFAGHLPRGSPVTTREPTPRLQEGSLSSSKASQDRKLTSTPREIAKSPHSTVPEHHPHPISPYEHLLRGVSGVDLYRSHIPLAFDPTSIPRGIPLDAAAAYYLPRHLAPNPTYPHLYPPYLIRGYPDTAALENRQTIINDYITSQQMHHNAATAMAQRADMLRGLSPRESSLALNYAAGPRGIIDLSQVPHLPVLVPPTPGTPATAMDRLAYLPTAPQPFSSRHSSSPLSPGGPTHLTKPTTTSSSERERDRDRERDRDREREKSILTSTTTVEHAPIWRPGTEQSSGSSGSSGGGGGSSSRPASHSHAHQHSPISPRTQDALQQRPSVLHNTGMKGIITAVEPSTPTVLRSTSTSSPVRPAATFPPATHCPLGGTLDGVYPTLMEPVLLPKEAPRVARPERPRADTGHAFLAKPPARSGLEPASSPSKGSEPRPLVPPVSGHATIARTPAKNLAPHHASPDPPAPPASASDPHREKTQSKPFSIQELELRSLGYHGSSYSPEGVEPVSPVSSPSLTHDKGLPKHLEELDKSHLEGELRPKQPGPVKLGGEAAHLPHLRPLPESQPSSSPLLQTAPGVKGHQRVVTLAQHISEVITQDYTRHHPQQLSAPLPAPLYSFPGASCPVLDLRRPPSDLYLPPPDHGAPARGSPHSEGGKRSPEPNKTSVLGGGEDGIEPVSPPEGMTEPGHSRSAVYPLLYRDGEQTEPSRMGSKSPGNTSQPPAFFSKLTESNSAMVKSKKQEINKKLNTHNRNEPEYNISQPGTEIFNMPAITGTGLMTYRSQAVQEHASTNMGLEAIIRKALMGKYDQWEESPPLSANAFNPLNASASLPAAMPITAADGRSDHTLTSPGGGGKAKVSGRPSSRKAKSPAPGLASGDRPPSVSSVHSEGDCNRRTPLTNRVWEDRPSSAGSTPFPYNPLIMRLQAGVMASPPPPGLPAGSGPLAGPHHAWDEEPKPLLCSQYETLSDSE

>NP_068810.2

MDELFPLIFPAEPAQASGPYVEIIEQPKQRGMRFRYKCEGRSAGSIPGERSTDTTKTHPTIKINGYTGPGTVRISLVTKDPPHRPHPHELVGKDCRDGFYEAELCPDRCIHSFQNLGIQCVKKRDLEQAISQRIQTNNNPFQVPIEEQRGDYDLNAVRLCFQVTVRDPSGRPLRLPPVLPHPIFDNRAPNTAELKICRVNRNSGSCLGGDEIFLLCDKVQKEDIEVYFTGPGWEARGSFSQADVHRQVAIVFRTPPYADPSLQAPVRVSMQLRRPSDRELSEPMEFQYLPDTDDRHRIEEKRKRTYETFKSIMKKSPFSGPTDPRPPPRRIAVPSRSSASVPKPAPQPYPFTSSLSTINYDEFPTMVFPSGQISQASALAPAPPQVLPQAPAPAPAPAMVSALAQAPAPVPVLAPGPPQAVAPPAPKPTQAGEGTLSEALLQLQFDDEDLGALLGNSTDPAVFTDLASVDNSEFQQLLNQGIPVAPHTTEPMLMEYPEAITRLVTGAQRPPDPAPAPLGAPGLPNGLLSGDEDFSSIADMDFSALLSQISS

1424 NP_056981.2 NP_005893.1

>NP_056981.2

MAAELVEAKNMVMSFRVSDLQMLLGFVGRSKSGLKHELVTRALQLVQFDCSPELFKKIKELYETRYAKKNSEPAPQPHRPLDPLTMHSTYDRAGAVPRTPLAGPNIDYPVLYGKYLNGLGRLPAKTLKPEVRLVKLPFFNMLDELLKPTELVPQNNEKLQESPCIFALTPRQVELIRNSRELQPGVKAVQVVLRICYSDTSCPQEDQYPPNIAVKVNHSYCSVPGYYPSNKPGVEPKRPCRPINLTHLMYLSSATNRITVTWGNYGKSYSVALYLVRQLTSSELLQRLKTIGVKHPELCKALVKEKLRLDPDSEIATTGVRVSLICPLVKMRLSVPCRAETCAHLQCFDAVFYLQMNEKKPTWMCPVCDKPAPYDQLIIDGLLSKILSECEDADEIEYLVDGSWCPIRAEKERSCSPQGAILVLGPSDANGLLPAPSVNGSGALGSTGGGGPVGSMENGKPGADVVDLTLDSSSSSEDEEEEEEEEEDEDEEGPRPKRRCPFQKGLVPAC

>NP_005893.1

MSSILPFTPPIVKRLLGWKKGEQNGQEEKWCEKAVKSLVKKLKKTGQLDELEKAITTQNVNTKCITIPRSLDGRLQVSHRKGLPHVIYCRLWRWPDLHSHHELRAMELCEFAFNMKKDEVCVNPYHYQRVETPVLPPVLVPRHTEIPAEFPPLDDYSHSIPENTNFPAGIEPQSNIPETPPPGYLSEDGETSDHQMNHSMDAGSPNLSPNPMSPAHNNLDLQPVTYCEPAFWCSISYYELNQRVGETFHASQPSMTVDGFTDPSNSERFCLGLLSNVNRNAAVELTRRHIGRGVRLYYIGGEVFAECLSDSAIFVQSPNCNQRYGWHPATVCKIPPGCNLKIFNNQEFAALLAQSVNQGFEAVYQLTRMCTIRMSFVKGWGAEYRRQTVTSTPCWIELHLNGPLQWLDKVLTQMGSPSIRCSSVS

1425 NP_002721.1 NP_003591.2

>NP_002721.1

MGNAAAAKKGSEQESVKEFLAKAKEDFLKKWESPAQNTAHLDQFERIKTLGTGSFGRVMLVKHKETGNHYAMKILDKQKVVKLKQIEHTLNEKRILQAVNFPFLVKLEFSFKDNSNLYMVMEYVPGGEMFSHLRRIGRFSEPHARFYAAQIVLTFEYLHSLDLIYRDLKPENLLIDQQGYIQVTDFGFAKRVKGRTWTLCGTPEYLAPEIILSKGYNKAVDWWALGVLIYEMAAGYPPFFADQPIQIYEKIVSGKVRFPSHFSSDLKDLLRNLLQVDLTKRFGNLKNGVNDIKNHKWFATTDWIAIYQRKVEAPFIPKFKGPGDTSNFDDYEEEEIRVSINEKCGKEFSEF

>NP_003591.2
[truncated: 49,554,568 more chars]
