# Supplementary material for: A novel matrix of sequence descriptors for predicting protein-protein interactions from amino acid sequences
Source: PLoS One. 2019 Jun 7;14(6):e0217312. doi: 10.1371/journal.pone.0217312 (PMC6555512; doi:10.1371/journal.pone.0217312)
Supplement: S2 File — There are 36,480 protein-protein pairs from total 2184 proteins, and the first column is protein ID, the second column is the other protein ID and the two proteins constitute the negative Protein-protein interaction. (DOC) [file pone.0217312.s002.doc]

Online support information B

There are 36,480 protein-protein pairs from total 2184 proteins, and the first column is protein ID, the second column is the other protein ID and the two proteins constitute the negative Protein-protein interaction.

Index	Protein_1_ID	protein_2_ID

1 Q8NCN2 P14598

>Q8NCN2

MSVEMDSSSFIQFDVPEYSSTVLSQLNELRLQGKLCDIIVHIQGQPFRAHKAVLAASSPYFRDHSALSTMSGLSISVIKNPNVFEQLLSFCYTGRMSLQLKDVVSFLTAASFLQMQCVIDKCTQILESIHSKISVGDVDSVTVGAEENPESRNGVKDSSFFANPVEISPPYCSQGRQPTASSDLRMETTPSKALRSRLQEEGHSDRGSSGSVSEYEIQIEGDHEQGDLLVRESQITEVKVKMEKSDRPSCSDSSSLGDDGYHTEMVDGEQVVAVNVGSYGSVLQHAYSYSQAASQPTNVSEAFGSLSNSSPSRSMLSCFRGGRARQKRALSVHLHSDLQGLVQGSDSEAMMNNPGYESSPRERSARGHWYPYNERLICIYCGKSFNQKGSLDRHMRLHMGITPFVCKFCGKKYTRKDQLEYHIRGHTDDKPFRCEICGKCFPFQGTLNQHLRKNHPGVAEVRSRIESPERTDVYVEQKLENDASASEMGLDSRMEIHTVSDAPD

>P14598

MGDTFIRHIALLGFEKRFVPSQHYVYMFLVKWQDLSEKVVYRRFTEIYEFHKTLKEMFPIEAGAINPENRIIPHLPAPKWFDGQRAAENRQGTLTEYCSTLMSLPTKISRCPHLLDFFKVRPDDLKLPTDNQTKKPETYLMPKDGKSTATDITGPIILQTYRAIADYEKTSGSEMALSTGDVVEVVEKSESGWWFCQMKAKRGWIPASFLEPLDSPDETEDPEPNYAGEPYVAIKAYTAVEGDEVSLLEGEAVEVIHKLLDGWWVIRKDDVTGYFPSMYLQKSGQDVSQAQRQIKRGAPPRRSSIRNAHSIHQRSRKRLSQDAYRRNSVRFLQQRRRQARPGPQSPGSPLEEERQTQRSKPQPAVPPRPSADLILNRCSESTKRKLASAV

2 NP_002189.1 NP_004160.3

>NP_002189.1

MPITRMRMRPWLEMQINSNQIPGLIWINKEEMIFQIPWKHAAKHGWDINKDACLFRSWAIHTGRYKAGEKEPDPKTWKANFRCAMNSLPDIEEVKDQSRNKGSSAVRVYRMLPPLTKNQRKERKSKSSRDAKSKAKRKSCGDSSPDTFSDGLSSSTLPDDHSSYTVPGYMQDLEVEQALTPALSPCAVSSTLPDWHIPVEVVPDSTSDLYNFQVSPMPSTSEATTDEDEEGKLPEDIMKLLEQSEWQPTNVDGKGYLLNEPGVQPTSVYGDFSCKEEPEIDSPGGDIGLSLQRVFTDLKNMDATWLDSLLTPVRLPSIQAIPCAP

>NP_004160.3

MTMPVNGAHKDADLWSSHDKMLAQPLKDSDVEVYNIIKKESNRQRVGLELIASENFASRAVLEALGSCLNNKYSEGYPGQRYYGGTEFIDELETLCQKRALQAYKLDPQCWGVNVQPYSGSPANFAVYTALVEPHGRIMGLDLPDGGHLTHGFMTDKKKISATSIFFESMPYKVNPDTGYINYDQLEENARLFHPKLIIAGTSCYSRNLEYARLRKIADENGAYLMADMAHISGLVAAGVVPSPFEHCHVVTTTTHKTLRGCRAGMIFYRKGVKSVDPKTGKEILYNLESLINSAVFPGLQGGPHNHAIAGVAVALKQAMTLEFKVYQHQVVANCRALSEALTELGYKIVTGGSDNHLILVDLRSKGTDGGRAEKVLEACSIACNKNTCPGDRSALRPSGLRLGTPALTSRGLLEKDFQKVAHFIHRGIELTLQIQSDTGVRATLKEFKERLAGDKYQAAVQALREEVESFASLFPLPGLPDF

3 P39880 NP_002305.1

>P39880

MLCVRGARLKRELDATATVLANRQDESEQSRKRLIEQSREFKKNTPEDLRKQVAPLLKSFQGEIDALSKRSKEAEAAFLNVYKRLIDVPDPVPALDLGQQLQLKVQRLHDIETENQKLRETLEEYNKEFAEVKNQEVTIKALKEKIREYEQTLKNQAETIALEKEQKLQNDFAEKERKLQETQMSTTSKLEEAEHKVQSLQTALEKTRTELFDLKTKYDEETTAKADEIEMIMTDLERANQRAEVAQREAETLREQLSSANHSLQLASQIQKAPDVEQAIEVLTRSSLEVELAAKEREIAQLVEDVQRLQASLTKLRENSASQISQLEQQLSAKNSTLKQLEEKLKGQADYEEVKKELNILKSMEFAPSEGAGTQDAAKPLEVLLLEKNRSLQSENAALRISNSDLSGSARRKGKDQPESRRPGSLPAPPPSQLPRNPGEQASNTNGTHQFSPAGLSQDFFSSSLASPSLPLASTGKFALNSLLQRQLMQSFYSKAMQEAGSTSMIFSTGPYSTNSISSQSPLQQSPDVNGMAPSPSQSESAGSVSEGEEMDTAEIARQVKEQLIKHNIGQRIFGHYVLGLSQGSVSEILARPKPWNKLTVRGKEPFHKMKQFLSDEQNILALRSIQGRQRENPGQSLNRLFQEVPKRRNGSEGNITTRIRASETGSDEAIKSILEQAKRELQVQKTAEPAQPSSASGSGNSDDAIRSILQQARREMEAQQAALDPALKQAPLSQSDITILTPKLLSTSPMPTVSSYPPLAISLKKPSAAPEAGASALPNPPALKKEAQDAPGLDPQGAADCAQGVLRQVKNEVGRSGAWKDHWWSAVQPERRNAASSEEAKAEETGGGKEKGSGGSGGGSQPRAERSQLQGPSSSEYWKEWPSAESPYSQSSELSLTGASRSETPQNSPLPSSPIVPMSKPTKPSVPPLTPEQYEVYMYQEVDTIELTRQVKEKLAKNGICQRIFGEKVLGLSQGSVSDMLSRPKPWSKLTQKGREPFIRMQLWLNGELGQGVLPVQGQQQGPVLHSVTSLQDPLQQGCVSSESTPKTSASCSPAPESPMSSSESVKSLTELVQQPCPPIEASKDSKPPEPSDPPASDSQPTTPLPLSGHSALSIQELVAMSPELDTYGITKRVKEVLTDNNLGQRLFGETILGLTQGSVSDLLARPKPWHKLSLKGREPFVRMQLWLNDPNNVEKLMDMKRMEKKAYMKRRHSSVSDSQPCEPPSVGTEYSQGASPQPQHQLKKPRVVLAPEEKEALKRAYQQKPYPSPKTIEDLATQLNLKTSTVINWFHNYRSRIRRELFIEEIQAGSQGQAGASDSPSARSGRAAPSSEGDSCDGVEATEGPGSADTEEPKSQGEAEREEVPRPAEQTEPPPSGTPGPDDARDDDHEGGPVEGPGPLPSPASATATAAPAAPEDAATSAAAAPGEGPAAPSSAPPPSNSSSSSAPRRPSSLQSLFGLPEAAGARDSRDNPLRKKKAANLNSIIHRLEKAASREEPIEWEF

>NP_002305.1

MRLTLLCCTWREERMGEEGSELPVCASCGQRIYDGQYLQALNADWHADCFRCCDCSASLSHQYYEKDGQLFCKKDYWARYGESCHGCSEQITKGLVMVAGELKYHPECFICLTCGTFIGDGDTYTLVEHSKLYCGHCYYQTVVTPVIEQILPDSPGSHLPHTVTLVSIPASSHGKRGLSVSIDPPHGPPGCGTEHSHTVRVQGVDPGCMSPDVKNSIHVGDRILEINGTPIRNVPLDEIDLLIQETSRLLQLTLEHDPHDTLGHGLGPETSPLSSPAYTPSGEAGSSARQKPVLRSCSIDRSPGAGSLGSPASQRKDLGRSESLRVVCRPHRIFRPSDLIHGEVLGKGCFGQAIKVTHRETGEVMVMKELIRFDEETQRTFLKEVKVMRCLEHPNVLKFIGVLYKDKRLNFITEYIKGGTLRGIIKSMDSQYPWSQRVSFAKDIASGMAYLHSMNIIHRDLNSHNCLVRENKNVVVADFGLARLMVDEKTQPEGLRSLKKPDRKKRYTVVGNPYWMAPEMINGRSYDEKVDVFSFGIVLCEIIGRVNADPDYLPRTMDFGLNVRGFLDRYCPPNCPPSFFPITVRCCDLDPEKRPSFVKLEHWLETLRMHLAGHLPLGPQLEQLDRGFWETYRRGESGLPAHPEVPD

4 NP_001511.2 Q08495

>NP_001511.2

MDALESLLDEVALEGLDGLCLPALWSRLETRVPPFPLPLEPCTQEFLWRALATHPGISFYEEPRERPDLQLQDRYEEIDLETGILESRRDPVALEDVYPIHMILENKDGIQGSCRYFKERKNITNDIRTKSLQPRCTMVEAFDRWGKKLIIVASQAMRYRALIGQEGDPDLKLPDFSYCILERLGRSRWQGELQRDLHTTAFKVDAGKLHYHRKILNKNGLITMQSHVIRLPTGAQQHSILLLLNRFHVDRRSKYDILMEKLSVMLSTRTNHIETLGKLREELGLCERTFKRLYQYMLNAGLAKVVSLRLQEIHPECGPCKTKKGTDVMVRCLKLLKEFKRNDHDDDEDEEVISKTVPPVDIVFERDMLTQTYDLIERRGTKGISQAEIRVAMNVGKLEARMLCRLLQRFKVVKGFMEDEGRQRTTKYISCVFAEESDLSRQYQREKARSELLTTVSLASMQEESLLPEGEDTFLSESDSEEERSSSKRRGRGSQKDTRASANLRPKTQPHHSTPTKGGWKVVNLHPLKKQPPSFPGAAEERACQSLASRDSLLDTSSVSEPNVSFVSHCADSNSGDIAVIEEVRMENPKESSSSLKTGRHSSGQDKPHETYRLLKRRNLIIEAVTNLRLIESLFTIQKMIMDQEKQEGVSTKCCKKSIVRLVRNLSEEGLLRLYRTTVIQDGIKKKVDLVVHPSMDQNDPLVRSAIEQVRFRISNSSTANRVKTSQPPVPQGEAEEDSQGKEGPSGSGDSQLSASSRSESGRMKKSDNKMGITPLRNYHPIVVPGLGRSLGFLPKMPRLRVVHMFLWYLIYGHPASNTVEKPSFISERRTIKQESGRAGVRPSSSGSAWEACSEAPSKGSQDGVTWEAEVELATETVYVDDASWMRYIPPIPVHRDFGFGWALVSDILLCLPLSIFIQIVQVSYKVDNLEEFLNDPLKKHTLIRFLPRPIRQQLLYKRRYIFSVVENLQRLCYMGLLQFGPTEKFQDKDQVFIFLKKNAVIVDTTICDPHYNLARSSRPFERRLYVLNSMQDVENYWFDLQCVCLNTPLGVVRCPRVRKNSSTDQGSDEEGSLQKEQESAMDKHNLERKCAMLEYTTGSREVVDEGLIPGDGLGAAGLDSSFYGHLKRNWIWTSYIINQAKKENTAAENGLTVRLQTFLSKRPMPLSARGNSRLNIWGEARVGSELCAGWEEQFEVDREPSLDRNRRVRGGKSQKRKRLKKDPGKKIKRKKKGEFPGEKSKRLRYHDEADQSALQRMTRLRVTWSMQEDGLLVLCRIASNVLNTKVKGPFVTWQVVRDILHATFEESLDKTSHSVGRRARYIVKNPQAYLNYKVCLAEVYQDKALVGDFMNRRGDYDDPKVCANEFKEFVEKLKEKFSSALRNSNLEIPDTLQELFARYRVLAIGDEKDQTRKEDELNSVDDIHFLVLQNLIQSTLALSDSQMKSYQSFQTFRLYREYKDHVLVKAFMECQKRSLVNRRRVNHTLGPKKNRALPFVPMSYQLSQTYYRIFTWRFPSTICTESFQFLDRMRAAGKLDQPDRFSFKDQDNNEPTNDMVAFSLDGPGGNCVAVLTLFSLGLISVDVRIPEQIIVVDSSMVENEVIKSLGKDGSLEDDEDEEDDLDEGVGGKRRSMEVKPAQASHTNYLLMRGYYSPGIVSTRNLNPNDSIVVNSCQMKFQLRCTPVPARLRPAAAPLEELTMGTSCLPDTFTKLINPQENTCSLEEFVLQLELSGYSPEDLTAALEILEAIIATGCFGIDKEELRRRFSALEKAGGGRTRTFADCIQALLEQHQVLEVGGNTARLVAMGSAWPWLLHSVRLKDREDADIQREDPQARPLEGSSSEDSPPEGQAPPSHSPRGTKRRASWASENGETDAEGTQMTPAKRPALQDSNLAPSLGPGAEDGAEAQAPSPPPALEDTAAAGAAQEDQEGVGEFSSPGQEQLSGQAQPPEGSEDPRGFTESFGAANISQAARERDCESVCFIGRPWRVVDGHLNLPVCKGMMEAMLYHIMTRPGIPESSLLRHYQGVLQPVAVLELLQGLESLGCIRKRWLRKPRPVSLFSTPVVEEVEVPSSLDESPMAFYEPTLDCTLRLGRVFPHEVNWNKWIHL

>Q08495

MERLQKQPLTSPGSVSPSRDSSVPGSPSSIVAKMDNQVLGYKDLAAIPKDKAILDIERPDLMIYEPHFTYSLLEHVELPRSRERSLSPKSTSPPPSPEVWADSRSPGIISQASAPRTTGTPRTSLPHFHHPETSRPDSNIYKKPPIYKQRESVGGSPQTKHLIEDLIIESSKFPAAQPPDPNQPAKIETDYWPCPPSLAVVETEWRKRKASRRGAEEEEEEEDDDSGEEMKALRERQREELSKVTSNLGKMILKEEMEKSLPIRRKTRSLPDRTPFHTSLHQGTSKSSSLPAYGRTTLSRLQSTEFSPSGSETGSPGLQNGEGQRGRMDRGNSLPCVLEQKIYPYEMLVVTNKGRTKLPPGVDRMRLERHLSAEDFSRVFAMSPEEFGKLALWKRNELKKKASLF

5 NP_005490.1 Q9Y2D0

>NP_005490.1

MALSRGLPRELAEAVAGGRVLVVGAGGIGCELLKNLVLTGFSHIDLIDLDTIDVSNLNRQFLFQKKHVGRSKAQVAKESVLQFYPKANIVAYHDSIMNPDYNVEFFRQFILVMNALDNRAARNHVNRMCLAADVPLIESGTAGYLGQVTTIKKGVTECYECHPKPTQRTFPGCTIRNTPSEPIHCIVWAKYLFNQLFGEEDADQEVSPDRADPEAAWEPTEAEARARASNEDGDIKRISTKEWAKSTGYDPVKLFTKLFKDDIRYLLTMDKLWRKRKPPVPLDWAEVQSQGEETNASDQQNEPQLGLKDQQVLDVKSYARLFSKSIETLRVHLAEKGDGAELIWDKDDPSAMDFVTSAANLRMHIFSMNMKSRFDIKSMAGNIIPAIATTNAVIAGLIVLEGLKILSGKIDQCRTIFLNKQPNPRKKLLVPCALDPPNPNCYVCASKPEVTVRLNVHKVTVLTLQDKIVKEKFAMVAPDVQIEDGKGTILISSEEGETEANNHKKLSEFGIRNGSRLQADDFLQDYTLLINILHSEDLGKDVEFEVVGDAPEKVGPKQAEDAAKSITNGSDDGAQPSTSTAQEQDDVLIVDSDEEDSSNNADVSEEERSRKRKLDEKENLSAKRSRIEQKEELDDVIALD

>Q9Y2D0

MVVMNSLRVILQASPGKLLWRKFQIPRFMPARPCSLYTCTYKTRNRALHPLWESVDLVPGGDRQSPINIRWRDSVYDPGLKPLTISYDPATCLHVWNNGYSFLVEFEDSTDKSVIKGGPLEHNYRLKQFHFHWGAIDAWGSEHTVDSKCFPAELHLVHWNAVRFENFEDAALEENGLAVIGVFLKLGKHHKELQKLVDTLPSIKHKDALVEFGSFDPSCLMPTCPDYWTYSGSLTTPPLSESVTWIIKKQPVEVDHDQLEQFRTLLFTSEGEKEKRMVDNFRPLQPLMNRTVRSSFRHDYVLNVQAKPKPATSQATP

6 NP_004070.3 NP_000846.1

>NP_004070.3

MKRLVCVLLVCSSAVAQLHKDPTLDHHWHLWKKTYGKQYKEKNEEAVRRLIWEKNLKFVMLHNLEHSMGMHSYDLGMNHLGDMTSEEVMSLMSSLRVPSQWQRNITYKSNPNRILPDSVDWREKGCVTEVKYQGSCGACWAFSAVGALEAQLKLKTGKLVSLSAQNLVDCSTEKYGNKGCNGGFMTTAFQYIIDNKGIDSDASYPYKAMDQKCQYDSKYRAATCSKYTELPYGREDVLKEAVANKGPVSVGVDARHPSFFLYRSGVYYEPSCTQNVNHGVLVVGYGDLNGKEYWLVKNSWGHNFGEEGYIRMARNKGNHCGIASFPSYPEI

>NP_000846.1

MSRRKISSESFSSLGSDYLETSPEEEGECPLSRLCWNGSRSPPGPLEPSPAAAAAAAAPAPTPAASAAAAAATAGARRVQRRRRVNLDSLGESISRLTAPSPQTIQQTLKRTLQYYEHQVIGYRDAEKNFHNISNRCSYADHSNKEEIEDVSGILQCTANILGLKFEEIQKRFGEEFFNICFHENERVLRAVGGTLQDFFNGFDALLEHIRTSFGKQATLESPSFLCKELPEGTLMLHYFHPHHIVGFAMLGMIKAAGKKIYRLDVEVEQVANEKLCSDVSNPGNCSCLTFLIKECENTNIMKNLPQGTSQVPADLRISINTFCRAFPFHLMFDPSMSVLQLGEGLRKQLRCDTHKVLKFEDCFEIVSPKVNATFERVLLRLSTPFVIRTKPEASGSENKDKVMEVKGQMIHVPESNSILFLGSPCVDKLDELMGRGLHLSDIPIHDATRDVILVGEQAKAQDGLKKRMDKLKATLERTHQALEEEKKKTVDLLYSIFPGDVAQQLWQGQQVQARKFDDVTMLFSDIVGFTAICAQCTPMQVISMLNELYTRFDHQCGFLDIYKVETIGDAYCVAAGLHRKSLCHAKPIALMALKMMELSEEVLTPDGRPIQMRIGIHSGSVLAGVVGVRMPRYCLFGNNVTLASKFESGSHPRRINVSPTTYQLLKREESFTFIPRSREELPDNFPKEIPGICYFLEVRTGPKPPKPSLSSSRIKKVSYNIGTMFLRETSL

7 NP_003073.1 NP_000365.3

>NP_003073.1

MGTPPGLQTDCEALLSRFQETDSVRFEDFTELWRNMKFGTIFCGRMRNLEKNMFTKEALALAWRYFLPPYTFQIRVGALYLLYGLYNTQLCQPKQKIRVALKDWDEVLKFQQDLVNAQHFDAAYIFRKLRLDRAFHFTAMPKLLSYRMKKKIHRAEVTEEFKDPSDRVMKLITSDVLEEMLNVHDHYQNMKHVISVDKSKPDKALSLIKDDFFDNIKNIVLEHQQWHKDRKNPSLKSKTNDGEEKMEGNSQETERCERAESLAKIKSKAFSVVIQASKSRRHRQVKLDSSDSDSASGQGQVKATRKKEKKERLKPAGRKMSLRNKGNVQNIHKEDKPLSLSMPVITEEEENESLSGTEFTASKKRRKH

>NP_000365.3

MEANGLGPQGFPELKNDTFLRAAWGEETDYTPVWCMRQAGRYLPEFRETRAAQDFFSTCRSPEACCELTLQPLRRFPLDAAIIFSDILVVPQALGMEVTMVPGKGPSFPEPLREEQDLERLRDPEVVASELGYVFQAITLTRQRLAGRVPLIGFAGAPWTLMTYMVEGGGSSTMAQAKRWLYQRPQASHQLLRILTDALVPYLVGQVVAGAQALQLFESHAGHLGPQLFNKFALPYIRDVAKQVKARLREAGLAPVPMIIFAKDGHFALEELAQAGYEVVGLDWTVAPKKARECVGKTVTLQGNLDPCALYASEEEIGQLVKQMLDDFGPHRYIANLGHGLYPDMDPEHVGAFVDAVHKHSRLLRQN

8 NP_055206.2 Q8N183

>NP_055206.2

MMVLKVEELVTGKKNGNGEAGEFLPEDFRDGEYEAAVTLEKQEDLKTLLAHPVTLGEQQWKSEKQREAELKKKKLEQRSKLENLEDLEIIIQLKKRKKYRKTKVPVVKEPEPEIITEPVDVPTFLKAALENKLPVVEKFLSDKNNPDVCDEYKRTALHRACLEGHLAIVEKLMEAGAQIEFRDMLESTAIHWASRGGNLDVLKLLLNKGAKISARDKLLSTALHVAVRTGHYECAEHLIACEADLNAKDREGDTPLHDAVRLNRYKMIRLLIMYGADLNIKNCAGKTPMDLVLHWQNGTKAIFDSLRENSYKTSRIATF

>Q8N183

MGWSQDLFRALWRSLSREVKEHVGTDQFGNKYYYIPQYKNWRGQTIREKRIVEAANKKEVDYEAGDIPTEWEAWIRRTRKTPPTMEEILKNEKHREEIKIKSQDFYEKEKLLSKETSEELLPPPVQTQIKGHASAPYFGKEEPSVAPSSTGKTFQPGSWMPRDGKSHNQ

9 NP_000624.2 NP_068815.2

>NP_000624.2

MAHAGRTGYDNREIVMKYIHYKLSQRGYEWDAGDVGAAPPGAAPAPGIFSSQPGHTPHPAASRDPVARTSPLQTPAAPGAAAGPALSPVPPVVHLTLRQAGDDFSRRYRRDFAEMSSQLHLTPFTARGRFATVVEELFRDGVNWGRIVAFFEFGGVMCVESVNREMSPLVDNIALWMTEYLNRHLHTWIQDNGGWDAFVELYGPSMRPLFDFSWLSLKTLLSLALVGACITLGAYLGHK

>NP_068815.2

MSHQPLSCLTEKEDSPSESTGNGPPHLAHPNLDTFTPEELLQQMKELLTENHQLKEAMKLNNQAMKGRFEELSAWTEKQKEERQFFEIQSKEAKERLMALSHENEKLKEELGKLKGKSERSSEDPTDDSRLPRAEAEQEKDQLRTQVVRLQAEKADLLGIVSELQLKLNSSGSSEDSFVEIRMAEGEAEGSVKEIKHSPGPTRTVSTGTALSKYRSRSADGAKNYFEHEELTVSQLLLCLREGNQKVERLEVALKEAKERVSDFEKKTSNRSEIETQTEGSTEKENDEEKGPETVGSEVEALNLQVTSLFKELQEAHTKLSEAELMKKRLQEKCQALERKNSAIPSELNEKQELVYTNKKLELQVESMLSEIKMEQAKTEDEKSKLTVLQMTHNKLLQEHNNALKTIEELTRKESEKVDRAVLKELSEKLELAEKALASKQLQMDEMKQTIAKQEEDLETMTILRAQMEVYCSDFHAERAAREKIHEEKEQLALQLAVLLKENDAFEDGGRQSLMEMQSRHGARTSDSDQQAYLVQRGAEDRDWRQQRNIPIHSCPKCGEVLPDIDTLQIHVMDCII

10 NP_009171.1 P57723

>NP_009171.1

MLEAPGPSDGCELSNPSASRVSCAGQMLEVQPGLYFGGAAAVAEPDHLREAGITAVLTVDSEEPSFKAGPGVEDLWRLFVPALDKPETDLLSHLDRCVAFIGQARAEGRAVLVHCHAGVSRSVAIITAFLMKTDQLPFEKAYEKLQILKPEAKMNEGFEWQLKLYQAMGYEVDTSSAIYKQYRLQKVTEKYPELQNLPQELFAVDPTTVSQGLKDEVLYKCRKCRRSLFRSSSILDHREGSGPIAFAHKRMTPSSMLTTGRQAQCTSYFIEPVQWMESALLGVMDGQLLCPKCSAKLGSFNWYGEQCSCGRWITPAFQIHKNRVDEMKILPVLGSQTGKI

>P57723

MSGSDGGLEEEPELSITLTLRMLMHGKEVGSIIGKKGETVKRIREQSSARITISEGSCPERITTITGSTAAVFHAVSMIAFKLDEDLCAAPANGGNVSRPPVTLRLVIPASQCGSLIGKAGTKIKEIRETTGAQVQVAGDLLPNSTERAVTVSGVPDAIILCVRQICAVILESPPKGATIPYHPSLSLGTVLLSANQGFSVQGQYGAVTPAEVTKLQQLSSHAVPFATPSVVPGLDPGTQTSSQEFLVPNDLIGCVIGRQGSKISEIRQMSGAHIKIGNQAEGAGERHVTITGSPVSIALAQYLITACLETAKSTSGGTPSSAPADLPAPFSPPLTALPTAPPGLLGTPYAISLSNFIGLKPMPFLALPPASPGPPPGLAAYTAKMAAANGSKKAERQKFSPY

11 NP_004102.1 Q9Y291

>NP_004102.1

MGIQGLAKLIADVAPSAIRENDIKSYFGRKVAIDASMSIYQFLIAVRQGGDVLQNEEGETTSHLMGMFYRTIRMMENGIKPVYVFDGKPPQLKSGELAKRSERRAEAEKQLQQAQAAGAEQEVEKFTKRLVKVTKQHNDECKHLLSLMGIPYLDAPSEAEASCAALVKAGKVYAAATEDMDCLTFGSPVLMRHLTASEAKKLPIQEFHLSRILQELGLNQEQFVDLCILLGSDYCESIRGIGPKRAVDLIQKHKSIEEIVRRLDPNKYPVPENWLHKEAHQLFLEPEVLDPESVELKWSEPNEEELIKFMCGEKQFSEERIRSGVKRLSKSRQGSTQGRLDDFFKVTGSLSSAKRKEPEPKGSTKKKAKTGAAGKFKRGK

>Q9Y291

MSSLSEYAFRMSRLSARLFGEVTRPTNSKSMKVVKLFSELPLAKKKETYDWYPNHHTYAELMQTLRFLGLYRDEHQDFMDEQKRLKKLRGKEKPKKGEGKRAAKRK

12 Q5VUA4 NP_055165.2

>Q5VUA4

MYRSSARSSVSSHRPKDDGGGGPRSGRSSGSSSGPARRSSPPPPPSGSSSRTPARRPRSPSGHRGRRASPSPPRGRRVSPSPPRARRGSPSPPRGRRLFPPGPAGFRGSSRGESRADYARDGRGDHPGDSGSRRRSPGLCSDSLEKSLRITVGNDHFCVSTPERRRLSDRLGSPVDNLEDMDRDDLTDDSVFTRSSQCSRGLERYISQEEGPLSPFLGQLDEDYRTKETFLHRSDYSPHISCHDELLRGTERNREKLKGYSIRSEERSREAKRPRYDDTVKINSMGGDHPSFTSGTRNYRQRRRSPSPRFLDPEFRELDLARRKREEEEERSRSLSQELVGVDGGGTGCSIPGLSGVLTASEPGYSLHRPEEVSVMPKKSILKKRIEVDIMEPSMQLESFSSSTSSSQDHPLYSGHPSLPLSGAIAAFASEIENKGTMVETALKEPQGNLYQWGPLPGIPKDNSPLREKFGSFLCHKDNLDLKAEGPERHTDFLLPHERASQDGSGFSRILSMLADSTSTQEKRRRSFPDIEDEEKFLYGDEEEDLKAESVPKPLGSSESEVMRQKASSLPSSAPAVKLESLEETNPEYAKIHDLLKTIGLDIGVAEISQLAARTQERLHGKKPSLRSSADRRSSVDRYFSADHCSSVDHRFSADRCSSVDHCFSADRRSSDPHRLESREAHHSNTHSPEVSHPHPPSPVDPYLLTKNSPPFLKSDHPVGHISGPEVVGSGFQSSVAVRCMLPSAPSAPIRLPHTAALSQFHMPRASQFAAARIPPNYQGPAIPPASFDAYRHYMAYAASRWPMYPTSQPSNHPVPEPHRIMPITKQATRSRPNLRVIPTVTPDKPKQKESLRGSIPAAQVPVQVSIPSLIRYNPEKISDEKNRASQKQKVIEEREKLKNDREARQKKMYYLRTELERLHKQQGEMLRKKRREKDGHKDPLLVEVSRLQDNIMKDIAELRQEAEEAEKKQSELDKVAQILGINIFDKSQKSLSDSREPTEKPGKAEKSKSPEKVSSFSNSSSNKESKVNNEKFRTKSPKPAESPQSATKQLDQPTAAYEYYDAGNHWCKDCNTICGTMFDFFTHMHNKKHTQTLDPYNRPWASKTQSEAKQDAIKRTDKITVPAKGSEFLVPISGFYCQLCEEFLGDPISGEQHVKGHQHNEKYKKYVDENPLYEERRNLDRQAGLAVVLETERRRQSELKRKLSEKPKEEKKEKKAKAVKEVKEDDKVSEKLEDQLSEGRNSPEKAENKRNTGIKLQLKEEVKKESPTSSSFGKFSWKKPEKEEEKSSLVTPSISKEEILESSKDKEDGKTEAGKAKPIKIKLSGKTVVAHTSPWMPVVTTSTQTKIRPNLPIPSTVLRKSCSATMSKPAPLNTFLSIKSSGTTAKPLPVVKESSADLLLPPDIISKAFGGEEVILKGSPEEKVVLAEKSEPSHLPEQILPPPPPPPPPPPPPPPVIPHPAAPSAAQANAILAPVKSNPVVSQTLSPGFVGPNILNPVLPVAIMASAQPAAIPSDETAPGVSESDRDQTLFSVLVRPPPPLSSVFSEQAKKLEKRNSCLATANAKDLYDIFYSSGGKGAPETKGAPETKLSGGPLANGENSNLSRTKSSDTSSTSPLNSSASQEELHQDEGLVAAPIVSNSEKPIAKTLVALGKWSVVEHVGPKSTGSTYGFLQPLTRLCQSRPYETITPKTDTLAIWTSSSFQSDTSRDISPEKSELDLGEPGPPGVEPPPQLLDIQCKESQKLVEIHLRESVNQDKESQELRKSEDCRESEIETNTELKERVKELSEGIVDEGVSTSIGPHSIDDSNLNHGNRYMWEGEVKQPNLLMIDKEAEQSNKLMTGSETPSKVVIKLSPQACSFTKAKLDSFLSEARSLLNPQDTPVKISAPELLLHSPARSAMCLTGSPQEQGVSVVSEEGLENSAPESASRTSRYRSLKLKRERSKDFQVKKIYELAVWDENKKRPETWESPEKPKTEALELQDVHPELTVTIESKALEDFEATDLKVEELTALGNLGDMPVDFCTTRVSPAHRSPTVLCQKVCEENSVSPIGCNSSDPADFEPIPSFSGFPLDSPKTLVLDFETEGERNSPNPRSVRIPSPNILKTGLTENVDRGLGGLEGTHQALDLLAGGMMPEEVKESSQLDKQESLGLELKTINSAGLGPSPCLPDLVDFVTRTSGVQKDKLCSPLSEPGDPSKCSSLELGPLQLEISNASTTEVAILQVDDDSGDPLNLVKAPVSRSPPREQVIEDNMVPQGMPEQETTVGAIQDHTESSVHN

>NP_055165.2

MHSEAEESKEVATDVFNSKNLAVQAQKKILGKMVSKSIATTLIDDTSSEVLDELYRVTREYTQNKKEAEKIIKNLIKTVIKLAILYRNNQFNQDELALMEKFKKKVHQLAMTVVSFHQVDYTFDRNVLSRLLNECREMLHQIIQRHLTAKSHGRVNNVFDHFSDCEFLAALYNPFGNFKPHLQKLCDGINKMLDEENI

13 NP_001941.2 NP_002717.3

>NP_001941.2

MAEAGPQAPPPPGTPSRHEKSLGLLTTKFVSLLQEAKDGVLDLKLAADTLAVRQKRRIYDITNVLEGIGLIEKKSKNSIQWKGVGPGCNTREIADKLIELKAEIEELQQREQELDQHKVWVQQSIRNVTEDVQNSCLAYVTHEDICRCFAGDTLLAIRAPSGTSLEVPIPEGLNGQKKYQIHLKSVSGPIEVLLVNKEAWSSPPVAVPVPPPEDLLQSPSAVSTPPPLPKPALAQSQEASRPNSPQLTPTAVPGSAEVQGMAGPAAEITVSGGPGTDSKDSGELSSLPLGPTTLDTRPLQSSALLDSSSSSSSSSSSSSNSNSSSSSGPNPSTSFEPIKADPTGVLELPKELSEIFDPTRECMSSELLEELMSSEVFAPLLRLSPPPGDHDYIYNLDESEGVCDLFDVPVLNL

>NP_002717.3

MLSLQYPDVYRDETAVQDYHGHKICDPYAWLEDPDSEQTKAFVEAQNKITVPFLEQCPIRGLYKERMTELYDYPKYSCHFKKGKRYFYFYNTGLQNQRVLYVQDSLEGEARVFLDPNILSDDGTVALRGYAFSEDGEYFAYGLSASGSDWVTIKFMKVDGAKELPDVLERVKFSCMAWTHDGKGMFYNSYPQQDGKSDGTETSTNLHQKLYYHVLGTDQSEDILCAEFPDEPKWMGGAELSDDGRYVLLSIREGCDPVNRLWYCDLQQESSGIAGILKWVKLIDNFEGEYDYVTNEGTVFTFKTNRQSPNYRVINIDFRDPEESKWKVLVPEHEKDVLEWIACVRSNFLVLCYLHDVKNILQLHDLTTGALLKTFPLDVGSIVGYSGQKKDTEIFYQFTSFLSPGIIYHCDLTKEELEPRVFREVTVKGIDASDYQTVQIFYPSKDGTKIPMFIVHKKGIKLDGSHPAFLYGYGGFNISITPNYSVSRLIFVRHMGGILAVANIRGGGEYGETWHKGGILANKQNCFDDFQCAAEYLIKEGYTSPKRLTINGGSNGGLLVAACANQRPDLFGCVIAQVGVMDMLKFHKYTIGHAWTTDYGCSDSKQHFEWLVKYSPLHNVKLPEADDIQYPSMLLLTADHDDRVVPLHSLKFIATLQYIVGRSRKQSNPLLIHVDTKAGHGAGKPTAKVIEEVSDMFAFIARCLNVDWIP

14 Q9BWT1 O75251

>Q9BWT1

MDARRVPQKDLRVKKNLKKFRYVKLISMETSSSSDDSCDSFASDNFANTRLQSVREGCRTRSQCRHSGPLRVAMKFPARSTRGATNKKAESRQPSENSVTDSNSDSEDESGMNFLEKRALNIKQNKAMLAKLMSELESFPGSFRGRHPLPGSDSQSRRPRRRTFPGVASRRNPERRARPLTRSRSRILGSLDALPMEEEEEEDKYMLVRKRKTVDGYMNEDDLPRSRRSRSSVTLPHIIRPVEEITEEELENVCSNSREKIYNRSLGSTCHQCRQKTIDTKTNCRNPDCWGVRGQFCGPCLRNRYGEEVRDALLDPNWHCPPCRGICNCSFCRQRDGRCATGVLVYLAKYHGFGNVHAYLKSLKQEFEMQA

>O75251

MAVLSAPGLRGFRILGLRSSVGPAVQARGVHQSVATDGPSSTQPALPKARAVAPKPSSRGEYVVAKLDDLVNWARRSSLWPMTFGLACCAVEMMHMAAPRYDMDRFGVVFRASPRQSDVMIVAGTLTNKMAPALRKVYDQMPEPRYVVSMGSCANGGGYYHYSYSVVRGCDRIVPVDIYIPGCPPTAEALLYGILQLQRKIKRERRLQIWYRR

15 NP_004134.1 NP_000317.1

>NP_004134.1

MPTTSRPALDVKGGTSPAKEDANQEMSSVAYSNLAVKDRKAVAILHYPGVASNGTKASGAPTSSSGSPIGSPTTTPPTKPPSFNLHPAPHLLASMQLQKLNSQYQGMAAATPGQPGEAGPLQNWDFGAQAGGAESLSPSAGAQSPAIIDSDPVDEEVLMSLVVELGLDRANELPELWLGQNEFDFTADFPSSC

>NP_000317.1

MSEGVGTFRMVPEEEQELRAQLEQLTTKDHGPVFGPCSQLPRHTLQKAKDELNEREETREEAVRELQEMVQAQAASGEELAVAVAERVQEKDSGFFLRFIRARKFNVGRAYELLRGYVNFRLQYPELFDSLSPEAVRCTIEAGYPGVLSSRDKYGRVVMLFNIENWQSQEITFDEILQAYCFILEKLLENEETQINGFCIIENFKGFTMQQAASLRTSDLRKMVDMLQDSFPARFKAIHFIHQPWYFTTTYNVVKPFLKSKLLERVFVHGDDLSGFYQEIDENILPSDFGGTLPKYDGKAVAEQLFGPQAQAENTAF

16 P36402 NP_000058.1

>P36402

MPQLDSGGGGAGGGDDLGAPDELLAFQDEGEEQDDKSRDSAAGPERDLAELKSSLVNESEGAAGGAGIPGVPGAGAGARGEAEALGREHAAQRLFPDKLPEPLEDGLKAPECTSGMYKETVYSAFNLLMHYPPPSGAGQHPQPQPPLHKANQPPHGVPQLSLYEHFNSPHPTPAPADISQKQVHRPLQTPDLSGFYSLTSGSMGQLPHTVSWFTHPSLMLGSGVPGHPAAIPHPAIVPPSGKQELQPFDRNLKTQAESKAEKEAKKPTIKKPLNAFMLYMKEMRAKVIAECTLKESAAINQILGRRWHALSREEQAKYYELARKERQLHMQLYPGWSARDNYGKKKRRSREKHQESTTETNWPRELKDGNGQESLSMSSSSSPA

>NP_000058.1

MSHHWGYGKHNGPEHWHKDFPIAKGERQSPVDIDTHTAKYDPSLKPLSVSYDQATSLRILNNGHAFNVEFDDSQDKAVLKGGPLDGTYRLIQFHFHWGSLDGQGSEHTVDKKKYAAELHLVHWNTKYGDFGKAVQQPDGLAVLGIFLKVGSAKPGLQKVVDVLDSIKTKGKSADFTNFDPRGLLPESLDYWTYPGSLTTPPLLECVTWIVLKEPISVSSEQVLKFRKLNFNGEGEPEELMVDNWRPAQPLKNRQIKASFK

17 NP_006684.1 NP_006635.2

>NP_006684.1

MQEIIASVDHIKFDLEIAVEQQLGAQPLPFPGMDKSGAAVCEFFLKAACGKGGMCPFRHISGEKTVVCKHWLRGLCKKGDQCEFLHEYDMTKMPECYFYSKFGECSNKECPFLHIDPESKIKDCPWYDRGFCKHGPLCRHRHTRRVICVNYLVGFCPEGPSCKFMHPRFELPMGTTEQPPLPQQTQPPAKQSNNPPLQRSSSLIQLTSQNSSPNQQRTPQVIGVMQSQNSSAGNRGPRPLEQVTCYKCGEKGHYANRCTKGHLAFLSGQ

>NP_006635.2

MSVVGLDVGSQSCYIAVARAGGIETIANEFSDRCTPSVISFGSKNRTIGVAAKNQQITHANNTVSNFKRFHGRAFNDPFIQKEKENLSYDLVPLKNGGVGIKVMYMGEEHLFSVEQITAMLLTKLKETAENSLKKPVTDCVISVPSFFTDAERRSVLDAAQIVGLNCLRLMNDMTAVALNYGIYKQDLPSLDEKPRIVVFVDMGHSAFQVSACAFNKGKLKVLGTAFDPFLGGKNFDEKLVEHFCAEFKTKYKLDAKSKIRALLRLYQECEKLKKLMSSNSTDLPLNIECFMNDKDVSGKMNRSQFEELCAELLQKIEVPLYSLLEQTHLKVEDVSAVEIVGGATRIPAVKERIAKFFGKDISTTLNADEAVARGCALQCAILSPAFKVREFSVTDAVPFPISLIWNHDSEDTEGVHEVFSRNHAAPFSKVLTFLRRGPFELEAFYSDPQGVPYPEAKIGRFVVQNVSAQKDGEKSRVKVKVRVNTHGIFTISTASMVEKVPTEENEMSSEADMECLNQRPPENPDTDKNVQQDNSEAGTQPQVQTDAQQTSQSPPSPELTSEENKIPDADKANEKKVDQPPEAKKPKIKVVNVELPIEANLVWQLGKDLLNMYIETEGKMIMQDKLEKERNDAKNAVEEYVYEFRDKLCGPYEKFICEQDHQNFLRLLTETEDWLYEEGEDQAKQAYVDKLEELMKIGTPVKVRFQEAEERPKMFEELGQRLQHYAKIAADFRNKDEKYNHIDESEMKKVEKSVNEVMEWMNNVMNAQAKKSLDQDPVVRAQEIKTKIKELNNTCEPVVTQPKPKIESPKLERTPNGPNIDKKEEDLEDKNNFGAEPPHQNGECYPNEKNSVNMDLD

18 NP_009181.2 Q96GX9

>NP_009181.2

MVDMDKLINNLEVQLNSEGGSMQVFKQVTASVRNRDPPEIEYRSNMTSPTLLDANPMENPALFNDIKIEPPEELLASDFSLPQVEPVDLSFHKPKAPLQPASMLQAPIRPPKPQSSPQTLVVSTSTSDMSTSANIPTVLTPGSVLTSSQSTGSQQILHVIHTIPSVSLPNKMGGLKTIPVVVQSLPMVYTTLPADGGPAAITVPLIGGDGKNAGSVKVDPTSMSPLEIPSDSEESTIESGSSALQSLQGLQQEPAAMAQMQGEESLDLKRRRIHQCDFAGCSKVYTKSSHLKAHRRIHTGEKPYKCTWDGCSWKFARSDELTRHFRKHTGIKPFRCTDCNRSFSRSDHLSLHRRRHDTM

>Q96GX9

MSGCDAREGDCCSRRCGAQDKEHPRYLIPELCKQFYHLGWVTGTGGGISLKHGDEIYIAPSGVQKERIQPEDMFVCDINEKDISGPSPSKKLKKSQCTPLFMNAYTMRGAGAVIHTHSKAAVMATLLFPGREFKITHQEMIKGIKKCTSGGYYRYDDMLVVPIIENTPEEKDLKDRMAHAMNEYPDSCAVLVRRHGVYVWGETWEKAKTMCECYDYLFDIAVSMKKVGLDPSQLPVGENGIV

19 NP_005230.1 O75880

>NP_005230.1

MNDFGIKNMDQVAPVANSYRGTLKRQPAFDTFDGSLFAVFPSLNEEQTLQEVPTGLDSISHDSANCELPLLTPCSKAVMSQALKATFSGFKKEQRRLGIPKNPWLWSEQQVCQWLLWATNEFSLVNVNLQRFGMNGQMLCNLGKERFLELAPDFVGDILWEHLEQMIKENQEKTEDQYEENSHLTSVPHWINSNTLGFGTEQAPYGMQTQNYPKGGLLDSMCPASTPSVLSSEQEFQMFPKSRLSSVSVTYCSVSQDFPGSNLNLLTNNSGTPKDHDSPENGADSFESSDSLLQSWNSQSSLLDVQRVPSFESFEDDCSQSLCLNKPTMSFKDYIQERSDPVEQGKPVIPAAVLAGFTGSGPIQLWQFLLELLSDKSCQSFISWTGDGWEFKLADPDEVARRWGKRKNKPKMNYEKLSRGLRYYYDKNIIHKTSGKRYVYRFVCDLQNLLGFTPEELHAILGVQPDTED

>O75880

MAMLVLVPGRVMRPLGGQLWRFLPRGLEFWGPAEGTARVLLRQFCARQAEAWRASGRPGYCLGTRPLSTARPPPPWSQKGPGDSTRPSKPGPVSWKSLAITFAIGGALLAGMKHVKKEKAEKLEKERQRHIGKPLLGGPFSLTTHTGERKTDKDYLGQWLLIYFGFTHCPDVCPEELEKMIQVVDEIDSITTLPDLTPLFISIDPERDTKEAIANYVKEFSPKLVGLTGTREEVDQVARAYRVYYSPGPKDEDEDYIVDHTIIMYLIGPDGEFLDYFGQNKRKGEIAASIATHMRPYRKKS

20 NP_004102.1 NP_000680.2

>NP_004102.1

MGIQGLAKLIADVAPSAIRENDIKSYFGRKVAIDASMSIYQFLIAVRQGGDVLQNEEGETTSHLMGMFYRTIRMMENGIKPVYVFDGKPPQLKSGELAKRSERRAEAEKQLQQAQAAGAEQEVEKFTKRLVKVTKQHNDECKHLLSLMGIPYLDAPSEAEASCAALVKAGKVYAAATEDMDCLTFGSPVLMRHLTASEAKKLPIQEFHLSRILQELGLNQEQFVDLCILLGSDYCESIRGIGPKRAVDLIQKHKSIEEIVRRLDPNKYPVPENWLHKEAHQLFLEPEVLDPESVELKWSEPNEEELIKFMCGEKQFSEERIRSGVKRLSKSRQGSTQGRLDDFFKVTGSLSSAKRKEPEPKGSTKKKAKTGAAGKFKRGK

>NP_000680.2

MSSSGTPDLPVLLTDLKIQYTKIFINNEWHDSVSGKKFPVFNPATEEELCQVEEGDKEDVDKAVKAARQAFQIGSPWRTMDASERGRLLYKLADLIERDRLLLATMESMNGGKLYSNAYLNDLAGCIKTLRYCAGWADKIQGRTIPIDGNFFTYTRHEPIGVCGQIIPWNFPLVMLIWKIGPALSCGNTVVVKPAEQTPLTALHVASLIKEAGFPPGVVNIVPGYGPTAGAAISSHMDIDKVAFTGSTEVGKLIKEAAGKSNLKRVTLELGGKSPCIVLADADLDNAVEFAHHGVFYHQGQCCIAASRIFVEESIYDEFVRRSVERAKKYILGNPLTPGVTQGPQIDKEQYDKILDLIESGKKEGAKLECGGGPWGNKGYFVQPTVFSNVTDEMRIAKEEIFGPVQQIMKFKSLDDVIKRANNTFYGLSAGVFTKDIDKAITISSALQAGTVWVNCYGVVSAQCPFGGFKMSGNGRELGEYGFHEYTEVKTVTVKISQKNS

21 Q96T88 NP_006235.1

>Q96T88

MWIQVRTMDGRQTHTVDSLSRLTKVEELRRKIQELFHVEPGLQRLFYRGKQMEDGHTLFDYEVRLNDTIQLLVRQSLVLPHSTKERDSELSDTDSGCCLGQSESDKSSTHGEAAAETDSRPADEDMWDETELGLYKVNEYVDARDTNMGAWFEAQVVRVTRKAPSRDEPCSSTSRPALEEDVIYHVKYDDYPENGVVQMNSRDVRARARTIIKWQDLEVGQVVMLNYNPDNPKERGFWYDAEISRKRETRTARELYANVVLGDDSLNDCRIIFVDEVFKIERPGEGSPMVDNPMRRKSGPSCKHCKDDVNRLCRVCACHLCGGRQDPDKQLMCDECDMAFHIYCLDPPLSSVPSEDEWYCPECRNDASEVVLAGERLRESKKKAKMASATSSSQRDWGKGMACVGRTKECTIVPSNHYGPIPGIPVGTMWRFRVQVSESGVHRPHVAGIHGRSNDGAYSLVLAGGYEDDVDHGNFFTYTGSGGRDLSGNKRTAEQSCDQKLTNTNRALALNCFAPINDQEGAEAKDWRSGKPVRVVRNVKGGKNSKYAPAEGNRYDGIYKVVKYWPEKGKSGFLVWRYLLRRDDDEPGPWTKEGKDRIKKLGLTMQYPEGYLEALANREREKENSKREEEEQQEGGFASPRTGKGKWKRKSAGGGPSRAGSPRRTSKKTKVEPYSLTAQQSSLIREDKSNAKLWNEVLASLKDRPASGSPFQLFLSKVEETFQCICCQELVFRPITTVCQHNVCKDCLDRSFRAQVFSCPACRYDLGRSYAMQVNQPLQTVLNQLFPGYGNGR

>NP_006235.1

METKLPPASTPTSPSSPGLSPVPPPDKVDGFSRRSLRRARPRRSHSSSQFRYQSNQQELTPLPLLKDVPASELHELLSRKLAQCGVMFDFLDCVADLKGKEVKRAALNELVECVGSTRGVLIEPVYPDIIRMISVNIFRTLPPSENPEFDPEEDEPNLEPSWPHLQLVYEFFLRFLESPDFQPSVAKRYVDQKFVLMLLELFDSEDPREREYLKTILHRVYGKFLGLRAYIRKQCNHIFLRFIYEFEHFNGVAELLEILGSIINGFALPLKTEHKQFLVRVLIPLHSVKSLSVFHAQLAYCVVQFLEKDATLTEHVIRGLLKYWPKTCTQKEVMFLGEMEEILDVIEPSQFVKIQEPLFKQVARCVSSPHFQVAERALYFWNNEYILSLIEDNCHTVLPAVFGTLYQVSKEHWNQTIVSLIYNVLKTFMEMNGKLFDELTASYKLEKQQEQQKAQERQELWQGLEELRLRRLQGTQGAKEAPLQRLTPQVAASGGQS

22 NP_006707.1 NP_000476.1

>NP_006707.1

MNSGAMRIHSKGHFQGGIQVKNEKNRPSLKSLKTDNRPEKSKCKPLWGKVFYLDLPSVTISEKLQKDIKDLGGRVEEFLSKDISYLISNKKEAKFAQTLGRISPVPSPESAYTAETTSPHPSHDGSSFKSPDTVCLSRGKLLVEKAIKDHDFIPSNSILSNALSWGVKILHIDDIRYYIEQKKKELYLLKKSSTSVRDGGKRVGSGAQKTRTGRLKKPFVKVEDMSQLYRPFYLQLTNMPFINYSIQKPCSPFDVDKPSSMQKQTQVKLRIQTDGDKYGGTSIQLQLKEKKKKGYCECCLQKYEDLETHLLSEQHRNFAQSNQYQVVDDIVSKLVFDFVEYEKDTPKKKRIKYSVGSLSPVSASVLKKTEQKEKVELQHISQKDCQEDDTTVKEQNFLYKETQETEKKLLFISEPIPHPSNELRGLNEKMSNKCSMLSTAEDDIRQNFTQLPLHKNKQECILDISEHTLSENDLEELRVDHYKCNIQASVHVSDFSTDNSGSQPKQKSDTVLFPAKDLKEKDLHSIFTHDSGLITINSSQEHLTVQAKAPFHTPPEEPNECDFKNMDSLPSGKIHRKVKIILGRNRKENLEPNAEFDKRTEFITQEENRICSSPVQSLLDLFQTSEEKSEFLGFTSYTEKSGICNVLDIWEEENSDNLLTAFFSSPSTSTFTGF

>NP_000476.1

MADSELQLVEQRIRSFPDFPTPGVVFRDISPVLKDPASFRAAIGLLARHLKATHGGRIDYIAGLDSRGFLFGPSLAQELGLGCVLIRKRGKLPGPTLWASYSLEYGKAELEIQKDALEPGQRVVVVDDLLATGGTMNAACELLGRLQAEVLECVSLVELTSLKGREKLAPVPFFSLLQYE

23 O60284 Q07020

>O60284

MDAEAEDKTLRTRSKGTEVPMDSLIQELSVAYDCSMAKKRTAEDQALGVPVNKRKSLLMKPRHYSPKADCQEDRSDRTEDDGPLETHGHSTAEEIMIKPMDESLLSTAQENSSRKEDRYSCYQELMVKSLMHLGKFEKNVSVQTVSENLNDSGIQSLKAESDEADECFLIHSDDGRDKIDDSQPPFCSSDDNESNSESAENGWDSGSNFSEETKPPRVPKYVLTDHKKDLLEVPEIKTEGDKFIPCENRCDSETERKDPQNALAEPLDGNAQPSFPDVEEEDSESLAVMTEEGSDLEKAKGNLSLLEQAIALQAERGCVFHNTYKELDRFLLEHLAGERRQTKVIDMGGRQIFNNKHSPRPEKRETKCPIPGCDGTGHVTGLYPHHRSLSGCPHKVRVPLEILAMHENVLKCPTPGCTGRGHVNSNRNTHRSLSGCPIAAAEKLAMSQDKNQLDSPQTGQCPDQAHRTSLVKQIEFNFPSQAITSPRATVSKEQEKFGKVPFDYASFDAQVFGKRPLIQTVQGRKTPPFPESKHFPNPVKFPNRLPSAGAHTQSPGRASSYSYGQCSEDTHIAAAAAILNLSTRCREATDILSNKPQSLHAKGAEIEVDENGTLDLSMKKNRILDKSAPLTSSNTSIPTPSSSPFKTSSILVNAAFYQALCDQEGWDTPINYSKTHGKTEEEKEKDPVSSLENLEEKKFPGEASIPSPKPKLHARDLKKELITCPTPGCDGSGHVTGNYASHRSVSGCPLADKTLKSLMAANSQELKCPTPGCDGSGHVTGNYASHRSLSGCPRARKGGVKMTPTKEEKEDPELKCPVIGCDGQGHISGKYTSHRTASGCPLAAKRQKENPLNGASLSWKLNKQELPHCPLPGCNGLGHVNNVFVTHRSLSGCPLNAQVIKKGKVSEELMTIKLKATGGIESDEEIRHLDEEIKELNESNLKIEADMMKLQTQITSMESNLKTIEEENKLIEQNNESLLKELAGLSQALISSLADIQLPQMGPISEQNFEAYVNTLTDMYSNLERDYSPECKALLESIKQAVKGIHV

>Q07020

MGVDIRHNKDRKVRRKEPKSQDIYLRLLVKLYRFLARRTNSTFNQVVLKRLFMSRTNRPPLSLSRMIRKMKLPGRENKTAVVVGTITDDVRVQEVPKLKVCALRVTSRARSRILRAGGKILTFDQLALDSPKGCGTVLLSGPRKGREVYRHFGKAPGTPHSHTKPYVRSKGRKFERARGRRASRGYKN

24 NP_009060.2 NP_003671.1

>NP_009060.2

MLLDAGPQFPAIGVGSFARHHHHSAAAAAAAAAEMQDRELSLAAAQNGFVDSAAAHMGAFKLNPGAHELSPGQSSAFTSQGPGAYPGSAAAAAAAAALGPHAAHVGSYSGPPFNSTRDFLFRSRGFGDSAPGGGQHGLFGPGAGGLHHAHSDAQGHLLFPGLPEQHGPHGSQNVLNGQMRLGLPGEVFGRSEQYRQVASPRTDPYSAAQLHNQYGPMNMNMGMNMAAAAAHHHHHHHHHPGAFFRYMRQQCIKQELICKWIDPEQLSNPKKSCNKTFSTMHELVTHVSVEHVGGPEQSNHVCFWEECPREGKPFKAKYKLVNHIRVHTGEKPFPCPFPGCGKVFARSENLKIHKRTHTGEKPFQCEFEGCDRRFANSSDRKKHMHVHTSDKPYLCKMCDKSYTHPSSLRKHMKVHESSPQGSESSPAASSGYESSTPPGLVSPSAEPQSSSNLSPAAAAAAAAAAAAAAAVSAVHRGGGSGSGGAGGGSGGGSGSGGGGGGAGGGGGGSSGGGSGTAGGHSGLSSNFNEWYV

>NP_003671.1

MGDAPSPEEKLHLITRNLQEVLGEEKLKEILKERELKIYWGTATTGKPHVAYFVPMSKIADFLKAGCEVTILFADLHAYLDNMKAPWELLELRVSYYENVIKAMLESIGVPLEKLKFIKGTDYQLSKEYTLDVYRLSSVVTQHDSKKAGAEVVKQVEHPLLSGLLYPGLQALDEEYLKVDAQFGGIDQRKIFTFAEKYLPALGYSKRVHLMNPMVPGLTGSKMSSSEEESKIDLLDRKEDVKKKLKKAFCEPGNVENNGVLSFIKHVLFPLKSEFVILRDEKWGGNKTYTAYVDLEKDFAAEVVHPGDLKNSVEVALNKLLDPIREKFNTPALKKLASAAYPDPSKQKPMAKGPAKNSEPEEVIPSRLDIRVGKIITVEKHPDADSLYVEKIDVGEAEPRTVVSGLVQFVPKEELQDRLVVVLCNLKPQKMRGVESQGMLLCASIEGINRQVEPLDPPAGSAPGEHVFVKGYEKGQPDEELKPKKKVFEKLQADFKISEECIAQWKQTNFMTKLGSISCKSLKGGNIS

25 NP_001007227.1 NP_203526.1

>NP_001007227.1

MSRVPSPPPPAEMSSGPVAESWCYTQIKVVKFSYMWTINNFSFCREEMGEVIKSSTFSSGANDKLKWCLRVNPKGLDEESKDYLSLYLLLVSCPKSEVRAKFKFSILNAKGEETKAMESQRAYRFVQGKDWGFKKFIRRDFLLDEANGLLPDDKLTLFCEVSVVQDSVNISGQNTMNMVKVPECRLADELGGLWENSRFTDCCLCVAGQEFQAHKAILAARSPVFSAMFEHEMEESKKNRVEINDVEPEVFKEMMCFIYTGKAPNLDKMADDLLAAADKYALERLKVMCEDALCSNLSVENAAEILILADLHSADQLKTQAVDFINYHASDVLETSGWKSMVVSHPHLVAEAYRSLASAQCPFLGPPRKRLKQS

>NP_203526.1

MSWSGLLHGLNTSLTCGPALVPRLWATCSMATLNQMHRLGPPKRPPRKLGPTEGRPQLKGVVLCTFTRKPKKPNSANRKCCRVRLSTGREAVCFIPGEGHTLQEHQIVLVEGGRTQDLPGVKLTVVRGKYDCGHVQKK

26 Q5XKR4 NP_004171.2

>Q5XKR4

MLSHADLLDARLGMKDAAELLGHREAVKCRLGVGGSDPGGHPGDLAPNSDPVEGATLLPGEDITTVGSTPASLAVSAKDPDKQPGPQGGPNPSQAGQQQGQQKQKRHRTRFTPAQLNELERSFAKTHYPDIFMREELALRIGLTESRVQVWFQNRRAKWKKRKKTTNVFRAPGTLLPTPGLPQFPSAAAAAAAAMGDSLCSFHANDTRWAAAAMPGVSQLPLPPALGRQQAMAQSLSQCSLAAGPPPNSMGLSNSLAGSNGAGLQSHLYQPAFPGMVPASLPGPSNVSGSPQLCSSPDSSDVWRGTSIASLRRKALEHTVSMSFT

>NP_004171.2

MDKNIGEQLNKAYEAFRQACMDRDSAVKELQQKTENYEQRIREQQEQLSLQQTIIDKLKSQLLLVNSTQDNNYGCVPLLEDSETRKNNLTLDQPQDKVISGIAREKLPKVRRQEVSSPRKETSARSLGSPLLHERGNIEKTFWDLKEEFHKICMLAKAQKDHLSKLNIPDTATETQCSVPIQCTDKTDKQEALFKPQAKDDINRGAPSITSVTPRGLCRDEEDTSFESLSKFNVKFPPMDNDSTFLHSTPERPGILSPATSEAVCQEKFNMEFRDNPGNFVKTEETLFEIQGIDPIASAIQNLKTTDKTKPSNLVNTCIRTTLDRAACLPPGDHNALYVNSFPLLDPSDAPFPSLDSPGKAIRGPQQPIWKPFPNQDSDSVVLSGTDSELHIPRVCEFCQAVFPPSITSRGDFLRHLNSHFNGET

27 Q8IZT9 NP_064632.2

>Q8IZT9

MAAKDQLEVQVMAAQEMELAGKDPVSHEHEERKPVTETKEGDVTDEHGERGSFAETDEHTGVDTKELEDIAADIKEHLAAKRKRIEKIAKACSEIKNRIKNVLRTTQLKRQKRDYRISLKLPNVLEEFITDEQKDEEGDGEKEEQIKIFQEQQKRWQQDGKGTERD

>NP_064632.2

MAAILGDTIMVAKGLVKLTQAAVETHLQHLGIGGELIMAARALQSTAVEQIGMFLGKVQGQDKHEEYFAENFGGPEGEFHFSVPHAAGASTDFSSASAPDQSAPPSLGHAHSEGPAPAYVASGPFREAGFPGQASSPLGRANGRLFANPRDSFSAMGFQRRFFHQDQSPVGGLTAEDIEKARQAKARPENKQHKQTLSEHARERKVPVTRIGRLANFGGLAVGLGFGALAEVAKKSLRSEDPSGKKAVLGSSPFLSEANAERIVRTLCKVRGAALKLGQMLSIQDDAFINPHLAKIFERVRQSADFMPLKQMMKTLNNDLGPNWRDKLEYFEERPFAAASIGQVHLARMKGGREVAMKIQYPGVAQSINSDVNNLMAVLNMSNMLPEGLFPEHLIDVLRRELALECDYQREAACARKFRDLLKGHPFFYVPEIVDELCSPHVLTTELVSGFPLDQAEGLSQEIRNEICYNILVLCLRELFEFHFMQTDPNWSNFFYDPQQHKVALLDFGATREYDRSFTDLYIQIIRAAADRDRETVRAKSIEMKFLTGYEVKVMEDAHLDAILILGEAFASDEPFDFGTQSTTEKIHNLIPVMLRHRLVPPPEETYSLHRKMGGSFLICSKLKARFPCKAMFEEAYSNYCKRQAQQ

28 NP_005515.1 Q9BYG8

>NP_005515.1

MPADIMEKNSSSPVAATPASVNTTPDKPKTASEHRKSSKPIMEKRRRARINESLSQLKTLILDALKKDSSRHSKLEKADILEMTVKHLRNLQRAQMTAALSTDPSVLGKYRAGFSECMNEVTRFLSTCEGVNTEVRTRLLGHLANCMTQINAMTYPGQPHPALQAPPPPPPGPGGPQHAPFAPPPPLVPIPGGAAPPPGGAPCKLGSQAGEAAKVFGGFQVVPAPDGQFAFLIPNGAFAHSGPVIPVYTSNSGTSVGPNAVSPSSGPSLTADSMWRPWRN

>Q9BYG8

MPSMLERISKNLVKEIGSKDLTPVKYLLSATKLRQFVILRKKKDSRSSFWEQSDYVPVEFSLNDILEPSSSVLETVVTGPFHFSDIMIQKHKADMGVNVGIEVSVSGEASVDHGCSLEFQIVTIPSPNLEDFQKRKLLDPEPSFLKECRRRGDNLYVVTEAVELINNTVLYDSSSVNILGKIALWITYGKGQGQGESLRVKKKALTLQKGMVMAYKRKQLVIKEKAILISDDDEQRTFQDEYEISEMVGYCAARSEGLLPSFHTISPTLFNASSNDMKLKPELFLTQQFLSGHLPKYEQVHILPVGRIEEPFWQNFKHLQEEVFQKIKTLAQLSKDVQDVMFYSILAMLRDRGALQDLMNMLELDSSGHLDGPGGAILKKLQQDSNHAWFNPKDPILYLLEAIMVLSDFQHDLLACSMEKRILLQQQELVRSILEPNFRYPWSIPFTLKPELLAPLQSEGLAITYGLLEECGLRTELDNPRSTWDVEAKMPLSALYGTLSLLQQLAEA

29 Q9BWX1 Q8TCS8

>Q9BWX1

MKTVKEKKECQRLRKSAKTRRVTQRKPSSGPVCWLCLREPGDPEKLGEFLQKDNISVHYFCLILSSKLPQRGQSNRGFHGFLPEDIKKEAARASRKICFVCKKKGAAINCQKDQCLRNFHLPCGQERGCLSQFFGEYKSFCDKHRPTQNIQHGHVGEESCILCCEDLSQQSVENIQSPCCSQAIYHRKCIQKYAHTSAKHFFKCPQCNNRKEFPQEMLRMGIHIPDRDAAWELEPGAFSDLYQRYQHCDAPICLYEQGRDSFEDEGRWCLILCATCGSHGTHRDCSSLRSNSKKWECEECSPAAATDYIPENSGDIPCCSSTFHPEEHFCRDNTLEENPGLSWTDWPEPSLLEKPESSRGRRSYSWRSKGVRITNSCKKSK

>Q8TCS8

MAACRYCCSCLRLRPLSDGPFLLPRRDRALTQLQVRALWSSAGSRAVAVDLGNRKLEISSGKLARFADGSAVVQSGDTAVMVTAVSKTKPSPSQFMPLVVDYRQKAAAAGRIPTNYLRREIGTSDKEILTSRIIDRSIRPLFPAGYFYDTQVLCNLLAVDGVNEPDVLAINGASVALSLSDIPWNGPVGAVRIGIIDGEYVVNPTRKEMSSSTLNLVVAGAPKSQIVMLEASAENILQQDFCHAIKVGVKYTQQIIQGIQQLVKETGVTKRTPQKLFTPSPEIVKYTHKLAMERLYAVFTDYEHDKVSRDEAVNKIRLDTEEQLKEKFPEADPYEIIESFNVVAKEVFRSIVLNEYKRCDGRDLTSLRNVSCEVDMFKTLHGSALFQRGQTQVLCTVTFDSLESGIKSDQVITAINGIKDKNFMLHYEFPPYATNEIGKVTGLNRRELGHGALAEKALYPVIPRDFPFTIRVTSEVLESNGSSSMASACGGSLALMDSGVPISSAVAGVAIGLVTKTDPEKGEIEDYRLLTDILGIEDYNGDMDFKIAGTNKGITALQADIKLPGIPIKIVMEAIQQASVAKKEILQIMNKTISKPRASRKENGPVVETVQVPLSKRAKFVGPGGYNLKKLQAETGVTISQVDEETFSVFAPTPSAMHEARDFITEICKDDQEQQLEFGAVYTATITEIRDTGVMVKLYPNMTAVLLHNTQLDQRKIKHPTALGLEVGQEIQVKYFGRDPADGRMRLSRKVLQSPATTVVRTLNDRSSIVMGEPISQSSSNSQ

30 NP_002096.1 NP_060383.2

>NP_002096.1

MSGRGKTGGKARAKAKSRSSRAGLQFPVGRVHRLLRKGHYAERVGAGAPVYLAAVLEYLTAEILELAGNAARDNKKTRIIPRHLQLAIRNDEELNKLLGGVTIAQGGVLPNIQAVLLPKKTSATVGPKAPSGGKKATQASQEY

>NP_060383.2

MEQPWPPPGPWSLPRAEGEAEEESDFDVFPSSPRCPQLPGGGAQMYSHGIELACQKQKEFVKSSVACKWNLAEAQQKLGSLALHNSESLDQEHAKAQTAVSELRQREEEWRQKEEALVQREKMCLWSTDAISKDVFNKSFINQDKRKDTEDEDKSESFMQKYEQKIRHFGMLSRWDDSQRFLSDHPYLVCEETAKYLILWCFHLEAEKKGALMEQIAHQAVVMQFIMEMAKNCNVDPRGCFRLFFQKAKAEEEGYFEAFKNELEAFKSRVRLYSQSQSFQPMTVQNHVPHSGVGSIGLLESLPQNPDYLQYSISTALCSLNSVVHKEDDEPKMMDTV

31 P33076 NP_037386.1

>P33076

MRCLAPRPAGSYLSEPQGSSQCATMELGPLEGGYLELLNSDADPLCLYHFYDQMDLAGEEEIELYSEPDTDTINCDQFSRLLCDMEGDEETREAYANIAELDQYVFQDSQLEGLSKDIFKHIGPDEVIGESMEMPAEVGQKSQKRPFPEELPADLKHWKPAEPPTVVTGSLLVGPVSDCSTLPCLPLPALFNQEPASGQMRLEKTDQIPMPFSSSSLSCLNLPEGPIQFVPTISTLPHGLWQISEAGTGVSSIFIYHGEVPQASQVPPPSGFTVHGLPTSPDRPGSTSPFAPSATDLPSMPEPALTSRANMTEHKTSPTQCPAAGEVSNKLPKWPEPVEQFYRSLQDTYGAEPAGPDGILVEVDLVQARLERSSSKSLERELATPDWAERQLAQGGLAEVLLAAKEHRRPRETRVIAVLGKAGQGKSYWAGAVSRAWACGRLPQYDFVFSVPCHCLNRPGDAYGLQDLLFSLGPQPLVAADEVFSHILKRPDRVLLILDGFEELEAQDGFLHSTCGPAPAEPCSLRGLLAGLFQKKLLRGCTLLLTARPRGRLVQSLSKADALFELSGFSMEQAQAYVMRYFESSGMTEHQDRALTLLRDRPLLLSHSHSPTLCRAVCQLSEALLELGEDAKLPSTLTGLYVGLLGRAALDSPPGALAELAKLAWELGRRHQSTLQEDQFPSADVRTWAMAKGLVQHPPRAAESELAFPSFLLQCFLGALWLALSGEIKDKELPQYLALTPRKKRPYDNWLEGVPRFLAGLIFQPPARCLGALLGPSAAASVDRKQKVLARYLKRLQPGTLRARQLLELLHCAHEAEEAGIWQHVVQELPGRLSFLGTRLTPPDAHVLGKALEAAGQDFSLDLRSTGICPSGLGSLVGLSCVTRFRAALSDTVALWESLRQHGETKLLQAAEEKFTIEPFKAKSLKDVEDLGKLVQTQRTRSSSEDTAGELPAVRDLKKLEFALGPVSGPQAFPKLVRILTAFSSLQHLDLDALSENKIGDEGVSQLSATFPQLKSLETLNLSQNNITDLGAYKLAEALPSLAASLLRLSLYNNCICDVGAESLARVLPDMVSLRVMDVQYNKFTAAGAQQLAASLRRCPHVETLAMWTPTIPFSVQEHLQQQDSRISLR

>NP_037386.1

MQSTSNHLWLLSDILGQGATANVFRGRHKKTGDLFAIKVFNNISFLRPVDVQMREFEVLKKLNHKNIVKLFAIEEETTTRHKVLIMEFCPCGSLYTVLEEPSNAYGLPESEFLIVLRDVVGGMNHLRENGIVHRDIKPGNIMRVIGEDGQSVYKLTDFGAARELEDDEQFVSLYGTEEYLHPDMYERAVLRKDHQKKYGATVDLWSIGVTFYHAATGSLPFRPFEGPRRNKEVMYKIITGKPSGAISGVQKAENGPIDWSGDMPVSCSLSRGLQVLLTPVLANILEADQEKCWGFDQFFAETSDILHRMVIHVFSLQQMTAHKIYIHSYNTATIFHELVYKQTKIISSNQELIYEGRRLVLEPGRLAQHFPKTTEENPIFVVSREPLNTIGLIYEKISLPKVHPRYDLDGDASMAKAITGVVCYACRIASTLLLYQELMRKGIRWLIELIKDDYNETVHKKTEVVITLDFCIRNIEKTVKVYEKLMKINLEAAELGEISDIHTKLLRLSSSQGTIETSLQDIDSRLSPGGSLADAWAHQEGTHPKDRNVEKLQVLLNCMTEIYYQFKKDKAERRLAYNEEQIHKFDKQKLYYHATKAMTHFTDECVKKYEAFLNKSEEWIRKMLHLRKQLLSLTNQCFDIEEEVSKYQEYTNELQETLPQKMFTASSGIKHTMTPIYPSSNTLVEMTLGMKKLKEEMEGVVKELAENNHILERFGSLTMDGGLRNVDCL

32 Q14839 NP_061833.1

>Q14839

MASGLGSPSPCSAGSEEEDMDALLNNSLPPPHPENEEDPEEDLSETETPKLKKKKKPKKPRDPKIPKSKRQKKERMLLCRQLGDSSGEGPEFVEEEEEVALRSDSEGSDYTPGKKKKKKLGPKKEKKSKSKRKEEEEEDDDDDDSKEPKSSAQLLEDWGMEDIDHVFSEEDYRTLTNYKAFSQFVRPLIAAKNPKIAVSKMMMVLGAKWREFSTNNPFKGSSGASVAAAAAAAVAVVESMVTATEVAPPPPPVEVPIRKAKTKEGKGPNARRKPKGSPRVPDAKKPKPKKVAPLKIKLGGFGSKRKRSSSEDDDLDVESDFDDASINSYSVSDGSTSRSSRSRKKLRTTKKKKKGEEEVTAVDGYETDHQDYCEVCQQGGEIILCDTCPRAYHMVCLDPDMEKAPEGKWSCPHCEKEGIQWEAKEDNSEGEEILEEVGGDLEEEDDHHMEFCRVCKDGGELLCCDTCPSSYHIHCLNPPLPEIPNGEWLCPRCTCPALKGKVQKILIWKWGQPPSPTPVPRPPDADPNTPSPKPLEGRPERQFFVKWQGMSYWHCSWVSELQLELHCQVMFRNYQRKNDMDEPPSGDFGGDEEKSRKRKNKDPKFAEMEERFYRYGIKPEWMMIHRILNHSVDKKGHVHYLIKWRDLPYDQASWESEDVEIQDYDLFKQSYWNHRELMRGEEGRPGKKLKKVKLRKLERPPETPTVDPTVKYERQPEYLDATGGTLHPYQMEGLNWLRFSWAQGTDTILADEMGLGKTVQTAVFLYSLYKEGHSKGPFLVSAPLSTIINWEREFEMWAPDMYVVTYVGDKDSRAIIRENEFSFEDNAIRGGKKASRMKKEASVKFHVLLTSYELITIDMAILGSIDWACLIVDEAHRLKNNQSKFFRVLNGYSLQHKLLLTGTPLQNNLEELFHLLNFLTPERFHNLEGFLEEFADIAKEDQIKKLHDMLGPHMLRRLKADVFKNMPSKTELIVRVELSPMQKKYYKYILTRNFEALNARGGGNQVSLLNVVMDLKKCCNHPYLFPVAAMEAPKMPNGMYDGSALIRASGKLLLLQKMLKNLKEGGHRVLIFSQMTKMLDLLEDFLEHEGYKYERIDGGITGNMRQEAIDRFNAPGAQQFCFLLSTRAGGLGINLATADTVIIYDSDWNPHNDIQAFSRAHRIGQNKKVMIYRFVTRASVEERITQVAKKKMMLTHLVVRPGLGSKTGSMSKQELDDILKFGTEELFKDEATDGGGDNKEGEDSSVIHYDDKAIERLLDRNQDETEDTELQGMNEYLSSFKVAQYVVREEEMGEEEEVEREIIKQEESVDPDYWEKLLRHHYEQQQEDLARNLGKGKRIRKQVNYNDGSQEDRDWQDDQSDNQSDYSVASEEGDEDFDERSEAPRRPSRKGLRNDKDKPLPPLLARVGGNIEVLGFNARQRKAFLNAIMRYGMPPQDAFTTQWLVRDLRGKSEKEFKAYVSLFMRHLCEPGADGAETFADGVPREGLSRQHVLTRIGVMSLIRKKVQEFEHVNGRWSMPELAEVEENKKMSQPGSPSPKTPTPSTPGDTQPNTPAPVPPAEDGIKIEENSLKEEESIEGEKEVKSTAPETAIECTQAPAPASEDEKVVVEPPEGEEKVEKAEVKERTEEPMETEPKGAADVEKVEEKSAIDLTPIVVEDKEEKKEEEEKKEVMLQNGETPKDLNDEKQKKNIKQRFMFNIADGGFTELHSLWQNEERAATVTKKTYEIWHRRHDYWLLAGIINHGYARWQDIQNDPRYAILNEPFKGEMNRGNFLEIKNKFLARRFKLLEQALVIEEQLRRAAYLNMSEDPSHPSMALNTRFAEVECLAESHQHLSKESMAGNKPANAVLHKVLKQLEELLSDMKADVTRLPATIARIPPVAVRLQMSERNILSRLANRAPEPTPQQVAQQQ

>NP_061833.1

MVDSVYRTRSLGVAAEGLPDQYADGEAARVWQLYIGDTRSRTAEYKAWLLGLLRQHGCQRVLDVACGTGVDSIMLVEEGFSVTSVDASDKMLKYALKERWNRRHEPAFDKWVIEEANWMTLDKDVPQSAEGGFDAVICLGNSFAHLPDCKGDQSEHRLALKNIASMVRAGGLLVIDHRNYDHILSTGCAPPGKNIYYKSDLTKDVTTSVLIVNNKAHMVTLDYTVQVPGAGQDGSPGLSKFRLSYYPHCLASFTELLQAAFGGKCQHSVLGDFKPYKPGQTYIPCYFIHVLKRTD

33 NP_001428.1 Q9Y2D0

>NP_001428.1

MDIKNSPSSLNSPSSYNCSQSILPLEHGSIYIPSSYVDSHHEYPAMTFYSPAVMNYSIPSNVTNLEGGPGRQTTSPNVLWPTPGHLSPLVVHRQLSHLYAEPQKSPWCEARSLEHTLPVNRETLKRKVSGNRCASPVTGPGSKRDAHFCAVCSDYASGYHYGVWSCEGCKAFFKRSIQGHNDYICPATNQCTIDKNRRKSCQACRLRKCYEVGMVKCGSRRERCGYRLVRRQRSADEQLHCAGKAKRSGGHAPRVRELLLDALSPEQLVLTLLEAEPPHVLISRPSAPFTEASMMMSLTKLADKELVHMISWAKKIPGFVELSLFDQVRLLESCWMEVLMMGLMWRSIDHPGKLIFAPDLVLDRDEGKCVEGILEIFDMLLATTSRFRELKLQHKEYLCVKAMILLNSSMYPLVTATQDADSSRKLAHLLNAVTDALVWVIAKSGISSQQQSMRLANLLMLLSHVRHASNKGMEHLLNMKCKNVVPVYDLLLEMLNAHVLRGCKSSITGSECSPAEDSKSKEGSQNPQSQ

>Q9Y2D0

MVVMNSLRVILQASPGKLLWRKFQIPRFMPARPCSLYTCTYKTRNRALHPLWESVDLVPGGDRQSPINIRWRDSVYDPGLKPLTISYDPATCLHVWNNGYSFLVEFEDSTDKSVIKGGPLEHNYRLKQFHFHWGAIDAWGSEHTVDSKCFPAELHLVHWNAVRFENFEDAALEENGLAVIGVFLKLGKHHKELQKLVDTLPSIKHKDALVEFGSFDPSCLMPTCPDYWTYSGSLTTPPLSESVTWIIKKQPVEVDHDQLEQFRTLLFTSEGEKEKRMVDNFRPLQPLMNRTVRSSFRHDYVLNVQAKPKPATSQATP

34 NP_631938.1 NP_056273.2

>NP_631938.1

MATGTGKHKLLSTGPTEPWSIREKLCLASSVMRSGDQNWVSVSRAIKPFAEPGRPPDWFSQKHCASQYSELLETTETPKRKRGEKGEVVETVEDVIVRKLTAERVEELKKVIKETQERYRRLKRDAELIQAGHMDSRLDELCNDIATKKKLEEEEAEVKRKATDAAYQARQAVKTPPRRLPTVMVRSPIDSASPGGDYPLGDLTPTTMEEATSGVNESEMAVASGHLNSTGVLLEVGGVLPMIHGGEIQQTPNTVAASPAASGAPTLSRLLEAGPTQFTTPLASFTTVASEPPVKLVPPPVESVSQATIVMMPALPAPSSAPAVSTTESVAPVSQPDNCVPMEAVGDPHTVTVSMDSSEISMIINSIKEECFRSGVAEAPVGSKAPSIDGKEELDLAEKMDIAVSYTGEELDFETVGDIIAIIEDKVDDHPEVLDVAAVEAALSFCEENDDPQSLPGPWEHPIQQERDKPVPLPAPEMTVKQERLDFEETENKGIHELVDIREPSAEIKVEPAEPEPVISGAEIVAGVVPATSMEPPELRSQDLDEELGSTAAGEIVEADVAIGKGDETPLTNVKTEASPESMLSPSHGSNPIEDPLEAETQHKFEMSDSLKEESGTIFGSQIKDAPGEDEEEDGVSEAASLEEPKEEDQGEGYLSEMDNEPPVSESDDGFSIHNATLQSHTLADSIPSSPASSQFSVCSEDQEAIQAQKIWKKAIMLVWRAAANHRYANVFLQPVTDDIAPGYHSIVQRPMDLSTIKKNIENGLIRSTAEFQRDIMLMFQNAVMYNSSDHDVYHMAVEMQRDVLEQIQQFLATQLIMQTSESGISAKSLRGRDSTRKQDASEKDSVPMGSPAFLLSLFMGHEWVWLDSEQDHPNDSELSNDCRSLFSSWDSSLDLDVGNWRETEDPEAEELEESSPEREPSELLVGDGGSEESQEAARKASHQNLLHFLSEVAYLMEPLCISSNESSEGCCPPSGTRQEGREIKASEGERELCRETEELSAKGDPLVAEKPLGENGKPEVASAPSVICTVQGLLTESEEGEAQQESKGEDQGEVYVSEMEDQPPSGECDDAFNIKETPLVDTLFSHATSSKLTDLSQDDPVQDHLLFKKTLLPVWKMIASHRFSSPFLKPVSERQAPGYKDVVKRPMDLTSLKRNLSKGRIRTMAQFLRDLMLMFQNAVMYNDSDHHVYHMAVEMRREVLEQIQVLNIWLDKRKGSSSLEGEPANPVDDGKPVF

>NP_056273.2

MEFAELIKTPRVDNVVLHRPFYPAVEGTLCLTGHHLILSSRQDNTEELWLLHSNIDAIDKRFVGSLGTIIIKCKDFRIIQLDIPGMEECLNIASSIEALSTLDSITLMYPFFYRPMFEVIEDGWHSFLPEQEFELYSSATSEWRLSYVNKEFAVCPSYPPIVTVPKSIDDEALRKVATFRHGGRFPVLSYYHKKNGMVIMRSGQPLTGTNGRRCKEDEKLINATLRAGKRGYIIDTRSLNVAQQTRAKGGGFEQEAHYPQWRRIHKSIERYHILQESLIKLVEACNDQTHNMDRWLSKLEASNWLTHIKEILTTACLAAQCIDREGASILIHGTEGTDSTLQVTSLAQIILEPRSRTIRGFEALIEREWLQAGHPFQQRCAQSAYCNTKQKWEAPVFLLFLDCVWQILRQFPCSFEFNENFLIMLFEHAYASQFGTFLGNNESERCKLKLQQKTMSLWSWVNQPSELSKFTNPLFEANNLVIWPSVAPQSLPLWEGIFLRWNRSSKYLDEAYEEMVNIIEYNKELQAKVNILRRQLAELETEDGMQESP

35 NP_057953.1 Q9UBR1

>NP_057953.1

MDLEKNYPTPRTSRTGHGGVNQLGGVFVNGRPLPDVVRQRIVELAHQGVRPCDISRQLRVSHGCVSKILGRYYETGSIKPGVIGGSKPKVATPKVVEKIAEYKRQNPTMFAWEIRDRLLAERVCDNDTVPSVSSINRIIRTKVQQPPNQPVPASSHSIVSTGSVTQVSSVSTDSAGSSYSISGILGITSPSADTNKRKRDEGIQESPVPNGHSLPGRDFLRKQMRGDLFTQQQLEVLDRVFERQHYSDIFTTTEPIKPEQTTEYSAMASLAGGLDDMKANLASPTPADIGSSVPGPQSYPIVTGRDLASTTLPGYPPHVPPAGQGSYSAPTLTGMVPGSEFSGSPYSHPQYSSYNDSWRFPNPGLLGSPYYYSAAARGAAPPAAATAYDRH

>Q9UBR1

MAGAEWKSLEECLEKHLPLPDLQEVKRVLYGKELRKLDLPREAFEAASREDFELQGYAFEAAEEQLRRPRIVHVGLVQNRIPLPANAPVAEQVSALHRRIKAIVEVAAMCGVNIICFQEAWTMPFAFCTREKLPWTEFAESAEDGPTTRFCQKLAKNHDMVVVSPILERDSEHGDVLWNTAVVISNSGAVLGKTRKNHIPRVGDFNESTYYMEGNLGHPVFQTQFGRIAVNICYGRHHPLNWLMYSINGAEIIFNPSATIGALSESLWPIEARNAAIANHCFTCAINRVGTEHFPNEFTSGDGKKAHQDFGYFYGSSYVAAPDSSRTPGLSRSRDGLLVAKLDLNLCQQVNDVWNFKMTGRYEMYARELAEAVKSNYSPTIVKE

36 NP_060274.3 O00338

>NP_060274.3

MKEPRIFPRERPTPWTRAPLPPRGRLDGSLGPQGGPVLNTGHPLGVNSDPFLMAAGSLGGNLTPFPRNPSPFPASSGSLASNPAPFPAGARDPSMASFPRGMNPTGTGAVSFPRPGGLLGPGPGPGPTLNPRTGALPGPGPLSNPRLGGLPGPGPMSNPRAGGLLGAGPDPRGGGPMGPGSGPNLRAGVLLTSGNGPPNPRPVGLGPGPNPNLRSGFLGTNPAPRSGVFPGPGLGPNPRPSGLGPGPNLDARAGGLLGTGSGLNLRMAGPQGLDLAPILRAAGLLGANSASFSQASGNMGTSPSSMARVPGPMGPNSGPSSRGIGLPGPNPSPMSRAPGPIGPNSAHFSRPVGPMGVNANPFPRGAGSSAFSQSSGTLASNPATFQRSAGLQGSNPTIFPRASGPLGPNPANFPRATGLQGPSPTTFPRSTGPLGPGQVTFPRPAAGHLGPSPAGPVGINPAPFTRPTGTLGLNPASFPRMNGPAGKSFVPFPRVGSLPGTNPAAFPRPGGPMAAMYPNGMLPP

>O00338

MALTSDLGKQIKLKEVEGTLLQPATVDNWSQIQSFEAKPDDLLICTYPKAGTTWIQEIVDMIEQNGDVEKCQRAIIQHRHPFIEWARPPQPSGVEKAKAMPSPRILKTHLSTQLLPPSFWENNCKFLYVARNAKDCMVSYYHFQRMNHMLPDPGTWEEYFETFINGKVVWGSWFDHVKGWWEMKDRHQILFLFYEDIKRDPKHEIRKVMQFMGKKVDETVLDKIVQETSFEKMKENPMTNRSTVSKSILDQSISSFMRKGTVGDWKNHFTVAQNERFDEIYRRKMEGTSINFCMEL

37 Q3YBR2 Q8TCS8

>Q3YBR2

MSLLDGLASSPRAPLQSSKARMKKLPKKSQNEKYRLKYLRLRKAAKATVFENAAICDEIARLEEKFLKAKEERRYLLKKLLQLQALTEGEVQAAAPSHSSSLPLTYGVASSVGTIQGAGPISGPSTGAEEPFGKKTKKEKKEKGKENNKLEVLKKTCKKKKMAGGARKLVQPIALDPSGRPVFPIGLGGLTVYSLGEIITDRPGFHDESAIYPVGYCSTRIYASMKCPDQKCLYTCQIKDGGVQPQFEIVPEDDPQNAIVSSSADACHAELLRTISTTMGKLMPNLLPAGADFFGFSHPAIHNLIQSCPGARKCINYQWVKFDVCKPGDGQLPEGLPENDAAMSFEAFQRQIFDEDQNDPLLPGSLDLPELQPAAFVSSYQPMYLTHEPLVDTHLQHLKSPSQGSPIQSSD

>Q8TCS8

MAACRYCCSCLRLRPLSDGPFLLPRRDRALTQLQVRALWSSAGSRAVAVDLGNRKLEISSGKLARFADGSAVVQSGDTAVMVTAVSKTKPSPSQFMPLVVDYRQKAAAAGRIPTNYLRREIGTSDKEILTSRIIDRSIRPLFPAGYFYDTQVLCNLLAVDGVNEPDVLAINGASVALSLSDIPWNGPVGAVRIGIIDGEYVVNPTRKEMSSSTLNLVVAGAPKSQIVMLEASAENILQQDFCHAIKVGVKYTQQIIQGIQQLVKETGVTKRTPQKLFTPSPEIVKYTHKLAMERLYAVFTDYEHDKVSRDEAVNKIRLDTEEQLKEKFPEADPYEIIESFNVVAKEVFRSIVLNEYKRCDGRDLTSLRNVSCEVDMFKTLHGSALFQRGQTQVLCTVTFDSLESGIKSDQVITAINGIKDKNFMLHYEFPPYATNEIGKVTGLNRRELGHGALAEKALYPVIPRDFPFTIRVTSEVLESNGSSSMASACGGSLALMDSGVPISSAVAGVAIGLVTKTDPEKGEIEDYRLLTDILGIEDYNGDMDFKIAGTNKGITALQADIKLPGIPIKIVMEAIQQASVAKKEILQIMNKTISKPRASRKENGPVVETVQVPLSKRAKFVGPGGYNLKKLQAETGVTISQVDEETFSVFAPTPSAMHEARDFITEICKDDQEQQLEFGAVYTATITEIRDTGVMVKLYPNMTAVLLHNTQLDQRKIKHPTALGLEVGQEIQVKYFGRDPADGRMRLSRKVLQSPATTVVRTLNDRSSIVMGEPISQSSSNSQ

38 NP_005925.2 O00142

>NP_005925.2

MDNQCTVQVRLELGHRAQLRKKPTTEGFTHDWMVFVRGPEQCDIQHFVEKVVFWLHDSFPKPRRVCKEPPYKVEESGYAGFIMPIEVHFKNKEEPRKVCFTYDLFLNLEGNPPVNHLRCEKLTFNNPTTEFRYKLLRAGGVMVMPEGADTVSRPSPDYPMLPTIPLSAFSDPKKTKPSHGSKDANKESSKTSKPHKVTKEHRERPRKDSESKSSSKELEREQAKSSKDTSRKLGEGRLPKEEKAPPPKAAFKEPKMALKETKLESTSPKGGPPPPPPPPPRASSKRPATADSPKPSAKKQKKSSSKGSRSAPGTSPRTSSSSSFSDKKPAKDKSSTRGEKVKAESEPREAKKALEVEESNSEDEASFKSESAQSSPSNSSSSSDSSSDSDFEPSQNHSQGPLRSMVEDLQSEESDEDDSSSGEEAAGKTNPGRDSRLSFSDSESDNSADSSLPSREPPPPQKPPPPNSKVSGRRSPESCSKPEKILKKGTYDKAYTDELVELHRRLMALRERNVLQQIVNLIEETGHFNVTNTTFDFDLFSLDETTVRKLQSCLEAVAT

>O00142

MLLWPLRGWAARALRCFGPGSRGSPASGPGPRRVQRRAWPPDKEQEKEKKSVICVEGNIASGKTTCLEFFSNATDVEVLTEPVSKWRNVRGHNPLGLMYHDASRWGLTLQTYVQLTMLDRHTRPQVSSVRLMERSIHSARYIFVENLYRSGKMPEVDYVVLSEWFDWILRNMDVSVDLIVYLRTNPETCYQRLKKRCREEEKVIPLEYLEAIHHLHEEWLIKGSLFPMAAPVLVIEADHHMERMLELFEQNRDRILTPENRKHCP

39 NP_002097.1 NP_008996.1

>NP_002097.1

MAGGKAGKDSGKAKTKAVSRSQRAGLQFPVGRIHRHLKSRTTSHGRVGATAAVYSAAILEYLTAEVLELAGNASKDLKVKRITPRHLQLAIRGDEELDSLIKATIAGGGVIPHIHKSLIGKKGQQKTV

>NP_008996.1

MVDYSVWDHIEVSDDEDETHPNIDTASLFRWRHQARVERMEQFQKEKEELDRGCRECKRKVAECQRKLKELEVAEGGKAELERLQAEAQQLRKEERSWEQKLEEMRKKEKSMPWNVDTLSKDGFSKSMVNTKPEKTEEDSEEVREQKHKTFVEKYEKQIKHFGMLRRWDDSQKYLSDNVHLVCEETANYLVIWCIDLEVEEKCALMEQVAHQTIVMQFILELAKSLKVDPRACFRQFFTKIKTADRQYMEGFNDELEAFKERVRGRAKLRIEKAMKEYEEEERKKRLGPGGLDPVEVYESLPEELQKCFDVKDVQMLQDAISKMDPTDAKYHMQRCIDSGLWVPNSKASEAKEGEEAGPGDPLLEAVPKTGDEKDVSV

40 NP_004069.1 NP_612393.2

>NP_004069.1

MPNWGGGKKCGVCQKTVYFAEEVQCEGNSFHKSCFLCMVCKKNLDSTTVAVHGEEIYCKSCYGKKYGPKGYGYGQGAGTLSTDKGESLGIKHEEAPGHRPTTNPNASKFAQKIGGSERCPRCSQAVYAAEKVIGAGKSWHKACFRCAKCGKGLESTTLADKDGEIYCKGCYAKNFGPKGFGFGQGAGALVHSE

>NP_612393.2

MRLTPRALCSAAQAAWRENFPLCGRDVARWFPGHMAKGLKKMQSSLKLVDCIIEVHDARIPLSGRNPLFQETLGLKPHLLVLNKMDLADLTEQQKIMQHLEGEGLKNVIFTNCVKDENVKQIIPMVTELIGRSHRYHRKENLEYCIMVIGVPNVGKSSLINSLRRQHLRKGKATRVGGEPGITRAVMSKIQVSERPLMFLLDTPGVLAPRIESVETGLKLALCGTVLDHLVGEETMADYLLYTLNKHQRFGYVQHYGLGSACDNVERVLKSVAVKLGKTQKVKVLTGTGNVNIIQPNYPAAARDFLQTFRRGLLGSVMLDLDVLRGHPPAETLP

41 NP_996670.1 Q9Y238

>NP_996670.1

MAPKQDPKPKFQEGERVLCFHGPLLYEAKCVKVAIKDKQVKYFIHYSGWNKKSAVRPRRSEKSLKTHEDIVALFPVPEGAPSVHHPLLTSSWDEWVPESRVLKYVDTNLQKQRELQKANQEQYAEGKMRGAAPGKKTSGLQQKNVEVKTKKNKQKTPGNGDGGSTSETPQPPRKKRARVDPTVENEETFMNRVEVKVKIPEELKPWLVDDWDLITRQKQLFYLPAKKNVDSILEDYANYKKSRGNTDNKEYAVNEVVAGIKEYFNVMLGTQLLYKFERPQYAEILADHPDAPMSQVYGAPHLLRLFVRIGAMLAYTPLDEKSLALLLNYLHDFLKYLAKNSATLFSASDYEVAPPEYHRKAV

>Q9Y238

METRSSKTRRSLASRTNECQGTMWAPTSPPAGSSSPSQPTWKSSLYSSLAYSEAFHYSFAARPRRLTQLALAQRPEPQLLRLRPSSLRTQDISHLLTGVFRNLYSAEVIGDEVSASLIKARGSENERHEEFVDQLQQIRELYKQRLDEFEMLERHITQAQARAIAENERVMSQAGVQDLESLVRLPPVKSVSRWCIDSELLRKHHLISPEDYYTDTVPFHSAPKGISLPGCSKLTFSCEKRSVQKKELNKKLEDSCRKKLAEFEDELDHTVDSLTWNLTPKAKERTREPLKKASQPRNKNWMNHLRVPQRELDRLLLARMESRNHFLKNPRFFPPNTRYGGKSLVFPPKKPAPIGEFQSTEPEQSCADTPVFLAKPPIGFFTDYEIGPVYEMVIALQNTTTTSRYLRVLPPSTPYFALGLGMFPGKGGMVAPGMTCQYIVQFFPDCLGDFDDFILVETQSAHTLLIPLQARRPPPVLTLSPVLDCGYCLIGGVKMTRFICKNVGFSVGRFCIMPKTSWPPLSFKAIATVGFVEQPPFGILPSVFELAPGHAILVEVLFSPKSLGKAEQTFIIMCDNCQIKELVTIGIGQLIALDLIYISGEKSQPDPGELTDLTAQHFIRFEPENLRSTARKQLIIRNATHVELAFYWQIMKPNLQPLMPGETFSMDSIKCYPDKETAFSIMPRKGVLSPHTDHEFILSFSPHELRDFHSVLQMVLEEVPEPVSSEAESLGHSSYSVDDVIVLEIEVKGSVEPFQVLLEPYALIIPGENYIGINVKKAFKMWNNSKSPIRYLWGKISDCHIIEVEPGTGVIEPSEVGDFELNFTGGVPGPTSQDLLCEIEDSPSPVVLHIEAVFKGPALIINVSALQFGLLRLGQKATNSIQIRNVSQLPATWRMKESPVSLQERPEDVSPFDIEPSSGQLHSLGECRVDITLEALHCQHLETVLELEVENGAWSYLPVYAEVQKPHVYLQSSQVEVRNLYLGVPTKTTITLINGTLLPTQFHWGKLLGHQAEFCMVTVSPKHGLLGPSEECQLKLELTAHTQEELTHLALPCHVSGMKKPLVLGISGKPQGLQVAITISKESSDCSTEQWPGHPKELRLDFGSAVPLRTRVTRQLILTNRSPIRTRFSLKFEYFGSPQNSLSKKTSLPNMPPALLKTVRMQEHLAKREQLDFMESMLSHGKGAAFFPHFSQGMLGPYQQLCIDITGCANMWGEYWDNLICTVGDLLPEVIPVHMAAVGCPISSLRTTSYTIDQAQKEPAMRFGTQVSGGDTVTRTLRLNNSSPCDIRLDWETYVPEDKEDRLVELLVFYGPPFPLRDQAGNELVCPDTPEGGCLLWSPGPSSSSEFSHETDSSVEGSSSASNRVAQKLISVILQAHEGVPSGHLYCISPKQVVVPAGGSSTIYISFTPMVLSPEILHKVECTGYALGFMSLDSKVEREIPRKRHRLQDFAVGPLKLDLHSYVRPAQLSVELDYGGSMEFQCQASDLIPEQPCSGVLSELVTTHHLKLTNTTEIPHYFRLMVSRPFSVSQDGASQDHRAPGPGQKQECEEETASADKQLVLQAQENMLVNVSFSLSLELLSYQKLPADQTLPGVDIQQSASGEREMVFTQNLLLEYTNQTTQVVPLRAVVAVPELQLSTSWVDFGTCFVSQQRVREVYLMNLSGCRSYWTMLMGQQEPAKAAVAFRVSPNSGLLEARSANAPPTSIALQVFFTARSSELYESTMVVEGVLGEKSCTLRLRGQGSYDERYMLPHQP

42 P51826 Q96P20

>P51826

MDSFDLALLQEWDLESLCVYEPDRNALRRKERERRNQETQQDDGTFNSSYSLFSEPYKTNKGDELSNRIQNTLGNYDEMKDFLTDRSNQSHLVGVPKPGVPQTPVNKIDEHFVADSRAQNQPSSICSTTTSTPAAVPVQQSKRGTMGWQKAGHPPSDGQQRATQQGSLRTLLGDGVGRQQPRAKQVCNVEVGLQTQERPPAMAAKHSSSGHCVQNFPPSLASKPSLVQQKPTAYVRPMDGQDQAPDESPKLKSSSETSVHCTSYRGVPASKPEPARAKAKLSKFSIPKQGEESRSGETNSCVEEIIREMTWLPPLSAIQAPGKVEPTKFPFPNKDSQLVSSGHNNPKKGDAEPESPDSGTSNTSMLEDDLKLSSDEEENEQQAAQRTALRALSDSAVVQQPNCRTSVPSSKGSSSSSSSGSSSSSSDSESSSGSDSETESSSSESEGSKPPHFSSPEAEPASSNKWQLDKWLNKVNPHKPPILIQNESHGSESNQYYNPVKEDVQDCGKVPDVCQPSLREKEIKSTCKEEQRPRTANKAPGSKGVKQKSPPAAVAVAVSAAAPPPAVPCAPAENAPAPARRSAGKKPTRRTERTSAGDGANCHRPEEPAAADALGTSVVVPPEPTKTRPCGNNRASHRKELRSSVTCEKRRTRGLSRIVPKSKEFIETESSSSSSSSDSDLESEQEEYPLSKAQTVAASASSGNDQRLKEAAANGGSGPRAPVGSINARTTSDIAKELEEQFYTLVPFGRNELLSPLKDSDEIRSLWVKIDLTLLSRIPEHLPQEPGVLSAPATKDSESAPPSHTSDTPAEKALPKSKRKRKCDNEDDYREIKKSQGEKDSSSRLATSTSNTLSANHCNMNINSVAIPINKNEKMLRSPISPLSDASKHKYTSEDLTSSSRPNGNSLFTSASSSKKPKADSQLQPHGGDLTKAAHNNSENIPLHKSRPQTKPWSPGSNGHRDCKRQKLVFDDMPRSADYFMQEAKRMKHKADAMVEKFGKALNYAEAALSFIECGNAMEQGPMESKSPYTMYSETVELIRYAMRLKTHSGPNATPEDKQLAALCYRCLALLYWRMFRLKRDHAVKYSKALIDYFKNSSKAAQAPSPWGASGKSTGTPSPMSPNPSPASSVGSQGSLSNASALSPSTIVSIPQRIHQMAANHVSITNSILHSYDYWEMADNLAKENREFFNDLDLLMGPVTLHSSMEHLVQYSQQGLHWLRNSAHLS

>Q96P20

MASTRCKLARYLEDLEDVDLKKFKMHLEDYPPQKGCIPLPRGQTEKADHVDLATLMIDFNGEEKAWAMAVWIFAAINRRDLYEKAKRDEPKWGSDNARVSNPTVICQEDSIEEEWMGLLEYLSRISICKMKKDYRKKYRKYVRSRFQCIEDRNARLGESVSLNKRYTRLRLIKEHRSQQEREQELLAIGKTKTCESPVSPIKMELLFDPDDEHSEPVHTVVFQGAAGIGKTILARKMMLDWASGTLYQDRFDYLFYIHCREVSLVTQRSLGDLIMSCCPDPNPPIHKIVRKPSRILFLMDGFDELQGAFDEHIGPLCTDWQKAERGDILLSSLIRKKLLPEASLLITTRPVALEKLQHLLDHPRHVEILGFSEAKRKEYFFKYFSDEAQARAAFSLIQENEVLFTMCFIPLVCWIVCTGLKQQMESGKSLAQTSKTTTAVYVFFLSSLLQPRGGSQEHGLCAHLWGLCSLAADGIWNQKILFEESDLRNHGLQKADVSAFLRMNLFQKEVDCEKFYSFIHMTFQEFFAAMYYLLEEEKEGRTNVPGSRLKLPSRDVTVLLENYGKFEKGYLIFVVRFLFGLVNQERTSYLEKKLSCKISQQIRLELLKWIEVKAKAKKLQIQPSQLELFYCLYEMQEEDFVQRAMDYFPKIEINLSTRMDHMVSSFCIENCHRVESLSLGFLHNMPKEEEEEEKEGRHLDMVQCVLPSSSHAACSHGLVNSHLTSSFCRGLFSVLSTSQSLTELDLSDNSLGDPGMRVLCETLQHPGCNIRRLWLGRCGLSHECCFDISLVLSSNQKLVELDLSDNALGDFGIRLLCVGLKHLLCNLKKLWLVSCCLTSACCQDLASVLSTSHSLTRLYVGENALGDSGVAILCEKAKNPQCNLQKLGLVNSGLTSVCCSALSSVLSTNQNLTHLYLRGNTLGDKGIKLLCEGLLHPDCKLQVLELDNCNLTSHCCWDLSTLLTSSQSLRKLSLGNNDLGDLGVMMFCEVLKQQSCLLQNLGLSEMYFNYETKSALETLQEEKPELTVVFEPSW

43 NP_003336.1 NP_004093.1

>NP_003336.1

MSGIALSRLAQERKAWRKDHPFGFVAVPTKNPDGTMNLMNWECAIPGKKGTPWEGGLFKLRMLFKDDYPSSPPKCKFEPPLFHPNVYPSGTVCLSILEEDKDWRPAITIKQILLGIQELLNEPNIQDPAQAEAYTIYCQNRVEYEKRVRAQAKKFAPS

>NP_004093.1

MVDAFLGTWKLVDSKNFDDYMKSLGVGFATRQVASMTKPTTIIEKNGDILTLKTHSTFKNTEISFKLGVEFDETTADDRKVKSIVTLDGGKLVHLQKWDGQETTLVRELIDGKLILTLTHGTAVCTRTYEKEA

44 NP_003911.1 Q6PGN9

>NP_003911.1

MDLHMMNCELLATCSALGYLEGDTYHKEPDCLESVKDLIRYLRHEDETRDVRQQLGAAQILQSDLLPILTQHHQDKPLFDAVIRLMVNLTQPALLCFGNLPKEPSFRHHFLQVLTYLQAYKEAFASEKAFGVLSETLYELLQLGWEERQEEDNLLIERILLLVRNILHVPADLDQEKKIDDDASAHDQLLWAIHLSGLDDLLLFLASSSAEEQWSLHVLEIVSLMFRDQNPEQLAGVGQGRLAQERSADFAELEVLRQREMAEKKTRALQRGNRHSRFGGSYIVQGLKSIGERDLIFHKGLHNLRNYSSDLGKQPKKVPKRRQAARELSIQRRSALNVRLFLRDFCSEFLENCYNRLMGSVKDHLLREKAQQHDETYYMWALAFFMAFNRAASFRPGLVSETLSVRTFHFIEQNLTNYYEMMLTDRKEAASWARRMHLALKAYQELLATVNEMDLSPDEAVRESSRIIKNNIFYVMEYRELFLALFRKFDERCQPRSFLRDLVETTHLFLKMLERFCRSRGNLVVQNKQKKRRKKKKKVLDQAIVSGNVPSSPEEVEAVWPALAEQLQCCAQNSELSMDSVVPFDAASEVPVEEQRAEAMVRIQDCLLAGQAPQALTLLRSAREVWPEGDVFGSQDISPEEEIQLLKQILSAPLPRQQGPEERGAEEEEEEEEEEEEELQVVQVSEKEFNFLDYLKRFACSTVVRAYVLLLRSYQQNSAHTNHCIVKMLHRLAHDLKMEALLFQLSVFCLFNRLLSDPAAGAYKELVTFAKYILGKFFALAAVNQKAFVELLFWKNTAVVREMTEGYGSLDDRSSSRRAPTWSPEEEAHLQELYLANKDVEGQDVVEAILAHLNTVPRTRKQIIHHLVQMGLADSVKDFQRKGTHIVLWTGDQELELQRLFEEFRDSDDVLGHIMKNITAKRSRARIVDKLLALGLVAERRELYKKRQKKLASSILPNGAESLKDFCQEDLEEEENLPEEDSEEEEEGGSEAEQVQGSLVLSNENLGQSLHQEGFSIPLLWLQNCLIRAADDREEDGCSQAVPLVPLTEENEEAMENEQFQQLLRKLGVRPPASGQETFWRIPAKLSPTQLRRAAASLSQPEEEQKLQPELQPKVPGEQGSDEEHCKEHRAQALRALLLAHKKKAGLASPEEEDAVGKEPLKAAPKKRQLLDSDEEQEEDEGRNRAPELGAPGIQKKKRYQIEDDEDD

>Q6PGN9

MEDLEEDVRFIVDETLDFGGLSPSDSREEEDITVLVTPEKPLRRGLSHRSDPNAVAPAPQGVRLSLGPLSPEKLEEILDEANRLAAQLEQCALQDRESAGEGLGPRRVKPSPRRETFVLKDSPVRDLLPTVNSLTRSTPSPSSLTPRLRSNDRKGSVRALRATSGKRPSNMKRESPTCNLFPASKSPASSPLTRSTPPVRGRAGPSGRAAASEETRAAKLRVSGSGEFVGLTLKFLHPSPPGPPTPIRSVLAPQPSTSNSQRLPRPQGAAAKSSSQLPIPSAIPRPASRMPLTSRSVPPGRGALPPDSLSTRKGLPRPSTAGHRVRESGHKVPVSQRLNLPVMGATRSNLQPPRKVAVPGPTR

45 NP_006222.2 NP_006750.3

>NP_006222.2

MSLRSGGRRRADPGADGEASRDDGATSSVSALKRLERSQWTDKMDLRFGFERLKEPGEKTGWLINMHPTEILDEDKRLGSAVDYYFIQDDGSRFKVALPYKPYFYIATRKGCEREVSSFLSKKFQGKIAKVETVPKEDLDLPNHLVGLKRNYIRLSFHTVEDLVKVRKEISPAVKKNREQDHASDAYTALLSSVLQRGGVITDEEETSKKIADQLDNIVDMREYDVPYHIRLSIDLKIHVAHWYNVRYRGNAFPVEITRRDDLVERPDPVVLAFDIETTKLPLKFPDAETDQIMMISYMIDGQGYLITNREIVSEDIEDFEFTPKPEYEGPFCVFNEPDEAHLIQRWFEHVQETKPTIMVTYNGDFFDWPFVEARAAVHGLSMQQEIGFQKDSQGEYKAPQCIHMDCLRWVKRDSYLPVGSHNLKAAAKAKLGYDPVELDPEDMCRMATEQPQTLATYSVSDAVATYYLYMKYVHPFIFALCTIIPMEPDEVLRKGSGTLCEALLMVQAFHANIIFPNKQEQEFNKLTDDGHVLDSETYVGGHVEALESGVFRSDIPCRFRMNPAAFDFLLQRVEKTLRHALEEEEKVPVEQVTNFEEVCDEIKSKLASLKDVPSRIECPLIYHLDVGAMYPNIILTNRLQPSAMVDEATCAACDFNKPGANCQRKMAWQWRGEFMPASRSEYHRIQHQLESEKFPPLFPEGPARAFHELSREEQAKYEKRRLADYCRKAYKKIHITKVEERLTTICQRENSFYVDTVRAFRDRRYEFKGLHKVWKKKLSAAVEVGDAAEVKRCKNMEVLYDSLQLAHKCILNSFYGYVMRKGARWYSMEMAGIVCFTGANIITQARELIEQIGRPLELDTDGIWCVLPNSFPENFVFKTTNVKKPKVTISYPGAMLNIMVKEGFTNDQYQELAEPSSLTYVTRSENSIFFEVDGPYLAMILPASKEEGKKLKKRYAVFNEDGSLAELKGFEVKRRGELQLIKIFQSSVFEAFLKGSTLEEVYGSVAKVADYWLDVLYSKAANMPDSELFELISENRSMSRKLEDYGEQKSTSISTAKRLAEFLGDQMVKDAGLSCRYIISRKPEGSPVTERAIPLAIFQAEPTVRKHFLRKWLKSSSLQDFDIRAILDWDYYIERLGSAIQKIITIPAALQQVKNPVPRVKHPDWLHKKLLEKNDVYKQKKISELFTLEGRRQVTMAEASEDSPRPSAPDMEDFGLVKLPHPAAPVTVKRKRVLWESQEESQDLTPTVPWQEILGQPPALGTSQEEWLVWLRFHKKKWQLQARQRLARRKRQRLESAEGVLRPGAIRDGPATGLGSFLRRTARSILDLPWQIVQISETSQAGLFRLWALVGSDLHCIRLSIPRVFYVNQRVAKAEEGASYRKVNRVLPRSNMVYNLYEYSVPEDMYQEHINEINAELSAPDIEGVYETQVPLLFRALVHLGCVCVVNKQLVRHLSGWEAETFALEHLEMRSLAQFSYLEPGSIRHIYLYHHAQAHKALFGIFIPSQRRASVFVLDTVRSNQMPSLGALYSAEHGLLLEKVGPELLPPPKHTFEVRAETDLKTICRAIQRFLLAYKEERRGPTLIAVQSSWELKRLASEIPVLEEFPLVPICVADKINYGVLDWQRHGARRMIRHYLNLDTCLSQAFEMSRYFHIPIGNLPEDISTFGSDLFFARHLQRHNHLLWLSPTARPDLGGKEADDNCLVMEFDDQATVEINSSGCYSTVCVELDLQNLAVNTILQSHHVNDMEGADSMGISFDVIQQASLEDMITGGQAASAPASYDETALCSNTFRILKSMVVGWVKEITQYHNIYADNQVMHFYRWLRSPSSLLHDPALHRTLHNMMKKLFLQLIAEFKRLGSSVIYANFNRIILCTKKRRVEDAIAYVEYITSSIHSKETFHSLTISFSRCWEFLLWMDPSNYGGIKGKVSSRIHCGLQDSQKAGGAEDEQENEDDEEERDGEEEEEAEESNVEDLLENNWNILQFLPQAASCQNYFLMIVSAYIVAVYHCMKDGLRRSAPGSTPVRRRGASQLSQEAEGAVGALPGMITFSQDYVANELTQSFFTITQKIQKKVTGSRNSTELSEMFPVLPGSHLLLNNPALEFIKYVCKVLSLDTNITNQVNKLNRDLLRLVDVGEFSEEAQFRDPCRSYVLPEVICRSCNFCRDLDLCKDSSFSEDGAVLPQWLCSNCQAPYDSSAIEMTLVEVLQKKLMAFTLQDLVCLKCRGVKETSMPVYCSCAGDFALTIHTQVFMEQIGIFRNIAQHYGMSYLLETLEWLLQKNPQLGH

>NP_006750.3

MSRFVQDLSKAMSQDGASQFQEVIRQELELSVKKELEKILTTASSHEFEHTKKDLDGFRKLFHRFLQEKGPSVDWGKIQRPPEDSIQPYEKIKARGLPDNISSVLNKLVVVKLNGGLGTSMGCKGPKSLIGVRNENTFLDLTVQQIEHLNKTYNTDVPLVLMNSFNTDEDTKKILQKYNHCRVKIYTFNQSRYPRINKESLLPVAKDVSYSGENTEAWYPPGHGDIYASFYNSGLLDTFIGEGKEYIFVSNIDNLGATVDLYILNHLMNPPNGKRCEFVMEVTNKTRADVKGGTLTQYEGKLRLVEIAQVPKAHVDEFKSVSKFKIFNTNNLWISLAAVKRLQEQNAIDMEIIVNAKTLDGGLNVIQLETAVGAAIKSFENSLGINVPRSRFLPVKTTSDLLLVMSNLYSLNAGSLTMSEKREFPTVPLVKLGSSFTKVQDYLRRFESIPDMLELDHLTVSGDVTFGKNVSLKGTVIIIANHGDRIDIPPGAVLENKIVSGNLRILDH

46 NP_005644.2 NP_057356.1

>NP_005644.2

MAWALKLPLADEVIESGLVQDFDASLSGIGQELGAGAYSMSDVLALPIFKQEESSLPPDNENKILPFQYVLCAATSPAVKLHDETLTYLNQGQSYEIRMLDNRKLGELPEINGKLVKSIFRVVFHDRRLQYTEHQQLEGWRWNRPGDRILDIDIPMSVGIIDPRANPTQLNTVEFLWDPAKRTSVFIQVHCISTEFTMRKHGGEKGVPFRVQIDTFKENENGEYTEHLHSASCQIKVFKPKGADRKQKTDREKMEKRTPHEKEKYQPSYETTILTECSPWPEITYVNNSPSPGFNSSHSSFSLGEGNGSPNHQPEPPPPVTDNLLPTTTPQEAQQWLHRNRFSTFTRLFTNFSGADLLKLTRDDVIQICGPADGIRLFNALKGRMVRPRLTIYVCQESLQLREQQQQQQQQQQKHEDGDSNGTFFVYHAIYLEELTAVELTEKIAQLFSISPCQISQIYKQGPTGIHVLISDEMIQNFQEEACFILDTMKAETNDSYHIILK

>NP_057356.1

MQLEIKVALNFIISYLYNKLPRRRADLFGEELERLLKKKYEGHWYPEKPLKGSGFRCVHIGEMVDPVVELAAKRSGLAVEDVRANVPEELSVWIDPFEVSYQIGEKGAVKVLYLDDSEGCGAPELDKEIKSSFNPDAQVFVPIGSQDSSLSNSPSPSFGQSPSPTFIPRSAQPITFTTASFAATKFGSTKMKKGGGAASGGGVASSGAGGQQPPQQPRMARSPTNSLLKHKSLSLSMHSLNFITANPAPQSQLSPNAKEFVYNGGGSPSLFFDAADGQGSGTPGPFGGSGAGTCNSSSFDMAQVFGGGANSLFLEKTPFVEGLSYNLNTMQYPSQQFQPVVLAN

47 NP_005924.2 NP_060177.2

>NP_005924.2

MAHSCRWRFPARPGTTGGGGGGGRRGLGGAPRQRVPALLLPPGPPVGGGGPGAPPSPPAVAAAAAAAGSSGAGVPGGAAAASAASSSSASSSSSSSSSASSGPALLRVGPGFDAALQVSAAIGTNLRRFRAVFGESGGGGGSGEDEQFLGFGSDEEVRVRSPTRSPSVKTSPRKPRGRPRSGSDRNSAILSDPSVFSPLNKSETKSGDKIKKKDSKSIEKKRGRPPTFPGVKIKITHGKDISELPKGNKEDSLKKIKRTPSATFQQATKIKKLRAGKLSPLKSKFKTGKLQIGRKGVQIVRRRGRPPSTERIKTPSGLLINSELEKPQKVRKDKEGTPPLTKEDKTVVRQSPRRIKPVRIIPSSKRTDATIAKQLLQRAKKGAQKKIEKEAAQLQGRKVKTQVKNIRQFIMPVVSAISSRIIKTPRRFIEDEDYDPPIKIARLESTPNSRFSAPSCGSSEKSSAASQHSSQMSSDSSRSSSPSVDTSTDSQASEEIQVLPEERSDTPEVHPPLPISQSPENESNDRRSRRYSVSERSFGSRTTKKLSTLQSAPQQQTSSSPPPPLLTPPPPLQPASSISDHTPWLMPPTIPLASPFLPASTAPMQGKRKSILREPTFRWTSLKHSRSEPQYFSSAKYAKEGLIRKPIFDNFRPPPLTPEDVGFASGFSASGTAASARLFSPLHSGTRFDMHKRSPLLRAPRFTPSEAHSRIFESVTLPSNRTSAGTSSSGVSNRKRKRKVFSPIRSEPRSPSHSMRTRSGRLSSSELSPLTPPSSVSSSLSISVSPLATSALNPTFTFPSHSLTQSGESAEKNQRPRKQTSAPAEPFSSSSPTPLFPWFTPGSQTERGRNKDKAPEELSKDRDADKSVEKDKSRERDREREKENKRESRKEKRKKGSEIQSSSALYPVGRVSKEKVVGEDVATSSSAKKATGRKKSSSHDSGTDITSVTLGDTTAVKTKILIKKGRGNLEKTNLDLGPTAPSLEKEKTLCLSTPSSSTVKHSTSSIGSMLAQADKLPMTDKRVASLLKKAKAQLCKIEKSKSLKQTDQPKAQGQESDSSETSVRGPRIKHVCRRAAVALGRKRAVFPDDMPTLSALPWEEREKILSSMGNDDKSSIAGSEDAEPLAPPIKPIKPVTRNKAPQEPPVKKGRRSRRCGQCPGCQVPEDCGVCTNCLDKPKFGGRNIKKQCCKMRKCQNLQWMPSKAYLQKQAKAVKKKEKKSKTSEKKDSKESSVVKNVVDSSQKPTPSAREDPAPKKSSSEPPPRKPVEEKSEEGNVSAPGPESKQATTPASRKSSKQVSQPALVIPPQPPTTGPPRKEVPKTTPSEPKKKQPPPPESGPEQSKQKKVAPRPSIPVKQKPKEKEKPPPVNKQENAGTLNILSTLSNGNSSKQKIPADGVHRIRVDFKEDCEAENVWEMGGLGILTSVPITPRVVCFLCASSGHVEFVYCQVCCEPFHKFCLEENERPLEDQLENWCCRRCKFCHVCGRQHQATKQLLECNKCRNSYHPECLGPNYPTKPTKKKKVWICTKCVRCKSCGSTTPGKGWDAQWSHDFSLCHDCAKLFAKGNFCPLCDKCYDDDDYESKMMQCGKCDRWVHSKCENLSDEMYEILSNLPESVAYTCVNCTERHPAEWRLALEKELQISLKQVLTALLNSRTTSHLLRYRQAAKPPDLNPETEESIPSRSSPEGPDPPVLTEVSKQDDQQPLDLEGVKRKMDQGNYTSVLEFSDDIVKIIQAAINSDGGQPEIKKANSMVKSFFIRQMERVFPWFSVKKSRFWEPNKVSSNSGMLPNAVLPPSLDHNYAQWQEREENSHTEQPPLMKKIIPAPKPKGPGEPDSPTPLHPPTPPILSTDRSREDSPELNPPPGIEDNRQCALCLTYGDDSANDAGRLLYIGQNEWTHVNCALWSAEVFEDDDGSLKNVHMAVIRGKQLRCEFCQKPGATVGCCLTSCTSNYHFMCSRAKNCVFLDDKKVYCQRHRDLIKGEVVPENGFEVFRRVFVDFEGISLRRKFLNGLEPENIHMMIGSMTIDCLGILNDLSDCEDKLFPIGYQCSRVYWSTTDARKRCVYTCKIVECRPPVVEPDINSTVEHDENRTIAHSPTSFTESSSKESQNTAEIISPPSPDRPPHSQTSGSCYYHVISKVPRIRTPSYSPTQRSPGCRPLPSAGSPTPTTHEIVTVGDPLLSSGLRSIGSRRHSTSSLSPQRSKLRIMSPMRTGNTYSRNNVSSVSTTGTATDLESSAKVVDHVLGPLNSSTSLGQNTSTSSNLQRTVVTVGNKNSHLDGSSSSEMKQSSASDLVSKSSSLKGEKTKVLSSKSSEGSAHNVAYPGIPKLAPQVHNTTSRELNVSKIGSFAEPSSVSFSSKEALSFPHLHLRGQRNDRDQHTDSTQSANSSPDEDTEVKTLKLSGMSNRSSIINEHMGSSSRDRRQKGKKSCKETFKEKHSSKSFLEPGQVTTGEEGNLKPEFMDEVLTPEYMGQRPCNNVSSDKIGDKGLSMPGVPKAPPMQVEGSAKELQAPRKRTVKVTLTPLKMENESQSKNALKESSPASPLQIESTSPTEPISASENPGDGPVAQPSPNNTSCQDSQSNNYQNLPVQDRNLMLPDGPKPQEDGSFKRRYPRRSARARSNMFFGLTPLYGVRSYGEEDIPFYSSSTGKKRGKRSAEGQVDGADDLSTSDEDDLYYYNFTRTVISSGGEERLASHNLFREEEQCDLPKISQLDGVDDGTESDTSVTATTRKSSQIPKRNGKENGTENLKIDRPEDAGEKEHVTKSSVGHKNEPKMDNCHSVSRVKTQGQDSLEAQLSSLESSRRVHTSTPSDKNLLDTYNTELLKSDSDNNNSDDCGNILPSDIMDFVLKNTPSMQALGESPESSSSELLNLGEGLGLDSNREKDMGLFEVFSQQLPTTEPVDSSVSSSISAEEQFELPLELPSDLSVLTTRSPTVPSQNPSRLAVISDSGEKRVTITEKSVASSESDPALLSPGVDPTPEGHMTPDHFIQGHMDADHISSPPCGSVEQGHGNNQDLTRNSSTPGLQVPVSPTVPIQNQKYVPNSTDSPGPSQISNAAVQTTPPHLKPATEKLIVVNQNMQPLYVLQTLPNGVTQKIQLTSSVSSTPSVMETNTSVLGPMGGGLTLTTGLNPSLPTSQSLFPSASKGLLPMSHHQHLHSFPAATQSSFPPNISNPPSGLLIGVQPPPDPQLLVSESSQRTDLSTTVATPSSGLKKRPISRLQTRKNKKLAPSSTPSNIAPSDVVSNMTLINFTPSQLPNHPSLLDLGSLNTSSHRTVPNIIKRSKSSIMYFEPAPLLPQSVGGTAATAAGTSTISQDTSHLTSGSVSGLASSSSVLNVVSMQTTTTPTSSASVPGHVTLTNPRLLGTPDIGSISNLLIKASQQSLGIQDQPVALPPSSGMFPQLGTSQTPSTAAITAASSICVLPSTQTTGITAASPSGEADEHYQLQHVNQLLASKTGIHSSQRDLDSASGPQVSNFTQTVDAPNSMGLEQNKALSSAVQASPTSPGGSPSSPSSGQRSASPSVPGPTKPKPKTKRFQLPLDKGNGKKHKVSHLRTSSSEAHIPDQETTSLTSGTGTPGAEAEQQDTASVEQSSQKECGQPAGQVAVLPEVQVTQNPANEQESAEPKTVEEEESNFSSPLMLWLQQEQKRKESITEKKPKKGLVFEISSDDGFQICAESIEDAWKSLTDKVQEARSNARLKQLSFAGVNGLRMLGILHDAVVFLIEQLSGAKHCRNYKFRFHKPEEANEPPLNPHGSARAEVHLRKSAFDMFNFLASKHRQPPEYNPNDEEEEEVQLKSARRATSMDLPMPMRFRHLKKTSKEAVGVYRSPIHGRGLFCKRNIDAGEMVIEYAGNVIRSIQTDKREKYYDSKGIGCYMFRIDDSEVVDATMHGNAARFINHSCEPNCYSRVINIDGQKHIVIFAMRKIYRGEELTYDYKFPIEDASNKLPCNCGAKKCRKFLN

>NP_060177.2

MPEQFSVAEFLAVTAEDLSSPAGAAAFAAKMPRYRGAALAREEILEGDQAILQRIKKAVRAIHSSGLGHVENEEQYREAVESLGNSHLSQNSHELSTGFLNLAVFTREVAALFKNLIQNLNNIVSFPLDSLMKGQLRDGRQDSKKQLEKAWKDYEAKMAKLEKERDRARVTGGIPGEVAQDMQRERRIFQLHMCEYLLKAGESQMKQGPDFLQSLIKFFHAQHNFFQDGWKAAQSLFPFIEKLAASVHALHQAQEDELQKLTQLRDSLRGTLQLESREEHLSRKNSGCGYSIHQHQGNKQFGTEKVGFLYKKSDGIRRVWQKRKCGVKYGCLTISHSTINRPPVKLTLLTCQVRPNPEEKKCFDLVTHNRTYHFQAEDEHECEAWVSVLQNSKDEALSSAFLGEPSAGPGSWGSAGHDGEPHDLTKLLIAEVKSRPGNSQCCDCGAADPTWLSTNLGVLTCIQCSGVHRELGVRFSRMQSLTLDLLGPSELLLALNMGNTSFNEVMEAQLPSHGGPKPSAESDMGTRRDYIMAKYVEHRFARRCTPEPQRLWTAICNRDLLSVLEAFANGQDFGQPLPGPDAQAPEELVLHLAVKVANQASLPLVDFIIQNGGHLDAKAADGNTALHYAALYNQPDCLKLLLKGRALVGTVNEAGETALDIARKKHHKECEELLEQAQAGTFAFPLHVDYSWVISTEPGSDSEEDEEEKRCLLKLPAQAHWASGRLDISNKTYETVASLGAATPQGESEDCPPPLPVKNSSRTLVQGCARHASGDRSEVSSLSSEAPETPESLGSPASSSSLMSPLEPGDPSQAPPNSEEGLREPPGTSRPSLTSGTTPSEMYLPVRFSSESTRSYRRGARSPEDGPSARQPLPRRNVPVGITEGDGSRTGSLPASSVQLLQD

48 P62807 Q14914

>P62807

MPEPAKSAPAPKKGSKKAVTKAQKKDGKKRKRSRKESYSVYVYKVLKQVHPDTGISSKAMGIMNSFVNDIFERIAGEASRLAHYNKRSTITSREIQTAVRLLLPGELAKHAVSEGTKAVTKYTSK

>Q14914

MVRTKTWTLKKHFVGYPTNSDFELKTAELPPLKNGEVLLEALFLTVDPYMRVAAKRLKEGDTMMGQQVAKVVESKNVALPKGTIVLASPGWTTHSISDGKDLEKLLTEWPDTIPLSLALGTVGMPGLTAYFGLLEICGVKGGETVMVNAAAGAVGSVVGQIAKLKGCKVVGAVGSDEKVAYLQKLGFDVVFNYKTVESLEETLKKASPDGYDCYFDNVGGEFSNTVIGQMKKFGRIAICGAISTYNRTGPLPPGPPPEIVIYQELRMEAFVVYRWQGDARQKALKDLLKWVLEGKIQYKEYIIEGFENMPAAFMGMLKGDNLGKTIVKA

49 Q14562 NP_060177.2

>Q14562

MAVAVAMAGALIGSEPGPAEELAKLEYLSLVSKVCTELDNHLGINDKDLAEFVISLAEKNTTFDTFKASLVKNGAEFTDSLISNLLRLIQTMRPPAKPSTSKDPVVKPKTEKEKLKELFPVLCQPDNPSVRTMLDEDDVKVAVDVLKELEALMPSAAGQEKQRDAEHRDRTKKKKRSRSRDRNRDRDRDRERNRDRDHKRRHRSRSRSRSRTRERNKVKSRYRSRSRSQSPPKDRKDRDKYGERNLDRWRDKHVDRPPPEEPTIGDIYNGKVTSIMQFGCFVQLEGLRKRWEGLVHISELRREGRVANVADVVSKGQRVKVKVLSFTGTKTSLSMKDVDQETGEDLNPNRRRNLVGETNEETSMRNPDRPTHLSLVSAPEVEDDSLERKRLTRISDPEKWEIKQMIAANVLSKEEFPDFDEETGILPKVDDEEDEDLEIELVEEEPPFLRGHTKQSMDMSPIKIVKNPDGSLSQAAMMQSALAKERRELKQAQREAEMDSIPMGLNKHWVDPLPDAEGRQIAANMRGIGMMPNDIPEWKKHAFGGNKASYGKKTQMSILEQRESLPIYKLKEQLVQAVHDNQILIVIGETGSGKTTQITQYLAEAGYTSRGKIGCTQPRRVAAMSVAKRVSEEFGCCLGQEVGYTIRFEDCTSPETVIKYMTDGMLLRECLIDPDLTQYAIIMLDEAHERTIHTDVLFGLLKKTVQKRQDMKLIVTSATLDAVKFSQYFYEAPIFTIPGRTYPVEILYTKEPETDYLDASLITVMQIHLTEPPGDILVFLTGQEEIDTACEILYERMKSLGPDVPELIILPVYSALPSEMQTRIFDPAPPGSRKVVIATNIAETSLTIDGIYYVVDPGFVKQKVYNSKTGIDQLVVTPISQAQAKQRAGRAGRTGPGKCYRLYTERAYRDEMLTTNVPEIQRTNLASTVLSLKAMGINDLLSFDFMDAPPMETLITAMEQLYTLGALDDEGLLTRLGRRMAEFPLEPMLCKMLIMSVHLGCSEEMLTIVSMLSVQNVFYRPKDKQALADQKKAKFHQTEGDHLTLLAVYNSWKNNKFSNPWCYENFIQARSLRRAQDIRKQMLGIMDRHKLDVVSCGKSTVRVQKAICSGFFRNAAKKDPQEGYRTLIDQQVVYIHPSSALFNRQPEWVVYHELVLTTKEYMREVTTIDPRWLVEFAPAFFKVSDPTKLSKQKKQQRLEPLYNRYEEPNAWRISRAFRRR

>NP_060177.2

MPEQFSVAEFLAVTAEDLSSPAGAAAFAAKMPRYRGAALAREEILEGDQAILQRIKKAVRAIHSSGLGHVENEEQYREAVESLGNSHLSQNSHELSTGFLNLAVFTREVAALFKNLIQNLNNIVSFPLDSLMKGQLRDGRQDSKKQLEKAWKDYEAKMAKLEKERDRARVTGGIPGEVAQDMQRERRIFQLHMCEYLLKAGESQMKQGPDFLQSLIKFFHAQHNFFQDGWKAAQSLFPFIEKLAASVHALHQAQEDELQKLTQLRDSLRGTLQLESREEHLSRKNSGCGYSIHQHQGNKQFGTEKVGFLYKKSDGIRRVWQKRKCGVKYGCLTISHSTINRPPVKLTLLTCQVRPNPEEKKCFDLVTHNRTYHFQAEDEHECEAWVSVLQNSKDEALSSAFLGEPSAGPGSWGSAGHDGEPHDLTKLLIAEVKSRPGNSQCCDCGAADPTWLSTNLGVLTCIQCSGVHRELGVRFSRMQSLTLDLLGPSELLLALNMGNTSFNEVMEAQLPSHGGPKPSAESDMGTRRDYIMAKYVEHRFARRCTPEPQRLWTAICNRDLLSVLEAFANGQDFGQPLPGPDAQAPEELVLHLAVKVANQASLPLVDFIIQNGGHLDAKAADGNTALHYAALYNQPDCLKLLLKGRALVGTVNEAGETALDIARKKHHKECEELLEQAQAGTFAFPLHVDYSWVISTEPGSDSEEDEEEKRCLLKLPAQAHWASGRLDISNKTYETVASLGAATPQGESEDCPPPLPVKNSSRTLVQGCARHASGDRSEVSSLSSEAPETPESLGSPASSSSLMSPLEPGDPSQAPPNSEEGLREPPGTSRPSLTSGTTPSEMYLPVRFSSESTRSYRRGARSPEDGPSARQPLPRRNVPVGITEGDGSRTGSLPASSVQLLQD

50 NP_009171.1 NP_003105.2

>NP_009171.1

MLEAPGPSDGCELSNPSASRVSCAGQMLEVQPGLYFGGAAAVAEPDHLREAGITAVLTVDSEEPSFKAGPGVEDLWRLFVPALDKPETDLLSHLDRCVAFIGQARAEGRAVLVHCHAGVSRSVAIITAFLMKTDQLPFEKAYEKLQILKPEAKMNEGFEWQLKLYQAMGYEVDTSSAIYKQYRLQKVTEKYPELQNLPQELFAVDPTTVSQGLKDEVLYKCRKCRRSLFRSSSILDHREGSGPIAFAHKRMTPSSMLTTGRQAQCTSYFIEPVQWMESALLGVMDGQLLCPKCSAKLGSFNWYGEQCSCGRWITPAFQIHKNRVDEMKILPVLGSQTGKI

>NP_003105.2

MTTKDYPSLWGFGTTKTFKIPIEHLDFKYIEKCSDVKHLEKILCVLRSGEEGYYPELTEFCEKHLQALAPESRALRKDKPAATAASFTAEEWEKIDGDIKSWVSEIKKEEDKMHFHETETFPAMKDNLPPVRGSNSCLHVGKEKYSKRPTKKKTPRDYAEWDKFDVEKECLKIDEDYKEKTVIDKSHLSKIETRIDTAGLTEKEKDFLATREKEKGNEAFNSGDYEEAVMYYTRSISALPTVVAYNNRAQAEIKLQNWNSAFQDCEKVLELEPGNVKALLRRATTYKHQNKLREATEDLSKVLDVEPDNDLAKKTLSEVERDLKNSEAASETQTKGKRMVIQEIENSEDEEGKSGRKHEDGGGDKKPAEPAGAARAAQPCVMGNIQKKLTGKAEGGKRPARGAPQRGQTPEAGADKRSPRRASAAAAAGGGATGHPGGGQGAENPAGLKSQGNELFRSGQFAEAAGKYSAAIALLEPAGSEIADDLSILYSNRAACYLKEGNCSGCIQDCNRALELHPFSMKPLLRRAMAYETLEQYGKAYVDYKTVLQIDCGLQLANDSVNRLSRILMELDGPNWREKLSPIPAVPASVPLQAWHPAKEMISKQAGDSSSHRQQGITDEKTFKALKEEGNQCVNDKNYKDALSKYSECLKINNKECAIYTNRALCYLKLCQFEEAKQDCDQALQLADGNVKAFYRRALAHKGLKNYQKSLIDLNKVILLDPSIIEAKMELEEVTRLLNLKDKTAPFNKEKERRKIEIQEVNEGKEEPGRPAGEVSMGCLASEKGGKSSRSPEDPEKLPIAKPNNAYEFGQIINALSTRKDKEACAHLLAITAPKDLPMFLSNKLEGDTFLLLIQSLKNNLIEKDPSLVYQHLLYLSKAERFKMMLTLISKGQKELIEQLFEDLSDTPNNHFTLEDIQALKRQYEL

51 NP_005578.1 Q8N183

>NP_005578.1

MGRKKIQITRIMDERNRQVTFTKRKFGLMKKAYELSVLCDCEIALIIFNSSNKLFQYASTDMDKVLLKYTEYNEPHESRTNSDIVEALNKKEHRGCDSPDPDTSYVLTPHTEEKYKKINEEFDNMMRNHKIAPGLPPQNFSMSVTVPVTSPNALSYTNPGSSLVSPSLAASSTLTDSSMLSPPQTTLHRNVSPGAPQRPPSTGNAGGMLSTTDLTVPNGAGSSPVGNGFVNSRASPNLIGATGANSLGKVMPTKSPPPPGGGNLGMNSRKPDLRVVIPPSSKGMMPPLSEEEELELNTQRISSSQATQPLATPVVSVTTPSLPPQGLVYSAMPTAYNTDYSLTSADLSALQGFNSPGMLSLGQVSAWQQHHLGQAALSSLVAGGQLSQGSNLSINTNQNISIKSEPISPPRDRMTPSGFQQQQQQQQQQQPPPPPQPQPQPPQPQPRQEMGRSPVDSLSSSSSSYDGSDREDPRGDFHSPIVLGRPPNTEDRESPSVKRMRMDAWVT

>Q8N183

MGWSQDLFRALWRSLSREVKEHVGTDQFGNKYYYIPQYKNWRGQTIREKRIVEAANKKEVDYEAGDIPTEWEAWIRRTRKTPPTMEEILKNEKHREEIKIKSQDFYEKEKLLSKETSEELLPPPVQTQIKGHASAPYFGKEEPSVAPSSTGKTFQPGSWMPRDGKSHNQ

52 NP_004082.1 NP_038203.2

>NP_004082.1

MLQGPRALASAAGQTPKVVPAMSPTELWPSGLSSPQLCPATATYYTPLYPQTAPPAAAPGTCLDATPHGPEGQVVRCLPAGRLPAKRKLDLEGIGRPVVPEFPTPKGKCIRVDGLPSPKTPKSPGEKTRYDTSLGLLTKKFIYLLSESEDGVLDLNWAAEVLDVQKRRIYDITNVLEGIQLIRKKAKNNIQWVGRGMFEDPTRPGKQQQLGQELKELMNTEQALDQLIQSCSLSFKHLTEDKANKRLAYVTYQDIRAVGNFKEQTVIAVKAPPQTRLEVPDRTEDNLQIYLKSTQGPIEVYLCPEEVQEPDSPSEEPLPSTSTLCPSPDSAQPSSSTDPSIMEPTASSVPAPAPTPQQAPPPPSLVPLEATDSLLELPHPLLQQTEDQFLSPTLACSSPLISFSPSLDQDDYLWGLEAGEGISDLFDSYDLGDLLIN

>NP_038203.2

MLQQVPENINFPAEEEKILEFWTEFNCFQECLKQSKHKPKFTFYDGPPFATGLPHYGHILAGTIKDIVTRYAHQSGFHVDRRFGWDCHGLPVEYEIDKTLGIRGPEDVAKMGITEYNNQCRAIVMRYSAEWKSTVSRLGRWIDFDNDYKTLYPQFMESVWWVFKQLYDKGLVYRGVKVMPFSTACNTPLSNFESHQNYKDVQDPSVFVTFPLEEDETVSLVAWTTTPWTLPSNLAVCVNPEMQYVKIKDVARGRLLILMEARLSALYKLESDYEILERFPGAYLKGKKYRPLFDYFLKCKENGAFTVLVDNYVKEEEGTGVVHQAPYFGAEDYRVCMDFNIIRKDSLPVCPVDASGCFTTEVTDFAGQYVKDADKSIIRTLKEQGRLLVATTFTHSYPFCWRSDTPLIYKAVPSWFVRVENMVDQLLRNNDLCYWVPELVREKRFGNWLKDARDWTISRNRYWGTPIPLWVSDDFEEVVCIGSVAELEELSGAKISDLHRESVDHLTIPSRCGKGSLHRISEVFDCWFESGSMPYAQVHYPFENKREFEDAFPADFIAEGIDQTRGWFYTLLVLATALFGQPPFKNVIVNGLVLASDGQKMSKRKKNYPDPVSIIQKYGADALRLYLINSPVVRAENLRFKEEGVRDVLKDVLLPWYNAYRFLIQNVLRLQKEEEIEFLYNENTVRESPNITDRWILSFMQSLIGFFETEMAAYRLYTVVPRLVKFVDILTNWYVRMNRRRLKGENGMEDCVMALETLFSVLLSLCRLMAPYTPFLTELMYQNLKVLIDPVSVQDKDTLSIHYLMLPRVREELIDKKTESAVSQMQSVIELGRVIRDRKTIPIKYPLKEIVVIHQDPEALKDIKSLEKYIIEELNVRKVTLSTDKNKYGIRLRAEPDHMVLGKRLKGAFKAVMTSIKQLSSEELEQFQKTGTIVVEGHELHDEDIRLMYTFDQATGGTAQFEAHSDAQALVLLDVTPDQSMVDEGMAREVINRIQKLRKKCNLVPTDEITVYYKAKSEGTYLNSVIESHTEFIFTTIKAPLKPYPVSPSDKVLIQEKTQLKGSELEITLTRGSSLPGPACAYVNLNICANGSEQGGVLLLENPKGDNRLDLLKLKSVVTSIFGVKNTELAVFHDETEIQNQTDLLSLSGKTLCVTAGSAPSLINSSSTLLCQYINLQLLNAKPQECLMGTVGTLLLENPLGQNGLTHQGLLYEAAKVFGLRSRKLKLFLNETQTQEITEDIPVKTLNMKTVYVSVLPTTADF

53 NP_004217.1 NP_005993.1

>NP_004217.1

MSRRRFDCRSISGLLTTTPQIPIKMENFNNFYILTSKELGRGKFAVVRQCISKSTGQEYAAKFLKKRRRGQDCRAEILHEIAVLELAKSCPRVINLHEVYENTSEIILILEYAAGGEIFSLCLPELAEMVSENDVIRLIKQILEGVYYLHQNNIVHLDLKPQNILLSSIYPLGDIKIVDFGMSRKIGHACELREIMGTPEYLAPEILNYDPITTATDMWNIGIIAYMLLTHTSPFVGEDNQETYLNISQVNVDYSEETFSSVSQLATDFIQSLLVKNPEKRPTAEICLSHSWLQQWDFENLFHPEETSSSSQTQDHSVRSSEDKTSKSSCNGTCGDREDKENIPEDSSMVSKRFRFDDSLPNPHELVSDLLC

>NP_005993.1

MEGQRWLPLEANPEVTNQFLKQLGLHPNWQFVDVYGMDPELLSMVPRPVCAVLLLFPITEKYEVFRTEEEEKIKSQGQDVTSSVYFMKQTISNACGTIGLIHAIANNKDKMHFESGSTLKKFLEESVSMSPEERARYLENYDAIRVTHETSAHEGQTEAPSIDEKVDLHFIALVHVDGHLYELDGRKPFPINHGETSDETLLEDAIEVCKKFMERDPDELRFNAIALSAA

54 NP_002120.1 NP_000036.2

>NP_002120.1

MGKGDPNKPRGKMSSYAFFVQTCREEHKKKHPDSSVNFAEFSKKCSERWKTMSAKEKSKFEDMAKSDKARYDREMKNYVPPKGDKKGKKKDPNAPKRPPSAFFLFCSEHRPKIKSEHPGLSIGDTAKKLGEMWSEQSAKDKQPYEQKAAKLKEKYEKDIAAYRAKGKSEAGKKGPGRPTGSKKKNEPEDEEEEEEEEDEDEEEEDEDEE

>NP_000036.2

MSAKSRTIGIIGAPFSKGQPRGGVEEGPTVLRKAGLLEKLKEQECDVKDYGDLPFADIPNDSPFQIVKNPRSVGKASEQLAGKVAEVKKNGRISLVLGGDHSLAIGSISGHARVHPDLGVIWVDAHTDINTPLTTTSGNLHGQPVSFLLKELKGKIPDVPGFSWVTPCISAKDIVYIGLRDVDPGEHYILKTLGIKYFSMTEVDRLGIGKVMEETLSYLLGRKKRPIHLSFDVDGLDPSFTPATGTPVVGGLTYREGLYITEEIYKTGLLSGLDIMEVNPSLGKTPEEVTRTVNTAVAITLACFGLAREGNHKPIDYLNPPK

55 NP_055126.1 NP_612393.2

>NP_055126.1

MPQAFLLGSIHEPAGALMEPQPCPGSLAESFLEEELRLNAELSQLQFSEPVGIIYNPVEYAWEPHRNYVTRYCQGPKEVLFLGMNPGPFGMAQTGVPFGEVSMVRDWLGIVGPVLTPPQEHPKRPVLGLECPQSEVSGARFWGFFRNLCGQPEVFFHHCFVHNLCPLLFLAPSGRNLTPAELPAKQREQLLGICDAALCRQVQLLGVRLVVGVGRLAEQRARRALAGLMPEVQVEGLLHPSPRNPQANKGWEAVAKERLNELGLLPLLLK

>NP_612393.2

MRLTPRALCSAAQAAWRENFPLCGRDVARWFPGHMAKGLKKMQSSLKLVDCIIEVHDARIPLSGRNPLFQETLGLKPHLLVLNKMDLADLTEQQKIMQHLEGEGLKNVIFTNCVKDENVKQIIPMVTELIGRSHRYHRKENLEYCIMVIGVPNVGKSSLINSLRRQHLRKGKATRVGGEPGITRAVMSKIQVSERPLMFLLDTPGVLAPRIESVETGLKLALCGTVLDHLVGEETMADYLLYTLNKHQRFGYVQHYGLGSACDNVERVLKSVAVKLGKTQKVKVLTGTGNVNIIQPNYPAAARDFLQTFRRGLLGSVMLDLDVLRGHPPAETLP

56 O95766 Q12882

>O95766

MAAAAAGAGSGPWAAQEKQFPPALLSFFIYNPRFGPREGQEENKILFYHPNEVEKNEKIRNVGLCEAIVQFTRTFSPSKPAKSLHTQKNRQFFNEPEENFWMVMVVRNPIIEKQSKDGKPVIEYQEEELLDKVYSSVLRQCYSMYKLFNGTFLKAMEDGGVKLLKERLEKFFHRYLQTLHLQSCDLLDIFGGISFFPLDKMTYLKIQSFINRMEESLNIVKYTAFLYNDQLIWSGLEQDDMRILYKYLTTSLFPRHIEPELAGRDSPIRAEMPGNLQHYGRFLTGPLNLNDPDAKCRFPKIFVNTDDTYEELHLIVYKAMSAAVCFMIDASVHPTLDFCRRLDSIVGPQLTVLASDICEQFNINKRMSGSEKEPQFKFIYFNHMNLAEKSTVHMRKTPSVSLTSVHPDLMKILGDINSDFTRVDEDEEIIVKAMSDYWVVGKKSDRRELYVILNQKNANLIEVNEEVKKLCATQFNNIFFLD

>Q12882

MAPVLSKDSADIESILALNPRTQTHATLCSTSAKKLDKKHWKRNPDKNCFNCEKLENNFDDIKHTTLGERGALREAMRCLKCADAPCQKSCPTNLDIKSFITSIANKNYYGAAKMIFSDNPLGLTCGMVCPTSDLCVGGCNLYATEEGPINIGGLQQFATEVFKAMSIPQIRNPSLPPPEKMSEAYSAKIALFGAGPASISCASFLARLGYSDITIFEKQEYVGGLSTSEIPQFRLPYDVVNFEIELMKDLGVKIICGKSLSVNEMTLSTLKEKGYKAAFIGIGLPEPNKDAIFQGLTQDQGFYTSKDFLPLVAKGSKAGMCACHSPLPSIRGVVIVLGAGDTAFDCATSALRCGARRVFIVFRKGFVNIRAVPEEMELAKEEKCEFLPFLSPRKVIVKGGRIVAMQFVRTEQDETGKWNEDEDQMVHLKADVVISAFGSVLSDPKVKEALSPIKFNRWGLPEVDPETMQTSEAWVFAGGDVVGLANTTVESVNDGKQASWYIHKYVQSQYGASVSAKPELPLFYTPIDLVDISVEMAGLKFINPFGLASATPATSTSMIRRAFEAGWGFALTKTFSLDKDIVTNVSPRIIRGTTSGPMYGPGQSSFLNIELISEKTAAYWCQSVTELKADFPDNIVIASIMCSYNKNDWTELAKKSEDSGADALELNLSCPHGMGERGMGLACGQDPELVRNICRWVRQAVQIPFFAKLTPNVTDIVSIARAAKEGGANGVTATNTVSGLMGLKSDGTPWPAVGIAKRTTYGGVSGTAIRPIALRAVTSIARALPGFPILATGGIDSAESGLQFLHSGASVLQVCSAIQNQDFTVIEDYCTGLKALLYLKSIEELQDWDGQSPATVSHQKGKPVPRIAELMDKKLPSFGPYLEQRKKIIAENKIRLKEQNVAFSPLKRNCFIPKRPIPTIKDVIGKALQYLGTFGELSNVEQVVAMIDEEMCINCGKCYMTCNDSGYQAIQFDPETHLPTITDTCTGCTLCLSVCPIVDCIKMVSRTTPYEPKRGVPLSVNPVC

57 NP_005345.3 NP_001087.2

>NP_005345.3

METPFYGDEALSGLGGGASGSGGSFASPGRLFPGAPPTAAAGSMMKKDALTLSLSEQVAAALKPAAAPPPTPLRADGAPSAAPPDGLLASPDLGLLKLASPELERLIIQSNGLVTTTPTSSQFLYPKVAASEEQEFAEGFVKALEDLHKQNQLGAGAAAAAAAAAAGGPSGTATGSAPPGELAPAAAAPEAPVYANLSSYAGGAGGAGGAATVAFAAEPVPFPPPPPPGALGPPRLAALKDEPQTVPDVPSFGESPPLSPIDMDTQERIKAERKRLRNRIAASKCRKRKLERISRLEEKVKTLKSQNTELASTASLLREQVAQLKQKVLSHVNSGCQLLPQHQVPAY

>NP_001087.2

MSAKAISEQTGKELLYKFICTTSAIQNRFKYARVTPDTDWARLLQDHPWLLSQNLVVKPDQLIKRRGKLGLVGVNLTLDGVKSWLKPRLGQEATVGKATGFLKNFLIEPFVPHSQAEEFYVCIYATREGDYVLFHHEGGVDVGDVDAKAQKLLVGVDEKLNPEDIKKHLLVHAPEDKKEILASFISGLFNFYEDLYFTYLEINPLVVTKDGVYVLDLAAKVDATADYICKVKWGDIEFPPPFGREAYPEEAYIADLDAKSGASLKLTLLNPKGRIWTMVAGGGASVVYSDTICDLGGVNELANYGEYSGAPSEQQTYDYAKTILSLMTREKHPDGKILIIGGSIANFTNVAATFKGIVRAIRDYQGPLKEHEVTIFVRRGGPNYQEGLRVMGEVGKTTGIPIHVFGTETHMTAIVGMALGHRPIPNQPPTAAHTANFLLNASGSTSTPAPSRTASFSESRADEVAPAKKAKPAMPQDSVPSPRSLQGKSTTLFSRHTKAIVWGMQTRAVQGMLDFDYVCSRDEPSVAAMVYPFTGDHKQKFYWGHKEILIPVFKNMADAMRKHPEVDVLINFASLRSAYDSTMETMNYAQIRTIAIIAEGIPEALTRKLIKKADQKGVTIIGPATVGGIKPGCFKIGNTGGMLDNILASKLYRPGSVAYVSRSGGMSNELNNIISRTTDGVYEGVAIGGDRYPGSTFMDHVLRYQDTPGVKMIVVLGEIGGTEEYKICRGIKEGRLTKPIVCWCIGTCATMFSSEVQFGHAGACANQASETAVAKNQALKEAGVFVPRSFDELGEIIQSVYEDLVANGVIVPAQEVPPPTVPMDYSWARELGLIRKPASFMTSICDERGQELIYAGMPITEVFKEEMGIGGVLGLLWFQKRLPKYSCQFIEMCLMVTADHGPAVSGAHNTIICARAGKDLVSSLTSGLLTIGDRFGGALDAAAKMFSKAFDSGIIPMEFVNKMKKEGKLIMGIGHRVKSINNPDMRVQILKDYVRQHFPATPLLDYALEVEKITTSKKPNLILNVDGLIGVAFVDMLRNCGSFTREEADEYIDIGALNGIFVLGRSMGFIGHYLDQKRLKQGLYRHPWDDISYVLPEHMSM

58 NP_690856.1 NP_002564.1

>NP_690856.1

MWIQVRTIDGSKTCTIEDVSRKATIEELRERVWALFDVRPECQRLFYRGKQLENGYTLFDYDVGLNDIIQLLVRPDPDHLPGTSTQIEAKPCSNSPPKVKKAPRVGPSNQPSTSARARLIDPGFGIYKVNELVDARDVGLGAWFEAHIHSVTRASDGQSRGKTPLKNGSSCKRTNGNIKHKSKENTNKLDSVPSTSNSDCVAADEDVIYHIQYDEYPESGTLEMNVKDLRPRARTILKWNELNVGDVVMVNYNVESPGQRGFWFDAEITTLKTISRTKKELRVKIFLGGSEGTLNDCKIISVDEIFKIERPGAHPLSFADGKFLRRNDPECDLCGGDPEKKCHSCSCRVCGGKHEPNMQLLCDECNVAYHIYCLNPPLDKVPEEEYWYCPSCKTDSSEVVKAGERLKMSKKKAKMPSASTESRRDWGRGMACVGRTRECTIVPSNHYGPIPGIPVGSTWRFRVQVSEAGVHRPHVGGIHGRSNDGAYSLVLAGGFADEVDRGDEFTYTGSGGKNLAGNKRIGAPSADQTLTNMNRALALNCDAPLDDKIGAESRNWRAGKPVRVIRSFKGRKISKYAPEEGNRYDGIYKVVKYWPEISSSHGFLVWRYLLRRDDVEPAPWTSEGIERSRRLCLRLQYPAGYPSDKEGKKPKGQSKKQPSGTTKRPISDDDCPSASKVYKASDSAEAIEAFQLTPQQQHLIREDCQNQKLWDEVLSHLVEGPNFLKKLEQSFMCVCCQELVYQPVTTECFHNVCKDCLQRSFKAQVFSCPACRHDLGQNYIMIPNEILQTLLDLFFPGYSKGR

>NP_002564.1

MSGEENPASKPTPVQDVQGDGRWMSLHHRFVADSKDKEPEVVFIGDSLVQLMHQCEIWRELFSPLHALNFGIGGDGTQHVLWRLENGELEHIRPKIVVVWVGTNNHGHTAEQVTGGIKAIVQLVNERQPQARVVVLGLLPRGQHPNPLREKNRQVNELVRAALAGHPRAHFLDADPGFVHSDGTISHHDMYDYLHLSRLGYTPVCRALHSLLLRLLAQDQGQGAPLLEPAP

59 Q01664 Q9H6S3

>Q01664

MEYFMVPTQKVPSLQHFRKTEKEVIGGLCSLANIPLTPETQRDQERRIRREIANSNERRRMQSINAGFQSLKTLIPHTDGEKLSKAAILQQTAEYIFSLEQEKTRLLQQNTQLKRFIQELSGSSPKRRRAEDKDEGIGSPDIWEDEKAEDLRREMIELRQQLDKERSVRMMLEEQVRSLEAHMYPEKLKVIAQQVQLQQQQEQVRLLHQEKLEREQQQLRTQLLPPPAPTHHPTVIVPAPPPPPSHHINVVTMGPSSVINSVSTSRQNLDTIVQAIQHIEGTQEKQELEEEQRRAVIVKPVRSCPEAPTSDTASDSEASDSDAMDQSREEPSGDGELP

>Q9H6S3

MSQSGAVSCCPGATNGSLGRSDGVAKMSPKDLFEQRKKYSNSNVIMHETSQYHVQHLATFIMDKSEAITSVDDAIRKLVQLSSKEKIWTQEMLLQVNDQSLRLLDIESQEELEDFPLPTVQRSQTVLNQLRYPSVLLLVCQDSEQSKPDVHFFHCDEVEAELVHEDIESALADCRLGKKMRPQTLKGHQEKIRQRQSILPPPQGPAPIPFQHRGGDSPEAKNRVGPQVPLSEPGFRRRESQEEPRAVLAQKIEKETQILNCALDDIEWFVARLQKAAEAFKQLNQRKKGKKKGKKAPAEGVLTLRARPPSEGEFIDCFQKIKLAINLLAKLQKHIQNPSAAELVHFLFGPLDLIVNTCSGPDIARSVSCPLLSRDAVDFLRGHLVPKEMSLWESLGESWMRPRSEWPREPQVPLYVPKFHSGWEPPVDVLQEAPWEVEGLASAPIEEVSPVSRQSIRNSQKHSPTSEPTPPGDALPPVSSPHTHRGYQPTPAMAKYVKILYDFTARNANELSVLKDEVLEVLEDGRQWWKLRSRSGQAGYVPCNILGEARPEDAGAPFEQAGQKYWGPASPTHKLPPSFPGNKDELMQHMDEVNDELIRKISNIRAQPQRHFRVERSQPVSQPLTYESGPDEVRAWLEAKAFSPRIVENLGILTGPQLFSLNKEELKKVCGEEGVRVYSQLTMQKAFLEKQQSGSELEELMNKFHSMNQRRGEDS

60 P01106 NP_006003.1

>P01106

MPLNVSFTNRNYDLDYDSVQPYFYCDEEENFYQQQQQSELQPPAPSEDIWKKFELLPTPPLSPSRRSGLCSPSYVAVTPFSLRGDNDGGGGSFSTADQLEMVTELLGGDMVNQSFICDPDDETFIKNIIIQDCMWSGFSAAAKLVSEKLASYQAARKDSGSPNPARGHSVCSTSSLYLQDLSAAASECIDPSVVFPYPLNDSSSPKSCASQDSSAFSPSSDSLLSSTESSPQGSPEPLVLHEETPPTTSSDSEEEQEDEEEIDVVSVEKRQAPGKRSESGSPSAGGHSKPPHSPLVLKRCHVSTHQHNYAAPPSTRKDYPAAKRVKLDSVRVLRQISNNRKCTSPRSSDTEENVKRRTHNVLERQRRNELKRSFFALRDQIPELENNEKAPKVVILKKATAYILSVQAEEQKLISEEDLLRKRREQLKHKLEQLRNSCA

>NP_006003.1

MWPGILVGGARVASCRYPALGPRLAAHFPAQRPPQRTLQNGLALQRCLHATATRALPLIPIVVEQTGRGERAYDIYSRLLRERIVCVMGPIDDSVASLVIAQLLFLQSESNKKPIHMYINSPGGVVTAGLAIYDTMQYILNPICTWCVGQAASMGSLLLAAGTPGMRHSLPNSRIMIHQPSGGARGQATDIAIQAEEIMKLKKQLYNIYAKHTKQSLQVIESAMERDRYMSPMEAQEFGILDKVLVHPPQDGEDEPTLVQKEPVEAAPAAEPVPAST

61 NP_003197.2 Q6UB35

>NP_003197.2

MSTGSLSDVEDLQEVEMLECDGLKMDSNKEFVTSNESTEESSNCENGSPQKGRGGLGKRRKAPTKKSPLSGVSQEGKQVQRNAANARERARMRVLSKAFSRLKTTLPWVPPDTKLSKLDTLRLASSYIAHLRQILANDKYENGYIHPVNLTWPFMVAGKPESDLKEVVTASRLCGTTAS

>Q6UB35

MGTRLPLVLRQLRRPPQPPGPPRRLRVPCRASSGGGGGGGGGREGLLGQRRPQDGQARSSCSPGGRTPAARDSIVREVIQNSKEVLSLLQEKNPAFKPVLAIIQAGDDNLMQEINQNLAEEAGLNITHICLPPDSSEAEIIDEILKINEDTRVHGLALQISENLFSNKVLNALKPEKDVDGVTDINLGKLVRGDAHECFVSPVAKAVIELLEKSGVNLDGKKILVVGAHGSLEAALQCLFQRKGSMTMSIQWKTRQLQSKLHEADIVVLGSPKPEEIPLTWIQPGTTVLNCSHDFLSGKVGCGSPRIHFGGLIEEDDVILLAAALRIQNMVSSGRRWLREQQHRRWRLHCLKLQPLSPVPSDIEISRGQTPKAVDVLAKEIGLLADEIEIYGKSKAKVRLSVLERLKDQADGKYVLVAGITPTPLGEGKSTVTIGLVQALTAHLNVNSFACLRQPSQGPTFGVKGGAAGGGYAQVIPMEEFNLHLTGDIHAITAANNLLAAAIDTRILHENTQTDKALYNRLVPLVNGVREFSEIQLARLKKLGINKTDPSTLTEEEVSKFARLDIDPSTITWQRVLDTNDRFLRKITIGQGNTEKGHYRQAQFDIAVASEIMAVLALTDSLADMKARLGRMVVASDKSGQPVTADDLGVTGALTVLMKDAIKPNLMQTLEGTPVFVHAGPFANIAHGNSSVLADKIALKLVGEEGFVVTEAGFGADIGMEKFFNIKCRASGLVPNVVVLVATVRALKMHGGGPSVTAGVPLKKEYTEENIQLVADGCCNLQKQIQITQLFGVPVVVALNVFKTDTRAEIDLVCELAKRAGAFDAVPCYHWSVGGKGSVDLARAVREAASKRSRFQFLYDVQVPIVDKIRTIAQAVYGAKDIELSPEAQAKIDRYTQQGFGNLPICMAKTHLSLSHQPDKKGVPRDFILPISDVRASIGAGFIYPLVGTMSTMPGLPTRPCFYDIDLDTETEQVKGLF

62 Q14119 Q9Y3B7

>Q14119

MEANWTAFLFQAHEASHHQQQAAQNSLLPLLSSAVEPPDQKPLLPIPITQKPQGAPETLKDAIGIKKEKPKTSFVCTYCSKAFRDSYHLRRHESCHTGIKLVSRPKKTPTTVVPLISTIAGDSSRTSLVSTIAGILSTVTTSSSGTNPSSSASTTAMPVTQSVKKPSKPVKKNHACEMCGKAFRDVYHLNRHKLSHSDEKPFECPICNQRFKRKDRMTYHVRSHEGGITKPYTCSVCGKGFSRPDHLSCHVKHVHSTERPFKCQTCTAAFATKDRLRTHMVRHEGKVSCNICGKLLSAAYITSHLKTHGQSQSINCNTCKQGISKTCMSEETSNQKQQQQQQQQQQQQQHVTSWPGKQVETLRLWEEAVKARKKEAANLCQTSTAATTPVTLTTPFSITSSVSSETMSNPVTVAAAMSMRSPVNVSSAVNITSPMNIGHPVTITSPLSMTSPLTLTTPVNLPTPVTAPVNIAHPVTITSPMNLPTPMTLAAPLNIAMRPVESMPFLPQALPTSPPW

>Q9Y3B7

MSKLGRAARGLRKPEVGGVIRAIVRAGLAMPGPPLGPVLGQRGVSINQFCKEFNERTKDIKEGIPLPTKILVKPDRTFEIKIGQPTVSYFLKAAAGIEKGARQTGKEVAGLVTLKHVYEIARIKAQDEAFALQDVPLSSVVRSIIGSARSLGIRVVKDLSSEELAAFQKERAIFLAAQKEADLAAQEEAAKK

63 NP_055367.2 Q06278

>NP_055367.2

MTQEYDNKRPVLVLQNEALYPQRRSYTSEDEAWKSFLENPLTAATKAMMSINGDEDSAAALGLLYDYYKVPRERRSSTAKPEVEHPEPDHSKRNSIPIVTEQPLISAGENRVQVLKNVPFNIVLPHGNQLGIDKRGHLTAPDTTVTVSIATMPTHSIKTETQPHGFAVGIPPAVYHPEPTERVVVFDRNLNTDQFSSGAQAPNAQRRTPDSTFSETFKEGVQEVFFPSDLSLRMPGMNSEDYVFDSVSGNNFEYTLEASKSLRQKPGDSTMTYLNKGQFYPITLKEVSSSEGIHHPISKVRSVIMVVFAEDKSREDQLRHWKYWHSRQHTAKQRCIDIADYKESFNTISNIEEIAYNAISFTWDINDEAKVFISVNCLSTDFSSQKGVKGLPLNIQVDTYSYNNRSNKPVHRAYCQIKVFCDKGAERKIRDEERKQSKRKVSDVKVPLLPSHKRMDITVFKPFIDLDTQPVLFIPDVHFANLQRGTHVLPIASEELEGEGSVLKRGPYGTEDDFAVPPSTKLARIEEPKRVLLYVRKESEEVFDALMLKTPSLKGLMEAISDKYDVPHDKIGKIFKKCKKGILVNMDDNIVKHYSNEDTFQLQIEEAGGSYKLTLTEI

>Q06278

MDRASELLFYVNGRKVIEKNVDPETMLLPYLRKKLRLTGTKYGCGGGGCGACTVMISRYNPITKRIRHHPANACLIPICSLYGAAVTTVEGIGSTHTRIHPVQERIAKCHGTQCGFCTPGMVMSIYTLLRNHPEPTLDQLTDALGGNLCRCTGYRPIIDACKTFCKTSGCCQSKENGVCCLDQGINGLPEFEEGSKTSPKLFAEEEFLPLDPTQELIFPPELMIMAEKQSQRTRVFGSERMMWFSPVTLKELLEFKFKYPQAPVIMGNTSVGPEVKFKGVFHPVIISPDRIEELSVVNHAYNGLTLGAGLSLAQVKDILADVVQKLPEEKTQMYHALLKHLGTLAGSQIRNMASLGGHIISRHPDSDLNPILAVGNCTLNLLSKEGKRQIPLNEQFLSKCPNADLKPQEILVSVNIPYSRKWEFVSAFRQAQRQENALAIVNSGMRVFFGEGDGIIRELCISYGGVGPATICAKNSCQKLIGRHWNEQMLDIACRLILNEVSLLGSAPGGKVEFKRTLIISFLFKFYLEVSQILKKMDPVHYPSLADKYESALEDLHSKHHCSTLKYQNIGPKQHPEDPIGHPIMHLSGVKHATGEAIYCDDMPLVDQELFLTFVTSSRAHAKIVSIDLSEALSMPGVVDIMTAEHLSDVNSFCFFTEAEKFLATDKVFCVGQLVCAVLADSEVQAKRAAKRVKIVYQDLEPLILTIEESIQHNSSFKPERKLEYGNVDEAFKVVDQILEGEIHMGGQEHFYMETQSMLVVPKGEDQEMDVYVSTQFPKYIQDIVASTLKLPANKVMCHVRRVGGAFGGKVLKTGIIAAVTAFAANKHGRAVRCVLERGEDMLITGGRHPYLGKYKAGFMNDGRILALDMEHYSNAGASLDESLFVIEMGLLKMDNAYKFPNLRCRGWACRTNLPSNTAFRGFGFPQAALITESCITEVAAKCGLSPEKVRIINMYKEIDQTPYKQEINAKNLIQCWRECMAMSSYSLRKVAVEKFNAENYWKKKGLAMVPLKFPVGLGSRAAGQAAALVHIYLDGSVLVTHGGIEMGQGVHTKMIQVVSRELRMPMSNVHLRGTSTETVPNANISGGSVVADLNGLAVKDACQTLLKRLEPIISKNPKGTWKDWAQTAFDESINLSAVGYFRGYESDMNWEKGEGQPFEYFVYGAACSEVEIDCLTGDHKNIRTDIVMDVGCSINPAIDIGQIEGAFIQGMGLYTIEELNYSPQGILHTRGPDQYKIPAICDMPTELHIALLPPSQNSNTLYSSKGLGESGVFLGCSVFFAIHDAVSAARQERGLHGPLTLNSPLTPEKIRMACEDKFTKMIPRDEPGSYVPWNVPI

64 O43248 NP_001434.1

>O43248

MFNSVNLGNFCSPSRKERGADFGERGSCASNLYLPSCTYYMPEFSTVSSFLPQAPSRQISYPYSAQVPPVREVSYGLEPSGKWHHRNSYSSCYAAADELMHRECLPPSTVTEILMKNEGSYGGHHHPSAPHATPAGFYSSVNKNSVLPQAFDRFFDNAYCGGGDPPAEPPCSGKGEAKGEPEAPPASGLASRAEAGAEAEAEEENTNPSSSGSAHSVAKEPAKGAAPNAPRTRKKRCPYSKFQIRELEREFFFNVYINKEKRLQLSRMLNLTDRQVKIWFQNRRMKEKKLSRDRLQYFSGNPLL

>NP_001434.1

MSFSGKYQLQSQENFEAFMKAIGLPEELIQKGKDIKGVSEIVQNGKHFKFTITAGSKVIQNEFTVGEECELETMTGEKVKTVVQLEGDNKLVTTFKNIKSVTELNGDIITNTMTLGDIVFKRISKRI

65 P50548 NP_001379.1

>P50548

MKTPADTGFAFPDWAYKPESSPGSRQIQLWHFILELLRKEEYQGVIAWQGDYGEFVIKDPDEVARLWGVRKCKPQMNYDKLSRALRYYYNKRILHKTKGKRFTYKFNFNKLVLVNYPFIDVGLAGGAVPQSAPPVPSGGSHFRFPPSTPSEVLSPTEDPRSPPACSSSSSSLFSAVVARRLGRGSVSDCSDGTSELEEPLGEDPRARPPGPPDLGAFRGPPLARLPHDPGVFRVYPRPRGGPEPLSPFPVSPLAGPGSLLPPQLSPALPMTPTHLAYTPSPTLSPMYPSGGGGPSGSGGGSHFSFSPEDMKRYLQAHTQSVYNYHLSPRAFLHYPGLVVPQPQRPDKCPLPPMAPETPPVPSSASSSSSSSSSPFKFKLQPPPLGRRQRAAGEKAVAGADKSGGSAGGLAEGAGALAPPPPPPQIKVEPISEGESEEVEVTDISDEDEEDGEVFKTPRAPPAPPKPEPGEAPGASQCMPLKLRFKRRWSEDCRLEGGGGPAGGFEDEGEDKKVRGEGPGEAGGPLTPRRVSSDLQHATAQLSLEHRDS

>NP_001379.1

MGILEKISEIEKEIARTQKNKATEYHLGLLKAKLAKYRAQLLEPSKSASSKGEGFDVMKSGDARVALIGFPSVGKSTFLSLMTSTASEAASYEFTTLTCIPGVIEYKGANIQLLDLPGIIEGAAQGKGRGRQVIAVARTADVIIMMLDATKGEVQRSLLEKELESVGIRLNKHKPNIYFKPKKGGGISFNSTVTLTQCSEKLVQLILHEYKIFNAEVLFREDCSPDEFIDVIVGNRVYMPCLYVYNKIDQISMEEVDRLARKPNSVVISCGMKLNLDYLLEMLWEYLALTCIYTKKRGQRPDFTDAIILRKGASVEHVCHRIHRSLASQFKYALVWGTSTKYSPQRVGLTHTMEHEDVIQIVKK

66 NP_002157.2 NP_434700.2

>NP_002157.2

MKAFSPVRSVRKNSLSDHSLGISRSKTPVDDPMSLLYNMNDCYSKLKELVPSIPQNKKVSKMEILQHVIDYILDLQIALDSHPTIVSLHHQRPGQNQASRTPLTTLNTDISILSLQASEFPSELMSNDSKALCG

>NP_434700.2

MSDYENDDECWSVLEGFRVTLTSVIDPSRITPYLRQCKVLNPDDEEQVLSDPNLVIRKRKVGVLLDILQRTGHKGYVAFLESLELYYPQLYKKVTGKEPARVFSMIIDASGESGLTQLLMTEVMKLQKKVQDLTALLSSKDDFIKELRVKDSLLRKHQERVQRLKEECEAGSRELKRCKEENYDLAMRLAHQSEEKGAALMRNRDLQLEIDQLKHSLMKAEDDCKVERKHTLKLRHAMEQRPSQELLWELQQEKALLQARVQELEASVQEGKLDRSSPYIQVLEEDWRQALRDHQEQANTIFSLRKDLRQGEARRLRCMEEKEMFELQCLALRKDSKMYKDRIEAILLQMEEVAIERDQAIATREELHAQHARGLQEKDALRKQVRELGEKADELQLQVFQCEAQLLAVEGRLRRQQLETLVLSSDLEDGSPRRSQELSLPQDLEDTQLSDKGCLAGGGSPKQPFAALHQEQVLRNPHDAGLSSGEPPEKERRRLKESFENYRRKRALRKMQKGWRQGEEDRENTTGSDNTDTEGS

67 NP_005332.1 Q9UDX5

>NP_005332.1

MDGSFVQHSVRVLQELNKQREKGQYCDATLDVGGLVFKAHWSVLACCSHFFQSLYGDGSGGSVVLPAGFAEIFGLLLDFFYTGHLALTSGNRDQVLLAARELRVPEAVELCQSFKPKTSVGQAAGGQSGLGPPASQNVNSHVKEPAGLEEEEVSRTLGLVPRDQEPRGSHSPQRPQLHSPAQSEGPSSLCGKLKQALKPCPLEDKKPEDCKVPPRPLEAEGAQLQGGSNEWEVVVQVEDDGDGDYMSEPEAVLTRRKSNVIRKPCAAEPALSAGSLAAEPAENRKGTAVPVECPTCHKKFLSKYYLKVHNRKHTGEKPFECPKCGKCYFRKENLLEHEARNCMNRSEQVFTCSVCQETFRRRMELRVHMVSHTGEMPYKCSSCSQQFMQKKDLQSHMIKLHGAPKPHACPTCAKCFLSRTELQLHEAFKHRGEKLFVCEECGHRASSRNGLQMHIKAKHRNERPHVCEFCSHAFTQKANLNMHLRTHTGEKPFQCHLCGKTFRTQASLDKHNRTHTGERPFSCEFCEQRFTEKGPLLRHVASRHQEGRPHFCQICGKTFKAVEQLRVHVRRHKGVRKFECTECGYKFTRQAHLRRHMEIHDRVENYNPRQRKLRNLIIEDEKMVVVALQPPAELEVGSAEVIVESLAQGGLASQLPGQRLCAEESFTGPGVLEPSLIITAAVPEDCDT

>Q9UDX5

MSEPQPRGAERDLYRDTWVRYLGYANEVGEAFRSLVPAAVVWLSYGVASSYVLADAIDKGKKAGEVPSPEAGRSARVTVAVVDTFVWQALASVAIPGFTINRVCAASLYVLGTATRWPLAVRKWTTTALGLLTIPIIIHPIDRSVDFLLDSSLRKLYPTVGKPSSS

68 NP_006684.1 Q8N573

>NP_006684.1

MQEIIASVDHIKFDLEIAVEQQLGAQPLPFPGMDKSGAAVCEFFLKAACGKGGMCPFRHISGEKTVVCKHWLRGLCKKGDQCEFLHEYDMTKMPECYFYSKFGECSNKECPFLHIDPESKIKDCPWYDRGFCKHGPLCRHRHTRRVICVNYLVGFCPEGPSCKFMHPRFELPMGTTEQPPLPQQTQPPAKQSNNPPLQRSSSLIQLTSQNSSPNQQRTPQVIGVMQSQNSSAGNRGPRPLEQVTCYKCGEKGHYANRCTKGHLAFLSGQ

>Q8N573

MSFQKPKGTIEYTVESRDSLNSIALKFDTTPNELVQLNKLFSRAVVTGQVLYVPDPEYVSSVESSPSLSPVSPLSPTSSEAEFDKTTNPDVHPTEATPSSTFTGIRPARVVSSTSEEEEAFTEKFLKINCKYITSGKGTVSGVLLVTPNNIMFDPHKNDPLVQENGCEEYGIMCPMEEVMSAAMYKEILDSKIKESLPIDIDQLSGRDFCHSKKMTGSNTEEIDSRIRDAGNDSASTAPRSTEESLSEDVFTESELSPIREELVSSDELRQDKSSGASSESVQTVNQAEVESLTVKSESTGTPGHLRSDTEHSTNEVGTLCHKTDLNNLEMAIKEDQIADNFQGISGPKEDSTSIKGNSDQDSFLHENSLHQEESQKENMPCGETAEFKQKQSVNKGKQGKEQNQDSQTEAEELRKLWKTHTMQQTKQQRENIQQVSQKEAKHKITSADGHIESSALLKEKQRHRLHKFLCLRVGKPMRKTFVSQASATMQQYAQRDKKHEYWFAVPQERTDHLYAFFIQWSPEIYAEDTGEYTREPGFIVVKKIEESETIEDSSNQAAAREWEITTREDINSKQVATVKADLESESFRPNLSDPSELLLPDQIEKLTKHLPPRTIGYPWTLVYGTGKHGTSLKTLYRTMTGLDTPVLMVIKDSDGQVFGALASEPLKVSDGFYGTGETFVFTFCPEFEVFKWTGDNMFFIKGDMDSLAFGGGGGEFALWLDGDLYHGRSHSCKTFGNRTLSKKEDFFIQDIEIWAFE

69 P20671 NP_996895.1

>P20671

MSGRGKQGGKARAKAKTRSSRAGLQFPVGRVHRLLRKGNYSERVGAGAPVYLAAVLEYLTAEILELAGNAARDNKKTRIIPRHLQLAIRNDEELNKLLGKVTIAQGGVLPNIQAVLLPKKTESHHKAKGK

>NP_996895.1

MIHSLFLINCSGDIFLEKHWKSVVSQSVCDYFFEAQEKAADVENVPPVISTPHHYLISIYRDKLFFVSVIQTEVPPLFVIEFLHRVADTFQDYFGECSEAAIKDNVVIVYELLEEMLDNGFPLATESNILKELIKPPTILRSVVNSITGSSNVGDTLPTGQLSNIPWRRAGVKYTNNEAYFDVVEEIDAIIDKSGSTVFAEIQGVIDACIKLSGMPDLSLSFMNPRLLDDVSFHPCIRFKRWESERVLSFIPPDGNFRLISYRVSSQNLVAIPVYVKHSISFKENSSCGRFDITIGPKQNMGKTIEGITVTVHMPKVVLNMNLTPTQGSYTFDPVTKVLTWDVGKITPQKLPSLKGLVNLQSGAPKPEENPSLNIQFKIQQLAISGLKVNRLDMYGEKYKPFKGVKYVTKAGKFQVRT

70 Q14938 NP_003076.1

>Q14938

MYSPYCLTQDEFHPFIEALLPHVRAFSYTWFNLQARKRKYFKKHEKRMSKDEERAVKDELLGEKPEIKQKWASRLLAKLRKDIRPEFREDFVLTITGKKPPCCVLSNPDQKGKIRRIDCLRQADKVWRLDLVMVILFKGIPLESTDGERLYKSPQCSNPGLCVQPHHIGVTIKELDLYLAYFVHTPESGQSDSSNQQGDADIKPLPNGHLSFQDCFVTSGVWNVTELVRVSQTPVATASGPNFSLADLESPSYYNINQVTLGRRSITSPPSTSTTKRPKSIDDSEMESPVDDVFYPGTGRSPAAGSSQSSGWPNDVDAGPASLKKSGKLDFCSALSSQGSSPRMAFTHHPLPVLAGVRPGSPRATASALHFPSTSIIQQSSPYFTHPTIRYHHHHGQDSLKEFVQFVCSDGSGQATGQPNGSGQGKVPGSFLLPPPPPVARPVPLPMPDSKSTSTAPDGAALTPPSPSFATTGASSANRFVSIGPRDGNFLNIPQQSQSWFL

>NP_003076.1

MDVFMKGLSMAKEGVVAAAEKTKQGVTEAAEKTKEGVLYVGSKTREGVVQGVASVAEKTKEQASHLGGAVFSGAGNIAAATGLVKREEFPTDLKPEEVAQEAAEEPLIEPLMEPEGESYEDPPQEEYQEYEPEA

71 NP_065090.1 P50226

>NP_065090.1

MADGKAGDEKPEKSQRAGAAGGPEEEAEKPVKTKTVSSSNGGESSSRSAEKRSAEEEAADLPTKPTKISKFGFAIGSQTTKKASAISIKLGSSKPKETVPTLAPKTLSVAAAFNEDEDSEPEEMPPEAKMRMKNIGRDTPTSAGPNSFNKGKHGFSDNQKLWERNIKSHLGNVHDQDN

>P50226

MELIQDTSRPPLEYVKGVPLIKYFAEALGPLQSFQARPDDLLISTYPKSGTTWVSQILDMIYQGGDLEKCHRAPIFMRVPFLEFKVPGIPSGMETLKNTPAPRLLKTHLPLALLPQTLLDQKVKVVYVARNAKDVAVSYYHFYHMAKVYPHPGTWESFLEKFMAGEVSYGSWYQHVQEWWELSRTHPVLYLFYEDMKENPKREIQKILEFVGRSLPEETVDLMVEHTSFKEMKKTPMTNYTTVRREFMDHSISPFMRKGMAGDWKTTFTVAQNERFDADYAEKMAGCSLSFRSEL

72 P09630 NP_000964.1

>P09630

MNSYFTNPSLSCHLAGGQDVLPNVALNSTAYDPVRHFSTYGAAVAQNRIYSTPFYSPQENVVFSSSRGPYDYGSNSFYQEKDMLSNCRQNTLGHNTQTSIAQDFSSEQGRTAPQDQKASIQIYPWMQRMNSHSGVGYGADRRRGRQIYSRYQTLELEKEFHFNRYLTRRRRIEIANALCLTERQIKIWFQNRRMKWKKESNLTSTLSGGGGGATADSLGGKEEKREETEEEKQKE

>NP_000964.1

MGRVIRGQRKGAGSVFRAHVKHRKGAARLRAVDFAERHGYIKGIVKDIIHDPGRGAPLAKVVFRDPYRFKKRTELFIAAEGIHTGQFVYCGKKAQLNIGNVLPVGTMPEGTIVCCLEEKPGDRGKLARASGNYATVISHNPETKKTRVKLPSGSKKVISSANRAVVGVVAGGGRIDKPILKAGRAYHKYKAKRNCWPRVRGVAMNPVEHPFGGGNHQHIGKPSTIRRDAPAGRKVGLIAARRTGRLRGTKTVQEKEN

73 Q92698 NP_005989.3

>Q92698

MRRSLAPSQLAKRKPEGRSCDDEDWQPGLVTPRKRKSSSETQIQECFLSPFRKPLSQLTNQPPCLDSSQHEAFIRSILSKPFKVPIPNYQGPLGSRALGLKRAGVRRALHDPLEKDALVLYEPPPLSAHDQLKLDKEKLPVHVVVDPILSKVLRPHQREGVKFLWECVTSRRIPGSHGCIMADEMGLGKTLQCITLMWTLLRQSPECKPEIDKAVVVSPSSLVKNWYNEVGKWLGGRIQPLAIDGGSKDEIDQKLEGFMNQRGARVSSPILIISYETFRLHVGVLQKGSVGLVICDEGHRLKNSENQTYQALDSLNTSRRVLISGTPIQNDLLEYFSLVHFVNSGILGTAHEFKKHFELPILKGRDAAASEADRQLGEERLRELTSIVNRCLIRRTSDILSKYLPVKIEQVVCCRLTPLQTELYKRFLRQAKPAEELLEGKMSVSSLSSITSLKKLCNHPALIYDKCVEEEDGFVGALDLFPPGYSSKALEPQLSGKMLVLDYILAVTRSRSSDKVVLVSNYTQTLDLFEKLCRARRYLYVRLDGTMSIKKRAKVVERFNSPSSPDFVFMLSSKAGGCGLNLIGANRLVMFDPDWNPANDEQAMARVWRDGQKKTCYIYRLLSAGTIEEKIFQRQSHKKALSSCVVDEEQDVERHFSLGELKELFILDEASLSDTHDRLHCRRCVNSRQIRPPPDGSDCTSDLAGWNHCTDKWGLRDEVLQAAWDAASTAITFVFHQRSHEEQRGLR

>NP_005989.3

MMGHRPVLVLSQNTKRESGRKVQSGNINAAKTIADIIRTCLGPKSMMKMLLDPMGGIVMTNDGNAILREIQVQHPAAKSMIEISRTQDEEVGDGTTSVIILAGEMLSVAEHFLEQQMHPTVVISAYRKALDDMISTLKKISIPVDISDSDMMLNIINSSITTKAISRWSSLACNIALDAVKMVQFEENGRKEIDIKKYARVEKIPGGIIEDSCVLRGVMINKDVTHPRMRRYIKNPRIVLLDSSLEYKKGESQTDIEITREEDFTRILQMEEEYIQQLCEDIIQLKPDVVITEKGISDLAQHYLMRANITAIRRVRKTDNNRIARACGARIVSRPEELREDDVGTGAGLLEIKKIGDEYFTFITDCKDPKACTILLRGASKEILSEVERNLQDAMQVCRNVLLDPQLVPGGGASEMAVAHALTEKSKAMTGVEQWPYRAVAQALEVIPRTLIQNCGASTIRLLTSLRAKHTQENCETWGVNGETGTLVDMKELGIWEPLAVKLQTYKTAVETAVLLLRIDDIVSGHKKKGDDQSRQGGAPDAGQE

74 NP_002895.3 NP_872578.1

>NP_002895.3

MLVIPPGLSEEEEALQKKFNKLKKKKKALLALKKQSSSSTTSQGGVKRSLSEQPVMDTATATEQAKQLVKSGAISAIKAETKNSGFKRSRTLEGKLKDPEKGPVPTFQPFQRSISADDDLQESSRRPQRKSLYESFVSSSDRLRELGPDGEEAEGPGAGDGPPRSFDWGYEERSGAHSSASPPRSRSRDRSHERNRDRDRDRERDRDRDRDRDRERDRDRDRDRDRDRERDRDRERDRDRDREGPFRRSDSFPERRAPRKGNTLYVYGEDMTPTLLRGAFSPFGNIIDLSMDPPRNCAFVTYEKMESADQAVAELNGTQVESVQLKVNIARKQPMLDAATGKSVWGSLAVQNSPKGCHRDKRTQIVYSDDVYKENLVDGF

>NP_872578.1

MAAPCVSYGGAVSYRLLLWGRGSLARKQGLWKTAAPELQTNVRSQILRLRHTAFVIPKKNVPTSKRETYTEDFIKKQIEEFNIGKRHLANMMGEDPETFTQEDIDRAIAYLFPSGLFEKRARPVMKHPEQIFPRQRAIQWGEDGRPFHYLFYTGKQSYYSLMHDVYGMLLNLEKHQSHLQAKSLLPEKTVTRDVIGSRWLIKEELEEMLVEKLSDLDYMQFIRLLEKLLTSQCGAAEEEFVQRFRRSVTLESKKQLIEPVQYDEQGMAFSKSEGKRKTAKAEAIVYKHGSGRIKVNGIDYQLYFPITQDREQLMFPFHFVDRLGKHDVTCTVSGGGRSAQAGAIRLAMAKALCSFVTEDEVEWMRQAGLLTTDPRVRERKKPGQEGARRKFTWKKR

75 Q15415 Q8N6F7

>Q15415

MVEADHPGKLFIGGLNRETNEKMLKAVFGKHGPISEVLLIKDRTSKSRGFAFITFENPADAKNAAKDMNGTSLHGKAIKVEQAKKPSFQSGGRRRPPASSRNRSPSGSLRSARGSSGGTRGWLPSHEGHLDDGGYTPDLKMSYSRGLIPVKRGPSSRSGGPPPKKSAPSAVARSNSWMGSQGPMSQRRENYGVPPRRATISSWRNDRMSTRHDGYATNDGNHPSCQETRDYAPPSRGYAYRDNGHSNRDEHSSRGYRNHRSSRETRDYAPPSRGHAYRDYGHSRRDESYSRGYRNHRSSRETREYAPPSRGHGYRDYGHSRRHESYSRGYRNHPSSRETRDYAPPHRDYAYRDYGHSSWDEHSSRGYSYHDGYGEALGRDHSEHLSGSSYRDALQRYGTSHGAPPARGPRMSYGGSTCHAYSNTRDRYGRSWESYSSCGDFHYCDREHVCRKDQRNPPSLGRVLPDPREAYGSSSYVASIVDGGESRSEKGDSSRY

>Q8N6F7

MGNSLLRENRRQQNTQEMPWNVRMQSPKQRTSRCWDHHIAEGCFCLPWKKILIFEKRQDSQNENERMSSTPIQDNVDQTYSEELCYTLINHRVLCTRPSGNSAEEYYENVPCKAERPRESLGGTETEYSLLHMPSTDPRHARSPEDEYELLMPHRISSHFLQQPRPLMAPSETQFSHL

76 NP_000511.1 Q8N573

>NP_000511.1

MTSSRLWFSLLLAAAFAGRATALWPWPQNFQTSDQRYVLYPNNFQFQYDVSSAAQPGCSVLDEAFQRYRDLLFGSGSWPRPYLTGKRHTLEKNVLVVSVVTPGCNQLPTLESVENYTLTINDDQCLLLSETVWGALRGLETFSQLVWKSAEGTFFINKTEIEDFPRFPHRGLLLDTSRHYLPLSSILDTLDVMAYNKLNVFHWHLVDDPSFPYESFTFPELMRKGSYNPVTHIYTAQDVKEVIEYARLRGIRVLAEFDTPGHTLSWGPGIPGLLTPCYSGSEPSGTFGPVNPSLNNTYEFMSTFFLEVSSVFPDFYLHLGGDEVDFTCWKSNPEIQDFMRKKGFGEDFKQLESFYIQTLLDIVSSYGKGYVVWQEVFDNKVKIQPDTIIQVWREDIPVNYMKELELVTKAGFRALLSAPWYLNRISYGPDWKDFYVVEPLAFEGTPEQKALVIGGEACMWGEYVDNTNLVPRLWPRAGAVAERLWSNKLTSDLTFAYERLSHFRCELLRRGVQAQPLNVGFCEQEFEQT

>Q8N573

MSFQKPKGTIEYTVESRDSLNSIALKFDTTPNELVQLNKLFSRAVVTGQVLYVPDPEYVSSVESSPSLSPVSPLSPTSSEAEFDKTTNPDVHPTEATPSSTFTGIRPARVVSSTSEEEEAFTEKFLKINCKYITSGKGTVSGVLLVTPNNIMFDPHKNDPLVQENGCEEYGIMCPMEEVMSAAMYKEILDSKIKESLPIDIDQLSGRDFCHSKKMTGSNTEEIDSRIRDAGNDSASTAPRSTEESLSEDVFTESELSPIREELVSSDELRQDKSSGASSESVQTVNQAEVESLTVKSESTGTPGHLRSDTEHSTNEVGTLCHKTDLNNLEMAIKEDQIADNFQGISGPKEDSTSIKGNSDQDSFLHENSLHQEESQKENMPCGETAEFKQKQSVNKGKQGKEQNQDSQTEAEELRKLWKTHTMQQTKQQRENIQQVSQKEAKHKITSADGHIESSALLKEKQRHRLHKFLCLRVGKPMRKTFVSQASATMQQYAQRDKKHEYWFAVPQERTDHLYAFFIQWSPEIYAEDTGEYTREPGFIVVKKIEESETIEDSSNQAAAREWEITTREDINSKQVATVKADLESESFRPNLSDPSELLLPDQIEKLTKHLPPRTIGYPWTLVYGTGKHGTSLKTLYRTMTGLDTPVLMVIKDSDGQVFGALASEPLKVSDGFYGTGETFVFTFCPEFEVFKWTGDNMFFIKGDMDSLAFGGGGGEFALWLDGDLYHGRSHSCKTFGNRTLSKKEDFFIQDIEIWAFE

77 P31275 NP_002454.1

>P31275

MGEHNLLNPGFVGPLVNIHTGDTFYFPNFRASGAQLPGLPSLSYPRRDNVCSLSWPSAEPCNGYPQPYLGSPVSLNPPFGRTCELARVEDGKGYYREPCAEGGGGGLKREERGRDPGAGPGAALLPLEPSGPPALGFKYDYAAGGGGGDGGGGAGPPHDPPSCQSLESDSSSSLLNEGNKGAGAGDPGSLVSPLNPGGGLSASGAPWYPINSRSRKKRKPYSKLQLAELEGEFLVNEFITRQRRRELSDRLNLSDQQVKIWFQNRRMKKKRLLLREQALSFF

>NP_002454.1

MSKAHKPWPYRRRSQFSSRKYLKKEMNSFQQQPPPFGTVPPQMMFPPNWQGAEKDAAFLAKDFNFLTLNNQPPPGNRSQPRAMGPENNLYSQYEQKVRPCIDLIDSLRALGVEQDLALPAIAVIGDQSSGKSSVLEALSGVALPRGSGIVTRCPLVLKLKKQPCEAWAGRISYRNTELELQDPGQVEKEIHKAQNVMAGNGRGISHELISLEITSPEVPDLTIIDLPGITRVAVDNQPRDIGLQIKALIKKYIQRQQTINLVVVPCNVDIATTEALSMAHEVDPEGDRTIGILTKPDLMDRGTEKSVMNVVRNLTYPLKKGYMIVKCRGQQEITNRLSLAEATKKEITFFQTHPYFRVLLEEGSATVPRLAERLTTELIMHIQKSLPLLEGQIRESHQKATEELRRCGADIPSQEADKMFFLIEKIKMFNQDIEKLVEGEEVVRENETRLYNKIREDFKNWVGILATNTQKVKNIIHEEVEKYEKQYRGKELLGFVNYKTFEIIVHQYIQQLVEPALSMLQKAMEIIQQAFINVAKKHFGEFFNLNQTVQSTIEDIKVKHTAKAENMIQLQFRMEQMVFCQDQIYSVVLKKVREEIFNPLGTPSQNMKLNSHFPSNESSVSSFTEIGIHLNAYFLETSKRLANQIPFIIQYFMLRENGDSLQKAMMQILQEKNRYSWLLQEQSETATKRRILKERIYRLTQARHALCQFSSKEIH

78 NP_005230.1 NP_003126.1

>NP_005230.1

MNDFGIKNMDQVAPVANSYRGTLKRQPAFDTFDGSLFAVFPSLNEEQTLQEVPTGLDSISHDSANCELPLLTPCSKAVMSQALKATFSGFKKEQRRLGIPKNPWLWSEQQVCQWLLWATNEFSLVNVNLQRFGMNGQMLCNLGKERFLELAPDFVGDILWEHLEQMIKENQEKTEDQYEENSHLTSVPHWINSNTLGFGTEQAPYGMQTQNYPKGGLLDSMCPASTPSVLSSEQEFQMFPKSRLSSVSVTYCSVSQDFPGSNLNLLTNNSGTPKDHDSPENGADSFESSDSLLQSWNSQSSLLDVQRVPSFESFEDDCSQSLCLNKPTMSFKDYIQERSDPVEQGKPVIPAAVLAGFTGSGPIQLWQFLLELLSDKSCQSFISWTGDGWEFKLADPDEVARRWGKRKNKPKMNYEKLSRGLRYYYDKNIIHKTSGKRYVYRFVCDLQNLLGFTPEELHAILGVQPDTED

>NP_003126.1

MACAAARSPADQDRFICIYPAYLNNKKTIAEGRRIPISKAVENPTATEIQDVCSAVGLNVFLEKNKMYSREWNRDVQYRGRVRVQLKQEDGSLCLVQFPSRKSVMLYAAEMIPKLKTRTQKTGGADQSLQQGEGSKKGKGKKKK

79 NP_002710.2 NP_203526.1

>NP_002710.2

MLTCNKAGSRMVVDAANSNGPFQPVVLLHIRDVPPADQEKLFIQKLRQCCVLFDFVSDPLSDLKWKEVKRAALSEMVEYITHNRNVITEPIYPEVVHMFAVNMFRTLPPSSNPTGAEFDPEEDEPTLEAAWPHLQLVYEFFLRFLESPDFQPNIAKKYIDQKFVLQLLELFDSEDPRERDFLKTTLHRIYGKFLGLRAYIRKQINNIFYRFIYETEHHNGIAELLEILGSIINGFALPLKEEHKIFLLKVLLPLHKVKSLSVYHPQLAYCVVQFLEKDSTLTEPVVMALLKYWPKTHSPKEVMFLNELEEILDVIEPSEFVKIMEPLFRQLAKCVSSPHFQVAERALYYWNNEYIMSLISDNAAKILPIMFPSLYRNSKTHWNKTIHGLIYNALKLFMEMNQKLFDDCTQQFKAEKLKEKLKMKEREEAWVKIENLAKANPQYTVYSQASTMSIPVAMETDGPLFEDVQMLRKTVKDEAHQAQKDPKKDRPLARRKSELPQDPHTKKALEAHCRADELASQDGR

>NP_203526.1

MSWSGLLHGLNTSLTCGPALVPRLWATCSMATLNQMHRLGPPKRPPRKLGPTEGRPQLKGVVLCTFTRKPKKPNSANRKCCRVRLSTGREAVCFIPGEGHTLQEHQIVLVEGGRTQDLPGVKLTVVRGKYDCGHVQKK

80 NP_004082.1 NP_005787.1

>NP_004082.1

MLQGPRALASAAGQTPKVVPAMSPTELWPSGLSSPQLCPATATYYTPLYPQTAPPAAAPGTCLDATPHGPEGQVVRCLPAGRLPAKRKLDLEGIGRPVVPEFPTPKGKCIRVDGLPSPKTPKSPGEKTRYDTSLGLLTKKFIYLLSESEDGVLDLNWAAEVLDVQKRRIYDITNVLEGIQLIRKKAKNNIQWVGRGMFEDPTRPGKQQQLGQELKELMNTEQALDQLIQSCSLSFKHLTEDKANKRLAYVTYQDIRAVGNFKEQTVIAVKAPPQTRLEVPDRTEDNLQIYLKSTQGPIEVYLCPEEVQEPDSPSEEPLPSTSTLCPSPDSAQPSSSTDPSIMEPTASSVPAPAPTPQQAPPPPSLVPLEATDSLLELPHPLLQQTEDQFLSPTLACSSPLISFSPSLDQDDYLWGLEAGEGISDLFDSYDLGDLLIN

>NP_005787.1

MGDKPIWEQIGSSFIQHYYQLFDNDRTQLGAIYIDASCLTWEGQQFQGKAAIVEKLSSLPFQKIQHSITAQDHQPTPDSCIISMVVGQLKADEDPIMGFHQMFLLKNINDAWVCTNDMFRLALHNFG

81 Q9UDW3 NP_001952.1

>Q9UDW3

MGKRYFCDYCDRSFQDNLHNRKKHLNGLQHLKAKKVWYDMFRDAAAILLDEQNKRPCRKFLLTGQCDFGSNCRFSHMSERDLQELSIQVEEERRAREWLLDAPELPEGHLEDWLEKRAKRLSSAPSSRAEPIRTTVFQYPVGWPPVQELPPSLRAPPPGGWPLQPRVQWG

>NP_001952.1

MVNFTVDQIRAIMDKKANIRNMSVIAHVDHGKSTLTDSLVCKAGIIASARAGETRFTDTRKDEQERCITIKSTAISLFYELSENDLNFIKQSKDGAGFLINLIDSPGHVDFSSEVTAALRVTDGALVVVDCVSGVCVQTETVLRQAIAERIKPVLMMNKMDRALLELQLEPEELYQTFQRIVENVNVIISTYGEGESGPMGNIMIDPVLGTVGFGSGLHGWAFTLKQFAEMYVAKFAAKGEGQLGPAERAKKVEDMMKKLWGDRYFDPANGKFSKSATSPEGKKLPRTFCQLILDPIFKVFDAIMNFKKEETAKLIEKLDIKLDSEDKDKEGKPLLKAVMRRWLPAGDALLQMITIHLPSPVTAQKYRCELLYEGPPDDEAAMGIKSCDPKGPLMMYISKMVPTSDKGRFYAFGRVFSGLVSTGLKVRIMGPNYTPGKKEDLYLKPIQRTILMMGRYVEPIEDVPCGNIVGLVGVDQFLVKTGTITTFEHAHNMRVMKFSVSPVVRVAVEAKNPADLPKLVEGLKRLAKSDPMVQCIIEESGEHIIAGAGELHLEICLKDLEEDHACIPIKKSDPVVSYRETVSEESNVLCLSKSPNKHNRLYMKARPFPDGLAEDIDKGEVSARQELKQRARYLAEKYEWDVAEARKIWCFGPDGTGPNILTDITKGVQYLNEIKDSVVAGFQWATKEGALCEENMRGVRFDVHDVTLHADAIHRGGGQIIPTARRCLYASVLTAQPRLMEPIYLVEIQCPEQVVGGIYGVLNRKRGHVFEESQVAGTPMFVVKAYLPVNESFGFTADLRSNTGGQAFPQCVFDHWQILPGDPFDNSSRPSQVVAETRKRKGLKEGIPALDNFLDKL

82 NP_001370.1 NP_004171.2

>NP_001370.1

MPARTAPARVPTLAVPAISLPDDVRRRLKDLERDSLTEKECVKEKLNLLHEFLQTEIKNQLCDLETKLRKEELSEEGYLAKVKSLLNKDLSLENGAHAYNREVNGRLENGNQARSEARRVGMADANSPPKPLSKPRTPRRSKSDGEAKPEPSPSPRITRKSTRQTTITSHFAKGPAKRKPQEESERAKSDESIKEEDKDQDEKRRRVTSRERVARPLPAEEPERAKSGTRTEKEEERDEKEEKRLRSQTKEPTPKQKLKEEPDREARAGVQADEDEDGDEKDEKKHRSQPKDLAAKRRPEEKEPEKVNPQISDEKDEDEKEEKRRKTTPKEPTEKKMARAKTVMNSKTHPPKCIQCGQYLDDPDLKYGQHPPDAVDEPQMLTNEKLSIFDANESGFESYEALPQHKLTCFSVYCKHGHLCPIDTGLIEKNIELFFSGSAKPIYDDDPSLEGGVNGKNLGPINEWWITGFDGGEKALIGFSTSFAEYILMDPSPEYAPIFGLMQEKIYISKIVVEFLQSNSDSTYEDLINKIETTVPPSGLNLNRFTEDSLLRHAQFVVEQVESYDEAGDSDEQPIFLTPCMRDLIKLAGVTLGQRRAQARRQTIRHSTREKDRGPTKATTTKLVYQIFDTFFAEQIEKDDREDKENAFKRRRCGVCEVCQQPECGKCKACKDMVKFGGSGRSKQACQERRCPNMAMKEADDDEEVDDNIPEMPSPKKMHQGKKKKQNKNRISWVGEAVKTDGKKSYYKKVCIDAETLEVGDCVSVIPDDSSKPLYLARVTALWEDSSNGQMFHAHWFCAGTDTVLGATSDPLELFLVDECEDMQLSYIHSKVKVIYKAPSENWAMEGGMDPESLLEGDDGKTYFYQLWYDQDYARFESPPKTQPTEDNKFKFCVSCARLAEMRQKEIPRVLEQLEDLDSRVLYYSATKNGILYRVGDGVYLPPEAFTFNIKLSSPVKRPRKEPVDEDLYPEHYRKYSDYIKGSNLDAPEPYRIGRIKEIFCPKKSNGRPNETDIKIRVNKFYRPENTHKSTPASYHADINLLYWSDEEAVVDFKAVQGRCTVEYGEDLPECVQVYSMGGPNRFYFLEAYNAKSKSFEDPPNHARSPGNKGKGKGKGKGKPKSQACEPSEPEIEIKLPKLRTLDVFSGCGGLSEGFHQAGISDTLWAIEMWDPAAQAFRLNNPGSTVFTEDCNILLKLVMAGETTNSRGQRLPQKGDVEMLCGGPPCQGFSGMNRFNSRTYSKFKNSLVVSFLSYCDYYRPRFFLLENVRNFVSFKRSMVLKLTLRCLVRMGYQCTFGVLQAGQYGVAQTRRRAIILAAAPGEKLPLFPEPLHVFAPRACQLSVVVDDKKFVSNITRLSSGPFRTITVRDTMSDLPEVRNGASALEISYNGEPQSWFQRQLRGAQYQPILRDHICKDMSALVAARMRHIPLAPGSDWRDLPNIEVRLSDGTMARKLRYTHHDRKNGRSSSGALRGVCSCVEAGKACDPAARQFNTLIPWCLPHTGNRHNHWAGLYGRLEWDGFFSTTVTNPEPMGKQGRVLHPEQHRVVSVRECARSQGFPDTYRLFGNILDKHRQVGNAVPPPLAKAIGLEIKLCMLAKARESASAKIKEEEAAKD

>NP_004171.2

MDKNIGEQLNKAYEAFRQACMDRDSAVKELQQKTENYEQRIREQQEQLSLQQTIIDKLKSQLLLVNSTQDNNYGCVPLLEDSETRKNNLTLDQPQDKVISGIAREKLPKVRRQEVSSPRKETSARSLGSPLLHERGNIEKTFWDLKEEFHKICMLAKAQKDHLSKLNIPDTATETQCSVPIQCTDKTDKQEALFKPQAKDDINRGAPSITSVTPRGLCRDEEDTSFESLSKFNVKFPPMDNDSTFLHSTPERPGILSPATSEAVCQEKFNMEFRDNPGNFVKTEETLFEIQGIDPIASAIQNLKTTDKTKPSNLVNTCIRTTLDRAACLPPGDHNALYVNSFPLLDPSDAPFPSLDSPGKAIRGPQQPIWKPFPNQDSDSVVLSGTDSELHIPRVCEFCQAVFPPSITSRGDFLRHLNSHFNGET

83 P20823 NP_001386.1

>P20823

MVSKLSQLQTELLAALLESGLSKEALIQALGEPGPYLLAGEGPLDKGESCGGGRGELAELPNGLGETRGSEDETDDDGEDFTPPILKELENLSPEEAAHQKAVVETLLQEDPWRVAKMVKSYLQQHNIPQREVVDTTGLNQSHLSQHLNKGTPMKTQKRAALYTWYVRKQREVAQQFTHAGQGGLIEEPTGDELPTKKGRRNRFKWGPASQQILFQAYERQKNPSKEERETLVEECNRAECIQRGVSPSQAQGLGSNLVTEVRVYNWFANRRKEEAFRHKLAMDTYSGPPPGPGPGPALPAHSSPGLPPPALSPSKVHGVRYGQPATSETAEVPSSSGGPLVTVSTPLHQVSPTGLEPSHSLLSTEAKLVSAAGGPLPPVSTLTALHSLEQTSPGLNQQPQNLIMASLPGVMTIGPGEPASLGPTFTNTGASTLVIGLASTQAQSVPVINSMGSSLTTLQPVQFSQPLHPSYQQPLMPPVQSHVTQSPFMATMAQLQSPHALYSHKPEVAQYTHTGLLPQTMLITDTTNLSALASLTPTKQVFTSDTEASSESGLHTPASQATTLHVPSQDPAGIQHLQPAHRLSASPTVSSSSLVLYQSSDSSNGQSHLLPSNHSVIETFISTQMASSSQ

>NP_001386.1

MEGLGRSCLWLRRELSPPRPRLLLLDCRSRELYESARIGGALSVALPALLLRRLRRGSLSVRALLPGPPLQPPPPAPVLLYDQGGGRRRRGEAEAEAEEWEAESVLGTLLQKLREEGYLAYYLQGGFSRFQAECPHLCETSLAGRAGSSMAPVPGPVPVVGLGSLCLGSDCSDAESEADRDSMSCGLDSEGATPPPVGLRASFPVQILPNLYLGSARDSANLESLAKLGIRYILNVTPNLPNFFEKNGDFHYKQIPISDHWSQNLSRFFPEAIEFIDEALSQNCGVLVHCLAGVSRSVTVTVAYLMQKLHLSLNDAYDLVKRKKSNISPNFNFMGQLLDFERSLRLEERHSQEQGSGGQASAASNPPSFFTTPTSDGAFELAPT

84 Q8IXQ6 P50135

>Q8IXQ6

MDFSMVAGAAAYNEKSGRITSLSLLFQKVFAQIFPQWRKGNTEECLPYKCSETGALGENYSWQIPINHNDFKILKNNERQLCEVLQNKFGCISTLVSPVQEGNSKSLQVFRKMLTPRIELSVWKDDLTTHAVDAVVNAANEDLLHGGGLALALVKAGGFEIQEESKQFVARYGKVSAGEIAVTGAGRLPCKQIIHAVGPRWMEWDKQGCTGKLQRAIVSILNYVIYKNTHIKTVAIPALSSGIFQFPLNLCTKTIVETIRVSLQGKPMMSNLKEIHLVSNEDPTVAAFKAASEFILGKSELGQETTPSFNAMVVNNLTLQIVQGHIEWQTADVIVNSVNPHDITVGPVAKSILQQAGVEMKSEFLATKAKQFQRSQLVLVTKGFNLFCKYIYHVLWHSEFPKPQILKHAMKECLEKCIEQNITSISFPALGTGNMEIKKETAAEILFDEVLTFAKDHVKHQLTVKFVIFPTDLEIYKAFSSEMAKRSKMLSLNNYSVPQSTREEKRENGLEARSPAINLMGFNVEEMYEAHAWIQRILSLQNHHIIENNHILYLGRKEHDILSQLQKTSSVSITEIISPGRTELEIEGARADLIEVVMNIEDMLCKVQEEMARKKERGLWRSLGQWTIQQQKTQDEMKENIIFLKCPVPPTQELLDQKKQFEKCGLQVLKVEKIDNEVLMAAFQRKKKMMEEKLHRQPVSHRLFQQVPYQFCNVVCRVGFQRMYSTPCDPKYGAGIYFTKNLKNLAEKAKKISAADKLIYVFEAEVLTGFFCQGHPLNIVPPPLSPGAIDGHDSVVDNVSSPETFVIFSGMQAIPQYLWTCTQEYVQSQDYSSGPMRPFAQHPWRGFASGSPVD

>P50135

MASSMRSLFSDHGKYVESFRRFLNHSTEHQCMQEFMDKKLPGIIGRIGDTKSEIKILSIGGGAGEIDLQILSKVQAQYPGVCINNEVVEPSAEQIAKYKELVAKTSNLENVKFAWHKETSSEYQSRMLEKKELQKWDFIHMIQMLYYVKDIPATLKFFHSLLGTNAKMLIIVVSGSSGWDKLWKKYGSRFPQDDLCQYITSDDLTQMLDNLGLKYECYDLLSTMDISDCFIDGNENGDLLWDFLTETCNFNATAPPDLRAELGKDLQEPEFSAKKEGKVLFNNTLSFIVIEA

85 NP_006222.2 Q969P6

>NP_006222.2

MSLRSGGRRRADPGADGEASRDDGATSSVSALKRLERSQWTDKMDLRFGFERLKEPGEKTGWLINMHPTEILDEDKRLGSAVDYYFIQDDGSRFKVALPYKPYFYIATRKGCEREVSSFLSKKFQGKIAKVETVPKEDLDLPNHLVGLKRNYIRLSFHTVEDLVKVRKEISPAVKKNREQDHASDAYTALLSSVLQRGGVITDEEETSKKIADQLDNIVDMREYDVPYHIRLSIDLKIHVAHWYNVRYRGNAFPVEITRRDDLVERPDPVVLAFDIETTKLPLKFPDAETDQIMMISYMIDGQGYLITNREIVSEDIEDFEFTPKPEYEGPFCVFNEPDEAHLIQRWFEHVQETKPTIMVTYNGDFFDWPFVEARAAVHGLSMQQEIGFQKDSQGEYKAPQCIHMDCLRWVKRDSYLPVGSHNLKAAAKAKLGYDPVELDPEDMCRMATEQPQTLATYSVSDAVATYYLYMKYVHPFIFALCTIIPMEPDEVLRKGSGTLCEALLMVQAFHANIIFPNKQEQEFNKLTDDGHVLDSETYVGGHVEALESGVFRSDIPCRFRMNPAAFDFLLQRVEKTLRHALEEEEKVPVEQVTNFEEVCDEIKSKLASLKDVPSRIECPLIYHLDVGAMYPNIILTNRLQPSAMVDEATCAACDFNKPGANCQRKMAWQWRGEFMPASRSEYHRIQHQLESEKFPPLFPEGPARAFHELSREEQAKYEKRRLADYCRKAYKKIHITKVEERLTTICQRENSFYVDTVRAFRDRRYEFKGLHKVWKKKLSAAVEVGDAAEVKRCKNMEVLYDSLQLAHKCILNSFYGYVMRKGARWYSMEMAGIVCFTGANIITQARELIEQIGRPLELDTDGIWCVLPNSFPENFVFKTTNVKKPKVTISYPGAMLNIMVKEGFTNDQYQELAEPSSLTYVTRSENSIFFEVDGPYLAMILPASKEEGKKLKKRYAVFNEDGSLAELKGFEVKRRGELQLIKIFQSSVFEAFLKGSTLEEVYGSVAKVADYWLDVLYSKAANMPDSELFELISENRSMSRKLEDYGEQKSTSISTAKRLAEFLGDQMVKDAGLSCRYIISRKPEGSPVTERAIPLAIFQAEPTVRKHFLRKWLKSSSLQDFDIRAILDWDYYIERLGSAIQKIITIPAALQQVKNPVPRVKHPDWLHKKLLEKNDVYKQKKISELFTLEGRRQVTMAEASEDSPRPSAPDMEDFGLVKLPHPAAPVTVKRKRVLWESQEESQDLTPTVPWQEILGQPPALGTSQEEWLVWLRFHKKKWQLQARQRLARRKRQRLESAEGVLRPGAIRDGPATGLGSFLRRTARSILDLPWQIVQISETSQAGLFRLWALVGSDLHCIRLSIPRVFYVNQRVAKAEEGASYRKVNRVLPRSNMVYNLYEYSVPEDMYQEHINEINAELSAPDIEGVYETQVPLLFRALVHLGCVCVVNKQLVRHLSGWEAETFALEHLEMRSLAQFSYLEPGSIRHIYLYHHAQAHKALFGIFIPSQRRASVFVLDTVRSNQMPSLGALYSAEHGLLLEKVGPELLPPPKHTFEVRAETDLKTICRAIQRFLLAYKEERRGPTLIAVQSSWELKRLASEIPVLEEFPLVPICVADKINYGVLDWQRHGARRMIRHYLNLDTCLSQAFEMSRYFHIPIGNLPEDISTFGSDLFFARHLQRHNHLLWLSPTARPDLGGKEADDNCLVMEFDDQATVEINSSGCYSTVCVELDLQNLAVNTILQSHHVNDMEGADSMGISFDVIQQASLEDMITGGQAASAPASYDETALCSNTFRILKSMVVGWVKEITQYHNIYADNQVMHFYRWLRSPSSLLHDPALHRTLHNMMKKLFLQLIAEFKRLGSSVIYANFNRIILCTKKRRVEDAIAYVEYITSSIHSKETFHSLTISFSRCWEFLLWMDPSNYGGIKGKVSSRIHCGLQDSQKAGGAEDEQENEDDEEERDGEEEEEAEESNVEDLLENNWNILQFLPQAASCQNYFLMIVSAYIVAVYHCMKDGLRRSAPGSTPVRRRGASQLSQEAEGAVGALPGMITFSQDYVANELTQSFFTITQKIQKKVTGSRNSTELSEMFPVLPGSHLLLNNPALEFIKYVCKVLSLDTNITNQVNKLNRDLLRLVDVGEFSEEAQFRDPCRSYVLPEVICRSCNFCRDLDLCKDSSFSEDGAVLPQWLCSNCQAPYDSSAIEMTLVEVLQKKLMAFTLQDLVCLKCRGVKETSMPVYCSCAGDFALTIHTQVFMEQIGIFRNIAQHYGMSYLLETLEWLLQKNPQLGH

>Q969P6

MRVVRLLRLRAALTLLGEVPRRPASRGVPGSRRTQKGSGARWEKEKHEDGVKWRQLEHKGPYFAPPYEPLPDGVRFFYEGRPVRLSVAAEEVATFYGRMLDHEYTTKEVFRKNFFNDWRKEMAVEEREVIKSLDKCDFTEIHRYFVDKAAARKVLSREEKQKLKEEAEKLQQEFGYCILDGHQEKIGNFKIEPPGLFRGRGDHPKMGMLKRRITPEDVVINCSRDSKIPEPPAGHQWKEVRSDNTVTWLAAWTESVQNSIKYIMLNPCSKLKGETAWQKFETARRLRGFVDEIRSQYRADWKSREMKTRQRAVALYFIDKLALRAGNEKEDGEAADTVGCCSLRVEHVQLHPEADGCQHVVEFDFLGKDCIRYYNRVPVEKPVYKNLQLFMENKDPRDDLFDRLTTTSLNKHLQELMDGLTAKVFRTYNASITLQEQLRALTRAEDSIAAKILSYNRANRVVAILCNHQRATPSTFEKSMQNLQTKIQAKKEQVAEARAELRRARAEHKAQGDGKSRSVLEKKRRLLEKLQEQLAQLSVQATDKEENKQVALGTSKLNYLDPRISIAWCKRFRVPVEKIYSKTQRERFAWALAMAGEDFEF

86 NP_477097.1 NP_000552.2

>NP_477097.1

MSRRSSRLQAKQQPQPSQTESPQEAQIIQAKKRKTTQDVKKRREEVTKKHQYEIRNCWPPVLSGGISPCIIIETPHKEIGTSDFSRFTNYRFKNLFINPSPLPDLSWGCSKEVWLNMLKKESRYVHDKHFEVLHSDLEPQMRSILLDWLLEVCEVYTLHRETFYLAQDFFDRFMLTQKDINKNMLQLIGITSLFIASKLEEIYAPKLQEFAYVTDGACSEEDILRMELIILKALKWELCPVTIISWLNLFLQVDALKDAPKVLLPQYSQETFIQIAQLLDLCILAIDSLEFQYRILTAAALCHFTSIEVVKKASGLEWDSISECVDWMVPFVNVVKSTSPVKLKTFKKIPMEDRHNIQTHTNYLAMLEEVNYINTFRKGGQLSPVCNGGIMTPPKSTEKPPGKH

>NP_000552.2

MPMILGYWDIRGLAHAIRLLLEYTDSSYEEKKYTMGDAPDYDRSQWLNEKFKLGLDFPNLPYLIDGAHKITQSNAILCYIARKHNLCGETEEEKIRVDILENQTMDNHMQLGMICYNPEFEKLKPKYLEELPEKLKLYSEFLGKRPWFAGNKITFVDFLVYDVLDLHRIFEPKCLDAFPNLKDFISRFEGLEKISAYMKSSRFLPRPVFSKMAVWGNK

87 NP_002193.2 NP_064586.1

>NP_002193.2

MGDMGDPPKKKRLISLCVGCGNQIHDQYILRVSPDLEWHAACLKCAECNQYLDESCTCFVRDGKTYCKRDYIRLYGIKCAKCSIGFSKNDFVMRARSKVYHIECFRCVACSRQLIPGDEFALREDGLFCRADHDVVERASLGAGDPLSPLHPARPLQMAAEPISARQPALRPHVHKQPEKTTRVRTVLNEKQLHTLRTCYAANPRPDALMKEQLVEMTGLSPRVIRVWFQNKRCKDKKRSIMMKQLQQQQPNDKTNIQGMTGTPMVAASPERHDGGLQANPVEVQSYQPPWKVLSDFALQSDIDQPAFQQLVNFSEGGPGSNSTGSEVASMSSQLPDTPNSMVASPIEA

>NP_064586.1

MIRLGGWCARRLCSAAVPAGRRGAAGGLGLAGGRALRVLVDMDGVLADFEGGFLRKFRARFPDQPFIALEDRRGFWVSEQYGRLRPGLSEKAISIWESKNFFFELEPLPGAVEAVKEMASLQNTDVFICTSPIKMFKYCPYEKYAWVEKYFGPDFLEQIVLTRDKTVVSADLLIDDRPDITGAEPTPSWEHVLFTACHNQHLQLQPPRRRLHSWADDWKAILDSKRPC

88 NP_058633.2 NP_003126.1

>NP_058633.2

MAPVHGDDSLSDSGSFVSSRARREKKSKKGRQEALERLKKAKAGEKYKYEVEDFTGVYEEVDEEQYSKLVQARQDDDWIVDDDGIGYVEDGREIFDDDLEDDALDADEKGKDGKARNKDKRNVKKLAVTKPNNIKSMFIACAGKKTADKAVDLSKDGLLGDILQDLNTETPQITPPPVMILKKKRSIGASPNPFSVHTATAVPSGKIASPVSRKEPPLTPVPLKRAEFAGDDVQVESTEEEQESGAMEFEDGDFDEPMEVEEVDLEPMAAKAWDKESEPAEEVKQEADSGKGTVSYLGSFLPDVSCWDIDQEGDSSFSVQEVQVDSSHLPLVKGADEEQVFHFYWLDAYEDQYNQPGVVFLFGKVWIESAETHVSCCVMVKNIERTLYFLPREMKIDLNTGKETGTPISMKDVYEEFDEKIATKYKIMKFKSKPVEKNYAFEIPDVPEKSEYLEVKYSAEMPQLPQDLKGETFSHVFGTNTSSLELFLMNRKIKGPCWLEVKSPQLLNQPVSWCKVEAMALKPDLVNVIKDVSPPPLVVMAFSMKTMQNAKNHQNEIIAMAALVHHSFALDKAAPKPPFQSHFCVVSKPKDCIFPYAFKEVIEKKNVKVEVAATERTLLGFFLAKVHKIDPDIIVGHNIYGFELEVLLQRINVCKAPHWSKIGRLKRSNMPKLGGRSGFGERNATCGRMICDVEISAKELIRCKSYHLSELVQQILKTERVVIPMENIQNMYSESSQLLYLLEHTWKDAKFILQIMCELNVLPLALQITNIAGNIMSRTLMGGRSERNEFLLLHAFYENNYIVPDKQIFRKPQQKLGDEDEEIDGDTNKYKKGRKKAAYAGGLVLDPKVGFYDKFILLLDFNSLYPSIIQEFNICFTTVQRVASEAQKVTEDGEQEQIPELPDPSLEMGILPREIRKLVERRKQVKQLMKQQDLNPDLILQYDIRQKALKLTANSMYGCLGFSYSRFYAKPLAALVTYKGREILMHTKEMVQKMNLEVIYGDTDSIMINTNSTNLEEVFKLGNKVKSEVNKLYKLLEIDIDGVFKSLLLLKKKKYAALVVEPTSDGNYVTKQELKGLDIVRRDWCDLAKDTGNFVIGQILSDQSRDTIVENIQKRLIEIGENVLNGSVPVSQFEINKALTKDPQDYPDKKSLPHVHVALWINSQGGRKVKAGDTVSYVICQDGSNLTASQRAYAPEQLQKQDNLTIDTQYYLAQQIHPVVARICEPIDGIDAVLIATWLGLDPTQFRVHHYHKDEENDALLGGPAQLTDEEKYRDCERFKCPCPTCGTENIYDNVFDGSGTDMEPSLYRCSNIDCKASPLTFTVQLSNKLIMDIRRFIKKYYDGWLICEEPTCRNRTRHLPLQFSRTGPLCPACMKATLQPEYSDKSLYTQLCFYRYIFDAECALEKLTTDHEKDKLKKQFFTPKVLQDYRKLKNTAEQFLSRSGYSEVNLSKLFAGCAVKS

>NP_003126.1

MACAAARSPADQDRFICIYPAYLNNKKTIAEGRRIPISKAVENPTATEIQDVCSAVGLNVFLEKNKMYSREWNRDVQYRGRVRVQLKQEDGSLCLVQFPSRKSVMLYAAEMIPKLKTRTQKTGGADQSLQQGEGSKKGKGKKKK

89 Q03112 Q9NYK5

>Q03112

MKSEDYPHETMAPDIHEERQYRCEDCDQLFESKAELADHQKFPCSTPHSAFSMVEEDFQQKLESENDLQEIHTIQECKECDQVFPDLQSLEKHMLSHTEEREYKCDQCPKAFNWKSNLIRHQMSHDSGKHYECENCAKVFTDPSNLQRHIRSQHVGARAHACPECGKTFATSSGLKQHKHIHSSVKPFICEVCHKSYTQFSNLCRHKRMHADCRTQIKCKDCGQMFSTTSSLNKHRRFCEGKNHFAAGGFFGQGISLPGTPAMDKTSMVNMSHANPGLADYFGANRHPAGLTFPTAPGFSFSFPGLFPSGLYHRPPLIPASSPVKGLSSTEQTNKSQSPLMTHPQILPATQDILKALSKHPSVGDNKPVELQPERSSEERPFEKISDQSESSDLDDVSTPSGSDLETTSGSDLESDIESDKEKFKENGKMFKDKVSPLQNLASINNKKEYSNHSIFSPSLEEQTAVSGAVNDSIKAIASIAEKYFGSTGLVGLQDKKVGALPYPSMFPLPFFPAFSQSMYPFPDRDLRSLPLKMEPQSPGEVKKLQKGSSESPFDLTTKRKDEKPLTPVPSKPPVTPATSQDQPLDLSMGSRSRASGTKLTEPRKNHVFGGKKGSNVESRPASDGSLQHARPTPFFMDPIYRVEKRKLTDPLEALKEKYLRPSPGFLFHPQFQLPDQRTWMSAIENMAEKLESFSALKPEASELLQSVPSMFNFRAPPNALPENLLRKGKERYTCRYCGKIFPRSANLTRHLRTHTGEQPYRCKYCDRSFSISSNLQRHVRNIHNKEKPFKCHLCDRCFGQQTNLDRHLKKHENGNMSGTATSSPHSELESTGAILDDKEDAYFTEIRNFIGNSNHGSQSPRNVEERMNGSHFKDEKALVTSQNSDLLDDEEVEDEVLLDEEDEDNDITGKTGKEPVTSNLHEGNPEDDYEETSALEMSCKTSPVRYKEEEYKSGLSALDHIRHFTDSLKMRKMEDNQYSEAELSSFSTSHVPEELKQPLHRKSKSQAYAMMLSLSDKESLHSTSHSSSNVWHSMARAAAESSAIQSISHV

>Q9NYK5

MEALAMGSRALRLWLVAPGGGIKWRFIATSPASQLSPTELTEMRNDLFNKEKARQLSLTPRTEKIEVKHVGKTDPGTVFVMNKNISTPYSCAMHLSEWYCRKSILALVDGQPWDMYKPLTKSCEIKFLTFKDCDPGEVNKAYWRSCAMMMGCVIERAFKDEYMVNLVRAPEVPVISGAFCYDVVLDSKLDEWMPTKENLRSFTKDAHALIYKDLPFETLEVEAKVALEIFQHSKYKVDFIEEKASQNPERIVKLHRIGDFIDVSEGPLIPRTSICFQYEVSAVHNLQPTQPSLIRRFQGVSLPVHLRAHFTIWDKLLERSRKMVTEDQSKATEECTST

90 Q9P2Y4 O60783

>Q9P2Y4

MEGSRPRAPSGHLAPSPPAFDGELDLQRYSNGPAVSAGSLGMGAVSWSESRAGERRFPCPVCGKRFRFNSILALHLRAHPGAQAFQCPHCGHRAAQRALLRSHLRTHQPERPRSPAARLLLELEERALLREARLGRARSSGGMQATPATEGLARPQAPSSSAFRCPYCKGKFRTSAERERHLHILHRPWKCGLCSFGSSQEEELLHHSLTAHGAPERPLAATSAAPPPQPQPQPPPQPEPRSVPQPEPEPEPEREATPTPAPAAPEEPPAPPEFRCQVCGQSFTQSWFLKGHMRKHKASFDHACPVCGRCFKEPWFLKNHMKVHASKLGPLRAPGPASGPARAPQPPDLGLLAYEPLGPALLLAPAPTPAERREPPSLLGYLSLRAGEGRPNGEGAEPGPGRSFGGFRPLSSALPARARRHRAEEPEEEEEVVEAEEETWARGRSLGSLASLHPRPGEGPGHSASAAGAQARSTATQEENGLLVGGTRPEGGRGATGKDCPFCGKSFRSAHHLKVHLRVHTGERPYKCPHCDYAGTQSGSLKYHLQRHHREQRSGAGPGPPPEPPPPSQRGSAPQSGAKPSPQPATWVEGASSPRPPSSGAGPGSRRKPASPGRTLRNGRGGEAEPLDLSLRAGPGGEAGPGGALHRCLFCPFATGAPELMALHLQVHHSRRARGRRPPQADASPPYARVPSGETPPSPSQEGEEGSGLSRPGEAGLGGQER

>O60783

MAAFMLGSLLRTFKQMVPSSASGQVRSHYVDWRMWRDVKRRKMAYEYADERLRINSLRKNTILPKILQDVADEEIAALPRDSCPVRIRNRCVMTSRPRGVKRRWRLSRIVFRHLADHGQLSGIQRATW

91 NP_009181.2 NP_004802.1

>NP_009181.2

MVDMDKLINNLEVQLNSEGGSMQVFKQVTASVRNRDPPEIEYRSNMTSPTLLDANPMENPALFNDIKIEPPEELLASDFSLPQVEPVDLSFHKPKAPLQPASMLQAPIRPPKPQSSPQTLVVSTSTSDMSTSANIPTVLTPGSVLTSSQSTGSQQILHVIHTIPSVSLPNKMGGLKTIPVVVQSLPMVYTTLPADGGPAAITVPLIGGDGKNAGSVKVDPTSMSPLEIPSDSEESTIESGSSALQSLQGLQQEPAAMAQMQGEESLDLKRRRIHQCDFAGCSKVYTKSSHLKAHRRIHTGEKPYKCTWDGCSWKFARSDELTRHFRKHTGIKPFRCTDCNRSFSRSDHLSLHRRRHDTM

>NP_004802.1

MEELDALLEELERSTLQDSDEYSNPAPLPLDQHSRKETNLDETSEILSIQDNTSPLPAQLVYTTNIQELNVYSEAQEPKESPPPSKTSAAAQLDELMAHLTEMQAKVAVRADAGKKHLPDKQDHKASLDSMLGGLEQELQDLGIATVPKGHCASCQKPIAGKVIHALGQSWHPEHFVCTHCKEEIGSSPFFERSGLAYCPNDYHQLFSPRCAYCAAPILDKVLTAMNQTWHPEHFFCSHCGEVFGAEGFHEKDKKPYCRKDFLAMFSPKCGGCNRPVLENYLSAMDTVWHPECFVCGDCFTSFSTGSFFELDGRPFCELHYHHRRGTLCHGCGQPITGRCISAMGYKFHPEHFVCAFCLTQLSKGIFREQNDKTYCQPCFNKLFPL

92 NP_005312.1 NP_077006.1

>NP_005312.1

MSETAPAAPAAPAPAEKTPVKKKARKSAGAAKRKASGPPVSELITKAVAASKERSGVSLAALKKALAAAGYDVEKNNSRIKLGLKSLVSKGTLVQTKGTGASGSFKLNKKAASGEAKPKAKKAGAAKAKKPAGAAKKPKKATGAATPKKSAKKTPKKAKKPAAAAGAKKAKSPKKAKAAKPKKAPKSPAKAKAVKPKAAKPKTAKPKAAKPKKAAAKKK

>NP_077006.1

MGKKLDLSKLTDEEAQHVLEVVQRDFDLRRKEEERLEALKGKIKKESSKRELLSDTAHLNETHCARCLQPYQLLVNSKRQCLECGLFTCKSCGRVHPEEQGWICDPCHLARVVKIGSLEWYYEHVKARFKRFGSAKVIRSLHGRLQGGAGPELISEERSGDSDQTDEDGEPGSEAQAQAQPFGSKKKRLLSVHDFDFEGDSDDSTQPQGHSLHLSSVPEARDSPQSLTDESCSEKAAPHKAEGLEEADTGASGCHSHPEEQPTSISPSRHGALAELCPPGGSHRMALGTAAALGSNVIRNEQLPLQYLADVDTSDEESIRAHVMASHHSKRRGRASSESQIFELNKHISAVECLLTYLENTVVPPLAKGLGAGVRTEADVEEEALRRKLEELTSNVSDQETSSEEEEAKDEKAEPNRDKSVGPLPQADPEVGTAAHQTNRQEKSPQDPGDPVQYNRTTDEELSELEDRVAVTASEVQQAESEVSDIESRIAALRAAGLTVKPSGKPRRKSNLPIFLPRVAGKLGKRPEDPNADPSSEAKAMAVPYLLRRKFSNSLKSQGKDDDSFDRKSVYRGSLTQRNPNARKGMASHTFAKPVVAHQS

93 NP_004069.1 NP_000282.1

>NP_004069.1

MPNWGGGKKCGVCQKTVYFAEEVQCEGNSFHKSCFLCMVCKKNLDSTTVAVHGEEIYCKSCYGKKYGPKGYGYGQGAGTLSTDKGESLGIKHEEAPGHRPTTNPNASKFAQKIGGSERCPRCSQAVYAAEKVIGAGKSWHKACFRCAKCGKGLESTTLADKDGEIYCKGCYAKNFGPKGFGFGQGAGALVHSE

>NP_000282.1

MSLSNKLTLDKLDVKGKRVVMRVDFNVPMKNNQITNNQRIKAAVPSIKFCLDNGAKSVVLMSHLGRPDGVPMPDKYSLEPVAVELKSLLGKDVLFLKDCVGPEVEKACANPAAGSVILLENLRFHVEEEGKGKDASGNKVKAEPAKIEAFRASLSKLGDVYVNDAFGTAHRAHSSMVGVNLPQKAGGFLMKKELNYFAKALESPERPFLAILGGAKVADKIQLINNMLDKVNEMIIGGGMAFTFLKVLNNMEIGTSLFDEEGAKIVKDLMSKAEKNGVKITLPVDFVTADKFDENAKTGQATVASGIPAGWMGLDCGPESSKKYAEAVTRAKQIVWNGPVGVFEWEAFARGTKALMDEVVKATSRGCITIIGGGDTATCCAKWNTEDKVSHVSTGGGASLELLEGKVLPGVDALSNI

94 NP_004119.1 NP_115568.2

>NP_004119.1

MAERGELDLTGAKQNTGVWLVKVPKYLSQQWAKASGRGEVGKLRIAKTQGRTEVSFTLNEDLANIHDIGGKPASVSAPREHPFVLQSVGGQTLTVFTESSSDKLSLEGIVVQRAECRPAASENYMRLKRLQIEESSKPVRLSQQLDKVVTTNYKPVANHQYNIEYERKKKEDGKRARADKQHVLDMLFSAFEKHQYYNLKDLVDITKQPVVYLKEILKEIGVQNVKGIHKNTWELKPEYRHYQGEEKSD

>NP_115568.2

MDPKDRKKIQFSVPAPPSQLDPRQVEMIRRRRPTPAMLFRLSEHSSPEEEASPHQRASGEGHHLKSKRPNPCAYTPPSLKAVQRIAESHLQSISNLNENQASEEEDELGELRELGYPREEDEEEEEDDEEEEEEEDSQAEVLKVIRQSAGQKTTCGQGLEGPWERPPPLDESERDGGSEDQVEDPALSEPGEEPQRPSPSEPGT

95 NP_001505.1 NP_057740.2

>NP_001505.1

MASTSRLDALPRVTCPNHPDAILVEDYRAGDMICPECGLVVGDRVIDVGSEWRTFSNDKATKDPSRVGDSQNPLLSDGDLSTMIGKGTGAASFDEFGNSKYQNRRTMSSSDRAMMNAFKEITTMADRINLPRNIVDRTNNLFKQVYEQKSLKGRANDAIASACLYIACRQEGVPRTFKEICAVSRISKKEIGRCFKLILKALETSVDLITTGDFMSRFCSNLCLPKQVQMAATHIARKAVELDLVPGRSPISVAAAAIYMASQASAEKRTQKEIGDIAGVADVTIRQSYRLIYPRAPDLFPTDFKFDTPVDKLPQL

>NP_057740.2

MEESDSEKTTEKENLGPRMDPPLGEPEGSLGWVLPNTAMKKKVLLMGKSGSGKTSMRSIIFANYIARDTRRLGATILDRIHSLQINSSLSTYSLVDSVGNTKTFDVEHSHVRFLGNLVLNLWDCGGQDTFMENYFTSQRDNIFRNVEVLIYVFDVESRELEKDMHYYQSCLEAILQNSPDAKIFCLVHKMDLVQEDQRDLIFKEREEDLRRLSRPLECSCFRTSIWDETLYKAWSSIVYQLIPNVQQLEMNLRNFAEIIEADEVLLFERATFLVISHYQCKEQRDAHRFEKISNIIKQFKLSCSKLAASFQSMEVRNSNFAAFIDIFTSNTYVMVVMSDPSIPSAATLINIRNARKHFEKLERVDGPKQCLLMR

96 Q5H9F3 Q06278

>Q5H9F3

MISTAPLYSGVHNWTSSDRIRMCGINEERRAPLSDEESTTGDCQHFGSQEFCVSSSFSKVELTAVGSGSNARGADPDGSATEKLGHKSEDKPDDPQPKMDYAGNVAEAEGFLVPLSSPGDGLKLPASDSAEASNSRADCSWTPLNTQMSKQVDCSPAGVKALDSRQGVGEKNTFILATLGTGVPVEGTLPLVTTNFSPLPAPICPPAPGSASVPHSVPDAFQVPLSVPAPVPHSGLVPVQVATSVPAPSPPLAPVPALAPAPPSVPTLISDSNPLSVSASVLVPVPASAPPSGPVPLSAPAPAPLSVPVSAPPLALIQAPVPPSAPTLVLAPVPTPVLAPMPASTPPAAPAPPSVPMPTPTPSSGPPSTPTLIPAFAPTPVPAPTPAPIFTPAPTPMPAATPAAIPTSAPIPASFSLSRVCFPAAQAPAMQKVPLSFQPGTVLTPSQPLVYIPPPSCGQPLSVATLPTTLGVSSTLTLPVLPSYLQDRCLPGVLASPELRSYPYAFSVARPLTSDSKLVSLEVNRLPCTSPSGSTTTQPAPDGVPGPLADTSLVTASAKVLPTPQPLLPAPSGSSAPPHPAKMPSGTEQQTEGTSVTFSPLKSPPQLEREMASPPECSEMPLDLSSKSNRQKLPLPNQRKTPPMPVLTPVHTSSKALLSTVLSRSQRTTQAAGGNVTSCLGSTSSPFVIFPEIVRNGDPSTWVKNSTALISTIPGTYVGVANPVPASLLLNKDPNLGLNRDPRHLPKQEPISIIDQGEPKGTGATCGKKGSQAGAEGQPSTVKRYTPARIAPGLPGCQTKELSLWKPTGPANIYPRCSVNGKPTSTQVLPVGWSPYHQASLLSIGISSAGQLTPSQGAPIRPTSVVSEFSGVPSLSSSEAVHGLPEGQPRPGGSFVPEQDPVTKNKTCRIAAKPYEEQVNPVLLTLSPQTGTLALSVQPSGGDIRMNQGPEESESHLCSDSTPKMEGPQGACGLKLAGDTKPKNQVLATYMSHELVLATPQNLPKMPELPLLPHDSHPKELILDVVPSSRRGSSTERPQLGSQVDLGRVKMEKVDGDVVFNLATCFRADGLPVAPQRGQAEVRAKAGQARVKQESVGVFACKNKWQPDDVTESLPPKKMKCGKEKDSEEQQLQPQAKAVVRSSHRPKCRKLPSDPQESTKKSPRGASDSGKEHNGVRGKHKHRKPTKPESQSPGKRADSHEEGSLEKKAKSSFRDFIPVVLSTRTRSQSGSICSSFAGMADSDMGSQEVFPTEEEEEVTPTPAKRRKVRKTQRDTQYRSHHAQDKSLLSQGRRHLWRAREMPWRTEAARQMWDTNEEEEEEEEEGLLKRKKRRRQKSRKYQTGEYLTEQEDEQRRKGRADLKARKQKTSSSQSLEHRLRNRNLLLPNKVQGISDSPNGFLPNNLEEPACLENSEKPSGKRKCKTKHMATVSEEAKDVVLYCLQKDSEDVNHRDNAGYTALHEACSRGWTDILNILLEHGANVNCSAQDGTRPVHDAVVNDNLETIWLLLSYGADPTLATYSGQTAMKLASSDTMKRFLSDHLSDLQGRAEGDPGVSWDFYSSSVLEEKDGFACDLLHNPPGSSDQEGDDPMEEDDFMFELSDKPLLPCYNLQVSVSRGPCNWFLFSDVLKRLKLSSRIFQARFPHFEITTMPKAEFYRQVASSQLLTPAERPGGLDDRSPPGSSETVELVRYEPDLLRLLGSEVEFQSCNS

>Q06278

MDRASELLFYVNGRKVIEKNVDPETMLLPYLRKKLRLTGTKYGCGGGGCGACTVMISRYNPITKRIRHHPANACLIPICSLYGAAVTTVEGIGSTHTRIHPVQERIAKCHGTQCGFCTPGMVMSIYTLLRNHPEPTLDQLTDALGGNLCRCTGYRPIIDACKTFCKTSGCCQSKENGVCCLDQGINGLPEFEEGSKTSPKLFAEEEFLPLDPTQELIFPPELMIMAEKQSQRTRVFGSERMMWFSPVTLKELLEFKFKYPQAPVIMGNTSVGPEVKFKGVFHPVIISPDRIEELSVVNHAYNGLTLGAGLSLAQVKDILADVVQKLPEEKTQMYHALLKHLGTLAGSQIRNMASLGGHIISRHPDSDLNPILAVGNCTLNLLSKEGKRQIPLNEQFLSKCPNADLKPQEILVSVNIPYSRKWEFVSAFRQAQRQENALAIVNSGMRVFFGEGDGIIRELCISYGGVGPATICAKNSCQKLIGRHWNEQMLDIACRLILNEVSLLGSAPGGKVEFKRTLIISFLFKFYLEVSQILKKMDPVHYPSLADKYESALEDLHSKHHCSTLKYQNIGPKQHPEDPIGHPIMHLSGVKHATGEAIYCDDMPLVDQELFLTFVTSSRAHAKIVSIDLSEALSMPGVVDIMTAEHLSDVNSFCFFTEAEKFLATDKVFCVGQLVCAVLADSEVQAKRAAKRVKIVYQDLEPLILTIEESIQHNSSFKPERKLEYGNVDEAFKVVDQILEGEIHMGGQEHFYMETQSMLVVPKGEDQEMDVYVSTQFPKYIQDIVASTLKLPANKVMCHVRRVGGAFGGKVLKTGIIAAVTAFAANKHGRAVRCVLERGEDMLITGGRHPYLGKYKAGFMNDGRILALDMEHYSNAGASLDESLFVIEMGLLKMDNAYKFPNLRCRGWACRTNLPSNTAFRGFGFPQAALITESCITEVAAKCGLSPEKVRIINMYKEIDQTPYKQEINAKNLIQCWRECMAMSSYSLRKVAVEKFNAENYWKKKGLAMVPLKFPVGLGSRAAGQAAALVHIYLDGSVLVTHGGIEMGQGVHTKMIQVVSRELRMPMSNVHLRGTSTETVPNANISGGSVVADLNGLAVKDACQTLLKRLEPIISKNPKGTWKDWAQTAFDESINLSAVGYFRGYESDMNWEKGEGQPFEYFVYGAACSEVEIDCLTGDHKNIRTDIVMDVGCSINPAIDIGQIEGAFIQGMGLYTIEELNYSPQGILHTRGPDQYKIPAICDMPTELHIALLPPSQNSNTLYSSKGLGESGVFLGCSVFFAIHDAVSAARQERGLHGPLTLNSPLTPEKIRMACEDKFTKMIPRDEPGSYVPWNVPI

97 NP_002008.2 Q14651

>NP_002008.2

MDGTIKEALSVVSDDQSLFDSAYGAAAHLPKADMTASGSPDYGQPHKINPLPPQQEWINQPVRVNVKREYDHMNGSRESPVDCSVSKCSKLVGGGESNPMNYNSYMDEKNGPPPPNMTTNERRVIVPADPTLWTQEHVRQWLEWAIKEYSLMEIDTSFFQNMDGKELCKMNKEDFLRATTLYNTEVLLSHLSYLRESSLLAYNTTSHTDQSSRLSVKEDPSYDSVRRGAWGNNMNSGLNKSPPLGGAQTISKNTEQRPQPDPYQILGPTSSRLANPGSGQIQLWQFLLELLSDSANASCITWEGTNGEFKMTDPDEVARRWGERKSKPNMNYDKLSRALRYYYDKNIMTKVHGKRYAYKFDFHGIAQALQPHPTESSMYKYPSDISYMPSYHAHQQKVNFVPPHPSSMPVTSSSFFGAASQYWTSPTGGIYPNPNVPRHPNTHVPSHLGSYY

>Q14651

MENSTTTISREELEELQEAFNKIDIDNSGYVSDYELQDLFKEASLPLPGYKVREIVEKILSVADSNKDGKISFEEFVSLMQELKSKDISKTFRKIINKREGITAIGGTSTISSEGTQHSYSEEEKVAFVNWINKALENDPDCKHLIPMNPNDDSLFKSLADGILLCKMINLSEPDTIDERAINKKKLTPFTISENLNLALNSASAIGCTVVNIGASDLKEGKPHLVLGLLWQIIKVGLFADIEISRNEALIALLNEGEELEELMKLSPEELLLRWVNYHLTNAGWHTISNFSQDIKDSRAYFHLLNQIAPKGGEDGPAIAIDLSGINETNDLKRAGLMLQEADKLGCKQFVTPADVVSGNPKLNLAFVANLFNTYPCLHKPNNNDIDMNLLEGESKEERTFRNWMNSLGVNPYINHLYSDLADALVIFQLYEMIRVPVNWSHVNKPPYPALGGNMKKIENCNYAVELGKNKAKFSLVGIAGQDLNEGNSTLTLALVWQLMRRYTLNVLSDLGEGEKVNDEIIIKWVNQTLKSANKKTSISSFKDKSISTSLPVLDLIDAIAPNAVRQEMIRRENLSDEDKLNNAKYAISVARKIGARIYALPDDLVEVKPKMVMTVFACLMGKGLNRIK

98 NP_005514.1 NP_055428.1

>NP_005514.1

MDFDERGPCSSNMYLPSCTYYVSGPDFSSLPSFLPQTPSSRPMTYSYSSNLPQVQPVREVTFREYAIEPATKWHPRGNLAHCYSAEELVHRDCLQAPSAAGVPGDVLAKSSANVYHHPTPAVSSNFYSTVGRNGVLPQAFDQFFETAYGTPENLASSDYPGDKSAEKGPPAATATSAAAAAAATGAPATSSSDSGGGGGCRETAAAAEEKERRRRPESSSSPESSSGHTEDKAGGSSGQRTRKKRCPYTKYQIRELEREFFFSVYINKEKRLQLSRMLNLTDRQVKIWFQNRRMKEKKINRDRLQYYSANPLL

>NP_055428.1

MAAPEERDLTQEQTEKLLQFQDLTGIESMDQCRHTLEQHNWNIEAAVQDRLNEQEGVPSVFNPPPSRPLQVNTADHRIYSYVVSRPQPRGLLGWGYYLIMLPFRFTYYTILDIFRFALRFIRPDPRSRVTDPVGDIVSFMHSFEEKYGRAHPVFYQGTYSQALNDAKRELRFLLVYLHGDDHQDSDEFCRNTLCAPEVISLINTRMLFWACSTNKPEGYRVSQALRENTYPFLAMIMLKDRRMTVVGRLEGLIQPDDLINQLTFIMDANQTYLVSERLEREERNQTQVLRQQQDEAYLASLRADQEKERKKREERERKRRKEEEVQQQKLAEERRRQNLQEEKERKLECLPPEPSPDDPESVKIIFKLPNDSRVERRFHFSQSLTVIHDFLFSLKESPEKFQIEANFPRRVLPCIPSEEWPNPPTLQEAGLSHTEVLFVQDLTDE

99 NP_008828.1 Q9NX20

>NP_008828.1

MSATGPISNYYVDSLISHDNEDLLASRFPATGAHPAAARPSGLVPDCSDFPSCSFAPKPAVFSTSWAPVPSQSSVVYHPYGPQPHLGADTRYMRTWLEPLSGAVSFPSFPAGGRHYALKPDAYPGRRADCGPGEGRSYPDYMYGSPGELRDRAPQTLPSPEADALAGSKHKEEKADLDPSNPVANWIHARSTRKKRCPYTKYQTLELEKEFLFNMYLTRDRRYEVARVLNLTERQVKIWFQNRRMKMKKMNKEKTDKEQS

>Q9NX20

MWRLLARASAPLLRVPLSDSWALLPASAGVKTLLPVPSFEDVSIPEKPKLRFIERAPLVPKVRREPKNLSDIRGPSTEATEFTEGNFAILALGGGYLHWGHFEMMRLTINRSMDPKNMFAIWRVPAPFKPITRKSVGHRMGGGKGAIDHYVTPVKAGRLVVEMGGRCEFEEVQGFLDQVAHKLPFAAKAVSRGTLEKMRKDQEERERNNQNPWTFERIATANMLGIRKVLSPYDLTHKGKYWGKFYMPKRV

100 NP_002126.2 NP_115865.1

>NP_002126.2

MPCIQAQYGTPAPSPGPRDHLASDPLTPEFIKPTMDLASPEAAPAAPTALPSFSTFMDGYTGEFDTFLYQLPGTVQPCSSASSSASSTSSSSATSPASASFKFEDFQVYGCYPGPLSGPVDEALSSSGSDYYGSPCSAPSPSTPSFQPPQLSPWDGSFGHFSPSQTYEGLRAWTEQLPKASGPPQPPAFFSFSPPTGPSPSLAQSPLKLFPSQATHQLGEGESYSMPTAFPGLAPTSPHLEGSGILDTPVTSTKARSGAPGGSEGRCAVCGDNASCQHYGVRTCEGCKGFFKRTVQKNAKYICLANKDCPVDKRRRNRCQFCRFQKCLAVGMVKEVVRTDSLKGRRGRLPSKPKQPPDASPANLLTSLVRAHLDSGPSTAKLDYSKFQELVLPHFGKEDAGDVQQFYDLLSGSLEVIRKWAEKIPGFAELSPADQDLLLESAFLELFILRLAYRSKPGEGKLIFCSGLVLHRLQCARGFGDWIDSILAFSRSLHSLLVDVPAFACLSALVLITDRHGLQEPRRVEELQNRIASCLKEHVAAVAGEPQPASCLSRLLGKLPELRTLCTQGLQRIFYLKLEDLVPPPPIIDKIFMDTLPF

>NP_115865.1

MPRYELALILKAMQRPETAATLKRTIEALMDRGAIVRDLENLGERALPYRISAHSQQHNRGGYFLVDFYAPTAAVESMVEHLSRDIDVIRGNIVKHPLTQELKECEGIVPVPLAEKLYSTKKRKK

101 P09017 Q8WVN8

>P09017

MIMSSYLMDSNYIDPKFPPCEEYSQNSYIPEHSPEYYGRTRESGFQHHHQELYPPPPPRPSYPERQYSCTSLQGPGNSRGHGPAQAGHHHPEKSQSLCEPAPLSGASASPSPAPPACSQPAPDHPSSAASKQPIVYPWMKKIHVSTVNPNYNGGEPKRSRAAYTRQQVLELEKEFHYNRYLTRRRRIEIAHSLCLSERQIKIWFQNRRMKWKKDHRLPNTKVRSAPPAGAAPSTLSAATPGTSEDHSQSATPPEQQRAEDITRL

>Q8WVN8

MSVSGLKAELKFLASIFDKNHERFRIVSWKLDELHCQFLVPQQGSPHSLPPPLTLHCNITESYPSSSPIWFVDSEDPNLTSVLERLEDTKNNNLLRQQLKWLICELCSLYNLPKHLDVEMLDQPLPTGQNGTTEEVTSEEEEEEEEMAEDIEDLDHYEMKEEEPISGKKSEDEGIEKENLAILEKIRKTQRQDHLNGAVSGSVQASDRLMKELRDIYRSQSYKTGIYSVELINDSLYDWHVKLQKVDPDSPLHSDLQILKEKEGIEYILLNFSFKDNFPFDPPFVRVVLPVLSGGYVLGGGALCMELLTKQGWSSAYSIESVIMQINATLVKGKARVQFGANKNQYNLARAQQSYNSIVQIHEKNGWYTPPKEDG

102 NP_003784.2 NP_066953.1

>NP_003784.2

MAPWLQLLSLLGLLPGAVAAPAQPRAASFQAWGPPSPELLAPTRFALEMFNRGRAAGTRAVLGLVRGRVRRAGQGSLYSLEATLEEPPCNDPMVCRLPVSKKTLLCSFQVLDELGRHVLLRKDCGPVDTKVPGAGEPKSAFTQGSAMISSLSQNHPDNRNETFSSVISLLNEDPLSQDLPVKMASIFKNFVITYNRTYESKEEARWRLSVFVNNMVRAQKIQALDRGTAQYGVTKFSDLTEEEFRTIYLNTLLRKEPGNKMKQAKSVGDLAPPEWDWRSKGAVTKVKDQGMCGSCWAFSVTGNVEGQWFLNQGTLLSLSEQELLDCDKMDKACMGGLPSNAYSAIKNLGGLETEDDYSYQGHMQSCNFSAEKAKVYINDSVELSQNEQKLAAWLAKRGPISVAINAFGMQFYRHGISRPLRPLCSPWLIDHAVLLVGYGNRSDVPFWAIKNSWGTDWGEKGYYYLHRGSGACGVNTMASSAVVD

>NP_066953.1

MVNPTVFFDIAVDGEPLGRVSFELFADKVPKTAENFRALSTGEKGFGYKGSCFHRIIPGFMCQGGDFTRHNGTGGKSIYGEKFEDENFILKHTGPGILSMANAGPNTNGSQFFICTAKTEWLDGKHVVFGKVKEGMNIVEAMERFGSRNGKTSKKITIADCGQLE

103 NP_002583.1 Q96PC2

>NP_002583.1

MFEARLVQGSILKKVLEALKDLINEACWDISSSGVNLQSMDSSHVSLVQLTLRSEGFDTYRCDRNLAMGVNLTSMSKILKCAGNEDIITLRAEDNADTLALVFEAPNQEKVSDYEMKLMDLDVEQLGIPEQEYSCVVKMPSGEFARICRDLSHIGDAVVISCAKDGVKFSASGELGNGNIKLSQTSNVDKEEEAVTIEMNEPVQLTFALRYLNFFTKATPLSSTVTLSMSADVPLVVEYKIADMGHLKYYLAPKIEDEEGS

>Q96PC2

MVVQNSADAGDMRAGVQLEPFLHQVGGHMSVMKYDEHTVCKPLVSREQRFYESLPLAMKRFTPQYKGTVTVHLWKDSTGHLSLVANPVKESQEPFKVSTESAAVAIWQTLQQTTGSNGSDCTLAQWPHAQLARSPKESPAKALLRSEPHLNTPAFSLVEDTNGNQVERKSFNPWGLQCHQAHLTRLCSEYPENKRHRFLLLENVVSQYTHPCVLDLKMGTRQHGDDASEEKKARHMRKCAQSTSACLGVRICGMQVYQTDKKYFLCKDKYYGRKLSVEGFRQALYQFLHNGSHLRRELLEPILHQLRALLSVIRSQSSYRFYSSSLLVIYDGQEPPERAPGSPHPHEAPQAAHGSSPGGLTKVDIRMIDFAHTTYKGYWNEHTTYDGPDPGYIFGLENLIRILQDIQEGE

104 NP_005369.2 NP_000445.1

>NP_005369.2

MPSCSTSTMPGMICKNPDLEFDSLQPCFYPDEDDFYFGGPDSTPPGEDIWKKFELLPTPPLSPSRGFAEHSSEPPSWVTEMLLENELWGSPAEEDAFGLGGLGGLTPNPVILQDCMWSGFSAREKLERAVSEKLQHGRGPPTAGSTAQSPGAGAASPAGRGHGGAAGAGRAGAALPAELAHPAAECVDPAVVFPFPVNKREPAPVPAAPASAPAAGPAVASGAGIAAPAGAPGVAPPRPGGRQTSGGDHKALSTSGEDTLSDSDDEDDEEEDEEEEIDVVTVEKRRSSSNTKAVTTFTITVRPKNAALGPGRAQSSELILKRCLPIHQQHNYAAPSPYVESEDAPPQKKIKSEASPRPLKSVIPPKAKSLSPRNSDSEDSERRRNHNILERQRRNDLRSSFLTLRDHVPELVKNEKAAKVVILKKATEYVHSLQAEEHQLLLEKEKLQARQQQLLKKIEHARTC

>NP_000445.1

MATKAVCVLKGDGPVQGIINFEQKESNGPVKVWGSIKGLTEGLHGFHVHEFGDNTAGCTSAGPHFNPLSRKHGGPKDEERHVGDLGNVTADKDGVADVSIEDSVISLSGDHCIIGRTLVVHEKADDLGKGGNEESTKTGNAGSRLACGVIGIAQ

105 Q9ULG1 NP_001367.2

>Q9ULG1

MASELGARDDGGCTELAKPLYLQYLERALRLDHFLRQTSAIFNRNISSDDSEDGLDDSNPLLPQSGDPLIQVKEEPPNSLLGETSGAGSSGMLNTYSLNGVLQSESKCDKGNLYNFSKLKKSRKWLKSILLSDESSEADSQSEDDDEEELNLSREELHNMLRLHKYKKLHQNKYSKDKELQQYQYYSAGLLSTYDPFYEQQRHLLGPKKKKFKEEKKLKAKLKKVKKKRRRDEELSSEESPRRHHHQTKVFAKFSHDAPPPGTKKKHLSIEQLNARRRKVWLSIVKKELPKANKQKASARNLFLTNSRKLAHQCMKEVRRAALQAQKNCKETLPRARRLTKEMLLYWKKYEKVEKEHRKRAEKEALEQRKLDEEMREAKRQQRKLNFLITQTELYAHFMSRKRDMGHDGIQEEILRKLEDSSTQRQIDIGGGVVVNITQEDYDSNHFKAQALKNAENAYHIHQARTRSFDEDAKESRAAALRAANKSGTGFGESYSLANPSIRAGEDIPQPTIFNGKLKGYQLKGMNWLANLYEQGINGILADEMGLGKTVQSIALLAHLAERENIWGPFLIISPASTLNNWHQEFTRFVPKFKVLPYWGNPHDRKVIRRFWSQKTLYTQDAPFHVVITSYQLVVQDVKYFQRVKWQYMVLDEAQALKSSSSVRWKILLQFQCRNRLLLTGTPIQNTMAELWALLHFIMPTLFDSHEEFNEWFSKDIESHAENKSAIDENQLSRLHMILKPFMLRRIKKDVENELSDKIEILMYCQLTSRQKLLYQALKNKISIEDLLQSSMGSTQQAQNTTSSLMNLVMQFRKVCNHPELFERQETWSPFHISLKPYHISKFIYRHGQIRVFNHSRDRWLRVLSPFAPDYIQRSLFHRKGINEESCFSFLRFIDISPAEMANLMLQGLLARWLALFLSLKASYRLHQLRSWGAPEGESHQRYLRNKDFLLGVNFPLSFPNLCSCPLLKSLVFSSHCKAVSGYSDQVVHQRRSATSSLRRCLLTELPSFLCVASPRVTAVPLDSYCNDRSAEYERRVLKEGGSLAAKQCLLNGAPELAADWLNRRSQFFPEPAGGLWSIRPQNGWSFIRIPGKESLITDSGKLYALDVLLTRLKSQGHRVLIYSQMTRMIDLLEEYMVYRKHTYMRLDGSSKISERRDMVADFQNRNDIFVFLLSTRAGGLGINLTAADTVIFYDSDWNPTVDQQAMDRAHRLGQTKQVTVYRLICKGTIEERILQRAKEKSEIQRMVISGGNFKPDTLKPKEVVSLLLDDEELEKKLRLRQEEKRQQEETNRVKERKRKREKYAEKKKKEDELDGKRRKEGVNLVIPFVPSADNSNLSADGDDSFISVDSAMPSPFSEISISSELHTGSIPLDESSSDMLVIVDDPASSAPQSRATNSPASITGSVSDTVNGISIQEMPAAGRGHSARSRGRPKGSGSTAKGAGKGRSRKSTAGSAAAMAGAKAGAAAASAAAYAAYGYNVSKGISASSPLQTSLVRPAGLADFGPSSASSPLSSPLSKGNNVPGNPKNLHMTSSLAPDSLVRKQGKGTNPSGGR

>NP_001367.2

MSEPGGGGGEDGSAGLEVSAVQNVADVSVLQKHLRKLVPLLLEDGGEAPAALEAALEEKSALEQMRKFLSDPQVHTVLVERSTLKEDVGDEGEEEKEFISYNINIDIHYGVKSNSLAFIKRTPVIDADKPVSSQLRVLTLSEDSPYETLHSFISNAVAPFFKSYIRESGKADRDGDKMAPSVEKKIAELEMGLLHLQQNIEIPEISLPIHPMITNVAKQCYERGEKPKVTDFGDKVEDPTFLNQLQSGVNRWIREIQKVTKLDRDPASGTALQEISFWLNLERALYRIQEKRESPEVLLTLDILKHGKRFHATVSFDTDTGLKQALETVNDYNPLMKDFPLNDLLSATELDKIRQALVAIFTHLRKIRNTKYPIQRALRLVEAISRDLSSQLLKVLGTRKLMHVAYEEFEKVMVACFEVFQTWDDEYEKLQVLLRDIVKRKREENLKMVWRINPAHRKLQARLDQMRKFRRQHEQLRAVIVRVLRPQVTAVAQQNQGEVPEPQDMKVAEVLFDAADANAIEEVNLAYENVKEVDGLDVSKEGTEAWEAAMKRYDERIDRVETRITARLRDQLGTAKNANEMFRIFSRFNALFVRPHIRGAIREYQTQLIQRVKDDIESLHDKFKVQYPQSQACKMSHVRDLPPVSGSIIWAKQIDRQLTAYMKRVEDVLGKGWENHVEGQKLKQDGDSFRMKLNTQEIFDDWARKVQQRNLGVSGRIFTIESTRVRGRTGNVLKLKVNFLPEIITLSKEVRNLKWLGFRVPLAIVNKAHQANQLYPFAISLIESVRTYERTCEKVEERNTISLLVAGLKKEVQALIAEGIALVWESYKLDPYVQRLAETVFNFQEKVDDLLIIEEKIDLEVRSLETCMYDHKTFSEILNRVQKAVDDLNLHSYSNLPIWVNKLDMEIERILGVRLQAGLRAWTQVLLGQAEDKAEVDMDTDAPQVSHKPGGEPKIKNVVHELRITNQVIYLNPPIEECRYKLYQEMFAWKMVVLSLPRIQSQRYQVGVHYELTEEEKFYRNALTRMPDGPVALEESYSAVMGIVSEVEQYVKVWLQYQCLWDMQAENIYNRLGEDLNKWQALLVQIRKARGTFDNAETKKEFGPVVIDYGKVQSKVNLKYDSWHKEVLSKFGQMLGSNMTEFHSQISKSRQELEQHSVDTASTSDAVTFITYVQSLKRKIKQFEKQVELYRNGQRLLEKQRFQFPPSWLYIDNIEGEWGAFNDIMRRKDSAIQQQVANLQMKIVQEDRAVESRTTDLLTDWEKTKPVTGNLRPEEALQALTIYEGKFGRLKDDREKCAKAKEALELTDTGLLSGSEERVQVALEELQDLKGVWSELSKVWEQIDQMKEQPWVSVQPRKLRQNLDALLNQLKSFPARLRQYASYEFVQRLLKGYMKINMLVIELKSEALKDRHWKQLMKRLHVNWVVSELTLGQIWDVDLQKNEAIVKDVLLVAQGEMALEEFLKQIREVWNTYELDLVNYQNKCRLIRGWDDLFNKVKEHINSVSAMKLSPYYKVFEEDALSWEDKLNRIMALFDVWIDVQRRWVYLEGIFTGSADIKHLLPVETQRFQSISTEFLALMKKVSKSPLVMDVLNIQGVQRSLERLADLLGKIQKALGEYLERERSSFPRFYFVGDEDLLEIIGNSKNVAKLQKHFKKMFAGVSSIILNEDNSVVLGISSREGEEVMFKTPVSITEHPKINEWLTLVEKEMRVTLAKLLAESVTEVEIFGKATSIDPNTYITWIDKYQAQLVVLSAQIAWSENVETALSSMGGGGDAAPLHSVLSNVEVTLNVLADSVLMEQPPLRRRKLEHLITELVHQRDVTRSLIKSKIDNAKSFEWLSQMRFYFDPKQTDVLQQLSIQMANAKFNYGFEYLGVQDKLVQTPLTDRCYLTMTQALEARLGGSPFGPAGTGKTESVKALGHQLGRFVLVFNCDETFDFQAMGRIFVGLCQVGAWGCFDEFNRLEERMLSAVSQQVQCIQEALREHSNPNYDKTSAPITCELLNKQVKVSPDMAIFITMNPGYAGRSNLPDNLKKLFRSLAMTKPDRQLIAQVMLYSQGFRTAEVLANKIVPFFKLCDEQLSSQSHYDFGLRALKSVLVSAGNVKRERIQKIKREKEERGEAVDEGEIAENLPEQEILIQSVCETMVPKLVAEDIPLLFSLLSDVFPGVQYHRGEMTALREELKKVCQEMYLTYGDGEEVGGMWVEKVLQLYQITQINHGLMMVGPSGSGKSMAWRVLLKALERLEGVEGVAHIIDPKAISKDHLYGTLDPNTREWTDGLFTHVLRKIIDSVRGELQKRQWIVFDGDVDPEWVENLNSVLDDNKLLTLPNGERLSLPPNVRIMFEVQDLKYATLATVSRCGMVWFSEDVLSTDMIFNNFLARLRSIPLDEGEDEAQRRRKGKEDEGEEAASPMLQIQRDAATIMQPYFTSNGLVTKALEHAFQLEHIMDLTRLRCLGSLFSMLHQACRNVAQYNANHPDFPMQIEQLERYIQRYLVYAILWSLSGDSRLKMRAELGEYIRRITTVPLPTAPNIPIIDYEVSISGEWSPWQAKVPQIEVETHKVAAPDVVVPTLDTVRHEALLYTWLAEHKPLVLCGPPGSGKTMTLFSALRALPDMEVVGLNFSSATTPELLLKTFDHYCEYRRTPNGVVLAPVQLGKWLVLFCDEINLPDMDKYGTQRVISFIRQMVEHGGFYRTSDQTWVKLERIQFVGACNPPTDPGRKPLSHRFLRHVPVVYVDYPGPASLTQIYGTFNRAMLRLIPSLRTYAEPLTAAMVEFYTMSQERFTQDTQPHYIYSPREMTRWVRGIFEALRPLETLPVEGLIRIWAHEALRLFQDRLVEDEERRWTDENIDTVALKHFPNIDREKAMSRPILYSNWLSKDYIPVDQEELRDYVKARLKVFYEEELDVPLVLFNEVLDHVLRIDRIFRQPQGHLLLIGVSGAGKTTLSRFVAWMNGLSVYQIKVHRKYTGEDFDEDLRTVLRRSGCKNEKIAFIMDESNVLDSGFLERMNTLLANGEVPGLFEGDEYATLMTQCKEGAQKEGLMLDSHEELYKWFTSQVIRNLHVVFTMNPSSEGLKDRAATSPALFNRCVLNWFGDWSTEALYQVGKEFTSKMDLEKPNYIVPDYMPVVYDKLPQPPSHREAIVNSCVFVHQTLHQANARLAKRGGRTMAITPRHYLDFINHYANLFHEKRSELEEQQMHLNVGLRKIKETVDQVEELRRDLRIKSQELEVKNAAANDKLKKMVKDQQEAEKKKVMSQEIQEQLHKQQEVIADKQMSVKEDLDKVEPAVIEAQNAVKSIKKQHLVEVRSMANPPAAVKLALESICLLLGESTTDWKQIRSIIMRENFIPTIVNFSAEEISDAIREKMKKNYMSNPSYNYEIVNRASLACGPMVKWAIAQLNYADMLKRVEPLRNELQKLEDDAKDNQQKANEVEQMIRDLEASIARYKEEYAVLISEAQAIKADLAAVEAKVNRSTALLKSLSAERERWEKTSETFKNQMSTIAGDCLLSAAFIAYAGYFDQQMRQNLFTTWSHHLQQANIQFRTDIARTEYLSNADERLRWQASSLPADDLCTENAIMLKRFNRYPLIIDPSGQATEFIMNEYKDRKITRTSFLDDAFRKNLESALRFGNPLLVQDVESYDPVLNPVLNREVRRTGGRVLITLGDQDIDLSPSFVIFLSTRDPTVEFPPDLCSRVTFVNFTVTRSSLQSQCLNEVLKAERPDVDEKRSDLLKLQGEFQLRLRQLEKSLLQALNEVKGRILDDDTIITTLENLKREAAEVTRKVEETDIVMQEVETVSQQYLPLSTACSSIYFTMESLKQIHFLYQYSLQFFLDIYHNVLYENPNLKGVTDHTQRLSIITKDLFQVAFNRVARGMLHQDHITFAMLLARIKLKGTVGEPTYDAEFQHFLRGNEIVLSAGSTPRIQGLTVEQAEAVVRLSCLPAFKDLIAKVQADEQFGIWLDSSSPEQTVPYLWSEETPATPIGQAIHRLLLIQAFRPDRLLAMAHMFVSTNLGESFMSIMEQPLDLTHIVGTEVKPNTPVLMCSVPGYDASGHVEDLAAEQNTQITSIAIGSAEGFNQADKAINTAVKSGRWVMLKNVHLAPGWLMQLEKKLHSLQPHACFRLFLTMEINPKVPVNLLRAGRIFVFEPPPGVKANMLRTFSSIPVSRICKSPNERARLYFLLAWFHAIIQERLRYAPLGWSKKYEFGESDLRSACDTVDTWLDDTAKGRQNISPDKIPWSALKTLMAQSIYGGRVDNEFDQRLLNTFLERLFTTRSFDSEFKLACKVDGHKDIQMPDGIRREEFVQWVELLPDTQTPSWLGLPNNAERVLLTTQGVDMISKMLKMQMLEDEDDLAYAETEKKTRTDSTSDGRPAWMRTLHTTASNWLHLIPQTLSHLKRTVENIKDPLFRFFEREVKMGAKLLQDVRQDLADVVQVCEGKKKQTNYLRTLINELVKGILPRSWSHYTVPAGMTVIQWVSDFSERIKQLQNISLAAASGGAKELKNIHVCLGGLFVPEAYITATRQYVAQANSWSLEELCLEVNVTTSQGATLDACSFGVTGLKLQGATCNNNKLSLSNAISTALPLTQLRWVKQTNTEKKASVVTLPVYLNFTRADLIFTVDFEIATKEDPRSFYERGVAVLCTE

106 NP_001943.1 P40394

>NP_001943.1

MSQQRPARKLPSLLLDPTEETVRRRCRDPINVEGLLPSKIRINLEDNVQYVSMRKALKVKRPRFDVSLVYLTRKFMDLVRSAPGGILDLNKVATKLGVRKRRVYDITNVLDGIDLVEKKSKNHIRWIGSDLSNFGAVPQQKKLQEELSDLSAMEDALDELIKDCAQQLFELTDDKENERLAYVTYQDIHSIQAFHEQIVIAVKAPAETRLDVPAPREDSITVHIRSTNGPIDVYLCEVEQGQTSNKRSEGVGTSSSESTHPEGPEEEENPQQSEELLEVSN

>P40394

MGTAGKVIKCKAAVLWEQKQPFSIEEIEVAPPKTKEVRIKILATGICRTDDHVIKGTMVSKFPVIVGHEATGIVESIGEGVTTVKPGDKVIPLFLPQCRECNACRNPDGNLCIRSDITGRGVLADGTTRFTCKGKPVHHFMNTSTFTEYTVVDESSVAKIDDAAPPEKVCLIGCGFSTGYGAAVKTGKVKPGSTCVVFGLGGVGLSVIMGCKSAGASRIIGIDLNKDKFEKAMAVGATECISPKDSTKPISEVLSEMTGNNVGYTFEVIGHLETMIDALASCHMNYGTSVVVGVPPSAKMLTYDPMLLFTGRTWKGCVFGGLKSRDDVPKLVTEFLAKKFDLDQLITHVLPFKKISEGFELLNSGQSIRTVLTF

107 NP_003160.2 Q9UDY6

>NP_003160.2

MSDSEDSNFSEEEDSERSSDGEEAEVDEERRSAAGSEKEEEPEDEEEEEEEEEYDEEEEEEDDDRPPKKPRHGGFILDEADVDDEYEDEDQWEDGAEDILEKEEIEASNIDNVVLDEDRSGARRLQNLWRDQREEELGEYYMKKYAKSSVGETVYGGSDELSDDITQQQLLPGVKDPNLWTVKCKIGEERATAISLMRKFIAYQFTDTPLQIKSVVAPEHVKGYIYVEAYKQTHVKQAIEGVGNLRLGYWNQQMVPIKEMTDVLKVVKEVANLKPKSWVRLKRGIYKDDIAQVDYVEPSQNTISLKMIPRIDYDRIKARMSLKDWFAKRKKFKRPPQRLFDAEKIRSLGGDVASDGDFLIFEGNRYSRKGFLFKSFAMSAVITEGVKPTLSELEKFEDQPEGIDLEVVTESTGKEREHNFQPGDNVEVCEGELINLQGKILSVDGNKITIMPKHEDLKDMLEFPAQELRKYFKMGDHVKVIAGRFEGDTGLIVRVEENFVILFSDLTMHELKVLPRDLQLCSETASGVDVGGQHEWGELVQLDPQTVGVIVRLERETFQVLNMYGKVVTVRHQAVTRKKDNRFAVALDSEQNNIHVKDIVKVIDGPHSGREGEIRHLFRSFAFLHCKKLVENGGMFVCKTRHLVLAGGSKPRDVTNFTVGGFAPMSPRISSPMHPSAGGQRGGFGSPGGGSGGMSRGRGRRDNELIGQTVRISQGPYKGYIGVVKDATESTARVELHSTCQTISVDRQRLTTVGSRRPGGMTSTYGRTPMYGSQTPMYGSGSRTPMYGSQTPLQDGSRTPHYGSQTPLHDGSRTPAQSGAWDPNNPNTPSRAEEEYEYAFDDEPTPSPQAYGGTPNPQTPGYPDPSSPQVNPQYNPQTPGTPAMYNTDQFSPYAAPSPQGSYQPSPSPQSYHQVAPSPAGYQNTHSPASYHPTPSPMAYQASPSPSPVGYSPMTPGAPSPGGYNPHTPGSGIEQNSSDWVTTDIQVKVRDTYLDTQVVGQTGVIRSVTGGMCSVYLKDSEKVVSISSEHLEPITPTKNNKVKVILGEDREATGVLLSIDGEDGIVRMDLDEQLKILNLRFLGKLLEA

>Q9UDY6

MASAASVTSLADEVNCPICQGTLREPVTIDCGHNFCRACLTRYCEIPGPDLEESPTCPLCKEPFRPGSFRPNWQLANVVENIERLQLVSTLGLGEEDVCQEHGEKIYFFCEDDEMQLCVVCREAGEHATHTMRFLEDAAAPYREQIHKCLKCLRKEREEIQEIQSRENKRMQVLLTQVSTKRQQVISEFAHLRKFLEEQQSILLAQLESQDGDILRQRDEFDLLVAGEICRFSALIEELEEKNERPARELLTDIRSTLIRCETRKCRKPVAVSPELGQRIRDFPQQALPLQREMKMFLEKLCFELDYEPAHISLDPQTSHPKLLLSEDHQRAQFSYKWQNSPDNPQRFDRATCVLAHTGITGGRHTWVVSIDLAHGGSCTVGVVSEDVQRKGELRLRPEEGVWAVRLAWGFVSALGSFPTRLTLKEQPRQVRVSLDYEVGWVTFTNAVTREPIYTFTASFTRKVIPFFGLWGRGSSFSLSS

108 Q8NFU5 NP_056273.2

>Q8NFU5

MATEPPSPLRVEAPGPPEMRTSPAIESTPEGTPQPAGGRLRFLNGCVPLSHQVAGHMYGKDKVGILQHPDGTVLKQLQPPPRGPRELEFYNMVYAADCFDGVLLELRKYLPKYYGIWSPPTAPNDLYLKLEDVTHKFNKPCIMDVKIGQKSYDPFASSEKIQQQVSKYPLMEEIGFLVLGMRVYHVHSDSYETENQHYGRSLTKETIKDGVSRFFHNGYCLRKDAVAASIQKIEKILQWFENQKQLNFYASSLLFVYEGSSQPTTTKLNDRTLAEKFLSKGQLSDTEVLEYNNNFHVLSSTANGKIESSVGKSLSKMYARHRKIYTKKHHSQTSLKVENLEQDNGWKSMSQEHLNGNVLSQLEKVFYHLPTGCQEIAEVEVRMIDFAHVFPSNTIDEGYVYGLKHLISVLRSILDN

>NP_056273.2

MEFAELIKTPRVDNVVLHRPFYPAVEGTLCLTGHHLILSSRQDNTEELWLLHSNIDAIDKRFVGSLGTIIIKCKDFRIIQLDIPGMEECLNIASSIEALSTLDSITLMYPFFYRPMFEVIEDGWHSFLPEQEFELYSSATSEWRLSYVNKEFAVCPSYPPIVTVPKSIDDEALRKVATFRHGGRFPVLSYYHKKNGMVIMRSGQPLTGTNGRRCKEDEKLINATLRAGKRGYIIDTRSLNVAQQTRAKGGGFEQEAHYPQWRRIHKSIERYHILQESLIKLVEACNDQTHNMDRWLSKLEASNWLTHIKEILTTACLAAQCIDREGASILIHGTEGTDSTLQVTSLAQIILEPRSRTIRGFEALIEREWLQAGHPFQQRCAQSAYCNTKQKWEAPVFLLFLDCVWQILRQFPCSFEFNENFLIMLFEHAYASQFGTFLGNNESERCKLKLQQKTMSLWSWVNQPSELSKFTNPLFEANNLVIWPSVAPQSLPLWEGIFLRWNRSSKYLDEAYEEMVNIIEYNKELQAKVNILRRQLAELETEDGMQESP

109 NP_005514.1 NP_004865.1

>NP_005514.1

MDFDERGPCSSNMYLPSCTYYVSGPDFSSLPSFLPQTPSSRPMTYSYSSNLPQVQPVREVTFREYAIEPATKWHPRGNLAHCYSAEELVHRDCLQAPSAAGVPGDVLAKSSANVYHHPTPAVSSNFYSTVGRNGVLPQAFDQFFETAYGTPENLASSDYPGDKSAEKGPPAATATSAAAAAAATGAPATSSSDSGGGGGCRETAAAAEEKERRRRPESSSSPESSSGHTEDKAGGSSGQRTRKKRCPYTKYQIRELEREFFFSVYINKEKRLQLSRMLNLTDRQVKIWFQNRRMKEKKINRDRLQYYSANPLL

>NP_004865.1

MSALRRSGYGPSDGPSYGRYYGPGGGDVPVHPPPPLYPLRPEPPQPPISWRVRGGGPAETTWLGEGGGGDGYYPSGGAWPEPGRAGGSHQEQPPYPSYNSNYWNSTARSRAPYPSTYPVRPELQGQSLNSYTNGAYGPTYPPGPGANTASYSGAYYAPGYTQTSYSTEVPSTYRSSGNSPTPVSRWIYPQQDCQTEAPPLRGQVPGYPPSQNPGMTLPHYPYGDGNRSVPQSGPTVRPQEDAWASPGAYGMGGRYPWPSSAPSAPPGNLYMTESTSPWPSSGSPQSPPSPPVQQPKDSSYPYSQSDQSMNRHNFPCSVHQYESSGTVNNDDSDLLDSQVQYSAEPQLYGNATSDHPNNQDQSSSLPEECVPSDESTPPSIKKIIHVLEKVQYLEQEVEEFVGKKTDKAYWLLEEMLTKELLELDSVETGGQDSVRQARKEAVCKIQAILEKLEKKGL

110 NP_776248.1 Q9H7D7

>NP_776248.1

MPASMFSIDNILAARPRCKDSVLPVAHSAAAPVVFPALHGDSLYGASGGASSDYGAFYPRPVAPGGAGLPAAVSGSRLGYNNYFYGQLHVQAAPVGPACCGAVPPLGAQQCSCVPTPPGYEGPGSVLVSPVPHQMLPYMNVGTLSRTELQLLNQLHCRRKRRHRTIFTDEQLEALENLFQETKYPDVGTREQLARKVHLREEKVEVWFKNRRAKWRRQKRSSSEESENAEKWNKTSSSKASPEKREEEGKSDLDSDS

>Q9H7D7

MQANGAGGGGGGGGGGGGGGGGGGGQGQTPELACLSAQNGESSPSSSSSAGDLAHANGLLPSAPSAASNNSNSLNVNNGVPGGAAAASSATVAAASATTAASSSLATPELGSSLKKKKRLSQSDEDVIRLIGQHLNGLGLNQTVDLLMQESGCRLEHPSATKFRNHVMEGDWDKAENDLNELKPLVHSPHAIVVRGALEISQTLLGIIVRMKFLLLQQKYLEYLEDGKVLEALQVLRCELTPLKYNTERIHVLSGYLMCSHAEDLRAKAEWEGKGTASRSKLLDKLQTYLPPSVMLPPRRLQTLLRQAVELQRDRCLYHNTKLDNNLDSVSLLIDHVCSRRQFPCYTQQILTEHCNEVWFCKFSNDGTKLATGSKDTTVIIWQVDPDTHLLKLLKTLEGHAYGVSYIAWSPDDNYLVACGPDDCSELWLWNVQTGELRTKMSQSHEDSLTSVAWNPDGKRFVTGGQRGQFYQCDLDGNLLDSWEGVRVQCLWCLSDGKTVLASDTHQRIRGYNFEDLTDRNIVQEDHPIMSFTISKNGRLALLNVATQGVHLWDLQDRVLVRKYQGVTQGFYTIHSCFGGHNEDFIASGSEDHKVYIWHKRSELPIAELTGHTRTVNCVSWNPQIPSMMASASDDGTVRIWGPAPFIDHQNIEEECSSMDS

111 Q6ISB3 P57723

>Q6ISB3

MSQESDNNKRLVALVPMPSDPPFNTRRAYTSEDEAWKSYLENPLTAATKAMMSINGDEDSAAALGLLYDYYKVPRDKRLLSVSKASDSQEDQEKRNCLGTSEAQSNLSGGENRVQVLKTVPVNLSLNQDHLENSKREQYSISFPESSAIIPVSGITVVKAEDFTPVFMAPPVHYPRGDGEEQRVVIFEQTQYDVPSLATHSAYLKDDQRSTPDSTYSESFKDAATEKFRSASVGAEEYMYDQTSSGTFQYTLEATKSLRQKQGEGPMTYLNKGQFYAITLSETGDNKCFRHPISKVRSVVMVVFSEDKNRDEQLKYWKYWHSRQHTAKQRVLDIADYKESFNTIGNIEEIAYNAVSFTWDVNEEAKIFITVNCLSTDFSSQKGVKGLPLMIQIDTYSYNNRSNKPIHRAYCQIKVFCDKGAERKIRDEERKQNRKKGKGQASQTQCNSSSDGKLAAIPLQKKSDITYFKTMPDLHSQPVLFIPDVHFANLQRTGQVYYNTDDEREGGSVLVKRMFRPMEEEFGPVPSKQMKEEGTKRVLLYVRKETDDVFDALMLKSPTVKGLMEAISEKYGLPVEKIAKLYKKSKKGILVNMDDNIIEHYSNEDTFILNMESMVEGFKVTLMEI

>P57723

MSGSDGGLEEEPELSITLTLRMLMHGKEVGSIIGKKGETVKRIREQSSARITISEGSCPERITTITGSTAAVFHAVSMIAFKLDEDLCAAPANGGNVSRPPVTLRLVIPASQCGSLIGKAGTKIKEIRETTGAQVQVAGDLLPNSTERAVTVSGVPDAIILCVRQICAVILESPPKGATIPYHPSLSLGTVLLSANQGFSVQGQYGAVTPAEVTKLQQLSSHAVPFATPSVVPGLDPGTQTSSQEFLVPNDLIGCVIGRQGSKISEIRQMSGAHIKIGNQAEGAGERHVTITGSPVSIALAQYLITACLETAKSTSGGTPSSAPADLPAPFSPPLTALPTAPPGLLGTPYAISLSNFIGLKPMPFLALPPASPGPPPGLAAYTAKMAAANGSKKAERQKFSPY

112 NP_006697.2 NP_001395.1

>NP_006697.2

MAERGGDGGESERFNPGELRMAQQQALRFRGPAPPPNAVMRGPPPLMRPPPPFGMMRGPPPPPRPPFGRPPFDPNMPPMPPPGGIPPPMGPPHLQRPPFMPPPMSSMPPPPGMMFPPGMPPVTAPGTPALPPTEEIWVENKTPDGKVYYYNARTRESAWTKPDGVKVIQQSELTPMLAAQAQVQAQAQAQAQAQAQAQAQAQAQAQAQAQAQAQAQAQAQAQAQAQAQAQAQAQAQAQAQAQVQAQVQAQVQAQAVGASTPTTSSPAPAVSTSTSSSTPSSTTSTTTTATSVAQTVSTPTTQDQTPSSAVSVATPTVSVSTPAPTATPVQTVPQPHPQTLPPAVPHSVPQPTTAIPAFPPVMVPPFRVPLPGMPIPLPGVAMMQIVSCPYVKTVATTKTGVLPGMAPPIVPMIHPQVAIAASPATLAGATAVSEWTEYKTADGKTYYYNNRTLESTWEKPQELKEKEKLEEKIKEPIKEPSEEPLPMETEEEDPKEEPIKEIKEEPKEEEMTEEEKAAQKAKPVATAPIPGTPWCVVWTGDERVFFYNPTTRLSMWDRPDDLIGRADVDKIIQEPPHKKGMEELKKLRHPTPTMLSIQKWQFSMSAIKEEQELMEEINEDEPVKAKKRKRDDNKDIDSEKEAAMEAEIKAARERAIVPLEARMKQFKDMLLERGVSAFSTWEKELHKIVFDPRYLLLNPKERKQVFDQYVKTRAEEERREKKNKIMQAKEDFKKMMEEAKFNPRATFSEFAAKHAKDSRFKAIEKMKDREALFNEFVAAARKKEKEDSKTRGEKIKSDFFELLSNHHLDSQSRWSKVKDKVESDPRYKAVDSSSMREDLFKQYIEKIAKNLDSEKEKELERQARIEASLREREREVQKARSEQTKEIDREREQHKREEAIQNFKALLSDMVRSSDVSWSDTRRTLRKDHRWESGSLLEREEKEKLFNEHIEALTKKKREHFRQLLDETSAITLTSTWKEVKKIIKEDPRCIKFSSSDRKKQREFEEYIRDKYITAKADFRTLLKETKFITYRSKKLIQESDQHLKDVEKILQNDKRYLVLDCVPEERRKLIVAYVDDLDRRGPPPPPTASEPTRRSTK

>NP_001395.1

MAAGTLYTYPENWRAFKALIAAQYSGAQVRVLSAPPHFHFGQTNRTPEFLRKFPAGKVPAFEGDDGFCVFESNAIAYYVSNEELRGSTPEAAAQVVQWVSFADSDIVPPASTWVFPTLGIMHHNKQATENAKEEVRRILGLLDAYLKTRTFLVGERVTLADITVVCTLLWLYKQVLEPSFRQAFPNTNRWFLTCINQPQFRAVLGEVKLCEKMAQFDAKKFAETQPKKDTPRKEKGSREEKQKPQAERKEEKKAAAPAPEEEMDECEQALAAEPKAKDPFAHLPKSTFVLDEFKRKYSNEDTLSVALPYFWEHFDKDGWSLWYSEYRFPEELTQTFMSCNLITGMFQRLDKLRKNAFASVILFGTNNSSSISGVWVFRGQELAFPLSPDWQVDYESYTWRKLDPGSEETQTLVREYFSWEGAFQHVGKAFNQGKIFK

113 P07992 Q9NX20

>P07992

MDPGKDKEGVPQPSGPPARKKFVIPLDEDEVPPGVAKPLFRSTQSLPTVDTSAQAAPQTYAEYAISQPLEGAGATCPTGSEPLAGETPNQALKPGAKSNSIIVSPRQRGNPVLKFVRNVPWEFGDVIPDYVLGQSTCALFLSLRYHNLHPDYIHGRLQSLGKNFALRVLLVQVDVKDPQQALKELAKMCILADCTLILAWSPEEAGRYLETYKAYEQKPADLLMEKLEQDFVSRVTECLTTVKSVNKTDSQTLLTTFGSLEQLIAASREDLALCPGLGPQKARRLFDVLHEPFLKVP

>Q9NX20

MWRLLARASAPLLRVPLSDSWALLPASAGVKTLLPVPSFEDVSIPEKPKLRFIERAPLVPKVRREPKNLSDIRGPSTEATEFTEGNFAILALGGGYLHWGHFEMMRLTINRSMDPKNMFAIWRVPAPFKPITRKSVGHRMGGGKGAIDHYVTPVKAGRLVVEMGGRCEFEEVQGFLDQVAHKLPFAAKAVSRGTLEKMRKDQEERERNNQNPWTFERIATANMLGIRKVLSPYDLTHKGKYWGKFYMPKRV

114 NP_003070.3 Q6PRD7

>NP_003070.3

MSKRPSYAPPPTPAPATQMPSTPGFVGYNPYSHLAYNNYRLGGNPGTNSRVTASSGITIPKPPKPPDKPLMPYMRYSRKVWDQVKASNPDLKLWEIGKIIGGMWRDLTDEEKQEYLNEYEAEKIEYNESMKAYHNSPAYLAYINAKSRAEAALEEESRQRQSRMEKGEPYMSIQPAEDPDDYDDGFSMKHTATARFQRNHRLISEILSESVVPDVRSVVTTARMQVLKRQVQSLMVHQRKLEAELLQIEERHQEKKRKFLESTDSFNNELKRLCGLKVEVDMEKIAAEIAQAEEQARKRQEEREKEAAEQAERSQSSIVPEEEQAANKGEEKKDDENIPMETEETHLEETTESQQNGEEGTSTPEDKESGQEGVDSMAEEGTSDSNTGSESNSATVEEPPTDPIPEDEKKE

>Q6PRD7

MGTSSTDSQQAGHRRCSTSNTSAENLTCLSLPGSPGKTAPLPGPAQAGAGQPLPKGCAAVKAEVGIPAPHTSQEVRIHIRRLLSWAAPGACGLRSTPCALPQALPQARPCPGRWFFPGCSLPTGGAQTILSLWTWRHFLNWALQQREENSGRARRVPPVPRTAPVSKGEGSHPPQNSNGEKVKTITPDVGLHQSLTSDPTVAVLRAKRAPEAHPPRSCSGSLTARVCHMGVCQGQGDTEDGRMTLMG

115 Q8NFU5 NP_005300.1

>Q8NFU5

MATEPPSPLRVEAPGPPEMRTSPAIESTPEGTPQPAGGRLRFLNGCVPLSHQVAGHMYGKDKVGILQHPDGTVLKQLQPPPRGPRELEFYNMVYAADCFDGVLLELRKYLPKYYGIWSPPTAPNDLYLKLEDVTHKFNKPCIMDVKIGQKSYDPFASSEKIQQQVSKYPLMEEIGFLVLGMRVYHVHSDSYETENQHYGRSLTKETIKDGVSRFFHNGYCLRKDAVAASIQKIEKILQWFENQKQLNFYASSLLFVYEGSSQPTTTKLNDRTLAEKFLSKGQLSDTEVLEYNNNFHVLSSTANGKIESSVGKSLSKMYARHRKIYTKKHHSQTSLKVENLEQDNGWKSMSQEHLNGNVLSQLEKVFYHLPTGCQEIAEVEVRMIDFAHVFPSNTIDEGYVYGLKHLISVLRSILDN

>NP_005300.1

MASSTGDRSQAVRHGLRAKVLTLDGMNPRVRRVEYAVRGPIVQRALELEQELRQGVKKPFTEVIRANIGDAQAMGQRPITFLRQVLALCVNPDLLSSPNFPDDAKKRAERILQACGGHSLGAYSVSSGIQLIREDVARYIERRDGGIPADPNNVFLSTGASDAIVTVLKLLVAGEGHTRTGVLIPIPQYPLYSATLAELGAVQVDYYLDEERAWALDVAELHRALGQARDHCRPRALCVINPGNPTGQVQTRECIEAVIRFAFEERLFLLADEVYQDNVYAAGSQFHSFKKVLMEMGPPYAGQQELASFHSTSKGYMGECGFRGGYVEVVNMDAAVQQQMLKLMSVRLCPPVPGQALLDLVVSPPAPTDPSFAQFQAEKQAVLAELAAKAKLTEQVFNEAPGISCNPVQGAMYSFPRVQLPPRAVERAQELGLAPDMFFCLRLLEETGICVVPGSGFGQREGTYHFRMTILPPLEKLRLLLEKLSRFHAKFTLEYS

116 Q99581 NP_001547.1

>Q99581

MRQSGASQPLLINMYLPDPVGDGLFKDGKNPSWGPLSPAVQKGSGQIQLWQFLLELLADRANAGCIAWEGGHGEFKLTDPDEVARRWGERKSKPNMNYDKLSRALRYYYDKNIMSKVHGKRYAYRFDFQGLAQACQPPPAHAHAAAAAAAAAAAAQDGALYKLPAGLAPLPFPGLSKLNLMAASAGVAPAGFSYWPGPGPAATAAAATAALYPSPSLQPPPGPFGAVAAASHLGGHYH

>NP_001547.1

MSWSPSLTTQTCGAWEMKERLGTGGFGNVIRWHNQETGEQIAIKQCRQELSPRNRERWCLEIQIMRRLTHPNVVAARDVPEGMQNLAPNDLPLLAMEYCQGGDLRKYLNQFENCCGLREGAILTLLSDIASALRYLHENRIIHRDLKPENIVLQQGEQRLIHKIIDLGYAKELDQGSLCTSFVGTLQYLAPELLEQQKYTVTVDYWSFGTLAFECITGFRPFLPNWQPVQWHSKVRQKSEVDIVVSEDLNGTVKFSSSLPYPNNLNSVLAERLEKWLQLMLMWHPRQRGTDPTYGPNGCFKALDDILNLKLVHILNMVTGTIHTYPVTEDESLQSLKARIQQDTGIPEEDQELLQEAGLALIPDKPATQCISDGKLNEGHTLDMDLVFLFDNSKITYETQISPRPQPESVSCILQEPKRNLAFFQLRKVWGQVWHSIQTLKEDCNRLQQGQRAAMMNLLRNNSCLSKMKNSMASMSQQLKAKLDFFKTSIQIDLEKYSEQTEFGITSDKLLLAWREMEQAVELCGRENEVKLLVERMMALQTDIVDLQRSPMGRKQGGTLDDLEEQARELYRRLREKPRDQRTEGDSQEMVRLLLQAIQSFEKKVRVIYTQLSKTVVCKQKALELLPKVEEVVSLMNEDEKTVVRLQEKRQKELWNLLKIACSKVRGPVSGSPDSMNASRLSQPGQLMSQPSTASNSLPEPAKKSEELVAEAHNLCTLLENAIQDTVREQDQSFTALDWSWLQTEEEEHSCLEQAS

117 O95926 Q9NR20

>O95926

MAAIAASEVLVDSAEEGSLAAAAELAAQKREQRLRKFRELHLMRNEARKLNHQEVVEEDKRLKLPANWEAKKARLEWELKEEEKKKECAARGEDYEKVKLLEISAEDAERWERKKKRKNPDLGFSDYAAAQLRQYHRLTKQIKPDMETYERLREKHGEEFFPTSNSLLHGTHVPSTEEIDRMVIDLEKQIEKRDKYSRRRPYNDDADIDYINERNAKFNKKAERFYGKYTAEIKQNLERGTAV

>Q9NR20

MPASELKASEIPFHPSIKTQDPKAEEKSPKKQKVTLTAAEALKLFKNQLSPYEQSEILGYAELWFLGLEAKKLDTAPEKFSKTSFDDEHGFYLKVLHDHIAYRYEVLETIGKGSFGQVAKCLDHKNNELVALKIIRNKKRFHQQALMELKILEALRKKDKDNTYNVVHMKDFFYFRNHFCITFELLGINLYELMKNNNFQGFSLSIVRRFTLSVLKCLQMLSVEKIIHCDLKPENIVLYQKGQASVKVIDFGSSCYEHQKVYTYIQSRFYRSPEVILGHPYDVAIDMWSLGCITAELYTGYPLFPGENEVEQLACIMEVLGLPPAGFIQTASRRQTFFDSKGFPKNITNNRGKKRYPDSKDLTMVLKTYDTSFLDFLRRCLVWEPSLRMTPDQALKHAWIHQSRNLKPQPRPQTLRKSNSFFPSETRKDKVQGCHHSSRKADEITKETTEKTKDSPTKHVQHSGDQQDCLQHGADTVQLPQLVDAPKKSEAAVGAEVSMTSPGQSKNFSLKNTNVLPPIV

118 NP_690048.1 P49753

>NP_690048.1

MVSSQPKYDLIREVGRGSYGVVYEAVIRKTSARVAVKKIRCHAPENVELALREFWALSSIKSQHPNVIHLEECILQKDGMVQKMSHGSNSSLYLQLVETSLKGEIAFDPRSAYYLWFVMDFCDGGDMNEYLLSRKPNRKTNTSFMLQLSSALAFLHKNQIIHRDLKPDNILISQTRLDTSDLEPTLKVADFGLSKVCSASGQNPEEPVSVNKCFLSTACGTDFYMAPEVWEGHYTAKADIFALGIIIWAMLERITFIDTETKKELLGSYVKQGTEIVPVGEALLENPKMELLIPVKKKSMNGRMKQLIKEMLAANPQDRPDAFELELRLVQIAFKDSSWET

>P49753

MSNKLLSPHPHSVVLRSEFKMASSPAVLRASRLYQWSLKSSAQFLGSPQLRQVGQIIRVPARMAATLILEPAGRCCWDEPVRIAVRGLAPEQPVTLRASLRDEKGALFQAHARYRADTLGELDLERAPALGGSFAGLEPMGLLWALEPEKPLVRLVKRDVRTPLAVELEVLDGHDPDPGRLLCQTRHERYFLPPGVRREPVRVGRVRGTLFLPPEPGPFPGIVDMFGTGGGLLEYRASLLAGKGFAVMALAYYNYEDLPKTMETLHLEYFEEAMNYLLSHPEVKGPGVGLLGISKGGELCLSMASFLKGITAAVVINGSVANVGGTLRYKGETLPPVGVNRNRIKVTKDGYADIVDVLNSPLEGPDQKSFIPVERAESTFLFLVGQDDHNWKSEFYANEACKRLQAHGRRKPQIICYPETGHYIEPPYFPLCRASLHALVGSPIIWGGEPRAHAMAQVDAWKQLQTFFHKHLGGHEGTIPSKV

119 O60231 NP_001434.1

>O60231

MATPAGLERWVQDELHSVLGLSERHVAQFLIGTAQRCTSAEEFVQRLRDTDTLDLSGPARDFALRLWNKVPRKAVVEKPARAAEREARALLEKNRSYRLLEDSEESSEETVSRAGSSLQKKRKKRKHLRKKREEEEEEEASEKGKKKTGGSKQQTEKPESEDEWERTERERLQDLEERDAFAERVRQRDKDRTRNVLERSDKKAYEEAQKRLKMAEEDRKAMVPELRKKSRREYLAKREREKLEDLEAELADEEFLFGDVELSRHERQELKYKRRVRDLAREYRAAGEQEKLEATNRYHMPKETRGQPARAVDLVEEESGAPGEEQRRWEEARLGAASLKFGARDAASQEPKYQLVLEEEETIEFVRATQLQGDEEPSAPPTSTQAQQKESIQAVRRSLPVFPFREELLAAIANHQVLIIEGETGSGKTTQIPQYLFEEGYTNKGMKIACTQPRRVAAMSVAARVAREMGVKLGNEVGYSIRFEDCTSERTVLRYMTDGMLLREFLSEPDLASYSVVMVDEAHERTLHTDILFGLIKDVARFRPELKVLVASATMDTARFSTFFDDAPVFRIPGRRFPVDIFYTKAPEADYLEACVVSVLQIHVTQPPGDILVFLTGQEEIEAACEMLQDRCRRLGSKIRELLVLPIYANLPSDMQARIFQPTPPGARKVVVATNIAETSLTIEGIIYVLDPGFCKQKSYNPRTGMESLTVTPCSKASANQRAGRAGRVAAGKCFRLYTAWAYQHELEETTVPEIQRTSLGNVVLLLKSLGIHDLMHFDFLDPPPYETLLLALEQLYALGALNHLGELTTSGRKMAELPVDPMLSKMILASEKYSCSEEILTVAAMLSVNNSIFYRPKDKVVHADNARVNFFLPGGDHLVLLNVYTQWAESGYSSQWCYENFVQFRSMRRARDVREQLEGLLERVEVGLSSCQGDYIRVRKAITAGYFYHTARLTRSGYRTVKQQQTVFIHPNSSLFEQQPRWLLYHELVLTTKEFMRQVLEIESSWLLEVAPHYYKAKELEDPHAKKMPKKIGKTREELG

>NP_001434.1

MSFSGKYQLQSQENFEAFMKAIGLPEELIQKGKDIKGVSEIVQNGKHFKFTITAGSKVIQNEFTVGEECELETMTGEKVKTVVQLEGDNKLVTTFKNIKSVTELNGDIITNTMTLGDIVFKRISKRI

120 Q9UL03 P09210

>Q9UL03

MPILLFLIDTSASMNQRSHLGTTYLDTAKGAVETFMKLRARDPASRGDRYMLVTFEEPPYAIKAGWKENHATFMNELKNLQAEGLTTLGQSLRTAFDLLNLNRLVTGIDNYGQGRNPFFLEPAIIITITDGSKLTTTSGVQDELHLPLNSPLPGSELTKEPFRWDQRLFALVLRLPGTMSVESEQLTGVPLDDSAITPMCEVTGGRSYSVCSPRMLNQCLESLVQKVQSGVVINFEKAGPDPSPVEDGQPDISRPFGSQPWHSCHKLIYVRPNPKTGVPIGHWPVPESFWPDQNSPTLPPRTSHPVVKFSCTDCEPMVIDKLPFDKYELEPSPLTQFILERKSPQTCWQVYVSNSAKYSELGHPFGYLKASTALNCVNLFVMPYNYPVLLPLLDDLFKVHKAKPTLKWRQSFESYLKTMPPYYLGPLKKAVRMMGAPNLIADSMEYGLSYSVISYLKKLSQQAKIESDRVIGSVGKKVVQETGIKVRSRSHGLSMAYRKDFQQLLQGISEDVPHRLLDLNMKEYTGFQVALLNKDLKPQTFRNAYDIPRRNLLDHLTRMRSNLLKSTRRFLKGQDEDQVHSVPIAQMGNYQEYLKQVPSPLRELDPDQPRRLHTFGNPFKLDKKGMMIDEADEFVAGPQNKHKRPGEPNMQGIPKRRRCMSPLLRGRQQNPVVNNHIGGKGPPAPTTQAQPDLIKPLPLHKISETTNDSIIHDVVENHVADQLSSDITPNAMDTEFSASSPASLLERPTNHMEALGHDHLGTNDLTVGGFLENHEEPRDKEQCAEENIPASSLNKGKKLMHCRSHEEVNTELKAQIMKEIRKPGRKYERIFTLLKHVQGSLQTRLIFLQNVIKEASRFKKRMLIEQLENFLDEIHRRANQINHINSN

>P09210

MAEKPKLHYSNIRGRMESIRWLLAAAGVEFEEKFIKSAEDLDKLRNDGYLMFQQVPMVEIDGMKLVQTRAILNYIASKYNLYGKDIKEKALIDMYIEGIADLGEMILLLPFSQPEEQDAKLALIQEKTKNRYFPAFEKVLKSHGQDYLVGNKLSRADIHLVELLYYVEELDSSLISSFPLLKALKTRISNLPTVKKFLQPGSPRKPPMDEKSLEESRKIFRF

121 NP_997001.1 NP_004332.2

>NP_997001.1

MVNVLKGVLIECDPAMKQFLLYLDESNALGKKFIIQDIDDTHVFVIAELVNVLQERVGELMDQNAFSLTQK

>NP_004332.2

MAALVLEDGSVLRGQPFGAAVSTAGEVVFQTGMVGYPEALTDPSYKAQILVLTYPLIGNYGIPPDEMDEFGLCKWFESSGIHVAALVVGECCPTPSHWSATRTLHEWLQQHGIPGLQGVDTRELTKKLREQGSLLGKLVQNGTEPSSLPFLDPNARPLVPEVSIKTPRVFNTGGAPRILALDCGLKYNQIRCLCQRGAEVTVVPWDHALDSQEYEGLFLSNGPGDPASYPSVVSTLSRVLSEPNPRPVFGICLGHQLLALAIGAKTYKMRYGNRGHNQPCLLVGSGRCFLTSQNHGFAVETDSLPADWAPLFTNANDGSNEGIVHNSLPFFSVQFHPEHQAGPSDMELLFDIFLETVKEATAGNPGGQTVRERLTERLCPPGIPTPGSGLPPPRKVLILGSGGLSIGQAGEFDYSGSQAIKALKEENIQTLLINPNIATVQTSQGLADKVYFLPITPHYVTQVIRNERPDGVLLTFGGQTALNCGVELTKAGVLARYGVRVLGTPVETIELTEDRRAFAARMAEIGEHVAPSEAANSLEQAQAAAERLGYPVLVRAAFALGGLGSGFASNREELSALVAPAFAHTSQVLVDKSLKGWKEIEYEVVRDAYGNCVTVCNMENLDPLGIHTGESIVVAPSQTLNDREYQLLRQTAIKVTQHLGIVGECNVQYALNPESEQYYIIEVNARLSRSSALASKATGYPLAYVAAKLALGIPLPELRNSVTGGTAAFEPSVDYCVVKIPRWDLSKFLRVSTKIGSCMKSVGEVMGIGRSFEEAFQKALRMVDENCVGFDHTVKPVSDMELETPTDKRIFVVAAALWAGYSVDRLYELTRIDRWFLHRMKRIIAHAQLLEQHRGQPLPPDLLQQAKCLGFSDKQIALAVLSTELAVRKLRQELGICPAVKQIDTVAAEWPAQTNYLYLTYWGTTHDLTFRTPHVLVLGSGVYRIGSSVEFDWCAVGCIQQLRKMGYKTIMVNYNPETVSTDYDMCDRLYFDEISFEVVMDIYELENPEGVILSMGGQLPNNMAMALHRQQCRVLGTSPEAIDSAENRFKFSRLLDTIGISQPQWRELSDLESARQFCQTVGYPCVVRPSYVLSGAAMNVAYTDGDLERFLSSAAAVSKEHPVVISKFIQEAKEIDVDAVASDGVVAAIAISEHVENAGVHSGDATLVTPPQDITAKTLERIKAIVHAVGQELQVTGPFNLQLIAKDDQLKVIECNVRVSRSFPFVSKTLGVDLVALATRVIMGEEVEPVGLMTGSGVVGVKVPQFSFSRLAGADVVLGVEMTSTGEVAGFGESRCEAYLKAMLSTGFKIPKKNILLTIGSYKNKSELLPTVRLLESLGYSLYASLGTADFYTEHGVKVTAVDWHFEEAVDGECPPQRSILEQLAEKNFELVINLSMRGAGGRRLSSFVTKGYRTRRLAADFSVPLIIDIKCTKLFVEALGQIGPAPPLKVHVDCMTSQKLVRLPGLIDVHVHLREPGGTHKEDFASGTAAALAGGITMVCAMPNTRPPIIDAPALALAQKLAEAGARCDFALFLGASSENAGTLGTVAGSAAGLKLYLNETFSELRLDSVVQWMEHFETWPSHLPIVAHAEQQTVAAVLMVAQLTQRSVHICHVARKEEILLIKAAKARGLPVTCEVAPHHLFLSHDDLERLGPGKGEVRPELGSRQDVEALWENMAVIDCFASDHAPHTLEEKCGSRPPPGFPGLETMLPLLLTAVSEGRLSLDDLLQRLHHNPRRIFHLPPQEDTYVEVDLEHEWTIPSHMPFSKAHWTPFEGQKVKGTVRRVVLRGEVAYIDGQVLVPPGYGQDVRKWPQGAVPQLPPSAPATSEMTTTPERPRRGIPGLPDGRFHLPPRIHRASDPGLPAEEPKEKSSRKVAEPELMGTPDGTCYPPPPVPRQASPQNLGTPGLLHPQTSPLLHSLVGQHILSVQQFTKDQMSHLFNVAHTLRMMVQKERSLDILKGKVMASMFYEVSTRTSSSFAAAMARLGGAVLSFSEATSSVQKGESLADSVQTMSCYADVVVLRHPQPGAVELAAKHCRRPVINAGDGVGEHPTQALLDIFTIREELGTVNGMTITMVGDLKHGRTVHSLACLLTQYRVSLRYVAPPSLRMPPTVRAFVASRGTKQEEFESIEEALPDTDVLYMTRIQKERFGSTQEYEACFGQFILTPHIMTRAKKKMVVMHPMPRVNEISVEVDSDPRAAYFRQAENGMYIRMALLATVLGRF

122 NP_005576.3 Q92871

>NP_005576.3

MFRSKRSGLVRRLWRSRVVPDREEGGSGGGGGGDEDGSLGSRAEPAPRAREGGGCGRSEVRPVAPRRPRDAVGQRGAQGAGRRRRAGGPPRPMSEPGAGAGSSLLDVAEPGGPGWLPESDCETVTCCLFSERDAAGAPRDASDPLAGAALEPAGGGRSREARSRLLLLEQELKTVTYSLLKRLKERSLDTLLEAVESRGGVPGGCVLVPRADLRLGGQPAPPQLLLGRLFRWPDLQHAVELKPLCGCHSFAAAADGPTVCCNPYHFSRLCGPESPPPPYSRLSPRDEYKPLDLSDSTLSYTETEATNSLITAPGEFSDASMSPDATKPSHWCSVAYWEHRTRVGRLYAVYDQAVSIFYDLPQGSGFCLGQLNLEQRSESVRRTRSKIGFGILLSKEPDGVWAYNRGEHPIFVNSPTLDAPGGRALVVRKVPPGYSIKVFDFERSGLQHAPEPDAADGPYDPNSVRISFAKGWGPCYSRQFITSCPCWLEILLNNPR

>Q92871

MAVTAQAARRKERVLCLFDVDGTLTPARQKIDPEVAAFLQKLRSRVQIGVVGGSDYCKIAEQLGDGDEVIEKFDYVFAENGTVQYKHGRLLSKQTIQNHLGEELLQDLINFCLSYMALLRLPKKRGTFIEFRNGMLNISPIGRSCTLEERIEFSELDKKEKIREKFVEALKTEFAGKGLRFSRGGMISFDVFPEGWDKRYCLDSLDQDSFDTIHFFGNETSPGGNDFEIFADPRTVGHSVVSPQDTVQRCREIFFPETAHEA

123 Q8IVL1 Q99551

>Q8IVL1

MPAILVASKMKSGLPKPVHSAAPILHVPPARAGPQPCYLKLGSKVEVSKTTYPSQIPLKSQVLQGLQEPAGEGLPLRKSGSVENGFDTQIYTDWANHYLTKSGHKRLIKDLQQDVTDGVLLAQIIQVVANEKIEDINGCPKNRSQMIENIDACLNFLAAKGINIQGLSAEEIRNGNLKAILGLFFSLSRYKQQQQQPQKQHLSSPLPPAVSQVAGAPSQCQAGTPQQQVPVTPQAPCQPHQPAPHQQSKAQAEMQSSASSKDSSQSKIIRFTLGQKKISRLSGPTARVSAAGSEAKTRGGSTTANNRRSQSFNNYDKSKPVTSPPPPPSSHEKEPLASSASSHPGMSDNAPASLESGSSSTPTNCSTSSAIPQPGAATKPWRSKSLSVKHSATVSMLSVKPPGPEAPRPTPEAMKPAPNNQKSMLEKLKLFNSKGGSKAGEGPGSRDTSCERLETLPSFEESEELEAASRMLTTVGPASSSPKIALKGIAQRTFSRALTNKKSSLKGNEKEKEKQQREKDKEKSKDLAKRASVTERLDLKEEPKEDPSGAAVPEMPKKSSKIASFIPKGGKLNSAKKEPMAPSHSGIPKPGMKSMPGKSPSAPAPSKEGERSRSGKLSSGLPQQKPQLDGRHSSSSSSLASSEGKGPGGTTLNHSISSQTVSGSVGTTQTTGSNTVSVQLPQPQQQYNHPNTATVAPFLYRSQTDTEGNVTAESSSTGVSVEPSHFTKTGQPALEELTGEDPEARRLRTVKNIADLRQNLEETMSSLRGTQVTHSTLETTFDTNVTTEMSGRSILSLTGRPTPLSWRLGQSSPRLQAGDAPSMGNGYPPRANASRFINTESGRYVYSAPLRRQLASRGSSVCHVDVSDKAGDEMDLEGISMDAPGYMSDGDVLSKNIRTDDITSGYMTDGGLGLYTRRLNRLPDGMAVVRETLQRNTSLGLGDADSWDDSSSVSSGISDTIDNLSTDDINTSSSISSYANTPASSRKNLDVQTDAEKHSQVERNSLWSGDDVKKSDGGSDSGIKMEPGSKWRRNPSDVSDESDKSTSGKKNPVISQTGSWRRGMTAQVGITMPRTKASAPAGALKTPGTGKTDDAKVSEKGRLSPKASQVKRSPSDAGRSSGDESKKPLPSSSRTPTANANSFGFKKQSGSAAGLAMITASGVTVTSRSATLGKIPKSSALVSRSAGRKSSMDGAQNQDDGYLALSSRTNLQYRSLPRPSKSNSRNGAGNRSSTSSIDSNISSKSAGLPVPKLREPSKTALGSSLPGLVNQTDKEKGISSDNESVASCNSVKVNPAAQPVSSPAQTSLQPGAKYPDVASPTLRRLFGGKPTKQVPIATAENMKNSVVISNPHATMTQQGNLDSPSGSGVLSSGSSSPLYSKNVDLNQSPLASSPSSAHSAPSNSLTWGTNASSSSAVSKDGLGFQSVSSLHTSCESIDISLSSGGVPSHNSSTGLIASSKDDSLTPFVRTNSVKTTLSESPLSSPAASPKFCRSTLPRKQDSDPHLDRNTLPKKGLRYTPTSQLRTQEDAKEWLRSHSAGGLQDTAANSPFSSGSSVTSPSGTRFNFSQLASPTTVTQMSLSNPTMLRTHSLSNADGQYDPYTDSRFRNSSMSLDEKSRTMSRSGSFRDGFEEESWEKSSVDNFVSRLHSSLHFSLPLFHHARYELVHGSSLSLVSSTSSVYSTPEEKCQSEIRKLRRELDASQEKVSALTTQLTANAHLVAAFEQSLGNMTIRLQSLTMTAEQKDSELNELRKTIELLKKQNAAAQAAINGVINTPELNCKGNGTAQSADLRIRRQHSSDSVSSINSATSHSSVGSNIESDSKKKKRKNWVNELRSSFKQAFGKKKSPKSASSHSDIEEMTDSSLPSSPKLPHNGSTGSTPLLRNSHSNSLISECMDSEAETVMQLRNELRDKEMKLTDIRLEALSSAHQLDQLREAMNRMQSEIEKLKAENDRLKSESQGSGCSRAPSQVSISASPRQSMGLSQHSLNLTESTSLDMLLDDTGECSARKEGGRHVKIVVSFQEEMKWKEDSRPHLFLIGCIGVSGKTKWDVLDGVVRRLFKEYIIHVDPVSQLGLNSDSVLGYSIGEIKRSNTSETPELLPCGYLVGENTTISVTVKGLAENSLDSLVFESLIPKPILQRYVSLLIEHRRIILSGPSGTGKTYLANRLSEYIVLREGRELTDGVIATFNVDHKSSKELRQYLSNLADQCNSENNAVDMPLVIILDNLHHVSSLGEIFNGLLNCKYHKCPYIIGTMNQATSSTPNLQLHHNFRWVLCANHTEPVKGFLGRFLRRKLMETEISGRVRNMELVKIIDWIPKVWHHLNRFLEAHSSSDVTIGPRLFLSCPIDVDGSRVWFTDLWNYSIIPYLLEAVREGLQLYGRRAPWEDPAKWVMDTYPWAASPQQHEWPPLLQLRPEDVGFDGYSMPREGSTSKQMPPSDAEGDPLMNMLMRLQEAANYSSPQSYDSDSNSNSHHDDILDSSLESTL

>Q99551

MQSLSLGQTSISKGLNYLTIMAPGNLWHMRNNFLFGSRCWMTRFSAENIFKSVSFRLFGVKCHNTDSEPLKNEDLLKNLLTMGVDIDMARKRQPGVFHRMITNEQDLKMFLLSKGASKEVIASIISRYPRAITRTPENLSKRWDLWRKIVTSDLEIVNILERSPESFFRSNNNLNLENNIKFLYSVGLTRKCLCRLLTNAPRTFSNSLDLNKQMVEFLQAAGLSLGHNDPADFVRKIIFKNPFILIQSTKRVKANIEFLRSTFNLNSEELLVLICGPGAEILDLSNDYARRSYANIKEKLFSLGCTEEEVQKFVLSYPDVIFLAEKKFNDKIDCLMEENISISQIIENPRVLDSSISTLKSRIKELVNAGCNLSTLNITLLSWSKKRYEAKLKKLSRFA

124 Q03923 O15540

>Q03923

MGPLTFRDVAIEFSLKEWQCLDTAQRNLYRNVMLENYRNLVFLGITVSKPDLITCLEQGKEAWSMKRHEIMVAKPTVMCSHFAQDLWPEQNIKDSFQKVTLKRYGKCRHENLPLRKGCESMDECKMHKGGCNGLNQCLTATQSKIFQCDKYVKVAHKFSNSNRHEIRHTKKKPFKCTKCGKSFGMISCLTEHSRIHTRVNFYKCEECGKAFNWSSTLTKHKRIHTGEKPYKCEECGKAFNQSSNLIKHKKIHTGEKPYKCEECGKTFNRFSTLTTHKIIHTGEKPYKCKECGKAFNRSSTLTTHRKIHTGEKPYKCEECGKAFKQSSNLTTHKIIHTGEKPYKCKKCGKAFNQSAHLTTHEVIHTGEKPYKCEKCGKAFNHFSHLTTHKIIHTGEKPYKCKECGKAFKHSSTLTKHKIIHTGEKPYKCKECEKAFNQSSKLTEHKKIHTGEKPYECEKCGKAFNQSSNLTRHKKSHTEEKPYKCEECGKGFKWPSTLTIHKIIHTGEKPYKCEECGKAFNQSSKLTKHKKIHTGEKPYTCEECGKAFNQSSNLTKHKRIHTGEKPYKCEECDKAFKWSSVLTKHKIIHTGEKLQI

>O15540

MVEAFCATWKLTNSQNFDEYMKALGVGFATRQVGNVTKPTVIISQEGDKVVIRTLSTFKNTEISFQLGEEFDETTADDRNCKSVVSLDGDKLVHIQKWDGKETNFVREIKDGKMVMTLTFGDVVAVRHYEKA

125 O43474 Q9UGK3

>O43474

MRQPPGESDMAVSDALLPSFSTFASGPAGREKTLRQAGAPNNRWREELSHMKRLPPVLPGRPYDLAAATVATDLESGGAGAACGGSNLAPLPRRETEEFNDLLDLDFILSNSLTHPPESVAATVSSSASASSSSSPSSSGPASAPSTCSFTYPIRAGNDPGVAPGGTGGGLLYGRESAPPPTAPFNLADINDVSPSGGFVAELLRPELDPVYIPPQQPQPPGGGLMGKFVLKASLSAPGSEYGSPSVISVSKGSPDGSHPVVVAPYNGGPPRTCPKIKQEAVSSCTHLGAGPPLSNGHRPAAHDFPLGRQLPSRTTPTLGLEEVLSSRDCHPALPLPPGFHPHPGPNYPSFLPDQMQPQVPPLHYQGQSRGFVARAGEPCVCWPHFGTHGMMLTPPSSPLELMPPGSCMPEEPKPKRGRRSWPRKRTATHTCDYAGCGKTYTKSSHLKAHLRTHTGEKPYHCDWDGCGWKFARSDELTRHYRKHTGHRPFQCQKCDRAFSRSDHLALHMKRHF

>Q9UGK3

MASALRPPRVPKPKGVLPSHYYESFLEKKGPCDRDYKKFWAGLQGLTIYFYNSNRDFQHVEKLNLGAFEKLTDEIPWGSSRDPGTHFSLILRDQEIKFKVETLECREMWKGFILTVVELRVPTDLTLLPGHLYMMSEVLAKEEARRALETPSCFLKVSRLEAQLLLERYPECGNLLLRPSGDGADGVSVTTRQMHNGTHVVRHYKVKREGPKYVIDVEQPFSCTSLDAVVNYFVSHTKKALVPFLLDEDYEKVLGYVEADKENGENVWVAPSAPGPGPAPCTGGPKPLSPASSQDKLPPLPPLPNQEENYVTPIGDGPAVDYENQDVASSSWPVILKPKKLPKPPAKLPKPPVGPKPEPKVFNGGLGRKLPVSSAQPLFPTAGLADMTAELQKKLEKRRALEH

126 NP_003661.1 NP_434700.2

>NP_003661.1

MERIPSAQPPPACLPKAPGLEHGDLPGMYPAHMYQVYKSRRGIKRSEDSKETYKLPHRLIEKKRRDRINECIAQLKDLLPEHLKLTTLGHLEKAVVLELTLKHVKALTNLIDQQQQKIIALQSGLQAGELSGRNVETGQEMFCSGFQTCAREVLQYLAKHENTRDLKSSQLVTHLHRVVSELLQGGTSRKPSDPAPKVMDFKEKPSSPAKGSEGPGKNCVPVIQRTFAHSSGEQSGSDTDTDSGYGGESEKGDLRSEQPCFKSDHGRRFTMGERIGAIKQESEEPPTKKNRMQLSDDEGHFTSSDLISSPFLGPHPHQPPFCLPFYLIPPSATAYLPMLEKCWYPTSVPVLYPGLNASAAALSSFMNPDKISAPLLMPQRLPSPLPAHPSVDSSVLLQALKPIPPLNLETKD

>NP_434700.2

MSDYENDDECWSVLEGFRVTLTSVIDPSRITPYLRQCKVLNPDDEEQVLSDPNLVIRKRKVGVLLDILQRTGHKGYVAFLESLELYYPQLYKKVTGKEPARVFSMIIDASGESGLTQLLMTEVMKLQKKVQDLTALLSSKDDFIKELRVKDSLLRKHQERVQRLKEECEAGSRELKRCKEENYDLAMRLAHQSEEKGAALMRNRDLQLEIDQLKHSLMKAEDDCKVERKHTLKLRHAMEQRPSQELLWELQQEKALLQARVQELEASVQEGKLDRSSPYIQVLEEDWRQALRDHQEQANTIFSLRKDLRQGEARRLRCMEEKEMFELQCLALRKDSKMYKDRIEAILLQMEEVAIERDQAIATREELHAQHARGLQEKDALRKQVRELGEKADELQLQVFQCEAQLLAVEGRLRRQQLETLVLSSDLEDGSPRRSQELSLPQDLEDTQLSDKGCLAGGGSPKQPFAALHQEQVLRNPHDAGLSSGEPPEKERRRLKESFENYRRKRALRKMQKGWRQGEEDRENTTGSDNTDTEGS

127 NP_002681.1 NP_004777.1

>NP_002681.1

MSKRKAPQETLNGGITDMLTELANFEKNVSQAIHKYNAYRKAASVIAKYPHKIKSGAEAKKLPGVGTKIAEKIDEFLATGKLRKLEKIRQDDTSSSINFLTRVSGIGPSAARKFVDEGIKTLEDLRKNEDKLNHHQRIGLKYFGDFEKRIPREEMLQMQDIVLNEVKKVDSEYIATVCGSFRRGAESSGDMDVLLTHPSFTSESTKQPKLLHQVVEQLQKVHFITDTLSKGETKFMGVCQLPSKNDEKEYPHRRIDIRLIPKDQYYCGVLYFTGSDIFNKNMRAHALEKGFTINEYTIRPLGVTGVAGEPLPVDSEKDIFDYIQWKYREPKDRSE

>NP_004777.1

MVGVKPVGSDPDFQPELSGAGSRLAVVKFTMRGCGPCLRIAPAFSSMSNKYPQAVFLEVDVHQCQGTAATNNISATPTFLFFRNKVRIDQYQGADAVGLEEKIKQHLENDPGSNEDTDIPKGYMDLMPFINKAGCECLNESDEHGFDNCLRKDTTFLESDCDEQLLITVAFNQPVKLYSMKFQGPDNGQGPKYVKIFINLPRSMDFEEAERSEPTQALELTEDDIKEDGIVPLRYVKFQNVNSVTIFVQSNQGEEETTRISYFTFIGTPVQATNMNDFKRVVGKKGESH

128 NP_056271.2 NP_055973.2

>NP_056271.2

MFAGLQDLGVANGEDLKETLTNCTEPLKAIEQFQTENGVLLPSLQSALPFLDLHGTPRLEFHQSVFDELRDKLLERVSAIASEGKAEERYKKLEDLLEKSFSLVKMPSLQPVVMCVMKHLPKVPEKKLKLVMADKELYRACAVEVKRQIWQDNQALFGDEVSPLLKQYILEKESALFSTELSVLHNFFSPSPKTRRQGEVVQRLTRMVGKNVKLYDMVLQFLRTLFLRTRNVHYCTLRAELLMSLHDLDVGEICTVDPCHKFTWCLDACIRERFVDSKRARELQGFLDGVKKGQEQVLGDLSMILCDPFAINTLALSTVRHLQELVGQETLPRDSPDLLLLLRLLALGQGAWDMIDSQVFKEPKMEVELITRFLPMLMSFLVDDYTFNVDQKLPAEEKAPVSYPNTLPESFTKFLQEQRMACEVGLYYVLHITKQRNKNALLRLLPGLVETFGDLAFGDIFLHLLTGNLALLADEFALEDFCSSLFDGFFLTASPRKENVHRHALRLLIHLHPRVAPSKLEALQKALEPTGQSGEAVKELYSQLGEKLEQLDHRKPSPAQAAETPALELPLPSVPAPAPL

>NP_055973.2

MAHTTKVNGSASGKAGDILSGDQDKEQKDPYFVETPYGYQLDLDFLKYVDDIQKGNTIKRLNIQKRRKPSVPCPEPRTTSGQQGIWTSTESLSSSNSDDNKQCPNFLIARSQVTSTPISKPPPPLETSLPFLTIPENRQLPPPSPQLPKHNLHVTKTLMETRRRLEQERATMQMTPGEFRRPRLASFGGMGTTSSLPSFVGSGNHNPAKHQLQNGYQGNGDYGSYAPAAPTTSSMGSSIRHSPLSSGISTPVTNVSPMHLQHIREQMAIALKRLKELEEQVRTIPVLQVKISVLQEEKRQLVSQLKNQRAASQINVCGVRKRSYSAGNASQLEQLSRARRSGGELYIDYEEEEMETVEQSTQRIKEFRQLTADMQALEQKIQDSSCEASSELRENGECRSVAVGAEENMNDIVVYHRGSRSCKDAAVGTLVEMRNCGVSVTEAMLGVMTEADKEIELQQQTIESLKEKIYRLEVQLRETTHDREMTKLKQELQAAGSRKKVDKATMAQPLVFSKVVEAVVQTRDQMVGSHMDLVDTCVGTSVETNSVGISCQPECKNKVVGPELPMNWWIVKERVEMHDRCAGRSVEMCDKSVSVEVSVCETGSNTEESVNDLTLLKTNLNLKEVRSIGCGDCSVDVTVCSPKECASRGVNTEAVSQVEAAVMAVPRTADQDTSTDLEQVHQFTNTETATLIESCTNTCLSTLDKQTSTQTVETRTVAVGEGRVKDINSSTKTRSIGVGTLLSGHSGFDRPSAVKTKESGVGQININDNYLVGLKMRTIACGPPQLTVGLTASRRSVGVGDDPVGESLENPQPQAPLGMMTGLDHYIERIQKLLAEQQTLLAENYSELAEAFGEPHSQMGSLNSQLISTLSSINSVMKSASTEELRNPDFQKTSLGKITGNYLGYTCKCGGLQSGSPLSSQTSQPEQEVGTSEGKPISSLDAFPTQEGTLSPVNLTDDQIAAGLYACTNNESTLKSIMKKKDGNKDSNGAKKNLQFVGINGGYETTSSDDSSSDESSSSESDDECDVIEYPLEEEEEEEDEDTRGMAEGHHAVNIEGLKSARVEDEMQVQECEPEKVEIRERYELSEKMLSACNLLKNTINDPKALTSKDMRFCLNTLQHEWFRVSSQKSAIPAMVGDYIAAFEAISPDVLRYVINLADGNGNTALHYSVSHSNFEIVKLLLDADVCNVDHQNKAGYTPIMLAALAAVEAEKDMRIVEELFGCGDVNAKASQAGQTALMLAVSHGRIDMVKGLLACGADVNIQDDEGSTALMCASEHGHVEIVKLLLAQPGCNGHLEDNDGSTALSIALEAGHKDIAVLLYAHVNFAKAQSPGTPRLGRKTSPGPTHRGSFD

129 NP_612211.1 NP_005078.2

>NP_612211.1

MFVLVEMVDTVRIPPWQFERKLNDSIAEELNKKLANKVVYNVGLCICLFDITKLEDAYVFPGDGASHTKVHFRCVVFHPFLDEILIGKIKGCSPEGVHVSLGFFDDILIPPESLQQPAKFDEAEQVWVWEYETEEGAHDLYMDTGEEIRFRVVDESFVDTSPTGPSSADATTSSEELPKKEAPYTLVGSISEPGLGLLSWWTSN

>NP_005078.2

MAELTVEVRGSNGAFYKGFIKDVHEDSLTVVFENNWQPERQVPFNEVRLPPPPDIKKEISEGDEVEVYSRANDQEPCGWWLAKVRMMKGEFYVIEYAACDATYNEIVTFERLRPVNQNKTVKKNTFFKCTVDVPEDLREACANENAHKDFKKAVGACRIFYHPETTQLMILSASEATVKRVNILSDMHLRSIRTKLMLMSRNEEATKHLECTKQLAAAFHEEFVVREDLMGLAIGTHGSNIQQARKVPGVTAIELDEDTGTFRIYGESADAVKKARGFLEFVEDFIQVPRNLVGKVIGKNGKVIQEIVDKSGVVRVRIEGDNENKLPREDGMVPFVFVGTKESIGNVQVLLEYHIAYLKEVEQLRMERLQIDEQLRQIGSRSYSGRGRGRRGPNYTSGYGTNSELSNPSETESERKDELSDWSLAGEDDRDSRHQRDSRRRPGGRGRSVSGGRGRGGPRGGKSSISSVLKDPDSNPYSLLDNTESDQTADTDASESHHSTNRRRRSRRRRTDEDAVLMDGMTESDTASVNENGLVTVADYISRAESQSRQRNLPRETLAKNKKEMAKDVIEEHGPSEKAINGPTSASGDDISKLQRTPGEEKINTLKEENTQEAAVLNGVS

130 Q70Z53 NP_006268.1

>Q70Z53

MHGHGGYDSDFSDDEHCGESSKRKKRTVEDDLLLQKPFQKEKHGKVAHKQVAAELLDREEARNRRFHLIAMDAYQRHRKFVNDYILYYGGKKEDFKRLGENDKTDLDVIRENHRFLWNEEDEMDMTWEKRLAKKYYDKLFKEYCIADLSKYKENKFGFRWRVEKEVISGKGQFFCGNKYCDKKEGLKSWEVNFGYIEHGEKRNALVKLRLCQECSIKLNFHHRRKEIKSKKRKDKTKKDCEESSHKKSRLSSAEEASKKKDKGHSSSKKSEDSLLRNSDEEESASESELWKGPLPETDEKSQEEEFDEYFQDLFL

>NP_006268.1

MAQFPTAMNGGPNMWAITSEERTKHDRQFDNLKPSGGYITGDQARNFFLQSGLPAPVLAEIWALSDLNKDGKMDQQEFSIAMKLIKLKLQGQQLPVVLPPIMKQPPMFSPLISARFGMGSMPNLSIPQPLPPAAPITSLSSATSGTNLPPLMMPTPLVPSVSTSSLPNGTASLIQPLPIPYSSSTLPHGSSYSLMMGGFGGASIQKAQSLIDLGSSSSTSSTASLSGNSPKTGTSEWAVPQPTRLKYRQKFNTLDKSMSGYLSGFQARNALLQSNLSQTQLATIWTLADVDGDGQLKAEEFILAMHLTDMAKAGQPLPLTLPPELVPPSFRGGKQIDSINGTLPSYQKMQEEEPQKKLPVTFEDKRKANYERGNMELEKRRQALMEQQQREAERKAQKEKEEWERKQRELQEQEWKKQLELEKRLEKQRELERQREEERRKDIERREAAKQELERQRRLEWERIRRQELLNQKNREQEEIVRLNSKKKNLHLELEALNGKHQQISGRLQDVRLKKQTQKTELEVLDKQCDLEIMEIKQLQQELQEYQNKLIYLVPEKQLLNERIKNMQFSNTPDSGVSLLHKKSLEKEELCQRLKEQLDALEKETASKLSEMDSFNNQLKCGNMDDSVLQCLLSLLSCLNNLFLLLKELRETYNTQQLALEQLYKIKRDKLKEIERKRLELMQKKKLEDEAARKAKQGKENLWKENLRKEEEEKQKRLQEEKTQEKIQEEERKAEEKQRKDKDTLKAEEKKRETASVLVNYRALYPFEARNHDEMSFNSGDIIQVDEKTVGEPGWLYGSFQGNFGWFPCNYVEKMPSSENEKAVSPKKALLPPTVSLSATSTSSEPLSSNQPASVTDYQNVSFSNLTVNTSWQKKSAFTRTVSPGSVSPIHGQGQVVENLKAQALCSWTAKKDNHLNFSKHDIITVLEQQENWWFGEVHGGRGWFPKSYVKIIPGSEVKREEPEALYAAVNKKPTSAAYSVGEEYIALYPYSSVEPGDLTFTEGEEILVTQKDGEWWTGSIGDRSGIFPSNYVKPKDQESFGSASKSGASNKKPEIAQVTSAYVASGSEQLSLAPGQLILILKKNTSGWWQGELQARGKKRQKGWFPASHVKLLGPSSERATPAFHPVCQVIAMYDYAANNEDELSFSKGQLINVMNKDDPDWWQGEINGVTGLFPSNYVKMTTDSDPSQQWCADLQTLDTMQPIERKRQGYIHELIQTEERYMADLQLVVEVFQKRMAESGFLTEGEMALIFVNWKELIMSNTKLLKALRVRKKTGGEKMPVQMIGDILAAELSHMQAYIRFCSCQLNGAALLQQKTDEDTDFKEFLKKLASDPRCKGMPLSSFLLKPMQRITRYPLLIRSILENTPESHADHSSLKLALERAEELCSQVNEGVREKENSDRLEWIQAHVQCEGLAEQLIFNSLTNCLGPRKLLHSGKLYKTKSNKELHGFLFNDFLLLTYMVKQFAVSSGSEKLFSSKSNAQFKMYKTPIFLNEVLVKLPTDPSSDEPVFHISHIDRVYTLRTDNINERTAWVQKIKAASEQYIDTEKKKREKAYQARSQKTSGIGRLMVHVIEATELKACKPNGKSNPYCEISMGSQSYTTRTIQDTLNPKWNFNCQFFIKDLYQDVLCLTLFDRDQFSPDDFLGRTEIPVAKIRTEQESKGPMTRRLLLHEVPTGEVWVRFDLQLFEQKTLL

131 NP_777584.1 NP_057395.1

>NP_777584.1

MDLLWMPLLLVAACVSAVHSSPEVNAGVSSIHITKPVHILEERSLLVLTPAGLTQMLNQTRFLMVLFHNPSSKQSRNLAEELGKAVEIMGKGKNGIGFGKVDITIEKELQQEFGITKAPELKLFFEGNRSEPISCKGVVESAALVVWLRRQISQKAFLFNSSEQVAEFVISRPLVIVGFFQDLEEEVAELFYDVIKDFPELTFGVITIGNVIGRFHVTLDSVLVFKKGKIVNRQKLINDSTNKQELNRVIKQHLTDFVIEYNTENKDLISELHIMSHMLLFVSKSSESYGIIIQHYKLASKEFQNKILFILVDADEPRNGRVFKYFRVTEVDIPSVQILNLSSDARYKMPSDDITYESLKKFGRSFLSKNATKHQSSEEIPKYWDQGLVKQLVGKNFNVVVFDKEKDVFVMFYAPWSKKCKMLFPLLEELGRKYQNHSTIIIAKIDVTANDIQLMYLDRYPFFRLFPSGSQQAVLYKGEHTLKGFSDFLESHIKTKIEDEDELLSVEQNEVIEEEVLAEEKEVPMMRKGLPEQQSPELENMTKYVSKLEEPAGKKKTSEEVVVVVAKPKGPPVQKKKPKVKEEL

>NP_057395.1

MAVTALAARTWLGVWGVRTMQARGFGSDQSENVDRGAGSIREAGGAFGKREQAEEERYFRAQSREQLAALKKHHEEEIVHHKKEIERLQKEIERHKQKIKMLKHDD

132 NP_006617.1 NP_203526.1

>NP_006617.1

MAAESDVLHFQFEQQGDVVLQKMNLLRQQNLFCDVSIYINDTEFQGHKVILAACSTFMRDQFLLTQSKHVRITILQSAEVGRKLLLSCYTGALEVKRKELLKYLTAASYLQMVHIVEKCTEALSKYLEIDLSMKNNNQHTDLCQSSDPDVKNEDENSDKDCEIIEISEDSPVNIDFHVKEEESNALQSTVESLTSERKEMKSPELSTVDIGFKDNEICILHVESISTAGVENGQFSQPCTSSKASMYFSETQHSLINSTVESRVAEVPGNQDQGLFCENTEGSYGTVSEIQNLEEGYSLRHQCPRCPRGFLHVENYLRHLKMHKLFLCLQCGKTFTQKKNLNRHIRGHMGIRPFQCTVCLKTFTAKSTLQDHLNIHSGDRPYKCHCCDMDFKHKSALKKHLTSVHGRSSGEKLSRPDLKRQSLL

>NP_203526.1

MSWSGLLHGLNTSLTCGPALVPRLWATCSMATLNQMHRLGPPKRPPRKLGPTEGRPQLKGVVLCTFTRKPKKPNSANRKCCRVRLSTGREAVCFIPGEGHTLQEHQIVLVEGGRTQDLPGVKLTVVRGKYDCGHVQKK

133 NP_001385.1 P22531

>NP_001385.1

MVTMEELREMDCSVLKRLMNRDENGGGAGGSGSHGTLGLPSGGKCLLLDCRPFLAHSAGYILGSVNVRCNTIVRRRAKGSVSLEQILPAEEEVRARLRSGLYSAVIVYDERSPRAESLREDSTVSLVVQALRRNAERTDICLLKGGYERFSSEYPEFCSKTKALAAIPPPVPPSATEPLDLGCSSCGTPLHDQGGPVEILPFLYLGSAYHAARRDMLDALGITALLNVSSDCPNHFEGHYQYKCIPVEDNHKADISSWFMEAIEYIDAVKDCRGRVLVHCQAGISRSATICLAYLMMKKRVRLEEAFEFVKQRRSIISPNFSFMGQLLQFESQVLATSCAAEAASPSGPLRERGKTPATPTSQFVFSFPVSVGVHSAPSSLPYLHSPITTSPSC

>P22531

MSYQQQQCKQPCQPPPVCPTPKCPEPCPPPKCPEPCPPPKCPQPCPPQQCQQKCPPVTPSPPCQPKCPPKSK

134 NP_008924.1 NP_689953.1

>NP_008924.1

MAAGTAAALAFLSQESRTRAGGVGGLRVPAPVTMDSFFFGCELSGHTRSFTFKVEEEDDAEHVLALTMLCLTEGAKDECNVVEVVARNHDHQEIAVPVANLKLSCQPMLSLDDFQLQPPVTFRLKSGSGPVRITGRHQIVTMSNDVSEEESEEEEEDSDEEEVELCPILPAKKQGGRP

>NP_689953.1

MAASLRLLGAASGLRYWSRRLRPAAGSFAAVCSRSVASKTPVGFIGLGNMGNPMAKNLMKHGYPLIIYDVFPDACKEFQDAGEQVVSSPADVAEKADRIITMLPTSINAIEAYSGANGILKKVKKGSLLIDSSTIDPAVSKELAKEVEKMGAVFMDAPVSGGVGAARSGNLTFMVGGVEDEFAAAQELLGCMGSNVVYCGAVGTGQAAKICNNMLLAISMIGTAEAMNLGIRLGLDPKLLAKILNMSSGRCWSSDTYNPVPGVMDGVPSANNYQGGFGTTLMAKDLGLAQDSATSTKSPILLGSLAHQIYRMMCAKGYSKKDFSSVFQFLREEETF

135 O75164 P25398

>O75164

MASESETLNPSARIMTFYPTMEEFRNFSRYIAYIESQGAHRAGLAKVVPPKEWKPRASYDDIDDLVIPAPIQQLVTGQSGLFTQYNIQKKAMTVREFRKIANSDKYCTPRYSEFEELERKYWKNLTFNPPIYGADVNGTLYEKHVDEWNIGRLRTILDLVEKESGITIEGVNTPYLYFGMWKTSFAWHTEDMDLYSINYLHFGEPKSWYSVPPEHGKRLERLAKGFFPGSAQSCEAFLRHKMTLISPLMLKKYGIPFDKVTQEAGEFMITFPYGYHAGFNHGFNCAESTNFATRRWIEYGKQAVLCSCRKDMVKISMDVFVRKFQPERYKLWKAGKDNTVIDHTLPTPEAAEFLKESELPPRAGNEEECPEEDMEGVEDGEEGDLKTSLAKHRIGTKRHRVCLEIPQEVSQSELFPKEDLSSEQYEMTECPAALAPVRPTHSSVRQVEDGLTFPDYSDSTEVKFEELKNVKLEEEDEEEEQEAAALDLSVNPASVGGRLVFSGSKKKSSSSLGSGSSRDSISSDSETSEPLSCRAQGQTGVLTVHSYAKGDGRVTVGEPCTRKKGSAARSFSERELAEVADEYMFSLEENKKSKGRRQPLSKLPRHHPLVLQECVSDDETSEQLTPEEEAEETEAWAKPLSQLWQNRPPNFEAEKEFNETMAQQAPHCAVCMIFQTYHQVEFGGFNQNCGNASDLAPQKQRTKPLIPEMCFTSTGCSTDINLSTPYLEEDGTSILVSCKKCSVRVHASCYGVPPAKASEDWMCSRCSANALEEDCCLCSLRGGALQRANDDRWVHVSCAVAILEARFVNIAERSPVDVSKIPLPRFKLKCIFCKKRRKRTAGCCVQCSHGRCPTAFHVSCAQAAGVMMQPDDWPFVVFITCFRHKIPNLERAKGALQSITAGQKVISKHKNGRFYQCEVVRLTTETFYEVNFDDGSFSDNLYPEDIVSQDCLQFGPPAEGEVVQVRWTDGQVYGAKFVASHPIQMYQVEFEDGSQLVVKRDDVYTLDEELPKRVKSRLSVASDMRFNEIFTEKEVKQEKKRQRVINSRYREDYIEPALYRAIME

>P25398

MAEEGIAAGGVMDVNTALQEVLKTALIHDGLARGIREAAKALDKRQAHLCVLASNCDEPMYVKLVEALCAEHQINLIKVDDNKKLGEWVGLCKIDREGKPRKVVGCSCVVVKDYGKESQAKDVIEEYFKCKK

136 P55895 Q8TE04

>P55895

MSLQMVTVSNNIALIQPGFSLMNFDGQVFFFGQKGWPKRSCPTGVFHLDVKHNHVKLKPTIFSKDSCYLPPLRYPATCTFKGSLESEKHQYIIHGGKTPNNEVSDKIYVMSIVCKNNKKVTFRCTEKDLVGDVPEARYGHSINVVYSRGKSMGVLFGGRSYMPSTHRTTEKWNSVADCLPCVFLVDFEFGCATSYILPELQDGLSFHVSIAKNDTIYILGGHSLANNIRPANLYRIRVDLPLGSPAVNCTVLPGGISVSSAILTQTNNDEFVIVGGYQLENQKRMICNIISLEDNKIEIREMETPDWTPDIKHSKIWFGSNMGNGTVFLGIPGDNKQVVSEGFYFYMLKCAEDDTNEEQTTFTNSQTSTEDPGDSTPFEDSEEFCFSAEANSFDGDDEFDTYNEDDEEDESETGYWITCCPTCDVDINTWVPFYSTELNKPAMIYCSHGDGHWVHAQCMDLAERTLIHLSAGSNKYYCNEHVEIARALHTPQRVLPLKKPPMKSLRKKGSGKILTPAKKSFLRRLFD

>Q8TE04

MLKLVGGGGGQDWACSVAGTSLGGEEAAFEVARPGDQGKAGGGSPGWGCAGIPDSAPGAGVLQAGAVGPARGGQGAEEVGESAGGGEERRVRHPQAPALRLLNRKPQGGSGEIKTPENDLQRGRLSRGPRTAPPAPGMGDRSGQQERSVPHSPGAPVGTSAAAVNGLLHNGFHPPPVQPPHVCSRGPVGGSDAAPQRLPLLPELQPQPLLPQHDSPAKKCRLRRRMDSGRKNRPPFPWFGMDIGGTLVKLVYFEPKDITAEEEQEEVENLKSIRKYLTSNTAYGKTGIRDVHLELKNLTMCGRKGNLHFIRFPSCAMHRFIQMGSEKNFSSLHTTLCATGGGAFKFEEDFRMIADLQLHKLDELDCLIQGLLYVDSVGFNGKPECYYFENPTNPELCQKKPYCLDNPYPMLLVNMGSGVSILAVYSKDNYKRVTGTSLGGGTFLGLCCLLTGCETFEEALEMAAKGDSTNVDKLVKDIYGGDYERFGLQGSAVASSFGNMMSKEKRDSISKEDLARATLVTITNNIGSIARMCALNENIDRVVFVGNFLRINMVSMKLLAYAMDFWSKGQLKALFLEHEGYFGAVGALLELFKMTDDK

137 NP_005641.1 P35558

>NP_005641.1

MQSFREQSSYHGNQQSYPQEVHGSSRLEEFSPRQAQMFQNFGGTGGSSGSSGSGSGGGRRGAAAAAAAMASETSGHQGYQGFRKEAGDFYYMAGNKDPVTTGTPQPPQRRPSGPVQSYGPPQGSSFGNQYGSEGHVGQFQAQHSGLGGVSHYQQDYTGPFSPGSAQYQQQASSQQQQQQVQQLRQQLYQSHQPLPQATGQPASSSSHLQPMQRPSTLPSSAAGYQLRVGQFGQHYQSSASSSSSSSFPSPQRFSQSGQSYDGSYNVNAGSQYEGHNVGSNAQAYGTQSNYSYQPQSMKNFEQAKIPQGTQQGQQQQQPQQQQHPSQHVMQYTNAATKLPLQSQVGQYNQPEVPVRSPMQFHQNFSPISNPSPAASVVQSPSCSSTPSPLMQTGENLQCGQGSVPMGSRNRILQLMPQLSPTPSMMPSPNSHAAGFKGFGLEGVPEKRLTDPGLSSLSALSTQVANLPNTVQHMLLSDALTPQKKTSKRPSSSKKADSCTNSEGSSQPEEQLKSPMAESLDGGCSSSSEDQGERVRQLSGQSTSSDTTYKGGASEKAGSSPAQGAQNEPPRLNASPAAREEATSPGAKDMPLSSDGNPKVNEKTVGVIVSREAMTGRVEKPGGQDKGSQEDDPAATQRPPSNGGAKETSHASLPQPEPPGGGGSKGNKNGDNNSNHNGEGNGQSGHSAAGPGFTSRTEPSKSPGSLRYSYKDSFGSAVPRNVSGFPQYPTGQEKGDFTGHGERKGRNEKFPSLLQEVLQGYHHHPDRRYSRSTQEHQGMAGSLEGTTRPNVLVSQTNELASRGLLNKSIGSLLENPHWGPWERKSSSTAPEMKQINLTDYPIPRKFEIEPQSSAHEPGGSLSERRSVICDISPLRQIVRDPGAHSLGHMSADTRIGRNDRLNPTLSQSVILPGGLVSMETKLKSQSGQIKEEDFEQSKSQASFNNKKSGDHCHPPSIKHESYRGNASPGAATHDSLSDYGPQDSRPTPMRRVPGRVGGREGMRGRSPSQYHDFAEKLKMSPGRSRGPGGDPHHMNPHMTFSERANRSSLHTPFSPNSETLASAYHANTRAHAYGDPNAGLNSQLHYKRQMYQQQPEEYKDWSSGSAQGVIAAAQHRQEGPRKSPRQQQFLDRVRSPLKNDKDGMMYGPPVGTYHDPSAQEAGRCLMSSDGLPNKGMELKHGSQKLQESCWDLSRQTSPAKSSGPPGMSSQKRYGPPHETDGHGLAEATQSSKPGSVMLRLPGQEDHSSQNPLIMRRRVRSFISPIPSKRQSQDVKNSSTEDKGRLLHSSKEGADKAFNSYAHLSHSQDIKSIPKRDSSKDLPSPDSRNCPAVTLTSPAKTKILPPRKGRGLKLEAIVQKITSPNIRRSASSNSAEAGGDTVTLDDILSLKSGPPEGGSVAVQDADIEKRKGEVASDLVSPANQELHVEKPLPRSSEEWRGSVDDKVKTETHAETVTAGKEPPGAMTSTTSQKPGSNQGRPDGSLGGTAPLIFPDSKNVPPVGILAPEANPKAEEKENDTVTISPKQEGFPPKGYFPSGKKKGRPIGSVNKQKKQQQPPPPPPQPPQIPEGSADGEPKPKKQRQRRERRKPGAQPRKRKTKQAVPIVEPQEPEIKLKYATQPLDKTDAKNKSFYPYIHVVNKCELGAVCTIINAEEEEQTKLVRGRKGQRSLTPPPSSTESKALPASSFMLQGPVVTESSVMGHLVCCLCGKWASYRNMGDLFGPFYPQDYAATLPKNPPPKRATEMQSKVKVRHKSASNGSKTDTEEEEEQQQQQKEQRSLAAHPRFKRRHRSEDCGGGPRSLSRGLPCKKAATEGSSEKTVLDSKPSVPTTSEGGPELELQIPELPLDSNEFWVHEGCILWANGIYLVCGRLYGLQEALEIAREMKCSHCQEAGATLGCYNKGCSFRYHYPCAIDADCLLHEENFSVRCPKHKPPLPCPLPPLQNKTAKGSLSTEQSERG

>P35558

MPPQLQNGLNLSAKVVQGSLDSLPQAVREFLENNAELCQPDHIHICDGSEEENGRLLGQMEEEGILRRLKKYDNCWLALTDPRDVARIESKTVIVTQEQRDTVPIPKTGLSQLGRWMSEEDFEKAFNARFPGCMKGRTMYVIPFSMGPLGSPLSKIGIELTDSPYVVASMRIMTRMGTPVLEAVGDGEFVKCLHSVGCPLPLQKPLVNNWPCNPELTLIAHLPDRREIISFGSGYGGNSLLGKKCFALRMASRLAKEEGWLAEHMLILGITNPEGEKKYLAAAFPSACGKTNLAMMNPSLPGWKVECVGDDIAWMKFDAQGHLRAINPENGFFGVAPGTSVKTNPNAIKTIQKNTIFTNVAETSDGGVYWEGIDEPLASGVTITSWKNKEWSSEDGEPCAHPNSRFCTPASQCPIIDAAWESPEGVPIEGIIFGGRRPAGVPLVYEALSWQHGVFVGAAMRSEATAAAEHKGKIIMHDPFAMRPFFGYNFGKYLAHWLSMAQHPAAKLPKIFHVNWFRKDKEGKFLWPGFGENSRVLEWMFNRIDGKASTKLTPIGYIPKEDALNLKGLGHINMMELFSISKEFWEKEVEDIEKYLEDQVNADLPCEIEREILALKQRISQM

138 NP_077021.1 Q9H2J4

>NP_077021.1

MDKSGIDSLDHVTSDAVELANRSDNSSDSSLFKTQCIPYSPKGEKRNPIRKFVRTPESVHASDSSSDSSFEPIPLTIKAIFERFKNRKKRYKKKKKRRYQPTGRPRGRPEGRRNPIYSLIDKKKQFRSRGSGFPFLESENEKNAPWRKILTFEQAVARGFFNYIEKLKYEHHLKESLKQMNVGEDLENEDFDSRRYKFLDDDGSISPIEESTAEDEDATHLEDNECDIKLAGDSFIVSSEFPVRLSVYLEEEDITEEAALSKKRATKAKNTGQRGLKM

>Q9H2J4

MQDPNADTEWNDILRKKGILPPKESLKELEEEAEEEQRILQQSVVKTYEDMTLEELEDHEDEFNEEDERAIEMYRRRRLAEWKATKLKNKFGEVLEISGKDYVQEVTKAGEGLWVILHLYKQGIPLCALINQHLSGLARKFPDVKFIKAISTTCIPNYPDRNLPTIFVYLEGDIKAQFIGPLVFGGMNLTRDELEWKLSESGAIMTDLEENPKKPIEDVLLSSVRRSVLMKRDSDSEGD

139 NP_778225.1 Q8N573

>NP_778225.1

MPDPSKSAPAPKKGSKKAVTKAQKKDGKKRKRGRKESYSIYVYKVLKQVHPDTGISSKAMGIMNSFVNDIFERIASEASRLAHYNKRSTITSREVQTAVRLLLPGELAKHAVSEGTKAVTKYTSSK

>Q8N573

MSFQKPKGTIEYTVESRDSLNSIALKFDTTPNELVQLNKLFSRAVVTGQVLYVPDPEYVSSVESSPSLSPVSPLSPTSSEAEFDKTTNPDVHPTEATPSSTFTGIRPARVVSSTSEEEEAFTEKFLKINCKYITSGKGTVSGVLLVTPNNIMFDPHKNDPLVQENGCEEYGIMCPMEEVMSAAMYKEILDSKIKESLPIDIDQLSGRDFCHSKKMTGSNTEEIDSRIRDAGNDSASTAPRSTEESLSEDVFTESELSPIREELVSSDELRQDKSSGASSESVQTVNQAEVESLTVKSESTGTPGHLRSDTEHSTNEVGTLCHKTDLNNLEMAIKEDQIADNFQGISGPKEDSTSIKGNSDQDSFLHENSLHQEESQKENMPCGETAEFKQKQSVNKGKQGKEQNQDSQTEAEELRKLWKTHTMQQTKQQRENIQQVSQKEAKHKITSADGHIESSALLKEKQRHRLHKFLCLRVGKPMRKTFVSQASATMQQYAQRDKKHEYWFAVPQERTDHLYAFFIQWSPEIYAEDTGEYTREPGFIVVKKIEESETIEDSSNQAAAREWEITTREDINSKQVATVKADLESESFRPNLSDPSELLLPDQIEKLTKHLPPRTIGYPWTLVYGTGKHGTSLKTLYRTMTGLDTPVLMVIKDSDGQVFGALASEPLKVSDGFYGTGETFVFTFCPEFEVFKWTGDNMFFIKGDMDSLAFGGGGGEFALWLDGDLYHGRSHSCKTFGNRTLSKKEDFFIQDIEIWAFE

140 NP_003084.1 P50224

>NP_003084.1

MPKFYCDYCDTYLTHDSPSVRKTHCSGRKHKENVKDYYQKWMEEQAQSLIDKTTAAFQQGKIPPTPFSAPPPAGAMIPPPPSLPGPPRPGMMPAPHMGGPPMMPMMGPPPPGMMPVGPAPGMRPPMGGHMPMMPGPPMMRPPARPMMVPTRPGMTRPDR

>P50224

MELIQDTSRPPLEYVKGVPLIKYFAEALGPLQSFQARPDDLLINTYPKSGTTWVSQILDMIYQGGDLEKCNRAPIYVRVPFLEVNDPGEPSGLETLKDTPPPRLIKSHLPLALLPQTLLDQKVKVVYVARNPKDVAVSYYHFHRMEKAHPEPGTWDSFLEKFMAGEVSYGSWYQHVQEWWELSRTHPVLYLFYEDMKENPKREIQKILEFVGRSLPEETMDFMVQHTSFKEMKKNPMTNYTTVPQELMDHSISPFMRKGMAGDWKTTFTVAQNERFDADYAEKMAGCSLSFRSEL

141 Q15562 NP_004324.2

>Q15562

MGEPRAGAALDDGSGWTGSEEGSEEGTGGSEGAGGDGGPDAEGVWSPDIEQSFQEALAIYPPCGRRKIILSDEGKMYGRNELIARYIKLRTGKTRTRKQVSSHIQVLARRKSREIQSKLKDQVSKDKAFQTMATMSSAQLISAPSLQAKLGPTGPQASELFQFWSGGSGPPWNVPDVKPFSQTPFTLSLTPPSTDLPGYEPPQALSPLPPPTPSPPAWQARGLGTARLQLVEFSAFVEPPDAVDSYQRHLFVHISQHCPSPGAPPLESVDVRQIYDKFPEKKGGLRELYDRGPPHAFFLVKFWADLNWGPSGEEAGAGGSISSGGFYGVSSQYESLEHMTLTCSSKVCSFGKQVVEKVETERAQLEDGRFVYRLLRSPMCEYLVNFLHKLRQLPERYMMNSVLENFTILQVVTNRDTQELLLCTAYVFEVSTSERGAQHHIYRLVRD

>NP_004324.2

MAALSGGGGGGAEPGQALFNGDMEPEAGAGAGAAASSAADPAIPEEVWNIKQMIKLTQEHIEALLDKFGGEHNPPSIYLEAYEEYTSKLDALQQREQQLLESLGNGTDFSVSSSASMDTVTSSSSSSLSVLPSSLSVFQNPTDVARSNPKSPQKPIVRVFLPNKQRTVVPARCGVTVRDSLKKALMMRGLIPECCAVYRIQDGEKKPIGWDTDISWLTGEELHVEVLENVPLTTHNFVRKTFFTLAFCDFCRKLLFQGFRCQTCGYKFHQRCSTEVPLMCVNYDQLDLLFVSKFFEHHPIPQEEASLAETALTSGSSPSAPASDSIGPQILTSPSPSKSIPIPQPFRPADEDHRNQFGQRDRSSSAPNVHINTIEPVNIDDLIRDQGFRGDGGSTTGLSATPPASLPGSLTNVKALQKSPGPQRERKSSSSSEDRNRMKTLGRRDSSDDWEIPDGQITVGQRIGSGSFGTVYKGKWHGDVAVKMLNVTAPTPQQLQAFKNEVGVLRKTRHVNILLFMGYSTKPQLAIVTQWCEGSSLYHHLHIIETKFEMIKLIDIARQTAQGMDYLHAKSIIHRDLKSNNIFLHEDLTVKIGDFGLATVKSRWSGSHQFEQLSGSILWMAPEVIRMQDKNPYSFQSDVYAFGIVLYELMTGQLPYSNINNRDQIIFMVGRGYLSPDLSKVRSNCPKAMKRLMAECLKKKRDERPLFPQILASIELLARSLPKIHRSASEPSLNRAGFQTEDFSLYACASPKTPIQAGGYGAFPVH

142 NP_002936.1 NP_001268.2

>NP_002936.1

MVGQLSEGAIAAIMQKGDTNIKPILQVINIRPITTGNSPPRYRLLMSDGLNTLSSFMLATQLNPLVEEEQLSSNCVCQIHRFIVNTLKDGRRVVILMELEVLKSAEAVGVKIGNPVPYNEGLGQPQVAPPAPAASPAASSRPQPQNGSSGMGSTVSKAYGASKTFGKAAGPSLSHTSGGTQSKVVPIASLTPYQSKWTICARVTNKSQIRTWSNSRGEGKLFSLELVDESGEIRATAFNEQVDKFFPLIEVNKVYYFSKGTLKIANKQFTAVKNDYEMTFNNETSVMPCEDDHHLPTVQFDFTGIDDLENKSKDSLVDIIGICKSYEDATKITVRSNNREVAKRNIYLMDTSGKVVTATLWGEDADKFDGSRQPVLAIKGARVSDFGGRSLSVLSSSTIIANPDIPEAYKLRGWFDAEGQALDGVSISDLKSGGVGGSNTNWKTLYEVKSENLGQGDKPDYFSSVATVVYLRKENCMYQACPTQDCNKKVIDQQNGLYRCEKCDTEFPNFKYRMILSVNIADFQENQWVTCFQESAEAILGQNAAYLGELKDKNEQAFEEVFQNANFRSFIFRVRVKVETYNDESRIKATVMDVKPVDYREYGRRLVMSIRRSALM

>NP_001268.2

MKTKFCTGGEAEPSPLGLLLSCGSGSAAPAPGVGQQRDAASDLESKQLGGQQPPLALPPPPPLPLPLPLPQPPPPQPPADEQPEPRTRRRAYLWCKEFLPGAWRGLREDEFHISVIRGGLSNMLFQCSLPDTTATLGDEPRKVLLRLYGAILQMRSCNKEGSEQAQKENEFQGAEAMVLESVMFAILAERSLGPKLYGIFPQGRLEQFIPSRRLDTEELSLPDISAEIAEKMATFHGMKMPFNKEPKWLFGTMEKYLKEVLRIKFTEESRIKKLHKLLSYNLPLELENLRSLLESTPSPVVFCHNDCQEGNILLLEGRENSEKQKLMLIDFEYSSYNYRGFDIGNHFCEWMYDYSYEKYPFFRANIRKYPTKKQQLHFISSYLPAFQNDFENLSTEEKSIIKEEMLLEVNRFALASHFLWGLWSIVQAKISSIEFGYMDYAQARFDAYFHQKRKLGV

143 P51688 Q9GZZ1

>P51688

MSCPVPACCALLLVLGLCRARPRNALLLLADDGGFESGAYNNSAIATPHLDALARRSLLFRNAFTSVSSCSPSRASLLTGLPQHQNGMYGLHQDVHHFNSFDKVRSLPLLLSQAGVRTGIIGKKHVGPETVYPFDFAYTEENGSVLQVGRNITRIKLLVRKFLQTQDDRPFFLYVAFHDPHRCGHSQPQYGTFCEKFGNGESGMGRIPDWTPQAYDPLDVLVPYFVPNTPAARADLAAQYTTVGRMDQGVGLVLQELRDAGVLNDTLVIFTSDNGIPFPSGRTNLYWPGTAEPLLVSSPEHPKRWGQVSEAYVSLLDLTPTILDWFSIPYPSYAIFGSKTIHLTGRSLLPALEAEPLWATVFGSQSHHEVTMSYPMRSVQHRHFRLVHNLNFKMPFPIDQDFYVSPTFQDLLNRTTAGQPTGWYKDLRHYYYRARWELYDRSRDPHETQNLATDPRFAQLLEMLRDQLAKWQWETHDPWVCAPDGVLEEKLSPQCQPLHNEL

>Q9GZZ1

MKGSRIELGDVTPHNIKQLKRLNQVIFPVSYNDKFYKDVLEVGELAKLAYFNDIAVGAVCCRVDHSQNQKRLYIMTLGCLAPYRRLGIGTKMLNHVLNICEKDGTFDNIYLHVQISNESAIDFYRKFGFEIIETKKNYYKRIEPADAHVLQKNLKVPSGQNADVQKTDN

144 P30260 Q12931

>P30260

MTVLQEPVQAAIWQALNHYAYRDAVFLAERLYAEVHSEEALFLLATCYYRSGKAYKAYRLLKGHSCTTPQCKYLLAKCCVDLSKLAEGEQILSGGVFNKQKSHDDIVTEFGDSACFTLSLLGHVYCKTDRLAKGSECYQKSLSLNPFLWSPFESLCEIGEKPDPDQTFKFTSLQNFSNCLPNSCTTQVPNHSLSHRQPETVLTETPQDTIELNRLNLESSNSKYSLNTDSSVSYIDSAVISPDTVPLGTGTSILSKQVQNKPKTGRSLLGGPAALSPLTPSFGILPLETPSPGDGSYLQNYTNTPPVIDVPSTGAPSKKSVARIGQTGTKSVFSQSGNSREVTPILAQTQSSGPQTSTTPQVLSPTITSPPNALPRRSSRLFTSDSSTTKENSKKLKMKFPPKIPNRKTKSKTNKGGITQPNINDSLEITKLDSSIISEGKISTITPQIQAFNLQKAAAEGLMSLLREMGKGYLALCSYNCKEAINILSHLPSHHYNTGWVLCQIGRAYFELSEYMQAERIFSEVRRIENYRVEGMEIYSTTLWHLQKDVALSVLSKDLTDMDKNSPEAWCAAGNCFSLQREHDIAIKFFQRAIQVDPNYAYAYTLLGHEFVLTEELDKALACFRNAIRVNPRHYNAWYGLGMIYYKQEKFSLAEMHFQKALDINPQSSVLLCHIGVVQHALKKSEKALDTLNKAIVIDPKNPLCKFHRASVLFANEKYKSALQELEELKQIVPKESLVYFLIGKVYKKLGQTHLALMNFSWAMDLDPKGANNQIKEAIDKRYLPDDEEPITQEEQIMGTDESQESSMTDADDTQLHAAESDEF

>Q12931

MARELRALLLWGRRLRPLLRAPALAAVPGGKPILCPRRTTAQLGPRRNPAWSLQAGRLFSTQTAEDKEEPLHSIISSTESVQGSTSKHEFQAETKKLLDIVARSLYSEKEVFIRELISNASDALEKLRHKLVSDGQALPEMEIHLQTNAEKGTITIQDTGIGMTQEELVSNLGTIARSGSKAFLDALQNQAEASSKIIGQFGVGFYSAFMVADRVEVYSRSAAPGSLGYQWLSDGSGVFEIAEASGVRTGTKIIIHLKSDCKEFSSEARVRDVVTKYSNFVSFPLYLNGRRMNTLQAIWMMDPKDVREWQHEEFYRYVAQAHDKPRYTLHYKTDAPLNIRSIFYVPDMKPSMFDVSRELGSSVALYSRKVLIQTKATDILPKWLRFIRGVVDSEDIPLNLSRELLQESALIRKLRDVLQQRLIKFFIDQSKKDAEKYAKFFEDYGLFMREGIVTATEQEVKEDIAKLLRYESSALPSGQLTSLSEYASRMRAGTRNIYYLCAPNRHLAEHSPYYEAMKKKDTEVLFCFEQFDELTLLHLREFDKKKLISVETDIVVDHYKEEKFEDRSPAAECLSEKETEELMAWMRNVLGSRVTNVKVTLRLDTHPAMVTVLEMGAARHFLRMQQLAKTQEERAQLLQPTLEINPRHALIKKLNQLRASEPGLAQLLVDQIYENAMIAAGLVDDPRAMVGRLNELLVKALERH

145 NP_005060.1 NP_056442.2

>NP_005060.1

MKEKSKNAAKTRREKENGEFYELAKLLPLPSAITSQLDKASIIRLTTSYLKMRAVFPEGLGDAWGQPSRAGPLDGVAKELGSHLLQTLDGFVFVVASDGKIMYISETASVHLGLSQVELTGNSIYEYIHPSDHDEMTAVLTAHQPLHHHLLQEYEIERSFFLRMKCVLAKRNAGLTCSGYKVIHCSGYLKIRQYMLDMSLYDSCYQIVGLVAVGQSLPPSAITEIKLYSNMFMFRASLDLKLIFLDSRVTEVTGYEPQDLIEKTLYHHVHGCDVFHLRYAHHLLLVKGQVTTKYYRLLSKRGGWVWVQSYATVVHNSRSSRPHCIVSVNYVLTEIEYKELQLSLEQVSTAKSQDSWRTALSTSQETRKLVKPKNTKMKTKLRTNPYPPQQYSSFQMDKLECGQLGNWRASPPASAAAPPELQPHSESSDLLYTPSYSLPFSYHYGHFPLDSHVFSSKKPMLPAKFGQPQGSPCEVARFFLSTLPASGECQWHYANPLVPSSSSPAKNPPEPPANTARHSLVPSYEAPAAAVRRFGEDTAPPSFPSCGHYREEPALGPAKAARQAARDGARLALARAAPECCAPPTPEAPGAPAQLPFVLLNYHRVLARRGPLGGAAPAASGLACAPGGPEAATGALRLRHPSPAATSPPGAPLPHYLGASVIITNGR

>NP_056442.2

MDALKSAGRALIRSPSLAKQSWGGGGRHRKLPENWTDTRETLLEGMLFSLKYLGMTLVEQPKGEELSAAAIKRIVATAKASGKKLQKVTLKVSPRGIILTDNLTNQLIENVSIYRISYCTADKMHDKVFAYIAQSQHNQSLECHAFLCTKRKMAQAVTLTVAQAFKVAFEFWQVSKEEKEKRDKASQEGGDVLGARQDCTPSLKSLVATGNLLDLEETAKAPLSTVSANTTNMDEVPRPQALSGSSVVWELDDGLDEAFSRLAQSRTNPQVLDTGLTAQDMHYAQCLSPVDWDKPDSSGTEQDDLFSF

146 AAA93070.1 NP_660198.1

>AAA93070.1

MSGCRVFIGRLNPAAREKDVERFFKGYGRIRDIDLKRGFGFVEFEDPRDADDAVYELDGKELCSERVTIEHARARSRGGRGRGRYSDRFSSRRPRNDRRNAPPVRTENRLIVENLSSRVSWQDLKDFMRQAGEVTFADAHRPKLNEGVVEFASYGDLKNAIEKLSGKEINGRKIKLIEGSKRHSRSRSRSRSRTRSSSRSRSRSRSRSRKSYSRSRSRSRSRSRSKSRSVSRSPVPEKSQKRGSSSRSKSPASVDRQRSRSRSRSRSVDSGN

>NP_660198.1

MLIPFSMKNCFQLLCNCQVPAAGFKKTVKNGLILQSISNDVYQNLAVEDWIHDHMNLEGKPILFFWQNSPSVVIGRHQNPWQECNLNLMREEGIKLARRRSGGGTVYHDMGNINLTFFTTKKKYDRMENLKLIVRALNAVQPQLDVQATKRFDLLLDGQFKISGTASKIGRTTAYHHCTLLCSTDGTFLSSLLKSPYQGIRSNATASIPSLVKNLLEKDPTLTCEVLMNAVATEYAAYHQIDNHIHLINPTDETLFPGINSKAKELQTWEWIYGKTPKFSINTSFHVLYEQSHLEIKVFIDIKNGRIEICNIEAPDHWLPLEIRDKLNSSLIGSKFCPTETTMLTNILLRTCPQDHKLNSKWNILCEKIKGIM

147 NP_003784.2 NP_003977.1

>NP_003784.2

MAPWLQLLSLLGLLPGAVAAPAQPRAASFQAWGPPSPELLAPTRFALEMFNRGRAAGTRAVLGLVRGRVRRAGQGSLYSLEATLEEPPCNDPMVCRLPVSKKTLLCSFQVLDELGRHVLLRKDCGPVDTKVPGAGEPKSAFTQGSAMISSLSQNHPDNRNETFSSVISLLNEDPLSQDLPVKMASIFKNFVITYNRTYESKEEARWRLSVFVNNMVRAQKIQALDRGTAQYGVTKFSDLTEEEFRTIYLNTLLRKEPGNKMKQAKSVGDLAPPEWDWRSKGAVTKVKDQGMCGSCWAFSVTGNVEGQWFLNQGTLLSLSEQELLDCDKMDKACMGGLPSNAYSAIKNLGGLETEDDYSYQGHMQSCNFSAEKAKVYINDSVELSQNEQKLAAWLAKRGPISVAINAFGMQFYRHGISRPLRPLCSPWLIDHAVLLVGYGNRSDVPFWAIKNSWGTDWGEKGYYYLHRGSGACGVNTMASSAVVD

>NP_003977.1

MACTIQKAEALDGAHLMQILWYDEEESLYPAVWLRDNCPCSDCYLDSAKARKLLVEALDVNIGIKGLIFDRKKVYITWPDEHYSEFQADWLKKRCFSKQARAKLQRELFFPECQYWGSELQLPTLDFEDVLRYDEHAYKWLSTLKKVGIVRLTGASDKPGEVSKLGKRMGFLYLTFYGHTWQVQDKIDANNVAYTTGKLSFHTDYPALHHPPGVQLLHCIKQTVTGGDSEIVDGFNVCQKLKKNNPQAFQILSSTFVDFTDIGVDYCDFSVQSKHKIIELDDKGQVVRINFNNATRDTIFDVPVERVQPFYAALKEFVDLMNSKESKFTFKMNPGDVITFDNWRLLHGRRSYEAGTEISRHLEGAYADWDVVMSRLRILRQRVENGN

148 NP_056953.2 NP_004146.1

>NP_056953.2

MGETLGDSPIDPESDSFTDTLSANISQEMTMVDTEMPFWPTNFGISSVDLSVMEDHSHSFDIKPFTTVDFSSISTPHYEDIPFTRTDPVVADYKYDLKLQEYQSAIKVEPASPPYYSEKTQLYNKPHEEPSNSLMAIECRVCGDKASGFHYGVHACEGCKGFFRRTIRLKLIYDRCDLNCRIHKKSRNKCQYCRFQKCLAVGMSHNAIRFGRMPQAEKEKLLAEISSDIDQLNPESADLRALAKHLYDSYIKSFPLTKAKARAILTGKTTDKSPFVIYDMNSLMMGEDKIKFKHITPLQEQSKEVAIRIFQGCQFRSVEAVQEITEYAKSIPGFVNLDLNDQVTLLKYGVHEIIYTMLASLMNKDGVLISEGQGFMTREFLKSLRKPFGDFMEPKFEFAVKFNALELDDSDLAIFIAVIILSGDRPGLLNVKPIEDIQDNLLQALELQLKLNHPESSQLFAKLLQKMTDLRQIVTEHVQLLQVIKKTETDMSLHPLLQEIYKDLY

>NP_004146.1

METLSNASGTFAIRLLKILCQDNPSHNVFCSPVSISSALAMVLLGAKGNTATQMAQALSLNTEEDIHRAFQSLLTEVNKAGTQYLLRTANRLFGEKTCQFLSTFKESCLQFYHAELKELSFIRAAEESRKHINTWVSKKTEGKIEELLPGSSIDAETRLVLVNAIYFKGKWNEPFDETYTREMPFKINQEEQRPVQMMYQEATFKLAHVGEVRAQLLELPYARKELSLLVLLPDDGVELSTVEKSLTFEKLTAWTKPDCMKSTEVEVLLPKFKLQEDYDMESVLRHLGIVDAFQQGKADLSAMSAERDLCLSKFVHKSFVEVNEEGTEAAAASSCFVVAECCMESGPRFCADHPFLFFIRHNRANSILFCGRFSSP

149 NP_004166.1 Q15046

>NP_004166.1

MSIGVPIKVLHEAEGHIVTCETNTGEVYRGKLIEAEDNMNCQMSNITVTYRDGRVAQLEQVYIRGSKIRFLILPDMLKNAPMLKSMKNKNQGSGAGRGKAAILKAQVAARGRGRGMGRGNIFQKRR

>Q15046

MAAVQAAEVKVDGSEPKLSKNELKRRLKAEKKVAEKEAKQKELSEKQLSQATAAATNHTTDNGVGPEEESVDPNQYYKIRSQAIHQLKVNGEDPYPHKFHVDISLTDFIQKYSHLQPGDHLTDITLKVAGRIHAKRASGGKLIFYDLRGEGVKLQVMANSRNYKSEEEFIHINNKLRRGDIIGVQGNPGKTKKGELSIIPYEITLLSPCLHMLPHLHFGLKDKETRYRQRYLDLILNDFVRQKFIIRSKIITYIRSFLDELGFLEIETPMMNIIPGGAVAKPFITYHNELDMNLYMRIAPELYHKMLVVGGIDRVYEIGRQFRNEGIDLTHNPEFTTCEFYMAYADYHDLMEITEKMVSGMVKHITGSYKVTYHPDGPEGQAYDVDFTPPFRRINMVEELEKALGMKLPETNLFETEETRKILDDICVAKAVECPPPRTTARLLDKLVGEFLEVTCINPTFICDHPQIMSPLAKWHRSKEGLTERFELFVMKKEICNAYTELNDPMRQRQLFEEQAKAKAAGDDEAMFIDENFCTALEYGLPPTAGWGMGIDRVAMFLTDSNNIKEVLLFPAMKPEDKKENVATTDTLESTTVGTSV

150 BAB62909.1 Q13287

>BAB62909.1

MDRSKRNSIAGFPPRVERLEEFEGGGGGEGNVSQVGRVWPSSYRALISAFSRLTRLDDFTCEKIGSGFFSEVFKVRHRASGQVMALKMNTLSSNRANMLKEVQLMNRLSHPNILRFMGVCVHQGQLHALTEYINSGNLEQLLDSNLHLPWTVRVKLAYDIAVGLSYLHFKGIFHRDLTSKNCLIKRDENGYSAVVADFGLAEKIPDVSMGSEKLAVVGSPFWMAPEVLRDEPYNEKADVFSYGIILCEIIARIQADPDYLPRTENFGLDYDAFQHMVGDCPPDFLQLTFNCCNMDPKLRPSFVEIGKTLEEILSRLQEEEQERDRKLQPTARGLLEKAPGVKRLSSLDDKIPHKSPCPRRTIWLSRSQSDIFSRKPPRTVSVLDPYYRPRDGAARTPKVNPFSARQDLMGGKIKFFDLPSKSVISLVFDLDAPGPGTMPLADWQEPLAPPIRRWRSLPGSPEFLHQEACPFVGREESLSDGPPPRLSSLKYRVKEIPPFRASALPAAQAHEAMDCSILQEENGFGSRPQGTSPCPAGASEEMEVEERPAGSTPATFSTSGIGLQTQGKQDG

>Q13287

MEADKDDTQQILKEHSPDEFIKDEQNKGLIDEITKKNIQLKKEIQKLETELQEATKEFQIKEDIPETKMKFLSVETPENDSQLSNISCSFQVSSKVPYEIQKGQALITFEKEEVAQNVVSMSKHHVQIKDVNLEVTAKPVPLNSGVRFQVYVEVSKMKINVTEIPDTLREDQMRDKLELSFSKSRNGGGEVDRVDYDRQSGSAVITFVEIGVADKILKKKEYPLYINQTCHRVTVSPYTEIHLKKYQIFSGTSKRTVLLTGMEGIQMDEEIVEDLINIHFQRAKNGGGEVDVVKCSLGQPHIAYFEE

151 O94953 P48163

>O94953

MGSEDHGAQNPSCKIMTFRPTMEEFKDFNKYVAYIESQGAHRAGLAKIIPPKEWKPRQTYDDIDDVVIPAPIQQVVTGQSGLFTQYNIQKKAMTVGEYRRLANSEKYCTPRHQDFDDLERKYWKNLTFVSPIYGADISGSLYDDDVAQWNIGSLRTILDMVERECGTIIEGVNTPYLYFGMWKTTFAWHTEDMDLYSINYLHFGEPKSWYAIPPEHGKRLERLAIGFFPGSSQGCDAFLRHKMTLISPIILKKYGIPFSRITQEAGEFMITFPYGYHAGFNHGFNCAESTNFATLRWIDYGKVATQCTCRKDMVKISMDVFVRILQPERYELWKQGKDLTVLDHTRPTALTSPELSSWSASRASLKAKLLRRSHRKRSQPKKPKPEDPKFPGEGTAGAALLEEAGGSVKEEAGPEVDPEEEEEEPQPLPHGREAEGAEEDGRGKLRPTKAKSERKKKSFGLLPPQLPPPPAHFPSEEALWLPSPLEPPVLGPGPAAMEESPLPAPLNVVPPEVPSEELEAKPRPIIPMLYVVPRPGKAAFNQEHVSCQQAFEHFAQKGPTWKEPVSPMELTGPEDGAASSGAGRMETKARAGEGQAPSTFSKLKMEIKKSRRHPLGRPPTRSPLSVVKQEASSDEEASPFSGEEDVSDPDALRPLLSLQWKNRAASFQAERKFNAAAARTEPYCAICTLFYPYCQALQTEKEAPIASLGKGCPATLPSKSRQKTRPLIPEMCFTSGGENTEPLPANSYIGDDGTSPLIACGKCCLQVHASCYGIRPELVNEGWTCSRCAAHAWTAECCLCNLRGGALQMTTDRRWIHVICAIAVPEARFLNVIERHPVDISAIPEQRWKLKCVYCRKRMKKVSGACIQCSYEHCSTSFHVTCAHAAGVLMEPDDWPYVVSITCLKHKSGGHAVQLLRAVSLGQVVITKNRNGLYYRCRVIGAASQTCYEVNFDDGSYSDNLYPESITSRDCVQLGPPSEGELVELRWTDGNLYKAKFISSVTSHIYQVEFEDGSQLTVKRGDIFTLEEELPKRVRSRLSLSTGAPQEPAFSGEEAKAAKRPRVGTPLATEDSGRSQDYVAFVESLLQVQGRPGAPF

>P48163

MEPEAPRRRHTHQRGYLLTRNPHLNKDLAFTLEERQQLNIHGLLPPSFNSQEIQVLRVVKNFEHLNSDFDRYLLLMDLQDRNEKLFYRVLTSDIEKFMPIVYTPTVGLACQQYSLVFRKPRGLFITIHDRGHIASVLNAWPEDVIKAIVVTDGERILGLGDLGCNGMGIPVGKLALYTACGGMNPQECLPVILDVGTENEELLKDPLYIGLRQRRVRGSEYDDFLDEFMEAVSSKYGMNCLIQFEDFANVNAFRLLNKYRNQYCTFNDDIQGTASVAVAGLLAALRITKNKLSDQTILFQGAGEAALGIAHLIVMALEKEGLPKEKAIKKIWLVDSKGLIVKGRASLTQEKEKFAHEHEEMKNLEAIVQEIKPTALIGVAAIGGAFSEQILKDMAAFNERPIIFALSNPTSKAECSAEQCYKITKGRAIFASGSPFDPVTLPNGQTLYPGQGNNSYVFPGVALGVVACGLRQITDNIFLTTAEVIAQQVSDKHLEEGRLYPPLNTIRDVSLKIAEKIVKDAYQEKTATVYPEPQNKEAFVRSQMYSTDYDQILPDCYSWPEEVQKIQTKVDQ

152 P10619 O75879

>P10619

MIRAAPPPLFLLLLLLLLLVSWASRGEAAPDQDEIQRLPGLAKQPSFRQYSGYLKGSGSKHLHYWFVESQKDPENSPVVLWLNGGPGCSSLDGLLTEHGPFLVQPDGVTLEYNPYSWNLIANVLYLESPAGVGFSYSDDKFYATNDTEVAQSNFEALQDFFRLFPEYKNNKLFLTGESYAGIYIPTLAVLVMQDPSMNLQGLAVGNGLSSYEQNDNSLVYFAYYHGLLGNRLWSSLQTHCCSQNKCNFYDNKDLECVTNLQEVARIVGNSGLNIYNLYAPCAGGVPSHFRYEKDTVVVQDLGNIFTRLPLKRMWHQALLRSGDKVRMDPPCTNTTAASTYLNNPYVRKALNIPEQLPQWDMCNFLVNLQYRRLYRSMNSQYLKLLSSQKYQILLYNGDVDMACNFMGDEWFVDSLNQKMEVQRRPWLVKYGDSGEQIAGFVKEFSHIAFLTIKGAGHMVPTDKPLAAFTMFSRFLNKQPY

>O75879

MAAPMLRWGCRGRRWAFARVDGGSCHRRGAPTGSTSNQIRGESSVAQQPLHTAQKTRKGEHKWAAVVGLEIHAQISSNSKLFSGSQVRFSAPPNSLVSFFDASLPGTLPVLNRRCVEAAVMTGLALNCHINKKSLFDRKHYFYADLPAGYQITQQRLPIAVNGSLIYGVCAGKKQSQVIPKTVRIKQIQLEQDSGKSLHDNLRSQTLIDLNRAGVGLLEVVLEPDMSCGEEAATAVRELQLILQALGTSQANMAEGQLRVDANISVHHPGEPLGVRTEVKNLNSIRFLAKAIDYEIQRQINELENGGEILNETRSFHHKLGCTMSMRDKEGKQDYRFMPEPNLPPLVLYDATSLPAGADPQQVINIDQIRETLPELPSVTREKLVQQYGMLLEHSFTLLNEVGLLEFFQNVIKETRAEPKKVTSWVLNTFLGYLKQQNLAVSESPVTPSALAELLDLLDSRTISSSAAKQVFEELWKREGKTPGQIVSEKQLELMQDQGALEQLCHSVMEAHPQVVMDVKNRNPRAINKLIGLVRKATQSRADPVMIKEILEKKLSL

153 NP_002137.4 NP_001435.1

>NP_002137.4

MQKATYYDNAAAALFGGYSSYPGSNGFGFDVPPQPPFQAATHLEGDYQRSACSLQSLGNAAPHAKSKELNGSCMRPGLAPEPLSAPPGSPPPSAAPTSATSNSSNGGGPSKSGPPKCGPGTNSTLTKQIFPWMKESRQTSKLKNNSPGTAEGCGGGGGGGGGGGSGGSGGGGGGGGGGDKSPPGSAASKRARTAYTSAQLVELEKEFHFNRYLCRPRRVEMANLLNLSERQIKIWFQNRRMKYKKDQKAKGLASSSGGPSPAGSPPQPMQSTAGFMNALHSMTPSYESPSPPAFGKAHQNAYALPSNYQPPLKGCGAPQKYPPTPAPEYEPHVLQANGGAYGTPTMQGSPVYVGGGGYADPLPPPAGPSLYGLNHLSHHPSGNLDYNGAPPMAPSQHHGPCEPHPTYTDLSSHHAPPPQGRIQEAPKLTHL

>NP_001435.1

MATVQQLEGRWRLVDSKGFDEYMKELGVGIALRKMGAMAKPDCIITCDGKNLTIKTESTLKTTQFSCTLGEKFEETTADGRKTQTVCNFTDGALVQHQEWDGKESTITRKLKDGKLVVECVMNNVTCTRIYEKVE

154 P33076 P14598

>P33076

MRCLAPRPAGSYLSEPQGSSQCATMELGPLEGGYLELLNSDADPLCLYHFYDQMDLAGEEEIELYSEPDTDTINCDQFSRLLCDMEGDEETREAYANIAELDQYVFQDSQLEGLSKDIFKHIGPDEVIGESMEMPAEVGQKSQKRPFPEELPADLKHWKPAEPPTVVTGSLLVGPVSDCSTLPCLPLPALFNQEPASGQMRLEKTDQIPMPFSSSSLSCLNLPEGPIQFVPTISTLPHGLWQISEAGTGVSSIFIYHGEVPQASQVPPPSGFTVHGLPTSPDRPGSTSPFAPSATDLPSMPEPALTSRANMTEHKTSPTQCPAAGEVSNKLPKWPEPVEQFYRSLQDTYGAEPAGPDGILVEVDLVQARLERSSSKSLERELATPDWAERQLAQGGLAEVLLAAKEHRRPRETRVIAVLGKAGQGKSYWAGAVSRAWACGRLPQYDFVFSVPCHCLNRPGDAYGLQDLLFSLGPQPLVAADEVFSHILKRPDRVLLILDGFEELEAQDGFLHSTCGPAPAEPCSLRGLLAGLFQKKLLRGCTLLLTARPRGRLVQSLSKADALFELSGFSMEQAQAYVMRYFESSGMTEHQDRALTLLRDRPLLLSHSHSPTLCRAVCQLSEALLELGEDAKLPSTLTGLYVGLLGRAALDSPPGALAELAKLAWELGRRHQSTLQEDQFPSADVRTWAMAKGLVQHPPRAAESELAFPSFLLQCFLGALWLALSGEIKDKELPQYLALTPRKKRPYDNWLEGVPRFLAGLIFQPPARCLGALLGPSAAASVDRKQKVLARYLKRLQPGTLRARQLLELLHCAHEAEEAGIWQHVVQELPGRLSFLGTRLTPPDAHVLGKALEAAGQDFSLDLRSTGICPSGLGSLVGLSCVTRFRAALSDTVALWESLRQHGETKLLQAAEEKFTIEPFKAKSLKDVEDLGKLVQTQRTRSSSEDTAGELPAVRDLKKLEFALGPVSGPQAFPKLVRILTAFSSLQHLDLDALSENKIGDEGVSQLSATFPQLKSLETLNLSQNNITDLGAYKLAEALPSLAASLLRLSLYNNCICDVGAESLARVLPDMVSLRVMDVQYNKFTAAGAQQLAASLRRCPHVETLAMWTPTIPFSVQEHLQQQDSRISLR

>P14598

MGDTFIRHIALLGFEKRFVPSQHYVYMFLVKWQDLSEKVVYRRFTEIYEFHKTLKEMFPIEAGAINPENRIIPHLPAPKWFDGQRAAENRQGTLTEYCSTLMSLPTKISRCPHLLDFFKVRPDDLKLPTDNQTKKPETYLMPKDGKSTATDITGPIILQTYRAIADYEKTSGSEMALSTGDVVEVVEKSESGWWFCQMKAKRGWIPASFLEPLDSPDETEDPEPNYAGEPYVAIKAYTAVEGDEVSLLEGEAVEVIHKLLDGWWVIRKDDVTGYFPSMYLQKSGQDVSQAQRQIKRGAPPRRSSIRNAHSIHQRSRKRLSQDAYRRNSVRFLQQRRRQARPGPQSPGSPLEEERQTQRSKPQPAVPPRPSADLILNRCSESTKRKLASAV

155 NP_003855.1 O75251

>NP_003855.1

MNGFTPDEMSRGGDAAAAVAAVVAAAAAAASAGNGTGAGTGAEVPGAGAVSAAGPPGAAGPGPGQLCCLREDGERCGRAAGNASFSKRIQKSISQKKVKIELDKSARHLYICDYHKNLIQSVRNRRKRKGSDDDGGDSPVQDIDTPEVDLYQLQVNTLRRYKRHFKLPTRPGLNKAQLVEIVGCHFRSIPVNEKDTLTYFIYSVKNDKNKSDLKVDSGVH

>O75251

MAVLSAPGLRGFRILGLRSSVGPAVQARGVHQSVATDGPSSTQPALPKARAVAPKPSSRGEYVVAKLDDLVNWARRSSLWPMTFGLACCAVEMMHMAAPRYDMDRFGVVFRASPRQSDVMIVAGTLTNKMAPALRKVYDQMPEPRYVVSMGSCANGGGYYHYSYSVVRGCDRIVPVDIYIPGCPPTAEALLYGILQLQRKIKRERRLQIWYRR

156 NP_002948.1 NP_002056.2

>NP_002948.1

MDTKHFLPLDFSTQVNSSLTSPTGRGSMAAPSLHPSLGPGIGSPGQLHSPISTLSSPINGMGPPFSVISSPMGPHSMSVPTTPTLGFSTGSPQLSSPMNPVSSSEDIKPPLGLNGVLKVPAHPSGNMASFTKHICAICGDRSSGKHYGVYSCEGCKGFFKRTVRKDLTYTCRDNKDCLIDKRQRNRCQYCRYQKCLAMGMKREAVQEERQRGKDRNENEVESTSSANEDMPVERILEAELAVEPKTETYVEANMGLNPSSPNDPVTNICQAADKQLFTLVEWAKRIPHFSELPLDDQVILLRAGWNELLIASFSHRSIAVKDGILLATGLHVHRNSAHSAGVGAIFDRVLTELVSKMRDMQMDKTELGCLRAIVLFNPDSKGLSNPAEVEALREKVYASLEAYCKHKYPEQPGRFAKLLLRLPALRSIGLKCLEHLFFFKLIGDTPIDTFLMEMLEAPHQMT

>NP_002056.2

MTTSASSHLNKGIKQVYMSLPQGEKVQAMYIWIDGTGEGLRCKTRTLDSEPKCVEELPEWNFDGSSTLQSEGSNSDMYLVPAAMFRDPFRKDPNKLVLCEVFKYNRRPAETNLRHTCKRIMDMVSNQHPWFGMEQEYTLMGTDGHPFGWPSNGFPGPQGPYYCGVGADRAYGRDIVEAHYRACLYAGVKIAGTNAEVMPAQWEFQIGPCEGISMGDHLWVARFILHRVCEDFGVIATFDPKPIPGNWNGAGCHTNFSTKAMREENGLKYIEEAIEKLSKRHQYHIRAYDPKGGLDNARRLTGFHETSNINDFSAGVANRSASIRIPRTVGQEKKGYFEDRRPSANCDPFSVTEALIRTCLLNETGDEPFQYKN

157 NP_004836.2 NP_000340.2

>NP_004836.2

MPVVTTDAESETGIPKSLSNEPPSETMEEIEHTCPQPRLTLTAPAPFADETNCQCQAPHEKLTIAQARLGTPADRPVRVYADGIFDLFHSGHARALMQAKTLFPNSYLLVGVCSDDLTHKFKGFTVMNEAERYEALRHCRYVDEVIRDAPWTLTPEFLEKHKIDFVAHDDIPYSSAGSDDVYKHIKEAGMFVPTQRTEGISTSDIITRIVRDYDVYARRNLQRGYTAKELNVSFINEKRYRFQNQVDKMKEKVKNVEERSKEFVNRVEEKSHDLIQKWEEKSREFIGNFLELFGPDGAWKQMFQERSSRMLQALSPKQSPVSSPTRSRSPSRSPSPTFSWLPLKTSPPSSPKAASASISSMSEGDEDEK

>NP_000340.2

MLLATFKLCAGSSYRHMRNMKGLRQQAVMAISQELNRRALGGPTPSTWINQVRRRSSLLGSRLEETLYSDQELAYLQQGEEAMQKALGILSNQEGWKKESQQDNGDKVMSKVVPDVGKVFRLEVVVDQPMERLYEELVERMEAMGEWNPNVKEIKVLQKIGKDTFITHELAAEAAGNLVGPRDFVSVRCAKRRGSTCVLAGMATDFGNMPEQKGVIRAEHGPTCMVLHPLAGSPSKTKLTWLLSIDLKGWLPKSIINQVLSQTQVDFANHLRKRLESHPASEARC

158 NP_056975.1 NP_003921.2

>NP_056975.1

MSAAIAALAASYGSGSGSESDSDSESSRCPLPAADSLMHLTKSPSSKPSLAVAVDSAPEVAVKEDLETGVHLDPAVKEVQYNPTYETMFAPEFGPENPFRTQQMAAPRNMLSGYAEPAHINDFMFEQQRRTFATYGYALDPSLDNHQVSAKYIGSVEEAEKNQGLTVFETGQKKTEKRKKFKENDASNIDGFLGPWAKYVDEKDVAKPSEEEQKELDEITAKRQKKGKQEEEKPGEEKTILHVKEMYDYQGRSYLHIPQDVGVNLRSTMPPEKCYLPKKQIHVWSGHTKGVSAVRLFPLSGHLLLSCSMDCKIKLWEVYGERRCLRTFIGHSKAVRDICFNTAGTQFLSAAYDRYLKLWDTETGQCISRFTNRKVPYCVKFNPDEDKQNLFVAGMSDKKIVQWDIRSGEIVQEYDRHLGAVNTIVFVDENRRFVSTSDDKSLRVWEWDIPVDFKYIAEPSMHSMPAVTLSPNGKWLACQSMDNQILIFGAQNRFRLNKKKIFKGHMVAGYACQVDFSPDMSYVISGDGNGKLNIWDWKTTKLYSRFKAHDKVCIGAVWHPHETSKVITCGWDGLIKLWD

>NP_003921.2

MPNPSSTSSPYPLPEEIRNLLADVETFVADILKGENLSKKAKEKRESLIKKIKDVKSIYLQEFQDKGDAEDGEEYDDPFAGPPDTISLASERYDKDDEAPSDGAQFPPIAAQDLPFVLKAGYLEKRRKDHSFLGFEWQKRWCALSKTVFYYYGSDKDKQQKGEFAIDGYSVRMNNTLRKDGKKDCCFEISAPDKRIYQFTAASPKDAEEWVQQLKFVLQDMESDIIPEDYDERGELYDDVDHPLPISNPLTSSQPIDDEIYEELPEEEEDSAPVKVEEQRKMSQDSVHHTSGDKSTDYANFYQGLWDCTGAFSDELSFKRGDVIYILSKEYNRYGWWVGEMKGAIGLVPKAYIMEMYDI

159 NP_037425.1 NP_001386.1

>NP_037425.1

MSDVEENNFEGRESRSQSKSPTGTPARVKSESRSGSRSPSRVSKHSESHSRSRSKSRSRSRRHSHRRYTRSRSHSHSHRRRSRSRSYTPEYRRRRSRSHSPMSNRRRHTGSRANPDPNTCLGVFGLSLYTTERDLREVFSRYGPLSGVNVVYDQRTGRSRGFAFVYFERIDDSKEAMERANGMELDGRRIRVDYSITKRAHTPTPGIYMGRPTHSGGGGGGGGGGGGGGGGRRRDSYYDRGYDRGYDRYEDYDYRYRRRSPSPYYSRYRSRSRSRSYSPRRY

>NP_001386.1

MEGLGRSCLWLRRELSPPRPRLLLLDCRSRELYESARIGGALSVALPALLLRRLRRGSLSVRALLPGPPLQPPPPAPVLLYDQGGGRRRRGEAEAEAEEWEAESVLGTLLQKLREEGYLAYYLQGGFSRFQAECPHLCETSLAGRAGSSMAPVPGPVPVVGLGSLCLGSDCSDAESEADRDSMSCGLDSEGATPPPVGLRASFPVQILPNLYLGSARDSANLESLAKLGIRYILNVTPNLPNFFEKNGDFHYKQIPISDHWSQNLSRFFPEAIEFIDEALSQNCGVLVHCLAGVSRSVTVTVAYLMQKLHLSLNDAYDLVKRKKSNISPNFNFMGQLLDFERSLRLEERHSQEQGSGGQASAASNPPSFFTTPTSDGAFELAPT

160 NP_002958.2 P23434

>NP_002958.2

MAETLSGLGDSGAAGAAALSSASSETGTRRLSDLRVIDLRAELRKRNVDSSGNKSVLMERLKKAIEDEGGNPDEIEITSEGNKKTSKRSSKGRKPEEEGVEDNGLEENSGDGQEDVETSLENLQDIDIMDISVLDEAEIDNGSVADCVEDDDADNLQESLSDSRELVEGEMKELPEQLQEHAIEDKETINNLDTSSSDFTILQEIEEPSLEPENEKILDILGETCKSEPVKEESSELEQPFAQDTSSVGPDRKLAEEEDLFDSAHPEEGDLDLASESTAHAQSSKADSLLAVVKREPAEQPGDGERTDCEPVGLEPAVEQSSAASELAEASSEELAEAPTEAPSPEARDSKEDGRKFDFDACNEVPPAPKESSTSEGADQKMSSPEDDSDTKRLSKEEKGRSSCGRNFWVSGLSSTTRATDLKNLFSKYGKVVGAKVVTNARSPGARCYGFVTMSTAEEATKCINHLHKTELHGKMISVEKAKNEPVGKKTSDKRDSDGKKEKSSNSDRSTNLKRDDKCDRKDDAKKGDDGSGEKSKDQDDQKPGPSERSRATKSGSRGTERTVVMDKSKGVPVISVKTSGSKERASKSQDRKSASREKRSVVSFDKVKEPRKSRDSESHSRVRERSEREQRMQAQWEREERERLEIARERLAFQRQRLERERMERERLERERMHVEHERRREQERIHREREELRRQQELRYEQERRPAVRRPYDLDRRDDAYWPEAKRAALDERYHSDFNRQDRFHDFDHRDRGRYPDHSVDRREGSRSMMGEREGQHYPERHGGPERHGRDSRDGWGGYGSDKRMSEGRGLPPPPRRDWGDHGRREDDRSWQGTADGGMMDRDHKRWQGGERSMSGHSGPGHMMNRGGMSGRGSFAPGGASRGHPIPHGGMQGGFGGQSRGSRPSDARFTRRY

>P23434

MALRVVRSVRALLCTLRAVPLPAAPCPPRPWQLGVGAVRTLRTGPALLSVRKFTEKHEWVTTENGIGTVGISNFAQEALGDVVYCSLPEVGTKLNKQDEFGALESVKAASELYSPLSGEVTEINEALAENPGLVNKSCYEDGWLIKMTLSNPSELDELMSEEAYEKYIKSIEE

161 NP_005185.2 Q96GX9

>NP_005185.2

MQRLVAWDPACLPLPPPPPAFKSMEVANFYYEADCLAAAYGGKAAPAAPPAARPGPRPPAGELGSIGDHERAIDFSPYLEPLGAPQAPAPATATDTFEAAPPAPAPAPASSGQHHDFLSDLFSDDYGGKNCKKPAEYGYVSLGRLGAAKGALHPGCFAPLHPPPPPPPPPAELKAEPGFEPADCKRKEEAGAPGGGAGMAAGFPYALRAYLGYQAVPSGSSGSLSTSSSSSPPGTPSPADAKAPPTACYAGAAPAPSQVKSKAKKTVDKHSDEYKIRRERNNIAVRKSRDKAKMRNLETQHKVLELTAENERLQKKVEQLSRELSTLRNLFKQLPEPLLASSGHC

>Q96GX9

MSGCDAREGDCCSRRCGAQDKEHPRYLIPELCKQFYHLGWVTGTGGGISLKHGDEIYIAPSGVQKERIQPEDMFVCDINEKDISGPSPSKKLKKSQCTPLFMNAYTMRGAGAVIHTHSKAAVMATLLFPGREFKITHQEMIKGIKKCTSGGYYRYDDMLVVPIIENTPEEKDLKDRMAHAMNEYPDSCAVLVRRHGVYVWGETWEKAKTMCECYDYLFDIAVSMKKVGLDPSQLPVGENGIV

162 NP_000277.1 NP_003621.1

>NP_000277.1

MAEHGAHFTAASVADDQPSIFEVVAQDSLMTAVRPALQHVVKVLAESNPTHYGFLWRWFDEIFTLLDLLLQQHYLSRTSASFSENFYGLKRIVMGDTHKSQRLASAGLPKQQLWKSIMFLVLLPYLKVKLEKLVSSLREEDEYSIHPPSSRWKRFYRAFLAAYPFVNMAWEGWFLVQQLRYILGKAQHHSPLLRLAGVQLGRLTVQDIQALEHKPAKASMMQQPARSVSEKINSALKKAVGGVALSLSTGLSVGVFFLQFLDWWYSSENQETIKSLTALPTPPPPVHLDYNSDSPLLPKMKTVCPLCRKTRVNDTVLATSGYVFCYRCVFHYVRSHQACPITGYPTEVQHLIKLYSPEN

>NP_003621.1

MLRSVWNFLKRHKKKCIFLGTVLGGVYILGKYGQKKIREIQEREAAEYIAQARRQYHFESNQRTCNMTVLSMLPTLREALMQQLNSESLTALLKNRPSNKLEIWEDLKIISFTRSTVAVYSTCMLVVLLRVQLNIIGGYIYLDNAAVGKNGTTILAPPDVQQQYLSSIQHLLGDGLTELITVIKQAVQKVLGSVSLKHSLSLLDLEQKLKEIRNLVEQHKSSSWINKDGSKPLLCHYMMPDEETPLAVQACGLSPRDITTIKLLNETRDMLESPDFSTVLNTCLNRGFSRLLDNMAEFFRPTEQDLQHGNSMNSLSSVSLPLAKIIPIVNGQIHSVCSETPSHFVQDLLTMEQVKDFAANVYEAFSTPQQLEK

163 Q9H0W9 P09001

>Q9H0W9

MACAEFSFHVPSLEELAGVMQKGLKDNFADVQVSVVDCPDLTKEPFTFPVKGICGKTRIAEVGGVPYLLPLVNQKKVYDLNKIAKEIKLPGAFILGAGAGPFQTLGFNSEFMPVIQTESEHKPPVNGSYFAHVNPADGGCLLEKYSEKCHDFQCALLANLFASEGQPGKVIEVKAKRRTGPLNFVTCMRETLEKHYGNKPIGMGGTFIIQKGKVKSHIMPAEFSSCPLNSDEEVNKWLHFYEMKAPLVCLPVFVSRDPGFDLRLEHTHFFSRHGEGGHYHYDTTPDIVEYLGYFLPAEFLYRIDQPKETHSIGRD

>P09001

MPGWRLLTQVGAQVLGRLGDGLGAALGPGNRTHIWLFVRGLHGKSGTWWDEHLSEENVPFIKQLVSDEDKAQLASKLCPLKDEPWPIHPWEPGSFRVGLIALKLGMMPLWTKDGQKHVVTLLQVQDCHVLKYTSKENCNGKMATLSVGGKTVSRFRKATSILEFYRELGLPPKQTVKIFNITDNAAIKPGTPLYAAHFRPGQYVDVTAKTIGKGFQGVMKRWGFKGQPATHGQTKTHRRPGAVATGDIGRVWPGTKMPGKMGNIYRTEYGLKVWRINTKHNIIYVNGSVPGHKNCLVKVKDSKLPAYKDLGKNLPFPTYFPDGDEEELPEDLYDENVCQPGAPSITFA

164 NP_004355.2 NP_000846.1

>NP_004355.2

MESADFYEAEPRPPMSSHLQSPPHAPSSAAFGFPRGAGPAQPPAPPAAPEPLGGICEHETSIDISAYIDPAAFNDEFLADLFQHSRQQEKAKAAVGPTGGGGGGDFDYPGAPAGPGGAVMPGGAHGPPPGYGCAAAGYLDGRLEPLYERVGAPALRPLVIKQEPREEDEAKQLALAGLFPYQPPPPPPPSHPHPHPPPAHLAAPHLQFQIAHCGQTTMHLQPGHPTPPPTPVPSPHPAPALGAAGLPGPGSALKGLGAAHPDLRASGGSGAGKAKKSVDKNSNEYRVRRERNNIAVRKSRDKAKQRNVETQQKVLELTSDNDRLRKRVEQLSRELDTLRGIFRQLPESSLVKAMGNCA

>NP_000846.1

MSRRKISSESFSSLGSDYLETSPEEEGECPLSRLCWNGSRSPPGPLEPSPAAAAAAAAPAPTPAASAAAAAATAGARRVQRRRRVNLDSLGESISRLTAPSPQTIQQTLKRTLQYYEHQVIGYRDAEKNFHNISNRCSYADHSNKEEIEDVSGILQCTANILGLKFEEIQKRFGEEFFNICFHENERVLRAVGGTLQDFFNGFDALLEHIRTSFGKQATLESPSFLCKELPEGTLMLHYFHPHHIVGFAMLGMIKAAGKKIYRLDVEVEQVANEKLCSDVSNPGNCSCLTFLIKECENTNIMKNLPQGTSQVPADLRISINTFCRAFPFHLMFDPSMSVLQLGEGLRKQLRCDTHKVLKFEDCFEIVSPKVNATFERVLLRLSTPFVIRTKPEASGSENKDKVMEVKGQMIHVPESNSILFLGSPCVDKLDELMGRGLHLSDIPIHDATRDVILVGEQAKAQDGLKKRMDKLKATLERTHQALEEEKKKTVDLLYSIFPGDVAQQLWQGQQVQARKFDDVTMLFSDIVGFTAICAQCTPMQVISMLNELYTRFDHQCGFLDIYKVETIGDAYCVAAGLHRKSLCHAKPIALMALKMMELSEEVLTPDGRPIQMRIGIHSGSVLAGVVGVRMPRYCLFGNNVTLASKFESGSHPRRINVSPTTYQLLKREESFTFIPRSREELPDNFPKEIPGICYFLEVRTGPKPPKPSLSSSRIKKVSYNIGTMFLRETSL

165 NP_003588.1 NP_003215.1

>NP_003588.1

MHTPDFAGPDDARAVDIMDICESILERKRHDSERSTCSILEQTDMEAVEALVCMSSWGQRSQKGDLLRIRPLTPVSDSGDVTTTVHMDAATPELPKDFHSLSTLCITPPQSPDLVEPSTRTPVSPQVTDSKACTATDVLQSSAVVARALSGGAERGLLGLEPVPSSPCRAKGTSVIRHTGESPAACFPTIQTPDCRLSDSREGEEQLLGHFETLQDTHLTDSLLSTNLVSCQPCLHKSGGLLLTDKGQQAGWPGAVQTCSPKNYENDLPRKTTPLISVSVPAPPVLCQMIPVTGQSSMLPAFLKPPPQLSVGTVRPILAQAAPAPQPVFVGPAVPQGAVMLVLPQGALPPPAPCAANVMAAGNTKLLPLAPAPVFITSSQNCVPQVDFSRRRNYVCSFPGCRKTYFKSSHLKAHLRTHTGEKPFNCSWDGCDKKFARSDELSRHRRTHTGEKKFVCPVCDRRFMRSDHLTKHARRHMTTKKIPGWQAEVGKLNRIASAESPGSPLVSMPASA

>NP_003215.1

MYTLLSGLYKYMFQKDEYCILILGLDNAGKTTFLEQSKTRFNKNYKGMSLSKITTTVGLNIGTVDVGKARLMFWDLGGQEELQSLWDKYYAECHGVIYVIDSTDEERLAESKQAFEKVVTSEALCGVPVLVLANKQDVETCLSIPDIKTAFSDCTSKIGRRDCLTQACSALTGKGVREGIEWMVKCVVRNVHRPPRQRDIT

166 Q0VG06 P48163

>Q0VG06

MAGAAPRVRYLAGFCCPLGGLAAGKPRVLCHEAEVFLSTGSELVYVYDQEGGLLTAAFRFPDQVWHLELLAPRRLLYALCARRGLYCLSLDHPGRSRSTSQDDRDSEDGDQPSPVIPVDPDACILPDAALCAFTLLDSVLVTLVQGPARWKMQLFEQPCPGEDPRPGGQIGEVELSSYTPPAGVPGKPAAPHFLPVLCSVSPSGSRVPHDLLGGSGGFTLEDALFGLLFGADATLLQSPVVLCGLPDGQLCCVILKALVTSRSAPGDPNALVKILHHLEEPVIFIGALKTEPQAAEAAENFLPDEDVHCDCLVAFGHHGRMLAIKASWDESGKLVPELREYCLPGPVLCAACGGGGRVYHSTPSDLCVVDLSRGSTPLGPEQPEEGPGGLPPMLCPASLNICSVVSLSASPRTHEGGTKLLALSAKGRLMTCSLDLDSEMPGPARMTTESAGQKIKELLSGIGNISERVSFLKKAVDQRNKALTSLNEAMNVSCALLSSGTGPRPISCTTSTTWSRLQTQDVLMATCVLENSSSFSLDQGWTLCIQVLTSSCALDLDSACSAITYTIPVDQLGPGARREVTLPLGPGENGGLDLPVTVSCTLFYSLREVVGGALAPSDSEDPFLDECPSDVLPEQEGVCLPLSRHTVDMLQCLRFPGLALPHTRAPSPLGPTRDPVATFLETCREPGSQPAGPASLRAEYLPPSVASIKVSAELLRAALKDGHSGVPLCCATLQWLLAENAAVDVVRARALSSIQGVAPDGANVHLIVREVAMTDLCPAGPIQAVEIQVESSSLADICRAHHAVVGRMQTMVTEQAAQGSSAPDLRVQYLRQIHANHETLLREVQTLRDRLCTEDEASSCATAQRLLQVYRQLRHPSLILL

>P48163

MEPEAPRRRHTHQRGYLLTRNPHLNKDLAFTLEERQQLNIHGLLPPSFNSQEIQVLRVVKNFEHLNSDFDRYLLLMDLQDRNEKLFYRVLTSDIEKFMPIVYTPTVGLACQQYSLVFRKPRGLFITIHDRGHIASVLNAWPEDVIKAIVVTDGERILGLGDLGCNGMGIPVGKLALYTACGGMNPQECLPVILDVGTENEELLKDPLYIGLRQRRVRGSEYDDFLDEFMEAVSSKYGMNCLIQFEDFANVNAFRLLNKYRNQYCTFNDDIQGTASVAVAGLLAALRITKNKLSDQTILFQGAGEAALGIAHLIVMALEKEGLPKEKAIKKIWLVDSKGLIVKGRASLTQEKEKFAHEHEEMKNLEAIVQEIKPTALIGVAAIGGAFSEQILKDMAAFNERPIIFALSNPTSKAECSAEQCYKITKGRAIFASGSPFDPVTLPNGQTLYPGQGNNSYVFPGVALGVVACGLRQITDNIFLTTAEVIAQQVSDKHLEEGRLYPPLNTIRDVSLKIAEKIVKDAYQEKTATVYPEPQNKEAFVRSQMYSTDYDQILPDCYSWPEEVQKIQTKVDQ

167 NP_002496.1 NP_004790.2

>NP_002496.1

MEQYTANSNSSTEQIVVQAGQIQQQQQGGVTAVQLQTEAQVASASGQQVQTLQVVQGQPLMVQVSGGQLITSTGQPIMVQAVPGGQGQTIMQVPVSGTQGLQQIQLVPPGQIQIQGGQAVQVQGQQGQTQQIIIQQPQTAVTAGQTQTQQQIAVQGQQVAQTAEGQTIVYQPVNADGTILQQVTVPVSGMITIPAASLAGAQIVQTGANTNTTSSGQGTVTVTLPVAGNVVNSGGMVMMVPGAGSVPAIQRIPLPGAEMLEEEPLYVNAKQYHRILKRRQARAKLEAEGKIPKERRKYLHESRHRHAMARKRGEGGRFFSPKEKDSPHMQDPNQADEEAMTQIIRVS

>NP_004790.2

MENYFQAEAYNLDKVLDEFEQNEDETVSSTLLDTKWNKILDPPSHRLSFNPTLASVNESAVSNESQPQLKVFSLAHSAPLTTEEEDHCANGQDCNLNPEIATMWIDENAVAEDQLIKRNYSWDDQCSAVEVGEKKCGNLACLPDEKNVLVVAVMHNCDKRTLQNDLQDCNNYNSQSLMDAFSCSLDNENRQTDQFSFSINESTEKDMNSEKQMDPLNRPKTEGRSVNHLCPTSSDSLASVCSPSQLKDDGSIGRDPSMSAITSLTVDSVISSQGTDGCPAVKKQENYIPDEDLTGKISSPRTDLGSPNSFSHMSEGILMKKEPAEESTTEESLRSGLPLLLKPDMPNGSGRNNDCERCSDCLVPNEVRADENEGYEHEETLGTTEFLNMTEHFSESQDMTNWKLTKLNEMNDSQVNEEKEKFLQISQPEDTNGDSGGQCVGLADAGLDLKGTCISESEECDFSTVIDTPAANYLSNGCDSYGMQDPGVSFVPKTLPSKEDSVTEEKEIEESKSECYSNIYEQRGNEATEGSGLLLNSTGDLMKKNYLHNFCSQVPSVLGQSSPKVVASLPSISVPFGGARPKQPSNLKLQIPKPLSDHLQNDFPANSGNNTKNKNDILGKAKLGENSATNVCSPSLGNISNVDTNGEHLESYEAEISTRPCLALAPDSPDNDLRAGQFGISARKPFTTLGEVAPVWVPDSQAPNCMKCEARFTFTKRRHHCRACGKVFCASCCSLKCKLLYMDRKEARVCVICHSVLMNAQAWENMMSASSQSPNPNNPAEYCSTIPPLQQAQASGALSSPPPTVMVPVGVLKHPGAEVAQPREQRRVWFADGILPNGEVADAAKLTMNGTSSAGTLAVSHDPVKPVTTSPLPAETDICLFSGSITQVGSPVGSAMNLIPEDGLPPILISTGVKGDYAVEEKPSQISVMQQLEDGGPDPLVFVLNANLLSMVKIVNYVNRKCWCFTTKGMHAVGQSEIVILLQCLPDEKCLPKDIFNHFVQLYRDALAGNVVSNLGHSFFSQSFLGSKEHGGFLYVTSTYQSLQDLVLPTPPYLFGILIQKWETPWAKVFPIRLMLRLGAEYRLYPCPLFSVRFRKPLFGETGHTIMNLLADFRNYQYTLPVVQGLVVDMEVRKTSIKIPSNRYNEMMKAMNKSNEHVLAGGACFNEKADSHLVCVQNDDGNYQTQAISIHNQPRKVTGASFFVFSGALKSSSGYLAKSSIVEDGVMVQITAENMDSLRQALREMKDFTITCGKADAEEPQEHIHIQWVDDDKNVSKGVVSPIDGKSMETITNVKIFHGSEYKANGKVIRWTEVFFLENDDQHNCLSDPADHSRLTEHVAKAFCLALCPHLKLLKEDGMTKLGLRVTLDSDQVGYQAGSNGQPLPSQYMNDLDSALVPVIHGGACQLSEGPVVMELIFYILENIV

168 P08048 NP_006635.2

>P08048

MDEDEFELQPQEPNSFFDGIGADATHMDGDQIVVEIQEAVFVSNIVDSDITVHNFVPDDPDSVVIQDVVEDVVIEEDVQCSDILEEADVSENVIIPEQVLDSDVTEEVSLPHCTVPDDVLASDITSTSMSMPEHVLTSESMHVCDIGHVEHMVHDSVVEAEIITDPLTSDIVSEEVLVADCAPEAVIDASGISVDQQDNDKASCEDYLMISLDDAGKIEHDGSTGVTIDAESEMDPCKVDSTCPEVIKVYIFKADPGEDDLGGTVDIVESEPENDHGVELLDQNSSIRVPREKMVYMTVNDSQQEDEDLNVAEIADEVYMEVIVGEEDAAVAAAAAAVHEQQIDEDEMKTFVPIAWAAAYGNNSDGIENRNGTASALLHIDESAGLGRLAKQKPKKKRRPDSRQYQTAIIIGPDGHPLTVYPCMICGKKFKSRGFLKRHMKNHPEHLAKKKYHCTDCDYTTNKKISLHNHLESHKLTSKAEKAIECDECGKHFSHAGALFTHKMVHKEKGANKMHKCKFCEYETAEQGLLNRHLLAVHSKNFPHICVECGKGFRHPSELRKHMRIHTGEKPYQCQYCEYRSADSSNLKTHIKTKHSKEMPFKCDICLLTFSDTKEVQQHTLVHQESKTHQCLHCDHKSSNSSDLKRHVISVHTKDYPHKCEMCEKGFHRPSELKKHVAVHKGKKMHQCRHCDFKIADPFVLSRHILSVHTKDLPFRCKRCRKGFRQQNELKKHMKTHSGRKVYQCEYCEYSTTDASGFKRHVISIHTKDYPHRCEYCKKGFRRPSEKNQHIMRHHKEVGLP

>NP_006635.2

MSVVGLDVGSQSCYIAVARAGGIETIANEFSDRCTPSVISFGSKNRTIGVAAKNQQITHANNTVSNFKRFHGRAFNDPFIQKEKENLSYDLVPLKNGGVGIKVMYMGEEHLFSVEQITAMLLTKLKETAENSLKKPVTDCVISVPSFFTDAERRSVLDAAQIVGLNCLRLMNDMTAVALNYGIYKQDLPSLDEKPRIVVFVDMGHSAFQVSACAFNKGKLKVLGTAFDPFLGGKNFDEKLVEHFCAEFKTKYKLDAKSKIRALLRLYQECEKLKKLMSSNSTDLPLNIECFMNDKDVSGKMNRSQFEELCAELLQKIEVPLYSLLEQTHLKVEDVSAVEIVGGATRIPAVKERIAKFFGKDISTTLNADEAVARGCALQCAILSPAFKVREFSVTDAVPFPISLIWNHDSEDTEGVHEVFSRNHAAPFSKVLTFLRRGPFELEAFYSDPQGVPYPEAKIGRFVVQNVSAQKDGEKSRVKVKVRVNTHGIFTISTASMVEKVPTEENEMSSEADMECLNQRPPENPDTDKNVQQDNSEAGTQPQVQTDAQQTSQSPPSPELTSEENKIPDADKANEKKVDQPPEAKKPKIKVVNVELPIEANLVWQLGKDLLNMYIETEGKMIMQDKLEKERNDAKNAVEEYVYEFRDKLCGPYEKFICEQDHQNFLRLLTETEDWLYEEGEDQAKQAYVDKLEELMKIGTPVKVRFQEAEERPKMFEELGQRLQHYAKIAADFRNKDEKYNHIDESEMKKVEKSVNEVMEWMNNVMNAQAKKSLDQDPVVRAQEIKTKIKELNNTCEPVVTQPKPKIESPKLERTPNGPNIDKKEEDLEDKNNFGAEPPHQNGECYPNEKNSVNMDLD

169 NP_000241.1 NP_071445.1

>NP_000241.1

MGVPFFSSLRCMVDLGPCWAGGLTAEMKLLLALAGLLAILATPQPSEGAAPAVLGEVDTSLVLSSMEEAKQLVDKAYKERRESIKQRLRSGSASPMELLSYFKQPVAATRTAVRAADYLHVALDLLERKLRSLWRRPFNVTDVLTPAQLNVLSKSSGCAYQDVGVTCPEQDKYRTITGMCNNRRSPTLGASNRAFVRWLPAEYEDGFSLPYGWTPGVKRNGFPVALARAVSNEIVRFPTDQLTPDQERSLMFMQWGQLLDHDLDFTPEPAARASFVTGVNCETSCVQQPPCFPLKIPPNDPRIKNQADCIPFFRSCPACPGSNITIRNQINALTSFVDASMVYGSEEPLARNLRNMSNQLGLLAVNQRFQDNGRALLPFDNLHDDPCLLTNRSARIPCFLAGDTRSSEMPELTSMHTLLLREHNRLATELKSLNPRWDGERLYQEARKIVGAMVQIITYRDYLPLVLGPTAMRKYLPTYRSYNDSVDPRIANVFTNAFRYGHTLIQPFMFRLDNRYQPMEPNPRVPLSRVFFASWRVVLEGGIDPILRGLMATPAKLNRQNQIAVDEIRERLFEQVMRIGLDLPALNMQRSRDHGLPGYNAWRRFCGLPQPETVGQLGTVLRNLKLARKLMEQYGTPNNIDIWMGGVSEPLKRKGRVGPLLACIIGTQFRKLRDGDRFWWENEGVFSMQQRQALAQISLPRIICDNTGITTVSKNNIFMSNSYPRDFVNCSTLPALNLASWREAS

>NP_071445.1

MGEEGGSASHDEEERASVLLGHSPGCEMCSQEAFQAQRSQLVELLVSGSLEGFESVLDWLLSWEVLSWEDYEGFHLLGQPLSHLARRLLDTVWNKGTWACQKLIAAAQEAQADSQSPKLHGCWDPHSLHPARDLQSHRPAIVRRLHSHVENMLDLAWERGFVSQYECDEIRLPIFTPSQRARRLLDLATVKANGLAAFLLQHVQELPVPLALPLEAATCKKYMAKLRTTVSAQSRFLSTYDGAETLCLEDIYTENVLEVWADVGMAGPPQKSPATLGLEELFSTPGHLNDDADTVLVVGEAGSGKSTLLQRLHLLWAAGQDFQEFLFVFPFSCRQLQCMAKPLSVRTLLFEHCCWPDVGQEDIFQLLLDHPDRVLLTFDGFDEFKFRFTDRERHCSPTDPTSVQTLLFNLLQGNLLKNARKVVTSRPAAVSAFLRKYIRTEFNLKGFSEQGIELYLRKRHHEPGVADRLIRLLQETSALHGLCHLPVFSWMVSKCHQELLLQEGGSPKTTTDMYLLILQHFLLHATPPDSASQGLGPSLLRGRLPTLLHLGRLALWGLGMCCYVFSAQQLQAAQVSPDDISLGFLVRAKGVVPGSTAPLEFLHITFQCFFAAFYLALSADVPPALLRHLFNCGRPGNSPMARLLPTMCIQASEGKDSSVAALLQKAEPHNLQITAAFLAGLLSREHWGLLAECQTSEKALLRRQACARWCLARSLRKHFHSIPPAAPGEAKSVHAMPGFIWLIRSLYEMQEERLARKAARGLNVGHLKLTFCSVGPTECAALAFVLQHLRRPVALQLDYNSVGDIGVEQLLPCLGVCKALYLRDNNISDRGICKLIECALHCEQLQKLALFNNKLTDGCAHSMAKLLACRQNFLALRLGNNYITAAGAQVLAEGLRGNTSLQFLGFWGNRVGDEGAQALAEALGDHQSLRWLSLVGNNIGSVGAQALALMLAKNVMLEELCLEENHLQDEGVCSLAEGLKKNSSLKILKLSNNCITYLGAEALLQALERNDTILEVWLRGNTFSLEEVDKLGCRDTRLLL

170 NP_055206.2 NP_060441.2

>NP_055206.2

MMVLKVEELVTGKKNGNGEAGEFLPEDFRDGEYEAAVTLEKQEDLKTLLAHPVTLGEQQWKSEKQREAELKKKKLEQRSKLENLEDLEIIIQLKKRKKYRKTKVPVVKEPEPEIITEPVDVPTFLKAALENKLPVVEKFLSDKNNPDVCDEYKRTALHRACLEGHLAIVEKLMEAGAQIEFRDMLESTAIHWASRGGNLDVLKLLLNKGAKISARDKLLSTALHVAVRTGHYECAEHLIACEADLNAKDREGDTPLHDAVRLNRYKMIRLLIMYGADLNIKNCAGKTPMDLVLHWQNGTKAIFDSLRENSYKTSRIATF

>NP_060441.2

MVFLTAQLWLRNRVTDRYFRIQEVLKHARHFRGRKNRCYRLAVRTVIRAFVKCTKARYLKKKNMRTLWINRITAASQEHGLKYPALIGNLVKCQVELNRKVLADLAIYEPKTFKSLAALASRRRHEGFAAALGDGKEPEGIFSRVVQYH

171 Q9NZI7 P40261

>Q9NZI7

MAWVLKMDEVIESGLVHDFDASLSGIGQELGAGAYSMSDVLALPIFKQEDSSLPLDGETEHPPFQYVMCAATSPAVKLHDETLTYLNQGQSYEIRMLDNRKMGDMPEINGKLVKSIIRVVFHDRRLQYTEHQQLEGWKWNRPGDRLLDLDIPMSVGIIDTRTNPSQLNAVEFLWDPAKRTSAFIQVHCISTEFTPRKHGGEKGVPFRIQVDTFKQNENGEYTDHLHSASCQIKVFKPKGADRKQKTDREKMEKRTAHEKEKYQPSYDTTILTEMRLEPIIEDAVEHEQKKSSKRTLPADYGDSLAKRGSCSPWPDAPTAYVNNSPSPAPTFTSPQQSTCSVPDSNSSSPNHQGDGASQTSGEQIQPSATIQETQQWLLKNRFSSYTRLFSNFSGADLLKLTKEDLVQICGAADGIRLYNSLKSRSVRPRLTIYVCREQPSSTVLQGQQQAASSASENGSGAPYVYHAIYLEEMIASEVARKLALVFNIPLHQINQVYRQGPTGIHILVSDQMVQNFQDESCFLFSTVKAESSDGIHIILK

>P40261

MESGFTSKDTYLSHFNPRDYLEKYYKFGSRHSAESQILKHLLKNLFKIFCLDGVKGDLLIDIGSGPTIYQLLSACESFKEIVVTDYSDQNLQELEKWLKKEPEAFDWSPVVTYVCDLEGNRVKGPEKEEKLRQAVKQVLKCDVTQSQPLGAVPLPPADCVLSTLCLDAACPDLPTYCRALRNLGSLLKPGGFLVIMDALKSSYYMIGEQKFSSLPLGREAVEAAVKEAGYTIEWFEVISQSYSSTMANNEGLFSLVARKLSRPL

172 Q04727 NP_659491.4

>Q04727

MIRDLSKMYPQTRHPAPHQPAQPFKFTISESCDRIKEEFQFLQAQYHSLKLECEKLASEKTEMQRHYVMYYEMSYGLNIEMHKQAEIVKRLNAICAQVIPFLSQEHQQQVVQAVERAKQVTMAELNAIIGQQLQAQHLSHGHGLPVPLTPHPSGLQPPAIPPIGSSAGLLALSSALGGQSHLPIKDEKKHHDNDHQRDRDSIKSSSVSPSASFRGAEKHRNSADYSSESKKQKTEEKEIAARYDSDGEKSDDNLVVDVSNEDPSSPRGSPAHSPRENGLDKTRLLKKDAPISPASIASSSSTPSSKSKELSLNEKSTTPVSKSNTPTPRTDAPTPGSNSTPGLRPVPGKPPGVDPLASSLRTPMAVPCPYPTPFGIVPHAGMNGELTSPGAAYAGLHNISPQMSAAAAAAAAAAAYGRSPVVGFDPHHHMRVPAIPPNLTGIPGGKPAYSFHVSADGQMQPVPFPPDALIGPGIPRHARQINTLNHGEVVCAVTISNPTRHVYTGGKGCVKVWDISHPGNKSPVSQLDCLNRDNYIRSCRLLPDGRTLIVGGEASTLSIWDLAAPTPRIKAELTSSAPACYALAISPDSKVCFSCCSDGNIAVWDLHNQTLVRQFQGHTDGASCIDISNDGTKLWTGGLDNTVRSWDLREGRQLQQHDFTSQIFSLGYCPTGEWLAVGMENSNVEVLHVTKPDKYQLHLHESCVLSLKFAHCGKWFVSTGKDNLLNAWRTPYGASIFQSKESSSVLSCDISVDDKYIVTGSGDKKATVYEVIY

>NP_659491.4

MDNKISPEAQVAELELDAVIGFNGHVPTGLKCHPDQEHMIYPLGCTVLIQAINTKEQNFLQGHGNNVSCLAISRSGEYIASGQVTFMGFKADIILWDYKNRELLARLSLHKGKIEALAFSPNDLYLVSLGGPDDGSVVVWSIAKRDAICGSPAAGLNVGNATNVIFSRCRDEMFMTAGNGTIRVWELDLPNRKIWPTECQTGQLKRIVMSIGVDDDDSFFYLGTTTGDILKMNPRTKLLTDVGPAKDKFSLGVSAIRCLKMGGLLVGSGAGLLVFCKSPGYKPIKKIQLQGGITSITLRGEGHQFLVGTEESHIYRVSFTDFKETLIATCHFDAVEDIVFPFGTAELFATCAKKDIRVWHTSSNRELLRITVPNMTCHGIDFMRDGKSIISAWNDGKIRAFAPETGRLMYVINNAHRIGVTAIATTSDCKRVISGGGEGEVRVWQIGCQTQKLEEALKEHKSSVSCIRVKRNNEECVTASTDGTCIIWDLVRLRRNQMILANTLFQCVCYHPEEFQIITSGTDRKIAYWEVFDGTVIRELEGSLSGSINGMDITQEGVHFVTGGNDHLVKVWDYNEGEVTHVGVGHSGNITRIRISPGNQYIVSVSADGAILRWKYPYTS

173 NP_006089.1 Q08493

>NP_006089.1

MTEQMTLRGTLKGHNGWVTQIATTPQFPDMILSASRDKTIIMWKLTRDETNYGIPQRALRGHSHFVSDVVISSDGQFALSGSWDGTLRLWDLTTGTTTRRFVGHTKDVLSVAFSSDNRQIVSGSRDKTIKLWNTLGVCKYTVQDESHSEWVSCVRFSPNSSNPIIVSCGWDKLVKVWNLANCKLKTNHIGHTGYLNTVTVSPDGSLCASGGKDGQAMLWDLNEGKHLYTLDGGDIINALCFSPNRYWLCAATGPSIKIWDLEGKIIVDELKQEVISTSSKAEPPQCTSLAWSADGQTLFAGYTDNLVRVWQVTIGTR

>Q08493

MENLGVGEGAEACSRLSRSRGRHSMTRAPKHLWRQPRRPIRIQQRFYSDPDKSAGCRERDLSPRPELRKSRLSWPVSSCRRFDLENGLSCGRRALDPQSSPGLGRIMQAPVPHSQRRESFLYRSDSDYELSPKAMSRNSSVASDLHGEDMIVTPFAQVLASLRTVRSNVAALARQQCLGAAKQGPVGNPSSSNQLPPAEDTGQKLALETLDELDWCLDQLETLQTRHSVGEMASNKFKRILNRELTHLSETSRSGNQVSEYISRTFLDQQTEVELPKVTAEEAPQPMSRISGLHGLCHSASLSSATVPRFGVQTDQEEQLAKELEDTNKWGLDVFKVAELSGNRPLTAIIFSIFQERDLLKTFQIPADTLATYLLMLEGHYHANVAYHNSLHAADVAQSTHVLLATPALEAVFTDLEILAALFASAIHDVDHPGVSNQFLINTNSELALMYNDASVLENHHLAVGFKLLQAENCDIFQNLSAKQRLSLRRMVIDMVLATDMSKHMNLLADLKTMVETKKVTSLGVLLLDNYSDRIQVLQNLVHCADLSNPTKPLPLYRQWTDRIMAEFFQQGDRERESGLDISPMCDKHTASVEKSQVGFIDYIAHPLWETWADLVHPDAQDLLDTLEDNREWYQSKIPRSPSDLTNPERDGPDRFQFELTLEEAEEEDEEEEEEGEETALAKEALELPDTELLSPEAGPDPGDLPLDNQRT

174 P17010 NP_061833.1

>P17010

MDEDGLELQQEPNSFFDATGADGTHMDGDQIVVEVQETVFVSDVVDSDITVHNFVPDDPDSVVIQDVIEDVVIEDVQCPDIMEEADVSETVIIPEQVLDSDVTEEVSLAHCTVPDDVLASDITSASMSMPEHVLTGDSIHVSDVGHVGHVGHVEHVVHDSVVEAEIVTDPLTTDVVSEEVLVADCASEAVIDANGIPVDQQDDDKGNCEDYLMISLDDAGKIEHDGSSGMTMDTESEIDPCKVDGTCPEVIKVYIFKADPGEDDLGGTVDIVESEPENDHGVELLDQNSSIRVPREKMVYMTVNDSQPEDEDLNVAEIADEVYMEVIVGEEDAAAAAAAAAVHEQQMDDNEIKTFMPIAWAAAYGNNSDGIENRNGTASALLHIDESAGLGRLAKQKPKKRRRPDSRQYQTAIIIGPDGHPLTVYPCMICGKKFKSRGFLKRHMKNHPEHLAKKKYRCTDCDYTTNKKISLHNHLESHKLTSKAEKAIECDECGKHFSHAGALFTHKMVHKEKGANKMHKCKFCEYETAEQGLLNRHLLAVHSKNFPHICVECGKGFRHPSELKKHMRIHTGEKPYQCQYCEYRSADSSNLKTHVKTKHSKEMPFKCDICLLTFSDTKEVQQHALIHQESKTHQCLHCDHKSSNSSDLKRHIISVHTKDYPHKCDMCDKGFHRPSELKKHVAAHKGKKMHQCRHCDFKIADPFVLSRHILSVHTKDLPFRCKRCRKGFRQQSELKKHMKTHSGRKVYQCEYCEYSTTDASGFKRHVISIHTKDYPHRCEYCKKGFRRPSEKNQHIMRHHKEVGLP

>NP_061833.1

MVDSVYRTRSLGVAAEGLPDQYADGEAARVWQLYIGDTRSRTAEYKAWLLGLLRQHGCQRVLDVACGTGVDSIMLVEEGFSVTSVDASDKMLKYALKERWNRRHEPAFDKWVIEEANWMTLDKDVPQSAEGGFDAVICLGNSFAHLPDCKGDQSEHRLALKNIASMVRAGGLLVIDHRNYDHILSTGCAPPGKNIYYKSDLTKDVTTSVLIVNNKAHMVTLDYTVQVPGAGQDGSPGLSKFRLSYYPHCLASFTELLQAAFGGKCQHSVLGDFKPYKPGQTYIPCYFIHVLKRTD

175 Q6PL18 Q8N573

>Q6PL18

MVVLRSSLELHNHSAASATGSLDLSSDFLSLEHIGRRRLRSAGAAQKKPAATTAKAGDGSSVKEVETYHRTRALRSLRKDAQNSSDSSFEKNVEITEQLANGRHFTRQLARQQADKKKEEHREDKVIPVTRSLRARNIVQSTEHLHEDNGDVEVRRSCRIRSRYSGVNQSMLFDKLITNTAEAVLQKMDDMKKMRRQRMRELEDLGVFNETEESNLNMYTRGKQKDIQRTDEETTDNQEGSVESSEEGEDQEHEDDGEDEDDEDDDDDDDDDDDDDDEDDEDEEDGEEENQKRYYLRQRKATVYYQAPLEKPRHQRKPNIFYSGPASPARPRYRLSSAGPRSPYCKRMNRRRHAIHSSDSTSSSSSEDEQHFERRRKRSRNRAINRCLPLNFRKDELKGIYKDRMKIGASLADVDPMQLDSSVRFDSVGGLSNHIAALKEMVVFPLLYPEVFEKFKIQPPRGCLFYGPPGTGKTLVARALANECSQGDKRVAFFMRKGADCLSKWVGESERQLRLLFDQAYQMRPSIIFFDEIDGLAPVRSSRQDQIHSSIVSTLLALMDGLDSRGEIVVIGATNRLDSIDPALRRPGRFDREFLFSLPDKEARKEILKIHTRDWNPKPLDTFLEELAENCVGYCGADIKSICAEAALCALRRRYPQIYTTSEKLQLDLSSINISAKDFEVAMQKMIPASQRAVTSPGQALSTVVKPLLQNTVDKILEALQRVFPHAEFRTNKTLDSDISCPLLESDLAYSDDDVPSVYENGLSQKSSHKAKDNFNFLHLNRNACYQPMSFRPRILIVGEPGFGQGSHLAPAVIHALEKFTVYTLDIPVLFGVSTTSPEETCAQVIREAKRTAPSIVYVPHIHVWWEIVGPTLKATFTTLLQNIPSFAPVLLLATSDKPHSALPEEVQELFIRDYGEIFNVQLPDKEERTKFFEDLILKQAAKPPISKKKAVLQALEVLPVAPPPEPRSLTAEEVKRLEEQEEDTFRELRIFLRNVTHRLAIDKRFRVFTKPVDPDEVPDYVTVIKQPMDLSSVISKIDLHKYLTVKDYLRDIDLICSNALEYNPDRDPGDRLIRHRACALRDTAYAIIKEELDEDFEQLCEEIQESRKKRGCSSSKYAPSYYHVMPKQNSTLVGDKRSDPEQNEKLKTPSTPVACSTPAQLKRKIRKKSNWYLGTIKKRRKISQAKDDSQNAIDHKIESDTEETQDTSVDHNETGNTGESSVEENEKQQNASESKLELRNNSNTCNIENELEDSRKTTACTELRDKIACNGDASSSQIIHISDENEGKEMCVLRMTRARRSQVEQQQLITVEKALAILSQPTPSLVVDHERLKNLLKTVVKKSQNYNIFQLENLYAVISQCIYRHRKDHDKTSLIQKMEQEVENFSCSR

>Q8N573

MSFQKPKGTIEYTVESRDSLNSIALKFDTTPNELVQLNKLFSRAVVTGQVLYVPDPEYVSSVESSPSLSPVSPLSPTSSEAEFDKTTNPDVHPTEATPSSTFTGIRPARVVSSTSEEEEAFTEKFLKINCKYITSGKGTVSGVLLVTPNNIMFDPHKNDPLVQENGCEEYGIMCPMEEVMSAAMYKEILDSKIKESLPIDIDQLSGRDFCHSKKMTGSNTEEIDSRIRDAGNDSASTAPRSTEESLSEDVFTESELSPIREELVSSDELRQDKSSGASSESVQTVNQAEVESLTVKSESTGTPGHLRSDTEHSTNEVGTLCHKTDLNNLEMAIKEDQIADNFQGISGPKEDSTSIKGNSDQDSFLHENSLHQEESQKENMPCGETAEFKQKQSVNKGKQGKEQNQDSQTEAEELRKLWKTHTMQQTKQQRENIQQVSQKEAKHKITSADGHIESSALLKEKQRHRLHKFLCLRVGKPMRKTFVSQASATMQQYAQRDKKHEYWFAVPQERTDHLYAFFIQWSPEIYAEDTGEYTREPGFIVVKKIEESETIEDSSNQAAAREWEITTREDINSKQVATVKADLESESFRPNLSDPSELLLPDQIEKLTKHLPPRTIGYPWTLVYGTGKHGTSLKTLYRTMTGLDTPVLMVIKDSDGQVFGALASEPLKVSDGFYGTGETFVFTFCPEFEVFKWTGDNMFFIKGDMDSLAFGGGGGEFALWLDGDLYHGRSHSCKTFGNRTLSKKEDFFIQDIEIWAFE

176 Q92841 NP_003134.1

>Q92841

MRGGGFGDRDRDRDRGGFGARGGGGLPPKKFGNPGERLRKKKWDLSELPKFEKNFYVEHPEVARLTPYEVDELRRKKEITVRGGDVCPKPVFAFHHANFPQYVMDVLMDQHFTEPTPIQCQGFPLALSGRDMVGIAQTGSGKTLAYLLPAIVHINHQPYLERGDGPICLVLAPTRELAQQVQQVADDYGKCSRLKSTCIYGGAPKGPQIRDLERGVEICIATPGRLIDFLESGKTNLRRCTYLVLDEADRMLDMGFEPQIRKIVDQIRPDRQTLMWSATWPKEVRQLAEDFLRDYTQINVGNLELSANHNILQIVDVCMESEKDHKLIQLMEEIMAEKENKTIIFVETKRRCDDLTRRMRRDGWPAMCIHGDKSQPERDWVLNEFRSGKAPILIATDVASRGLDVEDVKFVINYDYPNSSEDYVHRIGRTARSTNKGTAYTFFTPGNLKQARELIKVLEEANQAINPKLMQLVDHRGGGGGGGGRSRYRTTSSANNPNLMYQDECDRRLRGVKDGGRRDSASYRDRSETDRAGYANGSGYGSPNSAFGAQAGQYTYGQGTYGAAAYGTSSYTAQEYGAGTYGASSTTSTGRSSQSSSQQFSGIGRSGQQPQPLMSQQFAQPPGATNMIGYMGQTAYQYPPPPPPPPPSRK

>NP_003134.1

MFRRPVLQVLRQFVRHESETTTSLVLERSLNRVHLLGRVGQDPVLRQVEGKNPVTIFSLATNEMWRSGDSEVYQLGDVSQKTTWHRISVFRPGLRDVAYQYVKKGSRIYLEGKIDYGEYMDKNNVRRQATTIIADNIIFLSDQTKEKE

177 NP_004417.2 Q9UI17

>NP_004417.2

METESEQNSNSTNGSSSSGGSSRPQIAQMSLYERQAVQALQALQRQPNAAQYFHQFMLQQQLSNAQLHSLAAVQQATIAASRQASSPNTSTTQQQTTTTQASINLATTSAAQLISRSQSVSSPSATTLTQSVLLGNTTSPPLNQSQAQMYLRPQLGNLLQVNRTLGRNVPLASQLILMPNGAVAAVQQEVPSAQSPGVHADADQVQNLAVRNQQASAQGPQMQGSTQKAIPPGASPVSSLSQASSQALAVAQASSGATNQSLNLSQAGGGSGNSIPGSMGPGGGGQAHGGLGQLPSSGMGGGSCPRKGTGVVQPLPAAQTVTVSQGSQTEAESAAAKKAEADGSGQQNVGMNLTRTATPAPSQTLISSATYTQIQPHSLIQQQQQIHLQQKQVVIQQQIAIHHQQQFQHRQSQLLHTATHLQLAQQQQQQQQQQQQQQQPQATTLTAPQPPQVPPTQQVPPSQSQQQAQTLVVQPMLQSSPLSLPPDAAPKPPIPIQSKPPVAPIKPPQLGAAKMSAAQQPPPHIPVQVVGTRQPGTAQAQALGLAQLAAAVPTSRGMPGTVQSGQAHLASSPPSSQAPGALQECPPTLAPGMTLAPVQGTAHVVKGGATTSSPVVAQVPAAFYMQSVHLPGKPQTLAVKRKADSEEERDDVSTLGSMLPAKASPVAESPKVMDEKSSLGEKAESVANVNANTPSSELVALTPAPSVPPPTLAMVSRQMGDSKPPQAIVKPQILTHIIEGFVIQEGAEPFPVGCSQLLKESEKPLQTGLPTGLTENQSGGPLGVDSPSAELDKKANLLKCEYCGKYAPAEQFRGSKRFCSMTCAKRYNVSCSHQFRLKRKKMKEFQEANYARVRRRGPRRSSSDIARAKIQGKCHRGQEDSSRGSDNSSYDEALSPTSPGPLSVRAGHGERDLGNPNTAPPTPELHGINPVFLSSNPSRWSVEEVYEFIASLQGCQEIAEEFRSQEIDGQALLLLKEEHLMSAMNIKLGPALKICAKINVLKET

>Q9UI17

MLRPGAQLLRGLLLRSCPLQGSPGRPRSVCGREGEEKPPLSAETQWKDRAETVIIGGGCVGVSLAYHLAKAGMKDVVLLEKSELTAGSTWHAAGLTTYFHPGINLKKIHYDSIKLYEKLEEETGQVVGFHQPGSIRLATTPVRVDEFKYQMTRTGWHATEQYLIEPEKIQEMFPLLNMNKVLAGLYNPGDGHIDPYSLTMALAAGARKCGALLKYPAPVTSLKARSDGTWDVETPQGSMRANRIVNAAGFWAREVGKMIGLEHPLIPVQHQYVVTSTISEVKALKRELPVLRDLEGSYYLRQERDGLLFGPYESQEKMKVQDSWVTNGVPPGFGKELFESDLDRIMEHIKAAMEMVPVLKKADIINVVNGPITYSPDILPMVGPHQGVRNYWVAIGFGYGIIHAGGVGKYLSDWILHGEPPFDLIELDPNRYGKWTTTQYTEAKARESYGFNNIVGYPKEERFAGRPTQRVSGLYQRLESKCSMGFHAGWEQPHWFYKPGQDTQYRPSFRRTNWFEPVGSEYKQVMQRVGVTDLSPFGKFNIKGQDSIRLLDHLFANVIPKVGFTNISHMLTPKGRVYAELTVSHQSPGEFLLITGSGSELHDLRWIEEEAVKGGYDVEIKNITDELGVLGVAGPQARKVLQKLTPEDLSDDVFKFLQTKSLKVSNIPVTAIRISYTGELGWELYHRREDSVALYDAIMNAGQEEGIDNFGTYAMNALRLEKAFRAWGLEMNCDTNPLEAGLEYFVKLNKPADFIGKQALKQIKAKGLKRRLVCLTLATDDVDPEGNESIWYNGKVVGNTTSGSYSYSIQKSLAFAYVPVQLSEVGQQVEVELLGKNYPAVIIQEPLVLTEPTRNRLQKKGGKDKT

178 P29558 NP_001631.3

>P29558

MGKVWKQQMYPQYATYYYPQYLQAKQSLVPAHPMAPPSPSTTSSNNNSSSSSNSGWDQLSKTNLYIRGLPPHTTDQDLVKLCQPYGKIVSTKAILDKTTNKCKGYGFVDFDSPAAAQKAVSALKASGVQAQMAKQQEQDPTNLYISNLPLSMDEQELENMLKPFGQVISTRILRDSSGTSRGVGFARMESTEKCEAVIGHFNGKFIKTPPGVSAPTEPLLCKFADGGQKKRQNPNKYIPNGRPWHREGEVRLAGMTLTYDPTTAAIQNGFYPSPYSIATNRMITQTSITPYIASPVSAYQVQSPSWMQPQPYILQHPGAVLTPSMEHTMSLQPASMISPLAQQMSHLSLGSTGTYMPATSAMQGAYLPQYAHMQTTAVPVEEASGQQQVAVETSNDHSPYTFQPNK

>NP_001631.3

MERQVLLSEPEEAAALYRGLSRQPALSAACLGPEVTTQYGGQYRTVHTEWTQRDLERMENIRFCRQYLVFHDGDSVVFAGPAGNSVETRGELLSRESPSGTMKAVLRKAGGTGPGEEKQFLEVWEKNRKLKSFNLSALEKHGPVYEDDCFGCLSWSHSETHLLYVAEKKRPKAESFFQTKALDVSASDDEIARLKKPDQAIKGDQFVFYEDWGENMVSKSIPVLCVLDVESGNISVLEGVPENVSPGQAFWAPGDAGVVFVGWWHEPFRLGIRFCTNRRSALYYVDLIGGKCELLSDDSLAVSSPRLSPDQCRIVYLQYPSLIPHHQCSQLCLYDWYTKVTSVVVDVVPRQLGENFSGIYCSLLPLGCWSADSQRVVFDSAQRSRQDLFAVDTQVGTVTSLTAGGSGGSWKLLTIDQDLMVAQFSTPSLPPTLKVGFLPSAGKEQSVLWVSLEEAEPIPDIHWGIRVLQPPPEQENVQYAGLDFEAILLQPGSPPDKTQVPMVVMPHGGPHSSFVTAWMLFPAMLCKMGFAVLLVNYRGSTGFGQDSILSLPGNVGHQDVKDVQFAVEQVLQEEHFDASHVALMGGSHGGFISCHLIGQYPETYRACVARNPVINIASMLGSTDIPDWCVVEAGFPFSSDCLPDLSVWAEMLDKSPIRYIPQVKTPLLLMLGQEDRRVPFKQGMEYYRALKTRNVPVRLLLYPKSTHALSEVEVESDSFMNAVLWLRTHLGS

179 P09067 NP_000894.1

>P09067

MSSYFVNSFSGRYPNGPDYQLLNYGSGSSLSGSYRDPAAMHTGSYGYNYNGMDLSVNRSSASSSHFGAVGESSRAFPAPAQEPRFRQAASSCSLSSPESLPCTNGDSHGAKPSASSPSDQATSASSSANFTEIDEASASSEPEEAASQLSSPSLARAQPEPMATSTAAPEGQTPQIFPWMRKLHISHDMTGPDGKRARTAYTRYQTLELEKEFHFNRYLTRRRRIEIAHALCLSERQIKIWFQNRRMKWKKDNKLKSMSLATAGSAFQP

>NP_000894.1

MVGRRALIVLAHSERTSFNYAMKEAAAAALKKKGWEVVESDLYAMNFNPIISRKDITGKLKDPANFQYPAESVLAYKEGHLSPDIVAEQKKLEAADLVIFQFPLQWFGVPAILKGWFERVFIGEFAYTYAAMYDKGPFRSKKAVLSITTGGSGSMYSLQGIHGDMNVILWPIQSGILHFCGFQVLEPQLTYSIGHTPADARIQILEGWKKRLENIWDETPLYFAPSSLFDLNFQAGFLMKKEVQDEEKNKKFGLSVGHHLGKSIPTDNQIKARK

180 Q15723 NP_003105.2

>Q15723

MTSAVVDSGGTILELSSNGVENQEESEKVSEYPAVIVEPVPSARLEQGYAAQVLVYDDETYMMQDVAEEQEVETENVETVEASVHSSNAHCTDKTIEAAEALLHMESPTCLRDSRSPVEVFVPPCVSTPEFIHAAMRPDVITETVVEVSTEESEPMDTSPIPTSPDSHEPMKKKKVGRKPKTQQSPISNGSPELGIKKKPREGKGNTTYLWEFLLDLLQDKNTCPRYIKWTQREKGIFKLVDSKAVSKLWGKHKNKPDMNYETMGRALRYYYQRGILAKVEGQRLVYQFKDMPKNIVVIDDDKSETCNEDLAGTTDEKSLERVSLSAESLLKAASSVRSGKNSSPINCSRAEKGVARVVNITSPGHDASSRSPTTTASVSATAAPRTVRVAMQVPVVMTSLGQKISTVAVQSVNAGAPLITSTSPTTATSPKVVIQTIPTVMPASTENGDKITMQPAKIITIPATQLAQCQLQTKSNLTGSGSINIVGTPLAVRALTPVSIAHGTPVMRLSMPTQQASGQTPPRVISAVIKGPEVKSEAVAKKQEHDVKTLQLVEEKPADGNKTVTHVVVVSAPSAIALPVTMKTEGLVTCEK

>NP_003105.2

MTTKDYPSLWGFGTTKTFKIPIEHLDFKYIEKCSDVKHLEKILCVLRSGEEGYYPELTEFCEKHLQALAPESRALRKDKPAATAASFTAEEWEKIDGDIKSWVSEIKKEEDKMHFHETETFPAMKDNLPPVRGSNSCLHVGKEKYSKRPTKKKTPRDYAEWDKFDVEKECLKIDEDYKEKTVIDKSHLSKIETRIDTAGLTEKEKDFLATREKEKGNEAFNSGDYEEAVMYYTRSISALPTVVAYNNRAQAEIKLQNWNSAFQDCEKVLELEPGNVKALLRRATTYKHQNKLREATEDLSKVLDVEPDNDLAKKTLSEVERDLKNSEAASETQTKGKRMVIQEIENSEDEEGKSGRKHEDGGGDKKPAEPAGAARAAQPCVMGNIQKKLTGKAEGGKRPARGAPQRGQTPEAGADKRSPRRASAAAAAGGGATGHPGGGQGAENPAGLKSQGNELFRSGQFAEAAGKYSAAIALLEPAGSEIADDLSILYSNRAACYLKEGNCSGCIQDCNRALELHPFSMKPLLRRAMAYETLEQYGKAYVDYKTVLQIDCGLQLANDSVNRLSRILMELDGPNWREKLSPIPAVPASVPLQAWHPAKEMISKQAGDSSSHRQQGITDEKTFKALKEEGNQCVNDKNYKDALSKYSECLKINNKECAIYTNRALCYLKLCQFEEAKQDCDQALQLADGNVKAFYRRALAHKGLKNYQKSLIDLNKVILLDPSIIEAKMELEEVTRLLNLKDKTAPFNKEKERRKIEIQEVNEGKEEPGRPAGEVSMGCLASEKGGKSSRSPEDPEKLPIAKPNNAYEFGQIINALSTRKDKEACAHLLAITAPKDLPMFLSNKLEGDTFLLLIQSLKNNLIEKDPSLVYQHLLYLSKAERFKMMLTLISKGQKELIEQLFEDLSDTPNNHFTLEDIQALKRQYEL

181 NP_004647.1 Q9NWF9

>NP_004647.1

MNKGWLELESDPGLFTLLVEDFGVKGVQVEEIYDLQSKCQGPVYGFIFLFKWIEERRSRRKVSTLVDDTSVIDDDIVNNMFFAHQLIPNSCATHALLSVLLNCSSVDLGPTLSRMKDFTKGFSPESKGYAIGNAPELAKAHNSHARPEPRHLPEKQNGLSAVRTMEAFHFVSYVPITGRLFELDGLKVYPIDHGPWGEDEEWTDKARRVIMERIGLATAGEPYHDIRFNLMAVVPDRRIKYEARLHVLKVNRQTVLEALQQLIRVTQPELIQTHKSQESQLPEESKSASNKSPLVLEANRAPAASEGNHTDGAEEAAGSCAQAPSHSPPNKPKLVVKPPGSSLNGVHPNPTPIVQRLPAFLDNHNYAKSPMQEEEDLAAGVGRSRVPVRPPQQYSDDEDDYEDDEEDDVQNTNSALRYKGKGTGKPGALSGSADGQLSVLQPNTINVLAEKLKESQKDLSIPLSIKTSSGAGSPAVAVPTHSQPSPTPSNESTDTASEIGSAFNSPLRSPIRSANPTRPSSPVTSHISKVLFGEDDSLLRVDCIRYNRAVRDLGPVISTGLLHLAEDGVLSPLALTEGGKGSSPSIRPIQGSQGSSSPVEKEVVEATDSREKTGMVRPGEPLSGEKYSPKELLALLKCVEAEIANYEACLKEEVEKRKKFKIDDQRRTHNYDEFICTFISMLAQEGMLANLVEQNISVRRRQGVSIGRLHKQRKPDRRKRSRPYKAKRQ

>Q9NWF9

MEEGNNNEEVIHLNNFHCHRGQEWINLRDGPITISDSSDEERIPMLVTPAPQQHEEEDLDDDVILTEDDSEDDYGEFLDLGPPGISEFTKPSGQTEREPKPGPSHNQAANDIVNPRSEQKVIILEEGSLLYTESDPLETQNQSSEDSETELLSNLGESAALADDQAIEEDCWLDHPYFQSLNQQPREITNQVVPQERQPEAELGRLLFQHEFPGPAFPRPEPQQGGISGPSSPQPAHPLGEFEDQQLASDDEEPGPAFPMQESQEPNLENIWGQEAAEVDQELVELLVKETEARFPDVANGFIEEIIHFKNYYDLNVLCNFLLENPDYPKREDRIIINPSSSLLASQDETKLPKIDFFDYSKLTPLDQRCFIQAADLLMADFKVLSSQDIKWALHELKGHYAITRKALSDAIKKWQELSPETSGKRKKRKQMNQYSYIDFKFEQGDIKIEKRMFFLENKRRHCRSYDRRALLPAVQQEQEFYEQKIKEMAEHEDFLLALQMNEEQYQKDGQLIECRCCYGEFPFEELTQCADAHLFCKECLIRYAQEAVFGSGKLELSCMEGSCTCSFPTSELEKVLPQTILYKYYERKAEEEVAAAYADELVRCPSCSFPALLDSDVKRFSCPNPHCRKETCRKCQGLWKEHNGLTCEELAEKDDIKYRTSIEEKMTAARIRKCHKCGTGLIKSEGCNRMSCRCGAQMCYLCRVSINGYDHFCQHPRSPGAPCQECSRCSLWTDPTEDDEKLIEEIQKEAEEEQKRKNGENTFKRIGPPLEKPVEKVQRVEALPRPVPQNLPQPQMPPYAFAHPPFPLPPVRPVFNNFPLNMGPIPAPYVPPLPNVRVNYDFGPIHMPLEHNLPMHFGPQPRHRF

182 O60356 NP_005179.2

>O60356

MATFPPATSAPQQPPGPEDEDSSLDESDLYSLAHSYLGGGGRKGRTKREAAANTNRPSPGGHERKLVTKLQNSERKKRGARR

>NP_005179.2

MAGNVKKSSGAGGGSGSGGSGSGGLIGLMKDAFQPHHHHHHHLSPHPPGTVDKKMVEKCWKLMDKVVRLCQNPKLALKNSPPYILDLLPDTYQHLRTILSRYEGKMETLGENEYFRVFMENLMKKTKQTISLFKEGKERMYEENSQPRRNLTKLSLIFSHMLAELKGIFPSGLFQGDTFRITKADAAEFWRKAFGEKTIVPWKSFRQALHEVHPISSGLEAMALKSTIDLTCNDYISVFEFDIFTRLFQPWSSLLRNWNSLAVTHPGYMAFLTYDEVKARLQKFIHKPGSYIFRLSCTRLGQWAIGYVTADGNILQTIPHNKPLFQALIDGFREGFYLFPDGRNQNPDLTGLCEPTPQDHIKVTQEQYELYCEMGSTFQLCKICAENDKDVKIEPCGHLMCTSCLTSWQESEGQGCPFCRCEIKGTEPIVVDPFDPRGSGSLLRQGAEGAPSPNYDDDDDERADDTLFMMKELAGAKVERPPSPFSMAPQASLPPVPPRLDLLPQRVCVPSSASALGTASKAASGSLHKDKPLPVPPTLRDLPPPPPPDRPYSVGAESRPQRRPLPCTPGDCPSRDKLPPVPSSRLGDSWLPRPIPKVPVSAPSSSDPWTGRELTNRHSLPFSLPSQMEPRPDVPRLGSTFSLDTSMSMNSSPLVGPECDHPKIKPSSSANAIYSLAARPLPVPKLPPGEQCEGEEDTEYMTPSSRPLRPLDTSQSSRACDCDQQIDSCTYEAMYNIQSQAPSITESSTFGEGNLAAAHANTGPEESENEDDGYDVPKPPVPAVLARRTLSDISNASSSFGWLSLDGDPTTNVTEGSQVPERPPKPFPRRINSERKAGSCQQGSGPAASAATASPQLSSEIENLMSQGYSYQDIQKALVIAQNNIEMAKNILREFVSISSPAHVAT

183 NP_003098.1 NP_006635.2

>NP_003098.1

MVQQTNNAENTEALLAGESSDSGAGLELGIASSPTPGSTASTGGKADDPSWCKTPSGHIKRPMNAFMVWSQIERRKIMEQSPDMHNAEISKRLGKRWKLLKDSDKIPFIREAERLRLKHMADYPDYKYRPRKKVKSGNANSSSSAAASSKPGEKGDKVGGSGGGGHGGGGGGGSSNAGGGGGGASGGGANSKPAQKKSCGSKVAGGAGGGVSKPHAKLILAGGGGGGKAAAAAAASFAAEQAGAAALLPLGAAADHHSLYKARTPSASASASSAASASAALAAPGKHLAEKKVKRVYLFGGLGTSSSPVGGVGAGADPSDPLGLYEEEGAGCSPDAPSLSGRSSAASSPAAGRSPADHRGYASLRAASPAPSSAPSHASSSASSHSSSSSSSGSSSSDDEFEDDLLDLNPSSNFESMSLGSFSSSSALDRDLDFNFEPGSGSHFEFPDYCTPEVSEMISGDWLESSISNLVFTY

>NP_006635.2

MSVVGLDVGSQSCYIAVARAGGIETIANEFSDRCTPSVISFGSKNRTIGVAAKNQQITHANNTVSNFKRFHGRAFNDPFIQKEKENLSYDLVPLKNGGVGIKVMYMGEEHLFSVEQITAMLLTKLKETAENSLKKPVTDCVISVPSFFTDAERRSVLDAAQIVGLNCLRLMNDMTAVALNYGIYKQDLPSLDEKPRIVVFVDMGHSAFQVSACAFNKGKLKVLGTAFDPFLGGKNFDEKLVEHFCAEFKTKYKLDAKSKIRALLRLYQECEKLKKLMSSNSTDLPLNIECFMNDKDVSGKMNRSQFEELCAELLQKIEVPLYSLLEQTHLKVEDVSAVEIVGGATRIPAVKERIAKFFGKDISTTLNADEAVARGCALQCAILSPAFKVREFSVTDAVPFPISLIWNHDSEDTEGVHEVFSRNHAAPFSKVLTFLRRGPFELEAFYSDPQGVPYPEAKIGRFVVQNVSAQKDGEKSRVKVKVRVNTHGIFTISTASMVEKVPTEENEMSSEADMECLNQRPPENPDTDKNVQQDNSEAGTQPQVQTDAQQTSQSPPSPELTSEENKIPDADKANEKKVDQPPEAKKPKIKVVNVELPIEANLVWQLGKDLLNMYIETEGKMIMQDKLEKERNDAKNAVEEYVYEFRDKLCGPYEKFICEQDHQNFLRLLTETEDWLYEEGEDQAKQAYVDKLEELMKIGTPVKVRFQEAEERPKMFEELGQRLQHYAKIAADFRNKDEKYNHIDESEMKKVEKSVNEVMEWMNNVMNAQAKKSLDQDPVVRAQEIKTKIKELNNTCEPVVTQPKPKIESPKLERTPNGPNIDKKEEDLEDKNNFGAEPPHQNGECYPNEKNSVNMDLD

184 NP_005595.2 NP_001503.1

>NP_005595.2

MATAASNHYSLLTSSASIVHAEPPGGMQQGAGGYREAQSLVQGDYGALQSNGHPLSHAHQWITALSHGGGGGGGGGGGGGGGGGGGGGDGSPWSTSPLGQPDIKPSVVVQQGGRGDELHGPGALQQQHQQQQQQQQQQQQQQQQQQQQQRPPHLVHHAANHHPGPGAWRSAAAAAHLPPSMGASNGGLLYSQPSFTVNGMLGAGGQPAGLHHHGLRDAHDEPHHADHHPHPHSHPHQQPPPPPPPQGPPGHPGAHHDPHSDEDTPTSDDLEQFAKQFKQRRIKLGFTQADVGLALGTLYGNVFSQTTICRFEALQLSFKNMCKLKPLLNKWLEEADSSSGSPTSIDKIAAQGRKRKKRTSIEVSVKGALESHFLKCPKPSAQEITSLADSLQLEKEVVRVWFCNRRQKEKRMTPPGGTLPGAEDVYGGSRDTPPHHGVQTPVQ

>NP_001503.1

MAARPKLHYPNGRGRMESVRWVLAAAGVEFDEEFLETKEQLYKLQDGNHLLFQQVPMVEIDGMKLVQTRSILHYIADKHNLFGKNLKERTLIDMYVEGTLDLLELLIMHPFLKPDDQQKEVVNMAQKAIIRYFPVFEKILRGHGQSFLVGNQLSLADVILLQTILALEEKIPNILSAFPFLQEYTVKLSNIPTIKRFLEPGSKKKPPPDEIYVRTVYNIFRP

185 NP_071910.3 NP_056273.2

>NP_071910.3

MHTPPALPRRFQGGGRVRTPGSHRQGKDNLERDPSGGCVPDFLPQAQDSNHFIMESLFCESSGDSSLEKEFLGAPVGPSVSTPNSQHSSPSRSLSANSIKVEMYSDEESSRLLGPDERLLEKDDSVIVEDSLSEPLGYCDGSGPEPHSPGGIRLPNGKLKCDVCGMVCIGPNVLMVHKRSHTGERPFHCNQCGASFTQKGNLLRHIKLHSGEKPFKCPFCNYACRRRDALTGHLRTHSVSSPTVGKPYKCNYCGRSYKQQSTLEEHKERCHNYLQSLSTEAQALAGQPGDEIRDLEMVPDSMLHSSSERPTFIDRLANSLTKRKRSTPQKFVGEKQMRFSLSDLPYDVNSGGYEKDVELVAHHSLEPGFGSSLAFVGAEHLRPLRLPPTNCISELTPVISSVYTQMQPLPGRLELPGSREAGEGPEDLADGGPLLYRPRGPLTDPGASPSNGCQDSTDTESNHEDRVAGVVSLPQGPPPQPPPTIVVGRHSPAYAKEDPKPQEGLLRGTPGPSKEVLRVVGESGEPVKAFKCEHCRILFLDHVMFTIHMGCHGFRDPFECNICGYHSQDRYEFSSHIVRGEHKVG

>NP_056273.2

MEFAELIKTPRVDNVVLHRPFYPAVEGTLCLTGHHLILSSRQDNTEELWLLHSNIDAIDKRFVGSLGTIIIKCKDFRIIQLDIPGMEECLNIASSIEALSTLDSITLMYPFFYRPMFEVIEDGWHSFLPEQEFELYSSATSEWRLSYVNKEFAVCPSYPPIVTVPKSIDDEALRKVATFRHGGRFPVLSYYHKKNGMVIMRSGQPLTGTNGRRCKEDEKLINATLRAGKRGYIIDTRSLNVAQQTRAKGGGFEQEAHYPQWRRIHKSIERYHILQESLIKLVEACNDQTHNMDRWLSKLEASNWLTHIKEILTTACLAAQCIDREGASILIHGTEGTDSTLQVTSLAQIILEPRSRTIRGFEALIEREWLQAGHPFQQRCAQSAYCNTKQKWEAPVFLLFLDCVWQILRQFPCSFEFNENFLIMLFEHAYASQFGTFLGNNESERCKLKLQQKTMSLWSWVNQPSELSKFTNPLFEANNLVIWPSVAPQSLPLWEGIFLRWNRSSKYLDEAYEEMVNIIEYNKELQAKVNILRRQLAELETEDGMQESP

186 NP_892017.2 NP_000964.1

>NP_892017.2

MAAAVAVAAASRRQSCYLCDLPRMPWAMIWDFTEPVCRGCVNYEGADRVEFVIETARQLKRAHGCFPEGRSPPGAAASAAAKPPPLSAKDILLQQQQQLGHGGPEAAPRAPQALERYPLAAAAERPPRLGSDFGSSRPAASLAQPPTPQPPPVNGILVPNGFSKLEEPPELNRQSPNPRRGHAVPPTLVPLMNGSATPLPTALGLGGRAAASLAAVSGTAAASLGSAQPTDLGAHKRPASVSSSAAVEHEQREAAAKEKQPPPPAHRGPADSLSTAAGAAELSAEGAGKSRGSGEQDWVNRPKTVRDTLLALHQHGHSGPFESKFKKEPALTAGRLLGFEANGANGSKAVARTARKRKPSPEPEGEVGPPKINGEAQPWLSTSTEGLKIPMTPTSSFVSPPPPTASPHSNRTTPPEAAQNGQSPMAALILVADNAGGSHASKDANQVHSTTRRNSNSPPSPSSMNQRRLGPREVGGQGAGNTGGLEPVHPASLPDSSLATSAPLCCTLCHERLEDTHFVQCPSVPSHKFCFPCSRQSIKQQGASGEVYCPSGEKCPLVGSNVPWAFMQGEIATILAGDVKVKKERDS

>NP_000964.1

MGRVIRGQRKGAGSVFRAHVKHRKGAARLRAVDFAERHGYIKGIVKDIIHDPGRGAPLAKVVFRDPYRFKKRTELFIAAEGIHTGQFVYCGKKAQLNIGNVLPVGTMPEGTIVCCLEEKPGDRGKLARASGNYATVISHNPETKKTRVKLPSGSKKVISSANRAVVGVVAGGGRIDKPILKAGRAYHKYKAKRNCWPRVRGVAMNPVEHPFGGGNHQHIGKPSTIRRDAPAGRKVGLIAARRTGRLRGTKTVQEKEN

187 NP_003085.1 Q92526

>NP_003085.1

MAYRGQGQKVQKVMVQPINLIFRYLQNRSRIQVWLYEQVNMRIEGCIIGFDEYMNLVLDDAEEIHSKTKSRKQLGRIMLKGDNITLLQSVSN

>Q92526

MAAIKAVNSKAEVARAQAALAVNICAARGLQDVLRTNLGPKGTMKMLASGAGDIKLTKDGNVLLDEMQIQHPTASLIAKVATAQDDVTGDGTTSNVLIIGELLKQADLYISEGLHPRIIAEGFEAAKIKALEVLEEVKVTKEMKRKILLDVARTSLQTKVHAELADVLTEVVVDSVLAVRRPGYPIDLFMVEIMEMKHKLGTDTKLIQGLVLDHGARHPDMKKRVEDAFILICNVSLEYEKTEVNSGFFYKTAEEKEKLVKAERKFIEDRVQKIIDLKDKVCAQSNKGFVVINQKGIDPFSLDSLAKHGIVALRRAKRRNMERLSLACGGMAVNSFEDLTVDCLGHAGLVYEYTLGEEKFTFIEECVNPCSVTLLVKGPNKHTLTQVKDAIRDGLRAIKNAIEDGCMVPGAGAIEVAMAEALVTYKNSIKGRARLGVQAFADALLIIPKVLAQNAGYDPQETLVKVQAEHVESKQLVGVDLNTGEPMVAADAGVWDNYCVKKQLLHSCTVIATNILLVDEIMRAGMSSLK

188 NP_003358.1 NP_006432.1

>NP_003358.1

MDMLDPGLDPAASATAAAAASHDKGPEAEEGVELQEGGDGPGAEEQTAVAITSVQQAAFGDHNIQYQFRTETNGGQVTYRVVQVTDGQLDGQGDTAGAVSVVSTAAFAGGQQAVTQVGVDGAAQRPGPAAASVPPGPAAPFPLAVIQNPFSNGGSPAAEAVSGEARFAYFPASSVGDTTAVSVQTTDQSLQAGGQFYVMMTPQDVLQTGTQRTIAPRTHPYSPKIDGTRTPRDERRRAQHNEVERRRRDKINNWIVQLSKIIPDCNADNSKTGASKGGILSKACDYIRELRQTNQRMQETFKEAERLQMDNELLRQQIEELKNENALLRAQLQQHNLEMVGEGTRQ

>NP_006432.1

MAAAAVSSAKRSLRGELKQRLRAMSAEERLRQSRVLSQKVIAHSEYQKSKRISIFLSMQDEIETEEIIKDIFQRGKICFIPRYRFQSNHMDMVRIESPEEISLLPKTSWNIPQPGEGDVREEALSTGGLDLIFMPGLGFDKHGNRLGRGKGYYDAYLKRCLQHQEVKPYTLALAFKEQICLQVPVNENDMKVDEVLYEDSSTA

189 NP_002119.1 NP_038203.2

>NP_002119.1

MGKGDPKKPRGKMSSYAFFVQTCREEHKKKHPDASVNFSEFSKKCSERWKTMSAKEKGKFEDMAKADKARYEREMKTYIPPKGETKKKFKDPNAPKRPPSAFFLFCSEYRPKIKGEHPGLSIGDVAKKLGEMWNNTAADDKQPYEKKAAKLKEKYEKDIAAYRAKGKPDAAKKGVVKAEKSKKKKEEEEDEEDEEDEEEEEDEEDEDEEEDDDDE

>NP_038203.2

MLQQVPENINFPAEEEKILEFWTEFNCFQECLKQSKHKPKFTFYDGPPFATGLPHYGHILAGTIKDIVTRYAHQSGFHVDRRFGWDCHGLPVEYEIDKTLGIRGPEDVAKMGITEYNNQCRAIVMRYSAEWKSTVSRLGRWIDFDNDYKTLYPQFMESVWWVFKQLYDKGLVYRGVKVMPFSTACNTPLSNFESHQNYKDVQDPSVFVTFPLEEDETVSLVAWTTTPWTLPSNLAVCVNPEMQYVKIKDVARGRLLILMEARLSALYKLESDYEILERFPGAYLKGKKYRPLFDYFLKCKENGAFTVLVDNYVKEEEGTGVVHQAPYFGAEDYRVCMDFNIIRKDSLPVCPVDASGCFTTEVTDFAGQYVKDADKSIIRTLKEQGRLLVATTFTHSYPFCWRSDTPLIYKAVPSWFVRVENMVDQLLRNNDLCYWVPELVREKRFGNWLKDARDWTISRNRYWGTPIPLWVSDDFEEVVCIGSVAELEELSGAKISDLHRESVDHLTIPSRCGKGSLHRISEVFDCWFESGSMPYAQVHYPFENKREFEDAFPADFIAEGIDQTRGWFYTLLVLATALFGQPPFKNVIVNGLVLASDGQKMSKRKKNYPDPVSIIQKYGADALRLYLINSPVVRAENLRFKEEGVRDVLKDVLLPWYNAYRFLIQNVLRLQKEEEIEFLYNENTVRESPNITDRWILSFMQSLIGFFETEMAAYRLYTVVPRLVKFVDILTNWYVRMNRRRLKGENGMEDCVMALETLFSVLLSLCRLMAPYTPFLTELMYQNLKVLIDPVSVQDKDTLSIHYLMLPRVREELIDKKTESAVSQMQSVIELGRVIRDRKTIPIKYPLKEIVVIHQDPEALKDIKSLEKYIIEELNVRKVTLSTDKNKYGIRLRAEPDHMVLGKRLKGAFKAVMTSIKQLSSEELEQFQKTGTIVVEGHELHDEDIRLMYTFDQATGGTAQFEAHSDAQALVLLDVTPDQSMVDEGMAREVINRIQKLRKKCNLVPTDEITVYYKAKSEGTYLNSVIESHTEFIFTTIKAPLKPYPVSPSDKVLIQEKTQLKGSELEITLTRGSSLPGPACAYVNLNICANGSEQGGVLLLENPKGDNRLDLLKLKSVVTSIFGVKNTELAVFHDETEIQNQTDLLSLSGKTLCVTAGSAPSLINSSSTLLCQYINLQLLNAKPQECLMGTVGTLLLENPLGQNGLTHQGLLYEAAKVFGLRSRKLKLFLNETQTQEITEDIPVKTLNMKTVYVSVLPTTADF

190 NP_055620.1 NP_689508.3

>NP_055620.1

MWMTPKRSKMEVDEALVFRPEWTQRYLVVEPPEGDGALCLVCRRLIVATRERDVRRHYEAEHEYYERYVADGERAALVERLRQGDLPVASFTPEERAARAGLGLCRLLALKGRGWGEGDFVYQCMEVLLREVLPEHVSVLQGVDLSPDITRQRILSIDRNLRNQLFNRARDFKAYSLALDDQAFVAYENYLLVFIRGVGPELEVQEDLLTIINLTHHFSVGALMSAILESLQTAGLSLQRMVGLTTTHTLRMIGENSGLVSYMREKAVSPNCWNVIHYSGFLHLELLSSYDVDVNQIINTISEWIVLIKTRGVRRPEFQTLLTESESEHGERVNGRCLNNWLRRGKTLKLIFSLRKEMEAFLVSVGATTVHFSDKQWLCDFGFLVDIMEHLRELSEELRVSKVFAAAAFDHICTFEVKLNLFQRHIEEKNLTDFPALREVVDELKQQNKEDEKIFDPDRYQMVICRLQKEFERHFKDLRFIKKDLELFSNPFNFKPEYAPISVRVELTKLQANTNLWNEYRIKDLGQFYAGLSAESYPIIKGVACKVASLFDSNQICEKAFSYLTRNQHTLSQPLTDEHLQALFRVATTEMEPGWDDLVRERNESNP

>NP_689508.3

MFEEKASSPSGKMGGEEKPIGAGEEKQKEGGKKKNKEGSGDGGRAELNPWPEYIYTRLEMYNILKAEHDSILAEKAEKDSKPIKVTLPDGKQVDAESWKTTPYQIACGISQGLADNTVIAKVNNVVWDLDRPLEEDCTLELLKFEDEEAQAVYWHSSAHIMGEAMERVYGGCLCYGPPIENGFYYDMYLEEGGVSSNDFSSLEALCKKIIKEKQAFERLEVKKETLLAMFKYNKFKCRILNEKVNTPTTTVYRCGPLIDLCRGPHVRHTGKIKALKIHKNSSTYWEGKADMETLQRIYGISFPDPKMLKEWEKFQEEAKNRDHRKIGRDQELYFFHELSPGSCFFLPKGAYIYNALIEFIRSEYRKRGFQEVVTPNIFNSRLWMTSGHWQHYSENMFSFEVEKELFALKPMNCPGHCLMFDHRPRSWRELPLRLADFGVLHRNELSGALTGLTRVRRFQQDDAHIFCAMEQIEDEIKGCLDFLRTVYSVFGFSFKLNLSTRPEKFLGDIEVWDQAEKQLENSLNEFGEKWELNSGDGAFYGPKIDIQIKDAIGRYHQCATIQLDFQLPIRFNLTYVSHDGDDKKRPVIVHRAILGSVERMIAILTENYGGKWPFWLSPRQVMVVPVGPTCDEYAQKVRQQFHDAKFMADIDLDPGCTLNKKIRNAQLAQYNFILVVGEKEKISGTVNIRTRDNKVHGERTISETIERLQQLKEFRSKQAEEEF

191 NP_005422.1 P28838

>NP_005422.1

MCSAFHRAESGTELLARLEGRSSLKEIEPNLFADEDSPVHGDILEFHGPEGTGKTEMLYHLTARCILPKSEGGLEVEVLFIDTDYHFDMLRLVTILEHRLSQSSEEIIKYCLGRFFLVYCSSSTHLLLTLYSLESMFCSHPSLCLLILDSLSAFYWIDRVNGGESVNLQESTLRKCSQCLEKLVNDYRLVLFATTQTIMQKASSSSEEPSHASRRLCDVDIDYRPYLCKAWQQLVKHRMFFSKQDDSQSSNQFSLVSRCLKSNSLKKHFFIIGESGVEFC

>P28838

MFLLPLPAAGRVVVRRLAVRRFGSRSLSTADMTKGLVLGIYSKEKEDDVPQFTSAGENFDKLLAGKLRETLNISGPPLKAGKTRTFYGLHQDFPSVVLVGLGKKAAGIDEQENWHEGKENIRAAVAAGCRQIQDLELSSVEVDPCGDAQAAAEGAVLGLYEYDDLKQKKKMAVSAKLYGSGDQEAWQKGVLFASGQNLARQLMETPANEMTPTRFAEIIEKNLKSASSKTEVHIRPKSWIEEQAMGSFLSVAKGSDEPPVFLEIHYKGSPNANEPPLVFVGKGITFDSGGISIKASANMDLMRADMGGAATICSAIVSAAKLNLPINIIGLAPLCENMPSGKANKPGDVVRAKNGKTIQVDNTDAEGRLILADALCYAHTFNPKVILNAATLTGAMDVALGSGATGVFTNSSWLWNKLFEASIETGDRVWRMPLFEHYTRQVVDCQLADVNNIGKYRSAGACTAAAFLKEFVTHPKWAHLDIAGVMTNKDEVPYLRKGMTGRPTRTLIEFLLRFSQDNA

192 NP_005634.1 P43304

>NP_005634.1

MDDAHESPSDKGGETGESDETAAVPGDPGATDTDGIPEETDGDADVDLKEAAAEEGELESQDVSDLTTVEREDSSLLNPAAKKLKIDTKEKKEKKQKVDEDEIQKMQILVSSFSEEQLNRYEMYRRSAFPKAAIKRLIQSITGTSVSQNVVIAMSGISKVFVGEVVEEALDVCEKWGEMPPLQPKHMREAVRRLKSKGQIPNSKHKKIIFF

>P43304

MAFQKAVKGTILVGGGALATVLGLSQFAHYRRKQMNLAYVKAADCISEPVNREPPSREAQLLTLQNTSEFDILVIGGGATGSGCALDAVTRGLKTALVERDDFSSGTSSRSTKLIHGGVRYLQKAIMKLDIEQYRMVKEALHERANLLEIAPHLSAPLPIMLPVYKWWQLPYYWVGIKLYDLVAGSNCLKSSYVLSKSRALEHFPMLQKDKLVGAIVYYDGQHNDARMNLAIALTAARYGAATANYMEVVSLLKKTDPQTGKVRVSGARCKDVLTGQEFDVRAKCVINATGPFTDSVRKMDDKDAAAICQPSAGVHIVMPGYYSPESMGLLDPATSDGRVIFFLPWQKMTIAGTTDTPTDVTHHPIPSEEDINFILNEVRNYLSCDVEVRRGDVLAAWSGIRPLVTDPKSADTQSISRNHVVDISESGLITIAGGKWTTYRSMAEDTINAAVKTHNLKAGPSRTVGLFLQGGKDWSPTLYIRLVQDYGLESEVAQHLAATYGDKAFEVAKMASVTGKRWPIVGVRLVSEFPYIEAEVKYGIKEYACTAVDMISRRTRLAFLNVQAAEEALPRIVELMGRELNWDDYKKQEQLETARKFLYYEMGYKSRSEQLTDRSEISLLPSDIDRYKKRFHKFDADQKGFITIVDVQRVLESINVQMDENTLHEILNEVDLNKNGQVELNEFLQLMSAIQKGRVSGSRLAILMKTAEENLDRRVPIPVDRSCGGL

193 NP_036384.1 Q8WVN8

>NP_036384.1

MTLDHQIINPTLKWSQPAVPSGGPLVQHAHTTLDSDAGLTENPLTKLLAIGKEDDNAQWHMEDVIEDIIGMESSFKEEGADSPLLMQRTLSGSILDVYSGEQGISPINMGLTSASCPSSLPMKREITETDTRALAKERQKKDNHNLIERRRRYNINYRIKELGTLIPKSNDPDMRWNKGTILKASVEYIKWLQKEQQRARELEHRQKKLEQANRRLLLRIQELEIQARTHGLPTLASLGTVDLGAHVTKQQSHPEQNSVDYCQQLTVSQGPSPELCDQAIAFSDPLSYFTDLSFSAALKEEQRLDGMLLDDTISPFGTDPLLSATSPAVSKESSRRSSFSSDDGDEL

>Q8WVN8

MSVSGLKAELKFLASIFDKNHERFRIVSWKLDELHCQFLVPQQGSPHSLPPPLTLHCNITESYPSSSPIWFVDSEDPNLTSVLERLEDTKNNNLLRQQLKWLICELCSLYNLPKHLDVEMLDQPLPTGQNGTTEEVTSEEEEEEEEMAEDIEDLDHYEMKEEEPISGKKSEDEGIEKENLAILEKIRKTQRQDHLNGAVSGSVQASDRLMKELRDIYRSQSYKTGIYSVELINDSLYDWHVKLQKVDPDSPLHSDLQILKEKEGIEYILLNFSFKDNFPFDPPFVRVVLPVLSGGYVLGGGALCMELLTKQGWSSAYSIESVIMQINATLVKGKARVQFGANKNQYNLARAQQSYNSIVQIHEKNGWYTPPKEDG

194 NP_002457.1 P14598

>NP_002457.1

MSRRTRCEDLDELHYQDTDSDVPEQRDSKCKVKWTHEEDEQLRALVRQFGQQDWKFLASHFPNRTDQQCQYRWLRVLNPDLVKGPWTKEEDQKVIELVKKYGTKQWTLIAKHLKGRLGKQCRERWHNHLNPEVKKSCWTEEEDRIICEAHKVLGNRWAEIAKMLPGRTDNAVKNHWNSTIKRKVDTGGFLSESKDCKPPVYLLLELEDKDGLQSAQPTEGQGSLLTNWPSVPPTIKEEENSEEELAAATTSKEQEPIGTDLDAVRTPEPLEEFPKREDQEGSPPETSLPYKWVVEAANLLIPAVGSSLSEALDLIESDPDAWCDLSKFDLPEEPSAEDSINNSLVQLQASHQQQVLPPRQPSALVPSVTEYRLDGHTISDLSRSSRGELIPISPSTEVGGSGIGTPPSVLKRQRKRRVALSPVTENSTSLSFLDSCNSLTPKSTPVKTLPFSPSQFLNFWNKQDTLELESPSLTSTPVCSQKVVVTTPLHRDKTPLHQKHAAFVTPDQKYSMDNTPHTPTPFKNALEKYGPLKPLPQTPHLEEDLKEVLRSEAGIELIIEDDIRPEKQKRKPGLRRSPIKKVRKSLALDIVDEDVKLMMSTLPKSLSLPTTAPSNSSSLTLSGIKEDNSLLNQGFLQAKPEKAAVAQKPRSHFTTPAPMSSAWKTVACGGTRDQLFMQEKARQLLGRLKPSHTSRTLILS

>P14598

MGDTFIRHIALLGFEKRFVPSQHYVYMFLVKWQDLSEKVVYRRFTEIYEFHKTLKEMFPIEAGAINPENRIIPHLPAPKWFDGQRAAENRQGTLTEYCSTLMSLPTKISRCPHLLDFFKVRPDDLKLPTDNQTKKPETYLMPKDGKSTATDITGPIILQTYRAIADYEKTSGSEMALSTGDVVEVVEKSESGWWFCQMKAKRGWIPASFLEPLDSPDETEDPEPNYAGEPYVAIKAYTAVEGDEVSLLEGEAVEVIHKLLDGWWVIRKDDVTGYFPSMYLQKSGQDVSQAQRQIKRGAPPRRSSIRNAHSIHQRSRKRLSQDAYRRNSVRFLQQRRRQARPGPQSPGSPLEEERQTQRSKPQPAVPPRPSADLILNRCSESTKRKLASAV

195 NP_004489.1 NP_006235.1

>NP_004489.1

MNAQLTMEAIGELHGVSHEPVPAPADLLGGSPHARSSVAHRGSHLPPAHPRSMGMASLLDGGSGGGDYHHHHRAPEHSLAGPLHPTMTMACETPPGMSMPTTYTTLTPLQPLPPISTVSDKFPHHHHHHHHHHHPHHHQRLAGNVSGSFTLMRDERGLASMNNLYTPYHKDVAGMGQSLSPLSSSGLGSIHNSQQGLPHYAHPGAAMPTDKMLTPNGFEAHHPAMLGRHGEQHLTPTSAGMVPINGLPPHHPHAHLNAQGHGQLLGTAREPNPSVTGAQVSNGSNSGQMEEINTKEVAQRITTELKRYSIPQAIFAQRVLCRSQGTLSDLLRNPKPWSKLKSGRETFRRMWKWLQEPEFQRMSALRLAACKRKEQEHGKDRGNTPKKPRLVFTDVQRRTLHAIFKENKRPSKELQITISQQLGLELSTVSNFFMNARRRSLDKWQDEGSSNSGNSSSSSSTCTKA

>NP_006235.1

METKLPPASTPTSPSSPGLSPVPPPDKVDGFSRRSLRRARPRRSHSSSQFRYQSNQQELTPLPLLKDVPASELHELLSRKLAQCGVMFDFLDCVADLKGKEVKRAALNELVECVGSTRGVLIEPVYPDIIRMISVNIFRTLPPSENPEFDPEEDEPNLEPSWPHLQLVYEFFLRFLESPDFQPSVAKRYVDQKFVLMLLELFDSEDPREREYLKTILHRVYGKFLGLRAYIRKQCNHIFLRFIYEFEHFNGVAELLEILGSIINGFALPLKTEHKQFLVRVLIPLHSVKSLSVFHAQLAYCVVQFLEKDATLTEHVIRGLLKYWPKTCTQKEVMFLGEMEEILDVIEPSQFVKIQEPLFKQVARCVSSPHFQVAERALYFWNNEYILSLIEDNCHTVLPAVFGTLYQVSKEHWNQTIVSLIYNVLKTFMEMNGKLFDELTASYKLEKQQEQQKAQERQELWQGLEELRLRRLQGTQGAKEAPLQRLTPQVAASGGQS

196 NP_066018.1 Q96I99

>NP_066018.1

MNTAPSRPSPTRRDPYGFGDSRDSRRDRSPIRGSPRREPRDGRNGRDARDSRDIRDPRDLRDHRHSRDLRDHRDSRSVRDVRDVRDLRDFRDLRDSRDFRDQRDPMYDRYRDMRDSRDPMYRREGSYDRYLRMDDYCRRKDDSYFDRYRDSFDGRGPPGPESQSRAKERLKREERRREELYRQYFEEIQRRFDAERPVDCSVIVVNKQTKDYAESVGRKVRDLGMVVDLIFLNTEVSLSQALEDVSRGGSPFAIVITQQHQIHRSCTVNIMFGTPQEHRNMPQADAMVLVARNYERYKNECREKEREEIARQAAKMADEAILQERERGGPEEGVRGGHPPAIQSLINLLADNRYLTAEETDKIINYLRERKERLMRSSTDSLPGPISRQPLGATSGASLKTQPSSQPLQSGQVLPSATPTPSAPPTSQQELQAKILSLFNSGTVTANSSSASPSVAAGNTPNQNFSTAANSQPQQRSQASGNQPPSILGQGGSAQNMGPRPGAPSQGLFGQPSSRLAPASNMTSQRPVSSTGINFDNPSVQKALDTLIQSGPALSHLVSQTTAQMGQPQAPMGSYQRHY

>Q96I99

MASPVAAQAGKLLRALALRPRFLAAGSQAVQLTSRRWLNLQEYQSKKLMSDNGVRVQRFFVADTANEALEAAKRLNAKEIVLKAQILAGGRGKGVFNSGLKGGVHLTKDPNVVGQLAKQMIGYNLATKQTPKEGVKVNKVMVAEALDISRETYLAILMDRSCNGPVLVGSPQGGVDIEEVAASNPELIFKEQIDIFEGIKDSQAQRMAENLGFVGPLKSQAADQITKLYNLFLKIDATQVEVNPFGETPEGQVVCFDAKINFDDNAEFRQKDIFAMDDKSENEPIENEAAKYDLKYIGLDGNIACFVNGAGLAMATCDIIFLNGGKPANFLDLGGGVKEAQVYQAFKLLTADPKVEAILVNIFGGIVNCAIIANGITKACRELELKVPLVVRLEGTNVQEAQKILNNSGLPITSAIDLEDAAKKAVASVAKK

197 NP_612482.2 NP_061833.1

>NP_612482.2

MSDQDHSMDEMTAVVKIEKGVGGNNGGNGNGGGAFSQARSSSTGSSSSTGGGGQESQPSPLALLAATCSRIESPNENSNNSQGPSQSGGTGELDLTATQLSQGANGWQIISSSSGATPTSKEQSGSSTNGSNGSESSKNRTVSGGQYVVAAAPNLQNQQVLTGLPGVMPNIQYQVIPQFQTVDGQQLQFAATGAQVQQDGSGQIQIIPGANQQIITNRGSGGNIIAAMPNLLQQAVPLQGLANNVLSGQTQYVTNVPVALNGNITLLPVNSVSAATLTPSSQAVTISSSGSQESGSQPVTSGTTISSASLVSSQASSSSFFTNANSYSTTTTTSNMGIMNFTTSGSSGTNSQGQTPQRVSGLQGSDALNIQQNQTSGGSLQAGQQKEGEQNQQTQQQQILIQPQLVQGGQALQALQAAPLSGQTFTTQAISQETLQNLQLQAVPNSGPIIIRTPTVGPNGQVSWQTLQLQNLQVQNPQAQTITLAPMQGVSLGQTSSSNTTLTPIASAASIPAGTVTVNAAQLSSMPGLQTINLSALGTSGIQVHPIQGLPLAIANAPGDHGAQLGLHGAGGDGIHDDTAGGEEGENSPDAQPQAGRRTRREACTCPYCKDSEGRGSGDPGKKKQHICHIQGCGKVYGKTSHLRAHLRWHTGERPFMCTWSYCGKRFTRSDELQRHKRTHTGEKKFACPECPKRFMRSDHLSKHIKTHQNKKGGPGVALSVGTLPLDSGAGSEGSGTATPSALITTNMVAMEAICPEGIARLANSGINVMQVADLQSINISGNGF

>NP_061833.1

MVDSVYRTRSLGVAAEGLPDQYADGEAARVWQLYIGDTRSRTAEYKAWLLGLLRQHGCQRVLDVACGTGVDSIMLVEEGFSVTSVDASDKMLKYALKERWNRRHEPAFDKWVIEEANWMTLDKDVPQSAEGGFDAVICLGNSFAHLPDCKGDQSEHRLALKNIASMVRAGGLLVIDHRNYDHILSTGCAPPGKNIYYKSDLTKDVTTSVLIVNNKAHMVTLDYTVQVPGAGQDGSPGLSKFRLSYYPHCLASFTELLQAAFGGKCQHSVLGDFKPYKPGQTYIPCYFIHVLKRTD

198 P09067 P22531

>P09067

MSSYFVNSFSGRYPNGPDYQLLNYGSGSSLSGSYRDPAAMHTGSYGYNYNGMDLSVNRSSASSSHFGAVGESSRAFPAPAQEPRFRQAASSCSLSSPESLPCTNGDSHGAKPSASSPSDQATSASSSANFTEIDEASASSEPEEAASQLSSPSLARAQPEPMATSTAAPEGQTPQIFPWMRKLHISHDMTGPDGKRARTAYTRYQTLELEKEFHFNRYLTRRRRIEIAHALCLSERQIKIWFQNRRMKWKKDNKLKSMSLATAGSAFQP

>P22531

MSYQQQQCKQPCQPPPVCPTPKCPEPCPPPKCPEPCPPPKCPQPCPPQQCQQKCPPVTPSPPCQPKCPPKSK

199 O95926 Q9BXI3

>O95926

MAAIAASEVLVDSAEEGSLAAAAELAAQKREQRLRKFRELHLMRNEARKLNHQEVVEEDKRLKLPANWEAKKARLEWELKEEEKKKECAARGEDYEKVKLLEISAEDAERWERKKKRKNPDLGFSDYAAAQLRQYHRLTKQIKPDMETYERLREKHGEEFFPTSNSLLHGTHVPSTEEIDRMVIDLEKQIEKRDKYSRRRPYNDDADIDYINERNAKFNKKAERFYGKYTAEIKQNLERGTAV

>Q9BXI3

MEPGQPREPQEPREPGPGAETAAAPVWEEAKIFYDNLAPKKKPKSPKPQNAVTIAVSSRALFRMDEEQQIYTEQGVEEYVRYQLEHENEPFSPGPAFPFVKALEAVNRRLRELYPDSEDVFDIVLMTNNHAQVGVRLINSINHYDLFIERFCMTGGNSPICYLKAYHTNLYLSADAEKVREAIDEGIAAATIFSPSRDVVVSQSQLRVAFDGDAVLFSDESERIVKAHGLDRFFEHEKAHENKPLAQGPLKGFLEALGRLQKKFYSKGLRLECPIRTYLVTARSAASSGARALKTLRSWGLETDEALFLAGAPKGPLLEKIRPHIFFDDQMFHVAGAQEMGTVAAHVPYGVAQTPRRTAPAKQAPSAQ

200 NP_002157.2 P49753

>NP_002157.2

MKAFSPVRSVRKNSLSDHSLGISRSKTPVDDPMSLLYNMNDCYSKLKELVPSIPQNKKVSKMEILQHVIDYILDLQIALDSHPTIVSLHHQRPGQNQASRTPLTTLNTDISILSLQASEFPSELMSNDSKALCG

>P49753

MSNKLLSPHPHSVVLRSEFKMASSPAVLRASRLYQWSLKSSAQFLGSPQLRQVGQIIRVPARMAATLILEPAGRCCWDEPVRIAVRGLAPEQPVTLRASLRDEKGALFQAHARYRADTLGELDLERAPALGGSFAGLEPMGLLWALEPEKPLVRLVKRDVRTPLAVELEVLDGHDPDPGRLLCQTRHERYFLPPGVRREPVRVGRVRGTLFLPPEPGPFPGIVDMFGTGGGLLEYRASLLAGKGFAVMALAYYNYEDLPKTMETLHLEYFEEAMNYLLSHPEVKGPGVGLLGISKGGELCLSMASFLKGITAAVVINGSVANVGGTLRYKGETLPPVGVNRNRIKVTKDGYADIVDVLNSPLEGPDQKSFIPVERAESTFLFLVGQDDHNWKSEFYANEACKRLQAHGRRKPQIICYPETGHYIEPPYFPLCRASLHALVGSPIIWGGEPRAHAMAQVDAWKQLQTFFHKHLGGHEGTIPSKV

201 NP_066951.1 P0C7P0

>NP_066951.1

MIIPVRCFTCGKIVGNKWEAYLGLLQAEYTEGDALDALGLKRYCCRRMLLAHVDLIEKLLNYAPLEK

>P0C7P0

MRGAGAILRPAARGARDLNPRRDISSWLAQWFPRTPARSVVALKTPIKVELVAGKTYRWCVCGRSKKQPFCDGSHFFQRTGLSPLKFKAQETRMVALCTCKATQRPPYCDGTHRSERVQKAEVGSPL

202 NP_036455.1 NP_660198.1

>NP_036455.1

MSVDPLSSKALKIKRELSENTPHLSDEALMGLSVRELNRHLRGLSAEEVTRLKQRRRTLKNRGYAASCRVKRVCQKEELQKQKSELEREVDKLARENAAMRLELDALRGKCEALQGFARSVAAARGPATLVAPASVITIVKSTPGSGSGPAHGPDPAHGPASCS

>NP_660198.1

MLIPFSMKNCFQLLCNCQVPAAGFKKTVKNGLILQSISNDVYQNLAVEDWIHDHMNLEGKPILFFWQNSPSVVIGRHQNPWQECNLNLMREEGIKLARRRSGGGTVYHDMGNINLTFFTTKKKYDRMENLKLIVRALNAVQPQLDVQATKRFDLLLDGQFKISGTASKIGRTTAYHHCTLLCSTDGTFLSSLLKSPYQGIRSNATASIPSLVKNLLEKDPTLTCEVLMNAVATEYAAYHQIDNHIHLINPTDETLFPGINSKAKELQTWEWIYGKTPKFSINTSFHVLYEQSHLEIKVFIDIKNGRIEICNIEAPDHWLPLEIRDKLNSSLIGSKFCPTETTMLTNILLRTCPQDHKLNSKWNILCEKIKGIM

203 NP_005646.1 Q99469

>NP_005646.1

MLNFGASLQQTAEERMEMISERPKESMYSWNKTAEKSDFEAVEALMSMSCSWKSDFKKYVENRPVTPVSDLSEEENLLPGTPDFHTIPAFCLTPPYSPSDFEPSQVSNLMAPAPSTVHFKSLSDTAKPHIAAPFKEEEKSPVSAPKLPKAQATSVIRHTADAQLCNHQTCPMKAASILNYQNNSFRRRTHLNVEAARKNIPCAAVSPNRSKCERNTVADVDEKASAALYDFSVPSSETVICRSQPAPVSPQQKSVLVSPPAVSAGGVPPMPVICQMVPLPANNPVVTTVVPSTPPSQPPAVCPPVVFMGTQVPKGAVMFVVPQPVVQSSKPPVVSPNGTRLSPIAPAPGFSPSAAKVTPQIDSSRIRSHICSHPGCGKTYFKSSHLKAHTRTHTGEKPFSCSWKGCERRFARSDELSRHRRTHTGEKKFACPMCDRRFMRSDHLTKHARRHLSAKKLPNWQMEVSKLNDIALPPTPAPTQ

>Q99469

MIPPSSPREDGVDGLPKEAVGAEQPPSPASTSSQESKLQKLKRSLSFKTKSLRSKSADNFFQRTNSEDMKLQAHMVAEISPSSSPLPAPGSLTSTPARAGLHPGGKAHAFQEYIFKKPTFCDVCNHMIVGTNAKHGLRCKACKMSIHHKCTDGLAPQRCMGKLPKGFRRYYSSPLLIHEQFGCIKEVMPIACGNKVDPVYETLRFGTSLAQRTKKGSSGSGSDSPHRTSTSDLVEVPEEANGPGGGYDLRKRSNSVFTYPENGTDDFRDPAKNINHQGSLSKDPLQMNTYVALYKFVPQENEDLEMRPGDIITLLEDSNEDWWKGKIQDRIGFFPANFVQRLQQNEKIFRCVRTFIGCKEQGQITLKENQICVSSEEEQDGFIRVLSGKKKGLIPLDVLENI

204 NP_005988.1 Q13287

>NP_005988.1

MSLAGGRAPRKTAGNRLSGLLEAEEEDEFYQTTYGGFTEESGDDEYQGDQSDTEDEVDSDFDIDEGDEPSSDGEAEEPRRKRRVVTKAYKEPLKSLRPRKVNTPAGSSQKAREEKALLPLELQDDGSDSRKSMRQSTAEHTRQTFLRVQERQGQSRRRKGPHCERPLTQEELLREAKITEELNLRSLETYERLEADKKKQVHKKRKCPGPIITYHSVTVPLVGEPGPKEENVDIEGLDPAPSVSALTPHAGTGPVNPPARCSRTFITFSDDATFEEWFPQGRPPKVPVREVCPVTHRPALYRDPVTDIPYATARAFKIIREAYKKYITAHGLPPTASALGPGPPPPEPLPGSGPRALRQKIVIK

>Q13287

MEADKDDTQQILKEHSPDEFIKDEQNKGLIDEITKKNIQLKKEIQKLETELQEATKEFQIKEDIPETKMKFLSVETPENDSQLSNISCSFQVSSKVPYEIQKGQALITFEKEEVAQNVVSMSKHHVQIKDVNLEVTAKPVPLNSGVRFQVYVEVSKMKINVTEIPDTLREDQMRDKLELSFSKSRNGGGEVDRVDYDRQSGSAVITFVEIGVADKILKKKEYPLYINQTCHRVTVSPYTEIHLKKYQIFSGTSKRTVLLTGMEGIQMDEEIVEDLINIHFQRAKNGGGEVDVVKCSLGQPHIAYFEE

205 NP_004621.2 NP_037399.2

>NP_004621.2

MATGANATPLDFPSKKRKRSRWNQDTMEQKTVIPGMPTVIPPGLTREQERAYIVQLQIEDLTRKLRTGDLGIPPNPEDRSPSPEPIYNSEGKRLNTREFRTRKKLEEERHNLITEMVALNPDFKPPADYKPPATRVSDKVMIPQDEYPEINFVGLLIGPRGNTLKNIEKECNAKIMIRGKGSVKEGKVGRKDGQMLPGEDEPLHALVTANTMENVKKAVEQIRNILKQGIETPEDQNDLRKMQLRELARLNGTLREDDNRILRPWQSSETRSITNTTVCTKCGGAGHIASDCKFQRPGDPQSAQDKARMDKEYLSLMAELGEAPVPASVGSTSGPATTPLASAPRPAAPANNPPPPSLMSTTQSRPPWMNSGPSESRPYHGMHGGGPGGPGGGPHSFPHPLPSLTGGHGGHPMQHNPNGPPPPWMQPPPPPMNQGPHPPGHHGPPPMDQYLGSTPVGSGVYRLHQGKGMMPPPPMGMMPPPPPPPSGQPPPPPSGPLPPWQQQQQQPPPPPPPSSSMASSTPLPWQQNTTTTTTSAGTGSIPPWQQQQAAAAASPGAPQMQGNPTMVPLPPGVQPPLPPGAPPPPPPPPPGSAGMMYAPPPPPPPPMDPSNFVTMMGMGVAGMPPFGMPPAPPPPPPQN

>NP_037399.2

MRSMKALQKALSRAGSHCGRGGWGHPSRSPLLGGGVRHHLSEAAAQGRETPHSHQPQHQDHDSSESGMLSRLGDLLFYTIAEGQERIPIHKFTTALKATGLQTSDPRLRDCMSEMHRVVQESSSGGLLDRDLFRKCVSSNIVLLTQAFRKKFVIPDFEEFTGHVDRIFEDVKELTGGKVAAYIPQLAKSNPDLWGVSLCTVDGQRHSVGHTKIPFCLQSCVKPLTYAISISTLGTDYVHKFVGKEPSGLRYNKLSLNEEGIPHNPMVNAGAIVVSSLIKMDCNKAEKFDFVLQYLNKMAGNEYMGFSNATFQSEKETGDRNYAIGYYLKEKKCFPKGVDMMAALDLYFQLCSVEVTCESGSVMAATLANGGICPITGESVLSAEAVRNTLSLMHSCGMYDFSGQFAFHVGLPAKSAVSGAILLVVPNVMGMMCLSPPLDKLGNSHRGTSFCQKLVSLFNFHNYDNLRHCARKLDPRREGAEIRNKTVVNLLFAAYSGDVSALRRFALSAMDMEQKDYDSRTALHVAAAEGHIEVVKFLIEACKVNPFAKDRWGNIPLDDAVQFNHLEVVKLLQDYQDSYTLSETQAEAAAEALSKENLESMV

206 NP_001443.1 Q92871

>NP_001443.1

MTTEGGPPPAPLRRACSPVPGALQAALMSPPPAAAAAAAAAPETTSSSSSSSSASCASSSSSSNSASAPSAACKSAGGGGAGAGSGGAKKASSGLRRPEKPPYSYIALIVMAIQSSPSKRLTLSEIYQFLQARFPFFRGAYQGWKNSVRHNLSLNECFIKLPKGLGRPGKGHYWTIDPASEFMFEEGSFRRRPRGFRRKCQALKPMYHRVVSGLGFGASLLPQGFDFQAPPSAPLGCHSQGGYGGLDMMPAGYDAGAGAPSHAHPHHHHHHHVPHMSPNPGSTYMASCPVPAGPGGVGAAGGGGGGDYGPDSSSSPVPSSPAMASAIECHSPYTSPAAHWSSPGASPYLKQPPALTPSSNPAASAGLHSSMSSYSLEQSYLHQNAREDLSVGLPRYQHHSTPVCDRKDFVLNFNGISSFHPSASGSYYHHHHQSVCQDIKPCVM

>Q92871

MAVTAQAARRKERVLCLFDVDGTLTPARQKIDPEVAAFLQKLRSRVQIGVVGGSDYCKIAEQLGDGDEVIEKFDYVFAENGTVQYKHGRLLSKQTIQNHLGEELLQDLINFCLSYMALLRLPKKRGTFIEFRNGMLNISPIGRSCTLEERIEFSELDKKEKIREKFVEALKTEFAGKGLRFSRGGMISFDVFPEGWDKRYCLDSLDQDSFDTIHFFGNETSPGGNDFEIFADPRTVGHSVVSPQDTVQRCREIFFPETAHEA

207 NP_005625.2 Q9ULC6

>NP_005625.2

MRPVRENSSGARSPRVPADLARSILISLPFPPDSLAHRPPSSAPTESQGLFTVAAPAPGAPSPPATLAHLLPAPAMYSLLETELKNPVGTPTQAAGTGGPAAPGGAGKSSANAAGGANSGGGSSGGASGGGGGTDQDRVKRPMNAFMVWSRGQRRKMALENPKMHNSEISKRLGADWKLLTDAEKRPFIDEAKRLRAVHMKEYPDYKYRPRRKTKTLLKKDKYSLPSGLLPPGAAAAAAAAAAAAAAASSPVGVGQRLDTYTHVNGWANGAYSLVQEQLGYAQPPSMSSPPPPPALPPMHRYDMAGLQYSPMMPPGAQSYMNVAAAAAAASGYGGMAPSATAAAAAAYGQQPATAAAAAAAAAAMSLGPMGSVVKSEPSSPPPAIASHSQRACLGDLRDMISMYLPPGGDAADAASPLPGGRLHGVHQHYQGAGTAVNGTVPLTHI

>Q9ULC6

MAPKRVVQLSLKMPTHAVCVVGVEAHVDIHSDVPKGANSFRVSGSSGVEVFMVYNRTRVKEPIGKARWPLDTDADMVVSVGTASKELKDFKVRVSYFGEQEDQALGRSVLYLTGVDISLEVDTGRTGKVKRSQGDKKTWRWGPEGYGAILLVNCDRDNHRSAEPDLTHSWLMSLADLQDMSPMLLSCNGPDKLFDSHKLVLNVPFSDSKRVRVFCARGGNSLSDYKQVLGPQCLSYEVERQPGEQEIKFYVEGLTFPDADFLGLVSLSVSLVDPGTLPEVTLFTDTVGFRMAPWIMTPNTQPPEELYVCRVMDTHGSNEKFLEDMSYLTLKANCKLTICPQVENRNDRWIQDEMEFGYIEAPHKSFPVVFDSPRNRGLKDFPYKRILGPDFGYVTREIPLPGPSSLDSFGNLDVSPPVTVGGTEYPLGRILIGSSFPKSGGRQMARAVRNFLKAQQVQAPVELYSDWLSVGHVDEFLTFVPTSDQKGFRLLLASPSACLKLFQEKKEEGYGEAAQFDGLKHQAKRSINEMLADRHLQRDNLHAQKCIDWNRNVLKRELGLAESDIVDIPQLFFLKNFYAEAFFPDMVNMVVLGKYLGIPKPYGPIINGRCCLEEKVQSLLEPLGLHCIFIDDYLSYHELQGEIHCGTNVRRKPFPFKWWNMVP

208 NP_001092755.1 NP_076425.1

>NP_001092755.1

MSDAAVDTSSEITTKDLKEKKEVVEEAENGRDAPANGNAENEENGEQEADNEVDEEEEEGGEEEEEEEEGDGEEEDGDEDEEAESATGKRAAEDDEDDDVDTKKQKTDEDD

>NP_076425.1

MARKKVRPRLIAELARRVRALREQLNRPRDSQLYAVDYETLTRPFSGRRLPVRAWADVRRESRLLQLLGRLPLFGLGRLVTRKSWLWQHDEPCYWRLTRVRPDYTAQNLDHGKAWGILTFKGKTESEAREIEHVMYHDWRLVPKHEEEAFTAFTPAPEDSLASVPYPPLLRAMIIAERQKNGDTSTEEPMLNVQRIRMEPWDYPAKQEDKGRAKGTPV

209 NP_061134.1 P15428

>NP_061134.1

MSQGLPAAGSVLQRSVAAPGNQPQPQPQQQSPEDDDRKVRRREKNRVAAQRSRKKQTQKADKLHEEYESLEQENTMLRREIGKLTEELKHLTEALKEHEKMCPLLLCPMNFVPVPPRPDPVAGCLPR

>P15428

MHVNGKVALVTGAAQGIGRAFAEALLLKGAKVALVDWNLEAGVQCKAALDEQFEPQKTLFIQCDVADQQQLRDTFRKVVDHFGRLDILVNNAGVNNEKNWEKTLQINLVSVISGTYLGLDYMSKQNGGEGGIIINMSSLAGLMPVAQQPVYCASKHGIVGFTRSAALAANLMNSGVRLNAICPGFVNTAILESIEKEENMGQYIEYKDHIKDMIKYYGILDPPLIANGLITLIEDDALNGAIMKITTSKGIHFQDYDTTPFQAKTQ

210 NP_848511.1 NP_863651.1

>NP_848511.1

MERARPEPPPQPRPLRPAPPPLPVEGTSFWAAAMEPPPSSPTLSAAASATLASSCGEAVASGLQPAVRRLLQVKPEQVLLLPQPQAQNEEAAASSAQARLLQFRPDLRLLQPPTASDGATSRPELHPVQPLALHVKAKKQKLGPSLDQSVGPRGAVETGPRASRVVKLEGPGPALGYFRGDEKGKLEAEEVMRDSMQGGAGKSPAAIREGVIKTEEPERLLEDCRLGAEPASNGLVHGSAEVILAPTSGAFGPHQQDLRIPLTLHTVPPGARIQFQGAPPSELIRLTKVPLTPVPTKMQSLLEPSVKIETKDVPLTVLPSDAGIPDTPFSKDRNGHVKRPMNAFMVWARIHRPALAKANPAANNAEISVQLGLEWNKLSEEQKKPYYDEAQKIKEKHREEFPGWVYQPRPGKRKRFPLSVSNVFSGTTQNIISTNPTTVYPYRSPTYSVVIPSLQNPITHPVGETSPAIQLPTPAVQSPSPVTLFQPSVSSAAQVAVQDPSLPVYPALPPQRFTGPSQTDTHQLHSEATHTVKQPTPVSLESANRISSSASTAHARFATSTIQPPREYSSVSPCPRSAPIPQASPIPHPHVYQPPPLGHPATLFGTPPRFSFHHPYFLPGPHYFPSSTCPYSRPPFGYGNFPSSMPECLSYYEDRYPKHEGIFSTLNRDYSFRDYSSECTHSENSRSCENMNGTSYYNSHSHSGEENLNPVPQLDIGTLENVFTAPTSTPSSIQQVNVTDSDEEEEEKVLRDL

>NP_863651.1

MDAKARNCLLQHREALEKDIKTSYIMDHMISDGFLTISEEEKVRNEPTQQQRAAMLIKMILKKDNDSYVSFYNALLHEGYKDLAALLHDGIPVVSSSSGKDSVSGITSYVRTVLCEGGVPQRPVVFVTRKKLVNAIQQKLSKLKGEPGWVTIHGMAGCGKSVLAAEAVRDHSLLEGCFPGGVHWVSVGKQDKSGLLMKLQNLCTRLDQDESFSQRLPLNIEEAKDRLRILMLRKHPRSLLILDDVWDSWVLKAFDSQCQILLTTRDKSVTDSVMGPKYVVPVESSLGKEKGLEILSLFVNMKKADLPEQAHSIIKECKGSPLVVSLIGALLRDFPNRWEYYLKQLQNKQFKRIRKSSSYDYEALDEAMSISVEMLREDIKDYYTDLSILQKDVKVPTKVLCILWDMETEEVEDILQEFVNKSLLFCDRNGKSFRYYLHDLQVDFLTEKNCSQLQDLHKKIITQFQRYHQPHTLSPDQEDCMYWYNFLAYHMASAKMHKELCALMFSLDWIKAKTELVGPAHLIHEFVEYRHILDEKDCAVSENFQEFLSLNGHLLGRQPFPNIVQLGLCEPETSEVYQQAKLQAKQEVDNGMLYLEWINKKNITNLSRLVVRPHTDAVYHACFSEDGQRIASCGADKTLQVFKAETGEKLLEIKAHEDEVLCCAFSTDDRFIATCSVDKKVKIWNSMTGELVHTYDEHSEQVNCCHFTNSSHHLLLATGSSDCFLKLWDLNQKECRNTMFGHTNSVNHCRFSPDDKLLASCSADGTLKLWDATSANERKSINVKQFFLNLEDPQEDMEVIVKCCSWSADGARIMVAAKNKIFLFDIHTSGLLGEIHTGHHSTIQYCDFSPQNHLAVVALSQYCVELWNTDSRSKVADCRGHLSWVHGVMFSPDGSSFLTSSDDQTIRLWETKKVCKNSAVMLKQEVDVVFQENEVMVLAVDHIRRLQLINGRTGQIDYLTEAQVSCCCLSPHLQYIAFGDENGAIEILELVNNRIFQSRFQHKKTVWHIQFTADEKTLISSSDDAEIQVWNWQLDKCIFLRGHQETVKDFRLLKNSRLLSWSFDGTVKVWNIITGNKEKDFVCHQGTVLSCDISHDATKFSSTSADKTAKIWSFDLLLPLHELRGHNGCVRCSAFSVDSTLLATGDDNGEIRIWNVSNGELLHLCAPLSEEGAATHGGWVTDLCFSPDGKMLISAGGYIKWWNVVTGESSQTFYTNGTNLKKIHVSPDFKTYVTVDNLGILYILQTLE

211 NP_002135.2 Q16540

>NP_002135.2

MDYNRMNSFLEYPLCNRGPSAYSAHSAPTSFPPSSAQAVDSYASEGRYGGGLSSPAFQQNSGYPAQQPPSTLGVPFPSSAPSGYAPAACSPSYGPSQYYPLGQSEGDGGYFHPSSYGAQLGGLSDGYGAGGAGPGPYPPQHPPYGNEQTASFAPAYADLLSEDKETPCPSEPNTPTARTFDWMKVKRNPPKTAKVSEPGLGSPSGLRTNFTTRQLTELEKEFHFNKYLSRARRVEIAATLELNETQVKIWFQNRRMKQKKREREEGRVPPAPPGCPKEAAGDASDQSTCTSPEASPSSVTS

>Q16540

MARNVVYPLYRLGGPQLRVFRTNFFIQLVRPGVAQPEDTVQFRIPMEMTRVDLRNYLEGIYNVPVAAVRTRVQHGSNKRRDHRNVRIKKPDYKVAYVQLAHGQTFTFPDLFPEKDESPEGSAADDLYSMLEEERQQRQSSDPRRGGVPSWFGL

212 NP_003191.1 NP_659451.1

>NP_003191.1

MNQPQRMAPVGTDKELSDLLDFSMMFPLPVTNGKGRPASLAGAQFGGSGLEDRPSSGSWGSGDQSSSSFDPSRTFSEGTHFTESHSSLSSSTFLGPGLGGKSGERGAYASFGRDAGVGGLTQAGFLSGELALNSPGPLSPSGMKGTSQYYPSYSGSSRRRAADGSLDTQPKKVRKVPPGLPSSVYPPSSGEDYGRDATAYPSAKTPSSTYPAPFYVADGSLHPSAELWSPPGQAGFGPMLGGGSSPLPLPPGSGPVGSSGSSSTFGGLHQHERMGYQLHGAEVNGGLPSASSFSSAPGATYGGVSSHTPPVSGADSLLGSRGTTAGSSGDALGKALASIYSPDHSSNNFSSSPSTPVGSPQGLAGTSQWPRAGAPGALSPSYDGGLHGLQSKIEDHLDEAIHVLRSHAVGTAGDMHTLLPGHGALASGFTGPMSLGGRHAGLVGGSHPEDGLAGSTSLMHNHAALPSQPGTLPDLSRPPDSYSGLGRAGATAAASEIKREEKEDEENTSAADHSEEEKKELKAPRARTSPDEDEDDLLPPEQKAEREKERRVANNARERLRVRDINEAFKELGRMCQLHLNSEKPQTKLLILHQAVSVILNLEQQVRERNLNPKAACLKRREEEKVSGVVGDPQMVLSAPHPGLSEAHNPAGHM

>NP_659451.1

MEELLPDGQIWANMDPEERMLAAATAFTHICAGQGEGDVRREAQSIQYDPYSKASVAPGKRPALPVQLQYPHVESNVPSETVSEASQRLRKPVMKRKVLRRKPDGEVLVTDESIISESESGTENDQDLWDLRQRLMNVQFQEDKESSFDVSQKFNLPHEYQGISQDQLICSLQREGMGSPAYEQDLIVASRPKSFILPKLDQLSRNRGKTDRVARYFEYKRDWDSIRLPGEDHRKELRWGVREQMLCRAEPQSKPQHIYVPNNYLVPTEKKRSALRWGVRCDLANGVIPRKLPFPLSPS

213 Q02962 P18054

>Q02962

MDMHCKADPFSAMHPGHGGVNQLGGVFVNGRPLPDVVRQRIVELAHQGVRPCDISRQLRVSHGCVSKILGRYYETGSIKPGVIGGSKPKVATPKVVDKIAEYKRQNPTMFAWEIRDRLLAEGICDNDTVPSVSSINRIIRTKVQQPFHPTPDGAGTGVTAPGHTIVPSTASPPVSSASNDPVGSYSINGILGIPRSNGEKRKRDEVEVYTDPAHIRGGGGLHLVWTLRDVSEGSVPNGDSQSGVDSLRKHLRADTFTQQQLEALDRVFERPSYPDVFQASEHIKSEQGNEYSLPALTPGLDEVKSSLSASTNPELGSNVSGTQTYPVVTGRDMASTTLPGYPPHVPPTGQGSYPTSTLAGMVPGSEFSGNPYSHPQYTAYNEAWRFSNPALLSSPYYYSAAPRGSAPAAAAAAYDRH

>P18054

MGRYRIRVATGAWLFSGSYNRVQLWLVGTRGEAELELQLRPARGEEEEFDHDVAEDLGLLQFVRLRKHHWLVDDAWFCDRITVQGPGACAEVAFPCYRWVQGEDILSLPEGTARLPGDNALDMFQKHREKELKDRQQIYCWATWKEGLPLTIAADRKDDLPPNMRFHEEKRLDFEWTLKAGALEMALKRVYTLLSSWNCLEDFDQIFWGQKSALAEKVRQCWQDDELFSYQFLNGANPMLLRRSTSLPSRLVLPSGMEELQAQLEKELQNGSLFEADFILLDGIPANVIRGEKQYLAAPLVMLKMEPNGKLQPMVIQIQPPNPSSPTPTLFLPSDPPLAWLLAKSWVRNSDFQLHEIQYHLLNTHLVAEVIAVATMRCLPGLHPIFKFLIPHIRYTMEINTRARTQLISDGGIFDKAVSTGGGGHVQLLRRAAAQLTYCSLCPPDDLADRGLLGLPGALYAHDALRLWEIIARYVEGIVHLFYQRDDIVKGDPELQAWCREITEVGLCQAQDRGFPVSFQSQSQLCHFLTMCVFTCTAQHAAINQGQLDWYAWVPNAPCTMRMPPPTTKEDVTMATVMGSLPDVRQACLQMAISWHLSRRQPDMVPLGHHKEKYFSGPKPKAVLNQFRTDLEKLEKEITARNEQLDWPYEYLKPSCIENSVTI

214 Q9H7Z6 NP_004560.1

>Q9H7Z6

MAAQGAAAAVAAGTSGVAGEGEPGPGENAAAEGTAPSPGRVSPPTPARGEPEVTVEIGETYLCRRPDSTWHSAEVIQSRVNDQEGREEFYVHYVGFNRRLDEWVDKNRLALTKTVKDAVQKNSEKYLSELAEQPERKITRNQKRKHDEINHVQKTYAEMDPTTAALEKEHEAITKVKYVDKIHIGNYEIDAWYFSPFPEDYGKQPKLWLCEYCLKYMKYEKSYRFHLGQCQWRQPPGKEIYRKSNISVYEVDGKDHKIYCQNLCLLAKLFLDHKTLYFDVEPFVFYILTEVDRQGAHIVGYFSKEKESPDGNNVACILTLPPYQRRGYGKFLIAFSYELSKLESTVGSPEKPLSDLGKLSYRSYWSWVLLEILRDFRGTLSIKDLSQMTSITQNDIISTLQSLNMVKYWKGQHVICVTPKLVEEHLKSAQYKKPPITVDSVCLKWAPPKHKQVKLSKK

>NP_004560.1

MEDERSFSDICGGRLALQRRYYSPSCREFCLSCPRLSLRSLTAVTCTVWLAAYGLFTLCENSMILSAAIFITLLGLLGYLHFVKIDQETLLIIDSLGIQMTSSYASGKESTTFIEMGKVKDIVINEAIYMQKVIYYLCILLKDPVEPHGISQVVPVFQSAKPRLDCLIEVYRSCQEILAHQKATSTSP

215 P31249 NP_112176.1

>P31249

MLFEQGQQALELPECTMQKAAYYENPGLFGGYGYSKTTDTYGYSTPHQPYPPPAAASSLDTDYPGSACSIQSSAPLRAPAHKGAELNGSCMRPGTGNSQGGGGGSQPPGLNSEQQPPQPPPPPPTLPPSSPTNPGGGVPAKKPKGGPNASSSSATISKQIFPWMKESRQNSKQKNSCATAGESCEDKSPPGPASKRVRTAYTSAQLVELEKEFHFNRYLCRPRRVEMANLLNLTERQIKIWFQNRRMKYKKDQKAKGILHSPASQSPERSPPLGGAAGHVAYSGQLPPVPGLAYDAPSPPAFAKSQPNMYGLAAYTAPLSSCLPQQKRYAAPEFEPHPMASNGGGFASANLQGSPVYVGGNFVESMAPASGPVFNLGHLSHPSSASVDYSCAAQIPGNHHHGPCDPHPTYTDLSAHHSSQGRLPEAPKLTHL

>NP_112176.1

MAAPLSVEVEFGGGAELLFDGIKKHRVTLPGQEEPWDIRNLLIWIKKNLLKERPELFIQGDSVRPGILVLINDADWELLGELDYQLQDQDSVLFISTLHGG

216 NP_001484.1 Q8TBX8

>NP_001484.1

MDEEYDVIVLGTGLTECILSGIMSVNGKKVLHMDRNPYYGGESSSITPLEELYKRFQLLEGPPESMGRGRDWNVDLIPKFLMANGQLVKMLLYTEVTRYLDFKVVEGSFVYKGGKIYKVPSTETEALASNLMGMFEKRRFRKFLVFVANFDENDPKTFEGVDPQTTSMRDVYRKFDLGQDVIDFTGHALALYRTDDYLDQPCLETVNRIKLYSESLARYGKSPYLYPLYGLGELPQGFARLSAIYGGTYMLNKPVDDIIMENGKVVGVKSEGEVARCKQLICDPSYIPDRVRKAGQVIRIICILSHPIKNTNDANSCQIIIPQNQVNRKSDIYVCMISYAHNVAAQGKYIAIASTTVETTDPEKEVEPALELLEPIDQKFVAISDLYEPIDDGCESQVFCSCSYDATTHFETTCNDIKDIYKRMAGTAFDFENMKRKQNDVFGEAEQ

>Q8TBX8

MASSSVPPATVSAATAGPGPGFGFASKTKKKHFVQQKVKVFRAADPLVGVFLWGVAHSINELSQVPPPVMLLPDDFKASSKIKVNNHLFHRENLPSHFKFKEYCPQVFRNLRDRFGIDDQDYLVSLTRNPPSESEGSDGRFLISYDRTLVIKEVSSEDIADMHSNLSNYHQYIVKCHGNTLLPQFLGMYRVSVDNEDSYMLVMRNMFSHRLPVHRKYDLKGSLVSREASDKEKVKELPTLKDMDFLNKNQKVYIGEEEKKIFLEKLKRDVEFLVQLKIMDYSLLLGIHDIIRGSEPEEEGPVREDESEVDGDCSLTGPPALVGSYGTSPEGIGGYIHSHRPLGPGEFESFIDVYAIRSAEGAPQKEVYFMGLIDILTQYDAKKKAAHAAKTVKHGAGAEISTVHPEQYAKRFLDFITNIFA

217 Q8IUE6 P20591

>Q8IUE6

MSGRGKQGGKARAKAKSRSSRAGLQFPVGRVHRLLRKGNYAERVGAGAPVYLAAVLEYLTAEILELAGNAARDNKKTRIIPRHLQLAVRNDEELNKLLGGVTIAQGGVLPNIQAVLLPKKTESHKPGKNK

>P20591

MVVSEVDIAKADPAAASHPLLLNGDATVAQKNPGSVAENNLCSQYEEKVRPCIDLIDSLRALGVEQDLALPAIAVIGDQSSGKSSVLEALSGVALPRGSGIVTRCPLVLKLKKLVNEDKWRGKVSYQDYEIEISDASEVEKEINKAQNAIAGEGMGISHELITLEISSRDVPDLTLIDLPGITRVAVGNQPADIGYKIKTLIKKYIQRQETISLVVVPSNVDIATTEALSMAQEVDPEGDRTIGILTKPDLVDKGTEDKVVDVVRNLVFHLKKGYMIVKCRGQQEIQDQLSLSEALQREKIFFENHPYFRDLLEEGKATVPCLAEKLTSELITHICKSLPLLENQIKETHQRITEELQKYGVDIPEDENEKMFFLIDKVNAFNQDITALMQGEETVGEEDIRLFTRLRHEFHKWSTIIENNFQEGHKILSRKIQKFENQYRGRELPGFVNYRTFETIVKQQIKALEEPAVDMLHTVTDMVRLAFTDVSIKNFEEFFNLHRTAKSKIEDIRAEQEREGEKLIRLHFQMEQIVYCQDQVYRGALQKVREKELEEEKKKKSWDFGAFQSSSATDSSMEEIFQHLMAYHQEASKRISSHIPLIIQFFMLQTYGQQLQKAMLQLLQDKDTYSWLLKERSDTSDKRKFLKERLARLTQARRRLAQFPG

218 NP_006222.2 Q99707

>NP_006222.2

MSLRSGGRRRADPGADGEASRDDGATSSVSALKRLERSQWTDKMDLRFGFERLKEPGEKTGWLINMHPTEILDEDKRLGSAVDYYFIQDDGSRFKVALPYKPYFYIATRKGCEREVSSFLSKKFQGKIAKVETVPKEDLDLPNHLVGLKRNYIRLSFHTVEDLVKVRKEISPAVKKNREQDHASDAYTALLSSVLQRGGVITDEEETSKKIADQLDNIVDMREYDVPYHIRLSIDLKIHVAHWYNVRYRGNAFPVEITRRDDLVERPDPVVLAFDIETTKLPLKFPDAETDQIMMISYMIDGQGYLITNREIVSEDIEDFEFTPKPEYEGPFCVFNEPDEAHLIQRWFEHVQETKPTIMVTYNGDFFDWPFVEARAAVHGLSMQQEIGFQKDSQGEYKAPQCIHMDCLRWVKRDSYLPVGSHNLKAAAKAKLGYDPVELDPEDMCRMATEQPQTLATYSVSDAVATYYLYMKYVHPFIFALCTIIPMEPDEVLRKGSGTLCEALLMVQAFHANIIFPNKQEQEFNKLTDDGHVLDSETYVGGHVEALESGVFRSDIPCRFRMNPAAFDFLLQRVEKTLRHALEEEEKVPVEQVTNFEEVCDEIKSKLASLKDVPSRIECPLIYHLDVGAMYPNIILTNRLQPSAMVDEATCAACDFNKPGANCQRKMAWQWRGEFMPASRSEYHRIQHQLESEKFPPLFPEGPARAFHELSREEQAKYEKRRLADYCRKAYKKIHITKVEERLTTICQRENSFYVDTVRAFRDRRYEFKGLHKVWKKKLSAAVEVGDAAEVKRCKNMEVLYDSLQLAHKCILNSFYGYVMRKGARWYSMEMAGIVCFTGANIITQARELIEQIGRPLELDTDGIWCVLPNSFPENFVFKTTNVKKPKVTISYPGAMLNIMVKEGFTNDQYQELAEPSSLTYVTRSENSIFFEVDGPYLAMILPASKEEGKKLKKRYAVFNEDGSLAELKGFEVKRRGELQLIKIFQSSVFEAFLKGSTLEEVYGSVAKVADYWLDVLYSKAANMPDSELFELISENRSMSRKLEDYGEQKSTSISTAKRLAEFLGDQMVKDAGLSCRYIISRKPEGSPVTERAIPLAIFQAEPTVRKHFLRKWLKSSSLQDFDIRAILDWDYYIERLGSAIQKIITIPAALQQVKNPVPRVKHPDWLHKKLLEKNDVYKQKKISELFTLEGRRQVTMAEASEDSPRPSAPDMEDFGLVKLPHPAAPVTVKRKRVLWESQEESQDLTPTVPWQEILGQPPALGTSQEEWLVWLRFHKKKWQLQARQRLARRKRQRLESAEGVLRPGAIRDGPATGLGSFLRRTARSILDLPWQIVQISETSQAGLFRLWALVGSDLHCIRLSIPRVFYVNQRVAKAEEGASYRKVNRVLPRSNMVYNLYEYSVPEDMYQEHINEINAELSAPDIEGVYETQVPLLFRALVHLGCVCVVNKQLVRHLSGWEAETFALEHLEMRSLAQFSYLEPGSIRHIYLYHHAQAHKALFGIFIPSQRRASVFVLDTVRSNQMPSLGALYSAEHGLLLEKVGPELLPPPKHTFEVRAETDLKTICRAIQRFLLAYKEERRGPTLIAVQSSWELKRLASEIPVLEEFPLVPICVADKINYGVLDWQRHGARRMIRHYLNLDTCLSQAFEMSRYFHIPIGNLPEDISTFGSDLFFARHLQRHNHLLWLSPTARPDLGGKEADDNCLVMEFDDQATVEINSSGCYSTVCVELDLQNLAVNTILQSHHVNDMEGADSMGISFDVIQQASLEDMITGGQAASAPASYDETALCSNTFRILKSMVVGWVKEITQYHNIYADNQVMHFYRWLRSPSSLLHDPALHRTLHNMMKKLFLQLIAEFKRLGSSVIYANFNRIILCTKKRRVEDAIAYVEYITSSIHSKETFHSLTISFSRCWEFLLWMDPSNYGGIKGKVSSRIHCGLQDSQKAGGAEDEQENEDDEEERDGEEEEEAEESNVEDLLENNWNILQFLPQAASCQNYFLMIVSAYIVAVYHCMKDGLRRSAPGSTPVRRRGASQLSQEAEGAVGALPGMITFSQDYVANELTQSFFTITQKIQKKVTGSRNSTELSEMFPVLPGSHLLLNNPALEFIKYVCKVLSLDTNITNQVNKLNRDLLRLVDVGEFSEEAQFRDPCRSYVLPEVICRSCNFCRDLDLCKDSSFSEDGAVLPQWLCSNCQAPYDSSAIEMTLVEVLQKKLMAFTLQDLVCLKCRGVKETSMPVYCSCAGDFALTIHTQVFMEQIGIFRNIAQHYGMSYLLETLEWLLQKNPQLGH

>Q99707

MSPALQDLSQPEGLKKTLRDEINAILQKRIMVLDGGMGTMIQREKLNEEHFRGQEFKDHARPLKGNNDILSITQPDVIYQIHKEYLLAGADIIETNTFSSTSIAQADYGLEHLAYRMNMCSAGVARKAAEEVTLQTGIKRFVAGALGPTNKTLSVSPSVERPDYRNITFDELVEAYQEQAKGLLDGGVDILLIETIFDTANAKAALFALQNLFEEKYAPRPIFISGTIVDKSGRTLSGQTGEGFVISVSHGEPLCIGLNCALGAAEMRPFIEIIGKCTTAYVLCYPNAGLPNTFGDYDETPSMMAKHLKDFAMDGLVNIVGGCCGSTPDHIREIAEAVKNCKPRVPPATAFEGHMLLSGLEPFRIGPYTNFVNIGERCNVAGSRKFAKLIMAGNYEEALCVAKVQVEMGAQVLDVNMDDGMLDGPSAMTRFCNLIASEPDIAKVPLCIDSSNFAVIEAGLKCCQGKCIVNSISLKEGEDDFLEKARKIKKYGAAMVVMAFDEEGQATETDTKIRVCTRAYHLLVKKLGFNPNDIIFDPNILTIGTGMEEHNLYAINFIHATKVIKETLPGARISGGLSNLSFSFRGMEAIREAMHGVFLYHAIKSGMDMGIVNAGNLPVYDDIHKELLQLCEDLIWNKDPEATEKLLRYAQTQGTGGKKVIQTDEWRNGPVEERLEYALVKGIEKHIIEDTEEARLNQKKYPRPLNIIEGPLMNGMKIVGDLFGAGKMFLPQVIKSARVMKKAVGHLIPFMEKEREETRVLNGTVEEEDPYQGTIVLATVKGDVHDIGKNIVGVVLGCNNFRVIDLGVMTPCDKILKAALDHKADIIGLSGLITPSLDEMIFVAKEMERLAIRIPLLIGGATTSKTHTAVKIAPRYSAPVIHVLDASKSVVVCSQLLDENLKDEYFEEIMEEYEDIRQDHYESLKERRYLPLSQARKSGFQMDWLSEPHPVKPTFIGTQVFEDYDLQKLVDYIDWKPFFDVWQLRGKYPNRGFPKIFNDKTVGGEARKVYDDAHNMLNTLISQKKLRARGVVGFWPAQSIQDDIHLYAEAAVPQAAEPIATFYGLRQQAEKDSASTEPYYCLSDFIAPLHSGIRDYLGLFAVACFGVEELSKAYEDDGDDYSSIMVKALGDRLAEAFAEELHERVRRELWAYCGSEQLDVADLRRLRYKGIRPAPGYPSQPDHTEKLTMWRLADIEQSTGIRLTESLAMAPASAVSGLYFSNLKSKYFAVGKISKDQVEDYALRKNISVAEVEKWLGPILGYDTD

219 NP_001001998.1 Q06265

>NP_001001998.1

MAPPSTREPRVLSATSATKSDGEMVLPGFPDADSFVKFALGSVVAVTKASGGLPQFGDEYDFYRSFPGFQAFCETQGDRLLQCMSRVMQYHGCRSNIKDRSKVTELEDKFDLLVDANDVILERVGILLDEASGVNKNQQPVLPAGLQVPKTVVSSWNRKAAEYGKKAKSETFRLLHAKNIIRPQLKFREKIDNSNTPFLPKIFIKPNAQKPLPQALSKERRERPQDRPEDLDVPPALADFIHQQRTQQVEQDMFAHPYQYELNHFTPADAVLQKPQPQLYRPIEETPCHFISSLDELVELNEKLLNCQEFAVDLEHHSYRSFLGLTCLMQISTRTEDFIIDTLELRSDMYILNESLTDPAIVKVFHGADSDIEWLQKDFGLYVVNMFDTHQAARLLNLGRHSLDHLLKLYCNVDSNKQYQLADWRIRPLPEEMLSYARDDTHYLLYIYDKMRLEMWERGNGQPVQLQVVWQRSRDICLKKFIKPIFTDESYLELYRKQKKHLNTQQLTAFQLLFAWRDKTARREDESYGYVLPNHMMLKIAEELPKEPQGIIACCNPVPPLVRQQINEMHLLIQQAREMPLLKSEVAAGVKKSGPLPSAERLENVLFGPHDCSHAPPDGYPIIPTSGSVPVQKQASLFPDEKEDNLLGTTCLIATAVITLFNEPSAEDSKKGPLTVAQKKAQNIMESFENPFRMFLPSLGHRAPVSQAAKFDPSTKIYEISNRWKLAQVQVQKDSKEAVKKKAAEQTAAREQAKEACKAAAEQAISVRQQVVLENAAKKRERATSDPRTTEQKQEKKRLKISKKPKDPEPPEKEFTPYDYSQSDFKAFAGNSKSKVSSQFDPNKQTPSGKKCIAAKKIKQSVGNKSMSFPTGKSDRGFRYNWPQR

>Q06265

MKETPLSNCERRFLLRAIEEKKRLDGRQTYDYRNIRISFGTDYGCCIVELGKTRVLGQVSCELVSPKLNRATEGILFFNLELSQMAAPAFEPGRQSDLLVKLNRLMERCLRNSKCIDTESLCVVAGEKVWQIRVDLHLLNHDGNIIDAASIAAIVALCHFRRPDVSVQGDEVTLYTPEERDPVPLSIHHMPICVSFAFFQQGTYLLVDPNEREERVMDGLLVIAMNKHREICTIQSSGGIMLLKDQVLRCSKIAGVKVAEITELILKALENDQKVRKEGGKFGFAESIANQRITAFKMEKAPIDTSDVEEKAEEIIAEAEPPSEVVSTPVLWTPGTAQIGEGVENSWGDLEDSEKEDDEGGGDQAIILDGIKMDTGVEVSDIGSQDAPIILSDSEEEEMIILEPDKNPKKIRTQTTSAKQEKAPSKKPVKRRKKKRAAN

220 NP_001418.2 NP_005800.3

>NP_001418.2

MEENDPKPGEAAAAVEGQRQPESSPGGGSGGGGGSSPGEADTGRRRALMLPAVLQAPGNHQHPHRITNFFIDNILRPEFGRRKDAGTCCAGAGGGRGGGAGGEGGASGAEGGGGAGGSEQLLGSGSREPRQNPPCAPGAGGPLPAAGSDSPGDGEGGSKTLSLHGGAKKGGDPGGPLDGSLKARGLGGGDLSVSSDSDSSQAGANLGAQPMLWPAWVYCTRYSDRPSSGPRSRKPKKKNPNKEDKRPRTAFTAEQLQRLKAEFQTNRYLTEQRRQSLAQELSLNESQIKIWFQNKRAKIKKATGNKNTLAVHLMAQGLYNHSTTAKEGKSDSE

>NP_005800.3

MASGNARIGKPAPDFKATAVVDGAFKEVKLSDYKGKYVVLFFYPLDFTFVCPTEIIAFSNRAEDFRKLGCEVLGVSVDSQFTHLAWINTPRKEGGLGPLNIPLLADVTRRLSEDYGVLKTDEGIAYRGLFIIDGKGVLRQITVNDLPVGRSVDEALRLVQAFQYTDEHGEVCPAGWKPGSDTIKPNVDDSKEYFSKHN

221 Q8NHV9 NP_004332.2

>Q8NHV9

MARSLVHDTVFYCLSVYQVKISPTPQLGAASSAEGHVGQGAPGLMGNMNPEGGVNHENGMNRDGGMIPEGGGGNQEPRQQPQPPPEEPAQAAMEGPQPENMQPRTRRTKFTLLQVEELESVFRHTQYPDVPTRRELAENLGVTEDKVRVWFKNKRARCRRHQRELMLANELRADPDDCVYIVVD

>NP_004332.2

MAALVLEDGSVLRGQPFGAAVSTAGEVVFQTGMVGYPEALTDPSYKAQILVLTYPLIGNYGIPPDEMDEFGLCKWFESSGIHVAALVVGECCPTPSHWSATRTLHEWLQQHGIPGLQGVDTRELTKKLREQGSLLGKLVQNGTEPSSLPFLDPNARPLVPEVSIKTPRVFNTGGAPRILALDCGLKYNQIRCLCQRGAEVTVVPWDHALDSQEYEGLFLSNGPGDPASYPSVVSTLSRVLSEPNPRPVFGICLGHQLLALAIGAKTYKMRYGNRGHNQPCLLVGSGRCFLTSQNHGFAVETDSLPADWAPLFTNANDGSNEGIVHNSLPFFSVQFHPEHQAGPSDMELLFDIFLETVKEATAGNPGGQTVRERLTERLCPPGIPTPGSGLPPPRKVLILGSGGLSIGQAGEFDYSGSQAIKALKEENIQTLLINPNIATVQTSQGLADKVYFLPITPHYVTQVIRNERPDGVLLTFGGQTALNCGVELTKAGVLARYGVRVLGTPVETIELTEDRRAFAARMAEIGEHVAPSEAANSLEQAQAAAERLGYPVLVRAAFALGGLGSGFASNREELSALVAPAFAHTSQVLVDKSLKGWKEIEYEVVRDAYGNCVTVCNMENLDPLGIHTGESIVVAPSQTLNDREYQLLRQTAIKVTQHLGIVGECNVQYALNPESEQYYIIEVNARLSRSSALASKATGYPLAYVAAKLALGIPLPELRNSVTGGTAAFEPSVDYCVVKIPRWDLSKFLRVSTKIGSCMKSVGEVMGIGRSFEEAFQKALRMVDENCVGFDHTVKPVSDMELETPTDKRIFVVAAALWAGYSVDRLYELTRIDRWFLHRMKRIIAHAQLLEQHRGQPLPPDLLQQAKCLGFSDKQIALAVLSTELAVRKLRQELGICPAVKQIDTVAAEWPAQTNYLYLTYWGTTHDLTFRTPHVLVLGSGVYRIGSSVEFDWCAVGCIQQLRKMGYKTIMVNYNPETVSTDYDMCDRLYFDEISFEVVMDIYELENPEGVILSMGGQLPNNMAMALHRQQCRVLGTSPEAIDSAENRFKFSRLLDTIGISQPQWRELSDLESARQFCQTVGYPCVVRPSYVLSGAAMNVAYTDGDLERFLSSAAAVSKEHPVVISKFIQEAKEIDVDAVASDGVVAAIAISEHVENAGVHSGDATLVTPPQDITAKTLERIKAIVHAVGQELQVTGPFNLQLIAKDDQLKVIECNVRVSRSFPFVSKTLGVDLVALATRVIMGEEVEPVGLMTGSGVVGVKVPQFSFSRLAGADVVLGVEMTSTGEVAGFGESRCEAYLKAMLSTGFKIPKKNILLTIGSYKNKSELLPTVRLLESLGYSLYASLGTADFYTEHGVKVTAVDWHFEEAVDGECPPQRSILEQLAEKNFELVINLSMRGAGGRRLSSFVTKGYRTRRLAADFSVPLIIDIKCTKLFVEALGQIGPAPPLKVHVDCMTSQKLVRLPGLIDVHVHLREPGGTHKEDFASGTAAALAGGITMVCAMPNTRPPIIDAPALALAQKLAEAGARCDFALFLGASSENAGTLGTVAGSAAGLKLYLNETFSELRLDSVVQWMEHFETWPSHLPIVAHAEQQTVAAVLMVAQLTQRSVHICHVARKEEILLIKAAKARGLPVTCEVAPHHLFLSHDDLERLGPGKGEVRPELGSRQDVEALWENMAVIDCFASDHAPHTLEEKCGSRPPPGFPGLETMLPLLLTAVSEGRLSLDDLLQRLHHNPRRIFHLPPQEDTYVEVDLEHEWTIPSHMPFSKAHWTPFEGQKVKGTVRRVVLRGEVAYIDGQVLVPPGYGQDVRKWPQGAVPQLPPSAPATSEMTTTPERPRRGIPGLPDGRFHLPPRIHRASDPGLPAEEPKEKSSRKVAEPELMGTPDGTCYPPPPVPRQASPQNLGTPGLLHPQTSPLLHSLVGQHILSVQQFTKDQMSHLFNVAHTLRMMVQKERSLDILKGKVMASMFYEVSTRTSSSFAAAMARLGGAVLSFSEATSSVQKGESLADSVQTMSCYADVVVLRHPQPGAVELAAKHCRRPVINAGDGVGEHPTQALLDIFTIREELGTVNGMTITMVGDLKHGRTVHSLACLLTQYRVSLRYVAPPSLRMPPTVRAFVASRGTKQEEFESIEEALPDTDVLYMTRIQKERFGSTQEYEACFGQFILTPHIMTRAKKKMVVMHPMPRVNEISVEVDSDPRAAYFRQAENGMYIRMALLATVLGRF

222 Q04727 O00142

>Q04727

MIRDLSKMYPQTRHPAPHQPAQPFKFTISESCDRIKEEFQFLQAQYHSLKLECEKLASEKTEMQRHYVMYYEMSYGLNIEMHKQAEIVKRLNAICAQVIPFLSQEHQQQVVQAVERAKQVTMAELNAIIGQQLQAQHLSHGHGLPVPLTPHPSGLQPPAIPPIGSSAGLLALSSALGGQSHLPIKDEKKHHDNDHQRDRDSIKSSSVSPSASFRGAEKHRNSADYSSESKKQKTEEKEIAARYDSDGEKSDDNLVVDVSNEDPSSPRGSPAHSPRENGLDKTRLLKKDAPISPASIASSSSTPSSKSKELSLNEKSTTPVSKSNTPTPRTDAPTPGSNSTPGLRPVPGKPPGVDPLASSLRTPMAVPCPYPTPFGIVPHAGMNGELTSPGAAYAGLHNISPQMSAAAAAAAAAAAYGRSPVVGFDPHHHMRVPAIPPNLTGIPGGKPAYSFHVSADGQMQPVPFPPDALIGPGIPRHARQINTLNHGEVVCAVTISNPTRHVYTGGKGCVKVWDISHPGNKSPVSQLDCLNRDNYIRSCRLLPDGRTLIVGGEASTLSIWDLAAPTPRIKAELTSSAPACYALAISPDSKVCFSCCSDGNIAVWDLHNQTLVRQFQGHTDGASCIDISNDGTKLWTGGLDNTVRSWDLREGRQLQQHDFTSQIFSLGYCPTGEWLAVGMENSNVEVLHVTKPDKYQLHLHESCVLSLKFAHCGKWFVSTGKDNLLNAWRTPYGASIFQSKESSSVLSCDISVDDKYIVTGSGDKKATVYEVIY

>O00142

MLLWPLRGWAARALRCFGPGSRGSPASGPGPRRVQRRAWPPDKEQEKEKKSVICVEGNIASGKTTCLEFFSNATDVEVLTEPVSKWRNVRGHNPLGLMYHDASRWGLTLQTYVQLTMLDRHTRPQVSSVRLMERSIHSARYIFVENLYRSGKMPEVDYVVLSEWFDWILRNMDVSVDLIVYLRTNPETCYQRLKKRCREEEKVIPLEYLEAIHHLHEEWLIKGSLFPMAAPVLVIEADHHMERMLELFEQNRDRILTPENRKHCP

223 P09668 Q9HD40

>P09668

MWATLPLLCAGAWLLGVPVCGAAELSVNSLEKFHFKSWMSKHRKTYSTEEYHHRLQTFASNWRKINAHNNGNHTFKMALNQFSDMSFAEIKHKYLWSEPQNCSATKSNYLRGTGPYPPSVDWRKKGNFVSPVKNQGACGSCWTFSTTGALESAIAIATGKMLSLAEQQLVDCAQDFNNHGCQGGLPSQAFEYILYNKGIMGEDTYPYQGKDGYCKFQPGKAIGFVKDVANITIYDEEAMVEAVALYNPVSFAFEVTQDFMMYRTGIYSSTSCHKTPDKVNHAVLAVGYGEKNGIPYWIVKNSWGPQWGMNGYFLIERGKNMCGLAACASYPIPLV

>Q9HD40

MNRESFAAGERLVSPAYVRQGCEARRSHEHLIRLLLEKGKCPENGWDESTLELFLHELAIMDSNNFLGNCGVGEREGRVASALVARRHYRFIHGIGRSGDISAVQPKAAGSSLLNKITNSLVLDIIKLAGVHTVANCFVVPMATGMSLTLCFLTLRHKRPKAKYIIWPRIDQKSCFKSMITAGFEPVVIENVLEGDELRTDLKAVEAKVQELGPDCILCIHSTTSCFAPRVPDRLEELAVICANYDIPHIVNNAYGVQSSKCMHLIQQGARVGRIDAFVQSLDKNFMVPVGGAIIAGFNDSFIQEISKMYPGRASASPSLDVLITLLSLGSNGYKKLLKERKEMFSYLSNQIKKLSEAYNERLLHTPHNPISLAMTLKTLDEHRDKAVTQLGSMLFTRQVSGARVVPLGSMQTVSGYTFRGFMSHTNNYPCAYLNAASAIGMKMQDVDLFIKRLDRCLKAVRKERSKESDDNYDKTEDVDIEEMALKLDNVLLDTYQDASS

224 Q96T37 P17706

>Q96T37

MRTAGRDPVPRRSPRWRRAVPLCETSAGRRVTQLRGDDLRRPATMKGKERSPVKAKRSRGGEDSTSRGERSKKLGGSGGSNGSSSGKTDSGGGSRRSLHLDKSSSRGGSREYDTGGGSSSSRLHSYSSPSTKNSSGGGESRSSSRGGGGESRSSGAASSAPGGGDGAEYKTLKISELGSQLSDEAVEDGLFHEFKRFGDVSVKISHLSGSGSGDERVAFVNFRRPEDARAAKHARGRLVLYDRPLKIEAVYVSRRRSRSPLDKDTYPPSASVVGASVGGHRHPPGGGGGQRSLSPGGAALGYRDYRLQQLALGRLPPPPPPPLPRDLERERDYPFYERVRPAYSLEPRVGAGAGAAPFREVDEISPEDDQRANRTLFLGNLDITVTESDLRRAFDRFGVITEVDIKRPSRGQTSTYGFLKFENLDMSHRAKLAMSGKIIIRNPIKIGYGKATPTTRLWVGGLGPWVPLAALAREFDRFGTIRTIDYRKGDSWAYIQYESLDAAHAAWTHMRGFPLGGPDRRLRVDFADTEHRYQQQYLQPLPLTHYELVTDAFGHRAPDPLRGARDRTPPLLYRDRDRDLYPDSDWVPPPPPVRERSTRTAATSVPAYEPLDSLDRRRDGWSLDRDRGDRDLPSSRDQPRKRRLPEESGGRHLDRSPESDRPRKRHCAPSPDRSPELSSSRDRYNSDNDRSSRLLLERPSPIRDRRGSLEKSQGDKRDRKNSASAERDRKHRTTAPTEGKSPLKKEDRSDGSAPSTSTASSKLKSPSQKQDGGTAPVASASPKLCLAWQGMLLLKNSNFPSNMHLLQGDLQVASSLLVEGSTGGKVAQLKITQRLRLDQPKLDEVTRRIKVAGPNGYAILLAVPGSSDSRSSSSSAASDTATSTQRPLRNLVSYLKQKQAAGVISLPVGGNKDKENTGVLHAFPPCEFSQQFLDSPAKALAKSEEDYLVMIIVRGFGFQIGVRYENKKRENLALTLL

>P17706

MPTTIEREFEELDTQRRWQPLYLEIRNESHDYPHRVAKFPENRNRNRYRDVSPYDHSRVKLQNAENDYINASLVDIEEAQRSYILTQGPLPNTCCHFWLMVWQQKTKAVVMLNRIVEKESVKCAQYWPTDDQEMLFKETGFSVKLLSEDVKSYYTVHLLQLENINSGETRTISHFHYTTWPDFGVPESPASFLNFLFKVRESGSLNPDHGPAVIHCSAGIGRSGTFSLVDTCLVLMEKGDDINIKQVLLNMRKYRMGLIQTPDQLRFSYMAIIEGAKCIKGDSSIQKRWKELSKEDLSPAFDHSPNKIMTEKYNGNRIGLEEEKLTGDRCTGLSSKMQDTMEENSESALRKRIREDRKATTAQKVQQMKQRLNENERKRKRWLYWQPILTKMGFMSVILVGAFVGWTLFFQQNAL

225 Q8WVY7 NP_002826.2

>Q8WVY7

MALPIIVKWGGQEYSVTTLSEDDTVLDLKQFLKTLTGVLPERQKLLGLKVKGKPAENDVKLGALKLKPNTKIMMMGTREESLEDVLGPPPDNDDVVNDFDIEDEVVEVENREENLLKISRRVKEYKVEILNPPREGKKLLVLDVDYTLFDHRSCAETGVELMRPYLHEFLTSAYEDYDIVIWSATNMKWIEAKMKELGVSTNANYKITFMLDSAAMITVHTPRRGLIDVKPLGVIWGKFSEFYSKKNTIMFDDIGRNFLMNPQNGLKIRPFMKAHLNRDKDKELLKLTQYLKEIAKLDDFLDLNHKYWERYLSKKQGQ

>NP_002826.2

MEQVEILRKFIQRVQAMKSPDHNGEDNFARDFMRLRRLSTKYRTEKIYPTATGEKEENVKKNRYKDILPFDHSRVKLTLKTPSQDSDYINANFIKGVYGPKAYVATQGPLANTVIDFWRMIWEYNVVIIVMACREFEMGRKKCERYWPLYGEDPITFAPFKISCEDEQARTDYFIRTLLLEFQNESRRLYQFHYVNWPDHDVPSSFDSILDMISLMRKYQEHEDVPICIHCSAGCGRTGAICAIDYTWNLLKAGKIPEEFNVFNLIQEMRTQRHSAVQTKEQYELVHRAIAQLFEKQLQLYEIHGAQKIADGVNEINTENMISSIEPEKQDSPPPKPPRTRSCLVEGDAKEEILQPPEPHPVPPILTPSPPSAFPTVTTVWQDNDRYHPKPVLHMVSSEQHSADLNRNYSKSTELPGKNESTIEQIDKKLERNLSFEIKKVPLQEGPKSFDGNTLLNRGHAIKIKSASPCIADKISKPQELSSDLNVGDTSQNSCVDCSVTQSNKVSVTPPEESQNSDTPPRPDRLPLDEKGHVTWSFHGPENAIPIPDLSEGNSSDINYQTRKTVSLTPSPTTQVETPDLVDHDNTSPLFRTPLSFTNPLHSDDSDSDERNSDGAVTQNKTNISTASATVSAATSTESISTRKVLPMSIARHNIAGTTHSGAEKDVDVSEDSPPPLPERTPESFVLASEHNTPVRSEWSELQSQERSEQKKSEGLITSENEKCDHPAGGIHYEMCIECPPTFSDKREQISENPTEATDIGFGNRCGKPKGPRDPPSEWT

226 NP_005241.1 NP_001815.2

>NP_005241.1

MSHLFDPRLPALAASPMLYLYGPERPGLPLAFAPAAALAASGRAETPQKPPYSYIALIAMAIQDAPEQRVTLNGIYQFIMDRFPFYHDNRQGWQNSIRHNLSLNDCFVKVPREKGRPGKGSYWTLDPRCLDMFENGNYRRRKRKPKPGPGAPEAKRPRAETHQRSAEAQPEAGSGAGGSGPAISRLQAAPAGPSPLLDGPSPPAPLHWPGTASPNEDAGDAAQGAAAVAVGQAARTGDGPGSPLRPASRSSPKSSDKSKSFSIDSILAGKQGQKPPSGDELLGGAKPGPGGRLGASLLAASSSLRPPFNASLMLDPHVQGGFYQLGIPFLSYFPLQVPDTVLHFQ

>NP_001815.2

MPFGNTHNKFKLNYKPEEEYPDLSKHNNHMAKVLTLELYKKLRDKETPSGFTVDDVIQTGVDNPGHPFIMTVGCVAGDEESYEVFKELFDPIISDRHGGYKPTDKHKTDLNHENLKGGDDLDPNYVLSSRVRTGRSIKGYTLPPHCSRGERRAVEKLSVEALNSLTGEFKGKYYPLKSMTEKEQQQLIDDHFLFDKPVSPLLLASGMARDWPDARGIWHNDNKSFLVWVNEEDHLRVISMEKGGNMKEVFRRFCVGLQKIEEIFKKAGHPFMWNQHLGYVLTCPSNLGTGLRGGVHVKLAHLSKHPKFEEILTRLRLQKRGTGGVDTAAVGSVFDVSNADRLGSSEVEQVQLVVDGVKLMVEMEKKLEKGQSIDDMIPAQK

227 NP_004134.1 P35237

>NP_004134.1

MPTTSRPALDVKGGTSPAKEDANQEMSSVAYSNLAVKDRKAVAILHYPGVASNGTKASGAPTSSSGSPIGSPTTTPPTKPPSFNLHPAPHLLASMQLQKLNSQYQGMAAATPGQPGEAGPLQNWDFGAQAGGAESLSPSAGAQSPAIIDSDPVDEEVLMSLVVELGLDRANELPELWLGQNEFDFTADFPSSC

>P35237

MDVLAEANGTFALNLLKTLGKDNSKNVFFSPMSMSCALAMVYMGAKGNTAAQMAQILSFNKSGGGGDIHQGFQSLLTEVNKTGTQYLLRMANRLFGEKSCDFLSSFRDSCQKFYQAEMEELDFISAVEKSRKHINTWVAEKTEGKIAELLSPGSVDPLTRLVLVNAVYFRGNWDEQFDKENTEERLFKVSKNEEKPVQMMFKQSTFKKTYIGEIFTQILVLPYVGKELNMIIMLPDETTDLRTVEKELTYEKFVEWTRLDMMDEEEVEVSLPRFKLEESYDMESVLRNLGMTDAFELGKADFSGMSQTDLSLSKVVHKSFVEVNEEGTEAAAATAAIMMMRCARFVPRFCADHPFLFFIQHSKTNGILFCGRFSSP

228 NP_005895.1 NP_689952.1

>NP_005895.1

MFRTKRSALVRRLWRSRAPGGEDEEEGAGGGGGGGELRGEGATDSRAHGAGGGGPGRAGCCLGKAVRGAKGHHHPHPPAAGAGAAGGAEADLKALTHSVLKKLKERQLELLLQAVESRGGTRTACLLLPGRLDCRLGPGAPAGAQPAQPPSSYSLPLLLCKVFRWPDLRHSSEVKRLCCCESYGKINPELVCCNPHHLSRLCELESPPPPYSRYPMDFLKPTADCPDAVPSSAETGGTNYLAPGGLSDSQLLLEPGDRSHWCVVAYWEEKTRVGRLYCVQEPSLDIFYDLPQGNGFCLGQLNSDNKSQLVQKVRSKIGCGIQLTREVDGVWVYNRSSYPIFIKSATLDNPDSRTLLVHKVFPGFSIKAFDYEKAYSLQRPNDHEFMQQPWTGFTVQISFVKGWGQCYTRQFISSCPCWLEVIFNSR

>NP_689952.1

MATTGALGNYYVDSFLLGADAADELSVGRYAPGTLGQPPRQAATLAEHPDFSPCSFQSKATVFGASWNPVHAAGANAVPAAVYHHHHHHPYVHPQAPVAAAAPDGRYMRSWLEPTPGALSFAGLPSSRPYGIKPEPLSARRGDCPTLDTHTLSLTDYACGSPPVDREKQPSEGAFSENNAENESGGDKPPIDPNNPAANWLHARSTRKKRCPYTKHQTLELEKEFLFNMYLTRDRRYEVARLLNLTERQVKIWFQNRRMKMKKINKDRAKDE

229 NP_653209.1 Q9H4K7

>NP_653209.1

MMATPNQTACNAESPVALEEAKTSGAPGSPQTPPERHDSGGSLPLTPRMESHSEDEDLAGAVGGLGWNSRSPRTQSPGGCSAEAVLARKKHRRRPSKRKRHWRPYLELSWAEKQQRDERQSQRASRVREEMFAKGQPVAPYNTTQFLMNDRDPEEPNLDVPHGISHPGSSGESEAGDSDGRGRAHGEFQRKDFSETYERFHTESLQGRSKQELVRDYLELEKRLSQAEEETRRLQQLQACTGQQSCRQVEELAAEVQRLRTENQRLRQENQMWNREGCRCDEEPGT

>Q9H4K7

MAPARCFSARLRTVFQGVGHWALSTWAGLKPSRLLPQRASPRLLSVGRADLAKHQELPGKKLLSEKKLKRYFVDYRRVLVCGGNGGAGASCFHSEPRKEFGGPDGGDGGNGGHVILRVDQQVKSLSSVLSRYQGFSGEDGGSKNCFGRSGAVLYIRVPVGTLVKEGGRVVADLSCVGDEYIAALGGAGGKGNRFFLANNNRAPVTCTPGQPGQQRVLHLELKTVAHAGMVGFPNAGKSSLLRAISNARPAVASYPFTTLKPHVGIVHYEGHLQIAVADIPGIIRGAHQNRGLGSAFLRHIERCRFLLFVVDLSQPEPWTQVDDLKYELEMYEKGLSARPHAIVANKIDLPEAQANLSQLRDHLGQEVIVLSALTGENLEQLLLHLKVLYDAYAEAELGQGRQPLRW

230 NP_001036111.1 Q9P278

>NP_001036111.1

MALVFQFGQPVRAQPLPGLCHGKLIRTNACDVCNSTDLPEVEIISLLEEQLPHYKLRADTIYGYDHDDWLHTPLISPDANIDLTTEQIEETLKYFLLCAERVGQMTKTYNDIDAVTRLLEEKERDLELAARIGQSLLKKNKTLTERNELLEEQVEHIREEVSQLRHELSMKDELLQFYTSAAEESEPESVCSTPLKRNESSSSVQNYFHLDSLQKKLKDLEEENVVLRSEASQLKTETITYEEKEQQLVNDCVKELRDANVQIASISEELAKKTEDAARQQEEITHLLSQIVDLQKKAKACAVENEELVQHLGAAKDAQRQLTAELRELEDKYAECMEMLHEAQEELKNLRNKTMPNTTSRRYHSLGLFPMDSLAAEIEGTMRKELQLEEAESPDITHQKRVFETVRNINQVVKQRSLTPSPMNIPGSNQSSAMNSLLSSCVSTPRSSFYGSDIGNVVLDNKTNSIILETEAADLGNDERSKKPGTPGTPGSHDLETALRRLSLRRENYLSERRFFEEEQERKLQELAEKGELRSGSLTPTESIMSLGTHSRFSEFTGFSGMSFSSRSYLPEKLQIVKPLEGSATLHHWQQLAQPHLGGILDPRPGVVTKGFRTLDVDLDEVYCLNDFEEDDTGDHISLPRLATSTPVQHPETSAHHPGKCMSQTNSTFTFTTCRILHPSDELTRVTPSLNSAPTPACGSTSHLKSTPVATPCTPRRLSLAESFTNTRESTTTMSTSLGLVWLLKERGISAAVYDPQSWDRAGRGSLLHSYTPKMAVIPSTPPNSPMQTPTSSPPSFEFKCTSPPYDNFLASKPASSILREVREKNVRSSESQTDVSVSNLNLVDKVRRFGVAKVVNSGRAHVPTLTEEQGPLLCGPPGPAPALVPRGLVPEGLPLRCPTVTSAIGGLQLNSGIRRNRSFPTMVGSSMQMKAPVTLTSGILMGAKLSKQTSLR

>Q9P278

MAPTLLQKLFNKRGSSGSSAAASAQGRAPKEGPAFSWSCSEFDLNEIRLIVYQDCDRRGRQVLFDSKAVQKIEEVTAQKTEDVPIKISAKCCQGSSSVSSSSSSSISSHSSSGGSSHHAKEQLPKYQYTRPASDVNMLGEMMFGSVAMSYKGSTLKIHYIRSPPQLMISKVFSARMGSFCGSTNNLQDSFEYINQDPNLGKLNTNQNSLGPCRTGSNLAHSTPVDMPSRGQNEDRDSGIARSASLSSLLITPFPSPSSSTSSSSSYQRRWLRSQTTSLENGIIPRRSTDETFSLAEETCSSNPAMVRRKKIAISIIFSLCEKEEAQRNFQDFFFSHFPLFESHMNRLKSAIEKAMISCRKIAESSLRVQFYVSRLMEALGEFRGTIWNLYSVPRIAEPVWLTMMSGTLEKNQLCQRFLKEFTLLIEQINKNQFFAALLTAVLTYHLAWVPTVMPVDHPPIKAFSEKRTSQSVNMLAKTHPYNPLWAQLGDLYGAIGSPVRLTRTVVVGKQKDLVQRILYVLTYFLRCSELQENQLTWSGNHGEGDQVLNGSKIITALEKGEVEESEYVVITVRNEPALVPPILPPTAAERHNPWPTGFPECPEGTDSRDLGLKPDKEANRRPEQGSEACSAGCLGPASDASWKPQNAFCGDEKNKEAPQDGSSRLPSCEVLGAGMKMDQQAVCELLKVEMPTRLPDRSVAWPCPDRHLREKPSLEKVTFQIGSFASPESDFESRMKKMEERVKACGPSLEASEAADVAQDPQVSRSPFKPGFQENVCCPQNRLSEGDEGESDKGFAEDRGSRNDMAADIAGQLSHAADLGTASHGAGGTGGRRLEATRGLYVKAAEGPVLEPVAPRCVQRGPGLVAGANIPCGDDNKKANFRTEGDIPRNESSDSALGDSDDEACASAMLDLGHGGDRTGGSLEVELPLPRSQSISTQNVRNFGRSLLAGYCPTYMPDLVLHGTGSDEKLKQCLVADLVHTVHHPVLDEPIAEAVCIIADTDKWSVQVATSQRKVTDNMKLGQDVLVSSQVSSLLQSILQLYKLHLPADFCIMHLEDRLQEMYLKSKMLSEYLRGHTRVHVKELGVVLGIESNDLPLLTAIASTHSPYVAQILL

231 NP_001231.2 P50226

>NP_001231.2

MEGERKNNNKRWYFTREQLENSPSRRFGVDPDKELSYRQQAANLLQDMGQRLNVSQLTINTAIVYMHRFYMIQSFTQFPGNSVAPAALFLAAKVEEQPKKLEHVIKVAHTCLHPQESLPDTRSEAYLQQVQDLVILESIILQTLGFELTIDHPHTHVVKCTQLVRASKDLAQTSYFMATNSLHLTTFSLQYTPPVVACVCIHLACKWSNWEIPVSTDGKHWWEYVDATVTLELLDELTHEFLQILEKTPNRLKRIWNWRACEAAKKTKADDRGTDEKTSEQTILNMISQSSSDTTIAGLMSMSTSTTSAVPSLPVSEESSSNLTSVEMLPGKRWLSSQPSFKLEPTQGHRTSENLALTGVDHSLPQDGSNAFISQKQNSKSVPSAKVSLKEYRAKHAEELAAQKRQLENMEANVKSQYAYAAQNLLSHHDSHSSVILKMPIEGSENPERPFLEKADKTALKMRIPVAGGDKAASSKPEEIKMRIKVHAAADKHNSVEDSVTKSREHKEKHKTHPSNHHHHHNHHSHKHSHSQLPVGTGNKRPGDPKHSSQTSNLAHKTYSLSSSFSSSSSTRKRGPSEETGGAVFDHPAKIAKSTKSSSLNFSFPSLPTMGQMPGHSSDTSGLSFSQPSCKTRVPHSKLDKGPTGANGHNTTQTIDYQDTVNMLHSLLSAQGVQPTQPTAFEFVRPYSDYLNPRSGGISSRSGNTDKPRPPPLPSEPPPPLPPLPK

>P50226

MELIQDTSRPPLEYVKGVPLIKYFAEALGPLQSFQARPDDLLISTYPKSGTTWVSQILDMIYQGGDLEKCHRAPIFMRVPFLEFKVPGIPSGMETLKNTPAPRLLKTHLPLALLPQTLLDQKVKVVYVARNAKDVAVSYYHFYHMAKVYPHPGTWESFLEKFMAGEVSYGSWYQHVQEWWELSRTHPVLYLFYEDMKENPKREIQKILEFVGRSLPEETVDLMVEHTSFKEMKKTPMTNYTTVRREFMDHSISPFMRKGMAGDWKTTFTVAQNERFDADYAEKMAGCSLSFRSEL

232 Q99697 NP_003795.2

>Q99697

METNCRKLVSACVQLGVQPAAVECLFSKDSEIKKVEFTDSPESRKEAASSKFFPRQHPGANEKDKSQQGKNEDVGAEDPSKKKRQRRQRTHFTSQQLQELEATFQRNRYPDMSTREEIAVWTNLTEARVRVWFKNRRAKWRKRERNQQAELCKNGFGPQFNGLMQPYDDMYPGYSYNNWAAKGLTSASLSTKSFPFFNSMNVNPLSSQSMFSPPNSISSMSMSSSMVPSAVTGVPGSSLNSLNNLNNLSSPSLNSAVPTPACPYAPPTPPYVYRDTCNSSLASLRLKAKQHSSFGYASVQNPASNLSACQYAVDRPV

>NP_003795.2

MQPDMSLNVIKMKSSDFLESAELDSGGFGKVSLCFHRTQGLMIMKTVYKGPNCIEHNEALLEEAKMMNRLRHSRVVKLLGVIIEEGKYSLVMEYMEKGNLMHVLKAEMSTPLSVKGRIILEIIEGMCYLHGKGVIHKDLKPENILVDNDFHIKIADLGLASFKMWSKLNNEEHNELREVDGTAKKNGGTLYYMAPEHLNDVNAKPTEKSDVYSFAVVLWAIFANKEPYENAICEQQLIMCIKSGNRPDVDDITEYCPREIISLMKLCWEANPEARPTFPGIEEKFRPFYLSQLEESVEEDVKSLKKEYSNENAVVKRMQSLQLDCVAVPSSRSNSATEQPGSLHSSQGLGMGPVEESWFAPSLEHPQEENEPSLQSKLQDEANYHLYGSRMDRQTKQQPRQNVAYNREEERRRRVSHDPFAQQRPYENFQNTEGKGTAYSSAASHGNAVHQPSGLTSQPQVLYQNNGLYSSHGFGTRPLDPGTAGPRVWYRPIPSHMPSLHNIPVPETNYLGNTPTMPFSSLPPTDESIKYTIYNSTGIQIGAYNYMEIGGTSSSLLDSTNTNFKEEPAAKYQAIFDNTTSLTDKHLDPIRENLGKHWKNCARKLGFTQSQIDEIDHDYERDGLKEKVYQMLQKWVMREGIKGATVGKLAQALHQCSRIDLLSSLIYVSQN

233 NP_001417.3 NP_071445.1

>NP_001417.3

MEEQQPEPKSQRDSALGAAAAATPGGLSLSLSPGASGSSGSGSDGDSVPVSPQPAPPSPPAAPCLPPLAHHPHLPPHPPPPPPQHLAAPAHQPQPAAQLHRTTNFFIDNILRPDFGCKKEQPPPQLLVAAAARGGAGGGGRVERDRGQTAAGRDPVHPLGTRAPGAASLLCAPDANCGPPDGSQPAAAGAGASKAGNPAAAAAAAAAAVAAAAAAAAAKPSDTGGGGSGGGAGSPGAQGTKYPEHGNPAILLMGSANGGPVVKTDSQQPLVWPAWVYCTRYSDRPSSGPRTRKLKKKKNEKEDKRPRTAFTAEQLQRLKAEFQANRYITEQRRQTLAQELSLNESQIKIWFQNKRAKIKKATGIKNGLALHLMAQGLYNHSTTTVQDKDESE

>NP_071445.1

MGEEGGSASHDEEERASVLLGHSPGCEMCSQEAFQAQRSQLVELLVSGSLEGFESVLDWLLSWEVLSWEDYEGFHLLGQPLSHLARRLLDTVWNKGTWACQKLIAAAQEAQADSQSPKLHGCWDPHSLHPARDLQSHRPAIVRRLHSHVENMLDLAWERGFVSQYECDEIRLPIFTPSQRARRLLDLATVKANGLAAFLLQHVQELPVPLALPLEAATCKKYMAKLRTTVSAQSRFLSTYDGAETLCLEDIYTENVLEVWADVGMAGPPQKSPATLGLEELFSTPGHLNDDADTVLVVGEAGSGKSTLLQRLHLLWAAGQDFQEFLFVFPFSCRQLQCMAKPLSVRTLLFEHCCWPDVGQEDIFQLLLDHPDRVLLTFDGFDEFKFRFTDRERHCSPTDPTSVQTLLFNLLQGNLLKNARKVVTSRPAAVSAFLRKYIRTEFNLKGFSEQGIELYLRKRHHEPGVADRLIRLLQETSALHGLCHLPVFSWMVSKCHQELLLQEGGSPKTTTDMYLLILQHFLLHATPPDSASQGLGPSLLRGRLPTLLHLGRLALWGLGMCCYVFSAQQLQAAQVSPDDISLGFLVRAKGVVPGSTAPLEFLHITFQCFFAAFYLALSADVPPALLRHLFNCGRPGNSPMARLLPTMCIQASEGKDSSVAALLQKAEPHNLQITAAFLAGLLSREHWGLLAECQTSEKALLRRQACARWCLARSLRKHFHSIPPAAPGEAKSVHAMPGFIWLIRSLYEMQEERLARKAARGLNVGHLKLTFCSVGPTECAALAFVLQHLRRPVALQLDYNSVGDIGVEQLLPCLGVCKALYLRDNNISDRGICKLIECALHCEQLQKLALFNNKLTDGCAHSMAKLLACRQNFLALRLGNNYITAAGAQVLAEGLRGNTSLQFLGFWGNRVGDEGAQALAEALGDHQSLRWLSLVGNNIGSVGAQALALMLAKNVMLEELCLEENHLQDEGVCSLAEGLKKNSSLKILKLSNNCITYLGAEALLQALERNDTILEVWLRGNTFSLEEVDKLGCRDTRLLL

234 NP_001929.1 P12104

>NP_001929.1

MASSSGNDDDLTIPRAAINKMIKETLPNVRVANDARELVVNCCTEFIHLISSEANEICNKSEKKTISPEHVIQALESLGFGSYISEVKEVLQECKTVALKRRKASSRLENLGIPEEELLRQQQELFAKARQQQAELAQQEWLQMQQAAQQAQLAAASASASNQAGSSQDEEDDDDI

>P12104

MAFDSTWKVDRSENYDKFMEKMGVNIVKRKLAAHDNLKLTITQEGNKFTVKESSAFRNIEVVFELGVTFNYNLADGTELRGTWSLEGNKLIGKFKRTDNGNELNTVREIIGDELVQTYVYEGVEAKRIFKKD

235 P20290 Q8WVN8

>P20290

MRRTGAPAQADSRGRGRARGGCPGGEATLSQPPPRGGTRGQEPQMKETIMNQEKLAKLQAQVRIGGKGTARRKKKVVHRTATADDKKLQFSLKKLGVNNISGIEEVNMFTNQGTVIHFNNPKVQASLAANTFTITGHAETKQLTEMLPSILNQLGADSLTSLRRLAEALPKQSVDGKAPLATGEDDDDEVPDLVENFDEASKNEAN

>Q8WVN8

MSVSGLKAELKFLASIFDKNHERFRIVSWKLDELHCQFLVPQQGSPHSLPPPLTLHCNITESYPSSSPIWFVDSEDPNLTSVLERLEDTKNNNLLRQQLKWLICELCSLYNLPKHLDVEMLDQPLPTGQNGTTEEVTSEEEEEEEEMAEDIEDLDHYEMKEEEPISGKKSEDEGIEKENLAILEKIRKTQRQDHLNGAVSGSVQASDRLMKELRDIYRSQSYKTGIYSVELINDSLYDWHVKLQKVDPDSPLHSDLQILKEKEGIEYILLNFSFKDNFPFDPPFVRVVLPVLSGGYVLGGGALCMELLTKQGWSSAYSIESVIMQINATLVKGKARVQFGANKNQYNLARAQQSYNSIVQIHEKNGWYTPPKEDG

236 NP_068804.1 NP_000424.2

>NP_068804.1

MSTSQPGACPCQGAASRPAILYALLSSSLKAVPRPRSRCLCRQHRPVQLCAPHRTCREALDVLAKTVAFLRNLPSFWQLPPQDQRRLLQGCWGPLFLLGLAQDAVTFEVAEAPVPSILKKILLEEPSSSGGSGQLPDRPQPSLAAVQWLQCCLESFWSLELSPKEYACLKGTILFNPDVPGLQAASHIGHLQQEAHWVLCEVLEPWCPAAQGRLTRVLLTASTLKSIPTSLLGDLFFRPIIGDVDIAGLLGDMLLLR

>NP_000424.2

MSLVEAISLWNEGVLAADKKDWKGALDAFSAVQDPHSRICFNIGCMYTILKNMTEAEKAFTRSINRDKHLAVAYFQRGMLYYQTEKYDLAIKDLKEALIQLRGNQLIDYKILGLQFKLFACEVLYNIAFMYAKKEEWKKAEEQLALATSMKSEPRHSKIDKAMECVWKQKLYEPVVIPVGKLFRPNERQVAQLAKKDYLGKATVVASVVDQDSFSGFAPLQPQAAEPPPRPKTPEIFRALEGEAHRVLFGFVPETKEELQVMPGNIVFVLKKGNDNWATVMFNGQKGLVPCNYLEPVELRIHPQQQPQEESSPQSDIPAPPSSKAPGRPQLSPGQKQKEEPKEVKLSVPMPYTLKVHYKYTVVMKTQPGLPYSQVRDMVSKKLELRLEHTKLSYRPRDSNELVPLSEDSMKDAWGQVKNYCLTLWCENTVGDQGFPDEPKESEKADANNQTTEPQLKKGSQVEALFSYEATQPEDLEFQEGDIILVLSKVNEEWLEGECKGKVGIFPKVFVEDCATTDLESTRREV

237 Q15349 NP_055683.3

>Q15349

MDLSMKKFAVRRFFSVYLRRKSRSKSSSLSRLEEEGVVKEIDISHHVKEGFEKADPSQFELLKVLGQGSYGKVFLVRKVKGSDAGQLYAMKVLKKATLKVRDRVRSKMERDILAEVNHPFIVKLHYAFQTEGKLYLILDFLRGGDLFTRLSKEVMFTEEDVKFYLAELALALDHLHSLGIIYRDLKPENILLDEEGHIKITDFGLSKEAIDHDKRAYSFCGTIEYMAPEVVNRRGHTQSADWWSFGVLMFEMLTGSLPFQGKDRKETMALILKAKLGMPQFLSGEAQSLLRALFKRNPCNRLGAGIDGVEEIKRHPFFVTIDWNTLYRKEIKPPFKPAVGRPEDTFHFDPEFTARTPTDSPGVPPSANAHHLFRGFSFVASSLIQEPSQQDLHKVPVHPIVQQLHGNNIHFTDGYEIKEDIGVGSYSVCKRCVHKATDTEYAVKIIDKSKRDPSEEIEILLRYGQHPNIITLKDVYDDGKFVYLVMELMRGGELLDRILRQRYFSEREASDVLCTITKTMDYLHSQGVVHRDLKPSNILYRDESGSPESIRVCDFGFAKQLRAGNGLLMTPCYTANFVAPEVLKRQGYDAACDIWSLGILLYTMLAGFTPFANGPDDTPEEILARIGSGKYALSGGNWDSISDAAKDVVSKMLHVDPHQRLTAMQVLKHPWVVNREYLSPNQLSRQDVHLVKGAMAATYFALNRTPQAPRLEPVLSSNLAQRRGMKRLTSTRL

>NP_055683.3

MPLSSPNAAATASDMDKNSGSNSSSASSGSSKGQQPPRSASAGPAGESKPKSDGKNSSGSKRYNRKRELSYPKNESFNNQSRRSSSQKSKTFNKMPPQRGGGSSKLFSSSFNGGRRDEVAEAQRAEFSPAQFSGPKKINLNHLLNFTFEPRGQTGHFEGSGHGSWGKRNKWGHKPFNKELFLQANCQFVVSEDQDYTAHFADPDTLVNWDFVEQVRICSHEVPSCPICLYPPTAAKITRCGHIFCWACILHYLSLSEKTWSKCPICYSSVHKKDLKSVVATESHQYVVGDTITMQLMKREKGVLVALPKSKWMNVDHPIHLGDEQHSQYSKLLLASKEQVLHRVVLEEKVALEQQLAEEKHTPESCFIEAAIQELKTREEALSGLAGSRREVTGVVAALEQLVLMAPLAKESVFQPRKGVLEYLSAFDEETTEVCSLDTPSRPLALPLVEEEEAVSEPEPEGLPEACDDLELADDNLKEGTICTESSQQEPITKSGFTRLSSSPCYYFYQAEDGQHMFLHPVNVRCLVREYGSLERSPEKISATVVEIAGYSMSEDVRQRHRYLSHLPLTCEFSICELALQPPVVSKETLEMFSDDIEKRKRQRQKKAREERRRERRIEIEENKKQGKYPEVHIPLENLQQFPAFNSYTCSSDSALGPTSTEGHGALSISPLSRSPGSHADFLLTPLSPTASQGSPSFCVGSLEEDSPFPSFAQMLRVGKAKADVWPKTAPKKDENSLVPPAPVDSDGESDNSDRVPVPSFQNSFSQAIEAAFMKLDTPATSDPLSEEKGGKKRKKQKQKLLFSTSVVHTK

238 NP_060612.2 Q9BRJ2

>NP_060612.2

MSAQGDCEFLVQRARELVPQDLWAAKAWLITARSLYPADFNIQYEMYTIERNAERTATAGRLLYDMFVNFPDQPVVWREISIITSALRNDSQDKQTQFLRSLFETLPGRVQCEMLLKVTEQCFNTLERSEMLLLLLRRFPETVVQHGVGLGEALLEAETIEEQESPVNCFRKLFVCDVLPLIINNHDVRLPANLLYKYLNKAAEFYINYVTRSTQIENQHQGAQDTSDLMSPSKRSSQKYIIEGLTEKSSQIVDPWERLFKILNVVGMRCEWQMDKGRRSYGDILHRMKDLCRYMNNFDSEAHAKYKNQVVYSTMLVFFKNAFQYVNSIQPSLFQGPNAPSQVPLVLLEDVSNVYGDVEIDRNKHIHKKRKLAEGREKTMSSDDEDCSAKGRNRHIVVNKAELANSTEVLESFKLARESWELLYSLEFLDKEFTRICLAWKTDTWLWLRIFLTDMIIYQGQYKKAIASLHHLAALQGSISQPQITGQGTLEHQRALIQLATCHFALGEYRMTCEKVLDLMCYMVLPIQDGGKSQEEPSKVKPKFRKGSDLKLLPCTSKAIMPYCLHLMLACFKLRAFTDNRDDMALGHVIVLLQQEWPRGENLFLKAVNKICQQGNFQYENFFNYVTNIDMLEEFAYLRTQEGGKIHLELLPNQGMLIKHHTVTRGITKGVKEDFRLAMERQVSRCGENLMVVLHRFCINEKILLLQTLT

>Q9BRJ2

MAAPIPQGFSCLSRFLGWWFRQPVLVTQSAAIVPVRTKKRFTPPIYQPKFKTEKEFMQHARKAGLVIPPEKSDRSIHLACTAGIFDAYVPPEGDARISSLSKEGLIERTERMKKTMASQVSIRRIKDYDANFKIKDFPEKAKDIFIEAHLCLNNSDHDRLHTLVTEHCFPDMTWDIKYKTVRWSFVESLEPSHVVQVRCSSMMNQGNVYGQITVRMHTRQTLAIYDRFGRLMYGQEDVPKDVLEYVVFEKQLTNPYGSWRMHTKIVPPWAPPKQPILKTVMIPGPQLKPEEEYEEAQGEAQKPQLA

239 Q5XKR4 NP_006825.1

>Q5XKR4

MLSHADLLDARLGMKDAAELLGHREAVKCRLGVGGSDPGGHPGDLAPNSDPVEGATLLPGEDITTVGSTPASLAVSAKDPDKQPGPQGGPNPSQAGQQQGQQKQKRHRTRFTPAQLNELERSFAKTHYPDIFMREELALRIGLTESRVQVWFQNRRAKWKKRKKTTNVFRAPGTLLPTPGLPQFPSAAAAAAAAMGDSLCSFHANDTRWAAAAMPGVSQLPLPPALGRQQAMAQSLSQCSLAAGPPPNSMGLSNSLAGSNGAGLQSHLYQPAFPGMVPASLPGPSNVSGSPQLCSSPDSSDVWRGTSIASLRRKALEHTVSMSFT

>NP_006825.1

MAGGGAGDPGLGAAAAPAPETREHLFKVLVIGELGVGKTSIIKRYVHQLFSQHYRATIGVDFALKVLNWDSRTLVRLQLWDIAGQERFGNMTRVYYKEAVGAFVVFDISRSSTFEAVLKWKSDLDSKVHLPNGSPIPAVLLANKCDQNKDSSQSPSQVDQFCKEHGFAGWFETSAKDNINIEEAARFLVEKILVNHQSFPNEENDVDKIKLDQETLRAENKSQCC

240 NP_003064.2 NP_055595.2

>NP_003064.2

MMMMALSKTFGQKPVKFQLEDDGEFYMIGSEVGNYLRMFRGSLYKRYPSLWRRLATVEERKKIVASSHGKKTKPNTKDHGYTTLATSVTLLKASEVEEILDGNDEKYKAVSISTEPPTYLREQKAKRNSQWVPTLPNSSHHLDAVPCSTTINRNRMGRDKKRTFPLCFDDHDPAVIHENASQPEVLVPIRLDMEIDGQKLRDAFTWNMNEKLMTPEMFSEILCDDLDLNPLTFVPAIASAIRQQIESYPTDSILEDQSDQRVIIKLNIHVGNISLVDQFEWDMSEKENSPEKFALKLCSELGLGGEFVTTIAYSIRGQLSWHQKTYAFSENPLPTVEIAIRNTGDADQWCPLLETLTDAEMEKKIRDQDRNTRRMRRLANTAPAW

>NP_055595.2

MVGELRYREFRVPLGPGLHAYPDELIRQRVGHDGHPEYQIRWLILRRGDEGDGGSGQVDCKAEHILLWMSKDEIYANCHKMLGEDGQVIGPSQESAGEVGALDKSVLEEMETDVKSLIQRALRQLEECVGTIPPAPLLHTVHVLSAYASIEPLTGVFKDPRVLDLLMHMLSSPDYQIRWSAGRMIQALSSHDAGTRTQILLSLSQQEAIEKHLDFDSRCALLALFAQATLSEHPMSFEGIQLPQVPGRVLFSLVKRYLHVTSLLDQLNDSAAEPGAQNTSAPEELSGERGQLELEFSMAMGTLISELVQAMRWDQASDRPRSSARSPGSIFQPQLADVSPGLPAAQAQPSFRRSRRFRPRSEFASGNTYALYVRDTLQPGMRVRMLDDYEEISAGDEGEFRQSNNGVPPVQVFWESTGRTYWVHWHMLEILGFEEDIEDMVEADEYQGAVASRVLGRALPAWRWRPMTELYAVPYVLPEDEDTEECEHLTLAEWWELLFFIKKLDGPDHQEVLQILQENLDGEILDDEILAELAVPIELAQDLLLTLPQRLNDSALRDLINCHVYKKYGPEALAGNQAYPSLLEAQEDVLLLDAQAQAKDSEDAAKVEAKEPPSQSPNTPLQRLVEGYGPAGKILLDLEQALSSEGTQENKVKPLLLQLQRQPQPFLALMQSLDTPETNRTLHLTVLRILKQLVDFPEALLLPWHEAVDACMACLRSPNTDREVLQELIFFLHRLTSVSRDYAVVLNQLGARDAISKALEKHLGKLELAQELRDMVFKCEKHAHLYRKLITNILGGCIQMVLGQIEDHRRTHQPINIPFFDVFLRYLCQGSSVEVKEDKCWEKVEVSSNPHRASKLTDHNPKTYWESNGSAGSHYITLHMRRGILIRQLTLLVASEDSSYMPARVVVCGGDSTSSLHTELNSVNVMPSASRVILLENLTRFWPIIQIRIKRCQQGGIDTRIRGLEILGPKPTFWPVFREQLCRHTRLFYMVRAQAWSQDMAEDRRSLLHLSSRLNGALRQEQNFADRFLPDDEAAQALGKTCWEALVSPVVQNITSPDEDGISPLGWLLDQYLECQEAVFNPQSRGPAFFSRVRRLTHLLVHVEPCEAPPPVVATPRPKGRNRSHDWSSLATRGLPSSIMRNLTRCWRAVVEKQVNNFLTSSWRDDDFVPRYCEHFNILQNSSSELFGPRAAFLLALQNGCAGALLKLPFLKAAHVSEQFARHIDQQIQGSRIGGAQEMERLAQLQQCLQAVLIFSGLEIATTFEHYYQHYMADRLLGVVSSWLEGAVLEQIGPCFPNRLPQQMLQSLSTSKELQRQFHVYQLQQLDQELLKLEDTEKKIQVGLGASGKEHKSEKEEEAGAAAVVDVAEGEEEEEENEDLYYEGAMPEVSVLVLSRHSWPVASICHTLNPRTCLPSYLRGTLNRYSNFYNKSQSHPALERGSQRRLQWTWLGWAELQFGNQTLHVSTVQMWLLLYLNDLKAVSVESLLAFSGLSADMLNQAIGPLTSSRGPLDLHEQKDIPGGVLKIRDGSKEPRSRWDIVRLIPPQTYLQAEGEDGQNLEKRRNLLNCLIVRILKAHGDEGLHIDQLVCLVLEAWQKGPCPPRGLVSSLGKGSACSSTDVLSCILHLLGKGTLRRHDDRPQVLSYAVPVTVMEPHTESLNPGSSGPNPPLTFHTLQIRSRGVPYASCTATQSFSTFR

241 NP_000018.1 NP_000847.2

>NP_000018.1

MARKSNLPVLLVPFLLCQALVRCSSPLPLVVNTWPFKNATEAAWRALASGGSALDAVESGCAMCEREQCDGSVGFGGSPDELGETTLDAMIMDGTTMDVGAVGDLRRIKNAIGVARKVLEHTTHTLLVGESATTFAQSMGFINEDLSTSASQALHSDWLARNCQPNYWRNVIPDPSKYCGPYKPPGILKQDIPIHKETEDDRGHDTIGMVVIHKTGHIAAGTSTNGIKFKIHGRVGDSPIPGAGAYADDTAGAAAATGNGDILMRFLPSYQAVEYMRRGEDPTIACQKVISRIQKHFPEFFGAVICANVTGSYGAACNKLSTFTQFSFMVYNSEKNQPTEEKVDCI

>NP_000847.2

MFCTKLKDLKITGECPFSLLAPGQVPNESSEEAAGSSESCKATVPICQDIPEKNIQESLPQRKTSRSRVYLHTLAESICKLIFPEFERLNVALQRTLAKHKIKESRKSLEREDFEKTIAEQAVAAGVPVEVIKESLGEEVFKICYEEDENILGVVGGTLKDFLNSFSTLLKQSSHCQEAGKRGRLEDASILCLDKEDDFLHVYYFFPKRTTSLILPGIIKAAAHVLYETEVEVSLMPPCFHNDCSEFVNQPYLLYSVHMKSTKPSLSPSKPQSSLVIPTSLFCKTFPFHFMFDKDMTILQFGNGIRRLMNRRDFQGKPNFEEYFEILTPKINQTFSGIMTMLNMQFVVRVRRWDNSVKKSSRVMDLKGQMIYIVESSAILFLGSPCVDRLEDFTGRGLYLSDIPIHNALRDVVLIGEQARAQDGLKKRLGKLKATLEQAHQALEEEKKKTVDLLCSIFPCEVAQQLWQGQVVQAKKFSNVTMLFSDIVGFTAICSQCSPLQVITMLNALYTRFDQQCGELDVYKVETIGDAYCVAGGLHKESDTHAVQIALMALKMMELSDEVMSPHGEPIKMRIGLHSGSVFAGVVGVKMPRYCLFGNNVTLANKFESCSVPRKINVSPTTYRLLKDCPGFVFTPRSREELPPNFPSEIPGICHFLDAYQQGTNSKPCFQKKDVEDGNANFLGKASGID

242 Q9BWT3 P09001

>Q9BWT3

MKEMSANTVLDSQRQQKHYGITSPISLASPKEIDHIYTQKLIDAMKPFGVFEDEEELNHRLVVLGKLNNLVKEWISDVSESKNLPPSVVATVGGKIFTFGSYRLGVHTKGADIDALCVAPRHVERSDFFQSFFEKLKHQDGIRNLRAVEDAFVPVIKFEFDGIEIDLVFARLAIQTISDNLDLRDDSRLRSLDIRCIRSLNGCRVTDEILHLVPNKETFRLTLRAVKLWAKRRGIYSNMLGFLGGVSWAMLVARTCQLYPNAAASTLVHKFFLVFSKWEWPNPVLLKQPEESNLNLPVWDPRVNPSDRYHLMPIITPAYPQQNSTYNVSTSTRTVMVEEFKQGLAVTDEILQGKSDWSKLLEPPNFFQKYRHYIVLTASASTEENHLEWVGLVESKIRVLVGNLERNEFITLAHVNPQSFPGNKEHHKDNNYVSMWFLGIIFRRVENAESVNIDLTYDIQSFTDTVYRQANNINMLKEGMKIEATHVKKKQLHHYLPAEILQKKKKQSLSDVNRSSGGLQSKRLSLDSSCLDSSRDTDNGTPFNSPASKSDSPSVGETERNSAEPAAVIVEKPLSVPPAQGLSIPVIGAKVDSTVKTVSPPTVCTIPTVVGRNVIPRITTPHNPAQGQPHLNGMSNITKTVTPKRSHSPSIDGTPKRLKDVEKFIRLESTFKDPRTAEERKRKSVDAIGGESMPIPTIDTSRKKRLPSKELPDSSSPVPANNIRVIKNSIRLTLNR

>P09001

MPGWRLLTQVGAQVLGRLGDGLGAALGPGNRTHIWLFVRGLHGKSGTWWDEHLSEENVPFIKQLVSDEDKAQLASKLCPLKDEPWPIHPWEPGSFRVGLIALKLGMMPLWTKDGQKHVVTLLQVQDCHVLKYTSKENCNGKMATLSVGGKTVSRFRKATSILEFYRELGLPPKQTVKIFNITDNAAIKPGTPLYAAHFRPGQYVDVTAKTIGKGFQGVMKRWGFKGQPATHGQTKTHRRPGAVATGDIGRVWPGTKMPGKMGNIYRTEYGLKVWRINTKHNIIYVNGSVPGHKNCLVKVKDSKLPAYKDLGKNLPFPTYFPDGDEEELPEDLYDENVCQPGAPSITFA

243 Q15349 NP_071369.4

>Q15349

MDLSMKKFAVRRFFSVYLRRKSRSKSSSLSRLEEEGVVKEIDISHHVKEGFEKADPSQFELLKVLGQGSYGKVFLVRKVKGSDAGQLYAMKVLKKATLKVRDRVRSKMERDILAEVNHPFIVKLHYAFQTEGKLYLILDFLRGGDLFTRLSKEVMFTEEDVKFYLAELALALDHLHSLGIIYRDLKPENILLDEEGHIKITDFGLSKEAIDHDKRAYSFCGTIEYMAPEVVNRRGHTQSADWWSFGVLMFEMLTGSLPFQGKDRKETMALILKAKLGMPQFLSGEAQSLLRALFKRNPCNRLGAGIDGVEEIKRHPFFVTIDWNTLYRKEIKPPFKPAVGRPEDTFHFDPEFTARTPTDSPGVPPSANAHHLFRGFSFVASSLIQEPSQQDLHKVPVHPIVQQLHGNNIHFTDGYEIKEDIGVGSYSVCKRCVHKATDTEYAVKIIDKSKRDPSEEIEILLRYGQHPNIITLKDVYDDGKFVYLVMELMRGGELLDRILRQRYFSEREASDVLCTITKTMDYLHSQGVVHRDLKPSNILYRDESGSPESIRVCDFGFAKQLRAGNGLLMTPCYTANFVAPEVLKRQGYDAACDIWSLGILLYTMLAGFTPFANGPDDTPEEILARIGSGKYALSGGNWDSISDAAKDVVSKMLHVDPHQRLTAMQVLKHPWVVNREYLSPNQLSRQDVHLVKGAMAATYFALNRTPQAPRLEPVLSSNLAQRRGMKRLTSTRL

>NP_071369.4

MPPPSDIVKVAIEWPGANAQLLEIDQKRPLASIIKEVCDGWSLPNPEYYTLRYADGPQLYITEQTRSDIKNGTILQLAISPSRAARQLMERTQSSNMETRLDAMKELAKLSADVTFATEFINMDGIIVLTRLVESGTKLLSHYSEMLAFTLTAFLELMDHGIVSWDMVSITFIKQIAGYVSQPMVDVSILQRSLAILESMVLNSQSLYQKIAEEITVGQLISHLQVSNQEIQTYAIALINALFLKAPEDKRQDMANAFAQKHLRSIILNHVIRGNRPIKTEMAHQLYVLQVLTFNLLEERMMTKMDPNDQAQRDIIFELRRIAFDAESDPSNAPGSGTEKRKAMYTKDYKMLGFTNHINPAMDFTQTPPGMLALDNMLYLAKVHQDTYIRIVLENSSREDKHECPFGRSAIELTKMLCEILQVGELPNEGRNDYHPMFFTHDRAFEELFGICIQLLNKTWKEMRATAEDFNKVMQVVREQITRALPSKPNSLDQFKSKLRSLSYSEILRLRQSERMSQDDFQSPPIVELREKIQPEILELIKQQRLNRLCEGSSFRKIGNRRRQERFWYCRLALNHKVLHYGDLDDNPQGEVTFESLQEKIPVADIKAIVTGKDCPHMKEKSALKQNKEVLELAFSILYDPDETLNFIAPNKYEYCIWIDGLSALLGKDMSSELTKSDLDTLLSMEMKLRLLDLENIQIPEAPPPIPKEPSSYDFVYHYG

244 Q69YI7 Q7Z2X4

>Q69YI7

MAVPAKKRKMNFSEREVEIIVEELELKKHLLVNHFNAGVPLAAKSAAWHGILRRVNAVATCRRELPEVKKKWSDLKTEVRRKVAQVRAAVEGGEAPGPTEEDGAGGPGTGGGSGGGGPAVAPVLLTPMQQRICNLLGEATIISLPSTTEIHPVALGPSATAAAATVTLTQIPTETTYHTLEEGVVEYCTAEAPPPLPPETPVDMMAQHADTSVKPQALKSRIALNSAKLIQEQRVTNLHVKEIAQHLEQQNDLLQMIRRSQEVQACAQERQAQAMEGTQAALSVLIQVLRPMIKDFRRYLQSNTANPAPASDPGQVAQNGQPDSIIQ

>Q7Z2X4

MFSLPLSLPLCEDTAFLPSKCCSSHKTIKQARTLIMIFLASGTHFQTMLKSKLNVLTLKKEPLPAVIFHEPEAIELCTTTPLMKTRTHSGCKVTYLGKVSTTGMQFLSGCTEKPVIELWKKHTLAREDVFPANALLEIRPFQVWLHHLDHKGEATVHMDTFQVARIAYCTADHNVSPNIFAWVYREINDDLSYQMDCHAVECESKLEAKKLAHAMMEAFRKTFHSMKSDGRIHSNSSSEEVSQELESDDG

245 P13378 NP_055963.2

>P13378

MSSYFVNPLYSKYKAAAAAAAAAGEAINPTYYDCHFAPEVGGRHAAAAAALQLYGNSAAGFPHAPPQAHAHPHPSPPPSGTGCGGREGRGQEYFHPGGGSPAAAYQAAPPPPPHPPPPPPPPPCGGIACHGEPAKFYGYDNLQRQPIFTTQQEAELVQYPDCKSSSGNIGEDPDHLNQSSSPSQMFPWMRPQAAPGRRRGRQTYSRFQTLELEKEFLFNPYLTRKRRIEVSHALALTERQVKIWFQNRRMKWKKENNKDKFPVSRQEVKDGETKKEAQELEEDRAEGLTN

>NP_055963.2

MEDGGLTAFEEDQRCLSQSLPLPVSAEGPAAQTTAEPSRSFSSAHRHLSRRNGLSRLCQSRTALSEDRWSSYCLSSLAAQNICTSKLHCPAAPEHTDPSEPRGSVSCCSLLRGLSSGWSSPLLPAPVCNPNKAIFTVDAKTTEILVANDKACGLLGYSSQDLIGQKLTQFFLRSDSDVVEALSEEHMEADGHAAVVFGTVVDIISRSGEKIPVSVWMKRMRQERRLCCVVVLEPVERVSTWVAFQSDGTVTSCDSLFAHLHGYVSGEDVAGQHITDLIPSVQLPPSGQHIPKNLKIQRSVGRARDGTTFPLSLKLKSQPSSEEATTGEAAPVSGYRASVWVFCTISGLITLLPDGTIHGINHSFALTLFGYGKTELLGKNITFLIPGFYSYMDLAYNSSLQLPDLASCLDVGNESGCGERTLDPWQGQDPAEGGQDPRINVVLAGGHVVPRDEIRKLMESQDIFTGTQTELIAGGQLLSCLSPQPAPGVDNVPEGSLPVHGEQALPKDQQITALGREEPVAIESPGQDLLGESRSEPVDVKPFASCEDSEAPVPAEDGGSDAGMCGLCQKAQLERMGVSGPSGSDLWAGAAVAKPQAKGQLAGGSLLMHCPCYGSEWGLWWRSQDLAPSPSGMAGLSFGTPTLDEPWLGVENDREELQTCLIKEQLSQLSLAGALDVPHAELVPTECQAVTAPVSSCDLGGRDLCGGCTGSSSACYALATDLPGGLEAVEAQEVDVNSFSWNLKELFFSDQTDQTSSNCSCATSELRETPSSLAVGSDPDVGSLQEQGSCVLDDRELLLLTGTCVDLGQGRRFRESCVGHDPTEPLEVCLVSSEHYAASDRESPGHVPSTLDAGPEDTCPSAEEPRLNVQVTSTPVIVMRGAAGLQREIQEGAYSGSCYHRDGLRLSIQFEVRRVELQGPTPLFCCWLVKDLLHSQRDSAARTRLFLASLPGSTHSTAAELTGPSLVEVLRARPWFEEPPKAVELEGLAACEGEYSQKYSTMSPLGSGAFGFVWTAVDKEKNKEVVVKFIKKEKVLEDCWIEDPKLGKVTLEIAILSRVEHANIIKVLDIFENQGFFQLVMEKHGSGLDLFAFIDRHPRLDEPLASYIFRQLVSAVGYLRLKDIIHRDIKDENIVIAEDFTIKLIDFGSAAYLERGKLFYTFCGTIEYCAPEVLMGNPYRGPELEMWSLGVTLYTLVFEENPFCELEETVEAAIHPPYLVSKELMSLVSGLLQPVPERRTTLEKLVTDPWVTQPVNLADYTWEEVFRVNKPESGVLSAASLEMGNRSLSDVAQAQELCGGPVPGEAPNGQGCLHPGDPRLLTS

246 P50897 NP_872578.1

>P50897

MASPGCLWLLAVALLPWTCASRALQHLDPPAPLPLVIWHGMGDSCCNPLSMGAIKKMVEKKIPGIYVLSLEIGKTLMEDVENSFFLNVNSQVTTVCQALAKDPKLQQGYNAMGFSQGGQFLRAVAQRCPSPPMINLISVGGQHQGVFGLPRCPGESSHICDFIRKTLNAGAYSKVVQERLVQAEYWHDPIKEDVYRNHSIFLADINQERGINESYKKNLMALKKFVMVKFLNDSIVDPVDSEWFGFYRSGQAKETIPLQETSLYTQDRLGLKEMDNAGQLVFLATEGDHLQLSEEWFYAHIIPFLG

>NP_872578.1

MAAPCVSYGGAVSYRLLLWGRGSLARKQGLWKTAAPELQTNVRSQILRLRHTAFVIPKKNVPTSKRETYTEDFIKKQIEEFNIGKRHLANMMGEDPETFTQEDIDRAIAYLFPSGLFEKRARPVMKHPEQIFPRQRAIQWGEDGRPFHYLFYTGKQSYYSLMHDVYGMLLNLEKHQSHLQAKSLLPEKTVTRDVIGSRWLIKEELEEMLVEKLSDLDYMQFIRLLEKLLTSQCGAAEEEFVQRFRRSVTLESKKQLIEPVQYDEQGMAFSKSEGKRKTAKAEAIVYKHGSGRIKVNGIDYQLYFPITQDREQLMFPFHFVDRLGKHDVTCTVSGGGRSAQAGAIRLAMAKALCSFVTEDEVEWMRQAGLLTTDPRVRERKKPGQEGARRKFTWKKR

247 NP_005457.2 NP_004127.1

>NP_005457.2

MAAVDIRDNLLGISWVDSSWIPILNSGSVLDYFSERSNPFYDRTCNNEVVKMQRLTLEHLNQMVGIEYILLHAQEPILFIIRKQQRQSPAQVIPLADYYIIAGVIYQAPDLGSVINSRVLTAVHGIQSAFDEAMSYCRYHPSKGYWWHFKDHEEQDKVRPKAKRKEEPSSIFQRQRVDALLLDLRQKFPPKFVQLKPGEKPVPVDQTKKEAEPIPETVKPEEKETTKNVQQTVSAKGPPEKRMRLQ

>NP_004127.1

MDAPKAGYAFEYLIETLNDSSHKKFFDVSKLGTKYDVLPYSIRVLLEAAVRNCDGFLMKKEDVMNILDWKTKQSNVEVPFFPARVLLQDFTGIPAMVDFAAMREAVKTLGGDPEKVHPACPTDLTVDHSLQIDFSKCAIQNAPNPGGGDLQKAGKLSPLKVQPKKLPCRGQTTCRGSCDSGELGRNSGTFSSQIENTPILCPFHLQPVPEPETVLKNQEVEFGRNRERLQFFKWSSRVFKNVAVIPPGTGMAHQINLEYLSRVVFEEKDLLFPDSVVGTDSHITMVNGLGILGWGVGGIETEAVMLGLPVSLTLPEVVGCELTGSSNPFVTSIDVVLGITKHLRQVGVAGKFVEFFGSGVSQLSIVDRTTIANMCPEYGAILSFFPVDNVTLKHLEHTGFSKAKLESMETYLKAVKLFRNDQNSSGEPEYSQVIQINLNSIVPSVSGPKRPQDRVAVTDMKSDFQACLNEKVGFKGFQIAAEKQKDIVSIHYEGSEYKLSHGSVVIAAVISCTNNCNPSVMLAAGLLAKKAVEAGLRVKPYIRTSLSPGSGMVTHYLSSSGVLPYLSKLGFEIVGYGCSTCVGNTAPLSDAVLNAVKQGDLVTCGILSGNKNFEGRLCDCVRANYLASPPLVVAYAIAGTVNIDFQTEPLGTDPTGKNIYLHDIWPSREEVHRVEEEHVILSMFKALKDKIEMGNKRWNSLEAPDSVLFPWDLKSTYIRCPSFFDKLTKEPIALQAIENAHVLLYLGDSVTTDHISPAGSIARNSAAAKYLTNRGLTPREFNSYGARRGNDAVMTRGTFANIKLFNKFIGKPAPKTIHFPSGQTLDVFEAAELYQKEGIPLIILAGKKYGSGNSRDWAAKGPYLLGVKAVLAESYEKIHKDHLIGIGIAPLQFLPGENADSLGLSGRETFSLTFPEELSPGITLNIQTSTGKVFSVIASFEDDVEITLYKHGGLLNFVARKFS

248 Q9NPC7 P53677

>Q9NPC7

MQYSHHCEHLLERLNKQREAGFLCDCTIVIGEFQFKAHRNVLASFSEYFGAIYRSTSENNVFLDQSQVKADGFQKLLEFIYTGTLNLDSWNVKEIHQAADYLKVEEVVTKCKIKMEDFAFIANPSSTEISSITGNIELNQQTCLLTLRDYNNREKSEVSTDLIQANPKQGALAKKSSQTKKKKKAFNSPKTGQNKTVQYPSDILENASVELFLDANKLPTPVVEQVAQINDNSELELTSVVENTFPAQDIVHTVTVKRKRGKSQPNCALKEHSMSNIASVKSPYEAENSGEELDQRYSKAKPMCNTCGKVFSEASSLRRHMRIHKGVKPYVCHLCGKAFTQCNQLKTHVRTHTGEKPYKCELCDKGFAQKCQLVFHSRMHHGEEKPYKCDVCNLQFATSSNLKIHARKHSGEKPYVCDRCGQRFAQASTLTYHVRRHTGEKPYVCDTCGKAFAVSSSLITHSRKHTGEKPYICGICGKSFISSGELNKHFRSHTGERPFICELCGNSYTDIKNLKKHKTKVHSGADKTLDSSAEDHTLSEQDSIQKSPLSETMDVKPSDMTLPLALPLGTEDHHMLLPVTDTQSPTSDTLLRSTVNGYSEPQLIFLQQLY

>P53677

MIHSLFLINSSGDIFLEKHWKSVVSRSVCDYFFEAQERATEAENVPPVIPTPHHYLLSVYRHKIFFVAVIQTEVPPLFVIEFLHRVVDTFQDYFGVCSEPVIKDNVVVVYEVLEEMLDNGFPLATESNILKELIKPPTILRTVVNTITGSTNVGDQLPTGQLSVVPWRRTGVKYTNNEAYFDVIEEIDAIIDKSGSTITAEIQGVIDACVKLTGMPDLTLSFMNPRLLDDVSFHPCVRFKRWESERILSFIPPDGNFRLLSYHVSAQNLVAIPVYVKHNISFRDSSSLGRFEITVGPKQTMGKTIEGVTVTSQMPKGVLNMSLTPSQGTHTFDPVTKMLSWDVGKINPQKLPSLKGTMSLQAGASKPDENPTINLQFKIQQLAISGLKVNRLDMYGEKYKPFKGIKYMTKAGKFQVRT

249 Q9Y6X0 NP_002159.2

>Q9Y6X0

MEPEEEDELGSGRDVDSNSNADSEKWVAGDGLEEQEFSIKEANFTEGSLKLKIQTTKRAKKPPKNLENYICPPEIKITIKQSGDQKVSRAGKNSKATKEEERSHSKKKLLTASDLAASDLKGFQPQAYERPQKHSTLHYDTGLPQDFTGDTLKPKHQQKSSSQNHMDWSTNSDSGPVTQNCFISPESGRETASTSKIPALEPVASFAKAQGKKGSAGNTWSQLSNNNKDLLLGGVAPSPSSHSSPAPPSSSAECNGLQPLVDQDGGGTKEPPEPPTVGSKKKSSKKDVISQTIPNPDLDWVKNAQKAFDNTEGKREGYSADSAQEASPARQNVSSASNPENDSSHVRITIPIKAPSLDPTNHKRKKRQSIKAVVEKIMPEKALASGITMSSEVVNRILSNSEGNKKDPRVPKLSKMIENESPSVGLETGGNAEKVIPGGVSKPRKPPMVMTPPTCTDHSPSRKLPEIQHPKFAAKRRWTCSKPKPSTMLREAVMATSDKLMLEPPSAYPITPSSPLYTNTDSLTVITPVKKKRGRPKKQPLLTVETIHEGTSTSPVSPISREFPGTKKRKRRRNLAKLAQLVPGEDKPMSEMKFHKKVGKLGVLDKKTIKTINKMKTLKRKNILNQILSCSSSVALKAKAPPETSPGAAAIESKLGKQINVSKRGTIYIGKKRGRKPRAELPPPSEEPKTAIKHPRPVSSQPDVPAVPSNFQSLVASSPAAMHPLSTQLGGSNGNLSPASTETNFSELKTMPNLQPISALPTKTQKGIHSGTWKLSPPRLMANSPSHLCEIGSLKEITLSPVSESHSEETIPSDSGIGTDNNSTSDQAEKSSESRRRYSFDFCSLDNPEAIPSDTSTKNRHGHRQKHLIVDNFLAHESLKKPKHKRKRKSLQNRDDLQFLADLEELITKFQVFRISHRSYTFYHENPYPSIFRINFDHYYPVPYIQYDPLLYLRRTSDLKSKKKRGRPAKTNDTMTKVPFLQGFSYPIPSGSYYAPYGMPYTSMPMMNLGYYGQYPAPLYLSHTLGAASPFMRPTVPPPQFHTNSHVKMSGAAKHKAKHGVHLQGPVSMGLGDMQPSLNPPKVGSASLSSGRLHKRKHKHKHKHKEDRILGTHDNLSGLFAGKATGFSSHILSERLSSADKELPLVSEKNKHKEKQKHQHSEAGHKASKNNFEVDTLSTLSLSDAQHWTQAKEKGDLSSEPVDSCTKRYSGSGGDGGSTRSENLDVFSEMNPSNDKWDSDVSGSKRRSYEGFGTYREKDIQAFKMNRKERSSYDSSMSPGMPSPHLKVDQTAVHSKNEGSVPTMMTRKKPAAVDSVTIPPAPVLSLLAASAATSDAVGSSLKKRFKRREIEAIQCEVRKMCNYTKILSTKKNLDHVNKILKAKRLQRQSKTGNNFVKKRRGRPRKQPTQFDEDSRDQMPVLEKCIDLPSKRGQKPSLSPLVLEPAASQDTIMATIEAVIHMAREAPPLPPPPPPPLPPPPPPPLPPPPPLPKTPRGGKRKHKPQAPAQPPQQSPPQQPLPQEEEVKAKRQRKSRGSESEVLP

>NP_002159.2

MAGYLRVVRSLCRASGSRPAWAPAALTAPTSQEQPRRHYADKRIKVAKPVVEMDGDEMTRIIWQFIKEKLILPHVDIQLKYFDLGLPNRDQTDDQVTIDSALATQKYSVAVKCATITPDEARVEEFKLKKMWKSPNGTIRNILGGTVFREPIICKNIPRLVPGWTKPITIGRHAHGDQYKATDFVADRAGTFKMVFTPKDGSGVKEWEVYNFPAGGVGMGMYNTDESISGFAHSCFQYAIQKKWPLYMSTKNTILKAYDGRFKDIFQEIFDKHYKTDFDKNKIWYEHRLIDDMVAQVLKSSGGFVWACKNYDGDVQSDILAQGFGSLGLMTSVLVCPDGKTIEAEAAHGTVTRHYREHQKGRPTSTNPIASIFAWTRGLEHRGKLDGNQDLIRFAQMLEKVCVETVESGAMTKDLAGCIHGLSNVKLNEHFLNTTDFLDTIKSNLDRALGRQ

250 NP_056986.2 NP_002289.1

>NP_056986.2

MTSIHFVVHPLPGTEDQLNDRLREVSEKLNKYNLNSHPPLNVLEQATIKQCVVGPNHAAFLLEDGRVCRIGFSVQPDRLELGKPDNNDGSKLNSNSGAGRTSRPGRTSDSPWFLSGSETLGRLAGNTLGSRWSSGVGGSGGGSSGRSSAGARDSRRQTRVIRTGRDRGSGLLGSQPQPVIPASVIPEELISQAQVVLQGKSRSVIIRELQRTNLDVNLAVNNLLSRDDEDGDDGDDTASESYLPGEDLMSLLDADIHSAHPSVIIDADAMFSEDISYFGYPSFRRSSLSRLGSSRVLLLPLERDSELLRERESVLRLRERRWLDGASFDNERGSTSKEGEPNLDKKNTPVQSPVSLGEDLQWWPDKDGTKFICIGALYSELLAVSSKGELYQWKWSESEPYRNAQNPSLHHPRATFLGLTNEKIVLLSANSIRATVATENNKVATWVDETLSSVASKLEHTAQTYSELQGERIVSLHCCALYTCAQLENSLYWWGVVPFSQRKKMLEKARAKNKKPKSSAGISSMPNITVGTQVCLRNNPLYHAGAVAFSISAGIPKVGVLMESVWNMNDSCRFQLRSPESLKNMEKASKTTEAKPESKQEPVKTEMGPPPSPASTCSDASSIASSASMPYKRRRSTPAPKEEEKVNEEQWSLREVVFVEDVKNVPVGKVLKVDGAYVAVKFPGTSSNTNCQNSSGPDADPSSLLQDCRLLRIDELQVVKTGGTPKVPDCFQRTPKKLCIPEKTEILAVNVDSKGVHAVLKTGNWVRYCIFDLATGKAEQENNFPTSSIAFLGQNERNVAIFTAGQESPIILRDGNGTIYPMAKDCMGGIRDPDWLDLPPISSLGMGVHSLINLPANSTIKKKAAVIIMAVEKQTLMQHILRCDYEACRQYLMNLEQAVVLEQNLQMLQTFISHRCDGNRNILHACVSVCFPTSNKETKEEEEAERSERNTFAERLSAVEAIANAISVVSSNGPGNRAGSSSSRSLRLREMMRRSLRAAGLGRHEAGASSSDHQDPVSPPIAPPSWVPDPPAMDPDGDIDFILAPAVGSLTTAATGTGQGPSTSTIPGPSTEPSVVESKDRKANAHFILKLLCDSVVLQPYLRELLSAKDARGMTPFMSAVSGRAYPAAITILETAQKIAKAEISSSEKEEDVFMGMVCPSGTNPDDSPLYVLCCNDTCSFTWTGAEHINQDIFECRTCGLLESLCCCTECARVCHKGHDCKLKRTSPTAYCDCWEKCKCKTLIAGQKSARLDLLYRLLTATNLVTLPNSRGEHLLLFLVQTVARQTVEHCQYRPPRIREDRNRKTASPEDSDMPDHDLEPPRFAQLALERVLQDWNALKSMIMFGSQENKDPLSASSRIGHLLPEEQVYLNQQSGTIRLDCFTHCLIVKCTADILLLDTLLGTLVKELQNKYTPGRREEAIAVTMRFLRSVARVFVILSVEMASSKKKNNFIPQPIGKCKRVFQALLPYAVEELCNVAESLIVPVRMGIARPTAPFTLASTSIDAMQGSEELFSVEPLPPRPSSDQSSSSSQSQSSYIIRNPQQRRISQSQPVRGRDEEQDDIVSADVEEVEVVEGVAGEEDHHDEQEEHGEENAEAEGQHDEHDEDGSDMELDLLAAAETESDSESNHSNQDNASGRRSVVTAATAGSEAGASSVPAFFSEDDSQSNDSSDSDSSSSQSDDIEQETFMLDEPLERTTNSSHANGAAQAPRSMQWAVRNTQHQRAASTAPSSTSTPAASSAGLIYIDPSNLRRSGTISTSAAAAAAALEASNASSYLTSASSLARAYSIVIRQISDLMGLIPKYNHLVYSQIPAAVKLTYQDAVNLQNYVEEKLIPTWNWMVSIMDSTEAQLRYGSALASAGDPGHPNHPLHASQNSARRERMTAREEASLRTLEGRRRATLLSARQGMMSARGDFLNYALSLMRSHNDEHSDVLPVLDVCSLKHVAYVFQALIYWIKAMNQQTTLDTPQLERKRTRELLELGIDNEDSEHENDDDTNQSATLNDKDDDSLPAETGQNHPFFRRSDSMTFLGCIPPNPFEVPLAEAIPLADQPHLLQPNARKEDLFGRPSQGLYSSSASSGKCLMEVTVDRNCLEVLPTKMSYAANLKNVMNMQNRQKKEGEEQPVLPEETESSKPGPSAHDLAAQLKSSLLAEIGLTESEGPPLTSFRPQCSFMGMVISHDMLLGRWRLSLELFGRVFMEDVGAEPGSILTELGGFEVKESKFRREMEKLRNQQSRDLSLEVDRDRDLLIQQTMRQLNNHFGRRCATTPMAVHRVKVTFKDEPGEGSGVARSFYTAIAQAFLSNEKLPNLECIQNANKGTHTSLMQRLRNRGERDREREREREMRRSSGLRAGSRRDRDRDFRRQLSIDTRPFRPASEGNPSDDPEPLPAHRQALGERLYPRVQAMQPAFASKITGMLLELSPAQLLLLLASEDSLRARVDEAMELIIAHGRENGADSILDLGLVDSSEKVQQENRKRHGSSRSVVDMDLDDTDDGDDNAPLFYQPGKRGFYTPRPGKNTEARLNCFRNIGRILGLCLLQNELCPITLNRHVIKVLLGRKVNWHDFAFFDPVMYESLRQLILASQSSDADAVFSAMDLAFAIDLCKEEGGGQVELIPNGVNIPVTPQNVYEYVRKYAEHRMLVVAEQPLHAMRKGLLDVLPKNSLEDLTAEDFRLLVNGCGEVNVQMLISFTSFNDESGENAEKLLQFKRWFWSIVEKMSMTERQDLVYFWTSSPSLPASEEGFQPMPSITIRPPDDQHLPTANTCISRLYVPLYSSKQILKQKLLLAIKTKNFGFV

>NP_002289.1

MARGSVSDEEMMELREAFAKVDTDGNGYISFNELNDLFKAACLPLPGYRVREITENLMATGDLDQDGRISFDEFIKIFHGLKSTDVAKTFRKAINKKEGICAIGGTSEQSSVGTQHSYSEEEKYAFVNWINKALENDPDCRHVIPMNPNTNDLFNAVGDGIVLCKMINLSVPDTIDERTINKKKLTPFTIQENLNLALNSASAIGCHVVNIGAEDLKEGKPYLVLGLLWQVIKIGLFADIELSRNEALIALLREGESLEDLMKLSPEELLLRWANYHLENAGCNKIGNFSTDIKDSKAYYHLLEQVAPKGDEEGVPAVVIDMSGLREKDDIQRAECMLQQAERLGCRQFVTATDVVRGNPKLNLAFIANLFNRYPALHKPENQDIDWGALEGETREERTFRNWMNSLGVNPRVNHLYSDLSDALVIFQLYEKIKVPVDWNRVNKPPYPKLGGNMKKLENCNYAVELGKNQAKFSLVGIGGQDLNEGNRTLTLALIWQLMRRYTLNILEEIGGGQKVNDDIIVNWVNETLREAEKSSSISSFKDPKISTSLPVLDLIDAIQPGSINYDLLKTENLNDDEKLNNAKYAISMARKIGARVYALPEDLVEVNPKMVMTVFACLMGKGMKRV

251 NP_055048.1 NP_006535.1

>NP_055048.1

MNGEADCPTDLEMAAPKGQDRWSQEDMLTLLECMKNNLPSNDSSKFKTTESHMDWEKVAFKDFSGDMCKLKWVEISNEVRKFRTLTELILDAQEHVKNPYKGKKLKKHPDFPKKPLTPYFRFFMEKRAKYAKLHPEMSNLDLTKILSKKYKELPEKKKMKYIQDFQREKQEFERNLARFREDHPDLIQNAKKSDIPEKPKTPQQLWYTHEKKVYLKVRPDATTKEVKDSLGKQWSQLSDKKRLKWIHKALEQRKEYEEIMRDYIQKHPELNISEEGITKSTLTKAERQLKDKFDGRPTKPPPNSYSLYCAELMANMKDVPSTERMVLCSQQWKLLSQKEKDAYHKKCDQKKKDYEVELLRFLESLPEEEQQRVLGEEKMLNINKKQATSPASKKPAQEGGKGGSEKPKRPVSAMFIFSEEKRRQLQEERPELSESELTRLLARMWNDLSEKKKAKYKAREAALKAQSERKPGGEREERGKLPESPKRAEEIWQQSVIGDYLARFKNDRVKALKAMEMTWNNMEKKEKLMWIKKAAEDQKRYERELSEMRAPPAATNSSKKMKFQGEPKKPPMNGYQKFSQELLSNGELNHLPLKERMVEIGSRWQRISQSQKEHYKKLAEEQQKQYKVHLDLWVKSLSPQDRAAYKEYISNKRKSMTKLRGPNPKSSRTTLQSKSESEEDDEEDEDDEDEDEEEEDDENGDSSEDGGDSSESSSEDESEDGDENEEDDEDEDDDEDDDEDEDNESEGSSSSSSSSGDSSDSDSN

>NP_006535.1

MATTAELFEEPFVADEYIERLVWRTPGGGSRGGPEAFDPKRLLEEFVNHIQELQIMDERIQRKVEKLEQQCQKEAKEFAKKVQELQKSNQVAFQHFQELDEHISYVATKVCHLGDQLEGVNTPRQRAVEAQKLMKYFNEFLDGELKSDVFTNSEKIKEAADIIQKLHLIAQELPFDRFSEVKSKIASKYHDLECQLIQEFTSAQRRGEISRMREVAAVLLHFKGYSHCVDVYIKQCQEGAYLRNDIFEDAGILCQRVNKQVGDIFSNPETVLAKLIQNVFEIKLQSFVKEQLEECRKSDAEQYLKNLYDLYTRTTNLSSKLMEFNLGTDKQTFLSKLIKSIFISYLENYIEVETGYLKSRSAMILQRYYDSKNHQKRSIGTGGIQDLKERIRQRTNLPLGPSIDTHGETFLSQEVVVNLLQETKQAFERCHRLSDPSDLPRNAFRIFTILVEFLCIEHIDYALETGLAGIPSSDSRNANLYFLDVVQQANTIFHLFDKQFNDHLMPLISSSPKLSECLQKKKEIIEQMEMKLDTGIDRTLNCMIGQMKHILAAEQKKTDFKPEDENNVLIQYTNACVKVCAYVRKQVEKIKNSMDGKNVDTVLMELGVRFHRLIYEHLQQYSYSCMGGMLAICDVAEYRKCAKDFKIPMVLHLFDTLHALCNLLVVAPDNLKQVCSGEQLANLDKNILHSFVQLRADYRSARLARHFS

252 NP_055610.1 NP_004802.1

>NP_055610.1

MKQPIMADGPRCKRRKQANPRRKNVVNYDNVVDTGSETDEEDKLHIAEDDGIANPLDQETSPASVPNHESSPHVSQALLPREEEEDEIREGGVEHPWHNNEILQASVDGPEEMKEDYDTMGPEATIQTAINNGTVKNANCTSDFEEYFAKRKLEERDGHAVSIEEYLQRSDTAIIYPEAPEELSRLGTPEANGQEENDLPPGTPDAFAQLLTCPYCDRGYKRLTSLKEHIKYRHEKNEENFSCPLCSYTFAYRTQLERHMVTHKPGTDQHQMLTQGAGNRKFKCTECGKAFKYKHHLKEHLRIHSGEKPYECPNCKKRFSHSGSYSSHISSKKCIGLISVNGRMRNNIKTGSSPNSVSSSPTNSAITQLRNKLENGKPLSMSEQTGLLKIKTEPLDFNDYKVLMATHGFSGTSPFMNGGLGATSPLGVHPSAQSPMQHLGVGMEAPLLGFPTMNSNLSEVQKVLQIVDNTVSRQKMDCKAEEISKLKGYHMKDPCSQPEEQGVTSPNIPPVGLPVVSHNGATKSIIDYTLEKVNEAKACLQSLTTDSRRQISNIKKEKLRTLIDLVTDDKMIENHNISTPFSCQFCKESFPGPIPLHQHERYLCKMNEEIKAVLQPHENIVPNKAGVFVDNKALLLSSVLSEKGMTSPINPYKDHMSVLKAYYAMNMEPNSDELLKISIAVGLPQEFVKEWFEQRKVYQYSNSRSPSLERSSKPLAPNSNPPTKDSLLPRSPVKPMDSITSPSIAELHNSVTNCDPPLRLTKPSHFTNIKPVEKLDHSRSNTPSPLNLSSTSSKNSHSSSYTPNSFSSEELQAEPLDLSLPKQMKEPKSIIATKNKTKASSISLDHNSVSSSSENSDEPLNLTFIKKEFSNSNNLDNKSTNPVFSMNPFSAKPLYTALPPQSAFPPATFMPPVQTSIPGLRPYPGLDQMSFLPHMAYTYPTGAATFADMQQRRKYQRKQGFQGELLDGAQDYMSGLDDMTDSDSCLSRKKIKKTESGMYACDLCDKTFQKSSSLLRHKYEHTGKRPHQCQICKKAFKHKHHLIEHSRLHSGEKPYQCDKCGKRFSHSGSYSQHMNHRYSYCKREAEEREAAEREAREKGHLEPTELLMNRAYLQSITPQGYSDSEERESMPRDGESEKEHEKEGEDGYGKLGRQDGDEEFEEEEEESENKSMDTDPETIRDEEETGDHSMDDSSEDGKMETKSDHEEDNMEDGM

>NP_004802.1

MEELDALLEELERSTLQDSDEYSNPAPLPLDQHSRKETNLDETSEILSIQDNTSPLPAQLVYTTNIQELNVYSEAQEPKESPPPSKTSAAAQLDELMAHLTEMQAKVAVRADAGKKHLPDKQDHKASLDSMLGGLEQELQDLGIATVPKGHCASCQKPIAGKVIHALGQSWHPEHFVCTHCKEEIGSSPFFERSGLAYCPNDYHQLFSPRCAYCAAPILDKVLTAMNQTWHPEHFFCSHCGEVFGAEGFHEKDKKPYCRKDFLAMFSPKCGGCNRPVLENYLSAMDTVWHPECFVCGDCFTSFSTGSFFELDGRPFCELHYHHRRGTLCHGCGQPITGRCISAMGYKFHPEHFVCAFCLTQLSKGIFREQNDKTYCQPCFNKLFPL

253 NP_073598.1 P49589

>NP_073598.1

MAASQCLCCSKFLFQRQNLACFLTNPHCGSLVNADGHGEVWTDWNNMSKFFQYGWRCTTNENTYSNRTLMGNWNQERYDLRNIVQPKPLPSQFGHYFETTYDTSYNNKMPLSTHRFKREPHWFPGHQPELDPPRYKCTEKSTYMNSYSKP

>P49589

MADSSGQQGKGRRVQPQWSPPAGTQPCRLHLYNSLTRNKEVFIPQDGKKVTWYCCGPTVYDASHMGHARSYISFDILRRVLKDYFKFDVFYCMNITDIDDKIIKRARQNHLFEQYREKRPEAAQLLEDVQAALKPFSVKLNETTDPDKKQMLERIQHAVQLATEPLEKAVQSRLTGEEVNSCVEVLLEEAKDLLSDWLDSTLGCDVTDNSIFSKLPKFWEGDFHRDMEALNVLPPDVLTRVSEYVPEIVNFVQKIVDNGYGYVSNGSVYFDTAKFASSEKHSYGKLVPEAVGDQKALQEGEGDLSISADRLSEKRSPNDFALWKASKPGEPSWPCPWGKGRPGWHIECSAMAGTLLGASMDIHGGGFDLRFPHHDNELAQSEAYFENDCWVRYFLHTGHLTIAGCKMSKSLKNFITIKDALKKHSARQLRLAFLMHSWKDTLDYSSNTMESALQYEKFLNEFFLNVKDILRAPVDITGQFEKWGEEEAELNKNFYDKKTAIHKALCDNVDTRTVMEEMRALVSQCNLYMAARKAVRKRPNQALLENIALYLTHMLKIFGAVEEDSSLGFPVGGPGTSLSLEATVMPYLQVLSEFREGVRKIAREQKVPEILQLSDALRDNILPELGVRFEDHEGLPTVVKLVDRNTLLKEREEKRRVEEEKRKKKEEAARRKQEQEAAKLAKMKIPPSEMFLSETDKYSKFDENGLPTHDMEGKELSKGQAKKLKKLFEAQEKLYKEYLQMAQNGSFQ

254 NP_110517.2 Q9UBR1

>NP_110517.2

MDVGELLSYQPNRGTKRPRDDEEEEQKMRRKQTGTRERGRYREEEMTVVEEADDDKKRLLQIIDRDGEEEEEEEEPLDESSVKKMILTFEKRSYKNQELRIKFPDNPEKFMESELDLNDIIQEMHVVATMPDLYHLLVELNAVQSLLGLLGHDNTDVSIAVVDLLQELTDIDTLHESEEGAEVLIDALVDGQVVALLVQNLERLDESVKEEADGVHNTLAIVENMAEFRPEMCTEGAQQGLLQWLLKRLKAKMPFDANKLYCSEVLAILLQDNDENRELLGELDGIDVLLQQLSVFKRHNPSTAEEQEMMENLFDSLCSCLMLSSNRERFLKGEGLQLMNLMLREKKISRSSALKVLDHAMIGPEGTDNCHKFVDILGLRTIFPLFMKSPRKIKKVGTTEKEHEEHVCSILASLLRNLRGQQRTRLLNKFTENDSEKVDRLMELHFKYLGAMQVADKKIEGEKHDMVRRGEIIDNDTEEEFYLRRLDAGLFVLQHICYIMAEICNANVPQIRQRVHQILNMRGSSIKIVRHIIKEYAENIGDGRSPEFRENEQKRILGLLENF

>Q9UBR1

MAGAEWKSLEECLEKHLPLPDLQEVKRVLYGKELRKLDLPREAFEAASREDFELQGYAFEAAEEQLRRPRIVHVGLVQNRIPLPANAPVAEQVSALHRRIKAIVEVAAMCGVNIICFQEAWTMPFAFCTREKLPWTEFAESAEDGPTTRFCQKLAKNHDMVVVSPILERDSEHGDVLWNTAVVISNSGAVLGKTRKNHIPRVGDFNESTYYMEGNLGHPVFQTQFGRIAVNICYGRHHPLNWLMYSINGAEIIFNPSATIGALSESLWPIEARNAAIANHCFTCAINRVGTEHFPNEFTSGDGKKAHQDFGYFYGSSYVAAPDSSRTPGLSRSRDGLLVAKLDLNLCQQVNDVWNFKMTGRYEMYARELAEAVKSNYSPTIVKE

255 NP_000452.2 Q02252

>NP_000452.2

MTPNSMTENGLTAWDKPKHCPDREHDWKLVGMSEACLHRKSHSERRSTLKNEQSSPHLIQTTWTSSIFHLDHDDVNDQSVSSAQTFQTEEKKCKGYIPSYLDKDELCVVCGDKATGYHYRCITCEGCKGFFRRTIQKNLHPSYSCKYEGKCVIDKVTRNQCQECRFKKCIYVGMATDLVLDDSKRLAKRKLIEENREKRRREELQKSIGHKPEPTDEEWELIKTVTEAHVATNAQGSHWKQKRKFLPEDIGQAPIVNAPEGGKVDLEAFSHFTKIITPAITRVVDFAKKLPMFCELPCEDQIILLKGCCMEIMSLRAAVRYDPESETLTLNGEMAVTRGQLKNGGLGVVSDAIFDLGMSLSSFNLDDTEVALLQAVLLMSSDRPGLACVERIEKYQDSFLLAFEHYINYRKHHVTHFWPKLLMKVTDLRMIGACHASRFLHMKVECPTELFPPLFLEVFED

>Q02252

MAALLAAAAVRARILQVSSKVKSSPTWYSASSFSSSVPTVKLFIGGKFVESKSDKWIDIHNPATNEVIGRVPQATKAEMDAAIASCKRAFPAWADTSVLSRQQVLLRYQQLIKENLKEIAKLITLEQGKTLADAEGDVFRGLQVVEHACSVTSLMMGETMPSITKDMDLYSYRLPLGVCAGIAPFNFPAMIPLWMFPMAMVCGNTFLMKPSERVPGATMLLAKLLQDSGAPDGTLNIIHGQHEAVNFICDHPDIKAISFVGSNKAGEYIFERGSRHGKRVQANMGAKNHGVVMPDANKENTLNQLVGAAFGAAGQRCMALSTAVLVGEAKKWLPELVEHAKNLRVNAGDQPGADLGPLITPQAKERVCNLIDSGTKEGASILLDGRKIKVKGYENGNFVGPTIISNVKPNMTCYKEEIFGPVLVVLETETLDEAIQIVNNNPYGNGTAIFTTNGATARKYAHLVDVGQVGVNVPIPVPLPMFSFTGSRSSFRGDTNFYGKQGIQFYTQLKTITSQWKEEDATLSSPAVVMPTMGR

256 Q96QV6 NP_115568.2

>Q96QV6

MSGRGKQGGKARAKSKSRSSRAGLQFPVGRIHRLLRKGNYAERIGAGAPVYLAAVLEYLTAEILELAGNASRDNKKTRIIPRHLQLAIRNDEELNKLLGGVTIAQGGVLPNIQAVLLPKKTESHHHKAQSK

>NP_115568.2

MDPKDRKKIQFSVPAPPSQLDPRQVEMIRRRRPTPAMLFRLSEHSSPEEEASPHQRASGEGHHLKSKRPNPCAYTPPSLKAVQRIAESHLQSISNLNENQASEEEDELGELRELGYPREEDEEEEEDDEEEEEEEDSQAEVLKVIRQSAGQKTTCGQGLEGPWERPPPLDESERDGGSEDQVEDPALSEPGEEPQRPSPSEPGT

257 Q9BWE0 Q9Y2Q9

>Q9BWE0

MLERRCRGPLAMGLAQPRLLSGPSQESPQTLGKESRGLRQQGTSVAQSGAQAPGRAHRCAHCRRHFPGWVALWLHTRRCQARLPLPCPECGRRFRHAPFLALHRQVHAAATPDLGFACHLCGQSFRGWVALVLHLRAHSAAKRPIACPKCERRFWRRKQLRAHLRRCHPPAPEARPFICGNCGRSFAQWDQLVAHKRVHVAEALEEAAAKALGPRPRGRPAVTAPRPGGDAVDRPFQCACCGKRFRHKPNLIAHRRVHTGERPHQCPECGKRFTNKPYLTSHRRIHTGEKPYPCKECGRRFRHKPNLLSHSKIHKRSEGSAQAAPGPGSPQLPAGPQESAAEPTPAVPLKPAQEPPPGAPPEHPQDPIEAPPSLYSCDDCGRSFRLERFLRAHQRQHTGERPFTCAECGKNFGKKTHLVAHSRVHSGERPFACEECGRRFSQGSHLAAHRRDHAPDRPFVCPDCGKAFRHKPYLAAHRRIHTGEKPYVCPDCGKAFSQKSNLVSHRRIHTGERPYACPDCDRSFSQKSNLITHRKSHIRDGAFCCAICGQTFDDEERLLAHQKKHDV

>Q9Y2Q9

MAALCRTRAVAAESHFLRVFLFFRPFRGVGTESGSESGSSNAKEPKTRAGGFASALERHSELLQKVEPLQKGSPKNVESFASMLRHSPLTQMGPAKDKLVIGRIFHIVENDLYIDFGGKFHCVCRRPEVDGEKYQKGTRVRLRLLDLELTSRFLGATTDTTVLEANAVLLGIQESKDSRSKEEHHEK

258 P82970 NP_071761.1

>P82970

MPKRKAAGQGDMRQEPKRRSARLSAMLVPVTPEVKPKRTSSSRKMKTKSDMMEENIDTSAQAVAETKQEAVVEEDYNENAKNGEAKITEAPASEKEIVEVKEENIEDATEKGGEKKEAVAAEVKNEEEDQKEDEEDQNEEKGEAGKEDKDEKGEEDGKEDKNGNEKGEDAKEKEDGKKGEDGKGNGEDGKEKGEDEKEEEDRKETGDGKENEDGKEKGDKKEGKDVKVKEDEKEREDGKEDEGGNEEEAGKEKEDLKEEEEGKEEDEIKEDDGKKEEPQSIV

>NP_071761.1

MWIPVVGLPRRLRLSALAGAGRFCILGSEAATRKHLPARNHCGLSDSSPQLWPEPDFRNPPRKASKASLDFKRYVTDRRLAETLAQIYLGKPSRPPHLLLECNPGPGILTQALLEAGAKVVALESDKTFIPHLESLGKNLDGKLRVIHCDFFKLDPRSGGVIKPPAMSSRGLFKNLGIEAVPWTADIPLKVVGMFPSRGEKRALWKLAYDLYSCTSIYKFGRIEVNMFIGEKEFQKLMADPGNPDLYHVLSVIWQLACEIKVLHMEPWSSFDIYTRKGPLENPKRRELLDQLQQKLYLIQMIPRQNLFTKNLTPMNYNIFFHLLKHCFGRRSATVIDHLRSLTPLDARDILMQIGKQEDEKVVNMHPQDFKTLFETIERSKDCAYKWLYDETLEDR

259 NP_055856.1 O00170

>NP_055856.1

MSFRDLRNFTEMMRALGYPRHISMENFRTPNFGLVSEVLLWLVKRYEPQTDIPPDVDTEQDRVFFIKAIAQFMATKAHIKLNTKKLYQADGYAVKELLKITSVLYNAMKTKGMEGSEIVEEDVNKFKFDLGSKIADLKAARQLASEITSKGASLYDLLGMEVELREMRTEAIARPLEINETEKVMRIAIKEILTQVQKTKDLLNNVASDEANLEAKIEKRKLELERNRKRLETLQSVRPCFMDEYEKTEEELQKQYDTYLEKFQNLTYLEQQLEDHHRMEQERFEEAKNTLCLIQNKLKEEEKRLLKSGSNDDSDIDIQEDDESDSELEERRLPKPQTAMEMLMQGRPGKRIVGTMQGGDSDDNEDSEESEIDMEDDDDEDDDLEDESISLSPTKPNRRVRKSEPLDESDNDF

>O00170

MADIIARLREDGIQKRVIQEGRGELPDFQDGTKATFHYRTLHSDDEGTVLDDSRARGKPMELIIGKKFKLPVWETIVCTMREGEIAQFLCDIKHVVLYPLVAKSLRNIAVGKDPLEGQRHCCGVAQMREHSSLGHADLDALQQNPQPLIFHMEMLKVESPGTYQQDPWAMTDEEKAKAVPLIHQEGNRLYREGHVKEAAAKYYDAIACLKNLQMKEQPGSPEWIQLDQQITPLLLNYCQCKLVVEEYYEVLDHCSSILNKYDDNVKAYFKRGKAHAAVWNAQEAQADFAKVLELDPALAPVVSRELQALEARIRQKDEEDKARFRGIFSH

260 NP_005135.2 NP_001163.1

>NP_005135.2

MESTPSFLKGTPTWEKTAPENGIVRQEPGSPPRDGLHHGPLCLGEPAPFWRGVLSTPDSWLPPGFPQGPKDMLPLVEGEGPQNGERKVNWLGSKEGLRWKEAMLTHPLAFCGPACPPRCGPLMPEHSGGHLKSDPVAFRPWHCPFLLETKILERAPFWVPTCLPPYLVSGLPPEHPCDWPLTPHPWVYSGGQPKVPSAFSLGSKGFYYKDPSIPRLAKEPLAAAEPGLFGLNSGGHLQRAGEAERPSLHQRDGEMGAGRQQNPCPLFLGQPDTVPWTSWPACPPGLVHTLGNVWAGPGDGNLGYQLGPPATPRCPSPEPPVTQRGCCSSYPPTKGGGLGPCGKCQEGLEGGASGASEPSEEVNKASGPRACPPSHHTKLKKTWLTRHSEQFECPRGCPEVEERPVARLRALKRAGSPEVQGAMGSPAPKRPPDPFPGTAEQGAGGWQEVRDTSIGNKDVDSGQHDEQKGPQDGQASLQDPGLQDIPCLALPAKLAQCQSCAQAAGEGGGHACHSQQVRRSPLGGELQQEEDTATNSSSEEGPGSGPDSRLSTGLAKHLLSGLGDRLCRLLRREREALAWAQREGQGPAVTEDSPGIPRCCSRCHHGLFNTHWRCPRCSHRLCVACGRVAGTGRAREKAGFQEQSAEECTQEAGHAACSLMLTQFVSSQALAELSTAMHQVWVKFDIRGHCPCQADARVWAPGDAGQQKESTQKTPPTPQPSCNGDTHRTKSIKEETPDSAETPAEDRAGRGPLPCPSLCELLASTAVKLCLGHERIHMAFAPVTPALPSDDRITNILDSIIAQVVERKIQEKALGPGLRAGPGLRKGLGLPLSPVRPRLPPPGALLWLQEPQPCPRRGFHLFQEHWRQGQPVLVSGIQRTLQGNLWGTEALGALGGQVQALSPLGPPQPSSLGSTTFWEGFSWPELRPKSDEGSVLLLHRALGDEDTSRVENLAASLPLPEYCALHGKLNLASYLPPGLALRPLEPQLWAAYGVSPHRGHLGTKNLCVEVADLVSILVHADTPLPAWHRAQKDFLSGLDGEGLWSPGSQVSTVWHVFRAQDAQRIRRFLQMVCPAGAGALEPGAPGSCYLDAGLRRRLREEWGVSCWTLLQAPGEAVLVPAGAPHQVQGLVSTVSVTQHFLSPETSALSAQLCHQGPSLPPDCHLLYAQMDWAVFQAVKVAVGTLQEAK

>NP_001163.1

MSLRGSLSRLLQTRVHSILKKSVHSVAVIGAPFSQGQKRKGVEHGPAAIREAGLMKRLSSLGCHLKDFGDLSFTPVPKDDLYNNLIVNPRSVGLANQELAEVVSRAVSDGYSCVTLGGDHSLAIGTISGHARHCPDLCVVWVDAHADINTPLTTSSGNLHGQPVSFLLRELQDKVPQLPGFSWIKPCISSASIVYIGLRDVDPPEHFILKNYDIQYFSMRDIDRLGIQKVMERTFDLLIGKRQRPIHLSFDIDAFDPTLAPATGTPVVGGLTYREGMYIAEEIHNTGLLSALDLVEVNPQLATSEEEAKTTANLAVDVIASSFGQTREGGHIVYDQLPTPSSPDESENQARVRI

261 Q9C0F3 Q9HCP0

>Q9C0F3

MAATLLMAGSQAPVTFEDMAMYLTREEWRPLDAAQRDLYRDVMQENYGNVVSLDFEIRSENEVNPKQEISEDVQFGTTSERPAENAEENPESEEGFESGDRSERQWGDLTAEEWVSYPLQPVTDLLVHKEVHTGIRYHICSHCGKAFSQISDLNRHQKTHTGDRPYKCYECGKGFSRSSHLIQHQRTHTGERPYDCNECGKSFGRSSHLIQHQTIHTGEKPHKCNECGKSFCRLSHLIQHQRTHSGEKPYECEECGKSFSRSSHLAQHQRTHTGEKPYECNECGRGFSERSDLIKHYRVHTGERPYKCDECGKNFSQNSDLVRHRRAHTGEKPYHCNECGENFSRISHLVQHQRTHTGEKPYECNACGKSFSRSSHLITHQKIHTGEKPYECNECWRSFGERSDLIKHQRTHTGEKPYECVQCGKGFTQSSNLITHQRVHTGEKPYECTECEKSFSRSSALIKHKRVHTD

>Q9HCP0

MDHPSREKDERQRTTKPMAQRSAHCSRPSGSSSSSGVLMVGPNFRVGKKIGCGNFGELRLGKNLYTNEYVAIKLEPIKSRAPQLHLEYRFYKQLGSAGEGLPQVYYFGPCGKYNAMVLELLGPSLEDLFDLCDRTFTLKTVLMIAIQLLSRMEYVHSKNLIYRDVKPENFLIGRQGNKKEHVIHIIDFGLAKEYIDPETKKHIPYREHKSLTGTARYMSINTHLGKEQSRRDDLEALGHMFMYFLRGSLPWQGLKADTLKERYQKIGDTKRNTPIEALCENFPEEMATYLRYVRRLDFFEKPDYEYLRTLFTDLFEKKGYTFDYAYDWVGRPIPTPVGSVHVDSGASAITRESHTHRDRPSQQQPLRNQVVSSTNGELNVDDPTGAHSNAPITAHAEVEVVEEAKCCCFFKRKRKKTAQRHK

262 NP_065090.1 O00142

>NP_065090.1

MADGKAGDEKPEKSQRAGAAGGPEEEAEKPVKTKTVSSSNGGESSSRSAEKRSAEEEAADLPTKPTKISKFGFAIGSQTTKKASAISIKLGSSKPKETVPTLAPKTLSVAAAFNEDEDSEPEEMPPEAKMRMKNIGRDTPTSAGPNSFNKGKHGFSDNQKLWERNIKSHLGNVHDQDN

>O00142

MLLWPLRGWAARALRCFGPGSRGSPASGPGPRRVQRRAWPPDKEQEKEKKSVICVEGNIASGKTTCLEFFSNATDVEVLTEPVSKWRNVRGHNPLGLMYHDASRWGLTLQTYVQLTMLDRHTRPQVSSVRLMERSIHSARYIFVENLYRSGKMPEVDYVVLSEWFDWILRNMDVSVDLIVYLRTNPETCYQRLKKRCREEEKVIPLEYLEAIHHLHEEWLIKGSLFPMAAPVLVIEADHHMERMLELFEQNRDRILTPENRKHCP

263 NP_003911.1 NP_002454.1

>NP_003911.1

MDLHMMNCELLATCSALGYLEGDTYHKEPDCLESVKDLIRYLRHEDETRDVRQQLGAAQILQSDLLPILTQHHQDKPLFDAVIRLMVNLTQPALLCFGNLPKEPSFRHHFLQVLTYLQAYKEAFASEKAFGVLSETLYELLQLGWEERQEEDNLLIERILLLVRNILHVPADLDQEKKIDDDASAHDQLLWAIHLSGLDDLLLFLASSSAEEQWSLHVLEIVSLMFRDQNPEQLAGVGQGRLAQERSADFAELEVLRQREMAEKKTRALQRGNRHSRFGGSYIVQGLKSIGERDLIFHKGLHNLRNYSSDLGKQPKKVPKRRQAARELSIQRRSALNVRLFLRDFCSEFLENCYNRLMGSVKDHLLREKAQQHDETYYMWALAFFMAFNRAASFRPGLVSETLSVRTFHFIEQNLTNYYEMMLTDRKEAASWARRMHLALKAYQELLATVNEMDLSPDEAVRESSRIIKNNIFYVMEYRELFLALFRKFDERCQPRSFLRDLVETTHLFLKMLERFCRSRGNLVVQNKQKKRRKKKKKVLDQAIVSGNVPSSPEEVEAVWPALAEQLQCCAQNSELSMDSVVPFDAASEVPVEEQRAEAMVRIQDCLLAGQAPQALTLLRSAREVWPEGDVFGSQDISPEEEIQLLKQILSAPLPRQQGPEERGAEEEEEEEEEEEEELQVVQVSEKEFNFLDYLKRFACSTVVRAYVLLLRSYQQNSAHTNHCIVKMLHRLAHDLKMEALLFQLSVFCLFNRLLSDPAAGAYKELVTFAKYILGKFFALAAVNQKAFVELLFWKNTAVVREMTEGYGSLDDRSSSRRAPTWSPEEEAHLQELYLANKDVEGQDVVEAILAHLNTVPRTRKQIIHHLVQMGLADSVKDFQRKGTHIVLWTGDQELELQRLFEEFRDSDDVLGHIMKNITAKRSRARIVDKLLALGLVAERRELYKKRQKKLASSILPNGAESLKDFCQEDLEEEENLPEEDSEEEEEGGSEAEQVQGSLVLSNENLGQSLHQEGFSIPLLWLQNCLIRAADDREEDGCSQAVPLVPLTEENEEAMENEQFQQLLRKLGVRPPASGQETFWRIPAKLSPTQLRRAAASLSQPEEEQKLQPELQPKVPGEQGSDEEHCKEHRAQALRALLLAHKKKAGLASPEEEDAVGKEPLKAAPKKRQLLDSDEEQEEDEGRNRAPELGAPGIQKKKRYQIEDDEDD

>NP_002454.1

MSKAHKPWPYRRRSQFSSRKYLKKEMNSFQQQPPPFGTVPPQMMFPPNWQGAEKDAAFLAKDFNFLTLNNQPPPGNRSQPRAMGPENNLYSQYEQKVRPCIDLIDSLRALGVEQDLALPAIAVIGDQSSGKSSVLEALSGVALPRGSGIVTRCPLVLKLKKQPCEAWAGRISYRNTELELQDPGQVEKEIHKAQNVMAGNGRGISHELISLEITSPEVPDLTIIDLPGITRVAVDNQPRDIGLQIKALIKKYIQRQQTINLVVVPCNVDIATTEALSMAHEVDPEGDRTIGILTKPDLMDRGTEKSVMNVVRNLTYPLKKGYMIVKCRGQQEITNRLSLAEATKKEITFFQTHPYFRVLLEEGSATVPRLAERLTTELIMHIQKSLPLLEGQIRESHQKATEELRRCGADIPSQEADKMFFLIEKIKMFNQDIEKLVEGEEVVRENETRLYNKIREDFKNWVGILATNTQKVKNIIHEEVEKYEKQYRGKELLGFVNYKTFEIIVHQYIQQLVEPALSMLQKAMEIIQQAFINVAKKHFGEFFNLNQTVQSTIEDIKVKHTAKAENMIQLQFRMEQMVFCQDQIYSVVLKKVREEIFNPLGTPSQNMKLNSHFPSNESSVSSFTEIGIHLNAYFLETSKRLANQIPFIIQYFMLRENGDSLQKAMMQILQEKNRYSWLLQEQSETATKRRILKERIYRLTQARHALCQFSSKEIH

264 Q14526 NP_000934.1

>Q14526

MTFPEADILLKSGECAGQTMLDTMEAPGHSRQLLLQLNNQRTKGFLCDVIIVVQNALFRAHKNVLAASSAYLKSLVVHDNLLNLDHDMVSPAVFRLVLDFIYTGRLADGAEAAAAAAVAPGAEPSLGAVLAAASYLQIPDLVALCKKRLKRHGKYCHLRGGGGGGGGYAPYGRPGRGLRAATPVIQACYRSPVGPPPPPAAEPPSGPEAAVNTHCAELYASGPGPAAALCASERRCSPLCGLDLSKKSPPGSAAPERPLAERELPPRPDSPPSAGPAAYKEPPLALPSLPPLPFQKLEEAAPPSDPFRGGSGSPGPEPPGRPDGPSLLYRWMKHEPGLGSYGDELGRERGSPSERCEERGGDAAVSPGGPPLGLAPPPRYPGSLDGPGAGGDGDDYKSSSEETGSSEDPSPPGGHLEGYPCPHLAYGEPESFGDNLYVCIPCGKGFPSSEQLNAHVEAHVEEEEALYGRAEAAEVAAGAAGLGPPFGGGGDKVAGAPGGLGELLRPYRCASCDKSYKDPATLRQHEKTHWLTRPYPCTICGKKFTQRGTMTRHMRSHLGLKPFACDACGMRFTRQYRLTEHMRIHSGEKPYECQVCGGKFAQQRNLISHMKMHAVGGAAGAAGALAGLGGLPGVPGPDGKGKLDFPEGVFAVARLTAEQLSLKQQDKAAAAELLAQTTHFLHDPKVALESLYPLAKFTAELGLSPDKAAEVLSQGAHLAAGPDGGTIDRFSPT

>NP_000934.1

MGPGPRLLLPLVLCVGLGALVFSSGAEGFRKRGPSVTAKVFFDVRIGDKDVGRIVIGLFGKVVPKTVENFVALATGEKGYGYKGSKFHRVIKDFMIQGGDITTGDGTGGVSIYGETFPDENFKLKHYGIGWVSMANAGPDTNGSQFFITLTKPTWLDGKHVVFGKVIDGMTVVHSIELQATDGHDRPLTNCSIINSGKIDVKTPFVVEIADW

265 NP_055884.2 NP_001001936.1

>NP_055884.2

MHKKRVEEGEASDFSLAWDSSVTAAGGLEGEPECDQKTSRALEDRNSVTSQEERNEDDEDMEDESIYTCDHCQQDFESLADLTDHRAHRCPGDGDDDPQLSWVASSPSSKDVASPTQMIGDGCDLGLGEEEGGTGLPYPCQFCDKSFIRLSYLKRHEQIHSDKLPFKCTYCSRLFKHKRSRDRHIKLHTGDKKYHCHECEAAFSRSDHLKIHLKTHSSSKPFKCTVCKRGFSSTSSLQSHMQAHKKNKEHLAKSEKEAKKDDFMCDYCEDTFSQTEELEKHVLTRHPQLSEKADLQCIHCPEVFVDENTLLAHIHQAHANQKHKCPMCPEQFSSVEGVYCHLDSHRQPDSSNHSVSPDPVLGSVASMSSATPDSSASVERGSTPDSTLKPLRGQKKMRDDGQGWTKVVYSCPYCSKRDFNSLAVLEIHLKTIHADKPQQSHTCQICLDSMPTLYNLNEHVRKLHKNHAYPVMQFGNISAFHCNYCPEMFADINSLQEHIRVSHCGPNANPSDGNNAFFCNQCSMGFLTESSLTEHIQQAHCSVGSAKLESPVVQPTQSFMEVYSCPYCTNSPIFGSILKLTKHIKENHKNIPLAHSKKSKAEQSPVSSDVEVSSPKRQRLSASANSISNGEYPCNQCDLKFSNFESFQTHLKLHLELLLRKQACPQCKEDFDSQESLLQHLTVHYMTTSTHYVCESCDKQFSSVDDLQKHLLDMHTFVLYHCTLCQEVFDSKVSIQVHLAVKHSNEKKMYRCTACNWDFRKEADLQVHVKHSHLGNPAKAHKCIFCGETFSTEVELQCHITTHSKKYNCKFCSKAFHAIILLEKHLREKHCVFDAATENGTANGVPPMATKKAEPADLQGMLLKNPEAPNSHEASEDDVDASEPMYGCDICGAAYTMEVLLQNHRLRDHNIRPGEDDGSRKKAEFIKGSHKCNVCSRTFFSENGLREHLQTHRGPAKHYMCPICGERFPSLLTLTEHKVTHSKSLDTGTCRICKMPLQSEEEFIEHCQMHPDLRNSLTGFRCVVCMQTVTSTLELKIHGTFHMQKLAGSSAASSPNGQGLQKLYKCALCLKEFRSKQDLVKLDVNGLPYGLCAGCMARSANGQVGGLAPPEPADRPCAGLRCPECSVKFESAEDLESHMQVDHRDLTPETSGPRKGTQTSPVPRKKTYQCIKCQMTFENEREIQIHVANHMIEEGINHECKLCNQMFDSPAKLLCHLIEHSFEGMGGTFKCPVCFTVFVQANKLQQHIFAVHGQEDKIYDCSQCPQKFFFQTELQNHTMSQHAQ

>NP_001001936.1

MERYKALEQLLTELDDFLKILDQENLSSTALVKKSCLAELLRLYTKSSSSDEEYIYMNKVTINKQQNAESQGKAPEEQGLLPNGEPSQHSSAPQKSLPDLPPPKMIPERKQLAIPKTESPEGYYEEAEPYDTSLNEDGEAVSSSYESYDEEDGSKGKSAPYQWPSPEAGIELMRDARICAFLWRKKWLGQWAKQLCVIKDNRLLCYKSSKDHSPQLDVNLLGSSVIHKEKQVRKKEHKLKITPMNADVIVLGLQSKDQAEQWLRVIQEVSGLPSEGASEGNQYTPDAQRFNCQKPDIAEKYLSASEYGSSVDGHPEVPETKDVKKKCSAGLKLSNLMNLGRKKSTSLEPVERSLETSSYLNVLVNSQWKSRWCSVRDNHLHFYQDRNRSKVAQQPLSLVGCEVVPDPSPDHLYSFRILHKGEELAKLEAKSSEEMGHWLGLLLSESGSKTDPEEFTYDYVDADRVSCIVSAAKNSLLLMQRKFSEPNTYIDGLPSQDRQEELYDDVDLSELTAAVEPTEEATPVADDPNERESDRVYLDLTPVKSFLHGPSSAQAQASSPTLSCLDNATEALPADSGPGPTPDEPCIKCPENLGEQQLESLEPEDPSLRITTVKIQTEQQRISFPPSCPDAVVATPPGASPPVKDRLRVTSAEIKLGKNRTEAEVKRYTEEKERLEKKKEEIRGHLAQLRKEKRELKETLLKCTDKEVLASLEQKLKEIDEECRGEESRRVDLELSIMEVKDNLKKAEAGPVTLGTTVDTTHLENVSPRPKAVTPASAPDCTPVNSATTLKNRPLSVVVTGKGTVLQKAKEWEKKGAS

266 Q9BSH3 NP_001815.2

>Q9BSH3

MSRVLVPCHVKGSVALQVGDVRTSQGRPGVLVIDVTFPSVAPFELQEITFKNYYTAFLSIRVRQYTSAHTPAKWVTCLRDYCLMPDPHSEEGAQEYVSLFKHQMLCDMARISELRLILRQPSPLWLSFTVEELQIYQQGPKSPSVTFPKWLSHPVPCEQPALLREGLPDPSRVSSEVQQMWALTEMIRASHTSARIGRFDVDGCYDLNLLSYT

>NP_001815.2

MPFGNTHNKFKLNYKPEEEYPDLSKHNNHMAKVLTLELYKKLRDKETPSGFTVDDVIQTGVDNPGHPFIMTVGCVAGDEESYEVFKELFDPIISDRHGGYKPTDKHKTDLNHENLKGGDDLDPNYVLSSRVRTGRSIKGYTLPPHCSRGERRAVEKLSVEALNSLTGEFKGKYYPLKSMTEKEQQQLIDDHFLFDKPVSPLLLASGMARDWPDARGIWHNDNKSFLVWVNEEDHLRVISMEKGGNMKEVFRRFCVGLQKIEEIFKKAGHPFMWNQHLGYVLTCPSNLGTGLRGGVHVKLAHLSKHPKFEEILTRLRLQKRGTGGVDTAAVGSVFDVSNADRLGSSEVEQVQLVVDGVKLMVEMEKKLEKGQSIDDMIPAQK

267 NP_003064.2 Q9NVS2

>NP_003064.2

MMMMALSKTFGQKPVKFQLEDDGEFYMIGSEVGNYLRMFRGSLYKRYPSLWRRLATVEERKKIVASSHGKKTKPNTKDHGYTTLATSVTLLKASEVEEILDGNDEKYKAVSISTEPPTYLREQKAKRNSQWVPTLPNSSHHLDAVPCSTTINRNRMGRDKKRTFPLCFDDHDPAVIHENASQPEVLVPIRLDMEIDGQKLRDAFTWNMNEKLMTPEMFSEILCDDLDLNPLTFVPAIASAIRQQIESYPTDSILEDQSDQRVIIKLNIHVGNISLVDQFEWDMSEKENSPEKFALKLCSELGLGGEFVTTIAYSIRGQLSWHQKTYAFSENPLPTVEIAIRNTGDADQWCPLLETLTDAEMEKKIRDQDRNTRRMRRLANTAPAW

>Q9NVS2

MAALKALVSGCGRLLRGLLAGPAATSWSRLPARGFREVVETQEGKTTIIEGRITATPKESPNPPNPSGQCPICRWNLKHKYNYDDVLLLSQFIRPHGGMLPRKITGLCQEEHRKIEECVKMAHRAGLLPNHRPRLPEGVVPKSKPQLNRYLTRWAPGSVKPIYKKGPRWNRVRMPVGSPLLRDNVCYSRTPWKLYH

268 NP_003176.2 Q8N183

>NP_003176.2

MAAGSDLLDEVFFNSEVDEKVVSDLVGSLESQLAASAAHHHHLAPRTPEVRAAAAGALGNHVVSGSPAGAAGAGPAAPAEGAPGAAPEPPPAGRARPGGGGPQRPGPPSPRRPLVPAGPAPPAAKLRPPPEGSAGSCAPVPAAAAVAAGPEPAPAGPAKPAGPAALAARAGPGPGPGPGPGPGPGPGKPAGPGAAQTLNGSAALLNSHHAAAPAVSLVNNGPAALLPLPKPAAPGTVIQTPPFVGAAAPPAPAAPSPPAAPAPAAPAAAPPPPPPAPATLARPPGHPAGPPTAAPAVPPPAAAQNGGSAGAAPAPAPAAGGPAGVSGQPGPGAAAAAPAPGVKAESPKRVVQAAPPAAQTLAASGPASTAASMVIGPTMQGALPSPAAVPPPAPGTPTGLPKGAAGAVTQSLSRTPTATTSGIRATLTPTVLAPRLPQPPQNPTNIQNFQLPPGMVLVRSENGQLLMIPQQALAQMQAQAHAQPQTTMAPRPATPTSAPPVQISTVQAPGTPIIARQVTPTTIIKQVSQAQTTVQPSATLQRSPGVQPQLVLGGAAQTASLGTATAVQTGTPQRTVPGATTTSSAATETMENVKKCKNFLSTLIKLASSGKQSTETAANVKELVQNLLDGKIEAEDFTSRLYRELNSSPQPYLVPFLKRSLPALRQLTPDSAAFIQQSQQQPPPPTSQATTALTAVVLSSSVQRTAGKTAATVTSALQPPVLSLTQPTQVGVGKQGQPTPLVIQQPPKPGALIRPPQVTLTQTPMVALRQPHNRIMLTTPQQIQLNPLQPVPVVKPAVLPGTKALSAVSAQAAAAQKNKLKEPGGGSFRDDDDINDVASMAGVNLSEESARILATNSELVGTLTRSCKDETFLLQAPLQRRILEIGKKHGITELHPDVVSYVSHATQQRLQNLVEKISETAQQKNFSYKDDDRYEQASDVRAQLKFFEQLDQIEKQRKDEQEREILMRAAKSRSRQEDPEQLRLKQKAKEMQQQELAQMRQRDANLTALAAIGPRKKRKVDCPGPGSGAEGSGPGSVVPGSSGVGTPRQFTRQRITRVNLRDLIFCLENERETSHSLLLYKAFLK

>Q8N183

MGWSQDLFRALWRSLSREVKEHVGTDQFGNKYYYIPQYKNWRGQTIREKRIVEAANKKEVDYEAGDIPTEWEAWIRRTRKTPPTMEEILKNEKHREEIKIKSQDFYEKEKLLSKETSEELLPPPVQTQIKGHASAPYFGKEEPSVAPSSTGKTFQPGSWMPRDGKSHNQ

269 NP_065885.1 NP_000895.1

>NP_065885.1

MPPNKEASGLSSSPAGLICLPPISEELQLVWTQAAQTSELDSNEHLLKTFSYFPYPSLADIALLCLRYGLQMEKVKTWFMAQRLRCGISWSSEEIEETRARVVYRRDQLHFKSLLSFTHHAGRPPEEVPPPPVPAPEQVGIGIGPPTLSKPTQTKGLKVEPEEPSQMPPLPQSHQKLKESLMTPGSGAFPYQSDFWQHLQSSGLSKEQAGRGPNQSHGIGTASWNHSTTVPQPQARDKPPPIALIASSCKEESASSVTPSSSSTSSSFQVLANGATAASKPLQPLGCVPQSVSPSEQALPPHLEPAWPQGLRHNSVPGRVGPTEYLSPDMQRQRKTKRKTKEQLAILKSFFLQCQWARREDYQKLEQITGLPRPEIIQWFGDTRYALKHGQLKWFRDNAVPGAPSFQDPAIPTPPPSTRSLNERAETPPLPIPPPPPDIQPLERYWAAHQQLRETDIPQLSQASRLSTQQVLDWFDSRLPQPAEVVVCLDEEEEEEEEELPEDDEEEEEEEEDDDDDDDDVIIQD

>NP_000895.1

MAGKKVLIVYAHQEPKSFNGSLKNVAVDELSRQGCTVTVSDLYAMNFEPRATDKDITGTLSNPEVFNYGVETHEAYKQRSLASDITDEQKKVREADLVIFQFPLYWFSVPAILKGWMDRVLCQGFAFDIPGFYDSGLLQGKLALLSVTTGGTAEMYTKTGVNGDSRYFLWPLQHGTLHFCGFKVLAPQISFAPEIASEEERKGMVAAWSQRLQTIWKEEPIPCTAHWHFGQ

270 NP_004489.1 NP_004324.2

>NP_004489.1

MNAQLTMEAIGELHGVSHEPVPAPADLLGGSPHARSSVAHRGSHLPPAHPRSMGMASLLDGGSGGGDYHHHHRAPEHSLAGPLHPTMTMACETPPGMSMPTTYTTLTPLQPLPPISTVSDKFPHHHHHHHHHHHPHHHQRLAGNVSGSFTLMRDERGLASMNNLYTPYHKDVAGMGQSLSPLSSSGLGSIHNSQQGLPHYAHPGAAMPTDKMLTPNGFEAHHPAMLGRHGEQHLTPTSAGMVPINGLPPHHPHAHLNAQGHGQLLGTAREPNPSVTGAQVSNGSNSGQMEEINTKEVAQRITTELKRYSIPQAIFAQRVLCRSQGTLSDLLRNPKPWSKLKSGRETFRRMWKWLQEPEFQRMSALRLAACKRKEQEHGKDRGNTPKKPRLVFTDVQRRTLHAIFKENKRPSKELQITISQQLGLELSTVSNFFMNARRRSLDKWQDEGSSNSGNSSSSSSTCTKA

>NP_004324.2

MAALSGGGGGGAEPGQALFNGDMEPEAGAGAGAAASSAADPAIPEEVWNIKQMIKLTQEHIEALLDKFGGEHNPPSIYLEAYEEYTSKLDALQQREQQLLESLGNGTDFSVSSSASMDTVTSSSSSSLSVLPSSLSVFQNPTDVARSNPKSPQKPIVRVFLPNKQRTVVPARCGVTVRDSLKKALMMRGLIPECCAVYRIQDGEKKPIGWDTDISWLTGEELHVEVLENVPLTTHNFVRKTFFTLAFCDFCRKLLFQGFRCQTCGYKFHQRCSTEVPLMCVNYDQLDLLFVSKFFEHHPIPQEEASLAETALTSGSSPSAPASDSIGPQILTSPSPSKSIPIPQPFRPADEDHRNQFGQRDRSSSAPNVHINTIEPVNIDDLIRDQGFRGDGGSTTGLSATPPASLPGSLTNVKALQKSPGPQRERKSSSSSEDRNRMKTLGRRDSSDDWEIPDGQITVGQRIGSGSFGTVYKGKWHGDVAVKMLNVTAPTPQQLQAFKNEVGVLRKTRHVNILLFMGYSTKPQLAIVTQWCEGSSLYHHLHIIETKFEMIKLIDIARQTAQGMDYLHAKSIIHRDLKSNNIFLHEDLTVKIGDFGLATVKSRWSGSHQFEQLSGSILWMAPEVIRMQDKNPYSFQSDVYAFGIVLYELMTGQLPYSNINNRDQIIFMVGRGYLSPDLSKVRSNCPKAMKRLMAECLKKKRDERPLFPQILASIELLARSLPKIHRSASEPSLNRAGFQTEDFSLYACASPKTPIQAGGYGAFPVH

271 Q08050 P42679

>Q08050

MKTSPRRPLILKRRRLPLPVQNAPSETSEEEPKRSPAQQESNQAEASKEVAESNSCKFPAGIKIINHPTMPNTQVVAIPNNANIHSIITALTAKGKESGSSGPNKFILISCGGAPTQPPGLRPQTQTSYDAKRTEVTLETLGPKPAARDVNLPRPPGALCEQKRETCADGEAAGCTINNSLSNIQWLRKMSSDGLGSRSIKQEMEEKENCHLEQRQVKVEEPSRPSASWQNSVSERPPYSYMAMIQFAINSTERKRMTLKDIYTWIEDHFPYFKHIAKPGWKNSIRHNLSLHDMFVRETSANGKVSFWTIHPSANRYLTLDQVFKPLDPGSPQLPEHLESQQKRPNPELRRNMTIKTELPLGARRKMKPLLPRVSSYLVPIQFPVNQSLVLQPSVKVPLPLAASLMSSELARHSKRVRIAPKVLLAEEGIAPLSSAGPGKEEKLLFGEGFSPLLPVQTIKEEEIQPGEEMPHLARPIKVESPPLEEWPSPAPSFKEESSHSWEDSSQSPTPRPKKSYSGLRSPTRCVSEMLVIQHRERRERSRSRRKQHLLPPCVDEPELLFSEGPSTSRWAAELPFPADSSDPASQLSYSQEVGGPFKTPIKETLPISSTPSKSVLPRTPESWRLTPPAKVGGLDFSPVQTSQGASDPLPDPLGLMDLSTTPLQSAPPLESPQRLLSSEPLDLISVPFGNSSPSDIDVPKPGSPEPQVSGLAANRSLTEGLVLDTMNDSLSKILLDISFPGLDEDPLGPDNINWSQFIPELQ

>P42679

MAGRGSLVSWRAFHGCDSAEELPRVSPRFLRAWHPPPVSARMPTRRWAPGTQCITKCEHTRPKPGELAFRKGDVVTILEACENKSWYRVKHHTSGQEGLLAAGALREREALSADPKLSLMPWFHGKISGQEAVQQLQPPEDGLFLVRESARHPGDYVLCVSFGRDVIHYRVLHRDGHLTIDEAVFFCNLMDMVEHYSKDKGAICTKLVRPKRKHGTKSAEEELARAGWLLNLQHLTLGAQIGEGEFGAVLQGEYLGQKVAVKNIKCDVTAQAFLDETAVMTKMQHENLVRLLGVILHQGLYIVMEHVSKGNLVNFLRTRGRALVNTAQLLQFSLHVAEGMEYLESKKLVHRDLAARNILVSEDLVAKVSDFGLAKAERKGLDSSRLPVKWTAPEALKHGKFTSKSDVWSFGVLLWEVFSYGRAPYPKMSLKEVSEAVEKGYRMEPPEGCPGPVHVLMSSCWEAEPARRPPFRKLAEKLARELRSAGAPASVSGQDADGSTSPRSQEP

272 NP_001460.1 P28332

>NP_001460.1

MSGWESYYKTEGDEEAEEEQEENLEASGDYKYSGRDSLIFLVDASKAMFESQSEDELTPFDMSIQCIQSVYISKIISSDRDLLAVVFYGTEKDKNSVNFKNIYVLQELDNPGAKRILELDQFKGQQGQKRFQDMMGHGSDYSLSEVLWVCANLFSDVQFKMSHKRIMLFTNEDNPHGNDSAKASRARTKAGDLRDTGIFLDLMHLKKPGGFDISLFYRDIISIAEDEDLRVHFEESSKLEDLLRKVRAKETRKRALSRLKLKLNKDIVISVGIYNLVQKALKPPPIKLYRETNEPVKTKTRTFNTSTGGLLLPSDTKRSQIYGSRQIILEKEETEELKRFDDPGLMLMGFKPLVLLKKHHYLRPSLFVYPEESLVIGSSTLFSALLIKCLEKEVAALCRYTPRRNIPPYFVALVPQEEELDDQKIQVTPPGFQLVFLPFADDKRKMPFTEKIMATPEQVGKMKAIVEKLRFTYRSDSFENPVLQQHFRNLEALALDLMEPEQAVDLTLPKVEAMNKRLGSLVDEFKELVYPPDYNPEGKVTKRKHDNEGSGSKRPKVEYSEEELKTHISKGTLGKFTVPMLKEACRAYGLKSGLKKQELLEALTKHFQD

>P28332

MCTTGQVIRCKAAILWKPGAPFSIEEVEVAPPKAKEVRIKVVATGLCGTEMKVLGSKHLDLLYPTILGHEGAGIVESIGEGVSTVKPGDKVITLFLPQCGECTSCLNSEGNFCIQFKQSKTQLMSDGTSRFTCKGKSIYHFGNTSTFCEYTVIKEISVAKIDAVAPLEKVCLISCGFSTGFGAAINTAKVTPGSTCAVFGLGGVGLSVVMGCKAAGAARIIGVDVNKEKFKKAQELGATECLNPQDLKKPIQEVLFDMTDAGIDFCFEAIGNLDVLAAALASCNESYGVCVVVGVLPASVQLKISGQLFFSGRSLKGSVFGGWKSRQHIPKLVADYMAEKLNLDPLITHTLNLDKINEAVELMKTGKW

273 NP_009060.2 P0C7P0

>NP_009060.2

MLLDAGPQFPAIGVGSFARHHHHSAAAAAAAAAEMQDRELSLAAAQNGFVDSAAAHMGAFKLNPGAHELSPGQSSAFTSQGPGAYPGSAAAAAAAAALGPHAAHVGSYSGPPFNSTRDFLFRSRGFGDSAPGGGQHGLFGPGAGGLHHAHSDAQGHLLFPGLPEQHGPHGSQNVLNGQMRLGLPGEVFGRSEQYRQVASPRTDPYSAAQLHNQYGPMNMNMGMNMAAAAAHHHHHHHHHPGAFFRYMRQQCIKQELICKWIDPEQLSNPKKSCNKTFSTMHELVTHVSVEHVGGPEQSNHVCFWEECPREGKPFKAKYKLVNHIRVHTGEKPFPCPFPGCGKVFARSENLKIHKRTHTGEKPFQCEFEGCDRRFANSSDRKKHMHVHTSDKPYLCKMCDKSYTHPSSLRKHMKVHESSPQGSESSPAASSGYESSTPPGLVSPSAEPQSSSNLSPAAAAAAAAAAAAAAAVSAVHRGGGSGSGGAGGGSGGGSGSGGGGGGAGGGGGGSSGGGSGTAGGHSGLSSNFNEWYV

>P0C7P0

MRGAGAILRPAARGARDLNPRRDISSWLAQWFPRTPARSVVALKTPIKVELVAGKTYRWCVCGRSKKQPFCDGSHFFQRTGLSPLKFKAQETRMVALCTCKATQRPPYCDGTHRSERVQKAEVGSPL

274 NP_066285.1 NP_055141.2

>NP_066285.1

MAMVVSTWRDPQDEVPGSQGSQASQAPPVPGPPPGAPHTPQTPGQGGPASTPAQTAAGGQGGPGGPGSDKQQQQQHIECVVCGDKSSGKHYGQFTCEGCKSFFKRSVRRNLSYTCRANRNCPIDQHHRNQCQYCRLKKCLKVGMRREAVQRGRMPPTQPTHGQFALTNGDPLNCHSYLSGYISLLLRAEPYPTSRFGSQCMQPNNIMGIENICELAARMLFSAVEWARNIPFFPDLQITDQVALLRLTWSELFVLNAAQCSMPLHVAPLLAAAGLHASPMSADRVVAFMDHIRIFQEQVEKLKALHVDSAEYSCLKAIVLFTSDACGLSDVAHVESLQEKSQCALEEYVRSQYPNQPTRFGKLLLRLPSLRTVSSSVIEQLFFVRLVGKTPIETLIRDMLLSGSSFNWPYMAIQ

>NP_055141.2

MFQASMRSPNMEPFKQQKVEDFYDIGEELGSGQFAIVKKCREKSTGLEYAAKFIKKRQSRASRRGVSREEIEREVSILRQVLHHNVITLHDVYENRTDVVLILELVSGGELFDFLAQKESLSEEEATSFIKQILDGVNYLHTKKIAHFDLKPENIMLLDKNIPIPHIKLIDFGLAHEIEDGVEFKNIFGTPEFVAPEIVNYEPLGLEADMWSIGVITYILLSGASPFLGDTKQETLANITAVSYDFDEEFFSQTSELAKDFIRKLLVKETRKRLTIQEALRHPWITPVDNQQAMVRRESVVNLENFRKQYVRRRWKLSFSIVSLCNHLTRSLMKKVHLRPDEDLRNCESDTEEDIARRKALHPRRRSSTS

275 P41229 Q9NR20

>P41229

MEPGSDDFLPPPECPVFEPSWAEFRDPLGYIAKIRPIAEKSGICKIRPPADWQPPFAVEVDNFRFTPRIQRLNELEAQTRVKLNYLDQIAKFWEIQGSSLKIPNVERRILDLYSLSKIVVEEGGYEAICKDRRWARVAQRLNYPPGKNIGSLLRSHYERIVYPYEMYQSGANLVQCNTRPFDNEEKDKEYKPHSIPLRQSVQPSKFNSYGRRAKRLQPDPEPTEEDIEKNPELKKLQIYGAGPKMMGLGLMAKDKTLRKKDKEGPECPPTVVVKEELGGDVKVESTSPKTFLESKEELSHSPEPCTKMTMRLRRNHSNAQFIESYVCRMCSRGDEDDKLLLCDGCDDNYHIFCLLPPLPEIPKGVWRCPKCVMAECKRPPEAFGFEQATREYTLQSFGEMADSFKADYFNMPVHMVPTELVEKEFWRLVNSIEEDVTVEYGADIHSKEFGSGFPVSDSKRHLTPEEEEYATSGWNLNVMPVLEQSVLCHINADISGMKVPWLYVGMVFSAFCWHIEDHWSYSINYLHWGEPKTWYGVPSLAAEHLEEVMKKLTPELFDSQPDLLHQLVTLMNPNTLMSHGVPVVRTNQCAGEFVITFPRAYHSGFNQGYNFAEAVNFCTADWLPAGRQCIEHYRRLRRYCVFSHEELICKMAACPEKLDLNLAAAVHKEMFIMVQEERRLRKALLEKGITEAEREAFELLPDDERQCIKCKTTCFLSALACYDCPDGLVCLSHINDLCKCSSSRQYLRYRYTLDELPAMLHKLKVRAESFDTWANKVRVALEVEDGRKRSLEELRALESEARERRFPNSELLQQLKNCLSEAEACVSRALGLVSGQEAGPHRVAGLQMTLTELRAFLDQMNNLPCAMHQIGDVKGVLEQVEAYQAEAREALASLPSSPGLLQSLLERGRQLGVEVPEAQQLQRQVEQARWLDEVKRTLAPSARRGTLAVMRGLLVAGASVAPSPAVDKAQAELQELLTIAERWEEKAHLCLEARQKHPPATLEAIIREAENIPVHLPNIQALKEALAKARAWIADVDEIQNGDHYPCLDDLEGLVAVGRDLPVGLEELRQLELQVLTAHSWREKASKTFLKKNSCYTLLEVLCPCADAGSDSTKRSRWMEKELGLYKSDTELLGLSAQDLRDPGSVIVAFKEGEQKEKEGILQLRRTNSAKPSPLASSSTASSTTSICVCGQVLAGAGALQCDLCQDWFHGRCVSVPRLLSSPRPNPTSSPLLAWWEWDTKFLCPLCMRSRRPRLETILALLVALQRLPVRLPEGEALQCLTERAISWQGRARQALASEDVTALLGRLAELRQRLQAEPRPEEPPNYPAAPASDPLREGSGKDMPKVQGLLENGDSVTSPEKVAPEEGSGKRDLELLSSLLPQLTGPVLELPEATRAPLEELMMEGDLLEVTLDENHSIWQLLQAGQPPDLERIRTLLELEKAERHGSRARGRALERRRRRKVDRGGEGDDPAREELEPKRVRSSGPEAEEVQEEEELEEETGGEGPPAPIPTTGSPSTQENQNGLEPAEGTTSGPSAPFSTLTPRLHLPCPQQPPQQQL

>Q9NR20

MPASELKASEIPFHPSIKTQDPKAEEKSPKKQKVTLTAAEALKLFKNQLSPYEQSEILGYAELWFLGLEAKKLDTAPEKFSKTSFDDEHGFYLKVLHDHIAYRYEVLETIGKGSFGQVAKCLDHKNNELVALKIIRNKKRFHQQALMELKILEALRKKDKDNTYNVVHMKDFFYFRNHFCITFELLGINLYELMKNNNFQGFSLSIVRRFTLSVLKCLQMLSVEKIIHCDLKPENIVLYQKGQASVKVIDFGSSCYEHQKVYTYIQSRFYRSPEVILGHPYDVAIDMWSLGCITAELYTGYPLFPGENEVEQLACIMEVLGLPPAGFIQTASRRQTFFDSKGFPKNITNNRGKKRYPDSKDLTMVLKTYDTSFLDFLRRCLVWEPSLRMTPDQALKHAWIHQSRNLKPQPRPQTLRKSNSFFPSETRKDKVQGCHHSSRKADEITKETTEKTKDSPTKHVQHSGDQQDCLQHGADTVQLPQLVDAPKKSEAAVGAEVSMTSPGQSKNFSLKNTNVLPPIV

276 NP_000512.1 P63302

>NP_000512.1

MELCGLGLPRPPMLLALLLATLLAAMLALLTQVALVVQVAEAARAPSVSAKPGPALWPLPLSVKMTPNLLHLAPENFYISHSPNSTAGPSCTLLEEAFRRYHGYIFGFYKWHHEPAEFQAKTQVQQLLVSITLQSECDAFPNISSDESYTLLVKEPVAVLKANRVWGALRGLETFSQLVYQDSYGTFTINESTIIDSPRFSHRGILIDTSRHYLPVKIILKTLDAMAFNKFNVLHWHIVDDQSFPYQSITFPELSNKGSYSLSHVYTPNDVRMVIEYARLRGIRVLPEFDTPGHTLSWGKGQKDLLTPCYSRQNKLDSFGPINPTLNTTYSFLTTFFKEISEVFPDQFIHLGGDEVEFKCWESNPKIQDFMRQKGFGTDFKKLESFYIQKVLDIIATINKGSIVWQEVFDDKAKLAPGTIVEVWKDSAYPEELSRVTASGFPVILSAPWYLDLISYGQDWRKYYKVEPLDFGGTQKQKQLFIGGEACLWGEYVDATNLTPRLWPRASAVGERLWSSKDVRDMDDAYDRLTRHRCRMVERGIAAQPLYAGYCNHENM

>P63302

MALAVRVVYCGAUGYKSKYLQLKKKLEDEFPGRLDICGEGTPQATGFFEVMVAGKLIHSKKKGDGYVDTESKFLKLVAAIKAALAQG

277 Q9NR56 P24752

>Q9NR56

MAVSVTPIRDTKWLTLEVCREFQRGTCSRPDTECKFAHPSKSCQVENGRVIACFDSLKGRCSRENCKYLHPPPHLKTQLEINGRNNLIQQKNMAMLAQQMQLANAMMPGAPLQPVPMFSVAPSLATNASAAAFNPYLGPVSPSLVPAEILPTAPMLVTGNPGVPVPAAAAAAAQKLMRTDRLEVCREYQRGNCNRGENDCRFAHPADSTMIDTNDNTVTVCMDYIKGRCSREKCKYFHPPAHLQAKIKAAQYQVNQAAAAQAAATAAAMTQSAVKSLKRPLEATFDLGIPQAVLPPLPKRPALEKTNGATAVFNTGIFQYQQALANMQLQQHTAFLPPVPMVHGATPATVSAATTSATSVPFAATATANQIPIISAEHLTSHKYVTQM

>P24752

MAVLAALLRSGARSRSPLLRRLVQEIRYVERSYVSKPTLKEVVIVSATRTPIGSFLGSLSLLPATKLGSIAIQGAIEKAGIPKEEVKEAYMGNVLQGGEGQAPTRQAVLGAGLPISTPCTTINKVCASGMKAIMMASQSLMCGHQDVMVAGGMESMSNVPYVMNRGSTPYGGVKLEDLIVKDGLTDVYNKIHMGSCAENTAKKLNIARNEQDAYAINSYTRSKAAWEAGKFGNEVIPVTVTVKGQPDVVVKEDEEYKRVDFSKVPKLKTVFQKENGTVTAANASTLNDGAAALVLMTADAAKRLNVTPLARIVAFADAAVEPIDFPIAPVYAASMVLKDVGLKKEDIAMWEVNEAFSLVVLANIKMLEIDPQKVNINGGAVSLGHPIGMSGARIVGHLTHALKQGEYGLASICNGGGGASAMLIQKL

278 Q9BWT1 NP_000036.2

>Q9BWT1

MDARRVPQKDLRVKKNLKKFRYVKLISMETSSSSDDSCDSFASDNFANTRLQSVREGCRTRSQCRHSGPLRVAMKFPARSTRGATNKKAESRQPSENSVTDSNSDSEDESGMNFLEKRALNIKQNKAMLAKLMSELESFPGSFRGRHPLPGSDSQSRRPRRRTFPGVASRRNPERRARPLTRSRSRILGSLDALPMEEEEEEDKYMLVRKRKTVDGYMNEDDLPRSRRSRSSVTLPHIIRPVEEITEEELENVCSNSREKIYNRSLGSTCHQCRQKTIDTKTNCRNPDCWGVRGQFCGPCLRNRYGEEVRDALLDPNWHCPPCRGICNCSFCRQRDGRCATGVLVYLAKYHGFGNVHAYLKSLKQEFEMQA

>NP_000036.2

MSAKSRTIGIIGAPFSKGQPRGGVEEGPTVLRKAGLLEKLKEQECDVKDYGDLPFADIPNDSPFQIVKNPRSVGKASEQLAGKVAEVKKNGRISLVLGGDHSLAIGSISGHARVHPDLGVIWVDAHTDINTPLTTTSGNLHGQPVSFLLKELKGKIPDVPGFSWVTPCISAKDIVYIGLRDVDPGEHYILKTLGIKYFSMTEVDRLGIGKVMEETLSYLLGRKKRPIHLSFDVDGLDPSFTPATGTPVVGGLTYREGLYITEEIYKTGLLSGLDIMEVNPSLGKTPEEVTRTVNTAVAITLACFGLAREGNHKPIDYLNPPK

279 Q64ET8 NP_665683.1

>Q64ET8

MGKGNEDSDLHCSSIQCSTDQPPFQQISFTEKGSDEKKPFKEKGKTAFSHSSEKHIQRQGSEPNPNKENSEETKLKAGNSTAGSEPESSSYRENCRKRKMSSKDSCQDTAGNCPEKECSLSLNKKSRSSTAVHNSEIQETCDAHHRGHSRACTGHSKRHRSRALGVQTPSIRKSLVTSVRAMSEAVYQDLAQVWAQQIHSPLTCEQLTLLTRLRGPLCAQVQTLYSMATQAAYVFPAESWLVPATLPGPGESALDREAHPFPGQEITETVSGSDEAKL

>NP_665683.1

MAEKPKLHYFNARGRMESTRWLLAAAGVEFEEKFIKSAEDLDKLRNDGYLMFQQVPMVEIDGMKLVQTRAILNYIASKYNLYGKDIKERALIDMYIEGIADLGEMILLLPVCPPEEKDAKLALIKEKIKNRYFPAFEKVLKSHGQDYLVGNKLSRADIHLVELLYYVEELDSSLISSFPLLKALKTRISNLPTVKKFLQPGSPRKPPMDEKSLEEARKIFRF

280 Q12950 NP_002961.1

>Q12950

MNLPRAERLRSTPQRSLRDSDGEDGKIDVLGEEEDEDEEEAASQQFLEQSLQPGLQVARWGGVALPREHIEGGGGPSDPSEFGTEFRAPPRSAAASEDARQPAKPPSSYIALITMAILQSPHKRLTLSGICAFISGRFPYYRRKFPAWQNSIRHNLSLNDCFVKIPREPGRPGKGNYWSLDPASQDMFDNGSFLRRRKRFQRHQPTPGAHLPHPFPLPAAHAALHNPRPGPLLGAPAPPQPVPGAYPNTGPGRRPYALLHPHPPRYLLLSAPAYAGAPKKAEGADLATPAPFPCCSPHLVLSLGRRARVWRRHREADASLSALRVSCKGSGERVQGLRRVCPRPRGATAPCSSDRQACRTILQQQQRHQEEDCANGCAPTKGAVLGGHLSAASALLRYQAVAEGSGLTSLAAPLGGEGTSPVFLVSPTPSSLAESAGPS

>NP_002961.1

MAKFVIRPATAADCSDILRLIKELAKYEYMEEQVILTEKDLLEDGFGEHPFYHCLVAEVPKEHWTPEGHSIVGFAMYYFTYDPWIGKLLYLEDFFVMSDYRGFGIGSEILKNLSQVAMRCRCSSMHFLVAEWNEPSINFYKRRGASDLSSEEGWRLFKIDKEYLLKMATEE

281 NP_002958.2 NP_006268.1

>NP_002958.2

MAETLSGLGDSGAAGAAALSSASSETGTRRLSDLRVIDLRAELRKRNVDSSGNKSVLMERLKKAIEDEGGNPDEIEITSEGNKKTSKRSSKGRKPEEEGVEDNGLEENSGDGQEDVETSLENLQDIDIMDISVLDEAEIDNGSVADCVEDDDADNLQESLSDSRELVEGEMKELPEQLQEHAIEDKETINNLDTSSSDFTILQEIEEPSLEPENEKILDILGETCKSEPVKEESSELEQPFAQDTSSVGPDRKLAEEEDLFDSAHPEEGDLDLASESTAHAQSSKADSLLAVVKREPAEQPGDGERTDCEPVGLEPAVEQSSAASELAEASSEELAEAPTEAPSPEARDSKEDGRKFDFDACNEVPPAPKESSTSEGADQKMSSPEDDSDTKRLSKEEKGRSSCGRNFWVSGLSSTTRATDLKNLFSKYGKVVGAKVVTNARSPGARCYGFVTMSTAEEATKCINHLHKTELHGKMISVEKAKNEPVGKKTSDKRDSDGKKEKSSNSDRSTNLKRDDKCDRKDDAKKGDDGSGEKSKDQDDQKPGPSERSRATKSGSRGTERTVVMDKSKGVPVISVKTSGSKERASKSQDRKSASREKRSVVSFDKVKEPRKSRDSESHSRVRERSEREQRMQAQWEREERERLEIARERLAFQRQRLERERMERERLERERMHVEHERRREQERIHREREELRRQQELRYEQERRPAVRRPYDLDRRDDAYWPEAKRAALDERYHSDFNRQDRFHDFDHRDRGRYPDHSVDRREGSRSMMGEREGQHYPERHGGPERHGRDSRDGWGGYGSDKRMSEGRGLPPPPRRDWGDHGRREDDRSWQGTADGGMMDRDHKRWQGGERSMSGHSGPGHMMNRGGMSGRGSFAPGGASRGHPIPHGGMQGGFGGQSRGSRPSDARFTRRY

>NP_006268.1

MAQFPTAMNGGPNMWAITSEERTKHDRQFDNLKPSGGYITGDQARNFFLQSGLPAPVLAEIWALSDLNKDGKMDQQEFSIAMKLIKLKLQGQQLPVVLPPIMKQPPMFSPLISARFGMGSMPNLSIPQPLPPAAPITSLSSATSGTNLPPLMMPTPLVPSVSTSSLPNGTASLIQPLPIPYSSSTLPHGSSYSLMMGGFGGASIQKAQSLIDLGSSSSTSSTASLSGNSPKTGTSEWAVPQPTRLKYRQKFNTLDKSMSGYLSGFQARNALLQSNLSQTQLATIWTLADVDGDGQLKAEEFILAMHLTDMAKAGQPLPLTLPPELVPPSFRGGKQIDSINGTLPSYQKMQEEEPQKKLPVTFEDKRKANYERGNMELEKRRQALMEQQQREAERKAQKEKEEWERKQRELQEQEWKKQLELEKRLEKQRELERQREEERRKDIERREAAKQELERQRRLEWERIRRQELLNQKNREQEEIVRLNSKKKNLHLELEALNGKHQQISGRLQDVRLKKQTQKTELEVLDKQCDLEIMEIKQLQQELQEYQNKLIYLVPEKQLLNERIKNMQFSNTPDSGVSLLHKKSLEKEELCQRLKEQLDALEKETASKLSEMDSFNNQLKCGNMDDSVLQCLLSLLSCLNNLFLLLKELRETYNTQQLALEQLYKIKRDKLKEIERKRLELMQKKKLEDEAARKAKQGKENLWKENLRKEEEEKQKRLQEEKTQEKIQEEERKAEEKQRKDKDTLKAEEKKRETASVLVNYRALYPFEARNHDEMSFNSGDIIQVDEKTVGEPGWLYGSFQGNFGWFPCNYVEKMPSSENEKAVSPKKALLPPTVSLSATSTSSEPLSSNQPASVTDYQNVSFSNLTVNTSWQKKSAFTRTVSPGSVSPIHGQGQVVENLKAQALCSWTAKKDNHLNFSKHDIITVLEQQENWWFGEVHGGRGWFPKSYVKIIPGSEVKREEPEALYAAVNKKPTSAAYSVGEEYIALYPYSSVEPGDLTFTEGEEILVTQKDGEWWTGSIGDRSGIFPSNYVKPKDQESFGSASKSGASNKKPEIAQVTSAYVASGSEQLSLAPGQLILILKKNTSGWWQGELQARGKKRQKGWFPASHVKLLGPSSERATPAFHPVCQVIAMYDYAANNEDELSFSKGQLINVMNKDDPDWWQGEINGVTGLFPSNYVKMTTDSDPSQQWCADLQTLDTMQPIERKRQGYIHELIQTEERYMADLQLVVEVFQKRMAESGFLTEGEMALIFVNWKELIMSNTKLLKALRVRKKTGGEKMPVQMIGDILAAELSHMQAYIRFCSCQLNGAALLQQKTDEDTDFKEFLKKLASDPRCKGMPLSSFLLKPMQRITRYPLLIRSILENTPESHADHSSLKLALERAEELCSQVNEGVREKENSDRLEWIQAHVQCEGLAEQLIFNSLTNCLGPRKLLHSGKLYKTKSNKELHGFLFNDFLLLTYMVKQFAVSSGSEKLFSSKSNAQFKMYKTPIFLNEVLVKLPTDPSSDEPVFHISHIDRVYTLRTDNINERTAWVQKIKAASEQYIDTEKKKREKAYQARSQKTSGIGRLMVHVIEATELKACKPNGKSNPYCEISMGSQSYTTRTIQDTLNPKWNFNCQFFIKDLYQDVLCLTLFDRDQFSPDDFLGRTEIPVAKIRTEQESKGPMTRRLLLHEVPTGEVWVRFDLQLFEQKTLL

282 Q92925 NP_001547.1

>Q92925

MSPGNRMPMARLAGGTPCWLPIWCSSSASTWHPTHHDGSIPKTPACAPAQPPMPAQRRGLKRRKMADKVLPQRIRELVPESQAYMDLLAFERKLDQTIARKRMEIQEAIKKPLTQKRKLRIYISNTFSPSKAEGDSAGTAGTPGGTPAGDKVASWELRVEGKLLDDPSKQKRKFSSFFKSLVIELDKELYGPDGHLVEWYWMPTTQETDGFQVKRPGDLNVKCTLLLMLDHQPPQYKLDPRLARLLGVHTQTRAAIMQALWLYIKHNQLQDGHEREYINCNRYFRQIFSCGRLRFSEIPMKLAGLLQHPDPIVINHVISVDPNDQKKTACYDIDVEVDDPLKAQMSNFLASTTNQQEIASLDVKIHETIESINQLKTQRDFMLSFSTDPQDFIQEWLRSQRRDLKIITDVIGNPEEERRAAFYHQPWAQEAVGRHIFAKVQQRRQELEQVLGIRLT

>NP_001547.1

MSWSPSLTTQTCGAWEMKERLGTGGFGNVIRWHNQETGEQIAIKQCRQELSPRNRERWCLEIQIMRRLTHPNVVAARDVPEGMQNLAPNDLPLLAMEYCQGGDLRKYLNQFENCCGLREGAILTLLSDIASALRYLHENRIIHRDLKPENIVLQQGEQRLIHKIIDLGYAKELDQGSLCTSFVGTLQYLAPELLEQQKYTVTVDYWSFGTLAFECITGFRPFLPNWQPVQWHSKVRQKSEVDIVVSEDLNGTVKFSSSLPYPNNLNSVLAERLEKWLQLMLMWHPRQRGTDPTYGPNGCFKALDDILNLKLVHILNMVTGTIHTYPVTEDESLQSLKARIQQDTGIPEEDQELLQEAGLALIPDKPATQCISDGKLNEGHTLDMDLVFLFDNSKITYETQISPRPQPESVSCILQEPKRNLAFFQLRKVWGQVWHSIQTLKEDCNRLQQGQRAAMMNLLRNNSCLSKMKNSMASMSQQLKAKLDFFKTSIQIDLEKYSEQTEFGITSDKLLLAWREMEQAVELCGRENEVKLLVERMMALQTDIVDLQRSPMGRKQGGTLDDLEEQARELYRRLREKPRDQRTEGDSQEMVRLLLQAIQSFEKKVRVIYTQLSKTVVCKQKALELLPKVEEVVSLMNEDEKTVVRLQEKRQKELWNLLKIACSKVRGPVSGSPDSMNASRLSQPGQLMSQPSTASNSLPEPAKKSEELVAEAHNLCTLLENAIQDTVREQDQSFTALDWSWLQTEEEEHSCLEQAS

283 Q96LT9 NP_003240.1

>Q96LT9

MAAPEQPLAISRGCTSSSSLSPPRGDRTLLVRHLPAELTAEEKEDLLKYFGAQSVRVLSDKGRLKHTAFATFPNEKAAIKALTRLHQLKLLGHTLVVEFAKEQDRVHSPCPTSGSEKKKRSDDPVEDDKEKKELGYLTVENGIAPNHGLTFPLNSCLKYMYPPPSSTILANIVNALASVPKFYVQVLHLMNKMNLPTPFGPITARPPMYEDYMPLHAPLPPTSPQPPEEPPLPDEDEELSSEESEYESTDDEDRQRMNKLMELANLQPKRPKTIKQRHVRKKRKIKDMLNTPLCPSHSSLHPVLLPSDVFDQPQPVGNKRIEFHISTDMPAAFKKDLEKEQNCEEKNHDLPATEVDASNIGFGKIFPKPNLDITEEIKEDSDEMPSECISRRELEKGRISREEMETLSVFRSYEPGEPNCRIYVKNLAKHVQEKDLKYIFGRYVDFSSETQRIMFDIRLMKEGRMKGQAFIGLPNEKAAAKALKEANGYVLFGKPMVVQFARSARPKQDPKEGKRKC

>NP_003240.1

MKPPAACAGDMADAASPCSVVNDLRWDLSAQQIEERTRELIEQTKRVYDQVGTQEFEDVSYESTLKALADVEVTYTVQRNILDFPQHVSPSKDIRTASTEADKKLSEFDVEMSMREDVYQRIVWLQEKVQKDSLRPEAARYLERLIKLGRRNGLHLPRETQENIKRIKKKLSLLCIDFNKNLNEDTTFLPFTLQELGGLPEDFLNSLEKMEDGKLKVTLKYPHYFPLLKKCHVPETRRKVEEAFNCRCKEENCAILKELVTLRAQKSRLLGFHTHADYVLEMNMAKTSQTVATFLDELAQKLKPLGEQERAVILELKRAECERRGLPFDGRIRAWDMRYYMNQVEETRYCVDQNLLKEYFPVQVVTHGLLGIYQELLGLAFHHEEGASAWHEDVRLYTARDAASGEVVGKFYLDLYPREGKYGHAACFGLQPGCLRQDGSRQIAIAAMVANFTKPTADAPSLLQHDEVETYFHEFGHVMHQLCSQAEFAMFSGTHVERDFVEAPSQMLENWVWEQEPLLRMSRHYRTGSAVPRELLEKLIESRQANTGLFNLRQIVLAKVDQALHTQTDADPAEEYARLCQEILGVPATPGTNMPATFGHLAGGYDAQYYGYLWSEVYSMDMFHTRFKQEGVLNSKVGMDYRSCILRPGGSEDASAMLRRFLGRDPKQDAFLLSKGLQVGGCEPEPQVC

284 NP_066390.1 NP_057564.3

>NP_066390.1

MSGRGKQGGKARAKAKTRSSRAGLQFPVGRVHRLLRKGNYSERVGAGAPVYLAAVLEYLTAEILELAGNAARDNKKTRIIPRHLQLAIRNDEELNKLLGRVTIAQGGVLPNIQAVLLPKKTESHHKAKGK

>NP_057564.3

MKDPSRSSTSPSIINEDVIINGHSHEDDNPFAEYMWMENEEEFNRQIEEELWEEEFIERCFQEMLEEEEEHEWFIPARDLPQTMDQIQDQFNDLVISDGSSLEDLVVKSNLNPNAKEFVPGVKYGNI

285 Q15319 NP_006651.2

>Q15319

MMAMNSKQPFGMHPVLQEPKFSSLHSGSEAMRRVCLPAPQLQGNIFGSFDESLLARAEALAAVDIVSHGKNHPFKPDATYHTMSSVPCTSTSSTVPISHPAALTSHPHHAVHQGLEGDLLEHISPTLSVSGLGAPEHSVMPAQIHPHHLGAMGHLHQAMGMSHPHTVAPHSAMPACLSDVESDPRELEAFAERFKQRRIKLGVTQADVGAALANLKIPGVGSLSQSTICRFESLTLSHNNMIALKPVLQAWLEEAEAAYREKNSKPELFNGSERKRKRTSIAAPEKRSLEAYFAIQPRPSSEKIAAIAEKLDLKKNVVRVWFCNQRQKQKRMKYSAVH

>NP_006651.2

MPSCGACTCGAAAVRLITSSLASAQRGISGGRIHMSVLGRLGTFETQILQRAPLRSFTETPAYFASKDGISKDGSGDGNKKSASEGSSKKSGSGNSGKGGNQLRCPKCGDLCTHVETFVSSTRFVKCEKCHHFFVVLSEADSKKSIIKEPESAAEAVKLAFQQKPPPPPKKIYNYLDKYVVGQSFAKKVLSVAVYNHYKRIYNNIPANLRQQAEVEKQTSLTPRELEIRRREDEYRFTKLLQIAGISPHGNALGASMQQQVNQQIPQEKRGGEVLDSSHDDIKLEKSNILLLGPTGSGKTLLAQTLAKCLDVPFAICDCTTLTQAGYVGEDIESVIAKLLQDANYNVEKAQQGIVFLDEVDKIGSVPGIHQLRDVGGEGVQQGLLKLLEGTIVNVPEKNSRKLRGETVQVDTTNILFVASGAFNGLDRIISRRKNEKYLGFGTPSNLGKGRRAAAAADLANRSGESNTHQDIEEKDRLLRHVEARDLIEFGMIPEFVGRLPVVVPLHSLDEKTLVQILTEPRNAVIPQYQALFSMDKCELNVTEDALKAIARLALERKTGARGLRSIMEKLLLEPMFEVPNSDIVCVEVDKEVVEGKKEPGYIRAPTKESSEEEYDSGVEEEGWPRQADAANS

286 P22304 NP_005411.1

>P22304

MPPPRTGRGLLWLGLVLSSVCVALGSETQANSTTDALNVLLIIVDDLRPSLGCYGDKLVRSPNIDQLASHSLLFQNAFAQQAVCAPSRVSFLTGRRPDTTRLYDFNSYWRVHAGNFSTIPQYFKENGYVTMSVGKVFHPGISSNHTDDSPYSWSFPPYHPSSEKYENTKTCRGPDGELHANLLCPVDVLDVPEGTLPDKQSTEQAIQLLEKMKTSASPFFLAVGYHKPHIPFRYPKEFQKLYPLENITLAPDPEVPDGLPPVAYNPWMDIRQREDVQALNISVPYGPIPVDFQRKIRQSYFASVSYLDTQVGRLLSALDDLQLANSTIIAFTSDHGWALGEHGEWAKYSNFDVATHVPLIFYVPGRTASLPEAGEKLFPYLDPFDSASQLMEPGRQSMDLVELVSLFPTLAGLAGLQVPPRCPVPSFHVELCREGKNLLKHFRFRDLEEDPYLPGNPRELIAYSQYPRPSDIPQWNSDKPSLKDIKIMGYSIRTIDYRYTVWVGFNPDEFLANFSDIHAGELYFVDSDPLQDHNMYNDSQGGDLFQLLMP

>NP_005411.1

MNSELDYYEKFEEVHGILMYKDFVKYWDNVEAFQARPDDLVIATYPKSGTTWVSEIVYMIYKEGDVEKCKEDVIFNRIPFLECRKENLMNGVKQLDEMNSPRIVKTHLPPELLPASFWEKDCKIIYLCRNAKDVAVSFYYFFLMVAGHPNPGSFPEFVEKFMQGQVPYGSWYKHVKSWWEKGKSPRVLFLFYEDLKEDIRKEVIKLIHFLERKPSEELVDRIIHHTSFQEMKNNPSTNYTTLPDEIMNQKLSPFMRKGITGDWKNHFTVALNEKFDKHYEQQMKESTLKFRTEI

287 NP_004074.2 NP_001386.1

>NP_004074.2

MAAESLPFSFGTLSSWELEAWYEDLQEVLSSDENGGTYVSPPGNEEEESKIFTTLDPASLAWLTEEEPEPAEVTSTSQSPHSPDSSQSSLAQEEEEEDQGRTRKRKQSGHSPARAGKQRMKEKEQENERKVAQLAEENERLKQEIERLTREVEATRRALIDRMVNLHQA

>NP_001386.1

MEGLGRSCLWLRRELSPPRPRLLLLDCRSRELYESARIGGALSVALPALLLRRLRRGSLSVRALLPGPPLQPPPPAPVLLYDQGGGRRRRGEAEAEAEEWEAESVLGTLLQKLREEGYLAYYLQGGFSRFQAECPHLCETSLAGRAGSSMAPVPGPVPVVGLGSLCLGSDCSDAESEADRDSMSCGLDSEGATPPPVGLRASFPVQILPNLYLGSARDSANLESLAKLGIRYILNVTPNLPNFFEKNGDFHYKQIPISDHWSQNLSRFFPEAIEFIDEALSQNCGVLVHCLAGVSRSVTVTVAYLMQKLHLSLNDAYDLVKRKKSNISPNFNFMGQLLDFERSLRLEERHSQEQGSGGQASAASNPPSFFTTPTSDGAFELAPT

288 Q9UDW3 Q9BZQ8

>Q9UDW3

MGKRYFCDYCDRSFQDNLHNRKKHLNGLQHLKAKKVWYDMFRDAAAILLDEQNKRPCRKFLLTGQCDFGSNCRFSHMSERDLQELSIQVEEERRAREWLLDAPELPEGHLEDWLEKRAKRLSSAPSSRAEPIRTTVFQYPVGWPPVQELPPSLRAPPPGGWPLQPRVQWG

>Q9BZQ8

MGGSASSQLDEGKCAYIRGKTEAAIKNFSPYYSRQYSVAFCNHVRTEVEQQRDLTSQFLKTKPPLAPGTILYEAELSQFSEDIKKWKERYVVVKNDYAVESYENKEAYQRGAAPKCRILPAGGKVLTSEDEYNLLSDRHFPDPLASSEKENTQPFVVLPKEFPVYLWQPFFRHGYFCFHEAADQKRFSALLSDCVRHLNHDYMKQMTFEAQAFLEAVQFFRQEKGHYGSWEMITGDEIQILSNLVMEELLPTLQTDLLPKMKGKKNDRKRTWLGLLEEAYTLVQHQVSEGLSALKEECRALTKGLEGTIRSDMDQIVNSKNYLIGKIKAMVAQPAEKSCLESVQPFLASILEELMGPVSSGFSEVRVLFEKEVNEVSQNFQTTKDSVQLKEHLDRLMNLPLHSVKMEPCYTKVNLLHERLQDLKSRFRFPHIDLVVQRTQNYMQELMENAVFTFEQLLSPHLQGEASKTAVAIEKVKLRVLKQYDYDSSTIRKKIFQEALVQITLPTVQKALASTCKPELQKYEQFIFADHTNMIHVENVYEEILHQILLDETLKVIKEAAILKKHNLFEDNMALPSESVSSLTDLKPPTGSNQASPARRASAILPGVLGSETLSNEVFQESEEEKQPEVPSSLAKGESLSLPGPSPPPDGTEQVIISRVDDPVVNPVATEDTAGLPGTCSSELEFGGTLEDEEPAQEEPEPITASGSLKALRKLLTASVEVPVDSAPVMEEDTNGESHVPQENEEEEEKEPSQAAAIHPDNCEESEVSEREAQPPCPEAHGEELGGFPEVGSPASPPASGGLTEEPLGPMEGELPGEACTLTAHEGRGGKCTEEGDASQQEGCTLGSDPICLSESQVSEEQEEMGGQSSAAQATASVNAEEIKVARIHECQWVVEDAPNPDVLLSHKDDVKEGEGGQESFPELPSEE

289 NP_002348.1 Q02252

>NP_002348.1

MAAAVRMNIQMLLEAADYLERREREAEHGYASMLPYNNKDRDALKRRNKSKKNNSSSRSTHNEMEKNRRAHLRLCLEKLKGLVPLGPESSRHTTLSLLTKAKLHIKKLEDCDRKAVHQIDQLQREQRHLKRQLEKLGIERIRMDSIGSTVSSERSDSDREEIDVDVESTDYLTGDLDWSSSSVSDSDERGSMQSLGSDEGYSSTSIKRIKLQDSHKACLGL

>Q02252

MAALLAAAAVRARILQVSSKVKSSPTWYSASSFSSSVPTVKLFIGGKFVESKSDKWIDIHNPATNEVIGRVPQATKAEMDAAIASCKRAFPAWADTSVLSRQQVLLRYQQLIKENLKEIAKLITLEQGKTLADAEGDVFRGLQVVEHACSVTSLMMGETMPSITKDMDLYSYRLPLGVCAGIAPFNFPAMIPLWMFPMAMVCGNTFLMKPSERVPGATMLLAKLLQDSGAPDGTLNIIHGQHEAVNFICDHPDIKAISFVGSNKAGEYIFERGSRHGKRVQANMGAKNHGVVMPDANKENTLNQLVGAAFGAAGQRCMALSTAVLVGEAKKWLPELVEHAKNLRVNAGDQPGADLGPLITPQAKERVCNLIDSGTKEGASILLDGRKIKVKGYENGNFVGPTIISNVKPNMTCYKEEIFGPVLVVLETETLDEAIQIVNNNPYGNGTAIFTTNGATARKYAHLVDVGQVGVNVPIPVPLPMFSFTGSRSSFRGDTNFYGKQGIQFYTQLKTITSQWKEEDATLSSPAVVMPTMGR

290 NP_000439.1 NP_008996.1

>NP_000439.1

MAASFPPTLGLSSAPDEIQHPHIKFSEWKFKLFRVRSFEKTPEEAQKEKKDSFEGKPSLEQSPAVLDKADGQKPVPTQPLLKAHPKFSKKFHDNEKARGKAIHQANLRHLCRICGNSFRADEHNRRYPVHGPVDGKTLGLLRKKEKRATSWPDLIAKVFRIDVKADVDSIHPTEFCHNCWSIMHRKFSSAPCEVYFPRNVTMEWHPHTPSCDICNTARRGLKRKSLQPNLQLSKKLKTVLDQARQARQRKRRAQARISSKDVMKKIANCSKIHLSTKLLAVDFPEHFVKSISCQICEHILADPVETNCKHVFCRVCILRCLKVMGSYCPSCRYPCFPTDLESPVKSFLSVLNSLMVKCPAKECNEEVSLEKYNHHISSHKESKEIFVHINKGGRPRQHLLSLTRRAQKHRLRELKLQVKAFADKEEGGDVKSVCMTLFLLALRARNEHRQADELEAIMQGKGSGLQPAVCLAIRVNTFLSCSQYHKMYRTVKAITGRQIFQPLHALRNAEKVLLPGYHHFEWQPPLKNVSSSTDVGIIDGLSGLSSSVDDYPVDTIAKRFRYDSALVSALMDMEEDILEGMRSQDLDDYLNGPFTVVVKESCDGMGDVSEKHGSGPVVPEKAVRFSFTIMKITIAHSSQNVKVFEEAKPNSELCCKPLCLMLADESDHETLTAILSPLIAEREAMKSSELMLELGGILRTFKFIFRGTGYDEKLVREVEGLEASGSVYICTLCDATRLEASQNLVFHSITRSHAENLERYEVWRSNPYHESVEELRDRVKGVSAKPFIETVPSIDALHCDIGNAAEFYKIFQLEIGEVYKNPNASKEERKRWQATLDKHLRKKMNLKPIMRMNGNFARKLMTKETVDAVCELIPSEERHEALRELMDLYLKMKPVWRSSCPAKECPESLCQYSFNSQRFAELLSTKFKYRYEGKITNYFHKTLAHVPEIIERDGSIGAWASEGNESGNKLFRRFRKMNARQSKCYEMEDVLKHHWLYTSKYLQKFMNAHNALKTSGFTMNPQASLGDPLGIEDSLESQDSMEF

>NP_008996.1

MVDYSVWDHIEVSDDEDETHPNIDTASLFRWRHQARVERMEQFQKEKEELDRGCRECKRKVAECQRKLKELEVAEGGKAELERLQAEAQQLRKEERSWEQKLEEMRKKEKSMPWNVDTLSKDGFSKSMVNTKPEKTEEDSEEVREQKHKTFVEKYEKQIKHFGMLRRWDDSQKYLSDNVHLVCEETANYLVIWCIDLEVEEKCALMEQVAHQTIVMQFILELAKSLKVDPRACFRQFFTKIKTADRQYMEGFNDELEAFKERVRGRAKLRIEKAMKEYEEEERKKRLGPGGLDPVEVYESLPEELQKCFDVKDVQMLQDAISKMDPTDAKYHMQRCIDSGLWVPNSKASEAKEGEEAGPGDPLLEAVPKTGDEKDVSV

291 NP_055367.2 NP_110438.1

>NP_055367.2

MTQEYDNKRPVLVLQNEALYPQRRSYTSEDEAWKSFLENPLTAATKAMMSINGDEDSAAALGLLYDYYKVPRERRSSTAKPEVEHPEPDHSKRNSIPIVTEQPLISAGENRVQVLKNVPFNIVLPHGNQLGIDKRGHLTAPDTTVTVSIATMPTHSIKTETQPHGFAVGIPPAVYHPEPTERVVVFDRNLNTDQFSSGAQAPNAQRRTPDSTFSETFKEGVQEVFFPSDLSLRMPGMNSEDYVFDSVSGNNFEYTLEASKSLRQKPGDSTMTYLNKGQFYPITLKEVSSSEGIHHPISKVRSVIMVVFAEDKSREDQLRHWKYWHSRQHTAKQRCIDIADYKESFNTISNIEEIAYNAISFTWDINDEAKVFISVNCLSTDFSSQKGVKGLPLNIQVDTYSYNNRSNKPVHRAYCQIKVFCDKGAERKIRDEERKQSKRKVSDVKVPLLPSHKRMDITVFKPFIDLDTQPVLFIPDVHFANLQRGTHVLPIASEELEGEGSVLKRGPYGTEDDFAVPPSTKLARIEEPKRVLLYVRKESEEVFDALMLKTPSLKGLMEAISDKYDVPHDKIGKIFKKCKKGILVNMDDNIVKHYSNEDTFQLQIEEAGGSYKLTLTEI

>NP_110438.1

MLRALSRLGAGTPCRPRAPLVLPARGRKTRHDPLAKSKIERVNMPPAVDPAEFFVLMERYQHYRQTVRALRMEFVSEVQRKVHEARAGVLAERKALKDAAEHRELMAWNQAENRRLHELRIARLRQEEREQEQRQALEQARKAEEVQAWAQRKEREVLQLQEEVKNFITRENLEARVEAALDSRKNYNWAITREGLVVRPQRRDS

292 NP_004261.1 NP_872578.1

>NP_004261.1

MGEPQQVSALPPPPMQYIKEYTDENIQEGLAPKPPPPIKDSYMMFGNQFQCDDLIIRPLESQGIERLHPMQFDHKKELRKLNMSILINFLDLLDILIRSPGSIKREEKLEDLKLLFVHVHHLINEYRPHQARETLRVMMEVQKRQRLETAERFQKHLERVIEMIQNCLASLPDDLPHSEAGMRVKTEPMDADDSNNCTGQNEHQRENSGHRRDQIIEKDAALCVLIDEMNERP

>NP_872578.1

MAAPCVSYGGAVSYRLLLWGRGSLARKQGLWKTAAPELQTNVRSQILRLRHTAFVIPKKNVPTSKRETYTEDFIKKQIEEFNIGKRHLANMMGEDPETFTQEDIDRAIAYLFPSGLFEKRARPVMKHPEQIFPRQRAIQWGEDGRPFHYLFYTGKQSYYSLMHDVYGMLLNLEKHQSHLQAKSLLPEKTVTRDVIGSRWLIKEELEEMLVEKLSDLDYMQFIRLLEKLLTSQCGAAEEEFVQRFRRSVTLESKKQLIEPVQYDEQGMAFSKSEGKRKTAKAEAIVYKHGSGRIKVNGIDYQLYFPITQDREQLMFPFHFVDRLGKHDVTCTVSGGGRSAQAGAIRLAMAKALCSFVTEDEVEWMRQAGLLTTDPRVRERKKPGQEGARRKFTWKKR

293 NP_005162.1 Q9GZZ1

>NP_005162.1

MEDSHKSTTSETAPQPGSAVQGAHISHIAQQVSSLSESEESQDSSDSIGSSQKAHGILARRPSYRKILKDLSSEDTRGRKGDGENSGVSAAVTSMSVPTPIYQTSSGQYIAIAPNGALQLASPGTDGVQGLQTLTMTNSGSTQQGTTILQYAQTSDGQQILVPSNQVVVQTASGDMQTYQIRTTPSATSLPQTVVMTSPVTLTSQTTKTDDPQLKREIRLMKNREAARECRRKKKEYVKCLENRVAVLENQNKTLIEELKTLKDLYSNKSV

>Q9GZZ1

MKGSRIELGDVTPHNIKQLKRLNQVIFPVSYNDKFYKDVLEVGELAKLAYFNDIAVGAVCCRVDHSQNQKRLYIMTLGCLAPYRRLGIGTKMLNHVLNICEKDGTFDNIYLHVQISNESAIDFYRKFGFEIIETKKNYYKRIEPADAHVLQKNLKVPSGQNADVQKTDN

294 NP_006183.1 NP_005882.2

>NP_006183.1

MEQTYGEVNQLGGVFVNGRPLPNAIRLRIVELAQLGIRPCDISRQLRVSHGCVSKILARYNETGSILPGAIGGSKPRVTTPNVVKHIRDYKQGDPGIFAWEIRDRLLADGVCDKYNVPSVSSISRILRNKIGSLAQPGPYEASKQPPSQPTLPYNHIYQYPYPSPVSPTGAKMGSHPGVPGTAGHVSIPRSWPSAHSVSNILGIRTFMEQTGALAGSEGTAYSPKMEDWAGVNRTAFPATPAVNGLEKPALEADIKYTQSASTLSAVGGFLPACAYPASNQHGVYSAPGGGYLAPGPPWPPAQGPPLAPPGAGVAVHGGELAAAMTFKHPSREGSLPAPAARPRTPSVAYTDCPSRPRPPRGSSPRTRARRERQADPGAQVCAAAPAIGTGRIGGLAEEEASAGPRGARPASPQAQPCLWPDPPHFLYWSGFLGFSELGF

>NP_005882.2

MNAGSDPVVIVSAARTIIGSFNGALAAVPVQDLGSTVIKEVLKRATVAPEDVSEVIFGHVLAAGCGQNPVRQASVGAGIPYSVPAWSCQMICGSGLKAVCLAVQSIGIGDSSIVVAGGMENMSKAPHLAYLRTGVKIGEMPLTDSILCDGLTDAFHNCHMGITAENVAKKWQVSREDQDKVAVLSQNRTENAQKAGHFDKEIVPVLVSTRKGLIEVKTDEFPRHGSNIEAMSKLKPYFLTDGTGTVTPANASGINDGAAAVVLMKKSEADKRGLTPLARIVSWSQVGVEPSIMGIGPIPAIKQAVTKAGWSLEDVDIFEINEAFAAVSAAIVKELGLNPEKVNIEGGAIALGHPLGASGCRILVTLLHTLERMGRSRGVAALCIGGGMGIAMCVQRE

295 NP_002681.1 P28838

>NP_002681.1

MSKRKAPQETLNGGITDMLTELANFEKNVSQAIHKYNAYRKAASVIAKYPHKIKSGAEAKKLPGVGTKIAEKIDEFLATGKLRKLEKIRQDDTSSSINFLTRVSGIGPSAARKFVDEGIKTLEDLRKNEDKLNHHQRIGLKYFGDFEKRIPREEMLQMQDIVLNEVKKVDSEYIATVCGSFRRGAESSGDMDVLLTHPSFTSESTKQPKLLHQVVEQLQKVHFITDTLSKGETKFMGVCQLPSKNDEKEYPHRRIDIRLIPKDQYYCGVLYFTGSDIFNKNMRAHALEKGFTINEYTIRPLGVTGVAGEPLPVDSEKDIFDYIQWKYREPKDRSE

>P28838

MFLLPLPAAGRVVVRRLAVRRFGSRSLSTADMTKGLVLGIYSKEKEDDVPQFTSAGENFDKLLAGKLRETLNISGPPLKAGKTRTFYGLHQDFPSVVLVGLGKKAAGIDEQENWHEGKENIRAAVAAGCRQIQDLELSSVEVDPCGDAQAAAEGAVLGLYEYDDLKQKKKMAVSAKLYGSGDQEAWQKGVLFASGQNLARQLMETPANEMTPTRFAEIIEKNLKSASSKTEVHIRPKSWIEEQAMGSFLSVAKGSDEPPVFLEIHYKGSPNANEPPLVFVGKGITFDSGGISIKASANMDLMRADMGGAATICSAIVSAAKLNLPINIIGLAPLCENMPSGKANKPGDVVRAKNGKTIQVDNTDAEGRLILADALCYAHTFNPKVILNAATLTGAMDVALGSGATGVFTNSSWLWNKLFEASIETGDRVWRMPLFEHYTRQVVDCQLADVNNIGKYRSAGACTAAAFLKEFVTHPKWAHLDIAGVMTNKDEVPYLRKGMTGRPTRTLIEFLLRFSQDNA

296 NP_002895.3 Q9BUE6

>NP_002895.3

MLVIPPGLSEEEEALQKKFNKLKKKKKALLALKKQSSSSTTSQGGVKRSLSEQPVMDTATATEQAKQLVKSGAISAIKAETKNSGFKRSRTLEGKLKDPEKGPVPTFQPFQRSISADDDLQESSRRPQRKSLYESFVSSSDRLRELGPDGEEAEGPGAGDGPPRSFDWGYEERSGAHSSASPPRSRSRDRSHERNRDRDRDRERDRDRDRDRDRERDRDRDRDRDRDRERDRDRERDRDRDREGPFRRSDSFPERRAPRKGNTLYVYGEDMTPTLLRGAFSPFGNIIDLSMDPPRNCAFVTYEKMESADQAVAELNGTQVESVQLKVNIARKQPMLDAATGKSVWGSLAVQNSPKGCHRDKRTQIVYSDDVYKENLVDGF

>Q9BUE6

MSASLVRATVRAVSKRKLQPTRAALTLTPSAVNKIKQLLKDKPEHVGVKVGVRTRGCNGLSYTLEYTKTKGDSDEEVIQDGVRVFIEKKAQLTLLGTEMDYVEDKLSSEFVFNNPNIKGTCGCGESFNI

297 NP_005635.1 P25325

>NP_005635.1

MNQFGPSALINLSNFSSIKPEPASTPPQGSMANSTAVVKIPGTPGAGGRLSPENNQVLTKKKLQDLVREVDPNEQLDEDVEEMLLQIADDFIESVVTAACQLARHRKSSTLEVKDVQLHLERQWNMWIPGFGSEEIRPYKKACTTEAHKQRMALIRKTTKK

>P25325

MASPQLCRALVSAQWVAEALRAPRAGQPLQLLDASWYLPKLGRDARREFEERHIPGAAFFDIDQCSDRTSPYDHMLPGAEHFAEYAGRLGVGAATHVVIYDASDQGLYSAPRVWWMFRAFGHHAVSLLDGGLRHWLRQNLPLSSGKSQPAPAEFRAQLDPAFIKTYEDIKENLESRRFQVVDSRATGRFRGTEPEPRDGIEPGHIPGTVNIPFTDFLSQEGLEKSPEEIRHLFQEKKVDLSKPLVATCGSGVTACHVALGAYLCGKPDVPIYDGSWVEWYMRARPEDVISEGRGKTH

298 NP_055315.2 NP_001217.2

>NP_055315.2

MSGTNLDGNDEFDEQLRMQELYGDGKDGDTQTDAGGEPDSLGQQPTDTPYEWDLDKKAWFPKITEDFIATYQANYGFSNDGASSSTANVEDVHARTAEEPPQEKAPEPTDARKKGEKRKAESGWFHVEEDRNTNVYVSGLPPDITVDEFIQLMSKFGIIMRDPQTEEFKVKLYKDNQGNLKGDGLCCYLKRESVELALKLLDEDEIRGYKLHVEVAKFQLKGEYDASKKKKKCKDYKKKLSMQQKQLDWRPERRAGPSRMRHERVVIIKNMFHPMDFEDDPLVLNEIREDLRVECSKFGQIRKLLLFDRHPDGVASVSFRDPEEADYCIQTLDGRWFGGRQITAQAWDGTTDYQVEETSREREERLRGWEAFLNAPEANRGLRRSDSVSASERAGPSRARHFSEHPSTSKMNAQETATGMAFEEPIDEKKFEKTEDGGEFEEGASENNAKESSPEKEAEEGCPEKESEEGCPKRGFEGSCSQKESEEGNPVRGSEEDSPKKESKKKTLKNDCEENGLAKESEDDLNKESEEEVGPTKESEEDDSEKESDEDCSEKQSEDGSEREFEENGLEKDLDEEGSEKELHENVLDKELEENDSENSEFEDDGSEKVLDEEGSEREFDEDSDEKEEEEDTYEKVFDDESDEKEDEEYADEKGLEAADKKAEEGDADEKLFEESDDKEDEDADGKEVEDADEKLFEDDDSNEKLFDEEEDSSEKLFDDSDERGTLGGFGSVEEGPLSTGSSFILSSDDDDDDI

>NP_001217.2

MSSASGLRRGHPAGGEENMTETDAFYKREMFDPAEKYKMDHRRRGIALIFNHERFFWHLTLPERRGTCADRDNLTRRFSDLGFEVKCFNDLKAEELLLKIHEVSTVSHADADCFVCVFLSHGEGNHIYAYDAKIEIQTLTGLFKGDKCHSLVGKPKIFIIQACRGNQHDVPVIPLDVVDNQTEKLDTNITEVDAASVYTLPAGADFLMCYSVAEGYYSHRETVNGSWYIQDLCEMLGKYGSSLEFTELLTLVNRKVSQRRVDFCKDPSAIGKKQVPCFASMLTKKLHFFPKSN

299 Q969J2 Q9HBU6

>Q969J2

MAREPRKNAALDAQSAEDQTGLLTVKVEKEEASALTAEVRAPCSPARGPERSRQRFRGFRYPEAAGPREALSRLRELCGQWLQPEMHSKEQILELLVLEQFLTILPGNLQSWVREQHPESGEEVVVLLEYLERQLDEPAPQVPVGDQGQELLCCKMALLTQTQGSQSSQCQPMKALFKHESLGSQPLHDRVLQVPGLAQGGCCREDAMVASRLTPGSQGLLKMEDVALTLTPGWTQLDSSQVNLYRDEKQENHSSLVSLGGEIQTKSRDLPPVKKLPEKEHGKICHLREDIAQIPTHAEAGEQEGRLQRKQKNAIGSRRHYCHECGKSFAQSSGLTKHRRIHTGEKPYECEDCGKTFIGSSALVIHQRVHTGEKPYECEECGKVFSHSSNLIKHQRTHTGEKPYECDDCGKTFSQSCSLLEHHKIHTGEKPYQCNMCGKAFRRNSHLLRHQRIHGDKNVQNPEHGESWESQGRTESQWENTEAPVSYKCNECERSFTRNRSLIEHQKIHTGEKPYQCDTCGKGFTRTSYLVQHQRSHVGKKTLSQ

>Q9HBU6

MLCGRPRSSSDNRNFLRERAGLSSAAVQTRIGNSAASRRSPAARPPVPAPPALPRGRPGTEGSTSLSAPAVLVVAVAVVVVVVSAVAWAMANYIHVPPGSPEVPKLNVTVQDQEEHRCREGALSLLQHLRPHWDPQEVTLQLFTDGITNKLIGCYVGNTMEDVVLVRIYGNKTELLVDRDEEVKSFRVLQAHGCAPQLYCTFNNGLCYEFIQGEALDPKHVCNPAIFRLIARQLAKIHAIHAHNGWIPKSNLWLKMGKYFSLIPTGFADEDINKRFLSDIPSSQILQEEMTWMKEILSNLGSPVVLCHNDLLCKNIIYNEKQGDVQFIDYEYSGYNYLAYDIGNHFNEFAGVSDVDYSLYPDRELQSQWLRAYLEAYKEFKGFGTEVTEKEVEILFIQVNQFALASHFFWGLWALIQAKYSTIEFDFLGYAIVRFNQYFKMKPEVTALKVPE

300 NP_892017.2 P07864

>NP_892017.2

MAAAVAVAAASRRQSCYLCDLPRMPWAMIWDFTEPVCRGCVNYEGADRVEFVIETARQLKRAHGCFPEGRSPPGAAASAAAKPPPLSAKDILLQQQQQLGHGGPEAAPRAPQALERYPLAAAAERPPRLGSDFGSSRPAASLAQPPTPQPPPVNGILVPNGFSKLEEPPELNRQSPNPRRGHAVPPTLVPLMNGSATPLPTALGLGGRAAASLAAVSGTAAASLGSAQPTDLGAHKRPASVSSSAAVEHEQREAAAKEKQPPPPAHRGPADSLSTAAGAAELSAEGAGKSRGSGEQDWVNRPKTVRDTLLALHQHGHSGPFESKFKKEPALTAGRLLGFEANGANGSKAVARTARKRKPSPEPEGEVGPPKINGEAQPWLSTSTEGLKIPMTPTSSFVSPPPPTASPHSNRTTPPEAAQNGQSPMAALILVADNAGGSHASKDANQVHSTTRRNSNSPPSPSSMNQRRLGPREVGGQGAGNTGGLEPVHPASLPDSSLATSAPLCCTLCHERLEDTHFVQCPSVPSHKFCFPCSRQSIKQQGASGEVYCPSGEKCPLVGSNVPWAFMQGEIATILAGDVKVKKERDS

>P07864

MSTVKEQLIEKLIEDDENSQCKITIVGTGAVGMACAISILLKDLADELALVDVALDKLKGEMMDLQHGSLFFSTSKITSGKDYSVSANSRIVIVTAGARQQEGETRLALVQRNVAIMKSIIPAIVHYSPDCKILVVSNPVDILTYIVWKISGLPVTRVIGSGCNLDSARFRYLIGEKLGVHPTSCHGWIIGEHGDSSVPLWSGVNVAGVALKTLDPKLGTDSDKEHWKNIHKQVIQSAYEIIKLKGYTSWAIGLSVMDLVGSILKNLRRVHPVSTMVKGLYGIKEELFLSIPCVLGRNGVSDVVKINLNSEEEALFKKSAETLWNIQKDLIF

301 NP_150093.1 O75879

>NP_150093.1

MADGDSGSERGGGGGPCGFQPASRGGGEQETQELASKRLDIQNKRFYLDVKQNAKGRFLKIAEVGAGGSKSRLTLSMAVAAEFRDSLGDFIEHYAQLGPSSPEQLAAGAEEGGGPRRALKSEFLVRENRKYYLDLKENQRGRFLRIRQTVNRGGGGFGAGPGPGGLQSGQTIALPAQGLIEFRDALAKLIDDYGGEDDELAGGPGGGAGGPGGGLYGELPEGTSITVDSKRFFFDVGCNKYGVFLRVSEVKPSYRNAITVPFKAWGKFGGAFCRYADEMKEIQERQRDKLYERRGGGSGGGEESEGEEVDED

>O75879

MAAPMLRWGCRGRRWAFARVDGGSCHRRGAPTGSTSNQIRGESSVAQQPLHTAQKTRKGEHKWAAVVGLEIHAQISSNSKLFSGSQVRFSAPPNSLVSFFDASLPGTLPVLNRRCVEAAVMTGLALNCHINKKSLFDRKHYFYADLPAGYQITQQRLPIAVNGSLIYGVCAGKKQSQVIPKTVRIKQIQLEQDSGKSLHDNLRSQTLIDLNRAGVGLLEVVLEPDMSCGEEAATAVRELQLILQALGTSQANMAEGQLRVDANISVHHPGEPLGVRTEVKNLNSIRFLAKAIDYEIQRQINELENGGEILNETRSFHHKLGCTMSMRDKEGKQDYRFMPEPNLPPLVLYDATSLPAGADPQQVINIDQIRETLPELPSVTREKLVQQYGMLLEHSFTLLNEVGLLEFFQNVIKETRAEPKKVTSWVLNTFLGYLKQQNLAVSESPVTPSALAELLDLLDSRTISSSAAKQVFEELWKREGKTPGQIVSEKQLELMQDQGALEQLCHSVMEAHPQVVMDVKNRNPRAINKLIGLVRKATQSRADPVMIKEILEKKLSL

302 Q9UIL8 NP_004171.2

>Q9UIL8

MAQASPPRPERVLGASSPEARPAQEALLLPTGVFQVAEKMEKRTCALCPKDVEYNVLYFAQSENIAAHENCLLYSSGLVECEDQDPLNPDRSFDVESVKKEIQRGRKLKCKFCHKRGATVGCDLKNCNKNYHFFCAKKDDAVPQSDGVRGIYKLLCQQHAQFPIIAQSAKFSGVKRKRGRKKPLSGNHVQPPETMKCNTFIRQVKEEHGRHTDATVKVPFLKKCKEAGLLNYLLEEILDKVHSIPEKLMDETTSESDYEEIGSALFDCRLFEDTFVNFQAAIEKKIHASQQRWQQLKEEIELLQDLKQTLCSFQENRDLMSSSTSISSLSY

>NP_004171.2

MDKNIGEQLNKAYEAFRQACMDRDSAVKELQQKTENYEQRIREQQEQLSLQQTIIDKLKSQLLLVNSTQDNNYGCVPLLEDSETRKNNLTLDQPQDKVISGIAREKLPKVRRQEVSSPRKETSARSLGSPLLHERGNIEKTFWDLKEEFHKICMLAKAQKDHLSKLNIPDTATETQCSVPIQCTDKTDKQEALFKPQAKDDINRGAPSITSVTPRGLCRDEEDTSFESLSKFNVKFPPMDNDSTFLHSTPERPGILSPATSEAVCQEKFNMEFRDNPGNFVKTEETLFEIQGIDPIASAIQNLKTTDKTKPSNLVNTCIRTTLDRAACLPPGDHNALYVNSFPLLDPSDAPFPSLDSPGKAIRGPQQPIWKPFPNQDSDSVVLSGTDSELHIPRVCEFCQAVFPPSITSRGDFLRHLNSHFNGET

303 NP_005515.1 NP_006101.1

>NP_005515.1

MPADIMEKNSSSPVAATPASVNTTPDKPKTASEHRKSSKPIMEKRRRARINESLSQLKTLILDALKKDSSRHSKLEKADILEMTVKHLRNLQRAQMTAALSTDPSVLGKYRAGFSECMNEVTRFLSTCEGVNTEVRTRLLGHLANCMTQINAMTYPGQPHPALQAPPPPPPGPGGPQHAPFAPPPPLVPIPGGAAPPPGGAPCKLGSQAGEAAKVFGGFQVVPAPDGQFAFLIPNGAFAHSGPVIPVYTSNSGTSVGPNAVSPSSGPSLTADSMWRPWRN

>NP_006101.1

MPKRKVTFQGVGDEEDEDEIIVPKKKLVDPVAGSGGPGSRFKGKHSLDSDEEEDDDDGGSSKYDILASEDVEGQEAATLPSEGGVRITPFNLQEEMEEGHFDADGNYFLNRDAQIRDSWLDNIDWVKIRERPPGQRQASDSEEEDSLGQTSMSAQALLEGLLELLLPRETVAGALRRLGARGGGKGRKGPGQPSSPQRLDRLSGLADQMVARGNLGVYQETRERLAMRLKGLGCQTLGPHNPTPPPSLDMFAEELAEEELETPTPTQRGEAESRGDGLVDVMWEYKWENTGDAELYGPFTSAQMQTWVSEGYFPDGVYCRKLDPPGGQFYNSKRIDFDLYT

304 NP_003097.1 NP_055300.1

>NP_003097.1

MYNMMETELKPPGPQQTSGGGGGNSTAAAAGGNQKNSPDRVKRPMNAFMVWSRGQRRKMAQENPKMHNSEISKRLGAEWKLLSETEKRPFIDEAKRLRALHMKEHPDYKYRPRRKTKTLMKKDKYTLPGGLLAPGGNSMASGVGVGAGLGAGVNQRMDSYAHMNGWSNGSYSMMQDQLGYPQHPGLNAHGAAQMQPMHRYDVSALQYNSMTSSQTYMNGSPTYSMSYSQQGTPGMALGSMGSVVKSEASSSPPVVTSSSHSRAPCQAGDLRDMISMYLPGAEVPEPAAPSRLHMSQHYQSGPVPGTAINGTLPLSHM

>NP_055300.1

MPNYKLTYFNMRGRAEIIRYIFAYLDIQYEDHRIEQADWPEIKSTLPFGKIPILEVDGLTLHQSLAIARYLTKNTDLAGNTEMEQCHVDAIVDTLDDFMSCFPWAEKKQDVKEQMFNELLTYNAPHLMQDLDTYLGGREWLIGNSVTWADFYWEICSTTLLVFKPDLLDNHPRLVTLRKKVQAIPAVANWIKRRPQTKL

305 NP_036558.3 O75880

>NP_036558.3

MFLYNLTLQRATGISFAIHGNFSGTKQQEIVVSRGKILELLRPDPNTGKVHTLLTVEVFGVIRSLMAFRLTGGTKDYIVVGSDSGRIVILEYQPSKNMFEKIHQETFGKSGCRRIVPGQFLAVDPKGRAVMISAIEKQKLVYILNRDAAARLTISSPLEAHKANTLVYHVVGVDVGFENPMFACLEMDYEEADNDPTGEAAANTQQTLTFYELDLGLNHVVRKYSEPLEEHGNFLITVPGGSDGPSGVLICSENYITYKNFGDQPDIRCPIPRRRNDLDDPERGMIFVCSATHKTKSMFFFLAQTEQGDIFKITLETDEDMVTEIRLKYFDTVPVAAAMCVLKTGFLFVASEFGNHYLYQIAHLGDDDEEPEFSSAMPLEEGDTFFFQPRPLKNLVLVDELDSLSPILFCQIADLANEDTPQLYVACGRGPRSSLRVLRHGLEVSEMAVSELPGNPNAVWTVRRHIEDEFDAYIIVSFVNATLVLSIGETVEEVTDSGFLGTTPTLSCSLLGDDALVQVYPDGIRHIRADKRVNEWKTPGKKTIVKCAVNQRQVVIALTGGELVYFEMDPSGQLNEYTERKEMSADVVCMSLANVPPGEQRSRFLAVGLVDNTVRIISLDPSDCLQPLSMQALPAQPESLCIVEMGGTEKQDELGERGSIGFLYLNIGLQNGVLLRTVLDPVTGDLSDTRTRYLGSRPVKLFRVRMQGQEAVLAMSSRSWLSYSYQSRFHLTPLSYETLEFASGFASEQCPEGIVAISTNTLRILALEKLGAVFNQVAFPLQYTPRKFVIHPESNNLIIIETDHNAYTEATKAQRKQQMAEEMVEAAGEDERELAAEMAAAFLNENLPESIFGAPKAGNGQWASVIRVMNPIQGNTLDLVQLEQNEAAFSVAVCRFSNTGEDWYVLVGVAKDLILNPRSVAGGFVYTYKLVNNGEKLEFLHKTPVEEVPAAIAPFQGRVLIGVGKLLRVYDLGKKKLLRKCENKHIANYISGIQTIGHRVIVSDVQESFIWVRYKRNENQLIIFADDTYPRWVTTASLLDYDTVAGADKFGNICVVRLPPNTNDEVDEDPTGNKALWDRGLLNGASQKAEVIMNYHVGETVLSLQKTTLIPGGSESLVYTTLSGGIGILVPFTSHEDHDFFQHVEMHLRSEHPPLCGRDHLSFRSYYFPVKNVIDGDLCEQFNSMEPNKQKNVSEELDRTPPEVSKKLEDIRTRYAF

>O75880

MAMLVLVPGRVMRPLGGQLWRFLPRGLEFWGPAEGTARVLLRQFCARQAEAWRASGRPGYCLGTRPLSTARPPPPWSQKGPGDSTRPSKPGPVSWKSLAITFAIGGALLAGMKHVKKEKAEKLEKERQRHIGKPLLGGPFSLTTHTGERKTDKDYLGQWLLIYFGFTHCPDVCPEELEKMIQVVDEIDSITTLPDLTPLFISIDPERDTKEAIANYVKEFSPKLVGLTGTREEVDQVARAYRVYYSPGPKDEDEDYIVDHTIIMYLIGPDGEFLDYFGQNKRKGEIAASIATHMRPYRKKS

306 NP_003855.1 NP_057564.3

>NP_003855.1

MNGFTPDEMSRGGDAAAAVAAVVAAAAAAASAGNGTGAGTGAEVPGAGAVSAAGPPGAAGPGPGQLCCLREDGERCGRAAGNASFSKRIQKSISQKKVKIELDKSARHLYICDYHKNLIQSVRNRRKRKGSDDDGGDSPVQDIDTPEVDLYQLQVNTLRRYKRHFKLPTRPGLNKAQLVEIVGCHFRSIPVNEKDTLTYFIYSVKNDKNKSDLKVDSGVH

>NP_057564.3

MKDPSRSSTSPSIINEDVIINGHSHEDDNPFAEYMWMENEEEFNRQIEEELWEEEFIERCFQEMLEEEEEHEWFIPARDLPQTMDQIQDQFNDLVISDGSSLEDLVVKSNLNPNAKEFVPGVKYGNI

307 NP_004647.1 P22061

>NP_004647.1

MNKGWLELESDPGLFTLLVEDFGVKGVQVEEIYDLQSKCQGPVYGFIFLFKWIEERRSRRKVSTLVDDTSVIDDDIVNNMFFAHQLIPNSCATHALLSVLLNCSSVDLGPTLSRMKDFTKGFSPESKGYAIGNAPELAKAHNSHARPEPRHLPEKQNGLSAVRTMEAFHFVSYVPITGRLFELDGLKVYPIDHGPWGEDEEWTDKARRVIMERIGLATAGEPYHDIRFNLMAVVPDRRIKYEARLHVLKVNRQTVLEALQQLIRVTQPELIQTHKSQESQLPEESKSASNKSPLVLEANRAPAASEGNHTDGAEEAAGSCAQAPSHSPPNKPKLVVKPPGSSLNGVHPNPTPIVQRLPAFLDNHNYAKSPMQEEEDLAAGVGRSRVPVRPPQQYSDDEDDYEDDEEDDVQNTNSALRYKGKGTGKPGALSGSADGQLSVLQPNTINVLAEKLKESQKDLSIPLSIKTSSGAGSPAVAVPTHSQPSPTPSNESTDTASEIGSAFNSPLRSPIRSANPTRPSSPVTSHISKVLFGEDDSLLRVDCIRYNRAVRDLGPVISTGLLHLAEDGVLSPLALTEGGKGSSPSIRPIQGSQGSSSPVEKEVVEATDSREKTGMVRPGEPLSGEKYSPKELLALLKCVEAEIANYEACLKEEVEKRKKFKIDDQRRTHNYDEFICTFISMLAQEGMLANLVEQNISVRRRQGVSIGRLHKQRKPDRRKRSRPYKAKRQ

>P22061

MAWKSGGASHSELIHNLRKNGIIKTDKVFEVMLATDRSHYAKCNPYMDSPQSIGFQATISAPHMHAYALELLFDQLHEGAKALDVGSGSGILTACFARMVGCTGKVIGIDHIKELVDDSINNVRKDDPTLLSSGRVQLVVGDGRMGYAEEAPYDAIHVGAAAPVVPQALIDQLKPGGRLILPVGPAGGNQMLEQYDKLQDGSIKMKPLMGVIYVPLTDKEKQWSRWK

308 NP_002384.2 NP_005557.1

>NP_002384.2

MTSFSTSAQCSTSDSACRISPGQINQVRPKLPLLKILHAAGAQGEMFTVKEVMHYLGQYIMVKQLYDQQEQHMVYCGGDLLGELLGRQSFSVKDPSPLYDMLRKNLVTLATATTDAAQTLALAQDHSMDIPSQDQLKQSAEESSTSRKRTTEDDIPTLPTSEHKCIHSREDEDLIENLAQDETSRLDLGFEEWDVAGLPWWFLGNLRSNYTPRSNGSTDLQTNQDVGTAIVSDTTDDLWFLNESVSEQLGVGIKVEAADTEQTSEEVGKVSDKKVIEVGKNDDLEDSKSLSDDTDVEVTSEDEWQCTECKKFNSPSKRYCFRCWALRKDWYSDCSKLTHSLSTSDITAIPEKENEGNDVPDCRRTISAPVVRPKDAYIKKENSKLFDPCNSVEFLDLAHSSESQETISSMGEQLDNLSEQRTDTENMEDCQNLLKPCSLCEKRPRDGNIIHGRTGHLVTCFHCARRLKKAGASCPICKKEIQLVIKVFIA

>NP_005557.1

MATLKDQLIYNLLKEEQTPQNKITVVGVGAVGMACAISILMKDLADELALVDVIEDKLKGEMMDLQHGSLFLRTPKIVSGKDYNVTANSKLVIITAGARQQEGESRLNLVQRNVNIFKFIIPNVVKYSPNCKLLIVSNPVDILTYVAWKISGFPKNRVIGSGCNLDSARFRYLMGERLGVHPLSCHGWVLGEHGDSSVPVWSGMNVAGVSLKTLHPDLGTDKDKEQWKEVHKQVVESAYEVIKLKGYTSWAIGLSVADLAESIMKNLRRVHPVSTMIKGLYGIKDDVFLSVPCILGQNGISDLVKVTLTSEEEARLKKSADTLWGIQKELQF

309 Q5QNW6 Q9UGK3

>Q5QNW6

MPDPAKSAPAPKKGSKKAVTKVQKKDGKKRKRSRKESYSVYVYKVLKQVHPDTGISSKAMGIMNSFVNDIFERIAGEASRLAHYNKRSTITSREIQTAVRLLLPGELAKHAVSEGTKAVTKYTSSK

>Q9UGK3

MASALRPPRVPKPKGVLPSHYYESFLEKKGPCDRDYKKFWAGLQGLTIYFYNSNRDFQHVEKLNLGAFEKLTDEIPWGSSRDPGTHFSLILRDQEIKFKVETLECREMWKGFILTVVELRVPTDLTLLPGHLYMMSEVLAKEEARRALETPSCFLKVSRLEAQLLLERYPECGNLLLRPSGDGADGVSVTTRQMHNGTHVVRHYKVKREGPKYVIDVEQPFSCTSLDAVVNYFVSHTKKALVPFLLDEDYEKVLGYVEADKENGENVWVAPSAPGPGPAPCTGGPKPLSPASSQDKLPPLPPLPNQEENYVTPIGDGPAVDYENQDVASSSWPVILKPKKLPKPPAKLPKPPVGPKPEPKVFNGGLGRKLPVSSAQPLFPTAGLADMTAELQKKLEKRRALEH

310 NP_003210.1 NP_004039.1

>NP_003210.1

MPRAPRCRAVRSLLRSHYREVLPLATFVRRLGPQGWRLVQRGDPAAFRALVAQCLVCVPWDARPPPAAPSFRQVSCLKELVARVLQRLCERGAKNVLAFGFALLDGARGGPPEAFTTSVRSYLPNTVTDALRGSGAWGLLLRRVGDDVLVHLLARCALFVLVAPSCAYQVCGPPLYQLGAATQARPPPHASGPRRRLGCERAWNHSVREAGVPLGLPAPGARRRGGSASRSLPLPKRPRRGAAPEPERTPVGQGSWAHPGRTRGPSDRGFCVVSPARPAEEATSLEGALSGTRHSHPSVGRQHHAGPPSTSRPPRPWDTPCPPVYAETKHFLYSSGDKEQLRPSFLLSSLRPSLTGARRLVETIFLGSRPWMPGTPRRLPRLPQRYWQMRPLFLELLGNHAQCPYGVLLKTHCPLRAAVTPAAGVCAREKPQGSVAAPEEEDTDPRRLVQLLRQHSSPWQVYGFVRACLRRLVPPGLWGSRHNERRFLRNTKKFISLGKHAKLSLQELTWKMSVRDCAWLRRSPGVGCVPAAEHRLREEILAKFLHWLMSVYVVELLRSFFYVTETTFQKNRLFFYRKSVWSKLQSIGIRQHLKRVQLRELSEAEVRQHREARPALLTSRLRFIPKPDGLRPIVNMDYVVGARTFRREKRAERLTSRVKALFSVLNYERARRPGLLGASVLGLDDIHRAWRTFVLRVRAQDPPPELYFVKVDVTGAYDTIPQDRLTEVIASIIKPQNTYCVRRYAVVQKAAHGHVRKAFKSHVSTLTDLQPYMRQFVAHLQETSPLRDAVVIEQSSSLNEASSGLFDVFLRFMCHHAVRIRGKSYVQCQGIPQGSILSTLLCSLCYGDMENKLFAGIRRDGLLLRLVDDFLLVTPHLTHAKTFLRTLVRGVPEYGCVVNLRKTVVNFPVEDEALGGTAFVQMPAHGLFPWCGLLLDTRTLEVQSDYSSYARTSIRASLTFNRGFKAGRNMRRKLFGVLRLKCHSLFLDLQVNSLQTVCTNIYKILLLQAYRFHACVLQLPFHQQVWKNPTFFLRVISDTASLCYSILKAKNAGMSLGAKGAAGPLPSEAVQWLCHQAFLLKLTRHRVTYVPLLGSLRTAQTQLSRKLPGTTLTALEAAANPALPSDFKTILD

>NP_004039.1

MSRSVALAVLALLSLSGLEAIQRTPKIQVYSRHPAENGKSNFLNCYVSGFHPSDIEVDLLKNGERIEKVEHSDLSFSKDWSFYLLYYTEFTPTEKDEYACRVNHVTLSQPKIVKWDRDM

311 NP_002348.1 NP_004865.1

>NP_002348.1

MAAAVRMNIQMLLEAADYLERREREAEHGYASMLPYNNKDRDALKRRNKSKKNNSSSRSTHNEMEKNRRAHLRLCLEKLKGLVPLGPESSRHTTLSLLTKAKLHIKKLEDCDRKAVHQIDQLQREQRHLKRQLEKLGIERIRMDSIGSTVSSERSDSDREEIDVDVESTDYLTGDLDWSSSSVSDSDERGSMQSLGSDEGYSSTSIKRIKLQDSHKACLGL

>NP_004865.1

MSALRRSGYGPSDGPSYGRYYGPGGGDVPVHPPPPLYPLRPEPPQPPISWRVRGGGPAETTWLGEGGGGDGYYPSGGAWPEPGRAGGSHQEQPPYPSYNSNYWNSTARSRAPYPSTYPVRPELQGQSLNSYTNGAYGPTYPPGPGANTASYSGAYYAPGYTQTSYSTEVPSTYRSSGNSPTPVSRWIYPQQDCQTEAPPLRGQVPGYPPSQNPGMTLPHYPYGDGNRSVPQSGPTVRPQEDAWASPGAYGMGGRYPWPSSAPSAPPGNLYMTESTSPWPSSGSPQSPPSPPVQQPKDSSYPYSQSDQSMNRHNFPCSVHQYESSGTVNNDDSDLLDSQVQYSAEPQLYGNATSDHPNNQDQSSSLPEECVPSDESTPPSIKKIIHVLEKVQYLEQEVEEFVGKKTDKAYWLLEEMLTKELLELDSVETGGQDSVRQARKEAVCKIQAILEKLEKKGL

312 NP_004836.2 Q58A44

>NP_004836.2

MPVVTTDAESETGIPKSLSNEPPSETMEEIEHTCPQPRLTLTAPAPFADETNCQCQAPHEKLTIAQARLGTPADRPVRVYADGIFDLFHSGHARALMQAKTLFPNSYLLVGVCSDDLTHKFKGFTVMNEAERYEALRHCRYVDEVIRDAPWTLTPEFLEKHKIDFVAHDDIPYSSAGSDDVYKHIKEAGMFVPTQRTEGISTSDIITRIVRDYDVYARRNLQRGYTAKELNVSFINEKRYRFQNQVDKMKEKVKNVEERSKEFVNRVEEKSHDLIQKWEEKSREFIGNFLELFGPDGAWKQMFQERSSRMLQALSPKQSPVSSPTRSRSPSRSPSPTFSWLPLKTSPPSSPKAASASISSMSEGDEDEK

>Q58A44

MWILSNLMGTSEEGNLLSTVSPTVKALFGKTRVSPIFPFSPRSPFQPLIPRTPGSPWGPVGPASPLGPGFPIGPMGPGKPVGPKGPMLPLGPSGPVGPTSPLFPFCP

313 NP_002469.2 P57721

>NP_002469.2

MELLSPPLRDVDLTAPDGSLCSFATTDDFYDDPCFDSPDLRFFEDLDPRLMHVGALLKPEEHSHFPAAVHPAPGAREDEHVRAPSGHHQAGRCLLWACKACKRKTTNADRRKAATMRERRRLSKVNEAFETLKRCTSSNPNQRLPKVEILRNAIRYIEGLQALLRDQDAAPPGAAAAFYAPGPLPPGRGGEHYSGDSDASSPRSNCSDGMMDYSGPPSGARRRNCYEGAYYNEAPSEPRPGKSAAVSSLDCLSSIVERISTESPAAPALLLADVPSESPPRRQEAAAPSEGESSGDPTQSPDAAPQCPAGANPNPIYQVL

>P57721

MESKVSEGGLNVTLTIRLLMHGKEVGSIIGKKGETVKKMREESGARINISEGNCPERIVTITGPTDAIFKAFAMIAYKFEEDIINSMSNSPATSKPPVTLRLVVPASQCGSLIGKGGSKIKEIRESTGAQVQVAGDMLPNSTERAVTISGTPDAIIQCVKQICVVMLESPPKGATIPYRPKPASTPVIFAGGQAYTIQGQYAIPHPDQLTKLHQLAMQQTPFPPLGQTNPAFPGEKLPLHSSEEAQNLMGQSSGLDASPPASTHELTIPNDLIGCIIGRQGTKINEIRQMSGAQIKIANATEGSSERQITITGTPANISLAQYLINARLTSEVTGMGTL

314 Q92949 A2A3N6

>Q92949

MAESWLRLSGAGPAEEAGPEGGLEEPDALDDSLTSLQWLQEFSILNAKAPALPPGGTDPHGYHQVPGSAAPGSPLAADPACLGQPHTPGKPTSSCTSRSAPPGLQAPPPDDVDYATNPHVKPPYSYATLICMAMQASKATKITLSAIYKWITDNFCYFRHADPTWQNSIRHNLSLNKCFIKVPREKDEPGKGGFWRIDPQYAERLLSGAFKKRRLPPVHIHPAFARQAAQEPSAVPRAGPLTVNTEAQQLLREFEEATGEAGWGAGEGRLGHKRKQPLPKRVAKVPRPPSTLLPTPEEQGELEPLKGNFDWEAIFDAGTLGGELGALEALELSPPLSPASHVDVDLTIHGRHIDCPATWGPSVEQAADSLDFDETFLATSFLQHPWDESGSGCLPPEPLFEAGDATLASDLQDWASVGAFL

>A2A3N6

MASEVPYASGMPIKKIGHRSVDSSGGTTSSALKGAIQLGITHTVGSLSTKPESDVLMQDFHMVESIFFPSEGSNLTPAHHYNAFRFKTYAPVAFRYFWELFGIRPDDYLYSLCSEPLIELCSSGASGSLFYVSSDDEFIVKTVRHKEAEFLQKLLPGYYINLNQNPRTLLPKFYGLYCVQTGGKNIRIVVMNNLLPRSVKMHIKYDLKGSTYRRRASQKEREKPLPTFKDLDFLQDIPDGLFLDADVHNALCKTLQRDCLVLQSFKIMDYSLLMSIHNIDHAQREPLSSETQYSVDTRRPAPQKALYSTAMESIQGEARRGGTMETDDHMGGIPARNSKGERLLLYIGIIDILQSYRFVKKLEHSWKALIHDGDTVSVHRPGFYAEWFQRFMCNTVFKKIPLKPSPSKKLRSGSSFSQRAGSSGNSCITYQPLVSGEHKAQVTTKAEVEPGVHLGCPDVLPQTPPLEEISEGSPTPDPSFSPLVEETLQMLTTSVDNSEYMGNGDFLPTRLQAQQDAVNTVCHSKTRSNPENNVGLITLDNDCEVLTTLTPDTGRILSKLHTVQPKGKITFCMGIHVAHLALKHRQGNNHKIRIIAFVGNPVEDNEKNLVKLAKCLKKEKVNVDIINFGEEEVNTEKLTAFVNTLNGKDGTGSHLVTVPPGPSLADALISFPILAGEGGAMMGLGASDFEFGVDPSADPELALVLRVFMEEQRQRQEEEARQAAAASAAEAGIATTGTEDSDDALLKMTISQQEFGHTGLPDLSSMTEEEKIVCAMQMSLQGAEFGLAESADIDASSAMDTSEPAKEEDDYDVMQDPEFLQSVLENLPGVDPNNEAIRNAVGSLASQATKDSKKDKKEEDKK

315 NP_109377.1 NP_006635.2

>NP_109377.1

MQKATYYDSSAIYGGYPYQAANGFAYNANQQPYPASAALGADGEYHRPACSLQSPSSAGGHPKAHELSEACLRTLSAPPSQPPSLGEPPLHPPPPQAAPPAPQPPQPAPQPPAPTPAAPPPPSSASPPQNASNNPTPANAAKSPLLNSPTVAKQIFPWMKESRQNTKQKTSSSSSGESCAGDKSPPGQASSKRARTAYTSAQLVELEKEFHFNRYLCRPRRVEMANLLNLTERQIKIWFQNRRMKYKKDQKGKGMLTSSGGQSPSRSPVPPGAGGYLNSMHSLVNSVPYEPQSPPPFSKPPQGTYGLPPASYPASLPSCAPPPPPQKRYTAAGAGAGGTPDYDPHAHGLQGNGSYGTPHIQGSPVFVGGSYVEPMSNSGPALFGLTHLPHAASGAMDYGGAGPLGSGHHHGPGPGEPHPTYTDLTGHHPSQGRIQEAPKLTHL

>NP_006635.2

MSVVGLDVGSQSCYIAVARAGGIETIANEFSDRCTPSVISFGSKNRTIGVAAKNQQITHANNTVSNFKRFHGRAFNDPFIQKEKENLSYDLVPLKNGGVGIKVMYMGEEHLFSVEQITAMLLTKLKETAENSLKKPVTDCVISVPSFFTDAERRSVLDAAQIVGLNCLRLMNDMTAVALNYGIYKQDLPSLDEKPRIVVFVDMGHSAFQVSACAFNKGKLKVLGTAFDPFLGGKNFDEKLVEHFCAEFKTKYKLDAKSKIRALLRLYQECEKLKKLMSSNSTDLPLNIECFMNDKDVSGKMNRSQFEELCAELLQKIEVPLYSLLEQTHLKVEDVSAVEIVGGATRIPAVKERIAKFFGKDISTTLNADEAVARGCALQCAILSPAFKVREFSVTDAVPFPISLIWNHDSEDTEGVHEVFSRNHAAPFSKVLTFLRRGPFELEAFYSDPQGVPYPEAKIGRFVVQNVSAQKDGEKSRVKVKVRVNTHGIFTISTASMVEKVPTEENEMSSEADMECLNQRPPENPDTDKNVQQDNSEAGTQPQVQTDAQQTSQSPPSPELTSEENKIPDADKANEKKVDQPPEAKKPKIKVVNVELPIEANLVWQLGKDLLNMYIETEGKMIMQDKLEKERNDAKNAVEEYVYEFRDKLCGPYEKFICEQDHQNFLRLLTETEDWLYEEGEDQAKQAYVDKLEELMKIGTPVKVRFQEAEERPKMFEELGQRLQHYAKIAADFRNKDEKYNHIDESEMKKVEKSVNEVMEWMNNVMNAQAKKSLDQDPVVRAQEIKTKIKELNNTCEPVVTQPKPKIESPKLERTPNGPNIDKKEEDLEDKNNFGAEPPHQNGECYPNEKNSVNMDLD

316 NP_009108.1 Q9UKF7

>NP_009108.1

MYSEIQRERADIGGLMARPEYREWNPELIKPKKLLNPVKASRSHQELHRELLMNHRRGLGVDSKPELQRVLEHRRRNQLIKKKKEELEAKRLQCPFEQELLRRQQRLNQLEKPPEKEEDHAPEFIKVRENLRRIATLTSEEREL

>Q9UKF7

MLLKEYRICMPLTVDEYKIGQLYMISKHSHEQSDRGEGVEVVQNEPFEDPHHGNGQFTEKRVYLNSKLPSWARAVVPKIFYVTEKAWNYYPYTITEYTCSFLPKFSIHIETKYEDNKGSNDTIFDNEAKDVEREVCFIDIACDEIPERYYKESEDPKHFKSEKTGRGQLREGWRDSHQPIMCSYKLVTVKFEVWGLQTRVEQFVHKVVRDILLIGHRQAFAWVDEWYDMTMDDVREFERATQEATNKKIGIFPPAISISSIPLLPSSVRSAPSSAPSTPLSTDAPEFLSVPKDRPRKKSAPETLTLPDPEKKATLNLPGMHSSDKPCRPKSE

317 NP_000240.1 Q12815

>NP_000240.1

MSFVAGVIRRLDETVVNRIAAGEVIQRPANAIKEMIENCLDAKSTSIQVIVKEGGLKLIQIQDNGTGIRKEDLDIVCERFTTSKLQSFEDLASISTYGFRGEALASISHVAHVTITTKTADGKCAYRASYSDGKLKAPPKPCAGNQGTQITVEDLFYNIATRRKALKNPSEEYGKILEVVGRYSVHNAGISFSVKKQGETVADVRTLPNASTVDNIRSIFGNAVSRELIEIGCEDKTLAFKMNGYISNANYSVKKCIFLLFINHRLVESTSLRKAIETVYAAYLPKNTHPFLYLSLEISPQNVDVNVHPTKHEVHFLHEESILERVQQHIESKLLGSNSSRMYFTQTLLPGLAGPSGEMVKSTTSLTSSSTSGSSDKVYAHQMVRTDSREQKLDAFLQPLSKPLSSQPQAIVTEDKTDISSGRARQQDEEMLELPAPAEVAAKNQSLEGDTTKGTSEMSEKRGPTSSNPRKRHREDSDVEMVEDDSRKEMTAACTPRRRIINLTSVLSLQEEINEQGHEVLREMLHNHSFVGCVNPQWALAQHQTKLYLLNTTKLSEELFYQILIYDFANFGVLRLSEPAPLFDLAMLALDSPESGWTEEDGPKEGLAEYIVEFLKKKAEMLADYFSLEIDEEGNLIGLPLLIDNYVPPLEGLPIFILRLATEVNWDEEKECFESLSKECAMFYSIRKQYISEESTLSGQQSEVPGSIPNSWKWTVEHIVYKALRSHILPPKHFTEDGNILQLANLPDLYKVFERC

>Q12815

MTTRQATKDPLLRGVSPTPSKIPVRSQKRTPFPTVTSCAVDQENQDPRRWVQKPPLNIQRPLVDSAGPRPKARHQAETSQRLVGISQPRNPLEELRPSPRGQNVGPGPPAQTEAPGTIEFVADPAALATILSGEGVKSCHLGRQPSLAKRVLVRGSQGGTTQRVQGVRASAYLAPRTPTHRLDPARASCFSRLEGPGPRGRTLCPQRLQALISPSGPSFHPSTRPSFQELRRETAGSSRTSVSQASGLLLETPVQPAFSLPKGEREVVTHSDEGGVASLGLAQRVPLRENREMSHTRDSHDSHLMPSPAPVAQPLPGHVVPCPSPFGRAQRVPSPGPPTLTSYSVLRRLTVQPKTRFTPMPSTPRVQQAQWLRGVSPQSCSEDPALPWEQVAVRLFDQESCIRSLEGSGKPPVATPSGPHSNRTPSLQEVKIQRIGILQQLLRQEVEGLVGGQCVPLNGGSSLDMVELQPLLTEISRTLNATEHNSGTSHLPGLLKHSGLPKPCLPEECGEPQPCPPAEPGPPEAFCRSEPEIPEPSLQEQLEVPEPYPPAEPRPLESCCRSEPEIPESSRQEQLEVPEPCPPAEPRPLESYCRIEPEIPESSRQEQLEVPEPCPPAEPGPLQPSTQGQSGPPGPCPRVELGASEPCTLEHRSLESSLPPCCSQWAPATTSLIFSSQHPLCASPPICSLQSLRPPAGQAGLSNLAPRTLALRERLKSCLTAIHCFHEARLDDECAFYTSRAPPSGPTRVCTNPVATLLEWQDALCFIPVGSAAPQGSP

318 NP_000160.1 NP_006635.2

>NP_000160.1

MQLRNPELHLGCALALRFLALVSWDIPGARALDNGLARTPTMGWLHWERFMCNLDCQEEPDSCISEKLFMEMAELMVSEGWKDAGYEYLCIDDCWMAPQRDSEGRLQADPQRFPHGIRQLANYVHSKGLKLGIYADVGNKTCAGFPGSFGYYDIDAQTFADWGVDLLKFDGCYCDSLENLADGYKHMSLALNRTGRSIVYSCEWPLYMWPFQKPNYTEIRQYCNHWRNFADIDDSWKSIKSILDWTSFNQERIVDVAGPGGWNDPDMLVIGNFGLSWNQQVTQMALWAIMAAPLFMSNDLRHISPQAKALLQDKDVIAINQDPLGKQGYQLRQGDNFEVWERPLSGLAWAVAMINRQEIGGPRSYTIAVASLGKGVACNPACFITQLLPVKRKLGFYEWTSRLRSHINPTGTVLLQLENTMQMSLKDLL

>NP_006635.2

MSVVGLDVGSQSCYIAVARAGGIETIANEFSDRCTPSVISFGSKNRTIGVAAKNQQITHANNTVSNFKRFHGRAFNDPFIQKEKENLSYDLVPLKNGGVGIKVMYMGEEHLFSVEQITAMLLTKLKETAENSLKKPVTDCVISVPSFFTDAERRSVLDAAQIVGLNCLRLMNDMTAVALNYGIYKQDLPSLDEKPRIVVFVDMGHSAFQVSACAFNKGKLKVLGTAFDPFLGGKNFDEKLVEHFCAEFKTKYKLDAKSKIRALLRLYQECEKLKKLMSSNSTDLPLNIECFMNDKDVSGKMNRSQFEELCAELLQKIEVPLYSLLEQTHLKVEDVSAVEIVGGATRIPAVKERIAKFFGKDISTTLNADEAVARGCALQCAILSPAFKVREFSVTDAVPFPISLIWNHDSEDTEGVHEVFSRNHAAPFSKVLTFLRRGPFELEAFYSDPQGVPYPEAKIGRFVVQNVSAQKDGEKSRVKVKVRVNTHGIFTISTASMVEKVPTEENEMSSEADMECLNQRPPENPDTDKNVQQDNSEAGTQPQVQTDAQQTSQSPPSPELTSEENKIPDADKANEKKVDQPPEAKKPKIKVVNVELPIEANLVWQLGKDLLNMYIETEGKMIMQDKLEKERNDAKNAVEEYVYEFRDKLCGPYEKFICEQDHQNFLRLLTETEDWLYEEGEDQAKQAYVDKLEELMKIGTPVKVRFQEAEERPKMFEELGQRLQHYAKIAADFRNKDEKYNHIDESEMKKVEKSVNEVMEWMNNVMNAQAKKSLDQDPVVRAQEIKTKIKELNNTCEPVVTQPKPKIESPKLERTPNGPNIDKKEEDLEDKNNFGAEPPHQNGECYPNEKNSVNMDLD

319 Q03923 NP_002100.2

>Q03923

MGPLTFRDVAIEFSLKEWQCLDTAQRNLYRNVMLENYRNLVFLGITVSKPDLITCLEQGKEAWSMKRHEIMVAKPTVMCSHFAQDLWPEQNIKDSFQKVTLKRYGKCRHENLPLRKGCESMDECKMHKGGCNGLNQCLTATQSKIFQCDKYVKVAHKFSNSNRHEIRHTKKKPFKCTKCGKSFGMISCLTEHSRIHTRVNFYKCEECGKAFNWSSTLTKHKRIHTGEKPYKCEECGKAFNQSSNLIKHKKIHTGEKPYKCEECGKTFNRFSTLTTHKIIHTGEKPYKCKECGKAFNRSSTLTTHRKIHTGEKPYKCEECGKAFKQSSNLTTHKIIHTGEKPYKCKKCGKAFNQSAHLTTHEVIHTGEKPYKCEKCGKAFNHFSHLTTHKIIHTGEKPYKCKECGKAFKHSSTLTKHKIIHTGEKPYKCKECEKAFNQSSKLTEHKKIHTGEKPYECEKCGKAFNQSSNLTRHKKSHTEEKPYKCEECGKGFKWPSTLTIHKIIHTGEKPYKCEECGKAFNQSSKLTKHKKIHTGEKPYTCEECGKAFNQSSNLTKHKRIHTGEKPYKCEECDKAFKWSSVLTKHKIIHTGEKLQI

>NP_002100.2

MAERAALEELVKLQGERVRGLKQQKASAELIEEEVAKLLKLKAQLGPDESKQKFVLKTPKGTRDYSPRQMAVREKVFDVIIRCFKRHGAEVIDTPVFELKETLMGKYGEDSKLIYDLKDQGGELLSLRYDLTVPFARYLAMNKLTNIKRYHIAKVYRRDNPAMTRGRYREFYQCDFDIAGNFDPMIPDAECLKIMCEILSSLQIGDFLVKVNDRRILDGMFAICGVSDSKFRTICSSVDKLDKVSWEEVKNEMVGEKGLAPEVADRIGDYVQQHGGVSLVEQLLQDPKLSQNKQALEGLGDLKLLFEYLTLFGIDDKISFDLSLARGLDYYTGVIYEAVLLQTPAQAGEEPLGVGSVAAGGRYDGLVGMFDPKGRKVPCVGLSIGVERIFSIVEQRLEALEEKIRTTETQVLVASAQKKLLEERLKLVSELWDAGIKAELLYKKNPKLLNQLQYCEEAGIPLVAIIGEQELKDGVIKLRSVTSREEVDVRREDLVEEIKRRTGQPLCIC

320 NP_001420.2 NP_005852.2

>NP_001420.2

MAENVVEPGPPSAKRPKLSSPALSASASDGTDFGSLFDLEHDLPDELINSTELGLTNGGDINQLQTSLGMVQDAASKHKQLSELLRSGSSPNLNMGVGGPGQVMASQAQQSSPGLGLINSMVKSPMTQAGLTSPNMGMGTSGPNQGPTQSTGMMNSPVNQPAMGMNTGMNAGMNPGMLAAGNGQGIMPNQVMNGSIGAGRGRQNMQYPNPGMGSAGNLLTEPLQQGSPQMGGQTGLRGPQPLKMGMMNNPNPYGSPYTQNPGQQIGASGLGLQIQTKTVLSNNLSPFAMDKKAVPGGGMPNMGQQPAPQVQQPGLVTPVAQGMGSGAHTADPEKRKLIQQQLVLLLHAHKCQRREQANGEVRQCNLPHCRTMKNVLNHMTHCQSGKSCQVAHCASSRQIISHWKNCTRHDCPVCLPLKNAGDKRNQQPILTGAPVGLGNPSSLGVGQQSAPNLSTVSQIDPSSIERAYAALGLPYQVNQMPTQPQVQAKNQQNQQPGQSPQGMRPMSNMSASPMGVNGGVGVQTPSLLSDSMLHSAINSQNPMMSENASVPSLGPMPTAAQPSTTGIRKQWHEDITQDLRNHLVHKLVQAIFPTPDPAALKDRRMENLVAYARKVEGDMYESANNRAEYYHLLAEKIYKIQKELEEKRRTRLQKQNMLPNAAGMVPVSMNPGPNMGQPQPGMTSNGPLPDPSMIRGSVPNQMMPRITPQSGLNQFGQMSMAQPPIVPRQTPPLQHHGQLAQPGALNPPMGYGPRMQQPSNQGQFLPQTQFPSQGMNVTNIPLAPSSGQAPVSQAQMSSSSCPVNSPIMPPGSQGSHIHCPQLPQPALHQNSPSPVPSRTPTPHHTPPSIGAQQPPATTIPAPVPTPPAMPPGPQSQALHPPPRQTPTPPTTQLPQQVQPSLPAAPSADQPQQQPRSQQSTAASVPTPTAPLLPPQPATPLSQPAVSIEGQVSNPPSTSSTEVNSQAIAEKQPSQEVKMEAKMEVDQPEPADTQPEDISESKVEDCKMESTETEERSTELKTEIKEEEDQPSTSATQSSPAPGQSKKKIFKPEELRQALMPTLEALYRQDPESLPFRQPVDPQLLGIPDYFDIVKSPMDLSTIKRKLDTGQYQEPWQYVDDIWLMFNNAWLYNRKTSRVYKYCSKLSEVFEQEIDPVMQSLGYCCGRKLEFSPQTLCCYGKQLCTIPRDATYYSYQNRYHFCEKCFNEIQGESVSLGDDPSQPQTTINKEQFSKRKNDTLDPELFVECTECGRKMHQICVLHHEIIWPAGFVCDGCLKKSARTRKENKFSAKRLPSTRLGTFLENRVNDFLRRQNHPESGEVTVRVVHASDKTVEVKPGMKARFVDSGEMAESFPYRTKALFAFEEIDGVDLCFFGMHVQEYGSDCPPPNQRRVYISYLDSVHFFRPKCLRTAVYHEILIGYLEYVKKLGYTTGHIWACPPSEGDDYIFHCHPPDQKIPKPKRLQEWYKKMLDKAVSERIVHDYKDIFKQATEDRLTSAKELPYFEGDFWPNVLEESIKELEQEEEERKREENTSNESTDVTKGDSKNAKKKNNKKTSKNKSSLSRGNKKKPGMPNVSNDLSQKLYATMEKHKEVFFVIRLIAGPAANSLPPIVDPDPLIPCDLMDGRDAFLTLARDKHLEFSSLRRAQWSTMCMLVELHTQSQDRFVYTCNECKHHVETRWHCTVCEDYDLCITCYNTKNHDHKMEKLGLGLDDESNNQQAAATQSPGDSRRLSIQRCIQSLVHACQCRNANCSLPSCQKMKRVVQHTKGCKRKTNGGCPICKQLIALCCYHAKHCQENKCPVPFCLNIKQKLRQQQLQHRLQQAQMLRRRMASMQRTGVVGQQQGLPSPTPATPTTPTGQQPTTPQTPQPTSQPQPTPPNSMPPYLPRTQAAGPVSQGKAAGQVTPPTPPQTAQPPLPGPPPAAVEMAMQIQRAAETQRQMAHVQIFQRPIQHQMPPMTPMAPMGMNPPPMTRGPSGHLEPGMGPTGMQQQPPWSQGGLPQPQQLQSGMPRPAMMSVAQHGQPLNMAPQPGLGQVGISPLKPGTVSQQALQNLLRTLRSPSSPLQQQQVLSILHANPQLLAAFIKQRAAKYANSNPQPIPGQPGMPQGQPGLQPPTMPGQQGVHSNPAMQNMNPMQAGVQRAGLPQQQPQQQLQPPMGGMSPQAQQMNMNHNTMPSQFRDILRRQQMMQQQQQQGAGPGIGPGMANHNQFQQPQGVGYPPQQQQRMQHHMQQMQQGNMGQIGQLPQALGAEAGASLQAYQQRLLQQQMGSPVQPNPMSPQQHMLPNQAQSPHLQGQQIPNSLSNQVRSPQPVPSPRPQSQPPHSSPSPRMQPQPSPHHVSPQTSSPHPGLVAAQANPMEQGHFASPDQNSMLSQLASNPGMANLHGASATDLGLSTDNSDLNSNLSQSTLDIH

>NP_005852.2

MKGKEEKEGGARLGAGGGSPEKSPSAQELKEQGNRLFVGRKYPEAAACYGRAITRNPLVAVYYTNRALCYLKMQQHEQALADCRRALELDGQSVKAHFFLGQCQLEMESYDEAIANLQRAYSLAKEQRLNFGDDIPSALRIAKKKRWNSIEERRIHQESELHSYLSRLIAAERERELEECQRNHEGDEDDSHVRAQQACIEAKHDKYMADMDELFSQVDEKRKKRDIPDYLCGKISFELMREPCITPSGITYDRKDIEEHLQRVGHFDPVTRSPLTQEQLIPNLAMKEVIDAFISENGWVEDY

321 NP_001443.1 NP_056520.2

>NP_001443.1

MTTEGGPPPAPLRRACSPVPGALQAALMSPPPAAAAAAAAAPETTSSSSSSSSASCASSSSSSNSASAPSAACKSAGGGGAGAGSGGAKKASSGLRRPEKPPYSYIALIVMAIQSSPSKRLTLSEIYQFLQARFPFFRGAYQGWKNSVRHNLSLNECFIKLPKGLGRPGKGHYWTIDPASEFMFEEGSFRRRPRGFRRKCQALKPMYHRVVSGLGFGASLLPQGFDFQAPPSAPLGCHSQGGYGGLDMMPAGYDAGAGAPSHAHPHHHHHHHVPHMSPNPGSTYMASCPVPAGPGGVGAAGGGGGGDYGPDSSSSPVPSSPAMASAIECHSPYTSPAAHWSSPGASPYLKQPPALTPSSNPAASAGLHSSMSSYSLEQSYLHQNAREDLSVGLPRYQHHSTPVCDRKDFVLNFNGISSFHPSASGSYYHHHHQSVCQDIKPCVM

>NP_056520.2

MSGSHTPACGPFSALTPSIWPQEILAKYTQKEESAEQPEFYYDEFGFRVYKEEGDEPGSSLLANSPLMEDAPQRLRWQAHLEFTHNHDVGDLTWDKIAVSLPRSEKLRSLVLAGIPHGMRPQLWMRLSGALQKKRNSELSYREIVKNSSNDETIAAKQIEKDLLRTMPSNACFASMGSIGVPRLRRVLRALAWLYPEIGYCQGTGMVAACLLLFLEEEDAFWMMSAIIEDLLPASYFSTTLLGVQTDQRVLRHLIVQYLPRLDKLLQEHDIELSLITLHWFLTAFASVVDIKLLLRIWDLFFYEGSRVLFQLTLGMLHLKEEELIQSENSASIFNTLSDIPSQMEDAELLLGVAMRLAGSLTDVAVETQRRKHLAYLIADQGQLLGAGTLTNLSQVVRRRTQRRKSTITALLFGEDDLEALKAKNIKQTELVADLREAILRVARHFQCTDPKNCSVELTPDYSMESHQRDHENYVACSRSHRRRAKALLDFERHDDDELGFRKNDIITIVSQKDEHCWVGELNGLRGWFPAKFVEVLDERSKEYSIAGDDSVTEGVTDLVRGTLCPALKALFEHGLKKPSLLGGACHPWLFIEEAAGREVERDFASVYSRLVLCKTFRLDEDGKVLTPEELLYRAVQSVNVTHDAVHAQMDVKLRSLICVGLNEQVLHLWLEVLCSSLPTVEKWYQPWSFLRSPGWVQIKCELRVLCCFAFSLSQDWELPAKREAQQPLKEGVRDMLVKHHLFSWDVDG

322 NP_660328.2 Q9NQX6

>NP_660328.2

MMLPSPVTSTPFSVKDILNLEQQHQHFHGAHLQADLEHHFHSAPCMLAAAEGTQFSDGGEEDEEDEGEKLSYLNSLAAADGHGDSGLCPQGYVHTVLRDSCSEPKEHEEEPEVVRDRSQKSCQLKKSLETAGDCKAAEESERPKPRSRRKPRVLFSQAQVFELERRFKQQRYLSAPEREHLASSLKLTSTQVKIWFQNRRYKCKRQRQDKSLELGAHAPPPPPRRVAVPVLVRDGKPCVTPSAQAYGAPYSVGASAYSYNSFPAYGYGNSAAAAAAAAAAAAAAAAYSSSYGCAYPAGGGGGGGGTSAATTAMQPACSAAGGGPFVNVSNLGGFGSGGSAQPLHQGTAAGAACAQGTLQGIRAW

>Q9NQX6

MAQGLVTFADVAIDFSQEEWACLNSAQRDLYWDVMLENYSNLVSLDLESAYENKSLPTEKNIHEIRASKRNSDRRSKSLGRNWICEGTLERPQRSRGRYVNQMIINYVKRPATREGTPPRTHQRHHKENSFECKDCGKAFSRGYQLSQHQKIHTGEKPYECKECKKAFRWGNQLTQHQKIHTGEKPYECKDCGKAFRWGSSLVIHKRIHTGEKPYECKDCGKAFRRGDELTQHQRFHTGEKDYECKDCGKTFSRVYKLIQHKRIHSGEKPYECKDCGKAFICGSSLIQHKRIHTGEKPYECQECGKAFTRVNYLTQHQKIHTGEKPHECKECGKAFRWGSSLVKHERIHTGEKPYKCTECGKAFNCGYHLTQHERIHTGETPYKCKECGKAFIYGSSLVKHERIHTGVKPYGCTECGKSFSHGHQLTQHQKTHSGAKSYECKECGKACNHLNHLREHQRIHNS

323 NP_003063.2 NP_002890.1

>NP_003063.2

MSTPDPPLGGTPRPGPSPGPGPSPGAMLGPSPGPSPGSAHSMMGPSPGPPSAGHPIPTQGPGGYPQDNMHQMHKPMESMHEKGMSDDPRYNQMKGMGMRSGGHAGMGPPPSPMDQHSQGYPSPLGGSEHASSPVPASGPSSGPQMSSGPGGAPLDGADPQALGQQNRGPTPFNQNQLHQLRAQIMAYKMLARGQPLPDHLQMAVQGKRPMPGMQQQMPTLPPPSVSATGPGPGPGPGPGPGPGPAPPNYSRPHGMGGPNMPPPGPSGVPPGMPGQPPGGPPKPWPEGPMANAAAPTSTPQKLIPPQPTGRPSPAPPAVPPAASPVMPPQTQSPGQPAQPAPMVPLHQKQSRITPIQKPRGLDPVEILQEREYRLQARIAHRIQELENLPGSLAGDLRTKATIELKALRLLNFQRQLRQEVVVCMRRDTALETALNAKAYKRSKRQSLREARITEKLEKQQKIEQERKRRQKHQEYLNSILQHAKDFKEYHRSVTGKIQKLTKAVATYHANTEREQKKENERIEKERMRRLMAEDEEGYRKLIDQKKDKRLAYLLQQTDEYVANLTELVRQHKAAQVAKEKKKKKKKKKAENAEGQTPAIGPDGEPLDETSQMSDLPVKVIHVESGKILTGTDAPKAGQLEAWLEMNPGYEVAPRSDSEESGSEEEEEEEEEEQPQAAQPPTLPVEEKKKIPDPDSDDVSEVDARHIIENAKQDVDDEYGVSQALARGLQSYYAVAHAVTERVDKQSALMVNGVLKQYQIKGLEWLVSLYNNNLNGILADEMGLGKTIQTIALITYLMEHKRINGPFLIIVPLSTLSNWAYEFDKWAPSVVKVSYKGSPAARRAFVPQLRSGKFNVLLTTYEYIIKDKHILAKIRWKYMIVDEGHRMKNHHCKLTQVLNTHYVAPRRLLLTGTPLQNKLPELWALLNFLLPTIFKSCSTFEQWFNAPFAMTGEKVDLNEEETILIIRRLHKVLRPFLLRRLKKEVEAQLPEKVEYVIKCDMSALQRVLYRHMQAKGVLLTDGSEKDKKGKGGTKTLMNTIMQLRKICNHPYMFQHIEESFSEHLGFTGGIVQGLDLYRASGKFELLDRILPKLRATNHKVLLFCQMTSLMTIMEDYFAYRGFKYLRLDGTTKAEDRGMLLKTFNEPGSEYFIFLLSTRAGGLGLNLQSADTVIIFDSDWNPHQDLQAQDRAHRIGQQNEVRVLRLCTVNSVEEKILAAAKYKLNVDQKVIQAGMFDQKSSSHERRAFLQAILEHEEQDESRHCSTGSGSASFAHTAPPPAGVNPDLEEPPLKEEDEVPDDETVNQMIARHEEEFDLFMRMDLDRRREEARNPKRKPRLMEEDELPSWIIKDDAEVERLTCEEEEEKMFGRGSRHRKEVDYSDSLTEKQWLKAIEEGTLEEIEEEVRQKKSSRKRKRDSDAGSSTPTTSTRSRDKDDESKKQKKRGRPPAEKLSPNPPNLTKKMKKIVDAVIKYKDSSSGRQLSEVFIQLPSRKELPEYYELIRKPVDFKKIKERIRNHKYRSLNDLEKDVMLLCQNAQTFNLEGSLIYEDSIVLQSVFTSVRQKIEKEDDSEGEESEEEEEGEEEGSESESRSVKVKIKLGRKEKAQDRLKGGRRRPSRGSRAKPVVSDDDSEEEQEEDRSGSGSEED

>NP_002890.1

MPVDFTGYWKMLVNENFEEYLRALDVNVALRKIANLLKPDKEIVQDGDHMIIRTLSTFRNYIMDFQVGKEFEEDLTGIDDRKCMTTVSWDGDKLQCVQKGEKEGRGWTQWIEGDELHLEMRVEGVVCKQVFKKVQ

324 Q5T6S3 Q96PC2

>Q5T6S3

MENRALDPGTRDSYGATSHLPNKGALAKVKNNFKDLMSKLTEGQYVLCRWTDGLYYLGKIKRVSSSKQSCLVTFEDNSKYWVLWKDIQHAGVPGEEPKCNICLGKTSGPLNEILICGKCGLGYHQQCHIPIAGSADQPLLTPWFCRRCIFALAVRKGGALKKGAIARTLQAVKMVLSYQPEELEWDSPHRTNQQQCYCYCGGPGEWYLRMLQCYRCRQWFHEACTQCLNEPMMFGDRFYLFFCSVCNQGPEYIERLPLRWVDVVHLALYNLGVQSKKKYFDFEEILAFVNHHWELLQLGKLTSTPVTDRGPHLLNALNSYKSRFLCGKEIKKKKCIFRLRIRVPPNPPGKLLPDKGLLPNENSASSELRKRGKSKPGLLPHEFQQQKRRVYRRKRSKFLLEDAIPSSDFTSAWSTNHHLASIFDFTLDEIQSLKSASSGQTFFSDVDSTDAASTSGSASTSLSYDSRWTVGSRKRKLAAKAYMPLRAKRWAAELDGRCPSDSSAEGASVPERPDEGIDSHTFESISEDDSSLSHLKSSITNYFGAAGRLACGEKYQVLARRVTPEGKVQYLVEWEGTTPY

>Q96PC2

MVVQNSADAGDMRAGVQLEPFLHQVGGHMSVMKYDEHTVCKPLVSREQRFYESLPLAMKRFTPQYKGTVTVHLWKDSTGHLSLVANPVKESQEPFKVSTESAAVAIWQTLQQTTGSNGSDCTLAQWPHAQLARSPKESPAKALLRSEPHLNTPAFSLVEDTNGNQVERKSFNPWGLQCHQAHLTRLCSEYPENKRHRFLLLENVVSQYTHPCVLDLKMGTRQHGDDASEEKKARHMRKCAQSTSACLGVRICGMQVYQTDKKYFLCKDKYYGRKLSVEGFRQALYQFLHNGSHLRRELLEPILHQLRALLSVIRSQSSYRFYSSSLLVIYDGQEPPERAPGSPHPHEAPQAAHGSSPGGLTKVDIRMIDFAHTTYKGYWNEHTTYDGPDPGYIFGLENLIRILQDIQEGE

325 Q9UKN8 Q7RTR2

>Q9UKN8

MNTADQARVGPADDGPAPSGEEEGEGGGEAGGKEPAADAAPGPSAAFRLMVTRREPAVKLQYAVSGLEPLAWSEDHRVSVSTARSIAVLELICDVHNPGQDLVIHRTSVPAPLNSCLLKVGSKTEVAECKEKFAASKDPTVSQTFMLDRVFNPEGKALPPMRGFKYTSWSPMGCDANGRCLLAALTMDNRLTIQANLNRLQWVQLVDLTEIYGERLYETSYRLSKNEAPEGNLGDFAEFQRRHSMQTPVRMEWSGICTTQQVKHNNECRDVGSVLLAVLFENGNIAVWQFQLPFVGKESISSCNTIESGITSPSVLFWWEYEHNNRKMSGLIVGSAFGPIKILPVNLKAVKGYFTLRQPVILWKEMDQLPVHSIKCVPLYHPYQKCSCSLVVAARGSYVFWCLLLISKAGLNVHNSHVTGLHSLPIVSMTADKQNGTVYTCSSDGKVRQLIPIFTDVALKFEHQLIKLSDVFGSVRTHGIAVSPCGAYLAIITTEGMINGLHPVNKNYQVQFVTLKTFEEAAAQLLESSVQNLFKQVDLIDLVRWKILKDKHIPQFLQEALEKKIESSGVTYFWRFKLFLLRILYQSMQKTPSEALWKPTHEDSKILLVDSPGMGNADDEQQEEGTSSKQVVKQGLQERSKEGDVEEPTDDSLPTTGDAGGREPMEEKLLEIQGKIEAVEMHLTREHMKRVLGEVYLHTWITENTSIPTRGLCNFLMSDEEYDDRTARVLIGHISKKMNKQTFPEHCSLCKEILPFTDRKQAVCSNGHIWLRCFLTYQSCQSLIYRRCLLHDSIARHPAPEDPDWIKRLLQSPCPFCDSPVF

>Q7RTR2

MRKQEVRTGREAGQGHGTGSPAEQVKALMDLLAGKGSQGSQAPQALDRTPDAPLGPCSNDSRIQRHRKALLSKVGGGPELGGPWHRLASLLLVEGLTDLQLREHDFTQVEATRGGGHPARTVALDRLFLPLSRVSVPPRVSITIGVAGMGKTTLVRHFVRLWAHGQVGKDFSLVLPLTFRDLNTHEKLCADRLICSVFPHVGEPSLAVAVPARALLILDGLDECRTPLDFSNTVACTDPKKEIPVDHLITNIIRGNLFPEVSIWITSRPSASGQIPGGLVDRMTEIRGFNEEEIKVCLEQMFPEDQALLGWMLSQVQADRALYLMCTVPAFCRLTGMALGHLWRSRTGPQDAELWPPRTLCELYSWYFRMALSGEGQEKGKASPRIEQVAHGGRKMVGTLGRLAFHGLLKKKYVFYEQDMKAFGVDLALLQGAPCSCFLQREETLASSVAYCFTHLSLQEFVAAAYYYGASRRAIFDLFTESGVSWPRLGFLTHFRSAAQRAMQAEDGRLDVFLRFLSGLLSPRVNALLAGSLLAQGEHQAYRTQVAELLQGCLRPDAAVCARAINVLHCLHELQHTELARSVEEAMESGALARLTGPAHRAALAYLLQVSDACAQEANLSLSLSQGVLQSLLPQLLYCRKLRLDTNQFQDPVMELLGSVLSGKDCRIQKISLAENQISNKGAKALARSLLVNRSLTSLDLRGNSIGPQGAKALADALKINRTLTSLSLQGNTVRDDGARSMAEALASNRTLSMLHLQKNSIGPMGAQRMADALKQNRSLKELMFSSNSIGDGGAKALAEALKVNQGLESLDLQSNSISDAGVAALMGALCTNQTLLSLSLRENSISPEGAQAIAHALCANSTLKNLDLTANLLHDQGARAIAVAVRENRTLTSLHLQWNFIQAGAAQALGQALQLNRSLTSLDLQENAIGDDGACAVARALKVNTALTALYLQVASIGASGAQVLGEALAVNRTLEILDLRGNAIGVAGAKALANALKVNSSLRRLNLQENSLGMDGAICIATALSGNHRLQHINLQGNHIGDSGARMISEAIKTNAPTCTVEM

326 NP_075555.1 Q9BXI3

>NP_075555.1

MMASYPEPEDAAGALLAPETGRTVKEPEGPPPSPGKGGGGGGGTAPEKPDPAQKPPYSYVALIAMAIRESAEKRLTLSGIYQYIIAKFPFYEKNKKGWQNSIRHNLSLNECFIKVPREGGGERKGNYWTLDPACEDMFEKGNYRRRRRMKRPFRPPPAHFQPGKGLFGAGGAAGGCGVAGAGADGYGYLAPPKYLQSGFLNNSWPLPQPPSPMPYASCQMAAAAAAAAAAAAAAGPGSPGAAAVVKGLAGPAASYGPYTRVQSMALPPGVVNSYNGLGGPPAAPPPPPHPHPHPHAHHLHAAAAPPPAPPHHGAAAPPPGQLSPASPATAAPPAPAPTSAPGLQFACARQPELAMMHCSYWDHDSKTGALHSRLDL

>Q9BXI3

MEPGQPREPQEPREPGPGAETAAAPVWEEAKIFYDNLAPKKKPKSPKPQNAVTIAVSSRALFRMDEEQQIYTEQGVEEYVRYQLEHENEPFSPGPAFPFVKALEAVNRRLRELYPDSEDVFDIVLMTNNHAQVGVRLINSINHYDLFIERFCMTGGNSPICYLKAYHTNLYLSADAEKVREAIDEGIAAATIFSPSRDVVVSQSQLRVAFDGDAVLFSDESERIVKAHGLDRFFEHEKAHENKPLAQGPLKGFLEALGRLQKKFYSKGLRLECPIRTYLVTARSAASSGARALKTLRSWGLETDEALFLAGAPKGPLLEKIRPHIFFDDQMFHVAGAQEMGTVAAHVPYGVAQTPRRTAPAKQAPSAQ

327 Q08050 P63302

>Q08050

MKTSPRRPLILKRRRLPLPVQNAPSETSEEEPKRSPAQQESNQAEASKEVAESNSCKFPAGIKIINHPTMPNTQVVAIPNNANIHSIITALTAKGKESGSSGPNKFILISCGGAPTQPPGLRPQTQTSYDAKRTEVTLETLGPKPAARDVNLPRPPGALCEQKRETCADGEAAGCTINNSLSNIQWLRKMSSDGLGSRSIKQEMEEKENCHLEQRQVKVEEPSRPSASWQNSVSERPPYSYMAMIQFAINSTERKRMTLKDIYTWIEDHFPYFKHIAKPGWKNSIRHNLSLHDMFVRETSANGKVSFWTIHPSANRYLTLDQVFKPLDPGSPQLPEHLESQQKRPNPELRRNMTIKTELPLGARRKMKPLLPRVSSYLVPIQFPVNQSLVLQPSVKVPLPLAASLMSSELARHSKRVRIAPKVLLAEEGIAPLSSAGPGKEEKLLFGEGFSPLLPVQTIKEEEIQPGEEMPHLARPIKVESPPLEEWPSPAPSFKEESSHSWEDSSQSPTPRPKKSYSGLRSPTRCVSEMLVIQHRERRERSRSRRKQHLLPPCVDEPELLFSEGPSTSRWAAELPFPADSSDPASQLSYSQEVGGPFKTPIKETLPISSTPSKSVLPRTPESWRLTPPAKVGGLDFSPVQTSQGASDPLPDPLGLMDLSTTPLQSAPPLESPQRLLSSEPLDLISVPFGNSSPSDIDVPKPGSPEPQVSGLAANRSLTEGLVLDTMNDSLSKILLDISFPGLDEDPLGPDNINWSQFIPELQ

>P63302

MALAVRVVYCGAUGYKSKYLQLKKKLEDEFPGRLDICGEGTPQATGFFEVMVAGKLIHSKKKGDGYVDTESKFLKLVAAIKAALAQG

328 NP_004409.1 NP_057461.1

>NP_004409.1

MGLEAARELECAALGTLLRDPREAERTLLLDCRPFLAFCRRHVRAARPVPWNALLRRRARGPPAAVLACLLPDRALRTRLVRGELARAVVLDEGSASVAELRPDSPAHVLLAALLHETRAGPTAVYFLRGGFDGFQGCCPDLCSEAPAPALPPTGDKTSRSDSRAPVYDQGGPVEILPYLFLGSCSHSSDLQGLQACGITAVLNVSASCPNHFEGLFRYKSIPVEDNQMVEISAWFQEAIGFIDWVKNSGGRVLVHCQAGISRSATICLAYLMQSRRVRLDEAFDFVKQRRGVISPNFSFMGQLLQFETQVLCH

>NP_057461.1

MSEEFEANTMDSLVDMPFATVDIQDDCGITDEPQINLKRSQENEWVKSDQVKKRKKKRKDYQPNYFLSIPITNKEIIKGIKILQNAIIQQDERLAKAMVSDGSFHITLLVMQLLNEDEVNIGIDALLELKPFIEELLQGKHLTLPFQGIGTFGNQVGFVKLAEGDHVNSLLEIAETANRTFQEKGILVGESRSFKPHLTFMKLSKSPWLRKNGVKKIDPDLYEKFISHRFGEEILYRIDLCSMLKKKQSNGYYHCESSIVIGEKNGGEPDDAELVRLSKRLVENAVLKAVQQYLEETQNKNKPGEGSSVKTEAADQNGNDNENNRK

329 Q9Y6X0 P49411

>Q9Y6X0

MEPEEEDELGSGRDVDSNSNADSEKWVAGDGLEEQEFSIKEANFTEGSLKLKIQTTKRAKKPPKNLENYICPPEIKITIKQSGDQKVSRAGKNSKATKEEERSHSKKKLLTASDLAASDLKGFQPQAYERPQKHSTLHYDTGLPQDFTGDTLKPKHQQKSSSQNHMDWSTNSDSGPVTQNCFISPESGRETASTSKIPALEPVASFAKAQGKKGSAGNTWSQLSNNNKDLLLGGVAPSPSSHSSPAPPSSSAECNGLQPLVDQDGGGTKEPPEPPTVGSKKKSSKKDVISQTIPNPDLDWVKNAQKAFDNTEGKREGYSADSAQEASPARQNVSSASNPENDSSHVRITIPIKAPSLDPTNHKRKKRQSIKAVVEKIMPEKALASGITMSSEVVNRILSNSEGNKKDPRVPKLSKMIENESPSVGLETGGNAEKVIPGGVSKPRKPPMVMTPPTCTDHSPSRKLPEIQHPKFAAKRRWTCSKPKPSTMLREAVMATSDKLMLEPPSAYPITPSSPLYTNTDSLTVITPVKKKRGRPKKQPLLTVETIHEGTSTSPVSPISREFPGTKKRKRRRNLAKLAQLVPGEDKPMSEMKFHKKVGKLGVLDKKTIKTINKMKTLKRKNILNQILSCSSSVALKAKAPPETSPGAAAIESKLGKQINVSKRGTIYIGKKRGRKPRAELPPPSEEPKTAIKHPRPVSSQPDVPAVPSNFQSLVASSPAAMHPLSTQLGGSNGNLSPASTETNFSELKTMPNLQPISALPTKTQKGIHSGTWKLSPPRLMANSPSHLCEIGSLKEITLSPVSESHSEETIPSDSGIGTDNNSTSDQAEKSSESRRRYSFDFCSLDNPEAIPSDTSTKNRHGHRQKHLIVDNFLAHESLKKPKHKRKRKSLQNRDDLQFLADLEELITKFQVFRISHRSYTFYHENPYPSIFRINFDHYYPVPYIQYDPLLYLRRTSDLKSKKKRGRPAKTNDTMTKVPFLQGFSYPIPSGSYYAPYGMPYTSMPMMNLGYYGQYPAPLYLSHTLGAASPFMRPTVPPPQFHTNSHVKMSGAAKHKAKHGVHLQGPVSMGLGDMQPSLNPPKVGSASLSSGRLHKRKHKHKHKHKEDRILGTHDNLSGLFAGKATGFSSHILSERLSSADKELPLVSEKNKHKEKQKHQHSEAGHKASKNNFEVDTLSTLSLSDAQHWTQAKEKGDLSSEPVDSCTKRYSGSGGDGGSTRSENLDVFSEMNPSNDKWDSDVSGSKRRSYEGFGTYREKDIQAFKMNRKERSSYDSSMSPGMPSPHLKVDQTAVHSKNEGSVPTMMTRKKPAAVDSVTIPPAPVLSLLAASAATSDAVGSSLKKRFKRREIEAIQCEVRKMCNYTKILSTKKNLDHVNKILKAKRLQRQSKTGNNFVKKRRGRPRKQPTQFDEDSRDQMPVLEKCIDLPSKRGQKPSLSPLVLEPAASQDTIMATIEAVIHMAREAPPLPPPPPPPLPPPPPPPLPPPPPLPKTPRGGKRKHKPQAPAQPPQQSPPQQPLPQEEEVKAKRQRKSRGSESEVLP

>P49411

MAAATLLRATPHFSGLAAGRTFLLQGLLRLLKAPALPLLCRGLAVEAKKTYVRDKPHVNVGTIGHVDHGKTTLTAAITKILAEGGGAKFKKYEEIDNAPEERARGITINAAHVEYSTAARHYAHTDCPGHADYVKNMITGTAPLDGCILVVAANDGPMPQTREHLLLARQIGVEHVVVYVNKADAVQDSEMVELVELEIRELLTEFGYKGEETPVIVGSALCALEGRDPELGLKSVQKLLDAVDTYIPVPARDLEKPFLLPVEAVYSVPGRGTVVTGTLERGILKKGDECELLGHSKNIRTVVTGIEMFHKSLERAEAGDNLGALVRGLKREDLRRGLVMVKPGSIKPHQKVEAQVYILSKEEGGRHKPFVSHFMPVMFSLTWDMACRIILPPEKELAMPGEDLKFNLILRQPMILEKGQRFTLRDGNRTIGTGLVTNTLAMTEEEKNIKWG

330 NP_055331.1 NP_005264.2

>NP_055331.1

MADKRKLQGEIDRCLKKVSEGVEQFEDIWQKLHNAANANQKEKYEADLKKEIKKLQRLRDQIKTWVASNEIKDKRQLIDNRKLIETQMERFKVVERETKTKAYSKEGLGLAQKVDPAQKEKEEVGQWLTNTIDTLNMQVDQFESEVESLSVQTRKKKGDKDKQDRIEGLKRHIEKHRYHVRMLETILRMLDNDSILVDAIRKIKDDVEYYVDSSQDPDFEENEFLYDDLDLEDIPQALVATSPPSHSHMEDEIFNQSSSTPTSTTSSSPIPPSPANCTTENSEDDKKRGRSTDSEVSQSPAKNGSKPVHSNQHPQSPAVPPTYPSGPPPAASALSTTPGNNGVPAPAAPPSALGPKASPAPSHNSGTPAPYAQAVAPPAPSGPSTTQPRPPSVQPSGGGGGGSGGGGSSSSSNSSAGGGAGKQNGATSYSSVVADSPAEVALSSSGGNNASSQALGPPSGPHNPPPSTSKEPSAAAPTGAGGVAPGSGNNSGGPSLLVPLPVNPPSSPTPSFSDAKAAGALLNGPPQFSTAPEIKAPEPLSSLKSMAERAAISSGIEDPVPTLHLTERDIILSSTSAPPASAQPPLQLSEVNIPLSLGVCPLGPVPLTKEQLYQQAMEEAAWHHMPHPSDSERIRQYLPRNPCPTPPYHHQMPPPHSDTVEFYQRLSTETLFFIFYYLEGTKAQYLAAKALKKQSWRFHTKYMMWFQRHEEPKTITDEFEQGTYIYFDYEKWGQRKKEGFTFEYRYLEDRDLQ

>NP_005264.2

MSELEQLRQEAEQLRNQIRDARKACGDSTLTQITAGLDPVGRIQMRTRRTLRGHLAKIYAMHWGTDSRLLVSASQDGKLIIWDSYTTNKVHAIPLRSSWVMTCAYAPSGNFVACGGLDNICSIYSLKTREGNVRVSRELPGHTGYLSCCRFLDDNQIITSSGDTTCALWDIETGQQTVGFAGHSGDVMSLSLAPDGRTFVSGACDASIKLWDVRDSMCRQTFIGHESDINAVAFFPNGYAFTTGSDDATCRLFDLRADQELLMYSHDNIICGITSVAFSRSGRLLLAGYDDFNCNIWDAMKGDRAGVLAGHDNRVSCLGVTDDGMAVATGSWDSFLKIWN

331 NP_002158.2 NP_004324.2

>NP_002158.2

MKALSPVRGCYEAVCCLSERSLAIARGRGKGPAAEEPLSLLDDMNHCYSRLRELVPGVPRGTQLSQVEILQRVIDYILDLQVVLAEPAPGPPDGPHLPIQTAELAPELVISNDKRSFCH

>NP_004324.2

MAALSGGGGGGAEPGQALFNGDMEPEAGAGAGAAASSAADPAIPEEVWNIKQMIKLTQEHIEALLDKFGGEHNPPSIYLEAYEEYTSKLDALQQREQQLLESLGNGTDFSVSSSASMDTVTSSSSSSLSVLPSSLSVFQNPTDVARSNPKSPQKPIVRVFLPNKQRTVVPARCGVTVRDSLKKALMMRGLIPECCAVYRIQDGEKKPIGWDTDISWLTGEELHVEVLENVPLTTHNFVRKTFFTLAFCDFCRKLLFQGFRCQTCGYKFHQRCSTEVPLMCVNYDQLDLLFVSKFFEHHPIPQEEASLAETALTSGSSPSAPASDSIGPQILTSPSPSKSIPIPQPFRPADEDHRNQFGQRDRSSSAPNVHINTIEPVNIDDLIRDQGFRGDGGSTTGLSATPPASLPGSLTNVKALQKSPGPQRERKSSSSSEDRNRMKTLGRRDSSDDWEIPDGQITVGQRIGSGSFGTVYKGKWHGDVAVKMLNVTAPTPQQLQAFKNEVGVLRKTRHVNILLFMGYSTKPQLAIVTQWCEGSSLYHHLHIIETKFEMIKLIDIARQTAQGMDYLHAKSIIHRDLKSNNIFLHEDLTVKIGDFGLATVKSRWSGSHQFEQLSGSILWMAPEVIRMQDKNPYSFQSDVYAFGIVLYELMTGQLPYSNINNRDQIIFMVGRGYLSPDLSKVRSNCPKAMKRLMAECLKKKRDERPLFPQILASIELLARSLPKIHRSASEPSLNRAGFQTEDFSLYACASPKTPIQAGGYGAFPVH

332 NP_003649.2 P43897

>NP_003649.2

MHCHAELRLSSPGQLKAARRRYKTFMIDEILSKETCDYFEKLSLYSVCPSLVVRPKPLHSCTGSPSLRAYPLLSVITRQPTVISHLVPATPGIAQALSCHQVTEAVSAEAPGGEALASSESETEQPTPRQKKPRRSRTIFTELQLMGLEKKFQKQKYLSTPDRLDLAQSLGLTQLQVKTWYQNRRMKWKKMVLKGGQEAPTKPKGRPKKNSIPTSEEIEAEEKMNSQAQGQEQLEPSQGQEELCEAQEPKARDVPLEMAEPPDPPQELPIPSSEPPPLS

>P43897

MSLLRSLRVFLVARTGSYPAGSLLRQSPQPRHTFYAGPRLSASASSKELLMKLRRKTGYSFVNCKKALETCGGDLKQAEIWLHKEAQKEGWSKAAKLQGRKTKEGLIGLLQEGNTTVLVEVNCETDFVSRNLKFQLLVQQVALGTMMHCQTLKDQPSAYSKGFLNSSELSGLPAGPDREGSLKDQLALAIGKLGENMILKRAAWVKVPSGFYVGSYVHGAMQSPSLHKLVLGKYGALVICETSEQKTNLEDVGRRLGQHVVGMAPLSVGSLDDEPGGEAETKMLSQPYLLDPSITLGQYVQPQGVSVVDFVRFECGEGEEAAETE

333 Q7Z5Q5 NP_001167.2

>Q7Z5Q5

MENYEALVGFDLCNTPLSSVAQKIMSAMHSGDLVDSKTWGKSTETMEVINKSSVKYSVQLEDRKTQSPEKKDLKSLRSQTSRGSAKLSPQSFSVRLTDQLSADQKQKSISSLTLSSCLIPQYNQEASVLQKKGHKRKHFLMENINNENKGSINLKRKHITYNNLSEKTSKQMALEEDTDDAEGYLNSGNSGALKKHFCDIRHLDDWAKSQLIEMLKQAAALVITVMYTDGSTQLGADQTPVSSVRGIVVLVKRQAEGGHGCPDAPACGPVLEGFVSDDPCIYIQIEHSAIWDQEQEAHQQFARNVLFQTMKCKCPVICFNAKDFVRIVLQFFGNDGSWKHVADFIGLDPRIAAWLIDPSDATPSFEDLVEKYCEKSITVKVNSTYGNSSRNIVNQNVRENLKTLYRLTMDLCSKLKDYGLWQLFRTLELPLIPILAVMESHAIQVNKEEMEKTSALLGARLKELEQEAHFVAGERFLITSNNQLREILFGKLKLHLLSQRNSLPRTGLQKYPSTSEAVLNALRDLHPLPKIILEYRQVHKIKSTFVDGLLACMKKGSISSTWNQTGTVTGRLSAKHPNIQGISKHPIQITTPKNFKGKEDKILTISPRAMFVSSKGHTFLAADFSQIELRILTHLSGDPELLKLFQESERDDVFSTLTSQWKDVPVEQVTHADREQTKKVVYAVVYGAGKERLAACLGVPIQEAAQFLESFLQKYKKIKDFARAAIAQCHQTGCVVSIMGRRRPLPRIHAHDQQLRAQAERQAVNFVVQGSAADLCKLAMIHVFTAVAASHTLTARLVAQIHDELLFEVEDPQIPECAALVRRTMESLEQVQALELQLQVPLKVSLSAGRSWGHLVPLQEAWGPPPGPCRTESPSNSLAAPGSPASTQPPPLHFSPSFCL

>NP_001167.2

MLGLDACELGAQLLELLRLALCARVLLADKEGGPPAVDEVLDEAVPEYRAPGRKSLLEIRQLDPDDRSLAKYKRVLLGPLPPAVDPSLPNVQVTRLTLLSEQAPGPVVMDLTGDLAVLKDQVFVLKEGVDYRVKISFKVHREIVSGLKCLHHTYRRGLRVDKTVYMVGSYGPSAQEYEFVTPVEEAPRGALVRGPYLVVSLFTDDDRTHHLSWEWGLCICQDWKD

334 P41229 Q9UI17

>P41229

MEPGSDDFLPPPECPVFEPSWAEFRDPLGYIAKIRPIAEKSGICKIRPPADWQPPFAVEVDNFRFTPRIQRLNELEAQTRVKLNYLDQIAKFWEIQGSSLKIPNVERRILDLYSLSKIVVEEGGYEAICKDRRWARVAQRLNYPPGKNIGSLLRSHYERIVYPYEMYQSGANLVQCNTRPFDNEEKDKEYKPHSIPLRQSVQPSKFNSYGRRAKRLQPDPEPTEEDIEKNPELKKLQIYGAGPKMMGLGLMAKDKTLRKKDKEGPECPPTVVVKEELGGDVKVESTSPKTFLESKEELSHSPEPCTKMTMRLRRNHSNAQFIESYVCRMCSRGDEDDKLLLCDGCDDNYHIFCLLPPLPEIPKGVWRCPKCVMAECKRPPEAFGFEQATREYTLQSFGEMADSFKADYFNMPVHMVPTELVEKEFWRLVNSIEEDVTVEYGADIHSKEFGSGFPVSDSKRHLTPEEEEYATSGWNLNVMPVLEQSVLCHINADISGMKVPWLYVGMVFSAFCWHIEDHWSYSINYLHWGEPKTWYGVPSLAAEHLEEVMKKLTPELFDSQPDLLHQLVTLMNPNTLMSHGVPVVRTNQCAGEFVITFPRAYHSGFNQGYNFAEAVNFCTADWLPAGRQCIEHYRRLRRYCVFSHEELICKMAACPEKLDLNLAAAVHKEMFIMVQEERRLRKALLEKGITEAEREAFELLPDDERQCIKCKTTCFLSALACYDCPDGLVCLSHINDLCKCSSSRQYLRYRYTLDELPAMLHKLKVRAESFDTWANKVRVALEVEDGRKRSLEELRALESEARERRFPNSELLQQLKNCLSEAEACVSRALGLVSGQEAGPHRVAGLQMTLTELRAFLDQMNNLPCAMHQIGDVKGVLEQVEAYQAEAREALASLPSSPGLLQSLLERGRQLGVEVPEAQQLQRQVEQARWLDEVKRTLAPSARRGTLAVMRGLLVAGASVAPSPAVDKAQAELQELLTIAERWEEKAHLCLEARQKHPPATLEAIIREAENIPVHLPNIQALKEALAKARAWIADVDEIQNGDHYPCLDDLEGLVAVGRDLPVGLEELRQLELQVLTAHSWREKASKTFLKKNSCYTLLEVLCPCADAGSDSTKRSRWMEKELGLYKSDTELLGLSAQDLRDPGSVIVAFKEGEQKEKEGILQLRRTNSAKPSPLASSSTASSTTSICVCGQVLAGAGALQCDLCQDWFHGRCVSVPRLLSSPRPNPTSSPLLAWWEWDTKFLCPLCMRSRRPRLETILALLVALQRLPVRLPEGEALQCLTERAISWQGRARQALASEDVTALLGRLAELRQRLQAEPRPEEPPNYPAAPASDPLREGSGKDMPKVQGLLENGDSVTSPEKVAPEEGSGKRDLELLSSLLPQLTGPVLELPEATRAPLEELMMEGDLLEVTLDENHSIWQLLQAGQPPDLERIRTLLELEKAERHGSRARGRALERRRRRKVDRGGEGDDPAREELEPKRVRSSGPEAEEVQEEEELEEETGGEGPPAPIPTTGSPSTQENQNGLEPAEGTTSGPSAPFSTLTPRLHLPCPQQPPQQQL

>Q9UI17

MLRPGAQLLRGLLLRSCPLQGSPGRPRSVCGREGEEKPPLSAETQWKDRAETVIIGGGCVGVSLAYHLAKAGMKDVVLLEKSELTAGSTWHAAGLTTYFHPGINLKKIHYDSIKLYEKLEEETGQVVGFHQPGSIRLATTPVRVDEFKYQMTRTGWHATEQYLIEPEKIQEMFPLLNMNKVLAGLYNPGDGHIDPYSLTMALAAGARKCGALLKYPAPVTSLKARSDGTWDVETPQGSMRANRIVNAAGFWAREVGKMIGLEHPLIPVQHQYVVTSTISEVKALKRELPVLRDLEGSYYLRQERDGLLFGPYESQEKMKVQDSWVTNGVPPGFGKELFESDLDRIMEHIKAAMEMVPVLKKADIINVVNGPITYSPDILPMVGPHQGVRNYWVAIGFGYGIIHAGGVGKYLSDWILHGEPPFDLIELDPNRYGKWTTTQYTEAKARESYGFNNIVGYPKEERFAGRPTQRVSGLYQRLESKCSMGFHAGWEQPHWFYKPGQDTQYRPSFRRTNWFEPVGSEYKQVMQRVGVTDLSPFGKFNIKGQDSIRLLDHLFANVIPKVGFTNISHMLTPKGRVYAELTVSHQSPGEFLLITGSGSELHDLRWIEEEAVKGGYDVEIKNITDELGVLGVAGPQARKVLQKLTPEDLSDDVFKFLQTKSLKVSNIPVTAIRISYTGELGWELYHRREDSVALYDAIMNAGQEEGIDNFGTYAMNALRLEKAFRAWGLEMNCDTNPLEAGLEYFVKLNKPADFIGKQALKQIKAKGLKRRLVCLTLATDDVDPEGNESIWYNGKVVGNTTSGSYSYSIQKSLAFAYVPVQLSEVGQQVEVELLGKNYPAVIIQEPLVLTEPTRNRLQKKGGKDKT

335 Q03923 Q9UBW5

>Q03923

MGPLTFRDVAIEFSLKEWQCLDTAQRNLYRNVMLENYRNLVFLGITVSKPDLITCLEQGKEAWSMKRHEIMVAKPTVMCSHFAQDLWPEQNIKDSFQKVTLKRYGKCRHENLPLRKGCESMDECKMHKGGCNGLNQCLTATQSKIFQCDKYVKVAHKFSNSNRHEIRHTKKKPFKCTKCGKSFGMISCLTEHSRIHTRVNFYKCEECGKAFNWSSTLTKHKRIHTGEKPYKCEECGKAFNQSSNLIKHKKIHTGEKPYKCEECGKTFNRFSTLTTHKIIHTGEKPYKCKECGKAFNRSSTLTTHRKIHTGEKPYKCEECGKAFKQSSNLTTHKIIHTGEKPYKCKKCGKAFNQSAHLTTHEVIHTGEKPYKCEKCGKAFNHFSHLTTHKIIHTGEKPYKCKECGKAFKHSSTLTKHKIIHTGEKPYKCKECEKAFNQSSKLTEHKKIHTGEKPYECEKCGKAFNQSSNLTRHKKSHTEEKPYKCEECGKGFKWPSTLTIHKIIHTGEKPYKCEECGKAFNQSSKLTKHKKIHTGEKPYTCEECGKAFNQSSNLTKHKRIHTGEKPYKCEECDKAFKWSSVLTKHKIIHTGEKLQI

>Q9UBW5

MAEGKAGGAAGLFAKQVQKKFSRAQEKVLQKLGKAVETKDERFEQSASNFYQQQAEGHKLYKDLKNFLSAVKVMHESSKRVSETLQEIYSSEWDGHEELKAIVWNNDLLWEDYEEKLADQAVRTMEIYVAQFSEIKERIAKRGRKLVDYDSARHHLEAVQNAKKKDEAKTAKAEEEFNKAQTVFEDLNQELLEELPILYNSRIGCYVTIFQNISNLRDVFYREMSKLNHNLYEVMSKLEKQHSNKVFVVKGLSSSSRRSLVISPPVRTATVSSPLTSPTSPSTLSLKSESESVSATEDLAPDAAQGEDNSEIKELLEEEEIEKEGSEASSSEEDEPLPACNGPAQAQPSPTTERAKSQEEVLPSSTTPSPGGALSPSGQPSSSATEVVLRTRTASEGSEQPKKRASIQRTSAPPSRPPPPRATASPRPSSGNIPSSPTASGGGSPTSPRASLGTGTASPRTSLEVSPNPEPPEKPVRTPEAKENENIHNQNPEELCTSPTLMTSQVASEPGEAKKMEDKEKDNKLISANSSEGQDQLQVSMVPENNNLTAPEPQEEVSTSENPQL

336 NP_037425.1 P20132

>NP_037425.1

MSDVEENNFEGRESRSQSKSPTGTPARVKSESRSGSRSPSRVSKHSESHSRSRSKSRSRSRRHSHRRYTRSRSHSHSHRRRSRSRSYTPEYRRRRSRSHSPMSNRRRHTGSRANPDPNTCLGVFGLSLYTTERDLREVFSRYGPLSGVNVVYDQRTGRSRGFAFVYFERIDDSKEAMERANGMELDGRRIRVDYSITKRAHTPTPGIYMGRPTHSGGGGGGGGGGGGGGGGRRRDSYYDRGYDRGYDRYEDYDYRYRRRSPSPYYSRYRSRSRSRSYSPRRY

>P20132

MMSGEPLHVKTPIRDSMALSKMAGTSVYLKMDSAQPSGSFKIRGIGHFCKRWAKQGCAHFVCSSAGNAGMAAAYAARQLGVPATIVVPSTTPALTIERLKNEGATVKVVGELLDEAFELAKALAKNNPGWVYIPPFDDPLIWEGHASIVKELKETLWEKPGAIALSVGGGGLLCGVVQGLQEVGWGDVPVIAMETFGAHSFHAATTAGKLVSLPKITSVAKALGVKTVGAQALKLFQEHPIFSEVISDQEAVAAIEKFVDDEKILVEPACGAALAAVYSHVIQKLQLEGNLRTPLPSLVVIVCGGSNISLAQLRALKEQLGMTNRLPK

337 NP_006464.1 NP_005800.3

>NP_006464.1

MSEREERRFVEIPRESVRLMAESTGLELSDEVAALLAEDVCYRLREATQNSSQFMKHTKRRKLTVEDFNRALRWSSVEAVCGYGSQEALPMRPAREGELYFPEDREVNLVELALATNIPKGCAETAVRVHVSYLDGKGNLAPQGSVPSAVSSLTDDLLKYYHQVTRAVLGDDPQLMKVALQDLQTNSKIGALLPYFVYVVSGVKSVSHDLEQLHRLLQVARSLFRNPHLCLGPYVRCLVGSVLYCVLEPLAASINPLNDHWTLRDGAALLLSHIFWTHGDLVSGLYQHILLSLQKILADPVRPLCCHYGAVVGLHALGWKAVERVLYPHLSTYWTNLQAVLDDYSVSNAQVKADGHKVYGAILVAVERLLKMKAQAAEPNRGGPGGRGCRRLDDLPWDSLLFQESSSGGGAEPSFGSGLPLPPGGAGPEDPSLSVTLADIYRELYAFFGDSLATRFGTGQPAPTAPRPPGDKKEPAAAPDSVRKMPQLTASAIVSPHGDESPRGSGGGGPASASGPAASESRPLPRVHRARGAPRQQGPGTGTRDVFQKSRFAPRGAPHFRFIIAGRQAGRRCRGRLFQTAFPAPYGPSPASRYVQKLPMIGRTSRPARRWALSDYSLYLPL

>NP_005800.3

MASGNARIGKPAPDFKATAVVDGAFKEVKLSDYKGKYVVLFFYPLDFTFVCPTEIIAFSNRAEDFRKLGCEVLGVSVDSQFTHLAWINTPRKEGGLGPLNIPLLADVTRRLSEDYGVLKTDEGIAYRGLFIIDGKGVLRQITVNDLPVGRSVDEALRLVQAFQYTDEHGEVCPAGWKPGSDTIKPNVDDSKEYFSKHN

338 NP_005185.2 NP_000445.1

>NP_005185.2

MQRLVAWDPACLPLPPPPPAFKSMEVANFYYEADCLAAAYGGKAAPAAPPAARPGPRPPAGELGSIGDHERAIDFSPYLEPLGAPQAPAPATATDTFEAAPPAPAPAPASSGQHHDFLSDLFSDDYGGKNCKKPAEYGYVSLGRLGAAKGALHPGCFAPLHPPPPPPPPPAELKAEPGFEPADCKRKEEAGAPGGGAGMAAGFPYALRAYLGYQAVPSGSSGSLSTSSSSSPPGTPSPADAKAPPTACYAGAAPAPSQVKSKAKKTVDKHSDEYKIRRERNNIAVRKSRDKAKMRNLETQHKVLELTAENERLQKKVEQLSRELSTLRNLFKQLPEPLLASSGHC

>NP_000445.1

MATKAVCVLKGDGPVQGIINFEQKESNGPVKVWGSIKGLTEGLHGFHVHEFGDNTAGCTSAGPHFNPLSRKHGGPKDEERHVGDLGNVTADKDGVADVSIEDSVISLSGDHCIIGRTLVVHEKADDLGKGGNEESTKTGNAGSRLACGVIGIAQ

339 P08236 NP_004125.3

>P08236

MARGSAVAWAALGPLLWGCALGLQGGMLYPQESPSRECKELDGLWSFRADFSDNRRRGFEEQWYRRPLWESGPTVDMPVPSSFNDISQDWRLRHFVGWVWYEREVILPERWTQDLRTRVVLRIGSAHSYAIVWVNGVDTLEHEGGYLPFEADISNLVQVGPLPSRLRITIAINNTLTPTTLPPGTIQYLTDTSKYPKGYFVQNTYFDFFNYAGLQRSVLLYTTPTTYIDDITVTTSVEQDSGLVNYQISVKGSNLFKLEVRLLDAENKVVANGTGTQGQLKVPGVSLWWPYLMHERPAYLYSLEVQLTAQTSLGPVSDFYTLPVGIRTVAVTKSQFLINGKPFYFHGVNKHEDADIRGKGFDWPLLVKDFNLLRWLGANAFRTSHYPYAEEVMQMCDRYGIVVIDECPGVGLALPQFFNNVSLHHHMQVMEEVVRRDKNHPAVVMWSVANEPASHLESAGYYLKMVIAHTKSLDPSRPVTFVSNSNYAADKGAPYVDVICLNSYYSWYHDYGHLELIQLQLATQFENWYKKYQKPIIQSEYGAETIAGFHQDPPLMFTEEYQKSLLEQYHLGLDQKRRKYVVGELIWNFADFMTEQSPTRVLGNKKGIFTRQRQPKSAAFLLRERYWKIANETRYPHSVAKSQCLENSLFT

>NP_004125.3

MISASRAAAARLVGAAASRGPTAARHQDSWNGLSHEAFRLVSRRDYASEAIKGAVVGIDLGTTNSCVAVMEGKQAKVLENAEGARTTPSVVAFTADGERLVGMPAKRQAVTNPNNTFYATKRLIGRRYDDPEVQKDIKNVPFKIVRASNGDAWVEAHGKLYSPSQIGAFVLMKMKETAENYLGHTAKNAVITVPAYFNDSQRQATKDAGQISGLNVLRVINEPTAAALAYGLDKSEDKVIAVYDLGGGTFDISILEIQKGVFEVKSTNGDTFLGGEDFDQALLRHIVKEFKRETGVDLTKDNMALQRVREAAEKAKCELSSSVQTDINLPYLTMDSSGPKHLNMKLTRAQFEGIVTDLIRRTIAPCQKAMQDAEVSKSDIGEVILVGGMTRMPKVQQTVQDLFGRAPSKAVNPDEAVAIGAAIQGGVLAGDVTDVLLLDVTPLSLGIETLGGVFTKLINRNTTIPTKKSQVFSTAADGQTQVEIKVCQGEREMAGDNKLLGQFTLIGIPPAPRGVPQIEVTFDIDANGIVHVSAKDKGTGREQQIVIQSSGGLSKDDIENMVKNAEKYAEEDRRKKERVEAVNMAEGIIHDTETKMEEFKDQLPADECNKLKEEISKMRELLARKDSETGENIRQAASSLQQASLKLFEMAYKKMASEREGSGSSGTGEQKEDQKEEKQ

340 NP_009075.1 Q8N6F7

>NP_009075.1

MHRTTRIKITELNPHLMCALCGGYFIDATTIVECLHSFCKTCIVRYLETNKYCPMCDVQVHKTRPLLSIRSDKTLQDIVYKLVPGLFKDEMKRRRDFYAAYPLTEVPNGSNEDRGEVLEQEKGALSDDEIVSLSIEFYEGARDRDEKKGPLENGDGDKEKTGVRFLRCPAAMTVMHLAKFLRNKMDVPSKYKVEVLYEDEPLKEYYTLMDIAYIYPWRRNGPLPLKYRVQPACKRLTLATVPTPSEGTNTSGASECESVSDKAPSPATLPATSSSLPSPATPSHGSPSSHGPPATHPTSPTPPSTASGATTAANGGSLNCLQTPSSTSRGRKMTVNGAPVPPLT

>Q8N6F7

MGNSLLRENRRQQNTQEMPWNVRMQSPKQRTSRCWDHHIAEGCFCLPWKKILIFEKRQDSQNENERMSSTPIQDNVDQTYSEELCYTLINHRVLCTRPSGNSAEEYYENVPCKAERPRESLGGTETEYSLLHMPSTDPRHARSPEDEYELLMPHRISSHFLQQPRPLMAPSETQFSHL

341 NP_003790.1 Q15080

>NP_003790.1

MANSAKAEEYEKMSLEQAKASVNSETESSFNINENTTASGTGLSEKTSVCRQVDIARKRKEFEDDLVKESSSCGKDTPSKKRKLDPEIVPEEKDCGDAEGNSKKRKRETEDVPKDKSSTGDGTQNKRKIALEDVPEKQKNLEEGHSSTVAAHYNELQEVGLEKRSQSRIFYLRNFNNWMKSVLIGEFLEKVRQKKKRDITVLDLGCGKGGDLLKWKKGRINKLVCTDIADVSVKQCQQRYEDMKNRRDSEYIFSAEFITADSSKELLIDKFRDPQMCFDICSCQFVCHYSFESYEQADMMLRNACERLSPGGYFIGTTPNSFELIRRLEASETESFGNEIYTVKFQKKGDYPLFGCKYDFNLEGVVDVPEFLVYFPLLNEMAKKYNMKLVYKKTFLEFYEEKIKNNENKMLLKRMQALEPYPANESSKLVSEKVDDYEHAAKYMKNSQVRLPLGTLSKSEWEATSIYLVFAFEKQQ

>Q15080

MAVAQQLRAESDFEQLPDDVAISANIADIEEKRGFTSHFVFVIEVKTKGGSKYLIYRRYRQFHALQSKLEERFGPDSKSSALACTLPTLPAKVYVGVKQEIAEMRIPALNAYMKSLLSLPVWVLMDEDVRIFFYQSPYDSEQVPQALRRLRPRTRKVKSVSPQGNSVDRMAAPRAEALFDFTGNSKLELNFKAGDVIFLLSRINKDWLEGTVRGATGIFPLSFVKILKDFPEEDDPTNWLRCYYYEDTISTIKDIAVEEDLSSTPLLKDLLELTRREFQREDIALNYRDAEGDLVRLLSDEDVALMVRQARGLPSQKRLFPWKLHITQKDNYRVYNTMP

342 Q6IEG0 P40939

>Q6IEG0

MEGEPPPVEERRRLQEELNEFVESGCRTLEEVTASLGWDLDSLDPGEEEAAEDEVVICPYDSNHHMPKSSLAKHMASCRLRKMGYTKEEEDEMYNPEFFYENVKIPSITLNKDSQFQIIKQARTAVGKDSDCYNQRIYSSLPVEVPLNHKRFVCDLTQADRLALYDFVVEETKKKRSDSQIIENDSDLFVDLAAKINQDNSRKSPKSYLEILAEVRDYKRRRQSYRAKNVHITKKSYTEVIRDVINVHMEELSNHWQEEQEKAEDDAEKNEERRSASVDSRQSGGSYLDAECSRHRRDRSRSPHKRKRNKDKDKNCESRRRKERDGERHHSHKRRKQKI

>P40939

MVACRAIGILSRFSAFRILRSRGYICRNFTGSSALLTRTHINYGVKGDVAVVRINSPNSKVNTLSKELHSEFSEVMNEIWASDQIRSAVLISSKPGCFIAGADINMLAACKTLQEVTQLSQEAQRIVEKLEKSTKPIVAAINGSCLGGGLEVAISCQYRIATKDRKTVLGTPEVLLGALPGAGGTQRLPKMVGVPAALDMMLTGRSIRADRAKKMGLVDQLVEPLGPGLKPPEERTIEYLEEVAITFAKGLADKKISPKRDKGLVEKLTAYAMTIPFVRQQVYKKVEEKVRKQTKGLYPAPLKIIDVVKTGIEQGSDAGYLCESQKFGELVMTKESKALMGLYHGQVLCKKNKFGAPQKDVKHLAILGAGLMGAGIAQVSVDKGLKTILKDATLTALDRGQQQVFKGLNDKVKKKALTSFERDSIFSNLTGQLDYQGFEKADMVIEAVFEDLSLKHRVLKEVEAVIPDHCIFASNTSALPISEIAAVSKRPEKVIGMHYFSPVDKMQLLEIITTEKTSKDTSASAVAVGLKQGKVIIVVKDGPGFYTTRCLAPMMSEVIRILQEGVDPKKLDSLTTSFGFPVGAATLVDEVGVDVAKHVAEDLGKVFGERFGGGNPELLTQMVSKGFLGRKSGKGFYIYQEGVKRKDLNSDMDSILASLKLPPKSEVSSDEDIQFRLVTRFVNEAVMCLQEGILATPAEGDIGAVFGLGFPPCLGGPFRFVDLYGAQKIVDRLKKYEAAYGKQFTPCQLLADHANSPNKKFYQ

343 Q9Y6W3 P50135

>Q9Y6W3

MDATALERDAVQFARLAVQRDHEGRYSEAVFYYKEAAQALIYAEMAGSSLENIQEKITEYLERVQALHSAVQSKSADPLKSKHQLDLERAHFLVTQAFDEDEKENVEDAIELYTEAVDLCLKTSYETADKVLQNKLKQLARQALDRAEALSEPLTKPVGKISSTSVKPKPPPVRAHFPLGANPFLERPQSFISPQSCDAQGQRYTAEEIEVLRTTSKINGIEYVPFMNVDLRERFAYPMPFCDRWGKLPLSPKQKTTFSKWVRPEDLTNNPTMIYTVSSFSIKQTIVSDCSFVASLAISAAYERRFNKKLITGIIYPQNKDGEPEYNPCGKYMVKLHLNGVPRKVIIDDQLPVDHKGELLCSYSNNKSELWVSLIEKAYMKVMGGYDFPGSNSNIDLHALTGWIPERIAMHSDSQTFSKDNSFRMLYQRFHKGDVLITASTGMMTEAEGEKWGLVPTHAYAVLDIREFKGLRFIQLKNPWSHLRWKGRYSENDVKNWTPELQKYLNFDPRTAQKIDNGIFWISWDDLCQYYDVIYLSWNPGLFKESTCIHSTWDAKQGPVKDAYSLANNPQYKLEVQCPQGGAAVWVLLSRHITDKDDFANNREFITMVVYKTDGKKVYYPADPPPYIDGIRINSPHYLTKIKLTTPGTHTFTLVVSQYEKQNTIHYTVRVYSACSFTFSKIPSPYTLSKRINGKWSGQSAGGCGNFQETHKNNPIYQFHIEKTGPLLIELRGPRQYSVGFEVVTVSTLGDPGPHGFLRKSSGDYRCGFCYLELENIPSGIFNIIPSTFLPKQEGPFFLDFNSIIPIKITQLQ

>P50135

MASSMRSLFSDHGKYVESFRRFLNHSTEHQCMQEFMDKKLPGIIGRIGDTKSEIKILSIGGGAGEIDLQILSKVQAQYPGVCINNEVVEPSAEQIAKYKELVAKTSNLENVKFAWHKETSSEYQSRMLEKKELQKWDFIHMIQMLYYVKDIPATLKFFHSLLGTNAKMLIIVVSGSSGWDKLWKKYGSRFPQDDLCQYITSDDLTQMLDNLGLKYECYDLLSTMDISDCFIDGNENGDLLWDFLTETCNFNATAPPDLRAELGKDLQEPEFSAKKEGKVLFNNTLSFIVIEA

344 NP_001805.1 NP_055165.2

>NP_001805.1

MGAGPSLLLAALLLLLSGDGAVRCDTPANCTYLDLLGTWVFQVGSSGSQRDVNCSVMGPQEKKVVVYLQKLDTAYDDLGNSGHFTIIYNQGFEIVLNDYKWFAFFKYKEEGSKVTTYCNETMTGWVHDVLGRNWACFTGKKVGTASENVYVNTAHLKNSQEKYSNRLYKYDHNFVKAINAIQKSWTATTYMEYETLTLGDMIRRSGGHSRKIPRPKPAPLTAEIQQKILHLPTSWDWRNVHGINFVSPVRNQASCGSCYSFASMGMLEARIRILTNNSQTPILSPQEVVSCSQYAQGCEGGFPYLIAGKYAQDFGLVEEACFPYTGTDSPCKMKEDCFRYYSSEYHYVGGFYGGCNEALMKLELVHHGPMAVAFEVYDDFLHYKKGIYHHTGLRDPFNPFELTNHAVLLVGYGTDSASGMDYWIVKNSWGTGWGENGYFRIRRGTDECAIESIAVAATPIPKL

>NP_055165.2

MHSEAEESKEVATDVFNSKNLAVQAQKKILGKMVSKSIATTLIDDTSSEVLDELYRVTREYTQNKKEAEKIIKNLIKTVIKLAILYRNNQFNQDELALMEKFKKKVHQLAMTVVSFHQVDYTFDRNVLSRLLNECREMLHQIIQRHLTAKSHGRVNNVFDHFSDCEFLAALYNPFGNFKPHLQKLCDGINKMLDEENI

345 NP_000452.2 Q99469

>NP_000452.2

MTPNSMTENGLTAWDKPKHCPDREHDWKLVGMSEACLHRKSHSERRSTLKNEQSSPHLIQTTWTSSIFHLDHDDVNDQSVSSAQTFQTEEKKCKGYIPSYLDKDELCVVCGDKATGYHYRCITCEGCKGFFRRTIQKNLHPSYSCKYEGKCVIDKVTRNQCQECRFKKCIYVGMATDLVLDDSKRLAKRKLIEENREKRRREELQKSIGHKPEPTDEEWELIKTVTEAHVATNAQGSHWKQKRKFLPEDIGQAPIVNAPEGGKVDLEAFSHFTKIITPAITRVVDFAKKLPMFCELPCEDQIILLKGCCMEIMSLRAAVRYDPESETLTLNGEMAVTRGQLKNGGLGVVSDAIFDLGMSLSSFNLDDTEVALLQAVLLMSSDRPGLACVERIEKYQDSFLLAFEHYINYRKHHVTHFWPKLLMKVTDLRMIGACHASRFLHMKVECPTELFPPLFLEVFED

>Q99469

MIPPSSPREDGVDGLPKEAVGAEQPPSPASTSSQESKLQKLKRSLSFKTKSLRSKSADNFFQRTNSEDMKLQAHMVAEISPSSSPLPAPGSLTSTPARAGLHPGGKAHAFQEYIFKKPTFCDVCNHMIVGTNAKHGLRCKACKMSIHHKCTDGLAPQRCMGKLPKGFRRYYSSPLLIHEQFGCIKEVMPIACGNKVDPVYETLRFGTSLAQRTKKGSSGSGSDSPHRTSTSDLVEVPEEANGPGGGYDLRKRSNSVFTYPENGTDDFRDPAKNINHQGSLSKDPLQMNTYVALYKFVPQENEDLEMRPGDIITLLEDSNEDWWKGKIQDRIGFFPANFVQRLQQNEKIFRCVRTFIGCKEQGQITLKENQICVSSEEEQDGFIRVLSGKKKGLIPLDVLENI

346 NP_003075.1 NP_110395.1

>NP_003075.1

MAEGSRGGPTCSGVGGRQDPVSGSGGCNFPEYELPELNTRAFHVGAFGELWRGRLRGAGDLSLREPPASALPGSQAADSDREDAAVARDLDCSLEAAAELRAVCGLDKLKCLEDGEDPEVIPENTDLVTLGVRKRFLEHREETITIDRACRQETFVYEMESHAIGKKPENSADMIEEGELILSVNILYPVIFHKHKEHKPYQTMLVLGSQKLTQLRDSIRCVSDLQIGGEFSNTPDQAPEHISKDLYKSAFFYFEGTFYNDKRYPECRDLSRTIIEWSESHDRGYGKFQTARMEDFTFNDLCIKLGFPYLYCHQGDCEHVIVITDIRLVHHDDCLDRTLYPLLIKKHWLWTRKCFVCKMYTARWVTNNDSFAPEDPCFFCDVCFRMLHYDSEGNKLGEFLAYPYVDPGTFN

>NP_110395.1

MDLFGDLPEPERSPRPAAGKEAQKGPLLFDDLPPASSTDSGSGGPLLFDDLPPASSGDSGSLATSISQMVKTEGKGAKRKTSEEEKNGSEELVEKKVCKASSVIFGLKGYVAERKGEREEMQDAHVILNDITEECRPPSSLITRVSYFAVFDGHGGIRASKFAAQNLHQNLIRKFPKGDVISVEKTVKRCLLDTFKHTDEEFLKQASSQKPAWKDGSTATCVLAVDNILYIANLGDSRAILCRYNEESQKHAALSLSKEHNPTQYEERMRIQKAGGNVRDGRVLGVLEVSRSIGDGQYKRCGVTSVPDIRRCQLTPNDRFILLACDGLFKVFTPEEAVNFILSCLEDEKIQTREGKSAADARYEAACNRLANKAVQRGSADNVTVMVVRIGH

347 Q9BSH3 Q8WUN7

>Q9BSH3

MSRVLVPCHVKGSVALQVGDVRTSQGRPGVLVIDVTFPSVAPFELQEITFKNYYTAFLSIRVRQYTSAHTPAKWVTCLRDYCLMPDPHSEEGAQEYVSLFKHQMLCDMARISELRLILRQPSPLWLSFTVEELQIYQQGPKSPSVTFPKWLSHPVPCEQPALLREGLPDPSRVSSEVQQMWALTEMIRASHTSARIGRFDVDGCYDLNLLSYT

>Q8WUN7

MGGCVGAQHDSSGSLNENSEGTGVALGRNQPLKKEKPKWKSDYPMTDGQLRSKRDEFWDTAPAFEGRKEIWDALKAAAHAFESNDHELAQAIIDGANITLPHGALTECYDELGNRYQLPVYCLAPPINMIEEKSDIETLDIPEPPPNSGYECQLRLRLSTGKDLKLVVRSTDTVFHMKRRLHAAEGVEPGSQRWFFSGRPLTDKMKFEELKIPKDYVVQVIVSQPVQNPTPVEN

348 NP_004731.2 NP_000058.1

>NP_004731.2

MSSPSSPFREQSFLCAAGDAGEESRVQVLKNEVRRGSPVLLGWVEQAYADKCVCGPSAPPAPTPPSLSQRVMCNDLFKVNPFQLQQFRADPSTASLLLCPGGLDHKLNLRGKAWG

>NP_000058.1

MSHHWGYGKHNGPEHWHKDFPIAKGERQSPVDIDTHTAKYDPSLKPLSVSYDQATSLRILNNGHAFNVEFDDSQDKAVLKGGPLDGTYRLIQFHFHWGSLDGQGSEHTVDKKKYAAELHLVHWNTKYGDFGKAVQQPDGLAVLGIFLKVGSAKPGLQKVVDVLDSIKTKGKSADFTNFDPRGLLPESLDYWTYPGSLTTPPLLECVTWIVLKEPISVSSEQVLKFRKLNFNGEGEPEELMVDNWRPAQPLKNRQIKASFK

349 NP_004261.1 NP_000058.1

>NP_004261.1

MGEPQQVSALPPPPMQYIKEYTDENIQEGLAPKPPPPIKDSYMMFGNQFQCDDLIIRPLESQGIERLHPMQFDHKKELRKLNMSILINFLDLLDILIRSPGSIKREEKLEDLKLLFVHVHHLINEYRPHQARETLRVMMEVQKRQRLETAERFQKHLERVIEMIQNCLASLPDDLPHSEAGMRVKTEPMDADDSNNCTGQNEHQRENSGHRRDQIIEKDAALCVLIDEMNERP

>NP_000058.1

MSHHWGYGKHNGPEHWHKDFPIAKGERQSPVDIDTHTAKYDPSLKPLSVSYDQATSLRILNNGHAFNVEFDDSQDKAVLKGGPLDGTYRLIQFHFHWGSLDGQGSEHTVDKKKYAAELHLVHWNTKYGDFGKAVQQPDGLAVLGIFLKVGSAKPGLQKVVDVLDSIKTKGKSADFTNFDPRGLLPESLDYWTYPGSLTTPPLLECVTWIVLKEPISVSSEQVLKFRKLNFNGEGEPEELMVDNWRPAQPLKNRQIKASFK

350 NP_061156.1 Q8NFH8

>NP_061156.1

MDSVEKGAATSVSNPRGRPSRGRPPKLQRNSRGGQGRGVEKPPHLAALILARGGSKGIPLKNIKHLAGVPLIGWVLRAALDSGAFQSVWVSTDHDEIENVAKQFGAQVHRRSSEVSKDSSTSLDAIIEFLNYHNEVDIVGNIQATSPCLHPTDLQKVAEMIREEGYDSVFSVVRRHQFRWSEIQKGVREVTEPLNLNPAKRPRRQDWDGELYENGSFYFAKRHLIEMGYLQGGKMAYYEMRAEHSVDIDVDIDWPIAEQRVLRYGYFGKEKLKEIKLLVCNIDGCLTNGHIYVSGDQKEIISYDVKDAIGISLLKKSGIEVRLISERACSKQTLSSLKLDCKMEVSVSDKLAVVDEWRKEMGLCWKEVAYLGNEVSDEECLKRVGLSGAPADACSTAQKAVGYICKCNGGRGAIREFAEHICLLMEKVNNSCQK

>Q8NFH8

MEAAAAAAAAAAAAAAAGGGCGSGPPPLLLSEGEQQCYSELFARCAGAAGGGPGSGPPEAARVAPGTATAAAGPVADLFRASQLPAETLHQITELCGAKRVGYFGPTQFYIALKLIAAAQSGLPVRIESIKCELPLPRFMMSKNDGEIRFGNPAELHGTKVQIPYLTTEKNSFKRMDDEDKQQETQSPTMSPLASPPSSPPHYQRVPLSHGYSKLRSSAEQMHPAPYEARQPLVQPEGSSSGGPGTKPLRHQASLIRSFSVERELQDNSSYPDEPWRITEEQREYYVNQFRSLQPDPSSFISGSVAKNFFTKSKLSIPELSYIWELSDADCDGALTLPEFCAAFHLIVARKNGYPLPEGLPPTLQPEYLQAAFPKPKWDCQLFDSYSESLPANQQPRDLNRMEKTSVKDMADLPVPNQDVTSDDKQALKSTINEALPKDVSEDPATPKDSNSLKARPRSRSYSSTSIEEAMKRGEDPPTPPPRPQKTHSRASSLDLNKVFQPSVPATKSGLLPPPPALPPRPCPSQSEQVSEAELLPQLSRAPSQAAESSPAKKDVLYSQPPSKPIRRKFRPENQATENQEPSTAASGPASAATMKPHPTVQKQSSKQKKAIQTAIRKNKEANAVLARLNSELQQQLKEVHQERIALENQLEQLRPVTVL

351 NP_003098.1 NP_000365.3

>NP_003098.1

MVQQTNNAENTEALLAGESSDSGAGLELGIASSPTPGSTASTGGKADDPSWCKTPSGHIKRPMNAFMVWSQIERRKIMEQSPDMHNAEISKRLGKRWKLLKDSDKIPFIREAERLRLKHMADYPDYKYRPRKKVKSGNANSSSSAAASSKPGEKGDKVGGSGGGGHGGGGGGGSSNAGGGGGGASGGGANSKPAQKKSCGSKVAGGAGGGVSKPHAKLILAGGGGGGKAAAAAAASFAAEQAGAAALLPLGAAADHHSLYKARTPSASASASSAASASAALAAPGKHLAEKKVKRVYLFGGLGTSSSPVGGVGAGADPSDPLGLYEEEGAGCSPDAPSLSGRSSAASSPAAGRSPADHRGYASLRAASPAPSSAPSHASSSASSHSSSSSSSGSSSSDDEFEDDLLDLNPSSNFESMSLGSFSSSSALDRDLDFNFEPGSGSHFEFPDYCTPEVSEMISGDWLESSISNLVFTY

>NP_000365.3

MEANGLGPQGFPELKNDTFLRAAWGEETDYTPVWCMRQAGRYLPEFRETRAAQDFFSTCRSPEACCELTLQPLRRFPLDAAIIFSDILVVPQALGMEVTMVPGKGPSFPEPLREEQDLERLRDPEVVASELGYVFQAITLTRQRLAGRVPLIGFAGAPWTLMTYMVEGGGSSTMAQAKRWLYQRPQASHQLLRILTDALVPYLVGQVVAGAQALQLFESHAGHLGPQLFNKFALPYIRDVAKQVKARLREAGLAPVPMIIFAKDGHFALEELAQAGYEVVGLDWTVAPKKARECVGKTVTLQGNLDPCALYASEEEIGQLVKQMLDDFGPHRYIANLGHGLYPDMDPEHVGAFVDAVHKHSRLLRQN

352 O60264 NP_055683.3

>O60264

MSSAAEPPPPPPPESAPSKPAASIASGGSNSSNKGGPEGVAAQAVASAASAGPADAEMEEIFDDASPGKQKEIQEPDPTYEEKMQTDRANRFEYLLKQTELFAHFIQPAAQKTPTSPLKMKPGRPRIKKDEKQNLLSVGDYRHRRTEQEEDEELLTESSKATNVCTRFEDSPSYVKWGKLRDYQVRGLNWLISLYENGINGILADEMGLGKTLQTISLLGYMKHYRNIPGPHMVLVPKSTLHNWMSEFKRWVPTLRSVCLIGDKEQRAAFVRDVLLPGEWDVCVTSYEMLIKEKSVFKKFNWRYLVIDEAHRIKNEKSKLSEIVREFKTTNRLLLTGTPLQNNLHELWSLLNFLLPDVFNSADDFDSWFDTNNCLGDQKLVERLHMVLRPFLLRRIKADVEKSLPPKKEVKIYVGLSKMQREWYTRILMKDIDILNSAGKMDKMRLLNILMQLRKCCNHPYLFDGAEPGPPYTTDMHLVTNSGKMVVLDKLLPKLKEQGSRVLIFSQMTRVLDILEDYCMWRNYEYCRLDGQTPHDERQDSINAYNEPNSTKFVFMLSTRAGGLGINLATADVVILYDSDWNPQVDLQAMDRAHRIGQTKTVRVFRFITDNTVEERIVERAEMKLRLDSIVIQQGRLVDQNLNKIGKDEMLQMIRHGATHVFASKESEITDEDIDGILERGAKKTAEMNEKLSKMGESSLRNFTMDTESSVYNFEGEDYREKQKIAFTEWIEPPKRERKANYAVDAYFREALRVSEPKAPKAPRPPKQPNVQDFQFFPPRLFELLEKEILFYRKTIGYKVPRNPELPNAAQAQKEEQLKIDEAESLNDEELEEKEKLLTQGFTNWNKRDFNQFIKANEKWGRDDIENIAREVEGKTPEEVIEYSAVFWERCNELQDIEKIMAQIERGEARIQRRISIKKALDTKIGRYKAPFHQLRISYGTNKGKNYTEEEDRFLICMLHKLGFDKENVYDELRQCIRNSPQFRFDWFLKSRTAMELQRRCNTLITLIERENMELEEKEKAEKKKRGPKPSTQKRKMDGAPDGRGRKKKLKL

>NP_055683.3

MPLSSPNAAATASDMDKNSGSNSSSASSGSSKGQQPPRSASAGPAGESKPKSDGKNSSGSKRYNRKRELSYPKNESFNNQSRRSSSQKSKTFNKMPPQRGGGSSKLFSSSFNGGRRDEVAEAQRAEFSPAQFSGPKKINLNHLLNFTFEPRGQTGHFEGSGHGSWGKRNKWGHKPFNKELFLQANCQFVVSEDQDYTAHFADPDTLVNWDFVEQVRICSHEVPSCPICLYPPTAAKITRCGHIFCWACILHYLSLSEKTWSKCPICYSSVHKKDLKSVVATESHQYVVGDTITMQLMKREKGVLVALPKSKWMNVDHPIHLGDEQHSQYSKLLLASKEQVLHRVVLEEKVALEQQLAEEKHTPESCFIEAAIQELKTREEALSGLAGSRREVTGVVAALEQLVLMAPLAKESVFQPRKGVLEYLSAFDEETTEVCSLDTPSRPLALPLVEEEEAVSEPEPEGLPEACDDLELADDNLKEGTICTESSQQEPITKSGFTRLSSSPCYYFYQAEDGQHMFLHPVNVRCLVREYGSLERSPEKISATVVEIAGYSMSEDVRQRHRYLSHLPLTCEFSICELALQPPVVSKETLEMFSDDIEKRKRQRQKKAREERRRERRIEIEENKKQGKYPEVHIPLENLQQFPAFNSYTCSSDSALGPTSTEGHGALSISPLSRSPGSHADFLLTPLSPTASQGSPSFCVGSLEEDSPFPSFAQMLRVGKAKADVWPKTAPKKDENSLVPPAPVDSDGESDNSDRVPVPSFQNSFSQAIEAAFMKLDTPATSDPLSEEKGGKKRKKQKQKLLFSTSVVHTK

353 Q9NZI7 NP_001167.2

>Q9NZI7

MAWVLKMDEVIESGLVHDFDASLSGIGQELGAGAYSMSDVLALPIFKQEDSSLPLDGETEHPPFQYVMCAATSPAVKLHDETLTYLNQGQSYEIRMLDNRKMGDMPEINGKLVKSIIRVVFHDRRLQYTEHQQLEGWKWNRPGDRLLDLDIPMSVGIIDTRTNPSQLNAVEFLWDPAKRTSAFIQVHCISTEFTPRKHGGEKGVPFRIQVDTFKQNENGEYTDHLHSASCQIKVFKPKGADRKQKTDREKMEKRTAHEKEKYQPSYDTTILTEMRLEPIIEDAVEHEQKKSSKRTLPADYGDSLAKRGSCSPWPDAPTAYVNNSPSPAPTFTSPQQSTCSVPDSNSSSPNHQGDGASQTSGEQIQPSATIQETQQWLLKNRFSSYTRLFSNFSGADLLKLTKEDLVQICGAADGIRLYNSLKSRSVRPRLTIYVCREQPSSTVLQGQQQAASSASENGSGAPYVYHAIYLEEMIASEVARKLALVFNIPLHQINQVYRQGPTGIHILVSDQMVQNFQDESCFLFSTVKAESSDGIHIILK

>NP_001167.2

MLGLDACELGAQLLELLRLALCARVLLADKEGGPPAVDEVLDEAVPEYRAPGRKSLLEIRQLDPDDRSLAKYKRVLLGPLPPAVDPSLPNVQVTRLTLLSEQAPGPVVMDLTGDLAVLKDQVFVLKEGVDYRVKISFKVHREIVSGLKCLHHTYRRGLRVDKTVYMVGSYGPSAQEYEFVTPVEEAPRGALVRGPYLVVSLFTDDDRTHHLSWEWGLCICQDWKD

354 NP_005578.1 Q9UGC7

>NP_005578.1

MGRKKIQITRIMDERNRQVTFTKRKFGLMKKAYELSVLCDCEIALIIFNSSNKLFQYASTDMDKVLLKYTEYNEPHESRTNSDIVEALNKKEHRGCDSPDPDTSYVLTPHTEEKYKKINEEFDNMMRNHKIAPGLPPQNFSMSVTVPVTSPNALSYTNPGSSLVSPSLAASSTLTDSSMLSPPQTTLHRNVSPGAPQRPPSTGNAGGMLSTTDLTVPNGAGSSPVGNGFVNSRASPNLIGATGANSLGKVMPTKSPPPPGGGNLGMNSRKPDLRVVIPPSSKGMMPPLSEEEELELNTQRISSSQATQPLATPVVSVTTPSLPPQGLVYSAMPTAYNTDYSLTSADLSALQGFNSPGMLSLGQVSAWQQHHLGQAALSSLVAGGQLSQGSNLSINTNQNISIKSEPISPPRDRMTPSGFQQQQQQQQQQQPPPPPQPQPQPPQPQPRQEMGRSPVDSLSSSSSSYDGSDREDPRGDFHSPIVLGRPPNTEDRESPSVKRMRMDAWVT

>Q9UGC7

MRSRVLWGAARWLWPRRAVGPARRPLSSGSPPLEELFTRGGPLRTFLERQAGSEAHLKVRRPELLAVIKLLNEKERELRETEHLLHDENEDLRKLAENEITLCQKEITQLKHQIILLLVPSEETDENDLILEVTAGVGGQEAMLFTSEIFDMYQQYAAFKRWHFETLEYFPSELGGLRHASASIGGSEAYRHMKFEGGVHRVQRVPKTEKQGRVHTSTMTVAILPQPTEINLVINPKDLRIDTKRASGAGGQHVNTTDSAVRIVHLPTGVVSECQQERSQLKNKELAMTKLRAKLYSMHLEEEINKRQNARKIQIGSKGRSEKIRTYNFPQNRVTDHRINKTLHDLETFMQGDYLLDELVQSLKEYADYESLVEIISQKV

355 P31268 Q9BYD1

>P31268

MSSSYYVNALFSKYTAGTSLFQNAEPTSCSFAPNSQRSGYGAGAGAFASTVPGLYNVNSPLYQSPFASGYGLGADAYGNLPCASYDQNIPGLCSDLAKGACDKTDEGALHGAAEANFRIYPWMRSSGPDRKRGRQTYTRYQTLELEKEFHFNRYLTRRRRIEIAHALCLTERQIKIWFQNRRMKWKKEHKDEGPTAAAAPEGAVPSAAATAAADKADEEDDDEEEEDEEE

>Q9BYD1

MSSFSRAPQQWATFARIWYLLDGKMQPPGKLAAMASIRLQGLHKPVYHALSDCGDHVVIMNTRHIAFSGNKWEQKVYSSHTGYPGGFRQVTAAQLHLRDPVAIVKLAIYGMLPKNLHRRTMMERLHLFPDEYIPEDILKNLVEELPQPRKIPKRLDEYTQEEIDAFPRLWTPPEDYRL

356 NP_003712.1 Q9BYN0

>NP_003712.1

MELTQPAEDLIQTQQTPASELGDPEDPGEEAADGSDTVVLSLFPCTPEPVNPEPDASVSSPQAGSSLKHSTTLTNRQRGNEVSALPATLDSLSIHQLAAQGELDQLKEHLRKGDNLVNKPDERGFTPLIWASAFGEIETVRFLLEWGADPHILAKERESALSLASTGGYTDIVGLLLERDVDINIYDWNGGTPLLYAVRGNHVKCVEALLARGADLTTEADSGYTPMDLAVALGYRKVQQVIENHILKLFQSNLVPADPE

>Q9BYN0

MGLRAGGTLGRAGAGRGAPEGPGPSGGAQGGSIHSGRIAAVHNVPLSVLIRPLPSVLDPAKVQSLVDTIREDPDSVPPIDVLWIKGAQGGDYFYSFGGCHRYAAYQQLQRETIPAKLVQSTLSDLRVYLGASTPDLQ

357 Q3ZCT1 NP_653288.1

>Q3ZCT1

MIGMLESLQHESDLLQHDQIHTGEKPYECNECRKTFSLKQNLVEHKKMHTGEKSHECTECGKVCSRVSSLTLHLRSHTGKKAYKCNKCGKAFSQKENFLSHQKHHTGEKPYECEKVSIQMPTIIRHQKNHTGTKPYACKECGKAFNGKAYLTEHEKIHTGEKPFECNQCGRAFSQKQYLIKHQNIHTGKKPFKCSECGKAFSQKENLIIHQRIHTGEKPYECKGCGKAFIQKSSLIRHQRSHTGEKPYTCKECGKAFSGKSNLTEHEKIHIGEKPYKCNECGTIFRQKQYLIKHHNIHTGEKPYECNKCGKAFSRITSLIVHVRIHTGDKPYECKVCGKAFCQSSSLTVHMRSHTGEKPYGCNECGKAFSQFSTLALHMRIHTGEKPYQCSECGKAFSQKSHHIRHQRIHTH

>NP_653288.1

MLRTAGRDGLCRLSTYLEELEAVELKKFKLYLGTATELGEGKIPWGSMEKAGPLEMAQLLITHFGPEEAWRLALSTFERINRKDLWERGQREDLVRDTPPGGPSSLGNQSTCLLEVSLVTPRKDPQETYRDYVRRKFRLMEDRNARLGECVNLSHRYTRLLLVKEHSNPMQVQQQLLDTGRGHARTVGHQASPIKIETLFEPDEERPEPPRTVVMQGAAGIGKSMLAHKVMLDWADGKLFQGRFDYLFYINCREMNQSATECSMQDLIFSCWPEPSAPLQELIRVPERLLFIIDGFDELKPSFHDPQGPWCLCWEEKRPTELLLNSLIRKKLLPELSLLITTRPTALEKLHRLLEHPRHVEILGFSEAERKEYFYKYFHNAEQAGQVFNYVRDNEPLFTMCFVPLVCWVVCTCLQQQLEGGGLLRQTSRTTTAVYMLYLLSLMQPKPGAPRLQPPPNQRGLCSLAADGLWNQKILFEEQDLRKHGLDGEDVSAFLNMNIFQKDINCERYYSFIHLSFQEFFAAMYYILDEGEGGAGPDQDVTRLLTEYAFSERSFLALTSRFLFGLLNEETRSHLEKSLCWKVSPHIKMDLLQWIQSKAQSDGSTLQQGSLEFFSCLYEIQEEEFIQQALSHFQVIVVSNIASKMEHMVSSFCLKRCRSAQVLHLYGATYSADGEDRARCSAGAHTLLVQLPERTVLLDAYSEHLAAALCTNPNLIELSLYRNALGSRGVKLLCQGLRHPNCKLQNLRLKRCRISSSACEDLSAALIANKNLTRMDLSGNGVGFPGMMLLCEGLRHPQCRLQMIQLRKCQLESGACQEMASVLGTNPHLVELDLTGNALEDLGLRLLCQGLRHPVCRLRTLWLKICRLTAAACDELASTLSVNQSLRELDLSLNELGDLGVLLLCEGLRHPTCKLQTLRLGICRLGSAACEGLSVVLQANHNLRELDLSFNDLGDWGLWLLAEGLQHPACRLQKLWLDSCGLTAKACENLYFTLGINQTLTDLYLTNNALGDTGVRLLCKRLSHPGCKLRVLWLFGMDLNKMTHSRLAALRVTKPYLDIGC

358 Q9NQ33 Q99487

>Q9NQ33

MDNRGNSSLPDKLPIFPDSARLPLTRSFYLEPMVTFHVHPEAPVSSPYSEELPRLPFPSDSLILGNYSEPCPFSFPMPYPNYRGCEYSYGPAFTRKRNERERQRVKCVNEGYAQLRHHLPEEYLEKRLSKVETLRAAIKYINYLQSLLYPDKAETKNNPGKVSSMIATTSHHADPMFRIV

>Q99487

MGVNQSVGFPPVTGPHLVGCGDVMEGQNLQGSFFRLFYPCQKAEETMEQPLWIPRYEYCTGLAEYLQFNKRCGGLLFNLAVGSCRLPVSWNGPFKTKDSGYPLIIFSHGLGAFRTLYSAFCMELASRGFVVAVPEHRDRSAATTYFCKQAPEENQPTNESLQEEWIPFRRVEEGEKEFHVRNPQVHQRVSECLRVLKILQEVTAGQTVFNILPGGLDLMTLKGNIDMSRVAVMGHSFGGATAILALAKETQFRCAVALDAWMFPLERDFYPKARGPVFFINTEKFQTMESVNLMKKICAQHEQSRIITVLGSVHRSQTDFAFVTGNLIGKFFSTETRGSLDPYEGQEVMVRAMLAFLQKHLDLKEDYNQWNNLIEGIGPSLTPGAPHHLSSL

359 Q03923 Q14651

>Q03923

MGPLTFRDVAIEFSLKEWQCLDTAQRNLYRNVMLENYRNLVFLGITVSKPDLITCLEQGKEAWSMKRHEIMVAKPTVMCSHFAQDLWPEQNIKDSFQKVTLKRYGKCRHENLPLRKGCESMDECKMHKGGCNGLNQCLTATQSKIFQCDKYVKVAHKFSNSNRHEIRHTKKKPFKCTKCGKSFGMISCLTEHSRIHTRVNFYKCEECGKAFNWSSTLTKHKRIHTGEKPYKCEECGKAFNQSSNLIKHKKIHTGEKPYKCEECGKTFNRFSTLTTHKIIHTGEKPYKCKECGKAFNRSSTLTTHRKIHTGEKPYKCEECGKAFKQSSNLTTHKIIHTGEKPYKCKKCGKAFNQSAHLTTHEVIHTGEKPYKCEKCGKAFNHFSHLTTHKIIHTGEKPYKCKECGKAFKHSSTLTKHKIIHTGEKPYKCKECEKAFNQSSKLTEHKKIHTGEKPYECEKCGKAFNQSSNLTRHKKSHTEEKPYKCEECGKGFKWPSTLTIHKIIHTGEKPYKCEECGKAFNQSSKLTKHKKIHTGEKPYTCEECGKAFNQSSNLTKHKRIHTGEKPYKCEECDKAFKWSSVLTKHKIIHTGEKLQI

>Q14651

MENSTTTISREELEELQEAFNKIDIDNSGYVSDYELQDLFKEASLPLPGYKVREIVEKILSVADSNKDGKISFEEFVSLMQELKSKDISKTFRKIINKREGITAIGGTSTISSEGTQHSYSEEEKVAFVNWINKALENDPDCKHLIPMNPNDDSLFKSLADGILLCKMINLSEPDTIDERAINKKKLTPFTISENLNLALNSASAIGCTVVNIGASDLKEGKPHLVLGLLWQIIKVGLFADIEISRNEALIALLNEGEELEELMKLSPEELLLRWVNYHLTNAGWHTISNFSQDIKDSRAYFHLLNQIAPKGGEDGPAIAIDLSGINETNDLKRAGLMLQEADKLGCKQFVTPADVVSGNPKLNLAFVANLFNTYPCLHKPNNNDIDMNLLEGESKEERTFRNWMNSLGVNPYINHLYSDLADALVIFQLYEMIRVPVNWSHVNKPPYPALGGNMKKIENCNYAVELGKNKAKFSLVGIAGQDLNEGNSTLTLALVWQLMRRYTLNVLSDLGEGEKVNDEIIIKWVNQTLKSANKKTSISSFKDKSISTSLPVLDLIDAIAPNAVRQEMIRRENLSDEDKLNNAKYAISVARKIGARIYALPDDLVEVKPKMVMTVFACLMGKGLNRIK

360 P49184 Q9HD34

>P49184

MHYPTALLFLILANGAQAFRICAFNAQRLTLAKVAREQVMDTLVRILARCDIMVLQEVVDSSGSAIPLLLRELNRFDGSGPYSTLSSPQLGRSTYMETYVYFYRSHKTQVLSSYVYNDEDDVFAREPFVAQFSLPSNVLPSLVLVPLHTTPKAVEKELNALYDVFLEVSQHWQSKDVILLGDFNADCASLTKKRLDKLELRTEPGFHWVIADGEDTTVRASTHCTYDRVVLHGERCRSLLHTAAAFDFPTSFQLTEEEALNISDHYPVEVELKLSQAHSVQPLSLTVLLLLSLLSPQLCPAA

>Q9HD34

MAASSRAQVLSLYRAMLRESKRFSAYNYRTYAVRRIRDAFRENKNVKDPVEIQTLVNKAKRDLGVIRRQVHIGQLYSTDKLIIENRDMPRT

361 NP_079224.1 NP_061833.1

>NP_079224.1

MSELTKELMELVWGTKSSPGLSDTIFCRWTQGFVFSESEGSALEQFEGGPCAVIAPVQAFLLKKLLFSSEKSSWRDCSEEEQKELLCHTLCDILESACCDHSGSYCLVSWLRGKTTEETASISGSPAESSCQVEHSSALAVEELGFERFHALIQKRSFRSLPELKDAVLDQYSMWGNKFGVLLFLYSVLLTKGIENIKNEIEDASEPLIDPVYGHGSQSLINLLLTGHAVSNVWDGDRECSGMKLLGIHEQAAVGFLTLMEALRYCKVGSYLKSPKFPIWIVGSETHLTVFFAKDMALVAPEAPSEQARRVFQTYDPEDNGFIPDSLLEDVMKALDLVSDPEYINLMKNKLDPEGLGIILLGPFLQEFFPDQGSSGPESFTVYHYNGLKQSNYNEKVMYVEGTAVVMGFEDPMLQTDDTPIKRCLQTKWPYIELLWTTDRSPSLN

>NP_061833.1

MVDSVYRTRSLGVAAEGLPDQYADGEAARVWQLYIGDTRSRTAEYKAWLLGLLRQHGCQRVLDVACGTGVDSIMLVEEGFSVTSVDASDKMLKYALKERWNRRHEPAFDKWVIEEANWMTLDKDVPQSAEGGFDAVICLGNSFAHLPDCKGDQSEHRLALKNIASMVRAGGLLVIDHRNYDHILSTGCAPPGKNIYYKSDLTKDVTTSVLIVNNKAHMVTLDYTVQVPGAGQDGSPGLSKFRLSYYPHCLASFTELLQAAFGGKCQHSVLGDFKPYKPGQTYIPCYFIHVLKRTD

362 NP_000312.2 NP_001866.2

>NP_000312.2

MPPKTPRKTAATAAAAAAEPPAPPPPPPPEEDPEQDSGPEDLPLVRLEFEETEEPDFTALCQKLKIPDHVRERAWLTWEKVSSVDGVLGGYIQKKKELWGICIFIAAVDLDEMSFTFTELQKNIEISVHKFFNLLKEIDTSTKVDNAMSRLLKKYDVLFALFSKLERTCELIYLTQPSSSISTEINSALVLKVSWITFLLAKGEVLQMEDDLVISFQLMLCVLDYFIKLSPPMLLKEPYKTAVIPINGSPRTPRRGQNRSARIAKQLENDTRIIEVLCKEHECNIDEVKNVYFKNFIPFMNSLGLVTSNGLPEVENLSKRYEEIYLKNKDLDARLFLDHDKTLQTDSIDSFETQRTPRKSNLDEEVNVIPPHTPVRTVMNTIQQLMMILNSASDQPSENLISYFNNCTVNPKESILKRVKDIGYIFKEKFAKAVGQGCVEIGSQRYKLGVRLYYRVMESMLKSEEERLSIQNFSKLLNDNIFHMSLLACALEVVMATYSRSTSQNLDSGTDLSFPWILNVLNLKAFDFYKVIESFIKAEGNLTREMIKHLERCEHRIMESLAWLSDSPLFDLIKQSKDREGPTDHLESACPLNLPLQNNHTAADMYLSPVRSPKKKGSTTRVNSTANAETQATSAFQTQKPLKSTSLSLFYKKVYRLAYLRLNTLCERLLSEHPELEHIIWTLFQHTLQNEYELMRDRHLDQIMMCSMYGICKVKNIDLKFKIIVTAYKDLPHAVQETFKRVLIKEEEYDSIIVFYNSVFMQRLKTNILQYASTRPPTLSPIPHIPRSPYKFPSSPLRIPGGNIYISPLKSPYKISEGLPTPTKMTPRSRILVSIGESFGTSEKFQKINQMVCNSDRVLKRSAEGSNPPKPLKKLRFDIEGSDEADGSKHLPGESKFQQKLAEMTSTRTRMQKQKMNDSMDTSNKEEK

>NP_001866.2

MTRILTAFKVVRTLKTGFGFTNVTAHQKWKFSRPGIRLLSVKAQTAHIVLEDGTKMKGYSFGHPSSVAGEVVFNTGLGGYPEAITDPAYKGQILTMANPIIGNGGAPDTTALDELGLSKYLESNGIKVSGLLVLDYSKDYNHWLATKSLGQWLQEEKVPAIYGVDTRMLTKIIRDKGTMLGKIEFEGQPVDFVDPNKQNLIAEVSTKDVKVYGKGNPTKVVAVDCGIKNNVIRLLVKRGAEVHLVPWNHDFTKMEYDGILIAGGPGNPALAEPLIQNVRKILESDRKEPLFGISTGNLITGLAAGAKTYKMSMANRGQNQPVLNITNKQAFITAQNHGYALDNTLPAGWKPLFVNVNDQTNEGIMHESKPFFAVQFHPEVTPGPIDTEYLFDSFFSLIKKGKATTITSVLPKPALVASRVEVSKVLILGSGGLSIGQAGEFDYSGSQAVKAMKEENVKTVLMNPNIASVQTNEVGLKQADTVYFLPITPQFVTEVIKAEQPDGLILGMGGQTALNCGVELFKRGVLKEYGVKVLGTSVESIMATEDRQLFSDKLNEINEKIAPSFAVESIEDALKAADTIGYPVMIRSAYALGGLGSGICPNRETLMDLSTKAFAMTNQILVEKSVTGWKEIEYEVVRDADDNCVTVCNMENVDAMGVHTGDSVVVAPAQTLSNAEFQMLRRTSINVVRHLGIVGECNIQFALHPTSMEYCIIEVNARLSRSSALASKATGYPLAFIAAKIALGIPLPEIKNVVSGKTSACFEPSLDYMVTKIPRWDLDRFHGTSSRIGSSMKSVGEVMAIGRTFEESFQKALRMCHPSIEGFTPRLPMNKEWPSNLDLRKELSEPSSTRIYAIAKAIDDNMSLDEIEKLTYIDKWFLYKMRDILNMEKTLKGLNSESMTEETLKRAKEIGFSDKQISKCLGLTEAQTRELRLKKNIHPWVKQIDTLAAEYPSVTNYLYVTYNGQEHDVNFDDHGMMVLGCGPYHIGSSVEFDWCAVSSIRTLRQLGKKTVVVNCNPETVSTDFDECDKLYFEELSLERILDIYHQEACGGCIISVGGQIPNNLAVPLYKNGVKIMGTSPLQIDRAEDRSIFSAVLDELKVAQAPWKAVNTLNEALEFAKSVDYPCLLRPSYVLSGSAMNVVFSEDEMKKFLEEATRVSQEHPVVLTKFVEGAREVEMDAVGKDGRVISHAISEHVEDAGVHSGDATLMLPTQTISQGAIEKVKDATRKIAKAFAISGPFNVQFLVKGNDVLVIECNLRASRSFPFVSKTLGVDFIDVATKVMIGENVDEKHLPTLDHPIIPADYVAIKAPMFSWPRLRDADPILRCEMASTGEVACFGEGIHTAFLKAMLSTGFKIPQKGILIGIQQSFRPRFLGVAEQLHNEGFKLFATEATSDWLNANNVPATPVAWPSQEGQNPSLSSIRKLIRDGSIDLVINLPNNNTKFVHDNYVIRRTAVDSGIPLLTNFQVTKLFAEAVQKSRKVDSKSLFHYRQYSAGKAA

363 Q5QNW6 NP_002878.2

>Q5QNW6

MPDPAKSAPAPKKGSKKAVTKVQKKDGKKRKRSRKESYSVYVYKVLKQVHPDTGISSKAMGIMNSFVNDIFERIAGEASRLAHYNKRSTITSREIQTAVRLLLPGELAKHAVSEGTKAVTKYTSSK

>NP_002878.2

MDVLVSECSARLLQQEEEIKSLTAEIDRLKNCGCLGASPNLEQLQEENLKLKYRLNILRKSLQAERNKPTKNMINIISRLQEVFGHAIKAAYPDLENPPLLVTPSQQAKFGDYQCNSAMGISQMLKTKEQKVNPREIAENITKHLPDNECIEKVEIAGPGFINVHLRKDFVSEQLTSLLVNGVQLPALGENKKVIVDFSSPNIAKEMHVGHLRSTIIGESISRLFEFAGYDVLRLNHVGDWGTQFGMLIAHLQDKFPDYLTVSPPIGDLQVFYKESKKRFDTEEEFKKRAYQCVVLLQGKNPDITKAWKLICDVSRQELNKIYDALDVSLIERGESFYQDRMNDIVKEFEDRGFVQVDDGRKIVFVPGCSIPLTIVKSDGGYTYDTSDLAAIKQRLFEEKADMIIYVVDNGQSVHFQTIFAAAQMIGWYDPKVTRVFHAGFGVVLGEDKKKFKTRSGETVRLMDLLGEGLKRSMDKLKEKERDKVLTAEELNAAQTSVAYGCIKYADLSHNRLNDYIFSFDKMLDDRGNTAAYLLYAFTRIRSIARLANIDEEMLQKAARETKILLDHEKEWKLGRCILRFPEILQKILDDLFLHTLCDYIYELATAFTEFYDSCYCVEKDRQTGKILKVNMWRMLLCEAVAAVMAKGFDILGIKPVQRM

364 Q9UJC5 Q96Q11

>Q9UJC5

MVIRVFIASSSGFVAIKKKQQDVVRFLEANKIEFEEVDITMSEEQRQWMYKNVPPEKKPTQGNPLPPQIFNGDRYCGDYDSFFESKESNTVFSFLGLKPRLASKAEP

>Q96Q11

MLRCLYHWHRPVLNRRWSRLCLLKQYLFTMKLQSPEFQSLFTEGLKSLTELFVKENHELRIAGGAVRDLLNGVKPQDIDFATTATPTQMKEMFQSAGIRMINNRGEKHGTITARLHEENFEITTLRIDVTTDGRHAEVEFTTDWQKDAERRDLTINSMFLGFDGTLFDYFNGYEDLKNKKVRFVGHAKQRIQEDYLRILRYFRFYGRIVDKPGDHDPETLEAIAENAKGLAGISGERIWVELKKILVGNHVNHLIHLIYDLDVAPYIGLPANASLEEFDKVSKNVDGFSPKPVTLLASLFKVQDDVTKLDLRLKIAKEEKNLGLFIVKNRKDLIKATDSSDPLKPYQDFIIDSREPDATTRVCELLKYQGEHCLLKEMQQWSIPPFPVSGHDIRKVGISSGKEIGALLQQLREQWKKSGYQMEKDELLSYIKKT

365 NP_001385.1 O00142

>NP_001385.1

MVTMEELREMDCSVLKRLMNRDENGGGAGGSGSHGTLGLPSGGKCLLLDCRPFLAHSAGYILGSVNVRCNTIVRRRAKGSVSLEQILPAEEEVRARLRSGLYSAVIVYDERSPRAESLREDSTVSLVVQALRRNAERTDICLLKGGYERFSSEYPEFCSKTKALAAIPPPVPPSATEPLDLGCSSCGTPLHDQGGPVEILPFLYLGSAYHAARRDMLDALGITALLNVSSDCPNHFEGHYQYKCIPVEDNHKADISSWFMEAIEYIDAVKDCRGRVLVHCQAGISRSATICLAYLMMKKRVRLEEAFEFVKQRRSIISPNFSFMGQLLQFESQVLATSCAAEAASPSGPLRERGKTPATPTSQFVFSFPVSVGVHSAPSSLPYLHSPITTSPSC

>O00142

MLLWPLRGWAARALRCFGPGSRGSPASGPGPRRVQRRAWPPDKEQEKEKKSVICVEGNIASGKTTCLEFFSNATDVEVLTEPVSKWRNVRGHNPLGLMYHDASRWGLTLQTYVQLTMLDRHTRPQVSSVRLMERSIHSARYIFVENLYRSGKMPEVDYVVLSEWFDWILRNMDVSVDLIVYLRTNPETCYQRLKKRCREEEKVIPLEYLEAIHHLHEEWLIKGSLFPMAAPVLVIEADHHMERMLELFEQNRDRILTPENRKHCP

366 NP_003963.1 NP_000839.1

>NP_003963.1

MAVSRLDRLFILLDTGTTPVTRKAAAQQLGEVVKLHPHELNNLLSKVLIYLRSANWDTRIAAGQAVEAIVKNVPEWNPVPRTRQEPTSESSMEDSPTTERLNFDRFDICRLLQHGASLLGSAGAEFEVQDEKSGEVDPKERIARQRKLLQKKLGLNMGEAIGMSTEELFNDEDLDYTPTSASFVNKQPTLQAAELIDSEFRAGMSNRQKNKAKRMAKLFAKQRSRDAVETNEKSNDSTDGEPEEKRRKIANVVINQSANDSKVLIDNIPDSSSLIEETNEWPLESFCEELCNDLFNPSWEVRHGAGTGLREILKAHGKSGGKMGDSTLEEMIQQHQEWLEDLVIRLLCVFALDRFGDFVSDEVVAPVRETCAQTLGVVLKHMNETGVHKTVDVLLKLLTQEQWEVRHGGLLGIKYALAVRQDVINTLLPKVLTRIIEGLQDLDDDVRAVAAASLVPVVESLVYLQTQKVPFIINTLWDALLELDDLTASTNSIMTLLSSLLTYPQVQQCSIQQSLTVLVPRVWPFLHHTISSVRRAALETLFTLLSTQDQNSSSWLIPILPDMLRHIFQFCVLESSQEILDLIHKVWMELLSKASVQYVVAAACPWMGAWLCLMMQPSHLPIDLNMLLEVKARAKEKTGGKVRQGQSQNKEVLQEYIAGADTIMEDPATRDFVVMRARMMAAKLLGALCCCICDPGVNVVTQEIKPAESLGQLLLFHLNSKSALQRISVALVICEWAALQKECKAVTLAVQPRLLDILSEHLYYDEIAVPFTRMQNECKQLISSLADVHIEVGNRVNNNVLTIDQASDLVTTVFNEATSSFDLNPQVLQQLDSKRQQVQMTVTETNQEWQVLQLRVHTFAACAVVSLQQLPEKLNPIIKPLMETIKKEENTLVQNYAAQCIAKLLQQCTTRTPCPNSKIIKNLCSSLCVDPYLTPCVTCPVPTQSGQENSKGSTSEKDGMHHTVTKHRGIITLYRHQKAAFAITSRRGPTPKAVKAQIADLPAGSSGNILVELDEAQKPYLVQRRGAEFALTTIVKHFGGEMAVKLPHLWDAMVGPLRNTIDINNFDGKSLLDKGDSPAQELVNSLQVFETAAASMDSELHPLLVQHLPHLYMCLQYPSTAVRHMAARCVGVMSKIATMETMNIFLEKVLPWLGAIDDSVKQEGAIEALACVMEQLDVGIVPYIVLLVVPVLGRMSDQTDSVRFMATQCFATLIRLMPLEAGIPDPPNMSAELIQLKAKERHFLEQLLDGKKLENYKIPVPINAELRKYQQDGVNWLAFLNKYKLHGILCDDMGLGKTLQSICILAGDHCHRAQEYARSKLAECMPLPSLVVCPPTLTGHWVDEVGKFCSREYLNPLHYTGPPTERIRLQHQVKRHNLIVASYDVVRNDIDFFRNIKFNYCILDEGHVIKNGKTKLSKAVKQLTANYRIILSGTPIQNNVLELWSLFDFLMPGFLGTERQFAARYGKPILASRDARSSSREQEAGVLAMDALHRQVLPFLLRRMKEDVLQDLPPKIIQDYYCTLSPLQVQLYEDFAKSRAKCDVDETVSSATLSEETEKPKLKATGHVFQALQYLRKLCNHPALVLTPQHPEFKTTAEKLAVQNSSLHDIQHAPKLSALKQLLLDCGLGNGSTSESGTESVVAQHRILIFCQLKSMLDIVEHDLLKPHLPSVTYLRLDGSIPPGQRHSIVSRFNNDPSIDVLLLTTHVGGLGLNLTGADTVVFVEHDWNPMRDLQAMDRAHRIGQKRVVNVYRLITRGTLEEKIMGLQKFKMNIANTVISQENSSLQSMGTDQLLDLFTLDKDGKAEKADTSTSGKASMKSILENLSDLWDQEQYDSEYSLENFMHSLK

>NP_000839.1

MPMTLGYWNIRGLAHSIRLLLEYTDSSYEEKKYTMGDAPDYDRSQWLNEKFKLGLDFPNLPYLIDGTHKITQSNAILRYIARKHNLCGESEKEQIREDILENQFMDSRMQLAKLCYDPDFEKLKPEYLQALPEMLKLYSQFLGKQPWFLGDKITFVDFIAYDVLERNQVFEPSCLDAFPNLKDFISRFEGLEKISAYMKSSRFLPRPVFTKMAVWGNK

367 Q8TE85 NP_006576.2

>Q8TE85

MSNELDFRSVRLLKNDPVNLQKFSYTSEDEAWKTYLENPLTAATKAMMRVNGDDDSVAALSFLYDYYMGPKEKRILSSSTGGRNDQGKRYYHGMEYETDLTPLESPTHLMKFLTENVSGTPEYPDLLKKNNLMSLEGALPTPGKAAPLPAGPSKLEAGSVDSYLLPTTDMYDNGSLNSLFESIHGVPPTQRWQPDSTFKDDPQESMLFPDILKTSPEPPCPEDYPSLKSDFEYTLGSPKAIHIKSGESPMAYLNKGQFYPVTLRTPAGGKGLALSSNKVKSVVMVVFDNEKVPVEQLRFWKHWHSRQPTAKQRVIDVADCKENFNTVEHIEEVAYNALSFVWNVNEEAKVFIGVNCLSTDFSSQKGVKGVPLNLQIDTYDCGLGTERLVHRAVCQIKIFCDKGAERKMRDDERKQFRRKVKCPDSSNSGVKGCLLSGFRGNETTYLRPETDLETPPVLFIPNVHFSSLQRSGGAAPSAGPSSSNRLPLKRTCSPFTEEFEPLPSKQAKEGDLQRVLLYVRRETEEVFDALMLKTPDLKGLRNAISEKYGFPEENIYKVYKKCKRGETSLLHPRLSRHPPPDCLECSHPVTQVRNMGFGDGFWRQRDLDSNPSPTTVNSLHFTVNSE

>NP_006576.2

MALHVPKAPGFAQMLKEGAKHFSGLEEAVYRNIQACKELAQTTRTAYGPNGMNKMVINHLEKLFVTNDAATILRELEVQHPAAKMIVMASHMQEQEVGDGTNFVLVFAGALLELAEELLRIGLSVSEVIEGYEIACRKAHEILPNLVCCSAKNLRDIDEVSSLLRTSIMSKQYGNEVFLAKLIAQACVSIFPDSGHFNVDNIRVCKILGSGISSSSVLHGMVFKKETEGDVTSVKDAKIAVYSCPFDGMITETKGTVLIKTAEELMNFSKGEENLMDAQVKAIADTGANVVVTGGKVADMALHYANKYNIMLVRLNSKWDLRRLCKTVGATALPRLTPPVLEEMGHCDSVYLSEVGDTQVVVFKHEKEDGAISTIVLRGSTDNLMDDIERAVDDGVNTFKVLTRDKRLVPGGGATEIELAKQITSYGETCPGLEQYAIKKFAEAFEAIPRALAENSGVKANEVISKLYAVHQEGNKNVGLDIEAEVPAVKDMLEAGILDTYLGKYWAIKLATNAAVTVLRVDQIIMAKPAGGPKPPSGKKDWDDDQND

368 NP_002008.2 Q6P1L8

>NP_002008.2

MDGTIKEALSVVSDDQSLFDSAYGAAAHLPKADMTASGSPDYGQPHKINPLPPQQEWINQPVRVNVKREYDHMNGSRESPVDCSVSKCSKLVGGGESNPMNYNSYMDEKNGPPPPNMTTNERRVIVPADPTLWTQEHVRQWLEWAIKEYSLMEIDTSFFQNMDGKELCKMNKEDFLRATTLYNTEVLLSHLSYLRESSLLAYNTTSHTDQSSRLSVKEDPSYDSVRRGAWGNNMNSGLNKSPPLGGAQTISKNTEQRPQPDPYQILGPTSSRLANPGSGQIQLWQFLLELLSDSANASCITWEGTNGEFKMTDPDEVARRWGERKSKPNMNYDKLSRALRYYYDKNIMTKVHGKRYAYKFDFHGIAQALQPHPTESSMYKYPSDISYMPSYHAHQQKVNFVPPHPSSMPVTSSSFFGAASQYWTSPTGGIYPNPNVPRHPNTHVPSHLGSYY

>Q6P1L8

MAFFTGLWGPFTCVSRVLSHHCFSTTGSLSAIQKMTRVRVVDNSALGNSPYHRAPRCIHVYKKNGVGKVGDQILLAIKGQKKKALIVGHCMPGPRMTPRFDSNNVVLIEDNGNPVGTRIKTPIPTSLRKREGEYSKVLAIAQNFV

369 NP_001659.1 NP_000895.1

>NP_001659.1

MAATTANPEMTSDVPSLGPAIASGNSGPGIQGGGAIVQRAIKRRPGLDFDDDGEGNSKFLRCDDDQMSNDKERFARSDDEQSSADKERLARENHSEIERRRRNKMTAYITELSDMVPTCSALARKPDKLTILRMAVSHMKSLRGTGNTSTDGSYKPSFLTDQELKHLILEAADGFLFIVSCETGRVVYVSDSVTPVLNQPQSEWFGSTLYDQVHPDDVDKLREQLSTSENALTGRILDLKTGTVKKEGQQSSMRMCMGSRRSFICRMRCGSSSVDPVSVNRLSFVRNRCRNGLGSVKDGEPHFVVVHCTGYIKAWPPAGVSLPDDDPEAGQGSKFCLVAIGRLQVTSSPNCTDMSNVCQPTEFISRHNIEGIFTFVDHRCVATVGYQPQELLGKNIVEFCHPEDQQLLRDSFQQVVKLKGQVLSVMFRFRSKNQEWLWMRTSSFTFQNPYSDEIEYIICTNTNVKNSSQEPRPTLSNTIQRPQLGPTANLPLEMGSGQLAPRQQQQQTELDMVPGRDGLASYNHSQVVQPVTTTGPEHSKPLEKSDGLFAQDRDPRFSEIYHNINADQSKGISSSTVPATQQLFSQGNTFPPTPRPAENFRNSGLAPPVTIVQPSASAGQMLAQISRHSNPTQGATPTWTPTTRSGFSAQQVATQATAKTRTSQFGVGSFQTPSSFSSMSLPGAPTASPGAAAYPSLTNRGSNFAPETGQTAGQFQTRTAEGVGVWPQWQGQQPHHRSSSSEQHVQQPPAQQPGQPEVFQEMLSMLGDQSNSYNNEEFPDLTMFPPFSE

>NP_000895.1

MAGKKVLIVYAHQEPKSFNGSLKNVAVDELSRQGCTVTVSDLYAMNFEPRATDKDITGTLSNPEVFNYGVETHEAYKQRSLASDITDEQKKVREADLVIFQFPLYWFSVPAILKGWMDRVLCQGFAFDIPGFYDSGLLQGKLALLSVTTGGTAEMYTKTGVNGDSRYFLWPLQHGTLHFCGFKVLAPQISFAPEIASEEERKGMVAAWSQRLQTIWKEEPIPCTAHWHFGQ

370 NP_003207.1 Q16719

>NP_003207.1

MSDAGGGKKPPVDPQAGPGPGPGRAAGERGLSGSFPLVLKKLMENPPREARLDKEKGKEKLEEDEAAAASTMAVSASLMPPIWDKTIPYDGESFHLEYMDLDEFLLENGIPASPTHLAHNLLLPVAELEGKESASSSTASPPSSSTAIFQPSETVSSTESSLEKERETPSPIDPNCVEVDVNFNPDPADLVLSSVPGGELFNPRKHKFAEEDLKPQPMIKKAKKVFVPDEQKDEKYWTRRKKNNVAAKRSRDARRLKENQITIRAAFLEKENTALRTEVAELRKEVGKCKTIVSKYETKYGPL

>Q16719

MEPSSLELPADTVQRIAAELKCHPTDERVALHLDEEDKLRHFRECFYIPKIQDLPPVDLSLVNKDENAIYFLGNSLGLQPKMVKTYLEEELDKWAKIAAYGHEVGKRPWITGDESIVGLMKDIVGANEKEIALMNALTVNLHLLMLSFFKPTPKRYKILLEAKAFPSDHYAIESQLQLHGLNIEESMRMIKPREGEETLRIEDILEVIEKEGDSIAVILFSGVHFYTGQHFNIPAITKAGQAKGCYVGFDLAHAVGNVELYLHDWGVDFACWCSYKYLNAGAGGIAGAFIHEKHAHTIKPALVGWFGHELSTRFKMDNKLQLIPGVCGFRISNPPILLVCSLHASLEIFKQATMKALRKKSVLLTGYLEYLIKHNYGKDKAATKKPVVNIITPSHVEERGCQLTITFSVPNKDVFQELEKRGVVCDKRNPNGIRVAPVPLYNSFHDVYKFTNLLTSILDSAETKN

371 Q15562 NP_003360.2

>Q15562

MGEPRAGAALDDGSGWTGSEEGSEEGTGGSEGAGGDGGPDAEGVWSPDIEQSFQEALAIYPPCGRRKIILSDEGKMYGRNELIARYIKLRTGKTRTRKQVSSHIQVLARRKSREIQSKLKDQVSKDKAFQTMATMSSAQLISAPSLQAKLGPTGPQASELFQFWSGGSGPPWNVPDVKPFSQTPFTLSLTPPSTDLPGYEPPQALSPLPPPTPSPPAWQARGLGTARLQLVEFSAFVEPPDAVDSYQRHLFVHISQHCPSPGAPPLESVDVRQIYDKFPEKKGGLRELYDRGPPHAFFLVKFWADLNWGPSGEEAGAGGSISSGGFYGVSSQYESLEHMTLTCSSKVCSFGKQVVEKVETERAQLEDGRFVYRLLRSPMCEYLVNFLHKLRQLPERYMMNSVLENFTILQVVTNRDTQELLLCTAYVFEVSTSERGAQHHIYRLVRD

>NP_003360.2

MSASASVGGPVPQPPPGPAAALPPGSAARALHVELPSQQRRLRHLRNIAARNIVNRNGHQLLDTYFTLHLCSTEKIYKEFYRSEVIKNSLNPTWRSLDFGIMPDRLDTSVSCFVVKIWGGKENIYQLLIEWKVCLDGLKYLGQQIHARNQNEIIFGLNDGYYGAPFEHKGYSNAQKTILLQVDQNCVRNSYDVFSLLRLHRAQCAIKQTQVTVQKIGKEIEEKLRLTSTSNELKKKSECLQLKILVLQNELERQKKALGREVALLHKQQIALQDKGSAFSAEHLKLQLQKESLNELRKECTAKRELFLKTNAQLTIRCRQLLSELSYIYPIDLNEHKDYFVCGVKLPNSEDFQAKDDGSIAVALGYTAHLVSMISFFLQVPLRYPIIHKGSRSTIKDNINDKLTEKEREFPLYPKGGEKLQFDYGVYLLNKNIAQLRYQHGLGTPDLRQTLPNLKNFMEHGLMVRCDRHHTSSAIPVPKRQSSIFGGADVGFSGGIPSPDKGHRKRASSENERLQYKTPPPSYNSALAQPVTTVPSMGETERKITSLSSSLDTSLDFSKENKKKGEDLVGSLNGGHANVHPSQEQGEALSGHRATVNGTLLPSEQAGSASVQLPGEFHPVSEAELCCTVEQAEEIIGLEATGFASGDQLEAFNCIPVDSAVAVECDEQVLGEFEEFSRRIYALNENVSSFRRPRRSSDK

372 Q9GZP8 O75251

>Q9GZP8

MEFDLGAALEPTSQKPGVGAGHGGDPKLSPHKVQGRSEAGAGPGPKQGHHSSSDSSSSSSDSDTDVKSHAAGSKQHESIPGKAKKPKVKKKEKGKKEKGKKKEAPH

>O75251

MAVLSAPGLRGFRILGLRSSVGPAVQARGVHQSVATDGPSSTQPALPKARAVAPKPSSRGEYVVAKLDDLVNWARRSSLWPMTFGLACCAVEMMHMAAPRYDMDRFGVVFRASPRQSDVMIVAGTLTNKMAPALRKVYDQMPEPRYVVSMGSCANGGGYYHYSYSVVRGCDRIVPVDIYIPGCPPTAEALLYGILQLQRKIKRERRLQIWYRR

373 Q12946 NP_004865.1

>Q12946

MSSAPEKQQPPHGGGGGGGGGGGAAMDPASSGPSKAKKTNAGIRRPEKPPYSYIALIVMAIQSSPTKRLTLSEIYQFLQSRFPFFRGSYQGWKNSVRHNLSLNECFIKLPKGLGRPGKGHYWTIDPASEFMFEEGSFRRRPRGFRRKCQALKPMYSMMNGLGFNHLPDTYGFQGSAGGLSCPPNSLALEGGLGMMNGHLPGNVDGMALPSHSVPHLPSNGGHSYMGGCGGAAAGEYPHHDSSVPASPLLPTGAGGVMEPHAVYSGSAAAWPPSASAALNSGASYIKQQPLSPCNPAANPLSGSLSTHSLEQPYLHQNSHNAPAELQGIPRYHSQSPSMCDRKEFVFSFNAMASSSMHSAGGGSYYHQQVTYQDIKPCVM

>NP_004865.1

MSALRRSGYGPSDGPSYGRYYGPGGGDVPVHPPPPLYPLRPEPPQPPISWRVRGGGPAETTWLGEGGGGDGYYPSGGAWPEPGRAGGSHQEQPPYPSYNSNYWNSTARSRAPYPSTYPVRPELQGQSLNSYTNGAYGPTYPPGPGANTASYSGAYYAPGYTQTSYSTEVPSTYRSSGNSPTPVSRWIYPQQDCQTEAPPLRGQVPGYPPSQNPGMTLPHYPYGDGNRSVPQSGPTVRPQEDAWASPGAYGMGGRYPWPSSAPSAPPGNLYMTESTSPWPSSGSPQSPPSPPVQQPKDSSYPYSQSDQSMNRHNFPCSVHQYESSGTVNNDDSDLLDSQVQYSAEPQLYGNATSDHPNNQDQSSSLPEECVPSDESTPPSIKKIIHVLEKVQYLEQEVEEFVGKKTDKAYWLLEEMLTKELLELDSVETGGQDSVRQARKEAVCKIQAILEKLEKKGL

374 NP_004716.1 Q8N573

>NP_004716.1

MTGSNEFKLNQPPEDGISSVKFSPNTSQFLLVSSWDTSVRLYDVPANSMRLKYQHTGAVLDCAFYDPTHAWSGGLDHQLKMHDLNTDQENLVGTHDAPIRCVEYCPEVNVMVTGSWDQTVKLWDPRTPCNAGTFSQPEKVYTLSVSGDRLIVGTAGRRVLVWDLRNMGYVQQRRESSLKYQTRCIRAFPNKQGYVLSSIEGRVAVEYLDPSPEVQKKKYAFKCHRLKENNIEQIYPVNAISFHNIHNTFATGGSDGFVNIWDPFNKKRLCQFHRYPTSIASLAFSNDGTTLAIASSYMYEMDDTEHPEDGIFIRQVTDAETKPKSPCT

>Q8N573

MSFQKPKGTIEYTVESRDSLNSIALKFDTTPNELVQLNKLFSRAVVTGQVLYVPDPEYVSSVESSPSLSPVSPLSPTSSEAEFDKTTNPDVHPTEATPSSTFTGIRPARVVSSTSEEEEAFTEKFLKINCKYITSGKGTVSGVLLVTPNNIMFDPHKNDPLVQENGCEEYGIMCPMEEVMSAAMYKEILDSKIKESLPIDIDQLSGRDFCHSKKMTGSNTEEIDSRIRDAGNDSASTAPRSTEESLSEDVFTESELSPIREELVSSDELRQDKSSGASSESVQTVNQAEVESLTVKSESTGTPGHLRSDTEHSTNEVGTLCHKTDLNNLEMAIKEDQIADNFQGISGPKEDSTSIKGNSDQDSFLHENSLHQEESQKENMPCGETAEFKQKQSVNKGKQGKEQNQDSQTEAEELRKLWKTHTMQQTKQQRENIQQVSQKEAKHKITSADGHIESSALLKEKQRHRLHKFLCLRVGKPMRKTFVSQASATMQQYAQRDKKHEYWFAVPQERTDHLYAFFIQWSPEIYAEDTGEYTREPGFIVVKKIEESETIEDSSNQAAAREWEITTREDINSKQVATVKADLESESFRPNLSDPSELLLPDQIEKLTKHLPPRTIGYPWTLVYGTGKHGTSLKTLYRTMTGLDTPVLMVIKDSDGQVFGALASEPLKVSDGFYGTGETFVFTFCPEFEVFKWTGDNMFFIKGDMDSLAFGGGGGEFALWLDGDLYHGRSHSCKTFGNRTLSKKEDFFIQDIEIWAFE

375 NP_004069.1 NP_004790.2

>NP_004069.1

MPNWGGGKKCGVCQKTVYFAEEVQCEGNSFHKSCFLCMVCKKNLDSTTVAVHGEEIYCKSCYGKKYGPKGYGYGQGAGTLSTDKGESLGIKHEEAPGHRPTTNPNASKFAQKIGGSERCPRCSQAVYAAEKVIGAGKSWHKACFRCAKCGKGLESTTLADKDGEIYCKGCYAKNFGPKGFGFGQGAGALVHSE

>NP_004790.2

MENYFQAEAYNLDKVLDEFEQNEDETVSSTLLDTKWNKILDPPSHRLSFNPTLASVNESAVSNESQPQLKVFSLAHSAPLTTEEEDHCANGQDCNLNPEIATMWIDENAVAEDQLIKRNYSWDDQCSAVEVGEKKCGNLACLPDEKNVLVVAVMHNCDKRTLQNDLQDCNNYNSQSLMDAFSCSLDNENRQTDQFSFSINESTEKDMNSEKQMDPLNRPKTEGRSVNHLCPTSSDSLASVCSPSQLKDDGSIGRDPSMSAITSLTVDSVISSQGTDGCPAVKKQENYIPDEDLTGKISSPRTDLGSPNSFSHMSEGILMKKEPAEESTTEESLRSGLPLLLKPDMPNGSGRNNDCERCSDCLVPNEVRADENEGYEHEETLGTTEFLNMTEHFSESQDMTNWKLTKLNEMNDSQVNEEKEKFLQISQPEDTNGDSGGQCVGLADAGLDLKGTCISESEECDFSTVIDTPAANYLSNGCDSYGMQDPGVSFVPKTLPSKEDSVTEEKEIEESKSECYSNIYEQRGNEATEGSGLLLNSTGDLMKKNYLHNFCSQVPSVLGQSSPKVVASLPSISVPFGGARPKQPSNLKLQIPKPLSDHLQNDFPANSGNNTKNKNDILGKAKLGENSATNVCSPSLGNISNVDTNGEHLESYEAEISTRPCLALAPDSPDNDLRAGQFGISARKPFTTLGEVAPVWVPDSQAPNCMKCEARFTFTKRRHHCRACGKVFCASCCSLKCKLLYMDRKEARVCVICHSVLMNAQAWENMMSASSQSPNPNNPAEYCSTIPPLQQAQASGALSSPPPTVMVPVGVLKHPGAEVAQPREQRRVWFADGILPNGEVADAAKLTMNGTSSAGTLAVSHDPVKPVTTSPLPAETDICLFSGSITQVGSPVGSAMNLIPEDGLPPILISTGVKGDYAVEEKPSQISVMQQLEDGGPDPLVFVLNANLLSMVKIVNYVNRKCWCFTTKGMHAVGQSEIVILLQCLPDEKCLPKDIFNHFVQLYRDALAGNVVSNLGHSFFSQSFLGSKEHGGFLYVTSTYQSLQDLVLPTPPYLFGILIQKWETPWAKVFPIRLMLRLGAEYRLYPCPLFSVRFRKPLFGETGHTIMNLLADFRNYQYTLPVVQGLVVDMEVRKTSIKIPSNRYNEMMKAMNKSNEHVLAGGACFNEKADSHLVCVQNDDGNYQTQAISIHNQPRKVTGASFFVFSGALKSSSGYLAKSSIVEDGVMVQITAENMDSLRQALREMKDFTITCGKADAEEPQEHIHIQWVDDDKNVSKGVVSPIDGKSMETITNVKIFHGSEYKANGKVIRWTEVFFLENDDQHNCLSDPADHSRLTEHVAKAFCLALCPHLKLLKEDGMTKLGLRVTLDSDQVGYQAGSNGQPLPSQYMNDLDSALVPVIHGGACQLSEGPVVMELIFYILENIV

376 NP_006345.1 Q9H2W6

>NP_006345.1

MSELKDCPLQFHDFKSVDHLKVCPRYTAVLARSEDDGIGIEELDTLQLELETLLSSASRRLRVLEAETQILTDWQDKKGDRRFLKLGRDHELGAPPKHGKPKKQKLEGKAGHGPGPGPGRPKSKNLQPKIQEYEFTDDPIDVPRIPKNDAPNRFWASVEPYCADITSEEVRTLEELLKPPEDEAEHYKIPPLGKHYSQRWAQEDLLEEQKDGARAAAVADKKKGLMGPLTELDTKDVDALLKKSEAQHEQPEDGCPFGALTQRLLQALVEENIISPMEDSPIPDMSGKESGADGASTSPRNQNKPFSVPHTKSLESRIKEELIAQGLLESEDRPAEDSEDEVLAELRKRQAELKALSAHNRTKKHDLLRLAKEEVSRQELRQRVRMADNEVMDAFRKIMAARQKKRTPTKKEKDQAWKTLKERESILKLLDG

>Q9H2W6

MAAPVRRTLLGVAGGWRRFERLWAGSLSSRSLALAAAPSSNGSPWRLLGALCLQRPPVVSKPLTPLQEEMASLLQQIEIERSLYSDHELRALDENQRLAKKKADLHDEEDEQDILLAQDLEDMWEQKFLQFKLGARITEADEKNDRTSLNRKLDRNLVLLVREKFGDQDVWILPQAEWQPGETLRGTAERTLATLSENNMEAKFLGNAPCGHYTFKFPQAMRTESNLGAKVFFFKALLLTGDFSQAGNKGHHVWVTKDELGDYLKPKYLAQVRRFVSDL

377 NP_000240.1 Q8WYK0

>NP_000240.1

MSFVAGVIRRLDETVVNRIAAGEVIQRPANAIKEMIENCLDAKSTSIQVIVKEGGLKLIQIQDNGTGIRKEDLDIVCERFTTSKLQSFEDLASISTYGFRGEALASISHVAHVTITTKTADGKCAYRASYSDGKLKAPPKPCAGNQGTQITVEDLFYNIATRRKALKNPSEEYGKILEVVGRYSVHNAGISFSVKKQGETVADVRTLPNASTVDNIRSIFGNAVSRELIEIGCEDKTLAFKMNGYISNANYSVKKCIFLLFINHRLVESTSLRKAIETVYAAYLPKNTHPFLYLSLEISPQNVDVNVHPTKHEVHFLHEESILERVQQHIESKLLGSNSSRMYFTQTLLPGLAGPSGEMVKSTTSLTSSSTSGSSDKVYAHQMVRTDSREQKLDAFLQPLSKPLSSQPQAIVTEDKTDISSGRARQQDEEMLELPAPAEVAAKNQSLEGDTTKGTSEMSEKRGPTSSNPRKRHREDSDVEMVEDDSRKEMTAACTPRRRIINLTSVLSLQEEINEQGHEVLREMLHNHSFVGCVNPQWALAQHQTKLYLLNTTKLSEELFYQILIYDFANFGVLRLSEPAPLFDLAMLALDSPESGWTEEDGPKEGLAEYIVEFLKKKAEMLADYFSLEIDEEGNLIGLPLLIDNYVPPLEGLPIFILRLATEVNWDEEKECFESLSKECAMFYSIRKQYISEESTLSGQQSEVPGSIPNSWKWTVEHIVYKALRSHILPPKHFTEDGNILQLANLPDLYKVFERC

>Q8WYK0

MERPAPGEVVMSQAIQPAHATARGELSAGQLLKWIDTTACLAAEKHAGVSCVTASVDDIQFEETARVGQVITIKAKVTRAFSTSMEISIKVMVQDMLTGIEKLVSVAFSTFVAKPVGKEKIHLKPVTLLTEQDHVEHNLAAERRKVRLQHEDTFNNLMKESSKFDDLIFDEEEGAVSTRGTSVQSIELVLPPHANHHGNTFGGQIMAWMETVATISASRLCWAHPFLKSVDMFKFRGPSTVGDRLVFTAIVNNTFQTCVEVGVRVEAFDCQEWAEGRGRHINSAFLIYNAADDKENLITFPRIQPISKDDFRRYRGAIARKRIRLGRKYVISHKEEVPLCIHWDISKQASLSDSNVEALKKLAAKRGWEVTSTVEKIKIYTLEEHDVLSVWVEKHVGSPAHLAYRLLSDFTKRPLWDPHFVSCEVIDWVSEDDQLYHITCPILNDDKPKDLVVLVSRRKPLKDGNTYTVAVKSVILPSVPPSPQYIRSEIICAGFLIHAIDSNSCIVSYFNHMSASILPYFAGNLGGWSKSIEETAASCIQFLENPPDDGFVSTF

378 NP_060353.2 NP_002930.2

>NP_060353.2

MAAVWQQVLAVDARYNAYRTPTFPQFRTQYIRRRSQLLRENAKAGHPPALRRQYLRLRGQLLGQRYGPLSEPGSARAYSNSIVRSSRTTLDRMEDFEDDPRALGARGHRRSVSRGSYQLQAQMNRAVYEDRPPGSVVPTSAAEASRAMAGDTSLSENYAFAGMYHVFDQHVDEAVPRVRFANDDRHRLACCSLDGSISLCQLVPAPPTVLRVLRGHTRGVSDFAWSLSNDILVSTSLDATMRIWASEDGRCIREIPDPDSAELLCCTFQPVNNNLTVVGNAKHNVHVMNISTGKKVKGGSSKLTGRVLALSFDAPGRLLWAGDDRGSVFSFLFDMATGKLTKAKRLVVHEGSPVTSISARSWVSREARDPSLLINACLNKLLLYRVVDNEGTLQLKRSFPIEQSSHPVRSIFCPLMSFRQGACVVTGSEDMCVHFFDVERAAKAAVNKLQGHSAPVLDVSFNCDESLLASSDASGMVIVWRREQK

>NP_002930.2

MSLDIQSLDIQCEELSDARWAELLPLLQQCQVVRLDDCGLTEARCKDISSALRVNPALAELNLRSNELGDVGVHCVLQGLQTPSCKIQKLSLQNCCLTGAGCGVLSSTLRTLPTLQELHLSDNLLGDAGLQLLCEGLLDPQCRLEKLQLEYCSLSAASCEPLASVLRAKPDFKELTVSNNDINEAGVRVLCQGLKDSPCQLEALKLESCGVTSDNCRDLCGIVASKASLRELALGSNKLGDVGMAELCPGLLHPSSRLRTLWIWECGITAKGCGDLCRVLRAKESLKELSLAGNELGDEGARLLCETLLEPGCQLESLWVKSCSFTAACCSHFSSVLAQNRFLLELQISNNRLEDAGVRELCQGLGQPGSVLRVLWLADCDVSDSSCSSLAATLLANHSLRELDLSNNCLGDAGILQLVESVRQPGCLLEQLVLYDIYWSEEMEDRLQALEKDKPSLRVIS

379 P28715 P07203

>P28715

MGVQGLWKLLECSGRQVSPEALEGKILAVDISIWLNQALKGVRDRHGNSIENPHLLTLFHRLCKLLFFRIRPIFVFDGDAPLLKKQTLVKRRQRKDLASSDSRKTTEKLLKTFLKRQAIKTAFRSKRDEALPSLTQVRRENDLYVLPPLQEEEKHSSEEEDEKEWQERMNQKQALQEEFFHNPQAIDIESEDFSSLPPEVKHEILTDMKEFTKRRRTLFEAMPEESDDFSQYQLKGLLKKNYLNQHIEHVQKEMNQQHSGHIRRQYEDEGGFLKEVESRRVVSEDTSHYILIKGIQAKTVAEVDSESLPSSSKMHGMSFDVKSSPCEKLKTEKEPDATPPSPRTLLAMQAALLGSSSEEELESENRRQARGRNAPAAVDEGSISPRTLSAIKRALDDDEDVKVCAGDDVQTGGPGAEEMRINSSTENSDEGLKVRDGKGIPFTATLASSSVNSAEEHVASTNEGREPTDSVPKEQMSLVHVGTEAFPISDESMIKDRKDRLPLESAVVRHSDAPGLPNGRELTPASPTCTNSVSKNETHAEVLEQQNELCPYESKFDSSLLSSDDETKCKPNSASEVIGPVSLQETSSIVSVPSEAVDNVENVVSFNAKEHENFLETIQEQQTTESAGQDLISIPKAVEPMEIDSEESESDGSFIEVQSVISDEELQAEFPETSKPPSEQGEEELVGTREGEAPAESESLLRDNSERDDVDGEPQEAEKDAEDSLHEWQDINLEELETLESNLLAQQNSLKAQKQQQERIAATVTGQMFLESQELLRLFGIPYIQAPMEAEAQCAILDLTDQTSGTITDDSDIWLFGARHVYRNFFNKNKFVEYYQYVDFHNQLGLDRNKLINLAYLLGSDYTEGIPTVGCVTAMEILNEFPGHGLEPLLKFSEWWHEAQKNPKIRPNPHDTKVKKKLRTLQLTPGFPNPAVAEAYLKPVVDDSKGSFLWGKPDLDKIREFCQRYFGWNRTKTDESLFPVLKQLDAQQTQLRIDSFFRLAQQEKEDAKRIKSQRLNRAVTCMLRKEKEAAASEIEAVSVAMEKEFELLDKAKGKTQKRGITNTLEESSSLKRKRLSDSKGKNTCGGFLGETCLSESSDGSSSEDAESSSLMNVQRRTAAKEPKTSASDSQNSVKEAPVKNGGATTSSSSDSDDDGGKEKMVLVTARSVFGKKRRKLRRARGRKRKT

>P07203

MCAARLAAAAAQSVYAFSARPLAGGEPVSLGSLRGKVLLIENVASLUGTTVRDYTQMNELQRRLGPRGLVVLGFPCNQFGHQENAKNEEILNSLKYVRPGGGFEPNFMLFEKCEVNGAGAHPLFAFLREALPAPSDDATALMTDPKLITWSPVCRNDVAWNFEKFLVGPDGVPLRRYSRRFQTIDIEPDIEALLSQGPSCA

380 Q12857 Q16698

>Q12857

MYSPLCLTQDEFHPFIEALLPHVRAFAYTWFNLQARKRKYFKKHEKRMSKEEERAVKDELLSEKPEVKQKWASRLLAKLRKDIRPEYREDFVLTVTGKKPPCCVLSNPDQKGKMRRIDCLRQADKVWRLDLVMVILFKGIPLESTDGERLVKSPQCSNPGLCVQPHHIGVSVKELDLYLAYFVHAADSSQSESPSQPSDADIKDQPENGHLGFQDSFVTSGVFSVTELVRVSQTPIAAGTGPNFSLSDLESSSYYSMSPGAMRRSLPSTSSTSSTKRLKSVEDEMDSPGEEPFYTGQGRSPGSGSQSSGWHEVEPGMPSPTTLKKSEKSGFSSPSPSQTSSLGTAFTQHHRPVITGPRASPHATPSTLHFPTSPIIQQPGPYFSHPAIRYHPQETLKEFVQLVCPDAGQQAGQVGFLNPNGSSQGKVHNPFLPTPMLPPPPPPPMARPVPLPVPDTKPPTTSTEGGAASPTSPTYSTPSTSPANRFVSVGPRDPSFVNIPQQTQSWYLG

>Q16698

MKLPARVFFTLGSRLPCGLAPRRFFSYGTKILYQNTEALQSKFFSPLQKAMLPPNSFQGKVAFITGGGTGLGKGMTTLLSSLGAQCVIASRKMDVLKATAEQISSQTGNKVHAIQCDVRDPDMVQNTVSELIKVAGHPNIVINNAAGNFISPTERLSPNAWKTITDIVLNGTAFVTLEIGKQLIKAQKGAAFLSITTIYAETGSGFVVPSASAKAGVEAMSKSLAAEWGKYGMRFNVIQPGPIKTKGAFSRLDPTGTFEKEMIGRIPCGRLGTVEELANLAAFLCSDYASWINGAVIKFDGGEEVLISGEFNDLRKVTKEQWDTIEELIRKTKGS

381 Q9UNY5 NP_075066.1

>Q9UNY5

MAVSLTAAETLALQGTQGQEKMMMMGPKEEEQSCEYETRLPGNHSTSQEIFRQRFRHLRYQETPGPREALSQLRVLCCEWLRPEKHTKEQILEFLVLEQFLTILPEELQSWVRGHHPKSGEEAVTVLEDLEKGLEPEPQVPGPAHGPAQEEPWEKKESLGAAQEALSIQLQPKETQPFPKSEQVYLHFLSVVTEDGPEPKDKGSLPQPPITEVESQVFSEKLATDTSTFEATSEGTLELQQRNPKAERLRWSPAQEESFRQMVVIHKEIPTGKKDHECSECGKTFIYNSHLVVHQRVHSGEKPYKCSDCGKTFKQSSNLGQHQRIHTGEKPFECNECGKAFRWGAHLVQHQRIHSGEKPYECNECGKAFSQSSYLSQHRRIHSGEKPFICKECGKAYGWCSELIRHRRVHARKEPSH

>NP_075066.1

MASGLVRLLQQGHRCLLAPVAPKLVPPVRGVKKGFRAAFRFQKELERQRLLRCPPPPVRRSEKPNWDYHAEIQAFGHRLQENFSLDLLKTAFVNSCYIKSEEAKRQQLGIEKEAVLLNLKSNQELSEQGTSFSQTCLTQFLEDEYPDMPTEGIKNLVDFLTGEEVVCHVARNLAVEQLTLSEEFPVPPAVLQQTFFAVIGALLQSSGPERTALFIRDFLITQMTGKELFEMWKIINPMGLLVEELKKRNVSAPESRLTRQSGGTTALPLYFVGLYCDKKLIAEGPGETVLVAEEEAARVALRKLYGFTENRRPWNYSKPKETLRAEKSITAS

382 NP_003457.1 NP_006432.1

>NP_003457.1

MPHNSIRSGHGGLNQLGGAFVNGRPLPEVVRQRIVDLAHQGVRPCDISRQLRVSHGCVSKILGRYYETGSIRPGVIGGSKPKVATPKVVEKIGDYKRQNPTMFAWEIRDRLLAEGVCDNDTVPSVSSINRIIRTKVQQPFNLPMDSCVATKSLSPGHTLIPSSAVTPPESPQSDSLGSTYSINGLLGIAQPGSDKRKMDDSDQDSCRLSIDSQSSSSGPRKHLRTDAFSQHHLEPLECPFERQHYPEAYASPSHTKGEQGLYPLPLLNSTLDDGKATLTPSNTPLGRNLSTHQTYPVVADPHSPFAIKQETPEVSSSSSTPSSLSSSAFLDLQQVGSGVPPFNAFPHAASVYGQFTGQALLSGREMVGPTLPGYPPHIPTSGQGSYASSAIAGMVAGSEYSGNAYGHTPYSSYSEAWRFPNSSLLSSPYYYSSTSRPSAPPTTATAFDHL

>NP_006432.1

MAAAAVSSAKRSLRGELKQRLRAMSAEERLRQSRVLSQKVIAHSEYQKSKRISIFLSMQDEIETEEIIKDIFQRGKICFIPRYRFQSNHMDMVRIESPEEISLLPKTSWNIPQPGEGDVREEALSTGGLDLIFMPGLGFDKHGNRLGRGKGYYDAYLKRCLQHQEVKPYTLALAFKEQICLQVPVNENDMKVDEVLYEDSSTA

383 NP_002137.4 P22531

>NP_002137.4

MQKATYYDNAAAALFGGYSSYPGSNGFGFDVPPQPPFQAATHLEGDYQRSACSLQSLGNAAPHAKSKELNGSCMRPGLAPEPLSAPPGSPPPSAAPTSATSNSSNGGGPSKSGPPKCGPGTNSTLTKQIFPWMKESRQTSKLKNNSPGTAEGCGGGGGGGGGGGSGGSGGGGGGGGGGDKSPPGSAASKRARTAYTSAQLVELEKEFHFNRYLCRPRRVEMANLLNLSERQIKIWFQNRRMKYKKDQKAKGLASSSGGPSPAGSPPQPMQSTAGFMNALHSMTPSYESPSPPAFGKAHQNAYALPSNYQPPLKGCGAPQKYPPTPAPEYEPHVLQANGGAYGTPTMQGSPVYVGGGGYADPLPPPAGPSLYGLNHLSHHPSGNLDYNGAPPMAPSQHHGPCEPHPTYTDLSSHHAPPPQGRIQEAPKLTHL

>P22531

MSYQQQQCKQPCQPPPVCPTPKCPEPCPPPKCPEPCPPPKCPQPCPPQQCQQKCPPVTPSPPCQPKCPPKSK

384 NP_003160.2 NP_005908.1

>NP_003160.2

MSDSEDSNFSEEEDSERSSDGEEAEVDEERRSAAGSEKEEEPEDEEEEEEEEEYDEEEEEEDDDRPPKKPRHGGFILDEADVDDEYEDEDQWEDGAEDILEKEEIEASNIDNVVLDEDRSGARRLQNLWRDQREEELGEYYMKKYAKSSVGETVYGGSDELSDDITQQQLLPGVKDPNLWTVKCKIGEERATAISLMRKFIAYQFTDTPLQIKSVVAPEHVKGYIYVEAYKQTHVKQAIEGVGNLRLGYWNQQMVPIKEMTDVLKVVKEVANLKPKSWVRLKRGIYKDDIAQVDYVEPSQNTISLKMIPRIDYDRIKARMSLKDWFAKRKKFKRPPQRLFDAEKIRSLGGDVASDGDFLIFEGNRYSRKGFLFKSFAMSAVITEGVKPTLSELEKFEDQPEGIDLEVVTESTGKEREHNFQPGDNVEVCEGELINLQGKILSVDGNKITIMPKHEDLKDMLEFPAQELRKYFKMGDHVKVIAGRFEGDTGLIVRVEENFVILFSDLTMHELKVLPRDLQLCSETASGVDVGGQHEWGELVQLDPQTVGVIVRLERETFQVLNMYGKVVTVRHQAVTRKKDNRFAVALDSEQNNIHVKDIVKVIDGPHSGREGEIRHLFRSFAFLHCKKLVENGGMFVCKTRHLVLAGGSKPRDVTNFTVGGFAPMSPRISSPMHPSAGGQRGGFGSPGGGSGGMSRGRGRRDNELIGQTVRISQGPYKGYIGVVKDATESTARVELHSTCQTISVDRQRLTTVGSRRPGGMTSTYGRTPMYGSQTPMYGSGSRTPMYGSQTPLQDGSRTPHYGSQTPLHDGSRTPAQSGAWDPNNPNTPSRAEEEYEYAFDDEPTPSPQAYGGTPNPQTPGYPDPSSPQVNPQYNPQTPGTPAMYNTDQFSPYAAPSPQGSYQPSPSPQSYHQVAPSPAGYQNTHSPASYHPTPSPMAYQASPSPSPVGYSPMTPGAPSPGGYNPHTPGSGIEQNSSDWVTTDIQVKVRDTYLDTQVVGQTGVIRSVTGGMCSVYLKDSEKVVSISSEHLEPITPTKNNKVKVILGEDREATGVLLSIDGEDGIVRMDLDEQLKILNLRFLGKLLEA

>NP_005908.1

MSEPIRVLVTGAAGQIAYSLLYSIGNGSVFGKDQPIILVLLDITPMMGVLDGVLMELQDCALPLLKDVIATDKEDVAFKDLDVAILVGSMPRREGMERKDLLKANVKIFKSQGAALDKYAKKSVKVIVVGNPANTNCLTASKSAPSIPKENFSCLTRLDHNRAKAQIALKLGVTANDVKNVIIWGNHSSTQYPDVNHAKVKLQGKEVGVYEALKDDSWLKGEFVTTVQQRGAAVIKARKLSSAMSAAKAICDHVRDIWFGTPEGEFVSMGVISDGNSYGVPDDLLYSFPVVIKNKTWKFVEGLPINDFSREKMDLTAKELTEEKESAFEFLSSA

385 NP_055596.3 Q6P1L8

>NP_055596.3

MKLYVFLVNTGTTLTFDTELTVQTVADLKHAIQSKYKIAIQHQVLVVNGGECMAADRRVCTYSAGTDTNPIFLFNKEMILCDRPPAIPKTTFSTENDMEIKVEESLMMPAVFHTVASRTQLALEMYEVAKKLCSFCEGLVHDEHLQHQGWAAIMANLEDCSNSYQKLLFKFESIYSNYLQSIEDIKLKLTHLGTAVSVMAKIPLLECLTRHSYRECLGRLDSLPEHEDSEKAEMKRSTELVLSPDMPRTTNESLLTSFPKSVEHVSPDTADAESGKEIRESCQSTVHQQDETTIDTKDGDLPFFNVSLLDWINVQDRPNDVESLVRKCFDSMSRLDPRIIRPFIAECRQTIAKLDNQNMKAIKGLEDRLYALDQMIASCGRLVNEQKELAQGFLANQKRAENLKDASVLPDLCLSHANQLMIMLQNHRKLLDIKQKCTTAKQELANNLHVRLKWCCFVMLHADQDGEKLQALLRLVIELLERVKIVEALSTVPQMYCLAVVEVVRRKMFIKHYREWAGALVKDGKRLYEAEKSKRESFGKLFRKSFLRNRLFRGLDSWPPSFCTQKPRKFDCELPDISLKDLQFLQSFCPSEVQPFLRVPLLCDFEPLHQHVLALHNLVKAAQSLDEMSQTITDLLSEQKASVSQTSPQSASSPRMESTAGITTTTSPRTPPPLTVQDPLCPAVCPLEELSPDSIDAHTFDFETIPHPNIEQTIHQVSLDLDSLAESPESDFMSAVNEFVIEENLSSPNPISDPQSPEMMVESLYSSVINAIDSRRMQDTNVCGKEDFGDHTSLNVQLERCRVVAQDSHFSIQTIKEDLCHFRTFVQKEQCDFSNSLKCTAVEIRNIIEKVKCSLEITLKEKHQKELLSLKNEYEGKLDGLIKETEENENKIKKLKGELVCLEEVLQNKDNEFALVKHEKEAVICLQNEKDQKLLEMENIMHSQNCEIKELKQSREIVLEDLKKLHVENDEKLQLLRAELQSLEQSHLKELEDTLQVRHIQEFEKVMTDHRVSLEELKKENQQIINQIQESHAEIIQEKEKQLQELKLKVSDLSDTRCKLEVELALKEAETDEIKILLEESRAQQKETLKSLLEQETENLRTEISKLNQKIQDNNENYQVGLAELRTLMTIEKDQCISELISRHEEESNILKAELNKVTSLHNQAFEIEKNLKEQIIELQSKLDSELSALERQKDEKITQQEEKYEAIIQNLEKDRQKLVSSQEQDREQLIQKLNCEKDEAIQTALKEFKLEREVVEKELLEKVKHLENQIAKSPAIDSTRGDSSSLVAELQEKLQEEKAKFLEQLEEQEKRKNEEMQNVRTSLIAEQQTNFNTVLTREKMRKENIINDLSDKLKSTMQQQERDKDLIESLSEDRARLLEEKKKLEEEVSKLRSSSFVPSPYVATAPELYGACAPELPGESDRSAVETADEGRVDSAMETSMMSVQENIHMLSEEKQRIMLLERTLQLKEEENKRLNQRLMSQSMSSVSSRHSEKIAIRDFQVGDLVLIILDERHDNYVLFTVSPTLYFLHSESLPALDLKPGEGASGASRRPWVLGKVMEKEYCQAKKAQNRFKVPLGTKFYRVKAVSWNKKV

>Q6P1L8

MAFFTGLWGPFTCVSRVLSHHCFSTTGSLSAIQKMTRVRVVDNSALGNSPYHRAPRCIHVYKKNGVGKVGDQILLAIKGQKKKALIVGHCMPGPRMTPRFDSNNVVLIEDNGNPVGTRIKTPIPTSLRKREGEYSKVLAIAQNFV

386 Q9Y6W3 O94788

>Q9Y6W3

MDATALERDAVQFARLAVQRDHEGRYSEAVFYYKEAAQALIYAEMAGSSLENIQEKITEYLERVQALHSAVQSKSADPLKSKHQLDLERAHFLVTQAFDEDEKENVEDAIELYTEAVDLCLKTSYETADKVLQNKLKQLARQALDRAEALSEPLTKPVGKISSTSVKPKPPPVRAHFPLGANPFLERPQSFISPQSCDAQGQRYTAEEIEVLRTTSKINGIEYVPFMNVDLRERFAYPMPFCDRWGKLPLSPKQKTTFSKWVRPEDLTNNPTMIYTVSSFSIKQTIVSDCSFVASLAISAAYERRFNKKLITGIIYPQNKDGEPEYNPCGKYMVKLHLNGVPRKVIIDDQLPVDHKGELLCSYSNNKSELWVSLIEKAYMKVMGGYDFPGSNSNIDLHALTGWIPERIAMHSDSQTFSKDNSFRMLYQRFHKGDVLITASTGMMTEAEGEKWGLVPTHAYAVLDIREFKGLRFIQLKNPWSHLRWKGRYSENDVKNWTPELQKYLNFDPRTAQKIDNGIFWISWDDLCQYYDVIYLSWNPGLFKESTCIHSTWDAKQGPVKDAYSLANNPQYKLEVQCPQGGAAVWVLLSRHITDKDDFANNREFITMVVYKTDGKKVYYPADPPPYIDGIRINSPHYLTKIKLTTPGTHTFTLVVSQYEKQNTIHYTVRVYSACSFTFSKIPSPYTLSKRINGKWSGQSAGGCGNFQETHKNNPIYQFHIEKTGPLLIELRGPRQYSVGFEVVTVSTLGDPGPHGFLRKSSGDYRCGFCYLELENIPSGIFNIIPSTFLPKQEGPFFLDFNSIIPIKITQLQ

>O94788

MTSSKIEMPGEVKADPAALMASLHLLPSPTPNLEIKYTKIFINNEWQNSESGRVFPVYNPATGEQVCEVQEADKADIDKAVQAARLAFSLGSVWRRMDASERGRLLDKLADLVERDRAVLATMESLNGGKPFLQAFYVDLQGVIKTFRYYAGWADKIHGMTIPVDGDYFTFTRHEPIGVCGQIIPWNFPLLMFAWKIAPALCCGNTVVIKPAEQTPLSALYMGALIKEAGFPPGVINILPGYGPTAGAAIASHIGIDKIAFTGSTEVGKLIQEAAGRSNLKRVTLELGGKSPNIIFADADLDYAVEQAHQGVFFNQGQCCTAGSRIFVEESIYEEFVRRSVERAKRRVVGSPFDPTTEQGPQIDKKQYNKILELIQSGVAEGAKLECGGKGLGRKGFFIEPTVFSNVTDDMRIAKEEIFGPVQEILRFKTMDEVIERANNSDFGLVAAVFTNDINKALTVSSAMQAGTVWINCYNALNAQSPFGGFKMSGNGREMGEFGLREYSEVKTVTVKIPQKNS

387 NP_036449.1 NP_057330.2

>NP_036449.1

MVDELVLLLHALLMRHRALSIENSQLMEQLRLLVCERASLLRQVRPPSCPVPFPETFNGESSRLPEFIVQTASYMLVNENRFCNDAMKVAFLISLLTGEAEEWVVPYIEMDSPILGDYRAFLDEMKQCFGWDDDEDDDDEEEEDDY

>NP_057330.2

MATGTRYAGKVVVVTGGGRGIGAGIVRAFVNSGARVVICDKDESGGRALEQELPGAVFILCDVTQEDDVKTLVSETIRRFGRLDCVVNNAGHHPPPQRPEETSAQGFRQLLELNLLGTYTLTKLALPYLRKSQGNVINISSLVGAIGQAQAVPYVATKGAVTAMTKALALDESPYGVRVNCISPGNIWTPLWEELAALMPDPRATIREGMLAQPLGRMGQPAEVGAAAVFLASEANFCTGIELLVTGGAELGYGCKASRSTPVDAPDIPS

388 Q9HAZ2 Q9HD34

>Q9HAZ2

MRSKARARKLAKSDGDVVNNMYEPNRDLLASHSAEDEAEDSAMSPIPVGPPSPFPTSEDFTPKEGSPYEAPVYIPEDIPIPADFELRESSIPGAGLGVWAKRKMEAGERLGPCVVVPRAAAKETDFGWEQILTDVEVSPQEGCITKISEDLGSEKFCVDANQAGAGSWLKYIRVACSCDDQNLTMCQISEQIYYKVIKDIEPGEELLVHVKEGVYPLGTVPPGLDEEPTFRCDECDELFQSKLDLRRHKKYTCGSVGAALYEGLAEELKPEGLGGGSGQAHECKDCERMFPNKYSLEQHMVIHTEEREYKCDQCPKAFNWKSNLIRHQMSHDSGKRFECENCVKVFTDPSNLQRHIRSQHVGARAHACPDCGKTFATSSGLKQHKHIHSTVKPFICEVCHKSYTQFSNLCRHKRMHADCRTQIKCKDCGQMFSTTSSLNKHRRFCEGKNHYTPGGIFAPGLPLTPSPMMDKAKPSPSLNHASLGFNEYFPSRPHPGSLPFSTAPPTFPALTPGFPGIFPPSLYPRPPLLPPTSLLKSPLNHTQDAKLPSPLGNPALPLVSAVSNSSQGTTAAAGPEEKFESRLEDSCVEKLKTRSSDMSDGSDFEDVNTTTGTDLDTTTGTGSDLDSDVDSDPDKDKGKGKSAEGQPKFGGGLAPPGAPNSVAEVPVFYSQHSFFPPPDEQLLTATGAAGDSIKAIASIAEKYFGPGFMGMQEKKLGSLPYHSAFPFQFLPNFPHSLYPFTDRALAHNLLVKAEPKSPRDALKVGGPSAECPFDLTTKPKDVKPILPMPKGPSAPASGEEQPLDLSIGSRARASQNGGGREPRKNHVYGERKLGAGEGLPQVCPARMPQQPPLHYAKPSPFFMDPIYSRVEKRKVTDPVGALKEKYLRPSPLLFHPQMSAIETMTEKLESFAAMKADSGSSLQPLPHHPFNFRSPPPTLSDPILRKGKERYTCRYCGKIFPRSANLTRHLRTHTGEQPYRCKYCDRSFSISSNLQRHVRNIHNKEKPFKCHLCNRCFGQQTNLDRHLKKHEHENAPVSQHPGVLTNHLGTSASSPTSESDNHALLDEKEDSYFSEIRNFIANSEMNQASTRTEKRADMQIVDGSAQCPGLASEKQEDVEEEDDDDLEEDDEDSLAGKSQDDTVSPAPEPQAAYEDEEDEEPAASLAVGFDHTRRCAEDHEGGLLALEPMPTFGKGLDLRRAAEEAFEVKDVLNSTLDSEALKHTLCRQAKNQAYAMMLSLSEDTPLHTPSQGSLDAWLKVTGATSESGAFHPINHL

>Q9HD34

MAASSRAQVLSLYRAMLRESKRFSAYNYRTYAVRRIRDAFRENKNVKDPVEIQTLVNKAKRDLGVIRRQVHIGQLYSTDKLIIENRDMPRT

389 Q9NPE2 P78368

>Q9NPE2

MEAPGAPPRTLTWEAMEQIRYLHEEFPESWSVPRLAEGFDVSTDVIRRVLKSKFLPTLEQKLKQDQKVLKKAGLAHSLQHLRGSGNTSKLLPAGHSVSGSLLMPGHEASSKDPNHSTALKVIESDTHRTNTPRRRKGRNKEIQDLEESFVPVAAPLGHPRELQKYSSDSESPRGTGSGALPSGQKLEELKAEEPDNFSSKVVQRGREFFDSNGNFLYRI

>P78368

MDFDKKGGKGETEEGRRMSKAGGGRSSHGIRSSGTSSGVLMVGPNFRVGKKIGCGNFGELRLGKNLYTNEYVAIKLEPIKSRAPQLHLEYRFYKQLSATEGVPQVYYFGPCGKYNAMVLELLGPSLEDLFDLCDRTFTLKTVLMIAIQLITRMEYVHTKSLIYRDVKPENFLVGRPGTKRQHAIHIIDFGLAKEYIDPETKKHIPYREHKSLTGTARYMSINTHLGKEQSRRDDLEALGHMFMYFLRGSLPWQGLKADTLKERYQKIGDTKRATPIEVLCENFPEEMATYLRYVRRLDFFEKPDYDYLRKLFTDLFDRSGFVFDYEYDWAGKPLPTPIGTVHTDLPSQPQLRDKTQPHSKNQALNSTNGELNADDPTAGHSNAPITAPAEVEVADETKCCCFFKRRKRKSLQRHK

390 NP_006222.2 NP_000958.1

>NP_006222.2

MSLRSGGRRRADPGADGEASRDDGATSSVSALKRLERSQWTDKMDLRFGFERLKEPGEKTGWLINMHPTEILDEDKRLGSAVDYYFIQDDGSRFKVALPYKPYFYIATRKGCEREVSSFLSKKFQGKIAKVETVPKEDLDLPNHLVGLKRNYIRLSFHTVEDLVKVRKEISPAVKKNREQDHASDAYTALLSSVLQRGGVITDEEETSKKIADQLDNIVDMREYDVPYHIRLSIDLKIHVAHWYNVRYRGNAFPVEITRRDDLVERPDPVVLAFDIETTKLPLKFPDAETDQIMMISYMIDGQGYLITNREIVSEDIEDFEFTPKPEYEGPFCVFNEPDEAHLIQRWFEHVQETKPTIMVTYNGDFFDWPFVEARAAVHGLSMQQEIGFQKDSQGEYKAPQCIHMDCLRWVKRDSYLPVGSHNLKAAAKAKLGYDPVELDPEDMCRMATEQPQTLATYSVSDAVATYYLYMKYVHPFIFALCTIIPMEPDEVLRKGSGTLCEALLMVQAFHANIIFPNKQEQEFNKLTDDGHVLDSETYVGGHVEALESGVFRSDIPCRFRMNPAAFDFLLQRVEKTLRHALEEEEKVPVEQVTNFEEVCDEIKSKLASLKDVPSRIECPLIYHLDVGAMYPNIILTNRLQPSAMVDEATCAACDFNKPGANCQRKMAWQWRGEFMPASRSEYHRIQHQLESEKFPPLFPEGPARAFHELSREEQAKYEKRRLADYCRKAYKKIHITKVEERLTTICQRENSFYVDTVRAFRDRRYEFKGLHKVWKKKLSAAVEVGDAAEVKRCKNMEVLYDSLQLAHKCILNSFYGYVMRKGARWYSMEMAGIVCFTGANIITQARELIEQIGRPLELDTDGIWCVLPNSFPENFVFKTTNVKKPKVTISYPGAMLNIMVKEGFTNDQYQELAEPSSLTYVTRSENSIFFEVDGPYLAMILPASKEEGKKLKKRYAVFNEDGSLAELKGFEVKRRGELQLIKIFQSSVFEAFLKGSTLEEVYGSVAKVADYWLDVLYSKAANMPDSELFELISENRSMSRKLEDYGEQKSTSISTAKRLAEFLGDQMVKDAGLSCRYIISRKPEGSPVTERAIPLAIFQAEPTVRKHFLRKWLKSSSLQDFDIRAILDWDYYIERLGSAIQKIITIPAALQQVKNPVPRVKHPDWLHKKLLEKNDVYKQKKISELFTLEGRRQVTMAEASEDSPRPSAPDMEDFGLVKLPHPAAPVTVKRKRVLWESQEESQDLTPTVPWQEILGQPPALGTSQEEWLVWLRFHKKKWQLQARQRLARRKRQRLESAEGVLRPGAIRDGPATGLGSFLRRTARSILDLPWQIVQISETSQAGLFRLWALVGSDLHCIRLSIPRVFYVNQRVAKAEEGASYRKVNRVLPRSNMVYNLYEYSVPEDMYQEHINEINAELSAPDIEGVYETQVPLLFRALVHLGCVCVVNKQLVRHLSGWEAETFALEHLEMRSLAQFSYLEPGSIRHIYLYHHAQAHKALFGIFIPSQRRASVFVLDTVRSNQMPSLGALYSAEHGLLLEKVGPELLPPPKHTFEVRAETDLKTICRAIQRFLLAYKEERRGPTLIAVQSSWELKRLASEIPVLEEFPLVPICVADKINYGVLDWQRHGARRMIRHYLNLDTCLSQAFEMSRYFHIPIGNLPEDISTFGSDLFFARHLQRHNHLLWLSPTARPDLGGKEADDNCLVMEFDDQATVEINSSGCYSTVCVELDLQNLAVNTILQSHHVNDMEGADSMGISFDVIQQASLEDMITGGQAASAPASYDETALCSNTFRILKSMVVGWVKEITQYHNIYADNQVMHFYRWLRSPSSLLHDPALHRTLHNMMKKLFLQLIAEFKRLGSSVIYANFNRIILCTKKRRVEDAIAYVEYITSSIHSKETFHSLTISFSRCWEFLLWMDPSNYGGIKGKVSSRIHCGLQDSQKAGGAEDEQENEDDEEERDGEEEEEAEESNVEDLLENNWNILQFLPQAASCQNYFLMIVSAYIVAVYHCMKDGLRRSAPGSTPVRRRGASQLSQEAEGAVGALPGMITFSQDYVANELTQSFFTITQKIQKKVTGSRNSTELSEMFPVLPGSHLLLNNPALEFIKYVCKVLSLDTNITNQVNKLNRDLLRLVDVGEFSEEAQFRDPCRSYVLPEVICRSCNFCRDLDLCKDSSFSEDGAVLPQWLCSNCQAPYDSSAIEMTLVEVLQKKLMAFTLQDLVCLKCRGVKETSMPVYCSCAGDFALTIHTQVFMEQIGIFRNIAQHYGMSYLLETLEWLLQKNPQLGH

>NP_000958.1

MSHRKFSAPRHGSLGFLPRKRSSRHRGKVKSFPKDDPSKPVHLTAFLGYKAGMTHIVREVDRPGSKVNKKEVVEAVTIVETPPMVVVGIVGYVETPRGLRTFKTVFAEHISDECKRRFYKNWHKSKKKAFTKYCKKWQDEDGKKQLEKDFSSMKKYCQVIRVIAHTQMRLLPLRQKKAHLMEIQVNGGTVAEKLDWARERLEQQVPVNQVFGQDEMIDVIGVTKGKGYKGVTSRWHTKKLPRKTHRGLRKVACIGAWHPARVAFSVARAGQKGYHHRTEINKKIYKIGQGYLIKDGKLIKNNASTDYDLSDKSINPLGGFVHYGEVTNDFVMLKGCVVGTKKRVLTLRKSLLVQTKRRALEKIDLKFIDTTSKFGHGRFQTMEEKKAFMGPLKKDRIAKEEGA

391 P35548 NP_036529.1

>P35548

MASPSKGNDLFSPDEEGPAVVAGPGPGPGGAEGAAEERRVKVSSLPFSVEALMSDKKPPKEASPLPAESASAGATLRPLLLSGHGAREAHSPGPLVKPFETASVKSENSEDGAAWMQEPGRYSPPPRHTSPTTCTLRKHKTNRKPRTPFTTSQLLALERKFRQKQYLSIAERAEFSSSLNLTETQVKIWFQNRRAKAKRLQEAELEKLKMAAKPMLPSSFSLPFPISSPLQAASIYGASYPFHRPVLPIPPVGLYATPVGYGMYHLS

>NP_036529.1

MDSLGAVSTRLGFDLFKELKKTNDGNIFFSPVGILTAIGMVLLGTRGATASQLEEVFHSEKETKSSRIKAEEKEVIENTEAVHQQFQKFLTEISKLTNDYELNITNRLFGEKTYLFLQKYLDYVEKYYHASLEPVDFVNAADESRKKINSWVESKTNEKIKDLFPDGSISSSTKLVLVNMVYFKGQWDREFKKENTKEEKFWMNKSTSKSVQMMTQSHSFSFTFLEDLQAKILGIPYKNNDLSMFVLLPNDIDGLEKIIDKISPEKLVEWTSPGHMEERKVNLHLPRFEVEDGYDLEAVLAAMGMGDAFSEHKADYSGMSSGSGLYAQKFLHSSFVAVTEEGTEAAAATGIGFTVTSAPGHENVHCNHPFLFFIRHNESNSILFFGRFSSP

392 NP_005422.1 NP_001503.1

>NP_005422.1

MCSAFHRAESGTELLARLEGRSSLKEIEPNLFADEDSPVHGDILEFHGPEGTGKTEMLYHLTARCILPKSEGGLEVEVLFIDTDYHFDMLRLVTILEHRLSQSSEEIIKYCLGRFFLVYCSSSTHLLLTLYSLESMFCSHPSLCLLILDSLSAFYWIDRVNGGESVNLQESTLRKCSQCLEKLVNDYRLVLFATTQTIMQKASSSSEEPSHASRRLCDVDIDYRPYLCKAWQQLVKHRMFFSKQDDSQSSNQFSLVSRCLKSNSLKKHFFIIGESGVEFC

>NP_001503.1

MAARPKLHYPNGRGRMESVRWVLAAAGVEFDEEFLETKEQLYKLQDGNHLLFQQVPMVEIDGMKLVQTRSILHYIADKHNLFGKNLKERTLIDMYVEGTLDLLELLIMHPFLKPDDQQKEVVNMAQKAIIRYFPVFEKILRGHGQSFLVGNQLSLADVILLQTILALEEKIPNILSAFPFLQEYTVKLSNIPTIKRFLEPGSKKKPPPDEIYVRTVYNIFRP

393 NP_037479.1 NP_647473.1

>NP_037479.1

MSKSGFGSYGSISAADGASGGSDQLCERDATPAIKTQRPKVRIQDVVPCNVNQLLSSTVFDPVFKVRGIIVSQVSIVGVIRGAEKASNHICYKIDDMTAKPIEARQWFGREKVKQVTPLSVGVYVKVFGILKCPTGTKSLEVLKIHVLEDMNEFTVHILETVNAHMMLDKARRDTTVESVPVSPSEVNDAGDNDESHRNFIQDEVLRLIHECPHQEGKSIHELRAQLCDLSVKAIKEAIDYLTVEGHIYPTVDREHFKSAD

>NP_647473.1

MFSLSSTVQPQVTVPLSHLINAFHTPKNTSVSLSGVSVSQNQHRDVVPEHEAPSSECMFSDFLTKLNIVSIGKGKIFEGYRSMFMEPAKRMKKSLDTTDNWHIRPEPFSLSIPPSLNLRDLGLSELKIGQIDQLVENLLPGFCKGKNISSHWHTSHVSAQSFFENKYGNLDIFSTLRSSCLYRHHSRALQSICSDLQYWPVFIQSRGFKTLKSRTRRLQSTSERLAETQNIAPSFVKGFLLRDRGSDVESLDKLMKTKNIPEAHQDAFKTGFAEGFLKAQALTQKTNDSLRRTRLILFVLLLFGIYGLLKNPFLSVRFRTTTGLDSAVDPVQMKNVTFEHVKGVEEAKQELQEVVEFLKNPQKFTILGGKLPKGILLVGPPGTGKTLLARAVAGEADVPFYYASGSEFDEMFVGVGASRIRNLFREAKANAPCVIFIDELDSVGGKRIESPMHPYSRQTINQLLAEMDGFKPNEGVIIIGATNFPEALDNALIRPGRFDMQVTVPRPDVKGRTEILKWYLNKIKFDQSVDPEIIARGTVGFSGAELENLVNQAALKAAVDGKEMVTMKELEFSKDKILMGPERRSVEIDNKNKTITAYHESGHAIIAYYTKDAMPINKATIMPRGPTLGHVSLLPENDRWNETRAQLLAQMDVSMGGRVAEELIFGTDHITTGASSDFDNATKIAKRMVTKFGMSEKLGVMTYSDTGKLSPETQSAIEQEIRILLRDSYERAKHILKTHAKEHKNLAEALLTYETLDAKEIQIVLEGKKLEVR

394 NP_000116.2 NP_071369.4

>NP_000116.2

MTMTLHTKASGMALLHQIQGNELEPLNRPQLKIPLERPLGEVYLDSSKPAVYNYPEGAAYEFNAAAAANAQVYGQTGLPYGPGSEAAAFGSNGLGGFPPLNSVSPSPLMLLHPPPQLSPFLQPHGQQVPYYLENEPSGYTVREAGPPAFYRPNSDNRRQGGRERLASTNDKGSMAMESAKETRYCAVCNDYASGYHYGVWSCEGCKAFFKRSIQGHNDYMCPATNQCTIDKNRRKSCQACRLRKCYEVGMMKGGIRKDRRGGRMLKHKRQRDDGEGRGEVGSAGDMRAANLWPSPLMIKRSKKNSLALSLTADQMVSALLDAEPPILYSEYDPTRPFSEASMMGLLTNLADRELVHMINWAKRVPGFVDLTLHDQVHLLECAWLEILMIGLVWRSMEHPGKLLFAPNLLLDRNQGKCVEGMVEIFDMLLATSSRFRMMNLQGEEFVCLKSIILLNSGVYTFLSSTLKSLEEKDHIHRVLDKITDTLIHLMAKAGLTLQQQHQRLAQLLLILSHIRHMSNKGMEHLYSMKCKNVVPLYDLLLEMLDAHRLHAPTSRGGASVEETDQSHLATAGSTSSHSLQKYYITGEAEGFPATV

>NP_071369.4

MPPPSDIVKVAIEWPGANAQLLEIDQKRPLASIIKEVCDGWSLPNPEYYTLRYADGPQLYITEQTRSDIKNGTILQLAISPSRAARQLMERTQSSNMETRLDAMKELAKLSADVTFATEFINMDGIIVLTRLVESGTKLLSHYSEMLAFTLTAFLELMDHGIVSWDMVSITFIKQIAGYVSQPMVDVSILQRSLAILESMVLNSQSLYQKIAEEITVGQLISHLQVSNQEIQTYAIALINALFLKAPEDKRQDMANAFAQKHLRSIILNHVIRGNRPIKTEMAHQLYVLQVLTFNLLEERMMTKMDPNDQAQRDIIFELRRIAFDAESDPSNAPGSGTEKRKAMYTKDYKMLGFTNHINPAMDFTQTPPGMLALDNMLYLAKVHQDTYIRIVLENSSREDKHECPFGRSAIELTKMLCEILQVGELPNEGRNDYHPMFFTHDRAFEELFGICIQLLNKTWKEMRATAEDFNKVMQVVREQITRALPSKPNSLDQFKSKLRSLSYSEILRLRQSERMSQDDFQSPPIVELREKIQPEILELIKQQRLNRLCEGSSFRKIGNRRRQERFWYCRLALNHKVLHYGDLDDNPQGEVTFESLQEKIPVADIKAIVTGKDCPHMKEKSALKQNKEVLELAFSILYDPDETLNFIAPNKYEYCIWIDGLSALLGKDMSSELTKSDLDTLLSMEMKLRLLDLENIQIPEAPPPIPKEPSSYDFVYHYG

395 NP_055136.1 NP_000846.1

>NP_055136.1

MGSELIGRLAPRLGLAEPDMLRKAEEYLRLSRVKCVGLSARTTETSSAVMCLDLAASWMKCPLDRAYLIKLSGLNKETYQSCLKSFECLLGLNSNIGIRDLAVQFSCIEAVNMASKILKSYESSLPQTQQVDLDLSRPLFTSAALLSACKILKLKVDKNKMVATSGVKKAIFDRLCKQLEKIGQQVDREPGDVATPPRKRKKIVVEAPAKEMEKVEEMPHKPQKDEDLTQDYEEWKRKILENAASAQKATAE

>NP_000846.1

MSRRKISSESFSSLGSDYLETSPEEEGECPLSRLCWNGSRSPPGPLEPSPAAAAAAAAPAPTPAASAAAAAATAGARRVQRRRRVNLDSLGESISRLTAPSPQTIQQTLKRTLQYYEHQVIGYRDAEKNFHNISNRCSYADHSNKEEIEDVSGILQCTANILGLKFEEIQKRFGEEFFNICFHENERVLRAVGGTLQDFFNGFDALLEHIRTSFGKQATLESPSFLCKELPEGTLMLHYFHPHHIVGFAMLGMIKAAGKKIYRLDVEVEQVANEKLCSDVSNPGNCSCLTFLIKECENTNIMKNLPQGTSQVPADLRISINTFCRAFPFHLMFDPSMSVLQLGEGLRKQLRCDTHKVLKFEDCFEIVSPKVNATFERVLLRLSTPFVIRTKPEASGSENKDKVMEVKGQMIHVPESNSILFLGSPCVDKLDELMGRGLHLSDIPIHDATRDVILVGEQAKAQDGLKKRMDKLKATLERTHQALEEEKKKTVDLLYSIFPGDVAQQLWQGQQVQARKFDDVTMLFSDIVGFTAICAQCTPMQVISMLNELYTRFDHQCGFLDIYKVETIGDAYCVAAGLHRKSLCHAKPIALMALKMMELSEEVLTPDGRPIQMRIGIHSGSVLAGVVGVRMPRYCLFGNNVTLASKFESGSHPRRINVSPTTYQLLKREESFTFIPRSREELPDNFPKEIPGICYFLEVRTGPKPPKPSLSSSRIKKVSYNIGTMFLRETSL

396 NP_001316.1 NP_203526.1

>NP_001316.1

MAGLTVRDPAVDRSLRSVFVGNIPYEATEEQLKDIFSEVGPVVSFRLVYDRETGKPKGYGFCEYQDQETALSAMRNLNGREFSGRALRVDNAASEKNKEELKSLGTGAPVIESPYGETISPEDAPESISKAVASLPPEQMFELMKQMKLCVQNSPQEARNMLLQNPQLAYALLQAQVVMRIVDPEIALKILHRQTNIPTLIAGNPQPVHGAGPGSGSNVSMNQQNPQAPQAQSLGGMHVNGAPPLMQASMQGGVPAPGQMPAAVTGPGPGSLAPGGGMQAQVGMPGSGPVSMERGQVPMQDPRAAMQRGSLPANVPTPRGLLGDAPNDPRGGTLLSVTGEVEPRGYLGPPHQGPPMHHVPGHESRGPPPHELRGGPLPEPRPLMAEPRGPMLDQRGPPLDGRGGRDPRGIDARGMEARAMEARGLDARGLEARAMEARAMEARAMEARAMEARAMEVRGMEARGMDTRGPVPGPRGPIPSGMQGPSPINMGAVVPQGSRQVPVMQGTGMQGASIQGGSQPGGFSPGQNQVTPQDHEKAALIMQVLQLTADQIAMLPPEQRQSILILKEQIQKSTGAP

>NP_203526.1

MSWSGLLHGLNTSLTCGPALVPRLWATCSMATLNQMHRLGPPKRPPRKLGPTEGRPQLKGVVLCTFTRKPKKPNSANRKCCRVRLSTGREAVCFIPGEGHTLQEHQIVLVEGGRTQDLPGVKLTVVRGKYDCGHVQKK

397 NP_003007.2 NP_004093.1

>NP_003007.2

MSYGRPPPDVEGMTSLKVDNLTYRTSPDTLRRVFEKYGRVGDVYIPRDRYTKESRGFAFVRFHDKRDAEDAMDAMDGAVLDGRELRVQMARYGRPPDSHHSRRGPPPRRYGGGGYGRRSRSPRRRRRSRSRSRSRSRSRSRSRYSRSKSRSRTRSRSRSTSKSRSARRSKSKSSSVSRSRSRSRSRSRSRSPPPVSKRESKSRSRSKSPPKSPEEEGAVSS

>NP_004093.1

MVDAFLGTWKLVDSKNFDDYMKSLGVGFATRQVASMTKPTTIIEKNGDILTLKTHSTFKNTEISFKLGVEFDETTADDRKVKSIVTLDGGKLVHLQKWDGQETTLVRELIDGKLILTLTHGTAVCTRTYEKEA

398 NP_005729.1 Q14651

>NP_005729.1

MGNGLSDQTSILSNLPSFQSFHIVILGLDCAGKTTVLYRLQFNEFVNTVPTKGFNTEKIKVTLGNSKTVTFHFWDVGGQEKLRPLWKSYTRCTDGIVFVVDSVDVERMEEAKTELHKITRISENQGVPVLIVANKQDLRNSLSLSEIEKLLAMGELSSSTPWHLQPTCAIIGDGLKEGLEKLHDMIIKRRKMLRQQKKKR

>Q14651

MENSTTTISREELEELQEAFNKIDIDNSGYVSDYELQDLFKEASLPLPGYKVREIVEKILSVADSNKDGKISFEEFVSLMQELKSKDISKTFRKIINKREGITAIGGTSTISSEGTQHSYSEEEKVAFVNWINKALENDPDCKHLIPMNPNDDSLFKSLADGILLCKMINLSEPDTIDERAINKKKLTPFTISENLNLALNSASAIGCTVVNIGASDLKEGKPHLVLGLLWQIIKVGLFADIEISRNEALIALLNEGEELEELMKLSPEELLLRWVNYHLTNAGWHTISNFSQDIKDSRAYFHLLNQIAPKGGEDGPAIAIDLSGINETNDLKRAGLMLQEADKLGCKQFVTPADVVSGNPKLNLAFVANLFNTYPCLHKPNNNDIDMNLLEGESKEERTFRNWMNSLGVNPYINHLYSDLADALVIFQLYEMIRVPVNWSHVNKPPYPALGGNMKKIENCNYAVELGKNKAKFSLVGIAGQDLNEGNSTLTLALVWQLMRRYTLNVLSDLGEGEKVNDEIIIKWVNQTLKSANKKTSISSFKDKSISTSLPVLDLIDAIAPNAVRQEMIRRENLSDEDKLNNAKYAISVARKIGARIYALPDDLVEVKPKMVMTVFACLMGKGLNRIK

399 Q96N77 NP_000934.1

>Q96N77

MQAEDRSQFGSAAEMLSEQTAALGTGWESMNVQLDGAEPQVERGSQEERPWRTVPGPLEHLCCDLEEEPQSLQEKAQSAPWVPAIPQEGNTGDWEMAAALLAAGSQGLVTIKDVSLCFSQEEWRSLDPSQTDFYGEYVMQENCGIVVSLRFPIPKLDMLSQLEGGEEQWVPDPQDLEERDILRVTYTGDGSEHEGDTPELEAEPPRMLSSVSEDTVLWNPEHDESWDSMPSSSRGMLLGPPFLQEDSFSNLLCSTEMDSLLRPHTCPQCGKQFVWGSHLARHQQTHTGERPYSCLKCEKTFGRRHHLIRHQKTHLHDKTSRCSECGKNFRCNSHLASHQRVHAEGKSCKGQEVGESPGTRKRQRAPPVPKCHVCTECGKSFGRRHHLVRHWLTHTGEKPFQCPRCEKSFGRKHHLDRHLLTHQGQSPRNSWDRGTSVF

>NP_000934.1

MGPGPRLLLPLVLCVGLGALVFSSGAEGFRKRGPSVTAKVFFDVRIGDKDVGRIVIGLFGKVVPKTVENFVALATGEKGYGYKGSKFHRVIKDFMIQGGDITTGDGTGGVSIYGETFPDENFKLKHYGIGWVSMANAGPDTNGSQFFITLTKPTWLDGKHVVFGKVIDGMTVVHSIELQATDGHDRPLTNCSIINSGKIDVKTPFVVEIADW

400 NP_996670.1 NP_001815.2

>NP_996670.1

MAPKQDPKPKFQEGERVLCFHGPLLYEAKCVKVAIKDKQVKYFIHYSGWNKKSAVRPRRSEKSLKTHEDIVALFPVPEGAPSVHHPLLTSSWDEWVPESRVLKYVDTNLQKQRELQKANQEQYAEGKMRGAAPGKKTSGLQQKNVEVKTKKNKQKTPGNGDGGSTSETPQPPRKKRARVDPTVENEETFMNRVEVKVKIPEELKPWLVDDWDLITRQKQLFYLPAKKNVDSILEDYANYKKSRGNTDNKEYAVNEVVAGIKEYFNVMLGTQLLYKFERPQYAEILADHPDAPMSQVYGAPHLLRLFVRIGAMLAYTPLDEKSLALLLNYLHDFLKYLAKNSATLFSASDYEVAPPEYHRKAV

>NP_001815.2

MPFGNTHNKFKLNYKPEEEYPDLSKHNNHMAKVLTLELYKKLRDKETPSGFTVDDVIQTGVDNPGHPFIMTVGCVAGDEESYEVFKELFDPIISDRHGGYKPTDKHKTDLNHENLKGGDDLDPNYVLSSRVRTGRSIKGYTLPPHCSRGERRAVEKLSVEALNSLTGEFKGKYYPLKSMTEKEQQQLIDDHFLFDKPVSPLLLASGMARDWPDARGIWHNDNKSFLVWVNEEDHLRVISMEKGGNMKEVFRRFCVGLQKIEEIFKKAGHPFMWNQHLGYVLTCPSNLGTGLRGGVHVKLAHLSKHPKFEEILTRLRLQKRGTGGVDTAAVGSVFDVSNADRLGSSEVEQVQLVVDGVKLMVEMEKKLEKGQSIDDMIPAQK

401 BAB62909.1 NP_872578.1

>BAB62909.1

MDRSKRNSIAGFPPRVERLEEFEGGGGGEGNVSQVGRVWPSSYRALISAFSRLTRLDDFTCEKIGSGFFSEVFKVRHRASGQVMALKMNTLSSNRANMLKEVQLMNRLSHPNILRFMGVCVHQGQLHALTEYINSGNLEQLLDSNLHLPWTVRVKLAYDIAVGLSYLHFKGIFHRDLTSKNCLIKRDENGYSAVVADFGLAEKIPDVSMGSEKLAVVGSPFWMAPEVLRDEPYNEKADVFSYGIILCEIIARIQADPDYLPRTENFGLDYDAFQHMVGDCPPDFLQLTFNCCNMDPKLRPSFVEIGKTLEEILSRLQEEEQERDRKLQPTARGLLEKAPGVKRLSSLDDKIPHKSPCPRRTIWLSRSQSDIFSRKPPRTVSVLDPYYRPRDGAARTPKVNPFSARQDLMGGKIKFFDLPSKSVISLVFDLDAPGPGTMPLADWQEPLAPPIRRWRSLPGSPEFLHQEACPFVGREESLSDGPPPRLSSLKYRVKEIPPFRASALPAAQAHEAMDCSILQEENGFGSRPQGTSPCPAGASEEMEVEERPAGSTPATFSTSGIGLQTQGKQDG

>NP_872578.1

MAAPCVSYGGAVSYRLLLWGRGSLARKQGLWKTAAPELQTNVRSQILRLRHTAFVIPKKNVPTSKRETYTEDFIKKQIEEFNIGKRHLANMMGEDPETFTQEDIDRAIAYLFPSGLFEKRARPVMKHPEQIFPRQRAIQWGEDGRPFHYLFYTGKQSYYSLMHDVYGMLLNLEKHQSHLQAKSLLPEKTVTRDVIGSRWLIKEELEEMLVEKLSDLDYMQFIRLLEKLLTSQCGAAEEEFVQRFRRSVTLESKKQLIEPVQYDEQGMAFSKSEGKRKTAKAEAIVYKHGSGRIKVNGIDYQLYFPITQDREQLMFPFHFVDRLGKHDVTCTVSGGGRSAQAGAIRLAMAKALCSFVTEDEVEWMRQAGLLTTDPRVRERKKPGQEGARRKFTWKKR

402 O00472 P82675

>O00472

MAAGGTGGLREEQRYGLSCGRLGQDNITVLHVKLTETAIRALETYQSHKNLIPFRPSIQFQGLHGLVKIPKNDPLNEVHNFNFYLSNVGKDNPQGSFDCIQQTFSSSGASQLNCLGFIQDKITVCATNDSYQMTRERMTQAEEESRNRSTKVIKPGGPYVGKRVQIRKAPQAVSDTVPERKRSTPMNPANTIRKTHSSSTISQRPYRDRVIHLLALKAYKKPELLARLQKDGVNQKDKNSLGAILQQVANLNSKDLSYTLKDYVFKELQRDWPGYSEIDRRSLESVLSRKLNPSQNAAGTSRSESPVCSSRDAVSSPQKRLLDSEFIDPLMNKKARISHLTNRVPPTLNGHLNPTSEKSAAGLPLPPAAAAIPTPPPLPSTYLPISHPPQIVNSNSNSPSTPEGRGTQDLPVDSFSQNDSIYEDQQDKYTSRTSLETLPPGSVLLKCPKPMEENHSMSHKKSKKKSKKHKEKDQIKKHDIETIEEKEEDLKREEEIAKLNNSSPNSSGGVKEDCTASMEPSAIELPDYLIKYIAIVSYEQRQNYKDDFNAEYDEYRALHARMETVARRFIKLDAQRKRLSPGSKEYQNVHEEVLQEYQKIKQSSPNYHEEKYRCEYLHNKLAHIKRLIGEFDQQQAESWS

>P82675

MATAVRAVGCLPVLCSGTAGHLLGRQCSLNTLPAASILAWKSVLGNGHLSSLGTRDTHPYASLSRALQTQCCISSPSHLMSQQYRPYSFFTKLTADELWKGALAETGAGAKKGRGKRTKKKKRKDLNRGQIIGEGRYGFLWPGLNVPLMKNGAVQTIAQRSKEEQEKVEADMIQQREEWDRKKKMKVKRERGWSGNSWGGISLGPPDPGPCGETYEDFDTRILEVRNVFTMTAKEGRKKSIRVLVAVGNGKGAAGFSIGKATDRMDAFRKAKNRAVHHLHYIERYEDHTIFHDISLRFKRTHIKMKKQPKGYGLRCHRAIITICRLIGIKDMYAKVSGSINMLSLTQGLFRGLSRQETHQQLADKKGLHVVEIREECGPLPIVVASPRGPLRKDPEPEDEVPDVKLDWEDVKTAQGMKRSVWSNLKRAAT

403 NP_037425.1 NP_001087.2

>NP_037425.1

MSDVEENNFEGRESRSQSKSPTGTPARVKSESRSGSRSPSRVSKHSESHSRSRSKSRSRSRRHSHRRYTRSRSHSHSHRRRSRSRSYTPEYRRRRSRSHSPMSNRRRHTGSRANPDPNTCLGVFGLSLYTTERDLREVFSRYGPLSGVNVVYDQRTGRSRGFAFVYFERIDDSKEAMERANGMELDGRRIRVDYSITKRAHTPTPGIYMGRPTHSGGGGGGGGGGGGGGGGRRRDSYYDRGYDRGYDRYEDYDYRYRRRSPSPYYSRYRSRSRSRSYSPRRY

>NP_001087.2

MSAKAISEQTGKELLYKFICTTSAIQNRFKYARVTPDTDWARLLQDHPWLLSQNLVVKPDQLIKRRGKLGLVGVNLTLDGVKSWLKPRLGQEATVGKATGFLKNFLIEPFVPHSQAEEFYVCIYATREGDYVLFHHEGGVDVGDVDAKAQKLLVGVDEKLNPEDIKKHLLVHAPEDKKEILASFISGLFNFYEDLYFTYLEINPLVVTKDGVYVLDLAAKVDATADYICKVKWGDIEFPPPFGREAYPEEAYIADLDAKSGASLKLTLLNPKGRIWTMVAGGGASVVYSDTICDLGGVNELANYGEYSGAPSEQQTYDYAKTILSLMTREKHPDGKILIIGGSIANFTNVAATFKGIVRAIRDYQGPLKEHEVTIFVRRGGPNYQEGLRVMGEVGKTTGIPIHVFGTETHMTAIVGMALGHRPIPNQPPTAAHTANFLLNASGSTSTPAPSRTASFSESRADEVAPAKKAKPAMPQDSVPSPRSLQGKSTTLFSRHTKAIVWGMQTRAVQGMLDFDYVCSRDEPSVAAMVYPFTGDHKQKFYWGHKEILIPVFKNMADAMRKHPEVDVLINFASLRSAYDSTMETMNYAQIRTIAIIAEGIPEALTRKLIKKADQKGVTIIGPATVGGIKPGCFKIGNTGGMLDNILASKLYRPGSVAYVSRSGGMSNELNNIISRTTDGVYEGVAIGGDRYPGSTFMDHVLRYQDTPGVKMIVVLGEIGGTEEYKICRGIKEGRLTKPIVCWCIGTCATMFSSEVQFGHAGACANQASETAVAKNQALKEAGVFVPRSFDELGEIIQSVYEDLVANGVIVPAQEVPPPTVPMDYSWARELGLIRKPASFMTSICDERGQELIYAGMPITEVFKEEMGIGGVLGLLWFQKRLPKYSCQFIEMCLMVTADHGPAVSGAHNTIICARAGKDLVSSLTSGLLTIGDRFGGALDAAAKMFSKAFDSGIIPMEFVNKMKKEGKLIMGIGHRVKSINNPDMRVQILKDYVRQHFPATPLLDYALEVEKITTSKKPNLILNVDGLIGVAFVDMLRNCGSFTREEADEYIDIGALNGIFVLGRSMGFIGHYLDQKRLKQGLYRHPWDDISYVLPEHMSM

404 NP_000465.1 O00170

>NP_000465.1

MMQDVSSSPVSPADDSLSNSEEEPDRQQPPSGKRGGRKRRSSRRSAGGGAGPGGAAGGGVGGGDEPGSPAQGKRGKKSAGCGGGGGAGGGGGSSSGGGSPQSYEELQTQRVMANVRERQRTQSLNEAFAALRKIIPTLPSDKLSKIQTLKLAARYIDFLYQVLQSDELDSKMASCSYVAHERLSYAFSVWRMEGAWSMSASH

>O00170

MADIIARLREDGIQKRVIQEGRGELPDFQDGTKATFHYRTLHSDDEGTVLDDSRARGKPMELIIGKKFKLPVWETIVCTMREGEIAQFLCDIKHVVLYPLVAKSLRNIAVGKDPLEGQRHCCGVAQMREHSSLGHADLDALQQNPQPLIFHMEMLKVESPGTYQQDPWAMTDEEKAKAVPLIHQEGNRLYREGHVKEAAAKYYDAIACLKNLQMKEQPGSPEWIQLDQQITPLLLNYCQCKLVVEEYYEVLDHCSSILNKYDDNVKAYFKRGKAHAAVWNAQEAQADFAKVLELDPALAPVVSRELQALEARIRQKDEEDKARFRGIFSH

405 NP_002350.1 Q99707

>NP_002350.1

MTTPNKGNKALKVKREPGENGTSLTDEELVTMSVRELNQHLRGLSKEEIVQLKQRRRTLKNRGYAASCRVKRVTQKEELEKQKAELQQEVEKLASENASMKLELDALRSKYEALQTFARTVARSPVAPARGPLAAGLGPLVPGKVAATSVITIVKSKTDARS

>Q99707

MSPALQDLSQPEGLKKTLRDEINAILQKRIMVLDGGMGTMIQREKLNEEHFRGQEFKDHARPLKGNNDILSITQPDVIYQIHKEYLLAGADIIETNTFSSTSIAQADYGLEHLAYRMNMCSAGVARKAAEEVTLQTGIKRFVAGALGPTNKTLSVSPSVERPDYRNITFDELVEAYQEQAKGLLDGGVDILLIETIFDTANAKAALFALQNLFEEKYAPRPIFISGTIVDKSGRTLSGQTGEGFVISVSHGEPLCIGLNCALGAAEMRPFIEIIGKCTTAYVLCYPNAGLPNTFGDYDETPSMMAKHLKDFAMDGLVNIVGGCCGSTPDHIREIAEAVKNCKPRVPPATAFEGHMLLSGLEPFRIGPYTNFVNIGERCNVAGSRKFAKLIMAGNYEEALCVAKVQVEMGAQVLDVNMDDGMLDGPSAMTRFCNLIASEPDIAKVPLCIDSSNFAVIEAGLKCCQGKCIVNSISLKEGEDDFLEKARKIKKYGAAMVVMAFDEEGQATETDTKIRVCTRAYHLLVKKLGFNPNDIIFDPNILTIGTGMEEHNLYAINFIHATKVIKETLPGARISGGLSNLSFSFRGMEAIREAMHGVFLYHAIKSGMDMGIVNAGNLPVYDDIHKELLQLCEDLIWNKDPEATEKLLRYAQTQGTGGKKVIQTDEWRNGPVEERLEYALVKGIEKHIIEDTEEARLNQKKYPRPLNIIEGPLMNGMKIVGDLFGAGKMFLPQVIKSARVMKKAVGHLIPFMEKEREETRVLNGTVEEEDPYQGTIVLATVKGDVHDIGKNIVGVVLGCNNFRVIDLGVMTPCDKILKAALDHKADIIGLSGLITPSLDEMIFVAKEMERLAIRIPLLIGGATTSKTHTAVKIAPRYSAPVIHVLDASKSVVVCSQLLDENLKDEYFEEIMEEYEDIRQDHYESLKERRYLPLSQARKSGFQMDWLSEPHPVKPTFIGTQVFEDYDLQKLVDYIDWKPFFDVWQLRGKYPNRGFPKIFNDKTVGGEARKVYDDAHNMLNTLISQKKLRARGVVGFWPAQSIQDDIHLYAEAAVPQAAEPIATFYGLRQQAEKDSASTEPYYCLSDFIAPLHSGIRDYLGLFAVACFGVEELSKAYEDDGDDYSSIMVKALGDRLAEAFAEELHERVRRELWAYCGSEQLDVADLRRLRYKGIRPAPGYPSQPDHTEKLTMWRLADIEQSTGIRLTESLAMAPASAVSGLYFSNLKSKYFAVGKISKDQVEDYALRKNISVAEVEKWLGPILGYDTD

406 NP_006181.1 NP_002003.1

>NP_006181.1

MSKPELKEDKMLEVHFVGDDDVLNHILDREGGAKLKKERAQLLVNPKKIIKKPEYDLEEDDQEVLKDQNYVEIMGRDVQESLKNGSATGGGNKVYSFQNRKHSEKMAKLASELAKTPQKSVSFSLKNDPEITINVPQSSKGHSASDKVQPKNNDKSEFLSTAPRSLRKRLIVPRSHSDSESEYSASNSEDDEGVAQEHEEDTNAVIFSQKIQAQNRVVSAPVGKETPSKRMKRDKTSDLVEEYFEAHSSSKVLTSDRTLQKLKRAKLDQQTLRNLLSKVSPSFSAELKQLNQQYEKLFHKWMLQLHLGFNIVLYGLGSKRDLLERFRTTMLQDSIHVVINGFFPGISVKSVLNSITEEVLDHMGTFRSILDQLDWIVNKFKEDSSLELFLLIHNLDSQMLRGEKSQQIIGQLSSLHNIYLIASIDHLNAPLMWDHAKQSLFNWLWYETTTYSPYTEETSYENSLLVKQSGSLPLSSLTHVLRSLTPNARGIFRLLIKYQLDNQDNPSYIGLSFQDFYQQCREAFLVNSDLTLRAQLTEFRDHKLIRTKKGTDGVEYLLIPVDNGTLTDFLEKEEEEA

>NP_002003.1

MSFRFGQHLIKPSVVFLKTELSFALVNRKPVVPGHVLVCPLRPVERFHDLRPDEVADLFQTTQRVGTVVEKHFHGTSLTFSMQDGPEAGQTVKHVHVHVLPRKAGDFHRNDSIYEELQKHDKEDFPASWRSEEEMAAEAAALRVYFQ

407 NP_004950.2 NP_038203.2

>NP_004950.2

MDYSYDEDLDELCPVCGDKVSGYHYGLLTCESCKGFFKRTVQNNKHYTCTESQSCKIDKTQRKRCPFCRFQKCLTVGMRLEAVRADRMRGGRNKFGPMYKRDRALKQQKKAQIRANGFKLETGPPMGVPPPPPPAPDYVLPPSLHGPEPKGLAAGPPAGPLGDFGAPALPMAVPGAHGPLAGYLYPAFPGRAIKSEYPEPYASPPQPGLPYGYPEPFSGGPNVPELILQLLQLEPDEDQVRARILGCLQEPTKSRPDQPAAFGLLCRMADQTFISIVDWARRCMVFKELEVADQMTLLQNCWSELLVFDHIYRQVQHGKEGSILLVTGQEVELTTVATQAGSLLHSLVLRAQELVLQLLALQLDRQEFVCLKFIILFSLDLKFLNNHILVKDAQEKANAALLDYTLCHYPHCGDKFQQLLLCLVEVRALSMQAKEYLYHKHLGNEMPRNNLLIEMLQAKQT

>NP_038203.2

MLQQVPENINFPAEEEKILEFWTEFNCFQECLKQSKHKPKFTFYDGPPFATGLPHYGHILAGTIKDIVTRYAHQSGFHVDRRFGWDCHGLPVEYEIDKTLGIRGPEDVAKMGITEYNNQCRAIVMRYSAEWKSTVSRLGRWIDFDNDYKTLYPQFMESVWWVFKQLYDKGLVYRGVKVMPFSTACNTPLSNFESHQNYKDVQDPSVFVTFPLEEDETVSLVAWTTTPWTLPSNLAVCVNPEMQYVKIKDVARGRLLILMEARLSALYKLESDYEILERFPGAYLKGKKYRPLFDYFLKCKENGAFTVLVDNYVKEEEGTGVVHQAPYFGAEDYRVCMDFNIIRKDSLPVCPVDASGCFTTEVTDFAGQYVKDADKSIIRTLKEQGRLLVATTFTHSYPFCWRSDTPLIYKAVPSWFVRVENMVDQLLRNNDLCYWVPELVREKRFGNWLKDARDWTISRNRYWGTPIPLWVSDDFEEVVCIGSVAELEELSGAKISDLHRESVDHLTIPSRCGKGSLHRISEVFDCWFESGSMPYAQVHYPFENKREFEDAFPADFIAEGIDQTRGWFYTLLVLATALFGQPPFKNVIVNGLVLASDGQKMSKRKKNYPDPVSIIQKYGADALRLYLINSPVVRAENLRFKEEGVRDVLKDVLLPWYNAYRFLIQNVLRLQKEEEIEFLYNENTVRESPNITDRWILSFMQSLIGFFETEMAAYRLYTVVPRLVKFVDILTNWYVRMNRRRLKGENGMEDCVMALETLFSVLLSLCRLMAPYTPFLTELMYQNLKVLIDPVSVQDKDTLSIHYLMLPRVREELIDKKTESAVSQMQSVIELGRVIRDRKTIPIKYPLKEIVVIHQDPEALKDIKSLEKYIIEELNVRKVTLSTDKNKYGIRLRAEPDHMVLGKRLKGAFKAVMTSIKQLSSEELEQFQKTGTIVVEGHELHDEDIRLMYTFDQATGGTAQFEAHSDAQALVLLDVTPDQSMVDEGMAREVINRIQKLRKKCNLVPTDEITVYYKAKSEGTYLNSVIESHTEFIFTTIKAPLKPYPVSPSDKVLIQEKTQLKGSELEITLTRGSSLPGPACAYVNLNICANGSEQGGVLLLENPKGDNRLDLLKLKSVVTSIFGVKNTELAVFHDETEIQNQTDLLSLSGKTLCVTAGSAPSLINSSSTLLCQYINLQLLNAKPQECLMGTVGTLLLENPLGQNGLTHQGLLYEAAKVFGLRSRKLKLFLNETQTQEITEDIPVKTLNMKTVYVSVLPTTADF

408 Q15022 Q9P032

>Q15022

MAPQKHGGGGGGGSGPSAGSGGGGFGGSAAVAAATASGGKSGGGSCGGGGSYSASSSSSAAAAAGAAVLPVKKPKMEHVQADHELFLQAFEKPTQIYRFLRTRNLIAPIFLHRTLTYMSHRNSRTNIKRKTFKVDDMLSKVEKMKGEQESHSLSAHLQLTFTGFFHKNDKPSPNSENEQNSVTLEVLLVKVCHKKRKDVSCPIRQVPTGKKQVPLNPDLNQTKPGNFPSLAVSSNEFEPSNSHMVKSYSLLFRVTRPGRREFNGMINGETNENIDVNEELPARRKRNREDGEKTFVAQMTVFDKNRRLQLLDGEYEVAMQEMEECPISKKRATWETILDGKRLPPFETFSQGPTLQFTLRWTGETNDKSTAPIAKPLATRNSESLHQENKPGSVKPTQTIAVKESLTTDLQTRKEKDTPNENRQKLRIFYQFLYNNNTRQQTEARDDLHCPWCTLNCRKLYSLLKHLKLCHSRFIFNYVYHPKGARIDVSINECYDGSYAGNPQDIHRQPGFAFSRNGPVKRTPITHILVCRPKRTKASMSEFLESEDGEVEQQRTYSSGHNRLYFHSDTCLPLRPQEMEVDSEDEKDPEWLREKTITQIEEFSDVNEGEKEVMKLWNLHVMKHGFIADNQMNHACMLFVENYGQKIIKKNLCRNFMLHLVSMHDFNLISIMSIDKAVTKLREMQQKLEKGESASPANEEITEEQNGTANGFSEINSKEKALETDSVSGVSKQSKKQKL

>Q9P032

MGALVIRGIRNFNLENRAEREISKMKPSVAPRHPSTNSLLREQISLYPEVKGEIARKDEKLLSFLKDVYVDSKDPVSSLQVKAAETCQEPKEFRLPKDHHFDMINIKSIPKGKISIVEALTLLNNHKLFPETWTAEKIMQEYQLEQKDVNSLLKYFVTFEVEIFPPEDKKAIRSK

409 NP_000387.1 NP_004802.1

>NP_000387.1

MWGLKVLLLPVVSFALYPEEILDTHWELWKKTHRKQYNNKVDEISRRLIWEKNLKYISIHNLEASLGVHTYELAMNHLGDMTSEEVVQKMTGLKVPLSHSRSNDTLYIPEWEGRAPDSVDYRKKGYVTPVKNQGQCGSCWAFSSVGALEGQLKKKTGKLLNLSPQNLVDCVSENDGCGGGYMTNAFQYVQKNRGIDSEDAYPYVGQEESCMYNPTGKAAKCRGYREIPEGNEKALKRAVARVGPVSVAIDASLTSFQFYSKGVYYDESCNSDNLNHAVLAVGYGIQKGNKHWIIKNSWGENWGNKGYILMARNKNNACGIANLASFPKM

>NP_004802.1

MEELDALLEELERSTLQDSDEYSNPAPLPLDQHSRKETNLDETSEILSIQDNTSPLPAQLVYTTNIQELNVYSEAQEPKESPPPSKTSAAAQLDELMAHLTEMQAKVAVRADAGKKHLPDKQDHKASLDSMLGGLEQELQDLGIATVPKGHCASCQKPIAGKVIHALGQSWHPEHFVCTHCKEEIGSSPFFERSGLAYCPNDYHQLFSPRCAYCAAPILDKVLTAMNQTWHPEHFFCSHCGEVFGAEGFHEKDKKPYCRKDFLAMFSPKCGGCNRPVLENYLSAMDTVWHPECFVCGDCFTSFSTGSFFELDGRPFCELHYHHRRGTLCHGCGQPITGRCISAMGYKFHPEHFVCAFCLTQLSKGIFREQNDKTYCQPCFNKLFPL

410 P35452 NP_055433.2

>P35452

MCERSLYRAGYVGSLLNLQSPDSFYFSNLRPNGGQLAALPPISYPRGALPWAATPASCAPAQPAGATAFGGFSQPYLAGSGPLGLQPPTAKDGPEEQAKFYAPEAAAGPEERGRTRPSFAPESSLAPAVAALKAAKYDYAGVGRATPGSTTLLQGAPCAPGFKDDTKGPLNLNMTVQAAGVASCLRPSLPDGLPWGAAPGRARKKRKPYTKQQIAELENEFLVNEFINRQKRKELSNRLNLSDQQVKIWFQNRRMKKKRVVLREQALALY

>NP_055433.2

MNWRFVELLYFLFIWGRISVQPSHQEPAGTDQHVSKEFDWLISDRGPFHHSRSYLSFVERHRQGFTTRYKIYREFARWKVRNTAIERRDLVRHPVPLMPEFQRSIRLLGRRPTTQQFIDTIIKKYGTHLLISATLGGEEALTMYMDKSRLDRKSGNATQSVEALHQLASSYFVDRDGTMRRLHEIQISTGAIKVTETRTGPLGCNSYDNLDSVSSVLLQSTESKLHLQGLQIIFPQYLQEKFVQSALSYIMCNGEGEYLCQNSQCRCQCAEEFPQCNCPITDIQIMEYTLANMAKSWAEAYKDLENSDEFKSFMKRLPSNHFLTIGSIHQHWGNDWDLQNRYKLLQSATEAQRQKIQRTARKLFGLSVRCRHNPNHQLPRERTIQQWLARVQSLLYCNENGFWGTFLESQRSCVCHGSTTLCQRPIPCVIGGNNSCAMCSLANISLCGSCNKGYKLYRGRCEPQNVDSERSEQFISFETDLDFQDLELKYLLQKMDSRLYVHTTFISNEIRLDTFFDPRWRKRMSLTLKSNKNRMDFIHMVIGMSMRICQMRNSSLDPMFFVYVNPFSGSHSEGWNMPFGEFGYPRWEKIRLQNSQCYNWTLLLGNRWKTFFETVHIYLRSRTRLPTLLRNETGQGPVDLSDPSKRQFYIKISDVQVFGYSLRFNADLLRSAVQQVNQSYTQGGQFYSSSSVMLLLLDIRDRINRLAPPVAPGKPQLDLFSCMLKHRLKLTNSEIIRVNHALDLYNTEILKQSDQMTAKLC

411 Q01851 P40261

>Q01851

MMSMNSKQPHFAMHPTLPEHKYPSLHSSSEAIRRACLPTPPLQSNLFASLDETLLARAEALAAVDIAVSQGKSHPFKPDATYHTMNSVPCTSTSTVPLAHHHHHHHHHQALEPGDLLDHISSPSLALMAGAGGAGAAAGGGGAHDGPGGGGGPGGGGGPGGGGPGGGGGGGPGGGGGGPGGGLLGGSAHPHPHMHSLGHLSHPAAAAAMNMPSGLPHPGLVAAAAHHGAAAAAAAAAAGQVAAASAAAAVVGAAGLASICDSDTDPRELEAFAERFKQRRIKLGVTQADVGSALANLKIPGVGSLSQSTICRFESLTLSHNNMIALKPILQAWLEEAEGAQREKMNKPELFNGGEKKRKRTSIAAPEKRSLEAYFAVQPRPSSEKIAAIAEKLDLKKNVVRVWFCNQRQKQKRMKFSATY

>P40261

MESGFTSKDTYLSHFNPRDYLEKYYKFGSRHSAESQILKHLLKNLFKIFCLDGVKGDLLIDIGSGPTIYQLLSACESFKEIVVTDYSDQNLQELEKWLKKEPEAFDWSPVVTYVCDLEGNRVKGPEKEEKLRQAVKQVLKCDVTQSQPLGAVPLPPADCVLSTLCLDAACPDLPTYCRALRNLGSLLKPGGFLVIMDALKSSYYMIGEQKFSSLPLGREAVEAAVKEAGYTIEWFEVISQSYSSTMANNEGLFSLVARKLSRPL

412 NP_009060.2 NP_001024.1

>NP_009060.2

MLLDAGPQFPAIGVGSFARHHHHSAAAAAAAAAEMQDRELSLAAAQNGFVDSAAAHMGAFKLNPGAHELSPGQSSAFTSQGPGAYPGSAAAAAAAAALGPHAAHVGSYSGPPFNSTRDFLFRSRGFGDSAPGGGQHGLFGPGAGGLHHAHSDAQGHLLFPGLPEQHGPHGSQNVLNGQMRLGLPGEVFGRSEQYRQVASPRTDPYSAAQLHNQYGPMNMNMGMNMAAAAAHHHHHHHHHPGAFFRYMRQQCIKQELICKWIDPEQLSNPKKSCNKTFSTMHELVTHVSVEHVGGPEQSNHVCFWEECPREGKPFKAKYKLVNHIRVHTGEKPFPCPFPGCGKVFARSENLKIHKRTHTGEKPFQCEFEGCDRRFANSSDRKKHMHVHTSDKPYLCKMCDKSYTHPSSLRKHMKVHESSPQGSESSPAASSGYESSTPPGLVSPSAEPQSSSNLSPAAAAAAAAAAAAAAAVSAVHRGGGSGSGGAGGGSGGGSGSGGGGGGAGGGGGGSSGGGSGTAGGHSGLSSNFNEWYV

>NP_001024.1

MHVIKRDGRQERVMFDKITSRIQKLCYGLNMDFVDPAQITMKVIQGLYSGVTTVELDTLAAETAATLTTKHPDYAILAARIAVSNLHKETKKVFSDVMEDLYNYINPHNGKHSPMVAKSTLDIVLANKDRLNSAIIYDRDFSYNYFGFKTLERSYLLKINGKVAERPQHMLMRVSVGIHKEDIDAAIETYNLLSERWFTHASPTLFNAGTNRPQLSSCFLLSMKDDSIEGIYDTLKQCALISKSAGGIGVAVSCIRATGSYIAGTNGNSNGLVPMLRVYNNTARYVDQGGNKRPGAFAIYLEPWHLDIFEFLDLKKNTGKEEQRARDLFFALWIPDLFMKRVETNQDWSLMCPNECPGLDEVWGEEFEKLYASYEKQGRVRKVVKAQQLWYAIIESQTETGTPYMLYKDSCNRKSNQQNLGTIKCSNLCTEIVEYTSKDEVAVCNLASLALNMYVTSEHTYDFKKLAEVTKVVVRNLNKIIDINYYPVPEACLSNKRHRPIGIGVQGLADAFILMRYPFESAEAQLLNKQIFETIYYGALEASCDLAKEQGPYETYEGSPVSKGILQYDMWNVTPTDLWDWKVLKEKIAKYGIRNSLLIAPMPTASTAQILGNNESIEPYTSNIYTRRVLSGEFQIVNPHLLKDLTERGLWHEEMKNQIIACNGSIQSIPEIPDDLKQLYKTVWEISQKTVLKMAAERGAFIDQSQSLNIHIAEPNYGKLTSMHFYGWKQGLKTGMYYLRTRPAANPIQFTLNKEKLKDKEKVSKEEEEKERNTAAMVCSLENRDECLMCGS

413 NP_003874.2 Q8TF09

>NP_003874.2

MAKTVAYFYDPDVGNFHYGAGHPMKPHRLALTHSLVLHYGLYKKMIVFKPYQASQHDMCRFHSEDYIDFLQRVSPTNMQGFTKSLNAFNVGDDCPVFPGLFEFCSRYTGASLQGATQLNNKICDIAINWAGGLHHAKKFEASGFCYVNDIVIGILELLKYHPRVLYIDIDIHHGDGVQEAFYLTDRVMTVSFHKYGNYFFPGTGDMYEVGAESGRYYCLNVPLRDGIDDQSYKHLFQPVINQVVDFYQPTCIVLQCGADSLGCDRLGCFNLSIRGHGECVEYVKSFNIPLLVLGGGGYTVRNVARCWTYETSLLVEEAISEELPYSEYFEYFAPDFTLHPDVSTRIENQNSRQYLDQIRQTIFENLKMLNHAPSVQIHDVPADLLTYDRTDEADAEERGPEENYSRPEAPNEFYDGDHDNDKESDVEI

>Q8TF09

MAEVEETLKRIQSHKGVIGTMVVNAEGIPIRTTLDNSTTVQYAGLLHHLTMKAKSTVRDIDPQNDLTFLRIRSKKHEIMVAPDKEYLLIVIQNPCE

414 NP_066406.1 NP_001163.1

>NP_066406.1

MPEPSKSAPAPKKGSKKAITKAQKKDGKKRKRSRKESYSIYVYKVLKQVHPDTGISSKAMGIMNSFVNDIFERIAGEASRLAHYNKRSTITSREIQTAVRLLLPGELAKHAVSEGTKAVTKYTSSK

>NP_001163.1

MSLRGSLSRLLQTRVHSILKKSVHSVAVIGAPFSQGQKRKGVEHGPAAIREAGLMKRLSSLGCHLKDFGDLSFTPVPKDDLYNNLIVNPRSVGLANQELAEVVSRAVSDGYSCVTLGGDHSLAIGTISGHARHCPDLCVVWVDAHADINTPLTTSSGNLHGQPVSFLLRELQDKVPQLPGFSWIKPCISSASIVYIGLRDVDPPEHFILKNYDIQYFSMRDIDRLGIQKVMERTFDLLIGKRQRPIHLSFDIDAFDPTLAPATGTPVVGGLTYREGMYIAEEIHNTGLLSALDLVEVNPQLATSEEEAKTTANLAVDVIASSFGQTREGGHIVYDQLPTPSSPDESENQARVRI

415 Q8WVY7 Q8TF40

>Q8WVY7

MALPIIVKWGGQEYSVTTLSEDDTVLDLKQFLKTLTGVLPERQKLLGLKVKGKPAENDVKLGALKLKPNTKIMMMGTREESLEDVLGPPPDNDDVVNDFDIEDEVVEVENREENLLKISRRVKEYKVEILNPPREGKKLLVLDVDYTLFDHRSCAETGVELMRPYLHEFLTSAYEDYDIVIWSATNMKWIEAKMKELGVSTNANYKITFMLDSAAMITVHTPRRGLIDVKPLGVIWGKFSEFYSKKNTIMFDDIGRNFLMNPQNGLKIRPFMKAHLNRDKDKELLKLTQYLKEIAKLDDFLDLNHKYWERYLSKKQGQ

>Q8TF40

MAPTLFQKLFSKRTGLGAPGRDARDPDCGFSWPLPEFDPSQIRLIVYQDCERRGRNVLFDSSVKRRNEDISVSKLCSDAQVKVFGKCCQLKPGGDSSSSLDSSVTSSSDIKDQCLKYQGSRCSSDANMLGEMMFGSVAMSYKGSTLKIHQIRSPPQLMLSKVFTARTGSSICGSLNTLQDSLEFINQDNNTLKADNNTVINGLLGNIGLSQFCSPRRAFSEQGPLRLIRSASFFAVHSNPMDMPGRELNEDRDSGIARSASLSSLLITPFPSPNSSLTRSCASSYQRRWRRSQTTSLENGVFPRWSIEESFNLSDESCGPNPGIVRKKKIAIGVIFSLSKDEDENNKFNEFFFSHFPLFESHMNKLKSAIEQAMKMSRRSADASQRSLAYNRIVDALNEFRTTICNLYTMPRIGEPVWLTMMSGTPEKNHLCYRFMKEFTFLMENASKNQFLPALITAVLTNHLAWVPTVMPNGQPPIKIFLEKHSSQSVDMLAKTHPYNPLWAQLGDLYGAIGSPVRLARTVVVGKRQDMVQRLLYFLTYFIRCSELQETHLLENGEDEAIVMPGTVITTTLEKGEIEESEYVLVTMHRNKSSLLFKESEEIRTPNCNCKYCSHPLLGQNVENISQQEREDIQNSSKELLGISDECQMISPSDCQEENAVDVKQYRDKLRTCFDAKLETVVCTGSVPVDKCALSESGLESTEETWQSEKLLDSDSHTGKAMRSTGMVVEKKPPDKIVPASFSCEAAQTKVTFLIGDSMSPDSDTELRSQAVVDQITRHHTKPLKEERGAIDQHQETKQTTKDQSGESDTQNMVSEEPCELPCWNHSDPESMSLFDEYFNDDSIETRTIDDVPFKTSTDSKDHCCMLEFSKILCTKNNKQNNEFCKCIETVPQDSCKTCFPQQDQRDTLSILVPHGDKESSDKKIAVGTEWDIPRNESSDSALGDSESEDTGHDMTRQVSSYYGGEQEDWAEEDEIPFPGSKLIEVSAVQPNIANFGRSLLGGYCSSYVPDFVLQGIGSDERFRQCLMSDLSHAVQHPVLDEPIAEAVCIIADMDKWTVQVASSQRRVTDNKLGKEVLVSSLVSNLLHSTLQLYKHNLSPNFCVMHLEDRLQELYFKSKMLSEYLRGQMRVHVKELGVVLGIESSDLPLLAAVASTHSPYVAQILL

416 NP_005633.2 Q9NWF9

>NP_005633.2

MSKSKDDAPHELESQFILRLPPEYASTVRRAVQSGHVNLKDRLTIELHPDGRHGIVRVDRVPLASKLVDLPCVMESLKTIDKKTFYKTADICQMLVSTVDGDLYPPVEEPVASTDPKASKKKDKDKEKKFIWNHGITLPLKNVRKRRFRKTAKKKYIESPDVEKEVKRLLSTDAEAVSTRWEIIAEDETKEAENQGLDISSPGMSGHRQGHDSLEHDELREIFNDLSSSSEDEDETQHQDEEDINIIDTEEDLERQLQDKLNESDEQHQENEGTNQLVMGIQKQIDNMKGKLQETQDRAKRQEDLIMKVENLALKNRFQAVLDELKQKEDREKEQLSSLQEELESLLEK

>Q9NWF9

MEEGNNNEEVIHLNNFHCHRGQEWINLRDGPITISDSSDEERIPMLVTPAPQQHEEEDLDDDVILTEDDSEDDYGEFLDLGPPGISEFTKPSGQTEREPKPGPSHNQAANDIVNPRSEQKVIILEEGSLLYTESDPLETQNQSSEDSETELLSNLGESAALADDQAIEEDCWLDHPYFQSLNQQPREITNQVVPQERQPEAELGRLLFQHEFPGPAFPRPEPQQGGISGPSSPQPAHPLGEFEDQQLASDDEEPGPAFPMQESQEPNLENIWGQEAAEVDQELVELLVKETEARFPDVANGFIEEIIHFKNYYDLNVLCNFLLENPDYPKREDRIIINPSSSLLASQDETKLPKIDFFDYSKLTPLDQRCFIQAADLLMADFKVLSSQDIKWALHELKGHYAITRKALSDAIKKWQELSPETSGKRKKRKQMNQYSYIDFKFEQGDIKIEKRMFFLENKRRHCRSYDRRALLPAVQQEQEFYEQKIKEMAEHEDFLLALQMNEEQYQKDGQLIECRCCYGEFPFEELTQCADAHLFCKECLIRYAQEAVFGSGKLELSCMEGSCTCSFPTSELEKVLPQTILYKYYERKAEEEVAAAYADELVRCPSCSFPALLDSDVKRFSCPNPHCRKETCRKCQGLWKEHNGLTCEELAEKDDIKYRTSIEEKMTAARIRKCHKCGTGLIKSEGCNRMSCRCGAQMCYLCRVSINGYDHFCQHPRSPGAPCQECSRCSLWTDPTEDDEKLIEEIQKEAEEEQKRKNGENTFKRIGPPLEKPVEKVQRVEALPRPVPQNLPQPQMPPYAFAHPPFPLPPVRPVFNNFPLNMGPIPAPYVPPLPNVRVNYDFGPIHMPLEHNLPMHFGPQPRHRF

417 P13284 Q8N3H0

>P13284

MDSRHTFAPAAMTLSPLLLFLPPLLLLLDVPTAAVQASPLQALDFFGNGPPVNYKTGNLYLRGPLKKSNAPLVNVTLYYEALCGGCRAFLIRELFPTWLLVMEILNVTLVPYGNAQEQNVSGRWEFKCQHGEEECKFNKVEACVLDELDMELAFLTIVCMEEFEDMERSLPLCLQLYAPGLSPDTIMECAMGDRGMQLMHANAQRTDALQPPHEYVPWVTVNGKPLEDQTQLLTLVCQLYQGKKPDVCPSSTSSLRSVCFK

>Q8N3H0

MSKRYLQKATKGKLLIIIFIVTLWGKVVSSANHHKAHHVKTGTCEVVALHRCCNKNKIEERSQTVKCSCFPGQVAGTTRAAPSCVDASIVEQKWWCHMQPCLEGEECKVLPDRKGWSCSSGNKVKTTRVTH

418 NP_003073.1 Q9NVV4

>NP_003073.1

MGTPPGLQTDCEALLSRFQETDSVRFEDFTELWRNMKFGTIFCGRMRNLEKNMFTKEALALAWRYFLPPYTFQIRVGALYLLYGLYNTQLCQPKQKIRVALKDWDEVLKFQQDLVNAQHFDAAYIFRKLRLDRAFHFTAMPKLLSYRMKKKIHRAEVTEEFKDPSDRVMKLITSDVLEEMLNVHDHYQNMKHVISVDKSKPDKALSLIKDDFFDNIKNIVLEHQQWHKDRKNPSLKSKTNDGEEKMEGNSQETERCERAESLAKIKSKAFSVVIQASKSRRHRQVKLDSSDSDSASGQGQVKATRKKEKKERLKPAGRKMSLRNKGNVQNIHKEDKPLSLSMPVITEEEENESLSGTEFTASKKRRKH

>Q9NVV4

MAVPGVGLLTRLNLCARRRTRVQRPIVRLLSCPGTVAKDLRRDEQPSGSVETGFEDKIPKRRFSEMQNERREQAQRTVLIHCPEKISENKFLKYLSQFGPINNHFFYESFGLYAVVEFCQKESIGSLQNGTHTPSTAMETAIPFRSRFFNLKLKNQTSERSRVRSSNQLPRSNKQLFELLCYAESIDDQLNTLLKEFQLTEENTKLRYLTCSLIEDMAAAYFPDCIVRPFGSSVNTFGKLGCDLDMFLDLDETRNLSAHKISGNFLMEFQVKNVPSERIATQKILSVLGECLDHFGPGCVGVQKILNARCPLVRFSHQASGFQCDLTTNNRIALTSSELLYIYGALDSRVRALVFSVRCWARAHSLTSSIPGAWITNFSLTMMVIFFLQRRSPPILPTLDSLKTLADAEDKCVIEGNNCTFVRDLSRIKPSQNTETLELLLKEFFEYFGNFAFDKNSINIRQGREQNKPDSSPLYIQNPFETSLNISKNVSQSQLQKFVDLARESAWILQQEDTDRPSISSNRPWGLVSLLLPSAPNRKSFTKKKSNKFAIETVKNLLESLKGNRTENFTKTSGKRTISTQT

419 NP_570722.2 Q9UKF7

>NP_570722.2

MPLQVSDYSWQQTKTAVFLSLPLKGVCVRDTDVFCTENYLKVNFPPFLFEAFLYAPIDDESSKAKIGNDTIVFTLYKKEAAMWETLSVTGVDKEMMQRIREKSILQAQERAKEATEAKAAAKREDQKYALSVMMKIEEEERKKIEDMKENERIKATKALEAWKEYQRKAEEQKKIQREEKLCQKEKQIKEERKKIKYKSLTRNLASRNLAPKGRNSENIFTEKLKEDSIPAPRSVGSIKINFTPRVFPTALRESQVAEEEEWLHKQAEARRAMNTDIAELCDLKEEEKNPEWLKDKGNKLFATENYLAAINAYNLAIRLNNKMPLLYLNRAACHLKLKNLHKAIEDSSKALELLMPPVTDNANARMKAHVRRGTAFCQLELYVEGLQDYEAALKIDPSNKIVQIDAEKIRNVIQGTELKS

>Q9UKF7

MLLKEYRICMPLTVDEYKIGQLYMISKHSHEQSDRGEGVEVVQNEPFEDPHHGNGQFTEKRVYLNSKLPSWARAVVPKIFYVTEKAWNYYPYTITEYTCSFLPKFSIHIETKYEDNKGSNDTIFDNEAKDVEREVCFIDIACDEIPERYYKESEDPKHFKSEKTGRGQLREGWRDSHQPIMCSYKLVTVKFEVWGLQTRVEQFVHKVVRDILLIGHRQAFAWVDEWYDMTMDDVREFERATQEATNKKIGIFPPAISISSIPLLPSSVRSAPSSAPSTPLSTDAPEFLSVPKDRPRKKSAPETLTLPDPEKKATLNLPGMHSSDKPCRPKSE

420 P07992 P50135

>P07992

MDPGKDKEGVPQPSGPPARKKFVIPLDEDEVPPGVAKPLFRSTQSLPTVDTSAQAAPQTYAEYAISQPLEGAGATCPTGSEPLAGETPNQALKPGAKSNSIIVSPRQRGNPVLKFVRNVPWEFGDVIPDYVLGQSTCALFLSLRYHNLHPDYIHGRLQSLGKNFALRVLLVQVDVKDPQQALKELAKMCILADCTLILAWSPEEAGRYLETYKAYEQKPADLLMEKLEQDFVSRVTECLTTVKSVNKTDSQTLLTTFGSLEQLIAASREDLALCPGLGPQKARRLFDVLHEPFLKVP

>P50135

MASSMRSLFSDHGKYVESFRRFLNHSTEHQCMQEFMDKKLPGIIGRIGDTKSEIKILSIGGGAGEIDLQILSKVQAQYPGVCINNEVVEPSAEQIAKYKELVAKTSNLENVKFAWHKETSSEYQSRMLEKKELQKWDFIHMIQMLYYVKDIPATLKFFHSLLGTNAKMLIIVVSGSSGWDKLWKKYGSRFPQDDLCQYITSDDLTQMLDNLGLKYECYDLLSTMDISDCFIDGNENGDLLWDFLTETCNFNATAPPDLRAELGKDLQEPEFSAKKEGKVLFNNTLSFIVIEA

421 NP_055138.2 NP_056273.2

>NP_055138.2

MERVNDASCGPSGCYTYQVSRHSTEMLHNLNQQRKNGGRFCDVLLRVGDESFPAHRAVLAACSEYFESVFSAQLGDGGAADGGPADVGGATAAPGGGAGGSRELEMHTISSKVFGDILDFAYTSRIVVRLESFPELMTAAKFLLMRSVIEICQEVIKQSNVQILVPPARADIMLFRPPGTSDLGFPLDMTNGAALAANSNGIAGSMQPEEEAARAAGAAIAGQASLPVLPGVDRLPMVAGPLSPQLLTSPFPSVASSAPPLTGKRGRGRPRKANLLDSMFGSPGGLREAGILPCGLCGKVFTDANRLRQHEAQHGVTSLQLGYIDLPPPRLGENGLPISEDPDGPRKRSRTRKQVACEICGKIFRDVYHLNRHKLSHSGEKPYSCPVCGLRFKRKDRMSYHVRSHDGSVGKPYICQSCGKGFSRPDHLNGHIKQVHTSERPHKCQTCNASFATRDRLRSHLACHEDKVPCQVCGKYLRAAYMADHLKKHSEGPSNFCSICNRGFSSASYLKVHVKTHHGVPLPQVSRHQEPILNGGAAFHCARTYGNKEGQKCSHQDPIESSDSYGDLSDASDLKTPEKQSANGSFSCDMAVPKNKMESDGEKKYPCPECGSFFRSKSYLNKHIQKVHVRALGGPLGDLGPALGSPFSPQQNMSLLESFGFQIVQSAFASSLVDPEVDQQPMGPEGK

>NP_056273.2

MEFAELIKTPRVDNVVLHRPFYPAVEGTLCLTGHHLILSSRQDNTEELWLLHSNIDAIDKRFVGSLGTIIIKCKDFRIIQLDIPGMEECLNIASSIEALSTLDSITLMYPFFYRPMFEVIEDGWHSFLPEQEFELYSSATSEWRLSYVNKEFAVCPSYPPIVTVPKSIDDEALRKVATFRHGGRFPVLSYYHKKNGMVIMRSGQPLTGTNGRRCKEDEKLINATLRAGKRGYIIDTRSLNVAQQTRAKGGGFEQEAHYPQWRRIHKSIERYHILQESLIKLVEACNDQTHNMDRWLSKLEASNWLTHIKEILTTACLAAQCIDREGASILIHGTEGTDSTLQVTSLAQIILEPRSRTIRGFEALIEREWLQAGHPFQQRCAQSAYCNTKQKWEAPVFLLFLDCVWQILRQFPCSFEFNENFLIMLFEHAYASQFGTFLGNNESERCKLKLQQKTMSLWSWVNQPSELSKFTNPLFEANNLVIWPSVAPQSLPLWEGIFLRWNRSSKYLDEAYEEMVNIIEYNKELQAKVNILRRQLAELETEDGMQESP

422 NP_056271.2 Q8N6F7

>NP_056271.2

MFAGLQDLGVANGEDLKETLTNCTEPLKAIEQFQTENGVLLPSLQSALPFLDLHGTPRLEFHQSVFDELRDKLLERVSAIASEGKAEERYKKLEDLLEKSFSLVKMPSLQPVVMCVMKHLPKVPEKKLKLVMADKELYRACAVEVKRQIWQDNQALFGDEVSPLLKQYILEKESALFSTELSVLHNFFSPSPKTRRQGEVVQRLTRMVGKNVKLYDMVLQFLRTLFLRTRNVHYCTLRAELLMSLHDLDVGEICTVDPCHKFTWCLDACIRERFVDSKRARELQGFLDGVKKGQEQVLGDLSMILCDPFAINTLALSTVRHLQELVGQETLPRDSPDLLLLLRLLALGQGAWDMIDSQVFKEPKMEVELITRFLPMLMSFLVDDYTFNVDQKLPAEEKAPVSYPNTLPESFTKFLQEQRMACEVGLYYVLHITKQRNKNALLRLLPGLVETFGDLAFGDIFLHLLTGNLALLADEFALEDFCSSLFDGFFLTASPRKENVHRHALRLLIHLHPRVAPSKLEALQKALEPTGQSGEAVKELYSQLGEKLEQLDHRKPSPAQAAETPALELPLPSVPAPAPL

>Q8N6F7

MGNSLLRENRRQQNTQEMPWNVRMQSPKQRTSRCWDHHIAEGCFCLPWKKILIFEKRQDSQNENERMSSTPIQDNVDQTYSEELCYTLINHRVLCTRPSGNSAEEYYENVPCKAERPRESLGGTETEYSLLHMPSTDPRHARSPEDEYELLMPHRISSHFLQQPRPLMAPSETQFSHL

423 NP_006390.1 NP_005557.1

>NP_006390.1

MPHSSDSSDSSFSRSPPPGKQDSSDDVRRVQRREKNRIAAQKSRQRQTQKADTLHLESEDLEKQNAALRKEIKQLTEELKYFTSVLNSHEPLCSVLAASTPSPPEVVYSAHAFHQPHVSSPRFQP

>NP_005557.1

MATLKDQLIYNLLKEEQTPQNKITVVGVGAVGMACAISILMKDLADELALVDVIEDKLKGEMMDLQHGSLFLRTPKIVSGKDYNVTANSKLVIITAGARQQEGESRLNLVQRNVNIFKFIIPNVVKYSPNCKLLIVSNPVDILTYVAWKISGFPKNRVIGSGCNLDSARFRYLMGERLGVHPLSCHGWVLGEHGDSSVPVWSGMNVAGVSLKTLHPDLGTDKDKEQWKEVHKQVVESAYEVIKLKGYTSWAIGLSVADLAESIMKNLRRVHPVSTMIKGLYGIKDDVFLSVPCILGQNGISDLVKVTLTSEEEARLKKSADTLWGIQKELQF

424 NP_076921.1 Q14249

>NP_076921.1

MSSYFVNSLFSKYKTGESLRPNYYDCGFAQDLGGRPTVVYGPSSGGSFQHPSQIQEFYHGPSSLSTAPYQQNPCAVACHGDPGNFYGYDPLQRQSLFGAQDPDLVQYADCKLAAASGLGEEAEGSEQSPSPTQLFPWMRPQAAAGRRRGRQTYSRYQTLELEKEFLFNPYLTRKRRIEVSHALGLTERQVKIWFQNRRMKWKKENNKDKFPSSKCEQEELEKQKLERAPEAADEGDAQKGDKK

>Q14249

MRALRAGLTLALGAGLGAVVEGWRRRREDARAAPGLLGRLPVLPVAAAAELPPVPGGPRGPGELAKYGLPGLAQLKSRESYVLCYDPRTRGALWVVEQLRPERLRGDGDRRECDFREDDSVHAYHRATNADYRGSGFDRGHLAAAANHRWSQKAMDDTFYLSNVAPQVPHLNQNAWNNLEKYSRSLTRSYQNVYVCTGPLFLPRTEADGKSYVKYQVIGKNHVAVPTHFFKVLILEAAGGQIELRTYVMPNAPVDEAIPLERFLVPIESIERASGLLFVPNILARAGSLKAITAGSK

425 Q64ET8 P36957

>Q64ET8

MGKGNEDSDLHCSSIQCSTDQPPFQQISFTEKGSDEKKPFKEKGKTAFSHSSEKHIQRQGSEPNPNKENSEETKLKAGNSTAGSEPESSSYRENCRKRKMSSKDSCQDTAGNCPEKECSLSLNKKSRSSTAVHNSEIQETCDAHHRGHSRACTGHSKRHRSRALGVQTPSIRKSLVTSVRAMSEAVYQDLAQVWAQQIHSPLTCEQLTLLTRLRGPLCAQVQTLYSMATQAAYVFPAESWLVPATLPGPGESALDREAHPFPGQEITETVSGSDEAKL

>P36957

MLSRSRCVSRAFSRSLSAFQKGNCPLGRRSLPGVSLCQGPGYPNSRKVVINNSVFSVRFFRTTAVCKDDLVTVKTPAFAESVTEGDVRWEKAVGDTVAEDEVVCEIETDKTSVQVPSPANGVIEALLVPDGGKVEGGTPLFTLRKTGAAPAKAKPAEAPAAAAPKAEPTAAAVPPPAAPIPTQMPPVPSPSQPPSGKPVSAVKPTVAPPLAEAGAGKGLRSEHREKMNRMRQRIAQRLKEAQNTCAMLTTFNEIDMSNIQEMRARHKEAFLKKHNLKLGFMSAFVKASAFALQEQPVVNAVIDDTTKEVVYRDYIDISVAVATPRGLVVPVIRNVEAMNFADIERTITELGEKARKNELAIEDMDGGTFTISNGGVFGSLFGTPIINPPQSAILGMHGIFDRPVAIGGKVEVRPMMYVALTYDHRLIDGREAVTFLRKIKAAVEDPRVLLLDL

426 NP_073622.2 Q9HBU6

>NP_073622.2

MCKMAIIPDWLRSHPHTRKFTHSRPHSSPCRVYSRNGSPNKFRSSSTTAVANPTLSSLDVKRILFQKITDRGDELQKAFQLLDTGQNLTVSKSELRRIITDFLMPLTREQFQDVLAQIPLSTSGTVPYLAFLSRFGGIDLYINGIKRGGGNEMNCCRTLRELEIQVGEKVFKNIKTVMKAFELIDVNKTGLVRPQELRRVLETFCMKLRDEEYEKFSKHYNIHKDTAVDYNVFLKNLSINNDLNLRYCMGNQEVSLENQQAKNSKKERLLGSASSEDIWRNYSLDEIERNFCLQLSKSYEKVEKALSAGDPCKGGYVSFNYLKIVLDTFVYQIPRRIFIQLMKRFGLKATTKINWKQFLTSFHEPQGLQVSSKGPLTKRNSINSRNESHKENIITKLFRHTEDHSASLKKALLIINTKPDGPITREEFRYILNCMAVKLSDSEFKELMQMLDPGDTGVVNTSMFIDLIEENCRMRKTSPCTDAKTPFLLAWDSVEEIVHDTITRNLQAFYNMLRSYDLGDTGRIGRNNFKKIMHVFCPFLTNAHFIKLCSKIQDIGSGRILYKKLLACIGIDGPPTVSPVLVPKDQLLSEHLQKDEQQQPDLSERTKLTEDKTTLTKKMTTEEVIEKFKKCIQQQDPAFKKRFLDFSKEPNGKINVHDFKKVLEDTGMPMDDDQYALLTTKIGFEKEGMSYLDFAAGFEDPPMRGPETTPPQPPTPSKSYVNSHFITAEECLKLFPRRLKESFRDPYSAFFKTDADRDGIINMHDLHRLLLHLLLNLKDDEFERFLGLLGLRLSVTLNFREFQNLCEKRPWRTDEAPQRLIRPKQKVADSELACEQAHQYLVTKAKNRWSDLSKNFLETDNEGNGILRRRDIKNALYGFDIPLTPREFEKLWARYDTEGKGHITYQEFLQKLGINYSPAVHRPCAEDYFNFMGHFTKPQQLQEEMKELQQSTEKAVAARDKLMDRHQDISKAFTKTDQSKTNYISICKMQEVLEECGCSLTEGELTHLLNSWGVSRHDNAINYLDFLRAVENSKSTGAQPKEKEESMPINFATLNPQEAVRKIQEVVESSQLALSTAFSALDKEDTGFVKATEFGQVLKDFCYKLTDNQYHYFLRKLRIHLTPYINWKYFLQNFSCFLEETADEWAEKMPKGPPPTSPKATADRDILARLHKAVTSHYHAITQEFENFDTMKTNTISREEFRAICNRRVQILTDEQFDRLWNEMPVNAKGRLKYPDFLSRFSSETAATPMATGDSAVAQRGSSVPDVSEGTRSALSLPTQELRPGSKSQSHPCTPASTTVIPGTPPLQNCDPIESRLRKRIQGCWRQLLKECKEKDVARQGDINASDFLALVEKFNLDISKEECQQLIIKYDLKSNGKFAYCDFIQSCVLLLKAKESSLMHRMKIQNAHKMKEAGAETPSFYSALLRIQPKIVHCWRPMRRTFKSYDEAGTGLLSVADFRTVLRQYSINLSEEEFFHILEYYDKTLSSKISYNDFLRAFLQ

>Q9HBU6

MLCGRPRSSSDNRNFLRERAGLSSAAVQTRIGNSAASRRSPAARPPVPAPPALPRGRPGTEGSTSLSAPAVLVVAVAVVVVVVSAVAWAMANYIHVPPGSPEVPKLNVTVQDQEEHRCREGALSLLQHLRPHWDPQEVTLQLFTDGITNKLIGCYVGNTMEDVVLVRIYGNKTELLVDRDEEVKSFRVLQAHGCAPQLYCTFNNGLCYEFIQGEALDPKHVCNPAIFRLIARQLAKIHAIHAHNGWIPKSNLWLKMGKYFSLIPTGFADEDINKRFLSDIPSSQILQEEMTWMKEILSNLGSPVVLCHNDLLCKNIIYNEKQGDVQFIDYEYSGYNYLAYDIGNHFNEFAGVSDVDYSLYPDRELQSQWLRAYLEAYKEFKGFGTEVTEKEVEILFIQVNQFALASHFFWGLWALIQAKYSTIEFDFLGYAIVRFNQYFKMKPEVTALKVPE

427 Q96KK5 P53004

>Q96KK5

MSGRGKQGGKARAKAKTRSSRAGLQFPVGRVHRLLRKGNYAERVGAGAPVYLAAVLEYLTAEILELAGNAARDNKKTRIIPRHLQLAIRNDEELNKLLGKVTIAQGGVLPNIQAVLLPKKTESHHKAK

>P53004

MNAEPERKFGVVVVGVGRAGSVRMRDLRNPHPSSAFLNLIGFVSRRELGSIDGVQQISLEDALSSQEVEVAYICSESSSHEDYIRQFLNAGKHVLVEYPMTLSLAAAQELWELAEQKGKVLHEEHVELLMEEFAFLKKEVVGKDLLKGSLLFTAGPLEEERFGFPAFSGISRLTWLVSLFGELSLVSATLEERKEDQYMKMTVCLETEKKSPLSWIEEKGPGLKRNRYLSFHFKSGSLENVPNVGVNKNIFLKDQNIFVQKLLGQFSEKELAAEKKRILHCLGLAEEIQKYCCSRK

428 NP_005366.2 NP_203526.1

>NP_005366.2

MARRPRHSIYSSDEDDEDFEMCDHDYDGLLPKSGKRHLGKTRWTREEDEKLKKLVEQNGTDDWKVIANYLPNRTDVQCQHRWQKVLNPELIKGPWTKEEDQRVIELVQKYGPKRWSVIAKHLKGRIGKQCRERWHNHLNPEVKKTSWTEEEDRIIYQAHKRLGNRWAEIAKLLPGRTDNAIKNHWNSTMRRKVEQEGYLQESSKASQPAVATSFQKNSHLMGFAQAPPTAQLPATGQPTVNNDYSYYHISEAQNVSSHVPYPVALHVNIVNVPQPAAAAIQRHYNDEDPEKEKRIKELELLLMSTENELKGQQVLPTQNHTCSYPGWHSTTIADHTRPHGDSAPVSCLGEHHSTPSLPADPGSLPEESASPARCMIVHQGTILDNVKNLLEFAETLQFIDSFLNTSSNHENSDLEMPSLTSTPLIGHKLTVTTPFHRDQTVKTQKENTVFRTPAIKRSILESSPRTPTPFKHALAAQEIKYGPLKMLPQTPSHLVEDLQDVIKQESDESGIVAEFQENGPPLLKKIKQEVESPTDKSGNFFCSHHWEGDSLNTQLFTQTSPVADAPNILTSSVLMAPASEDEDNVLKAFTVPKNRSLASPLQPCSSTWEPASCGKMEEQMTSSSQARKYVNAFSARTLVM

>NP_203526.1

MSWSGLLHGLNTSLTCGPALVPRLWATCSMATLNQMHRLGPPKRPPRKLGPTEGRPQLKGVVLCTFTRKPKKPNSANRKCCRVRLSTGREAVCFIPGEGHTLQEHQIVLVEGGRTQDLPGVKLTVVRGKYDCGHVQKK

429 NP_004442.3 Q92526

>NP_004442.3

MSSQVVGIEPLYIKAEPASPDSPKGSSETETEPPVALAPGPAPTRCLPGHKEEEDGEGAGPGEQGGGKLVLSSLPKRLCLVCGDVASGYHYGVASCEACKAFFKRTIQGSIEYSCPASNECEITKRRRKACQACRFTKCLRVGMLKEGVRLDRVRGGRQKYKRRPEVDPLPFPGPFPAGPLAVAGGPRKTAAPVNALVSHLLVVEPEKLYAMPDPAGPDGHLPAVATLCDLFDREIVVTISWAKSIPGFSSLSLSDQMSVLQSVWMEVLVLGVAQRSLPLQDELAFAEDLVLDEEGARAAGLGELGAALLQLVRRLQALRLEREEYVLLKALALANSDSVHIEDAEAVEQLREALHEALLEYEAGRAGPGGGAERRRAGRLLLTLPLLRQTAGKVLAHFYGVKLEGKVPMHKLFLEMLEAMMD

>Q92526

MAAIKAVNSKAEVARAQAALAVNICAARGLQDVLRTNLGPKGTMKMLASGAGDIKLTKDGNVLLDEMQIQHPTASLIAKVATAQDDVTGDGTTSNVLIIGELLKQADLYISEGLHPRIIAEGFEAAKIKALEVLEEVKVTKEMKRKILLDVARTSLQTKVHAELADVLTEVVVDSVLAVRRPGYPIDLFMVEIMEMKHKLGTDTKLIQGLVLDHGARHPDMKKRVEDAFILICNVSLEYEKTEVNSGFFYKTAEEKEKLVKAERKFIEDRVQKIIDLKDKVCAQSNKGFVVINQKGIDPFSLDSLAKHGIVALRRAKRRNMERLSLACGGMAVNSFEDLTVDCLGHAGLVYEYTLGEEKFTFIEECVNPCSVTLLVKGPNKHTLTQVKDAIRDGLRAIKNAIEDGCMVPGAGAIEVAMAEALVTYKNSIKGRARLGVQAFADALLIIPKVLAQNAGYDPQETLVKVQAEHVESKQLVGVDLNTGEPMVAADAGVWDNYCVKKQLLHSCTVIATNILLVDEIMRAGMSSLK

430 Q15562 NP_060383.2

>Q15562

MGEPRAGAALDDGSGWTGSEEGSEEGTGGSEGAGGDGGPDAEGVWSPDIEQSFQEALAIYPPCGRRKIILSDEGKMYGRNELIARYIKLRTGKTRTRKQVSSHIQVLARRKSREIQSKLKDQVSKDKAFQTMATMSSAQLISAPSLQAKLGPTGPQASELFQFWSGGSGPPWNVPDVKPFSQTPFTLSLTPPSTDLPGYEPPQALSPLPPPTPSPPAWQARGLGTARLQLVEFSAFVEPPDAVDSYQRHLFVHISQHCPSPGAPPLESVDVRQIYDKFPEKKGGLRELYDRGPPHAFFLVKFWADLNWGPSGEEAGAGGSISSGGFYGVSSQYESLEHMTLTCSSKVCSFGKQVVEKVETERAQLEDGRFVYRLLRSPMCEYLVNFLHKLRQLPERYMMNSVLENFTILQVVTNRDTQELLLCTAYVFEVSTSERGAQHHIYRLVRD

>NP_060383.2

MEQPWPPPGPWSLPRAEGEAEEESDFDVFPSSPRCPQLPGGGAQMYSHGIELACQKQKEFVKSSVACKWNLAEAQQKLGSLALHNSESLDQEHAKAQTAVSELRQREEEWRQKEEALVQREKMCLWSTDAISKDVFNKSFINQDKRKDTEDEDKSESFMQKYEQKIRHFGMLSRWDDSQRFLSDHPYLVCEETAKYLILWCFHLEAEKKGALMEQIAHQAVVMQFIMEMAKNCNVDPRGCFRLFFQKAKAEEEGYFEAFKNELEAFKSRVRLYSQSQSFQPMTVQNHVPHSGVGSIGLLESLPQNPDYLQYSISTALCSLNSVVHKEDDEPKMMDTV

431 NP_006464.1 P78368

>NP_006464.1

MSEREERRFVEIPRESVRLMAESTGLELSDEVAALLAEDVCYRLREATQNSSQFMKHTKRRKLTVEDFNRALRWSSVEAVCGYGSQEALPMRPAREGELYFPEDREVNLVELALATNIPKGCAETAVRVHVSYLDGKGNLAPQGSVPSAVSSLTDDLLKYYHQVTRAVLGDDPQLMKVALQDLQTNSKIGALLPYFVYVVSGVKSVSHDLEQLHRLLQVARSLFRNPHLCLGPYVRCLVGSVLYCVLEPLAASINPLNDHWTLRDGAALLLSHIFWTHGDLVSGLYQHILLSLQKILADPVRPLCCHYGAVVGLHALGWKAVERVLYPHLSTYWTNLQAVLDDYSVSNAQVKADGHKVYGAILVAVERLLKMKAQAAEPNRGGPGGRGCRRLDDLPWDSLLFQESSSGGGAEPSFGSGLPLPPGGAGPEDPSLSVTLADIYRELYAFFGDSLATRFGTGQPAPTAPRPPGDKKEPAAAPDSVRKMPQLTASAIVSPHGDESPRGSGGGGPASASGPAASESRPLPRVHRARGAPRQQGPGTGTRDVFQKSRFAPRGAPHFRFIIAGRQAGRRCRGRLFQTAFPAPYGPSPASRYVQKLPMIGRTSRPARRWALSDYSLYLPL

>P78368

MDFDKKGGKGETEEGRRMSKAGGGRSSHGIRSSGTSSGVLMVGPNFRVGKKIGCGNFGELRLGKNLYTNEYVAIKLEPIKSRAPQLHLEYRFYKQLSATEGVPQVYYFGPCGKYNAMVLELLGPSLEDLFDLCDRTFTLKTVLMIAIQLITRMEYVHTKSLIYRDVKPENFLVGRPGTKRQHAIHIIDFGLAKEYIDPETKKHIPYREHKSLTGTARYMSINTHLGKEQSRRDDLEALGHMFMYFLRGSLPWQGLKADTLKERYQKIGDTKRATPIEVLCENFPEEMATYLRYVRRLDFFEKPDYDYLRKLFTDLFDRSGFVFDYEYDWAGKPLPTPIGTVHTDLPSQPQLRDKTQPHSKNQALNSTNGELNADDPTAGHSNAPITAPAEVEVADETKCCCFFKRRKRKSLQRHK

432 NP_071801.1 NP_659451.1

>NP_071801.1

MERKISRIHLVSEPSITHFLQVSWEKTLESGFVITLTDGHSAWTGTVSESEISQEADDMAMEKGKYVGELRKALLSGAGPADVYTFNFSKESCYFFFEKNLKDVSFRLGSFNLEKVENPAEVIRELICYCLDTIAENQAKNEHLQKENERLLRDWNDVQGRFEKCVSAKEALETDLYKRFILVLNEKKTKIRSLHNKLLNAAQEREKDIKQEGETAICSEMTADRDPVYDESTDEESENQTDLSGLASAAVSKDDSIISSLDVTDIAPSRKRRQRMQRNLGTEPKMAPQENQLQEKENSRPDSSLPETSKKEHISAENMSLETLRNSSPEDLFDEI

>NP_659451.1

MEELLPDGQIWANMDPEERMLAAATAFTHICAGQGEGDVRREAQSIQYDPYSKASVAPGKRPALPVQLQYPHVESNVPSETVSEASQRLRKPVMKRKVLRRKPDGEVLVTDESIISESESGTENDQDLWDLRQRLMNVQFQEDKESSFDVSQKFNLPHEYQGISQDQLICSLQREGMGSPAYEQDLIVASRPKSFILPKLDQLSRNRGKTDRVARYFEYKRDWDSIRLPGEDHRKELRWGVREQMLCRAEPQSKPQHIYVPNNYLVPTEKKRSALRWGVRCDLANGVIPRKLPFPLSPS

433 Q9BWE0 NP_002159.2

>Q9BWE0

MLERRCRGPLAMGLAQPRLLSGPSQESPQTLGKESRGLRQQGTSVAQSGAQAPGRAHRCAHCRRHFPGWVALWLHTRRCQARLPLPCPECGRRFRHAPFLALHRQVHAAATPDLGFACHLCGQSFRGWVALVLHLRAHSAAKRPIACPKCERRFWRRKQLRAHLRRCHPPAPEARPFICGNCGRSFAQWDQLVAHKRVHVAEALEEAAAKALGPRPRGRPAVTAPRPGGDAVDRPFQCACCGKRFRHKPNLIAHRRVHTGERPHQCPECGKRFTNKPYLTSHRRIHTGEKPYPCKECGRRFRHKPNLLSHSKIHKRSEGSAQAAPGPGSPQLPAGPQESAAEPTPAVPLKPAQEPPPGAPPEHPQDPIEAPPSLYSCDDCGRSFRLERFLRAHQRQHTGERPFTCAECGKNFGKKTHLVAHSRVHSGERPFACEECGRRFSQGSHLAAHRRDHAPDRPFVCPDCGKAFRHKPYLAAHRRIHTGEKPYVCPDCGKAFSQKSNLVSHRRIHTGERPYACPDCDRSFSQKSNLITHRKSHIRDGAFCCAICGQTFDDEERLLAHQKKHDV

>NP_002159.2

MAGYLRVVRSLCRASGSRPAWAPAALTAPTSQEQPRRHYADKRIKVAKPVVEMDGDEMTRIIWQFIKEKLILPHVDIQLKYFDLGLPNRDQTDDQVTIDSALATQKYSVAVKCATITPDEARVEEFKLKKMWKSPNGTIRNILGGTVFREPIICKNIPRLVPGWTKPITIGRHAHGDQYKATDFVADRAGTFKMVFTPKDGSGVKEWEVYNFPAGGVGMGMYNTDESISGFAHSCFQYAIQKKWPLYMSTKNTILKAYDGRFKDIFQEIFDKHYKTDFDKNKIWYEHRLIDDMVAQVLKSSGGFVWACKNYDGDVQSDILAQGFGSLGLMTSVLVCPDGKTIEAEAAHGTVTRHYREHQKGRPTSTNPIASIFAWTRGLEHRGKLDGNQDLIRFAQMLEKVCVETVESGAMTKDLAGCIHGLSNVKLNEHFLNTTDFLDTIKSNLDRALGRQ

434 NP_064706.1 NP_689953.1

>NP_064706.1

MSIETLLEAARFLEWQAQQQQRAREEQERLRLEQEREQEQKKANSLARLAHTLPVEEPRMEAPPLPLSPPAPPPAPPPPLATPAPLTVIPIPVVTNSPQPLPPPPPLPAAAQPLPLAPRQPALVGAPGLSIKEPAPLPSRPQVPTPAPLLPDSKATIPPNGSPKPLQPLPTPVLTIAPHPGVQPQLAPQQPPPPTLGTLKLAPAEEVKSSEQKKRPGGIGTREVHNKLEKNRRAHLKECFETLKRNIPNVDDKKTSNLSVLRTALRYIQSLKRKEKEYEHEMERLAREKIATQQRLAELKHELSQWMDVLEIDRVLRQTGQPEDDQASTSTASEGEDNIDEDMEEDRAGLGPPKLSHRPQPELLKSTLPPPSTTPAPLPPHPHPHPHSVALPPAHLPVQQQQPQQKTPLPAPPPPPAAPAQTLVPAPAHLVATAGGGSTVIAHTATTHASVIQTVNHVLQGPGGKHIAHIAPSAPSPAVQLAPATPPIGHITVHPATLNHVAHLGSQLPLYPQPVAVSHIAHTLSHQQVNGTAGLGPPATVMAKPAVGAQVVHHPQLVGQTVLNPVTMVTMPSFPVSTLKLA

>NP_689953.1

MAASLRLLGAASGLRYWSRRLRPAAGSFAAVCSRSVASKTPVGFIGLGNMGNPMAKNLMKHGYPLIIYDVFPDACKEFQDAGEQVVSSPADVAEKADRIITMLPTSINAIEAYSGANGILKKVKKGSLLIDSSTIDPAVSKELAKEVEKMGAVFMDAPVSGGVGAARSGNLTFMVGGVEDEFAAAQELLGCMGSNVVYCGAVGTGQAAKICNNMLLAISMIGTAEAMNLGIRLGLDPKLLAKILNMSSGRCWSSDTYNPVPGVMDGVPSANNYQGGFGTTLMAKDLGLAQDSATSTKSPILLGSLAHQIYRMMCAKGYSKKDFSSVFQFLREEETF

435 NP_001291.3 O75897

>NP_001291.3

MDVLPMCSIFQELQIVHETGYFSALPSLEEYWQQTCLELERYLQSEPCYVSASEIKFDSQEDLWTKIILAREKKEESELKISSSPPEDTLISPSFCYNLETNSLNSDVSSESSDSSEELSPTAKFTSDPIGEVLVSSGKLSSSVTSTPPSSPELSREPSQLWGCVPGELPSPGKVRSGTSGKPGDKGNGDASPDGRRRVHRCHFNGCRKVYTKSSHLKAHQRTHTGEKPYRCSWEGCEWRFARSDELTRHFRKHTGAKPFKCSHCDRCFSRSDHLALHMKRHL

>O75897

MALHDMEDFTFDGTKRLSVNYVKGILQPTDTCDIWDKIWNFQAKPDDLLISTYPKAGTTWTQEIVELIQNEGDVEKSKRAPTHQRFPFLEMKIPSLGSGLEQAHAMPSPRILKTHLPFHLLPPSLLEKNCKIIYVARNPKDNMVSYYHFQRMNKALPAPGTWEEYFETFLAGKVCWGSWHEHVKGWWEAKDKHRILYLFYEDMKKNPKHEIQKLAEFIGKKLDDKVLDKIVHYTSFDVMKQNPMANYSSIPAEIMDHSISPFMRKGAVGDWKKHFTVAQNERFDEDYKKKMTDTRLTFHFQF

436 NP_003064.2 P59044

>NP_003064.2

MMMMALSKTFGQKPVKFQLEDDGEFYMIGSEVGNYLRMFRGSLYKRYPSLWRRLATVEERKKIVASSHGKKTKPNTKDHGYTTLATSVTLLKASEVEEILDGNDEKYKAVSISTEPPTYLREQKAKRNSQWVPTLPNSSHHLDAVPCSTTINRNRMGRDKKRTFPLCFDDHDPAVIHENASQPEVLVPIRLDMEIDGQKLRDAFTWNMNEKLMTPEMFSEILCDDLDLNPLTFVPAIASAIRQQIESYPTDSILEDQSDQRVIIKLNIHVGNISLVDQFEWDMSEKENSPEKFALKLCSELGLGGEFVTTIAYSIRGQLSWHQKTYAFSENPLPTVEIAIRNTGDADQWCPLLETLTDAEMEKKIRDQDRNTRRMRRLANTAPAW

>P59044

MDQPEAPCSSTGPRLAVARELLLAALEELSQEQLKRFRHKLRDVGPDGRSIPWGRLERADAVDLAEQLAQFYGPEPALEVARKTLKRADARDVAAQLQERRLQRLGLGSGTLLSVSEYKKKYREHVLQLHARVKERNARSVKITKRFTKLLIAPESAAPEEAMGPAEEPEPGRARRSDTHTFNRLFRRDEEGRRPLTVVLQGPAGIGKTMAAKKILYDWAAGKLYQGQVDFAFFMPCGELLERPGTRSLADLILDQCPDRGAPVPQMLAQPQRLLFILDGADELPALGGPEAAPCTDPFEAASGARVLGGLLSKALLPTALLLVTTRAAAPGRLQGRLCSPQCAEVRGFSDKDKKKYFYKYFRDERRAERAYRFVKENETLFALCFVPFVCWIVCTVLRQQLELGRDLSRTSKTTTSVYLLFITSVLSSAPVADGPRLQGDLRNLCRLAREGVLGRRAQFAEKELEQLELRGSKVQTLFLSKKELPGVLETEVTYQFIDQSFQEFLAALSYLLEDGGVPRTAAGGVGTLLRGDAQPHSHLVLTTRFLFGLLSAERMRDIERHFGCMVSERVKQEALRWVQGQGQGCPGVAPEVTEGAKGLEDTEEPEEEEEGEEPNYPLELLYCLYETQEDAFVRQALCRFPELALQRVRFCRMDVAVLSYCVRCCPAGQALRLISCRLVAAQEKKKKSLGKRLQASLGGGSSSQGTTKQLPASLLHPLFQAMTDPLCHLSSLTLSHCKLPDAVCRDLSEALRAAPALTELGLLHNRLSEAGLRMLSEGLAWPQCRVQTVRVQLPDPQRGLQYLVGMLRQSPALTTLDLSGCQLPAPMVTYLCAVLQHQGCGLQTLSLASVELSEQSLQELQAVKRAKPDLVITHPALDGHPQPPKELISTF

437 NP_001443.1 NP_006422.1

>NP_001443.1

MTTEGGPPPAPLRRACSPVPGALQAALMSPPPAAAAAAAAAPETTSSSSSSSSASCASSSSSSNSASAPSAACKSAGGGGAGAGSGGAKKASSGLRRPEKPPYSYIALIVMAIQSSPSKRLTLSEIYQFLQARFPFFRGAYQGWKNSVRHNLSLNECFIKLPKGLGRPGKGHYWTIDPASEFMFEEGSFRRRPRGFRRKCQALKPMYHRVVSGLGFGASLLPQGFDFQAPPSAPLGCHSQGGYGGLDMMPAGYDAGAGAPSHAHPHHHHHHHVPHMSPNPGSTYMASCPVPAGPGGVGAAGGGGGGDYGPDSSSSPVPSSPAMASAIECHSPYTSPAAHWSSPGASPYLKQPPALTPSSNPAASAGLHSSMSSYSLEQSYLHQNAREDLSVGLPRYQHHSTPVCDRKDFVLNFNGISSFHPSASGSYYHHHHQSVCQDIKPCVM

>NP_006422.1

MASLSLAPVNIFKAGADEERAETARLTSFIGAIAIGDLVKSTLGPKGMDKILLSSGRDASLMVTNDGATILKNIGVDNPAAKVLVDMSRVQDDEVGDGTTSVTVLAAELLREAESLIAKKIHPQTIIAGWREATKAAREALLSSAVDHGSDEVKFRQDLMNIAGTTLSSKLLTHHKDHFTKLAVEAVLRLKGSGNLEAIHIIKKLGGSLADSYLDEGFLLDKKIGVNQPKRIENAKILIANTGMDTDKIKIFGSRVRVDSTAKVAEIEHAEKEKMKEKVERILKHGINCFINRQLIYNYPEQLFGAAGVMAIEHADFAGVERLALVTGGEIASTFDHPELVKLGSCKLIEEVMIGEDKLIHFSGVALGEACTIVLRGATQQILDEAERSLHDALCVLAQTVKDSRTVYGGGCSEMLMAHAVTQLANRTPGKEAVAMESYAKALRMLPTIIADNAGYDSADLVAQLRAAHSEGNTTAGLDMREGTIGDMAILGITESFQVKRQVLLSAAEAAEVILRVDNIIKAAPRKRVPDHHPC

438 NP_056943.1 NP_060311.1

>NP_056943.1

MANSANTNTVPKLYRSVIEDVINDVRDIFLDDGVDEQVLMELKTLWENKLMQSRAVDGFHSEEQQLLLQVQQQHQPQQQQHHHHHHHQQAQPQQTVPQQAQTQQVLIPASQQATAPQVIVPDSKLIQHMNASNMSAAATAATLALPAGVTPVQQILTNSGQLLQVVRAANGAQYIFQPQQSVVLQQQVIPQMQPGGVQAPVIQQVLAPLPGGISPQTGVIIQPQQILFTGNKTQVIPTTVAAPTPAQAQITATGQQQPQAQPAQTQAPLVLQVDGTGDTSSEEDEDEEEDYDDDEEEDKEKDGAEDGQVEEEPLNSEDDVSDEEGQELFDTENVVVCQYDKIHRSKNKWKFHLKDGIMNLNGRDYIFSKAIGDAEW

>NP_060311.1

MAVSTVFSTSSLMLALSRHSLLSPLLSVTSFRRFYRGDSPTDSQKDMIEIPLPPWQERTDESIETKRARLLYESRKRGMLENCILLSLFAKEHLQHMTEKQLNLYDRLINEPSNDWDIYYWATEAKPAPEIFENEVMALLRDFAKNKNKEQRLRAPDLEYLFEKPR

439 NP_006726.1 NP_872578.1

>NP_006726.1

MNYEFEREIGFINSQPSLAECLTSFPPVADTFQSSSIKTSTLSHSTLIPPPFEQTIPSLNPGSHPRHGAGGRPKPSPAGSRGSPVPAGALQPPEYPWMKEKKAAKKTALLPAAAAAATAAATGPACLSHKESLEIADGSGGGSRRLRTAYTNTQLLELEKEFHFNKYLCRPRRVEIAALLDLTERQVKVWFQNRRMKHKRQTQCKENQNSEGKCKSLEDSEKVEEDEEEKTLFEQALSVSGALLEREGYTFQQNALSQQQAPNGHNGDSQSFPVSPLTSNEKNLKHFQHQSPTVPNCLSTMGQNCGAGLNNDSPEALEVPSLQDFSVFSTDSCLQLSDAVSPSLPGSLDSPVDISADSLDFFTDTLTTIDLQHLNY

>NP_872578.1

MAAPCVSYGGAVSYRLLLWGRGSLARKQGLWKTAAPELQTNVRSQILRLRHTAFVIPKKNVPTSKRETYTEDFIKKQIEEFNIGKRHLANMMGEDPETFTQEDIDRAIAYLFPSGLFEKRARPVMKHPEQIFPRQRAIQWGEDGRPFHYLFYTGKQSYYSLMHDVYGMLLNLEKHQSHLQAKSLLPEKTVTRDVIGSRWLIKEELEEMLVEKLSDLDYMQFIRLLEKLLTSQCGAAEEEFVQRFRRSVTLESKKQLIEPVQYDEQGMAFSKSEGKRKTAKAEAIVYKHGSGRIKVNGIDYQLYFPITQDREQLMFPFHFVDRLGKHDVTCTVSGGGRSAQAGAIRLAMAKALCSFVTEDEVEWMRQAGLLTTDPRVRERKKPGQEGARRKFTWKKR

440 NP_036427.1 NP_066953.1

>NP_036427.1

MIRIAALNASSTIEDDHEGSFKSHKTQTKEAQEAEAFALYHKALDLQKHDRFEESAKAYHELLEASLLREAVSSGDEKEGLKHPGLILKYSTYKNLAQLAAQREDLETAMEFYLEAVMLDSTDVNLWYKIGHVALRLIRIPLARHAFEEGLRCNPDHWPCLDNLITVLYTLSDYTTCLYFICKALEKDCRYSKGLVLKEKIFEEQPCLRKDSLRMFLKCDMSIHDVSVSAAETQAIVDEALGLRKKRQALIVREKEPDLKLVQPIPFFTWKCLGESLLAMYNHLTTCEPPRPSLGKRIDLSDYQDPSQPLESSMVVTPVNVIQPSTVSTNPAVAVAEPVVSYTSVATTSFPLHSPGLLETGAPVGDISGGDKSKKGVKRKKISEESGETAKRRSARVRNTKCKKEEKVDFQELLMKFLPSRLRKLDPEEEDDSFNNYEVQSEAKLESFPSIGPQRLSFDSATFMESEKQDVHEFLLENLTNGGILELMMRYLKAMGHKFLVRWPPGLAEVVLSVYHSWRRHSTSLPNPLLRDCSNKHIKDMMLMSLSCMELQLDQWLLTKGRSSAVSPRNCPAGMVNGRFGPDFPGTHCLGDLLQLSFASSQRDLFEDGWLEFVVRVYWLKARFLALQGDMEQALENYDICTEMLQSSTAIQVEAGAERRDIVIRLPNLHNDSVVSLEEIDKNLKSLERCQSLEEIQRLYEAGDYKAVVHLLRPTLCTSGFDRAKHLEFMTSIPERPAQLLLLQDSLLRLKDYRQCFECSDVALNEAVQQMVNSGEAAAKEEWVATVTQLLMGIEQALSADSSGSILKVSSSTTGLVRLTNNLIQVIDCSMAVQEEAKEPHVSSVLPWIILHRIIWQEEDTFHSLCHQQQLQNPAEEGMSETPMLPSSLMLLNTAHEYLGRRSWCCNSDGALLRFYVRVLQKELAASTSEDTHPYKEELETALEQCFYCLYSFPSKKSKARYLEEHSAQQVDLIWEDALFMFEYFKPKTLPEFDSYKTSTVSADLANLLKRIATIVPRTERPALSLDKVSAYIEGTSTEVPCLPEGADPSPPVVNELYYLLADYHFKNKEQSKAIKFYMHDICICPNRFDSWAGMALARASRIQDKLNSNELKSDGPIWKHATPVLNCFRRALEIDSSNLSLWIEYGTMSYALHSFASRQLKQWRGELPPELVQQMEGRRDSMLETAKHCFTSAARCEGDGDEEEWLIHYMLGKVAEKQQQPPTVYLLHYRQAGHYLHEEAARYPKKIHYHNPPELAMEALEVYFRLHASILKLLGKPDSGVGAEVLVNFMKEAAEGPFARGEEKNTPKASEKEKACLVDEDSHSSAGTLPGPGASLPSSSGPGLTSPPYTATPIDHDYVKCKKPHQQATPDDRSQDSTAVALSDSSSTQDFFNEPTSLLEGSRKSYTEKRLPILSSQAGATGKDLQGATEERGKNEESLESTEGFRAAEQGVQKPAAETPASACIPGKPSASTPTLWDGKKRGDLPGEPVAFPQGLPAGAEEQRQFLTEQCIASFRLCLSRFPQHYKSLYRLAFLYTYSKTHRNLQWARDVLLGSSIPWQQLQHMPAQGLFCERNKTNFFNGIWRIPVDEIDRPGSFAWHMNRSIVLLLKVLAQLRDHSTLLKVSSMLQRTPDQGKKYLRDADRQVLAQRAFILTVKVLEDTLSELAEGSERPGPKVCGLPGARMTTDVSHKASPEDGQEGLPQPKKPPLADGSGPGPEPGGKVGLLNHRPVAMDAGDSADQSGERKDKESPRAGPTEPMDTSEATVCHSDLERTPPLLPGRPARDRGPESRPTELSLEELSISARQQPTPLTPAQPAPAPAPATTTGTRAGGHPEEPLSRLSRKRKLLEDTESGKTLLLDAYRVWQQGQKGVAYDLGRVERIMSETYMLIKQVDEEAALEQAVKFCQVHLGAAAQRQASGDTPTTPKHPKDSRENFFPVTVVPTAPDPVPADSVQRPSDAHTKPRPALAAATTIITCPPSASASTLDQSKDPGPPRPHRPEATPSMASLGPEGEELARVAEGTSFPPQEPRHSPQVKMAPTSSPAEPHCWPAEAALGTGAEPTCSQEGKLRPEPRRDGEAQEAASETQPLSSPPTAASSKAPSSGSAQPPEGHPGKPEPSRAKSRPLPNMPKLVIPSAATKFPPEITVTPPTPTLLSPKGSISEETKQKLKSAILSAQSAANVRKESLCQPALEVLETSSQESSLESETDEDDDYMDI

>NP_066953.1

MVNPTVFFDIAVDGEPLGRVSFELFADKVPKTAENFRALSTGEKGFGYKGSCFHRIIPGFMCQGGDFTRHNGTGGKSIYGEKFEDENFILKHTGPGILSMANAGPNTNGSQFFICTAKTEWLDGKHVVFGKVKEGMNIVEAMERFGSRNGKTSKKITIADCGQLE

441 NP_057640.1 NP_055595.2

>NP_057640.1

MSKGRAEAAAGAAGILLRYLQEQNRPYSSQDVFGNLQREHGLGKAVVVKTLEQLAQQGKIKEKMYGKQKIYFADQDQFDMVSDADLQVLDGKIVALTAKVQSLQQSCRYMEAELKELSSALTTPEMQKEIQELKKECAGYRERLKNIKAATNHVTPEEKEQVYRERQKYCKEWRKRKRMATELSDAILEGYPKSKKQFFEEVGIETDEDYNVTLPDP

>NP_055595.2

MVGELRYREFRVPLGPGLHAYPDELIRQRVGHDGHPEYQIRWLILRRGDEGDGGSGQVDCKAEHILLWMSKDEIYANCHKMLGEDGQVIGPSQESAGEVGALDKSVLEEMETDVKSLIQRALRQLEECVGTIPPAPLLHTVHVLSAYASIEPLTGVFKDPRVLDLLMHMLSSPDYQIRWSAGRMIQALSSHDAGTRTQILLSLSQQEAIEKHLDFDSRCALLALFAQATLSEHPMSFEGIQLPQVPGRVLFSLVKRYLHVTSLLDQLNDSAAEPGAQNTSAPEELSGERGQLELEFSMAMGTLISELVQAMRWDQASDRPRSSARSPGSIFQPQLADVSPGLPAAQAQPSFRRSRRFRPRSEFASGNTYALYVRDTLQPGMRVRMLDDYEEISAGDEGEFRQSNNGVPPVQVFWESTGRTYWVHWHMLEILGFEEDIEDMVEADEYQGAVASRVLGRALPAWRWRPMTELYAVPYVLPEDEDTEECEHLTLAEWWELLFFIKKLDGPDHQEVLQILQENLDGEILDDEILAELAVPIELAQDLLLTLPQRLNDSALRDLINCHVYKKYGPEALAGNQAYPSLLEAQEDVLLLDAQAQAKDSEDAAKVEAKEPPSQSPNTPLQRLVEGYGPAGKILLDLEQALSSEGTQENKVKPLLLQLQRQPQPFLALMQSLDTPETNRTLHLTVLRILKQLVDFPEALLLPWHEAVDACMACLRSPNTDREVLQELIFFLHRLTSVSRDYAVVLNQLGARDAISKALEKHLGKLELAQELRDMVFKCEKHAHLYRKLITNILGGCIQMVLGQIEDHRRTHQPINIPFFDVFLRYLCQGSSVEVKEDKCWEKVEVSSNPHRASKLTDHNPKTYWESNGSAGSHYITLHMRRGILIRQLTLLVASEDSSYMPARVVVCGGDSTSSLHTELNSVNVMPSASRVILLENLTRFWPIIQIRIKRCQQGGIDTRIRGLEILGPKPTFWPVFREQLCRHTRLFYMVRAQAWSQDMAEDRRSLLHLSSRLNGALRQEQNFADRFLPDDEAAQALGKTCWEALVSPVVQNITSPDEDGISPLGWLLDQYLECQEAVFNPQSRGPAFFSRVRRLTHLLVHVEPCEAPPPVVATPRPKGRNRSHDWSSLATRGLPSSIMRNLTRCWRAVVEKQVNNFLTSSWRDDDFVPRYCEHFNILQNSSSELFGPRAAFLLALQNGCAGALLKLPFLKAAHVSEQFARHIDQQIQGSRIGGAQEMERLAQLQQCLQAVLIFSGLEIATTFEHYYQHYMADRLLGVVSSWLEGAVLEQIGPCFPNRLPQQMLQSLSTSKELQRQFHVYQLQQLDQELLKLEDTEKKIQVGLGASGKEHKSEKEEEAGAAAVVDVAEGEEEEEENEDLYYEGAMPEVSVLVLSRHSWPVASICHTLNPRTCLPSYLRGTLNRYSNFYNKSQSHPALERGSQRRLQWTWLGWAELQFGNQTLHVSTVQMWLLLYLNDLKAVSVESLLAFSGLSADMLNQAIGPLTSSRGPLDLHEQKDIPGGVLKIRDGSKEPRSRWDIVRLIPPQTYLQAEGEDGQNLEKRRNLLNCLIVRILKAHGDEGLHIDQLVCLVLEAWQKGPCPPRGLVSSLGKGSACSSTDVLSCILHLLGKGTLRRHDDRPQVLSYAVPVTVMEPHTESLNPGSSGPNPPLTFHTLQIRSRGVPYASCTATQSFSTFR

442 Q14863 Q9NP31

>Q14863

MPGISSQILTNAQGQVIGTLPWVVNSASVAAPAPAQSLQVQAVTPQLLLNAQGQVIATLASSPLPPPVAVRKPSTPESPAKSEVQPIQPTPTVPQPAVVIASPAPAAKPSASAPIPITCSETPTVSQLVSKPHTPSLDEDGINLEEIREFAKNFKIRRLSLGLTQTQVGQALTATEGPAYSQSAICRFEKLDITPKSAQKLKPVLEKWLNEAELRNQEGQQNLMEFVGGEPSKKRKRRTSFTPQAIEALNAYFEKNPLPTGQEITEIAKELNYDREVVRVWFCNRRQTLKNTSKLNVFQIP

>Q9NP31

MEFPLAQICPQGSHEAPIPTFSTFQITDMTRRSCQNLGYTAASPQAPEAASNTGNAERAEEVPGEGSLFLQAETRAWFQKTQAHWLLQHGAAPAWFHGFITRREAERLLEPKPQGCYLVRFSESAVTFVLTYRSRTCCRHFLLAQLRDGRHVVLGEDSAHARLQDLLLHYTAHPLSPYGETLTEPLARQTPEPAGLSLRTEESNFGSKSQDPNPQYSPIIKQGQAPVPMQKEGAGEKEPSQLLRPKPPIPAKPQLPPEVYTIPVPRHRPAPRPKPSNPIYNEPDEPIAFYAMGRGSPGEAPSNIYVEVEDEGLPATLGHPVLRKSWSRPVPGGQNTGGSQLHSENSVIGQGPPLPHQPPPAWRHTLPHNLSRQVLQDRGQAWLPLGPPQ

443 Q70Z53 Q58A44

>Q70Z53

MHGHGGYDSDFSDDEHCGESSKRKKRTVEDDLLLQKPFQKEKHGKVAHKQVAAELLDREEARNRRFHLIAMDAYQRHRKFVNDYILYYGGKKEDFKRLGENDKTDLDVIRENHRFLWNEEDEMDMTWEKRLAKKYYDKLFKEYCIADLSKYKENKFGFRWRVEKEVISGKGQFFCGNKYCDKKEGLKSWEVNFGYIEHGEKRNALVKLRLCQECSIKLNFHHRRKEIKSKKRKDKTKKDCEESSHKKSRLSSAEEASKKKDKGHSSSKKSEDSLLRNSDEEESASESELWKGPLPETDEKSQEEEFDEYFQDLFL

>Q58A44

MWILSNLMGTSEEGNLLSTVSPTVKALFGKTRVSPIFPFSPRSPFQPLIPRTPGSPWGPVGPASPLGPGFPIGPMGPGKPVGPKGPMLPLGPSGPVGPTSPLFPFCP

444 NP_004292.1 Q9BZE1

>NP_004292.1

MSGGVYGGDEVGALVFDIGSYTVRAGYAGEDCPKVDFPTAIGMVVERDDGSTLMEIDGDKGKQGGPTYYIDTNALRVPRENMEAISPLKNGMVEDWDSFQAILDHTYKMHVKSEASLHPVLMSEAPWNTRAKREKLTELMFEHYNIPAFFLCKTAVLTAFANGRSTGLILDSGATHTTAIPVHDGYVLQQGIVKSPLAGDFITMQCRELFQEMNIELVPPYMIASKEAVREGSPANWKRKEKLPQVTRSWHNYMCNCVIQDFQASVLQVSDSTYDEQVAAQMPTVHYEFPNGYNCDFGAERLKIPEGLFDPSNVKGLSGNTMLGVSHVVTTSVGMCDIDIRPGLYGSVIVAGGNTLIQSFTDRLNRELSQKTPPSMRLKLIANNTTVERRFSSWIGGSILASLGTFQQMWISKQEYEEGGKQCVERKCP

>Q9BZE1

MALASGPARRALAGSGQLGLGGFGAPRRGAYEWGVRSTRKSEPPPLDRVYEIPGLEPITFAGKMHFVPWLARPIFPPWDRGYKDPRFYRSPPLHEHPLYKDQACYIFHHRCRLLEGVKQALWLTKTKLIEGLPEKVLSLVDDPRNHIENQDECVLNVISHARLWQTTEEIPKRETYCPVIVDNLIQLCKSQILKHPSLARRICVQNSTFSATWNRESLLLQVRGSGGARLSTKDPLPTIASREEIEATKNHVLETFYPISPIIDLHECNIYDVKNDTGFQEGYPYPYPHTLYLLDKANLRPHRLQPDQLRAKMILFAFGSALAQARLLYGNDAKVLEQPVVVQSVGTDGRVFHFLVFQLNTTDLDCNEGVKNLAWVDSDQLLYQHFWCLPVIKKRVVVEPVGPVGFKPETFRKFLALYLHGAA

445 Q9UI36 NP_660198.1

>Q9UI36

MAVPAALIPPTQLVPPQPPISTSASSSGTTTSTSSATSSPAPSIGPPASSGPTLFRPEPIASAAAAAATVTSTGGGGGGGGSGGGGGSSGNGGGGGGGGGGSNCNPNLAAASNGSGGGGGGISAGGGVASSTPINASTGSSSSSSSSSSSSSSSSSSSSSSSSCGPLPGKPVYSTPSPVENTPQNNECKMVDLRGAKVASFTVEGCELICLPQAFDLFLKHLVGGLHTVYTKLKRLEITPVVCNVEQVRILRGLGAIQPGVNRCKLISRKDFETLYNDCTNASSRPGRPPKRTQSVTSPENSHIMPHSVPGLMSPGIIPPTGLTAAAAAAAAATNAAIAEAMKVKKIKLEAMSNYHASNNQHGADSENGDMNSSVGSSDGSWDKETLPSSPSQGPQASITHPRMPGARSLPLSHPLNHLQQSHLLPNGLELPFMMMPHPLIPVSLPPASVTMAMSQMNHLSTIANMAAAAQVQSPPSRVETSVIKERVPDSPSPAPSLEEGRRPGSHPSSHRSSSVSSSPARTESSSDRIPVHQNGLSMNQMLMGLSPNVLPGPKEGDLAGHDMGHESKRMHIEKDETPLSTPTARDSLDKLSLTGHGQPLPPGFPSPFLFPDGLSSIETLLTNIQGLLKVAIDNARAQEKQVQLEKTELKMDFLRERELRETLEKQLAMEQKNRAIVQKRLKKEKKAKRKLQEALEFETKRREQAEQTLKQAASTDSLRVLNDSLTPEIEADRSGGRTDAERTIQDGRLYLKTTVMY

>NP_660198.1

MLIPFSMKNCFQLLCNCQVPAAGFKKTVKNGLILQSISNDVYQNLAVEDWIHDHMNLEGKPILFFWQNSPSVVIGRHQNPWQECNLNLMREEGIKLARRRSGGGTVYHDMGNINLTFFTTKKKYDRMENLKLIVRALNAVQPQLDVQATKRFDLLLDGQFKISGTASKIGRTTAYHHCTLLCSTDGTFLSSLLKSPYQGIRSNATASIPSLVKNLLEKDPTLTCEVLMNAVATEYAAYHQIDNHIHLINPTDETLFPGINSKAKELQTWEWIYGKTPKFSINTSFHVLYEQSHLEIKVFIDIKNGRIEICNIEAPDHWLPLEIRDKLNSSLIGSKFCPTETTMLTNILLRTCPQDHKLNSKWNILCEKIKGIM

446 NP_061824.2 NP_071761.1

>NP_061824.2

MSCSESPAANSFLVDSLISSGRGEAGGGGGGAGGGGGGGYYAHGGVYLPPAADLPYGLQSCGLFPTLGGKRNEAASPGSGGGGGGLGPGAHGYGPSPIDLWLDAPRSCRMEPPDGPPPPPQQQPPPPPQPPQPAPQATSCSFAQNIKEESSYCLYDSADKCPKVSATAAELAPFPRGPPPDGCALGTSSGVPVPGYFRLSQAYGTAKGYGSGGGGAQQLGAGPFPAQPPGRGFDLPPALASGSADAARKERALDSPPPPTLACGSGGGSQGDEEAHASSSAAEELSPAPSESSKASPEKDSLGNSKGENAANWLTAKSGRKKRCPYTKHQTLELEKEFLFNMYLTRERRLEISRSVHLTDRQVKIWFQNRRMKLKKMNRENRIRELTANFNFS

>NP_071761.1

MWIPVVGLPRRLRLSALAGAGRFCILGSEAATRKHLPARNHCGLSDSSPQLWPEPDFRNPPRKASKASLDFKRYVTDRRLAETLAQIYLGKPSRPPHLLLECNPGPGILTQALLEAGAKVVALESDKTFIPHLESLGKNLDGKLRVIHCDFFKLDPRSGGVIKPPAMSSRGLFKNLGIEAVPWTADIPLKVVGMFPSRGEKRALWKLAYDLYSCTSIYKFGRIEVNMFIGEKEFQKLMADPGNPDLYHVLSVIWQLACEIKVLHMEPWSSFDIYTRKGPLENPKRRELLDQLQQKLYLIQMIPRQNLFTKNLTPMNYNIFFHLLKHCFGRRSATVIDHLRSLTPLDARDILMQIGKQEDEKVVNMHPQDFKTLFETIERSKDCAYKWLYDETLEDR

447 NP_006212.1 P49753

>NP_006212.1

MADEEKLPPGWEKRMSRSSGRVYYFNHITNASQWERPSGNSSSGGKNGQGEPARVRCSHLLVKHSQSRRPSSWRQEKITRTKEEALELINGYIQKIKSGEEDFESLASQFSDCSSAKARGDLGAFSRGQMQKPFEDASFALRTGEMSGPVFTDSGIHIILRTE

>P49753

MSNKLLSPHPHSVVLRSEFKMASSPAVLRASRLYQWSLKSSAQFLGSPQLRQVGQIIRVPARMAATLILEPAGRCCWDEPVRIAVRGLAPEQPVTLRASLRDEKGALFQAHARYRADTLGELDLERAPALGGSFAGLEPMGLLWALEPEKPLVRLVKRDVRTPLAVELEVLDGHDPDPGRLLCQTRHERYFLPPGVRREPVRVGRVRGTLFLPPEPGPFPGIVDMFGTGGGLLEYRASLLAGKGFAVMALAYYNYEDLPKTMETLHLEYFEEAMNYLLSHPEVKGPGVGLLGISKGGELCLSMASFLKGITAAVVINGSVANVGGTLRYKGETLPPVGVNRNRIKVTKDGYADIVDVLNSPLEGPDQKSFIPVERAESTFLFLVGQDDHNWKSEFYANEACKRLQAHGRRKPQIICYPETGHYIEPPYFPLCRASLHALVGSPIIWGGEPRAHAMAQVDAWKQLQTFFHKHLGGHEGTIPSKV

448 P20290 P49638

>P20290

MRRTGAPAQADSRGRGRARGGCPGGEATLSQPPPRGGTRGQEPQMKETIMNQEKLAKLQAQVRIGGKGTARRKKKVVHRTATADDKKLQFSLKKLGVNNISGIEEVNMFTNQGTVIHFNNPKVQASLAANTFTITGHAETKQLTEMLPSILNQLGADSLTSLRRLAEALPKQSVDGKAPLATGEDDDDEVPDLVENFDEASKNEAN

>P49638

MAEARSQPSAGPQLNALPDHSPLLQPGLAALRRRAREAGVPLAPLPLTDSFLLRFLRARDFDLDLAWRLLKNYYKWRAECPEISADLHPRSIIGLLKAGYHGVLRSRDPTGSKVLIYRIAHWDPKVFTAYDVFRVSLITSELIVQEVETQRNGIKAIFDLEGWQFSHAFQITPSVAKKIAAVLTDSFPLKVRGIHLINEPVIFHAVFSMIKPFLTEKIKERIHMHGNNYKQSLLQHFPDILPLEYGGEEFSMEDICQEWTNFIMKSEDYLSSISESIQ

449 Q16670 P57721

>Q16670

MATALVSAHSLAPLNLKKEGLRVVREDHYSTWEQGFKLQGNSKGLGQEPLCKQFRQLRYEETTGPREALSRLRELCQQWLQPETHTKEQILELLVLEQFLIILPKELQARVQEHHPESREDVVVVLEDLQLDLGETGQQDPDQPKKQKILVEEMAPLKGVQEQQVRHECEVTKPEKEKGEETRIENGKLIVVTDSCGRVESSGKISEPMEAHNEGSNLERHQAKPKEKIEYKCSEREQRFIQHLDLIEHASTHTGKKLCESDVCQSSSLTGHKKVLSREKGHQCHECGKAFQRSSHLVRHQKIHLGEKPYQCNECGKVFSQNAGLLEHLRIHTGEKPYLCIHCGKNFRRSSHLNRHQRIHSQEEPCECKECGKTFSQALLLTHHQRIHSHSKSHQCNECGKAFSLTSDLIRHHRIHTGEKPFKCNICQKAFRLNSHLAQHVRIHNEEKPYQCSECGEAFRQRSGLFQHQRYHHKDKLA

>P57721

MESKVSEGGLNVTLTIRLLMHGKEVGSIIGKKGETVKKMREESGARINISEGNCPERIVTITGPTDAIFKAFAMIAYKFEEDIINSMSNSPATSKPPVTLRLVVPASQCGSLIGKGGSKIKEIRESTGAQVQVAGDMLPNSTERAVTISGTPDAIIQCVKQICVVMLESPPKGATIPYRPKPASTPVIFAGGQAYTIQGQYAIPHPDQLTKLHQLAMQQTPFPPLGQTNPAFPGEKLPLHSSEEAQNLMGQSSGLDASPPASTHELTIPNDLIGCIIGRQGTKINEIRQMSGAQIKIANATEGSSERQITITGTPANISLAQYLINARLTSEVTGMGTL

450 NP_002909.3 Q9UPN7

>NP_002909.3

MATQAYTELQAAPPPSQPPQAPPQAQPQPPPPPPPAAPQPPQPPTAAATPQPQYVTELQSPQPQAQPPGGQKQYVTELPAVPAPSQPTGAPTPSPAPQQYIVVTVSEGAMRASETVSEASPGSTASQTGVPTQVVQQVQGTQQRLLVQTSVQAKPGHVSPLQLTNIQVPQQALPTQRLVVQSAAPGSKGGQVSLTVHGTQQVHSPPEQSPVQANSSSSKTAGAPTGTVPQQLQVHGVQQSVPVTQERSVVQATPQAPKPGPVQPLTVQGLQPVHVAQEVQQLQQVPVPHVYSSQVQYVEGGDASYTASAIRSSTYSYPETPLYTQTASTSYYEAAGTATQVSTPATSQAVASSGSMPMYVSGSQVVASSASTGAGASNSSGGGGSGGGGGGGGGGGGGGSGSTGGGGSGAGTYVIQGGYMLGSASQSYSHTTRASPATVQWLLDNYETAEGVSLPRSTLYCHYLLHCQEQKLEPVNAASFGKLIRSVFMGLRTRRLGTRGNSKYHYYGLRIKASSPLLRLMEDQQHMAMRGQPFSQKQRLKPIQKMEGMTNGVAVGQQPSTGLSDISAQVQQYQQFLDASRSLPDFTELDLQGKVLPEGVGPGDIKAFQVLYREHCEAIVDVMVNLQFTLVETLWKTFWRYNLSQPSEAPPLAVHDEAEKRLPKAILVLLSKFEPVLQWTKHCDNVLYQGLVEILIPDVLRPIPSALTQAIRNFAKSLESWLTHAMVNIPEEMLRVKVAAAGAFAQTLRRYTSLNHLAQAARAVLQNTAQINQMLSDLNRVDFANVQEQASWVCRCEDRVVQRLEQDFKVTLQQQNSLEQWAAWLDGVVSQVLKPYQGSAGFPKAAKLFLLKWSFYSSMVIRDLTLRSAASFGSFHLIRLLYDEYMYYLIEHRVAQAKGETPIAVMGEFANLATSLNPLDPDKDEEEEEEEESEDELPQDISLAAGGESPALGPETLEPPAKLARTDARGLFVQALPSS

>Q9UPN7

MEAVSLRAVPGRGRAQHEAEGWDLGRGCAQGGHSAPVVSASSSGDSLGRLTRRWLCLCLQGAMFWKFDLHTSSHLDTLLEREDLSLPELLDEEDVLQECKVVNRKLLDFLLQPPHLQAMVAWVTQEPPDSGEERLRYKYPSVACEILTSDVPQINDALGADESLLNRLYGFLQSTGSLNPLLASFFSKVMGILINRKTDQLVSFLRKKDDFVDLLLQHIGTSAIMDLLLRLLTCVERPQLRQDVVNWLNEEKIVQRLIEQIHPSKDENQHSNASQSLCDIIRLSREQMIQVQDSPEPDQLLATLEKQETIEQLLSNMFEGEQSQSVIVSGIQVLLTLLEPRRPRSESVTVNSFFSSVDGQLELLAQGALESTVSSVGALHALRPRLSCFHQLLLEPPKLEPLQMTWGMLAPPLGNTRLHVVKLLASALSANDAALTHELLALDVPNTMLDLFFHYVFNNFLHAQVEGCVSTMLSLGPPPDSSPETPIQNPVVKHLLQQCRLVERILTSWEENDRVQCAGGPRKGYMGHLTRVAGALVQNTEKGPNAEQLRQLLKELPSEQQEQWEAFVSGPLAETNKKNMVDLVNTHHLHSSSDDEDDRLKEFNFPEEAVLQQAFMDFQMQRMTSAFIDHFGFNDEEFGEQEESVNAPFDKTANITFSLNADDENPNANLLEICYKDRIQQFDDDEEEEDEEEAQGSGESDGEDGAWQGSQLARGARLGQPPGVRSGGSTDSEDEEEEDEEEEEDEEGIGCAARGGATPLSYPSPGPQPPGPSWTATFDPVPTDAPTSPRVSGEEELHTGPPAPQGPLSVPQGLPTQSLASPPARDALQLRSQDPTPPSAPQEATEGSKVTEPSAPCQALVSIGDLQATFHGIRSAPSSSDSATRDPSTSVPASGAHQPPQTTEGEKSPEPLGLPQSQSAQALTPPPIPNGSAPEGPASPGSQ

451 NP_003205.2 P57721

>NP_003205.2

MASNSWNASSSPGEAREDGPEGLDKGLDNDAEGVWSPDIEQSFQEALAIYPPCGRRKIILSDEGKMYGRNELIARYIKLRTGKTRTRKQVSSHIQVLARKKVREYQVGIKAMNLDQVSKDKALQSMASMSSAQIVSASVLQNKFSPPSPLPQAVFSTSSRFWSSPPLLGQQPGPSQDIKPFAQPAYPIQPPLPPTLSSYEPLAPLPSAAASVPVWQDRTIASSRLRLLEYSAFMEVQRDPDTYSKHLFVHIGQTNPAFSDPPLEAVDVRQIYDKFPEKKGGLKELYEKGPPNAFFLVKFWADLNSTIQEGPGAFYGVSSQYSSADSMTISVSTKVCSFGKQVVEKVETEYARLENGRFVYRIHRSPMCEYMINFIHKLKHLPEKYMMNSVLENFTILQVVTSRDSQETLLVIAFVFEVSTSEHGAQHHVYKLVKD

>P57721

MESKVSEGGLNVTLTIRLLMHGKEVGSIIGKKGETVKKMREESGARINISEGNCPERIVTITGPTDAIFKAFAMIAYKFEEDIINSMSNSPATSKPPVTLRLVVPASQCGSLIGKGGSKIKEIRESTGAQVQVAGDMLPNSTERAVTISGTPDAIIQCVKQICVVMLESPPKGATIPYRPKPASTPVIFAGGQAYTIQGQYAIPHPDQLTKLHQLAMQQTPFPPLGQTNPAFPGEKLPLHSSEEAQNLMGQSSGLDASPPASTHELTIPNDLIGCIIGRQGTKINEIRQMSGAQIKIANATEGSSERQITITGTPANISLAQYLINARLTSEVTGMGTL

452 NP_006038.2 NP_000552.2

>NP_006038.2

MAVVIRLQGLPIVAGTMDIRHFFSGLTIPDGGVHIVGGELGEAFIVFATDEDARLGMMRTGGTIKGSKVTLLLSSKTEMQNMIELSRRRFETANLDIPPANASRSGPPPSSGMSSRVNLPTTVSNFNNPSPSVVTATTSVHESNKNIQTFSTASVGTAPPNMGASFGSPTFSSTVPSTASPMNTVPPPPIPPIPAMPSLPPMPSIPPIPVPPPVPTLPPVPPVPPIPPVPSVPPMTPLPPMSGMPPLNPPPVAPLPAGMNGSGAPMNLNNNLNPMFLGPLNPVNPIQMNSQSSVKPLPINPDDLYVSVHGMPFSAMENDVRDFFHGLRVDAVHLLKDHVGRNNGNGLVKFLSPQDTFEALKRNRMLMIQRYVEVSPATERQWVAAGGHITFKQNMGPSGQTHPPPQTLPRSKSPSGQKRSRSRSPHEAGFCVYLKGLPFEAENKHVIDFFKKLDIVEDSIYIAYGPNGKATGEGFVEFRNEADYKAALCRHKQYMGNRFIQVHPITKKGMLEKIDMIRKRLQNFSYDQREMILNPEGDVNSAKVCAHITNIPFSITKMDVLQFLEGIPVDENAVHVLVDNNGQGLGQALVQFKNEDDARKSERLHRKKLNGREAFVHVVTLEDMREIEKNPPAQGKKGLKMPVPGNPAVPGMPNAGLPGVGLPSAGLPGAGLPSTGLPGSAITSAGLPGAGMPSAGIPSAGGEEHAFLTVGSKEANNGPPFNFPGNFGGSNAFGPPIPPPGLGGGAFGDARPGMPSVGNSGLPGLGLDVPGFGGGPNNLSGPSGFGGGPQNFGNGPGSLGGPPGFGSGPPGLGSAPGHLGGPPAFGPGPGPGPGPGPIHIGGPPGFASSSGKPGPTVIKVQNMPFTVSIDEILDFFYGYQVIPGSVCLKYNEKGMPTGEAMVAFESRDEATAAVIDLNDRPIGSRKVKLVLG

>NP_000552.2

MPMILGYWDIRGLAHAIRLLLEYTDSSYEEKKYTMGDAPDYDRSQWLNEKFKLGLDFPNLPYLIDGAHKITQSNAILCYIARKHNLCGETEEEKIRVDILENQTMDNHMQLGMICYNPEFEKLKPKYLEELPEKLKLYSEFLGKRPWFAGNKITFVDFLVYDVLDLHRIFEPKCLDAFPNLKDFISRFEGLEKISAYMKSSRFLPRPVFSKMAVWGNK

453 NP_006684.1 Q9UI17

>NP_006684.1

MQEIIASVDHIKFDLEIAVEQQLGAQPLPFPGMDKSGAAVCEFFLKAACGKGGMCPFRHISGEKTVVCKHWLRGLCKKGDQCEFLHEYDMTKMPECYFYSKFGECSNKECPFLHIDPESKIKDCPWYDRGFCKHGPLCRHRHTRRVICVNYLVGFCPEGPSCKFMHPRFELPMGTTEQPPLPQQTQPPAKQSNNPPLQRSSSLIQLTSQNSSPNQQRTPQVIGVMQSQNSSAGNRGPRPLEQVTCYKCGEKGHYANRCTKGHLAFLSGQ

>Q9UI17

MLRPGAQLLRGLLLRSCPLQGSPGRPRSVCGREGEEKPPLSAETQWKDRAETVIIGGGCVGVSLAYHLAKAGMKDVVLLEKSELTAGSTWHAAGLTTYFHPGINLKKIHYDSIKLYEKLEEETGQVVGFHQPGSIRLATTPVRVDEFKYQMTRTGWHATEQYLIEPEKIQEMFPLLNMNKVLAGLYNPGDGHIDPYSLTMALAAGARKCGALLKYPAPVTSLKARSDGTWDVETPQGSMRANRIVNAAGFWAREVGKMIGLEHPLIPVQHQYVVTSTISEVKALKRELPVLRDLEGSYYLRQERDGLLFGPYESQEKMKVQDSWVTNGVPPGFGKELFESDLDRIMEHIKAAMEMVPVLKKADIINVVNGPITYSPDILPMVGPHQGVRNYWVAIGFGYGIIHAGGVGKYLSDWILHGEPPFDLIELDPNRYGKWTTTQYTEAKARESYGFNNIVGYPKEERFAGRPTQRVSGLYQRLESKCSMGFHAGWEQPHWFYKPGQDTQYRPSFRRTNWFEPVGSEYKQVMQRVGVTDLSPFGKFNIKGQDSIRLLDHLFANVIPKVGFTNISHMLTPKGRVYAELTVSHQSPGEFLLITGSGSELHDLRWIEEEAVKGGYDVEIKNITDELGVLGVAGPQARKVLQKLTPEDLSDDVFKFLQTKSLKVSNIPVTAIRISYTGELGWELYHRREDSVALYDAIMNAGQEEGIDNFGTYAMNALRLEKAFRAWGLEMNCDTNPLEAGLEYFVKLNKPADFIGKQALKQIKAKGLKRRLVCLTLATDDVDPEGNESIWYNGKVVGNTTSGSYSYSIQKSLAFAYVPVQLSEVGQQVEVELLGKNYPAVIIQEPLVLTEPTRNRLQKKGGKDKT

454 Q99879 NP_000340.2

>Q99879

MPEPVKSAPVPKKGSKKAINKAQKKDGKKRKRSRKESYSVYVYKVLKQVHPDTGISSKAMGIMNSFVNDIFERIAGEASRLAHYNKRSTITSREIQTAVRLLLPGELAKHAVSEGTKAVTKYTSSK

>NP_000340.2

MLLATFKLCAGSSYRHMRNMKGLRQQAVMAISQELNRRALGGPTPSTWINQVRRRSSLLGSRLEETLYSDQELAYLQQGEEAMQKALGILSNQEGWKKESQQDNGDKVMSKVVPDVGKVFRLEVVVDQPMERLYEELVERMEAMGEWNPNVKEIKVLQKIGKDTFITHELAAEAAGNLVGPRDFVSVRCAKRRGSTCVLAGMATDFGNMPEQKGVIRAEHGPTCMVLHPLAGSPSKTKLTWLLSIDLKGWLPKSIINQVLSQTQVDFANHLRKRLESHPASEARC

455 NP_004710.2 NP_003921.2

>NP_004710.2

MKKKTVCTLNMGDKKYEDMEGEENGDNTISTGLLYSEADRCPICLNCLLEKEVGFPESCNHVFCMTCILKWAETLASCPIDRKPFQAVFKFSALEGYVKVQVKKQLRETKDKKNENSFEKQVSCHENSKSCIRRKAIVREDLLSAKVCDLKWIHRNSLYSETGGKKNAAIKINKPQRSNWSTNQCFRNFFSNMFSSVSHSGESSFTYRAYCTEFIEASEISALIRQKRHELELSWFPDTLPGIGRIGFIPWNVETEVLPLISSVLPRTIFPTSTISFEHFGTSCKGYALAHTQEGEEKKQTSGTSNTRGSRRKPAMTTPTRRSTRNTRAETASQSQRSPISDNSGCDAPGNSNPSLSVPSSAESEKQTRQAPKRKSVRRGRKPPLLKKKLRSSVAAPEKSSSNDSVDEETAESDTSPVLEKEHQPDVDSSNICTVQTHVENQSANCLKSCNEQIEESEKHTANYDTEERVGSSSSESCAQDLPVLVGEEGEVKKLENTGIEANVLCLESEISENILEKGGDPLEKQDQISGLSQSEVKTDVCTVHLPNDFPTCLTSESKVYQPVSCPLSDLSENVESVVNEEKITESSLVEITEHKDFTLKTEELIESPKLESSEGEIIQTVDRQSVKSPEVQLLGHVETEDVEIIATCDTFGNEDFNNIQDSENNLLKNNLLNTKLEKSLEEKNESLTEHPRSTELPKTHIEQIQKHFSEDNNEMIPMECDSFCSDQNESEVEPSVNADLKQMNENSVTHCSENNMPSSDLADEKVETVSQPSESPKDTIDKTKKPRTRRSRFHSPSTTWSPNKDTPQEKKRPQSPSPRRETGKESRKSQSPSPKNESARGRKKSRSQSPKKDIARERRQSQSRSPKRDTTRESRRSESLSPRRETSRENKRSQPRVKDSSPGEKSRSQSRERESDRDGQRRERERRTRKWSRSRSHSRSPSRCRTKSKSSSFGRIDRDSYSPRWKGRWANDGWRCPRGNDRYRKNDPEKQNENTRKEKNDIHLDADDPNSADKHRNDCPNWITEKINSGPDPRTRNPEKLKESHWEENRNENSGNSWNKNFGSGWVSNRGRGRGNRGRGTYRSSFAYKDQNENRWQNRKPLSGNSNSSGSESFKFVEQQSYKRKSEQEFSFDTPADRSGWTSASSWAVRKTLPADVQNYYSRRGRNSSGPQSGWMKQEEETSGQDSSLKDQTNQQVDGSQLPINMMQPQMNVMQQQMNAQHQPMNIFPYPVGVHAPLMNIQRNPFNIHPQLPLHLHTGVPLMQVATPTSVSQGLPPPPPPPPPSQQVNYIASQPDGKQLQGIPSSSHVSNNMSTPVLPAPTAAPGNTGMVQGPSSGNTSSSSHSKASNAAVKLAESKVSVAVEASADSSKTDKKLQIQEKAAQEVKLAIKPFYQNKDITKEEYKEIVRKAVDKVCHSKSGEVNSTKVANLVKAYVDKYKYSRKGSQKKTLEEPVSTEKNIG

>NP_003921.2

MPNPSSTSSPYPLPEEIRNLLADVETFVADILKGENLSKKAKEKRESLIKKIKDVKSIYLQEFQDKGDAEDGEEYDDPFAGPPDTISLASERYDKDDEAPSDGAQFPPIAAQDLPFVLKAGYLEKRRKDHSFLGFEWQKRWCALSKTVFYYYGSDKDKQQKGEFAIDGYSVRMNNTLRKDGKKDCCFEISAPDKRIYQFTAASPKDAEEWVQQLKFVLQDMESDIIPEDYDERGELYDDVDHPLPISNPLTSSQPIDDEIYEELPEEEEDSAPVKVEEQRKMSQDSVHHTSGDKSTDYANFYQGLWDCTGAFSDELSFKRGDVIYILSKEYNRYGWWVGEMKGAIGLVPKAYIMEMYDI

456 NP_000240.1 NP_000660.1

>NP_000240.1

MSFVAGVIRRLDETVVNRIAAGEVIQRPANAIKEMIENCLDAKSTSIQVIVKEGGLKLIQIQDNGTGIRKEDLDIVCERFTTSKLQSFEDLASISTYGFRGEALASISHVAHVTITTKTADGKCAYRASYSDGKLKAPPKPCAGNQGTQITVEDLFYNIATRRKALKNPSEEYGKILEVVGRYSVHNAGISFSVKKQGETVADVRTLPNASTVDNIRSIFGNAVSRELIEIGCEDKTLAFKMNGYISNANYSVKKCIFLLFINHRLVESTSLRKAIETVYAAYLPKNTHPFLYLSLEISPQNVDVNVHPTKHEVHFLHEESILERVQQHIESKLLGSNSSRMYFTQTLLPGLAGPSGEMVKSTTSLTSSSTSGSSDKVYAHQMVRTDSREQKLDAFLQPLSKPLSSQPQAIVTEDKTDISSGRARQQDEEMLELPAPAEVAAKNQSLEGDTTKGTSEMSEKRGPTSSNPRKRHREDSDVEMVEDDSRKEMTAACTPRRRIINLTSVLSLQEEINEQGHEVLREMLHNHSFVGCVNPQWALAQHQTKLYLLNTTKLSEELFYQILIYDFANFGVLRLSEPAPLFDLAMLALDSPESGWTEEDGPKEGLAEYIVEFLKKKAEMLADYFSLEIDEEGNLIGLPLLIDNYVPPLEGLPIFILRLATEVNWDEEKECFESLSKECAMFYSIRKQYISEESTLSGQQSEVPGSIPNSWKWTVEHIVYKALRSHILPPKHFTEDGNILQLANLPDLYKVFERC

>NP_000660.1

MSTAGKVIKCKAAVLWELKKPFSIEEVEVAPPKAHEVRIKMVAAGICRSDEHVVSGNLVTPLPVILGHEAAGIVESVGEGVTTVKPGDKVIPLFTPQCGKCRICKNPESNYCLKNDLGNPRGTLQDGTRRFTCSGKPIHHFVGVSTFSQYTVVDENAVAKIDAASPLEKVCLIGCGFSTGYGSAVKVAKVTPGSTCAVFGLGGVGLSVVMGCKAAGAARIIAVDINKDKFAKAKELGATECINPQDYKKPIQEVLKEMTDGGVDFSFEVIGRLDTMMASLLCCHEACGTSVIVGVPPDSQNLSINPMLLLTGRTWKGAIFGGFKSKESVPKLVADFMAKKFSLDALITNILPFEKINEGFDLLRSGKSIRTVLTF

457 Q02386 Q9BSE5

>Q02386

MTKSKEAVTFKDVAVVFSEEELQLLDLAQRKLYRDVMLENFRNVVSVGHQSTPDGLPQLEREEKLWMMKMATQRDNSSGAKNLKEMETLQEVGLRYLPHEELFCSQIWQQITRELIKYQDSVVNIQRTGCQLEKRDDLHYKDEGFSNQSSHLQVHRVHTGEKPYKGEHCVKSFSWSSHLQINQRAHAGEKPYKCEKCDNAFRRFSSLQAHQRVHSRAKSYTNDASYRSFSQRSHLPHHQRVPTGENPYKYEECGRNVGKSSHCQAPLIVHTGEKPYKCEECGVGFSQRSYLQVHLKVHTGKKPYKCEECGKSFSWRSRLQAHERIHTGEKPYKCNACGKSFSYSSHLNIHCRIHTGEKPYKCEECGKGFSVGSHLQAHQISHTGEKPYKCEECGKGFCRASNLLDHQRGHTGEKPYQCDACGKGFSRSSDFNIHFRVHTGEKPYKCEECGKGFSQASNLLAHQRGHTGEKPYKCGTCGKGFSRSSDLNVHCRIHTGEKPYKCERCGKAFSQFSSLQVHQRVHTGEKPYQCAECGKGFSVGSQLQAHQRCHTGEKPYQCEECGKGFCRASNFLAHRGVHTGEKPYRCDVCGKRFRQRSYLQAHQRVHTGERPYKCEECGKVFSWSSYLQAHQRVHTGEKPYKCEECGKGFSWSSSLIIHQRVHADDEGDKDFPSSEDSHRKTR

>Q9BSE5

MLRLLASGCARGPGPGVGARPAAGLFHPGRRQSRQASDAPRNQPPSPEFVARPVGVCSMMRLPVQTSPEGLDAAFIGVPLDTGTSNRPGARFGPRRIREESVMLGTVNPSTGALPFQSLMVADLGDVNVNLYNLQDSCRRIQEAYEKIVAAGCIPLTLGGDHTITYPILQAMAKKHGPVGLLHVDAHTDTTDKALGEKLYHGAPFRRCVDEGLLDCKRVVQIGIRGSSTTLDPYRYNRSQGFRVVLAEDCWMKSLVPLMGEVRQQMGGKPIYISFDIDALDPAYAPGTGTPEIAGLTPSQALEIIRGCQGLNVMGCDLVEVSPPYDLSGNTALLAANLLFEMLCALPKVTTV

458 NP_003933.1 Q13287

>NP_003933.1

MGDEDDDESCAVELRITEANLTGHEEKVSVENFELLKVLGTGAYGKVFLVRKAGGHDAGKLYAMKVLRKAALVQRAKTQEHTRTERSVLELVRQAPFLVTLHYAFQTDAKLHLILDYVSGGEMFTHLYQRQYFKEAEVRVYGGEIVLALEHLHKLGIIYRDLKLENVLLDSEGHIVLTDFGLSKEFLTEEKERTFSFCGTIEYMAPEIIRSKTGHGKAVDWWSLGILLFELLTGASPFTLEGERNTQAEVSRRILKCSPPFPPRIGPVAQDLLQRLLCKDPKKRLGAGPQGAQEVRNHPFFQGLDWVALAARKIPAPFRPQIRSELDVGNFAEEFTRLEPVYSPPGSPPPGDPRIFQGYSFVAPSILFDHNNAVMTDGLEAPGAGDRPGRAAVARSAMMQDSPFFQQYELDLREPALGQGSFSVCRRCRQRQSGQEFAVKILSRRLEANTQREVAALRLCQSHPNVVNLHEVHHDQLHTYLVLELLRGGELLEHIRKKRHFSESEASQILRSLVSAVSFMHEEAGVVHRDLKPENILYADDTPGAPVKIIDFGFARLRPQSPGVPMQTPCFTLQYAAPELLAQQGYDESCDLWSLGVILYMMLSGQVPFQGASGQGGQSQAAEIMCKIREGRFSLDGEAWQGVSEEAKELVRGLLTVDPAKRLKLEGLRGSSWLQDGSARSSPPLRTPDVLESSGPAVRSGLNATFMAFNRGKREGFFLKSVENAPLAKRRKQKLRSATASRRGSPAPANPGRAPVASKGAPRRANGPLPPS

>Q13287

MEADKDDTQQILKEHSPDEFIKDEQNKGLIDEITKKNIQLKKEIQKLETELQEATKEFQIKEDIPETKMKFLSVETPENDSQLSNISCSFQVSSKVPYEIQKGQALITFEKEEVAQNVVSMSKHHVQIKDVNLEVTAKPVPLNSGVRFQVYVEVSKMKINVTEIPDTLREDQMRDKLELSFSKSRNGGGEVDRVDYDRQSGSAVITFVEIGVADKILKKKEYPLYINQTCHRVTVSPYTEIHLKKYQIFSGTSKRTVLLTGMEGIQMDEEIVEDLINIHFQRAKNGGGEVDVVKCSLGQPHIAYFEE

459 Q7Z7J5 NP_620448.1

>Q7Z7J5

MSDANLDSSKKNFLEGEVDDEESVILTLVPVKDDANMEQMEPSVSSTSDVKLEKPKKYNPGHLLQTNEQFTAPQKARCKIPALPLPTILPPINKVCRDTLRDWCQQLGLSTNGKKIEVYLRLHRHAYPEQRQDMPEMSQETRLQRCSRKRKAVTKRARLQRSYEMNERAEETNTVEVITSAPGAMLASWARIAARAVQPKALNSCSIPVSVEAFLMQASGVRWCVVHGRLLSADTKGWVRLQFHAGQAWVPTTHRRMISLFLLPACIFPSPGIEDNMLCPDCAKRNKKMMKRLMTVEK

>NP_620448.1

MSLHFLYYCSEPTLDVKIAFCQGFDKQVDVSYIAKHYNMSKSKVDNQFYSVEVGDSTFTVLKRYQNLKPIGSGAQGIVCAAYDAVLDRNVAIKKLSRPFQNQTHAKRAYRELVLMKCVNHKNIISLLNVFTPQKTLEEFQDVYLVMELMDANLCQVIQMELDHERMSYLLYQMLCGIKHLHSAGIIHRDLKPSNIVVKSDCTLKILDFGLARTAGTSFMMTPYVVTRYYRAPEVILGMGYKENVDIWSVGCIMGEMVRHKILFPGRDYIDQWNKVIEQLGTPCPEFMKKLQPTVRNYVENRPKYAGLTFPKLFPDSLFPADSEHNKLKASQARDLLSKMLVIDPAKRISVDDALQHPYINVWYDPAEVEAPPPQIYDKQLDEREHTIEEWKELIYKEVMNSEEKTKNGVVKGQPSPSGAAVNSSESLPPSSSVNDISSMSTDQTLASDTDSSLEASAGPLGCCR

460 NP_055586.1 Q6PRD7

>NP_055586.1

MSGPGNKRAAGDGGSGPPEKKLSREEKTTTTLIEPIRLGGISSTEEMDLKVLQFKNKKLAERLEQRQACEDELRERIEKLEKRQATDDATLLIVNRYWAQLDETVEALLRCHESQGELSSAPEAPGTQEGPTCDGTPLPEPGTSELRDPLLMQLRPPLSEPALAFVVALGASSSEEVELELQGRMEFSKAAVSRVVEASDRLQRRVEELCQRVYSRGDSEPLSEAAQAHTRELGRENRRLQDLATQLQEKHHRISLEYSELQDKVTSAETKVLEMETTVEDLQWDIEKLRKREQKLNKHLAEALEQLNSGYYVSGSSSGFQGGQITLSMQKFEMLNAELEENQELANSRMAELEKLQAELQGAVRTNERLKVALRSLPEEVVRETGEYRMLQAQFSLLYNESLQVKTQLDEARGLLLATKNSHLRHIEHMESDELGLQKKLRTEVIQLEDTLAQVRKEYEMLRIEFEQNLAANEQAGPINREMRHLISSLQNHNHQLKGDAQRYKRKLREVQAEIGKLRAQASGSAHSTPNLGHPEDSGVSAPAPGKEEGGPGPVSTPDNRKEMAPVPGTTTTTTSVKKEELVPSEEDFQGITPGAQGPSSRGREPEARPKRELREREGPSLGPPPVASALSRADREKAKVEETKRKESELLKGLRAELKKAQESQKEMKLLLDMYKSAPKEQRDKVQLMAAERKAKAEVDELRSRIRELEERDRRESKKIADEDALRRIRQAEEQIEHLQRKLGATKQEEEALLSEMDVTGQAFEDMQEQNGRLLQQLREKDDANFKLMSERIKANQIHKLLREEKDELGEQVLGLKSQVDAQLLTVQKLEEKERALQGSLGGVEKELTLRSQALELNKRKAVEAAQLAEDLKVQLEHVQTRLREIQPCLAESRAAREKESFNLKRAQEDISRLRRKLEKQRKVEVYADADEILQEEIKEYKARLTCPCCNTRKKDAVLTKCFHVFCFECVRGRYEARQRKCPKCNAAFGAHDFHRIYIS

>Q6PRD7

MGTSSTDSQQAGHRRCSTSNTSAENLTCLSLPGSPGKTAPLPGPAQAGAGQPLPKGCAAVKAEVGIPAPHTSQEVRIHIRRLLSWAAPGACGLRSTPCALPQALPQARPCPGRWFFPGCSLPTGGAQTILSLWTWRHFLNWALQQREENSGRARRVPPVPRTAPVSKGEGSHPPQNSNGEKVKTITPDVGLHQSLTSDPTVAVLRAKRAPEAHPPRSCSGSLTARVCHMGVCQGQGDTEDGRMTLMG

461 Q96LA8 P51857

>Q96LA8

MSQPKKRKLESGGGGEGGEGTEEEDGAEREAALERPRRTKRERDQLYYECYSDVSVHEEMIADRVRTDAYRLGILRNWAALRGKTVLDVGAGTGILSIFCAQAGARRVYAVEASAIWQQAREVVRFNGLEDRVHVLPGPVETVELPEQVDAIVSEWMGYGLLHESMLSSVLHARTKWLKEGGLLLPASAELFIAPISDQMLEWRLGFWSQVKQHYGVDMSCLEGFATRCLMGHSEIVVQGLSGEDVLARPQRFAQLELSRAGLEQELEAGVGGRFRCSCYGSAPMHGFAIWFQVTFPGGESEKPLVLSTSPFHPATHWKQALLYLNEPVQVEQDTDVSGEITLLPSRDNPRRLRVLLRYKVGDQEEKTKDFAMED

>P51857

MDLSAASHRIPLSDGNSIPIIGLGTYSEPKSTPKGACATSVKVAIDTGYRHIDGAYIYQNEHEVGEAIREKIAEGKVRREDIFYCGKLWATNHVPEMVRPTLERTLRVLQLDYVDLYIIEVPMAFKPGDEIYPRDENGKWLYHKSNLCATWEAMEACKDAGLVKSLGVSNFNRRQLELILNKPGLKHKPVSNQVECHPYFTQPKLLKFCQQHDIVITAYSPLGTSRNPIWVNVSSPPLLKDALLNSLGKRYNKTAAQIVLRFNIQRGVVVIPKSFNLERIKENFQIFDFSLTEEEMKDIEALNKNVRFVELLMWRDHPEYPFHDEY

462 P54803 NP_004721.1

>P54803

MAEWLLSASWQRRAKAMTAAAGSAGRAAVPLLLCALLAPGGAYVLDDSDGLGREFDGIGAVSGGGATSRLLVNYPEPYRSQILDYLFKPNFGASLHILKVEIGGDGQTTDGTEPSHMHYALDENYFRGYEWWLMKEAKKRNPNITLIGLPWSFPGWLGKGFDWPYVNLQLTAYYVVTWIVGAKRYHDLDIDYIGIWNERSYNANYIKILRKMLNYQGLQRVKIIASDNLWESISASMLLDAELFKVVDVIGAHYPGTHSAKDAKLTGKKLWSSEDFSTLNSDMGAGCWGRILNQNYINGYMTSTIAWNLVASYYEQLPYGRCGLMTAQEPWSGHYVVESPVWVSAHTTQFTQPGWYYLKTVGHLEKGGSYVALTDGLGNLTIIIETMSHKHSKCIRPFLPYFNVSQQFATFVLKGSFSEIPELQVWYTKLGKTSERFLFKQLDSLWLLDSDGSFTLSLHEDELFTLTTLTTGRKGSYPLPPKSQPFPSTYKDDFNVDYPFFSEAPNFADQTGVFEYFTNIEDPGEHHFTLRQVLNQRPITWAADASNTISIIGDYNWTNLTIKCDVYIETPDTGGVFIAGRVNKGGILIRSARGIFFWIFANGSYRVTGDLAGWIIYALGRVEVTAKKWYTLTLTIKGHFASGMLNDKSLWTDIPVNFPKNGWAAIGTHSFEFAQFDNFLVEATR

>NP_004721.1

MADDPSAADRNVEIWKIKKLIKSLEAARGNGTSMISLIIPPKDQISRVAKMLADEFGTASNIKSRVNRLSVLGAITSVQQRLKLYNKVPPNGLVVYCGTIVTEEGKEKKVNIDFEPFKPINTSLYLCDNKFHTEALTALLSDDSKFGFIVIDGSGALFGTLQGNTREVLHKFTVDLPKKHGRGGQSALRFARLRMEKRHNYVRKVAETAVQLFISGDKVNVAGLVLAGSADFKTELSQSDMFDQRLQSKVLKLVDISYGGENGFNQAIELSTEVLSNVKFIQEKKLIGRYFDEISQDTGKYCFGVEDTLKALEMGAVEILIVYENLDIMRYVLHCQGTEEEKILYLTPEQEKDKSHFTDKETGQEHELIESMPLLEWFANNYKKFGATLEIVTDKSQEGSQFVKGFGGIGGILRYRVDFQGMEYQGGDDEFFDLDDY

463 NP_000955.1 O75570

>NP_000955.1

MASNSSSCPTPGGGHLNGYPVPPYAFFFPPMLGGLSPPGALTTLQHQLPVSGYSTPSPATIETQSSSSEEIVPSPPSPPPLPRIYKPCFVCQDKSSGYHYGVSACEGCKGFFRRSIQKNMVYTCHRDKNCIINKVTRNRCQYCRLQKCFEVGMSKESVRNDRNKKKKEVPKPECSESYTLTPEVGELIEKVRKAHQETFPALCQLGKYTTNNSSEQRVSLDIDLWDKFSELSTKCIIKTVEFAKQLPGFTTLTIADQITLLKAACLDILILRICTRYTPEQDTMTFSDGLTLNRTQMHNAGFGPLTDLVFAFANQLLPLEMDDAETGLLSAICLICGDRQDLEQPDRVDMLQEPLLEALKVYVRKRRPSRPHMFPKMLMKITDLRSISAKGAERVITLKMEIPGSMPPLIQEMLENSEGLDTLSGQPGGGGRDGGGLAPPPGSCSPSLSPSSNRSSPATHSP

>O75570

MNRHLCVWLFRHPSLNGYLQCHIQLHSHQFRQIHLDTRLQVFRQNRNCILHLLSKNWSRRYCHQDTKMLWKHKALQKYMENLSKEYQTLEQCLQHIPVNEENRRSLNRRHAELAPLAAIYQEIQETEQAIEELESMCKSLNKQDEKQLQELALEERQTIDQKINMLYNELFQSLVPKEKYDKNDVILEVTAGRTTGGDICQQFTREIFDMYQNYSCYKHWQFELLNYTPADYGGLHHAAARISGDGVYKHLKYEGGIHRVQRIPEVGLSSRMQRIHTGTMSVIVLPQPDEVDVKLDPKDLRIDTFRAKGAGGQHVNKTDSAVRLVHIPTGLVVECQQERSQIKNKEIAFRVLRARLYQQIIEKDKRQQQSARKLQVGTRAQSERIRTYNFTQDRVSDHRIAYEVRDIKEFLCGGKGLDQLIQRLLQSADEEAIAELLDEHLKSAK

464 Q6UXN9 Q9NZE8

>Q6UXN9

MKLTDSVLRSFRVAKVFRENSDKINCFDFSPNGETVISSSDDDSIVLYDCQEGKPKRTLYSKKYGVDLIRYTHAANTVVYSSNKIDDTIRYLSLHDNKYIRYFPGHSKRVVALSMSPVDDTFISGSLDKTIRLWDLRSPNCQGLMHLQGKPVCSFDPEGLIFAAGVNSEMVKLYDLRSFDKGPFATFKMQYDRTCEWTGLKFSNDGKLILISTNGSFIRLIDAFKGVVMHTFGGYANSKAVTLEASFTPDSQFIMIGSEDGKIHVWNGESGIKVAVLDGKHTGPITCLQFNPKFMTFASACSNMAFWLPTIDD

>Q9NZE8

MAASAFAGAVRAASGILRPLNILASSTYRNCVKNASLISALSTGRFSHIQTPVVSSTPRLTTSERNLTCGHTSVILNRMAPVLPSVLKLPVRSLTYFSARKGKRKTVKAVIDRFLRLHCGLWVRRKAGYKKKLWKKTPARKKRLREFVFCNKTQSKLLDKMTTSFWKRRNWYVDDPYQKYHDRTNLKV

465 NP_006345.1 NP_005078.2

>NP_006345.1

MSELKDCPLQFHDFKSVDHLKVCPRYTAVLARSEDDGIGIEELDTLQLELETLLSSASRRLRVLEAETQILTDWQDKKGDRRFLKLGRDHELGAPPKHGKPKKQKLEGKAGHGPGPGPGRPKSKNLQPKIQEYEFTDDPIDVPRIPKNDAPNRFWASVEPYCADITSEEVRTLEELLKPPEDEAEHYKIPPLGKHYSQRWAQEDLLEEQKDGARAAAVADKKKGLMGPLTELDTKDVDALLKKSEAQHEQPEDGCPFGALTQRLLQALVEENIISPMEDSPIPDMSGKESGADGASTSPRNQNKPFSVPHTKSLESRIKEELIAQGLLESEDRPAEDSEDEVLAELRKRQAELKALSAHNRTKKHDLLRLAKEEVSRQELRQRVRMADNEVMDAFRKIMAARQKKRTPTKKEKDQAWKTLKERESILKLLDG

>NP_005078.2

MAELTVEVRGSNGAFYKGFIKDVHEDSLTVVFENNWQPERQVPFNEVRLPPPPDIKKEISEGDEVEVYSRANDQEPCGWWLAKVRMMKGEFYVIEYAACDATYNEIVTFERLRPVNQNKTVKKNTFFKCTVDVPEDLREACANENAHKDFKKAVGACRIFYHPETTQLMILSASEATVKRVNILSDMHLRSIRTKLMLMSRNEEATKHLECTKQLAAAFHEEFVVREDLMGLAIGTHGSNIQQARKVPGVTAIELDEDTGTFRIYGESADAVKKARGFLEFVEDFIQVPRNLVGKVIGKNGKVIQEIVDKSGVVRVRIEGDNENKLPREDGMVPFVFVGTKESIGNVQVLLEYHIAYLKEVEQLRMERLQIDEQLRQIGSRSYSGRGRGRRGPNYTSGYGTNSELSNPSETESERKDELSDWSLAGEDDRDSRHQRDSRRRPGGRGRSVSGGRGRGGPRGGKSSISSVLKDPDSNPYSLLDNTESDQTADTDASESHHSTNRRRRSRRRRTDEDAVLMDGMTESDTASVNENGLVTVADYISRAESQSRQRNLPRETLAKNKKEMAKDVIEEHGPSEKAINGPTSASGDDISKLQRTPGEEKINTLKEENTQEAAVLNGVS

466 NP_073624.2 NP_003134.1

>NP_073624.2

MENSEKTEVVLLACGSFNPITNMHLRLFELAKDYMNGTGRYTVVKGIISPVGDAYKKKGLIPAYHRVIMAELATKNSKWVEVDTWESLQKEWKETLKVLRHHQEKLEASDCDHQQNSPTLERPGRKRKWTETQDSSQKKSLEPKTKAVPKVKLLCGADLLESFAVPNLWKSEDITQIVANYGLICVTRAGNDAQKFIYESDVLWKHRSNIHVVNEWIANDISSTKIRRALRRGQSIRYLVPDLVQEYIEKHNLYSSESEDRNAGVILAPLQRNTAEAKT

>NP_003134.1

MFRRPVLQVLRQFVRHESETTTSLVLERSLNRVHLLGRVGQDPVLRQVEGKNPVTIFSLATNEMWRSGDSEVYQLGDVSQKTTWHRISVFRPGLRDVAYQYVKKGSRIYLEGKIDYGEYMDKNNVRRQATTIIADNIIFLSDQTKEKE

467 NP_036389.2 Q6XUX3

>NP_036389.2

MVWEVKTNQMPNAVQKLLLVMDKRASGMNDSLELLQCNENLPSSPGYNSCDEHMELDDLPELQAVQSDPTQSGMYQLSSDVSHQEYPRSSWNQNTSDIPETTYRENEVDWLTELANIATSPQSPLMQCSFYNRSSPVHIIATSKSLHSYARPPPVSSSSKSEPAFPHHHWKEETPVRHERANSESESGIFCMSSLSDDDDLGWCNSWPSTVWHCFLKGTRLCFHKGSNKEWQDVEDFARAEGCDNEEDLQMGIHKGYGSDGLKLLSHEESVSFGESVLKLTFDPGTVEDGLLTVECKLDHPFYVKNKGWSSFYPSLTVVQHGIPCCEVHIGDVCLPPGHPDAINFDDSGVFDTFKSYDFTPMDSSAVYVLSSMARQRRASLSCGGPGGQDFARSGFSKNCGSPGSSQLSSNSLYAKAVKNHSSGTVSATSPNKCKRPMNAFMLFAKKYRVEYTQMYPGKDNRAISVILGDRWKKMKNEERRMYTLEAKALAEEQKRLNPDCWKRKRTNSGSQQH

>Q6XUX3

MEGDGVPWGSEPVSGPGPGGGGMIRELCRGFGRYRRYLGRLRQNLRETQKFFRDIKCSHNHTCLSSLTGGGGAERGPAGDVAETGLQAGQLSCISFPPKEEKYLQQIVDCLPCILILGQDCNVKCQLLNLLLGVQVLPTTKLGSEESCKLRRLRFTYGTQTRVSLALPGQYELVHTLVAHQGNWETIPEEDLEVQENNEDAAHVLAELEVTMHHALLQEVDVVVAPCQGLRPTVDVLGDLVNDFLPVITYALHKDELSERDEQELQEIRKYFSFPVFFFKVPKLGSEIIDSSTRRMESERSPLYRQLIDLGYLSSSHWNCGAPGQDTKAQSMLVEQSEKLRHLSTFSHQVLQTRLVDAAKALNLVHCHCLDIFINQAFDMQRDLQITPKRLEYTRKKENELYESLMNIANRKQEEMKDMIVETLNTMKEELLDDATNMEFKDVIVPENGEPVGTREIKCCIRQIQELIISRLNQAVANKLISSVDYLRESFVGTLERCLQSLEKSQDVSVHITSNYLKQILNAAYHVEVTFHSGSSVTRMLWEQIKQIIQRITWVSPPAITLEWKRKVAQEAIESLSASKLAKSICSQFRTRLNSSHEAFAASLRQLEAGHSGRLEKTEDLWLRVRKDHAPRLARLSLESRSLQDVLLHRKPKLGQELGRGQYGVVYLCDNWGGHFPCALKSVVPPDEKHWNDLALEFHYMRSLPKHERLVDLHGSVIDYNYGGGSSIAVLLIMERLHRDLYTGLKAGLTLETRLQIALDVVEGIRFLHSQGLVHRDIKLKNVLLDKQNRAKITDLGFCKPEAMMSGSIVGTPIHMAPELFTGKYDNSVDVYAFGILFWYICSGSVKLPEAFERCASKDHLWNNVRRGARPERLPVFDEECWQLMEACWDGDPLKRPLLGIVQPMLQGIMNRLCKSNSEQPNRGLDDST

468 NP_001941.2 Q01581

>NP_001941.2

MAEAGPQAPPPPGTPSRHEKSLGLLTTKFVSLLQEAKDGVLDLKLAADTLAVRQKRRIYDITNVLEGIGLIEKKSKNSIQWKGVGPGCNTREIADKLIELKAEIEELQQREQELDQHKVWVQQSIRNVTEDVQNSCLAYVTHEDICRCFAGDTLLAIRAPSGTSLEVPIPEGLNGQKKYQIHLKSVSGPIEVLLVNKEAWSSPPVAVPVPPPEDLLQSPSAVSTPPPLPKPALAQSQEASRPNSPQLTPTAVPGSAEVQGMAGPAAEITVSGGPGTDSKDSGELSSLPLGPTTLDTRPLQSSALLDSSSSSSSSSSSSSNSNSSSSSGPNPSTSFEPIKADPTGVLELPKELSEIFDPTRECMSSELLEELMSSEVFAPLLRLSPPPGDHDYIYNLDESEGVCDLFDVPVLNL

>Q01581

MPGSLPLNAEACWPKDVGIVALEIYFPSQYVDQAELEKYDGVDAGKYTIGLGQAKMGFCTDREDINSLCMTVVQNLMERNNLSYDCIGRLEVGTETIIDKSKSVKTNLMQLFEESGNTDIEGIDTTNACYGGTAAVFNAVNWIESSSWDGRYALVVAGDIAVYATGNARPTGGVGAVALLIGPNAPLIFERGLRGTHMQHAYDFYKPDMLSEYPIVDGKLSIQCYLSALDRCYSVYCKKIHAQWQKEGNDKDFTLNDFGFMIFHSPYCKLVQKSLARMLLNDFLNDQNRDKNSIYSGLEAFGDVKLEDTYFDRDVEKAFMKASSELFSQKTKASLLVSNQNGNMYTSSVYGSLASVLAQYSPQQLAGKRIGVFSYGSGLAATLYSLKVTQDATPGSALDKITASLCDLKSRLDSRTGVAPDVFAENMKLREDTHHLVNYIPQGSIDSLFEGTWYLVRVDEKHRRTYARRPTPNDDTLDEGVGLVHSNIATEHIPSPAKKVPRLPATAAEPEAAVISNGEH

469 NP_008924.1 NP_002631.3

>NP_008924.1

MAAGTAAALAFLSQESRTRAGGVGGLRVPAPVTMDSFFFGCELSGHTRSFTFKVEEEDDAEHVLALTMLCLTEGAKDECNVVEVVARNHDHQEIAVPVANLKLSCQPMLSLDDFQLQPPVTFRLKSGSGPVRITGRHQIVTMSNDVSEEESEEEEEDSDEEEVELCPILPAKKQGGRP

>NP_002631.3

MDDLCEANGTFAISLFKILGEEDNSRNVFFSPMSISSALAMVFMGAKGSTAAQMSQALCLYKDGDIHRGFQSLLSEVNRTGTQYLLRTANRLFGEKTCDFLPDFKEYCQKFYQAELEELSFAEDTEECRKHINDWVAEKTEGKISEVLDAGTVDPLTKLVLVNAIYFKGKWNEQFDRKYTRGMLFKTNEEKKTVQMMFKEAKFKMGYADEVHTQVLELPYVEEELSMVILLPDDNTDLAVVEKALTYEKFKAWTNSEKLTKSKVQVFLPRLKLEESYDLEPFLRRLGMIDAFDEAKADFSGMSTEKNVPLSKVAHKCFVEVNEEGTEAAAATAVVRNSRCSRMEPRFCADHPFLFFIRHHKTNCILFCGRFSSP

470 NP_008882.2 Q969Y2

>NP_008882.2

MAALAEEQTEVAVKLEPEGPPTLLPPQAGDGAGEGSGGTTNNGPNGGGGNVAASSSTGGDGGTPKPTVAVSAAAPAGAAPVPAAAPDAGAPHDRQTLLAVLQFLRQSKLREAEEALRREAGLLEEAVAGSGAPGEVDSAGAEVTSALLSRVTASAPGPAAPDPPGTGASGATVVSGSASGPAAPGKVGSVAVEDQPDVSAVLSAYNQQGDPTMYEEYYSGLKHFIECSLDCHRAELSQLFYPLFVHMYLELVYNQHENEAKSFFEKFHGDQECYYQDDLRVLSSLTKKEHMKGNETMLDFRTSKFVLRISRDSYQLLKRHLQEKQNNQIWNIVQEHLYIDIFDGMPRSKQQIDAMVGSLAGEAKREANKSKVFFGLLKEPEIEVPLDDEDEEGENEEGKPKKKKPKKDSIGSKSKKQDPNAPPQNRIPLPELKDSDKLDKIMNMKETTKRVRLGPDCLPSICFYTFLNAYQGLTAVDVTDDSSLIAGGFADSTVRVWSVTPKKLRSVKQASDLSLIDKESDDVLERIMDEKTASELKILYGHSGPVYGASFSPDRNYLLSSSEDGTVRLWSLQTFTCLVGYKGHNYPVWDTQFSPYGYYFVSGGHDRVARLWATDHYQPLRIFAGHLADVNCTRFHPNSNYVATGSADRTVRLWDVLNGNCVRIFTGHKGPIHSLTFSPNGRFLATGATDGRVLLWDIGHGLMVGELKGHTDTVCSLRFSRDGEILASGSMDNTVRLWDAIKAFEDLETDDFTTATGHINLPENSQELLLGTYMTKSTPVVHLHFTRRNLVLAAGAYSPQ

>Q969Y2

MWRGLWTLAAQAARGPRRLCTRRSSGAPAPGSGATIFALSSGQGRCGIAVIRTSGPASGHALRILTAPRDLPLARHASLRLLSDPRSGEPLDRALVLWFPGPQSFTGEDCVEFHVHGGPAVVSGVLQALGSVPGLRPAEAGEFTRRAFANGKLNLTEVEGLADLIHAETEAQRRQALRQLDGELGHLCRGWAETLTKALAHVEAYIDFGEDDNLEEGVLEQADIEVRALQVALGAHLRDARRGQRLRSGAHVVVTGPPNAGKSSLVNLLSRKPVSIVSPEPGTTRDVLETPVDLAGFPVLLSDTAGLREGVGPVEQEGVRRARERLEQADLILAMLDASDLASPSSCNFLATVVASVGAQSPSDSSQRLLLVLNKSDLLSPEGPGPGPDLPPHLLLSCLTGEGLDGLLEALRKELAAVCGDPSTDPPLLTRARHQHHLQGCLDALGHYKQSKDLALAAEALRVARGHLTRLTGGGGTEEILDIIFQDFCVGK

471 P28347 NP_065078.1

>P28347

MEPSSWSGSESPAENMERMSDSADKPIDNDAEGVWSPDIEQSFQEALAIYPPCGRRKIILSDEGKMYGRNELIARYIKLRTGKTRTRKQVSSHIQVLARRKSRDFHSKLKDQTAKDKALQHMAAMSSAQIVSATAIHNKLGLPGIPRPTFPGAPGFWPGMIQTGQPGSSQDVKPFVQQAYPIQPAVTAPIPGFEPASAPAPSVPAWQGRSIGTTKLRLVEFSAFLEQQRDPDSYNKHLFVHIGHANHSYSDPLLESVDIRQIYDKFPEKKGGLKELFGKGPQNAFFLVKFWADLNCNIQDDAGAFYGVTSQYESSENMTVTCSTKVCSFGKQVVEKVETEYARFENGRFVYRINRSPMCEYMINFIHKLKHLPEKYMMNSVLENFTILLVVTNRDTQETLLCMACVFEVSNSEHGAQHHIYRLVKD

>NP_065078.1

MGKGCKVVVCGLLSVGKTAILEQLLYGNHTIGMEDCETMEDVYMASVETDRGVKEQLHLYDTRGLQEGVELPKHYFSFADGFVLVYSVNNLESFQRVELLKKEIDKFKDKKEVAIVVLGNKIDLSEQRQVDAEVAQQWAKSEKVRLWEVTVTDRKTLIEPFTLLASKLSQPQSKSSFPLPGRKNKGNSNSEN

472 NP_006006.3 P40394

>NP_006006.3

MAAQVAPAAASSLGNPPPPPPSELKKAEQQQREEAGGEAAAAAAAERGEMKAAAGQESEGPAVGPPQPLGKELQDGAESNGGGGGGGAGSGGGPGAEPDLKNSNGNAGPRPALNNNLTEPPGGGGGGSSDGVGAPPHSAAAALPPPAYGFGQPYGRSPSAVAAAAAAVFHQQHGGQQSPGLAALQSGGGGGLEPYAGPQQNSHDHGFPNHQYNSYYPNRSAYPPPAPAYALSSPRGGTPGSGAAAAAGSKPPPSSSASASSSSSSFAQQRFGAMGGGGPSAAGGGTPQPTATPTLNQLLTSPSSARGYQGYPGGDYSGGPQDGGAGKGPADMASQCWGAAAAAAAAAAASGGAQQRSHHAPMSPGSSGGGGQPLARTPQPSSPMDQMGKMRPQPYGGTNPYSQQQGPPSGPQQGHGYPGQPYGSQTPQRYPMTMQGRAQSAMGGLSYTQQIPPYGQQGPSGYGQQGQTPYYNQQSPHPQQQQPPYSQQPPSQTPHAQPSYQQQPQSQPPQLQSSQPPYSQQPSQPPHQQSPAPYPSQQSTTQQHPQSQPPYSQPQAQSPYQQQQPQQPAPSTLSQQAAYPQPQSQQSQQTAYSQQRFPPPQELSQDSFGSQASSAPSMTSSKGGQEDMNLSLQSRPSSLPDLSGSIDDLPMGTEGALSPGVSTSGISSSQGEQSNPAQSPFSPHTSPHLPGIRGPSPSPVGSPASVAQSRSGPLSPAAVPGNQMPPRPPSGQSDSIMHPSMNQSSIAQDRGYMQRNPQMPQYSSPQPGSALSPRQPSGGQIHTGMGSYQQNSMGSYGPQGGQYGPQGGYPRQPNYNALPNANYPSAGMAGGINPMGAGGQMHGQPGIPPYGTLPPGRMSHASMGNRPYGPNMANMPPQVGSGMCPPPGGMNRKTQETAVAMHVAANSIQNRPPGYPNMNQGGMMGTGPPYGQGINSMAGMINPQGPPYSMGGTMANNSAGMAASPEMMGLGDVKLTPATKMNNKADGTPKTESKSKKSSSSTTTNEKITKLYELGGEPERKMWVDRYLAFTEEKAMGMTNLPAVGRKPLDLYRLYVSVKEIGGLTQVNKNKKWRELATNLNVGTSSSAASSLKKQYIQCLYAFECKIERGEDPPPDIFAAADSKKSQPKIQPPSPAGSGSMQGPQTPQSTSSSMAEGGDLKPPTPASTPHSQIPPLPGMSRSNSVGIQDAFNDGSDSTFQKRNSMTPNPGYQPSMNTSDMMGRMSYEPNKDPYGSMRKAPGSDPFMSSGQGPNGGMGDPYSRAAGPGLGNVAMGPRQHYPYGGPYDRVRTEPGIGPEGNMSTGAPQPNLMPSNPDSGMYSPSRYPPQQQQQQQQRHDSYGNQFSTQGTPSGSPFPSQQTTMYQQQQQNYKRPMDGTYGPPAKRHEGEMYSVPYSTGQGQPQQQQLPPAQPQPASQQQAAQPSPQQDVYNQYGNAYPATATAATERRPAGGPQNQFPFQFGRDRVSAPPGTNAQQNMPPQMMGGPIQASAEVAQQGTMWQGRNDMTYNYANRQSTGSAPQGPAYHGVNRTDEMLHTDQRANHEGSWPSHGTRQPPYGPSAPVPPMTRPPPSNYQPPPSMQNHIPQVSSPAPLPRPMENRTSPSKSPFLHSGMKMQKAGPPVPASHIAPAPVQPPMIRRDITFPPGSVEATQPVLKQRRRLTMKDIGTPEAWRVMMSLKSGLLAESTWALDTINILLYDDNSIMTFNLSQLPGLLELLVEYFRRCLIEIFGILKEYEVGDPGQRTLLDPGRFSKVSSPAPMEGGEEEEELLGPKLEEEEEEEVVENDEEIAFSGKDKPASENSEEKLISKFDKLPVKIVQKNDPFVVDCSDKLGRVQEFDSGLLHWRIGGGDTTEHIQTHFESKTELLPSRPHAPCPPAPRKHVTTAEGTPGTTDQEGPPPDGPPEKRITATMDDMLSTRSSTLTEDGAKSSEAIKESSKFPFGISPAQSHRNIKILEDEPHSKDETPLCTLLDWQDSLAKRCVCVSNTIRSLSFVPGNDFEMSKHPGLLLILGKLILLHHKHPERKQAPLTYEKEEEQDQGVSCNKVEWWWDCLEMLRENTLVTLANISGQLDLSPYPESICLPVLDGLLHWAVCPSAEAQDPFSTLGPNAVLSPQRLVLETLSKLSIQDNNVDLILATPPFSRLEKLYSTMVRFLSDRKNPVCREMAVVLLANLAQGDSLAARAIAVQKGSIGNLLGFLEDSLAATQFQQSQASLLHMQNPPFEPTSVDMMRRAARALLALAKVDENHSEFTLYESRLLDISVSPLMNSLVSQVICDVLFLIGQS

>P40394

MGTAGKVIKCKAAVLWEQKQPFSIEEIEVAPPKTKEVRIKILATGICRTDDHVIKGTMVSKFPVIVGHEATGIVESIGEGVTTVKPGDKVIPLFLPQCRECNACRNPDGNLCIRSDITGRGVLADGTTRFTCKGKPVHHFMNTSTFTEYTVVDESSVAKIDDAAPPEKVCLIGCGFSTGYGAAVKTGKVKPGSTCVVFGLGGVGLSVIMGCKSAGASRIIGIDLNKDKFEKAMAVGATECISPKDSTKPISEVLSEMTGNNVGYTFEVIGHLETMIDALASCHMNYGTSVVVGVPPSAKMLTYDPMLLFTGRTWKGCVFGGLKSRDDVPKLVTEFLAKKFDLDQLITHVLPFKKISEGFELLNSGQSIRTVLTF

473 Q96LT9 P57723

>Q96LT9

MAAPEQPLAISRGCTSSSSLSPPRGDRTLLVRHLPAELTAEEKEDLLKYFGAQSVRVLSDKGRLKHTAFATFPNEKAAIKALTRLHQLKLLGHTLVVEFAKEQDRVHSPCPTSGSEKKKRSDDPVEDDKEKKELGYLTVENGIAPNHGLTFPLNSCLKYMYPPPSSTILANIVNALASVPKFYVQVLHLMNKMNLPTPFGPITARPPMYEDYMPLHAPLPPTSPQPPEEPPLPDEDEELSSEESEYESTDDEDRQRMNKLMELANLQPKRPKTIKQRHVRKKRKIKDMLNTPLCPSHSSLHPVLLPSDVFDQPQPVGNKRIEFHISTDMPAAFKKDLEKEQNCEEKNHDLPATEVDASNIGFGKIFPKPNLDITEEIKEDSDEMPSECISRRELEKGRISREEMETLSVFRSYEPGEPNCRIYVKNLAKHVQEKDLKYIFGRYVDFSSETQRIMFDIRLMKEGRMKGQAFIGLPNEKAAAKALKEANGYVLFGKPMVVQFARSARPKQDPKEGKRKC

>P57723

MSGSDGGLEEEPELSITLTLRMLMHGKEVGSIIGKKGETVKRIREQSSARITISEGSCPERITTITGSTAAVFHAVSMIAFKLDEDLCAAPANGGNVSRPPVTLRLVIPASQCGSLIGKAGTKIKEIRETTGAQVQVAGDLLPNSTERAVTVSGVPDAIILCVRQICAVILESPPKGATIPYHPSLSLGTVLLSANQGFSVQGQYGAVTPAEVTKLQQLSSHAVPFATPSVVPGLDPGTQTSSQEFLVPNDLIGCVIGRQGSKISEIRQMSGAHIKIGNQAEGAGERHVTITGSPVSIALAQYLITACLETAKSTSGGTPSSAPADLPAPFSPPLTALPTAPPGLLGTPYAISLSNFIGLKPMPFLALPPASPGPPPGLAAYTAKMAAANGSKKAERQKFSPY

474 NP_002008.2 P40939

>NP_002008.2

MDGTIKEALSVVSDDQSLFDSAYGAAAHLPKADMTASGSPDYGQPHKINPLPPQQEWINQPVRVNVKREYDHMNGSRESPVDCSVSKCSKLVGGGESNPMNYNSYMDEKNGPPPPNMTTNERRVIVPADPTLWTQEHVRQWLEWAIKEYSLMEIDTSFFQNMDGKELCKMNKEDFLRATTLYNTEVLLSHLSYLRESSLLAYNTTSHTDQSSRLSVKEDPSYDSVRRGAWGNNMNSGLNKSPPLGGAQTISKNTEQRPQPDPYQILGPTSSRLANPGSGQIQLWQFLLELLSDSANASCITWEGTNGEFKMTDPDEVARRWGERKSKPNMNYDKLSRALRYYYDKNIMTKVHGKRYAYKFDFHGIAQALQPHPTESSMYKYPSDISYMPSYHAHQQKVNFVPPHPSSMPVTSSSFFGAASQYWTSPTGGIYPNPNVPRHPNTHVPSHLGSYY

>P40939

MVACRAIGILSRFSAFRILRSRGYICRNFTGSSALLTRTHINYGVKGDVAVVRINSPNSKVNTLSKELHSEFSEVMNEIWASDQIRSAVLISSKPGCFIAGADINMLAACKTLQEVTQLSQEAQRIVEKLEKSTKPIVAAINGSCLGGGLEVAISCQYRIATKDRKTVLGTPEVLLGALPGAGGTQRLPKMVGVPAALDMMLTGRSIRADRAKKMGLVDQLVEPLGPGLKPPEERTIEYLEEVAITFAKGLADKKISPKRDKGLVEKLTAYAMTIPFVRQQVYKKVEEKVRKQTKGLYPAPLKIIDVVKTGIEQGSDAGYLCESQKFGELVMTKESKALMGLYHGQVLCKKNKFGAPQKDVKHLAILGAGLMGAGIAQVSVDKGLKTILKDATLTALDRGQQQVFKGLNDKVKKKALTSFERDSIFSNLTGQLDYQGFEKADMVIEAVFEDLSLKHRVLKEVEAVIPDHCIFASNTSALPISEIAAVSKRPEKVIGMHYFSPVDKMQLLEIITTEKTSKDTSASAVAVGLKQGKVIIVVKDGPGFYTTRCLAPMMSEVIRILQEGVDPKKLDSLTTSFGFPVGAATLVDEVGVDVAKHVAEDLGKVFGERFGGGNPELLTQMVSKGFLGRKSGKGFYIYQEGVKRKDLNSDMDSILASLKLPPKSEVSSDEDIQFRLVTRFVNEAVMCLQEGILATPAEGDIGAVFGLGFPPCLGGPFRFVDLYGAQKIVDRLKKYEAAYGKQFTPCQLLADHANSPNKKFYQ

475 P23760 NP_004777.1

>P23760

MTTLAGAVPRMMRPGPGQNYPRSGFPLEVSTPLGQGRVNQLGGVFINGRPLPNHIRHKIVEMAHHGIRPCVISRQLRVSHGCVSKILCRYQETGSIRPGAIGGSKPKQVTTPDVEKKIEEYKRENPGMFSWEIRDKLLKDAVCDRNTVPSVSSISRILRSKFGKGEEEEADLERKEAEESEKKAKHSIDGILSERASAPQSDEGSDIDSEPDLPLKRKQRRSRTTFTAEQLEELERAFERTHYPDIYTREELAQRAKLTEARVQVWFSNRRARWRKQAGANQLMAFNHLIPGGFPPTAMPTLPTYQLSETSYQPTSIPQAVSDPSSTVHRPQPLPPSTVHQSTIPSNPDSSSAYCLPSTRHGFSSYTDSFVPPSGPSNPMNPTIGNGLSPQVMGLLTNHGGVPHQPQTDYALSPLTGGLEPTTTVSASCSQRLDHMKSLDSLPTSQSYCPPTYSTTGYSMDPVTGYQYGQYGQSKPWTF

>NP_004777.1

MVGVKPVGSDPDFQPELSGAGSRLAVVKFTMRGCGPCLRIAPAFSSMSNKYPQAVFLEVDVHQCQGTAATNNISATPTFLFFRNKVRIDQYQGADAVGLEEKIKQHLENDPGSNEDTDIPKGYMDLMPFINKAGCECLNESDEHGFDNCLRKDTTFLESDCDEQLLITVAFNQPVKLYSMKFQGPDNGQGPKYVKIFINLPRSMDFEEAERSEPTQALELTEDDIKEDGIVPLRYVKFQNVNSVTIFVQSNQGEEETTRISYFTFIGTPVQATNMNDFKRVVGKKGESH

476 NP_068811.1 NP_003840.2

>NP_068811.1

MSWAARPPFLPQRHAAGQCGPVGVRKEMHCGVASRWRRRRPWLDPAAAAAAAVAGGEQQTPEPEPGEAGRDGMGDSGRDSRSPDSSSPNPLPQGVPPPSPPGPPLPPSTAPSLGGSGAPPPPPMPPPPLGSPFPVISSSMGSPGLPPPAPPGFSGPVSSPQINSTVSLPGGGSGPPEDVKPPVLGVRGLHCPPPPGGPGAGKRLCAICGDRSSGKHYGVYSCEGCKGFFKRTIRKDLTYSCRDNKDCTVDKRQRNRCQYCRYQKCLATGMKREAVQEERQRGKDKDGDGEGAGGAPEEMPVDRILEAELAVEQKSDQGVEGPGGTGGSGSSPNDPVTNICQAADKQLFTLVEWAKRIPHFSSLPLDDQVILLRAGWNELLIASFSHRSIDVRDGILLATGLHVHRNSAHSAGVGAIFDRVLTELVSKMRDMRMDKTELGCLRAIILFNPDAKGLSNPSEVEVLREKVYASLETYCKQKYPEQQGRFAKLLLRLPALRSIGLKCLEHLFFFKLIGDTPIDTFLMEMLEAPHQLA

>NP_003840.2

MTATLAAAADIATMVSGSSGLAAARLLSRSFLLPQNGIRHCSYTASRQHLYVDKNTKIICQGFTGKQGTFHSQQALEYGTKLVGGTTPGKGGQTHLGLPVFNTVKEAKEQTGATASVIYVPPPFAAAAINEAIEAEIPLVVCITEGIPQQDMVRVKHKLLRQEKTRLIGPNCPGVINPGECKIGIMPGHIHKKGRIGIVSRSGTLTYEAVHQTTQVGLGQSLCVGIGGDPFNGTDFIDCLEIFLNDSATEGIILIGEIGGNAEENAAEFLKQHNSGPNSKPVVSFIAGLTAPPGRRMGHAGAIIAGGKGGAKEKISALQSAGVVVSMSPAQLGTTIYKEFEKRKML

477 Q9UK76 NP_000894.1

>Q9UK76

MTTTTTFKGVDPNSRNSSRVLRPPGGGSNFSLGFDEPTEQPVRKNKMASNIFGTPEENQASWAKSAGAKSSGGREDLESSGLQRRNSSEASSGDFLDLKGEGDIHENVDTDLPGSLGQSEEKPVPAAPVPSPVAPAPVPSRRNPPGGKSSLVLG

>NP_000894.1

MVGRRALIVLAHSERTSFNYAMKEAAAAALKKKGWEVVESDLYAMNFNPIISRKDITGKLKDPANFQYPAESVLAYKEGHLSPDIVAEQKKLEAADLVIFQFPLQWFGVPAILKGWFERVFIGEFAYTYAAMYDKGPFRSKKAVLSITTGGSGSMYSLQGIHGDMNVILWPIQSGILHFCGFQVLEPQLTYSIGHTPADARIQILEGWKKRLENIWDETPLYFAPSSLFDLNFQAGFLMKKEVQDEEKNKKFGLSVGHHLGKSIPTDNQIKARK

478 NP_005366.2 NP_055129.2

>NP_005366.2

MARRPRHSIYSSDEDDEDFEMCDHDYDGLLPKSGKRHLGKTRWTREEDEKLKKLVEQNGTDDWKVIANYLPNRTDVQCQHRWQKVLNPELIKGPWTKEEDQRVIELVQKYGPKRWSVIAKHLKGRIGKQCRERWHNHLNPEVKKTSWTEEEDRIIYQAHKRLGNRWAEIAKLLPGRTDNAIKNHWNSTMRRKVEQEGYLQESSKASQPAVATSFQKNSHLMGFAQAPPTAQLPATGQPTVNNDYSYYHISEAQNVSSHVPYPVALHVNIVNVPQPAAAAIQRHYNDEDPEKEKRIKELELLLMSTENELKGQQVLPTQNHTCSYPGWHSTTIADHTRPHGDSAPVSCLGEHHSTPSLPADPGSLPEESASPARCMIVHQGTILDNVKNLLEFAETLQFIDSFLNTSSNHENSDLEMPSLTSTPLIGHKLTVTTPFHRDQTVKTQKENTVFRTPAIKRSILESSPRTPTPFKHALAAQEIKYGPLKMLPQTPSHLVEDLQDVIKQESDESGIVAEFQENGPPLLKKIKQEVESPTDKSGNFFCSHHWEGDSLNTQLFTQTSPVADAPNILTSSVLMAPASEDEDNVLKAFTVPKNRSLASPLQPCSSTWEPASCGKMEEQMTSSSQARKYVNAFSARTLVM

>NP_055129.2

MTTEQRRSLQAFQDYIRKTLDPTYILSYMAPWFREEEVQYIQAEKNNKGPMEAATLFLKFLLELQEEGWFRGFLDALDHAGYSGLYEAIESWDFKKIEKLEEYRLLLKRLQPEFKTRIIPTDIISDLSECLINQECEEILQICSTKGMMAGAEKLVECLLRSDKENWPKTLKLALEKERNKFSELWIVEKGIKDVETEDLEDKMETSDIQIFYQEDPECQNLSENSCPPSEVSDTNLYSPFKPRNYQLELALPAMKGKNTIICAPTGCGKTFVSLLICEHHLKKFPQGQKGKVVFFANQIPVYEQQKSVFSKYFERHGYRVTGISGATAENVPVEQIVENNDIIILTPQILVNNLKKGTIPSLSIFTLMIFDECHNTSKQHPYNMIMFNYLDQKLGGSSGPLPQVIGLTASVGVGDAKNTDEALDYICKLCASLDASVIATVKHNLEELEQVVYKPQKFFRKVESRISDKFKYIIAQLMRDTESLAKRICKDLENLSQIQNREFGTQKYEQWIVTVQKACMVFQMPDKDEESRICKALFLYTSHLRKYNDALIISEHARMKDALDYLKDFFSNVRAAGFDEIEQDLTQRFEEKLQELESVSRDPSNENPKLEDLCFILQEEYHLNPETITILFVKTRALVDALKNWIEGNPKLSFLKPGILTGRGKTNQNTGMTLPAQKCILDAFKASGDHNILIATSVADEGIDIAQCNLVILYEYVGNVIKMIQTRGRGRARGSKCFLLTSNAGVIEKEQINMYKEKMMNDSILRLQTWDEAVFREKILHIQTHEKFIRDSQEKPKPVPDKENKKLLCRKCKALACYTADVRVIEECHYTVLGDAFKECFVSRPHPKPKQFSSFEKRAKIFCARQNCSHDWGIHVKYKTFEIPVIKIESFVVEDIATGVQTLYSKWKDFHFEKIPFDPAEMSK

479 NP_002098.1 Q9Y238

>NP_002098.1

MARTKQTARKSTGGKAPRKQLATKAARKSAPSTGGVKKPHRYRPGTVALREIRRYQKSTELLIRKLPFQRLVREIAQDFKTDLRFQSAAIGALQEASEAYLVGLFEDTNLCAIHAKRVTIMPKDIQLARRIRGERA

>Q9Y238

METRSSKTRRSLASRTNECQGTMWAPTSPPAGSSSPSQPTWKSSLYSSLAYSEAFHYSFAARPRRLTQLALAQRPEPQLLRLRPSSLRTQDISHLLTGVFRNLYSAEVIGDEVSASLIKARGSENERHEEFVDQLQQIRELYKQRLDEFEMLERHITQAQARAIAENERVMSQAGVQDLESLVRLPPVKSVSRWCIDSELLRKHHLISPEDYYTDTVPFHSAPKGISLPGCSKLTFSCEKRSVQKKELNKKLEDSCRKKLAEFEDELDHTVDSLTWNLTPKAKERTREPLKKASQPRNKNWMNHLRVPQRELDRLLLARMESRNHFLKNPRFFPPNTRYGGKSLVFPPKKPAPIGEFQSTEPEQSCADTPVFLAKPPIGFFTDYEIGPVYEMVIALQNTTTTSRYLRVLPPSTPYFALGLGMFPGKGGMVAPGMTCQYIVQFFPDCLGDFDDFILVETQSAHTLLIPLQARRPPPVLTLSPVLDCGYCLIGGVKMTRFICKNVGFSVGRFCIMPKTSWPPLSFKAIATVGFVEQPPFGILPSVFELAPGHAILVEVLFSPKSLGKAEQTFIIMCDNCQIKELVTIGIGQLIALDLIYISGEKSQPDPGELTDLTAQHFIRFEPENLRSTARKQLIIRNATHVELAFYWQIMKPNLQPLMPGETFSMDSIKCYPDKETAFSIMPRKGVLSPHTDHEFILSFSPHELRDFHSVLQMVLEEVPEPVSSEAESLGHSSYSVDDVIVLEIEVKGSVEPFQVLLEPYALIIPGENYIGINVKKAFKMWNNSKSPIRYLWGKISDCHIIEVEPGTGVIEPSEVGDFELNFTGGVPGPTSQDLLCEIEDSPSPVVLHIEAVFKGPALIINVSALQFGLLRLGQKATNSIQIRNVSQLPATWRMKESPVSLQERPEDVSPFDIEPSSGQLHSLGECRVDITLEALHCQHLETVLELEVENGAWSYLPVYAEVQKPHVYLQSSQVEVRNLYLGVPTKTTITLINGTLLPTQFHWGKLLGHQAEFCMVTVSPKHGLLGPSEECQLKLELTAHTQEELTHLALPCHVSGMKKPLVLGISGKPQGLQVAITISKESSDCSTEQWPGHPKELRLDFGSAVPLRTRVTRQLILTNRSPIRTRFSLKFEYFGSPQNSLSKKTSLPNMPPALLKTVRMQEHLAKREQLDFMESMLSHGKGAAFFPHFSQGMLGPYQQLCIDITGCANMWGEYWDNLICTVGDLLPEVIPVHMAAVGCPISSLRTTSYTIDQAQKEPAMRFGTQVSGGDTVTRTLRLNNSSPCDIRLDWETYVPEDKEDRLVELLVFYGPPFPLRDQAGNELVCPDTPEGGCLLWSPGPSSSSEFSHETDSSVEGSSSASNRVAQKLISVILQAHEGVPSGHLYCISPKQVVVPAGGSSTIYISFTPMVLSPEILHKVECTGYALGFMSLDSKVEREIPRKRHRLQDFAVGPLKLDLHSYVRPAQLSVELDYGGSMEFQCQASDLIPEQPCSGVLSELVTTHHLKLTNTTEIPHYFRLMVSRPFSVSQDGASQDHRAPGPGQKQECEEETASADKQLVLQAQENMLVNVSFSLSLELLSYQKLPADQTLPGVDIQQSASGEREMVFTQNLLLEYTNQTTQVVPLRAVVAVPELQLSTSWVDFGTCFVSQQRVREVYLMNLSGCRSYWTMLMGQQEPAKAAVAFRVSPNSGLLEARSANAPPTSIALQVFFTARSSELYESTMVVEGVLGEKSCTLRLRGQGSYDERYMLPHQP

480 Q96JN0 NP_005407.1

>Q96JN0

MQRMIQQFAAEYTSKNSSTQDPSQPNSTKNQSLPKASPVTTSPTAATTQNPVLSKLLMADQDSPLDLTVRKSQSEPSEQDGVLDLSTKKSPCAGSTSLSHSPGCSSTQGNGRPGRPSQYRPDGLRSGDGVPPRSLQDGTREGFGHSTSLKVPLARSLQISEELLSRNQLSTAASLGPSGLQNHGQHLILSREASWAKPHYEFNLSRMKFRGNGALSNISDLPFLAENSAFPKMALQAKQDGKKDVSHSSPVDLKIPQVRGMDLSWESRTGDQYSYSSLVMGSQTESALSKKLRAILPKQSRKSMLDAGPDSWGSDAEQSTSGQPYPTSDQEGDPGSKQPRKKRGRYRQYNSEILEEAISVVMSGKMSVSKAQSIYGIPHSTLEYKVKERLGTLKNPPKKKMKLMRSEGPDVSVKIELDPQGEAAQSANESKNE

>NP_005407.1

MSSYQQKQTFTPPPQLQQQQVKQPSQPPPQEIFVPTTKEPCHSKVPQPGNTKIPEPGCTKVPEPGCTKVPEPGCTKVPEPGCTKVPEPGCTKVPEPGCTKVPEPGYTKVPEPGSIKVPDQGFIKFPEPGAIKVPEQGYTKVPVPGYTKLPEPCPSTVTPGPAQQKTKQK

481 NP_003454.1 NP_037392.1

>NP_003454.1

MARMNRPAPVEVTYKNMRFLITHNPTNATLNKFIEELKKYGVTTIVRVCEATYDTTLVEKEGIHVLDWPFDDGAPPSNQIVDDWLSLVKIKFREEPGCCIAVHCVAGLGRAPVLVALALIEGGMKYEDAVQFIRQKRRGAFNSKQLLYLEKYRPKMRLRFKDSNGHRNNCCIQ

>NP_037392.1

MAGKKNVLSSLAVYAEDSEPESDGEAGIEAVGSAAEEKGGLVSDAYGEDDFSRLGGDEDGYEEEEDENSRQSEDDDSETEKPEADDPKDNTEAEKRDPQELVASFSERVRNMSPDEIKIPPEPPGRCSNHLQDKIQKLYERKIKEGMDMNYIIQRKKEFRNPSIYEKLIQFCAIDELGTNYPKDMFDPHGWSEDSYYEALAKAQKIEMDKLEKAKKERTKIEFVTGTKKGTTTNATSTTTTTASTAVADAQKRKSKWDSAIPVTTIAQPTILTTTATLPAVVTVTTSASGSKTTVISAVGTIVKKAKQ

482 NP_060624.1 Q9UDY6

>NP_060624.1

MAKVSVLNVAVLENPSPFHSPFRFEISFECSEALADDLEWKIIYVGSAESEEFDQILDSVLVGPVPAGRHMFVFQADAPNPSLIPETDAVGVTVVLITCTYHGQEFIRVGYYVNNEYLNPELRENPPMKPDFSQLQRNILASNPRVTRFHINWDNNMDRLEAIETQDPSLGCGLPLNCTPIKGLGLPGCIPGLLPENSMDCI

>Q9UDY6

MASAASVTSLADEVNCPICQGTLREPVTIDCGHNFCRACLTRYCEIPGPDLEESPTCPLCKEPFRPGSFRPNWQLANVVENIERLQLVSTLGLGEEDVCQEHGEKIYFFCEDDEMQLCVVCREAGEHATHTMRFLEDAAAPYREQIHKCLKCLRKEREEIQEIQSRENKRMQVLLTQVSTKRQQVISEFAHLRKFLEEQQSILLAQLESQDGDILRQRDEFDLLVAGEICRFSALIEELEEKNERPARELLTDIRSTLIRCETRKCRKPVAVSPELGQRIRDFPQQALPLQREMKMFLEKLCFELDYEPAHISLDPQTSHPKLLLSEDHQRAQFSYKWQNSPDNPQRFDRATCVLAHTGITGGRHTWVVSIDLAHGGSCTVGVVSEDVQRKGELRLRPEEGVWAVRLAWGFVSALGSFPTRLTLKEQPRQVRVSLDYEVGWVTFTNAVTREPIYTFTASFTRKVIPFFGLWGRGSSFSLSS

483 NP_665906.1 P82664

>NP_665906.1

MSESSSKSSQPLASKQEKDGTEKRGRGRPRKQPPVSPGTALVGSQKEPSEVPTPKRPRGRPKGSKNKGAAKTRKTTTTPGRKPRGRPKKLEKEEEEGISQESSEEEQ

>P82664

MAARTAFGAVCRRLWQGLGNFSVNTSKGNTAKNGGLLLSTNMKWVQFSNLHVDVPKDLTKPVVTISDEPDILYKRLSVLVKGHDKAVLDSYEYFAVLAAKELGISIKVHEPPRKIERFTLLQSVHIYKKHRVQYEMRTLYRCLELEHLTGSTADVYLEYIQRNLPEGVAMEVTKTQLEQLPEHIKEPIWETLSEEKEESKS

484 NP_001978.1 Q8TF40

>NP_001978.1

MSETPAQCSIKQERISYTPPESPVPSYASSTPLHVPVPRALRMEEDSIRLPAHLRLQPIYWSRDDVAQWLKWAENEFSLRPIDSNTFEMNGKALLLLTKEDFRYRSPHSGDVLYELLQHILKQRKPRILFSPFFHPGNSIHTQPEVILHQNHEEDNCVQRTPRPSVDNVHHNPPTIELLHRSRSPITTNHRPSPDPEQRPLRSPLDNMIRRLSPAERAQGPRPHQENNHQESYPLSVSPMENNHCPASSESHPKPSSPRQESTRVIQLMPSPIMHPLILNPRHSVDFKQSRLSEDGLHREGKPINLSHREDLAYMNHIMVSVSPPEEHAMPIGRIADCRLLWDYVYQLLSDSRYENFIRWEDKESKIFRIVDPNGLARLWGNHKNRTNMTYEKMSRALRHYYKLNIIRKEPGQRLLFRFMKTPDEIMSGRTDRLEHLESQELDEQIYQEDEC

>Q8TF40

MAPTLFQKLFSKRTGLGAPGRDARDPDCGFSWPLPEFDPSQIRLIVYQDCERRGRNVLFDSSVKRRNEDISVSKLCSDAQVKVFGKCCQLKPGGDSSSSLDSSVTSSSDIKDQCLKYQGSRCSSDANMLGEMMFGSVAMSYKGSTLKIHQIRSPPQLMLSKVFTARTGSSICGSLNTLQDSLEFINQDNNTLKADNNTVINGLLGNIGLSQFCSPRRAFSEQGPLRLIRSASFFAVHSNPMDMPGRELNEDRDSGIARSASLSSLLITPFPSPNSSLTRSCASSYQRRWRRSQTTSLENGVFPRWSIEESFNLSDESCGPNPGIVRKKKIAIGVIFSLSKDEDENNKFNEFFFSHFPLFESHMNKLKSAIEQAMKMSRRSADASQRSLAYNRIVDALNEFRTTICNLYTMPRIGEPVWLTMMSGTPEKNHLCYRFMKEFTFLMENASKNQFLPALITAVLTNHLAWVPTVMPNGQPPIKIFLEKHSSQSVDMLAKTHPYNPLWAQLGDLYGAIGSPVRLARTVVVGKRQDMVQRLLYFLTYFIRCSELQETHLLENGEDEAIVMPGTVITTTLEKGEIEESEYVLVTMHRNKSSLLFKESEEIRTPNCNCKYCSHPLLGQNVENISQQEREDIQNSSKELLGISDECQMISPSDCQEENAVDVKQYRDKLRTCFDAKLETVVCTGSVPVDKCALSESGLESTEETWQSEKLLDSDSHTGKAMRSTGMVVEKKPPDKIVPASFSCEAAQTKVTFLIGDSMSPDSDTELRSQAVVDQITRHHTKPLKEERGAIDQHQETKQTTKDQSGESDTQNMVSEEPCELPCWNHSDPESMSLFDEYFNDDSIETRTIDDVPFKTSTDSKDHCCMLEFSKILCTKNNKQNNEFCKCIETVPQDSCKTCFPQQDQRDTLSILVPHGDKESSDKKIAVGTEWDIPRNESSDSALGDSESEDTGHDMTRQVSSYYGGEQEDWAEEDEIPFPGSKLIEVSAVQPNIANFGRSLLGGYCSSYVPDFVLQGIGSDERFRQCLMSDLSHAVQHPVLDEPIAEAVCIIADMDKWTVQVASSQRRVTDNKLGKEVLVSSLVSNLLHSTLQLYKHNLSPNFCVMHLEDRLQELYFKSKMLSEYLRGQMRVHVKELGVVLGIESSDLPLLAAVASTHSPYVAQILL

485 NP_002097.1 Q16540

>NP_002097.1

MAGGKAGKDSGKAKTKAVSRSQRAGLQFPVGRIHRHLKSRTTSHGRVGATAAVYSAAILEYLTAEVLELAGNASKDLKVKRITPRHLQLAIRGDEELDSLIKATIAGGGVIPHIHKSLIGKKGQQKTV

>Q16540

MARNVVYPLYRLGGPQLRVFRTNFFIQLVRPGVAQPEDTVQFRIPMEMTRVDLRNYLEGIYNVPVAAVRTRVQHGSNKRRDHRNVRIKKPDYKVAYVQLAHGQTFTFPDLFPEKDESPEGSAADDLYSMLEEERQQRQSSDPRRGGVPSWFGL

486 NP_004588.1 BAC04299.1

>NP_004588.1

MSLLNKPKSEMTPEELQKREEEEFNTGPLSVLTQSVKNNTQVLINCRNNKKLLGRVKAFDRHCNMVLENVKEMWTEVPKSGKGKKKSKPVNKDRYISKMFLRGDSVIVVLRNPLIAGK

>BAC04299.1

MEVLRPQLIRIDGRNYRKNPVQEQTYQHEEDEEDFYQGSMECADEPCDAYEVEQTPQGFRSTLRAPSLLYNLIHLNTSNDCGFQKITLDCQNIYTWKSRHIVGKRGDTRKKIEMETKTSISIPKPGQDGEIVITGQHRNGVISARTRIDVLLDTFRRKQPFTHFLAFFLNEVEVQEGFLRFQEEVLAKCSMDHGVDSSIFQNPKKLHLTIGMLVLLSEEEIQQTCEMLQQCKEEFINDISGGKPLEVEMAGIEYMNDDPGMVDVLYAKVHMKDGSNRLQELVDRVLERFQASGLIVKEWNSVKLHATVMNTLFRKDPNAEGRYNLYTAEGKYIFKERESFDGRNILKSFALLPRLEYNDAISAHCNLCLPGSSDSPASASQVAGITGVSDAYSQSLPGKS

487 NP_000240.1 NP_620448.1

>NP_000240.1

MSFVAGVIRRLDETVVNRIAAGEVIQRPANAIKEMIENCLDAKSTSIQVIVKEGGLKLIQIQDNGTGIRKEDLDIVCERFTTSKLQSFEDLASISTYGFRGEALASISHVAHVTITTKTADGKCAYRASYSDGKLKAPPKPCAGNQGTQITVEDLFYNIATRRKALKNPSEEYGKILEVVGRYSVHNAGISFSVKKQGETVADVRTLPNASTVDNIRSIFGNAVSRELIEIGCEDKTLAFKMNGYISNANYSVKKCIFLLFINHRLVESTSLRKAIETVYAAYLPKNTHPFLYLSLEISPQNVDVNVHPTKHEVHFLHEESILERVQQHIESKLLGSNSSRMYFTQTLLPGLAGPSGEMVKSTTSLTSSSTSGSSDKVYAHQMVRTDSREQKLDAFLQPLSKPLSSQPQAIVTEDKTDISSGRARQQDEEMLELPAPAEVAAKNQSLEGDTTKGTSEMSEKRGPTSSNPRKRHREDSDVEMVEDDSRKEMTAACTPRRRIINLTSVLSLQEEINEQGHEVLREMLHNHSFVGCVNPQWALAQHQTKLYLLNTTKLSEELFYQILIYDFANFGVLRLSEPAPLFDLAMLALDSPESGWTEEDGPKEGLAEYIVEFLKKKAEMLADYFSLEIDEEGNLIGLPLLIDNYVPPLEGLPIFILRLATEVNWDEEKECFESLSKECAMFYSIRKQYISEESTLSGQQSEVPGSIPNSWKWTVEHIVYKALRSHILPPKHFTEDGNILQLANLPDLYKVFERC

>NP_620448.1

MSLHFLYYCSEPTLDVKIAFCQGFDKQVDVSYIAKHYNMSKSKVDNQFYSVEVGDSTFTVLKRYQNLKPIGSGAQGIVCAAYDAVLDRNVAIKKLSRPFQNQTHAKRAYRELVLMKCVNHKNIISLLNVFTPQKTLEEFQDVYLVMELMDANLCQVIQMELDHERMSYLLYQMLCGIKHLHSAGIIHRDLKPSNIVVKSDCTLKILDFGLARTAGTSFMMTPYVVTRYYRAPEVILGMGYKENVDIWSVGCIMGEMVRHKILFPGRDYIDQWNKVIEQLGTPCPEFMKKLQPTVRNYVENRPKYAGLTFPKLFPDSLFPADSEHNKLKASQARDLLSKMLVIDPAKRISVDDALQHPYINVWYDPAEVEAPPPQIYDKQLDEREHTIEEWKELIYKEVMNSEEKTKNGVVKGQPSPSGAAVNSSESLPPSSSVNDISSMSTDQTLASDTDSSLEASAGPLGCCR

488 NP_003404.1 Q9BZE1

>NP_003404.1

MTMLLDGGPQFPGLGVGSFGAPRHHEMPNREPAGMGLNPFGDSTHAAAAAAAAAAFKLSPAAAHDLSSGQSSAFTPQGSGYANALGHHHHHHHHHHHTSQVPSYGGAASAAFNSTREFLFRQRSSGLSEAASGGGQHGLFAGSASSLHAPAGIPEPPSYLLFPGLHEQGAGHPSPTGHVDNNQVHLGLRGELFGRADPYRPVASPRTDPYAAGAQFPNYSPMNMNMGVNVAAHHGPGAFFRYMRQPIKQELSCKWIDEAQLSRPKKSCDRTFSTMHELVTHVTMEHVGGPEQNNHVCYWEECPREGKSFKAKYKLVNHIRVHTGEKPFPCPFPGCGKIFARSENLKIHKRTHTGEKPFKCEFEGCDRRFANSSDRKKHMHVHTSDKPYICKVCDKSYTHPSSLRKHMKVHESQGSDSSPAASSGYESSTPPAIASANSKDTTKTPSAVQTSTSHNPGLPPNFNEWYV

>Q9BZE1

MALASGPARRALAGSGQLGLGGFGAPRRGAYEWGVRSTRKSEPPPLDRVYEIPGLEPITFAGKMHFVPWLARPIFPPWDRGYKDPRFYRSPPLHEHPLYKDQACYIFHHRCRLLEGVKQALWLTKTKLIEGLPEKVLSLVDDPRNHIENQDECVLNVISHARLWQTTEEIPKRETYCPVIVDNLIQLCKSQILKHPSLARRICVQNSTFSATWNRESLLLQVRGSGGARLSTKDPLPTIASREEIEATKNHVLETFYPISPIIDLHECNIYDVKNDTGFQEGYPYPYPHTLYLLDKANLRPHRLQPDQLRAKMILFAFGSALAQARLLYGNDAKVLEQPVVVQSVGTDGRVFHFLVFQLNTTDLDCNEGVKNLAWVDSDQLLYQHFWCLPVIKKRVVVEPVGPVGFKPETFRKFLALYLHGAA

489 NP_005672.1 NP_004369.1

>NP_005672.1

MSDFSEELKGPVTDDEEVETSVLSGAGMHFPWLQTYVETVAIGGKRRKDFAQTTSACLSFIQEALLKHQWQQAAEYMYSYFQTLEDSDSYKRQAAPEIIWKLGSEILFYHPKSNMESFNTFANRMKNIGVMNYLKISLQHALYLLHHGMLKDAKRNLSEAETWRHGENTSSREILINLIQAYKGLLQYYTWSEKKMELSKLDKDDYAYNAVAQDVFNHSWKTSANISALIKIPGVWDPFVKSYVEMLEFYGDRDGAQEVLTNYAYDEKFPSNPNAHIYLYNFLKRQKAPRSKLISVLKILYQIVPSHKLMLEFHTLLRKSEKEEHRKLGLEVLFGVLDFAGCTKNITAWKYLAKYLKNILMGNHLAWVQEEWNSRKNWWPGFHFSYFWAKSDWKEDTALACEKAFVAGLLLGKGCRYFRYILKQDHQILGKKIKRMKRSVKKYSIVNPRL

>NP_004369.1

MPNFAGTWKMRSSENFDELLKALGVNAMLRKVAVAAASKPHVEIRQDGDQFYIKTSTTVRTTEINFKVGEGFEEETVDGRKCRSLATWENENKIHCTQTLLEGDGPKTYWTRELANDELILTFGADDVVCTRIYVRE

490 NP_003381.1 Q8WXC3

>NP_003381.1

MSFLSRQQPPPPRRAGAACTLRQKLIFSPCSDCEEEEEEEEEEGSGHSTGEDSAFQEPDSPLPPARSPTEPGPERRRSPGPAPGSPGELEEDLLLPGACPGADEAGGGAEGDSWEEEGFGSSSPVKSPAAPYFLGSSFSPVRCGGPGDASPRGCGARRAGEGRRSPRPDHPGTPPHKTFRKLRLFDTPHTPKSLLSKARGIDSSSVKLRGSSLFMDTEKSGKREFDVRQTPQVNINPFTPDSLLLHSSGQCRRRKRTYWNDSCGEDMEASDYELEDETRPAKRITITESNMKSRYTTEFHELEKIGSGEFGSVFKCVKRLDGCIYAIKRSKKPLAGSVDEQNALREVYAHAVLGQHSHVVRYFSAWAEDDHMLIQNEYCNGGSLADAISENYRIMSYFKEAELKDLLLQVGRGLRYIHSMSLVHMDIKPSNIFISRTSIPNAASEEGDEDDWASNKVMFKIGDLGHVTRISSPQVEEGDSRFLANEVLQENYTHLPKADIFALALTVVCAAGAEPLPRNGDQWHEIRQGRLPRIPQVLSQEFTELLKVMIHPDPERRPSAMALVKHSVLLSASRKSAEQLRIELNAEKFKNSLLQKELKKAQMAKAAAEERALFTDRMATRSTTQSNRTSRLIGKKMNRSVSLTIY

>Q8WXC3

MGTKREAILKVLENLTPEELKKFKMKLGTVPLREGFERIPRGALGQLDIVDLTDKLVASYYEDYAAELVVAVLRDMRMLEEAARLQRAA

491 NP_004587.1 NP_055904.1

>NP_004587.1

MAVPETRPNHTIYINNLNEKIKKDELKKSLYAIFSQFGQILDILVSRSLKMRGQAFVIFKEVSSATNALRSMQGFPFYDKPMRIQYAKTDSDIIAKMKGTFVERDRKREKRKPKSQETPATKKAVQGGGATPVVGAVQGPVPGMPPMTQAPRIMHHMPGQPPYMPPPGMIPPPGLAPGQIPPGAMPPQQLMPGQMPPAQPLSENPPNHILFLTNLPEETNELMLSMLFNQFPGFKEVRLVPGRHDIAFVEFDNEVQAGAARDALQGFKITQNNAMKISFAKK

>NP_055904.1

MVGERHAGDLMVPLGPRLQAYPEELIRQRPGHDGHPEYLIRWSVLKCGEVGKVGVEEGKAEHILMWLSAPEVYANCPGLLGERALSKGLQHEPAGVSGSFPRDPGGLDEVAMGEMEADVQALVRRAARQLAESGTPSLTAAVLHTIHVLSAYASIGPLTGVFRETGALDLLMHMLCNPEPQIRRSAGKMLQALAAHDAGSRAHVLLSLSQQDGIEQHMDFDSRYTLLELFAETTSSEEHCMAFEGIHLPQIPGKLLFSLVKRYLCVTSLLDQLNSSPELGAGDQSSPCATREKSRGQRELEFSMAVGNLISELVRSMGWARNLSEQGMSPPRPTRSIFQPYISGPSLLLPTIVTTPRRQGWVFRQRSEFSSRSGYGEYVQQTLQPGMRVRMLDDYEEISAGDEGEFRQSNNGIPPVQVFWQSTGRTYWVHWHMLEILGPEEATEDKASAAVEKGAGATVLGTAFPSWDWNPMDGLYPLPYLQPEPQKNERVGYLTQAEWWELLFFIKKLDLCEQQPIFQNLWKNLDETLGEKALGEISVSVEMAESLLQVLSSRFEGSTLNDLLNSQIYTKYGLLSNEPSSSSTSRNHSCTPDPEEESKSEASFSEEETESLKAKAEAPKTEAEPTKTRTETPMAQSDSQLFNQLLVTEGMTLPTEMKEAASEMARALRGPGPRSSLDQHVAAVVATVQISSLDTNLQLSGLSALSQAVEEVTERDHPLVRPDRSLREKLVKMLVELLTNQVGEKMVVVQALRLLYLLMTKHEWRPLFAREGGIYAVLVCMQEYKTSVLVQQAGLAALKMLAVASSSEIPTFVTGRDSIHSLFDAQMTREIFASIDSATRPGSESLLLTVPAAVILMLNTEGCSSAARNGLLLLNLLLCNHHTLGDQIITQELRDTLFRHSGIAPRTEPMPTTRTILMMLLNRYSEPPGSPERAALETPIIQGQDGSPELLIRSLVGGPSAELLLDLERVLCREGSPGGAVRPLLKRLQQETQPFLLLLRTLDAPGPNKTLLLSVLRVITRLLDFPEAMVLPWHEVLEPCLNCLSGPSSDSEIVQELTCFLHRLASMHKDYAVVLCCLGAKEILSKVLDKHSAQLLLGCELRDLVTECEKYAQLYSNLTSSILAGCIQMVLGQIEDHRRTHQPINIPFFDVFLRHLCQGSSVEVKEDKCWEKVEVSSNPHRASKLTDHNPKTYWESNGSTGSHYITLHMHRGVLVRQLTLLVASEDSSYMPARVVVFGGDSTSCIGTELNTVNVMPSASRVILLENLNRFWPIIQIRIKRCQQGGIDTRVRGVEVLGPKPTFWPLFREQLCRRTCLFYTIRAQAWSRDIAEDHRRLLQLCPRLNRVLRHEQNFADRFLPDDEAAQALGKTCWEALVSPLVQNITSPDAEGVSALGWLLDQYLEQRETSRNPLSRAASFASRVRRLCHLLVHVEPPPGPSPEPSTRPFSKNSKGRDRSPAPSPVLPSSSLRNITQCWLSVVQEQVSRFLAAAWRAPDFVPRYCKLYEHLQRAGSELFGPRAAFMLALRSGFSGALLQQSFLTAAHMSEQFARYIDQQIQGGLIGGAPGVEMLGQLQRHLEPIMVLSGLELATTFEHFYQHYMADRLLSFGSSWLEGAVLEQIGLCFPNRLPQLMLQSLSTSEELQRQFHLFQLQRLDKLFLEQEDEEEKRLEEEEEEEEEEEAEKELFIEDPSPAISILVLSPRCWPVSPLCYLYHPRKCLPTEFCDALDRFSSFYSQSQNHPVLDMGPHRRLQWTWLGRAELQFGKQILHVSTVQMWLLLKFNQTEEVSVETLLKDSDLSPELLLQALVPLTSGNGPLTLHEGQDFPHGGVLRLHEPGPQRSGEALWLIPPQAYLNVEKDEGRTLEQKRNLLSCLLVRILKAHGEKGLHIDQLVCLVLEAWQKGPNPPGTLGHTVAGGVACTSTDVLSCILHLLGQGYVKRRDDRPQILMYAAPEPMGPCRGQADVPFCGSQSETSKPSPEAVATLASLQLPAGRTMSPQEVEGLMKQTVRQVQETLNLEPDVAQHLLAHSHWGAEQLLQSYSEDPEPLLLAAGLCVHQAQAVPVRPDHCPVCVSPLGCDDDLPSLCCMHYCCKSCWNEYLTTRIEQNLVLNCTCPIADCPAQPTGAFIRAIVSSPEVISKYEKALLRGYVESCSNLTWCTNPQGCDRILCRQGLGCGTTCSKCGWASCFNCSFPEAHYPASCGHMSQWVDDGGYYDGMSVEAQSKHLAKLISKRCPSCQAPIEKNEGCLHMTCAKCNHGFCWRCLKSWKPNHKDYYNCSAMVSKAARQEKRFQDYNERCTFHHQAREFAVNLRNRVSAIHEVPPPRSFTFLNDACQGLEQARKVLAYACVYSFYSQDAEYMDVVEQQTENLELHTNALQILLEETLLRCRDLASSLRLLRADCLSTGMELLRRIQERLLAILQHSAQDFRVGLQSPSVEAWEAKGPNMPGSQPQASSGPEAEEEEEDDEDDVPEWQQDEFDEELDNDSFSYDESENLDQETFFFGDEEEDEDEAYD

492 NP_001349.2 NP_002878.2

>NP_001349.2

MSKRHRLDLGEDYPSGKKRAGTDGKDRDRDRDREDRSKDRDRERDRGDREREREKEKEKELRASTNAMLISAGLPPLKASHSAHSTHSAHSTHSTHSAHSTHAGHAGHTSLPQCINPFTNLPHTPRYYDILKKRLQLPVWEYKDRFTDILVRHQSFVLVGETGSGKTTQIPQWCVEYMRSLPGPKRGVACTQPRRVAAMSVAQRVADEMDVMLGQEVGYSIRFEDCSSAKTILKYMTDGMLLREAMNDPLLERYGVIILDEAHERTLATDILMGVLKEVVRQRSDLKVIVMSATLDAGKFQIYFDNCPLLTIPGRTHPVEIFYTPEPERDYLEAAIRTVIQIHMCEEEEGDLLLFLTGQEEIDEACKRIKREVDDLGPEVGDIKIIPLYSTLPPQQQQRIFEPPPPKKQNGAIGRKVVVSTNIAETSLTIDGVVFVIDPGFAKQKVYNPRIRVESLLVTAISKASAQQRAGRAGRTRPGKCFRLYTEKAYKTEMQDNTYPEILRSNLGSVVLQLKKLGIDDLVHFDFMDPPAPETLMRALELLNYLAALNDDGDLTELGSMMAEFPLDPQLAKMVIASCDYNCSNEVLSITAMLSVPQCFVRPTEAKKAADEAKMRFAHIDGDHLTLLNVYHAFKQNHESVQWCYDNFINYRSLMSADNVRQQLSRIMDRFNLPRRSTDFTSRDYYINIRKALVTGYFMQVAHLERTGHYLTVKDNQVVQLHPSTVLDHKPEWVLYNEFVLTTKNYIRTCTDIKPEWLVKIAPQYYDMSNFPQCEAKRQLDRIIAKLQSKEYSQY

>NP_002878.2

MDVLVSECSARLLQQEEEIKSLTAEIDRLKNCGCLGASPNLEQLQEENLKLKYRLNILRKSLQAERNKPTKNMINIISRLQEVFGHAIKAAYPDLENPPLLVTPSQQAKFGDYQCNSAMGISQMLKTKEQKVNPREIAENITKHLPDNECIEKVEIAGPGFINVHLRKDFVSEQLTSLLVNGVQLPALGENKKVIVDFSSPNIAKEMHVGHLRSTIIGESISRLFEFAGYDVLRLNHVGDWGTQFGMLIAHLQDKFPDYLTVSPPIGDLQVFYKESKKRFDTEEEFKKRAYQCVVLLQGKNPDITKAWKLICDVSRQELNKIYDALDVSLIERGESFYQDRMNDIVKEFEDRGFVQVDDGRKIVFVPGCSIPLTIVKSDGGYTYDTSDLAAIKQRLFEEKADMIIYVVDNGQSVHFQTIFAAAQMIGWYDPKVTRVFHAGFGVVLGEDKKKFKTRSGETVRLMDLLGEGLKRSMDKLKEKERDKVLTAEELNAAQTSVAYGCIKYADLSHNRLNDYIFSFDKMLDDRGNTAAYLLYAFTRIRSIARLANIDEEMLQKAARETKILLDHEKEWKLGRCILRFPEILQKILDDLFLHTLCDYIYELATAFTEFYDSCYCVEKDRQTGKILKVNMWRMLLCEAVAAVMAKGFDILGIKPVQRM

493 NP_005089.2 Q96HA7

>NP_005089.2

MSTGSVSDPEEMELRGLQREYPVPASKRPPLRGVERSYASPSDNSSAEEEDPDGEEERCALGTAGSAEGCKRKRPRVAGGGGAGGSAGGGGKKPLPAKGSAAECKQSQRNAANARERARMRVLSKAFSRLKTSLPWVPPDTKLSKLDTLRLASSYIAHLRQLLQEDRYENGYVHPVNLTWPFVVSGRPDSDTKEVSAANRLCGTTA

>Q96HA7

MSLERELRQLSKAKAKAQRAGQRREEAALCHQLGELLAGHGRYAEALEQHWQELQLRERADDPLGCAVAHRKIGERLAEMEDYPAALQHQHQYLELAHSLRNHTELQRAWATIGRTHLDIYDHCQSRDALLQAQAAFEKSLAIVDEELEGTLAQGELNEMRTRLYLNLGLTFESLQQTALCNDYFRKSIFLAEQNHLYEDLFRARYNLGTIHWRAGQHSQAMRCLEGARECAHTMRKRFMESECCVVIAQVLQDLGDFLAAKRALKKAYRLGSQKPVQRAAICQNLQHVLAVVRLQQQLEEAEGRDPQGAMVICEQLGDLFSKAGDFPRAAEAYQKQLRFAELLDRPGAERAIIHVSLATTLGDMKDHHGAVRHYEEELRLRSGNVLEEAKTWLNIALSREEAGDAYELLAPCFQKALSCAQQAQRPQLQRQVLQHLHTVQLRLQPQEAPETETRLRELSVAEDEDEEEEAEEAAATAESEALEAGEVELSEGEDDTDGLTPQLEEDEELQGHLGRRKGSKWNRRNDMGETLLHRACIEGQLRRVQDLVRQGHPLNPRDYCGWTPLHEACNYGHLEIVRFLLDHGAAVDDPGGQGCEGITPLHDALNCGHFEVAELLLERGASVTLRTRKGLSPLETLQQWVKLYRRDLDLETRQKARAMEMLLQAAASGQDPHSSQAFHTPSSLLFDPETSPPLSPCPEPPSNSTRLPEASQAHVRVSPGQAAPAMARPRRSRHGPASSSSSSEGEDSAGPARPSQKRPRCSATAQRVAAWTPGPASNREAATASTSRAAYQAAIRGVGSAQSRLGPGPPRGHSKALAPQAALIPEEECLAGDWLELDMPLTRSRRPRPRGTGDNRRPSSTSGSDSEESRPRARAKQVRLTCMQSCSAPVNAGPSSLASEPPGSPSTPRVSEPSGDSSAAGQPLGPAPPPPIRVRVQVQDHLFLIPVPHSSDTHSVAWLAEQAAQRYYQTCGLLPRLTLRKEGALLAPQDLIPDVLQSNDEVLAEVTSWDLPPLTDRYRRACQSLGQGEHQQVLQAVELQGLGLSFSACSLALDQAQLTPLLRALKLHTALRELRLAGNRLGDKCVAELVAALGTMPSLALLDLSSNHLGPEGLRQLAMGLPGQATLQSLEELDLSMNPLGDGCGQSLASLLHACPLLSTLRLQACGFGPSFFLSHQTALGSAFQDAEHLKTLSLSYNALGAPALARTLQSLPAGTLLHLELSSVAAGKGDSDLMEPVFRYLAKEGCALAHLTLSANHLGDKAVRDLCRCLSLCPSLISLDLSANPEISCASLEELLSTLQKRPQGLSFLGLSGCAVQGPLGLGLWDKIAAQLRELQLCSRRLCAEDRDALRQLQPSRPGPGECTLDHGSKLFFRRL

494 NP_003084.1 Q13287

>NP_003084.1

MPKFYCDYCDTYLTHDSPSVRKTHCSGRKHKENVKDYYQKWMEEQAQSLIDKTTAAFQQGKIPPTPFSAPPPAGAMIPPPPSLPGPPRPGMMPAPHMGGPPMMPMMGPPPPGMMPVGPAPGMRPPMGGHMPMMPGPPMMRPPARPMMVPTRPGMTRPDR

>Q13287

MEADKDDTQQILKEHSPDEFIKDEQNKGLIDEITKKNIQLKKEIQKLETELQEATKEFQIKEDIPETKMKFLSVETPENDSQLSNISCSFQVSSKVPYEIQKGQALITFEKEEVAQNVVSMSKHHVQIKDVNLEVTAKPVPLNSGVRFQVYVEVSKMKINVTEIPDTLREDQMRDKLELSFSKSRNGGGEVDRVDYDRQSGSAVITFVEIGVADKILKKKEYPLYINQTCHRVTVSPYTEIHLKKYQIFSGTSKRTVLLTGMEGIQMDEEIVEDLINIHFQRAKNGGGEVDVVKCSLGQPHIAYFEE

495 P09668 NP_002100.2

>P09668

MWATLPLLCAGAWLLGVPVCGAAELSVNSLEKFHFKSWMSKHRKTYSTEEYHHRLQTFASNWRKINAHNNGNHTFKMALNQFSDMSFAEIKHKYLWSEPQNCSATKSNYLRGTGPYPPSVDWRKKGNFVSPVKNQGACGSCWTFSTTGALESAIAIATGKMLSLAEQQLVDCAQDFNNHGCQGGLPSQAFEYILYNKGIMGEDTYPYQGKDGYCKFQPGKAIGFVKDVANITIYDEEAMVEAVALYNPVSFAFEVTQDFMMYRTGIYSSTSCHKTPDKVNHAVLAVGYGEKNGIPYWIVKNSWGPQWGMNGYFLIERGKNMCGLAACASYPIPLV

>NP_002100.2

MAERAALEELVKLQGERVRGLKQQKASAELIEEEVAKLLKLKAQLGPDESKQKFVLKTPKGTRDYSPRQMAVREKVFDVIIRCFKRHGAEVIDTPVFELKETLMGKYGEDSKLIYDLKDQGGELLSLRYDLTVPFARYLAMNKLTNIKRYHIAKVYRRDNPAMTRGRYREFYQCDFDIAGNFDPMIPDAECLKIMCEILSSLQIGDFLVKVNDRRILDGMFAICGVSDSKFRTICSSVDKLDKVSWEEVKNEMVGEKGLAPEVADRIGDYVQQHGGVSLVEQLLQDPKLSQNKQALEGLGDLKLLFEYLTLFGIDDKISFDLSLARGLDYYTGVIYEAVLLQTPAQAGEEPLGVGSVAAGGRYDGLVGMFDPKGRKVPCVGLSIGVERIFSIVEQRLEALEEKIRTTETQVLVASAQKKLLEERLKLVSELWDAGIKAELLYKKNPKLLNQLQYCEEAGIPLVAIIGEQELKDGVIKLRSVTSREEVDVRREDLVEEIKRRTGQPLCIC

496 NP_057412.1 P78368

>NP_057412.1

MALLGKRCDVPTNGCGPDRWNSAFTRKDEIITSLVSALDSMCSALSKLNAEVACVAVHDESAFVVGTEKGRMFLNARKELQSDFLRFCRGPPWKDPEAEHPKKVQRGEGGGRSLPRSSLEHGSDVYLLRKMVEEVFDVLYSEALGRASVVPLPYERLLREPGLLAVQGLPEGLAFRRPAEYDPKALMAILEHSHRIRFKLKRPLEDGGRDSKALVELNGVSLIPKGSRDCGLHGQAPKVPPQDLPPTATSSSMASFLYSTALPNHAIRELKQEAPSCPLAPSDLGLSRPMPEPKATGAQDFSDCCGQKPTGPGGPLIQNVHASKRILFSIVHDKSEKWDAFIKETEDINTLRECVQILFNSRYAEALGLDHMVPVPYRKIACDPEAVEIVGIPDKIPFKRPCTYGVPKLKRILEERHSIHFIIKRMFDERIFTGNKFTKDTTKLEPASPPEDTSAEVSRATVLDLAGNARSDKGSMSEDCGPGTSGELGGLRPIKIEPEDLDIIQVTVPDPSPTSEEMTDSMPGHLPSEDSGYGMEMLTDKGLSEDARPEERPVEDSHGDVIRPLRKQVELLFNTRYAKAIGISEPVKVPYSKFLMHPEELFVVGLPEGISLRRPNCFGIAKLRKILEASNSIQFVIKRPELLTEGVKEPIMDSQGTASSLGFSPPALPPERDSGDPLVDESLKRQGFQENYDARLSRIDIANTLREQVQDLFNKKYGEALGIKYPVQVPYKRIKSNPGSVIIEGLPPGIPFRKPCTFGSQNLERILAVADKIKFTVTRPFQGLIPKPDEDDANRLGEKVILREQVKELFNEKYGEALGLNRPVLVPYKLIRDSPDAVEVTGLPDDIPFRNPNTYDIHRLEKILKAREHVRMVIINQLQPFAEICNDAKVPAKDSSIPKRKRKRVSEGNSVSSSSSSSSSSSSNPDSVASANQISLVQWPMYMVDYAGLNVQLPGPLNY

>P78368

MDFDKKGGKGETEEGRRMSKAGGGRSSHGIRSSGTSSGVLMVGPNFRVGKKIGCGNFGELRLGKNLYTNEYVAIKLEPIKSRAPQLHLEYRFYKQLSATEGVPQVYYFGPCGKYNAMVLELLGPSLEDLFDLCDRTFTLKTVLMIAIQLITRMEYVHTKSLIYRDVKPENFLVGRPGTKRQHAIHIIDFGLAKEYIDPETKKHIPYREHKSLTGTARYMSINTHLGKEQSRRDDLEALGHMFMYFLRGSLPWQGLKADTLKERYQKIGDTKRATPIEVLCENFPEEMATYLRYVRRLDFFEKPDYDYLRKLFTDLFDRSGFVFDYEYDWAGKPLPTPIGTVHTDLPSQPQLRDKTQPHSKNQALNSTNGELNADDPTAGHSNAPITAPAEVEVADETKCCCFFKRRKRKSLQRHK

497 NP_003185.1 Q9Y2J8

>NP_003185.1

MDQNNSLPPYAQGLASPQGAMTPGIPIFSPMMPYGTGLTPQPIQNTNSLSILEEQQRQQQQQQQQQQQQQQQQQQQQQQQQQQQQQQQQQQQQQQAVAAAAVQQSTSQQATQGTSGQAPQLFHSQTLTTAPLPGTTPLYPSPMTPMTPITPATPASESSGIVPQLQNIVSTVNLGCKLDLKTIALRARNAEYNPKRFAAVIMRIREPRTTALIFSSGKMVCTGAKSEEQSRLAARKYARVVQKLGFPAKFLDFKIQNMVGSCDVKFPIRLEGLVLTHQQFSSYEPELFPGLIYRMIKPRIVLLIFVSGKVVLTGAKVRAEIYEAFENIYPILKGFRKTT

>Q9Y2J8

MLRERTVRLQYGSRVEAVYVLGTYLWTDVYSAAPAGAQTFSLKHSEHVWVEVVRDGEAEEVATNGKQRWLLSPSTTLRVTMSQASTEASSDKVTVNYYDEEGSIPIDQAGLFLTAIEISLDVDADRDGVVEKNNPKKASWTWGPEGQGAILLVNCDRETPWLPKEDCRDEKVYSKEDLKDMSQMILRTKGPDRLPAGYEIVLYISMSDSDKVGVFYVENPFFGQRYIHILGRRKLYHVVKYTGGSAELLFFVEGLCFPDEGFSGLVSIHVSLLEYMAQDIPLTPIFTDTVIFRIAPWIMTPNILPPVSVFVCCMKDNYLFLKEVKNLVEKTNCELKVCFQYLNRGDRWIQDEIEFGYIEAPHKGFPVVLDSPRDGNLKDFPVKELLGPDFGYVTREPLFESVTSLDSFGNLEVSPPVTVNGKTYPLGRILIGSSFPLSGGRRMTKVVRDFLKAQQVQAPVELYSDWLTVGHVDEFMSFVPIPGTKKFLLLMASTSACYKLFREKQKDGHGEAIMFKGLGGMSSKRITINKILSNESLVQENLYFQRCLDWNRDILKKELGLTEQDIIDLPALFKMDEDHRARAFFPNMVNMIVLDKDLGIPKPFGPQVEEECCLEMHVRGLLEPLGLECTFIDDISAYHKFLGEVHCGTNVRRKPFTFKWWHMVP

498 NP_003008.1 Q969Y2

>NP_003008.1

MHRDSCPLDCKVYVGNLGNNGNKTELERAFGYYGPLRSVWVARNPPGFAFVEFEDPRDAADAVRELDGRTLCGCRVRVELSNGEKRSRNRGPPPSWGRRPRDDYRRRSPPPRRRSPRRRSFSRSRSRSLSRDRRRERSLSRERNHKPSRSFSRSRSRSRSNERK

>Q969Y2

MWRGLWTLAAQAARGPRRLCTRRSSGAPAPGSGATIFALSSGQGRCGIAVIRTSGPASGHALRILTAPRDLPLARHASLRLLSDPRSGEPLDRALVLWFPGPQSFTGEDCVEFHVHGGPAVVSGVLQALGSVPGLRPAEAGEFTRRAFANGKLNLTEVEGLADLIHAETEAQRRQALRQLDGELGHLCRGWAETLTKALAHVEAYIDFGEDDNLEEGVLEQADIEVRALQVALGAHLRDARRGQRLRSGAHVVVTGPPNAGKSSLVNLLSRKPVSIVSPEPGTTRDVLETPVDLAGFPVLLSDTAGLREGVGPVEQEGVRRARERLEQADLILAMLDASDLASPSSCNFLATVVASVGAQSPSDSSQRLLLVLNKSDLLSPEGPGPGPDLPPHLLLSCLTGEGLDGLLEALRKELAAVCGDPSTDPPLLTRARHQHHLQGCLDALGHYKQSKDLALAAEALRVARGHLTRLTGGGGTEEILDIIFQDFCVGK

499 NP_006227.1 NP_115865.1

>NP_006227.1

MATAASNPYLPGNSLLAAGSIVHSDAAGAGGGGGGGGGGGGGGAGGGGGGMQPGSAAVTSGAYRGDPSSVKMVQSDFMQGAMAASNGGHMLSHAHQWVTALPHAAAAAAAAAAAAVEASSPWSGSAVGMAGSPQQPPQPPPPPPQGPDVKGGAGRDDLHAGTALHHRGPPHLGPPPPPPHQGHPGGWGAAAAAAAAAAAAAAAAHLPSMAGGQQPPPQSLLYSQPGGFTVNGMLSAPPGPGGGGGGAGGGAQSLVHPGLVRGDTPELAEHHHHHHHHAHPHPPHPHHAQGPPHHGGGGGGAGPGLNSHDPHSDEDTPTSDDLEQFAKQFKQRRIKLGFTQADVGLALGTLYGNVFSQTTICRFEALQLSFKNMCKLKPLLNKWLEEADSSTGSPTSIDKIAAQGRKRKKRTSIEVSVKGALESHFLKCPKPSAQEITNLADSLQLEKEVVRVWFCNRRQKEKRMTPPGIQQQTPDDVYSQVGTVSADTPPPHHGLQTSVQ

>NP_115865.1

MPRYELALILKAMQRPETAATLKRTIEALMDRGAIVRDLENLGERALPYRISAHSQQHNRGGYFLVDFYAPTAAVESMVEHLSRDIDVIRGNIVKHPLTQELKECEGIVPVPLAEKLYSTKKRKK

500 P04554 NP_004324.2

>P04554

MVRYRVRSLSERSHEVYRQQLHGQEQGHHGQEEQGLSPEHVEVYERTHGQSHYRRRHCSRRRLHRIHRRQHRSCRRRKRRSCRHRRRHRRGCRTRKRTCRRH

>NP_004324.2

MAALSGGGGGGAEPGQALFNGDMEPEAGAGAGAAASSAADPAIPEEVWNIKQMIKLTQEHIEALLDKFGGEHNPPSIYLEAYEEYTSKLDALQQREQQLLESLGNGTDFSVSSSASMDTVTSSSSSSLSVLPSSLSVFQNPTDVARSNPKSPQKPIVRVFLPNKQRTVVPARCGVTVRDSLKKALMMRGLIPECCAVYRIQDGEKKPIGWDTDISWLTGEELHVEVLENVPLTTHNFVRKTFFTLAFCDFCRKLLFQGFRCQTCGYKFHQRCSTEVPLMCVNYDQLDLLFVSKFFEHHPIPQEEASLAETALTSGSSPSAPASDSIGPQILTSPSPSKSIPIPQPFRPADEDHRNQFGQRDRSSSAPNVHINTIEPVNIDDLIRDQGFRGDGGSTTGLSATPPASLPGSLTNVKALQKSPGPQRERKSSSSSEDRNRMKTLGRRDSSDDWEIPDGQITVGQRIGSGSFGTVYKGKWHGDVAVKMLNVTAPTPQQLQAFKNEVGVLRKTRHVNILLFMGYSTKPQLAIVTQWCEGSSLYHHLHIIETKFEMIKLIDIARQTAQGMDYLHAKSIIHRDLKSNNIFLHEDLTVKIGDFGLATVKSRWSGSHQFEQLSGSILWMAPEVIRMQDKNPYSFQSDVYAFGIVLYELMTGQLPYSNINNRDQIIFMVGRGYLSPDLSKVRSNCPKAMKRLMAECLKKKRDERPLFPQILASIELLARSLPKIHRSASEPSLNRAGFQTEDFSLYACASPKTPIQAGGYGAFPVH

501 P50219 Q9Y2Q9

>P50219

MEKSKNFRIDALLAVDPPRAASAQSAPLALVTSLAAAASGTGGGGGGGGASGGTSGSCSPASSEPPAAPADRLRAESPSPPRLLAAHCALLPKPGFLGAGGGGGGTGGGHGGPHHHAHPGAAAAAAAAAAAAAAGGLALGLHPGGAQGGAGLPAQAALYGHPVYGYSAAAAAAALAGQHPALSYSYPQVQGAHPAHPADPIKLGAGTFQLDQWLRASTAGMILPKMPDFNSQAQSNLLGKCRRPRTAFTSQQLLELEHQFKLNKYLSRPKRFEVATSLMLTETQVKIWFQNRRMKWKRSKKAKEQAAQEAEKQKGGGGGAGKGGAEEPGAEELLGPPAPGDKGSGRRLRDLRDSDPEEDEDEDDEDHFPYSNGASVHAASSDCSSEDDSPPPRPSHQPAPQ

>Q9Y2Q9

MAALCRTRAVAAESHFLRVFLFFRPFRGVGTESGSESGSSNAKEPKTRAGGFASALERHSELLQKVEPLQKGSPKNVESFASMLRHSPLTQMGPAKDKLVIGRIFHIVENDLYIDFGGKFHCVCRRPEVDGEKYQKGTRVRLRLLDLELTSRFLGATTDTTVLEANAVLLGIQESKDSRSKEEHHEK

502 Q9Y2V3 Q9BW04

>Q9Y2V3

MHLPGCAPAMADGSFSLAGHLLRSPGGSTSRLHSIEAILGFTKDDGILGTFPAERGARGAKERDRRLGARPACPKAPEEGSEPSPPPAPAPAPEYEAPRPYCPKEPWEARPSPGLPVGPATGEAKLSEEEQPKKKHRRNRTTFTTYQLHELERAFEKSHYPDVYSREELAGKVNLPEVRVQVWFQNRRAKWRRQEKLEVSSMKLQDSPLLSFSRSPPSATLSPLGAGPGSGGGPAGGALPLESWLGPPLPGGGATALQSLPGFGPPAQSLPASYTPPPPPPPFLNSPPLGPGLQPLAPPPPSYPCGPGFGDKFPLDEADPRNSSIAALRLKAKEHIQAIGKPWQAL

>Q9BW04

MPERELWPAGTGSEPVTRVGSCDSMMSSTSTRSGSSDSSYDFLSTEEKECLLFLEETIGSLDTEADSGLSTDESEPATTPRGFRALPITQPTPRGGPEETITQQGRTPRTVTESSSSHPPEPQGLGLRSGSYSLPRNIHIARSQNFRKSTTQASSHNPGEPGRLAPEPEKEQVSQSSQPRQAPASPQEAALDLDVVLIPPPEAFRDTQPEQCREASLPEGPGQQGHTPQLHTPSSSQEREQTPSEAMSQKAKETVSTRYTQPQPPPAGLPQNARAEDAPLSSGEDPNSRLAPLTTPKPRKLPPNIVLKSSRSSFHSDPQHWLSRHTEAAPGDSGLISCSLQEQRKARKEALEKLGLPQDQDEPGLHLSKPTSSIRPKETRAQHLSPAPGLAQPAAPAQASAAIPAAGKALAQAPAPAPGPAQGPLPMKSPAPGNVAASKSMPIPIPKAPRANSALTPPKPESGLTLQESNTPGLRQMNFKSNTLERSGVGLSSYLSTEKDASPKTSTSLGKGSFLDKISPSVLRNSRPRPASLGTGKDFAGIQVGKLADLEQEQSSKRLSYQGQSRDKLPRPPCVSVKISPKGVPNEHRREALKKLGLLKE

503 NP_075555.1 P29144

>NP_075555.1

MMASYPEPEDAAGALLAPETGRTVKEPEGPPPSPGKGGGGGGGTAPEKPDPAQKPPYSYVALIAMAIRESAEKRLTLSGIYQYIIAKFPFYEKNKKGWQNSIRHNLSLNECFIKVPREGGGERKGNYWTLDPACEDMFEKGNYRRRRRMKRPFRPPPAHFQPGKGLFGAGGAAGGCGVAGAGADGYGYLAPPKYLQSGFLNNSWPLPQPPSPMPYASCQMAAAAAAAAAAAAAAGPGSPGAAAVVKGLAGPAASYGPYTRVQSMALPPGVVNSYNGLGGPPAAPPPPPHPHPHPHAHHLHAAAAPPPAPPHHGAAAPPPGQLSPASPATAAPPAPAPTSAPGLQFACARQPELAMMHCSYWDHDSKTGALHSRLDL

>P29144

MATAATEEPFPFHGLLPKKETGAASFLCRYPEYDGRGVLIAVLDTGVDPGAPGMQVTTDGKPKIVDIIDTTGSGDVNTATEVEPKDGEIVGLSGRVLKIPASWTNPSGKYHIGIKNGYDFYPKALKERIQKERKEKIWDPVHRVALAEACRKQEEFDVANNGSSQANKLIKEELQSQVELLNSFEKKYSDPGPVYDCLVWHDGEVWRACIDSNEDGDLSKSTVLRNYKEAQEYGSFGTAEMLNYSVNIYDDGNLLSIVTSGGAHGTHVASIAAGHFPEEPERNGVAPGAQILSIKIGDTRLSTMETGTGLIRAMIEVINHKCDLVNYSYGEATHWPNSGRICEVINEAVWKHNIIYVSSAGNNGPCLSTVGCPGGTTSSVIGVGAYVSPDMMVAEYSLREKLPANQYTWSSRGPSADGALGVSISAPGGAIASVPNWTLRGTQLMNGTSMSSPNACGGIALILSGLKANNIDYTVHSVRRALENTAVKADNIEVFAQGHGIIQVDKAYDYLVQNTSFANKLGFTVTVGNNRGIYLRDPVQVAAPSDHGVGIEPVFPENTENSEKISLQLHLALTSNSSWVQCPSHLELMNQCRHINIRVDPRGLREGLHYTEVCGYDIASPNAGPLFRVPITAVIAAKVNESSHYDLAFTDVHFKPGQIRRHFIEVPEGATWAEVTVCSCSSEVSAKFVLHAVQLVKQRAYRSHEFYKFCSLPEKGTLTEAFPVLGGKAIEFCIARWWASLSDVNIDYTISFHGIVCTAPQLNIHASEGINRFDVQSSLKYEDLAPCITLKNWVQTLRPVSAKTKPLGSRDVLPNNRQLYEMVLTYNFHQPKSGEVTPSCPLLCELLYESEFDSQLWIIFDQNKRQMGSGDAYPHQYSLKLEKGDYTIRLQIRHEQISDLERLKDLPFIVSHRLSNTLSLDIHENHSFALLGKKKSSNLTLPPKYNQPFFVTSLPDDKIPKGAGPGCYLAGSLTLSKTELGKKADVIPVHYYLIPPPTKTKNGSKDKEKDSEKEKDLKEEFTEALRDLKIQWMTKLDSSDIYNELKETYPNYLPLYVARLHQLDAEKERMKRLNEIVDAANAVISHIDQTALAVYIAMKTDPRPDAATIKNDMDKQKSTLVDALCRKGCALADHLLHTQAQDGAISTDAEGKEEEGESPLDSLAETFWETTKWTDLFDNKVLTFAYKHALVNKMYGRGLKFATKLVEEKPTKENWKNCIQLMKLLGWTHCASFTENWLPIMYPPDYCVF

504 P35453 P22532

>P35453

MSRAGSWDMDGLRADGGGAGGAPASSSSSSVAAAAASGQCRGFLSAPVFAGTHSGRAAAAAAAAAAAAAAASGFAYPGTSERTGSSSSSSSSAVVAARPEAPPAKECPAPTPAAAAAAPPSAPALGYGYHFGNGYYSCRMSHGVGLQQNALKSSPHASLGGFPVEKYMDVSGLASSSVPANEVPARAKEVSFYQGYTSPYQHVPGYIDMVSTFGSGEPRHEAYISMEGYQSWTLANGWNSQVYCTKDQPQGSHFWKSSFPGDVALNQPDMCVYRRGRKKRVPYTKLQLKELENEYAINKFINKDKRRRISAATNLSERQVTIWFQNRRVKDKKIVSKLKDTVS

>P22532

MSYQQQQCKQPCQPPPVCPTPKCPEPCPPPKCPEPCPSPKCPQPCPPQQCQQKYPPVTPSPPCQPKCPPKSK

505 NP_056943.1 NP_004125.3

>NP_056943.1

MANSANTNTVPKLYRSVIEDVINDVRDIFLDDGVDEQVLMELKTLWENKLMQSRAVDGFHSEEQQLLLQVQQQHQPQQQQHHHHHHHQQAQPQQTVPQQAQTQQVLIPASQQATAPQVIVPDSKLIQHMNASNMSAAATAATLALPAGVTPVQQILTNSGQLLQVVRAANGAQYIFQPQQSVVLQQQVIPQMQPGGVQAPVIQQVLAPLPGGISPQTGVIIQPQQILFTGNKTQVIPTTVAAPTPAQAQITATGQQQPQAQPAQTQAPLVLQVDGTGDTSSEEDEDEEEDYDDDEEEDKEKDGAEDGQVEEEPLNSEDDVSDEEGQELFDTENVVVCQYDKIHRSKNKWKFHLKDGIMNLNGRDYIFSKAIGDAEW

>NP_004125.3

MISASRAAAARLVGAAASRGPTAARHQDSWNGLSHEAFRLVSRRDYASEAIKGAVVGIDLGTTNSCVAVMEGKQAKVLENAEGARTTPSVVAFTADGERLVGMPAKRQAVTNPNNTFYATKRLIGRRYDDPEVQKDIKNVPFKIVRASNGDAWVEAHGKLYSPSQIGAFVLMKMKETAENYLGHTAKNAVITVPAYFNDSQRQATKDAGQISGLNVLRVINEPTAAALAYGLDKSEDKVIAVYDLGGGTFDISILEIQKGVFEVKSTNGDTFLGGEDFDQALLRHIVKEFKRETGVDLTKDNMALQRVREAAEKAKCELSSSVQTDINLPYLTMDSSGPKHLNMKLTRAQFEGIVTDLIRRTIAPCQKAMQDAEVSKSDIGEVILVGGMTRMPKVQQTVQDLFGRAPSKAVNPDEAVAIGAAIQGGVLAGDVTDVLLLDVTPLSLGIETLGGVFTKLINRNTTIPTKKSQVFSTAADGQTQVEIKVCQGEREMAGDNKLLGQFTLIGIPPAPRGVPQIEVTFDIDANGIVHVSAKDKGTGREQQIVIQSSGGLSKDDIENMVKNAEKYAEEDRRKKERVEAVNMAEGIIHDTETKMEEFKDQLPADECNKLKEEISKMRELLARKDSETGENIRQAASSLQQASLKLFEMAYKKMASEREGSGSSGTGEQKEDQKEEKQ

506 P15586 Q16772

>P15586

MRLLPLAPGRLRRGSPRHLPSCSPALLLLVLGGCLGVFGVAAGTRRPNVVLLLTDDQDEVLGGMTPLKKTKALIGEMGMTFSSAYVPSALCCPSRASILTGKYPHNHHVVNNTLEGNCSSKSWQKIQEPNTFPAILRSMCGYQTFFAGKYLNEYGAPDAGGLEHVPLGWSYWYALEKNSKYYNYTLSINGKARKHGENYSVDYLTDVLANVSLDFLDYKSNFEPFFMMIATPAPHSPWTAAPQYQKAFQNVFAPRNKNFNIHGTNKHWLIRQAKTPMTNSSIQFLDNAFRKRWQTLLSVDDLVEKLVKRLEFTGELNNTYIFYTSDNGYHTGQFSLPIDKRQLYEFDIKVPLLVRGPGIKPNQTSKMLVANIDLGPTILDIAGYDLNKTQMDGMSLLPILRGASNLTWRSDVLVEYQGEGRNVTDPTCPSLSPGVSQCFPDCVCEDAYNNTYACVRTMSALWNLQYCEFDDQEVFVEVYNLTADPDQITNIAKTIDPELLGKMNYRLMMLQSCSGPTCRTPGVFDPGYRFDPRLMFSNRGSVRTRRFSKHLL

>Q16772

MAGKPKLHYFNGRGRMEPIRWLLAAAGVEFEEKFIGSAEDLGKLRNDGSLMFQQVPMVEIDGMKLVQTRAILNYIASKYNLYGKDIKERALIDMYTEGMADLNEMILLLPLCRPEEKDAKIALIKEKTKSRYFPAFEKVLQSHGQDYLVGNKLSRADISLVELLYYVEELDSSLISNFPLLKALKTRISNLPTVKKFLQPGSPRKPPADAKALEEARKIFRF

507 NP_115696.2 P16050

>NP_115696.2

MAGRPHPYDGNSSDPENWDRKLHSRPRKLYKHSSTSSRIAKGGVDHTKMSLHGASGGHERSRDRRRSSDRSRDSSHERTESQLTPCIRNVTSPTRQHHVEREKDHSSSRPSSPRPQKASPNGSISSAGNSSRNSSQSSSDGSCKTAGEMVFVYENAKEGARNIRTSERVTLIVDNTRFVVDPSIFTAQPNTMLGRMFGSGREHNFTRPNEKGEYEVAEGIGSTVFRAILDYYKTGIIRCPDGISIPELREACDYLCISFEYSTIKCRDLSALMHELSNDGARRQFEFYLEEMILPLMVASAQSGERECHIVVLTDDDVVDWDEEYPPQMGEEYSQIIYSTKLYRFFKYIENRDVAKSVLKERGLKKIRLGIEGYPTYKEKVKKRPGGRPEVIYNYVQRPFIRMSWEKEEGKSRHVDFQCVKSKSITNLAAAAADIPQDQLVVMHPTPQVDELDILPIHPPSGNSDLDPDAQNPML

>P16050

MGLYRIRVSTGASLYAGSNNQVQLWLVGQHGEAALGKRLWPARGKETELKVEVPEYLGPLLFVKLRKRHLLKDDAWFCNWISVQGPGAGDEVRFPCYRWVEGNGVLSLPEGTGRTVGEDPQGLFQKHREEELEERRKLYRWGNWKDGLILNMAGAKLYDLPVDERFLEDKRVDFEVSLAKGLADLAIKDSLNVLTCWKDLDDFNRIFWCGQSKLAERVRDSWKEDALFGYQFLNGANPVVLRRSAHLPARLVFPPGMEELQAQLEKELEGGTLFEADFSLLDGIKANVILCSQQHLAAPLVMLKLQPDGKLLPMVIQLQLPRTGSPPPPLFLPTDPPMAWLLAKCWVRSSDFQLHELQSHLLRGHLMAEVIVVATMRCLPSIHPIFKLIIPHLRYTLEINVRARTGLVSDMGIFDQIMSTGGGGHVQLLKQAGAFLTYSSFCPPDDLADRGLLGVKSSFYAQDALRLWEIIYRYVEGIVSLHYKTDVAVKDDPELQTWCREITEIGLQGAQDRGFPVSLQARDQVCHFVTMCIFTCTGQHASVHLGQLDWYSWVPNAPCTMRLPPPTTKDATLETVMATLPNFHQASLQMSITWQLGRRQPVMVAVGQHEEEYFSGPEPKAVLKKFREELAALDKEIEIRNAKLDMPYEYLRPSVVENSVAI

508 Q9HCG8 P18054

>Q9HCG8

MKSSVAQIKPSSGHDRRENLNSYQRNSSPEDRYEEQERSPRDRDYFDYSRSDYEHSRRGRSYDSSMESRNRDREKRRERERDTDRKRSRKSPSPGRRNPETSVTQSSSAQDEPATKKKKDELDPLLTRTGGAYIPPAKLRMMQEQITDKNSLAYQRMSWEALKKSINGLINKVNISNISIIIQELLQENIVRGRGLLSRSVLQAQSASPIFTHVYAALVAIINSKFPQIGELILKRLILNFRKGYRRNDKQLCLTASKFVAHLINQNVAHEVLCLEMLTLLLERPTDDSVEVAIGFLKECGLKLTQVSPRGINAIFERLRNILHESEIDKRVQYMIEVMFAVRKDGFKDHPIILEGLDLVEEDDQFTHMLPLEDDYNPEDVLNVFKMDPNFMENEEKYKAIKKEILDEGDTDSNTDQDAGSSEEDEEEEEEEGEEDEEGQKVTIHDKTEINLVSFRRTIYLAIQSSLDFEECAHKLLKMEFPESQTKELCNMILDCCAQQRTYEKFFGLLAGRFCMLKKEYMESFEGIFKEQYDTIHRLETNKLRNVAKMFAHLLYTDSLPWSVLECIKLSEETTTSSSRIFVKIFFQELCEYMGLPKLNARLKDETLQPFFEGLLPRDNPRNTRFAINFFTSIGLGGLTDELREHLKNTPKVIVAQKPDVEQNKSSPSSSSSASSSSESDSSDSDSDSSDSSSESSSEESDSSSISSHSSASANDVRKKGHGKTRSKEVDKLIRNQQTNDRKQKERRQEHGHQETRTERERRSEKHRDQNSRGSNWRDPITKYTSDKDVPSERNNYSRVANDRDQEMHIDLENKHGDPKKKRGERRNSFSENEKHTHRIKDSENFRRKDRSKSKEMNRKHSGSRSDEDRYQNGAERRWEKSSRYSEQSRESKKNQDRRREKSPAKQK

>P18054

MGRYRIRVATGAWLFSGSYNRVQLWLVGTRGEAELELQLRPARGEEEEFDHDVAEDLGLLQFVRLRKHHWLVDDAWFCDRITVQGPGACAEVAFPCYRWVQGEDILSLPEGTARLPGDNALDMFQKHREKELKDRQQIYCWATWKEGLPLTIAADRKDDLPPNMRFHEEKRLDFEWTLKAGALEMALKRVYTLLSSWNCLEDFDQIFWGQKSALAEKVRQCWQDDELFSYQFLNGANPMLLRRSTSLPSRLVLPSGMEELQAQLEKELQNGSLFEADFILLDGIPANVIRGEKQYLAAPLVMLKMEPNGKLQPMVIQIQPPNPSSPTPTLFLPSDPPLAWLLAKSWVRNSDFQLHEIQYHLLNTHLVAEVIAVATMRCLPGLHPIFKFLIPHIRYTMEINTRARTQLISDGGIFDKAVSTGGGGHVQLLRRAAAQLTYCSLCPPDDLADRGLLGLPGALYAHDALRLWEIIARYVEGIVHLFYQRDDIVKGDPELQAWCREITEVGLCQAQDRGFPVSFQSQSQLCHFLTMCVFTCTAQHAAINQGQLDWYAWVPNAPCTMRMPPPTTKEDVTMATVMGSLPDVRQACLQMAISWHLSRRQPDMVPLGHHKEKYFSGPKPKAVLNQFRTDLEKLEKEITARNEQLDWPYEYLKPSCIENSVTI

509 NP_006225.1 NP_056520.2

>NP_006225.1

MNAPPAFESFLLFEGEKKITINKDTKVPNACLFTINKEDHTLGNIIKSQLLKDPQVLFAGYKVPHPLEHKIIIRVQTTPDYSPQEAFTNAITDLISELSLLEERFRVAIKDKQEGIE

>NP_056520.2

MSGSHTPACGPFSALTPSIWPQEILAKYTQKEESAEQPEFYYDEFGFRVYKEEGDEPGSSLLANSPLMEDAPQRLRWQAHLEFTHNHDVGDLTWDKIAVSLPRSEKLRSLVLAGIPHGMRPQLWMRLSGALQKKRNSELSYREIVKNSSNDETIAAKQIEKDLLRTMPSNACFASMGSIGVPRLRRVLRALAWLYPEIGYCQGTGMVAACLLLFLEEEDAFWMMSAIIEDLLPASYFSTTLLGVQTDQRVLRHLIVQYLPRLDKLLQEHDIELSLITLHWFLTAFASVVDIKLLLRIWDLFFYEGSRVLFQLTLGMLHLKEEELIQSENSASIFNTLSDIPSQMEDAELLLGVAMRLAGSLTDVAVETQRRKHLAYLIADQGQLLGAGTLTNLSQVVRRRTQRRKSTITALLFGEDDLEALKAKNIKQTELVADLREAILRVARHFQCTDPKNCSVELTPDYSMESHQRDHENYVACSRSHRRRAKALLDFERHDDDELGFRKNDIITIVSQKDEHCWVGELNGLRGWFPAKFVEVLDERSKEYSIAGDDSVTEGVTDLVRGTLCPALKALFEHGLKKPSLLGGACHPWLFIEEAAGREVERDFASVYSRLVLCKTFRLDEDGKVLTPEELLYRAVQSVNVTHDAVHAQMDVKLRSLICVGLNEQVLHLWLEVLCSSLPTVEKWYQPWSFLRSPGWVQIKCELRVLCCFAFSLSQDWELPAKREAQQPLKEGVRDMLVKHHLFSWDVDG

510 NP_000439.1 NP_001619.1

>NP_000439.1

MAASFPPTLGLSSAPDEIQHPHIKFSEWKFKLFRVRSFEKTPEEAQKEKKDSFEGKPSLEQSPAVLDKADGQKPVPTQPLLKAHPKFSKKFHDNEKARGKAIHQANLRHLCRICGNSFRADEHNRRYPVHGPVDGKTLGLLRKKEKRATSWPDLIAKVFRIDVKADVDSIHPTEFCHNCWSIMHRKFSSAPCEVYFPRNVTMEWHPHTPSCDICNTARRGLKRKSLQPNLQLSKKLKTVLDQARQARQRKRRAQARISSKDVMKKIANCSKIHLSTKLLAVDFPEHFVKSISCQICEHILADPVETNCKHVFCRVCILRCLKVMGSYCPSCRYPCFPTDLESPVKSFLSVLNSLMVKCPAKECNEEVSLEKYNHHISSHKESKEIFVHINKGGRPRQHLLSLTRRAQKHRLRELKLQVKAFADKEEGGDVKSVCMTLFLLALRARNEHRQADELEAIMQGKGSGLQPAVCLAIRVNTFLSCSQYHKMYRTVKAITGRQIFQPLHALRNAEKVLLPGYHHFEWQPPLKNVSSSTDVGIIDGLSGLSSSVDDYPVDTIAKRFRYDSALVSALMDMEEDILEGMRSQDLDDYLNGPFTVVVKESCDGMGDVSEKHGSGPVVPEKAVRFSFTIMKITIAHSSQNVKVFEEAKPNSELCCKPLCLMLADESDHETLTAILSPLIAEREAMKSSELMLELGGILRTFKFIFRGTGYDEKLVREVEGLEASGSVYICTLCDATRLEASQNLVFHSITRSHAENLERYEVWRSNPYHESVEELRDRVKGVSAKPFIETVPSIDALHCDIGNAAEFYKIFQLEIGEVYKNPNASKEERKRWQATLDKHLRKKMNLKPIMRMNGNFARKLMTKETVDAVCELIPSEERHEALRELMDLYLKMKPVWRSSCPAKECPESLCQYSFNSQRFAELLSTKFKYRYEGKITNYFHKTLAHVPEIIERDGSIGAWASEGNESGNKLFRRFRKMNARQSKCYEMEDVLKHHWLYTSKYLQKFMNAHNALKTSGFTMNPQASLGDPLGIEDSLESQDSMEF

>NP_001619.1

MASRLLLNNGAKMPILGLGTWKSPPGQVTEAVKVAIDVGYRHIDCAHVYQNENEVGVAIQEKLREQVVKREELFIVSKLWCTYHEKGLVKGACQKTLSDLKLDYLDLYLIHWPTGFKPGKEFFPLDESGNVVPSDTNILDTWAAMEELVDEGLVKAIGISNFNHLQVEMILNKPGLKYKPAVNQIECHPYLTQEKLIQYCQSKGIVVTAYSPLGSPDRPWAKPEDPSLLEDPRIKAIAAKHNKTTAQVLIRFPMQRNLVVIPKSVTPERIAENFKVFDFELSSQDMTTLLSYNRNWRVCALLSCTSHKDYPFHEEF

511 NP_877433.2 P25325

>NP_877433.2

MRHNQMCCETPPTVTVYVKSGSNRSHQPKKPITLKRPICKDNWQAFEKNTHNNNKSKRPKGPCLVIQRQDMTAFFKLFDDDLIQDFLWMDCCCKIADKYLLAMTFVYFKRAKFTISEHTRINFFIALYLANTVEEDEEETKYEIFPWALGKNWRKLFPNFLKLRDQLWDRIDYRAIVSRRCCEEVMAIAPTHYIWQRERSVHHSGAVRNYNRDEVQLPRGPSATPVDCSLCGKKRRYVRLGLSSSSSLSSHTAGVTEKHSQDSYNSLSMDIIGDPSQAYTGSEVVNDHQSNKGKKTNFLKKDKSMEWFTGSEE

>P25325

MASPQLCRALVSAQWVAEALRAPRAGQPLQLLDASWYLPKLGRDARREFEERHIPGAAFFDIDQCSDRTSPYDHMLPGAEHFAEYAGRLGVGAATHVVIYDASDQGLYSAPRVWWMFRAFGHHAVSLLDGGLRHWLRQNLPLSSGKSQPAPAEFRAQLDPAFIKTYEDIKENLESRRFQVVDSRATGRFRGTEPEPRDGIEPGHIPGTVNIPFTDFLSQEGLEKSPEEIRHLFQEKKVDLSKPLVATCGSGVTACHVALGAYLCGKPDVPIYDGSWVEWYMRARPEDVISEGRGKTH

512 Q9UHV7 NP_004291.1

>Q9UHV7

MSASFVPNGASLEDCHCNLFCLADLTGIKWKKYVWQGPTSAPILFPVTEEDPILSSFSRCLKADVLGVWRRDQRPGRRELWIFWWGEDPVLLTLFTMTYQKKKMECGRMDFPMNAVLCFSKAVHNLLERCLMNRNFVRIGKWFVKPYEKDEKPINKSEHLSCSFTFFLHGDSNVCTSVEINQHQPVYLLSEEHITLAQQSNSPFQVILCPFGLNGTLTGQAFKMSDSATKKLIGEWKQFYPISCCLKEMSEEKQEDMDWEDDSLAAVEVLVAGVRMIYPACFVLVPQSDIPTPSPVGSTHCSSSCLGVHQVPASTRDPAMSSVTLTPPTSPEEVQTVDPQSVQKWVKFSSVSDGFNSDSTSHHGGKIPRKLANHVVDRVWQECNMNRAQNKRKYSASSGGLCEEATAAKVASWDFVEATQRTNCSCLRHKNLKSRNAGQQGQAPSLGQQQQILPKHKTNEKQEKSEKPQKRPLTPFHHRVSVSDDVGMDADSASQRLVISAPDSQVRFSNIRTNDVAKTPQMHGTEMANSPQPPPLSPHPCDVVDEGVTKTPSTPQSQHFYQMPTPDPLVPSKPMEDRIDSLSQSFPPQYQEAVEPTVYVGTAVNLEEDEANIAWKYYKFPKKKDVEFLPPQLPSDKFKDDPVGPFGQESVTSVTELMVQCKKPLKVSDELVQQYQIKNQCLSAIASDAEQEPKIDPYAFVEGDEEFLFPDKKDRQNSEREAGKKHKVEDGTSSVTVLSHEEDAMSLFSPSIKQDAPRPTSHARPPSTSLIYDSDLAVSYTDLDNLFNSDEDELTPGSKKSANGSDDKASCKESKTGNLDPLSCISTADLHKMYPTPPSLEQHIMGFSPMNMNNKEYGSMDTTPGGTVLEGNSSSIGAQFKIEVDEGFCSPKPSEIKDFSYVYKPENCQILVGCSMFAPLKTLPSQYLPPIKLPEECIYRQSWTVGKLELLSSGPSMPFIKEGDGSNMDQEYGTAYTPQTHTSFGMPPSSAPPSNSGAGILPSPSTPRFPTPRTPRTPRTPRGAGGPASAQGSVKYENSDLYSPASTPSTCRPLNSVEPATVPSIPEAHSLYVNLILSESVMNLFKDCNFDSCCICVCNMNIKGADVGVYIPDPTQEAQYRCTCGFSAVMNRKFGNNSGLFLEDELDIIGRNTDCGKEAEKRFEALRATSAEHVNGGLKESEKLSDDLILLLQDQCTNLFSPFGAADQDPFPKSGVISNWVRVEERDCCNDCYLALEHGRQFMDNMSGGKVDEALVKSSCLHPWSKRNDVSMQCSQDILRMLLSLQPVLQDAIQKKRTVRPWGVQGPLTWQQFHKMAGRGSYGTDESPEPLPIPTFLLGYDYDYLVLSPFALPYWERLMLEPYGSQRDIAYVVLCPENEALLNGAKSFFRDLTAIYESCRLGQHRPVSRLLTDGIMRVGSTASKKLSEKLVAEWFSQAADGNNEAFSKLKLYAQVCRYDLGPYLASLPLDSSLLSQPNLVAPTSQSLITPPQMTNTGNANTPSATLASAASSTMTVTSGVAISTSVATANSTLTTASTSSSSSSNLNSGVSSNKLPSFPPFGSMNSNAAGSMSTQANTVQSGQLGGQQTSALQTAGISGESSSLPTQPHPDVSESTMDRDKVGIPTDGDSHAVTYPPAIVVYIIDPFTYENTDESTNSSSVWTLGLLRCFLEMVQTLPPHIKSTVSVQIIPCQYLLQPVKHEDREIYPQHLKSLAFSAFTQCRRPLPTSTNVKTLTGFGPGLAMETALRSPDRPECIRLYAPPFILAPVKDKQTELGETFGEAGQKYNVLFVGYCLSHDQRWILASCTDLYGELLETCIINIDVPNRARRKKSSARKFGLQKLWEWCLGLVQMSSLPWRVVIGRLGRIGHGELKDWSCLLSRRNLQSLSKRLKDMCRMCGISAADSPSILSACLVAMEPQGSFVIMPDSVSTGSVFGRSTTLNMQTSQLNTPQDTSCTHILVFPTSASVQVASATYTTENLDLAFNPNNDGADGMGIFDLLDTGDDLDPDIINILPASPTGSPVHSPGSHYPHGGDAGKGQSTDRLLSTEPHEEVPNILQQPLALGYFVSTAKAGPLPDWFWSACPQAQYQCPLFLKASLHLHVPSVQSDELLHSKHSHPLDSNQTSDVLRFVLEQYNALSWLTCDPATQDRRSCLPIHFVVLNQLYNFIMNML

>NP_004291.1

MAEQATKSVLFVCLGNICRSPIAEAVFRKLVTDQNISENWRVDSAATSGYEIGNPPDYRGQSCMKRHGIPMSHVARQITKEDFATFDYILCMDESNLRDLNRKSNQVKTCKAKIELLGSYDPQKQLIIEDPYYGNDSDFETVYQQCVRCCRAFLEKAH

513 NP_055586.1 NP_000476.1

>NP_055586.1

MSGPGNKRAAGDGGSGPPEKKLSREEKTTTTLIEPIRLGGISSTEEMDLKVLQFKNKKLAERLEQRQACEDELRERIEKLEKRQATDDATLLIVNRYWAQLDETVEALLRCHESQGELSSAPEAPGTQEGPTCDGTPLPEPGTSELRDPLLMQLRPPLSEPALAFVVALGASSSEEVELELQGRMEFSKAAVSRVVEASDRLQRRVEELCQRVYSRGDSEPLSEAAQAHTRELGRENRRLQDLATQLQEKHHRISLEYSELQDKVTSAETKVLEMETTVEDLQWDIEKLRKREQKLNKHLAEALEQLNSGYYVSGSSSGFQGGQITLSMQKFEMLNAELEENQELANSRMAELEKLQAELQGAVRTNERLKVALRSLPEEVVRETGEYRMLQAQFSLLYNESLQVKTQLDEARGLLLATKNSHLRHIEHMESDELGLQKKLRTEVIQLEDTLAQVRKEYEMLRIEFEQNLAANEQAGPINREMRHLISSLQNHNHQLKGDAQRYKRKLREVQAEIGKLRAQASGSAHSTPNLGHPEDSGVSAPAPGKEEGGPGPVSTPDNRKEMAPVPGTTTTTTSVKKEELVPSEEDFQGITPGAQGPSSRGREPEARPKRELREREGPSLGPPPVASALSRADREKAKVEETKRKESELLKGLRAELKKAQESQKEMKLLLDMYKSAPKEQRDKVQLMAAERKAKAEVDELRSRIRELEERDRRESKKIADEDALRRIRQAEEQIEHLQRKLGATKQEEEALLSEMDVTGQAFEDMQEQNGRLLQQLREKDDANFKLMSERIKANQIHKLLREEKDELGEQVLGLKSQVDAQLLTVQKLEEKERALQGSLGGVEKELTLRSQALELNKRKAVEAAQLAEDLKVQLEHVQTRLREIQPCLAESRAAREKESFNLKRAQEDISRLRRKLEKQRKVEVYADADEILQEEIKEYKARLTCPCCNTRKKDAVLTKCFHVFCFECVRGRYEARQRKCPKCNAAFGAHDFHRIYIS

>NP_000476.1

MADSELQLVEQRIRSFPDFPTPGVVFRDISPVLKDPASFRAAIGLLARHLKATHGGRIDYIAGLDSRGFLFGPSLAQELGLGCVLIRKRGKLPGPTLWASYSLEYGKAELEIQKDALEPGQRVVVVDDLLATGGTMNAACELLGRLQAEVLECVSLVELTSLKGREKLAPVPFFSLLQYE

514 O60341 NP_036205.1

>O60341

MLSGKKAAAAAAAAAAAATGTEAGPGTAGGSENGSEVAAQPAGLSGPAEVGPGAVGERTPRKKEPPRASPPGGLAEPPGSAGPQAGPTVVPGSATPMETGIAETPEGRRTSRRKRAKVEYREMDESLANLSEDEYYSEEERNAKAEKEKKLPPPPPQAPPEEENESEPEEPSGVEGAAFQSRLPHDRMTSQEAACFPDIISGPQQTQKVFLFIRNRTLQLWLDNPKIQLTFEATLQQLEAPYNSDTVLVHRVHSYLERHGLINFGIYKRIKPLPTKKTGKVIIIGSGVSGLAAARQLQSFGMDVTLLEARDRVGGRVATFRKGNYVADLGAMVVTGLGGNPMAVVSKQVNMELAKIKQKCPLYEANGQAVPKEKDEMVEQEFNRLLEATSYLSHQLDFNVLNNKPVSLGQALEVVIQLQEKHVKDEQIEHWKKIVKTQEELKELLNKMVNLKEKIKELHQQYKEASEVKPPRDITAEFLVKSKHRDLTALCKEYDELAETQGKLEEKLQELEANPPSDVYLSSRDRQILDWHFANLEFANATPLSTLSLKHWDQDDDFEFTGSHLTVRNGYSCVPVALAEGLDIKLNTAVRQVRYTASGCEVIAVNTRSTSQTFIYKCDAVLCTLPLGVLKQQPPAVQFVPPLPEWKTSAVQRMGFGNLNKVVLCFDRVFWDPSVNLFGHVGSTTASRGELFLFWNLYKAPILLALVAGEAAGIMENISDDVIVGRCLAILKGIFGSSAVPQPKETVVSRWRADPWARGSYSYVAAGSSGNDYDLMAQPITPGPSIPGAPQPIPRLFFAGEHTIRNYPATVHGALLSGLREAGRIADQFLGAMYTLPRQATPGVPAQQSPSM

>NP_036205.1

MASMGTLAFDEYGRPFLIIKDQDRKSRLMGLEALKSHIMAAKAVANTMRTSLGPNGLDKMMVDKDGDVTVTNDGATILSMMDVDHQIAKLMVELSKSQDDEIGDGTTGVVVLAGALLEEAEQLLDRGIHPIRIADGYEQAARVAIEHLDKISDSVLVDIKDTEPLIQTAKTTLGSKVVNSCHRQMAEIAVNAVLTVADMERRDVDFELIKVEGKVGGRLEDTKLIKGVIVDKDFSHPQMPKKVEDAKIAILTCPFEPPKPKTKHKLDVTSVEDYKALQKYEKEKFEEMIQQIKETGANLAICQWGFDDEANHLLLQNNLPAVRWVGGPEIELIAIATGGRIVPRFSELTAEKLGFAGLVQEISFGTTKDKMLVIEQCKNSRAVTIFIRGGNKMIIEEAKRSLHDALCVIRNLIRDNRVVYGGGAAEISCALAVSQEADKCPTLEQYAMRAFADALEVIPMALSENSGMNPIQTMTEVRARQVKEMNPALGIDCLHKGTNDMKQQHVIETLIGKKQQISLATQMVRMILKIDDIRKPGESEE

515 Q8WXA9 Q58A44

>Q8WXA9

MTSLMPGAGLLPIPTPNPLTTLGVSLSSLGAIPAAALDPNIATLGEIPQPPLMGNVDPSKIDEIRRTVYVGNLNSQTTTADQLLEFFKQVGEVKFVRMAGDETQPTRFAFVEFADQNSVPRALAFNGVMFGDRPLKINHSNNAIVKPPEMTPQAAAKELEEVMKRVREAQSFISAAIEPESGKSNERKGGRSRSHTRSKSRSSSKSHSRRKRSQSKHRSRSHNRSRSRQKDRRRSKSPHKKRSKSRERRKSRSRSHSRDKRKDTREKIKEKERVKEKDREKEREREKEREKEKERGKNKDRDKEREKDREKDKEKDREREREKEHEKDRDKEKEKEQDKEKEREKDRSKEIDEKRKKDKKSRTPPRSYNASRRSRSSSRERRRRRSRSSSRSPRTSKTIKRKSSRSPSPRSRNKKDKKREKERDHISERRERERSTSMRKSSNDRDGKEKLEKNSTSLKEKEHNKEPDSSVSKEVDDKDAPRTEENKIQHNGNCQLNEENLSTKTEAV

>Q58A44

MWILSNLMGTSEEGNLLSTVSPTVKALFGKTRVSPIFPFSPRSPFQPLIPRTPGSPWGPVGPASPLGPGFPIGPMGPGKPVGPKGPMLPLGPSGPVGPTSPLFPFCP

516 NP_776248.1 NP_004823.1

>NP_776248.1

MPASMFSIDNILAARPRCKDSVLPVAHSAAAPVVFPALHGDSLYGASGGASSDYGAFYPRPVAPGGAGLPAAVSGSRLGYNNYFYGQLHVQAAPVGPACCGAVPPLGAQQCSCVPTPPGYEGPGSVLVSPVPHQMLPYMNVGTLSRTELQLLNQLHCRRKRRHRTIFTDEQLEALENLFQETKYPDVGTREQLARKVHLREEKVEVWFKNRRAKWRRQKRSSSEESENAEKWNKTSSSKASPEKREEEGKSDLDSDS

>NP_004823.1

MSGESARSLGKGSAPPGPVPEGSIRIYSMRFCPFAERTRLVLKAKGIRHEVININLKNKPEWFFKKNPFGLVPVLENSQGQLIYESAITCEYLDEAYPGKKLLPDDPYEKACQKMILELFSKVPSLVGSFIRSQNKEDYAGLKEEFRKEFTKLEEVLTNKKTTFFGGNSISMIDYLIWPWFERLEAMKLNECVDHTPKLKLWMAAMKEDPTVSALLTSEKDWQGFLELYLQNSPEACDYGL

517 NP_005180.1 NP_003320.2

>NP_005180.1

MEELSSVGEQVFAAECILSKRLRKGKLEYLVKWRGWSSKHNSWEPEENILDPRLLLAFQKKEHEKEVQNRKRGKRPRGRPRKLTAMSSCSRRSKLKEPDAPSKSKSSSSSSSSTSSSSSSDEEDDSDLDAKRGPRGRETHPVPQKKAQILVAKPELKDPIRKKRGRKPLPPEQKATRRPVSLAKVLKTARKDLGAPASKLPPPLSAPVAGLAALKAHAKEACGGPSAMATPENLASLMKGMASSPGRGGISWQSSIVHYMNRMTQSQAQAASRLALKAQATNKCGLGLDLKVRTQKGELGMSPPGSKIPKAPSGGAVEQKVGNTGGPPHTHGASRVPAGCPGPQPAPTQELSLQVLDLQSVKNGMPGVGLLARHATATKGVPATNPAPGKGTGSGLIGASGATMPTDTSKSEKLASRAVAPPTPASKRDCVKGSATPSGQESRTAPGEARKAATLPEMSAGEESSSSDSDPDSASPPSTGQNPSVSVQTSQDWKPTRSLIEHVFVTDVTANLITVTVKESPTSVGFFNLRHY

>NP_003320.2

MVKQIESKTAFQEALDAAGDKLVVVDFSATWCGPCKMIKPFFHSLSEKYSNVIFLEVDVDDCQDVASECEVKCMPTFQFFKKGQKVGEFSGANKEKLEATINELV

518 NP_066390.1 Q9NVV4

>NP_066390.1

MSGRGKQGGKARAKAKTRSSRAGLQFPVGRVHRLLRKGNYSERVGAGAPVYLAAVLEYLTAEILELAGNAARDNKKTRIIPRHLQLAIRNDEELNKLLGRVTIAQGGVLPNIQAVLLPKKTESHHKAKGK

>Q9NVV4

MAVPGVGLLTRLNLCARRRTRVQRPIVRLLSCPGTVAKDLRRDEQPSGSVETGFEDKIPKRRFSEMQNERREQAQRTVLIHCPEKISENKFLKYLSQFGPINNHFFYESFGLYAVVEFCQKESIGSLQNGTHTPSTAMETAIPFRSRFFNLKLKNQTSERSRVRSSNQLPRSNKQLFELLCYAESIDDQLNTLLKEFQLTEENTKLRYLTCSLIEDMAAAYFPDCIVRPFGSSVNTFGKLGCDLDMFLDLDETRNLSAHKISGNFLMEFQVKNVPSERIATQKILSVLGECLDHFGPGCVGVQKILNARCPLVRFSHQASGFQCDLTTNNRIALTSSELLYIYGALDSRVRALVFSVRCWARAHSLTSSIPGAWITNFSLTMMVIFFLQRRSPPILPTLDSLKTLADAEDKCVIEGNNCTFVRDLSRIKPSQNTETLELLLKEFFEYFGNFAFDKNSINIRQGREQNKPDSSPLYIQNPFETSLNISKNVSQSQLQKFVDLARESAWILQQEDTDRPSISSNRPWGLVSLLLPSAPNRKSFTKKKSNKFAIETVKNLLESLKGNRTENFTKTSGKRTISTQT

519 P55157 NP_064586.1

>P55157

MILLAVLFLCFISSYSASVKGHTTGLSLNNDRLYKLTYSTEVLLDRGKGKLQDSVGYRISSNVDVALLWRNPDGDDDQLIQITMKDVNVENVNQQRGEKSIFKGKSPSKIMGKENLEALQRPTLLHLIHGKVKEFYSYQNEAVAIENIKRGLASLFQTQLSSGTTNEVDISGNCKVTYQAHQDKVIKIKALDSCKIARSGFTTPNQVLGVSSKATSVTTYKIEDSFVIAVLAEETHNFGLNFLQTIKGKIVSKQKLELKTTEAGPRLMSGKQAAAIIKAVDSKYTAIPIVGQVFQSHCKGCPSLSELWRSTRKYLQPDNLSKAEAVRNFLAFIQHLRTAKKEEILQILKMENKEVLPQLVDAVTSAQTSDSLEAILDFLDFKSDSSIILQERFLYACGFASHPNEELLRALISKFKGSIGSSDIRETVMIITGTLVRKLCQNEGCKLKAVVEAKKLILGGLEKAEKKEDTRMYLLALKNALLPEGIPSLLKYAEAGEGPISHLATTALQRYDLPFITDEVKKTLNRIYHQNRKVHEKTVRTAAAAIILNNNPSYMDVKNILLSIGELPQEMNKYMLAIVQDILRFEMPASKIVRRVLKEMVAHNYDRFSRSGSSSAYTGYIERSPRSASTYSLDILYSGSGILRRSNLNIFQYIGKAGLHGSQVVIEAQGLEALIAATPDEGEENLDSYAGMSAILFDVQLRPVTFFNGYSDLMSKMLSASGDPISVVKGLILLIDHSQELQLQSGLKANIEVQGGLAIDISGAMEFSLWYRESKTRVKNRVTVVITTDITVDSSFVKAGLETSTETEAGLEFISTVQFSQYPFLVCMQMDKDEAPFRQFEKKYERLSTGRGYVSQKRKESVLAGCEFPLHQENSEMCKVVFAPQPDSTSSGWF

>NP_064586.1

MIRLGGWCARRLCSAAVPAGRRGAAGGLGLAGGRALRVLVDMDGVLADFEGGFLRKFRARFPDQPFIALEDRRGFWVSEQYGRLRPGLSEKAISIWESKNFFFELEPLPGAVEAVKEMASLQNTDVFICTSPIKMFKYCPYEKYAWVEKYFGPDFLEQIVLTRDKTVVSADLLIDDRPDITGAEPTPSWEHVLFTACHNQHLQLQPPRRRLHSWADDWKAILDSKRPC

520 NP_073568.2 NP_056273.2

>NP_073568.2

MSRPVRNRKVVDYSQFQESDDADEDYGRDSGPPTKKIRSSPREAKNKRRSGKNSQEDSEDSEDKDVKTKKDDSHSAEDSEDEKEDHKNVRQQRQAASKAASKQREMLMEDVGSEEEQEEEDEAPFQEKDSGSDEDFLMEDDDDSDYGSSKKKNKKMVKKSKPERKEKKMPKPRLKATVTPSPVKGKGKVGRPTASKASKEKTPSPKEEDEEPESPPEKKTSTSPPPEKSGDEGSEDEAPSGED

>NP_056273.2

MEFAELIKTPRVDNVVLHRPFYPAVEGTLCLTGHHLILSSRQDNTEELWLLHSNIDAIDKRFVGSLGTIIIKCKDFRIIQLDIPGMEECLNIASSIEALSTLDSITLMYPFFYRPMFEVIEDGWHSFLPEQEFELYSSATSEWRLSYVNKEFAVCPSYPPIVTVPKSIDDEALRKVATFRHGGRFPVLSYYHKKNGMVIMRSGQPLTGTNGRRCKEDEKLINATLRAGKRGYIIDTRSLNVAQQTRAKGGGFEQEAHYPQWRRIHKSIERYHILQESLIKLVEACNDQTHNMDRWLSKLEASNWLTHIKEILTTACLAAQCIDREGASILIHGTEGTDSTLQVTSLAQIILEPRSRTIRGFEALIEREWLQAGHPFQQRCAQSAYCNTKQKWEAPVFLLFLDCVWQILRQFPCSFEFNENFLIMLFEHAYASQFGTFLGNNESERCKLKLQQKTMSLWSWVNQPSELSKFTNPLFEANNLVIWPSVAPQSLPLWEGIFLRWNRSSKYLDEAYEEMVNIIEYNKELQAKVNILRRQLAELETEDGMQESP

521 Q86XK2 P40394

>Q86XK2

MNSVRAANRRPRRVSRPRPVQQQQQQPPQQPPPQPPQQQPPQQQPPPPPQQQQQQQPPPPPPPPPPLPQERNNVGERDDDVPADMVAEESGPGAQNSPYQLRRKTLLPKRTACPTKNSMEGASTSTTENFGHRAKRARVSGKSQDLSAAPAEQYLQEKLPDEVVLKIFSYLLEQDLCRAACVCKRFSELANDPILWKRLYMEVFEYTRPMMHPEPGKFYQINPEEYEHPNPWKESFQQLYKGAHVKPGFAEHFYSNPARYKGRENMLYYDTIEDALGGVQEAHFDGLIFVHSGIYTDEWIYIESPITMIGAAPGKVADKVIIENTRDSTFVFMEGSEDAYVGYMTIRFNPDDKSAQHHNAHHCLEITVNCSPIIDHCIIRSTCTVGSAVCVSGQGACPTIKHCNISDCENVGLYITDHAQGIYEDNEISNNALAGIWVKNHGNPIIRRNHIHHGRDVGVFTFDHGMGYFESCNIHRNRIAGFEVKAYANPTVVRCEIHHGQTGGIYVHEKGRGQFIENKIYANNFAGVWITSNSDPTIRGNSIFNGNQGGVYIFGDGRGLIEGNDIYGNALAGIQIRTNSCPIVRHNKIHDGQHGGIYVHEKGQGVIEENEVYSNTLAGVWVTTGSTPVLRRNRIHSGKQVGVYFYDNGHGVLEDNDIYNHMYSGVQIRTGSNPKIRRNKIWGGQNGGILVYNSGLGCIEDNEIFDNAMAGVWIKTDSNPTLRRNKIHDGRDGGICIFNGGRGLLEENDIFRNAQAGVLISTNSHPILRKNRIFDGFAAGIEITNHATATLEGNQIFNNRFGGLFLASGVNVTMKDNKIMNNQDAIEKAVSRGQCLYKISSYTSYPMHDFYRCHTCNTTDRNAICVNCIKKCHQGHDVEFIRHDRFFCDCGAGTLSNPCTLAGEPTHDTDTLYDSAPPIESNTLQHN

>P40394

MGTAGKVIKCKAAVLWEQKQPFSIEEIEVAPPKTKEVRIKILATGICRTDDHVIKGTMVSKFPVIVGHEATGIVESIGEGVTTVKPGDKVIPLFLPQCRECNACRNPDGNLCIRSDITGRGVLADGTTRFTCKGKPVHHFMNTSTFTEYTVVDESSVAKIDDAAPPEKVCLIGCGFSTGYGAAVKTGKVKPGSTCVVFGLGGVGLSVIMGCKSAGASRIIGIDLNKDKFEKAMAVGATECISPKDSTKPISEVLSEMTGNNVGYTFEVIGHLETMIDALASCHMNYGTSVVVGVPPSAKMLTYDPMLLFTGRTWKGCVFGGLKSRDDVPKLVTEFLAKKFDLDQLITHVLPFKKISEGFELLNSGQSIRTVLTF

522 NP_003205.2 NP_002291.1

>NP_003205.2

MASNSWNASSSPGEAREDGPEGLDKGLDNDAEGVWSPDIEQSFQEALAIYPPCGRRKIILSDEGKMYGRNELIARYIKLRTGKTRTRKQVSSHIQVLARKKVREYQVGIKAMNLDQVSKDKALQSMASMSSAQIVSASVLQNKFSPPSPLPQAVFSTSSRFWSSPPLLGQQPGPSQDIKPFAQPAYPIQPPLPPTLSSYEPLAPLPSAAASVPVWQDRTIASSRLRLLEYSAFMEVQRDPDTYSKHLFVHIGQTNPAFSDPPLEAVDVRQIYDKFPEKKGGLKELYEKGPPNAFFLVKFWADLNSTIQEGPGAFYGVSSQYSSADSMTISVSTKVCSFGKQVVEKVETEYARLENGRFVYRIHRSPMCEYMINFIHKLKHLPEKYMMNSVLENFTILQVVTSRDSQETLLVIAFVFEVSTSEHGAQHHVYKLVKD

>NP_002291.1

MATLKEKLIAPVAEEEATVPNNKITVVGVGQVGMACAISILGKSLADELALVDVLEDKLKGEMMDLQHGSLFLQTPKIVADKDYSVTANSKIVVVTAGVRQQEGESRLNLVQRNVNVFKFIIPQIVKYSPDCIIIVVSNPVDILTYVTWKLSGLPKHRVIGSGCNLDSARFRYLMAEKLGIHPSSCHGWILGEHGDSSVAVWSGVNVAGVSLQELNPEMGTDNDSENWKEVHKMVVESAYEVIKLKGYTNWAIGLSVADLIESMLKNLSRIHPVSTMVKGMYGIENEVFLSLPCILNARGLTSVINQKLKDDEVAQLKKSADTLWDIQKDLKDL

523 P01106 NP_005521.1

>P01106

MPLNVSFTNRNYDLDYDSVQPYFYCDEEENFYQQQQQSELQPPAPSEDIWKKFELLPTPPLSPSRRSGLCSPSYVAVTPFSLRGDNDGGGGSFSTADQLEMVTELLGGDMVNQSFICDPDDETFIKNIIIQDCMWSGFSAAAKLVSEKLASYQAARKDSGSPNPARGHSVCSTSSLYLQDLSAAASECIDPSVVFPYPLNDSSSPKSCASQDSSAFSPSSDSLLSSTESSPQGSPEPLVLHEETPPTTSSDSEEEQEDEEEIDVVSVEKRQAPGKRSESGSPSAGGHSKPPHSPLVLKRCHVSTHQHNYAAPPSTRKDYPAAKRVKLDSVRVLRQISNNRKCTSPRSSDTEENVKRRTHNVLERQRRNELKRSFFALRDQIPELENNEKAPKVVILKKATAYILSVQAEEQKLISEEDLLRKRREQLKHKLEQLRNSCA

>NP_005521.1

MAGPAWISKVSRLLGAFHNPKQVTRGFTGGVQTVTLIPGDGIGPEISAAVMKIFDAAKAPIQWEERNVTAIQGPGGKWMIPSEAKESMDKNKMGLKGPLKTPIAAGHPSMNLLLRKTFDLYANVRPCVSIEGYKTPYTDVNIVTIRENTEGEYSGIEHVIVDGVVQSIKLITEGASKRIAEFAFEYARNNHRSNVTAVHKANIMRMSDGLFLQKCREVAESCKDIKFNEMYLDTVCLNMVQDPSQFDVLVMPNLYGDILSDLCAGLIGGLGVTPSGNIGANGVAIFESVHGTAPDIAGKDMANPTALLLSAVMMLRHMGLFDHAARIEAACFATIKDGKSLTKDLGGNAKCSDFTEEICRRVKDLD

524 NP_976225.1 NP_002475.2

>NP_976225.1

MSTKNFRVSDGDWICPDKKCGNVNFARRTSCNRCGREKTTEAKMMKAGGTEIGKTLAEKSRGLFSANDWQCKTCSNVNWARRSECNMCNTPKYAKLEERTGYGGGFNERENVEYIEREESDGEYDEFGRKKKKYRGKAVGPASILKEVEDKESEGEEEDEDEDLSKYKLDEDEDEDDADLSKYNLDASEEEDSNKKKSNRRSRSKSRSSHSRSSSRSSSPSSSRSRSRSRSRSSSSSQSRSRSSSRERSRSRGSKSRSSSRSHRGSSSPRKRSYSSSSSSPERNRKRSRSRSSSSGDRKKRRTRSRSPERRHRSSSGSSHSGSRSSSKKK

>NP_002475.2

MEEVPHDCPGADSAQAGRGASCQGCPNQRLCASGAGATPDTAIEEIKEKMKTVKHKILVLSGKGGVGKSTFSAHLAHGLAEDENTQIALLDIDICGPSIPKIMGLEGEQVHQSGSGWSPVYVEDNLGVMSVGFLLSSPDDAVIWRGPKKNGMIKQFLRDVDWGEVDYLIVDTPPGTSDEHLSVVRYLATAHIDGAVIITTPQEVSLQDVRKEINFCRKVKLPIIGVVENMSGFICPKCKKESQIFPPTTGGAELMCQDLEVPLLGRVPLDPLIGKNCDKGQSFFIDAPDSPATLAYRSIIQRIQEFCNLHQSKEENLISS

525 P51688 Q96LI5

>P51688

MSCPVPACCALLLVLGLCRARPRNALLLLADDGGFESGAYNNSAIATPHLDALARRSLLFRNAFTSVSSCSPSRASLLTGLPQHQNGMYGLHQDVHHFNSFDKVRSLPLLLSQAGVRTGIIGKKHVGPETVYPFDFAYTEENGSVLQVGRNITRIKLLVRKFLQTQDDRPFFLYVAFHDPHRCGHSQPQYGTFCEKFGNGESGMGRIPDWTPQAYDPLDVLVPYFVPNTPAARADLAAQYTTVGRMDQGVGLVLQELRDAGVLNDTLVIFTSDNGIPFPSGRTNLYWPGTAEPLLVSSPEHPKRWGQVSEAYVSLLDLTPTILDWFSIPYPSYAIFGSKTIHLTGRSLLPALEAEPLWATVFGSQSHHEVTMSYPMRSVQHRHFRLVHNLNFKMPFPIDQDFYVSPTFQDLLNRTTAGQPTGWYKDLRHYYYRARWELYDRSRDPHETQNLATDPRFAQLLEMLRDQLAKWQWETHDPWVCAPDGVLEEKLSPQCQPLHNEL

>Q96LI5

MRLIGMPKEKYDPPDPRRIYTIMSAEEVANGKKSHWAELEISGRVRSLSTSLWSLTHLTALHLNDNYLSRIPPDIAKLHNLVYLDLSSNKLRSLPAELGNMVSLRELLLNNNLLRVLPYELGRLFQLQTLGLKGNPLSQDILNLYQDPDGTRKLLNFMLDNLAVHPEQLPPRPWITLKERDQILPSASFTVMCYNVLCDKYATRQLYGYCPSWALNWEYRKKGIMEEIVNCDADIISLQEVETEQYFTLFLPALKERGYDGFFSPKSRAKIMSEQERKHVDGCAIFFKTEKFTLVQKHTVEFNQVAMANSDGSEAMLNRVMTKDNIGVAVVLEVHKELFGAGMKPIHAADKQLLIVANAHMHWDPEYSDVKLIQTMMFVSEVKNILEKASSRPGSPTADPNSIPLVLCADLNSLPDSGVVEYLSNGGVADNHKDFKELRYNECLMNFSCNGKNGSSEGRITHGFQLKSAYENNLMPYTNYTFDFKGVIDYIFYSKTHMNVLGVLGPLDPQWLVENNITGCPHPHIPSDHFSLLTQLELHPPLLPLVNGVHLPNRR

526 Q9Y6P5 Q9BYC8

>Q9Y6P5

MRLAAAANEAYTAPLAVSGLLGCKQCGGGRDQDEELGIRIPRPLGQGPSRFIPEKEILQVGSEDAQMHALFADSFAALGRLDNITLVMVFHPQYLESFLKTQHYLLQMDGPLPLHYRHYIGIMAAARHQCSYLVNLHVNDFLHVGGDPKWLNGLENAPQKLQNLGELNKVLAHRPWLITKEHIEGLLKAEEHSWSLAELVHAVVLLTHYHSLASFTFGCGISPEIHCDGGHTFRPPSVSNYCICDITNGNHSVDEMPVNSAENVSVSDSFFEVEALMEKMRQLQECRDEEEASQEEMASRFEIEKRESMFVFSSDDEEVTPARAVSRHFEDTSYGYKDFSRHGMHVPTFRVQDYCWEDHGYSLVNRLYPDVGQLIDEKFHIAYNLTYNTMAMHKDVDTSMLRRAIWNYIHCMFGIRYDDYDYGEINQLLDRSFKVYIKTVVCTPEKVTKRMYDSFWRQFKHSEKVHVNLLLIEARMQAELLYALRAITRYMT

>Q9BYC8

MALAMLVLVVSPWSAARGVLRNYWERLLRKLPQSRPGFPSPPWGPALAVQGPAMFTEPANDTSGSKENSSLLDSIFWMAAPKNRRTIEVNRCRRRNPQKLIKVKNNIDVCPECGHLKQKHVLCAYCYEKVCKETAEIRRQIGKQEGGPFKAPTIETVVLYTGETPSEQDQGKRIIERDRKRPSWFTQN

527 P09874 Q96HA7

>P09874

MAESSDKLYRVEYAKSGRASCKKCSESIPKDSLRMAIMVQSPMFDGKVPHWYHFSCFWKVGHSIRHPDVEVDGFSELRWDDQQKVKKTAEAGGVTGKGQDGIGSKAEKTLGDFAAEYAKSNRSTCKGCMEKIEKGQVRLSKKMVDPEKPQLGMIDRWYHPGCFVKNREELGFRPEYSASQLKGFSLLATEDKEALKKQLPGVKSEGKRKGDEVDGVDEVAKKKSKKEKDKDSKLEKALKAQNDLIWNIKDELKKVCSTNDLKELLIFNKQQVPSGESAILDRVADGMVFGALLPCEECSGQLVFKSDAYYCTGDVTAWTKCMVKTQTPNRKEWVTPKEFREISYLKKLKVKKQDRIFPPETSASVAATPPPSTASAPAAVNSSASADKPLSNMKILTLGKLSRNKDEVKAMIEKLGGKLTGTANKASLCISTKKEVEKMNKKMEEVKEANIRVVSEDFLQDVSASTKSLQELFLAHILSPWGAEVKAEPVEVVAPRGKSGAALSKKSKGQVKEEGINKSEKRMKLTLKGGAAVDPDSGLEHSAHVLEKGGKVFSATLGLVDIVKGTNSYYKLQLLEDDKENRYWIFRSWGRVGTVIGSNKLEQMPSKEDAIEHFMKLYEEKTGNAWHSKNFTKYPKKFYPLEIDYGQDEEAVKKLTVNPGTKSKLPKPVQDLIKMIFDVESMKKAMVEYEIDLQKMPLGKLSKRQIQAAYSILSEVQQAVSQGSSDSQILDLSNRFYTLIPHDFGMKKPPLLNNADSVQAKVEMLDNLLDIEVAYSLLRGGSDDSSKDPIDVNYEKLKTDIKVVDRDSEEAEIIRKYVKNTHATTHNAYDLEVIDIFKIEREGECQRYKPFKQLHNRRLLWHGSRTTNFAGILSQGLRIAPPEAPVTGYMFGKGIYFADMVSKSANYCHTSQGDPIGLILLGEVALGNMYELKHASHISKLPKGKHSVKGLGKTTPDPSANISLDGVDVPLGTGISSGVNDTSLLYNEYIVYDIAQVNLKYLLKLKFNFKTSLW

>Q96HA7

MSLERELRQLSKAKAKAQRAGQRREEAALCHQLGELLAGHGRYAEALEQHWQELQLRERADDPLGCAVAHRKIGERLAEMEDYPAALQHQHQYLELAHSLRNHTELQRAWATIGRTHLDIYDHCQSRDALLQAQAAFEKSLAIVDEELEGTLAQGELNEMRTRLYLNLGLTFESLQQTALCNDYFRKSIFLAEQNHLYEDLFRARYNLGTIHWRAGQHSQAMRCLEGARECAHTMRKRFMESECCVVIAQVLQDLGDFLAAKRALKKAYRLGSQKPVQRAAICQNLQHVLAVVRLQQQLEEAEGRDPQGAMVICEQLGDLFSKAGDFPRAAEAYQKQLRFAELLDRPGAERAIIHVSLATTLGDMKDHHGAVRHYEEELRLRSGNVLEEAKTWLNIALSREEAGDAYELLAPCFQKALSCAQQAQRPQLQRQVLQHLHTVQLRLQPQEAPETETRLRELSVAEDEDEEEEAEEAAATAESEALEAGEVELSEGEDDTDGLTPQLEEDEELQGHLGRRKGSKWNRRNDMGETLLHRACIEGQLRRVQDLVRQGHPLNPRDYCGWTPLHEACNYGHLEIVRFLLDHGAAVDDPGGQGCEGITPLHDALNCGHFEVAELLLERGASVTLRTRKGLSPLETLQQWVKLYRRDLDLETRQKARAMEMLLQAAASGQDPHSSQAFHTPSSLLFDPETSPPLSPCPEPPSNSTRLPEASQAHVRVSPGQAAPAMARPRRSRHGPASSSSSSEGEDSAGPARPSQKRPRCSATAQRVAAWTPGPASNREAATASTSRAAYQAAIRGVGSAQSRLGPGPPRGHSKALAPQAALIPEEECLAGDWLELDMPLTRSRRPRPRGTGDNRRPSSTSGSDSEESRPRARAKQVRLTCMQSCSAPVNAGPSSLASEPPGSPSTPRVSEPSGDSSAAGQPLGPAPPPPIRVRVQVQDHLFLIPVPHSSDTHSVAWLAEQAAQRYYQTCGLLPRLTLRKEGALLAPQDLIPDVLQSNDEVLAEVTSWDLPPLTDRYRRACQSLGQGEHQQVLQAVELQGLGLSFSACSLALDQAQLTPLLRALKLHTALRELRLAGNRLGDKCVAELVAALGTMPSLALLDLSSNHLGPEGLRQLAMGLPGQATLQSLEELDLSMNPLGDGCGQSLASLLHACPLLSTLRLQACGFGPSFFLSHQTALGSAFQDAEHLKTLSLSYNALGAPALARTLQSLPAGTLLHLELSSVAAGKGDSDLMEPVFRYLAKEGCALAHLTLSANHLGDKAVRDLCRCLSLCPSLISLDLSANPEISCASLEELLSTLQKRPQGLSFLGLSGCAVQGPLGLGLWDKIAAQLRELQLCSRRLCAEDRDALRQLQPSRPGPGECTLDHGSKLFFRRL

528 Q9BSH3 O75251

>Q9BSH3

MSRVLVPCHVKGSVALQVGDVRTSQGRPGVLVIDVTFPSVAPFELQEITFKNYYTAFLSIRVRQYTSAHTPAKWVTCLRDYCLMPDPHSEEGAQEYVSLFKHQMLCDMARISELRLILRQPSPLWLSFTVEELQIYQQGPKSPSVTFPKWLSHPVPCEQPALLREGLPDPSRVSSEVQQMWALTEMIRASHTSARIGRFDVDGCYDLNLLSYT

>O75251

MAVLSAPGLRGFRILGLRSSVGPAVQARGVHQSVATDGPSSTQPALPKARAVAPKPSSRGEYVVAKLDDLVNWARRSSLWPMTFGLACCAVEMMHMAAPRYDMDRFGVVFRASPRQSDVMIVAGTLTNKMAPALRKVYDQMPEPRYVVSMGSCANGGGYYHYSYSVVRGCDRIVPVDIYIPGCPPTAEALLYGILQLQRKIKRERRLQIWYRR

529 NP_002220.1 P49591

>NP_002220.1

MCTKMEQPFYHDDSYTATGYGRAPGGLSLHDYKLLKPSLAVNLADPYRSLKAPGARGPGPEGGGGGSYFSGQGSDTGASLKLASSELERLIVPNSNGVITTTPTPPGQYFYPRGGGSGGGAGGAGGGVTEEQEGFADGFVKALDDLHKMNHVTPPNVSLGATGGPPAGPGGVYAGPEPPPVYTNLSSYSPASASSGGAGAAVGTGSSYPTTTISYLPHAPPFAGGHPAQLGLGRGASTFKEEPQTVPEARSRDATPPVSPINMEDQERIKVERKRLRNRLAATKCRKRKLERIARLEDKVKTLKAENAGLSSTAGLLREQVAQLKQKVMTHVSNGCQLLLGVKGHAF

>P49591

MVLDLDLFRVDKGGDPALIRETQEKRFKDPGLVDQLVKADSEWRRCRFRADNLNKLKNLCSKTIGEKMKKKEPVGDDESVPENVLSFDDLTADALANLKVSQIKKVRLLIDEAILKCDAERIKLEAERFENLREIGNLLHPSVPISNDEDVDNKVERIWGDCTVRKKYSHVDLVVMVDGFEGEKGAVVAGSRGYFLKGVLVFLEQALIQYALRTLGSRGYIPIYTPFFMRKEVMQEVAQLSQFDEELYKVIGKGSEKSDDNSYDEKYLIATSEQPIAALHRDEWLRPEDLPIKYAGLSTCFRQEVGSHGRDTRGIFRVHQFEKIEQFVYSSPHDNKSWEMFEEMITTAEEFYQSLGIPYHIVNIVSGSLNHAASKKLDLEAWFPGSGAFRELVSCSNCTDYQARRLRIRYGQTKKMMDKVEFVHMLNATMCATTRTICAILENYQTEKGITVPEKLKEFMPPGLQELIPFVKPAPIEQEPSKKQKKQHEGSKKKAAARDVTLENRLQNMEVTDA

530 NP_005654.2 NP_001893.2

>NP_005654.2

MASMRESDTGLWLHNKLGATDELWAPPSIASLLTAAVIDNIRLCFHGLSSAVKLKLLLGTLHLPRRTVDEMKGALMEIIQLASLDSDPWVLMVADILKSFPDTGSLNLELEEQNPNVQDILGELREKVGECEASAMLPLECQYLNKNALTTLAGPLTPPVKHFQLKRKPKSATLRAELLQKSTETAQQLKRSAGVPFHAKGRGLLRKMDTTTPLKGIPKQAPFRSPTAPSVFSPTGNRTPIPPSRTLLRKERGVKLLDISELDMVGAGREAKRRRKTLDAEVVEKPAKEETVVENATPDYAAGLVSTQKLGSLNNEPALPSTSYLPSTPSVVPASSYIPSSETPPAPSSREASRPPEEPSAPSPTLPAQFKQRAPMYNSGLSPATPTPAAPTSPLTPTTPPAVAPTTQTPPVAMVAPQTQAPAQQQPKKNLSLTREQMFAAQEMFKTANKVTRPEKALILGFMAGSRENPCQEQGDVIQIKLSEHTEDLPKADGQGSTTMLVDTVFEMNYATGQWTRFKKYKPMTNVS

>NP_001893.2

MQEKDASSQGFLPHFQHFATQAIHVGQDPEQWTSRAVVPPISLSTTFKQGAPGQHSGFEYSRSGNPTRNCLEKAVAALDGAKYCLAFASGLAATVTITHLLKAGDQIICMDDVYGGTNRYFRQVASEFGLKISFVDCSKIKLLEAAITPETKLVWIETPTNPTQKVIDIEGCAHIVHKHGDIILVVDNTFMSPYFQRPLALGADISMYSATKYMNGHSDVVMGLVSVNCESLHNRLRFLQNSLGAVPSPIDCYLCNRGLKTLHVRMEKHFKNGMAVAQFLESNPWVEKVIYPGLPSHPQHELVKRQCTGCTGMVTFYIKGTLQHAEIFLKNLKLFTLAESLGGFESLAELPAIMTHASVLKNDRDVLGISDTLIRLSVGLEDEEDLLEDLDQALKAAHPPSGSHS

531 P35269 O60783

>P35269

MAALGPSSQNVTEYVVRVPKNTTKKYNIMAFNAADKVNFATWNQARLERDLSNKKIYQEEEMPESGAGSEFNRKLREEARRKKYGIVLKEFRPEDQPWLLRVNGKSGRKFKGIKKGGVTENTSYYIFTQCPDGAFEAFPVHNWYNFTPLARHRTLTAEEAEEEWERRNKVLNHFSIMQQRRLKDQDQDEDEEEKEKRGRRKASELRIHDLEDDLEMSSDASDASGEEGGRVPKAKKKAPLAKGGRKKKKKKGSDDEAFEDSDDGDFEGQEVDYMSDGSSSSQEEPESKAKAPQQEEGPKGVDEQSDSSEESEEEKPPEEDKEEEEEKKAPTPQEKKRRKDSSEESDSSEESDIDSEASSALFMAKKKTPPKRERKPSGGSSRGNSRPGTPSAEGGSTSSTLRAAASKLEQGKRVSEMPAAKRLRLDTGPQSLSGKSTPQPPSGKTTPNSGDVQVTEDAVRRYLTRKPMTTKDLLKKFQTKKTGLSSEQTVNVLAQILKRLNPERKMINDKMHFSLKE

>O60783

MAAFMLGSLLRTFKQMVPSSASGQVRSHYVDWRMWRDVKRRKMAYEYADERLRINSLRKNTILPKILQDVADEEIAALPRDSCPVRIRNRCVMTSRPRGVKRRWRLSRIVFRHLADHGQLSGIQRATW

532 P08048 NP_004125.3

>P08048

MDEDEFELQPQEPNSFFDGIGADATHMDGDQIVVEIQEAVFVSNIVDSDITVHNFVPDDPDSVVIQDVVEDVVIEEDVQCSDILEEADVSENVIIPEQVLDSDVTEEVSLPHCTVPDDVLASDITSTSMSMPEHVLTSESMHVCDIGHVEHMVHDSVVEAEIITDPLTSDIVSEEVLVADCAPEAVIDASGISVDQQDNDKASCEDYLMISLDDAGKIEHDGSTGVTIDAESEMDPCKVDSTCPEVIKVYIFKADPGEDDLGGTVDIVESEPENDHGVELLDQNSSIRVPREKMVYMTVNDSQQEDEDLNVAEIADEVYMEVIVGEEDAAVAAAAAAVHEQQIDEDEMKTFVPIAWAAAYGNNSDGIENRNGTASALLHIDESAGLGRLAKQKPKKKRRPDSRQYQTAIIIGPDGHPLTVYPCMICGKKFKSRGFLKRHMKNHPEHLAKKKYHCTDCDYTTNKKISLHNHLESHKLTSKAEKAIECDECGKHFSHAGALFTHKMVHKEKGANKMHKCKFCEYETAEQGLLNRHLLAVHSKNFPHICVECGKGFRHPSELRKHMRIHTGEKPYQCQYCEYRSADSSNLKTHIKTKHSKEMPFKCDICLLTFSDTKEVQQHTLVHQESKTHQCLHCDHKSSNSSDLKRHVISVHTKDYPHKCEMCEKGFHRPSELKKHVAVHKGKKMHQCRHCDFKIADPFVLSRHILSVHTKDLPFRCKRCRKGFRQQNELKKHMKTHSGRKVYQCEYCEYSTTDASGFKRHVISIHTKDYPHRCEYCKKGFRRPSEKNQHIMRHHKEVGLP

>NP_004125.3

MISASRAAAARLVGAAASRGPTAARHQDSWNGLSHEAFRLVSRRDYASEAIKGAVVGIDLGTTNSCVAVMEGKQAKVLENAEGARTTPSVVAFTADGERLVGMPAKRQAVTNPNNTFYATKRLIGRRYDDPEVQKDIKNVPFKIVRASNGDAWVEAHGKLYSPSQIGAFVLMKMKETAENYLGHTAKNAVITVPAYFNDSQRQATKDAGQISGLNVLRVINEPTAAALAYGLDKSEDKVIAVYDLGGGTFDISILEIQKGVFEVKSTNGDTFLGGEDFDQALLRHIVKEFKRETGVDLTKDNMALQRVREAAEKAKCELSSSVQTDINLPYLTMDSSGPKHLNMKLTRAQFEGIVTDLIRRTIAPCQKAMQDAEVSKSDIGEVILVGGMTRMPKVQQTVQDLFGRAPSKAVNPDEAVAIGAAIQGGVLAGDVTDVLLLDVTPLSLGIETLGGVFTKLINRNTTIPTKKSQVFSTAADGQTQVEIKVCQGEREMAGDNKLLGQFTLIGIPPAPRGVPQIEVTFDIDANGIVHVSAKDKGTGREQQIVIQSSGGLSKDDIENMVKNAEKYAEEDRRKKERVEAVNMAEGIIHDTETKMEEFKDQLPADECNKLKEEISKMRELLARKDSETGENIRQAASSLQQASLKLFEMAYKKMASEREGSGSSGTGEQKEDQKEEKQ

533 NP_002884.1 Q16822

>NP_002884.1

MASKEMFEDTVEERVINEEYKIWKKNTPFLYDLVMTHALQWPSLTVQWLPEVTKPEGKDYALHWLVLGTHTSDEQNHLVVARVHIPNDDAQFDASHCDSDKGEFGGFGSVTGKIECEIKINHEGEVNRARYMPQNPHIIATKTPSSDVLVFDYTKHPAKPDPSGECNPDLRLRGHQKEGYGLSWNSNLSGHLLSASDDHTVCLWDINAGPKEGKIVDAKAIFTGHSAVVEDVAWHLLHESLFGSVADDQKLMIWDTRSNTTSKPSHLVDAHTAEVNCLSFNPYSEFILATGSADKTVALWDLRNLKLKLHTFESHKDEIFQVHWSPHNETILASSGTDRRLNVWDLSKIGEEQSAEDAEDGPPELLFIHGGHTAKISDFSWNPNEPWVICSVSEDNIMQIWQMAENIYNDEESDVTTSELEGQGS

>Q16822

MAALYRPGLRLNWHGLSPLGWPSCRSIQTLRVLSGDLGQLPTGIRDFVEHSARLCQPEGIHICDGTEAENTATLTLLEQQGLIRKLPKYNNCWLARTDPKDVARVESKTVIVTPSQRDTVPLPPGGARGQLGNWMSPADFQRAVDERFPGCMQGRTMYVLPFSMGPVGSPLSRIGVQLTDSAYVVASMRIMTRLGTPVLQALGDGDFVKCLHSVGQPLTGQGEPVSQWPCNPEKTLIGHVPDQREIISFGSGYGGNSLLGKKCFALRIASRLARDEGWLAEHMLILGITSPAGKKALCAAAFPSACGKTNLAMMRPALPGWKVECVGDDIAWMRFDSEGRLRAINPENGFFGVAPGTSATTNPNAMATIQSNTIFTNVAETSDGGVYWEGIDQPLPPGVTVTSWLGKPWKPGDKEPCAHPNSRFCAPARQCPIMDPAWEAPEGVPIDAIIFGGRRPKGVPLVYEAFNWRHGVFVGRAMRSESTAAAEHKGKIIMHDPFAMRPFFGYNFGHYLEHWLSMEGRKGAQLPRIFHVNWFRRDEAGHFLWPGFGENARVLDWICRRLEGEDSARETPIGLVPKEGALDLSGLRAIDTTQLFSLPKDFWEQEVRDIRSYLTEQVNQDLPKEVLAELEALERRVHKM

534 NP_003186.1 NP_000894.1

>NP_003186.1

MMGKEEEIARIARRLDKMVTKKSAEGAMDLLRELKAMPITLHLLQSTRVGMSVNALRKQSSDEEVIALAKSLIKSWKKLLDASDAKARERGRGMPLPTSSRDASEAPDPSRKRPELPRAPSTPRITTFPPVPVTCDAVRNKCREMLTAALQTDHDHVAIGADCERLSAQIEECIFRDVGNTDMKYKNRVRSRISNLKDAKNPDLRRNVLCGAITPQQIAVMTSEEMASDELKEIRKAMTKEAIREHQMARTGGTQTDLFTCGKCRKKNCTYTQVQTRSSDEPMTTFVVCNECGNRWKFC

>NP_000894.1

MVGRRALIVLAHSERTSFNYAMKEAAAAALKKKGWEVVESDLYAMNFNPIISRKDITGKLKDPANFQYPAESVLAYKEGHLSPDIVAEQKKLEAADLVIFQFPLQWFGVPAILKGWFERVFIGEFAYTYAAMYDKGPFRSKKAVLSITTGGSGSMYSLQGIHGDMNVILWPIQSGILHFCGFQVLEPQLTYSIGHTPADARIQILEGWKKRLENIWDETPLYFAPSSLFDLNFQAGFLMKKEVQDEEKNKKFGLSVGHHLGKSIPTDNQIKARK

535 NP_006708.2 Q96T66

>NP_006708.2

MKTPFGKTPGQRSRADAGHAGVSANMMKKRTSHKKHRSSVGPSKPVSQPRRNIVGCRIQHGWKEGNGPVTQWKGTVLDQVPVNPSLYLIKYDGFDCVYGLELNKDERVSALEVLPDRVATSRISDAHLADTMIGKAVEHMFETEDGSKDEWRGMVLARAPVMNTWFYITYEKDPVLYMYQLLDDYKEGDLRIMPDSNDSPPAEREPGEVVDSLVGKQVEYAKEDGSKRTGMVIHQVEAKPSVYFIKFDDDFHIYVYDLVKTS

>Q96T66

MKSRIPVVLLACGSFNPITNMHLRMFEVARDHLHQTGMYQVIQGIISPVNDTYGKKDLAASHHRVAMARLALQTSDWIRVDPWESEQAQWMETVKVLRHHHSKLLRSPPQMEGPDHGKALFSTPAAVPELKLLCGADVLKTFQTPNLWKDAHIQEIVEKFGLVCVGRVGHDPKGYIAESPILRMHQHNIHLAKEPVQNEISATYIRRALGQGQSVKYLIPDAVITYIKDHGLYTKGSTWKGKSTQSTEGKTS

536 NP_149020.2 Q9HD34

>NP_149020.2

MLLPLPPQSSKPVPKKSQSSKIVPSHHDPSEKTGENCQTKISPSSLQESPSSLQGALKKRSAFEDLTNASQCQPVQPKKEANKEFVKVVSKKINRNTHALGLAKKNKRNLKWHKLEVTPVVASTTVVPNIMEKPLILDISTTSKTPNTEEASLFRKPLVLKEEPTIEDETLINKSLSLKKCSNHEEVSLLEKLQPLQEESDSDDAFVIEPMTFKKTHKTEEAAITKKTLSLKKKMCASQRKQSCQEESLAVQDVNMEEDSFFMESMSFKKKPKTEESIPTHKLSSLKKKCTIYGKICHFRKPPVLQTTICGAMSSIKKPTTEKETLFQELSVLQEKHTTEHEMSILKKSLALQKTNFKEDSLVKESLAFKKKPSTEEAIMMPVILKEQCMTEGKRSRLKPLVLQEITSGEKSLIMKPLSIKEKPSTEKESFSQEPSALQKKHTTQEEVSILKEPSSLLKSPTEESPFDEALAFTKKCTIEEAPPTKKPLILKRKHATQGTMSHLKKPLILQTTSGEKSLIKEPLPFKEEKVSLKKKCTTQEMMSICPELLDFQDMIGEDKNSFFMEPMSFRKNPTTEETVLTKTSLSLQEKKITQGKMSHLKKPLVLQKITSEEESFYKKLLPFKMKSTTEEKFLSQEPSALKEKHTTLQEVSLSKESLAIQEKATTEEEFSQELFSLHVKHTNKSGSLFQEALVLQEKTDAEEDSLKNLLALQEKSTMEEESLINKLLALKEELSAEAATNIQTQLSLKKKSTSHGKVFFLKKQLALNETINEEEFLNKQPLALEGYPSIAEGETLFKKLLAMQEEPSIEKEAVLKEPTIDTEAHFKEPLALQEEPSTEKEAVLKEPSVDTEAHFKETLALQEKPSIEQEALFKRHSALWEKPSTEKETIFKESLDLQEKPSIKKETLLKKPLALKMSTINEAVLFEDMIALNEKPTTGKELSFKEPLALQESPTYKEDTFLKTLLVPQVGTSPNVSSTAPESITSKSSIATMTSVGKSGTINEAFLFEDMITLNEKPTTGKELSFKEPLALQESPTCKEDTFLETFLIPQIGTSPYVFSTTPESITEKSSIATMTSVGKSRTTTESSACESASDKPVSPQAKGTPKEITPREDIDEDSSDPSFNPMYAKEIFSYMKEREEQFILTDYMNRQIEITSDMRAILVDWLVEVQVSFEMTHETLYLAVKLVDLYLMKAVCKKDKLQLLGATAFMIAAKFEEHNSPRVDDFVYICDDNYQRSEVLSMEINILNVLKCDINIPIAYHFLRRYARCIHTNMKTLTLSRYICEMTLQEYHYVQEKASKLAAASLLLALYMKKLGYWVPFLEHYSGYSISELHPLVRQLNKLLTFSSYDSLKAVYYKYSHPVFFEVAKIPALDMLKLEEILNCDCEAQGLVL

>Q9HD34

MAASSRAQVLSLYRAMLRESKRFSAYNYRTYAVRRIRDAFRENKNVKDPVEIQTLVNKAKRDLGVIRRQVHIGQLYSTDKLIIENRDMPRT

537 NP_937859.1 Q9Y216

>NP_937859.1

MTVGKSSKMLQHIDYRMRCILQDGRIFIGTFKAFDKHMNLILCDCDEFRKIKPKNSKQAEREEKRVLGLVLLRGENLVSMTVEGPPPKDTGIARVPLAGAAGGPGIGRAAGRGIPAGVPMPQAPAGLAGPVRGVGGPSQQVMTPQGRGTVAAAAAAATASIAGAPTQYPPGRGGPPPPMGRGAPPPGMMGPPPGMRPPMGPPMGIPPGRGTPMGMPPPGMRPPPPGMRGPPPPGMRPPRP

>Q9Y216

MEHIRTPKVENVRLVDRVSPKKAALGTLYLTATHVIFVENSPDARKETWILHSQISTIEKQATTATGCPLLIRCKNFQIIQLIIPQERDCHDVYISLIRLARPVKYEELYCFSFNPMLDKEEREQGWVLIDLSEEYTRMGLPNHYWQLSDVNRDYRVCDSYPTELYVPKSATAHIIVGSSKFRSRRRFPVLSYYYKDNHASICRSSQPLSGFSARCLEDEQMLQAIRKANPGSDFVYVVDTRPKLNAMANRAAGKGYENEDNYSNIKFQFIGIENIHVMRNSLQKMLEVCELKSPSMSDFLWGLENSGWLRHIKAIMDAGIFIAKAVSEEGASVLVHCSDGWDRTAQVCSVASLLLDPHYRTLKGFMVLIEKDWISFGHKFNHRYGNLDGDPKEISPVIDQFIECVWQLMEQFPCAFEFNERFLIHIQHHIYSCQFGNFLCNSQKERRELKIQERTYSLWAHLWKNRADYLNPLFRADHSQTQGTLHLPTTPCNFMYKFWSGMYNRFEKGMQPRQSVTDYLMAVKEETQQLEEELEALEERLEKIQKVQLNCTKVKSKQSEPSKHSGFSTSDNSIANTPQDYSGNMKSFPSRSPSQGDEDSALILTQDNLKSSDPDLSANSDQESGVEDLSCRSPSGGEHAPSEDSGKDRDSDEAVFLTA

538 Q96JN0 Q9HD33

>Q96JN0

MQRMIQQFAAEYTSKNSSTQDPSQPNSTKNQSLPKASPVTTSPTAATTQNPVLSKLLMADQDSPLDLTVRKSQSEPSEQDGVLDLSTKKSPCAGSTSLSHSPGCSSTQGNGRPGRPSQYRPDGLRSGDGVPPRSLQDGTREGFGHSTSLKVPLARSLQISEELLSRNQLSTAASLGPSGLQNHGQHLILSREASWAKPHYEFNLSRMKFRGNGALSNISDLPFLAENSAFPKMALQAKQDGKKDVSHSSPVDLKIPQVRGMDLSWESRTGDQYSYSSLVMGSQTESALSKKLRAILPKQSRKSMLDAGPDSWGSDAEQSTSGQPYPTSDQEGDPGSKQPRKKRGRYRQYNSEILEEAISVVMSGKMSVSKAQSIYGIPHSTLEYKVKERLGTLKNPPKKKMKLMRSEGPDVSVKIELDPQGEAAQSANESKNE

>Q9HD33

MAAAGLALLCRRVSSALKSSRSLITPQVPACTGFFLSLLPKSTPNVTSFHQYRLLHTTLSRKGLEEFFDDPKNWGQEKVKSGAAWTCQQLRNKSNEDLHKLWYVLLKERNMLLTLEQEAKRQRLPMPSPERLDKVVDSMDALDKVVQEREDALRLLQTGQERARPGAWRRDIFGRIIWHKFKQWVIPWHLNKRYNRKRFFALPYVDHFLRLEREKRARIKARKENLERKKAKILLKKFPHLAEAQKSSLV

539 NP_055586.1 O75394

>NP_055586.1

MSGPGNKRAAGDGGSGPPEKKLSREEKTTTTLIEPIRLGGISSTEEMDLKVLQFKNKKLAERLEQRQACEDELRERIEKLEKRQATDDATLLIVNRYWAQLDETVEALLRCHESQGELSSAPEAPGTQEGPTCDGTPLPEPGTSELRDPLLMQLRPPLSEPALAFVVALGASSSEEVELELQGRMEFSKAAVSRVVEASDRLQRRVEELCQRVYSRGDSEPLSEAAQAHTRELGRENRRLQDLATQLQEKHHRISLEYSELQDKVTSAETKVLEMETTVEDLQWDIEKLRKREQKLNKHLAEALEQLNSGYYVSGSSSGFQGGQITLSMQKFEMLNAELEENQELANSRMAELEKLQAELQGAVRTNERLKVALRSLPEEVVRETGEYRMLQAQFSLLYNESLQVKTQLDEARGLLLATKNSHLRHIEHMESDELGLQKKLRTEVIQLEDTLAQVRKEYEMLRIEFEQNLAANEQAGPINREMRHLISSLQNHNHQLKGDAQRYKRKLREVQAEIGKLRAQASGSAHSTPNLGHPEDSGVSAPAPGKEEGGPGPVSTPDNRKEMAPVPGTTTTTTSVKKEELVPSEEDFQGITPGAQGPSSRGREPEARPKRELREREGPSLGPPPVASALSRADREKAKVEETKRKESELLKGLRAELKKAQESQKEMKLLLDMYKSAPKEQRDKVQLMAAERKAKAEVDELRSRIRELEERDRRESKKIADEDALRRIRQAEEQIEHLQRKLGATKQEEEALLSEMDVTGQAFEDMQEQNGRLLQQLREKDDANFKLMSERIKANQIHKLLREEKDELGEQVLGLKSQVDAQLLTVQKLEEKERALQGSLGGVEKELTLRSQALELNKRKAVEAAQLAEDLKVQLEHVQTRLREIQPCLAESRAAREKESFNLKRAQEDISRLRRKLEKQRKVEVYADADEILQEEIKEYKARLTCPCCNTRKKDAVLTKCFHVFCFECVRGRYEARQRKCPKCNAAFGAHDFHRIYIS

>O75394

MFLSAVFFAKSKSKNILVRMVSEAGTGFCFNTKRNRLREKLTLLHYDPVVKQRVLFVEKKKIRSL

540 NP_001460.1 P49753

>NP_001460.1

MSGWESYYKTEGDEEAEEEQEENLEASGDYKYSGRDSLIFLVDASKAMFESQSEDELTPFDMSIQCIQSVYISKIISSDRDLLAVVFYGTEKDKNSVNFKNIYVLQELDNPGAKRILELDQFKGQQGQKRFQDMMGHGSDYSLSEVLWVCANLFSDVQFKMSHKRIMLFTNEDNPHGNDSAKASRARTKAGDLRDTGIFLDLMHLKKPGGFDISLFYRDIISIAEDEDLRVHFEESSKLEDLLRKVRAKETRKRALSRLKLKLNKDIVISVGIYNLVQKALKPPPIKLYRETNEPVKTKTRTFNTSTGGLLLPSDTKRSQIYGSRQIILEKEETEELKRFDDPGLMLMGFKPLVLLKKHHYLRPSLFVYPEESLVIGSSTLFSALLIKCLEKEVAALCRYTPRRNIPPYFVALVPQEEELDDQKIQVTPPGFQLVFLPFADDKRKMPFTEKIMATPEQVGKMKAIVEKLRFTYRSDSFENPVLQQHFRNLEALALDLMEPEQAVDLTLPKVEAMNKRLGSLVDEFKELVYPPDYNPEGKVTKRKHDNEGSGSKRPKVEYSEEELKTHISKGTLGKFTVPMLKEACRAYGLKSGLKKQELLEALTKHFQD

>P49753

MSNKLLSPHPHSVVLRSEFKMASSPAVLRASRLYQWSLKSSAQFLGSPQLRQVGQIIRVPARMAATLILEPAGRCCWDEPVRIAVRGLAPEQPVTLRASLRDEKGALFQAHARYRADTLGELDLERAPALGGSFAGLEPMGLLWALEPEKPLVRLVKRDVRTPLAVELEVLDGHDPDPGRLLCQTRHERYFLPPGVRREPVRVGRVRGTLFLPPEPGPFPGIVDMFGTGGGLLEYRASLLAGKGFAVMALAYYNYEDLPKTMETLHLEYFEEAMNYLLSHPEVKGPGVGLLGISKGGELCLSMASFLKGITAAVVINGSVANVGGTLRYKGETLPPVGVNRNRIKVTKDGYADIVDVLNSPLEGPDQKSFIPVERAESTFLFLVGQDDHNWKSEFYANEACKRLQAHGRRKPQIICYPETGHYIEPPYFPLCRASLHALVGSPIIWGGEPRAHAMAQVDAWKQLQTFFHKHLGGHEGTIPSKV

541 NP_006464.1 Q9H2J4

>NP_006464.1

MSEREERRFVEIPRESVRLMAESTGLELSDEVAALLAEDVCYRLREATQNSSQFMKHTKRRKLTVEDFNRALRWSSVEAVCGYGSQEALPMRPAREGELYFPEDREVNLVELALATNIPKGCAETAVRVHVSYLDGKGNLAPQGSVPSAVSSLTDDLLKYYHQVTRAVLGDDPQLMKVALQDLQTNSKIGALLPYFVYVVSGVKSVSHDLEQLHRLLQVARSLFRNPHLCLGPYVRCLVGSVLYCVLEPLAASINPLNDHWTLRDGAALLLSHIFWTHGDLVSGLYQHILLSLQKILADPVRPLCCHYGAVVGLHALGWKAVERVLYPHLSTYWTNLQAVLDDYSVSNAQVKADGHKVYGAILVAVERLLKMKAQAAEPNRGGPGGRGCRRLDDLPWDSLLFQESSSGGGAEPSFGSGLPLPPGGAGPEDPSLSVTLADIYRELYAFFGDSLATRFGTGQPAPTAPRPPGDKKEPAAAPDSVRKMPQLTASAIVSPHGDESPRGSGGGGPASASGPAASESRPLPRVHRARGAPRQQGPGTGTRDVFQKSRFAPRGAPHFRFIIAGRQAGRRCRGRLFQTAFPAPYGPSPASRYVQKLPMIGRTSRPARRWALSDYSLYLPL

>Q9H2J4

MQDPNADTEWNDILRKKGILPPKESLKELEEEAEEEQRILQQSVVKTYEDMTLEELEDHEDEFNEEDERAIEMYRRRRLAEWKATKLKNKFGEVLEISGKDYVQEVTKAGEGLWVILHLYKQGIPLCALINQHLSGLARKFPDVKFIKAISTTCIPNYPDRNLPTIFVYLEGDIKAQFIGPLVFGGMNLTRDELEWKLSESGAIMTDLEENPKKPIEDVLLSSVRRSVLMKRDSDSEGD

542 NP_055675.1 Q9NX20

>NP_055675.1

MNLQRYWGEIPISSSQTNRSSFDLLPREFRLVEVHDPPLHQPSANKPKPPTMLDIPSEPCSLTIHTIQLIQHNRRLRNLIATAQAQNQQQTEGVKTEESEPLPSCPGSPPLPDDLLPLDCKNPNAPFQIRHSDPESDFYRGKGEPVTELSWHSCRQLLYQAVATILAHAGFDCANESVLETLTDVAHEYCLKFTKLLRFAVDREARLGQTPFPDVMEQVFHEVGIGSVLSLQKFWQHRIKDYHSYMLQISKQLSEEYERIVNPEKATEDAKPVKIKEEPVSDITFPVSEELEADLASGDQSLPMGVLGAQSERFPSNLEVEASPQASSAEVNASPLWNLAHVKMEPQESEEGNVSGHGVLGSDVFEEPMSGMSEAGIPQSPDDSDSSYGSHSTDSLMGSSPVFNQRCKKRMRKI

>Q9NX20

MWRLLARASAPLLRVPLSDSWALLPASAGVKTLLPVPSFEDVSIPEKPKLRFIERAPLVPKVRREPKNLSDIRGPSTEATEFTEGNFAILALGGGYLHWGHFEMMRLTINRSMDPKNMFAIWRVPAPFKPITRKSVGHRMGGGKGAIDHYVTPVKAGRLVVEMGGRCEFEEVQGFLDQVAHKLPFAAKAVSRGTLEKMRKDQEERERNNQNPWTFERIATANMLGIRKVLSPYDLTHKGKYWGKFYMPKRV

543 NP_000170.1 NP_061883.1

>NP_000170.1

MSRQSTLYSFFPKSPALSDANKASARASREGGRAAAAPGASPSPGGDAAWSEAGPGPRPLARSASPPKAKNLNGGLRRSVAPAAPTSCDFSPGDLVWAKMEGYPWWPCLVYNHPFDGTFIREKGKSVRVHVQFFDDSPTRGWVSKRLLKPYTGSKSKEAQKGGHFYSAKPEILRAMQRADEALNKDKIKRLELAVCDEPSEPEEEEEMEVGTTYVTDKSEEDNEIESEEEVQPKTQGSRRSSRQIKKRRVISDSESDIGGSDVEFKPDTKEEGSSDEISSGVGDSESEGLNSPVKVARKRKRMVTGNGSLKRKSSRKETPSATKQATSISSETKNTLRAFSAPQNSESQAHVSGGGDDSSRPTVWYHETLEWLKEEKRRDEHRRRPDHPDFDASTLYVPEDFLNSCTPGMRKWWQIKSQNFDLVICYKVGKFYELYHMDALIGVSELGLVFMKGNWAHSGFPEIAFGRYSDSLVQKGYKVARVEQTETPEMMEARCRKMAHISKYDRVVRREICRIITKGTQTYSVLEGDPSENYSKYLLSLKEKEEDSSGHTRAYGVCFVDTSLGKFFIGQFSDDRHCSRFRTLVAHYPPVQVLFEKGNLSKETKTILKSSLSCSLQEGLIPGSQFWDASKTLRTLLEEEYFREKLSDGIGVMLPQVLKGMTSESDSIGLTPGEKSELALSALGGCVFYLKKCLIDQELLSMANFEEYIPLDSDTVSTTRSGAIFTKAYQRMVLDAVTLNNLEIFLNGTNGSTEGTLLERVDTCHTPFGKRLLKQWLCAPLCNHYAINDRLDAIEDLMVVPDKISEVVELLKKLPDLERLLSKIHNVGSPLKSQNHPDSRAIMYEETTYSKKKIIDFLSALEGFKVMCKIIGIMEEVADGFKSKILKQVISLQTKNPEGRFPDLTVELNRWDTAFDHEKARKTGLITPKAGFDSDYDQALADIRENEQSLLEYLEKQRNRIGCRTIVYWGIGRNRYQLEIPENFTTRNLPEEYELKSTKKGCKRYWTKTIEKKLANLINAEERRDVSLKDCMRRLFYNFDKNYKDWQSAVECIAVLDVLLCLANYSRGGDGPMCRPVILLPEDTPPFLELKGSRHPCITKTFFGDDFIPNDILIGCEEEEQENGKAYCVLVTGPNMGGKSTLMRQAGLLAVMAQMGCYVPAEVCRLTPIDRVFTRLGASDRIMSGESTFFVELSETASILMHATAHSLVLVDELGRGTATFDGTAIANAVVKELAETIKCRTLFSTHYHSLVEDYSQNVAVRLGHMACMVENECEDPSQETITFLYKFIKGACPKSYGFNAARLANLPEEVIQKGHRKAREFEKMNQSLRLFREVCLASERSTVDAEAVHKLLTLIKEL

>NP_061883.1

MDFSRRSFHRSLSSSLQAPVVSTVGMQRLGTTPSVYGGAGGRGIRISNSRHTVNYGSDLTGGGDLFVGNEKMAMQNLNDRLASYLEKVRTLEQSNSKLEVQIKQWYETNAPRAGRDYSAYYRQIEELRSQIKDAQLQNARCVLQIDNAKLAAEDFRLKYETERGIRLTVEADLQGLNKVFDDLTLHKTDLEIQIEELNKDLALLKKEHQEEVDGLHKHLGNTVNVEVDAAPGLNLGVIMNEMRQKYEVMAQKNLQEAKEQFERQTAVLQQQVTVNTEELKGTEVQLTELRRTSQSLEIELQSHLSMKESLEHTLEETKARYSSQLANLQSLLSSLEAQLMQIRSNMERQNNEYHILLDIKTRLEQEIATYRRLLEGEDVKTTEYQLSTLEERDIKKTRKIKTVVQEVVDGKVVSSEVKEVEENI

544 NP_055130.1 NP_056177.3

>NP_055130.1

MADGNEDLRADDLPGPAFESYESMELACPAERSGHVAVSDGRHMFVWGGYKSNQVRGLYDFYLPREELWIYNMETGRWKKINTEGDVPPSMSGSCAVCVDRVLYLFGGHHSRGNTNKFYMLDSRSTDRVLQWERIDCQGIPPSSKDKLGVWVYKNKLIFFGGYGYLPEDKVLGTFEFDETSFWNSSHPRGWNDHVHILDTETFTWSQPITTGKAPSPRAAHACATVGNRGFVFGGRYRDARMNDLHYLNLDTWEWNELIPQGICPVGRSWHSLTPVSSDHLFLFGGFTTDKQPLSDAWTYCISKNEWIQFNHPYTEKPRLWHTACASDEGEVIVFGGCANNLLVHHRAAHSNEILIFSVQPKSLVRLSLEAVICFKEMLANSWNCLPKHLLHSVNQRFGSNNTSGS

>NP_056177.3

MTPSEGARAGTGRELEMLDSLLALGGLVLLRDSVEWEGRSLLKALVKKSALCGEQVHILGCEVSEEEFREGFDSDINNRLVYHDFFRDPLNWSKTEEAFPGGPLGALRAMCKRTDPVPVTIALDSLSWLLLRLPCTTLCQVLHAVSHQDSCPGDSSSVGKVSVLGLLHEELHGPGPVGALSSLAQTEVTLGGTMGQASAHILCRRPRQRPTDQTQWFSILPDFSLDLQEGPSVESQPYSDPHIPPVDPTTHLTFNLHLSKKEREARDSLILPFQFSSEKQQALLRPRPGQATSHIFYEPDAYDDLDQEDPDDDLDI

545 Q6EKJ0 Q14651

>Q6EKJ0

MAQVAVSTLPVEEESSSETRMVVTFLVSALESMCKELAKSKAEVACIAVYETDVFVVGTERGCAFVNARTDFQKDFAKYCVAEGLCEVKPPCPVNGMQVHSGETEILRKAVEDYFCFCYGKALGTTVMVPVPYEKMLRDQSAVVVQGLPEGVAFQHPENYDLATLKWILENKAGISFIINRPFLGPESQLGGPGMVTDAERSIVSPSESCGPINVKTEPMEDSGISLKAEAVSVKKESEDPNYYQYNMQGSHPSSTSNEVIEMELPMEDSTPLVPSEEPNEDPEAEVKIEGNTNSSSVTNSAAGVEDLNIVQVTVPDNEKERLSSIEKIKQLREQVNDLFSRKFGEAIGVDFPVKVPYRKITFNPGCVVIDGMPPGVVFKAPGYLEISSMRRILEAAEFIKFTVIRPLPGLELSNVGKRKIDQEGRVFQEKWERAYFFVEVQNIPTCLICKQSMSVSKEYNLRRHYQTNHSKHYDQYTERMRDEKLHELKKGLRKYLLGSSDTECPEQKQVFANPSPTQKSPVQPVEDLAGNLWEKLREKIRSFVAYSIAIDEITDINNTTQLAIFIRGVDENFDVSEELLDTVPMTGTKSGNEIFLRVEKSLKKFCINWSRLVSVASTGTPAMVDANNGLVTKLKSRVATFCKGAELKSICCIIHPESLCAQKLKMDHVMDVVVKSVNWICSRGLNHSEFTTLLYELDSQYGSLLYYTEIKWLSRGLVLKRFFESLEEIDSFMSSRGKPLPQLSSIDWIRDLAFLVDMTMHLNALNISLQGHSQIVTQMYDLIRAFLAKLCLWETHLTRNNLAHFPTLKLVSRNESDGLNYIPKIAELKTEFQKRLSDFKLYESELTLFSSPFSTKIDSVHEELQMEVIDLQCNTVLKTKYDKVGIPEFYKYLWGSYPKYKHHCAKILSMFGSTYICEQLFSIMKLSKTKYCSQLKDSQWDSVLHIAT

>Q14651

MENSTTTISREELEELQEAFNKIDIDNSGYVSDYELQDLFKEASLPLPGYKVREIVEKILSVADSNKDGKISFEEFVSLMQELKSKDISKTFRKIINKREGITAIGGTSTISSEGTQHSYSEEEKVAFVNWINKALENDPDCKHLIPMNPNDDSLFKSLADGILLCKMINLSEPDTIDERAINKKKLTPFTISENLNLALNSASAIGCTVVNIGASDLKEGKPHLVLGLLWQIIKVGLFADIEISRNEALIALLNEGEELEELMKLSPEELLLRWVNYHLTNAGWHTISNFSQDIKDSRAYFHLLNQIAPKGGEDGPAIAIDLSGINETNDLKRAGLMLQEADKLGCKQFVTPADVVSGNPKLNLAFVANLFNTYPCLHKPNNNDIDMNLLEGESKEERTFRNWMNSLGVNPYINHLYSDLADALVIFQLYEMIRVPVNWSHVNKPPYPALGGNMKKIENCNYAVELGKNKAKFSLVGIAGQDLNEGNSTLTLALVWQLMRRYTLNVLSDLGEGEKVNDEIIIKWVNQTLKSANKKTSISSFKDKSISTSLPVLDLIDAIAPNAVRQEMIRRENLSDEDKLNNAKYAISVARKIGARIYALPDDLVEVKPKMVMTVFACLMGKGLNRIK

546 NP_004134.1 P50135

>NP_004134.1

MPTTSRPALDVKGGTSPAKEDANQEMSSVAYSNLAVKDRKAVAILHYPGVASNGTKASGAPTSSSGSPIGSPTTTPPTKPPSFNLHPAPHLLASMQLQKLNSQYQGMAAATPGQPGEAGPLQNWDFGAQAGGAESLSPSAGAQSPAIIDSDPVDEEVLMSLVVELGLDRANELPELWLGQNEFDFTADFPSSC

>P50135

MASSMRSLFSDHGKYVESFRRFLNHSTEHQCMQEFMDKKLPGIIGRIGDTKSEIKILSIGGGAGEIDLQILSKVQAQYPGVCINNEVVEPSAEQIAKYKELVAKTSNLENVKFAWHKETSSEYQSRMLEKKELQKWDFIHMIQMLYYVKDIPATLKFFHSLLGTNAKMLIIVVSGSSGWDKLWKKYGSRFPQDDLCQYITSDDLTQMLDNLGLKYECYDLLSTMDISDCFIDGNENGDLLWDFLTETCNFNATAPPDLRAELGKDLQEPEFSAKKEGKVLFNNTLSFIVIEA

547 Q13356 NP_000662.3

>Q13356

MGKRQHQKDKMYITCAEYTHFYGGKKPDLPQTNFRRLPFDHCSLSLQPFVYPVCTPDGIVFDLLNIVPWLKKYGTNPSNGEKLDGRSLIKLNFSKNSEGKYHCPVLFTVFTNNTHIVAVRTTGNVYAYEAVEQLNIKAKNFRDLLTDEPFSRQDIITLQDPTNLDKFNVSNFYHVKNNMKIIDPDEEKAKQDPSYYLKNTNAETRETLQELYKEFKGDEILAATMKAPEKKKVDKLNAAHYSTGKVSASFTSTAMVPETTHEAAAIDEDVLRYQFVKKKGYVRLHTNKGDLNLELHCDLTPKTCENFIRLCKKHYYDGTIFHRSIRNFVIQGGDPTGTGTGGESYWGKPFKDEFRPNLSHTGRGILSMANSGPNSNRSQFFITFRSCAYLDKKHTIFGRVVGGFDVLTAMENVESDPKTDRPKEEIRIDATTVFVDPYEEADAQIAQERKTQLKVAPETKVKSSQPQAGSQGPQTFRQGVGKYINPAATKRAAEEEPSTSATVPMSKKKPSRGFGDFSSW

>NP_000662.3

MANEVIKCKAAVAWEAGKPLSIEEIEVAPPKAHEVRIKIIATAVCHTDAYTLSGADPEGCFPVILGHEGAGIVESVGEGVTKLKAGDTVIPLYIPQCGECKFCLNPKTNLCQKIRVTQGKGLMPDGTSRFTCKGKTILHYMGTSTFSEYTVVADISVAKIDPLAPLDKVCLLGCGISTGYGAAVNTAKLEPGSVCAVFGLGGVGLAVIMGCKVAGASRIIGVDINKDKFARAKEFGATECINPQDFSKPIQEVLIEMTDGGVDYSFECIGNVKVMRAALEACHKGWGVSVVVGVAASGEEIATRPFQLVTGRTWKGTAFGGWKSVESVPKLVSEYMSKKIKVDEFVTHNLSFDEINKAFELMHSGKSIRTVVKI

548 NP_060740.1 NP_077268.1

>NP_060740.1

MGEAEVGGGGAAGDKGPGEAATSPAEETVVWSPEVEVCLFHAMLGHKPVGVNRHFHMICIRDKFSQNIGRQVPSKVIWDHLSTMYDMQALHESEILPFPNPERNFVLPEEIIQEVREGKVMIEEEMKEEMKEDVDPHNGADDVFSSSGSLGKASEKSSKDKEKNSSDLGCKEGADKRKRSRVTDKVLTANSNPSSPSAAKRRRT

>NP_077268.1

MIEVVCNDRLGKKVRVKCNTDDTIGDLKKLIAAQTGTRWNKIVLKKWYTIFKDHVSLGDYEIHDGMNLELYYQ

549 NP_001281.1 Q13938

>NP_001281.1

MSSTPHDPFYSSPFGPFYRRHTPYMVQPEYRIYEMNKRLQSRTEDSDNLWWDAFATEFFEDDATLTLSFCLEDGPKRYTIGRTLIPRYFSTVFEGGVTDLYYILKHSKESYHNSSITVDCDQCTMVTQHGKPMFTKVCTEGRLILEFTFDDLMRIKTWHFTIRQYRELVPRSILAMHAQDPQVLDQLSKNITRMGLTNFTLNYLRLCVILEPMQELMSRHKTYNLSPRDCLKTCLFQKWQRMVAPPAEPTRQPTTKRRKRKNSTSSTSNSSAGNNANSTGSKKKTTAANLSLSSQVPDVMVVGEPTLMGGEFGDEDERLITRLENTQYDAANGMDDEEDFNNSPALGNNSPWNSKPPATQETKSENPPPQASQ

>Q13938

MDAVDATMEKLRAQCLSRGASGIQGLARFFRQLDRDGSRSLDADEFRQGLAKLGLVLDQAEAEGVCRKWDRNGSGTLDLEEFLRALRPPMSQAREAVIAAAFAKLDRSGDGVVTVDDLRGVYSGRAHPKVRSGEWTEDEVLRRFLDNFDSSEKDGQVTLAEFQDYYSGVSASMNTDEEFVAMMTSAWQL

550 NP_005312.1 Q969Y2

>NP_005312.1

MSETAPAAPAAPAPAEKTPVKKKARKSAGAAKRKASGPPVSELITKAVAASKERSGVSLAALKKALAAAGYDVEKNNSRIKLGLKSLVSKGTLVQTKGTGASGSFKLNKKAASGEAKPKAKKAGAAKAKKPAGAAKKPKKATGAATPKKSAKKTPKKAKKPAAAAGAKKAKSPKKAKAAKPKKAPKSPAKAKAVKPKAAKPKTAKPKAAKPKKAAAKKK

>Q969Y2

MWRGLWTLAAQAARGPRRLCTRRSSGAPAPGSGATIFALSSGQGRCGIAVIRTSGPASGHALRILTAPRDLPLARHASLRLLSDPRSGEPLDRALVLWFPGPQSFTGEDCVEFHVHGGPAVVSGVLQALGSVPGLRPAEAGEFTRRAFANGKLNLTEVEGLADLIHAETEAQRRQALRQLDGELGHLCRGWAETLTKALAHVEAYIDFGEDDNLEEGVLEQADIEVRALQVALGAHLRDARRGQRLRSGAHVVVTGPPNAGKSSLVNLLSRKPVSIVSPEPGTTRDVLETPVDLAGFPVLLSDTAGLREGVGPVEQEGVRRARERLEQADLILAMLDASDLASPSSCNFLATVVASVGAQSPSDSSQRLLLVLNKSDLLSPEGPGPGPDLPPHLLLSCLTGEGLDGLLEALRKELAAVCGDPSTDPPLLTRARHQHHLQGCLDALGHYKQSKDLALAAEALRVARGHLTRLTGGGGTEEILDIIFQDFCVGK

551 NP_003080.2 Q92665

>NP_003080.2

MTQFLPPNLLALFAPRDPIPYLPPLEKLPHEKHHNQPYCGIAPYIREFEDPRDAPPPTRAETREERMERKRREKIERRQQEVETELKMWDPHNDPNAQGDAFKTLFVARVNYDTTESKLRREFEVYGPIKRIHMVYSKRSGKPRGYAFIEYEHERDMHSAYKHADGKKIDGRRVLVDVERGRTVKGWRPRRLGGGLGGTRRGGADVNIRHSGRDDTSRYDERPGPSPLPHRDRDRDRERERRERSRERDKERERRRSRSRDRRRRSRSRDKEERRRSRERSKDKDRDRKRRSSRSRERARRERERKEELRGGGGDMAEPSEAGDAPPDDGPPGELGPDGPDGPEEKGRDRDRERRRSHRSERERRRDRDRDRDRDREHKRGERGSERGRDEARGGGGGQDNGLEGLGNDSRDMYMESEGGDGYLAPENGYLMEAAPE

>Q92665

MFPRVSTFLPLRPLSRHPLSSGSPETSAAAIMLLTVRHGTVRYRSSALLARTKNNIQRYFGTNSVICSKKDKQSVRTEETSKETSESQDSEKENTKKDLLGIIKGMKVELSTVNVRTTKPPKRRPLKSLEATLGRLRRATEYAPKKRIEPLSPELVAAASAVADSLPFDKQTTKSELLSQLQQHEEESRAQRDAKRPKISFSNIISDMKVARSATARVRSRPELRIQFDEGYDNYPGQEKTDDLKKRKNIFTGKRLNIFDMMAVTKEAPETDTSPSLWDVEFAKQLATVNEQPLQNGFEELIQWTKEGKLWEFPINNEAGFDDDGSEFHEHIFLEKHLESFPKQGPIRHFMELVTCGLSKNPYLSVKQKVEHIEWFRNYFNEKKDILKESNIQFN

552 P21675 P25325

>P21675

MGPGCDLLLRTAATITAAAIMSDTDSDEDSAGGGPFSLAGFLFGNINGAGQLEGESVLDDECKKHLAGLGALGLGSLITELTANEELTGTDGALVNDEGWVRSTEDAVDYSDINEVAEDESRRYQQTMGSLQPLCHSDYDEDDYDADCEDIDCKLMPPPPPPPGPMKKDKDQDSITGEKVDFSSSSDSESEMGPQEATQAESEDGKLTLPLAGIMQHDATKLLPSVTELFPEFRPGKVLRFLRLFGPGKNVPSVWRSARRKRKKKHRELIQEEQIQEVECSVESEVSQKSLWNYDYAPPPPPEQCLSDDEITMMAPVESKFSQSTGDIDKVTDTKPRVAEWRYGPARLWYDMLGVPEDGSGFDYGFKLRKTEHEPVIKSRMIEEFRKLEENNGTDLLADENFLMVTQLHWEDDIIWDGEDVKHKGTKPQRASLAGWLPSSMTRNAMAYNVQQGFAATLDDDKPWYSIFPIDNEDLVYGRWEDNIIWDAQAMPRLLEPPVLTLDPNDENLILEIPDEKEEATSNSPSKESKKESSLKKSRILLGKTGVIKEEPQQNMSQPEVKDPWNLSNDEYYYPKQQGLRGTFGGNIIQHSIPAVELRQPFFPTHMGPIKLRQFHRPPLKKYSFGALSQPGPHSVQPLLKHIKKKAKMREQERQASGGGEMFFMRTPQDLTGKDGDLILAEYSEENGPLMMQVGMATKIKNYYKRKPGKDPGAPDCKYGETVYCHTSPFLGSLHPGQLLQAFENNLFRAPIYLHKMPETDFLIIRTRQGYYIRELVDIFVVGQQCPLFEVPGPNSKRANTHIRDFLQVFIYRLFWKSKDRPRRIRMEDIKKAFPSHSESSIRKRLKLCADFKRTGMDSNWWVLKSDFRLPTEEEIRAMVSPEQCCAYYSMIAAEQRLKDAGYGEKSFFAPEEENEEDFQMKIDDEVRTAPWNTTRAFIAAMKGKCLLEVTGVADPTGCGEGFSYVKIPNKPTQQKDDKEPQPVKKTVTGTDADLRRLSLKNAKQLLRKFGVPEEEIKKLSRWEVIDVVRTMSTEQARSGEGPMSKFARGSRFSVAEHQERYKEECQRIFDLQNKVLSSTEVLSTDTDSSSAEDSDFEEMGKNIENMLQNKKTSSQLSREREEQERKELQRMLLAAGSAASGNNHRDDDTASVTSLNSSATGRCLKIYRTFRDEEGKEYVRCETVRKPAVIDAYVRIRTTKDEEFIRKFALFDEQHREEMRKERRRIQEQLRRLKRNQEKEKLKGPPEKKPKKMKERPDLKLKCGACGAIGHMRTNKFCPLYYQTNAPPSNPVAMTEEQEEELEKTVIHNDNEELIKVEGTKIVLGKQLIESADEVRRKSLVLKFPKQQLPPKKKRRVGTTVHCDYLNRPHKSIHRRRTDPMVTLSSILESIINDMRDLPNTYPFHTPVNAKVVKDYYKIITRPMDLQTLRENVRKRLYPSREEFREHLELIVKNSATYNGPKHSLTQISQSMLDLCDEKLKEKEDKLARLEKAINPLLDDDDQVAFSFILDNIVTQKMMAVPDSWPFHHPVNKKFVPDYYKVIVNPMDLETIRKNISKHKYQSRESFLDDVNLILANSVKYNGPESQYTKTAQEIVNVCYQTLTEYDEHLTQLEKDICTAKEAALEEAELESLDPMTPGPYTPQPPDLYDTNTSLSMSRDASVFQDESNMSVLDIPSATPEKQVTQEGEDGDGDLADEEEGTVQQPQASVLYEDLLMSEGEDDEEDAGSDEEGDNPFSAIQLSESGSDSDVGSGGIRPKQPRMLQENTRMDMENEESMMSYEGDGGEASHGLEDSNISYGSYEEPDPKSNTQDTSFSSIGGYEVSEEEEDEEEEEQRSGPSVLSQVHLSEDEEDSEDFHSIAGDSDLDSDE

>P25325

MASPQLCRALVSAQWVAEALRAPRAGQPLQLLDASWYLPKLGRDARREFEERHIPGAAFFDIDQCSDRTSPYDHMLPGAEHFAEYAGRLGVGAATHVVIYDASDQGLYSAPRVWWMFRAFGHHAVSLLDGGLRHWLRQNLPLSSGKSQPAPAEFRAQLDPAFIKTYEDIKENLESRRFQVVDSRATGRFRGTEPEPRDGIEPGHIPGTVNIPFTDFLSQEGLEKSPEEIRHLFQEKKVDLSKPLVATCGSGVTACHVALGAYLCGKPDVPIYDGSWVEWYMRARPEDVISEGRGKTH

553 Q99873 NP_036429.2

>Q99873

MENFVATLANGMSLQPPLEEVSCGQAESSEKPNAEDMTSKDYYFDSYAHFGIHEEMLKDEVRTLTYRNSMFHNRHLFKDKVVLDVGSGTGILCMFAAKAGARKVIGIECSSISDYAVKIVKANKLDHVVTIIKGKVEEVELPVEKVDIIISEWMGYCLFYESMLNTVLYARDKWLAPDGLIFPDRATLYVTAIEDRQYKDYKIHWWENVYGFDMSCIKDVAIKEPLVDVVDPKQLVTNACLIKEVDIYTVKVEDLTFTSPFCLQVKRNDYVHALVAYFNIEFTRCHKRTGFSTSPESPYTHWKQTVFYMEDYLTVKTGEEIFGTIGMRPNAKNNRDLDFTIDLDFKGQLCELSCSTDYRMR

>NP_036429.2

MVMEKPSPLLVGREFVRQYYTLLNKAPEYLHRFYGRNSSYVHGGVDASGKPQEAVYGQNDIHHKVLSLNFSECHTKIRHVDAHATLSDGVVVQVMGLLSNSGQPERKFMQTFVLAPEGSVPNKFYVHNDMFRYEDEVFGDSEPELDEESEDEVEEEQEERQPSPEPVQENANSGYYEAHPVTNGIEEPLEESSHEPEPEPESETKTEELKPQVEEKNLEELEEKSTTPPPAEPVSLPQEPPKAFSWASVTSKNLPPSGTVSSSGIPPHVKAPVSQPRVEAKPEVQSQPPRVREQRPRERPGFPPRGPRPGRGDMEQNDSDNRRIIRYPDSHQLFVGNLPHDIDENELKEFFMSFGNVVELRINTKGVGGKLPNFGFVVFDDSEPVQRILIAKPIMFRGEVRLNVEEKKTRAARERETRGGGDDRRDIRRNDRGPGGPRGIVGGGMMRDRDGRGPPPRGGMAQKLGSGRGTGQMEGRFTGQRR

554 NP_114432.1 P17516

>NP_114432.1

MSSMWSEYTIGGVKIYFPYKAYPSQLAMMNSILRGLNSKQHCLLESPTGSGKSLALLCSALAWQQSLSGKPADEGVSEKAEVQLSCCCACHSKDFTNNDMNQGTSRHFNYPSTPPSERNGTSSTCQDSPEKTTLAAKLSAKKQASIYRDENDDFQVEKKRIRPLETTQQIRKRHCFGTEVHNLDAKVDSGKTVKLNSPLEKINSFSPQKPPGHCSRCCCSTKQGNSQESSNTIKKDHTGKSKIPKIYFGTRTHKQIAQITRELRRTAYSGVPMTILSSRDHTCVHPEVVGNFNRNEKCMELLDGKNGKSCYFYHGVHKISDQHTLQTFQGMCKAWDIEELVSLGKKLKACPYYTARELIQDADIIFCPYNYLLDAQIRESMDLNLKEQVVILDEAHNIEDCARESASYSVTEVQLRFARDELDSMVNNNIRKKDHEPLRAVCCSLINWLEANAEYLVERDYESACKIWSGNEMLLTLHKMGITTATFPILQGHFSAVLQKEEKISPIYGKEEAREVPVISASTQIMLKGLFMVLDYLFRQNSRFADDYKIAIQQTYSWTNQIDISDKNGLLVLPKNKKRSRQKTAVHVLNFWCLNPAVAFSDINGKVQTIVLTSGTLSPMKSFSSELGVTFTIQLEANHIIKNSQVWVGTIGSGPKGRNLCATFQNTETFEFQDEVGALLLSVCQTVSQGILCFLPSYKLLEKLKERWLSTGLWHNLELVKTVIVEPQGGEKTNFDELLQVYYDAIKYKGEKDGALLVAVCRGKVSEGLDFSDDNARAVITIGIPFPNVKDLQVELKRQYNDHHSKLRGLLPGRQWYEIQAYRALNQALGRCIRHRNDWGALILVDDRFRNNPSRYISGLSKWVRQQIQHHSTFESALESLAEFSKKHQKVLNVSIKDRTNIQDNESTLEVTSLKYSTPPYLLEAASHLSPENFVEDEAKICVQELQCPKIITKNSPLPSSIISRKEKNDPVFLEEAGKAEKIVISRSTSPTFNKQTKRVSWSSFNSLGQYFTGKIPKATPELGSSENSASSPPRFKTEKMESKTVLPFTDKCESSNLTVNTSFGSCPQSETIISSLKIDATLTRKNHSEHPLCSEEALDPDIELSLVSEEDKQSTSNRDFETEAEDESIYFTPELYDPEDTDEEKNDLAETDRGNRLANNSDCILAKDLFEIRTIKEVDSAREVKAEDCIDTKLNGILHIEESKIDDIDGNVKTTWINELELGKTHEIEIKNFKPSPSKNKGMFPGFK

>P17516

MDPKYQRVELNDGHFMPVLGFGTYAPPEVPRNRAVEVTKLAIEAGFRHIDSAYLYNNEEQVGLAIRSKIADGSVKREDIFYTSKLWCTFFQPQMVQPALESSLKKLQLDYVDLYLLHFPMALKPGETPLPKDENGKVIFDTVDLSATWEVMEKCKDAGLAKSIGVSNFNCRQLEMILNKPGLKYKPVCNQVECHPYLNQSKLLDFCKSKDIVLVAHSALGTQRHKLWVDPNSPVLLEDPVLCALAKKHKRTPALIALRYQLQRGVVVLAKSYNEQRIRENIQVFEFQLTSEDMKVLDGLNRNYRYVVMDFLMDHPDYPFSDEY

555 NP_005162.1 NP_689953.1

>NP_005162.1

MEDSHKSTTSETAPQPGSAVQGAHISHIAQQVSSLSESEESQDSSDSIGSSQKAHGILARRPSYRKILKDLSSEDTRGRKGDGENSGVSAAVTSMSVPTPIYQTSSGQYIAIAPNGALQLASPGTDGVQGLQTLTMTNSGSTQQGTTILQYAQTSDGQQILVPSNQVVVQTASGDMQTYQIRTTPSATSLPQTVVMTSPVTLTSQTTKTDDPQLKREIRLMKNREAARECRRKKKEYVKCLENRVAVLENQNKTLIEELKTLKDLYSNKSV

>NP_689953.1

MAASLRLLGAASGLRYWSRRLRPAAGSFAAVCSRSVASKTPVGFIGLGNMGNPMAKNLMKHGYPLIIYDVFPDACKEFQDAGEQVVSSPADVAEKADRIITMLPTSINAIEAYSGANGILKKVKKGSLLIDSSTIDPAVSKELAKEVEKMGAVFMDAPVSGGVGAARSGNLTFMVGGVEDEFAAAQELLGCMGSNVVYCGAVGTGQAAKICNNMLLAISMIGTAEAMNLGIRLGLDPKLLAKILNMSSGRCWSSDTYNPVPGVMDGVPSANNYQGGFGTTLMAKDLGLAQDSATSTKSPILLGSLAHQIYRMMCAKGYSKKDFSSVFQFLREEETF

556 NP_000449.1 NP_003493.1

>NP_000449.1

MVSKLTSLQQELLSALLSSGVTKEVLVQALEELLPSPNFGVKLETLPLSPGSGAEPDTKPVFHTLTNGHAKGRLSGDEGSEDGDDYDTPPILKELQALNTEEAAEQRAEVDRMLSEDPWRAAKMIKGYMQQHNIPQREVVDVTGLNQSHLSQHLNKGTPMKTQKRAALYTWYVRKQREILRQFNQTVQSSGNMTDKSSQDQLLFLFPEFSQQSHGPGQSDDACSEPTNKKMRRNRFKWGPASQQILYQAYDRQKNPSKEEREALVEECNRAECLQRGVSPSKAHGLGSNLVTEVRVYNWFANRRKEEAFRQKLAMDAYSSNQTHSLNPLLSHGSPHHQPSSSPPNKLSGVRYSQQGNNEITSSSTISHHGNSAMVTSQSVLQQVSPASLDPGHNLLSPDGKMISVSGGGLPPVSTLTNIHSLSHHNPQQSQNLIMTPLSGVMAIAQSLNTSQAQSVPVINSVAGSLAALQPVQFSQQLHSPHQQPLMQQSPGSHMAQQPFMAAVTQLQNSHMYAHKQEPPQYSHTSRFPSAMVVTDTSSISTLTNMSSSKQCPLQAW

>NP_003493.1

MNIQEQGFPLDLGASFTEDAPRPPVPGEEGELVSTDPRPASYSFCSGKGVGIKGETSTATPRRSDLDLGYEPEGSASPTPPYLKWAESLHSLLDDQDGISLFRTFLKQEGCADLLDFWFACTGFRKLEPCDSNEEKRLKLARAIYRKYILDNNGIVSRQTKPATKSFIKGCIMKQLIDPAMFDQAQTEIQATMEENTYPSFLKSDIYLEYTRTGSESPKVCSDQSSGSGTGKGISGYLPTLNEDEEWKCDQDMDEDDGRDAAPPGRLPQKLLLETAAPRVSSSRRYSEGREFRYGSWREPVNPYYVNAGYALAPATSANDSEQQSLSSDADTLSLTDSSVDGIPPYRIRKQHRREMQESVQVNGRVPLPHIPRTYRVPKEVRVEPQKFAEELIHRLEAVQRTREAEEKLEERLKRVRMEEEGEDGDPSSGPPGPCHKLPPAPAWHHFPPRCVDMGCAGLRDAHEENPESILDEHVQRVLRTPGRQSPGPGHRSPDSGHVAKMPVALGGAASGHGKHVPKSGAKLDAAGLHHHRHVHHHVHHSTARPKEQVEAEATRRAQSSFAWGLEPHSHGARSRGYSESVGAAPNASDGLAHSGKVGVACKRNAKKAESGKSASTEVPGASEDAEKNQKIMQWIIEGEKEISRHRRTGHGSSGTRKPQPHENSRPLSLEHPWAGPQLRTSVQPSHLFIQDPTMPPHPAPNPLTQLEEARRRLEEEEKRASRAPSKQRYVQEVMRRGRACVRPACAPVLHVVPAVSDMELSETETRSQRKVGGGSAQPCDSIVVAYYFCGEPIPYRTLVRGRAVTLGQFKELLTKKGSYRYYFKKVSDEFDCGVVFEEVREDEAVLPVFEEKIIGKVEKVD

557 Q99697 NP_056449.1

>Q99697

METNCRKLVSACVQLGVQPAAVECLFSKDSEIKKVEFTDSPESRKEAASSKFFPRQHPGANEKDKSQQGKNEDVGAEDPSKKKRQRRQRTHFTSQQLQELEATFQRNRYPDMSTREEIAVWTNLTEARVRVWFKNRRAKWRKRERNQQAELCKNGFGPQFNGLMQPYDDMYPGYSYNNWAAKGLTSASLSTKSFPFFNSMNVNPLSSQSMFSPPNSISSMSMSSSMVPSAVTGVPGSSLNSLNNLNNLSSPSLNSAVPTPACPYAPPTPPYVYRDTCNSSLASLRLKAKQHSSFGYASVQNPASNLSACQYAVDRPV

>NP_056449.1

MANVPWAEVCEKFQAALALSRVELHKNPEKEPYKSKYSARALLEEVKALLGPAPEDEDERPEAEDGPGAGDHALGLPAEVVEPEGPVAQRAVRLAVIEFHLGVNHIDTEELSAGEEHLVKCLRLLRRYRLSHDCISLCIQAQNNLGILWSEREEIETAQAYLESSEALYNQYMKEVGSPPLDPTERFLPEEEKLTEQERSKRFEKVYTHNLYYLAQVYQHLEMFEKAAHYCHSTLKRQLEHNAYHPIEWAINAATLSQFYINKLCFMEARHCLSAANVIFGQTGKISATEDTPEAEGEVPELYHQRKGEIARCWIKYCLTLMQNAQLSMQDNIGELDLDKQSELRALRKKELDEEESIRKKAVQFGTGELCDAISAVEEKVSYLRPLDFEEARELFLLGQHYVFEAKEFFQIDGYVTDHIEVVQDHSALFKVLAFFETDMERRCKMHKRRIAMLEPLTVDLNPQYYLLVNRQIQFEIAHAYYDMMDLKVAIADRLRDPDSHIVKKINNLNKSALKYYQLFLDSLRDPNKVFPEHIGEDVLRPAMLAKFRVARLYGKIITADPKKELENLATSLEHYKFIVDYCEKHPEAAQEIEVELELSKEMVSLLPTKMERFRTKMALT

558 Q8WV37 NP_036199.2

>Q8WV37

MALPQGHLTFRDVAIEFSQAEWKCLDPAQRALYKDVMLENYRNLVSLGISLPDLNINSMLEQRREPWSGESEVKIAKNSDGRECIKGVNTGSSYALGSNAEDKPIKKQLGVSFHLHLSELELFPDERVINGCNQVENFINHSSSVSCLQEMSSSVKTPIFNRNDFDDSSFLPQEQKVHLREKPYECNEHSKVFRVSSSLTKHQVIHTVEKPYKCNSCGKVFSRNSHLAEHCRIHTGEKPYKCNVCGKVFSYNSNFARHQRIHTREKPYECNECGKVFSNNSYLARHQRIHAEEKPYKCNECGKGFSHKSSLANHWRIYTGEKPYKCDECGKAFYRIALLVRHQKIHTGEKPYKCNECGKVFIQNSHLAQHWRIHTGEKPYKCNECGKVFNQLSNLARHRRIHTGEKPYKCNECGKAFSEYSGLSAHLVIHTGEKPYKCSECGKAFRHKLSLTNHQRIHTGERPYKCNECGKVFNRIAHLARHRKIHTGEKPYKCNECGKAFSRISYLAQHWTIHMG

>NP_036199.2

MSRQLSRARPATVLGAMEMGRRMDAPTSAAVTRAFLERGHTEIDTAFVYSEGQSETILGGLGLRLGGSDCRVKIDTKAIPLFGNSLKPDSLRFQLETSLKRLQCPRVDLFYLHMPDHSTPVEETLRACHQLHQEGKFVELGLSNYAAWEVAEICTLCKSNGWILPTVYQGMYNAITRQVETELFPCLRHFGLRFYAFNPLAGGLLTGKYKYEDKNGKQPVGRFFGNTWAEMYRNRYWKEHHFEGIALVEKALQAAYGASAPSMTSATLRWMYHHSQLQGAHGDAVILGMSSLEQLEQNLAAAEEGPLEPAVVDAFNQAWHLVTHECPNYFR

559 P09017 Q9Y4B6

>P09017

MIMSSYLMDSNYIDPKFPPCEEYSQNSYIPEHSPEYYGRTRESGFQHHHQELYPPPPPRPSYPERQYSCTSLQGPGNSRGHGPAQAGHHHPEKSQSLCEPAPLSGASASPSPAPPACSQPAPDHPSSAASKQPIVYPWMKKIHVSTVNPNYNGGEPKRSRAAYTRQQVLELEKEFHYNRYLTRRRRIEIAHSLCLSERQIKIWFQNRRMKWKKDHRLPNTKVRSAPPAGAAPSTLSAATPGTSEDHSQSATPPEQQRAEDITRL

>Q9Y4B6

MTTVVVHVDSKAELTTLLEQWEKEHGSGQDMVPILTRMSQLIEKETEEYRKGDPDPFDDRHPGRADPECMLGHLLRILFKNDDFMNALVNAYVMTSREPPLNTAACRLLLDIMPGLETAVVFQEKEGIVENLFKWAREADQPLRTYSTGLLGGAMENQDIAANYRDENSQLVAIVLRRLRELQLQEVALRQENKRPSPRKLSSEPLLPLDEEAVDMDYGDMAVDVVDGDQEEASGDMEISFHLDSGHKTSSRVNSTTKPEDGGLKKNKSAKQGDRENFRKAKQKLGFSSSDPDRMFVELSNSSWSEMSPWVIGTNYTLYPMTPAIEQRLILQYLTPLGEYQELLPIFMQLGSRELMMFYIDLKQTNDVLLTFEALKHLASLLLHNKFATEFVAHGGVQKLLEIPRPSMAATGVSMCLYYLSYNQDAMERVCMHPHNVLSDVVNYTLWLMECSHASGCCHATMFFSICFSFRAVLELFDRYDGLRRLVNLISTLEILNLEDQGALLSDDEIFASRQTGKHTCMALRKYFEAHLAIKLEQVKQSLQRTEGGILVHPQPPYKACSYTHEQIVEMMEFLIEYGPAQLYWEPAEVFLKLSCVQLLLQLISIACNWKTYYARNDTVRFALDVLAILTVVPKIQLQLAESVDVLDEAGSTVSTVGISIILGVAEGEFFIHDAEIQKSALQIIINCVCGPDNRISSIGKFISGTPRRKLPQNPKSSEHTLAKMWNVVQSNNGIKVLLSLLSIKMPITDADQIRALACKALVGLSRSSTVRQIISKLPLFSSCQIQQLMKEPVLQDKRSDHVKFCKYAAELIERVSGKPLLIGTDVSLARLQKADVVAQSRISFPEKELLLLIRNHLISKGLGETATVLTKEADLPMTAASHSSAFTPVTAAASPVSLPRTPRIANGIATRLGSHAAVGASAPSAPTAHPQPRPPQGPLALPGPSYAGNSPLIGRISFIRERPSPCNGRKIRVLRQKSDHGAYSQSPAIKKQLDRHLPSPPTLDSIITEYLREQHARCKNPVATCPPFSLFTPHQCPEPKQRRQAPINFTSRLNRRASFPKYGGVDGGCFDRHLIFSRFRPISVFREANEDESGFTCCAFSARERFLMLGTCTGQLKLYNVFSGQEEASYNCHNSAITHLEPSRDGSLLLTSATWSQPLSALWGMKSVFDMKHSFTEDHYVEFSKHSQDRVIGTKGDIAHIYDIQTGNKLLTLFNPDLANNYKRNCATFNPTDDLVLNDGVLWDVRSAQAIHKFDKFNMNISGVFHPNGLEVIINTEIWDLRTFHLLHTVPALDQCRVVFNHTGTVMYGAMLQADDEDDLMEERMKSPFGSSFRTFNATDYKPIATIDVKRNIFDLCTDTKDCYLAVIENQGSMDALNMDTVCRLYEVGRQRLAEDEDEEEDQEEEEQEEEDDDEDDDDTDDLDELDTDQLLEAELEEDDNNENAGEDGDNDFSPSDEELANLLEEGEDGEDEDSDADEEVELILGDTDSSDNSDLEDDIILSLNE

560 NP_004917.2 O43182

>NP_004917.2

MTTTLVSATIFDLSEVLCKGNKMLNYSAPSAGGCLLDRKAVGTPAGGGFPRRHSVTLPSSKFHQNQLLSSLKGEPAPALSSRDSRFRDRSFSEGGERLLPTQKQPGGGQVNSSRYKTELCRPFEENGACKYGDKCQFAHGIHELRSLTRHPKYKTELCRTFHTIGFCPYGPRCHFIHNAEERRALAGARDLSADRPRLQHSFSFAGFPSAAATAAATGLLDSPTSITPPPILSADDLLGSPTLPDGTNNPFAFSSQELASLFAPSMGLPGGGSPTTFLFRPMSESPHMFDSPPSPQDSLSDQEGYLSSSSSSHSGSDSPTLDNSRRLPIFSRLSISDD

>O43182

MSAQSLLHSVFSCSSPASSSAASAKGFSKRKLRQTRSLDPALIGGCGSDEAGAEGSARGATAGRLYSPSLPAESLGPRLASSSRGPPPRATRLPPPGPLCSSFSTPSTPQEKSPSGSFHFDYEVPLGRGGLKKSMAWDLPSVLAGPASSRSASSILCSSGGGPNGIFASPRRWLQQRKFQSPPDSRGHPYVVWKSEGDFTWNSMSGRSVRLRSVPIQSLSELERARLQEVAFYQLQQDCDLSCQITIPKDGQKRKKSLRKKLDSLGKEKNKDKEFIPQAFGMPLSQVIANDRAYKLKQDLQRDEQKDASDFVASLLPFGNKRQNKELSSSNSSLSSTSETPNESTSPNTPEPAPRARRRGAMSVDSITDLDDNQSRLLEALQLSLPAEAQSKKEKARDKKLSLNPIYRQVPRLVDSCCQHLEKHGLQTVGIFRVGSSKKRVRQLREEFDRGIDVSLEEEHSVHDVAALLKEFLRDMPDPLLTRELYTAFINTLLLEPEEQLGTLQLLIYLLPPCNCDTLHRLLQFLSIVARHADDNISKDGQEVTGNKMTSLNLATIFGPNLLHKQKSSDKEFSVQSSARAEESTAIIAVVQKMIENYEALFMVPPDLQNEVLISLLETDPDVVDYLLRRKASQSSSPDMLQSEVSFSVGGRHSSTDSNKASSGDISPYDNNSPVLSERSLLAMQEDAAPGGSEKLYRVPGQFMLVGHLSSSKSRESSPGPRLGKDLSEEPFDIWGTWHSTLKSGSKDPGMTGSSGDIFESSSLRAGPCSLSQGNLSPNWPRWQGSPAELDSDTQGARRTQAAAPATEGRAHPAVSRACSTPHVQVAGKAERPTARSEQYLTLSGAHDLSESELDVAGLQSRATPQCQRPHGSGRDDKRPPPPYPGPGKPAAAAAWIQGPPEGVETPTDQGGQAAEREQQVTQKKLSSANSLPAGEQDSPRLGDAGWLDWQRERWQIWELLSTDNPDALPETLV

561 NP_001942.2 Q8TAK5

>NP_001942.2

MAAAEPASSGQQAPAGQGQGQRPPPQPPQAQAPQPPPPPQLGGAGGGSSRHEKSLGLLTTKFVSLLQEAKDGVLDLKAAADTLAVRQKRRIYDITNVLEGIDLIEKKSKNSIQWKGVGAGCNTKEVIDRLRYLKAEIEDLELKERELDQQKLWLQQSIKNVMDDSINNRFSYVTHEDICNCFNGDTLLAIQAPSGTQLEVPIPEMGQNGQKKYQINLKSHSGPIHVLLINKESSSSKPVVFPVPPPDDLTQPSSQSLTPVTPQKSSMATQNLPEQHVSERSQALQQTSATDISSAGSISGDIIDELMSSDVFPLLRLSPTPADDYNFNLDDNEGVCDLFDVQILNY

>Q8TAK5

MSLVDLGKRLLEAARKGQDDEVRTLMANGAPFTTDWLGTSPLHLAAQYGHYSTAEVLLRAGVSRDARTKVDRTPLHMAAADGHAHIVELLVRNGADVNAKDMLKMTALHWATERHHRDVVELLIKYGADVHAFSKFDKSAFDIALEKNNAEILVILQEAMQNQVNVNPERANPVTDPVSMAAPFIFTSGEVVNLASLISSTNTKTTSGDPHASTVQFSNSTTSVLATLAALAEASVPLSNSHRATANTEEIIEGNSVDSSIQQVMGSGGQRVITIVTDGVPLGNIQTSIPTGGIGQPFIVTVQDGQQVLTVPAGKVAEETVIKEEEEEKLPLTKKPRIGEKTNSVEESKEGNERELLQQQLQEANRRAQEYRHQLLKKEQEAEQYRLKLEAIARQQPNGVDFTMVEEVAEVDAVVVTEGELEERETKVTGSAGTTEPHTRVSMATVSS

562 NP_065988.1 NP_001893.2

>NP_065988.1

MSGRQRTLFQTWGSSISRSSGTPGCSSGTERPQSPGSSKAPLPAAAEAQLESDDDVLLVAAYEAERQLCLENGGFCTSAGALWIYPTNCPVRDYQLHISRAALFCNTLVCLPTGLGKTFIAAVVMYNFYRWFPSGKVVFMAPTKPLVTQQIEACYQVMGIPQSHMAEMTGSTQASTRKEIWCSKRVLFLTPQVMVNDLSRGACPAAEIKCLVIDEAHKALGNYAYCQVVRELVKYTNHFRILALSATPGSDIKAVQQVITNLLIGQIELRSEDSPDILTYSHERKVEKLIVPLGEELAAIQKTYIQILESFARSLIQRNVLMRRDIPNLTKYQIILARDQFRKNPSPNIVGIQQGIIEGEFAICISLYHGYELLQQMGMRSLYFFLCGIMDGTKGMTRSKNELGRNEDFMKLYNHLECMFARTRSTSANGISAIQQGDKNKKFVYSHPKLKKLEEVVIEHFKSWNAENTTEKKRDETRVMIFSSFRDSVQEIAEMLSQHQPIIRVMTFVGHASGKSTKGFTQKEQLEVVKQFRDGGYNTLVSTCVGEEGLDIGEVDLIICFDSQKSPIRLVQRMGRTGRKRQGRIVIILSEGREERIYNQSQSNKRSIYKAISSNRQVLHFYQRSPRMVPDGINPKLHKMFITHGVYEPEKPSRNLQRKSSIFSYRDGMRQSSLKKDWFLSEEEFKLWNRLYRLRDSDEIKEITLPQVQFSSLQNEENKPAQESTTGIHQLSLSEWRLWQDHPLPTHQVDHSDRCRHFIGLMQMIEGMRHEEGECSYELEVESYLQMEDVTSTFIAPRNESNNLASDTFITHKKSSFIKNINQGSSSSVIESDEECAEIVKQTHIKPTKIVSLKKKVSKEIKKDQLKKENNHGIIDSVDNDRNSTVENIFQEDLPNDKRTSDTDEIAATCTINENVIKEPCVLLTECQFTNKSTSSLAGNVLDSGYNSFNDEKSVSSNLFLPFEEELYIVRTDDQFYNCHSLTKEVLANVERFLSYSPPPLSGLSDLEYEIAKGTALENLLFLPCAEHLRSDKCTCLLSHSAVNSQQNLELNSLKCINYPSEKSCLYDIPNDNISDEPSLCDCDVHKHNQNENLVPNNRVQIHRSPAQNLVGENNHDVDNSDLPVLSTDQDESLLLFEDVNTEFDDVSLSPLNSKSESLPVSDKTAISETPLVSQFLISDELLLDNNSELQDQITRDANSFKSRDQRGVQEEKVKNHEDIFDCSRDLFSVTFDLGFCSPDSDDEILEHTSDSNRPLDDLYGRYLEIKEISDANYVSNQALIPRDHSKNFTSGTVIIPSNEDMQNPNYVHLPLSAAKNEELLSPGYSQFSLPVQKKVMSTPLSKSNTLNSFSKIRKEILKTPDSSKEKVNLQRFKEALNSTFDYSEFSLEKSKSSGPMYLHKSCHSVEDGQLLTSNESEDDEIFRRKVKRAKGNVLNSPEDQKNSEVDSPLHAVKKRRFPINRSELSSSDESENFPKPCSQLEDFKVCNGNARRGIKVPKRQSHLKHVARKFLDDEAELSEEDAEYVSSDENDESENEQDSSLLDFLNDETQLSQAINDSEMRAIYMKSLRSPMMNNKYKMIHKTHKNINIFSQIPEQDETYLEDSFCVDEEESCKGQSSEEEVCVDFNLITDDCFANSKKYKTRRAVMLKEMMEQNCAHSKKKLSRIILPDDSSEEENNVNDKRESNIAVNPSTVKKNKQQDHCLNSVPSGSSAQSKVRSTPRVNPLAKQSKQTSLNLKDTISEVSDFKPQNHNEVQSTTPPFTTVDSQKDCRKFPVPQKDGSALEDSSTSGASCSKSRPHLAGTHTSLRLPQEGKGTCILVGGHEITSGLEVISSLRAIHGLQVEVCPLNGCDYIVSNRMVVERRSQSEMLNSVNKNKFIEQIQHLQSMFERICVIVEKDREKTGDTSRMFRRTKSYDSLLTTLIGAGIRILFSSCQEETADLLKELSLVEQRKNVGIHVPTVVNSNKSEALQFYLSIPNISYITALNMCHQFSSVKRMANSSLQEISMYAQVTHQKAEEIYRYIHYVFDIQMLPNDLNQDRLKSDI

>NP_001893.2

MQEKDASSQGFLPHFQHFATQAIHVGQDPEQWTSRAVVPPISLSTTFKQGAPGQHSGFEYSRSGNPTRNCLEKAVAALDGAKYCLAFASGLAATVTITHLLKAGDQIICMDDVYGGTNRYFRQVASEFGLKISFVDCSKIKLLEAAITPETKLVWIETPTNPTQKVIDIEGCAHIVHKHGDIILVVDNTFMSPYFQRPLALGADISMYSATKYMNGHSDVVMGLVSVNCESLHNRLRFLQNSLGAVPSPIDCYLCNRGLKTLHVRMEKHFKNGMAVAQFLESNPWVEKVIYPGLPSHPQHELVKRQCTGCTGMVTFYIKGTLQHAEIFLKNLKLFTLAESLGGFESLAELPAIMTHASVLKNDRDVLGISDTLIRLSVGLEDEEDLLEDLDQALKAAHPPSGSHS

563 Q14863 Q9Y3E5

>Q14863

MPGISSQILTNAQGQVIGTLPWVVNSASVAAPAPAQSLQVQAVTPQLLLNAQGQVIATLASSPLPPPVAVRKPSTPESPAKSEVQPIQPTPTVPQPAVVIASPAPAAKPSASAPIPITCSETPTVSQLVSKPHTPSLDEDGINLEEIREFAKNFKIRRLSLGLTQTQVGQALTATEGPAYSQSAICRFEKLDITPKSAQKLKPVLEKWLNEAELRNQEGQQNLMEFVGGEPSKKRKRRTSFTPQAIEALNAYFEKNPLPTGQEITEIAKELNYDREVVRVWFCNRRQTLKNTSKLNVFQIP

>Q9Y3E5

MPSKSLVMEYLAHPSTLGLAVGVACGMCLGWSLRVCFGMLPKSKTSKTHTDTESEASILGDSGEYKMILVVRNDLKMGKGKVAAQCSHAAVSAYKQIQRRNPEMLKQWEYCGQPKVVVKAPDEETLIALLAHAKMLGLTVSLIQDAGRTQIAPGSQTVLGIGPGPADLIDKVTGHLKLY

564 NP_002936.1 NP_037399.2

>NP_002936.1

MVGQLSEGAIAAIMQKGDTNIKPILQVINIRPITTGNSPPRYRLLMSDGLNTLSSFMLATQLNPLVEEEQLSSNCVCQIHRFIVNTLKDGRRVVILMELEVLKSAEAVGVKIGNPVPYNEGLGQPQVAPPAPAASPAASSRPQPQNGSSGMGSTVSKAYGASKTFGKAAGPSLSHTSGGTQSKVVPIASLTPYQSKWTICARVTNKSQIRTWSNSRGEGKLFSLELVDESGEIRATAFNEQVDKFFPLIEVNKVYYFSKGTLKIANKQFTAVKNDYEMTFNNETSVMPCEDDHHLPTVQFDFTGIDDLENKSKDSLVDIIGICKSYEDATKITVRSNNREVAKRNIYLMDTSGKVVTATLWGEDADKFDGSRQPVLAIKGARVSDFGGRSLSVLSSSTIIANPDIPEAYKLRGWFDAEGQALDGVSISDLKSGGVGGSNTNWKTLYEVKSENLGQGDKPDYFSSVATVVYLRKENCMYQACPTQDCNKKVIDQQNGLYRCEKCDTEFPNFKYRMILSVNIADFQENQWVTCFQESAEAILGQNAAYLGELKDKNEQAFEEVFQNANFRSFIFRVRVKVETYNDESRIKATVMDVKPVDYREYGRRLVMSIRRSALM

>NP_037399.2

MRSMKALQKALSRAGSHCGRGGWGHPSRSPLLGGGVRHHLSEAAAQGRETPHSHQPQHQDHDSSESGMLSRLGDLLFYTIAEGQERIPIHKFTTALKATGLQTSDPRLRDCMSEMHRVVQESSSGGLLDRDLFRKCVSSNIVLLTQAFRKKFVIPDFEEFTGHVDRIFEDVKELTGGKVAAYIPQLAKSNPDLWGVSLCTVDGQRHSVGHTKIPFCLQSCVKPLTYAISISTLGTDYVHKFVGKEPSGLRYNKLSLNEEGIPHNPMVNAGAIVVSSLIKMDCNKAEKFDFVLQYLNKMAGNEYMGFSNATFQSEKETGDRNYAIGYYLKEKKCFPKGVDMMAALDLYFQLCSVEVTCESGSVMAATLANGGICPITGESVLSAEAVRNTLSLMHSCGMYDFSGQFAFHVGLPAKSAVSGAILLVVPNVMGMMCLSPPLDKLGNSHRGTSFCQKLVSLFNFHNYDNLRHCARKLDPRREGAEIRNKTVVNLLFAAYSGDVSALRRFALSAMDMEQKDYDSRTALHVAAAEGHIEVVKFLIEACKVNPFAKDRWGNIPLDDAVQFNHLEVVKLLQDYQDSYTLSETQAEAAAEALSKENLESMV

565 Q9Y2V3 Q96M27

>Q9Y2V3

MHLPGCAPAMADGSFSLAGHLLRSPGGSTSRLHSIEAILGFTKDDGILGTFPAERGARGAKERDRRLGARPACPKAPEEGSEPSPPPAPAPAPEYEAPRPYCPKEPWEARPSPGLPVGPATGEAKLSEEEQPKKKHRRNRTTFTTYQLHELERAFEKSHYPDVYSREELAGKVNLPEVRVQVWFQNRRAKWRRQEKLEVSSMKLQDSPLLSFSRSPPSATLSPLGAGPGSGGGPAGGALPLESWLGPPLPGGGATALQSLPGFGPPAQSLPASYTPPPPPPPFLNSPPLGPGLQPLAPPPPSYPCGPGFGDKFPLDEADPRNSSIAALRLKAKEHIQAIGKPWQAL

>Q96M27

MMEESGIETTPPGTPPPNPAGLAATAMSSTPVPLAATSSFSSPNVSSMESFPPLAYSTPQPPLPPVRPSAPLPFVPPPAVPSVPPLVTSMPPPVSPSTAAAFGNPPVSHFPPSTSAPNTLLPAPPSGPPISGFSVGSTYDITRGHAGRAPQTPLMPSFSAPSGTGLLPTPITQQASLTSLAQGTGTTSAITFPEEQEDPRITRGQDEASAGGIWGFIKGVAGNPMVKSVLDKTKHSVESMITTLDPGMAPYIKSGGELDIVVTSNKEVKVAAVRDAFQEVFGLAVVVGEAGQSNIAPQPVGYAAGLKGAQERIDSLRRTGVIHEKQTAVSVENFIAELLPDKWFDIGCLVVEDPVHGIHLETFTQATPVPLEFVQQAQSLTPQDYNLRWSGLLVTVGEVLEKSLLNVSRTDWHMAFTGMSRRQMIYSAARAIAGMYKQRLPPRTV

566 NP_005048.2 Q9NR19

>NP_005048.2

MNLELLESFGQNYPEEADGTLDCISMALTCTFNRWGTLLAVGCNDGRIVIWDFLTRGIAKIISAHIHPVCSLCWSRDGHKLVSASTDNIVSQWDVLSGDCDQRFRFPSPILKVQYHPRDQNKVLVCPMKSAPVMLTLSDSKHVVLPVDDDSDLNVVASFDRRGEYIYTGNAKGKILVLKTDSQDLVASFRVTTGTSNTTAIKSIEFARKGSCFLINTADRIIRVYDGREILTCGRDGEPEPMQKLQDLVNRTPWKKCCFSGDGEYIVAGSARQHALYIWEKSIGNLVKILHGTRGELLLDVAWHPVRPIIASISSGVVSIWAQNQVENWSAFAPDFKELDENVEYEERESEFDIEDEDKSEPEQTGADAAEDEEVDVTSVDPIAAFCSSDEELEDSKALLYLPIAPEVEDPEENPYGPPPDAVQTSLMDEGASSEKKRQSSADGSQPPKKKPKTTNIELQGVPNDEVHPLLGVKGDGKSKKKQAGRPKGSKGKEKDSPFKPKLYKGDRGLPLEGSAKGKVQAELSQPLTAGGAISELL

>Q9NR19

MGLPEERVRSGSGSRGQEEAGAGGRARSWSPPPEVSRSAHVPSLQRYRELHRRSVEEPREFWGDIAKEFYWKTPCPGPFLRYNFDVTKGKIFIEWMKGATTNICYNVLDRNVHEKKLGDKVAFYWEGNEPGETTQITYHQLLVQVCQFSNVLRKQGIQKGDRVAIYMPMIPELVVAMLACARIGALHSIVFAGFSSESLCERILDSSCSLLITTDAFYRGEKLVNLKELADEALQKCQEKGFPVRCCIVVKHLGRAELGMGDSTSQSPPIKRSCPDVQISWNQGIDLWWHELMQEAGDECEPEWCDAEDPLFILYTSGSTGKPKGVVHTVGGYMLYVATTFKYVFDFHAEDVFWCTADIGWITGHSYVTYGPLANGATSVLFEGIPTYPDVNRLWSIVDKYKVTKFYTAPTAIRLLMKFGDEPVTKHSRASLQVLGTVGEPINPEAWLWYHRVVGAQRCPIVDTFWQTETGGHMLTPLPGATPMKPGSATFPFFGVAPAILNESGEELEGEAEGYLVFKQPWPGIMRTVYGNHERFETTYFKKFPGYYVTGDGCQRDQDGYYWITGRIDDMLNVSGHLLSTAEVESALVEHEAVAEAAVVGHPHPVKGECLYCFVTLCDGHTFSPKLTEELKKQIREKIGPIATPDYIQNAPGLPKTRSGKIMRRVLRKIAQNDHDLGDMSTVADPSVISHLFSHRCLTIQ

567 Q15170 NP_004623.1

>Q15170

MDKPRKENEEEPQSRPRPMRRGLRWSTLPKSSPPRSSLRRSSPRRRSSFLRSSCLSSCLRCSSRRTPSAGLSRKDLFEGRPPMEQPPCGVGKHKLEEGSFKERLARSRPQFRGDIHGRNLSNEEMIQAADELEEMKRVRNKLMIMHWKAKRSRPYPI

>NP_004623.1

MMLKGITRLISRIHKLDPGRFLHMGTQARQSIAAHLDNQVPVESPRAISRTNENDPAKHGDQHEGQHYNISPQDLETVFPHGLPPRFVMQVKTFSEACLMVRKPALELLHYLKNTSFAYPAIRYLLYGEKGTGKTLSLCHVIHFCAKQDWLILHIPDAHLWVKNCRDLLQSSYNKQRFDQPLEASTWLKNFKTTNERFLNQIKVQEKYVWNKRESTEKGSPLGEVVEQGITRVRNATDAVGIVLKELKRQSSLGMFHLLVAVDGINALWGRTTLKREDKSPIAPEELALVHNLRKMMKNDWHGGAIVSALSQTGSLFKPRKAYLPQELLGKEGFDALDPFIPILVSNYNPKEFESCIQYYLENNWLQHEKAPTEEGKKELLFLSNANPSLLERHCAYL

568 NP_000391.1 NP_000174.1

>NP_000391.1

MKLNVDGLLVYFPYDYIYPEQFSYMRELKRTLDAKGHGVLEMPSGTGKTVSLLALIMAYQRAYPLEVTKLIYCSRTVPEIEKVIEELRKLLNFYEKQEGEKLPFLGLALSSRKNLCIHPEVTPLRFGKDVDGKCHSLTASYVRAQYQHDTSLPHCRFYEEFDAHGREVPLPAGIYNLDDLKALGRRQGWCPYFLARYSILHANVVVYSYHYLLDPKIADLVSKELARKAVVVFDEAHNIDNVCIDSMSVNLTRRTLDRCQGNLETLQKTVLRIKETDEQRLRDEYRRLVEGLREASAARETDAHLANPVLPDEVLQEAVPGSIRTAEHFLGFLRRLLEYVKWRLRVQHVVQESPPAFLSGLAQRVCIQRKPLRFCAERLRSLLHTLEITDLADFSPLTLLANFATLVSTYAKGFTIIIEPFDDRTPTIANPILHFSCMDASLAIKPVFERFQSVIITSGTLSPLDIYPKILDFHPVTMATFTMTLARVCLCPMIIGRGNDQVAISSKFETREDIAVIRNYGNLLLEMSAVVPDGIVAFFTSYQYMESTVASWYEQGILENIQRNKLLFIETQDGAETSVALEKYQEACENGRGAILLSVARGKVSEGIDFVHHYGRAVIMFGVPYVYTQSRILKARLEYLRDQFQIRENDFLTFDAMRHAAQCVGRAIRGKTDYGLMVFADKRFARGDKRGKLPRWIQEHLTDANLNLTVDEGVQVAKYFLRQMAQPFHREDQLGLSLLSLEQLESEETLKRIEQIAQQL

>NP_000174.1

MTILTYPFKNLPTASKWALRFSIRPLSCSSQLRAAPAVQTKTKKTLAKPNIRNVVVVDGVRTPFLLSGTSYKDLMPHDLARAALTGLLHRTSVPKEVVDYIIFGTVIQEVKTSNVAREAALGAGFSDKTPAHTVTMACISANQAMTTGVGLIASGQCDVIVAGGVELMSDVPIRHSRKMRKLMLDLNKAKSMGQRLSLISKFRFNFLAPELPAVSEFSTSETMGHSADRLAAAFAVSRLEQDEYALRSHSLAKKAQDEGLLSDVVPFKVPGKDTVTKDNGIRPSSLEQMAKLKPAFIKPYGTVTAANSSFLTDGASAMLIMAEEKALAMGYKPKAYLRDFMYVSQDPKDQLLLGPTYATPKVLEKAGLTMNDIDAFEFHEAFSGQILANFKAMDSDWFAENYMGRKTKVGLPPLEKFNNWGGSLSLGHPFGATGCRLVMAAANRLRKEGGQYGLVAACAAGGQGHAMIVEAYPK

569 NP_003588.1 NP_005978.2

>NP_003588.1

MHTPDFAGPDDARAVDIMDICESILERKRHDSERSTCSILEQTDMEAVEALVCMSSWGQRSQKGDLLRIRPLTPVSDSGDVTTTVHMDAATPELPKDFHSLSTLCITPPQSPDLVEPSTRTPVSPQVTDSKACTATDVLQSSAVVARALSGGAERGLLGLEPVPSSPCRAKGTSVIRHTGESPAACFPTIQTPDCRLSDSREGEEQLLGHFETLQDTHLTDSLLSTNLVSCQPCLHKSGGLLLTDKGQQAGWPGAVQTCSPKNYENDLPRKTTPLISVSVPAPPVLCQMIPVTGQSSMLPAFLKPPPQLSVGTVRPILAQAAPAPQPVFVGPAVPQGAVMLVLPQGALPPPAPCAANVMAAGNTKLLPLAPAPVFITSSQNCVPQVDFSRRRNYVCSFPGCRKTYFKSSHLKAHLRTHTGEKPFNCSWDGCDKKFARSDELSRHRRTHTGEKKFVCPVCDRRFMRSDHLTKHARRHMTTKKIPGWQAEVGKLNRIASAESPGSPLVSMPASA

>NP_005978.2

MNSQQQKQPCTPPPQPQQQQVKQPCQPPPQEPCIPKTKEPCHPKVPEPCHPKVPEPCQPKVPEPCQPKVPEPCPSTVTPAPAQQKTKQK

570 NP_055758.1 P82909

>NP_055758.1

MASKRKSTTPCMVRTSQVVEQDVPEEVDRAKEKGIGTPQPDVAKDSWAAELENSSKENEVIEVKSMGESQSKKLQGGYECKYCPYSTQNLNEFTEHVDMQHPNVILNPLYVCAECNFTTKKYDSLSDHNSKFHPGEANFKLKLIKRNNQTVLEQSIETTNHVVSITTSGPGTGDSDSGISVSKTPIMKPGKPKADAKKVPKKPEEITPENHVEGTARLVTDTAEILSRLGGVELLQDTLGHVMPSVQLPPNINLVPKVPVPLNTTKYNSALDTNATMINSFNKFPYPTQAELSWLTAASKHPEEHIRIWFATQRLKHGISWSPEEVEEARKKMFNGTIQSVPPTITVLPAQLAPTKVTQPILQTALPCQILGQTSLVLTQVTSGSTTVSCSPITLAVAGVTNHGQKRPLVTPQAAPEPKRPHIAQVPEPPPKVANPPLTPASDRKKTKEQIAHLKASFLQSQFPDDAEVYRLIEVTGLARSEIKKWFSDHRYRCQRGIVHITSESLAKDQLAIAASRHGRTYHAYPDFAPQKFKEKTQGQVKILEDSFLKSSFPTQAELDRLRVETKLSRREIDSWFSERRKLRDSMEQAVLDSMGSGKKGQDVGAPNGALSRLDQLSGAQLTSSLPSPSPAIAKSQEQVHLLRSTFARTQWPTPQEYDQLAAKTGLVRTEIVRWFKENRCLLKTGTVKWMEQYQHQPMADDHGYDAVARKATKPMAESPKNGGDVVPQYYKDPKKLCEEDLEKLVTRVKVGSEPAKDCLPAKPSEATSDRSEGSSRDGQGSDENEESSVVDYVEVTVGEEDAISDRSDSWSQAAAEGVSELAESDSDCVPAEAGQA

>P82909

MMGSKMASASRVVQVVKPHTPLIRFPDRRDNPKPNVSEALRSAGLPSHSSVISQHSKGSKSPDLLMYQGPPDTAEIIKTLPQKYRRKLVSQEEMEFIQRGGPE

571 P62807 P57721

>P62807

MPEPAKSAPAPKKGSKKAVTKAQKKDGKKRKRSRKESYSVYVYKVLKQVHPDTGISSKAMGIMNSFVNDIFERIAGEASRLAHYNKRSTITSREIQTAVRLLLPGELAKHAVSEGTKAVTKYTSK

>P57721

MESKVSEGGLNVTLTIRLLMHGKEVGSIIGKKGETVKKMREESGARINISEGNCPERIVTITGPTDAIFKAFAMIAYKFEEDIINSMSNSPATSKPPVTLRLVVPASQCGSLIGKGGSKIKEIRESTGAQVQVAGDMLPNSTERAVTISGTPDAIIQCVKQICVVMLESPPKGATIPYRPKPASTPVIFAGGQAYTIQGQYAIPHPDQLTKLHQLAMQQTPFPPLGQTNPAFPGEKLPLHSSEEAQNLMGQSSGLDASPPASTHELTIPNDLIGCIIGRQGTKINEIRQMSGAQIKIANATEGSSERQITITGTPANISLAQYLINARLTSEVTGMGTL

572 NP_003159.1 Q3BBV0

>NP_003159.1

MALETVPKDLRHLRACLLCSLVKTIDQFEYDGCDNCDAYLQMKGNREMVYDCTSSSFDGIIAMMSPEDSWVSKWQRVSNFKPGVYAVSVTGRLPQGIVRELKSRGVAYKSRDTAIKT

>Q3BBV0

MVVSAGPWSSEKAETNILEINEKLRPQLAENKQQFRNLKEKCFVTQLAGFLANRQKKYKYEECKDLIKFMLRNERQFKEEKLAEQLKQAEELRQYKVLVHSQERELTQLREKLREGRDASRSLNQHLQALLTPDEPDKSQGQDLQEQLAEGCRLAQHLVQKLSPENDEDEDEDVQVEEAEKVLESSAPREVQKAEESKVPEDSLEECAITCSNSHGPCDSNQPHKNINITFEEDKVNSTLVVDRESSHDECQDAVNILPVPGPTSSATNVSMVVSAGPLSSEKAEMNILEMNEKLRPQLAEKKQQFRNLKEKCFVTQLAGFLANQQNKYKYEECEDLIKSMLRNERQFKEEKLAEQLKQAEELRQYKVLVHSQERELTQLREKLREGRDASRSLNQHLQALLTPDEPDKSQGQDLQEQLAEGCRLAQHLVQKLSPENDEDEDEDVQVEEAEKVLESSAPREVQKAEESKVPEDSLEECAITCSNSHGPCDSNQPHKNINITFEEDKVNSALVVDRESSHDECQDAVNILPVPGPTSSATNVSMVVSAGPLSSEKAEMNILEMNEKLRPQLAEKKQQFRNLKEKCFVTQLACFLANQQNKYKYEECKDLIKSVLRNERQFKEEKLAEQLKQAEELRQYKVLVHSQERELTQLREKLREGRDASRSLNQHLQALLTPDEPDKSQGQDLQEQLAEGCRLAQQLFQKLSPENDNDHDEDVQVEVAEKVQKSSAPREMQKAEEKEVPEDSLEECAITCSNSHGPYDSNQPHRKTKITFEEDKVDSTLIGSSSHVEWEDAVHIIPENESDDEEEEEKGPVSPRNLQESEEEEVPQESWDEGYSTLSIPPEMLASYQSYSGTFHSLEEQQVCMAVDIGGHRWDQVKKEDQEATGPRLSRELLDEKGPEVLQDSLDRCYSTPSGYLELTDSCQPYRSAFYILEQQRVGWALDMDEIEKYQEVEEDQDPSCPRLSRELLDEKEPEVLQDSLDRCYSTPSGYLELPDLGQPYRSAVYSLEEQYLGLALDVDRIKKDQEEEEDQGPPCPRLSRELLEAVEPEVLQDSLDRCYSTPSSCLEQPDSCLPYGSSFYALEEKHVGFSLDVGEIEKKGKGKKRRGRRSTKKRRRRGRKEGEEDQNPPCPRLSGMLMEVEEPEVLQDSLDRCYSTPSMYFELPDSFQHYRSVFYSFEEQHISFALDVDNRFLTLMGTSLHLVFQMGVIFPQ

573 Q6NYC1 Q96P20

>Q6NYC1

MNHKSKKRIREAKRSARPELKDSLDWTRHNYYESFSLSPAAVADNVERADALQLSVEEFVERYERPYKPVVLLNAQEGWSAQEKWTLERLKRKYRNQKFKCGEDNDGYSVKMKMKYYIEYMESTRDDSPLYIFDSSYGEHPKRRKLLEDYKVPKFFTDDLFQYAGEKRRPPYRWFVMGPPRSGTGIHIDPLGTSAWNALVQGHKRWCLFPTSTPRELIKVTRDEGGNQQDEAITWFNVIYPRTQLPTWPPEFKPLEILQKPGETVFVPGGWWHVVLNLDTTIAITQNFASSTNFPVVWHKTVRGRPKLSRKWYRILKQEHPELAVLADSVDLQESTGIASDSSSDSSSSSSSSSSDSDSECESGSEGDGTVHRRKKRRTCSMVGNGDTTSQDDCVSKERSSSR

>Q96P20

MASTRCKLARYLEDLEDVDLKKFKMHLEDYPPQKGCIPLPRGQTEKADHVDLATLMIDFNGEEKAWAMAVWIFAAINRRDLYEKAKRDEPKWGSDNARVSNPTVICQEDSIEEEWMGLLEYLSRISICKMKKDYRKKYRKYVRSRFQCIEDRNARLGESVSLNKRYTRLRLIKEHRSQQEREQELLAIGKTKTCESPVSPIKMELLFDPDDEHSEPVHTVVFQGAAGIGKTILARKMMLDWASGTLYQDRFDYLFYIHCREVSLVTQRSLGDLIMSCCPDPNPPIHKIVRKPSRILFLMDGFDELQGAFDEHIGPLCTDWQKAERGDILLSSLIRKKLLPEASLLITTRPVALEKLQHLLDHPRHVEILGFSEAKRKEYFFKYFSDEAQARAAFSLIQENEVLFTMCFIPLVCWIVCTGLKQQMESGKSLAQTSKTTTAVYVFFLSSLLQPRGGSQEHGLCAHLWGLCSLAADGIWNQKILFEESDLRNHGLQKADVSAFLRMNLFQKEVDCEKFYSFIHMTFQEFFAAMYYLLEEEKEGRTNVPGSRLKLPSRDVTVLLENYGKFEKGYLIFVVRFLFGLVNQERTSYLEKKLSCKISQQIRLELLKWIEVKAKAKKLQIQPSQLELFYCLYEMQEEDFVQRAMDYFPKIEINLSTRMDHMVSSFCIENCHRVESLSLGFLHNMPKEEEEEEKEGRHLDMVQCVLPSSSHAACSHGLVNSHLTSSFCRGLFSVLSTSQSLTELDLSDNSLGDPGMRVLCETLQHPGCNIRRLWLGRCGLSHECCFDISLVLSSNQKLVELDLSDNALGDFGIRLLCVGLKHLLCNLKKLWLVSCCLTSACCQDLASVLSTSHSLTRLYVGENALGDSGVAILCEKAKNPQCNLQKLGLVNSGLTSVCCSALSSVLSTNQNLTHLYLRGNTLGDKGIKLLCEGLLHPDCKLQVLELDNCNLTSHCCWDLSTLLTSSQSLRKLSLGNNDLGDLGVMMFCEVLKQQSCLLQNLGLSEMYFNYETKSALETLQEEKPELTVVFEPSW

574 Q12873 NP_004530.1

>Q12873

MKAADTVILWARSKNDQLRISFPPGLCWGDRMPDKDDIRLLPSALGVKKRKRGPKKQKENKPGKPRKRKKRDSEEEFGSERDEYREKSESGGSEYGTGPGRKRRRKHREKKEKKTKRRKKGEGDGGQKQVEQKSSATLLLTWGLEDVEHVFSEEDYHTLTNYKAFSQFMRPLIAKKNPKIPMSKMMTILGAKWREFSANNPFKGSAAAVAAAAAAAAAAVAEQVSAAVSSATPIAPSGPPALPPPPAADIQPPPIRRAKTKEGKGPGHKRRSKSPRVPDGRKKLRGKKMAPLKIKLGLLGGKRKKGGSYVFQSDEGPEPEAEESDLDSGSVHSASGRPDGPVRTKKLKRGRPGRKKKKVLGCPAVAGEEEVDGYETDHQDYCEVCQQGGEIILCDTCPRAYHLVCLDPELDRAPEGKWSCPHCEKEGVQWEAKEEEEEYEEEGEEEGEKEEEDDHMEYCRVCKDGGELLCCDACISSYHIHCLNPPLPDIPNGEWLCPRCTCPVLKGRVQKILHWRWGEPPVAVPAPQQADGNPDVPPPRPLQGRSEREFFVKWVGLSYWHCSWAKELQLEIFHLVMYRNYQRKNDMDEPPPLDYGSGEDDGKSDKRKVKDPHYAEMEEKYYRFGIKPEWMTVHRIINHSVDKKGNYHYLVKWRDLPYDQSTWEEDEMNIPEYEEHKQSYWRHRELIMGEDPAQPRKYKKKKKELQGDGPPSSPTNDPTVKYETQPRFITATGGTLHMYQLEGLNWLRFSWAQGTDTILADEMGLGKTIQTIVFLYSLYKEGHTKGPFLVSAPLSTIINWEREFQMWAPKFYVVTYTGDKDSRAIIRENEFSFEDNAIKGGKKAFKMKREAQVKFHVLLTSYELITIDQAALGSIRWACLVVDEAHRLKNNQSKFFRVLNGYKIDHKLLLTGTPLQNNLEELFHLLNFLTPERFNNLEGFLEEFADISKEDQIKKLHDLLGPHMLRRLKADVFKNMPAKTELIVRVELSPMQKKYYKYILTRNFEALNSRGGGNQVSLLNIMMDLKKCCNHPYLFPVAAMESPKLPSGAYEGGALIKSSGKLMLLQKMLRKLKEQGHRVLIFSQMTKMLDLLEDFLDYEGYKYERIDGGITGALRQEAIDRFNAPGAQQFCFLLSTRAGGLGINLATADTVIIFDSDWNPHNDIQAFSRAHRIGQANKVMIYRFVTRASVEERITQVAKRKMMLTHLVVRPGLGSKAGSMSKQELDDILKFGTEELFKDENEGENKEEDSSVIHYDNEAIARLLDRNQDATEDTDVQNMNEYLSSFKVAQYVVREEDKIEEIEREIIKQEENVDPDYWEKLLRHHYEQQQEDLARNLGKGKRVRKQVNYNDAAQEDQDNQSEYSVGSEEEDEDFDERPEGRRQSKRQLRNEKDKPLPPLLARVGGNIEVLGFNTRQRKAFLNAVMRWGMPPQDAFTTQWLVRDLRGKTEKEFKAYVSLFMRHLCEPGADGSETFADGVPREGLSRQQVLTRIGVMSLVKKKVQEFEHINGRWSMPELMPDPSADSKRSSRASSPTKTSPTTPEASATNSPCTSKPATPAPSEKGEGIRTPLEKEEAENQEEKPEKNSRIGEKMETEADAPSPAPSLGERLEPRKIPLEDEVPGVPGEMEPEPGYRGDREKSATESTPGERGEEKPLDGQEHRERPEGETGDLGKREDVKGDRELRPGPRDEPRSNGRREEKTEKPRFMFNIADGGFTELHTLWQNEERAAISSGKLNEIWHRRHDYWLLAGIVLHGYARWQDIQNDAQFAIINEPFKTEANKGNFLEMKNKFLARRFKLLEQALVIEEQLRRAAYLNLSQEPAHPAMALHARFAEAECLAESHQHLSKESLAGNKPANAVLHKVLNQLEELLSDMKADVTRLPATLSRIPPIAARLQMSERSILSRLASKGTEPHPTPAYPPGPYATPPGYGAAFSAAPVGALAAAGANYSQMPAGSFITAATNGPPVLVKKEKEMVGALVSDGLDRKEPRAGEVICIDD

>NP_004530.1

MVLAELYVSDREGSDATGDGTKEKPFKTGLKALMTVGKEPFPTIYVDSQKENERWNVISKSQLKNIKKMWHREQMKSESREKKEAEDSLRREKNLEEAKKITIKNDPSLPEPKCVKIGALEGYRGQRVKVFGWVHRLRRQGKNLMFLVLRDGTGYLQCVLADELCQCYNGVLLSTESSVAVYGMLNLTPKGKQAPGGHELSCDFWELIGLAPAGGADNLINEESDVDVQLNNRHMMIRGENMSKILKARSMVTRCFRDHFFDRGYYEVTPPTLVQTQVEGGATLFKLDYFGEEAFLTQSSQLYLETCLPALGDVFCIAQSYRAEQSRTRRHLAEYTHVEAECPFLTFDDLLNRLEDLVCDVVDRILKSPAGSIVHELNPNFQPPKRPFKRMNYSDAIVWLKEHDVKKEDGTFYEFGEDIPEAPERLMTDTINEPILLCRFPVEIKSFYMQRCPEDSRLTESVDVLMPNVGEIVGGSMRIFDSEEILAGYKREGIDPTPYYWYTDQRKYGTCPHGGYGLGLERFLTWILNRYHIRDVCLYPRFVQRCTP

575 O75529 P83881

>O75529

MKRVRTEQIQMAVSCYLKRRQYVDSDGPLKQGLRLSQTAEEMAANLTVQSESGCANIVSAAPCQAEPQQYEVQFGRLRNFLTDSDSQHSHEVMPLLYPLFVYLHLNLVQNSPKSTVESFYSRFHGMFLQNASQKDVIEQLQTTQTIQDILSNFKLRAFLDNKYVVRLQEDSYNYLIRYLQSDNNTALCKVLTLHIHLDVQPAKRTDYQLYASGSSSRSENNGLEPPDMPSPILQNEAALEVLQESIKRVKDGPPSLTTICFYAFYNTEQLLNTAEISPDSKLLAAGFDNSCIKLWSLRSKKLKSEPHQVDVSRIHLACDILEEEDDEDDNAGTEMKILRGHCGPVYSTRFLADSSGLLSCSEDMSIRYWDLGSFTNTVLYQGHAYPVWDLDISPYSLYFASGSHDRTARLWSFDRTYPLRIYAGHLADVDCVKFHPNSNYLATGSTDKTVRLWSAQQGNSVRLFTGHRGPVLSLAFSPNGKYLASAGEDQRLKLWDLASGTLYKELRGHTDNITSLTFSPDSGLIASASMDNSVRVWDIRNTYCSAPADGSSSELVGVYTGQMSNVLSVQFMACNLLLVTGITQENQEH

>P83881

MVNVPKTRRTFCKKCGKHQPHKVTQYKKGKDSLYAQGKRRYDRKQSGYGGQTKPIFRKKAKTTKKIVLRLECVEPNCRSKRMLAIKRCKHFELGGDKKRKGQVIQF

576 NP_001871.2 P35237

>NP_001871.2

MKFKLHVNSARQYKDLWNMSDDKPFLCTAPGCGQRFTNEDHLAVHKHKHEMTLKFGPARNDSVIVADQTPTPTRFLKNCEEVGLFNELASPFENEFKKASEDDIKKMPLDLSPLATPIIRSKIEEPSVVETTHQDSPLPHPESTTSDEKEVPLAQTAQPTSAIVRPASLQVPNVLLTSSDSSVIIQQAVPSPTSSTVITQAPSSNRPIVPVPGPFPLLLHLPNGQTMPVAIPASITSSNVHVPAAVPLVRPVTMVPSVPGIPGPSSPQPVQSEAKMRLKAALTQQHPPVTNGDTVKGHGSGLVRTQSEESRPQSLQQPATSTTETPASPAHTTPQTQSTSGRRRRAANEDPDEKRRKFLERNRAAASRCRQKRKVWVQSLEKKAEDLSSLNGQLQSEVTLLRNEVAQLKQLLLAHKDCPVTAMQKKSGYHTADKDDSSEDISVPSSPHTEAIQHSSVSTSNGVSSTSKAEAVATSVLTQMADQSTEPALSQIVMAPSSQSQPSGS

>P35237

MDVLAEANGTFALNLLKTLGKDNSKNVFFSPMSMSCALAMVYMGAKGNTAAQMAQILSFNKSGGGGDIHQGFQSLLTEVNKTGTQYLLRMANRLFGEKSCDFLSSFRDSCQKFYQAEMEELDFISAVEKSRKHINTWVAEKTEGKIAELLSPGSVDPLTRLVLVNAVYFRGNWDEQFDKENTEERLFKVSKNEEKPVQMMFKQSTFKKTYIGEIFTQILVLPYVGKELNMIIMLPDETTDLRTVEKELTYEKFVEWTRLDMMDEEEVEVSLPRFKLEESYDMESVLRNLGMTDAFELGKADFSGMSQTDLSLSKVVHKSFVEVNEEGTEAAAATAAIMMMRCARFVPRFCADHPFLFFIQHSKTNGILFCGRFSSP

577 NP_055206.2 NP_550438.1

>NP_055206.2

MMVLKVEELVTGKKNGNGEAGEFLPEDFRDGEYEAAVTLEKQEDLKTLLAHPVTLGEQQWKSEKQREAELKKKKLEQRSKLENLEDLEIIIQLKKRKKYRKTKVPVVKEPEPEIITEPVDVPTFLKAALENKLPVVEKFLSDKNNPDVCDEYKRTALHRACLEGHLAIVEKLMEAGAQIEFRDMLESTAIHWASRGGNLDVLKLLLNKGAKISARDKLLSTALHVAVRTGHYECAEHLIACEADLNAKDREGDTPLHDAVRLNRYKMIRLLIMYGADLNIKNCAGKTPMDLVLHWQNGTKAIFDSLRENSYKTSRIATF

>NP_550438.1

MAAGRLFLSRLRAPFSSMAKSPLEGVSSSRGLHAGRGPRRLSIEGNIAVGKSTFVKLLTKTYPEWHVATEPVATWQNIQAAGTQKACTAQSLGNLLDMMYREPARWSYTFQTFSFLSRLKVQLEPFPEKLLQARKPVQIFERSVYSDRYIFAKNLFENGSLSDIEWHIYQDWHSFLLWEFASRITLHGFIYLQASPQVCLKRLYQRAREEEKGIELAYLEQLHGQHEAWLIHKTTKLHFEALMNIPVLVLDVNDDFSEEVTKQEDLMREVNTFVKNL

578 NP_000018.1 NP_758958.3

>NP_000018.1

MARKSNLPVLLVPFLLCQALVRCSSPLPLVVNTWPFKNATEAAWRALASGGSALDAVESGCAMCEREQCDGSVGFGGSPDELGETTLDAMIMDGTTMDVGAVGDLRRIKNAIGVARKVLEHTTHTLLVGESATTFAQSMGFINEDLSTSASQALHSDWLARNCQPNYWRNVIPDPSKYCGPYKPPGILKQDIPIHKETEDDRGHDTIGMVVIHKTGHIAAGTSTNGIKFKIHGRVGDSPIPGAGAYADDTAGAAAATGNGDILMRFLPSYQAVEYMRRGEDPTIACQKVISRIQKHFPEFFGAVICANVTGSYGAACNKLSTFTQFSFMVYNSEKNQPTEEKVDCI

>NP_758958.3

MLEKLMGADSLQLFRSRYTLGKIYFIGFQRSILLSKSENSLNSIAKETEEGRETVTRKEGWKRRHEDGYLEMAQRHLQRSLCPWVSYLPQPYAELEEVSSHVGKVFMARNYEFLAYEASKDRRQPLERMWTCNYNQQKDQSCNHKEITSTKAE

579 Q6EKJ0 NP_008830.2

>Q6EKJ0

MAQVAVSTLPVEEESSSETRMVVTFLVSALESMCKELAKSKAEVACIAVYETDVFVVGTERGCAFVNARTDFQKDFAKYCVAEGLCEVKPPCPVNGMQVHSGETEILRKAVEDYFCFCYGKALGTTVMVPVPYEKMLRDQSAVVVQGLPEGVAFQHPENYDLATLKWILENKAGISFIINRPFLGPESQLGGPGMVTDAERSIVSPSESCGPINVKTEPMEDSGISLKAEAVSVKKESEDPNYYQYNMQGSHPSSTSNEVIEMELPMEDSTPLVPSEEPNEDPEAEVKIEGNTNSSSVTNSAAGVEDLNIVQVTVPDNEKERLSSIEKIKQLREQVNDLFSRKFGEAIGVDFPVKVPYRKITFNPGCVVIDGMPPGVVFKAPGYLEISSMRRILEAAEFIKFTVIRPLPGLELSNVGKRKIDQEGRVFQEKWERAYFFVEVQNIPTCLICKQSMSVSKEYNLRRHYQTNHSKHYDQYTERMRDEKLHELKKGLRKYLLGSSDTECPEQKQVFANPSPTQKSPVQPVEDLAGNLWEKLREKIRSFVAYSIAIDEITDINNTTQLAIFIRGVDENFDVSEELLDTVPMTGTKSGNEIFLRVEKSLKKFCINWSRLVSVASTGTPAMVDANNGLVTKLKSRVATFCKGAELKSICCIIHPESLCAQKLKMDHVMDVVVKSVNWICSRGLNHSEFTTLLYELDSQYGSLLYYTEIKWLSRGLVLKRFFESLEEIDSFMSSRGKPLPQLSSIDWIRDLAFLVDMTMHLNALNISLQGHSQIVTQMYDLIRAFLAKLCLWETHLTRNNLAHFPTLKLVSRNESDGLNYIPKIAELKTEFQKRLSDFKLYESELTLFSSPFSTKIDSVHEELQMEVIDLQCNTVLKTKYDKVGIPEFYKYLWGSYPKYKHHCAKILSMFGSTYICEQLFSIMKLSKTKYCSQLKDSQWDSVLHIAT

>NP_008830.2

MAALSGVRWLTRALVSAGNPGAWRGLSTSAAAHAASRSQAEDVRVEGSFPVTMLPGDGVGPELMHAVKEVFKAAAVPVEFQEHHLSEVQNMASEEKLEQVLSSMKENKVAIIGKIHTPMEYKGELASYDMRLRRKLDLFANVVHVKSLPGYMTRHNNLDLVIIREQTEGEYSSLEHESARGVIECLKIVTRAKSQRIAKFAFDYATKKGRGKVTAVHKANIMKLGDGLFLQCCEEVAELYPKIKFETMIIDNCCMQLVQNPYQFDVLVMPNLYGNIIDNLAAGLVGGAGVVPGESYSAEYAVFETGARHPFAQAVGRNIANPTAMLLSASNMLRHLNLEYHSSMIADAVKKVIKVGKVRTRDMGGYSTTTDFIKSVIGHLQTKGS

580 P04554 NP_000839.1

>P04554

MVRYRVRSLSERSHEVYRQQLHGQEQGHHGQEEQGLSPEHVEVYERTHGQSHYRRRHCSRRRLHRIHRRQHRSCRRRKRRSCRHRRRHRRGCRTRKRTCRRH

>NP_000839.1

MPMTLGYWNIRGLAHSIRLLLEYTDSSYEEKKYTMGDAPDYDRSQWLNEKFKLGLDFPNLPYLIDGTHKITQSNAILRYIARKHNLCGESEKEQIREDILENQFMDSRMQLAKLCYDPDFEKLKPEYLQALPEMLKLYSQFLGKQPWFLGDKITFVDFIAYDVLERNQVFEPSCLDAFPNLKDFISRFEGLEKISAYMKSSRFLPRPVFTKMAVWGNK

581 NP_004731.2 Q9UBR1

>NP_004731.2

MSSPSSPFREQSFLCAAGDAGEESRVQVLKNEVRRGSPVLLGWVEQAYADKCVCGPSAPPAPTPPSLSQRVMCNDLFKVNPFQLQQFRADPSTASLLLCPGGLDHKLNLRGKAWG

>Q9UBR1

MAGAEWKSLEECLEKHLPLPDLQEVKRVLYGKELRKLDLPREAFEAASREDFELQGYAFEAAEEQLRRPRIVHVGLVQNRIPLPANAPVAEQVSALHRRIKAIVEVAAMCGVNIICFQEAWTMPFAFCTREKLPWTEFAESAEDGPTTRFCQKLAKNHDMVVVSPILERDSEHGDVLWNTAVVISNSGAVLGKTRKNHIPRVGDFNESTYYMEGNLGHPVFQTQFGRIAVNICYGRHHPLNWLMYSINGAEIIFNPSATIGALSESLWPIEARNAAIANHCFTCAINRVGTEHFPNEFTSGDGKKAHQDFGYFYGSSYVAAPDSSRTPGLSRSRDGLLVAKLDLNLCQQVNDVWNFKMTGRYEMYARELAEAVKSNYSPTIVKE

582 NP_004951.1 P23434

>NP_004951.1

MASNDYTQQATQSYGAYPTQPGQGYSQQSSQPYGQQSYSGYSQSTDTSGYGQSSYSSYGQSQNTGYGTQSTPQGYGSTGGYGSSQSSQSSYGQQSSYPGYGQQPAPSSTSGSYGSSSQSSSYGQPQSGSYSQQPSYGGQQQSYGQQQSYNPPQGYGQQNQYNSSSGGGGGGGGGGNYGQDQSSMSSGGGSGGGYGNQDQSGGGGSGGYGQQDRGGRGRGGSGGGGGGGGGGYNRSSGGYEPRGRGGGRGGRGGMGGSDRGGFNKFGGPRDQGSRHDSEQDNSDNNTIFVQGLGENVTIESVADYFKQIGIIKTNKKTGQPMINLYTDRETGKLKGEATVSFDDPPSAKAAIDWFDGKEFSGNPIKVSFATRRADFNRGGGNGRGGRGRGGPMGRGGYGGGGSGGGGRGGFPSGGGGGGGQQRAGDWKCPNPTCENMNFSWRNECNQCKAPKPDGPGGGPGGSHMGGNYGDDRRGGRGGYDRGGYRGRGGDRGGFRGGRGGGDRGGFGPGKMDSRGEHRQDRRERPY

>P23434

MALRVVRSVRALLCTLRAVPLPAAPCPPRPWQLGVGAVRTLRTGPALLSVRKFTEKHEWVTTENGIGTVGISNFAQEALGDVVYCSLPEVGTKLNKQDEFGALESVKAASELYSPLSGEVTEINEALAENPGLVNKSCYEDGWLIKMTLSNPSELDELMSEEAYEKYIKSIEE

583 Q9ULV5 NP_002291.1

>Q9ULV5

MVQEAPAALPTEPGPSPVPAFLGKLWALVGDPGTDHLIRWSPSGTSFLVSDQSRFAKEVLPQYFKHSNMASFVRQLNMYGFRKVVSIEQGGLLRPERDHVEFQHPSFVRGREQLLERVRRKVPALRGDDGRWRPEDLGRLLGEVQALRGVQESTEARLRELRQQNEILWREVVTLRQSHGQQHRVIGKLIQCLFGPLQAGPSNAGGKRKLSLMLDEGSSCPTPAKFNTCPLPGALLQDPYFIQSPLPETNLGLSPHRARGPIISDIPEDSPSPEGTRLSPSSDGRREKGLALLKEEPASPGGDGEAGLALAPNECDFCVTAPPPLPVAVVQAILEGKGSFSPEGPRNAQQPEPGDPREIPDRGPLGLESGDRSPESLLPPMLLQPPQESVEPAGPLDVLGPSLQGREWTLMDLDMELSLMQPLVPERGEPELAVKGLNSPSPGKDPTLGAPLLLDVQAALGGPALGLPGALTIYSTPESRTASYLGPEASPSP

>NP_002291.1

MATLKEKLIAPVAEEEATVPNNKITVVGVGQVGMACAISILGKSLADELALVDVLEDKLKGEMMDLQHGSLFLQTPKIVADKDYSVTANSKIVVVTAGVRQQEGESRLNLVQRNVNVFKFIIPQIVKYSPDCIIIVVSNPVDILTYVTWKLSGLPKHRVIGSGCNLDSARFRYLMAEKLGIHPSSCHGWILGEHGDSSVAVWSGVNVAGVSLQELNPEMGTDNDSENWKEVHKMVVESAYEVIKLKGYTNWAIGLSVADLIESMLKNLSRIHPVSTMVKGMYGIENEVFLSLPCILNARGLTSVINQKLKDDEVAQLKKSADTLWDIQKDLKDL

584 Q04727 NP_002563.1

>Q04727

MIRDLSKMYPQTRHPAPHQPAQPFKFTISESCDRIKEEFQFLQAQYHSLKLECEKLASEKTEMQRHYVMYYEMSYGLNIEMHKQAEIVKRLNAICAQVIPFLSQEHQQQVVQAVERAKQVTMAELNAIIGQQLQAQHLSHGHGLPVPLTPHPSGLQPPAIPPIGSSAGLLALSSALGGQSHLPIKDEKKHHDNDHQRDRDSIKSSSVSPSASFRGAEKHRNSADYSSESKKQKTEEKEIAARYDSDGEKSDDNLVVDVSNEDPSSPRGSPAHSPRENGLDKTRLLKKDAPISPASIASSSSTPSSKSKELSLNEKSTTPVSKSNTPTPRTDAPTPGSNSTPGLRPVPGKPPGVDPLASSLRTPMAVPCPYPTPFGIVPHAGMNGELTSPGAAYAGLHNISPQMSAAAAAAAAAAAYGRSPVVGFDPHHHMRVPAIPPNLTGIPGGKPAYSFHVSADGQMQPVPFPPDALIGPGIPRHARQINTLNHGEVVCAVTISNPTRHVYTGGKGCVKVWDISHPGNKSPVSQLDCLNRDNYIRSCRLLPDGRTLIVGGEASTLSIWDLAAPTPRIKAELTSSAPACYALAISPDSKVCFSCCSDGNIAVWDLHNQTLVRQFQGHTDGASCIDISNDGTKLWTGGLDNTVRSWDLREGRQLQQHDFTSQIFSLGYCPTGEWLAVGMENSNVEVLHVTKPDKYQLHLHESCVLSLKFAHCGKWFVSTGKDNLLNAWRTPYGASIFQSKESSSVLSCDISVDDKYIVTGSGDKKATVYEVIY

>NP_002563.1

MSQGDSNPAAIPHAAEDIQGDDRWMSQHNRFVLDCKDKEPDVLFVGDSMVQLMQQYEIWRELFSPLHALNFGIGGDTTRHVLWRLKNGELENIKPKVIVVWVGTNNHENTAEEVAGGIEAIVQLINTRQPQAKIIVLGLLPRGEKPNPLRQKNAKVNQLLKVSLPKLANVQLLDTDGGFVHSDGAISCHDMFDFLHLTGGGYAKICKPLHELIMQLLEETPEEKQTTIA

585 NP_001797.1 Q9NP74

>NP_001797.1

MSKISQQNSTPGVNGISVIHTQAHASGLQQVPQLVPAGPGGGGKAVAPSKQSKKSSPMDRNSDEYRQRRERNNMAVKKSRLKSKQKAQDTLQRVNQLKEENERLEAKIKLLTKELSVLKDLFLEHAHNLADNVQSISTENTTADGDNAGQ

>Q9NP74

MEEAELVKGRLQAITDKRKIQEEISQKRLKIEEDKLKHQHLKKKALREKWLLDGISSGKEQEEMKKQNQQDQHQIQVLEQSILRLEKEIQDLEKAELQISTKEEAILKKLKSIERTTEDIIRSVKVEREERAEESIEDIYANIPDLPKSYIPSRLRKEINEEKEDDEQNRKALYAMEIKVEKDLKTGESTVLSSIPLPSDDFKGTGIKVYDDGQKSVYAVSSNHSAAYNGTDGLAPVEVEELLRQASERNSKSPTEYHEPVYANPFYRPTTPQRETVTPGPNFQERIKIKTNGLGIGVNESIHNMGNGLSEERGNNFNHISPIPPVPHPRSVIQQAEEKLHTPQKRLMTPWEESNVMQDKDAPSPKPRLSPRETIFGKSEHQNSSPTCQEDEEDVRYNIVHSLPPDINDTEPVTMIFMGYQQAEDSEEDKKFLTGYDGIIHAELVVIDDEEEEDEGEAEKPSYHPIAPHSQVYQPAKPTPLPRKRSEASPHENTNHKSPHKNSISLKEQEESLGSPVHHSPFDAQTTGDGTEDPSLTALRMRMAKLGKKVI

586 NP_009053.1 P35237

>NP_009053.1

MKGQQKTAETEEGTVQIQEGAVATGEDPTSVAIASIQSAATFPDPNVKYVFRTENGGQVMYRVIQVSEGQLDGQTEGTGAISGYPATQSMTQAVIQGAFTSDDAVDTEGTAAETHYTYFPSTAVGDGAGGTTSGSTAAVVTTQGSEALLGQATPPGTGQFFVMMSPQEVLQGGSQRSIAPRTHPYSPKSEAPRTTRDEKRRAQHNEVERRRRDKINNWIVQLSKIIPDCSMESTKSGQSKGGILSKACDYIQELRQSNHRLSEELQGLDQLQLDNDVLRQQVEDLKNKNLLLRAQLRHHGLEVVIKNDSN

>P35237

MDVLAEANGTFALNLLKTLGKDNSKNVFFSPMSMSCALAMVYMGAKGNTAAQMAQILSFNKSGGGGDIHQGFQSLLTEVNKTGTQYLLRMANRLFGEKSCDFLSSFRDSCQKFYQAEMEELDFISAVEKSRKHINTWVAEKTEGKIAELLSPGSVDPLTRLVLVNAVYFRGNWDEQFDKENTEERLFKVSKNEEKPVQMMFKQSTFKKTYIGEIFTQILVLPYVGKELNMIIMLPDETTDLRTVEKELTYEKFVEWTRLDMMDEEEVEVSLPRFKLEESYDMESVLRNLGMTDAFELGKADFSGMSQTDLSLSKVVHKSFVEVNEEGTEAAAATAAIMMMRCARFVPRFCADHPFLFFIQHSKTNGILFCGRFSSP

587 Q12951 Q9H6S3

>Q12951

MSSFDLPAPSPPRCSPQFPSIGQEPPEMNLYYENFFHPQGVPSPQRPSFEGGGEYGATPNPYLWFNGPTMTPPPYLPGPNASPFLPQAYGVQRPLLPSVSGLGGSDLGWLPIPSQEELMKLVRPPYSYSALIAMAIHGAPDKRLTLSQIYQYVADNFPFYNKSKAGWQNSIRHNLSLNDCFKKVPRDEDDPGKGNYWTLDPNCEKMFDNGNFRRKRKRKSDVSSSTASLALEKTESSLPVDSPKTTEPQDILDGASPGGTTSSPEKRPSPPPSGAPCLNSFLSSMTAYVSGGSPTSHPLVTPGLSPEPSDKTGQNSLTFNSFSPLTNLSNHSGGGDWANPMPTNMLSYGGSVLSQFSPHFYNSVNTSGVLYPREGTEV

>Q9H6S3

MSQSGAVSCCPGATNGSLGRSDGVAKMSPKDLFEQRKKYSNSNVIMHETSQYHVQHLATFIMDKSEAITSVDDAIRKLVQLSSKEKIWTQEMLLQVNDQSLRLLDIESQEELEDFPLPTVQRSQTVLNQLRYPSVLLLVCQDSEQSKPDVHFFHCDEVEAELVHEDIESALADCRLGKKMRPQTLKGHQEKIRQRQSILPPPQGPAPIPFQHRGGDSPEAKNRVGPQVPLSEPGFRRRESQEEPRAVLAQKIEKETQILNCALDDIEWFVARLQKAAEAFKQLNQRKKGKKKGKKAPAEGVLTLRARPPSEGEFIDCFQKIKLAINLLAKLQKHIQNPSAAELVHFLFGPLDLIVNTCSGPDIARSVSCPLLSRDAVDFLRGHLVPKEMSLWESLGESWMRPRSEWPREPQVPLYVPKFHSGWEPPVDVLQEAPWEVEGLASAPIEEVSPVSRQSIRNSQKHSPTSEPTPPGDALPPVSSPHTHRGYQPTPAMAKYVKILYDFTARNANELSVLKDEVLEVLEDGRQWWKLRSRSGQAGYVPCNILGEARPEDAGAPFEQAGQKYWGPASPTHKLPPSFPGNKDELMQHMDEVNDELIRKISNIRAQPQRHFRVERSQPVSQPLTYESGPDEVRAWLEAKAFSPRIVENLGILTGPQLFSLNKEELKKVCGEEGVRVYSQLTMQKAFLEKQQSGSELEELMNKFHSMNQRRGEDS

588 NP_003588.1 NP_005204.1

>NP_003588.1

MHTPDFAGPDDARAVDIMDICESILERKRHDSERSTCSILEQTDMEAVEALVCMSSWGQRSQKGDLLRIRPLTPVSDSGDVTTTVHMDAATPELPKDFHSLSTLCITPPQSPDLVEPSTRTPVSPQVTDSKACTATDVLQSSAVVARALSGGAERGLLGLEPVPSSPCRAKGTSVIRHTGESPAACFPTIQTPDCRLSDSREGEEQLLGHFETLQDTHLTDSLLSTNLVSCQPCLHKSGGLLLTDKGQQAGWPGAVQTCSPKNYENDLPRKTTPLISVSVPAPPVLCQMIPVTGQSSMLPAFLKPPPQLSVGTVRPILAQAAPAPQPVFVGPAVPQGAVMLVLPQGALPPPAPCAANVMAAGNTKLLPLAPAPVFITSSQNCVPQVDFSRRRNYVCSFPGCRKTYFKSSHLKAHLRTHTGEKPFNCSWDGCDKKFARSDELSRHRRTHTGEKKFVCPVCDRRFMRSDHLTKHARRHMTTKKIPGWQAEVGKLNRIASAESPGSPLVSMPASA

>NP_005204.1

MIPGGLSEAKPATPEIQEIVDKVKPQLEEKTNETYGKLEAVQYKTQVVAGTNYYIKVRAGDNKYMHLKVFKSLPGQNEDLVLTGYQVDKNKDDELTGF

589 NP_061825.2 P46459

>NP_061825.2

MSSYFVNSTFPVTLASGQESFLGQLPLYSSGYADPLRHYPAPYGPGPGQDKGFATSSYYPPAGGGYGRAAPCDYGPAPAFYREKESACALSGADEQPPFHPEPRKSDCAQDKSVFGETEEQKCSTPVYPWMQRMNSCNSSSFGPSGRRGRQTYTRYQTLELEKEFHYNRYLTRRRRIEIAHALCLTERQIKIWFQNRRMKWKKESKLLSASQLSAEEEEEKQAE

>P46459

MAGRSMQAARCPTDELSLTNCAVVNEKDFQSGQHVIVRTSPNHRYTFTLKTHPSVVPGSIAFSLPQRKWAGLSIGQEIEVSLYTFDKAKQCIGTMTIEIDFLQKKSIDSNPYDTDKMAAEFIQQFNNQAFSVGQQLVFSFNEKLFGLLVKDIEAMDPSILKGEPATGKRQKIEVGLVVGNSQVAFEKAENSSLNLIGKAKTKENRQSIINPDWNFEKMGIGGLDKEFSDIFRRAFASRVFPPEIVEQMGCKHVKGILLYGPPGCGKTLLARQIGKMLNAREPKVVNGPEILNKYVGESEANIRKLFADAEEEQRRLGANSGLHIIIFDEIDAICKQRGSMAGSTGVHDTVVNQLLSKIDGVEQLNNILVIGMTNRPDLIDEALLRPGRLEVKMEIGLPDEKGRLQILHIHTARMRGHQLLSADVDIKELAVETKNFSGAELEGLVRAAQSTAMNRHIKASTKVEVDMEKAESLQVTRGDFLASLENDIKPAFGTNQEDYASYIMNGIIKWGDPVTRVLDDGELLVQQTKNSDRTPLVSVLLEGPPHSGKTALAAKIAEESNFPFIKICSPDKMIGFSETAKCQAMKKIFDDAYKSQLSCVVVDDIERLLDYVPIGPRFSNLVLQALLVLLKKAPPQGRKLLIIGTTSRKDVLQEMEMLNAFSTTIHVPNIATGEQLLEALELLGNFKDKERTTIAQQVKGKKVWIGIKKLLMLIEMSLQMDPEYRVRKFLALLREEGASPLDFD

590 O60231 NP_005300.1

>O60231

MATPAGLERWVQDELHSVLGLSERHVAQFLIGTAQRCTSAEEFVQRLRDTDTLDLSGPARDFALRLWNKVPRKAVVEKPARAAEREARALLEKNRSYRLLEDSEESSEETVSRAGSSLQKKRKKRKHLRKKREEEEEEEASEKGKKKTGGSKQQTEKPESEDEWERTERERLQDLEERDAFAERVRQRDKDRTRNVLERSDKKAYEEAQKRLKMAEEDRKAMVPELRKKSRREYLAKREREKLEDLEAELADEEFLFGDVELSRHERQELKYKRRVRDLAREYRAAGEQEKLEATNRYHMPKETRGQPARAVDLVEEESGAPGEEQRRWEEARLGAASLKFGARDAASQEPKYQLVLEEEETIEFVRATQLQGDEEPSAPPTSTQAQQKESIQAVRRSLPVFPFREELLAAIANHQVLIIEGETGSGKTTQIPQYLFEEGYTNKGMKIACTQPRRVAAMSVAARVAREMGVKLGNEVGYSIRFEDCTSERTVLRYMTDGMLLREFLSEPDLASYSVVMVDEAHERTLHTDILFGLIKDVARFRPELKVLVASATMDTARFSTFFDDAPVFRIPGRRFPVDIFYTKAPEADYLEACVVSVLQIHVTQPPGDILVFLTGQEEIEAACEMLQDRCRRLGSKIRELLVLPIYANLPSDMQARIFQPTPPGARKVVVATNIAETSLTIEGIIYVLDPGFCKQKSYNPRTGMESLTVTPCSKASANQRAGRAGRVAAGKCFRLYTAWAYQHELEETTVPEIQRTSLGNVVLLLKSLGIHDLMHFDFLDPPPYETLLLALEQLYALGALNHLGELTTSGRKMAELPVDPMLSKMILASEKYSCSEEILTVAAMLSVNNSIFYRPKDKVVHADNARVNFFLPGGDHLVLLNVYTQWAESGYSSQWCYENFVQFRSMRRARDVREQLEGLLERVEVGLSSCQGDYIRVRKAITAGYFYHTARLTRSGYRTVKQQQTVFIHPNSSLFEQQPRWLLYHELVLTTKEFMRQVLEIESSWLLEVAPHYYKAKELEDPHAKKMPKKIGKTREELG

>NP_005300.1

MASSTGDRSQAVRHGLRAKVLTLDGMNPRVRRVEYAVRGPIVQRALELEQELRQGVKKPFTEVIRANIGDAQAMGQRPITFLRQVLALCVNPDLLSSPNFPDDAKKRAERILQACGGHSLGAYSVSSGIQLIREDVARYIERRDGGIPADPNNVFLSTGASDAIVTVLKLLVAGEGHTRTGVLIPIPQYPLYSATLAELGAVQVDYYLDEERAWALDVAELHRALGQARDHCRPRALCVINPGNPTGQVQTRECIEAVIRFAFEERLFLLADEVYQDNVYAAGSQFHSFKKVLMEMGPPYAGQQELASFHSTSKGYMGECGFRGGYVEVVNMDAAVQQQMLKLMSVRLCPPVPGQALLDLVVSPPAPTDPSFAQFQAEKQAVLAELAAKAKLTEQVFNEAPGISCNPVQGAMYSFPRVQLPPRAVERAQELGLAPDMFFCLRLLEETGICVVPGSGFGQREGTYHFRMTILPPLEKLRLLLEKLSRFHAKFTLEYS

591 NP_003205.2 Q96A58

>NP_003205.2

MASNSWNASSSPGEAREDGPEGLDKGLDNDAEGVWSPDIEQSFQEALAIYPPCGRRKIILSDEGKMYGRNELIARYIKLRTGKTRTRKQVSSHIQVLARKKVREYQVGIKAMNLDQVSKDKALQSMASMSSAQIVSASVLQNKFSPPSPLPQAVFSTSSRFWSSPPLLGQQPGPSQDIKPFAQPAYPIQPPLPPTLSSYEPLAPLPSAAASVPVWQDRTIASSRLRLLEYSAFMEVQRDPDTYSKHLFVHIGQTNPAFSDPPLEAVDVRQIYDKFPEKKGGLKELYEKGPPNAFFLVKFWADLNSTIQEGPGAFYGVSSQYSSADSMTISVSTKVCSFGKQVVEKVETEYARLENGRFVYRIHRSPMCEYMINFIHKLKHLPEKYMMNSVLENFTILQVVTSRDSQETLLVIAFVFEVSTSEHGAQHHVYKLVKD

>Q96A58

MAKSAEVKLAIFGRAGVGKSALVVRFLTKRFIWEYDPTLESTYRHQATIDDEVVSMEILDTAGQEDTIQREGHMRWGEGFVLVYDITDRGSFEEVLPLKNILDEIKKPKNVTLILVGNKADLDHSRQVSTEEGEKLATELACAFYECSACTGEGNITEIFYELCREVRRRRMVQGKTRRRSSTTHVKQAINKMLTKISS

592 Q9UBD5 Q9HBG6

>Q9UBD5

MATSSMSKGCFVFKPNSKKRKISLPIEDYFNKGKNEPEDSKLRFETYQLIWQQMKSENERLQEELNKNLFDNLIEFLQKSHSGFQKNSRDLGGQIKLREIPTAALVLGVNVTDHDLTFGSLTEALQNNVTPYVVSLQAKDCPDMKHFLQKLISQLMDCCVDIKSKEEESVHVTQRKTHYSMDSLSSWYMTVTQKTDPKMLSKKRTTSSQWQSPPVVVILKDMESFATKVLQDFIIISSQHLHEFPLILIFGIATSPIIIHRLLPHAVSSLLCIELFQSLSCKEHLTTVLDKLLLTTQFPFKINEKVLQVLTNIFLYHDFSVQNFIKGLQLSLLEHFYSQPLSVLCCNLPEAKRRINFLSNNQCENIRRLPSFRRYVEKQASEKQVALLTNERYLKEETQLLLENLHVYHMNYFLVLRCLHKFTSSLPKYPLGRQIRELYCTCLEKNIWDSEEYASVLQLLRMLAKDELMTILEKCFKVFKSYCENHLGSTAKRIEEFLAQFQSLDETKEEEDASGSQPKGLQKTDLYHLQKSLLEMKELRRSKKQTKFEVLRENVVNFIDCLVREYLLPPETQPLHEVVYFSAAHALREHLNAAPRIALHTALNNPYYYLKNEALKSEEGCIPNIAPDICIAYKLHLECSRLINLVDWSEAFATVVTAAEKMDANSATSEEMNEIIHARFIRAVSELELLGFIKPTKQKTDHVARLTWGGC

>Q9HBG6

MRAVLTWRDKAEHCINDIAFKPDGTQLILAAGSRLLVYDTSDGTLLQPLKGHKDTVYCVAYAKDGKRFASGSADKSVIIWTSKLEGILKYTHNDAIQCVSYNPITHQLASCSSSDFGLWSPEQKSVSKHKSSSKIICCSWTNDGQYLALGMFNGIISIRNKNGEEKVKIERPGGSLSPIWSICWNPSSRWESFWMNRENEDAEDVIVNRYIQEIPSTLKSAVYSSQGSEAEEEEPEEEDDSPRDDNLEERNDILAVADWGQKVSFYQLSGKQIGKDRALNFDPCCISYFTKGEYILLGGSDKQVSLFTKDGVRLGTVGEQNSWVWTCQAKPDSNYVVVGCQDGTISFYQLIFSTVHGLYKDRYAYRDSMTDVIVQHLITEQKVRIKCKELVKKIAIYRNRLAIQLPEKILIYELYSEDLSDMHYRVKEKIIKKFECNLLVVCANHIILCQEKRLQCLSFSGVKEREWQMESLIRYIKVIGGPPGREGLLVGLKNGQILKIFVDNLFAIVLLKQATAVRCLDMSASRKKLAVVDENDTCLVYDIDTKELLFQEPNANSVAWNTQCEDMLCFSGGGYLNIKASTFPVHRQKLQGFVVGYNGSKIFCLHVFSISAVEVPQSAPMYQYLDRKLFKEAYQIACLGVTDTDWRELAMEALEGLDFETAKKAFIRVQDLRYLELISSIEERKKRGETNNDLFLADVFSYQGKFHEAAKLYKRSGHENLALEMYTDLCMFEYAKDFLGSGDPKETKMLITKQADWARNIKEPKAAVEMYISAGEHVKAIEICGDHGWVDMLIDIARKLDKAEREPLLLCATYLKKLDSPGYAAETYLKMGDLKSLVQLHVETQRWDEAFALGEKHPEFKDDIYMPYAQWLAENDRFEEAQKAFHKAGRQREAVQVLEQLTNNAVAESRFNDAAYYYWMLSMQCLDIAQDPAQKDTMLGKFYHFQRLAELYHGYHAIHRHTEDPFSVHRPETLFNISRFLLHSLPKDTPSGISKVKILFTLAKQSKALGAYRLARHAYDKLRGLYIPARFQKSIELGTLTIRAKPFHDSEELVPLCYRCSTNNPLLNNLGNVCINCRQPFIFSASSYDVLHLVEFYLEEGITDEEAISLIDLEVLRPKRDDRQLEIANNSSQILRLVETKDSIGDEDPFTAKLSFEQGGSEFVPVVVSRLVLRSMSRRDVLIKRWPPPLRWQYFRSLLPDASITMCPSCFQMFHSEDYELLVLQHGCCPYCRRCKDDPGP

593 NP_036377.1 NP_689953.1

>NP_036377.1

MALTSFLPAPTQLSQDQLEAEEKARSQRSRQTSLVSSRREPPPYGYRKGWIPRLLEDFGDGGAFPEIHVAQYPLDMGRKKKMSNALAIQVDSEGKIKYDAIARQGQSKDKVIYSKYTDLVPKEVMNADDPDLQRPDEEAIKEITEKTRVALEKSVSQKVAAAMPVRAADKLAPAQYIRYTPSQQGVAFNSGAKQRVIRMVEMQKDPMEPPRFKINKKIPRGPPSPPAPVMHSPSRKMTVKEQQEWKIPPCISNWKNAKGYTIPLDKRLAADGRGLQTVHINENFAKLAEALYIADRKAREAVEMRAQVERKMAQKEKEKHEEKLREMAQKARERRAGIKTHVEKEDGEARERDEIRHDRRKERQHDRNLSRAAPDKRSKLQRNENRDISEVIALGVPNPRTSNEVQYDQRLFNQSKGMDSGFAGGEDEIYNVYDQAWRGGKDMAQSIYRPSKNLDKDMYGDDLEARIKTNRFVPDKEFSGSDRRQRGREGPVQFEEDPFGLDKFLEEAKQHGGSKRPSDSSRPKEHEHEGKKRRKE

>NP_689953.1

MAASLRLLGAASGLRYWSRRLRPAAGSFAAVCSRSVASKTPVGFIGLGNMGNPMAKNLMKHGYPLIIYDVFPDACKEFQDAGEQVVSSPADVAEKADRIITMLPTSINAIEAYSGANGILKKVKKGSLLIDSSTIDPAVSKELAKEVEKMGAVFMDAPVSGGVGAARSGNLTFMVGGVEDEFAAAQELLGCMGSNVVYCGAVGTGQAAKICNNMLLAISMIGTAEAMNLGIRLGLDPKLLAKILNMSSGRCWSSDTYNPVPGVMDGVPSANNYQGGFGTTLMAKDLGLAQDSATSTKSPILLGSLAHQIYRMMCAKGYSKKDFSSVFQFLREEETF

594 NP_008828.1 Q99707

>NP_008828.1

MSATGPISNYYVDSLISHDNEDLLASRFPATGAHPAAARPSGLVPDCSDFPSCSFAPKPAVFSTSWAPVPSQSSVVYHPYGPQPHLGADTRYMRTWLEPLSGAVSFPSFPAGGRHYALKPDAYPGRRADCGPGEGRSYPDYMYGSPGELRDRAPQTLPSPEADALAGSKHKEEKADLDPSNPVANWIHARSTRKKRCPYTKYQTLELEKEFLFNMYLTRDRRYEVARVLNLTERQVKIWFQNRRMKMKKMNKEKTDKEQS

>Q99707

MSPALQDLSQPEGLKKTLRDEINAILQKRIMVLDGGMGTMIQREKLNEEHFRGQEFKDHARPLKGNNDILSITQPDVIYQIHKEYLLAGADIIETNTFSSTSIAQADYGLEHLAYRMNMCSAGVARKAAEEVTLQTGIKRFVAGALGPTNKTLSVSPSVERPDYRNITFDELVEAYQEQAKGLLDGGVDILLIETIFDTANAKAALFALQNLFEEKYAPRPIFISGTIVDKSGRTLSGQTGEGFVISVSHGEPLCIGLNCALGAAEMRPFIEIIGKCTTAYVLCYPNAGLPNTFGDYDETPSMMAKHLKDFAMDGLVNIVGGCCGSTPDHIREIAEAVKNCKPRVPPATAFEGHMLLSGLEPFRIGPYTNFVNIGERCNVAGSRKFAKLIMAGNYEEALCVAKVQVEMGAQVLDVNMDDGMLDGPSAMTRFCNLIASEPDIAKVPLCIDSSNFAVIEAGLKCCQGKCIVNSISLKEGEDDFLEKARKIKKYGAAMVVMAFDEEGQATETDTKIRVCTRAYHLLVKKLGFNPNDIIFDPNILTIGTGMEEHNLYAINFIHATKVIKETLPGARISGGLSNLSFSFRGMEAIREAMHGVFLYHAIKSGMDMGIVNAGNLPVYDDIHKELLQLCEDLIWNKDPEATEKLLRYAQTQGTGGKKVIQTDEWRNGPVEERLEYALVKGIEKHIIEDTEEARLNQKKYPRPLNIIEGPLMNGMKIVGDLFGAGKMFLPQVIKSARVMKKAVGHLIPFMEKEREETRVLNGTVEEEDPYQGTIVLATVKGDVHDIGKNIVGVVLGCNNFRVIDLGVMTPCDKILKAALDHKADIIGLSGLITPSLDEMIFVAKEMERLAIRIPLLIGGATTSKTHTAVKIAPRYSAPVIHVLDASKSVVVCSQLLDENLKDEYFEEIMEEYEDIRQDHYESLKERRYLPLSQARKSGFQMDWLSEPHPVKPTFIGTQVFEDYDLQKLVDYIDWKPFFDVWQLRGKYPNRGFPKIFNDKTVGGEARKVYDDAHNMLNTLISQKKLRARGVVGFWPAQSIQDDIHLYAEAAVPQAAEPIATFYGLRQQAEKDSASTEPYYCLSDFIAPLHSGIRDYLGLFAVACFGVEELSKAYEDDGDDYSSIMVKALGDRLAEAFAEELHERVRRELWAYCGSEQLDVADLRRLRYKGIRPAPGYPSQPDHTEKLTMWRLADIEQSTGIRLTESLAMAPASAVSGLYFSNLKSKYFAVGKISKDQVEDYALRKNISVAEVEKWLGPILGYDTD

595 NP_006345.1 Q8N142

>NP_006345.1

MSELKDCPLQFHDFKSVDHLKVCPRYTAVLARSEDDGIGIEELDTLQLELETLLSSASRRLRVLEAETQILTDWQDKKGDRRFLKLGRDHELGAPPKHGKPKKQKLEGKAGHGPGPGPGRPKSKNLQPKIQEYEFTDDPIDVPRIPKNDAPNRFWASVEPYCADITSEEVRTLEELLKPPEDEAEHYKIPPLGKHYSQRWAQEDLLEEQKDGARAAAVADKKKGLMGPLTELDTKDVDALLKKSEAQHEQPEDGCPFGALTQRLLQALVEENIISPMEDSPIPDMSGKESGADGASTSPRNQNKPFSVPHTKSLESRIKEELIAQGLLESEDRPAEDSEDEVLAELRKRQAELKALSAHNRTKKHDLLRLAKEEVSRQELRQRVRMADNEVMDAFRKIMAARQKKRTPTKKEKDQAWKTLKERESILKLLDG

>Q8N142

MSGTRASNDRPPGAGGVKRGRLQQEAAATGSRVTVVLGAQWGDEGKGKVVDLLATDADIISRCQGGNNAGHTVVVDGKEYDFHLLPSGIINTKAVSFIGNGVVIHLPGLFEEAEKNEKKGLKDWEKRLIISDRAHLVFDFHQAVDGLQEVQRQAQEGKNIGTTKKGIGPTYSSKAARTGLRICDLLSDFDEFSSRFKNLAHQHQSMFPTLEIDIEGQLKRLKGFAERIRPMVRDGVYFMYEALHGPPKKILVEGANAALLDIDFGTYPFVTSSNCTVGGVCTGLGIPPQNIGDVYGVVKAYTTRVGIGAFPTEQINEIGGLLQTRGHEWGVTTGRKRRCGWLDLMILRYAHMVNGFTALALTKLDILDVLGEVKVGVSYKLNGKRIPYFPANQEMLQKVEVEYETLPGWKADTTGARRWEDLPPQAQNYIRFVENHVGVAVKWVGVGKSRESMIQLF

596 NP_115285.1 P59942

>NP_115285.1

MSCGRPPPDVDGMITLKVDNLTYRTSPDSLRRVFEKYGRVGDVYIPREPHTKAPRGFAFVRFHDRRDAQDAEAAMDGAELDGRELRVQVARYGRRDLPRSRQGEPRGRSRGGGYGRRSRSYGRRSRSPRRRHRSRSRGPSCSRSRSRSRYRGSRYSRSPYSRSPYSRSRYSRSPYSRSRYRESRYGGSHYSSSGYSNSRYSRYHSSRSHSKSGSSTSSRSASTSKSSSARRSKSSSVSRSRSRSRSSSMTRSPPRVSKRKSKSRSRSKRPPKSPEEEGQMSS

>P59942

MVLPLPWLSRYHFLRLLLPSWSLAPQGSHGCCSQNPKASMEEQTNSRGNGKMTSPPRGPGTHRTAELARAEELLEQQLELYQALLEGQEGAWEAQALVLKIQKLKEQMRRHQESLGGGA

597 NP_005625.2 P59942

>NP_005625.2

MRPVRENSSGARSPRVPADLARSILISLPFPPDSLAHRPPSSAPTESQGLFTVAAPAPGAPSPPATLAHLLPAPAMYSLLETELKNPVGTPTQAAGTGGPAAPGGAGKSSANAAGGANSGGGSSGGASGGGGGTDQDRVKRPMNAFMVWSRGQRRKMALENPKMHNSEISKRLGADWKLLTDAEKRPFIDEAKRLRAVHMKEYPDYKYRPRRKTKTLLKKDKYSLPSGLLPPGAAAAAAAAAAAAAAASSPVGVGQRLDTYTHVNGWANGAYSLVQEQLGYAQPPSMSSPPPPPALPPMHRYDMAGLQYSPMMPPGAQSYMNVAAAAAAASGYGGMAPSATAAAAAAYGQQPATAAAAAAAAAAMSLGPMGSVVKSEPSSPPPAIASHSQRACLGDLRDMISMYLPPGGDAADAASPLPGGRLHGVHQHYQGAGTAVNGTVPLTHI

>P59942

MVLPLPWLSRYHFLRLLLPSWSLAPQGSHGCCSQNPKASMEEQTNSRGNGKMTSPPRGPGTHRTAELARAEELLEQQLELYQALLEGQEGAWEAQALVLKIQKLKEQMRRHQESLGGGA

598 NP_001443.1 NP_055433.2

>NP_001443.1

MTTEGGPPPAPLRRACSPVPGALQAALMSPPPAAAAAAAAAPETTSSSSSSSSASCASSSSSSNSASAPSAACKSAGGGGAGAGSGGAKKASSGLRRPEKPPYSYIALIVMAIQSSPSKRLTLSEIYQFLQARFPFFRGAYQGWKNSVRHNLSLNECFIKLPKGLGRPGKGHYWTIDPASEFMFEEGSFRRRPRGFRRKCQALKPMYHRVVSGLGFGASLLPQGFDFQAPPSAPLGCHSQGGYGGLDMMPAGYDAGAGAPSHAHPHHHHHHHVPHMSPNPGSTYMASCPVPAGPGGVGAAGGGGGGDYGPDSSSSPVPSSPAMASAIECHSPYTSPAAHWSSPGASPYLKQPPALTPSSNPAASAGLHSSMSSYSLEQSYLHQNAREDLSVGLPRYQHHSTPVCDRKDFVLNFNGISSFHPSASGSYYHHHHQSVCQDIKPCVM

>NP_055433.2

MNWRFVELLYFLFIWGRISVQPSHQEPAGTDQHVSKEFDWLISDRGPFHHSRSYLSFVERHRQGFTTRYKIYREFARWKVRNTAIERRDLVRHPVPLMPEFQRSIRLLGRRPTTQQFIDTIIKKYGTHLLISATLGGEEALTMYMDKSRLDRKSGNATQSVEALHQLASSYFVDRDGTMRRLHEIQISTGAIKVTETRTGPLGCNSYDNLDSVSSVLLQSTESKLHLQGLQIIFPQYLQEKFVQSALSYIMCNGEGEYLCQNSQCRCQCAEEFPQCNCPITDIQIMEYTLANMAKSWAEAYKDLENSDEFKSFMKRLPSNHFLTIGSIHQHWGNDWDLQNRYKLLQSATEAQRQKIQRTARKLFGLSVRCRHNPNHQLPRERTIQQWLARVQSLLYCNENGFWGTFLESQRSCVCHGSTTLCQRPIPCVIGGNNSCAMCSLANISLCGSCNKGYKLYRGRCEPQNVDSERSEQFISFETDLDFQDLELKYLLQKMDSRLYVHTTFISNEIRLDTFFDPRWRKRMSLTLKSNKNRMDFIHMVIGMSMRICQMRNSSLDPMFFVYVNPFSGSHSEGWNMPFGEFGYPRWEKIRLQNSQCYNWTLLLGNRWKTFFETVHIYLRSRTRLPTLLRNETGQGPVDLSDPSKRQFYIKISDVQVFGYSLRFNADLLRSAVQQVNQSYTQGGQFYSSSSVMLLLLDIRDRINRLAPPVAPGKPQLDLFSCMLKHRLKLTNSEIIRVNHALDLYNTEILKQSDQMTAKLC

599 NP_006259.1 NP_071910.3

>NP_006259.1

MAAVVENVVKLLGEQYYKDAMEQCHNYNARLCAERSVRLPFLDSQTGVAQSNCYIWMEKRHRGPGLASGQLYSYPARRWRKKRRAHPPEDPRLSFPSIKPDTDQTLKKEGLISQDGSSLEALLRTDPLEKRGAPDPRVDDDSLGEFPVTNSRARKRILEPDDFLDDLDDEDYEEDTPKRRGKGKSKGKGVGSARKKLDASILEDRDKPYACDICGKRYKNRPGLSYHYAHSHLAEEEGEDKEDSQPPTPVSQRSEEQKSKKGPDGLALPNNYCDFCLGDSKINKKTGQPEELVSCSDCGRSGHPSCLQFTPVMMAAVKTYRWQCIECKCCNICGTSENDDQLLFCDDCDRGYHMYCLTPSMSEPPEGSWSCHLCLDLLKEKASIYQNQNSS

>NP_071910.3

MHTPPALPRRFQGGGRVRTPGSHRQGKDNLERDPSGGCVPDFLPQAQDSNHFIMESLFCESSGDSSLEKEFLGAPVGPSVSTPNSQHSSPSRSLSANSIKVEMYSDEESSRLLGPDERLLEKDDSVIVEDSLSEPLGYCDGSGPEPHSPGGIRLPNGKLKCDVCGMVCIGPNVLMVHKRSHTGERPFHCNQCGASFTQKGNLLRHIKLHSGEKPFKCPFCNYACRRRDALTGHLRTHSVSSPTVGKPYKCNYCGRSYKQQSTLEEHKERCHNYLQSLSTEAQALAGQPGDEIRDLEMVPDSMLHSSSERPTFIDRLANSLTKRKRSTPQKFVGEKQMRFSLSDLPYDVNSGGYEKDVELVAHHSLEPGFGSSLAFVGAEHLRPLRLPPTNCISELTPVISSVYTQMQPLPGRLELPGSREAGEGPEDLADGGPLLYRPRGPLTDPGASPSNGCQDSTDTESNHEDRVAGVVSLPQGPPPQPPPTIVVGRHSPAYAKEDPKPQEGLLRGTPGPSKEVLRVVGESGEPVKAFKCEHCRILFLDHVMFTIHMGCHGFRDPFECNICGYHSQDRYEFSSHIVRGEHKVG

600 NP_005089.2 Q9BU02

>NP_005089.2

MSTGSVSDPEEMELRGLQREYPVPASKRPPLRGVERSYASPSDNSSAEEEDPDGEEERCALGTAGSAEGCKRKRPRVAGGGGAGGSAGGGGKKPLPAKGSAAECKQSQRNAANARERARMRVLSKAFSRLKTSLPWVPPDTKLSKLDTLRLASSYIAHLRQLLQEDRYENGYVHPVNLTWPFVVSGRPDSDTKEVSAANRLCGTTA

>Q9BU02

MAQGLIEVERKFLPGPGTEERLQELGGTLEYRVTFRDTYYDTPELSLMQADHWLRRREDSGWELKCPGAAGVLGPHTEYKELTAEPTIVAQLCKVLRADGLGAGDVAAVLGPLGLQEVASFVTKRSAWKLVLLGADEEEPQLRVDLDTADFGYAVGEVEALVHEEAEVPTALEKIHRLSSMLGVPAQETAPAKLIVYLQRFRPQDYQRLLEVNSSRERPQETEDPDHCLG

601 NP_000512.1 Q9NVV4

>NP_000512.1

MELCGLGLPRPPMLLALLLATLLAAMLALLTQVALVVQVAEAARAPSVSAKPGPALWPLPLSVKMTPNLLHLAPENFYISHSPNSTAGPSCTLLEEAFRRYHGYIFGFYKWHHEPAEFQAKTQVQQLLVSITLQSECDAFPNISSDESYTLLVKEPVAVLKANRVWGALRGLETFSQLVYQDSYGTFTINESTIIDSPRFSHRGILIDTSRHYLPVKIILKTLDAMAFNKFNVLHWHIVDDQSFPYQSITFPELSNKGSYSLSHVYTPNDVRMVIEYARLRGIRVLPEFDTPGHTLSWGKGQKDLLTPCYSRQNKLDSFGPINPTLNTTYSFLTTFFKEISEVFPDQFIHLGGDEVEFKCWESNPKIQDFMRQKGFGTDFKKLESFYIQKVLDIIATINKGSIVWQEVFDDKAKLAPGTIVEVWKDSAYPEELSRVTASGFPVILSAPWYLDLISYGQDWRKYYKVEPLDFGGTQKQKQLFIGGEACLWGEYVDATNLTPRLWPRASAVGERLWSSKDVRDMDDAYDRLTRHRCRMVERGIAAQPLYAGYCNHENM

>Q9NVV4

MAVPGVGLLTRLNLCARRRTRVQRPIVRLLSCPGTVAKDLRRDEQPSGSVETGFEDKIPKRRFSEMQNERREQAQRTVLIHCPEKISENKFLKYLSQFGPINNHFFYESFGLYAVVEFCQKESIGSLQNGTHTPSTAMETAIPFRSRFFNLKLKNQTSERSRVRSSNQLPRSNKQLFELLCYAESIDDQLNTLLKEFQLTEENTKLRYLTCSLIEDMAAAYFPDCIVRPFGSSVNTFGKLGCDLDMFLDLDETRNLSAHKISGNFLMEFQVKNVPSERIATQKILSVLGECLDHFGPGCVGVQKILNARCPLVRFSHQASGFQCDLTTNNRIALTSSELLYIYGALDSRVRALVFSVRCWARAHSLTSSIPGAWITNFSLTMMVIFFLQRRSPPILPTLDSLKTLADAEDKCVIEGNNCTFVRDLSRIKPSQNTETLELLLKEFFEYFGNFAFDKNSINIRQGREQNKPDSSPLYIQNPFETSLNISKNVSQSQLQKFVDLARESAWILQQEDTDRPSISSNRPWGLVSLLLPSAPNRKSFTKKKSNKFAIETVKNLLESLKGNRTENFTKTSGKRTISTQT

602 NP_003191.1 NP_001814.2

>NP_003191.1

MNQPQRMAPVGTDKELSDLLDFSMMFPLPVTNGKGRPASLAGAQFGGSGLEDRPSSGSWGSGDQSSSSFDPSRTFSEGTHFTESHSSLSSSTFLGPGLGGKSGERGAYASFGRDAGVGGLTQAGFLSGELALNSPGPLSPSGMKGTSQYYPSYSGSSRRRAADGSLDTQPKKVRKVPPGLPSSVYPPSSGEDYGRDATAYPSAKTPSSTYPAPFYVADGSLHPSAELWSPPGQAGFGPMLGGGSSPLPLPPGSGPVGSSGSSSTFGGLHQHERMGYQLHGAEVNGGLPSASSFSSAPGATYGGVSSHTPPVSGADSLLGSRGTTAGSSGDALGKALASIYSPDHSSNNFSSSPSTPVGSPQGLAGTSQWPRAGAPGALSPSYDGGLHGLQSKIEDHLDEAIHVLRSHAVGTAGDMHTLLPGHGALASGFTGPMSLGGRHAGLVGGSHPEDGLAGSTSLMHNHAALPSQPGTLPDLSRPPDSYSGLGRAGATAAASEIKREEKEDEENTSAADHSEEEKKELKAPRARTSPDEDEDDLLPPEQKAEREKERRVANNARERLRVRDINEAFKELGRMCQLHLNSEKPQTKLLILHQAVSVILNLEQQVRERNLNPKAACLKRREEEKVSGVVGDPQMVLSAPHPGLSEAHNPAGHM

>NP_001814.2

MPFSNSHNALKLRFPAEDEFPDLSAHNNHMAKVLTPELYAELRAKSTPSGFTLDDVIQTGVDNPGHPYIMTVGCVAGDEESYEVFKDLFDPIIEDRHGGYKPSDEHKTDLNPDNLQGGDDLDPNYVLSSRVRTGRSIRGFCLPPHCSRGERRAIEKLAVEALSSLDGDLAGRYYALKSMTEAEQQQLIDDHFLFDKPVSPLLLASGMARDWPDARGIWHNDNKTFLVWVNEEDHLRVISMQKGGNMKEVFTRFCTGLTQIETLFKSKDYEFMWNPHLGYILTCPSNLGTGLRAGVHIKLPNLGKHEKFSEVLKRLRLQKRGTGGVDTAAVGGVFDVSNADRLGFSEVELVQMVVDGVKLLIEMEQRLEQGQAIDDLMPAQK

603 P16402 NP_000476.1

>P16402

MSETAPLAPTIPAPAEKTPVKKKAKKAGATAGKRKASGPPVSELITKAVAASKERSGVSLAALKKALAAAGYDVEKNNSRIKLGLKSLVSKGTLVQTKGTGASGSFKLNKKAASGEGKPKAKKAGAAKPRKPAGAAKKPKKVAGAATPKKSIKKTPKKVKKPATAAGTKKVAKSAKKVKTPQPKKAAKSPAKAKAPKPKAAKPKSGKPKVTKAKKAAPKKK

>NP_000476.1

MADSELQLVEQRIRSFPDFPTPGVVFRDISPVLKDPASFRAAIGLLARHLKATHGGRIDYIAGLDSRGFLFGPSLAQELGLGCVLIRKRGKLPGPTLWASYSLEYGKAELEIQKDALEPGQRVVVVDDLLATGGTMNAACELLGRLQAEVLECVSLVELTSLKGREKLAPVPFFSLLQYE

604 P31271 Q9Y3D5

>P31271

MTASVLLHPRWIEPTVMFLYDNGGGLVADELNKNMEGAAAAAAAAAAAAAAGAGGGGFPHPAAAAAGGNFSVAAAAAAAAAAAANQCRNLMAHPAPLAPGAASAYSSAPGEAPPSAAAAAAAAAAAAAAAAAASSSGGPGPAGPAGAEAAKQCSPCSAAAQSSSGPAALPYGYFGSGYYPCARMGPHPNAIKSCAQPASAAAAAAFADKYMDTAGPAAEEFSSRAKEFAFYHQGYAAGPYHHHQPMPGYLDMPVVPGLGGPGESRHEPLGLPMESYQPWALPNGWNGQMYCPKEQAQPPHLWKSTLPDVVSHPSDASSYRRGRKKRVPYTKVQLKELEREYATNKFITKDKRRRISATTNLSERQVTIWFQNRRVKEKKVINKLKTTS

>Q9Y3D5

MAAVVAVCGGLGRKKLTHLVTAAVSLTHPGTHTVLWRRGCSQQVSSNEDLPISMENPYKEPLKKCILCGKHVDYKNVQLLSQFVSPFTGCIYGRHITGLCGKKQKEITKAIKRAQIMGFMPVTYKDPAYLKDPKVCNIRYRE

605 NP_005243.1 Q9HD34

>NP_005243.1

MMFSGFNADYEASSSRCSSASPAGDSLSYYHSPADSFSSMGSPVNAQDFCTDLAVSSANFIPTVTAISTSPDLQWLVQPALVSSVAPSQTRAPHPFGVPAPSAGAYSRAGVVKTMTGGRAQSIGRRGKVEQLSPEEEEKRRIRRERNKMAAAKCRNRRRELTDTLQAETDQLEDEKSALQTEIANLLKEKEKLEFILAAHRPACKIPDDLGFPEEMSVASLDLTGGLPEVATPESEEAFTLPLLNDPEPKPSVEPVKSISSMELKTEPFDDFLFPASSRPSGSETARSVPDMDLSGSFYAADWEPLHSGSLGMGPMATELEPLCTPVVTCTPSCTAYTSSFVFTYPEADSFPSCAAAHRKGSSSNEPSSDSLSSPTLLAL

>Q9HD34

MAASSRAQVLSLYRAMLRESKRFSAYNYRTYAVRRIRDAFRENKNVKDPVEIQTLVNKAKRDLGVIRRQVHIGQLYSTDKLIIENRDMPRT

606 Q8TCE9 Q9UHN1

>Q8TCE9

MSSLPVPYTLPVSLPVGSCVIITGTPILTFVKDPQLEVNFYTGMDEDSDIAFQFRLHFGHPAIMNSCVFGIWRYEEKCYYLPFEDGKPFELCIYVRHKEYKVMVNGQRIYNFAHRFPPASVKMLQVFRDISLTRVLISD

>Q9UHN1

MRSRVAVRACHKVCRCLLSGFGGRVDAGQPELLTERSSPKGGHVKSHAELEGNGEHPEAPGSGEGSEALLEICQRRHFLSGSKQQLSRDSLLSGCHPGFGPLGVELRKNLAAEWWTSVVVFREQVFPVDALHHKPGPLLPGDSAFRLVSAETLREILQDKELSKEQLVAFLENVLKTSGKLRENLLHGALEHYVNCLDLVNKRLPYGLAQIGVCFHPVFDTKQIRNGVKSIGEKTEASLVWFTPPRTSNQWLDFWLRHRLQWWRKFAMSPSNFSSSDCQDEEGRKGNKLYYNFPWGKELIETLWNLGDHELLHMYPGNVSKLHGRDGRKNVVPCVLSVNGDLDRGMLAYLYDSFQLTENSFTRKKNLHRKVLKLHPCLAPIKVALDVGRGPTLELRQVCQGLFNELLENGISVWPGYLETMQSSLEQLYSKYDEMSILFTVLVTETTLENGLIHLRSRDTTMKEMMHISKLKDFLIKYISSAKNV

607 NP_055368.1 P82664

>NP_055368.1

MLFWHTQPEHYNQHNSGSYLRDVLALPIFKQEEPQLSPENEARLPPLQYVLCAATSPAVKLHEETLTYLNQGQSYEIRLLENRKLGDFQDLNTKYVKSIIRVVFHDRRLQYTEHQQLEGWRWSRPGDRILDIDIPLSVGILDPRASPTQLNAVEFLWDPAKRASAFIQVHCISTEFTPRKHGGEKGVPFRVQIDTFKQNENGEYTEHLHSASCQIKVFKPKGADRKQKTDREKMEKRTAQEKEKYQPSYETTILTECSPWPDVAYQVNSAPSPSYNGSPNSFGLGEGNASPTHPVEALPVGSDHLLPSASIQDAQQWLHRNRFSQFCRLFASFSGADLLKMSRDDLVQICGPADGIRLFNAIKGRNVRPKMTIYVCQELEQNRVPLQQKRDGSGDSNLSVYHAIFLEELTTLELIEKIANLYSISPQHIHRVYRQGPTGIHVVVSNEMVQNFQDESCFVLSTIKAESNDGYHIILKCGL

>P82664

MAARTAFGAVCRRLWQGLGNFSVNTSKGNTAKNGGLLLSTNMKWVQFSNLHVDVPKDLTKPVVTISDEPDILYKRLSVLVKGHDKAVLDSYEYFAVLAAKELGISIKVHEPPRKIERFTLLQSVHIYKKHRVQYEMRTLYRCLELEHLTGSTADVYLEYIQRNLPEGVAMEVTKTQLEQLPEHIKEPIWETLSEEKEESKS

608 Q96RE9 P21399

>Q96RE9

MMKSQGLVSFKDVAVDFTQEEWQQLDPSQRTLYRDVMLENYSHLVSMGYPVSKPDVISKLEQGEEPWIIKGDISNWIYPDEYQADGRQDRKSNLHNSQSCILGTVSFHHKILKGVTRDGSLCSILKVCQGDGQLQRFLENQDKLFRQVTFVNSKTVTEASGHKYNPLGKIFQECIETDISIQRFHKYDAFKKNLKPNIDLPSCYKSNSRKKPDQSFGGGKSSSQSEPNSNLEKIHNGVIPFDDNQCGNVFRNTQSLIQYQNVETKEKSCVCVTCGKAFAKKSQLIVHQRIHTGKKPYDCGACGKAFSEKFHLVVHQRTHTGEKPYDCSECGKAFSQKSSLIIHQRVHTGEKPYECSECGKAFSQKSPLIIHQRIHTGEKPYECRECGKAFSQKSQLIIHHRAHTGEKPYECTECGKAFCEKSHLIIHKRIHTGEKPYKCAQCEEAFSRKTELITHQLVHTGEKPYECTECGKTFSRKSQLIIHQRTHTGEKPYKCSECGKAFCQKSHLIGHQRIHTGEKPYICTECGKAFSQKSHLPGHQRIHTGEKPYICAECGKAFSQKSDLVLHQRIHTGERPYQCAICGKAFIQKSQLTVHQRIHTVVKS

>P21399

MSNPFAHLAEPLDPVQPGKKFFNLNKLEDSRYGRLPFSIRVLLEAAIRNCDEFLVKKQDIENILHWNVTQHKNIEVPFKPARVILQDFTGVPAVVDFAAMRDAVKKLGGDPEKINPVCPADLVIDHSIQVDFNRRADSLQKNQDLEFERNRERFEFLKWGSQAFHNMRIIPPGSGIIHQVNLEYLARVVFDQDGYYYPDSLVGTDSHTTMIDGLGILGWGVGGIEAEAVMLGQPISMVLPQVIGYRLMGKPHPLVTSTDIVLTITKHLRQVGVVGKFVEFFGPGVAQLSIADRATIANMCPEYGATAAFFPVDEVSITYLVQTGRDEEKLKYIKKYLQAVGMFRDFNDPSQDPDFTQVVELDLKTVVPCCSGPKRPQDKVAVSDMKKDFESCLGAKQGFKGFQVAPEHHNDHKTFIYDNTEFTLAHGSVVIAAITSCTNTSNPSVMLGAGLLAKKAVDAGLNVMPYIKTSLSPGSGVVTYYLQESGVMPYLSQLGFDVVGYGCMTCIGNSGPLPEPVVEAITQGDLVAVGVLSGNRNFEGRVHPNTRANYLASPPLVIAYAIAGTIRIDFEKEPLGVNAKGQQVFLKDIWPTRDEIQAVERQYVIPGMFKEVYQKIETVNESWNALATPSDKLFFWNSKSTYIKSPPFFENLTLDLQPPKSIVDAYVLLNLGDSVTTDHISPAGNIARNSPAARYLTNRGLTPREFNSYGSRRGNDAVMARGTFANIRLLNRFLNKQAPQTIHLPSGEILDVFDAAERYQQAGLPLIVLAGKEYGAGSSRDWAAKGPFLLGIKAVLAESYERIHRSNLVGMGVIPLEYLPGENADALGLTGQERYTIIIPENLKPQMKVQVKLDTGKTFQAVMRFDTDVELTYFLNGGILNYMIRKMAK

609 NP_002948.1 Q92526

>NP_002948.1

MDTKHFLPLDFSTQVNSSLTSPTGRGSMAAPSLHPSLGPGIGSPGQLHSPISTLSSPINGMGPPFSVISSPMGPHSMSVPTTPTLGFSTGSPQLSSPMNPVSSSEDIKPPLGLNGVLKVPAHPSGNMASFTKHICAICGDRSSGKHYGVYSCEGCKGFFKRTVRKDLTYTCRDNKDCLIDKRQRNRCQYCRYQKCLAMGMKREAVQEERQRGKDRNENEVESTSSANEDMPVERILEAELAVEPKTETYVEANMGLNPSSPNDPVTNICQAADKQLFTLVEWAKRIPHFSELPLDDQVILLRAGWNELLIASFSHRSIAVKDGILLATGLHVHRNSAHSAGVGAIFDRVLTELVSKMRDMQMDKTELGCLRAIVLFNPDSKGLSNPAEVEALREKVYASLEAYCKHKYPEQPGRFAKLLLRLPALRSIGLKCLEHLFFFKLIGDTPIDTFLMEMLEAPHQMT

>Q92526

MAAIKAVNSKAEVARAQAALAVNICAARGLQDVLRTNLGPKGTMKMLASGAGDIKLTKDGNVLLDEMQIQHPTASLIAKVATAQDDVTGDGTTSNVLIIGELLKQADLYISEGLHPRIIAEGFEAAKIKALEVLEEVKVTKEMKRKILLDVARTSLQTKVHAELADVLTEVVVDSVLAVRRPGYPIDLFMVEIMEMKHKLGTDTKLIQGLVLDHGARHPDMKKRVEDAFILICNVSLEYEKTEVNSGFFYKTAEEKEKLVKAERKFIEDRVQKIIDLKDKVCAQSNKGFVVINQKGIDPFSLDSLAKHGIVALRRAKRRNMERLSLACGGMAVNSFEDLTVDCLGHAGLVYEYTLGEEKFTFIEECVNPCSVTLLVKGPNKHTLTQVKDAIRDGLRAIKNAIEDGCMVPGAGAIEVAMAEALVTYKNSIKGRARLGVQAFADALLIIPKVLAQNAGYDPQETLVKVQAEHVESKQLVGVDLNTGEPMVAADAGVWDNYCVKKQLLHSCTVIATNILLVDEIMRAGMSSLK

610 NP_076921.1 NP_060441.2

>NP_076921.1

MSSYFVNSLFSKYKTGESLRPNYYDCGFAQDLGGRPTVVYGPSSGGSFQHPSQIQEFYHGPSSLSTAPYQQNPCAVACHGDPGNFYGYDPLQRQSLFGAQDPDLVQYADCKLAAASGLGEEAEGSEQSPSPTQLFPWMRPQAAAGRRRGRQTYSRYQTLELEKEFLFNPYLTRKRRIEVSHALGLTERQVKIWFQNRRMKWKKENNKDKFPSSKCEQEELEKQKLERAPEAADEGDAQKGDKK

>NP_060441.2

MVFLTAQLWLRNRVTDRYFRIQEVLKHARHFRGRKNRCYRLAVRTVIRAFVKCTKARYLKKKNMRTLWINRITAASQEHGLKYPALIGNLVKCQVELNRKVLADLAIYEPKTFKSLAALASRRRHEGFAAALGDGKEPEGIFSRVVQYH

611 Q9Y3Z3 Q13287

>Q9Y3Z3

MQRADSEQPSKRPRCDDSPRTPSNTPSAEADWSPGLELHPDYKTWGPEQVCSFLRRGGFEEPVLLKNIRENEITGALLPCLDESRFENLGVSSLGERKKLLSYIQRLVQIHVDTMKVINDPIHGHIELHPLLVRIIDTPQFQRLRYIKQLGGGYYVFPGASHNRFEHSLGVGYLAGCLVHALGEKQPELQISERDVLCVQIAGLCHDLGHGPFSHMFDGRFIPLARPEVKWTHEQGSVMMFEHLINSNGIKPVMEQYGLIPEEDICFIKEQIVGPLESPVEDSLWPYKGRPENKSFLYEIVSNKRNGIDVDKWDYFARDCHHLGIQNNFDYKRFIKFARVCEVDNELRICARDKEVGNLYDMFHTRNSLHRRAYQHKVGNIIDTMITDAFLKADDYIEITGAGGKKYRISTAIDDMEAYTKLTDNIFLEILYSTDPKLKDAREILKQIEYRNLFKYVGETQPTGQIKIKREDYESLPKEVASAKPKVLLDVKLKAEDFIVDVINMDYGMQEKNPIDHVSFYCKTAPNRAIRITKNQVSQLLPEKFAEQLIRVYCKKVDRKSLYAARQYFVQWCADRNFTKPQDGDVIAPLITPQKKEWNDSTSVQNPTRLREASKSRVQLFKDDPM

>Q13287

MEADKDDTQQILKEHSPDEFIKDEQNKGLIDEITKKNIQLKKEIQKLETELQEATKEFQIKEDIPETKMKFLSVETPENDSQLSNISCSFQVSSKVPYEIQKGQALITFEKEEVAQNVVSMSKHHVQIKDVNLEVTAKPVPLNSGVRFQVYVEVSKMKINVTEIPDTLREDQMRDKLELSFSKSRNGGGEVDRVDYDRQSGSAVITFVEIGVADKILKKKEYPLYINQTCHRVTVSPYTEIHLKKYQIFSGTSKRTVLLTGMEGIQMDEEIVEDLINIHFQRAKNGGGEVDVVKCSLGQPHIAYFEE

612 NP_000241.1 NP_002631.3

>NP_000241.1

MGVPFFSSLRCMVDLGPCWAGGLTAEMKLLLALAGLLAILATPQPSEGAAPAVLGEVDTSLVLSSMEEAKQLVDKAYKERRESIKQRLRSGSASPMELLSYFKQPVAATRTAVRAADYLHVALDLLERKLRSLWRRPFNVTDVLTPAQLNVLSKSSGCAYQDVGVTCPEQDKYRTITGMCNNRRSPTLGASNRAFVRWLPAEYEDGFSLPYGWTPGVKRNGFPVALARAVSNEIVRFPTDQLTPDQERSLMFMQWGQLLDHDLDFTPEPAARASFVTGVNCETSCVQQPPCFPLKIPPNDPRIKNQADCIPFFRSCPACPGSNITIRNQINALTSFVDASMVYGSEEPLARNLRNMSNQLGLLAVNQRFQDNGRALLPFDNLHDDPCLLTNRSARIPCFLAGDTRSSEMPELTSMHTLLLREHNRLATELKSLNPRWDGERLYQEARKIVGAMVQIITYRDYLPLVLGPTAMRKYLPTYRSYNDSVDPRIANVFTNAFRYGHTLIQPFMFRLDNRYQPMEPNPRVPLSRVFFASWRVVLEGGIDPILRGLMATPAKLNRQNQIAVDEIRERLFEQVMRIGLDLPALNMQRSRDHGLPGYNAWRRFCGLPQPETVGQLGTVLRNLKLARKLMEQYGTPNNIDIWMGGVSEPLKRKGRVGPLLACIIGTQFRKLRDGDRFWWENEGVFSMQQRQALAQISLPRIICDNTGITTVSKNNIFMSNSYPRDFVNCSTLPALNLASWREAS

>NP_002631.3

MDDLCEANGTFAISLFKILGEEDNSRNVFFSPMSISSALAMVFMGAKGSTAAQMSQALCLYKDGDIHRGFQSLLSEVNRTGTQYLLRTANRLFGEKTCDFLPDFKEYCQKFYQAELEELSFAEDTEECRKHINDWVAEKTEGKISEVLDAGTVDPLTKLVLVNAIYFKGKWNEQFDRKYTRGMLFKTNEEKKTVQMMFKEAKFKMGYADEVHTQVLELPYVEEELSMVILLPDDNTDLAVVEKALTYEKFKAWTNSEKLTKSKVQVFLPRLKLEESYDLEPFLRRLGMIDAFDEAKADFSGMSTEKNVPLSKVAHKCFVEVNEEGTEAAAATAVVRNSRCSRMEPRFCADHPFLFFIRHHKTNCILFCGRFSSP

613 Q96S96 NP_001815.2

>Q96S96

MGWTMRLVTAALLLGLMMVVTGDEDENSPCAHEALLDEDTLFCQGLEVFYPELGNIGCKVVPDCNNYRQKITSWMEPIVKFPGAVDGATYILVMVDPDAPSRAEPRQRFWRHWLVTDIKGADLKKGKIQGQELSAYQAPSPPAHSGFHRYQFFVYLQEGKVISLLPKENKTRGSWKMDRFLNRFHLGEPEASTQFMTQNYQDSPTLQAPRERASEPKHKNQAEIAAC

>NP_001815.2

MPFGNTHNKFKLNYKPEEEYPDLSKHNNHMAKVLTLELYKKLRDKETPSGFTVDDVIQTGVDNPGHPFIMTVGCVAGDEESYEVFKELFDPIISDRHGGYKPTDKHKTDLNHENLKGGDDLDPNYVLSSRVRTGRSIKGYTLPPHCSRGERRAVEKLSVEALNSLTGEFKGKYYPLKSMTEKEQQQLIDDHFLFDKPVSPLLLASGMARDWPDARGIWHNDNKSFLVWVNEEDHLRVISMEKGGNMKEVFRRFCVGLQKIEEIFKKAGHPFMWNQHLGYVLTCPSNLGTGLRGGVHVKLAHLSKHPKFEEILTRLRLQKRGTGGVDTAAVGSVFDVSNADRLGSSEVEQVQLVVDGVKLMVEMEKKLEKGQSIDDMIPAQK

614 NP_777584.1 Q9NY74

>NP_777584.1

MDLLWMPLLLVAACVSAVHSSPEVNAGVSSIHITKPVHILEERSLLVLTPAGLTQMLNQTRFLMVLFHNPSSKQSRNLAEELGKAVEIMGKGKNGIGFGKVDITIEKELQQEFGITKAPELKLFFEGNRSEPISCKGVVESAALVVWLRRQISQKAFLFNSSEQVAEFVISRPLVIVGFFQDLEEEVAELFYDVIKDFPELTFGVITIGNVIGRFHVTLDSVLVFKKGKIVNRQKLINDSTNKQELNRVIKQHLTDFVIEYNTENKDLISELHIMSHMLLFVSKSSESYGIIIQHYKLASKEFQNKILFILVDADEPRNGRVFKYFRVTEVDIPSVQILNLSSDARYKMPSDDITYESLKKFGRSFLSKNATKHQSSEEIPKYWDQGLVKQLVGKNFNVVVFDKEKDVFVMFYAPWSKKCKMLFPLLEELGRKYQNHSTIIIAKIDVTANDIQLMYLDRYPFFRLFPSGSQQAVLYKGEHTLKGFSDFLESHIKTKIEDEDELLSVEQNEVIEEEVLAEEKEVPMMRKGLPEQQSPELENMTKYVSKLEEPAGKKKTSEEVVVVVAKPKGPPVQKKKPKVKEEL

>Q9NY74

MSRRRKHDDSPSPKKTPHKTVAAEECGSVVEPGRRRLRSARGSWPCGAREGPPGPVRQREQPPTAALCSKSNPEERYETPKRALKMDSLSSSFSSPNDPDGQNDIFWDQNSPLTKQLGKGRKKQIYTTDSDEISHIVNRIAPQDEKPTTNSMLDMWIGETAIPCTPSVAKGKSRAKISCTKLKTQSQEEELMKLAKQFDKNMEELDVIQEQNKRNYDFTQMISETEILSNYKDNIQMWSLHNIVPEIDNATKKPIKGNTKISVANNQNSSQKPFDQIAEAAFNAIFDGSTQKCSGQLSQELPEAFWSTSNTTFVKTNALKEEKIITNETLVIEKLSNKTPRSLSSQVDTPIMTKSCVTSCTKEPETSNKYIDAFTTSDFEDDWENLLGSEPFAMQNIDMPELFPSKTAHVTDQKEICTFNSKTVKNTSRANTSPDARLGDSKVLQDLSSKTYDRELIDAEYRFSPNSNKSNKLSTGNKMKFENSSNKIVIQDEIQNCIVTSNLTKIKEDILTNSTEASERKSALNTRYSNEQKNKCILNQSIKAPVNTDLFGSANLGSKTSVSNPNQTSASKVGSFFDDWNDPSFANEIIKACHQLDNTWEADDVDDDLLYQACDDIERLTQQQDIRKDSKTSESICEINNNSEHGAKLTQQQDIRKDSKTSESICEINNNSEHGAKNMFAISKQGSNLVQSKHLNPGSISVQTSLTNSSQIDKPMKMEKGEMYGNSPRFLGATNLTMYSKISNCQINNLHVSYTNTDVPIQVNSSKLVLPGSSSLNVTSDHMNTEITTYKKKLSTNQPCHKTVTDEAQSNLNTTVGFSKFTFTRMKNSQILSQFNQNCITGSMSDTKITQGVEKKKGVNPLLEEAVGQQSLVKLSESLKQSSKEEEEKNRKCSPEEIQRKRQEALVRRMAKARASSVNAAPTSFL

615 NP_065885.1 P20132

>NP_065885.1

MPPNKEASGLSSSPAGLICLPPISEELQLVWTQAAQTSELDSNEHLLKTFSYFPYPSLADIALLCLRYGLQMEKVKTWFMAQRLRCGISWSSEEIEETRARVVYRRDQLHFKSLLSFTHHAGRPPEEVPPPPVPAPEQVGIGIGPPTLSKPTQTKGLKVEPEEPSQMPPLPQSHQKLKESLMTPGSGAFPYQSDFWQHLQSSGLSKEQAGRGPNQSHGIGTASWNHSTTVPQPQARDKPPPIALIASSCKEESASSVTPSSSSTSSSFQVLANGATAASKPLQPLGCVPQSVSPSEQALPPHLEPAWPQGLRHNSVPGRVGPTEYLSPDMQRQRKTKRKTKEQLAILKSFFLQCQWARREDYQKLEQITGLPRPEIIQWFGDTRYALKHGQLKWFRDNAVPGAPSFQDPAIPTPPPSTRSLNERAETPPLPIPPPPPDIQPLERYWAAHQQLRETDIPQLSQASRLSTQQVLDWFDSRLPQPAEVVVCLDEEEEEEEEELPEDDEEEEEEEEDDDDDDDDVIIQD

>P20132

MMSGEPLHVKTPIRDSMALSKMAGTSVYLKMDSAQPSGSFKIRGIGHFCKRWAKQGCAHFVCSSAGNAGMAAAYAARQLGVPATIVVPSTTPALTIERLKNEGATVKVVGELLDEAFELAKALAKNNPGWVYIPPFDDPLIWEGHASIVKELKETLWEKPGAIALSVGGGGLLCGVVQGLQEVGWGDVPVIAMETFGAHSFHAATTAGKLVSLPKITSVAKALGVKTVGAQALKLFQEHPIFSEVISDQEAVAAIEKFVDDEKILVEPACGAALAAVYSHVIQKLQLEGNLRTPLPSLVVIVCGGSNISLAQLRALKEQLGMTNRLPK

616 Q9UGL1 Q93008

>Q9UGL1

MEAATTLHPGPRPALPLGGPGPLGEFLPPPECPVFEPSWEEFADPFAFIHKIRPIAEQTGICKVRPPPDWQPPFACDVDKLHFTPRIQRLNELEAQTRVKLNFLDQIAKYWELQGSTLKIPHVERKILDLFQLNKLVAEEGGFAVVCKDRKWTKIATKMGFAPGKAVGSHIRGHYERILNPYNLFLSGDSLRCLQKPNLTTDTKDKEYKPHDIPQRQSVQPSETCPPARRAKRMRAEAMNIKIEPEETTEARTHNLRRRMGCPTPKCENEKEMKSSIKQEPIERKDYIVENEKEKPKSRSKKATNAVDLYVCLLCGSGNDEDRLLLCDGCDDSYHTFCLIPPLHDVPKGDWRCPKCLAQECSKPQEAFGFEQAARDYTLRTFGEMADAFKSDYFNMPVHMVPTELVEKEFWRLVSTIEEDVTVEYGADIASKEFGSGFPVRDGKIKLSPEEEEYLDSGWNLNNMPVMEQSVLAHITADICGMKLPWLYVGMCFSSFCWHIEDHWSYSINYLHWGEPKTWYGVPGYAAEQLENVMKKLAPELFVSQPDLLHQLVTIMNPNTLMTHEVPVYRTNQCAGEFVITFPRAYHSGFNQGFNFAEAVNFCTVDWLPLGRQCVEHYRLLHRYCVFSHDEMICKMASKADVLDVVVASTVQKDMAIMIEDEKALRETVRKLGVIDSERMDFELLPDDERQCVKCKTTCFMSAISCSCKPGLLVCLHHVKELCSCPPYKYKLRYRYTLDDLYPMMNALKLRAESYNEWALNVNEALEAKITKKKSLVSFKALIEESEMKKFPDNDLLRHLRLVTQDAEKCASVAQQLLNGKRQTRYRSGGGKSQNQLTVNELRQFVTQLYALPCVLSQTPLLKDLLNRVEDFQQHSQKLLSEETPSAAELQDLLDVSFEFDVELPQLAEMRIRLEQARWLEEVQQACLDPSSLTLDDMRRLIDLGVGLAPYSAVEKAMARLQELLTVSEHWDDKAKSLLKARPRHSLNSLATAVKEIEEIPAYLPNGAALKDSVQRARDWLQDVEGLQAGGRVPVLDTLIELVTRGRSIPVHLNSLPRLETLVAEVQAWKECAVNTFLTENSPYSLLEVLCPRCDIGLLGLKRKQRKLKEPLPNGKKKSTKLESLSDLERALTESKETASAMATLGEARLREMEALQSLRLANEGKLLSPLQDVDIKICLCQKAPAAPMIQCELCRDAFHTSCVAVPSISQGLRIWLCPHCRRSEKPPLEKILPLLASLQRIRVRLPEGDALRYMIERTVNWQHRAQQLLSSGNLKFVQDRVGSGLLYSRWQASAGQVSDTNKVSQPPGTTSFSLPDDWDNRTSYLHSPFSTGRSCIPLHGVSPEVNELLMEAQLLQVSLPEIQELYQTLLAKPSPAQQTDRSSPVRPSSEKNDCCRGKRDGINSLERKLKRRLEREGLSSERWERVKKMRTPKKKKIKLSHPKDMNNFKLERERSYELVRSAETHSLPSDTSYSEQEDSEDEDAICPAVSCLQPEGDEVDWVQCDGSCNQWFHQVCVGVSPEMAEKEDYICVRCTVKDAPSRK

>Q93008

MTATTRGSPVGGNDNQGQAPDGQSQPPLQQNQTSSPDSSNENSPATPPDEQGQGDAPPQLEDEEPAFPHTDLAKLDDMINRPRWVVPVLPKGELEVLLEAAIDLSKKGLDVKSEACQRFFRDGLTISFTKILTDEAVSGWKFEIHVSLVELCVAKLSQDWFPLLELLAMALNPHCKFHIYNGTRPCESVSSSVQLPEDELFARSPDPRSPKGWLVDLLNKFGTLNGFQILHDRFINGSALNVQIIAALIKPFGQCYEFLTLHTVKKYFLPIIEMVPQFLENLTDEELKKEAKNEAKNDALSMIIKSLKNLASRVPGQEETVKNLEIFRLKMILRLLQISSFNGKMNALNEVNKVISSVSYYTHRHGNPEEEEWLTAERMAEWIQQNNILSIVLRDSLHQPQYVEKLEKILRFVIKEKALTLQDLDNIWAAQAGKHEAIVKNVHDLLAKLAWDFSPEQLDHLFDCFKASWTNASKKQREKLLELIRRLAEDDKDGVMAHKVLNLLWNLAHSDDVPVDIMDLALSAHIKILDYSCSQDRDTQKIQWIDRFIEELRTNDKWVIPALKQIREICSLFGEAPQNLSQTQRSPHVFYRHDLINQLQHNHALVTLVAENLATYMESMRLYARDHEDYDPQTVRLGSRYSHVQEVQERLNFLRFLLKDGQLWLCAPQAKQIWKCLAENAVYLCDREACFKWYSKLMGDEPDLDPDINKDFFESNVLQLDPSLLTENGMKCFERFFKAVNCREGKLVAKRRAYMMDDLELIGLDYLWRVVIQSNDDIASRAIDLLKEIYTNLGPRLQVNQVVIHEDFIQSCFDRLKASYDTLCVLDGDKDSVNCARQEAVRMVRVLTVLREYINECDSDYHEERTILPMSRAFRGKHLSFVVRFPNQGRQVDDLEVWSHTNDTIGSVRRCILNRIKANVAHTKIELFVGGELIDPADDRKLIGQLNLKDKSLITAKLTQISSNMPSSPDSSSDSSTGSPGNHGNHYSDGPNPEVESCLPGVIMSLHPRYISFLWQVADLGSSLNMPPLRDGARVLMKLMPPDSTTIEKLRAICLDHAKLGESSLSPSLDSLFFGPSASQVLYLTEVVYALLMPAGAPLADDSSDFQFHFLKSGGLPLVLSMLTRNNFLPNADMETRRGAYLNALKIAKLLLTAIGYGHVRAVAEACQPGVEGVNPMTQINQVTHDQAVVLQSALQSIPNPSSECMLRNVSVRLAQQISDEASRYMPDICVIRAIQKIIWASGCGSLQLVFSPNEEITKIYEKTNAGNEPDLEDEQVCCEALEVMTLCFALIPTALDALSKEKAWQTFIIDLLLHCHSKTVRQVAQEQFFLMCTRCCMGHRPLLFFITLLFTVLGSTARERAKHSGDYFTLLRHLLNYAYNSNINVPNAEVLLNNEIDWLKRIRDDVKRTGETGIEETILEGHLGVTKELLAFQTSEKKFHIGCEKGGANLIKELIDDFIFPASNVYLQYMRNGELPAEQAIPVCGSPPTINAGFELLVALAVGCVRNLKQIVDSLTEMYYIGTAITTCEALTEWEYLPPVGPRPPKGFVGLKNAGATCYMNSVIQQLYMIPSIRNGILAIEGTGSDVDDDMSGDEKQDNESNVDPRDDVFGYPQQFEDKPALSKTEDRKEYNIGVLRHLQVIFGHLAASRLQYYVPRGFWKQFRLWGEPVNLREQHDALEFFNSLVDSLDEALKALGHPAMLSKVLGGSFADQKICQGCPHRYECEESFTTLNVDIRNHQNLLDSLEQYVKGDLLEGANAYHCEKCNKKVDTVKRLLIKKLPPVLAIQLKRFDYDWERECAIKFNDYFEFPRELDMEPYTVAGVAKLEGDNVNPESQLIQQSEQSESETAGSTKYRLVGVLVHSGQASGGHYYSYIIQRNGGDGERNRWYKFDDGDVTECKMDDDEEMKNQCFGGEYMGEVFDHMMKRMSYRRQKRWWNAYILFYERMDTIDQDDELIRYISELAITTRPHQIIMPSAIERSVRKQNVQFMHNRMQYSMEYFQFMKKLLTCNGVYLNPPPGQDHLLPEAEEITMISIQLAARFLFTTGFHTKKVVRGSASDWYDALCILLRHSKNVRFWFAHNVLFNVSNRFSEYLLECPSAEVRGAFAKLIVFIAHFSLQDGPCPSPFASPGPSSQAYDNLSLSDHLLRAVLNLLRREVSEHGRHLQQYFNLFVMYANLGVAEKTQLLKLSVPATFMLVSLDEGPGPPIKYQYAELGKLYSVVSQLIRCCNVSSRMQSSINGNPPLPNPFGDPNLSQPIMPIQQNVADILFVRTSYVKKIIEDCSNSEETVKLLRFCCWENPQFSSTVLSELLWQVAYSYTYELRPYLDLLLQILLIEDSWQTHRIHNALKGIPDDRDGLFDTIQRSKNHYQKRAYQCIKCMVALFSNCPVAYQILQGNGDLKRKWTWAVEWLGDELERRPYTGNPQYTYNNWSPPVQSNETSNGYFLERSHSARMTLAKACELCPEEEPDDQDAPDEHESPPPEDAPLYPHSPGSQYQQNNHVHGQPYTGPAAHHMNNPQRTGQRAQENYEGSEEVSPPQTKDQ

617 NP_056023.3 P49411

>NP_056023.3

MPKSGFTKPIQSENSDSDSNMVEKPYGRKSKDKIASYSKTPKIERSDVSKEMKEKSSMKRKLPFTISPSRNEERDSDTDSDPGHTSENWGERLISSYRTYSEKEGPEKKKTKKEAGNKKSTPVSILFGYPLSERKQMALLMQMTARDNSPDSTPNHPSQTTPAQKKTPSSSSRQKDKVNKRNERGETPLHMAAIRGDVKQVKELISLGANVNVKDFAGWTPLHEACNVGYYDVAKILIAAGADVNTQGLDDDTPLHDSASSGHRDIVKLLLRHGGNPFQANKHGERPVDVAETEELELLLKREVPLSDDDESYTDSEEAQSVNPSSVDENIDSETEKDSLICESKQILPSKTPLPSALDEYEFKDDDDEEINKMIDDRHILRKEQRKENEPEAEKTHLFAKQEKAFYPKSFKSKKQKPSRVLYSSTESSDEEALQNKKISTSCSVIPETSNSDMQTKKEYVVSGEHKQKGKVKRKLKNQNKNKENQELKQEKEGKENTRITNLTVNTGLDCSEKTREEGNFRKSFSPKDDTSLHLFHISTGKSPKHSCGLSEKQSTPLKQEHTKTCLSPGSSEMSLQPDLVRYDNTESEFLPESSSVKSCKHKEKSKHQKDFHLEFGEKSNAKIKDEDHSPTFENSDCTLKKMDKEGKTLKKHKLKHKEREKEKHKKEIEGEKEKYKTKDSAKELQRSVEFDREFWKENFFKSDETEDLFLNMEHESLTLEKKSKLEKNIKDDKSTKEKHVSKERNFKEERDKIKKESEKSFREEKIKDLKEERENIPTDKDSEFTSLGMSAIEESIGLHLVEKEIDIEKQEKHIKESKEKPEKRSQIKEKDIEKMERKTFEKEKKIKHEHKSEKDKLDLSECVDKIKEKDKLYSHHTEKCHKEGEKSKNTAAIKKTDDREKSREKMDRKHDKEKPEKERHLAESKEKHLMEKKNKQSDNSEYSKSEKGKNKEKDRELDKKEKSRDKESINITNSKHIQEEKKSSIVDGNKAQHEKPLSLKEKTKDEPLKTPDGKEKDKKDKDIDRYKERDKHKDKIQINSLLKLKSEADKPKPKSSPASKDTRPKEKRLVNDDLMQTSFERMLSLKDLEIEQWHKKHKEKIKQKEKERLRNRNCLELKIKDKEKTKHTPTESKNKELTRSKSSEVTDAYTKEKQPKDAVSNRSQSVDTKNVMTLGKSSFVSDNSLNRSPRSENEKPGLSSRSVSMISVASSEDSCHTTVTTPRPPVEYDSDFMLESSESQMSFSQSPFLSIAKSPALHERELDSLADLPERIKPPYANRLSTSHLRSSSVEDVKLIISEGRPTIEVRRCSMPSVICEHTKQFQTISEESNQGSLLTVPGDTSPSPKPEVFSNVPERDLSNVSNIHSSFATSPTGASNSKYVSADRNLIKNTAPVNTVMDSPVHLEPSSQVGVIQNKSWEMPVDRLETLSTRDFICPNSNIPDQESSLQSFCNSENKVLKENADFLSLRQTELPGNSCAQDPASFMPPQQPCSFPSQSLSDAESISKHMSLSYVANQEPGILQQKNAVQIISSALDTDNESTKDTENTFVLGDVQKTDAFVPVYSDSTIQEASPNFEKAYTLPVLPSEKDFNGSDASTQLNTHYAFSKLTYKSSSGHEVENSTTDTQVISHEKENKLESLVLTHLSRCDSDLCEMNAGMPKGNLNEQDPKHCPESEKCLLSIEDEESQQSILSSLENHSQQSTQPEMHKYGQLVKVELEENAEDDKTENQIPQRMTRNKANTMANQSKQILASCTLLSEKDSESSSPRGRIRLTEDDDPQIHHPRKRKVSRVPQPVQVSPSLLQAKEKTQQSLAAIVDSLKLDEIQPYSSERANPYFEYLHIRKKIEEKRKLLCSVIPQAPQYYDEYVTFNGSYLLDGNPLSKICIPTITPPPSLSDPLKELFRQQEVVRMKLRLQHSIEREKLIVSNEQEVLRVHYRAARTLANQTLPFSACTVLLDAEVYNVPLDSQSDDSKTSVRDRFNARQFMSWLQDVDDKFDKLKTCLLMRQQHEAAALNAVQRLEWQLKLQELDPATYKSISIYEIQEFYVPLVDVNDDFELTPI

>P49411

MAAATLLRATPHFSGLAAGRTFLLQGLLRLLKAPALPLLCRGLAVEAKKTYVRDKPHVNVGTIGHVDHGKTTLTAAITKILAEGGGAKFKKYEEIDNAPEERARGITINAAHVEYSTAARHYAHTDCPGHADYVKNMITGTAPLDGCILVVAANDGPMPQTREHLLLARQIGVEHVVVYVNKADAVQDSEMVELVELEIRELLTEFGYKGEETPVIVGSALCALEGRDPELGLKSVQKLLDAVDTYIPVPARDLEKPFLLPVEAVYSVPGRGTVVTGTLERGILKKGDECELLGHSKNIRTVVTGIEMFHKSLERAEAGDNLGALVRGLKREDLRRGLVMVKPGSIKPHQKVEAQVYILSKEEGGRHKPFVSHFMPVMFSLTWDMACRIILPPEKELAMPGEDLKFNLILRQPMILEKGQRFTLRDGNRTIGTGLVTNTLAMTEEEKNIKWG

618 Q96DT0 NP_003320.2

>Q96DT0

MSQPSGGRAPGTRIYSWSCPTVMSPGEKLDPIPDSFILQPPVFHPVVPYVTTIFGGLHAGKMVMLQGVVPLDAHRFQVDFQCGCSLCPRPDIAFHFNPRFHTTKPHVICNTLHGGRWQREARWPHLALRRGSSFLILFLFGNEEVKVSVNGQHFLHFRYRLPLSHVDTLGIFGDILVEAVGFLNINPFVEGSREYPAGHPFLLMSPRLEVPCSHALPQGLSPGQVIIVRGLVLQEPKHFTVSLRDQAAHAPVTLRASFADRTLAWISRWGQKKLISAPFLFYPQRFFEVLLLFQEGGLKLALNGQGLGATSMNQQALEQLRELRISGSVQLYCVHS

>NP_003320.2

MVKQIESKTAFQEALDAAGDKLVVVDFSATWCGPCKMIKPFFHSLSEKYSNVIFLEVDVDDCQDVASECEVKCMPTFQFFKKGQKVGEFSGANKEKLEATINELV

619 P51826 P36957

>P51826

MDSFDLALLQEWDLESLCVYEPDRNALRRKERERRNQETQQDDGTFNSSYSLFSEPYKTNKGDELSNRIQNTLGNYDEMKDFLTDRSNQSHLVGVPKPGVPQTPVNKIDEHFVADSRAQNQPSSICSTTTSTPAAVPVQQSKRGTMGWQKAGHPPSDGQQRATQQGSLRTLLGDGVGRQQPRAKQVCNVEVGLQTQERPPAMAAKHSSSGHCVQNFPPSLASKPSLVQQKPTAYVRPMDGQDQAPDESPKLKSSSETSVHCTSYRGVPASKPEPARAKAKLSKFSIPKQGEESRSGETNSCVEEIIREMTWLPPLSAIQAPGKVEPTKFPFPNKDSQLVSSGHNNPKKGDAEPESPDSGTSNTSMLEDDLKLSSDEEENEQQAAQRTALRALSDSAVVQQPNCRTSVPSSKGSSSSSSSGSSSSSSDSESSSGSDSETESSSSESEGSKPPHFSSPEAEPASSNKWQLDKWLNKVNPHKPPILIQNESHGSESNQYYNPVKEDVQDCGKVPDVCQPSLREKEIKSTCKEEQRPRTANKAPGSKGVKQKSPPAAVAVAVSAAAPPPAVPCAPAENAPAPARRSAGKKPTRRTERTSAGDGANCHRPEEPAAADALGTSVVVPPEPTKTRPCGNNRASHRKELRSSVTCEKRRTRGLSRIVPKSKEFIETESSSSSSSSDSDLESEQEEYPLSKAQTVAASASSGNDQRLKEAAANGGSGPRAPVGSINARTTSDIAKELEEQFYTLVPFGRNELLSPLKDSDEIRSLWVKIDLTLLSRIPEHLPQEPGVLSAPATKDSESAPPSHTSDTPAEKALPKSKRKRKCDNEDDYREIKKSQGEKDSSSRLATSTSNTLSANHCNMNINSVAIPINKNEKMLRSPISPLSDASKHKYTSEDLTSSSRPNGNSLFTSASSSKKPKADSQLQPHGGDLTKAAHNNSENIPLHKSRPQTKPWSPGSNGHRDCKRQKLVFDDMPRSADYFMQEAKRMKHKADAMVEKFGKALNYAEAALSFIECGNAMEQGPMESKSPYTMYSETVELIRYAMRLKTHSGPNATPEDKQLAALCYRCLALLYWRMFRLKRDHAVKYSKALIDYFKNSSKAAQAPSPWGASGKSTGTPSPMSPNPSPASSVGSQGSLSNASALSPSTIVSIPQRIHQMAANHVSITNSILHSYDYWEMADNLAKENREFFNDLDLLMGPVTLHSSMEHLVQYSQQGLHWLRNSAHLS

>P36957

MLSRSRCVSRAFSRSLSAFQKGNCPLGRRSLPGVSLCQGPGYPNSRKVVINNSVFSVRFFRTTAVCKDDLVTVKTPAFAESVTEGDVRWEKAVGDTVAEDEVVCEIETDKTSVQVPSPANGVIEALLVPDGGKVEGGTPLFTLRKTGAAPAKAKPAEAPAAAAPKAEPTAAAVPPPAAPIPTQMPPVPSPSQPPSGKPVSAVKPTVAPPLAEAGAGKGLRSEHREKMNRMRQRIAQRLKEAQNTCAMLTTFNEIDMSNIQEMRARHKEAFLKKHNLKLGFMSAFVKASAFALQEQPVVNAVIDDTTKEVVYRDYIDISVAVATPRGLVVPVIRNVEAMNFADIERTITELGEKARKNELAIEDMDGGTFTISNGGVFGSLFGTPIINPPQSAILGMHGIFDRPVAIGGKVEVRPMMYVALTYDHRLIDGREAVTFLRKIKAAVEDPRVLLLDL

620 Q9UNN4 NP_004332.2

>Q9UNN4

MACLNPVPKLYRSVIEDVIEGVRNLFAEEGIEEQVLKDLKQLWETKVLQSKATEDFFRNSIQSPLFTLQLPHSLHQTLQSSTASLVIPAGRTLPSFTTAELGTSNSSANFTFPGYPIHVPAGVTLQTVSGHLYKVNVPIMVTETSGRAGILQHPIQQVFQQLGQPSVIQTSVPQLNPWSLQATTEKSQRIETVLQQPAILPSGPVDRKHLENATSDILVSPGNEHKIVPEALLCHQESSHYISLPGVVFSPQVSQTNSDVESVLSGSASMAQNLHDESLSTSPHGALHQHVTDIQLHILKNRMYGCDSVKQPRNIEEPSNIPVSEKDSNSQVDLSIRVTDDDIGEIIQVDGSGDTSSNEEIGSTRDADENEFLGNIDGGDLKVPEEEADSISNEDSATNSSDNEDPQVNIVEEDPLNSGDDVSEQDVPDLFDTDNVIVCQYDKIHRSKNKWKFYLKDGVMCFGGRDYVFAKAIGDAEW

>NP_004332.2

MAALVLEDGSVLRGQPFGAAVSTAGEVVFQTGMVGYPEALTDPSYKAQILVLTYPLIGNYGIPPDEMDEFGLCKWFESSGIHVAALVVGECCPTPSHWSATRTLHEWLQQHGIPGLQGVDTRELTKKLREQGSLLGKLVQNGTEPSSLPFLDPNARPLVPEVSIKTPRVFNTGGAPRILALDCGLKYNQIRCLCQRGAEVTVVPWDHALDSQEYEGLFLSNGPGDPASYPSVVSTLSRVLSEPNPRPVFGICLGHQLLALAIGAKTYKMRYGNRGHNQPCLLVGSGRCFLTSQNHGFAVETDSLPADWAPLFTNANDGSNEGIVHNSLPFFSVQFHPEHQAGPSDMELLFDIFLETVKEATAGNPGGQTVRERLTERLCPPGIPTPGSGLPPPRKVLILGSGGLSIGQAGEFDYSGSQAIKALKEENIQTLLINPNIATVQTSQGLADKVYFLPITPHYVTQVIRNERPDGVLLTFGGQTALNCGVELTKAGVLARYGVRVLGTPVETIELTEDRRAFAARMAEIGEHVAPSEAANSLEQAQAAAERLGYPVLVRAAFALGGLGSGFASNREELSALVAPAFAHTSQVLVDKSLKGWKEIEYEVVRDAYGNCVTVCNMENLDPLGIHTGESIVVAPSQTLNDREYQLLRQTAIKVTQHLGIVGECNVQYALNPESEQYYIIEVNARLSRSSALASKATGYPLAYVAAKLALGIPLPELRNSVTGGTAAFEPSVDYCVVKIPRWDLSKFLRVSTKIGSCMKSVGEVMGIGRSFEEAFQKALRMVDENCVGFDHTVKPVSDMELETPTDKRIFVVAAALWAGYSVDRLYELTRIDRWFLHRMKRIIAHAQLLEQHRGQPLPPDLLQQAKCLGFSDKQIALAVLSTELAVRKLRQELGICPAVKQIDTVAAEWPAQTNYLYLTYWGTTHDLTFRTPHVLVLGSGVYRIGSSVEFDWCAVGCIQQLRKMGYKTIMVNYNPETVSTDYDMCDRLYFDEISFEVVMDIYELENPEGVILSMGGQLPNNMAMALHRQQCRVLGTSPEAIDSAENRFKFSRLLDTIGISQPQWRELSDLESARQFCQTVGYPCVVRPSYVLSGAAMNVAYTDGDLERFLSSAAAVSKEHPVVISKFIQEAKEIDVDAVASDGVVAAIAISEHVENAGVHSGDATLVTPPQDITAKTLERIKAIVHAVGQELQVTGPFNLQLIAKDDQLKVIECNVRVSRSFPFVSKTLGVDLVALATRVIMGEEVEPVGLMTGSGVVGVKVPQFSFSRLAGADVVLGVEMTSTGEVAGFGESRCEAYLKAMLSTGFKIPKKNILLTIGSYKNKSELLPTVRLLESLGYSLYASLGTADFYTEHGVKVTAVDWHFEEAVDGECPPQRSILEQLAEKNFELVINLSMRGAGGRRLSSFVTKGYRTRRLAADFSVPLIIDIKCTKLFVEALGQIGPAPPLKVHVDCMTSQKLVRLPGLIDVHVHLREPGGTHKEDFASGTAAALAGGITMVCAMPNTRPPIIDAPALALAQKLAEAGARCDFALFLGASSENAGTLGTVAGSAAGLKLYLNETFSELRLDSVVQWMEHFETWPSHLPIVAHAEQQTVAAVLMVAQLTQRSVHICHVARKEEILLIKAAKARGLPVTCEVAPHHLFLSHDDLERLGPGKGEVRPELGSRQDVEALWENMAVIDCFASDHAPHTLEEKCGSRPPPGFPGLETMLPLLLTAVSEGRLSLDDLLQRLHHNPRRIFHLPPQEDTYVEVDLEHEWTIPSHMPFSKAHWTPFEGQKVKGTVRRVVLRGEVAYIDGQVLVPPGYGQDVRKWPQGAVPQLPPSAPATSEMTTTPERPRRGIPGLPDGRFHLPPRIHRASDPGLPAEEPKEKSSRKVAEPELMGTPDGTCYPPPPVPRQASPQNLGTPGLLHPQTSPLLHSLVGQHILSVQQFTKDQMSHLFNVAHTLRMMVQKERSLDILKGKVMASMFYEVSTRTSSSFAAAMARLGGAVLSFSEATSSVQKGESLADSVQTMSCYADVVVLRHPQPGAVELAAKHCRRPVINAGDGVGEHPTQALLDIFTIREELGTVNGMTITMVGDLKHGRTVHSLACLLTQYRVSLRYVAPPSLRMPPTVRAFVASRGTKQEEFESIEEALPDTDVLYMTRIQKERFGSTQEYEACFGQFILTPHIMTRAKKKMVVMHPMPRVNEISVEVDSDPRAAYFRQAENGMYIRMALLATVLGRF

621 NP_001506.1 P04183

>NP_001506.1

MDEEPERTKRWEGGYERTWEILKEDESGSLKATIEDILFKAKRKRVFEHHGQVRLGMMRHLYVVVDGSRTMEDQDLKPNRLTCTLKLLEYFVEEYFDQNPISQIGIIVTKSKRAEKLTELSGNPRKHITSLKKAVDMTCHGEPSLYNSLSIAMQTLKHMPGHTSREVLIIFSSLTTCDPSNIYDLIKTLKAAKIRVSVIGLSAEVRVCTVLARETGGTYHVILDESHYKELLTHHVSPPPASSSSECSLIRMGFPQHTIASLSDQDAKPSFSMAHLDGNTEPGLTLGGYFCPQCRAKYCELPVECKICGLTLVSAPHLARSYHHLFPLDAFQEIPLEEYNGERFCYGCQGELKDQHVYVCAVCQNVFCVDCDVFVHDSLHCCPGCIHKIPAPSGV

>P04183

MSCINLPTVLPGSPSKTRGQIQVILGPMFSGKSTELMRRVRRFQIAQYKCLVIKYAKDTRYSSSFCTHDRNTMEALPACLLRDVAQEALGVAVIGIDEGQFFPDIVEFCEAMANAGKTVIVAALDGTFQRKPFGAILNLVPLAESVVKLTAVCMECFREAAYTKRLGTEKEVEVIGGADKYHSVCRLCYFKKASGQPAGPDNKENCPVPGKPGEAVAARKLFAPQQILQCSPAN

622 NP_000170.1 Q16540

>NP_000170.1

MSRQSTLYSFFPKSPALSDANKASARASREGGRAAAAPGASPSPGGDAAWSEAGPGPRPLARSASPPKAKNLNGGLRRSVAPAAPTSCDFSPGDLVWAKMEGYPWWPCLVYNHPFDGTFIREKGKSVRVHVQFFDDSPTRGWVSKRLLKPYTGSKSKEAQKGGHFYSAKPEILRAMQRADEALNKDKIKRLELAVCDEPSEPEEEEEMEVGTTYVTDKSEEDNEIESEEEVQPKTQGSRRSSRQIKKRRVISDSESDIGGSDVEFKPDTKEEGSSDEISSGVGDSESEGLNSPVKVARKRKRMVTGNGSLKRKSSRKETPSATKQATSISSETKNTLRAFSAPQNSESQAHVSGGGDDSSRPTVWYHETLEWLKEEKRRDEHRRRPDHPDFDASTLYVPEDFLNSCTPGMRKWWQIKSQNFDLVICYKVGKFYELYHMDALIGVSELGLVFMKGNWAHSGFPEIAFGRYSDSLVQKGYKVARVEQTETPEMMEARCRKMAHISKYDRVVRREICRIITKGTQTYSVLEGDPSENYSKYLLSLKEKEEDSSGHTRAYGVCFVDTSLGKFFIGQFSDDRHCSRFRTLVAHYPPVQVLFEKGNLSKETKTILKSSLSCSLQEGLIPGSQFWDASKTLRTLLEEEYFREKLSDGIGVMLPQVLKGMTSESDSIGLTPGEKSELALSALGGCVFYLKKCLIDQELLSMANFEEYIPLDSDTVSTTRSGAIFTKAYQRMVLDAVTLNNLEIFLNGTNGSTEGTLLERVDTCHTPFGKRLLKQWLCAPLCNHYAINDRLDAIEDLMVVPDKISEVVELLKKLPDLERLLSKIHNVGSPLKSQNHPDSRAIMYEETTYSKKKIIDFLSALEGFKVMCKIIGIMEEVADGFKSKILKQVISLQTKNPEGRFPDLTVELNRWDTAFDHEKARKTGLITPKAGFDSDYDQALADIRENEQSLLEYLEKQRNRIGCRTIVYWGIGRNRYQLEIPENFTTRNLPEEYELKSTKKGCKRYWTKTIEKKLANLINAEERRDVSLKDCMRRLFYNFDKNYKDWQSAVECIAVLDVLLCLANYSRGGDGPMCRPVILLPEDTPPFLELKGSRHPCITKTFFGDDFIPNDILIGCEEEEQENGKAYCVLVTGPNMGGKSTLMRQAGLLAVMAQMGCYVPAEVCRLTPIDRVFTRLGASDRIMSGESTFFVELSETASILMHATAHSLVLVDELGRGTATFDGTAIANAVVKELAETIKCRTLFSTHYHSLVEDYSQNVAVRLGHMACMVENECEDPSQETITFLYKFIKGACPKSYGFNAARLANLPEEVIQKGHRKAREFEKMNQSLRLFREVCLASERSTVDAEAVHKLLTLIKEL

>Q16540

MARNVVYPLYRLGGPQLRVFRTNFFIQLVRPGVAQPEDTVQFRIPMEMTRVDLRNYLEGIYNVPVAAVRTRVQHGSNKRRDHRNVRIKKPDYKVAYVQLAHGQTFTFPDLFPEKDESPEGSAADDLYSMLEEERQQRQSSDPRRGGVPSWFGL

623 NP_001978.1 Q14CX7

>NP_001978.1

MSETPAQCSIKQERISYTPPESPVPSYASSTPLHVPVPRALRMEEDSIRLPAHLRLQPIYWSRDDVAQWLKWAENEFSLRPIDSNTFEMNGKALLLLTKEDFRYRSPHSGDVLYELLQHILKQRKPRILFSPFFHPGNSIHTQPEVILHQNHEEDNCVQRTPRPSVDNVHHNPPTIELLHRSRSPITTNHRPSPDPEQRPLRSPLDNMIRRLSPAERAQGPRPHQENNHQESYPLSVSPMENNHCPASSESHPKPSSPRQESTRVIQLMPSPIMHPLILNPRHSVDFKQSRLSEDGLHREGKPINLSHREDLAYMNHIMVSVSPPEEHAMPIGRIADCRLLWDYVYQLLSDSRYENFIRWEDKESKIFRIVDPNGLARLWGNHKNRTNMTYEKMSRALRHYYKLNIIRKEPGQRLLFRFMKTPDEIMSGRTDRLEHLESQELDEQIYQEDEC

>Q14CX7

MATRGHVQDPNDRRLRPIYDYLDNGNNKMAIQQADKLLKKHKDLHCAKVLKAIGLQRTGKQEEAFTLAQEVAALEPTDDNSLQALTILYREMHRPELVTKLYEAAVKKVPNSEEYHSHLFMAYARVGEYKKMQQAGMALYKIVPKNPYYFWSVMSLIMQSISAQDENLSKTMFLPLAERMVEKMVKEDKIEAEAEVELYYMILERLGKYQEALDVIRGKLGEKLTSEIQSRENKCMAMYKKLSRWPECNALSRRLLLKNSDDWQFYLTYFDSVFRLIEEAWSPPAEGEHSLEGEVHYSAEKAVKFIEDRITEESKSSRHLRGPHLAKLELIRRLRSQGCNDEYKLGDPEELMFQYFKKFGDKPCCFTDLKVFVDLLPATQCTKFINQLLGVVPLSTPTEDKLALPADIRALQQHLCVVQLTRLLGLYHTMDKNQKLSVVRELMLRYQHGLEFGKTCLKTELQFSDYYCLLAVHALIDVWRETGDETTVWQALTLLEEGLTHSPSNAQFKLLLVRIYCMLGAFEPVVDLYSSLDAKHIQHDTIGYLLTRYAESLGQYAAASQSCNFALRFFHSNQKDTSEYIIQAYKYGAFEKIPEFIAFRNRLNNSLHFAQVRTERMLLDLLLEANISTSLAESIKSMNLRPEEDDIPWEDLRDNRDLNVFFSWDPKDRDVSEEHKKLSLEEETLWLRIRSLTLRLISGLPSLNHPVEPKNSEKTAENGVSSRIDILRLLLQQLEATLETGKRFIEKDIQYPFLGPVPTRMGGFFNSGCSQCQISSFYLVNDIYELDTSGLEDTMEIQERIENSFKSLLDQLKDVFSKCKGDLLEVKDGNLKTHPTLLENLVFFVETISVILWVSSYCESVLRPYKLNLQKKKKKKKETSIIMPPVFTSFQDYVTGLQTLISNVVDHIKGLETHLIALKLEELILEDTSLSPEERKFSKTVQGKVQSSYLHSLLEMGELLKKRLETTKKLKI

624 Q9HCG8 NP_006262.1

>Q9HCG8

MKSSVAQIKPSSGHDRRENLNSYQRNSSPEDRYEEQERSPRDRDYFDYSRSDYEHSRRGRSYDSSMESRNRDREKRRERERDTDRKRSRKSPSPGRRNPETSVTQSSSAQDEPATKKKKDELDPLLTRTGGAYIPPAKLRMMQEQITDKNSLAYQRMSWEALKKSINGLINKVNISNISIIIQELLQENIVRGRGLLSRSVLQAQSASPIFTHVYAALVAIINSKFPQIGELILKRLILNFRKGYRRNDKQLCLTASKFVAHLINQNVAHEVLCLEMLTLLLERPTDDSVEVAIGFLKECGLKLTQVSPRGINAIFERLRNILHESEIDKRVQYMIEVMFAVRKDGFKDHPIILEGLDLVEEDDQFTHMLPLEDDYNPEDVLNVFKMDPNFMENEEKYKAIKKEILDEGDTDSNTDQDAGSSEEDEEEEEEEGEEDEEGQKVTIHDKTEINLVSFRRTIYLAIQSSLDFEECAHKLLKMEFPESQTKELCNMILDCCAQQRTYEKFFGLLAGRFCMLKKEYMESFEGIFKEQYDTIHRLETNKLRNVAKMFAHLLYTDSLPWSVLECIKLSEETTTSSSRIFVKIFFQELCEYMGLPKLNARLKDETLQPFFEGLLPRDNPRNTRFAINFFTSIGLGGLTDELREHLKNTPKVIVAQKPDVEQNKSSPSSSSSASSSSESDSSDSDSDSSDSSSESSSEESDSSSISSHSSASANDVRKKGHGKTRSKEVDKLIRNQQTNDRKQKERRQEHGHQETRTERERRSEKHRDQNSRGSNWRDPITKYTSDKDVPSERNNYSRVANDRDQEMHIDLENKHGDPKKKRGERRNSFSENEKHTHRIKDSENFRRKDRSKSKEMNRKHSGSRSDEDRYQNGAERRWEKSSRYSEQSRESKKNQDRRREKSPAKQK

>NP_006262.1

MGSELETAMETLINVFHAHSGKEGDKYKLSKKELKELLQTELSGFLDAQKDVDAVDKVMKELDENGDGEVDFQEYVVLVAALTVACNNFFWENS

625 NP_055850.1 NP_005267.2

>NP_055850.1

MASKRKSTTPCMIPVKTVVLQDASMEAQPAETLPEGPQQDLPPEASAASSEAAQNPSSTDGSTLANGHRSTLDGYLYSCKYCDFRSHDMTQFVGHMNSEHTDFNKDPTFVCSGCSFLAKTPEGLSLHNATCHSGEASFVWNVAKPDNHVVVEQSIPESTSTPDLAGEPSAEGADGQAEIIITKTPIMKIMKGKAEAKKIHTLKENVPSQPVGEALPKLSTGEMEVREGDHSFINGAVPVSQASASSAKNPHAANGPLIGTVPVLPAGIAQFLSLQQQPPVHAQHHVHQPLPTAKALPKVMIPLSSIPTYNAAMDSNSFLKNSFHKFPYPTKAELCYLTVVTKYPEEQLKIWFTAQRLKQGISWSPEEIEDARKKMFNTVIQSVPQPTITVLNTPLVASAGNVQHLIQAALPGHVVGQPEGTGGGLLVTQPLMANGLQATSSPLPLTVTSVPKQPGVAPINTVCSNTTSAVKVVNAAQSLLTACPSITSQAFLDASIYKNKKSHEQLSALKGSFCRNQFPGQSEVEHLTKVTGLSTREVRKWFSDRRYHCRNLKGSRAMIPGDHSSIIIDSVPEVSFSPSSKVPEVTCIPTTATLATHPSAKRQSWHQTPDFTPTKYKERAPEQLRALESSFAQNPLPLDEELDRLRSETKMTRREIDSWFSERRKKVNAEETKKAEENASQEEEEAAEDEGGEEDLASELRVSGENGSLEMPSSHILAERKVSPIKINLKNLRVTEANGRNEIPGLGACDPEDDESNKLAEQLPGKVSCKKTAQQRHLLRQLFVQTQWPSNQDYDSIMAQTGLPRPEVVRWFGDSRYALKNGQLKWYEDYKRGNFPPGLLVIAPGNRELLQDYYMTHKMLYEEDLQNLCDKTQMSSQQVKQWFAEKMGEETRAVADTGSEDQGPGTGELTAVHKGMGDTYSEVSENSESWEPRVPEASSEPFDTSSPQAGRQLETD

>NP_005267.2

MASKKVCIVGSGNWGSAIAKIVGGNAAQLAQFDPRVTMWVFEEDIGGKKLTEIINTQHENVKYLPGHKLPPNVVAVPDVVQAAEDADILIFVVPHQFIGKICDQLKGHLKANATGISLIKGVDEGPNGLKLISEVIGERLGIPMSVLMGANIASEVADEKFCETTIGCKDPAQGQLLKELMQTPNFRITVVQEVDTVEICGALKNVVAVGAGFCDGLGFGDNTKAAVIRLGLMEMIAFAKLFCSGPVSSATFLESCGVADLITTCYGGRNRKVAEAFARTGKSIEQLEKELLNGQKLQGPETARELYSILQHKGLVDKFPLFMAVYKVCYEGQPVGEFIHCLQNHPEHM

626 NP_056953.2 NP_005407.1

>NP_056953.2

MGETLGDSPIDPESDSFTDTLSANISQEMTMVDTEMPFWPTNFGISSVDLSVMEDHSHSFDIKPFTTVDFSSISTPHYEDIPFTRTDPVVADYKYDLKLQEYQSAIKVEPASPPYYSEKTQLYNKPHEEPSNSLMAIECRVCGDKASGFHYGVHACEGCKGFFRRTIRLKLIYDRCDLNCRIHKKSRNKCQYCRFQKCLAVGMSHNAIRFGRMPQAEKEKLLAEISSDIDQLNPESADLRALAKHLYDSYIKSFPLTKAKARAILTGKTTDKSPFVIYDMNSLMMGEDKIKFKHITPLQEQSKEVAIRIFQGCQFRSVEAVQEITEYAKSIPGFVNLDLNDQVTLLKYGVHEIIYTMLASLMNKDGVLISEGQGFMTREFLKSLRKPFGDFMEPKFEFAVKFNALELDDSDLAIFIAVIILSGDRPGLLNVKPIEDIQDNLLQALELQLKLNHPESSQLFAKLLQKMTDLRQIVTEHVQLLQVIKKTETDMSLHPLLQEIYKDLY

>NP_005407.1

MSSYQQKQTFTPPPQLQQQQVKQPSQPPPQEIFVPTTKEPCHSKVPQPGNTKIPEPGCTKVPEPGCTKVPEPGCTKVPEPGCTKVPEPGCTKVPEPGCTKVPEPGYTKVPEPGSIKVPDQGFIKFPEPGAIKVPEQGYTKVPVPGYTKLPEPCPSTVTPGPAQQKTKQK

627 P17947 NP_061821.1

>P17947

MLQACKMEGFPLVPPPSEDLVPYDTDLYQRQTHEYYPYLSSDGESHSDHYWDFHPHHVHSEFESFAENNFTELQSVQPPQLQQLYRHMELEQMHVLDTPMVPPHPSLGHQVSYLPRMCLQYPSLSPAQPSSDEEEGERQSPPLEVSDGEADGLEPGPGLLPGETGSKKKIRLYQFLLDLLRSGDMKDSIWWVDKDKGTFQFSSKHKEALAHRWGIQKGNRKKMTYQKMARALRNYGKTGEVKKVKKKLTYQFSGEVLGRGGLAERRHPPH

>NP_061821.1

MSIAGVAAQEIRVPLKTGFLHNGRAMGNMRKTYWSSRSEFKNNFLNIDPITMAYSLNSSAQERLIPLGHASKSAPMNGHCFAENGPSQKSSLPPLLIPPSENLGPHEEDQVVCGFKKLTVNGVCASTPPLTPIKNSPSLFPCAPLCERGSRPLPPLPISEALSLDDTDCEVEFLTSSDTDFLLEDSTLSDFKYDVPGRRSFRGCGQINYAYFDTPAVSAADLSYVSDQNGGVPDPNPPPPQTHRRLRRSHSGPAGSFNKPAIRISNCCIHRASPNSDEDKPEVPPRVPIPPRPVKPDYRRWSAEVTSSTYSDEDRPPKVPPREPLSPSNSRTPSPKSLPSYLNGVMPPTQSFAPDPKYVSSKALQRQNSEGSASKVPCILPIIENGKKVSSTHYYLLPERPPYLDKYEKFFREAEETNGGAQIQPLPADCGISSATEKPDSKTKMDLGGHVKRKHLSYVVSP

628 NP_006103.1 O43504

>NP_006103.1

MATTKRVLYVGGLAEEVDDKVLHAAFIPFGDITDIQIPLDYETEKHRGFAFVEFELAEDAAAAIDNMNESELFGRTIRVNLAKPMRIKEGSSRPVWSDDDWLKKFSGKTLEENKEEEGSEPPKAETQEGEPIAKKARSNPQVYMDIKIGNKPAGRIQMLLRSDVVPMTAENFRCLCTHEKGFGFKGSSFHRIIPQFMCQGGDFTNHNGTGGKSIYGKKFDDENFILKHTGPGLLSMANSGPNTNGSQFFLTCDKTDWLDGKHVVFGEVTEGLDVLRQIEAQGSKDGKPKQKVIIADCGEYV

>O43504

MEATLEQHLEDTMKNPSIVGVLCTDSQGLNLGCRGTLSDEHAGVISVLAQQAAKLTSDPTDIPVVCLESDNGNIMIQKHDGITVAVHKMAS

629 NP_006222.2 NP_004291.1

>NP_006222.2

MSLRSGGRRRADPGADGEASRDDGATSSVSALKRLERSQWTDKMDLRFGFERLKEPGEKTGWLINMHPTEILDEDKRLGSAVDYYFIQDDGSRFKVALPYKPYFYIATRKGCEREVSSFLSKKFQGKIAKVETVPKEDLDLPNHLVGLKRNYIRLSFHTVEDLVKVRKEISPAVKKNREQDHASDAYTALLSSVLQRGGVITDEEETSKKIADQLDNIVDMREYDVPYHIRLSIDLKIHVAHWYNVRYRGNAFPVEITRRDDLVERPDPVVLAFDIETTKLPLKFPDAETDQIMMISYMIDGQGYLITNREIVSEDIEDFEFTPKPEYEGPFCVFNEPDEAHLIQRWFEHVQETKPTIMVTYNGDFFDWPFVEARAAVHGLSMQQEIGFQKDSQGEYKAPQCIHMDCLRWVKRDSYLPVGSHNLKAAAKAKLGYDPVELDPEDMCRMATEQPQTLATYSVSDAVATYYLYMKYVHPFIFALCTIIPMEPDEVLRKGSGTLCEALLMVQAFHANIIFPNKQEQEFNKLTDDGHVLDSETYVGGHVEALESGVFRSDIPCRFRMNPAAFDFLLQRVEKTLRHALEEEEKVPVEQVTNFEEVCDEIKSKLASLKDVPSRIECPLIYHLDVGAMYPNIILTNRLQPSAMVDEATCAACDFNKPGANCQRKMAWQWRGEFMPASRSEYHRIQHQLESEKFPPLFPEGPARAFHELSREEQAKYEKRRLADYCRKAYKKIHITKVEERLTTICQRENSFYVDTVRAFRDRRYEFKGLHKVWKKKLSAAVEVGDAAEVKRCKNMEVLYDSLQLAHKCILNSFYGYVMRKGARWYSMEMAGIVCFTGANIITQARELIEQIGRPLELDTDGIWCVLPNSFPENFVFKTTNVKKPKVTISYPGAMLNIMVKEGFTNDQYQELAEPSSLTYVTRSENSIFFEVDGPYLAMILPASKEEGKKLKKRYAVFNEDGSLAELKGFEVKRRGELQLIKIFQSSVFEAFLKGSTLEEVYGSVAKVADYWLDVLYSKAANMPDSELFELISENRSMSRKLEDYGEQKSTSISTAKRLAEFLGDQMVKDAGLSCRYIISRKPEGSPVTERAIPLAIFQAEPTVRKHFLRKWLKSSSLQDFDIRAILDWDYYIERLGSAIQKIITIPAALQQVKNPVPRVKHPDWLHKKLLEKNDVYKQKKISELFTLEGRRQVTMAEASEDSPRPSAPDMEDFGLVKLPHPAAPVTVKRKRVLWESQEESQDLTPTVPWQEILGQPPALGTSQEEWLVWLRFHKKKWQLQARQRLARRKRQRLESAEGVLRPGAIRDGPATGLGSFLRRTARSILDLPWQIVQISETSQAGLFRLWALVGSDLHCIRLSIPRVFYVNQRVAKAEEGASYRKVNRVLPRSNMVYNLYEYSVPEDMYQEHINEINAELSAPDIEGVYETQVPLLFRALVHLGCVCVVNKQLVRHLSGWEAETFALEHLEMRSLAQFSYLEPGSIRHIYLYHHAQAHKALFGIFIPSQRRASVFVLDTVRSNQMPSLGALYSAEHGLLLEKVGPELLPPPKHTFEVRAETDLKTICRAIQRFLLAYKEERRGPTLIAVQSSWELKRLASEIPVLEEFPLVPICVADKINYGVLDWQRHGARRMIRHYLNLDTCLSQAFEMSRYFHIPIGNLPEDISTFGSDLFFARHLQRHNHLLWLSPTARPDLGGKEADDNCLVMEFDDQATVEINSSGCYSTVCVELDLQNLAVNTILQSHHVNDMEGADSMGISFDVIQQASLEDMITGGQAASAPASYDETALCSNTFRILKSMVVGWVKEITQYHNIYADNQVMHFYRWLRSPSSLLHDPALHRTLHNMMKKLFLQLIAEFKRLGSSVIYANFNRIILCTKKRRVEDAIAYVEYITSSIHSKETFHSLTISFSRCWEFLLWMDPSNYGGIKGKVSSRIHCGLQDSQKAGGAEDEQENEDDEEERDGEEEEEAEESNVEDLLENNWNILQFLPQAASCQNYFLMIVSAYIVAVYHCMKDGLRRSAPGSTPVRRRGASQLSQEAEGAVGALPGMITFSQDYVANELTQSFFTITQKIQKKVTGSRNSTELSEMFPVLPGSHLLLNNPALEFIKYVCKVLSLDTNITNQVNKLNRDLLRLVDVGEFSEEAQFRDPCRSYVLPEVICRSCNFCRDLDLCKDSSFSEDGAVLPQWLCSNCQAPYDSSAIEMTLVEVLQKKLMAFTLQDLVCLKCRGVKETSMPVYCSCAGDFALTIHTQVFMEQIGIFRNIAQHYGMSYLLETLEWLLQKNPQLGH

>NP_004291.1

MAEQATKSVLFVCLGNICRSPIAEAVFRKLVTDQNISENWRVDSAATSGYEIGNPPDYRGQSCMKRHGIPMSHVARQITKEDFATFDYILCMDESNLRDLNRKSNQVKTCKAKIELLGSYDPQKQLIIEDPYYGNDSDFETVYQQCVRCCRAFLEKAH

630 NP_009181.2 NP_066565.1

>NP_009181.2

MVDMDKLINNLEVQLNSEGGSMQVFKQVTASVRNRDPPEIEYRSNMTSPTLLDANPMENPALFNDIKIEPPEELLASDFSLPQVEPVDLSFHKPKAPLQPASMLQAPIRPPKPQSSPQTLVVSTSTSDMSTSANIPTVLTPGSVLTSSQSTGSQQILHVIHTIPSVSLPNKMGGLKTIPVVVQSLPMVYTTLPADGGPAAITVPLIGGDGKNAGSVKVDPTSMSPLEIPSDSEESTIESGSSALQSLQGLQQEPAAMAQMQGEESLDLKRRRIHQCDFAGCSKVYTKSSHLKAHRRIHTGEKPYKCTWDGCSWKFARSDELTRHFRKHTGIKPFRCTDCNRSFSRSDHLSLHRRRHDTM

>NP_066565.1

MADESETAVKPPAPPLPQMMEGNGNGHEHCSDCENEEDNSYNRGGLSPANDTGAKKKKKKQKKKKEKGSETDSAQDQPVKMNSLPAERIQEIQKAIELFSVGQGPAKTMEEASKRSYQFWDTQPVPKLGEVVNTHGPVEPDKDNIRQEPYTLPQGFTWDALDLGDRGVLKELYTLLNENYVEDDDNMFRFDYSPEFLLWALRPPGWLPQWHCGVRVVSSRKLVGFISAIPANIHIYDTEKKMVEINFLCVHKKLRSKRVAPVLIREITRRVHLEGIFQAVYTAGVVLPKPVGTCRYWHRSLNPRKLIEVKFSHLSRNMTMQRTMKLYRLPETPKTAGLRPMETKDIPVVHQLLTRYLKQFHLTPVMSQEEVEHWFYPQENIIDTFVVENANGEVTDFLSFYTLPSTIMNHPTHKSLKAAYSFYNVHTQTPLLDLMSDALVLAKMKGFDVFNALDLMENKTFLEKLKFGIGDGNLQYYLYNWKCPSMGAEKVGLVLQ

631 NP_057272.1 NP_004155.2

>NP_057272.1

MSGGVYGGDEVGALVFDIGSFSVRAGYAGEDCPKADFPTTVGLLAAEEGGGLELEGDKEKKGKIFHIDTNALHVPRDGAEVMSPLKNGMIEDWECFRAILDHTYSKHVKSEPNLHPVLMSEAPWNTRAKREKLTELMFEQYNIPAFFLCKTAVLTAFANGRSTGLVLDSGATHTTAIPVHDGYVLQQGIVKSPLAGDFISMQCRELFQEMAIDIIPPYMIAAKEPVREGAPPNWKKKEKLPQVSKSWHNYMCNEVIQDFQASVLQVSDSPYDEQVAAQMPTVHYEMPNGYNTDYGAERLRIPEGLFDPSNVKGLSGNTMLGVGHVVTTSIGMCDIDIRPGLYGSVIVTGGNTLLQGFTDRLNRELSQKTPPSMRLKLIASNSTMERKFSPWIGGSILASLGTFQQMWISKQEYEEGGKQCVERKCP

>NP_004155.2

MTRDQNGTWEMESNENFEGYMKALDIDFATRKIAVRLTQTKVIDQDGDNFKTKTTSTFRNYDVDFTVGVEFDEYTKSLDNRHVKALVTWEGDVLVCVQKGEKENRGWKQWIEGDKLYLELTCGDQVCRQVFKKK

632 NP_001665.1 NP_065390.1

>NP_001665.1

MMLQHPGQVSASEVSASAIVPCLSPPGSLVFEDFANLTPFVKEELRFAIQNKHLCHRMSSALESVTVSDRPLGVSITKAEVAPEEDERKKRRRERNKIAAAKCRNKKKEKTECLQKESEKLESVNAELKAQIEELKNEKQHLIYMLNLHRPTCIVRAQNGRTPEDERNLFIQQIKEGTLQS

>NP_065390.1

MFQAAERPQEWAMEGPRDGLKKERLLDDRHDSGLDSMKDEEYEQMVKELQEIRLEPQEVPRGSEPWKQQLTEDGDSFLHLAIIHEEKALTMEVIRQVKGDLAFLNFQNNLQQTPLHLAVITNQPEIAEALLGAGCDPELRDFRGNTPLHLACEQGCLASVGVLTQSCTTPHLHSILKATNYNGHTCLHLASIHGYLGIVELLVSLGADVNAQEPCNGRTALHLAVDLQNPDLVSLLLKCGADVNRVTYQGYSPYQLTWGRPSTRIQQQLGQLTLENLQMLPESEDEESYDTESEFTEFTEDELPYDDCVFGGQRLTL

633 NP_073207.1 Q9BYD1

>NP_073207.1

MTSSYGHVLERQPALGGRLDSPGNLDTLQAKKNFSVSHLLDLEEAGDMVAAQADENVGEAGRSLLESPGLTSGSDTPQQDNDQLNSEEKKKRKQRRNRTTFNSSQLQALERVFERTHYPDAFVREDLARRVNLTEARVQVWFQNRRAKFRRNERAMLANKNASLLKSYSGDVTAVEQPIVPRPAPRPTDYLSWGTASPYSAMATYSATCANNSPAQGINMANSIANLRLKAKEYSLQRNQVPTVN

>Q9BYD1

MSSFSRAPQQWATFARIWYLLDGKMQPPGKLAAMASIRLQGLHKPVYHALSDCGDHVVIMNTRHIAFSGNKWEQKVYSSHTGYPGGFRQVTAAQLHLRDPVAIVKLAIYGMLPKNLHRRTMMERLHLFPDEYIPEDILKNLVEELPQPRKIPKRLDEYTQEEIDAFPRLWTPPEDYRL

634 Q12857 NP_057740.2

>Q12857

MYSPLCLTQDEFHPFIEALLPHVRAFAYTWFNLQARKRKYFKKHEKRMSKEEERAVKDELLSEKPEVKQKWASRLLAKLRKDIRPEYREDFVLTVTGKKPPCCVLSNPDQKGKMRRIDCLRQADKVWRLDLVMVILFKGIPLESTDGERLVKSPQCSNPGLCVQPHHIGVSVKELDLYLAYFVHAADSSQSESPSQPSDADIKDQPENGHLGFQDSFVTSGVFSVTELVRVSQTPIAAGTGPNFSLSDLESSSYYSMSPGAMRRSLPSTSSTSSTKRLKSVEDEMDSPGEEPFYTGQGRSPGSGSQSSGWHEVEPGMPSPTTLKKSEKSGFSSPSPSQTSSLGTAFTQHHRPVITGPRASPHATPSTLHFPTSPIIQQPGPYFSHPAIRYHPQETLKEFVQLVCPDAGQQAGQVGFLNPNGSSQGKVHNPFLPTPMLPPPPPPPMARPVPLPVPDTKPPTTSTEGGAASPTSPTYSTPSTSPANRFVSVGPRDPSFVNIPQQTQSWYLG

>NP_057740.2

MEESDSEKTTEKENLGPRMDPPLGEPEGSLGWVLPNTAMKKKVLLMGKSGSGKTSMRSIIFANYIARDTRRLGATILDRIHSLQINSSLSTYSLVDSVGNTKTFDVEHSHVRFLGNLVLNLWDCGGQDTFMENYFTSQRDNIFRNVEVLIYVFDVESRELEKDMHYYQSCLEAILQNSPDAKIFCLVHKMDLVQEDQRDLIFKEREEDLRRLSRPLECSCFRTSIWDETLYKAWSSIVYQLIPNVQQLEMNLRNFAEIIEADEVLLFERATFLVISHYQCKEQRDAHRFEKISNIIKQFKLSCSKLAASFQSMEVRNSNFAAFIDIFTSNTYVMVVMSDPSIPSAATLINIRNARKHFEKLERVDGPKQCLLMR

635 NP_003160.2 NP_002070.1

>NP_003160.2

MSDSEDSNFSEEEDSERSSDGEEAEVDEERRSAAGSEKEEEPEDEEEEEEEEEYDEEEEEEDDDRPPKKPRHGGFILDEADVDDEYEDEDQWEDGAEDILEKEEIEASNIDNVVLDEDRSGARRLQNLWRDQREEELGEYYMKKYAKSSVGETVYGGSDELSDDITQQQLLPGVKDPNLWTVKCKIGEERATAISLMRKFIAYQFTDTPLQIKSVVAPEHVKGYIYVEAYKQTHVKQAIEGVGNLRLGYWNQQMVPIKEMTDVLKVVKEVANLKPKSWVRLKRGIYKDDIAQVDYVEPSQNTISLKMIPRIDYDRIKARMSLKDWFAKRKKFKRPPQRLFDAEKIRSLGGDVASDGDFLIFEGNRYSRKGFLFKSFAMSAVITEGVKPTLSELEKFEDQPEGIDLEVVTESTGKEREHNFQPGDNVEVCEGELINLQGKILSVDGNKITIMPKHEDLKDMLEFPAQELRKYFKMGDHVKVIAGRFEGDTGLIVRVEENFVILFSDLTMHELKVLPRDLQLCSETASGVDVGGQHEWGELVQLDPQTVGVIVRLERETFQVLNMYGKVVTVRHQAVTRKKDNRFAVALDSEQNNIHVKDIVKVIDGPHSGREGEIRHLFRSFAFLHCKKLVENGGMFVCKTRHLVLAGGSKPRDVTNFTVGGFAPMSPRISSPMHPSAGGQRGGFGSPGGGSGGMSRGRGRRDNELIGQTVRISQGPYKGYIGVVKDATESTARVELHSTCQTISVDRQRLTTVGSRRPGGMTSTYGRTPMYGSQTPMYGSGSRTPMYGSQTPLQDGSRTPHYGSQTPLHDGSRTPAQSGAWDPNNPNTPSRAEEEYEYAFDDEPTPSPQAYGGTPNPQTPGYPDPSSPQVNPQYNPQTPGTPAMYNTDQFSPYAAPSPQGSYQPSPSPQSYHQVAPSPAGYQNTHSPASYHPTPSPMAYQASPSPSPVGYSPMTPGAPSPGGYNPHTPGSGIEQNSSDWVTTDIQVKVRDTYLDTQVVGQTGVIRSVTGGMCSVYLKDSEKVVSISSEHLEPITPTKNNKVKVILGEDREATGVLLSIDGEDGIVRMDLDEQLKILNLRFLGKLLEA

>NP_002070.1

MAPPSVFAEVPQAQPVLVFKLTADFREDPDPRKVNLGVGAYRTDDCHPWVLPVVKKVEQKIANDNSLNHEYLPILGLAEFRSCASRLALGDDSPALKEKRVGGVQSLGGTGALRIGADFLARWYNGTNNKNTPVYVSSPTWENHNAVFSAAGFKDIRSYRYWDAEKRGLDLQGFLNDLENAPEFSIVVLHACAHNPTGIDPTPEQWKQIASVMKHRFLFPFFDSAYQGFASGNLERDAWAIRYFVSEGFEFFCAQSFSKNFGLYNERVGNLTVVGKEPESILQVLSQMEKIVRITWSNPPAQGARIVASTLSNPELFEEWTGNVKTMADRILTMRSELRARLEALKTPGTWNHITDQIGMFSFTGLNPKQVEYLVNEKHIYLLPSGRINVSGLTTKNLDYVATSIHEAVTKIQ

636 NP_002909.3 P28838

>NP_002909.3

MATQAYTELQAAPPPSQPPQAPPQAQPQPPPPPPPAAPQPPQPPTAAATPQPQYVTELQSPQPQAQPPGGQKQYVTELPAVPAPSQPTGAPTPSPAPQQYIVVTVSEGAMRASETVSEASPGSTASQTGVPTQVVQQVQGTQQRLLVQTSVQAKPGHVSPLQLTNIQVPQQALPTQRLVVQSAAPGSKGGQVSLTVHGTQQVHSPPEQSPVQANSSSSKTAGAPTGTVPQQLQVHGVQQSVPVTQERSVVQATPQAPKPGPVQPLTVQGLQPVHVAQEVQQLQQVPVPHVYSSQVQYVEGGDASYTASAIRSSTYSYPETPLYTQTASTSYYEAAGTATQVSTPATSQAVASSGSMPMYVSGSQVVASSASTGAGASNSSGGGGSGGGGGGGGGGGGGGSGSTGGGGSGAGTYVIQGGYMLGSASQSYSHTTRASPATVQWLLDNYETAEGVSLPRSTLYCHYLLHCQEQKLEPVNAASFGKLIRSVFMGLRTRRLGTRGNSKYHYYGLRIKASSPLLRLMEDQQHMAMRGQPFSQKQRLKPIQKMEGMTNGVAVGQQPSTGLSDISAQVQQYQQFLDASRSLPDFTELDLQGKVLPEGVGPGDIKAFQVLYREHCEAIVDVMVNLQFTLVETLWKTFWRYNLSQPSEAPPLAVHDEAEKRLPKAILVLLSKFEPVLQWTKHCDNVLYQGLVEILIPDVLRPIPSALTQAIRNFAKSLESWLTHAMVNIPEEMLRVKVAAAGAFAQTLRRYTSLNHLAQAARAVLQNTAQINQMLSDLNRVDFANVQEQASWVCRCEDRVVQRLEQDFKVTLQQQNSLEQWAAWLDGVVSQVLKPYQGSAGFPKAAKLFLLKWSFYSSMVIRDLTLRSAASFGSFHLIRLLYDEYMYYLIEHRVAQAKGETPIAVMGEFANLATSLNPLDPDKDEEEEEEEESEDELPQDISLAAGGESPALGPETLEPPAKLARTDARGLFVQALPSS

>P28838

MFLLPLPAAGRVVVRRLAVRRFGSRSLSTADMTKGLVLGIYSKEKEDDVPQFTSAGENFDKLLAGKLRETLNISGPPLKAGKTRTFYGLHQDFPSVVLVGLGKKAAGIDEQENWHEGKENIRAAVAAGCRQIQDLELSSVEVDPCGDAQAAAEGAVLGLYEYDDLKQKKKMAVSAKLYGSGDQEAWQKGVLFASGQNLARQLMETPANEMTPTRFAEIIEKNLKSASSKTEVHIRPKSWIEEQAMGSFLSVAKGSDEPPVFLEIHYKGSPNANEPPLVFVGKGITFDSGGISIKASANMDLMRADMGGAATICSAIVSAAKLNLPINIIGLAPLCENMPSGKANKPGDVVRAKNGKTIQVDNTDAEGRLILADALCYAHTFNPKVILNAATLTGAMDVALGSGATGVFTNSSWLWNKLFEASIETGDRVWRMPLFEHYTRQVVDCQLADVNNIGKYRSAGACTAAAFLKEFVTHPKWAHLDIAGVMTNKDEVPYLRKGMTGRPTRTLIEFLLRFSQDNA

637 NP_060058.1 NP_620448.1

>NP_060058.1

MATEEKKPETEAARAQPTPSSSATQSKPTPVKPNYALKFTLAGHTKAVSSVKFSPNGEWLASSSADKLIKIWGAYDGKFEKTISGHKLGISDVAWSSDSNLLVSASDDKTLKIWDVSSGKCLKTLKGHSNYVFCCNFNPQSNLIVSGSFDESVRIWDVKTGKCLKTLPAHSDPVSAVHFNRDGSLIVSSSYDGLCRIWDTASGQCLKTLIDDDNPPVSFVKFSPNGKYILAATLDNTLKLWDYSKGKCLKTYTGHKNEKYCIFANFSVTGGKWIVSGSEDNLVYIWNLQTKEIVQKLQGHTDVVISTACHPTENIIASAALENDKTIKLWKSDC

>NP_620448.1

MSLHFLYYCSEPTLDVKIAFCQGFDKQVDVSYIAKHYNMSKSKVDNQFYSVEVGDSTFTVLKRYQNLKPIGSGAQGIVCAAYDAVLDRNVAIKKLSRPFQNQTHAKRAYRELVLMKCVNHKNIISLLNVFTPQKTLEEFQDVYLVMELMDANLCQVIQMELDHERMSYLLYQMLCGIKHLHSAGIIHRDLKPSNIVVKSDCTLKILDFGLARTAGTSFMMTPYVVTRYYRAPEVILGMGYKENVDIWSVGCIMGEMVRHKILFPGRDYIDQWNKVIEQLGTPCPEFMKKLQPTVRNYVENRPKYAGLTFPKLFPDSLFPADSEHNKLKASQARDLLSKMLVIDPAKRISVDDALQHPYINVWYDPAEVEAPPPQIYDKQLDEREHTIEEWKELIYKEVMNSEEKTKNGVVKGQPSPSGAAVNSSESLPPSSSVNDISSMSTDQTLASDTDSSLEASAGPLGCCR

638 NP_001941.2 NP_005882.2

>NP_001941.2

MAEAGPQAPPPPGTPSRHEKSLGLLTTKFVSLLQEAKDGVLDLKLAADTLAVRQKRRIYDITNVLEGIGLIEKKSKNSIQWKGVGPGCNTREIADKLIELKAEIEELQQREQELDQHKVWVQQSIRNVTEDVQNSCLAYVTHEDICRCFAGDTLLAIRAPSGTSLEVPIPEGLNGQKKYQIHLKSVSGPIEVLLVNKEAWSSPPVAVPVPPPEDLLQSPSAVSTPPPLPKPALAQSQEASRPNSPQLTPTAVPGSAEVQGMAGPAAEITVSGGPGTDSKDSGELSSLPLGPTTLDTRPLQSSALLDSSSSSSSSSSSSSNSNSSSSSGPNPSTSFEPIKADPTGVLELPKELSEIFDPTRECMSSELLEELMSSEVFAPLLRLSPPPGDHDYIYNLDESEGVCDLFDVPVLNL

>NP_005882.2

MNAGSDPVVIVSAARTIIGSFNGALAAVPVQDLGSTVIKEVLKRATVAPEDVSEVIFGHVLAAGCGQNPVRQASVGAGIPYSVPAWSCQMICGSGLKAVCLAVQSIGIGDSSIVVAGGMENMSKAPHLAYLRTGVKIGEMPLTDSILCDGLTDAFHNCHMGITAENVAKKWQVSREDQDKVAVLSQNRTENAQKAGHFDKEIVPVLVSTRKGLIEVKTDEFPRHGSNIEAMSKLKPYFLTDGTGTVTPANASGINDGAAAVVLMKKSEADKRGLTPLARIVSWSQVGVEPSIMGIGPIPAIKQAVTKAGWSLEDVDIFEINEAFAAVSAAIVKELGLNPEKVNIEGGAIALGHPLGASGCRILVTLLHTLERMGRSRGVAALCIGGGMGIAMCVQRE

639 O15156 NP_112176.1

>O15156

MGSPEDDLIGIPFPDHSSELLSCLNEQRQLGHLCDLTIRTQGLEYRTHRAVLAACSHYFKKLFTEGGGGAVMGAGGSGTATGGAGAGVCELDFVGPEALGALLEFAYTATLTTSSANMPAVLQAARLLEIPCVIAACMEILQGSGLEAPSPDEDDCERARQYLEAFATATASGVPNGEDSPPQVPLPPPPPPPPRPVARRSRKPRKAFLQTKGARANHLVPEVPTVPAHPLTYEEEEVAGRVGSSGGSGPGDSYSPPTGTASPPEGPQSYEPYEGEEEEEELVYPPAYGLAQGGGPPLSPEELGSDEDAIDPDLMAYLSSLHQDNLAPGLDSQDKLVRKRRSQMPQECPVCHKIIHGAGKLPRHMRTHTGEKPFACEVCGVRFTRNDKLKIHMRKHTGERPYSCPHCPARFLHSYDLKNHMHLHTGDRPYECHLCHKAFAKEDHLQRHLKGQNCLEVRTRRRRKDDAPPHYPPPSTAAASPAGLDLSNGHLDTFRLSLARFWEQSAPTGPPVSTPGPPDDDEEEGAPTTPQAEGAMESS

>NP_112176.1

MAAPLSVEVEFGGGAELLFDGIKKHRVTLPGQEEPWDIRNLLIWIKKNLLKERPELFIQGDSVRPGILVLINDADWELLGELDYQLQDQDSVLFISTLHGG

640 NP_004489.1 P40394

>NP_004489.1

MNAQLTMEAIGELHGVSHEPVPAPADLLGGSPHARSSVAHRGSHLPPAHPRSMGMASLLDGGSGGGDYHHHHRAPEHSLAGPLHPTMTMACETPPGMSMPTTYTTLTPLQPLPPISTVSDKFPHHHHHHHHHHHPHHHQRLAGNVSGSFTLMRDERGLASMNNLYTPYHKDVAGMGQSLSPLSSSGLGSIHNSQQGLPHYAHPGAAMPTDKMLTPNGFEAHHPAMLGRHGEQHLTPTSAGMVPINGLPPHHPHAHLNAQGHGQLLGTAREPNPSVTGAQVSNGSNSGQMEEINTKEVAQRITTELKRYSIPQAIFAQRVLCRSQGTLSDLLRNPKPWSKLKSGRETFRRMWKWLQEPEFQRMSALRLAACKRKEQEHGKDRGNTPKKPRLVFTDVQRRTLHAIFKENKRPSKELQITISQQLGLELSTVSNFFMNARRRSLDKWQDEGSSNSGNSSSSSSTCTKA

>P40394

MGTAGKVIKCKAAVLWEQKQPFSIEEIEVAPPKTKEVRIKILATGICRTDDHVIKGTMVSKFPVIVGHEATGIVESIGEGVTTVKPGDKVIPLFLPQCRECNACRNPDGNLCIRSDITGRGVLADGTTRFTCKGKPVHHFMNTSTFTEYTVVDESSVAKIDDAAPPEKVCLIGCGFSTGYGAAVKTGKVKPGSTCVVFGLGGVGLSVIMGCKSAGASRIIGIDLNKDKFEKAMAVGATECISPKDSTKPISEVLSEMTGNNVGYTFEVIGHLETMIDALASCHMNYGTSVVVGVPPSAKMLTYDPMLLFTGRTWKGCVFGGLKSRDDVPKLVTEFLAKKFDLDQLITHVLPFKKISEGFELLNSGQSIRTVLTF

641 NP_115701.2 Q92871

>NP_115701.2

MSQATKRKHVVKEVLGEHIVPSDQQQIVRVLRTPGNNLHEVETAQGQRFLVSMPSKYRKNIWIKRGDFLIVDPIEEGEKVKAEISFVLCKDHVRSLQKEGFWPEAFSEVAEKHNNRNRQTQPELPAEPQLSGEESSSEDDSDLFVNTNRRQYHESEEESEEEEAA

>Q92871

MAVTAQAARRKERVLCLFDVDGTLTPARQKIDPEVAAFLQKLRSRVQIGVVGGSDYCKIAEQLGDGDEVIEKFDYVFAENGTVQYKHGRLLSKQTIQNHLGEELLQDLINFCLSYMALLRLPKKRGTFIEFRNGMLNISPIGRSCTLEERIEFSELDKKEKIREKFVEALKTEFAGKGLRFSRGGMISFDVFPEGWDKRYCLDSLDQDSFDTIHFFGNETSPGGNDFEIFADPRTVGHSVVSPQDTVQRCREIFFPETAHEA

642 Q13461 Q14651

>Q13461

MAGRSDMDPPAAFSGFPALPAVAPSGPPPSPLAGAEPGREPEEAAAGRGEAAPTPAPGPGRRRRRPLQRGKPPYSYIALIAMALAHAPGRRLTLAAIYRFITERFAFYRDSPRKWQNSIRHNLTLNDCFVKVPREPGNPGKGNYWTLDPAAADMFDNGSFLRRRKRFKRAELPAHAAAAPGPPLPFPYAPYAPAPGPALLVPPPSAGPGPSPPARLFSVDSLVNLQPELAGLGAPEPPCCAAPDAAAAAFPPCAAAASPPLYSQVPDRLVLPATRPGPGPLPAEPLLALAGPAAALGPLSPGEAYLRQPGFASGLERYL

>Q14651

MENSTTTISREELEELQEAFNKIDIDNSGYVSDYELQDLFKEASLPLPGYKVREIVEKILSVADSNKDGKISFEEFVSLMQELKSKDISKTFRKIINKREGITAIGGTSTISSEGTQHSYSEEEKVAFVNWINKALENDPDCKHLIPMNPNDDSLFKSLADGILLCKMINLSEPDTIDERAINKKKLTPFTISENLNLALNSASAIGCTVVNIGASDLKEGKPHLVLGLLWQIIKVGLFADIEISRNEALIALLNEGEELEELMKLSPEELLLRWVNYHLTNAGWHTISNFSQDIKDSRAYFHLLNQIAPKGGEDGPAIAIDLSGINETNDLKRAGLMLQEADKLGCKQFVTPADVVSGNPKLNLAFVANLFNTYPCLHKPNNNDIDMNLLEGESKEERTFRNWMNSLGVNPYINHLYSDLADALVIFQLYEMIRVPVNWSHVNKPPYPALGGNMKKIENCNYAVELGKNKAKFSLVGIAGQDLNEGNSTLTLALVWQLMRRYTLNVLSDLGEGEKVNDEIIIKWVNQTLKSANKKTSISSFKDKSISTSLPVLDLIDAIAPNAVRQEMIRRENLSDEDKLNNAKYAISVARKIGARIYALPDDLVEVKPKMVMTVFACLMGKGLNRIK

643 NP_001796.2 NP_004484.1

>NP_001796.2

MSHGTYYECEPRGGQQPLEFSGGRAGPGELGDMCEHEASIDLSAYIESGEEQLLSDLFAVKPAPEARGLKGPGTPAFPHYLPPDPRPFAYPPHTFGPDRKALGPGIYSSPGSYDPRAVAVKEEPRGPEGSRAASRGSYNPLQYQVAHCGQTAMHLPPTLAAPGQPLRVLKAPLATAAPPCSPLLKAPSPAGPLHKGKKAVNKDSLEYRLRRERNNIAVRKSRDKAKRRILETQQKVLEYMAENERLRSRVEQLTQELDTLRNLFRQIPEAANLIKGVGGCS

>NP_004484.1

MAAACRSVKGLVAVITGGASGLGLATAERLVGQGASAVLLDLPNSGGEAQAKKLGNNCVFAPADVTSEKDVQTALALAKGKFGRVDVAVNCAGIAVASKTYNLKKGQTHTLEDFQRVLDVNLMGTFNVIRLVAGEMGQNEPDQGGQRGVIINTASVAAFEGQVGQAAYSASKGGIVGMTLPIARDLAPIGIRVMTIAPGLFGTPLLTSLPEKVCNFLASQVPFPSRLGDPAEYAHLVQAIIENPFLNGEVIRLDGAIRMQP

644 Q9Y4A5 Q9UDX5

>Q9Y4A5

MAFVATQGATVVDQTTLMKKYLQFVAALTDVNTPDETKLKMMQEVSENFENVTSSPQYSTFLEHIIPRFLTFLQDGEVQFLQEKPAQQLRKLVLEIIHRIPTNEHLRPHTKNVLSVMFRFLETENEENVLICLRIIIELHKQFRPPITQEIHHFLDFVKQIYKELPKVVNRYFENPQVIPENTVPPPEMVGMITTIAVKVNPEREDSETRTHSIIPRGSLSLKVLAELPIIVVLMYQLYKLNIHNVVAEFVPLIMNTIAIQVSAQARQHKLYNKELYADFIAAQIKTLSFLAYIIRIYQELVTKYSQQMVKGMLQLLSNCPAETAHLRKELLIAAKHILTTELRNQFIPCMDKLFDESILIGSGYTARETLRPLAYSTLADLVHHVRQHLPLSDLSLAVQLFAKNIDDESLPSSIQTMSCKLLLNLVDCIRSKSEQESGNGRDVLMRMLEVFVLKFHTIARYQLSAIFKKCKPQSELGAVEAALPGVPTAPAAPGPAPSPAPVPAPPPPPPPPPPATPVTPAPVPPFEKQGEKDKEDKQTFQVTDCRSLVKTLVCGVKTITWGITSCKAPGEAQFIPNKQLQPKETQIYIKLVKYAMQALDIYQVQIAGNGQTYIRVANCQTVRMKEEKEVLEHFAGVFTMMNPLTFKEIFQTTVPYMVERISKNYALQIVANSFLANPTTSALFATILVEYLLDRLPEMGSNVELSNLYLKLFKLVFGSVSLFAAENEQMLKPHLHKIVNSSMELAQTAKEPYNYFLLLRALFRSIGGGSHDLLYQEFLPLLPNLLQGLNMLQSGLHKQHMKDLFVELCLTVPVRLSSLLPYLPMLMDPLVSALNGSQTLVSQGLRTLELCVDNLQPDFLYDHIQPVRAELMQALWRTLRNPADSISHVAYRVLGKFGGSNRKMLKESQKLHYVVTEVQGPSITVEFSDCKASLQLPMEKAIETALDCLKSANTEPYYRRQAWEVIKCFLVAMMSLEDNKHALYQLLAHPNFTEKTIPNVIISHRYKAQDTPARKTFEQALTGAFMSAVIKDLRPSALPFVASLIRHYTMVAVAQQCGPFLLPCYQVGSQPSTAMFHSEENGSKGMDPLVLIDAIAICMAYEEKELCKIGEVALAVIFDVASIILGSKERACQLPLFSYIVERLCACCYEQAWYAKLGGVVSIKFLMERLPLTWVLQNQQTFLKALLFVMMDLTGEVSNGAVAMAKTTLEQLLMRCATPLKDEERAEEIVAAQEKSFHHVTHDLVREVTSPNSTVRKQAMHSLQVLAQVTGKSVTVIMEPHKEVLQDMVPPKKHLLRHQPANAQIGLMEGNTFCTTLQPRLFTMDLNVVEHKVFYTELLNLCEAEDSALTKLPCYKSLPSLVPLRIAALNALAACNYLPQSREKIIAALFKALNSTNSELQEAGEACMRKFLEGATIEVDQIHTHMRPLLMMLGDYRSLTLNVVNRLTSVTRLFPNSFNDKFCDQMMQHLRKWMEVVVITHKGGQRSDGNESISECGRCPLSPFCQFEEMKICSAIINLFHLIPAAPQTLVKPLLEVVMKTERAMLIEAGSPFREPLIKFLTRHPSQTVELFMMEATLNDPQWSRMFMSFLKHKDARPLRDVLAANPNRFITLLLPGGAQTAVRPGSPSTSTMRLDLQFQAIKIISIIVKNDDSWLASQHSLVSQLRRVWVSENFQERHRKENMAATNWKEPKLLAYCLLNYCKRNYGDIELLFQLLRAFTGRFLCNMTFLKEYMEEEIPKNYSIAQKRALFFRFVDFNDPNFGDELKAKVLQHILNPAFLYSFEKGEGEQLLGPPNPEGDNPESITSVFITKVLDPEKQADMLDSLRIYLLQYATLLVEHAPHHIHDNNKNRNSKLRRLMTFAWPCLLSKACVDPACKYSGHLLLAHIIAKFAIHKKIVLQVFHSLLKAHAMEARAIVRQAMAILTPAVPARMEDGHQMLTHWTRKIIVEEGHTVPQLVHILHLIVQHFKVYYPVRHHLVQHMVSAMQRLGFTPSVTIEQRRLAVDLSEVVIKWELQRIKDQQPDSDMDPNSSGEGVNSVSSSIKRGLSVDSAQEVKRFRTATGAISAVFGRSQSLPGADSLLAKPIDKQHTDTVVNFLIRVACQVNDNTNTAGSPGEVLSRRCVNLLKTALRPDMWPKSELKLQWFDKLLMTVEQPNQVNYGNICTGLEVLSFLLTVLQSPAILSSFKPLQRGIAACMTCGNTKVLRAVHSLLSRLMSIFPTEPSTSSVASKYEELECLYAAVGKVIYEGLTNYEKATNANPSQLFGTLMILKSACSNNPSYIDRLISVFMRSLQKMVREHLNPQAASGSTEATSGTSELVMLSLELVKTRLAVMSMEMRKNFIQAILTSLIEKSPDAKILRAVVKIVEEWVKNNSPMAANQTPTLREKSILLVKMMTYIEKRFPEDLELNAQFLDLVNYVYRDETLSGSELTAKLEPAFLSGLRCAQPLIRAKFFEVFDNSMKRRVYERLLYVTCSQNWEAMGNHFWIKQCIELLLAVCEKSTPIGTSCQGAMLPSITNVINLADSHDRAAFAMVTHVKQEPRERENSESKEEDVEIDIELAPGDQTSTPKTKELSEKDIGNQLHMLTNRHDKFLDTLREVKTGALLSAFVQLCHISTTLAEKTWVQLFPRLWKILSDRQQHALAGEISPFLCSGSHQVQRDCQPSALNCFVEAMSQCVPPIPIRPCVLKYLGKTHNLWFRSTLMLEHQAFEKGLSLQIKPKQTTEFYEQESITPPQQEILDSLAELYSLLQEEDMWAGLWQKRCKYSETATAIAYEQHGFFEQAQESYEKAMDKAKKEHERSNASPAIFPEYQLWEDHWIRCSKELNQWEALTEYGQSKGHINPYLVLECAWRVSNWTAMKEALVQVEVSCPKEMAWKVNMYRGYLAICHPEEQQLSFIERLVEMASSLAIREWRRLPHVVSHVHTPLLQAAQQIIELQEAAQINAGLQPTNLGRNNSLHDMKTVVKTWRNRLPIVSDDLSHWSSIFMWRQHHYQGKPTWSGMHSSSIVTAYENSSQHDPSSNNAMLGVHASASAIIQYGKIARKQGLVNVALDILSRIHTIPTVPIVDCFQKIRQQVKCYLQLAGVMGKNECMQGLEVIESTNLKYFTKEMTAEFYALKGMFLAQINKSEEANKAFSAAVQMHDVLVKAWAMWGDYLENIFVKERQLHLGVSAITCYLHACRHQNESKSRKYLAKVLWLLSFDDDKNTLADAVDKYCIGVPPIQWLAWIPQLLTCLVGSEGKLLLNLISQVGRVYPQAVYFPIRTLYLTLKIEQRERYKSDPGPIRATAPMWRCSRIMHMQRELHPTLLSSLEGIVDQMVWFRENWHEEVLRQLQQGLAKCYSVAFEKSGAVSDAKITPHTLNFVKKLVSTFGVGLENVSNVSTMFSSAASESLARRAQATAQDPVFQKLKGQFTTDFDFSVPGSMKLHNLISKLKKWIKILEAKTKQLPKFFLIEEKCRFLSNFSAQTAEVEIPGEFLMPKPTHYYIKIARFMPRVEIVQKHNTAARRLYIRGHNGKIYPYLVMNDACLTESRREERVLQLLRLLNPCLEKRKETTKRHLFFTVPRVVAVSPQMRLVEDNPSSLSLVEIYKQRCAKKGIEHDNPISRYYDRLATVQARGTQASHQVLRDILKEVQSNMVPRSMLKEWALHTFPNATDYWTFRKMFTIQLALIGFAEFVLHLNRLNPEMLQIAQDTGKLNVAYFRFDINDATGDLDANRPVPFRLTPNISEFLTTIGVSGPLTASMIAVARCFAQPNFKVDGILKTVLRDEIIAWHKKTQEDTSSPLSAAGQPENMDSQQLVSLVQKAVTAIMTRLHNLAQFEGGESKVNTLVAAANSLDNLCRMDPAWHPWL

>Q9UDX5

MSEPQPRGAERDLYRDTWVRYLGYANEVGEAFRSLVPAAVVWLSYGVASSYVLADAIDKGKKAGEVPSPEAGRSARVTVAVVDTFVWQALASVAIPGFTINRVCAASLYVLGTATRWPLAVRKWTTTALGLLTIPIIIHPIDRSVDFLLDSSLRKLYPTVGKPSSS

645 Q7RTV3 O75891

>Q7RTV3

MIRGFEAPMAENPPPPPPPVIFCHDSPKRVLVSVIRTTPIKPTCGGGGEPEPPPPLIPTSPGFSDFMVYPWRWGENAHNVTLSPGAAGAAASAALPAAAAAEHSGLRGRGAPPPAASASAAASGGEDEEEASSPDSGHLKDGIRRGRPRADTVRDLINEGEHSSSRIRCNICNRVFPREKSLQAHKRTHTGERPYLCDYPDCGKAFVQSGQLKTHQRLHTGEKPFVCSENGCLSRFTHANRHCPKHPYARLKREEPTDTLSKHQAADNKAAAEWLARYWEMREQRTPTLKGKLVQKADQEQQDPLEYLQSDEEDDEKRGAQRRLQEQRERLHGALALIELANLTGAPLRQ

>O75891

MKIAVIGQSLFGQEVYCHLRKEGHEVVGVFTVPDKDGKADPLGLEAEKDGVPVFKYSRWRAKGQALPDVVAKYQALGAELNVLPFCSQFIPMEIISAPRHGSIIYHPSLLPRHRGASAINWTLIHGDKKGGFSIFWADDGLDTGDLLLQKECEVLPDDTVSTLYNRFLFPEGIKGMVQAVRLIAEGKAPRLPQPEEGATYEGIQKKETAKINWDQPAEAIHNWIRGNDKVPGAWTEACEQKLTFFNSTLNTSGLVPEGDALPIPGAHRPGVVTKAGLILFGNDDKMLLVKNIQLEDGKMILASNFFKGAASSVLELTEAELVTAEAVRSVWQRILPKVLEVEDSTDFFKSGAASVDVVRLVEEVKELCDGLELENEDVYMASTFGDFIQLLVRKLRGDDEEGECSIDYVEMAVNKRTVRMPHQLFIGGEFVDAEGAKTSETINPTDGSVICQVSLAQVTDVDKAVAAAKDAFENGRWGKISARDRGRLMYRLADLMEQHQEELATIEALDAGAVYTLALKTHVGMSIQTFRYFAGWCDKIQGSTIPINQARPNRNLTLTRKEPVGVCGIIIPWNYPLMMLSWKTAACLAAGNTVVIKPAQVTPLTALKFAELTLKAGIPKGVVNVLPGSGSLVGQRLSDHPDVRKIGFTGSTEVGKHIMKSCAISNVKKVSLELGGKSPLIIFADCDLNKAVQMGMSSVFFNKGENCIAAGRLFVEDSIHDEFVRRVVEEVRKMKVGNPLDRDTDHGPQNHHAHLVKLMEYCQHGVKEGATLVCGGNQVPRPGFFFEPTVFTDVEDHMFIAKEESFGPVMIISRFADGDLDAVLSRANATEFGLASGVFTRDINKALYVSDKLQAGTVFVNTYNKTDVAAPFGGFKQSGFGKDLGEAALNEYLRVKTVTFEY

646 NP_006500.2 NP_002003.1

>NP_006500.2

MLRSGPASGPSVPTGRAMPSRRVARPPAAPELGALGSPDLSSLSLAVSRSTDELEIIDEYIKENGFGLDGGQPGPGEGLPRLVSRGAASLSTVTLGPVAPPATPPPWGCPLGRLVSPAPGPGPQPHLVITEQPKQRGMRFRYECEGRSAGSILGESSTEASKTLPAIELRDCGGLREVEVTACLVWKDWPHRVHPHSLVGKDCTDGICRVRLRPHVSPRHSFNNLGIQCVRKKEIEAAIERKIQLGIDPYNAGSLKNHQEVDMNVVRICFQASYRDQQGQMRRMDPVLSEPVYDKKSTNTSELRICRINKESGPCTGGEELYLLCDKVQKEDISVVFSRASWEGRADFSQADVHRQIAIVFKTPPYEDLEIVEPVTVNVFLQRLTDGVCSEPLPFTYLPRDHDSYGVDKKRKRGMPDVLGELNSSDPHGIESKRRKKKPAILDHFLPNHGSGPFLPPSALLPDPDFFSGTVSLPGLEPPGGPDLLDDGFAYDPTAPTLFTMLDLLPPAPPHASAVVCSGGAGAVVGETPGPEPLTLDSYQAPGPGDGGTASLVGSNMFPNHYREAAFGGGLLSPGPEAT

>NP_002003.1

MSFRFGQHLIKPSVVFLKTELSFALVNRKPVVPGHVLVCPLRPVERFHDLRPDEVADLFQTTQRVGTVVEKHFHGTSLTFSMQDGPEAGQTVKHVHVHVLPRKAGDFHRNDSIYEELQKHDKEDFPASWRSEEEMAAEAAALRVYFQ

647 NP_003007.2 NP_002475.2

>NP_003007.2

MSYGRPPPDVEGMTSLKVDNLTYRTSPDTLRRVFEKYGRVGDVYIPRDRYTKESRGFAFVRFHDKRDAEDAMDAMDGAVLDGRELRVQMARYGRPPDSHHSRRGPPPRRYGGGGYGRRSRSPRRRRRSRSRSRSRSRSRSRSRYSRSKSRSRTRSRSRSTSKSRSARRSKSKSSSVSRSRSRSRSRSRSRSPPPVSKRESKSRSRSKSPPKSPEEEGAVSS

>NP_002475.2

MEEVPHDCPGADSAQAGRGASCQGCPNQRLCASGAGATPDTAIEEIKEKMKTVKHKILVLSGKGGVGKSTFSAHLAHGLAEDENTQIALLDIDICGPSIPKIMGLEGEQVHQSGSGWSPVYVEDNLGVMSVGFLLSSPDDAVIWRGPKKNGMIKQFLRDVDWGEVDYLIVDTPPGTSDEHLSVVRYLATAHIDGAVIITTPQEVSLQDVRKEINFCRKVKLPIIGVVENMSGFICPKCKKESQIFPPTTGGAELMCQDLEVPLLGRVPLDPLIGKNCDKGQSFFIDAPDSPATLAYRSIIQRIQEFCNLHQSKEENLISS

648 Q9NPC7 Q9HD40

>Q9NPC7

MQYSHHCEHLLERLNKQREAGFLCDCTIVIGEFQFKAHRNVLASFSEYFGAIYRSTSENNVFLDQSQVKADGFQKLLEFIYTGTLNLDSWNVKEIHQAADYLKVEEVVTKCKIKMEDFAFIANPSSTEISSITGNIELNQQTCLLTLRDYNNREKSEVSTDLIQANPKQGALAKKSSQTKKKKKAFNSPKTGQNKTVQYPSDILENASVELFLDANKLPTPVVEQVAQINDNSELELTSVVENTFPAQDIVHTVTVKRKRGKSQPNCALKEHSMSNIASVKSPYEAENSGEELDQRYSKAKPMCNTCGKVFSEASSLRRHMRIHKGVKPYVCHLCGKAFTQCNQLKTHVRTHTGEKPYKCELCDKGFAQKCQLVFHSRMHHGEEKPYKCDVCNLQFATSSNLKIHARKHSGEKPYVCDRCGQRFAQASTLTYHVRRHTGEKPYVCDTCGKAFAVSSSLITHSRKHTGEKPYICGICGKSFISSGELNKHFRSHTGERPFICELCGNSYTDIKNLKKHKTKVHSGADKTLDSSAEDHTLSEQDSIQKSPLSETMDVKPSDMTLPLALPLGTEDHHMLLPVTDTQSPTSDTLLRSTVNGYSEPQLIFLQQLY

>Q9HD40

MNRESFAAGERLVSPAYVRQGCEARRSHEHLIRLLLEKGKCPENGWDESTLELFLHELAIMDSNNFLGNCGVGEREGRVASALVARRHYRFIHGIGRSGDISAVQPKAAGSSLLNKITNSLVLDIIKLAGVHTVANCFVVPMATGMSLTLCFLTLRHKRPKAKYIIWPRIDQKSCFKSMITAGFEPVVIENVLEGDELRTDLKAVEAKVQELGPDCILCIHSTTSCFAPRVPDRLEELAVICANYDIPHIVNNAYGVQSSKCMHLIQQGARVGRIDAFVQSLDKNFMVPVGGAIIAGFNDSFIQEISKMYPGRASASPSLDVLITLLSLGSNGYKKLLKERKEMFSYLSNQIKKLSEAYNERLLHTPHNPISLAMTLKTLDEHRDKAVTQLGSMLFTRQVSGARVVPLGSMQTVSGYTFRGFMSHTNNYPCAYLNAASAIGMKMQDVDLFIKRLDRCLKAVRKERSKESDDNYDKTEDVDIEEMALKLDNVLLDTYQDASS

649 NP_115285.1 NP_659451.1

>NP_115285.1

MSCGRPPPDVDGMITLKVDNLTYRTSPDSLRRVFEKYGRVGDVYIPREPHTKAPRGFAFVRFHDRRDAQDAEAAMDGAELDGRELRVQVARYGRRDLPRSRQGEPRGRSRGGGYGRRSRSYGRRSRSPRRRHRSRSRGPSCSRSRSRSRYRGSRYSRSPYSRSPYSRSRYSRSPYSRSRYRESRYGGSHYSSSGYSNSRYSRYHSSRSHSKSGSSTSSRSASTSKSSSARRSKSSSVSRSRSRSRSSSMTRSPPRVSKRKSKSRSRSKRPPKSPEEEGQMSS

>NP_659451.1

MEELLPDGQIWANMDPEERMLAAATAFTHICAGQGEGDVRREAQSIQYDPYSKASVAPGKRPALPVQLQYPHVESNVPSETVSEASQRLRKPVMKRKVLRRKPDGEVLVTDESIISESESGTENDQDLWDLRQRLMNVQFQEDKESSFDVSQKFNLPHEYQGISQDQLICSLQREGMGSPAYEQDLIVASRPKSFILPKLDQLSRNRGKTDRVARYFEYKRDWDSIRLPGEDHRKELRWGVREQMLCRAEPQSKPQHIYVPNNYLVPTEKKRSALRWGVRCDLANGVIPRKLPFPLSPS

650 Q6DRA6 NP_000467.1

>Q6DRA6

MPEPAKFAPAPKKGSKKAVTKAQKKDGKKRKRSRKESYSIYVYKVLKRVHPDTGIWCKAMGIMNSFLNDIFERIAGEASRLAHYNKRSTITSRRSRRPCACCCPASWPSTPCPRAPRRSPSTPAPSESLPGPGARSLPPSLPPRVAGCFVSKGSFQGHLTPLVK

>NP_000467.1

MEEKLKKTKIIFVVGGPGSGKGTQCEKIVQKYGYTHLSTGDLLRSEVSSGSARGKKLSEIMEKGQLVPLETVLDMLRDAMVAKVNTSKGFLIDGYPREVQQGEEFERRIGQPTLLLYVDAGPETMTQRLLKRGETSGRVDDNEETIKKRLETYYKATEPVIAFYEKRGIVRKVNAEGSVDSVFSQVCTHLDALK

651 NP_663628.1 NP_001753.1

>NP_663628.1

MAAGSTTLRAVGKLQVRLATKTEPKKLEKYLQKLSALPMTADILAETGIRKTVKRLRKHQHVGDFARDLAARWKKLVLVDRNTGPDPQDPEESASRQRFGEALQEREKAWGFPENATAPRSPSHSPEHRRTARRTPPGQQRPHPRSPSREPRAERKRPRMAPADSGPHRDPPTRTAPLPMPEGPEPAVPGEQPGRGHAHAAQGGPLLGQGCQGQPQGEAVGSHSKGHKSSRGASAQKSPPVQESQSERLQAAGADSAGPKTVPSHVFSELWDPSEAWMQANYDLLSAFEAMTSQANPEALCAPALQEEAAFPGRRVNAKMPVYSGSRPACQLQVPTLRQQCLRVPRNNPDALGDVEGVPYSVLEPVLEGWTPDQLYRTEKDNAALARETDELWRIHCLQDFKEEKPQEHESWRELYLRLRDAREQRLRVVTTKIRSARENKPSGRQTKMICFNSVAKTPYDASRRQEKSAGAADPGNGEMEPAPKPAGSSQAPSGLGDGDGGSVSGGGSSNRHAAPADKTRKQAAKKVAPLMAKAIRDYKGRFSRR

>NP_001753.1

MAAVKTLNPKAEVARAQAALAVNISAARGLQDVLRTNLGPKGTMKMLVSGAGDIKLTKDGNVLLHEMQIQHPTASLIAKVATAQDDITGDGTTSNVLIIGELLKQADLYISEGLHPRIITEGFEAAKEKALQFLEEVKVSREMDRETLIDVARTSLRTKVHAELADVLTEAVVDSILAIKKQDEPIDLFMIEIMEMKHKSETDTSLIRGLVLDHGARHPDMKKRVEDAYILTCNVSLEYEKTEVNSGFFYKSAEEREKLVKAERKFIEDRVKKIIELKRKVCGDSDKGFVVINQKGIDPFSLDALSKEGIVALRRAKRRNMERLTLACGGVALNSFDDLSPDCLGHAGLVYEYTLGEEKFTFIEKCNNPRSVTLLIKGPNKHTLTQIKDAVRDGLRAVKNAIDDGCVVPGAGAVEVAMAEALIKHKPSVKGRAQLGVQAFADALLIIPKVLAQNSGFDLQETLVKIQAEHSESGQLVGVDLNTGEPMVAAEVGVWDNYCVKKQLLHSCTVIATNILLVDEIMRAGMSSLKG

652 NP_006312.1 Q9P278

>NP_006312.1

MSVDMNSQGSDSNEEDYDPNCEEEEEEEEDDPGDIEDYYVGVASDVEQQGADAFDPEEYQFTCLTYKESEGALNEHMTSLASVLKVSHSVAKLILVNFHWQVSEILDRYKSNSAQLLVEARVQPNPSKHVPTSHPPHHCAVCMQFVRKENLLSLACQHQFCRSCWEQHCSVLVKDGVGVGVSCMAQDCPLRTPEDFVFPLLPNEELREKYRRYLFRDYVESHYQLQLCPGADCPMVIRVQEPRARRVQCNRCNEVFCFKCRQMYHAPTDCATIRKWLTKCADDSETANYISAHTKDCPKCNICIEKNGGCNHMQCSKCKHDFCWMCLGDWKTHGSEYYECSRYKENPDIVNQSQQAQAREALKKYLFYFERWENHNKSLQLEAQTYQRIHEKIQERVMNNLGTWIDWQYLQNAAKLLAKCRYTLQYTYPYAYYMESGPRKKLFEYQQAQLEAEIENLSWKVERADSYDRGDLENQMHIAEQRRRTLLKDFHDT

>Q9P278

MAPTLLQKLFNKRGSSGSSAAASAQGRAPKEGPAFSWSCSEFDLNEIRLIVYQDCDRRGRQVLFDSKAVQKIEEVTAQKTEDVPIKISAKCCQGSSSVSSSSSSSISSHSSSGGSSHHAKEQLPKYQYTRPASDVNMLGEMMFGSVAMSYKGSTLKIHYIRSPPQLMISKVFSARMGSFCGSTNNLQDSFEYINQDPNLGKLNTNQNSLGPCRTGSNLAHSTPVDMPSRGQNEDRDSGIARSASLSSLLITPFPSPSSSTSSSSSYQRRWLRSQTTSLENGIIPRRSTDETFSLAEETCSSNPAMVRRKKIAISIIFSLCEKEEAQRNFQDFFFSHFPLFESHMNRLKSAIEKAMISCRKIAESSLRVQFYVSRLMEALGEFRGTIWNLYSVPRIAEPVWLTMMSGTLEKNQLCQRFLKEFTLLIEQINKNQFFAALLTAVLTYHLAWVPTVMPVDHPPIKAFSEKRTSQSVNMLAKTHPYNPLWAQLGDLYGAIGSPVRLTRTVVVGKQKDLVQRILYVLTYFLRCSELQENQLTWSGNHGEGDQVLNGSKIITALEKGEVEESEYVVITVRNEPALVPPILPPTAAERHNPWPTGFPECPEGTDSRDLGLKPDKEANRRPEQGSEACSAGCLGPASDASWKPQNAFCGDEKNKEAPQDGSSRLPSCEVLGAGMKMDQQAVCELLKVEMPTRLPDRSVAWPCPDRHLREKPSLEKVTFQIGSFASPESDFESRMKKMEERVKACGPSLEASEAADVAQDPQVSRSPFKPGFQENVCCPQNRLSEGDEGESDKGFAEDRGSRNDMAADIAGQLSHAADLGTASHGAGGTGGRRLEATRGLYVKAAEGPVLEPVAPRCVQRGPGLVAGANIPCGDDNKKANFRTEGDIPRNESSDSALGDSDDEACASAMLDLGHGGDRTGGSLEVELPLPRSQSISTQNVRNFGRSLLAGYCPTYMPDLVLHGTGSDEKLKQCLVADLVHTVHHPVLDEPIAEAVCIIADTDKWSVQVATSQRKVTDNMKLGQDVLVSSQVSSLLQSILQLYKLHLPADFCIMHLEDRLQEMYLKSKMLSEYLRGHTRVHVKELGVVLGIESNDLPLLTAIASTHSPYVAQILL

653 NP_000391.1 Q12849

>NP_000391.1

MKLNVDGLLVYFPYDYIYPEQFSYMRELKRTLDAKGHGVLEMPSGTGKTVSLLALIMAYQRAYPLEVTKLIYCSRTVPEIEKVIEELRKLLNFYEKQEGEKLPFLGLALSSRKNLCIHPEVTPLRFGKDVDGKCHSLTASYVRAQYQHDTSLPHCRFYEEFDAHGREVPLPAGIYNLDDLKALGRRQGWCPYFLARYSILHANVVVYSYHYLLDPKIADLVSKELARKAVVVFDEAHNIDNVCIDSMSVNLTRRTLDRCQGNLETLQKTVLRIKETDEQRLRDEYRRLVEGLREASAARETDAHLANPVLPDEVLQEAVPGSIRTAEHFLGFLRRLLEYVKWRLRVQHVVQESPPAFLSGLAQRVCIQRKPLRFCAERLRSLLHTLEITDLADFSPLTLLANFATLVSTYAKGFTIIIEPFDDRTPTIANPILHFSCMDASLAIKPVFERFQSVIITSGTLSPLDIYPKILDFHPVTMATFTMTLARVCLCPMIIGRGNDQVAISSKFETREDIAVIRNYGNLLLEMSAVVPDGIVAFFTSYQYMESTVASWYEQGILENIQRNKLLFIETQDGAETSVALEKYQEACENGRGAILLSVARGKVSEGIDFVHHYGRAVIMFGVPYVYTQSRILKARLEYLRDQFQIRENDFLTFDAMRHAAQCVGRAIRGKTDYGLMVFADKRFARGDKRGKLPRWIQEHLTDANLNLTVDEGVQVAKYFLRQMAQPFHREDQLGLSLLSLEQLESEETLKRIEQIAQQL

>Q12849

MAGTRWVLGALLRGCGCNCSSCRRTGAACLPFYSAAGSIPSGVSGRRRLLLLLGAAAAAASQTRGLQTGPVPPGRLAGPPAVATSAAAAAAASYSALRASLLPQSLAAAAAVPTRSYSQESKTTYLEDLPPPPEYELAPSKLEEEVDDVFLIRAQGLPWSCTMEDVLNFFSDCRIRNGENGIHFLLNRDGKRRGDALIEMESEQDVQKALEKHRMYMGQRYVEVYEINNEDVDALMKSLQVKSSPVVNDGVVRLRGLPYSCNEKDIVDFFAGLNIVDITFVMDYRGRRKTGEAYVQFEEPEMANQALLKHREEIGNRYIEIFPSRRNEVRTHVGSYKGKKIASFPTAKYITEPEMVFEEHEVNEDIQPMTAFESEKEIELPKEVPEKLPEAADFGTTSSLHFVHMRGLPFQANAQDIINFFAPLKPVRITMEYSSSGKATGEADVHFETHEDAVAAMLKDRSHVHHRYIELFLNSCPKGK

654 NP_002909.3 Q13287

>NP_002909.3

MATQAYTELQAAPPPSQPPQAPPQAQPQPPPPPPPAAPQPPQPPTAAATPQPQYVTELQSPQPQAQPPGGQKQYVTELPAVPAPSQPTGAPTPSPAPQQYIVVTVSEGAMRASETVSEASPGSTASQTGVPTQVVQQVQGTQQRLLVQTSVQAKPGHVSPLQLTNIQVPQQALPTQRLVVQSAAPGSKGGQVSLTVHGTQQVHSPPEQSPVQANSSSSKTAGAPTGTVPQQLQVHGVQQSVPVTQERSVVQATPQAPKPGPVQPLTVQGLQPVHVAQEVQQLQQVPVPHVYSSQVQYVEGGDASYTASAIRSSTYSYPETPLYTQTASTSYYEAAGTATQVSTPATSQAVASSGSMPMYVSGSQVVASSASTGAGASNSSGGGGSGGGGGGGGGGGGGGSGSTGGGGSGAGTYVIQGGYMLGSASQSYSHTTRASPATVQWLLDNYETAEGVSLPRSTLYCHYLLHCQEQKLEPVNAASFGKLIRSVFMGLRTRRLGTRGNSKYHYYGLRIKASSPLLRLMEDQQHMAMRGQPFSQKQRLKPIQKMEGMTNGVAVGQQPSTGLSDISAQVQQYQQFLDASRSLPDFTELDLQGKVLPEGVGPGDIKAFQVLYREHCEAIVDVMVNLQFTLVETLWKTFWRYNLSQPSEAPPLAVHDEAEKRLPKAILVLLSKFEPVLQWTKHCDNVLYQGLVEILIPDVLRPIPSALTQAIRNFAKSLESWLTHAMVNIPEEMLRVKVAAAGAFAQTLRRYTSLNHLAQAARAVLQNTAQINQMLSDLNRVDFANVQEQASWVCRCEDRVVQRLEQDFKVTLQQQNSLEQWAAWLDGVVSQVLKPYQGSAGFPKAAKLFLLKWSFYSSMVIRDLTLRSAASFGSFHLIRLLYDEYMYYLIEHRVAQAKGETPIAVMGEFANLATSLNPLDPDKDEEEEEEEESEDELPQDISLAAGGESPALGPETLEPPAKLARTDARGLFVQALPSS

>Q13287

MEADKDDTQQILKEHSPDEFIKDEQNKGLIDEITKKNIQLKKEIQKLETELQEATKEFQIKEDIPETKMKFLSVETPENDSQLSNISCSFQVSSKVPYEIQKGQALITFEKEEVAQNVVSMSKHHVQIKDVNLEVTAKPVPLNSGVRFQVYVEVSKMKINVTEIPDTLREDQMRDKLELSFSKSRNGGGEVDRVDYDRQSGSAVITFVEIGVADKILKKKEYPLYINQTCHRVTVSPYTEIHLKKYQIFSGTSKRTVLLTGMEGIQMDEEIVEDLINIHFQRAKNGGGEVDVVKCSLGQPHIAYFEE

655 NP_004069.1 P20591

>NP_004069.1

MPNWGGGKKCGVCQKTVYFAEEVQCEGNSFHKSCFLCMVCKKNLDSTTVAVHGEEIYCKSCYGKKYGPKGYGYGQGAGTLSTDKGESLGIKHEEAPGHRPTTNPNASKFAQKIGGSERCPRCSQAVYAAEKVIGAGKSWHKACFRCAKCGKGLESTTLADKDGEIYCKGCYAKNFGPKGFGFGQGAGALVHSE

>P20591

MVVSEVDIAKADPAAASHPLLLNGDATVAQKNPGSVAENNLCSQYEEKVRPCIDLIDSLRALGVEQDLALPAIAVIGDQSSGKSSVLEALSGVALPRGSGIVTRCPLVLKLKKLVNEDKWRGKVSYQDYEIEISDASEVEKEINKAQNAIAGEGMGISHELITLEISSRDVPDLTLIDLPGITRVAVGNQPADIGYKIKTLIKKYIQRQETISLVVVPSNVDIATTEALSMAQEVDPEGDRTIGILTKPDLVDKGTEDKVVDVVRNLVFHLKKGYMIVKCRGQQEIQDQLSLSEALQREKIFFENHPYFRDLLEEGKATVPCLAEKLTSELITHICKSLPLLENQIKETHQRITEELQKYGVDIPEDENEKMFFLIDKVNAFNQDITALMQGEETVGEEDIRLFTRLRHEFHKWSTIIENNFQEGHKILSRKIQKFENQYRGRELPGFVNYRTFETIVKQQIKALEEPAVDMLHTVTDMVRLAFTDVSIKNFEEFFNLHRTAKSKIEDIRAEQEREGEKLIRLHFQMEQIVYCQDQVYRGALQKVREKELEEEKKKKSWDFGAFQSSSATDSSMEEIFQHLMAYHQEASKRISSHIPLIIQFFMLQTYGQQLQKAMLQLLQDKDTYSWLLKERSDTSDKRKFLKERLARLTQARRRLAQFPG

656 NP_002469.2 NP_004160.3

>NP_002469.2

MELLSPPLRDVDLTAPDGSLCSFATTDDFYDDPCFDSPDLRFFEDLDPRLMHVGALLKPEEHSHFPAAVHPAPGAREDEHVRAPSGHHQAGRCLLWACKACKRKTTNADRRKAATMRERRRLSKVNEAFETLKRCTSSNPNQRLPKVEILRNAIRYIEGLQALLRDQDAAPPGAAAAFYAPGPLPPGRGGEHYSGDSDASSPRSNCSDGMMDYSGPPSGARRRNCYEGAYYNEAPSEPRPGKSAAVSSLDCLSSIVERISTESPAAPALLLADVPSESPPRRQEAAAPSEGESSGDPTQSPDAAPQCPAGANPNPIYQVL

>NP_004160.3

MTMPVNGAHKDADLWSSHDKMLAQPLKDSDVEVYNIIKKESNRQRVGLELIASENFASRAVLEALGSCLNNKYSEGYPGQRYYGGTEFIDELETLCQKRALQAYKLDPQCWGVNVQPYSGSPANFAVYTALVEPHGRIMGLDLPDGGHLTHGFMTDKKKISATSIFFESMPYKVNPDTGYINYDQLEENARLFHPKLIIAGTSCYSRNLEYARLRKIADENGAYLMADMAHISGLVAAGVVPSPFEHCHVVTTTTHKTLRGCRAGMIFYRKGVKSVDPKTGKEILYNLESLINSAVFPGLQGGPHNHAIAGVAVALKQAMTLEFKVYQHQVVANCRALSEALTELGYKIVTGGSDNHLILVDLRSKGTDGGRAEKVLEACSIACNKNTCPGDRSALRPSGLRLGTPALTSRGLLEKDFQKVAHFIHRGIELTLQIQSDTGVRATLKEFKERLAGDKYQAAVQALREEVESFASLFPLPGLPDF

657 Q92949 NP_054902.1

>Q92949

MAESWLRLSGAGPAEEAGPEGGLEEPDALDDSLTSLQWLQEFSILNAKAPALPPGGTDPHGYHQVPGSAAPGSPLAADPACLGQPHTPGKPTSSCTSRSAPPGLQAPPPDDVDYATNPHVKPPYSYATLICMAMQASKATKITLSAIYKWITDNFCYFRHADPTWQNSIRHNLSLNKCFIKVPREKDEPGKGGFWRIDPQYAERLLSGAFKKRRLPPVHIHPAFARQAAQEPSAVPRAGPLTVNTEAQQLLREFEEATGEAGWGAGEGRLGHKRKQPLPKRVAKVPRPPSTLLPTPEEQGELEPLKGNFDWEAIFDAGTLGGELGALEALELSPPLSPASHVDVDLTIHGRHIDCPATWGPSVEQAADSLDFDETFLATSFLQHPWDESGSGCLPPEPLFEAGDATLASDLQDWASVGAFL

>NP_054902.1

MAEVEETLKRLQSQKGVQGIIVVNTEGIPIKSTMDNPTTTQYASLMHSFILKARSTVRDIDPQNDLTFLRIRSKKNEIMVAPDKDYFLIVIQNPTE

658 NP_006617.1 AAH30144.2

>NP_006617.1

MAAESDVLHFQFEQQGDVVLQKMNLLRQQNLFCDVSIYINDTEFQGHKVILAACSTFMRDQFLLTQSKHVRITILQSAEVGRKLLLSCYTGALEVKRKELLKYLTAASYLQMVHIVEKCTEALSKYLEIDLSMKNNNQHTDLCQSSDPDVKNEDENSDKDCEIIEISEDSPVNIDFHVKEEESNALQSTVESLTSERKEMKSPELSTVDIGFKDNEICILHVESISTAGVENGQFSQPCTSSKASMYFSETQHSLINSTVESRVAEVPGNQDQGLFCENTEGSYGTVSEIQNLEEGYSLRHQCPRCPRGFLHVENYLRHLKMHKLFLCLQCGKTFTQKKNLNRHIRGHMGIRPFQCTVCLKTFTAKSTLQDHLNIHSGDRPYKCHCCDMDFKHKSALKKHLTSVHGRSSGEKLSRPDLKRQSLL

>AAH30144.2

MAACIAAGHWAAMGLGRSFQAARTLLPPPASIACRVHAGPVRQQSTGPSEPGAFQPPPKPVIVDKHRPVEPERRFLSPEFIPRRGRTDPLKFQIERKDMLERRKVLHIPEFYVGSILRVTTADPYASGKISQFLGICIQRSGRGLGATFILRNVIEGQGVEICFELYNPRVQEIQVVKLEKRLDDSLLYLRDALPEYSTFDVNMKPVVQEPNQKVPVNELKVKMKPKPWSKRWERPNFNIKGIRFDLCLTEQQMKEAQKWNQPWLEFDMMREYDTSKIEAAIWKEIEASKRS

659 NP_003646.2 NP_060322.1

>NP_003646.2

MELPAVGEHVFAVESIEKKRIRKGRVEYLVKWRGWSPKYNTWEPEENILDPRLLIAFQNRERQEQLMGYRKRGPKPKPLVVQVPTFARRSNVLTGLQDSSTDNRAKLDLGAQGKGQGHQYELNSKKHHQYQPHSKERAGKPPPPGKSGKYYYQLNSKKHHPYQPDPKMYDLQYQGGHKEAPSPTCPDLGAKSHPPDKWAQGAGAKGYLGAVKPLAGAAGAPGKGSEKGPPNGMMPAPKEAVTGNGIGGKMKIVKNKNKNGRIVIVMSKYMENGMQAVKIKSGEVAEGEARSPSHKKRAADERHPPADRTFKKAAGAEEKKVEAPPKRREEEVSGVSDPQPQDAGSRKLSPTKEAFGEQPLQLTTKPDLLAWDPARNTHPPSHHPHPHPHHHHHHHHHHHHAVGLNLSHVRKRCLSETHGEREPCKKRLTARSISTPTCLGGSPAAERPADLPPAAALPQPEVILLDSDLDEPIDLRCVKTRSEAGEPPSSLQVKPETPASAAVAVAAAAAPTTTAEKPPAEAQDEPAESLSEFKPFFGNIIITDVTANCLTVTFKEYVTV

>NP_060322.1

MVSSAQMGFNLQALLEQLSQDELSKFKYLITTFSLAHELQKIPHKEVDKADGKQLVEILTTHCDSYWVEMASLQVFEKMHRMDLSERAKDEVREAALKSFNKRKPLSLGITRKERPPLDVDEMLERFKTEAQAFTETKGNVICLGKEVFKGKKPDKDNRCRYILKTKFREMWKSWPGDSKEVQVMAERYKMLIPFSNPRVLPGPFSYTVVLYGPAGLGKTTLAQKLMLDWAEDNLIHKFKYAFYLSCRELSRLGPCSFAELVFRDWPELQDDIPHILAQARKILFVIDGFDELGAAPGALIEDICGDWEKKKPVPVLLGSLLNRVMLPKAALLVTTRPRALRDLRILAEEPIYIRVEGFLEEDRRAYFLRHFGDEDQAMRAFELMRSNAALFQLGSAPAVCWIVCTTLKLQMEKGEDPVPTCLTRTGLFLRFLCSRFPQGAQLRGALRTLSLLAAQGLWAQTSVLHREDLERLGVQESDLRLFLDGDILRQDRVSKGCYSFIHLSFQQFLTALFYTLEKEEEEDRDGHTWDIGDVQKLLSGVERLRNPDLIQAGYYSFGLANEKRAKELEATFGCRMSPDIKQELLRCDISCKGGHSTVTDLQELLGCLYESQEEELVKEVMAQFKEISLHLNAVDVVPSSFCVKHCRNLQKMSLQVIKENLPENVTASESDAEVERSQDDQHMLPFWTDLCSIFGSNKDLMGLAINDSFLSASLVRILCEQIASDTCHLQRVVFKNISPADAHRNLCLALRGHKTVTYLTLQGNDQDDMFPALCEVLRHPECNLRYLGLVSCSATTQQWADLSLALEVNQSLTCVNLSDNELLDEGAKLLYTTLRHPKCFLQRLSLENCHLTEANCKDLAAVLVVSRELTHLCLAKNPIGNTGVKFLCEGLRYPECKLQTLVLWNCDITSDGCCDLTKLLQEKSSLLCLDLGLNHIGVKGMKFLCEALRKPLCNLRCLWLWGCSIPPFSCEDLCSALSCNQSLVTLDLGQNPLGSSGVKMLFETLTCSSGTLRTLRLKIDDFNDELNKLLEEIEEKNPQLIIDTEKHHPWAERPSSHDFMI

660 NP_006029.1 O15540

>NP_006029.1

MGKPSSMDTKFKDDLFRKYVQFHESKVDTTTSRQRPGSDECLRVAASTLLSLHKVDPFYRFRLIQFYEVVESSLRSLSSSSLRALHGAFSMLETVGINLFLYPWKKEFRSIKTYTGPFVYYVKSTLLEEDIRAILSCMGYTPELGTAYKLRELVETLQVKMVSFELFLAKVECEQMLEIHSQVKDKGYSELDIVSERKSSAEDVRGCSDALRRRAEGREHLTASMSRVALQKSASERAAKDYYKPRVTKPSRSVDAYDSYWESRKPPLKASLSLRKEPVATDVGDDLKDEIIRPSPSLLTMASSPHGSPDVLPPASPSNGPALLRGTYFSTQDDVDLYTDSEPRATYRRQDALRPDVWLLRNDAHSLYHKRSPPAKESALSKCQSCGLSCSSSLCQRCDSLLTCPPASKPSAFPSKASTHDSLAHGASLREKYPGQTQGLDRLPHLHSKSKPSTTPTSRCGFCNRPGATNTCTQCSKVSCDACLSAYHYDPCYKKSELHKFMPNNQLNYKSTQLSHLVYR

>O15540

MVEAFCATWKLTNSQNFDEYMKALGVGFATRQVGNVTKPTVIISQEGDKVVIRTLSTFKNTEISFQLGEEFDETTADDRNCKSVVSLDGDKLVHIQKWDGKETNFVREIKDGKMVMTLTFGDVVAVRHYEKA

661 NP_036601.2 Q9Y3B7

>NP_036601.2

MNKKKKPFLGMPAPLGYVPGLGRGATGFTTRSDIGPARDANDPVDDRHAPPGKRTVGDQMKKNQAADDDDEDLNDTNYDEFNGYAGSLFSSGPYEKDDEEADAIYAALDKRMDERRKERREQREKEEIEKYRMERPKIQQQFSDLKRKLAEVTEEEWLSIPEVGDARNKRQRNPRYEKLTPVPDSFFAKHLQTGENHTSVDPRQTQFGGLNTPYPGGLNTPYPGGMTPGLMTPGTGELDMRKIGQARNTLMDMRLSQVSDSVSGQTVVDPKGYLTDLNSMIPTHGGDINDIKKARLLLKSVRETNPHHPPAWIASARLEEVTGKLQVARNLIMKGTEMCPKSEDVWLEAARLQPGDTAKAVVAQAVRHLPQSVRIYIRAAELETDIRAKKRVLRKALEHVPNSVRLWKAAVELEEPEDARIMLSRAVECCPTSVELWLALARLETYENARKVLNKARENIPTDRHIWITAAKLEEANGNTQMVEKIIDRAITSLRANGVEINREQWIQDAEECDRAGSVATCQAVMRAVIGIGIEEEDRKHTWMEDADSCVAHNALECARAIYAYALQVFPSKKSVWLRAAYFEKNHGTRESLEALLQRAVAHCPKAEVLWLMGAKSKWLAGDVPAARSILALAFQANPNSEEIWLAAVKLESENDEYERARRLLAKARSSAPTARVFMKSVKLEWVQDNIRAAQDLCEEALRHYEDFPKLWMMKGQIEEQKEMMEKAREAYNQGLKKCPHSTPLWLLLSRLEEKIGQLTRARAILEKSRLKNPKNPGLWLESVRLEYRAGLKNIANTLMAKALQECPNSGILWSEAIFLEARPQRRTKSVDALKKCEHDPHVLLAVAKLFWSQRKITKAREWFHRTVKIDSDLGDAWAFFYKFELQHGTEEQQEEVRKRCESAEPRHGELWCAVSKDIANWQKKIGDILRLVAGRIKNTF

>Q9Y3B7

MSKLGRAARGLRKPEVGGVIRAIVRAGLAMPGPPLGPVLGQRGVSINQFCKEFNERTKDIKEGIPLPTKILVKPDRTFEIKIGQPTVSYFLKAAAGIEKGARQTGKEVAGLVTLKHVYEIARIKAQDEAFALQDVPLSSVVRSIIGSARSLGIRVVKDLSSEELAAFQKERAIFLAAQKEADLAAQEEAAKK

662 Q9UGN5 Q9HD40

>Q9UGN5

MAARRRRSTGGGRARALNESKRVNNGNTAPEDSSPAKKTRRCQRQESKKMPVAGGKANKDRTEDKQDGMPGRSWASKRVSESVKALLLKGKAPVDPECTAKVGKAHVYCEGNDVYDVMLNQTNLQFNNNKYYLIQLLEDDAQRNFSVWMRWGRVGKMGQHSLVACSGNLNKAKEIFQKKFLDKTKNNWEDREKFEKVPGKYDMLQMDYATNTQDEEETKKEESLKSPLKPESQLDLRVQELIKLICNVQAMEEMMMEMKYNTKKAPLGKLTVAQIKAGYQSLKKIEDCIRAGQHGRALMEACNEFYTRIPHDFGLRTPPLIRTQKELSEKIQLLEALGDIEIAIKLVKTELQSPEHPLDQHYRNLHCALRPLDHESYEFKVISQYLQSTHAPTHSDYTMTLLDLFEVEKDGEKEAFREDLHNRMLLWHGSRMSNWVGILSHGLRIAPPEAPITGYMFGKGIYFADMSSKSANYCFASRLKNTGLLLLSEVALGQCNELLEANPKAEGLLQGKHSTKGLGKMAPSSAHFVTLNGSTVPLGPASDTGILNPDGYTLNYNEYIVYNPNQVRMRYLLKVQFNFLQLW

>Q9HD40

MNRESFAAGERLVSPAYVRQGCEARRSHEHLIRLLLEKGKCPENGWDESTLELFLHELAIMDSNNFLGNCGVGEREGRVASALVARRHYRFIHGIGRSGDISAVQPKAAGSSLLNKITNSLVLDIIKLAGVHTVANCFVVPMATGMSLTLCFLTLRHKRPKAKYIIWPRIDQKSCFKSMITAGFEPVVIENVLEGDELRTDLKAVEAKVQELGPDCILCIHSTTSCFAPRVPDRLEELAVICANYDIPHIVNNAYGVQSSKCMHLIQQGARVGRIDAFVQSLDKNFMVPVGGAIIAGFNDSFIQEISKMYPGRASASPSLDVLITLLSLGSNGYKKLLKERKEMFSYLSNQIKKLSEAYNERLLHTPHNPISLAMTLKTLDEHRDKAVTQLGSMLFTRQVSGARVVPLGSMQTVSGYTFRGFMSHTNNYPCAYLNAASAIGMKMQDVDLFIKRLDRCLKAVRKERSKESDDNYDKTEDVDIEEMALKLDNVLLDTYQDASS

663 P10243 NP_000365.3

>P10243

MAKRSRSEDEDDDLQYADHDYEVPQQKGLKKLWNRVKWTRDEDDKLKKLVEQHGTDDWTLIASHLQNRSDFQCQHRWQKVLNPELIKGPWTKEEDQRVIELVQKYGPKRWSLIAKHLKGRIGKQCRERWHNHLNPEVKKSSWTEEEDRIIYEAHKRLGNRWAEIAKLLPGRTDNSIKNHWNSTMRRKVEQEGYLQDGIKSERSSSKLQHKPCAAMDHMQTQNQFYIPVQIPGYQYVSPEGNCIEHVQPTSAFIQQPFIDEDPDKEKKIKELEMLLMSAENEVRRKRIPSQPGSFSSWSGSFLMDDNMSNTLNSLDEHTSEFYSMDENQPVSAQQNSPTKFLAVEANAVLSSLQTIPEFAETLELIESDPVAWSDVTSFDISDAAASPIKSTPVKLMRIQHNEGAMECQFNVSLVLEGKKNTCNGGNSEAVPLTSPNIAKFSTPPAILRKKRKMRVGHSPGSELRDGSLNDGGNMALKHTPLKTLPFSPSQFFNTCPGNEQLNIENPSFTSTPICGQKALITTPLHKETTPKDQKENVGFRTPTIRRSILGTTPRTPTPFKNALAAQEKKYGPLKIVSQPLAFLEEDIREVLKEETGTDLFLKEEDEPAYKSCKQENTASGKKVRKSLVLDNWEKEESGTQLLTEDISDMQSENRFTTSLLMIPLLEIHDNRCNLIPEKQDINSTNKTYTLTKKKPNPNTSKVVKLEKNLQSNCEWETVVYGKTEDQLIMTEQARRYLSTYTATSSTSRALIL

>NP_000365.3

MEANGLGPQGFPELKNDTFLRAAWGEETDYTPVWCMRQAGRYLPEFRETRAAQDFFSTCRSPEACCELTLQPLRRFPLDAAIIFSDILVVPQALGMEVTMVPGKGPSFPEPLREEQDLERLRDPEVVASELGYVFQAITLTRQRLAGRVPLIGFAGAPWTLMTYMVEGGGSSTMAQAKRWLYQRPQASHQLLRILTDALVPYLVGQVVAGAQALQLFESHAGHLGPQLFNKFALPYIRDVAKQVKARLREAGLAPVPMIIFAKDGHFALEELAQAGYEVVGLDWTVAPKKARECVGKTVTLQGNLDPCALYASEEEIGQLVKQMLDDFGPHRYIANLGHGLYPDMDPEHVGAFVDAVHKHSRLLRQN

664 P35453 NP_003671.1

>P35453

MSRAGSWDMDGLRADGGGAGGAPASSSSSSVAAAAASGQCRGFLSAPVFAGTHSGRAAAAAAAAAAAAAAASGFAYPGTSERTGSSSSSSSSAVVAARPEAPPAKECPAPTPAAAAAAPPSAPALGYGYHFGNGYYSCRMSHGVGLQQNALKSSPHASLGGFPVEKYMDVSGLASSSVPANEVPARAKEVSFYQGYTSPYQHVPGYIDMVSTFGSGEPRHEAYISMEGYQSWTLANGWNSQVYCTKDQPQGSHFWKSSFPGDVALNQPDMCVYRRGRKKRVPYTKLQLKELENEYAINKFINKDKRRRISAATNLSERQVTIWFQNRRVKDKKIVSKLKDTVS

>NP_003671.1

MGDAPSPEEKLHLITRNLQEVLGEEKLKEILKERELKIYWGTATTGKPHVAYFVPMSKIADFLKAGCEVTILFADLHAYLDNMKAPWELLELRVSYYENVIKAMLESIGVPLEKLKFIKGTDYQLSKEYTLDVYRLSSVVTQHDSKKAGAEVVKQVEHPLLSGLLYPGLQALDEEYLKVDAQFGGIDQRKIFTFAEKYLPALGYSKRVHLMNPMVPGLTGSKMSSSEEESKIDLLDRKEDVKKKLKKAFCEPGNVENNGVLSFIKHVLFPLKSEFVILRDEKWGGNKTYTAYVDLEKDFAAEVVHPGDLKNSVEVALNKLLDPIREKFNTPALKKLASAAYPDPSKQKPMAKGPAKNSEPEEVIPSRLDIRVGKIITVEKHPDADSLYVEKIDVGEAEPRTVVSGLVQFVPKEELQDRLVVVLCNLKPQKMRGVESQGMLLCASIEGINRQVEPLDPPAGSAPGEHVFVKGYEKGQPDEELKPKKKVFEKLQADFKISEECIAQWKQTNFMTKLGSISCKSLKGGNIS

665 NP_000391.1 NP_001547.1

>NP_000391.1

MKLNVDGLLVYFPYDYIYPEQFSYMRELKRTLDAKGHGVLEMPSGTGKTVSLLALIMAYQRAYPLEVTKLIYCSRTVPEIEKVIEELRKLLNFYEKQEGEKLPFLGLALSSRKNLCIHPEVTPLRFGKDVDGKCHSLTASYVRAQYQHDTSLPHCRFYEEFDAHGREVPLPAGIYNLDDLKALGRRQGWCPYFLARYSILHANVVVYSYHYLLDPKIADLVSKELARKAVVVFDEAHNIDNVCIDSMSVNLTRRTLDRCQGNLETLQKTVLRIKETDEQRLRDEYRRLVEGLREASAARETDAHLANPVLPDEVLQEAVPGSIRTAEHFLGFLRRLLEYVKWRLRVQHVVQESPPAFLSGLAQRVCIQRKPLRFCAERLRSLLHTLEITDLADFSPLTLLANFATLVSTYAKGFTIIIEPFDDRTPTIANPILHFSCMDASLAIKPVFERFQSVIITSGTLSPLDIYPKILDFHPVTMATFTMTLARVCLCPMIIGRGNDQVAISSKFETREDIAVIRNYGNLLLEMSAVVPDGIVAFFTSYQYMESTVASWYEQGILENIQRNKLLFIETQDGAETSVALEKYQEACENGRGAILLSVARGKVSEGIDFVHHYGRAVIMFGVPYVYTQSRILKARLEYLRDQFQIRENDFLTFDAMRHAAQCVGRAIRGKTDYGLMVFADKRFARGDKRGKLPRWIQEHLTDANLNLTVDEGVQVAKYFLRQMAQPFHREDQLGLSLLSLEQLESEETLKRIEQIAQQL

>NP_001547.1

MSWSPSLTTQTCGAWEMKERLGTGGFGNVIRWHNQETGEQIAIKQCRQELSPRNRERWCLEIQIMRRLTHPNVVAARDVPEGMQNLAPNDLPLLAMEYCQGGDLRKYLNQFENCCGLREGAILTLLSDIASALRYLHENRIIHRDLKPENIVLQQGEQRLIHKIIDLGYAKELDQGSLCTSFVGTLQYLAPELLEQQKYTVTVDYWSFGTLAFECITGFRPFLPNWQPVQWHSKVRQKSEVDIVVSEDLNGTVKFSSSLPYPNNLNSVLAERLEKWLQLMLMWHPRQRGTDPTYGPNGCFKALDDILNLKLVHILNMVTGTIHTYPVTEDESLQSLKARIQQDTGIPEEDQELLQEAGLALIPDKPATQCISDGKLNEGHTLDMDLVFLFDNSKITYETQISPRPQPESVSCILQEPKRNLAFFQLRKVWGQVWHSIQTLKEDCNRLQQGQRAAMMNLLRNNSCLSKMKNSMASMSQQLKAKLDFFKTSIQIDLEKYSEQTEFGITSDKLLLAWREMEQAVELCGRENEVKLLVERMMALQTDIVDLQRSPMGRKQGGTLDDLEEQARELYRRLREKPRDQRTEGDSQEMVRLLLQAIQSFEKKVRVIYTQLSKTVVCKQKALELLPKVEEVVSLMNEDEKTVVRLQEKRQKELWNLLKIACSKVRGPVSGSPDSMNASRLSQPGQLMSQPSTASNSLPEPAKKSEELVAEAHNLCTLLENAIQDTVREQDQSFTALDWSWLQTEEEEHSCLEQAS

666 Q9UIL8 P57721

>Q9UIL8

MAQASPPRPERVLGASSPEARPAQEALLLPTGVFQVAEKMEKRTCALCPKDVEYNVLYFAQSENIAAHENCLLYSSGLVECEDQDPLNPDRSFDVESVKKEIQRGRKLKCKFCHKRGATVGCDLKNCNKNYHFFCAKKDDAVPQSDGVRGIYKLLCQQHAQFPIIAQSAKFSGVKRKRGRKKPLSGNHVQPPETMKCNTFIRQVKEEHGRHTDATVKVPFLKKCKEAGLLNYLLEEILDKVHSIPEKLMDETTSESDYEEIGSALFDCRLFEDTFVNFQAAIEKKIHASQQRWQQLKEEIELLQDLKQTLCSFQENRDLMSSSTSISSLSY

>P57721

MESKVSEGGLNVTLTIRLLMHGKEVGSIIGKKGETVKKMREESGARINISEGNCPERIVTITGPTDAIFKAFAMIAYKFEEDIINSMSNSPATSKPPVTLRLVVPASQCGSLIGKGGSKIKEIRESTGAQVQVAGDMLPNSTERAVTISGTPDAIIQCVKQICVVMLESPPKGATIPYRPKPASTPVIFAGGQAYTIQGQYAIPHPDQLTKLHQLAMQQTPFPPLGQTNPAFPGEKLPLHSSEEAQNLMGQSSGLDASPPASTHELTIPNDLIGCIIGRQGTKINEIRQMSGAQIKIANATEGSSERQITITGTPANISLAQYLINARLTSEVTGMGTL

667 P20290 Q9ULC6

>P20290

MRRTGAPAQADSRGRGRARGGCPGGEATLSQPPPRGGTRGQEPQMKETIMNQEKLAKLQAQVRIGGKGTARRKKKVVHRTATADDKKLQFSLKKLGVNNISGIEEVNMFTNQGTVIHFNNPKVQASLAANTFTITGHAETKQLTEMLPSILNQLGADSLTSLRRLAEALPKQSVDGKAPLATGEDDDDEVPDLVENFDEASKNEAN

>Q9ULC6

MAPKRVVQLSLKMPTHAVCVVGVEAHVDIHSDVPKGANSFRVSGSSGVEVFMVYNRTRVKEPIGKARWPLDTDADMVVSVGTASKELKDFKVRVSYFGEQEDQALGRSVLYLTGVDISLEVDTGRTGKVKRSQGDKKTWRWGPEGYGAILLVNCDRDNHRSAEPDLTHSWLMSLADLQDMSPMLLSCNGPDKLFDSHKLVLNVPFSDSKRVRVFCARGGNSLSDYKQVLGPQCLSYEVERQPGEQEIKFYVEGLTFPDADFLGLVSLSVSLVDPGTLPEVTLFTDTVGFRMAPWIMTPNTQPPEELYVCRVMDTHGSNEKFLEDMSYLTLKANCKLTICPQVENRNDRWIQDEMEFGYIEAPHKSFPVVFDSPRNRGLKDFPYKRILGPDFGYVTREIPLPGPSSLDSFGNLDVSPPVTVGGTEYPLGRILIGSSFPKSGGRQMARAVRNFLKAQQVQAPVELYSDWLSVGHVDEFLTFVPTSDQKGFRLLLASPSACLKLFQEKKEEGYGEAAQFDGLKHQAKRSINEMLADRHLQRDNLHAQKCIDWNRNVLKRELGLAESDIVDIPQLFFLKNFYAEAFFPDMVNMVVLGKYLGIPKPYGPIINGRCCLEEKVQSLLEPLGLHCIFIDDYLSYHELQGEIHCGTNVRRKPFPFKWWNMVP

668 NP_003085.1 Q9NQR4

>NP_003085.1

MAYRGQGQKVQKVMVQPINLIFRYLQNRSRIQVWLYEQVNMRIEGCIIGFDEYMNLVLDDAEEIHSKTKSRKQLGRIMLKGDNITLLQSVSN

>Q9NQR4

MTSFRLALIQLQISSIKSDNVTRACSFIREAATQGAKIVSLPECFNSPYGAKYFPEYAEKIPGESTQKLSEVAKECSIYLIGGSIPEEDAGKLYNTCAVFGPDGTLLAKYRKIHLFDIDVPGKITFQESKTLSPGDSFSTFDTPYCRVGLGICYDMRFAELAQIYAQRGCQLLVYPGAFNLTTGPAHWELLQRSRAVDNQVYVATASPARDDKASYVAWGHSTVVNPWGEVLAKAGTEEAIVYSDIDLKKLAEIRQQIPVFRQKRSDLYAVEMKKP

669 NP_006302.2 NP_068804.1

>NP_006302.2

MSSSGYPPNQGAFSTEQSRYPPHSVQYTFPNTRHQQEFAVPDYRSSHLEVSQASQLLQQQQQQQLRRRPSLLSEFHPGSDRPQERRTSYEPFHPGPSPVDHDSLESKRPRLEQVSDSHFQRVSAAVLPLVHPLPEGLRASADAKKDPAFGGKHEAPSSPISGQPCGDDQNASPSKLSKEELIQSMDRVDREIAKVEQQILKLKKKQQQLEEEAAKPPEPEKPVSPPPVEQKHRSIVQIIYDENRKKAEEAHKIFEGLGPKVELPLYNQPSDTKVYHENIKTNQVMRKKLILFFKRRNHARKQREQKICQRYDQLMEAWEKKVDRIENNPRRKAKESKTREYYEKQFPEIRKQREQQERFQRVGQRGAGLSATIARSEHEISEIIDGLSEQENNEKQMRQLSVIPPMMFDAEQRRVKFINMNGLMEDPMKVYKDRQFMNVWTDHEKEIFKDKFIQHPKNFGLIASYLERKSVPDCVLYYYLTKKNENYKALVRRNYGKRRGRNQQIARPSQEEKVEEKEEDKAEKTEKKEEEKKDEEEKDEKEDSKENTKEKDKIDGTAEETEEREQATPRGRKTANSQGRRKGRITRSMTNEAAAASAAAAAATEEPPPPLPPPPEPISTEPVETSRWTEEEMEVAKKGLVEHGRNWAAIAKMVGTKSEAQCKNFYFNYKRRHNLDNLLQQHKQKTSRKPREERDVSQCESVASTVSAQEDEDIEASNEEENPEDSEVEAVKPSEDSPENATSRGNTEPAVELEPTTETAPSTSPSLAVPSTKPAEDESVETQVNDSISAETAEQMDVDQQEHSAEEGSVCDPPPATKADSVDVEVRVPENHASKVEGDNTKERDLDRASEKVEPRDEDLVVAQQINAQRPEPQSDNDSSATCSADEDVDGEPERQRMFPMDSKPSLLNPTGSILVSSPLKPNPLDLPQLQHRAAVIPPMVSCTPCNIPIGTPVSGYALYQRHIKAMHESALLEEQRQRQEQIDLECRSSTSPCGTSKSPNREWEVLQPAPHQVITNLPEGVRLPTTRPTRPPPPLIPSSKTTVASEKPSFIMGGSISQGTPGTYLTSHNQASYTQETPKPSVGSISLGLPRQQESAKSATLPYIKQEEFSPRSQNSQPEGLLVRAQHEGVVRGTAGAIQEGSITRGTPTSKISVESIPSLRGSITQGTPALPQTGIPTEALVKGSISRMPIEDSSPEKGREEAASKGHVIYEGKSGHILSYDNIKNAREGTRSPRTAHEISLKRSYESVEGNIKQGMSMRESPVSAPLEGLICRALPRGSPHSDLKERTVLSGSIMQGTPRATTESFEDGLKYPKQIKRESPPIRAFEGAITKGKPYDGITTIKEMGRSIHEIPRQDILTQESRKTPEVVQSTRPIIEGSISQGTPIKFDNNSGQSAIKHNVKSLITGPSKLSRGMPPLEIVPENIKVVERGKYEDVKAGETVRSRHTSVVSSGPSVLRSTLHEAPKAQLSPGIYDDTSARRTPVSYQNTMSRGSPMMNRTSDVTISSNKSTNHERKSTLTPTQRESIPAKSPVPGVDPVVSHSPFDPHHRGSTAGEVYRSHLPTHLDPAMPFHRALDPAAAAYLFQRQLSPTPGYPSQYQLYAMENTRQTILNDYITSQQMQVNLRPDVARGLSPREQPLGLPYPATRGIIDLTNMPPTILVPHPGGTSTPPMDRITYIPGTQITFPPRPYNSASMSPGHPTHLAAAASAEREREREREKERERERIAAASSDLYLRPGSEQPGRPGSHGYVRSPSPSVRTQETMLQQRPSVFQGTNGTSVITPLDPTAQLRIMPLPAGGPSISQGLPASRYNTAADALAALVDAAASAPQMDVSKTKESKHEAARLEENLRSRSAAVSEQQQLEQKTLEVEKRSVQCLYTSSAFPSGKPQPHSSVVYSEAGKDKGPPPKSRYEEELRTRGKTTITAANFIDVIITRQIASDKDARERGSQSSDSSSSLSSHRYETPSDAIEVISPASSPAPPQEKLQTYQPEVVKANQAENDPTRQYEGPLHHYRPQQESPSPQQQLPPSSQAEGMGQVPRTHRLITLADHICQIITQDFARNQVSSQTPQQPPTSTFQNSPSALVSTPVRTKTSNRYSPESQAQSVHHQRPGSRVSPENLVDKSRGSRPGKSPERSHVSSEPYEPISPPQVPVVHEKQDSLLLLSQRGAEPAEQRNDARSPGSISYLPSFFTKLENTSPMVKSKKQEIFRKLNSSGGGDSDMAAAQPGTEIFNLPAVTTSGSVSSRGHSFADPASNLGLEDIIRKALMGSFDDKVEDHGVVMSQPMGVVPGTANTSVVTSGETRREEGDPSPHSGGVCKPKLISKSNSRKSKSPIPGQGYLGTERPSSVSSVHSEGDYHRQTPGWAWEDRPSSTGSTQFPYNPLTMRMLSSTPPTPIACAPSAVNQAAPHQQNRIWEREPAPLLSAQYETLSDSDD

>NP_068804.1

MSTSQPGACPCQGAASRPAILYALLSSSLKAVPRPRSRCLCRQHRPVQLCAPHRTCREALDVLAKTVAFLRNLPSFWQLPPQDQRRLLQGCWGPLFLLGLAQDAVTFEVAEAPVPSILKKILLEEPSSSGGSGQLPDRPQPSLAAVQWLQCCLESFWSLELSPKEYACLKGTILFNPDVPGLQAASHIGHLQQEAHWVLCEVLEPWCPAAQGRLTRVLLTASTLKSIPTSLLGDLFFRPIIGDVDIAGLLGDMLLLR

670 NP_079224.1 NP_006635.2

>NP_079224.1

MSELTKELMELVWGTKSSPGLSDTIFCRWTQGFVFSESEGSALEQFEGGPCAVIAPVQAFLLKKLLFSSEKSSWRDCSEEEQKELLCHTLCDILESACCDHSGSYCLVSWLRGKTTEETASISGSPAESSCQVEHSSALAVEELGFERFHALIQKRSFRSLPELKDAVLDQYSMWGNKFGVLLFLYSVLLTKGIENIKNEIEDASEPLIDPVYGHGSQSLINLLLTGHAVSNVWDGDRECSGMKLLGIHEQAAVGFLTLMEALRYCKVGSYLKSPKFPIWIVGSETHLTVFFAKDMALVAPEAPSEQARRVFQTYDPEDNGFIPDSLLEDVMKALDLVSDPEYINLMKNKLDPEGLGIILLGPFLQEFFPDQGSSGPESFTVYHYNGLKQSNYNEKVMYVEGTAVVMGFEDPMLQTDDTPIKRCLQTKWPYIELLWTTDRSPSLN

>NP_006635.2

MSVVGLDVGSQSCYIAVARAGGIETIANEFSDRCTPSVISFGSKNRTIGVAAKNQQITHANNTVSNFKRFHGRAFNDPFIQKEKENLSYDLVPLKNGGVGIKVMYMGEEHLFSVEQITAMLLTKLKETAENSLKKPVTDCVISVPSFFTDAERRSVLDAAQIVGLNCLRLMNDMTAVALNYGIYKQDLPSLDEKPRIVVFVDMGHSAFQVSACAFNKGKLKVLGTAFDPFLGGKNFDEKLVEHFCAEFKTKYKLDAKSKIRALLRLYQECEKLKKLMSSNSTDLPLNIECFMNDKDVSGKMNRSQFEELCAELLQKIEVPLYSLLEQTHLKVEDVSAVEIVGGATRIPAVKERIAKFFGKDISTTLNADEAVARGCALQCAILSPAFKVREFSVTDAVPFPISLIWNHDSEDTEGVHEVFSRNHAAPFSKVLTFLRRGPFELEAFYSDPQGVPYPEAKIGRFVVQNVSAQKDGEKSRVKVKVRVNTHGIFTISTASMVEKVPTEENEMSSEADMECLNQRPPENPDTDKNVQQDNSEAGTQPQVQTDAQQTSQSPPSPELTSEENKIPDADKANEKKVDQPPEAKKPKIKVVNVELPIEANLVWQLGKDLLNMYIETEGKMIMQDKLEKERNDAKNAVEEYVYEFRDKLCGPYEKFICEQDHQNFLRLLTETEDWLYEEGEDQAKQAYVDKLEELMKIGTPVKVRFQEAEERPKMFEELGQRLQHYAKIAADFRNKDEKYNHIDESEMKKVEKSVNEVMEWMNNVMNAQAKKSLDQDPVVRAQEIKTKIKELNNTCEPVVTQPKPKIESPKLERTPNGPNIDKKEEDLEDKNNFGAEPPHQNGECYPNEKNSVNMDLD

671 O75529 NP_003795.2

>O75529

MKRVRTEQIQMAVSCYLKRRQYVDSDGPLKQGLRLSQTAEEMAANLTVQSESGCANIVSAAPCQAEPQQYEVQFGRLRNFLTDSDSQHSHEVMPLLYPLFVYLHLNLVQNSPKSTVESFYSRFHGMFLQNASQKDVIEQLQTTQTIQDILSNFKLRAFLDNKYVVRLQEDSYNYLIRYLQSDNNTALCKVLTLHIHLDVQPAKRTDYQLYASGSSSRSENNGLEPPDMPSPILQNEAALEVLQESIKRVKDGPPSLTTICFYAFYNTEQLLNTAEISPDSKLLAAGFDNSCIKLWSLRSKKLKSEPHQVDVSRIHLACDILEEEDDEDDNAGTEMKILRGHCGPVYSTRFLADSSGLLSCSEDMSIRYWDLGSFTNTVLYQGHAYPVWDLDISPYSLYFASGSHDRTARLWSFDRTYPLRIYAGHLADVDCVKFHPNSNYLATGSTDKTVRLWSAQQGNSVRLFTGHRGPVLSLAFSPNGKYLASAGEDQRLKLWDLASGTLYKELRGHTDNITSLTFSPDSGLIASASMDNSVRVWDIRNTYCSAPADGSSSELVGVYTGQMSNVLSVQFMACNLLLVTGITQENQEH

>NP_003795.2

MQPDMSLNVIKMKSSDFLESAELDSGGFGKVSLCFHRTQGLMIMKTVYKGPNCIEHNEALLEEAKMMNRLRHSRVVKLLGVIIEEGKYSLVMEYMEKGNLMHVLKAEMSTPLSVKGRIILEIIEGMCYLHGKGVIHKDLKPENILVDNDFHIKIADLGLASFKMWSKLNNEEHNELREVDGTAKKNGGTLYYMAPEHLNDVNAKPTEKSDVYSFAVVLWAIFANKEPYENAICEQQLIMCIKSGNRPDVDDITEYCPREIISLMKLCWEANPEARPTFPGIEEKFRPFYLSQLEESVEEDVKSLKKEYSNENAVVKRMQSLQLDCVAVPSSRSNSATEQPGSLHSSQGLGMGPVEESWFAPSLEHPQEENEPSLQSKLQDEANYHLYGSRMDRQTKQQPRQNVAYNREEERRRRVSHDPFAQQRPYENFQNTEGKGTAYSSAASHGNAVHQPSGLTSQPQVLYQNNGLYSSHGFGTRPLDPGTAGPRVWYRPIPSHMPSLHNIPVPETNYLGNTPTMPFSSLPPTDESIKYTIYNSTGIQIGAYNYMEIGGTSSSLLDSTNTNFKEEPAAKYQAIFDNTTSLTDKHLDPIRENLGKHWKNCARKLGFTQSQIDEIDHDYERDGLKEKVYQMLQKWVMREGIKGATVGKLAQALHQCSRIDLLSSLIYVSQN

672 P01106 Q7RTR2

>P01106

MPLNVSFTNRNYDLDYDSVQPYFYCDEEENFYQQQQQSELQPPAPSEDIWKKFELLPTPPLSPSRRSGLCSPSYVAVTPFSLRGDNDGGGGSFSTADQLEMVTELLGGDMVNQSFICDPDDETFIKNIIIQDCMWSGFSAAAKLVSEKLASYQAARKDSGSPNPARGHSVCSTSSLYLQDLSAAASECIDPSVVFPYPLNDSSSPKSCASQDSSAFSPSSDSLLSSTESSPQGSPEPLVLHEETPPTTSSDSEEEQEDEEEIDVVSVEKRQAPGKRSESGSPSAGGHSKPPHSPLVLKRCHVSTHQHNYAAPPSTRKDYPAAKRVKLDSVRVLRQISNNRKCTSPRSSDTEENVKRRTHNVLERQRRNELKRSFFALRDQIPELENNEKAPKVVILKKATAYILSVQAEEQKLISEEDLLRKRREQLKHKLEQLRNSCA

>Q7RTR2

MRKQEVRTGREAGQGHGTGSPAEQVKALMDLLAGKGSQGSQAPQALDRTPDAPLGPCSNDSRIQRHRKALLSKVGGGPELGGPWHRLASLLLVEGLTDLQLREHDFTQVEATRGGGHPARTVALDRLFLPLSRVSVPPRVSITIGVAGMGKTTLVRHFVRLWAHGQVGKDFSLVLPLTFRDLNTHEKLCADRLICSVFPHVGEPSLAVAVPARALLILDGLDECRTPLDFSNTVACTDPKKEIPVDHLITNIIRGNLFPEVSIWITSRPSASGQIPGGLVDRMTEIRGFNEEEIKVCLEQMFPEDQALLGWMLSQVQADRALYLMCTVPAFCRLTGMALGHLWRSRTGPQDAELWPPRTLCELYSWYFRMALSGEGQEKGKASPRIEQVAHGGRKMVGTLGRLAFHGLLKKKYVFYEQDMKAFGVDLALLQGAPCSCFLQREETLASSVAYCFTHLSLQEFVAAAYYYGASRRAIFDLFTESGVSWPRLGFLTHFRSAAQRAMQAEDGRLDVFLRFLSGLLSPRVNALLAGSLLAQGEHQAYRTQVAELLQGCLRPDAAVCARAINVLHCLHELQHTELARSVEEAMESGALARLTGPAHRAALAYLLQVSDACAQEANLSLSLSQGVLQSLLPQLLYCRKLRLDTNQFQDPVMELLGSVLSGKDCRIQKISLAENQISNKGAKALARSLLVNRSLTSLDLRGNSIGPQGAKALADALKINRTLTSLSLQGNTVRDDGARSMAEALASNRTLSMLHLQKNSIGPMGAQRMADALKQNRSLKELMFSSNSIGDGGAKALAEALKVNQGLESLDLQSNSISDAGVAALMGALCTNQTLLSLSLRENSISPEGAQAIAHALCANSTLKNLDLTANLLHDQGARAIAVAVRENRTLTSLHLQWNFIQAGAAQALGQALQLNRSLTSLDLQENAIGDDGACAVARALKVNTALTALYLQVASIGASGAQVLGEALAVNRTLEILDLRGNAIGVAGAKALANALKVNSSLRRLNLQENSLGMDGAICIATALSGNHRLQHINLQGNHIGDSGARMISEAIKTNAPTCTVEM

673 NP_004074.2 Q96GX9

>NP_004074.2

MAAESLPFSFGTLSSWELEAWYEDLQEVLSSDENGGTYVSPPGNEEEESKIFTTLDPASLAWLTEEEPEPAEVTSTSQSPHSPDSSQSSLAQEEEEEDQGRTRKRKQSGHSPARAGKQRMKEKEQENERKVAQLAEENERLKQEIERLTREVEATRRALIDRMVNLHQA

>Q96GX9

MSGCDAREGDCCSRRCGAQDKEHPRYLIPELCKQFYHLGWVTGTGGGISLKHGDEIYIAPSGVQKERIQPEDMFVCDINEKDISGPSPSKKLKKSQCTPLFMNAYTMRGAGAVIHTHSKAAVMATLLFPGREFKITHQEMIKGIKKCTSGGYYRYDDMLVVPIIENTPEEKDLKDRMAHAMNEYPDSCAVLVRRHGVYVWGETWEKAKTMCECYDYLFDIAVSMKKVGLDPSQLPVGENGIV

674 Q6NYC1 O75570

>Q6NYC1

MNHKSKKRIREAKRSARPELKDSLDWTRHNYYESFSLSPAAVADNVERADALQLSVEEFVERYERPYKPVVLLNAQEGWSAQEKWTLERLKRKYRNQKFKCGEDNDGYSVKMKMKYYIEYMESTRDDSPLYIFDSSYGEHPKRRKLLEDYKVPKFFTDDLFQYAGEKRRPPYRWFVMGPPRSGTGIHIDPLGTSAWNALVQGHKRWCLFPTSTPRELIKVTRDEGGNQQDEAITWFNVIYPRTQLPTWPPEFKPLEILQKPGETVFVPGGWWHVVLNLDTTIAITQNFASSTNFPVVWHKTVRGRPKLSRKWYRILKQEHPELAVLADSVDLQESTGIASDSSSDSSSSSSSSSSDSDSECESGSEGDGTVHRRKKRRTCSMVGNGDTTSQDDCVSKERSSSR

>O75570

MNRHLCVWLFRHPSLNGYLQCHIQLHSHQFRQIHLDTRLQVFRQNRNCILHLLSKNWSRRYCHQDTKMLWKHKALQKYMENLSKEYQTLEQCLQHIPVNEENRRSLNRRHAELAPLAAIYQEIQETEQAIEELESMCKSLNKQDEKQLQELALEERQTIDQKINMLYNELFQSLVPKEKYDKNDVILEVTAGRTTGGDICQQFTREIFDMYQNYSCYKHWQFELLNYTPADYGGLHHAAARISGDGVYKHLKYEGGIHRVQRIPEVGLSSRMQRIHTGTMSVIVLPQPDEVDVKLDPKDLRIDTFRAKGAGGQHVNKTDSAVRLVHIPTGLVVECQQERSQIKNKEIAFRVLRARLYQQIIEKDKRQQQSARKLQVGTRAQSERIRTYNFTQDRVSDHRIAYEVRDIKEFLCGGKGLDQLIQRLLQSADEEAIAELLDEHLKSAK

675 NP_003087.1 NP_006235.1

>NP_003087.1

MSKAHPPELKKFMDKKLSLKLNGGRHVQGILRGFDPFMNLVIDECVEMATSGQQNNIGMVVIRGNSIIMLEALERV

>NP_006235.1

METKLPPASTPTSPSSPGLSPVPPPDKVDGFSRRSLRRARPRRSHSSSQFRYQSNQQELTPLPLLKDVPASELHELLSRKLAQCGVMFDFLDCVADLKGKEVKRAALNELVECVGSTRGVLIEPVYPDIIRMISVNIFRTLPPSENPEFDPEEDEPNLEPSWPHLQLVYEFFLRFLESPDFQPSVAKRYVDQKFVLMLLELFDSEDPREREYLKTILHRVYGKFLGLRAYIRKQCNHIFLRFIYEFEHFNGVAELLEILGSIINGFALPLKTEHKQFLVRVLIPLHSVKSLSVFHAQLAYCVVQFLEKDATLTEHVIRGLLKYWPKTCTQKEVMFLGEMEEILDVIEPSQFVKIQEPLFKQVARCVSSPHFQVAERALYFWNNEYILSLIEDNCHTVLPAVFGTLYQVSKEHWNQTIVSLIYNVLKTFMEMNGKLFDELTASYKLEKQQEQQKAQERQELWQGLEELRLRRLQGTQGAKEAPLQRLTPQVAASGGQS

676 P31267 Q9ULC6

>P31267

MSSYFVNPTFPGSLPSGQDSFLGQLPLYQAGYDALRPFPASYGASSLPDKTYTSPCFYQQSNSVLACNRASYEYGASCFYSDKDLSGASPSGSGKQRGPGDYLHFSPEQQYKPDSSSGQGKALHDEGADRKYTSPVYPWMQRMNSCAGAVYGSHGRRGRQTYTRYQTLELEKEFHFNRYLTRRRRIEIANALCLTERQIKIWFQNRRMKWKKENKLINSTQPSGEDSEAKAGE

>Q9ULC6

MAPKRVVQLSLKMPTHAVCVVGVEAHVDIHSDVPKGANSFRVSGSSGVEVFMVYNRTRVKEPIGKARWPLDTDADMVVSVGTASKELKDFKVRVSYFGEQEDQALGRSVLYLTGVDISLEVDTGRTGKVKRSQGDKKTWRWGPEGYGAILLVNCDRDNHRSAEPDLTHSWLMSLADLQDMSPMLLSCNGPDKLFDSHKLVLNVPFSDSKRVRVFCARGGNSLSDYKQVLGPQCLSYEVERQPGEQEIKFYVEGLTFPDADFLGLVSLSVSLVDPGTLPEVTLFTDTVGFRMAPWIMTPNTQPPEELYVCRVMDTHGSNEKFLEDMSYLTLKANCKLTICPQVENRNDRWIQDEMEFGYIEAPHKSFPVVFDSPRNRGLKDFPYKRILGPDFGYVTREIPLPGPSSLDSFGNLDVSPPVTVGGTEYPLGRILIGSSFPKSGGRQMARAVRNFLKAQQVQAPVELYSDWLSVGHVDEFLTFVPTSDQKGFRLLLASPSACLKLFQEKKEEGYGEAAQFDGLKHQAKRSINEMLADRHLQRDNLHAQKCIDWNRNVLKRELGLAESDIVDIPQLFFLKNFYAEAFFPDMVNMVVLGKYLGIPKPYGPIINGRCCLEEKVQSLLEPLGLHCIFIDDYLSYHELQGEIHCGTNVRRKPFPFKWWNMVP

677 NP_004062.2 NP_055904.1

>NP_004062.2

MRHSKRTYCPDWDDKDWDYGKWRSSSSHKRRKRSHSSAQENKRCKYNHSKMCDSHYLESRSINEKDYHSRRYIDEYRNDYTQGCEPGHRQRDHESRYQNHSSKSSGRSGRSSYKSKHRIHHSTSHRRSHGKSHRRKRTRSVEDDEEGHLICQSGDVLSARYEIVDTLGEGAFGKVVECIDHKAGGRHVAVKIVKNVDRYCEAARSEIQVLEHLNTTDPNSTFRCVQMLEWFEHHGHICIVFELLGLSTYDFIKENGFLPFRLDHIRKMAYQICKSVNFLHSNKLTHTDLKPENILFVQSDYTEAYNPKIKRDERTLINPDIKVVDFGSATYDDEHHSTLVSTRHYRAPEVILALGWSQPCDVWSIGCILIEYYLGFTVFPTHDSKEHLAMMERILGPLPKHMIQKTRKRKYFHHDRLDWDEHSSAGRYVSRRCKPLKEFMLSQDVEHERLFDLIQKMLEYDPAKRITLREALKHPFFDLLKKSI

>NP_055904.1

MVGERHAGDLMVPLGPRLQAYPEELIRQRPGHDGHPEYLIRWSVLKCGEVGKVGVEEGKAEHILMWLSAPEVYANCPGLLGERALSKGLQHEPAGVSGSFPRDPGGLDEVAMGEMEADVQALVRRAARQLAESGTPSLTAAVLHTIHVLSAYASIGPLTGVFRETGALDLLMHMLCNPEPQIRRSAGKMLQALAAHDAGSRAHVLLSLSQQDGIEQHMDFDSRYTLLELFAETTSSEEHCMAFEGIHLPQIPGKLLFSLVKRYLCVTSLLDQLNSSPELGAGDQSSPCATREKSRGQRELEFSMAVGNLISELVRSMGWARNLSEQGMSPPRPTRSIFQPYISGPSLLLPTIVTTPRRQGWVFRQRSEFSSRSGYGEYVQQTLQPGMRVRMLDDYEEISAGDEGEFRQSNNGIPPVQVFWQSTGRTYWVHWHMLEILGPEEATEDKASAAVEKGAGATVLGTAFPSWDWNPMDGLYPLPYLQPEPQKNERVGYLTQAEWWELLFFIKKLDLCEQQPIFQNLWKNLDETLGEKALGEISVSVEMAESLLQVLSSRFEGSTLNDLLNSQIYTKYGLLSNEPSSSSTSRNHSCTPDPEEESKSEASFSEEETESLKAKAEAPKTEAEPTKTRTETPMAQSDSQLFNQLLVTEGMTLPTEMKEAASEMARALRGPGPRSSLDQHVAAVVATVQISSLDTNLQLSGLSALSQAVEEVTERDHPLVRPDRSLREKLVKMLVELLTNQVGEKMVVVQALRLLYLLMTKHEWRPLFAREGGIYAVLVCMQEYKTSVLVQQAGLAALKMLAVASSSEIPTFVTGRDSIHSLFDAQMTREIFASIDSATRPGSESLLLTVPAAVILMLNTEGCSSAARNGLLLLNLLLCNHHTLGDQIITQELRDTLFRHSGIAPRTEPMPTTRTILMMLLNRYSEPPGSPERAALETPIIQGQDGSPELLIRSLVGGPSAELLLDLERVLCREGSPGGAVRPLLKRLQQETQPFLLLLRTLDAPGPNKTLLLSVLRVITRLLDFPEAMVLPWHEVLEPCLNCLSGPSSDSEIVQELTCFLHRLASMHKDYAVVLCCLGAKEILSKVLDKHSAQLLLGCELRDLVTECEKYAQLYSNLTSSILAGCIQMVLGQIEDHRRTHQPINIPFFDVFLRHLCQGSSVEVKEDKCWEKVEVSSNPHRASKLTDHNPKTYWESNGSTGSHYITLHMHRGVLVRQLTLLVASEDSSYMPARVVVFGGDSTSCIGTELNTVNVMPSASRVILLENLNRFWPIIQIRIKRCQQGGIDTRVRGVEVLGPKPTFWPLFREQLCRRTCLFYTIRAQAWSRDIAEDHRRLLQLCPRLNRVLRHEQNFADRFLPDDEAAQALGKTCWEALVSPLVQNITSPDAEGVSALGWLLDQYLEQRETSRNPLSRAASFASRVRRLCHLLVHVEPPPGPSPEPSTRPFSKNSKGRDRSPAPSPVLPSSSLRNITQCWLSVVQEQVSRFLAAAWRAPDFVPRYCKLYEHLQRAGSELFGPRAAFMLALRSGFSGALLQQSFLTAAHMSEQFARYIDQQIQGGLIGGAPGVEMLGQLQRHLEPIMVLSGLELATTFEHFYQHYMADRLLSFGSSWLEGAVLEQIGLCFPNRLPQLMLQSLSTSEELQRQFHLFQLQRLDKLFLEQEDEEEKRLEEEEEEEEEEEAEKELFIEDPSPAISILVLSPRCWPVSPLCYLYHPRKCLPTEFCDALDRFSSFYSQSQNHPVLDMGPHRRLQWTWLGRAELQFGKQILHVSTVQMWLLLKFNQTEEVSVETLLKDSDLSPELLLQALVPLTSGNGPLTLHEGQDFPHGGVLRLHEPGPQRSGEALWLIPPQAYLNVEKDEGRTLEQKRNLLSCLLVRILKAHGEKGLHIDQLVCLVLEAWQKGPNPPGTLGHTVAGGVACTSTDVLSCILHLLGQGYVKRRDDRPQILMYAAPEPMGPCRGQADVPFCGSQSETSKPSPEAVATLASLQLPAGRTMSPQEVEGLMKQTVRQVQETLNLEPDVAQHLLAHSHWGAEQLLQSYSEDPEPLLLAAGLCVHQAQAVPVRPDHCPVCVSPLGCDDDLPSLCCMHYCCKSCWNEYLTTRIEQNLVLNCTCPIADCPAQPTGAFIRAIVSSPEVISKYEKALLRGYVESCSNLTWCTNPQGCDRILCRQGLGCGTTCSKCGWASCFNCSFPEAHYPASCGHMSQWVDDGGYYDGMSVEAQSKHLAKLISKRCPSCQAPIEKNEGCLHMTCAKCNHGFCWRCLKSWKPNHKDYYNCSAMVSKAARQEKRFQDYNERCTFHHQAREFAVNLRNRVSAIHEVPPPRSFTFLNDACQGLEQARKVLAYACVYSFYSQDAEYMDVVEQQTENLELHTNALQILLEETLLRCRDLASSLRLLRADCLSTGMELLRRIQERLLAILQHSAQDFRVGLQSPSVEAWEAKGPNMPGSQPQASSGPEAEEEEEDDEDDVPEWQQDEFDEELDNDSFSYDESENLDQETFFFGDEEEDEDEAYD

678 O95766 O75880

>O95766

MAAAAAGAGSGPWAAQEKQFPPALLSFFIYNPRFGPREGQEENKILFYHPNEVEKNEKIRNVGLCEAIVQFTRTFSPSKPAKSLHTQKNRQFFNEPEENFWMVMVVRNPIIEKQSKDGKPVIEYQEEELLDKVYSSVLRQCYSMYKLFNGTFLKAMEDGGVKLLKERLEKFFHRYLQTLHLQSCDLLDIFGGISFFPLDKMTYLKIQSFINRMEESLNIVKYTAFLYNDQLIWSGLEQDDMRILYKYLTTSLFPRHIEPELAGRDSPIRAEMPGNLQHYGRFLTGPLNLNDPDAKCRFPKIFVNTDDTYEELHLIVYKAMSAAVCFMIDASVHPTLDFCRRLDSIVGPQLTVLASDICEQFNINKRMSGSEKEPQFKFIYFNHMNLAEKSTVHMRKTPSVSLTSVHPDLMKILGDINSDFTRVDEDEEIIVKAMSDYWVVGKKSDRRELYVILNQKNANLIEVNEEVKKLCATQFNNIFFLD

>O75880

MAMLVLVPGRVMRPLGGQLWRFLPRGLEFWGPAEGTARVLLRQFCARQAEAWRASGRPGYCLGTRPLSTARPPPPWSQKGPGDSTRPSKPGPVSWKSLAITFAIGGALLAGMKHVKKEKAEKLEKERQRHIGKPLLGGPFSLTTHTGERKTDKDYLGQWLLIYFGFTHCPDVCPEELEKMIQVVDEIDSITTLPDLTPLFISIDPERDTKEAIANYVKEFSPKLVGLTGTREEVDQVARAYRVYYSPGPKDEDEDYIVDHTIIMYLIGPDGEFLDYFGQNKRKGEIAASIATHMRPYRKKS

679 P13378 NP_076425.1

>P13378

MSSYFVNPLYSKYKAAAAAAAAAGEAINPTYYDCHFAPEVGGRHAAAAAALQLYGNSAAGFPHAPPQAHAHPHPSPPPSGTGCGGREGRGQEYFHPGGGSPAAAYQAAPPPPPHPPPPPPPPPCGGIACHGEPAKFYGYDNLQRQPIFTTQQEAELVQYPDCKSSSGNIGEDPDHLNQSSSPSQMFPWMRPQAAPGRRRGRQTYSRFQTLELEKEFLFNPYLTRKRRIEVSHALALTERQVKIWFQNRRMKWKKENNKDKFPVSRQEVKDGETKKEAQELEEDRAEGLTN

>NP_076425.1

MARKKVRPRLIAELARRVRALREQLNRPRDSQLYAVDYETLTRPFSGRRLPVRAWADVRRESRLLQLLGRLPLFGLGRLVTRKSWLWQHDEPCYWRLTRVRPDYTAQNLDHGKAWGILTFKGKTESEAREIEHVMYHDWRLVPKHEEEAFTAFTPAPEDSLASVPYPPLLRAMIIAERQKNGDTSTEEPMLNVQRIRMEPWDYPAKQEDKGRAKGTPV

680 NP_002710.2 Q9UBR1

>NP_002710.2

MLTCNKAGSRMVVDAANSNGPFQPVVLLHIRDVPPADQEKLFIQKLRQCCVLFDFVSDPLSDLKWKEVKRAALSEMVEYITHNRNVITEPIYPEVVHMFAVNMFRTLPPSSNPTGAEFDPEEDEPTLEAAWPHLQLVYEFFLRFLESPDFQPNIAKKYIDQKFVLQLLELFDSEDPRERDFLKTTLHRIYGKFLGLRAYIRKQINNIFYRFIYETEHHNGIAELLEILGSIINGFALPLKEEHKIFLLKVLLPLHKVKSLSVYHPQLAYCVVQFLEKDSTLTEPVVMALLKYWPKTHSPKEVMFLNELEEILDVIEPSEFVKIMEPLFRQLAKCVSSPHFQVAERALYYWNNEYIMSLISDNAAKILPIMFPSLYRNSKTHWNKTIHGLIYNALKLFMEMNQKLFDDCTQQFKAEKLKEKLKMKEREEAWVKIENLAKANPQYTVYSQASTMSIPVAMETDGPLFEDVQMLRKTVKDEAHQAQKDPKKDRPLARRKSELPQDPHTKKALEAHCRADELASQDGR

>Q9UBR1

MAGAEWKSLEECLEKHLPLPDLQEVKRVLYGKELRKLDLPREAFEAASREDFELQGYAFEAAEEQLRRPRIVHVGLVQNRIPLPANAPVAEQVSALHRRIKAIVEVAAMCGVNIICFQEAWTMPFAFCTREKLPWTEFAESAEDGPTTRFCQKLAKNHDMVVVSPILERDSEHGDVLWNTAVVISNSGAVLGKTRKNHIPRVGDFNESTYYMEGNLGHPVFQTQFGRIAVNICYGRHHPLNWLMYSINGAEIIFNPSATIGALSESLWPIEARNAAIANHCFTCAINRVGTEHFPNEFTSGDGKKAHQDFGYFYGSSYVAAPDSSRTPGLSRSRDGLLVAKLDLNLCQQVNDVWNFKMTGRYEMYARELAEAVKSNYSPTIVKE

681 Q9UBP6 P32322

>Q9UBP6

MAAETRNVAGAEAPPPQKRYYRQRAHSNPMADHTLRYPVKPEEMDWSELYPEFFAPLTQNQSHDDPKDKKEKRAQAQVEFADIGCGYGGLLVELSPLFPDTLILGLEIRVKVSDYVQDRIRALRAAPAGGFQNIACLRSNAMKHLPNFFYKGQLTKMFFLFPDPHFKRTKHKWRIISPTLLAEYAYVLRVGGLVYTITDVLELHDWMCTHFEEHPLFERVPLEDLSEDPVVGHLGTSTEEGKKVLRNGGKNFPAIFRRIQDPVLQAVTSQTSLPGH

>P32322

MSVGFIGAGQLAFALAKGFTAAGVLAAHKIMASSPDMDLATVSALRKMGVKLTPHNKETVQHSDVLFLAVKPHIIPFILDEIGADIEDRHIVVSCAAGVTISSIEKKLSAFRPAPRVIRCMTNTPVVVREGATVYATGTHAQVEDGRLMEQLLSSVGFCTEVEEDLIDAVTGLSGSGPAYAFTALDALADGGVKMGLPRRLAVRLGAQALLGAAKMLLHSEQHPGQLKDNVSSPGGATIHALHVLESGGFRSLLINAVEASCIRTRELQSMADQEQVSPAAIKKTILDKVKLDSPAGTALSPSGHTKLLPRSLAPAGKD

682 NP_077021.1 Q16643

>NP_077021.1

MDKSGIDSLDHVTSDAVELANRSDNSSDSSLFKTQCIPYSPKGEKRNPIRKFVRTPESVHASDSSSDSSFEPIPLTIKAIFERFKNRKKRYKKKKKRRYQPTGRPRGRPEGRRNPIYSLIDKKKQFRSRGSGFPFLESENEKNAPWRKILTFEQAVARGFFNYIEKLKYEHHLKESLKQMNVGEDLENEDFDSRRYKFLDDDGSISPIEESTAEDEDATHLEDNECDIKLAGDSFIVSSEFPVRLSVYLEEEDITEEAALSKKRATKAKNTGQRGLKM

>Q16643

MAGVSFSGHRLELLAAYEEVIREESAADWALYTYEDGSDDLKLAASGEGGLQELSGHFENQKVMYGFCSVKDSQAALPKYVLINWVGEDVPDARKCACASHVAKVAEFFQGVDVIVNASSVEDIDAGAIGQRLSNGLARLSSPVLHRLRLREDENAEPVGTTYQKTDAAVEMKRINREQFWEQAKKEEELRKEEERKKALDERLRFEQERMEQERQEQEERERRYREREQQIEEHRRKQQTLEAEEAKRRLKEQSIFGDHRDEEEETHMKKSESEVEEAAAIIAQRPDNPREFFKQQERVASASAGSCDVPSPFNHRPGSHLDSHRRMAPTPIPTRSPSDSSTASTPVAEQIERALDEVTSSQPPPLPPPPPPAQETQEPSPILDSEETRAAAPQAWAGPMEEPPQAQAPPRGPGSPAEDLMFMESAEQAVLAAPVEPATADATEIHDAADTIETDTATADTTVANNVPPAATSLIDLWPGNGEGASTLQGEPRAPTPPSGTEVTLAEVPLLDEVAPEPLLPAGEGCATLLNFDELPEPPATFCDPEEVEGESLAAPQTPTLPSALEELEQEQEPEPHLLTNGETTQKEGTQASEGYFSQSQEEEFAQSEELCAKAPPPVFYNKPPEIDITCWDADPVPEEEEGFEGGD

683 P53999 P49591

>P53999

MPKSKELVSSSSSGSDSDSEVDKKLKRKKQVAPEKPVKKQKTGETSRALSSSKQSSSSRDDNMFQIGKMRYVSVRDFKGKVLIDIREYWMDPEGEMKPGRKGISLNPEQWSQLKEQISDIDDAVRKL

>P49591

MVLDLDLFRVDKGGDPALIRETQEKRFKDPGLVDQLVKADSEWRRCRFRADNLNKLKNLCSKTIGEKMKKKEPVGDDESVPENVLSFDDLTADALANLKVSQIKKVRLLIDEAILKCDAERIKLEAERFENLREIGNLLHPSVPISNDEDVDNKVERIWGDCTVRKKYSHVDLVVMVDGFEGEKGAVVAGSRGYFLKGVLVFLEQALIQYALRTLGSRGYIPIYTPFFMRKEVMQEVAQLSQFDEELYKVIGKGSEKSDDNSYDEKYLIATSEQPIAALHRDEWLRPEDLPIKYAGLSTCFRQEVGSHGRDTRGIFRVHQFEKIEQFVYSSPHDNKSWEMFEEMITTAEEFYQSLGIPYHIVNIVSGSLNHAASKKLDLEAWFPGSGAFRELVSCSNCTDYQARRLRIRYGQTKKMMDKVEFVHMLNATMCATTRTICAILENYQTEKGITVPEKLKEFMPPGLQELIPFVKPAPIEQEPSKKQKKQHEGSKKKAAARDVTLENRLQNMEVTDA

684 Q6NXG1 NP_001631.3

>Q6NXG1

MTASPDYLVVLFGITAGATGAKLGSDEKELILLFWKVVDLANKKVGQLHEVLVRPDQLELTEDCKEETKIDVESLSSASQLDQALRQFNQSVSNELNIGVGTSFCLCTDGQLHVRQILHPEASKKNVLLPECFYSFFDLRKEFKKCCPGSPDIDKLDVATMTEYLNFEKSSSVSRYGASQVEDMGNIILAMISEPYNHRFSDPERVNYKFESGTCSKMELIDDNTVVRARGLPWQSSDQDIARFFKGLNIAKGGAALCLNAQGRRNGEALVRFVSEEHRDLALQRHKHHMGTRYIEVYKATGEDFLKIAGGTSNEVAQFLSKENQVIVRMRGLPFTATAEEVVAFFGQHCPITGGKEGILFVTYPDGRPTGDAFVLFACEEYAQNALRKHKDLLGKRYIELFRSTAAEVQQVLNRFSSAPLIPLPTPPIIPVLPQQFVPPTNVRDCIRLRGLPYAATIEDILDFLGEFATDIRTHGVHMVLNHQGRPSGDAFIQMKSADRAFMAAQKCHKKNMKDRYVEVFQCSAEEMNFVLMGGTLNRNGLSPPPCKLPCLSPPSYTFPAPAAVIPTEAAIYQPSVILNPRALQPSTAYYPAGTQLFMNYTAYYPSPPGSPNSLGYFPTAANLSGVPPQPGTVVRMQGLAYNTGVKEILNFFQGYQYATEDGLIHTNDQARTLPKEWVCI

>NP_001631.3

MERQVLLSEPEEAAALYRGLSRQPALSAACLGPEVTTQYGGQYRTVHTEWTQRDLERMENIRFCRQYLVFHDGDSVVFAGPAGNSVETRGELLSRESPSGTMKAVLRKAGGTGPGEEKQFLEVWEKNRKLKSFNLSALEKHGPVYEDDCFGCLSWSHSETHLLYVAEKKRPKAESFFQTKALDVSASDDEIARLKKPDQAIKGDQFVFYEDWGENMVSKSIPVLCVLDVESGNISVLEGVPENVSPGQAFWAPGDAGVVFVGWWHEPFRLGIRFCTNRRSALYYVDLIGGKCELLSDDSLAVSSPRLSPDQCRIVYLQYPSLIPHHQCSQLCLYDWYTKVTSVVVDVVPRQLGENFSGIYCSLLPLGCWSADSQRVVFDSAQRSRQDLFAVDTQVGTVTSLTAGGSGGSWKLLTIDQDLMVAQFSTPSLPPTLKVGFLPSAGKEQSVLWVSLEEAEPIPDIHWGIRVLQPPPEQENVQYAGLDFEAILLQPGSPPDKTQVPMVVMPHGGPHSSFVTAWMLFPAMLCKMGFAVLLVNYRGSTGFGQDSILSLPGNVGHQDVKDVQFAVEQVLQEEHFDASHVALMGGSHGGFISCHLIGQYPETYRACVARNPVINIASMLGSTDIPDWCVVEAGFPFSSDCLPDLSVWAEMLDKSPIRYIPQVKTPLLLMLGQEDRRVPFKQGMEYYRALKTRNVPVRLLLYPKSTHALSEVEVESDSFMNAVLWLRTHLGS

685 Q03060 NP_001547.1

>Q03060

MSKCARKKYIKTNPRQMTMETVESQHDGSITASLTESKSAHVQTQTGQNSIPALAQVSVAGSGTRRGSPAVTLVQLPSGQTIHVQGVIQTPQPWVIQSSEIHTVQVAAIAETDESAESEGVIDSHKRREILSRRPSYRKILNELSSDVPGVPKIEEERSEEEGTPPSIATMAVPTSIYQTSTGQYIAIAQGGTIQISNPGSDGVQGLQALTMTNSGAPPPGATIVQYAAQSADGTQQFFVPGSQVVVQDEETELAPSHMAAATGDMPTYQIRAPTAALPQGVVMAASPGSLHSPQQLAEEATRKRELRLMKNREAAKECRRRKKEYVKCLESRVAVLEVQNKKLIEELETLKDICSPKTDY

>NP_001547.1

MSWSPSLTTQTCGAWEMKERLGTGGFGNVIRWHNQETGEQIAIKQCRQELSPRNRERWCLEIQIMRRLTHPNVVAARDVPEGMQNLAPNDLPLLAMEYCQGGDLRKYLNQFENCCGLREGAILTLLSDIASALRYLHENRIIHRDLKPENIVLQQGEQRLIHKIIDLGYAKELDQGSLCTSFVGTLQYLAPELLEQQKYTVTVDYWSFGTLAFECITGFRPFLPNWQPVQWHSKVRQKSEVDIVVSEDLNGTVKFSSSLPYPNNLNSVLAERLEKWLQLMLMWHPRQRGTDPTYGPNGCFKALDDILNLKLVHILNMVTGTIHTYPVTEDESLQSLKARIQQDTGIPEEDQELLQEAGLALIPDKPATQCISDGKLNEGHTLDMDLVFLFDNSKITYETQISPRPQPESVSCILQEPKRNLAFFQLRKVWGQVWHSIQTLKEDCNRLQQGQRAAMMNLLRNNSCLSKMKNSMASMSQQLKAKLDFFKTSIQIDLEKYSEQTEFGITSDKLLLAWREMEQAVELCGRENEVKLLVERMMALQTDIVDLQRSPMGRKQGGTLDDLEEQARELYRRLREKPRDQRTEGDSQEMVRLLLQAIQSFEKKVRVIYTQLSKTVVCKQKALELLPKVEEVVSLMNEDEKTVVRLQEKRQKELWNLLKIACSKVRGPVSGSPDSMNASRLSQPGQLMSQPSTASNSLPEPAKKSEELVAEAHNLCTLLENAIQDTVREQDQSFTALDWSWLQTEEEEHSCLEQAS

686 NP_066940.1 Q8NHM5

>NP_066940.1

MAATVATAAAVAPAPAPGTDSASSVHWFRKGLRLHDNPALLAAVRGARCVRCVYILDPWFAASSSVGINRWRFLLQSLEDLDTSLRKLNSRLFVVRGQPADVFPRLFKEWGVTRLTFEYDSEPFGKERDAAIMKMAKEAGVEVVTENSHTLYDLDRIIELNGQKPPLTYKRFQAIISRMELPKKPVGLVTSQQMESCRAEIQENHDETYGVPSLEELGFPTEGLGPAVWQGGETEALARLDKHLERKAWVANYERPRMNANSLLASPTGLSPYLRFGCLSCRLFYYRLWDLYKKVKRNSTPPLSLFGQLLWREFFYTAATNNPRFDRMEGNPICIQIPWDRNPEALAKWAEGKTGFPWIDAIMTQLRQEGWIHHLARHAVACFLTRGDLWVSWESGVRVFDELLLDADFSVNAGSWMWLSCSAFFQQFFHCYCPVGFGRRTDPSGDYIRRYLPKLKAFPSRYIYEPWNAPESIQKAAKCIIGVDYPRPIVNHAETSRLNIERMKQIYQQLSRYRGLCLLASVPSCVEDLSHPVAEPSSSQAGSMSSAGPRPLPSGPASPKRKLEAAEEPPGEELSKRARVAELPTPELPSKDA

>Q8NHM5

MAGPQMGGSAEDHPPRKRHAAEKQKKKTVIYTKCFEFESATQRPIDRQRYDENEDLSDVEEIVSVRGFSLEEKLRSQLYQGDFVHAMEGKDFNYEYVQREALRVPLIFREKDGLGIKMPDPDFTVRDVKLLVGSRRLVDVMDVNTQKGTEMSMSQFVRYYETPEAQRDKLYNVISLEFSHTKLEHLVKRPTVVDLVDWVDNMWPQHLKEKQTEATNAIAEMKYPKVKKYCLMSVKGCFTDFHIDFGGTSVWYHVFRGGKIFWLIPPTLHNLALYEEWVLSGKQSDIFLGDRVERCQRIELKQGYTFFIPSGWIHAVYTPVDSLVFGGNILHSFNVPMQLRIYEIEDRTRVQPKFRYPFYYEMCWYVLERYVYCVTQRSHLTQEYQRESMLIDAPRKPSIDGFSSDSWLEMEEEACDQQPQEEEEKDEEGEGRDRAPKPPTDGSTSPTSTPSEDQEALGKKPKAPALRFLKRTLSNESEESVKSTTLAVDYPKTPTGSPATEVSAKWTHLTEFELKGLKALVEKLESLPENKKCVPEGIEDPQALLEGVKNVLKEHADDDPSLAITGVPVVTWPKKTPKNRAVGRPKGKLGPASAVKLAANRTTAGARRRRTRCRKCEACLRTECGECHFCKDMKKFGGPGRMKQSCIMRQCIAPVLPHTAVCLVCGEAGKEDTVEEEEGKFNLMLMECSICNEIIHPGCLKIKESEGVVNDELPNCWECPKCNHAGKTGKQKRGPGFKYASNLPGSLLKEQKMNRDNKEGQEPAKRRSECEEAPRRRSDEHSKKVPPDGLLRRKSDDVHLRKKRKYEKPQELSGRKRASSLQTSPGSSSHLSPRPPLGSSLSPWWRSSLTYFQQQLKPGKEDKLFRKKRRSWKNAEDRMALANKPLRRFKQEPEDELPEAPPKTRESDHSRSSSPTAGPSTEGAEGPEEKKKVKMRRKRRLPNKELSRELSKELNHEIQRTENSLANENQQPIKSEPESEGEEPKRPPGICERPHRFSKGLNGTPRELRHQLGPSLRSPPRVISRPPPSVSPPKCIQMERHVIRPPPISPPPDSLPLDDGAAHVMHREVWMAVFSYLSHQDLCVCMRVCRTWNRWCCDKRLWTRIDLNHCKSITPLMLSGIIRRQPVSLDLSWTNISKKQLSWLINRLPGLRDLVLSGCSWIAVSALCSSSCPLLRTLDVQWVEGLKDAQMRDLLSPPTDNRPGQMDNRSKLRNIVELRLAGLDITDASLRLIIRHMPLLSKLHLSYCNHVTDQSINLLTAVGTTTRDSLTEINLSDCNKVTDQCLSFFKRCGNICHIDLRYCKQVTKEGCEQFIAEMSVSVQFGQVEEKLLQKLS

687 NP_055436.2 NP_003977.1

>NP_055436.2

MVMSSYMVNSKYVDPKFPPCEEYLQGGYLGEQGADYYGGGAQGADFQPPGLYPRPDFGEQPFGGSGPGPGSALPARGHGQEPGGPGGHYAAPGEPCPAPPAPPPAPLPGARAYSQSDPKQPPSGTALKQPAVVYPWMKKVHVNSVNPNYTGGEPKRSRTAYTRQQVLELEKEFHFNRYLTRRRRIEIAHTLCLSERQIKIWFQNRRMKWKKDHKLPNTKGRSSSSSSSSSCSSSVAPSQHLQPMAKDHHTDLTTL

>NP_003977.1

MACTIQKAEALDGAHLMQILWYDEEESLYPAVWLRDNCPCSDCYLDSAKARKLLVEALDVNIGIKGLIFDRKKVYITWPDEHYSEFQADWLKKRCFSKQARAKLQRELFFPECQYWGSELQLPTLDFEDVLRYDEHAYKWLSTLKKVGIVRLTGASDKPGEVSKLGKRMGFLYLTFYGHTWQVQDKIDANNVAYTTGKLSFHTDYPALHHPPGVQLLHCIKQTVTGGDSEIVDGFNVCQKLKKNNPQAFQILSSTFVDFTDIGVDYCDFSVQSKHKIIELDDKGQVVRINFNNATRDTIFDVPVERVQPFYAALKEFVDLMNSKESKFTFKMNPGDVITFDNWRLLHGRRSYEAGTEISRHLEGAYADWDVVMSRLRILRQRVENGN

688 NP_002008.2 NP_003126.1

>NP_002008.2

MDGTIKEALSVVSDDQSLFDSAYGAAAHLPKADMTASGSPDYGQPHKINPLPPQQEWINQPVRVNVKREYDHMNGSRESPVDCSVSKCSKLVGGGESNPMNYNSYMDEKNGPPPPNMTTNERRVIVPADPTLWTQEHVRQWLEWAIKEYSLMEIDTSFFQNMDGKELCKMNKEDFLRATTLYNTEVLLSHLSYLRESSLLAYNTTSHTDQSSRLSVKEDPSYDSVRRGAWGNNMNSGLNKSPPLGGAQTISKNTEQRPQPDPYQILGPTSSRLANPGSGQIQLWQFLLELLSDSANASCITWEGTNGEFKMTDPDEVARRWGERKSKPNMNYDKLSRALRYYYDKNIMTKVHGKRYAYKFDFHGIAQALQPHPTESSMYKYPSDISYMPSYHAHQQKVNFVPPHPSSMPVTSSSFFGAASQYWTSPTGGIYPNPNVPRHPNTHVPSHLGSYY

>NP_003126.1

MACAAARSPADQDRFICIYPAYLNNKKTIAEGRRIPISKAVENPTATEIQDVCSAVGLNVFLEKNKMYSREWNRDVQYRGRVRVQLKQEDGSLCLVQFPSRKSVMLYAAEMIPKLKTRTQKTGGADQSLQQGEGSKKGKGKKKK

689 NP_008882.2 NP_001893.2

>NP_008882.2

MAALAEEQTEVAVKLEPEGPPTLLPPQAGDGAGEGSGGTTNNGPNGGGGNVAASSSTGGDGGTPKPTVAVSAAAPAGAAPVPAAAPDAGAPHDRQTLLAVLQFLRQSKLREAEEALRREAGLLEEAVAGSGAPGEVDSAGAEVTSALLSRVTASAPGPAAPDPPGTGASGATVVSGSASGPAAPGKVGSVAVEDQPDVSAVLSAYNQQGDPTMYEEYYSGLKHFIECSLDCHRAELSQLFYPLFVHMYLELVYNQHENEAKSFFEKFHGDQECYYQDDLRVLSSLTKKEHMKGNETMLDFRTSKFVLRISRDSYQLLKRHLQEKQNNQIWNIVQEHLYIDIFDGMPRSKQQIDAMVGSLAGEAKREANKSKVFFGLLKEPEIEVPLDDEDEEGENEEGKPKKKKPKKDSIGSKSKKQDPNAPPQNRIPLPELKDSDKLDKIMNMKETTKRVRLGPDCLPSICFYTFLNAYQGLTAVDVTDDSSLIAGGFADSTVRVWSVTPKKLRSVKQASDLSLIDKESDDVLERIMDEKTASELKILYGHSGPVYGASFSPDRNYLLSSSEDGTVRLWSLQTFTCLVGYKGHNYPVWDTQFSPYGYYFVSGGHDRVARLWATDHYQPLRIFAGHLADVNCTRFHPNSNYVATGSADRTVRLWDVLNGNCVRIFTGHKGPIHSLTFSPNGRFLATGATDGRVLLWDIGHGLMVGELKGHTDTVCSLRFSRDGEILASGSMDNTVRLWDAIKAFEDLETDDFTTATGHINLPENSQELLLGTYMTKSTPVVHLHFTRRNLVLAAGAYSPQ

>NP_001893.2

MQEKDASSQGFLPHFQHFATQAIHVGQDPEQWTSRAVVPPISLSTTFKQGAPGQHSGFEYSRSGNPTRNCLEKAVAALDGAKYCLAFASGLAATVTITHLLKAGDQIICMDDVYGGTNRYFRQVASEFGLKISFVDCSKIKLLEAAITPETKLVWIETPTNPTQKVIDIEGCAHIVHKHGDIILVVDNTFMSPYFQRPLALGADISMYSATKYMNGHSDVVMGLVSVNCESLHNRLRFLQNSLGAVPSPIDCYLCNRGLKTLHVRMEKHFKNGMAVAQFLESNPWVEKVIYPGLPSHPQHELVKRQCTGCTGMVTFYIKGTLQHAEIFLKNLKLFTLAESLGGFESLAELPAIMTHASVLKNDRDVLGISDTLIRLSVGLEDEEDLLEDLDQALKAAHPPSGSHS

690 Q8IUM7 Q9UKF7

>Q8IUM7

MYRSTKGASKARRDQINAEIRNLKELLPLAEADKVRLSYLHIMSLACIYTRKGVFFAGGTPLAGPTGLLSAQELEDIVAALPGFLLVFTAEGKLLYLSESVSEHLGHSMVDLVAQGDSIYDIIDPADHLTVRQQLTLPSALDTDRLFRCRFNTSKSLRRQSAGNKLVLIRGRFHAHPPGAYWAGNPVFTAFCAPLEPRPRPGPGPGPGPASLFLAMFQSRHAKDLALLDISESVLIYLGFERSELLCKSWYGLLHPEDLAHASAQHYRLLAESGDIQAEMVVRLQAKTGGWAWIYCLLYSEGPEGPITANNYPISDMEAWSLRQQLNSEDTQAAYVLGTPTMLPSFPENILSQEECSSTNPLFTAALGAPRSTSFPSAPELSVVSASEELPRPSKELDFSYLTFPSGPEPSLQAELSKDLVCTPPYTPHQPGGCAFLFSLHEPFQTHLPTPSSTLQEQLTPSTATFSDQLTPSSATFPDPLTSPLQGQLTETSVRSYEDQLTPCTSTFPDQLLPSTATFPEPLGSPAHEQLTPPSTAFQAHLDSPSQTFPEQLSPNPTKTYFAQEGCSFLYEKLPPSPSSPGNGDCTLLALAQLRGPLSVDVPLVPEGLLTPEASPVKQSFFHYSEKEQNEIDRLIQQISQLAQGMDRPFSAEAGTGGLEPLGGLEPLDSNLSLSGAGPPVLSLDLKPWKCQELDFLADPDNMFLEETPVEDIFMDLSTPDPSEEWGSGDPEAEGPGGAPSPCNNLSPEDHSFLEDLATYETAFETGVSAFPYDGFTDELHQLQSQVQDSFHEDGSGGEPTF

>Q9UKF7

MLLKEYRICMPLTVDEYKIGQLYMISKHSHEQSDRGEGVEVVQNEPFEDPHHGNGQFTEKRVYLNSKLPSWARAVVPKIFYVTEKAWNYYPYTITEYTCSFLPKFSIHIETKYEDNKGSNDTIFDNEAKDVEREVCFIDIACDEIPERYYKESEDPKHFKSEKTGRGQLREGWRDSHQPIMCSYKLVTVKFEVWGLQTRVEQFVHKVVRDILLIGHRQAFAWVDEWYDMTMDDVREFERATQEATNKKIGIFPPAISISSIPLLPSSVRSAPSSAPSTPLSTDAPEFLSVPKDRPRKKSAPETLTLPDPEKKATLNLPGMHSSDKPCRPKSE

691 NP_004575.1 NP_004865.1

>NP_004575.1

MKCLVTGGNVKVLGKAVHSLSRIGDELYLEPLEDGLSLRTVNSSRSAYACFLFAPLFFQQYQAATPGQDLLRCKILMKSFLSVFRSLAMLEKTVEKCCISLNGRSSRLVVQLHCKFGVRKTHNLSFQDCESLQAVFDPASCPHMLRAPARVLGEAVLPFSPALAEVTLGIGRGRRVILRSYHEEEADSTAKAMVTEMCLGEEDFQQLQAQEGVAITFCLKEFRGLLSFAESANLNLSIHFDAPGRPAIFTIKDSLLDGHFVLATLSDTDSHSQDLGSPERHQPVPQLQAHSTPHPDDFANDDIDSYMIAMETTIGNEGSRVLPSISLSPGPQPPKSPGPHSEEEDEAEPSTVPGTPPPKKFRSLFFGSILAPVRSPQGPSPVLAEDSEGEG

>NP_004865.1

MSALRRSGYGPSDGPSYGRYYGPGGGDVPVHPPPPLYPLRPEPPQPPISWRVRGGGPAETTWLGEGGGGDGYYPSGGAWPEPGRAGGSHQEQPPYPSYNSNYWNSTARSRAPYPSTYPVRPELQGQSLNSYTNGAYGPTYPPGPGANTASYSGAYYAPGYTQTSYSTEVPSTYRSSGNSPTPVSRWIYPQQDCQTEAPPLRGQVPGYPPSQNPGMTLPHYPYGDGNRSVPQSGPTVRPQEDAWASPGAYGMGGRYPWPSSAPSAPPGNLYMTESTSPWPSSGSPQSPPSPPVQQPKDSSYPYSQSDQSMNRHNFPCSVHQYESSGTVNNDDSDLLDSQVQYSAEPQLYGNATSDHPNNQDQSSSLPEECVPSDESTPPSIKKIIHVLEKVQYLEQEVEEFVGKKTDKAYWLLEEMLTKELLELDSVETGGQDSVRQARKEAVCKIQAILEKLEKKGL

692 NP_002379.2 Q6WQI6

>NP_002379.2

MAGTVVLDDVELREAQRDYLDFLDDEEDQGIYQSKVRELISDNQYRLIVNVNDLRRKNEKRANRLLNNAFEELVAFQRALKDFVASIDATYAKQYEEFYVGLEGSFGSKHVSPRTLTSCFLSCVVCVEGIVTKCSLVRPKVVRSVHYCPATKKTIERRYSDLTTLVAFPSSSVYPTKDEENNPLETEYGLSVYKDHQTITIQEMPEKAPAGQLPRSVDVILDDDLVDKAKPGDRVQVVGTYRCLPGKKGGYTSGTFRTVLIACNVKQMSKDAQPSFSAEDIAKIKKFSKTRSKDIFDQLAKSLAPSIHGHDYVKKAILCLLLGGVERDLENGSHIRGDINILLIGDPSVAKSQLLRYVLCTAPRAIPTTGRGSSGVGLTAAVTTDQETGERRLEAGAMVLADRGVVCIDEFDKMSDMDRTAIHEVMEQGRVTIAKAGIHARLNARCSVLAAANPVYGRYDQYKTPMENIGLQDSLLSRFDLLFIMLDQMDPEQDREISDHVLRMHRYRAPGEQDGDAMPLGSAVDILATDDPNFSQEDQQDTQIYEKHDNLLHGTKKKKEKMVSAAFMKKYIHVAKIIKPVLTQESATYIAEEYSRLRSQDSMSSDTARTSPVTARTLETLIRLATAHAKARMSKTVDLQDAEEAVELVQYAYFKKVLEKEKKRKKRSEDESETEDEEEKSQEDQEQKRKRRKTRQPDAKDGDSYDPYDFSDTEEEMPQVHTPKTADSQETKESQKVELSESRLKAFKVALLDVFREAHAQSIGMNRLTESINRDSEEPFSSVEIQAALSKMQDDNQVMVSEGIIFLI

>Q6WQI6

MGNWGLGIAPWVDGESELEFRRLGMQGPLEALRRREWNTQRASFSFSFLIALSPHTVDYCHSYELFNRRWHGHVLATQRPSLFILMLV

693 Q9NV88 P56277

>Q9NV88

MKLYCLSGHPTLPCNVLKFKSTTIMLDCGLDMTSTLNFLPLPLVQSPRLSNLPGWSLKDGNAFLDKELKECSGHVFVDSVPEFCLPETELIDLSTVDVILISNYHCMMALPYITEHTGFTGTVYATEPTVQIGRLLMEELVNFIERVPKAQSASLWKNKDIQRLLPSPLKDAVEVSTWRRCYTMQEVNSALSKIQLVGYSQKIELFGAVQVTPLSSGYALGSSNWIIQSHYEKVSYVSGSSLLTTHPQPMDQASLKNSDVLVLTGLTQIPTANPDGMVGEFCSNLALTVRNGGNVLVPCYPSGVIYDLLECLYQYIDSAGLSSVPLYFISPVANSSLEFSQIFAEWLCHNKQSKVYLPEPPFPHAELIQTNKLKHYPSIHGDFSNDFRQPCVVFTGHPSLRFGDVVHFMELWGKSSLNTVIFTEPDFSYLEALAPYQPLAMKCIYCPIDTRLNFIQVSKLLKEVQPLHVVCPEQYTQPPPAQSHRMDLMIDCQPPAMSYRRAEVLALPFKRRYEKIEIMPELADSLVPMEIKPGISLATVSAVLHTKDNKHLLQPPPRPAQPTSGKKRKRVSDDVPDCKVLKPLLSGSIPVEQFVQTLEKHGFSDIKVEDTAKGHIVLLQEAETLIQIEEDSTHIICDNDEMLRVRLRDLVLKFLQKF

>P56277

MPQKDPCQKQACEIQKCLQANSYMESKCQAVIQELRKCCAQYPKGRSVVCSGFEKEEEENLTRKSASK

694 NP_001507.2 P46439

>NP_001507.2

MVSDEDELNLLVIVVDANPIWWGKQALKESQFTLSKCIDAVMVLGNSHLFMNRSNKLAVIASHIQESRFLYPGKNGRLGDFFGDPGNPPEFNPSGSKDGKYELLTSANEVIVEEIKDLMTKSDIKGQHTETLLAGSLAKALCYIHRMNKEVKDNQEMKSRILVIKAAEDSALQYMNFMNVIFAAQKQNILIDACVLDSDSGLLQQACDITGGLYLKVPQMPSLLQYLLWVFLPDQDQRSQLILPPPVHVDYRAACFCHRNLIEIGYVCSVCLSIFCNFSPICTTCETAFKISLPPVLKAKKKKLKVSA

>P46439

MPMTLGYWDIRGLAHAIRLLLEYTDSSYVEKKYTLGDAPDYDRSQWLNEKFKLGLDFPNLPYLIDGAHKITQSNAILRYIARKHNLCGETEEEKIRVDILENQVMDNHMELVRLCYDPDFEKLKPKYLEELPEKLKLYSEFLGKRPWFAGDKITFVDFLAYDVLDMKRIFEPKCLDAFLNLKDFISRFEGLKKISAYMKSSQFLRGLLFGKSATWNSK

695 NP_071378.1 P09210

>NP_071378.1

MEENEVESSSDAAPGPGRPEEPSESGLGVGTSEAVSADSSDAAAAPGQAEADDSGVGQSSDRGSRSQEEVSESSSSADPLPNSYLPDSSSVSHGPVAGVTGGPPALVHSSALPDPNMLVSDCTASSSDLGSAIDKIIESTIGPDLIQNCITVTSAEDGGAETTRYLILQGPDDGAPMTSPMSSSTLAHSLAAIEALADGPTSTSTCLEAQGGPSSPVQLPPASGAEEPDLQSLEAMMEVVVVQQFKCKMCQYRSSTKATLLRHMRERHFRPVAAAAAAAGKKGRLRKWSTSTKSQEEEGPEEEDDDDIVDAGAIDDLEEDSDYNPAEDEPRGRQLRLQRPTPSTPRPRRRPGRPRKLPRLEISDLPDGVEGEPLVSSQSGQSPPEPQDPEAPSSSGPGHLVAMGKVSRTPVEAGVSQSDAENAAPSCPDEHDTLPRRRGRPSRRFLGKKYRKYYYKSPKPLLRPFLCRICGSRFLSHEDLRFHVNSHEAGDPQLFKCLQCSYRSRRWSSLKEHMFNHVGSKPYKCDECSYTSVYRKDVIRHAAVHSRDRKKRPDPTPKLSSFPCPVCGRVYPMQKRLTQHMKTHSTEKPHMCDKCGKSFKKRYTFKMHLLTHIQAVANRRFKCEFCEFVCEDKKALLNHQLSHVSDKPFKCSFCPYRTFREDFLLSHVAVKHTGAKPFACEYCHFSTRHKKNLRLHVRCRHASSFEEWGRRHPEEPPSRRRPFFSLQQIEELKQQHSAAPGPPPSSPGPPEIPPEATTFQSSEAPSLLCSDTLGGATIIYQQGAEESTAMATQTALDLLLNMSAQRELGGTALQVAVVKSEDVEAGLASPGGQPSPEGATPQVVTLHVAEPGGGAAAESQLGPPDLPQITLAPGPFGGTGYSVITAPPMEEGTSAPGTPYSEEPAGEAAQAVVVSDTLKEAGTHYIMATDGTQLHHIELTADGSISFPSPDALASGAKWPLLQCGGLPRDGPEPPSPAKTHCVGDSQSSASSPPATSKALGLAVPPSPPSAATAASKKFSCKICAEAFPGRAEMESHKRAHAGPGAFKCPDCPFSARQWPEVRAHMAQHSSLRPHQCSQCSFASKNKKDLRRHMLTHTKEKPFACHLCGQRFNRNGHLKFHIQRLHSPDGRKSGTPTARAPTQTPTQTIILNSDDETLATLHTALQSSHGVLGPERLQQALSQEHIIVAQEQTVTNQEEAAYIQEITTADGQTVQHLVTSDNQVQYIISQDGVQHLLPQEYVVVPEGHHIQVQEGQITHIQYEQGAPFLQESQIQYVPVSPGQQLVTQAQLEAAAHSAVTAVADAAMAQAQGLFGTDETVPEHIQQLQHQGIEYDVITLADD

>P09210

MAEKPKLHYSNIRGRMESIRWLLAAAGVEFEEKFIKSAEDLDKLRNDGYLMFQQVPMVEIDGMKLVQTRAILNYIASKYNLYGKDIKEKALIDMYIEGIADLGEMILLLPFSQPEEQDAKLALIQEKTKNRYFPAFEKVLKSHGQDYLVGNKLSRADIHLVELLYYVEELDSSLISSFPLLKALKTRISNLPTVKKFLQPGSPRKPPMDEKSLEESRKIFRF

696 NP_071394.2 NP_001001936.1

>NP_071394.2

MTGEVGSEVHLEINDPNVISQEEADSPSDSGQGSYETIGPLSEGDSDEEIFVSKKLKNRKVLQDSDSETEDTNASPEKTTYDSAEEENKENLYAGKNTKIKRIYKTVADSDESYMEKSLYQENLEAQVKPCLELSLQSGNSTDFTTDRKSSKKHIHDKEGTAGKAKVKSKRRLEKEERKMEKIRQLKKKETKNQEDDVEQPFNDSGCLLVDKDLFETGLEDENNSPLEDEESLESIRAAVKNKVKKHKKKEPSLESGVHSFEEGSELSKGTTRKERKAARLSKEALKQLHSETQRLIRESALNLPYHMPENKTIHDFFKRKPRPTCHGNAMALLKSSKYQSSHHKEIIDTANTTEMNSDHHSKGSEQTTGAENEVETNALPVVSKETQIITGSDESCRKDLVKNEELEIQEKQKQSDIRPSPGDSSVLQQESNFLGNNHSEECQVGGLVAFEPHALEGEGPQNPEETDEKVEEPEQQNKSSAVGPPEKVRRFTLDRLKQLGVDVSIKPRLGADEDSFVILEPETNRELEALKQRFWKHANPAAKPRAGQTVNVNVIVKDMGTDGKEELKADVVPVTLAPKKLDGASHTKPGEKLQVLKAKLQEAMKLRRFEERQKRQALFKLDNEDGFEEEEEEEEEMTDESEEDGEEKVEKEEKEEELEEEEEKEEEEEEEGNQETAEFLLSSEEIETKDEKEMDKENNDGSSEIGKAVGFLSVPKSLSSDSTLLLFKDSSSKMGYFPTEEKSETDENSGKQPSKLDEDDSCSLLTKESSHNSSFELIGSTIPSYQPCNRQTGRGTSFFPTAGGFRSPSPGLFRASLVSSASKSSGKLSEPSLPIEDSQDLYNASPEPKTLFLGAGDFQFCLEDDTQSQLLDADGFLNVRNHRNQYQALKPRLPLASMDENAMDANMDELLDLCTGKFTSQAEKHLPRKSDKKENMEELLNLCSGKFTSQDASTPASSELNKQEKESSMGDPMEEALALCSGSFPTDKEEEDEEEEFGDFRLVSNDNEFDSDEDEHSDSGNDLALEDHEDDDEEELLKRSEKLKRQMRLRKYLEDEAEVSGSDVGSEDEYDGEEIDEYEEDVIDEVLPSDEELQSQIKKIHMKTMLDDDKRQLRLYQERYLADGDLHSDGPGRMRKFRWKNIDDASQMDLFHRDSDDDQTEEQLDESEARWRKERIEREQWLRDMAQQGKITAEEEEEIGEDSQFMILAKKVTAKALQKNASRPMVIQESKSLLRNPFEAIRPGSAQQVKTGSLLNQPKAVLQKLAALSDHNPSAPRNSRNFVFHTLSPVKAEAAKESSKSQVKKRGPSFMTSPSPKHLKTDDSTSGLTRSIFKYLES

>NP_001001936.1

MERYKALEQLLTELDDFLKILDQENLSSTALVKKSCLAELLRLYTKSSSSDEEYIYMNKVTINKQQNAESQGKAPEEQGLLPNGEPSQHSSAPQKSLPDLPPPKMIPERKQLAIPKTESPEGYYEEAEPYDTSLNEDGEAVSSSYESYDEEDGSKGKSAPYQWPSPEAGIELMRDARICAFLWRKKWLGQWAKQLCVIKDNRLLCYKSSKDHSPQLDVNLLGSSVIHKEKQVRKKEHKLKITPMNADVIVLGLQSKDQAEQWLRVIQEVSGLPSEGASEGNQYTPDAQRFNCQKPDIAEKYLSASEYGSSVDGHPEVPETKDVKKKCSAGLKLSNLMNLGRKKSTSLEPVERSLETSSYLNVLVNSQWKSRWCSVRDNHLHFYQDRNRSKVAQQPLSLVGCEVVPDPSPDHLYSFRILHKGEELAKLEAKSSEEMGHWLGLLLSESGSKTDPEEFTYDYVDADRVSCIVSAAKNSLLLMQRKFSEPNTYIDGLPSQDRQEELYDDVDLSELTAAVEPTEEATPVADDPNERESDRVYLDLTPVKSFLHGPSSAQAQASSPTLSCLDNATEALPADSGPGPTPDEPCIKCPENLGEQQLESLEPEDPSLRITTVKIQTEQQRISFPPSCPDAVVATPPGASPPVKDRLRVTSAEIKLGKNRTEAEVKRYTEEKERLEKKKEEIRGHLAQLRKEKRELKETLLKCTDKEVLASLEQKLKEIDEECRGEESRRVDLELSIMEVKDNLKKAEAGPVTLGTTVDTTHLENVSPRPKAVTPASAPDCTPVNSATTLKNRPLSVVVTGKGTVLQKAKEWEKKGAS

697 O95766 NP_036429.2

>O95766

MAAAAAGAGSGPWAAQEKQFPPALLSFFIYNPRFGPREGQEENKILFYHPNEVEKNEKIRNVGLCEAIVQFTRTFSPSKPAKSLHTQKNRQFFNEPEENFWMVMVVRNPIIEKQSKDGKPVIEYQEEELLDKVYSSVLRQCYSMYKLFNGTFLKAMEDGGVKLLKERLEKFFHRYLQTLHLQSCDLLDIFGGISFFPLDKMTYLKIQSFINRMEESLNIVKYTAFLYNDQLIWSGLEQDDMRILYKYLTTSLFPRHIEPELAGRDSPIRAEMPGNLQHYGRFLTGPLNLNDPDAKCRFPKIFVNTDDTYEELHLIVYKAMSAAVCFMIDASVHPTLDFCRRLDSIVGPQLTVLASDICEQFNINKRMSGSEKEPQFKFIYFNHMNLAEKSTVHMRKTPSVSLTSVHPDLMKILGDINSDFTRVDEDEEIIVKAMSDYWVVGKKSDRRELYVILNQKNANLIEVNEEVKKLCATQFNNIFFLD

>NP_036429.2

MVMEKPSPLLVGREFVRQYYTLLNKAPEYLHRFYGRNSSYVHGGVDASGKPQEAVYGQNDIHHKVLSLNFSECHTKIRHVDAHATLSDGVVVQVMGLLSNSGQPERKFMQTFVLAPEGSVPNKFYVHNDMFRYEDEVFGDSEPELDEESEDEVEEEQEERQPSPEPVQENANSGYYEAHPVTNGIEEPLEESSHEPEPEPESETKTEELKPQVEEKNLEELEEKSTTPPPAEPVSLPQEPPKAFSWASVTSKNLPPSGTVSSSGIPPHVKAPVSQPRVEAKPEVQSQPPRVREQRPRERPGFPPRGPRPGRGDMEQNDSDNRRIIRYPDSHQLFVGNLPHDIDENELKEFFMSFGNVVELRINTKGVGGKLPNFGFVVFDDSEPVQRILIAKPIMFRGEVRLNVEEKKTRAARERETRGGGDDRRDIRRNDRGPGGPRGIVGGGMMRDRDGRGPPPRGGMAQKLGSGRGTGQMEGRFTGQRR

698 Q8TAP9 Q9BSE5

>Q8TAP9

MQRQNFRPPTPPYPGPGGGGWGSGSSFRGTPGGGGPRPPSPRDGYGSPHHTPPYGPRSRPYGSSHSPRHGGSFPGGRFGSPSPGGYPGSYSRSPAGSQQQFGYSPGQQQTHPQGSPRTSTPFGSGRVREKRMSNELENYFKPSMLEDPWAGLEPVSVVDISQQYSNTQTFTGKKGRYFC

>Q9BSE5

MLRLLASGCARGPGPGVGARPAAGLFHPGRRQSRQASDAPRNQPPSPEFVARPVGVCSMMRLPVQTSPEGLDAAFIGVPLDTGTSNRPGARFGPRRIREESVMLGTVNPSTGALPFQSLMVADLGDVNVNLYNLQDSCRRIQEAYEKIVAAGCIPLTLGGDHTITYPILQAMAKKHGPVGLLHVDAHTDTTDKALGEKLYHGAPFRRCVDEGLLDCKRVVQIGIRGSSTTLDPYRYNRSQGFRVVLAEDCWMKSLVPLMGEVRQQMGGKPIYISFDIDALDPAYAPGTGTPEIAGLTPSQALEIIRGCQGLNVMGCDLVEVSPPYDLSGNTALLAANLLFEMLCALPKVTTV

699 NP_003358.1 NP_000662.3

>NP_003358.1

MDMLDPGLDPAASATAAAAASHDKGPEAEEGVELQEGGDGPGAEEQTAVAITSVQQAAFGDHNIQYQFRTETNGGQVTYRVVQVTDGQLDGQGDTAGAVSVVSTAAFAGGQQAVTQVGVDGAAQRPGPAAASVPPGPAAPFPLAVIQNPFSNGGSPAAEAVSGEARFAYFPASSVGDTTAVSVQTTDQSLQAGGQFYVMMTPQDVLQTGTQRTIAPRTHPYSPKIDGTRTPRDERRRAQHNEVERRRRDKINNWIVQLSKIIPDCNADNSKTGASKGGILSKACDYIRELRQTNQRMQETFKEAERLQMDNELLRQQIEELKNENALLRAQLQQHNLEMVGEGTRQ

>NP_000662.3

MANEVIKCKAAVAWEAGKPLSIEEIEVAPPKAHEVRIKIIATAVCHTDAYTLSGADPEGCFPVILGHEGAGIVESVGEGVTKLKAGDTVIPLYIPQCGECKFCLNPKTNLCQKIRVTQGKGLMPDGTSRFTCKGKTILHYMGTSTFSEYTVVADISVAKIDPLAPLDKVCLLGCGISTGYGAAVNTAKLEPGSVCAVFGLGGVGLAVIMGCKVAGASRIIGVDINKDKFARAKEFGATECINPQDFSKPIQEVLIEMTDGGVDYSFECIGNVKVMRAALEACHKGWGVSVVVGVAASGEEIATRPFQLVTGRTWKGTAFGGWKSVESVPKLVSEYMSKKIKVDEFVTHNLSFDEINKAFELMHSGKSIRTVVKI

700 O15178 P28838

>O15178

MSSPGTESAGKSLQYRVDHLLSAVENELQAGSEKGDPTERELRVGLEESELWLRFKELTNEMIVTKNGRRMFPVLKVNVSGLDPNAMYSFLLDFVAADNHRWKYVNGEWVPGGKPEPQAPSCVYIHPDSPNFGAHWMKAPVSFSKVKLTNKLNGGGQIMLNSLHKYEPRIHIVRVGGPQRMITSHCFPETQFIAVTAYQNEEITALKIKYNPFAKAFLDAKERSDHKEMMEEPGDSQQPGYSQWGWLLPGTSTLCPPANPHPQFGGALSLPSTHSCDRYPTLRSHRSSPYPSPYAHRNNSPTYSDNSPACLSMLQSHDNWSSLGMPAHPSMLPVSHNASPPTSSSQYPSLWSVSNGAVTPGSQAAAVSNGLGAQFFRGSPAHYTPLTHPVSAPSSSGSPLYEGAAAATDIVDSQYDAAAQGRLIASWTPVSPPSM

>P28838

MFLLPLPAAGRVVVRRLAVRRFGSRSLSTADMTKGLVLGIYSKEKEDDVPQFTSAGENFDKLLAGKLRETLNISGPPLKAGKTRTFYGLHQDFPSVVLVGLGKKAAGIDEQENWHEGKENIRAAVAAGCRQIQDLELSSVEVDPCGDAQAAAEGAVLGLYEYDDLKQKKKMAVSAKLYGSGDQEAWQKGVLFASGQNLARQLMETPANEMTPTRFAEIIEKNLKSASSKTEVHIRPKSWIEEQAMGSFLSVAKGSDEPPVFLEIHYKGSPNANEPPLVFVGKGITFDSGGISIKASANMDLMRADMGGAATICSAIVSAAKLNLPINIIGLAPLCENMPSGKANKPGDVVRAKNGKTIQVDNTDAEGRLILADALCYAHTFNPKVILNAATLTGAMDVALGSGATGVFTNSSWLWNKLFEASIETGDRVWRMPLFEHYTRQVVDCQLADVNNIGKYRSAGACTAAAFLKEFVTHPKWAHLDIAGVMTNKDEVPYLRKGMTGRPTRTLIEFLLRFSQDNA

701 P16402 NP_001619.1

>P16402

MSETAPLAPTIPAPAEKTPVKKKAKKAGATAGKRKASGPPVSELITKAVAASKERSGVSLAALKKALAAAGYDVEKNNSRIKLGLKSLVSKGTLVQTKGTGASGSFKLNKKAASGEGKPKAKKAGAAKPRKPAGAAKKPKKVAGAATPKKSIKKTPKKVKKPATAAGTKKVAKSAKKVKTPQPKKAAKSPAKAKAPKPKAAKPKSGKPKVTKAKKAAPKKK

>NP_001619.1

MASRLLLNNGAKMPILGLGTWKSPPGQVTEAVKVAIDVGYRHIDCAHVYQNENEVGVAIQEKLREQVVKREELFIVSKLWCTYHEKGLVKGACQKTLSDLKLDYLDLYLIHWPTGFKPGKEFFPLDESGNVVPSDTNILDTWAAMEELVDEGLVKAIGISNFNHLQVEMILNKPGLKYKPAVNQIECHPYLTQEKLIQYCQSKGIVVTAYSPLGSPDRPWAKPEDPSLLEDPRIKAIAAKHNKTTAQVLIRFPMQRNLVVIPKSVTPERIAENFKVFDFELSSQDMTTLLSYNRNWRVCALLSCTSHKDYPFHEEF

702 NP_115285.1 P48728

>NP_115285.1

MSCGRPPPDVDGMITLKVDNLTYRTSPDSLRRVFEKYGRVGDVYIPREPHTKAPRGFAFVRFHDRRDAQDAEAAMDGAELDGRELRVQVARYGRRDLPRSRQGEPRGRSRGGGYGRRSRSYGRRSRSPRRRHRSRSRGPSCSRSRSRSRYRGSRYSRSPYSRSPYSRSRYSRSPYSRSRYRESRYGGSHYSSSGYSNSRYSRYHSSRSHSKSGSSTSSRSASTSKSSSARRSKSSSVSRSRSRSRSSSMTRSPPRVSKRKSKSRSRSKRPPKSPEEEGQMSS

>P48728

MQRAVSVVARLGFRLQAFPPALCRPLSCAQEVLRRTPLYDFHLAHGGKMVAFAGWSLPVQYRDSHTDSHLHTRQHCSLFDVSHMLQTKILGSDRVKLMESLVVGDIAELRPNQGTLSLFTNEAGGILDDLIVTNTSEGHLYVVSNAGCWEKDLALMQDKVRELQNQGRDVGLEVLDNALLALQGPTAAQVLQAGVADDLRKLPFMTSAVMEVFGVSGCRVTRCGYTGEDGVEISVPVAGAVHLATAILKNPEVKLAGLAARDSLRLEAGLCLYGNDIDEHTTPVEGSLSWTLGKRRRAAMDFPGAKVIVPQLKGRVQRRRVGLMCEGAPMRAHSPILNMEGTKIGTVTSGCPSPSLKKNVAMGYVPCEYSRPGTMLLVEVRRKQQMAVVSKMPFVPTNYYTLK

703 P22492 NP_056520.2

>P22492

MSETVPAASASAGVAAMEKLPTKKRGRKPAGLISASRKVPNLSVSKLITEALSVSQERVGMSLVALKKALAAAGYDVEKNNSRIKLSLKSLVNKGILVQTRGTGASGSFKLSKKVIPKSTRSKAKKSVSAKTKKLVLSRDSKSPKTAKTNKRAKKPRATTPKTVRSGRKAKGAKGKQQQKSPVKARASKSKLTQHHEVNVRKATSKK

>NP_056520.2

MSGSHTPACGPFSALTPSIWPQEILAKYTQKEESAEQPEFYYDEFGFRVYKEEGDEPGSSLLANSPLMEDAPQRLRWQAHLEFTHNHDVGDLTWDKIAVSLPRSEKLRSLVLAGIPHGMRPQLWMRLSGALQKKRNSELSYREIVKNSSNDETIAAKQIEKDLLRTMPSNACFASMGSIGVPRLRRVLRALAWLYPEIGYCQGTGMVAACLLLFLEEEDAFWMMSAIIEDLLPASYFSTTLLGVQTDQRVLRHLIVQYLPRLDKLLQEHDIELSLITLHWFLTAFASVVDIKLLLRIWDLFFYEGSRVLFQLTLGMLHLKEEELIQSENSASIFNTLSDIPSQMEDAELLLGVAMRLAGSLTDVAVETQRRKHLAYLIADQGQLLGAGTLTNLSQVVRRRTQRRKSTITALLFGEDDLEALKAKNIKQTELVADLREAILRVARHFQCTDPKNCSVELTPDYSMESHQRDHENYVACSRSHRRRAKALLDFERHDDDELGFRKNDIITIVSQKDEHCWVGELNGLRGWFPAKFVEVLDERSKEYSIAGDDSVTEGVTDLVRGTLCPALKALFEHGLKKPSLLGGACHPWLFIEEAAGREVERDFASVYSRLVLCKTFRLDEDGKVLTPEELLYRAVQSVNVTHDAVHAQMDVKLRSLICVGLNEQVLHLWLEVLCSSLPTVEKWYQPWSFLRSPGWVQIKCELRVLCCFAFSLSQDWELPAKREAQQPLKEGVRDMLVKHHLFSWDVDG

704 NP_005244.1 Q7L0Y3

>NP_005244.1

MYQDYPGNFDTSSRGSSGSPAHAESYSSGGGGQQKFRVDMPGSGSAFIPTINAITTSQDLQWMVQPTVITSMSNPYPRSHPYSPLPGLASVPGHMALPRPGVIKTIGTTVGRRRRDEQLSPEEEEKRRIRRERNKLAAAKCRNRRRELTEKLQAETEELEEEKSGLQKEIAELQKEKEKLEFMLVAHGPVCKISPEERRSPPAPGLQPMRSGGGSVGAVVVKQEPLEEDSPSSSSAGLDKAQRSVIKPISIAGGFYGEEPLHTPIVVTSTPAVTPGTSNLVFTYPSVLEQESPASPSESCSKAHRRSSSSGDQSSDSLNSPTLLAL

>Q7L0Y3

MAAFLKMSVSVNFFRPFTRFLVPFTLHRKRNNLTILQRYMSSKIPAVTYPKNESTPPSEELELDKWKTTMKSSVQEECVSTISSSKDEDPLAATREFIEMWRLLGREVPEHITEEELKTLMECVSNTAKKKYLKYLYTKEKVKKARQIKKEMKAAAREEAKNIKLLETTEEDKQKNFLFLRLWDRNMDIAMGWKGAQAMQFGQPLVFDMAYENYMKRKELQNTVSQLLESEGWNRRNVDPFHIYFCNLKIDGALHRELVKRYQEKWDKLLLTSTEKSHVDLFPKDSIIYLTADSPNVMTTFRHDKVYVIGSFVDKSMQPGTSLAKAKRLNLATECLPLDKYLQWEIGNKNLTLDQMIRILLCLKNNGNWQEALQFVPKRKHTGFLEISQHSQEFINRLKKAKT

705 NP_077021.1 NP_060383.2

>NP_077021.1

MDKSGIDSLDHVTSDAVELANRSDNSSDSSLFKTQCIPYSPKGEKRNPIRKFVRTPESVHASDSSSDSSFEPIPLTIKAIFERFKNRKKRYKKKKKRRYQPTGRPRGRPEGRRNPIYSLIDKKKQFRSRGSGFPFLESENEKNAPWRKILTFEQAVARGFFNYIEKLKYEHHLKESLKQMNVGEDLENEDFDSRRYKFLDDDGSISPIEESTAEDEDATHLEDNECDIKLAGDSFIVSSEFPVRLSVYLEEEDITEEAALSKKRATKAKNTGQRGLKM

>NP_060383.2

MEQPWPPPGPWSLPRAEGEAEEESDFDVFPSSPRCPQLPGGGAQMYSHGIELACQKQKEFVKSSVACKWNLAEAQQKLGSLALHNSESLDQEHAKAQTAVSELRQREEEWRQKEEALVQREKMCLWSTDAISKDVFNKSFINQDKRKDTEDEDKSESFMQKYEQKIRHFGMLSRWDDSQRFLSDHPYLVCEETAKYLILWCFHLEAEKKGALMEQIAHQAVVMQFIMEMAKNCNVDPRGCFRLFFQKAKAEEEGYFEAFKNELEAFKSRVRLYSQSQSFQPMTVQNHVPHSGVGSIGLLESLPQNPDYLQYSISTALCSLNSVVHKEDDEPKMMDTV

706 Q86WB0 NP_003977.1

>Q86WB0

MAAPCEGQAFAVGVEKNWGAVVRSPEGTPQKIRQLIDEGIAPEEGGVDAKDTSATSQSVNGSPQAEQPSLESTSKEAFFSRVETFSSLKWAGKPFELSPLVCAKYGWVTVECDMLKCSSCQAFLCASLQPAFDFDRYKQRCAELKKALCTAHEKFCFWPDSPSPDRFGMLPLDEPAILVSEFLDRFQSLCHLDLQLPSLRPEDLKTMCLTEDKISLLLHLLEDELDHRTDERKTTIKLGSDIQVHVTACILSVCGWACSSSLESMQLSLITCSQCMRKVGLWGFQQIESSMTDLDASFGLTSSPIPGLEGRPERLPLVPESPRRMMTRSQDATFSPGSEQAEKSPGPIVSRTRSWDSSSPVDRPEPEAASPTTRTRPVTRSMGTGDTPGLEVPSSPLRKAKRARLCSSSSSDTSSRSFFDPTSQHRDWCPWVNITLGKESRENGGTEPDASAPAEPGWKAVLTILLAHKQSSQPAETDSMSLSEKSRKVFRIFRQWESLCSC

>NP_003977.1

MACTIQKAEALDGAHLMQILWYDEEESLYPAVWLRDNCPCSDCYLDSAKARKLLVEALDVNIGIKGLIFDRKKVYITWPDEHYSEFQADWLKKRCFSKQARAKLQRELFFPECQYWGSELQLPTLDFEDVLRYDEHAYKWLSTLKKVGIVRLTGASDKPGEVSKLGKRMGFLYLTFYGHTWQVQDKIDANNVAYTTGKLSFHTDYPALHHPPGVQLLHCIKQTVTGGDSEIVDGFNVCQKLKKNNPQAFQILSSTFVDFTDIGVDYCDFSVQSKHKIIELDDKGQVVRINFNNATRDTIFDVPVERVQPFYAALKEFVDLMNSKESKFTFKMNPGDVITFDNWRLLHGRRSYEAGTEISRHLEGAYADWDVVMSRLRILRQRVENGN

707 NP_001507.2 NP_066953.1

>NP_001507.2

MVSDEDELNLLVIVVDANPIWWGKQALKESQFTLSKCIDAVMVLGNSHLFMNRSNKLAVIASHIQESRFLYPGKNGRLGDFFGDPGNPPEFNPSGSKDGKYELLTSANEVIVEEIKDLMTKSDIKGQHTETLLAGSLAKALCYIHRMNKEVKDNQEMKSRILVIKAAEDSALQYMNFMNVIFAAQKQNILIDACVLDSDSGLLQQACDITGGLYLKVPQMPSLLQYLLWVFLPDQDQRSQLILPPPVHVDYRAACFCHRNLIEIGYVCSVCLSIFCNFSPICTTCETAFKISLPPVLKAKKKKLKVSA

>NP_066953.1

MVNPTVFFDIAVDGEPLGRVSFELFADKVPKTAENFRALSTGEKGFGYKGSCFHRIIPGFMCQGGDFTRHNGTGGKSIYGEKFEDENFILKHTGPGILSMANAGPNTNGSQFFICTAKTEWLDGKHVVFGKVKEGMNIVEAMERFGSRNGKTSKKITIADCGQLE

708 Q86V15 NP_001547.1

>Q86V15

MDLGTAEGTRCTDPPAGKPAMAPKRKGGLKLNAICAKLSRQVVVEKRADAGSHTEGSPSQPRDQERSGPESGAARAPRSEEDKRRAVIEKWVNGEYSEEPAPTPVLGRIAREGLELPPEGVYMVQPQGCSDEEDHAEEPSKDGGALEEKDSDGAASKEDSGPSTRQASGEASSLRDYAASTMTEFLGMFGYDDQNTRDELARKISFEKLHAGSTPEAATSSMLPTSEDTLSKRARFSKYEEYIRKLKAGEQLSWPAPSTKTEERVGKEVVGTLPGLRLPSSTAHLETKATILPLPSHSSVQMQNLVARASKYDFFIQKLKTGENLRPQNGSTYKKPSKYDLENVKYLHLFKPGEGSPDMGGAIAFKTGKVGRPSKYDVRGIQKPGPAKVPPTPSLAPAPLASVPSAPSAPGPGPEPPASLSFNTPEYLKSTFSKTDSITTGTVSTVKNGLPTDKPAVTEDVNIYQKYIARFSGSQHCGHIHCAYQYREHYHCLDPECNYQRFTSKQDVIRHYNMHKKRDNSLQHGFMRFSPLDDCSVYYHGCHLNGKSTHYHCMQVGCNKVYTSTSDVMTHENFHKKNTQLINDGFQRFRATEDCGTADCQFYGQKTTHFHCRRPGCTFTFKNKCDIEKHKSYHIKDDAYAKDGFKKFYKYEECKYEGCVYSKATNHFHCIRAGCGFTFTSTSQMTSHKRKHERRHIRSSGALGLPPSLLGAKDTEHEESSNDDLVDFSALSSKNSSLSASPTSQQSSASLAAATAATEAGPSATKPPNSKISGLLPQGLPGSIPLALALSNSGLPTPTPYFPILAGRGSTSLPVGTPSLLGAVSSGSAASATPDTPTLVASGAGDSAPVAAASVPAPPASIMERISASKGLISPMMARLAAAALKPSATFDPGSGQQVTPARFPPAQVKPEPGESTGAPGPHEASQDRSLDLTVKEPSNESNGHAVPANSSLLSSLMNKMSQGNPGLGSLLNIKAEAEGSPAAEPSPFLGKAVKALVQEKLAEPWKVYLRRFGTKDFCDGQCDFLHKAHFHCVVEECGALFSTLDGAIKHANFHFRTEGGAAKGNTEAAFPASAAETKPPMAPSSPPVPPVTTATVSSLEGPAPSPASVPSTPTLLAWKQLASTIPQMPQIPASVPHLPASPLATTSLENAKPQVKPGFLQFQENDPCLATDCKYANKFHFHCLFGNCKYVCKTSGKAESHCLDHINPNNNLVNVRDQFAYYSLQCLCPNQHCEFRMRGHYHCLRTGCYFVTNITTKLPWHIKKHEKAERRAANGFKYFTKREECGRLGCKYNQVSSHFHCIREGCQFSFLLKHQMTSHARKHMRRMLGKNFDRVPPSQGPPGLMDAETDECMDYTGCSPGAMSSESSTMDRSCSSTPVGNESTAAGNTISMPTASGAKKRFWIIEDMSPFGKRRKTASSRKMLDEGMMLEGFRRFDLYEDCKDAACQFSLKVTHYHCTRENCGYKFCGRTHMYKHAQHHDRVDNLVLDDFKRFKASLSRHFADCPFSGTSTHFHCLRCRFRCTDSTKVTAHRKHHGKQDVISAAGFRQFSSSADCAVPDCKYKLKCSHFHCTFPGCRHTVVGMSQMDSHKRKHEKQERGEPAAEGPAPGPPISLDGSLSLGAEPGSLLFLQSAAAGLGLALGDAGDPGPPDAAAPGPREGAAAAAAAAGESSQEDEEEELELPEEEAEDDEDEDDDEDDDDEDDDEDDDDEDLRTDSEESLPEAAAEAAGAGARTPALAALAALGAPGPAPTAASSP

>NP_001547.1

MSWSPSLTTQTCGAWEMKERLGTGGFGNVIRWHNQETGEQIAIKQCRQELSPRNRERWCLEIQIMRRLTHPNVVAARDVPEGMQNLAPNDLPLLAMEYCQGGDLRKYLNQFENCCGLREGAILTLLSDIASALRYLHENRIIHRDLKPENIVLQQGEQRLIHKIIDLGYAKELDQGSLCTSFVGTLQYLAPELLEQQKYTVTVDYWSFGTLAFECITGFRPFLPNWQPVQWHSKVRQKSEVDIVVSEDLNGTVKFSSSLPYPNNLNSVLAERLEKWLQLMLMWHPRQRGTDPTYGPNGCFKALDDILNLKLVHILNMVTGTIHTYPVTEDESLQSLKARIQQDTGIPEEDQELLQEAGLALIPDKPATQCISDGKLNEGHTLDMDLVFLFDNSKITYETQISPRPQPESVSCILQEPKRNLAFFQLRKVWGQVWHSIQTLKEDCNRLQQGQRAAMMNLLRNNSCLSKMKNSMASMSQQLKAKLDFFKTSIQIDLEKYSEQTEFGITSDKLLLAWREMEQAVELCGRENEVKLLVERMMALQTDIVDLQRSPMGRKQGGTLDDLEEQARELYRRLREKPRDQRTEGDSQEMVRLLLQAIQSFEKKVRVIYTQLSKTVVCKQKALELLPKVEEVVSLMNEDEKTVVRLQEKRQKELWNLLKIACSKVRGPVSGSPDSMNASRLSQPGQLMSQPSTASNSLPEPAKKSEELVAEAHNLCTLLENAIQDTVREQDQSFTALDWSWLQTEEEEHSCLEQAS

709 NP_003654.3 Q9H4K7

>NP_003654.3

MERFVVTAPPARNRSKTALYVTPLDRVTEFGGELHEDGGKLFCTSCNVVLNHVRKSAISDHLKSKTHTKRKAEFEEQNVRKKQRPLTASLQCNSTAQTEKVSVIQDFVKMCLEANIPLEKADHPAVRAFLSRHVKNGGSIPKSDQLRRAYLPDGYENENQLLNSQDC

>Q9H4K7

MAPARCFSARLRTVFQGVGHWALSTWAGLKPSRLLPQRASPRLLSVGRADLAKHQELPGKKLLSEKKLKRYFVDYRRVLVCGGNGGAGASCFHSEPRKEFGGPDGGDGGNGGHVILRVDQQVKSLSSVLSRYQGFSGEDGGSKNCFGRSGAVLYIRVPVGTLVKEGGRVVADLSCVGDEYIAALGGAGGKGNRFFLANNNRAPVTCTPGQPGQQRVLHLELKTVAHAGMVGFPNAGKSSLLRAISNARPAVASYPFTTLKPHVGIVHYEGHLQIAVADIPGIIRGAHQNRGLGSAFLRHIERCRFLLFVVDLSQPEPWTQVDDLKYELEMYEKGLSARPHAIVANKIDLPEAQANLSQLRDHLGQEVIVLSALTGENLEQLLLHLKVLYDAYAEAELGQGRQPLRW

710 O00472 NP_003192.1

>O00472

MAAGGTGGLREEQRYGLSCGRLGQDNITVLHVKLTETAIRALETYQSHKNLIPFRPSIQFQGLHGLVKIPKNDPLNEVHNFNFYLSNVGKDNPQGSFDCIQQTFSSSGASQLNCLGFIQDKITVCATNDSYQMTRERMTQAEEESRNRSTKVIKPGGPYVGKRVQIRKAPQAVSDTVPERKRSTPMNPANTIRKTHSSSTISQRPYRDRVIHLLALKAYKKPELLARLQKDGVNQKDKNSLGAILQQVANLNSKDLSYTLKDYVFKELQRDWPGYSEIDRRSLESVLSRKLNPSQNAAGTSRSESPVCSSRDAVSSPQKRLLDSEFIDPLMNKKARISHLTNRVPPTLNGHLNPTSEKSAAGLPLPPAAAAIPTPPPLPSTYLPISHPPQIVNSNSNSPSTPEGRGTQDLPVDSFSQNDSIYEDQQDKYTSRTSLETLPPGSVLLKCPKPMEENHSMSHKKSKKKSKKHKEKDQIKKHDIETIEEKEEDLKREEEIAKLNNSSPNSSGGVKEDCTASMEPSAIELPDYLIKYIAIVSYEQRQNYKDDFNAEYDEYRALHARMETVARRFIKLDAQRKRLSPGSKEYQNVHEEVLQEYQKIKQSSPNYHEEKYRCEYLHNKLAHIKRLIGEFDQQQAESWS

>NP_003192.1

MAFLRSMWGVLSALGRSGAELCTGCGSRLRSPFSFVYLPRWFSSVLASCPKKPVSSYLRFSKEQLPIFKAQNPDAKTTELIRRIAQRWRELPDSKKKIYQDAYRAEWQVYKEEISRFKEQLTPSQIMSLEKEIMDKHLKRKAMTKKKELTLLGKPKRPRSAYNVYVAERFQEAKGDSPQEKLKTVKENWKNLSDSEKELYIQHAKEDETRYHNEMKSWEEQMIEVGRKDLLRRTIKKQRKYGAEEC

711 NP_055130.1 Q8N9W6

>NP_055130.1

MADGNEDLRADDLPGPAFESYESMELACPAERSGHVAVSDGRHMFVWGGYKSNQVRGLYDFYLPREELWIYNMETGRWKKINTEGDVPPSMSGSCAVCVDRVLYLFGGHHSRGNTNKFYMLDSRSTDRVLQWERIDCQGIPPSSKDKLGVWVYKNKLIFFGGYGYLPEDKVLGTFEFDETSFWNSSHPRGWNDHVHILDTETFTWSQPITTGKAPSPRAAHACATVGNRGFVFGGRYRDARMNDLHYLNLDTWEWNELIPQGICPVGRSWHSLTPVSSDHLFLFGGFTTDKQPLSDAWTYCISKNEWIQFNHPYTEKPRLWHTACASDEGEVIVFGGCANNLLVHHRAAHSNEILIFSVQPKSLVRLSLEAVICFKEMLANSWNCLPKHLLHSVNQRFGSNNTSGS

>Q8N9W6

MQTDSLSPSPNPVSPVPLNNPTSAPRYGTVIPNRIFVGGIDFKTNESDLRKFFSQYGSVKEVKIVNDRAGVSKGYGFVTFETQEDAQKILQEAEKLNYKDKKLNIGPAIRKQQVGIPRSSIMPAAGTMYLTTSTGYPYTYHNGVAYFHTPEVTSVPPPWPSRSVCSSPVMVAQPIYQQPAYHYQATTQYLPGQWQWSVPQPSASSAPFLYLQPSEVIYQPVEIAQDGGCVPPPLSLMETSVPEPYSDHGVQATYHQVYAPSAITMPAPVMQPEPIKTVWSIHY

712 P13349 NP_002070.1

>P13349

MDVMDGCQFSPSEYFYDGSCIPSPEGEFGDEFVPRVAAFGAHKAELQGSDEDEHVRAPTGHHQAGHCLMWACKACKRKSTTMDRRKAATMRERRRLKKVNQAFETLKRCTTTNPNQRLPKVEILRNAIRYIESLQELLREQVENYYSLPGQSCSEPTSPTSNCSDGMPECNSPVWSRKSSTFDSIYCPDVSNVYATDKNSLSSLDCLSNIVDRITSSEQPGLPLQDLASLSPVASTDSQPATPGASSSRLIYHVL

>NP_002070.1

MAPPSVFAEVPQAQPVLVFKLTADFREDPDPRKVNLGVGAYRTDDCHPWVLPVVKKVEQKIANDNSLNHEYLPILGLAEFRSCASRLALGDDSPALKEKRVGGVQSLGGTGALRIGADFLARWYNGTNNKNTPVYVSSPTWENHNAVFSAAGFKDIRSYRYWDAEKRGLDLQGFLNDLENAPEFSIVVLHACAHNPTGIDPTPEQWKQIASVMKHRFLFPFFDSAYQGFASGNLERDAWAIRYFVSEGFEFFCAQSFSKNFGLYNERVGNLTVVGKEPESILQVLSQMEKIVRITWSNPPAQGARIVASTLSNPELFEEWTGNVKTMADRILTMRSELRARLEALKTPGTWNHITDQIGMFSFTGLNPKQVEYLVNEKHIYLLPSGRINVSGLTTKNLDYVATSIHEAVTKIQ

713 NP_003027.1 NP_001079.1

>NP_003027.1

MEAAAGGRGCFQPHPGLQKTLEQFHLSSMSSLGGPAAFSARWAQEAYKKESAKEAGAAAVPAPVPAATEPPPVLHLPAIQPPPPVLPGPFFMPSDRSTERCETVLEGETISCFVVGGEKRLCLPQILNSVLRDFSLQQINAVCDELHIYCSRCTADQLEILKVMGILPFSAPSCGLITKTDAERLCNALLYGGAYPPPCKKELAASLALGLELSERSVRVYHECFGKCKGLLVPELYSSPSAACIQCLDCRLMYPPHKFVVHSHKALENRTCHWGFDSANWRAYILLSQDYTGKEEQARLGRCLDDVKEKFDYGNKYKRRVPRVSSEPPASIRPKTDDTSSQSPAPSEKDKPSSWLRTLAGSSNKSLGCVHPRQRLSAFRPWSPAVSASEKELSPHLPALIRDSFYSYKSFETAVAPNVALAPPAQQKVVSSPPCAAAVSRAPEPLATCTQPRKRKLTVDTPGAPETLAPVAAPEEDKDSEAEVEVESREEFTSSLSSLSSPSFTSSSSAKDLGSPGARALPSAVPDAAAPADAPSGLEAELEHLRQALEGGLDTKEAKEKFLHEVVKMRVKQEEKLSAALQAKRSLHQELEFLRVAKKEKLREATEAKRNLRKEIERLRAENEKKMKEANESRLRLKRELEQARQARVCDKGCEAGRLRAKYSAQIEDLQVKLQHAEADREQLRADLLREREAREHLEKVVKELQEQLWPRARPEAAGSEGAAELEP

>NP_001079.1

MSTQSTHPLKPEAPRLPPGIPESPSCQRRHTLPASEFRCLTPEDAVSAFEIEREAFISVLGVCPLYLDEIRHFLTLCPELSLGWFEEGCLVAFIIGSLWDKERLMQESLTLHRSGGHIAHLHVLAVHRAFRQQGRGPILLWRYLHHLGSQPAVRRAALMCEDALVPFYERFSFHAVGPCAITVGSLTFMELHCSLRGHPFLRRNSGC

714 NP_002403.1 Q38SD2

>NP_002403.1

MDKDCEMKRTTLDSPLGKLELSGCEQGLHEIKLLGKGTSAADAVEVPAPAAVLGGPEPLMQCTAWLNAYFHQPEAIEEFPVPALHHPVFQQESFTRQVLWKLLKVVKFGEVISYQQLAALAGNPKAARAVGGAMRGNPVPILIPCHRVVCSSGAVGNYSGGLAVKEWLLAHEGHRLGKPGLGGSSGLAGAWLKGAGATSGSPPAGRN

>Q38SD2

MSQRPPSMYWCVGPEESAVCPERAMETLNGAGDTGGKPSTRGGDPAARSRRTEGIRAAYRRGDRGGARDLLEEACDQCASQLEKGQLLSIPAAYGDLEMVRYLLSKRLVELPTEPTDDNPAVVAAYFGHTAVVQNTLPTEPTDDNPAVVAAYFGHTAVVQELLESLPGPCSPQRLLNWMLALACQRGHLGVVKLLVLTHGADPESYAVRKNEFPVIVRLPLYAAIKSGNEDIAIFLLRHGAYFCSYILLDSPDPSKHLLRKYFIEASPLPSSYPGKTALRVKWSHLRLPWVDLDWLIDISCQITELDLSANCLATLPSVIPWGLINLRKLNLSDNHLGELPGVQSSDEIICSRLLEIDISSNKLSHLPPGFLHLSKLQKLTASKNCLEKLFEEENATNWIGLRKLQELDISDNKLTELPALFLHSFKSLNSLNVSRNNLKVFPDPWACPLKCCKASRNALECLPDKMAVFWKNHLKDVDFSENALKEVPLGLFQLDALMFLRLQGNQLAALPPQEKWTCRQLKTLDLSRNQLGKNEDGLKTKRIAFFTTRGRQRSGTEAASVLEFPAFLSESLEVLCLNDNHLDTVPPSVCLLKSLSELYLGNNPGLRELPPELGQLGNLWQLDTEDLTISNVPAEIQKEGPKAMLSYLRAQLRKAEKCKLMKMIIVGPPRQGKSTLLEILQTGRAPQVVHGEATIRTTKWELQRPAGSRAKVESVEFNVWDIGGPASMATVNQCFFTDKALYVVVWNLALGEEAVANLQFWLLNIEAKAPNAVVLVVGTHLDLIEAKFRVERIATLRAYVLALCRSPSGSRATGFPDITFKHLHEISCKSLEGQEGLRQLIFHVTCSMKDVGSTIGCQRLAGRLIPRSYLSLQEAVLAEQQRRSRDDDVQYLTDRQLEQLVEQTPDNDIKDYEDLQSAISFLIETGTLLHFPDTSHGLRNLYFLDPIWLSECLQRIFNIKGSRSVAKNGVIRAEDLRMLLVGTGFTQQTEEQYFQFLAKFEIALPVANDSYLLPHLLPSKPGLDTHGMRHPTANTIQRVFKMSFVPVGFWQRFIARMLISLAEMDLQLFENKKNTKSRNRKVTIYSFTGNQRNRCSTFRVKRNQTIYWQEGLLVTFDGGYLSVESSDVNWKKKKSGGMKIVCQSEVRDFSAMAFITDHVNSLIDQWFPALTATESDGTPLMEQYVPCPVCETAWAQHTDPSEKSEDVQYFDMEDCVLTAIERDFISCPRHPDLPVPLQELVPELFMTDFPARLFLENSKLEHSEDEGSVLGQGGSGTVIYRARYQGQPVAVKRFHIKKFKNFANVPADTMLRHLRATDAMKNFSEFRQEASMLHALQHPCIVALIGISIHPLCFALELAPLSSLNTVLSENARDSSFIPLGHMLTQKIAYQIASGLAYLHKKNIIFCDLKSDNILVWSLDVKEHINIKLSDYGISRQSFHEGALGVEGTPGYQAPEIRPRIVYDEKVDMFSYGMVLYELLSGQRPALGHHQLQIAKKLSKGIRPVLGQPEEVQFRRLQALMMECWDTKPEKRPLALSVVSQMKDPTFATFMYELCCGKQTAFFSSQGQEYTVVFWDGKEESRNYTVVNTEKGLMEVQRMCCPGMKVSCQLQVQRSLWTATEDQKIYIYTLKGMCPLNTPQQALDTPAVVTCFLAVPVIKKNSYLVLAGLADGLVAVFPVVRGTPKDSCSYLCSHTANRSKFSIADEDARQNPYPVKAMEVVNSGSEVWYSNGPGLLVIDCASLEICRRLEPYMAPSMVTSVVCSSEGRGEEVVWCLDDKANSLVMYHSTTYQLCARYFCGVPSPLRDMFPVRPLDTEPPAASHTANPKVPEGDSIADVSIMYSEELGTQILIHQESLTDYCSMSSYSSSPPRQAARSPSSLPSSPASSSSVPFSTDCEDSDMLHTPGAASDRSEHDLTPMDGETFSQHLQAVKILAVRDLIWVPRRGGDVIVIGLEKDSGAQRGRVIAVLKARELTPHGVLVDAAVVAKDTVVCTFENENTEWCLAVWRGWGAREFDIFYQSYEELGRLEACTRKRR

715 NP_000116.2 NP_001753.1

>NP_000116.2

MTMTLHTKASGMALLHQIQGNELEPLNRPQLKIPLERPLGEVYLDSSKPAVYNYPEGAAYEFNAAAAANAQVYGQTGLPYGPGSEAAAFGSNGLGGFPPLNSVSPSPLMLLHPPPQLSPFLQPHGQQVPYYLENEPSGYTVREAGPPAFYRPNSDNRRQGGRERLASTNDKGSMAMESAKETRYCAVCNDYASGYHYGVWSCEGCKAFFKRSIQGHNDYMCPATNQCTIDKNRRKSCQACRLRKCYEVGMMKGGIRKDRRGGRMLKHKRQRDDGEGRGEVGSAGDMRAANLWPSPLMIKRSKKNSLALSLTADQMVSALLDAEPPILYSEYDPTRPFSEASMMGLLTNLADRELVHMINWAKRVPGFVDLTLHDQVHLLECAWLEILMIGLVWRSMEHPGKLLFAPNLLLDRNQGKCVEGMVEIFDMLLATSSRFRMMNLQGEEFVCLKSIILLNSGVYTFLSSTLKSLEEKDHIHRVLDKITDTLIHLMAKAGLTLQQQHQRLAQLLLILSHIRHMSNKGMEHLYSMKCKNVVPLYDLLLEMLDAHRLHAPTSRGGASVEETDQSHLATAGSTSSHSLQKYYITGEAEGFPATV

>NP_001753.1

MAAVKTLNPKAEVARAQAALAVNISAARGLQDVLRTNLGPKGTMKMLVSGAGDIKLTKDGNVLLHEMQIQHPTASLIAKVATAQDDITGDGTTSNVLIIGELLKQADLYISEGLHPRIITEGFEAAKEKALQFLEEVKVSREMDRETLIDVARTSLRTKVHAELADVLTEAVVDSILAIKKQDEPIDLFMIEIMEMKHKSETDTSLIRGLVLDHGARHPDMKKRVEDAYILTCNVSLEYEKTEVNSGFFYKSAEEREKLVKAERKFIEDRVKKIIELKRKVCGDSDKGFVVINQKGIDPFSLDALSKEGIVALRRAKRRNMERLTLACGGVALNSFDDLSPDCLGHAGLVYEYTLGEEKFTFIEKCNNPRSVTLLIKGPNKHTLTQIKDAVRDGLRAVKNAIDDGCVVPGAGAVEVAMAEALIKHKPSVKGRAQLGVQAFADALLIIPKVLAQNSGFDLQETLVKIQAEHSESGQLVGVDLNTGEPMVAAEVGVWDNYCVKKQLLHSCTVIATNILLVDEIMRAGMSSLKG

716 NP_005997.2 Q9UKF7

>NP_005997.2

MDLTKMGMIQLQNPSHPTGLLCKANQMRLAGTLCDVVIMVDSQEFHAHRTVLACTSKMFEILFHRNSQHYTLDFLSPKTFQQILEYAYTATLQAKAEDLDDLLYAAEILEIEYLEEQCLKMLETIQASDDNDTEATMADGGAEEEEDRKARYLKNIFISKHSSEESGYASVAGQSLPGPMVDQSPSVSTSFGLSAMSPTKAAVDSLMTIGQSLLQGTLQPPAGPEEPTLAGGGRHPGVAEVKTEMMQVDEVPSQDSPGAAESSISGGMGDKVEERGKEGPGTPTRSSVITSARELHYGREESAEQVPPPAEAGQAPTGRPEHPAPPPEKHLGIYSVLPNHKADAVLSMPSSVTSGLHVQPALAVSMDFSTYGGLLPQGFIQRELFSKLGELAVGMKSESRTIGEQCSVCGVELPDNEAVEQHRKLHSGMKTYGCELCGKRFLDSLRLRMHLLAHSAGAKAFVCDQCGAQFSKEDALETHRQTHTGTDMAVFCLLCGKRFQAQSALQQHMEVHAGVRSYICSECNRTFPSHTALKRHLRSHTGDHPYECEFCGSCFRDESTLKSHKRIHTGEKPYECNGCGKKFSLKHQLETHYRVHTGEKPFECKLCHQRSRDYSAMIKHLRTHNGASPYQCTICTEYCPSLSSMQKHMKGHKPEEIPPDWRIEKTYLYLCYV

>Q9UKF7

MLLKEYRICMPLTVDEYKIGQLYMISKHSHEQSDRGEGVEVVQNEPFEDPHHGNGQFTEKRVYLNSKLPSWARAVVPKIFYVTEKAWNYYPYTITEYTCSFLPKFSIHIETKYEDNKGSNDTIFDNEAKDVEREVCFIDIACDEIPERYYKESEDPKHFKSEKTGRGQLREGWRDSHQPIMCSYKLVTVKFEVWGLQTRVEQFVHKVVRDILLIGHRQAFAWVDEWYDMTMDDVREFERATQEATNKKIGIFPPAISISSIPLLPSSVRSAPSSAPSTPLSTDAPEFLSVPKDRPRKKSAPETLTLPDPEKKATLNLPGMHSSDKPCRPKSE

717 NP_067015.2 NP_003121.1

>NP_067015.2

MNDFDECGQSAASMYLPGCAYYVAPSDFASKPSFLSQPSSCQMTFPYSSNLAPHVQPVREVAFRDYGLERAKWPYRGGGGGGSAGGGSSGGGPGGGGGGAGGYAPYYAAAAAAAAAAAAAEEAAMQRELLPPAGRRPDVLFKAPEPVCAAPGPPHGPAGAASNFYSAVGRNGILPQGFDQFYEAAPGPPFAGPQPPPPPAPPQPEGAADKGDPRTGAGGGGGSPCTKATPGSEPKGAAEGSGGDGEGPPGEAGAEKSSSAVAPQRSRKKRCPYTKYQIRELEREFFFNVYINKEKRLQLSRMLNLTDRQVKIWFQNRRMKEKKLNRDRLQYFTGNPLF

>NP_003121.1

MAYPGHPGAGGGYYPGGYGGAPGGPAFPGQTQDPLYGYFAAVAGQDGQIDADELQRCLTQSGIAGGYKPFNLETCRLMVSMLDRDMSGTMGFNEFKELWAVLNGWRQHFISFDTDRSGTVDPQELQKALTTMGFRLSPQAVNSIAKRYSTNGKITFDDYIACCVKLRALTDSFRRRDTAQQGVVNFPYDDFIQCVMSV

718 NP_055792.1 NP_001166.3

>NP_055792.1

MWRRKHPRTSGGTRGVLSGNRGVEYGSGRGHLGTFEGRWRKLPKMPEAVGTDPSTSRKMAELEEVTLDGKPLQALRVTDLKAALEQRGLAKSGQKSALVKRLKGALMLENLQKHSTPHAAFQPNSQIGEEMSQNSFIKQYLEKQQELLRQRLEREAREAAELEEASAESEDEMIHPEGVASLLPPDFQSSLERPELELSRHSPRKSSSISEEKGDSDDEKPRKGERRSSRVRQARAAKLSEGSQPAEEEEDQETPSRNLRVRADRNLKTEEEEEEEEEEEEDDEEEEGDDEGQKSREAPILKEFKEEGEEIPRVKPEEMMDERPKTRSQEQEVLERGGRFTRSQEEARKSHLARQQQEKEMKTTSPLEEEEREIKSSQGLKEKSKSPSPPRLTEDRKKASLVALPEQTASEEETPPPLLTKEASSPPPHPQLHSEEEIEPMEGPAPPVLIQLSPPNTDADTRELLVSQHTVQLVGGLSPLSSPSDTKAESPAEKVPEESVLPLVQKSTLADYSAQKDLEPESDRSAQPLPLKIEELALAKGITEECLKQPSLEQKEGRRASHTLLPSHRLKQSADSSSSRSSSSSSSSSRSRSRSPDSSGSRSHSPLRSKQRDVAQARTHANPRGRPKMGSRSTSESRSRSRSRSRSASSNSRKSLSPGVSRDSSTSYTETKDPSSGQEVATPPVPQLQVCEPKERTSTSSSSVQARRLSQPESAEKHVTQRLQPERGSPKKCEAEEAEPPAATQPQTSETQTSHLPESERIHHTVEEKEEVTMDTSENRPENDVPEPPMPIADQVSNDDRPEGSVEDEEKKESSLPKSFKRKISVVSATKGVPAGNSDTEGGQPGRKRRWGASTATTQKKPSISITTESLKSLIPDIKPLAGQEAVVDLHADDSRISEDETERNGDDGTHDKGLKICRTVTQVVPAEGQENGQREEEEEEKEPEAEPPVPPQVSVEVALPPPAEHEVKKVTLGDTLTRRSISQQKSGVSITIDDPVRTAQVPSPPRGKISNIVHISNLVRPFTLGQLKELLGRTGTLVEEAFWIDKIKSHCFVTYSTVEEAVATRTALHGVKWPQSNPKFLCADYAEQDELDYHRGLLVDRPSETKTEEQGIPRPLHPPPPPPVQPPQHPRAEQREQERAVREQWAEREREMERRERTRSEREWDRDKVREGPRSRSRSRDRRRKERAKSKEKKSEKKEKAQEEPPAKLLDDLFRKTKAAPCIYWLPLTDSQIVQKEAERAERAKEREKRRKEQEEEEQKEREKEAERERNRQLEREKRREHSRERDRERERERERDRGDRDRDRERDRERGRERDRRDTKRHSRSRSRSTPVRDRGGRR

>NP_001166.3

MTEKAPEPHVEEDDDDELDSKLNYKPPPQKSLKELQEMDKDDESLIKYKKTLLGDGPVVTDPKAPNVVVTRLTLVCESAPGPITMDLTGDLEALKKETIVLKEGSEYRVKIHFKVNRDIVSGLKYVQHTYRTGVKVDKATFMVGSYGPRPEEYEFLTPVEEAPKGMLARGTYHNKSFFTDDDKQDHLSWEWNLSIKKEWTE

719 NP_006590.1 NP_056053.1

>NP_006590.1

MPSDFISLLSADLDLESPKSLYSRESVYDLLPKELQLPPSRETSVASMSQTSGGEAGSPPPAVVAADASSAPSSSSMGGACSSFTTSSSPTIYSTSVTDSKAMQVESCSSAVGVSNRGVSEKQLTSNTVQQHPSTPKRHTVLYISPPPEDLLDNSRMSCQDEGCGLESEQSCSMWMEDSPSNFSNMSTSSYNDNTEVPRKSRKRNPKQRPGVKRRDCEESNMDIFDADSAKAPHYVLSQLTTDNKGNSKAGNGTLENQKGTGVKKSPMLCGQYPVKSEGKELKIVVQPETQHRARYLTEGSRGSVKDRTQQGFPTVKLEGHNEPVVLQVFVGNDSGRVKPHGFYQACRVTGRNTTPCKEVDIEGTTVIEVGLDPSNNMTLAVDCVGILKLRNADVEARIGIAGSKKKSTRARLVFRVNIMRKDGSTLTLQTPSSPILCTQPAGVPEILKKSLHSCSVKGEEEVFLIGKNFLKGTKVIFQENVSDENSWKSEAEIDMELFHQNHLIVKVPPYHDQHITLPVSVGIYVVTNAGRSHDVQPFTYTPDPAAAGALNVNVKKEISSPARPCSFEEAMKAMKTTGCNLDKVNIIPNALMTPLIPSSMIKSEDVTPMEVTAEKRSSTIFKTTKSVGSTQQTLENISNIAGNGSFSSPSSSHLPSENEKQQQIQPKAYNPETLTTIQTQDISQPGTFPAVSASSQLPNSDALLQQATQFQTRETQSREILQSDGTVVNLSQLTEASQQQQQSPLQEQAQTLQQQISSNIFPSPNSVSQLQNTIQQLQAGSFTGSTASGSSGSVDLVQQVLEAQQQLSSVLFSAPDGNENVQEQLSADIFQQVSQIQSGVSPGMFSSTEPTVHTRPDNLLPGRAESVHPQSENTLSNQQQQQQQQQQVMESSAAMVMEMQQSICQAAAQIQSELFPSTASANGNLQQSPVYQQTSHMMSALSTNEDMQMQCELFSSPPAVSGNETSTTTTQQVATPGTTMFQTSSSGDGEETGTQAKQIQNSVFQTMVQMQHSGDNQPQVNLFSSTKSMMSVQNSGTQQQGNGLFQQGNEMMSLQSGNFLQQSSHSQAQLFHPQNPIADAQNLSQETQGSLFHSPNPIVHSQTSTTSSEQMQPPMFHSQSTIAVLQGSSVPQDQQSTNIFLSQSPMNNLQTNTVAQEAFFAAPNSISPLQSTSNSEQQAAFQQQAPISHIQTPMLSQEQAQPPQQGLFQPQVALGSLPPNPMPQSQQGTMFQSQHSIVAMQSNSPSQEQQQQQQQQQQQQQQQQQSILFSNQNTMATMASPKQPPPNMIFNPNQNPMANQEQQNQSIFHQQSNMAPMNQEQQPMQFQSQSTVSSLQNPGPTQSESSQTPLFHSSPQIQLVQGSPSSQEQQVTLFLSPASMSALQTSINQQDMQQSPLYSPQNNMPGIQGATSSPQPQATLFHNTAGGTMNQLQNSPGSSQQTSGMFLFGIQNNCSQLLTSGPATLPDQLMAISQPGQPQNEGQPPVTTLLSQQMPENSPLASSINTNQNIEKIDLLVSLQNQGNNLTGSF

>NP_056053.1

MPRPELPLPEGWEEARDFDGKVYYIDHTNRTTSWIDPRDRYTKPLTFADCISDELPLGWEEAYDPQVGDYFIDHNTKTTQIEDPRVQWRREQEHMLKDYLVVAQEALSAQKEIYQVKQQRLELAQQEYQQLHAVWEHKLGSQVSLVSGSSSSSKYDPEILKAEIATAKSRVNKLKREMVHLQHELQFKERGFQTLKKIDKKMSDAQGSYKLDEAQAVLRETKAIKKAITCGEKEKQDLIKSLAMLKDGFRTDRGSHSDLWSSSSSLESSSFPLPKQYLDVSSQTDISGSFGINSNNQLAEKVRLRLRYEEAKRRIANLKIQLAKLDSEAWPGVLDSERDRLILINEKEELLKEMRFISPRKWTQGEVEQLEMARKRLEKDLQAARDTQSKALTERLKLNSKRNQLVRELEEATRQVATLHSQLKSLSSSMQSLSSGSSPGSLTSSRGSLVASSLDSSTSASFTDLYYDPFEQLDSELQSKVEFLLLEGATGFRPSGCITTIHEDEVAKTQKAEGGGRLQALRSLSGTPKSMTSLSPRSSLSSPSPPCSPLMADPLLAGDAFLNSLEFEDPELSATLCELSLGNSAQERYRLEEPGTEGKQLGQAVNTAQGCGLKVACVSAAVSDESVAGDSGVYEASVQRLGASEAAAFDSDESEAVGATRIQIALKYDEKNKQFAILIIQLSNLSALLQQQDQKVNIRVAVLPCSESTTCLFRTRPLDASDTLVFNEVFWVSMSYPALHQKTLRVDVCTTDRSHLEECLGGAQISLAEVCRSGERSTRWYNLLSYKYLKKQSRELKPVGVMAPASGPASTDAVSALLEQTAVELEKRQEGRSSTQTLEDSWRYEETSENEAVAEEEEEEVEEEEGEEDVFTEKASPDMDGYPALKVDKETNTETPAPSPTVVRPKDRRVGTPSQGPFLRGSTIIRSKTFSPGPQSQYVCRLNRSDSDSSTLSKKPPFVRNSLERRSVRMKRPSSVKSLRSERLIRTSLDLELDLQATRTWHSQLTQEISVLKELKEQLEQAKSHGEKELPQWLREDERFRLLLRMLEKRQMDRAEHKGELQTDKMMRAAAKDVHRLRGQSCKEPPEVQSFREKMAFFTRPRMNIPALSADDV

720 Q8IZT9 NP_000658.1

>Q8IZT9

MAAKDQLEVQVMAAQEMELAGKDPVSHEHEERKPVTETKEGDVTDEHGERGSFAETDEHTGVDTKELEDIAADIKEHLAAKRKRIEKIAKACSEIKNRIKNVLRTTQLKRQKRDYRISLKLPNVLEEFITDEQKDEEGDGEKEEQIKIFQEQQKRWQQDGKGTERD

>NP_000658.1

MSTAGKVIKCKAAVLWELKKPFSIEEVEVAPPKAHEVRIKMVAVGICGTDDHVVSGTMVTPLPVILGHEAAGIVESVGEGVTTVKPGDKVIPLAIPQCGKCRICKNPESNYCLKNDVSNPQGTLQDGTSRFTCRRKPIHHFLGISTFSQYTVVDENAVAKIDAASPLEKVCLIGCGFSTGYGSAVNVAKVTPGSTCAVFGLGGVGLSAIMGCKAAGAARIIAVDINKDKFAKAKELGATECINPQDYKKPIQEVLKEMTDGGVDFSFEVIGRLDTMMASLLCCHEACGTSVIVGVPPDSQNLSMNPMLLLTGRTWKGAILGGFKSKECVPKLVADFMAKKFSLDALITHVLPFEKINEGFDLLHSGKSIRTILMF

721 Q9UK76 Q9NRV9

>Q9UK76

MTTTTTFKGVDPNSRNSSRVLRPPGGGSNFSLGFDEPTEQPVRKNKMASNIFGTPEENQASWAKSAGAKSSGGREDLESSGLQRRNSSEASSGDFLDLKGEGDIHENVDTDLPGSLGQSEEKPVPAAPVPSPVAPAPVPSRRNPPGGKSSLVLG

>Q9NRV9

MLGMIKNSLFGSVETWPWQVLSKGDKEEVAYEERACEGGKFATVEVTDKPVDEALREAMPKVAKYAGGTNDKGIGMGMTVPISFAVFPNEDGSLQKKLKVWFRIPNQFQSDPPAPSDKSVKIEEREGITVYSMQFGGYAKEADYVAQATRLRAALEGTATYRGDIYFCTGYDPPMKPYGRRNEIWLLKT

722 NP_003191.1 P08729

>NP_003191.1

MNQPQRMAPVGTDKELSDLLDFSMMFPLPVTNGKGRPASLAGAQFGGSGLEDRPSSGSWGSGDQSSSSFDPSRTFSEGTHFTESHSSLSSSTFLGPGLGGKSGERGAYASFGRDAGVGGLTQAGFLSGELALNSPGPLSPSGMKGTSQYYPSYSGSSRRRAADGSLDTQPKKVRKVPPGLPSSVYPPSSGEDYGRDATAYPSAKTPSSTYPAPFYVADGSLHPSAELWSPPGQAGFGPMLGGGSSPLPLPPGSGPVGSSGSSSTFGGLHQHERMGYQLHGAEVNGGLPSASSFSSAPGATYGGVSSHTPPVSGADSLLGSRGTTAGSSGDALGKALASIYSPDHSSNNFSSSPSTPVGSPQGLAGTSQWPRAGAPGALSPSYDGGLHGLQSKIEDHLDEAIHVLRSHAVGTAGDMHTLLPGHGALASGFTGPMSLGGRHAGLVGGSHPEDGLAGSTSLMHNHAALPSQPGTLPDLSRPPDSYSGLGRAGATAAASEIKREEKEDEENTSAADHSEEEKKELKAPRARTSPDEDEDDLLPPEQKAEREKERRVANNARERLRVRDINEAFKELGRMCQLHLNSEKPQTKLLILHQAVSVILNLEQQVRERNLNPKAACLKRREEEKVSGVVGDPQMVLSAPHPGLSEAHNPAGHM

>P08729

MSIHFSSPVFTSRSAAFSGRGAQVRLSSARPGGLGSSSLYGLGASRPRVAVRSAYGGPVGAGIREVTINQSLLAPLRLDADPSLQRVRQEESEQIKTLNNKFASFIDKVRFLEQQNKLLETKWTLLQEQKSAKSSRLPDIFEAQIAGLRGQLEALQVDGGRLEQGLRTMQDVVEDFKNKYEDEINRRTAAENEFVVLKKDVDAAYMSKVELEAKVDALNDEINFLRTLNETELTELQSQISDTSVVLSMDNSRSLDLDGIIAEVKAQYEEMAKCSRAEAEAWYQTKFETLQAQAGKHGDDLRNTRNEISEMNRAIQRLQAEIDNIKNQRAKLEAAIAEAEERGELALKDARAKQEELEAALQRAKQDMARQLREYQELMSVKLALDIEIATYRKLLEGEESRLAGDGVGAVNISVMNSTGGSSSGGGIGLTLGGTMGSNALSFSSSAGPGLLKAYSIRTASASRRSARD

723 Q9GZZ0 NP_000680.2

>Q9GZZ0

MSSYLEYVSCSSSGGVGGDVLSLAPKFCRSDARPVALQPAFPLGNGDGAFVSCLPLAAARPSPSPPAAPARPSVPPPAAPQYAQCTLEGAYEPGAAPAAAAGGADYGFLGSGPAYDFPGVLGRAADDGGSHVHYATSAVFSGGGSFLLSGQVDYAAFGEPGPFPACLKASADGHPGAFQTASPAPGTYPKSVSPASGLPAAFSTFEWMKVKRNASKKGKLAEYGAASPSSAIRTNFSTKQLTELEKEFHFNKYLTRARRIEIANCLHLNDTQVKIWFQNRRMKQKKREREGLLATAIPVAPLQLPLSGTTPTKFIKNPGSPSQSQEPS

>NP_000680.2

MSSSGTPDLPVLLTDLKIQYTKIFINNEWHDSVSGKKFPVFNPATEEELCQVEEGDKEDVDKAVKAARQAFQIGSPWRTMDASERGRLLYKLADLIERDRLLLATMESMNGGKLYSNAYLNDLAGCIKTLRYCAGWADKIQGRTIPIDGNFFTYTRHEPIGVCGQIIPWNFPLVMLIWKIGPALSCGNTVVVKPAEQTPLTALHVASLIKEAGFPPGVVNIVPGYGPTAGAAISSHMDIDKVAFTGSTEVGKLIKEAAGKSNLKRVTLELGGKSPCIVLADADLDNAVEFAHHGVFYHQGQCCIAASRIFVEESIYDEFVRRSVERAKKYILGNPLTPGVTQGPQIDKEQYDKILDLIESGKKEGAKLECGGGPWGNKGYFVQPTVFSNVTDEMRIAKEEIFGPVQQIMKFKSLDDVIKRANNTFYGLSAGVFTKDIDKAITISSALQAGTVWVNCYGVVSAQCPFGGFKMSGNGRELGEYGFHEYTEVKTVTVKISQKNS

724 NP_004489.1 NP_150634.1

>NP_004489.1

MNAQLTMEAIGELHGVSHEPVPAPADLLGGSPHARSSVAHRGSHLPPAHPRSMGMASLLDGGSGGGDYHHHHRAPEHSLAGPLHPTMTMACETPPGMSMPTTYTTLTPLQPLPPISTVSDKFPHHHHHHHHHHHPHHHQRLAGNVSGSFTLMRDERGLASMNNLYTPYHKDVAGMGQSLSPLSSSGLGSIHNSQQGLPHYAHPGAAMPTDKMLTPNGFEAHHPAMLGRHGEQHLTPTSAGMVPINGLPPHHPHAHLNAQGHGQLLGTAREPNPSVTGAQVSNGSNSGQMEEINTKEVAQRITTELKRYSIPQAIFAQRVLCRSQGTLSDLLRNPKPWSKLKSGRETFRRMWKWLQEPEFQRMSALRLAACKRKEQEHGKDRGNTPKKPRLVFTDVQRRTLHAIFKENKRPSKELQITISQQLGLELSTVSNFFMNARRRSLDKWQDEGSSNSGNSSSSSSTCTKA

>NP_150634.1

MADKVLKEKRKLFIRSMGEGTINGLLDELLQTRVLNKEEMEKVKRENATVMDKTRALIDSVIPKGAQACQICITYICEEDSYLAGTLGLSADQTSGNYLNMQDSQGVLSSFPAPQAVQDNPAMPTSSGSEGNVKLCSLEEAQRIWKQKSAEIYPIMDKSSRTRLALIICNEEFDSIPRRTGAEVDITGMTMLLQNLGYSVDVKKNLTASDMTTELEAFAHRPEHKTSDSTFLVFMSHGIREGICGKKHSEQVPDILQLNAIFNMLNTKNCPSLKDKPKVIIIQACRGDSPGVVWFKDSVGVSGNLSLPTTEEFEDDAIKKAHIEKDFIAFCSSTPDNVSWRHPTMGSVFIGRLIEHMQEYACSCDVEEIFRKVRFSFEQPDGRAQMPTTERVTLTRCFYLFPGH

725 NP_003916.1 NP_005116.1

>NP_003916.1

MGTTGLESLSLGDRGAAPTVTSSERLVPDPPNDLRKEDVAMELERVGEDEEQMMIKRSSECNPLLQEPIASAQFGATAGTECRKSVPCGWERVVKQRLFGKTAGRFDVYFISPQGLKFRSKSSLANYLHKNGETSLKPEDFDFTVLSKRGIKSRYKDCSMAALTSHLQNQSNNSNWNLRTRSKCKKDVFMPPSSSSELQESRGLSNFTSTHLLLKEDEGVDDVNFRKVRKPKGKVTILKGIPIKKTKKGCRKSCSGFVQSDSKRESVCNKADAESEPVAQKSQLDRTVCISDAGACGETLSVTSEENSLVKKKERSLSSGSNFCSEQKTSGIINKFCSAKDSEHNEKYEDTFLESEEIGTKVEVVERKEHLHTDILKRGSEMDNNCSPTRKDFTGEKIFQEDTIPRTQIERRKTSLYFSSKYNKEALSPPRRKAFKKWTPPRSPFNLVQETLFHDPWKLLIATIFLNRTSGKMAIPVLWKFLEKYPSAEVARTADWRDVSELLKPLGLYDLRAKTIVKFSDEYLTKQWKYPIELHGIGKYGNDSYRIFCVNEWKQVHPEDHKLNKYHDWLWENHEKLSLS

>NP_005116.1

MASDSGNQGTLCTLEFAVQMTCQSCVDAVRKSLQGVAGVQDVEVHLEDQMVLVHTTLPSQEVQALLEGTGRQAVLKGMGSGQLQNLGAAVAILGGPGTVQGVVRFLQLTPERCLIEGTIDGLEPGLHGLHVHQYGDLTNNCNSCGNHFNPDGASHGGPQDSDRHRGDLGNVRADADGRAIFRMEDEQLKVWDVIGRSLIIDEGEDDLGRGGHPLSKITGNSGERLACGIIARSAGLFQNPKQICSCDGLTIWEERGRPIAGKGRKESAQPPAHL

726 NP_002154.1 P40394

>NP_002154.1

MCDRNGGRRLRQWLIEQIDSSMYPGLIWENEEKSMFRIPWKHAGKQDYNQEVDASIFKAWAVFKGKFKEGDKAEPATWKTRLRCALNKSPDFEEVTDRSQLDISEPYKVYRIVPEEEQKCKLGVATAGCVNEVTEMECGRSEIDELIKEPSVDDYMGMIKRSPSPPEACRSQLLPDWWAQQPSTGVPLVTGYTTYDAHHSAFSQMVISFYYGGKLVGQATTTCPEGCRLSLSQPGLPGTKLYGPEGLELVRFPPADAIPSERQRQVTRKLFGHLERGVLLHSSRQGVFVKRLCQGRVFCSGNAVVCKGRPNKLERDEVVQVFDTSQFFRELQQFYNSQGRLPDGRVVLCFGEEFPDMAPLRSKLILVQIEQLYVRQLAEEAGKSCGAGSVMQAPEEPPPDQVFRMFPDICASHQRSFFRENQQITV

>P40394

MGTAGKVIKCKAAVLWEQKQPFSIEEIEVAPPKTKEVRIKILATGICRTDDHVIKGTMVSKFPVIVGHEATGIVESIGEGVTTVKPGDKVIPLFLPQCRECNACRNPDGNLCIRSDITGRGVLADGTTRFTCKGKPVHHFMNTSTFTEYTVVDESSVAKIDDAAPPEKVCLIGCGFSTGYGAAVKTGKVKPGSTCVVFGLGGVGLSVIMGCKSAGASRIIGIDLNKDKFEKAMAVGATECISPKDSTKPISEVLSEMTGNNVGYTFEVIGHLETMIDALASCHMNYGTSVVVGVPPSAKMLTYDPMLLFTGRTWKGCVFGGLKSRDDVPKLVTEFLAKKFDLDQLITHVLPFKKISEGFELLNSGQSIRTVLTF

727 NP_005310.1 NP_005557.1

>NP_005310.1

MSETAPAAPAAAPPAEKAPVKKKAAKKAGGTPRKASGPPVSELITKAVAASKERSGVSLAALKKALAAAGYDVEKNNSRIKLGLKSLVSKGTLVQTKGTGASGSFKLNKKAASGEAKPKVKKAGGTKPKKPVGAAKKPKKAAGGATPKKSAKKTPKKAKKPAAATVTKKVAKSPKKAKVAKPKKAAKSAAKAVKPKAAKPKVVKPKKAAPKKK

>NP_005557.1

MATLKDQLIYNLLKEEQTPQNKITVVGVGAVGMACAISILMKDLADELALVDVIEDKLKGEMMDLQHGSLFLRTPKIVSGKDYNVTANSKLVIITAGARQQEGESRLNLVQRNVNIFKFIIPNVVKYSPNCKLLIVSNPVDILTYVAWKISGFPKNRVIGSGCNLDSARFRYLMGERLGVHPLSCHGWVLGEHGDSSVPVWSGMNVAGVSLKTLHPDLGTDKDKEQWKEVHKQVVESAYEVIKLKGYTSWAIGLSVADLAESIMKNLRRVHPVSTMIKGLYGIKDDVFLSVPCILGQNGISDLVKVTLTSEEEARLKKSADTLWGIQKELQF

728 O00462 Q01064

>O00462

MRLHLLLLLALCGAGTTAAELSYSLRGNWSICNGNGSLELPGAVPGCVHSALFQQGLIQDSYYRFNDLNYRWVSLDNWTYSKEFKIPFEISKWQKVNLILEGVDTVSKILFNEVTIGETDNMFNRYSFDITNVVRDVNSIELRFQSAVLYAAQQSKAHTRYQVPPDCPPLVQKGECHVNFVRKEQCSFSWDWGPSFPTQGIWKDVRIEAYNICHLNYFTFSPIYDKSAQEWNLEIESTFDVVSSKPVGGQVIVAIPKLQTQQTYSIELQPGKRIVELFVNISKNITVETWWPHGHGNQTGYNMTVLFELDGGLNIEKSAKVYFRTVELIEEPIKGSPGLSFYFKINGFPIFLKGSNWIPADSFQDRVTSELLRLLLQSVVDANMNTLRVWGGGIYEQDEFYELCDELGIMVWQDFMFACALYPTDQGFLDSVTAEVAYQIKRLKSHPSIIIWSGNNENEEALMMNWYHISFTDRPIYIKDYVTLYVKNIRELVLAGDKSRPFITSSPTNGAETVAEAWVSQNPNSNYFGDVHFYDYISDCWNWKVFPKARFASEYGYQSWPSFSTLEKVSSTEDWSFNSKFSLHRQHHEGGNKQMLYQAGLHFKLPQSTDPLRTFKDTIYLTQVMQAQCVKTETEFYRRSRSEIVDQQGHTMGALYWQLNDIWQAPSWASLEYGGKWKMLHYFAQNFFAPLLPVGFENENTFYIYGVSDLHSDYSMTLSVRVHTWSSLEPVCSRVTERFVMKGGEAVCLYEEPVSELLRRCGNCTRESCVVSFYLSADHELLSPTNYHFLSSPKEAVGLCKAQITAIISQQGDIFVFDLETSAVAPFVWLDVGSIPGRFSDNGFLMTEKTRTILFYPWEPTSKNELEQSFHVTSLTDIY

>Q01064

MELSPRSPPEMLEESDCPSPLELKSAPSKKMWIKLRSLLRYMVKQLENGEINIEELKKNLEYTASLLEAVYIDETRQILDTEDELQELRSDAVPSEVRDWLASTFTQQARAKGRRAEEKPKFRSIVHAVQAGIFVERMFRRTYTSVGPTYSTAVLNCLKNLDLWCFDVFSLNQAADDHALRTIVFELLTRHNLISRFKIPTVFLMSFLDALETGYGKYKNPYHNQIHAADVTQTVHCFLLRTGMVHCLSEIELLAIIFAAAIHDYEHTGTTNSFHIQTKSECAIVYNDRSVLENHHISSVFRLMQDDEMNIFINLTKDEFVELRALVIEMVLATDMSCHFQQVKTMKTALQQLERIDKPKALSLLLHAADISHPTKQWLVHSRWTKALMEEFFRQGDKEAELGLPFSPLCDRTSTLVAQSQIGFIDFIVEPTFSVLTDVAEKSVQPLADEDSKSKNQPSFQWRQPSLDVEVGDPNPDVVSFRSTWVKRIQENKQKWKERAASGITNQMSIDELSPCEEEAPPSPAEDEHNQNGNLD

729 NP_002031.2 NP_064530.1

>NP_002031.2

MTKREAEELIEIEIDGTEKAECTEESIVEQTYAPAECVSQAIDINEPIGNLKKLLEPRLQCSLDAHEICLQDIQLDPERSLFDQGVKTDGTVQLSVQVISYQGIEPKLNILEIVKPADTVEVVIDPDAHHAESEAHLVEEAQVITLDGTKHITTISDETSEQVTRWAAALEGYRKEQERLGIPYDPIQWSTDQVLHWVVWVMKEFSMTDIDLTTLNISGRELCSLNQEDFFQRVPRGEILWSHLELLRKYVLASQEQQMNEIVTIDQPVQIIPASVQSATPTTIKVINSSAKAAKVQRAPRISGEDRSSPGNRTGNNGQIQLWQFLLELLTDKDARDCISWVGDEGEFKLNQPELVAQKWGQRKNKPTMNYEKLSRALRYYYDGDMICKVQGKRFVYKFVCDLKTLIGYSAAELNRLVTECEQKKLAKMQLHGIAQPVTAVALATASLQTEKDN

>NP_064530.1

MDFNMKKLASDAGIFFTRAVQFTEEKFGQAEKTELDAHFENLLARADSTKNWTEKILRQTEVLLQPNPSARVEEFLYEKLDRKVPSRVTNGELLAQYMADAASELGPTTPYGKTLIKVAEAEKQLGAAERDFIHTASISFLTPLRNFLEGDWKTISKERRLLQNRRLDLDACKARLKKAKAAEAKATTVPDFQETRPRNYILSASASALWNDEVDKAEQELRVAQTEFDRQAEVTRLLLEGISSTHVNHLRCLHEFVKSQTTYYAQCYRHMLDLQKQLGRFPGTFVGTTEPASPPLSSTSPTTAAATMPVVPSVASLAPPGEASLCLEEVAPPASGTRKARVLYDYEAADSSELALLADELITVYSLPGMDPDWLIGERGNKKGKVPVTYLELLS

730 Q8IVW6 NP_004050.3

>Q8IVW6

MEPLQQQQQQQQQQQKQPHLAPLQMDAREKQGQQMREAQFLYAQKLVTQPTLLSATAGRPSGSTPLGPLARVPPTAAVAQVFERGNMNSEPEEEDGGLEDEDGDDEVAEVAEKETQAASKYFHVQKVARQDPRVAPMSNLLPAPGLPPHGQQAKEDHTKDASKASPSVSTAGQPNWNLDEQLKQNGGLAWSDDADGGRGREISRDFAKLYELDGDPERKEFLDDLFVFMQKRGTPINRIPIMAKQILDLYMLYKLVTEKGGLVEIINKKIWREITKGLNLPTSITSAAFTLRTQYMKYLYAYECEKKALSSPAELQAAIDGNRREGRRPSYSSSLFGYSPAAATAAAAAGAPALLSPPKIRFPILGLGSSSGTNTSSPRISPATTLRKGDGAPVTTVPVPNRLAVPVTLASQQAGTRTAALEQLRERLESGEPAEKKASRLSEEEQRLVQQAFQRNFFSMARQLPMKIRINGRAEDRAEASAAALNLTTSSIGSINMSVDIDGTTYAGVLFAQKPVVHLITGSAPQSLGSSASSSSSSHCSPSPTSSRGTPSAEPSTSWSL

>NP_004050.3

MAKQLQARRLDGIDYNPWVEFVKLASEHDVVNLGQGFPDFPPPDFAVEAFQHAVSGDFMLNQYTKTFGYPPLTKILASFFGELLGQEIDPLRNVLVTVGGYGALFTAFQALVDEGDEVIIIEPFFDCYEPMTMMAGGRPVFVSLKPGPIQNGELGSSSNWQLDPMELAGKFTSRTKALVLNTPNNPLGKVFSREELELVASLCQQHDVVCITDEVYQWMVYDGHQHISIASLPGMWERTLTIGSAGKTFSATGWKVGWVLGPDHIMKHLRTVHQNSVFHCPTQSQAAVAESFEREQLLFRQPSSYFVQFPQAMQRCRDHMIRSLQSVGLKPIIPQGSYFLITDISDFKRKMPDLPGAVDEPYDRRFVKWMIKNKGLVAIPVSIFYSVPHQKHFDHYIRFCFVKDEATLQAMDEKLRKWKVEL

731 NP_004489.1 NP_005172.1

>NP_004489.1

MNAQLTMEAIGELHGVSHEPVPAPADLLGGSPHARSSVAHRGSHLPPAHPRSMGMASLLDGGSGGGDYHHHHRAPEHSLAGPLHPTMTMACETPPGMSMPTTYTTLTPLQPLPPISTVSDKFPHHHHHHHHHHHPHHHQRLAGNVSGSFTLMRDERGLASMNNLYTPYHKDVAGMGQSLSPLSSSGLGSIHNSQQGLPHYAHPGAAMPTDKMLTPNGFEAHHPAMLGRHGEQHLTPTSAGMVPINGLPPHHPHAHLNAQGHGQLLGTAREPNPSVTGAQVSNGSNSGQMEEINTKEVAQRITTELKRYSIPQAIFAQRVLCRSQGTLSDLLRNPKPWSKLKSGRETFRRMWKWLQEPEFQRMSALRLAACKRKEQEHGKDRGNTPKKPRLVFTDVQRRTLHAIFKENKRPSKELQITISQQLGLELSTVSNFFMNARRRSLDKWQDEGSSNSGNSSSSSSTCTKA

>NP_005172.1

MAKEWGYASHNGPDHWHELFPNAKGENQSPVELHTKDIRHDPSLQPWSVSYDGGSAKTILNNGKTCRVVFDDTYDRSMLRGGPLPGPYRLRQFHLHWGSSDDHGSEHTVDGVKYAAELHLVHWNPKYNTFKEALKQRDGIAVIGIFLKIGHENGEFQIFLDALDKIKTKGKEAPFTKFDPSCLFPACRDYWTYQGSFTTPPCEECIVWLLLKEPMTVSSDQMAKLRSLLSSAENEPPVPLVSNWRPPQPINNRVVRASFK

732 P49184 P28332

>P49184

MHYPTALLFLILANGAQAFRICAFNAQRLTLAKVAREQVMDTLVRILARCDIMVLQEVVDSSGSAIPLLLRELNRFDGSGPYSTLSSPQLGRSTYMETYVYFYRSHKTQVLSSYVYNDEDDVFAREPFVAQFSLPSNVLPSLVLVPLHTTPKAVEKELNALYDVFLEVSQHWQSKDVILLGDFNADCASLTKKRLDKLELRTEPGFHWVIADGEDTTVRASTHCTYDRVVLHGERCRSLLHTAAAFDFPTSFQLTEEEALNISDHYPVEVELKLSQAHSVQPLSLTVLLLLSLLSPQLCPAA

>P28332

MCTTGQVIRCKAAILWKPGAPFSIEEVEVAPPKAKEVRIKVVATGLCGTEMKVLGSKHLDLLYPTILGHEGAGIVESIGEGVSTVKPGDKVITLFLPQCGECTSCLNSEGNFCIQFKQSKTQLMSDGTSRFTCKGKSIYHFGNTSTFCEYTVIKEISVAKIDAVAPLEKVCLISCGFSTGFGAAINTAKVTPGSTCAVFGLGGVGLSVVMGCKAAGAARIIGVDVNKEKFKKAQELGATECLNPQDLKKPIQEVLFDMTDAGIDFCFEAIGNLDVLAAALASCNESYGVCVVVGVLPASVQLKISGQLFFSGRSLKGSVFGGWKSRQHIPKLVADYMAEKLNLDPLITHTLNLDKINEAVELMKTGKW

733 O75164 P83881

>O75164

MASESETLNPSARIMTFYPTMEEFRNFSRYIAYIESQGAHRAGLAKVVPPKEWKPRASYDDIDDLVIPAPIQQLVTGQSGLFTQYNIQKKAMTVREFRKIANSDKYCTPRYSEFEELERKYWKNLTFNPPIYGADVNGTLYEKHVDEWNIGRLRTILDLVEKESGITIEGVNTPYLYFGMWKTSFAWHTEDMDLYSINYLHFGEPKSWYSVPPEHGKRLERLAKGFFPGSAQSCEAFLRHKMTLISPLMLKKYGIPFDKVTQEAGEFMITFPYGYHAGFNHGFNCAESTNFATRRWIEYGKQAVLCSCRKDMVKISMDVFVRKFQPERYKLWKAGKDNTVIDHTLPTPEAAEFLKESELPPRAGNEEECPEEDMEGVEDGEEGDLKTSLAKHRIGTKRHRVCLEIPQEVSQSELFPKEDLSSEQYEMTECPAALAPVRPTHSSVRQVEDGLTFPDYSDSTEVKFEELKNVKLEEEDEEEEQEAAALDLSVNPASVGGRLVFSGSKKKSSSSLGSGSSRDSISSDSETSEPLSCRAQGQTGVLTVHSYAKGDGRVTVGEPCTRKKGSAARSFSERELAEVADEYMFSLEENKKSKGRRQPLSKLPRHHPLVLQECVSDDETSEQLTPEEEAEETEAWAKPLSQLWQNRPPNFEAEKEFNETMAQQAPHCAVCMIFQTYHQVEFGGFNQNCGNASDLAPQKQRTKPLIPEMCFTSTGCSTDINLSTPYLEEDGTSILVSCKKCSVRVHASCYGVPPAKASEDWMCSRCSANALEEDCCLCSLRGGALQRANDDRWVHVSCAVAILEARFVNIAERSPVDVSKIPLPRFKLKCIFCKKRRKRTAGCCVQCSHGRCPTAFHVSCAQAAGVMMQPDDWPFVVFITCFRHKIPNLERAKGALQSITAGQKVISKHKNGRFYQCEVVRLTTETFYEVNFDDGSFSDNLYPEDIVSQDCLQFGPPAEGEVVQVRWTDGQVYGAKFVASHPIQMYQVEFEDGSQLVVKRDDVYTLDEELPKRVKSRLSVASDMRFNEIFTEKEVKQEKKRQRVINSRYREDYIEPALYRAIME

>P83881

MVNVPKTRRTFCKKCGKHQPHKVTQYKKGKDSLYAQGKRRYDRKQSGYGGQTKPIFRKKAKTTKKIVLRLECVEPNCRSKRMLAIKRCKHFELGGDKKRKGQVIQF

734 P15586 P11245

>P15586

MRLLPLAPGRLRRGSPRHLPSCSPALLLLVLGGCLGVFGVAAGTRRPNVVLLLTDDQDEVLGGMTPLKKTKALIGEMGMTFSSAYVPSALCCPSRASILTGKYPHNHHVVNNTLEGNCSSKSWQKIQEPNTFPAILRSMCGYQTFFAGKYLNEYGAPDAGGLEHVPLGWSYWYALEKNSKYYNYTLSINGKARKHGENYSVDYLTDVLANVSLDFLDYKSNFEPFFMMIATPAPHSPWTAAPQYQKAFQNVFAPRNKNFNIHGTNKHWLIRQAKTPMTNSSIQFLDNAFRKRWQTLLSVDDLVEKLVKRLEFTGELNNTYIFYTSDNGYHTGQFSLPIDKRQLYEFDIKVPLLVRGPGIKPNQTSKMLVANIDLGPTILDIAGYDLNKTQMDGMSLLPILRGASNLTWRSDVLVEYQGEGRNVTDPTCPSLSPGVSQCFPDCVCEDAYNNTYACVRTMSALWNLQYCEFDDQEVFVEVYNLTADPDQITNIAKTIDPELLGKMNYRLMMLQSCSGPTCRTPGVFDPGYRFDPRLMFSNRGSVRTRRFSKHLL

>P11245

MDIEAYFERIGYKNSRNKLDLETLTDILEHQIRAVPFENLNMHCGQAMELGLEAIFDHIVRRNRGGWCLQVNQLLYWALTTIGFQTTMLGGYFYIPPVNKYSTGMVHLLLQVTIDGRNYIVDAGSGSSSQMWQPLELISGKDQPQVPCIFCLTEERGIWYLDQIRREQYITNKEFLNSHLLPKKKHQKIYLFTLEPRTIEDFESMNTYLQTSPTSSFITTSFCSLQTPEGVYCLVGFILTYRKFNYKDNTDLVEFKTLTEEEVEEVLKNIFKISLGRNLVPKPGDGSLTI

735 NP_066951.1 Q9BU02

>NP_066951.1

MIIPVRCFTCGKIVGNKWEAYLGLLQAEYTEGDALDALGLKRYCCRRMLLAHVDLIEKLLNYAPLEK

>Q9BU02

MAQGLIEVERKFLPGPGTEERLQELGGTLEYRVTFRDTYYDTPELSLMQADHWLRRREDSGWELKCPGAAGVLGPHTEYKELTAEPTIVAQLCKVLRADGLGAGDVAAVLGPLGLQEVASFVTKRSAWKLVLLGADEEEPQLRVDLDTADFGYAVGEVEALVHEEAEVPTALEKIHRLSSMLGVPAQETAPAKLIVYLQRFRPQDYQRLLEVNSSRERPQETEDPDHCLG

736 Q3YBR2 NP_005882.2

>Q3YBR2

MSLLDGLASSPRAPLQSSKARMKKLPKKSQNEKYRLKYLRLRKAAKATVFENAAICDEIARLEEKFLKAKEERRYLLKKLLQLQALTEGEVQAAAPSHSSSLPLTYGVASSVGTIQGAGPISGPSTGAEEPFGKKTKKEKKEKGKENNKLEVLKKTCKKKKMAGGARKLVQPIALDPSGRPVFPIGLGGLTVYSLGEIITDRPGFHDESAIYPVGYCSTRIYASMKCPDQKCLYTCQIKDGGVQPQFEIVPEDDPQNAIVSSSADACHAELLRTISTTMGKLMPNLLPAGADFFGFSHPAIHNLIQSCPGARKCINYQWVKFDVCKPGDGQLPEGLPENDAAMSFEAFQRQIFDEDQNDPLLPGSLDLPELQPAAFVSSYQPMYLTHEPLVDTHLQHLKSPSQGSPIQSSD

>NP_005882.2

MNAGSDPVVIVSAARTIIGSFNGALAAVPVQDLGSTVIKEVLKRATVAPEDVSEVIFGHVLAAGCGQNPVRQASVGAGIPYSVPAWSCQMICGSGLKAVCLAVQSIGIGDSSIVVAGGMENMSKAPHLAYLRTGVKIGEMPLTDSILCDGLTDAFHNCHMGITAENVAKKWQVSREDQDKVAVLSQNRTENAQKAGHFDKEIVPVLVSTRKGLIEVKTDEFPRHGSNIEAMSKLKPYFLTDGTGTVTPANASGINDGAAAVVLMKKSEADKRGLTPLARIVSWSQVGVEPSIMGIGPIPAIKQAVTKAGWSLEDVDIFEINEAFAAVSAAIVKELGLNPEKVNIEGGAIALGHPLGASGCRILVTLLHTLERMGRSRGVAALCIGGGMGIAMCVQRE

737 Q16666 Q9Y291

>Q16666

MGKKYKNIVLLKGLEVINDYHFRMVKSLLSNDLKLNLKMREEYDKIQIADLMEEKFRGDAGLGKLIKIFEDIPTLEDLAETLKKEKLKVKGPALSRKRKKEVDATSPAPSTSSTVKTEGAEATPGAQKRKKSTKEKAGPKGSKVSEEQTQPPSPAGAGMSTAMGRSPSPKTSLSAPPNSSSTENPKTVAKCQVTPRRNVLQKRPVIVKVLSTTKPFEYETPEMEKKIMFHATVATQTQFFHVKVLNTSLKEKFNGKKIIIISDYLEYDSLLEVNEESTVSEAGPNQTFEVPNKIINRAKETLKIDILHKQASGNIVYGVFMLHKKTVNQKTTIYEIQDDRGKMDVVGTGQCHNIPCEEGDKLQLFCFRLRKKNQMSKLISEMHSFIQIKKKTNPRNNDPKSMKLPQEQRQLPYPSEASTTFPESHLRTPQMPPTTPSSSFFTKKSEDTISKMNDFMRMQILKEGSHFPGPFMTSIGPAESHPHTPQMPPSTPSSSFLTTKSEDTISKMNDFMRMQILKEGSHFPGPFMTSIGPAESHPHTPQMPPSTPSSSFLTTLKPRLKTEPEEVSIEDSAQSDLKEVMVLNATESFVYEPKEQKKMFHATVATENEVFRVKVFNIDLKEKFTPKKIIAIANYVCRNGFLEVYPFTLVADVNADRNMEIPKGLIRSASVTPKINQLCSQTKGSFVNGVFEVHKKNVRGEFTYYEIQDNTGKMEVVVHGRLTTINCEEGDKLKLTCFELAPKSGNTGELRSVIHSHIKVIKTRKNKKDILNPDSSMETSPDFFF

>Q9Y291

MSSLSEYAFRMSRLSARLFGEVTRPTNSKSMKVVKLFSELPLAKKKETYDWYPNHHTYAELMQTLRFLGLYRDEHQDFMDEQKRLKKLRGKEKPKKGEGKRAAKRK

738 P15586 Q6PRD7

>P15586

MRLLPLAPGRLRRGSPRHLPSCSPALLLLVLGGCLGVFGVAAGTRRPNVVLLLTDDQDEVLGGMTPLKKTKALIGEMGMTFSSAYVPSALCCPSRASILTGKYPHNHHVVNNTLEGNCSSKSWQKIQEPNTFPAILRSMCGYQTFFAGKYLNEYGAPDAGGLEHVPLGWSYWYALEKNSKYYNYTLSINGKARKHGENYSVDYLTDVLANVSLDFLDYKSNFEPFFMMIATPAPHSPWTAAPQYQKAFQNVFAPRNKNFNIHGTNKHWLIRQAKTPMTNSSIQFLDNAFRKRWQTLLSVDDLVEKLVKRLEFTGELNNTYIFYTSDNGYHTGQFSLPIDKRQLYEFDIKVPLLVRGPGIKPNQTSKMLVANIDLGPTILDIAGYDLNKTQMDGMSLLPILRGASNLTWRSDVLVEYQGEGRNVTDPTCPSLSPGVSQCFPDCVCEDAYNNTYACVRTMSALWNLQYCEFDDQEVFVEVYNLTADPDQITNIAKTIDPELLGKMNYRLMMLQSCSGPTCRTPGVFDPGYRFDPRLMFSNRGSVRTRRFSKHLL

>Q6PRD7

MGTSSTDSQQAGHRRCSTSNTSAENLTCLSLPGSPGKTAPLPGPAQAGAGQPLPKGCAAVKAEVGIPAPHTSQEVRIHIRRLLSWAAPGACGLRSTPCALPQALPQARPCPGRWFFPGCSLPTGGAQTILSLWTWRHFLNWALQQREENSGRARRVPPVPRTAPVSKGEGSHPPQNSNGEKVKTITPDVGLHQSLTSDPTVAVLRAKRAPEAHPPRSCSGSLTARVCHMGVCQGQGDTEDGRMTLMG

739 NP_061975.2 NP_001705.2

>NP_061975.2

MSSYFVNSFCGRYPNGPDYQLHNYGDHSSVSEQFRDSASMHSGRYGYGYNGMDLSVGRSGSGHFGSGERARSYAASASAAPAEPRYSQPATSTHSPQPDPLPCSAVAPSPGSDSHHGGKNSLSNSSGASADAGSTHISSREGVGTASGAEEDAPASSEQASAQSEPSPAPPAQPQIYPWMRKLHISHDNIGGPEGKRARTAYTRYQTLELEKEFHFNRYLTRRRRIEIAHALCLSERQIKIWFQNRRMKWKKDNKLKSMSMAAAGGAFRP

>NP_001705.2

MAAEEVLQTVDHYKTEIERLTKELTETTHEKIQAAEYGLVVLEEKLTLKQQYDELEAEYDSLKQELEQLKEAFGQSFSIHRKVAEDGETREETLLQESASKEAYYLGKILEMQNELKQSRAVVTNVQAENERLTAVVQDLKENNEMVELQRIRMKDEIREYKFREARLLQDYTELEEENITLQKLVSTLKQNQVEYEGLKHEIKRFEEETVLLNSQLEDAIRLKEIAEHQLEEALETLKNEREQKNNLRKELSQYISLNDNHISISVDGLKFAEDGSEPNNDDKMNGHIHGPLVKLNGDYRTPTLRKGESLNPVSDLFSELNISEIQKLKQQLMQVEREKAILLANLQESQTQLEHTKGALTEQHERVHRLTEHVNAMRGLQSSKELKAELDGEKGRDSGEEAHDYEVDINGLEILECKYRVAVTEVIDLKAEIKALKEKYNKSVENYTDEKAKYESKIQMYDEQVTSLEKTTKESGEKMAHMEKELQKMTSIANENHSTLNTAQDELVTFSEELAQLYHHVCLCNNETPNRVMLDYYRQSRVTRSGSLKGPDDPRGLLSPRLARRGVSSPVETRTSSEPVAKESTEASKEPSPTKTPTISPVITAPPSSPVLDTSDIRKEPMNIYNLNAIIRDQIKHLQKAVDRSLQLSRQRAAARELAPMIDKDKEALMEEILKLKSLLSTKREQIATLRAVLKANKQTAEVALANLKNKYENEKAMVTETMTKLRNELKALKEDAATFSSLRAMFATRCDEYVTQLDEMQRQLAAAEDEKKTLNTLLRMAIQQKLALTQRLEDLEFDHEQSRRSKGKLGKSKIGSPKVSGEASVTVPTIDTYLLHSQGPQTPNIRVSSGTQRKRQFSPSLCDQSRPRTSGASYLQNLLRVPPDPTSTESFLLKGPPSMSEFIQGHRLSKEKRLTVAPPDCQQPAASVPPQCSQLAGRQDCPTVSPDTALPEEQPHSSSQCAPLHCLSKPPHP

740 NP_002422.1 NP_001340.2

>NP_002422.1

MDDQGCPRCKTTKYRNPSLKLMVNVCGHTLCESCVDLLFVRGAGNCPECGTPLRKSNFRVQLFEDPTVDKEVEIRKKVLKIYNKREEDFPSLREYNDFLEEVEEIVFNLTNNVDLDNTKKKMEIYQKENKDVIQKNKLKLTREQEELEEALEVERQENEQRRLFIQKEEQLQQILKRKNKQAFLDELESSDLPVALLLAQHKDRSTQLEMQLEKPKPVKPVTFSTGIKMGQHISLAPIHKLEEALYEYQPLQIETYGPHVPELEMLGRLGYLNHVRAASPQDLAGGYTSSLACHRALQDAFSGLFWQPS

>NP_001340.2

MPSASASRKSQEKPREIMDAAEDYAKERYGISSMIQSQEKPDRVLVRVRDLTIQKADEVVWVRARVHTSRAKGKQCFLVLRQQQFNVQALVAVGDHASKQMVKFAANINKESIVDVEGVVRKVNQKIGSCTQQDVELHVQKIYVISLAEPRLPLQLDDAVRPEAEGEEEGRATVNQDTRLDNRVIDLRTSTSQAVFRLQSGICHLFRETLINKGFVEIQTPKIISAASEGGANVFTVSYFKNNAYLAQSPQLYKQMCICADFEKVFSIGPVFRAEDSNTHRHLTEFVGLDIEMAFNYHYHEVMEEIADTMVQIFKGLQERFQTEIQTVNKQFPCEPFKFLEPTLRLEYCEALAMLREAGVEMGDEDDLSTPNEKLLGHLVKEKYDTDFYILDKYPLAVRPFYTMPDPRNPKQSNSYDMFMRGEEILSGAQRIHDPQLLTERALHHGIDLEKIKAYIDSFRFGAPPHAGGGIGLERVTMLFLGLHNVRQTSMFPRDPKRLTP

741 NP_054733.2 NP_071761.1

>NP_054733.2

MADVTARSLQYEYKANSNLVLQADRSLIDRTRRDEPTGEVLSLVGKLEGTRMGDKAQRTKPQMQEERRAKRRKRDEDRHDINKMKGYTLLSEGIDEMVGIIYKPKTKETRETYEVLLSFIQAALGDQPRDILCGAADEVLAVLKNEKLRDKERRKEIDLLLGQTDDTRYHVLVNLGKKITDYGGDKEIQNMDDNIDETYGVNVQFESDEEEGDEDVYGEVREEASDDDMEGDEAVVRCTLSANLVASGELMSSKKKDLHPRDIDAFWLQRQLSRFYDDAIVSQKKADEVLEILKTASDDRECENQLVLLLGFNTFDFIKVLRQHRMMILYCTLLASAQSEAEKERIMGKMEADPELSKFLYQLHETEKEDLIREERSRRERVRQSRMDTDLETMDLDQGGEALAPRQVLDLEDLVFTQGSHFMANKRCQLPDGSFRRQRKGYEEVHVPALKPKPFGSEEQLLPVEKLPKYAQAGFEGFKTLNRIQSKLYRAALETDENLLLCAPTGAGKTNVALMCMLREIGKHINMDGTINVDDFKIIYIAPMRSLVQEMVGSFGKRLATYGITVAELTGDHQLCKEEISATQIIVCTPEKWDIITRKGGERTYTQLVRLIILDEIHLLHDDRGPVLEALVARAIRNIEMTQEDVRLIGLSATLPNYEDVATFLRVDPAKGLFYFDNSFRPVPLEQTYVGITEKKAIKRFQIMNEIVYEKIMEHAGKNQVLVFVHSRKETGKTARAIRDMCLEKDTLGLFLREGSASTEVLRTEAEQCKNLELKDLLPYGFAIHHAGMTRVDRTLVEDLFADKHIQVLVSTATLAWGVNLPAHTVIIKGTQVYSPEKGRWTELGALDILQMLGRAGRPQYDTKGEGILITSHGELQYYLSLLNQQLPIESQMVSKLPDMLNAEIVLGNVQNAKDAVNWLGYAYLYIRMLRSPTLYGISHDDLKGDPLLDQRRLDLVHTAALMLDKNNLVKYDKKTGNFQVTELGRIASHYYITNDTVQTYNQLLKPTLSEIELFRVFSLSSEFKNITVREEEKLELQKLLERVPIPVKESIEEPSAKINVLLQAFISQLKLEGFALMADMVYVTQSAGRLMRAIFEIVLNRGWAQLTDKTLNLCKMIDKRMWQSMCPLRQFRKLPEEVVKKIEKKNFPFERLYDLNHNEIGELIRMPKMGKTIHKYVHLFPKLELSVHLQPITRSTLKVELTITPDFQWDEKVHGSSEAFWILVEDVDSEVILHHEYFLLKAKYAQDEHLITFFVPVFEPLPPQYFIRVVSDRWLSCETQLPVSFRHLILPEKYPPPTELLDLQPLPVSALRNSAFESLYQDKFPFFNPIQTQVFNTVYNSDDNVFVGAPTGSGKTICAEFAILRMLLQSSEGRCVYITPMEALAEQVYMDWYEKFQDRLNKKVVLLTGETSTDLKLLGKGNIIISTPEKWDILSRRWKQRKNVQNINLFVVDEVHLIGGENGPVLEVICSRMRYISSQIERPIRIVALSSSLSNAKDVAHWLGCSATSTFNFHPNVRPVPLELHIQGFNISHTQTRLLSMAKPVYHAITKHSPKKPVIVFVPSRKQTRLTAIDILTTCAADIQRQRFLHCTEKDLIPYLEKLSDSTLKETLLNGVGYLHEGLSPMERRLVEQLFSSGAIQVVVASRSLCWGMNVAAHLVIIMDTQYYNGKIHAYVDYPIYDVLQMVGHANRPLQDDEGRCVIMCQGSKKDFFKKFLYEPLPVESHLDHCMHDHFNAEIVTKTIENKQDAVDYLTWTFLYRRMTQNPNYYNLQGISHRHLSDHLSELVEQTLSDLEQSKCISIEDEMDVAPLNLGMIAAYYYINYTTIELFSMSLNAKTKVRGLIEIISNAAEYENIPIRHHEDNLLRQLAQKVPHKLNNPKFNDPHVKTNLLLQAHLSRMQLSAELQSDTEEILSKAIRLIQACVDVLSSNGWLSPALAAMELAQMVTQAMWSKDSYLKQLPHFTSEHIKRCTDKGVESVFDIMEMEDEERNALLQLTDSQIADVARFCNRYPNIELSYEVVDKDSIRSGGPVVVLVQLEREEEVTGPVIAPLFPQKREEGWWVVIGDAKSNSLISIKRLTLQQKAKVKLDFVAPATGAHNYTLYFMSDAYMGCDQEYKFSVDVKEAETDSDSD

>NP_071761.1

MWIPVVGLPRRLRLSALAGAGRFCILGSEAATRKHLPARNHCGLSDSSPQLWPEPDFRNPPRKASKASLDFKRYVTDRRLAETLAQIYLGKPSRPPHLLLECNPGPGILTQALLEAGAKVVALESDKTFIPHLESLGKNLDGKLRVIHCDFFKLDPRSGGVIKPPAMSSRGLFKNLGIEAVPWTADIPLKVVGMFPSRGEKRALWKLAYDLYSCTSIYKFGRIEVNMFIGEKEFQKLMADPGNPDLYHVLSVIWQLACEIKVLHMEPWSSFDIYTRKGPLENPKRRELLDQLQQKLYLIQMIPRQNLFTKNLTPMNYNIFFHLLKHCFGRRSATVIDHLRSLTPLDARDILMQIGKQEDEKVVNMHPQDFKTLFETIERSKDCAYKWLYDETLEDR

742 NP_036339.1 NP_003810.1

>NP_036339.1

MDWVMKHNGPNDASDGTVRLRGLPFGCSKEEIVQFFQGLEIVPNGITLTMDYQGRSTGEAFVQFASKEIAENALGKHKERIGHRYIEIFRSSRSEIKGFYDPPRRLLGQRPGPYDRPIGGRGGYYGAGRGSMYDRMRRGGDGYDGGYGGFDDYGGYNNYGYGNDGFDDRMRDGRGMGGHGYGGAGDASSGFHGGHFVHMRGLPFRATENDIANFFSPLNPIRVHIDIGADGRATGEADVEFVTHEDAVAAMSKDKNNMQHRYIELFLNSTPGGGSGMGGSGMGGYGRDGMDNQGGYGSVGRMGMGNNYSGGYGTPDGLGGYGRGGGGSGGYYGQGGMSGGGWRGMY

>NP_003810.1

MNAAASSYPMASLYVGDLHSDVTEAMLYEKFSPAGPVLSIRVCRDMITRRSLGYAYVNFQQPADAERALDTMNFDVIKGKPIRIMWSQRDPSLRKSGVGNVFIKNLDKSIDNKALYDTFSAFGNILSCKVVCDENGSKGYAFVHFETQEAADKAIEKMNGMLLNDRKVFVGRFKSRKEREAELGAKAKEFTNVYIKNFGEEVDDESLKELFSQFGKTLSVKVMRDPNGKSKGFGFVSYEKHEDANKAVEEMNGKEISGKIIFVGRAQKKVERQAELKRKFEQLKQERISRYQGVNLYIKNLDDTIDDEKLRKEFSPFGSITSAKVMLEDGRSKGFGFVCFSSPEEATKAVTEMNGRIVGSKPLYVALAQRKEERKAHLTNQYMQRVAGMRALPANAILNQFQPAAGGYFVPAVPQAQGRPPYYTPNQLAQMRPNPRWQQGGRPQGFQGMPSAIRQSGPRPTLRHLAPTGSECPDRLAMDFGGAGAAQQGLTDSCQSGGVPTAVQNLAPRAAVAAAAPRAVAPYKYASSVRSPHPAIQPLQAPQPAVHVQGQEPLTASMLAAAPPQEQKQMLGERLFPLIQTMHSNLAGKITGMLLEIDNSELLHMLESPESLRSKVDEAVAVLQAHHAKKEAAQKVGAVAAATS

743 NP_003850.1 NP_001340.2

>NP_003850.1

MASLEVSRSPRRSRRELEVRSPRQNKYSVLLPTYNERENLPLIVWLLVKSFSESGINYEIIIIDDGSPDGTRDVAEQLEKIYGSDRILLRPREKKLGLGTAYIHGMKHATGNYIIIMDADLSHHPKFIPEFIRKQKEGNFDIVSGTRYKGNGGVYGWDLKRKIISRGANFLTQILLRPGASDLTGSFRLYRKEVLEKLIEKCVSKGYVFQMEMIVRARQLNYTIGEVPISFVDRVYGESKLGGNEIVSFLKGLLTLFATT

>NP_001340.2

MPSASASRKSQEKPREIMDAAEDYAKERYGISSMIQSQEKPDRVLVRVRDLTIQKADEVVWVRARVHTSRAKGKQCFLVLRQQQFNVQALVAVGDHASKQMVKFAANINKESIVDVEGVVRKVNQKIGSCTQQDVELHVQKIYVISLAEPRLPLQLDDAVRPEAEGEEEGRATVNQDTRLDNRVIDLRTSTSQAVFRLQSGICHLFRETLINKGFVEIQTPKIISAASEGGANVFTVSYFKNNAYLAQSPQLYKQMCICADFEKVFSIGPVFRAEDSNTHRHLTEFVGLDIEMAFNYHYHEVMEEIADTMVQIFKGLQERFQTEIQTVNKQFPCEPFKFLEPTLRLEYCEALAMLREAGVEMGDEDDLSTPNEKLLGHLVKEKYDTDFYILDKYPLAVRPFYTMPDPRNPKQSNSYDMFMRGEEILSGAQRIHDPQLLTERALHHGIDLEKIKAYIDSFRFGAPPHAGGGIGLERVTMLFLGLHNVRQTSMFPRDPKRLTP

744 NP_037406.1 NP_057256.2

>NP_037406.1

MDPRGILKAFPKRQKIHADASSKVLAKIPRREEGEEAEEWLSSLRAHVVRTGIGRARAELFEKQIVQHGGQLCPAQGPGVTHIVVDEGMDYERALRLLRLPQLPPGAQLVKSAWLSLCLQERRLVDVAGFSIFIPSRYLDHPQPSKAEQDASIPPGTHEALLQTALSPPPPPTRPVSPPQKAKEAPNTQAQPISDDEASDGEETQVSAADLEALISGHYPTSLEGDCEPSPAPAVLDKWVCAQPSSQKATNHNLHITEKLEVLAKAYSVQGDKWRALGYAKAINALKSFHKPVTSYQEACSIPGIGKRMAEKIIEILESGHLRKLDHISESVPVLELFSNIWGAGTKTAQMWYQQGFRSLEDIRSQASLTTQQAIGLKHYSDFLERMPREEATEIEQTVQKAAQAFNSGLLCVACGSYRRGKATCGDVDVLITHPDGRSHRGIFSRLLDSLRQEGFLTDDLVSQEENGQQQKYLGVCRLPGPGRRHRRLDIIVVPYSEFACALLYFTGSAHFNRSMRALAKTKGMSLSEHALSTAVVRNTHGCKVGPGRVLPTPTEKDVFRLLGLPYREPAERDW

>NP_057256.2

MFVQEEKIFAGKVLRLHICASDGAEWLEEATEDTSVEKLKERCLKHCAHGSLEDPKSITHHKLIHAASERVLSDARTILEENIQDQDVLLLIKKRAPSPLPKMADVSAEEKKKQDQKAPDKEAILRATANLPSYNMDRAAVQTNMRDFQTELRKILVSLIEVAQKLLALNPDAVELFKKANAMLDEDEDERVDEAALRQLTEMGFPENRATKALQLNHMSVPQAMEWLIEHAEDPTIDTPLPGQAPPEAEGATAAASEAAAGASATDEEARDELTEIFKKIRRKREFRADARAVISLMEMGFDEKEVIDALRVNNNQQNAACEWLLGDRKPSPEELDKGIDPDSPLFQAILDNPVVQLGLTNPKTLLAFEDMLENPLNSTQWMNDPETGPVMLQISRIFQTLNRT

745 P01106 NP_002305.1

>P01106

MPLNVSFTNRNYDLDYDSVQPYFYCDEEENFYQQQQQSELQPPAPSEDIWKKFELLPTPPLSPSRRSGLCSPSYVAVTPFSLRGDNDGGGGSFSTADQLEMVTELLGGDMVNQSFICDPDDETFIKNIIIQDCMWSGFSAAAKLVSEKLASYQAARKDSGSPNPARGHSVCSTSSLYLQDLSAAASECIDPSVVFPYPLNDSSSPKSCASQDSSAFSPSSDSLLSSTESSPQGSPEPLVLHEETPPTTSSDSEEEQEDEEEIDVVSVEKRQAPGKRSESGSPSAGGHSKPPHSPLVLKRCHVSTHQHNYAAPPSTRKDYPAAKRVKLDSVRVLRQISNNRKCTSPRSSDTEENVKRRTHNVLERQRRNELKRSFFALRDQIPELENNEKAPKVVILKKATAYILSVQAEEQKLISEEDLLRKRREQLKHKLEQLRNSCA

>NP_002305.1

MRLTLLCCTWREERMGEEGSELPVCASCGQRIYDGQYLQALNADWHADCFRCCDCSASLSHQYYEKDGQLFCKKDYWARYGESCHGCSEQITKGLVMVAGELKYHPECFICLTCGTFIGDGDTYTLVEHSKLYCGHCYYQTVVTPVIEQILPDSPGSHLPHTVTLVSIPASSHGKRGLSVSIDPPHGPPGCGTEHSHTVRVQGVDPGCMSPDVKNSIHVGDRILEINGTPIRNVPLDEIDLLIQETSRLLQLTLEHDPHDTLGHGLGPETSPLSSPAYTPSGEAGSSARQKPVLRSCSIDRSPGAGSLGSPASQRKDLGRSESLRVVCRPHRIFRPSDLIHGEVLGKGCFGQAIKVTHRETGEVMVMKELIRFDEETQRTFLKEVKVMRCLEHPNVLKFIGVLYKDKRLNFITEYIKGGTLRGIIKSMDSQYPWSQRVSFAKDIASGMAYLHSMNIIHRDLNSHNCLVRENKNVVVADFGLARLMVDEKTQPEGLRSLKKPDRKKRYTVVGNPYWMAPEMINGRSYDEKVDVFSFGIVLCEIIGRVNADPDYLPRTMDFGLNVRGFLDRYCPPNCPPSFFPITVRCCDLDPEKRPSFVKLEHWLETLRMHLAGHLPLGPQLEQLDRGFWETYRRGESGLPAHPEVPD

746 NP_001752.2 NP_550438.1

>NP_001752.2

MGSGGVVHCRCAKCFCYPTKRRIRRRPRNLTILSLPEDVLFHILKWLSVEDILAVRAVHSQLKDLVDNHASVWACASFQELWPSPGNLKLFERAAEKGNFEAAVKLGIAYLYNEGLSVSDEARAEVNGLKASRFFSLAERLNVGAAPFIWLFIRPPWSVSGSCCKAVVHESLRAECQLQRTHKASILHCLGRVLSLFEDEEKQQQAHDLFEEAAHQGCLTSSYLLWESDRRTDVSDPGRCLHSFRKLRDYAAKGCWEAQLSLAKACANANQLGLEVRASSEIVCQLFQASQAVSKQQVFSVQKGLNDTMRYILIDWLVEVATMKDFTSLCLHLTVECVDRYLRRRLVPRYRLQLLGIACMVICTRFISKEILTIREAVWLTDNTYKYEDLVRMMGEIVSALEGKIRVPTVVDYKEVLLTLVPVELRTQHLCSFLCELSLLHTSLSAYAPARLAAAALLLARLTHGQTQPWTTQLWDLTGFSYEDLIPCVLSLHKKCFHDDAPKDYRQVSLTAVKQRFEDKRYGEISQEEVLSYSQLCAALGVTQDSPDPPTFLSTGEIHAFLSSPSGRRTKRKRENSLQEDRGSFVTTPTAELSSQEETLLGSFLDWSLDCCSGYEGDQESEGEKEGDVTAPSGILDVTVVYLNPEQHCCQESSDEEACPEDKGPQDPQALALDTQIPATPGPKPLVRTSREPGKDVTTSGYSSVSTASPTSSVDGGLGALPQPTSVLSLDSDSHTQPCHHQARKSCLQCRPPSPPESSVPQQQVKRINLCIHSEEEDMNLGLVRL

>NP_550438.1

MAAGRLFLSRLRAPFSSMAKSPLEGVSSSRGLHAGRGPRRLSIEGNIAVGKSTFVKLLTKTYPEWHVATEPVATWQNIQAAGTQKACTAQSLGNLLDMMYREPARWSYTFQTFSFLSRLKVQLEPFPEKLLQARKPVQIFERSVYSDRYIFAKNLFENGSLSDIEWHIYQDWHSFLLWEFASRITLHGFIYLQASPQVCLKRLYQRAREEEKGIELAYLEQLHGQHEAWLIHKTTKLHFEALMNIPVLVLDVNDDFSEEVTKQEDLMREVNTFVKNL

747 NP_006615.1 O75394

>NP_006615.1

MSRVHGMHPKETTRQLSLAVKDGLIVETLTVGCKGSKAGIEQEGYWLPGDEIDWETENHDWYCFECHLPGEVLICDLCFRVYHSKCLSDEFRLRDSSSPWQCPVCRSIKKKNTNKQEMGTYLRFIVSRMKERAIDLNKKGKDNKHPMYRRLVHSAVDVPTIQEKVNEGKYRSYEEFKADAQLLLHNTVIFYGADSEQADIARMLYKDTCHELDELQLCKNCFYLSNARPDNWFCYPCIPNHELVWAKMKGFGFWPAKVMQKEDNQVDVRFFGHHHQRAWIPSENIQDITVNIHRLHVKRSMGWKKACDELELHQRFLREGRFWKSKNEDRGEEEAESSISSTSNEQLKVTQEPRAKKGRRNQSVEPKKEEPEPETEAVSSSQEIPTMPQPIEKVSVSTQTKKLSASSPRMLHRSTQTTNDGVCQSMCHDKYTKIFNDFKDRMKSDHKRETERVVREALEKLRSEMEEEKRQAVNKAVANMQGEMDRKCKQVKEKCKEEFVEEIKKLATQHKQLISQTKKKQWCYNCEEEAMYHCCWNTSYCSIKCQQEHWHAEHKRTCRRKR

>O75394

MFLSAVFFAKSKSKNILVRMVSEAGTGFCFNTKRNRLREKLTLLHYDPVVKQRVLFVEKKKIRSL

748 Q96LA8 Q93015

>Q96LA8

MSQPKKRKLESGGGGEGGEGTEEEDGAEREAALERPRRTKRERDQLYYECYSDVSVHEEMIADRVRTDAYRLGILRNWAALRGKTVLDVGAGTGILSIFCAQAGARRVYAVEASAIWQQAREVVRFNGLEDRVHVLPGPVETVELPEQVDAIVSEWMGYGLLHESMLSSVLHARTKWLKEGGLLLPASAELFIAPISDQMLEWRLGFWSQVKQHYGVDMSCLEGFATRCLMGHSEIVVQGLSGEDVLARPQRFAQLELSRAGLEQELEAGVGGRFRCSCYGSAPMHGFAIWFQVTFPGGESEKPLVLSTSPFHPATHWKQALLYLNEPVQVEQDTDVSGEITLLPSRDNPRRLRVLLRYKVGDQEEKTKDFAMED

>Q93015

MELILSTSPAELTLDPACQPKLPLDSTCQPEMTFNPGPTELTLDPEHQPEETPAPSLAELTLEPVHRRPELLDACADLINDQWPRSRTSRLHSLGQSSDAFPLCLMLLSPHPTLEAAPVVVGHARLSRVLNQPQSLLVETVVVARALRGRGFGRRLMEGLEVFARARGFRKLHLTTHDQVHFYTHLGYQLGEPVQGLVFTSRRLPATLLNAFPTAPSPRPPRKAPNLTAQAAPRGPKGPPLPPPPPLPECLTISPPVPSGPPSKSLLETQYQNVRGRPIFWMEKDI

749 O75529 NP_006397.1

>O75529

MKRVRTEQIQMAVSCYLKRRQYVDSDGPLKQGLRLSQTAEEMAANLTVQSESGCANIVSAAPCQAEPQQYEVQFGRLRNFLTDSDSQHSHEVMPLLYPLFVYLHLNLVQNSPKSTVESFYSRFHGMFLQNASQKDVIEQLQTTQTIQDILSNFKLRAFLDNKYVVRLQEDSYNYLIRYLQSDNNTALCKVLTLHIHLDVQPAKRTDYQLYASGSSSRSENNGLEPPDMPSPILQNEAALEVLQESIKRVKDGPPSLTTICFYAFYNTEQLLNTAEISPDSKLLAAGFDNSCIKLWSLRSKKLKSEPHQVDVSRIHLACDILEEEDDEDDNAGTEMKILRGHCGPVYSTRFLADSSGLLSCSEDMSIRYWDLGSFTNTVLYQGHAYPVWDLDISPYSLYFASGSHDRTARLWSFDRTYPLRIYAGHLADVDCVKFHPNSNYLATGSTDKTVRLWSAQQGNSVRLFTGHRGPVLSLAFSPNGKYLASAGEDQRLKLWDLASGTLYKELRGHTDNITSLTFSPDSGLIASASMDNSVRVWDIRNTYCSAPADGSSSELVGVYTGQMSNVLSVQFMACNLLLVTGITQENQEH

>NP_006397.1

MEALPLLAATTPDHGRHRRLLLLPLLLFLLPAGAVQGWETEERPRTREEECHFYAGGQVYPGEASRVSVADHSLHLSKAKISKPAPYWEGTAVIDGEFKELKLTDYRGKYLVFFFYPLDFTFVCPTEIIAFGDRLEEFRSINTEVVACSVDSQFTHLAWINTPRRQGGLGPIRIPLLSDLTHQISKDYGVYLEDSGHTLRGLFIIDDKGILRQITLNDLPVGRSVDETLRLVQAFQYTDKHGEVCPAGWKPGSETIIPDPAGKLKYFDKLN

750 Q9UKI8 NP_005495.2

>Q9UKI8

MSVQSSSGSLEGPPSWSQLSTSPTPGSAAAARSLLNHTPPSGRPREGAMDELHSLDPRRQELLEARFTGVASGSTGSTGSCSVGAKASTNNESSNHSFGSLGSLSDKESETPEKKQSESSRGRKRKAENQNESSQGKSIGGRGHKISDYFEYQGGNGSSPVRGIPPAIRSPQNSHSHSTPSSSVRPNSPSPTALAFGDHPIVQPKQLSFKIIQTDLTMLKLAALESNKIQDLEKKEGRIDDLLRANCDLRRQIDEQQKLLEKYKERLNKCISMSKKLLIEKSTQEKLSSREKSMQDRLRLGHFTTVRHGASFTEQWTDGFAFQNLVKQQEWVNQQREDIERQRKLLAKRKPPTANNSQAPSTNSEPKQRKNKAVNGAENDPFVRPNLPQLLTLAEYHEQEEIFKLRLGHLKKEEAEIQAELERLERVRNLHIRELKRINNEDNSQFKDHPTLNERYLLLHLLGRGGFSEVYKAFDLYEQRYAAVKIHQLNKSWRDEKKENYHKHACREYRIHKELDHPRIVKLYDYFSLDTDTFCTVLEYCEGNDLDFYLKQHKLMSEKEARSIVMQIVNALRYLNEIKPPIIHYDLKPGNILLVDGTACGEIKITDFGLSKIMDDDSYGVDGMDLTSQGAGTYWYLPPECFVVGKEPPKISNKVDVWSVGVIFFQCLYGRKPFGHNQSQQDILQENTILKATEVQFPVKPVVSSEAKAFIRRCLAYRKEDRFDVHQLANDPYLLPHMRRSNSSGNLHMAGLTASPTPPSSSIITY

>NP_005495.2

MKDCSNGCSAECTGEGGSKEVVGTFKAKDLIVTPATILKEKPDPNNLVFGTVFTDHMLTVEWSSEFGWEKPHIKPLQNLSLHPGSSALHYAVELFEGLKAFRGVDNKIRLFQPNLNMDRMYRSAVRATLPVFDKEELLECIQQLVKLDQEWVPYSTSASLYIRPTFIGTEPSLGVKKPTKALLFVLLSPVGPYFSSGTFNPVSLWANPKYVRAWKGGTGDCKMGGNYGSSLFAQCEAVDNGCQQVLWLYGEDHQITEVGTMNLFLYWINEDGEEELATPPLDGIILPGVTRRCILDLAHQWGEFKVSERYLTMDDLTTALEGNRVREMFGSGTACVVCPVSDILYKGETIHIPTMENGPKLASRILSKLTDIQYGREESDWTIVLS

751 O00115 NP_064530.1

>O00115

MIPLLLAALLCVPAGALTCYGDSGQPVDWFVVYKLPALRGSGEAAQRGLQYKYLDESSGGWRDGRALINSPEGAVGRSLQPLYRSNTSQLAFLLYNDQPPQPSKAQDSSMRGHTKGVLLLDHDGGFWLVHSVPNFPPPASSAAYSWPHSACTYGQTLLCVSFPFAQFSKMGKQLTYTYPWVYNYQLEGIFAQEFPDLENVVKGHHVSQEPWNSSITLTSQAGAVFQSFAKFSKFGDDLYSGWLAAALGTNLQVQFWHKTVGILPSNCSDIWQVLNVNQIAFPGPAGPSFNSTEDHSKWCVSPKGPWTCVGDMNRNQGEEQRGGGTLCAQLPALWKAFQPLVKNYQPCNGMARKPSRAYKI

>NP_064530.1

MDFNMKKLASDAGIFFTRAVQFTEEKFGQAEKTELDAHFENLLARADSTKNWTEKILRQTEVLLQPNPSARVEEFLYEKLDRKVPSRVTNGELLAQYMADAASELGPTTPYGKTLIKVAEAEKQLGAAERDFIHTASISFLTPLRNFLEGDWKTISKERRLLQNRRLDLDACKARLKKAKAAEAKATTVPDFQETRPRNYILSASASALWNDEVDKAEQELRVAQTEFDRQAEVTRLLLEGISSTHVNHLRCLHEFVKSQTTYYAQCYRHMLDLQKQLGRFPGTFVGTTEPASPPLSSTSPTTAAATMPVVPSVASLAPPGEASLCLEEVAPPASGTRKARVLYDYEAADSSELALLADELITVYSLPGMDPDWLIGERGNKKGKVPVTYLELLS

752 NP_005307.1 O43182

>NP_005307.1

MATSSEEVLLIVKKVRQKKQDGALYLMAERIAWAPEGKDRFTISHMYADIKCQKISPEGKAKIQLQLVLHAGDTTNFHFSNESTAVKERDAVKDLLQQLLPKFKRKANKELEEKNRMLQEDPVLFQLYKDLVVSQVISAEEFWANRLNVNATDSSSTSNHKQDVGISAAFLADVRPQTDGCNGLRYNLTSDIIESIFRTYPAVKMKYAENVPHNMTEKEFWTRFFQSHYFHRDRLNTGSKDLFAECAKIDEKGLKTMVSLGVKNPLLDLTALEDKPLDEGYGISSVPSASNSKSIKENSNAAIIKRFNHHSAMVLAAGLRKQEAQNEQTSEPSNMDGNSGDADCFQPAVKRAKLQESIEYEDLGKNNSVKTIALNLKKSDRYYHGPTPIQSLQYATSQDIINSFQSIRQEMEAYTPKLTQVLSSSAASSTITALSPGGALMQGGTQQAINQMVPNDIQSELKHLYVAVGELLRHFWSCFPVNTPFLEEKVVKMKSNLERFQVTKLCPFQEKIRRQYLSTNLVSHIEEMLQTAYNKLHTWQSRRLMKKT

>O43182

MSAQSLLHSVFSCSSPASSSAASAKGFSKRKLRQTRSLDPALIGGCGSDEAGAEGSARGATAGRLYSPSLPAESLGPRLASSSRGPPPRATRLPPPGPLCSSFSTPSTPQEKSPSGSFHFDYEVPLGRGGLKKSMAWDLPSVLAGPASSRSASSILCSSGGGPNGIFASPRRWLQQRKFQSPPDSRGHPYVVWKSEGDFTWNSMSGRSVRLRSVPIQSLSELERARLQEVAFYQLQQDCDLSCQITIPKDGQKRKKSLRKKLDSLGKEKNKDKEFIPQAFGMPLSQVIANDRAYKLKQDLQRDEQKDASDFVASLLPFGNKRQNKELSSSNSSLSSTSETPNESTSPNTPEPAPRARRRGAMSVDSITDLDDNQSRLLEALQLSLPAEAQSKKEKARDKKLSLNPIYRQVPRLVDSCCQHLEKHGLQTVGIFRVGSSKKRVRQLREEFDRGIDVSLEEEHSVHDVAALLKEFLRDMPDPLLTRELYTAFINTLLLEPEEQLGTLQLLIYLLPPCNCDTLHRLLQFLSIVARHADDNISKDGQEVTGNKMTSLNLATIFGPNLLHKQKSSDKEFSVQSSARAEESTAIIAVVQKMIENYEALFMVPPDLQNEVLISLLETDPDVVDYLLRRKASQSSSPDMLQSEVSFSVGGRHSSTDSNKASSGDISPYDNNSPVLSERSLLAMQEDAAPGGSEKLYRVPGQFMLVGHLSSSKSRESSPGPRLGKDLSEEPFDIWGTWHSTLKSGSKDPGMTGSSGDIFESSSLRAGPCSLSQGNLSPNWPRWQGSPAELDSDTQGARRTQAAAPATEGRAHPAVSRACSTPHVQVAGKAERPTARSEQYLTLSGAHDLSESELDVAGLQSRATPQCQRPHGSGRDDKRPPPPYPGPGKPAAAAAWIQGPPEGVETPTDQGGQAAEREQQVTQKKLSSANSLPAGEQDSPRLGDAGWLDWQRERWQIWELLSTDNPDALPETLV

753 NP_054885.1 NP_004835.2

>NP_054885.1

MAASSSGEKEKERLGGGLGVAGGNSTRERLLSALEDLEVLSRELIEMLAISRNQKLLQAGEENQVLELLIHRDGEFQELMKLALNQGKIHHEMQVLEKEVEKRDSDIQQLQKQLKEAEQILATAVYQAKEKLKSIEKARKGAISSEEIIKYAHRISASNAVCAPLTWVPGDPRRPYPTDLEMRSGLLGQMNNPSTNGVNGHLPGDALAAGRLPDVLAPQYPWQSNDMSMNMLPPNHSSDFLLEPPGHNKENEDDVEIMSTDSSSSSSESD

>NP_004835.2

MDAALKRSRSEEPAEILPPARDEEEEEEEGMEQGLEEEEEVDPRIQGELEKLNQSTDDINRRETELEDARQKFRSVLVEATVKLDELVKKIGKAVEDSKPYWEARRVARQAQLEAQKATQDFQRATEVLRAAKETISLAEQRLLEDDKRQFDSAWQEMLNHATQRVMEAEQTKTRSELVHKETAARYNAAMGRMRQLEKKLKRAINKSKPYFELKAKYYVQLEQLKKTVDDLQAKLTLAKGEYKMALKNLEMISDEIHERRRSSAMGPRGCGVGAEGSSTSVEDLPGSKPEPDAISVASEAFEDDSCSNFVSEDDSETQSVSSFSSGPTSPSEMPDQFPAVVRPGSLDLPSPVSLSEFGMMFPVLGPRSECSGASSPECEVERGDRAEGAENKTSDKANNNRGLSSSSGSGGSSKSQSSTSPEGQALENRMKQLSLQCSKGRDGIIADIKMVQIG

754 NP_004951.1 Q9BRV8

>NP_004951.1

MASNDYTQQATQSYGAYPTQPGQGYSQQSSQPYGQQSYSGYSQSTDTSGYGQSSYSSYGQSQNTGYGTQSTPQGYGSTGGYGSSQSSQSSYGQQSSYPGYGQQPAPSSTSGSYGSSSQSSSYGQPQSGSYSQQPSYGGQQQSYGQQQSYNPPQGYGQQNQYNSSSGGGGGGGGGGNYGQDQSSMSSGGGSGGGYGNQDQSGGGGSGGYGQQDRGGRGRGGSGGGGGGGGGGYNRSSGGYEPRGRGGGRGGRGGMGGSDRGGFNKFGGPRDQGSRHDSEQDNSDNNTIFVQGLGENVTIESVADYFKQIGIIKTNKKTGQPMINLYTDRETGKLKGEATVSFDDPPSAKAAIDWFDGKEFSGNPIKVSFATRRADFNRGGGNGRGGRGRGGPMGRGGYGGGGSGGGGRGGFPSGGGGGGGQQRAGDWKCPNPTCENMNFSWRNECNQCKAPKPDGPGGGPGGSHMGGNYGDDRRGGRGGYDRGGYRGRGGDRGGFRGGRGGGDRGGFGPGKMDSRGEHRQDRRERPY

>Q9BRV8

MSCTIEKILTDAKTLLERLREHDAAAESLVDQSAALHRRVAAMREAGTALPDQYQEDASDMKDMSKYKPHILLSQENTQIRDLQQENRELWISLEEHQDALELIMSKYRKQMLQLMVAKKAVDAEPVLKAHQSHSAEIESQIDRICEMGEVMRKAVQVDDDQFCKIQEKLAQLELENKELRELLSISSESLQARKENSMDTASQAIK

755 Q13461 NP_000377.1

>Q13461

MAGRSDMDPPAAFSGFPALPAVAPSGPPPSPLAGAEPGREPEEAAAGRGEAAPTPAPGPGRRRRRPLQRGKPPYSYIALIAMALAHAPGRRLTLAAIYRFITERFAFYRDSPRKWQNSIRHNLTLNDCFVKVPREPGNPGKGNYWTLDPAAADMFDNGSFLRRRKRFKRAELPAHAAAAPGPPLPFPYAPYAPAPGPALLVPPPSAGPGPSPPARLFSVDSLVNLQPELAGLGAPEPPCCAAPDAAAAAFPPCAAAASPPLYSQVPDRLVLPATRPGPGPLPAEPLLALAGPAAALGPLSPGEAYLRQPGFASGLERYL

>NP_000377.1

MSSSGLNSEKVAALIQKLNSDPQFVLAQNVGTTHDLLDICLKRATVQRAQHVFQHAVPQEGKPITNQKSSGRCWIFSCLNVMRLPFMKKLNIEEFEFSQSYLFFWDKVERCYFFLSAFVDTAQRKEPEDGRLVQFLLMNPANDGGQWDMLVNIVEKYGVIPKKCFPESYTTEATRRMNDILNHKMREFCIRLRNLVHSGATKGEISATQDVMMEEIFRVVCICLGNPPETFTWEYRDKDKNYQKIGPITPLEFYREHVKPLFNMEDKICLVNDPRPQHKYNKLYTVEYLSNMVGGRKTLYNNQPIDFLKKMVAASIKDGEAVWFGCDVGKHFNSKLGLSDMNLYDHELVFGVSLKNMNKAERLTFGESLMTHAMTFTAVSEKDDQDGAFTKWRVENSWGEDHGHKGYLCMTDEWFSEYVYEVVVDRKHVPEEVLAVLEQEPIILPAWDPMGALAE

756 Q9UEE5 P17516

>Q9UEE5

MIPLEKPGSGGSSPGATSGSGRAGRGLSGPCRPPPPPQARGLLTEIRAVVRTEPFQDGYSLCPGRELGRGKFAVVRKCIKKDSGKEFAAKFMRKRRKGQDCRMEIIHEIAVLELAQDNPWVINLHEVYETASEMILVLEYAAGGEIFDQCVADREEAFKEKDVQRLMRQILEGVHFLHTRDVVHLDLKPQNILLTSESPLGDIKIVDFGLSRILKNSEELREIMGTPEYVAPEILSYDPISMATDMWSIGVLTYVMLTGISPFLGNDKQETFLNISQMNLSYSEEEFDVLSESAVDFIRTLLVKKPEDRATAEECLKHPWLTQSSIQEPSFRMEKALEEANALQEGHSVPEINSDTDKSETEESIVTEELIVVTSYTLGQCRQSEKEKMEQKAISKRFKFEEPLLQEIPGEFIY

>P17516

MDPKYQRVELNDGHFMPVLGFGTYAPPEVPRNRAVEVTKLAIEAGFRHIDSAYLYNNEEQVGLAIRSKIADGSVKREDIFYTSKLWCTFFQPQMVQPALESSLKKLQLDYVDLYLLHFPMALKPGETPLPKDENGKVIFDTVDLSATWEVMEKCKDAGLAKSIGVSNFNCRQLEMILNKPGLKYKPVCNQVECHPYLNQSKLLDFCKSKDIVLVAHSALGTQRHKLWVDPNSPVLLEDPVLCALAKKHKRTPALIALRYQLQRGVVVLAKSYNEQRIRENIQVFEFQLTSEDMKVLDGLNRNYRYVVMDFLMDHPDYPFSDEY

757 NP_002139.2 P50135

>NP_002139.2

MSFPNSSPAANTFLVDSLISACRSDSFYSSSASMYMPPPSADMGTYGMQTCGLLPSLAKREVNHQNMGMNVHPYIPQVDSWTDPNRSCRIEQPVTQQVPTCSFTTNIKEESNCCMYSDKRNKLISAEVPSYQRLVPESCPVENPEVPVPGYFRLSQTYATGKTQEYNNSPEGSSTVMLQLNPRGAAKPQLSAAQLQMEKKMNEPVSGQEPTKVSQVESPEAKGGLPEERSCLAEVSVSSPEVQEKESKEEIKSDTPTSNWLTAKSGRKKRCPYTKHQTLELEKEFLFNMYLTRERRLEISKSVNLTDRQVKIWFQNRRMKLKKMSRENRIRELTANLTFS

>P50135

MASSMRSLFSDHGKYVESFRRFLNHSTEHQCMQEFMDKKLPGIIGRIGDTKSEIKILSIGGGAGEIDLQILSKVQAQYPGVCINNEVVEPSAEQIAKYKELVAKTSNLENVKFAWHKETSSEYQSRMLEKKELQKWDFIHMIQMLYYVKDIPATLKFFHSLLGTNAKMLIIVVSGSSGWDKLWKKYGSRFPQDDLCQYITSDDLTQMLDNLGLKYECYDLLSTMDISDCFIDGNENGDLLWDFLTETCNFNATAPPDLRAELGKDLQEPEFSAKKEGKVLFNNTLSFIVIEA

758 NP_071900.2 Q8N6F7

>NP_071900.2

MDQTCELPRRNCLLPFSNPVNLDAPEDKDSPFGNGQSNFSEPLNGCTMQLSTVSGTSQNAYGQDSPSCYIPLRRLQDLASMINVEYLNGSADGSESFQDPEKSDSRAQTPIVCTSLSPGGPTALAMKQEPSCNNSPELQVKVTKTIKNGFLHFENFTCVDDADVDSEMDPEQPVTEDESIEEIFEETQTNATCNYETKSENGVKVAMGSEQDSTPESRHGAVKSPFLPLAPQTETQKNKQRNEVDGSNEKAALLPAPFSLGDTNITIEEQLNSINLSFQDDPDSSTSTLGNMLELPGTSSSSTSQELPFCQPKKKSTPLKYEVGDLIWAKFKRRPWWPCRICSDPLINTHSKMKVSNRRPYRQYYVEAFGDPSERAWVAGKAIVMFEGRHQFEELPVLRRRGKQKEKGYRHKVPQKILSKWEASVGLAEQYDVPKGSKNRKCIPGSIKLDSEEDMPFEDCTNDPESEHDLLLNGCLKSLAFDSEHSADEKEKPCAKSRARKSSDNPKRTSVKKGHIQFEAHKDERRGKIPENLGLNFISGDISDTQASNELSRIANSLTGSNTAPGSFLFSSCGKNTAKKEFETSNGDSLLGLPEGALISKCSREKNKPQRSLVCGSKVKLCYIGAGDEEKRSDSISICTTSDDGSSDLDPIEHSSESDNSVLEIPDAFDRTENMLSMQKNEKIKYSRFAATNTRVKAKQKPLISNSHTDHLMGCTKSAEPGTETSQVNLSDLKASTLVHKPQSDFTNDALSPKFNLSSSISSENSLIKGGAANQALLHSKSKQPKFRSIKCKHKENPVMAEPPVINEECSLKCCSSDTKGSPLASISKSGKVDGLKLLNNMHEKTRDSSDIETAVVKHVLSELKELSYRSLGEDVSDSGTSKPSKPLLFSSASSQNHIPIEPDYKFSTLLMMLKDMHDSKTKEQRLMTAQNLVSYRSPGRGDCSTNSPVGVSKVLVSGGSTHNSEKKGDGTQNSANPSPSGGDSALSGELSASLPGLLSDKRDLPASGKSRSDCVTRRNCGRSKPSSKLRDAFSAQMVKNTVNRKALKTERKRKLNQLPSVTLDAVLQGDRERGGSLRGGAEDPSKEDPLQIMGHLTSEDGDHFSDVHFDSKVKQSDPGKISEKGLSFENGKGPELDSVMNSENDELNGVNQVVPKKRWQRLNQRRTKPRKRMNRFKEKENSECAFRVLLPSDPVQEGRDEFPEHRTPSASILEEPLTEQNHADCLDSAGPRLNVCDKSSASIGDMEKEPGIPSLTPQAELPEPAVRSEKKRLRKPSKWLLEYTEEYDQIFAPKKKQKKVQEQVHKVSSRCEEESLLARGRSSAQNKQVDENSLISTKEEPPVLEREAPFLEGPLAQSELGGGHAELPQLTLSVPVAPEVSPRPALESEELLVKTPGNYESKRQRKPTKKLLESNDLDPGFMPKKGDLGLSKKCYEAGHLENGITESCATSYSKDFGGGTTKIFDKPRKRKRQRHAAAKMQCKKVKNDDSSKEIPGSEGELMPHRTATSPKETVEEGVEHDPGMPASKKMQGERGGGAALKENVCQNCEKLGELLLCEAQCCGAFHLECLGLTEMPRGKFICNECRTGIHTCFVCKQSGEDVKRCLLPLCGKFYHEECVQKYPPTVMQNKGFRCSLHICITCHAANPANVSASKGRLMRCVRCPVAYHANDFCLAAGSKILASNSIICPNHFTPRRGCRNHEHVNVSWCFVCSEGGSLLCCDSCPAAFHRECLNIDIPEGNWYCNDCKAGKKPHYREIVWVKVGRYRWWPAEICHPRAVPSNIDKMRHDVGEFPVLFFGSNDYLWTHQARVFPYMEGDVSSKDKMGKGVDGTYKKALQEAAARFEELKAQKELRQLQEDRKNDKKPPPYKHIKVNRPIGRVQIFTADLSEIPRCNCKATDENPCGIDSECINRMLLYECHPTVCPAGGRCQNQCFSKRQYPEVEIFRTLQRGWGLRTKTDIKKGEFVNEYVGELIDEEECRARIRYAQEHDITNFYMLTLDKDRIIDAGPKGNYARFMNHCCQPNCETQKWSVNGDTRVGLFALSDIKAGTELTFNYNLECLGNGKTVCKCGAPNCSGFLGVRPKNQPIATEEKSKKFKKKQQGKRRTQGEITKEREDECFSCGDAGQLVSCKKPGCPKVYHADCLNLTKRPAGKWECPWHQCDICGKEAASFCEMCPSSFCKQHREGMLFISKLDGRLSCTEHDPCGPNPLEPGEIREYVPPPVPLPPGPSTHLAEQSTGMAAQAPKMSDKPPADTNQMLSLSKKALAGTCQRPLLPERPLERTDSRPQPLDKVRDLAGSGTKSQSLVSSQRPLDRPPAVAGPRPQLSDKPSPVTSPSSSPSVRSQPLERPLGTADPRLDKSIGAASPRPQSLEKTSVPTGLRLPPPDRLLITSSPKPQTSDRPTDKPHASLSQRLPPPEKVLSAVVQTLVAKEKALRPVDQNTQSKNRAALVMDLIDLTPRQKERAASPHQVTPQADEKMPVLESSSWPASKGLGHMPRAVEKGCVSDPLQTSGKAAAPSEDPWQAVKSLTQARLLSQPPAKAFLYEPTTQASGRASAGAEQTPGPLSQSPGLVKQAKQMVGGQQLPALAAKSGQSFRSLGKAPASLPTEEKKLVTTEQSPWALGKASSRAGLWPIVAGQTLAQSCWSAGSTQTLAQTCWSLGRGQDPKPEQNTLPALNQAPSSHKCAESEQK

>Q8N6F7

MGNSLLRENRRQQNTQEMPWNVRMQSPKQRTSRCWDHHIAEGCFCLPWKKILIFEKRQDSQNENERMSSTPIQDNVDQTYSEELCYTLINHRVLCTRPSGNSAEEYYENVPCKAERPRESLGGTETEYSLLHMPSTDPRHARSPEDEYELLMPHRISSHFLQQPRPLMAPSETQFSHL

759 NP_001417.3 P08319

>NP_001417.3

MEEQQPEPKSQRDSALGAAAAATPGGLSLSLSPGASGSSGSGSDGDSVPVSPQPAPPSPPAAPCLPPLAHHPHLPPHPPPPPPQHLAAPAHQPQPAAQLHRTTNFFIDNILRPDFGCKKEQPPPQLLVAAAARGGAGGGGRVERDRGQTAAGRDPVHPLGTRAPGAASLLCAPDANCGPPDGSQPAAAGAGASKAGNPAAAAAAAAAAVAAAAAAAAAKPSDTGGGGSGGGAGSPGAQGTKYPEHGNPAILLMGSANGGPVVKTDSQQPLVWPAWVYCTRYSDRPSSGPRTRKLKKKKNEKEDKRPRTAFTAEQLQRLKAEFQANRYITEQRRQTLAQELSLNESQIKIWFQNKRAKIKKATGIKNGLALHLMAQGLYNHSTTTVQDKDESE

>P08319

MGTKGKVIKCKAAIAWEAGKPLCIEEVEVAPPKAHEVRIQIIATSLCHTDATVIDSKFEGLAFPVIVGHEAAGIVESIGPGVTNVKPGDKVIPLYAPLCRKCKFCLSPLTNLCGKISNLKSPASDQQLMEDKTSRFTCKGKPVYHFFGTSTFSQYTVVSDINLAKIDDDANLERVCLLGCGFSTGYGAAINNAKVTPGSTCAVFGLGGVGLSAVMGCKAAGASRIIGIDINSEKFVKAKALGATDCLNPRDLHKPIQEVIIELTKGGVDFALDCAGGSETMKAALDCTTAGWGSCTFIGVAAGSKGLTVFPEELIIGRTINGTFFGGWKSVDSIPKLVTDYKNKKFNLDALVTHTLPFDKISEAFDLMNQGKSIRTILIF

760 Q12873 Q16719

>Q12873

MKAADTVILWARSKNDQLRISFPPGLCWGDRMPDKDDIRLLPSALGVKKRKRGPKKQKENKPGKPRKRKKRDSEEEFGSERDEYREKSESGGSEYGTGPGRKRRRKHREKKEKKTKRRKKGEGDGGQKQVEQKSSATLLLTWGLEDVEHVFSEEDYHTLTNYKAFSQFMRPLIAKKNPKIPMSKMMTILGAKWREFSANNPFKGSAAAVAAAAAAAAAAVAEQVSAAVSSATPIAPSGPPALPPPPAADIQPPPIRRAKTKEGKGPGHKRRSKSPRVPDGRKKLRGKKMAPLKIKLGLLGGKRKKGGSYVFQSDEGPEPEAEESDLDSGSVHSASGRPDGPVRTKKLKRGRPGRKKKKVLGCPAVAGEEEVDGYETDHQDYCEVCQQGGEIILCDTCPRAYHLVCLDPELDRAPEGKWSCPHCEKEGVQWEAKEEEEEYEEEGEEEGEKEEEDDHMEYCRVCKDGGELLCCDACISSYHIHCLNPPLPDIPNGEWLCPRCTCPVLKGRVQKILHWRWGEPPVAVPAPQQADGNPDVPPPRPLQGRSEREFFVKWVGLSYWHCSWAKELQLEIFHLVMYRNYQRKNDMDEPPPLDYGSGEDDGKSDKRKVKDPHYAEMEEKYYRFGIKPEWMTVHRIINHSVDKKGNYHYLVKWRDLPYDQSTWEEDEMNIPEYEEHKQSYWRHRELIMGEDPAQPRKYKKKKKELQGDGPPSSPTNDPTVKYETQPRFITATGGTLHMYQLEGLNWLRFSWAQGTDTILADEMGLGKTIQTIVFLYSLYKEGHTKGPFLVSAPLSTIINWEREFQMWAPKFYVVTYTGDKDSRAIIRENEFSFEDNAIKGGKKAFKMKREAQVKFHVLLTSYELITIDQAALGSIRWACLVVDEAHRLKNNQSKFFRVLNGYKIDHKLLLTGTPLQNNLEELFHLLNFLTPERFNNLEGFLEEFADISKEDQIKKLHDLLGPHMLRRLKADVFKNMPAKTELIVRVELSPMQKKYYKYILTRNFEALNSRGGGNQVSLLNIMMDLKKCCNHPYLFPVAAMESPKLPSGAYEGGALIKSSGKLMLLQKMLRKLKEQGHRVLIFSQMTKMLDLLEDFLDYEGYKYERIDGGITGALRQEAIDRFNAPGAQQFCFLLSTRAGGLGINLATADTVIIFDSDWNPHNDIQAFSRAHRIGQANKVMIYRFVTRASVEERITQVAKRKMMLTHLVVRPGLGSKAGSMSKQELDDILKFGTEELFKDENEGENKEEDSSVIHYDNEAIARLLDRNQDATEDTDVQNMNEYLSSFKVAQYVVREEDKIEEIEREIIKQEENVDPDYWEKLLRHHYEQQQEDLARNLGKGKRVRKQVNYNDAAQEDQDNQSEYSVGSEEEDEDFDERPEGRRQSKRQLRNEKDKPLPPLLARVGGNIEVLGFNTRQRKAFLNAVMRWGMPPQDAFTTQWLVRDLRGKTEKEFKAYVSLFMRHLCEPGADGSETFADGVPREGLSRQQVLTRIGVMSLVKKKVQEFEHINGRWSMPELMPDPSADSKRSSRASSPTKTSPTTPEASATNSPCTSKPATPAPSEKGEGIRTPLEKEEAENQEEKPEKNSRIGEKMETEADAPSPAPSLGERLEPRKIPLEDEVPGVPGEMEPEPGYRGDREKSATESTPGERGEEKPLDGQEHRERPEGETGDLGKREDVKGDRELRPGPRDEPRSNGRREEKTEKPRFMFNIADGGFTELHTLWQNEERAAISSGKLNEIWHRRHDYWLLAGIVLHGYARWQDIQNDAQFAIINEPFKTEANKGNFLEMKNKFLARRFKLLEQALVIEEQLRRAAYLNLSQEPAHPAMALHARFAEAECLAESHQHLSKESLAGNKPANAVLHKVLNQLEELLSDMKADVTRLPATLSRIPPIAARLQMSERSILSRLASKGTEPHPTPAYPPGPYATPPGYGAAFSAAPVGALAAAGANYSQMPAGSFITAATNGPPVLVKKEKEMVGALVSDGLDRKEPRAGEVICIDD

>Q16719

MEPSSLELPADTVQRIAAELKCHPTDERVALHLDEEDKLRHFRECFYIPKIQDLPPVDLSLVNKDENAIYFLGNSLGLQPKMVKTYLEEELDKWAKIAAYGHEVGKRPWITGDESIVGLMKDIVGANEKEIALMNALTVNLHLLMLSFFKPTPKRYKILLEAKAFPSDHYAIESQLQLHGLNIEESMRMIKPREGEETLRIEDILEVIEKEGDSIAVILFSGVHFYTGQHFNIPAITKAGQAKGCYVGFDLAHAVGNVELYLHDWGVDFACWCSYKYLNAGAGGIAGAFIHEKHAHTIKPALVGWFGHELSTRFKMDNKLQLIPGVCGFRISNPPILLVCSLHASLEIFKQATMKALRKKSVLLTGYLEYLIKHNYGKDKAATKKPVVNIITPSHVEERGCQLTITFSVPNKDVFQELEKRGVVCDKRNPNGIRVAPVPLYNSFHDVYKFTNLLTSILDSAETKN

761 NP_005973.1 NP_001995.1

>NP_005973.1

MSMLPSFGFTQEQVACVCEVLQQGGNLERLGRFLWSLPACDHLHKNESVLKAKAVVAFHRGNFRELYKILESHQFSPHNHPKLQQLWLKAHYVEAEKLRGRPLGAVGKYRVRRKFPLPRTIWDGEETSYCFKEKSRGVLREWYAHNPYPSPREKRELAEATGLTTTQVSNWFKNRRQRDRAAEAKERENTENNNSSSNKQNQLSPLEGGKPLMSSSEEEFSPPQSPDQNSVLLLQGNMGHARSSNYSLPGLTASQPSHGLQTHQHQLQDSLLGPLTSSLVDLGS

>NP_001995.1

MPLSRWLRSVGVFLLPAPYWAPRERWLGSLRRPSLVHGYPVLAWHSARCWCQAWTEEPRALCSSLRMNGDQNSDVYAQEKQDFVQHFSQIVRVLTEDEMGHPEIGDAIARLKEVLEYNAIGGKYNRGLTVVVAFRELVEPRKQDADSLQRAWTVGWCVELLQAFFLVADDIMDSSLTRRGQICWYQKPGVGLDAINDANLLEACIYRLLKLYCREQPYYLNLIELFLQSSYQTEIGQTLDLLTAPQGNVDLVRFTEKRYKSIVKYKTAFYSFYLPIAAAMYMAGIDGEKEHANAKKILLEMGEFFQIQDDYLDLFGDPSVTGKIGTDIQDNKCSWLVVQCLQRATPEQYQILKENYGQKEAEKVARVKALYEELDLPAVFLQYEEDSYSHIMALIEQYAAPLPPAVFLGLARKIYKRRK

762 NP_660328.2 NP_055285.1

>NP_660328.2

MMLPSPVTSTPFSVKDILNLEQQHQHFHGAHLQADLEHHFHSAPCMLAAAEGTQFSDGGEEDEEDEGEKLSYLNSLAAADGHGDSGLCPQGYVHTVLRDSCSEPKEHEEEPEVVRDRSQKSCQLKKSLETAGDCKAAEESERPKPRSRRKPRVLFSQAQVFELERRFKQQRYLSAPEREHLASSLKLTSTQVKIWFQNRRYKCKRQRQDKSLELGAHAPPPPPRRVAVPVLVRDGKPCVTPSAQAYGAPYSVGASAYSYNSFPAYGYGNSAAAAAAAAAAAAAAAAYSSSYGCAYPAGGGGGGGGTSAATTAMQPACSAAGGGPFVNVSNLGGFGSGGSAQPLHQGTAAGAACAQGTLQGIRAW

>NP_055285.1

MKERRAPQPVVARCKLVLVGDVQCGKTAMLQVLAKDCYPETYVPTVFENYTACLETEEQRVELSLWDTSGSPYYDNVRPLCYSDSDAVLLCFDISRPETVDSALKKWRTEILDYCPSTRVLLIGCKTDLRTDLSTLMELSHQKQAPISYEQGCAIAKQLGAEIYLEGSAFTSEKSIHSIFRTASMLCLNKPSPLPQKSPVRSLSKRLLHLPSRSELISSTFKKEKAKSCSIM

763 Q9ULG1 O43182

>Q9ULG1

MASELGARDDGGCTELAKPLYLQYLERALRLDHFLRQTSAIFNRNISSDDSEDGLDDSNPLLPQSGDPLIQVKEEPPNSLLGETSGAGSSGMLNTYSLNGVLQSESKCDKGNLYNFSKLKKSRKWLKSILLSDESSEADSQSEDDDEEELNLSREELHNMLRLHKYKKLHQNKYSKDKELQQYQYYSAGLLSTYDPFYEQQRHLLGPKKKKFKEEKKLKAKLKKVKKKRRRDEELSSEESPRRHHHQTKVFAKFSHDAPPPGTKKKHLSIEQLNARRRKVWLSIVKKELPKANKQKASARNLFLTNSRKLAHQCMKEVRRAALQAQKNCKETLPRARRLTKEMLLYWKKYEKVEKEHRKRAEKEALEQRKLDEEMREAKRQQRKLNFLITQTELYAHFMSRKRDMGHDGIQEEILRKLEDSSTQRQIDIGGGVVVNITQEDYDSNHFKAQALKNAENAYHIHQARTRSFDEDAKESRAAALRAANKSGTGFGESYSLANPSIRAGEDIPQPTIFNGKLKGYQLKGMNWLANLYEQGINGILADEMGLGKTVQSIALLAHLAERENIWGPFLIISPASTLNNWHQEFTRFVPKFKVLPYWGNPHDRKVIRRFWSQKTLYTQDAPFHVVITSYQLVVQDVKYFQRVKWQYMVLDEAQALKSSSSVRWKILLQFQCRNRLLLTGTPIQNTMAELWALLHFIMPTLFDSHEEFNEWFSKDIESHAENKSAIDENQLSRLHMILKPFMLRRIKKDVENELSDKIEILMYCQLTSRQKLLYQALKNKISIEDLLQSSMGSTQQAQNTTSSLMNLVMQFRKVCNHPELFERQETWSPFHISLKPYHISKFIYRHGQIRVFNHSRDRWLRVLSPFAPDYIQRSLFHRKGINEESCFSFLRFIDISPAEMANLMLQGLLARWLALFLSLKASYRLHQLRSWGAPEGESHQRYLRNKDFLLGVNFPLSFPNLCSCPLLKSLVFSSHCKAVSGYSDQVVHQRRSATSSLRRCLLTELPSFLCVASPRVTAVPLDSYCNDRSAEYERRVLKEGGSLAAKQCLLNGAPELAADWLNRRSQFFPEPAGGLWSIRPQNGWSFIRIPGKESLITDSGKLYALDVLLTRLKSQGHRVLIYSQMTRMIDLLEEYMVYRKHTYMRLDGSSKISERRDMVADFQNRNDIFVFLLSTRAGGLGINLTAADTVIFYDSDWNPTVDQQAMDRAHRLGQTKQVTVYRLICKGTIEERILQRAKEKSEIQRMVISGGNFKPDTLKPKEVVSLLLDDEELEKKLRLRQEEKRQQEETNRVKERKRKREKYAEKKKKEDELDGKRRKEGVNLVIPFVPSADNSNLSADGDDSFISVDSAMPSPFSEISISSELHTGSIPLDESSSDMLVIVDDPASSAPQSRATNSPASITGSVSDTVNGISIQEMPAAGRGHSARSRGRPKGSGSTAKGAGKGRSRKSTAGSAAAMAGAKAGAAAASAAAYAAYGYNVSKGISASSPLQTSLVRPAGLADFGPSSASSPLSSPLSKGNNVPGNPKNLHMTSSLAPDSLVRKQGKGTNPSGGR

>O43182

MSAQSLLHSVFSCSSPASSSAASAKGFSKRKLRQTRSLDPALIGGCGSDEAGAEGSARGATAGRLYSPSLPAESLGPRLASSSRGPPPRATRLPPPGPLCSSFSTPSTPQEKSPSGSFHFDYEVPLGRGGLKKSMAWDLPSVLAGPASSRSASSILCSSGGGPNGIFASPRRWLQQRKFQSPPDSRGHPYVVWKSEGDFTWNSMSGRSVRLRSVPIQSLSELERARLQEVAFYQLQQDCDLSCQITIPKDGQKRKKSLRKKLDSLGKEKNKDKEFIPQAFGMPLSQVIANDRAYKLKQDLQRDEQKDASDFVASLLPFGNKRQNKELSSSNSSLSSTSETPNESTSPNTPEPAPRARRRGAMSVDSITDLDDNQSRLLEALQLSLPAEAQSKKEKARDKKLSLNPIYRQVPRLVDSCCQHLEKHGLQTVGIFRVGSSKKRVRQLREEFDRGIDVSLEEEHSVHDVAALLKEFLRDMPDPLLTRELYTAFINTLLLEPEEQLGTLQLLIYLLPPCNCDTLHRLLQFLSIVARHADDNISKDGQEVTGNKMTSLNLATIFGPNLLHKQKSSDKEFSVQSSARAEESTAIIAVVQKMIENYEALFMVPPDLQNEVLISLLETDPDVVDYLLRRKASQSSSPDMLQSEVSFSVGGRHSSTDSNKASSGDISPYDNNSPVLSERSLLAMQEDAAPGGSEKLYRVPGQFMLVGHLSSSKSRESSPGPRLGKDLSEEPFDIWGTWHSTLKSGSKDPGMTGSSGDIFESSSLRAGPCSLSQGNLSPNWPRWQGSPAELDSDTQGARRTQAAAPATEGRAHPAVSRACSTPHVQVAGKAERPTARSEQYLTLSGAHDLSESELDVAGLQSRATPQCQRPHGSGRDDKRPPPPYPGPGKPAAAAAWIQGPPEGVETPTDQGGQAAEREQQVTQKKLSSANSLPAGEQDSPRLGDAGWLDWQRERWQIWELLSTDNPDALPETLV

764 NP_004517.2 Q9NRJ4

>NP_004517.2

MAESSESFTMASSPAQRRRGNDPLTSSPGRSSRRTDALTSSPGRDLPPFEDESEGLLGTEGPLEEEEDGEELIGDGMERDYRAIPELDAYEAEGLALDDEDVEELTASQREAAERAMRQRDREAGRGLGRMRRGLLYDSDEEDEERPARKRRQVERATEDGEEDEEMIESIENLEDLKGHSVREWVSMAGPRLEIHHRFKNFLRTHVDSHGHNVFKERISDMCKENRESLVVNYEDLAAREHVLAYFLPEAPAELLQIFDEAALEVVLAMYPKYDRITNHIHVRISHLPLVEELRSLRQLHLNQLIRTSGVVTSCTGVLPQLSMVKYNCNKCNFVLGPFCQSQNQEVKPGSCPECQSAGPFEVNMEETIYQNYQRIRIQESPGKVAAGRLPRSKDAILLADLVDSCKPGDEIELTGIYHNNYDGSLNTANGFPVFATVILANHVAKKDNKVAVGELTDEDVKMITSLSKDQQIGEKIFASIAPSIYGHEDIKRGLALALFGGEPKNPGGKHKVRGDINVLLCGDPGTAKSQFLKYIEKVSSRAIFTTGQGASAVGLTAYVQRHPVSREWTLEAGALVLADRGVCLIDEFDKMNDQDRTSIHEAMEQQSISISKAGIVTSLQARCTVIAAANPIGGRYDPSLTFSENVDLTEPIISRFDILCVVRDTVDPVQDEMLARFVVGSHVRHHPSNKEEEGLANGSAAEPAMPNTYGVEPLPQEVLKKYIIYAKERVHPKLNQMDQDKVAKMYSDLRKESMATGSIPITVRHIESMIRMAEAHARIHLRDYVIEDDVNMAIRVMLESFIDTQKFSVMRSMRKTFARYLSFRRDNNELLLFILKQLVAEQVTYQRNRFGAQQDTIEVPEKDLVDKARQINIHNLSAFYDSELFRMNKFSHDLKRKMILQQF

>Q9NRJ4

MYAAVEHGPVLCSDSNILCLSWKGRVPKSEKEKPVCRRRYYEEGWLATGNGRGVVGVTFTSSHCRRDRSTPQRINFNLRGHNSEVVLVRWNEPYQKLATCDADGGIFVWIQYEGRWSVELVNDRGAQVSDFTWSHDGTQALISYRDGFVLVGSVSGQRHWSSEINLESQITCGIWTPDDQQVLFGTADGQVIVMDCHGRMLAHVLLHESDGVLGMSWNYPIFLVEDSSESDTDSDDYAPPQDGPAAYPIPVQNIKPLLTVSFTSGDISLMNNYDDLSPTVIRSGLKEVVAQWCTQGDLLAVAGMERQTQLGELPNGPLLKSAMVKFYNVRGEHIFTLDTLVQRPIISICWGHRDSRLLMASGPALYVVRVEHRVSSLQLLCQQAIASTLREDKDVSKLTLPPRLCSYLSTAFIPTIKPPIPDPNNMRDFVSYPSAGNERLHCTMKRTEDDPEVGGPCYTLYLEYLGGLVPILKGRRISKLRPEFVIMDPRTDSKPDEIYGNSLISTVIDSCNCSDSSDIELSDDWAAKKSPKISRASKSPKLPRISIEARKSPKLPRAAQELSRSPRLPLRKPSVGSPSLTRREFPFEDITQHNYLAQVTSNIWGTKFKIVGLAAFLPTNLGAVIYKTSLLHLQPRQMTIYLPEVRKISMDYINLPVFNPNVFSEDEDDLPVTGASGVPENSPPCTVNIPIAPIHSSAQAMSPTQSIGLVQSLLANQNVQLDVLTNQTTAVGTAEHAGDSATQYPVSNRYSNPGQVIFGSVEMGRIIQNPPPLSLPPPPQGPMQLSTVGHGDRDHEHLQKSAKALRPTPQLAAEGDAVVFSAPQEVQVTKINPPPPYPGTIPAAPTTAAPPPPLPPPQPPVDVCLKKGDFSLYPTSVHYQTPLGYERITTFDSSGNVEEVCRPRTRMLCSQNTYTLPGPGSSATLRLTATEKKVPQPCSSATLNRLTVPRYSIPTGDPPPYPEIASQLAQGRGAAQRSDNSLIHATLRRNNREATLKMAQLADSPRAPLQPLAKSKGGPGGVVTQLPARPPPALYTCSQCSGTGPSSQPGASLAHTASASPLASQSSYSLLSPPDSARDRTDYVNSAFTEDEALSQHCQLEKPLRHPPLPEAAVTLKRPPPYQWDPMLGEDVWVPQERTAQTSGPNPLKLSSLMLSQGQHLDVSRLPFISPKSPASPTATFQTGYGMGVPYPGSYNNPPLPGVQAPCSPKDALSPTQFAQQEPAVVLQPLYPPSLSYCTLPPMYPGSSTCSSLQLPPVALHPWSSYSACPPMQNPQGTLPPKPHLVVEKPLVSPPPADLQSHLGTEVMVETADNFQEVLSLTESPVPQRTEKFGKKNRKRLDSRAEEGSVQAITEGKVKKEARTLSDFNSLISSPHLGREKKKVKSQKDQLKSKKLNKTNEFQDSSESEPELFISGDELMNQSQGSRKGWKSKRSPRAAGELEEAKCRRASEKEDGRLGSQGFVYVMANKQPLWNEATQVYQLDFGGRVTQESAKNFQIELEGRQVMQFGRIDGSAYILDFQYPFSAVQAFAVALANVTQRLK

765 P51513 P49638

>P51513

MMAAAPIQQNGTHTGVPIDLDPPDSRKRPLEAPPEAGSTKRTNTGEDGQYFLKVLIPSYAAGSIIGKGGQTIVQLQKETGATIKLSKLSKSKDFYPGTTERVCLIQGTVEALNAVHGFIAEKIREMPQNVAKTEPVSILQPQTTVNPDRIKQTLPSSPTTTKSSPSDPMTTSRANQVKIIVPNSTAGLIIGKGGATVKAVMEQSGAWVQLSQKPDGINLQERVVTVSGEPEQNRKAVELIIQKIQEDPQSGSCLNISYANVTGPVANSNPTGSPYANTAEVLPTAAAAAGLLGHANLAGVAAFPAVLSGFTGNDLVAITSALNTLASYGYNLNTLGLGLSQAAATGALAAAAASANPAAAAANLLATYASEASASGSTAGGTAGTFALGSLAAATAATNGYFGAASPLAASAILGTEKSTDGSKDVVEIAVPENLVGAILGKGGKTLVEYQELTGARIQISKKGEFVPGTRNRKVTITGTPAATQAAQYLITQRITYEQGVRAANPQKVG

>P49638

MAEARSQPSAGPQLNALPDHSPLLQPGLAALRRRAREAGVPLAPLPLTDSFLLRFLRARDFDLDLAWRLLKNYYKWRAECPEISADLHPRSIIGLLKAGYHGVLRSRDPTGSKVLIYRIAHWDPKVFTAYDVFRVSLITSELIVQEVETQRNGIKAIFDLEGWQFSHAFQITPSVAKKIAAVLTDSFPLKVRGIHLINEPVIFHAVFSMIKPFLTEKIKERIHMHGNNYKQSLLQHFPDILPLEYGGEEFSMEDICQEWTNFIMKSEDYLSSISESIQ

766 NP_001231.2 P17706

>NP_001231.2

MEGERKNNNKRWYFTREQLENSPSRRFGVDPDKELSYRQQAANLLQDMGQRLNVSQLTINTAIVYMHRFYMIQSFTQFPGNSVAPAALFLAAKVEEQPKKLEHVIKVAHTCLHPQESLPDTRSEAYLQQVQDLVILESIILQTLGFELTIDHPHTHVVKCTQLVRASKDLAQTSYFMATNSLHLTTFSLQYTPPVVACVCIHLACKWSNWEIPVSTDGKHWWEYVDATVTLELLDELTHEFLQILEKTPNRLKRIWNWRACEAAKKTKADDRGTDEKTSEQTILNMISQSSSDTTIAGLMSMSTSTTSAVPSLPVSEESSSNLTSVEMLPGKRWLSSQPSFKLEPTQGHRTSENLALTGVDHSLPQDGSNAFISQKQNSKSVPSAKVSLKEYRAKHAEELAAQKRQLENMEANVKSQYAYAAQNLLSHHDSHSSVILKMPIEGSENPERPFLEKADKTALKMRIPVAGGDKAASSKPEEIKMRIKVHAAADKHNSVEDSVTKSREHKEKHKTHPSNHHHHHNHHSHKHSHSQLPVGTGNKRPGDPKHSSQTSNLAHKTYSLSSSFSSSSSTRKRGPSEETGGAVFDHPAKIAKSTKSSSLNFSFPSLPTMGQMPGHSSDTSGLSFSQPSCKTRVPHSKLDKGPTGANGHNTTQTIDYQDTVNMLHSLLSAQGVQPTQPTAFEFVRPYSDYLNPRSGGISSRSGNTDKPRPPPLPSEPPPPLPPLPK

>P17706

MPTTIEREFEELDTQRRWQPLYLEIRNESHDYPHRVAKFPENRNRNRYRDVSPYDHSRVKLQNAENDYINASLVDIEEAQRSYILTQGPLPNTCCHFWLMVWQQKTKAVVMLNRIVEKESVKCAQYWPTDDQEMLFKETGFSVKLLSEDVKSYYTVHLLQLENINSGETRTISHFHYTTWPDFGVPESPASFLNFLFKVRESGSLNPDHGPAVIHCSAGIGRSGTFSLVDTCLVLMEKGDDINIKQVLLNMRKYRMGLIQTPDQLRFSYMAIIEGAKCIKGDSSIQKRWKELSKEDLSPAFDHSPNKIMTEKYNGNRIGLEEEKLTGDRCTGLSSKMQDTMEENSESALRKRIREDRKATTAQKVQQMKQRLNENERKRKRWLYWQPILTKMGFMSVILVGAFVGWTLFFQQNAL

767 P04066 NP_112198.1

>P04066

MRAPGMRSRPAGPALLLLLLFLGAAESVRRAQPPRRYTPDWPSLDSRPLPAWFDEAKFGVFIHWGVFSVPAWGSEWFWWHWQGEGRPQYQRFMRDNYPPGFSYADFGPQFTARFFHPEEWADLFQAAGAKYVVLTTKHHEGFTNWPSPVSWNWNSKDVGPHRDLVGELGTALRKRNIRYGLYHSLLEWFHPLYLLDKKNGFKTQHFVSAKTMPELYDLVNSYKPDLIWSDGEWECPDTYWNSTNFLSWLYNDSPVKDEVVVNDRWGQNCSCHHGGYYNCEDKFKPQSLPDHKWEMCTSIDKFSWGYRRDMALSDVTEESEIISELVQTVSLGGNYLLNIGPTKDGLIVPIFQERLLAVGKWLSINGEAIYASKPWRVQWEKNTTSVWYTSKGSAVYAIFLHWPENGVLNLESPITTSTTKITMLGIQGDLKWSTDPDKGLFISLPQLPPSAVPAEFAWTIKLTGVK

>NP_112198.1

MLKNKGHSSKKDNLAVNAVALQDHILHDLQLRNLSVADHSKTQVQKKENKSLKRDTKAIIDTGLKKTTQCPKLEDSEKEYVLDPKPPPLTLAQKLGLIGPPPPPLSSDEWEKVKQRSLLQGDSVQPCPICKEEFELRPQVLLSCSHVFHKACLQAFEKFTNKKTCPLCRKNQYQTRVIHDGARLFRIKCVTRIQAYWRGCVVRKWYRNLRKTVPPTDAKLRKKFFEKKFTEISHRILCSYNTNIEELFAEIDQCLAINRSVLQQLEEKCGHEITEEEWEKIQVQALRRETHECSICLAPLSAAGGQRVGAGRRSREMALLSCSHVFHHACLLALEEFSVGDRPPFHACPLCRSCYQKKILEC

768 NP_001036111.1 P78368

>NP_001036111.1

MALVFQFGQPVRAQPLPGLCHGKLIRTNACDVCNSTDLPEVEIISLLEEQLPHYKLRADTIYGYDHDDWLHTPLISPDANIDLTTEQIEETLKYFLLCAERVGQMTKTYNDIDAVTRLLEEKERDLELAARIGQSLLKKNKTLTERNELLEEQVEHIREEVSQLRHELSMKDELLQFYTSAAEESEPESVCSTPLKRNESSSSVQNYFHLDSLQKKLKDLEEENVVLRSEASQLKTETITYEEKEQQLVNDCVKELRDANVQIASISEELAKKTEDAARQQEEITHLLSQIVDLQKKAKACAVENEELVQHLGAAKDAQRQLTAELRELEDKYAECMEMLHEAQEELKNLRNKTMPNTTSRRYHSLGLFPMDSLAAEIEGTMRKELQLEEAESPDITHQKRVFETVRNINQVVKQRSLTPSPMNIPGSNQSSAMNSLLSSCVSTPRSSFYGSDIGNVVLDNKTNSIILETEAADLGNDERSKKPGTPGTPGSHDLETALRRLSLRRENYLSERRFFEEEQERKLQELAEKGELRSGSLTPTESIMSLGTHSRFSEFTGFSGMSFSSRSYLPEKLQIVKPLEGSATLHHWQQLAQPHLGGILDPRPGVVTKGFRTLDVDLDEVYCLNDFEEDDTGDHISLPRLATSTPVQHPETSAHHPGKCMSQTNSTFTFTTCRILHPSDELTRVTPSLNSAPTPACGSTSHLKSTPVATPCTPRRLSLAESFTNTRESTTTMSTSLGLVWLLKERGISAAVYDPQSWDRAGRGSLLHSYTPKMAVIPSTPPNSPMQTPTSSPPSFEFKCTSPPYDNFLASKPASSILREVREKNVRSSESQTDVSVSNLNLVDKVRRFGVAKVVNSGRAHVPTLTEEQGPLLCGPPGPAPALVPRGLVPEGLPLRCPTVTSAIGGLQLNSGIRRNRSFPTMVGSSMQMKAPVTLTSGILMGAKLSKQTSLR

>P78368

MDFDKKGGKGETEEGRRMSKAGGGRSSHGIRSSGTSSGVLMVGPNFRVGKKIGCGNFGELRLGKNLYTNEYVAIKLEPIKSRAPQLHLEYRFYKQLSATEGVPQVYYFGPCGKYNAMVLELLGPSLEDLFDLCDRTFTLKTVLMIAIQLITRMEYVHTKSLIYRDVKPENFLVGRPGTKRQHAIHIIDFGLAKEYIDPETKKHIPYREHKSLTGTARYMSINTHLGKEQSRRDDLEALGHMFMYFLRGSLPWQGLKADTLKERYQKIGDTKRATPIEVLCENFPEEMATYLRYVRRLDFFEKPDYDYLRKLFTDLFDRSGFVFDYEYDWAGKPLPTPIGTVHTDLPSQPQLRDKTQPHSKNQALNSTNGELNADDPTAGHSNAPITAPAEVEVADETKCCCFFKRRKRKSLQRHK

769 P41235 NP_002961.1

>P41235

MRLSKTLVDMDMADYSAALDPAYTTLEFENVQVLTMGNDTSPSEGTNLNAPNSLGVSALCAICGDRATGKHYGASSCDGCKGFFRRSVRKNHMYSCRFSRQCVVDKDKRNQCRYCRLKKCFRAGMKKEAVQNERDRISTRRSSYEDSSLPSINALLQAEVLSRQITSPVSGINGDIRAKKIASIADVCESMKEQLLVLVEWAKYIPAFCELPLDDQVALLRAHAGEHLLLGATKRSMVFKDVLLLGNDYIVPRHCPELAEMSRVSIRILDELVLPFQELQIDDNEYAYLKAIIFFDPDAKGLSDPGKIKRLRSQVQVSLEDYINDRQYDSRGRFGELLLLLPTLQSITWQMIEQIQFIKLFGMAKIDNLLQEMLLGGSPSDAPHAHHPLHPHLMQEHMGTNVIVANTMPTHLSNGQMCEWPRPRGQAATPETPQPSPPGGSGSEPYKLLPGAVATIVKPLSAIPQPTITKQEVI

>NP_002961.1

MAKFVIRPATAADCSDILRLIKELAKYEYMEEQVILTEKDLLEDGFGEHPFYHCLVAEVPKEHWTPEGHSIVGFAMYYFTYDPWIGKLLYLEDFFVMSDYRGFGIGSEILKNLSQVAMRCRCSSMHFLVAEWNEPSINFYKRRGASDLSSEEGWRLFKIDKEYLLKMATEE

770 NP_079441.1 Q8N142

>NP_079441.1

MEELQEPLRGQLRLCFTQAARTSLLLLRLNDAALRALQECQRQQVRPVIAFQGHRGYLRLPGPGWSCLFSFIVSQCCQEGAGGSLDLVCQRFLRSGPNSLHCLGSLRERLIIWAAMDSIPAPSSVQGHNLTEDARHPESWQNTGGYSEGDAVSQPQMALEEVSVSDPLASNQGQSLPGSSREHMAQWEVRSQTHVPNREPVQALPSSASRKRLDKKRSVPVATVELEEKRFRTLPLVPSPLQGLTNQDLQEGEDWEQEDEDMDPRLEHSSSVQEDSESPSPEDIPDYLLQYRAIHSAEQQHAYEQDFETDYAEYRILHARVGTASQRFIELGAEIKRVRRGTPEYKVLEDKIIQEYKKFRKQYPSYREEKRRCEYLHQKLSHIKGLILEFEEKNRGS

>Q8N142

MSGTRASNDRPPGAGGVKRGRLQQEAAATGSRVTVVLGAQWGDEGKGKVVDLLATDADIISRCQGGNNAGHTVVVDGKEYDFHLLPSGIINTKAVSFIGNGVVIHLPGLFEEAEKNEKKGLKDWEKRLIISDRAHLVFDFHQAVDGLQEVQRQAQEGKNIGTTKKGIGPTYSSKAARTGLRICDLLSDFDEFSSRFKNLAHQHQSMFPTLEIDIEGQLKRLKGFAERIRPMVRDGVYFMYEALHGPPKKILVEGANAALLDIDFGTYPFVTSSNCTVGGVCTGLGIPPQNIGDVYGVVKAYTTRVGIGAFPTEQINEIGGLLQTRGHEWGVTTGRKRRCGWLDLMILRYAHMVNGFTALALTKLDILDVLGEVKVGVSYKLNGKRIPYFPANQEMLQKVEVEYETLPGWKADTTGARRWEDLPPQAQNYIRFVENHVGVAVKWVGVGKSRESMIQLF

771 NP_054790.2 NP_006432.1

>NP_054790.2

MVLDDLPNLEDIYTSLCSSTMEDSEMDFDSGLEDDDTKSDSILEDSTIFVAFKGNIDDKDFKWKLDAILKNVPNLLHMESSKLKVQKVEPWNSVRVTFNIPREAAERLRILAQSNNQQLRDLGILSVQIEGEGAINLALAQNRSQDVRMNGPMGAGNSVRMEAGFPMASGPGIIRMNNPATVMIPPGGNVSSSMMAPGPNPELQPRTPRPASQSDAMDPLLSGLHIQQQSHPSGSLAPPHHPMQPVSVNRQMNPANFPQLQQQQQQQQQQQQQQQQQQQQQQQQQLQARPPQQHQQQQPQGIRPQFTAPTQVPVPPGWNQLPSGALQPPPAQGSLGTMTANQGWKKAPLPGPMQQQLQARPSLATVQTPSHPPPPYPFGSQQASQAHTNFPQMSNPGQFTAPQMKSLQGGPSRVPTPLQQPHLTNKSPASSPSSFQQGSPASSPTVNQTQQQMGPRPPQNNPLPQGFQQPVSSPGRNPMVQQGNVPPNFMVMQQQPPNQGPQSLHPGLGGMPKRLPPGFSAGQANPNFMQGQVPSTTATTPGNSGAPQLQANQNVQHAGGQGAGPPQNQMQVSHGPPNMMQPSLMGIHGNMNNQQAGTSGVPQVNLSNMQGQPQQGPPSQLMGMHQQIVPSQGQMVQQQGTLNPQNPMILSRAQLMPQGQMMVNPPSQNLGPSPQRMTPPKQMLSQQGPQMMAPHNQMMGPQGQVLLQQNPMIEQIMTNQMQGNKQQFNTQNQSNVMPGPAQIMRGPTPNMQGNMVQFTGQMSGQMLPQQGPVNNSPSQVMGIQGQVLRPPGPSPHMAQQHGDPATTANNDVSLSQMMPDVSIQQTNMVPPHVQAMQGNSASGNHFSGHGMSFNAPFSGAPNGNQMSCGQNPGFPVNKDVTLTSPLLVNLLQSDISAGHFGVNNKQNNTNANKPKKKKPPRKKKNSQQDLNTPDTRPAGLEEADQPPLPGEQGINLDNSGPKLPEFSNRPPGYPSQPVEQRPLQQMPPQLMQHVAPPPQPPQQQPQPQLPQQQQPPPPSQPQSQQQQQQQQQMMMMLMMQQDPKSVRLPVSQNVHPPRGPLNPDSQRMPMQQSGSVPVMVSLQGPASVPPSPDKQRMPMPVNTPLGSNSRKMVYQESPQNPSSSPLAEMASLPEASGSEAPSVPGGPNNMPSHVVLPQNQLMMTGPKPGPSPLSATQGATPQQPPVNSLPSSHGHHFPNVAAPTQTSRPKTPNRASPRPYYPQTPNNRPPSTEPSEISLSPERLNASIAGLFPPQINIPLPPRPNLNRGFDQQGLNPTTLKAIGQAPSNLTMNPSNFATPQTHKLDSVVVNSGKQSNSGATKRASPSNSRRSSPGSSRKTTPSPGRQNSKAPKLTLASQTNAALLQNVELPRNVLVSPTPLANPPVPGSFPNNSGLNPQNSTVSVAAVGGVVEDNKESLNVPQDSDCQNSQSRKEQVNIELKAVPAQEVKMVVPEDQSKKDGQPSDPNKLPSVEENKNLVSPAMREAPTSLSQLLDNSGAPNVTIKPPGLTDLEVTPPVVSGEDLKKASVIPTLQDLSSSKEPSNSLNLPHSNELCSSLVHPELSEVSSNVAPSIPPVMSRPVSSSSISTPLPPNQITVFVTSNPITTSANTSAALPTHLQSALMSTVVTMPNAGSKVMVSEGQSAAQSNARPQFITPVFINSSSIIQVMKGSQPSTIPAAPLTTNSGLMPPSVAVVGPLHIPQNIKFSSAPVPPNALSSSPAPNIQTGRPLVLSSRATPVQLPSPPCTSSPVVPSHPPVQQVKELNPDEASPQVNTSADQNTLPSSQSTTMVSPLLTNSPGSSGNRRSPVSSSKGKGKVDKIGQILLTKACKKVTGSLEKGEEQYGADGETEGQGLDTTAPGLMGTEQLSTELDSKTPTPPAPTLLKMTSSPVGPGTASAGPSLPGGALPTSVRSIVTTLVPSELISAVPTTKSNHGGIASESLAGGLVEEKVGSHPELLPSIAPSQNLVSKETSTTALQASVARPELEVNAAIVSGQSSEPKEIVEKSKIPGRRNSRTEEPTVASESVENGHRKRSSRPASASSSTKDITSAVQSKRRKSK

>NP_006432.1

MAAAAVSSAKRSLRGELKQRLRAMSAEERLRQSRVLSQKVIAHSEYQKSKRISIFLSMQDEIETEEIIKDIFQRGKICFIPRYRFQSNHMDMVRIESPEEISLLPKTSWNIPQPGEGDVREEALSTGGLDLIFMPGLGFDKHGNRLGRGKGYYDAYLKRCLQHQEVKPYTLALAFKEQICLQVPVNENDMKVDEVLYEDSSTA

772 NP_002544.1 Q7RTR2

>NP_002544.1

MPHLENVVLCRESQVSILQSLFGERHHFSFPSIFIYGHTASGKTYVTQTLLKTLELPHVFVNCVECFTLRLLLEQILNKLNHLSSSEDGCSTEITCETFNDFVRLFKQVTTAENLKDQTVYIVLDKAEYLRDMEANLLPGFLRLQELADRNVTVLFLSEIVWEKFRPNTGCFEPFVLYFPDYSIGNLQKILSHDHPPEYSADFYAAYINILLGVFYTVCRDLKELRHLAVLNFPKYCEPVVKGEASERDTRKLWRNIEPHLKKAMQTVYLREISSSQWEKLQKDDTDPGQLKGLSAHTHVELPYYSKFILIAAYLASYNPARTDKRFFLKHHGKIKKTNFLKKHEKTSNHLLGPKPFPLDRLLAILYSIVDSRVAPTANIFSQITSLVTLQLLTLVGHDDQLDGPKYKCTVSLDFIRAIARTVNFDIIKYLYDFL

>Q7RTR2

MRKQEVRTGREAGQGHGTGSPAEQVKALMDLLAGKGSQGSQAPQALDRTPDAPLGPCSNDSRIQRHRKALLSKVGGGPELGGPWHRLASLLLVEGLTDLQLREHDFTQVEATRGGGHPARTVALDRLFLPLSRVSVPPRVSITIGVAGMGKTTLVRHFVRLWAHGQVGKDFSLVLPLTFRDLNTHEKLCADRLICSVFPHVGEPSLAVAVPARALLILDGLDECRTPLDFSNTVACTDPKKEIPVDHLITNIIRGNLFPEVSIWITSRPSASGQIPGGLVDRMTEIRGFNEEEIKVCLEQMFPEDQALLGWMLSQVQADRALYLMCTVPAFCRLTGMALGHLWRSRTGPQDAELWPPRTLCELYSWYFRMALSGEGQEKGKASPRIEQVAHGGRKMVGTLGRLAFHGLLKKKYVFYEQDMKAFGVDLALLQGAPCSCFLQREETLASSVAYCFTHLSLQEFVAAAYYYGASRRAIFDLFTESGVSWPRLGFLTHFRSAAQRAMQAEDGRLDVFLRFLSGLLSPRVNALLAGSLLAQGEHQAYRTQVAELLQGCLRPDAAVCARAINVLHCLHELQHTELARSVEEAMESGALARLTGPAHRAALAYLLQVSDACAQEANLSLSLSQGVLQSLLPQLLYCRKLRLDTNQFQDPVMELLGSVLSGKDCRIQKISLAENQISNKGAKALARSLLVNRSLTSLDLRGNSIGPQGAKALADALKINRTLTSLSLQGNTVRDDGARSMAEALASNRTLSMLHLQKNSIGPMGAQRMADALKQNRSLKELMFSSNSIGDGGAKALAEALKVNQGLESLDLQSNSISDAGVAALMGALCTNQTLLSLSLRENSISPEGAQAIAHALCANSTLKNLDLTANLLHDQGARAIAVAVRENRTLTSLHLQWNFIQAGAAQALGQALQLNRSLTSLDLQENAIGDDGACAVARALKVNTALTALYLQVASIGASGAQVLGEALAVNRTLEILDLRGNAIGVAGAKALANALKVNSSLRRLNLQENSLGMDGAICIATALSGNHRLQHINLQGNHIGDSGARMISEAIKTNAPTCTVEM

773 Q01851 Q92526

>Q01851

MMSMNSKQPHFAMHPTLPEHKYPSLHSSSEAIRRACLPTPPLQSNLFASLDETLLARAEALAAVDIAVSQGKSHPFKPDATYHTMNSVPCTSTSTVPLAHHHHHHHHHQALEPGDLLDHISSPSLALMAGAGGAGAAAGGGGAHDGPGGGGGPGGGGGPGGGGPGGGGGGGPGGGGGGPGGGLLGGSAHPHPHMHSLGHLSHPAAAAAMNMPSGLPHPGLVAAAAHHGAAAAAAAAAAGQVAAASAAAAVVGAAGLASICDSDTDPRELEAFAERFKQRRIKLGVTQADVGSALANLKIPGVGSLSQSTICRFESLTLSHNNMIALKPILQAWLEEAEGAQREKMNKPELFNGGEKKRKRTSIAAPEKRSLEAYFAVQPRPSSEKIAAIAEKLDLKKNVVRVWFCNQRQKQKRMKFSATY

>Q92526

MAAIKAVNSKAEVARAQAALAVNICAARGLQDVLRTNLGPKGTMKMLASGAGDIKLTKDGNVLLDEMQIQHPTASLIAKVATAQDDVTGDGTTSNVLIIGELLKQADLYISEGLHPRIIAEGFEAAKIKALEVLEEVKVTKEMKRKILLDVARTSLQTKVHAELADVLTEVVVDSVLAVRRPGYPIDLFMVEIMEMKHKLGTDTKLIQGLVLDHGARHPDMKKRVEDAFILICNVSLEYEKTEVNSGFFYKTAEEKEKLVKAERKFIEDRVQKIIDLKDKVCAQSNKGFVVINQKGIDPFSLDSLAKHGIVALRRAKRRNMERLSLACGGMAVNSFEDLTVDCLGHAGLVYEYTLGEEKFTFIEECVNPCSVTLLVKGPNKHTLTQVKDAIRDGLRAIKNAIEDGCMVPGAGAIEVAMAEALVTYKNSIKGRARLGVQAFADALLIIPKVLAQNAGYDPQETLVKVQAEHVESKQLVGVDLNTGEPMVAADAGVWDNYCVKKQLLHSCTVIATNILLVDEIMRAGMSSLK

774 Q96EG1 P15428

>Q96EG1

MGWLFLKVLLAGVSFSGFLYPLVDFCISGKTRGQKPNFVIILADDMGWGDLGANWAETKDTANLDKMASEGMRFVDFHAAASTCSPSRASLLTGRLGLRNGVTRNFAVTSVGGLPLNETTLAEVLQQAGYVTGIIGKWHLGHHGSYHPNFRGFDYYFGIPYSHDMGCTDTPGYNHPPCPACPQGDGPSRNLQRDCYTDVALPLYENLNIVEQPVNLSSLAQKYAEKATQFIQRASTSGRPFLLYVALAHMHVPLPVTQLPAAPRGRSLYGAGLWEMDSLVGQIKDKVDHTVKENTFLWFTGDNGPWAQKCELAGSVGPFTGFWQTRQGGSPAKQTTWEGGHRVPALAYWPGRVPVNVTSTALLSVLDIFPTVVALAQASLPQGRRFDGVDVSEVLFGRSQPGHRVLFHPNSGAAGEFGALQTVRLERYKAFYITGGARACDGSTGPELQHKFPLIFNLEDDTAEAVPLERGGAEYQAVLPEVRKVLADVLQDIANDNISSADYTQDPSVTPCCNPYQIACRCQAA

>P15428

MHVNGKVALVTGAAQGIGRAFAEALLLKGAKVALVDWNLEAGVQCKAALDEQFEPQKTLFIQCDVADQQQLRDTFRKVVDHFGRLDILVNNAGVNNEKNWEKTLQINLVSVISGTYLGLDYMSKQNGGEGGIIINMSSLAGLMPVAQQPVYCASKHGIVGFTRSAALAANLMNSGVRLNAICPGFVNTAILESIEKEENMGQYIEYKDHIKDMIKYYGILDPPLIANGLITLIEDDALNGAIMKITTSKGIHFQDYDTTPFQAKTQ

775 P31271 NP_665683.1

>P31271

MTASVLLHPRWIEPTVMFLYDNGGGLVADELNKNMEGAAAAAAAAAAAAAAGAGGGGFPHPAAAAAGGNFSVAAAAAAAAAAAANQCRNLMAHPAPLAPGAASAYSSAPGEAPPSAAAAAAAAAAAAAAAAAASSSGGPGPAGPAGAEAAKQCSPCSAAAQSSSGPAALPYGYFGSGYYPCARMGPHPNAIKSCAQPASAAAAAAFADKYMDTAGPAAEEFSSRAKEFAFYHQGYAAGPYHHHQPMPGYLDMPVVPGLGGPGESRHEPLGLPMESYQPWALPNGWNGQMYCPKEQAQPPHLWKSTLPDVVSHPSDASSYRRGRKKRVPYTKVQLKELEREYATNKFITKDKRRRISATTNLSERQVTIWFQNRRVKEKKVINKLKTTS

>NP_665683.1

MAEKPKLHYFNARGRMESTRWLLAAAGVEFEEKFIKSAEDLDKLRNDGYLMFQQVPMVEIDGMKLVQTRAILNYIASKYNLYGKDIKERALIDMYIEGIADLGEMILLLPVCPPEEKDAKLALIKEKIKNRYFPAFEKVLKSHGQDYLVGNKLSRADIHLVELLYYVEELDSSLISSFPLLKALKTRISNLPTVKKFLQPGSPRKPPMDEKSLEEARKIFRF

776 Q01196 O75170

>Q01196

MRIPVDASTSRRFTPPSTALSPGKMSEALPLGAPDAGAALAGKLRSGDRSMVEVLADHPGELVRTDSPNFLCSVLPTHWRCNKTLPIAFKVVALGDVPDGTLVTVMAGNDENYSAELRNATAAMKNQVARFNDLRFVGRSGRGKSFTLTITVFTNPPQVATYHRAIKITVDGPREPRRHRQKLDDQTKPGSLSFSERLSELEQLRRTAMRVSPHHPAPTPNPRASLNHSTAFNPQPQSQMQDTRQIQPSPPWSYDQSYQYLGSIASPSVHPATPISPGRASGMTTLSAELSSRLSTAPDLTAFSDPRQFPALPSISDPRMHYPGAFTYSPTPVTSGIGIGMSAMGSATRYHTYLPPPYPGSSQAQGGPFQASSPSYHLYYGASAGSYQFSMVGGERSPPRILPPCTNASTGSALLNPSLPNQSDVVEAEGSHSNSPTNMAPSARLEEAVWRPY

>O75170

MFWKFDLNTTSHVDKLLDKEHVTLQELMDEDDILQECKAQNQKLLDFLCRQQCMEELVSLITQDPPLDMEEKVRFKYPNTACELLTCDVPQISDRLGGDESLLSLLYDFLDHEPPLNPLLASFFSKTIGNLIARKTEQVITFLKKKDKFISLVLKHIGTSALMDLLLRLVSCVEPAGLRQDVLHWLNEEKVIQRLVELIHPSQDEDRQSNASQTLCDIVRLGRDQGSQLQEALEPDPLLTALESQDCVEQLLKNMFDGDRTESCLVSGTQVLLTLLETRRVGTEGLVDSFSQGLERSYAVSSSVLHGIEPRLKDFHQLLLNPPKKKAILTTIGVLEEPLGNARLHGARLMAALLHTNTPSINQELCRLNTMDLLLDLFFKYTWNNFLHFQVELCIAAILSHAAREERTEASGSESRVEPPHENGNRSLETPQPAASLPDNTMVTHLFQKCCLVQRILEAWEANDHTQAAGGMRRGNMGHLTRIANAVVQNLERGPVQTHISEVIRGLPADCRGRWESFVEETLTETNRRNTVDLVSTHHLHSSSEDEDIEGAFPNELSLQQAFSDYQIQQMTANFVDQFGFNDEEFADQDDNINAPFDRIAEINFNIDADEDSPSAALFEACCSDRIQPFDDDEDEDIWEDSDTRCAARVMARPRFGAPHASESCSKNGPERGGQDGKASLEAHRDAPGAGAPPAPGKKEAPPVEGDSEGAMWTAVFDEPANSTPTAPGVVRDVGSSVWAAGTSAPEEKGWAKFTDFQPFCCSESGPRCSSPVDTECSHAEGSRSQGPEKASQASYFAVSPASPCAWNVCVTRKAPLLASDSSSSGGSHSEDGDQKAASAMDAVSRGPGREAPPLPTVARTEEAVGRVGCADSRLLSPACPAPKEVTAAPAVAVPPEATVAITTALSKAGPAIPTPAVSSALAVAVPLGPIMAVTAAPAMVATLGTVTKDGKTDAPPEGAALNGPV

777 NP_003400.2 NP_002940.2

>NP_003400.2

MEFFISMSETIKYNDDDHKTLFLKTLNEQRLEGEFCDIAIVVEDVKFRAHRCVLAACSTYFKKLFKKLEVDSSSVIEIDFLRSDIFEEVLNYMYTAKISVKKEDVNLMMSSGQILGIRFLDKLCSQKRDVSSPDENNGQSKSKYCLKINRPIGDAADTQDDDVEEIGDQDDSPSDDTVEGTPPSQEDGKSPTTTLRVQEAILKELGSEEVRKVNCYGQEVESMETPESKDLGSQTPQALTFNDGMSEVKDEQTPGWTTAASDMKFEYLLYGHHREQIACQACGKTFSDEGRLRKHEKLHTADRPFVCEMCTKGFTTQAHLKEHLKIHTGYKPYSCEVCGKSFIRAPDLKKHERVHSNERPFACHMCDKAFKHKSHLKDHERRHRGEKPFVCGSCTKAFAKASDLKRHENNMHSERKQVTPSAIQSETEQLQAAAMAAEAEQQLETIACS

>NP_002940.2

MLPAAARPLWGPCLGLRAAAFRLARRQVPCVCAVRHMRSSGHQRCEALAGAPLDNAPKEYPPKIQQLVQDIASLTLLEISDLNELLKKTLKIQDVGLVPMGGVMSGAVPAAAAQEAVEEDIPIAKERTHFTVRLTEAKPVDKVKLIKEIKNYIQGINLVQAKKLVESLPQEIKANVAKAEAEKIKAALEAVGGTVVLE

778 Q9UI36 Q6P1L8

>Q9UI36

MAVPAALIPPTQLVPPQPPISTSASSSGTTTSTSSATSSPAPSIGPPASSGPTLFRPEPIASAAAAAATVTSTGGGGGGGGSGGGGGSSGNGGGGGGGGGGSNCNPNLAAASNGSGGGGGGISAGGGVASSTPINASTGSSSSSSSSSSSSSSSSSSSSSSSSCGPLPGKPVYSTPSPVENTPQNNECKMVDLRGAKVASFTVEGCELICLPQAFDLFLKHLVGGLHTVYTKLKRLEITPVVCNVEQVRILRGLGAIQPGVNRCKLISRKDFETLYNDCTNASSRPGRPPKRTQSVTSPENSHIMPHSVPGLMSPGIIPPTGLTAAAAAAAAATNAAIAEAMKVKKIKLEAMSNYHASNNQHGADSENGDMNSSVGSSDGSWDKETLPSSPSQGPQASITHPRMPGARSLPLSHPLNHLQQSHLLPNGLELPFMMMPHPLIPVSLPPASVTMAMSQMNHLSTIANMAAAAQVQSPPSRVETSVIKERVPDSPSPAPSLEEGRRPGSHPSSHRSSSVSSSPARTESSSDRIPVHQNGLSMNQMLMGLSPNVLPGPKEGDLAGHDMGHESKRMHIEKDETPLSTPTARDSLDKLSLTGHGQPLPPGFPSPFLFPDGLSSIETLLTNIQGLLKVAIDNARAQEKQVQLEKTELKMDFLRERELRETLEKQLAMEQKNRAIVQKRLKKEKKAKRKLQEALEFETKRREQAEQTLKQAASTDSLRVLNDSLTPEIEADRSGGRTDAERTIQDGRLYLKTTVMY

>Q6P1L8

MAFFTGLWGPFTCVSRVLSHHCFSTTGSLSAIQKMTRVRVVDNSALGNSPYHRAPRCIHVYKKNGVGKVGDQILLAIKGQKKKALIVGHCMPGPRMTPRFDSNNVVLIEDNGNPVGTRIKTPIPTSLRKREGEYSKVLAIAQNFV

779 NP_005185.2 NP_002881.1

>NP_005185.2

MQRLVAWDPACLPLPPPPPAFKSMEVANFYYEADCLAAAYGGKAAPAAPPAARPGPRPPAGELGSIGDHERAIDFSPYLEPLGAPQAPAPATATDTFEAAPPAPAPAPASSGQHHDFLSDLFSDDYGGKNCKKPAEYGYVSLGRLGAAKGALHPGCFAPLHPPPPPPPPPAELKAEPGFEPADCKRKEEAGAPGGGAGMAAGFPYALRAYLGYQAVPSGSSGSLSTSSSSSPPGTPSPADAKAPPTACYAGAAPAPSQVKSKAKKTVDKHSDEYKIRRERNNIAVRKSRDKAKMRNLETQHKVLELTAENERLQKKVEQLSRELSTLRNLFKQLPEPLLASSGHC

>NP_002881.1

MMAAEAGSEEGGPVTAGAGGGGAAAGSSAYPAVCRVKIPAALPVAAAPYPGLVETGVAGTLGGGAALGSEFLGAGSVAGALGGAGLTGGGTAAGVAGAAAGVAGAAVAGPSGDMALTKLPTSLLAETLGPGGGFPPLPPPPYLPPLGAGLGTVDEGDSLDGPEYEEEEVAIPLTAPPTNQWYHGKLDRTIAEERLRQAGKSGSYLIRESDRRPGSFVLSFLSQMNVVNHFRIIAMCGDYYIGGRRFSSLSDLIGYYSHVSCLLKGEKLLYPVAPPEPVEDRRRVRAILPYTKVPDTDEISFLKGDMFIVHNELEDGWMWVTNLRTDEQGLIVEDLVEEVGREEDPHEGKIWFHGKISKQEAYNLLMTVGQVCSFLVRPSDNTPGDYSLYFRTNENIQRFKICPTPNNQFMMGGRYYNSIGDIIDHYRKEQIVEGYYLKEPVPMQDQEQVLNDTVDGKEIYNTIRRKTKDAFYKNIVKKGYLLKKGKGKRWKNLYFILEGSDAQLIYFESEKRATKPKGLIDLSVCSVYVVHDSLFGRPNCFQIVVQHFSEEHYIFYFAGETPEQAEDWMKGLQAFCNLRKSSPGTSNKRLRQVSSLVLHIEEAHKLPVKHFTNPYCNIYLNSVQVAKTHAREGQNPVWSEEFVFDDLPPDINRFEITLSNKTKKSKDPDILFMRCQLSRLQKGHATDEWFLLSSHIPLKGIEPGSLRVRARYSMEKIMPEEEYSEFKELILQKELHVVYALSHVCGQDRTLLASILLRIFLHEKLESLLLCTLNDREISMEDEATTLFRATTLASTLMEQYMKATATQFVHHALKDSILKIMESKQSCELSPSKLEKNEDVNTNLTHLLNILSELVEKIFMASEILPPTLRYIYGCLQKSVQHKWPTNTTMRTRVVSGFVFLRLICPAILNPRMFNIISDSPSPIAARTLILVAKSVQNLANLVEFGAKEPYMEGVNPFIKSNKHRMIMFLDELGNVPELPDTTEHSRTDLSRDLAALHEICVAHSDELRTLSNERGAQQHVLKKLLAITELLQQKQNQYTKTNDVR

780 Q86UP8 NP_003945.2

>Q86UP8

MAQVAVSTLPVEEESSSETRMVVTFLVSALESMCKELAKSKAEVACIAVYETDVFVVGTERGCAFVNARTDFQKDFAKYCVAEGLCEVKPPCPVNGMQVHSGETEILRKAVEDYFCFCYGKALGTTVMVPVPYEKMLRDQSAVVVQGLPEGVAFQHPENYDLATLKWILENKAGISFIINRPFLGPESQLGGPGMVTDAERSIVSPSESCGPINVKTEPMEDSGISLKAEAVSVKKESEDPNYYQYNMQGSHPSSTSNEVIEMELPMEDSTPLVPSEEPNEDPEAEVKIEGNTNSSSVTNSAAGVEDLNIVQVTVPDNEKERLSSIEKIKQLREQVNDLFSRKFGEAIGVDFPVKVPYRKITFNPGCVVIDGMPPGVVFKAPGYLEISSMRRILEAAEFIKFTVIRPLPGLELSNVGKRKIDQEGRVFQEKWERAYFFVEVQNIPTCLICKQSMSVSKEYNLRRHYQTNHSKHYDQYMERMRDEKLHELKKGLRKYLLGLSDTECPEQKQVFAHPSPTQKSPVQPVEDLAGNLWEKLREKIRSFVAYSIAIDEITDINNTTQLAIFIRGVDENFDVSEELLDTVPMTGTKSGNEIFSRVEKSLKKFCIDWSKLVSVASTGTPAMVDANNGLVTKLKSRVATFCKGAELKSICCIIHPESLCAQKLKMDHVMDVVVKSVNWICSRGLNHSEFTTLLYELDSQYGSLLYYTEIKWLSRGLVLKRFFESLEEIDSFMSSRGKPLPQLSSIDWIRDLAFLVDMTMHLNALNISLQGHSQIVTQMYDLIRAFLAKLCLWETHLTRNNLAHFPTLKLASRNESDGLNYIPKIAELKTEFQKRLSDFKLYESELTLFSSPFSTKIDSVHEELQMEVIDLQCNTVLKTKYDKVGIPEFYKYLWGSYPKYKHHCAKILSMFGSTYICEQLFSIMKLSKTKYCSQLKDSQWDSVLHIAT

>NP_003945.2

MAVMEMACPGAPGSAVGQQKELPKAKEKTPPLGKKQSSVYKLEAVEKSPVFCGKWEILNDVITKGTAKEGSEAGPAAISIIAQAECENSQEFSPTFSERIFIAGSKQYSQSESLDQIPNNVAHATEGKMARVCWKGKRRSKARKKRKKKSSKSLAHAGVALAKPLPRTPEQESCTIPVQEDESPLGAPYVRNTPQFTKPLKEPGLGQLCFKQLGEGLRPALPRSELHKLISPLQCLNHVWKLHHPQDGGPLPLPTHPFPYSRLPHPFPFHPLQPWKPHPLESFLGKLACVDSQKPLPDPHLSKLACVDSPKPLPGPHLEPSCLSRGAHEKFSVEEYLVHALQGSVSSGQAHSLTSLAKTWAARGSRSREPSPKTEDNEGVLLTEKLKPVDYEYREEVHWATHQLRLGRGSFGEVHRMEDKQTGFQCAVKKVRLEVFRAEELMACAGLTSPRIVPLYGAVREGPWVNIFMELLEGGSLGQLVKEQGCLPEDRALYYLGQALEGLEYLHSRRILHGDVKADNVLLSSDGSHAALCDFGHAVCLQPDGLGKSLLTGDYIPGTETHMAPEVVLGRSCDAKVDVWSSCCMMLHMLNGCHPWTQFFRGPLCLKIASEPPPVREIPPSCAPLTAQAIQEGLRKEPIHRVSAAELGGKVNRALQQVGGLKSPWRGEYKEPRHPPPNQANYHQTLHAQPRELSPRAPGPRPAEETTGRAPKLQPPLPPEPPEPNKSPPLTLSKEESGMWEPLPLSSLEPAPARNPSSPERKATVPEQELQQLEIELFLNSLSQPFSLEEQEQILSCLSIDSLSLSDDSEKNPSKASQSSRDTLSSGVHSWSSQAEARSSSWNMVLARGRPTDTPSYFNGVKVQIQSLNGEHLHIREFHRVKVGDIATGISSQIPAAAFSLVTKDGQPVRYDMEVPDSGIDLQCTLAPDGSFAWSWRVKHGQLENRP

781 NP_997001.1 NP_054902.1

>NP_997001.1

MVNVLKGVLIECDPAMKQFLLYLDESNALGKKFIIQDIDDTHVFVIAELVNVLQERVGELMDQNAFSLTQK

>NP_054902.1

MAEVEETLKRLQSQKGVQGIIVVNTEGIPIKSTMDNPTTTQYASLMHSFILKARSTVRDIDPQNDLTFLRIRSKKNEIMVAPDKDYFLIVIQNPTE

782 P0C7P1 Q58A45

>P0C7P1

MVEADHPGKLFIGGLNRETNEKMLKAVFGKHGPISEVLLIKDRTSKSRGFAFITFENPADAKNAAKDMNGKSLHGKAIKVEQAKKPSFQSGGRRRPPASSRNRSPSGSLRSARGSRGGTRGWLPSQEGHLDDGGYTPDLKMSYSRGLIPVKRGPSSRSGGPPPKKSAPSAVARSNSWMGSQGPMSQRRENYGVPPRRATISSWRNDRMSTRHDGYATNDGNHPSCQETRDYAPPSRGYAYRDNGHSNRDEHSSRGYRNHRSSRETRDYAPPSRGHAYRDYGHSRRDESYSRGYRNRRSSRETREYAPPSRGHGYRDYGHSRRHESYSRGYRNHPSSRETRDYAPPHRDYAYRDYGHSSWDEHSSRGYSYHDGYGEALGRDHSEHLSGSSYRDALQRYGTSHGAPPARGPRMSYGGSTCHAYSNTRDRYGRSWESYSSCGDFHYCDREHVCRKDQRNPPSLGRVLPDPREACGSSSYVASIVDGGESRSEKGDSSRY

>Q58A45

MDGGALTDTSLTDSYFSTSFIGVNGFGSPVETKYPLMQRMTNSSSSPSLLNDSAKPYSAHDPLTSPASSLFNDFGALNISQRRKPRKYRLGMLEERLVPMGSKARKAKNPIGCLADRCKSGVPINMVWWNRVTENNLQTPNPTASEFIPKGGSTSRLSNVSQSNMSAFSQVFSHPSMGSPATAGLAPGMSLSAGSSPLHSPKITPHTSPAPRRRSHTPNPASYMVPSSASTSVNNPVSQTPSSGQVIQKETVGGTTYFYTDTTPAPLTGMVFPNYHIYPPTAPHVAYMQPKANAPSFFMADELRQELINRHLITMAQIDQADMPAVPTEVDSYHSLFPLEPLPPPNRIQKSSNFGYITSCYKAVNSKDDLPYCLRRIHGFRLVNTKCMVLVDMWKKIQHSNIVTLREVFTTKAFAEPSLVFAYDFHAGGETMMSRHFNDPNADAYFTKRKWGQHEGPLPRQHAGLLPESLIWAYIVQLSSALRTIHTAGLACRVMDPTKILITGKTRLRVNCVGVFDVLTFDNSQNNNPLALMAQYQQADLISLGKVVLALACNSLAGIQRENLQKAMELVTINYSSDLKNLILYLLTDQNRMRSVNDIMPMIGARFYTQLDAAQMRNDVIEEDLAKEVQNGRLFRLLAKLGTINERPEFQKDPTWSETGDRYLLKLFRDHLFHQVTEAGAPWIDLSHIISCLNKLDAGVPEKISLISRDEKSVLVVTYSDLKRCFENTFQELIAAANGQL

783 NP_060853.3 Q96RP9

>NP_060853.3

MATEIGSPPRFFHMPRFQHQAPRQLFYKRPDFAQQQAMQQLTFDGKRMRKAVNRKTIDYNPSVIKYLENRIWQRDQRDMRAIQPDAGYYNDLVPPIGMLNNPMNAVTTKFVRTSTNKVKCPVFVVRWTPEGRRLVTGASSGEFTLWNGLTFNFETILQAHDSPVRAMTWSHNDMWMLTADHGGYVKYWQSNMNNVKMFQAHKEAIREASFSPTDNKFATCSDDGTVRIWDFLRCHEERILRGHGADVKCVDWHPTKGLVVSGSKDSQQPIKFWDPKTGQSLATLHAHKNTVMEVKLNLNGNWLLTASRDHLCKLFDIRNLKEELQVFRGHKKEATAVAWHPVHEGLFASGGSDGSLLFWHVGVEKEVGGMEMAHEGMIWSLAWHPLGHILCSGSNDHTSKFWTRNRPGDKMRDRYNLNLLPGMSEDGVEYDDLEPNSLAVIPGMGIPEQLKLAMEQEQMGKDESNEIEMTIPGLDWGMEEVMQKDQKKVPQKKVPYAKPIPAQFQQAWMQNKVPIPAPNEVLNDRKEDIKLEEKKKTQAEIEQEMATLQYTNPQLLEQLKIERLAQKQVEQIQPPPSSGTPLLGPQPFPGQGPMSQIPQGFQQPHPSQQMPMNMAQMGPPGPQGQFRPPGPQGQMGPQGPPLHQGGGGPQGFMGPQGPQGPPQGLPRPQDMHGPQGMQRHPGPHGPLGPQGPPGPQGSSGPQGHMGPQGPPGPQGHIGPQGPPGPQGHLGPQGPPGTQGMQGPPGPRGMQGPPHPHGIQGGPGSQGIQGPVSQGPLMGLNPRGMQGPPGPRENQGPAPQGMIMGHPPQEMRGPHPPGGLLGHGPQEMRGPQEIRGMQGPPPQGSMLGPPQELRGPPGSQSQQGPPQGSLGPPPQGGMQGPPGPQGQQNPARGPHPSQGPIPFQQQKTPLLGDGPRAPFNQEGQSTGPPPLIPGLGQQGAQGRIPPLNPGQGPGPNKGDSRGPPNHHMGPMSERRHEQSGGPEHGPERGPFRGGQDCRGPPDRRGPHPDFPDDFSRPDDFHPDKRFGHRLREFEGRGGPLPQEEKWRRGGPGPPFPPDHREFSEGDGRGAARGPPGAWEGRRPGDERFPRDPEDPRFRGRREESFRRGAPPRHEGRAPPRGRDGFPGPEDFGPEENFDASEEAARGRDLRGRGRGTPRGGRKGLLPTPDEFPRFEGGRKPDSWDGNREPGPGHEHFRDTPRPDHPPHDGHSPASRERSSSLQGMDMASLPPRKRPWHDGPGTSEHREMEAPGGPSEDRGGKGRGGPGPAQRVPKSGRSSSLDGEHHDGYHRDEPFGGPPGSGTPSRGGRSGSNWGRGSNMNSGPPRRGASRGGGRGR

>Q96RP9

MRLLGAAAVAALGRGRAPASLGWQRKQVNWKACRWSSSGVIPNEKIRNIGISAHIDSGKTTLTERVLYYTGRIAKMHEVKGKDGVGAVMDSMELERQRGITIQSAATYTMWKDVNINIIDTPGHVDFTIEVERALRVLDGAVLVLCAVGGVQCQTMTVNRQMKRYNVPFLTFINKLDRMGSNPARALQQMRSKLNHNAAFMQIPMGLEGNFKGIVDLIEERAIYFDGDFGQIVRYGEIPAELRAAATDHRQELIECVANSDEQLGEMFLEEKIPSISDLKLAIRRATLKRSFTPVFLGSALKNKGVQPLLDAVLEYLPNPSEVQNYAILNKEDDSKEKTKILMNSSRDNSHPFVGLAFKLEVGRFGQLTYVRSYQGELKKGDTIYNTRTRKKVRLQRLARMHADMMEDVEEVYAGDICALFGIDCASGDTFTDKANSGLSMESIHVPDPVISIAMKPSNKNDLEKFSKGIGRFTREDPTFKVYFDTENKETVISGMGELHLEIYAQRLEREYGCPCITGKPKVAFRETITAPVPFDFTHKKQSGGAGQYGKVIGVLEPLDPEDYTKLEFSDETFGSNIPKQFVPAVEKGFLDACEKGPLSGHKLSGLRFVLQDGAHHMVDSNEISFIRAGEGALKQALANATLCILEPIMAVEVVAPNEFQGQVIAGINRRHGVITGQDGVEDYFTLYADVPLNDMFGYSTELRSCTEGKGEYTMEYSRYQPCLPSTQEDVINKYLEATGQLPVKKGKAKN

784 NP_009153.3 NP_055463.1

>NP_009153.3

MASRRKSTTPCMVLASEQDPDLELISDLDEGPPVLTPVENTRAESISSDEEVHESVDSDNQQNKKVEGGYECKYCTFQTPDLNMFTFHVDSEHPNVVLNSSYVCVECNFLTKRYDALSEHNLKYHPGEENFKLTMVKRNNQTIFEQTINDLTFDGSFVKEENAEQAESTEVSSSGISISKTPIMKMMKNKVENKRIAVHHNSVEDVPEEKENEIKPDREEIVENPSSSASESNTSTSIVNRIHPSTASTVVTPAAVLPGLAQVITAVSAQQNSNLIPKVLIPVNSIPTYNAALDNNPLLLNTYNKFPYPTMSEITVLSAQAKYTEEQIKIWFSAQRLKHGVSWTPEEVEEARRKQFNGTVHTVPQTITVIPTHISTGSNGLPSILQTCQIVGQPGLVLTQVAGTNTLPVTAPIALTVAGVPSQNNIQKSQVPAAQPTAETKPATAAVPTSQSVKHETALVNPDSFGIRAKKTKEQLAELKVSYLKNQFPHDSEIIRLMKITGLTKGEIKKWFSDTRYNQRNSKSNQCLHLNNDSSTTIIIDSSDETTESPTVGTAQPKQSWNPFPDFTPQKFKEKTAEQLRVLQASFLNSSVLTDEELNRLRAQTKLTRREIDAWFTEKKKSKALKEEKMEIDESNAGSSKEEAGETSPADESGAPKSGSTGKICKKTPEQLHMLKSAFVRTQWPSPEEYDKLAKESGLARTDIVSWFGDTRYAWKNGNLKWYYYYQSANSSSMNGLSSLRKRGRGRPKGRGRGRPRGRPRGSKRINNWDRGPSLIKFKTGTAILKDYYLKHKFLNEQDLDELVNKSHMGYEQVREWFAERQRRSELGIELFEENEEEDEVIDDQEEDEEETDDSDTWEPPRHVKRKLSKSDD

>NP_055463.1

MDSLPDEFFVRHPAVEDQRKEETENKLEKSSGQLNKQENDIPTDLVPVNLLLEVKKLLNAINTLPKGVVPHIKKFLQEDFSFQTMQREVAANSQNGEEIVPALTLRFLITQLEAALRNIQAGNYTAHQINIGYYLTLLFLYGVALTERGKKEDYTEAENKFLVMKMMIQENEICENFMSLVYFGRGLLRCAQKRYNGGLLEFHKSLQEIGDKNDHWFDIDPTEDEDLPTTFKDLLNNFIKTTESNIMKQTICSYLDCERSCEADILKNTSYKGFFQLMCSKSCCVYFHKICWKKFKNLKYPGENDQSFSGKKCLKEGCTGDMVRMLQCDVPGIVKILFEVVRKDEYITIENLGASYRKLISLKITDTDIRPKISLKFNTKDEMPIFKLDYNYFYHLLHIIIISGTDIVRQIFDEAMPPPLLKKELLIHKNVLESYYNHLWTNHPLGGSWHLLYPPNKELPQSKQFDLCLLLALIKHLNVFPAPKKGWNMEPPSSDISKSADILRLCKYRDILLSEILMNGLTESQFNSIWKKVSDILLRLGMMQEDIDKVKENPIENISLDYHQLSVYLGIPVPEIIQRMLSCYQQGIALQSITGSQRIEIEELQNEEEELSPPLMEYNINVKSHPEIQFAEINKDGTSIPSESSTESLKDLQEVKSKQRKKKKTKNKKNKDSKEDQVPYVVEKEEQLRKEQANPHSVSRLIKDDASDVQEDSAMEDKFYSLDELHILDMIEQGSAGKVTTDYGETEKERLARQRQLYKLHYQCEDFKRQLRTVTFRWQENQMQIKKKDKIIASLNQQVAFGINKVSKLQRQIHAKDNEIKNLKEQLSMKRSQWEMEKHNLESTMKTYVSKLNAETSRALTAEVYFLQCRRDFGLLHLEQTEKECLNQLARVTHMAASNLESLQLKAAVDSWNAIVADVRNKIAFLRTQYNEQINKVKQGFALSTLPPVQLPPPPPSPEILMQQFLGRPLVKESFFRPILTVPQMPAVCPGVVSATGQPRAPLMTGIAWALPAPVGDAVPPSAGLRSDPSIMNWERITDRLKTAFPQQTRKELTDFLRKLKDAYGKSLSELTFDEIVCKISQFIDPKKSQSQGKSVSNVNCVSPSHSPSQPDAAQPPKPAWRPLTSQGPATWEGASNPDEEEEEEEPCVICHENLSPENLSVLPCAHKFHAQCIRPWLMQQGTCPTCRLHVLLPEEFPGHPSRQLPKI

785 Q7Z5Q5 NP_037386.1

>Q7Z5Q5

MENYEALVGFDLCNTPLSSVAQKIMSAMHSGDLVDSKTWGKSTETMEVINKSSVKYSVQLEDRKTQSPEKKDLKSLRSQTSRGSAKLSPQSFSVRLTDQLSADQKQKSISSLTLSSCLIPQYNQEASVLQKKGHKRKHFLMENINNENKGSINLKRKHITYNNLSEKTSKQMALEEDTDDAEGYLNSGNSGALKKHFCDIRHLDDWAKSQLIEMLKQAAALVITVMYTDGSTQLGADQTPVSSVRGIVVLVKRQAEGGHGCPDAPACGPVLEGFVSDDPCIYIQIEHSAIWDQEQEAHQQFARNVLFQTMKCKCPVICFNAKDFVRIVLQFFGNDGSWKHVADFIGLDPRIAAWLIDPSDATPSFEDLVEKYCEKSITVKVNSTYGNSSRNIVNQNVRENLKTLYRLTMDLCSKLKDYGLWQLFRTLELPLIPILAVMESHAIQVNKEEMEKTSALLGARLKELEQEAHFVAGERFLITSNNQLREILFGKLKLHLLSQRNSLPRTGLQKYPSTSEAVLNALRDLHPLPKIILEYRQVHKIKSTFVDGLLACMKKGSISSTWNQTGTVTGRLSAKHPNIQGISKHPIQITTPKNFKGKEDKILTISPRAMFVSSKGHTFLAADFSQIELRILTHLSGDPELLKLFQESERDDVFSTLTSQWKDVPVEQVTHADREQTKKVVYAVVYGAGKERLAACLGVPIQEAAQFLESFLQKYKKIKDFARAAIAQCHQTGCVVSIMGRRRPLPRIHAHDQQLRAQAERQAVNFVVQGSAADLCKLAMIHVFTAVAASHTLTARLVAQIHDELLFEVEDPQIPECAALVRRTMESLEQVQALELQLQVPLKVSLSAGRSWGHLVPLQEAWGPPPGPCRTESPSNSLAAPGSPASTQPPPLHFSPSFCL

>NP_037386.1

MQSTSNHLWLLSDILGQGATANVFRGRHKKTGDLFAIKVFNNISFLRPVDVQMREFEVLKKLNHKNIVKLFAIEEETTTRHKVLIMEFCPCGSLYTVLEEPSNAYGLPESEFLIVLRDVVGGMNHLRENGIVHRDIKPGNIMRVIGEDGQSVYKLTDFGAARELEDDEQFVSLYGTEEYLHPDMYERAVLRKDHQKKYGATVDLWSIGVTFYHAATGSLPFRPFEGPRRNKEVMYKIITGKPSGAISGVQKAENGPIDWSGDMPVSCSLSRGLQVLLTPVLANILEADQEKCWGFDQFFAETSDILHRMVIHVFSLQQMTAHKIYIHSYNTATIFHELVYKQTKIISSNQELIYEGRRLVLEPGRLAQHFPKTTEENPIFVVSREPLNTIGLIYEKISLPKVHPRYDLDGDASMAKAITGVVCYACRIASTLLLYQELMRKGIRWLIELIKDDYNETVHKKTEVVITLDFCIRNIEKTVKVYEKLMKINLEAAELGEISDIHTKLLRLSSSQGTIETSLQDIDSRLSPGGSLADAWAHQEGTHPKDRNVEKLQVLLNCMTEIYYQFKKDKAERRLAYNEEQIHKFDKQKLYYHATKAMTHFTDECVKKYEAFLNKSEEWIRKMLHLRKQLLSLTNQCFDIEEEVSKYQEYTNELQETLPQKMFTASSGIKHTMTPIYPSSNTLVEMTLGMKKLKEEMEGVVKELAENNHILERFGSLTMDGGLRNVDCL

786 NP_055596.3 NP_001434.1

>NP_055596.3

MKLYVFLVNTGTTLTFDTELTVQTVADLKHAIQSKYKIAIQHQVLVVNGGECMAADRRVCTYSAGTDTNPIFLFNKEMILCDRPPAIPKTTFSTENDMEIKVEESLMMPAVFHTVASRTQLALEMYEVAKKLCSFCEGLVHDEHLQHQGWAAIMANLEDCSNSYQKLLFKFESIYSNYLQSIEDIKLKLTHLGTAVSVMAKIPLLECLTRHSYRECLGRLDSLPEHEDSEKAEMKRSTELVLSPDMPRTTNESLLTSFPKSVEHVSPDTADAESGKEIRESCQSTVHQQDETTIDTKDGDLPFFNVSLLDWINVQDRPNDVESLVRKCFDSMSRLDPRIIRPFIAECRQTIAKLDNQNMKAIKGLEDRLYALDQMIASCGRLVNEQKELAQGFLANQKRAENLKDASVLPDLCLSHANQLMIMLQNHRKLLDIKQKCTTAKQELANNLHVRLKWCCFVMLHADQDGEKLQALLRLVIELLERVKIVEALSTVPQMYCLAVVEVVRRKMFIKHYREWAGALVKDGKRLYEAEKSKRESFGKLFRKSFLRNRLFRGLDSWPPSFCTQKPRKFDCELPDISLKDLQFLQSFCPSEVQPFLRVPLLCDFEPLHQHVLALHNLVKAAQSLDEMSQTITDLLSEQKASVSQTSPQSASSPRMESTAGITTTTSPRTPPPLTVQDPLCPAVCPLEELSPDSIDAHTFDFETIPHPNIEQTIHQVSLDLDSLAESPESDFMSAVNEFVIEENLSSPNPISDPQSPEMMVESLYSSVINAIDSRRMQDTNVCGKEDFGDHTSLNVQLERCRVVAQDSHFSIQTIKEDLCHFRTFVQKEQCDFSNSLKCTAVEIRNIIEKVKCSLEITLKEKHQKELLSLKNEYEGKLDGLIKETEENENKIKKLKGELVCLEEVLQNKDNEFALVKHEKEAVICLQNEKDQKLLEMENIMHSQNCEIKELKQSREIVLEDLKKLHVENDEKLQLLRAELQSLEQSHLKELEDTLQVRHIQEFEKVMTDHRVSLEELKKENQQIINQIQESHAEIIQEKEKQLQELKLKVSDLSDTRCKLEVELALKEAETDEIKILLEESRAQQKETLKSLLEQETENLRTEISKLNQKIQDNNENYQVGLAELRTLMTIEKDQCISELISRHEEESNILKAELNKVTSLHNQAFEIEKNLKEQIIELQSKLDSELSALERQKDEKITQQEEKYEAIIQNLEKDRQKLVSSQEQDREQLIQKLNCEKDEAIQTALKEFKLEREVVEKELLEKVKHLENQIAKSPAIDSTRGDSSSLVAELQEKLQEEKAKFLEQLEEQEKRKNEEMQNVRTSLIAEQQTNFNTVLTREKMRKENIINDLSDKLKSTMQQQERDKDLIESLSEDRARLLEEKKKLEEEVSKLRSSSFVPSPYVATAPELYGACAPELPGESDRSAVETADEGRVDSAMETSMMSVQENIHMLSEEKQRIMLLERTLQLKEEENKRLNQRLMSQSMSSVSSRHSEKIAIRDFQVGDLVLIILDERHDNYVLFTVSPTLYFLHSESLPALDLKPGEGASGASRRPWVLGKVMEKEYCQAKKAQNRFKVPLGTKFYRVKAVSWNKKV

>NP_001434.1

MSFSGKYQLQSQENFEAFMKAIGLPEELIQKGKDIKGVSEIVQNGKHFKFTITAGSKVIQNEFTVGEECELETMTGEKVKTVVQLEGDNKLVTTFKNIKSVTELNGDIITNTMTLGDIVFKRISKRI

787 P42785 Q5EBM0

>P42785

MGRRALLLLLLSFLAPWATIALRPALRALGSLHLPTNPTSLPAVAKNYSVLYFQQKVDHFGFNTVKTFNQRYLVADKYWKKNGGSILFYTGNEGDIIWFCNNTGFMWDVAEELKAMLVFAEHRYYGESLPFGDNSFKDSRHLNFLTSEQALADFAELIKHLKRTIPGAENQPVIAIGGSYGGMLAAWFRMKYPHMVVGALAASAPIWQFEDLVPCGVFMKIVTTDFRKSGPHCSESIHRSWDAINRLSNTGSGLQWLTGALHLCSPLTSQDIQHLKDWISETWVNLAMVDYPYASNFLQPLPAWPIKVVCQYLKNPNVSDSLLLQNIFQALNVYYNYSGQVKCLNISETATSSLGTLGWSYQACTEVVMPFCTNGVDDMFEPHSWNLKELSDDCFQQWGVRPRPSWITTMYGGKNISSHTNIVFSNGELDPWSGGGVTKDITDTLVAVTISEGAHHLDLRTKNALDPMSVLLARSLEVRHMKNWIRDFYDSAGKQH

>Q5EBM0

MAFARRLLRGPLSGPLLGRRGVCAGAMAPPRRFVLELPDCTLAHFALGADAPGDADAPDPRLAALLGPPERSYSLCVPVTPDAGCGARVRAARLHQRLLHQLRRGPFQRCQLLRLLCYCPGGQAGGAQQGFLLRDPLDDPDTRQALLELLGACQEAPRPHLGEFEADPRGQLWQRLWEVQDGRRLQVGCAQVVPVPEPPLHPVVPDLPSSVVFPDREAARAVLEECTSFIPEARAVLDLVDQCPKQIQKGKFQVVAIEGLDATGKTTVTQSVADSLKAVLLKSPPSCIGQWRKIFDDEPTIIRRAFYSLGNYIVASEIAKESAKSPVIVDRYWHSTATYAIATEVSGGLQHLPPAHHPVYQWPEDLLKPDLILLLTVSPEERLQRLQGRGMEKTREEAELEANSVFRQKVEMSYQRMENPGCHVVDASPSREKVLQTVLSLIQNSFSGP

788 Q9NQX1 NP_659491.4

>Q9NQX1

MLGMYVPDRFSLKSSRVQDGMGLYTARRVRKGEKFGPFAGEKRMPEDLDENMDYRLMWEVRGSKGEVLYILDATNPRHSNWLRFVHEAPSQEQKNLAAIQEGENIFYLAVEDIETDTELLIGYLDSDMEAEEEEQQIMTVIKEGEVENSRRQSTAGRKDRLGCKEDYACPQCESSFTSEDILAEHLQTLHQKPTEEKEFKCKNCGKKFPVKQALQRHVLQCTAKSSLKESSRSFQCSVCNSSFSSASSFEQHQETCRGDARFVCKADSCGKRLKSKDALKRHQENVHTGDPKKKLICSVCNKKCSSASSLQEHRKIHEIFDCQECMKKFISANQLKRHMITHSEKRPYNCEICNKSFKRLDQVGAHKVIHSEDKPYKCKLCGKGFAHRNVYKNHKKTHSEERPFQCEECKALFRTPFSLQRHLLIHNSERTFKCHHCDATFKRKDTLNVHVQVVHERHKKYRCELCNKAFVTPSVLRSHKKTHTGEKEKICPYCGQKFASSGTLRVHIRSHTGERPYQCPYCEKGFSKNDGLKMHIRTHTREKPYKCSECSKAFSQKRGLDEHKRTHTGEKPFQCDVCDLAFSLKKMLIRHKMTHNPNRPLAECQFCHKKFTRNDYLKVHMDNIHGVADS

>NP_659491.4

MDNKISPEAQVAELELDAVIGFNGHVPTGLKCHPDQEHMIYPLGCTVLIQAINTKEQNFLQGHGNNVSCLAISRSGEYIASGQVTFMGFKADIILWDYKNRELLARLSLHKGKIEALAFSPNDLYLVSLGGPDDGSVVVWSIAKRDAICGSPAAGLNVGNATNVIFSRCRDEMFMTAGNGTIRVWELDLPNRKIWPTECQTGQLKRIVMSIGVDDDDSFFYLGTTTGDILKMNPRTKLLTDVGPAKDKFSLGVSAIRCLKMGGLLVGSGAGLLVFCKSPGYKPIKKIQLQGGITSITLRGEGHQFLVGTEESHIYRVSFTDFKETLIATCHFDAVEDIVFPFGTAELFATCAKKDIRVWHTSSNRELLRITVPNMTCHGIDFMRDGKSIISAWNDGKIRAFAPETGRLMYVINNAHRIGVTAIATTSDCKRVISGGGEGEVRVWQIGCQTQKLEEALKEHKSSVSCIRVKRNNEECVTASTDGTCIIWDLVRLRRNQMILANTLFQCVCYHPEEFQIITSGTDRKIAYWEVFDGTVIRELEGSLSGSINGMDITQEGVHFVTGGNDHLVKVWDYNEGEVTHVGVGHSGNITRIRISPGNQYIVSVSADGAILRWKYPYTS

789 O60902 Q9P2W6

>O60902

MEELTAFVSKSFDQKVKEKKEAITYREVLESGPLRGAKEPTGCTEAGRDDRSSPAVRAAGGGGGGGGGGGGGGGGGGVGGGGAGGGAGGGRSPVRELDMGAAERSREPGSPRLTEVSPELKDRKEDAKGMEDEGQTKIKQRRSRTNFTLEQLNELERLFDETHYPDAFMREELSQRLGLSEARVQVWFQNRRAKCRKQENQLHKGVLIGAASQFEACRVAPYVNVGALRMPFQQDSHCNVTPLSFQVQAQLQLDSAVAHAHHHLHPHLAAHAPYMMFPAPPFGLPLATLAADSASAASVVAAAAAAKTTSKNSSIADLRLKAKKHAAALGL

>Q9P2W6

MGRTWCGMWRRRRPGRRSAVPRWPHLSSQSGVEPPDRWTGTPGWPSRDQEAPGSMMPPAAAQPSAHGALVPPATAHEPVDHPALHWLACCCCLSLPGQLPLAIRLGWDLDLEAGPSSGKLCPRARRWQPLPS

790 NP_005850.1 Q9NX20

>NP_005850.1

MADRDSGSEQGGAALGSGGSLGHPGSGSGSGGGGGGGGGGGGSGGGGGGAPGGLQHETQELASKRVDIQNKRFYLDVKQNAKGRFLKIAEVGAGGNKSRLTLSMSVAVEFRDYLGDFIEHYAQLGPSQPPDLAQAQDEPRRALKSEFLVRENRKYYMDLKENQRGRFLRIRQTVNRGPGLGSTQGQTIALPAQGLIEFRDALAKLIDDYGVEEEPAELPEGTSLTVDNKRFFFDVGSNKYGVFMRVSEVKPTYRNSITVPYKVWAKFGHTFCKYSEEMKKIQEKQREKRAACEQLHQQQQQQQEETAAATLLLQGEEEGEED

>Q9NX20

MWRLLARASAPLLRVPLSDSWALLPASAGVKTLLPVPSFEDVSIPEKPKLRFIERAPLVPKVRREPKNLSDIRGPSTEATEFTEGNFAILALGGGYLHWGHFEMMRLTINRSMDPKNMFAIWRVPAPFKPITRKSVGHRMGGGKGAIDHYVTPVKAGRLVVEMGGRCEFEEVQGFLDQVAHKLPFAAKAVSRGTLEKMRKDQEERERNNQNPWTFERIATANMLGIRKVLSPYDLTHKGKYWGKFYMPKRV

791 NP_003787.2 NP_002631.3

>NP_003787.2

MEAPTVETPPDPSPPSAPAPALVPLRAPDVARLREEQEKVVTNCQERIQHWKKVDNDYNALRERLSTLPDKLSYNIMVPFGPFAFMPGKLVHTNEVTVLLGDNWFAKCSAKQAVGLVEHRKEHVRKTIDDLKKVMKNFESRVEFTEDLQKMSDAAGDIVDIREEIKCDFEFKAKHRIAHKPHSKPKTSDIFEADIANDVKSKDLLADKELWARLEELERQEELLGELDSKPDTVIANGEDTTSSEEEKEDRNTNVNAMHQVTDSHTPCHKDVASSEPFSGQVNSQLNCSVNGSSSYHSDDDDDDDDDDDDDNIDDDDGDNDHEALGVGDNSIPTIYFSHTVEPKRVRINTGKNTTLKFSEKKEEAKRKRKNSTGSGHSAQELPTIRTPADIYRAFVDVVNGEYVPRKSILKSRSRENSVCSDTSESSAAEFDDRRGVLRSISCEEATCSDTSESILEEEPQENQKKLLPLSVTPEAFSGTVIEKEFVSPSLTPPPAIAHPALPTIPERKEVLLEASEETGKRVSKFKAARLQQKD

>NP_002631.3

MDDLCEANGTFAISLFKILGEEDNSRNVFFSPMSISSALAMVFMGAKGSTAAQMSQALCLYKDGDIHRGFQSLLSEVNRTGTQYLLRTANRLFGEKTCDFLPDFKEYCQKFYQAELEELSFAEDTEECRKHINDWVAEKTEGKISEVLDAGTVDPLTKLVLVNAIYFKGKWNEQFDRKYTRGMLFKTNEEKKTVQMMFKEAKFKMGYADEVHTQVLELPYVEEELSMVILLPDDNTDLAVVEKALTYEKFKAWTNSEKLTKSKVQVFLPRLKLEESYDLEPFLRRLGMIDAFDEAKADFSGMSTEKNVPLSKVAHKCFVEVNEEGTEAAAATAVVRNSRCSRMEPRFCADHPFLFFIRHHKTNCILFCGRFSSP

792 NP_060370.1 O43182

>NP_060370.1

MLLGRLTSQLLRAVPWAGGRPPWPVSGVLGSRVCGPLYSTSPAGPGRAASLPRKGAQLELEEMLVPRKMSVSPLESWLTARCFLPRLDTGTAGTVAPPQSYQCPPSQIGEGAEQGDEGVADAPQIQCKNVLKIRRRKMNHHKYRKLVKKTRFLRRKVQEGRLRRKQIKFEKDLRRIWLKAGLKEAPEGWQTPKIYLRGK

>O43182

MSAQSLLHSVFSCSSPASSSAASAKGFSKRKLRQTRSLDPALIGGCGSDEAGAEGSARGATAGRLYSPSLPAESLGPRLASSSRGPPPRATRLPPPGPLCSSFSTPSTPQEKSPSGSFHFDYEVPLGRGGLKKSMAWDLPSVLAGPASSRSASSILCSSGGGPNGIFASPRRWLQQRKFQSPPDSRGHPYVVWKSEGDFTWNSMSGRSVRLRSVPIQSLSELERARLQEVAFYQLQQDCDLSCQITIPKDGQKRKKSLRKKLDSLGKEKNKDKEFIPQAFGMPLSQVIANDRAYKLKQDLQRDEQKDASDFVASLLPFGNKRQNKELSSSNSSLSSTSETPNESTSPNTPEPAPRARRRGAMSVDSITDLDDNQSRLLEALQLSLPAEAQSKKEKARDKKLSLNPIYRQVPRLVDSCCQHLEKHGLQTVGIFRVGSSKKRVRQLREEFDRGIDVSLEEEHSVHDVAALLKEFLRDMPDPLLTRELYTAFINTLLLEPEEQLGTLQLLIYLLPPCNCDTLHRLLQFLSIVARHADDNISKDGQEVTGNKMTSLNLATIFGPNLLHKQKSSDKEFSVQSSARAEESTAIIAVVQKMIENYEALFMVPPDLQNEVLISLLETDPDVVDYLLRRKASQSSSPDMLQSEVSFSVGGRHSSTDSNKASSGDISPYDNNSPVLSERSLLAMQEDAAPGGSEKLYRVPGQFMLVGHLSSSKSRESSPGPRLGKDLSEEPFDIWGTWHSTLKSGSKDPGMTGSSGDIFESSSLRAGPCSLSQGNLSPNWPRWQGSPAELDSDTQGARRTQAAAPATEGRAHPAVSRACSTPHVQVAGKAERPTARSEQYLTLSGAHDLSESELDVAGLQSRATPQCQRPHGSGRDDKRPPPPYPGPGKPAAAAAWIQGPPEGVETPTDQGGQAAEREQQVTQKKLSSANSLPAGEQDSPRLGDAGWLDWQRERWQIWELLSTDNPDALPETLV

793 NP_060780.2 NP_036205.1

>NP_060780.2

MPGRGRCPDCGSTELVEDSHYSQSQLVCSDCGCVVTEGVLTTTFSDEGNLREVTYSRSTGENEQVSRSQQRGLRRVRDLCRVLQLPPTFEDTAVAYYQQAYRHSGIRAARLQKKEVLVGCCVLITCRQHNWPLTMGAICTLLYADLDVFSSTYMQIVKLLGLDVPSLCLAELVKTYCSSFKLFQASPSVPAKYVEDKEKMLSRTMQLVELANETWLVTGRHPLPVITAATFLAWQSLQPADRLSCSLARFCKLANVDLPYPASSRLQELLAVLLRMAEQLAWLRVLRLDKRSVVKHIGDLLQHRQSLVRSAFRDGTAEVETREKEPPGWGQGQGEGEVGNNSLGLPQGKRPASPALLLPPCMLKSPKRICPVPPVSTVTGDENISDSEIEQYLRTPQEVRDFQRAQAARQAATSVPNPP

>NP_036205.1

MASMGTLAFDEYGRPFLIIKDQDRKSRLMGLEALKSHIMAAKAVANTMRTSLGPNGLDKMMVDKDGDVTVTNDGATILSMMDVDHQIAKLMVELSKSQDDEIGDGTTGVVVLAGALLEEAEQLLDRGIHPIRIADGYEQAARVAIEHLDKISDSVLVDIKDTEPLIQTAKTTLGSKVVNSCHRQMAEIAVNAVLTVADMERRDVDFELIKVEGKVGGRLEDTKLIKGVIVDKDFSHPQMPKKVEDAKIAILTCPFEPPKPKTKHKLDVTSVEDYKALQKYEKEKFEEMIQQIKETGANLAICQWGFDDEANHLLLQNNLPAVRWVGGPEIELIAIATGGRIVPRFSELTAEKLGFAGLVQEISFGTTKDKMLVIEQCKNSRAVTIFIRGGNKMIIEEAKRSLHDALCVIRNLIRDNRVVYGGGAAEISCALAVSQEADKCPTLEQYAMRAFADALEVIPMALSENSGMNPIQTMTEVRARQVKEMNPALGIDCLHKGTNDMKQQHVIETLIGKKQQISLATQMVRMILKIDDIRKPGESEE

794 NP_006312.1 Q9BUT1

>NP_006312.1

MSVDMNSQGSDSNEEDYDPNCEEEEEEEEDDPGDIEDYYVGVASDVEQQGADAFDPEEYQFTCLTYKESEGALNEHMTSLASVLKVSHSVAKLILVNFHWQVSEILDRYKSNSAQLLVEARVQPNPSKHVPTSHPPHHCAVCMQFVRKENLLSLACQHQFCRSCWEQHCSVLVKDGVGVGVSCMAQDCPLRTPEDFVFPLLPNEELREKYRRYLFRDYVESHYQLQLCPGADCPMVIRVQEPRARRVQCNRCNEVFCFKCRQMYHAPTDCATIRKWLTKCADDSETANYISAHTKDCPKCNICIEKNGGCNHMQCSKCKHDFCWMCLGDWKTHGSEYYECSRYKENPDIVNQSQQAQAREALKKYLFYFERWENHNKSLQLEAQTYQRIHEKIQERVMNNLGTWIDWQYLQNAAKLLAKCRYTLQYTYPYAYYMESGPRKKLFEYQQAQLEAEIENLSWKVERADSYDRGDLENQMHIAEQRRRTLLKDFHDT

>Q9BUT1

MGRLDGKVIILTAAAQGIGQAAALAFAREGAKVIATDINESKLQELEKYPGIQTRVLDVTKKKQIDQFANEVERLDVLFNVAGFVHHGTVLDCEEKDWDFSMNLNVRSMYLMIKAFLPKMLAQKSGNIINMSSVASSVKGVVNRCVYSTTKAAVIGLTKSVAADFIQQGIRCNCVCPGTVDTPSLQERIQARGNPEEARNDFLKRQKTGRFATAEEIAMLCVYLASDESAYVTGNPVIIDGGWSL

795 Q08999 Q16698

>Q08999

MPSGGDQSPPPPPPPPAAAASDEEEEDDGEAEDAAPPAESPTPQIQQRFDELCSRLNMDEAARAEAWDSYRSMSESYTLEGNDLHWLACALYVACRKSVPTVSKGTVEGNYVSLTRILKCSEQSLIEFFNKMKKWEDMANLPPHFRERTERLERNFTVSAVIFKKYEPIFQDIFKYPQEEQPRQQRGRKQRRQPCTVSEIFHFCWVLFIYAKGNFPMISDDLVNSYHLLLCALDLVYGNALQCSNRKELVNPNFKGLSEDFHAKDSKPSSDPPCIIEKLCSLHDGLVLEAKGIKEHFWKPYIRKLYEKKLLKGKEENLTGFLEPGNFGESFKAINKAYEEYVLSVGNLDERIFLGEDAEEEIGTLSRCLNAGSGTETAERVQMKNILQQHFDKSKALRISTPLTGVRYIKENSPCVTPVSTATHSLSRLHTMLTGLRNAPSEKLEQILRTCSRDPTQAIANRLKEMFEIYSQHFQPDEDFSNCAKEIASKHFRFAEMLYYKVLESVIEQEQKRLGDMDLSGILEQDAFHRSLLACCLEVVTFSYKPPGNFPFITEIFDVPLYHFYKVIEVFIRAEDGLCREVVKHLNQIEEQILDHLAWKPESPLWEKIRDNENRVPTCEEVMPPQNLERADEICIAGSPLTPRRVTEVRADTGGLGRSITSPTTLYDRYSSPPASTTRRRLFVENDSPSDGGTPGRMPPQPLVNAVPVQNVSGETVSVTPVPGQTLVTMATATVTANNGQTVTIPVQGIANENGGITFFPVQVNVGGQAQAVTGSIQPLSAQALAGSLSSQQVTGTTLQVPGQVAIQQISPGGQQQKQGQSVTSSSNRPRKTSSLSLFFRKVYHLAAVRLRDLCAKLDISDELRKKIWTCFEFSIIQCPELMMDRHLDQLLMCAIYVMAKVTKEDKSFQNIMRCYRTQPQARSQVYRSVLIKGKRKRRNSGSSDSRSHQNSPTELNKDRTSRDSSPVMRSSSTLPVPQPSSAPPTPTRLTGANSDMEEEERGDLIQFYNNIYIKQIKTFAMKYSQANMDAPPLSPYPFVRTGSPRRIQLSQNHPVYISPHKNETMLSPREKIFYYFSNSPSKRLREINSMIRTGETPTKKRGILLEDGSESPAKRICPENHSALLRRLQDVANDRGSH

>Q16698

MKLPARVFFTLGSRLPCGLAPRRFFSYGTKILYQNTEALQSKFFSPLQKAMLPPNSFQGKVAFITGGGTGLGKGMTTLLSSLGAQCVIASRKMDVLKATAEQISSQTGNKVHAIQCDVRDPDMVQNTVSELIKVAGHPNIVINNAAGNFISPTERLSPNAWKTITDIVLNGTAFVTLEIGKQLIKAQKGAAFLSITTIYAETGSGFVVPSASAKAGVEAMSKSLAAEWGKYGMRFNVIQPGPIKTKGAFSRLDPTGTFEKEMIGRIPCGRLGTVEELANLAAFLCSDYASWINGAVIKFDGGEEVLISGEFNDLRKVTKEQWDTIEELIRKTKGS

796 Q8IZU0 NP_075066.1

>Q8IZU0

MAAWGKKHAGKDPVRDECEERNRFTETREEDVTDEHGEREPFAETDEHTGANTKKPEDTAEDLTAKRKRMKMDKTCSKTKNKSKHALRKKQLKRQKRDYIHSLKLLNVLEEYITDEQKEEEEEEGEEEELIRIFQEQQKKWQQYRSVRRERLKEMKLLRDQFVKALEDFEDLCDRVFSDEDSELDN

>NP_075066.1

MASGLVRLLQQGHRCLLAPVAPKLVPPVRGVKKGFRAAFRFQKELERQRLLRCPPPPVRRSEKPNWDYHAEIQAFGHRLQENFSLDLLKTAFVNSCYIKSEEAKRQQLGIEKEAVLLNLKSNQELSEQGTSFSQTCLTQFLEDEYPDMPTEGIKNLVDFLTGEEVVCHVARNLAVEQLTLSEEFPVPPAVLQQTFFAVIGALLQSSGPERTALFIRDFLITQMTGKELFEMWKIINPMGLLVEELKKRNVSAPESRLTRQSGGTTALPLYFVGLYCDKKLIAEGPGETVLVAEEEAARVALRKLYGFTENRRPWNYSKPKETLRAEKSITAS

797 P02545 Q8NFG4

>P02545

METPSQRRATRSGAQASSTPLSPTRITRLQEKEDLQELNDRLAVYIDRVRSLETENAGLRLRITESEEVVSREVSGIKAAYEAELGDARKTLDSVAKERARLQLELSKVREEFKELKARNTKKEGDLIAAQARLKDLEALLNSKEAALSTALSEKRTLEGELHDLRGQVAKLEAALGEAKKQLQDEMLRRVDAENRLQTMKEELDFQKNIYSEELRETKRRHETRLVEIDNGKQREFESRLADALQELRAQHEDQVEQYKKELEKTYSAKLDNARQSAERNSNLVGAAHEELQQSRIRIDSLSAQLSQLQKQLAAKEAKLRDLEDSLARERDTSRRLLAEKEREMAEMRARMQQQLDEYQELLDIKLALDMEIHAYRKLLEGEEERLRLSPSPTSQRSRGRASSHSSQTQGGGSVTKKRKLESTESRSSFSQHARTSGRVAVEEVDEEGKFVRLRNKSNEDQSMGNWQIKRQNGDDPLLTYRFPPKFTLKAGQVVTIWAAGAGATHSPPTDLVWKAQNTWGCGNSLRTALINSTGEEVAMRKLVRSVTVVEDDEDEDGDDLLHHHHGSHCSSSGDPAEYNLRSRTVLCGTCGQPADKASASGSGAQVGGPISSGSSASSVTVTRSYRSVGGSGGGSFGDNLVTRSYLLGNSSPRTQSPQNCSIM

>Q8NFG4

MNAIVALCHFCELHGPRTLFCTEVLHAPLPQGDGNEDSPGQGEQAEEEEGGIQMNSRMRAHSPAEGASVESSSPGPKKSDMCEGCRSLAAGHPGYISHDKETSIKYVSHQHPSHPQLFSIVRQACVRSLSCEVCPGREGPIFFGDEQHGFVFSHTFFIKDSLARGFQRWYSIITIMMDRIYLINSWPFLLGKVRGIIDELQGKALKVFEAEQFGCPQRAQRMNTAFTPFLHQRNGNAARSLTSLTSDDNLWACLHTSFAWLLKACGSRLTEKLLEGAPTEDTLVQMEKLADLEEESESWDNSEAEEEEKAPVLPESTEGRELTQGPAESSSLSGCGSWQPRKLPVFKSLRHMRQVLGAPSFRMLAWHVLMGNQVIWKSRDVDLVQSAFEVLRTMLPVGCVRIIPYSSQYEEAYRCNFLGLSPHVQIPPHVLSSEFAVIVEVHAAARSTLHPVGCEDDQSLSKYEFVVTSGSPVAADRVGPTILNKIEAALTNQNLSVDVVDQCLVCLKEEWMNKVKVLFKFTKVDSRPKEDTQKLLSILGASEEDNVKLLKFWMTGLSKTYKSHLMSTVRSPTASESRN

798 NP_078805.3 Q9P0R6

>NP_078805.3

MADVLSVLRQYNIQKKEIVVKGDEVIFGEFSWPKNVKTNYVVWGTGKEGQPREYYTLDSILFLLNNVHLSHPVYVRRAATENIPVVRRPDRKDLLGYLNGEASTSASIDRSAPLEIGLQRSTQVKRAADEVLAEAKKPRIEDEECVRLDKERLAARLEGHKEGIVQTEQIRSLSEAMSVEKIAAIKAKIMAKKRSTIKTDLDDDITALKQRSFVDAEVDVTRDIVSRERVWRTRTTILQSTGKNFSKNIFAILQSVKAREEGRAPEQRPAPNAAPVDPTLRTKQPIPAAYNRYDQERFKGKEETEGFKIDTMGTYHGMTLKSVTEGASARKTQTPAAQPVPRPVSQARPPPNQKKGSRTPIIIIPAATTSLITMLNAKDLLQDLKFVPSDEKKKQGCQRENETLIQRRKDQMQPGGTAISVTVPYRVVDQPLKLMPQDWDRVVAVFVQGPAWQFKGWPWLLPDGSPVDIFAKIKAFHLKYDEVRLDPNVQKWDVTVLELSYHKRHLDRPVFLRFWETLDRYMVKHKSHLRF

>Q9P0R6

METDCNPMELSSMSGFEEGSELNGFEGTDMKDMRLEAEAVVNDVLFAVNNMFVSKSLRCADDVAYINVETKERNRYCLELTEAGLKVVGYAFDQVDDHLQTPYHETVYSLLDTLSPAYREAFGNALLQRLEALKRDGQS

799 P49184 Q14790

>P49184

MHYPTALLFLILANGAQAFRICAFNAQRLTLAKVAREQVMDTLVRILARCDIMVLQEVVDSSGSAIPLLLRELNRFDGSGPYSTLSSPQLGRSTYMETYVYFYRSHKTQVLSSYVYNDEDDVFAREPFVAQFSLPSNVLPSLVLVPLHTTPKAVEKELNALYDVFLEVSQHWQSKDVILLGDFNADCASLTKKRLDKLELRTEPGFHWVIADGEDTTVRASTHCTYDRVVLHGERCRSLLHTAAAFDFPTSFQLTEEEALNISDHYPVEVELKLSQAHSVQPLSLTVLLLLSLLSPQLCPAA

>Q14790

MDFSRNLYDIGEQLDSEDLASLKFLSLDYIPQRKQEPIKDALMLFQRLQEKRMLEESNLSFLKELLFRINRLDLLITYLNTRKEEMERELQTPGRAQISAYRVMLYQISEEVSRSELRSFKFLLQEEISKCKLDDDMNLLDIFIEMEKRVILGEGKLDILKRVCAQINKSLLKIINDYEEFSKERSSSLEGSPDEFSNGEELCGVMTISDSPREQDSESQTLDKVYQMKSKPRGYCLIINNHNFAKAREKVPKLHSIRDRNGTHLDAGALTTTFEELHFEIKPHDDCTVEQIYEILKIYQLMDHSNMDCFICCILSHGDKGIIYGTDGQEAPIYELTSQFTGLKCPSLAGKPKVFFIQACQGDNYQKGIPVETDSEEQPYLEMDLSSPQTRYIPDEADFLLGMATVNNCVSYRNPAEGTWYIQSLCQSLRERCPRGDDILTILTEVNYEVSNKDDKKNMGKQMPQPTFTLRKKLVFPSD

800 NP_001964.2 NP_002055.1

>NP_001964.2

MDSAITLWQFLLQLLQKPQNKHMICWTSNDGQFKLLQAEEVARLWGIRKNKPNMNYDKLSRALRYYYVKNIIKKVNGQKFVYKFVSYPEILNMDPMTVGRIEGDCESLNFSEVSSSSKDVENGGKDKPPQPGAKTSSRNDYIHSGLYSSFTLNSLNSSNVKLFKLIKTENPAEKLAEKKSPQEPTPSVIKFVTTPSKKPPVEPVAATISIGPSISPSSEETIQALETLVSPKLPSLEAPTSASNVMTAFATTPPISSIPPLQEPPRTPSPPLSSHPDIDTDIDSVASQPMELPENLSLEPKDQDSVLLEKDKVNNSSRSKKPKGLELAPTLVITSSDPSPLGILSPSLPTASLTPAFFSQTPIILTPSPLLSSIHFWSTLSPVAPLSPARLQGANTLFQFPSVLNSHGPFTLSGLDGPSTPGPFSPDLQKT

>NP_002055.1

MAQEFVNCKIQPGKVVVFIKPTCPYCRRAQEILSQLPIKQGLLEFVDITATNHTNEIQDYLQQLTGARTVPRVFIGKDCIGGCSDLVSLQQSGELLTRLKQIGALQ

801 NP_001507.2 NP_003493.1

>NP_001507.2

MVSDEDELNLLVIVVDANPIWWGKQALKESQFTLSKCIDAVMVLGNSHLFMNRSNKLAVIASHIQESRFLYPGKNGRLGDFFGDPGNPPEFNPSGSKDGKYELLTSANEVIVEEIKDLMTKSDIKGQHTETLLAGSLAKALCYIHRMNKEVKDNQEMKSRILVIKAAEDSALQYMNFMNVIFAAQKQNILIDACVLDSDSGLLQQACDITGGLYLKVPQMPSLLQYLLWVFLPDQDQRSQLILPPPVHVDYRAACFCHRNLIEIGYVCSVCLSIFCNFSPICTTCETAFKISLPPVLKAKKKKLKVSA

>NP_003493.1

MNIQEQGFPLDLGASFTEDAPRPPVPGEEGELVSTDPRPASYSFCSGKGVGIKGETSTATPRRSDLDLGYEPEGSASPTPPYLKWAESLHSLLDDQDGISLFRTFLKQEGCADLLDFWFACTGFRKLEPCDSNEEKRLKLARAIYRKYILDNNGIVSRQTKPATKSFIKGCIMKQLIDPAMFDQAQTEIQATMEENTYPSFLKSDIYLEYTRTGSESPKVCSDQSSGSGTGKGISGYLPTLNEDEEWKCDQDMDEDDGRDAAPPGRLPQKLLLETAAPRVSSSRRYSEGREFRYGSWREPVNPYYVNAGYALAPATSANDSEQQSLSSDADTLSLTDSSVDGIPPYRIRKQHRREMQESVQVNGRVPLPHIPRTYRVPKEVRVEPQKFAEELIHRLEAVQRTREAEEKLEERLKRVRMEEEGEDGDPSSGPPGPCHKLPPAPAWHHFPPRCVDMGCAGLRDAHEENPESILDEHVQRVLRTPGRQSPGPGHRSPDSGHVAKMPVALGGAASGHGKHVPKSGAKLDAAGLHHHRHVHHHVHHSTARPKEQVEAEATRRAQSSFAWGLEPHSHGARSRGYSESVGAAPNASDGLAHSGKVGVACKRNAKKAESGKSASTEVPGASEDAEKNQKIMQWIIEGEKEISRHRRTGHGSSGTRKPQPHENSRPLSLEHPWAGPQLRTSVQPSHLFIQDPTMPPHPAPNPLTQLEEARRRLEEEEKRASRAPSKQRYVQEVMRRGRACVRPACAPVLHVVPAVSDMELSETETRSQRKVGGGSAQPCDSIVVAYYFCGEPIPYRTLVRGRAVTLGQFKELLTKKGSYRYYFKKVSDEFDCGVVFEEVREDEAVLPVFEEKIIGKVEKVD

802 Q16385 Q8N142

>Q16385

MNGDDAFARRPTVGAQIPEKIQKAFDDIAKYFSKEEWEKMKASEKIFYVYMKRKYEAMTKLGFKATLPPFMCNKRAEDFQGNDLDNDPNRGNQVERPQMTFGRLQGISPKIMPKKPAEEGNDSEEVPEASGPQNDGKELCPPGKPTTSEKIHERSGPKRGEHAWTHRLRERKQLVIYEEISDPEEDDE

>Q8N142

MSGTRASNDRPPGAGGVKRGRLQQEAAATGSRVTVVLGAQWGDEGKGKVVDLLATDADIISRCQGGNNAGHTVVVDGKEYDFHLLPSGIINTKAVSFIGNGVVIHLPGLFEEAEKNEKKGLKDWEKRLIISDRAHLVFDFHQAVDGLQEVQRQAQEGKNIGTTKKGIGPTYSSKAARTGLRICDLLSDFDEFSSRFKNLAHQHQSMFPTLEIDIEGQLKRLKGFAERIRPMVRDGVYFMYEALHGPPKKILVEGANAALLDIDFGTYPFVTSSNCTVGGVCTGLGIPPQNIGDVYGVVKAYTTRVGIGAFPTEQINEIGGLLQTRGHEWGVTTGRKRRCGWLDLMILRYAHMVNGFTALALTKLDILDVLGEVKVGVSYKLNGKRIPYFPANQEMLQKVEVEYETLPGWKADTTGARRWEDLPPQAQNYIRFVENHVGVAVKWVGVGKSRESMIQLF

803 Q9H9S0 Q9Y2J8

>Q9H9S0

MSVDPACPQSLPCFEASDCKESSPMPVICGPEENYPSLQMSSAEMPHTETVSPLPSSMDLLIQDSPDSSTSPKGKQPTSAEKSVAKKEDKVPVKKQKTRTVFSSTQLCVLNDRFQRQKYLSLQQMQELSNILNLSYKQVKTWFQNQRMKSKRWQKNNWPKNSNGVTQKASAPTYPSLYSSYHQGCLVNPTGNLPMWSNQTWNNSTWSNQTQNIQSWSNHSWNTQTWCTQSWNNQAWNSPFYNCGEESLQSCMQFQPNSPASDLEAALEAAGEGLNVIQQTTRYFSTPQTMDLFLNYSMNMQPEDV

>Q9Y2J8

MLRERTVRLQYGSRVEAVYVLGTYLWTDVYSAAPAGAQTFSLKHSEHVWVEVVRDGEAEEVATNGKQRWLLSPSTTLRVTMSQASTEASSDKVTVNYYDEEGSIPIDQAGLFLTAIEISLDVDADRDGVVEKNNPKKASWTWGPEGQGAILLVNCDRETPWLPKEDCRDEKVYSKEDLKDMSQMILRTKGPDRLPAGYEIVLYISMSDSDKVGVFYVENPFFGQRYIHILGRRKLYHVVKYTGGSAELLFFVEGLCFPDEGFSGLVSIHVSLLEYMAQDIPLTPIFTDTVIFRIAPWIMTPNILPPVSVFVCCMKDNYLFLKEVKNLVEKTNCELKVCFQYLNRGDRWIQDEIEFGYIEAPHKGFPVVLDSPRDGNLKDFPVKELLGPDFGYVTREPLFESVTSLDSFGNLEVSPPVTVNGKTYPLGRILIGSSFPLSGGRRMTKVVRDFLKAQQVQAPVELYSDWLTVGHVDEFMSFVPIPGTKKFLLLMASTSACYKLFREKQKDGHGEAIMFKGLGGMSSKRITINKILSNESLVQENLYFQRCLDWNRDILKKELGLTEQDIIDLPALFKMDEDHRARAFFPNMVNMIVLDKDLGIPKPFGPQVEEECCLEMHVRGLLEPLGLECTFIDDISAYHKFLGEVHCGTNVRRKPFTFKWWHMVP

804 NP_004102.1 NP_000662.3

>NP_004102.1

MGIQGLAKLIADVAPSAIRENDIKSYFGRKVAIDASMSIYQFLIAVRQGGDVLQNEEGETTSHLMGMFYRTIRMMENGIKPVYVFDGKPPQLKSGELAKRSERRAEAEKQLQQAQAAGAEQEVEKFTKRLVKVTKQHNDECKHLLSLMGIPYLDAPSEAEASCAALVKAGKVYAAATEDMDCLTFGSPVLMRHLTASEAKKLPIQEFHLSRILQELGLNQEQFVDLCILLGSDYCESIRGIGPKRAVDLIQKHKSIEEIVRRLDPNKYPVPENWLHKEAHQLFLEPEVLDPESVELKWSEPNEEELIKFMCGEKQFSEERIRSGVKRLSKSRQGSTQGRLDDFFKVTGSLSSAKRKEPEPKGSTKKKAKTGAAGKFKRGK

>NP_000662.3

MANEVIKCKAAVAWEAGKPLSIEEIEVAPPKAHEVRIKIIATAVCHTDAYTLSGADPEGCFPVILGHEGAGIVESVGEGVTKLKAGDTVIPLYIPQCGECKFCLNPKTNLCQKIRVTQGKGLMPDGTSRFTCKGKTILHYMGTSTFSEYTVVADISVAKIDPLAPLDKVCLLGCGISTGYGAAVNTAKLEPGSVCAVFGLGGVGLAVIMGCKVAGASRIIGVDINKDKFARAKEFGATECINPQDFSKPIQEVLIEMTDGGVDYSFECIGNVKVMRAALEACHKGWGVSVVVGVAASGEEIATRPFQLVTGRTWKGTAFGGWKSVESVPKLVSEYMSKKIKVDEFVTHNLSFDEINKAFELMHSGKSIRTVVKI

805 NP_116062.2 NP_004155.2

>NP_116062.2

MASPQGGQIAIAMRLRNQLQSVYKMDPLRNEEEVRVKIKDLNEHIVCCLCAGYFVDATTITECLHTFCKSCIVKYLQTSKYCPMCNIKIHETQPLLNLKLDRVMQDIVYKLVPGLQDSEEKRIREFYQSRGLDRVTQPTGEEPALSNLGLPFSSFDHSKAHYYRYDEQLNLCLERLSSGKDKNKSVLQNKYVRCSVRAEVRHLRRVLCHRLMLNPQHVQLLFDNEVLPDHMTMKQIWLSRWFGKPSPLLLQYSVKEKRR

>NP_004155.2

MTRDQNGTWEMESNENFEGYMKALDIDFATRKIAVRLTQTKVIDQDGDNFKTKTTSTFRNYDVDFTVGVEFDEYTKSLDNRHVKALVTWEGDVLVCVQKGEKENRGWKQWIEGDKLYLELTCGDQVCRQVFKKK

806 NP_006185.1 NP_002717.3

>NP_006185.1

MEPAFGEVNQLGGVFVNGRPLPNAIRLRIVELAQLGIRPCDISRQLRVSHGCVSKILARYNETGSILPGAIGGSKPRVTTPTVVKHIRTYKQRDPGIFAWEIRDRLLADGVCDKYNVPSVSSISRILRNKIGNLAQQGHYDSYKQHQPTPQPALPYNHIYSYPSPITAAAAKVPTPPGVPAIPGSVAMPRTWPSSHSVTDILGIRSITDQVSDSSPYHSPKVEEWSSLGRNNFPAAAPHAVNGLEKGALEQEAKYGQAPNGLPAVGSFVSASSMAPYPTPAQVSPYMTYSAAPSGYVAGHGWQHAGGTSLSPHNCDIPASLAFKGMQAAREGSHSVTASAL

>NP_002717.3

MLSLQYPDVYRDETAVQDYHGHKICDPYAWLEDPDSEQTKAFVEAQNKITVPFLEQCPIRGLYKERMTELYDYPKYSCHFKKGKRYFYFYNTGLQNQRVLYVQDSLEGEARVFLDPNILSDDGTVALRGYAFSEDGEYFAYGLSASGSDWVTIKFMKVDGAKELPDVLERVKFSCMAWTHDGKGMFYNSYPQQDGKSDGTETSTNLHQKLYYHVLGTDQSEDILCAEFPDEPKWMGGAELSDDGRYVLLSIREGCDPVNRLWYCDLQQESSGIAGILKWVKLIDNFEGEYDYVTNEGTVFTFKTNRQSPNYRVINIDFRDPEESKWKVLVPEHEKDVLEWIACVRSNFLVLCYLHDVKNILQLHDLTTGALLKTFPLDVGSIVGYSGQKKDTEIFYQFTSFLSPGIIYHCDLTKEELEPRVFREVTVKGIDASDYQTVQIFYPSKDGTKIPMFIVHKKGIKLDGSHPAFLYGYGGFNISITPNYSVSRLIFVRHMGGILAVANIRGGGEYGETWHKGGILANKQNCFDDFQCAAEYLIKEGYTSPKRLTINGGSNGGLLVAACANQRPDLFGCVIAQVGVMDMLKFHKYTIGHAWTTDYGCSDSKQHFEWLVKYSPLHNVKLPEADDIQYPSMLLLTADHDDRVVPLHSLKFIATLQYIVGRSRKQSNPLLIHVDTKAGHGAGKPTAKVIEEVSDMFAFIARCLNVDWIP

807 NP_002139.2 NP_068707.1

>NP_002139.2

MSFPNSSPAANTFLVDSLISACRSDSFYSSSASMYMPPPSADMGTYGMQTCGLLPSLAKREVNHQNMGMNVHPYIPQVDSWTDPNRSCRIEQPVTQQVPTCSFTTNIKEESNCCMYSDKRNKLISAEVPSYQRLVPESCPVENPEVPVPGYFRLSQTYATGKTQEYNNSPEGSSTVMLQLNPRGAAKPQLSAAQLQMEKKMNEPVSGQEPTKVSQVESPEAKGGLPEERSCLAEVSVSSPEVQEKESKEEIKSDTPTSNWLTAKSGRKKRCPYTKHQTLELEKEFLFNMYLTRERRLEISKSVNLTDRQVKIWFQNRRMKLKKMSRENRIRELTANLTFS

>NP_068707.1

MIEESGNKRKTMAEKRQLFIEMRAQNFDVIRLSTYRTACKLRFVQKRCNLHLVDIWNMIEAFRDNGLNTLDHTTEISVSRLETVISSIYYQLNKRLPSTHQISVEQSISLLLNFMIAAYDSEGRGKLTVFSVKAMLATMCGGKMLDKLRYVFSQMSDSNGLMIFSKFDQFLKEVLKLPTAVFEGPSFGYTEHSVRTCFPQQRKIMLNMFLDTMMADPPPQCLVWLPLMHRLAHVENVFHPVECSYCRCESMMGFRYRCQQCHNYQLCQNCFWRGHAGGPHSNQHQMKEHSSWKSPAKKLSHAISKSLGCVPTREPPHPVFPEQPEKPLDLAHIVPPRPLTNMNDTMVSHMSSGVPTPTKRLQYSQDIPSHLADEHALIASYVARLQHCARVLDSPSRLDEEHRLIARYAARLAAEAGNVTRPPTDLSFNFDANKQQRQLIAELENKNREILQEIQRLRLEHEQASQPTPEKAQQNPTLLAELRLLRQRKDELEQRMSALQESRRELMVQLEELMKLLKEEEQKQAAQATGSPHTSPTHGGGRPMPMPVRSTSAGSTPTHCPQDSLSGVGGDVQEAFAQGTRRNLRNDLLVAADSITNTMSSLVKELHSAEEGAEEEEEKMQNGKDRG

808 NP_001007227.1 NP_056238.2

>NP_001007227.1

MSRVPSPPPPAEMSSGPVAESWCYTQIKVVKFSYMWTINNFSFCREEMGEVIKSSTFSSGANDKLKWCLRVNPKGLDEESKDYLSLYLLLVSCPKSEVRAKFKFSILNAKGEETKAMESQRAYRFVQGKDWGFKKFIRRDFLLDEANGLLPDDKLTLFCEVSVVQDSVNISGQNTMNMVKVPECRLADELGGLWENSRFTDCCLCVAGQEFQAHKAILAARSPVFSAMFEHEMEESKKNRVEINDVEPEVFKEMMCFIYTGKAPNLDKMADDLLAAADKYALERLKVMCEDALCSNLSVENAAEILILADLHSADQLKTQAVDFINYHASDVLETSGWKSMVVSHPHLVAEAYRSLASAQCPFLGPPRKRLKQS

>NP_056238.2

MVFPAKRFCLVPSMEGVRWAFSCGTWLPSRAEWLLAVRSIQPEEKERIGQFVFARDAKAAMAGRLMIRKLVAEKLNIPWNHIRLQRTAKGKPVLAKDSSNPYPNFNFNISHQGDYAVLAAEPELQVGIDIMKTSFPGRGSIPEFFHIMKRKFTNKEWETIRSFKDEWTQLDMFYRNWALKESFIKAIGVGLGFELQRLEFDLSPLNLDIGQVYKETRLFLDGEEEKEWAFEESKIDEHHFVAVALRKPDGSRHQDVPSQDDSKPTQRQFTILNFNDLMSSAVPMTPEDPSFWDCFCFTEEIPIRNGTKS

809 NP_006183.1 NP_001001936.1

>NP_006183.1

MEQTYGEVNQLGGVFVNGRPLPNAIRLRIVELAQLGIRPCDISRQLRVSHGCVSKILARYNETGSILPGAIGGSKPRVTTPNVVKHIRDYKQGDPGIFAWEIRDRLLADGVCDKYNVPSVSSISRILRNKIGSLAQPGPYEASKQPPSQPTLPYNHIYQYPYPSPVSPTGAKMGSHPGVPGTAGHVSIPRSWPSAHSVSNILGIRTFMEQTGALAGSEGTAYSPKMEDWAGVNRTAFPATPAVNGLEKPALEADIKYTQSASTLSAVGGFLPACAYPASNQHGVYSAPGGGYLAPGPPWPPAQGPPLAPPGAGVAVHGGELAAAMTFKHPSREGSLPAPAARPRTPSVAYTDCPSRPRPPRGSSPRTRARRERQADPGAQVCAAAPAIGTGRIGGLAEEEASAGPRGARPASPQAQPCLWPDPPHFLYWSGFLGFSELGF

>NP_001001936.1

MERYKALEQLLTELDDFLKILDQENLSSTALVKKSCLAELLRLYTKSSSSDEEYIYMNKVTINKQQNAESQGKAPEEQGLLPNGEPSQHSSAPQKSLPDLPPPKMIPERKQLAIPKTESPEGYYEEAEPYDTSLNEDGEAVSSSYESYDEEDGSKGKSAPYQWPSPEAGIELMRDARICAFLWRKKWLGQWAKQLCVIKDNRLLCYKSSKDHSPQLDVNLLGSSVIHKEKQVRKKEHKLKITPMNADVIVLGLQSKDQAEQWLRVIQEVSGLPSEGASEGNQYTPDAQRFNCQKPDIAEKYLSASEYGSSVDGHPEVPETKDVKKKCSAGLKLSNLMNLGRKKSTSLEPVERSLETSSYLNVLVNSQWKSRWCSVRDNHLHFYQDRNRSKVAQQPLSLVGCEVVPDPSPDHLYSFRILHKGEELAKLEAKSSEEMGHWLGLLLSESGSKTDPEEFTYDYVDADRVSCIVSAAKNSLLLMQRKFSEPNTYIDGLPSQDRQEELYDDVDLSELTAAVEPTEEATPVADDPNERESDRVYLDLTPVKSFLHGPSSAQAQASSPTLSCLDNATEALPADSGPGPTPDEPCIKCPENLGEQQLESLEPEDPSLRITTVKIQTEQQRISFPPSCPDAVVATPPGASPPVKDRLRVTSAEIKLGKNRTEAEVKRYTEEKERLEKKKEEIRGHLAQLRKEKRELKETLLKCTDKEVLASLEQKLKEIDEECRGEESRRVDLELSIMEVKDNLKKAEAGPVTLGTTVDTTHLENVSPRPKAVTPASAPDCTPVNSATTLKNRPLSVVVTGKGTVLQKAKEWEKKGAS

810 Q15306 Q9UDX5

>Q15306

MNLEGGGRGGEFGMSAVSCGNGKLRQWLIDQIDSGKYPGLVWENEEKSIFRIPWKHAGKQDYNREEDAALFKAWALFKGKFREGIDKPDPPTWKTRLRCALNKSNDFEELVERSQLDISDPYKVYRIVPEGAKKGAKQLTLEDPQMSMSHPYTMTTPYPSLPAQQVHNYMMPPLDRSWRDYVPDQPHPEIPYQCPMTFGPRGHHWQGPACENGCQVTGTFYACAPPESQAPGVPTEPSIRSAEALAFSDCRLHICLYYREILVKELTTSSPEGCRISHGHTYDASNLDQVLFPYPEDNGQRKNIEKLLSHLERGVVLWMAPDGLYAKRLCQSRIYWDGPLALCNDRPNKLERDQTCKLFDTQQFLSELQAFAHHGRSLPRFQVTLCFGEEFPDPQRQRKLITAHVEPLLARQLYYFAQQNSGHFLRGYDLPEHISNPEDYHRSIRHSSIQE

>Q9UDX5

MSEPQPRGAERDLYRDTWVRYLGYANEVGEAFRSLVPAAVVWLSYGVASSYVLADAIDKGKKAGEVPSPEAGRSARVTVAVVDTFVWQALASVAIPGFTINRVCAASLYVLGTATRWPLAVRKWTTTALGLLTIPIIIHPIDRSVDFLLDSSLRKLYPTVGKPSSS

811 NP_008993.1 NP_060177.2

>NP_008993.1

MNRSRQVTCVAWVRCGVAKETPDKVELSKEEVKRLIAEAKEKLQEEGGGSDEEETGSPSEDGMQSARTQARPREPLEDGDPEDDRTLDDDELAEYDLDKYDEEGDPDAETLGESLLGLTVYGSNDQDPYVTLKDTEQYEREDFLIKPSDNLIVCGRAEQDQCNLEVHVYNQEEDSFYVHHDILLSAYPLSVEWLNFDPSPDDSTGNYIAVGNMTPVIEVWDLDIVDSLEPVFTLGSKLSKKKKKKGKKSSSAEGHTDAVLDLSWNKLIRNVLASASADNTVILWDMSLGKPAASLAVHTDKVQTLQFHPFEAQTLISGSYDKSVALYDCRSPDESHRMWRFSGQIERVTWNHFSPCHFLASTDDGFVYNLDARSDKPIFTLNAHNDEISGLDLSSQIKGCLVTASADKYVKIWDILGDRPSLVHSRDMKMGVLFCSSCCPDLPFIYAFGGQKEGLRVWDISTVSSVNEAFGRRERLVLGSARNSSISGPFGSRSSDTPMES

>NP_060177.2

MPEQFSVAEFLAVTAEDLSSPAGAAAFAAKMPRYRGAALAREEILEGDQAILQRIKKAVRAIHSSGLGHVENEEQYREAVESLGNSHLSQNSHELSTGFLNLAVFTREVAALFKNLIQNLNNIVSFPLDSLMKGQLRDGRQDSKKQLEKAWKDYEAKMAKLEKERDRARVTGGIPGEVAQDMQRERRIFQLHMCEYLLKAGESQMKQGPDFLQSLIKFFHAQHNFFQDGWKAAQSLFPFIEKLAASVHALHQAQEDELQKLTQLRDSLRGTLQLESREEHLSRKNSGCGYSIHQHQGNKQFGTEKVGFLYKKSDGIRRVWQKRKCGVKYGCLTISHSTINRPPVKLTLLTCQVRPNPEEKKCFDLVTHNRTYHFQAEDEHECEAWVSVLQNSKDEALSSAFLGEPSAGPGSWGSAGHDGEPHDLTKLLIAEVKSRPGNSQCCDCGAADPTWLSTNLGVLTCIQCSGVHRELGVRFSRMQSLTLDLLGPSELLLALNMGNTSFNEVMEAQLPSHGGPKPSAESDMGTRRDYIMAKYVEHRFARRCTPEPQRLWTAICNRDLLSVLEAFANGQDFGQPLPGPDAQAPEELVLHLAVKVANQASLPLVDFIIQNGGHLDAKAADGNTALHYAALYNQPDCLKLLLKGRALVGTVNEAGETALDIARKKHHKECEELLEQAQAGTFAFPLHVDYSWVISTEPGSDSEEDEEEKRCLLKLPAQAHWASGRLDISNKTYETVASLGAATPQGESEDCPPPLPVKNSSRTLVQGCARHASGDRSEVSSLSSEAPETPESLGSPASSSSLMSPLEPGDPSQAPPNSEEGLREPPGTSRPSLTSGTTPSEMYLPVRFSSESTRSYRRGARSPEDGPSARQPLPRRNVPVGITEGDGSRTGSLPASSVQLLQD

812 NP_036389.2 Q8N573

>NP_036389.2

MVWEVKTNQMPNAVQKLLLVMDKRASGMNDSLELLQCNENLPSSPGYNSCDEHMELDDLPELQAVQSDPTQSGMYQLSSDVSHQEYPRSSWNQNTSDIPETTYRENEVDWLTELANIATSPQSPLMQCSFYNRSSPVHIIATSKSLHSYARPPPVSSSSKSEPAFPHHHWKEETPVRHERANSESESGIFCMSSLSDDDDLGWCNSWPSTVWHCFLKGTRLCFHKGSNKEWQDVEDFARAEGCDNEEDLQMGIHKGYGSDGLKLLSHEESVSFGESVLKLTFDPGTVEDGLLTVECKLDHPFYVKNKGWSSFYPSLTVVQHGIPCCEVHIGDVCLPPGHPDAINFDDSGVFDTFKSYDFTPMDSSAVYVLSSMARQRRASLSCGGPGGQDFARSGFSKNCGSPGSSQLSSNSLYAKAVKNHSSGTVSATSPNKCKRPMNAFMLFAKKYRVEYTQMYPGKDNRAISVILGDRWKKMKNEERRMYTLEAKALAEEQKRLNPDCWKRKRTNSGSQQH

>Q8N573

MSFQKPKGTIEYTVESRDSLNSIALKFDTTPNELVQLNKLFSRAVVTGQVLYVPDPEYVSSVESSPSLSPVSPLSPTSSEAEFDKTTNPDVHPTEATPSSTFTGIRPARVVSSTSEEEEAFTEKFLKINCKYITSGKGTVSGVLLVTPNNIMFDPHKNDPLVQENGCEEYGIMCPMEEVMSAAMYKEILDSKIKESLPIDIDQLSGRDFCHSKKMTGSNTEEIDSRIRDAGNDSASTAPRSTEESLSEDVFTESELSPIREELVSSDELRQDKSSGASSESVQTVNQAEVESLTVKSESTGTPGHLRSDTEHSTNEVGTLCHKTDLNNLEMAIKEDQIADNFQGISGPKEDSTSIKGNSDQDSFLHENSLHQEESQKENMPCGETAEFKQKQSVNKGKQGKEQNQDSQTEAEELRKLWKTHTMQQTKQQRENIQQVSQKEAKHKITSADGHIESSALLKEKQRHRLHKFLCLRVGKPMRKTFVSQASATMQQYAQRDKKHEYWFAVPQERTDHLYAFFIQWSPEIYAEDTGEYTREPGFIVVKKIEESETIEDSSNQAAAREWEITTREDINSKQVATVKADLESESFRPNLSDPSELLLPDQIEKLTKHLPPRTIGYPWTLVYGTGKHGTSLKTLYRTMTGLDTPVLMVIKDSDGQVFGALASEPLKVSDGFYGTGETFVFTFCPEFEVFKWTGDNMFFIKGDMDSLAFGGGGGEFALWLDGDLYHGRSHSCKTFGNRTLSKKEDFFIQDIEIWAFE

813 Q86V15 Q9Y6G3

>Q86V15

MDLGTAEGTRCTDPPAGKPAMAPKRKGGLKLNAICAKLSRQVVVEKRADAGSHTEGSPSQPRDQERSGPESGAARAPRSEEDKRRAVIEKWVNGEYSEEPAPTPVLGRIAREGLELPPEGVYMVQPQGCSDEEDHAEEPSKDGGALEEKDSDGAASKEDSGPSTRQASGEASSLRDYAASTMTEFLGMFGYDDQNTRDELARKISFEKLHAGSTPEAATSSMLPTSEDTLSKRARFSKYEEYIRKLKAGEQLSWPAPSTKTEERVGKEVVGTLPGLRLPSSTAHLETKATILPLPSHSSVQMQNLVARASKYDFFIQKLKTGENLRPQNGSTYKKPSKYDLENVKYLHLFKPGEGSPDMGGAIAFKTGKVGRPSKYDVRGIQKPGPAKVPPTPSLAPAPLASVPSAPSAPGPGPEPPASLSFNTPEYLKSTFSKTDSITTGTVSTVKNGLPTDKPAVTEDVNIYQKYIARFSGSQHCGHIHCAYQYREHYHCLDPECNYQRFTSKQDVIRHYNMHKKRDNSLQHGFMRFSPLDDCSVYYHGCHLNGKSTHYHCMQVGCNKVYTSTSDVMTHENFHKKNTQLINDGFQRFRATEDCGTADCQFYGQKTTHFHCRRPGCTFTFKNKCDIEKHKSYHIKDDAYAKDGFKKFYKYEECKYEGCVYSKATNHFHCIRAGCGFTFTSTSQMTSHKRKHERRHIRSSGALGLPPSLLGAKDTEHEESSNDDLVDFSALSSKNSSLSASPTSQQSSASLAAATAATEAGPSATKPPNSKISGLLPQGLPGSIPLALALSNSGLPTPTPYFPILAGRGSTSLPVGTPSLLGAVSSGSAASATPDTPTLVASGAGDSAPVAAASVPAPPASIMERISASKGLISPMMARLAAAALKPSATFDPGSGQQVTPARFPPAQVKPEPGESTGAPGPHEASQDRSLDLTVKEPSNESNGHAVPANSSLLSSLMNKMSQGNPGLGSLLNIKAEAEGSPAAEPSPFLGKAVKALVQEKLAEPWKVYLRRFGTKDFCDGQCDFLHKAHFHCVVEECGALFSTLDGAIKHANFHFRTEGGAAKGNTEAAFPASAAETKPPMAPSSPPVPPVTTATVSSLEGPAPSPASVPSTPTLLAWKQLASTIPQMPQIPASVPHLPASPLATTSLENAKPQVKPGFLQFQENDPCLATDCKYANKFHFHCLFGNCKYVCKTSGKAESHCLDHINPNNNLVNVRDQFAYYSLQCLCPNQHCEFRMRGHYHCLRTGCYFVTNITTKLPWHIKKHEKAERRAANGFKYFTKREECGRLGCKYNQVSSHFHCIREGCQFSFLLKHQMTSHARKHMRRMLGKNFDRVPPSQGPPGLMDAETDECMDYTGCSPGAMSSESSTMDRSCSSTPVGNESTAAGNTISMPTASGAKKRFWIIEDMSPFGKRRKTASSRKMLDEGMMLEGFRRFDLYEDCKDAACQFSLKVTHYHCTRENCGYKFCGRTHMYKHAQHHDRVDNLVLDDFKRFKASLSRHFADCPFSGTSTHFHCLRCRFRCTDSTKVTAHRKHHGKQDVISAAGFRQFSSSADCAVPDCKYKLKCSHFHCTFPGCRHTVVGMSQMDSHKRKHEKQERGEPAAEGPAPGPPISLDGSLSLGAEPGSLLFLQSAAAGLGLALGDAGDPGPPDAAAPGPREGAAAAAAAAGESSQEDEEEELELPEEEAEDDEDEDDDEDDDDEDDDEDDDDEDLRTDSEESLPEAAAEAAGAGARTPALAALAALGAPGPAPTAASSP

>Q9Y6G3

MAVAAVKWVMSKRTILKHLFPVQNGALYCVCHKSTYSPLPDDYNCNVELALTSDGRTIVCYHPSVDIPYEHTKPIPRPDPVHNNEETHDQVLKTRLEEKVEHLEEGPMIEQLSKMFFTTKHRWYPHGRYHRCRKNLNPPKDR

814 NP_002938.1 P30838

>NP_002938.1

MVDMMDLPRSRINAGMLAQFIDKPVCFVGRLEKIHPTGKMFILSDGEGKNGTIELMEPLDEEISGIVEVVGRVTAKATILCTSYVQFKEDSHPFDLGLYNEAVKIIHDFPQFYPLGIVQHD

>P30838

MSKISEAVKRARAAFSSGRTRPLQFRIQQLEALQRLIQEQEQELVGALAADLHKNEWNAYYEEVVYVLEEIEYMIQKLPEWAADEPVEKTPQTQQDELYIHSEPLGVVLVIGTWNYPFNLTIQPMVGAIAAGNAVVLKPSELSENMASLLATIIPQYLDKDLYPVINGGVPETTELLKERFDHILYTGSTGVGKIIMTAAAKHLTPVTLELGGKSPCYVDKNCDLDVACRRIAWGKFMNSGQTCVAPDYILCDPSIQNQIVEKLKKSLKEFYGEDAKKSRDYGRIISARHFQRVMGLIEGQKVAYGGTGDAATRYIAPTILTDVDPQSPVMQEEIFGPVLPIVCVRSLEEAIQFINQREKPLALYMFSSNDKVIKKMIAETSSGGVAANDVIVHITLHSLPFGGVGNSGMGSYHGKKSFETFSHRRSCLVRPLMNDEGLKVRYPPSPAKMTQH

815 P20671 NP_001166.3

>P20671

MSGRGKQGGKARAKAKTRSSRAGLQFPVGRVHRLLRKGNYSERVGAGAPVYLAAVLEYLTAEILELAGNAARDNKKTRIIPRHLQLAIRNDEELNKLLGKVTIAQGGVLPNIQAVLLPKKTESHHKAKGK

>NP_001166.3

MTEKAPEPHVEEDDDDELDSKLNYKPPPQKSLKELQEMDKDDESLIKYKKTLLGDGPVVTDPKAPNVVVTRLTLVCESAPGPITMDLTGDLEALKKETIVLKEGSEYRVKIHFKVNRDIVSGLKYVQHTYRTGVKVDKATFMVGSYGPRPEEYEFLTPVEEAPKGMLARGTYHNKSFFTDDDKQDHLSWEWNLSIKKEWTE

816 NP_001795.2 NP_000845.1

>NP_001795.2

MYVGYVLDKDSPVYPGPARPASLGLGPQAYGPPAPPPAPPQYPDFSSYSHVEPAPAPPTAWGAPFPAPKDDWAAAYGPGPAAPAASPASLAFGPPPDFSPVPAPPGPGPGLLAQPLGGPGTPSSPGAQRPTPYEWMRRSVAAGGGGGSGKTRTKDKYRVVYTDHQRLELEKEFHYSRYITIRRKSELAANLGLTERQVKIWFQNRRAKERKVNKKKQQQQQPPQPPMAHDITATPAGPSLGGLCPSNTSLLATSSPMPVKEEFLP

>NP_000845.1

MGLELFLDLVSQPSRAVYIFAKKNGIPLELRTVDLVKGQHKSKEFLQINSLGKLPTLKDGDFILTESSAILIYLSCKYQTPDHWYPSDLQARARVHEYLGWHADCIRGTFGIPLWVQVLGPLIGVQVPEEKVERNRTAMDQALQWLEDKFLGDRPFLAGQQVTLADLMALEELMQPVALGYELFEGRPRLAAWRGRVEAFLGAELCQEAHSIILSILEQAAKKTLPTPSPEAYQAMLLRIARIP

817 NP_003911.1 Q7Z7A3

>NP_003911.1

MDLHMMNCELLATCSALGYLEGDTYHKEPDCLESVKDLIRYLRHEDETRDVRQQLGAAQILQSDLLPILTQHHQDKPLFDAVIRLMVNLTQPALLCFGNLPKEPSFRHHFLQVLTYLQAYKEAFASEKAFGVLSETLYELLQLGWEERQEEDNLLIERILLLVRNILHVPADLDQEKKIDDDASAHDQLLWAIHLSGLDDLLLFLASSSAEEQWSLHVLEIVSLMFRDQNPEQLAGVGQGRLAQERSADFAELEVLRQREMAEKKTRALQRGNRHSRFGGSYIVQGLKSIGERDLIFHKGLHNLRNYSSDLGKQPKKVPKRRQAARELSIQRRSALNVRLFLRDFCSEFLENCYNRLMGSVKDHLLREKAQQHDETYYMWALAFFMAFNRAASFRPGLVSETLSVRTFHFIEQNLTNYYEMMLTDRKEAASWARRMHLALKAYQELLATVNEMDLSPDEAVRESSRIIKNNIFYVMEYRELFLALFRKFDERCQPRSFLRDLVETTHLFLKMLERFCRSRGNLVVQNKQKKRRKKKKKVLDQAIVSGNVPSSPEEVEAVWPALAEQLQCCAQNSELSMDSVVPFDAASEVPVEEQRAEAMVRIQDCLLAGQAPQALTLLRSAREVWPEGDVFGSQDISPEEEIQLLKQILSAPLPRQQGPEERGAEEEEEEEEEEEEELQVVQVSEKEFNFLDYLKRFACSTVVRAYVLLLRSYQQNSAHTNHCIVKMLHRLAHDLKMEALLFQLSVFCLFNRLLSDPAAGAYKELVTFAKYILGKFFALAAVNQKAFVELLFWKNTAVVREMTEGYGSLDDRSSSRRAPTWSPEEEAHLQELYLANKDVEGQDVVEAILAHLNTVPRTRKQIIHHLVQMGLADSVKDFQRKGTHIVLWTGDQELELQRLFEEFRDSDDVLGHIMKNITAKRSRARIVDKLLALGLVAERRELYKKRQKKLASSILPNGAESLKDFCQEDLEEEENLPEEDSEEEEEGGSEAEQVQGSLVLSNENLGQSLHQEGFSIPLLWLQNCLIRAADDREEDGCSQAVPLVPLTEENEEAMENEQFQQLLRKLGVRPPASGQETFWRIPAKLSPTQLRRAAASLSQPEEEQKLQPELQPKVPGEQGSDEEHCKEHRAQALRALLLAHKKKAGLASPEEEDAVGKEPLKAAPKKRQLLDSDEEQEEDEGRNRAPELGAPGIQKKKRYQIEDDEDD

>Q7Z7A3

MPAPPCASCHAARAALRRPLSGQALCGACFCAAFEAEVLHTVLAGRLLPPGAVVAVGASGGKDSTVLAHVLRALAPRLGISLQLVAVDEGIGGYRDAALAAVRRQAARWELPLTVVAYEDLFGGWTMDAVARSTAGSGRSRSCCTFCGVLRRRALEEGARRVGATHIVTGHNADDMAETVLMNFLRGDAGRLARGGGLGSPGEGGALPRCRPLQFASQKEVVLYAHFRRLDYFSEECVYAPEAFRGHARDLLKRLEAARPSAVLDLVHSAERLALAPAARPPRPGACSRCGALASRALCQACALLDGLNRGRPRLAIGKGRRGLDEEATPGTPGDPARPPASKAVPTF

818 NP_006181.1 Q9BYG8

>NP_006181.1

MSKPELKEDKMLEVHFVGDDDVLNHILDREGGAKLKKERAQLLVNPKKIIKKPEYDLEEDDQEVLKDQNYVEIMGRDVQESLKNGSATGGGNKVYSFQNRKHSEKMAKLASELAKTPQKSVSFSLKNDPEITINVPQSSKGHSASDKVQPKNNDKSEFLSTAPRSLRKRLIVPRSHSDSESEYSASNSEDDEGVAQEHEEDTNAVIFSQKIQAQNRVVSAPVGKETPSKRMKRDKTSDLVEEYFEAHSSSKVLTSDRTLQKLKRAKLDQQTLRNLLSKVSPSFSAELKQLNQQYEKLFHKWMLQLHLGFNIVLYGLGSKRDLLERFRTTMLQDSIHVVINGFFPGISVKSVLNSITEEVLDHMGTFRSILDQLDWIVNKFKEDSSLELFLLIHNLDSQMLRGEKSQQIIGQLSSLHNIYLIASIDHLNAPLMWDHAKQSLFNWLWYETTTYSPYTEETSYENSLLVKQSGSLPLSSLTHVLRSLTPNARGIFRLLIKYQLDNQDNPSYIGLSFQDFYQQCREAFLVNSDLTLRAQLTEFRDHKLIRTKKGTDGVEYLLIPVDNGTLTDFLEKEEEEA

>Q9BYG8

MPSMLERISKNLVKEIGSKDLTPVKYLLSATKLRQFVILRKKKDSRSSFWEQSDYVPVEFSLNDILEPSSSVLETVVTGPFHFSDIMIQKHKADMGVNVGIEVSVSGEASVDHGCSLEFQIVTIPSPNLEDFQKRKLLDPEPSFLKECRRRGDNLYVVTEAVELINNTVLYDSSSVNILGKIALWITYGKGQGQGESLRVKKKALTLQKGMVMAYKRKQLVIKEKAILISDDDEQRTFQDEYEISEMVGYCAARSEGLLPSFHTISPTLFNASSNDMKLKPELFLTQQFLSGHLPKYEQVHILPVGRIEEPFWQNFKHLQEEVFQKIKTLAQLSKDVQDVMFYSILAMLRDRGALQDLMNMLELDSSGHLDGPGGAILKKLQQDSNHAWFNPKDPILYLLEAIMVLSDFQHDLLACSMEKRILLQQQELVRSILEPNFRYPWSIPFTLKPELLAPLQSEGLAITYGLLEECGLRTELDNPRSTWDVEAKMPLSALYGTLSLLQQLAEA

819 NP_055331.1 Q9NQX6

>NP_055331.1

MADKRKLQGEIDRCLKKVSEGVEQFEDIWQKLHNAANANQKEKYEADLKKEIKKLQRLRDQIKTWVASNEIKDKRQLIDNRKLIETQMERFKVVERETKTKAYSKEGLGLAQKVDPAQKEKEEVGQWLTNTIDTLNMQVDQFESEVESLSVQTRKKKGDKDKQDRIEGLKRHIEKHRYHVRMLETILRMLDNDSILVDAIRKIKDDVEYYVDSSQDPDFEENEFLYDDLDLEDIPQALVATSPPSHSHMEDEIFNQSSSTPTSTTSSSPIPPSPANCTTENSEDDKKRGRSTDSEVSQSPAKNGSKPVHSNQHPQSPAVPPTYPSGPPPAASALSTTPGNNGVPAPAAPPSALGPKASPAPSHNSGTPAPYAQAVAPPAPSGPSTTQPRPPSVQPSGGGGGGSGGGGSSSSSNSSAGGGAGKQNGATSYSSVVADSPAEVALSSSGGNNASSQALGPPSGPHNPPPSTSKEPSAAAPTGAGGVAPGSGNNSGGPSLLVPLPVNPPSSPTPSFSDAKAAGALLNGPPQFSTAPEIKAPEPLSSLKSMAERAAISSGIEDPVPTLHLTERDIILSSTSAPPASAQPPLQLSEVNIPLSLGVCPLGPVPLTKEQLYQQAMEEAAWHHMPHPSDSERIRQYLPRNPCPTPPYHHQMPPPHSDTVEFYQRLSTETLFFIFYYLEGTKAQYLAAKALKKQSWRFHTKYMMWFQRHEEPKTITDEFEQGTYIYFDYEKWGQRKKEGFTFEYRYLEDRDLQ

>Q9NQX6

MAQGLVTFADVAIDFSQEEWACLNSAQRDLYWDVMLENYSNLVSLDLESAYENKSLPTEKNIHEIRASKRNSDRRSKSLGRNWICEGTLERPQRSRGRYVNQMIINYVKRPATREGTPPRTHQRHHKENSFECKDCGKAFSRGYQLSQHQKIHTGEKPYECKECKKAFRWGNQLTQHQKIHTGEKPYECKDCGKAFRWGSSLVIHKRIHTGEKPYECKDCGKAFRRGDELTQHQRFHTGEKDYECKDCGKTFSRVYKLIQHKRIHSGEKPYECKDCGKAFICGSSLIQHKRIHTGEKPYECQECGKAFTRVNYLTQHQKIHTGEKPHECKECGKAFRWGSSLVKHERIHTGEKPYKCTECGKAFNCGYHLTQHERIHTGETPYKCKECGKAFIYGSSLVKHERIHTGVKPYGCTECGKSFSHGHQLTQHQKTHSGAKSYECKECGKACNHLNHLREHQRIHNS

820 Q04727 O75897

>Q04727

MIRDLSKMYPQTRHPAPHQPAQPFKFTISESCDRIKEEFQFLQAQYHSLKLECEKLASEKTEMQRHYVMYYEMSYGLNIEMHKQAEIVKRLNAICAQVIPFLSQEHQQQVVQAVERAKQVTMAELNAIIGQQLQAQHLSHGHGLPVPLTPHPSGLQPPAIPPIGSSAGLLALSSALGGQSHLPIKDEKKHHDNDHQRDRDSIKSSSVSPSASFRGAEKHRNSADYSSESKKQKTEEKEIAARYDSDGEKSDDNLVVDVSNEDPSSPRGSPAHSPRENGLDKTRLLKKDAPISPASIASSSSTPSSKSKELSLNEKSTTPVSKSNTPTPRTDAPTPGSNSTPGLRPVPGKPPGVDPLASSLRTPMAVPCPYPTPFGIVPHAGMNGELTSPGAAYAGLHNISPQMSAAAAAAAAAAAYGRSPVVGFDPHHHMRVPAIPPNLTGIPGGKPAYSFHVSADGQMQPVPFPPDALIGPGIPRHARQINTLNHGEVVCAVTISNPTRHVYTGGKGCVKVWDISHPGNKSPVSQLDCLNRDNYIRSCRLLPDGRTLIVGGEASTLSIWDLAAPTPRIKAELTSSAPACYALAISPDSKVCFSCCSDGNIAVWDLHNQTLVRQFQGHTDGASCIDISNDGTKLWTGGLDNTVRSWDLREGRQLQQHDFTSQIFSLGYCPTGEWLAVGMENSNVEVLHVTKPDKYQLHLHESCVLSLKFAHCGKWFVSTGKDNLLNAWRTPYGASIFQSKESSSVLSCDISVDDKYIVTGSGDKKATVYEVIY

>O75897

MALHDMEDFTFDGTKRLSVNYVKGILQPTDTCDIWDKIWNFQAKPDDLLISTYPKAGTTWTQEIVELIQNEGDVEKSKRAPTHQRFPFLEMKIPSLGSGLEQAHAMPSPRILKTHLPFHLLPPSLLEKNCKIIYVARNPKDNMVSYYHFQRMNKALPAPGTWEEYFETFLAGKVCWGSWHEHVKGWWEAKDKHRILYLFYEDMKKNPKHEIQKLAEFIGKKLDDKVLDKIVHYTSFDVMKQNPMANYSSIPAEIMDHSISPFMRKGAVGDWKKHFTVAQNERFDEDYKKKMTDTRLTFHFQF

821 NP_005654.2 NP_071906.1

>NP_005654.2

MASMRESDTGLWLHNKLGATDELWAPPSIASLLTAAVIDNIRLCFHGLSSAVKLKLLLGTLHLPRRTVDEMKGALMEIIQLASLDSDPWVLMVADILKSFPDTGSLNLELEEQNPNVQDILGELREKVGECEASAMLPLECQYLNKNALTTLAGPLTPPVKHFQLKRKPKSATLRAELLQKSTETAQQLKRSAGVPFHAKGRGLLRKMDTTTPLKGIPKQAPFRSPTAPSVFSPTGNRTPIPPSRTLLRKERGVKLLDISELDMVGAGREAKRRRKTLDAEVVEKPAKEETVVENATPDYAAGLVSTQKLGSLNNEPALPSTSYLPSTPSVVPASSYIPSSETPPAPSSREASRPPEEPSAPSPTLPAQFKQRAPMYNSGLSPATPTPAAPTSPLTPTTPPAVAPTTQTPPVAMVAPQTQAPAQQQPKKNLSLTREQMFAAQEMFKTANKVTRPEKALILGFMAGSRENPCQEQGDVIQIKLSEHTEDLPKADGQGSTTMLVDTVFEMNYATGQWTRFKKYKPMTNVS

>NP_071906.1

MDALVEDDICILNHEKAHKRDTVTPVSIYSGDESVASHFALVTAYEDIKKRLKDSEKENSLLKKRIRFLEEKLIARFEEETSSVGREQVNKAYHAYREVCIDRDNLKSKLDKMNKDNSESLKVLNEQLQSKEVELLQLRTEVETQQVMRNLNPPSSNWEVEKLSCDLKIHGLEQELELMRKECSDLKIELQKAKQTDPYQEDNLKSRDLQKLSISSDNMQHAYWELKREMSNLHLVTQVQAELLRKLKTSTAIKKACAPVGCSEDLGRDSTKLHLMNFTATYTRHPPLLPNGKALCHTTSSPLPGDVKVLSEKAILQSWTDNERSIPNDGTCFQEHSSYGRNSLEDNSWVFPSPPKSSETAFGETKTKTLPLPNLPPLHYLDQHNQNCLYKN

822 NP_659480.1 P17706

>NP_659480.1

MPEGPLVRKFHHLVSPFVGQQVVKTGGSSKKLQPASLQSLWLQDTQVHGKKLFLRFDLDEEMGPPGSSPTPEPPQKEVQKEGAADPKQVGEPSGQKTLDGSSRSAELVPQGEDDSEYLERDAPAGDAGRWLRVSFGLFGSVWVNDFSRAKKANKRGDWRDPSPRLVLHFGGGGFLAFYNCQLSWSSSPVVTPTCDILSEKFHRGQALEALGQAQPVCYTLLDQRYFSGLGNIIKNEALYRAGIHPLSLGSVLSASRREVLVDHVVEFSTAWLQGKFQGRPQHTQVYQKEQCPAGHQVMKEAFGPEDGLQRLTWWCPQCQPQLSEEPEQCQFS

>P17706

MPTTIEREFEELDTQRRWQPLYLEIRNESHDYPHRVAKFPENRNRNRYRDVSPYDHSRVKLQNAENDYINASLVDIEEAQRSYILTQGPLPNTCCHFWLMVWQQKTKAVVMLNRIVEKESVKCAQYWPTDDQEMLFKETGFSVKLLSEDVKSYYTVHLLQLENINSGETRTISHFHYTTWPDFGVPESPASFLNFLFKVRESGSLNPDHGPAVIHCSAGIGRSGTFSLVDTCLVLMEKGDDINIKQVLLNMRKYRMGLIQTPDQLRFSYMAIIEGAKCIKGDSSIQKRWKELSKEDLSPAFDHSPNKIMTEKYNGNRIGLEEEKLTGDRCTGLSSKMQDTMEENSESALRKRIREDRKATTAQKVQQMKQRLNENERKRKRWLYWQPILTKMGFMSVILVGAFVGWTLFFQQNAL

823 NP_003855.1 NP_004291.1

>NP_003855.1

MNGFTPDEMSRGGDAAAAVAAVVAAAAAAASAGNGTGAGTGAEVPGAGAVSAAGPPGAAGPGPGQLCCLREDGERCGRAAGNASFSKRIQKSISQKKVKIELDKSARHLYICDYHKNLIQSVRNRRKRKGSDDDGGDSPVQDIDTPEVDLYQLQVNTLRRYKRHFKLPTRPGLNKAQLVEIVGCHFRSIPVNEKDTLTYFIYSVKNDKNKSDLKVDSGVH

>NP_004291.1

MAEQATKSVLFVCLGNICRSPIAEAVFRKLVTDQNISENWRVDSAATSGYEIGNPPDYRGQSCMKRHGIPMSHVARQITKEDFATFDYILCMDESNLRDLNRKSNQVKTCKAKIELLGSYDPQKQLIIEDPYYGNDSDFETVYQQCVRCCRAFLEKAH

824 NP_443082.2 P29144

>NP_443082.2

MAASVRQARSLLGVAATLAPGSRGYRARPPPRRRPGPRWPDPEDLLTPRWQLGPRYAAKQFARYGAASGVVPGSLWPSPEQLRELEAEEREWYPSLATMQESLRVKQLAEEQKRREREQHIAECMAKMPQMIVNWQQQQRENWEKAQADKERRARLQAEAQELLGYQVDPRSARFQELLQDLEKKERKRLKEEKQKRKKEARAAALAAAVAQDPAASGAPSS

>P29144

MATAATEEPFPFHGLLPKKETGAASFLCRYPEYDGRGVLIAVLDTGVDPGAPGMQVTTDGKPKIVDIIDTTGSGDVNTATEVEPKDGEIVGLSGRVLKIPASWTNPSGKYHIGIKNGYDFYPKALKERIQKERKEKIWDPVHRVALAEACRKQEEFDVANNGSSQANKLIKEELQSQVELLNSFEKKYSDPGPVYDCLVWHDGEVWRACIDSNEDGDLSKSTVLRNYKEAQEYGSFGTAEMLNYSVNIYDDGNLLSIVTSGGAHGTHVASIAAGHFPEEPERNGVAPGAQILSIKIGDTRLSTMETGTGLIRAMIEVINHKCDLVNYSYGEATHWPNSGRICEVINEAVWKHNIIYVSSAGNNGPCLSTVGCPGGTTSSVIGVGAYVSPDMMVAEYSLREKLPANQYTWSSRGPSADGALGVSISAPGGAIASVPNWTLRGTQLMNGTSMSSPNACGGIALILSGLKANNIDYTVHSVRRALENTAVKADNIEVFAQGHGIIQVDKAYDYLVQNTSFANKLGFTVTVGNNRGIYLRDPVQVAAPSDHGVGIEPVFPENTENSEKISLQLHLALTSNSSWVQCPSHLELMNQCRHINIRVDPRGLREGLHYTEVCGYDIASPNAGPLFRVPITAVIAAKVNESSHYDLAFTDVHFKPGQIRRHFIEVPEGATWAEVTVCSCSSEVSAKFVLHAVQLVKQRAYRSHEFYKFCSLPEKGTLTEAFPVLGGKAIEFCIARWWASLSDVNIDYTISFHGIVCTAPQLNIHASEGINRFDVQSSLKYEDLAPCITLKNWVQTLRPVSAKTKPLGSRDVLPNNRQLYEMVLTYNFHQPKSGEVTPSCPLLCELLYESEFDSQLWIIFDQNKRQMGSGDAYPHQYSLKLEKGDYTIRLQIRHEQISDLERLKDLPFIVSHRLSNTLSLDIHENHSFALLGKKKSSNLTLPPKYNQPFFVTSLPDDKIPKGAGPGCYLAGSLTLSKTELGKKADVIPVHYYLIPPPTKTKNGSKDKEKDSEKEKDLKEEFTEALRDLKIQWMTKLDSSDIYNELKETYPNYLPLYVARLHQLDAEKERMKRLNEIVDAANAVISHIDQTALAVYIAMKTDPRPDAATIKNDMDKQKSTLVDALCRKGCALADHLLHTQAQDGAISTDAEGKEEEGESPLDSLAETFWETTKWTDLFDNKVLTFAYKHALVNKMYGRGLKFATKLVEEKPTKENWKNCIQLMKLLGWTHCASFTENWLPIMYPPDYCVF

825 NP_036218.1 NP_036199.2

>NP_036218.1

MSGFSPELIDYLEGKISFEEFERRREERKTREKKSLQEKGKLSAEENPDDSEVPSSSGINSTKSQDKDVNEGETSDGVRKSVHKVFASMLGENEDDEEEEEEEEEEEEEEETPEQPTAGDVFVLEMVLNRETKKMMKEKRPRSKLPRALRGLMGEANIRFARGEREEAILMCMEIIRQAPLAYEPFSTLAMIYEDQGDMEKSLQFELIAAHLNPSDTEEWVRLAEMSLEQDNIKQAIFCYTKALKYEPTNVRYLWERSSLYEQMGDHKMAMDGYRRILNLLSPSDGERFMQLARDMAKSYYEANDVTSAINIIDEAFSKHQGLVSMEDVNIAAELYISNKQYDKALEIITDFSGIVLEKKTSEEGTSEENKAPENVTCTIPDGVPIDITVKLMVCLVHLNILEPLNPLLTTLVEQNPEDMGDLYLDVAEAFLDVGEYNSALPLLSALVCSERYNLAVVWLRHAECLKALGYMERAAESYGKVVDLAPLHLDARISLSTLQQQLGQPEKALEALEPMYDPDTLAQDANAAQQELKLLLHRSTLLFSQGKMYGYVDTLLTMLAMLLKVAMNRAQVCLISSSKSGERHLYLIKVSRDKISDSNDQESANCDAKAIFAVLTSVLTKDDWWNLLLKAIYSLCDLSRFQEAELLVDSSLEYYSFYDDRQKRKELEYFGLSAAILDKNFRKAYNYIRIMVMENVNKPQLWNIFNQVTMHSQDVRHHRFCLRLMLKNPENHALCVLNGHNAFVSGSFKHALGQYVQAFRTHPDEPLYSFCIGLTFIHMASQKYVLRRHALIVQGFSFLNRYLSLRGPCQESFYNLGRGLHQLGLIHLAIHYYQKALELPPLVVEGIELDQLDLRRDIAYNLSLIYQSSGNTGMAQTLLYTYCSI

>NP_036199.2

MSRQLSRARPATVLGAMEMGRRMDAPTSAAVTRAFLERGHTEIDTAFVYSEGQSETILGGLGLRLGGSDCRVKIDTKAIPLFGNSLKPDSLRFQLETSLKRLQCPRVDLFYLHMPDHSTPVEETLRACHQLHQEGKFVELGLSNYAAWEVAEICTLCKSNGWILPTVYQGMYNAITRQVETELFPCLRHFGLRFYAFNPLAGGLLTGKYKYEDKNGKQPVGRFFGNTWAEMYRNRYWKEHHFEGIALVEKALQAAYGASAPSMTSATLRWMYHHSQLQGAHGDAVILGMSSLEQLEQNLAAAEEGPLEPAVVDAFNQAWHLVTHECPNYFR

826 Q9UHV7 NP_000894.1

>Q9UHV7

MSASFVPNGASLEDCHCNLFCLADLTGIKWKKYVWQGPTSAPILFPVTEEDPILSSFSRCLKADVLGVWRRDQRPGRRELWIFWWGEDPVLLTLFTMTYQKKKMECGRMDFPMNAVLCFSKAVHNLLERCLMNRNFVRIGKWFVKPYEKDEKPINKSEHLSCSFTFFLHGDSNVCTSVEINQHQPVYLLSEEHITLAQQSNSPFQVILCPFGLNGTLTGQAFKMSDSATKKLIGEWKQFYPISCCLKEMSEEKQEDMDWEDDSLAAVEVLVAGVRMIYPACFVLVPQSDIPTPSPVGSTHCSSSCLGVHQVPASTRDPAMSSVTLTPPTSPEEVQTVDPQSVQKWVKFSSVSDGFNSDSTSHHGGKIPRKLANHVVDRVWQECNMNRAQNKRKYSASSGGLCEEATAAKVASWDFVEATQRTNCSCLRHKNLKSRNAGQQGQAPSLGQQQQILPKHKTNEKQEKSEKPQKRPLTPFHHRVSVSDDVGMDADSASQRLVISAPDSQVRFSNIRTNDVAKTPQMHGTEMANSPQPPPLSPHPCDVVDEGVTKTPSTPQSQHFYQMPTPDPLVPSKPMEDRIDSLSQSFPPQYQEAVEPTVYVGTAVNLEEDEANIAWKYYKFPKKKDVEFLPPQLPSDKFKDDPVGPFGQESVTSVTELMVQCKKPLKVSDELVQQYQIKNQCLSAIASDAEQEPKIDPYAFVEGDEEFLFPDKKDRQNSEREAGKKHKVEDGTSSVTVLSHEEDAMSLFSPSIKQDAPRPTSHARPPSTSLIYDSDLAVSYTDLDNLFNSDEDELTPGSKKSANGSDDKASCKESKTGNLDPLSCISTADLHKMYPTPPSLEQHIMGFSPMNMNNKEYGSMDTTPGGTVLEGNSSSIGAQFKIEVDEGFCSPKPSEIKDFSYVYKPENCQILVGCSMFAPLKTLPSQYLPPIKLPEECIYRQSWTVGKLELLSSGPSMPFIKEGDGSNMDQEYGTAYTPQTHTSFGMPPSSAPPSNSGAGILPSPSTPRFPTPRTPRTPRTPRGAGGPASAQGSVKYENSDLYSPASTPSTCRPLNSVEPATVPSIPEAHSLYVNLILSESVMNLFKDCNFDSCCICVCNMNIKGADVGVYIPDPTQEAQYRCTCGFSAVMNRKFGNNSGLFLEDELDIIGRNTDCGKEAEKRFEALRATSAEHVNGGLKESEKLSDDLILLLQDQCTNLFSPFGAADQDPFPKSGVISNWVRVEERDCCNDCYLALEHGRQFMDNMSGGKVDEALVKSSCLHPWSKRNDVSMQCSQDILRMLLSLQPVLQDAIQKKRTVRPWGVQGPLTWQQFHKMAGRGSYGTDESPEPLPIPTFLLGYDYDYLVLSPFALPYWERLMLEPYGSQRDIAYVVLCPENEALLNGAKSFFRDLTAIYESCRLGQHRPVSRLLTDGIMRVGSTASKKLSEKLVAEWFSQAADGNNEAFSKLKLYAQVCRYDLGPYLASLPLDSSLLSQPNLVAPTSQSLITPPQMTNTGNANTPSATLASAASSTMTVTSGVAISTSVATANSTLTTASTSSSSSSNLNSGVSSNKLPSFPPFGSMNSNAAGSMSTQANTVQSGQLGGQQTSALQTAGISGESSSLPTQPHPDVSESTMDRDKVGIPTDGDSHAVTYPPAIVVYIIDPFTYENTDESTNSSSVWTLGLLRCFLEMVQTLPPHIKSTVSVQIIPCQYLLQPVKHEDREIYPQHLKSLAFSAFTQCRRPLPTSTNVKTLTGFGPGLAMETALRSPDRPECIRLYAPPFILAPVKDKQTELGETFGEAGQKYNVLFVGYCLSHDQRWILASCTDLYGELLETCIINIDVPNRARRKKSSARKFGLQKLWEWCLGLVQMSSLPWRVVIGRLGRIGHGELKDWSCLLSRRNLQSLSKRLKDMCRMCGISAADSPSILSACLVAMEPQGSFVIMPDSVSTGSVFGRSTTLNMQTSQLNTPQDTSCTHILVFPTSASVQVASATYTTENLDLAFNPNNDGADGMGIFDLLDTGDDLDPDIINILPASPTGSPVHSPGSHYPHGGDAGKGQSTDRLLSTEPHEEVPNILQQPLALGYFVSTAKAGPLPDWFWSACPQAQYQCPLFLKASLHLHVPSVQSDELLHSKHSHPLDSNQTSDVLRFVLEQYNALSWLTCDPATQDRRSCLPIHFVVLNQLYNFIMNML

>NP_000894.1

MVGRRALIVLAHSERTSFNYAMKEAAAAALKKKGWEVVESDLYAMNFNPIISRKDITGKLKDPANFQYPAESVLAYKEGHLSPDIVAEQKKLEAADLVIFQFPLQWFGVPAILKGWFERVFIGEFAYTYAAMYDKGPFRSKKAVLSITTGGSGSMYSLQGIHGDMNVILWPIQSGILHFCGFQVLEPQLTYSIGHTPADARIQILEGWKKRLENIWDETPLYFAPSSLFDLNFQAGFLMKKEVQDEEKNKKFGLSVGHHLGKSIPTDNQIKARK

827 P49640 NP_005078.2

>P49640

MESRKDMVVFLDGGQLGTLVGKRVSNLSEAVGSPLPEPPEKMVPRGCLSPRAVPPATRERGGGGPEEEPVDGLAGSAAGPGAEPQVAGAAMLGPGPPAPSVDSLSGQGQPSSSDTESDFYEEIEVSCTPDCATGNAEYQHSKGSGSEALVGSPNGGSETPKSNGGSGGGGSQGTLACSASDQMRRYRTAFTREQIARLEKEFYRENYVSRPRRCELAAALNLPETTIKVWFQNRRMKDKRQRLAMTWPHPADPAFYTYMMSHAAAAGGLPYPFPSHLPLPYYSPVGLGAASAASAAASPFSGSLRPLDTFRVLSQPYPRPELLCAFRHPPLYPGPAHGLGASAGGPCSCLACHSGPANGLAPRAAAASDFTCASTSRSDSFLTFAPSVLSKASSVALDQREEVPLTR

>NP_005078.2

MAELTVEVRGSNGAFYKGFIKDVHEDSLTVVFENNWQPERQVPFNEVRLPPPPDIKKEISEGDEVEVYSRANDQEPCGWWLAKVRMMKGEFYVIEYAACDATYNEIVTFERLRPVNQNKTVKKNTFFKCTVDVPEDLREACANENAHKDFKKAVGACRIFYHPETTQLMILSASEATVKRVNILSDMHLRSIRTKLMLMSRNEEATKHLECTKQLAAAFHEEFVVREDLMGLAIGTHGSNIQQARKVPGVTAIELDEDTGTFRIYGESADAVKKARGFLEFVEDFIQVPRNLVGKVIGKNGKVIQEIVDKSGVVRVRIEGDNENKLPREDGMVPFVFVGTKESIGNVQVLLEYHIAYLKEVEQLRMERLQIDEQLRQIGSRSYSGRGRGRRGPNYTSGYGTNSELSNPSETESERKDELSDWSLAGEDDRDSRHQRDSRRRPGGRGRSVSGGRGRGGPRGGKSSISSVLKDPDSNPYSLLDNTESDQTADTDASESHHSTNRRRRSRRRRTDEDAVLMDGMTESDTASVNENGLVTVADYISRAESQSRQRNLPRETLAKNKKEMAKDVIEEHGPSEKAINGPTSASGDDISKLQRTPGEEKINTLKEENTQEAAVLNGVS

828 NP_055368.1 Q8NEL9

>NP_055368.1

MLFWHTQPEHYNQHNSGSYLRDVLALPIFKQEEPQLSPENEARLPPLQYVLCAATSPAVKLHEETLTYLNQGQSYEIRLLENRKLGDFQDLNTKYVKSIIRVVFHDRRLQYTEHQQLEGWRWSRPGDRILDIDIPLSVGILDPRASPTQLNAVEFLWDPAKRASAFIQVHCISTEFTPRKHGGEKGVPFRVQIDTFKQNENGEYTEHLHSASCQIKVFKPKGADRKQKTDREKMEKRTAQEKEKYQPSYETTILTECSPWPDVAYQVNSAPSPSYNGSPNSFGLGEGNASPTHPVEALPVGSDHLLPSASIQDAQQWLHRNRFSQFCRLFASFSGADLLKMSRDDLVQICGPADGIRLFNAIKGRNVRPKMTIYVCQELEQNRVPLQQKRDGSGDSNLSVYHAIFLEELTTLELIEKIANLYSISPQHIHRVYRQGPTGIHVVVSNEMVQNFQDESCFVLSTIKAESNDGYHIILKCGL

>Q8NEL9

MNYPGRGSPRSPEHNGRGGGGGAWELGSDARPAFGGGVCCFEHLPGGDPDDGDVPLALLRGEPGLHLAPGTDDHNHHLALDPCLSDENYDFSSAESGSSLRYYSEGESGGGGSSLSLHPPQQPPLVPTNSGGGGATGGSPGERKRTRLGGPAARHRYEVVTELGPEEVRWFYKEDKKTWKPFIGYDSLRIELAFRTLLQTTGARPQGGDRDGDHVCSPTGPASSSGEDDDEDRACGFCQSTTGHEPEMVELVNIEPVCVRGGLYEVDVTQGECYPVYWNQADKIPVMRGQWFIDGTWQPLEEEESNLIEQEHLNCFRGQQMQENFDIEVSKSIDGKDAVHSFKLSRNHVDWHSVDEVYLYSDATTSKIARTVTQKLGFSKASSSGTRLHRGYVEEATLEDKPSQTTHIVFVVHGIGQKMDQGRIIKNTAMMREAARKIEERHFSNHATHVEFLPVEWRSKLTLDGDTVDSITPDKVRGLRDMLNSSAMDIMYYTSPLYRDELVKGLQQELNRLYSLFCSRNPDFEEKGGKVSIVSHSLGCVITYDIMTGWNPVRLYEQLLQKEEELPDERWMSYEERHLLDELYITKRRLKEIEERLHGLKASSMTQTPALKFKVENFFCMGSPLAVFLALRGIRPGNTGSQDHILPREICNRLLNIFHPTDPVAYRLEPLILKHYSNISPVQIHWYNTSNPLPYEHMKPSFLNPAKEPTSVSENEGISTIPSPVTSPVLSRRHYGESITNIGKASILGAASIGKGLGGMLFSRFGRSSTTQSSETSKDSMEDEKKPVASPSATTVGTQTLPHSSSGFLDSAYFRLQESFFNLPQLLFPENVMQNKDNALVELDHRIDFELREGLVESRYWSAVTSHTAYWSSLDVALFLLTFMYKHEHDDDAKPNLDPI

829 NP_001317.1 NP_434700.2

>NP_001317.1

MSGDGATEQAAEYVPEKVKKAEKKLEENPYDLDAWSILIREAQNQPIDKARKTYERLVAQFPSSGRFWKLYIEAEIKAKNYDKVEKLFQRCLMKVLHIDLWKCYLSYVRETKGKLPSYKEKMAQAYDFALDKIGMEIMSYQIWVDYINFLKGVEAVGSYAENQRITAVRRVYQRGCVNPMINIEQLWRDYNKYEEGINIHLAKKMIEDRSRDYMNARRVAKEYETVMKGLDRNAPSVPPQNTPQEAQQVDMWKKYIQWEKSNPLRTEDQTLITKRVMFAYEQCLLVLGHHPDIWYEAAQYLEQSSKLLAEKGDMNNAKLFSDEAANIYERAISTLLKKNMLLYFAYADYEESRMKYEKVHSIYNRLLAIEDIDPTLVYIQYMKFARRAEGIKSGRMIFKKAREDTRTRHHVYVTAALMEYYCSKDKSVAFKIFELGLKKYGDIPEYVLAYIDYLSHLNEDNNTRVLFERVLTSGSLPPEKSGEIWARFLAFESNIGDLASILKVEKRRFTAFKEEYEGKETALLVDRYKFMDLYPCSASELKALGYKDVSRAKLAAIIPDPVVAPSIVPVLKDEVDRKPEYPKPDTQQMIPFQPRHLAPPGLHPVPGGVFPVPPAAVVLMKLLPPPICFQGPFVQVDELMEIFRRCKIPNTVEEAVRIITGGAPELAVEGNGPVESNAVLTKAVKRPNEDSDEDEEKGAVVPPVHDIYRARQQKRIR

>NP_434700.2

MSDYENDDECWSVLEGFRVTLTSVIDPSRITPYLRQCKVLNPDDEEQVLSDPNLVIRKRKVGVLLDILQRTGHKGYVAFLESLELYYPQLYKKVTGKEPARVFSMIIDASGESGLTQLLMTEVMKLQKKVQDLTALLSSKDDFIKELRVKDSLLRKHQERVQRLKEECEAGSRELKRCKEENYDLAMRLAHQSEEKGAALMRNRDLQLEIDQLKHSLMKAEDDCKVERKHTLKLRHAMEQRPSQELLWELQQEKALLQARVQELEASVQEGKLDRSSPYIQVLEEDWRQALRDHQEQANTIFSLRKDLRQGEARRLRCMEEKEMFELQCLALRKDSKMYKDRIEAILLQMEEVAIERDQAIATREELHAQHARGLQEKDALRKQVRELGEKADELQLQVFQCEAQLLAVEGRLRRQQLETLVLSSDLEDGSPRRSQELSLPQDLEDTQLSDKGCLAGGGSPKQPFAALHQEQVLRNPHDAGLSSGEPPEKERRRLKESFENYRRKRALRKMQKGWRQGEEDRENTTGSDNTDTEGS

830 NP_003063.2 O15091

>NP_003063.2

MSTPDPPLGGTPRPGPSPGPGPSPGAMLGPSPGPSPGSAHSMMGPSPGPPSAGHPIPTQGPGGYPQDNMHQMHKPMESMHEKGMSDDPRYNQMKGMGMRSGGHAGMGPPPSPMDQHSQGYPSPLGGSEHASSPVPASGPSSGPQMSSGPGGAPLDGADPQALGQQNRGPTPFNQNQLHQLRAQIMAYKMLARGQPLPDHLQMAVQGKRPMPGMQQQMPTLPPPSVSATGPGPGPGPGPGPGPGPAPPNYSRPHGMGGPNMPPPGPSGVPPGMPGQPPGGPPKPWPEGPMANAAAPTSTPQKLIPPQPTGRPSPAPPAVPPAASPVMPPQTQSPGQPAQPAPMVPLHQKQSRITPIQKPRGLDPVEILQEREYRLQARIAHRIQELENLPGSLAGDLRTKATIELKALRLLNFQRQLRQEVVVCMRRDTALETALNAKAYKRSKRQSLREARITEKLEKQQKIEQERKRRQKHQEYLNSILQHAKDFKEYHRSVTGKIQKLTKAVATYHANTEREQKKENERIEKERMRRLMAEDEEGYRKLIDQKKDKRLAYLLQQTDEYVANLTELVRQHKAAQVAKEKKKKKKKKKAENAEGQTPAIGPDGEPLDETSQMSDLPVKVIHVESGKILTGTDAPKAGQLEAWLEMNPGYEVAPRSDSEESGSEEEEEEEEEEQPQAAQPPTLPVEEKKKIPDPDSDDVSEVDARHIIENAKQDVDDEYGVSQALARGLQSYYAVAHAVTERVDKQSALMVNGVLKQYQIKGLEWLVSLYNNNLNGILADEMGLGKTIQTIALITYLMEHKRINGPFLIIVPLSTLSNWAYEFDKWAPSVVKVSYKGSPAARRAFVPQLRSGKFNVLLTTYEYIIKDKHILAKIRWKYMIVDEGHRMKNHHCKLTQVLNTHYVAPRRLLLTGTPLQNKLPELWALLNFLLPTIFKSCSTFEQWFNAPFAMTGEKVDLNEEETILIIRRLHKVLRPFLLRRLKKEVEAQLPEKVEYVIKCDMSALQRVLYRHMQAKGVLLTDGSEKDKKGKGGTKTLMNTIMQLRKICNHPYMFQHIEESFSEHLGFTGGIVQGLDLYRASGKFELLDRILPKLRATNHKVLLFCQMTSLMTIMEDYFAYRGFKYLRLDGTTKAEDRGMLLKTFNEPGSEYFIFLLSTRAGGLGLNLQSADTVIIFDSDWNPHQDLQAQDRAHRIGQQNEVRVLRLCTVNSVEEKILAAAKYKLNVDQKVIQAGMFDQKSSSHERRAFLQAILEHEEQDESRHCSTGSGSASFAHTAPPPAGVNPDLEEPPLKEEDEVPDDETVNQMIARHEEEFDLFMRMDLDRRREEARNPKRKPRLMEEDELPSWIIKDDAEVERLTCEEEEEKMFGRGSRHRKEVDYSDSLTEKQWLKAIEEGTLEEIEEEVRQKKSSRKRKRDSDAGSSTPTTSTRSRDKDDESKKQKKRGRPPAEKLSPNPPNLTKKMKKIVDAVIKYKDSSSGRQLSEVFIQLPSRKELPEYYELIRKPVDFKKIKERIRNHKYRSLNDLEKDVMLLCQNAQTFNLEGSLIYEDSIVLQSVFTSVRQKIEKEDDSEGEESEEEEEGEEEGSESESRSVKVKIKLGRKEKAQDRLKGGRRRPSRGSRAKPVVSDDDSEEEQEEDRSGSGSEED

>O15091

MTFYLFGIRSFPKLWKSPYLGLGPGHSYVSLFLADRCGIRNQQRLFSLKTMSPQNTKATNLIAKARYLRKDEGSNKQVYSVPHFFLAGAAKERSQMNSQTEDHALAPVRNTIQLPTQPLNSEEWDKLKEDLKENTGKTSFESWIISQMAGCHSSIDVAKSLLAWVAAKNNGIVSYDLLVKYLYLCVFHMQTSEVIDVFEIMKARYKTLEPRGYSLLIRGLIHSDRWREALLLLEDIKKVITPSKKNYNDCIQGALLHQDVNTAWNLYQELLGHDIVPMLETLKAFFDFGKDIKDDNYSNKLLDILSYLRNNQLYPGESFAHSIKTWFESVPGKQWKGQFTTVRKSGQCSGCGKTIESIQLSPEEYECLKGKIMRDVIDGGDQYRKTTPQELKRFENFIKSRPPFDVVIDGLNVAKMFPKVRESQLLLNVVSQLAKRNLRLLVLGRKHMLRRSSQWSRDEMEEVQKQASCFFADDISEDDPFLLYATLHSGNHCRFITRDLMRDHKACLPDAKTQRLFFKWQQGHQLAIVNRFPGSKLTFQRILSYDTVVQTTGDSWHIPYDEDLVERCSCEVPTKWLCLHQKT

831 NP_002681.1 NP_003977.1

>NP_002681.1

MSKRKAPQETLNGGITDMLTELANFEKNVSQAIHKYNAYRKAASVIAKYPHKIKSGAEAKKLPGVGTKIAEKIDEFLATGKLRKLEKIRQDDTSSSINFLTRVSGIGPSAARKFVDEGIKTLEDLRKNEDKLNHHQRIGLKYFGDFEKRIPREEMLQMQDIVLNEVKKVDSEYIATVCGSFRRGAESSGDMDVLLTHPSFTSESTKQPKLLHQVVEQLQKVHFITDTLSKGETKFMGVCQLPSKNDEKEYPHRRIDIRLIPKDQYYCGVLYFTGSDIFNKNMRAHALEKGFTINEYTIRPLGVTGVAGEPLPVDSEKDIFDYIQWKYREPKDRSE

>NP_003977.1

MACTIQKAEALDGAHLMQILWYDEEESLYPAVWLRDNCPCSDCYLDSAKARKLLVEALDVNIGIKGLIFDRKKVYITWPDEHYSEFQADWLKKRCFSKQARAKLQRELFFPECQYWGSELQLPTLDFEDVLRYDEHAYKWLSTLKKVGIVRLTGASDKPGEVSKLGKRMGFLYLTFYGHTWQVQDKIDANNVAYTTGKLSFHTDYPALHHPPGVQLLHCIKQTVTGGDSEIVDGFNVCQKLKKNNPQAFQILSSTFVDFTDIGVDYCDFSVQSKHKIIELDDKGQVVRINFNNATRDTIFDVPVERVQPFYAALKEFVDLMNSKESKFTFKMNPGDVITFDNWRLLHGRRSYEAGTEISRHLEGAYADWDVVMSRLRILRQRVENGN

832 NP_008835.5 P82914

>NP_008835.5

MAGSGAGVRCSLLRLQETLSAADRCGAALAGHQLIRGLGQECVLSSSPAVLALQTSLVFSRDFGLLVFVRKSLNSIEFRECREEILKFLCIFLEKMGQKIAPYSVEIKNTCTSVYTKDRAAKCKIPALDLLIKLLQTFRSSRLMDEFKIGELFSKFYGELALKKKIPDTVLEKVYELLGLLGEVHPSEMINNAENLFRAFLGELKTQMTSAVREPKLPVLAGCLKGLSSLLCNFTKSMEEDPQTSREIFNFVLKAIRPQIDLKRYAVPSAGLRLFALHASQFSTCLLDNYVSLFEVLLKWCAHTNVELKKAALSALESFLKQVSNMVAKNAEMHKNKLQYFMEQFYGIIRNVDSNNKELSIAIRGYGLFAGPCKVINAKDVDFMYVELIQRCKQMFLTQTDTGDDRVYQMPSFLQSVASVLLYLDTVPEVYTPVLEHLVVMQIDSFPQYSPKMQLVCCRAIVKVFLALAAKGPVLRNCISTVVHQGLIRICSKPVVLPKGPESESEDHRASGEVRTGKWKVPTYKDYVDLFRHLLSSDQMMDSILADEAFFSVNSSSESLNHLLYDEFVKSVLKIVEKLDLTLEIQTVGEQENGDEAPGVWMIPTSDPAANLHPAKPKDFSAFINLVEFCREILPEKQAEFFEPWVYSFSYELILQSTRLPLISGFYKLLSITVRNAKKIKYFEGVSPKSLKHSPEDPEKYSCFALFVKFGKEVAVKMKQYKDELLASCLTFLLSLPHNIIELDVRAYVPALQMAFKLGLSYTPLAEVGLNALEEWSIYIDRHVMQPYYKDILPCLDGYLKTSALSDETKNNWEVSALSRAAQKGFNKVVLKHLKKTKNLSSNEAISLEEIRIRVVQMLGSLGGQINKNLLTVTSSDEMMKSYVAWDREKRLSFAVPFREMKPVIFLDVFLPRVTELALTASDRQTKVAACELLHSMVMFMLGKATQMPEGGQGAPPMYQLYKRTFPVLLRLACDVDQVTRQLYEPLVMQLIHWFTNNKKFESQDTVALLEAILDGIVDPVDSTLRDFCGRCIREFLKWSIKQITPQQQEKSPVNTKSLFKRLYSLALHPNAFKRLGASLAFNNIYREFREEESLVEQFVFEALVIYMESLALAHADEKSLGTIQQCCDAIDHLCRIIEKKHVSLNKAKKRRLPRGFPPSASLCLLDLVKWLLAHCGRPQTECRHKSIELFYKFVPLLPGNRSPNLWLKDVLKEEGVSFLINTFEGGGCGQPSGILAQPTLLYLRGPFSLQATLCWLDLLLAALECYNTFIGERTVGALQVLGTEAQSSLLKAVAFFLESIAMHDIIAAEKCFGTGAAGNRTSPQEGERYNYSKCTVVVRIMEFTTTLLNTSPEGWKLLKKDLCNTHLMRVLVQTLCEPASIGFNIGDVQVMAHLPDVCVNLMKALKMSPYKDILETHLREKITAQSIEELCAVNLYGPDAQVDRSRLAAVVSACKQLHRAGLLHNILPSQSTDLHHSVGTELLSLVYKGIAPGDERQCLPSLDLSCKQLASGLLELAFAFGGLCERLVSLLLNPAVLSTASLGSSQGSVIHFSHGEYFYSLFSETINTELLKNLDLAVLELMQSSVDNTKMVSAVLNGMLDQSFRERANQKHQGLKLATTILQHWKKCDSWWAKDSPLETKMAVLALLAKILQIDSSVSFNTSHGSFPEVFTTYISLLADTKLDLHLKGQAVTLLPFFTSLTGGSLEELRRVLEQLIVAHFPMQSREFPPGTPRFNNYVDCMKKFLDALELSQSPMLLELMTEVLCREQQHVMEELFQSSFRRIARRGSCVTQVGLLESVYEMFRKDDPRLSFTRQSFVDRSLLTLLWHCSLDALREFFSTIVVDAIDVLKSRFTKLNESTFDTQITKKMGYYKILDVMYSRLPKDDVHAKESKINQVFHGSCITEGNELTKTLIKLCYDAFTENMAGENQLLERRRLYHCAAYNCAISVICCVFNELKFYQGFLFSEKPEKNLLIFENLIDLKRRYNFPVEVEVPMERKKKYIEIRKEAREAANGDSDGPSYMSSLSYLADSTLSEEMSQFDFSTGVQSYSYSSQDPRPATGRFRRREQRDPTVHDDVLELEMDELNRHECMAPLTALVKHMHRSLGPPQGEEDSVPRDLPSWMKFLHGKLGNPIVPLNIRLFLAKLVINTEEVFRPYAKHWLSPLLQLAASENNGGEGIHYMVVEIVATILSWTGLATPTGVPKDEVLANRLLNFLMKHVFHPKRAVFRHNLEIIKTLVECWKDCLSIPYRLIFEKFSGKDPNSKDNSVGIQLLGIVMANDLPPYDPQCGIQSSEYFQALVNNMSFVRYKEVYAAAAEVLGLILRYVMERKNILEESLCELVAKQLKQHQNTMEDKFIVCLNKVTKSFPPLADRFMNAVFFLLPKFHGVLKTLCLEVVLCRVEGMTELYFQLKSKDFVQVMRHRDDERQKVCLDIIYKMMPKLKPVELRELLNPVVEFVSHPSTTCREQMYNILMWIHDNYRDPESETDNDSQEIFKLAKDVLIQGLIDENPGLQLIIRNFWSHETRLPSNTLDRLLALNSLYSPKIEVHFLSLATNFLLEMTSMSPDYPNPMFEHPLSECEFQEYTIDSDWRFRSTVLTPMFVETQASQGTLQTRTQEGSLSARWPVAGQIRATQQQHDFTLTQTADGRSSFDWLTGSSTDPLVDHTSPSSDSLLFAHKRSERLQRAPLKSVGPDFGKKRLGLPGDEVDNKVKGAAGRTDLLRLRRRFMRDQEKLSLMYARKGVAEQKREKEIKSELKMKQDAQVVLYRSYRHGDLPDIQIKHSSLITPLQAVAQRDPIIAKQLFSSLFSGILKEMDKFKTLSEKNNITQKLLQDFNRFLNTTFSFFPPFVSCIQDISCQHAALLSLDPAAVSAGCLASLQQPVGIRLLEEALLRLLPAELPAKRVRGKARLPPDVLRWVELAKLYRSIGEYDVLRGIFTSEIGTKQITQSALLAEARSDYSEAAKQYDEALNKQDWVDGEPTEAEKDFWELASLDCYNHLAEWKSLEYCSTASIDSENPPDLNKIWSEPFYQETYLPYMIRSKLKLLLQGEADQSLLTFIDKAMHGELQKAILELHYSQELSLLYLLQDDVDRAKYYIQNGIQSFMQNYSSIDVLLHQSRLTKLQSVQALTEIQEFISFISKQGNLSSQVPLKRLLNTWTNRYPDAKMDPMNIWDDIITNRCFFLSKIEEKLTPLPEDNSMNVDQDGDPSDRMEVQEQEEDISSLIRSCKFSMKMKMIDSARKQNNFSLAMKLLKELHKESKTRDDWLVSWVQSYCRLSHCRSRSQGCSEQVLTVLKTVSLLDENNVSSYLSKNILAFRDQNILLGTTYRIIANALSSEPACLAEIEEDKARRILELSGSSSEDSEKVIAGLYQRAFQHLSEAVQAAEEEAQPPSWSCGPAAGVIDAYMTLADFCDQQLRKEEENASVIDSAELQAYPALVVEKMLKALKLNSNEARLKFPRLLQIIERYPEETLSLMTKEISSVPCWQFISWISHMVALLDKDQAVAVQHSVEEITDNYPQAIVYPFIISSESYSFKDTSTGHKNKEFVARIKSKLDQGGVIQDFINALDQLSNPELLFKDWSNDVRAELAKTPVNKKNIEKMYERMYAALGDPKAPGLGAFRRKFIQTFGKEFDKHFGKGGSKLLRMKLSDFNDITNMLLLKMNKDSKPPGNLKECSPWMSDFKVEFLRNELEIPGQYDGRGKPLPEYHVRIAGFDERVTVMASLRRPKRIIIRGHDEREHPFLVKGGEDLRQDQRVEQLFQVMNGILAQDSACSQRALQLRTYSVVPMTSRLGLIEWLENTVTLKDLLLNTMSQEEKAAYLSDPRAPPCEYKDWLTKMSGKHDVGAYMLMYKGANRTETVTSFRKRESKVPADLLKRAFVRMSTSPEAFLALRSHFASSHALICISHWILGIGDRHLNNFMVAMETGGVIGIDFGHAFGSATQFLPVPELMPFRLTRQFINLMLPMKETGLMYSIMVHALRAFRSDPGLLTNTMDVFVKEPSFDWKNFEQKMLKKGGSWIQEINVAEKNWYPRQKICYAKRKLAGANPAVITCDELLLGHEKAPAFRDYVAVARGSKDHNIRAQEPESGLSEETQVKCLMDQATDPNILGRTWEGWEPWM

>P82914

MLRVAWRTLSLIRTRAVTQVLVPGLPGGGSAKFPFNQWGLQPRSLLLQAARGYVVRKPAQSRLDDDPPPSTLLKDYQNVPGIEKVDDVVKRLLSLEMANKKEMLKIKQEQFMKKIVANPEDTRSLEARIIALSVKIRSYEEHLEKHRKDKAHKRYLLMSIDQRKKMLKNLRNTNYDVFEKICWGLGIEYTFPPLYYRRAHRRFVTKKALCIRVFQETQKLKKRRRALKAAAAAQKQAKRRNPDSPAKAIPKTLKDSQ

833 Q15562 NP_055525.3

>Q15562

MGEPRAGAALDDGSGWTGSEEGSEEGTGGSEGAGGDGGPDAEGVWSPDIEQSFQEALAIYPPCGRRKIILSDEGKMYGRNELIARYIKLRTGKTRTRKQVSSHIQVLARRKSREIQSKLKDQVSKDKAFQTMATMSSAQLISAPSLQAKLGPTGPQASELFQFWSGGSGPPWNVPDVKPFSQTPFTLSLTPPSTDLPGYEPPQALSPLPPPTPSPPAWQARGLGTARLQLVEFSAFVEPPDAVDSYQRHLFVHISQHCPSPGAPPLESVDVRQIYDKFPEKKGGLRELYDRGPPHAFFLVKFWADLNWGPSGEEAGAGGSISSGGFYGVSSQYESLEHMTLTCSSKVCSFGKQVVEKVETERAQLEDGRFVYRLLRSPMCEYLVNFLHKLRQLPERYMMNSVLENFTILQVVTNRDTQELLLCTAYVFEVSTSERGAQHHIYRLVRD

>NP_055525.3

MTGAEIESGAQVKPEKKPGEEVVGGAEIENDVPLVVRPKVRTQAQIMPGARPKNKSKVMPGASTKVETSAVGGARPKSKAKAIPVSRFKEEAQMWAQPRFGAERLSKTERNSQTNIIASPLVSTDSVLVAKTKYLSEDRELVNTDTESFPRRKAHYQAGFQPSFRSKEETNMGSWCCPRPTSKQEASPNSDFKWVDKSVSSLFWSGDEVTAKFHPGNRVKDSNRSMHMANQEANTMSRSQTNQELYIASSSGSEDESVKTPWFWARDKTNTWSGPREDPNSRSRFRSKKEVYVESSSGSEHEDHLESWFGAGKEAKFRSKMRAGKEANNRARHRAKREACIDFMPGSIDVIKKESCFWPEENANTFSRPMIKKEARARAMTKEEAKTKARARAKQEARSEEEALIGTWFWATDESSMADEASIESSLQVEDESIIGSWFWTEEEASMGTGASSKSRPRTDGERIGDSLFGAREKTSMKTGAEATSESILAADDEQVIIGSWFWAGEEVNQEAEEETIFGSWFWVIDAASVESGVGVSCESRTRSEEEEVIGPWFWSGEQVDIEAGIGEEARPGAEEETIFGSWFWAENQTYMDCRAETSCDTMQGAEEEEPIIGSWFWTRVEACVEGDVNSKSSLEDKEEAMIPCFGAKEEVSMKHGTGVRCRFMAGAEETNNKSCFWAEKEPCMYPAGGGSWKSRPEEEEDIVNSWFWSRKYTKPEAIIGSWLWATEESNIDGTGEKAKLLTEEETIINSWFWKEDEAISEATDREESRPEAEEGDIIGSWFWAGEEDRLEPAAETREEDRLAAEKEGIVGSWFGAREETIRREAGSCSKSSPKAEEEEVIIGSWFWEEEASPEAVAGVGFESKPGTEEEEITVGSWFWPEEEASIQAGSQAVEEMESETEEETIFGSWFWDGKEVSEEAGPCCVSKPEDDEEMIVESWFWSRDKAIKETGTVATCESKPENEEGAIVGSWFEAEDEVDNRTDNGSNCGSRTLADEDEAIVGSWFWAGDEAHFESNPSPVFRAICRSTCSVEQEPDPSRRPQSWEEVTVQFKPGPWGRVGFPSISPFRFPKEAASLFCEMFGGKPRNMVLSPEGEDQESLLQPDQPSPEFPFQYDPSYRSVQEIREHLRAKESTEPESSSCNCIQCELKIGSEEFEELLLLMEKIRDPFIHEISKIAMGMRSASQFTRDFIRDSGVVSLIETLLNYPSSRVRTSFLENMIRMAPPYPNLNIIQTYICKVCEETLAYSVDSPEQLSGIRMIRHLTTTTDYHTLVANYMSGFLSLLATGNAKTRFHVLKMLLNLSENLFMTKELLSAEAVSEFIGLFNREETNDNIQIVLAIFENIGNNIKKETVFSDDDFNIEPLISAFHKVEKFAKELQGKTDNQNDPEGDQEN

834 NP_849194.1 NP_002291.1

>NP_849194.1

MSAEASGPAAAAAPSLEAPKPSGLEPGPAAYGLKPLTPNSKYVKLNVGGSLHYTTLRTLTGQDTMLKAMFSGRVEVLTDAGGWVLIDRSGRHFGTILNYLRDGSVPLPESTRELGELLGEARYYLVQGLIEDCQLALQQKRETLSPLCLIPMVTSPREEQQLLASTSKPVVKLLHNRSNNKYSYTSTSDDNLLKNIELFDKLALRFHGRLLFLKDVLGDEICCWSFYGQGRKIAEVCCTSIVYATEKKQTKVEFPEARIFEETLNILIYETPRGPDPALLEATGGAAGAGGAGRGEDEENREHRVRRIHVRRHITHDERPHGQQIVFKD

>NP_002291.1

MATLKEKLIAPVAEEEATVPNNKITVVGVGQVGMACAISILGKSLADELALVDVLEDKLKGEMMDLQHGSLFLQTPKIVADKDYSVTANSKIVVVTAGVRQQEGESRLNLVQRNVNVFKFIIPQIVKYSPDCIIIVVSNPVDILTYVTWKLSGLPKHRVIGSGCNLDSARFRYLMAEKLGIHPSSCHGWILGEHGDSSVAVWSGVNVAGVSLQELNPEMGTDNDSENWKEVHKMVVESAYEVIKLKGYTNWAIGLSVADLIESMLKNLSRIHPVSTMVKGMYGIENEVFLSLPCILNARGLTSVINQKLKDDEVAQLKKSADTLWDIQKDLKDL

835 NP_003575.1 O00764

>NP_003575.1

MSQWHHPRSGWGRRRDFSGRSSAKKKGGNHIPERWKDYLPVGQRMPGTRFIAFKVPLQKSFEKKLAPEECFSPLDLFNKIREQNEELGLIIDLTYTQRYYKPEDLPETVPYLKIFTVGHQVPDDETIFKFKHAVNGFLKENKDNDKLIGVHCTHGLNRTGYLICRYLIDVEGVRPDDAIELFNRCRGHCLERQNYIEDLQNGPIRKNWNSSVPRSSDFEDSAHLMQPVHNKPVKQGPRYNLHQIQGHSAPRHFHTQTQSLQQSVRKFSENPHVYQRHHLPPPGPPGEDYSHRRYSWNVKPNASRAAQDRRRWYPYNYSRLSYPACWEWTQ

>O00764

MEEECRVLSIQSHVIRGYVGNRAATFPLQVLGFEIDAVNSVQFSNHTGYAHWKGQVLNSDELQELYEGLRLNNMNKYDYVLTGYTRDKSFLAMVVDIVQELKQQNPRLVYVCDPVLGDKWDGEGSMYVPEDLLPVYKEKVVPLADIITPNQFEAELLSGRKIHSQEEALRVMDMLHSMGPDTVVITSSDLPSPQGSNYLIVLGSQRRRNPAGSVVMERIRMDIRKVDAVFVGTGDLFAAMLLAWTHKHPNNLKVACEKTVSTLHHVLQRTIQCAKAQAGEGVRPSPMQLELRMVQSKRDIEDPEIVVQATVL

836 NP_001230.1 NP_056520.2

>NP_001230.1

MYHNSSQKRHWTFSSEEQLARLRADANRKFRCKAVANGKVLPNDPVFLEPHEEMTLCKYYEKRLLEFCSVFKPAMPRSVVGTACMYFKRFYLNNSVMEYHPRIIMLTCAFLACKVDEFNVSSPQFVGNLRESPLGQEKALEQILEYELLLIQQLNFHLIVHNPYRPFEGFLIDLKTRYPILENPEILRKTADDFLNRIALTDAYLLYTPSQIALTAILSSASRAGITMESYLSESLMLKENRTCLSQLLDIMKSMRNLVKKYEPPRSEEVAVLKQKLERCHSAELALNVITKKRKGYEDDDYVSKKSKHEEEEWTDDDLVESL

>NP_056520.2

MSGSHTPACGPFSALTPSIWPQEILAKYTQKEESAEQPEFYYDEFGFRVYKEEGDEPGSSLLANSPLMEDAPQRLRWQAHLEFTHNHDVGDLTWDKIAVSLPRSEKLRSLVLAGIPHGMRPQLWMRLSGALQKKRNSELSYREIVKNSSNDETIAAKQIEKDLLRTMPSNACFASMGSIGVPRLRRVLRALAWLYPEIGYCQGTGMVAACLLLFLEEEDAFWMMSAIIEDLLPASYFSTTLLGVQTDQRVLRHLIVQYLPRLDKLLQEHDIELSLITLHWFLTAFASVVDIKLLLRIWDLFFYEGSRVLFQLTLGMLHLKEEELIQSENSASIFNTLSDIPSQMEDAELLLGVAMRLAGSLTDVAVETQRRKHLAYLIADQGQLLGAGTLTNLSQVVRRRTQRRKSTITALLFGEDDLEALKAKNIKQTELVADLREAILRVARHFQCTDPKNCSVELTPDYSMESHQRDHENYVACSRSHRRRAKALLDFERHDDDELGFRKNDIITIVSQKDEHCWVGELNGLRGWFPAKFVEVLDERSKEYSIAGDDSVTEGVTDLVRGTLCPALKALFEHGLKKPSLLGGACHPWLFIEEAAGREVERDFASVYSRLVLCKTFRLDEDGKVLTPEELLYRAVQSVNVTHDAVHAQMDVKLRSLICVGLNEQVLHLWLEVLCSSLPTVEKWYQPWSFLRSPGWVQIKCELRVLCCFAFSLSQDWELPAKREAQQPLKEGVRDMLVKHHLFSWDVDG

837 Q9NQ92 NP_003121.1

>Q9NQ92

MDLQAAGAQAQGAAEPSRGPPLPSARGAPPSPEAGFATADHSSQERETEKAMDRLARGTQSIPNDSPARGEGTHSEEEGFAMDEEDSDGELNTWELSEGTNCPPKEQPGDLFNEDWDSELKADQGNPYDADDIQESISQELKPWVCCAPQGDMIYDPSWHHPPPLIPYYSKMVFETGQFDDAED

>NP_003121.1

MAYPGHPGAGGGYYPGGYGGAPGGPAFPGQTQDPLYGYFAAVAGQDGQIDADELQRCLTQSGIAGGYKPFNLETCRLMVSMLDRDMSGTMGFNEFKELWAVLNGWRQHFISFDTDRSGTVDPQELQKALTTMGFRLSPQAVNSIAKRYSTNGKITFDDYIACCVKLRALTDSFRRRDTAQQGVVNFPYDDFIQCVMSV

838 NP_878314.1 NP_006101.1

>NP_878314.1

MTGKAGEALSKPKSETVAKSTSGGAPARCTGFGIQEILGLNKEPPSSHPRAALDGLAPGHLLAARSVLSPAGVGGMGLLGPGGLPGFYTQPTFLEVLSDPQSVHLQPLGRASGPLDTSQTASSDSEDVSSSDRKMSKSALNQTKKRKKRRHRTIFTSYQLEELEKAFNEAHYPDVYAREMLAMKTELPEDRIQVWFQNRRAKWRKREKCWGRSSVMAEYGLYGAMVRHSIPLPESILKSAKDGIMDSCAPWLLGMHKKSLEAAAESGRKPEGERQALPKLDKMEQDERGPDAQAAISQEELRENSIAVLRAKAQEHSTKVLGTVSGPDSLARSTEKPEEEEAMDEDRPAERLSPPQLEDMA

>NP_006101.1

MPKRKVTFQGVGDEEDEDEIIVPKKKLVDPVAGSGGPGSRFKGKHSLDSDEEEDDDDGGSSKYDILASEDVEGQEAATLPSEGGVRITPFNLQEEMEEGHFDADGNYFLNRDAQIRDSWLDNIDWVKIRERPPGQRQASDSEEEDSLGQTSMSAQALLEGLLELLLPRETVAGALRRLGARGGGKGRKGPGQPSSPQRLDRLSGLADQMVARGNLGVYQETRERLAMRLKGLGCQTLGPHNPTPPPSLDMFAEELAEEELETPTPTQRGEAESRGDGLVDVMWEYKWENTGDAELYGPFTSAQMQTWVSEGYFPDGVYCRKLDPPGGQFYNSKRIDFDLYT

839 Q96DT0 NP_001340.2

>Q96DT0

MSQPSGGRAPGTRIYSWSCPTVMSPGEKLDPIPDSFILQPPVFHPVVPYVTTIFGGLHAGKMVMLQGVVPLDAHRFQVDFQCGCSLCPRPDIAFHFNPRFHTTKPHVICNTLHGGRWQREARWPHLALRRGSSFLILFLFGNEEVKVSVNGQHFLHFRYRLPLSHVDTLGIFGDILVEAVGFLNINPFVEGSREYPAGHPFLLMSPRLEVPCSHALPQGLSPGQVIIVRGLVLQEPKHFTVSLRDQAAHAPVTLRASFADRTLAWISRWGQKKLISAPFLFYPQRFFEVLLLFQEGGLKLALNGQGLGATSMNQQALEQLRELRISGSVQLYCVHS

>NP_001340.2

MPSASASRKSQEKPREIMDAAEDYAKERYGISSMIQSQEKPDRVLVRVRDLTIQKADEVVWVRARVHTSRAKGKQCFLVLRQQQFNVQALVAVGDHASKQMVKFAANINKESIVDVEGVVRKVNQKIGSCTQQDVELHVQKIYVISLAEPRLPLQLDDAVRPEAEGEEEGRATVNQDTRLDNRVIDLRTSTSQAVFRLQSGICHLFRETLINKGFVEIQTPKIISAASEGGANVFTVSYFKNNAYLAQSPQLYKQMCICADFEKVFSIGPVFRAEDSNTHRHLTEFVGLDIEMAFNYHYHEVMEEIADTMVQIFKGLQERFQTEIQTVNKQFPCEPFKFLEPTLRLEYCEALAMLREAGVEMGDEDDLSTPNEKLLGHLVKEKYDTDFYILDKYPLAVRPFYTMPDPRNPKQSNSYDMFMRGEEILSGAQRIHDPQLLTERALHHGIDLEKIKAYIDSFRFGAPPHAGGGIGLERVTMLFLGLHNVRQTSMFPRDPKRLTP

840 NP_006521.1 NP_055963.2

>NP_006521.1

MFKRMAEFGPDSGGRVKGVTIVKPIVYGNVARYFGKKREEDGHTHQWTVYVKPYRNEDMSAYVKKIQFKLHESYGNPLRVVTKPPYEITETGWGEFEIIIKIFFIDPNERPVTLYHLLKLFQSDTNAMLGKKTVVSEFYDEMIFQDPTAMMQQLLTTSRQLTLGAYKHETEFAELEVKTREKLEAAKKKTSFEIAELKERLKASRETINCLKNEIRKLEEDDQAKDI

>NP_055963.2

MEDGGLTAFEEDQRCLSQSLPLPVSAEGPAAQTTAEPSRSFSSAHRHLSRRNGLSRLCQSRTALSEDRWSSYCLSSLAAQNICTSKLHCPAAPEHTDPSEPRGSVSCCSLLRGLSSGWSSPLLPAPVCNPNKAIFTVDAKTTEILVANDKACGLLGYSSQDLIGQKLTQFFLRSDSDVVEALSEEHMEADGHAAVVFGTVVDIISRSGEKIPVSVWMKRMRQERRLCCVVVLEPVERVSTWVAFQSDGTVTSCDSLFAHLHGYVSGEDVAGQHITDLIPSVQLPPSGQHIPKNLKIQRSVGRARDGTTFPLSLKLKSQPSSEEATTGEAAPVSGYRASVWVFCTISGLITLLPDGTIHGINHSFALTLFGYGKTELLGKNITFLIPGFYSYMDLAYNSSLQLPDLASCLDVGNESGCGERTLDPWQGQDPAEGGQDPRINVVLAGGHVVPRDEIRKLMESQDIFTGTQTELIAGGQLLSCLSPQPAPGVDNVPEGSLPVHGEQALPKDQQITALGREEPVAIESPGQDLLGESRSEPVDVKPFASCEDSEAPVPAEDGGSDAGMCGLCQKAQLERMGVSGPSGSDLWAGAAVAKPQAKGQLAGGSLLMHCPCYGSEWGLWWRSQDLAPSPSGMAGLSFGTPTLDEPWLGVENDREELQTCLIKEQLSQLSLAGALDVPHAELVPTECQAVTAPVSSCDLGGRDLCGGCTGSSSACYALATDLPGGLEAVEAQEVDVNSFSWNLKELFFSDQTDQTSSNCSCATSELRETPSSLAVGSDPDVGSLQEQGSCVLDDRELLLLTGTCVDLGQGRRFRESCVGHDPTEPLEVCLVSSEHYAASDRESPGHVPSTLDAGPEDTCPSAEEPRLNVQVTSTPVIVMRGAAGLQREIQEGAYSGSCYHRDGLRLSIQFEVRRVELQGPTPLFCCWLVKDLLHSQRDSAARTRLFLASLPGSTHSTAAELTGPSLVEVLRARPWFEEPPKAVELEGLAACEGEYSQKYSTMSPLGSGAFGFVWTAVDKEKNKEVVVKFIKKEKVLEDCWIEDPKLGKVTLEIAILSRVEHANIIKVLDIFENQGFFQLVMEKHGSGLDLFAFIDRHPRLDEPLASYIFRQLVSAVGYLRLKDIIHRDIKDENIVIAEDFTIKLIDFGSAAYLERGKLFYTFCGTIEYCAPEVLMGNPYRGPELEMWSLGVTLYTLVFEENPFCELEETVEAAIHPPYLVSKELMSLVSGLLQPVPERRTTLEKLVTDPWVTQPVNLADYTWEEVFRVNKPESGVLSAASLEMGNRSLSDVAQAQELCGGPVPGEAPNGQGCLHPGDPRLLTS

841 Q9NZI7 NP_000792.1

>Q9NZI7

MAWVLKMDEVIESGLVHDFDASLSGIGQELGAGAYSMSDVLALPIFKQEDSSLPLDGETEHPPFQYVMCAATSPAVKLHDETLTYLNQGQSYEIRMLDNRKMGDMPEINGKLVKSIIRVVFHDRRLQYTEHQQLEGWKWNRPGDRLLDLDIPMSVGIIDTRTNPSQLNAVEFLWDPAKRTSAFIQVHCISTEFTPRKHGGEKGVPFRIQVDTFKQNENGEYTDHLHSASCQIKVFKPKGADRKQKTDREKMEKRTAHEKEKYQPSYDTTILTEMRLEPIIEDAVEHEQKKSSKRTLPADYGDSLAKRGSCSPWPDAPTAYVNNSPSPAPTFTSPQQSTCSVPDSNSSSPNHQGDGASQTSGEQIQPSATIQETQQWLLKNRFSSYTRLFSNFSGADLLKLTKEDLVQICGAADGIRLYNSLKSRSVRPRLTIYVCREQPSSTVLQGQQQAASSASENGSGAPYVYHAIYLEEMIASEVARKLALVFNIPLHQINQVYRQGPTGIHILVSDQMVQNFQDESCFLFSTVKAESSDGIHIILK

>NP_000792.1

MGVQVETISPGDGRTFPKRGQTCVVHYTGMLEDGKKFDSSRDRNKPFKFMLGKQEVIRGWEEGVAQMSVGQRAKLTISPDYAYGATGHPGIIPPHATLVFDVELLKLE

842 Q15910 Q9P2W6

>Q15910

MGQTGKKSEKGPVCWRKRVKSEYMRLRQLKRFRRADEVKSMFSSNRQKILERTEILNQEWKQRRIQPVHILTSVSSLRGTRECSVTSDLDFPTQVIPLKTLNAVASVPIMYSWSPLQQNFMVEDETVLHNIPYMGDEVLDQDGTFIEELIKNYDGKVHGDRECGFINDEIFVELVNALGQYNDDDDDDDGDDPEEREEKQKDLEDHRDDKESRPPRKFPSDKIFEAISSMFPDKGTAEELKEKYKELTEQQLPGALPPECTPNIDGPNAKSVQREQSLHSFHTLFCRRCFKYDCFLHPFHATPNTYKRKNTETALDNKPCGPQCYQHLEGAKEFAAALTAERIKTPPKRPGGRRRGRLPNNSSRPSTPTINVLESKDTDSDREAGTETGGENNDKEEEEKKDETSSSSEANSRCQTPIKMKPNIEPPENVEWSGAEASMFRVLIGTYYDNFCAIARLIGTKTCRQVYEFRVKESSIIAPAPAEDVDTPPRKKKRKHRLWAAHCRKIQLKKDGSSNHVYNYQPCDHPRQPCDSSCPCVIAQNFCEKFCQCSSECQNRFPGCRCKAQCNTKQCPCYLAVRECDPDLCLTCGAADHWDSKNVSCKNCSIQRGSKKHLLLAPSDVAGWGIFIKDPVQKNEFISEYCGEIISQDEADRRGKVYDKYMCSFLFNLNNDFVVDATRKGNKIRFANHSVNPNCYAKVMMVNGDHRIGIFAKRAIQTGEELFFDYRYSQADALKYVGIEREMEIP

>Q9P2W6

MGRTWCGMWRRRRPGRRSAVPRWPHLSSQSGVEPPDRWTGTPGWPSRDQEAPGSMMPPAAAQPSAHGALVPPATAHEPVDHPALHWLACCCCLSLPGQLPLAIRLGWDLDLEAGPSSGKLCPRARRWQPLPS

843 NP_001512.1 NP_004799.1

>NP_001512.1

MDTCGVGYVALGEAGPVGNMTVVDSPGQEVLNQLDVKTSSEMTSAEASVEMSLPTPLPGFEDSPDQRRLPPEQESLSRLEQPDLSSEMSKVSKPRASKPGRKRGGRTRKGPKRPQQPNPPSAPLVPGLLDQSNPLSTPMPKKRGRKSKAELLLLKLSKDLDRPESQSPKRPPEDFETPSGERPRRRAAQVALLYLQELAEELSTALPAPVSCPEGPKVSSPTKPKKIRQPAACPGGEEVDGAPRDEDFFLQVEAEDVEESEGPSESSSEPEPVVPRSTPRGSTSGKQKPHCRGMAPNGLPNHIMAPVWKCLHLTKDFREQKHSYWEFAEWIPLAWKWHLLSELEAAPYLPQEEKSPLFSVQREGLPEDGTLYRINRFSSITAHPERWDVSFFTGGPLWALDWCPVPEGAGASQYVALFSSPDMNETHPLSQLHSGPGLLQLWGLGTLQQESCPGNRAHFVYGIACDNGCIWDLKFCPSGAWELPGTPRKAPLLPRLGLLALACSDGKVLLFSLPHPEALLAQQPPDAVKPAIYKVQCVATLQVGSMQATDPSECGQCLSLAWMPTRPHQHLAAGYYNGMVVFWNLPTNSPLQRIRLSDGSLKLYPFQCFLAHDQAVRTLQWCKANSHFLVSAGSDRKIKFWDLRRPYEPINSIKRFLSTELAWLLPYNGVTVAQDNCYASYGLCGIHYIDAGYLGFKAYFTAPRKGTVWSLSGSDWLGTIAAGDISGELIAAILPDMALNPINVKRPVERRFPIYKADLIPYQDSPEGPDHSSASSGVPNPPKARTYTETVNHHYLLFQDTDLGSFHDLLRREPMLRMQEGEGHSQLCLDRLQLEAIHKVRFSPNLDSYGWLVSGGQSGLVRIHFVRGLASPLGHRMQLESRAHFNAMFQPSSPTRRPGFSPTSHRLLPTP

>NP_004799.1

MAEDSESAASQQSLELDDQDTCGIDGDNEEETEHAKGSPGGYLGAKKKKKKQKRKKEKPNSGGTKSDSASDSQEIKIQQPSKNPSVPMQKLQDIQRAMELLSACQGPARNIDEAAKHRYQFWDTQPVPKLDEVITSHGAIEPDKDNVRQEPYSLPQGFMWDTLDLSDAEVLKELYTLLNENYVEDDDNMFRFDYSPEFLLWALRPPGWLLQWHCGVRVSSNKKLVGFISAIPANIRIYDSVKKMVEINFLCVHKKLRSKRVAPVLIREITRRVNLEGIFQAVYTAGVVLPKPIATCRYWHRSLNPRKLVEVKFSHLSRNMTLQRTMKLYRLPDVTKTSGLRPMEPKDIKSVRELINTYLKQFHLAPVMDEEEVAHWFLPREHIIDTFVVESPNGKLTDFLSFYTLPSTVMHHPAHKSLKAAYSFYNIHTETPLLDLMSDALILAKSKGFDVFNALDLMENKTFLEKLKFGIGDGNLQYYLYNWRCPGTDSEKVGLVLQ

844 Q86UE8 NP_006180.1

>Q86UE8

MMEELHSLDPRRQELLEARFTGVGVSKGPLNSESSNQSLCSVGSLSDKEVETPEKKQNDQRNRKRKAEPYETSQGKGTPRGHKISDYFEFAGGSAPGTSPGRSVPPVARSSPQHSLSNPLPRRVEQPLYGLDGSAAKEATEEQSALPTLMSVMLAKPRLDTEQLAQRGAGLCFTFVSAQQNSPSSTGSGNTEHSCSSQKQISIQHRQTQSDLTIEKISALENSKNSDLEKKEGRIDDLLRANCDLRRQIDEQQKMLEKYKERLNRCVTMSKKLLIEKSKQEKMACRDKSMQDRLRLGHFTTVRHGASFTEQWTDGYAFQNLIKQQERINSQREEIERQRKMLAKRKPPAMGQAPPATNEQKQRKSKTNGAENETPSSGNTELKDTAPALGAHSLLRLTLAEYHEQEEIFKLRLGHLKKEEAEIQAELERLERVRNLHIRELKRIHNEDNSQFKDHPTLNDRYLLLHLLGRGGFSEVYKAFDLTEQRYVAVKIHQLNKNWRDEKKENYHKHACREYRIHKELDHPRIVKLYDYFSLDTDSFCTVLEYCEGNDLDFYLKQHKLMSEKEARSIIMQIVNALKYLNEIKPPIIHYDLKPGNILLVNGTACGEIKITDFGLSKIMDDDSYNSVDGMELTSQGAGTYWYLPPECFVVGKEPPKISNKVDVWSVGVIFYQCLYGRKPFGHNQSQQDILQENTILKATEVQFPPKPVVTPEAKAFIRRCLAYRKEDRIDVQQLACDPYLLPHIRKSVSTSSPAGAAIASTSGASNNSSSN

>NP_006180.1

MAEDRPQQPQLDMPLVLDQGLTRQMRLRVESLKQRGEKRQDGEKLLQPAESVYRLNFTQQQRLQFERWNVVLDKPGKVTITGTSQNWTPDLTNLMTRQLLDPTAIFWRKEDSDAIDWNEADALEFGERLSDLAKIRKVMYFLVTFGEGVEPANLKASVVFNQL

845 NP_002139.2 NP_006635.2

>NP_002139.2

MSFPNSSPAANTFLVDSLISACRSDSFYSSSASMYMPPPSADMGTYGMQTCGLLPSLAKREVNHQNMGMNVHPYIPQVDSWTDPNRSCRIEQPVTQQVPTCSFTTNIKEESNCCMYSDKRNKLISAEVPSYQRLVPESCPVENPEVPVPGYFRLSQTYATGKTQEYNNSPEGSSTVMLQLNPRGAAKPQLSAAQLQMEKKMNEPVSGQEPTKVSQVESPEAKGGLPEERSCLAEVSVSSPEVQEKESKEEIKSDTPTSNWLTAKSGRKKRCPYTKHQTLELEKEFLFNMYLTRERRLEISKSVNLTDRQVKIWFQNRRMKLKKMSRENRIRELTANLTFS

>NP_006635.2

MSVVGLDVGSQSCYIAVARAGGIETIANEFSDRCTPSVISFGSKNRTIGVAAKNQQITHANNTVSNFKRFHGRAFNDPFIQKEKENLSYDLVPLKNGGVGIKVMYMGEEHLFSVEQITAMLLTKLKETAENSLKKPVTDCVISVPSFFTDAERRSVLDAAQIVGLNCLRLMNDMTAVALNYGIYKQDLPSLDEKPRIVVFVDMGHSAFQVSACAFNKGKLKVLGTAFDPFLGGKNFDEKLVEHFCAEFKTKYKLDAKSKIRALLRLYQECEKLKKLMSSNSTDLPLNIECFMNDKDVSGKMNRSQFEELCAELLQKIEVPLYSLLEQTHLKVEDVSAVEIVGGATRIPAVKERIAKFFGKDISTTLNADEAVARGCALQCAILSPAFKVREFSVTDAVPFPISLIWNHDSEDTEGVHEVFSRNHAAPFSKVLTFLRRGPFELEAFYSDPQGVPYPEAKIGRFVVQNVSAQKDGEKSRVKVKVRVNTHGIFTISTASMVEKVPTEENEMSSEADMECLNQRPPENPDTDKNVQQDNSEAGTQPQVQTDAQQTSQSPPSPELTSEENKIPDADKANEKKVDQPPEAKKPKIKVVNVELPIEANLVWQLGKDLLNMYIETEGKMIMQDKLEKERNDAKNAVEEYVYEFRDKLCGPYEKFICEQDHQNFLRLLTETEDWLYEEGEDQAKQAYVDKLEELMKIGTPVKVRFQEAEERPKMFEELGQRLQHYAKIAADFRNKDEKYNHIDESEMKKVEKSVNEVMEWMNNVMNAQAKKSLDQDPVVRAQEIKTKIKELNNTCEPVVTQPKPKIESPKLERTPNGPNIDKKEEDLEDKNNFGAEPPHQNGECYPNEKNSVNMDLD

846 P09668 NP_002564.1

>P09668

MWATLPLLCAGAWLLGVPVCGAAELSVNSLEKFHFKSWMSKHRKTYSTEEYHHRLQTFASNWRKINAHNNGNHTFKMALNQFSDMSFAEIKHKYLWSEPQNCSATKSNYLRGTGPYPPSVDWRKKGNFVSPVKNQGACGSCWTFSTTGALESAIAIATGKMLSLAEQQLVDCAQDFNNHGCQGGLPSQAFEYILYNKGIMGEDTYPYQGKDGYCKFQPGKAIGFVKDVANITIYDEEAMVEAVALYNPVSFAFEVTQDFMMYRTGIYSSTSCHKTPDKVNHAVLAVGYGEKNGIPYWIVKNSWGPQWGMNGYFLIERGKNMCGLAACASYPIPLV

>NP_002564.1

MSGEENPASKPTPVQDVQGDGRWMSLHHRFVADSKDKEPEVVFIGDSLVQLMHQCEIWRELFSPLHALNFGIGGDGTQHVLWRLENGELEHIRPKIVVVWVGTNNHGHTAEQVTGGIKAIVQLVNERQPQARVVVLGLLPRGQHPNPLREKNRQVNELVRAALAGHPRAHFLDADPGFVHSDGTISHHDMYDYLHLSRLGYTPVCRALHSLLLRLLAQDQGQGAPLLEPAP

847 Q5TB30 NP_620448.1

>Q5TB30

MESQGVPPGPYRATKLWNEVTTSFRAGMPLRKHRQHFKKYGNCFTAGEAVDWLYDLLRNNSNFGPEVTRQQTIQLLRKFLKNHVIEDIKGRWGSENVDDNNQLFRFPATSPLKTLPRRYPELRKNNIENFSKDKDSIFKLRNLSRRTPKRHGLHLSQENGEKIKHEIINEDQENAIDNRELSQEDVEEVWRYVILIYLQTILGVPSLEEVINPKQVIPQYIMYNMANTSKRGVVILQNKSDDLPHWVLSAMKCLANWPRSNDMNNPTYVGFERDVFRTIADYFLDLPEPLLTFEYYELFVNILVVCGYITVSDRSSGIHKIQDDPQSSKFLHLNNLNSFKSTECLLLSLLHREKNKEESDSTERLQISNPGFQERCAKKMQLVNLRNRRVSANDIMGGSCHNLIGLSNMHDLSSNSKPRCCSLEGIVDVPGNSSKEASSVFHQSFPNIEGQNNKLFLESKPKQEFLLNLHSEENIQKPFSAGFKRTSTLTVQDQEELCNGKCKSKQLCRSQSLLLRSSTRRNSYINTPVAEIIMKPNVGQGSTSVQTAMESELGESSATINKRLCKSTIELSENSLLPASSMLTGTQSLLQPHLERVAIDALQLCCLLLPPPNRRKLQLLMRMISRMSQNVDMPKLHDAMGTRSLMIHTFSRCVLCCAEEVDLDELLAGRLVSFLMDHHQEILQVPSYLQTAVEKHLDYLKKGHVSIVSLKILCNSQLQVANSSQNSLIKKGDFISSHKQPLKVRNVDRIWDSNSYMTLSIPPSLISVLILCLTMAPSHDEECG

>NP_620448.1

MSLHFLYYCSEPTLDVKIAFCQGFDKQVDVSYIAKHYNMSKSKVDNQFYSVEVGDSTFTVLKRYQNLKPIGSGAQGIVCAAYDAVLDRNVAIKKLSRPFQNQTHAKRAYRELVLMKCVNHKNIISLLNVFTPQKTLEEFQDVYLVMELMDANLCQVIQMELDHERMSYLLYQMLCGIKHLHSAGIIHRDLKPSNIVVKSDCTLKILDFGLARTAGTSFMMTPYVVTRYYRAPEVILGMGYKENVDIWSVGCIMGEMVRHKILFPGRDYIDQWNKVIEQLGTPCPEFMKKLQPTVRNYVENRPKYAGLTFPKLFPDSLFPADSEHNKLKASQARDLLSKMLVIDPAKRISVDDALQHPYINVWYDPAEVEAPPPQIYDKQLDEREHTIEEWKELIYKEVMNSEEKTKNGVVKGQPSPSGAAVNSSESLPPSSSVNDISSMSTDQTLASDTDSSLEASAGPLGCCR

848 NP_003085.1 P51857

>NP_003085.1

MAYRGQGQKVQKVMVQPINLIFRYLQNRSRIQVWLYEQVNMRIEGCIIGFDEYMNLVLDDAEEIHSKTKSRKQLGRIMLKGDNITLLQSVSN

>P51857

MDLSAASHRIPLSDGNSIPIIGLGTYSEPKSTPKGACATSVKVAIDTGYRHIDGAYIYQNEHEVGEAIREKIAEGKVRREDIFYCGKLWATNHVPEMVRPTLERTLRVLQLDYVDLYIIEVPMAFKPGDEIYPRDENGKWLYHKSNLCATWEAMEACKDAGLVKSLGVSNFNRRQLELILNKPGLKHKPVSNQVECHPYFTQPKLLKFCQQHDIVITAYSPLGTSRNPIWVNVSSPPLLKDALLNSLGKRYNKTAAQIVLRFNIQRGVVVIPKSFNLERIKENFQIFDFSLTEEEMKDIEALNKNVRFVELLMWRDHPEYPFHDEY

849 Q5VUA4 NP_002100.2

>Q5VUA4

MYRSSARSSVSSHRPKDDGGGGPRSGRSSGSSSGPARRSSPPPPPSGSSSRTPARRPRSPSGHRGRRASPSPPRGRRVSPSPPRARRGSPSPPRGRRLFPPGPAGFRGSSRGESRADYARDGRGDHPGDSGSRRRSPGLCSDSLEKSLRITVGNDHFCVSTPERRRLSDRLGSPVDNLEDMDRDDLTDDSVFTRSSQCSRGLERYISQEEGPLSPFLGQLDEDYRTKETFLHRSDYSPHISCHDELLRGTERNREKLKGYSIRSEERSREAKRPRYDDTVKINSMGGDHPSFTSGTRNYRQRRRSPSPRFLDPEFRELDLARRKREEEEERSRSLSQELVGVDGGGTGCSIPGLSGVLTASEPGYSLHRPEEVSVMPKKSILKKRIEVDIMEPSMQLESFSSSTSSSQDHPLYSGHPSLPLSGAIAAFASEIENKGTMVETALKEPQGNLYQWGPLPGIPKDNSPLREKFGSFLCHKDNLDLKAEGPERHTDFLLPHERASQDGSGFSRILSMLADSTSTQEKRRRSFPDIEDEEKFLYGDEEEDLKAESVPKPLGSSESEVMRQKASSLPSSAPAVKLESLEETNPEYAKIHDLLKTIGLDIGVAEISQLAARTQERLHGKKPSLRSSADRRSSVDRYFSADHCSSVDHRFSADRCSSVDHCFSADRRSSDPHRLESREAHHSNTHSPEVSHPHPPSPVDPYLLTKNSPPFLKSDHPVGHISGPEVVGSGFQSSVAVRCMLPSAPSAPIRLPHTAALSQFHMPRASQFAAARIPPNYQGPAIPPASFDAYRHYMAYAASRWPMYPTSQPSNHPVPEPHRIMPITKQATRSRPNLRVIPTVTPDKPKQKESLRGSIPAAQVPVQVSIPSLIRYNPEKISDEKNRASQKQKVIEEREKLKNDREARQKKMYYLRTELERLHKQQGEMLRKKRREKDGHKDPLLVEVSRLQDNIMKDIAELRQEAEEAEKKQSELDKVAQILGINIFDKSQKSLSDSREPTEKPGKAEKSKSPEKVSSFSNSSSNKESKVNNEKFRTKSPKPAESPQSATKQLDQPTAAYEYYDAGNHWCKDCNTICGTMFDFFTHMHNKKHTQTLDPYNRPWASKTQSEAKQDAIKRTDKITVPAKGSEFLVPISGFYCQLCEEFLGDPISGEQHVKGHQHNEKYKKYVDENPLYEERRNLDRQAGLAVVLETERRRQSELKRKLSEKPKEEKKEKKAKAVKEVKEDDKVSEKLEDQLSEGRNSPEKAENKRNTGIKLQLKEEVKKESPTSSSFGKFSWKKPEKEEEKSSLVTPSISKEEILESSKDKEDGKTEAGKAKPIKIKLSGKTVVAHTSPWMPVVTTSTQTKIRPNLPIPSTVLRKSCSATMSKPAPLNTFLSIKSSGTTAKPLPVVKESSADLLLPPDIISKAFGGEEVILKGSPEEKVVLAEKSEPSHLPEQILPPPPPPPPPPPPPPPVIPHPAAPSAAQANAILAPVKSNPVVSQTLSPGFVGPNILNPVLPVAIMASAQPAAIPSDETAPGVSESDRDQTLFSVLVRPPPPLSSVFSEQAKKLEKRNSCLATANAKDLYDIFYSSGGKGAPETKGAPETKLSGGPLANGENSNLSRTKSSDTSSTSPLNSSASQEELHQDEGLVAAPIVSNSEKPIAKTLVALGKWSVVEHVGPKSTGSTYGFLQPLTRLCQSRPYETITPKTDTLAIWTSSSFQSDTSRDISPEKSELDLGEPGPPGVEPPPQLLDIQCKESQKLVEIHLRESVNQDKESQELRKSEDCRESEIETNTELKERVKELSEGIVDEGVSTSIGPHSIDDSNLNHGNRYMWEGEVKQPNLLMIDKEAEQSNKLMTGSETPSKVVIKLSPQACSFTKAKLDSFLSEARSLLNPQDTPVKISAPELLLHSPARSAMCLTGSPQEQGVSVVSEEGLENSAPESASRTSRYRSLKLKRERSKDFQVKKIYELAVWDENKKRPETWESPEKPKTEALELQDVHPELTVTIESKALEDFEATDLKVEELTALGNLGDMPVDFCTTRVSPAHRSPTVLCQKVCEENSVSPIGCNSSDPADFEPIPSFSGFPLDSPKTLVLDFETEGERNSPNPRSVRIPSPNILKTGLTENVDRGLGGLEGTHQALDLLAGGMMPEEVKESSQLDKQESLGLELKTINSAGLGPSPCLPDLVDFVTRTSGVQKDKLCSPLSEPGDPSKCSSLELGPLQLEISNASTTEVAILQVDDDSGDPLNLVKAPVSRSPPREQVIEDNMVPQGMPEQETTVGAIQDHTESSVHN

>NP_002100.2

MAERAALEELVKLQGERVRGLKQQKASAELIEEEVAKLLKLKAQLGPDESKQKFVLKTPKGTRDYSPRQMAVREKVFDVIIRCFKRHGAEVIDTPVFELKETLMGKYGEDSKLIYDLKDQGGELLSLRYDLTVPFARYLAMNKLTNIKRYHIAKVYRRDNPAMTRGRYREFYQCDFDIAGNFDPMIPDAECLKIMCEILSSLQIGDFLVKVNDRRILDGMFAICGVSDSKFRTICSSVDKLDKVSWEEVKNEMVGEKGLAPEVADRIGDYVQQHGGVSLVEQLLQDPKLSQNKQALEGLGDLKLLFEYLTLFGIDDKISFDLSLARGLDYYTGVIYEAVLLQTPAQAGEEPLGVGSVAAGGRYDGLVGMFDPKGRKVPCVGLSIGVERIFSIVEQRLEALEEKIRTTETQVLVASAQKKLLEERLKLVSELWDAGIKAELLYKKNPKLLNQLQYCEEAGIPLVAIIGEQELKDGVIKLRSVTSREEVDVRREDLVEEIKRRTGQPLCIC

850 P29558 NP_056238.2

>P29558

MGKVWKQQMYPQYATYYYPQYLQAKQSLVPAHPMAPPSPSTTSSNNNSSSSSNSGWDQLSKTNLYIRGLPPHTTDQDLVKLCQPYGKIVSTKAILDKTTNKCKGYGFVDFDSPAAAQKAVSALKASGVQAQMAKQQEQDPTNLYISNLPLSMDEQELENMLKPFGQVISTRILRDSSGTSRGVGFARMESTEKCEAVIGHFNGKFIKTPPGVSAPTEPLLCKFADGGQKKRQNPNKYIPNGRPWHREGEVRLAGMTLTYDPTTAAIQNGFYPSPYSIATNRMITQTSITPYIASPVSAYQVQSPSWMQPQPYILQHPGAVLTPSMEHTMSLQPASMISPLAQQMSHLSLGSTGTYMPATSAMQGAYLPQYAHMQTTAVPVEEASGQQQVAVETSNDHSPYTFQPNK

>NP_056238.2

MVFPAKRFCLVPSMEGVRWAFSCGTWLPSRAEWLLAVRSIQPEEKERIGQFVFARDAKAAMAGRLMIRKLVAEKLNIPWNHIRLQRTAKGKPVLAKDSSNPYPNFNFNISHQGDYAVLAAEPELQVGIDIMKTSFPGRGSIPEFFHIMKRKFTNKEWETIRSFKDEWTQLDMFYRNWALKESFIKAIGVGLGFELQRLEFDLSPLNLDIGQVYKETRLFLDGEEEKEWAFEESKIDEHHFVAVALRKPDGSRHQDVPSQDDSKPTQRQFTILNFNDLMSSAVPMTPEDPSFWDCFCFTEEIPIRNGTKS

851 NP_006707.1 NP_066565.1

>NP_006707.1

MNSGAMRIHSKGHFQGGIQVKNEKNRPSLKSLKTDNRPEKSKCKPLWGKVFYLDLPSVTISEKLQKDIKDLGGRVEEFLSKDISYLISNKKEAKFAQTLGRISPVPSPESAYTAETTSPHPSHDGSSFKSPDTVCLSRGKLLVEKAIKDHDFIPSNSILSNALSWGVKILHIDDIRYYIEQKKKELYLLKKSSTSVRDGGKRVGSGAQKTRTGRLKKPFVKVEDMSQLYRPFYLQLTNMPFINYSIQKPCSPFDVDKPSSMQKQTQVKLRIQTDGDKYGGTSIQLQLKEKKKKGYCECCLQKYEDLETHLLSEQHRNFAQSNQYQVVDDIVSKLVFDFVEYEKDTPKKKRIKYSVGSLSPVSASVLKKTEQKEKVELQHISQKDCQEDDTTVKEQNFLYKETQETEKKLLFISEPIPHPSNELRGLNEKMSNKCSMLSTAEDDIRQNFTQLPLHKNKQECILDISEHTLSENDLEELRVDHYKCNIQASVHVSDFSTDNSGSQPKQKSDTVLFPAKDLKEKDLHSIFTHDSGLITINSSQEHLTVQAKAPFHTPPEEPNECDFKNMDSLPSGKIHRKVKIILGRNRKENLEPNAEFDKRTEFITQEENRICSSPVQSLLDLFQTSEEKSEFLGFTSYTEKSGICNVLDIWEEENSDNLLTAFFSSPSTSTFTGF

>NP_066565.1

MADESETAVKPPAPPLPQMMEGNGNGHEHCSDCENEEDNSYNRGGLSPANDTGAKKKKKKQKKKKEKGSETDSAQDQPVKMNSLPAERIQEIQKAIELFSVGQGPAKTMEEASKRSYQFWDTQPVPKLGEVVNTHGPVEPDKDNIRQEPYTLPQGFTWDALDLGDRGVLKELYTLLNENYVEDDDNMFRFDYSPEFLLWALRPPGWLPQWHCGVRVVSSRKLVGFISAIPANIHIYDTEKKMVEINFLCVHKKLRSKRVAPVLIREITRRVHLEGIFQAVYTAGVVLPKPVGTCRYWHRSLNPRKLIEVKFSHLSRNMTMQRTMKLYRLPETPKTAGLRPMETKDIPVVHQLLTRYLKQFHLTPVMSQEEVEHWFYPQENIIDTFVVENANGEVTDFLSFYTLPSTIMNHPTHKSLKAAYSFYNVHTQTPLLDLMSDALVLAKMKGFDVFNALDLMENKTFLEKLKFGIGDGNLQYYLYNWKCPSMGAEKVGLVLQ

852 NP_002098.1 NP_056053.1

>NP_002098.1

MARTKQTARKSTGGKAPRKQLATKAARKSAPSTGGVKKPHRYRPGTVALREIRRYQKSTELLIRKLPFQRLVREIAQDFKTDLRFQSAAIGALQEASEAYLVGLFEDTNLCAIHAKRVTIMPKDIQLARRIRGERA

>NP_056053.1

MPRPELPLPEGWEEARDFDGKVYYIDHTNRTTSWIDPRDRYTKPLTFADCISDELPLGWEEAYDPQVGDYFIDHNTKTTQIEDPRVQWRREQEHMLKDYLVVAQEALSAQKEIYQVKQQRLELAQQEYQQLHAVWEHKLGSQVSLVSGSSSSSKYDPEILKAEIATAKSRVNKLKREMVHLQHELQFKERGFQTLKKIDKKMSDAQGSYKLDEAQAVLRETKAIKKAITCGEKEKQDLIKSLAMLKDGFRTDRGSHSDLWSSSSSLESSSFPLPKQYLDVSSQTDISGSFGINSNNQLAEKVRLRLRYEEAKRRIANLKIQLAKLDSEAWPGVLDSERDRLILINEKEELLKEMRFISPRKWTQGEVEQLEMARKRLEKDLQAARDTQSKALTERLKLNSKRNQLVRELEEATRQVATLHSQLKSLSSSMQSLSSGSSPGSLTSSRGSLVASSLDSSTSASFTDLYYDPFEQLDSELQSKVEFLLLEGATGFRPSGCITTIHEDEVAKTQKAEGGGRLQALRSLSGTPKSMTSLSPRSSLSSPSPPCSPLMADPLLAGDAFLNSLEFEDPELSATLCELSLGNSAQERYRLEEPGTEGKQLGQAVNTAQGCGLKVACVSAAVSDESVAGDSGVYEASVQRLGASEAAAFDSDESEAVGATRIQIALKYDEKNKQFAILIIQLSNLSALLQQQDQKVNIRVAVLPCSESTTCLFRTRPLDASDTLVFNEVFWVSMSYPALHQKTLRVDVCTTDRSHLEECLGGAQISLAEVCRSGERSTRWYNLLSYKYLKKQSRELKPVGVMAPASGPASTDAVSALLEQTAVELEKRQEGRSSTQTLEDSWRYEETSENEAVAEEEEEEVEEEEGEEDVFTEKASPDMDGYPALKVDKETNTETPAPSPTVVRPKDRRVGTPSQGPFLRGSTIIRSKTFSPGPQSQYVCRLNRSDSDSSTLSKKPPFVRNSLERRSVRMKRPSSVKSLRSERLIRTSLDLELDLQATRTWHSQLTQEISVLKELKEQLEQAKSHGEKELPQWLREDERFRLLLRMLEKRQMDRAEHKGELQTDKMMRAAAKDVHRLRGQSCKEPPEVQSFREKMAFFTRPRMNIPALSADDV

853 NP_009042.1 NP_008878.3

>NP_009042.1

MAKDAGLIEANGELKVFIDQNLSPGKGVVSLVAVHPSTVNPLGKQLLPKTFGQSNVNIAQQVVIGTPQRPAASNTLVVGSPHTPSTHFASQNQPSDSSPWSAGKRNRKGEKNGKGLRHFSMKVCEKVQRKGTTSYNEVADELVAEFSAADNHILPNESAYDQKNIRRRVYDALNVLMAMNIISKEKKEIKWIGLPTNSAQECQNLEVERQRRLERIKQKQSQLQELILQQIAFKNLVQRNRHAEQQASRPPPPNSVIHLPFIIVNTSKKTVIDCSISNDKFEYLFNFDNTFEIHDDIEVLKRMGMACGLESGSCSAEDLKMARSLVPKALEPYVTEMAQGTVGGVFITTAGSTSNGTRFSASDLTNGADGMLATSSNGSQYSGSRVETPVSYVGEDDEEDDDFNENDEDD

>NP_008878.3

MASGGSGGVSVPALWSEVNRYGQNGDFTRALKTVNKILQINKDDVTALHCKVVCLIQNGSFKEALNVINTHTKVLANNSLSFEKAYCEYRLNRIENALKTIESANQQTDKLKELYGQVLYRLERYDECLAVYRDLVRNSQDDYDEERKTNLSAVVAAQSNWEKVVPENLGLQEGTHELCYNTACALIGQGQLNQAMKILQKAEDLCRRSLSEDTDGTEEDPQAELAIIHGQMAYILQLQGRTEEALQLYNQIIKLKPTDVGLLAVIANNIITINKDQNVFDSKKKVKLTNAEGVEFKLSKKQLQAIEFNKALLAMYTNQAEQCRKISASLQSQSPEHLLPVLIQAAQLCREKQHTKAIELLQEFSDQHPENAAEIKLTMAQLKISQGNISKACLILRSIEELKHKPGMVSALVTMYSHEEDIDSAIEVFTQAIQWYQNHQPKSPAHLSLIREAANFKLKYGRKKEAISDLQQLWKQNPKDIHTLAQLISAYSLVDPEKAKALSKHLPSSDSMSLKVDVEALENSAGATYIRKKGGKVTGDSQPKEQGQGDLKKKKKKKKGKLPKNYDPKVTPDPERWLPMRERSYYRGRKKGKKKDQIGKGTQGATAGASSELDASKTVSSPPTSPRPGSAATVSASTSNIIPPRHQKPAGAPATKKKQQQKKKKGGKGGW

854 Q13510 NP_006784.1

>Q13510

MPGRSCVALVLLAAAVSCAVAQHAPPWTEDCRKSTYPPSGPTYRGAVPWYTINLDLPPYKRWHELMLDKAPVLKVIVNSLKNMINTFVPSGKIMQVVDEKLPGLLGNFPGPFEEEMKGIAAVTDIPLGEIISFNIFYELFTICTSIVAEDKKGHLIHGRNMDFGVFLGWNINNDTWVITEQLKPLTVNLDFQRNNKTVFKASSFAGYVGMLTGFKPGLFSLTLNERFSINGGYLGILEWILGKKDVMWIGFLTRTVLENSTSYEEAKNLLTKTKILAPAYFILGGNQSGEGCVITRDRKESLDVYELDAKQGRWYVVQTNYDRWKHPFFLDDRRTPAKMCLNRTSQENISFETMYDVLSTKPVLNKLTVYTTLIDVTKGQFETYLRDCPDPCIGW

>NP_006784.1

MAAAVGRLLRASVARHVSAIPWGISATAALRPAACGRTSLTNLLCSGSSQAKLFSTSSSCHAPAVTQHAPYFKGTAVVNGEFKDLSLDDFKGKYLVLFFYPLDFTFVCPTEIVAFSDKANEFHDVNCEVVAVSVDSHFSHLAWINTPRKNGGLGHMNIALLSDLTKQISRDYGVLLEGSGLALRGLFIIDPNGVIKHLSVNDLPVGRSVEETLRLVKAFQYVETHGEVCPANWTPDSPTIKPSPAASKEYFQKVNQ

855 P0C7P1 NP_036361.1

>P0C7P1

MVEADHPGKLFIGGLNRETNEKMLKAVFGKHGPISEVLLIKDRTSKSRGFAFITFENPADAKNAAKDMNGKSLHGKAIKVEQAKKPSFQSGGRRRPPASSRNRSPSGSLRSARGSRGGTRGWLPSQEGHLDDGGYTPDLKMSYSRGLIPVKRGPSSRSGGPPPKKSAPSAVARSNSWMGSQGPMSQRRENYGVPPRRATISSWRNDRMSTRHDGYATNDGNHPSCQETRDYAPPSRGYAYRDNGHSNRDEHSSRGYRNHRSSRETRDYAPPSRGHAYRDYGHSRRDESYSRGYRNRRSSRETREYAPPSRGHGYRDYGHSRRHESYSRGYRNHPSSRETRDYAPPHRDYAYRDYGHSSWDEHSSRGYSYHDGYGEALGRDHSEHLSGSSYRDALQRYGTSHGAPPARGPRMSYGGSTCHAYSNTRDRYGRSWESYSSCGDFHYCDREHVCRKDQRNPPSLGRVLPDPREACGSSSYVASIVDGGESRSEKGDSSRY

>NP_036361.1

MSTSWSDRLQNAADMPANMDKHALKKYRREAYHRVFVNRSLAMEKIKCFGFDMDYTLAVYKSPEYESLGFELTVERLVSIGYPQELLSFAYDSTFPTRGLVFDTLYGNLLKVDAYGNLLVCAHGFNFIRGPETREQYPNKFIQRDDTERFYILNTLFNLPETYLLACLVDFFTNCPRYTSCETGFKDGDLFMSYRSMFQDVRDAVDWVHYKGSLKEKTVENLEKYVVKDGKLPLLLSRMKEVGKVFLATNSDYKYTDKIMTYLFDFPHGPKPGSSHRPWQSYFDLILVDARKPLFFGEGTVLRQVDTKTGKLKIGTYTGPLQHGIVYSGGSSDTICDLLGAKGKDILYIGDHIFGDILKSKKRQGWRTFLVIPELAQELHVWTDKSSLFEELQSLDIFLAELYKHLDSSSNERPDISSIQRRIKKVTHDMDMCYGMMGSLFRSGSRQTLFASQVMRYADLYAASFINLLYYPFSYLFRAAHVLMPHESTVEHTHVDINEMESPLATRNRTSVDFKDTDYKRHQLTRSISEIKPPNLFPLAPQEITHCHDEDDDEEEEEEEE

856 NP_002096.1 NP_004127.1

>NP_002096.1

MSGRGKTGGKARAKAKSRSSRAGLQFPVGRVHRLLRKGHYAERVGAGAPVYLAAVLEYLTAEILELAGNAARDNKKTRIIPRHLQLAIRNDEELNKLLGGVTIAQGGVLPNIQAVLLPKKTSATVGPKAPSGGKKATQASQEY

>NP_004127.1

MDAPKAGYAFEYLIETLNDSSHKKFFDVSKLGTKYDVLPYSIRVLLEAAVRNCDGFLMKKEDVMNILDWKTKQSNVEVPFFPARVLLQDFTGIPAMVDFAAMREAVKTLGGDPEKVHPACPTDLTVDHSLQIDFSKCAIQNAPNPGGGDLQKAGKLSPLKVQPKKLPCRGQTTCRGSCDSGELGRNSGTFSSQIENTPILCPFHLQPVPEPETVLKNQEVEFGRNRERLQFFKWSSRVFKNVAVIPPGTGMAHQINLEYLSRVVFEEKDLLFPDSVVGTDSHITMVNGLGILGWGVGGIETEAVMLGLPVSLTLPEVVGCELTGSSNPFVTSIDVVLGITKHLRQVGVAGKFVEFFGSGVSQLSIVDRTTIANMCPEYGAILSFFPVDNVTLKHLEHTGFSKAKLESMETYLKAVKLFRNDQNSSGEPEYSQVIQINLNSIVPSVSGPKRPQDRVAVTDMKSDFQACLNEKVGFKGFQIAAEKQKDIVSIHYEGSEYKLSHGSVVIAAVISCTNNCNPSVMLAAGLLAKKAVEAGLRVKPYIRTSLSPGSGMVTHYLSSSGVLPYLSKLGFEIVGYGCSTCVGNTAPLSDAVLNAVKQGDLVTCGILSGNKNFEGRLCDCVRANYLASPPLVVAYAIAGTVNIDFQTEPLGTDPTGKNIYLHDIWPSREEVHRVEEEHVILSMFKALKDKIEMGNKRWNSLEAPDSVLFPWDLKSTYIRCPSFFDKLTKEPIALQAIENAHVLLYLGDSVTTDHISPAGSIARNSAAAKYLTNRGLTPREFNSYGARRGNDAVMTRGTFANIKLFNKFIGKPAPKTIHFPSGQTLDVFEAAELYQKEGIPLIILAGKKYGSGNSRDWAAKGPYLLGVKAVLAESYEKIHKDHLIGIGIAPLQFLPGENADSLGLSGRETFSLTFPEELSPGITLNIQTSTGKVFSVIASFEDDVEITLYKHGGLLNFVARKFS

857 NP_006158.2 NP_001217.2

>NP_006158.2

MLRVPEPRPGEAKAEGAAPPTPSKPLTSFLIQDILRDGAQRQGGRTSSQRQRDPEPEPEPEPEGGRSRAGAQNDQLSTGPRAAPEEAETLAETEPERHLGSYLLDSENTSGALPRLPQTPKQPQKRSRAAFSHTQVIELERKFSHQKYLSAPERAHLAKNLKLTETQVKIWFQNRRYKTKRKQLSSELGDLEKHSSLPALKEEAFSRASLVSVYNSYPYYPYLYCVGSWSPAFW

>NP_001217.2

MSSASGLRRGHPAGGEENMTETDAFYKREMFDPAEKYKMDHRRRGIALIFNHERFFWHLTLPERRGTCADRDNLTRRFSDLGFEVKCFNDLKAEELLLKIHEVSTVSHADADCFVCVFLSHGEGNHIYAYDAKIEIQTLTGLFKGDKCHSLVGKPKIFIIQACRGNQHDVPVIPLDVVDNQTEKLDTNITEVDAASVYTLPAGADFLMCYSVAEGYYSHRETVNGSWYIQDLCEMLGKYGSSLEFTELLTLVNRKVSQRRVDFCKDPSAIGKKQVPCFASMLTKKLHFFPKSN

858 O95926 NP_653288.1

>O95926

MAAIAASEVLVDSAEEGSLAAAAELAAQKREQRLRKFRELHLMRNEARKLNHQEVVEEDKRLKLPANWEAKKARLEWELKEEEKKKECAARGEDYEKVKLLEISAEDAERWERKKKRKNPDLGFSDYAAAQLRQYHRLTKQIKPDMETYERLREKHGEEFFPTSNSLLHGTHVPSTEEIDRMVIDLEKQIEKRDKYSRRRPYNDDADIDYINERNAKFNKKAERFYGKYTAEIKQNLERGTAV

>NP_653288.1

MLRTAGRDGLCRLSTYLEELEAVELKKFKLYLGTATELGEGKIPWGSMEKAGPLEMAQLLITHFGPEEAWRLALSTFERINRKDLWERGQREDLVRDTPPGGPSSLGNQSTCLLEVSLVTPRKDPQETYRDYVRRKFRLMEDRNARLGECVNLSHRYTRLLLVKEHSNPMQVQQQLLDTGRGHARTVGHQASPIKIETLFEPDEERPEPPRTVVMQGAAGIGKSMLAHKVMLDWADGKLFQGRFDYLFYINCREMNQSATECSMQDLIFSCWPEPSAPLQELIRVPERLLFIIDGFDELKPSFHDPQGPWCLCWEEKRPTELLLNSLIRKKLLPELSLLITTRPTALEKLHRLLEHPRHVEILGFSEAERKEYFYKYFHNAEQAGQVFNYVRDNEPLFTMCFVPLVCWVVCTCLQQQLEGGGLLRQTSRTTTAVYMLYLLSLMQPKPGAPRLQPPPNQRGLCSLAADGLWNQKILFEEQDLRKHGLDGEDVSAFLNMNIFQKDINCERYYSFIHLSFQEFFAAMYYILDEGEGGAGPDQDVTRLLTEYAFSERSFLALTSRFLFGLLNEETRSHLEKSLCWKVSPHIKMDLLQWIQSKAQSDGSTLQQGSLEFFSCLYEIQEEEFIQQALSHFQVIVVSNIASKMEHMVSSFCLKRCRSAQVLHLYGATYSADGEDRARCSAGAHTLLVQLPERTVLLDAYSEHLAAALCTNPNLIELSLYRNALGSRGVKLLCQGLRHPNCKLQNLRLKRCRISSSACEDLSAALIANKNLTRMDLSGNGVGFPGMMLLCEGLRHPQCRLQMIQLRKCQLESGACQEMASVLGTNPHLVELDLTGNALEDLGLRLLCQGLRHPVCRLRTLWLKICRLTAAACDELASTLSVNQSLRELDLSLNELGDLGVLLLCEGLRHPTCKLQTLRLGICRLGSAACEGLSVVLQANHNLRELDLSFNDLGDWGLWLLAEGLQHPACRLQKLWLDSCGLTAKACENLYFTLGINQTLTDLYLTNNALGDTGVRLLCKRLSHPGCKLRVLWLFGMDLNKMTHSRLAALRVTKPYLDIGC

859 NP_003063.2 NP_002003.1

>NP_003063.2

MSTPDPPLGGTPRPGPSPGPGPSPGAMLGPSPGPSPGSAHSMMGPSPGPPSAGHPIPTQGPGGYPQDNMHQMHKPMESMHEKGMSDDPRYNQMKGMGMRSGGHAGMGPPPSPMDQHSQGYPSPLGGSEHASSPVPASGPSSGPQMSSGPGGAPLDGADPQALGQQNRGPTPFNQNQLHQLRAQIMAYKMLARGQPLPDHLQMAVQGKRPMPGMQQQMPTLPPPSVSATGPGPGPGPGPGPGPGPAPPNYSRPHGMGGPNMPPPGPSGVPPGMPGQPPGGPPKPWPEGPMANAAAPTSTPQKLIPPQPTGRPSPAPPAVPPAASPVMPPQTQSPGQPAQPAPMVPLHQKQSRITPIQKPRGLDPVEILQEREYRLQARIAHRIQELENLPGSLAGDLRTKATIELKALRLLNFQRQLRQEVVVCMRRDTALETALNAKAYKRSKRQSLREARITEKLEKQQKIEQERKRRQKHQEYLNSILQHAKDFKEYHRSVTGKIQKLTKAVATYHANTEREQKKENERIEKERMRRLMAEDEEGYRKLIDQKKDKRLAYLLQQTDEYVANLTELVRQHKAAQVAKEKKKKKKKKKAENAEGQTPAIGPDGEPLDETSQMSDLPVKVIHVESGKILTGTDAPKAGQLEAWLEMNPGYEVAPRSDSEESGSEEEEEEEEEEQPQAAQPPTLPVEEKKKIPDPDSDDVSEVDARHIIENAKQDVDDEYGVSQALARGLQSYYAVAHAVTERVDKQSALMVNGVLKQYQIKGLEWLVSLYNNNLNGILADEMGLGKTIQTIALITYLMEHKRINGPFLIIVPLSTLSNWAYEFDKWAPSVVKVSYKGSPAARRAFVPQLRSGKFNVLLTTYEYIIKDKHILAKIRWKYMIVDEGHRMKNHHCKLTQVLNTHYVAPRRLLLTGTPLQNKLPELWALLNFLLPTIFKSCSTFEQWFNAPFAMTGEKVDLNEEETILIIRRLHKVLRPFLLRRLKKEVEAQLPEKVEYVIKCDMSALQRVLYRHMQAKGVLLTDGSEKDKKGKGGTKTLMNTIMQLRKICNHPYMFQHIEESFSEHLGFTGGIVQGLDLYRASGKFELLDRILPKLRATNHKVLLFCQMTSLMTIMEDYFAYRGFKYLRLDGTTKAEDRGMLLKTFNEPGSEYFIFLLSTRAGGLGLNLQSADTVIIFDSDWNPHQDLQAQDRAHRIGQQNEVRVLRLCTVNSVEEKILAAAKYKLNVDQKVIQAGMFDQKSSSHERRAFLQAILEHEEQDESRHCSTGSGSASFAHTAPPPAGVNPDLEEPPLKEEDEVPDDETVNQMIARHEEEFDLFMRMDLDRRREEARNPKRKPRLMEEDELPSWIIKDDAEVERLTCEEEEEKMFGRGSRHRKEVDYSDSLTEKQWLKAIEEGTLEEIEEEVRQKKSSRKRKRDSDAGSSTPTTSTRSRDKDDESKKQKKRGRPPAEKLSPNPPNLTKKMKKIVDAVIKYKDSSSGRQLSEVFIQLPSRKELPEYYELIRKPVDFKKIKERIRNHKYRSLNDLEKDVMLLCQNAQTFNLEGSLIYEDSIVLQSVFTSVRQKIEKEDDSEGEESEEEEEGEEEGSESESRSVKVKIKLGRKEKAQDRLKGGRRRPSRGSRAKPVVSDDDSEEEQEEDRSGSGSEED

>NP_002003.1

MSFRFGQHLIKPSVVFLKTELSFALVNRKPVVPGHVLVCPLRPVERFHDLRPDEVADLFQTTQRVGTVVEKHFHGTSLTFSMQDGPEAGQTVKHVHVHVLPRKAGDFHRNDSIYEELQKHDKEDFPASWRSEEEMAAEAAALRVYFQ

860 P07602 Q9NYK5

>P07602

MYALFLLASLLGAALAGPVLGLKECTRGSAVWCQNVKTASDCGAVKHCLQTVWNKPTVKSLPCDICKDVVTAAGDMLKDNATEEEILVYLEKTCDWLPKPNMSASCKEIVDSYLPVILDIIKGEMSRPGEVCSALNLCESLQKHLAELNHQKQLESNKIPELDMTEVVAPFMANIPLLLYPQDGPRSKPQPKDNGDVCQDCIQMVTDIQTAVRTNSTFVQALVEHVKEECDRLGPGMADICKNYISQYSEIAIQMMMHMQPKEICALVGFCDEVKEMPMQTLVPAKVASKNVIPALELVEPIKKHEVPAKSDVYCEVCEFLVKEVTKLIDNNKTEKEILDAFDKMCSKLPKSLSEECQEVVDTYGSSILSILLEEVSPELVCSMLHLCSGTRLPALTVHVTQPKDGGFCEVCKKLVGYLDRNLEKNSTKQEILAALEKGCSFLPDPYQKQCDQFVAEYEPVLIEILVEVMDPSFVCLKIGACPSAHKPLLGTEKCIWGPSYWCQNTETAAQCNAVEHCKRHVWN

>Q9NYK5

MEALAMGSRALRLWLVAPGGGIKWRFIATSPASQLSPTELTEMRNDLFNKEKARQLSLTPRTEKIEVKHVGKTDPGTVFVMNKNISTPYSCAMHLSEWYCRKSILALVDGQPWDMYKPLTKSCEIKFLTFKDCDPGEVNKAYWRSCAMMMGCVIERAFKDEYMVNLVRAPEVPVISGAFCYDVVLDSKLDEWMPTKENLRSFTKDAHALIYKDLPFETLEVEAKVALEIFQHSKYKVDFIEEKASQNPERIVKLHRIGDFIDVSEGPLIPRTSICFQYEVSAVHNLQPTQPSLIRRFQGVSLPVHLRAHFTIWDKLLERSRKMVTEDQSKATEECTST

861 P08236 NP_001344.2

>P08236

MARGSAVAWAALGPLLWGCALGLQGGMLYPQESPSRECKELDGLWSFRADFSDNRRRGFEEQWYRRPLWESGPTVDMPVPSSFNDISQDWRLRHFVGWVWYEREVILPERWTQDLRTRVVLRIGSAHSYAIVWVNGVDTLEHEGGYLPFEADISNLVQVGPLPSRLRITIAINNTLTPTTLPPGTIQYLTDTSKYPKGYFVQNTYFDFFNYAGLQRSVLLYTTPTTYIDDITVTTSVEQDSGLVNYQISVKGSNLFKLEVRLLDAENKVVANGTGTQGQLKVPGVSLWWPYLMHERPAYLYSLEVQLTAQTSLGPVSDFYTLPVGIRTVAVTKSQFLINGKPFYFHGVNKHEDADIRGKGFDWPLLVKDFNLLRWLGANAFRTSHYPYAEEVMQMCDRYGIVVIDECPGVGLALPQFFNNVSLHHHMQVMEEVVRRDKNHPAVVMWSVANEPASHLESAGYYLKMVIAHTKSLDPSRPVTFVSNSNYAADKGAPYVDVICLNSYYSWYHDYGHLELIQLQLATQFENWYKKYQKPIIQSEYGAETIAGFHQDPPLMFTEEYQKSLLEQYHLGLDQKRRKYVVGELIWNFADFMTEQSPTRVLGNKKGIFTRQRQPKSAAFLLRERYWKIANETRYPHSVAKSQCLENSLFT

>NP_001344.2

MDSKYQCVKLNDGHFMPVLGFGTYAPAEVPKSKALEATKLAIEAGFRHIDSAHLYNNEEQVGLAIRSKIADGSVKREDIFYTSKLWCNSHRPELVRPALERSLKNLQLDYVDLYLIHFPVSVKPGEEVIPKDENGKILFDTVDLCATWEAVEKCKDAGLAKSIGVSNFNRRQLEMILNKPGLKYKPVCNQVECHPYFNQRKLLDFCKSKDIVLVAYSALGSHREEPWVDPNSPVLLEDPVLCALAKKHKRTPALIALRYQLQRGVVVLAKSYNEQRIRQNVQVFEFQLTSEEMKAIDGLNRNVRYLTLDIFAGPPNYPFSDEY

862 Q9BS31 NP_005882.2

>Q9BS31

MTKAQESLTLEDVAVDFTWEEWQFLSPAQKDLYRDVMLENYSNLVSVGYQAGKPDALTKLEQGEPLWTLEDEIHSPAHPEIEKADDHLQQPLQNQKILKRTGQRYEHGRTLKSYLGLTNQSRRYNRKEPAEFNGDGAFLHDNHEQMPTEIEFPESRKPISTKSQFLKHQQTHNIEKAHECTDCGKAFLKKSQLTEHKRIHTGKKPHVCSLCGKAFYKKYRLTEHERAHRGEKPHGCSLCGKAFYKRYRLTEHERAHKGEKPYGCSECGKAFPRKSELTEHQRIHTGIKPHQCSECGRAFSRKSLLVVHQRTHTGEKPHTCSECGKGFIQKGNLNIHQRTHTGEKPYGCIDCGKAFSQKSCLVAHQRYHTGKTPFVCPECGQPCSQKSGLIRHQKIHSGEKPYKCSDCGKAFLTKTMLIVHHRTHTGERPYGCDECEKAYFYMSCLVKHKRIHSREKRGDSVKVENPSTASHSLSPSEHVQGKSPVNMVTVAMVAGQCEFAHILHS

>NP_005882.2

MNAGSDPVVIVSAARTIIGSFNGALAAVPVQDLGSTVIKEVLKRATVAPEDVSEVIFGHVLAAGCGQNPVRQASVGAGIPYSVPAWSCQMICGSGLKAVCLAVQSIGIGDSSIVVAGGMENMSKAPHLAYLRTGVKIGEMPLTDSILCDGLTDAFHNCHMGITAENVAKKWQVSREDQDKVAVLSQNRTENAQKAGHFDKEIVPVLVSTRKGLIEVKTDEFPRHGSNIEAMSKLKPYFLTDGTGTVTPANASGINDGAAAVVLMKKSEADKRGLTPLARIVSWSQVGVEPSIMGIGPIPAIKQAVTKAGWSLEDVDIFEINEAFAAVSAAIVKELGLNPEKVNIEGGAIALGHPLGASGCRILVTLLHTLERMGRSRGVAALCIGGGMGIAMCVQRE

863 Q15349 O00170

>Q15349

MDLSMKKFAVRRFFSVYLRRKSRSKSSSLSRLEEEGVVKEIDISHHVKEGFEKADPSQFELLKVLGQGSYGKVFLVRKVKGSDAGQLYAMKVLKKATLKVRDRVRSKMERDILAEVNHPFIVKLHYAFQTEGKLYLILDFLRGGDLFTRLSKEVMFTEEDVKFYLAELALALDHLHSLGIIYRDLKPENILLDEEGHIKITDFGLSKEAIDHDKRAYSFCGTIEYMAPEVVNRRGHTQSADWWSFGVLMFEMLTGSLPFQGKDRKETMALILKAKLGMPQFLSGEAQSLLRALFKRNPCNRLGAGIDGVEEIKRHPFFVTIDWNTLYRKEIKPPFKPAVGRPEDTFHFDPEFTARTPTDSPGVPPSANAHHLFRGFSFVASSLIQEPSQQDLHKVPVHPIVQQLHGNNIHFTDGYEIKEDIGVGSYSVCKRCVHKATDTEYAVKIIDKSKRDPSEEIEILLRYGQHPNIITLKDVYDDGKFVYLVMELMRGGELLDRILRQRYFSEREASDVLCTITKTMDYLHSQGVVHRDLKPSNILYRDESGSPESIRVCDFGFAKQLRAGNGLLMTPCYTANFVAPEVLKRQGYDAACDIWSLGILLYTMLAGFTPFANGPDDTPEEILARIGSGKYALSGGNWDSISDAAKDVVSKMLHVDPHQRLTAMQVLKHPWVVNREYLSPNQLSRQDVHLVKGAMAATYFALNRTPQAPRLEPVLSSNLAQRRGMKRLTSTRL

>O00170

MADIIARLREDGIQKRVIQEGRGELPDFQDGTKATFHYRTLHSDDEGTVLDDSRARGKPMELIIGKKFKLPVWETIVCTMREGEIAQFLCDIKHVVLYPLVAKSLRNIAVGKDPLEGQRHCCGVAQMREHSSLGHADLDALQQNPQPLIFHMEMLKVESPGTYQQDPWAMTDEEKAKAVPLIHQEGNRLYREGHVKEAAAKYYDAIACLKNLQMKEQPGSPEWIQLDQQITPLLLNYCQCKLVVEEYYEVLDHCSSILNKYDDNVKAYFKRGKAHAAVWNAQEAQADFAKVLELDPALAPVVSRELQALEARIRQKDEEDKARFRGIFSH

864 Q08999 AAH30144.2

>Q08999

MPSGGDQSPPPPPPPPAAAASDEEEEDDGEAEDAAPPAESPTPQIQQRFDELCSRLNMDEAARAEAWDSYRSMSESYTLEGNDLHWLACALYVACRKSVPTVSKGTVEGNYVSLTRILKCSEQSLIEFFNKMKKWEDMANLPPHFRERTERLERNFTVSAVIFKKYEPIFQDIFKYPQEEQPRQQRGRKQRRQPCTVSEIFHFCWVLFIYAKGNFPMISDDLVNSYHLLLCALDLVYGNALQCSNRKELVNPNFKGLSEDFHAKDSKPSSDPPCIIEKLCSLHDGLVLEAKGIKEHFWKPYIRKLYEKKLLKGKEENLTGFLEPGNFGESFKAINKAYEEYVLSVGNLDERIFLGEDAEEEIGTLSRCLNAGSGTETAERVQMKNILQQHFDKSKALRISTPLTGVRYIKENSPCVTPVSTATHSLSRLHTMLTGLRNAPSEKLEQILRTCSRDPTQAIANRLKEMFEIYSQHFQPDEDFSNCAKEIASKHFRFAEMLYYKVLESVIEQEQKRLGDMDLSGILEQDAFHRSLLACCLEVVTFSYKPPGNFPFITEIFDVPLYHFYKVIEVFIRAEDGLCREVVKHLNQIEEQILDHLAWKPESPLWEKIRDNENRVPTCEEVMPPQNLERADEICIAGSPLTPRRVTEVRADTGGLGRSITSPTTLYDRYSSPPASTTRRRLFVENDSPSDGGTPGRMPPQPLVNAVPVQNVSGETVSVTPVPGQTLVTMATATVTANNGQTVTIPVQGIANENGGITFFPVQVNVGGQAQAVTGSIQPLSAQALAGSLSSQQVTGTTLQVPGQVAIQQISPGGQQQKQGQSVTSSSNRPRKTSSLSLFFRKVYHLAAVRLRDLCAKLDISDELRKKIWTCFEFSIIQCPELMMDRHLDQLLMCAIYVMAKVTKEDKSFQNIMRCYRTQPQARSQVYRSVLIKGKRKRRNSGSSDSRSHQNSPTELNKDRTSRDSSPVMRSSSTLPVPQPSSAPPTPTRLTGANSDMEEEERGDLIQFYNNIYIKQIKTFAMKYSQANMDAPPLSPYPFVRTGSPRRIQLSQNHPVYISPHKNETMLSPREKIFYYFSNSPSKRLREINSMIRTGETPTKKRGILLEDGSESPAKRICPENHSALLRRLQDVANDRGSH

>AAH30144.2

MAACIAAGHWAAMGLGRSFQAARTLLPPPASIACRVHAGPVRQQSTGPSEPGAFQPPPKPVIVDKHRPVEPERRFLSPEFIPRRGRTDPLKFQIERKDMLERRKVLHIPEFYVGSILRVTTADPYASGKISQFLGICIQRSGRGLGATFILRNVIEGQGVEICFELYNPRVQEIQVVKLEKRLDDSLLYLRDALPEYSTFDVNMKPVVQEPNQKVPVNELKVKMKPKPWSKRWERPNFNIKGIRFDLCLTEQQMKEAQKWNQPWLEFDMMREYDTSKIEAAIWKEIEASKRS

865 P08651 NP_004125.3

>P08651

MYSSPLCLTQDEFHPFIEALLPHVRAFAYTWFNLQARKRKYFKKHEKRMSKDEERAVKDELLGEKPEVKQKWASRLLAKLRKDIRPECREDFVLSITGKKAPGCVLSNPDQKGKMRRIDCLRQADKVWRLDLVMVILFKGIPLESTDGERLVKAAQCGHPVLCVQPHHIGVAVKELDLYLAYFVRERDAEQSGSPRTGMGSDQEDSKPITLDTTDFQESFVTSGVFSVTELIQVSRTPVVTGTGPNFSLGELQGHLAYDLNPASTGLRRTLPSTSSSGSKRHKSGSMEEDVDTSPGGDYYTSPSSPTSSSRNWTEDMEGGISSPVKKTEMDKSPFNSPSPQDSPRLSSFTQHHRPVIAVHSGIARSPHPSSALHFPTTSILPQTASTYFPHTAIRYPPHLNPQDPLKDLVSLACDPASQQPGPLNGSGQLKMPSHCLSAQMLAPPPPGLPRLALPPATKPATTSEGGATSPTSPSYSPPDTSPANRSFVGLGPRDPAGIYQAQSWYLG

>NP_004125.3

MISASRAAAARLVGAAASRGPTAARHQDSWNGLSHEAFRLVSRRDYASEAIKGAVVGIDLGTTNSCVAVMEGKQAKVLENAEGARTTPSVVAFTADGERLVGMPAKRQAVTNPNNTFYATKRLIGRRYDDPEVQKDIKNVPFKIVRASNGDAWVEAHGKLYSPSQIGAFVLMKMKETAENYLGHTAKNAVITVPAYFNDSQRQATKDAGQISGLNVLRVINEPTAAALAYGLDKSEDKVIAVYDLGGGTFDISILEIQKGVFEVKSTNGDTFLGGEDFDQALLRHIVKEFKRETGVDLTKDNMALQRVREAAEKAKCELSSSVQTDINLPYLTMDSSGPKHLNMKLTRAQFEGIVTDLIRRTIAPCQKAMQDAEVSKSDIGEVILVGGMTRMPKVQQTVQDLFGRAPSKAVNPDEAVAIGAAIQGGVLAGDVTDVLLLDVTPLSLGIETLGGVFTKLINRNTTIPTKKSQVFSTAADGQTQVEIKVCQGEREMAGDNKLLGQFTLIGIPPAPRGVPQIEVTFDIDANGIVHVSAKDKGTGREQQIVIQSSGGLSKDDIENMVKNAEKYAEEDRRKKERVEAVNMAEGIIHDTETKMEEFKDQLPADECNKLKEEISKMRELLARKDSETGENIRQAASSLQQASLKLFEMAYKKMASEREGSGSSGTGEQKEDQKEEKQ

866 A6NEQ0 Q8N427

>A6NEQ0

MVEADHPGKLFIGGLNRETNEKMLKAVFGKHGPISEVLLIKDRTSKSRGFAFITFENPADAKNAAKDMNGKSLHGKAIKVEQAKKPSFQSGGRRRPPASSRNRSPSGSLRSARGSRGGTRGWLPSQEGHLDDGGYTPDLKMSYSRGLIPVKRGPSSRSGGPPPKKSAPSAVARSNSWMGSQGPMSQRRENYGVPPRRATISSWRNDRMSTRHDGYATNDGNHPSCQETRDYAPPSRGYAYRDNGHSNRDEHSSRGYRNHRSSRETRDYAPPSRGHAYRDYGHSRRDESYSRGYRNRRSSRETREYAPPSRGHGYRDYGHSRRHESYSRGYRNHPSSRETRDYAPPHRDYAYRDYGHSSWDEHSSRGYSYHDGYGEALGRDHSEHLSGSSYRDALQRYGTAHGAPPARGPRMSYGGSTCHAYSNTRDRYGRSWESYSSCGDFHYCDREHVCRKDQRNPPSLGRVLPDPREACGSSSYVASIVDGGESRSEKGDSSRY

>Q8N427

MASKKREVQLQTVINNQSLWDEMLQNKGLTVIDVYQAWCGPCRAMQPLFRKLKNELNEDEILHFAVAEADNIVTLQPFRDKCEPVFLFSVNGKIIEKIQGANAPLVNKKVINLIDEERKIAAGEMARPQYPEIPLVDSDSEVSEESPCESVQELYSIAIIKPDAVISKKVLEIKRKITKAGFIIEAEHKTVLTEEQVVNFYSRIADQCDFEEFVSFMTSGLSYILVVSQGSKHNPPSEETEPQTDTEPNERSEDQPEVEAQVTPGMMKNKQDSLQEYLERQHLAQLCDIEEDAANVAKFMDAFFPDFKKMKSMKLEKTLALLRPNLFHERKDDVLRIIKDEDFKILEQRQVVLSEKEAQALCKEYENEDYFNKLIENMTSGPSLALVLLRDNGLQYWKQLLGPRTVEEAIEYFPESLCAQFAMDSLPVNQLYGSDSLETAEREIQHFFPLQSTLGLIKPHATSEQREQILKIVKEAGFDLTQVKKMFLTPEQIEKIYPKVTGKDFYKDLLEMLSVGPSMVMILTKWNAVAEWRRLMGPTDPEEAKLLSPDSIRAQFGISKLKNIVHGASNAYEAKEVVNRLFEDPEEN

867 Q9C002 NP_003945.2

>Q9C002

MSFFQLLMKRKELIPLVVFMTVAAGGASSFAVYSLWKTDVILDRKKNPEPWETVDPTVPQKLITINQQWKPIEELQNVQRVTK

>NP_003945.2

MAVMEMACPGAPGSAVGQQKELPKAKEKTPPLGKKQSSVYKLEAVEKSPVFCGKWEILNDVITKGTAKEGSEAGPAAISIIAQAECENSQEFSPTFSERIFIAGSKQYSQSESLDQIPNNVAHATEGKMARVCWKGKRRSKARKKRKKKSSKSLAHAGVALAKPLPRTPEQESCTIPVQEDESPLGAPYVRNTPQFTKPLKEPGLGQLCFKQLGEGLRPALPRSELHKLISPLQCLNHVWKLHHPQDGGPLPLPTHPFPYSRLPHPFPFHPLQPWKPHPLESFLGKLACVDSQKPLPDPHLSKLACVDSPKPLPGPHLEPSCLSRGAHEKFSVEEYLVHALQGSVSSGQAHSLTSLAKTWAARGSRSREPSPKTEDNEGVLLTEKLKPVDYEYREEVHWATHQLRLGRGSFGEVHRMEDKQTGFQCAVKKVRLEVFRAEELMACAGLTSPRIVPLYGAVREGPWVNIFMELLEGGSLGQLVKEQGCLPEDRALYYLGQALEGLEYLHSRRILHGDVKADNVLLSSDGSHAALCDFGHAVCLQPDGLGKSLLTGDYIPGTETHMAPEVVLGRSCDAKVDVWSSCCMMLHMLNGCHPWTQFFRGPLCLKIASEPPPVREIPPSCAPLTAQAIQEGLRKEPIHRVSAAELGGKVNRALQQVGGLKSPWRGEYKEPRHPPPNQANYHQTLHAQPRELSPRAPGPRPAEETTGRAPKLQPPLPPEPPEPNKSPPLTLSKEESGMWEPLPLSSLEPAPARNPSSPERKATVPEQELQQLEIELFLNSLSQPFSLEEQEQILSCLSIDSLSLSDDSEKNPSKASQSSRDTLSSGVHSWSSQAEARSSSWNMVLARGRPTDTPSYFNGVKVQIQSLNGEHLHIREFHRVKVGDIATGISSQIPAAAFSLVTKDGQPVRYDMEVPDSGIDLQCTLAPDGSFAWSWRVKHGQLENRP

868 Q03112 NP_000174.1

>Q03112

MKSEDYPHETMAPDIHEERQYRCEDCDQLFESKAELADHQKFPCSTPHSAFSMVEEDFQQKLESENDLQEIHTIQECKECDQVFPDLQSLEKHMLSHTEEREYKCDQCPKAFNWKSNLIRHQMSHDSGKHYECENCAKVFTDPSNLQRHIRSQHVGARAHACPECGKTFATSSGLKQHKHIHSSVKPFICEVCHKSYTQFSNLCRHKRMHADCRTQIKCKDCGQMFSTTSSLNKHRRFCEGKNHFAAGGFFGQGISLPGTPAMDKTSMVNMSHANPGLADYFGANRHPAGLTFPTAPGFSFSFPGLFPSGLYHRPPLIPASSPVKGLSSTEQTNKSQSPLMTHPQILPATQDILKALSKHPSVGDNKPVELQPERSSEERPFEKISDQSESSDLDDVSTPSGSDLETTSGSDLESDIESDKEKFKENGKMFKDKVSPLQNLASINNKKEYSNHSIFSPSLEEQTAVSGAVNDSIKAIASIAEKYFGSTGLVGLQDKKVGALPYPSMFPLPFFPAFSQSMYPFPDRDLRSLPLKMEPQSPGEVKKLQKGSSESPFDLTTKRKDEKPLTPVPSKPPVTPATSQDQPLDLSMGSRSRASGTKLTEPRKNHVFGGKKGSNVESRPASDGSLQHARPTPFFMDPIYRVEKRKLTDPLEALKEKYLRPSPGFLFHPQFQLPDQRTWMSAIENMAEKLESFSALKPEASELLQSVPSMFNFRAPPNALPENLLRKGKERYTCRYCGKIFPRSANLTRHLRTHTGEQPYRCKYCDRSFSISSNLQRHVRNIHNKEKPFKCHLCDRCFGQQTNLDRHLKKHENGNMSGTATSSPHSELESTGAILDDKEDAYFTEIRNFIGNSNHGSQSPRNVEERMNGSHFKDEKALVTSQNSDLLDDEEVEDEVLLDEEDEDNDITGKTGKEPVTSNLHEGNPEDDYEETSALEMSCKTSPVRYKEEEYKSGLSALDHIRHFTDSLKMRKMEDNQYSEAELSSFSTSHVPEELKQPLHRKSKSQAYAMMLSLSDKESLHSTSHSSSNVWHSMARAAAESSAIQSISHV

>NP_000174.1

MTILTYPFKNLPTASKWALRFSIRPLSCSSQLRAAPAVQTKTKKTLAKPNIRNVVVVDGVRTPFLLSGTSYKDLMPHDLARAALTGLLHRTSVPKEVVDYIIFGTVIQEVKTSNVAREAALGAGFSDKTPAHTVTMACISANQAMTTGVGLIASGQCDVIVAGGVELMSDVPIRHSRKMRKLMLDLNKAKSMGQRLSLISKFRFNFLAPELPAVSEFSTSETMGHSADRLAAAFAVSRLEQDEYALRSHSLAKKAQDEGLLSDVVPFKVPGKDTVTKDNGIRPSSLEQMAKLKPAFIKPYGTVTAANSSFLTDGASAMLIMAEEKALAMGYKPKAYLRDFMYVSQDPKDQLLLGPTYATPKVLEKAGLTMNDIDAFEFHEAFSGQILANFKAMDSDWFAENYMGRKTKVGLPPLEKFNNWGGSLSLGHPFGATGCRLVMAAANRLRKEGGQYGLVAACAAGGQGHAMIVEAYPK

869 Q96T76 NP_065076.2

>Q96T76

MAAAAAVEAAAPMGALWGLVHDFVVGQQEGPADQVAADVKSGNYTVLQVVEALGSSLENPEPRTRARAIQLLSQVLLHCHTLLLEKEVVHLILFYENRLKDHHLVIPSVLQGLKALSLCVALPPGLAVSVLKAIFQEVHVQSLPQVDRHTVYNIITNFMRTREEELKSLGADFTFGFIQVMDGEKDPRNLLVAFRIVHDLISRDYSLGPFVEELFEVTSCYFPIDFTPPPNDPHGIQREDLILSLRAVLASTPRFAEFLLPLLIEKVDSEVLSAKLDSLQTLNACCAVYGQKELKDFLPSLWASIRREVFQTASERVEAEGLAALHSLTACLSRSVLRADAEDLLDSFLSNILQDCRHHLCEPDMKLVWPSAKLLQAAAGASARACDSVTSNVLPLLLEQFHKHSQSSQRRTILEMLLGFLKLQQKWSYEDKDQRPLNGFKDQLCSLVFMALTDPSTQLQLVGIRTLTVLGAQPDLLSYEDLELAVGHLYRLSFLKEDSQSCRVAALEASGTLAALYPVAFSSHLVPKLAEELRVGESNLTNGDEPTQCSRHLCCLQALSAVSTHPSIVKETLPLLLQHLWQVNRGNMVAQSSDVIAVCQSLRQMAEKCQQDPESCWYFHQTAIPCLLALAVQASMPEKEPSVLRKVLLEDEVLAAMVSVIGTATTHLSPELAAQSVTHIVPLFLDGNVSFLPENSFPSRFQPFQDGSSGQRRLIALLMAFVCSLPRNVEIPQLNQLMRELLELSCCHSCPFSSTAAAKCFAGLLNKHPAGQQLDEFLQLAVDKVEAGLGSGPCRSQAFTLLLWVTKALVLRYHPLSSCLTARLMGLLSDPELGPAAADGFSLLMSDCTDVLTRAGHAEVRIMFRQRFFTDNVPALVQGFHAAPQDVKPNYLKGLSHVLNRLPKPVLLPELPTLLSLLLEALSCPDCVVQLSTLSCLQPLLLEAPQVMSLHVDTLVTKFLNLSSSPSMAVRIAALQCMHALTRLPTPVLLPYKPQVIRALAKPLDDKKRLVRKEAVSARGEWFLLGSPGS

>NP_065076.2

MFSRRSHGDVKKSTQKVLDPKKDVLTRLKHLRALLDNVDANDLKQFFETNYSQIYFIFYENFIALENSLKLKGNNKSQREELDSILFLFEKILQFLPERIFFRWHYQSIGSTLKKLLHTGNSIKIRCEGIRLFLLWLQALQTNCAEEQVLIFACLVPGFPAVMSSRGPCTLETLINPSPSVADVKIYPEEITPLLPAISGEKIAEDQTCFFLQILLKYMVIQAASLEWKNKENQDTGFKFLFTLFRKYYLPHLFPSFTKLTNIYKPVLDIPHLRPKPVYITTTRDNENIYSTKIPYMAARVVFIKWIVTFFLEKKYLTATQNTKNGVDVLPKIIQTVGGGAVQERAPELDGGGPTEQDKSHSNSSTLSDRRLSNSSLCSIEEEHRMVYEMVQRILLSTRGYVNFVNEVFHQAFLLPSCEIAVTRKVVQVYRKWILQDKPVFMEEPDRKDVAQEDAEKLGFSETDSKEASSESSGHKRSSSWGRTYSFTSAMSRGCVTEEENTNVKAGVQALLQVFLTNSANIFLLEPCAEVPVLLKEQVDACKAVLIIFRRMIMELTMNKKTWEQMLQILLRITEAVMQKPKDKQIKDLFAQSLAGLLFRTLMVAWIRANLCVYISRELWDDFLGVLSSLTEWEELINEWANIMDSLTAVLARTVYGVEMTNLPLDKLSEQKEKKQRGKGCVLDPQKGTTVGRSFSLSWRSHPDVTEPMRFRSATTSGAPGVEKARNIVRQKATEVEECQQSENAPAAGSGHLTVGQQQQVLRSSSTSDIPEPLCSDSSQGQKAENTQNSSSSEPQPIQENKGHVKREHEGITILVRRSSSPAELDLKDDLQQTQGKCRERQKSESTNSDTTLGCTNEAELSMGPWQTCEEDPELNTPTDVVADADARHWLQLSPTDASNLTDSSECLTDDCSIIAGGSLTGWHPDSAAVLWRRVLGILGDVNNIQSPKIHARVFCYLYELWYKLAKIRDNLAISLDNQSSPSPPVLIPPLRMFASWLFKAATLPNEYKEGKLQAYRLICAMMTRRQDVLPNSDFLVHFYLVMHLGLTSEDQDILNTIIRHCPPRFFSLGFPGFSMLVGDFITAAARVLSTDILTAPRSEAVTVLGSLVCFPNTYQEIPLLQSVPEVNEAITGTEDVKHYLINILLKNATEEPNEYARCIAVCSLGVWICEELAQCTSHPQVKEAINVIGVTLKFPNKIVAQVACDVLQLLVSYWEKLQMFETSLPRKMAEILVATVAFLLPSAEYSSVETDKKFIVSLLLCLLDWCMALPVSVLLHPVSTAVLEEQHSARAPLLDYIYRVLHCCVCGSSTYTQQSHYILTLADLSSTDYDPFLPLANVKSSEPVQYHSSAELGNLLTVEEEKKRRSLELIPLTARMVMAHLVNHLGHYPLSGGPAILHSLVSENHDNAHVEGSELSFEVFRSPNLQLFVFNDSTLISYLQTPTEGPVGGSPVGSLSDVRVIVRDISGKYSWDGKVLYGPLEGCLAPNGRNPSFLISSWHRDTFGPQKDSSQVEEGDDVLDKLLENIGHTSPECLLPSQLNLNEPSLTPCGMNYDQEKEIIEVILRQNAQEDEYIQSHNFDSAMKVTSQGQPSPVEPRGPFYFCRLLLDDLGMNSWDRRKNFHLLKKNSKLLRELKNLDSRQCRETHKIAVFYIAEGQEDKCSILSNERGSQAYEDFVAGLGWEVDLSTHCGFMGGLQRNGSTGQTAPYYATSTVEVIFHVSTRMPSDSDDSLTKKLRHLGNDEVHIVWSEHSRDYRRGIIPTAFGDVSIIIYPMKNHMFFIAITKKPEVPFFGPLFDGAIVSGKLLPSLVCATCINASRAVKCLIPLYQSFYEERALYLEAIIQNHREVMTFEDFAAQVFSPSPSYSLSGTD

870 NP_004955.2 NP_001503.1

>NP_004955.2

MAQTQGTRRKVCYYYDGDVGNYYYGQGHPMKPHRIRMTHNLLLNYGLYRKMEIYRPHKANAEEMTKYHSDDYIKFLRSIRPDNMSEYSKQMQRFNVGEDCPVFDGLFEFCQLSTGGSVASAVKLNKQQTDIAVNWAGGLHHAKKSEASGFCYVNDIVLAILELLKYHQRVLYIDIDIHHGDGVEEAFYTTDRVMTVSFHKYGEYFPGTGDLRDIGAGKGKYYAVNYPLRDGIDDESYEAIFKPVMSKVMEMFQPSAVVLQCGSDSLSGDRLGCFNLTIKGHAKCVEFVKSFNLPMLMLGGGGYTIRNVARCWTYETAVALDTEIPNELPYNDYFEYFGPDFKLHISPSNMTNQNTNEYLEKIKQRLFENLRMLPHAPGVQMQAIPEDAIPEESGDEDEDDPDKRISICSSDKRIACEEEFSDSEEEGEGGRKNSSNFKKAKRVKTEDEKEKDPEEKKEVTEEEKTKEEKPEAKGVKEEVKLA

>NP_001503.1

MAARPKLHYPNGRGRMESVRWVLAAAGVEFDEEFLETKEQLYKLQDGNHLLFQQVPMVEIDGMKLVQTRSILHYIADKHNLFGKNLKERTLIDMYVEGTLDLLELLIMHPFLKPDDQQKEVVNMAQKAIIRYFPVFEKILRGHGQSFLVGNQLSLADVILLQTILALEEKIPNILSAFPFLQEYTVKLSNIPTIKRFLEPGSKKKPPPDEIYVRTVYNIFRP

871 O94953 NP_000467.1

>O94953

MGSEDHGAQNPSCKIMTFRPTMEEFKDFNKYVAYIESQGAHRAGLAKIIPPKEWKPRQTYDDIDDVVIPAPIQQVVTGQSGLFTQYNIQKKAMTVGEYRRLANSEKYCTPRHQDFDDLERKYWKNLTFVSPIYGADISGSLYDDDVAQWNIGSLRTILDMVERECGTIIEGVNTPYLYFGMWKTTFAWHTEDMDLYSINYLHFGEPKSWYAIPPEHGKRLERLAIGFFPGSSQGCDAFLRHKMTLISPIILKKYGIPFSRITQEAGEFMITFPYGYHAGFNHGFNCAESTNFATLRWIDYGKVATQCTCRKDMVKISMDVFVRILQPERYELWKQGKDLTVLDHTRPTALTSPELSSWSASRASLKAKLLRRSHRKRSQPKKPKPEDPKFPGEGTAGAALLEEAGGSVKEEAGPEVDPEEEEEEPQPLPHGREAEGAEEDGRGKLRPTKAKSERKKKSFGLLPPQLPPPPAHFPSEEALWLPSPLEPPVLGPGPAAMEESPLPAPLNVVPPEVPSEELEAKPRPIIPMLYVVPRPGKAAFNQEHVSCQQAFEHFAQKGPTWKEPVSPMELTGPEDGAASSGAGRMETKARAGEGQAPSTFSKLKMEIKKSRRHPLGRPPTRSPLSVVKQEASSDEEASPFSGEEDVSDPDALRPLLSLQWKNRAASFQAERKFNAAAARTEPYCAICTLFYPYCQALQTEKEAPIASLGKGCPATLPSKSRQKTRPLIPEMCFTSGGENTEPLPANSYIGDDGTSPLIACGKCCLQVHASCYGIRPELVNEGWTCSRCAAHAWTAECCLCNLRGGALQMTTDRRWIHVICAIAVPEARFLNVIERHPVDISAIPEQRWKLKCVYCRKRMKKVSGACIQCSYEHCSTSFHVTCAHAAGVLMEPDDWPYVVSITCLKHKSGGHAVQLLRAVSLGQVVITKNRNGLYYRCRVIGAASQTCYEVNFDDGSYSDNLYPESITSRDCVQLGPPSEGELVELRWTDGNLYKAKFISSVTSHIYQVEFEDGSQLTVKRGDIFTLEEELPKRVRSRLSLSTGAPQEPAFSGEEAKAAKRPRVGTPLATEDSGRSQDYVAFVESLLQVQGRPGAPF

>NP_000467.1

MEEKLKKTKIIFVVGGPGSGKGTQCEKIVQKYGYTHLSTGDLLRSEVSSGSARGKKLSEIMEKGQLVPLETVLDMLRDAMVAKVNTSKGFLIDGYPREVQQGEEFERRIGQPTLLLYVDAGPETMTQRLLKRGETSGRVDDNEETIKKRLETYYKATEPVIAFYEKRGIVRKVNAEGSVDSVFSQVCTHLDALK

872 Q9UNY5 P15428

>Q9UNY5

MAVSLTAAETLALQGTQGQEKMMMMGPKEEEQSCEYETRLPGNHSTSQEIFRQRFRHLRYQETPGPREALSQLRVLCCEWLRPEKHTKEQILEFLVLEQFLTILPEELQSWVRGHHPKSGEEAVTVLEDLEKGLEPEPQVPGPAHGPAQEEPWEKKESLGAAQEALSIQLQPKETQPFPKSEQVYLHFLSVVTEDGPEPKDKGSLPQPPITEVESQVFSEKLATDTSTFEATSEGTLELQQRNPKAERLRWSPAQEESFRQMVVIHKEIPTGKKDHECSECGKTFIYNSHLVVHQRVHSGEKPYKCSDCGKTFKQSSNLGQHQRIHTGEKPFECNECGKAFRWGAHLVQHQRIHSGEKPYECNECGKAFSQSSYLSQHRRIHSGEKPFICKECGKAYGWCSELIRHRRVHARKEPSH

>P15428

MHVNGKVALVTGAAQGIGRAFAEALLLKGAKVALVDWNLEAGVQCKAALDEQFEPQKTLFIQCDVADQQQLRDTFRKVVDHFGRLDILVNNAGVNNEKNWEKTLQINLVSVISGTYLGLDYMSKQNGGEGGIIINMSSLAGLMPVAQQPVYCASKHGIVGFTRSAALAANLMNSGVRLNAICPGFVNTAILESIEKEENMGQYIEYKDHIKDMIKYYGILDPPLIANGLITLIEDDALNGAIMKITTSKGIHFQDYDTTPFQAKTQ

873 NP_000929.1 NP_077015.1

>NP_000929.1

MYDADEDMQYDEDDDEITPDLWQEACWIVISSYFDEKGLVRQQLDSFDEFIQMSVQRIVEDAPPIDLQAEAQHASGEVEEPPRYLLKFEQIYLSKPTHWERDGAPSPMMPNEARLRNLTYSAPLYVDITKTVIKEGEEQLQTQHQKTFIGKIPIMLRSTYCLLNGLTDRDLCELNECPLDPGGYFIINGSEKVLIAQEKMATNTVYVFAKKDSKYAYTGECRSCLENSSRPTSTIWVSMLARGGQGAKKSAIGQRIVATLPYIKQEVPIIIVFRALGFVSDRDILEHIIYDFEDPEMMEMVKPSLDEAFVIQEQNVALNFIGSRGAKPGVTKEKRIKYAKEVLQKEMLPHVGVSDFCETKKAYFLGYMVHRLLLAALGRRELDDRDHYGNKRLDLAGPLLAFLFRGMFKNLLKEVRIYAQKFIDRGKDFNLELAIKTRIISDGLKYSLATGNWGDQKKAHQARAGVSQVLNRLTFASTLSHLRRLNSPIGRDGKLAKPRQLHNTLWGMVCPAETPEGHAVGLVKNLALMAYISVGSQPSPILEFLEEWSMENLEEISPAAIADATKIFVNGCWVGIHKDPEQLMNTLRKLRRQMDIIVSEVSMIRDIREREIRIYTDAGRICRPLLIVEKQKLLLKKRHIDQLKEREYNNYSWQDLVASGVVEYIDTLEEETVMLAMTPDDLQEKEVAYCSTYTHCEIHPSMILGVCASIIPFPDHNQSPRNTYQSAMGKQAMGVYITNFHVRMDTLAHVLYYPQKPLVTTRSMEYLRFRELPAGINSIVAIASYTGYNQEDSVIMNRSAVDRGFFRSVFYRSYKEQESKKGFDQEEVFEKPTRETCQGMRHAIYDKLDDDGLIAPGVRVSGDDVIIGKTVTLPENEDELESTNRRYTKRDCSTFLRTSETGIVDQVMVTLNQEGYKFCKIRVRSVRIPQIGDKFASRHGQKGTCGIQYRQEDMPFTCEGITPDIIINPHAIPSRMTIGHLIECLQGKVSANKGEIGDATPFNDAVNVQKISNLLSDYGYHLRGNEVLYNGFTGRKITSQIFIGPTYYQRLKHMVDDKIHSRARGPIQILNRQPMEGRSRDGGLRFGEMERDCQIAHGAAQFLRERLFEASDPYQVHVCNLCGIMAIANTRTHTYECRGCRNKTQISLVRMPYACKLLFQELMSMSIAPRMMSV

>NP_077015.1

MGELCRRDSALTALDEETLWEMMESHRHRIVRCICPSRLTPYLRQAKVLCQLDEEEVLHSPRLTNSAMRAGHLLDLLKTRGKNGAIAFLESLKFHNPDVYTLVTGLQPDVDFSNFSGLMETSKLTECLAGAIGSLQEELNQEKGQKEVLLRRCQQLQEHLGLAETRAEGLHQLEADHSRMKREVSAHFHEVLRLKDEMLSLSLHYSNALQEKELAASRCRSLQEELYLLKQELQRANMVSSCELELQEQSLRTASDQESGDEELNRLKEENEKLRSLTFSLAEKDILEQSLDEARGSRQELVERIHSLRERAVAAERQREQYWEEKEQTLLQFQKSKMACQLYREKVNALQAQVCELQKERDQAYSARDSAQREISQSLVEKDSLRRQVFELTDQVCELRTQLRQLQAEPPGVLKQEARTREPCPREKQRLVRMHAICPRDDSDCSLVSSTESQLLSDLSATSSRELVDSFRSSSPAPPSQQSLYKRVAEDFGEEPWSFSSCLEIPEGDPGALPGAKAGDPHLDYELLDTADLPQLESSLQPVSPGRLDVSESGVLMRRRPARRILSQVTMLAFQGDALLEQISVIGGNLTGIFIHRVTPGSAADQMALRPGTQIVMVDYEASEPLFKAVLEDTTLEEAVGLLRRVDGFCCLSVKVNTDGYKRLLQDLEAKVATSGDSFYIRVNLAMEGRAKGELQVHCNEVLHVTDTMFQGCGCWHAHRVNSYTMKDTAAHGTIPNYSRAQQQLIALIQDMTQQCTVTRKPSSGGPQKLVRIVSMDKAKASPLRLSFDRGQLDPSRMEGSSTCFWAESCLTLVPYTLVWPHRPARPRPVLLVPRAVGKILSEKLCLLQGFKKCLAEYLSQEEYEAWSQRGDIIQEGEVSGGRCWVTRHAVESLMEKNTHALLDVQLDSVCTLHRMDIFPIVIHVSVNEKMAKKLKKGLQRLGTSEEQLLEAARQEEGDLDRAPCLYSSLAPDGWSDLDGLLSCVRQAIADEQKKVVWTEQSPR

874 NP_001460.1 NP_006432.1

>NP_001460.1

MSGWESYYKTEGDEEAEEEQEENLEASGDYKYSGRDSLIFLVDASKAMFESQSEDELTPFDMSIQCIQSVYISKIISSDRDLLAVVFYGTEKDKNSVNFKNIYVLQELDNPGAKRILELDQFKGQQGQKRFQDMMGHGSDYSLSEVLWVCANLFSDVQFKMSHKRIMLFTNEDNPHGNDSAKASRARTKAGDLRDTGIFLDLMHLKKPGGFDISLFYRDIISIAEDEDLRVHFEESSKLEDLLRKVRAKETRKRALSRLKLKLNKDIVISVGIYNLVQKALKPPPIKLYRETNEPVKTKTRTFNTSTGGLLLPSDTKRSQIYGSRQIILEKEETEELKRFDDPGLMLMGFKPLVLLKKHHYLRPSLFVYPEESLVIGSSTLFSALLIKCLEKEVAALCRYTPRRNIPPYFVALVPQEEELDDQKIQVTPPGFQLVFLPFADDKRKMPFTEKIMATPEQVGKMKAIVEKLRFTYRSDSFENPVLQQHFRNLEALALDLMEPEQAVDLTLPKVEAMNKRLGSLVDEFKELVYPPDYNPEGKVTKRKHDNEGSGSKRPKVEYSEEELKTHISKGTLGKFTVPMLKEACRAYGLKSGLKKQELLEALTKHFQD

>NP_006432.1

MAAAAVSSAKRSLRGELKQRLRAMSAEERLRQSRVLSQKVIAHSEYQKSKRISIFLSMQDEIETEEIIKDIFQRGKICFIPRYRFQSNHMDMVRIESPEEISLLPKTSWNIPQPGEGDVREEALSTGGLDLIFMPGLGFDKHGNRLGRGKGYYDAYLKRCLQHQEVKPYTLALAFKEQICLQVPVNENDMKVDEVLYEDSSTA

875 P50539 NP_000847.2

>P50539

MERVKMINVQRLLEAAEFLERRERECEHGYASSFPSMPSPRLQHSKPPRRLSRAQKHSSGSSNTSTANRSTHNELEKNRRAHLRLCLERLKVLIPLGPDCTRHTTLGLLNKAKAHIKKLEEAERKSQHQLENLEREQRFLKWRLEQLQGPQEMERIRMDSIGSTISSDRSDSEREEIEVDVESTEFSHGEVDNISTTSISDIDDHSSLPSIGSDEGYSSASVKLSFTS

>NP_000847.2

MFCTKLKDLKITGECPFSLLAPGQVPNESSEEAAGSSESCKATVPICQDIPEKNIQESLPQRKTSRSRVYLHTLAESICKLIFPEFERLNVALQRTLAKHKIKESRKSLEREDFEKTIAEQAVAAGVPVEVIKESLGEEVFKICYEEDENILGVVGGTLKDFLNSFSTLLKQSSHCQEAGKRGRLEDASILCLDKEDDFLHVYYFFPKRTTSLILPGIIKAAAHVLYETEVEVSLMPPCFHNDCSEFVNQPYLLYSVHMKSTKPSLSPSKPQSSLVIPTSLFCKTFPFHFMFDKDMTILQFGNGIRRLMNRRDFQGKPNFEEYFEILTPKINQTFSGIMTMLNMQFVVRVRRWDNSVKKSSRVMDLKGQMIYIVESSAILFLGSPCVDRLEDFTGRGLYLSDIPIHNALRDVVLIGEQARAQDGLKKRLGKLKATLEQAHQALEEEKKKTVDLLCSIFPCEVAQQLWQGQVVQAKKFSNVTMLFSDIVGFTAICSQCSPLQVITMLNALYTRFDQQCGELDVYKVETIGDAYCVAGGLHKESDTHAVQIALMALKMMELSDEVMSPHGEPIKMRIGLHSGSVFAGVVGVKMPRYCLFGNNVTLANKFESCSVPRKINVSPTTYRLLKDCPGFVFTPRSREELPPNFPSEIPGICHFLDAYQQGTNSKPCFQKKDVEDGNANFLGKASGID

876 O75486 P20591

>O75486

MYSPRGSQGRGTAEATANSPSPPIAPSHSRVTFSLSTLHTLSPPPRPFPSVSRAAAQKPHHLHPHILLAGSAAVPPRVLKAEMNNTAASPMSTATSSSGRSTGKSISFATELQSMMYSLGDARRPLHETAVLVEDVVHTQLINLLQQAAEVSQLRGARVITPEDLLFLMRKDKKKLRRLLKYMFIRDYKSKIVKGIDEDDLLEDKLSGSNNANKRQKIAQDFLNSIDQTGELLAMFEDDEIDEVKQERMERAERQTRIMDSAQYAEFCESRQLSFSKKASKFRDWLDCSSMEIKPNVVAMEILAYLAYETVAQLVDLALLVRQDMVTKAGDPFSHAISATFIQYHNSAESTAACGVEAHSDAIQPCHIREAIRRYSHRIGPLSPFTNAYRRNGMAFLAC

>P20591

MVVSEVDIAKADPAAASHPLLLNGDATVAQKNPGSVAENNLCSQYEEKVRPCIDLIDSLRALGVEQDLALPAIAVIGDQSSGKSSVLEALSGVALPRGSGIVTRCPLVLKLKKLVNEDKWRGKVSYQDYEIEISDASEVEKEINKAQNAIAGEGMGISHELITLEISSRDVPDLTLIDLPGITRVAVGNQPADIGYKIKTLIKKYIQRQETISLVVVPSNVDIATTEALSMAQEVDPEGDRTIGILTKPDLVDKGTEDKVVDVVRNLVFHLKKGYMIVKCRGQQEIQDQLSLSEALQREKIFFENHPYFRDLLEEGKATVPCLAEKLTSELITHICKSLPLLENQIKETHQRITEELQKYGVDIPEDENEKMFFLIDKVNAFNQDITALMQGEETVGEEDIRLFTRLRHEFHKWSTIIENNFQEGHKILSRKIQKFENQYRGRELPGFVNYRTFETIVKQQIKALEEPAVDMLHTVTDMVRLAFTDVSIKNFEEFFNLHRTAKSKIEDIRAEQEREGEKLIRLHFQMEQIVYCQDQVYRGALQKVREKELEEEKKKKSWDFGAFQSSSATDSSMEEIFQHLMAYHQEASKRISSHIPLIIQFFMLQTYGQQLQKAMLQLLQDKDTYSWLLKERSDTSDKRKFLKERLARLTQARRRLAQFPG

877 NP_002544.1 P15428

>NP_002544.1

MPHLENVVLCRESQVSILQSLFGERHHFSFPSIFIYGHTASGKTYVTQTLLKTLELPHVFVNCVECFTLRLLLEQILNKLNHLSSSEDGCSTEITCETFNDFVRLFKQVTTAENLKDQTVYIVLDKAEYLRDMEANLLPGFLRLQELADRNVTVLFLSEIVWEKFRPNTGCFEPFVLYFPDYSIGNLQKILSHDHPPEYSADFYAAYINILLGVFYTVCRDLKELRHLAVLNFPKYCEPVVKGEASERDTRKLWRNIEPHLKKAMQTVYLREISSSQWEKLQKDDTDPGQLKGLSAHTHVELPYYSKFILIAAYLASYNPARTDKRFFLKHHGKIKKTNFLKKHEKTSNHLLGPKPFPLDRLLAILYSIVDSRVAPTANIFSQITSLVTLQLLTLVGHDDQLDGPKYKCTVSLDFIRAIARTVNFDIIKYLYDFL

>P15428

MHVNGKVALVTGAAQGIGRAFAEALLLKGAKVALVDWNLEAGVQCKAALDEQFEPQKTLFIQCDVADQQQLRDTFRKVVDHFGRLDILVNNAGVNNEKNWEKTLQINLVSVISGTYLGLDYMSKQNGGEGGIIINMSSLAGLMPVAQQPVYCASKHGIVGFTRSAALAANLMNSGVRLNAICPGFVNTAILESIEKEENMGQYIEYKDHIKDMIKYYGILDPPLIANGLITLIEDDALNGAIMKITTSKGIHFQDYDTTPFQAKTQ

878 Q06265 NP_057130.1

>Q06265

MKETPLSNCERRFLLRAIEEKKRLDGRQTYDYRNIRISFGTDYGCCIVELGKTRVLGQVSCELVSPKLNRATEGILFFNLELSQMAAPAFEPGRQSDLLVKLNRLMERCLRNSKCIDTESLCVVAGEKVWQIRVDLHLLNHDGNIIDAASIAAIVALCHFRRPDVSVQGDEVTLYTPEERDPVPLSIHHMPICVSFAFFQQGTYLLVDPNEREERVMDGLLVIAMNKHREICTIQSSGGIMLLKDQVLRCSKIAGVKVAEITELILKALENDQKVRKEGGKFGFAESIANQRITAFKMEKAPIDTSDVEEKAEEIIAEAEPPSEVVSTPVLWTPGTAQIGEGVENSWGDLEDSEKEDDEGGGDQAIILDGIKMDTGVEVSDIGSQDAPIILSDSEEEEMIILEPDKNPKKIRTQTTSAKQEKAPSKKPVKRRKKKRAAN

>NP_057130.1

MAPPVRYCIPGERLCNLEEGSPGSGTYTRHGYIFSSLAGCLMKSSENGALPVVSVVRETESQLLPDVGAIVTCKVSSINSRFAKVHILYVGSMPLKNSFRGTIRKEDVRATEKDKVEIYKSFRPGDIVLAKVISLGDAQSNYLLTTAENELGVVVAHSESGIQMVPISWCEMQCPKTHTKEFRKVARVQPEFLQT

879 Q16587 NP_005267.2

>Q16587

MEIPAPEPEKTALSSQDPALSLKENLEDISGWGLPEARSKESVSFKDVAVDFTQEEWGQLDSPQRALYRDVMLENYQNLLALGPPLHKPDVISHLERGEEPWSMQREVPRGPCPEWELKAVPSQQQGICKEEPAQEPIMERPLGGAQAWGRQAGALQRSQAAPGRRTCHGLGRPVEEFPLRCPLFAQQRVPEGGPLLDTRKNVQATEGRTKAPARLCAGENASTPSEPEKFPQVRRQRGAGAGEGEFVCGECGKAFRQSSSLTLHRRWHSREKAYKCDECGKAFTWSTNLLEHRRIHTGEKPFFCGECGKAFSCHSSLNVHQRIHTGERPYKCSACEKAFSCSSLLSMHLRVHTGEKPYRCGECGKAFNQRTHLTRHHRIHTGEKPYQCGSCGKAFTCHSSLTVHEKIHSGDKPFKCSDCEKAFNSRSRLTLHQRTHTGEKPFKCADCGKGFSCHAYLLVHRRIHSGEKPFKCNECGKAFSSHAYLIVHRRIHTGEKPFDCSQCWKAFSCHSSLIMHQRIHTGEKPYKCSECGRAFSQNHCLIKHQKIHSGEKSFKCEKCGEMFNWSSHLTEHQRLHSEGKPLAIQFNKHLLSTYYVPGSLLGAGDAGLRDVDPIDALDVAKLLCVVPPRAGRNFSLGSKPRN

>NP_005267.2

MASKKVCIVGSGNWGSAIAKIVGGNAAQLAQFDPRVTMWVFEEDIGGKKLTEIINTQHENVKYLPGHKLPPNVVAVPDVVQAAEDADILIFVVPHQFIGKICDQLKGHLKANATGISLIKGVDEGPNGLKLISEVIGERLGIPMSVLMGANIASEVADEKFCETTIGCKDPAQGQLLKELMQTPNFRITVVQEVDTVEICGALKNVVAVGAGFCDGLGFGDNTKAAVIRLGLMEMIAFAKLFCSGPVSSATFLESCGVADLITTCYGGRNRKVAEAFARTGKSIEQLEKELLNGQKLQGPETARELYSILQHKGLVDKFPLFMAVYKVCYEGQPVGEFIHCLQNHPEHM

880 NP_005307.1 NP_004747.1

>NP_005307.1

MATSSEEVLLIVKKVRQKKQDGALYLMAERIAWAPEGKDRFTISHMYADIKCQKISPEGKAKIQLQLVLHAGDTTNFHFSNESTAVKERDAVKDLLQQLLPKFKRKANKELEEKNRMLQEDPVLFQLYKDLVVSQVISAEEFWANRLNVNATDSSSTSNHKQDVGISAAFLADVRPQTDGCNGLRYNLTSDIIESIFRTYPAVKMKYAENVPHNMTEKEFWTRFFQSHYFHRDRLNTGSKDLFAECAKIDEKGLKTMVSLGVKNPLLDLTALEDKPLDEGYGISSVPSASNSKSIKENSNAAIIKRFNHHSAMVLAAGLRKQEAQNEQTSEPSNMDGNSGDADCFQPAVKRAKLQESIEYEDLGKNNSVKTIALNLKKSDRYYHGPTPIQSLQYATSQDIINSFQSIRQEMEAYTPKLTQVLSSSAASSTITALSPGGALMQGGTQQAINQMVPNDIQSELKHLYVAVGELLRHFWSCFPVNTPFLEEKVVKMKSNLERFQVTKLCPFQEKIRRQYLSTNLVSHIEEMLQTAYNKLHTWQSRRLMKKT

>NP_004747.1

MSRSAAASGGPRRPERHLPPAPCGAPGPPETCRTEPDGAGTMNKLRQSLRRRKPAYVPEASRPHQWQADEDAVRKGTCSFPVRYLGHVEVEESRGMHVCEDAVKKLKAMGRKSVKSVLWVSADGLRVVDDKTKDLLVDQTIEKVSFCAPDRNLDKAFSYICRDGTTRRWICHCFLALKDSGERLSHAVGCAFAACLERKQRREKECGVTAAFDASRTSFAREGSFRLSGGGRPAEREAPDKKKAEAAAAPTVAPGPAQPGHVSPTPATTSPGEKGEAGTPVAAGTTAAAIPRRHAPLEQLVRQGSFRGFPALSQKNSPFKRQLSLRLNELPSTLQRRTDFQVKGTVPEMEPPGAGDSDSINALCTQISSSFASAGAPAPGPPPATTGTSAWGEPSVPPAAAFQPGHKRTPSEAERWLEEVSQVAKAQQQQQQQQQQQQQQQQQQQQAASVAPVPTMPPALQPFPAPVGPFDAAPAQVAVFLPPPHMQPPFVPAYPGLGYPPMPRVPVVGITPSQMVANAFCSAAQLQPQPATLLGKAGAFPPPAIPSAPGSQARPRPNGAPWPPEPAPAPAPELDPFEAQWAALEGKATVEKPSNPFSGDLQKTFEIEL

881 Q96S96 NP_037386.1

>Q96S96

MGWTMRLVTAALLLGLMMVVTGDEDENSPCAHEALLDEDTLFCQGLEVFYPELGNIGCKVVPDCNNYRQKITSWMEPIVKFPGAVDGATYILVMVDPDAPSRAEPRQRFWRHWLVTDIKGADLKKGKIQGQELSAYQAPSPPAHSGFHRYQFFVYLQEGKVISLLPKENKTRGSWKMDRFLNRFHLGEPEASTQFMTQNYQDSPTLQAPRERASEPKHKNQAEIAAC

>NP_037386.1

MQSTSNHLWLLSDILGQGATANVFRGRHKKTGDLFAIKVFNNISFLRPVDVQMREFEVLKKLNHKNIVKLFAIEEETTTRHKVLIMEFCPCGSLYTVLEEPSNAYGLPESEFLIVLRDVVGGMNHLRENGIVHRDIKPGNIMRVIGEDGQSVYKLTDFGAARELEDDEQFVSLYGTEEYLHPDMYERAVLRKDHQKKYGATVDLWSIGVTFYHAATGSLPFRPFEGPRRNKEVMYKIITGKPSGAISGVQKAENGPIDWSGDMPVSCSLSRGLQVLLTPVLANILEADQEKCWGFDQFFAETSDILHRMVIHVFSLQQMTAHKIYIHSYNTATIFHELVYKQTKIISSNQELIYEGRRLVLEPGRLAQHFPKTTEENPIFVVSREPLNTIGLIYEKISLPKVHPRYDLDGDASMAKAITGVVCYACRIASTLLLYQELMRKGIRWLIELIKDDYNETVHKKTEVVITLDFCIRNIEKTVKVYEKLMKINLEAAELGEISDIHTKLLRLSSSQGTIETSLQDIDSRLSPGGSLADAWAHQEGTHPKDRNVEKLQVLLNCMTEIYYQFKKDKAERRLAYNEEQIHKFDKQKLYYHATKAMTHFTDECVKKYEAFLNKSEEWIRKMLHLRKQLLSLTNQCFDIEEEVSKYQEYTNELQETLPQKMFTASSGIKHTMTPIYPSSNTLVEMTLGMKKLKEEMEGVVKELAENNHILERFGSLTMDGGLRNVDCL

882 P10619 P49411

>P10619

MIRAAPPPLFLLLLLLLLLVSWASRGEAAPDQDEIQRLPGLAKQPSFRQYSGYLKGSGSKHLHYWFVESQKDPENSPVVLWLNGGPGCSSLDGLLTEHGPFLVQPDGVTLEYNPYSWNLIANVLYLESPAGVGFSYSDDKFYATNDTEVAQSNFEALQDFFRLFPEYKNNKLFLTGESYAGIYIPTLAVLVMQDPSMNLQGLAVGNGLSSYEQNDNSLVYFAYYHGLLGNRLWSSLQTHCCSQNKCNFYDNKDLECVTNLQEVARIVGNSGLNIYNLYAPCAGGVPSHFRYEKDTVVVQDLGNIFTRLPLKRMWHQALLRSGDKVRMDPPCTNTTAASTYLNNPYVRKALNIPEQLPQWDMCNFLVNLQYRRLYRSMNSQYLKLLSSQKYQILLYNGDVDMACNFMGDEWFVDSLNQKMEVQRRPWLVKYGDSGEQIAGFVKEFSHIAFLTIKGAGHMVPTDKPLAAFTMFSRFLNKQPY

>P49411

MAAATLLRATPHFSGLAAGRTFLLQGLLRLLKAPALPLLCRGLAVEAKKTYVRDKPHVNVGTIGHVDHGKTTLTAAITKILAEGGGAKFKKYEEIDNAPEERARGITINAAHVEYSTAARHYAHTDCPGHADYVKNMITGTAPLDGCILVVAANDGPMPQTREHLLLARQIGVEHVVVYVNKADAVQDSEMVELVELEIRELLTEFGYKGEETPVIVGSALCALEGRDPELGLKSVQKLLDAVDTYIPVPARDLEKPFLLPVEAVYSVPGRGTVVTGTLERGILKKGDECELLGHSKNIRTVVTGIEMFHKSLERAEAGDNLGALVRGLKREDLRRGLVMVKPGSIKPHQKVEAQVYILSKEEGGRHKPFVSHFMPVMFSLTWDMACRIILPPEKELAMPGEDLKFNLILRQPMILEKGQRFTLRDGNRTIGTGLVTNTLAMTEEEKNIKWG

883 O75030 NP_005800.3

>O75030

MQSESGIVPDFEVGEEFHEEPKTYYELKSQPLKSSSSAEHPGASKPPISSSSMTSRILLRQQLMREQMQEQERREQQQKLQAAQFMQQRVPVSQTPAINVSVPTTLPSATQVPMEVLKVQTHLENPTKYHIQQAQRQQVKQYLSTTLANKHANQVLSLPCPNQPGDHVMPPVPGSSAPNSPMAMLTLNSNCEKEGFYKFEEQNRAESECPGMNTHSRASCMQMDDVIDDIISLESSYNEEILGLMDPALQMANTLPVSGNLIDLYGNQGLPPPGLTISNSCPANLPNIKRELTACIFPTESEARALAKERQKKDNHNLIERRRRFNINDRIKELGTLIPKSNDPDMRWNKGTILKASVDYIRKLQREQQRAKELENRQKKLEHANRHLLLRIQELEMQARAHGLSLIPSTGLCSPDLVNRIIKQEPVLENCSQDLLQHHADLTCTTTLDLTDGTITFNNNLGTGTEANQAYSVPTKMGSKLEDILMDDTLSPVGVTDPLLSSVSPGASKTSSRRSSMSMEETEHTC

>NP_005800.3

MASGNARIGKPAPDFKATAVVDGAFKEVKLSDYKGKYVVLFFYPLDFTFVCPTEIIAFSNRAEDFRKLGCEVLGVSVDSQFTHLAWINTPRKEGGLGPLNIPLLADVTRRLSEDYGVLKTDEGIAYRGLFIIDGKGVLRQITVNDLPVGRSVDEALRLVQAFQYTDEHGEVCPAGWKPGSDTIKPNVDDSKEYFSKHN

884 Q9NPC8 NP_000036.2

>Q9NPC8

MSMLPTFGFTQEQVACVCEVLQQGGNIERLGRFLWSLPACEHLHKNESVLKAKAVVAFHRGNFRELYKILESHQFSPHNHAKLQQLWLKAHYIEAEKLRGRPLGAVGKYRVRRKFPLPRSIWDGEETSYCFKEKSRSVLREWYAHNPYPSPREKRELAEATGLTTTQVSNWFKNRRQRDRAAEAKERENNENSNSNSHNPLNGSGKSVLGSSEDEKTPSGTPDHSSSSPALLLSPPPPGLPSLHSLGHPPGPSAVPVPVPGGGGADPLQHHHGLQDSILNPMSANLVDLGS

>NP_000036.2

MSAKSRTIGIIGAPFSKGQPRGGVEEGPTVLRKAGLLEKLKEQECDVKDYGDLPFADIPNDSPFQIVKNPRSVGKASEQLAGKVAEVKKNGRISLVLGGDHSLAIGSISGHARVHPDLGVIWVDAHTDINTPLTTTSGNLHGQPVSFLLKELKGKIPDVPGFSWVTPCISAKDIVYIGLRDVDPGEHYILKTLGIKYFSMTEVDRLGIGKVMEETLSYLLGRKKRPIHLSFDVDGLDPSFTPATGTPVVGGLTYREGLYITEEIYKTGLLSGLDIMEVNPSLGKTPEEVTRTVNTAVAITLACFGLAREGNHKPIDYLNPPK

885 Q70Z53 Q8WVM0

>Q70Z53

MHGHGGYDSDFSDDEHCGESSKRKKRTVEDDLLLQKPFQKEKHGKVAHKQVAAELLDREEARNRRFHLIAMDAYQRHRKFVNDYILYYGGKKEDFKRLGENDKTDLDVIRENHRFLWNEEDEMDMTWEKRLAKKYYDKLFKEYCIADLSKYKENKFGFRWRVEKEVISGKGQFFCGNKYCDKKEGLKSWEVNFGYIEHGEKRNALVKLRLCQECSIKLNFHHRRKEIKSKKRKDKTKKDCEESSHKKSRLSSAEEASKKKDKGHSSSKKSEDSLLRNSDEEESASESELWKGPLPETDEKSQEEEFDEYFQDLFL

>Q8WVM0

MAASGKLSTCRLPPLPTIREIIKLLRLQAAKQLSQNFLLDLRLTDKIVRKAGNLTNAYVYEVGPGPGGITRSILNADVAELLVVEKDTRFIPGLQMLSDAAPGKLRIVHGDVLTFKVEKAFSESLKRPWEDDPPNVHIIGNLPFSVSTPLIIKWLENISCRDGPFVYGRTQMTLTFQKEVAERLAANTGSKQRSRLSVMAQYLCNVRHIFTIPGQAFVPKPEVDVGVVHFTPLIQPKIEQPFKLVEKVVQNVFQFRRKYCHRGLRMLFPEAQRLESTGRLLELADIDPTLRPRQLSISHFKSLCDVYRKMCDEDPQLFAYNFREELKRRKSKNEEKEEDDAENYRL

886 Q9NQ33 NP_001952.1

>Q9NQ33

MDNRGNSSLPDKLPIFPDSARLPLTRSFYLEPMVTFHVHPEAPVSSPYSEELPRLPFPSDSLILGNYSEPCPFSFPMPYPNYRGCEYSYGPAFTRKRNERERQRVKCVNEGYAQLRHHLPEEYLEKRLSKVETLRAAIKYINYLQSLLYPDKAETKNNPGKVSSMIATTSHHADPMFRIV

>NP_001952.1

MVNFTVDQIRAIMDKKANIRNMSVIAHVDHGKSTLTDSLVCKAGIIASARAGETRFTDTRKDEQERCITIKSTAISLFYELSENDLNFIKQSKDGAGFLINLIDSPGHVDFSSEVTAALRVTDGALVVVDCVSGVCVQTETVLRQAIAERIKPVLMMNKMDRALLELQLEPEELYQTFQRIVENVNVIISTYGEGESGPMGNIMIDPVLGTVGFGSGLHGWAFTLKQFAEMYVAKFAAKGEGQLGPAERAKKVEDMMKKLWGDRYFDPANGKFSKSATSPEGKKLPRTFCQLILDPIFKVFDAIMNFKKEETAKLIEKLDIKLDSEDKDKEGKPLLKAVMRRWLPAGDALLQMITIHLPSPVTAQKYRCELLYEGPPDDEAAMGIKSCDPKGPLMMYISKMVPTSDKGRFYAFGRVFSGLVSTGLKVRIMGPNYTPGKKEDLYLKPIQRTILMMGRYVEPIEDVPCGNIVGLVGVDQFLVKTGTITTFEHAHNMRVMKFSVSPVVRVAVEAKNPADLPKLVEGLKRLAKSDPMVQCIIEESGEHIIAGAGELHLEICLKDLEEDHACIPIKKSDPVVSYRETVSEESNVLCLSKSPNKHNRLYMKARPFPDGLAEDIDKGEVSARQELKQRARYLAEKYEWDVAEARKIWCFGPDGTGPNILTDITKGVQYLNEIKDSVVAGFQWATKEGALCEENMRGVRFDVHDVTLHADAIHRGGGQIIPTARRCLYASVLTAQPRLMEPIYLVEIQCPEQVVGGIYGVLNRKRGHVFEESQVAGTPMFVVKAYLPVNESFGFTADLRSNTGGQAFPQCVFDHWQILPGDPFDNSSRPSQVVAETRKRKGLKEGIPALDNFLDKL

887 NP_003649.2 NP_001005.1

>NP_003649.2

MHCHAELRLSSPGQLKAARRRYKTFMIDEILSKETCDYFEKLSLYSVCPSLVVRPKPLHSCTGSPSLRAYPLLSVITRQPTVISHLVPATPGIAQALSCHQVTEAVSAEAPGGEALASSESETEQPTPRQKKPRRSRTIFTELQLMGLEKKFQKQKYLSTPDRLDLAQSLGLTQLQVKTWYQNRRMKWKKMVLKGGQEAPTKPKGRPKKNSIPTSEEIEAEEKMNSQAQGQEQLEPSQGQEELCEAQEPKARDVPLEMAEPPDPPQELPIPSSEPPPLS

>NP_001005.1

MLMPKKNRIAIYELLFKEGVMVAKKDVHMPKHPELADKNVPNLHVMKAMQSLKSRGYVKEQFAWRHFYWYLTNEGIQYLRDYLHLPPEIVPATLRRSRPETGRPRPKGLEGERPARLTRGEADRDTYRRSAVPPGADKKAEAGAGSATEFQFRGGFGRGRGQPPQ

888 NP_002136.1 Q99551

>NP_002136.1

MNFEFEREIGFINSQPSLAECLTSFPAVLETFQTSSIKESTLIPPPPPFEQTFPSLQPGASTLQRPRSQKRAEDGPALPPPPPPPLPAAPPAPEFPWMKEKKSAKKPSQSATSPSPAASAVPASGVGSPADGLGLPEAGGGGARRLRTAYTNTQLLELEKEFHFNKYLCRPRRVEIAALLDLTERQVKVWFQNRRMKHKRQTQHREPPDGEPACPGALEDICDPAEEPAASPGGPSASRAAWEACCHPPEVVPGALSADPRPLAVRLEGAGASSPGCALRGAGGLEPGPLPEDVFSGRQDSPFLPDLNFFAADSCLQLSGGLSPSLQGSLDSPVPFSEEELDFFTSTLCAIDLQFP

>Q99551

MQSLSLGQTSISKGLNYLTIMAPGNLWHMRNNFLFGSRCWMTRFSAENIFKSVSFRLFGVKCHNTDSEPLKNEDLLKNLLTMGVDIDMARKRQPGVFHRMITNEQDLKMFLLSKGASKEVIASIISRYPRAITRTPENLSKRWDLWRKIVTSDLEIVNILERSPESFFRSNNNLNLENNIKFLYSVGLTRKCLCRLLTNAPRTFSNSLDLNKQMVEFLQAAGLSLGHNDPADFVRKIIFKNPFILIQSTKRVKANIEFLRSTFNLNSEELLVLICGPGAEILDLSNDYARRSYANIKEKLFSLGCTEEEVQKFVLSYPDVIFLAEKKFNDKIDCLMEENISISQIIENPRVLDSSISTLKSRIKELVNAGCNLSTLNITLLSWSKKRYEAKLKKLSRFA

889 Q9BUQ8 NP_057740.2

>Q9BUQ8

MAGELADKKDRDASPSKEERKRSRTPDRERDRDRDRKSSPSKDRKRHRSRDRRRGGSRSRSRSRSKSAERERRHKERERDKERDRNKKDRDRDKDGHRRDKDRKRSSLSPGRGKDFKSRKDRDSKKDEEDEHGDKKPKAQPLSLEELLAKKKAEEEAEAKPKFLSKAEREAEALKRRQQEVEERQRMLEEERKKRKQFQDLGRKMLEDPQERERRERRERMERETNGNEDEEGRQKIREEKDKSKELHAIKERYLGGIKKRRRTRHLNDRKFVFEWDASEDTSIDYNPLYKERHQVQLLGRGFIAGIDLKQQKREQSRFYGDLMEKRRTLEEKEQEEARLRKLRKKEAKQRWDDRHWSQKKLDEMTDRDWRIFREDYSITTKGGKIPNPIRSWKDSSLPPHILEVIDKCGYKEPTPIQRQAIPIGLQNRDIIGVAETGSGKTAAFLIPLLVWITTLPKIDRIEESDQGPYAIILAPTRELAQQIEEETIKFGKPLGIRTVAVIGGISREDQGFRLRMGCEIVIATPGRLIDVLENRYLVLSRCTYVVLDEADRMIDMGFEPDVQKILEHMPVSNQKPDTDEAEDPEKMLANFESGKHKYRQTVMFTATMPPAVERLARSYLRRPAVVYIGSAGKPHERVEQKVFLMSESEKRKKLLAILEQGFDPPIIIFVNQKKGCDVLAKSLEKMGYNACTLHGGKGQEQREFALSNLKAGAKDILVATDVAGRGIDIQDVSMVVNYDMAKNIEDYIHRIGRTGRAGKSGVAITFLTKEDSAVFYELKQAILESPVSSCPPELANHPDAQHKPGTILTKKRREETIFA

>NP_057740.2

MEESDSEKTTEKENLGPRMDPPLGEPEGSLGWVLPNTAMKKKVLLMGKSGSGKTSMRSIIFANYIARDTRRLGATILDRIHSLQINSSLSTYSLVDSVGNTKTFDVEHSHVRFLGNLVLNLWDCGGQDTFMENYFTSQRDNIFRNVEVLIYVFDVESRELEKDMHYYQSCLEAILQNSPDAKIFCLVHKMDLVQEDQRDLIFKEREEDLRRLSRPLECSCFRTSIWDETLYKAWSSIVYQLIPNVQQLEMNLRNFAEIIEADEVLLFERATFLVISHYQCKEQRDAHRFEKISNIIKQFKLSCSKLAASFQSMEVRNSNFAAFIDIFTSNTYVMVVMSDPSIPSAATLINIRNARKHFEKLERVDGPKQCLLMR

890 NP_001978.1 NP_006003.1

>NP_001978.1

MSETPAQCSIKQERISYTPPESPVPSYASSTPLHVPVPRALRMEEDSIRLPAHLRLQPIYWSRDDVAQWLKWAENEFSLRPIDSNTFEMNGKALLLLTKEDFRYRSPHSGDVLYELLQHILKQRKPRILFSPFFHPGNSIHTQPEVILHQNHEEDNCVQRTPRPSVDNVHHNPPTIELLHRSRSPITTNHRPSPDPEQRPLRSPLDNMIRRLSPAERAQGPRPHQENNHQESYPLSVSPMENNHCPASSESHPKPSSPRQESTRVIQLMPSPIMHPLILNPRHSVDFKQSRLSEDGLHREGKPINLSHREDLAYMNHIMVSVSPPEEHAMPIGRIADCRLLWDYVYQLLSDSRYENFIRWEDKESKIFRIVDPNGLARLWGNHKNRTNMTYEKMSRALRHYYKLNIIRKEPGQRLLFRFMKTPDEIMSGRTDRLEHLESQELDEQIYQEDEC

>NP_006003.1

MWPGILVGGARVASCRYPALGPRLAAHFPAQRPPQRTLQNGLALQRCLHATATRALPLIPIVVEQTGRGERAYDIYSRLLRERIVCVMGPIDDSVASLVIAQLLFLQSESNKKPIHMYINSPGGVVTAGLAIYDTMQYILNPICTWCVGQAASMGSLLLAAGTPGMRHSLPNSRIMIHQPSGGARGQATDIAIQAEEIMKLKKQLYNIYAKHTKQSLQVIESAMERDRYMSPMEAQEFGILDKVLVHPPQDGEDEPTLVQKEPVEAAPAAEPVPAST

891 P28715 NP_071933.2

>P28715

MGVQGLWKLLECSGRQVSPEALEGKILAVDISIWLNQALKGVRDRHGNSIENPHLLTLFHRLCKLLFFRIRPIFVFDGDAPLLKKQTLVKRRQRKDLASSDSRKTTEKLLKTFLKRQAIKTAFRSKRDEALPSLTQVRRENDLYVLPPLQEEEKHSSEEEDEKEWQERMNQKQALQEEFFHNPQAIDIESEDFSSLPPEVKHEILTDMKEFTKRRRTLFEAMPEESDDFSQYQLKGLLKKNYLNQHIEHVQKEMNQQHSGHIRRQYEDEGGFLKEVESRRVVSEDTSHYILIKGIQAKTVAEVDSESLPSSSKMHGMSFDVKSSPCEKLKTEKEPDATPPSPRTLLAMQAALLGSSSEEELESENRRQARGRNAPAAVDEGSISPRTLSAIKRALDDDEDVKVCAGDDVQTGGPGAEEMRINSSTENSDEGLKVRDGKGIPFTATLASSSVNSAEEHVASTNEGREPTDSVPKEQMSLVHVGTEAFPISDESMIKDRKDRLPLESAVVRHSDAPGLPNGRELTPASPTCTNSVSKNETHAEVLEQQNELCPYESKFDSSLLSSDDETKCKPNSASEVIGPVSLQETSSIVSVPSEAVDNVENVVSFNAKEHENFLETIQEQQTTESAGQDLISIPKAVEPMEIDSEESESDGSFIEVQSVISDEELQAEFPETSKPPSEQGEEELVGTREGEAPAESESLLRDNSERDDVDGEPQEAEKDAEDSLHEWQDINLEELETLESNLLAQQNSLKAQKQQQERIAATVTGQMFLESQELLRLFGIPYIQAPMEAEAQCAILDLTDQTSGTITDDSDIWLFGARHVYRNFFNKNKFVEYYQYVDFHNQLGLDRNKLINLAYLLGSDYTEGIPTVGCVTAMEILNEFPGHGLEPLLKFSEWWHEAQKNPKIRPNPHDTKVKKKLRTLQLTPGFPNPAVAEAYLKPVVDDSKGSFLWGKPDLDKIREFCQRYFGWNRTKTDESLFPVLKQLDAQQTQLRIDSFFRLAQQEKEDAKRIKSQRLNRAVTCMLRKEKEAAASEIEAVSVAMEKEFELLDKAKGKTQKRGITNTLEESSSLKRKRLSDSKGKNTCGGFLGETCLSESSDGSSSEDAESSSLMNVQRRTAAKEPKTSASDSQNSVKEAPVKNGGATTSSSSDSDDDGGKEKMVLVTARSVFGKKRRKLRRARGRKRKT

>NP_071933.2

MQNVINTVKGKALEVAEYLTPVLKESKFKETGVITPEEFVAAGDHLVHHCPTWQWATGEELKVKAYLPTGKQFLVTKNVPCYKRCKQMEYSDELEAIIEEDDGDGGWVDTYHNTGITGITEAVKEITLENKDNIRLQDCSALCEEEEDEDEGEAADMEEYEESGLLETDEATLDTRKIVEACKAKTDAGGEDAILQTRTYDLYITYDKYYQTPRLWLFGYDEQRQPLTVEHMYEDISQDHVKKTVTIENHPHLPPPPMCSVHPCRHAEVMKKIIETVAEGGGELGVHMYLLIFLKFVQAVIPTIEYDYTRHFTM

892 Q15319 O14561

>Q15319

MMAMNSKQPFGMHPVLQEPKFSSLHSGSEAMRRVCLPAPQLQGNIFGSFDESLLARAEALAAVDIVSHGKNHPFKPDATYHTMSSVPCTSTSSTVPISHPAALTSHPHHAVHQGLEGDLLEHISPTLSVSGLGAPEHSVMPAQIHPHHLGAMGHLHQAMGMSHPHTVAPHSAMPACLSDVESDPRELEAFAERFKQRRIKLGVTQADVGAALANLKIPGVGSLSQSTICRFESLTLSHNNMIALKPVLQAWLEEAEAAYREKNSKPELFNGSERKRKRTSIAAPEKRSLEAYFAIQPRPSSEKIAAIAEKLDLKKNVVRVWFCNQRQKQKRMKYSAVH

>O14561

MASRVLSAYVSRLPAAFAPLPRVRMLAVARPLSTALCSAGTQTRLGTLQPALVLAQVPGRVTQLCRQYSDMPPLTLEGIQDRVLYVLKLYDKIDPEKLSVNSHFMKDLGLDSLDQVEIIMAMEDEFGFEIPDIDAEKLMCPQEIVDYIADKKDVYE

893 NP_663628.1 NP_620448.1

>NP_663628.1

MAAGSTTLRAVGKLQVRLATKTEPKKLEKYLQKLSALPMTADILAETGIRKTVKRLRKHQHVGDFARDLAARWKKLVLVDRNTGPDPQDPEESASRQRFGEALQEREKAWGFPENATAPRSPSHSPEHRRTARRTPPGQQRPHPRSPSREPRAERKRPRMAPADSGPHRDPPTRTAPLPMPEGPEPAVPGEQPGRGHAHAAQGGPLLGQGCQGQPQGEAVGSHSKGHKSSRGASAQKSPPVQESQSERLQAAGADSAGPKTVPSHVFSELWDPSEAWMQANYDLLSAFEAMTSQANPEALCAPALQEEAAFPGRRVNAKMPVYSGSRPACQLQVPTLRQQCLRVPRNNPDALGDVEGVPYSVLEPVLEGWTPDQLYRTEKDNAALARETDELWRIHCLQDFKEEKPQEHESWRELYLRLRDAREQRLRVVTTKIRSARENKPSGRQTKMICFNSVAKTPYDASRRQEKSAGAADPGNGEMEPAPKPAGSSQAPSGLGDGDGGSVSGGGSSNRHAAPADKTRKQAAKKVAPLMAKAIRDYKGRFSRR

>NP_620448.1

MSLHFLYYCSEPTLDVKIAFCQGFDKQVDVSYIAKHYNMSKSKVDNQFYSVEVGDSTFTVLKRYQNLKPIGSGAQGIVCAAYDAVLDRNVAIKKLSRPFQNQTHAKRAYRELVLMKCVNHKNIISLLNVFTPQKTLEEFQDVYLVMELMDANLCQVIQMELDHERMSYLLYQMLCGIKHLHSAGIIHRDLKPSNIVVKSDCTLKILDFGLARTAGTSFMMTPYVVTRYYRAPEVILGMGYKENVDIWSVGCIMGEMVRHKILFPGRDYIDQWNKVIEQLGTPCPEFMKKLQPTVRNYVENRPKYAGLTFPKLFPDSLFPADSEHNKLKASQARDLLSKMLVIDPAKRISVDDALQHPYINVWYDPAEVEAPPPQIYDKQLDEREHTIEEWKELIYKEVMNSEEKTKNGVVKGQPSPSGAAVNSSESLPPSSSVNDISSMSTDQTLASDTDSSLEASAGPLGCCR

894 NP_005654.2 NP_004332.2

>NP_005654.2

MASMRESDTGLWLHNKLGATDELWAPPSIASLLTAAVIDNIRLCFHGLSSAVKLKLLLGTLHLPRRTVDEMKGALMEIIQLASLDSDPWVLMVADILKSFPDTGSLNLELEEQNPNVQDILGELREKVGECEASAMLPLECQYLNKNALTTLAGPLTPPVKHFQLKRKPKSATLRAELLQKSTETAQQLKRSAGVPFHAKGRGLLRKMDTTTPLKGIPKQAPFRSPTAPSVFSPTGNRTPIPPSRTLLRKERGVKLLDISELDMVGAGREAKRRRKTLDAEVVEKPAKEETVVENATPDYAAGLVSTQKLGSLNNEPALPSTSYLPSTPSVVPASSYIPSSETPPAPSSREASRPPEEPSAPSPTLPAQFKQRAPMYNSGLSPATPTPAAPTSPLTPTTPPAVAPTTQTPPVAMVAPQTQAPAQQQPKKNLSLTREQMFAAQEMFKTANKVTRPEKALILGFMAGSRENPCQEQGDVIQIKLSEHTEDLPKADGQGSTTMLVDTVFEMNYATGQWTRFKKYKPMTNVS

>NP_004332.2

MAALVLEDGSVLRGQPFGAAVSTAGEVVFQTGMVGYPEALTDPSYKAQILVLTYPLIGNYGIPPDEMDEFGLCKWFESSGIHVAALVVGECCPTPSHWSATRTLHEWLQQHGIPGLQGVDTRELTKKLREQGSLLGKLVQNGTEPSSLPFLDPNARPLVPEVSIKTPRVFNTGGAPRILALDCGLKYNQIRCLCQRGAEVTVVPWDHALDSQEYEGLFLSNGPGDPASYPSVVSTLSRVLSEPNPRPVFGICLGHQLLALAIGAKTYKMRYGNRGHNQPCLLVGSGRCFLTSQNHGFAVETDSLPADWAPLFTNANDGSNEGIVHNSLPFFSVQFHPEHQAGPSDMELLFDIFLETVKEATAGNPGGQTVRERLTERLCPPGIPTPGSGLPPPRKVLILGSGGLSIGQAGEFDYSGSQAIKALKEENIQTLLINPNIATVQTSQGLADKVYFLPITPHYVTQVIRNERPDGVLLTFGGQTALNCGVELTKAGVLARYGVRVLGTPVETIELTEDRRAFAARMAEIGEHVAPSEAANSLEQAQAAAERLGYPVLVRAAFALGGLGSGFASNREELSALVAPAFAHTSQVLVDKSLKGWKEIEYEVVRDAYGNCVTVCNMENLDPLGIHTGESIVVAPSQTLNDREYQLLRQTAIKVTQHLGIVGECNVQYALNPESEQYYIIEVNARLSRSSALASKATGYPLAYVAAKLALGIPLPELRNSVTGGTAAFEPSVDYCVVKIPRWDLSKFLRVSTKIGSCMKSVGEVMGIGRSFEEAFQKALRMVDENCVGFDHTVKPVSDMELETPTDKRIFVVAAALWAGYSVDRLYELTRIDRWFLHRMKRIIAHAQLLEQHRGQPLPPDLLQQAKCLGFSDKQIALAVLSTELAVRKLRQELGICPAVKQIDTVAAEWPAQTNYLYLTYWGTTHDLTFRTPHVLVLGSGVYRIGSSVEFDWCAVGCIQQLRKMGYKTIMVNYNPETVSTDYDMCDRLYFDEISFEVVMDIYELENPEGVILSMGGQLPNNMAMALHRQQCRVLGTSPEAIDSAENRFKFSRLLDTIGISQPQWRELSDLESARQFCQTVGYPCVVRPSYVLSGAAMNVAYTDGDLERFLSSAAAVSKEHPVVISKFIQEAKEIDVDAVASDGVVAAIAISEHVENAGVHSGDATLVTPPQDITAKTLERIKAIVHAVGQELQVTGPFNLQLIAKDDQLKVIECNVRVSRSFPFVSKTLGVDLVALATRVIMGEEVEPVGLMTGSGVVGVKVPQFSFSRLAGADVVLGVEMTSTGEVAGFGESRCEAYLKAMLSTGFKIPKKNILLTIGSYKNKSELLPTVRLLESLGYSLYASLGTADFYTEHGVKVTAVDWHFEEAVDGECPPQRSILEQLAEKNFELVINLSMRGAGGRRLSSFVTKGYRTRRLAADFSVPLIIDIKCTKLFVEALGQIGPAPPLKVHVDCMTSQKLVRLPGLIDVHVHLREPGGTHKEDFASGTAAALAGGITMVCAMPNTRPPIIDAPALALAQKLAEAGARCDFALFLGASSENAGTLGTVAGSAAGLKLYLNETFSELRLDSVVQWMEHFETWPSHLPIVAHAEQQTVAAVLMVAQLTQRSVHICHVARKEEILLIKAAKARGLPVTCEVAPHHLFLSHDDLERLGPGKGEVRPELGSRQDVEALWENMAVIDCFASDHAPHTLEEKCGSRPPPGFPGLETMLPLLLTAVSEGRLSLDDLLQRLHHNPRRIFHLPPQEDTYVEVDLEHEWTIPSHMPFSKAHWTPFEGQKVKGTVRRVVLRGEVAYIDGQVLVPPGYGQDVRKWPQGAVPQLPPSAPATSEMTTTPERPRRGIPGLPDGRFHLPPRIHRASDPGLPAEEPKEKSSRKVAEPELMGTPDGTCYPPPPVPRQASPQNLGTPGLLHPQTSPLLHSLVGQHILSVQQFTKDQMSHLFNVAHTLRMMVQKERSLDILKGKVMASMFYEVSTRTSSSFAAAMARLGGAVLSFSEATSSVQKGESLADSVQTMSCYADVVVLRHPQPGAVELAAKHCRRPVINAGDGVGEHPTQALLDIFTIREELGTVNGMTITMVGDLKHGRTVHSLACLLTQYRVSLRYVAPPSLRMPPTVRAFVASRGTKQEEFESIEEALPDTDVLYMTRIQKERFGSTQEYEACFGQFILTPHIMTRAKKKMVVMHPMPRVNEISVEVDSDPRAAYFRQAENGMYIRMALLATVLGRF

895 NP_006582.1 NP_057256.2

>NP_006582.1

MADQLYLENIDEFVTDQNKIVTYKWLSYTLGVHVNQAKQMLYDYVERKRKENSGAQLHVTYLVSGSLIQNGHSCHKVAVVREDKLEAVKSKLAVTASIHVYSIQKAMLKDSGPLFNTDYDILKSNLQNCSKFSAIQCAAAVPRAPAESSSSSKKFEQSHLHMSSETQANNELTTNGHGPPASKQVSQQPKGIMGMFASKAAAKTQETNKETKTEAKEVTNASAAGNKAPGKGNMMSNFFGKAAMNKFKVNLDSEQAVKEEKIVEQPTVSVTEPKLATPAGLKKSSKKAEPVKVLQKEKKRGKRVALSDDETKETENMRKKRRRIKLPESDSSEDEVFPDSPGAYEAESPSPPPPPSPPLEPVPKTEPEPPSVKSSSGENKRKRKRVLKSKTYLDGEGCIVTEKVYESESCTDSEEELNMKTSSVHRPPAMTVKKEPREERKGPKKGTAALGKANRQVSITGFFQRK

>NP_057256.2

MFVQEEKIFAGKVLRLHICASDGAEWLEEATEDTSVEKLKERCLKHCAHGSLEDPKSITHHKLIHAASERVLSDARTILEENIQDQDVLLLIKKRAPSPLPKMADVSAEEKKKQDQKAPDKEAILRATANLPSYNMDRAAVQTNMRDFQTELRKILVSLIEVAQKLLALNPDAVELFKKANAMLDEDEDERVDEAALRQLTEMGFPENRATKALQLNHMSVPQAMEWLIEHAEDPTIDTPLPGQAPPEAEGATAAASEAAAGASATDEEARDELTEIFKKIRRKREFRADARAVISLMEMGFDEKEVIDALRVNNNQQNAACEWLLGDRKPSPEELDKGIDPDSPLFQAILDNPVVQLGLTNPKTLLAFEDMLENPLNSTQWMNDPETGPVMLQISRIFQTLNRT

896 NP_490595.1 NP_002078.1

>NP_490595.1

MASGRGASSRWFFTREQLENTPSRRCGVEADKELSCRQQAANLIQEMGQRLNVSQLTINTAIVYMHRFYMHHSFTKFNKNIISSTALFLAAKVEEQARKLEHVIKVAHACLHPLEPLLDTKCDAYLQQTQELVILETIMLQTLGFEITIEHPHTDVVKCTQLVRASKDLAQTSYFMATNSLHLTTFCLQYKPTVIACVCIHLACKWSNWEIPVSTDGKHWWEYVDPTVTLELLDELTHEFLQILEKTPNRLKKIRNWRANQAARKPKVDGQVSETPLLGSSLVQNSILVDSVTGVPTNPSFQKPSTSAFPAPVPLNSGNISVQDSHTSDNLSMLATGMPSTSYGLSSHQEWPQHQDSARTEQLYSQKQETSLSGSQYNINFQQGPSISLHSGLHHRPDKISDHSSVKQEYTHKAGSSKHHGPISTTPGIIPQKMSLDKYREKRKLETLDLDVRDHYIAAQVEQQHKQGQSQAASSSSVTSPIKMKIPIANTEKYMADKKEKSGSLKLRIPIPPTDKSASKEELKMKIKVSSSERHSSSDEGSGKSKHSSPHISRDHKEKHKEHPSSRHHTSSHKHSHSHSGSSSGGSKHSADGIPPTVLRSPVGLSSDGISSSSSSSRKRLHVNDASHNHHSKMSKSSKSSGSSSSSSSSVKQYISSHNSVFNHPLPPPPPVTYQVGYGHLSTLVKLDKKPVETNGPDANHEYSTSSQHMDYKDTFDMLDSLLSAQGMNM

>NP_002078.1

MWTLVSWVALTAGLVAGTRCPDGQFCPVACCLDPGGASYSCCRPLLDKWPTTLSRHLGGPCQVDAHCSAGHSCIFTVSGTSSCCPFPEAVACGDGHHCCPRGFHCSADGRSCFQRSGNNSVGAIQCPDSQFECPDFSTCCVMVDGSWGCCPMPQASCCEDRVHCCPHGAFCDLVHTRCITPTGTHPLAKKLPAQRTNRAVALSSSVMCPDARSRCPDGSTCCELPSGKYGCCPMPNATCCSDHLHCCPQDTVCDLIQSKCLSKENATTDLLTKLPAHTVGDVKCDMEVSCPDGYTCCRLQSGAWGCCPFTQAVCCEDHIHCCPAGFTCDTQKGTCEQGPHQVPWMEKAPAHLSLPDPQALKRDVPCDNVSSCPSSDTCCQLTSGEWGCCPIPEAVCCSDHQHCCPQGYTCVAEGQCQRGSEIVAGLEKMPARRASLSHPRDIGCDQHTSCPVGQTCCPSLGGSWACCQLPHAVCCEDRQHCCPAGYTCNVKARSCEKEVVSAQPATFLARSPHVGVKDVECGEGHFCHDNQTCCRDNRQGWACCPYRQGVCCADRRHCCPAGFRCAARGTKCLRREAPRWDAPLRDPALRQLL

897 O00115 Q6PRD7

>O00115

MIPLLLAALLCVPAGALTCYGDSGQPVDWFVVYKLPALRGSGEAAQRGLQYKYLDESSGGWRDGRALINSPEGAVGRSLQPLYRSNTSQLAFLLYNDQPPQPSKAQDSSMRGHTKGVLLLDHDGGFWLVHSVPNFPPPASSAAYSWPHSACTYGQTLLCVSFPFAQFSKMGKQLTYTYPWVYNYQLEGIFAQEFPDLENVVKGHHVSQEPWNSSITLTSQAGAVFQSFAKFSKFGDDLYSGWLAAALGTNLQVQFWHKTVGILPSNCSDIWQVLNVNQIAFPGPAGPSFNSTEDHSKWCVSPKGPWTCVGDMNRNQGEEQRGGGTLCAQLPALWKAFQPLVKNYQPCNGMARKPSRAYKI

>Q6PRD7

MGTSSTDSQQAGHRRCSTSNTSAENLTCLSLPGSPGKTAPLPGPAQAGAGQPLPKGCAAVKAEVGIPAPHTSQEVRIHIRRLLSWAAPGACGLRSTPCALPQALPQARPCPGRWFFPGCSLPTGGAQTILSLWTWRHFLNWALQQREENSGRARRVPPVPRTAPVSKGEGSHPPQNSNGEKVKTITPDVGLHQSLTSDPTVAVLRAKRAPEAHPPRSCSGSLTARVCHMGVCQGQGDTEDGRMTLMG

898 NP_005241.1 NP_004172.2

>NP_005241.1

MSHLFDPRLPALAASPMLYLYGPERPGLPLAFAPAAALAASGRAETPQKPPYSYIALIAMAIQDAPEQRVTLNGIYQFIMDRFPFYHDNRQGWQNSIRHNLSLNDCFVKVPREKGRPGKGSYWTLDPRCLDMFENGNYRRRKRKPKPGPGAPEAKRPRAETHQRSAEAQPEAGSGAGGSGPAISRLQAAPAGPSPLLDGPSPPAPLHWPGTASPNEDAGDAAQGAAAVAVGQAARTGDGPGSPLRPASRSSPKSSDKSKSFSIDSILAGKQGQKPPSGDELLGGAKPGPGGRLGASLLAASSSLRPPFNASLMLDPHVQGGFYQLGIPFLSYFPLQVPDTVLHFQ

>NP_004172.2

MQLKPMEINPEMLNKVLSRLGVAGQWRFVDVLGLEEESLGSVPAPACALLLLFPLTAQHENFRKKQIEELKGQEVSPKVYFMKQTIGNSCGTIGLIHAVANNQDKLGFEDGSVLKQFLSETEKMSPEDRAKCFEKNEAIQAAHDAVAQEGQCRVDDKVNFHFILFNNVDGHLYELDGRMPFPVNHGASSEDTLLKDAAKVCREFTEREQGEVRFSAVALCKAA

899 NP_003086.1 P50135

>NP_003086.1

MSLPLNPKPFLNGLTGKPVMVKLKWGMEYKGYLVSVDGYMNMQLANTEEYIDGALSGHLGEVLIRCNNVLYIRGVEEEEEDGEMRE

>P50135

MASSMRSLFSDHGKYVESFRRFLNHSTEHQCMQEFMDKKLPGIIGRIGDTKSEIKILSIGGGAGEIDLQILSKVQAQYPGVCINNEVVEPSAEQIAKYKELVAKTSNLENVKFAWHKETSSEYQSRMLEKKELQKWDFIHMIQMLYYVKDIPATLKFFHSLLGTNAKMLIIVVSGSSGWDKLWKKYGSRFPQDDLCQYITSDDLTQMLDNLGLKYECYDLLSTMDISDCFIDGNENGDLLWDFLTETCNFNATAPPDLRAELGKDLQEPEFSAKKEGKVLFNNTLSFIVIEA

900 Q9GZZ0 P78368

>Q9GZZ0

MSSYLEYVSCSSSGGVGGDVLSLAPKFCRSDARPVALQPAFPLGNGDGAFVSCLPLAAARPSPSPPAAPARPSVPPPAAPQYAQCTLEGAYEPGAAPAAAAGGADYGFLGSGPAYDFPGVLGRAADDGGSHVHYATSAVFSGGGSFLLSGQVDYAAFGEPGPFPACLKASADGHPGAFQTASPAPGTYPKSVSPASGLPAAFSTFEWMKVKRNASKKGKLAEYGAASPSSAIRTNFSTKQLTELEKEFHFNKYLTRARRIEIANCLHLNDTQVKIWFQNRRMKQKKREREGLLATAIPVAPLQLPLSGTTPTKFIKNPGSPSQSQEPS

>P78368

MDFDKKGGKGETEEGRRMSKAGGGRSSHGIRSSGTSSGVLMVGPNFRVGKKIGCGNFGELRLGKNLYTNEYVAIKLEPIKSRAPQLHLEYRFYKQLSATEGVPQVYYFGPCGKYNAMVLELLGPSLEDLFDLCDRTFTLKTVLMIAIQLITRMEYVHTKSLIYRDVKPENFLVGRPGTKRQHAIHIIDFGLAKEYIDPETKKHIPYREHKSLTGTARYMSINTHLGKEQSRRDDLEALGHMFMYFLRGSLPWQGLKADTLKERYQKIGDTKRATPIEVLCENFPEEMATYLRYVRRLDFFEKPDYDYLRKLFTDLFDRSGFVFDYEYDWAGKPLPTPIGTVHTDLPSQPQLRDKTQPHSKNQALNSTNGELNADDPTAGHSNAPITAPAEVEVADETKCCCFFKRRKRKSLQRHK

901 Q14839 Q9NX20

>Q14839

MASGLGSPSPCSAGSEEEDMDALLNNSLPPPHPENEEDPEEDLSETETPKLKKKKKPKKPRDPKIPKSKRQKKERMLLCRQLGDSSGEGPEFVEEEEEVALRSDSEGSDYTPGKKKKKKLGPKKEKKSKSKRKEEEEEDDDDDDSKEPKSSAQLLEDWGMEDIDHVFSEEDYRTLTNYKAFSQFVRPLIAAKNPKIAVSKMMMVLGAKWREFSTNNPFKGSSGASVAAAAAAAVAVVESMVTATEVAPPPPPVEVPIRKAKTKEGKGPNARRKPKGSPRVPDAKKPKPKKVAPLKIKLGGFGSKRKRSSSEDDDLDVESDFDDASINSYSVSDGSTSRSSRSRKKLRTTKKKKKGEEEVTAVDGYETDHQDYCEVCQQGGEIILCDTCPRAYHMVCLDPDMEKAPEGKWSCPHCEKEGIQWEAKEDNSEGEEILEEVGGDLEEEDDHHMEFCRVCKDGGELLCCDTCPSSYHIHCLNPPLPEIPNGEWLCPRCTCPALKGKVQKILIWKWGQPPSPTPVPRPPDADPNTPSPKPLEGRPERQFFVKWQGMSYWHCSWVSELQLELHCQVMFRNYQRKNDMDEPPSGDFGGDEEKSRKRKNKDPKFAEMEERFYRYGIKPEWMMIHRILNHSVDKKGHVHYLIKWRDLPYDQASWESEDVEIQDYDLFKQSYWNHRELMRGEEGRPGKKLKKVKLRKLERPPETPTVDPTVKYERQPEYLDATGGTLHPYQMEGLNWLRFSWAQGTDTILADEMGLGKTVQTAVFLYSLYKEGHSKGPFLVSAPLSTIINWEREFEMWAPDMYVVTYVGDKDSRAIIRENEFSFEDNAIRGGKKASRMKKEASVKFHVLLTSYELITIDMAILGSIDWACLIVDEAHRLKNNQSKFFRVLNGYSLQHKLLLTGTPLQNNLEELFHLLNFLTPERFHNLEGFLEEFADIAKEDQIKKLHDMLGPHMLRRLKADVFKNMPSKTELIVRVELSPMQKKYYKYILTRNFEALNARGGGNQVSLLNVVMDLKKCCNHPYLFPVAAMEAPKMPNGMYDGSALIRASGKLLLLQKMLKNLKEGGHRVLIFSQMTKMLDLLEDFLEHEGYKYERIDGGITGNMRQEAIDRFNAPGAQQFCFLLSTRAGGLGINLATADTVIIYDSDWNPHNDIQAFSRAHRIGQNKKVMIYRFVTRASVEERITQVAKKKMMLTHLVVRPGLGSKTGSMSKQELDDILKFGTEELFKDEATDGGGDNKEGEDSSVIHYDDKAIERLLDRNQDETEDTELQGMNEYLSSFKVAQYVVREEEMGEEEEVEREIIKQEESVDPDYWEKLLRHHYEQQQEDLARNLGKGKRIRKQVNYNDGSQEDRDWQDDQSDNQSDYSVASEEGDEDFDERSEAPRRPSRKGLRNDKDKPLPPLLARVGGNIEVLGFNARQRKAFLNAIMRYGMPPQDAFTTQWLVRDLRGKSEKEFKAYVSLFMRHLCEPGADGAETFADGVPREGLSRQHVLTRIGVMSLIRKKVQEFEHVNGRWSMPELAEVEENKKMSQPGSPSPKTPTPSTPGDTQPNTPAPVPPAEDGIKIEENSLKEEESIEGEKEVKSTAPETAIECTQAPAPASEDEKVVVEPPEGEEKVEKAEVKERTEEPMETEPKGAADVEKVEEKSAIDLTPIVVEDKEEKKEEEEKKEVMLQNGETPKDLNDEKQKKNIKQRFMFNIADGGFTELHSLWQNEERAATVTKKTYEIWHRRHDYWLLAGIINHGYARWQDIQNDPRYAILNEPFKGEMNRGNFLEIKNKFLARRFKLLEQALVIEEQLRRAAYLNMSEDPSHPSMALNTRFAEVECLAESHQHLSKESMAGNKPANAVLHKVLKQLEELLSDMKADVTRLPATIARIPPVAVRLQMSERNILSRLANRAPEPTPQQVAQQQ

>Q9NX20

MWRLLARASAPLLRVPLSDSWALLPASAGVKTLLPVPSFEDVSIPEKPKLRFIERAPLVPKVRREPKNLSDIRGPSTEATEFTEGNFAILALGGGYLHWGHFEMMRLTINRSMDPKNMFAIWRVPAPFKPITRKSVGHRMGGGKGAIDHYVTPVKAGRLVVEMGGRCEFEEVQGFLDQVAHKLPFAAKAVSRGTLEKMRKDQEERERNNQNPWTFERIATANMLGIRKVLSPYDLTHKGKYWGKFYMPKRV

902 O14862 NP_203526.1

>O14862

MESKYKEILLLTGLDNITDEELDRFKFFLSDEFNIATGKLHTANRIQVATLMIQNAGAVSAVMKTIRIFQKLNYMLLAKRLQEEKEKVDKQYKSVTKPKPLSQAEMSPAASAAIRNDVAKQRAAPKVSPHVKPEQKQMVAQQESIREGFQKRCLPVMVLKAKKPFTFETQEGKQEMFHATVATEKEFFFVKVFNTLLKDKFIPKRIIIIARYYRHSGFLEVNSASRVLDAESDQKVNVPLNIIRKAGETPKINTLQTQPLGTIVNGLFVVQKVTEKKKNILFDLSDNTGKMEVLGVRNEDTMKCKEGDKVRLTFFTLSKNGEKLQLTSGVHSTIKVIKAKKKT

>NP_203526.1

MSWSGLLHGLNTSLTCGPALVPRLWATCSMATLNQMHRLGPPKRPPRKLGPTEGRPQLKGVVLCTFTRKPKKPNSANRKCCRVRLSTGREAVCFIPGEGHTLQEHQIVLVEGGRTQDLPGVKLTVVRGKYDCGHVQKK

903 NP_001092755.1 Q16540

>NP_001092755.1

MSDAAVDTSSEITTKDLKEKKEVVEEAENGRDAPANGNAENEENGEQEADNEVDEEEEEGGEEEEEEEEGDGEEEDGDEDEEAESATGKRAAEDDEDDDVDTKKQKTDEDD

>Q16540

MARNVVYPLYRLGGPQLRVFRTNFFIQLVRPGVAQPEDTVQFRIPMEMTRVDLRNYLEGIYNVPVAAVRTRVQHGSNKRRDHRNVRIKKPDYKVAYVQLAHGQTFTFPDLFPEKDESPEGSAADDLYSMLEEERQQRQSSDPRRGGVPSWFGL

904 NP_006521.1 NP_001651.1

>NP_006521.1

MFKRMAEFGPDSGGRVKGVTIVKPIVYGNVARYFGKKREEDGHTHQWTVYVKPYRNEDMSAYVKKIQFKLHESYGNPLRVVTKPPYEITETGWGEFEIIIKIFFIDPNERPVTLYHLLKLFQSDTNAMLGKKTVVSEFYDEMIFQDPTAMMQQLLTTSRQLTLGAYKHETEFAELEVKTREKLEAAKKKTSFEIAELKERLKASRETINCLKNEIRKLEEDDQAKDI

>NP_001651.1

MGLTISSLFSRLFGKKQMRILMVGLDAAGKTTILYKLKLGEIVTTIPTIGFNVETVEYKNICFTVWDVGGQDRIRPLWKHYFQNTQGLIFVVDSNDRERIQEVADELQKMLLVDELRDAVLLLFANKQDLPNAMAISEMTDKLGLQSLRNRTWYVQATCATQGTGLYEGLDWLSNELSKR

905 Q13523 NP_001937.2

>Q13523

MAAAETQSLREQPEMEDANSEKSINEENGEVSEDQSQNKHSRHKKKKHKHRSKHKKHKHSSEEDKDKKHKHKHKHKKHKRKEVIDASDKEGMSPAKRTKLDDLALLEDLEKQRALIKAELDNELMEGKVQSGMGLILQGYESGSEEEGEIHEKARNGNRSSTRSSSTKGKLELVDNKITTKKRSKSRSKERTRHRSDKKKSKGGIEIVKEKTTRSKSKERKKSKSPSKRSKSQDQARKSKSPTLRRRSQEKIGKARSPTDDKVKIEDKSKSKDRKKSPIINESRSRDRGKKSRSPVDLRGKSKDRRSRSKERKSKRSETDKEKKPIKSPSKDASSGKENRSPSRRPGRSPKRRSLSPKPRDKSRRSRSPLLNDRRSKQSKSPSRTLSPGRRAKSRSLERKRREPERRRLSSPRTRPRDDILSRRERSKDASPINRWSPTRRRSRSPIRRRSRSPLRRSRSPRRRSRSPRRRDRGRRSRSRLRRRSRSRGGRRRRSRSKVKEDKFKGSLSEGMKVEQESSSDDNLEDFDVEEEDEEALIEQRRIQRQAIVQKYKYLAEDSNMSVPSEPSSPQSSTRTRSPSPDDILERVAADVKEYERENVDTFEASVKAKHNLMTVEQNNGSSQKKLLAPDMFTESDDMFAAYFDSARLRAAGIGKDFKENPNLRDNWTDAEGYYRVNIGEVLDKRYNVYGYTGQGVFSNVVRARDNARANQEVAVKIIRNNELMQKTGLKELEFLKKLNDADPDDKFHCLRLFRHFYHKQHLCLVFEPLSMNLREVLKKYGKDVGLHIKAVRSYSQQLFLALKLLKRCNILHADIKPDNILVNESKTILKLCDFGSASHVADNDITPYLVSRFYRAPEIIIGKSYDYGIDMWSVGCTLYELYTGKILFPGKTNNHMLKLAMDLKGKMPNKMIRKGVFKDQHFDQNLNFMYIEVDKVTEREKVTVMSTINPTKDLLADLIGCQRLPEDQRKKVHQLKDLLDQILMLDPAKRISINQALQHAFIQEKI

>NP_001937.2

MIDTLRPVPFASEMAISKTVAWLNEQLELGNERLLLMDCRPQELYESSHIESAINVAIPGIMLRRLQKGNLPVRALFTRGEDRDRFTRRCGTDTVVLYDESSSDWNENTGGESVLGLLLKKLKDEGCRAFYLEGGFSKFQAEFSLHCETNLDGSCSSSSPPLPVLGLGGLRISSDSSSDIESDLDRDPNSATDSDGSPLSNSQPSFPVEILPFLYLGCAKDSTNLDVLEEFGIKYILNVTPNLPNLFENAGEFKYKQIPISDHWSQNLSQFFPEAISFIDEARGKNCGVLVHCLAGISRSVTVTVAYLMQKLNLSMNDAYDIVKMKKSNISPNFNFMGQLLDFERTLGLSSPCDNRVPAQQLYFTTPSNQNVYQVDSLQST

906 Q92698 NP_002055.1

>Q92698

MRRSLAPSQLAKRKPEGRSCDDEDWQPGLVTPRKRKSSSETQIQECFLSPFRKPLSQLTNQPPCLDSSQHEAFIRSILSKPFKVPIPNYQGPLGSRALGLKRAGVRRALHDPLEKDALVLYEPPPLSAHDQLKLDKEKLPVHVVVDPILSKVLRPHQREGVKFLWECVTSRRIPGSHGCIMADEMGLGKTLQCITLMWTLLRQSPECKPEIDKAVVVSPSSLVKNWYNEVGKWLGGRIQPLAIDGGSKDEIDQKLEGFMNQRGARVSSPILIISYETFRLHVGVLQKGSVGLVICDEGHRLKNSENQTYQALDSLNTSRRVLISGTPIQNDLLEYFSLVHFVNSGILGTAHEFKKHFELPILKGRDAAASEADRQLGEERLRELTSIVNRCLIRRTSDILSKYLPVKIEQVVCCRLTPLQTELYKRFLRQAKPAEELLEGKMSVSSLSSITSLKKLCNHPALIYDKCVEEEDGFVGALDLFPPGYSSKALEPQLSGKMLVLDYILAVTRSRSSDKVVLVSNYTQTLDLFEKLCRARRYLYVRLDGTMSIKKRAKVVERFNSPSSPDFVFMLSSKAGGCGLNLIGANRLVMFDPDWNPANDEQAMARVWRDGQKKTCYIYRLLSAGTIEEKIFQRQSHKKALSSCVVDEEQDVERHFSLGELKELFILDEASLSDTHDRLHCRRCVNSRQIRPPPDGSDCTSDLAGWNHCTDKWGLRDEVLQAAWDAASTAITFVFHQRSHEEQRGLR

>NP_002055.1

MAQEFVNCKIQPGKVVVFIKPTCPYCRRAQEILSQLPIKQGLLEFVDITATNHTNEIQDYLQQLTGARTVPRVFIGKDCIGGCSDLVSLQQSGELLTRLKQIGALQ

907 NP_002120.1 NP_004160.3

>NP_002120.1

MGKGDPNKPRGKMSSYAFFVQTCREEHKKKHPDSSVNFAEFSKKCSERWKTMSAKEKSKFEDMAKSDKARYDREMKNYVPPKGDKKGKKKDPNAPKRPPSAFFLFCSEHRPKIKSEHPGLSIGDTAKKLGEMWSEQSAKDKQPYEQKAAKLKEKYEKDIAAYRAKGKSEAGKKGPGRPTGSKKKNEPEDEEEEEEEEDEDEEEEDEDEE

>NP_004160.3

MTMPVNGAHKDADLWSSHDKMLAQPLKDSDVEVYNIIKKESNRQRVGLELIASENFASRAVLEALGSCLNNKYSEGYPGQRYYGGTEFIDELETLCQKRALQAYKLDPQCWGVNVQPYSGSPANFAVYTALVEPHGRIMGLDLPDGGHLTHGFMTDKKKISATSIFFESMPYKVNPDTGYINYDQLEENARLFHPKLIIAGTSCYSRNLEYARLRKIADENGAYLMADMAHISGLVAAGVVPSPFEHCHVVTTTTHKTLRGCRAGMIFYRKGVKSVDPKTGKEILYNLESLINSAVFPGLQGGPHNHAIAGVAVALKQAMTLEFKVYQHQVVANCRALSEALTELGYKIVTGGSDNHLILVDLRSKGTDGGRAEKVLEACSIACNKNTCPGDRSALRPSGLRLGTPALTSRGLLEKDFQKVAHFIHRGIELTLQIQSDTGVRATLKEFKERLAGDKYQAAVQALREEVESFASLFPLPGLPDF

908 O43248 NP_001995.1

>O43248

MFNSVNLGNFCSPSRKERGADFGERGSCASNLYLPSCTYYMPEFSTVSSFLPQAPSRQISYPYSAQVPPVREVSYGLEPSGKWHHRNSYSSCYAAADELMHRECLPPSTVTEILMKNEGSYGGHHHPSAPHATPAGFYSSVNKNSVLPQAFDRFFDNAYCGGGDPPAEPPCSGKGEAKGEPEAPPASGLASRAEAGAEAEAEEENTNPSSSGSAHSVAKEPAKGAAPNAPRTRKKRCPYSKFQIRELEREFFFNVYINKEKRLQLSRMLNLTDRQVKIWFQNRRMKEKKLSRDRLQYFSGNPLL

>NP_001995.1

MPLSRWLRSVGVFLLPAPYWAPRERWLGSLRRPSLVHGYPVLAWHSARCWCQAWTEEPRALCSSLRMNGDQNSDVYAQEKQDFVQHFSQIVRVLTEDEMGHPEIGDAIARLKEVLEYNAIGGKYNRGLTVVVAFRELVEPRKQDADSLQRAWTVGWCVELLQAFFLVADDIMDSSLTRRGQICWYQKPGVGLDAINDANLLEACIYRLLKLYCREQPYYLNLIELFLQSSYQTEIGQTLDLLTAPQGNVDLVRFTEKRYKSIVKYKTAFYSFYLPIAAAMYMAGIDGEKEHANAKKILLEMGEFFQIQDDYLDLFGDPSVTGKIGTDIQDNKCSWLVVQCLQRATPEQYQILKENYGQKEAEKVARVKALYEELDLPAVFLQYEEDSYSHIMALIEQYAAPLPPAVFLGLARKIYKRRK

909 NP_005316.1 Q12882

>NP_005316.1

MSETVPPAPAASAAPEKPLAGKKAKKPAKAAAASKKKPAGPSVSELIVQAASSSKERGGVSLAALKKALAAAGYDVEKNNSRIKLGIKSLVSKGTLVQTKGTGASGSFKLNKKASSVETKPGASKVATKTKATGASKKLKKATGASKKSVKTPKKAKKPAATRKSSKNPKKPKTVKPKKVAKSPAKAKAVKPKAAKARVTKPKTAKPKKAAPKKK

>Q12882

MAPVLSKDSADIESILALNPRTQTHATLCSTSAKKLDKKHWKRNPDKNCFNCEKLENNFDDIKHTTLGERGALREAMRCLKCADAPCQKSCPTNLDIKSFITSIANKNYYGAAKMIFSDNPLGLTCGMVCPTSDLCVGGCNLYATEEGPINIGGLQQFATEVFKAMSIPQIRNPSLPPPEKMSEAYSAKIALFGAGPASISCASFLARLGYSDITIFEKQEYVGGLSTSEIPQFRLPYDVVNFEIELMKDLGVKIICGKSLSVNEMTLSTLKEKGYKAAFIGIGLPEPNKDAIFQGLTQDQGFYTSKDFLPLVAKGSKAGMCACHSPLPSIRGVVIVLGAGDTAFDCATSALRCGARRVFIVFRKGFVNIRAVPEEMELAKEEKCEFLPFLSPRKVIVKGGRIVAMQFVRTEQDETGKWNEDEDQMVHLKADVVISAFGSVLSDPKVKEALSPIKFNRWGLPEVDPETMQTSEAWVFAGGDVVGLANTTVESVNDGKQASWYIHKYVQSQYGASVSAKPELPLFYTPIDLVDISVEMAGLKFINPFGLASATPATSTSMIRRAFEAGWGFALTKTFSLDKDIVTNVSPRIIRGTTSGPMYGPGQSSFLNIELISEKTAAYWCQSVTELKADFPDNIVIASIMCSYNKNDWTELAKKSEDSGADALELNLSCPHGMGERGMGLACGQDPELVRNICRWVRQAVQIPFFAKLTPNVTDIVSIARAAKEGGANGVTATNTVSGLMGLKSDGTPWPAVGIAKRTTYGGVSGTAIRPIALRAVTSIARALPGFPILATGGIDSAESGLQFLHSGASVLQVCSAIQNQDFTVIEDYCTGLKALLYLKSIEELQDWDGQSPATVSHQKGKPVPRIAELMDKKLPSFGPYLEQRKKIIAENKIRLKEQNVAFSPLKRNCFIPKRPIPTIKDVIGKALQYLGTFGELSNVEQVVAMIDEEMCINCGKCYMTCNDSGYQAIQFDPETHLPTITDTCTGCTLCLSVCPIVDCIKMVSRTTPYEPKRGVPLSVNPVC

910 P22304 NP_005042.1

>P22304

MPPPRTGRGLLWLGLVLSSVCVALGSETQANSTTDALNVLLIIVDDLRPSLGCYGDKLVRSPNIDQLASHSLLFQNAFAQQAVCAPSRVSFLTGRRPDTTRLYDFNSYWRVHAGNFSTIPQYFKENGYVTMSVGKVFHPGISSNHTDDSPYSWSFPPYHPSSEKYENTKTCRGPDGELHANLLCPVDVLDVPEGTLPDKQSTEQAIQLLEKMKTSASPFFLAVGYHKPHIPFRYPKEFQKLYPLENITLAPDPEVPDGLPPVAYNPWMDIRQREDVQALNISVPYGPIPVDFQRKIRQSYFASVSYLDTQVGRLLSALDDLQLANSTIIAFTSDHGWALGEHGEWAKYSNFDVATHVPLIFYVPGRTASLPEAGEKLFPYLDPFDSASQLMEPGRQSMDLVELVSLFPTLAGLAGLQVPPRCPVPSFHVELCREGKNLLKHFRFRDLEEDPYLPGNPRELIAYSQYPRPSDIPQWNSDKPSLKDIKIMGYSIRTIDYRYTVWVGFNPDEFLANFSDIHAGELYFVDSDPLQDHNMYNDSQGGDLFQLLMP

>NP_005042.1

MAALDSLSLFTSLGLSEQKARETLKNSALSAQLREAATQAQQTLGSTIDKATGILLYGLASRLRDTRRLSFLVSYIASKKIHTEPQLSAALEYVRSHPLDPIDTVDFERECGVGVIVTPEQIEEAVEAAINRHRPQLLVERYHFNMGLLMGEARAVLKWADGKMIKNEVDMQVLHLLGPKLEADLEKKFKVAKARLEETDRRTAKDVVENGETADQTLSLMEQLRGEALKFHKPGENYKTPGYVVTPHTMNLLKQHLEITGGQVRTRFPPEPNGILHIGHAKAINFNFGYAKANNGICFLRFDDTNPEKEEAKFFTAICDMVAWLGYTPYKVTYASDYFDQLYAWAVELIRRGLAYVCHQRGEELKGHNTLPSPWRDRPMEESLLLFEAMRKGKFSEGEATLRMKLVMEDGKMDPVAYRVKYTPHHRTGDKWCIYPTYDYTHCLCDSIEHITHSLCTKEFQARRSSYFWLCNALDVYCPVQWEYGRLNLHYAVVSKRKILQLVATGAVRDWDDPRLFTLTALRRRGFPPEAINNFCARVGVTVAQTTMEPHLLEACVRDVLNDTAPRAMAVLESLRVIITNFPAAKSLDIQVPNFPADETKGFHQVPFAPIVFIERTDFKEEPEPGFKRLAWGQPVGLRHTGYVIELQHVVKGPSGCVESLEVTCRRADAGEKPKAFIHWVSQPLMCEVRLYERLFQHKNPEDPTEVPGGFLSDLNLASLHVVDAALVDCSVALAKPFDKFQFERLGYFSVDPDSHQGKLVFNRTVTLKEDPGKV

911 Q8IXQ5 Q9NZE8

>Q8IXQ5

MAASGVEKSSKKKTEKKLAAREEAKLLAGFMGVMNNMRKQKTLCDVILMVQERKIPAHRVVLAAASHFFNLMFTTNMLESKSFEVELKDAEPDIIEQLVEFAYTARISVNSNNVQSLLDAANQYQIEPVKKMCVDFLKEQVDASNCLGISVLAECLDCPELKATADDFIHQHFTEVYKTDEFLQLDVKRVTHLLNQDTLTVRAEDQVYDAAVRWLKYDEPNRQPFMVDILAKVRFPLISKNFLSKTVQAEPLIQDNPECLKMVISGMRYHLLSPEDREELVDGTRPRRKKHDYRIALFGGSQPQSCRYFNPKDYSWTDIRCPFEKRRDAACVFWDNVVYILGGSQLFPIKRMDCYNVVKDSWYSKLGPPTPRDSLAACAAEGKIYTSGGSEVGNSALYLFECYDTRTESWHTKPSMLTQRCSHGMVEANGLIYVCGGSLGNNVSGRVLNSCEVYDPATETWTELCPMIEARKNHGLVFVKDKIFAVGGQNGLGGLDNVEYYDIKLNEWKMVSPMPWKGVTVKCAAVGSIVYVLAGFQGVGRLGHILEYNTETDKWVANSKVRAFPVTSCLICVVDTCGANEETLET

>Q9NZE8

MAASAFAGAVRAASGILRPLNILASSTYRNCVKNASLISALSTGRFSHIQTPVVSSTPRLTTSERNLTCGHTSVILNRMAPVLPSVLKLPVRSLTYFSARKGKRKTVKAVIDRFLRLHCGLWVRRKAGYKKKLWKKTPARKKRLREFVFCNKTQSKLLDKMTTSFWKRRNWYVDDPYQKYHDRTNLKV

912 NP_079058.1 NP_004835.2

>NP_079058.1

MEELEQGLLMQPWAWLQLAENSLLAKVFITKQGYALLVSDLQQVWHEQVDTSVVSQRAKELNKRLTAPPAAFLCHLDNLLRPLLKDAAHPSEATFSCDCVADALILRVRSELSGLPFYWNFHCMLASPSLVSQHLIRPLMGMSLALQCQVRELATLLHMKDLEIQDYQESGATLIRDRLKTEPFEENSFLEQFMIEKLPEACSIGDGKPFVMNLQDLYMAVTTQEVQVGQKHQGAGDPHTSNSASLQGIDSQCVNQPEQLVSSAPTLSAPEKESTGTSGPLQRPQLSKVKRKKPRGLFS

>NP_004835.2

MDAALKRSRSEEPAEILPPARDEEEEEEEGMEQGLEEEEEVDPRIQGELEKLNQSTDDINRRETELEDARQKFRSVLVEATVKLDELVKKIGKAVEDSKPYWEARRVARQAQLEAQKATQDFQRATEVLRAAKETISLAEQRLLEDDKRQFDSAWQEMLNHATQRVMEAEQTKTRSELVHKETAARYNAAMGRMRQLEKKLKRAINKSKPYFELKAKYYVQLEQLKKTVDDLQAKLTLAKGEYKMALKNLEMISDEIHERRRSSAMGPRGCGVGAEGSSTSVEDLPGSKPEPDAISVASEAFEDDSCSNFVSEDDSETQSVSSFSSGPTSPSEMPDQFPAVVRPGSLDLPSPVSLSEFGMMFPVLGPRSECSGASSPECEVERGDRAEGAENKTSDKANNNRGLSSSSGSGGSSKSQSSTSPEGQALENRMKQLSLQCSKGRDGIIADIKMVQIG

913 O75478 NP_057330.2

>O75478

MDRLGSFSNDPSDKPPCRGCSSYLMEPYIKCAECGPPPFFLCLQCFTRGFEYKKHQSDHTYEIMTSDFPVLDPSWTAQEEMALLEAVMDCGFGNWQDVANQMCTKTKEECEKHYMKHFINNPLFASTLLNLKQAEEAKTADTAIPFHSTDDPPRPTFDSLLSRDMAGYMPARADFIEEFDNYAEWDLRDIDFVEDDSDILHALKMAVVDIYHSRLKERQRRKKIIRDHGLINLRKFQLMERRYPKEVQDLYETMRRFARIVGPVEHDKFIESHALEFELRREIKRLQEYRTAGITNFCSARTYDHLKKTREEERLKRTMLSEVLQYIQDSSACQQWLRRQADIDSGLSPSIPMASNSGRRSAPPLNLTGLPGTEKLNEKEKELCQMVRLVPGAYLEYKSALLNECNKQGGLRLAQARALIKIDVNKTRKIYDFLIREGYITKG

>NP_057330.2

MATGTRYAGKVVVVTGGGRGIGAGIVRAFVNSGARVVICDKDESGGRALEQELPGAVFILCDVTQEDDVKTLVSETIRRFGRLDCVVNNAGHHPPPQRPEETSAQGFRQLLELNLLGTYTLTKLALPYLRKSQGNVINISSLVGAIGQAQAVPYVATKGAVTAMTKALALDESPYGVRVNCISPGNIWTPLWEELAALMPDPRATIREGMLAQPLGRMGQPAEVGAAAVFLASEANFCTGIELLVTGGAELGYGCKASRSTPVDAPDIPS

914 AAG28389.1 Q3I5F7

>AAG28389.1

MSTEGGFGGTSSSDAQQSLQSFWPRVMEEIRNLTVKDFRVQELPLARIKKIMKLDEDVKMISAEAPVLFAKAAQIFITELTLRAWIHTEDNKRRTLQRNDIAMAITKFDQFDFLIDIVPRDELKPPKRQEEVRQSVTPAEPVQYYFTLAQQPTAVQVQGQQQGQQTTSSTTTIQPGQIIIAQPQQGQTTPVTMQVGEGQQVQIVQAQPQGQAQQAQSGTGQTMQVMQQIITNTGEIQQIPVQLNAGQLQYIRLAQPVSGTQVVQGQIQTLATNAQQGQRNASQGKPRRCLKETLQITQTEVQQGQQQFSQFTDGQRNSVQQARVSELTGEAEPREVKATGNSTPCTSSLPTTHPPSHRAGASCVCCSQPQQSSTSPPPSDALQWVVVEVSGTPNQLETHRELHAPLPGMTSLSPLHPSQQLYQIQQVTMPAGQDLAQPMFIQSANQPSDGQAPQVTGD

>Q3I5F7

MLQHPKVKGPSIALLGFSKGGDLCLSMASFLKGITATVLINACVANTVAPLHYKDMIIPKLVDDLGKVKITKSGFLTFMDTWSNPLEEHNHQSLVPLEKAQVPFLFIVGMDDQSWKSEFYAQIASERLQAHGKERPQIICYPETGHCIDPPYFPPSRASVHAVLGEAIFYGGEPKAHSKAQVDAWQQIQTFFHKHLNGKKSVKHSKI

915 NP_055028.2 NP_647473.1

>NP_055028.2

MSSSGTLSNYYVDSLIGHEGDEVFAARFGPPGPGAQGRPAGVADGPAATAAEFASCSFAPRSAVFSASWSAVPSQPPAAAAMSGLYHPYVPPPPLAASASEPGRYVRSWMEPLPGFPGGAGGGGGGGGGGPGRGPSPGPSGPANGRHYGIKPETRAAPAPATAASTTSSSSTSLSSSSKRTECSVARESQGSSGPEFSCNSFLQEKAAAATGGTGPGAGIGAATGTGGSSEPSACSDHPIPGCSLKEEEKQHSQPQQQQLDPNNPAANWIHARSTRKKRCPYTKYQTLELEKEFLFNMYLTRDRRYEVARILNLTERQVKIWFQNRRMKMKKMSKEKCPKGD

>NP_647473.1

MFSLSSTVQPQVTVPLSHLINAFHTPKNTSVSLSGVSVSQNQHRDVVPEHEAPSSECMFSDFLTKLNIVSIGKGKIFEGYRSMFMEPAKRMKKSLDTTDNWHIRPEPFSLSIPPSLNLRDLGLSELKIGQIDQLVENLLPGFCKGKNISSHWHTSHVSAQSFFENKYGNLDIFSTLRSSCLYRHHSRALQSICSDLQYWPVFIQSRGFKTLKSRTRRLQSTSERLAETQNIAPSFVKGFLLRDRGSDVESLDKLMKTKNIPEAHQDAFKTGFAEGFLKAQALTQKTNDSLRRTRLILFVLLLFGIYGLLKNPFLSVRFRTTTGLDSAVDPVQMKNVTFEHVKGVEEAKQELQEVVEFLKNPQKFTILGGKLPKGILLVGPPGTGKTLLARAVAGEADVPFYYASGSEFDEMFVGVGASRIRNLFREAKANAPCVIFIDELDSVGGKRIESPMHPYSRQTINQLLAEMDGFKPNEGVIIIGATNFPEALDNALIRPGRFDMQVTVPRPDVKGRTEILKWYLNKIKFDQSVDPEIIARGTVGFSGAELENLVNQAALKAAVDGKEMVTMKELEFSKDKILMGPERRSVEIDNKNKTITAYHESGHAIIAYYTKDAMPINKATIMPRGPTLGHVSLLPENDRWNETRAQLLAQMDVSMGGRVAEELIFGTDHITTGASSDFDNATKIAKRMVTKFGMSEKLGVMTYSDTGKLSPETQSAIEQEIRILLRDSYERAKHILKTHAKEHKNLAEALLTYETLDAKEIQIVLEGKKLEVR

916 Q06265 Q99547

>Q06265

MKETPLSNCERRFLLRAIEEKKRLDGRQTYDYRNIRISFGTDYGCCIVELGKTRVLGQVSCELVSPKLNRATEGILFFNLELSQMAAPAFEPGRQSDLLVKLNRLMERCLRNSKCIDTESLCVVAGEKVWQIRVDLHLLNHDGNIIDAASIAAIVALCHFRRPDVSVQGDEVTLYTPEERDPVPLSIHHMPICVSFAFFQQGTYLLVDPNEREERVMDGLLVIAMNKHREICTIQSSGGIMLLKDQVLRCSKIAGVKVAEITELILKALENDQKVRKEGGKFGFAESIANQRITAFKMEKAPIDTSDVEEKAEEIIAEAEPPSEVVSTPVLWTPGTAQIGEGVENSWGDLEDSEKEDDEGGGDQAIILDGIKMDTGVEVSDIGSQDAPIILSDSEEEEMIILEPDKNPKKIRTQTTSAKQEKAPSKKPVKRRKKKRAAN

>Q99547

MAAERKTKLSKNLLRMKFMQRGLDSETKKQLEEEEKKIISEEHWYLDLPELKEKESFIIEEQSFLLCEDLLYGRMSFRGFNPEVEKLMLQMNAKHKAEEVEDETVELDVSDEEMARRYETLVGTIGKKFARKRDHANYEEDENGDITPIKAKKMFLKPQD

917 NP_004166.1 NP_056442.2

>NP_004166.1

MSIGVPIKVLHEAEGHIVTCETNTGEVYRGKLIEAEDNMNCQMSNITVTYRDGRVAQLEQVYIRGSKIRFLILPDMLKNAPMLKSMKNKNQGSGAGRGKAAILKAQVAARGRGRGMGRGNIFQKRR

>NP_056442.2

MDALKSAGRALIRSPSLAKQSWGGGGRHRKLPENWTDTRETLLEGMLFSLKYLGMTLVEQPKGEELSAAAIKRIVATAKASGKKLQKVTLKVSPRGIILTDNLTNQLIENVSIYRISYCTADKMHDKVFAYIAQSQHNQSLECHAFLCTKRKMAQAVTLTVAQAFKVAFEFWQVSKEEKEKRDKASQEGGDVLGARQDCTPSLKSLVATGNLLDLEETAKAPLSTVSANTTNMDEVPRPQALSGSSVVWELDDGLDEAFSRLAQSRTNPQVLDTGLTAQDMHYAQCLSPVDWDKPDSSGTEQDDLFSF

918 Q13461 NP_004126.1

>Q13461

MAGRSDMDPPAAFSGFPALPAVAPSGPPPSPLAGAEPGREPEEAAAGRGEAAPTPAPGPGRRRRRPLQRGKPPYSYIALIAMALAHAPGRRLTLAAIYRFITERFAFYRDSPRKWQNSIRHNLTLNDCFVKVPREPGNPGKGNYWTLDPAAADMFDNGSFLRRRKRFKRAELPAHAAAAPGPPLPFPYAPYAPAPGPALLVPPPSAGPGPSPPARLFSVDSLVNLQPELAGLGAPEPPCCAAPDAAAAAFPPCAAAASPPLYSQVPDRLVLPATRPGPGPLPAEPLLALAGPAAALGPLSPGEAYLRQPGFASGLERYL

>NP_004126.1

MALKVATVAGSAAKAVLGPALLCRPWEVLGAHEVPSRNIFSEQTIPPSAKYGGRHTVTMIPGDGIGPELMLHVKSVFRHACVPVDFEEVHVSSNADEEDIRNAIMAIRRNRVALKGNIETNHNLPPSHKSRNNILRTSLDLYANVIHCKSLPGVVTRHKDIDILIVRENTEGEYSSLEHESVAGVVESLKIITKAKSLRIAEYAFKLAQESGRKKVTAVHKANIMKLGDGLFLQCCREVAARYPQITFENMIVDNTTMQLVSRPQQFDVMVMPNLYGNIVNNVCAGLVGGPGLVAGANYGHVYAVFETATRNTGKSIANKNIANPTATLLASCMMLDHLKLHSYATSIRKAVLASMDNENMHTPDIGGQGTTSEAIQDVIRHIRVINGRAVEA

919 Q16560 NP_005172.1

>Q16560

MNDWMPIAKEYDPLKAGSIDGTDEDPHDRAVWRAMLARYVPNKGVIGDPLLTLFVARLNLQTKEDKLKEVFSRYGDIRRLRLVRDLVTGFSKGYAFIEYKEERAVIKAYRDADGLVIDQHEIFVDYELERTLKGWIPRRLGGGLGGKKESGQLRFGGRDRPFRKPINLPVVKNDLYREGKRERRERSRSRERHWDSRTRDRDHDRGREKRWQEREPTRVWPDNDWERERDFRDDRIKGREKKERGK

>NP_005172.1

MAKEWGYASHNGPDHWHELFPNAKGENQSPVELHTKDIRHDPSLQPWSVSYDGGSAKTILNNGKTCRVVFDDTYDRSMLRGGPLPGPYRLRQFHLHWGSSDDHGSEHTVDGVKYAAELHLVHWNPKYNTFKEALKQRDGIAVIGIFLKIGHENGEFQIFLDALDKIKTKGKEAPFTKFDPSCLFPACRDYWTYQGSFTTPPCEECIVWLLLKEPMTVSSDQMAKLRSLLSSAENEPPVPLVSNWRPPQPINNRVVRASFK

920 NP_663628.1 NP_689953.1

>NP_663628.1

MAAGSTTLRAVGKLQVRLATKTEPKKLEKYLQKLSALPMTADILAETGIRKTVKRLRKHQHVGDFARDLAARWKKLVLVDRNTGPDPQDPEESASRQRFGEALQEREKAWGFPENATAPRSPSHSPEHRRTARRTPPGQQRPHPRSPSREPRAERKRPRMAPADSGPHRDPPTRTAPLPMPEGPEPAVPGEQPGRGHAHAAQGGPLLGQGCQGQPQGEAVGSHSKGHKSSRGASAQKSPPVQESQSERLQAAGADSAGPKTVPSHVFSELWDPSEAWMQANYDLLSAFEAMTSQANPEALCAPALQEEAAFPGRRVNAKMPVYSGSRPACQLQVPTLRQQCLRVPRNNPDALGDVEGVPYSVLEPVLEGWTPDQLYRTEKDNAALARETDELWRIHCLQDFKEEKPQEHESWRELYLRLRDAREQRLRVVTTKIRSARENKPSGRQTKMICFNSVAKTPYDASRRQEKSAGAADPGNGEMEPAPKPAGSSQAPSGLGDGDGGSVSGGGSSNRHAAPADKTRKQAAKKVAPLMAKAIRDYKGRFSRR

>NP_689953.1

MAASLRLLGAASGLRYWSRRLRPAAGSFAAVCSRSVASKTPVGFIGLGNMGNPMAKNLMKHGYPLIIYDVFPDACKEFQDAGEQVVSSPADVAEKADRIITMLPTSINAIEAYSGANGILKKVKKGSLLIDSSTIDPAVSKELAKEVEKMGAVFMDAPVSGGVGAARSGNLTFMVGGVEDEFAAAQELLGCMGSNVVYCGAVGTGQAAKICNNMLLAISMIGTAEAMNLGIRLGLDPKLLAKILNMSSGRCWSSDTYNPVPGVMDGVPSANNYQGGFGTTLMAKDLGLAQDSATSTKSPILLGSLAHQIYRMMCAKGYSKKDFSSVFQFLREEETF

921 O15370 Q9NR19

>O15370

MVQQRGARAKRDGGPPPPGPGPAEEGAREPGWCKTPSGHIKRPMNAFMVWSQHERRKIMDQWPDMHNAEISKRLGRRWQLLQDSEKIPFVREAERLRLKHMADYPDYKYRPRKKSKGAPAKARPRPPGGSGGGSRLKPGPQLPGRGGRRAAGGPLGGGAAAPEDDDEDDDEELLEVRLVETPGRELWRMVPAGRAARGQAERAQGPSGEGAAAAAAASPTPSEDEEPEEEEEEAAAAEEGEEETVASGEESLGFLSRLPPGPAGLDCSALDRDPDLQPPSGTSHFEFPDYCTPEVTEMIAGDWRPSSIADLVFTY

>Q9NR19

MGLPEERVRSGSGSRGQEEAGAGGRARSWSPPPEVSRSAHVPSLQRYRELHRRSVEEPREFWGDIAKEFYWKTPCPGPFLRYNFDVTKGKIFIEWMKGATTNICYNVLDRNVHEKKLGDKVAFYWEGNEPGETTQITYHQLLVQVCQFSNVLRKQGIQKGDRVAIYMPMIPELVVAMLACARIGALHSIVFAGFSSESLCERILDSSCSLLITTDAFYRGEKLVNLKELADEALQKCQEKGFPVRCCIVVKHLGRAELGMGDSTSQSPPIKRSCPDVQISWNQGIDLWWHELMQEAGDECEPEWCDAEDPLFILYTSGSTGKPKGVVHTVGGYMLYVATTFKYVFDFHAEDVFWCTADIGWITGHSYVTYGPLANGATSVLFEGIPTYPDVNRLWSIVDKYKVTKFYTAPTAIRLLMKFGDEPVTKHSRASLQVLGTVGEPINPEAWLWYHRVVGAQRCPIVDTFWQTETGGHMLTPLPGATPMKPGSATFPFFGVAPAILNESGEELEGEAEGYLVFKQPWPGIMRTVYGNHERFETTYFKKFPGYYVTGDGCQRDQDGYYWITGRIDDMLNVSGHLLSTAEVESALVEHEAVAEAAVVGHPHPVKGECLYCFVTLCDGHTFSPKLTEELKKQIREKIGPIATPDYIQNAPGLPKTRSGKIMRRVLRKIAQNDHDLGDMSTVADPSVISHLFSHRCLTIQ

922 NP_056986.2 NP_000660.1

>NP_056986.2

MTSIHFVVHPLPGTEDQLNDRLREVSEKLNKYNLNSHPPLNVLEQATIKQCVVGPNHAAFLLEDGRVCRIGFSVQPDRLELGKPDNNDGSKLNSNSGAGRTSRPGRTSDSPWFLSGSETLGRLAGNTLGSRWSSGVGGSGGGSSGRSSAGARDSRRQTRVIRTGRDRGSGLLGSQPQPVIPASVIPEELISQAQVVLQGKSRSVIIRELQRTNLDVNLAVNNLLSRDDEDGDDGDDTASESYLPGEDLMSLLDADIHSAHPSVIIDADAMFSEDISYFGYPSFRRSSLSRLGSSRVLLLPLERDSELLRERESVLRLRERRWLDGASFDNERGSTSKEGEPNLDKKNTPVQSPVSLGEDLQWWPDKDGTKFICIGALYSELLAVSSKGELYQWKWSESEPYRNAQNPSLHHPRATFLGLTNEKIVLLSANSIRATVATENNKVATWVDETLSSVASKLEHTAQTYSELQGERIVSLHCCALYTCAQLENSLYWWGVVPFSQRKKMLEKARAKNKKPKSSAGISSMPNITVGTQVCLRNNPLYHAGAVAFSISAGIPKVGVLMESVWNMNDSCRFQLRSPESLKNMEKASKTTEAKPESKQEPVKTEMGPPPSPASTCSDASSIASSASMPYKRRRSTPAPKEEEKVNEEQWSLREVVFVEDVKNVPVGKVLKVDGAYVAVKFPGTSSNTNCQNSSGPDADPSSLLQDCRLLRIDELQVVKTGGTPKVPDCFQRTPKKLCIPEKTEILAVNVDSKGVHAVLKTGNWVRYCIFDLATGKAEQENNFPTSSIAFLGQNERNVAIFTAGQESPIILRDGNGTIYPMAKDCMGGIRDPDWLDLPPISSLGMGVHSLINLPANSTIKKKAAVIIMAVEKQTLMQHILRCDYEACRQYLMNLEQAVVLEQNLQMLQTFISHRCDGNRNILHACVSVCFPTSNKETKEEEEAERSERNTFAERLSAVEAIANAISVVSSNGPGNRAGSSSSRSLRLREMMRRSLRAAGLGRHEAGASSSDHQDPVSPPIAPPSWVPDPPAMDPDGDIDFILAPAVGSLTTAATGTGQGPSTSTIPGPSTEPSVVESKDRKANAHFILKLLCDSVVLQPYLRELLSAKDARGMTPFMSAVSGRAYPAAITILETAQKIAKAEISSSEKEEDVFMGMVCPSGTNPDDSPLYVLCCNDTCSFTWTGAEHINQDIFECRTCGLLESLCCCTECARVCHKGHDCKLKRTSPTAYCDCWEKCKCKTLIAGQKSARLDLLYRLLTATNLVTLPNSRGEHLLLFLVQTVARQTVEHCQYRPPRIREDRNRKTASPEDSDMPDHDLEPPRFAQLALERVLQDWNALKSMIMFGSQENKDPLSASSRIGHLLPEEQVYLNQQSGTIRLDCFTHCLIVKCTADILLLDTLLGTLVKELQNKYTPGRREEAIAVTMRFLRSVARVFVILSVEMASSKKKNNFIPQPIGKCKRVFQALLPYAVEELCNVAESLIVPVRMGIARPTAPFTLASTSIDAMQGSEELFSVEPLPPRPSSDQSSSSSQSQSSYIIRNPQQRRISQSQPVRGRDEEQDDIVSADVEEVEVVEGVAGEEDHHDEQEEHGEENAEAEGQHDEHDEDGSDMELDLLAAAETESDSESNHSNQDNASGRRSVVTAATAGSEAGASSVPAFFSEDDSQSNDSSDSDSSSSQSDDIEQETFMLDEPLERTTNSSHANGAAQAPRSMQWAVRNTQHQRAASTAPSSTSTPAASSAGLIYIDPSNLRRSGTISTSAAAAAAALEASNASSYLTSASSLARAYSIVIRQISDLMGLIPKYNHLVYSQIPAAVKLTYQDAVNLQNYVEEKLIPTWNWMVSIMDSTEAQLRYGSALASAGDPGHPNHPLHASQNSARRERMTAREEASLRTLEGRRRATLLSARQGMMSARGDFLNYALSLMRSHNDEHSDVLPVLDVCSLKHVAYVFQALIYWIKAMNQQTTLDTPQLERKRTRELLELGIDNEDSEHENDDDTNQSATLNDKDDDSLPAETGQNHPFFRRSDSMTFLGCIPPNPFEVPLAEAIPLADQPHLLQPNARKEDLFGRPSQGLYSSSASSGKCLMEVTVDRNCLEVLPTKMSYAANLKNVMNMQNRQKKEGEEQPVLPEETESSKPGPSAHDLAAQLKSSLLAEIGLTESEGPPLTSFRPQCSFMGMVISHDMLLGRWRLSLELFGRVFMEDVGAEPGSILTELGGFEVKESKFRREMEKLRNQQSRDLSLEVDRDRDLLIQQTMRQLNNHFGRRCATTPMAVHRVKVTFKDEPGEGSGVARSFYTAIAQAFLSNEKLPNLECIQNANKGTHTSLMQRLRNRGERDREREREREMRRSSGLRAGSRRDRDRDFRRQLSIDTRPFRPASEGNPSDDPEPLPAHRQALGERLYPRVQAMQPAFASKITGMLLELSPAQLLLLLASEDSLRARVDEAMELIIAHGRENGADSILDLGLVDSSEKVQQENRKRHGSSRSVVDMDLDDTDDGDDNAPLFYQPGKRGFYTPRPGKNTEARLNCFRNIGRILGLCLLQNELCPITLNRHVIKVLLGRKVNWHDFAFFDPVMYESLRQLILASQSSDADAVFSAMDLAFAIDLCKEEGGGQVELIPNGVNIPVTPQNVYEYVRKYAEHRMLVVAEQPLHAMRKGLLDVLPKNSLEDLTAEDFRLLVNGCGEVNVQMLISFTSFNDESGENAEKLLQFKRWFWSIVEKMSMTERQDLVYFWTSSPSLPASEEGFQPMPSITIRPPDDQHLPTANTCISRLYVPLYSSKQILKQKLLLAIKTKNFGFV

>NP_000660.1

MSTAGKVIKCKAAVLWELKKPFSIEEVEVAPPKAHEVRIKMVAAGICRSDEHVVSGNLVTPLPVILGHEAAGIVESVGEGVTTVKPGDKVIPLFTPQCGKCRICKNPESNYCLKNDLGNPRGTLQDGTRRFTCSGKPIHHFVGVSTFSQYTVVDENAVAKIDAASPLEKVCLIGCGFSTGYGSAVKVAKVTPGSTCAVFGLGGVGLSVVMGCKAAGAARIIAVDINKDKFAKAKELGATECINPQDYKKPIQEVLKEMTDGGVDFSFEVIGRLDTMMASLLCCHEACGTSVIVGVPPDSQNLSINPMLLLTGRTWKGAIFGGFKSKESVPKLVADFMAKKFSLDALITNILPFEKINEGFDLLRSGKSIRTVLTF

923 Q99081 NP_054765.1

>Q99081

MNPQQQRMAAIGTDKELSDLLDFSAMFSPPVNSGKTRPTTLGSSQFSGSGIDERGGTTSWGTSGQPSPSYDSSRGFTDSPHYSDHLNDSRLGAHEGLSPTPFMNSNLMGKTSERGSFSLYSRDTGLPGCQSSLLRQDLGLGSPAQLSSSGKPGTAYYSFSATSSRRRPLHDSAALDPLQAKKVRKVPPGLPSSVYAPSPNSDDFNRESPSYPSPKPPTSMFASTFFMQDGTHNSSDLWSSSNGMSQPGFGGILGTSTSHMSQSSSYGNLHSHDRLSYPPHSVSPTDINTSLPPMSSFHRGSTSSSPYVAASHTPPINGSDSILGTRGNAAGSSQTGDALGKALASIYSPDHTSSSFPSNPSTPVGSPSPLTGTSQWPRPGGQAPSSPSYENSLHSLQSRMEDRLDRLDDAIHVLRNHAVGPSTSLPAGHSDIHSLLGPSHNAPIGSLNSNYGGSSLVASSRSASMVGTHREDSVSLNGNHSVLSSTVTTSSTDLNHKTQENYRGGLQSQSGTVVTTEIKTENKEKDENLHEPPSSDDMKSDDESSQKDIKVSSRGRTSSTNEDEDLNPEQKIEREKERRMANNARERLRVRDINEAFKELGRMCQLHLKSEKPQTKLLILHQAVAVILSLEQQVRERNLNPKAACLKRREEEKVSAVSAEPPTTLPGTHPGLSETTNPMGHM

>NP_054765.1

MAASVLNTVLRRLPMLSLFRGSHRVQVPLQTLCTKAPSEEDSLSSVPISPYKDEPWKYLESEEYQERYGSRPVWADYRRNHKGGVPPQRTRKTCIRRNKVVGNPCPICRDHKLHVDFRNVKLLEQFVCAHTGIIFYAPYTGVCVKQHKRLTQAIQKARDHGLLIYHIPQVEPRDLDFSTSHGAVSATPPAPTLVSGDPWYPWYNWKQPPERELSRLRRLYQGHLQEESGPPPESMPKMPPRTPAEASSTGQTGPQSAL

924 NP_055850.1 NP_000365.3

>NP_055850.1

MASKRKSTTPCMIPVKTVVLQDASMEAQPAETLPEGPQQDLPPEASAASSEAAQNPSSTDGSTLANGHRSTLDGYLYSCKYCDFRSHDMTQFVGHMNSEHTDFNKDPTFVCSGCSFLAKTPEGLSLHNATCHSGEASFVWNVAKPDNHVVVEQSIPESTSTPDLAGEPSAEGADGQAEIIITKTPIMKIMKGKAEAKKIHTLKENVPSQPVGEALPKLSTGEMEVREGDHSFINGAVPVSQASASSAKNPHAANGPLIGTVPVLPAGIAQFLSLQQQPPVHAQHHVHQPLPTAKALPKVMIPLSSIPTYNAAMDSNSFLKNSFHKFPYPTKAELCYLTVVTKYPEEQLKIWFTAQRLKQGISWSPEEIEDARKKMFNTVIQSVPQPTITVLNTPLVASAGNVQHLIQAALPGHVVGQPEGTGGGLLVTQPLMANGLQATSSPLPLTVTSVPKQPGVAPINTVCSNTTSAVKVVNAAQSLLTACPSITSQAFLDASIYKNKKSHEQLSALKGSFCRNQFPGQSEVEHLTKVTGLSTREVRKWFSDRRYHCRNLKGSRAMIPGDHSSIIIDSVPEVSFSPSSKVPEVTCIPTTATLATHPSAKRQSWHQTPDFTPTKYKERAPEQLRALESSFAQNPLPLDEELDRLRSETKMTRREIDSWFSERRKKVNAEETKKAEENASQEEEEAAEDEGGEEDLASELRVSGENGSLEMPSSHILAERKVSPIKINLKNLRVTEANGRNEIPGLGACDPEDDESNKLAEQLPGKVSCKKTAQQRHLLRQLFVQTQWPSNQDYDSIMAQTGLPRPEVVRWFGDSRYALKNGQLKWYEDYKRGNFPPGLLVIAPGNRELLQDYYMTHKMLYEEDLQNLCDKTQMSSQQVKQWFAEKMGEETRAVADTGSEDQGPGTGELTAVHKGMGDTYSEVSENSESWEPRVPEASSEPFDTSSPQAGRQLETD

>NP_000365.3

MEANGLGPQGFPELKNDTFLRAAWGEETDYTPVWCMRQAGRYLPEFRETRAAQDFFSTCRSPEACCELTLQPLRRFPLDAAIIFSDILVVPQALGMEVTMVPGKGPSFPEPLREEQDLERLRDPEVVASELGYVFQAITLTRQRLAGRVPLIGFAGAPWTLMTYMVEGGGSSTMAQAKRWLYQRPQASHQLLRILTDALVPYLVGQVVAGAQALQLFESHAGHLGPQLFNKFALPYIRDVAKQVKARLREAGLAPVPMIIFAKDGHFALEELAQAGYEVVGLDWTVAPKKARECVGKTVTLQGNLDPCALYASEEEIGQLVKQMLDDFGPHRYIANLGHGLYPDMDPEHVGAFVDAVHKHSRLLRQN

925 NP_002120.1 Q7Z569

>NP_002120.1

MGKGDPNKPRGKMSSYAFFVQTCREEHKKKHPDSSVNFAEFSKKCSERWKTMSAKEKSKFEDMAKSDKARYDREMKNYVPPKGDKKGKKKDPNAPKRPPSAFFLFCSEHRPKIKSEHPGLSIGDTAKKLGEMWSEQSAKDKQPYEQKAAKLKEKYEKDIAAYRAKGKSEAGKKGPGRPTGSKKKNEPEDEEEEEEEEDEDEEEEDEDEE

>Q7Z569

MSVSLVVIRLELAEHSPVPAGFGFSAAAGEMSDEEIKKTTLASAVACLEGKSPGEKVAIIHQHLGRREMTDVIIETMKSNPDELKTTVEERKSSEASPTAQRSKDHSKECINAAPDSPSKQLPDQISFFSGNPSVEIVHGIMHLYKTNKMTSLKEDVRRSAMLCILTVPAAMTSHDLMKFVAPFNDVIEQMKIIRDSTPNQYMVLIKFRAQADADSFYMTCNGRQFNSIEDDVCQLVYVERAEVLKSEDGASLPVMDLTELPKCTVCLERMDESVNGILTTLCNHSFHSQCLQRWDDTTCPVCRYCQTPEPVEENKCFECGVQENLWICLICGHIGCGRYVSRHAYKHFEETQHTYAMQLTNHRVWDYAGDNYVHRLVASKTDGKIVQYECEGDTCQEEKIDALQLEYSYLLTSQLESQRIYWENKIVRIEKDTAEEINNMKTKFKETIEKCDNLEHKLNDLLKEKQSVERKCTQLNTKVAKLTNELKEEQEMNKCLRANQVLLQNKLKEEERVLKETCDQKDLQITEIQEQLRDVMFYLETQQKINHLPAETRQEIQEGQINIAMASASSPASSGGSGKLPSRKGRSKRGK

926 Q6DN03 P23434

>Q6DN03

MPEPAKFAPAPKKGSKKAVTKAQKKDGKKRKRSRKESYSIYVYKVLKRVHPDTGIWCKAMGIMNSFLNDIFERIAGEASRLAHYNKRSTITSRRSRRPCACCCPASWPSTPCPRAPRRSPSTPAPSESLPGPGARSLPPSLPPRVAGCFVSKGSFQGHLTTSVKESFLCCQSQLMFLASRLVNFRRAHNTKHR

>P23434

MALRVVRSVRALLCTLRAVPLPAAPCPPRPWQLGVGAVRTLRTGPALLSVRKFTEKHEWVTTENGIGTVGISNFAQEALGDVVYCSLPEVGTKLNKQDEFGALESVKAASELYSPLSGEVTEINEALAENPGLVNKSCYEDGWLIKMTLSNPSELDELMSEEAYEKYIKSIEE

927 P09430 NP_002717.3

>P09430

MSTSRKLKSHGMRRSKSRSPHKGVKRGGSKRKYRKGNLKSRKRGDDANRNYRSHL

>NP_002717.3

MLSLQYPDVYRDETAVQDYHGHKICDPYAWLEDPDSEQTKAFVEAQNKITVPFLEQCPIRGLYKERMTELYDYPKYSCHFKKGKRYFYFYNTGLQNQRVLYVQDSLEGEARVFLDPNILSDDGTVALRGYAFSEDGEYFAYGLSASGSDWVTIKFMKVDGAKELPDVLERVKFSCMAWTHDGKGMFYNSYPQQDGKSDGTETSTNLHQKLYYHVLGTDQSEDILCAEFPDEPKWMGGAELSDDGRYVLLSIREGCDPVNRLWYCDLQQESSGIAGILKWVKLIDNFEGEYDYVTNEGTVFTFKTNRQSPNYRVINIDFRDPEESKWKVLVPEHEKDVLEWIACVRSNFLVLCYLHDVKNILQLHDLTTGALLKTFPLDVGSIVGYSGQKKDTEIFYQFTSFLSPGIIYHCDLTKEELEPRVFREVTVKGIDASDYQTVQIFYPSKDGTKIPMFIVHKKGIKLDGSHPAFLYGYGGFNISITPNYSVSRLIFVRHMGGILAVANIRGGGEYGETWHKGGILANKQNCFDDFQCAAEYLIKEGYTSPKRLTINGGSNGGLLVAACANQRPDLFGCVIAQVGVMDMLKFHKYTIGHAWTTDYGCSDSKQHFEWLVKYSPLHNVKLPEADDIQYPSMLLLTADHDDRVVPLHSLKFIATLQYIVGRSRKQSNPLLIHVDTKAGHGAGKPTAKVIEEVSDMFAFIARCLNVDWIP

928 NP_006708.2 NP_006784.1

>NP_006708.2

MKTPFGKTPGQRSRADAGHAGVSANMMKKRTSHKKHRSSVGPSKPVSQPRRNIVGCRIQHGWKEGNGPVTQWKGTVLDQVPVNPSLYLIKYDGFDCVYGLELNKDERVSALEVLPDRVATSRISDAHLADTMIGKAVEHMFETEDGSKDEWRGMVLARAPVMNTWFYITYEKDPVLYMYQLLDDYKEGDLRIMPDSNDSPPAEREPGEVVDSLVGKQVEYAKEDGSKRTGMVIHQVEAKPSVYFIKFDDDFHIYVYDLVKTS

>NP_006784.1

MAAAVGRLLRASVARHVSAIPWGISATAALRPAACGRTSLTNLLCSGSSQAKLFSTSSSCHAPAVTQHAPYFKGTAVVNGEFKDLSLDDFKGKYLVLFFYPLDFTFVCPTEIVAFSDKANEFHDVNCEVVAVSVDSHFSHLAWINTPRKNGGLGHMNIALLSDLTKQISRDYGVLLEGSGLALRGLFIIDPNGVIKHLSVNDLPVGRSVEETLRLVKAFQYVETHGEVCPANWTPDSPTIKPSPAASKEYFQKVNQ

929 Q9ULG1 Q9NR19

>Q9ULG1

MASELGARDDGGCTELAKPLYLQYLERALRLDHFLRQTSAIFNRNISSDDSEDGLDDSNPLLPQSGDPLIQVKEEPPNSLLGETSGAGSSGMLNTYSLNGVLQSESKCDKGNLYNFSKLKKSRKWLKSILLSDESSEADSQSEDDDEEELNLSREELHNMLRLHKYKKLHQNKYSKDKELQQYQYYSAGLLSTYDPFYEQQRHLLGPKKKKFKEEKKLKAKLKKVKKKRRRDEELSSEESPRRHHHQTKVFAKFSHDAPPPGTKKKHLSIEQLNARRRKVWLSIVKKELPKANKQKASARNLFLTNSRKLAHQCMKEVRRAALQAQKNCKETLPRARRLTKEMLLYWKKYEKVEKEHRKRAEKEALEQRKLDEEMREAKRQQRKLNFLITQTELYAHFMSRKRDMGHDGIQEEILRKLEDSSTQRQIDIGGGVVVNITQEDYDSNHFKAQALKNAENAYHIHQARTRSFDEDAKESRAAALRAANKSGTGFGESYSLANPSIRAGEDIPQPTIFNGKLKGYQLKGMNWLANLYEQGINGILADEMGLGKTVQSIALLAHLAERENIWGPFLIISPASTLNNWHQEFTRFVPKFKVLPYWGNPHDRKVIRRFWSQKTLYTQDAPFHVVITSYQLVVQDVKYFQRVKWQYMVLDEAQALKSSSSVRWKILLQFQCRNRLLLTGTPIQNTMAELWALLHFIMPTLFDSHEEFNEWFSKDIESHAENKSAIDENQLSRLHMILKPFMLRRIKKDVENELSDKIEILMYCQLTSRQKLLYQALKNKISIEDLLQSSMGSTQQAQNTTSSLMNLVMQFRKVCNHPELFERQETWSPFHISLKPYHISKFIYRHGQIRVFNHSRDRWLRVLSPFAPDYIQRSLFHRKGINEESCFSFLRFIDISPAEMANLMLQGLLARWLALFLSLKASYRLHQLRSWGAPEGESHQRYLRNKDFLLGVNFPLSFPNLCSCPLLKSLVFSSHCKAVSGYSDQVVHQRRSATSSLRRCLLTELPSFLCVASPRVTAVPLDSYCNDRSAEYERRVLKEGGSLAAKQCLLNGAPELAADWLNRRSQFFPEPAGGLWSIRPQNGWSFIRIPGKESLITDSGKLYALDVLLTRLKSQGHRVLIYSQMTRMIDLLEEYMVYRKHTYMRLDGSSKISERRDMVADFQNRNDIFVFLLSTRAGGLGINLTAADTVIFYDSDWNPTVDQQAMDRAHRLGQTKQVTVYRLICKGTIEERILQRAKEKSEIQRMVISGGNFKPDTLKPKEVVSLLLDDEELEKKLRLRQEEKRQQEETNRVKERKRKREKYAEKKKKEDELDGKRRKEGVNLVIPFVPSADNSNLSADGDDSFISVDSAMPSPFSEISISSELHTGSIPLDESSSDMLVIVDDPASSAPQSRATNSPASITGSVSDTVNGISIQEMPAAGRGHSARSRGRPKGSGSTAKGAGKGRSRKSTAGSAAAMAGAKAGAAAASAAAYAAYGYNVSKGISASSPLQTSLVRPAGLADFGPSSASSPLSSPLSKGNNVPGNPKNLHMTSSLAPDSLVRKQGKGTNPSGGR

>Q9NR19

MGLPEERVRSGSGSRGQEEAGAGGRARSWSPPPEVSRSAHVPSLQRYRELHRRSVEEPREFWGDIAKEFYWKTPCPGPFLRYNFDVTKGKIFIEWMKGATTNICYNVLDRNVHEKKLGDKVAFYWEGNEPGETTQITYHQLLVQVCQFSNVLRKQGIQKGDRVAIYMPMIPELVVAMLACARIGALHSIVFAGFSSESLCERILDSSCSLLITTDAFYRGEKLVNLKELADEALQKCQEKGFPVRCCIVVKHLGRAELGMGDSTSQSPPIKRSCPDVQISWNQGIDLWWHELMQEAGDECEPEWCDAEDPLFILYTSGSTGKPKGVVHTVGGYMLYVATTFKYVFDFHAEDVFWCTADIGWITGHSYVTYGPLANGATSVLFEGIPTYPDVNRLWSIVDKYKVTKFYTAPTAIRLLMKFGDEPVTKHSRASLQVLGTVGEPINPEAWLWYHRVVGAQRCPIVDTFWQTETGGHMLTPLPGATPMKPGSATFPFFGVAPAILNESGEELEGEAEGYLVFKQPWPGIMRTVYGNHERFETTYFKKFPGYYVTGDGCQRDQDGYYWITGRIDDMLNVSGHLLSTAEVESALVEHEAVAEAAVVGHPHPVKGECLYCFVTLCDGHTFSPKLTEELKKQIREKIGPIATPDYIQNAPGLPKTRSGKIMRRVLRKIAQNDHDLGDMSTVADPSVISHLFSHRCLTIQ

930 Q9H0W9 P13995

>Q9H0W9

MACAEFSFHVPSLEELAGVMQKGLKDNFADVQVSVVDCPDLTKEPFTFPVKGICGKTRIAEVGGVPYLLPLVNQKKVYDLNKIAKEIKLPGAFILGAGAGPFQTLGFNSEFMPVIQTESEHKPPVNGSYFAHVNPADGGCLLEKYSEKCHDFQCALLANLFASEGQPGKVIEVKAKRRTGPLNFVTCMRETLEKHYGNKPIGMGGTFIIQKGKVKSHIMPAEFSSCPLNSDEEVNKWLHFYEMKAPLVCLPVFVSRDPGFDLRLEHTHFFSRHGEGGHYHYDTTPDIVEYLGYFLPAEFLYRIDQPKETHSIGRD

>P13995

MAATSLMSALAARLLQPAHSCSLRLRPFHLAAVRNEAVVISGRKLAQQIKQEVRQEVEEWVASGNKRPHLSVILVGENPASHSYVLNKTRAAAVVGINSETIMKPASISEEELLNLINKLNNDDNVDGLLVQLPLPEHIDERRICNAVSPDKDVDGFHVINVGRMCLDQYSMLPATPWGVWEIIKRTGIPTLGKNVVVAGRSKNVGMPIAMLLHTDGAHERPGGDATVTISHRYTPKEQLKKHTILADIVISAAGIPNLITADMIKEGAAVIDVGINRVHDPVTAKPKLVGDVDFEGVRQKAGYITPVPGGVGPMTVAMLMKNTIIAAKKVLRLEEREVLKSKELGVATN

931 Q9H0W9 NP_006180.1

>Q9H0W9

MACAEFSFHVPSLEELAGVMQKGLKDNFADVQVSVVDCPDLTKEPFTFPVKGICGKTRIAEVGGVPYLLPLVNQKKVYDLNKIAKEIKLPGAFILGAGAGPFQTLGFNSEFMPVIQTESEHKPPVNGSYFAHVNPADGGCLLEKYSEKCHDFQCALLANLFASEGQPGKVIEVKAKRRTGPLNFVTCMRETLEKHYGNKPIGMGGTFIIQKGKVKSHIMPAEFSSCPLNSDEEVNKWLHFYEMKAPLVCLPVFVSRDPGFDLRLEHTHFFSRHGEGGHYHYDTTPDIVEYLGYFLPAEFLYRIDQPKETHSIGRD

>NP_006180.1

MAEDRPQQPQLDMPLVLDQGLTRQMRLRVESLKQRGEKRQDGEKLLQPAESVYRLNFTQQQRLQFERWNVVLDKPGKVTITGTSQNWTPDLTNLMTRQLLDPTAIFWRKEDSDAIDWNEADALEFGERLSDLAKIRKVMYFLVTFGEGVEPANLKASVVFNQL

932 NP_001428.1 NP_001001936.1

>NP_001428.1

MDIKNSPSSLNSPSSYNCSQSILPLEHGSIYIPSSYVDSHHEYPAMTFYSPAVMNYSIPSNVTNLEGGPGRQTTSPNVLWPTPGHLSPLVVHRQLSHLYAEPQKSPWCEARSLEHTLPVNRETLKRKVSGNRCASPVTGPGSKRDAHFCAVCSDYASGYHYGVWSCEGCKAFFKRSIQGHNDYICPATNQCTIDKNRRKSCQACRLRKCYEVGMVKCGSRRERCGYRLVRRQRSADEQLHCAGKAKRSGGHAPRVRELLLDALSPEQLVLTLLEAEPPHVLISRPSAPFTEASMMMSLTKLADKELVHMISWAKKIPGFVELSLFDQVRLLESCWMEVLMMGLMWRSIDHPGKLIFAPDLVLDRDEGKCVEGILEIFDMLLATTSRFRELKLQHKEYLCVKAMILLNSSMYPLVTATQDADSSRKLAHLLNAVTDALVWVIAKSGISSQQQSMRLANLLMLLSHVRHASNKGMEHLLNMKCKNVVPVYDLLLEMLNAHVLRGCKSSITGSECSPAEDSKSKEGSQNPQSQ

>NP_001001936.1

MERYKALEQLLTELDDFLKILDQENLSSTALVKKSCLAELLRLYTKSSSSDEEYIYMNKVTINKQQNAESQGKAPEEQGLLPNGEPSQHSSAPQKSLPDLPPPKMIPERKQLAIPKTESPEGYYEEAEPYDTSLNEDGEAVSSSYESYDEEDGSKGKSAPYQWPSPEAGIELMRDARICAFLWRKKWLGQWAKQLCVIKDNRLLCYKSSKDHSPQLDVNLLGSSVIHKEKQVRKKEHKLKITPMNADVIVLGLQSKDQAEQWLRVIQEVSGLPSEGASEGNQYTPDAQRFNCQKPDIAEKYLSASEYGSSVDGHPEVPETKDVKKKCSAGLKLSNLMNLGRKKSTSLEPVERSLETSSYLNVLVNSQWKSRWCSVRDNHLHFYQDRNRSKVAQQPLSLVGCEVVPDPSPDHLYSFRILHKGEELAKLEAKSSEEMGHWLGLLLSESGSKTDPEEFTYDYVDADRVSCIVSAAKNSLLLMQRKFSEPNTYIDGLPSQDRQEELYDDVDLSELTAAVEPTEEATPVADDPNERESDRVYLDLTPVKSFLHGPSSAQAQASSPTLSCLDNATEALPADSGPGPTPDEPCIKCPENLGEQQLESLEPEDPSLRITTVKIQTEQQRISFPPSCPDAVVATPPGASPPVKDRLRVTSAEIKLGKNRTEAEVKRYTEEKERLEKKKEEIRGHLAQLRKEKRELKETLLKCTDKEVLASLEQKLKEIDEECRGEESRRVDLELSIMEVKDNLKKAEAGPVTLGTTVDTTHLENVSPRPKAVTPASAPDCTPVNSATTLKNRPLSVVVTGKGTVLQKAKEWEKKGAS

933 NP_005672.1 NP_116119.2

>NP_005672.1

MSDFSEELKGPVTDDEEVETSVLSGAGMHFPWLQTYVETVAIGGKRRKDFAQTTSACLSFIQEALLKHQWQQAAEYMYSYFQTLEDSDSYKRQAAPEIIWKLGSEILFYHPKSNMESFNTFANRMKNIGVMNYLKISLQHALYLLHHGMLKDAKRNLSEAETWRHGENTSSREILINLIQAYKGLLQYYTWSEKKMELSKLDKDDYAYNAVAQDVFNHSWKTSANISALIKIPGVWDPFVKSYVEMLEFYGDRDGAQEVLTNYAYDEKFPSNPNAHIYLYNFLKRQKAPRSKLISVLKILYQIVPSHKLMLEFHTLLRKSEKEEHRKLGLEVLFGVLDFAGCTKNITAWKYLAKYLKNILMGNHLAWVQEEWNSRKNWWPGFHFSYFWAKSDWKEDTALACEKAFVAGLLLGKGCRYFRYILKQDHQILGKKIKRMKRSVKKYSIVNPRL

>NP_116119.2

MEFLKTCVLRRNACTAVCFWRSKVVQKPSVRRISTTSPRSTVMPAWVIDKYGKNEVLRFTQNMMMPIIHYPNEVIVKVHAASVNPIDVNMRSGYGATALNMKRDPLHVKIKGEEFPLTLGRDVSGVVMECGLDVKYFKPGDEVWAAVPPWKQGTLSEFVVVSGNEVSHKPKSLTHTQAASLPYVALTAWSAINKVGGLNDKNCTGKRVLILGASGGVGTFAIQVMKAWDAHVTAVCSQDASELVRKLGADDVIDYKSGSVEEQLKSLKPFDFILDNVGGSTETWAPDFLKKWSGATYVTLVTPFLLNMDRLGIADGMLQTGVTVGSKALKHFWKGVHYRWAFFMASGPCLDDIAELVDAGKIRPVIEQTFPFSKVPEAFLKVERGHARGKTVINVV

934 O60902 NP_001880.2

>O60902

MEELTAFVSKSFDQKVKEKKEAITYREVLESGPLRGAKEPTGCTEAGRDDRSSPAVRAAGGGGGGGGGGGGGGGGGGVGGGGAGGGAGGGRSPVRELDMGAAERSREPGSPRLTEVSPELKDRKEDAKGMEDEGQTKIKQRRSRTNFTLEQLNELERLFDETHYPDAFMREELSQRLGLSEARVQVWFQNRRAKCRKQENQLHKGVLIGAASQFEACRVAPYVNVGALRMPFQQDSHCNVTPLSFQVQAQLQLDSAVAHAHHHLHPHLAAHAPYMMFPAPPFGLPLATLAADSASAASVVAAAAAAKTTSKNSSIADLRLKAKKHAAALGL

>NP_001880.2

MATGQKLMRAVRVFEFGGPEVLKLRSDIAVPIPKDHQVLIKVHACGVNPVETYIRSGTYSRKPLLPYTPGSDVAGVIEAVGDNASAFKKGDRVFTSSTISGGYAEYALAADHTVYKLPEKLDFKQGAAIGIPYFTAYRALIHSACVKAGESVLVHGASGGVGLAACQIARAYGLKILGTAGTEEGQKIVLQNGAHEVFNHREVNYIDKIKKYVGEKGIDIIIEMLANVNLSKDLSLLSHGGRVIVVGSRGTIEINPRDTMAKESSIIGVTLFSSTKEEFQQYAAALQAGMEIGWLKPVIGSQYPLEKVAEAHENIIHGSGATGKMILLL

935 NP_003790.1 P35237

>NP_003790.1

MANSAKAEEYEKMSLEQAKASVNSETESSFNINENTTASGTGLSEKTSVCRQVDIARKRKEFEDDLVKESSSCGKDTPSKKRKLDPEIVPEEKDCGDAEGNSKKRKRETEDVPKDKSSTGDGTQNKRKIALEDVPEKQKNLEEGHSSTVAAHYNELQEVGLEKRSQSRIFYLRNFNNWMKSVLIGEFLEKVRQKKKRDITVLDLGCGKGGDLLKWKKGRINKLVCTDIADVSVKQCQQRYEDMKNRRDSEYIFSAEFITADSSKELLIDKFRDPQMCFDICSCQFVCHYSFESYEQADMMLRNACERLSPGGYFIGTTPNSFELIRRLEASETESFGNEIYTVKFQKKGDYPLFGCKYDFNLEGVVDVPEFLVYFPLLNEMAKKYNMKLVYKKTFLEFYEEKIKNNENKMLLKRMQALEPYPANESSKLVSEKVDDYEHAAKYMKNSQVRLPLGTLSKSEWEATSIYLVFAFEKQQ

>P35237

MDVLAEANGTFALNLLKTLGKDNSKNVFFSPMSMSCALAMVYMGAKGNTAAQMAQILSFNKSGGGGDIHQGFQSLLTEVNKTGTQYLLRMANRLFGEKSCDFLSSFRDSCQKFYQAEMEELDFISAVEKSRKHINTWVAEKTEGKIAELLSPGSVDPLTRLVLVNAVYFRGNWDEQFDKENTEERLFKVSKNEEKPVQMMFKQSTFKKTYIGEIFTQILVLPYVGKELNMIIMLPDETTDLRTVEKELTYEKFVEWTRLDMMDEEEVEVSLPRFKLEESYDMESVLRNLGMTDAFELGKADFSGMSQTDLSLSKVVHKSFVEVNEEGTEAAAATAAIMMMRCARFVPRFCADHPFLFFIQHSKTNGILFCGRFSSP

936 NP_036455.1 NP_060177.2

>NP_036455.1

MSVDPLSSKALKIKRELSENTPHLSDEALMGLSVRELNRHLRGLSAEEVTRLKQRRRTLKNRGYAASCRVKRVCQKEELQKQKSELEREVDKLARENAAMRLELDALRGKCEALQGFARSVAAARGPATLVAPASVITIVKSTPGSGSGPAHGPDPAHGPASCS

>NP_060177.2

MPEQFSVAEFLAVTAEDLSSPAGAAAFAAKMPRYRGAALAREEILEGDQAILQRIKKAVRAIHSSGLGHVENEEQYREAVESLGNSHLSQNSHELSTGFLNLAVFTREVAALFKNLIQNLNNIVSFPLDSLMKGQLRDGRQDSKKQLEKAWKDYEAKMAKLEKERDRARVTGGIPGEVAQDMQRERRIFQLHMCEYLLKAGESQMKQGPDFLQSLIKFFHAQHNFFQDGWKAAQSLFPFIEKLAASVHALHQAQEDELQKLTQLRDSLRGTLQLESREEHLSRKNSGCGYSIHQHQGNKQFGTEKVGFLYKKSDGIRRVWQKRKCGVKYGCLTISHSTINRPPVKLTLLTCQVRPNPEEKKCFDLVTHNRTYHFQAEDEHECEAWVSVLQNSKDEALSSAFLGEPSAGPGSWGSAGHDGEPHDLTKLLIAEVKSRPGNSQCCDCGAADPTWLSTNLGVLTCIQCSGVHRELGVRFSRMQSLTLDLLGPSELLLALNMGNTSFNEVMEAQLPSHGGPKPSAESDMGTRRDYIMAKYVEHRFARRCTPEPQRLWTAICNRDLLSVLEAFANGQDFGQPLPGPDAQAPEELVLHLAVKVANQASLPLVDFIIQNGGHLDAKAADGNTALHYAALYNQPDCLKLLLKGRALVGTVNEAGETALDIARKKHHKECEELLEQAQAGTFAFPLHVDYSWVISTEPGSDSEEDEEEKRCLLKLPAQAHWASGRLDISNKTYETVASLGAATPQGESEDCPPPLPVKNSSRTLVQGCARHASGDRSEVSSLSSEAPETPESLGSPASSSSLMSPLEPGDPSQAPPNSEEGLREPPGTSRPSLTSGTTPSEMYLPVRFSSESTRSYRRGARSPEDGPSARQPLPRRNVPVGITEGDGSRTGSLPASSVQLLQD

937 Q8IUM7 Q9Y6G3

>Q8IUM7

MYRSTKGASKARRDQINAEIRNLKELLPLAEADKVRLSYLHIMSLACIYTRKGVFFAGGTPLAGPTGLLSAQELEDIVAALPGFLLVFTAEGKLLYLSESVSEHLGHSMVDLVAQGDSIYDIIDPADHLTVRQQLTLPSALDTDRLFRCRFNTSKSLRRQSAGNKLVLIRGRFHAHPPGAYWAGNPVFTAFCAPLEPRPRPGPGPGPGPASLFLAMFQSRHAKDLALLDISESVLIYLGFERSELLCKSWYGLLHPEDLAHASAQHYRLLAESGDIQAEMVVRLQAKTGGWAWIYCLLYSEGPEGPITANNYPISDMEAWSLRQQLNSEDTQAAYVLGTPTMLPSFPENILSQEECSSTNPLFTAALGAPRSTSFPSAPELSVVSASEELPRPSKELDFSYLTFPSGPEPSLQAELSKDLVCTPPYTPHQPGGCAFLFSLHEPFQTHLPTPSSTLQEQLTPSTATFSDQLTPSSATFPDPLTSPLQGQLTETSVRSYEDQLTPCTSTFPDQLLPSTATFPEPLGSPAHEQLTPPSTAFQAHLDSPSQTFPEQLSPNPTKTYFAQEGCSFLYEKLPPSPSSPGNGDCTLLALAQLRGPLSVDVPLVPEGLLTPEASPVKQSFFHYSEKEQNEIDRLIQQISQLAQGMDRPFSAEAGTGGLEPLGGLEPLDSNLSLSGAGPPVLSLDLKPWKCQELDFLADPDNMFLEETPVEDIFMDLSTPDPSEEWGSGDPEAEGPGGAPSPCNNLSPEDHSFLEDLATYETAFETGVSAFPYDGFTDELHQLQSQVQDSFHEDGSGGEPTF

>Q9Y6G3

MAVAAVKWVMSKRTILKHLFPVQNGALYCVCHKSTYSPLPDDYNCNVELALTSDGRTIVCYHPSVDIPYEHTKPIPRPDPVHNNEETHDQVLKTRLEEKVEHLEEGPMIEQLSKMFFTTKHRWYPHGRYHRCRKNLNPPKDR

938 NP_001005360.1 P52735

>NP_001005360.1

MGNRGMEELIPLVNKLQDAFSSIGQSCHLDLPQIAVVGGQSAGKSSVLENFVGRDFLPRGSGIVTRRPLILQLIFSKTEHAEFLHCKSKKFTDFDEVRQEIEAETDRVTGTNKGISPVPINLRVYSPHVLNLTLIDLPGITKVPVGDQPPDIEYQIKDMILQFISRESSLILAVTPANMDLANSDALKLAKEVDPQGLRTIGVITKLDLMDEGTDARDVLENKLLPLRRGYIGVVNRSQKDIEGKKDIRAALAAERKFFLSHPAYRHMADRMGTPHLQKTLNQQLTNHIRESLPALRSKLQSQLLSLEKEVEEYKNFRPDDPTRKTKALLQMVQQFGVDFEKRIEGSGDQVDTLELSGGARINRIFHERFPFELVKMEFDEKDLRREISYAIKNIHGVRTGLFTPDLAFEAIVKKQVVKLKEPCLKCVDLVIQELINTVRQCTSKLSSYPRLREETERIVTTYIREREGRTKDQILLLIDIEQSYINTNHEDFIGFANAQQRSTQLNKKRAIPNQGEILVIRRGWLTINNISLMKGGSKEYWFVLTAESLSWYKDEEEKEKKYMLPLDNLKIRDVEKGFMSNKHVFAIFNTEQRNVYKDLRQIELACDSQEDVDSWKASFLRAGVYPEKDQAENEDGAQENTFSMDPQLERQVETIRNLVDSYVAIINKSIRDLMPKTIMHLMINNTKAFIHHELLAYLYSSADQSSLMEESADQAQRRDDMLRMYHALKEALNIIGDISTSTVSTPVPPPVDDTWLQSASSHSPTPQRRPVSSIHPPGRPPAVRGPTPGPPLIPVPVGAAASFSAPPIPSRPGPQSVFANSDLFPAPPQIPSRPVRIPPGIPPGVPSRRPPAAPSRPTIIRPAEPSLLD

>P52735

MEQWRQCGRWLIDCKVLPPNHRVVWPSAVVFDLAQALRDGVLLCQLLHNLSPGSIDLKDINFRPQMSQFLCLKNIRTFLKVCHDKFGLRNSELFDPFDLFDVRDFGKVISAVSRLSLHSIAQNKGIRPFPSEETTENDDDVYRSLEELADEHDLGEDIYDCVPCEDGGDDIYEDIIKVEVQQPMIRYMQKMGMTEDDKRNCCLLEIQETEAKYYRTLEDIEKNYMSPLRLVLSPADMAAVFINLEDLIKVHHSFLRAIDVSVMVGGSTLAKVFLDFKERLLIYGEYCSHMEHAQNTLNQLLASREDFRQKVEECTLKVQDGKFKLQDLLVVPMQRVLKYHLLLKELLSHSAERPERQQLKEALEAMQDLAMYINEVKRDKETLRKISEFQSSIENLQVKLEEFGRPKIDGELKVRSIVNHTKQDRYLFLFDKVVIVCKRKGYSYELKEIIELLFHKMTDDPMNNKDVKKSHGKMWSYGFYLIHLQGKQGFQFFCKTEDMKRKWMEQFEMAMSNIKPDKANANHHSFQMYTFDKTTNCKACKMFLRGTFYQGYMCTKCGVGAHKECLEVIPPCKFTSPADLDASGAGPGPKMVAMQNYHGNPAPPGKPVLTFQTGDVLELLRGDPESPWWEGRLVQTRKSGYFPSSSVKPCPVDGRPPISRPPSREIDYTAYPWFAGNMERQQTDNLLKSHASGTYLIRERPAEAERFAISIKFNDEVKHIKVVEKDNWIHITEAKKFDSLLELVEYYQCHSLKESFKQLDTTLKYPYKSRERSASRASSRSPASCASYNFSFLSPQGLSFASQGPSAPFWSVFTPRVIGTAVARYNFAARDMRELSLREGDVVRIYSRIGGDQGWWKGETNGRIGWFPSTYVEEEGIQ

939 NP_000297.1 Q9H9P8

>NP_000297.1

MSCQAFTSADTFIPLNSDASATLPLIMHHSAAECLPVSNHATNVMSTATGLHYSVPSCHYGNQPSTYGVMAGSLTPCLYKFPDHTLSHGFPPIHQPLLAEDPTAADFKQELRRKSKLVEEPIDMDSPEIRELEKFANEFKVRRIKLGYTQTNVGEALAAVHGSEFSQTTICRFENLQLSFKNACKLKAILSKWLEEAEQVGALYNEKVGANERKRKRRTTISIAAKDALERHFGEQNKPSSQEIMRMAEELNLEKEVVRVWFCNRRQREKRVKTSLNQSLFSISKEHLECR

>Q9H9P8

MVPALRYLVGACGRARGRFAGGSPGACGFASGRPRPLCGGSRSASTSSFDIVIVGGGIVGLASARALILRHPSLSIGVLEKEKDLAVHQTGHNSGVIHSGIYYKPESLKAKLCVQGAALLYEYCQQKGISYKQCGKLIVAVEQEEIPRLQALYEKGLQNGVPGLRLIQQEDIKKKEPYCRGLMAIDCPHTGIVDYRQVALSFAQDFQEAGGSVLTNFEVKGIEMAKESPSRSIDGMQYPIVIKNTKGEEIRCQYVVTCAGLYSDRISELSGCTPDPRIVPFRGDYLLLKPEKCYLVKGNIYPVPDSRFPFLGVHFTPRMDGSIWLGPNAVLAFKREGYRPFDFSATDVMDIIINSGLIKLASQNFSYGVTEMYKACFLGATVKYLQKFIPEITISDILRGPAGVRAQALDRDGNLVEDFVFDAGVGDIGNRILHVRNAPSPAATSSIAISGMIADEVQQRFEL

940 Q9BXL8 NP_060441.2

>Q9BXL8

MFARGLKRKCVGHEEDVEGALAGLKTVSSYSLQRQSLLDMSLVKLQLCHMLVEPNLCRSVLIANTVRQIQEEMTQDGTWRTVAPQAAERAPLDRLVSTEILCRAAWGQEGAHPAPGLGDGHTQGPVSDLCPVTSAQAPRHLQSSAWEMDGPRENRGSFHKSLDQIFETLETKNPSCMEELFSDVDSPYYDLDTVLTGMMGGARPGPCEGLEGLAPATPGPSSSCKSDLGELDHVVEILVET

>NP_060441.2

MVFLTAQLWLRNRVTDRYFRIQEVLKHARHFRGRKNRCYRLAVRTVIRAFVKCTKARYLKKKNMRTLWINRITAASQEHGLKYPALIGNLVKCQVELNRKVLADLAIYEPKTFKSLAALASRRRHEGFAAALGDGKEPEGIFSRVVQYH

941 NP_477097.1 Q9HCP0

>NP_477097.1

MSRRSSRLQAKQQPQPSQTESPQEAQIIQAKKRKTTQDVKKRREEVTKKHQYEIRNCWPPVLSGGISPCIIIETPHKEIGTSDFSRFTNYRFKNLFINPSPLPDLSWGCSKEVWLNMLKKESRYVHDKHFEVLHSDLEPQMRSILLDWLLEVCEVYTLHRETFYLAQDFFDRFMLTQKDINKNMLQLIGITSLFIASKLEEIYAPKLQEFAYVTDGACSEEDILRMELIILKALKWELCPVTIISWLNLFLQVDALKDAPKVLLPQYSQETFIQIAQLLDLCILAIDSLEFQYRILTAAALCHFTSIEVVKKASGLEWDSISECVDWMVPFVNVVKSTSPVKLKTFKKIPMEDRHNIQTHTNYLAMLEEVNYINTFRKGGQLSPVCNGGIMTPPKSTEKPPGKH

>Q9HCP0

MDHPSREKDERQRTTKPMAQRSAHCSRPSGSSSSSGVLMVGPNFRVGKKIGCGNFGELRLGKNLYTNEYVAIKLEPIKSRAPQLHLEYRFYKQLGSAGEGLPQVYYFGPCGKYNAMVLELLGPSLEDLFDLCDRTFTLKTVLMIAIQLLSRMEYVHSKNLIYRDVKPENFLIGRQGNKKEHVIHIIDFGLAKEYIDPETKKHIPYREHKSLTGTARYMSINTHLGKEQSRRDDLEALGHMFMYFLRGSLPWQGLKADTLKERYQKIGDTKRNTPIEALCENFPEEMATYLRYVRRLDFFEKPDYEYLRTLFTDLFEKKGYTFDYAYDWVGRPIPTPVGSVHVDSGASAITRESHTHRDRPSQQQPLRNQVVSSTNGELNVDDPTGAHSNAPITAHAEVEVVEEAKCCCFFKRKRKKTAQRHK

942 NP_006730.2 NP_004337.2

>NP_006730.2

MSGFDDPGIFYSDSFGGDAQADEGQARKSQLQRRFKEFLRQYRVGTDRTGFTFKYRDELKRHYNLGEYWIEVEMEDLASFDEDLADYLYKQPAEHLQLLEEAAKEVADEVTRPRPSGEEVLQDIQVMLKSDASPSSIRSLKSDMMSHLVKIPGIIIAASAVRAKATRISIQCRSCRNTLTNIAMRPGLEGYALPRKCNTDQAGRPKCPLDPYFIMPDKCKCVDFQTLKLQELPDAVPHGEMPRHMQLYCDRYLCDKVVPGNRVTIMGIYSIKKFGLTTSRGRDRVGVGIRSSYIRVLGIQVDTDGSGRSFAGAVSPQEEEEFRRLAALPNVYEVISKSIAPSIFGGTDMKKAIACLLFGGSRKRLPDGLTRRGDINLLMLGDPGTAKSQLLKFVEKCSPIGVYTSGKGSSAAGLTASVMRDPSSRNFIMEGGAMVLADGGVVCIDEFDKMREDDRVAIHEAMEQQTISIAKAGITTTLNSRCSVLAAANSVFGRWDETKGEDNIDFMPTILSRFDMIFIVKDEHNEERDVMLAKHVITLHVSALTQTQAVEGEIDLAKLKKFIAYCRVKCGPRLSAEAAEKLKNRYIIMRSGARQHERDSDRRSSIPITVRQLEAIVRIAEALSKMKLQPFATEADVEEALRLFQVSTLDAALSGTLSGVEGFTSQEDQEMLSRIEKQLKRRFAIGSQVSEHSIIKDFTKQKYPEHAIHKVLQLMLRRGEIQHRMQRKVLYRLK

>NP_004337.2

MENTENSVDSKSIKNLEPKIIHGSESMDSGISLDNSYKMDYPEMGLCIIINNKNFHKSTGMTSRSGTDVDAANLRETFRNLKYEVRNKNDLTREEIVELMRDVSKEDHSKRSSFVCVLLSHGEEGIIFGTNGPVDLKKITNFFRGDRCRSLTGKPKLFIIQACRGTELDCGIETDSGVDDDMACHKIPVEADFLYAYSTAPGYYSWRNSKDGSWFIQSLCAMLKQYADKLEFMHILTRVNRKVATEFESFSFDATFHAKKQIPCIVSMLTKELYFYH

943 NP_006266.2 NP_001393.1

>NP_006266.2

MPRVYIGRLSYNVREKDIQRFFSGYGRLLEVDLKNGYGFVEFEDSRDADDAVYELNGKELCGERVIVEHARGPRRDRDGYSYGSRSGGGGYSSRRTSGRDKYGPPVRTEYRLIVENLSSRCSWQDLKDFMRQAGEVTYADAHKERTNEGVIEFRSYSDMKRALDKLDGTEINGRNIRLIEDKPRTSHRRSYSGSRSRSRSRRRSRSRSRRSSRSRSRSISKSRSRSRSRSKGRSRSRSKGRKSRSKSKSKPKSDRGSHSHSRSRSKDEYEKSRSRSRSRSPKENGKGDIKSKSRSRSQSRSNSPLPVPPSKARSVSPPPKRATSRSRSRSRSKSRSRSRSSSRD

>NP_001393.1

MGKEKTHINIVVIGHVDSGKSTTTGHLIYKCGGIDKRTIEKFEKEAAEMGKGSFKYAWVLDKLKAERERGITIDISLWKFETSKYYVTIIDAPGHRDFIKNMITGTSQADCAVLIVAAGVGEFEAGISKNGQTREHALLAYTLGVKQLIVGVNKMDSTEPPYSQKRYEEIVKEVSTYIKKIGYNPDTVAFVPISGWNGDNMLEPSANMPWFKGWKVTRKDGNASGTTLLEALDCILPPTRPTDKPLRLPLQDVYKIGGIGTVPVGRVETGVLKPGMVVTFAPVNVTTEVKSVEMHHEALSEALPGDNVGFNVKNVSVKDVRRGNVAGDSKNDPPMEAAGFTAQVIILNHPGQISAGYAPVLDCHTAHIACKFAELKEKIDRRSGKKLEDGPKFLKSGDAAIVDMVPGKPMCVESFSDYPPLGRFAVRDMRQTVAVGVIKAVDKKAAGAGKVTKSAQKAQKAK

944 NP_872363.1 NP_001619.1

>NP_872363.1

MRILANKTRLPHPRRREAPGSPPLSPRGHCPPAPAKPMHPENKLTNHGKTGNGGAQSQHQNVNQGPTCNVGSKGVGAGNHGAKANQISPSNSSLKNPQAGVPPFSSLKGKVKRDRSVSVDSGEQREAGTPSLDSEAKEVAPRSKRRCVLERKQPYSGDEWCSGPDSEEDDKPIGATHNCNVADPAMAAPQLGPGQTTQLPLSESSVPGAPHGPPPGLRPDAPGGGGGGGGVPGKPPSQFVYVFTTHLANTAAEAVLQGRADSILAYHQQNVPRAKLDQAPKVPPTPEPLPLSTPSAGTPQSQPPPLPPPPPPAPGSAPPALPPEGPPEDSSQDLAPNSVGAASTGGGTGGTHPNTPTATTANNPLPPGGDPSSAPGPALLGEAAAPGNGQRSLVGSEGLSKEQLEHRERSLQTLRDIERLLLRSGETEPFLKGPPGGAGEGGPPAQAPPPPQQPPTAPPSGLKKYEEPLQSMISQTQSLGGPPLEHEVPGHPPGGDMGQQMNMMIQRLGQDSLTPEQVAWRKLQEEYYEEKRRKEEQIGLHGSRPLQDMMGMGGMMVRGPPPPYHSKPGDQWPPGMGAQLRGPMDVQDPMQLRGGPPFPGPRFPGNQIQRVPGFGGMQSMPMEVPMNAMQRPVRPGMGWTEDLPPMGGPSNFAQNTMPYPGGQGEAERFMTPRVREELLRHQLLEKRSMGMQRPLGMAGSGMGQSMEMERMMQAHRQMDPAMFPGQMAGGEGLAGTPMGMEFGGGRGLLSPPMGQSGLREVDPPMGPGNLNMNMNVNMNMNMNLNVQMTPQQQMLMSQKMRGPGDLMGPQGLSPEEMARVRAQNSSGVMGGPQKMLMPSQFPNQGQQGFSGGQGPYQAMSQDMGNTQDMFSPDQSSMPMSNVGTTRLSHMPLPPASNPPGTVHSAPNRGLGRRPSDLTISINQMGSPGMGHLKSPTLSQVHSPLVTSPSANLKSPQTPSQMVPLPSANPPGPLKSPQVLGSSLSVRSPTGSPSRLKSPSMAVPSPGWVASPKTAMPSPGVSQNKQPPLNMNSSTTLSNMEQGTLPPSGPRSSSSAPPANPPSGLMNPSLPFTSSPDPTPSQNPLSLMMTQMSKYAMPSSTPLYHNAIKTIATSDDELLPDRPLLPPPPPPQGSGPGISNSQPSQMHLNSAAAQSPMGMNLPGQQPLSHEPPPAMLPSPTPLGSNIPLHPNAQGTGGPPQNSMMMAPGGPDSLNAPCGPVPSSSQMMPFPPRLQQPHGAMAPTGGGGGGPGLQQHYPSGMALPPEDLPNQPPGPMPPQQHLMGKAMAGRMGDAYPPGVLPGVASVLNDPELSEVIRPTPTGIPEFDLSRIIPSEKPSSTLQYFPKSENQPPKAQPPNLHLMNLQNMMAEQTPSRPPNLPGQQGVQRGLNMSMCHPGQMSLLGRTGVPPQQGMVPHGLHQGVMSPPQGLMTQQNFMLMKQRGVGGEVYSQPPHMLSPQGSLMGPPPQQNLMVSHPLRQRSVSLDSQMGYLPAPGGMANLPF

>NP_001619.1

MASRLLLNNGAKMPILGLGTWKSPPGQVTEAVKVAIDVGYRHIDCAHVYQNENEVGVAIQEKLREQVVKREELFIVSKLWCTYHEKGLVKGACQKTLSDLKLDYLDLYLIHWPTGFKPGKEFFPLDESGNVVPSDTNILDTWAAMEELVDEGLVKAIGISNFNHLQVEMILNKPGLKYKPAVNQIECHPYLTQEKLIQYCQSKGIVVTAYSPLGSPDRPWAKPEDPSLLEDPRIKAIAAKHNKTTAQVLIRFPMQRNLVVIPKSVTPERIAENFKVFDFELSSQDMTTLLSYNRNWRVCALLSCTSHKDYPFHEEF

945 NP_001418.2 NP_005787.1

>NP_001418.2

MEENDPKPGEAAAAVEGQRQPESSPGGGSGGGGGSSPGEADTGRRRALMLPAVLQAPGNHQHPHRITNFFIDNILRPEFGRRKDAGTCCAGAGGGRGGGAGGEGGASGAEGGGGAGGSEQLLGSGSREPRQNPPCAPGAGGPLPAAGSDSPGDGEGGSKTLSLHGGAKKGGDPGGPLDGSLKARGLGGGDLSVSSDSDSSQAGANLGAQPMLWPAWVYCTRYSDRPSSGPRSRKPKKKNPNKEDKRPRTAFTAEQLQRLKAEFQTNRYLTEQRRQSLAQELSLNESQIKIWFQNKRAKIKKATGNKNTLAVHLMAQGLYNHSTTAKEGKSDSE

>NP_005787.1

MGDKPIWEQIGSSFIQHYYQLFDNDRTQLGAIYIDASCLTWEGQQFQGKAAIVEKLSSLPFQKIQHSITAQDHQPTPDSCIISMVVGQLKADEDPIMGFHQMFLLKNINDAWVCTNDMFRLALHNFG

946 NP_003097.1 NP_004299.1

>NP_003097.1

MYNMMETELKPPGPQQTSGGGGGNSTAAAAGGNQKNSPDRVKRPMNAFMVWSRGQRRKMAQENPKMHNSEISKRLGAEWKLLSETEKRPFIDEAKRLRALHMKEHPDYKYRPRRKTKTLMKKDKYTLPGGLLAPGGNSMASGVGVGAGLGAGVNQRMDSYAHMNGWSNGSYSMMQDQLGYPQHPGLNAHGAAQMQPMHRYDVSALQYNSMTSSQTYMNGSPTYSMSYSQQGTPGMALGSMGSVVKSEASSSPPVVTSSSHSRAPCQAGDLRDMISMYLPGAEVPEPAAPSRLHMSQHYQSGPVPGTAINGTLPLSHM

>NP_004299.1

MDPLSELQDDLTLDDTSEALNQLKLASIDEKNWPSDEMPDFPKSDDSKSSSPELVTHLKWDDPYYDIARHQIVEVAGDDKYGRKIIVFSACRMPPSHQLDHSKLLGYLKHTLDQYVESDYTLLYLHHGLTSDNKPSLSWLRDAYREFDRKYKKNIKALYIVHPTMFIKTLLILFKPLISFKFGQKIFYVNYLSELSEHVKLEQLGIPRQVLKYDDFLKSTQKSPATAPKPMPPRPPLPNQQFGVSLQHLQEKNPEQEPIPIVLRETVAYLQAHALTTEGIFRRSANTQVVREVQQKYNMGLPVDFDQYNELHLPAVILKTFLRELPEPLLTFDLYPHVVGFLNIDESQRVPATLQVLQTLPEENYQVLRFLTAFLVQISAHSDQNKMTNTNLAVVFGPNLLWAKDAAITLKAINPINTFTKFLLDHQGELFPSPDPSGL

947 NP_036427.1 NP_077006.1

>NP_036427.1

MIRIAALNASSTIEDDHEGSFKSHKTQTKEAQEAEAFALYHKALDLQKHDRFEESAKAYHELLEASLLREAVSSGDEKEGLKHPGLILKYSTYKNLAQLAAQREDLETAMEFYLEAVMLDSTDVNLWYKIGHVALRLIRIPLARHAFEEGLRCNPDHWPCLDNLITVLYTLSDYTTCLYFICKALEKDCRYSKGLVLKEKIFEEQPCLRKDSLRMFLKCDMSIHDVSVSAAETQAIVDEALGLRKKRQALIVREKEPDLKLVQPIPFFTWKCLGESLLAMYNHLTTCEPPRPSLGKRIDLSDYQDPSQPLESSMVVTPVNVIQPSTVSTNPAVAVAEPVVSYTSVATTSFPLHSPGLLETGAPVGDISGGDKSKKGVKRKKISEESGETAKRRSARVRNTKCKKEEKVDFQELLMKFLPSRLRKLDPEEEDDSFNNYEVQSEAKLESFPSIGPQRLSFDSATFMESEKQDVHEFLLENLTNGGILELMMRYLKAMGHKFLVRWPPGLAEVVLSVYHSWRRHSTSLPNPLLRDCSNKHIKDMMLMSLSCMELQLDQWLLTKGRSSAVSPRNCPAGMVNGRFGPDFPGTHCLGDLLQLSFASSQRDLFEDGWLEFVVRVYWLKARFLALQGDMEQALENYDICTEMLQSSTAIQVEAGAERRDIVIRLPNLHNDSVVSLEEIDKNLKSLERCQSLEEIQRLYEAGDYKAVVHLLRPTLCTSGFDRAKHLEFMTSIPERPAQLLLLQDSLLRLKDYRQCFECSDVALNEAVQQMVNSGEAAAKEEWVATVTQLLMGIEQALSADSSGSILKVSSSTTGLVRLTNNLIQVIDCSMAVQEEAKEPHVSSVLPWIILHRIIWQEEDTFHSLCHQQQLQNPAEEGMSETPMLPSSLMLLNTAHEYLGRRSWCCNSDGALLRFYVRVLQKELAASTSEDTHPYKEELETALEQCFYCLYSFPSKKSKARYLEEHSAQQVDLIWEDALFMFEYFKPKTLPEFDSYKTSTVSADLANLLKRIATIVPRTERPALSLDKVSAYIEGTSTEVPCLPEGADPSPPVVNELYYLLADYHFKNKEQSKAIKFYMHDICICPNRFDSWAGMALARASRIQDKLNSNELKSDGPIWKHATPVLNCFRRALEIDSSNLSLWIEYGTMSYALHSFASRQLKQWRGELPPELVQQMEGRRDSMLETAKHCFTSAARCEGDGDEEEWLIHYMLGKVAEKQQQPPTVYLLHYRQAGHYLHEEAARYPKKIHYHNPPELAMEALEVYFRLHASILKLLGKPDSGVGAEVLVNFMKEAAEGPFARGEEKNTPKASEKEKACLVDEDSHSSAGTLPGPGASLPSSSGPGLTSPPYTATPIDHDYVKCKKPHQQATPDDRSQDSTAVALSDSSSTQDFFNEPTSLLEGSRKSYTEKRLPILSSQAGATGKDLQGATEERGKNEESLESTEGFRAAEQGVQKPAAETPASACIPGKPSASTPTLWDGKKRGDLPGEPVAFPQGLPAGAEEQRQFLTEQCIASFRLCLSRFPQHYKSLYRLAFLYTYSKTHRNLQWARDVLLGSSIPWQQLQHMPAQGLFCERNKTNFFNGIWRIPVDEIDRPGSFAWHMNRSIVLLLKVLAQLRDHSTLLKVSSMLQRTPDQGKKYLRDADRQVLAQRAFILTVKVLEDTLSELAEGSERPGPKVCGLPGARMTTDVSHKASPEDGQEGLPQPKKPPLADGSGPGPEPGGKVGLLNHRPVAMDAGDSADQSGERKDKESPRAGPTEPMDTSEATVCHSDLERTPPLLPGRPARDRGPESRPTELSLEELSISARQQPTPLTPAQPAPAPAPATTTGTRAGGHPEEPLSRLSRKRKLLEDTESGKTLLLDAYRVWQQGQKGVAYDLGRVERIMSETYMLIKQVDEEAALEQAVKFCQVHLGAAAQRQASGDTPTTPKHPKDSRENFFPVTVVPTAPDPVPADSVQRPSDAHTKPRPALAAATTIITCPPSASASTLDQSKDPGPPRPHRPEATPSMASLGPEGEELARVAEGTSFPPQEPRHSPQVKMAPTSSPAEPHCWPAEAALGTGAEPTCSQEGKLRPEPRRDGEAQEAASETQPLSSPPTAASSKAPSSGSAQPPEGHPGKPEPSRAKSRPLPNMPKLVIPSAATKFPPEITVTPPTPTLLSPKGSISEETKQKLKSAILSAQSAANVRKESLCQPALEVLETSSQESSLESETDEDDDYMDI

>NP_077006.1

MGKKLDLSKLTDEEAQHVLEVVQRDFDLRRKEEERLEALKGKIKKESSKRELLSDTAHLNETHCARCLQPYQLLVNSKRQCLECGLFTCKSCGRVHPEEQGWICDPCHLARVVKIGSLEWYYEHVKARFKRFGSAKVIRSLHGRLQGGAGPELISEERSGDSDQTDEDGEPGSEAQAQAQPFGSKKKRLLSVHDFDFEGDSDDSTQPQGHSLHLSSVPEARDSPQSLTDESCSEKAAPHKAEGLEEADTGASGCHSHPEEQPTSISPSRHGALAELCPPGGSHRMALGTAAALGSNVIRNEQLPLQYLADVDTSDEESIRAHVMASHHSKRRGRASSESQIFELNKHISAVECLLTYLENTVVPPLAKGLGAGVRTEADVEEEALRRKLEELTSNVSDQETSSEEEEAKDEKAEPNRDKSVGPLPQADPEVGTAAHQTNRQEKSPQDPGDPVQYNRTTDEELSELEDRVAVTASEVQQAESEVSDIESRIAALRAAGLTVKPSGKPRRKSNLPIFLPRVAGKLGKRPEDPNADPSSEAKAMAVPYLLRRKFSNSLKSQGKDDDSFDRKSVYRGSLTQRNPNARKGMASHTFAKPVVAHQS

948 NP_001349.2 NP_002712.1

>NP_001349.2

MSKRHRLDLGEDYPSGKKRAGTDGKDRDRDRDREDRSKDRDRERDRGDREREREKEKEKELRASTNAMLISAGLPPLKASHSAHSTHSAHSTHSTHSAHSTHAGHAGHTSLPQCINPFTNLPHTPRYYDILKKRLQLPVWEYKDRFTDILVRHQSFVLVGETGSGKTTQIPQWCVEYMRSLPGPKRGVACTQPRRVAAMSVAQRVADEMDVMLGQEVGYSIRFEDCSSAKTILKYMTDGMLLREAMNDPLLERYGVIILDEAHERTLATDILMGVLKEVVRQRSDLKVIVMSATLDAGKFQIYFDNCPLLTIPGRTHPVEIFYTPEPERDYLEAAIRTVIQIHMCEEEEGDLLLFLTGQEEIDEACKRIKREVDDLGPEVGDIKIIPLYSTLPPQQQQRIFEPPPPKKQNGAIGRKVVVSTNIAETSLTIDGVVFVIDPGFAKQKVYNPRIRVESLLVTAISKASAQQRAGRAGRTRPGKCFRLYTEKAYKTEMQDNTYPEILRSNLGSVVLQLKKLGIDDLVHFDFMDPPAPETLMRALELLNYLAALNDDGDLTELGSMMAEFPLDPQLAKMVIASCDYNCSNEVLSITAMLSVPQCFVRPTEAKKAADEAKMRFAHIDGDHLTLLNVYHAFKQNHESVQWCYDNFINYRSLMSADNVRQQLSRIMDRFNLPRRSTDFTSRDYYINIRKALVTGYFMQVAHLERTGHYLTVKDNQVVQLHPSTVLDHKPEWVLYNEFVLTTKNYIRTCTDIKPEWLVKIAPQYYDMSNFPQCEAKRQLDRIIAKLQSKEYSQY

>NP_002712.1

MAPLDLDKYVEIARLCKYLPENDLKRLCDYVCDLLLEESNVQPVSTPVTVCGDIHGQFYDLCELFRTGGQVPDTNYIFMGDFVDRGYYSLETFTYLLALKAKWPDRITLLRGNHESRQITQVYGFYDECQTKYGNANAWRYCTKVFDMLTVAALIDEQILCVHGGLSPDIKTLDQIRTIERNQEIPHKGAFCDLVWSDPEDVDTWAISPRGAGWLFGAKVTNEFVHINNLKLICRAHQLVHEGYKFMFDEKLVTVWSAPNYCYRCGNIASIMVFKDVNTREPKLFRAVPDSERVIPPRTTTPYFL

949 Q96LT9 NP_113608.1

>Q96LT9

MAAPEQPLAISRGCTSSSSLSPPRGDRTLLVRHLPAELTAEEKEDLLKYFGAQSVRVLSDKGRLKHTAFATFPNEKAAIKALTRLHQLKLLGHTLVVEFAKEQDRVHSPCPTSGSEKKKRSDDPVEDDKEKKELGYLTVENGIAPNHGLTFPLNSCLKYMYPPPSSTILANIVNALASVPKFYVQVLHLMNKMNLPTPFGPITARPPMYEDYMPLHAPLPPTSPQPPEEPPLPDEDEELSSEESEYESTDDEDRQRMNKLMELANLQPKRPKTIKQRHVRKKRKIKDMLNTPLCPSHSSLHPVLLPSDVFDQPQPVGNKRIEFHISTDMPAAFKKDLEKEQNCEEKNHDLPATEVDASNIGFGKIFPKPNLDITEEIKEDSDEMPSECISRRELEKGRISREEMETLSVFRSYEPGEPNCRIYVKNLAKHVQEKDLKYIFGRYVDFSSETQRIMFDIRLMKEGRMKGQAFIGLPNEKAAAKALKEANGYVLFGKPMVVQFARSARPKQDPKEGKRKC

>NP_113608.1

MAAPVVTAPGRALLRAGAGRLLRGGVQELLRPRHEGNAPDLACNFSLSQNRGTVIVERWWKVPLAGEGRKPRLHRRHRVYKLVEDTKHRPKENLELILTQSVENVGVRGDLVSVKKSLGRNRLLPQGLAVYASPENKKLFEEEKLLRQEGKLEKIQTKAGEATVKFLKSCRLEVGMKNNVKWELNPEIVARHFFKNLGVVVAPHTLKLPEEPITRWGEYWCEVTVNGLDTVRVPMSVVNFEKPKTKRYKYWLAQQAAKAMAPTSPQI

950 NP_076921.1 NP_006432.1

>NP_076921.1

MSSYFVNSLFSKYKTGESLRPNYYDCGFAQDLGGRPTVVYGPSSGGSFQHPSQIQEFYHGPSSLSTAPYQQNPCAVACHGDPGNFYGYDPLQRQSLFGAQDPDLVQYADCKLAAASGLGEEAEGSEQSPSPTQLFPWMRPQAAAGRRRGRQTYSRYQTLELEKEFLFNPYLTRKRRIEVSHALGLTERQVKIWFQNRRMKWKKENNKDKFPSSKCEQEELEKQKLERAPEAADEGDAQKGDKK

>NP_006432.1

MAAAAVSSAKRSLRGELKQRLRAMSAEERLRQSRVLSQKVIAHSEYQKSKRISIFLSMQDEIETEEIIKDIFQRGKICFIPRYRFQSNHMDMVRIESPEEISLLPKTSWNIPQPGEGDVREEALSTGGLDLIFMPGLGFDKHGNRLGRGKGYYDAYLKRCLQHQEVKPYTLALAFKEQICLQVPVNENDMKVDEVLYEDSSTA

951 O75164 NP_112198.1

>O75164

MASESETLNPSARIMTFYPTMEEFRNFSRYIAYIESQGAHRAGLAKVVPPKEWKPRASYDDIDDLVIPAPIQQLVTGQSGLFTQYNIQKKAMTVREFRKIANSDKYCTPRYSEFEELERKYWKNLTFNPPIYGADVNGTLYEKHVDEWNIGRLRTILDLVEKESGITIEGVNTPYLYFGMWKTSFAWHTEDMDLYSINYLHFGEPKSWYSVPPEHGKRLERLAKGFFPGSAQSCEAFLRHKMTLISPLMLKKYGIPFDKVTQEAGEFMITFPYGYHAGFNHGFNCAESTNFATRRWIEYGKQAVLCSCRKDMVKISMDVFVRKFQPERYKLWKAGKDNTVIDHTLPTPEAAEFLKESELPPRAGNEEECPEEDMEGVEDGEEGDLKTSLAKHRIGTKRHRVCLEIPQEVSQSELFPKEDLSSEQYEMTECPAALAPVRPTHSSVRQVEDGLTFPDYSDSTEVKFEELKNVKLEEEDEEEEQEAAALDLSVNPASVGGRLVFSGSKKKSSSSLGSGSSRDSISSDSETSEPLSCRAQGQTGVLTVHSYAKGDGRVTVGEPCTRKKGSAARSFSERELAEVADEYMFSLEENKKSKGRRQPLSKLPRHHPLVLQECVSDDETSEQLTPEEEAEETEAWAKPLSQLWQNRPPNFEAEKEFNETMAQQAPHCAVCMIFQTYHQVEFGGFNQNCGNASDLAPQKQRTKPLIPEMCFTSTGCSTDINLSTPYLEEDGTSILVSCKKCSVRVHASCYGVPPAKASEDWMCSRCSANALEEDCCLCSLRGGALQRANDDRWVHVSCAVAILEARFVNIAERSPVDVSKIPLPRFKLKCIFCKKRRKRTAGCCVQCSHGRCPTAFHVSCAQAAGVMMQPDDWPFVVFITCFRHKIPNLERAKGALQSITAGQKVISKHKNGRFYQCEVVRLTTETFYEVNFDDGSFSDNLYPEDIVSQDCLQFGPPAEGEVVQVRWTDGQVYGAKFVASHPIQMYQVEFEDGSQLVVKRDDVYTLDEELPKRVKSRLSVASDMRFNEIFTEKEVKQEKKRQRVINSRYREDYIEPALYRAIME

>NP_112198.1

MLKNKGHSSKKDNLAVNAVALQDHILHDLQLRNLSVADHSKTQVQKKENKSLKRDTKAIIDTGLKKTTQCPKLEDSEKEYVLDPKPPPLTLAQKLGLIGPPPPPLSSDEWEKVKQRSLLQGDSVQPCPICKEEFELRPQVLLSCSHVFHKACLQAFEKFTNKKTCPLCRKNQYQTRVIHDGARLFRIKCVTRIQAYWRGCVVRKWYRNLRKTVPPTDAKLRKKFFEKKFTEISHRILCSYNTNIEELFAEIDQCLAINRSVLQQLEEKCGHEITEEEWEKIQVQALRRETHECSICLAPLSAAGGQRVGAGRRSREMALLSCSHVFHHACLLALEEFSVGDRPPFHACPLCRSCYQKKILEC

952 Q5VVJ2 NP_071445.1

>Q5VVJ2

MAAEEADVDIEGDVVAAAGAQPGSGENTASVLQKDHYLDSSWRTENGLIPWTLDNTISEENRAVIEKMLLEEEYYLSKKSQPEKVWLDQKEDDKKYMKSLQKTAKIMVHSPTKPASYSVKWTIEEKELFEQGLAKFGRRWTKISKLIGSRTVLQVKSYARQYFKNKVKCGLDKETPNQKTGHNLQVKNEDKGTKAWTPSCLRGRADPNLNAVKIEKLSDDEEVDITDEVDELSSQTPQKNSSSDLLLDFPNSKMHETNQGEFITSDSQEALFSKSSRGCLQNEKQDETLSSSEITLWTEKQSNGDKKSIELNDQKFNELIKNCNKHDGRGIIVDARQLPSPEPCEIQKNLNDNEMLFHSCQMVEESHEEEELKPPEQEIEIDRNIIQEEEKQAIPEFFEGRQAKTPERYLKIRNYILDQWEICKPKYLNKTSVRPGLKNCGDVNCIGRIHTYLELIGAINFGCEQAVYNRPQTVDKVRIRDRKDAVEAYQLAQRLQSMRTRRRRVRDPWGNWCDAKDLEGQTFEHLSAEELAKRREEEKGRPVKSLKVPRPTKSSFDPFQLIPCNFFSEEKQEPFQVKVASEALLIMDLHAHVSMAEVIGLLGGRYSEVDKVVEVCAAEPCNSLSTGLQCEMDPVSQTQASETLAVRGFSVIGWYHSHPAFDPNPSLRDIDTQAKYQSYFSRGGAKFIGMIVSPYNRNNPLPYSQITCLVISEEISPDGSYRLPYKFEVQQMLEEPQWGLVFEKTRWIIEKYRLSHSSVPMDKIFRRDSDLTCLQKLLECMRKTLSKVTNCFMAEEFLTEIENLFLSNYKSNQENGVTEENCTKELLM

>NP_071445.1

MGEEGGSASHDEEERASVLLGHSPGCEMCSQEAFQAQRSQLVELLVSGSLEGFESVLDWLLSWEVLSWEDYEGFHLLGQPLSHLARRLLDTVWNKGTWACQKLIAAAQEAQADSQSPKLHGCWDPHSLHPARDLQSHRPAIVRRLHSHVENMLDLAWERGFVSQYECDEIRLPIFTPSQRARRLLDLATVKANGLAAFLLQHVQELPVPLALPLEAATCKKYMAKLRTTVSAQSRFLSTYDGAETLCLEDIYTENVLEVWADVGMAGPPQKSPATLGLEELFSTPGHLNDDADTVLVVGEAGSGKSTLLQRLHLLWAAGQDFQEFLFVFPFSCRQLQCMAKPLSVRTLLFEHCCWPDVGQEDIFQLLLDHPDRVLLTFDGFDEFKFRFTDRERHCSPTDPTSVQTLLFNLLQGNLLKNARKVVTSRPAAVSAFLRKYIRTEFNLKGFSEQGIELYLRKRHHEPGVADRLIRLLQETSALHGLCHLPVFSWMVSKCHQELLLQEGGSPKTTTDMYLLILQHFLLHATPPDSASQGLGPSLLRGRLPTLLHLGRLALWGLGMCCYVFSAQQLQAAQVSPDDISLGFLVRAKGVVPGSTAPLEFLHITFQCFFAAFYLALSADVPPALLRHLFNCGRPGNSPMARLLPTMCIQASEGKDSSVAALLQKAEPHNLQITAAFLAGLLSREHWGLLAECQTSEKALLRRQACARWCLARSLRKHFHSIPPAAPGEAKSVHAMPGFIWLIRSLYEMQEERLARKAARGLNVGHLKLTFCSVGPTECAALAFVLQHLRRPVALQLDYNSVGDIGVEQLLPCLGVCKALYLRDNNISDRGICKLIECALHCEQLQKLALFNNKLTDGCAHSMAKLLACRQNFLALRLGNNYITAAGAQVLAEGLRGNTSLQFLGFWGNRVGDEGAQALAEALGDHQSLRWLSLVGNNIGSVGAQALALMLAKNVMLEELCLEENHLQDEGVCSLAEGLKKNSSLKILKLSNNCITYLGAEALLQALERNDTILEVWLRGNTFSLEEVDKLGCRDTRLLL

953 NP_000519.2 NP_077268.1

>NP_000519.2

MGAYARASGVCARGCLDSAGPWTMSRALRPPLPPLCFFLLLLAAAGARAGGYETCPTVQPNMLNVHLLPHTHDDVGWLKTVDQYFYGIKNDIQHAGVQYILDSVISALLADPTRRFIYVEIAFFSRWWHQQTNATQEVVRDLVRQGRLEFANGGWVMNDEAATHYGAIVDQMTLGLRFLEDTFGNDGRPRVAWHIDPFGHSREQASLFAQMGFDGFFFGRLDYQDKWVRMQKLEMEQVWRASTSLKPPTADLFTGVLPNGYNPPRNLCWDVLCVDQPLVEDPRSPEYNAKELVDYFLNVATAQGRYYRTNHTVMTMGSDFQYENANMWFKNLDKLIRLVNAQQAKGSSVHVLYSTPACYLWELNKANLTWSVKHDDFFPYADGPHQFWTGYFSSRPALKRYERLSYNFLQVCNQLEALVGLAANVGPYGSGDSAPLNEAMAVLQHHDAVSGTSRQHVANDYARQLAAGWGPCEVLLSNALARLRGFKDHFTFCQQLNISICPLSQTAARFQVIVYNPLGRKVNWMVRLPVSEGVFVVKDPNGRTVPSDVVIFPSSDSQAHPPELLFSASLPALGFSTYSVAQVPRWKPQARAPQPIPRRSWSPALTIENEHIRATFDPDTGLLMEIMNMNQQLLLPVRQTFFWYNASIGDNESDQASGAYIFRPNQQKPLPVSRWAQIHLVKTPLVQEVHQNFSAWCSQVVRLYPGQRHLELEWSVGPIPVGDTWGKEVISRFDTPLETKGRFYTDSNGREILERRRDYRPTWKLNQTEPVAGNYYPVNTRIYITDGNMQLTVLTDRSQGGSSLRDGSLELMVHRRLLKDDGRGVSEPLMENGSGAWVRGRHLVLLDTAQAAAAGHRLLAEQEVLAPQVVLAPGGGAAYNLGAPPRTQFSGLRRDLPPSVHLLTLASWGPEMVLLRLEHQFAVGEDSGRNLSAPVTLNLRDLFSTFTITRLQETTLVANQLREAASRLKWTTNTGPTPHQTPYQLDPANITLEPMEIRTFLASVQWKEVDG

>NP_077268.1

MIEVVCNDRLGKKVRVKCNTDDTIGDLKKLIAAQTGTRWNKIVLKKWYTIFKDHVSLGDYEIHDGMNLELYYQ

954 P32314 Q13084

>P32314

MGPVIGMTPDKRAETPGAEKIAGLSQIYKMGSLPEAVDAARPKATLVDSESADDELTNLNWLHESTNLLTNFSLGSEGLPIVSPLYDIEGDDVPSFGPACYQNPEKKSATSKPPYSFSLLIYMAIEHSPNKCLPVKEIYSWILDHFPYFATAPTGWKNSVRHNLSLNKCFQKVERSHGKVNGKGSLWCVDPEYKPNLIQALKKQPFSSASSQNGSLSPHYLSSVIKQNQVRNLKESDIDAAAAMMLLNTSIEQGILECEKPLPLKTALQKKRSYGNAFHHPSAVRLQESDSLATSIDPKEDHNYSASSMAAQRCASRSSVSSLSSVDEVYEFIPKNSHVGSDGSEGFHSEEDTDVDYEDDPLGDSGYASQPCAKISEKGQSGKKMRKQTCQEIDEELKEAAGSLLHLAGIRTCLGSLISTAKTQNQKQRKK

>Q13084

MAFTASQRWEEGQARQIGFRFRWALNPERLAMPLHKYPVWLWKRLQLREGICSRLPGHYLRSLEEERTPTPVHYRPHGAKFKINPKNGQRERVEDVPIPIYFPPESQRGLWGGEGWILGQIYANNDKLSKRLKKVWKPQLFEREFYSEILDKKFTVTVTMRTLDLIDEAYGLDFYILKTPKEDLCSKFGMDLKRGMLLRLARQDPQLHPEDPERRAAIYDKYKEFAIPEEEAEWVGLTLEEAIEKQRLLEEKDPVPLFKIYVAELIQQLQQQALSEPAVVQKRASGQ

955 CAA58827.1 NP_001217.2

>CAA58827.1

MADEEEDPTFEEENEEIGGGAEGGQGKRKRLFSKELRCMMYGFGDDQNPYTESVDILEDLVIEFITEMTHKAMSIGRQGRVQVEDIVFLIRKDPRKFARVKDLLTMNEELKRARKAFDEANYGS

>NP_001217.2

MSSASGLRRGHPAGGEENMTETDAFYKREMFDPAEKYKMDHRRRGIALIFNHERFFWHLTLPERRGTCADRDNLTRRFSDLGFEVKCFNDLKAEELLLKIHEVSTVSHADADCFVCVFLSHGEGNHIYAYDAKIEIQTLTGLFKGDKCHSLVGKPKIFIIQACRGNQHDVPVIPLDVVDNQTEKLDTNITEVDAASVYTLPAGADFLMCYSVAEGYYSHRETVNGSWYIQDLCEMLGKYGSSLEFTELLTLVNRKVSQRRVDFCKDPSAIGKKQVPCFASMLTKKLHFFPKSN

956 NP_004621.2 NP_872578.1

>NP_004621.2

MATGANATPLDFPSKKRKRSRWNQDTMEQKTVIPGMPTVIPPGLTREQERAYIVQLQIEDLTRKLRTGDLGIPPNPEDRSPSPEPIYNSEGKRLNTREFRTRKKLEEERHNLITEMVALNPDFKPPADYKPPATRVSDKVMIPQDEYPEINFVGLLIGPRGNTLKNIEKECNAKIMIRGKGSVKEGKVGRKDGQMLPGEDEPLHALVTANTMENVKKAVEQIRNILKQGIETPEDQNDLRKMQLRELARLNGTLREDDNRILRPWQSSETRSITNTTVCTKCGGAGHIASDCKFQRPGDPQSAQDKARMDKEYLSLMAELGEAPVPASVGSTSGPATTPLASAPRPAAPANNPPPPSLMSTTQSRPPWMNSGPSESRPYHGMHGGGPGGPGGGPHSFPHPLPSLTGGHGGHPMQHNPNGPPPPWMQPPPPPMNQGPHPPGHHGPPPMDQYLGSTPVGSGVYRLHQGKGMMPPPPMGMMPPPPPPPSGQPPPPPSGPLPPWQQQQQQPPPPPPPSSSMASSTPLPWQQNTTTTTTSAGTGSIPPWQQQQAAAAASPGAPQMQGNPTMVPLPPGVQPPLPPGAPPPPPPPPPGSAGMMYAPPPPPPPPMDPSNFVTMMGMGVAGMPPFGMPPAPPPPPPQN

>NP_872578.1

MAAPCVSYGGAVSYRLLLWGRGSLARKQGLWKTAAPELQTNVRSQILRLRHTAFVIPKKNVPTSKRETYTEDFIKKQIEEFNIGKRHLANMMGEDPETFTQEDIDRAIAYLFPSGLFEKRARPVMKHPEQIFPRQRAIQWGEDGRPFHYLFYTGKQSYYSLMHDVYGMLLNLEKHQSHLQAKSLLPEKTVTRDVIGSRWLIKEELEEMLVEKLSDLDYMQFIRLLEKLLTSQCGAAEEEFVQRFRRSVTLESKKQLIEPVQYDEQGMAFSKSEGKRKTAKAEAIVYKHGSGRIKVNGIDYQLYFPITQDREQLMFPFHFVDRLGKHDVTCTVSGGGRSAQAGAIRLAMAKALCSFVTEDEVEWMRQAGLLTTDPRVRERKKPGQEGARRKFTWKKR

957 NP_005891.1 Q9UPN9

>NP_005891.1

MNVTSLFSFTSPAVKRLLGWKQGDEEEKWAEKAVDALVKKLKKKKGAMEELEKALSCPGQPSNCVTIPRSLDGRLQVSHRKGLPHVIYCRVWRWPDLQSHHELKPLECCEFPFGSKQKEVCINPYHYKRVESPVLPPVLVPRHSEYNPQHSLLAQFRNLGQNEPHMPLNATFPDSFQQPNSHPFPHSPNSSYPNSPGSSSSTYPHSPTSSDPGSPFQMPADTPPPAYLPPEDPMTQDGSQPMDTNMMAPPLPSEINRGDVQAVAYEEPKHWCSIVYYELNNRVGEAFHASSTSVLVDGFTDPSNNKNRFCLGLLSNVNRNSTIENTRRHIGKGVHLYYVGGEVYAECLSDSSIFVQSRNCNYHHGFHPTTVCKIPSGCSLKIFNNQEFAQLLAQSVNHGFETVYELTKMCTIRMSFVKGWGAEYHRQDVTSTPCWIEIHLHGPLQWLDKVLTQMGSPHNPISSVS

>Q9UPN9

MAENKGGGEAESGGGGSGSAPVTAGAAGPAAQEAEPPLTAVLVEEEEEEGGRAGAEGGAAGPDDGGVAAASSGSAQAASSPAASVGTGVAGGAVSTPAPAPASAPAPGPSAGPPPGPPASLLDTCAVCQQSLQSRREAEPKLLPCLHSFCLRCLPEPERQLSVPIPGGSNGDIQQVGVIRCPVCRQECRQIDLVDNYFVKDTSEAPSSSDEKSEQVCTSCEDNASAVGFCVECGEWLCKTCIEAHQRVKFTKDHLIRKKEDVSESVGASGQRPVFCPVHKQEQLKLFCETCDRLTCRDCQLLEHKEHRYQFLEEAFQNQKGAIENLLAKLLEKKNYVHFAATQVQNRIKEVNETNKRVEQEIKVAIFTLINEINKKGKSLLQQLENVTKERQMKLLQQQNDITGLSRQVKHVMNFTNWAIASGSSTALLYSKRLITFQLRHILKARCDPVPAANGAIRFHCDPTFWAKNVVNLGNLVIESKPAPGYTPNVVVGQVPPGTNHISKTPGQINLAQLRLQHMQQQVYAQKHQQLQQMRMQQPPAPVPTTTTTTQQHPRQAAPQMLQQQPPRLISVQTMQRGNMNCGAFQAHQMRLAQNAARIPGIPRHSGPQYSMMQPHLQRQHSNPGHAGPFPVVSVHNTTINPTSPTTATMANANRGPTSPSVTAIELIPSVTNPENLPSLPDIPPIQLEDAGSSSLDNLLSRYISGSHLPPQPTSTMNPSPGPSALSPGSSGLSNSHTPVRPPSTSSTGSRGSCGSSGRTAEKTSLSFKSDQVKVKQEPGTEDEICSFSGGVKQEKTEDGRRSACMLSSPESSLTPPLSTNLHLESELDALASLENHVKTEPADMNESCKQSGLSSLVNGKSPIRSLMHRSARIGGDGNNKDDDPNEDWCAVCQNGGDLLCCEKCPKVFHLTCHVPTLLSFPSGDWICTFCRDIGKPEVEYDCDNLQHSKKGKTAQGLSPVDQRKCERLLLYLYCHELSIEFQEPVPASIPNYYKIIKKPMDLSTVKKKLQKKHSQHYQIPDDFVADVRLIFKNCERFNEMMKVVQVYADTQEINLKADSEVAQAGKAVALYFEDKLTEIYSDRTFAPLPEFEQEEDDGEVTEDSDEDFIQPRRKRLKSDERPVHIK

958 Q8NDX5 Q8N7A1

>Q8NDX5

MDTEPNPGTSSVSTTTSSTTTTTITTSSSRMQQPQISVYSGSDRHAVQVIQQALHRPPSSAAQYLQQMYAAQQQHLMLHTAALQQQHLSSSQLQSLAAVQASLSSGRPSTSPTGSVTQQSSMSQTSINLSTSPTPAQLISRSQASSSTSGSITQQTMLLGSTSPTLTASQAQMYLRAQMLIFTPATTVAAVQSDIPVVSSSSSSSCQSAATQVQNLTLRSQKLGVLSSSQNGPPKSTSQTQSLTICHNKTTVTSSKISQRDPSPESNKKGESPSLESRSTAVTRTSSIHQLIAPASYSPIQPHSLIKHQQIPLHSPPSKVSHHQLILQQQQQQIQPITLQNSTQDPPPSQHCIPLQNHGLPPAPSNAQSQHCSPIQSHPSPLTVSPNQSQSAQQSVVVSPPPPHSPSQSPTIIIHPQALIQPHPLVSSALQPGPNLQQSTANQVQATAQLNLPSHLPLPASPVVHIGPVQQSALVSPGQQIVSPSHQQYSSLQSSPIPIASPPQMSTSPPAQIPPLPLQSMQSLQVQPEILSQGQVLVQNALVSEEELPAAEALVQLPFQTLPPPQTVAVNLQVQPPAPVDPPVVYQVEDVCEEEMPEESDECVRMDRTPPPPTLSPAAITVGRGEDLTSEHPLLEQVELPAVASVSASVIKSPSDPSHVSVPPPPLLLPAATTRSNSTSMHSSIPSIENKPPQAIVKPQILTHVIEGFVIQEGLEPFPVSRSSLLIEQPVKKRPLLDNQVINSVCVQPELQNNTKHADNSSDTEMEDMIAEETLEEMDSELLKCEFCGKMGYANEFLRSKRFCTMSCAKRYNVSCSKKFALSRWNRKPDNQSLGHRGRRPSGPDGAAREHILRQLPITYPSAEEDLASHEDSVPSAMTTRLRRQSERERERELRDVRIRKMPENSDLLPVAQTEPSIWTVDDVWAFIHSLPGCQDIADEFRAQEIDGQALLLLKEDHLMSAMNIKLGPALKICARINSLKES

>Q8N7A1

MADSQLFCVAEERSGHCAVVDGNFLYVWGGYVSIEDNEVYLPNDEIWTYDIDSGLWRMHLMEGELPASMSGSCGACINGKLYIFGGYDDKGYSNRLYFVNLRTRDETYIWEKITDFEGQPPTPRDKLSCWVYKDRLIYFGGYGCRRHSELQDCFDVHDASWEEQIFWGWHNDVHIFDTKTQTWFQPEIKGGVPPQPRAAHTCAVLGNKGYIFGGRVLQTRMNDLHYLNLDTWTWSGRITINGESPKHRSWHTLTPIADDKLFLCGGLSADNIPLSDGWIHNVTTNCWKQLTHLPKTRPRLWHTACLGKENEIMVFGGSKDDLLALDTGHCNDLLIFQTQPYSLLRSCLDCIGKNSIMLESQISLLPPKLLQQVLKKITFWAAANHREEQRVQKEETENKYQWISSN

959 O75132 NP_006784.1

>O75132

MENNLKTCPKEDGDFVSDKIKFKIEEEDDDGIPPDSLERMDFKSEQEDMKQTDSGGERAGLGGTGCSCKPPGKYLSAESEDDYGALFSQYSSTLYDVAMEAVTQSLLSSRNMSSRKKSPAWKHFFISPRDSTKAICMYCVKEFSRGKNEKDLSTSCLMRHVRRAHPTVLIQENGSVSAVSSFPSPSLLLPPQPADAGDLSTILSPIKLVQKVASKIPSPDRITEESVSVVSSEEISSDMSVSEKCGREEALVGSSPHLPALHYDEPAENLAEKSLPLPKSTSGSRRRSAVWKHFYLSPLDNSKAVCIHCMNEFSRGKNGKDLGTSCLIRHMWRAHRAIVLQENGGTGIPPLYSTPPTLLPSLLPPEGELSSVSSSPVKPVRESPSASSSPDRLTEDLQSHLNPGDGLMEDVAAFSSSDDVGEASASSPEKQQADGLSPRLFESGAIFQQNKKVMKRLKSEVWHHFSLAPMDSLKAECRYCGCAISRGKKGDVGTSCLMRHLYRRHPEVVGSQKGFLGASLANSPYATLASAESSSSKLTDLPTVVTKNNQVMFPVNSKKTSKLWNHFSICSADSTKVVCLHCGRTISRGKKPTNLGTSCLLRHLQRFHSNVLKTEVSETARPSSPDTRVPRGTELSGASSFDDTNEKFYDSHPVAKKITSLIAEMIALDLQPYSFVDNVGFNRLLEYLKPQYSLPAPSYFSRTAIPGMYDNVKQIIMSHLKEAESGVIHFTSGIWMSNQTREYLTLTAHWVSFESPARPRCDDHHCSALLDVSQVDCDYSGNSIQKQLECWWEAWVTSTGLQVGITVTDNASIGKTLNEGEHSSVQCFSHTVNLIVSEAIKSQRMVQNLLSLARKICERVHRSPKAKEKLAELQREYALPQHHLIQDVPSKWSTSFHMLERLIEQKRAINEMSVECNFRELISCDQWEVMQSVCRALKPFEAASREMSTQMSTLSQVIPMVHILNRKVEMLFEETMGIDTMLRSLKEAMVSRLSATLHDPRYVFATLLDPRYKASLFTEEEAEQYKQDLIRELELMNSTSEDVAASHRCDAGSPSKDSAAEENLWSLVAKVKKKDPREKLPEAMVLAYLEEEVLEHSCDPLTYWNLKKASWPGLSALAVRFLGCPPSIVPSEKLFNTPTENGSLGQSRLMMEHFEKLIFLKVNLPLIYFQY

>NP_006784.1

MAAAVGRLLRASVARHVSAIPWGISATAALRPAACGRTSLTNLLCSGSSQAKLFSTSSSCHAPAVTQHAPYFKGTAVVNGEFKDLSLDDFKGKYLVLFFYPLDFTFVCPTEIVAFSDKANEFHDVNCEVVAVSVDSHFSHLAWINTPRKNGGLGHMNIALLSDLTKQISRDYGVLLEGSGLALRGLFIIDPNGVIKHLSVNDLPVGRSVEETLRLVKAFQYVETHGEVCPANWTPDSPTIKPSPAASKEYFQKVNQ

960 NP_254280.1 Q96GX9

>NP_254280.1

MSGRGKQGGKARAKAKSRSSRAGLQFPVGRVHRLLRKGNYSERVGAGAPVYLAAVLEYLTAEILELAGNAARDNKKTRIIPRHLQLAIRNDEELNKLLGRVTIAQGGVLPNIQAVLLPKKTESHHKAKGK

>Q96GX9

MSGCDAREGDCCSRRCGAQDKEHPRYLIPELCKQFYHLGWVTGTGGGISLKHGDEIYIAPSGVQKERIQPEDMFVCDINEKDISGPSPSKKLKKSQCTPLFMNAYTMRGAGAVIHTHSKAAVMATLLFPGREFKITHQEMIKGIKKCTSGGYYRYDDMLVVPIIENTPEEKDLKDRMAHAMNEYPDSCAVLVRRHGVYVWGETWEKAKTMCECYDYLFDIAVSMKKVGLDPSQLPVGENGIV

961 NP_937872.1 P36957

>NP_937872.1

MFRAGEASKRPLPGPSPPRVRSVEVARGRAGYGFTLSGQAPCVLSCVMRGSPADFVGLRAGDQILAVNEINVKKASHEDVVKLIGKCSGVLHMVIAEGVGRFESCSSDEEGGLYEGKGWLKPKLDSKALGINRAERVVEEMQSGGIFNMIFENPSLCASNSEPLKLKQRSLSESAATRFDVGHESINNPNPNMLSKEEISKVIHDDSVFSIGLESHDDFALDASILNVAMIVGYLGSIELPSTSSNLESDSLQAIRGCMRRLRAEQKIHSLVTMKIMHDCVQLSTDKAGVVAEYPAEKLAFSAVCPDDRRFFGLVTMQTNDDGSLAQEEEGALRTSCHVFMVDPDLFNHKIHQGIARRFGFECTADPDTNGCLEFPASSLPVLQFISVLYRDMGELIEGMRARAFLDGDADAHQNNSTSSNSDSGIGNFHQEEKSNRVLVVDLGGSSSRHGPGGSAWDGVGGRGAQPWGAPWTGPFCPDPEGSPPFEAAHQTDRFWDLNKHLGPASPVEVPPASLRSSVPPSKRGTVGAGCGFNQRWLPVHVLREWQCGHTSDQDSYTDSTDGWSSINCGTLPPPMSKIPADRYRVEGSFAQPPLNAPKREWSRKAFGMQSIFGPHRNVRKTKEDKKGSKFGRGTGLTQPSQRTSARRSFGRSKRFSITRSLDDLESATVSDGELTGADLKDCVSNNSLSSNASLPSVQSCRRLRERRVASWAVSFERLLQDPVGVRYFSDFLRKEFSEENILFWQACEYFNHVPAHDKKELSYRAREIFSKFLCSKATTPVNIDSQAQLADDVLRAPHPDMFKEQQLQIFNLMKFDSYTRFLKSPLYQECILAEVEGRALPDSQQVPSSPASKHSLGSDHSSVSTPKKLSGKSKSGRSLNEELGDEDSEKKRKGAFFSWSRTRSTGRSQKKREHGDHADDALHANGGLCRRESQGSVSSAGSLDLSEACRTLAPEKDKATKHCCIHLPDGTSCVVAVKAGFSIKDILSGLCERHGINGAAADLFLVGGDKPLVLHQDSSILESRDLRLEKRTLFRLDLVPINRSVGLKAKPTKPVTEVLRPVVARYGLDLSGLLVRLSGEKEPLDLGAPISSLDGQRVVLEEKDPSRGKASADKQKGVPVKQNTAVNSSSRNHSATGEERTLGKSNSIKIKGENGKNARDPRLSKREESIAKIGKKKYQKINLDEAEEFFELISKAQSNRADDQRGLLRKEDLVLPEFLRLPPGSTELTLPTPAAVAKGFSKRSATGNGRESASQPGEQWEPVQESSDSPSTSPGSASSPPGPPGTTPPGQKSPSGPFCTPQSPVSLAQEGTAQIWKRQSQEVEAGGIQTVEDEHVAELTLMGEGDISSPNSTLLPPPSTPQEVPGPSRPGSGTHGSRDLPVNRIIDVDLVTGSAPGRDGGIAGAQAGPGRSQASGGPPTSDLPGLGPVPGEPAKPKTSAHHATFV

>P36957

MLSRSRCVSRAFSRSLSAFQKGNCPLGRRSLPGVSLCQGPGYPNSRKVVINNSVFSVRFFRTTAVCKDDLVTVKTPAFAESVTEGDVRWEKAVGDTVAEDEVVCEIETDKTSVQVPSPANGVIEALLVPDGGKVEGGTPLFTLRKTGAAPAKAKPAEAPAAAAPKAEPTAAAVPPPAAPIPTQMPPVPSPSQPPSGKPVSAVKPTVAPPLAEAGAGKGLRSEHREKMNRMRQRIAQRLKEAQNTCAMLTTFNEIDMSNIQEMRARHKEAFLKKHNLKLGFMSAFVKASAFALQEQPVVNAVIDDTTKEVVYRDYIDISVAVATPRGLVVPVIRNVEAMNFADIERTITELGEKARKNELAIEDMDGGTFTISNGGVFGSLFGTPIINPPQSAILGMHGIFDRPVAIGGKVEVRPMMYVALTYDHRLIDGREAVTFLRKIKAAVEDPRVLLLDL

962 NP_006747.1 P49753

>NP_006747.1

MEDEVVRFAKKMDKMVQKKNAAGALDLLKELKNIPMTLELLQSTRIGMSVNAIRKQSTDEEVTSLAKSLIKSWKKLLDGPSTEKDLDEKKKEPAITSQNSPEAREESTSSGNVSNRKDETNARDTYVSSFPRAPSTSDSVRLKCREMLAAALRTGDDYIAIGADEEELGSQIEEAIYQEIRNTDMKYKNRVRSRISNLKDAKNPNLRKNVLCGNIPPDLFARMTAEEMASDELKEMRKNLTKEAIREHQMAKTGGTQTDLFTCGKCKKKNCTYTQVQTRSADEPMTTFVVCNECGNRWKFC

>P49753

MSNKLLSPHPHSVVLRSEFKMASSPAVLRASRLYQWSLKSSAQFLGSPQLRQVGQIIRVPARMAATLILEPAGRCCWDEPVRIAVRGLAPEQPVTLRASLRDEKGALFQAHARYRADTLGELDLERAPALGGSFAGLEPMGLLWALEPEKPLVRLVKRDVRTPLAVELEVLDGHDPDPGRLLCQTRHERYFLPPGVRREPVRVGRVRGTLFLPPEPGPFPGIVDMFGTGGGLLEYRASLLAGKGFAVMALAYYNYEDLPKTMETLHLEYFEEAMNYLLSHPEVKGPGVGLLGISKGGELCLSMASFLKGITAAVVINGSVANVGGTLRYKGETLPPVGVNRNRIKVTKDGYADIVDVLNSPLEGPDQKSFIPVERAESTFLFLVGQDDHNWKSEFYANEACKRLQAHGRRKPQIICYPETGHYIEPPYFPLCRASLHALVGSPIIWGGEPRAHAMAQVDAWKQLQTFFHKHLGGHEGTIPSKV

963 O75529 Q06278

>O75529

MKRVRTEQIQMAVSCYLKRRQYVDSDGPLKQGLRLSQTAEEMAANLTVQSESGCANIVSAAPCQAEPQQYEVQFGRLRNFLTDSDSQHSHEVMPLLYPLFVYLHLNLVQNSPKSTVESFYSRFHGMFLQNASQKDVIEQLQTTQTIQDILSNFKLRAFLDNKYVVRLQEDSYNYLIRYLQSDNNTALCKVLTLHIHLDVQPAKRTDYQLYASGSSSRSENNGLEPPDMPSPILQNEAALEVLQESIKRVKDGPPSLTTICFYAFYNTEQLLNTAEISPDSKLLAAGFDNSCIKLWSLRSKKLKSEPHQVDVSRIHLACDILEEEDDEDDNAGTEMKILRGHCGPVYSTRFLADSSGLLSCSEDMSIRYWDLGSFTNTVLYQGHAYPVWDLDISPYSLYFASGSHDRTARLWSFDRTYPLRIYAGHLADVDCVKFHPNSNYLATGSTDKTVRLWSAQQGNSVRLFTGHRGPVLSLAFSPNGKYLASAGEDQRLKLWDLASGTLYKELRGHTDNITSLTFSPDSGLIASASMDNSVRVWDIRNTYCSAPADGSSSELVGVYTGQMSNVLSVQFMACNLLLVTGITQENQEH

>Q06278

MDRASELLFYVNGRKVIEKNVDPETMLLPYLRKKLRLTGTKYGCGGGGCGACTVMISRYNPITKRIRHHPANACLIPICSLYGAAVTTVEGIGSTHTRIHPVQERIAKCHGTQCGFCTPGMVMSIYTLLRNHPEPTLDQLTDALGGNLCRCTGYRPIIDACKTFCKTSGCCQSKENGVCCLDQGINGLPEFEEGSKTSPKLFAEEEFLPLDPTQELIFPPELMIMAEKQSQRTRVFGSERMMWFSPVTLKELLEFKFKYPQAPVIMGNTSVGPEVKFKGVFHPVIISPDRIEELSVVNHAYNGLTLGAGLSLAQVKDILADVVQKLPEEKTQMYHALLKHLGTLAGSQIRNMASLGGHIISRHPDSDLNPILAVGNCTLNLLSKEGKRQIPLNEQFLSKCPNADLKPQEILVSVNIPYSRKWEFVSAFRQAQRQENALAIVNSGMRVFFGEGDGIIRELCISYGGVGPATICAKNSCQKLIGRHWNEQMLDIACRLILNEVSLLGSAPGGKVEFKRTLIISFLFKFYLEVSQILKKMDPVHYPSLADKYESALEDLHSKHHCSTLKYQNIGPKQHPEDPIGHPIMHLSGVKHATGEAIYCDDMPLVDQELFLTFVTSSRAHAKIVSIDLSEALSMPGVVDIMTAEHLSDVNSFCFFTEAEKFLATDKVFCVGQLVCAVLADSEVQAKRAAKRVKIVYQDLEPLILTIEESIQHNSSFKPERKLEYGNVDEAFKVVDQILEGEIHMGGQEHFYMETQSMLVVPKGEDQEMDVYVSTQFPKYIQDIVASTLKLPANKVMCHVRRVGGAFGGKVLKTGIIAAVTAFAANKHGRAVRCVLERGEDMLITGGRHPYLGKYKAGFMNDGRILALDMEHYSNAGASLDESLFVIEMGLLKMDNAYKFPNLRCRGWACRTNLPSNTAFRGFGFPQAALITESCITEVAAKCGLSPEKVRIINMYKEIDQTPYKQEINAKNLIQCWRECMAMSSYSLRKVAVEKFNAENYWKKKGLAMVPLKFPVGLGSRAAGQAAALVHIYLDGSVLVTHGGIEMGQGVHTKMIQVVSRELRMPMSNVHLRGTSTETVPNANISGGSVVADLNGLAVKDACQTLLKRLEPIISKNPKGTWKDWAQTAFDESINLSAVGYFRGYESDMNWEKGEGQPFEYFVYGAACSEVEIDCLTGDHKNIRTDIVMDVGCSINPAIDIGQIEGAFIQGMGLYTIEELNYSPQGILHTRGPDQYKIPAICDMPTELHIALLPPSQNSNTLYSSKGLGESGVFLGCSVFFAIHDAVSAARQERGLHGPLTLNSPLTPEKIRMACEDKFTKMIPRDEPGSYVPWNVPI

964 Q9Y3D8 NP_005691.2

>Q9Y3D8

MLLPNILLTGTPGVGKTTLGKELASKSGLKYINVGDLAREEQLYDGYDEEYDCPILDEDRVVDELDNQMREGGVIVDYHGCDFFPERWFHIVFVLRTDTNVLYERLETRGYNEKKLTDNIQCEIFQVLYEEATASYKEEIVHQLPSNKPEELENNVDQILKWIEQWIKDHNS

>NP_005691.2

MADTQYILPNDIGVSSLDCREAFRLLSPTERLYAYHLSRAAWYGGLAVLLQTSPEAPYIYALLSRLFRAQDPDQLRQHALAEGLTEEEYQAFLVYAAGVYSNMGNYKSFGDTKFVPNLPKEKLERVILGSEAAQQHPEEVRGLWQTCGELMFSLEPRLRHLGLGKEGITTYFSGNCTMEDAKLAQDFLDSQNLSAYNTRLFKEVDGEGKPYYEVRLASVLGSEPSLDSEVTSKLKSYEFRGSPFQVTRGDYAPILQKVVEQLEKAKAYAANSHQGQMLAQYIESFTQGSIEAHKRGSRFWIQDKGPIVESYIGFIESYRDPFGSRGEFEGFVAVVNKAMSAKFERLVASAEQLLKELPWPPTFEKDKFLTPDFTSLDVLTFAGSGIPAGINIPNYDDLRQTEGFKNVSLGNVLAVAYATQREKLTFLEEDDKDLYILWKGPSFDVQVGLHELLGHGSGKLFVQDEKGAFNFDQETVINPETGEQIQSWYRSGETWDSKFSTIASSYEECRAESVGLYLCLHPQVLEIFGFEGADAEDVIYVNWLNMVRAGLLALEFYTPEAFNWRQAHMQARFVILRVLLEAGEGLVTITPTTGSDGRPDARVRLDRSKIRSVGKPALERFLRRLQVLKSTGDVAGGRALYEGYATVTDAPPECFLTLRDTVLLRKESRKLIVQPNTRLEGSDVQLLEYEASAAGLIRSFSERFPEDGPELEEILTQLATADARFWKGPSEAPSGQA

965 NP_005240.3 NP_000845.1

>NP_005240.3

MLDMGDRKEVKMIPKSSFSINSLVPEAVQNDNHHASHGHHNSHHPQHHHHHHHHHHHPPPPAPQPPPPPQQQQPPPPPPPAPQPPQTRGAPAADDDKGPQQLLLPPPPPPPPAAALDGAKADGLGGKGEPGGGPGELAPVGPDEKEKGAGAGGEEKKGAGEGGKDGEGGKEGEKKNGKYEKPPFSYNALIMMAIRQSPEKRLTLNGIYEFIMKNFPYYRENKQGWQNSIRHNLSLNKCFVKVPRHYDDPGKGNYWMLDPSSDDVFIGGTTGKLRRRSTTSRAKLAFKRGARLTSTGLTFMDRAGSLYWPMSPFLSLHHPRASSTLSYNGTTSAYPSHPMPYSSVLTQNSLGNNHSFSTANGLSVDRLVNGEIPYATHHLTAAALAASVPCGLSVPCSGTYSLNPCSVNLLAGQTSYFFPHVPHPSMTSQSSTSMSARAASSSTSPQAPSTLPCESLRPSLPSFTTGLSGGLSDYFTHQNQGSSSNPLIH

>NP_000845.1

MGLELFLDLVSQPSRAVYIFAKKNGIPLELRTVDLVKGQHKSKEFLQINSLGKLPTLKDGDFILTESSAILIYLSCKYQTPDHWYPSDLQARARVHEYLGWHADCIRGTFGIPLWVQVLGPLIGVQVPEEKVERNRTAMDQALQWLEDKFLGDRPFLAGQQVTLADLMALEELMQPVALGYELFEGRPRLAAWRGRVEAFLGAELCQEAHSIILSILEQAAKKTLPTPSPEAYQAMLLRIARIP

966 NP_056076.1 NP_004040.1

>NP_056076.1

MVALRGLGSGLQPWCPLDLRLEWVDTVWELDFTETEPLDPSIEAEIIETGLAAFTKLYESLLPFATGEHGSMESIWTFFIENNVSHSTLVALFYHFVQIVHKKNVSVQYREYGLHAAGLYFLLLEVPGSVANQVFHPVMFDKCIQTLKKSWPQESNLNRKRKKEQPKSSQANPGRHRKRGKPPRREDIEMDEIIEEQEDENICFSARDLSQIRNAIFHLLKNFLRLLPKFSLKEKPQCVQNCIEVFVSLTNFEPVLHECHVTQARALNQAKYIPELAYYGLYLLCSPIHGEGDKVISCVFHQMLSVILMLEVGEGSHRAPLAVTSQVINCRNQAVQFISALVDELKESIFPVVRILLQHICAKVVDKSEYRTFAAQSLVQLLSKLPCGEYAMFIAWLYKYSRSSKIPHRVFTLDVVLALLELPEREVDNTLSLEHQKFLKHKFLVQEIMFDRCLDKAPTVRSKALSSFAHCLELTVTSASESILELLINSPTFSVIESHPGTLLRNSSAFSYQRQTSNRSEPSGEINIDSSGETVGSGERCVMAMLRRRIRDEKTNVRKSALQVLVSILKHCDVSGMKEDLWILQDQCRDPAVSVRKQALQSLTELLMAQPRCVQIQKAWLRGVVPVVMDCESTVQEKALEFLDQLLLQNIRHHSHFHSGDDSQVLAWALLTLLTTESQELSRYLNKAFHIWSKKEKFSPTFINNVISHTGTEHSAPAWMLLSKIAGSSPRLDYSRIIQSWEKISSQQNPNSNTLGHILCVIGHIAKHLPKSTRDKVTDAVKCKLNGFQWSLEVISSAVDALQRLCRASAETPAEEQELLTQVCGDVLSTCEHRLSNIVLKENGTGNMDEDLLVKYIFTLGDIAQLCPARVEKRIFLLIQSVLASSADADHSPSSQGSSEAPASQPPPQVRGSVMPSVIRAHAIITLGKLCLQHEDLAKKSIPALVRELEVCEDVAVRNNVIIVMCDLCIRYTIMVDKYIPNISMCLKDSDPFIRKQTLILLTNLLQEEFVKWKGSLFFRFVSTLIDSHPDIASFGEFCLAHLLLKRNPVMFFQHFIECIFHFNNYEKHEKYNKFPQSEREKRLFSLKGKSNKERRMKIYKFLLEHFTDEQRFNITSKICLSILACFADGILPLDLDASELLSDTFEVLSSKEIKLLAMRSKPDKDLLMEEDDMALANVVMQEAQKKLISQVQKRNFIENIIPIIISLKTVLEKNKIPALRELMHYLREVMQDYRDELKDFFAVDKQLASELEYDMKKYQEQLVQEQELAKHADVAGTAGGAEVAPVAQVALCLETVPVPAGQENPAMSPAVSQPCTPRASAGHVAVSSPTPETGPLQRLLPKARPMSLSTIAILNSVKKAVESKSRHRSRSLGVLPFTLNSGSPEKTCSQVSSYSLEQESNGEIEHVTKRAISTPEKSISDVTFGAGVSYIGTPRTPSSAKEKIEGRSQGNDILCLSLPDKPPPQPQQWNVRSPARNKDTPACSRRSLRKTPLKTAN

>NP_004040.1

MTDCEFGYIYRLAQDYLQCVLQIPQPGSGPSKTSRVLQNVAFSVQKEVEKNLKSCLDNVNVVSVDTARTLFNQVMEKEFEDGIINWGRIVTIFAFEGILIKKLLRQQIAPDVDTYKEISYFVAEFIMNNTGEWIRQNGGWENGFVKKFEPKSGWMTFLEVTGKICEMLSLLKQYC

967 NP_005633.2 NP_002442.2

>NP_005633.2

MSKSKDDAPHELESQFILRLPPEYASTVRRAVQSGHVNLKDRLTIELHPDGRHGIVRVDRVPLASKLVDLPCVMESLKTIDKKTFYKTADICQMLVSTVDGDLYPPVEEPVASTDPKASKKKDKDKEKKFIWNHGITLPLKNVRKRRFRKTAKKKYIESPDVEKEVKRLLSTDAEAVSTRWEIIAEDETKEAENQGLDISSPGMSGHRQGHDSLEHDELREIFNDLSSSSEDEDETQHQDEEDINIIDTEEDLERQLQDKLNESDEQHQENEGTNQLVMGIQKQIDNMKGKLQETQDRAKRQEDLIMKVENLALKNRFQAVLDELKQKEDREKEQLSSLQEELESLLEK

>NP_002442.2

MASGTTTTAVKIGIIGGTGLDDPEILEGRTEKYVDTPFGKPSDALILGKIKNVDCVLLARHGRQHTIMPSKVNYQANIWALKEEGCTHVIVTTACGSLREEIQPGDIVIIDQFIDRTTMRPQSFYDGSHSCARGVCHIPMAEPFCPKTREVLIETAKKLGLRCHSKGTMVTIEGPRFSSRAESFMFRTWGADVINMTTVPEVVLAKEAGICYASIAMATDYDCWKEHEEAVSVDRVLKTLKENANKAKSLLLTTIPQIGSTEWSETLHNLKNMAQFSVLLPRH

968 NP_004575.1 NP_036199.2

>NP_004575.1

MKCLVTGGNVKVLGKAVHSLSRIGDELYLEPLEDGLSLRTVNSSRSAYACFLFAPLFFQQYQAATPGQDLLRCKILMKSFLSVFRSLAMLEKTVEKCCISLNGRSSRLVVQLHCKFGVRKTHNLSFQDCESLQAVFDPASCPHMLRAPARVLGEAVLPFSPALAEVTLGIGRGRRVILRSYHEEEADSTAKAMVTEMCLGEEDFQQLQAQEGVAITFCLKEFRGLLSFAESANLNLSIHFDAPGRPAIFTIKDSLLDGHFVLATLSDTDSHSQDLGSPERHQPVPQLQAHSTPHPDDFANDDIDSYMIAMETTIGNEGSRVLPSISLSPGPQPPKSPGPHSEEEDEAEPSTVPGTPPPKKFRSLFFGSILAPVRSPQGPSPVLAEDSEGEG

>NP_036199.2

MSRQLSRARPATVLGAMEMGRRMDAPTSAAVTRAFLERGHTEIDTAFVYSEGQSETILGGLGLRLGGSDCRVKIDTKAIPLFGNSLKPDSLRFQLETSLKRLQCPRVDLFYLHMPDHSTPVEETLRACHQLHQEGKFVELGLSNYAAWEVAEICTLCKSNGWILPTVYQGMYNAITRQVETELFPCLRHFGLRFYAFNPLAGGLLTGKYKYEDKNGKQPVGRFFGNTWAEMYRNRYWKEHHFEGIALVEKALQAAYGASAPSMTSATLRWMYHHSQLQGAHGDAVILGMSSLEQLEQNLAAAEEGPLEPAVVDAFNQAWHLVTHECPNYFR

969 P28340 NP_001995.1

>P28340

MDGKRRPGPGPGVPPKRARGGLWDDDDAPRPSQFEEDLALMEEMEAEHRLQEQEEEELQSVLEGVADGQVPPSAIDPRWLRPTPPALDPQTEPLIFQQLEIDHYVGPAQPVPGGPPPSRGSVPVLRAFGVTDEGFSVCCHIHGFAPYFYTPAPPGFGPEHMGDLQRELNLAISRDSRGGRELTGPAVLAVELCSRESMFGYHGHGPSPFLRITVALPRLVAPARRLLEQGIRVAGLGTPSFAPYEANVDFEIRFMVDTDIVGCNWLELPAGKYALRLKEKATQCQLEADVLWSDVVSHPPEGPWQRIAPLRVLSFDIECAGRKGIFPEPERDPVIQICSLGLRWGEPEPFLRLALTLRPCAPILGAKVQSYEKEEDLLQAWSTFIRIMDPDVITGYNIQNFDLPYLISRAQTLKVQTFPFLGRVAGLCSNIRDSSFQSKQTGRRDTKVVSMVGRVQMDMLQVLLREYKLRSYTLNAVSFHFLGEQKEDVQHSIITDLQNGNDQTRRRLAVYCLKDAYLPLRLLERLMVLVNAVEMARVTGVPLSYLLSRGQQVKVVSQLLRQAMHEGLLMPVVKSEGGEDYTGATVIEPLKGYYDVPIATLDFSSLYPSIMMAHNLCYTTLLRPGTAQKLGLTEDQFIRTPTGDEFVKTSVRKGLLPQILENLLSARKRAKAELAKETDPLRRQVLDGRQLALKVSANSVYGFTGAQVGKLPCLEISQSVTGFGRQMIEKTKQLVESKYTVENGYSTSAKVVYGDTDSVMCRFGVSSVAEAMALGREAADWVSGHFPSPIRLEFEKVYFPYLLISKKRYAGLLFSSRPDAHDRMDCKGLEAVRRDNCPLVANLVTASLRRLLIDRDPEGAVAHAQDVISDLLCNRIDISQLVITKELTRAASDYAGKQAHVELAERMRKRDPGSAPSLGDRVPYVIISAAKGVAAYMKSEDPLFVLEHSLPIDTQYYLEQQLAKPLLRIFEPILGEGRAEAVLLRGDHTRCKTVLTGKVGGLLAFAKRRNCCIGCRTVLSHQGAVCEFCQPRESELYQKEVSHLNALEERFSRLWTQCQRCQGSLHEDVICTSRDCPIFYMRKKVRKDLEDQEQLLRRFGPPGPEAW

>NP_001995.1

MPLSRWLRSVGVFLLPAPYWAPRERWLGSLRRPSLVHGYPVLAWHSARCWCQAWTEEPRALCSSLRMNGDQNSDVYAQEKQDFVQHFSQIVRVLTEDEMGHPEIGDAIARLKEVLEYNAIGGKYNRGLTVVVAFRELVEPRKQDADSLQRAWTVGWCVELLQAFFLVADDIMDSSLTRRGQICWYQKPGVGLDAINDANLLEACIYRLLKLYCREQPYYLNLIELFLQSSYQTEIGQTLDLLTAPQGNVDLVRFTEKRYKSIVKYKTAFYSFYLPIAAAMYMAGIDGEKEHANAKKILLEMGEFFQIQDDYLDLFGDPSVTGKIGTDIQDNKCSWLVVQCLQRATPEQYQILKENYGQKEAEKVARVKALYEELDLPAVFLQYEEDSYSHIMALIEQYAAPLPPAVFLGLARKIYKRRK

970 NP_005244.1 NP_001344.2

>NP_005244.1

MYQDYPGNFDTSSRGSSGSPAHAESYSSGGGGQQKFRVDMPGSGSAFIPTINAITTSQDLQWMVQPTVITSMSNPYPRSHPYSPLPGLASVPGHMALPRPGVIKTIGTTVGRRRRDEQLSPEEEEKRRIRRERNKLAAAKCRNRRRELTEKLQAETEELEEEKSGLQKEIAELQKEKEKLEFMLVAHGPVCKISPEERRSPPAPGLQPMRSGGGSVGAVVVKQEPLEEDSPSSSSAGLDKAQRSVIKPISIAGGFYGEEPLHTPIVVTSTPAVTPGTSNLVFTYPSVLEQESPASPSESCSKAHRRSSSSGDQSSDSLNSPTLLAL

>NP_001344.2

MDSKYQCVKLNDGHFMPVLGFGTYAPAEVPKSKALEATKLAIEAGFRHIDSAHLYNNEEQVGLAIRSKIADGSVKREDIFYTSKLWCNSHRPELVRPALERSLKNLQLDYVDLYLIHFPVSVKPGEEVIPKDENGKILFDTVDLCATWEAVEKCKDAGLAKSIGVSNFNRRQLEMILNKPGLKYKPVCNQVECHPYFNQRKLLDFCKSKDIVLVAYSALGSHREEPWVDPNSPVLLEDPVLCALAKKHKRTPALIALRYQLQRGVVVLAKSYNEQRIRQNVQVFEFQLTSEEMKAIDGLNRNVRYLTLDIFAGPPNYPFSDEY

971 NP_006615.1 Q8WXC3

>NP_006615.1

MSRVHGMHPKETTRQLSLAVKDGLIVETLTVGCKGSKAGIEQEGYWLPGDEIDWETENHDWYCFECHLPGEVLICDLCFRVYHSKCLSDEFRLRDSSSPWQCPVCRSIKKKNTNKQEMGTYLRFIVSRMKERAIDLNKKGKDNKHPMYRRLVHSAVDVPTIQEKVNEGKYRSYEEFKADAQLLLHNTVIFYGADSEQADIARMLYKDTCHELDELQLCKNCFYLSNARPDNWFCYPCIPNHELVWAKMKGFGFWPAKVMQKEDNQVDVRFFGHHHQRAWIPSENIQDITVNIHRLHVKRSMGWKKACDELELHQRFLREGRFWKSKNEDRGEEEAESSISSTSNEQLKVTQEPRAKKGRRNQSVEPKKEEPEPETEAVSSSQEIPTMPQPIEKVSVSTQTKKLSASSPRMLHRSTQTTNDGVCQSMCHDKYTKIFNDFKDRMKSDHKRETERVVREALEKLRSEMEEEKRQAVNKAVANMQGEMDRKCKQVKEKCKEEFVEEIKKLATQHKQLISQTKKKQWCYNCEEEAMYHCCWNTSYCSIKCQQEHWHAEHKRTCRRKR

>Q8WXC3

MGTKREAILKVLENLTPEELKKFKMKLGTVPLREGFERIPRGALGQLDIVDLTDKLVASYYEDYAAELVVAVLRDMRMLEEAARLQRAA

972 NP_000957.1 NP_112198.1

>NP_000957.1

MATNKERLFAAGALGPGSGYPGAGFPFAFPGALRGSPPFEMLSPSFRGLGQPDLPKEMASLSVETQSTSSEEMVPSSPSPPPPPRVYKPCFVCNDKSSGYHYGVSSCEGCKGFFRRSIQKNMVYTCHRDKNCIINKVTRNRCQYCRLQKCFEVGMSKEAVRNDRNKKKKEVKEEGSPDSYELSPQLEELITKVSKAHQETFPSLCQLGKYTTNSSADHRVQLDLGLWDKFSELATKCIIKIVEFAKRLPGFTGLSIADQITLLKAACLDILMLRICTRYTPEQDTMTFSDGLTLNRTQMHNAGFGPLTDLVFAFAGQLLPLEMDDTETGLLSAICLICGDRMDLEEPEKVDKLQEPLLEALRLYARRRRPSQPYMFPRMLMKITDLRGISTKGAERAITLKMEIPGPMPPLIREMLENPEMFEDDSSQPGPHPNASSEDEVPGGQGKGGLKSPA

>NP_112198.1

MLKNKGHSSKKDNLAVNAVALQDHILHDLQLRNLSVADHSKTQVQKKENKSLKRDTKAIIDTGLKKTTQCPKLEDSEKEYVLDPKPPPLTLAQKLGLIGPPPPPLSSDEWEKVKQRSLLQGDSVQPCPICKEEFELRPQVLLSCSHVFHKACLQAFEKFTNKKTCPLCRKNQYQTRVIHDGARLFRIKCVTRIQAYWRGCVVRKWYRNLRKTVPPTDAKLRKKFFEKKFTEISHRILCSYNTNIEELFAEIDQCLAINRSVLQQLEEKCGHEITEEEWEKIQVQALRRETHECSICLAPLSAAGGQRVGAGRRSREMALLSCSHVFHHACLLALEEFSVGDRPPFHACPLCRSCYQKKILEC

973 Q6PL18 Q96P20

>Q6PL18

MVVLRSSLELHNHSAASATGSLDLSSDFLSLEHIGRRRLRSAGAAQKKPAATTAKAGDGSSVKEVETYHRTRALRSLRKDAQNSSDSSFEKNVEITEQLANGRHFTRQLARQQADKKKEEHREDKVIPVTRSLRARNIVQSTEHLHEDNGDVEVRRSCRIRSRYSGVNQSMLFDKLITNTAEAVLQKMDDMKKMRRQRMRELEDLGVFNETEESNLNMYTRGKQKDIQRTDEETTDNQEGSVESSEEGEDQEHEDDGEDEDDEDDDDDDDDDDDDDDEDDEDEEDGEEENQKRYYLRQRKATVYYQAPLEKPRHQRKPNIFYSGPASPARPRYRLSSAGPRSPYCKRMNRRRHAIHSSDSTSSSSSEDEQHFERRRKRSRNRAINRCLPLNFRKDELKGIYKDRMKIGASLADVDPMQLDSSVRFDSVGGLSNHIAALKEMVVFPLLYPEVFEKFKIQPPRGCLFYGPPGTGKTLVARALANECSQGDKRVAFFMRKGADCLSKWVGESERQLRLLFDQAYQMRPSIIFFDEIDGLAPVRSSRQDQIHSSIVSTLLALMDGLDSRGEIVVIGATNRLDSIDPALRRPGRFDREFLFSLPDKEARKEILKIHTRDWNPKPLDTFLEELAENCVGYCGADIKSICAEAALCALRRRYPQIYTTSEKLQLDLSSINISAKDFEVAMQKMIPASQRAVTSPGQALSTVVKPLLQNTVDKILEALQRVFPHAEFRTNKTLDSDISCPLLESDLAYSDDDVPSVYENGLSQKSSHKAKDNFNFLHLNRNACYQPMSFRPRILIVGEPGFGQGSHLAPAVIHALEKFTVYTLDIPVLFGVSTTSPEETCAQVIREAKRTAPSIVYVPHIHVWWEIVGPTLKATFTTLLQNIPSFAPVLLLATSDKPHSALPEEVQELFIRDYGEIFNVQLPDKEERTKFFEDLILKQAAKPPISKKKAVLQALEVLPVAPPPEPRSLTAEEVKRLEEQEEDTFRELRIFLRNVTHRLAIDKRFRVFTKPVDPDEVPDYVTVIKQPMDLSSVISKIDLHKYLTVKDYLRDIDLICSNALEYNPDRDPGDRLIRHRACALRDTAYAIIKEELDEDFEQLCEEIQESRKKRGCSSSKYAPSYYHVMPKQNSTLVGDKRSDPEQNEKLKTPSTPVACSTPAQLKRKIRKKSNWYLGTIKKRRKISQAKDDSQNAIDHKIESDTEETQDTSVDHNETGNTGESSVEENEKQQNASESKLELRNNSNTCNIENELEDSRKTTACTELRDKIACNGDASSSQIIHISDENEGKEMCVLRMTRARRSQVEQQQLITVEKALAILSQPTPSLVVDHERLKNLLKTVVKKSQNYNIFQLENLYAVISQCIYRHRKDHDKTSLIQKMEQEVENFSCSR

>Q96P20

MASTRCKLARYLEDLEDVDLKKFKMHLEDYPPQKGCIPLPRGQTEKADHVDLATLMIDFNGEEKAWAMAVWIFAAINRRDLYEKAKRDEPKWGSDNARVSNPTVICQEDSIEEEWMGLLEYLSRISICKMKKDYRKKYRKYVRSRFQCIEDRNARLGESVSLNKRYTRLRLIKEHRSQQEREQELLAIGKTKTCESPVSPIKMELLFDPDDEHSEPVHTVVFQGAAGIGKTILARKMMLDWASGTLYQDRFDYLFYIHCREVSLVTQRSLGDLIMSCCPDPNPPIHKIVRKPSRILFLMDGFDELQGAFDEHIGPLCTDWQKAERGDILLSSLIRKKLLPEASLLITTRPVALEKLQHLLDHPRHVEILGFSEAKRKEYFFKYFSDEAQARAAFSLIQENEVLFTMCFIPLVCWIVCTGLKQQMESGKSLAQTSKTTTAVYVFFLSSLLQPRGGSQEHGLCAHLWGLCSLAADGIWNQKILFEESDLRNHGLQKADVSAFLRMNLFQKEVDCEKFYSFIHMTFQEFFAAMYYLLEEEKEGRTNVPGSRLKLPSRDVTVLLENYGKFEKGYLIFVVRFLFGLVNQERTSYLEKKLSCKISQQIRLELLKWIEVKAKAKKLQIQPSQLELFYCLYEMQEEDFVQRAMDYFPKIEINLSTRMDHMVSSFCIENCHRVESLSLGFLHNMPKEEEEEEKEGRHLDMVQCVLPSSSHAACSHGLVNSHLTSSFCRGLFSVLSTSQSLTELDLSDNSLGDPGMRVLCETLQHPGCNIRRLWLGRCGLSHECCFDISLVLSSNQKLVELDLSDNALGDFGIRLLCVGLKHLLCNLKKLWLVSCCLTSACCQDLASVLSTSHSLTRLYVGENALGDSGVAILCEKAKNPQCNLQKLGLVNSGLTSVCCSALSSVLSTNQNLTHLYLRGNTLGDKGIKLLCEGLLHPDCKLQVLELDNCNLTSHCCWDLSTLLTSSQSLRKLSLGNNDLGDLGVMMFCEVLKQQSCLLQNLGLSEMYFNYETKSALETLQEEKPELTVVFEPSW

974 NP_005241.1 NP_000174.1

>NP_005241.1

MSHLFDPRLPALAASPMLYLYGPERPGLPLAFAPAAALAASGRAETPQKPPYSYIALIAMAIQDAPEQRVTLNGIYQFIMDRFPFYHDNRQGWQNSIRHNLSLNDCFVKVPREKGRPGKGSYWTLDPRCLDMFENGNYRRRKRKPKPGPGAPEAKRPRAETHQRSAEAQPEAGSGAGGSGPAISRLQAAPAGPSPLLDGPSPPAPLHWPGTASPNEDAGDAAQGAAAVAVGQAARTGDGPGSPLRPASRSSPKSSDKSKSFSIDSILAGKQGQKPPSGDELLGGAKPGPGGRLGASLLAASSSLRPPFNASLMLDPHVQGGFYQLGIPFLSYFPLQVPDTVLHFQ

>NP_000174.1

MTILTYPFKNLPTASKWALRFSIRPLSCSSQLRAAPAVQTKTKKTLAKPNIRNVVVVDGVRTPFLLSGTSYKDLMPHDLARAALTGLLHRTSVPKEVVDYIIFGTVIQEVKTSNVAREAALGAGFSDKTPAHTVTMACISANQAMTTGVGLIASGQCDVIVAGGVELMSDVPIRHSRKMRKLMLDLNKAKSMGQRLSLISKFRFNFLAPELPAVSEFSTSETMGHSADRLAAAFAVSRLEQDEYALRSHSLAKKAQDEGLLSDVVPFKVPGKDTVTKDNGIRPSSLEQMAKLKPAFIKPYGTVTAANSSFLTDGASAMLIMAEEKALAMGYKPKAYLRDFMYVSQDPKDQLLLGPTYATPKVLEKAGLTMNDIDAFEFHEAFSGQILANFKAMDSDWFAENYMGRKTKVGLPPLEKFNNWGGSLSLGHPFGATGCRLVMAAANRLRKEGGQYGLVAACAAGGQGHAMIVEAYPK

975 NP_003122.1 NP_004790.2

>NP_003122.1

MLPTQAGAAAALGRGSALGGSLNRTPTGRPGGGGGTRGANGGRVPGNGAGLGPGRLEREAAAAAATTPAPTAGALYSGSEGDSESGEEEELGAERRGLKRSLSEMEIGMVVGGPEASAAATGGYGPVSGAVSGAKPGKKTRGRVKIKMEFIDNKLRRYTTFSKRKTGIMKKAYELSTLTGTQVLLLVASETGHVYTFATRKLQPMITSETGKALIQTCLNSPDSPPRSDPTTDQRMSATGFEETDLTYQVSESDSSGETKDTLKPAFTVTNLPGTTSTIQTAPSTSTTMQVSSGPSFPITNYLAPVSASVSPSAVSSANGTVLKSTGSGPVSSGGLMQLPTSFTLMPGGAVAQQVPVQAIQVHQAPQQASPSRDSSTDLTQTSSSGTVTLPATIMTSSVPTTVGGHMMYPSPHAVMYAPTSGLGDGSLTVLNAFSQAPSTMQVSHSQVQEPGGVPQVFLTASSGTVQIPVSAVQLHQMAVIGQQAGSSSNLTELQVVNLDTAHSTKSE

>NP_004790.2

MENYFQAEAYNLDKVLDEFEQNEDETVSSTLLDTKWNKILDPPSHRLSFNPTLASVNESAVSNESQPQLKVFSLAHSAPLTTEEEDHCANGQDCNLNPEIATMWIDENAVAEDQLIKRNYSWDDQCSAVEVGEKKCGNLACLPDEKNVLVVAVMHNCDKRTLQNDLQDCNNYNSQSLMDAFSCSLDNENRQTDQFSFSINESTEKDMNSEKQMDPLNRPKTEGRSVNHLCPTSSDSLASVCSPSQLKDDGSIGRDPSMSAITSLTVDSVISSQGTDGCPAVKKQENYIPDEDLTGKISSPRTDLGSPNSFSHMSEGILMKKEPAEESTTEESLRSGLPLLLKPDMPNGSGRNNDCERCSDCLVPNEVRADENEGYEHEETLGTTEFLNMTEHFSESQDMTNWKLTKLNEMNDSQVNEEKEKFLQISQPEDTNGDSGGQCVGLADAGLDLKGTCISESEECDFSTVIDTPAANYLSNGCDSYGMQDPGVSFVPKTLPSKEDSVTEEKEIEESKSECYSNIYEQRGNEATEGSGLLLNSTGDLMKKNYLHNFCSQVPSVLGQSSPKVVASLPSISVPFGGARPKQPSNLKLQIPKPLSDHLQNDFPANSGNNTKNKNDILGKAKLGENSATNVCSPSLGNISNVDTNGEHLESYEAEISTRPCLALAPDSPDNDLRAGQFGISARKPFTTLGEVAPVWVPDSQAPNCMKCEARFTFTKRRHHCRACGKVFCASCCSLKCKLLYMDRKEARVCVICHSVLMNAQAWENMMSASSQSPNPNNPAEYCSTIPPLQQAQASGALSSPPPTVMVPVGVLKHPGAEVAQPREQRRVWFADGILPNGEVADAAKLTMNGTSSAGTLAVSHDPVKPVTTSPLPAETDICLFSGSITQVGSPVGSAMNLIPEDGLPPILISTGVKGDYAVEEKPSQISVMQQLEDGGPDPLVFVLNANLLSMVKIVNYVNRKCWCFTTKGMHAVGQSEIVILLQCLPDEKCLPKDIFNHFVQLYRDALAGNVVSNLGHSFFSQSFLGSKEHGGFLYVTSTYQSLQDLVLPTPPYLFGILIQKWETPWAKVFPIRLMLRLGAEYRLYPCPLFSVRFRKPLFGETGHTIMNLLADFRNYQYTLPVVQGLVVDMEVRKTSIKIPSNRYNEMMKAMNKSNEHVLAGGACFNEKADSHLVCVQNDDGNYQTQAISIHNQPRKVTGASFFVFSGALKSSSGYLAKSSIVEDGVMVQITAENMDSLRQALREMKDFTITCGKADAEEPQEHIHIQWVDDDKNVSKGVVSPIDGKSMETITNVKIFHGSEYKANGKVIRWTEVFFLENDDQHNCLSDPADHSRLTEHVAKAFCLALCPHLKLLKEDGMTKLGLRVTLDSDQVGYQAGSNGQPLPSQYMNDLDSALVPVIHGGACQLSEGPVVMELIFYILENIV

976 Q9H6T0 P23434

>Q9H6T0

MTPPPPPPPPPGPDPAADPAADPCPWPGSLVVLFGATAGALGRDLGSDETDLILLVWQVVEPRSRQVGTLHKSLVRAEAAALSTQCREASGLSADSLARAEPLDKVLQQFSQLVNGDVALLGGGPYMLCTDGQQLLRQVLHPEASRKNLVLPDMFFSFYDLRREFHMQHPSTCPARDLTVATMAQGLGLETDATEDDFGVWEVKTMVAVILHLLKEPSSQLFSKPEVIKQKYETGPCSDSTVPCPYSSKADVVDSETVVRARGLPWQSSDQDVARFFKGLNVARGGVALCLNAQGRRNGEALIRFVDSEQRDLALQRHKHHMGVRYIEVYKATGEEFVKIAGGTSLEVARFLSREDQVILRLRGLPFSAGPTDVLGFLGPECPVTGGTEGLLFVRHPDGRPTGDAFALFACEELAQAALRRHKGMLGKRYIELFRSTAAEVQQVLNRYASGPLLPTLTAPLLPIPFPLAPGTGRDCVRLRGLPYTATIEDILSFLGEAAADIRPHGVHMVLNQQGRPSGDAFIQMTSAERALAAAQRCHKKVMKERYVEVVPCSTEEMSRVLMGGTLGRSGMSPPPCKLPCLSPPTYTTFQATPTLIPTETAALYPSSALLPAARVPAAPTPVAYYPGPATQLYLNYTAYYPSPPVSPTTVGYLTTPTAALASAPTSVLSQSGALVRMQGVPYTAGMKDLLSVFQAYQLPADDYTSLMPVGDPPRTVLQAPKEWVCL

>P23434

MALRVVRSVRALLCTLRAVPLPAAPCPPRPWQLGVGAVRTLRTGPALLSVRKFTEKHEWVTTENGIGTVGISNFAQEALGDVVYCSLPEVGTKLNKQDEFGALESVKAASELYSPLSGEVTEINEALAENPGLVNKSCYEDGWLIKMTLSNPSELDELMSEEAYEKYIKSIEE

977 NP_037393.1 P49638

>NP_037393.1

MAWDMCNQDSESVWSDIECAALVGEDQPLCPDLPELDLSELDVNDLDTDSFLGGLKWCSDQSEIISNQYNNEPSNIFEKIDEENEANLLAVLTETLDSLPVDEDGLPSFDALTDGDVTTDNEASPSSMPDGTPPPQEAEEPSLLKKLLLAPANTQLSYNECSGLSTQNHANHNHRIRTNPAIVKTENSWSNKAKSICQQQKPQRRPCSELLKYLTTNDDPPHTKPTENRNSSRDKCTSKKKSHTQSQSQHLQAKPTTLSLPLTPESPNDPKGSPFENKTIERTLSVELSGTAGLTPPTTPPHKANQDNPFRASPKLKSSCKTVVPPPSKKPRYSESSGTQGNNSTKKGPEQSELYAQLSKSSVLTGGHEERKTKRPSLRLFGDHDYCQSINSKTEILINISQELQDSRQLENKDVSSDWQGQICSSTDSDQCYLRETLEASKQVSPCSTRKQLQDQEIRAELNKHFGHPSQAVFDDEADKTGELRDSDFSNEQFSKLPMFINSGLAMDGLFDDSEDESDKLSYPWDGTQSYSLFNVSPSCSSFNSPCRDSVSPPKSLFSQRPQRMRSRSRSFSRHRSCSRSPYSRSRSRSPGSRSSSRSCYYYESSHYRHRTHRNSPLYVRSRSRSPYSRRPRYDSYEEYQHERLKREEYRREYEKRESERAKQRERQRQKAIEERRVIYVGKIRPDTTRTELRDRFEVFGEIEECTVNLRDDGDSYGFITYRYTCDAFAALENGYTLRRSNETDFELYFCGRKQFFKSNYADLDSNSDDFDPASTKSKYDSLDFDSLLKEAQRSLRR

>P49638

MAEARSQPSAGPQLNALPDHSPLLQPGLAALRRRAREAGVPLAPLPLTDSFLLRFLRARDFDLDLAWRLLKNYYKWRAECPEISADLHPRSIIGLLKAGYHGVLRSRDPTGSKVLIYRIAHWDPKVFTAYDVFRVSLITSELIVQEVETQRNGIKAIFDLEGWQFSHAFQITPSVAKKIAAVLTDSFPLKVRGIHLINEPVIFHAVFSMIKPFLTEKIKERIHMHGNNYKQSLLQHFPDILPLEYGGEEFSMEDICQEWTNFIMKSEDYLSSISESIQ

978 NP_068809.1 Q9NP74

>NP_068809.1

MSDNEDNFDGDDFDDVEEDEGLDDLENAEEEGQENVEILPSGERPQANQKRITTPYMTKYERARVLGTRALQIAMCAPVMVELEGETDPLLIAMKELKARKIPIIIRRYLPDGSYEDWGVDELIITD

>Q9NP74

MEEAELVKGRLQAITDKRKIQEEISQKRLKIEEDKLKHQHLKKKALREKWLLDGISSGKEQEEMKKQNQQDQHQIQVLEQSILRLEKEIQDLEKAELQISTKEEAILKKLKSIERTTEDIIRSVKVEREERAEESIEDIYANIPDLPKSYIPSRLRKEINEEKEDDEQNRKALYAMEIKVEKDLKTGESTVLSSIPLPSDDFKGTGIKVYDDGQKSVYAVSSNHSAAYNGTDGLAPVEVEELLRQASERNSKSPTEYHEPVYANPFYRPTTPQRETVTPGPNFQERIKIKTNGLGIGVNESIHNMGNGLSEERGNNFNHISPIPPVPHPRSVIQQAEEKLHTPQKRLMTPWEESNVMQDKDAPSPKPRLSPRETIFGKSEHQNSSPTCQEDEEDVRYNIVHSLPPDINDTEPVTMIFMGYQQAEDSEEDKKFLTGYDGIIHAELVVIDDEEEEDEGEAEKPSYHPIAPHSQVYQPAKPTPLPRKRSEASPHENTNHKSPHKNSISLKEQEESLGSPVHHSPFDAQTTGDGTEDPSLTALRMRMAKLGKKVI

979 Q99729 P22061

>Q99729

MSEAGEEQPMETTGATENGHEAVPEASRGRGWTGAAAGAGGATAAPPSGNQNGAEGDQINASKNEEDAGKMFVGGLSWDTSKKDLKDYFTKFGEVVDCTIKMDPNTGRSRGFGFILFKDAASVEKVLDQKEHRLDGRVIDPKKAMAMKKDPVKKIFVGGLNPESPTEEKIREYFGEFGEIEAIELPMDPKLNKRRGFVFITFKEEEPVKKVLEKKFHTVSGSKCEIKVAQPKEVYQQQQYGSGGRGNRNRGNRGSGGGGGGGGQSQSWNQGYGNYWNQGYGYQQGYGPGYGGYDYSPYGYYGYGPGYDYSQGSTNYGKSQRRGGHQNNYKPY

>P22061

MAWKSGGASHSELIHNLRKNGIIKTDKVFEVMLATDRSHYAKCNPYMDSPQSIGFQATISAPHMHAYALELLFDQLHEGAKALDVGSGSGILTACFARMVGCTGKVIGIDHIKELVDDSINNVRKDDPTLLSSGRVQLVVGDGRMGYAEEAPYDAIHVGAAAPVVPQALIDQLKPGGRLILPVGPAGGNQMLEQYDKLQDGSIKMKPLMGVIYVPLTDKEKQWSRWK

980 Q9NPC7 NP_203526.1

>Q9NPC7

MQYSHHCEHLLERLNKQREAGFLCDCTIVIGEFQFKAHRNVLASFSEYFGAIYRSTSENNVFLDQSQVKADGFQKLLEFIYTGTLNLDSWNVKEIHQAADYLKVEEVVTKCKIKMEDFAFIANPSSTEISSITGNIELNQQTCLLTLRDYNNREKSEVSTDLIQANPKQGALAKKSSQTKKKKKAFNSPKTGQNKTVQYPSDILENASVELFLDANKLPTPVVEQVAQINDNSELELTSVVENTFPAQDIVHTVTVKRKRGKSQPNCALKEHSMSNIASVKSPYEAENSGEELDQRYSKAKPMCNTCGKVFSEASSLRRHMRIHKGVKPYVCHLCGKAFTQCNQLKTHVRTHTGEKPYKCELCDKGFAQKCQLVFHSRMHHGEEKPYKCDVCNLQFATSSNLKIHARKHSGEKPYVCDRCGQRFAQASTLTYHVRRHTGEKPYVCDTCGKAFAVSSSLITHSRKHTGEKPYICGICGKSFISSGELNKHFRSHTGERPFICELCGNSYTDIKNLKKHKTKVHSGADKTLDSSAEDHTLSEQDSIQKSPLSETMDVKPSDMTLPLALPLGTEDHHMLLPVTDTQSPTSDTLLRSTVNGYSEPQLIFLQQLY

>NP_203526.1

MSWSGLLHGLNTSLTCGPALVPRLWATCSMATLNQMHRLGPPKRPPRKLGPTEGRPQLKGVVLCTFTRKPKKPNSANRKCCRVRLSTGREAVCFIPGEGHTLQEHQIVLVEGGRTQDLPGVKLTVVRGKYDCGHVQKK

981 NP_056943.1 NP_653288.1

>NP_056943.1

MANSANTNTVPKLYRSVIEDVINDVRDIFLDDGVDEQVLMELKTLWENKLMQSRAVDGFHSEEQQLLLQVQQQHQPQQQQHHHHHHHQQAQPQQTVPQQAQTQQVLIPASQQATAPQVIVPDSKLIQHMNASNMSAAATAATLALPAGVTPVQQILTNSGQLLQVVRAANGAQYIFQPQQSVVLQQQVIPQMQPGGVQAPVIQQVLAPLPGGISPQTGVIIQPQQILFTGNKTQVIPTTVAAPTPAQAQITATGQQQPQAQPAQTQAPLVLQVDGTGDTSSEEDEDEEEDYDDDEEEDKEKDGAEDGQVEEEPLNSEDDVSDEEGQELFDTENVVVCQYDKIHRSKNKWKFHLKDGIMNLNGRDYIFSKAIGDAEW

>NP_653288.1

MLRTAGRDGLCRLSTYLEELEAVELKKFKLYLGTATELGEGKIPWGSMEKAGPLEMAQLLITHFGPEEAWRLALSTFERINRKDLWERGQREDLVRDTPPGGPSSLGNQSTCLLEVSLVTPRKDPQETYRDYVRRKFRLMEDRNARLGECVNLSHRYTRLLLVKEHSNPMQVQQQLLDTGRGHARTVGHQASPIKIETLFEPDEERPEPPRTVVMQGAAGIGKSMLAHKVMLDWADGKLFQGRFDYLFYINCREMNQSATECSMQDLIFSCWPEPSAPLQELIRVPERLLFIIDGFDELKPSFHDPQGPWCLCWEEKRPTELLLNSLIRKKLLPELSLLITTRPTALEKLHRLLEHPRHVEILGFSEAERKEYFYKYFHNAEQAGQVFNYVRDNEPLFTMCFVPLVCWVVCTCLQQQLEGGGLLRQTSRTTTAVYMLYLLSLMQPKPGAPRLQPPPNQRGLCSLAADGLWNQKILFEEQDLRKHGLDGEDVSAFLNMNIFQKDINCERYYSFIHLSFQEFFAAMYYILDEGEGGAGPDQDVTRLLTEYAFSERSFLALTSRFLFGLLNEETRSHLEKSLCWKVSPHIKMDLLQWIQSKAQSDGSTLQQGSLEFFSCLYEIQEEEFIQQALSHFQVIVVSNIASKMEHMVSSFCLKRCRSAQVLHLYGATYSADGEDRARCSAGAHTLLVQLPERTVLLDAYSEHLAAALCTNPNLIELSLYRNALGSRGVKLLCQGLRHPNCKLQNLRLKRCRISSSACEDLSAALIANKNLTRMDLSGNGVGFPGMMLLCEGLRHPQCRLQMIQLRKCQLESGACQEMASVLGTNPHLVELDLTGNALEDLGLRLLCQGLRHPVCRLRTLWLKICRLTAAACDELASTLSVNQSLRELDLSLNELGDLGVLLLCEGLRHPTCKLQTLRLGICRLGSAACEGLSVVLQANHNLRELDLSFNDLGDWGLWLLAEGLQHPACRLQKLWLDSCGLTAKACENLYFTLGINQTLTDLYLTNNALGDTGVRLLCKRLSHPGCKLRVLWLFGMDLNKMTHSRLAALRVTKPYLDIGC

982 NP_005513.1 Q8WXI4

>NP_005513.1

MDNARMNSFLEYPILSSGDSGTCSARAYPSDHRITTFQSCAVSANSCGGDDRFLVGRGVQIGSPHHHHHHHHHHPQPATYQTSGNLGVSYSHSSCGPSYGSQNFSAPYSPYALNQEADVSGGYPQCAPAVYSGNLSSPMVQHHHHHQGYAGGAVGSPQYIHHSYGQEHQSLALATYNNSLSPLHASHQEACRSPASETSSPAQTFDWMKVKRNPPKTGKVGEYGYLGQPNAVRTNFTTKQLTELEKEFHFNKYLTRARRVEIAASLQLNETQVKIWFQNRRMKQKKREKEGLLPISPATPPGNDEKAEESSEKSSSSPCVPSPGSSTSDTLTTSH

>Q8WXI4

MIQNVGNHLRRGLASVFSNRTSRKSALRAGNDSAMADGEGYRNPTEVQMSQLVLPCHTNQRGELSVGQLLKWIDTTACLSAERHAGCPCVTASMDDIYFEHTISVGQVVNIKAKVNRAFNSSMEVGIQVASEDLCSEKQWNVCKALATFVARREITKVKLKQITPRTEEEKMEHSVAAERRRMRLVYADTIKDLLANCAIQGDLESRDCSRMVPAEKTRVESVELVLPPHANHQGNTFGGQIMAWMENVATIAASRLCRAHPTLKAIEMFHFRGPSQVGDRLVLKAIVNNAFKHSMEVGVCVEAYRQEAETHRRHINSAFMTFVVLDADDQPQLLPWIRPQPGDGERRYREASARKKIRLDRKYIVSCKQTEVPLSVPWDPSNQVYLSYNNVSSLKMLVAKDNWVLSSEISQVRLYTLEDDKFLSFHMEMVVHVDAAQAFLLLSDLRQRPEWDKHYRSVELVQQVDEDDAIYHVTSPALGGHTKPQDFVILASRRKPCDNGDPYVIALRSVTLPTHRETPEYRRGETLCSGFCLWREGDQLTKCCWVRVSLTELVSASGFYSWGLESRSKGRRSDGWNGKLAGGHLSTLKAIPVAKINSRFGYLQDT

983 NP_619790.1 Q9UGK3

>NP_619790.1

MPEPTKSAPAPKKGSKKAVTKAQKKDGKKRKRSRKESYSVYVYKVLKQVHPDTGISSKAMGIMNSFVNDIFERIAGEASRLAHYNKRSTITSREIQTAVRLLLPGELAKHAVSEGTKAVTKYTSSK

>Q9UGK3

MASALRPPRVPKPKGVLPSHYYESFLEKKGPCDRDYKKFWAGLQGLTIYFYNSNRDFQHVEKLNLGAFEKLTDEIPWGSSRDPGTHFSLILRDQEIKFKVETLECREMWKGFILTVVELRVPTDLTLLPGHLYMMSEVLAKEEARRALETPSCFLKVSRLEAQLLLERYPECGNLLLRPSGDGADGVSVTTRQMHNGTHVVRHYKVKREGPKYVIDVEQPFSCTSLDAVVNYFVSHTKKALVPFLLDEDYEKVLGYVEADKENGENVWVAPSAPGPGPAPCTGGPKPLSPASSQDKLPPLPPLPNQEENYVTPIGDGPAVDYENQDVASSSWPVILKPKKLPKPPAKLPKPPVGPKPEPKVFNGGLGRKLPVSSAQPLFPTAGLADMTAELQKKLEKRRALEH

984 B2CW77 P42765

>B2CW77

MDRPGPGSARPGRTVHVWGYRVEWKVRNGRKLQPSEWAGRGDLGGFKRRWKDTRATVGTTFRRRSRVSLVGELSKFPLPSDSSGGKSSSSFARGALAWCRQRNPNPSCAAAETGARTSLPKERCRGWRLGNWLHKHPHPNTCPRLPACWLPPILTERGERVPKLVPLLACYPKSKPKD

>P42765

MALLRGVFVVAAKRTPFGAYGGLLKDFTATDLSEFAAKAALSAGKVSPETVDSVIMGNVLQSSSDAIYLARHVGLRVGIPKETPALTINRLCGSGFQSIVNGCQEICVKEAEVVLCGGTESMSQAPYCVRNVRFGTKLGSDIKLEDSLWVSLTDQHVQLPMAMTAENLAVKHKISREECDKYALQSQQRWKAANDAGYFNDEMAPIEVKTKKGKQTMQVDEHARPQTTLEQLQKLPPVFKKDGTVTAGNASGVADGAGAVIIASEDAVKKHNFTPLARIVGYFVSGCDPSIMGIGPVPAISGALKKAGLSLKDMDLVEVNEAFAPQYLAVERSLDLDISKTNVNGGAIALGHPLGGSGSRITAHLVHELRRRGGKYAVGSACIGGGQGIAVIIQSTA

985 NP_002936.1 NP_065723.1

>NP_002936.1

MVGQLSEGAIAAIMQKGDTNIKPILQVINIRPITTGNSPPRYRLLMSDGLNTLSSFMLATQLNPLVEEEQLSSNCVCQIHRFIVNTLKDGRRVVILMELEVLKSAEAVGVKIGNPVPYNEGLGQPQVAPPAPAASPAASSRPQPQNGSSGMGSTVSKAYGASKTFGKAAGPSLSHTSGGTQSKVVPIASLTPYQSKWTICARVTNKSQIRTWSNSRGEGKLFSLELVDESGEIRATAFNEQVDKFFPLIEVNKVYYFSKGTLKIANKQFTAVKNDYEMTFNNETSVMPCEDDHHLPTVQFDFTGIDDLENKSKDSLVDIIGICKSYEDATKITVRSNNREVAKRNIYLMDTSGKVVTATLWGEDADKFDGSRQPVLAIKGARVSDFGGRSLSVLSSSTIIANPDIPEAYKLRGWFDAEGQALDGVSISDLKSGGVGGSNTNWKTLYEVKSENLGQGDKPDYFSSVATVVYLRKENCMYQACPTQDCNKKVIDQQNGLYRCEKCDTEFPNFKYRMILSVNIADFQENQWVTCFQESAEAILGQNAAYLGELKDKNEQAFEEVFQNANFRSFIFRVRVKVETYNDESRIKATVMDVKPVDYREYGRRLVMSIRRSALM

>NP_065723.1

MGQCRSANAEDAQEFSDVERAIETLIKNFHQYSVEGGKETLTPSELRDLVTQQLPHLMPSNCGLEEKIANLGSCNDSKLEFRSFWELIGEAAKSVKLERPVRGH

986 Q9Y6P5 NP_005495.2

>Q9Y6P5

MRLAAAANEAYTAPLAVSGLLGCKQCGGGRDQDEELGIRIPRPLGQGPSRFIPEKEILQVGSEDAQMHALFADSFAALGRLDNITLVMVFHPQYLESFLKTQHYLLQMDGPLPLHYRHYIGIMAAARHQCSYLVNLHVNDFLHVGGDPKWLNGLENAPQKLQNLGELNKVLAHRPWLITKEHIEGLLKAEEHSWSLAELVHAVVLLTHYHSLASFTFGCGISPEIHCDGGHTFRPPSVSNYCICDITNGNHSVDEMPVNSAENVSVSDSFFEVEALMEKMRQLQECRDEEEASQEEMASRFEIEKRESMFVFSSDDEEVTPARAVSRHFEDTSYGYKDFSRHGMHVPTFRVQDYCWEDHGYSLVNRLYPDVGQLIDEKFHIAYNLTYNTMAMHKDVDTSMLRRAIWNYIHCMFGIRYDDYDYGEINQLLDRSFKVYIKTVVCTPEKVTKRMYDSFWRQFKHSEKVHVNLLLIEARMQAELLYALRAITRYMT

>NP_005495.2

MKDCSNGCSAECTGEGGSKEVVGTFKAKDLIVTPATILKEKPDPNNLVFGTVFTDHMLTVEWSSEFGWEKPHIKPLQNLSLHPGSSALHYAVELFEGLKAFRGVDNKIRLFQPNLNMDRMYRSAVRATLPVFDKEELLECIQQLVKLDQEWVPYSTSASLYIRPTFIGTEPSLGVKKPTKALLFVLLSPVGPYFSSGTFNPVSLWANPKYVRAWKGGTGDCKMGGNYGSSLFAQCEAVDNGCQQVLWLYGEDHQITEVGTMNLFLYWINEDGEEELATPPLDGIILPGVTRRCILDLAHQWGEFKVSERYLTMDDLTTALEGNRVREMFGSGTACVVCPVSDILYKGETIHIPTMENGPKLASRILSKLTDIQYGREESDWTIVLS

987 NP_004442.3 NP_003320.2

>NP_004442.3

MSSQVVGIEPLYIKAEPASPDSPKGSSETETEPPVALAPGPAPTRCLPGHKEEEDGEGAGPGEQGGGKLVLSSLPKRLCLVCGDVASGYHYGVASCEACKAFFKRTIQGSIEYSCPASNECEITKRRRKACQACRFTKCLRVGMLKEGVRLDRVRGGRQKYKRRPEVDPLPFPGPFPAGPLAVAGGPRKTAAPVNALVSHLLVVEPEKLYAMPDPAGPDGHLPAVATLCDLFDREIVVTISWAKSIPGFSSLSLSDQMSVLQSVWMEVLVLGVAQRSLPLQDELAFAEDLVLDEEGARAAGLGELGAALLQLVRRLQALRLEREEYVLLKALALANSDSVHIEDAEAVEQLREALHEALLEYEAGRAGPGGGAERRRAGRLLLTLPLLRQTAGKVLAHFYGVKLEGKVPMHKLFLEMLEAMMD

>NP_003320.2

MVKQIESKTAFQEALDAAGDKLVVVDFSATWCGPCKMIKPFFHSLSEKYSNVIFLEVDVDDCQDVASECEVKCMPTFQFFKKGQKVGEFSGANKEKLEATINELV

988 NP_003511.1 Q07020

>NP_003511.1

MPEPSKSAPAPKKGSKKAVTKAQKKDGKKRKRSRKESYSVYVYKVLKQVHPDTGISSKAMGIMNSFVNDIFERIAGEASRLAHYNKRSTITSREIQTAVRLLLPGELAKHAVSEGTKAVTKYTSSK

>Q07020

MGVDIRHNKDRKVRRKEPKSQDIYLRLLVKLYRFLARRTNSTFNQVVLKRLFMSRTNRPPLSLSRMIRKMKLPGRENKTAVVVGTITDDVRVQEVPKLKVCALRVTSRARSRILRAGGKILTFDQLALDSPKGCGTVLLSGPRKGREVYRHFGKAPGTPHSHTKPYVRSKGRKFERARGRRASRGYKN

989 NP_076920.1 NP_005527.1

>NP_076920.1

MAMSSFLINSNYVDPKFPPCEEYSQSDYLPSDHSPGYYAGGQRRESSFQPEAGFGRRAACTVQRYAACRDPGPPPPPPPPPPPPPPPGLSPRAPAPPPAGALLPEPGQRCEAVSSSPPPPPCAQNPLHPSPSHSACKEPVVYPWMRKVHVSTVNPNYAGGEPKRSRTAYTRQQVLELEKEFHYNRYLTRRRRVEIAHALCLSERQIKIWFQNRRMKWKKDHKLPNTKIRSGGAAGSAGGPPGRPNGGPRAL

>NP_005527.1

MADPWQECMDYAVTLARQAGEVVCEAIKNEMNVMLKSSPVDLVTATDQKVEKMLISSIKEKYPSHSFIGEESVAAGEKSILTDNPTWIIDPIDGTTNFVHRFPFVAVSIGFAVNKKIEFGVVYSCVEGKMYTARKGKGAFCNGQKLQVSQQEDITKSLLVTELGSSRTPETVRMVLSNMEKLFCIPVHGIRSVGTAAVNMCLVATGGADAYYEMGIHCWDVAGAGIIVTEAGGVLMDVTGGPFDLMSRRVIAANNRILAERIAKEIQVIPLQRDDED

990 NP_003503.1 NP_006535.1

>NP_003503.1

MSGRGKQGGKARAKAKSRSSRAGLQFPVGRVHRLLRKGNYAERVGAGAPVYLAAVLEYLTAEILELAGNAARDNKKTRIIPRHLQLAIRNDEELNKLLGRVTIAQGGVLPNIQAVLLPKKTESHHKAKGK

>NP_006535.1

MATTAELFEEPFVADEYIERLVWRTPGGGSRGGPEAFDPKRLLEEFVNHIQELQIMDERIQRKVEKLEQQCQKEAKEFAKKVQELQKSNQVAFQHFQELDEHISYVATKVCHLGDQLEGVNTPRQRAVEAQKLMKYFNEFLDGELKSDVFTNSEKIKEAADIIQKLHLIAQELPFDRFSEVKSKIASKYHDLECQLIQEFTSAQRRGEISRMREVAAVLLHFKGYSHCVDVYIKQCQEGAYLRNDIFEDAGILCQRVNKQVGDIFSNPETVLAKLIQNVFEIKLQSFVKEQLEECRKSDAEQYLKNLYDLYTRTTNLSSKLMEFNLGTDKQTFLSKLIKSIFISYLENYIEVETGYLKSRSAMILQRYYDSKNHQKRSIGTGGIQDLKERIRQRTNLPLGPSIDTHGETFLSQEVVVNLLQETKQAFERCHRLSDPSDLPRNAFRIFTILVEFLCIEHIDYALETGLAGIPSSDSRNANLYFLDVVQQANTIFHLFDKQFNDHLMPLISSSPKLSECLQKKKEIIEQMEMKLDTGIDRTLNCMIGQMKHILAAEQKKTDFKPEDENNVLIQYTNACVKVCAYVRKQVEKIKNSMDGKNVDTVLMELGVRFHRLIYEHLQQYSYSCMGGMLAICDVAEYRKCAKDFKIPMVLHLFDTLHALCNLLVVAPDNLKQVCSGEQLANLDKNILHSFVQLRADYRSARLARHFS

991 NP_079485.1 P42765

>NP_079485.1

MSKLSFRARALDASKPLPVFRCEDLPDLHEYASINRAVPQMPTGMEKEEESEHHLQRAISAQQVYGEKRDNMVIPVPEAESNIAYYESIYPGEFKMPKQLIHIQPFSLDAEQPDYDLDSEDEVFVNKLKKKMDICPLQFEEMIDRLEKGSGQQPVSLQEAKLLLKEDDELIREVYEYWIKKRKNCRGPSLIPSVKQEKRDGSSTNDPYVAFRRRTEKMQTRKNRKNDEASYEKMLKLRRDLSRAVTILEMIKRREKSKRELLHLTLEIMEKRYNLGDYNGEIMSEVMAQRQPMKPTYAIPIIPITNSSQFKHQEAMDVKEFKVNKQDKADLIRPKRKYEKKPKVLPSSAAATPQQTSPAALPVFNAKDLNQYDFPSSDEEPLSQVLSGSSEAEEDNDPDGPFAFRRKAGCQYYAPHLDQTGNWPWTSPKDGGLGDVRYRYCLTTLTVPQRCIGFARRRVGRGGRVLLDRAHSDYDSVFHHLDLEMLSSPQHSPVNQFANTSETNTSDKSFSKDLSQILVNIKSCRWRHFRPRTPSLHDSDNDELSCRKLYRSINRTGTAQPGTQTCSTSTQSKSSSGSAHFAFTAEQYQQHQQQLALMQKQQLAQIQQQQANSNSSTNTSQNLASNQQKSGFRLNIQGLERTLQGFVSKTLDSASAQFAASALVTSEQLMGFKMKDDVVLGIGVNGVLPASGVYKGLHLSSTTPTALVHTSPSTAGSALLQPSNITQTSSSHSALSHQVTAANSATTQVLIGNNIRLTVPSSVATVNSIAPINARHIPRTLSAVPSSALKLAAAANCQVSKVPSSSSVDSVPRENHESEKPALNNIADNTVAMEVT

>P42765

MALLRGVFVVAAKRTPFGAYGGLLKDFTATDLSEFAAKAALSAGKVSPETVDSVIMGNVLQSSSDAIYLARHVGLRVGIPKETPALTINRLCGSGFQSIVNGCQEICVKEAEVVLCGGTESMSQAPYCVRNVRFGTKLGSDIKLEDSLWVSLTDQHVQLPMAMTAENLAVKHKISREECDKYALQSQQRWKAANDAGYFNDEMAPIEVKTKKGKQTMQVDEHARPQTTLEQLQKLPPVFKKDGTVTAGNASGVADGAGAVIIASEDAVKKHNFTPLARIVGYFVSGCDPSIMGIGPVPAISGALKKAGLSLKDMDLVEVNEAFAPQYLAVERSLDLDISKTNVNGGAIALGHPLGGSGSRITAHLVHELRRRGGKYAVGSACIGGGQGIAVIIQSTA

992 NP_002496.1 O14772

>NP_002496.1

MEQYTANSNSSTEQIVVQAGQIQQQQQGGVTAVQLQTEAQVASASGQQVQTLQVVQGQPLMVQVSGGQLITSTGQPIMVQAVPGGQGQTIMQVPVSGTQGLQQIQLVPPGQIQIQGGQAVQVQGQQGQTQQIIIQQPQTAVTAGQTQTQQQIAVQGQQVAQTAEGQTIVYQPVNADGTILQQVTVPVSGMITIPAASLAGAQIVQTGANTNTTSSGQGTVTVTLPVAGNVVNSGGMVMMVPGAGSVPAIQRIPLPGAEMLEEEPLYVNAKQYHRILKRRQARAKLEAEGKIPKERRKYLHESRHRHAMARKRGEGGRFFSPKEKDSPHMQDPNQADEEAMTQIIRVS

>O14772

MAAARDPPEVSLREATQRKLRRFSELRGKLVARGEFWDIVAITAADEKQELAYNQQLSEKLKRKELPLGVQYHVFVDPAGAKIGNGGSTLCALQCLEKLYGDKWNSFTILLIHSGGYSQRLPNASALGKIFTALPLGNPIYQMLELKLAMYIDFPLNMNPGILVTCADDIELYSIGEFEFIRFDKPGFTALAHPSSLTIGTTHGVFVLDPFDDLKHRDLEYRSCHRFLHKPSIEKMYQFNAVCRPGNFCQQDFAGGDIADLKLDSDYVYTDSLFYMDHKSAKMLLAFYEKIGTLSCEIDAYGDFLQALGPGATVEYTRNTSNVIKEESELVEMRQRIFHLLKGTSLNVVVLNNSKFYHIGTTEEYLFYFTSDNSLKSELGLQSITFSIFPDIPECSGKTSCIIQSILDSRCSVAPGSVVEYSRLGPDVSVGENCIISGSYILTKAALPAHSFVCSLSLKMNRCLKYATMAFGVQDNLKKSVKTLSDIKLLQFFGVCFLSCLDVWNLKVTEELFSGNKTCLSLWTARIFPVCSSLSDSVITSLKMLNAVKNKSAFSLNSYKLLSIEEMLIYKDVEDMITYREQIFLEISLKSSLM

993 Q9P2Y4 NP_060322.1

>Q9P2Y4

MEGSRPRAPSGHLAPSPPAFDGELDLQRYSNGPAVSAGSLGMGAVSWSESRAGERRFPCPVCGKRFRFNSILALHLRAHPGAQAFQCPHCGHRAAQRALLRSHLRTHQPERPRSPAARLLLELEERALLREARLGRARSSGGMQATPATEGLARPQAPSSSAFRCPYCKGKFRTSAERERHLHILHRPWKCGLCSFGSSQEEELLHHSLTAHGAPERPLAATSAAPPPQPQPQPPPQPEPRSVPQPEPEPEPEREATPTPAPAAPEEPPAPPEFRCQVCGQSFTQSWFLKGHMRKHKASFDHACPVCGRCFKEPWFLKNHMKVHASKLGPLRAPGPASGPARAPQPPDLGLLAYEPLGPALLLAPAPTPAERREPPSLLGYLSLRAGEGRPNGEGAEPGPGRSFGGFRPLSSALPARARRHRAEEPEEEEEVVEAEEETWARGRSLGSLASLHPRPGEGPGHSASAAGAQARSTATQEENGLLVGGTRPEGGRGATGKDCPFCGKSFRSAHHLKVHLRVHTGERPYKCPHCDYAGTQSGSLKYHLQRHHREQRSGAGPGPPPEPPPPSQRGSAPQSGAKPSPQPATWVEGASSPRPPSSGAGPGSRRKPASPGRTLRNGRGGEAEPLDLSLRAGPGGEAGPGGALHRCLFCPFATGAPELMALHLQVHHSRRARGRRPPQADASPPYARVPSGETPPSPSQEGEEGSGLSRPGEAGLGGQER

>NP_060322.1

MVSSAQMGFNLQALLEQLSQDELSKFKYLITTFSLAHELQKIPHKEVDKADGKQLVEILTTHCDSYWVEMASLQVFEKMHRMDLSERAKDEVREAALKSFNKRKPLSLGITRKERPPLDVDEMLERFKTEAQAFTETKGNVICLGKEVFKGKKPDKDNRCRYILKTKFREMWKSWPGDSKEVQVMAERYKMLIPFSNPRVLPGPFSYTVVLYGPAGLGKTTLAQKLMLDWAEDNLIHKFKYAFYLSCRELSRLGPCSFAELVFRDWPELQDDIPHILAQARKILFVIDGFDELGAAPGALIEDICGDWEKKKPVPVLLGSLLNRVMLPKAALLVTTRPRALRDLRILAEEPIYIRVEGFLEEDRRAYFLRHFGDEDQAMRAFELMRSNAALFQLGSAPAVCWIVCTTLKLQMEKGEDPVPTCLTRTGLFLRFLCSRFPQGAQLRGALRTLSLLAAQGLWAQTSVLHREDLERLGVQESDLRLFLDGDILRQDRVSKGCYSFIHLSFQQFLTALFYTLEKEEEEDRDGHTWDIGDVQKLLSGVERLRNPDLIQAGYYSFGLANEKRAKELEATFGCRMSPDIKQELLRCDISCKGGHSTVTDLQELLGCLYESQEEELVKEVMAQFKEISLHLNAVDVVPSSFCVKHCRNLQKMSLQVIKENLPENVTASESDAEVERSQDDQHMLPFWTDLCSIFGSNKDLMGLAINDSFLSASLVRILCEQIASDTCHLQRVVFKNISPADAHRNLCLALRGHKTVTYLTLQGNDQDDMFPALCEVLRHPECNLRYLGLVSCSATTQQWADLSLALEVNQSLTCVNLSDNELLDEGAKLLYTTLRHPKCFLQRLSLENCHLTEANCKDLAAVLVVSRELTHLCLAKNPIGNTGVKFLCEGLRYPECKLQTLVLWNCDITSDGCCDLTKLLQEKSSLLCLDLGLNHIGVKGMKFLCEALRKPLCNLRCLWLWGCSIPPFSCEDLCSALSCNQSLVTLDLGQNPLGSSGVKMLFETLTCSSGTLRTLRLKIDDFNDELNKLLEEIEEKNPQLIIDTEKHHPWAERPSSHDFMI

994 NP_006749.1 NP_000840.2

>NP_006749.1

MAEYLASIFGTEKDKVNCSFYFKIGACRHGDRCSRLHNKPTFSQTIALLNIYRNPQNSSQSADGLRCAVSDVEMQEHYDEFFEEVFTEMEEKYGEVEEMNVCDNLGDHLVGNVYVKFRREEDAEKAVIDLNNRWFNGQPIHAELSPVTDFREACCRQYEMGECTRGGFCNFMHLKPISRELRRELYGRRRKKHRSRSRSRERRSRSRDRGRGGGGGGGGGGGGRERDRRRSRDRERSGRF

>NP_000840.2

MSCESSMVLGYWDIRGLAHAIRLLLEFTDTSYEEKRYTCGEAPDYDRSQWLDVKFKLDLDFPNLPYLLDGKNKITQSNAILRYIARKHNMCGETEEEKIRVDIIENQVMDFRTQLIRLCYSSDHEKLKPQYLEELPGQLKQFSMFLGKFSWFAGEKLTFVDFLTYDILDQNRIFDPKCLDEFPNLKAFMCRFEALEKIAAYLQSDQFCKMPINNKMAQWGNKPVC

995 NP_004587.1 Q8N183

>NP_004587.1

MAVPETRPNHTIYINNLNEKIKKDELKKSLYAIFSQFGQILDILVSRSLKMRGQAFVIFKEVSSATNALRSMQGFPFYDKPMRIQYAKTDSDIIAKMKGTFVERDRKREKRKPKSQETPATKKAVQGGGATPVVGAVQGPVPGMPPMTQAPRIMHHMPGQPPYMPPPGMIPPPGLAPGQIPPGAMPPQQLMPGQMPPAQPLSENPPNHILFLTNLPEETNELMLSMLFNQFPGFKEVRLVPGRHDIAFVEFDNEVQAGAARDALQGFKITQNNAMKISFAKK

>Q8N183

MGWSQDLFRALWRSLSREVKEHVGTDQFGNKYYYIPQYKNWRGQTIREKRIVEAANKKEVDYEAGDIPTEWEAWIRRTRKTPPTMEEILKNEKHREEIKIKSQDFYEKEKLLSKETSEELLPPPVQTQIKGHASAPYFGKEEPSVAPSSTGKTFQPGSWMPRDGKSHNQ

996 NP_612482.2 Q07973

>NP_612482.2

MSDQDHSMDEMTAVVKIEKGVGGNNGGNGNGGGAFSQARSSSTGSSSSTGGGGQESQPSPLALLAATCSRIESPNENSNNSQGPSQSGGTGELDLTATQLSQGANGWQIISSSSGATPTSKEQSGSSTNGSNGSESSKNRTVSGGQYVVAAAPNLQNQQVLTGLPGVMPNIQYQVIPQFQTVDGQQLQFAATGAQVQQDGSGQIQIIPGANQQIITNRGSGGNIIAAMPNLLQQAVPLQGLANNVLSGQTQYVTNVPVALNGNITLLPVNSVSAATLTPSSQAVTISSSGSQESGSQPVTSGTTISSASLVSSQASSSSFFTNANSYSTTTTTSNMGIMNFTTSGSSGTNSQGQTPQRVSGLQGSDALNIQQNQTSGGSLQAGQQKEGEQNQQTQQQQILIQPQLVQGGQALQALQAAPLSGQTFTTQAISQETLQNLQLQAVPNSGPIIIRTPTVGPNGQVSWQTLQLQNLQVQNPQAQTITLAPMQGVSLGQTSSSNTTLTPIASAASIPAGTVTVNAAQLSSMPGLQTINLSALGTSGIQVHPIQGLPLAIANAPGDHGAQLGLHGAGGDGIHDDTAGGEEGENSPDAQPQAGRRTRREACTCPYCKDSEGRGSGDPGKKKQHICHIQGCGKVYGKTSHLRAHLRWHTGERPFMCTWSYCGKRFTRSDELQRHKRTHTGEKKFACPECPKRFMRSDHLSKHIKTHQNKKGGPGVALSVGTLPLDSGAGSEGSGTATPSALITTNMVAMEAICPEGIARLANSGINVMQVADLQSINISGNGF

>Q07973

MSSPISKSRSLAAFLQQLRSPRQPPRLVTSTAYTSPQPREVPVCPLTAGGETQNAAALPGPTSWPLLGSLLQILWKGGLKKQHDTLVEYHKKYGKIFRMKLGSFESVHLGSPCLLEALYRTESAYPQRLEIKPWKAYRDYRKEGYGLLILEGEDWQRVRSAFQKKLMKPGEVMKLDNKINEVLADFMGRIDELCDERGHVEDLYSELNKWSFESICLVLYEKRFGLLQKNAGDEAVNFIMAIKTMMSTFGRMMVTPVELHKSLNTKVWQDHTLAWDTIFKSVKACIDNRLEKYSQQPSADFLCDIYHQNRLSKKELYAAVTELQLAAVETTANSLMWILYNLSRNPQVQQKLLKEIQSVLPENQVPRAEDLRNMPYLKACLKESMRLTPSVPFTTRTLDKATVLGEYALPKGTVLMLNTQVLGSSEDNFEDSSQFRPERWLQEKEKINPFAHLPFGVGKRMCIGRRLAELQLHLALCWIVRKYDIQATDNEPVEMLHSGTLVPSRELPIAFCQR

997 NP_003073.1 NP_075066.1

>NP_003073.1

MGTPPGLQTDCEALLSRFQETDSVRFEDFTELWRNMKFGTIFCGRMRNLEKNMFTKEALALAWRYFLPPYTFQIRVGALYLLYGLYNTQLCQPKQKIRVALKDWDEVLKFQQDLVNAQHFDAAYIFRKLRLDRAFHFTAMPKLLSYRMKKKIHRAEVTEEFKDPSDRVMKLITSDVLEEMLNVHDHYQNMKHVISVDKSKPDKALSLIKDDFFDNIKNIVLEHQQWHKDRKNPSLKSKTNDGEEKMEGNSQETERCERAESLAKIKSKAFSVVIQASKSRRHRQVKLDSSDSDSASGQGQVKATRKKEKKERLKPAGRKMSLRNKGNVQNIHKEDKPLSLSMPVITEEEENESLSGTEFTASKKRRKH

>NP_075066.1

MASGLVRLLQQGHRCLLAPVAPKLVPPVRGVKKGFRAAFRFQKELERQRLLRCPPPPVRRSEKPNWDYHAEIQAFGHRLQENFSLDLLKTAFVNSCYIKSEEAKRQQLGIEKEAVLLNLKSNQELSEQGTSFSQTCLTQFLEDEYPDMPTEGIKNLVDFLTGEEVVCHVARNLAVEQLTLSEEFPVPPAVLQQTFFAVIGALLQSSGPERTALFIRDFLITQMTGKELFEMWKIINPMGLLVEELKKRNVSAPESRLTRQSGGTTALPLYFVGLYCDKKLIAEGPGETVLVAEEEAARVALRKLYGFTENRRPWNYSKPKETLRAEKSITAS

998 NP_003211.1 NP_006262.1

>NP_003211.1

MLWKLTDNIKYEDCEDRHDGTSNGTARLPQLGTVGQSPYTSAPPLSHTPNADFQPPYFPPPYQPIYPQSQDPYSHVNDPYSLNPLHAQPQPQHPGWPGQRQSQESGLLHTHRGLPHQLSGLDPRRDYRRHEDLLHGPHALSSGLGDLSIHSLPHAIEEVPHVEDPGINIPDQTVIKKGPVSLSKSNSNAVSAIPINKDNLFGGVVNPNEVFCSVPGRLSLLSSTSKYKVTVAEVQRRLSPPECLNASLLGGVLRRAKSKNGGRSLREKLDKIGLNLPAGRRKAANVTLLTSLVEGEAVHLARDFGYVCETEFPAKAVAEFLNRQHSDPNEQVTRKNMLLATKQICKEFTDLLAQDRSPLGNSRPNPILEPGIQSCLTHFNLISHGFGSPAVCAAVTALQNYLTEALKAMDKMYLSNNPNSHTDNNAKSSDKEEKHRK

>NP_006262.1

MGSELETAMETLINVFHAHSGKEGDKYKLSKKELKELLQTELSGFLDAQKDVDAVDKVMKELDENGDGEVDFQEYVVLVAALTVACNNFFWENS

999 NP_066964.1 NP_055867.3

>NP_066964.1

MVRSGNKAAVVLCMDVGFTMSNSIPGIESPFEQAKKVITMFVQRQVFAENKDEIALVLFGTDGTDNPLSGGDQYQNITVHRHLMLPDFDLLEDIESKIQPGSQQADFLDALIVSMDVIQHETIGKKFEKRHIEIFTDLSSRFSKSQLDIIIHSLKKCDISLQFFLPFSLGKEDGSGDRGDGPFRLGGHGPSFPLKGITEQQKEGLEIVKMVMISLEGEDGLDEIYSFSESLRKLCVFKKIERHSIHWPCRLTIGSNLSIRIAAYKSILQERVKKTWTVVDAKTLKKEDIQKETVYCLNDDDETEVLKEDIIQGFRYGSDIVPFSKVDEEQMKYKSEGKCFSVLGFCKSSQVQRRFFMGNQVLKVFAARDDEAAAVALSSLIHALDDLDMVAIVRYAYDKRANPQVGVAFPHIKHNYECLVYVQLPFMEDLRQYMFSSLKNSKKYAPTEAQLNAVDALIDSMSLAKKDEKTDTLEDLFPTTKIPNPRFQRLFQCLLHRALHPREPLPPIQQHIWNMLNPPAEVTTKSQIPLSKIKTLFPLIEAKKKDQVTAQEIFQDNHEDGPTAKKLKTEQGGAHFSVSSLAEGSVTSVGSVNPAENFRVLVKQKKASFEEASNQLINHIEQFLDTNETPYFMKSIDCIRAFREEAIKFSEEQRFNNFLKALQEKVEIKQLNHFWEIVVQDGITLITKEEASGSSVTAEEAKKFLAPKDKPSGDTAAVFEEGGDVDDLLDMI

>NP_055867.3

MLLHLCSVKNLYQNRFLGLAAMASPSRNSQSRRRCKEPLRYSYNPDQFHNMDLRGGPHDGVTIPRSTSDTDLVTSDSRSTLMVSSSYYSIGHSQDLVIHWDIKEEVDAGDWIGMYLIDEVLSENFLDYKNRGVNGSHRGQIIWKIDASSYFVEPETKICFKYYHGVSGALRATTPSVTVKNSAAPIFKSIGADETVQGQGSRRLISFSLSDFQAMGLKKGMFFNPDPYLKISIQPGKHSIFPALPHHGQERRSKIIGNTVNPIWQAEQFSFVSLPTDVLEIEVKDKFAKSRPIIKRFLGKLSMPVQRLLERHAIGDRVVSYTLGRRLPTDHVSGQLQFRFEITSSIHPDDEEISLSTEPESAQIQDSPMNNLMESGSGEPRSEAPESSESWKPEQLGEGSVPDGPGNQSIELSRPAEEAAVITEAGDQGMVSVGPEGAGELLAQVQKDIQPAPSAEELAEQLDLGEEASALLLEDGEAPASTKEEPLEEEATTQSRAGREEEEKEQEEEGDVSTLEQGEGRLQLRASVKRKSRPCSLPVSELETVIASACGDPETPRTHYIRIHTLLHSMPSAQGGSAAEEEDGAEEESTLKDSSEKDGLSEVDTVAADPSALEEDREEPEGATPGTAHPGHSGGHFPSLANGAAQDGDTHPSTGSESDSSPRQGGDHSCEGCDASCCSPSCYSSSCYSTSCYSSSCYSASCYSPSCYNGNRFASHTRFSSVDSAKISESTVFSSQDDEEEENSAFESVPDSMQSPELDPESTNGAGPWQDELAAPSGHVERSPEGLESPVAGPSNRREGECPILHNSQPVSQLPSLRPEHHHYPTIDEPLPPNWEARIDSHGRVFYVDHVNRTTTWQRPTAAATPDGMRRSGSIQQMEQLNRRYQNIQRTIATERSEEDSGSQSCEQAPAGGGGGGGSDSEAESSQSSLDLRREGSLSPVNSQKITLLLQSPAVKFITNPEFFTVLHANYSAYRVFTSSTCLKHMILKVRRDARNFERYQHNRDLVNFINMFADTRLELPRGWEIKTDQQGKSFFVDHNSRATTFIDPRIPLQNGRLPNHLTHRQHLQRLRSYSAGEASEVSRNRGASLLARPGHSLVAAIRSQHQHESLPLAYNDKIVAFLRQPNIFEMLQERQPSLARNHTLREKIHYIRTEGNHGLEKLSCDADLVILLSLFEEEIMSYVPLQAAFHPGYSFSPRCSPCSSPQNSPGLQRASARAPSPYRRDFEAKLRNFYRKLEAKGFGQGPGKIKLIIRRDHLLEGTFNQVMAYSRKELQRNKLYVTFVGEEGLDYSGPSREFFFLLSQELFNPYYGLFEYSANDTYTVQISPMSAFVENHLEWFRFSGRILGLALIHQYLLDAFFTRPFYKALLRLPCDLSDLEYLDEEFHQSLQWMKDNNITDILDLTFTVNEEVFGQVTERELKSGGANTQVTEKNKKEYIERMVKWRVERGVVQQTEALVRGFYEVVDSRLVSVFDARELELVIAGTAEIDLNDWRNNTEYRGGYHDGHLVIRWFWAAVERFNNEQRLRLLQFVTGTSSVPYEGFAALRGSNGLRRFCIEKWGKITSLPRAHTCFNRLDLPPYPSYSMLYEKLLTAVEETSTFGLE

1000 NP_001316.1 Q92665

>NP_001316.1

MAGLTVRDPAVDRSLRSVFVGNIPYEATEEQLKDIFSEVGPVVSFRLVYDRETGKPKGYGFCEYQDQETALSAMRNLNGREFSGRALRVDNAASEKNKEELKSLGTGAPVIESPYGETISPEDAPESISKAVASLPPEQMFELMKQMKLCVQNSPQEARNMLLQNPQLAYALLQAQVVMRIVDPEIALKILHRQTNIPTLIAGNPQPVHGAGPGSGSNVSMNQQNPQAPQAQSLGGMHVNGAPPLMQASMQGGVPAPGQMPAAVTGPGPGSLAPGGGMQAQVGMPGSGPVSMERGQVPMQDPRAAMQRGSLPANVPTPRGLLGDAPNDPRGGTLLSVTGEVEPRGYLGPPHQGPPMHHVPGHESRGPPPHELRGGPLPEPRPLMAEPRGPMLDQRGPPLDGRGGRDPRGIDARGMEARAMEARGLDARGLEARAMEARAMEARAMEARAMEARAMEVRGMEARGMDTRGPVPGPRGPIPSGMQGPSPINMGAVVPQGSRQVPVMQGTGMQGASIQGGSQPGGFSPGQNQVTPQDHEKAALIMQVLQLTADQIAMLPPEQRQSILILKEQIQKSTGAP

>Q92665

MFPRVSTFLPLRPLSRHPLSSGSPETSAAAIMLLTVRHGTVRYRSSALLARTKNNIQRYFGTNSVICSKKDKQSVRTEETSKETSESQDSEKENTKKDLLGIIKGMKVELSTVNVRTTKPPKRRPLKSLEATLGRLRRATEYAPKKRIEPLSPELVAAASAVADSLPFDKQTTKSELLSQLQQHEEESRAQRDAKRPKISFSNIISDMKVARSATARVRSRPELRIQFDEGYDNYPGQEKTDDLKKRKNIFTGKRLNIFDMMAVTKEAPETDTSPSLWDVEFAKQLATVNEQPLQNGFEELIQWTKEGKLWEFPINNEAGFDDDGSEFHEHIFLEKHLESFPKQGPIRHFMELVTCGLSKNPYLSVKQKVEHIEWFRNYFNEKKDILKESNIQFN

1001 P60008 NP_002305.1

>P60008

MLHASTIWHLRSTPPRRKQWGHCDPHRILVASEVTTEITSPTPAPRAQVCGGQPWVTVLDPLSGHTGREAERHFATVSISAVELKYCHGWRPAGQRVPSKTATGQRTCAKPCQKPSTSKVILRAVADKGTCKYVSLATLKKAVSTTGYDMARNAYHFKRVLKGLVDKGSAGSFTLGKKQASKSKLKVKRQRQQRWRSGQRPFGQHRSLLGSKQGHKRLIKGVRRVAKCHCN

>NP_002305.1

MRLTLLCCTWREERMGEEGSELPVCASCGQRIYDGQYLQALNADWHADCFRCCDCSASLSHQYYEKDGQLFCKKDYWARYGESCHGCSEQITKGLVMVAGELKYHPECFICLTCGTFIGDGDTYTLVEHSKLYCGHCYYQTVVTPVIEQILPDSPGSHLPHTVTLVSIPASSHGKRGLSVSIDPPHGPPGCGTEHSHTVRVQGVDPGCMSPDVKNSIHVGDRILEINGTPIRNVPLDEIDLLIQETSRLLQLTLEHDPHDTLGHGLGPETSPLSSPAYTPSGEAGSSARQKPVLRSCSIDRSPGAGSLGSPASQRKDLGRSESLRVVCRPHRIFRPSDLIHGEVLGKGCFGQAIKVTHRETGEVMVMKELIRFDEETQRTFLKEVKVMRCLEHPNVLKFIGVLYKDKRLNFITEYIKGGTLRGIIKSMDSQYPWSQRVSFAKDIASGMAYLHSMNIIHRDLNSHNCLVRENKNVVVADFGLARLMVDEKTQPEGLRSLKKPDRKKRYTVVGNPYWMAPEMINGRSYDEKVDVFSFGIVLCEIIGRVNADPDYLPRTMDFGLNVRGFLDRYCPPNCPPSFFPITVRCCDLDPEKRPSFVKLEHWLETLRMHLAGHLPLGPQLEQLDRGFWETYRRGESGLPAHPEVPD

1002 Q9Y6X0 NP_002444.2

>Q9Y6X0

MEPEEEDELGSGRDVDSNSNADSEKWVAGDGLEEQEFSIKEANFTEGSLKLKIQTTKRAKKPPKNLENYICPPEIKITIKQSGDQKVSRAGKNSKATKEEERSHSKKKLLTASDLAASDLKGFQPQAYERPQKHSTLHYDTGLPQDFTGDTLKPKHQQKSSSQNHMDWSTNSDSGPVTQNCFISPESGRETASTSKIPALEPVASFAKAQGKKGSAGNTWSQLSNNNKDLLLGGVAPSPSSHSSPAPPSSSAECNGLQPLVDQDGGGTKEPPEPPTVGSKKKSSKKDVISQTIPNPDLDWVKNAQKAFDNTEGKREGYSADSAQEASPARQNVSSASNPENDSSHVRITIPIKAPSLDPTNHKRKKRQSIKAVVEKIMPEKALASGITMSSEVVNRILSNSEGNKKDPRVPKLSKMIENESPSVGLETGGNAEKVIPGGVSKPRKPPMVMTPPTCTDHSPSRKLPEIQHPKFAAKRRWTCSKPKPSTMLREAVMATSDKLMLEPPSAYPITPSSPLYTNTDSLTVITPVKKKRGRPKKQPLLTVETIHEGTSTSPVSPISREFPGTKKRKRRRNLAKLAQLVPGEDKPMSEMKFHKKVGKLGVLDKKTIKTINKMKTLKRKNILNQILSCSSSVALKAKAPPETSPGAAAIESKLGKQINVSKRGTIYIGKKRGRKPRAELPPPSEEPKTAIKHPRPVSSQPDVPAVPSNFQSLVASSPAAMHPLSTQLGGSNGNLSPASTETNFSELKTMPNLQPISALPTKTQKGIHSGTWKLSPPRLMANSPSHLCEIGSLKEITLSPVSESHSEETIPSDSGIGTDNNSTSDQAEKSSESRRRYSFDFCSLDNPEAIPSDTSTKNRHGHRQKHLIVDNFLAHESLKKPKHKRKRKSLQNRDDLQFLADLEELITKFQVFRISHRSYTFYHENPYPSIFRINFDHYYPVPYIQYDPLLYLRRTSDLKSKKKRGRPAKTNDTMTKVPFLQGFSYPIPSGSYYAPYGMPYTSMPMMNLGYYGQYPAPLYLSHTLGAASPFMRPTVPPPQFHTNSHVKMSGAAKHKAKHGVHLQGPVSMGLGDMQPSLNPPKVGSASLSSGRLHKRKHKHKHKHKEDRILGTHDNLSGLFAGKATGFSSHILSERLSSADKELPLVSEKNKHKEKQKHQHSEAGHKASKNNFEVDTLSTLSLSDAQHWTQAKEKGDLSSEPVDSCTKRYSGSGGDGGSTRSENLDVFSEMNPSNDKWDSDVSGSKRRSYEGFGTYREKDIQAFKMNRKERSSYDSSMSPGMPSPHLKVDQTAVHSKNEGSVPTMMTRKKPAAVDSVTIPPAPVLSLLAASAATSDAVGSSLKKRFKRREIEAIQCEVRKMCNYTKILSTKKNLDHVNKILKAKRLQRQSKTGNNFVKKRRGRPRKQPTQFDEDSRDQMPVLEKCIDLPSKRGQKPSLSPLVLEPAASQDTIMATIEAVIHMAREAPPLPPPPPPPLPPPPPPPLPPPPPLPKTPRGGKRKHKPQAPAQPPQQSPPQQPLPQEEEVKAKRQRKSRGSESEVLP

>NP_002444.2

MNQKLLKLENLLRFHTIYRQLHSLCQRRALRQWRHGFSSAYPVWTAQLCAWPWPTDVLTGAALSQYRLLVTKKEEGPWKSQLSSTKSKKVVEVWIGMTIEELARAMEKNTDYVYEALLNTDIDIDSLEADSHLDEVWIKEVITKAGMKLKWSKLKQDKVRKNKDAVRRPQADPALLTPRSPVVTIMGHVDHGKTTLLDKFRKTQVAAVETGGITQHIGAFLVSLPSGEKITFLDTPGHAAFSAMRARGAQVTDIVVLVVAADDGVMKQTVESIQHAKDAQVPIILAVNKCDKAEADPEKVKKELLAYDVVCEDYGGDVQAVPVSALTGDNLMALAEATVALAEMLELKADPNGPVEGTVIESFTDKGRGLVTTAIIQRGTLRKGSVLVAGKCWAKVRLMFDENGKTIDEAYPSMPVGITGWRDLPSAGEEILEVESEPRAREVVDWRKYEQEQEKGQEDLKIIEEKRKEHKEAHQKAREKYGHLLWKKRSILRFLERKEQIPLKPKEKRERDSNVLSVIIKGDVDGSVEAILNIIDTYDASHECELELVHFGVGDVSANDVNLAETFDGVIYGFNVNAGNVIQQSAAKKGVKIKLHKIIYRLVEDLQEELSSRLPCAVEEHPVGEASILATFSVTEGKKKVPVAGCRVQKGQLEKQKKFKLTRNGHVIWKGSLTSLKHHKDDISIVKTGMDCGLSLDEDNMEFQVGDRIVCYEEKQIQAKTSWDPGF

1003 Q06416 Q9NQR4

>Q06416

MAGHLASDFAFSPPPGGGGDGPWGAEPGWVDPLTWLSFQGPPGGPGIGPGVGPGSEVWGIPPCPPPYELCGGMAYCGPQVGVGLVPQGGLETSQPESEAGVGVESNSNGASPEPCTVPPGAVKLEKEKLEQNPEKSQDIKALQKELEQFAKLLKQKRITLGYTQADVGLILGVLFGKVFSQKTICRFEALQLSFKNICKLRPLLQKWVEEADNNENLQEICKAETLMQARKRKRTSIENRVRGNLENLFLQCPKPTLQISHIAQQLGLEKDVVRVWFCNRRQKGKRSSSDYAQREDFEAAGSPFSGGPVSFPPAPGPHFGTPGYGSPHFTALYSSVPFPEGEVFPPVSVITLGSPMHSN

>Q9NQR4

MTSFRLALIQLQISSIKSDNVTRACSFIREAATQGAKIVSLPECFNSPYGAKYFPEYAEKIPGESTQKLSEVAKECSIYLIGGSIPEEDAGKLYNTCAVFGPDGTLLAKYRKIHLFDIDVPGKITFQESKTLSPGDSFSTFDTPYCRVGLGICYDMRFAELAQIYAQRGCQLLVYPGAFNLTTGPAHWELLQRSRAVDNQVYVATASPARDDKASYVAWGHSTVVNPWGEVLAKAGTEEAIVYSDIDLKKLAEIRQQIPVFRQKRSDLYAVEMKKP

1004 NP_001978.1 NP_004591.2

>NP_001978.1

MSETPAQCSIKQERISYTPPESPVPSYASSTPLHVPVPRALRMEEDSIRLPAHLRLQPIYWSRDDVAQWLKWAENEFSLRPIDSNTFEMNGKALLLLTKEDFRYRSPHSGDVLYELLQHILKQRKPRILFSPFFHPGNSIHTQPEVILHQNHEEDNCVQRTPRPSVDNVHHNPPTIELLHRSRSPITTNHRPSPDPEQRPLRSPLDNMIRRLSPAERAQGPRPHQENNHQESYPLSVSPMENNHCPASSESHPKPSSPRQESTRVIQLMPSPIMHPLILNPRHSVDFKQSRLSEDGLHREGKPINLSHREDLAYMNHIMVSVSPPEEHAMPIGRIADCRLLWDYVYQLLSDSRYENFIRWEDKESKIFRIVDPNGLARLWGNHKNRTNMTYEKMSRALRHYYKLNIIRKEPGQRLLFRFMKTPDEIMSGRTDRLEHLESQELDEQIYQEDEC

>NP_004591.2

MEESVNQMQPLNEKQIANSQDGYVWQVTDMNRLHRFLCFGSEGGTYYIKEQKLGLENAEALIRLIEDGRGCEVIQEIKSFSQEGRTTKQEPMLFALAICSQCSDISTKQAAFKAVSEVCRIPTHLFTFIQFKKDLKESMKCGMWGRALRKAIADWYNEKGGMALALAVTKYKQRNGWSHKDLLRLSHLKPSSEGLAIVTKYITKGWKEVHELYKEKALSVETEKLLKYLEAVEKVKRTRDELEVIHLIEEHRLVREHLLTNHLKSKEVWKALLQEMPLTALLRNLGKMTANSVLEPGNSEVSLVCEKLCNEKLLKKARIHPFHILIALETYKTGHGLRGKLKWRPDEEILKALDAAFYKTFKTVEPTGKRFLLAVDVSASMNQRVLGSILNASTVAAAMCMVVTRTEKDSYVVAFSDEMVPCPVTTDMTLQQVLMAMSQIPAGGTDCSLPMIWAQKTNTPADVFIVFTDNETFAGGVHPAIALREYRKKMDIPAKLIVCGMTSNGFTIADPDDRGMLDMCGFDTGALDVIRNFTLDMI

1005 Q69YI7 NP_110379.2

>Q69YI7

MAVPAKKRKMNFSEREVEIIVEELELKKHLLVNHFNAGVPLAAKSAAWHGILRRVNAVATCRRELPEVKKKWSDLKTEVRRKVAQVRAAVEGGEAPGPTEEDGAGGPGTGGGSGGGGPAVAPVLLTPMQQRICNLLGEATIISLPSTTEIHPVALGPSATAAAATVTLTQIPTETTYHTLEEGVVEYCTAEAPPPLPPETPVDMMAQHADTSVKPQALKSRIALNSAKLIQEQRVTNLHVKEIAQHLEQQNDLLQMIRRSQEVQACAQERQAQAMEGTQAALSVLIQVLRPMIKDFRRYLQSNTANPAPASDPGQVAQNGQPDSIIQ

>NP_110379.2

MEGPLSVFGDRSTGETIRSQNVMAAASIANIVKSSLGPVGLDKMLVDDIGDVTITNDGATILKLLEVEHPAAKVLCELADLQDKEVGDGTTSVVIIAAELLKNADELVKQKIHPTSVISGYRLACKEAVRYINENLIVNTDELGRDCLINAAKTSMSSKIIGINGDFFANMVVDAVLAIKYTDIRGQPRYPVNSVNILKAHGRSQMESMLISGYALNCVVGSQGMPKRIVNAKIACLDFSLQKTKMKLGVQVVITDPEKLDQIRQRESDITKERIQKILATGANVILTTGGIDDMCLKYFVEAGAMAVRRVLKRDLKRIAKASGATILSTLANLEGEETFEAAMLGQAEEVVQERICDDELILIKNTKARTSASIILRGANDFMCDEMERSLHDALCVVKRVLESKSVVPGGGAVEAALSIYLENYATSMGSREQLAIAEFARSLLVIPNTLAVNAAQDSTDLVAKLRAFHNEAQVNPERKNLKWIGLDLSNGKPRDNKQAGVFEPTIVKVKSLKFATEAAITILRIDDLIKLHPESKDDKHGSYEDAVHSGALND

1006 NP_003202.3 NP_000847.2

>NP_003202.3

MEAENAGSYSLQQAQAFYTFPFQQLMAEAPNMAVVNEQQMPEEVPAPAPAQEPVQEAPKGRKRKPRTTEPKQPVEPKKPVESKKSGKSAKSKEKQEKITDTFKVKRKVDRFNGVSEAELLTKTLPDILTFNLDIVIIGINPGLMAAYKGHHYPGPGNHFWKCLFMSGLSEVQLNHMDDHTLPGKYGIGFTNMVERTTPGSKDLSSKEFREGGRILVQKLQKYQPRIAVFNGKCIYEIFSKEVFGVKVKNLEFGLQPHKIPDTETLCYVMPSSSARCAQFPRAQDKVHYYIKLKDLRDQLKGIERNMDVQEVQYTFDLQLAQEDAKKMAVKEEKYDPGYEAAYGGAYGENPCSSEPCGFSSNGLIESVELRGESAFSGIPNGQWMTQSFTDQIPSFSNHCGTQEQEEESHA

>NP_000847.2

MFCTKLKDLKITGECPFSLLAPGQVPNESSEEAAGSSESCKATVPICQDIPEKNIQESLPQRKTSRSRVYLHTLAESICKLIFPEFERLNVALQRTLAKHKIKESRKSLEREDFEKTIAEQAVAAGVPVEVIKESLGEEVFKICYEEDENILGVVGGTLKDFLNSFSTLLKQSSHCQEAGKRGRLEDASILCLDKEDDFLHVYYFFPKRTTSLILPGIIKAAAHVLYETEVEVSLMPPCFHNDCSEFVNQPYLLYSVHMKSTKPSLSPSKPQSSLVIPTSLFCKTFPFHFMFDKDMTILQFGNGIRRLMNRRDFQGKPNFEEYFEILTPKINQTFSGIMTMLNMQFVVRVRRWDNSVKKSSRVMDLKGQMIYIVESSAILFLGSPCVDRLEDFTGRGLYLSDIPIHNALRDVVLIGEQARAQDGLKKRLGKLKATLEQAHQALEEEKKKTVDLLCSIFPCEVAQQLWQGQVVQAKKFSNVTMLFSDIVGFTAICSQCSPLQVITMLNALYTRFDQQCGELDVYKVETIGDAYCVAGGLHKESDTHAVQIALMALKMMELSDEVMSPHGEPIKMRIGLHSGSVFAGVVGVKMPRYCLFGNNVTLANKFESCSVPRKINVSPTTYRLLKDCPGFVFTPRSREELPPNFPSEIPGICHFLDAYQQGTNSKPCFQKKDVEDGNANFLGKASGID

1007 NP_000241.1 NP_001166.3

>NP_000241.1

MGVPFFSSLRCMVDLGPCWAGGLTAEMKLLLALAGLLAILATPQPSEGAAPAVLGEVDTSLVLSSMEEAKQLVDKAYKERRESIKQRLRSGSASPMELLSYFKQPVAATRTAVRAADYLHVALDLLERKLRSLWRRPFNVTDVLTPAQLNVLSKSSGCAYQDVGVTCPEQDKYRTITGMCNNRRSPTLGASNRAFVRWLPAEYEDGFSLPYGWTPGVKRNGFPVALARAVSNEIVRFPTDQLTPDQERSLMFMQWGQLLDHDLDFTPEPAARASFVTGVNCETSCVQQPPCFPLKIPPNDPRIKNQADCIPFFRSCPACPGSNITIRNQINALTSFVDASMVYGSEEPLARNLRNMSNQLGLLAVNQRFQDNGRALLPFDNLHDDPCLLTNRSARIPCFLAGDTRSSEMPELTSMHTLLLREHNRLATELKSLNPRWDGERLYQEARKIVGAMVQIITYRDYLPLVLGPTAMRKYLPTYRSYNDSVDPRIANVFTNAFRYGHTLIQPFMFRLDNRYQPMEPNPRVPLSRVFFASWRVVLEGGIDPILRGLMATPAKLNRQNQIAVDEIRERLFEQVMRIGLDLPALNMQRSRDHGLPGYNAWRRFCGLPQPETVGQLGTVLRNLKLARKLMEQYGTPNNIDIWMGGVSEPLKRKGRVGPLLACIIGTQFRKLRDGDRFWWENEGVFSMQQRQALAQISLPRIICDNTGITTVSKNNIFMSNSYPRDFVNCSTLPALNLASWREAS

>NP_001166.3

MTEKAPEPHVEEDDDDELDSKLNYKPPPQKSLKELQEMDKDDESLIKYKKTLLGDGPVVTDPKAPNVVVTRLTLVCESAPGPITMDLTGDLEALKKETIVLKEGSEYRVKIHFKVNRDIVSGLKYVQHTYRTGVKVDKATFMVGSYGPRPEEYEFLTPVEEAPKGMLARGTYHNKSFFTDDDKQDHLSWEWNLSIKKEWTE

1008 NP_001001930.1 Q9BY32

>NP_001001930.1

MVDTESPLCPLSPLEAGDLESPLSEEFLQEMGNIQEISQSIGEDSSGSFGFTEYQYLGSCPGSDGSVITDTLSPASSPSSVTYPVVPGSVDESPSGALNIECRICGDKASGYHYGVHACEGCKGFFRRTIRLKLVYDKCDRSCKIQKKNRNKCQYCRFHKCLSVGMSHNAIRFGRMPRSEKAKLKAEILTCEHDIEDSETADLKSLAKRIYEAYLKNFNMNKVKARVILSGKASNNPPFVIHDMETLCMAEKTLVAKLVANGIQNKEAEVRIFHCCQCTSVETVTELTEFAKAIPGFANLDLNDQVTLLKYGVYEAIFAMLSSVMNKDGMLVAYGNGFITREFLKSLRKPFCDIMEPKFDFAMKFNALELDDSDISLFVAAIICCGDRPGLLNVGHIEKMQEGIVHVLRLHLQSNHPDDIFLFPKLLQKMADLRQLVTEHAQLVQIIKKTESDAALHPLLQEIYRDMY

>Q9BY32

MAASLVGKKIVFVTGNAKKLEEVVQILGDKFPCTLVAQKIDLPEYQGEPDEISIQKCQEAVRQVQGPVLVEDTCLCFNALGGLPGPYIKWFLEKLKPEGLHQLLAGFEDKSAYALCTFALSTGDPSQPVRLFRGRTSGRIVAPRGCQDFGWDPCFQPDGYEQTYAEMPKAEKNAVSHRFRALLELQEYFGSLAA

1009 NP_009075.1 Q14914

>NP_009075.1

MHRTTRIKITELNPHLMCALCGGYFIDATTIVECLHSFCKTCIVRYLETNKYCPMCDVQVHKTRPLLSIRSDKTLQDIVYKLVPGLFKDEMKRRRDFYAAYPLTEVPNGSNEDRGEVLEQEKGALSDDEIVSLSIEFYEGARDRDEKKGPLENGDGDKEKTGVRFLRCPAAMTVMHLAKFLRNKMDVPSKYKVEVLYEDEPLKEYYTLMDIAYIYPWRRNGPLPLKYRVQPACKRLTLATVPTPSEGTNTSGASECESVSDKAPSPATLPATSSSLPSPATPSHGSPSSHGPPATHPTSPTPPSTASGATTAANGGSLNCLQTPSSTSRGRKMTVNGAPVPPLT

>Q14914

MVRTKTWTLKKHFVGYPTNSDFELKTAELPPLKNGEVLLEALFLTVDPYMRVAAKRLKEGDTMMGQQVAKVVESKNVALPKGTIVLASPGWTTHSISDGKDLEKLLTEWPDTIPLSLALGTVGMPGLTAYFGLLEICGVKGGETVMVNAAAGAVGSVVGQIAKLKGCKVVGAVGSDEKVAYLQKLGFDVVFNYKTVESLEETLKKASPDGYDCYFDNVGGEFSNTVIGQMKKFGRIAICGAISTYNRTGPLPPGPPPEIVIYQELRMEAFVVYRWQGDARQKALKDLLKWVLEGKIQYKEYIIEGFENMPAAFMGMLKGDNLGKTIVKA

1010 Q86WB0 NP_000886.1

>Q86WB0

MAAPCEGQAFAVGVEKNWGAVVRSPEGTPQKIRQLIDEGIAPEEGGVDAKDTSATSQSVNGSPQAEQPSLESTSKEAFFSRVETFSSLKWAGKPFELSPLVCAKYGWVTVECDMLKCSSCQAFLCASLQPAFDFDRYKQRCAELKKALCTAHEKFCFWPDSPSPDRFGMLPLDEPAILVSEFLDRFQSLCHLDLQLPSLRPEDLKTMCLTEDKISLLLHLLEDELDHRTDERKTTIKLGSDIQVHVTACILSVCGWACSSSLESMQLSLITCSQCMRKVGLWGFQQIESSMTDLDASFGLTSSPIPGLEGRPERLPLVPESPRRMMTRSQDATFSPGSEQAEKSPGPIVSRTRSWDSSSPVDRPEPEAASPTTRTRPVTRSMGTGDTPGLEVPSSPLRKAKRARLCSSSSSDTSSRSFFDPTSQHRDWCPWVNITLGKESRENGGTEPDASAPAEPGWKAVLTILLAHKQSSQPAETDSMSLSEKSRKVFRIFRQWESLCSC

>NP_000886.1

MPEIVDTCSLASPASVCRTKHLHLRCSVDFTRRTLTGTAALTVQSQEDNLRSLVLDTKDLTIEKVVINGQEVKYALGERQSYKGSPMEISLPIALSKNQEIVIEISFETSPKSSALQWLTPEQTSGKEHPYLFSQCQAIHCRAILPCQDTPSVKLTYTAEVSVPKELVALMSAIRDGETPDPEDPSRKIYKFIQKVPIPCYLIALVVGALESRQIGPRTLVWSEKEQVEKSAYEFSETESMLKIAEDLGGPYVWGQYDLLVLPPSFPYGGMENPCLTFVTPTLLAGDKSLSNVIAHEISHSWTGNLVTNKTWDHFWLNEGHTVYLERHICGRLFGEKFRHFNALGGWGELQNSVKTFGETHPFTKLVVDLTDIDPDVAYSSVPYEKGFALLFYLEQLLGGPEIFLGFLKAYVEKFSYKSITTDDWKDFLYSYFKDKVDVLNQVDWNAWLYSPGLPPIKPNYDMTLTNACIALSQRWITAKEDDLNSFNATDLKDLSSHQLNEFLAQTLQRAPLPLGHIKRMQEVYNFNAINNSEIRFRWLRLCIQSKWEDAIPLALKMATEQGRMKFTRPLFKDLAAFDKSHDQAVRTYQEHKASMHPVTAMLVGKDLKVD

1011 Q96T88 NP_055867.3

>Q96T88

MWIQVRTMDGRQTHTVDSLSRLTKVEELRRKIQELFHVEPGLQRLFYRGKQMEDGHTLFDYEVRLNDTIQLLVRQSLVLPHSTKERDSELSDTDSGCCLGQSESDKSSTHGEAAAETDSRPADEDMWDETELGLYKVNEYVDARDTNMGAWFEAQVVRVTRKAPSRDEPCSSTSRPALEEDVIYHVKYDDYPENGVVQMNSRDVRARARTIIKWQDLEVGQVVMLNYNPDNPKERGFWYDAEISRKRETRTARELYANVVLGDDSLNDCRIIFVDEVFKIERPGEGSPMVDNPMRRKSGPSCKHCKDDVNRLCRVCACHLCGGRQDPDKQLMCDECDMAFHIYCLDPPLSSVPSEDEWYCPECRNDASEVVLAGERLRESKKKAKMASATSSSQRDWGKGMACVGRTKECTIVPSNHYGPIPGIPVGTMWRFRVQVSESGVHRPHVAGIHGRSNDGAYSLVLAGGYEDDVDHGNFFTYTGSGGRDLSGNKRTAEQSCDQKLTNTNRALALNCFAPINDQEGAEAKDWRSGKPVRVVRNVKGGKNSKYAPAEGNRYDGIYKVVKYWPEKGKSGFLVWRYLLRRDDDEPGPWTKEGKDRIKKLGLTMQYPEGYLEALANREREKENSKREEEEQQEGGFASPRTGKGKWKRKSAGGGPSRAGSPRRTSKKTKVEPYSLTAQQSSLIREDKSNAKLWNEVLASLKDRPASGSPFQLFLSKVEETFQCICCQELVFRPITTVCQHNVCKDCLDRSFRAQVFSCPACRYDLGRSYAMQVNQPLQTVLNQLFPGYGNGR

>NP_055867.3

MLLHLCSVKNLYQNRFLGLAAMASPSRNSQSRRRCKEPLRYSYNPDQFHNMDLRGGPHDGVTIPRSTSDTDLVTSDSRSTLMVSSSYYSIGHSQDLVIHWDIKEEVDAGDWIGMYLIDEVLSENFLDYKNRGVNGSHRGQIIWKIDASSYFVEPETKICFKYYHGVSGALRATTPSVTVKNSAAPIFKSIGADETVQGQGSRRLISFSLSDFQAMGLKKGMFFNPDPYLKISIQPGKHSIFPALPHHGQERRSKIIGNTVNPIWQAEQFSFVSLPTDVLEIEVKDKFAKSRPIIKRFLGKLSMPVQRLLERHAIGDRVVSYTLGRRLPTDHVSGQLQFRFEITSSIHPDDEEISLSTEPESAQIQDSPMNNLMESGSGEPRSEAPESSESWKPEQLGEGSVPDGPGNQSIELSRPAEEAAVITEAGDQGMVSVGPEGAGELLAQVQKDIQPAPSAEELAEQLDLGEEASALLLEDGEAPASTKEEPLEEEATTQSRAGREEEEKEQEEEGDVSTLEQGEGRLQLRASVKRKSRPCSLPVSELETVIASACGDPETPRTHYIRIHTLLHSMPSAQGGSAAEEEDGAEEESTLKDSSEKDGLSEVDTVAADPSALEEDREEPEGATPGTAHPGHSGGHFPSLANGAAQDGDTHPSTGSESDSSPRQGGDHSCEGCDASCCSPSCYSSSCYSTSCYSSSCYSASCYSPSCYNGNRFASHTRFSSVDSAKISESTVFSSQDDEEEENSAFESVPDSMQSPELDPESTNGAGPWQDELAAPSGHVERSPEGLESPVAGPSNRREGECPILHNSQPVSQLPSLRPEHHHYPTIDEPLPPNWEARIDSHGRVFYVDHVNRTTTWQRPTAAATPDGMRRSGSIQQMEQLNRRYQNIQRTIATERSEEDSGSQSCEQAPAGGGGGGGSDSEAESSQSSLDLRREGSLSPVNSQKITLLLQSPAVKFITNPEFFTVLHANYSAYRVFTSSTCLKHMILKVRRDARNFERYQHNRDLVNFINMFADTRLELPRGWEIKTDQQGKSFFVDHNSRATTFIDPRIPLQNGRLPNHLTHRQHLQRLRSYSAGEASEVSRNRGASLLARPGHSLVAAIRSQHQHESLPLAYNDKIVAFLRQPNIFEMLQERQPSLARNHTLREKIHYIRTEGNHGLEKLSCDADLVILLSLFEEEIMSYVPLQAAFHPGYSFSPRCSPCSSPQNSPGLQRASARAPSPYRRDFEAKLRNFYRKLEAKGFGQGPGKIKLIIRRDHLLEGTFNQVMAYSRKELQRNKLYVTFVGEEGLDYSGPSREFFFLLSQELFNPYYGLFEYSANDTYTVQISPMSAFVENHLEWFRFSGRILGLALIHQYLLDAFFTRPFYKALLRLPCDLSDLEYLDEEFHQSLQWMKDNNITDILDLTFTVNEEVFGQVTERELKSGGANTQVTEKNKKEYIERMVKWRVERGVVQQTEALVRGFYEVVDSRLVSVFDARELELVIAGTAEIDLNDWRNNTEYRGGYHDGHLVIRWFWAAVERFNNEQRLRLLQFVTGTSSVPYEGFAALRGSNGLRRFCIEKWGKITSLPRAHTCFNRLDLPPYPSYSMLYEKLLTAVEETSTFGLE

1012 NP_068556.1 P00915

>NP_068556.1

MLGAVKMEGHEPSDWSSYYAEPEGYSSVSNMNAGLGMNGMNTYMSMSAAAMGSGSGNMSAGSMNMSSYVGAGMSPSLAGMSPGAGAMAGMGGSAGAAGVAGMGPHLSPSLSPLGGQAAGAMGGLAPYANMNSMSPMYGQAGLSRARDPKTYRRSYTHAKPPYSYISLITMAIQQSPNKMLTLSEIYQWIMDLFPFYRQNQQRWQNSIRHSLSFNDCFLKVPRSPDKPGKGSFWTLHPDSGNMFENGCYLRRQKRFKCEKQLALKEAAGAAGSGKKAAAGAQASQAQLGEAAGPASETPAGTESPHSSASPCQEHKRGGLGELKGTPAAALSPPEPAPSPGQQQQAAAHLLGPPHHPGLPPEAHLKPEHHYAFNHPFSINNLMSSEQQHHHSHHHHQPHKMDLKAYEQVMHYPGYGSPMPGSLAMGPVTNKTGLDASPLAADTSYYQGVYSRPIMNSS

>P00915

MASPDWGYDDKNGPEQWSKLYPIANGNNQSPVDIKTSETKHDTSLKPISVSYNPATAKEIINVGHSFHVNFEDNDNRSVLKGGPFSDSYRLFQFHFHWGSTNEHGSEHTVDGVKYSAELHVAHWNSAKYSSLAEAASKADGLAVIGVLMKVGEANPKLQKVLDALQAIKTKGKRAPFTNFDPSTLLPSSLDFWTYPGSLTHPPLYESVTWIICKESISVSSEQLAQFRSLLSNVEGDNAVPMQHNNRPTQPLKGRTVRASF

1013 NP_055238.1 Q8NFH8

>NP_055238.1

MNREDRNVLRMKERERRNQEIQQGEDAFPPSSPLFAEPYKVTSKEDKLSSRIQSMLGNYDEMKDFIGDRSIPKLVAIPKPTVPPSADEKSNPNFFEQRHGGSHQSSKWTPVGPAPSTSQSQKRSSGLQSGHSSQRTSAGSSSGTNSSGQRHDRESYNNSGSSSRKKGQHGSEHSKSRSSSPGKPQAVSSLNSSHSRSHGNDHHSKEHQRSKSPRDPDANWDSPSRVPFSSGQHSTQSFPPSLMSKSNSMLQKPTAYVRPMDGQESMEPKLSSEHYSSQSHGNSMTELKPSSKAHLTKLKIPSQPLDASASGDVSCVDEILKEMTHSWPPPLTAIHTPCKTEPSKFPFPTKESQQSNFGTGEQKRYNPSKTSNGHQSKSMLKDDLKLSSSEDSDGEQDCDKTMPRSTPGSNSEPSHHNSEGADNSRDDSSSHSGSESSSGSDSESESSSSDSEANEPSQSASPEPEPPPTNKWQLDNWLNKVNPHKVSPASSVDSNIPSSQGYKKEGREQGTGNSYTDTSGPKETSSATPGRDSKTIQKGSESGRGRQKSPAQSDSTTQRRTVGKKQPKKAEKAAAEEPRGGLKIESETPVDLASSMPSSRHKAATKGSRKPNIKKESKSSPRPTAEKKKYKSTSKSSQKSREIIETDTSSSDSDESESLPPSSQTPKYPESNRTPVKPSSVEEEDSFFRQRMFSPMEEKELLSPLSEPDDRYPLIVKIDLNLLTRIPGKPYKETEPPKGEKKNVPEKHTREAQKQASEKVSNKGKRKHKNEDDNRASESKKPKTEDKNSAGHKPSSNRESSKQSAAKEKDLLPSPAGPVPSKDPKTEHGSRKRTISQSSSLKSSSNSNKETSGSSKNSSSTSKQKKTEGKTSSSSKEVKEKAPSSSSNCPPSAPTLDSSKPRRTKLVFDDRNYSADHYLQEAKKLKHNADALSDRFEKAVYYLDAVVSFIECGNALEKNAQESKSPFPMYSETVDLIKYTMKLKNYLAPDATAADKRLTVLCLRCESLLYLRLFKLKKENALKYSKTLTEHLKNSYNNSQAPSPGLGSKAVGMPSPVSPKLSPGNSGNYSSGASSASASGSSVTIPQKIHQMAASYVQVTSNFLYATEIWDQAEQLSKEQKEFFAELDKVMGPLIFNASIMTDLVRYTRQGLHWLRQDAKLIS

>Q8NFH8

MEAAAAAAAAAAAAAAAGGGCGSGPPPLLLSEGEQQCYSELFARCAGAAGGGPGSGPPEAARVAPGTATAAAGPVADLFRASQLPAETLHQITELCGAKRVGYFGPTQFYIALKLIAAAQSGLPVRIESIKCELPLPRFMMSKNDGEIRFGNPAELHGTKVQIPYLTTEKNSFKRMDDEDKQQETQSPTMSPLASPPSSPPHYQRVPLSHGYSKLRSSAEQMHPAPYEARQPLVQPEGSSSGGPGTKPLRHQASLIRSFSVERELQDNSSYPDEPWRITEEQREYYVNQFRSLQPDPSSFISGSVAKNFFTKSKLSIPELSYIWELSDADCDGALTLPEFCAAFHLIVARKNGYPLPEGLPPTLQPEYLQAAFPKPKWDCQLFDSYSESLPANQQPRDLNRMEKTSVKDMADLPVPNQDVTSDDKQALKSTINEALPKDVSEDPATPKDSNSLKARPRSRSYSSTSIEEAMKRGEDPPTPPPRPQKTHSRASSLDLNKVFQPSVPATKSGLLPPPPALPPRPCPSQSEQVSEAELLPQLSRAPSQAAESSPAKKDVLYSQPPSKPIRRKFRPENQATENQEPSTAASGPASAATMKPHPTVQKQSSKQKKAIQTAIRKNKEANAVLARLNSELQQQLKEVHQERIALENQLEQLRPVTVL

1014 NP_009108.1 Q93015

>NP_009108.1

MYSEIQRERADIGGLMARPEYREWNPELIKPKKLLNPVKASRSHQELHRELLMNHRRGLGVDSKPELQRVLEHRRRNQLIKKKKEELEAKRLQCPFEQELLRRQQRLNQLEKPPEKEEDHAPEFIKVRENLRRIATLTSEEREL

>Q93015

MELILSTSPAELTLDPACQPKLPLDSTCQPEMTFNPGPTELTLDPEHQPEETPAPSLAELTLEPVHRRPELLDACADLINDQWPRSRTSRLHSLGQSSDAFPLCLMLLSPHPTLEAAPVVVGHARLSRVLNQPQSLLVETVVVARALRGRGFGRRLMEGLEVFARARGFRKLHLTTHDQVHFYTHLGYQLGEPVQGLVFTSRRLPATLLNAFPTAPSPRPPRKAPNLTAQAAPRGPKGPPLPPPPPLPECLTISPPVPSGPPSKSLLETQYQNVRGRPIFWMEKDI

1015 NP_003787.2 NP_000660.1

>NP_003787.2

MEAPTVETPPDPSPPSAPAPALVPLRAPDVARLREEQEKVVTNCQERIQHWKKVDNDYNALRERLSTLPDKLSYNIMVPFGPFAFMPGKLVHTNEVTVLLGDNWFAKCSAKQAVGLVEHRKEHVRKTIDDLKKVMKNFESRVEFTEDLQKMSDAAGDIVDIREEIKCDFEFKAKHRIAHKPHSKPKTSDIFEADIANDVKSKDLLADKELWARLEELERQEELLGELDSKPDTVIANGEDTTSSEEEKEDRNTNVNAMHQVTDSHTPCHKDVASSEPFSGQVNSQLNCSVNGSSSYHSDDDDDDDDDDDDDNIDDDDGDNDHEALGVGDNSIPTIYFSHTVEPKRVRINTGKNTTLKFSEKKEEAKRKRKNSTGSGHSAQELPTIRTPADIYRAFVDVVNGEYVPRKSILKSRSRENSVCSDTSESSAAEFDDRRGVLRSISCEEATCSDTSESILEEEPQENQKKLLPLSVTPEAFSGTVIEKEFVSPSLTPPPAIAHPALPTIPERKEVLLEASEETGKRVSKFKAARLQQKD

>NP_000660.1

MSTAGKVIKCKAAVLWELKKPFSIEEVEVAPPKAHEVRIKMVAAGICRSDEHVVSGNLVTPLPVILGHEAAGIVESVGEGVTTVKPGDKVIPLFTPQCGKCRICKNPESNYCLKNDLGNPRGTLQDGTRRFTCSGKPIHHFVGVSTFSQYTVVDENAVAKIDAASPLEKVCLIGCGFSTGYGSAVKVAKVTPGSTCAVFGLGGVGLSVVMGCKAAGAARIIAVDINKDKFAKAKELGATECINPQDYKKPIQEVLKEMTDGGVDFSFEVIGRLDTMMASLLCCHEACGTSVIVGVPPDSQNLSINPMLLLTGRTWKGAIFGGFKSKESVPKLVADFMAKKFSLDALITNILPFEKINEGFDLLRSGKSIRTVLTF

1016 Q9NV88 NP_000340.2

>Q9NV88

MKLYCLSGHPTLPCNVLKFKSTTIMLDCGLDMTSTLNFLPLPLVQSPRLSNLPGWSLKDGNAFLDKELKECSGHVFVDSVPEFCLPETELIDLSTVDVILISNYHCMMALPYITEHTGFTGTVYATEPTVQIGRLLMEELVNFIERVPKAQSASLWKNKDIQRLLPSPLKDAVEVSTWRRCYTMQEVNSALSKIQLVGYSQKIELFGAVQVTPLSSGYALGSSNWIIQSHYEKVSYVSGSSLLTTHPQPMDQASLKNSDVLVLTGLTQIPTANPDGMVGEFCSNLALTVRNGGNVLVPCYPSGVIYDLLECLYQYIDSAGLSSVPLYFISPVANSSLEFSQIFAEWLCHNKQSKVYLPEPPFPHAELIQTNKLKHYPSIHGDFSNDFRQPCVVFTGHPSLRFGDVVHFMELWGKSSLNTVIFTEPDFSYLEALAPYQPLAMKCIYCPIDTRLNFIQVSKLLKEVQPLHVVCPEQYTQPPPAQSHRMDLMIDCQPPAMSYRRAEVLALPFKRRYEKIEIMPELADSLVPMEIKPGISLATVSAVLHTKDNKHLLQPPPRPAQPTSGKKRKRVSDDVPDCKVLKPLLSGSIPVEQFVQTLEKHGFSDIKVEDTAKGHIVLLQEAETLIQIEEDSTHIICDNDEMLRVRLRDLVLKFLQKF

>NP_000340.2

MLLATFKLCAGSSYRHMRNMKGLRQQAVMAISQELNRRALGGPTPSTWINQVRRRSSLLGSRLEETLYSDQELAYLQQGEEAMQKALGILSNQEGWKKESQQDNGDKVMSKVVPDVGKVFRLEVVVDQPMERLYEELVERMEAMGEWNPNVKEIKVLQKIGKDTFITHELAAEAAGNLVGPRDFVSVRCAKRRGSTCVLAGMATDFGNMPEQKGVIRAEHGPTCMVLHPLAGSPSKTKLTWLLSIDLKGWLPKSIINQVLSQTQVDFANHLRKRLESHPASEARC

1017 NP_068809.1 Q8TCS8

>NP_068809.1

MSDNEDNFDGDDFDDVEEDEGLDDLENAEEEGQENVEILPSGERPQANQKRITTPYMTKYERARVLGTRALQIAMCAPVMVELEGETDPLLIAMKELKARKIPIIIRRYLPDGSYEDWGVDELIITD

>Q8TCS8

MAACRYCCSCLRLRPLSDGPFLLPRRDRALTQLQVRALWSSAGSRAVAVDLGNRKLEISSGKLARFADGSAVVQSGDTAVMVTAVSKTKPSPSQFMPLVVDYRQKAAAAGRIPTNYLRREIGTSDKEILTSRIIDRSIRPLFPAGYFYDTQVLCNLLAVDGVNEPDVLAINGASVALSLSDIPWNGPVGAVRIGIIDGEYVVNPTRKEMSSSTLNLVVAGAPKSQIVMLEASAENILQQDFCHAIKVGVKYTQQIIQGIQQLVKETGVTKRTPQKLFTPSPEIVKYTHKLAMERLYAVFTDYEHDKVSRDEAVNKIRLDTEEQLKEKFPEADPYEIIESFNVVAKEVFRSIVLNEYKRCDGRDLTSLRNVSCEVDMFKTLHGSALFQRGQTQVLCTVTFDSLESGIKSDQVITAINGIKDKNFMLHYEFPPYATNEIGKVTGLNRRELGHGALAEKALYPVIPRDFPFTIRVTSEVLESNGSSSMASACGGSLALMDSGVPISSAVAGVAIGLVTKTDPEKGEIEDYRLLTDILGIEDYNGDMDFKIAGTNKGITALQADIKLPGIPIKIVMEAIQQASVAKKEILQIMNKTISKPRASRKENGPVVETVQVPLSKRAKFVGPGGYNLKKLQAETGVTISQVDEETFSVFAPTPSAMHEARDFITEICKDDQEQQLEFGAVYTATITEIRDTGVMVKLYPNMTAVLLHNTQLDQRKIKHPTALGLEVGQEIQVKYFGRDPADGRMRLSRKVLQSPATTVVRTLNDRSSIVMGEPISQSSSNSQ

1018 NP_008828.1 NP_001344.2

>NP_008828.1

MSATGPISNYYVDSLISHDNEDLLASRFPATGAHPAAARPSGLVPDCSDFPSCSFAPKPAVFSTSWAPVPSQSSVVYHPYGPQPHLGADTRYMRTWLEPLSGAVSFPSFPAGGRHYALKPDAYPGRRADCGPGEGRSYPDYMYGSPGELRDRAPQTLPSPEADALAGSKHKEEKADLDPSNPVANWIHARSTRKKRCPYTKYQTLELEKEFLFNMYLTRDRRYEVARVLNLTERQVKIWFQNRRMKMKKMNKEKTDKEQS

>NP_001344.2

MDSKYQCVKLNDGHFMPVLGFGTYAPAEVPKSKALEATKLAIEAGFRHIDSAHLYNNEEQVGLAIRSKIADGSVKREDIFYTSKLWCNSHRPELVRPALERSLKNLQLDYVDLYLIHFPVSVKPGEEVIPKDENGKILFDTVDLCATWEAVEKCKDAGLAKSIGVSNFNRRQLEMILNKPGLKYKPVCNQVECHPYFNQRKLLDFCKSKDIVLVAYSALGSHREEPWVDPNSPVLLEDPVLCALAKKHKRTPALIALRYQLQRGVVVLAKSYNEQRIRQNVQVFEFQLTSEEMKAIDGLNRNVRYLTLDIFAGPPNYPFSDEY

1019 NP_005632.1 Q96G61

>NP_005632.1

MAEEKKLKLSNTVLPSESMKVVAESMGIAQIQEETCQLLTDEVSYRIKEIAQDALKFMHMGKRQKLTTSDIDYALKLKNVEPLYGFHAQEFIPFRFASGGGRELYFYEEKEVDLSDIINTPLPRVPLDVCLKAHWLSIEGCQPAIPENPPPAPKEQQKAEATEPLKSAKPGQEEDGPLKGKGQGATTADGKGKEKKAPPLLEGAPLRLKPRSIHELSVEQQLYYKEITEACVGSCEAKRAEALQSIATDPGLYQMLPRFSTFISEGVRVNVVQNNLALLIYLMRMVKALMDNPTLYLEKYVHELIPAVMTCIVSRQLCLRPDVDNHWALRDFAARLVAQICKHFSTTTNNIQSRITKTFTKSWVDEKTPWTTRYGSIAGLAELGHDVIKTLILPRLQQEGERIRSVLDGPVLSNIDRIGADHVQSLLLKHCAPVLAKLRPPPDNQDAYRAEFGSLGPLLCSQVVKARAQAALQAQQVNRTTLTITQPRPTLTLSQAPQPGPRTPGLLKVPGSIALPVQTLVSARAAAPPQPSPPPTKFIVMSSSSSAPSTQQVLSLSTSAPGSGSTTTSPVTTTVPSVQPIVKLVSTATTAPPSTAPSGPGSVQKYIVVSLPPTGEGKGGPTSHPSPVPPPASSPSPLSGSALCGGKQEAGDSPPPAPGTPKANGSQPNSGSPQPAP

>Q96G61

MKCKPNQTRTYDPEGFKKRAACLCFRSEREDEVLLVSSSRYPDRWIVPGGGMEPEEEPGGAAVREVYEEAGVKGKLGRLLGVFEQNQDRKHRTYVYVLTVTELLEDWEDSVSIGRKREWFKVEDAIKVLQCHKPVHAEYLEKLKLGGSPTNGNSMAPSSPDSDP

1020 NP_612211.1 NP_004300.1

>NP_612211.1

MFVLVEMVDTVRIPPWQFERKLNDSIAEELNKKLANKVVYNVGLCICLFDITKLEDAYVFPGDGASHTKVHFRCVVFHPFLDEILIGKIKGCSPEGVHVSLGFFDDILIPPESLQQPAKFDEAEQVWVWEYETEEGAHDLYMDTGEEIRFRVVDESFVDTSPTGPSSADATTSSEELPKKEAPYTLVGSISEPGLGLLSWWTSN

>NP_004300.1

MAEQEPTAEQLAQIAAENEEDEHSVNYKPPAQKSIQEIQELDKDDESLRKYKEALLGRVAVSADPNVPNVVVTGLTLVCSSAPGPLELDLTGDLESFKKQSFVLKEGVEYRIKISFRVNREIVSGMKYIQHTYRKGVKIDKTDYMVGSYGPRAEEYEFLTPVEEAPKGMLARGSYSIKSRFTDDDKTDHLSWEWNLTIKKDWKD

1021 NP_008823.1 O15540

>NP_008823.1

MKGDTRHLNGEEDAGGREDSILVNGACSDQSSDSPPILEAIRTPEIRGRRSSSRLSKREVSSLLSYTQDLTGDGDGEDGDGSDTPVMPKLFRETRTRSESPAVRTRNNNSVSSRERHRPSPRSTRGRQGRNHVDESPVEFPATRSLRRRATASAGTPWPSPPSSYLTIDLTDDTEDTHGTPQSSSTPYARLAQDSQQGGMESPQVEADSGDGDSSEYQDGKEFGIGDLVWGKIKGFSWWPAMVVSWKATSKRQAMSGMRWVQWFGDGKFSEVSADKLVALGLFSQHFNLATFNKLVSYRKAMYHALEKARVRAGKTFPSSPGDSLEDQLKPMLEWAHGGFKPTGIEGLKPNNTQPVVNKSKVRRAGSRKLESRKYENKTRRRTADDSATSDYCPAPKRLKTNCYNNGKDRGDEDQSREQMASDVANNKSSLEDGCLSCGRKNPVSFHPLFEGGLCQTCRDRFLELFYMYDDDGYQSYCTVCCEGRELLLCSNTSCCRCFCVECLEVLVGTGTAAEAKLQEPWSCYMCLPQRCHGVLRRRKDWNVRLQAFFTSDTGLEYEAPKLYPAIPAARRRPIRVLSLFDGIATGYLVLKELGIKVGKYVASEVCEESIAVGTVKHEGNIKYVNDVRNITKKNIEEWGPFDLVIGGSPCNDLSNVNPARKGLYEGTGRLFFEFYHLLNYSRPKEGDDRPFFWMFENVVAMKVGDKRDISRFLECNPVMIDAIKVSAAHRARYFWGNLPGMNRPVIASKNDKLELQDCLEYNRIAKLKKVQTITTKSNSIKQGKNQLFPVVMNGKEDVLWCTELERIFGFPVHYTDVSNMGRGARQKLLGRSWSVPVIRHLFAPLKDYFACE

>O15540

MVEAFCATWKLTNSQNFDEYMKALGVGFATRQVGNVTKPTVIISQEGDKVVIRTLSTFKNTEISFQLGEEFDETTADDRNCKSVVSLDGDKLVHIQKWDGKETNFVREIKDGKMVMTLTFGDVVAVRHYEKA

1022 NP_004442.3 Q5H9J7

>NP_004442.3

MSSQVVGIEPLYIKAEPASPDSPKGSSETETEPPVALAPGPAPTRCLPGHKEEEDGEGAGPGEQGGGKLVLSSLPKRLCLVCGDVASGYHYGVASCEACKAFFKRTIQGSIEYSCPASNECEITKRRRKACQACRFTKCLRVGMLKEGVRLDRVRGGRQKYKRRPEVDPLPFPGPFPAGPLAVAGGPRKTAAPVNALVSHLLVVEPEKLYAMPDPAGPDGHLPAVATLCDLFDREIVVTISWAKSIPGFSSLSLSDQMSVLQSVWMEVLVLGVAQRSLPLQDELAFAEDLVLDEEGARAAGLGELGAALLQLVRRLQALRLEREEYVLLKALALANSDSVHIEDAEAVEQLREALHEALLEYEAGRAGPGGGAERRRAGRLLLTLPLLRQTAGKVLAHFYGVKLEGKVPMHKLFLEMLEAMMD

>Q5H9J7

MENVPKENKVVEKAPVQNEAPALGGGEYQEPGGNVKGVWAPPAPGFGEDVPNRLVDNIDMIDGDGDDMERFMEEMRELRRKIRELQLRYSLRILIGDPPHHDHHDEFCLMP

1023 NP_002154.1 P22528

>NP_002154.1

MCDRNGGRRLRQWLIEQIDSSMYPGLIWENEEKSMFRIPWKHAGKQDYNQEVDASIFKAWAVFKGKFKEGDKAEPATWKTRLRCALNKSPDFEEVTDRSQLDISEPYKVYRIVPEEEQKCKLGVATAGCVNEVTEMECGRSEIDELIKEPSVDDYMGMIKRSPSPPEACRSQLLPDWWAQQPSTGVPLVTGYTTYDAHHSAFSQMVISFYYGGKLVGQATTTCPEGCRLSLSQPGLPGTKLYGPEGLELVRFPPADAIPSERQRQVTRKLFGHLERGVLLHSSRQGVFVKRLCQGRVFCSGNAVVCKGRPNKLERDEVVQVFDTSQFFRELQQFYNSQGRLPDGRVVLCFGEEFPDMAPLRSKLILVQIEQLYVRQLAEEAGKSCGAGSVMQAPEEPPPDQVFRMFPDICASHQRSFFRENQQITV

>P22528

MSSQQQKQPCTPPPQLQQQQVKQPCQPPPQEPCIPKTKEPCHPKVPEPCHPKVPEPCQPKVPEPCHPKVPEPCPSIVTPAPAQQKTKQK

1024 NP_006221.1 NP_060383.2

>NP_006221.1

MFSEQAAQRAHTLLSPPSANNATFARVPVATYTNSSQPFRLGERSFSRQYAHIYATRLIQMRPFLENRAQQHWGSGVGVKKLCELQPEEKCCVVGTLFKAMPLQPSILREVSEEHNLLPQPPRSKYIHPDDELVLEDELQRIKLKGTIDVSKLVTGTVLAVFGSVRDDGKFLVEDYCFADLAPQKPAPPLDTDRFVLLVSGLGLGGGGGESLLGTQLLVDVVTGQLGDEGEQCSAAHVSRVILAGNLLSHSTQSRDSINKAKYLTKKTQAASVEAVKMLDEILLQLSASVPVDVMPGEFDPTNYTLPQQPLHPCMFPLATAYSTLQLVTNPYQATIDGVRFLGTSGQNVSDIFRYSSMEDHLEILEWTLRVRHISPTAPDTLGCYPFYKTDPFIFPECPHVYFCGNTPSFGSKIIRGPEDQTVLLVTVPDFSATQTACLVNLRSLACQPISFSGFGAEDDDLGGLGLGP

>NP_060383.2

MEQPWPPPGPWSLPRAEGEAEEESDFDVFPSSPRCPQLPGGGAQMYSHGIELACQKQKEFVKSSVACKWNLAEAQQKLGSLALHNSESLDQEHAKAQTAVSELRQREEEWRQKEEALVQREKMCLWSTDAISKDVFNKSFINQDKRKDTEDEDKSESFMQKYEQKIRHFGMLSRWDDSQRFLSDHPYLVCEETAKYLILWCFHLEAEKKGALMEQIAHQAVVMQFIMEMAKNCNVDPRGCFRLFFQKAKAEEEGYFEAFKNELEAFKSRVRLYSQSQSFQPMTVQNHVPHSGVGSIGLLESLPQNPDYLQYSISTALCSLNSVVHKEDDEPKMMDTV

1025 Q8TAT5 Q9BYC8

>Q8TAT5

MVEGPGCTLNGEKIRARVLPGQAVTGVRGSALRSLQGRALRLAASTVVVSPQAAALNNDSSQNVLSLFNGYVYSGVETLGKELFMYFGPKALRIHFGMKGFIMINPLEYKYKNGASPVLEVQLTKDLICFFDSSVELRNSMESQQRIRMMKELDVCSPEFSFLRAESEVKKQKGRMLGDVLMDQNVLPGVGNIIKNEALFDSGLHPAVKVCQLTDEQIHHLMKMIRDFSILFYRCRKAGLALSKHYKVYKRPNCGQCHCRITVCRFGDNNRMTYFCPHCQKENPQHVDICKLPTRNTIISWTSSRVDHVMDSVARKSEEHWTCVVCTLINKPSSKACDACLTSRPIDSVLKSEENSTVFSHLMKYPCNTFGKPHTEVKINRKTAFGTTTLVLTDFSNKSSTLERKTKQNQILDEEFQNSPPASVCLNDIQHPSKKTTNDITQLSSKVNISPTISSESKLFSPAHKKPKTAHYSSPELKSCNPGYSNSELQINMTDGPRTLNPDSPRCSKHNRLCILRVVRKDGENKGRQFYACPLPREAQCGFFEWADLSFPFCNHGKRSTMKTVLKIGPNNGKNFFVCPLGKEKQCNFFQWAENGPGIKIIPGC

>Q9BYC8

MALAMLVLVVSPWSAARGVLRNYWERLLRKLPQSRPGFPSPPWGPALAVQGPAMFTEPANDTSGSKENSSLLDSIFWMAAPKNRRTIEVNRCRRRNPQKLIKVKNNIDVCPECGHLKQKHVLCAYCYEKVCKETAEIRRQIGKQEGGPFKAPTIETVVLYTGETPSEQDQGKRIIERDRKRPSWFTQN

1026 NP_005137.1 NP_006692.1

>NP_005137.1

MGSSKKHRGEKEAAGTTAAAGTGGATEQPPRHREHKKHKHRSGGSGGSGGERRKRSRERGGERGSGRRGAEAEARSSTHGRERSQAEPSERRVKREKRDDGYEAAASSKTSSGDASSLSIEETNKLRAKLGLKPLEVNAIKKEAGTKEEPVTADVINPMALRQREELREKLAAAKEKRLLNQKLGKIKTLGEDDPWLDDTAAWIERSRQLQKEKDLAEKRAKLLEEMDQEFGVSTLVEEEFGQRRQDLYSARDLQGLTVEHAIDSFREGETMILTLKDKGVLQEEEDVLVNVNLVDKERAEKNVELRKKKPDYLPYAEDESVDDLAQQKPRSILSKYDEELEGERPHSFRLEQGGTADGLRERELEEIRAKLRLQAQSLSTVGPRLASEYLTPEEMVTFKKTKRRVKKIRKKEKEVVVRADDLLPLGDQTQDGDFGSRLRGRGRRRVSEVEEEKEPVPQPLPSDDTRVENMDISDEEEGGAPPPGSPQVLEEDEAELELQKQLEKGRRLRQLQQLQQLRDSGEKVVEIVKKLESRQRGWEEDEDPERKGAIVFNATSEFCRTLGEIPTYGLAGNREEQEELMDFERDEERSANGGSESDGEENIGWSTVNLDEEKQQQDFSASSTTILDEEPIVNRGLAAALLLCQNKGLLETTVQKVARVKAPNKSLPSAVYCIEDKMAIDDKYSRREEYRGFTQDFKEKDGYKPDVKIEYVDETGRKLTPKEAFRQLSHRFHGKGSGKMKTERRMKKLDEEALLKKMSSSDTPLGTVALLQEKQKAQKTPYIVLSGSGKSMNANTITK

>NP_006692.1

MSYMLPHLHNGWQVDQAILSEEDRVVVIRFGHDWDPTCMKMDEVLYSIAEKVKNFAVIYLVDITEVPDFNKMYELYDPCTVMFFFRNKHIMIDLGTGNNNKINWAMEDKQEMVDIIETVYRGARKGRGLVVSPKDYSTKYRY

1027 P17010 NP_003921.2

>P17010

MDEDGLELQQEPNSFFDATGADGTHMDGDQIVVEVQETVFVSDVVDSDITVHNFVPDDPDSVVIQDVIEDVVIEDVQCPDIMEEADVSETVIIPEQVLDSDVTEEVSLAHCTVPDDVLASDITSASMSMPEHVLTGDSIHVSDVGHVGHVGHVEHVVHDSVVEAEIVTDPLTTDVVSEEVLVADCASEAVIDANGIPVDQQDDDKGNCEDYLMISLDDAGKIEHDGSSGMTMDTESEIDPCKVDGTCPEVIKVYIFKADPGEDDLGGTVDIVESEPENDHGVELLDQNSSIRVPREKMVYMTVNDSQPEDEDLNVAEIADEVYMEVIVGEEDAAAAAAAAAVHEQQMDDNEIKTFMPIAWAAAYGNNSDGIENRNGTASALLHIDESAGLGRLAKQKPKKRRRPDSRQYQTAIIIGPDGHPLTVYPCMICGKKFKSRGFLKRHMKNHPEHLAKKKYRCTDCDYTTNKKISLHNHLESHKLTSKAEKAIECDECGKHFSHAGALFTHKMVHKEKGANKMHKCKFCEYETAEQGLLNRHLLAVHSKNFPHICVECGKGFRHPSELKKHMRIHTGEKPYQCQYCEYRSADSSNLKTHVKTKHSKEMPFKCDICLLTFSDTKEVQQHALIHQESKTHQCLHCDHKSSNSSDLKRHIISVHTKDYPHKCDMCDKGFHRPSELKKHVAAHKGKKMHQCRHCDFKIADPFVLSRHILSVHTKDLPFRCKRCRKGFRQQSELKKHMKTHSGRKVYQCEYCEYSTTDASGFKRHVISIHTKDYPHRCEYCKKGFRRPSEKNQHIMRHHKEVGLP

>NP_003921.2

MPNPSSTSSPYPLPEEIRNLLADVETFVADILKGENLSKKAKEKRESLIKKIKDVKSIYLQEFQDKGDAEDGEEYDDPFAGPPDTISLASERYDKDDEAPSDGAQFPPIAAQDLPFVLKAGYLEKRRKDHSFLGFEWQKRWCALSKTVFYYYGSDKDKQQKGEFAIDGYSVRMNNTLRKDGKKDCCFEISAPDKRIYQFTAASPKDAEEWVQQLKFVLQDMESDIIPEDYDERGELYDDVDHPLPISNPLTSSQPIDDEIYEELPEEEEDSAPVKVEEQRKMSQDSVHHTSGDKSTDYANFYQGLWDCTGAFSDELSFKRGDVIYILSKEYNRYGWWVGEMKGAIGLVPKAYIMEMYDI

1028 P28698 NP_002070.1

>P28698

MRPAVLGSPDRAPPEDEGPVMVKLEDSEEEGEAALWDPGPEAARLRFRCFRYEEATGPQEALAQLRELCRQWLRPEVRSKEQMLELLVLEQFLGALPPEIQARVQGQRPGSPEEAAALVDGLRREPGGPRRWVTVQVQGQEVLSEKMEPSSFQPLPETEPPTPEPGPKTPPRTMQESPLGLQVKEESEVTEDSDFLESGPLAATQESVPTLLPEEAQRCGTVLDQIFPHSKTGPEGPSWREHPRALWHEEAGGIFSPGFALQLGSISAGPGSVSPHLHVPWDLGMAGLSGQIQSPSREGGFAHALLLPSDLRSEQDPTDEDPCRGVGPALITTRWRSPRGRSRGRPSTGGGVVRGGRCDVCGKVFSQRSNLLRHQKIHTGERPFVCSECGRSFSRSSHLLRHQLTHTEERPFVCGDCGQGFVRSARLEEHRRVHTGEQPFRCAECGQSFRQRSNLLQHQRIHGDPPGPGAKPPAPPGAPEPPGPFPCSECRESFARRAVLLEHQAVHTGDKSFGCVECGERFGRRSVLLQHRRVHSGERPFACAECGQSFRQRSNLTQHRRIHTGERPFACAECGKAFRQRPTLTQHLRVHTGEKPFACPECGQRFSQRLKLTRHQRTHTGEKPYHCGECGLGFTQVSRLTEHQRIHTGERPFACPECGQSFRQHANLTQHRRIHTGERPYACPECGKAFRQRPTLTQHLRTHRREKPFACQDCGRRFHQSTKLIQHQRVHSAE

>NP_002070.1

MAPPSVFAEVPQAQPVLVFKLTADFREDPDPRKVNLGVGAYRTDDCHPWVLPVVKKVEQKIANDNSLNHEYLPILGLAEFRSCASRLALGDDSPALKEKRVGGVQSLGGTGALRIGADFLARWYNGTNNKNTPVYVSSPTWENHNAVFSAAGFKDIRSYRYWDAEKRGLDLQGFLNDLENAPEFSIVVLHACAHNPTGIDPTPEQWKQIASVMKHRFLFPFFDSAYQGFASGNLERDAWAIRYFVSEGFEFFCAQSFSKNFGLYNERVGNLTVVGKEPESILQVLSQMEKIVRITWSNPPAQGARIVASTLSNPELFEEWTGNVKTMADRILTMRSELRARLEALKTPGTWNHITDQIGMFSFTGLNPKQVEYLVNEKHIYLLPSGRINVSGLTTKNLDYVATSIHEAVTKIQ

1029 NP_003850.1 Q16822

>NP_003850.1

MASLEVSRSPRRSRRELEVRSPRQNKYSVLLPTYNERENLPLIVWLLVKSFSESGINYEIIIIDDGSPDGTRDVAEQLEKIYGSDRILLRPREKKLGLGTAYIHGMKHATGNYIIIMDADLSHHPKFIPEFIRKQKEGNFDIVSGTRYKGNGGVYGWDLKRKIISRGANFLTQILLRPGASDLTGSFRLYRKEVLEKLIEKCVSKGYVFQMEMIVRARQLNYTIGEVPISFVDRVYGESKLGGNEIVSFLKGLLTLFATT

>Q16822

MAALYRPGLRLNWHGLSPLGWPSCRSIQTLRVLSGDLGQLPTGIRDFVEHSARLCQPEGIHICDGTEAENTATLTLLEQQGLIRKLPKYNNCWLARTDPKDVARVESKTVIVTPSQRDTVPLPPGGARGQLGNWMSPADFQRAVDERFPGCMQGRTMYVLPFSMGPVGSPLSRIGVQLTDSAYVVASMRIMTRLGTPVLQALGDGDFVKCLHSVGQPLTGQGEPVSQWPCNPEKTLIGHVPDQREIISFGSGYGGNSLLGKKCFALRIASRLARDEGWLAEHMLILGITSPAGKKALCAAAFPSACGKTNLAMMRPALPGWKVECVGDDIAWMRFDSEGRLRAINPENGFFGVAPGTSATTNPNAMATIQSNTIFTNVAETSDGGVYWEGIDQPLPPGVTVTSWLGKPWKPGDKEPCAHPNSRFCAPARQCPIMDPAWEAPEGVPIDAIIFGGRRPKGVPLVYEAFNWRHGVFVGRAMRSESTAAAEHKGKIIMHDPFAMRPFFGYNFGHYLEHWLSMEGRKGAQLPRIFHVNWFRRDEAGHFLWPGFGENARVLDWICRRLEGEDSARETPIGLVPKEGALDLSGLRAIDTTQLFSLPKDFWEQEVRDIRSYLTEQVNQDLPKEVLAELEALERRVHKM

1030 NP_003914.1 Q12815

>NP_003914.1

MGPCSGSRLGPPEAESPSQPPKRRKKRYLRHDKPPYTYLAMIALVIQAAPSRRLKLAQIIRQVQAVFPFFREDYEGWKDSIRHNLSSNRCFRKVPKDPAKPQAKGNFWAVDVSLIPAEALRLQNTALCRRWQNGGARGAFAKDLGPYVLHGRPYRPPSPPPPPSEGFSIKSLLGGSGEGAPWPGLAPQSSPVPAGTGNSGEEAVPTPPLPSSERPLWPLCPLPGPTRVEGETVQGGAIGPSTLSPEPRAWPLHLLQGTAVPGGRSSGGHRASLWGQLPTSYLPIYTPNVVMPLAPPPTSCPQCPSTSPAYWGVAPETRGPPGLLCDLDALFQGVPPNKSIYDVWVSHPRDLAAPGPGWLLSWCSL

>Q12815

MTTRQATKDPLLRGVSPTPSKIPVRSQKRTPFPTVTSCAVDQENQDPRRWVQKPPLNIQRPLVDSAGPRPKARHQAETSQRLVGISQPRNPLEELRPSPRGQNVGPGPPAQTEAPGTIEFVADPAALATILSGEGVKSCHLGRQPSLAKRVLVRGSQGGTTQRVQGVRASAYLAPRTPTHRLDPARASCFSRLEGPGPRGRTLCPQRLQALISPSGPSFHPSTRPSFQELRRETAGSSRTSVSQASGLLLETPVQPAFSLPKGEREVVTHSDEGGVASLGLAQRVPLRENREMSHTRDSHDSHLMPSPAPVAQPLPGHVVPCPSPFGRAQRVPSPGPPTLTSYSVLRRLTVQPKTRFTPMPSTPRVQQAQWLRGVSPQSCSEDPALPWEQVAVRLFDQESCIRSLEGSGKPPVATPSGPHSNRTPSLQEVKIQRIGILQQLLRQEVEGLVGGQCVPLNGGSSLDMVELQPLLTEISRTLNATEHNSGTSHLPGLLKHSGLPKPCLPEECGEPQPCPPAEPGPPEAFCRSEPEIPEPSLQEQLEVPEPYPPAEPRPLESCCRSEPEIPESSRQEQLEVPEPCPPAEPRPLESYCRIEPEIPESSRQEQLEVPEPCPPAEPGPLQPSTQGQSGPPGPCPRVELGASEPCTLEHRSLESSLPPCCSQWAPATTSLIFSSQHPLCASPPICSLQSLRPPAGQAGLSNLAPRTLALRERLKSCLTAIHCFHEARLDDECAFYTSRAPPSGPTRVCTNPVATLLEWQDALCFIPVGSAAPQGSP

1031 O60341 NP_009197.1

>O60341

MLSGKKAAAAAAAAAAAATGTEAGPGTAGGSENGSEVAAQPAGLSGPAEVGPGAVGERTPRKKEPPRASPPGGLAEPPGSAGPQAGPTVVPGSATPMETGIAETPEGRRTSRRKRAKVEYREMDESLANLSEDEYYSEEERNAKAEKEKKLPPPPPQAPPEEENESEPEEPSGVEGAAFQSRLPHDRMTSQEAACFPDIISGPQQTQKVFLFIRNRTLQLWLDNPKIQLTFEATLQQLEAPYNSDTVLVHRVHSYLERHGLINFGIYKRIKPLPTKKTGKVIIIGSGVSGLAAARQLQSFGMDVTLLEARDRVGGRVATFRKGNYVADLGAMVVTGLGGNPMAVVSKQVNMELAKIKQKCPLYEANGQAVPKEKDEMVEQEFNRLLEATSYLSHQLDFNVLNNKPVSLGQALEVVIQLQEKHVKDEQIEHWKKIVKTQEELKELLNKMVNLKEKIKELHQQYKEASEVKPPRDITAEFLVKSKHRDLTALCKEYDELAETQGKLEEKLQELEANPPSDVYLSSRDRQILDWHFANLEFANATPLSTLSLKHWDQDDDFEFTGSHLTVRNGYSCVPVALAEGLDIKLNTAVRQVRYTASGCEVIAVNTRSTSQTFIYKCDAVLCTLPLGVLKQQPPAVQFVPPLPEWKTSAVQRMGFGNLNKVVLCFDRVFWDPSVNLFGHVGSTTASRGELFLFWNLYKAPILLALVAGEAAGIMENISDDVIVGRCLAILKGIFGSSAVPQPKETVVSRWRADPWARGSYSYVAAGSSGNDYDLMAQPITPGPSIPGAPQPIPRLFFAGEHTIRNYPATVHGALLSGLREAGRIADQFLGAMYTLPRQATPGVPAQQSPSM

>NP_009197.1

MAASAAAAELQASGGPRHPVCLLVLGMAGSGKTTFVQRLTGHLHAQGTPPYVINLDPAVHEVPFPANIDIRDTVKYKEVMKQYGLGPNGGIVTSLNLFATRFDQVMKFIEKAQNMSKYVLIDTPGQIEVFTWSASGTIITEALASSFPTVVIYVMDTSRSTNPVTFMSNMLYACSILYKTKLPFIVVMNKTDIIDHSFAVEWMQDFEAFQDALNQETTYVSNLTRSMSLVLDEFYSSLRVVGVSAVLGTGLDELFVQVTSAAEEYEREYRPEYERLKKSLANAESQQQREQLERLRKDMGSVALDAGTAKDSLSPVLHPSDLILTRGTLDEEDEEADSDTDDIDHRVTEESHEEPAFQNFMQESMAQYWKRNNK

1032 NP_055368.1 NP_002159.2

>NP_055368.1

MLFWHTQPEHYNQHNSGSYLRDVLALPIFKQEEPQLSPENEARLPPLQYVLCAATSPAVKLHEETLTYLNQGQSYEIRLLENRKLGDFQDLNTKYVKSIIRVVFHDRRLQYTEHQQLEGWRWSRPGDRILDIDIPLSVGILDPRASPTQLNAVEFLWDPAKRASAFIQVHCISTEFTPRKHGGEKGVPFRVQIDTFKQNENGEYTEHLHSASCQIKVFKPKGADRKQKTDREKMEKRTAQEKEKYQPSYETTILTECSPWPDVAYQVNSAPSPSYNGSPNSFGLGEGNASPTHPVEALPVGSDHLLPSASIQDAQQWLHRNRFSQFCRLFASFSGADLLKMSRDDLVQICGPADGIRLFNAIKGRNVRPKMTIYVCQELEQNRVPLQQKRDGSGDSNLSVYHAIFLEELTTLELIEKIANLYSISPQHIHRVYRQGPTGIHVVVSNEMVQNFQDESCFVLSTIKAESNDGYHIILKCGL

>NP_002159.2

MAGYLRVVRSLCRASGSRPAWAPAALTAPTSQEQPRRHYADKRIKVAKPVVEMDGDEMTRIIWQFIKEKLILPHVDIQLKYFDLGLPNRDQTDDQVTIDSALATQKYSVAVKCATITPDEARVEEFKLKKMWKSPNGTIRNILGGTVFREPIICKNIPRLVPGWTKPITIGRHAHGDQYKATDFVADRAGTFKMVFTPKDGSGVKEWEVYNFPAGGVGMGMYNTDESISGFAHSCFQYAIQKKWPLYMSTKNTILKAYDGRFKDIFQEIFDKHYKTDFDKNKIWYEHRLIDDMVAQVLKSSGGFVWACKNYDGDVQSDILAQGFGSLGLMTSVLVCPDGKTIEAEAAHGTVTRHYREHQKGRPTSTNPIASIFAWTRGLEHRGKLDGNQDLIRFAQMLEKVCVETVESGAMTKDLAGCIHGLSNVKLNEHFLNTTDFLDTIKSNLDRALGRQ

1033 Q14562 NP_005267.2

>Q14562

MAVAVAMAGALIGSEPGPAEELAKLEYLSLVSKVCTELDNHLGINDKDLAEFVISLAEKNTTFDTFKASLVKNGAEFTDSLISNLLRLIQTMRPPAKPSTSKDPVVKPKTEKEKLKELFPVLCQPDNPSVRTMLDEDDVKVAVDVLKELEALMPSAAGQEKQRDAEHRDRTKKKKRSRSRDRNRDRDRDRERNRDRDHKRRHRSRSRSRSRTRERNKVKSRYRSRSRSQSPPKDRKDRDKYGERNLDRWRDKHVDRPPPEEPTIGDIYNGKVTSIMQFGCFVQLEGLRKRWEGLVHISELRREGRVANVADVVSKGQRVKVKVLSFTGTKTSLSMKDVDQETGEDLNPNRRRNLVGETNEETSMRNPDRPTHLSLVSAPEVEDDSLERKRLTRISDPEKWEIKQMIAANVLSKEEFPDFDEETGILPKVDDEEDEDLEIELVEEEPPFLRGHTKQSMDMSPIKIVKNPDGSLSQAAMMQSALAKERRELKQAQREAEMDSIPMGLNKHWVDPLPDAEGRQIAANMRGIGMMPNDIPEWKKHAFGGNKASYGKKTQMSILEQRESLPIYKLKEQLVQAVHDNQILIVIGETGSGKTTQITQYLAEAGYTSRGKIGCTQPRRVAAMSVAKRVSEEFGCCLGQEVGYTIRFEDCTSPETVIKYMTDGMLLRECLIDPDLTQYAIIMLDEAHERTIHTDVLFGLLKKTVQKRQDMKLIVTSATLDAVKFSQYFYEAPIFTIPGRTYPVEILYTKEPETDYLDASLITVMQIHLTEPPGDILVFLTGQEEIDTACEILYERMKSLGPDVPELIILPVYSALPSEMQTRIFDPAPPGSRKVVIATNIAETSLTIDGIYYVVDPGFVKQKVYNSKTGIDQLVVTPISQAQAKQRAGRAGRTGPGKCYRLYTERAYRDEMLTTNVPEIQRTNLASTVLSLKAMGINDLLSFDFMDAPPMETLITAMEQLYTLGALDDEGLLTRLGRRMAEFPLEPMLCKMLIMSVHLGCSEEMLTIVSMLSVQNVFYRPKDKQALADQKKAKFHQTEGDHLTLLAVYNSWKNNKFSNPWCYENFIQARSLRRAQDIRKQMLGIMDRHKLDVVSCGKSTVRVQKAICSGFFRNAAKKDPQEGYRTLIDQQVVYIHPSSALFNRQPEWVVYHELVLTTKEYMREVTTIDPRWLVEFAPAFFKVSDPTKLSKQKKQQRLEPLYNRYEEPNAWRISRAFRRR

>NP_005267.2

MASKKVCIVGSGNWGSAIAKIVGGNAAQLAQFDPRVTMWVFEEDIGGKKLTEIINTQHENVKYLPGHKLPPNVVAVPDVVQAAEDADILIFVVPHQFIGKICDQLKGHLKANATGISLIKGVDEGPNGLKLISEVIGERLGIPMSVLMGANIASEVADEKFCETTIGCKDPAQGQLLKELMQTPNFRITVVQEVDTVEICGALKNVVAVGAGFCDGLGFGDNTKAAVIRLGLMEMIAFAKLFCSGPVSSATFLESCGVADLITTCYGGRNRKVAEAFARTGKSIEQLEKELLNGQKLQGPETARELYSILQHKGLVDKFPLFMAVYKVCYEGQPVGEFIHCLQNHPEHM

1034 P31267 Q9P278

>P31267

MSSYFVNPTFPGSLPSGQDSFLGQLPLYQAGYDALRPFPASYGASSLPDKTYTSPCFYQQSNSVLACNRASYEYGASCFYSDKDLSGASPSGSGKQRGPGDYLHFSPEQQYKPDSSSGQGKALHDEGADRKYTSPVYPWMQRMNSCAGAVYGSHGRRGRQTYTRYQTLELEKEFHFNRYLTRRRRIEIANALCLTERQIKIWFQNRRMKWKKENKLINSTQPSGEDSEAKAGE

>Q9P278

MAPTLLQKLFNKRGSSGSSAAASAQGRAPKEGPAFSWSCSEFDLNEIRLIVYQDCDRRGRQVLFDSKAVQKIEEVTAQKTEDVPIKISAKCCQGSSSVSSSSSSSISSHSSSGGSSHHAKEQLPKYQYTRPASDVNMLGEMMFGSVAMSYKGSTLKIHYIRSPPQLMISKVFSARMGSFCGSTNNLQDSFEYINQDPNLGKLNTNQNSLGPCRTGSNLAHSTPVDMPSRGQNEDRDSGIARSASLSSLLITPFPSPSSSTSSSSSYQRRWLRSQTTSLENGIIPRRSTDETFSLAEETCSSNPAMVRRKKIAISIIFSLCEKEEAQRNFQDFFFSHFPLFESHMNRLKSAIEKAMISCRKIAESSLRVQFYVSRLMEALGEFRGTIWNLYSVPRIAEPVWLTMMSGTLEKNQLCQRFLKEFTLLIEQINKNQFFAALLTAVLTYHLAWVPTVMPVDHPPIKAFSEKRTSQSVNMLAKTHPYNPLWAQLGDLYGAIGSPVRLTRTVVVGKQKDLVQRILYVLTYFLRCSELQENQLTWSGNHGEGDQVLNGSKIITALEKGEVEESEYVVITVRNEPALVPPILPPTAAERHNPWPTGFPECPEGTDSRDLGLKPDKEANRRPEQGSEACSAGCLGPASDASWKPQNAFCGDEKNKEAPQDGSSRLPSCEVLGAGMKMDQQAVCELLKVEMPTRLPDRSVAWPCPDRHLREKPSLEKVTFQIGSFASPESDFESRMKKMEERVKACGPSLEASEAADVAQDPQVSRSPFKPGFQENVCCPQNRLSEGDEGESDKGFAEDRGSRNDMAADIAGQLSHAADLGTASHGAGGTGGRRLEATRGLYVKAAEGPVLEPVAPRCVQRGPGLVAGANIPCGDDNKKANFRTEGDIPRNESSDSALGDSDDEACASAMLDLGHGGDRTGGSLEVELPLPRSQSISTQNVRNFGRSLLAGYCPTYMPDLVLHGTGSDEKLKQCLVADLVHTVHHPVLDEPIAEAVCIIADTDKWSVQVATSQRKVTDNMKLGQDVLVSSQVSSLLQSILQLYKLHLPADFCIMHLEDRLQEMYLKSKMLSEYLRGHTRVHVKELGVVLGIESNDLPLLTAIASTHSPYVAQILL

1035 NP_037406.1 AAH07654.1

>NP_037406.1

MDPRGILKAFPKRQKIHADASSKVLAKIPRREEGEEAEEWLSSLRAHVVRTGIGRARAELFEKQIVQHGGQLCPAQGPGVTHIVVDEGMDYERALRLLRLPQLPPGAQLVKSAWLSLCLQERRLVDVAGFSIFIPSRYLDHPQPSKAEQDASIPPGTHEALLQTALSPPPPPTRPVSPPQKAKEAPNTQAQPISDDEASDGEETQVSAADLEALISGHYPTSLEGDCEPSPAPAVLDKWVCAQPSSQKATNHNLHITEKLEVLAKAYSVQGDKWRALGYAKAINALKSFHKPVTSYQEACSIPGIGKRMAEKIIEILESGHLRKLDHISESVPVLELFSNIWGAGTKTAQMWYQQGFRSLEDIRSQASLTTQQAIGLKHYSDFLERMPREEATEIEQTVQKAAQAFNSGLLCVACGSYRRGKATCGDVDVLITHPDGRSHRGIFSRLLDSLRQEGFLTDDLVSQEENGQQQKYLGVCRLPGPGRRHRRLDIIVVPYSEFACALLYFTGSAHFNRSMRALAKTKGMSLSEHALSTAVVRNTHGCKVGPGRVLPTPTEKDVFRLLGLPYREPAERDW

>AAH07654.1

MLSSCVRPVPTTVRFVDSLICNSSRSFMDLKALLSSLNDFASLSFAESWDNVGLLVEPSPPHTVNTLFLTNDLTEEVMEEVLQKKADLILSYHPPIFRPMKRITWNTWKERLVIRALENRVGIYSPHTAYDAAPQGVNNWLAKGLGACTSRPIHPSKAPNYPTEGNHRVEFNVNYTQDLDKVMSAVKGIDGVSVTSFSARTGNEEQTRINLNCTQKALMQVVDFLSRNKQLYQKTEILSLEKPLLLHTGMGRLCTLDESVSLATMIDRIKRHLKLSHIRLALGVGRTLESQVKVVALCAGSGSSVLQGVEADLYLTGEMSHHDTLDAASQGINVILCEHSNTERGFLSDLRDMLDSHLENKINIILSETDRDPLQVV

1036 NP_003588.1 NP_002070.1

>NP_003588.1

MHTPDFAGPDDARAVDIMDICESILERKRHDSERSTCSILEQTDMEAVEALVCMSSWGQRSQKGDLLRIRPLTPVSDSGDVTTTVHMDAATPELPKDFHSLSTLCITPPQSPDLVEPSTRTPVSPQVTDSKACTATDVLQSSAVVARALSGGAERGLLGLEPVPSSPCRAKGTSVIRHTGESPAACFPTIQTPDCRLSDSREGEEQLLGHFETLQDTHLTDSLLSTNLVSCQPCLHKSGGLLLTDKGQQAGWPGAVQTCSPKNYENDLPRKTTPLISVSVPAPPVLCQMIPVTGQSSMLPAFLKPPPQLSVGTVRPILAQAAPAPQPVFVGPAVPQGAVMLVLPQGALPPPAPCAANVMAAGNTKLLPLAPAPVFITSSQNCVPQVDFSRRRNYVCSFPGCRKTYFKSSHLKAHLRTHTGEKPFNCSWDGCDKKFARSDELSRHRRTHTGEKKFVCPVCDRRFMRSDHLTKHARRHMTTKKIPGWQAEVGKLNRIASAESPGSPLVSMPASA

>NP_002070.1

MAPPSVFAEVPQAQPVLVFKLTADFREDPDPRKVNLGVGAYRTDDCHPWVLPVVKKVEQKIANDNSLNHEYLPILGLAEFRSCASRLALGDDSPALKEKRVGGVQSLGGTGALRIGADFLARWYNGTNNKNTPVYVSSPTWENHNAVFSAAGFKDIRSYRYWDAEKRGLDLQGFLNDLENAPEFSIVVLHACAHNPTGIDPTPEQWKQIASVMKHRFLFPFFDSAYQGFASGNLERDAWAIRYFVSEGFEFFCAQSFSKNFGLYNERVGNLTVVGKEPESILQVLSQMEKIVRITWSNPPAQGARIVASTLSNPELFEEWTGNVKTMADRILTMRSELRARLEALKTPGTWNHITDQIGMFSFTGLNPKQVEYLVNEKHIYLLPSGRINVSGLTTKNLDYVATSIHEAVTKIQ

1037 Q13123 P11586

>Q13123

MPERDSEPFSNPLAPDGHDVDDPHSFHQSKLTNEDFRKLLMTPRAAPTSAPPSKSRHHEMPREYNEDEDPAARRRKKKSYYAKLRQQEIERERELAEKYRDRAKERRDGVNKDYEETELISTTANYRAVGPTAEADKSAAEKRRQLIQESKFLGGDMEHTHLVKGLDFALLQKVRLEIASKDKEEEELMEKPQKETKKDEDPENKIEFKTRLGRNVYRMLFKSKAYERNELFLPGRMAYVVDLDDEYADTDIPTTLIRSKADCPTMEAQTTLTTNDIVISKLTQILSYLRQGTRNKKLKKKDKGKLEEKKPPEADMNIFEDIGDYVPSTTKTPRDKERERYRERERDRERDRDRDRERERERDRERERERDREREEEKKRHSYFEKPKVDDEPMDVDKGPGSTKELIKSINEKFAGSAGWEGTESLKKPEDKKQLGDFFGMSNSYAECYPATMDDMAVDSDEEVDYSKMDQGNKKGPLGRWDFDTQEEYSEYMNNKEALPKAAFQYGIKMSEGRKTRRFKETNDKAELDRQWKKISAIIEKRKKMEADGVEVKRPKY

>P11586

MAPAEILNGKEISAQIRARLKNQVTQLKEQVPGFTPRLAILQVGNRDDSNLYINVKLKAAEEIGIKATHIKLPRTTTESEVMKYITSLNEDSTVHGFLVQLPLDSENSINTEEVINAIAPEKDVDGLTSINAGRLARGDLNDCFIPCTPKGCLELIKETGVPIAGRHAVVVGRSKIVGAPMHDLLLWNNATVTTCHSKTAHLDEEVNKGDILVVATGQPEMVKGEWIKPGAIVIDCGINYVPDDKKPNGRKVVGDVAYDEAKERASFITPVPGGVGPMTVAMLMQSTVESAKRFLEKFKPGKWMIQYNNLNLKTPVPSDIDISRSCKPKPIGKLAREIGLLSEEVELYGETKAKVLLSALERLKHRPDGKYVVVTGITPTPLGEGKSTTTIGLVQALGAHLYQNVFACVRQPSQGPTFGIKGGAAGGGYSQVIPMEEFNLHLTGDIHAITAANNLVAAAIDARIFHELTQTDKALFNRLVPSVNGVRRFSDIQIRRLKRLGIEKTDPTTLTDEEINRFARLDIDPETITWQRVLDTNDRFLRKITIGQAPTEKGHTRTAQFDISVASEIMAVLALTTSLEDMRERLGKMVVASSKKGEPVSAEDLGVSGALTVLMKDAIKPNLMQTLEGTPVFVHAGPFANIAHGNSSIIADRIALKLVGPEGFVVTEAGFGADIGMEKFFNIKCRYSGLCPHVVVLVATVRALKMHGGGPTVTAGLPLPKAYIQENLELVEKGFSNLKKQIENARMFGIPVVVAVNAFKTDTESELDLISRLSREHGAFDAVKCTHWAEGGKGALALAQAVQRAAQAPSSFQLLYDLKLPVEDKIRIIAQKIYGADDIELLPEAQHKAEVYTKQGFGNLPICMAKTHLSLSHNPEQKGVPTGFILPIRDIRASVGAGFLYPLVGTMSTMPGLPTRPCFYDIDLDPETEQVNGLF

1038 NP_005633.2 NP_060767.2

>NP_005633.2

MSKSKDDAPHELESQFILRLPPEYASTVRRAVQSGHVNLKDRLTIELHPDGRHGIVRVDRVPLASKLVDLPCVMESLKTIDKKTFYKTADICQMLVSTVDGDLYPPVEEPVASTDPKASKKKDKDKEKKFIWNHGITLPLKNVRKRRFRKTAKKKYIESPDVEKEVKRLLSTDAEAVSTRWEIIAEDETKEAENQGLDISSPGMSGHRQGHDSLEHDELREIFNDLSSSSEDEDETQHQDEEDINIIDTEEDLERQLQDKLNESDEQHQENEGTNQLVMGIQKQIDNMKGKLQETQDRAKRQEDLIMKVENLALKNRFQAVLDELKQKEDREKEQLSSLQEELESLLEK

>NP_060767.2

MAAAALGSSSGSASPAVAELCQNTPETFLEASKLLLTYADNILRNPNDEKYRSIRIGNTAFSTRLLPVRGAVECLFEMGFEEGETHLIFPKKASVEQLQKIRDLIAIERSSRLDGSNKSHKVKSSQQPAASTQLPTTPSSNPSGLNQHTRNRQGQSSDPPSASTVAADSAILEVLQSNIQHVLVYENPALQEKALACIPVQELKRKSQEKLSRARKLDKGINISDEDFLLLELLHWFKEEFFHWVNNVLCSKCGGQTRSRDRSLLPSDDELKWGAKEVEDHYCDACQFSNRFPRYNNPEKLLETRCGRCGEWANCFTLCCRAVGFEARYVWDYTDHVWTEVYSPSQQRWLHCDACEDVCDKPLLYEIGWGKKLSYVIAFSKDEVVDVTWRYSCKHEEVIARRTKVKEALLRDTINGLNKQRQLFLSENRRKELLQRIIVELVEFISPKTPKPGELGGRISGSVAWRVARGEMGLQRKETLFIPCENEKISKQLHLCYNIVKDRYVRVSNNNQTISGWENGVWKMESIFRKVETDWHMVYLARKEGSSFAYISWKFECGSVGLKVDSISIRTSSQTFQTGTVEWKLRSDTAQVELTGDNSLHSYADFSGATEVILEAELSRGDGDVAWQHTQLFRQSLNDHEENCLEIIIKFSDL

1039 NP_000519.2 NP_064632.2

>NP_000519.2

MGAYARASGVCARGCLDSAGPWTMSRALRPPLPPLCFFLLLLAAAGARAGGYETCPTVQPNMLNVHLLPHTHDDVGWLKTVDQYFYGIKNDIQHAGVQYILDSVISALLADPTRRFIYVEIAFFSRWWHQQTNATQEVVRDLVRQGRLEFANGGWVMNDEAATHYGAIVDQMTLGLRFLEDTFGNDGRPRVAWHIDPFGHSREQASLFAQMGFDGFFFGRLDYQDKWVRMQKLEMEQVWRASTSLKPPTADLFTGVLPNGYNPPRNLCWDVLCVDQPLVEDPRSPEYNAKELVDYFLNVATAQGRYYRTNHTVMTMGSDFQYENANMWFKNLDKLIRLVNAQQAKGSSVHVLYSTPACYLWELNKANLTWSVKHDDFFPYADGPHQFWTGYFSSRPALKRYERLSYNFLQVCNQLEALVGLAANVGPYGSGDSAPLNEAMAVLQHHDAVSGTSRQHVANDYARQLAAGWGPCEVLLSNALARLRGFKDHFTFCQQLNISICPLSQTAARFQVIVYNPLGRKVNWMVRLPVSEGVFVVKDPNGRTVPSDVVIFPSSDSQAHPPELLFSASLPALGFSTYSVAQVPRWKPQARAPQPIPRRSWSPALTIENEHIRATFDPDTGLLMEIMNMNQQLLLPVRQTFFWYNASIGDNESDQASGAYIFRPNQQKPLPVSRWAQIHLVKTPLVQEVHQNFSAWCSQVVRLYPGQRHLELEWSVGPIPVGDTWGKEVISRFDTPLETKGRFYTDSNGREILERRRDYRPTWKLNQTEPVAGNYYPVNTRIYITDGNMQLTVLTDRSQGGSSLRDGSLELMVHRRLLKDDGRGVSEPLMENGSGAWVRGRHLVLLDTAQAAAAGHRLLAEQEVLAPQVVLAPGGGAAYNLGAPPRTQFSGLRRDLPPSVHLLTLASWGPEMVLLRLEHQFAVGEDSGRNLSAPVTLNLRDLFSTFTITRLQETTLVANQLREAASRLKWTTNTGPTPHQTPYQLDPANITLEPMEIRTFLASVQWKEVDG

>NP_064632.2

MAAILGDTIMVAKGLVKLTQAAVETHLQHLGIGGELIMAARALQSTAVEQIGMFLGKVQGQDKHEEYFAENFGGPEGEFHFSVPHAAGASTDFSSASAPDQSAPPSLGHAHSEGPAPAYVASGPFREAGFPGQASSPLGRANGRLFANPRDSFSAMGFQRRFFHQDQSPVGGLTAEDIEKARQAKARPENKQHKQTLSEHARERKVPVTRIGRLANFGGLAVGLGFGALAEVAKKSLRSEDPSGKKAVLGSSPFLSEANAERIVRTLCKVRGAALKLGQMLSIQDDAFINPHLAKIFERVRQSADFMPLKQMMKTLNNDLGPNWRDKLEYFEERPFAAASIGQVHLARMKGGREVAMKIQYPGVAQSINSDVNNLMAVLNMSNMLPEGLFPEHLIDVLRRELALECDYQREAACARKFRDLLKGHPFFYVPEIVDELCSPHVLTTELVSGFPLDQAEGLSQEIRNEICYNILVLCLRELFEFHFMQTDPNWSNFFYDPQQHKVALLDFGATREYDRSFTDLYIQIIRAAADRDRETVRAKSIEMKFLTGYEVKVMEDAHLDAILILGEAFASDEPFDFGTQSTTEKIHNLIPVMLRHRLVPPPEETYSLHRKMGGSFLICSKLKARFPCKAMFEEAYSNYCKRQAQQ

1040 NP_659480.1 NP_000848.1

>NP_659480.1

MPEGPLVRKFHHLVSPFVGQQVVKTGGSSKKLQPASLQSLWLQDTQVHGKKLFLRFDLDEEMGPPGSSPTPEPPQKEVQKEGAADPKQVGEPSGQKTLDGSSRSAELVPQGEDDSEYLERDAPAGDAGRWLRVSFGLFGSVWVNDFSRAKKANKRGDWRDPSPRLVLHFGGGGFLAFYNCQLSWSSSPVVTPTCDILSEKFHRGQALEALGQAQPVCYTLLDQRYFSGLGNIIKNEALYRAGIHPLSLGSVLSASRREVLVDHVVEFSTAWLQGKFQGRPQHTQVYQKEQCPAGHQVMKEAFGPEDGLQRLTWWCPQCQPQLSEEPEQCQFS

>NP_000848.1

MYGFVNHALELLVIRNYGPEVWEDIKKEAQLDEEGQFLVRIIYDDSKTYDLVAAASKVLNLNAGEILQMFGKMFFVFCQESGYDTILRVLGSNVREFLQNLDALHDHLATIYPGMRAPSFRCTDAEKGKGLILHYYSEREGLQDIVIGIIKTVAQQIHGTEIDMKVIQQRNEECDHTQFLIEEKESKEEDFYEDLDRFEENGTQESRISPYTFCKAFPFHIIFDRDLVVTQCGNAIYRVLPQLQPGNCSLLSVFSLVRPHIDISFHGILSHINTVFVLRSKEGLLDVEKLECEDELTGTEISCLRLKGQMIYLPEADSILFLCSPSVMNLDDLTRRGLYLSDIPLHDATRDLVLLGEQFREEYKLTQELEILTDRLQLTLRALEDEKKKTDTLLYSVLPPSVANELRHKRPVPAKRYDNVTILFSGIVGFNAFCSKHASGEGAMKIVNLLNDLYTRFDTLTDSRKNPFVYKVETVGDKYMTVSGLPEPCIHHARSICHLALDMMEIAGQVQVDGESVQITIGIHTGEVVTGVIGQRMPRYCLFGNTVNLTSRTETTGEKGKINVSEYTYRCLMSPENSDPQFHLEHRGPVSMKGKKEPMQVWFLSRKNTGTEETKQDDD

1041 NP_055436.2 NP_057461.1

>NP_055436.2

MVMSSYMVNSKYVDPKFPPCEEYLQGGYLGEQGADYYGGGAQGADFQPPGLYPRPDFGEQPFGGSGPGPGSALPARGHGQEPGGPGGHYAAPGEPCPAPPAPPPAPLPGARAYSQSDPKQPPSGTALKQPAVVYPWMKKVHVNSVNPNYTGGEPKRSRTAYTRQQVLELEKEFHFNRYLTRRRRIEIAHTLCLSERQIKIWFQNRRMKWKKDHKLPNTKGRSSSSSSSSSCSSSVAPSQHLQPMAKDHHTDLTTL

>NP_057461.1

MSEEFEANTMDSLVDMPFATVDIQDDCGITDEPQINLKRSQENEWVKSDQVKKRKKKRKDYQPNYFLSIPITNKEIIKGIKILQNAIIQQDERLAKAMVSDGSFHITLLVMQLLNEDEVNIGIDALLELKPFIEELLQGKHLTLPFQGIGTFGNQVGFVKLAEGDHVNSLLEIAETANRTFQEKGILVGESRSFKPHLTFMKLSKSPWLRKNGVKKIDPDLYEKFISHRFGEEILYRIDLCSMLKKKQSNGYYHCESSIVIGEKNGGEPDDAELVRLSKRLVENAVLKAVQQYLEETQNKNKPGEGSSVKTEAADQNGNDNENNRK

1042 NP_002883.3 Q9NRJ4

>NP_002883.3

MKAADEPAYLTVGTDVSAKYRGAFCEAKIKTVKRLVKVKVLLKQDNTTQLVQDDQVKGPLRVGAIVETRTSDGSFQEAIISKLTDASWYTVVFDDGDERTLRRTSLCLKGERHFAESETLDQLPLTNPEHFGTPVIAKKTNRGRRSSLPVTEDEKEEESSEEEDEDKRRLNDELLGKVVSVVSATERTEWYPALVISPSCNDDITVKKDQCLVRSFIDSKFYSIARKDIKEVDILNLPESELSTKPGLQKASIFLKTRVVPDNWKMDISEILESSSSDDEDGPAEENDEEKEKEAKKTEEEVPEEELDPEERDNFLQQLYKFMEDRGTPINKPPVLGYKDLNLFKLFRLVYHQGGCDNIDSGAVWKQIYMDLGIPILNSAASYNVKTAYRKYLYGFEEYCRSANIQFRTVHHHEPKVKEEKKDLEESMEEALKLDQEMPLTEVKSEPEENIDSNSESEREEIELKSPRGRRRIARDVNSIKKEIEEEKTEDKLKDNDTENKDVDDDYETAEKKENELLLGRKNTPKQKEKKIKKQEDSDKDSDEEEEKSQEREETESKCDSEGEEDEEDMEPCLTGTKVKVKYGRGKTQKIYEASIKSTEIDDGEVLYLVHYYGWNVRYDEWVKADRIIWPLDKGGPKKKQKKKAKNKEDSEKDEKRDEERQKSKRGRPPLKSTLSSNMPYGLSKTANSEGKSDSCSSDSETEDALEKNLINEELSLKDELEKNENLNDDKLDEENPKISAHILKENDRTQMQPLETLKLEVGENEQIVQIFGNKMEKTEEVKKEAEKSPKGKGRRSKTKDLSLEIIKISSFGQNEAGSEPHIEAHSLELSSLDNKNFSSATEDEIDQCVKEKKLKRKILGQSSPEKKIRIENGMEMTNTVSQERTSDCIGSEGMKNLNFEQHFERENEGMPSLIAESNQCIQQLTSERFDSPAEETVNIPLKEDEDAMPLIGPETLVCHEVDLDDLDEKDKTSIEDVAVESSESNSLVSIPPALPPVVQHNFSVASPLTLSQDESRSVKSESDITIEVDSIAEESQEGLCERESANGFETNVASGTCSIIVQERESREKGQKRPSDGNSGLMAKKQKRTPKRTSAAAKNEKNGTGQSSDSEDLPVLDNSSKCTPVKHLNVSKPQKLARSPARISPHIKDGEKDKHREKHPNSSPRTYKWSFQLNELDNMNSTERISFLQEKLQEIRKYYMSLKSEVATIDRRRKRLKKKDREVSHAGASMSSASSDTGMSPSSSSPPQNVLAVECR

>Q9NRJ4

MYAAVEHGPVLCSDSNILCLSWKGRVPKSEKEKPVCRRRYYEEGWLATGNGRGVVGVTFTSSHCRRDRSTPQRINFNLRGHNSEVVLVRWNEPYQKLATCDADGGIFVWIQYEGRWSVELVNDRGAQVSDFTWSHDGTQALISYRDGFVLVGSVSGQRHWSSEINLESQITCGIWTPDDQQVLFGTADGQVIVMDCHGRMLAHVLLHESDGVLGMSWNYPIFLVEDSSESDTDSDDYAPPQDGPAAYPIPVQNIKPLLTVSFTSGDISLMNNYDDLSPTVIRSGLKEVVAQWCTQGDLLAVAGMERQTQLGELPNGPLLKSAMVKFYNVRGEHIFTLDTLVQRPIISICWGHRDSRLLMASGPALYVVRVEHRVSSLQLLCQQAIASTLREDKDVSKLTLPPRLCSYLSTAFIPTIKPPIPDPNNMRDFVSYPSAGNERLHCTMKRTEDDPEVGGPCYTLYLEYLGGLVPILKGRRISKLRPEFVIMDPRTDSKPDEIYGNSLISTVIDSCNCSDSSDIELSDDWAAKKSPKISRASKSPKLPRISIEARKSPKLPRAAQELSRSPRLPLRKPSVGSPSLTRREFPFEDITQHNYLAQVTSNIWGTKFKIVGLAAFLPTNLGAVIYKTSLLHLQPRQMTIYLPEVRKISMDYINLPVFNPNVFSEDEDDLPVTGASGVPENSPPCTVNIPIAPIHSSAQAMSPTQSIGLVQSLLANQNVQLDVLTNQTTAVGTAEHAGDSATQYPVSNRYSNPGQVIFGSVEMGRIIQNPPPLSLPPPPQGPMQLSTVGHGDRDHEHLQKSAKALRPTPQLAAEGDAVVFSAPQEVQVTKINPPPPYPGTIPAAPTTAAPPPPLPPPQPPVDVCLKKGDFSLYPTSVHYQTPLGYERITTFDSSGNVEEVCRPRTRMLCSQNTYTLPGPGSSATLRLTATEKKVPQPCSSATLNRLTVPRYSIPTGDPPPYPEIASQLAQGRGAAQRSDNSLIHATLRRNNREATLKMAQLADSPRAPLQPLAKSKGGPGGVVTQLPARPPPALYTCSQCSGTGPSSQPGASLAHTASASPLASQSSYSLLSPPDSARDRTDYVNSAFTEDEALSQHCQLEKPLRHPPLPEAAVTLKRPPPYQWDPMLGEDVWVPQERTAQTSGPNPLKLSSLMLSQGQHLDVSRLPFISPKSPASPTATFQTGYGMGVPYPGSYNNPPLPGVQAPCSPKDALSPTQFAQQEPAVVLQPLYPPSLSYCTLPPMYPGSSTCSSLQLPPVALHPWSSYSACPPMQNPQGTLPPKPHLVVEKPLVSPPPADLQSHLGTEVMVETADNFQEVLSLTESPVPQRTEKFGKKNRKRLDSRAEEGSVQAITEGKVKKEARTLSDFNSLISSPHLGREKKKVKSQKDQLKSKKLNKTNEFQDSSESEPELFISGDELMNQSQGSRKGWKSKRSPRAAGELEEAKCRRASEKEDGRLGSQGFVYVMANKQPLWNEATQVYQLDFGGRVTQESAKNFQIELEGRQVMQFGRIDGSAYILDFQYPFSAVQAFAVALANVTQRLK

1043 Q8N9N5 Q16822

>Q8N9N5

MMSEHDLADVVQIAVEDLSPDHPVVLENHVVTDEDEPALKRQRLEINCQDPSIKTICLRLDSIEAKLQALEATCKSLEEKLDLVTNKQHSPIQVPMVAGSPLGATQTCNKVRCVVPQTTVILNNDRQNAIVAKMEDPLSNRAPDSLENVISNAVPGRRQNTIVVKVPGQEDSHHEDGESGSEASDSVSSCGQAGSQSIGSNVTLITLNSEEDYPNGTWLGDENNPEMRVRCAIIPSDMLHISTNCRTAEKMALTLLDYLFHREVQAVSNLSGQGKHGKKQLDPLTIYGIRCHLFYKFGITESDWYRIKQSIDSKCRTAWRRKQRGQSLAVKSFSRRTPNSSSYCPSESMMSTPPPASELPQPQPQPQALHYALANAQQVQIHQIGEDGQVQVGHLHIAQVPQGEQVQITQDSEGNLQIHHVGQDGQLLEATRIPCLLAPSVFKASSGQVLQGAQLIAVASSDPAAAGVDGSPLQGSDIQVQYVQLAPVSDHTAGAQTAEALQPTLQPEMQLEHGAIQIQ

>Q16822

MAALYRPGLRLNWHGLSPLGWPSCRSIQTLRVLSGDLGQLPTGIRDFVEHSARLCQPEGIHICDGTEAENTATLTLLEQQGLIRKLPKYNNCWLARTDPKDVARVESKTVIVTPSQRDTVPLPPGGARGQLGNWMSPADFQRAVDERFPGCMQGRTMYVLPFSMGPVGSPLSRIGVQLTDSAYVVASMRIMTRLGTPVLQALGDGDFVKCLHSVGQPLTGQGEPVSQWPCNPEKTLIGHVPDQREIISFGSGYGGNSLLGKKCFALRIASRLARDEGWLAEHMLILGITSPAGKKALCAAAFPSACGKTNLAMMRPALPGWKVECVGDDIAWMRFDSEGRLRAINPENGFFGVAPGTSATTNPNAMATIQSNTIFTNVAETSDGGVYWEGIDQPLPPGVTVTSWLGKPWKPGDKEPCAHPNSRFCAPARQCPIMDPAWEAPEGVPIDAIIFGGRRPKGVPLVYEAFNWRHGVFVGRAMRSESTAAAEHKGKIIMHDPFAMRPFFGYNFGHYLEHWLSMEGRKGAQLPRIFHVNWFRRDEAGHFLWPGFGENARVLDWICRRLEGEDSARETPIGLVPKEGALDLSGLRAIDTTQLFSLPKDFWEQEVRDIRSYLTEQVNQDLPKEVLAELEALERRVHKM

1044 NP_001256.1 NP_003134.1

>NP_001256.1

MYVSYLLDKDVSMYPSSVRHSGGLNLAPQNFVSPPQYPDYGGYHVAAAAAAAANLDSAQSPGPSWPAAYGAPLREDWNGYAPGGAAAAANAVAHGLNGGSPAAAMGYSSPADYHPHHHPHHHPHHPAAAPSCASGLLQTLNPGPPGPAATAAAEQLSPGGQRRNLCEWMRKPAQQSLGSQVKTRTKDKYRVVYTDHQRLELEKEFHYSRYITIRRKAELAATLGLSERQVKIWFQNRRAKERKINKKKLQQQQQQQPPQPPPPPPQPPQPQPGPLRSVPEPLSPVSSLQASVSGSVPGVLGPTGGVLNPTVTQ

>NP_003134.1

MFRRPVLQVLRQFVRHESETTTSLVLERSLNRVHLLGRVGQDPVLRQVEGKNPVTIFSLATNEMWRSGDSEVYQLGDVSQKTTWHRISVFRPGLRDVAYQYVKKGSRIYLEGKIDYGEYMDKNNVRRQATTIIADNIIFLSDQTKEKE

1045 Q6EKJ0 NP_006825.1

>Q6EKJ0

MAQVAVSTLPVEEESSSETRMVVTFLVSALESMCKELAKSKAEVACIAVYETDVFVVGTERGCAFVNARTDFQKDFAKYCVAEGLCEVKPPCPVNGMQVHSGETEILRKAVEDYFCFCYGKALGTTVMVPVPYEKMLRDQSAVVVQGLPEGVAFQHPENYDLATLKWILENKAGISFIINRPFLGPESQLGGPGMVTDAERSIVSPSESCGPINVKTEPMEDSGISLKAEAVSVKKESEDPNYYQYNMQGSHPSSTSNEVIEMELPMEDSTPLVPSEEPNEDPEAEVKIEGNTNSSSVTNSAAGVEDLNIVQVTVPDNEKERLSSIEKIKQLREQVNDLFSRKFGEAIGVDFPVKVPYRKITFNPGCVVIDGMPPGVVFKAPGYLEISSMRRILEAAEFIKFTVIRPLPGLELSNVGKRKIDQEGRVFQEKWERAYFFVEVQNIPTCLICKQSMSVSKEYNLRRHYQTNHSKHYDQYTERMRDEKLHELKKGLRKYLLGSSDTECPEQKQVFANPSPTQKSPVQPVEDLAGNLWEKLREKIRSFVAYSIAIDEITDINNTTQLAIFIRGVDENFDVSEELLDTVPMTGTKSGNEIFLRVEKSLKKFCINWSRLVSVASTGTPAMVDANNGLVTKLKSRVATFCKGAELKSICCIIHPESLCAQKLKMDHVMDVVVKSVNWICSRGLNHSEFTTLLYELDSQYGSLLYYTEIKWLSRGLVLKRFFESLEEIDSFMSSRGKPLPQLSSIDWIRDLAFLVDMTMHLNALNISLQGHSQIVTQMYDLIRAFLAKLCLWETHLTRNNLAHFPTLKLVSRNESDGLNYIPKIAELKTEFQKRLSDFKLYESELTLFSSPFSTKIDSVHEELQMEVIDLQCNTVLKTKYDKVGIPEFYKYLWGSYPKYKHHCAKILSMFGSTYICEQLFSIMKLSKTKYCSQLKDSQWDSVLHIAT

>NP_006825.1

MAGGGAGDPGLGAAAAPAPETREHLFKVLVIGELGVGKTSIIKRYVHQLFSQHYRATIGVDFALKVLNWDSRTLVRLQLWDIAGQERFGNMTRVYYKEAVGAFVVFDISRSSTFEAVLKWKSDLDSKVHLPNGSPIPAVLLANKCDQNKDSSQSPSQVDQFCKEHGFAGWFETSAKDNINIEEAARFLVEKILVNHQSFPNEENDVDKIKLDQETLRAENKSQCC

1046 P60008 P46459

>P60008

MLHASTIWHLRSTPPRRKQWGHCDPHRILVASEVTTEITSPTPAPRAQVCGGQPWVTVLDPLSGHTGREAERHFATVSISAVELKYCHGWRPAGQRVPSKTATGQRTCAKPCQKPSTSKVILRAVADKGTCKYVSLATLKKAVSTTGYDMARNAYHFKRVLKGLVDKGSAGSFTLGKKQASKSKLKVKRQRQQRWRSGQRPFGQHRSLLGSKQGHKRLIKGVRRVAKCHCN

>P46459

MAGRSMQAARCPTDELSLTNCAVVNEKDFQSGQHVIVRTSPNHRYTFTLKTHPSVVPGSIAFSLPQRKWAGLSIGQEIEVSLYTFDKAKQCIGTMTIEIDFLQKKSIDSNPYDTDKMAAEFIQQFNNQAFSVGQQLVFSFNEKLFGLLVKDIEAMDPSILKGEPATGKRQKIEVGLVVGNSQVAFEKAENSSLNLIGKAKTKENRQSIINPDWNFEKMGIGGLDKEFSDIFRRAFASRVFPPEIVEQMGCKHVKGILLYGPPGCGKTLLARQIGKMLNAREPKVVNGPEILNKYVGESEANIRKLFADAEEEQRRLGANSGLHIIIFDEIDAICKQRGSMAGSTGVHDTVVNQLLSKIDGVEQLNNILVIGMTNRPDLIDEALLRPGRLEVKMEIGLPDEKGRLQILHIHTARMRGHQLLSADVDIKELAVETKNFSGAELEGLVRAAQSTAMNRHIKASTKVEVDMEKAESLQVTRGDFLASLENDIKPAFGTNQEDYASYIMNGIIKWGDPVTRVLDDGELLVQQTKNSDRTPLVSVLLEGPPHSGKTALAAKIAEESNFPFIKICSPDKMIGFSETAKCQAMKKIFDDAYKSQLSCVVVDDIERLLDYVPIGPRFSNLVLQALLVLLKKAPPQGRKLLIIGTTSRKDVLQEMEMLNAFSTTIHVPNIATGEQLLEALELLGNFKDKERTTIAQQVKGKKVWIGIKKLLMLIEMSLQMDPEYRVRKFLALLREEGASPLDFD

1047 NP_795361.1 Q93088

>NP_795361.1

MTHSLVCPETVSRVSSVLNRNTRQFGKKHLFDQDEETCWNSDQGPSQWVTLEFPQLIRVSQLQIQFQGGFSSRRGCLEGSQGTQALHKIVDFYPEDNNSLQTFPIPAAEVDRLKVTFEDATDFFGRVVIYHLRVLGEKV

>Q93088

MPPVGGKKAKKGILERLNAGEIVIGDGGFVFALEKRGYVKAGPWTPEAAVEHPEAVRQLHREFLRAGSNVMQTFTFYASEDKLENRGNYVLEKISGQEVNEAACDIARQVADEGDALVAGGVSQTPSYLSCKSETEVKKVFLQQLEVFMKKNVDFLIAEYFEHVEEAVWAVETLIASGKPVAATMCIGPEGDLHGVPPGECAVRLVKAGASIIGVNCHFDPTISLKTVKLMKEGLEAARLKAHLMSQPLAYHTPDCNKQGFIDLPEFPFGLEPRVATRWDIQKYAREAYNLGVRYIGGCCGFEPYHIRAIAEELAPERGFLPPASEKHGSWGSGLDMHTKPWVRARARKEYWENLRIASGRPYNPSMSKPDGWGVTKGTAELMQQKEATTEQQLKELFEKQKFKSQ

1048 O95926 O43182

>O95926

MAAIAASEVLVDSAEEGSLAAAAELAAQKREQRLRKFRELHLMRNEARKLNHQEVVEEDKRLKLPANWEAKKARLEWELKEEEKKKECAARGEDYEKVKLLEISAEDAERWERKKKRKNPDLGFSDYAAAQLRQYHRLTKQIKPDMETYERLREKHGEEFFPTSNSLLHGTHVPSTEEIDRMVIDLEKQIEKRDKYSRRRPYNDDADIDYINERNAKFNKKAERFYGKYTAEIKQNLERGTAV

>O43182

MSAQSLLHSVFSCSSPASSSAASAKGFSKRKLRQTRSLDPALIGGCGSDEAGAEGSARGATAGRLYSPSLPAESLGPRLASSSRGPPPRATRLPPPGPLCSSFSTPSTPQEKSPSGSFHFDYEVPLGRGGLKKSMAWDLPSVLAGPASSRSASSILCSSGGGPNGIFASPRRWLQQRKFQSPPDSRGHPYVVWKSEGDFTWNSMSGRSVRLRSVPIQSLSELERARLQEVAFYQLQQDCDLSCQITIPKDGQKRKKSLRKKLDSLGKEKNKDKEFIPQAFGMPLSQVIANDRAYKLKQDLQRDEQKDASDFVASLLPFGNKRQNKELSSSNSSLSSTSETPNESTSPNTPEPAPRARRRGAMSVDSITDLDDNQSRLLEALQLSLPAEAQSKKEKARDKKLSLNPIYRQVPRLVDSCCQHLEKHGLQTVGIFRVGSSKKRVRQLREEFDRGIDVSLEEEHSVHDVAALLKEFLRDMPDPLLTRELYTAFINTLLLEPEEQLGTLQLLIYLLPPCNCDTLHRLLQFLSIVARHADDNISKDGQEVTGNKMTSLNLATIFGPNLLHKQKSSDKEFSVQSSARAEESTAIIAVVQKMIENYEALFMVPPDLQNEVLISLLETDPDVVDYLLRRKASQSSSPDMLQSEVSFSVGGRHSSTDSNKASSGDISPYDNNSPVLSERSLLAMQEDAAPGGSEKLYRVPGQFMLVGHLSSSKSRESSPGPRLGKDLSEEPFDIWGTWHSTLKSGSKDPGMTGSSGDIFESSSLRAGPCSLSQGNLSPNWPRWQGSPAELDSDTQGARRTQAAAPATEGRAHPAVSRACSTPHVQVAGKAERPTARSEQYLTLSGAHDLSESELDVAGLQSRATPQCQRPHGSGRDDKRPPPPYPGPGKPAAAAAWIQGPPEGVETPTDQGGQAAEREQQVTQKKLSSANSLPAGEQDSPRLGDAGWLDWQRERWQIWELLSTDNPDALPETLV

1049 O60341 NP_006422.1

>O60341

MLSGKKAAAAAAAAAAAATGTEAGPGTAGGSENGSEVAAQPAGLSGPAEVGPGAVGERTPRKKEPPRASPPGGLAEPPGSAGPQAGPTVVPGSATPMETGIAETPEGRRTSRRKRAKVEYREMDESLANLSEDEYYSEEERNAKAEKEKKLPPPPPQAPPEEENESEPEEPSGVEGAAFQSRLPHDRMTSQEAACFPDIISGPQQTQKVFLFIRNRTLQLWLDNPKIQLTFEATLQQLEAPYNSDTVLVHRVHSYLERHGLINFGIYKRIKPLPTKKTGKVIIIGSGVSGLAAARQLQSFGMDVTLLEARDRVGGRVATFRKGNYVADLGAMVVTGLGGNPMAVVSKQVNMELAKIKQKCPLYEANGQAVPKEKDEMVEQEFNRLLEATSYLSHQLDFNVLNNKPVSLGQALEVVIQLQEKHVKDEQIEHWKKIVKTQEELKELLNKMVNLKEKIKELHQQYKEASEVKPPRDITAEFLVKSKHRDLTALCKEYDELAETQGKLEEKLQELEANPPSDVYLSSRDRQILDWHFANLEFANATPLSTLSLKHWDQDDDFEFTGSHLTVRNGYSCVPVALAEGLDIKLNTAVRQVRYTASGCEVIAVNTRSTSQTFIYKCDAVLCTLPLGVLKQQPPAVQFVPPLPEWKTSAVQRMGFGNLNKVVLCFDRVFWDPSVNLFGHVGSTTASRGELFLFWNLYKAPILLALVAGEAAGIMENISDDVIVGRCLAILKGIFGSSAVPQPKETVVSRWRADPWARGSYSYVAAGSSGNDYDLMAQPITPGPSIPGAPQPIPRLFFAGEHTIRNYPATVHGALLSGLREAGRIADQFLGAMYTLPRQATPGVPAQQSPSM

>NP_006422.1

MASLSLAPVNIFKAGADEERAETARLTSFIGAIAIGDLVKSTLGPKGMDKILLSSGRDASLMVTNDGATILKNIGVDNPAAKVLVDMSRVQDDEVGDGTTSVTVLAAELLREAESLIAKKIHPQTIIAGWREATKAAREALLSSAVDHGSDEVKFRQDLMNIAGTTLSSKLLTHHKDHFTKLAVEAVLRLKGSGNLEAIHIIKKLGGSLADSYLDEGFLLDKKIGVNQPKRIENAKILIANTGMDTDKIKIFGSRVRVDSTAKVAEIEHAEKEKMKEKVERILKHGINCFINRQLIYNYPEQLFGAAGVMAIEHADFAGVERLALVTGGEIASTFDHPELVKLGSCKLIEEVMIGEDKLIHFSGVALGEACTIVLRGATQQILDEAERSLHDALCVLAQTVKDSRTVYGGGCSEMLMAHAVTQLANRTPGKEAVAMESYAKALRMLPTIIADNAGYDSADLVAQLRAAHSEGNTTAGLDMREGTIGDMAILGITESFQVKRQVLLSAAEAAEVILRVDNIIKAAPRKRVPDHHPC

1050 NP_005243.1 NP_004040.1

>NP_005243.1

MMFSGFNADYEASSSRCSSASPAGDSLSYYHSPADSFSSMGSPVNAQDFCTDLAVSSANFIPTVTAISTSPDLQWLVQPALVSSVAPSQTRAPHPFGVPAPSAGAYSRAGVVKTMTGGRAQSIGRRGKVEQLSPEEEEKRRIRRERNKMAAAKCRNRRRELTDTLQAETDQLEDEKSALQTEIANLLKEKEKLEFILAAHRPACKIPDDLGFPEEMSVASLDLTGGLPEVATPESEEAFTLPLLNDPEPKPSVEPVKSISSMELKTEPFDDFLFPASSRPSGSETARSVPDMDLSGSFYAADWEPLHSGSLGMGPMATELEPLCTPVVTCTPSCTAYTSSFVFTYPEADSFPSCAAAHRKGSSSNEPSSDSLSSPTLLAL

>NP_004040.1

MTDCEFGYIYRLAQDYLQCVLQIPQPGSGPSKTSRVLQNVAFSVQKEVEKNLKSCLDNVNVVSVDTARTLFNQVMEKEFEDGIINWGRIVTIFAFEGILIKKLLRQQIAPDVDTYKEISYFVAEFIMNNTGEWIRQNGGWENGFVKKFEPKSGWMTFLEVTGKICEMLSLLKQYC

1051 NP_000225.1 NP_077268.1

>NP_000225.1

MQRSIMSFFHPKKEGKAKKPEKEASNSSRETEPPPKAALKEWNGVVSESDSPVKRPGRKAARVLGSEGEEEDEALSPAKGQKPALDCSQVSPPRPATSPENNASLSDTSPMDSSPSGIPKRRTARKQLPKRTIQEVLEEQSEDEDREAKRKKEEEEEETPKESLTEAEVATEKEGEDGDQPTTPPKPLKTSKAETPTESVSEPEVATKQELQEEEEQTKPPRRAPKTLSSFFTPRKPAVKKEVKEEEPGAPGKEGAAEGPLDPSGYNPAKNNYHPVEDACWKPGQKVPYLAVARTFEKIEEVSARLRMVETLSNLLRSVVALSPPDLLPVLYLSLNHLGPPQQGLELGVGDGVLLKAVAQATGRQLESVRAEAAEKGDVGLVAENSRSTQRLMLPPPPLTASGVFSKFRDIARLTGSASTAKKIDIIKGLFVACRHSEARFIARSLSGRLRLGLAEQSVLAALSQAVSLTPPGQEFPPAMVDAGKGKTAEARKTWLEEQGMILKQTFCEVPDLDRIIPVLLEHGLERLPEHCKLSPGIPLKPMLAHPTRGISEVLKRFEEAAFTCEYKYDGQRAQIHALEGGEVKIFSRNQEDNTGKYPDIISRIPKIKLPSVTSFILDTEAVAWDREKKQIQPFQVLTTRKRKEVDASEIQVQVCLYAFDLIYLNGESLVREPLSRRRQLLRENFVETEGEFVFATSLDTKDIEQIAEFLEQSVKDSCEGLMVKTLDVDATYEIAKRSHNWLKLKKDYLDGVGDTLDLVVIGAYLGRGKRAGRYGGFLLASYDEDSEELQAICKLGTGFSDEELEEHHQSLKALVLPSPRPYVRIDGAVIPDHWLDPSAVWEVKCADLSLSPIYPAARGLVDSDKGISLRFPRFIRVREDKQPEQATTSAQVACLYRKQSQIQNQQGEDSGSDPEDTY

>NP_077268.1

MIEVVCNDRLGKKVRVKCNTDDTIGDLKKLIAAQTGTRWNKIVLKKWYTIFKDHVSLGDYEIHDGMNLELYYQ

1052 NP_254280.1 NP_006592.3

>NP_254280.1

MSGRGKQGGKARAKAKSRSSRAGLQFPVGRVHRLLRKGNYSERVGAGAPVYLAAVLEYLTAEILELAGNAARDNKKTRIIPRHLQLAIRNDEELNKLLGRVTIAQGGVLPNIQAVLLPKKTESHHKAKGK

>NP_006592.3

MQPASAKWYDRRDYVFIEFCVEDSKDVNVNFEKSKLTFSCLGGSDNFKHLNEIDLFHCIDPNDSKHKRTDRSILCCLRKGESGQSWPRLTKERAKLNWLSVDFNNWKDWEDDSDEDMSNFDRFSEMMNNMGGDEDVDLPEVDGADDDSQDSDDEKMPDLE

1053 NP_037392.1 NP_005557.1

>NP_037392.1

MAGKKNVLSSLAVYAEDSEPESDGEAGIEAVGSAAEEKGGLVSDAYGEDDFSRLGGDEDGYEEEEDENSRQSEDDDSETEKPEADDPKDNTEAEKRDPQELVASFSERVRNMSPDEIKIPPEPPGRCSNHLQDKIQKLYERKIKEGMDMNYIIQRKKEFRNPSIYEKLIQFCAIDELGTNYPKDMFDPHGWSEDSYYEALAKAQKIEMDKLEKAKKERTKIEFVTGTKKGTTTNATSTTTTTASTAVADAQKRKSKWDSAIPVTTIAQPTILTTTATLPAVVTVTTSASGSKTTVISAVGTIVKKAKQ

>NP_005557.1

MATLKDQLIYNLLKEEQTPQNKITVVGVGAVGMACAISILMKDLADELALVDVIEDKLKGEMMDLQHGSLFLRTPKIVSGKDYNVTANSKLVIITAGARQQEGESRLNLVQRNVNIFKFIIPNVVKYSPNCKLLIVSNPVDILTYVAWKISGFPKNRVIGSGCNLDSARFRYLMGERLGVHPLSCHGWVLGEHGDSSVPVWSGMNVAGVSLKTLHPDLGTDKDKEQWKEVHKQVVESAYEVIKLKGYTSWAIGLSVADLAESIMKNLRRVHPVSTMIKGLYGIKDDVFLSVPCILGQNGISDLVKVTLTSEEEARLKKSADTLWGIQKELQF

1054 NP_000390.2 NP_055867.3

>NP_000390.2

MMTAKAVDKIPVTLSGFVHQLSDNIYPVEDLAATSVTIFPNAELGGPFDQMNGVAGDGMINIDMTGEKRSLDLPYPSSFAPVSAPRNQTFTYMGKFSIDPQYPGASCYPEGIINIVSAGILQGVTSPASTTASSSVTSASPNPLATGPLGVCTMSQTQPDLDHLYSPPPPPPPYSGCAGDLYQDPSAFLSAATTSTSSSLAYPPPPSYPSPKPATDPGLFPMIPDYPGFFPSQCQRDLHGTAGPDRKPFPCPLDTLRVPPPLTPLSTIRNFTLGGPSAGVTGPGASGGSEGPRLPGSSSAAAAAAAAAAYNPHHLPLRPILRPRKYPNRPSKTPVHERPYPCPAEGCDRRFSRSDELTRHIRIHTGHKPFQCRICMRNFSRSDHLTTHIRTHTGEKPFACDYCGRKFARSDERKRHTKIHLRQKERKSSAPSASVPAPSTASCSGGVQPGGTLCSSNSSSLGGGPLAPCSSRTRTP

>NP_055867.3

MLLHLCSVKNLYQNRFLGLAAMASPSRNSQSRRRCKEPLRYSYNPDQFHNMDLRGGPHDGVTIPRSTSDTDLVTSDSRSTLMVSSSYYSIGHSQDLVIHWDIKEEVDAGDWIGMYLIDEVLSENFLDYKNRGVNGSHRGQIIWKIDASSYFVEPETKICFKYYHGVSGALRATTPSVTVKNSAAPIFKSIGADETVQGQGSRRLISFSLSDFQAMGLKKGMFFNPDPYLKISIQPGKHSIFPALPHHGQERRSKIIGNTVNPIWQAEQFSFVSLPTDVLEIEVKDKFAKSRPIIKRFLGKLSMPVQRLLERHAIGDRVVSYTLGRRLPTDHVSGQLQFRFEITSSIHPDDEEISLSTEPESAQIQDSPMNNLMESGSGEPRSEAPESSESWKPEQLGEGSVPDGPGNQSIELSRPAEEAAVITEAGDQGMVSVGPEGAGELLAQVQKDIQPAPSAEELAEQLDLGEEASALLLEDGEAPASTKEEPLEEEATTQSRAGREEEEKEQEEEGDVSTLEQGEGRLQLRASVKRKSRPCSLPVSELETVIASACGDPETPRTHYIRIHTLLHSMPSAQGGSAAEEEDGAEEESTLKDSSEKDGLSEVDTVAADPSALEEDREEPEGATPGTAHPGHSGGHFPSLANGAAQDGDTHPSTGSESDSSPRQGGDHSCEGCDASCCSPSCYSSSCYSTSCYSSSCYSASCYSPSCYNGNRFASHTRFSSVDSAKISESTVFSSQDDEEEENSAFESVPDSMQSPELDPESTNGAGPWQDELAAPSGHVERSPEGLESPVAGPSNRREGECPILHNSQPVSQLPSLRPEHHHYPTIDEPLPPNWEARIDSHGRVFYVDHVNRTTTWQRPTAAATPDGMRRSGSIQQMEQLNRRYQNIQRTIATERSEEDSGSQSCEQAPAGGGGGGGSDSEAESSQSSLDLRREGSLSPVNSQKITLLLQSPAVKFITNPEFFTVLHANYSAYRVFTSSTCLKHMILKVRRDARNFERYQHNRDLVNFINMFADTRLELPRGWEIKTDQQGKSFFVDHNSRATTFIDPRIPLQNGRLPNHLTHRQHLQRLRSYSAGEASEVSRNRGASLLARPGHSLVAAIRSQHQHESLPLAYNDKIVAFLRQPNIFEMLQERQPSLARNHTLREKIHYIRTEGNHGLEKLSCDADLVILLSLFEEEIMSYVPLQAAFHPGYSFSPRCSPCSSPQNSPGLQRASARAPSPYRRDFEAKLRNFYRKLEAKGFGQGPGKIKLIIRRDHLLEGTFNQVMAYSRKELQRNKLYVTFVGEEGLDYSGPSREFFFLLSQELFNPYYGLFEYSANDTYTVQISPMSAFVENHLEWFRFSGRILGLALIHQYLLDAFFTRPFYKALLRLPCDLSDLEYLDEEFHQSLQWMKDNNITDILDLTFTVNEEVFGQVTERELKSGGANTQVTEKNKKEYIERMVKWRVERGVVQQTEALVRGFYEVVDSRLVSVFDARELELVIAGTAEIDLNDWRNNTEYRGGYHDGHLVIRWFWAAVERFNNEQRLRLLQFVTGTSSVPYEGFAALRGSNGLRRFCIEKWGKITSLPRAHTCFNRLDLPPYPSYSMLYEKLLTAVEETSTFGLE

1055 Q6NXT2 NP_060217.1

>Q6NXT2

MARTKQTARKSTGGKAPRKQLATKAARKSTPSTCGVKPHRYRPGTVALREIRRYQKSTELLIRKLPFQRLVREIAQDFNTDLRFQSAAVGALQEASEAYLVGLLEDTNLCAIHAKRVTIMPKDIQLARRIRGERA

>NP_060217.1

MLTDSGGGGTSFEEDLDSVAPRSAPAGASEPPPPGGVGLGIRTVRLFGEAGPASGVGSSGGGGSGSGTGGGDAALDFKLAAAVLRTGGGGGASGSDEDEVSEVESFILDQEDLDNPVLKTTSEIFLSSTAEGADLRTVDPETQARLEALLEAAGIGKLSTADGKAFADPEVLRRLTSSVSCALDEAAAALTRMKAENSHNAGQVDTRSLAEACSDGDVNAVRKLLDEGRSVNEHTEEGESLLCLACSAGYYELAQVLLAMHANVEDRGNKGDITPLMAASSGGYLDIVKLLLLHDADVNSQSATGNTALTYACAGGFVDIVKVLLNEGANIEDHNENGHTPLMEAASAGHVEVARVLLDHGAGINTHSNEFKESALTLACYKGHLDMVRFLLEAGADQEHKTDEMHTALMEACMDGHVEVARLLLDSGAQVNMPADSFESPLTLAACGGHVELAALLIERGANLEEVNDEGYTPLMEAAREGHEEMVALLLAQGANINAQTEETQETALTLACCGGFSEVADFLIKAGADIELGCSTPLMEASQEGHLELVKYLLASGANVHATTATGDTALTYACENGHTDVADVLLQAGADLEHESEGGRTPLMKAARAGHLCTVQFLISKGANVNRATANNDHTVVSLACAGGHLAVVELLLAHGADPTHRLKDGSTMLIEAAKGGHTNVVSYLLDYPNNVLSVPTTDVSQLPPPSQDQSQVPRVPTHTLAMVVPPQEPDRTSQENSPALLGVQKGTSKQKSSSLQVADQDLLPSFHPYQPLECIVEETEGKLNELGQRISAIEKAQLKSLELIQGEPLNKDKIEELKKNREEQVQKKKKILKELQKVERQLQMKTQQQFTKEYLETKGQKDTVSLHQQCSHRGVFPEGEGDGSLPEDHFSELPQVDTILFKDNDVDDEQQSPPSAEQIDFVPVQPLSSPQCNFSSDLGSNGTNSLELQKVSGNQQIVGQPQIAITGHDQGLLVQEPDGLMVATPAQTLTDTLDDLIAAVSTRVPTGSNSSSQTTECLTPESCSQTTSNVASQSMPPVYPSVDIDAHTESNHDTALTLACAGGHEELVSVLIARDAKIEHRDKKGFTPLILAATAGHVGVVEILLDKGGDIEAQSERTKDTPLSLACSGGRQEVVDLLLARGANKEHRNVSDYTPLSLAASGGYVNIIKILLNAGAEINSRTGSKLGISPLMLAAMNGHVPAVKLLLDMGSDINAQIETNRNTALTLACFQGRAEVVSLLLDRKANVEHRAKTGLTPLMEAASGGYAEVGRVLLDKGADVNAPPVPSSRDTALTIAADKGHYKFCELLIHRGAHIDVRNKKGNTPLWLASNGGHFDVVQLLVQAGADVDAADNRKITPLMSAFRKGHVKVVQYLVKEVNQFPSDIECMRYIATITDKELLKKCHQCVETIVKAKDQQAAEANKNASILLKELDLEKSREESRKQALAAKREKRKEKRKKKKEEQKRKQEEDEENKPKENSELPEDEDEEENDEDVEQEVPIEPPSATTTTTIGISATSATFTNVFGKKRANVVTTPSTNRKNKKNKTKETPPTAHLILPEQHMSLAQQKADKNKINGEPRGGGAGGNSDSDNLDSTDCNSESSSGGKSQELNFVMDVNSSKYPSLLLHSQEEKTSTATSKTQTRLEGEVTPNSLSTSYKTVSLPLSSPNIKLNLTSPKRGQKREEGWKEVVRRSKKLSVPASVVSRIMGRGGCNITAIQDVTGAHIDVDKQKDKNGERMITIRGGTESTRYAVQLINALIQDPAKELEDLIPKNHIRTPASTKSIHANFSSGVGTTAASSKNAFPLGAPTLVTSQATTLSTFQPANKLNKNVPTNVRSSFPVSLPLAYPHPHFALLAAQTMQQIRHPRLPMAQFGGTFSPSPNTWGPFPVRPVNPGNTNSSPKHNNTSRLPNQNGTVLPSESAGLATASCPITVSSVVAASQQLCVTNTRTPSSVRKQLFACVPKTSPPATVISSVTSTCSSLPSVSSAPITSGQAPTTFLPASTSQAQLSSQKMESFSAVPPTKEKVSTQDQPMANLCTPSSTANSCSSSASNTPGAPETHPSSSPTPTSSNTQEEAQPSSVSDLSPMSMPFASNSEPAPLTLTSPRMVAADNQDTSNLPQLAVPAPRVSHRMQPRGSFYSMVPNATIHQDPQSIFVTNPVTLTPPQGPPAAVQLSSAVNIMNGSQMHINPANKSLPPTFGPATLFNHFSSLFDSSQVPANQGWGDGPLSSRVATDASFTVQSAFLGNSVLGHLENMHPDNSKAPGFRPPSQRVSTSPVGLPSIDPSGSSPSSSSAPLASFSGIPGTRVFLQGPAPVGTPSFNRQHFSPHPWTSASNSSTSAPPTLGQPKGVSASQDRKIPPPIGTERLARIRQGGSVAQAPAGTSFVAPVGHSGIWSFGVNAVSEGLSGWSQSVMGNHPMHQQLSDPSTFSQHQPMERDDSGMVAPSNIFHQPMASGFVDFSKGLPISMYGGTIIPSHPQLADVPGGPLFNGLHNPDPAWNPMIKVIQNSTECTDAQQIWPGTWAPHIGNMHLKYVN

1056 NP_005332.1 NP_055867.3

>NP_005332.1

MDGSFVQHSVRVLQELNKQREKGQYCDATLDVGGLVFKAHWSVLACCSHFFQSLYGDGSGGSVVLPAGFAEIFGLLLDFFYTGHLALTSGNRDQVLLAARELRVPEAVELCQSFKPKTSVGQAAGGQSGLGPPASQNVNSHVKEPAGLEEEEVSRTLGLVPRDQEPRGSHSPQRPQLHSPAQSEGPSSLCGKLKQALKPCPLEDKKPEDCKVPPRPLEAEGAQLQGGSNEWEVVVQVEDDGDGDYMSEPEAVLTRRKSNVIRKPCAAEPALSAGSLAAEPAENRKGTAVPVECPTCHKKFLSKYYLKVHNRKHTGEKPFECPKCGKCYFRKENLLEHEARNCMNRSEQVFTCSVCQETFRRRMELRVHMVSHTGEMPYKCSSCSQQFMQKKDLQSHMIKLHGAPKPHACPTCAKCFLSRTELQLHEAFKHRGEKLFVCEECGHRASSRNGLQMHIKAKHRNERPHVCEFCSHAFTQKANLNMHLRTHTGEKPFQCHLCGKTFRTQASLDKHNRTHTGERPFSCEFCEQRFTEKGPLLRHVASRHQEGRPHFCQICGKTFKAVEQLRVHVRRHKGVRKFECTECGYKFTRQAHLRRHMEIHDRVENYNPRQRKLRNLIIEDEKMVVVALQPPAELEVGSAEVIVESLAQGGLASQLPGQRLCAEESFTGPGVLEPSLIITAAVPEDCDT

>NP_055867.3

MLLHLCSVKNLYQNRFLGLAAMASPSRNSQSRRRCKEPLRYSYNPDQFHNMDLRGGPHDGVTIPRSTSDTDLVTSDSRSTLMVSSSYYSIGHSQDLVIHWDIKEEVDAGDWIGMYLIDEVLSENFLDYKNRGVNGSHRGQIIWKIDASSYFVEPETKICFKYYHGVSGALRATTPSVTVKNSAAPIFKSIGADETVQGQGSRRLISFSLSDFQAMGLKKGMFFNPDPYLKISIQPGKHSIFPALPHHGQERRSKIIGNTVNPIWQAEQFSFVSLPTDVLEIEVKDKFAKSRPIIKRFLGKLSMPVQRLLERHAIGDRVVSYTLGRRLPTDHVSGQLQFRFEITSSIHPDDEEISLSTEPESAQIQDSPMNNLMESGSGEPRSEAPESSESWKPEQLGEGSVPDGPGNQSIELSRPAEEAAVITEAGDQGMVSVGPEGAGELLAQVQKDIQPAPSAEELAEQLDLGEEASALLLEDGEAPASTKEEPLEEEATTQSRAGREEEEKEQEEEGDVSTLEQGEGRLQLRASVKRKSRPCSLPVSELETVIASACGDPETPRTHYIRIHTLLHSMPSAQGGSAAEEEDGAEEESTLKDSSEKDGLSEVDTVAADPSALEEDREEPEGATPGTAHPGHSGGHFPSLANGAAQDGDTHPSTGSESDSSPRQGGDHSCEGCDASCCSPSCYSSSCYSTSCYSSSCYSASCYSPSCYNGNRFASHTRFSSVDSAKISESTVFSSQDDEEEENSAFESVPDSMQSPELDPESTNGAGPWQDELAAPSGHVERSPEGLESPVAGPSNRREGECPILHNSQPVSQLPSLRPEHHHYPTIDEPLPPNWEARIDSHGRVFYVDHVNRTTTWQRPTAAATPDGMRRSGSIQQMEQLNRRYQNIQRTIATERSEEDSGSQSCEQAPAGGGGGGGSDSEAESSQSSLDLRREGSLSPVNSQKITLLLQSPAVKFITNPEFFTVLHANYSAYRVFTSSTCLKHMILKVRRDARNFERYQHNRDLVNFINMFADTRLELPRGWEIKTDQQGKSFFVDHNSRATTFIDPRIPLQNGRLPNHLTHRQHLQRLRSYSAGEASEVSRNRGASLLARPGHSLVAAIRSQHQHESLPLAYNDKIVAFLRQPNIFEMLQERQPSLARNHTLREKIHYIRTEGNHGLEKLSCDADLVILLSLFEEEIMSYVPLQAAFHPGYSFSPRCSPCSSPQNSPGLQRASARAPSPYRRDFEAKLRNFYRKLEAKGFGQGPGKIKLIIRRDHLLEGTFNQVMAYSRKELQRNKLYVTFVGEEGLDYSGPSREFFFLLSQELFNPYYGLFEYSANDTYTVQISPMSAFVENHLEWFRFSGRILGLALIHQYLLDAFFTRPFYKALLRLPCDLSDLEYLDEEFHQSLQWMKDNNITDILDLTFTVNEEVFGQVTERELKSGGANTQVTEKNKKEYIERMVKWRVERGVVQQTEALVRGFYEVVDSRLVSVFDARELELVIAGTAEIDLNDWRNNTEYRGGYHDGHLVIRWFWAAVERFNNEQRLRLLQFVTGTSSVPYEGFAALRGSNGLRRFCIEKWGKITSLPRAHTCFNRLDLPPYPSYSMLYEKLLTAVEETSTFGLE

1057 O00462 Q99469

>O00462

MRLHLLLLLALCGAGTTAAELSYSLRGNWSICNGNGSLELPGAVPGCVHSALFQQGLIQDSYYRFNDLNYRWVSLDNWTYSKEFKIPFEISKWQKVNLILEGVDTVSKILFNEVTIGETDNMFNRYSFDITNVVRDVNSIELRFQSAVLYAAQQSKAHTRYQVPPDCPPLVQKGECHVNFVRKEQCSFSWDWGPSFPTQGIWKDVRIEAYNICHLNYFTFSPIYDKSAQEWNLEIESTFDVVSSKPVGGQVIVAIPKLQTQQTYSIELQPGKRIVELFVNISKNITVETWWPHGHGNQTGYNMTVLFELDGGLNIEKSAKVYFRTVELIEEPIKGSPGLSFYFKINGFPIFLKGSNWIPADSFQDRVTSELLRLLLQSVVDANMNTLRVWGGGIYEQDEFYELCDELGIMVWQDFMFACALYPTDQGFLDSVTAEVAYQIKRLKSHPSIIIWSGNNENEEALMMNWYHISFTDRPIYIKDYVTLYVKNIRELVLAGDKSRPFITSSPTNGAETVAEAWVSQNPNSNYFGDVHFYDYISDCWNWKVFPKARFASEYGYQSWPSFSTLEKVSSTEDWSFNSKFSLHRQHHEGGNKQMLYQAGLHFKLPQSTDPLRTFKDTIYLTQVMQAQCVKTETEFYRRSRSEIVDQQGHTMGALYWQLNDIWQAPSWASLEYGGKWKMLHYFAQNFFAPLLPVGFENENTFYIYGVSDLHSDYSMTLSVRVHTWSSLEPVCSRVTERFVMKGGEAVCLYEEPVSELLRRCGNCTRESCVVSFYLSADHELLSPTNYHFLSSPKEAVGLCKAQITAIISQQGDIFVFDLETSAVAPFVWLDVGSIPGRFSDNGFLMTEKTRTILFYPWEPTSKNELEQSFHVTSLTDIY

>Q99469

MIPPSSPREDGVDGLPKEAVGAEQPPSPASTSSQESKLQKLKRSLSFKTKSLRSKSADNFFQRTNSEDMKLQAHMVAEISPSSSPLPAPGSLTSTPARAGLHPGGKAHAFQEYIFKKPTFCDVCNHMIVGTNAKHGLRCKACKMSIHHKCTDGLAPQRCMGKLPKGFRRYYSSPLLIHEQFGCIKEVMPIACGNKVDPVYETLRFGTSLAQRTKKGSSGSGSDSPHRTSTSDLVEVPEEANGPGGGYDLRKRSNSVFTYPENGTDDFRDPAKNINHQGSLSKDPLQMNTYVALYKFVPQENEDLEMRPGDIITLLEDSNEDWWKGKIQDRIGFFPANFVQRLQQNEKIFRCVRTFIGCKEQGQITLKENQICVSSEEEQDGFIRVLSGKKKGLIPLDVLENI

1058 NP_037479.1 NP_612393.2

>NP_037479.1

MSKSGFGSYGSISAADGASGGSDQLCERDATPAIKTQRPKVRIQDVVPCNVNQLLSSTVFDPVFKVRGIIVSQVSIVGVIRGAEKASNHICYKIDDMTAKPIEARQWFGREKVKQVTPLSVGVYVKVFGILKCPTGTKSLEVLKIHVLEDMNEFTVHILETVNAHMMLDKARRDTTVESVPVSPSEVNDAGDNDESHRNFIQDEVLRLIHECPHQEGKSIHELRAQLCDLSVKAIKEAIDYLTVEGHIYPTVDREHFKSAD

>NP_612393.2

MRLTPRALCSAAQAAWRENFPLCGRDVARWFPGHMAKGLKKMQSSLKLVDCIIEVHDARIPLSGRNPLFQETLGLKPHLLVLNKMDLADLTEQQKIMQHLEGEGLKNVIFTNCVKDENVKQIIPMVTELIGRSHRYHRKENLEYCIMVIGVPNVGKSSLINSLRRQHLRKGKATRVGGEPGITRAVMSKIQVSERPLMFLLDTPGVLAPRIESVETGLKLALCGTVLDHLVGEETMADYLLYTLNKHQRFGYVQHYGLGSACDNVERVLKSVAVKLGKTQKVKVLTGTGNVNIIQPNYPAAARDFLQTFRRGLLGSVMLDLDVLRGHPPAETLP

1059 O43248 NP_055463.1

>O43248

MFNSVNLGNFCSPSRKERGADFGERGSCASNLYLPSCTYYMPEFSTVSSFLPQAPSRQISYPYSAQVPPVREVSYGLEPSGKWHHRNSYSSCYAAADELMHRECLPPSTVTEILMKNEGSYGGHHHPSAPHATPAGFYSSVNKNSVLPQAFDRFFDNAYCGGGDPPAEPPCSGKGEAKGEPEAPPASGLASRAEAGAEAEAEEENTNPSSSGSAHSVAKEPAKGAAPNAPRTRKKRCPYSKFQIRELEREFFFNVYINKEKRLQLSRMLNLTDRQVKIWFQNRRMKEKKLSRDRLQYFSGNPLL

>NP_055463.1

MDSLPDEFFVRHPAVEDQRKEETENKLEKSSGQLNKQENDIPTDLVPVNLLLEVKKLLNAINTLPKGVVPHIKKFLQEDFSFQTMQREVAANSQNGEEIVPALTLRFLITQLEAALRNIQAGNYTAHQINIGYYLTLLFLYGVALTERGKKEDYTEAENKFLVMKMMIQENEICENFMSLVYFGRGLLRCAQKRYNGGLLEFHKSLQEIGDKNDHWFDIDPTEDEDLPTTFKDLLNNFIKTTESNIMKQTICSYLDCERSCEADILKNTSYKGFFQLMCSKSCCVYFHKICWKKFKNLKYPGENDQSFSGKKCLKEGCTGDMVRMLQCDVPGIVKILFEVVRKDEYITIENLGASYRKLISLKITDTDIRPKISLKFNTKDEMPIFKLDYNYFYHLLHIIIISGTDIVRQIFDEAMPPPLLKKELLIHKNVLESYYNHLWTNHPLGGSWHLLYPPNKELPQSKQFDLCLLLALIKHLNVFPAPKKGWNMEPPSSDISKSADILRLCKYRDILLSEILMNGLTESQFNSIWKKVSDILLRLGMMQEDIDKVKENPIENISLDYHQLSVYLGIPVPEIIQRMLSCYQQGIALQSITGSQRIEIEELQNEEEELSPPLMEYNINVKSHPEIQFAEINKDGTSIPSESSTESLKDLQEVKSKQRKKKKTKNKKNKDSKEDQVPYVVEKEEQLRKEQANPHSVSRLIKDDASDVQEDSAMEDKFYSLDELHILDMIEQGSAGKVTTDYGETEKERLARQRQLYKLHYQCEDFKRQLRTVTFRWQENQMQIKKKDKIIASLNQQVAFGINKVSKLQRQIHAKDNEIKNLKEQLSMKRSQWEMEKHNLESTMKTYVSKLNAETSRALTAEVYFLQCRRDFGLLHLEQTEKECLNQLARVTHMAASNLESLQLKAAVDSWNAIVADVRNKIAFLRTQYNEQINKVKQGFALSTLPPVQLPPPPPSPEILMQQFLGRPLVKESFFRPILTVPQMPAVCPGVVSATGQPRAPLMTGIAWALPAPVGDAVPPSAGLRSDPSIMNWERITDRLKTAFPQQTRKELTDFLRKLKDAYGKSLSELTFDEIVCKISQFIDPKKSQSQGKSVSNVNCVSPSHSPSQPDAAQPPKPAWRPLTSQGPATWEGASNPDEEEEEEEPCVICHENLSPENLSVLPCAHKFHAQCIRPWLMQQGTCPTCRLHVLLPEEFPGHPSRQLPKI

1060 NP_003400.2 O15131

>NP_003400.2

MEFFISMSETIKYNDDDHKTLFLKTLNEQRLEGEFCDIAIVVEDVKFRAHRCVLAACSTYFKKLFKKLEVDSSSVIEIDFLRSDIFEEVLNYMYTAKISVKKEDVNLMMSSGQILGIRFLDKLCSQKRDVSSPDENNGQSKSKYCLKINRPIGDAADTQDDDVEEIGDQDDSPSDDTVEGTPPSQEDGKSPTTTLRVQEAILKELGSEEVRKVNCYGQEVESMETPESKDLGSQTPQALTFNDGMSEVKDEQTPGWTTAASDMKFEYLLYGHHREQIACQACGKTFSDEGRLRKHEKLHTADRPFVCEMCTKGFTTQAHLKEHLKIHTGYKPYSCEVCGKSFIRAPDLKKHERVHSNERPFACHMCDKAFKHKSHLKDHERRHRGEKPFVCGSCTKAFAKASDLKRHENNMHSERKQVTPSAIQSETEQLQAAAMAAEAEQQLETIACS

>O15131

MASPGKDNYRMKSYKNKALNPQEMRRRREEEGIQLRKQKREEQLFKRRNVYLPRNDESMLESPIQDPDISSTVPIPEEEVVTTDMVQMIFSNNADQQLTATQKFRKLLSKEPNPPIDQVIQKPGVVQRFVKFLERNENCTLQFEAAWALTNIASGTFLHTKVVIETGAVPIFIKLLNSEHEDVQEQAVWALGNIAGDNAECRDFVLNCEILPPLLELLTNSNRLTTTRNAVWALSNLCRGKNPPPNFSKVSPCLNVLSRLLFSSDPDVLADVCWALSYLSDGPNDKIQAVIDSGVCRRLVELLMHNDYKVVSPALRAVGNIVTGDDIQTQVILNCSALPCLLHLLSSPKESIRKEACWTVSNITAGNRAQIQAVIDANIFPVLIEILQKAEFRTRKEAAWAITNATSGGTPEQIRYLVALGCIKPLCDLLTVMDSKIVQVALNGLENILRLGEQESKQNGIGINPYCALIEEAYGLDKIEFLQSHENQEIYQKAFDLIEHYFGVEEDDPSIVPQVDENQQQFIFQQQEAPMDGFQL

1061 P0C1S8 NP_004754.1

>P0C1S8

MDDKDIDKELRQKLNFSYCEETEIEGQKKVEESREASSQTPEKGEVQDSEAKGTPPWTPLSNVHELDTSSEKDKESPDQILRTPVSHPLKCPETPAQPDSRSKLLPSDSPSTPKTMLSRLVISPTGKLPSRGPKHLKLTPAPLKDEMTSLALVNINPFTPESYKKLFLQSGGKRKIRGDLEEAGPEEGKGGLPAKRCVLRETNMASRYEKEFLEVEKIGVGEFGTVYKCIKRLDGCVYAIKRSMKTFTELSNENSALHEVYAHAVLGHHPHVVRYYSSWAEDDHMIIQNEYCNGGSLQAAISENTKSGNHFEEPKLKDILLQISLGLNYIHNSSMVHLDIKPSNIFICHKMQSESSGVIEEVENEADWFLSANVMYKIGDLGHATSINKPKVEEGDSRFLANEILQEDYRHLPKADIFALGLTIAVAAGAESLPTNGAAWHHIRKGNFPDVPQELSESFSSLLKNMIQPDAEQRPSAAALARNTVLRPSLGKTEELQQQLNLEKFKTATLERELREAQQAQSPQGYTHHGDTGVSGTHTGSRSTKRLVGGKSARSSSFTSGEREPLH

>NP_004754.1

MFRKGKKRHSSSSSQSSEISTKSKSVDSSLGGLSRSSTVASLDTDSTKSSGQSNNNSDTCAEFRIKYVGAIEKLKLSEGKGLEGPLDLINYIDVAQQDGKLPFVPPEEEFIMGVSKYGIKVSTSDQYDVLHRHALYLIIRMVCYDDGLGAGKSLLALKTTDASNEEYSLWVYQCNSLEQAQAICKVLSTAFDSVLTSEKP

1062 NP_001281.1 NP_003354.2

>NP_001281.1

MSSTPHDPFYSSPFGPFYRRHTPYMVQPEYRIYEMNKRLQSRTEDSDNLWWDAFATEFFEDDATLTLSFCLEDGPKRYTIGRTLIPRYFSTVFEGGVTDLYYILKHSKESYHNSSITVDCDQCTMVTQHGKPMFTKVCTEGRLILEFTFDDLMRIKTWHFTIRQYRELVPRSILAMHAQDPQVLDQLSKNITRMGLTNFTLNYLRLCVILEPMQELMSRHKTYNLSPRDCLKTCLFQKWQRMVAPPAEPTRQPTTKRRKRKNSTSSTSNSSAGNNANSTGSKKKTTAANLSLSSQVPDVMVVGEPTLMGGEFGDEDERLITRLENTQYDAANGMDDEEDFNNSPALGNNSPWNSKPPATQETKSENPPPQASQ

>NP_003354.2

MAEGGGCRERPDAETQKSELGPLMRTTLQRGAQWYLIDSRWFKQWKKYVGFDSWDMYNVGEHNLFPGPIDNSGLFSDPESQTLKEHLIDELDYVLVPTEAWNKLLNWYGCVEGQQPIVRKVVEHGLFVKHCKVEVYLLELKLCENSDPTNVLSCHFSKADTIATIEKEMRKLFNIPAERETRLWNKYMSNTYEQLSKLDNTVQDAGLYQGQVLVIEPQNEDGTWPRQTLQSKSSTAPSRNFTTSPKSSASPYSSVSASLIANGDSTSTCGMHSSGVSRGGSGFSASYNCQEPPSSHIQPGLCGLGNLGNTCFMNSALQCLSNTAPLTDYFLKDEYEAEINRDNPLGMKGEIAEAYAELIKQMWSGRDAHVAPRMFKTQVGRFAPQFSGYQQQDSQELLAFLLDGLHEDLNRVKKKPYLELKDANGRPDAVVAKEAWENHRLRNDSVIVDTFHGLFKSTLVCPECAKVSVTFDPFCYLTLPLPLKKDRVMEVFLVPADPHCRPTQYRVTVPLMGAVSDLCEALSRLSGIAAENMVVADVYNHRFHKIFQMDEGLNHIMPRDDIFVYEVCSTSVDGSECVTLPVYFRERKSRPSSTSSASALYGQPLLLSVPKHKLTLESLYQAVCDRISRYVKQPLPDEFGSSPLEPGACNGSRNSCEGEDEEEMEHQEEGKEQLSETEGSGEDEPGNDPSETTQKKIKGQPCPKRLFTFSLVNSYGTADINSLAADGKLLKLNSRSTLAMDWDSETRRLYYDEQESEAYEKHVSMLQPQKKKKTTVALRDCIELFTTMETLGEHDPWYCPNCKKHQQATKKFDLWSLPKILVVHLKRFSYNRYWRDKLDTVVEFPIRGLNMSEFVCNLSARPYVYDLIAVSNHYGAMGVGHYTAYAKNKLNGKWYYFDDSNVSLASEDQIVTKAAYVLFYQRRDDEFYKTPSLSSSGSSDGGTRPSSSQQGFGDDEACSMDTN

1063 NP_002097.1 Q13938

>NP_002097.1

MAGGKAGKDSGKAKTKAVSRSQRAGLQFPVGRIHRHLKSRTTSHGRVGATAAVYSAAILEYLTAEVLELAGNASKDLKVKRITPRHLQLAIRGDEELDSLIKATIAGGGVIPHIHKSLIGKKGQQKTV

>Q13938

MDAVDATMEKLRAQCLSRGASGIQGLARFFRQLDRDGSRSLDADEFRQGLAKLGLVLDQAEAEGVCRKWDRNGSGTLDLEEFLRALRPPMSQAREAVIAAAFAKLDRSGDGVVTVDDLRGVYSGRAHPKVRSGEWTEDEVLRRFLDNFDSSEKDGQVTLAEFQDYYSGVSASMNTDEEFVAMMTSAWQL

1064 Q9UHQ1 Q9NP92

>Q9UHQ1

MKCEHCTRKECSKKTKTDDQENVSADAPSPAQENGEKGEFHKLADAKIFLSDCLACDSCMTAEEGVQLSQQNAKDFFRVLNLNKKCDTSKHKVLVVSVCPQSLPYFAAKFNLSVTDASRRLCGFLKSLGVHYVFDTTIAADFSILESQKEFVRRYRQHSEEERTLPMLTSACPGWVRYAERVLGRPITAHLCTAKSPQQVMGSLVKDYFARQQNLSPEKIFHVIVAPCYDKKLEALQESLPPALHGSRGADCVLTSGEIAQIMEQGDLSVRDAAVDTLFGDLKEDKVTRHDGASSDGHLAHIFRHAAKELFNEDVEEVTYRALRNKDFQEVTLEKNGEVVLRFAAAYGFRNIQNMILKLKKGKFPFHFVEVLACAGGCLNGRGQAQTPDGHADKALLRQMEGIYADIPVRRPESSAHVQELYQEWLEGINSPKAREVLHTTYQSQERGTHSLDIKW

>Q9NP92

MAAARCWRPLLRGPRLSLHTAANAAATATETTCQDVAATPVARYPPIVASMTADSKAARLRRIERWQATVHAAESVDEKLRILTKMQFMKYMVYPQTFALNADRWYQYFTKTVFLSGLPPPPAEPEPEPEPEPEPALDLAALRAVACDCLLQEHFYLRRRRRVHRYEESEVISLPFLDQLVSTLVGLLSPHNPALAAAALDYRCPVHFYWVRGEEIIPRGHRRGRIDDLRYQIDDKPNNQIRISKQLAEFVPLDYSVPIEIPTIKCKPDKLPLFKRQYENHIFVGSKTADPCCYGHTQFHLLPDKLRRERLLRQNCADQIEVVFRANAIASLFAWTGAQAMYQGFWSEADVTRPFVSQAVITDGKYFSFFCYQLNTLALTTQADQNNPRKNICWGTQSKPLYETIEDNDVKGFNDDVLLQIVHFLLNRPKEEKSQLLEN

1065 P19388 NP_001631.3

>P19388

MDDEEETYRLWKIRKTIMQLCHDRGYLVTQDELDQTLEEFKAQSGDKPSEGRPRRTDLTVLVAHNDDPTDQMFVFFPEEPKVGIKTIKVYCQRMQEENITRALIVVQQGMTPSAKQSLVDMAPKYILEQFLQQELLINITEHELVPEHVVMTKEEVTELLARYKLRENQLPRIQAGDPVARYFGIKRGQVVKIIRPSETAGRYITYRLVQ

>NP_001631.3

MERQVLLSEPEEAAALYRGLSRQPALSAACLGPEVTTQYGGQYRTVHTEWTQRDLERMENIRFCRQYLVFHDGDSVVFAGPAGNSVETRGELLSRESPSGTMKAVLRKAGGTGPGEEKQFLEVWEKNRKLKSFNLSALEKHGPVYEDDCFGCLSWSHSETHLLYVAEKKRPKAESFFQTKALDVSASDDEIARLKKPDQAIKGDQFVFYEDWGENMVSKSIPVLCVLDVESGNISVLEGVPENVSPGQAFWAPGDAGVVFVGWWHEPFRLGIRFCTNRRSALYYVDLIGGKCELLSDDSLAVSSPRLSPDQCRIVYLQYPSLIPHHQCSQLCLYDWYTKVTSVVVDVVPRQLGENFSGIYCSLLPLGCWSADSQRVVFDSAQRSRQDLFAVDTQVGTVTSLTAGGSGGSWKLLTIDQDLMVAQFSTPSLPPTLKVGFLPSAGKEQSVLWVSLEEAEPIPDIHWGIRVLQPPPEQENVQYAGLDFEAILLQPGSPPDKTQVPMVVMPHGGPHSSFVTAWMLFPAMLCKMGFAVLLVNYRGSTGFGQDSILSLPGNVGHQDVKDVQFAVEQVLQEEHFDASHVALMGGSHGGFISCHLIGQYPETYRACVARNPVINIASMLGSTDIPDWCVVEAGFPFSSDCLPDLSVWAEMLDKSPIRYIPQVKTPLLLMLGQEDRRVPFKQGMEYYRALKTRNVPVRLLLYPKSTHALSEVEVESDSFMNAVLWLRTHLGS

1066 Q04727 NP_002930.2

>Q04727

MIRDLSKMYPQTRHPAPHQPAQPFKFTISESCDRIKEEFQFLQAQYHSLKLECEKLASEKTEMQRHYVMYYEMSYGLNIEMHKQAEIVKRLNAICAQVIPFLSQEHQQQVVQAVERAKQVTMAELNAIIGQQLQAQHLSHGHGLPVPLTPHPSGLQPPAIPPIGSSAGLLALSSALGGQSHLPIKDEKKHHDNDHQRDRDSIKSSSVSPSASFRGAEKHRNSADYSSESKKQKTEEKEIAARYDSDGEKSDDNLVVDVSNEDPSSPRGSPAHSPRENGLDKTRLLKKDAPISPASIASSSSTPSSKSKELSLNEKSTTPVSKSNTPTPRTDAPTPGSNSTPGLRPVPGKPPGVDPLASSLRTPMAVPCPYPTPFGIVPHAGMNGELTSPGAAYAGLHNISPQMSAAAAAAAAAAAYGRSPVVGFDPHHHMRVPAIPPNLTGIPGGKPAYSFHVSADGQMQPVPFPPDALIGPGIPRHARQINTLNHGEVVCAVTISNPTRHVYTGGKGCVKVWDISHPGNKSPVSQLDCLNRDNYIRSCRLLPDGRTLIVGGEASTLSIWDLAAPTPRIKAELTSSAPACYALAISPDSKVCFSCCSDGNIAVWDLHNQTLVRQFQGHTDGASCIDISNDGTKLWTGGLDNTVRSWDLREGRQLQQHDFTSQIFSLGYCPTGEWLAVGMENSNVEVLHVTKPDKYQLHLHESCVLSLKFAHCGKWFVSTGKDNLLNAWRTPYGASIFQSKESSSVLSCDISVDDKYIVTGSGDKKATVYEVIY

>NP_002930.2

MSLDIQSLDIQCEELSDARWAELLPLLQQCQVVRLDDCGLTEARCKDISSALRVNPALAELNLRSNELGDVGVHCVLQGLQTPSCKIQKLSLQNCCLTGAGCGVLSSTLRTLPTLQELHLSDNLLGDAGLQLLCEGLLDPQCRLEKLQLEYCSLSAASCEPLASVLRAKPDFKELTVSNNDINEAGVRVLCQGLKDSPCQLEALKLESCGVTSDNCRDLCGIVASKASLRELALGSNKLGDVGMAELCPGLLHPSSRLRTLWIWECGITAKGCGDLCRVLRAKESLKELSLAGNELGDEGARLLCETLLEPGCQLESLWVKSCSFTAACCSHFSSVLAQNRFLLELQISNNRLEDAGVRELCQGLGQPGSVLRVLWLADCDVSDSSCSSLAATLLANHSLRELDLSNNCLGDAGILQLVESVRQPGCLLEQLVLYDIYWSEEMEDRLQALEKDKPSLRVIS

1067 NP_036377.1 NP_004799.1

>NP_036377.1

MALTSFLPAPTQLSQDQLEAEEKARSQRSRQTSLVSSRREPPPYGYRKGWIPRLLEDFGDGGAFPEIHVAQYPLDMGRKKKMSNALAIQVDSEGKIKYDAIARQGQSKDKVIYSKYTDLVPKEVMNADDPDLQRPDEEAIKEITEKTRVALEKSVSQKVAAAMPVRAADKLAPAQYIRYTPSQQGVAFNSGAKQRVIRMVEMQKDPMEPPRFKINKKIPRGPPSPPAPVMHSPSRKMTVKEQQEWKIPPCISNWKNAKGYTIPLDKRLAADGRGLQTVHINENFAKLAEALYIADRKAREAVEMRAQVERKMAQKEKEKHEEKLREMAQKARERRAGIKTHVEKEDGEARERDEIRHDRRKERQHDRNLSRAAPDKRSKLQRNENRDISEVIALGVPNPRTSNEVQYDQRLFNQSKGMDSGFAGGEDEIYNVYDQAWRGGKDMAQSIYRPSKNLDKDMYGDDLEARIKTNRFVPDKEFSGSDRRQRGREGPVQFEEDPFGLDKFLEEAKQHGGSKRPSDSSRPKEHEHEGKKRRKE

>NP_004799.1

MAEDSESAASQQSLELDDQDTCGIDGDNEEETEHAKGSPGGYLGAKKKKKKQKRKKEKPNSGGTKSDSASDSQEIKIQQPSKNPSVPMQKLQDIQRAMELLSACQGPARNIDEAAKHRYQFWDTQPVPKLDEVITSHGAIEPDKDNVRQEPYSLPQGFMWDTLDLSDAEVLKELYTLLNENYVEDDDNMFRFDYSPEFLLWALRPPGWLLQWHCGVRVSSNKKLVGFISAIPANIRIYDSVKKMVEINFLCVHKKLRSKRVAPVLIREITRRVNLEGIFQAVYTAGVVLPKPIATCRYWHRSLNPRKLVEVKFSHLSRNMTLQRTMKLYRLPDVTKTSGLRPMEPKDIKSVRELINTYLKQFHLAPVMDEEEVAHWFLPREHIIDTFVVESPNGKLTDFLSFYTLPSTVMHHPAHKSLKAAYSFYNIHTETPLLDLMSDALILAKSKGFDVFNALDLMENKTFLEKLKFGIGDGNLQYYLYNWRCPGTDSEKVGLVLQ

1068 NP_001029027.1 Q9NY74

>NP_001029027.1

MSSFEGQMAEYPTISIDRFDRENLRARAYFLSHCHKDHMKGLRAPTLKRRLECSLKVYLYCSPVTKELLLTSPKYRFWKKRIISIEIETPTQISLVDEASGEKEEIVVTLLPAGHCPGSVMFLFQGNNGTVLYTGDFRLAQGEAARMELLHSGGRVKDIQSVYLDTTFCDPRFYQIPSREECLSGVLELVRSWITRSPYHVVWLNCKAAYGYEYLFTNLSEELGVQVHVNKLDMFRNMPEILHHLTTDRNTQIHACRHPKAEEYFQWSKLPCGITSRNRIPLHIISIKPSTMWFGERSRKTNVIVRTGESSYRACFSFHSSYSEIKDFLSYLCPVNAYPNVIPVGTTMDKVVEILKPLCRSSQSTEPKYKPLGKLKRARTVHRDSEEEDDYLFDDPLPIPLRHKVPYPETFHPEVFSMTAVSEKQPEKLRQTPGCCRAECMQSSRFTNFVDCEESNSESEEEVGIPASLQGDLGSVLHLQKADGDVPQWEVFFKRNDEITDESLENFPSSTVAGGSQSPKLFSDSDGESTHISSQNSSQSTHITEQGSQGWDSQSDTVLLSSQERNSGDITSLDKADYRPTIKENIPASLMEQNVICPKDTYSDLKSRDKDVTIVPSTGEPTTLSSETHIPEEKSLLNLSTNADSQSSSDFEVPSTPEAELPKREHLQYLYEKLATGESIAVKKRKCSLLDT

>Q9NY74

MSRRRKHDDSPSPKKTPHKTVAAEECGSVVEPGRRRLRSARGSWPCGAREGPPGPVRQREQPPTAALCSKSNPEERYETPKRALKMDSLSSSFSSPNDPDGQNDIFWDQNSPLTKQLGKGRKKQIYTTDSDEISHIVNRIAPQDEKPTTNSMLDMWIGETAIPCTPSVAKGKSRAKISCTKLKTQSQEEELMKLAKQFDKNMEELDVIQEQNKRNYDFTQMISETEILSNYKDNIQMWSLHNIVPEIDNATKKPIKGNTKISVANNQNSSQKPFDQIAEAAFNAIFDGSTQKCSGQLSQELPEAFWSTSNTTFVKTNALKEEKIITNETLVIEKLSNKTPRSLSSQVDTPIMTKSCVTSCTKEPETSNKYIDAFTTSDFEDDWENLLGSEPFAMQNIDMPELFPSKTAHVTDQKEICTFNSKTVKNTSRANTSPDARLGDSKVLQDLSSKTYDRELIDAEYRFSPNSNKSNKLSTGNKMKFENSSNKIVIQDEIQNCIVTSNLTKIKEDILTNSTEASERKSALNTRYSNEQKNKCILNQSIKAPVNTDLFGSANLGSKTSVSNPNQTSASKVGSFFDDWNDPSFANEIIKACHQLDNTWEADDVDDDLLYQACDDIERLTQQQDIRKDSKTSESICEINNNSEHGAKLTQQQDIRKDSKTSESICEINNNSEHGAKNMFAISKQGSNLVQSKHLNPGSISVQTSLTNSSQIDKPMKMEKGEMYGNSPRFLGATNLTMYSKISNCQINNLHVSYTNTDVPIQVNSSKLVLPGSSSLNVTSDHMNTEITTYKKKLSTNQPCHKTVTDEAQSNLNTTVGFSKFTFTRMKNSQILSQFNQNCITGSMSDTKITQGVEKKKGVNPLLEEAVGQQSLVKLSESLKQSSKEEEEKNRKCSPEEIQRKRQEALVRRMAKARASSVNAAPTSFL

1069 Q5XKR4 NP_000657.1

>Q5XKR4

MLSHADLLDARLGMKDAAELLGHREAVKCRLGVGGSDPGGHPGDLAPNSDPVEGATLLPGEDITTVGSTPASLAVSAKDPDKQPGPQGGPNPSQAGQQQGQQKQKRHRTRFTPAQLNELERSFAKTHYPDIFMREELALRIGLTESRVQVWFQNRRAKWKKRKKTTNVFRAPGTLLPTPGLPQFPSAAAAAAAAMGDSLCSFHANDTRWAAAAMPGVSQLPLPPALGRQQAMAQSLSQCSLAAGPPPNSMGLSNSLAGSNGAGLQSHLYQPAFPGMVPASLPGPSNVSGSPQLCSSPDSSDVWRGTSIASLRRKALEHTVSMSFT

>NP_000657.1

MTSKGPEEEHPSVTLFRQYLRIRTVQPKPDYGAAVAFFEETARQLGLGCQKVEVAPGYVVTVLTWPGTNPTLSSILLNSHTDVVPVFKEHWSHDPFEAFKDSEGYIYARGAQDMKCVSIQYLEAVRRLKVEGHRFPRTIHMTFVPDEEVGGHQGMELFVQRPEFHALRAGFALDEGIANPTDAFTVFYSERSPWWVRVTSTGRPGHASRFMEDTAAEKLHKVVNSILAFREKEWQRLQSNPHLKEGSVTSVNLTKLEGGVAYNVIPATMSASFDFRVAPDVDFKAFEEQLQSWCQAAGEGVTLEFAQKWMHPQVTPTDDSNPWWAAFSRVCKDMNLTLEPEIMPAATDNRYIRAVGVPALGFSPMNRTPVLLHDHDERLHEAVFLRGVDIYTRLLPALASVPALPSDS

1070 Q99879 Q9NWF9

>Q99879

MPEPVKSAPVPKKGSKKAINKAQKKDGKKRKRSRKESYSVYVYKVLKQVHPDTGISSKAMGIMNSFVNDIFERIAGEASRLAHYNKRSTITSREIQTAVRLLLPGELAKHAVSEGTKAVTKYTSSK

>Q9NWF9

MEEGNNNEEVIHLNNFHCHRGQEWINLRDGPITISDSSDEERIPMLVTPAPQQHEEEDLDDDVILTEDDSEDDYGEFLDLGPPGISEFTKPSGQTEREPKPGPSHNQAANDIVNPRSEQKVIILEEGSLLYTESDPLETQNQSSEDSETELLSNLGESAALADDQAIEEDCWLDHPYFQSLNQQPREITNQVVPQERQPEAELGRLLFQHEFPGPAFPRPEPQQGGISGPSSPQPAHPLGEFEDQQLASDDEEPGPAFPMQESQEPNLENIWGQEAAEVDQELVELLVKETEARFPDVANGFIEEIIHFKNYYDLNVLCNFLLENPDYPKREDRIIINPSSSLLASQDETKLPKIDFFDYSKLTPLDQRCFIQAADLLMADFKVLSSQDIKWALHELKGHYAITRKALSDAIKKWQELSPETSGKRKKRKQMNQYSYIDFKFEQGDIKIEKRMFFLENKRRHCRSYDRRALLPAVQQEQEFYEQKIKEMAEHEDFLLALQMNEEQYQKDGQLIECRCCYGEFPFEELTQCADAHLFCKECLIRYAQEAVFGSGKLELSCMEGSCTCSFPTSELEKVLPQTILYKYYERKAEEEVAAAYADELVRCPSCSFPALLDSDVKRFSCPNPHCRKETCRKCQGLWKEHNGLTCEELAEKDDIKYRTSIEEKMTAARIRKCHKCGTGLIKSEGCNRMSCRCGAQMCYLCRVSINGYDHFCQHPRSPGAPCQECSRCSLWTDPTEDDEKLIEEIQKEAEEEQKRKNGENTFKRIGPPLEKPVEKVQRVEALPRPVPQNLPQPQMPPYAFAHPPFPLPPVRPVFNNFPLNMGPIPAPYVPPLPNVRVNYDFGPIHMPLEHNLPMHFGPQPRHRF

1071 NP_004517.2 P57721

>NP_004517.2

MAESSESFTMASSPAQRRRGNDPLTSSPGRSSRRTDALTSSPGRDLPPFEDESEGLLGTEGPLEEEEDGEELIGDGMERDYRAIPELDAYEAEGLALDDEDVEELTASQREAAERAMRQRDREAGRGLGRMRRGLLYDSDEEDEERPARKRRQVERATEDGEEDEEMIESIENLEDLKGHSVREWVSMAGPRLEIHHRFKNFLRTHVDSHGHNVFKERISDMCKENRESLVVNYEDLAAREHVLAYFLPEAPAELLQIFDEAALEVVLAMYPKYDRITNHIHVRISHLPLVEELRSLRQLHLNQLIRTSGVVTSCTGVLPQLSMVKYNCNKCNFVLGPFCQSQNQEVKPGSCPECQSAGPFEVNMEETIYQNYQRIRIQESPGKVAAGRLPRSKDAILLADLVDSCKPGDEIELTGIYHNNYDGSLNTANGFPVFATVILANHVAKKDNKVAVGELTDEDVKMITSLSKDQQIGEKIFASIAPSIYGHEDIKRGLALALFGGEPKNPGGKHKVRGDINVLLCGDPGTAKSQFLKYIEKVSSRAIFTTGQGASAVGLTAYVQRHPVSREWTLEAGALVLADRGVCLIDEFDKMNDQDRTSIHEAMEQQSISISKAGIVTSLQARCTVIAAANPIGGRYDPSLTFSENVDLTEPIISRFDILCVVRDTVDPVQDEMLARFVVGSHVRHHPSNKEEEGLANGSAAEPAMPNTYGVEPLPQEVLKKYIIYAKERVHPKLNQMDQDKVAKMYSDLRKESMATGSIPITVRHIESMIRMAEAHARIHLRDYVIEDDVNMAIRVMLESFIDTQKFSVMRSMRKTFARYLSFRRDNNELLLFILKQLVAEQVTYQRNRFGAQQDTIEVPEKDLVDKARQINIHNLSAFYDSELFRMNKFSHDLKRKMILQQF

>P57721

MESKVSEGGLNVTLTIRLLMHGKEVGSIIGKKGETVKKMREESGARINISEGNCPERIVTITGPTDAIFKAFAMIAYKFEEDIINSMSNSPATSKPPVTLRLVVPASQCGSLIGKGGSKIKEIRESTGAQVQVAGDMLPNSTERAVTISGTPDAIIQCVKQICVVMLESPPKGATIPYRPKPASTPVIFAGGQAYTIQGQYAIPHPDQLTKLHQLAMQQTPFPPLGQTNPAFPGEKLPLHSSEEAQNLMGQSSGLDASPPASTHELTIPNDLIGCIIGRQGTKINEIRQMSGAQIKIANATEGSSERQITITGTPANISLAQYLINARLTSEVTGMGTL

1072 Q15415 NP_065845.1

>Q15415

MVEADHPGKLFIGGLNRETNEKMLKAVFGKHGPISEVLLIKDRTSKSRGFAFITFENPADAKNAAKDMNGTSLHGKAIKVEQAKKPSFQSGGRRRPPASSRNRSPSGSLRSARGSSGGTRGWLPSHEGHLDDGGYTPDLKMSYSRGLIPVKRGPSSRSGGPPPKKSAPSAVARSNSWMGSQGPMSQRRENYGVPPRRATISSWRNDRMSTRHDGYATNDGNHPSCQETRDYAPPSRGYAYRDNGHSNRDEHSSRGYRNHRSSRETRDYAPPSRGHAYRDYGHSRRDESYSRGYRNHRSSRETREYAPPSRGHGYRDYGHSRRHESYSRGYRNHPSSRETRDYAPPHRDYAYRDYGHSSWDEHSSRGYSYHDGYGEALGRDHSEHLSGSSYRDALQRYGTSHGAPPARGPRMSYGGSTCHAYSNTRDRYGRSWESYSSCGDFHYCDREHVCRKDQRNPPSLGRVLPDPREAYGSSSYVASIVDGGESRSEKGDSSRY

>NP_065845.1

MTTKRKIIGRLVPCRCFRGEEEIISVLDYSHCSLQQVPKEVFNFERTLEELYLDANQIEELPKQLFNCQALRKLSIPDNDLSNLPTTIASLVNLKELDISKNGVQEFPENIKCCKCLTIIEASVNPISKLPDGFTQLLNLTQLYLNDAFLEFLPANFGRLVKLRILELRENHLKTLPKSMHKLAQLERLDLGNNEFGELPEVLDQIQNLRELWMDNNALQVLPGSIGKLKMLVYLDMSKNRIETVDMDISGCEALEDLLLSSNMLQQLPDSIGLLKKLTTLKVDDNQLTMLPNTIGNLSLLEEFDCSCNELESLPSTIGYLHSLRTLAVDENFLPELPREIGSCKNVTVMSLRSNKLEFLPEEIGQMQKLRVLNLSDNRLKNLPFSFTKLKELAALWLSDNQSKALIPLQTEAHPETKQRVLTNYMFPQQPRGDEDFQSDSDSFNPTLWEEQRQQRMTVAFEFEDKKEDDENAGKVKDLSCQAPWERGQRGITLQPARLSGDCCTPWARCDQQIQDMPVPQNDPQLAWGCISGLQQERSMCTPLPVAAQSTTLPSLSGRQVEINLKRYPTPYPEDLKNMVKSVQNLVGKPSHGVRVENSNPTANTEQTVKEKYEHKWPVAPKEITVEDSFVHPANEMRIGELHPSLAETPLYPPKLVLLGKDKKESTDESEVDKTHCLNNSVSSGTYSDYSPSQASSGSSNTRVKVGSLQTTAKDAVHNSLWGNRIAPSFPQPLDSKPLLSQREAVPPGNIPQRPDRLPMSDTFTDNWTDGSHYDNTGFVAEETTAENANSNPLLSSKSRSTSSHGRRPLIRQDRIVGVPLELEQSTHRHTPETEVPPSNPWQNWTRTPSPFEDRTAFPSKLETTPTTSPLPERKEHIKESTEIPSPFSPGVPWEYHDSNPNRSLSNVFSQIHCRPESSKGVISISKSTERLSPLMKDIKSNKFKKSQSIDEIDIGTYKVYNIPLENYASGSDHLGSHERPDKMLGPEHGMSSMSRSQSVPMLDDEMLTYGSSKGPQQQKASMTKKVYQFDQSFNPQGSVEVKAEKRIPPPFQHNPEYVQQASKNIAKDLISPRAYRGYPPMEQMFSFSQPSVNEDAVVNAQFASQGARAGFLRRADSLVSATEMAMFRRVNEPHELPPTDRYGRPPYRGGLDRQSSVTVTESQFLKRNGRYEDEHPSYQEVKAQAGSFPVKNLTQRRPLSARSYSTESYGASQTRPVSARPTMAALLEKIPSDYNLGNYGDKPSDNSDLKTRPTPVKGEESCGKMPADWRQQLLRHIEARRLDRNAAYKHNTVNLGMLPYGGISAMHAGRSMTLNLQTKSKFDHQELPLQKTPSQQSNILDNGQEDVSPSGQWNPYPLGRRDVPPDTITKKAGSHIQTLMGSQSLQHRSREQQPYEGNINKVTIQQFQSPLPIQIPSSQATRGPQPGRCLIQTKGQRSMDGYPEQFCVRIEKNPGLGFSISGGISGQGNPFKPSDKGIFVTRVQPDGPASNLLQPGDKILQANGHSFVHMEHEKAVLLLKSFQNTVDLVIQRELTV

1073 NP_054781.1 NP_071347.2

>NP_054781.1

MAPVEHVVADAGAFLRHAALQDIGKNIYTIREVVTEIRDKATRRRLAVLPYELRFKEPLPEYVRLVTEFSKKTGDYPSLSATDIQVLALTYQLEAEFVGVSHLKQEPQKVKVSSSIQHPETPLHISGFHLPYKPKPPQETEKGHSACEPENLEFSSFMFWRNPLPNIDHELQELLIDRGEDVPSEEEEEEENGFEDRKDDSDDDGGGWITPSNIKQIQQELEQCDVPEDVRVGCLTTDFAMQNVLLQMGLHVLAVNGMLIREARSYILRCHGCFKTTSDMSRVFCSHCGNKTLKKVSVTVSDDGTLHMHFSRNPKVLNPRGLRYSLPTPKGGKYAINPHLTEDQRFPQLRLSQKARQKTNVFAPDYIAGVSPFVENDISSRSATLQVRDSTLGAGRRRLNPNASRKKFVKKR

>NP_071347.2

MASKGAGMSFSRKSYRLTSDAEKSRVTGIVQEKLLNDYLNRIFSSSEHAPPAATSRKPLNFQNLPEHLDQLLQVDNEEEESQGQVEGRLGPSTVVLDHTGGFEGLLLVDDDLLGVIGHSNFGTIRSTTCVYKGKWLYEVLISSQGLMQIGWCTISCRFNQEEGVGDTHNSYAYDGNRVRKWNVTTTNYGKAWAAGDIVSCLIDLDDGTLSFCLNGVSLGTAFENLSRGLGMAYFPAISLSFKESVAFNFGSRPLRYPVAGYRPLQDPPSADLVRAQRLLGCFRAVLSVELDPVEGRLLDKESSKWRLRGQPTVLLTLAHIFHHFAPLLRKVYLVEAVLMSFLLGIVEKGTPTQAQSVVHQVLDLLWLFMEDYEVQDCLKQLMMSLLRLYRFSPIVPDLGLQIHYLRLTIAILRHEKSRKFLLSNVLFDVLRSVVFFYIKSPLRVEEAGLQELIPTTWWPHCSSREGKESTEMKEETAEERLRRRAYERGCQRLRKRIEVVEELQVQILKLLLDNKDDNGGEASRYIFLTKFRKFLQENASGRGNMPMLCPPEYMVCFLHRLISALRYYWDEYKASNPHASFSEEAYIPPQVFYNGKVDYFDLQRLGGLLSHLRKTLKDDLASKANIVIDPLELQSTAMDDLDEDEEPAPAMAQRPMQALAVGGPLPLPRPGWLSSPTLGRANRFLSTAAVSLMTPRRPLSTSEKVKVRTLSVEQRTREDIEGSHWNEGLLLGRPPEEPEQPLTENSLLEVLDGAVMMYNLSVHQQLGKMVGVSDDVNEYAMALRDTEDKLRRCPKRRKDILAELTKSQKVFSEKLDHLSRRLAWVHATVYSQEKMLDIYWLLRVCLRTIEHGDRTGSLFAFMPEFYLSVAINSYSALKNYFGPVHSMEELPGYEETLTRLAAILAKHFADARIVGTDIRDSLMQALASYVCYPHSLRAVERIPEEQRIAMVRNLLAPYEQRPWAQTNWILVRLWRGCGFGYRYTRLPHLLKTKLEDANLPSLQKPCPSTLLQQHMADLLQQGPDVAPSFLNSVLNQLNWAFSEFIGMIQEIQQAAERLERNFVDSRQLKVCATCFDLSVSLLRVLEMTITLVPEIFLDWTRPTSEMLLRRLAQLLNQVLNRVTAERNLFDRVVTLRLPGLESVDHYPILVAVTGILVQLLVRGPASEREQATSVLLADPCFQLRSICYLLGQPEPPAPGTALPAPDRKRFSLQSYADYISADELAQVEQMLAHLTSASAQAAAASLPTSEEDLCPICYAHPISAVFQPCGHKSCKACINQHLMNNKDCFFCKTTIVSVEDWEKGANTSTTSSAA

1074 NP_075555.1 P14598

>NP_075555.1

MMASYPEPEDAAGALLAPETGRTVKEPEGPPPSPGKGGGGGGGTAPEKPDPAQKPPYSYVALIAMAIRESAEKRLTLSGIYQYIIAKFPFYEKNKKGWQNSIRHNLSLNECFIKVPREGGGERKGNYWTLDPACEDMFEKGNYRRRRRMKRPFRPPPAHFQPGKGLFGAGGAAGGCGVAGAGADGYGYLAPPKYLQSGFLNNSWPLPQPPSPMPYASCQMAAAAAAAAAAAAAAGPGSPGAAAVVKGLAGPAASYGPYTRVQSMALPPGVVNSYNGLGGPPAAPPPPPHPHPHPHAHHLHAAAAPPPAPPHHGAAAPPPGQLSPASPATAAPPAPAPTSAPGLQFACARQPELAMMHCSYWDHDSKTGALHSRLDL

>P14598

MGDTFIRHIALLGFEKRFVPSQHYVYMFLVKWQDLSEKVVYRRFTEIYEFHKTLKEMFPIEAGAINPENRIIPHLPAPKWFDGQRAAENRQGTLTEYCSTLMSLPTKISRCPHLLDFFKVRPDDLKLPTDNQTKKPETYLMPKDGKSTATDITGPIILQTYRAIADYEKTSGSEMALSTGDVVEVVEKSESGWWFCQMKAKRGWIPASFLEPLDSPDETEDPEPNYAGEPYVAIKAYTAVEGDEVSLLEGEAVEVIHKLLDGWWVIRKDDVTGYFPSMYLQKSGQDVSQAQRQIKRGAPPRRSSIRNAHSIHQRSRKRLSQDAYRRNSVRFLQQRRRQARPGPQSPGSPLEEERQTQRSKPQPAVPPRPSADLILNRCSESTKRKLASAV

1075 Q3ZCT1 NP_055867.3

>Q3ZCT1

MIGMLESLQHESDLLQHDQIHTGEKPYECNECRKTFSLKQNLVEHKKMHTGEKSHECTECGKVCSRVSSLTLHLRSHTGKKAYKCNKCGKAFSQKENFLSHQKHHTGEKPYECEKVSIQMPTIIRHQKNHTGTKPYACKECGKAFNGKAYLTEHEKIHTGEKPFECNQCGRAFSQKQYLIKHQNIHTGKKPFKCSECGKAFSQKENLIIHQRIHTGEKPYECKGCGKAFIQKSSLIRHQRSHTGEKPYTCKECGKAFSGKSNLTEHEKIHIGEKPYKCNECGTIFRQKQYLIKHHNIHTGEKPYECNKCGKAFSRITSLIVHVRIHTGDKPYECKVCGKAFCQSSSLTVHMRSHTGEKPYGCNECGKAFSQFSTLALHMRIHTGEKPYQCSECGKAFSQKSHHIRHQRIHTH

>NP_055867.3

MLLHLCSVKNLYQNRFLGLAAMASPSRNSQSRRRCKEPLRYSYNPDQFHNMDLRGGPHDGVTIPRSTSDTDLVTSDSRSTLMVSSSYYSIGHSQDLVIHWDIKEEVDAGDWIGMYLIDEVLSENFLDYKNRGVNGSHRGQIIWKIDASSYFVEPETKICFKYYHGVSGALRATTPSVTVKNSAAPIFKSIGADETVQGQGSRRLISFSLSDFQAMGLKKGMFFNPDPYLKISIQPGKHSIFPALPHHGQERRSKIIGNTVNPIWQAEQFSFVSLPTDVLEIEVKDKFAKSRPIIKRFLGKLSMPVQRLLERHAIGDRVVSYTLGRRLPTDHVSGQLQFRFEITSSIHPDDEEISLSTEPESAQIQDSPMNNLMESGSGEPRSEAPESSESWKPEQLGEGSVPDGPGNQSIELSRPAEEAAVITEAGDQGMVSVGPEGAGELLAQVQKDIQPAPSAEELAEQLDLGEEASALLLEDGEAPASTKEEPLEEEATTQSRAGREEEEKEQEEEGDVSTLEQGEGRLQLRASVKRKSRPCSLPVSELETVIASACGDPETPRTHYIRIHTLLHSMPSAQGGSAAEEEDGAEEESTLKDSSEKDGLSEVDTVAADPSALEEDREEPEGATPGTAHPGHSGGHFPSLANGAAQDGDTHPSTGSESDSSPRQGGDHSCEGCDASCCSPSCYSSSCYSTSCYSSSCYSASCYSPSCYNGNRFASHTRFSSVDSAKISESTVFSSQDDEEEENSAFESVPDSMQSPELDPESTNGAGPWQDELAAPSGHVERSPEGLESPVAGPSNRREGECPILHNSQPVSQLPSLRPEHHHYPTIDEPLPPNWEARIDSHGRVFYVDHVNRTTTWQRPTAAATPDGMRRSGSIQQMEQLNRRYQNIQRTIATERSEEDSGSQSCEQAPAGGGGGGGSDSEAESSQSSLDLRREGSLSPVNSQKITLLLQSPAVKFITNPEFFTVLHANYSAYRVFTSSTCLKHMILKVRRDARNFERYQHNRDLVNFINMFADTRLELPRGWEIKTDQQGKSFFVDHNSRATTFIDPRIPLQNGRLPNHLTHRQHLQRLRSYSAGEASEVSRNRGASLLARPGHSLVAAIRSQHQHESLPLAYNDKIVAFLRQPNIFEMLQERQPSLARNHTLREKIHYIRTEGNHGLEKLSCDADLVILLSLFEEEIMSYVPLQAAFHPGYSFSPRCSPCSSPQNSPGLQRASARAPSPYRRDFEAKLRNFYRKLEAKGFGQGPGKIKLIIRRDHLLEGTFNQVMAYSRKELQRNKLYVTFVGEEGLDYSGPSREFFFLLSQELFNPYYGLFEYSANDTYTVQISPMSAFVENHLEWFRFSGRILGLALIHQYLLDAFFTRPFYKALLRLPCDLSDLEYLDEEFHQSLQWMKDNNITDILDLTFTVNEEVFGQVTERELKSGGANTQVTEKNKKEYIERMVKWRVERGVVQQTEALVRGFYEVVDSRLVSVFDARELELVIAGTAEIDLNDWRNNTEYRGGYHDGHLVIRWFWAAVERFNNEQRLRLLQFVTGTSSVPYEGFAALRGSNGLRRFCIEKWGKITSLPRAHTCFNRLDLPPYPSYSMLYEKLLTAVEETSTFGLE

1076 Q99081 Q9H0B2

>Q99081

MNPQQQRMAAIGTDKELSDLLDFSAMFSPPVNSGKTRPTTLGSSQFSGSGIDERGGTTSWGTSGQPSPSYDSSRGFTDSPHYSDHLNDSRLGAHEGLSPTPFMNSNLMGKTSERGSFSLYSRDTGLPGCQSSLLRQDLGLGSPAQLSSSGKPGTAYYSFSATSSRRRPLHDSAALDPLQAKKVRKVPPGLPSSVYAPSPNSDDFNRESPSYPSPKPPTSMFASTFFMQDGTHNSSDLWSSSNGMSQPGFGGILGTSTSHMSQSSSYGNLHSHDRLSYPPHSVSPTDINTSLPPMSSFHRGSTSSSPYVAASHTPPINGSDSILGTRGNAAGSSQTGDALGKALASIYSPDHTSSSFPSNPSTPVGSPSPLTGTSQWPRPGGQAPSSPSYENSLHSLQSRMEDRLDRLDDAIHVLRNHAVGPSTSLPAGHSDIHSLLGPSHNAPIGSLNSNYGGSSLVASSRSASMVGTHREDSVSLNGNHSVLSSTVTTSSTDLNHKTQENYRGGLQSQSGTVVTTEIKTENKEKDENLHEPPSSDDMKSDDESSQKDIKVSSRGRTSSTNEDEDLNPEQKIEREKERRMANNARERLRVRDINEAFKELGRMCQLHLKSEKPQTKLLILHQAVAVILSLEQQVRERNLNPKAACLKRREEEKVSAVSAEPPTTLPGTHPGLSETTNPMGHM

>Q9H0B2

MRRSKADVERYVASVLGLTPSPRQKSMKGFYFAKLYYEAKEYDLAKKYICTYINVQERDPKAHRFLGLLYELEENTEKAVECYRRSVELNPTQKDLVLKIAELLCKNDVTDGRAKYWVERAAKLFPGSPAIYKLKEQLLDCEGEDGWNKLFDLIQSELYVRPDDVHVNIRLVELYRSTKRLKDAVAHCHEAERNIALRSSLEWNSCVVQTLKEYLESLQCLESDKSDWQATNTDLLLAYANLMLLTLSTRDVQENRELLESFDSALQSAKSSLGGNDELSATFLEMKGHFYMYAGSLLLKMGQHGNNVQWRALSELAALCYLIAFQVPRPKIKLREGKAGQNLLEMMACDRLSQSGHMLLSLSRGKQDFLKEVVETFANKIGQSALYDALFSSQSPKDTSFLGSDDIGKIDVQEPELEDLARYDVGAIRAHNGSLQHLTWLGLQWNSLPALPGIRKWLKQLFHRLPHETSRLETNAPESICILDLEVFLLGVVYTSHLQLKEKCNSHHSSYQPLCLPFPVCKQLCTERQKSWWDAVCTLIHRKAVPGNLAKLRLLVQHEINTLRAQEKHGLQPALLVHWAKYLQKTGSGLNSFYGQLEYIGRSVHYWKKVLPLLKIIKKNSIPEPIDPLFKHFHSVDIQASEIVEYEEDAHITFAMLDAVNGNIEDAVTAFESIKSVVSYWNLALIFHRKAEDIENDALSPEEQEECRNYLTKTRDYLIKIIDDGDSNLSVVKKLPVPLESVKQMLNSVMQELEDYSEGGPLYKNGSLRNADSEIKHSTPSPTKYSLSPSKSYKYSPETPPRWTEDRNSLLNMICQQVEAIKKEMQELKLNSSKSASRHRWPTENYGPDSVPDGYQGSQTFHGAPLTEAAHRHFTIEKHGDSKWIIYRFTKQLCGTERARAKIS

1077 NP_006275.1 P48729

>NP_006275.1

MSCSGSGADPEAAPASAASAPGPAPPVSAPAALPSSTAAENKASPAGTAGGPGAGAAAGGTGPLAARAGEPAERRGAAPVSAGGAAPPEGAISNGVYVLPSAANGDVKPVVSSTPLVDFLMQLEDYTPTIPDAVTGYYLNRAGFEASDPRIIRLISLAAQKFISDIANDALQHCKMKGTASGSSRSKSKDRKYTLTMEDLTPALSEYGINVKKPHYFT

>P48729

MASSSGSKAEFIVGGKYKLVRKIGSGSFGDIYLAINITNGEEVAVKLESQKARHPQLLYESKLYKILQGGVGIPHIRWYGQEKDYNVLVMDLLGPSLEDLFNFCSRRFTMKTVLMLADQMISRIEYVHTKNFIHRDIKPDNFLMGIGRHCNKLFLIDFGLAKKYRDNRTRQHIPYREDKNLTGTARYASINAHLGIEQSRRDDMESLGYVLMYFNRTSLPWQGLKAATKKQKYEKISEKKMSTPVEVLCKGFPAEFAMYLNYCRGLRFEEAPDYMYLRQLFRILFRTLNHQYDYTFDWTMLKQKAAQQAASSSGQGQQAQTPTGKQTDKTKSNMKGF

1078 NP_005641.1 NP_002878.2

>NP_005641.1

MQSFREQSSYHGNQQSYPQEVHGSSRLEEFSPRQAQMFQNFGGTGGSSGSSGSGSGGGRRGAAAAAAAMASETSGHQGYQGFRKEAGDFYYMAGNKDPVTTGTPQPPQRRPSGPVQSYGPPQGSSFGNQYGSEGHVGQFQAQHSGLGGVSHYQQDYTGPFSPGSAQYQQQASSQQQQQQVQQLRQQLYQSHQPLPQATGQPASSSSHLQPMQRPSTLPSSAAGYQLRVGQFGQHYQSSASSSSSSSFPSPQRFSQSGQSYDGSYNVNAGSQYEGHNVGSNAQAYGTQSNYSYQPQSMKNFEQAKIPQGTQQGQQQQQPQQQQHPSQHVMQYTNAATKLPLQSQVGQYNQPEVPVRSPMQFHQNFSPISNPSPAASVVQSPSCSSTPSPLMQTGENLQCGQGSVPMGSRNRILQLMPQLSPTPSMMPSPNSHAAGFKGFGLEGVPEKRLTDPGLSSLSALSTQVANLPNTVQHMLLSDALTPQKKTSKRPSSSKKADSCTNSEGSSQPEEQLKSPMAESLDGGCSSSSEDQGERVRQLSGQSTSSDTTYKGGASEKAGSSPAQGAQNEPPRLNASPAAREEATSPGAKDMPLSSDGNPKVNEKTVGVIVSREAMTGRVEKPGGQDKGSQEDDPAATQRPPSNGGAKETSHASLPQPEPPGGGGSKGNKNGDNNSNHNGEGNGQSGHSAAGPGFTSRTEPSKSPGSLRYSYKDSFGSAVPRNVSGFPQYPTGQEKGDFTGHGERKGRNEKFPSLLQEVLQGYHHHPDRRYSRSTQEHQGMAGSLEGTTRPNVLVSQTNELASRGLLNKSIGSLLENPHWGPWERKSSSTAPEMKQINLTDYPIPRKFEIEPQSSAHEPGGSLSERRSVICDISPLRQIVRDPGAHSLGHMSADTRIGRNDRLNPTLSQSVILPGGLVSMETKLKSQSGQIKEEDFEQSKSQASFNNKKSGDHCHPPSIKHESYRGNASPGAATHDSLSDYGPQDSRPTPMRRVPGRVGGREGMRGRSPSQYHDFAEKLKMSPGRSRGPGGDPHHMNPHMTFSERANRSSLHTPFSPNSETLASAYHANTRAHAYGDPNAGLNSQLHYKRQMYQQQPEEYKDWSSGSAQGVIAAAQHRQEGPRKSPRQQQFLDRVRSPLKNDKDGMMYGPPVGTYHDPSAQEAGRCLMSSDGLPNKGMELKHGSQKLQESCWDLSRQTSPAKSSGPPGMSSQKRYGPPHETDGHGLAEATQSSKPGSVMLRLPGQEDHSSQNPLIMRRRVRSFISPIPSKRQSQDVKNSSTEDKGRLLHSSKEGADKAFNSYAHLSHSQDIKSIPKRDSSKDLPSPDSRNCPAVTLTSPAKTKILPPRKGRGLKLEAIVQKITSPNIRRSASSNSAEAGGDTVTLDDILSLKSGPPEGGSVAVQDADIEKRKGEVASDLVSPANQELHVEKPLPRSSEEWRGSVDDKVKTETHAETVTAGKEPPGAMTSTTSQKPGSNQGRPDGSLGGTAPLIFPDSKNVPPVGILAPEANPKAEEKENDTVTISPKQEGFPPKGYFPSGKKKGRPIGSVNKQKKQQQPPPPPPQPPQIPEGSADGEPKPKKQRQRRERRKPGAQPRKRKTKQAVPIVEPQEPEIKLKYATQPLDKTDAKNKSFYPYIHVVNKCELGAVCTIINAEEEEQTKLVRGRKGQRSLTPPPSSTESKALPASSFMLQGPVVTESSVMGHLVCCLCGKWASYRNMGDLFGPFYPQDYAATLPKNPPPKRATEMQSKVKVRHKSASNGSKTDTEEEEEQQQQQKEQRSLAAHPRFKRRHRSEDCGGGPRSLSRGLPCKKAATEGSSEKTVLDSKPSVPTTSEGGPELELQIPELPLDSNEFWVHEGCILWANGIYLVCGRLYGLQEALEIAREMKCSHCQEAGATLGCYNKGCSFRYHYPCAIDADCLLHEENFSVRCPKHKPPLPCPLPPLQNKTAKGSLSTEQSERG

>NP_002878.2

MDVLVSECSARLLQQEEEIKSLTAEIDRLKNCGCLGASPNLEQLQEENLKLKYRLNILRKSLQAERNKPTKNMINIISRLQEVFGHAIKAAYPDLENPPLLVTPSQQAKFGDYQCNSAMGISQMLKTKEQKVNPREIAENITKHLPDNECIEKVEIAGPGFINVHLRKDFVSEQLTSLLVNGVQLPALGENKKVIVDFSSPNIAKEMHVGHLRSTIIGESISRLFEFAGYDVLRLNHVGDWGTQFGMLIAHLQDKFPDYLTVSPPIGDLQVFYKESKKRFDTEEEFKKRAYQCVVLLQGKNPDITKAWKLICDVSRQELNKIYDALDVSLIERGESFYQDRMNDIVKEFEDRGFVQVDDGRKIVFVPGCSIPLTIVKSDGGYTYDTSDLAAIKQRLFEEKADMIIYVVDNGQSVHFQTIFAAAQMIGWYDPKVTRVFHAGFGVVLGEDKKKFKTRSGETVRLMDLLGEGLKRSMDKLKEKERDKVLTAEELNAAQTSVAYGCIKYADLSHNRLNDYIFSFDKMLDDRGNTAAYLLYAFTRIRSIARLANIDEEMLQKAARETKILLDHEKEWKLGRCILRFPEILQKILDDLFLHTLCDYIYELATAFTEFYDSCYCVEKDRQTGKILKVNMWRMLLCEAVAAVMAKGFDILGIKPVQRM

1079 BAB62909.1 Q8WVN8

>BAB62909.1

MDRSKRNSIAGFPPRVERLEEFEGGGGGEGNVSQVGRVWPSSYRALISAFSRLTRLDDFTCEKIGSGFFSEVFKVRHRASGQVMALKMNTLSSNRANMLKEVQLMNRLSHPNILRFMGVCVHQGQLHALTEYINSGNLEQLLDSNLHLPWTVRVKLAYDIAVGLSYLHFKGIFHRDLTSKNCLIKRDENGYSAVVADFGLAEKIPDVSMGSEKLAVVGSPFWMAPEVLRDEPYNEKADVFSYGIILCEIIARIQADPDYLPRTENFGLDYDAFQHMVGDCPPDFLQLTFNCCNMDPKLRPSFVEIGKTLEEILSRLQEEEQERDRKLQPTARGLLEKAPGVKRLSSLDDKIPHKSPCPRRTIWLSRSQSDIFSRKPPRTVSVLDPYYRPRDGAARTPKVNPFSARQDLMGGKIKFFDLPSKSVISLVFDLDAPGPGTMPLADWQEPLAPPIRRWRSLPGSPEFLHQEACPFVGREESLSDGPPPRLSSLKYRVKEIPPFRASALPAAQAHEAMDCSILQEENGFGSRPQGTSPCPAGASEEMEVEERPAGSTPATFSTSGIGLQTQGKQDG

>Q8WVN8

MSVSGLKAELKFLASIFDKNHERFRIVSWKLDELHCQFLVPQQGSPHSLPPPLTLHCNITESYPSSSPIWFVDSEDPNLTSVLERLEDTKNNNLLRQQLKWLICELCSLYNLPKHLDVEMLDQPLPTGQNGTTEEVTSEEEEEEEEMAEDIEDLDHYEMKEEEPISGKKSEDEGIEKENLAILEKIRKTQRQDHLNGAVSGSVQASDRLMKELRDIYRSQSYKTGIYSVELINDSLYDWHVKLQKVDPDSPLHSDLQILKEKEGIEYILLNFSFKDNFPFDPPFVRVVLPVLSGGYVLGGGALCMELLTKQGWSSAYSIESVIMQINATLVKGKARVQFGANKNQYNLARAQQSYNSIVQIHEKNGWYTPPKEDG

1080 Q8IZL9 Q02252

>Q8IZL9

MDQYCILGRIGEGAHGIVFKAKHVETGEIVALKKVALRRLEDGFPNQALREIKALQEMEDNQYVVQLKAVFPHGGGFVLAFEFMLSDLAEVVRHAQRPLAQAQVKSYLQMLLKGVAFCHANNIVHRDLKPANLLISASGQLKIADFGLARVFSPDGSRLYTHQVATRWYRAPELLYGARQYDQGVDLWSVGCIMGELLNGSPLFPGKNDIEQLCYVLRILGTPNPQVWPELTELPDYNKISFKEQVPMPLEEVLPDVSPQALDLLGQFLLYPPHQRIAASKALLHQYFFTAPLPAHPSELPIPQRLGGPAPKAHPGPPHIHDFHVDRPLEESLLNPELIRPFILEG

>Q02252

MAALLAAAAVRARILQVSSKVKSSPTWYSASSFSSSVPTVKLFIGGKFVESKSDKWIDIHNPATNEVIGRVPQATKAEMDAAIASCKRAFPAWADTSVLSRQQVLLRYQQLIKENLKEIAKLITLEQGKTLADAEGDVFRGLQVVEHACSVTSLMMGETMPSITKDMDLYSYRLPLGVCAGIAPFNFPAMIPLWMFPMAMVCGNTFLMKPSERVPGATMLLAKLLQDSGAPDGTLNIIHGQHEAVNFICDHPDIKAISFVGSNKAGEYIFERGSRHGKRVQANMGAKNHGVVMPDANKENTLNQLVGAAFGAAGQRCMALSTAVLVGEAKKWLPELVEHAKNLRVNAGDQPGADLGPLITPQAKERVCNLIDSGTKEGASILLDGRKIKVKGYENGNFVGPTIISNVKPNMTCYKEEIFGPVLVVLETETLDEAIQIVNNNPYGNGTAIFTTNGATARKYAHLVDVGQVGVNVPIPVPLPMFSFTGSRSSFRGDTNFYGKQGIQFYTQLKTITSQWKEEDATLSSPAVVMPTMGR

1081 NP_002043.2 NP_006083.1

>NP_002043.2

MYQSLAMAANHGPPPGAYEAGGPGAFMHGAGAASSPVYVPTPRVPSSVLGLSYLQGGGAGSASGGASGGSSGGAASGAGPGTQQGSPGWSQAGADGAAYTPPPVSPRFSFPGTTGSLAAAAAAAAAREAAAYSSGGGAAGAGLAGREQYGRAGFAGSYSSPYPAYMADVGASWAAAAAASAGPFDSPVLHSLPGRANPAARHPNLDMFDDFSEGRECVNCGAMSTPLWRRDGTGHYLCNACGLYHKMNGINRPLIKPQRRLSASRRVGLSCANCQTTTTTLWRRNAEGEPVCNACGLYMKLHGVPRPLAMRKEGIQTRKRKPKNLNKSKTPAAPSGSESLPPASGASSNSSNATTSSSEEMRPIKTEPGLSSHYGHSSSVSQTFSVSAMSGHGPSIHPVLSALKLSPQGYASPVSQSPQTSSKQDSWNSLVLADSHGDIITA

>NP_006083.1

MEEQGHSEMEIIPSESHPHIQLLKSNRELLVTHIRNTQCLVDNLLKNDYFSAEDAEIVCACPTQPDKVRKILDLVQSKGEEVSEFFLYLLQQLADAYVDLRPWLLEIGFSPSLLTQSKVVVNTDPVSRYTQQLRHHLGRDSKFVLCYAQKEELLLEEIYMDTIMELVGFSNESLGSLNSLACLLDHTTGILNEQGETIFILGDAGVGKSMLLQRLQSLWATGRLDAGVKFFFHFRCRMFSCFKESDRLCLQDLLFKHYCYPERDPEEVFAFLLRFPHVALFTFDGLDELHSDLDLSRVPDSSCPWEPAHPLVLLANLLSGKLLKGASKLLTARTGIEVPRQFLRKKVLLRGFSPSHLRAYARRMFPERALQDRLLSQLEANPNLCSLCSVPLFCWIIFRCFQHFRAAFEGSPQLPDCTMTLTDVFLLVTEVHLNRMQPSSLVQRNTRSPVETLHAGRDTLCSLGQVAHRGMEKSLFVFTQEEVQASGLQERDMQLGFLRALPELGPGGDQQSYEFFHLTLQAFFTAFFLVLDDRVGTQELLRFFQEWMPPAGAATTSCYPPFLPFQCLQGSGPAREDLFKNKDHFQFTNLFLCGLLSKAKQKLLRHLVPAAALRRKRKALWAHLFSSLRGYLKSLPRVQVESFNQVQAMPTFIWMLRCIYETQSQKVGQLAARGICANYLKLTYCNACSADCSALSFVLHHFPKRLALDLDNNNLNDYGVRELQPCFSRLTVLRLSVNQITDGGVKVLSEELTKYKIVTYLGLYNNQITDVGARYVTKILDECKGLTHLKLGKNKITSEGGKYLALAVKNSKSISEVGMWGNQVGDEGAKAFAEALRNHPSLTTLSLASNGISTEGGKSLARALQQNTSLEILWLTQNELNDEVAESLAEMLKVNQTLKHLWLIQNQITAKGTAQLADALQSNTGITEICLNGNLIKPEEAKVYEDEKRIICF

1082 Q9ULV3 Q8N183

>Q9ULV3

MFSQQQQQQLQQQQQQLQQLQQQQLQQQQLQQQQLLQLQQLLQQSPPQAPLPMAVSRGLPPQQPQQPLLNLQGTNSASLLNGSMLQRALLLQQLQGLDQFAMPPATYDTAGLTMPTATLGNLRGYGMASPGLAAPSLTPPQLATPNLQQFFPQATRQSLLGPPPVGVPMNPSQFNLSGRNPQKQARTSSSTTPNRKDSSSQTMPVEDKSDPPEGSEEAAEPRMDTPEDQDLLPCPEDIAKEKRTPAPEPEPCEASELPAKRLRSSEEPTEKEPPGQLQVKAQPQARMTVPKQTQTPDLLPEALEAQVLPRFQPRVLQVQAQVQSQTQPRIPSTDTQVQPKLQKQAQTQTSPEHLVLQQKQVQPQLQQEAEPQKQVQPQVQPQAHSQGPRQVQLQQEAEPLKQVQPQVQPQAHSQPPRQVQLQLQKQVQTQTYPQVHTQAQPSVQPQEHPPAQVSVQPPEQTHEQPHTQPQVSLLAPEQTPVVVHVCGLEMPPDAVEAGGGMEKTLPEPVGTQVSMEEIQNESACGLDVGECENRAREMPGVWGAGGSLKVTILQGSDSRAFSTVPLTPVPRPSDSVSSTPAATSTPSKQALQFFCYICKASCSSQQEFQDHMSEPQHQQRLGEIQHMSQACLLSLLPVPRDVLETEDEEPPPRRWCNTCQLYYMGDLIQHRRTQDHKIAKQSLRPFCTVCNRYFKTPRKFVEHVKSQGHKDKAKELKSLEKEIAGQDEDHFITVDAVGCFEGDEEEEEDDEDEEEIEVEEELCKQVRSRDISREEWKGSETYSPNTAYGVDFLVPVMGYICRICHKFYHSNSGAQLSHCKSLGHFENLQKYKAAKNPSPTTRPVSRRCAINARNALTALFTSSGRPPSQPNTQDKTPSKVTARPSQPPLPRRSTRLKT

>Q8N183

MGWSQDLFRALWRSLSREVKEHVGTDQFGNKYYYIPQYKNWRGQTIREKRIVEAANKKEVDYEAGDIPTEWEAWIRRTRKTPPTMEEILKNEKHREEIKIKSQDFYEKEKLLSKETSEELLPPPVQTQIKGHASAPYFGKEEPSVAPSSTGKTFQPGSWMPRDGKSHNQ

1083 Q13761 NP_000658.1

>Q13761

MRIPVDPSTSRRFTPPSPAFPCGGGGGKMGENSGALSAQAAVGPGGRARPEVRSMVDVLADHAGELVRTDSPNFLCSVLPSHWRCNKTLPVAFKVVALGDVPDGTVVTVMAGNDENYSAELRNASAVMKNQVARFNDLRFVGRSGRGKSFTLTITVFTNPTQVATYHRAIKVTVDGPREPRRHRQKLEDQTKPFPDRFGDLERLRMRVTPSTPSPRGSLSTTSHFSSQPQTPIQGTSELNPFSDPRQFDRSFPTLPTLTESRFPDPRMHYPGAMSAAFPYSATPSGTSISSLSVAGMPATSRFHHTYLPPPYPGAPQNQSGPFQANPSPYHLYYGTSSGSYQFSMVAGSSSGGDRSPTRMLASCTSSAASVAAGNLMNPSLGGQSDGVEADGSHSNSPTALSTPGRMDEAVWRPY

>NP_000658.1

MSTAGKVIKCKAAVLWELKKPFSIEEVEVAPPKAHEVRIKMVAVGICGTDDHVVSGTMVTPLPVILGHEAAGIVESVGEGVTTVKPGDKVIPLAIPQCGKCRICKNPESNYCLKNDVSNPQGTLQDGTSRFTCRRKPIHHFLGISTFSQYTVVDENAVAKIDAASPLEKVCLIGCGFSTGYGSAVNVAKVTPGSTCAVFGLGGVGLSAIMGCKAAGAARIIAVDINKDKFAKAKELGATECINPQDYKKPIQEVLKEMTDGGVDFSFEVIGRLDTMMASLLCCHEACGTSVIVGVPPDSQNLSMNPMLLLTGRTWKGAILGGFKSKECVPKLVADFMAKKFSLDALITHVLPFEKINEGFDLLHSGKSIRTILMF

1084 NP_001940.1 Q9H4K7

>NP_001940.1

MRKGIQPALEQYLVTAGGGEGAAVVAAAAAASMDKRALLASPGFAAAAAAAAAPGAYIQILTTNTSTTSCSSSLQSGAVAAGPLLPSAPGAEQTAGSLLYTTPHGPSSRAGLLQQPPALGRGGSGGGGGPPAKRRLELGESGHQYLSDGLKTPKGKGRAALRSPDSPKTPKSPSEKTRYDTSLGLLTKKFIQLLSQSPDGVLDLNKAAEVLKVQKRRIYDITNVLEGIHLIKKKSKNNVQWMGCSLSEDGGMLAQCQGLSKEVTELSQEEKKLDELIQSCTLDLKLLTEDSENQRLAYVTYQDIRKISGLKDQTVIVVKAPPETRLEVPDSIESLQIHLASTQGPIEVYLCPEETETHSPMKTNNQDHNGNIPKPASKDLASTNSGHSDCSVSMGNLSPLASPANLLQQTEDQIPSNLEGPFVNLLPPLLQEDYLLSLGEEEGISDLFDAYDLEKLPLVEDFMCS

>Q9H4K7

MAPARCFSARLRTVFQGVGHWALSTWAGLKPSRLLPQRASPRLLSVGRADLAKHQELPGKKLLSEKKLKRYFVDYRRVLVCGGNGGAGASCFHSEPRKEFGGPDGGDGGNGGHVILRVDQQVKSLSSVLSRYQGFSGEDGGSKNCFGRSGAVLYIRVPVGTLVKEGGRVVADLSCVGDEYIAALGGAGGKGNRFFLANNNRAPVTCTPGQPGQQRVLHLELKTVAHAGMVGFPNAGKSSLLRAISNARPAVASYPFTTLKPHVGIVHYEGHLQIAVADIPGIIRGAHQNRGLGSAFLRHIERCRFLLFVVDLSQPEPWTQVDDLKYELEMYEKGLSARPHAIVANKIDLPEAQANLSQLRDHLGQEVIVLSALTGENLEQLLLHLKVLYDAYAEAELGQGRQPLRW

1085 NP_005422.1 Q9Y2J8

>NP_005422.1

MCSAFHRAESGTELLARLEGRSSLKEIEPNLFADEDSPVHGDILEFHGPEGTGKTEMLYHLTARCILPKSEGGLEVEVLFIDTDYHFDMLRLVTILEHRLSQSSEEIIKYCLGRFFLVYCSSSTHLLLTLYSLESMFCSHPSLCLLILDSLSAFYWIDRVNGGESVNLQESTLRKCSQCLEKLVNDYRLVLFATTQTIMQKASSSSEEPSHASRRLCDVDIDYRPYLCKAWQQLVKHRMFFSKQDDSQSSNQFSLVSRCLKSNSLKKHFFIIGESGVEFC

>Q9Y2J8

MLRERTVRLQYGSRVEAVYVLGTYLWTDVYSAAPAGAQTFSLKHSEHVWVEVVRDGEAEEVATNGKQRWLLSPSTTLRVTMSQASTEASSDKVTVNYYDEEGSIPIDQAGLFLTAIEISLDVDADRDGVVEKNNPKKASWTWGPEGQGAILLVNCDRETPWLPKEDCRDEKVYSKEDLKDMSQMILRTKGPDRLPAGYEIVLYISMSDSDKVGVFYVENPFFGQRYIHILGRRKLYHVVKYTGGSAELLFFVEGLCFPDEGFSGLVSIHVSLLEYMAQDIPLTPIFTDTVIFRIAPWIMTPNILPPVSVFVCCMKDNYLFLKEVKNLVEKTNCELKVCFQYLNRGDRWIQDEIEFGYIEAPHKGFPVVLDSPRDGNLKDFPVKELLGPDFGYVTREPLFESVTSLDSFGNLEVSPPVTVNGKTYPLGRILIGSSFPLSGGRRMTKVVRDFLKAQQVQAPVELYSDWLTVGHVDEFMSFVPIPGTKKFLLLMASTSACYKLFREKQKDGHGEAIMFKGLGGMSSKRITINKILSNESLVQENLYFQRCLDWNRDILKKELGLTEQDIIDLPALFKMDEDHRARAFFPNMVNMIVLDKDLGIPKPFGPQVEEECCLEMHVRGLLEPLGLECTFIDDISAYHKFLGEVHCGTNVRRKPFTFKWWHMVP

1086 NP_055315.2 Q9BZQ8

>NP_055315.2

MSGTNLDGNDEFDEQLRMQELYGDGKDGDTQTDAGGEPDSLGQQPTDTPYEWDLDKKAWFPKITEDFIATYQANYGFSNDGASSSTANVEDVHARTAEEPPQEKAPEPTDARKKGEKRKAESGWFHVEEDRNTNVYVSGLPPDITVDEFIQLMSKFGIIMRDPQTEEFKVKLYKDNQGNLKGDGLCCYLKRESVELALKLLDEDEIRGYKLHVEVAKFQLKGEYDASKKKKKCKDYKKKLSMQQKQLDWRPERRAGPSRMRHERVVIIKNMFHPMDFEDDPLVLNEIREDLRVECSKFGQIRKLLLFDRHPDGVASVSFRDPEEADYCIQTLDGRWFGGRQITAQAWDGTTDYQVEETSREREERLRGWEAFLNAPEANRGLRRSDSVSASERAGPSRARHFSEHPSTSKMNAQETATGMAFEEPIDEKKFEKTEDGGEFEEGASENNAKESSPEKEAEEGCPEKESEEGCPKRGFEGSCSQKESEEGNPVRGSEEDSPKKESKKKTLKNDCEENGLAKESEDDLNKESEEEVGPTKESEEDDSEKESDEDCSEKQSEDGSEREFEENGLEKDLDEEGSEKELHENVLDKELEENDSENSEFEDDGSEKVLDEEGSEREFDEDSDEKEEEEDTYEKVFDDESDEKEDEEYADEKGLEAADKKAEEGDADEKLFEESDDKEDEDADGKEVEDADEKLFEDDDSNEKLFDEEEDSSEKLFDDSDERGTLGGFGSVEEGPLSTGSSFILSSDDDDDDI

>Q9BZQ8

MGGSASSQLDEGKCAYIRGKTEAAIKNFSPYYSRQYSVAFCNHVRTEVEQQRDLTSQFLKTKPPLAPGTILYEAELSQFSEDIKKWKERYVVVKNDYAVESYENKEAYQRGAAPKCRILPAGGKVLTSEDEYNLLSDRHFPDPLASSEKENTQPFVVLPKEFPVYLWQPFFRHGYFCFHEAADQKRFSALLSDCVRHLNHDYMKQMTFEAQAFLEAVQFFRQEKGHYGSWEMITGDEIQILSNLVMEELLPTLQTDLLPKMKGKKNDRKRTWLGLLEEAYTLVQHQVSEGLSALKEECRALTKGLEGTIRSDMDQIVNSKNYLIGKIKAMVAQPAEKSCLESVQPFLASILEELMGPVSSGFSEVRVLFEKEVNEVSQNFQTTKDSVQLKEHLDRLMNLPLHSVKMEPCYTKVNLLHERLQDLKSRFRFPHIDLVVQRTQNYMQELMENAVFTFEQLLSPHLQGEASKTAVAIEKVKLRVLKQYDYDSSTIRKKIFQEALVQITLPTVQKALASTCKPELQKYEQFIFADHTNMIHVENVYEEILHQILLDETLKVIKEAAILKKHNLFEDNMALPSESVSSLTDLKPPTGSNQASPARRASAILPGVLGSETLSNEVFQESEEEKQPEVPSSLAKGESLSLPGPSPPPDGTEQVIISRVDDPVVNPVATEDTAGLPGTCSSELEFGGTLEDEEPAQEEPEPITASGSLKALRKLLTASVEVPVDSAPVMEEDTNGESHVPQENEEEEEKEPSQAAAIHPDNCEESEVSEREAQPPCPEAHGEELGGFPEVGSPASPPASGGLTEEPLGPMEGELPGEACTLTAHEGRGGKCTEEGDASQQEGCTLGSDPICLSESQVSEEQEEMGGQSSAAQATASVNAEEIKVARIHECQWVVEDAPNPDVLLSHKDDVKEGEGGQESFPELPSEE

1087 Q96BP2 O75570

>Q96BP2

MATPSLRGRLARFGNPRKPVLKPNKPLILANRVGERRREKGEATCITEMSVMMACWKQNEFRDDACRKEIQGFLDCAARAQEARKMRSIQETLGESGSLLPNKLNKLLQRFPNKPYLS

>O75570

MNRHLCVWLFRHPSLNGYLQCHIQLHSHQFRQIHLDTRLQVFRQNRNCILHLLSKNWSRRYCHQDTKMLWKHKALQKYMENLSKEYQTLEQCLQHIPVNEENRRSLNRRHAELAPLAAIYQEIQETEQAIEELESMCKSLNKQDEKQLQELALEERQTIDQKINMLYNELFQSLVPKEKYDKNDVILEVTAGRTTGGDICQQFTREIFDMYQNYSCYKHWQFELLNYTPADYGGLHHAAARISGDGVYKHLKYEGGIHRVQRIPEVGLSSRMQRIHTGTMSVIVLPQPDEVDVKLDPKDLRIDTFRAKGAGGQHVNKTDSAVRLVHIPTGLVVECQQERSQIKNKEIAFRVLRARLYQQIIEKDKRQQQSARKLQVGTRAQSERIRTYNFTQDRVSDHRIAYEVRDIKEFLCGGKGLDQLIQRLLQSADEEAIAELLDEHLKSAK

1088 NP_653209.1 NP_002684.1

>NP_653209.1

MMATPNQTACNAESPVALEEAKTSGAPGSPQTPPERHDSGGSLPLTPRMESHSEDEDLAGAVGGLGWNSRSPRTQSPGGCSAEAVLARKKHRRRPSKRKRHWRPYLELSWAEKQQRDERQSQRASRVREEMFAKGQPVAPYNTTQFLMNDRDPEEPNLDVPHGISHPGSSGESEAGDSDGRGRAHGEFQRKDFSETYERFHTESLQGRSKQELVRDYLELEKRLSQAEEETRRLQQLQACTGQQSCRQVEELAAEVQRLRTENQRLRQENQMWNREGCRCDEEPGT

>NP_002684.1

MSRLLWRKVAGATVGPGPVPAPGRWVSSSVPASDPSDGQRRRQQQQQQQQQQQQQPQQPQVLSSEGGQLRHNPLDIQMLSRGLHEQIFGQGGEMPGEAAVRRSVEHLQKHGLWGQPAVPLPDVELRLPPLYGDNLDQHFRLLAQKQSLPYLEAANLLLQAQLPPKPPAWAWAEGWTRYGPEGEAVPVAIPEERALVFDVEVCLAEGTCPTLAVAISPSAWYSWCSQRLVEERYSWTSQLSPADLIPLEVPTGASSPTQRDWQEQLVVGHNVSFDRAHIREQYLIQGSRMRFLDTMSMHMAISGLSSFQRSLWIAAKQGKHKVQPPTKQGQKSQRKARRGPAISSWDWLDISSVNSLAEVHRLYVGGPPLEKEPRELFVKGTMKDIRENFQDLMQYCAQDVWATHEVFQQQLPLFLERCPHPVTLAGMLEMGVSYLPVNQNWERYLAEAQGTYEELQREMKKSLMDLANDACQLLSGERYKEDPWLWDLEWDLQEFKQKKAKKVKKEPATASKLPIEGAGAPGDPMDQEDLGPCSEEEEFQQDVMARACLQKLKGTTELLPKRPQHLPGHPGWYRKLCPRLDDPAWTPGPSLLSLQMRVTPKLMALTWDGFPLHYSERHGWGYLVPGRRDNLAKLPTGTTLESAGVVCPYRAIESLYRKHCLEQGKQQLMPQEAGLAEEFLLTDNSAIWQTVEELDYLEVEAEAKMENLRAAVPGQPLALTARGGPKDTQPSYHHGNGPYNDVDIPGCWFFKLPHKDGNSCNVGSPFAKDFLPKMEDGTLQAGPGGASGPRALEINKMISFWRNAHKRISSQMVVWLPRSALPRAVIRHPDYDEEGLYGAILPQVVTAGTITRRAVEPTWLTASNARPDRVGSELKAMVQAPPGYTLVGADVDSQELWIAAVLGDAHFAGMHGCTAFGWMTLQGRKSRGTDLHSKTATTVGISREHAKIFNYGRIYGAGQPFAERLLMQFNHRLTQQEAAEKAQQMYAATKGLRWYRLSDEGEWLVRELNLPVDRTEGGWISLQDLRKVQRETARKSQWKKWEVVAERAWKGGTESEMFNKLESIATSDIPRTPVLGCCISRALEPSAVQEEFMTSRVNWVVQSSAVDYLHLMLVAMKWLFEEFAIDGRFCISIHDEVRYLVREEDRYRAALALQITNLLTRCMFAYKLGLNDLPQSVAFFSAVDIDRCLRKEVTMDCKTPSNPTGMERRYGIPQGEALDIYQIIELTKGSLEKRSQPGP

1089 Q5QNW6 NP_001893.2

>Q5QNW6

MPDPAKSAPAPKKGSKKAVTKVQKKDGKKRKRSRKESYSVYVYKVLKQVHPDTGISSKAMGIMNSFVNDIFERIAGEASRLAHYNKRSTITSREIQTAVRLLLPGELAKHAVSEGTKAVTKYTSSK

>NP_001893.2

MQEKDASSQGFLPHFQHFATQAIHVGQDPEQWTSRAVVPPISLSTTFKQGAPGQHSGFEYSRSGNPTRNCLEKAVAALDGAKYCLAFASGLAATVTITHLLKAGDQIICMDDVYGGTNRYFRQVASEFGLKISFVDCSKIKLLEAAITPETKLVWIETPTNPTQKVIDIEGCAHIVHKHGDIILVVDNTFMSPYFQRPLALGADISMYSATKYMNGHSDVVMGLVSVNCESLHNRLRFLQNSLGAVPSPIDCYLCNRGLKTLHVRMEKHFKNGMAVAQFLESNPWVEKVIYPGLPSHPQHELVKRQCTGCTGMVTFYIKGTLQHAEIFLKNLKLFTLAESLGGFESLAELPAIMTHASVLKNDRDVLGISDTLIRLSVGLEDEEDLLEDLDQALKAAHPPSGSHS

1090 Q8NFD5 NP_002475.2

>Q8NFD5

MAHNAGAAAAAGTHSAKSGGSEAALKEGGSAAALSSSSSSSAAAAAASSSSSSGPGSAMETGLLPNHKLKTVGEAPAAPPHQQHHHHHHAHHHHHHAHHLHHHHALQQQLNQFQQQQQQQQQQQQQQQQQQHPISNNNSLGGAGGGAPQPGPDMEQPQHGGAKDSAAGGQADPPGPPLLSKPGDEDDAPPKMGEPAGGRYEHPGLGALGTQQPPVAVPGGGGGPAAVPEFNNYYGSAAPASGGPGGRAGPCFDQHGGQQSPGMGMMHSASAAAAGAPGSMDPLQNSHEGYPNSQCNHYPGYSRPGAGGGGGGGGGGGGGSGGGGGGGGAGAGGAGAGAVAAAAAAAAAAAGGGGGGGYGGSSAGYGVLSSPRQQGGGMMMGPGGGGAASLSKAAAGSAAGGFQRFAGQNQHPSGATPTLNQLLTSPSPMMRSYGGSYPEYSSPSAPPPPPSQPQSQAAAAGAAAGGQQAAAGMGLGKDMGAQYAAASPAWAAAQQRSHPAMSPGTPGPTMGRSQGSPMDPMVMKRPQLYGMGSNPHSQPQQSSPYPGGSYGPPGPQRYPIGIQGRTPGAMAGMQYPQQQMPPQYGQQGVSGYCQQGQQPYYSQQPQPPHLPPQAQYLPSQSQQRYQPQQDMSQEGYGTRSQPPLAPGKPNHEDLNLIQQERPSSLPDLSGSIDDLPTGTEATLSSAVSASGSTSSQGDQSNPAQSPFSPHASPHLSSIPGGPSPSPVGSPVGSNQSRSGPISPASIPGSQMPPQPPGSQSESSSHPALSQSPMPQERGFMAGTQRNPQMAQYGPQQTGPSMSPHPSPGGQMHAGISSFQQSNSSGTYGPQMSQYGPQGNYSRPPAYSGVPSASYSGPGPGMGISANNQMHGQGPSQPCGAVPLGRMPSAGMQNRPFPGNMSSMTPSSPGMSQQGGPGMGPPMPTVNRKAQEAAAAVMQAAANSAQSRQGSFPGMNQSGLMASSSPYSQPMNNSSSLMNTQAPPYSMAPAMVNSSAASVGLADMMSPGESKLPLPLKADGKEEGTPQPESKSKKSSSSTTTGEKITKVYELGNEPERKLWVDRYLTFMEERGSPVSSLPAVGKKPLDLFRLYVCVKEIGGLAQVNKNKKWRELATNLNVGTSSSAASSLKKQYIQYLFAFECKIERGEEPPPEVFSTGDTKKQPKLQPPSPANSGSLQGPQTPQSTGSNSMAEVPGDLKPPTPASTPHGQMTPMQGGRSSTISVHDPFSDVSDSSFPKRNSMTPNAPYQQGMSMPDVMGRMPYEPNKDPFGGMRKVPGSSEPFMTQGQMPNSSMQDMYNQSPSGAMSNLGMGQRQQFPYGASYDRRHEPYGQQYPGQGPPSGQPPYGGHQPGLYPQQPNYKRHMDGMYGPPAKRHEGDMYNMQYSSQQQEMYNQYGGSYSGPDRRPIQGQYPYPYSRERMQGPGQIQTHGIPPQMMGGPLQSSSSEGPQQNMWAARNDMPYPYQNRQGPGGPTQAPPYPGMNRTDDMMVPDQRINHESQWPSHVSQRQPYMSSSASMQPITRPPQPSYQTPPSLPNHISRAPSPASFQRSLENRMSPSKSPFLPSMKMQKVMPTVPTSQVTGPPPQPPPIRREITFPPGSVEASQPVLKQRRKITSKDIVTPEAWRVMMSLKSGLLAESTWALDTINILLYDDSTVATFNLSQLSGFLELLVEYFRKCLIDIFGILMEYEVGDPSQKALDHNAARKDDSQSLADDSGKEEEDAECIDDDEEDEEDEEEDSEKTESDEKSSIALTAPDAAADPKEKPKQASKFDKLPIKIVKKNNLFVVDRSDKLGRVQEFNSGLLHWQLGGGDTTEHIQTHFESKMEIPPRRRPPPPLSSAGRKKEQEGKGDSEEQQEKSIIATIDDVLSARPGALPEDANPGPQTESSKFPFGIQQAKSHRNIKLLEDEPRSRDETPLCTIAHWQDSLAKRCICVSNIVRSLSFVPGNDAEMSKHPGLVLILGKLILLHHEHPERKRAPQTYEKEEDEDKGVACSKDEWWWDCLEVLRDNTLVTLANISGQLDLSAYTESICLPILDGLLHWMVCPSAEAQDPFPTVGPNSVLSPQRLVLETLCKLSIQDNNVDLILATPPFSRQEKFYATLVRYVGDRKNPVCREMSMALLSNLAQGDALAARAIAVQKGSIGNLISFLEDGVTMAQYQQSQHNLMHMQPPPLEPPSVDMMCRAAKALLAMARVDENRSEFLLHEGRLLDISISAVLNSLVASVICDVLFQIGQL

>NP_002475.2

MEEVPHDCPGADSAQAGRGASCQGCPNQRLCASGAGATPDTAIEEIKEKMKTVKHKILVLSGKGGVGKSTFSAHLAHGLAEDENTQIALLDIDICGPSIPKIMGLEGEQVHQSGSGWSPVYVEDNLGVMSVGFLLSSPDDAVIWRGPKKNGMIKQFLRDVDWGEVDYLIVDTPPGTSDEHLSVVRYLATAHIDGAVIITTPQEVSLQDVRKEINFCRKVKLPIIGVVENMSGFICPKCKKESQIFPPTTGGAELMCQDLEVPLLGRVPLDPLIGKNCDKGQSFFIDAPDSPATLAYRSIIQRIQEFCNLHQSKEENLISS

1091 NP_055206.2 NP_653288.1

>NP_055206.2

MMVLKVEELVTGKKNGNGEAGEFLPEDFRDGEYEAAVTLEKQEDLKTLLAHPVTLGEQQWKSEKQREAELKKKKLEQRSKLENLEDLEIIIQLKKRKKYRKTKVPVVKEPEPEIITEPVDVPTFLKAALENKLPVVEKFLSDKNNPDVCDEYKRTALHRACLEGHLAIVEKLMEAGAQIEFRDMLESTAIHWASRGGNLDVLKLLLNKGAKISARDKLLSTALHVAVRTGHYECAEHLIACEADLNAKDREGDTPLHDAVRLNRYKMIRLLIMYGADLNIKNCAGKTPMDLVLHWQNGTKAIFDSLRENSYKTSRIATF

>NP_653288.1

MLRTAGRDGLCRLSTYLEELEAVELKKFKLYLGTATELGEGKIPWGSMEKAGPLEMAQLLITHFGPEEAWRLALSTFERINRKDLWERGQREDLVRDTPPGGPSSLGNQSTCLLEVSLVTPRKDPQETYRDYVRRKFRLMEDRNARLGECVNLSHRYTRLLLVKEHSNPMQVQQQLLDTGRGHARTVGHQASPIKIETLFEPDEERPEPPRTVVMQGAAGIGKSMLAHKVMLDWADGKLFQGRFDYLFYINCREMNQSATECSMQDLIFSCWPEPSAPLQELIRVPERLLFIIDGFDELKPSFHDPQGPWCLCWEEKRPTELLLNSLIRKKLLPELSLLITTRPTALEKLHRLLEHPRHVEILGFSEAERKEYFYKYFHNAEQAGQVFNYVRDNEPLFTMCFVPLVCWVVCTCLQQQLEGGGLLRQTSRTTTAVYMLYLLSLMQPKPGAPRLQPPPNQRGLCSLAADGLWNQKILFEEQDLRKHGLDGEDVSAFLNMNIFQKDINCERYYSFIHLSFQEFFAAMYYILDEGEGGAGPDQDVTRLLTEYAFSERSFLALTSRFLFGLLNEETRSHLEKSLCWKVSPHIKMDLLQWIQSKAQSDGSTLQQGSLEFFSCLYEIQEEEFIQQALSHFQVIVVSNIASKMEHMVSSFCLKRCRSAQVLHLYGATYSADGEDRARCSAGAHTLLVQLPERTVLLDAYSEHLAAALCTNPNLIELSLYRNALGSRGVKLLCQGLRHPNCKLQNLRLKRCRISSSACEDLSAALIANKNLTRMDLSGNGVGFPGMMLLCEGLRHPQCRLQMIQLRKCQLESGACQEMASVLGTNPHLVELDLTGNALEDLGLRLLCQGLRHPVCRLRTLWLKICRLTAAACDELASTLSVNQSLRELDLSLNELGDLGVLLLCEGLRHPTCKLQTLRLGICRLGSAACEGLSVVLQANHNLRELDLSFNDLGDWGLWLLAEGLQHPACRLQKLWLDSCGLTAKACENLYFTLGINQTLTDLYLTNNALGDTGVRLLCKRLSHPGCKLRVLWLFGMDLNKMTHSRLAALRVTKPYLDIGC

1092 NP_073598.1 NP_996895.1

>NP_073598.1

MAASQCLCCSKFLFQRQNLACFLTNPHCGSLVNADGHGEVWTDWNNMSKFFQYGWRCTTNENTYSNRTLMGNWNQERYDLRNIVQPKPLPSQFGHYFETTYDTSYNNKMPLSTHRFKREPHWFPGHQPELDPPRYKCTEKSTYMNSYSKP

>NP_996895.1

MIHSLFLINCSGDIFLEKHWKSVVSQSVCDYFFEAQEKAADVENVPPVISTPHHYLISIYRDKLFFVSVIQTEVPPLFVIEFLHRVADTFQDYFGECSEAAIKDNVVIVYELLEEMLDNGFPLATESNILKELIKPPTILRSVVNSITGSSNVGDTLPTGQLSNIPWRRAGVKYTNNEAYFDVVEEIDAIIDKSGSTVFAEIQGVIDACIKLSGMPDLSLSFMNPRLLDDVSFHPCIRFKRWESERVLSFIPPDGNFRLISYRVSSQNLVAIPVYVKHSISFKENSSCGRFDITIGPKQNMGKTIEGITVTVHMPKVVLNMNLTPTQGSYTFDPVTKVLTWDVGKITPQKLPSLKGLVNLQSGAPKPEENPSLNIQFKIQQLAISGLKVNRLDMYGEKYKPFKGVKYVTKAGKFQVRT

1093 NP_077021.1 NP_003158.2

>NP_077021.1

MDKSGIDSLDHVTSDAVELANRSDNSSDSSLFKTQCIPYSPKGEKRNPIRKFVRTPESVHASDSSSDSSFEPIPLTIKAIFERFKNRKKRYKKKKKRRYQPTGRPRGRPEGRRNPIYSLIDKKKQFRSRGSGFPFLESENEKNAPWRKILTFEQAVARGFFNYIEKLKYEHHLKESLKQMNVGEDLENEDFDSRRYKFLDDDGSISPIEESTAEDEDATHLEDNECDIKLAGDSFIVSSEFPVRLSVYLEEEDITEEAALSKKRATKAKNTGQRGLKM

>NP_003158.2

MSDDFLWFEGIAFPTMGFRSETLRKVRDEFVIRDEDVIILTYPKSGTNWLAEILCLMHSKGDAKWIQSVPIWERSPWVESEIGYTALSETESPRLFSSHLPIQLFPKSFFSSKAKVIYLMRNPRDVLVSGYFFWKNMKFIKKPKSWEEYFEWFCQGTVLYGSWFDHIHGWMPMREEKNFLLLSYEELKQDTGRTIEKICQFLGKTLEPEELNLILKNSSFQSMKENKMSNYSLLSVDYVVDKAQLLRKGVSGDWKNHFTVAQAEDFDKLFQEKMADLPRELFPWE

1094 NP_005513.1 Q93008

>NP_005513.1

MDNARMNSFLEYPILSSGDSGTCSARAYPSDHRITTFQSCAVSANSCGGDDRFLVGRGVQIGSPHHHHHHHHHHPQPATYQTSGNLGVSYSHSSCGPSYGSQNFSAPYSPYALNQEADVSGGYPQCAPAVYSGNLSSPMVQHHHHHQGYAGGAVGSPQYIHHSYGQEHQSLALATYNNSLSPLHASHQEACRSPASETSSPAQTFDWMKVKRNPPKTGKVGEYGYLGQPNAVRTNFTTKQLTELEKEFHFNKYLTRARRVEIAASLQLNETQVKIWFQNRRMKQKKREKEGLLPISPATPPGNDEKAEESSEKSSSSPCVPSPGSSTSDTLTTSH

>Q93008

MTATTRGSPVGGNDNQGQAPDGQSQPPLQQNQTSSPDSSNENSPATPPDEQGQGDAPPQLEDEEPAFPHTDLAKLDDMINRPRWVVPVLPKGELEVLLEAAIDLSKKGLDVKSEACQRFFRDGLTISFTKILTDEAVSGWKFEIHVSLVELCVAKLSQDWFPLLELLAMALNPHCKFHIYNGTRPCESVSSSVQLPEDELFARSPDPRSPKGWLVDLLNKFGTLNGFQILHDRFINGSALNVQIIAALIKPFGQCYEFLTLHTVKKYFLPIIEMVPQFLENLTDEELKKEAKNEAKNDALSMIIKSLKNLASRVPGQEETVKNLEIFRLKMILRLLQISSFNGKMNALNEVNKVISSVSYYTHRHGNPEEEEWLTAERMAEWIQQNNILSIVLRDSLHQPQYVEKLEKILRFVIKEKALTLQDLDNIWAAQAGKHEAIVKNVHDLLAKLAWDFSPEQLDHLFDCFKASWTNASKKQREKLLELIRRLAEDDKDGVMAHKVLNLLWNLAHSDDVPVDIMDLALSAHIKILDYSCSQDRDTQKIQWIDRFIEELRTNDKWVIPALKQIREICSLFGEAPQNLSQTQRSPHVFYRHDLINQLQHNHALVTLVAENLATYMESMRLYARDHEDYDPQTVRLGSRYSHVQEVQERLNFLRFLLKDGQLWLCAPQAKQIWKCLAENAVYLCDREACFKWYSKLMGDEPDLDPDINKDFFESNVLQLDPSLLTENGMKCFERFFKAVNCREGKLVAKRRAYMMDDLELIGLDYLWRVVIQSNDDIASRAIDLLKEIYTNLGPRLQVNQVVIHEDFIQSCFDRLKASYDTLCVLDGDKDSVNCARQEAVRMVRVLTVLREYINECDSDYHEERTILPMSRAFRGKHLSFVVRFPNQGRQVDDLEVWSHTNDTIGSVRRCILNRIKANVAHTKIELFVGGELIDPADDRKLIGQLNLKDKSLITAKLTQISSNMPSSPDSSSDSSTGSPGNHGNHYSDGPNPEVESCLPGVIMSLHPRYISFLWQVADLGSSLNMPPLRDGARVLMKLMPPDSTTIEKLRAICLDHAKLGESSLSPSLDSLFFGPSASQVLYLTEVVYALLMPAGAPLADDSSDFQFHFLKSGGLPLVLSMLTRNNFLPNADMETRRGAYLNALKIAKLLLTAIGYGHVRAVAEACQPGVEGVNPMTQINQVTHDQAVVLQSALQSIPNPSSECMLRNVSVRLAQQISDEASRYMPDICVIRAIQKIIWASGCGSLQLVFSPNEEITKIYEKTNAGNEPDLEDEQVCCEALEVMTLCFALIPTALDALSKEKAWQTFIIDLLLHCHSKTVRQVAQEQFFLMCTRCCMGHRPLLFFITLLFTVLGSTARERAKHSGDYFTLLRHLLNYAYNSNINVPNAEVLLNNEIDWLKRIRDDVKRTGETGIEETILEGHLGVTKELLAFQTSEKKFHIGCEKGGANLIKELIDDFIFPASNVYLQYMRNGELPAEQAIPVCGSPPTINAGFELLVALAVGCVRNLKQIVDSLTEMYYIGTAITTCEALTEWEYLPPVGPRPPKGFVGLKNAGATCYMNSVIQQLYMIPSIRNGILAIEGTGSDVDDDMSGDEKQDNESNVDPRDDVFGYPQQFEDKPALSKTEDRKEYNIGVLRHLQVIFGHLAASRLQYYVPRGFWKQFRLWGEPVNLREQHDALEFFNSLVDSLDEALKALGHPAMLSKVLGGSFADQKICQGCPHRYECEESFTTLNVDIRNHQNLLDSLEQYVKGDLLEGANAYHCEKCNKKVDTVKRLLIKKLPPVLAIQLKRFDYDWERECAIKFNDYFEFPRELDMEPYTVAGVAKLEGDNVNPESQLIQQSEQSESETAGSTKYRLVGVLVHSGQASGGHYYSYIIQRNGGDGERNRWYKFDDGDVTECKMDDDEEMKNQCFGGEYMGEVFDHMMKRMSYRRQKRWWNAYILFYERMDTIDQDDELIRYISELAITTRPHQIIMPSAIERSVRKQNVQFMHNRMQYSMEYFQFMKKLLTCNGVYLNPPPGQDHLLPEAEEITMISIQLAARFLFTTGFHTKKVVRGSASDWYDALCILLRHSKNVRFWFAHNVLFNVSNRFSEYLLECPSAEVRGAFAKLIVFIAHFSLQDGPCPSPFASPGPSSQAYDNLSLSDHLLRAVLNLLRREVSEHGRHLQQYFNLFVMYANLGVAEKTQLLKLSVPATFMLVSLDEGPGPPIKYQYAELGKLYSVVSQLIRCCNVSSRMQSSINGNPPLPNPFGDPNLSQPIMPIQQNVADILFVRTSYVKKIIEDCSNSEETVKLLRFCCWENPQFSSTVLSELLWQVAYSYTYELRPYLDLLLQILLIEDSWQTHRIHNALKGIPDDRDGLFDTIQRSKNHYQKRAYQCIKCMVALFSNCPVAYQILQGNGDLKRKWTWAVEWLGDELERRPYTGNPQYTYNNWSPPVQSNETSNGYFLERSHSARMTLAKACELCPEEEPDDQDAPDEHESPPPEDAPLYPHSPGSQYQQNNHVHGQPYTGPAAHHMNNPQRTGQRAQENYEGSEEVSPPQTKDQ

1095 NP_899196.1 NP_055683.3

>NP_899196.1

MENSDSNDKGSGDQSAAQRRSQMDRLDREEAFYQFVNNLSEEDYRLMRDNNLLGTPGESTEEELLRRLQQIKEGPPPQNSDENRGGDSSDDVSNGDSIIDWLNSVRQTGNTTRSGQRGNQSWRAVSRTNPNSGDFRFSLEINVNRNNGSQNSENENEPSARRSSGENVENNSQRQVENPRSESTSARPSRSERNSTEALTEVPPTRGQRRARSRSPDHRRTRARAERSRSPLHPMSEIPRRSHHSISSQTFEHPLVNETEGSSRTRHHVTLRQQISGPELLSRGLFAASGTRNASQGAGSSDTAASGESTGSGQRPPTIVLDLQVRRVRPGEYRQRDSIASRTRSRSQTPNNTVTYESERGGFRRTFSRSERAGVRTYVSTIRIPIRRILNTGLSETTSVAIQTMLRQIMTGFGELSYFMYSDSDSEPTGSVSNRNMERAESRSGRGGSGGGSSSGSSSSSSSSSSSSSSSSSSSSPSSSSGGESSETSSDLFEGSNEGSSSSGSSGARREGRHRAPVTFDESGSLPFLSLAQFFLLNEDDDDQPRGLTKEQIDNLAMRSFGENDALKTCSVCITEYTEGNKLRKLPCSHEYHVHCIDRWLSENSTCPICRRAVLASGNRESVV

>NP_055683.3

MPLSSPNAAATASDMDKNSGSNSSSASSGSSKGQQPPRSASAGPAGESKPKSDGKNSSGSKRYNRKRELSYPKNESFNNQSRRSSSQKSKTFNKMPPQRGGGSSKLFSSSFNGGRRDEVAEAQRAEFSPAQFSGPKKINLNHLLNFTFEPRGQTGHFEGSGHGSWGKRNKWGHKPFNKELFLQANCQFVVSEDQDYTAHFADPDTLVNWDFVEQVRICSHEVPSCPICLYPPTAAKITRCGHIFCWACILHYLSLSEKTWSKCPICYSSVHKKDLKSVVATESHQYVVGDTITMQLMKREKGVLVALPKSKWMNVDHPIHLGDEQHSQYSKLLLASKEQVLHRVVLEEKVALEQQLAEEKHTPESCFIEAAIQELKTREEALSGLAGSRREVTGVVAALEQLVLMAPLAKESVFQPRKGVLEYLSAFDEETTEVCSLDTPSRPLALPLVEEEEAVSEPEPEGLPEACDDLELADDNLKEGTICTESSQQEPITKSGFTRLSSSPCYYFYQAEDGQHMFLHPVNVRCLVREYGSLERSPEKISATVVEIAGYSMSEDVRQRHRYLSHLPLTCEFSICELALQPPVVSKETLEMFSDDIEKRKRQRQKKAREERRRERRIEIEENKKQGKYPEVHIPLENLQQFPAFNSYTCSSDSALGPTSTEGHGALSISPLSRSPGSHADFLLTPLSPTASQGSPSFCVGSLEEDSPFPSFAQMLRVGKAKADVWPKTAPKKDENSLVPPAPVDSDGESDNSDRVPVPSFQNSFSQAIEAAFMKLDTPATSDPLSEEKGGKKRKKQKQKLLFSTSVVHTK

1096 NP_066951.1 NP_110379.2

>NP_066951.1

MIIPVRCFTCGKIVGNKWEAYLGLLQAEYTEGDALDALGLKRYCCRRMLLAHVDLIEKLLNYAPLEK

>NP_110379.2

MEGPLSVFGDRSTGETIRSQNVMAAASIANIVKSSLGPVGLDKMLVDDIGDVTITNDGATILKLLEVEHPAAKVLCELADLQDKEVGDGTTSVVIIAAELLKNADELVKQKIHPTSVISGYRLACKEAVRYINENLIVNTDELGRDCLINAAKTSMSSKIIGINGDFFANMVVDAVLAIKYTDIRGQPRYPVNSVNILKAHGRSQMESMLISGYALNCVVGSQGMPKRIVNAKIACLDFSLQKTKMKLGVQVVITDPEKLDQIRQRESDITKERIQKILATGANVILTTGGIDDMCLKYFVEAGAMAVRRVLKRDLKRIAKASGATILSTLANLEGEETFEAAMLGQAEEVVQERICDDELILIKNTKARTSASIILRGANDFMCDEMERSLHDALCVVKRVLESKSVVPGGGAVEAALSIYLENYATSMGSREQLAIAEFARSLLVIPNTLAVNAAQDSTDLVAKLRAFHNEAQVNPERKNLKWIGLDLSNGKPRDNKQAGVFEPTIVKVKSLKFATEAAITILRIDDLIKLHPESKDDKHGSYEDAVHSGALND

1097 NP_068809.1 NP_002684.1

>NP_068809.1

MSDNEDNFDGDDFDDVEEDEGLDDLENAEEEGQENVEILPSGERPQANQKRITTPYMTKYERARVLGTRALQIAMCAPVMVELEGETDPLLIAMKELKARKIPIIIRRYLPDGSYEDWGVDELIITD

>NP_002684.1

MSRLLWRKVAGATVGPGPVPAPGRWVSSSVPASDPSDGQRRRQQQQQQQQQQQQQPQQPQVLSSEGGQLRHNPLDIQMLSRGLHEQIFGQGGEMPGEAAVRRSVEHLQKHGLWGQPAVPLPDVELRLPPLYGDNLDQHFRLLAQKQSLPYLEAANLLLQAQLPPKPPAWAWAEGWTRYGPEGEAVPVAIPEERALVFDVEVCLAEGTCPTLAVAISPSAWYSWCSQRLVEERYSWTSQLSPADLIPLEVPTGASSPTQRDWQEQLVVGHNVSFDRAHIREQYLIQGSRMRFLDTMSMHMAISGLSSFQRSLWIAAKQGKHKVQPPTKQGQKSQRKARRGPAISSWDWLDISSVNSLAEVHRLYVGGPPLEKEPRELFVKGTMKDIRENFQDLMQYCAQDVWATHEVFQQQLPLFLERCPHPVTLAGMLEMGVSYLPVNQNWERYLAEAQGTYEELQREMKKSLMDLANDACQLLSGERYKEDPWLWDLEWDLQEFKQKKAKKVKKEPATASKLPIEGAGAPGDPMDQEDLGPCSEEEEFQQDVMARACLQKLKGTTELLPKRPQHLPGHPGWYRKLCPRLDDPAWTPGPSLLSLQMRVTPKLMALTWDGFPLHYSERHGWGYLVPGRRDNLAKLPTGTTLESAGVVCPYRAIESLYRKHCLEQGKQQLMPQEAGLAEEFLLTDNSAIWQTVEELDYLEVEAEAKMENLRAAVPGQPLALTARGGPKDTQPSYHHGNGPYNDVDIPGCWFFKLPHKDGNSCNVGSPFAKDFLPKMEDGTLQAGPGGASGPRALEINKMISFWRNAHKRISSQMVVWLPRSALPRAVIRHPDYDEEGLYGAILPQVVTAGTITRRAVEPTWLTASNARPDRVGSELKAMVQAPPGYTLVGADVDSQELWIAAVLGDAHFAGMHGCTAFGWMTLQGRKSRGTDLHSKTATTVGISREHAKIFNYGRIYGAGQPFAERLLMQFNHRLTQQEAAEKAQQMYAATKGLRWYRLSDEGEWLVRELNLPVDRTEGGWISLQDLRKVQRETARKSQWKKWEVVAERAWKGGTESEMFNKLESIATSDIPRTPVLGCCISRALEPSAVQEEFMTSRVNWVVQSSAVDYLHLMLVAMKWLFEEFAIDGRFCISIHDEVRYLVREEDRYRAALALQITNLLTRCMFAYKLGLNDLPQSVAFFSAVDIDRCLRKEVTMDCKTPSNPTGMERRYGIPQGEALDIYQIIELTKGSLEKRSQPGP

1098 NP_056464.1 Q8IUQ0

>NP_056464.1

MASVQASRRQWCYLCDLPKMPWAMVWDFSEAVCRGCVNFEGADRIELLIDAARQLKRSHVLPEGRSPGPPALKHPATKDLAAAAAQGPQLPPPQAQPQPSGTGGGVSGQDRYDRATSSGRLPLPSPALEYTLGSRLANGLGREEAVAEGARRALLGSMPGLMPPGLLAAAVSGLGSRGLTLAPGLSPARPLFGSDFEKEKQQRNADCLAELNEAMRGRAEEWHGRPKAVREQLLALSACAPFNVRFKKDHGLVGRVFAFDATARPPGYEFELKLFTEYPCGSGNVYAGVLAVARQMFHDALREPGKALASSGFKYLEYERRHGSGEWRQLGELLTDGVRSFREPAPAEALPQQYPEPAPAALCGPPPRAPSRNLAPTPRRRKASPEPEGEAAGKMTTEEQQQRHWVAPGGPYSAETPGVPSPIAALKNVAEALGHSPKDPGGGGGPVRAGGASPAASSTAQPPTQHRLVARNGEAEVSPTAGAEAVSGGGSGTGATPGAPLCCTLCRERLEDTHFVQCPSVPGHKFCFPCSREFIKAQGPAGEVYCPSGDKCPLVGSSVPWAFMQGEIATILAGDIKVKKERDP

>Q8IUQ0

MGPVSLLPKYQKLNTWNGDLAKMTHLQAGLSPETIEKARLELNENPDVLHQDIQQVRDMIITRPDIGFLRTDDAFILRFLRARKFHQADAFRLLAQYFQYRQLNLDMFKNFKADDPGIKRALIDGFPGVLENRDHYGRKILLLFAANWDQSRNSFTDILRAILLSLEVLIEDPELQINGFILIIDWSNFSFKQASKLTPSILKLAIEGLQDSFPARFGGVHFVNQPWYIHALYTLIKPFLKDKTRKRIFLHGNNLNSLHQLIHPEFLPSEFGGTLPPYDMGTWARTLLGPDYSDENDYTHTSYNAMHVKHTSSNLERECSPKLMKRSQSVVEAGTLKHEEKGENENTQPLLALD

1099 NP_054753.1 NP_001007554.1

>NP_054753.1

MAKVQVNNVVVLDNPSPFYNPFQFEITFECIEDLSEDLEWKIIYVGSAESEEYDQVLDSVLVGPVPAGRHMFVFQADAPNPGLIPDADAVGVTVVLITCTYRGQEFIRVGYYVNNEYTETELRENPPVKPDFSKLQRNILASNPRVTRFHINWEDNTEKLEDAESSNPNLQSLLSTDALPSASKGWSTSENSLNVMLESHMDCM

>NP_001007554.1

MSFDPNLLHNNGHNGYPNGTSAALRETGVIEKLLTSYGFIQCSERQARLFFHCSQYNGNLQDLKVGDDVEFEVSSDRRTGKPIAVKLVKIKQEILPEERMNGQVVCAVPHNLESKSPAAPGQSPTGSVCYERNGEVFYLTYTPEDVEGNVQLETGDKINFVIDNNKHTGAVSARNIMLLKKKQARCQGVVCAMKEAFGFIERGDVVKEIFFHYSEFKGDLETLQPGDDVEFTIKDRNGKEVATDVRLLPQGTVIFEDISIEHFEGTVTKVIPKVPSKNQNDPLPGRIKVDFVIPKELPFGDKDTKSKVTLLEGDHVRFNISTDRRDKLERATNIEVLSNTFQFTNEAREMGVIAAMRDGFGFIKCVDRDVRMFFHFSEILDGNQLHIADEVEFTVVPDMLSAQRNHAIRIKKLPKGTVSFHSHSDHRFLGTVEKEATFSNPKTTSPNKGKEKEAEDGIIAYDDCGVKLTIAFQAKDVEGSTSPQIGDKVEFSISDKQRPGQQVATCVRLLGRNSNSKRLLGYVATLKDNFGFIETANHDKEIFFHYSEFSGDVDSLELGDMVEYSLSKGKGNKVSAEKVNKTHSVNGITEEADPTIYSGKVIRPLRSVDPTQTEYQGMIEIVEEGDMKGEVYPFGIVGMANKGDCLQKGESVKFQLCVLGQNAQTMAYNITPLRRATVECVKDQFGFINYEVGDSKKLFFHVKEVQDGIELQAGDEVEFSVILNQRTGKCSACNVWRVCEGPKAVAAPRPDRLVNRLKNITLDDASAPRLMVLRQPRGPDNSMGFGAERKIRQAGVID

1100 Q13268 P11245

>Q13268

MSSTGIDRKGVLANRVAVVTGSTSGIGFAIARRLARDGAHVVISSRKQQNVDRAMAKLQGEGLSVAGIVCHVGKAEDREQLVAKALEHCGGVDFLVCSAGVNPLVGSTLGTSEQIWDKILSVNVKSPALLLSQLLPYMENRRGAVILVSSIAAYNPVVALGVYNVSKTALLGLTRTLALELAPKDIRVNCVVPGIIKTDFSKVFHGNESLWKNFKEHHQLQRIGESEDCAGIVSFLCSPDASYVNGENIAVAGYSTRL

>P11245

MDIEAYFERIGYKNSRNKLDLETLTDILEHQIRAVPFENLNMHCGQAMELGLEAIFDHIVRRNRGGWCLQVNQLLYWALTTIGFQTTMLGGYFYIPPVNKYSTGMVHLLLQVTIDGRNYIVDAGSGSSSQMWQPLELISGKDQPQVPCIFCLTEERGIWYLDQIRREQYITNKEFLNSHLLPKKKHQKIYLFTLEPRTIEDFESMNTYLQTSPTSSFITTSFCSLQTPEGVYCLVGFILTYRKFNYKDNTDLVEFKTLTEEEVEEVLKNIFKISLGRNLVPKPGDGSLTI

1101 P0C665 NP_057740.2

>P0C665

MDRRRMALRPGSRRPTAFFFHSRWLVPNLLAFFLGLSGAGPIHLPMPWPNGRRHRVLDPHTQLSTHEAPGRWKPVAPRRMKACPQVLLEW

>NP_057740.2

MEESDSEKTTEKENLGPRMDPPLGEPEGSLGWVLPNTAMKKKVLLMGKSGSGKTSMRSIIFANYIARDTRRLGATILDRIHSLQINSSLSTYSLVDSVGNTKTFDVEHSHVRFLGNLVLNLWDCGGQDTFMENYFTSQRDNIFRNVEVLIYVFDVESRELEKDMHYYQSCLEAILQNSPDAKIFCLVHKMDLVQEDQRDLIFKEREEDLRRLSRPLECSCFRTSIWDETLYKAWSSIVYQLIPNVQQLEMNLRNFAEIIEADEVLLFERATFLVISHYQCKEQRDAHRFEKISNIIKQFKLSCSKLAASFQSMEVRNSNFAAFIDIFTSNTYVMVVMSDPSIPSAATLINIRNARKHFEKLERVDGPKQCLLMR

1102 NP_005229.1 NP_037386.1

>NP_005229.1

MKAAVDLKPTLTIIKTEKVDLELFPSPDMECADVPLLTPSSKEMMSQALKATFSGFTKEQQRLGIPKDPRQWTETHVRDWVMWAVNEFSLKGVDFQKFCMNGAALCALGKDCFLELAPDFVGDILWEHLEILQKEDVKPYQVNGVNPAYPESRYTSDYFISYGIEHAQCVPPSEFSEPSFITESYQTLHPISSEELLSLKYENDYPSVILRDPLQTDTLQNDYFAIKQEVVTPDNMCMGRTSRGKLGGQDSFESIESYDSCDRLTQSWSSQSSFNSLQRVPSYDSFDSEDYPAALPNHKPKGTFKDYVRDRADLNKDKPVIPAAALAGYTGSGPIQLWQFLLELLTDKSCQSFISWTGDGWEFKLSDPDEVARRWGKRKNKPKMNYEKLSRGLRYYYDKNIIHKTAGKRYVYRFVCDLQSLLGYTPEELHAMLDVKPDADE

>NP_037386.1

MQSTSNHLWLLSDILGQGATANVFRGRHKKTGDLFAIKVFNNISFLRPVDVQMREFEVLKKLNHKNIVKLFAIEEETTTRHKVLIMEFCPCGSLYTVLEEPSNAYGLPESEFLIVLRDVVGGMNHLRENGIVHRDIKPGNIMRVIGEDGQSVYKLTDFGAARELEDDEQFVSLYGTEEYLHPDMYERAVLRKDHQKKYGATVDLWSIGVTFYHAATGSLPFRPFEGPRRNKEVMYKIITGKPSGAISGVQKAENGPIDWSGDMPVSCSLSRGLQVLLTPVLANILEADQEKCWGFDQFFAETSDILHRMVIHVFSLQQMTAHKIYIHSYNTATIFHELVYKQTKIISSNQELIYEGRRLVLEPGRLAQHFPKTTEENPIFVVSREPLNTIGLIYEKISLPKVHPRYDLDGDASMAKAITGVVCYACRIASTLLLYQELMRKGIRWLIELIKDDYNETVHKKTEVVITLDFCIRNIEKTVKVYEKLMKINLEAAELGEISDIHTKLLRLSSSQGTIETSLQDIDSRLSPGGSLADAWAHQEGTHPKDRNVEKLQVLLNCMTEIYYQFKKDKAERRLAYNEEQIHKFDKQKLYYHATKAMTHFTDECVKKYEAFLNKSEEWIRKMLHLRKQLLSLTNQCFDIEEEVSKYQEYTNELQETLPQKMFTASSGIKHTMTPIYPSSNTLVEMTLGMKKLKEEMEGVVKELAENNHILERFGSLTMDGGLRNVDCL

1103 O60814 NP_001025.1

>O60814

MPEPAKSAPAPKKGSKKAVTKAQKKDGKKRKRSRKESYSVYVYKVLKQVHPDTGISSKAMGIMNSFVNDIFERIAGEASRLAHYNKRSTITSREIQTAVRLLLPGELAKHAVSEGTKAVTKYTSAK

>NP_001025.1

MLSLRVPLAPITDPQQLQLSPLKGLSLVDKENTPPALSGTRVLASKTARRIFQEPTEPKTKAAAPGVEDEPLLRENPRRFVIFPIEYHDIWQMYKKAEASFWTAEEVDLSKDIQHWESLKPEERYFISHVLAFFAASDGIVNENLVERFSQEVQITEARCFYGFQIAMENIHSEMYSLLIDTYIKDPKEREFLFNAIETMPCVKKKADWALRWIGDKEATYGERVVAFAAVEGIFFSGSFASIFWLKKRGLMPGLTFSNELISRDEGLHCDFACLMFKHLVHKPSEERVREIIINAVRIEQEFLTEALPVKLIGMNCTLMKQYIEFVADRLMLELGFSKVFRVENPFDFMENISLEGKTNFFEKRVGEYQRMGVMSSPTENSFTLDADF

1104 Q9UDW3 NP_004530.1

>Q9UDW3

MGKRYFCDYCDRSFQDNLHNRKKHLNGLQHLKAKKVWYDMFRDAAAILLDEQNKRPCRKFLLTGQCDFGSNCRFSHMSERDLQELSIQVEEERRAREWLLDAPELPEGHLEDWLEKRAKRLSSAPSSRAEPIRTTVFQYPVGWPPVQELPPSLRAPPPGGWPLQPRVQWG

>NP_004530.1

MVLAELYVSDREGSDATGDGTKEKPFKTGLKALMTVGKEPFPTIYVDSQKENERWNVISKSQLKNIKKMWHREQMKSESREKKEAEDSLRREKNLEEAKKITIKNDPSLPEPKCVKIGALEGYRGQRVKVFGWVHRLRRQGKNLMFLVLRDGTGYLQCVLADELCQCYNGVLLSTESSVAVYGMLNLTPKGKQAPGGHELSCDFWELIGLAPAGGADNLINEESDVDVQLNNRHMMIRGENMSKILKARSMVTRCFRDHFFDRGYYEVTPPTLVQTQVEGGATLFKLDYFGEEAFLTQSSQLYLETCLPALGDVFCIAQSYRAEQSRTRRHLAEYTHVEAECPFLTFDDLLNRLEDLVCDVVDRILKSPAGSIVHELNPNFQPPKRPFKRMNYSDAIVWLKEHDVKKEDGTFYEFGEDIPEAPERLMTDTINEPILLCRFPVEIKSFYMQRCPEDSRLTESVDVLMPNVGEIVGGSMRIFDSEEILAGYKREGIDPTPYYWYTDQRKYGTCPHGGYGLGLERFLTWILNRYHIRDVCLYPRFVQRCTP

1105 P23771 NP_001503.1

>P23771

MEVTADQPRWVSHHHPAVLNGQHPDTHHPGLSHSYMDAAQYPLPEEVDVLFNIDGQGNHVPPYYGNSVRATVQRYPPTHHGSQVCRPPLLHGSLPWLDGGKALGSHHTASPWNLSPFSKTSIHHGSPGPLSVYPPASSSSLSGGHASPHLFTFPPTPPKDVSPDPSLSTPGSAGSARQDEKECLKYQVPLPDSMKLESSHSRGSMTALGGASSSTHHPITTYPPYVPEYSSGLFPPSSLLGGSPTGFGCKSRPKARSSTGRECVNCGATSTPLWRRDGTGHYLCNACGLYHKMNGQNRPLIKPKRRLSAARRAGTSCANCQTTTTTLWRRNANGDPVCNACGLYYKLHNINRPLTMKKEGIQTRNRKMSSKSKKCKKVHDSLEDFPKNSSFNPAALSRHMSSLSHISPFSHSSHMLTTPTPMHPPSSLSFGPHHPSSMVTAMG

>NP_001503.1

MAARPKLHYPNGRGRMESVRWVLAAAGVEFDEEFLETKEQLYKLQDGNHLLFQQVPMVEIDGMKLVQTRSILHYIADKHNLFGKNLKERTLIDMYVEGTLDLLELLIMHPFLKPDDQQKEVVNMAQKAIIRYFPVFEKILRGHGQSFLVGNQLSLADVILLQTILALEEKIPNILSAFPFLQEYTVKLSNIPTIKRFLEPGSKKKPPPDEIYVRTVYNIFRP

1106 O60264 P53677

>O60264

MSSAAEPPPPPPPESAPSKPAASIASGGSNSSNKGGPEGVAAQAVASAASAGPADAEMEEIFDDASPGKQKEIQEPDPTYEEKMQTDRANRFEYLLKQTELFAHFIQPAAQKTPTSPLKMKPGRPRIKKDEKQNLLSVGDYRHRRTEQEEDEELLTESSKATNVCTRFEDSPSYVKWGKLRDYQVRGLNWLISLYENGINGILADEMGLGKTLQTISLLGYMKHYRNIPGPHMVLVPKSTLHNWMSEFKRWVPTLRSVCLIGDKEQRAAFVRDVLLPGEWDVCVTSYEMLIKEKSVFKKFNWRYLVIDEAHRIKNEKSKLSEIVREFKTTNRLLLTGTPLQNNLHELWSLLNFLLPDVFNSADDFDSWFDTNNCLGDQKLVERLHMVLRPFLLRRIKADVEKSLPPKKEVKIYVGLSKMQREWYTRILMKDIDILNSAGKMDKMRLLNILMQLRKCCNHPYLFDGAEPGPPYTTDMHLVTNSGKMVVLDKLLPKLKEQGSRVLIFSQMTRVLDILEDYCMWRNYEYCRLDGQTPHDERQDSINAYNEPNSTKFVFMLSTRAGGLGINLATADVVILYDSDWNPQVDLQAMDRAHRIGQTKTVRVFRFITDNTVEERIVERAEMKLRLDSIVIQQGRLVDQNLNKIGKDEMLQMIRHGATHVFASKESEITDEDIDGILERGAKKTAEMNEKLSKMGESSLRNFTMDTESSVYNFEGEDYREKQKIAFTEWIEPPKRERKANYAVDAYFREALRVSEPKAPKAPRPPKQPNVQDFQFFPPRLFELLEKEILFYRKTIGYKVPRNPELPNAAQAQKEEQLKIDEAESLNDEELEEKEKLLTQGFTNWNKRDFNQFIKANEKWGRDDIENIAREVEGKTPEEVIEYSAVFWERCNELQDIEKIMAQIERGEARIQRRISIKKALDTKIGRYKAPFHQLRISYGTNKGKNYTEEEDRFLICMLHKLGFDKENVYDELRQCIRNSPQFRFDWFLKSRTAMELQRRCNTLITLIERENMELEEKEKAEKKKRGPKPSTQKRKMDGAPDGRGRKKKLKL

>P53677

MIHSLFLINSSGDIFLEKHWKSVVSRSVCDYFFEAQERATEAENVPPVIPTPHHYLLSVYRHKIFFVAVIQTEVPPLFVIEFLHRVVDTFQDYFGVCSEPVIKDNVVVVYEVLEEMLDNGFPLATESNILKELIKPPTILRTVVNTITGSTNVGDQLPTGQLSVVPWRRTGVKYTNNEAYFDVIEEIDAIIDKSGSTITAEIQGVIDACVKLTGMPDLTLSFMNPRLLDDVSFHPCVRFKRWESERILSFIPPDGNFRLLSYHVSAQNLVAIPVYVKHNISFRDSSSLGRFEITVGPKQTMGKTIEGVTVTSQMPKGVLNMSLTPSQGTHTFDPVTKMLSWDVGKINPQKLPSLKGTMSLQAGASKPDENPTINLQFKIQQLAISGLKVNRLDMYGEKYKPFKGIKYMTKAGKFQVRT

1107 NP_003933.1 Q8WVN8

>NP_003933.1

MGDEDDDESCAVELRITEANLTGHEEKVSVENFELLKVLGTGAYGKVFLVRKAGGHDAGKLYAMKVLRKAALVQRAKTQEHTRTERSVLELVRQAPFLVTLHYAFQTDAKLHLILDYVSGGEMFTHLYQRQYFKEAEVRVYGGEIVLALEHLHKLGIIYRDLKLENVLLDSEGHIVLTDFGLSKEFLTEEKERTFSFCGTIEYMAPEIIRSKTGHGKAVDWWSLGILLFELLTGASPFTLEGERNTQAEVSRRILKCSPPFPPRIGPVAQDLLQRLLCKDPKKRLGAGPQGAQEVRNHPFFQGLDWVALAARKIPAPFRPQIRSELDVGNFAEEFTRLEPVYSPPGSPPPGDPRIFQGYSFVAPSILFDHNNAVMTDGLEAPGAGDRPGRAAVARSAMMQDSPFFQQYELDLREPALGQGSFSVCRRCRQRQSGQEFAVKILSRRLEANTQREVAALRLCQSHPNVVNLHEVHHDQLHTYLVLELLRGGELLEHIRKKRHFSESEASQILRSLVSAVSFMHEEAGVVHRDLKPENILYADDTPGAPVKIIDFGFARLRPQSPGVPMQTPCFTLQYAAPELLAQQGYDESCDLWSLGVILYMMLSGQVPFQGASGQGGQSQAAEIMCKIREGRFSLDGEAWQGVSEEAKELVRGLLTVDPAKRLKLEGLRGSSWLQDGSARSSPPLRTPDVLESSGPAVRSGLNATFMAFNRGKREGFFLKSVENAPLAKRRKQKLRSATASRRGSPAPANPGRAPVASKGAPRRANGPLPPS

>Q8WVN8

MSVSGLKAELKFLASIFDKNHERFRIVSWKLDELHCQFLVPQQGSPHSLPPPLTLHCNITESYPSSSPIWFVDSEDPNLTSVLERLEDTKNNNLLRQQLKWLICELCSLYNLPKHLDVEMLDQPLPTGQNGTTEEVTSEEEEEEEEMAEDIEDLDHYEMKEEEPISGKKSEDEGIEKENLAILEKIRKTQRQDHLNGAVSGSVQASDRLMKELRDIYRSQSYKTGIYSVELINDSLYDWHVKLQKVDPDSPLHSDLQILKEKEGIEYILLNFSFKDNFPFDPPFVRVVLPVLSGGYVLGGGALCMELLTKQGWSSAYSIESVIMQINATLVKGKARVQFGANKNQYNLARAQQSYNSIVQIHEKNGWYTPPKEDG

1108 NP_055792.1 Q8NFG4

>NP_055792.1

MWRRKHPRTSGGTRGVLSGNRGVEYGSGRGHLGTFEGRWRKLPKMPEAVGTDPSTSRKMAELEEVTLDGKPLQALRVTDLKAALEQRGLAKSGQKSALVKRLKGALMLENLQKHSTPHAAFQPNSQIGEEMSQNSFIKQYLEKQQELLRQRLEREAREAAELEEASAESEDEMIHPEGVASLLPPDFQSSLERPELELSRHSPRKSSSISEEKGDSDDEKPRKGERRSSRVRQARAAKLSEGSQPAEEEEDQETPSRNLRVRADRNLKTEEEEEEEEEEEEDDEEEEGDDEGQKSREAPILKEFKEEGEEIPRVKPEEMMDERPKTRSQEQEVLERGGRFTRSQEEARKSHLARQQQEKEMKTTSPLEEEEREIKSSQGLKEKSKSPSPPRLTEDRKKASLVALPEQTASEEETPPPLLTKEASSPPPHPQLHSEEEIEPMEGPAPPVLIQLSPPNTDADTRELLVSQHTVQLVGGLSPLSSPSDTKAESPAEKVPEESVLPLVQKSTLADYSAQKDLEPESDRSAQPLPLKIEELALAKGITEECLKQPSLEQKEGRRASHTLLPSHRLKQSADSSSSRSSSSSSSSSRSRSRSPDSSGSRSHSPLRSKQRDVAQARTHANPRGRPKMGSRSTSESRSRSRSRSRSASSNSRKSLSPGVSRDSSTSYTETKDPSSGQEVATPPVPQLQVCEPKERTSTSSSSVQARRLSQPESAEKHVTQRLQPERGSPKKCEAEEAEPPAATQPQTSETQTSHLPESERIHHTVEEKEEVTMDTSENRPENDVPEPPMPIADQVSNDDRPEGSVEDEEKKESSLPKSFKRKISVVSATKGVPAGNSDTEGGQPGRKRRWGASTATTQKKPSISITTESLKSLIPDIKPLAGQEAVVDLHADDSRISEDETERNGDDGTHDKGLKICRTVTQVVPAEGQENGQREEEEEEKEPEAEPPVPPQVSVEVALPPPAEHEVKKVTLGDTLTRRSISQQKSGVSITIDDPVRTAQVPSPPRGKISNIVHISNLVRPFTLGQLKELLGRTGTLVEEAFWIDKIKSHCFVTYSTVEEAVATRTALHGVKWPQSNPKFLCADYAEQDELDYHRGLLVDRPSETKTEEQGIPRPLHPPPPPPVQPPQHPRAEQREQERAVREQWAEREREMERRERTRSEREWDRDKVREGPRSRSRSRDRRRKERAKSKEKKSEKKEKAQEEPPAKLLDDLFRKTKAAPCIYWLPLTDSQIVQKEAERAERAKEREKRRKEQEEEEQKEREKEAERERNRQLEREKRREHSRERDRERERERERDRGDRDRDRERDRERGRERDRRDTKRHSRSRSRSTPVRDRGGRR

>Q8NFG4

MNAIVALCHFCELHGPRTLFCTEVLHAPLPQGDGNEDSPGQGEQAEEEEGGIQMNSRMRAHSPAEGASVESSSPGPKKSDMCEGCRSLAAGHPGYISHDKETSIKYVSHQHPSHPQLFSIVRQACVRSLSCEVCPGREGPIFFGDEQHGFVFSHTFFIKDSLARGFQRWYSIITIMMDRIYLINSWPFLLGKVRGIIDELQGKALKVFEAEQFGCPQRAQRMNTAFTPFLHQRNGNAARSLTSLTSDDNLWACLHTSFAWLLKACGSRLTEKLLEGAPTEDTLVQMEKLADLEEESESWDNSEAEEEEKAPVLPESTEGRELTQGPAESSSLSGCGSWQPRKLPVFKSLRHMRQVLGAPSFRMLAWHVLMGNQVIWKSRDVDLVQSAFEVLRTMLPVGCVRIIPYSSQYEEAYRCNFLGLSPHVQIPPHVLSSEFAVIVEVHAAARSTLHPVGCEDDQSLSKYEFVVTSGSPVAADRVGPTILNKIEAALTNQNLSVDVVDQCLVCLKEEWMNKVKVLFKFTKVDSRPKEDTQKLLSILGASEEDNVKLLKFWMTGLSKTYKSHLMSTVRSPTASESRN

1109 NP_004119.1 Q2VPK5

>NP_004119.1

MAERGELDLTGAKQNTGVWLVKVPKYLSQQWAKASGRGEVGKLRIAKTQGRTEVSFTLNEDLANIHDIGGKPASVSAPREHPFVLQSVGGQTLTVFTESSSDKLSLEGIVVQRAECRPAASENYMRLKRLQIEESSKPVRLSQQLDKVVTTNYKPVANHQYNIEYERKKKEDGKRARADKQHVLDMLFSAFEKHQYYNLKDLVDITKQPVVYLKEILKEIGVQNVKGIHKNTWELKPEYRHYQGEEKSD

>Q2VPK5

MCQVGEDYGEPAPEEPPPAPRPSREQKCVKCKEAQPVVVIRAGDAFCRDCFKAFYVHKFRAMLGKNRLIFPGEKVLLAWSGGPSSSSMVWQVLEGLSQDSAKRLRFVAGVIFVDEGAACGQSLEERSKTLAEVKPILQATGFPWHVVALEEVFSLPPSVLWCSAQELVGSEGAYKAAVDSFLQQQHVLGAGGGPGPTQGEEQPPQPPLDPQNLARPPAPAQTEALSQLFCSVRTLTAKEELLQTLRTHLILHMARAHGYSKVMTGDSCTRLAIKLMTNLALGRGAFLAWDTGFSDERHGDVVVVRPMRDHTLKEVAFYNRLFSVPSVFTPAVDTKAPEKASIHRLMEAFILRLQTQFPSTVSTVYRTSEKLVKGPRDGPAAGDSGPRCLLCMCALDVDAADSATAFGAQTSSRLSQMQSPIPLTETRTPPGPCCSPGVGWAQRCGQGACRREDPQACIEEQLCYSCRVNMKDLPSLDPLPPYILAEAQLRTQRAWGLQEIRDCLIEDSDDEAGQS

1110 NP_079441.1 Q16540

>NP_079441.1

MEELQEPLRGQLRLCFTQAARTSLLLLRLNDAALRALQECQRQQVRPVIAFQGHRGYLRLPGPGWSCLFSFIVSQCCQEGAGGSLDLVCQRFLRSGPNSLHCLGSLRERLIIWAAMDSIPAPSSVQGHNLTEDARHPESWQNTGGYSEGDAVSQPQMALEEVSVSDPLASNQGQSLPGSSREHMAQWEVRSQTHVPNREPVQALPSSASRKRLDKKRSVPVATVELEEKRFRTLPLVPSPLQGLTNQDLQEGEDWEQEDEDMDPRLEHSSSVQEDSESPSPEDIPDYLLQYRAIHSAEQQHAYEQDFETDYAEYRILHARVGTASQRFIELGAEIKRVRRGTPEYKVLEDKIIQEYKKFRKQYPSYREEKRRCEYLHQKLSHIKGLILEFEEKNRGS

>Q16540

MARNVVYPLYRLGGPQLRVFRTNFFIQLVRPGVAQPEDTVQFRIPMEMTRVDLRNYLEGIYNVPVAAVRTRVQHGSNKRRDHRNVRIKKPDYKVAYVQLAHGQTFTFPDLFPEKDESPEGSAADDLYSMLEEERQQRQSSDPRRGGVPSWFGL

1111 NP_004066.1 NP_036309.1

>NP_004066.1

MGVNAVHWFRKGLRLHDNPALKECIQGADTIRCVYILDPWFAGSSNVGINRWRFLLQCLEDLDANLRKLNSRLFVIRGQPADVFPRLFKEWNITKLSIEYDSEPFGKERDAAIKKLATEAGVEVIVRISHTLYDLDKIIELNGGQPPLTYKRFQTLISKMEPLEIPVETITSEVIEKCTTPLSDDHDEKYGVPSLEELGFDTDGLSSAVWPGGETEALTRLERHLERKAWVANFERPRMNANSLLASPTGLSPYLRFGCLSCRLFYFKLTDLYKKVKKNSSPPLSLYGQLLWREFFYTAATNNPRFDKMEGNPICVQIPWDKNPEALAKWAEGRTGFPWIDAIMTQLRQEGWIHHLARHAVACFLTRGDLWISWEEGMKVFEELLLDADWSINAGSWMWLSCSSFFQQFFHCYCPVGFGRRTDPNGDYIRRYLPVLRGFPAKYIYDPWNAPEGIQKVAKCLIGVNYPKPMVNHAEASRLNIERMKQIYQQLSRYRGLGLLASVPSNPNGNGGFMGYSAENIPGCSSSGSCSQGSGILHYAHGDSQQTHLLKQGRSSMGTGLSGGKRPSQEEDTQSIGPKVQRQSTN

>NP_036309.1

MSRRPCSCALRPPRCSCSASPSAVTAAGRPRPSDSCKEESSTLSVKMKCDFNCNHVHSGLKLVKPDDIGRLVSYTPAYLEGSCKDCIKDYERLSCIGSPIVSPRIVQLETESKRLHNKENQHVQQTLNSTNEIEALETSRLYEDSGYSSFSLQSGLSEHEEGSLLEENFGDSLQSCLLQIQSPDQYPNKNLLPVLHFEKVVCSTLKKNAKRNPKVDREMLKEIIARGNFRLQNIIGRKMGLECVDILSELFRRGLRHVLATILAQLSDMDLINVSKVSTTWKKILEDDKGAFQLYSKAIQRVTENNNKFSPHASTREYVMFRTPLASVQKSAAQTSLKKDAQTKLSNQGDQKGSTYSRHNEFSEVAKTLKKNESLKACIRCNSPAKYDCYLQRATCKREGCGFDYCTKCLCNYHTTKDCSDGKLLKASCKIGPLPGTKKSKKNLRRL

1112 NP_005313.1 Q9NX20

>NP_005313.1

MSETAPAETATPAPVEKSPAKKKATKKAAGAGAAKRKATGPPVSELITKAVAASKERNGLSLAALKKALAAGGYDVEKNNSRIKLGLKSLVSKGTLVQTKGTGASGSFKLNKKAASGEAKPKAKKAGAAKAKKPAGATPKKAKKAAGAKKAVKKTPKKAKKPAAAGVKKVAKSPKKAKAAAKPKKATKSPAKPKAVKPKAAKPKAAKPKAAKPKAAKAKKAAAKKK

>Q9NX20

MWRLLARASAPLLRVPLSDSWALLPASAGVKTLLPVPSFEDVSIPEKPKLRFIERAPLVPKVRREPKNLSDIRGPSTEATEFTEGNFAILALGGGYLHWGHFEMMRLTINRSMDPKNMFAIWRVPAPFKPITRKSVGHRMGGGKGAIDHYVTPVKAGRLVVEMGGRCEFEEVQGFLDQVAHKLPFAAKAVSRGTLEKMRKDQEERERNNQNPWTFERIATANMLGIRKVLSPYDLTHKGKYWGKFYMPKRV

1113 NP_002189.1 P55210

>NP_002189.1

MPITRMRMRPWLEMQINSNQIPGLIWINKEEMIFQIPWKHAAKHGWDINKDACLFRSWAIHTGRYKAGEKEPDPKTWKANFRCAMNSLPDIEEVKDQSRNKGSSAVRVYRMLPPLTKNQRKERKSKSSRDAKSKAKRKSCGDSSPDTFSDGLSSSTLPDDHSSYTVPGYMQDLEVEQALTPALSPCAVSSTLPDWHIPVEVVPDSTSDLYNFQVSPMPSTSEATTDEDEEGKLPEDIMKLLEQSEWQPTNVDGKGYLLNEPGVQPTSVYGDFSCKEEPEIDSPGGDIGLSLQRVFTDLKNMDATWLDSLLTPVRLPSIQAIPCAP

>P55210

MADDQGCIEEQGVEDSANEDSVDAKPDRSSFVPSLFSKKKKNVTMRSIKTTRDRVPTYQYNMNFEKLGKCIIINNKNFDKVTGMGVRNGTDKDAEALFKCFRSLGFDVIVYNDCSCAKMQDLLKKASEEDHTNAACFACILLSHGEENVIYGKDGVTPIKDLTAHFRGDRCKTLLEKPKLFFIQACRGTELDDGIQADSGPINDTDANPRYKIPVEADFLFAYSTVPGYYSWRSPGRGSWFVQALCSILEEHGKDLEIMQILTRVNDRVARHFESQSDDPHFHEKKQIPCVVSMLTKELYFSQ

1114 NP_000519.2 NP_001748.1

>NP_000519.2

MGAYARASGVCARGCLDSAGPWTMSRALRPPLPPLCFFLLLLAAAGARAGGYETCPTVQPNMLNVHLLPHTHDDVGWLKTVDQYFYGIKNDIQHAGVQYILDSVISALLADPTRRFIYVEIAFFSRWWHQQTNATQEVVRDLVRQGRLEFANGGWVMNDEAATHYGAIVDQMTLGLRFLEDTFGNDGRPRVAWHIDPFGHSREQASLFAQMGFDGFFFGRLDYQDKWVRMQKLEMEQVWRASTSLKPPTADLFTGVLPNGYNPPRNLCWDVLCVDQPLVEDPRSPEYNAKELVDYFLNVATAQGRYYRTNHTVMTMGSDFQYENANMWFKNLDKLIRLVNAQQAKGSSVHVLYSTPACYLWELNKANLTWSVKHDDFFPYADGPHQFWTGYFSSRPALKRYERLSYNFLQVCNQLEALVGLAANVGPYGSGDSAPLNEAMAVLQHHDAVSGTSRQHVANDYARQLAAGWGPCEVLLSNALARLRGFKDHFTFCQQLNISICPLSQTAARFQVIVYNPLGRKVNWMVRLPVSEGVFVVKDPNGRTVPSDVVIFPSSDSQAHPPELLFSASLPALGFSTYSVAQVPRWKPQARAPQPIPRRSWSPALTIENEHIRATFDPDTGLLMEIMNMNQQLLLPVRQTFFWYNASIGDNESDQASGAYIFRPNQQKPLPVSRWAQIHLVKTPLVQEVHQNFSAWCSQVVRLYPGQRHLELEWSVGPIPVGDTWGKEVISRFDTPLETKGRFYTDSNGREILERRRDYRPTWKLNQTEPVAGNYYPVNTRIYITDGNMQLTVLTDRSQGGSSLRDGSLELMVHRRLLKDDGRGVSEPLMENGSGAWVRGRHLVLLDTAQAAAAGHRLLAEQEVLAPQVVLAPGGGAAYNLGAPPRTQFSGLRRDLPPSVHLLTLASWGPEMVLLRLEHQFAVGEDSGRNLSAPVTLNLRDLFSTFTITRLQETTLVANQLREAASRLKWTTNTGPTPHQTPYQLDPANITLEPMEIRTFLASVQWKEVDG

>NP_001748.1

MSSGIHVALVTGGNKGIGLAIVRDLCRLFSGDVVLTARDVTRGQAAVQQLQAEGLSPRFHQLDIDDLQSIRALRDFLRKEYGGLDVLVNNAGIAFKVADPTPFHIQAEVTMKTNFFGTRDVCTELLPLIKPQGRVVNVSSIMSVRALKSCSPELQQKFRSETITEEELVGLMNKFVEDTKKGVHQKEGWPSSAYGVTKIGVTVLSRIHARKLSEQRKGDKILLNACCPGWVRTDMAGPKATKSPEEGAETPVYLALLPPDAEGPHGQFVSEKRVEQW

1115 NP_005578.1 Q9NP31

>NP_005578.1

MGRKKIQITRIMDERNRQVTFTKRKFGLMKKAYELSVLCDCEIALIIFNSSNKLFQYASTDMDKVLLKYTEYNEPHESRTNSDIVEALNKKEHRGCDSPDPDTSYVLTPHTEEKYKKINEEFDNMMRNHKIAPGLPPQNFSMSVTVPVTSPNALSYTNPGSSLVSPSLAASSTLTDSSMLSPPQTTLHRNVSPGAPQRPPSTGNAGGMLSTTDLTVPNGAGSSPVGNGFVNSRASPNLIGATGANSLGKVMPTKSPPPPGGGNLGMNSRKPDLRVVIPPSSKGMMPPLSEEEELELNTQRISSSQATQPLATPVVSVTTPSLPPQGLVYSAMPTAYNTDYSLTSADLSALQGFNSPGMLSLGQVSAWQQHHLGQAALSSLVAGGQLSQGSNLSINTNQNISIKSEPISPPRDRMTPSGFQQQQQQQQQQQPPPPPQPQPQPPQPQPRQEMGRSPVDSLSSSSSSYDGSDREDPRGDFHSPIVLGRPPNTEDRESPSVKRMRMDAWVT

>Q9NP31

MEFPLAQICPQGSHEAPIPTFSTFQITDMTRRSCQNLGYTAASPQAPEAASNTGNAERAEEVPGEGSLFLQAETRAWFQKTQAHWLLQHGAAPAWFHGFITRREAERLLEPKPQGCYLVRFSESAVTFVLTYRSRTCCRHFLLAQLRDGRHVVLGEDSAHARLQDLLLHYTAHPLSPYGETLTEPLARQTPEPAGLSLRTEESNFGSKSQDPNPQYSPIIKQGQAPVPMQKEGAGEKEPSQLLRPKPPIPAKPQLPPEVYTIPVPRHRPAPRPKPSNPIYNEPDEPIAFYAMGRGSPGEAPSNIYVEVEDEGLPATLGHPVLRKSWSRPVPGGQNTGGSQLHSENSVIGQGPPLPHQPPPAWRHTLPHNLSRQVLQDRGQAWLPLGPPQ

1116 NP_009181.2 NP_036429.2

>NP_009181.2

MVDMDKLINNLEVQLNSEGGSMQVFKQVTASVRNRDPPEIEYRSNMTSPTLLDANPMENPALFNDIKIEPPEELLASDFSLPQVEPVDLSFHKPKAPLQPASMLQAPIRPPKPQSSPQTLVVSTSTSDMSTSANIPTVLTPGSVLTSSQSTGSQQILHVIHTIPSVSLPNKMGGLKTIPVVVQSLPMVYTTLPADGGPAAITVPLIGGDGKNAGSVKVDPTSMSPLEIPSDSEESTIESGSSALQSLQGLQQEPAAMAQMQGEESLDLKRRRIHQCDFAGCSKVYTKSSHLKAHRRIHTGEKPYKCTWDGCSWKFARSDELTRHFRKHTGIKPFRCTDCNRSFSRSDHLSLHRRRHDTM

>NP_036429.2

MVMEKPSPLLVGREFVRQYYTLLNKAPEYLHRFYGRNSSYVHGGVDASGKPQEAVYGQNDIHHKVLSLNFSECHTKIRHVDAHATLSDGVVVQVMGLLSNSGQPERKFMQTFVLAPEGSVPNKFYVHNDMFRYEDEVFGDSEPELDEESEDEVEEEQEERQPSPEPVQENANSGYYEAHPVTNGIEEPLEESSHEPEPEPESETKTEELKPQVEEKNLEELEEKSTTPPPAEPVSLPQEPPKAFSWASVTSKNLPPSGTVSSSGIPPHVKAPVSQPRVEAKPEVQSQPPRVREQRPRERPGFPPRGPRPGRGDMEQNDSDNRRIIRYPDSHQLFVGNLPHDIDENELKEFFMSFGNVVELRINTKGVGGKLPNFGFVVFDDSEPVQRILIAKPIMFRGEVRLNVEEKKTRAARERETRGGGDDRRDIRRNDRGPGGPRGIVGGGMMRDRDGRGPPPRGGMAQKLGSGRGTGQMEGRFTGQRR

1117 NP_001659.1 P35558

>NP_001659.1

MAATTANPEMTSDVPSLGPAIASGNSGPGIQGGGAIVQRAIKRRPGLDFDDDGEGNSKFLRCDDDQMSNDKERFARSDDEQSSADKERLARENHSEIERRRRNKMTAYITELSDMVPTCSALARKPDKLTILRMAVSHMKSLRGTGNTSTDGSYKPSFLTDQELKHLILEAADGFLFIVSCETGRVVYVSDSVTPVLNQPQSEWFGSTLYDQVHPDDVDKLREQLSTSENALTGRILDLKTGTVKKEGQQSSMRMCMGSRRSFICRMRCGSSSVDPVSVNRLSFVRNRCRNGLGSVKDGEPHFVVVHCTGYIKAWPPAGVSLPDDDPEAGQGSKFCLVAIGRLQVTSSPNCTDMSNVCQPTEFISRHNIEGIFTFVDHRCVATVGYQPQELLGKNIVEFCHPEDQQLLRDSFQQVVKLKGQVLSVMFRFRSKNQEWLWMRTSSFTFQNPYSDEIEYIICTNTNVKNSSQEPRPTLSNTIQRPQLGPTANLPLEMGSGQLAPRQQQQQTELDMVPGRDGLASYNHSQVVQPVTTTGPEHSKPLEKSDGLFAQDRDPRFSEIYHNINADQSKGISSSTVPATQQLFSQGNTFPPTPRPAENFRNSGLAPPVTIVQPSASAGQMLAQISRHSNPTQGATPTWTPTTRSGFSAQQVATQATAKTRTSQFGVGSFQTPSSFSSMSLPGAPTASPGAAAYPSLTNRGSNFAPETGQTAGQFQTRTAEGVGVWPQWQGQQPHHRSSSSEQHVQQPPAQQPGQPEVFQEMLSMLGDQSNSYNNEEFPDLTMFPPFSE

>P35558

MPPQLQNGLNLSAKVVQGSLDSLPQAVREFLENNAELCQPDHIHICDGSEEENGRLLGQMEEEGILRRLKKYDNCWLALTDPRDVARIESKTVIVTQEQRDTVPIPKTGLSQLGRWMSEEDFEKAFNARFPGCMKGRTMYVIPFSMGPLGSPLSKIGIELTDSPYVVASMRIMTRMGTPVLEAVGDGEFVKCLHSVGCPLPLQKPLVNNWPCNPELTLIAHLPDRREIISFGSGYGGNSLLGKKCFALRMASRLAKEEGWLAEHMLILGITNPEGEKKYLAAAFPSACGKTNLAMMNPSLPGWKVECVGDDIAWMKFDAQGHLRAINPENGFFGVAPGTSVKTNPNAIKTIQKNTIFTNVAETSDGGVYWEGIDEPLASGVTITSWKNKEWSSEDGEPCAHPNSRFCTPASQCPIIDAAWESPEGVPIEGIIFGGRRPAGVPLVYEALSWQHGVFVGAAMRSEATAAAEHKGKIIMHDPFAMRPFFGYNFGKYLAHWLSMAQHPAAKLPKIFHVNWFRKDKEGKFLWPGFGENSRVLEWMFNRIDGKASTKLTPIGYIPKEDALNLKGLGHINMMELFSISKEFWEKEVEDIEKYLEDQVNADLPCEIEREILALKQRISQM

1118 NP_002681.1 Q9Y4K4

>NP_002681.1

MSKRKAPQETLNGGITDMLTELANFEKNVSQAIHKYNAYRKAASVIAKYPHKIKSGAEAKKLPGVGTKIAEKIDEFLATGKLRKLEKIRQDDTSSSINFLTRVSGIGPSAARKFVDEGIKTLEDLRKNEDKLNHHQRIGLKYFGDFEKRIPREEMLQMQDIVLNEVKKVDSEYIATVCGSFRRGAESSGDMDVLLTHPSFTSESTKQPKLLHQVVEQLQKVHFITDTLSKGETKFMGVCQLPSKNDEKEYPHRRIDIRLIPKDQYYCGVLYFTGSDIFNKNMRAHALEKGFTINEYTIRPLGVTGVAGEPLPVDSEKDIFDYIQWKYREPKDRSE

>Q9Y4K4

MEAPLRPAADILRRNPQQDYELVQRVGSGTYGDVYKARNVHTGELAAVKIIKLEPGDDFSLIQQEIFMVKECKHCNIVAYFGSYLSREKLWICMEYCGGGSLQDIYHVTGPLSELQIAYVCRETLQGLAYLHTKGKMHRDIKGANILLTDHGDVKLADFGVAAKITATIAKRKSFIGTPYWMAPEVAAVEKNGGYNQLCDIWAVGITAIELGELQPPMFDLHPMRALFLMSKSNFQPPKLKDKTKWSSTFHNFVKIALTKNPKKRPTAERLLTHTFVAQPGLSRALAVELLDKVNNPDNHAHYTEADDDDFEPHAIIRHTIRSTNRNARAERTASEINFDKLQFEPPLRKETEARDEMGLSSDPNFMLQWNPFVDGANTGKSTSKRAIPPPLPPKPRISSYPEDNFPDEEKASTIKHCPDSESRAPQILRRQSSPSCGPVAETSSIGNGDGISKLMSENTEGSAQAPQLPRKNDKRDFPKPAINGLPPTPKVLMGACFSKVFDGCPLKINCATSWIHPDTKDQYIIFGTEDGIYTLNLNELHEATMEQLFPRKCTWLYVINNTLMSLSEGKTFQLYSHNLIALFEHAKKPGLAAHIQTHRFPDRILPRKFALTTKIPDTKGCHKCCIVRNPYTGHKYLCGALQSGIVLLQWYEPMQKFMLIKHFDFPLPSPLNVFEMLVIPEQEYPMVCVAISKGTESNQVVQFETINLNSASSWFTEIGAGSQQLDSIHVTQLERDTVLVCLDKFVKIVNLQGKLKSSKKLASELSFDFRIESVVCLQDSVLAFWKHGMQGKSFKSDEVTQEISDETRVFRLLGSDRVVVLESRPTENPTAHSNLYILAGHENSY

1119 NP_003508.1 P25325

>NP_003508.1

MSGRGKQGGKARAKAKSRSSRAGLQFPVGRVHRLLRKGNYAERVGAGAPVYMAAVLEYLTAEILELAGNAARDNKKTRIIPRHLQLAIRNDEELNKLLGKVTIAQGGVLPNIQAVLLPKKTESHKAKSK

>P25325

MASPQLCRALVSAQWVAEALRAPRAGQPLQLLDASWYLPKLGRDARREFEERHIPGAAFFDIDQCSDRTSPYDHMLPGAEHFAEYAGRLGVGAATHVVIYDASDQGLYSAPRVWWMFRAFGHHAVSLLDGGLRHWLRQNLPLSSGKSQPAPAEFRAQLDPAFIKTYEDIKENLESRRFQVVDSRATGRFRGTEPEPRDGIEPGHIPGTVNIPFTDFLSQEGLEKSPEEIRHLFQEKKVDLSKPLVATCGSGVTACHVALGAYLCGKPDVPIYDGSWVEWYMRARPEDVISEGRGKTH

1120 NP_115874.3 Q8N142

>NP_115874.3

MNGEYRGRGFGRGRFQSWKRGRGGGNFSGKWREREHRPDLSKTTGKRTSEQTPQFLLSTKTPQSMQSTLDRFIPYKGWKLYFSEVYSDSSPLIEKIQAFEKFFTRHIDLYDKDEIERKGSILVDFKELTEGGEVTNLIPDIATELRDAPEKTLACMGLAIHQVLTKDLERHAAELQAQEGLSNDGETMVNVPHIHARVYNYEPLTQLKNVRANYYGKYIALRGTVVRVSNIKPLCTKMAFLCAACGEIQSFPLPDGKYSLPTKCPVPVCRGRSFTALRSSPLTVTMDWQSIKIQELMSDDQREAGRIPRTIECELVHDLVDSCVPGDTVTITGIVKVSNAEEGSRNKNDKCMFLLYIEANSISNSKGQKTKSSEDGCKHGMLMEFSLKDLYAIQEIQAEENLFKLIVNSLCPVIFGHELVKAGLALALFGGSQKYADDKNRIPIRGDPHILVVGDPGLGKSQMLQAACNVAPRGVYVCGNTTTTSGLTVTLSKDSSSGDFALEAGALVLGDQGICGIDEFDKMGNQHQALLEAMEQQSISLAKAGVVCSLPARTSIIAAANPVGGHYNKAKTVSENLKMGSALLSRFDLVFILLDTPNEHHDHLLSEHVIAIRAGKQRTISSATVARMNSQDSNTSVLEVVSEKPLSERLKVVPGETIDPIPHQLLRKYIGYARQYVYPRLSTEAARVLQDFYLELRKQSQRLNSSPITTRQLESLIRLTEARARLELREEATKEDAEDIVEIMKYSMLGTYSDEFGNLDFERSQHGSGMSNRSTAKRFISALNNVAERTYNNIFQFHQLRQIAKELNIQVADFENFIGSLNDQGYLLKKGPKVYQLQTM

>Q8N142

MSGTRASNDRPPGAGGVKRGRLQQEAAATGSRVTVVLGAQWGDEGKGKVVDLLATDADIISRCQGGNNAGHTVVVDGKEYDFHLLPSGIINTKAVSFIGNGVVIHLPGLFEEAEKNEKKGLKDWEKRLIISDRAHLVFDFHQAVDGLQEVQRQAQEGKNIGTTKKGIGPTYSSKAARTGLRICDLLSDFDEFSSRFKNLAHQHQSMFPTLEIDIEGQLKRLKGFAERIRPMVRDGVYFMYEALHGPPKKILVEGANAALLDIDFGTYPFVTSSNCTVGGVCTGLGIPPQNIGDVYGVVKAYTTRVGIGAFPTEQINEIGGLLQTRGHEWGVTTGRKRRCGWLDLMILRYAHMVNGFTALALTKLDILDVLGEVKVGVSYKLNGKRIPYFPANQEMLQKVEVEYETLPGWKADTTGARRWEDLPPQAQNYIRFVENHVGVAVKWVGVGKSRESMIQLF

1121 Q16385 Q9BXL7

>Q16385

MNGDDAFARRPTVGAQIPEKIQKAFDDIAKYFSKEEWEKMKASEKIFYVYMKRKYEAMTKLGFKATLPPFMCNKRAEDFQGNDLDNDPNRGNQVERPQMTFGRLQGISPKIMPKKPAEEGNDSEEVPEASGPQNDGKELCPPGKPTTSEKIHERSGPKRGEHAWTHRLRERKQLVIYEEISDPEEDDE

>Q9BXL7

MPGGGPEMDDYMETLKDEEDALWENVECNRHMLSRYINPAKLTPYLRQCKVIDEQDEDEVLNAPMLPSKINRAGRLLDILHTKGQRGYVVFLESLEFYYPELYKLVTGKEPTRRFSTIVVEEGHEGLTHFLMNEVIKLQQQMKAKDLQRCELLARLRQLEDEKKQMTLTRVELLTFQERYYKMKEERDSYNDELVKVKDDNYNLAMRYAQLSEEKNMAVMRSRDLQLEIDQLKHRLNKMEEECKLERNQSLKLKNDIENRPKKEQVLELERENEMLKTKNQELQSIIQAGKRSLPDSDKAILDILEHDRKEALEDRQELVNRIYNLQEEARQAEELRDKYLEEKEDLELKCSTLGKDCEMYKHRMNTVMLQLEEVERERDQAFHSRDEAQTQYSQCLIEKDKYRKQIRELEEKNDEMRIEMVRREACIVNLESKLRRLSKDSNNLDQSLPRNLPVTIISQDFGDASPRTNGQEADDSSTSEESPEDSKYFLPYHPPQRRMNLKGIQLQRAKSPISLKRTSDFQAKGHEEEGTDASPSSCGSLPITNSFTKMQPPRSRSSIMSITAEPPGNDSIVRRYKEDAPHRSTVEEDNDSGGFDALDLDDDSHERYSFGPSSIHSSSSSHQSEGLDAYDLEQVNLMFRKFSLERPFRPSVTSVGHVRGPGPSVQHTTLNGDSLTSQLTLLGGNARGSFVHSVKPGSLAEKAGLREGHQLLLLEGCIRGERQSVPLDTCTKEEAHWTIQRCSGPVTLHYKVNHEGYRKLVKDMEDGLITSGDSFYIRLNLNISSQLDACTMSLKCDDVVHVRDTMYQDRHEWLCARVDPFTDHDLDMGTIPSYSRAQQLLLVKLQRLMHRGSREEVDGTHHTLRALRNTLQPEEALSTSDPRVSPRLSRASFLFGQLLQFVSRSENKYKRMNSNERVRIISGSPLGSLARSSLDATKLLTEKQEELDPESELGKNLSLIPYSLVRAFYCERRRPVLFTPTVLAKTLVQRLLNSGGAMEFTICKSDIVTRDEFLRRQKTETIIYSREKNPNAFECIAPANIEAVAAKNKHCLLEAGIGCTRDLIKSNIYPIVLFIRVCEKNIKRFRKLLPRPETEEEFLRVCRLKEKELEALPCLYATVEPDMWGSVEELLRVVKDKIGEEQRKTIWVDEDQL

1122 P20823 Q9P0M9

>P20823

MVSKLSQLQTELLAALLESGLSKEALIQALGEPGPYLLAGEGPLDKGESCGGGRGELAELPNGLGETRGSEDETDDDGEDFTPPILKELENLSPEEAAHQKAVVETLLQEDPWRVAKMVKSYLQQHNIPQREVVDTTGLNQSHLSQHLNKGTPMKTQKRAALYTWYVRKQREVAQQFTHAGQGGLIEEPTGDELPTKKGRRNRFKWGPASQQILFQAYERQKNPSKEERETLVEECNRAECIQRGVSPSQAQGLGSNLVTEVRVYNWFANRRKEEAFRHKLAMDTYSGPPPGPGPGPALPAHSSPGLPPPALSPSKVHGVRYGQPATSETAEVPSSSGGPLVTVSTPLHQVSPTGLEPSHSLLSTEAKLVSAAGGPLPPVSTLTALHSLEQTSPGLNQQPQNLIMASLPGVMTIGPGEPASLGPTFTNTGASTLVIGLASTQAQSVPVINSMGSSLTTLQPVQFSQPLHPSYQQPLMPPVQSHVTQSPFMATMAQLQSPHALYSHKPEVAQYTHTGLLPQTMLITDTTNLSALASLTPTKQVFTSDTEASSESGLHTPASQATTLHVPSQDPAGIQHLQPAHRLSASPTVSSSSLVLYQSSDSSNGQSHLLPSNHSVIETFISTQMASSSQ

>Q9P0M9

MASVVLALRTRTAVTSLLSPTPATALAVRYASKKSGGSSKNLGGKSSGRRQGIKKMEGHYVHAGNIIATQRHFRWHPGAHVGVGKNKCLYALEEGIVRYTKEVYVPHPRNTEAVDLITRLPKGAVLYKTFVHVVPAKPEGTFKLVAML

1123 Q9Y618 Q9Y2J8

>Q9Y618

MSGSTQPVAQTWRATEPRYPPHSLSYPVQIARTHTDVGLLEYQHHSRDYASHLSPGSIIQPQRRRPSLLSEFQPGNERSQELHLRPESHSYLPELGKSEMEFIESKRPRLELLPDPLLRPSPLLATGQPAGSEDLTKDRSLTGKLEPVSPPSPPHTDPELELVPPRLSKEELIQNMDRVDREITMVEQQISKLKKKQQQLEEEAAKPPEPEKPVSPPPIESKHRSLVQIIYDENRKKAEAAHRILEGLGPQVELPLYNQPSDTRQYHENIKINQAMRKKLILYFKRRNHARKQWEQKFCQRYDQLMEAWEKKVERIENNPRRRAKESKVREYYEKQFPEIRKQRELQERMQSRVGQRGSGLSMSAARSEHEVSEIIDGLSEQENLEKQMRQLAVIPPMLYDADQQRIKFINMNGLMADPMKVYKDRQVMNMWSEQEKETFREKFMQHPKNFGLIASFLERKTVAECVLYYYLTKKNENYKSLVRRSYRRRGKSQQQQQQQQQQQQQQQQQPMPRSSQEEKDEKEKEKEAEKEEEKPEVENDKEDLLKEKTDDTSGEDNDEKEAVASKGRKTANSQGRRKGRITRSMANEANSEEAITPQQSAELASMELNESSRWTEEEMETAKKGLLEHGRNWSAIARMVGSKTVSQCKNFYFNYKKRQNLDEILQQHKLKMEKERNARRKKKKAPAAASEEAAFPPVVEDEEMEASGVSGNEEEMVEEAEALHASGNEVPRGECSGPATVNNSSDTESIPSPHTEAAKDTGQNGPKPPATLGADGPPPGPPTPPPEDIPAPTEPTPASEATGAPTPPPAPPSPSAPPPVVPKEEKEEETAAAPPVEEGEEQKPPAAEELAVDTGKAEEPVKSECTEEAEEGPAKGKDAEAAEATAEGALKAEKKEGGSGRATTAKSSGAPQDSDSSATCSADEVDEAEGGDKNRLLSPRPSLLTPTGDPRANASPQKPLDLKQLKQRAAAIPPIQVTKVHEPPREDAAPTKPAPPAPPPPQNLQPESDAPQQPGSSPRGKSRSPAPPADKEAEKPVFFPAFAAEAQKLPGDPPCWTSGLPFPVPPREVIKASPHAPDPSAFSYAPPGHPLPLGLHDTARPVLPRPPTISNPPPLISSAKHPSVLERQIGAISQGMSVQLHVPYSEHAKAPVGPVTMGLPLPMDPKKLAPFSGVKQEQLSPRGQAGPPESLGVPTAQEASVLRGTALGSVPGGSITKGIPSTRVPSDSAITYRGSITHGTPADVLYKGTITRIIGEDSPSRLDRGREDSLPKGHVIYEGKKGHVLSYEGGMSVTQCSKEDGRSSSGPPHETAAPKRTYDMMEGRVGRAISSASIEGLMGRAIPPERHSPHHLKEQHHIRGSITQGIPRSYVEAQEDYLRREAKLLKREGTPPPPPPSRDLTEAYKTQALGPLKLKPAHEGLVATVKEAGRSIHEIPREELRHTPELPLAPRPLKEGSITQGTPLKYDTGASTTGSKKHDVRSLIGSPGRTFPPVHPLDVMADARALERACYEESLKSRPGTASSSGGSIARGAPVIVPELGKPRQSPLTYEDHGAPFAGHLPRGSPVTTREPTPRLQEGSLSSSKASQDRKLTSTPREIAKSPHSTVPEHHPHPISPYEHLLRGVSGVDLYRSHIPLAFDPTSIPRGIPLDAAAAYYLPRHLAPNPTYPHLYPPYLIRGYPDTAALENRQTIINDYITSQQMHHNAATAMAQRADMLRGLSPRESSLALNYAAGPRGIIDLSQVPHLPVLVPPTPGTPATAMDRLAYLPTAPQPFSSRHSSSPLSPGGPTHLTKPTTTSSSERERDRDRERDRDREREKSILTSTTTVEHAPIWRPGTEQSSGSSGSSGGGGGSSSRPASHSHAHQHSPISPRTQDALQQRPSVLHNTGMKGIITAVEPSTPTVLRSTSTSSPVRPAATFPPATHCPLGGTLDGVYPTLMEPVLLPKEAPRVARPERPRADTGHAFLAKPPARSGLEPASSPSKGSEPRPLVPPVSGHATIARTPAKNLAPHHASPDPPAPPASASDPHREKTQSKPFSIQELELRSLGYHGSSYSPEGVEPVSPVSSPSLTHDKGLPKHLEELDKSHLEGELRPKQPGPVKLGGEAAHLPHLRPLPESQPSSSPLLQTAPGVKGHQRVVTLAQHISEVITQDYTRHHPQQLSAPLPAPLYSFPGASCPVLDLRRPPSDLYLPPPDHGAPARGSPHSEGGKRSPEPNKTSVLGGGEDGIEPVSPPEGMTEPGHSRSAVYPLLYRDGEQTEPSRMGSKSPGNTSQPPAFFSKLTESNSAMVKSKKQEINKKLNTHNRNEPEYNISQPGTEIFNMPAITGTGLMTYRSQAVQEHASTNMGLEAIIRKALMGKYDQWEESPPLSANAFNPLNASASLPAAMPITAADGRSDHTLTSPGGGGKAKVSGRPSSRKAKSPAPGLASGDRPPSVSSVHSEGDCNRRTPLTNRVWEDRPSSAGSTPFPYNPLIMRLQAGVMASPPPPGLPAGSGPLAGPHHAWDEEPKPLLCSQYETLSDSE

>Q9Y2J8

MLRERTVRLQYGSRVEAVYVLGTYLWTDVYSAAPAGAQTFSLKHSEHVWVEVVRDGEAEEVATNGKQRWLLSPSTTLRVTMSQASTEASSDKVTVNYYDEEGSIPIDQAGLFLTAIEISLDVDADRDGVVEKNNPKKASWTWGPEGQGAILLVNCDRETPWLPKEDCRDEKVYSKEDLKDMSQMILRTKGPDRLPAGYEIVLYISMSDSDKVGVFYVENPFFGQRYIHILGRRKLYHVVKYTGGSAELLFFVEGLCFPDEGFSGLVSIHVSLLEYMAQDIPLTPIFTDTVIFRIAPWIMTPNILPPVSVFVCCMKDNYLFLKEVKNLVEKTNCELKVCFQYLNRGDRWIQDEIEFGYIEAPHKGFPVVLDSPRDGNLKDFPVKELLGPDFGYVTREPLFESVTSLDSFGNLEVSPPVTVNGKTYPLGRILIGSSFPLSGGRRMTKVVRDFLKAQQVQAPVELYSDWLTVGHVDEFMSFVPIPGTKKFLLLMASTSACYKLFREKQKDGHGEAIMFKGLGGMSSKRITINKILSNESLVQENLYFQRCLDWNRDILKKELGLTEQDIIDLPALFKMDEDHRARAFFPNMVNMIVLDKDLGIPKPFGPQVEEECCLEMHVRGLLEPLGLECTFIDDISAYHKFLGEVHCGTNVRRKPFTFKWWHMVP

1124 NP_254280.1 Q99707

>NP_254280.1

MSGRGKQGGKARAKAKSRSSRAGLQFPVGRVHRLLRKGNYSERVGAGAPVYLAAVLEYLTAEILELAGNAARDNKKTRIIPRHLQLAIRNDEELNKLLGRVTIAQGGVLPNIQAVLLPKKTESHHKAKGK

>Q99707

MSPALQDLSQPEGLKKTLRDEINAILQKRIMVLDGGMGTMIQREKLNEEHFRGQEFKDHARPLKGNNDILSITQPDVIYQIHKEYLLAGADIIETNTFSSTSIAQADYGLEHLAYRMNMCSAGVARKAAEEVTLQTGIKRFVAGALGPTNKTLSVSPSVERPDYRNITFDELVEAYQEQAKGLLDGGVDILLIETIFDTANAKAALFALQNLFEEKYAPRPIFISGTIVDKSGRTLSGQTGEGFVISVSHGEPLCIGLNCALGAAEMRPFIEIIGKCTTAYVLCYPNAGLPNTFGDYDETPSMMAKHLKDFAMDGLVNIVGGCCGSTPDHIREIAEAVKNCKPRVPPATAFEGHMLLSGLEPFRIGPYTNFVNIGERCNVAGSRKFAKLIMAGNYEEALCVAKVQVEMGAQVLDVNMDDGMLDGPSAMTRFCNLIASEPDIAKVPLCIDSSNFAVIEAGLKCCQGKCIVNSISLKEGEDDFLEKARKIKKYGAAMVVMAFDEEGQATETDTKIRVCTRAYHLLVKKLGFNPNDIIFDPNILTIGTGMEEHNLYAINFIHATKVIKETLPGARISGGLSNLSFSFRGMEAIREAMHGVFLYHAIKSGMDMGIVNAGNLPVYDDIHKELLQLCEDLIWNKDPEATEKLLRYAQTQGTGGKKVIQTDEWRNGPVEERLEYALVKGIEKHIIEDTEEARLNQKKYPRPLNIIEGPLMNGMKIVGDLFGAGKMFLPQVIKSARVMKKAVGHLIPFMEKEREETRVLNGTVEEEDPYQGTIVLATVKGDVHDIGKNIVGVVLGCNNFRVIDLGVMTPCDKILKAALDHKADIIGLSGLITPSLDEMIFVAKEMERLAIRIPLLIGGATTSKTHTAVKIAPRYSAPVIHVLDASKSVVVCSQLLDENLKDEYFEEIMEEYEDIRQDHYESLKERRYLPLSQARKSGFQMDWLSEPHPVKPTFIGTQVFEDYDLQKLVDYIDWKPFFDVWQLRGKYPNRGFPKIFNDKTVGGEARKVYDDAHNMLNTLISQKKLRARGVVGFWPAQSIQDDIHLYAEAAVPQAAEPIATFYGLRQQAEKDSASTEPYYCLSDFIAPLHSGIRDYLGLFAVACFGVEELSKAYEDDGDDYSSIMVKALGDRLAEAFAEELHERVRRELWAYCGSEQLDVADLRRLRYKGIRPAPGYPSQPDHTEKLTMWRLADIEQSTGIRLTESLAMAPASAVSGLYFSNLKSKYFAVGKISKDQVEDYALRKNISVAEVEKWLGPILGYDTD

1125 NP_055392.1 NP_003360.2

>NP_055392.1

MRRKGRCHRGSAARHPSSPCSVKHSPTRETLTYAQAQRMVEIEIEGRLHRISIFDPLEIILEDDLTAQEMSECNSNKENSERPPVCLRTKRHKNNRVKKKNEALPSAHGTPASASALPEPKVRIVEYSPPSAPRRPPVYYKFIEKSAEELDNEVEYDMDEEDYAWLEIVNEKRKGDCVPAVSQSMFEFLMDRFEKESHCENQKQGEQQSLIDEDAVCCICMDGECQNSNVILFCDMCNLAVHQECYGVPYIPEGQWLCRHCLQSRARPADCVLCPNKGGAFKKTDDDRWGHVVCALWIPEVGFANTVFIEPIDGVRNIPPARWKLTCYLCKQKGVGACIQCHKANCYTAFHVTCAQKAGLYMKMEPVKELTGGGTTFSVRKTAYCDVHTPPGCTRRPLNIYGDVEMKNGVCRKESSVKTVRSTSKVRKKAKKAKKALAEPCAVLPTVCAPYIPPQRLNRIANQVAIQRKKQFVERAHSYWLLKRLSRNGAPLLRRLQSSLQSQRSSQQRENDEEMKAAKEKLKYWQRLRHDLERARLLIELLRKREKLKREQVKVEQVAMELRLTPLTVLLRSVLDQLQDKDPARIFAQPVSLKEVPDYLDHIKHPMDFATMRKRLEAQGYKNLHEFEEDFDLIIDNCMKYNARDTVFYRAAVRLRDQGGVVLRQARREVDSIGLEEASGMHLPERPAAAPRRPFSWEDVDRLLDPANRAHLGLEEQLRELLDMLDLTCAMKSSGSRSKRAKLLKKEIALLRNKLSQQHSQPLPTGPGLEGFEEDGAALGPEAGEEVLPRLETLLQPRKRSRSTCGDSEVEEESPGKRLDAGLTNGFGGARSEQEPGGGLGRKATPRRRCASESSISSSNSPLCDSSFNAPKCGRGKPALVRRHTLEDRSELISCIENGNYAKAARIAAEVGQSSMWISTDAAASVLEPLKVVWAKCSGYPSYPALIIDPKMPRVPGHHNGVTIPAPPLDVLKIGEHMQTKSDEKLFLVLFFDNKRSWQWLPKSKMVPLGIDETIDKLKMMEGRNSSIRKAVRIAFDRAMNHLSRVHGEPTSDLSDID

>NP_003360.2

MSASASVGGPVPQPPPGPAAALPPGSAARALHVELPSQQRRLRHLRNIAARNIVNRNGHQLLDTYFTLHLCSTEKIYKEFYRSEVIKNSLNPTWRSLDFGIMPDRLDTSVSCFVVKIWGGKENIYQLLIEWKVCLDGLKYLGQQIHARNQNEIIFGLNDGYYGAPFEHKGYSNAQKTILLQVDQNCVRNSYDVFSLLRLHRAQCAIKQTQVTVQKIGKEIEEKLRLTSTSNELKKKSECLQLKILVLQNELERQKKALGREVALLHKQQIALQDKGSAFSAEHLKLQLQKESLNELRKECTAKRELFLKTNAQLTIRCRQLLSELSYIYPIDLNEHKDYFVCGVKLPNSEDFQAKDDGSIAVALGYTAHLVSMISFFLQVPLRYPIIHKGSRSTIKDNINDKLTEKEREFPLYPKGGEKLQFDYGVYLLNKNIAQLRYQHGLGTPDLRQTLPNLKNFMEHGLMVRCDRHHTSSAIPVPKRQSSIFGGADVGFSGGIPSPDKGHRKRASSENERLQYKTPPPSYNSALAQPVTTVPSMGETERKITSLSSSLDTSLDFSKENKKKGEDLVGSLNGGHANVHPSQEQGEALSGHRATVNGTLLPSEQAGSASVQLPGEFHPVSEAELCCTVEQAEEIIGLEATGFASGDQLEAFNCIPVDSAVAVECDEQVLGEFEEFSRRIYALNENVSSFRRPRRSSDK

1126 NP_006006.3 NP_550438.1

>NP_006006.3

MAAQVAPAAASSLGNPPPPPPSELKKAEQQQREEAGGEAAAAAAAERGEMKAAAGQESEGPAVGPPQPLGKELQDGAESNGGGGGGGAGSGGGPGAEPDLKNSNGNAGPRPALNNNLTEPPGGGGGGSSDGVGAPPHSAAAALPPPAYGFGQPYGRSPSAVAAAAAAVFHQQHGGQQSPGLAALQSGGGGGLEPYAGPQQNSHDHGFPNHQYNSYYPNRSAYPPPAPAYALSSPRGGTPGSGAAAAAGSKPPPSSSASASSSSSSFAQQRFGAMGGGGPSAAGGGTPQPTATPTLNQLLTSPSSARGYQGYPGGDYSGGPQDGGAGKGPADMASQCWGAAAAAAAAAAASGGAQQRSHHAPMSPGSSGGGGQPLARTPQPSSPMDQMGKMRPQPYGGTNPYSQQQGPPSGPQQGHGYPGQPYGSQTPQRYPMTMQGRAQSAMGGLSYTQQIPPYGQQGPSGYGQQGQTPYYNQQSPHPQQQQPPYSQQPPSQTPHAQPSYQQQPQSQPPQLQSSQPPYSQQPSQPPHQQSPAPYPSQQSTTQQHPQSQPPYSQPQAQSPYQQQQPQQPAPSTLSQQAAYPQPQSQQSQQTAYSQQRFPPPQELSQDSFGSQASSAPSMTSSKGGQEDMNLSLQSRPSSLPDLSGSIDDLPMGTEGALSPGVSTSGISSSQGEQSNPAQSPFSPHTSPHLPGIRGPSPSPVGSPASVAQSRSGPLSPAAVPGNQMPPRPPSGQSDSIMHPSMNQSSIAQDRGYMQRNPQMPQYSSPQPGSALSPRQPSGGQIHTGMGSYQQNSMGSYGPQGGQYGPQGGYPRQPNYNALPNANYPSAGMAGGINPMGAGGQMHGQPGIPPYGTLPPGRMSHASMGNRPYGPNMANMPPQVGSGMCPPPGGMNRKTQETAVAMHVAANSIQNRPPGYPNMNQGGMMGTGPPYGQGINSMAGMINPQGPPYSMGGTMANNSAGMAASPEMMGLGDVKLTPATKMNNKADGTPKTESKSKKSSSSTTTNEKITKLYELGGEPERKMWVDRYLAFTEEKAMGMTNLPAVGRKPLDLYRLYVSVKEIGGLTQVNKNKKWRELATNLNVGTSSSAASSLKKQYIQCLYAFECKIERGEDPPPDIFAAADSKKSQPKIQPPSPAGSGSMQGPQTPQSTSSSMAEGGDLKPPTPASTPHSQIPPLPGMSRSNSVGIQDAFNDGSDSTFQKRNSMTPNPGYQPSMNTSDMMGRMSYEPNKDPYGSMRKAPGSDPFMSSGQGPNGGMGDPYSRAAGPGLGNVAMGPRQHYPYGGPYDRVRTEPGIGPEGNMSTGAPQPNLMPSNPDSGMYSPSRYPPQQQQQQQQRHDSYGNQFSTQGTPSGSPFPSQQTTMYQQQQQNYKRPMDGTYGPPAKRHEGEMYSVPYSTGQGQPQQQQLPPAQPQPASQQQAAQPSPQQDVYNQYGNAYPATATAATERRPAGGPQNQFPFQFGRDRVSAPPGTNAQQNMPPQMMGGPIQASAEVAQQGTMWQGRNDMTYNYANRQSTGSAPQGPAYHGVNRTDEMLHTDQRANHEGSWPSHGTRQPPYGPSAPVPPMTRPPPSNYQPPPSMQNHIPQVSSPAPLPRPMENRTSPSKSPFLHSGMKMQKAGPPVPASHIAPAPVQPPMIRRDITFPPGSVEATQPVLKQRRRLTMKDIGTPEAWRVMMSLKSGLLAESTWALDTINILLYDDNSIMTFNLSQLPGLLELLVEYFRRCLIEIFGILKEYEVGDPGQRTLLDPGRFSKVSSPAPMEGGEEEEELLGPKLEEEEEEEVVENDEEIAFSGKDKPASENSEEKLISKFDKLPVKIVQKNDPFVVDCSDKLGRVQEFDSGLLHWRIGGGDTTEHIQTHFESKTELLPSRPHAPCPPAPRKHVTTAEGTPGTTDQEGPPPDGPPEKRITATMDDMLSTRSSTLTEDGAKSSEAIKESSKFPFGISPAQSHRNIKILEDEPHSKDETPLCTLLDWQDSLAKRCVCVSNTIRSLSFVPGNDFEMSKHPGLLLILGKLILLHHKHPERKQAPLTYEKEEEQDQGVSCNKVEWWWDCLEMLRENTLVTLANISGQLDLSPYPESICLPVLDGLLHWAVCPSAEAQDPFSTLGPNAVLSPQRLVLETLSKLSIQDNNVDLILATPPFSRLEKLYSTMVRFLSDRKNPVCREMAVVLLANLAQGDSLAARAIAVQKGSIGNLLGFLEDSLAATQFQQSQASLLHMQNPPFEPTSVDMMRRAARALLALAKVDENHSEFTLYESRLLDISVSPLMNSLVSQVICDVLFLIGQS

>NP_550438.1

MAAGRLFLSRLRAPFSSMAKSPLEGVSSSRGLHAGRGPRRLSIEGNIAVGKSTFVKLLTKTYPEWHVATEPVATWQNIQAAGTQKACTAQSLGNLLDMMYREPARWSYTFQTFSFLSRLKVQLEPFPEKLLQARKPVQIFERSVYSDRYIFAKNLFENGSLSDIEWHIYQDWHSFLLWEFASRITLHGFIYLQASPQVCLKRLYQRAREEEKGIELAYLEQLHGQHEAWLIHKTTKLHFEALMNIPVLVLDVNDDFSEEVTKQEDLMREVNTFVKNL

1127 Q8IZL9 NP_002055.1

>Q8IZL9

MDQYCILGRIGEGAHGIVFKAKHVETGEIVALKKVALRRLEDGFPNQALREIKALQEMEDNQYVVQLKAVFPHGGGFVLAFEFMLSDLAEVVRHAQRPLAQAQVKSYLQMLLKGVAFCHANNIVHRDLKPANLLISASGQLKIADFGLARVFSPDGSRLYTHQVATRWYRAPELLYGARQYDQGVDLWSVGCIMGELLNGSPLFPGKNDIEQLCYVLRILGTPNPQVWPELTELPDYNKISFKEQVPMPLEEVLPDVSPQALDLLGQFLLYPPHQRIAASKALLHQYFFTAPLPAHPSELPIPQRLGGPAPKAHPGPPHIHDFHVDRPLEESLLNPELIRPFILEG

>NP_002055.1

MAQEFVNCKIQPGKVVVFIKPTCPYCRRAQEILSQLPIKQGLLEFVDITATNHTNEIQDYLQQLTGARTVPRVFIGKDCIGGCSDLVSLQQSGELLTRLKQIGALQ

1128 P21675 NP_037386.1

>P21675

MGPGCDLLLRTAATITAAAIMSDTDSDEDSAGGGPFSLAGFLFGNINGAGQLEGESVLDDECKKHLAGLGALGLGSLITELTANEELTGTDGALVNDEGWVRSTEDAVDYSDINEVAEDESRRYQQTMGSLQPLCHSDYDEDDYDADCEDIDCKLMPPPPPPPGPMKKDKDQDSITGEKVDFSSSSDSESEMGPQEATQAESEDGKLTLPLAGIMQHDATKLLPSVTELFPEFRPGKVLRFLRLFGPGKNVPSVWRSARRKRKKKHRELIQEEQIQEVECSVESEVSQKSLWNYDYAPPPPPEQCLSDDEITMMAPVESKFSQSTGDIDKVTDTKPRVAEWRYGPARLWYDMLGVPEDGSGFDYGFKLRKTEHEPVIKSRMIEEFRKLEENNGTDLLADENFLMVTQLHWEDDIIWDGEDVKHKGTKPQRASLAGWLPSSMTRNAMAYNVQQGFAATLDDDKPWYSIFPIDNEDLVYGRWEDNIIWDAQAMPRLLEPPVLTLDPNDENLILEIPDEKEEATSNSPSKESKKESSLKKSRILLGKTGVIKEEPQQNMSQPEVKDPWNLSNDEYYYPKQQGLRGTFGGNIIQHSIPAVELRQPFFPTHMGPIKLRQFHRPPLKKYSFGALSQPGPHSVQPLLKHIKKKAKMREQERQASGGGEMFFMRTPQDLTGKDGDLILAEYSEENGPLMMQVGMATKIKNYYKRKPGKDPGAPDCKYGETVYCHTSPFLGSLHPGQLLQAFENNLFRAPIYLHKMPETDFLIIRTRQGYYIRELVDIFVVGQQCPLFEVPGPNSKRANTHIRDFLQVFIYRLFWKSKDRPRRIRMEDIKKAFPSHSESSIRKRLKLCADFKRTGMDSNWWVLKSDFRLPTEEEIRAMVSPEQCCAYYSMIAAEQRLKDAGYGEKSFFAPEEENEEDFQMKIDDEVRTAPWNTTRAFIAAMKGKCLLEVTGVADPTGCGEGFSYVKIPNKPTQQKDDKEPQPVKKTVTGTDADLRRLSLKNAKQLLRKFGVPEEEIKKLSRWEVIDVVRTMSTEQARSGEGPMSKFARGSRFSVAEHQERYKEECQRIFDLQNKVLSSTEVLSTDTDSSSAEDSDFEEMGKNIENMLQNKKTSSQLSREREEQERKELQRMLLAAGSAASGNNHRDDDTASVTSLNSSATGRCLKIYRTFRDEEGKEYVRCETVRKPAVIDAYVRIRTTKDEEFIRKFALFDEQHREEMRKERRRIQEQLRRLKRNQEKEKLKGPPEKKPKKMKERPDLKLKCGACGAIGHMRTNKFCPLYYQTNAPPSNPVAMTEEQEEELEKTVIHNDNEELIKVEGTKIVLGKQLIESADEVRRKSLVLKFPKQQLPPKKKRRVGTTVHCDYLNRPHKSIHRRRTDPMVTLSSILESIINDMRDLPNTYPFHTPVNAKVVKDYYKIITRPMDLQTLRENVRKRLYPSREEFREHLELIVKNSATYNGPKHSLTQISQSMLDLCDEKLKEKEDKLARLEKAINPLLDDDDQVAFSFILDNIVTQKMMAVPDSWPFHHPVNKKFVPDYYKVIVNPMDLETIRKNISKHKYQSRESFLDDVNLILANSVKYNGPESQYTKTAQEIVNVCYQTLTEYDEHLTQLEKDICTAKEAALEEAELESLDPMTPGPYTPQPPDLYDTNTSLSMSRDASVFQDESNMSVLDIPSATPEKQVTQEGEDGDGDLADEEEGTVQQPQASVLYEDLLMSEGEDDEEDAGSDEEGDNPFSAIQLSESGSDSDVGSGGIRPKQPRMLQENTRMDMENEESMMSYEGDGGEASHGLEDSNISYGSYEEPDPKSNTQDTSFSSIGGYEVSEEEEDEEEEEQRSGPSVLSQVHLSEDEEDSEDFHSIAGDSDLDSDE

>NP_037386.1

MQSTSNHLWLLSDILGQGATANVFRGRHKKTGDLFAIKVFNNISFLRPVDVQMREFEVLKKLNHKNIVKLFAIEEETTTRHKVLIMEFCPCGSLYTVLEEPSNAYGLPESEFLIVLRDVVGGMNHLRENGIVHRDIKPGNIMRVIGEDGQSVYKLTDFGAARELEDDEQFVSLYGTEEYLHPDMYERAVLRKDHQKKYGATVDLWSIGVTFYHAATGSLPFRPFEGPRRNKEVMYKIITGKPSGAISGVQKAENGPIDWSGDMPVSCSLSRGLQVLLTPVLANILEADQEKCWGFDQFFAETSDILHRMVIHVFSLQQMTAHKIYIHSYNTATIFHELVYKQTKIISSNQELIYEGRRLVLEPGRLAQHFPKTTEENPIFVVSREPLNTIGLIYEKISLPKVHPRYDLDGDASMAKAITGVVCYACRIASTLLLYQELMRKGIRWLIELIKDDYNETVHKKTEVVITLDFCIRNIEKTVKVYEKLMKINLEAAELGEISDIHTKLLRLSSSQGTIETSLQDIDSRLSPGGSLADAWAHQEGTHPKDRNVEKLQVLLNCMTEIYYQFKKDKAERRLAYNEEQIHKFDKQKLYYHATKAMTHFTDECVKKYEAFLNKSEEWIRKMLHLRKQLLSLTNQCFDIEEEVSKYQEYTNELQETLPQKMFTASSGIKHTMTPIYPSSNTLVEMTLGMKKLKEEMEGVVKELAENNHILERFGSLTMDGGLRNVDCL

1129 NP_005625.2 NP_056238.2

>NP_005625.2

MRPVRENSSGARSPRVPADLARSILISLPFPPDSLAHRPPSSAPTESQGLFTVAAPAPGAPSPPATLAHLLPAPAMYSLLETELKNPVGTPTQAAGTGGPAAPGGAGKSSANAAGGANSGGGSSGGASGGGGGTDQDRVKRPMNAFMVWSRGQRRKMALENPKMHNSEISKRLGADWKLLTDAEKRPFIDEAKRLRAVHMKEYPDYKYRPRRKTKTLLKKDKYSLPSGLLPPGAAAAAAAAAAAAAAASSPVGVGQRLDTYTHVNGWANGAYSLVQEQLGYAQPPSMSSPPPPPALPPMHRYDMAGLQYSPMMPPGAQSYMNVAAAAAAASGYGGMAPSATAAAAAAYGQQPATAAAAAAAAAAMSLGPMGSVVKSEPSSPPPAIASHSQRACLGDLRDMISMYLPPGGDAADAASPLPGGRLHGVHQHYQGAGTAVNGTVPLTHI

>NP_056238.2

MVFPAKRFCLVPSMEGVRWAFSCGTWLPSRAEWLLAVRSIQPEEKERIGQFVFARDAKAAMAGRLMIRKLVAEKLNIPWNHIRLQRTAKGKPVLAKDSSNPYPNFNFNISHQGDYAVLAAEPELQVGIDIMKTSFPGRGSIPEFFHIMKRKFTNKEWETIRSFKDEWTQLDMFYRNWALKESFIKAIGVGLGFELQRLEFDLSPLNLDIGQVYKETRLFLDGEEEKEWAFEESKIDEHHFVAVALRKPDGSRHQDVPSQDDSKPTQRQFTILNFNDLMSSAVPMTPEDPSFWDCFCFTEEIPIRNGTKS

1130 NP_036455.1 NP_005172.1

>NP_036455.1

MSVDPLSSKALKIKRELSENTPHLSDEALMGLSVRELNRHLRGLSAEEVTRLKQRRRTLKNRGYAASCRVKRVCQKEELQKQKSELEREVDKLARENAAMRLELDALRGKCEALQGFARSVAAARGPATLVAPASVITIVKSTPGSGSGPAHGPDPAHGPASCS

>NP_005172.1

MAKEWGYASHNGPDHWHELFPNAKGENQSPVELHTKDIRHDPSLQPWSVSYDGGSAKTILNNGKTCRVVFDDTYDRSMLRGGPLPGPYRLRQFHLHWGSSDDHGSEHTVDGVKYAAELHLVHWNPKYNTFKEALKQRDGIAVIGIFLKIGHENGEFQIFLDALDKIKTKGKEAPFTKFDPSCLFPACRDYWTYQGSFTTPPCEECIVWLLLKEPMTVSSDQMAKLRSLLSSAENEPPVPLVSNWRPPQPINNRVVRASFK

1131 NP_000200.1 Q6V1X1

>NP_000200.1

MNGEEQYYAATQLYKDPCAFQRGPAPEFSASPPACLYMGRQPPPPPPHPFPGALGALEQGSPPDISPYEVPPLADDPAVAHLHHHLPAQLALPHPPAGPFPEGAEPGVLEEPNRVQLPFPWMKSTKAHAWKGQWAGGAYAAEPEENKRTRTAYTRAQLLELEKEFLFNKYISRPRRVELAVMLNLTERHIKIWFQNRRMKWKKEEDKKRGGGTAVGGGGVAEPEQDCAVTSGEELLALPPPPPPGGAVPPAAPVAAREGRLPPGLSASPQPSSVAPRRPQEPR

>Q6V1X1

MWKRSEQMKIKSGKCNMAAAMETEQLGVEIFETADCEENIESQDRPKLEPFYVERYSWSQLKKLLADTRKYHGYMMAKAPHDFMFVKRNDPDGPHSDRIYYLAMSGENRENTLFYSEIPKTINRAAVLMLSWKPLLDLFQATLDYGMYSREEELLRERKRIGTVGIASYDYHQGSGTFLFQAGSGIYHVKDGGPQGFTQQPLRPNLVETSCPNIRMDPKLCPADPDWIAFIHSNDIWISNIVTREERRLTYVHNELANMEEDARSAGVATFVLQEEFDRYSGYWWCPKAETTPSGGKILRILYEENDESEVEIIHVTSPMLETRRADSFRYPKTGTANPKVTFKMSEIMIDAEGRIIDVIDKELIQPFEILFEGVEYIARAGWTPEGKYAWSILLDRSQTRLQIVLISPELFIPVEDDVMERQRLIESVPDSVTPLIIYEETTDIWINIHDIFHVFPQSHEEEIEFIFASECKTGFRHLYKITSILKESKYKRSSGGLPAPSDFKCPIKEEIAITSGEWEVLGRHGSNIQVDEVRRLVYFEGTKDSPLEHHLYVVSYVNPGEVTRLTDRGYSHSCCISQHCDFFISKYSNQKNPHCVSLYKLSSPEDDPTCKTKEFWATILDSAGPLPDYTPPEIFSFESTTGFTLYGMLYKPHDLQPGKKYPTVLFIYGGPQVQLVNNRFKGVKYFRLNTLASLGYVVVVIDNRGSCHRGLKFEGAFKYKMGQIEIDDQVEGLQYLASRYDFIDLDRVGIHGWSYGGYLSLMALMQRSDIFRVAIAGAPVTLWIFYDTGYTERYMGHPDQNEQGYYLGSVAMQAEKFPSEPNRLLLLHGFLDENVHFAHTSILLSFLVRAGKPYDLQIYPQERHSIRVPESGEHYELHLLHYLQENLGSRIAALKVI

1132 NP_001796.2 P50226

>NP_001796.2

MSHGTYYECEPRGGQQPLEFSGGRAGPGELGDMCEHEASIDLSAYIESGEEQLLSDLFAVKPAPEARGLKGPGTPAFPHYLPPDPRPFAYPPHTFGPDRKALGPGIYSSPGSYDPRAVAVKEEPRGPEGSRAASRGSYNPLQYQVAHCGQTAMHLPPTLAAPGQPLRVLKAPLATAAPPCSPLLKAPSPAGPLHKGKKAVNKDSLEYRLRRERNNIAVRKSRDKAKRRILETQQKVLEYMAENERLRSRVEQLTQELDTLRNLFRQIPEAANLIKGVGGCS

>P50226

MELIQDTSRPPLEYVKGVPLIKYFAEALGPLQSFQARPDDLLISTYPKSGTTWVSQILDMIYQGGDLEKCHRAPIFMRVPFLEFKVPGIPSGMETLKNTPAPRLLKTHLPLALLPQTLLDQKVKVVYVARNAKDVAVSYYHFYHMAKVYPHPGTWESFLEKFMAGEVSYGSWYQHVQEWWELSRTHPVLYLFYEDMKENPKREIQKILEFVGRSLPEETVDLMVEHTSFKEMKKTPMTNYTTVRREFMDHSISPFMRKGMAGDWKTTFTVAQNERFDADYAEKMAGCSLSFRSEL

1133 Q6NYC1 Q99469

>Q6NYC1

MNHKSKKRIREAKRSARPELKDSLDWTRHNYYESFSLSPAAVADNVERADALQLSVEEFVERYERPYKPVVLLNAQEGWSAQEKWTLERLKRKYRNQKFKCGEDNDGYSVKMKMKYYIEYMESTRDDSPLYIFDSSYGEHPKRRKLLEDYKVPKFFTDDLFQYAGEKRRPPYRWFVMGPPRSGTGIHIDPLGTSAWNALVQGHKRWCLFPTSTPRELIKVTRDEGGNQQDEAITWFNVIYPRTQLPTWPPEFKPLEILQKPGETVFVPGGWWHVVLNLDTTIAITQNFASSTNFPVVWHKTVRGRPKLSRKWYRILKQEHPELAVLADSVDLQESTGIASDSSSDSSSSSSSSSSDSDSECESGSEGDGTVHRRKKRRTCSMVGNGDTTSQDDCVSKERSSSR

>Q99469

MIPPSSPREDGVDGLPKEAVGAEQPPSPASTSSQESKLQKLKRSLSFKTKSLRSKSADNFFQRTNSEDMKLQAHMVAEISPSSSPLPAPGSLTSTPARAGLHPGGKAHAFQEYIFKKPTFCDVCNHMIVGTNAKHGLRCKACKMSIHHKCTDGLAPQRCMGKLPKGFRRYYSSPLLIHEQFGCIKEVMPIACGNKVDPVYETLRFGTSLAQRTKKGSSGSGSDSPHRTSTSDLVEVPEEANGPGGGYDLRKRSNSVFTYPENGTDDFRDPAKNINHQGSLSKDPLQMNTYVALYKFVPQENEDLEMRPGDIITLLEDSNEDWWKGKIQDRIGFFPANFVQRLQQNEKIFRCVRTFIGCKEQGQITLKENQICVSSEEEQDGFIRVLSGKKKGLIPLDVLENI

1134 NP_663628.1 NP_689508.3

>NP_663628.1

MAAGSTTLRAVGKLQVRLATKTEPKKLEKYLQKLSALPMTADILAETGIRKTVKRLRKHQHVGDFARDLAARWKKLVLVDRNTGPDPQDPEESASRQRFGEALQEREKAWGFPENATAPRSPSHSPEHRRTARRTPPGQQRPHPRSPSREPRAERKRPRMAPADSGPHRDPPTRTAPLPMPEGPEPAVPGEQPGRGHAHAAQGGPLLGQGCQGQPQGEAVGSHSKGHKSSRGASAQKSPPVQESQSERLQAAGADSAGPKTVPSHVFSELWDPSEAWMQANYDLLSAFEAMTSQANPEALCAPALQEEAAFPGRRVNAKMPVYSGSRPACQLQVPTLRQQCLRVPRNNPDALGDVEGVPYSVLEPVLEGWTPDQLYRTEKDNAALARETDELWRIHCLQDFKEEKPQEHESWRELYLRLRDAREQRLRVVTTKIRSARENKPSGRQTKMICFNSVAKTPYDASRRQEKSAGAADPGNGEMEPAPKPAGSSQAPSGLGDGDGGSVSGGGSSNRHAAPADKTRKQAAKKVAPLMAKAIRDYKGRFSRR

>NP_689508.3

MFEEKASSPSGKMGGEEKPIGAGEEKQKEGGKKKNKEGSGDGGRAELNPWPEYIYTRLEMYNILKAEHDSILAEKAEKDSKPIKVTLPDGKQVDAESWKTTPYQIACGISQGLADNTVIAKVNNVVWDLDRPLEEDCTLELLKFEDEEAQAVYWHSSAHIMGEAMERVYGGCLCYGPPIENGFYYDMYLEEGGVSSNDFSSLEALCKKIIKEKQAFERLEVKKETLLAMFKYNKFKCRILNEKVNTPTTTVYRCGPLIDLCRGPHVRHTGKIKALKIHKNSSTYWEGKADMETLQRIYGISFPDPKMLKEWEKFQEEAKNRDHRKIGRDQELYFFHELSPGSCFFLPKGAYIYNALIEFIRSEYRKRGFQEVVTPNIFNSRLWMTSGHWQHYSENMFSFEVEKELFALKPMNCPGHCLMFDHRPRSWRELPLRLADFGVLHRNELSGALTGLTRVRRFQQDDAHIFCAMEQIEDEIKGCLDFLRTVYSVFGFSFKLNLSTRPEKFLGDIEVWDQAEKQLENSLNEFGEKWELNSGDGAFYGPKIDIQIKDAIGRYHQCATIQLDFQLPIRFNLTYVSHDGDDKKRPVIVHRAILGSVERMIAILTENYGGKWPFWLSPRQVMVVPVGPTCDEYAQKVRQQFHDAKFMADIDLDPGCTLNKKIRNAQLAQYNFILVVGEKEKISGTVNIRTRDNKVHGERTISETIERLQQLKEFRSKQAEEEF

1135 NP_003661.1 NP_055300.1

>NP_003661.1

MERIPSAQPPPACLPKAPGLEHGDLPGMYPAHMYQVYKSRRGIKRSEDSKETYKLPHRLIEKKRRDRINECIAQLKDLLPEHLKLTTLGHLEKAVVLELTLKHVKALTNLIDQQQQKIIALQSGLQAGELSGRNVETGQEMFCSGFQTCAREVLQYLAKHENTRDLKSSQLVTHLHRVVSELLQGGTSRKPSDPAPKVMDFKEKPSSPAKGSEGPGKNCVPVIQRTFAHSSGEQSGSDTDTDSGYGGESEKGDLRSEQPCFKSDHGRRFTMGERIGAIKQESEEPPTKKNRMQLSDDEGHFTSSDLISSPFLGPHPHQPPFCLPFYLIPPSATAYLPMLEKCWYPTSVPVLYPGLNASAAALSSFMNPDKISAPLLMPQRLPSPLPAHPSVDSSVLLQALKPIPPLNLETKD

>NP_055300.1

MPNYKLTYFNMRGRAEIIRYIFAYLDIQYEDHRIEQADWPEIKSTLPFGKIPILEVDGLTLHQSLAIARYLTKNTDLAGNTEMEQCHVDAIVDTLDDFMSCFPWAEKKQDVKEQMFNELLTYNAPHLMQDLDTYLGGREWLIGNSVTWADFYWEICSTTLLVFKPDLLDNHPRLVTLRKKVQAIPAVANWIKRRPQTKL

1136 NP_004488.2 NP_060177.2

>NP_004488.2

MLGSVKMEAHDLAEWSYYPEAGEVYSPVTPVPTMAPLNSYMTLNPLSSPYPPGGLPASPLPSGPLAPPAPAAPLGPTFPGLGVSGGSSSSGYGAPGPGLVHGKEMPKGYRRPLAHAKPPYSYISLITMAIQQAPGKMLTLSEIYQWIMDLFPYYRENQQRWQNSIRHSLSFNDCFVKVARSPDKPGKGSYWALHPSSGNMFENGCYLRRQKRFKLEEKVKKGGSGAATTTRNGTGSAASTTTPAATVTSPPQPPPPAPEPEAQGGEDVGALDCGSPASSTPYFTGLELPGELKLDAPYNFNHPFSINNLMSEQTPAPPKLDVGFGGYGAEGGEPGVYYQGLYSRSLLNAS

>NP_060177.2

MPEQFSVAEFLAVTAEDLSSPAGAAAFAAKMPRYRGAALAREEILEGDQAILQRIKKAVRAIHSSGLGHVENEEQYREAVESLGNSHLSQNSHELSTGFLNLAVFTREVAALFKNLIQNLNNIVSFPLDSLMKGQLRDGRQDSKKQLEKAWKDYEAKMAKLEKERDRARVTGGIPGEVAQDMQRERRIFQLHMCEYLLKAGESQMKQGPDFLQSLIKFFHAQHNFFQDGWKAAQSLFPFIEKLAASVHALHQAQEDELQKLTQLRDSLRGTLQLESREEHLSRKNSGCGYSIHQHQGNKQFGTEKVGFLYKKSDGIRRVWQKRKCGVKYGCLTISHSTINRPPVKLTLLTCQVRPNPEEKKCFDLVTHNRTYHFQAEDEHECEAWVSVLQNSKDEALSSAFLGEPSAGPGSWGSAGHDGEPHDLTKLLIAEVKSRPGNSQCCDCGAADPTWLSTNLGVLTCIQCSGVHRELGVRFSRMQSLTLDLLGPSELLLALNMGNTSFNEVMEAQLPSHGGPKPSAESDMGTRRDYIMAKYVEHRFARRCTPEPQRLWTAICNRDLLSVLEAFANGQDFGQPLPGPDAQAPEELVLHLAVKVANQASLPLVDFIIQNGGHLDAKAADGNTALHYAALYNQPDCLKLLLKGRALVGTVNEAGETALDIARKKHHKECEELLEQAQAGTFAFPLHVDYSWVISTEPGSDSEEDEEEKRCLLKLPAQAHWASGRLDISNKTYETVASLGAATPQGESEDCPPPLPVKNSSRTLVQGCARHASGDRSEVSSLSSEAPETPESLGSPASSSSLMSPLEPGDPSQAPPNSEEGLREPPGTSRPSLTSGTTPSEMYLPVRFSSESTRSYRRGARSPEDGPSARQPLPRRNVPVGITEGDGSRTGSLPASSVQLLQD

1137 NP_002683.2 Q9Y291

>NP_002683.2

MAPERLRSRALSAFKLRGLLLRGEAIKYLTEALQSISELELEDKLEKIINAVEKQPLSSNMIERSVVEAAVQECSQSVDETIEHVFNIIGAFDIPRFVYNSERKKFLPLLMTNHPAPNLFGTPRDKAEMFRERYTILHQRTHRHELFTPPVIGSHPDESGSKFQLKTIETLLGSTTKIGDAIVLGMITQLKEGKFFLEDPTGTVQLDLSKAQFHSGLYTEACFVLAEGWFEDQVFHVNAFGFPPTEPSSTTRAYYGNINFFGGPSNTSVKTSAKLKQLEEENKDAMFVFLSDVWLDQVEVLEKLRIMFAGYSPAPPTCFILCGNFSSAPYGKNQVQALKDSLKTLADIICEYPDIHQSSRFVFVPGPEDPGFGSILPRPPLAESITNEFRQRVPFSVFTTNPCRIQYCTQEITVFREDLVNKMCRNCVRFPSSNLAIPNHFVKTILSQGHLTPLPLYVCPVYWAYDYALRVYPVPDLLVIADKYDPFTTTNTECLCINPGSFPRSGFSFKVFYPSNKTVEDSKLQGF

>Q9Y291

MSSLSEYAFRMSRLSARLFGEVTRPTNSKSMKVVKLFSELPLAKKKETYDWYPNHHTYAELMQTLRFLGLYRDEHQDFMDEQKRLKKLRGKEKPKKGEGKRAAKRK

1138 Q8IXQ6 Q6PGN9

>Q8IXQ6

MDFSMVAGAAAYNEKSGRITSLSLLFQKVFAQIFPQWRKGNTEECLPYKCSETGALGENYSWQIPINHNDFKILKNNERQLCEVLQNKFGCISTLVSPVQEGNSKSLQVFRKMLTPRIELSVWKDDLTTHAVDAVVNAANEDLLHGGGLALALVKAGGFEIQEESKQFVARYGKVSAGEIAVTGAGRLPCKQIIHAVGPRWMEWDKQGCTGKLQRAIVSILNYVIYKNTHIKTVAIPALSSGIFQFPLNLCTKTIVETIRVSLQGKPMMSNLKEIHLVSNEDPTVAAFKAASEFILGKSELGQETTPSFNAMVVNNLTLQIVQGHIEWQTADVIVNSVNPHDITVGPVAKSILQQAGVEMKSEFLATKAKQFQRSQLVLVTKGFNLFCKYIYHVLWHSEFPKPQILKHAMKECLEKCIEQNITSISFPALGTGNMEIKKETAAEILFDEVLTFAKDHVKHQLTVKFVIFPTDLEIYKAFSSEMAKRSKMLSLNNYSVPQSTREEKRENGLEARSPAINLMGFNVEEMYEAHAWIQRILSLQNHHIIENNHILYLGRKEHDILSQLQKTSSVSITEIISPGRTELEIEGARADLIEVVMNIEDMLCKVQEEMARKKERGLWRSLGQWTIQQQKTQDEMKENIIFLKCPVPPTQELLDQKKQFEKCGLQVLKVEKIDNEVLMAAFQRKKKMMEEKLHRQPVSHRLFQQVPYQFCNVVCRVGFQRMYSTPCDPKYGAGIYFTKNLKNLAEKAKKISAADKLIYVFEAEVLTGFFCQGHPLNIVPPPLSPGAIDGHDSVVDNVSSPETFVIFSGMQAIPQYLWTCTQEYVQSQDYSSGPMRPFAQHPWRGFASGSPVD

>Q6PGN9

MEDLEEDVRFIVDETLDFGGLSPSDSREEEDITVLVTPEKPLRRGLSHRSDPNAVAPAPQGVRLSLGPLSPEKLEEILDEANRLAAQLEQCALQDRESAGEGLGPRRVKPSPRRETFVLKDSPVRDLLPTVNSLTRSTPSPSSLTPRLRSNDRKGSVRALRATSGKRPSNMKRESPTCNLFPASKSPASSPLTRSTPPVRGRAGPSGRAAASEETRAAKLRVSGSGEFVGLTLKFLHPSPPGPPTPIRSVLAPQPSTSNSQRLPRPQGAAAKSSSQLPIPSAIPRPASRMPLTSRSVPPGRGALPPDSLSTRKGLPRPSTAGHRVRESGHKVPVSQRLNLPVMGATRSNLQPPRKVAVPGPTR

1139 NP_619790.1 Q6WQI6

>NP_619790.1

MPEPTKSAPAPKKGSKKAVTKAQKKDGKKRKRSRKESYSVYVYKVLKQVHPDTGISSKAMGIMNSFVNDIFERIAGEASRLAHYNKRSTITSREIQTAVRLLLPGELAKHAVSEGTKAVTKYTSSK

>Q6WQI6

MGNWGLGIAPWVDGESELEFRRLGMQGPLEALRRREWNTQRASFSFSFLIALSPHTVDYCHSYELFNRRWHGHVLATQRPSLFILMLV

1140 NP_002496.1 NP_002003.1

>NP_002496.1

MEQYTANSNSSTEQIVVQAGQIQQQQQGGVTAVQLQTEAQVASASGQQVQTLQVVQGQPLMVQVSGGQLITSTGQPIMVQAVPGGQGQTIMQVPVSGTQGLQQIQLVPPGQIQIQGGQAVQVQGQQGQTQQIIIQQPQTAVTAGQTQTQQQIAVQGQQVAQTAEGQTIVYQPVNADGTILQQVTVPVSGMITIPAASLAGAQIVQTGANTNTTSSGQGTVTVTLPVAGNVVNSGGMVMMVPGAGSVPAIQRIPLPGAEMLEEEPLYVNAKQYHRILKRRQARAKLEAEGKIPKERRKYLHESRHRHAMARKRGEGGRFFSPKEKDSPHMQDPNQADEEAMTQIIRVS

>NP_002003.1

MSFRFGQHLIKPSVVFLKTELSFALVNRKPVVPGHVLVCPLRPVERFHDLRPDEVADLFQTTQRVGTVVEKHFHGTSLTFSMQDGPEAGQTVKHVHVHVLPRKAGDFHRNDSIYEELQKHDKEDFPASWRSEEEMAAEAAALRVYFQ

1141 NP_006345.1 NP_055904.1

>NP_006345.1

MSELKDCPLQFHDFKSVDHLKVCPRYTAVLARSEDDGIGIEELDTLQLELETLLSSASRRLRVLEAETQILTDWQDKKGDRRFLKLGRDHELGAPPKHGKPKKQKLEGKAGHGPGPGPGRPKSKNLQPKIQEYEFTDDPIDVPRIPKNDAPNRFWASVEPYCADITSEEVRTLEELLKPPEDEAEHYKIPPLGKHYSQRWAQEDLLEEQKDGARAAAVADKKKGLMGPLTELDTKDVDALLKKSEAQHEQPEDGCPFGALTQRLLQALVEENIISPMEDSPIPDMSGKESGADGASTSPRNQNKPFSVPHTKSLESRIKEELIAQGLLESEDRPAEDSEDEVLAELRKRQAELKALSAHNRTKKHDLLRLAKEEVSRQELRQRVRMADNEVMDAFRKIMAARQKKRTPTKKEKDQAWKTLKERESILKLLDG

>NP_055904.1

MVGERHAGDLMVPLGPRLQAYPEELIRQRPGHDGHPEYLIRWSVLKCGEVGKVGVEEGKAEHILMWLSAPEVYANCPGLLGERALSKGLQHEPAGVSGSFPRDPGGLDEVAMGEMEADVQALVRRAARQLAESGTPSLTAAVLHTIHVLSAYASIGPLTGVFRETGALDLLMHMLCNPEPQIRRSAGKMLQALAAHDAGSRAHVLLSLSQQDGIEQHMDFDSRYTLLELFAETTSSEEHCMAFEGIHLPQIPGKLLFSLVKRYLCVTSLLDQLNSSPELGAGDQSSPCATREKSRGQRELEFSMAVGNLISELVRSMGWARNLSEQGMSPPRPTRSIFQPYISGPSLLLPTIVTTPRRQGWVFRQRSEFSSRSGYGEYVQQTLQPGMRVRMLDDYEEISAGDEGEFRQSNNGIPPVQVFWQSTGRTYWVHWHMLEILGPEEATEDKASAAVEKGAGATVLGTAFPSWDWNPMDGLYPLPYLQPEPQKNERVGYLTQAEWWELLFFIKKLDLCEQQPIFQNLWKNLDETLGEKALGEISVSVEMAESLLQVLSSRFEGSTLNDLLNSQIYTKYGLLSNEPSSSSTSRNHSCTPDPEEESKSEASFSEEETESLKAKAEAPKTEAEPTKTRTETPMAQSDSQLFNQLLVTEGMTLPTEMKEAASEMARALRGPGPRSSLDQHVAAVVATVQISSLDTNLQLSGLSALSQAVEEVTERDHPLVRPDRSLREKLVKMLVELLTNQVGEKMVVVQALRLLYLLMTKHEWRPLFAREGGIYAVLVCMQEYKTSVLVQQAGLAALKMLAVASSSEIPTFVTGRDSIHSLFDAQMTREIFASIDSATRPGSESLLLTVPAAVILMLNTEGCSSAARNGLLLLNLLLCNHHTLGDQIITQELRDTLFRHSGIAPRTEPMPTTRTILMMLLNRYSEPPGSPERAALETPIIQGQDGSPELLIRSLVGGPSAELLLDLERVLCREGSPGGAVRPLLKRLQQETQPFLLLLRTLDAPGPNKTLLLSVLRVITRLLDFPEAMVLPWHEVLEPCLNCLSGPSSDSEIVQELTCFLHRLASMHKDYAVVLCCLGAKEILSKVLDKHSAQLLLGCELRDLVTECEKYAQLYSNLTSSILAGCIQMVLGQIEDHRRTHQPINIPFFDVFLRHLCQGSSVEVKEDKCWEKVEVSSNPHRASKLTDHNPKTYWESNGSTGSHYITLHMHRGVLVRQLTLLVASEDSSYMPARVVVFGGDSTSCIGTELNTVNVMPSASRVILLENLNRFWPIIQIRIKRCQQGGIDTRVRGVEVLGPKPTFWPLFREQLCRRTCLFYTIRAQAWSRDIAEDHRRLLQLCPRLNRVLRHEQNFADRFLPDDEAAQALGKTCWEALVSPLVQNITSPDAEGVSALGWLLDQYLEQRETSRNPLSRAASFASRVRRLCHLLVHVEPPPGPSPEPSTRPFSKNSKGRDRSPAPSPVLPSSSLRNITQCWLSVVQEQVSRFLAAAWRAPDFVPRYCKLYEHLQRAGSELFGPRAAFMLALRSGFSGALLQQSFLTAAHMSEQFARYIDQQIQGGLIGGAPGVEMLGQLQRHLEPIMVLSGLELATTFEHFYQHYMADRLLSFGSSWLEGAVLEQIGLCFPNRLPQLMLQSLSTSEELQRQFHLFQLQRLDKLFLEQEDEEEKRLEEEEEEEEEEEAEKELFIEDPSPAISILVLSPRCWPVSPLCYLYHPRKCLPTEFCDALDRFSSFYSQSQNHPVLDMGPHRRLQWTWLGRAELQFGKQILHVSTVQMWLLLKFNQTEEVSVETLLKDSDLSPELLLQALVPLTSGNGPLTLHEGQDFPHGGVLRLHEPGPQRSGEALWLIPPQAYLNVEKDEGRTLEQKRNLLSCLLVRILKAHGEKGLHIDQLVCLVLEAWQKGPNPPGTLGHTVAGGVACTSTDVLSCILHLLGQGYVKRRDDRPQILMYAAPEPMGPCRGQADVPFCGSQSETSKPSPEAVATLASLQLPAGRTMSPQEVEGLMKQTVRQVQETLNLEPDVAQHLLAHSHWGAEQLLQSYSEDPEPLLLAAGLCVHQAQAVPVRPDHCPVCVSPLGCDDDLPSLCCMHYCCKSCWNEYLTTRIEQNLVLNCTCPIADCPAQPTGAFIRAIVSSPEVISKYEKALLRGYVESCSNLTWCTNPQGCDRILCRQGLGCGTTCSKCGWASCFNCSFPEAHYPASCGHMSQWVDDGGYYDGMSVEAQSKHLAKLISKRCPSCQAPIEKNEGCLHMTCAKCNHGFCWRCLKSWKPNHKDYYNCSAMVSKAARQEKRFQDYNERCTFHHQAREFAVNLRNRVSAIHEVPPPRSFTFLNDACQGLEQARKVLAYACVYSFYSQDAEYMDVVEQQTENLELHTNALQILLEETLLRCRDLASSLRLLRADCLSTGMELLRRIQERLLAILQHSAQDFRVGLQSPSVEAWEAKGPNMPGSQPQASSGPEAEEEEEDDEDDVPEWQQDEFDEELDNDSFSYDESENLDQETFFFGDEEEDEDEAYD

1142 NP_002350.1 NP_055410.1

>NP_002350.1

MTTPNKGNKALKVKREPGENGTSLTDEELVTMSVRELNQHLRGLSKEEIVQLKQRRRTLKNRGYAASCRVKRVTQKEELEKQKAELQQEVEKLASENASMKLELDALRSKYEALQTFARTVARSPVAPARGPLAAGLGPLVPGKVAATSVITIVKSKTDARS

>NP_055410.1

MARSVRVLVDMDGVLADFEAGLLRGFRRRFPEEPHVPLEQRRGFLAREQYRALRPDLADKVASVYEAPGFFLDLEPIPGALDAVREMNDLPDTQVFICTSPLLKYHHCVGEKYRWVEQHLGPQFVERIILTRDKTVVLGDLLIDDKDTVRGQEETPSWEHILFTCCHNRHLVLPPTRRRLLSWSDNWREILDSKRGAAQRE

1143 NP_002388.2 Q96P71

>NP_002388.2

MGRKKIQITRIMDERNRQVTFTKRKFGLMKKAYELSVLCDCEIALIIFNSTNKLFQYASTDMDKVLLKYTEYNEPHESRTNSDIVETLRKKGLNGCDSPDPDADDSVGHSPESEDKYRKINEDIDLMISRQRLCAVPPPNFEMPVSIPVSSHNSLVYSNPVSSLGNPNLLPLAHPSLQRNSMSPGVTHRPPSAGNTGGLMGGDLTSGAGTSAGNGYGNPRNSPGLLVSPGNLNKNMQAKSPPPMNLGMNNRKPDLRVLIPPGSKNTMPSVSEDVDLLLNQRINNSQSAQSLATPVVSVATPTLPGQGMGGYPSAISTTYGTEYSLSSADLSSLSGFNTASALHLGSVTGWQQQHLHNMPPSALSQLGACTSTHLSQSSNLSLPSTQSLNIKSEPVSPPRDRTTTPSRYPQHTRHEAGRSPVDSLSSCSSSYDGSDREDHRNEFHSPIGLTRPSPDERESPSVKRMRLSEGWAT

>Q96P71

MACAGLLTVCLLRPPAPQPQPQTPRHPQLAPDPGPAGHTLFQDVFRRADKNDDGKLSFEEFQNYFADGVLSLGELQELFSGIDGHLTDNLETEKLCDYFSEHLGVYRPVLAALESLNRAVLAAMDATKLEYERASKVDQFVTRFLLRETVSQLQALQSSLEGASDTLEAQAHGWRSDAESVEAQSRLCGSRRAGRRALRSVSRSSTWSPGSSDTGRSSEAEMQWRLQVNRLQELIDQLECKVRAVGPGPHKGGPSWYPPEPGPCWRPGPHSVPSQAPRLEPLREEDLAKGPDLHILMAQRQVQVAEEGLQDFHRALRCYVDFTGAQSHCLHVSAQKMLDGASFTLYEFWQDEASWRRHQQSPGSKAFQRILIDHLRAPDTLTTVFFPASWWIMNNN

1144 CAA58827.1 NP_005787.1

>CAA58827.1

MADEEEDPTFEEENEEIGGGAEGGQGKRKRLFSKELRCMMYGFGDDQNPYTESVDILEDLVIEFITEMTHKAMSIGRQGRVQVEDIVFLIRKDPRKFARVKDLLTMNEELKRARKAFDEANYGS

>NP_005787.1

MGDKPIWEQIGSSFIQHYYQLFDNDRTQLGAIYIDASCLTWEGQQFQGKAAIVEKLSSLPFQKIQHSITAQDHQPTPDSCIISMVVGQLKADEDPIMGFHQMFLLKNINDAWVCTNDMFRLALHNFG

1145 NP_005180.1 NP_659491.4

>NP_005180.1

MEELSSVGEQVFAAECILSKRLRKGKLEYLVKWRGWSSKHNSWEPEENILDPRLLLAFQKKEHEKEVQNRKRGKRPRGRPRKLTAMSSCSRRSKLKEPDAPSKSKSSSSSSSSTSSSSSSDEEDDSDLDAKRGPRGRETHPVPQKKAQILVAKPELKDPIRKKRGRKPLPPEQKATRRPVSLAKVLKTARKDLGAPASKLPPPLSAPVAGLAALKAHAKEACGGPSAMATPENLASLMKGMASSPGRGGISWQSSIVHYMNRMTQSQAQAASRLALKAQATNKCGLGLDLKVRTQKGELGMSPPGSKIPKAPSGGAVEQKVGNTGGPPHTHGASRVPAGCPGPQPAPTQELSLQVLDLQSVKNGMPGVGLLARHATATKGVPATNPAPGKGTGSGLIGASGATMPTDTSKSEKLASRAVAPPTPASKRDCVKGSATPSGQESRTAPGEARKAATLPEMSAGEESSSSDSDPDSASPPSTGQNPSVSVQTSQDWKPTRSLIEHVFVTDVTANLITVTVKESPTSVGFFNLRHY

>NP_659491.4

MDNKISPEAQVAELELDAVIGFNGHVPTGLKCHPDQEHMIYPLGCTVLIQAINTKEQNFLQGHGNNVSCLAISRSGEYIASGQVTFMGFKADIILWDYKNRELLARLSLHKGKIEALAFSPNDLYLVSLGGPDDGSVVVWSIAKRDAICGSPAAGLNVGNATNVIFSRCRDEMFMTAGNGTIRVWELDLPNRKIWPTECQTGQLKRIVMSIGVDDDDSFFYLGTTTGDILKMNPRTKLLTDVGPAKDKFSLGVSAIRCLKMGGLLVGSGAGLLVFCKSPGYKPIKKIQLQGGITSITLRGEGHQFLVGTEESHIYRVSFTDFKETLIATCHFDAVEDIVFPFGTAELFATCAKKDIRVWHTSSNRELLRITVPNMTCHGIDFMRDGKSIISAWNDGKIRAFAPETGRLMYVINNAHRIGVTAIATTSDCKRVISGGGEGEVRVWQIGCQTQKLEEALKEHKSSVSCIRVKRNNEECVTASTDGTCIIWDLVRLRRNQMILANTLFQCVCYHPEEFQIITSGTDRKIAYWEVFDGTVIRELEGSLSGSINGMDITQEGVHFVTGGNDHLVKVWDYNEGEVTHVGVGHSGNITRIRISPGNQYIVSVSADGAILRWKYPYTS

1146 P20823 Q9P2W6

>P20823

MVSKLSQLQTELLAALLESGLSKEALIQALGEPGPYLLAGEGPLDKGESCGGGRGELAELPNGLGETRGSEDETDDDGEDFTPPILKELENLSPEEAAHQKAVVETLLQEDPWRVAKMVKSYLQQHNIPQREVVDTTGLNQSHLSQHLNKGTPMKTQKRAALYTWYVRKQREVAQQFTHAGQGGLIEEPTGDELPTKKGRRNRFKWGPASQQILFQAYERQKNPSKEERETLVEECNRAECIQRGVSPSQAQGLGSNLVTEVRVYNWFANRRKEEAFRHKLAMDTYSGPPPGPGPGPALPAHSSPGLPPPALSPSKVHGVRYGQPATSETAEVPSSSGGPLVTVSTPLHQVSPTGLEPSHSLLSTEAKLVSAAGGPLPPVSTLTALHSLEQTSPGLNQQPQNLIMASLPGVMTIGPGEPASLGPTFTNTGASTLVIGLASTQAQSVPVINSMGSSLTTLQPVQFSQPLHPSYQQPLMPPVQSHVTQSPFMATMAQLQSPHALYSHKPEVAQYTHTGLLPQTMLITDTTNLSALASLTPTKQVFTSDTEASSESGLHTPASQATTLHVPSQDPAGIQHLQPAHRLSASPTVSSSSLVLYQSSDSSNGQSHLLPSNHSVIETFISTQMASSSQ

>Q9P2W6

MGRTWCGMWRRRRPGRRSAVPRWPHLSSQSGVEPPDRWTGTPGWPSRDQEAPGSMMPPAAAQPSAHGALVPPATAHEPVDHPALHWLACCCCLSLPGQLPLAIRLGWDLDLEAGPSSGKLCPRARRWQPLPS

1147 Q86UE8 NP_006819.2

>Q86UE8

MMEELHSLDPRRQELLEARFTGVGVSKGPLNSESSNQSLCSVGSLSDKEVETPEKKQNDQRNRKRKAEPYETSQGKGTPRGHKISDYFEFAGGSAPGTSPGRSVPPVARSSPQHSLSNPLPRRVEQPLYGLDGSAAKEATEEQSALPTLMSVMLAKPRLDTEQLAQRGAGLCFTFVSAQQNSPSSTGSGNTEHSCSSQKQISIQHRQTQSDLTIEKISALENSKNSDLEKKEGRIDDLLRANCDLRRQIDEQQKMLEKYKERLNRCVTMSKKLLIEKSKQEKMACRDKSMQDRLRLGHFTTVRHGASFTEQWTDGYAFQNLIKQQERINSQREEIERQRKMLAKRKPPAMGQAPPATNEQKQRKSKTNGAENETPSSGNTELKDTAPALGAHSLLRLTLAEYHEQEEIFKLRLGHLKKEEAEIQAELERLERVRNLHIRELKRIHNEDNSQFKDHPTLNDRYLLLHLLGRGGFSEVYKAFDLTEQRYVAVKIHQLNKNWRDEKKENYHKHACREYRIHKELDHPRIVKLYDYFSLDTDSFCTVLEYCEGNDLDFYLKQHKLMSEKEARSIIMQIVNALKYLNEIKPPIIHYDLKPGNILLVNGTACGEIKITDFGLSKIMDDDSYNSVDGMELTSQGAGTYWYLPPECFVVGKEPPKISNKVDVWSVGVIFYQCLYGRKPFGHNQSQQDILQENTILKATEVQFPPKPVVTPEAKAFIRRCLAYRKEDRIDVQQLACDPYLLPHIRKSVSTSSPAGAAIASTSGASNNSSSN

>NP_006819.2

MALPRLTGALRSFSNVTKQDNYNEEVADLKIKRSKLHEQVLDLGLTWKKIIKFLNEKLEKSKMQSINEDLKDILHAAKQIVGTDNGREAIESGAAFLFMTFHLKDSVGHKETKAIKQMFGPFPSSSATAACNATNRIISHFSQDDLTALVQMTEKEHGDRVFFGKNLAFSFDMHDLDHFDELPINGETQKTISLDYKKFLNEHLQEACTPELKPVEKTNGSFLWCEVEKYLNSTLKEMTEVPRVEDLCCTLYDMLASIKSGDELQDELFELLGPEGLELIEKLLQNRITIVDRFLNSSNDHRFQALQDNCKKILGENAKPNYGCQVTIQSEQEKQLMKQYRREEKRIARREKKAGEDLEVSEGLMCFDPKELRIQREQALLNARSVPILSRQRDADVEKIHYPHVYDSQAEAMKTSAFIAGAKMILPEGIQRENNKLYEEVRIPYSEPMPLSFEEKPVYIQDLDEIGQLAFKGMKRLNRIQSIVFETAYNTNENMLICAPTGAGKTNIAMLTVLHEIRQHFQQGVIKKNEFKIVYVAPMKALAAEMTDYFSRRLEPLGIIVKELTGDMQLSKSEILRTQMLVTTPEKWDVVTRKSVGDVALSQIVRLLILDEVHLLHEDRGPVLESIVARTLRQVESTQSMIRILGLSATLPNYLDVATFLHVNPYIGLFFFDGRFRPVPLGQTFLGIKCANKMQQLNNMDEVCYENVLKQVKAGHQVMVFVHARNATVRTAMSLIERAKNCGHIPFFFPTQGHDYVLAEKQVQRSRNKQVRELFPDGFSIHHAGMLRQDRNLVENLFSNGHIKVLVCTATLAWGVNLPAHAVIIKGTQIYAAKRGSFVDLGILDVMQIFGRAGRPQFDKFGEGIIITTHDKLSHYLTLLTQRNPIESQFLESLADNLNAEIALGTVTNVEEAVKWISYTYLYVRMRANPLAYGISHKAYQIDPTLRKHREQLVIEVGRKLDKAQMIRFEERTGYFSSTDLGRTASHYYIKYNTIETFNELFDAHKTEGDIFAIVSKAEEFDQIKVREEEIEELDTLLSNFCELSTPGGVENSYGKINILLQTYISRGEMDSFSLISDSAYVAQNAARIVRALFEIALRKRWPTMTYRLLNLSKVIDKRLWGWASPLRQFSILPPHILTRLEEKKLTVDKLKDMRKDEIGHILHHVNIGLKVKQCVHQIPSVMMEASIQPITRTVLRVTLSIYADFTWNDQVHGTVGEPWWIWVEDPTNDHIYHSEYFLALKKQVISKEAQLLVFTIPIFEPLPSQYYIRAVSDRWLGAEAVCIINFQHLILPERHPPHTELLDLQPLPITALGCKAYEALYNFSHFNPVQTQIFHTLYHTDCNVLLGAPTGSGKTVAAELAIFRVFNKYPTSKAVYIAPLKALVRERMDDWKVRIEEKLGKKVIELTGDVTPDMKSIAKADLIVTTPEKWDGVSRSWQNRNYVQQVTILIIDEIHLLGEERGPVLEVIVSRTNFISSHTEKPVRIVGLSTALANARDLADWLNIKQMGLFNFRPSVRPVPLEVHIQGFPGQHYCPRMASMNKPAFQAIRSHSPAKPVLIFVSSRRQTRLTALELIAFLATEEDPKQWLNMDEREMENIIATVRDSNLKLTLAFGIGMHHAGLHERDRKTVEELFVNCKVQVLIATSTLAWGVNFPAHLVIIKGTEYYDGKTRRYVDFPITDVLQMMGRAGRPQFDDQGKAVILVHDIKKDFYKKFLYEPFPVESSLLGVLSDHLNAEIAGGTITSKQDALDYITWTYFFRRLIMNPSYYNLGDVSHDSVNKFLSHLIEKSLIELELSYCIEIGEDNRSIEPLTYGRIASYYYLKHQTVKMFKDRLKPECSTEELLSILSDAEEYTDLPVRHNEDHMNSELAKCLPIESNPHSFDSPHTKAHLLLQAHLSRAMLPCPDYDTDTKTVLDQALRVCQAMLDVAANQGWLVTVLNITNLIQMVIQGRWLKDSSLLTLPNIENHHLHLFKKWKPIMKGPHARGRTSIESLPELIHACGGKDHVFSSMVESELHAAKTKQAWNFLSHLPVINVGISVKGSWDDLVEGHNELSVSTLTADKRDDNKWIKLHADQEYVLQVSLQRVHFGFHKGKPESCAVTPRFPKSKDEGWFLILGEVDKRELIALKRVGYIRNHHVASLSFYTPEIPGRYIYTLYFMSDCYLGLDQQYDIYLNVTQASLSAQVNTKVSDSLTDLALK

1148 NP_000452.2 Q9Y2T1

>NP_000452.2

MTPNSMTENGLTAWDKPKHCPDREHDWKLVGMSEACLHRKSHSERRSTLKNEQSSPHLIQTTWTSSIFHLDHDDVNDQSVSSAQTFQTEEKKCKGYIPSYLDKDELCVVCGDKATGYHYRCITCEGCKGFFRRTIQKNLHPSYSCKYEGKCVIDKVTRNQCQECRFKKCIYVGMATDLVLDDSKRLAKRKLIEENREKRRREELQKSIGHKPEPTDEEWELIKTVTEAHVATNAQGSHWKQKRKFLPEDIGQAPIVNAPEGGKVDLEAFSHFTKIITPAITRVVDFAKKLPMFCELPCEDQIILLKGCCMEIMSLRAAVRYDPESETLTLNGEMAVTRGQLKNGGLGVVSDAIFDLGMSLSSFNLDDTEVALLQAVLLMSSDRPGLACVERIEKYQDSFLLAFEHYINYRKHHVTHFWPKLLMKVTDLRMIGACHASRFLHMKVECPTELFPPLFLEVFED

>Q9Y2T1

MSSAMLVTCLPDPSSSFREDAPRPPVPGEEGETPPCQPGVGKGQVTKPMSVSSNTRRNEDGLGEPEGRASPDSPLTRWTKSLHSLLGDQDGAYLFRTFLEREKCVDTLDFWFACNGFRQMNLKDTKTLRVAKAIYKRYIENNSIVSKQLKPATKTYIRDGIKKQQIDSIMFDQAQTEIQSVMEENAYQMFLTSDIYLEYVRSGGENTAYMSNGGLGSLKVVCGYLPTLNEEEEWTCADFKCKLSPTVVGLSSKTLRATASVRSTETVDSGYRSFKRSDPVNPYHIGSGYVFAPATSANDSEISSDALTDDSMSMTDSSVDGIPPYRVGSKKQLQREMHRSVKANGQVSLPHFPRTHRLPKEMTPVEPATFAAELISRLEKLKLELESRHSLEERLQQIREDEEREGSELTLNSREGAPTQHPLSLLPSGSYEEDPQTILDDHLSRVLKTPGCQSPGVGRYSPRSRSPDHHHHHHSQYHSLLPPGGKLPPAAASPGACPLLGGKGFVTKQTTKHVHHHYIHHHAVPKTKEEIEAEATQRVHCFCPGGSEYYCYSKCKSHSKAPETMPSEQFGGSRGSTLPKRNGKGTEPGLALPAREGGAPGGAGALQLPREEGDRSQDVWQWMLESERQSKPKPHSAQSTKKAYPLESARSSPGERASRHHLWGGNSGHPRTTPRAHLFTQDPAMPPLTPPNTLAQLEEACRRLAEVSKPPKQRCCVASQQRDRNHSATVQTGATPFSNPSLAPEDHKEPKKLAGVHALQASELVVTYFFCGEEIPYRRMLKAQSLTLGHFKEQLSKKGNYRYYFKKASDEFACGAVFEEIWEDETVLPMYEGRILGKVERID

1149 NP_000529.1 Q93015

>NP_000529.1

MEAQGVAEGAGPGAASGVPHPAALAPAAAPTLAPASVAAAASQFTLLVMQPCAGQDEAAAPGGSVGAGKPVRYLCEGAGDGEEEAGEDEADLLDTSDPPGGGESAASLEDLEDEETHSGGEGSSGGARRRGSGGGSMSKTCTYEGCSETTSQVAKQRKPWMCKKHRNKMYKDKYKKKKSDQALNCGGTASTGSAGNVKLEESADNILSIVKQRTGSFGDRPARPTLLEQVLNQKRLSLLRSPEVVQFLQKQQQLLNQQVLEQRQQQFPGTSM

>Q93015

MELILSTSPAELTLDPACQPKLPLDSTCQPEMTFNPGPTELTLDPEHQPEETPAPSLAELTLEPVHRRPELLDACADLINDQWPRSRTSRLHSLGQSSDAFPLCLMLLSPHPTLEAAPVVVGHARLSRVLNQPQSLLVETVVVARALRGRGFGRRLMEGLEVFARARGFRKLHLTTHDQVHFYTHLGYQLGEPVQGLVFTSRRLPATLLNAFPTAPSPRPPRKAPNLTAQAAPRGPKGPPLPPPPPLPECLTISPPVPSGPPSKSLLETQYQNVRGRPIFWMEKDI

1150 P15586 NP_004146.1

>P15586

MRLLPLAPGRLRRGSPRHLPSCSPALLLLVLGGCLGVFGVAAGTRRPNVVLLLTDDQDEVLGGMTPLKKTKALIGEMGMTFSSAYVPSALCCPSRASILTGKYPHNHHVVNNTLEGNCSSKSWQKIQEPNTFPAILRSMCGYQTFFAGKYLNEYGAPDAGGLEHVPLGWSYWYALEKNSKYYNYTLSINGKARKHGENYSVDYLTDVLANVSLDFLDYKSNFEPFFMMIATPAPHSPWTAAPQYQKAFQNVFAPRNKNFNIHGTNKHWLIRQAKTPMTNSSIQFLDNAFRKRWQTLLSVDDLVEKLVKRLEFTGELNNTYIFYTSDNGYHTGQFSLPIDKRQLYEFDIKVPLLVRGPGIKPNQTSKMLVANIDLGPTILDIAGYDLNKTQMDGMSLLPILRGASNLTWRSDVLVEYQGEGRNVTDPTCPSLSPGVSQCFPDCVCEDAYNNTYACVRTMSALWNLQYCEFDDQEVFVEVYNLTADPDQITNIAKTIDPELLGKMNYRLMMLQSCSGPTCRTPGVFDPGYRFDPRLMFSNRGSVRTRRFSKHLL

>NP_004146.1

METLSNASGTFAIRLLKILCQDNPSHNVFCSPVSISSALAMVLLGAKGNTATQMAQALSLNTEEDIHRAFQSLLTEVNKAGTQYLLRTANRLFGEKTCQFLSTFKESCLQFYHAELKELSFIRAAEESRKHINTWVSKKTEGKIEELLPGSSIDAETRLVLVNAIYFKGKWNEPFDETYTREMPFKINQEEQRPVQMMYQEATFKLAHVGEVRAQLLELPYARKELSLLVLLPDDGVELSTVEKSLTFEKLTAWTKPDCMKSTEVEVLLPKFKLQEDYDMESVLRHLGIVDAFQQGKADLSAMSAERDLCLSKFVHKSFVEVNEEGTEAAAASSCFVVAECCMESGPRFCADHPFLFFIRHNRANSILFCGRFSSP

1151 Q03060 NP_060322.1

>Q03060

MSKCARKKYIKTNPRQMTMETVESQHDGSITASLTESKSAHVQTQTGQNSIPALAQVSVAGSGTRRGSPAVTLVQLPSGQTIHVQGVIQTPQPWVIQSSEIHTVQVAAIAETDESAESEGVIDSHKRREILSRRPSYRKILNELSSDVPGVPKIEEERSEEEGTPPSIATMAVPTSIYQTSTGQYIAIAQGGTIQISNPGSDGVQGLQALTMTNSGAPPPGATIVQYAAQSADGTQQFFVPGSQVVVQDEETELAPSHMAAATGDMPTYQIRAPTAALPQGVVMAASPGSLHSPQQLAEEATRKRELRLMKNREAAKECRRRKKEYVKCLESRVAVLEVQNKKLIEELETLKDICSPKTDY

>NP_060322.1

MVSSAQMGFNLQALLEQLSQDELSKFKYLITTFSLAHELQKIPHKEVDKADGKQLVEILTTHCDSYWVEMASLQVFEKMHRMDLSERAKDEVREAALKSFNKRKPLSLGITRKERPPLDVDEMLERFKTEAQAFTETKGNVICLGKEVFKGKKPDKDNRCRYILKTKFREMWKSWPGDSKEVQVMAERYKMLIPFSNPRVLPGPFSYTVVLYGPAGLGKTTLAQKLMLDWAEDNLIHKFKYAFYLSCRELSRLGPCSFAELVFRDWPELQDDIPHILAQARKILFVIDGFDELGAAPGALIEDICGDWEKKKPVPVLLGSLLNRVMLPKAALLVTTRPRALRDLRILAEEPIYIRVEGFLEEDRRAYFLRHFGDEDQAMRAFELMRSNAALFQLGSAPAVCWIVCTTLKLQMEKGEDPVPTCLTRTGLFLRFLCSRFPQGAQLRGALRTLSLLAAQGLWAQTSVLHREDLERLGVQESDLRLFLDGDILRQDRVSKGCYSFIHLSFQQFLTALFYTLEKEEEEDRDGHTWDIGDVQKLLSGVERLRNPDLIQAGYYSFGLANEKRAKELEATFGCRMSPDIKQELLRCDISCKGGHSTVTDLQELLGCLYESQEEELVKEVMAQFKEISLHLNAVDVVPSSFCVKHCRNLQKMSLQVIKENLPENVTASESDAEVERSQDDQHMLPFWTDLCSIFGSNKDLMGLAINDSFLSASLVRILCEQIASDTCHLQRVVFKNISPADAHRNLCLALRGHKTVTYLTLQGNDQDDMFPALCEVLRHPECNLRYLGLVSCSATTQQWADLSLALEVNQSLTCVNLSDNELLDEGAKLLYTTLRHPKCFLQRLSLENCHLTEANCKDLAAVLVVSRELTHLCLAKNPIGNTGVKFLCEGLRYPECKLQTLVLWNCDITSDGCCDLTKLLQEKSSLLCLDLGLNHIGVKGMKFLCEALRKPLCNLRCLWLWGCSIPPFSCEDLCSALSCNQSLVTLDLGQNPLGSSGVKMLFETLTCSSGTLRTLRLKIDDFNDELNKLLEEIEEKNPQLIIDTEKHHPWAERPSSHDFMI

1152 NP_004217.1 Q9NR20

>NP_004217.1

MSRRRFDCRSISGLLTTTPQIPIKMENFNNFYILTSKELGRGKFAVVRQCISKSTGQEYAAKFLKKRRRGQDCRAEILHEIAVLELAKSCPRVINLHEVYENTSEIILILEYAAGGEIFSLCLPELAEMVSENDVIRLIKQILEGVYYLHQNNIVHLDLKPQNILLSSIYPLGDIKIVDFGMSRKIGHACELREIMGTPEYLAPEILNYDPITTATDMWNIGIIAYMLLTHTSPFVGEDNQETYLNISQVNVDYSEETFSSVSQLATDFIQSLLVKNPEKRPTAEICLSHSWLQQWDFENLFHPEETSSSSQTQDHSVRSSEDKTSKSSCNGTCGDREDKENIPEDSSMVSKRFRFDDSLPNPHELVSDLLC

>Q9NR20

MPASELKASEIPFHPSIKTQDPKAEEKSPKKQKVTLTAAEALKLFKNQLSPYEQSEILGYAELWFLGLEAKKLDTAPEKFSKTSFDDEHGFYLKVLHDHIAYRYEVLETIGKGSFGQVAKCLDHKNNELVALKIIRNKKRFHQQALMELKILEALRKKDKDNTYNVVHMKDFFYFRNHFCITFELLGINLYELMKNNNFQGFSLSIVRRFTLSVLKCLQMLSVEKIIHCDLKPENIVLYQKGQASVKVIDFGSSCYEHQKVYTYIQSRFYRSPEVILGHPYDVAIDMWSLGCITAELYTGYPLFPGENEVEQLACIMEVLGLPPAGFIQTASRRQTFFDSKGFPKNITNNRGKKRYPDSKDLTMVLKTYDTSFLDFLRRCLVWEPSLRMTPDQALKHAWIHQSRNLKPQPRPQTLRKSNSFFPSETRKDKVQGCHHSSRKADEITKETTEKTKDSPTKHVQHSGDQQDCLQHGADTVQLPQLVDAPKKSEAAVGAEVSMTSPGQSKNFSLKNTNVLPPIV

1153 NP_061134.1 NP_002056.2

>NP_061134.1

MSQGLPAAGSVLQRSVAAPGNQPQPQPQQQSPEDDDRKVRRREKNRVAAQRSRKKQTQKADKLHEEYESLEQENTMLRREIGKLTEELKHLTEALKEHEKMCPLLLCPMNFVPVPPRPDPVAGCLPR

>NP_002056.2

MTTSASSHLNKGIKQVYMSLPQGEKVQAMYIWIDGTGEGLRCKTRTLDSEPKCVEELPEWNFDGSSTLQSEGSNSDMYLVPAAMFRDPFRKDPNKLVLCEVFKYNRRPAETNLRHTCKRIMDMVSNQHPWFGMEQEYTLMGTDGHPFGWPSNGFPGPQGPYYCGVGADRAYGRDIVEAHYRACLYAGVKIAGTNAEVMPAQWEFQIGPCEGISMGDHLWVARFILHRVCEDFGVIATFDPKPIPGNWNGAGCHTNFSTKAMREENGLKYIEEAIEKLSKRHQYHIRAYDPKGGLDNARRLTGFHETSNINDFSAGVANRSASIRIPRTVGQEKKGYFEDRRPSANCDPFSVTEALIRTCLLNETGDEPFQYKN

1154 Q9UNL4 Q93015

>Q9UNL4

MAAGMYLEHYLDSIENLPFELQRNFQLMRDLDQRTEDLKAEIDKLATEYMSSARSLSSEEKLALLKQIQEAYGKCKEFGDDKVQLAMQTYEMVDKHIRRLDTDLARFEADLKEKQIESSDYDSSSSKGKKKGRTQKEKKAARARSKGKNSDEEAPKTAQKKLKLVRTSPEYGMPSVTFGSVHPSDVLDMPVDPNEPTYCLCHQVSYGEMIGCDNPDCSIEWFHFACVGLTTKPRGKWFCPRCSQERKKK

>Q93015

MELILSTSPAELTLDPACQPKLPLDSTCQPEMTFNPGPTELTLDPEHQPEETPAPSLAELTLEPVHRRPELLDACADLINDQWPRSRTSRLHSLGQSSDAFPLCLMLLSPHPTLEAAPVVVGHARLSRVLNQPQSLLVETVVVARALRGRGFGRRLMEGLEVFARARGFRKLHLTTHDQVHFYTHLGYQLGEPVQGLVFTSRRLPATLLNAFPTAPSPRPPRKAPNLTAQAAPRGPKGPPLPPPPPLPECLTISPPVPSGPPSKSLLETQYQNVRGRPIFWMEKDI

1155 NP_003027.1 Q9Y3B7

>NP_003027.1

MEAAAGGRGCFQPHPGLQKTLEQFHLSSMSSLGGPAAFSARWAQEAYKKESAKEAGAAAVPAPVPAATEPPPVLHLPAIQPPPPVLPGPFFMPSDRSTERCETVLEGETISCFVVGGEKRLCLPQILNSVLRDFSLQQINAVCDELHIYCSRCTADQLEILKVMGILPFSAPSCGLITKTDAERLCNALLYGGAYPPPCKKELAASLALGLELSERSVRVYHECFGKCKGLLVPELYSSPSAACIQCLDCRLMYPPHKFVVHSHKALENRTCHWGFDSANWRAYILLSQDYTGKEEQARLGRCLDDVKEKFDYGNKYKRRVPRVSSEPPASIRPKTDDTSSQSPAPSEKDKPSSWLRTLAGSSNKSLGCVHPRQRLSAFRPWSPAVSASEKELSPHLPALIRDSFYSYKSFETAVAPNVALAPPAQQKVVSSPPCAAAVSRAPEPLATCTQPRKRKLTVDTPGAPETLAPVAAPEEDKDSEAEVEVESREEFTSSLSSLSSPSFTSSSSAKDLGSPGARALPSAVPDAAAPADAPSGLEAELEHLRQALEGGLDTKEAKEKFLHEVVKMRVKQEEKLSAALQAKRSLHQELEFLRVAKKEKLREATEAKRNLRKEIERLRAENEKKMKEANESRLRLKRELEQARQARVCDKGCEAGRLRAKYSAQIEDLQVKLQHAEADREQLRADLLREREAREHLEKVVKELQEQLWPRARPEAAGSEGAAELEP

>Q9Y3B7

MSKLGRAARGLRKPEVGGVIRAIVRAGLAMPGPPLGPVLGQRGVSINQFCKEFNERTKDIKEGIPLPTKILVKPDRTFEIKIGQPTVSYFLKAAAGIEKGARQTGKEVAGLVTLKHVYEIARIKAQDEAFALQDVPLSSVVRSIIGSARSLGIRVVKDLSSEELAAFQKERAIFLAAQKEADLAAQEEAAKK

1156 NP_000225.1 NP_659451.1

>NP_000225.1

MQRSIMSFFHPKKEGKAKKPEKEASNSSRETEPPPKAALKEWNGVVSESDSPVKRPGRKAARVLGSEGEEEDEALSPAKGQKPALDCSQVSPPRPATSPENNASLSDTSPMDSSPSGIPKRRTARKQLPKRTIQEVLEEQSEDEDREAKRKKEEEEEETPKESLTEAEVATEKEGEDGDQPTTPPKPLKTSKAETPTESVSEPEVATKQELQEEEEQTKPPRRAPKTLSSFFTPRKPAVKKEVKEEEPGAPGKEGAAEGPLDPSGYNPAKNNYHPVEDACWKPGQKVPYLAVARTFEKIEEVSARLRMVETLSNLLRSVVALSPPDLLPVLYLSLNHLGPPQQGLELGVGDGVLLKAVAQATGRQLESVRAEAAEKGDVGLVAENSRSTQRLMLPPPPLTASGVFSKFRDIARLTGSASTAKKIDIIKGLFVACRHSEARFIARSLSGRLRLGLAEQSVLAALSQAVSLTPPGQEFPPAMVDAGKGKTAEARKTWLEEQGMILKQTFCEVPDLDRIIPVLLEHGLERLPEHCKLSPGIPLKPMLAHPTRGISEVLKRFEEAAFTCEYKYDGQRAQIHALEGGEVKIFSRNQEDNTGKYPDIISRIPKIKLPSVTSFILDTEAVAWDREKKQIQPFQVLTTRKRKEVDASEIQVQVCLYAFDLIYLNGESLVREPLSRRRQLLRENFVETEGEFVFATSLDTKDIEQIAEFLEQSVKDSCEGLMVKTLDVDATYEIAKRSHNWLKLKKDYLDGVGDTLDLVVIGAYLGRGKRAGRYGGFLLASYDEDSEELQAICKLGTGFSDEELEEHHQSLKALVLPSPRPYVRIDGAVIPDHWLDPSAVWEVKCADLSLSPIYPAARGLVDSDKGISLRFPRFIRVREDKQPEQATTSAQVACLYRKQSQIQNQQGEDSGSDPEDTY

>NP_659451.1

MEELLPDGQIWANMDPEERMLAAATAFTHICAGQGEGDVRREAQSIQYDPYSKASVAPGKRPALPVQLQYPHVESNVPSETVSEASQRLRKPVMKRKVLRRKPDGEVLVTDESIISESESGTENDQDLWDLRQRLMNVQFQEDKESSFDVSQKFNLPHEYQGISQDQLICSLQREGMGSPAYEQDLIVASRPKSFILPKLDQLSRNRGKTDRVARYFEYKRDWDSIRLPGEDHRKELRWGVREQMLCRAEPQSKPQHIYVPNNYLVPTEKKRSALRWGVRCDLANGVIPRKLPFPLSPS

1157 P17010 NP_066950.1

>P17010

MDEDGLELQQEPNSFFDATGADGTHMDGDQIVVEVQETVFVSDVVDSDITVHNFVPDDPDSVVIQDVIEDVVIEDVQCPDIMEEADVSETVIIPEQVLDSDVTEEVSLAHCTVPDDVLASDITSASMSMPEHVLTGDSIHVSDVGHVGHVGHVEHVVHDSVVEAEIVTDPLTTDVVSEEVLVADCASEAVIDANGIPVDQQDDDKGNCEDYLMISLDDAGKIEHDGSSGMTMDTESEIDPCKVDGTCPEVIKVYIFKADPGEDDLGGTVDIVESEPENDHGVELLDQNSSIRVPREKMVYMTVNDSQPEDEDLNVAEIADEVYMEVIVGEEDAAAAAAAAAVHEQQMDDNEIKTFMPIAWAAAYGNNSDGIENRNGTASALLHIDESAGLGRLAKQKPKKRRRPDSRQYQTAIIIGPDGHPLTVYPCMICGKKFKSRGFLKRHMKNHPEHLAKKKYRCTDCDYTTNKKISLHNHLESHKLTSKAEKAIECDECGKHFSHAGALFTHKMVHKEKGANKMHKCKFCEYETAEQGLLNRHLLAVHSKNFPHICVECGKGFRHPSELKKHMRIHTGEKPYQCQYCEYRSADSSNLKTHVKTKHSKEMPFKCDICLLTFSDTKEVQQHALIHQESKTHQCLHCDHKSSNSSDLKRHIISVHTKDYPHKCDMCDKGFHRPSELKKHVAAHKGKKMHQCRHCDFKIADPFVLSRHILSVHTKDLPFRCKRCRKGFRQQSELKKHMKTHSGRKVYQCEYCEYSTTDASGFKRHVISIHTKDYPHRCEYCKKGFRRPSEKNQHIMRHHKEVGLP

>NP_066950.1

MPGKKARKNAQPSPARAPAELEVECATQLRRFGDKLNFRQKLLNLISKLFCSGT

1158 NP_005924.2 NP_004172.2

>NP_005924.2

MAHSCRWRFPARPGTTGGGGGGGRRGLGGAPRQRVPALLLPPGPPVGGGGPGAPPSPPAVAAAAAAAGSSGAGVPGGAAAASAASSSSASSSSSSSSSASSGPALLRVGPGFDAALQVSAAIGTNLRRFRAVFGESGGGGGSGEDEQFLGFGSDEEVRVRSPTRSPSVKTSPRKPRGRPRSGSDRNSAILSDPSVFSPLNKSETKSGDKIKKKDSKSIEKKRGRPPTFPGVKIKITHGKDISELPKGNKEDSLKKIKRTPSATFQQATKIKKLRAGKLSPLKSKFKTGKLQIGRKGVQIVRRRGRPPSTERIKTPSGLLINSELEKPQKVRKDKEGTPPLTKEDKTVVRQSPRRIKPVRIIPSSKRTDATIAKQLLQRAKKGAQKKIEKEAAQLQGRKVKTQVKNIRQFIMPVVSAISSRIIKTPRRFIEDEDYDPPIKIARLESTPNSRFSAPSCGSSEKSSAASQHSSQMSSDSSRSSSPSVDTSTDSQASEEIQVLPEERSDTPEVHPPLPISQSPENESNDRRSRRYSVSERSFGSRTTKKLSTLQSAPQQQTSSSPPPPLLTPPPPLQPASSISDHTPWLMPPTIPLASPFLPASTAPMQGKRKSILREPTFRWTSLKHSRSEPQYFSSAKYAKEGLIRKPIFDNFRPPPLTPEDVGFASGFSASGTAASARLFSPLHSGTRFDMHKRSPLLRAPRFTPSEAHSRIFESVTLPSNRTSAGTSSSGVSNRKRKRKVFSPIRSEPRSPSHSMRTRSGRLSSSELSPLTPPSSVSSSLSISVSPLATSALNPTFTFPSHSLTQSGESAEKNQRPRKQTSAPAEPFSSSSPTPLFPWFTPGSQTERGRNKDKAPEELSKDRDADKSVEKDKSRERDREREKENKRESRKEKRKKGSEIQSSSALYPVGRVSKEKVVGEDVATSSSAKKATGRKKSSSHDSGTDITSVTLGDTTAVKTKILIKKGRGNLEKTNLDLGPTAPSLEKEKTLCLSTPSSSTVKHSTSSIGSMLAQADKLPMTDKRVASLLKKAKAQLCKIEKSKSLKQTDQPKAQGQESDSSETSVRGPRIKHVCRRAAVALGRKRAVFPDDMPTLSALPWEEREKILSSMGNDDKSSIAGSEDAEPLAPPIKPIKPVTRNKAPQEPPVKKGRRSRRCGQCPGCQVPEDCGVCTNCLDKPKFGGRNIKKQCCKMRKCQNLQWMPSKAYLQKQAKAVKKKEKKSKTSEKKDSKESSVVKNVVDSSQKPTPSAREDPAPKKSSSEPPPRKPVEEKSEEGNVSAPGPESKQATTPASRKSSKQVSQPALVIPPQPPTTGPPRKEVPKTTPSEPKKKQPPPPESGPEQSKQKKVAPRPSIPVKQKPKEKEKPPPVNKQENAGTLNILSTLSNGNSSKQKIPADGVHRIRVDFKEDCEAENVWEMGGLGILTSVPITPRVVCFLCASSGHVEFVYCQVCCEPFHKFCLEENERPLEDQLENWCCRRCKFCHVCGRQHQATKQLLECNKCRNSYHPECLGPNYPTKPTKKKKVWICTKCVRCKSCGSTTPGKGWDAQWSHDFSLCHDCAKLFAKGNFCPLCDKCYDDDDYESKMMQCGKCDRWVHSKCENLSDEMYEILSNLPESVAYTCVNCTERHPAEWRLALEKELQISLKQVLTALLNSRTTSHLLRYRQAAKPPDLNPETEESIPSRSSPEGPDPPVLTEVSKQDDQQPLDLEGVKRKMDQGNYTSVLEFSDDIVKIIQAAINSDGGQPEIKKANSMVKSFFIRQMERVFPWFSVKKSRFWEPNKVSSNSGMLPNAVLPPSLDHNYAQWQEREENSHTEQPPLMKKIIPAPKPKGPGEPDSPTPLHPPTPPILSTDRSREDSPELNPPPGIEDNRQCALCLTYGDDSANDAGRLLYIGQNEWTHVNCALWSAEVFEDDDGSLKNVHMAVIRGKQLRCEFCQKPGATVGCCLTSCTSNYHFMCSRAKNCVFLDDKKVYCQRHRDLIKGEVVPENGFEVFRRVFVDFEGISLRRKFLNGLEPENIHMMIGSMTIDCLGILNDLSDCEDKLFPIGYQCSRVYWSTTDARKRCVYTCKIVECRPPVVEPDINSTVEHDENRTIAHSPTSFTESSSKESQNTAEIISPPSPDRPPHSQTSGSCYYHVISKVPRIRTPSYSPTQRSPGCRPLPSAGSPTPTTHEIVTVGDPLLSSGLRSIGSRRHSTSSLSPQRSKLRIMSPMRTGNTYSRNNVSSVSTTGTATDLESSAKVVDHVLGPLNSSTSLGQNTSTSSNLQRTVVTVGNKNSHLDGSSSSEMKQSSASDLVSKSSSLKGEKTKVLSSKSSEGSAHNVAYPGIPKLAPQVHNTTSRELNVSKIGSFAEPSSVSFSSKEALSFPHLHLRGQRNDRDQHTDSTQSANSSPDEDTEVKTLKLSGMSNRSSIINEHMGSSSRDRRQKGKKSCKETFKEKHSSKSFLEPGQVTTGEEGNLKPEFMDEVLTPEYMGQRPCNNVSSDKIGDKGLSMPGVPKAPPMQVEGSAKELQAPRKRTVKVTLTPLKMENESQSKNALKESSPASPLQIESTSPTEPISASENPGDGPVAQPSPNNTSCQDSQSNNYQNLPVQDRNLMLPDGPKPQEDGSFKRRYPRRSARARSNMFFGLTPLYGVRSYGEEDIPFYSSSTGKKRGKRSAEGQVDGADDLSTSDEDDLYYYNFTRTVISSGGEERLASHNLFREEEQCDLPKISQLDGVDDGTESDTSVTATTRKSSQIPKRNGKENGTENLKIDRPEDAGEKEHVTKSSVGHKNEPKMDNCHSVSRVKTQGQDSLEAQLSSLESSRRVHTSTPSDKNLLDTYNTELLKSDSDNNNSDDCGNILPSDIMDFVLKNTPSMQALGESPESSSSELLNLGEGLGLDSNREKDMGLFEVFSQQLPTTEPVDSSVSSSISAEEQFELPLELPSDLSVLTTRSPTVPSQNPSRLAVISDSGEKRVTITEKSVASSESDPALLSPGVDPTPEGHMTPDHFIQGHMDADHISSPPCGSVEQGHGNNQDLTRNSSTPGLQVPVSPTVPIQNQKYVPNSTDSPGPSQISNAAVQTTPPHLKPATEKLIVVNQNMQPLYVLQTLPNGVTQKIQLTSSVSSTPSVMETNTSVLGPMGGGLTLTTGLNPSLPTSQSLFPSASKGLLPMSHHQHLHSFPAATQSSFPPNISNPPSGLLIGVQPPPDPQLLVSESSQRTDLSTTVATPSSGLKKRPISRLQTRKNKKLAPSSTPSNIAPSDVVSNMTLINFTPSQLPNHPSLLDLGSLNTSSHRTVPNIIKRSKSSIMYFEPAPLLPQSVGGTAATAAGTSTISQDTSHLTSGSVSGLASSSSVLNVVSMQTTTTPTSSASVPGHVTLTNPRLLGTPDIGSISNLLIKASQQSLGIQDQPVALPPSSGMFPQLGTSQTPSTAAITAASSICVLPSTQTTGITAASPSGEADEHYQLQHVNQLLASKTGIHSSQRDLDSASGPQVSNFTQTVDAPNSMGLEQNKALSSAVQASPTSPGGSPSSPSSGQRSASPSVPGPTKPKPKTKRFQLPLDKGNGKKHKVSHLRTSSSEAHIPDQETTSLTSGTGTPGAEAEQQDTASVEQSSQKECGQPAGQVAVLPEVQVTQNPANEQESAEPKTVEEEESNFSSPLMLWLQQEQKRKESITEKKPKKGLVFEISSDDGFQICAESIEDAWKSLTDKVQEARSNARLKQLSFAGVNGLRMLGILHDAVVFLIEQLSGAKHCRNYKFRFHKPEEANEPPLNPHGSARAEVHLRKSAFDMFNFLASKHRQPPEYNPNDEEEEEVQLKSARRATSMDLPMPMRFRHLKKTSKEAVGVYRSPIHGRGLFCKRNIDAGEMVIEYAGNVIRSIQTDKREKYYDSKGIGCYMFRIDDSEVVDATMHGNAARFINHSCEPNCYSRVINIDGQKHIVIFAMRKIYRGEELTYDYKFPIEDASNKLPCNCGAKKCRKFLN

>NP_004172.2

MQLKPMEINPEMLNKVLSRLGVAGQWRFVDVLGLEEESLGSVPAPACALLLLFPLTAQHENFRKKQIEELKGQEVSPKVYFMKQTIGNSCGTIGLIHAVANNQDKLGFEDGSVLKQFLSETEKMSPEDRAKCFEKNEAIQAAHDAVAQEGQCRVDDKVNFHFILFNNVDGHLYELDGRMPFPVNHGASSEDTLLKDAAKVCREFTEREQGEVRFSAVALCKAA

1159 NP_005924.2 P42765

>NP_005924.2

MAHSCRWRFPARPGTTGGGGGGGRRGLGGAPRQRVPALLLPPGPPVGGGGPGAPPSPPAVAAAAAAAGSSGAGVPGGAAAASAASSSSASSSSSSSSSASSGPALLRVGPGFDAALQVSAAIGTNLRRFRAVFGESGGGGGSGEDEQFLGFGSDEEVRVRSPTRSPSVKTSPRKPRGRPRSGSDRNSAILSDPSVFSPLNKSETKSGDKIKKKDSKSIEKKRGRPPTFPGVKIKITHGKDISELPKGNKEDSLKKIKRTPSATFQQATKIKKLRAGKLSPLKSKFKTGKLQIGRKGVQIVRRRGRPPSTERIKTPSGLLINSELEKPQKVRKDKEGTPPLTKEDKTVVRQSPRRIKPVRIIPSSKRTDATIAKQLLQRAKKGAQKKIEKEAAQLQGRKVKTQVKNIRQFIMPVVSAISSRIIKTPRRFIEDEDYDPPIKIARLESTPNSRFSAPSCGSSEKSSAASQHSSQMSSDSSRSSSPSVDTSTDSQASEEIQVLPEERSDTPEVHPPLPISQSPENESNDRRSRRYSVSERSFGSRTTKKLSTLQSAPQQQTSSSPPPPLLTPPPPLQPASSISDHTPWLMPPTIPLASPFLPASTAPMQGKRKSILREPTFRWTSLKHSRSEPQYFSSAKYAKEGLIRKPIFDNFRPPPLTPEDVGFASGFSASGTAASARLFSPLHSGTRFDMHKRSPLLRAPRFTPSEAHSRIFESVTLPSNRTSAGTSSSGVSNRKRKRKVFSPIRSEPRSPSHSMRTRSGRLSSSELSPLTPPSSVSSSLSISVSPLATSALNPTFTFPSHSLTQSGESAEKNQRPRKQTSAPAEPFSSSSPTPLFPWFTPGSQTERGRNKDKAPEELSKDRDADKSVEKDKSRERDREREKENKRESRKEKRKKGSEIQSSSALYPVGRVSKEKVVGEDVATSSSAKKATGRKKSSSHDSGTDITSVTLGDTTAVKTKILIKKGRGNLEKTNLDLGPTAPSLEKEKTLCLSTPSSSTVKHSTSSIGSMLAQADKLPMTDKRVASLLKKAKAQLCKIEKSKSLKQTDQPKAQGQESDSSETSVRGPRIKHVCRRAAVALGRKRAVFPDDMPTLSALPWEEREKILSSMGNDDKSSIAGSEDAEPLAPPIKPIKPVTRNKAPQEPPVKKGRRSRRCGQCPGCQVPEDCGVCTNCLDKPKFGGRNIKKQCCKMRKCQNLQWMPSKAYLQKQAKAVKKKEKKSKTSEKKDSKESSVVKNVVDSSQKPTPSAREDPAPKKSSSEPPPRKPVEEKSEEGNVSAPGPESKQATTPASRKSSKQVSQPALVIPPQPPTTGPPRKEVPKTTPSEPKKKQPPPPESGPEQSKQKKVAPRPSIPVKQKPKEKEKPPPVNKQENAGTLNILSTLSNGNSSKQKIPADGVHRIRVDFKEDCEAENVWEMGGLGILTSVPITPRVVCFLCASSGHVEFVYCQVCCEPFHKFCLEENERPLEDQLENWCCRRCKFCHVCGRQHQATKQLLECNKCRNSYHPECLGPNYPTKPTKKKKVWICTKCVRCKSCGSTTPGKGWDAQWSHDFSLCHDCAKLFAKGNFCPLCDKCYDDDDYESKMMQCGKCDRWVHSKCENLSDEMYEILSNLPESVAYTCVNCTERHPAEWRLALEKELQISLKQVLTALLNSRTTSHLLRYRQAAKPPDLNPETEESIPSRSSPEGPDPPVLTEVSKQDDQQPLDLEGVKRKMDQGNYTSVLEFSDDIVKIIQAAINSDGGQPEIKKANSMVKSFFIRQMERVFPWFSVKKSRFWEPNKVSSNSGMLPNAVLPPSLDHNYAQWQEREENSHTEQPPLMKKIIPAPKPKGPGEPDSPTPLHPPTPPILSTDRSREDSPELNPPPGIEDNRQCALCLTYGDDSANDAGRLLYIGQNEWTHVNCALWSAEVFEDDDGSLKNVHMAVIRGKQLRCEFCQKPGATVGCCLTSCTSNYHFMCSRAKNCVFLDDKKVYCQRHRDLIKGEVVPENGFEVFRRVFVDFEGISLRRKFLNGLEPENIHMMIGSMTIDCLGILNDLSDCEDKLFPIGYQCSRVYWSTTDARKRCVYTCKIVECRPPVVEPDINSTVEHDENRTIAHSPTSFTESSSKESQNTAEIISPPSPDRPPHSQTSGSCYYHVISKVPRIRTPSYSPTQRSPGCRPLPSAGSPTPTTHEIVTVGDPLLSSGLRSIGSRRHSTSSLSPQRSKLRIMSPMRTGNTYSRNNVSSVSTTGTATDLESSAKVVDHVLGPLNSSTSLGQNTSTSSNLQRTVVTVGNKNSHLDGSSSSEMKQSSASDLVSKSSSLKGEKTKVLSSKSSEGSAHNVAYPGIPKLAPQVHNTTSRELNVSKIGSFAEPSSVSFSSKEALSFPHLHLRGQRNDRDQHTDSTQSANSSPDEDTEVKTLKLSGMSNRSSIINEHMGSSSRDRRQKGKKSCKETFKEKHSSKSFLEPGQVTTGEEGNLKPEFMDEVLTPEYMGQRPCNNVSSDKIGDKGLSMPGVPKAPPMQVEGSAKELQAPRKRTVKVTLTPLKMENESQSKNALKESSPASPLQIESTSPTEPISASENPGDGPVAQPSPNNTSCQDSQSNNYQNLPVQDRNLMLPDGPKPQEDGSFKRRYPRRSARARSNMFFGLTPLYGVRSYGEEDIPFYSSSTGKKRGKRSAEGQVDGADDLSTSDEDDLYYYNFTRTVISSGGEERLASHNLFREEEQCDLPKISQLDGVDDGTESDTSVTATTRKSSQIPKRNGKENGTENLKIDRPEDAGEKEHVTKSSVGHKNEPKMDNCHSVSRVKTQGQDSLEAQLSSLESSRRVHTSTPSDKNLLDTYNTELLKSDSDNNNSDDCGNILPSDIMDFVLKNTPSMQALGESPESSSSELLNLGEGLGLDSNREKDMGLFEVFSQQLPTTEPVDSSVSSSISAEEQFELPLELPSDLSVLTTRSPTVPSQNPSRLAVISDSGEKRVTITEKSVASSESDPALLSPGVDPTPEGHMTPDHFIQGHMDADHISSPPCGSVEQGHGNNQDLTRNSSTPGLQVPVSPTVPIQNQKYVPNSTDSPGPSQISNAAVQTTPPHLKPATEKLIVVNQNMQPLYVLQTLPNGVTQKIQLTSSVSSTPSVMETNTSVLGPMGGGLTLTTGLNPSLPTSQSLFPSASKGLLPMSHHQHLHSFPAATQSSFPPNISNPPSGLLIGVQPPPDPQLLVSESSQRTDLSTTVATPSSGLKKRPISRLQTRKNKKLAPSSTPSNIAPSDVVSNMTLINFTPSQLPNHPSLLDLGSLNTSSHRTVPNIIKRSKSSIMYFEPAPLLPQSVGGTAATAAGTSTISQDTSHLTSGSVSGLASSSSVLNVVSMQTTTTPTSSASVPGHVTLTNPRLLGTPDIGSISNLLIKASQQSLGIQDQPVALPPSSGMFPQLGTSQTPSTAAITAASSICVLPSTQTTGITAASPSGEADEHYQLQHVNQLLASKTGIHSSQRDLDSASGPQVSNFTQTVDAPNSMGLEQNKALSSAVQASPTSPGGSPSSPSSGQRSASPSVPGPTKPKPKTKRFQLPLDKGNGKKHKVSHLRTSSSEAHIPDQETTSLTSGTGTPGAEAEQQDTASVEQSSQKECGQPAGQVAVLPEVQVTQNPANEQESAEPKTVEEEESNFSSPLMLWLQQEQKRKESITEKKPKKGLVFEISSDDGFQICAESIEDAWKSLTDKVQEARSNARLKQLSFAGVNGLRMLGILHDAVVFLIEQLSGAKHCRNYKFRFHKPEEANEPPLNPHGSARAEVHLRKSAFDMFNFLASKHRQPPEYNPNDEEEEEVQLKSARRATSMDLPMPMRFRHLKKTSKEAVGVYRSPIHGRGLFCKRNIDAGEMVIEYAGNVIRSIQTDKREKYYDSKGIGCYMFRIDDSEVVDATMHGNAARFINHSCEPNCYSRVINIDGQKHIVIFAMRKIYRGEELTYDYKFPIEDASNKLPCNCGAKKCRKFLN

>P42765

MALLRGVFVVAAKRTPFGAYGGLLKDFTATDLSEFAAKAALSAGKVSPETVDSVIMGNVLQSSSDAIYLARHVGLRVGIPKETPALTINRLCGSGFQSIVNGCQEICVKEAEVVLCGGTESMSQAPYCVRNVRFGTKLGSDIKLEDSLWVSLTDQHVQLPMAMTAENLAVKHKISREECDKYALQSQQRWKAANDAGYFNDEMAPIEVKTKKGKQTMQVDEHARPQTTLEQLQKLPPVFKKDGTVTAGNASGVADGAGAVIIASEDAVKKHNFTPLARIVGYFVSGCDPSIMGIGPVPAISGALKKAGLSLKDMDLVEVNEAFAPQYLAVERSLDLDISKTNVNGGAIALGHPLGGSGSRITAHLVHELRRRGGKYAVGSACIGGGQGIAVIIQSTA

1160 NP_004070.3 NP_036205.1

>NP_004070.3

MKRLVCVLLVCSSAVAQLHKDPTLDHHWHLWKKTYGKQYKEKNEEAVRRLIWEKNLKFVMLHNLEHSMGMHSYDLGMNHLGDMTSEEVMSLMSSLRVPSQWQRNITYKSNPNRILPDSVDWREKGCVTEVKYQGSCGACWAFSAVGALEAQLKLKTGKLVSLSAQNLVDCSTEKYGNKGCNGGFMTTAFQYIIDNKGIDSDASYPYKAMDQKCQYDSKYRAATCSKYTELPYGREDVLKEAVANKGPVSVGVDARHPSFFLYRSGVYYEPSCTQNVNHGVLVVGYGDLNGKEYWLVKNSWGHNFGEEGYIRMARNKGNHCGIASFPSYPEI

>NP_036205.1

MASMGTLAFDEYGRPFLIIKDQDRKSRLMGLEALKSHIMAAKAVANTMRTSLGPNGLDKMMVDKDGDVTVTNDGATILSMMDVDHQIAKLMVELSKSQDDEIGDGTTGVVVLAGALLEEAEQLLDRGIHPIRIADGYEQAARVAIEHLDKISDSVLVDIKDTEPLIQTAKTTLGSKVVNSCHRQMAEIAVNAVLTVADMERRDVDFELIKVEGKVGGRLEDTKLIKGVIVDKDFSHPQMPKKVEDAKIAILTCPFEPPKPKTKHKLDVTSVEDYKALQKYEKEKFEEMIQQIKETGANLAICQWGFDDEANHLLLQNNLPAVRWVGGPEIELIAIATGGRIVPRFSELTAEKLGFAGLVQEISFGTTKDKMLVIEQCKNSRAVTIFIRGGNKMIIEEAKRSLHDALCVIRNLIRDNRVVYGGGAAEISCALAVSQEADKCPTLEQYAMRAFADALEVIPMALSENSGMNPIQTMTEVRARQVKEMNPALGIDCLHKGTNDMKQQHVIETLIGKKQQISLATQMVRMILKIDDIRKPGESEE

1161 BAB62909.1 NP_001217.2

>BAB62909.1

MDRSKRNSIAGFPPRVERLEEFEGGGGGEGNVSQVGRVWPSSYRALISAFSRLTRLDDFTCEKIGSGFFSEVFKVRHRASGQVMALKMNTLSSNRANMLKEVQLMNRLSHPNILRFMGVCVHQGQLHALTEYINSGNLEQLLDSNLHLPWTVRVKLAYDIAVGLSYLHFKGIFHRDLTSKNCLIKRDENGYSAVVADFGLAEKIPDVSMGSEKLAVVGSPFWMAPEVLRDEPYNEKADVFSYGIILCEIIARIQADPDYLPRTENFGLDYDAFQHMVGDCPPDFLQLTFNCCNMDPKLRPSFVEIGKTLEEILSRLQEEEQERDRKLQPTARGLLEKAPGVKRLSSLDDKIPHKSPCPRRTIWLSRSQSDIFSRKPPRTVSVLDPYYRPRDGAARTPKVNPFSARQDLMGGKIKFFDLPSKSVISLVFDLDAPGPGTMPLADWQEPLAPPIRRWRSLPGSPEFLHQEACPFVGREESLSDGPPPRLSSLKYRVKEIPPFRASALPAAQAHEAMDCSILQEENGFGSRPQGTSPCPAGASEEMEVEERPAGSTPATFSTSGIGLQTQGKQDG

>NP_001217.2

MSSASGLRRGHPAGGEENMTETDAFYKREMFDPAEKYKMDHRRRGIALIFNHERFFWHLTLPERRGTCADRDNLTRRFSDLGFEVKCFNDLKAEELLLKIHEVSTVSHADADCFVCVFLSHGEGNHIYAYDAKIEIQTLTGLFKGDKCHSLVGKPKIFIIQACRGNQHDVPVIPLDVVDNQTEKLDTNITEVDAASVYTLPAGADFLMCYSVAEGYYSHRETVNGSWYIQDLCEMLGKYGSSLEFTELLTLVNRKVSQRRVDFCKDPSAIGKKQVPCFASMLTKKLHFFPKSN

1162 Q9BXL8 NP_055141.2

>Q9BXL8

MFARGLKRKCVGHEEDVEGALAGLKTVSSYSLQRQSLLDMSLVKLQLCHMLVEPNLCRSVLIANTVRQIQEEMTQDGTWRTVAPQAAERAPLDRLVSTEILCRAAWGQEGAHPAPGLGDGHTQGPVSDLCPVTSAQAPRHLQSSAWEMDGPRENRGSFHKSLDQIFETLETKNPSCMEELFSDVDSPYYDLDTVLTGMMGGARPGPCEGLEGLAPATPGPSSSCKSDLGELDHVVEILVET

>NP_055141.2

MFQASMRSPNMEPFKQQKVEDFYDIGEELGSGQFAIVKKCREKSTGLEYAAKFIKKRQSRASRRGVSREEIEREVSILRQVLHHNVITLHDVYENRTDVVLILELVSGGELFDFLAQKESLSEEEATSFIKQILDGVNYLHTKKIAHFDLKPENIMLLDKNIPIPHIKLIDFGLAHEIEDGVEFKNIFGTPEFVAPEIVNYEPLGLEADMWSIGVITYILLSGASPFLGDTKQETLANITAVSYDFDEEFFSQTSELAKDFIRKLLVKETRKRLTIQEALRHPWITPVDNQQAMVRRESVVNLENFRKQYVRRRWKLSFSIVSLCNHLTRSLMKKVHLRPDEDLRNCESDTEEDIARRKALHPRRRSSTS

1163 NP_002543.2 BAC04299.1

>NP_002543.2

MSSRKSKSNSLIHTECLSQVQRILRERFCRQSPHSNLFGVQVQYKHLSELLKRTALHGESNSVLIIGPRGSGKTMLINHALKELMEIEEVSENVLQVHLNGLLQINDKIALKEITRQLNLENVVGDKVFGSFAENLSFLLEALKKGDRTSSCPVIFILDEFDLFAHHKNQTLLYNLFDISQSAQTPIAVIGLTCRLDILELLEKRVKSRFSHRQIHLMNSFGFPQYVKIFKEQLSLPAEFPDKVFAEKWNENVQYLSEDRSVQEVLQKHFNISKNLRSLHMLLMLALNRVTASHPFMTAVDLMEASQLCSMDSKANIVHGLSVLEICLIIAMKHLNDIYEEEPFNFQMVYNEFQKFVQRKAHSVYNFEKPVVMKAFEHLQQLELIKPMERTSGNSQREYQLMKLLLDNTQIMNALQKYPNCPTDVRQWATSSLSWL

>BAC04299.1

MEVLRPQLIRIDGRNYRKNPVQEQTYQHEEDEEDFYQGSMECADEPCDAYEVEQTPQGFRSTLRAPSLLYNLIHLNTSNDCGFQKITLDCQNIYTWKSRHIVGKRGDTRKKIEMETKTSISIPKPGQDGEIVITGQHRNGVISARTRIDVLLDTFRRKQPFTHFLAFFLNEVEVQEGFLRFQEEVLAKCSMDHGVDSSIFQNPKKLHLTIGMLVLLSEEEIQQTCEMLQQCKEEFINDISGGKPLEVEMAGIEYMNDDPGMVDVLYAKVHMKDGSNRLQELVDRVLERFQASGLIVKEWNSVKLHATVMNTLFRKDPNAEGRYNLYTAEGKYIFKERESFDGRNILKSFALLPRLEYNDAISAHCNLCLPGSSDSPASASQVAGITGVSDAYSQSLPGKS

1164 NP_005644.2 O95749

>NP_005644.2

MAWALKLPLADEVIESGLVQDFDASLSGIGQELGAGAYSMSDVLALPIFKQEESSLPPDNENKILPFQYVLCAATSPAVKLHDETLTYLNQGQSYEIRMLDNRKLGELPEINGKLVKSIFRVVFHDRRLQYTEHQQLEGWRWNRPGDRILDIDIPMSVGIIDPRANPTQLNTVEFLWDPAKRTSVFIQVHCISTEFTMRKHGGEKGVPFRVQIDTFKENENGEYTEHLHSASCQIKVFKPKGADRKQKTDREKMEKRTPHEKEKYQPSYETTILTECSPWPEITYVNNSPSPGFNSSHSSFSLGEGNGSPNHQPEPPPPVTDNLLPTTTPQEAQQWLHRNRFSTFTRLFTNFSGADLLKLTRDDVIQICGPADGIRLFNALKGRMVRPRLTIYVCQESLQLREQQQQQQQQQQKHEDGDSNGTFFVYHAIYLEELTAVELTEKIAQLFSISPCQISQIYKQGPTGIHVLISDEMIQNFQEEACFILDTMKAETNDSYHIILK

>O95749

MEKTQETVQRILLEPYKYLLQLPGKQVRTKLSQAFNHWLKVPEDKLQIIIEVTEMLHNASLLIDDIEDNSKLRRGFPVAHSIYGIPSVINSANYVYFLGLEKVLTLDHPDAVKLFTRQLLELHQGQGLDIYWRDNYTCPTEEEYKAMVLQKTGGLFGLAVGLMQLFSDYKEDLKPLLNTLGLFFQIRDDYANLHSKEYSENKSFCEDLTEGKFSFPTIHAIWSRPESTQVQNILRQRTENIDIKKYCVHYLEDVGSFEYTRNTLKELEAKAYKQIDARGGNPELVALVKHLSKMFKEENE

1165 Q7Z591 Q8IXM3

>Q7Z591

MASSETEIRWAEPGLGKGPQRRRWAWAEDKRDVDRSSSQSWEEERLFPNATSPELLEDFRLAQQHLPPLEWDPHPQPDGHQDSESGETSGEEAEAEDVDSPASSHEPLAWLPQQGRQLDMTEEEPDGTLGSLEVEEAGESSSRLGYEAGLSLEGHGNTSPMALGHGQARGWVASGEQASGDKLSEHSEVNPSVELSPARSWSSGTVSLDHPSDSLDSTWEGETDGPQPTALAETLPEGPSHHLLSPDGRTGGSVARATPMEFQDSSAPPAQSPQHATDRWRRETTRFFCPQPKEHIWKQTKTSPKPLPSRFIGSISPLNPQPRPTRQGRPLPRQGATLAGRSSSNAPKYGRGQLNYPLPDFSKVGPRVRFPKDESYRPPKSRSHNRKPQAPARPLIFKSPAEIVQEVLLSSGEAALAKDTPPAHPITRVPQEFQTPEQATELVHQLQEDYHRLLTKYAEAENTIDQLRLGAKVNLFSDPPQPNHSIHTGMVPQGTKVLSFTIPQPRSAEWWPGPAEDPQASAASGWPSARGDLSPSSLTSMPTLGWLPENRDISEDQSSAEQTQALASQASQFLAKVESFERLIQAGRLMPQDQVKGFQRLKAAHAALEEEYLKACREQHPAQPLAGSKGTPGRFDPRRELEAEIYRLGSCLEELKEHIDQTQQEPEPPGSDSALDSTPALPCLHQPTHLPAPSGQAPMPAIKTSCPEPATTTAAASTGPCPLHVNVEVSSGNSEVEDRPQDPLARLRHKELQMEQVYHGLMERYLSVKSLPEAMRMEEEEEGEEEEEEEGGGDSLEVDGVAATPGKAEATRVLPRQCPVQAEKSHGAPLEEATEKMVSMKPPGFQASLARDGHMSGLGKAEAAPPGPGVPPHPPGTKSAASHQSSMTSLEGSGISERLPQKPLHRGGGPHLEETWMASPETDSGFVGSETSRVSPLTQTPEHRLSHISTAGTLAQPFAASVPRDGASYPKARGSLIPRRATEPSTPRSQAQRYLSSPSGPLRQRAPNFSLERTLAAEMAVPGSEFEGHKRISEQPLPNKTISPPPAPAPAAAPLPCGPTETIPSFLLTRAGRDQAICELQEEVSRLRLRLEDSLHQPLQGSPTRPASAFDRPARTRGRPADSPATWGSHYGSKSTERLPGEPRGEEQIVPPGRQRARSSSVPREVLRLSLSSESELPSLPLFSEKSKTTKDSPQAARDGKRGVGSAGWPDRVTFRGQYTGHEYHVLSPKAVPKGNGTVSCPHCRPIRTQDAGGAVTGDPLGPPPADTLQCPLCGQVGSPPEADGPGSATSGAEKATTRRKASSTPSPKQRSKQAGSSPRPPPGLWYLATAPPAPAPPAFAYISSVPIMPYPPAAVYYAPAGPTSAQPAAKWPPTASPPPARRHRHSIQLDLGDLEELNKALSRAVQAAESVRSTTRQMRSSLSADLRQAHSLRGSCLF

>Q8IXM3

MGVLAAAARCLVRGADRMSKWTSKRGPRSFRGRKGRGAKGIGFLTSGWRFVQIKEMVPEFVVPDLTGFKLKPYVSYLAPESEETPLTAAQLFSEAVAPAIEKDFKDGTFDPDNLEKYGFEPTQEGKLFQLYPRNFLR

1166 Q9H6T0 NP_065842.1

>Q9H6T0

MTPPPPPPPPPGPDPAADPAADPCPWPGSLVVLFGATAGALGRDLGSDETDLILLVWQVVEPRSRQVGTLHKSLVRAEAAALSTQCREASGLSADSLARAEPLDKVLQQFSQLVNGDVALLGGGPYMLCTDGQQLLRQVLHPEASRKNLVLPDMFFSFYDLRREFHMQHPSTCPARDLTVATMAQGLGLETDATEDDFGVWEVKTMVAVILHLLKEPSSQLFSKPEVIKQKYETGPCSDSTVPCPYSSKADVVDSETVVRARGLPWQSSDQDVARFFKGLNVARGGVALCLNAQGRRNGEALIRFVDSEQRDLALQRHKHHMGVRYIEVYKATGEEFVKIAGGTSLEVARFLSREDQVILRLRGLPFSAGPTDVLGFLGPECPVTGGTEGLLFVRHPDGRPTGDAFALFACEELAQAALRRHKGMLGKRYIELFRSTAAEVQQVLNRYASGPLLPTLTAPLLPIPFPLAPGTGRDCVRLRGLPYTATIEDILSFLGEAAADIRPHGVHMVLNQQGRPSGDAFIQMTSAERALAAAQRCHKKVMKERYVEVVPCSTEEMSRVLMGGTLGRSGMSPPPCKLPCLSPPTYTTFQATPTLIPTETAALYPSSALLPAARVPAAPTPVAYYPGPATQLYLNYTAYYPSPPVSPTTVGYLTTPTAALASAPTSVLSQSGALVRMQGVPYTAGMKDLLSVFQAYQLPADDYTSLMPVGDPPRTVLQAPKEWVCL

>NP_065842.1

MPSTNRAGSLKDPEIAELFFKEDPEKLFTDLREIGHGSFGAVYFARDVRTNEVVAIKKMSYSGKQSTEKWQDIIKEVKFLQRIKHPNSIEYKGCYLREHTAWLVMEYCLGSASDLLEVHKKPLQEVEIAAITHGALQGLAYLHSHTMIHRDIKAGNILLTEPGQVKLADFGSASMASPANSFVGTPYWMAPEVILAMDEGQYDGKVDVWSLGITCIELAERKPPLFNMNAMSALYHIAQNESPTLQSNEWSDYFRNFVDSCLQKIPQDRPTSEELLKHIFVLRERPETVLIDLIQRTKDAVRELDNLQYRKMKKLLFQEAHNGPAVEAQEEEEEQDHGVGRTGTVNSVGSNQSIPSMSISASSQSSSVNSLPDVSDDKSELDMMEGDHTVMSNSSVIHLKPEEENYREEGDPRTRASDPQSPPQVSRHKSHYRNREHFATIRTASLVTRQMQEHEQDSELREQMSGYKRMRRQHQKQLMTLENKLKAEMDEHRLRLDKDLETQRNNFAAEMEKLIKKHQAAMEKEAKVMSNEEKKFQQHIQAQQKKELNSFLESQKREYKLRKEQLKEELNENQSTPKKEKQEWLSKQKENIQHFQAEEEANLLRRQRQYLELECRRFKRRMLLGRHNLEQDLVREELNKRQTQKDLEHAMLLRQHESMQELEFRHLNTIQKMRCELIRLQHQTELTNQLEYNKRRERELRRKHVMEVRQQPKSLKSKELQIKKQFQDTCKIQTRQYKALRNHLLETTPKSEHKAVLKRLKEEQTRKLAILAEQYDHSINEMLSTQALRLDEAQEAECQVLKMQLQQELELLNAYQSKIKMQAEAQHDRELRELEQRVSLRRALLEQKIEEEMLALQNERTERIRSLLERQAREIEAFDSESMRLGFSNMVLSNLSPEAFSHSYPGASGWSHNPTGGPGPHWGHPMGGPPQAWGHPMQGGPQPWGHPSGPMQGVPRGSSMGVRNSPQALRRTASGGRTEQGMSRSTSVTSQISNGSHMSYT

1167 Q6NSW7 NP_002055.1

>Q6NSW7

MSVDPACPQSLPCFEASDCKESSPMPVICGPEENYPSLQMSSAEMPHTETVSPLPSSMDLLIQDSPDSSTSPKGKQPTSAENSVAKKEDKVPVKKQKTRTVFSSTQLCVLNDRFQRQKYLSLQQMQELSNILNLSYKQVKTWFQNQRMKSKRWQKNNWPKNSNGVTQKASAPTYPSLYSSYHQGCLVNPTGNLPMWSNQTWNNSTWSNQTQNIQSWSNHSWNTQTWCTQSWNNQAWNSPFYNCGEESLQSCMHFQPNSPASDLEAALEAAGEGLNVIQQTTRYFSTPQTMDLFLNYSMNMQPEDV

>NP_002055.1

MAQEFVNCKIQPGKVVVFIKPTCPYCRRAQEILSQLPIKQGLLEFVDITATNHTNEIQDYLQQLTGARTVPRVFIGKDCIGGCSDLVSLQQSGELLTRLKQIGALQ

1168 O00462 Q8TE68

>O00462

MRLHLLLLLALCGAGTTAAELSYSLRGNWSICNGNGSLELPGAVPGCVHSALFQQGLIQDSYYRFNDLNYRWVSLDNWTYSKEFKIPFEISKWQKVNLILEGVDTVSKILFNEVTIGETDNMFNRYSFDITNVVRDVNSIELRFQSAVLYAAQQSKAHTRYQVPPDCPPLVQKGECHVNFVRKEQCSFSWDWGPSFPTQGIWKDVRIEAYNICHLNYFTFSPIYDKSAQEWNLEIESTFDVVSSKPVGGQVIVAIPKLQTQQTYSIELQPGKRIVELFVNISKNITVETWWPHGHGNQTGYNMTVLFELDGGLNIEKSAKVYFRTVELIEEPIKGSPGLSFYFKINGFPIFLKGSNWIPADSFQDRVTSELLRLLLQSVVDANMNTLRVWGGGIYEQDEFYELCDELGIMVWQDFMFACALYPTDQGFLDSVTAEVAYQIKRLKSHPSIIIWSGNNENEEALMMNWYHISFTDRPIYIKDYVTLYVKNIRELVLAGDKSRPFITSSPTNGAETVAEAWVSQNPNSNYFGDVHFYDYISDCWNWKVFPKARFASEYGYQSWPSFSTLEKVSSTEDWSFNSKFSLHRQHHEGGNKQMLYQAGLHFKLPQSTDPLRTFKDTIYLTQVMQAQCVKTETEFYRRSRSEIVDQQGHTMGALYWQLNDIWQAPSWASLEYGGKWKMLHYFAQNFFAPLLPVGFENENTFYIYGVSDLHSDYSMTLSVRVHTWSSLEPVCSRVTERFVMKGGEAVCLYEEPVSELLRRCGNCTRESCVVSFYLSADHELLSPTNYHFLSSPKEAVGLCKAQITAIISQQGDIFVFDLETSAVAPFVWLDVGSIPGRFSDNGFLMTEKTRTILFYPWEPTSKNELEQSFHVTSLTDIY

>Q8TE68

MSTTTGPEAAPKPSAKSIYEQRKRYSTVVMADVSQYPVNHLVTFCLGEDDGVHTVEDASRKLAVMDSQGRVWAQEMLLRVSPDHVTLLDPASKEELESYPLGAIVRCDAVMPPGRSRSLLLLVCQEPERAQPDVHFFQGLRLGAELIREDIQGALHNYRSGRGERRAAALRATQEELQRDRSPAAETPPLQRRPSVRAVISTVERGAGRGRPQAKPIPEAEEAQRPEPVGTSSNADSASPDLGPRGPDLAVLQAEREVDILNHVFDDVESFVSRLQKSAEAARVLEHRERGRRSRRRAAGEGLLTLRAKPPSEAEYTDVLQKIKYAFSLLARLRGNIADPSSPELLHFLFGPLQMIVNTSGGPEFASSVRRPHLTSDAVALLRDNVTPRENELWTSLGDSWTRPGLELSPEEGPPYRPEFFSGWEPPVTDPQSRAWEDPVEKQLQHERRRRQQSAPQVAVNGHRDLEPESEPQLESETAGKWVLCNYDFQARNSSELSVKQRDVLEVLDDSRKWWKVRDPAGQEGYVPYNILTPYPGPRLHHSQSPARSLNSTPPPPPAPAPAPPPALARPRWDRPRWDSCDSLNGLDPSEKEKFSQMLIVNEELQARLAQGRSGPSRAVPGPRAPEPQLSPGSDASEVRAWLQAKGFSSGTVDALGVLTGAQLFSLQREELRAVSPEEGARVYSQVTVQRSLLEDKEKVSELEAVMEKQKKKVEGEVEMEVI

1169 P41229 NP_001227.1

>P41229

MEPGSDDFLPPPECPVFEPSWAEFRDPLGYIAKIRPIAEKSGICKIRPPADWQPPFAVEVDNFRFTPRIQRLNELEAQTRVKLNYLDQIAKFWEIQGSSLKIPNVERRILDLYSLSKIVVEEGGYEAICKDRRWARVAQRLNYPPGKNIGSLLRSHYERIVYPYEMYQSGANLVQCNTRPFDNEEKDKEYKPHSIPLRQSVQPSKFNSYGRRAKRLQPDPEPTEEDIEKNPELKKLQIYGAGPKMMGLGLMAKDKTLRKKDKEGPECPPTVVVKEELGGDVKVESTSPKTFLESKEELSHSPEPCTKMTMRLRRNHSNAQFIESYVCRMCSRGDEDDKLLLCDGCDDNYHIFCLLPPLPEIPKGVWRCPKCVMAECKRPPEAFGFEQATREYTLQSFGEMADSFKADYFNMPVHMVPTELVEKEFWRLVNSIEEDVTVEYGADIHSKEFGSGFPVSDSKRHLTPEEEEYATSGWNLNVMPVLEQSVLCHINADISGMKVPWLYVGMVFSAFCWHIEDHWSYSINYLHWGEPKTWYGVPSLAAEHLEEVMKKLTPELFDSQPDLLHQLVTLMNPNTLMSHGVPVVRTNQCAGEFVITFPRAYHSGFNQGYNFAEAVNFCTADWLPAGRQCIEHYRRLRRYCVFSHEELICKMAACPEKLDLNLAAAVHKEMFIMVQEERRLRKALLEKGITEAEREAFELLPDDERQCIKCKTTCFLSALACYDCPDGLVCLSHINDLCKCSSSRQYLRYRYTLDELPAMLHKLKVRAESFDTWANKVRVALEVEDGRKRSLEELRALESEARERRFPNSELLQQLKNCLSEAEACVSRALGLVSGQEAGPHRVAGLQMTLTELRAFLDQMNNLPCAMHQIGDVKGVLEQVEAYQAEAREALASLPSSPGLLQSLLERGRQLGVEVPEAQQLQRQVEQARWLDEVKRTLAPSARRGTLAVMRGLLVAGASVAPSPAVDKAQAELQELLTIAERWEEKAHLCLEARQKHPPATLEAIIREAENIPVHLPNIQALKEALAKARAWIADVDEIQNGDHYPCLDDLEGLVAVGRDLPVGLEELRQLELQVLTAHSWREKASKTFLKKNSCYTLLEVLCPCADAGSDSTKRSRWMEKELGLYKSDTELLGLSAQDLRDPGSVIVAFKEGEQKEKEGILQLRRTNSAKPSPLASSSTASSTTSICVCGQVLAGAGALQCDLCQDWFHGRCVSVPRLLSSPRPNPTSSPLLAWWEWDTKFLCPLCMRSRRPRLETILALLVALQRLPVRLPEGEALQCLTERAISWQGRARQALASEDVTALLGRLAELRQRLQAEPRPEEPPNYPAAPASDPLREGSGKDMPKVQGLLENGDSVTSPEKVAPEEGSGKRDLELLSSLLPQLTGPVLELPEATRAPLEELMMEGDLLEVTLDENHSIWQLLQAGQPPDLERIRTLLELEKAERHGSRARGRALERRRRRKVDRGGEGDDPAREELEPKRVRSSGPEAEEVQEEEELEEETGGEGPPAPIPTTGSPSTQENQNGLEPAEGTTSGPSAPFSTLTPRLHLPCPQQPPQQQL

>NP_001227.1

MSSCSRVALVTGANRGIGLAIARELCRQFSGDVVLTARDVARGQAAVQQLQAEGLSPRFHQLDIDDLQSIRALRDFLRKEYGGLNVLVNNAAVAFKSDDPMPFDIKAEMTLKTNFFATRNMCNELLPIMKPHGRVVNISSLQCLRAFENCSEDLQERFHSETLTEGDLVDLMKKFVEDTKNEVHEREGWPNSPYGVSKLGVTVLSRILARRLDEKRKADRILVNACCPGPVKTDMDGKDSIRTVEEGAETPVYLALLPPDATEPQGQLVHDKVVQNW

1170 NP_059106.2 NP_004835.2

>NP_059106.2

MTTSLLLHPRWPESLMYVYEDSAAESGIGGGGGGGGGGTGGAGGGCSGASPGKAPSMDGLGSSCPASHCRDLLPHPVLGRPPAPLGAPQGAVYTDIPAPEAARQCAPPPAPPTSSSATLGYGYPFGGSYYGCRLSHNVNLQQKPCAYHPGDKYPEPSGALPGDDLSSRAKEFAFYPSFASSYQAMPGYLDVSVVPGISGHPEPRHDALIPVEGYQHWALSNGWDSQVYCSKEQSQSAHLWKSPFPDVVPLQPEVSSYRRGRKKRVPYTKVQLKELEKEYAASKFITKEKRRRISATTNLSERQVTIWFQNRRVKEKKVVSKSKAPHLHST

>NP_004835.2

MDAALKRSRSEEPAEILPPARDEEEEEEEGMEQGLEEEEEVDPRIQGELEKLNQSTDDINRRETELEDARQKFRSVLVEATVKLDELVKKIGKAVEDSKPYWEARRVARQAQLEAQKATQDFQRATEVLRAAKETISLAEQRLLEDDKRQFDSAWQEMLNHATQRVMEAEQTKTRSELVHKETAARYNAAMGRMRQLEKKLKRAINKSKPYFELKAKYYVQLEQLKKTVDDLQAKLTLAKGEYKMALKNLEMISDEIHERRRSSAMGPRGCGVGAEGSSTSVEDLPGSKPEPDAISVASEAFEDDSCSNFVSEDDSETQSVSSFSSGPTSPSEMPDQFPAVVRPGSLDLPSPVSLSEFGMMFPVLGPRSECSGASSPECEVERGDRAEGAENKTSDKANNNRGLSSSSGSGGSSKSQSSTSPEGQALENRMKQLSLQCSKGRDGIIADIKMVQIG

1171 NP_009210.1 P82664

>NP_009210.1

MSDFDEFERQLNENKQERDKENRHRKRSHSRSRSRDRKRRSRSRDRRNRDQRSASRDRRRRSKPLTRGAKEEHGGLIRSPRHEKKKKVRKYWDVPPPGFEHITPMQYKAMQAAGQIPATALLPTMTPDGLAVTPTPVPVVGSQMTRQARRLYVGNIPFGITEEAMMDFFNAQMRLGGLTQAPGNPVLAVQINQDKNFAFLEFRSVDETTQAMAFDGIIFQGQSLKIRRPHDYQPLPGMSENPSVYVPGVVSTVVPDSAHKLFIGGLPNYLNDDQVKELLTSFGPLKAFNLVKDSATGLSKGYAFCEYVDINVTDQAIAGLNGMQLGDKKLLVQRASVGAKNATLVSPPSTINQTPVTLQVPGLMSSQVQMGGHPTEVLCLMNMVLPEELLDDEEYEEIVEDVRDECSKYGLVKSIEIPRPVDGVEVPGCGKIFVEFTSVFDCQKAMQGLTGRKFANRVVVTKYCDPDSYHRRDFW

>P82664

MAARTAFGAVCRRLWQGLGNFSVNTSKGNTAKNGGLLLSTNMKWVQFSNLHVDVPKDLTKPVVTISDEPDILYKRLSVLVKGHDKAVLDSYEYFAVLAAKELGISIKVHEPPRKIERFTLLQSVHIYKKHRVQYEMRTLYRCLELEHLTGSTADVYLEYIQRNLPEGVAMEVTKTQLEQLPEHIKEPIWETLSEEKEESKS

1172 P17947 NP_003810.1

>P17947

MLQACKMEGFPLVPPPSEDLVPYDTDLYQRQTHEYYPYLSSDGESHSDHYWDFHPHHVHSEFESFAENNFTELQSVQPPQLQQLYRHMELEQMHVLDTPMVPPHPSLGHQVSYLPRMCLQYPSLSPAQPSSDEEEGERQSPPLEVSDGEADGLEPGPGLLPGETGSKKKIRLYQFLLDLLRSGDMKDSIWWVDKDKGTFQFSSKHKEALAHRWGIQKGNRKKMTYQKMARALRNYGKTGEVKKVKKKLTYQFSGEVLGRGGLAERRHPPH

>NP_003810.1

MNAAASSYPMASLYVGDLHSDVTEAMLYEKFSPAGPVLSIRVCRDMITRRSLGYAYVNFQQPADAERALDTMNFDVIKGKPIRIMWSQRDPSLRKSGVGNVFIKNLDKSIDNKALYDTFSAFGNILSCKVVCDENGSKGYAFVHFETQEAADKAIEKMNGMLLNDRKVFVGRFKSRKEREAELGAKAKEFTNVYIKNFGEEVDDESLKELFSQFGKTLSVKVMRDPNGKSKGFGFVSYEKHEDANKAVEEMNGKEISGKIIFVGRAQKKVERQAELKRKFEQLKQERISRYQGVNLYIKNLDDTIDDEKLRKEFSPFGSITSAKVMLEDGRSKGFGFVCFSSPEEATKAVTEMNGRIVGSKPLYVALAQRKEERKAHLTNQYMQRVAGMRALPANAILNQFQPAAGGYFVPAVPQAQGRPPYYTPNQLAQMRPNPRWQQGGRPQGFQGMPSAIRQSGPRPTLRHLAPTGSECPDRLAMDFGGAGAAQQGLTDSCQSGGVPTAVQNLAPRAAVAAAAPRAVAPYKYASSVRSPHPAIQPLQAPQPAVHVQGQEPLTASMLAAAPPQEQKQMLGERLFPLIQTMHSNLAGKITGMLLEIDNSELLHMLESPESLRSKVDEAVAVLQAHHAKKEAAQKVGAVAAATS

1173 NP_777584.1 NP_000895.1

>NP_777584.1

MDLLWMPLLLVAACVSAVHSSPEVNAGVSSIHITKPVHILEERSLLVLTPAGLTQMLNQTRFLMVLFHNPSSKQSRNLAEELGKAVEIMGKGKNGIGFGKVDITIEKELQQEFGITKAPELKLFFEGNRSEPISCKGVVESAALVVWLRRQISQKAFLFNSSEQVAEFVISRPLVIVGFFQDLEEEVAELFYDVIKDFPELTFGVITIGNVIGRFHVTLDSVLVFKKGKIVNRQKLINDSTNKQELNRVIKQHLTDFVIEYNTENKDLISELHIMSHMLLFVSKSSESYGIIIQHYKLASKEFQNKILFILVDADEPRNGRVFKYFRVTEVDIPSVQILNLSSDARYKMPSDDITYESLKKFGRSFLSKNATKHQSSEEIPKYWDQGLVKQLVGKNFNVVVFDKEKDVFVMFYAPWSKKCKMLFPLLEELGRKYQNHSTIIIAKIDVTANDIQLMYLDRYPFFRLFPSGSQQAVLYKGEHTLKGFSDFLESHIKTKIEDEDELLSVEQNEVIEEEVLAEEKEVPMMRKGLPEQQSPELENMTKYVSKLEEPAGKKKTSEEVVVVVAKPKGPPVQKKKPKVKEEL

>NP_000895.1

MAGKKVLIVYAHQEPKSFNGSLKNVAVDELSRQGCTVTVSDLYAMNFEPRATDKDITGTLSNPEVFNYGVETHEAYKQRSLASDITDEQKKVREADLVIFQFPLYWFSVPAILKGWMDRVLCQGFAFDIPGFYDSGLLQGKLALLSVTTGGTAEMYTKTGVNGDSRYFLWPLQHGTLHFCGFKVLAPQISFAPEIASEEERKGMVAAWSQRLQTIWKEEPIPCTAHWHFGQ

1174 NP_073568.2 Q8N427

>NP_073568.2

MSRPVRNRKVVDYSQFQESDDADEDYGRDSGPPTKKIRSSPREAKNKRRSGKNSQEDSEDSEDKDVKTKKDDSHSAEDSEDEKEDHKNVRQQRQAASKAASKQREMLMEDVGSEEEQEEEDEAPFQEKDSGSDEDFLMEDDDDSDYGSSKKKNKKMVKKSKPERKEKKMPKPRLKATVTPSPVKGKGKVGRPTASKASKEKTPSPKEEDEEPESPPEKKTSTSPPPEKSGDEGSEDEAPSGED

>Q8N427

MASKKREVQLQTVINNQSLWDEMLQNKGLTVIDVYQAWCGPCRAMQPLFRKLKNELNEDEILHFAVAEADNIVTLQPFRDKCEPVFLFSVNGKIIEKIQGANAPLVNKKVINLIDEERKIAAGEMARPQYPEIPLVDSDSEVSEESPCESVQELYSIAIIKPDAVISKKVLEIKRKITKAGFIIEAEHKTVLTEEQVVNFYSRIADQCDFEEFVSFMTSGLSYILVVSQGSKHNPPSEETEPQTDTEPNERSEDQPEVEAQVTPGMMKNKQDSLQEYLERQHLAQLCDIEEDAANVAKFMDAFFPDFKKMKSMKLEKTLALLRPNLFHERKDDVLRIIKDEDFKILEQRQVVLSEKEAQALCKEYENEDYFNKLIENMTSGPSLALVLLRDNGLQYWKQLLGPRTVEEAIEYFPESLCAQFAMDSLPVNQLYGSDSLETAEREIQHFFPLQSTLGLIKPHATSEQREQILKIVKEAGFDLTQVKKMFLTPEQIEKIYPKVTGKDFYKDLLEMLSVGPSMVMILTKWNAVAEWRRLMGPTDPEEAKLLSPDSIRAQFGISKLKNIVHGASNAYEAKEVVNRLFEDPEEN

1175 NP_003502.1 NP_000061.1

>NP_003502.1

MSGRGKQGGKARAKAKTRSSRAGLQFPVGRVHRLLRKGNYAERVGAGAPVYLAAVLEYLTAEILELAGNAARDNKKTRIIPRHLQLAIRNDEELNKLLGKVTIAQGGVLPNIQAVLLPKKTESHHKAKGK

>NP_000061.1

MPTVISASVAPRTAAEPRSPGPVPHPAQSKATEAGGGNPSGIYSAIISRNFPIIGVKEKTFEQLHKKCLEKKVLYVDPEFPPDETSLFYSQKFPIQFVWKRPPEICENPRFIIDGANRTDICQGELGDCWFLAAIACLTLNQHLLFRVIPHDQSFIENYAGIFHFQFWRYGEWVDVVIDDCLPTYNNQLVFTKSNHRNEFWSALLEKAYAKLHGSYEALKGGNTTEAMEDFTGGVAEFFEIRDAPSDMYKIMKKAIERGSLMGCSIDDGTNMTYGTSPSGLNMGELIARMVRNMDNSLLQDSDLDPRGSDERPTRTIIPVQYETRMACGLVRGHAYSVTGLDEVPFKGEKVKLVRLRNPWGQVEWNGSWSDRWKDWSFVDKDEKARLQHQVTEDGEFWMSYEDFIYHFTKLEICNLTADALQSDKLQTWTVSVNEGRWVRGCSAGGCRNFPDTFWTNPQYRLKLLEEDDDPDDSEVICSFLVALMQKNRRKDRKLGASLFTIGFAIYEVPKEMHGNKQHLQKDFFLYNASKARSKTYINMREVSQRFRLPPSEYVIVPSTYEPHQEGEFILRVFSEKRNLSEEVENTISVDRPVKKKKTKPIIFVSDRANSNKELGVDQESEEGKGKTSPDKQKQSPQPQPGSSDQESEEQQQFRNIFKQIAGDDMEICADELKKVLNTVVNKHKDLKTHGFTLESCRSMIALMDTDGSGKLNLQEFHHLWNKIKAWQKIFKHYDTDQSGTINSYEMRNAVNDAGFHLNNQLYDIITMRYADKHMNIDFDSFICCFVRLEGMFRAFHAFDKDGDGIIKLNVLEWLQLTMYA

1176 P35251 Q9BU02

>P35251

MDIRKFFGVIPSGKKLVSETVKKNEKTKSDEETLKAKKGIKEIKVNSSRKEDDFKQKQPSKKKRIIYDSDSESEETLQVKNAKKPPEKLPVSSKPGKISRQDPVTYISETDEEDDFMCKKAASKSKENGRSTNSHLGTSNMKKNEENTKTKNKPLSPIKLTPTSVLDYFGTGSVQRSNKKMVASKRKELSQNTDESGLNDEAIAKQLQLDEDAELERQLHEDEEFARTLAMLDEEPKTKKARKDTEAGETFSSVQANLSKAEKHKYPHKVKTAQVSDERKSYSPRKQSKYESSKESQQHSKSSADKIGEVSSPKASSKLAIMKRKEESSYKEIEPVASKRKENAIKLKGETKTPKKTKSSPAKKESVSPEDSEKKRTNYQAYRSYLNREGPKALGSKEIPKGAENCLEGLIFVITGVLESIERDEAKSLIERYGGKVTGNVSKKTNYLVMGRDSGQSKSDKAAALGTKIIDEDGLLNLIRTMPGKKSKYEIAVETEMKKESKLERTPQKNVQGKRKISPSKKESESKKSRPTSKRDSLAKTIKKETDVFWKSLDFKEQVAEETSGDSKARNLADDSSENKVENLLWVDKYKPTSLKTIIGQQGDQSCANKLLRWLRNWQKSSSEDKKHAAKFGKFSGKDDGSSFKAALLSGPPGVGKTTTASLVCQELGYSYVELNASDTRSKSSLKAIVAESLNNTSIKGFYSNGAASSVSTKHALIMDEVDGMAGNEDRGGIQELIGLIKHTKIPIICMCNDRNHPKIRSLVHYCFDLRFQRPRVEQIKGAMMSIAFKEGLKIPPPAMNEIILGANQDIRQVLHNLSMWCARSKALTYDQAKADSHRAKKDIKMGPFDVARKVFAAGEETAHMSLVDKSDLFFHDYSIAPLFVQENYIHVKPVAAGGDMKKHLMLLSRAADSICDGDLVDSQIRSKQNWSLLPAQAIYASVLPGELMRGYMTQFPTFPSWLGKHSSTGKHDRIVQDLALHMSLRTYSSKRTVNMDYLSLLRDALVQPLTSQGVDGVQDVVALMDTYYLMKEDFENIMEISSWGGKPSPFSKLDPKVKAAFTRAYNKEAHLTPYSLQAIKASRHSTSPSLDSEYNEELNEDDSQSDEKDQDAIETDAMIKKKTKSSKPSKPEKDKEPRKGKGKSSKK

>Q9BU02

MAQGLIEVERKFLPGPGTEERLQELGGTLEYRVTFRDTYYDTPELSLMQADHWLRRREDSGWELKCPGAAGVLGPHTEYKELTAEPTIVAQLCKVLRADGLGAGDVAAVLGPLGLQEVASFVTKRSAWKLVLLGADEEEPQLRVDLDTADFGYAVGEVEALVHEEAEVPTALEKIHRLSSMLGVPAQETAPAKLIVYLQRFRPQDYQRLLEVNSSRERPQETEDPDHCLG

1177 P20671 NP_000934.1

>P20671

MSGRGKQGGKARAKAKTRSSRAGLQFPVGRVHRLLRKGNYSERVGAGAPVYLAAVLEYLTAEILELAGNAARDNKKTRIIPRHLQLAIRNDEELNKLLGKVTIAQGGVLPNIQAVLLPKKTESHHKAKGK

>NP_000934.1

MGPGPRLLLPLVLCVGLGALVFSSGAEGFRKRGPSVTAKVFFDVRIGDKDVGRIVIGLFGKVVPKTVENFVALATGEKGYGYKGSKFHRVIKDFMIQGGDITTGDGTGGVSIYGETFPDENFKLKHYGIGWVSMANAGPDTNGSQFFITLTKPTWLDGKHVVFGKVIDGMTVVHSIELQATDGHDRPLTNCSIINSGKIDVKTPFVVEIADW

1178 NP_004445.1 NP_000934.1

>NP_004445.1

MDGFYDQQVPFMVPGKSRSEECRGRPVIDRKRKFLDTDLAHDSEELFQDLSQLQEAWLAEAQVPDDEQFVPDFQSDNLVLHAPPPTKIKRELHSPSSELSSCSHEQALGANYGEKCLYNYCAYDRKPPSGFKPLTPPTTPLSPTHQNPLFPPPQATLPTSGHAPAAGPVQGVGPAPAPHSLPEPGPQQQTFAVPRPPHQPLQMPKMMPENQYPSEQRFQRQLSEPCHPFPPQPGVPGDNRPSYHRQMSEPIVPAAPPPPQGFKQEYHDPLYEHGVPGMPGPPAHGFQSPMGIKQEPRDYCVDSEVPNCQSSYMRGGYFSSSHEGFSYEKDPRLYFDDTCVVPERLEGKVKQEPTMYREGPPYQRRGSLQLWQFLVTLLDDPANAHFIAWTGRGMEFKLIEPEEVARRWGIQKNRPAMNYDKLSRSLRYYYEKGIMQKVAGERYVYKFVCDPDALFSMAFPDNQRPFLKAESECHLSEEDTLPLTHFEDSPAYLLDMDRCSSLPYAEGFAY

>NP_000934.1

MGPGPRLLLPLVLCVGLGALVFSSGAEGFRKRGPSVTAKVFFDVRIGDKDVGRIVIGLFGKVVPKTVENFVALATGEKGYGYKGSKFHRVIKDFMIQGGDITTGDGTGGVSIYGETFPDENFKLKHYGIGWVSMANAGPDTNGSQFFITLTKPTWLDGKHVVFGKVIDGMTVVHSIELQATDGHDRPLTNCSIINSGKIDVKTPFVVEIADW

1179 NP_004526.1 NP_002717.3

>NP_004526.1

MSLENEDKRARTRSKALRGPPETTAADLSCPTPGCTGSGHVRGKYSRHRSLQSCPLAKKRKLEGAEAEHLVSKRKSHPLKLALDEGYGVDSDGSEDTEVKDASVSDESEGTLEGAEAETSGQDEIHRPETAEGRSPVKSHFGSNPIGSATASSKGSYSSYQGIIATSLLNLGQIAEETLVEEDLGQAAKPGPGIVHLLQEAAEGAASEEGEKGLFIQPEDAEEVVEVTTERSQDLCPQSLEDAASEESSKQKGILSHEEEDEEEEEEEEEEEEDEEEEEEEEEEEEEEEEEEEEEEEEEEEEEEEEAAPDVIFQEDTSHTSAQKAPELRGPESPSPKPEYSVIVEVRSDDDKDEDTHSRKSTVTDESEMQDMMTRGNLGLLEQAIALKAEQVRTVCEPGCPPAEQSQLGLGEPGKAAKPLDTVRKSYYSKDPSRAEKREIKCPTPGCDGTGHVTGLYPHHRSLSGCPHKDRIPPEILAMHENVLKCPTPGCTGQGHVNSNRNTHRSLSGCPIAAAEKLAKSHEKQQPQTGDPSKSSSNSDRILRPMCFVKQLEVPPYGSYRPNVAPATPRANLAKELEKFSKVTFDYASFDAQVFGKRMLAPKIQTSETSPKAFQCFDYSQDAEAAHMAATAILNLSTRCWEMPENLSTKPQDLPSKSVDIEVDENGTLDLSMHKHRKRENAFPSSSSCSSSPGVKSPDASQRHSSTSAPSSSMTSPQSSQASRQDEWDRPLDYTKPSRLREEEPEESEPAAHSFASSEADDQEVSEENFEERKYPGEVTLTNFKLKFLSKDIKKELLTCPTPGCDGSGHITGNYASHRSLSGCPLADKSLRNLMAAHSADLKCPTPGCDGSGHITGNYASHRSLSGCPRAKKSGVKVAPTKDDKEDPELMKCPVPGCVGLGHISGKYASHRSASGCPLAARRQKEGSLNGSSFSWKSLKNEGPTCPTPGCDGSGHANGSFLTHRSLSGCPRATFAGKKGKLSGDEVLSPKFKTSDVLENDEEIKQLNQEIRDLNESNSEMEAAMVQLQSQISSMEKNLKNIEEENKLIEEQNEALFLELSGLSQALIQSLANIRLPHMEPICEQNFDAYVSTLTDMYSNQDPENKDLLESIKQAVRGIQV

>NP_002717.3

MLSLQYPDVYRDETAVQDYHGHKICDPYAWLEDPDSEQTKAFVEAQNKITVPFLEQCPIRGLYKERMTELYDYPKYSCHFKKGKRYFYFYNTGLQNQRVLYVQDSLEGEARVFLDPNILSDDGTVALRGYAFSEDGEYFAYGLSASGSDWVTIKFMKVDGAKELPDVLERVKFSCMAWTHDGKGMFYNSYPQQDGKSDGTETSTNLHQKLYYHVLGTDQSEDILCAEFPDEPKWMGGAELSDDGRYVLLSIREGCDPVNRLWYCDLQQESSGIAGILKWVKLIDNFEGEYDYVTNEGTVFTFKTNRQSPNYRVINIDFRDPEESKWKVLVPEHEKDVLEWIACVRSNFLVLCYLHDVKNILQLHDLTTGALLKTFPLDVGSIVGYSGQKKDTEIFYQFTSFLSPGIIYHCDLTKEELEPRVFREVTVKGIDASDYQTVQIFYPSKDGTKIPMFIVHKKGIKLDGSHPAFLYGYGGFNISITPNYSVSRLIFVRHMGGILAVANIRGGGEYGETWHKGGILANKQNCFDDFQCAAEYLIKEGYTSPKRLTINGGSNGGLLVAACANQRPDLFGCVIAQVGVMDMLKFHKYTIGHAWTTDYGCSDSKQHFEWLVKYSPLHNVKLPEADDIQYPSMLLLTADHDDRVVPLHSLKFIATLQYIVGRSRKQSNPLLIHVDTKAGHGAGKPTAKVIEEVSDMFAFIARCLNVDWIP

1180 O75478 NP_001167.2

>O75478

MDRLGSFSNDPSDKPPCRGCSSYLMEPYIKCAECGPPPFFLCLQCFTRGFEYKKHQSDHTYEIMTSDFPVLDPSWTAQEEMALLEAVMDCGFGNWQDVANQMCTKTKEECEKHYMKHFINNPLFASTLLNLKQAEEAKTADTAIPFHSTDDPPRPTFDSLLSRDMAGYMPARADFIEEFDNYAEWDLRDIDFVEDDSDILHALKMAVVDIYHSRLKERQRRKKIIRDHGLINLRKFQLMERRYPKEVQDLYETMRRFARIVGPVEHDKFIESHALEFELRREIKRLQEYRTAGITNFCSARTYDHLKKTREEERLKRTMLSEVLQYIQDSSACQQWLRRQADIDSGLSPSIPMASNSGRRSAPPLNLTGLPGTEKLNEKEKELCQMVRLVPGAYLEYKSALLNECNKQGGLRLAQARALIKIDVNKTRKIYDFLIREGYITKG

>NP_001167.2

MLGLDACELGAQLLELLRLALCARVLLADKEGGPPAVDEVLDEAVPEYRAPGRKSLLEIRQLDPDDRSLAKYKRVLLGPLPPAVDPSLPNVQVTRLTLLSEQAPGPVVMDLTGDLAVLKDQVFVLKEGVDYRVKISFKVHREIVSGLKCLHHTYRRGLRVDKTVYMVGSYGPSAQEYEFVTPVEEAPRGALVRGPYLVVSLFTDDDRTHHLSWEWGLCICQDWKD

1181 NP_061139.2 NP_000886.1

>NP_061139.2

MAGSVGLALCGQTLVVRGGSRFLATSIASSDDDSLFIYDCSAAEKKSQENKGEDAPLDQGSGAILASTFSKSGSYFALTDDSKRLILFRTKPWQCLSVRTVARRCTALTFIASEEKVLVADKSGDVYSFSVLEPHGCGRLELGHLSMLLDVAVSPDDRFILTADRDEKIRVSWAAAPHSIESFCLGHTEFVSRISVVPTQPGLLLSSSGDGTLRLWEYRSGRQLHCCHLASLQELVDPQAPQKFAASRIAFWCQENCVALLCDGTPVVYIFQLDARRQQLVYRQQLAFQHQVWDVAFEETQGLWVLQDCQEAPLVLYRPVGDQWQSVPESTVLKKVSGVLRGNWAMLEGSAGADASFSSLYKATFDNVTSYLKKKEERLQQQLEKKQRRRSPPPGPDGHAKKMRPGEATLSC

>NP_000886.1

MPEIVDTCSLASPASVCRTKHLHLRCSVDFTRRTLTGTAALTVQSQEDNLRSLVLDTKDLTIEKVVINGQEVKYALGERQSYKGSPMEISLPIALSKNQEIVIEISFETSPKSSALQWLTPEQTSGKEHPYLFSQCQAIHCRAILPCQDTPSVKLTYTAEVSVPKELVALMSAIRDGETPDPEDPSRKIYKFIQKVPIPCYLIALVVGALESRQIGPRTLVWSEKEQVEKSAYEFSETESMLKIAEDLGGPYVWGQYDLLVLPPSFPYGGMENPCLTFVTPTLLAGDKSLSNVIAHEISHSWTGNLVTNKTWDHFWLNEGHTVYLERHICGRLFGEKFRHFNALGGWGELQNSVKTFGETHPFTKLVVDLTDIDPDVAYSSVPYEKGFALLFYLEQLLGGPEIFLGFLKAYVEKFSYKSITTDDWKDFLYSYFKDKVDVLNQVDWNAWLYSPGLPPIKPNYDMTLTNACIALSQRWITAKEDDLNSFNATDLKDLSSHQLNEFLAQTLQRAPLPLGHIKRMQEVYNFNAINNSEIRFRWLRLCIQSKWEDAIPLALKMATEQGRMKFTRPLFKDLAAFDKSHDQAVRTYQEHKASMHPVTAMLVGKDLKVD

1182 NP_004587.1 Q9HD34

>NP_004587.1

MAVPETRPNHTIYINNLNEKIKKDELKKSLYAIFSQFGQILDILVSRSLKMRGQAFVIFKEVSSATNALRSMQGFPFYDKPMRIQYAKTDSDIIAKMKGTFVERDRKREKRKPKSQETPATKKAVQGGGATPVVGAVQGPVPGMPPMTQAPRIMHHMPGQPPYMPPPGMIPPPGLAPGQIPPGAMPPQQLMPGQMPPAQPLSENPPNHILFLTNLPEETNELMLSMLFNQFPGFKEVRLVPGRHDIAFVEFDNEVQAGAARDALQGFKITQNNAMKISFAKK

>Q9HD34

MAASSRAQVLSLYRAMLRESKRFSAYNYRTYAVRRIRDAFRENKNVKDPVEIQTLVNKAKRDLGVIRRQVHIGQLYSTDKLIIENRDMPRT

1183 NP_003251.2 NP_003192.1

>NP_003251.2

MYPQGRHPTPLQSGQPFKFSILEICDRIKEEFQFLQAQYHSLKLECEKLASEKTEMQRHYVMYYEMSYGLNIEMHKQAEIVKRLSGICAQIIPFLTQEHQQQVLQAVERAKQVTVGELNSLIGQQLQPLSHHAPPVPLTPRPAGLVGGSATGLLALSGALAAQAQLAAAVKEDRAGVEAEGSRVERAPSRSASPSPPESLVEEERPSGPGGGGKQRADEKEPSGPYESDEDKSDYNLVVDEDQPSEPPSPATTPCGKVPICIPARRDLVDSPASLASSLGSPLPRAKELILNDLPASTPASKSCDSSPPQDASTPGPSSASHLCQLAAKPAPSTDSVALRSPLTLSSPFTTSFSLGSHSTLNGDLSVPSSYVSLHLSPQVSSSVVYGRSPVMAFESHPHLRGSSVSSSLPSIPGGKPAYSFHVSADGQMQPVPFPSDALVGAGIPRHARQLHTLAHGEVVCAVTISGSTQHVYTGGKGCVKVWDVGQPGAKTPVAQLDCLNRDNYIRSCKLLPDGRSLIVGGEASTLSIWDLAAPTPRIKAELTSSAPACYALAVSPDAKVCFSCCSDGNIVVWDLQNQTMVRQFQGHTDGASCIDISDYGTRLWTGGLDNTVRCWDLREGRQLQQHDFSSQIFSLGHCPNQDWLAVGMESSNVEILHVRKPEKYQLHLHESCVLSLKFASCGRWFVSTGKDNLLNAWRTPYGASIFQSKESSSVLSCDISRNNKYIVTGSGDKKATVYEVVY

>NP_003192.1

MAFLRSMWGVLSALGRSGAELCTGCGSRLRSPFSFVYLPRWFSSVLASCPKKPVSSYLRFSKEQLPIFKAQNPDAKTTELIRRIAQRWRELPDSKKKIYQDAYRAEWQVYKEEISRFKEQLTPSQIMSLEKEIMDKHLKRKAMTKKKELTLLGKPKRPRSAYNVYVAERFQEAKGDSPQEKLKTVKENWKNLSDSEKELYIQHAKEDETRYHNEMKSWEEQMIEVGRKDLLRRTIKKQRKYGAEEC

1184 NP_942595.1 P17516

>NP_942595.1

MKKFSRMPKSEGGSGGGAAGGGAGGAGAGAGCGSGGSSVGVRVFAVGRHQVTLEESLAEGGFSTVFLVRTHGGIRCALKRMYVNNMPDLNVCKREITIMKELSGHKNIVGYLDCAVNSISDNVWEVLILMEYCRAGQVVNQMNKKLQTGFTEPEVLQIFCDTCEAVARLHQCKTPIIHRDLKVENILLNDGGNYVLCDFGSATNKFLNPQKDGVNVVEEEIKKYTTLSYRAPEMINLYGGKPITTKADIWALGCLLYKLCFFTLPFGESQVAICDGNFTIPDNSRYSRNIHCLIRFMLEPDPEHRPDIFQVSYFAFKFAKKDCPVSNINNSSIPSALPEPMTASEAAARKSQIKARITDTIGPTETSIAPRQRPKANSATTATPSVLTIQSSATPVKVLAPGEFGNHRPKGALRPGNGPEILLGQGPPQQPPQQHRVLQQLQQGDWRLQQLHLQHRHPHQQQQQQQQQQQQQQQQQQQQQQQQQQQHHHHHHHHLLQDAYMQQYQHATQQQQMLQQQFLMHSVYQPQPSASQYPTMMPQYQQAFFQQQMLAQHQPSQQQASPEYLTSPQEFSPALVSYTSSLPAQVGTIMDSSYSANRSVADKEAIANFTNQKNISNPPDMSGWNPFGEDNFSKLTEEELLDREFDLLRSNRLEERASSDKNVDSLSAPHNHPPEDPFGSVPFISHSGSPEKKAEHSSINQENGTANPIKNGKTSPASKDQRTGKKTSVQGQVQKGNDESESDFESDPPSPKSSEEEEQDDEEVLQGEQGDFNDDDTEPENLGHRPLLMDSEDEEEEEKHSSDSDYEQAKAKYSDMSSVYRDRSGSGPTQDLNTILLTSAQLSSDVAVETPKQEFDVFGAVPFFAVRAQQPQQEKNEKNLPQHRFPAAGLEQEEFDVFTKAPFSKKVNVQECHAVGPEAHTIPGYPKSVDVFGSTPFQPFLTSTSKSESNEDLFGLVPFDEITGSQQQKVKQRSLQKLSSRQRRTKQDMSKSNGKRHHGTPTSTKKTLKPTYRTPERARRHKKVGRRDSQSSNEFLTISDSKENISVALTDGKDRGNVLQPEESLLDPFGAKPFHSPDLSWHPPHQGLSDIRADHNTVLPGRPRQNSLHGSFHSADVLKMDDFGAVPFTELVVQSITPHQSQQSQPVELDPFGAAPFPSKQ

>P17516

MDPKYQRVELNDGHFMPVLGFGTYAPPEVPRNRAVEVTKLAIEAGFRHIDSAYLYNNEEQVGLAIRSKIADGSVKREDIFYTSKLWCTFFQPQMVQPALESSLKKLQLDYVDLYLLHFPMALKPGETPLPKDENGKVIFDTVDLSATWEVMEKCKDAGLAKSIGVSNFNCRQLEMILNKPGLKYKPVCNQVECHPYLNQSKLLDFCKSKDIVLVAHSALGTQRHKLWVDPNSPVLLEDPVLCALAKKHKRTPALIALRYQLQRGVVVLAKSYNEQRIRENIQVFEFQLTSEDMKVLDGLNRNYRYVVMDFLMDHPDYPFSDEY

1185 NP_001036111.1 Q9NP92

>NP_001036111.1

MALVFQFGQPVRAQPLPGLCHGKLIRTNACDVCNSTDLPEVEIISLLEEQLPHYKLRADTIYGYDHDDWLHTPLISPDANIDLTTEQIEETLKYFLLCAERVGQMTKTYNDIDAVTRLLEEKERDLELAARIGQSLLKKNKTLTERNELLEEQVEHIREEVSQLRHELSMKDELLQFYTSAAEESEPESVCSTPLKRNESSSSVQNYFHLDSLQKKLKDLEEENVVLRSEASQLKTETITYEEKEQQLVNDCVKELRDANVQIASISEELAKKTEDAARQQEEITHLLSQIVDLQKKAKACAVENEELVQHLGAAKDAQRQLTAELRELEDKYAECMEMLHEAQEELKNLRNKTMPNTTSRRYHSLGLFPMDSLAAEIEGTMRKELQLEEAESPDITHQKRVFETVRNINQVVKQRSLTPSPMNIPGSNQSSAMNSLLSSCVSTPRSSFYGSDIGNVVLDNKTNSIILETEAADLGNDERSKKPGTPGTPGSHDLETALRRLSLRRENYLSERRFFEEEQERKLQELAEKGELRSGSLTPTESIMSLGTHSRFSEFTGFSGMSFSSRSYLPEKLQIVKPLEGSATLHHWQQLAQPHLGGILDPRPGVVTKGFRTLDVDLDEVYCLNDFEEDDTGDHISLPRLATSTPVQHPETSAHHPGKCMSQTNSTFTFTTCRILHPSDELTRVTPSLNSAPTPACGSTSHLKSTPVATPCTPRRLSLAESFTNTRESTTTMSTSLGLVWLLKERGISAAVYDPQSWDRAGRGSLLHSYTPKMAVIPSTPPNSPMQTPTSSPPSFEFKCTSPPYDNFLASKPASSILREVREKNVRSSESQTDVSVSNLNLVDKVRRFGVAKVVNSGRAHVPTLTEEQGPLLCGPPGPAPALVPRGLVPEGLPLRCPTVTSAIGGLQLNSGIRRNRSFPTMVGSSMQMKAPVTLTSGILMGAKLSKQTSLR

>Q9NP92

MAAARCWRPLLRGPRLSLHTAANAAATATETTCQDVAATPVARYPPIVASMTADSKAARLRRIERWQATVHAAESVDEKLRILTKMQFMKYMVYPQTFALNADRWYQYFTKTVFLSGLPPPPAEPEPEPEPEPEPALDLAALRAVACDCLLQEHFYLRRRRRVHRYEESEVISLPFLDQLVSTLVGLLSPHNPALAAAALDYRCPVHFYWVRGEEIIPRGHRRGRIDDLRYQIDDKPNNQIRISKQLAEFVPLDYSVPIEIPTIKCKPDKLPLFKRQYENHIFVGSKTADPCCYGHTQFHLLPDKLRRERLLRQNCADQIEVVFRANAIASLFAWTGAQAMYQGFWSEADVTRPFVSQAVITDGKYFSFFCYQLNTLALTTQADQNNPRKNICWGTQSKPLYETIEDNDVKGFNDDVLLQIVHFLLNRPKEEKSQLLEN

1186 Q8IXH6 Q96RP9

>Q8IXH6

MFQRLSSLFFSTPSPPEDPDCPRAFVSEEDEVDGWLIIDLPDSYAAPPSPGAAPAPAGRPPPAPSLMDESWFVTPPACFTAEGPGLGPARLQSSPLEDLLIEHPSMSVYVTGSTIVLEPGSPSPLPDAALPDGDLSEGELTPARREPRAARHAAPLPARAALLEKAGQVRRLQRARQRAERHALSAKAVQRQNRARESRPRRSKNQSSFIYQPCQRQFNY

>Q96RP9

MRLLGAAAVAALGRGRAPASLGWQRKQVNWKACRWSSSGVIPNEKIRNIGISAHIDSGKTTLTERVLYYTGRIAKMHEVKGKDGVGAVMDSMELERQRGITIQSAATYTMWKDVNINIIDTPGHVDFTIEVERALRVLDGAVLVLCAVGGVQCQTMTVNRQMKRYNVPFLTFINKLDRMGSNPARALQQMRSKLNHNAAFMQIPMGLEGNFKGIVDLIEERAIYFDGDFGQIVRYGEIPAELRAAATDHRQELIECVANSDEQLGEMFLEEKIPSISDLKLAIRRATLKRSFTPVFLGSALKNKGVQPLLDAVLEYLPNPSEVQNYAILNKEDDSKEKTKILMNSSRDNSHPFVGLAFKLEVGRFGQLTYVRSYQGELKKGDTIYNTRTRKKVRLQRLARMHADMMEDVEEVYAGDICALFGIDCASGDTFTDKANSGLSMESIHVPDPVISIAMKPSNKNDLEKFSKGIGRFTREDPTFKVYFDTENKETVISGMGELHLEIYAQRLEREYGCPCITGKPKVAFRETITAPVPFDFTHKKQSGGAGQYGKVIGVLEPLDPEDYTKLEFSDETFGSNIPKQFVPAVEKGFLDACEKGPLSGHKLSGLRFVLQDGAHHMVDSNEISFIRAGEGALKQALANATLCILEPIMAVEVVAPNEFQGQVIAGINRRHGVITGQDGVEDYFTLYADVPLNDMFGYSTELRSCTEGKGEYTMEYSRYQPCLPSTQEDVINKYLEATGQLPVKKGKAKN

1187 O60814 NP_065845.1

>O60814

MPEPAKSAPAPKKGSKKAVTKAQKKDGKKRKRSRKESYSVYVYKVLKQVHPDTGISSKAMGIMNSFVNDIFERIAGEASRLAHYNKRSTITSREIQTAVRLLLPGELAKHAVSEGTKAVTKYTSAK

>NP_065845.1

MTTKRKIIGRLVPCRCFRGEEEIISVLDYSHCSLQQVPKEVFNFERTLEELYLDANQIEELPKQLFNCQALRKLSIPDNDLSNLPTTIASLVNLKELDISKNGVQEFPENIKCCKCLTIIEASVNPISKLPDGFTQLLNLTQLYLNDAFLEFLPANFGRLVKLRILELRENHLKTLPKSMHKLAQLERLDLGNNEFGELPEVLDQIQNLRELWMDNNALQVLPGSIGKLKMLVYLDMSKNRIETVDMDISGCEALEDLLLSSNMLQQLPDSIGLLKKLTTLKVDDNQLTMLPNTIGNLSLLEEFDCSCNELESLPSTIGYLHSLRTLAVDENFLPELPREIGSCKNVTVMSLRSNKLEFLPEEIGQMQKLRVLNLSDNRLKNLPFSFTKLKELAALWLSDNQSKALIPLQTEAHPETKQRVLTNYMFPQQPRGDEDFQSDSDSFNPTLWEEQRQQRMTVAFEFEDKKEDDENAGKVKDLSCQAPWERGQRGITLQPARLSGDCCTPWARCDQQIQDMPVPQNDPQLAWGCISGLQQERSMCTPLPVAAQSTTLPSLSGRQVEINLKRYPTPYPEDLKNMVKSVQNLVGKPSHGVRVENSNPTANTEQTVKEKYEHKWPVAPKEITVEDSFVHPANEMRIGELHPSLAETPLYPPKLVLLGKDKKESTDESEVDKTHCLNNSVSSGTYSDYSPSQASSGSSNTRVKVGSLQTTAKDAVHNSLWGNRIAPSFPQPLDSKPLLSQREAVPPGNIPQRPDRLPMSDTFTDNWTDGSHYDNTGFVAEETTAENANSNPLLSSKSRSTSSHGRRPLIRQDRIVGVPLELEQSTHRHTPETEVPPSNPWQNWTRTPSPFEDRTAFPSKLETTPTTSPLPERKEHIKESTEIPSPFSPGVPWEYHDSNPNRSLSNVFSQIHCRPESSKGVISISKSTERLSPLMKDIKSNKFKKSQSIDEIDIGTYKVYNIPLENYASGSDHLGSHERPDKMLGPEHGMSSMSRSQSVPMLDDEMLTYGSSKGPQQQKASMTKKVYQFDQSFNPQGSVEVKAEKRIPPPFQHNPEYVQQASKNIAKDLISPRAYRGYPPMEQMFSFSQPSVNEDAVVNAQFASQGARAGFLRRADSLVSATEMAMFRRVNEPHELPPTDRYGRPPYRGGLDRQSSVTVTESQFLKRNGRYEDEHPSYQEVKAQAGSFPVKNLTQRRPLSARSYSTESYGASQTRPVSARPTMAALLEKIPSDYNLGNYGDKPSDNSDLKTRPTPVKGEESCGKMPADWRQQLLRHIEARRLDRNAAYKHNTVNLGMLPYGGISAMHAGRSMTLNLQTKSKFDHQELPLQKTPSQQSNILDNGQEDVSPSGQWNPYPLGRRDVPPDTITKKAGSHIQTLMGSQSLQHRSREQQPYEGNINKVTIQQFQSPLPIQIPSSQATRGPQPGRCLIQTKGQRSMDGYPEQFCVRIEKNPGLGFSISGGISGQGNPFKPSDKGIFVTRVQPDGPASNLLQPGDKILQANGHSFVHMEHEKAVLLLKSFQNTVDLVIQRELTV

1188 P20823 Q96P71

>P20823

MVSKLSQLQTELLAALLESGLSKEALIQALGEPGPYLLAGEGPLDKGESCGGGRGELAELPNGLGETRGSEDETDDDGEDFTPPILKELENLSPEEAAHQKAVVETLLQEDPWRVAKMVKSYLQQHNIPQREVVDTTGLNQSHLSQHLNKGTPMKTQKRAALYTWYVRKQREVAQQFTHAGQGGLIEEPTGDELPTKKGRRNRFKWGPASQQILFQAYERQKNPSKEERETLVEECNRAECIQRGVSPSQAQGLGSNLVTEVRVYNWFANRRKEEAFRHKLAMDTYSGPPPGPGPGPALPAHSSPGLPPPALSPSKVHGVRYGQPATSETAEVPSSSGGPLVTVSTPLHQVSPTGLEPSHSLLSTEAKLVSAAGGPLPPVSTLTALHSLEQTSPGLNQQPQNLIMASLPGVMTIGPGEPASLGPTFTNTGASTLVIGLASTQAQSVPVINSMGSSLTTLQPVQFSQPLHPSYQQPLMPPVQSHVTQSPFMATMAQLQSPHALYSHKPEVAQYTHTGLLPQTMLITDTTNLSALASLTPTKQVFTSDTEASSESGLHTPASQATTLHVPSQDPAGIQHLQPAHRLSASPTVSSSSLVLYQSSDSSNGQSHLLPSNHSVIETFISTQMASSSQ

>Q96P71

MACAGLLTVCLLRPPAPQPQPQTPRHPQLAPDPGPAGHTLFQDVFRRADKNDDGKLSFEEFQNYFADGVLSLGELQELFSGIDGHLTDNLETEKLCDYFSEHLGVYRPVLAALESLNRAVLAAMDATKLEYERASKVDQFVTRFLLRETVSQLQALQSSLEGASDTLEAQAHGWRSDAESVEAQSRLCGSRRAGRRALRSVSRSSTWSPGSSDTGRSSEAEMQWRLQVNRLQELIDQLECKVRAVGPGPHKGGPSWYPPEPGPCWRPGPHSVPSQAPRLEPLREEDLAKGPDLHILMAQRQVQVAEEGLQDFHRALRCYVDFTGAQSHCLHVSAQKMLDGASFTLYEFWQDEASWRRHQQSPGSKAFQRILIDHLRAPDTLTTVFFPASWWIMNNN

1189 NP_004575.1 NP_001753.1

>NP_004575.1

MKCLVTGGNVKVLGKAVHSLSRIGDELYLEPLEDGLSLRTVNSSRSAYACFLFAPLFFQQYQAATPGQDLLRCKILMKSFLSVFRSLAMLEKTVEKCCISLNGRSSRLVVQLHCKFGVRKTHNLSFQDCESLQAVFDPASCPHMLRAPARVLGEAVLPFSPALAEVTLGIGRGRRVILRSYHEEEADSTAKAMVTEMCLGEEDFQQLQAQEGVAITFCLKEFRGLLSFAESANLNLSIHFDAPGRPAIFTIKDSLLDGHFVLATLSDTDSHSQDLGSPERHQPVPQLQAHSTPHPDDFANDDIDSYMIAMETTIGNEGSRVLPSISLSPGPQPPKSPGPHSEEEDEAEPSTVPGTPPPKKFRSLFFGSILAPVRSPQGPSPVLAEDSEGEG

>NP_001753.1

MAAVKTLNPKAEVARAQAALAVNISAARGLQDVLRTNLGPKGTMKMLVSGAGDIKLTKDGNVLLHEMQIQHPTASLIAKVATAQDDITGDGTTSNVLIIGELLKQADLYISEGLHPRIITEGFEAAKEKALQFLEEVKVSREMDRETLIDVARTSLRTKVHAELADVLTEAVVDSILAIKKQDEPIDLFMIEIMEMKHKSETDTSLIRGLVLDHGARHPDMKKRVEDAYILTCNVSLEYEKTEVNSGFFYKSAEEREKLVKAERKFIEDRVKKIIELKRKVCGDSDKGFVVINQKGIDPFSLDALSKEGIVALRRAKRRNMERLTLACGGVALNSFDDLSPDCLGHAGLVYEYTLGEEKFTFIEKCNNPRSVTLLIKGPNKHTLTQIKDAVRDGLRAVKNAIDDGCVVPGAGAVEVAMAEALIKHKPSVKGRAQLGVQAFADALLIIPKVLAQNSGFDLQETLVKIQAEHSESGQLVGVDLNTGEPMVAAEVGVWDNYCVKKQLLHSCTVIATNILLVDEIMRAGMSSLKG

1190 Q86YN6 O75251

>Q86YN6

MAGNDCGALLDEELSSFFLNYLADTQGGGSGEEQLYADFPELDLSQLDASDFDSATCFGELQWCPENSETEPNQYSPDDSELFQIDSENEALLAELTKTLDDIPEDDVGLAAFPALDGGDALSCTSASPAPSSAPPSPAPEKPSAPAPEVDELSLLQKLLLATSYPTSSSDTQKEGTAWRQAGLRSKSQRPCVKADSTQDKKAPMMQSQSRSCTELHKHLTSAQCCLQDRGLQPPCLQSPRLPAKEDKEPGEDCPSPQPAPASPRDSLALGRADPGAPVSQEDMQAMVQLIRYMHTYCLPQRKLPPQTPEPLPKACSNPSQQVRSRPWSRHHSKASWAEFSILRELLAQDVLCDVSKPYRLATPVYASLTPRSRPRPPKDSQASPGRPSSVEEVRIAASPKSTGPRPSLRPLRLEVKREVRRPARLQQQEEEDEEEEEEEEEEEKEEEEEWGRKRPGRGLPWTKLGRKLESSVCPVRRSRRLNPELGPWLTFADEPLVPSEPQGALPSLCLAPKAYDVERELGSPTDEDSGQDQQLLRGPQIPALESPCESGCGDMDEDPSCPQLPPRDSPRCLMLALSQSDPTFGKKSFEQTLTVELCGTAGLTPPTTPPYKPTEEDPFKPDIKHSLGKEIALSLPSPEGLSLKATPGAAHKLPKKHPERSELLSHLRHATAQPASQAGQKRPFSCSFGDHDYCQVLRPEGVLQRKVLRSWEPSGVHLEDWPQQGAPWAEAQAPGREEDRSCDAGAPPKDSTLLRDHEIRASLTKHFGLLETALEEEDLASCKSPEYDTVFEDSSSSSGESSFLPEEEEEEGEEEEEDDEEEDSGVSPTCSDHCPYQSPPSKANRQLCSRSRSSSGSSPCHSWSPATRRNFRCESRGPCSDRTPSIRHARKRREKAIGEGRVVYIQNLSSDMSSRELKRRFEVFGEIEECEVLTRNRRGEKYGFITYRCSEHAALSLTKGAALRKRNEPSFQLSYGGLRHFCWPRYTDYDSNSEEALPASGKSKYEAMDFDSLLKEAQQSLH

>O75251

MAVLSAPGLRGFRILGLRSSVGPAVQARGVHQSVATDGPSSTQPALPKARAVAPKPSSRGEYVVAKLDDLVNWARRSSLWPMTFGLACCAVEMMHMAAPRYDMDRFGVVFRASPRQSDVMIVAGTLTNKMAPALRKVYDQMPEPRYVVSMGSCANGGGYYHYSYSVVRGCDRIVPVDIYIPGCPPTAEALLYGILQLQRKIKRERRLQIWYRR

1191 Q96S96 Q9NWU1

>Q96S96

MGWTMRLVTAALLLGLMMVVTGDEDENSPCAHEALLDEDTLFCQGLEVFYPELGNIGCKVVPDCNNYRQKITSWMEPIVKFPGAVDGATYILVMVDPDAPSRAEPRQRFWRHWLVTDIKGADLKKGKIQGQELSAYQAPSPPAHSGFHRYQFFVYLQEGKVISLLPKENKTRGSWKMDRFLNRFHLGEPEASTQFMTQNYQDSPTLQAPRERASEPKHKNQAEIAAC

>Q9NWU1

MSNCLQNFLKITSTRLLCSRLCQQLRSKRKFFGTVPISRLHRRVVITGIGLVTPLGVGTHLVWDRLIGGESGIVSLVGEEYKSIPCSVAAYVPRGSDEGQFNEQNFVSKSDIKSMSSPTIMAIGAAELAMKDSGWHPQSEADQVATGVAIGMGMIPLEVVSETALNFQTKGYNKVSPFFVPKILVNMAAGQVSIRYKLKGPNHAVSTACTTGAHAVGDSFRFIAHGDADVMVAGGTDSCISPLSLAGFSRARALSTNSDPKLACRPFHPKRDGFVMGEGAAVLVLEEYEHAVQRRARIYAEVLGYGLSGDAGHITAPDPEGEGALRCMAAALKDAGVQPEEISYINAHATSTPLGDAAENKAIKHLFKDHAYALAVSSTKGATGHLLGAAGAVEAAFTTLACYYQKLPPTLNLDCSEPEFDLNYVPLKAQEWKTEKRFIGLTNSFGFGGTNATLCIAGL

1192 Q96N77 NP_002056.2

>Q96N77

MQAEDRSQFGSAAEMLSEQTAALGTGWESMNVQLDGAEPQVERGSQEERPWRTVPGPLEHLCCDLEEEPQSLQEKAQSAPWVPAIPQEGNTGDWEMAAALLAAGSQGLVTIKDVSLCFSQEEWRSLDPSQTDFYGEYVMQENCGIVVSLRFPIPKLDMLSQLEGGEEQWVPDPQDLEERDILRVTYTGDGSEHEGDTPELEAEPPRMLSSVSEDTVLWNPEHDESWDSMPSSSRGMLLGPPFLQEDSFSNLLCSTEMDSLLRPHTCPQCGKQFVWGSHLARHQQTHTGERPYSCLKCEKTFGRRHHLIRHQKTHLHDKTSRCSECGKNFRCNSHLASHQRVHAEGKSCKGQEVGESPGTRKRQRAPPVPKCHVCTECGKSFGRRHHLVRHWLTHTGEKPFQCPRCEKSFGRKHHLDRHLLTHQGQSPRNSWDRGTSVF

>NP_002056.2

MTTSASSHLNKGIKQVYMSLPQGEKVQAMYIWIDGTGEGLRCKTRTLDSEPKCVEELPEWNFDGSSTLQSEGSNSDMYLVPAAMFRDPFRKDPNKLVLCEVFKYNRRPAETNLRHTCKRIMDMVSNQHPWFGMEQEYTLMGTDGHPFGWPSNGFPGPQGPYYCGVGADRAYGRDIVEAHYRACLYAGVKIAGTNAEVMPAQWEFQIGPCEGISMGDHLWVARFILHRVCEDFGVIATFDPKPIPGNWNGAGCHTNFSTKAMREENGLKYIEEAIEKLSKRHQYHIRAYDPKGGLDNARRLTGFHETSNINDFSAGVANRSASIRIPRTVGQEKKGYFEDRRPSANCDPFSVTEALIRTCLLNETGDEPFQYKN

1193 NP_056943.1 NP_006461.3

>NP_056943.1

MANSANTNTVPKLYRSVIEDVINDVRDIFLDDGVDEQVLMELKTLWENKLMQSRAVDGFHSEEQQLLLQVQQQHQPQQQQHHHHHHHQQAQPQQTVPQQAQTQQVLIPASQQATAPQVIVPDSKLIQHMNASNMSAAATAATLALPAGVTPVQQILTNSGQLLQVVRAANGAQYIFQPQQSVVLQQQVIPQMQPGGVQAPVIQQVLAPLPGGISPQTGVIIQPQQILFTGNKTQVIPTTVAAPTPAQAQITATGQQQPQAQPAQTQAPLVLQVDGTGDTSSEEDEDEEEDYDDDEEEDKEKDGAEDGQVEEEPLNSEDDVSDEEGQELFDTENVVVCQYDKIHRSKNKWKFHLKDGIMNLNGRDYIFSKAIGDAEW

>NP_006461.3

MAELDLMAPGPLPRATAQPPAPLSPDSGSPSPDSGSASPVEEEDVGSSEKLGRETEEQDSDSAEQGDPAGEGKEVLCDFCLDDTRRVKAVKSCLTCMVNYCEEHLQPHQVNIKLQSHLLTEPVKDHNWRYCPAHHSPLSAFCCPDQQCICQDCCQEHSGHTIVSLDAARRDKEAELQCTQLDLERKLKLNENAISRLQANQKSVLVSVSEVKAVAEMQFGELLAAVRKAQANVMLFLEEKEQAALSQANGIKAHLEYRSAEMEKSKQELERMAAISNTVQFLEEYCKFKNTEDITFPSVYVGLKDKLSGIRKVITESTVHLIQLLENYKKKLQEFSKEEEYDIRTQVSAVVQRKYWTSKPEPSTREQFLQYAYDITFDPDTAHKYLRLQEENRKVTNTTPWEHPYPDLPSRFLHWRQVLSQQSLYLHRYYFEVEIFGAGTYVGLTCKGIDRKGEERNSCISGNNFSWSLQWNGKEFTAWYSDMETPLKAGPFRRLGVYIDFPGGILSFYGVEYDTMTLVHKFACKFSEPVYAAFWLSKKENAIRIVDLGEEPEKPAPSLVGTAP

1194 NP_071801.1 NP_004050.3

>NP_071801.1

MERKISRIHLVSEPSITHFLQVSWEKTLESGFVITLTDGHSAWTGTVSESEISQEADDMAMEKGKYVGELRKALLSGAGPADVYTFNFSKESCYFFFEKNLKDVSFRLGSFNLEKVENPAEVIRELICYCLDTIAENQAKNEHLQKENERLLRDWNDVQGRFEKCVSAKEALETDLYKRFILVLNEKKTKIRSLHNKLLNAAQEREKDIKQEGETAICSEMTADRDPVYDESTDEESENQTDLSGLASAAVSKDDSIISSLDVTDIAPSRKRRQRMQRNLGTEPKMAPQENQLQEKENSRPDSSLPETSKKEHISAENMSLETLRNSSPEDLFDEI

>NP_004050.3

MAKQLQARRLDGIDYNPWVEFVKLASEHDVVNLGQGFPDFPPPDFAVEAFQHAVSGDFMLNQYTKTFGYPPLTKILASFFGELLGQEIDPLRNVLVTVGGYGALFTAFQALVDEGDEVIIIEPFFDCYEPMTMMAGGRPVFVSLKPGPIQNGELGSSSNWQLDPMELAGKFTSRTKALVLNTPNNPLGKVFSREELELVASLCQQHDVVCITDEVYQWMVYDGHQHISIASLPGMWERTLTIGSAGKTFSATGWKVGWVLGPDHIMKHLRTVHQNSVFHCPTQSQAAVAESFEREQLLFRQPSSYFVQFPQAMQRCRDHMIRSLQSVGLKPIIPQGSYFLITDISDFKRKMPDLPGAVDEPYDRRFVKWMIKNKGLVAIPVSIFYSVPHQKHFDHYIRFCFVKDEATLQAMDEKLRKWKVEL

1195 NP_066406.1 Q01064

>NP_066406.1

MPEPSKSAPAPKKGSKKAITKAQKKDGKKRKRSRKESYSIYVYKVLKQVHPDTGISSKAMGIMNSFVNDIFERIAGEASRLAHYNKRSTITSREIQTAVRLLLPGELAKHAVSEGTKAVTKYTSSK

>Q01064

MELSPRSPPEMLEESDCPSPLELKSAPSKKMWIKLRSLLRYMVKQLENGEINIEELKKNLEYTASLLEAVYIDETRQILDTEDELQELRSDAVPSEVRDWLASTFTQQARAKGRRAEEKPKFRSIVHAVQAGIFVERMFRRTYTSVGPTYSTAVLNCLKNLDLWCFDVFSLNQAADDHALRTIVFELLTRHNLISRFKIPTVFLMSFLDALETGYGKYKNPYHNQIHAADVTQTVHCFLLRTGMVHCLSEIELLAIIFAAAIHDYEHTGTTNSFHIQTKSECAIVYNDRSVLENHHISSVFRLMQDDEMNIFINLTKDEFVELRALVIEMVLATDMSCHFQQVKTMKTALQQLERIDKPKALSLLLHAADISHPTKQWLVHSRWTKALMEEFFRQGDKEAELGLPFSPLCDRTSTLVAQSQIGFIDFIVEPTFSVLTDVAEKSVQPLADEDSKSKNQPSFQWRQPSLDVEVGDPNPDVVSFRSTWVKRIQENKQKWKERAASGITNQMSIDELSPCEEEAPPSPAEDEHNQNGNLD

1196 P16402 NP_004823.1

>P16402

MSETAPLAPTIPAPAEKTPVKKKAKKAGATAGKRKASGPPVSELITKAVAASKERSGVSLAALKKALAAAGYDVEKNNSRIKLGLKSLVSKGTLVQTKGTGASGSFKLNKKAASGEGKPKAKKAGAAKPRKPAGAAKKPKKVAGAATPKKSIKKTPKKVKKPATAAGTKKVAKSAKKVKTPQPKKAAKSPAKAKAPKPKAAKPKSGKPKVTKAKKAAPKKK

>NP_004823.1

MSGESARSLGKGSAPPGPVPEGSIRIYSMRFCPFAERTRLVLKAKGIRHEVININLKNKPEWFFKKNPFGLVPVLENSQGQLIYESAITCEYLDEAYPGKKLLPDDPYEKACQKMILELFSKVPSLVGSFIRSQNKEDYAGLKEEFRKEFTKLEEVLTNKKTTFFGGNSISMIDYLIWPWFERLEAMKLNECVDHTPKLKLWMAAMKEDPTVSALLTSEKDWQGFLELYLQNSPEACDYGL

1197 NP_002388.2 NP_000839.1

>NP_002388.2

MGRKKIQITRIMDERNRQVTFTKRKFGLMKKAYELSVLCDCEIALIIFNSTNKLFQYASTDMDKVLLKYTEYNEPHESRTNSDIVETLRKKGLNGCDSPDPDADDSVGHSPESEDKYRKINEDIDLMISRQRLCAVPPPNFEMPVSIPVSSHNSLVYSNPVSSLGNPNLLPLAHPSLQRNSMSPGVTHRPPSAGNTGGLMGGDLTSGAGTSAGNGYGNPRNSPGLLVSPGNLNKNMQAKSPPPMNLGMNNRKPDLRVLIPPGSKNTMPSVSEDVDLLLNQRINNSQSAQSLATPVVSVATPTLPGQGMGGYPSAISTTYGTEYSLSSADLSSLSGFNTASALHLGSVTGWQQQHLHNMPPSALSQLGACTSTHLSQSSNLSLPSTQSLNIKSEPVSPPRDRTTTPSRYPQHTRHEAGRSPVDSLSSCSSSYDGSDREDHRNEFHSPIGLTRPSPDERESPSVKRMRLSEGWAT

>NP_000839.1

MPMTLGYWNIRGLAHSIRLLLEYTDSSYEEKKYTMGDAPDYDRSQWLNEKFKLGLDFPNLPYLIDGTHKITQSNAILRYIARKHNLCGESEKEQIREDILENQFMDSRMQLAKLCYDPDFEKLKPEYLQALPEMLKLYSQFLGKQPWFLGDKITFVDFIAYDVLERNQVFEPSCLDAFPNLKDFISRFEGLEKISAYMKSSRFLPRPVFTKMAVWGNK

1198 Q86UE8 NP_003192.1

>Q86UE8

MMEELHSLDPRRQELLEARFTGVGVSKGPLNSESSNQSLCSVGSLSDKEVETPEKKQNDQRNRKRKAEPYETSQGKGTPRGHKISDYFEFAGGSAPGTSPGRSVPPVARSSPQHSLSNPLPRRVEQPLYGLDGSAAKEATEEQSALPTLMSVMLAKPRLDTEQLAQRGAGLCFTFVSAQQNSPSSTGSGNTEHSCSSQKQISIQHRQTQSDLTIEKISALENSKNSDLEKKEGRIDDLLRANCDLRRQIDEQQKMLEKYKERLNRCVTMSKKLLIEKSKQEKMACRDKSMQDRLRLGHFTTVRHGASFTEQWTDGYAFQNLIKQQERINSQREEIERQRKMLAKRKPPAMGQAPPATNEQKQRKSKTNGAENETPSSGNTELKDTAPALGAHSLLRLTLAEYHEQEEIFKLRLGHLKKEEAEIQAELERLERVRNLHIRELKRIHNEDNSQFKDHPTLNDRYLLLHLLGRGGFSEVYKAFDLTEQRYVAVKIHQLNKNWRDEKKENYHKHACREYRIHKELDHPRIVKLYDYFSLDTDSFCTVLEYCEGNDLDFYLKQHKLMSEKEARSIIMQIVNALKYLNEIKPPIIHYDLKPGNILLVNGTACGEIKITDFGLSKIMDDDSYNSVDGMELTSQGAGTYWYLPPECFVVGKEPPKISNKVDVWSVGVIFYQCLYGRKPFGHNQSQQDILQENTILKATEVQFPPKPVVTPEAKAFIRRCLAYRKEDRIDVQQLACDPYLLPHIRKSVSTSSPAGAAIASTSGASNNSSSN

>NP_003192.1

MAFLRSMWGVLSALGRSGAELCTGCGSRLRSPFSFVYLPRWFSSVLASCPKKPVSSYLRFSKEQLPIFKAQNPDAKTTELIRRIAQRWRELPDSKKKIYQDAYRAEWQVYKEEISRFKEQLTPSQIMSLEKEIMDKHLKRKAMTKKKELTLLGKPKRPRSAYNVYVAERFQEAKGDSPQEKLKTVKENWKNLSDSEKELYIQHAKEDETRYHNEMKSWEEQMIEVGRKDLLRRTIKKQRKYGAEEC

1199 NP_001790.1 P30711

>NP_001790.1

MALDVKSRAKRYEKLDFLGEGQFATVYKARDKNTNQIVAIKKIKLGHRSEAKDGINRTALREIKLLQELSHPNIIGLLDAFGHKSNISLVFDFMETDLEVIIKDNSLVLTPSHIKAYMLMTLQGLEYLHQHWILHRDLKPNNLLLDENGVLKLADFGLAKSFGSPNRAYTHQVVTRWYRAPELLFGARMYGVGVDMWAVGCILAELLLRVPFLPGDSDLDQLTRIFETLGTPTEEQWPDMCSLPDYVTFKSFPGIPLHHIFSAAGDDLLDLIQGLFLFNPCARITATQALKMKYFSNRPGPTPGCQLPRPNCPVETLKEQSNPALAIKRKRTEALEQGGLPKKLIF

>P30711

MGLELYLDLLSQPCRAVYIFAKKNDIPFELRIVDLIKGQHLSDAFAQVNPLKKVPALKDGDFTLTESVAILLYLTRKYKVPDYWYPQDLQARARVDEYLAWQHTTLRRSCLRALWHKVMFPVFLGEPVSPQTLAATLAELDVTLQLLEDKFLQNKAFLTGPHISLADLVAITELMHPVGAGCQVFEGRPKLATWRQRVEAAVGEDLFQEAHEVILKAKDFPPADPTIKQKLMPWVLAMIR

1200 Q5XKR4 Q9NYK5

>Q5XKR4

MLSHADLLDARLGMKDAAELLGHREAVKCRLGVGGSDPGGHPGDLAPNSDPVEGATLLPGEDITTVGSTPASLAVSAKDPDKQPGPQGGPNPSQAGQQQGQQKQKRHRTRFTPAQLNELERSFAKTHYPDIFMREELALRIGLTESRVQVWFQNRRAKWKKRKKTTNVFRAPGTLLPTPGLPQFPSAAAAAAAAMGDSLCSFHANDTRWAAAAMPGVSQLPLPPALGRQQAMAQSLSQCSLAAGPPPNSMGLSNSLAGSNGAGLQSHLYQPAFPGMVPASLPGPSNVSGSPQLCSSPDSSDVWRGTSIASLRRKALEHTVSMSFT

>Q9NYK5

MEALAMGSRALRLWLVAPGGGIKWRFIATSPASQLSPTELTEMRNDLFNKEKARQLSLTPRTEKIEVKHVGKTDPGTVFVMNKNISTPYSCAMHLSEWYCRKSILALVDGQPWDMYKPLTKSCEIKFLTFKDCDPGEVNKAYWRSCAMMMGCVIERAFKDEYMVNLVRAPEVPVISGAFCYDVVLDSKLDEWMPTKENLRSFTKDAHALIYKDLPFETLEVEAKVALEIFQHSKYKVDFIEEKASQNPERIVKLHRIGDFIDVSEGPLIPRTSICFQYEVSAVHNLQPTQPSLIRRFQGVSLPVHLRAHFTIWDKLLERSRKMVTEDQSKATEECTST

1201 NP_002884.1 NP_006651.2

>NP_002884.1

MASKEMFEDTVEERVINEEYKIWKKNTPFLYDLVMTHALQWPSLTVQWLPEVTKPEGKDYALHWLVLGTHTSDEQNHLVVARVHIPNDDAQFDASHCDSDKGEFGGFGSVTGKIECEIKINHEGEVNRARYMPQNPHIIATKTPSSDVLVFDYTKHPAKPDPSGECNPDLRLRGHQKEGYGLSWNSNLSGHLLSASDDHTVCLWDINAGPKEGKIVDAKAIFTGHSAVVEDVAWHLLHESLFGSVADDQKLMIWDTRSNTTSKPSHLVDAHTAEVNCLSFNPYSEFILATGSADKTVALWDLRNLKLKLHTFESHKDEIFQVHWSPHNETILASSGTDRRLNVWDLSKIGEEQSAEDAEDGPPELLFIHGGHTAKISDFSWNPNEPWVICSVSEDNIMQIWQMAENIYNDEESDVTTSELEGQGS

>NP_006651.2

MPSCGACTCGAAAVRLITSSLASAQRGISGGRIHMSVLGRLGTFETQILQRAPLRSFTETPAYFASKDGISKDGSGDGNKKSASEGSSKKSGSGNSGKGGNQLRCPKCGDLCTHVETFVSSTRFVKCEKCHHFFVVLSEADSKKSIIKEPESAAEAVKLAFQQKPPPPPKKIYNYLDKYVVGQSFAKKVLSVAVYNHYKRIYNNIPANLRQQAEVEKQTSLTPRELEIRRREDEYRFTKLLQIAGISPHGNALGASMQQQVNQQIPQEKRGGEVLDSSHDDIKLEKSNILLLGPTGSGKTLLAQTLAKCLDVPFAICDCTTLTQAGYVGEDIESVIAKLLQDANYNVEKAQQGIVFLDEVDKIGSVPGIHQLRDVGGEGVQQGLLKLLEGTIVNVPEKNSRKLRGETVQVDTTNILFVASGAFNGLDRIISRRKNEKYLGFGTPSNLGKGRRAAAAADLANRSGESNTHQDIEEKDRLLRHVEARDLIEFGMIPEFVGRLPVVVPLHSLDEKTLVQILTEPRNAVIPQYQALFSMDKCELNVTEDALKAIARLALERKTGARGLRSIMEKLLLEPMFEVPNSDIVCVEVDKEVVEGKKEPGYIRAPTKESSEEEYDSGVEEEGWPRQADAANS

1202 P28360 NP_036232.1

>P28360

MTSLPLGVKVEDSAFGKPAGGGAGQAPSAAAATAAAMGADEEGAKPKVSPSLLPFSVEALMADHRKPGAKESALAPSEGVQAAGGSAQPLGVPPGSLGAPDAPSSPRPLGHFSVGGLLKLPEDALVKAESPEKPERTPWMQSPRFSPPPARRLSPPACTLRKHKTNRKPRTPFTTAQLLALERKFRQKQYLSIAERAEFSSSLSLTETQVKIWFQNRRAKAKRLQEAELEKLKMAAKPMLPPAAFGLSFPLGGPAAVAAAAGASLYGASGPFQRAALPVAPVGLYTAHVGYSMYHLT

>NP_036232.1

MQVAMNGKARKEAVQTAAKELLKFVNRSPSPFHAVAECRNRLLQAGFSELKETEKWNIKPESKYFMTRNSSTIIAFAVGGQYVPGNGFSLIGAHTDSPCLRVKRRSRRSQVGFQQVGVETYGGGIWSTWFDRDLTLAGRVIVKCPTSGRLEQQLVHVERPILRIPHLAIHLQRNINENFGPNTEMHLVPILATAIQEELEKGTPEPGPLNAVDERHHSVLMSLLCAHLGLSPKDIVEMELCLADTQPAVLGGAYDEFIFAPRLDNLHSCFCALQALIDSCAGPGSLATEPHVRMVTLYDNEEVGSESAQGAQSLLTELVLRRISASCQHPTAFEEAIPKSFMISADMAHAVHPNYLDKHEENHRPLFHKGPVIKVNSKQRYASNAVSEALIREVANKVKVPLQDLMVRNDTPCGTTIGPILASRLGLRVLDLGSPQLAMHSIREMACTTGVLQTLTLFKGFFELFPSLSHNLLVD

1203 Q01459 Q96PC2

>Q01459

MSRPQLRRWRLVSSPPSGVPGLALLALLALLALRLAAGTDCPCPEPELCRPIRHHPDFEVFVFDVGQKTWKSYDWSQITTVATFGKYDSELMCYAHSKGARVVLKGDVSLKDIIDPAFRASWIAQKLNLAKTQYMDGINIDIEQEVNCLSPEYDALTALVKETTDSFHREIEGSQVTFDVAWSPKNIDRRCYNYTGIADACDFLFVMSYDEQSQIWSECIAAANAPYNQTLTGYNDYIKMSINPKKLVMGVPWYGYDYTCLNLSEDHVCTIAKVPFRGAPCSDAAGRQVPYKTIMKQINSSISGNLWDKDQRAPYYNYKDPAGHFHQVWYDNPQSISLKATYIQNYRLRGIGMWNANCLDYSGDAVAKQQTEEMWEVLKPKLLQR

>Q96PC2

MVVQNSADAGDMRAGVQLEPFLHQVGGHMSVMKYDEHTVCKPLVSREQRFYESLPLAMKRFTPQYKGTVTVHLWKDSTGHLSLVANPVKESQEPFKVSTESAAVAIWQTLQQTTGSNGSDCTLAQWPHAQLARSPKESPAKALLRSEPHLNTPAFSLVEDTNGNQVERKSFNPWGLQCHQAHLTRLCSEYPENKRHRFLLLENVVSQYTHPCVLDLKMGTRQHGDDASEEKKARHMRKCAQSTSACLGVRICGMQVYQTDKKYFLCKDKYYGRKLSVEGFRQALYQFLHNGSHLRRELLEPILHQLRALLSVIRSQSSYRFYSSSLLVIYDGQEPPERAPGSPHPHEAPQAAHGSSPGGLTKVDIRMIDFAHTTYKGYWNEHTTYDGPDPGYIFGLENLIRILQDIQEGE

1204 NP_003098.1 P42679

>NP_003098.1

MVQQTNNAENTEALLAGESSDSGAGLELGIASSPTPGSTASTGGKADDPSWCKTPSGHIKRPMNAFMVWSQIERRKIMEQSPDMHNAEISKRLGKRWKLLKDSDKIPFIREAERLRLKHMADYPDYKYRPRKKVKSGNANSSSSAAASSKPGEKGDKVGGSGGGGHGGGGGGGSSNAGGGGGGASGGGANSKPAQKKSCGSKVAGGAGGGVSKPHAKLILAGGGGGGKAAAAAAASFAAEQAGAAALLPLGAAADHHSLYKARTPSASASASSAASASAALAAPGKHLAEKKVKRVYLFGGLGTSSSPVGGVGAGADPSDPLGLYEEEGAGCSPDAPSLSGRSSAASSPAAGRSPADHRGYASLRAASPAPSSAPSHASSSASSHSSSSSSSGSSSSDDEFEDDLLDLNPSSNFESMSLGSFSSSSALDRDLDFNFEPGSGSHFEFPDYCTPEVSEMISGDWLESSISNLVFTY

>P42679

MAGRGSLVSWRAFHGCDSAEELPRVSPRFLRAWHPPPVSARMPTRRWAPGTQCITKCEHTRPKPGELAFRKGDVVTILEACENKSWYRVKHHTSGQEGLLAAGALREREALSADPKLSLMPWFHGKISGQEAVQQLQPPEDGLFLVRESARHPGDYVLCVSFGRDVIHYRVLHRDGHLTIDEAVFFCNLMDMVEHYSKDKGAICTKLVRPKRKHGTKSAEEELARAGWLLNLQHLTLGAQIGEGEFGAVLQGEYLGQKVAVKNIKCDVTAQAFLDETAVMTKMQHENLVRLLGVILHQGLYIVMEHVSKGNLVNFLRTRGRALVNTAQLLQFSLHVAEGMEYLESKKLVHRDLAARNILVSEDLVAKVSDFGLAKAERKGLDSSRLPVKWTAPEALKHGKFTSKSDVWSFGVLLWEVFSYGRAPYPKMSLKEVSEAVEKGYRMEPPEGCPGPVHVLMSSCWEAEPARRPPFRKLAEKLARELRSAGAPASVSGQDADGSTSPRSQEP

1205 NP_001231.2 Q969Y2

>NP_001231.2

MEGERKNNNKRWYFTREQLENSPSRRFGVDPDKELSYRQQAANLLQDMGQRLNVSQLTINTAIVYMHRFYMIQSFTQFPGNSVAPAALFLAAKVEEQPKKLEHVIKVAHTCLHPQESLPDTRSEAYLQQVQDLVILESIILQTLGFELTIDHPHTHVVKCTQLVRASKDLAQTSYFMATNSLHLTTFSLQYTPPVVACVCIHLACKWSNWEIPVSTDGKHWWEYVDATVTLELLDELTHEFLQILEKTPNRLKRIWNWRACEAAKKTKADDRGTDEKTSEQTILNMISQSSSDTTIAGLMSMSTSTTSAVPSLPVSEESSSNLTSVEMLPGKRWLSSQPSFKLEPTQGHRTSENLALTGVDHSLPQDGSNAFISQKQNSKSVPSAKVSLKEYRAKHAEELAAQKRQLENMEANVKSQYAYAAQNLLSHHDSHSSVILKMPIEGSENPERPFLEKADKTALKMRIPVAGGDKAASSKPEEIKMRIKVHAAADKHNSVEDSVTKSREHKEKHKTHPSNHHHHHNHHSHKHSHSQLPVGTGNKRPGDPKHSSQTSNLAHKTYSLSSSFSSSSSTRKRGPSEETGGAVFDHPAKIAKSTKSSSLNFSFPSLPTMGQMPGHSSDTSGLSFSQPSCKTRVPHSKLDKGPTGANGHNTTQTIDYQDTVNMLHSLLSAQGVQPTQPTAFEFVRPYSDYLNPRSGGISSRSGNTDKPRPPPLPSEPPPPLPPLPK

>Q969Y2

MWRGLWTLAAQAARGPRRLCTRRSSGAPAPGSGATIFALSSGQGRCGIAVIRTSGPASGHALRILTAPRDLPLARHASLRLLSDPRSGEPLDRALVLWFPGPQSFTGEDCVEFHVHGGPAVVSGVLQALGSVPGLRPAEAGEFTRRAFANGKLNLTEVEGLADLIHAETEAQRRQALRQLDGELGHLCRGWAETLTKALAHVEAYIDFGEDDNLEEGVLEQADIEVRALQVALGAHLRDARRGQRLRSGAHVVVTGPPNAGKSSLVNLLSRKPVSIVSPEPGTTRDVLETPVDLAGFPVLLSDTAGLREGVGPVEQEGVRRARERLEQADLILAMLDASDLASPSSCNFLATVVASVGAQSPSDSSQRLLLVLNKSDLLSPEGPGPGPDLPPHLLLSCLTGEGLDGLLEALRKELAAVCGDPSTDPPLLTRARHQHHLQGCLDALGHYKQSKDLALAAEALRVARGHLTRLTGGGGTEEILDIIFQDFCVGK

1206 NP_006581.2 NP_006436.3

>NP_006581.2

MSGRSKRESRGSTRGKRESESRGSSGRVKRERDREREPEAASSRGSPVRVKREFEPASAREAPASVVPFVRVKREREVDEDSEPEREVRAKNGRVDSEDRRSRHCPYLDTINRSVLDFDFEKLCSISLSHINAYACLVCGKYFQGRGLKSHAYIHSVQFSHHVFLNLHTLKFYCLPDNYEIIDSSLEDITYVLKPTFTKQQIANLDKQAKLSRAYDGTTYLPGIVGLNNIKANDYANAVLQALSNVPPLRNYFLEEDNYKNIKRPPGDIMFLLVQRFGELMRKLWNPRNFKAHVSPHEMLQAVVLCSKKTFQITKQGDGVDFLSWFLNALHSALGGTKKKKKTIVTDVFQGSMRIFTKKLPHPDLPAEEKEQLLHNDEYQETMVESTFMYLTLDLPTAPLYKDEKEQLIIPQVPLFNILAKFNGITEKEYKTYKENFLKRFQLTKLPPYLIFCIKRFTKNNFFVEKNPTIVNFPITNVDLREYLSEEVQAVHKNTTYDLIANIVHDGKPSEGSYRIHVLHHGTGKWYELQDLQVTDILPQMITLSEAYIQIWKRRDNDETNQQGA

>NP_006436.3

MAGVFPYRGPGNPVPGPLAPLPDYMSEEKLQEKARKWQQLQAKRYAEKRKFGFVDAQKEDMPPEHVRKIIRDHGDMTNRKFRHDKRVYLGALKYMPHAVLKLLENMPMPWEQIRDVPVLYHITGAISFVNEIPWVIEPVYISQWGSMWIMMRREKRDRRHFKRMRFPPFDDEEPPLDYADNILDVEPLEAIQLELDPEEDAPVLDWFYDHQPLRDSRKYVNGSTYQRWQFTLPMMSTLYRLANQLLTDLVDDNYFYLFDLKAFFTSKALNMAIPGGPKFEPLVRDINLQDEDWNEFNDINKIIIRQPIRTEYKIAFPYLYNNLPHHVHLTWYHTPNVVFIKTEDPDLPAFYFDPLINPISHRHSVKSQEPLPDDDEEFELPEFVEPFLKDTPLYTDNTANGIALLWAPRPFNLRSGRTRRALDIPLVKNWYREHCPAGQPVKVRVSYQKLLKYYVLNALKHRPPKAQKKRYLFRSFKATKFFQSTKLDWVEVGLQVCRQGYNMLNLLIHRKNLNYLHLDYNFNLKPVKTLTTKERKKSRFGNAFHLCREVLRLTKLVVDSHVQYRLGNVDAFQLADGLQYIFAHVGQLTGMYRYKYKLMRQIRMCKDLKHLIYYRFNTGPVGKGPGCGFWAAGWRVWLFFMRGITPLLERWLGNLLARQFEGRHSKGVAKTVTKQRVESHFDLELRAAVMHDILDMMPEGIKQNKARTILQHLSEAWRCWKANIPWKVPGLPTPIENMILRYVKAKADWWTNTAHYNRERIRRGATVDKTVCKKNLGRLTRLYLKAEQERQHNYLKDGPYITAEEAVAVYTTTVHWLESRRFSPIPFPPLSYKHDTKLLILALERLKEAYSVKSRLNQSQREELGLIEQAYDNPHEALSRIKRHLLTQRAFKEVGIEFMDLYSHLVPVYDVEPLEKITDAYLDQYLWYEADKRRLFPPWIKPADTEPPPLLVYKWCQGINNLQDVWETSEGECNVMLESRFEKMYEKIDLTLLNRLLRLIVDHNIADYMTAKNNVVINYKDMNHTNSYGIIRGLQFASFIVQYYGLVMDLLVLGLHRASEMAGPPQMPNDFLSFQDIATEAAHPIRLFCRYIDRIHIFFRFTADEARDLIQRYLTEHPDPNNENIVGYNNKKCWPRDARMRLMKHDVNLGRAVFWDIKNRLPRSVTTVQWENSFVSVYSKDNPNLLFNMCGFECRILPKCRTSYEEFTHKDGVWNLQNEVTKERTAQCFLRVDDESMQRFHNRVRQILMASGSTTFTKIVNKWNTALIGLMTYFREAVVNTQELLDLLVKCENKIQTRIKIGLNSKMPSRFPPVVFYTPKELGGLGMLSMGHVLIPQSDLRWSKQTDVGITHFRSGMSHEEDQLIPNLYRYIQPWESEFIDSQRVWAEYALKRQEAIAQNRRLTLEDLEDSWDRGIPRINTLFQKDRHTLAYDKGWRVRTDFKQYQVLKQNPFWWTHQRHDGKLWNLNNYRTDMIQALGGVEGILEHTLFKGTYFPTWEGLFWEKASGFEESMKWKKLTNAQRSGLNQIPNRRFTLWWSPTINRANVYVGFQVQLDLTGIFMHGKIPTLKISLIQIFRAHLWQKIHESIVMDLCQVFDQELDALEIETVQKETIHPRKSYKMNSSCADILLFASYKWNVSRPSLLADSKDVMDSTTTQKYWIDIQLRWGDYDSHDIERYARAKFLDYTTDNMSIYPSPTGVLIAIDLAYNLHSAYGNWFPGSKPLIQQAMAKIMKANPALYVLRERIRKGLQLYSSEPTEPYLSSQNYGELFSNQIIWFVDDTNVYRVTIHKTFEGNLTTKPINGAIFIFNPRTGQLFLKIIHTSVWAGQKRLGQLAKWKTAEEVAALIRSLPVEEQPKQIIVTRKGMLDPLEVHLLDFPNIVIKGSELQLPFQACLKVEKFGDLILKATEPQMVLFNLYDDWLKTISSYTAFSRLILILRALHVNNDRAKVILKPDKTTITEPHHIWPTLTDEEWIKVEVQLKDLILADYGKKNNVNVASLTQSEIRDIILGMEISAPSQQRQQIAEIEKQTKEQSQLTATQTRTVNKHGDEIITSTTSNYETQTFSSKTEWRVRAISAANLHLRTNHIYVSSDDIKETGYTYILPKNVLKKFICISDLRAQIAGYLYGVSPPDNPQVKEIRCIVMVPQWGTHQTVHLPGQLPQHEYLKEMEPLGWIHTQPNESPQLSPQDVTTHAKIMADNPSWDGEKTIIITCSFTPGSCTLTAYKLTPSGYEWGRQNTDKGNNPKGYLPSHYERVQMLLSDRFLGFFMVPAQSSWNYNFMGVRHDPNMKYELQLANPKEFYHEVHRPSHFLNFALLQEGEVYSADREDLYA

1207 P0C1S8 Q9NP74

>P0C1S8

MDDKDIDKELRQKLNFSYCEETEIEGQKKVEESREASSQTPEKGEVQDSEAKGTPPWTPLSNVHELDTSSEKDKESPDQILRTPVSHPLKCPETPAQPDSRSKLLPSDSPSTPKTMLSRLVISPTGKLPSRGPKHLKLTPAPLKDEMTSLALVNINPFTPESYKKLFLQSGGKRKIRGDLEEAGPEEGKGGLPAKRCVLRETNMASRYEKEFLEVEKIGVGEFGTVYKCIKRLDGCVYAIKRSMKTFTELSNENSALHEVYAHAVLGHHPHVVRYYSSWAEDDHMIIQNEYCNGGSLQAAISENTKSGNHFEEPKLKDILLQISLGLNYIHNSSMVHLDIKPSNIFICHKMQSESSGVIEEVENEADWFLSANVMYKIGDLGHATSINKPKVEEGDSRFLANEILQEDYRHLPKADIFALGLTIAVAAGAESLPTNGAAWHHIRKGNFPDVPQELSESFSSLLKNMIQPDAEQRPSAAALARNTVLRPSLGKTEELQQQLNLEKFKTATLERELREAQQAQSPQGYTHHGDTGVSGTHTGSRSTKRLVGGKSARSSSFTSGEREPLH

>Q9NP74

MEEAELVKGRLQAITDKRKIQEEISQKRLKIEEDKLKHQHLKKKALREKWLLDGISSGKEQEEMKKQNQQDQHQIQVLEQSILRLEKEIQDLEKAELQISTKEEAILKKLKSIERTTEDIIRSVKVEREERAEESIEDIYANIPDLPKSYIPSRLRKEINEEKEDDEQNRKALYAMEIKVEKDLKTGESTVLSSIPLPSDDFKGTGIKVYDDGQKSVYAVSSNHSAAYNGTDGLAPVEVEELLRQASERNSKSPTEYHEPVYANPFYRPTTPQRETVTPGPNFQERIKIKTNGLGIGVNESIHNMGNGLSEERGNNFNHISPIPPVPHPRSVIQQAEEKLHTPQKRLMTPWEESNVMQDKDAPSPKPRLSPRETIFGKSEHQNSSPTCQEDEEDVRYNIVHSLPPDINDTEPVTMIFMGYQQAEDSEEDKKFLTGYDGIIHAELVVIDDEEEEDEGEAEKPSYHPIAPHSQVYQPAKPTPLPRKRSEASPHENTNHKSPHKNSISLKEQEESLGSPVHHSPFDAQTTGDGTEDPSLTALRMRMAKLGKKVI

1208 NP_663628.1 NP_005989.3

>NP_663628.1

MAAGSTTLRAVGKLQVRLATKTEPKKLEKYLQKLSALPMTADILAETGIRKTVKRLRKHQHVGDFARDLAARWKKLVLVDRNTGPDPQDPEESASRQRFGEALQEREKAWGFPENATAPRSPSHSPEHRRTARRTPPGQQRPHPRSPSREPRAERKRPRMAPADSGPHRDPPTRTAPLPMPEGPEPAVPGEQPGRGHAHAAQGGPLLGQGCQGQPQGEAVGSHSKGHKSSRGASAQKSPPVQESQSERLQAAGADSAGPKTVPSHVFSELWDPSEAWMQANYDLLSAFEAMTSQANPEALCAPALQEEAAFPGRRVNAKMPVYSGSRPACQLQVPTLRQQCLRVPRNNPDALGDVEGVPYSVLEPVLEGWTPDQLYRTEKDNAALARETDELWRIHCLQDFKEEKPQEHESWRELYLRLRDAREQRLRVVTTKIRSARENKPSGRQTKMICFNSVAKTPYDASRRQEKSAGAADPGNGEMEPAPKPAGSSQAPSGLGDGDGGSVSGGGSSNRHAAPADKTRKQAAKKVAPLMAKAIRDYKGRFSRR

>NP_005989.3

MMGHRPVLVLSQNTKRESGRKVQSGNINAAKTIADIIRTCLGPKSMMKMLLDPMGGIVMTNDGNAILREIQVQHPAAKSMIEISRTQDEEVGDGTTSVIILAGEMLSVAEHFLEQQMHPTVVISAYRKALDDMISTLKKISIPVDISDSDMMLNIINSSITTKAISRWSSLACNIALDAVKMVQFEENGRKEIDIKKYARVEKIPGGIIEDSCVLRGVMINKDVTHPRMRRYIKNPRIVLLDSSLEYKKGESQTDIEITREEDFTRILQMEEEYIQQLCEDIIQLKPDVVITEKGISDLAQHYLMRANITAIRRVRKTDNNRIARACGARIVSRPEELREDDVGTGAGLLEIKKIGDEYFTFITDCKDPKACTILLRGASKEILSEVERNLQDAMQVCRNVLLDPQLVPGGGASEMAVAHALTEKSKAMTGVEQWPYRAVAQALEVIPRTLIQNCGASTIRLLTSLRAKHTQENCETWGVNGETGTLVDMKELGIWEPLAVKLQTYKTAVETAVLLLRIDDIVSGHKKKGDDQSRQGGAPDAGQE

1209 NP_055909.2 NP_065078.1

>NP_055909.2

MVSGPLALRWCAWAGRGDMGPDMELPSHSKQLLLQLNQQRTKGFLCDVIIMVENSIFRAHKNVLAASSIYFKSLVLHDNLINLDTDMVSSTVFQQILDFIYTGKLLPSDQPAEPNFSTLLTAASYLQLPELAALCRRKLKRAGKPFGSGRAGSTGMGRPPRSQRLSTASVIQARYQGLVDGRKGAHAPQELPQAKGSDDELFLGGSNQDSVQGLGRAVCPAGGEAGLGGCSSSTNGSSGGCEQELGLDLSKKSPPLPPATPGPHLTPDDAAQLSDSQHGSPPAASAPPVANSASYSELGGTPDEPMDLEGAEDNHLSLLEAPGGQPRKSLRHSTRKKEWGKKEPVAGSPFERREAGPKGPCPGEEGEGVGDRVPNGILASGAGPSGPYGEPPYPCKEEEENGKDASEDSAQSGSEGGSGHASAHYMYRQEGYETVSYGDNLYVCIPCAKGFPSSEQLNAHVETHTEEELFIKEEGAYETGSGGAEEEAEDLSAPSAAYTAEPRPFKCSVCEKTYKDPATLRQHEKTHWLTRPFPCNICGKMFTQRGTMTRHMRSHLGLKPFACDECGMRFTRQYRLTEHMRVHSGEKPYECQLCGGKFTQQRNLISHLRMHTSPS

>NP_065078.1

MGKGCKVVVCGLLSVGKTAILEQLLYGNHTIGMEDCETMEDVYMASVETDRGVKEQLHLYDTRGLQEGVELPKHYFSFADGFVLVYSVNNLESFQRVELLKKEIDKFKDKKEVAIVVLGNKIDLSEQRQVDAEVAQQWAKSEKVRLWEVTVTDRKTLIEPFTLLASKLSQPQSKSSFPLPGRKNKGNSNSEN

1210 O75529 NP_003737.1

>O75529

MKRVRTEQIQMAVSCYLKRRQYVDSDGPLKQGLRLSQTAEEMAANLTVQSESGCANIVSAAPCQAEPQQYEVQFGRLRNFLTDSDSQHSHEVMPLLYPLFVYLHLNLVQNSPKSTVESFYSRFHGMFLQNASQKDVIEQLQTTQTIQDILSNFKLRAFLDNKYVVRLQEDSYNYLIRYLQSDNNTALCKVLTLHIHLDVQPAKRTDYQLYASGSSSRSENNGLEPPDMPSPILQNEAALEVLQESIKRVKDGPPSLTTICFYAFYNTEQLLNTAEISPDSKLLAAGFDNSCIKLWSLRSKKLKSEPHQVDVSRIHLACDILEEEDDEDDNAGTEMKILRGHCGPVYSTRFLADSSGLLSCSEDMSIRYWDLGSFTNTVLYQGHAYPVWDLDISPYSLYFASGSHDRTARLWSFDRTYPLRIYAGHLADVDCVKFHPNSNYLATGSTDKTVRLWSAQQGNSVRLFTGHRGPVLSLAFSPNGKYLASAGEDQRLKLWDLASGTLYKELRGHTDNITSLTFSPDSGLIASASMDNSVRVWDIRNTYCSAPADGSSSELVGVYTGQMSNVLSVQFMACNLLLVTGITQENQEH

>NP_003737.1

MCDRKAVIKNADMSEEMQQDSVECATQALEKYNIEKDIAAHIKKEFDKKYNPTWHCIVGRNFGSYVTHETKHFIYFYLGQVAILLFKSG

1211 NP_003122.1 Q96Q11

>NP_003122.1

MLPTQAGAAAALGRGSALGGSLNRTPTGRPGGGGGTRGANGGRVPGNGAGLGPGRLEREAAAAAATTPAPTAGALYSGSEGDSESGEEEELGAERRGLKRSLSEMEIGMVVGGPEASAAATGGYGPVSGAVSGAKPGKKTRGRVKIKMEFIDNKLRRYTTFSKRKTGIMKKAYELSTLTGTQVLLLVASETGHVYTFATRKLQPMITSETGKALIQTCLNSPDSPPRSDPTTDQRMSATGFEETDLTYQVSESDSSGETKDTLKPAFTVTNLPGTTSTIQTAPSTSTTMQVSSGPSFPITNYLAPVSASVSPSAVSSANGTVLKSTGSGPVSSGGLMQLPTSFTLMPGGAVAQQVPVQAIQVHQAPQQASPSRDSSTDLTQTSSSGTVTLPATIMTSSVPTTVGGHMMYPSPHAVMYAPTSGLGDGSLTVLNAFSQAPSTMQVSHSQVQEPGGVPQVFLTASSGTVQIPVSAVQLHQMAVIGQQAGSSSNLTELQVVNLDTAHSTKSE

>Q96Q11

MLRCLYHWHRPVLNRRWSRLCLLKQYLFTMKLQSPEFQSLFTEGLKSLTELFVKENHELRIAGGAVRDLLNGVKPQDIDFATTATPTQMKEMFQSAGIRMINNRGEKHGTITARLHEENFEITTLRIDVTTDGRHAEVEFTTDWQKDAERRDLTINSMFLGFDGTLFDYFNGYEDLKNKKVRFVGHAKQRIQEDYLRILRYFRFYGRIVDKPGDHDPETLEAIAENAKGLAGISGERIWVELKKILVGNHVNHLIHLIYDLDVAPYIGLPANASLEEFDKVSKNVDGFSPKPVTLLASLFKVQDDVTKLDLRLKIAKEEKNLGLFIVKNRKDLIKATDSSDPLKPYQDFIIDSREPDATTRVCELLKYQGEHCLLKEMQQWSIPPFPVSGHDIRKVGISSGKEIGALLQQLREQWKKSGYQMEKDELLSYIKKT

1212 NP_004102.1 NP_008996.1

>NP_004102.1

MGIQGLAKLIADVAPSAIRENDIKSYFGRKVAIDASMSIYQFLIAVRQGGDVLQNEEGETTSHLMGMFYRTIRMMENGIKPVYVFDGKPPQLKSGELAKRSERRAEAEKQLQQAQAAGAEQEVEKFTKRLVKVTKQHNDECKHLLSLMGIPYLDAPSEAEASCAALVKAGKVYAAATEDMDCLTFGSPVLMRHLTASEAKKLPIQEFHLSRILQELGLNQEQFVDLCILLGSDYCESIRGIGPKRAVDLIQKHKSIEEIVRRLDPNKYPVPENWLHKEAHQLFLEPEVLDPESVELKWSEPNEEELIKFMCGEKQFSEERIRSGVKRLSKSRQGSTQGRLDDFFKVTGSLSSAKRKEPEPKGSTKKKAKTGAAGKFKRGK

>NP_008996.1

MVDYSVWDHIEVSDDEDETHPNIDTASLFRWRHQARVERMEQFQKEKEELDRGCRECKRKVAECQRKLKELEVAEGGKAELERLQAEAQQLRKEERSWEQKLEEMRKKEKSMPWNVDTLSKDGFSKSMVNTKPEKTEEDSEEVREQKHKTFVEKYEKQIKHFGMLRRWDDSQKYLSDNVHLVCEETANYLVIWCIDLEVEEKCALMEQVAHQTIVMQFILELAKSLKVDPRACFRQFFTKIKTADRQYMEGFNDELEAFKERVRGRAKLRIEKAMKEYEEEERKKRLGPGGLDPVEVYESLPEELQKCFDVKDVQMLQDAISKMDPTDAKYHMQRCIDSGLWVPNSKASEAKEGEEAGPGDPLLEAVPKTGDEKDVSV

1213 Q14863 Q58A45

>Q14863

MPGISSQILTNAQGQVIGTLPWVVNSASVAAPAPAQSLQVQAVTPQLLLNAQGQVIATLASSPLPPPVAVRKPSTPESPAKSEVQPIQPTPTVPQPAVVIASPAPAAKPSASAPIPITCSETPTVSQLVSKPHTPSLDEDGINLEEIREFAKNFKIRRLSLGLTQTQVGQALTATEGPAYSQSAICRFEKLDITPKSAQKLKPVLEKWLNEAELRNQEGQQNLMEFVGGEPSKKRKRRTSFTPQAIEALNAYFEKNPLPTGQEITEIAKELNYDREVVRVWFCNRRQTLKNTSKLNVFQIP

>Q58A45

MDGGALTDTSLTDSYFSTSFIGVNGFGSPVETKYPLMQRMTNSSSSPSLLNDSAKPYSAHDPLTSPASSLFNDFGALNISQRRKPRKYRLGMLEERLVPMGSKARKAKNPIGCLADRCKSGVPINMVWWNRVTENNLQTPNPTASEFIPKGGSTSRLSNVSQSNMSAFSQVFSHPSMGSPATAGLAPGMSLSAGSSPLHSPKITPHTSPAPRRRSHTPNPASYMVPSSASTSVNNPVSQTPSSGQVIQKETVGGTTYFYTDTTPAPLTGMVFPNYHIYPPTAPHVAYMQPKANAPSFFMADELRQELINRHLITMAQIDQADMPAVPTEVDSYHSLFPLEPLPPPNRIQKSSNFGYITSCYKAVNSKDDLPYCLRRIHGFRLVNTKCMVLVDMWKKIQHSNIVTLREVFTTKAFAEPSLVFAYDFHAGGETMMSRHFNDPNADAYFTKRKWGQHEGPLPRQHAGLLPESLIWAYIVQLSSALRTIHTAGLACRVMDPTKILITGKTRLRVNCVGVFDVLTFDNSQNNNPLALMAQYQQADLISLGKVVLALACNSLAGIQRENLQKAMELVTINYSSDLKNLILYLLTDQNRMRSVNDIMPMIGARFYTQLDAAQMRNDVIEEDLAKEVQNGRLFRLLAKLGTINERPEFQKDPTWSETGDRYLLKLFRDHLFHQVTEAGAPWIDLSHIISCLNKLDAGVPEKISLISRDEKSVLVVTYSDLKRCFENTFQELIAAANGQL

1214 NP_001871.2 NP_002717.3

>NP_001871.2

MKFKLHVNSARQYKDLWNMSDDKPFLCTAPGCGQRFTNEDHLAVHKHKHEMTLKFGPARNDSVIVADQTPTPTRFLKNCEEVGLFNELASPFENEFKKASEDDIKKMPLDLSPLATPIIRSKIEEPSVVETTHQDSPLPHPESTTSDEKEVPLAQTAQPTSAIVRPASLQVPNVLLTSSDSSVIIQQAVPSPTSSTVITQAPSSNRPIVPVPGPFPLLLHLPNGQTMPVAIPASITSSNVHVPAAVPLVRPVTMVPSVPGIPGPSSPQPVQSEAKMRLKAALTQQHPPVTNGDTVKGHGSGLVRTQSEESRPQSLQQPATSTTETPASPAHTTPQTQSTSGRRRRAANEDPDEKRRKFLERNRAAASRCRQKRKVWVQSLEKKAEDLSSLNGQLQSEVTLLRNEVAQLKQLLLAHKDCPVTAMQKKSGYHTADKDDSSEDISVPSSPHTEAIQHSSVSTSNGVSSTSKAEAVATSVLTQMADQSTEPALSQIVMAPSSQSQPSGS

>NP_002717.3

MLSLQYPDVYRDETAVQDYHGHKICDPYAWLEDPDSEQTKAFVEAQNKITVPFLEQCPIRGLYKERMTELYDYPKYSCHFKKGKRYFYFYNTGLQNQRVLYVQDSLEGEARVFLDPNILSDDGTVALRGYAFSEDGEYFAYGLSASGSDWVTIKFMKVDGAKELPDVLERVKFSCMAWTHDGKGMFYNSYPQQDGKSDGTETSTNLHQKLYYHVLGTDQSEDILCAEFPDEPKWMGGAELSDDGRYVLLSIREGCDPVNRLWYCDLQQESSGIAGILKWVKLIDNFEGEYDYVTNEGTVFTFKTNRQSPNYRVINIDFRDPEESKWKVLVPEHEKDVLEWIACVRSNFLVLCYLHDVKNILQLHDLTTGALLKTFPLDVGSIVGYSGQKKDTEIFYQFTSFLSPGIIYHCDLTKEELEPRVFREVTVKGIDASDYQTVQIFYPSKDGTKIPMFIVHKKGIKLDGSHPAFLYGYGGFNISITPNYSVSRLIFVRHMGGILAVANIRGGGEYGETWHKGGILANKQNCFDDFQCAAEYLIKEGYTSPKRLTINGGSNGGLLVAACANQRPDLFGCVIAQVGVMDMLKFHKYTIGHAWTTDYGCSDSKQHFEWLVKYSPLHNVKLPEADDIQYPSMLLLTADHDDRVVPLHSLKFIATLQYIVGRSRKQSNPLLIHVDTKAGHGAGKPTAKVIEEVSDMFAFIARCLNVDWIP

1215 P35548 NP_008830.2

>P35548

MASPSKGNDLFSPDEEGPAVVAGPGPGPGGAEGAAEERRVKVSSLPFSVEALMSDKKPPKEASPLPAESASAGATLRPLLLSGHGAREAHSPGPLVKPFETASVKSENSEDGAAWMQEPGRYSPPPRHTSPTTCTLRKHKTNRKPRTPFTTSQLLALERKFRQKQYLSIAERAEFSSSLNLTETQVKIWFQNRRAKAKRLQEAELEKLKMAAKPMLPSSFSLPFPISSPLQAASIYGASYPFHRPVLPIPPVGLYATPVGYGMYHLS

>NP_008830.2

MAALSGVRWLTRALVSAGNPGAWRGLSTSAAAHAASRSQAEDVRVEGSFPVTMLPGDGVGPELMHAVKEVFKAAAVPVEFQEHHLSEVQNMASEEKLEQVLSSMKENKVAIIGKIHTPMEYKGELASYDMRLRRKLDLFANVVHVKSLPGYMTRHNNLDLVIIREQTEGEYSSLEHESARGVIECLKIVTRAKSQRIAKFAFDYATKKGRGKVTAVHKANIMKLGDGLFLQCCEEVAELYPKIKFETMIIDNCCMQLVQNPYQFDVLVMPNLYGNIIDNLAAGLVGGAGVVPGESYSAEYAVFETGARHPFAQAVGRNIANPTAMLLSASNMLRHLNLEYHSSMIADAVKKVIKVGKVRTRDMGGYSTTTDFIKSVIGHLQTKGS

1216 NP_005504.2 NP_000340.2

>NP_005504.2

MADPDVLTEVPAALKRLAKYVIRGFYGIEHALALDILIRNSCVKEEDMLELLKFDRKQLRSVLNNLKGDKFIKCRMRVETAADGKTTRHNYYFINYRTLVNVVKYKLDHMRRRIETDERDSTNRASFKCPVCSSTFTDLEANQLFDPMTGTFRCTFCHTEVEEDESAMPKKDARTLLARFNEQIEPIYALLRETEDVNLAYEILEPEPTEIPALKQSKDHAATTAGAASLAGGHHREAWATKGPSYEDLYTQNVVINMDDQEDLHRASLEGKSAKERPIWLRESTVQGAYGSEDMKEGGIDMDAFQEREEGHAGPDDNEEVMRALLIHEKKTSSAMAGSVGAAAPVTAANGSDSESETSESDDDSPPRPAAVAVHKREEDEEEDDEFEEVADDPIVMVAGRPFSYSEVSQRPELVAQMTPEEKEAYIAMGQRMFEDLFE

>NP_000340.2

MLLATFKLCAGSSYRHMRNMKGLRQQAVMAISQELNRRALGGPTPSTWINQVRRRSSLLGSRLEETLYSDQELAYLQQGEEAMQKALGILSNQEGWKKESQQDNGDKVMSKVVPDVGKVFRLEVVVDQPMERLYEELVERMEAMGEWNPNVKEIKVLQKIGKDTFITHELAAEAAGNLVGPRDFVSVRCAKRRGSTCVLAGMATDFGNMPEQKGVIRAEHGPTCMVLHPLAGSPSKTKLTWLLSIDLKGWLPKSIINQVLSQTQVDFANHLRKRLESHPASEARC

1217 Q96KF2 NP_005195.2

>Q96KF2

MLCAHFSDQGPAHLTTSKSAFLSNKKTSTLKHLLGETRSDGSACNSGISGGRGRKIP

>NP_005195.2

MEYMSTGSDNKEEIDLLIKHLNVSDVIDIMENLYASEEPAVYEPSLMTMCQDSNQNDERSKSLLLSGQEVPWLSSVRYGTVEDLLAFANHISNTAKHFYGQRPQESGILLNMVITPQNGRYQIDSDVLLIPWKLTYRNIGSDFIPRGAFGKVYLAQDIKTKKRMACKLIPVDQFKPSDVEIQACFRHENIAELYGAVLWGETVHLFMEAGEGGSVLEKLESCGPMREFEIIWVTKHVLKGLDFLHSKKVIHHDIKPSNIVFMSTKAVLVDFGLSVQMTEDVYFPKDLRGTEIYMSPEVILCRGHSTKADIYSLGATLIHMQTGTPPWVKRYPRSAYPSYLYIIHKQAPPLEDIADDCSPGMRELIEASLERNPNHRPRAADLLKHEALNPPREDQPRCQSLDSALLERKRLLSRKELELPENIADSSCTGSTEESEMLKRQRSLYIDLGALAGYFNLVRGPPTLEYG

1218 NP_008999.2 NP_149075.2

>NP_008999.2

MKEDQVVAEEPGFQDEEESLFQDIDLLQKHGINVADIKKLKSVGICTIKGIQMTTRRALCNVKGLSEAKVDKIKEAANKLIEPGFLTAFEYSEKRKMVFHITTGSQEFDKLLGGGIESMAITEAFGEFRTGKTQLSHTLCVTAQLPGAGGYPGGKIIFIDTENTFRPDRLRDIADRFNVDHDAVLDNVLYARAYTSEHQMELLDYVAAKFHEEAGIFKLLIIDSIMALFRVDFSGRGELAERQQKLAQMLSRLQKISEEYNVAVFVTNQMTADPGATMTFQADPKKPIGGHILAHASTTRISLRKGRGELRIAKIYDSPEMPENEATFAITAGGIGDAKE

>NP_149075.2

MVSKRRLSKSEDKESLTEDASKTRKQPLSKKTKKSHIANEVEENDSIFVKLLKISGIILKTGESQNQLAVDQIAFQKKLFQTLRRHPSYPKIIEEFVSGLESYIEDEDSFRNCLLSCERLQDEEASMGASYSKSLIKLLLGIDILQPAIIKTLFEKLPEYFFENKNSDEINIPRLIVSQLKWLDRVVDGKDLTTKIMQLISIAPENLQHDIITSLPEILGDSQHADVGKELSDLLIENTSLTVPILDVLSSLRLDPNFLLKVRQLVMDKLSSIRLEDLPVIIKFILHSVTAMDTLEVISELREKLDLQHCVLPSRLQASQVKLKSKGRASSSGNQESSGQSCIILLFDVIKSAIRYEKTISEAWIKAIENTASVSEHKVFDLVMLFIIYSTNTQTKKYIDRVLRNKIRSGCIQEQLLQSTFSVHYLVLKDMCSSILSLAQSLLHSLDQSIISFGSLLYKYAFKFFDTYCQQEVVGALVTHICSGNEAEVDTALDVLLELVVLNPSAMMMNAVFVKGILDYLDNISPQQIRKLFYVLSTLAFSKQNEASSHIQDDMHLVIRKQLSSTVFKYKLIGIIGAVTMAGIMAADRSESPSLTQERANLSDEQCTQVTSLLQLVHSCSEQSPQASALYYDEFANLIQHEKLDPKALEWVGHTICNDFQDAFVVDSCVVPEGDFPFPVKALYGLEEYDTQDGIAINLLPLLFSQDFAKDGGPVTSQESGQKLVSPLCLAPYFRLLRLCVERQHNGNLEEIDGLLDCPIFLTDLEPGEKLESMSAKERSFMCSLIFLTLNWFREIVNAFCQETSPEMKGKVLTRLKHIVELQIILEKYLAVTPDYVPPLGNFDVETLDITPHTVTAISAKIRKKGKIERKQKTDGSKTSSSDTLSEEKNSECDPTPSHRGQLNKEFTGKEEKTSLLLHNSHAFFRELDIEVFSILHCGLVTKFILDTEMHTEATEVVQLGPPELLFLLEDLSQKLESMLTPPIARRVPFLKNKGSRNIGFSHLQQRSAQEIVHCVFQLLTPMCNHLENIHNYFQCLAAENHGVVDGPGVKVQEYHIMSSCYQRLLQIFHGLFAWSGFSQPENQNLLYSALHVLSSRLKQGEHSQPLEELLSQSVHYLQNFHQSIPSFQCALYLIRLLMVILEKSTASAQNKEKIASLARQFLCRVWPSGDKEKSNISNDQLHALLCIYLEHTESILKAIEEIAGVGVPELINSPKDASSSTFPTLTRHTFVVFFRVMMAELEKTVKKIEPGTAADSQQIHEEKLLYWNMAVRDFSILINLIKVFDSHPVLHVCLKYGRLFVEAFLKQCMPLLDFSFRKHREDVLSLLETFQLDTRLLHHLCGHSKIHQDTRLTQHVPLLKKTLELLVCRVKAMLTLNNCREAFWLGNLKNRDLQGEEIKSQNSQESTADESEDDMSSQASKSKATEVSLQNPPESGTDGCILLIVLSWWSRTLPTYVYCQMLLCPFPFPP

1219 NP_005245.2 NP_004865.1

>NP_005245.2

MSLVDLGKKLLEAARAGQDDEVRILMANGAPFTTDWLGTSPLHLAAQYGHYSTTEVLLRAGVSRDARTKVDRTPLHMAASEGHASIVEVLLKHGADVNAKDMLKMTALHWATEHNHQEVVELLIKYGADVHTQSKFCKTAFDISIDNGNEDLAEILQIAMQNQINTNPESPDTVTIHAATPQFIIGPGGVVNLTGLVSSENSSKATDETGVSAVQFGNSSTSVLATLAALAEASAPLSNSSETPVVATEEVVTAESVDGAIQQVVSSGGQQVITIVTDGIQLGNLHSIPTSGIGQPIIVTMPDGQQVLTVPATDIAEETVISEEPPAKRQCIEIIENRVESAEIEEREALQKQLDEANREAQKYRQQLLKKEQEAEAYRQKLEAMTRLQTNKEAV

>NP_004865.1

MSALRRSGYGPSDGPSYGRYYGPGGGDVPVHPPPPLYPLRPEPPQPPISWRVRGGGPAETTWLGEGGGGDGYYPSGGAWPEPGRAGGSHQEQPPYPSYNSNYWNSTARSRAPYPSTYPVRPELQGQSLNSYTNGAYGPTYPPGPGANTASYSGAYYAPGYTQTSYSTEVPSTYRSSGNSPTPVSRWIYPQQDCQTEAPPLRGQVPGYPPSQNPGMTLPHYPYGDGNRSVPQSGPTVRPQEDAWASPGAYGMGGRYPWPSSAPSAPPGNLYMTESTSPWPSSGSPQSPPSPPVQQPKDSSYPYSQSDQSMNRHNFPCSVHQYESSGTVNNDDSDLLDSQVQYSAEPQLYGNATSDHPNNQDQSSSLPEECVPSDESTPPSIKKIIHVLEKVQYLEQEVEEFVGKKTDKAYWLLEEMLTKELLELDSVETGGQDSVRQARKEAVCKIQAILEKLEKKGL

1220 NP_001444.2 Q9P278

>NP_001444.2

MQARYSVSSPNSLGVVPYLGGEQSYYRAAAAAAGGGYTAMPAPMSVYSHPAHAEQYPGGMARAYGPYTPQPQPKDMVKPPYSYIALITMAIQNAPDKKITLNGIYQFIMDRFPFYRDNKQGWQNSIRHNLSLNECFVKVPRDDKKPGKGSYWTLDPDSYNMFENGSFLRRRRRFKKKDAVKDKEEKDRLHLKEPPPPGRQPPPAPPEQADGNAPGPQPPPVRIQDIKTENGTCPSPPQPLSPAAALGSGSAAAVPKIESPDSSSSSLSSGSSPPGSLPSARPLSLDGADSAPPPPAPSAPPPHHSQGFSVDNIMTSLRGSPQSAAAELSSGLLASAAASSRAGIAPPLALGAYSPGQSSLYSSPCSQTSSAGSSGGGGGGAGAAGGAGGAGTYHCNLQAMSLYAAGERGGHLQGAPGGAGGSAVDDPLPDYSLPPVTSSSSSSLSHGGGGGGGGGGQEAGHHPAAHQGRLTSWYLNQAGGDLGHLASAAAAAAAAGYPGQQQNFHSVREMFESQRIGLNNSPVNGNSSCQMAFPSSQSLYRTSGAFVYDCSKF

>Q9P278

MAPTLLQKLFNKRGSSGSSAAASAQGRAPKEGPAFSWSCSEFDLNEIRLIVYQDCDRRGRQVLFDSKAVQKIEEVTAQKTEDVPIKISAKCCQGSSSVSSSSSSSISSHSSSGGSSHHAKEQLPKYQYTRPASDVNMLGEMMFGSVAMSYKGSTLKIHYIRSPPQLMISKVFSARMGSFCGSTNNLQDSFEYINQDPNLGKLNTNQNSLGPCRTGSNLAHSTPVDMPSRGQNEDRDSGIARSASLSSLLITPFPSPSSSTSSSSSYQRRWLRSQTTSLENGIIPRRSTDETFSLAEETCSSNPAMVRRKKIAISIIFSLCEKEEAQRNFQDFFFSHFPLFESHMNRLKSAIEKAMISCRKIAESSLRVQFYVSRLMEALGEFRGTIWNLYSVPRIAEPVWLTMMSGTLEKNQLCQRFLKEFTLLIEQINKNQFFAALLTAVLTYHLAWVPTVMPVDHPPIKAFSEKRTSQSVNMLAKTHPYNPLWAQLGDLYGAIGSPVRLTRTVVVGKQKDLVQRILYVLTYFLRCSELQENQLTWSGNHGEGDQVLNGSKIITALEKGEVEESEYVVITVRNEPALVPPILPPTAAERHNPWPTGFPECPEGTDSRDLGLKPDKEANRRPEQGSEACSAGCLGPASDASWKPQNAFCGDEKNKEAPQDGSSRLPSCEVLGAGMKMDQQAVCELLKVEMPTRLPDRSVAWPCPDRHLREKPSLEKVTFQIGSFASPESDFESRMKKMEERVKACGPSLEASEAADVAQDPQVSRSPFKPGFQENVCCPQNRLSEGDEGESDKGFAEDRGSRNDMAADIAGQLSHAADLGTASHGAGGTGGRRLEATRGLYVKAAEGPVLEPVAPRCVQRGPGLVAGANIPCGDDNKKANFRTEGDIPRNESSDSALGDSDDEACASAMLDLGHGGDRTGGSLEVELPLPRSQSISTQNVRNFGRSLLAGYCPTYMPDLVLHGTGSDEKLKQCLVADLVHTVHHPVLDEPIAEAVCIIADTDKWSVQVATSQRKVTDNMKLGQDVLVSSQVSSLLQSILQLYKLHLPADFCIMHLEDRLQEMYLKSKMLSEYLRGHTRVHVKELGVVLGIESNDLPLLTAIASTHSPYVAQILL

1221 Q8IZL9 P42679

>Q8IZL9

MDQYCILGRIGEGAHGIVFKAKHVETGEIVALKKVALRRLEDGFPNQALREIKALQEMEDNQYVVQLKAVFPHGGGFVLAFEFMLSDLAEVVRHAQRPLAQAQVKSYLQMLLKGVAFCHANNIVHRDLKPANLLISASGQLKIADFGLARVFSPDGSRLYTHQVATRWYRAPELLYGARQYDQGVDLWSVGCIMGELLNGSPLFPGKNDIEQLCYVLRILGTPNPQVWPELTELPDYNKISFKEQVPMPLEEVLPDVSPQALDLLGQFLLYPPHQRIAASKALLHQYFFTAPLPAHPSELPIPQRLGGPAPKAHPGPPHIHDFHVDRPLEESLLNPELIRPFILEG

>P42679

MAGRGSLVSWRAFHGCDSAEELPRVSPRFLRAWHPPPVSARMPTRRWAPGTQCITKCEHTRPKPGELAFRKGDVVTILEACENKSWYRVKHHTSGQEGLLAAGALREREALSADPKLSLMPWFHGKISGQEAVQQLQPPEDGLFLVRESARHPGDYVLCVSFGRDVIHYRVLHRDGHLTIDEAVFFCNLMDMVEHYSKDKGAICTKLVRPKRKHGTKSAEEELARAGWLLNLQHLTLGAQIGEGEFGAVLQGEYLGQKVAVKNIKCDVTAQAFLDETAVMTKMQHENLVRLLGVILHQGLYIVMEHVSKGNLVNFLRTRGRALVNTAQLLQFSLHVAEGMEYLESKKLVHRDLAARNILVSEDLVAKVSDFGLAKAERKGLDSSRLPVKWTAPEALKHGKFTSKSDVWSFGVLLWEVFSYGRAPYPKMSLKEVSEAVEKGYRMEPPEGCPGPVHVLMSSCWEAEPARRPPFRKLAEKLARELRSAGAPASVSGQDADGSTSPRSQEP

1222 Q8NCN2 NP_003840.2

>Q8NCN2

MSVEMDSSSFIQFDVPEYSSTVLSQLNELRLQGKLCDIIVHIQGQPFRAHKAVLAASSPYFRDHSALSTMSGLSISVIKNPNVFEQLLSFCYTGRMSLQLKDVVSFLTAASFLQMQCVIDKCTQILESIHSKISVGDVDSVTVGAEENPESRNGVKDSSFFANPVEISPPYCSQGRQPTASSDLRMETTPSKALRSRLQEEGHSDRGSSGSVSEYEIQIEGDHEQGDLLVRESQITEVKVKMEKSDRPSCSDSSSLGDDGYHTEMVDGEQVVAVNVGSYGSVLQHAYSYSQAASQPTNVSEAFGSLSNSSPSRSMLSCFRGGRARQKRALSVHLHSDLQGLVQGSDSEAMMNNPGYESSPRERSARGHWYPYNERLICIYCGKSFNQKGSLDRHMRLHMGITPFVCKFCGKKYTRKDQLEYHIRGHTDDKPFRCEICGKCFPFQGTLNQHLRKNHPGVAEVRSRIESPERTDVYVEQKLENDASASEMGLDSRMEIHTVSDAPD

>NP_003840.2

MTATLAAAADIATMVSGSSGLAAARLLSRSFLLPQNGIRHCSYTASRQHLYVDKNTKIICQGFTGKQGTFHSQQALEYGTKLVGGTTPGKGGQTHLGLPVFNTVKEAKEQTGATASVIYVPPPFAAAAINEAIEAEIPLVVCITEGIPQQDMVRVKHKLLRQEKTRLIGPNCPGVINPGECKIGIMPGHIHKKGRIGIVSRSGTLTYEAVHQTTQVGLGQSLCVGIGGDPFNGTDFIDCLEIFLNDSATEGIILIGEIGGNAEENAAEFLKQHNSGPNSKPVVSFIAGLTAPPGRRMGHAGAIIAGGKGGAKEKISALQSAGVVVSMSPAQLGTTIYKEFEKRKML

1223 P43699 Q9P0R6

>P43699

MSMSPKHTTPFSVSDILSPLEESYKKVGMEGGGLGAPLAAYRQGQAAPPTAAMQQHAVGHHGAVTAAYHMTAAGVPQLSHSAVGGYCNGNLGNMSELPPYQDTMRNSASGPGWYGANPDPRFPAISRFMGPASGMNMSGMGGLGSLGDVSKNMAPLPSAPRRKRRVLFSQAQVYELERRFKQQKYLSAPEREHLASMIHLTPTQVKIWFQNHRYKMKRQAKDKAAQQQLQQDSGGGGGGGGTGCPQQQQAQQQSPRRVAVPVLVKDGKPCQAGAPAPGAASLQGHAQQQAQHQAQAAQAAAAAISVGSGGAGLGAHPGHQPGSAGQSPDLAHHAASPAALQGQVSSLSHLNSSGSDYGTMSCSTLLYGRTW

>Q9P0R6

METDCNPMELSSMSGFEEGSELNGFEGTDMKDMRLEAEAVVNDVLFAVNNMFVSKSLRCADDVAYINVETKERNRYCLELTEAGLKVVGYAFDQVDDHLQTPYHETVYSLLDTLSPAYREAFGNALLQRLEALKRDGQS

1224 Q9BS31 NP_037399.2

>Q9BS31

MTKAQESLTLEDVAVDFTWEEWQFLSPAQKDLYRDVMLENYSNLVSVGYQAGKPDALTKLEQGEPLWTLEDEIHSPAHPEIEKADDHLQQPLQNQKILKRTGQRYEHGRTLKSYLGLTNQSRRYNRKEPAEFNGDGAFLHDNHEQMPTEIEFPESRKPISTKSQFLKHQQTHNIEKAHECTDCGKAFLKKSQLTEHKRIHTGKKPHVCSLCGKAFYKKYRLTEHERAHRGEKPHGCSLCGKAFYKRYRLTEHERAHKGEKPYGCSECGKAFPRKSELTEHQRIHTGIKPHQCSECGRAFSRKSLLVVHQRTHTGEKPHTCSECGKGFIQKGNLNIHQRTHTGEKPYGCIDCGKAFSQKSCLVAHQRYHTGKTPFVCPECGQPCSQKSGLIRHQKIHSGEKPYKCSDCGKAFLTKTMLIVHHRTHTGERPYGCDECEKAYFYMSCLVKHKRIHSREKRGDSVKVENPSTASHSLSPSEHVQGKSPVNMVTVAMVAGQCEFAHILHS

>NP_037399.2

MRSMKALQKALSRAGSHCGRGGWGHPSRSPLLGGGVRHHLSEAAAQGRETPHSHQPQHQDHDSSESGMLSRLGDLLFYTIAEGQERIPIHKFTTALKATGLQTSDPRLRDCMSEMHRVVQESSSGGLLDRDLFRKCVSSNIVLLTQAFRKKFVIPDFEEFTGHVDRIFEDVKELTGGKVAAYIPQLAKSNPDLWGVSLCTVDGQRHSVGHTKIPFCLQSCVKPLTYAISISTLGTDYVHKFVGKEPSGLRYNKLSLNEEGIPHNPMVNAGAIVVSSLIKMDCNKAEKFDFVLQYLNKMAGNEYMGFSNATFQSEKETGDRNYAIGYYLKEKKCFPKGVDMMAALDLYFQLCSVEVTCESGSVMAATLANGGICPITGESVLSAEAVRNTLSLMHSCGMYDFSGQFAFHVGLPAKSAVSGAILLVVPNVMGMMCLSPPLDKLGNSHRGTSFCQKLVSLFNFHNYDNLRHCARKLDPRREGAEIRNKTVVNLLFAAYSGDVSALRRFALSAMDMEQKDYDSRTALHVAAAEGHIEVVKFLIEACKVNPFAKDRWGNIPLDDAVQFNHLEVVKLLQDYQDSYTLSETQAEAAAEALSKENLESMV

1225 Q13461 NP_005557.1

>Q13461

MAGRSDMDPPAAFSGFPALPAVAPSGPPPSPLAGAEPGREPEEAAAGRGEAAPTPAPGPGRRRRRPLQRGKPPYSYIALIAMALAHAPGRRLTLAAIYRFITERFAFYRDSPRKWQNSIRHNLTLNDCFVKVPREPGNPGKGNYWTLDPAAADMFDNGSFLRRRKRFKRAELPAHAAAAPGPPLPFPYAPYAPAPGPALLVPPPSAGPGPSPPARLFSVDSLVNLQPELAGLGAPEPPCCAAPDAAAAAFPPCAAAASPPLYSQVPDRLVLPATRPGPGPLPAEPLLALAGPAAALGPLSPGEAYLRQPGFASGLERYL

>NP_005557.1

MATLKDQLIYNLLKEEQTPQNKITVVGVGAVGMACAISILMKDLADELALVDVIEDKLKGEMMDLQHGSLFLRTPKIVSGKDYNVTANSKLVIITAGARQQEGESRLNLVQRNVNIFKFIIPNVVKYSPNCKLLIVSNPVDILTYVAWKISGFPKNRVIGSGCNLDSARFRYLMGERLGVHPLSCHGWVLGEHGDSSVPVWSGMNVAGVSLKTLHPDLGTDKDKEQWKEVHKQVVESAYEVIKLKGYTSWAIGLSVADLAESIMKNLRRVHPVSTMIKGLYGIKDDVFLSVPCILGQNGISDLVKVTLTSEEEARLKKSADTLWGIQKELQF

1226 Q96EG1 NP_001434.1

>Q96EG1

MGWLFLKVLLAGVSFSGFLYPLVDFCISGKTRGQKPNFVIILADDMGWGDLGANWAETKDTANLDKMASEGMRFVDFHAAASTCSPSRASLLTGRLGLRNGVTRNFAVTSVGGLPLNETTLAEVLQQAGYVTGIIGKWHLGHHGSYHPNFRGFDYYFGIPYSHDMGCTDTPGYNHPPCPACPQGDGPSRNLQRDCYTDVALPLYENLNIVEQPVNLSSLAQKYAEKATQFIQRASTSGRPFLLYVALAHMHVPLPVTQLPAAPRGRSLYGAGLWEMDSLVGQIKDKVDHTVKENTFLWFTGDNGPWAQKCELAGSVGPFTGFWQTRQGGSPAKQTTWEGGHRVPALAYWPGRVPVNVTSTALLSVLDIFPTVVALAQASLPQGRRFDGVDVSEVLFGRSQPGHRVLFHPNSGAAGEFGALQTVRLERYKAFYITGGARACDGSTGPELQHKFPLIFNLEDDTAEAVPLERGGAEYQAVLPEVRKVLADVLQDIANDNISSADYTQDPSVTPCCNPYQIACRCQAA

>NP_001434.1

MSFSGKYQLQSQENFEAFMKAIGLPEELIQKGKDIKGVSEIVQNGKHFKFTITAGSKVIQNEFTVGEECELETMTGEKVKTVVQLEGDNKLVTTFKNIKSVTELNGDIITNTMTLGDIVFKRISKRI

1227 NP_054885.1 P55210

>NP_054885.1

MAASSSGEKEKERLGGGLGVAGGNSTRERLLSALEDLEVLSRELIEMLAISRNQKLLQAGEENQVLELLIHRDGEFQELMKLALNQGKIHHEMQVLEKEVEKRDSDIQQLQKQLKEAEQILATAVYQAKEKLKSIEKARKGAISSEEIIKYAHRISASNAVCAPLTWVPGDPRRPYPTDLEMRSGLLGQMNNPSTNGVNGHLPGDALAAGRLPDVLAPQYPWQSNDMSMNMLPPNHSSDFLLEPPGHNKENEDDVEIMSTDSSSSSSESD

>P55210

MADDQGCIEEQGVEDSANEDSVDAKPDRSSFVPSLFSKKKKNVTMRSIKTTRDRVPTYQYNMNFEKLGKCIIINNKNFDKVTGMGVRNGTDKDAEALFKCFRSLGFDVIVYNDCSCAKMQDLLKKASEEDHTNAACFACILLSHGEENVIYGKDGVTPIKDLTAHFRGDRCKTLLEKPKLFFIQACRGTELDDGIQADSGPINDTDANPRYKIPVEADFLFAYSTVPGYYSWRSPGRGSWFVQALCSILEEHGKDLEIMQILTRVNDRVARHFESQSDDPHFHEKKQIPCVVSMLTKELYFSQ

1228 AAG28389.1 Q08495

>AAG28389.1

MSTEGGFGGTSSSDAQQSLQSFWPRVMEEIRNLTVKDFRVQELPLARIKKIMKLDEDVKMISAEAPVLFAKAAQIFITELTLRAWIHTEDNKRRTLQRNDIAMAITKFDQFDFLIDIVPRDELKPPKRQEEVRQSVTPAEPVQYYFTLAQQPTAVQVQGQQQGQQTTSSTTTIQPGQIIIAQPQQGQTTPVTMQVGEGQQVQIVQAQPQGQAQQAQSGTGQTMQVMQQIITNTGEIQQIPVQLNAGQLQYIRLAQPVSGTQVVQGQIQTLATNAQQGQRNASQGKPRRCLKETLQITQTEVQQGQQQFSQFTDGQRNSVQQARVSELTGEAEPREVKATGNSTPCTSSLPTTHPPSHRAGASCVCCSQPQQSSTSPPPSDALQWVVVEVSGTPNQLETHRELHAPLPGMTSLSPLHPSQQLYQIQQVTMPAGQDLAQPMFIQSANQPSDGQAPQVTGD

>Q08495

MERLQKQPLTSPGSVSPSRDSSVPGSPSSIVAKMDNQVLGYKDLAAIPKDKAILDIERPDLMIYEPHFTYSLLEHVELPRSRERSLSPKSTSPPPSPEVWADSRSPGIISQASAPRTTGTPRTSLPHFHHPETSRPDSNIYKKPPIYKQRESVGGSPQTKHLIEDLIIESSKFPAAQPPDPNQPAKIETDYWPCPPSLAVVETEWRKRKASRRGAEEEEEEEDDDSGEEMKALRERQREELSKVTSNLGKMILKEEMEKSLPIRRKTRSLPDRTPFHTSLHQGTSKSSSLPAYGRTTLSRLQSTEFSPSGSETGSPGLQNGEGQRGRMDRGNSLPCVLEQKIYPYEMLVVTNKGRTKLPPGVDRMRLERHLSAEDFSRVFAMSPEEFGKLALWKRNELKKKASLF

1229 NP_109377.1 Q12815

>NP_109377.1

MQKATYYDSSAIYGGYPYQAANGFAYNANQQPYPASAALGADGEYHRPACSLQSPSSAGGHPKAHELSEACLRTLSAPPSQPPSLGEPPLHPPPPQAAPPAPQPPQPAPQPPAPTPAAPPPPSSASPPQNASNNPTPANAAKSPLLNSPTVAKQIFPWMKESRQNTKQKTSSSSSGESCAGDKSPPGQASSKRARTAYTSAQLVELEKEFHFNRYLCRPRRVEMANLLNLTERQIKIWFQNRRMKYKKDQKGKGMLTSSGGQSPSRSPVPPGAGGYLNSMHSLVNSVPYEPQSPPPFSKPPQGTYGLPPASYPASLPSCAPPPPPQKRYTAAGAGAGGTPDYDPHAHGLQGNGSYGTPHIQGSPVFVGGSYVEPMSNSGPALFGLTHLPHAASGAMDYGGAGPLGSGHHHGPGPGEPHPTYTDLTGHHPSQGRIQEAPKLTHL

>Q12815

MTTRQATKDPLLRGVSPTPSKIPVRSQKRTPFPTVTSCAVDQENQDPRRWVQKPPLNIQRPLVDSAGPRPKARHQAETSQRLVGISQPRNPLEELRPSPRGQNVGPGPPAQTEAPGTIEFVADPAALATILSGEGVKSCHLGRQPSLAKRVLVRGSQGGTTQRVQGVRASAYLAPRTPTHRLDPARASCFSRLEGPGPRGRTLCPQRLQALISPSGPSFHPSTRPSFQELRRETAGSSRTSVSQASGLLLETPVQPAFSLPKGEREVVTHSDEGGVASLGLAQRVPLRENREMSHTRDSHDSHLMPSPAPVAQPLPGHVVPCPSPFGRAQRVPSPGPPTLTSYSVLRRLTVQPKTRFTPMPSTPRVQQAQWLRGVSPQSCSEDPALPWEQVAVRLFDQESCIRSLEGSGKPPVATPSGPHSNRTPSLQEVKIQRIGILQQLLRQEVEGLVGGQCVPLNGGSSLDMVELQPLLTEISRTLNATEHNSGTSHLPGLLKHSGLPKPCLPEECGEPQPCPPAEPGPPEAFCRSEPEIPEPSLQEQLEVPEPYPPAEPRPLESCCRSEPEIPESSRQEQLEVPEPCPPAEPRPLESYCRIEPEIPESSRQEQLEVPEPCPPAEPGPLQPSTQGQSGPPGPCPRVELGASEPCTLEHRSLESSLPPCCSQWAPATTSLIFSSQHPLCASPPICSLQSLRPPAGQAGLSNLAPRTLALRERLKSCLTAIHCFHEARLDDECAFYTSRAPPSGPTRVCTNPVATLLEWQDALCFIPVGSAAPQGSP

1230 NP_005625.2 P82909

>NP_005625.2

MRPVRENSSGARSPRVPADLARSILISLPFPPDSLAHRPPSSAPTESQGLFTVAAPAPGAPSPPATLAHLLPAPAMYSLLETELKNPVGTPTQAAGTGGPAAPGGAGKSSANAAGGANSGGGSSGGASGGGGGTDQDRVKRPMNAFMVWSRGQRRKMALENPKMHNSEISKRLGADWKLLTDAEKRPFIDEAKRLRAVHMKEYPDYKYRPRRKTKTLLKKDKYSLPSGLLPPGAAAAAAAAAAAAAAASSPVGVGQRLDTYTHVNGWANGAYSLVQEQLGYAQPPSMSSPPPPPALPPMHRYDMAGLQYSPMMPPGAQSYMNVAAAAAAASGYGGMAPSATAAAAAAYGQQPATAAAAAAAAAAMSLGPMGSVVKSEPSSPPPAIASHSQRACLGDLRDMISMYLPPGGDAADAASPLPGGRLHGVHQHYQGAGTAVNGTVPLTHI

>P82909

MMGSKMASASRVVQVVKPHTPLIRFPDRRDNPKPNVSEALRSAGLPSHSSVISQHSKGSKSPDLLMYQGPPDTAEIIKTLPQKYRRKLVSQEEMEFIQRGGPE

1231 NP_937872.1 NP_004790.2

>NP_937872.1

MFRAGEASKRPLPGPSPPRVRSVEVARGRAGYGFTLSGQAPCVLSCVMRGSPADFVGLRAGDQILAVNEINVKKASHEDVVKLIGKCSGVLHMVIAEGVGRFESCSSDEEGGLYEGKGWLKPKLDSKALGINRAERVVEEMQSGGIFNMIFENPSLCASNSEPLKLKQRSLSESAATRFDVGHESINNPNPNMLSKEEISKVIHDDSVFSIGLESHDDFALDASILNVAMIVGYLGSIELPSTSSNLESDSLQAIRGCMRRLRAEQKIHSLVTMKIMHDCVQLSTDKAGVVAEYPAEKLAFSAVCPDDRRFFGLVTMQTNDDGSLAQEEEGALRTSCHVFMVDPDLFNHKIHQGIARRFGFECTADPDTNGCLEFPASSLPVLQFISVLYRDMGELIEGMRARAFLDGDADAHQNNSTSSNSDSGIGNFHQEEKSNRVLVVDLGGSSSRHGPGGSAWDGVGGRGAQPWGAPWTGPFCPDPEGSPPFEAAHQTDRFWDLNKHLGPASPVEVPPASLRSSVPPSKRGTVGAGCGFNQRWLPVHVLREWQCGHTSDQDSYTDSTDGWSSINCGTLPPPMSKIPADRYRVEGSFAQPPLNAPKREWSRKAFGMQSIFGPHRNVRKTKEDKKGSKFGRGTGLTQPSQRTSARRSFGRSKRFSITRSLDDLESATVSDGELTGADLKDCVSNNSLSSNASLPSVQSCRRLRERRVASWAVSFERLLQDPVGVRYFSDFLRKEFSEENILFWQACEYFNHVPAHDKKELSYRAREIFSKFLCSKATTPVNIDSQAQLADDVLRAPHPDMFKEQQLQIFNLMKFDSYTRFLKSPLYQECILAEVEGRALPDSQQVPSSPASKHSLGSDHSSVSTPKKLSGKSKSGRSLNEELGDEDSEKKRKGAFFSWSRTRSTGRSQKKREHGDHADDALHANGGLCRRESQGSVSSAGSLDLSEACRTLAPEKDKATKHCCIHLPDGTSCVVAVKAGFSIKDILSGLCERHGINGAAADLFLVGGDKPLVLHQDSSILESRDLRLEKRTLFRLDLVPINRSVGLKAKPTKPVTEVLRPVVARYGLDLSGLLVRLSGEKEPLDLGAPISSLDGQRVVLEEKDPSRGKASADKQKGVPVKQNTAVNSSSRNHSATGEERTLGKSNSIKIKGENGKNARDPRLSKREESIAKIGKKKYQKINLDEAEEFFELISKAQSNRADDQRGLLRKEDLVLPEFLRLPPGSTELTLPTPAAVAKGFSKRSATGNGRESASQPGEQWEPVQESSDSPSTSPGSASSPPGPPGTTPPGQKSPSGPFCTPQSPVSLAQEGTAQIWKRQSQEVEAGGIQTVEDEHVAELTLMGEGDISSPNSTLLPPPSTPQEVPGPSRPGSGTHGSRDLPVNRIIDVDLVTGSAPGRDGGIAGAQAGPGRSQASGGPPTSDLPGLGPVPGEPAKPKTSAHHATFV

>NP_004790.2

MENYFQAEAYNLDKVLDEFEQNEDETVSSTLLDTKWNKILDPPSHRLSFNPTLASVNESAVSNESQPQLKVFSLAHSAPLTTEEEDHCANGQDCNLNPEIATMWIDENAVAEDQLIKRNYSWDDQCSAVEVGEKKCGNLACLPDEKNVLVVAVMHNCDKRTLQNDLQDCNNYNSQSLMDAFSCSLDNENRQTDQFSFSINESTEKDMNSEKQMDPLNRPKTEGRSVNHLCPTSSDSLASVCSPSQLKDDGSIGRDPSMSAITSLTVDSVISSQGTDGCPAVKKQENYIPDEDLTGKISSPRTDLGSPNSFSHMSEGILMKKEPAEESTTEESLRSGLPLLLKPDMPNGSGRNNDCERCSDCLVPNEVRADENEGYEHEETLGTTEFLNMTEHFSESQDMTNWKLTKLNEMNDSQVNEEKEKFLQISQPEDTNGDSGGQCVGLADAGLDLKGTCISESEECDFSTVIDTPAANYLSNGCDSYGMQDPGVSFVPKTLPSKEDSVTEEKEIEESKSECYSNIYEQRGNEATEGSGLLLNSTGDLMKKNYLHNFCSQVPSVLGQSSPKVVASLPSISVPFGGARPKQPSNLKLQIPKPLSDHLQNDFPANSGNNTKNKNDILGKAKLGENSATNVCSPSLGNISNVDTNGEHLESYEAEISTRPCLALAPDSPDNDLRAGQFGISARKPFTTLGEVAPVWVPDSQAPNCMKCEARFTFTKRRHHCRACGKVFCASCCSLKCKLLYMDRKEARVCVICHSVLMNAQAWENMMSASSQSPNPNNPAEYCSTIPPLQQAQASGALSSPPPTVMVPVGVLKHPGAEVAQPREQRRVWFADGILPNGEVADAAKLTMNGTSSAGTLAVSHDPVKPVTTSPLPAETDICLFSGSITQVGSPVGSAMNLIPEDGLPPILISTGVKGDYAVEEKPSQISVMQQLEDGGPDPLVFVLNANLLSMVKIVNYVNRKCWCFTTKGMHAVGQSEIVILLQCLPDEKCLPKDIFNHFVQLYRDALAGNVVSNLGHSFFSQSFLGSKEHGGFLYVTSTYQSLQDLVLPTPPYLFGILIQKWETPWAKVFPIRLMLRLGAEYRLYPCPLFSVRFRKPLFGETGHTIMNLLADFRNYQYTLPVVQGLVVDMEVRKTSIKIPSNRYNEMMKAMNKSNEHVLAGGACFNEKADSHLVCVQNDDGNYQTQAISIHNQPRKVTGASFFVFSGALKSSSGYLAKSSIVEDGVMVQITAENMDSLRQALREMKDFTITCGKADAEEPQEHIHIQWVDDDKNVSKGVVSPIDGKSMETITNVKIFHGSEYKANGKVIRWTEVFFLENDDQHNCLSDPADHSRLTEHVAKAFCLALCPHLKLLKEDGMTKLGLRVTLDSDQVGYQAGSNGQPLPSQYMNDLDSALVPVIHGGACQLSEGPVVMELIFYILENIV

1232 Q96JN0 NP_002890.1

>Q96JN0

MQRMIQQFAAEYTSKNSSTQDPSQPNSTKNQSLPKASPVTTSPTAATTQNPVLSKLLMADQDSPLDLTVRKSQSEPSEQDGVLDLSTKKSPCAGSTSLSHSPGCSSTQGNGRPGRPSQYRPDGLRSGDGVPPRSLQDGTREGFGHSTSLKVPLARSLQISEELLSRNQLSTAASLGPSGLQNHGQHLILSREASWAKPHYEFNLSRMKFRGNGALSNISDLPFLAENSAFPKMALQAKQDGKKDVSHSSPVDLKIPQVRGMDLSWESRTGDQYSYSSLVMGSQTESALSKKLRAILPKQSRKSMLDAGPDSWGSDAEQSTSGQPYPTSDQEGDPGSKQPRKKRGRYRQYNSEILEEAISVVMSGKMSVSKAQSIYGIPHSTLEYKVKERLGTLKNPPKKKMKLMRSEGPDVSVKIELDPQGEAAQSANESKNE

>NP_002890.1

MPVDFTGYWKMLVNENFEEYLRALDVNVALRKIANLLKPDKEIVQDGDHMIIRTLSTFRNYIMDFQVGKEFEEDLTGIDDRKCMTTVSWDGDKLQCVQKGEKEGRGWTQWIEGDELHLEMRVEGVVCKQVFKKVQ

1233 NP_848511.1 NP_004332.2

>NP_848511.1

MERARPEPPPQPRPLRPAPPPLPVEGTSFWAAAMEPPPSSPTLSAAASATLASSCGEAVASGLQPAVRRLLQVKPEQVLLLPQPQAQNEEAAASSAQARLLQFRPDLRLLQPPTASDGATSRPELHPVQPLALHVKAKKQKLGPSLDQSVGPRGAVETGPRASRVVKLEGPGPALGYFRGDEKGKLEAEEVMRDSMQGGAGKSPAAIREGVIKTEEPERLLEDCRLGAEPASNGLVHGSAEVILAPTSGAFGPHQQDLRIPLTLHTVPPGARIQFQGAPPSELIRLTKVPLTPVPTKMQSLLEPSVKIETKDVPLTVLPSDAGIPDTPFSKDRNGHVKRPMNAFMVWARIHRPALAKANPAANNAEISVQLGLEWNKLSEEQKKPYYDEAQKIKEKHREEFPGWVYQPRPGKRKRFPLSVSNVFSGTTQNIISTNPTTVYPYRSPTYSVVIPSLQNPITHPVGETSPAIQLPTPAVQSPSPVTLFQPSVSSAAQVAVQDPSLPVYPALPPQRFTGPSQTDTHQLHSEATHTVKQPTPVSLESANRISSSASTAHARFATSTIQPPREYSSVSPCPRSAPIPQASPIPHPHVYQPPPLGHPATLFGTPPRFSFHHPYFLPGPHYFPSSTCPYSRPPFGYGNFPSSMPECLSYYEDRYPKHEGIFSTLNRDYSFRDYSSECTHSENSRSCENMNGTSYYNSHSHSGEENLNPVPQLDIGTLENVFTAPTSTPSSIQQVNVTDSDEEEEEKVLRDL

>NP_004332.2

MAALVLEDGSVLRGQPFGAAVSTAGEVVFQTGMVGYPEALTDPSYKAQILVLTYPLIGNYGIPPDEMDEFGLCKWFESSGIHVAALVVGECCPTPSHWSATRTLHEWLQQHGIPGLQGVDTRELTKKLREQGSLLGKLVQNGTEPSSLPFLDPNARPLVPEVSIKTPRVFNTGGAPRILALDCGLKYNQIRCLCQRGAEVTVVPWDHALDSQEYEGLFLSNGPGDPASYPSVVSTLSRVLSEPNPRPVFGICLGHQLLALAIGAKTYKMRYGNRGHNQPCLLVGSGRCFLTSQNHGFAVETDSLPADWAPLFTNANDGSNEGIVHNSLPFFSVQFHPEHQAGPSDMELLFDIFLETVKEATAGNPGGQTVRERLTERLCPPGIPTPGSGLPPPRKVLILGSGGLSIGQAGEFDYSGSQAIKALKEENIQTLLINPNIATVQTSQGLADKVYFLPITPHYVTQVIRNERPDGVLLTFGGQTALNCGVELTKAGVLARYGVRVLGTPVETIELTEDRRAFAARMAEIGEHVAPSEAANSLEQAQAAAERLGYPVLVRAAFALGGLGSGFASNREELSALVAPAFAHTSQVLVDKSLKGWKEIEYEVVRDAYGNCVTVCNMENLDPLGIHTGESIVVAPSQTLNDREYQLLRQTAIKVTQHLGIVGECNVQYALNPESEQYYIIEVNARLSRSSALASKATGYPLAYVAAKLALGIPLPELRNSVTGGTAAFEPSVDYCVVKIPRWDLSKFLRVSTKIGSCMKSVGEVMGIGRSFEEAFQKALRMVDENCVGFDHTVKPVSDMELETPTDKRIFVVAAALWAGYSVDRLYELTRIDRWFLHRMKRIIAHAQLLEQHRGQPLPPDLLQQAKCLGFSDKQIALAVLSTELAVRKLRQELGICPAVKQIDTVAAEWPAQTNYLYLTYWGTTHDLTFRTPHVLVLGSGVYRIGSSVEFDWCAVGCIQQLRKMGYKTIMVNYNPETVSTDYDMCDRLYFDEISFEVVMDIYELENPEGVILSMGGQLPNNMAMALHRQQCRVLGTSPEAIDSAENRFKFSRLLDTIGISQPQWRELSDLESARQFCQTVGYPCVVRPSYVLSGAAMNVAYTDGDLERFLSSAAAVSKEHPVVISKFIQEAKEIDVDAVASDGVVAAIAISEHVENAGVHSGDATLVTPPQDITAKTLERIKAIVHAVGQELQVTGPFNLQLIAKDDQLKVIECNVRVSRSFPFVSKTLGVDLVALATRVIMGEEVEPVGLMTGSGVVGVKVPQFSFSRLAGADVVLGVEMTSTGEVAGFGESRCEAYLKAMLSTGFKIPKKNILLTIGSYKNKSELLPTVRLLESLGYSLYASLGTADFYTEHGVKVTAVDWHFEEAVDGECPPQRSILEQLAEKNFELVINLSMRGAGGRRLSSFVTKGYRTRRLAADFSVPLIIDIKCTKLFVEALGQIGPAPPLKVHVDCMTSQKLVRLPGLIDVHVHLREPGGTHKEDFASGTAAALAGGITMVCAMPNTRPPIIDAPALALAQKLAEAGARCDFALFLGASSENAGTLGTVAGSAAGLKLYLNETFSELRLDSVVQWMEHFETWPSHLPIVAHAEQQTVAAVLMVAQLTQRSVHICHVARKEEILLIKAAKARGLPVTCEVAPHHLFLSHDDLERLGPGKGEVRPELGSRQDVEALWENMAVIDCFASDHAPHTLEEKCGSRPPPGFPGLETMLPLLLTAVSEGRLSLDDLLQRLHHNPRRIFHLPPQEDTYVEVDLEHEWTIPSHMPFSKAHWTPFEGQKVKGTVRRVVLRGEVAYIDGQVLVPPGYGQDVRKWPQGAVPQLPPSAPATSEMTTTPERPRRGIPGLPDGRFHLPPRIHRASDPGLPAEEPKEKSSRKVAEPELMGTPDGTCYPPPPVPRQASPQNLGTPGLLHPQTSPLLHSLVGQHILSVQQFTKDQMSHLFNVAHTLRMMVQKERSLDILKGKVMASMFYEVSTRTSSSFAAAMARLGGAVLSFSEATSSVQKGESLADSVQTMSCYADVVVLRHPQPGAVELAAKHCRRPVINAGDGVGEHPTQALLDIFTIREELGTVNGMTITMVGDLKHGRTVHSLACLLTQYRVSLRYVAPPSLRMPPTVRAFVASRGTKQEEFESIEEALPDTDVLYMTRIQKERFGSTQEYEACFGQFILTPHIMTRAKKKMVVMHPMPRVNEISVEVDSDPRAAYFRQAENGMYIRMALLATVLGRF

1234 Q9Y618 NP_004337.2

>Q9Y618

MSGSTQPVAQTWRATEPRYPPHSLSYPVQIARTHTDVGLLEYQHHSRDYASHLSPGSIIQPQRRRPSLLSEFQPGNERSQELHLRPESHSYLPELGKSEMEFIESKRPRLELLPDPLLRPSPLLATGQPAGSEDLTKDRSLTGKLEPVSPPSPPHTDPELELVPPRLSKEELIQNMDRVDREITMVEQQISKLKKKQQQLEEEAAKPPEPEKPVSPPPIESKHRSLVQIIYDENRKKAEAAHRILEGLGPQVELPLYNQPSDTRQYHENIKINQAMRKKLILYFKRRNHARKQWEQKFCQRYDQLMEAWEKKVERIENNPRRRAKESKVREYYEKQFPEIRKQRELQERMQSRVGQRGSGLSMSAARSEHEVSEIIDGLSEQENLEKQMRQLAVIPPMLYDADQQRIKFINMNGLMADPMKVYKDRQVMNMWSEQEKETFREKFMQHPKNFGLIASFLERKTVAECVLYYYLTKKNENYKSLVRRSYRRRGKSQQQQQQQQQQQQQQQQQPMPRSSQEEKDEKEKEKEAEKEEEKPEVENDKEDLLKEKTDDTSGEDNDEKEAVASKGRKTANSQGRRKGRITRSMANEANSEEAITPQQSAELASMELNESSRWTEEEMETAKKGLLEHGRNWSAIARMVGSKTVSQCKNFYFNYKKRQNLDEILQQHKLKMEKERNARRKKKKAPAAASEEAAFPPVVEDEEMEASGVSGNEEEMVEEAEALHASGNEVPRGECSGPATVNNSSDTESIPSPHTEAAKDTGQNGPKPPATLGADGPPPGPPTPPPEDIPAPTEPTPASEATGAPTPPPAPPSPSAPPPVVPKEEKEEETAAAPPVEEGEEQKPPAAEELAVDTGKAEEPVKSECTEEAEEGPAKGKDAEAAEATAEGALKAEKKEGGSGRATTAKSSGAPQDSDSSATCSADEVDEAEGGDKNRLLSPRPSLLTPTGDPRANASPQKPLDLKQLKQRAAAIPPIQVTKVHEPPREDAAPTKPAPPAPPPPQNLQPESDAPQQPGSSPRGKSRSPAPPADKEAEKPVFFPAFAAEAQKLPGDPPCWTSGLPFPVPPREVIKASPHAPDPSAFSYAPPGHPLPLGLHDTARPVLPRPPTISNPPPLISSAKHPSVLERQIGAISQGMSVQLHVPYSEHAKAPVGPVTMGLPLPMDPKKLAPFSGVKQEQLSPRGQAGPPESLGVPTAQEASVLRGTALGSVPGGSITKGIPSTRVPSDSAITYRGSITHGTPADVLYKGTITRIIGEDSPSRLDRGREDSLPKGHVIYEGKKGHVLSYEGGMSVTQCSKEDGRSSSGPPHETAAPKRTYDMMEGRVGRAISSASIEGLMGRAIPPERHSPHHLKEQHHIRGSITQGIPRSYVEAQEDYLRREAKLLKREGTPPPPPPSRDLTEAYKTQALGPLKLKPAHEGLVATVKEAGRSIHEIPREELRHTPELPLAPRPLKEGSITQGTPLKYDTGASTTGSKKHDVRSLIGSPGRTFPPVHPLDVMADARALERACYEESLKSRPGTASSSGGSIARGAPVIVPELGKPRQSPLTYEDHGAPFAGHLPRGSPVTTREPTPRLQEGSLSSSKASQDRKLTSTPREIAKSPHSTVPEHHPHPISPYEHLLRGVSGVDLYRSHIPLAFDPTSIPRGIPLDAAAAYYLPRHLAPNPTYPHLYPPYLIRGYPDTAALENRQTIINDYITSQQMHHNAATAMAQRADMLRGLSPRESSLALNYAAGPRGIIDLSQVPHLPVLVPPTPGTPATAMDRLAYLPTAPQPFSSRHSSSPLSPGGPTHLTKPTTTSSSERERDRDRERDRDREREKSILTSTTTVEHAPIWRPGTEQSSGSSGSSGGGGGSSSRPASHSHAHQHSPISPRTQDALQQRPSVLHNTGMKGIITAVEPSTPTVLRSTSTSSPVRPAATFPPATHCPLGGTLDGVYPTLMEPVLLPKEAPRVARPERPRADTGHAFLAKPPARSGLEPASSPSKGSEPRPLVPPVSGHATIARTPAKNLAPHHASPDPPAPPASASDPHREKTQSKPFSIQELELRSLGYHGSSYSPEGVEPVSPVSSPSLTHDKGLPKHLEELDKSHLEGELRPKQPGPVKLGGEAAHLPHLRPLPESQPSSSPLLQTAPGVKGHQRVVTLAQHISEVITQDYTRHHPQQLSAPLPAPLYSFPGASCPVLDLRRPPSDLYLPPPDHGAPARGSPHSEGGKRSPEPNKTSVLGGGEDGIEPVSPPEGMTEPGHSRSAVYPLLYRDGEQTEPSRMGSKSPGNTSQPPAFFSKLTESNSAMVKSKKQEINKKLNTHNRNEPEYNISQPGTEIFNMPAITGTGLMTYRSQAVQEHASTNMGLEAIIRKALMGKYDQWEESPPLSANAFNPLNASASLPAAMPITAADGRSDHTLTSPGGGGKAKVSGRPSSRKAKSPAPGLASGDRPPSVSSVHSEGDCNRRTPLTNRVWEDRPSSAGSTPFPYNPLIMRLQAGVMASPPPPGLPAGSGPLAGPHHAWDEEPKPLLCSQYETLSDSE

>NP_004337.2

MENTENSVDSKSIKNLEPKIIHGSESMDSGISLDNSYKMDYPEMGLCIIINNKNFHKSTGMTSRSGTDVDAANLRETFRNLKYEVRNKNDLTREEIVELMRDVSKEDHSKRSSFVCVLLSHGEEGIIFGTNGPVDLKKITNFFRGDRCRSLTGKPKLFIIQACRGTELDCGIETDSGVDDDMACHKIPVEADFLYAYSTAPGYYSWRNSKDGSWFIQSLCAMLKQYADKLEFMHILTRVNRKVATEFESFSFDATFHAKKQIPCIVSMLTKELYFYH

1235 Q14562 NP_001937.2

>Q14562

MAVAVAMAGALIGSEPGPAEELAKLEYLSLVSKVCTELDNHLGINDKDLAEFVISLAEKNTTFDTFKASLVKNGAEFTDSLISNLLRLIQTMRPPAKPSTSKDPVVKPKTEKEKLKELFPVLCQPDNPSVRTMLDEDDVKVAVDVLKELEALMPSAAGQEKQRDAEHRDRTKKKKRSRSRDRNRDRDRDRERNRDRDHKRRHRSRSRSRSRTRERNKVKSRYRSRSRSQSPPKDRKDRDKYGERNLDRWRDKHVDRPPPEEPTIGDIYNGKVTSIMQFGCFVQLEGLRKRWEGLVHISELRREGRVANVADVVSKGQRVKVKVLSFTGTKTSLSMKDVDQETGEDLNPNRRRNLVGETNEETSMRNPDRPTHLSLVSAPEVEDDSLERKRLTRISDPEKWEIKQMIAANVLSKEEFPDFDEETGILPKVDDEEDEDLEIELVEEEPPFLRGHTKQSMDMSPIKIVKNPDGSLSQAAMMQSALAKERRELKQAQREAEMDSIPMGLNKHWVDPLPDAEGRQIAANMRGIGMMPNDIPEWKKHAFGGNKASYGKKTQMSILEQRESLPIYKLKEQLVQAVHDNQILIVIGETGSGKTTQITQYLAEAGYTSRGKIGCTQPRRVAAMSVAKRVSEEFGCCLGQEVGYTIRFEDCTSPETVIKYMTDGMLLRECLIDPDLTQYAIIMLDEAHERTIHTDVLFGLLKKTVQKRQDMKLIVTSATLDAVKFSQYFYEAPIFTIPGRTYPVEILYTKEPETDYLDASLITVMQIHLTEPPGDILVFLTGQEEIDTACEILYERMKSLGPDVPELIILPVYSALPSEMQTRIFDPAPPGSRKVVIATNIAETSLTIDGIYYVVDPGFVKQKVYNSKTGIDQLVVTPISQAQAKQRAGRAGRTGPGKCYRLYTERAYRDEMLTTNVPEIQRTNLASTVLSLKAMGINDLLSFDFMDAPPMETLITAMEQLYTLGALDDEGLLTRLGRRMAEFPLEPMLCKMLIMSVHLGCSEEMLTIVSMLSVQNVFYRPKDKQALADQKKAKFHQTEGDHLTLLAVYNSWKNNKFSNPWCYENFIQARSLRRAQDIRKQMLGIMDRHKLDVVSCGKSTVRVQKAICSGFFRNAAKKDPQEGYRTLIDQQVVYIHPSSALFNRQPEWVVYHELVLTTKEYMREVTTIDPRWLVEFAPAFFKVSDPTKLSKQKKQQRLEPLYNRYEEPNAWRISRAFRRR

>NP_001937.2

MIDTLRPVPFASEMAISKTVAWLNEQLELGNERLLLMDCRPQELYESSHIESAINVAIPGIMLRRLQKGNLPVRALFTRGEDRDRFTRRCGTDTVVLYDESSSDWNENTGGESVLGLLLKKLKDEGCRAFYLEGGFSKFQAEFSLHCETNLDGSCSSSSPPLPVLGLGGLRISSDSSSDIESDLDRDPNSATDSDGSPLSNSQPSFPVEILPFLYLGCAKDSTNLDVLEEFGIKYILNVTPNLPNLFENAGEFKYKQIPISDHWSQNLSQFFPEAISFIDEARGKNCGVLVHCLAGISRSVTVTVAYLMQKLNLSMNDAYDIVKMKKSNISPNFNFMGQLLDFERTLGLSSPCDNRVPAQQLYFTTPSNQNVYQVDSLQST

1236 P32314 NP_004337.2

>P32314

MGPVIGMTPDKRAETPGAEKIAGLSQIYKMGSLPEAVDAARPKATLVDSESADDELTNLNWLHESTNLLTNFSLGSEGLPIVSPLYDIEGDDVPSFGPACYQNPEKKSATSKPPYSFSLLIYMAIEHSPNKCLPVKEIYSWILDHFPYFATAPTGWKNSVRHNLSLNKCFQKVERSHGKVNGKGSLWCVDPEYKPNLIQALKKQPFSSASSQNGSLSPHYLSSVIKQNQVRNLKESDIDAAAAMMLLNTSIEQGILECEKPLPLKTALQKKRSYGNAFHHPSAVRLQESDSLATSIDPKEDHNYSASSMAAQRCASRSSVSSLSSVDEVYEFIPKNSHVGSDGSEGFHSEEDTDVDYEDDPLGDSGYASQPCAKISEKGQSGKKMRKQTCQEIDEELKEAAGSLLHLAGIRTCLGSLISTAKTQNQKQRKK

>NP_004337.2

MENTENSVDSKSIKNLEPKIIHGSESMDSGISLDNSYKMDYPEMGLCIIINNKNFHKSTGMTSRSGTDVDAANLRETFRNLKYEVRNKNDLTREEIVELMRDVSKEDHSKRSSFVCVLLSHGEEGIIFGTNGPVDLKKITNFFRGDRCRSLTGKPKLFIIQACRGTELDCGIETDSGVDDDMACHKIPVEADFLYAYSTAPGYYSWRNSKDGSWFIQSLCAMLKQYADKLEFMHILTRVNRKVATEFESFSFDATFHAKKQIPCIVSMLTKELYFYH

1237 NP_003066.2 NP_000895.1

>NP_003066.2

MAVRKKDGGPNVKYYEAADTVTQFDNVRLWLGKNYKKYIQAEPPTNKSLSSLVVQLLQFQEEVFGKHVSNAPLTKLPIKCFLDFKAGGSLCHILAAAYKFKSDQGWRRYDFQNPSRMDRNVEMFMTIEKSLVQNNCLSRPNIFLCPEIEPKLLGKLKDIIKRHQGTVTEDKNNASHVVYPVPGNLEEEEWVRPVMKRDKQVLLHWGYYPDSYDTWIPASEIEASVEDAPTPEKPRKVHAKWILDTDTFNEWMNEEDYEVNDDKNPVSRRKKISAKTLTDEVNSPDSDRRDKKGGNYKKRKRSPSPSPTPEAKKKNAKKGPSTPYTKSKRGHREEEQEDLTKDMDEPSPVPNVEEVTLPKTVNTKKDSESAPVKGGTMTDLDEQEDESMETTGKDEDENSTGNKGEQTKNPDLHEDNVTEQTHHIIIPSYAAWFDYNSVHAIERRALPEFFNGKNKSKTPEIYLAYRNFMIDTYRLNPQEYLTSTACRRNLAGDVCAIMRVHAFLEQWGLINYQVDAESRPTPMGPPPTSHFHVLADTPSGLVPLQPKTPQQTSASQQMLNFPDKGKEKPTDMQNFGLRTDMYTKKNVPSKSKAAASATREWTEQETLLLLEALEMYKDDWNKVSEHVGSRTQDECILHFLRLPIEDPYLEDSEASLGPLAYQPIPFSQSGNPVMSTVAFLASVVDPRVASAAAKSALEEFSKMKEEVPTALVEAHVRKVEEAAKVTGKADPAFGLESSGIAGTTSDEPERIEESGNDEARVEGQATDEKKEPKEPREGGGAIEEEAKEKTSEAPKKDEEKGKEGDSEKESEKSDGDPIVDPEKEKEPKEGQEEVLKEVVESEGERKTKVERDIGEGNLSTAAAAALAAAAVKAKHLAAVEERKIKSLVALLVETQMKKLEIKLRHFEELETIMDREREALEYQRQQLLADRQAFHMEQLKYAEMRARQQHFQQMHQQQQQPPPALPPGSQPIPPTGAAGPPAVHGLAVAPASVVPAPAGSGAPPGSLGPSEQIGQAGSTAGPQQQQPAGAPQPGAVPPGVPPPGPHGPSPFPNQQTPPSMMPGAVPGSGHPGVAGNAPLGLPFGMPPPPPPPAPSIIPFGSLADSISINLPAPPNLHGHHHHLPFAPGTLPPPNLPVSMANPLHPNLPATTTMPSSLPLGPGLGSAAAQSPAIVAAVQGNLLPSASPLPDPGTPLPPDPTAPSPGTVTPVPPPQ

>NP_000895.1

MAGKKVLIVYAHQEPKSFNGSLKNVAVDELSRQGCTVTVSDLYAMNFEPRATDKDITGTLSNPEVFNYGVETHEAYKQRSLASDITDEQKKVREADLVIFQFPLYWFSVPAILKGWMDRVLCQGFAFDIPGFYDSGLLQGKLALLSVTTGGTAEMYTKTGVNGDSRYFLWPLQHGTLHFCGFKVLAPQISFAPEIASEEERKGMVAAWSQRLQTIWKEEPIPCTAHWHFGQ

1238 NP_003194.3 P04183

>NP_003194.3

MAHRPKRTFRQRAADSSDSDGAEESPAEPGAPRELPVPGSAEEEPPSGGGRAQVAGLPHRVRGPRGRGRVWASSRRATKAAPRADEGSESRTLDVSTDEEDKIHHSSESKDDQGLSSDSSSSLGEKELSSTVKIPDAAFIQAARRKRELARAQDDYISLDVQHTSSISGMKRESEDDPESEPDDHEKRIPFTLRPQTLRQRMAEESISRNEETSEESQEDEKQDTWEQQQMRKAVKIIEERDIDLSCGNGSSKVKKFDTSISFPPVNLEIIKKQLNTRLTLLQETHRSHLREYEKYVQDVKSSKSTIQNLESSSNQALNCKFYKSMKIYVENLIDCLNEKIINIQEIESSMHALLLKQAMTFMKRRQDELKHESTYLQQLSRKDETSTSGNFSVDEKTQWILEEIESRRTKRRQARVLSGNCNHQEGTSSDDELPSAEMIDFQKSQGDILQKQKKVFEEVQDDFCNIQNILLKFQQWREKFPDSYYEAFISLCIPKLLNPLIRVQLIDWNPLKLESTGLKEMPWFKSVEEFMDSSVEDSKKESSSDKKVLSAIINKTIIPRLTDFVEFLWDPLSTSQTTSLITHCRVILEEHSTCENEVSKSRQDLLKSIVSRMKKAVEDDVFIPLYPKSAVENKTSPHSKFQERQFWSGLKLFRNILLWNGLLTDDTLQELGLGKLLNRYLIIALLNATPGPDVVKKCNQVAACLPEKWFENSAMRTSIPQLENFIQFLLQSAHKLSRSEFRDEVEEIILILVKIKALNQAESFIGEHHLDHLKSLIKED

>P04183

MSCINLPTVLPGSPSKTRGQIQVILGPMFSGKSTELMRRVRRFQIAQYKCLVIKYAKDTRYSSSFCTHDRNTMEALPACLLRDVAQEALGVAVIGIDEGQFFPDIVEFCEAMANAGKTVIVAALDGTFQRKPFGAILNLVPLAESVVKLTAVCMECFREAAYTKRLGTEKEVEVIGGADKYHSVCRLCYFKKASGQPAGPDNKENCPVPGKPGEAVAARKLFAPQQILQCSPAN

1239 Q96RE9 Q9NP86

>Q96RE9

MMKSQGLVSFKDVAVDFTQEEWQQLDPSQRTLYRDVMLENYSHLVSMGYPVSKPDVISKLEQGEEPWIIKGDISNWIYPDEYQADGRQDRKSNLHNSQSCILGTVSFHHKILKGVTRDGSLCSILKVCQGDGQLQRFLENQDKLFRQVTFVNSKTVTEASGHKYNPLGKIFQECIETDISIQRFHKYDAFKKNLKPNIDLPSCYKSNSRKKPDQSFGGGKSSSQSEPNSNLEKIHNGVIPFDDNQCGNVFRNTQSLIQYQNVETKEKSCVCVTCGKAFAKKSQLIVHQRIHTGKKPYDCGACGKAFSEKFHLVVHQRTHTGEKPYDCSECGKAFSQKSSLIIHQRVHTGEKPYECSECGKAFSQKSPLIIHQRIHTGEKPYECRECGKAFSQKSQLIIHHRAHTGEKPYECTECGKAFCEKSHLIIHKRIHTGEKPYKCAQCEEAFSRKTELITHQLVHTGEKPYECTECGKTFSRKSQLIIHQRTHTGEKPYKCSECGKAFCQKSHLIGHQRIHTGEKPYICTECGKAFSQKSHLPGHQRIHTGEKPYICAECGKAFSQKSDLVLHQRIHTGERPYQCAICGKAFIQKSQLTVHQRIHTVVKS

>Q9NP86

MQFPMGPACIFLRKGIAEKQRERPLGQDEIEELREAFLEFDKDRDGFISCKDLGNLMRTMGYMPTEMELIELGQQIRMNLGGRVDFDDFVELMTPKLLAETAGMIGVQEMRDAFKEFDTNGDGEITLVELQQAMQRLLGERLTPREISEVVREADVNGDGTVDFEEFVKMMSR

1240 NP_000160.1 Q96T66

>NP_000160.1

MQLRNPELHLGCALALRFLALVSWDIPGARALDNGLARTPTMGWLHWERFMCNLDCQEEPDSCISEKLFMEMAELMVSEGWKDAGYEYLCIDDCWMAPQRDSEGRLQADPQRFPHGIRQLANYVHSKGLKLGIYADVGNKTCAGFPGSFGYYDIDAQTFADWGVDLLKFDGCYCDSLENLADGYKHMSLALNRTGRSIVYSCEWPLYMWPFQKPNYTEIRQYCNHWRNFADIDDSWKSIKSILDWTSFNQERIVDVAGPGGWNDPDMLVIGNFGLSWNQQVTQMALWAIMAAPLFMSNDLRHISPQAKALLQDKDVIAINQDPLGKQGYQLRQGDNFEVWERPLSGLAWAVAMINRQEIGGPRSYTIAVASLGKGVACNPACFITQLLPVKRKLGFYEWTSRLRSHINPTGTVLLQLENTMQMSLKDLL

>Q96T66

MKSRIPVVLLACGSFNPITNMHLRMFEVARDHLHQTGMYQVIQGIISPVNDTYGKKDLAASHHRVAMARLALQTSDWIRVDPWESEQAQWMETVKVLRHHHSKLLRSPPQMEGPDHGKALFSTPAAVPELKLLCGADVLKTFQTPNLWKDAHIQEIVEKFGLVCVGRVGHDPKGYIAESPILRMHQHNIHLAKEPVQNEISATYIRRALGQGQSVKYLIPDAVITYIKDHGLYTKGSTWKGKSTQSTEGKTS

1241 NP_005634.1 O43182

>NP_005634.1

MDDAHESPSDKGGETGESDETAAVPGDPGATDTDGIPEETDGDADVDLKEAAAEEGELESQDVSDLTTVEREDSSLLNPAAKKLKIDTKEKKEKKQKVDEDEIQKMQILVSSFSEEQLNRYEMYRRSAFPKAAIKRLIQSITGTSVSQNVVIAMSGISKVFVGEVVEEALDVCEKWGEMPPLQPKHMREAVRRLKSKGQIPNSKHKKIIFF

>O43182

MSAQSLLHSVFSCSSPASSSAASAKGFSKRKLRQTRSLDPALIGGCGSDEAGAEGSARGATAGRLYSPSLPAESLGPRLASSSRGPPPRATRLPPPGPLCSSFSTPSTPQEKSPSGSFHFDYEVPLGRGGLKKSMAWDLPSVLAGPASSRSASSILCSSGGGPNGIFASPRRWLQQRKFQSPPDSRGHPYVVWKSEGDFTWNSMSGRSVRLRSVPIQSLSELERARLQEVAFYQLQQDCDLSCQITIPKDGQKRKKSLRKKLDSLGKEKNKDKEFIPQAFGMPLSQVIANDRAYKLKQDLQRDEQKDASDFVASLLPFGNKRQNKELSSSNSSLSSTSETPNESTSPNTPEPAPRARRRGAMSVDSITDLDDNQSRLLEALQLSLPAEAQSKKEKARDKKLSLNPIYRQVPRLVDSCCQHLEKHGLQTVGIFRVGSSKKRVRQLREEFDRGIDVSLEEEHSVHDVAALLKEFLRDMPDPLLTRELYTAFINTLLLEPEEQLGTLQLLIYLLPPCNCDTLHRLLQFLSIVARHADDNISKDGQEVTGNKMTSLNLATIFGPNLLHKQKSSDKEFSVQSSARAEESTAIIAVVQKMIENYEALFMVPPDLQNEVLISLLETDPDVVDYLLRRKASQSSSPDMLQSEVSFSVGGRHSSTDSNKASSGDISPYDNNSPVLSERSLLAMQEDAAPGGSEKLYRVPGQFMLVGHLSSSKSRESSPGPRLGKDLSEEPFDIWGTWHSTLKSGSKDPGMTGSSGDIFESSSLRAGPCSLSQGNLSPNWPRWQGSPAELDSDTQGARRTQAAAPATEGRAHPAVSRACSTPHVQVAGKAERPTARSEQYLTLSGAHDLSESELDVAGLQSRATPQCQRPHGSGRDDKRPPPPYPGPGKPAAAAAWIQGPPEGVETPTDQGGQAAEREQQVTQKKLSSANSLPAGEQDSPRLGDAGWLDWQRERWQIWELLSTDNPDALPETLV

1242 NP_000160.1 NP_001880.2

>NP_000160.1

MQLRNPELHLGCALALRFLALVSWDIPGARALDNGLARTPTMGWLHWERFMCNLDCQEEPDSCISEKLFMEMAELMVSEGWKDAGYEYLCIDDCWMAPQRDSEGRLQADPQRFPHGIRQLANYVHSKGLKLGIYADVGNKTCAGFPGSFGYYDIDAQTFADWGVDLLKFDGCYCDSLENLADGYKHMSLALNRTGRSIVYSCEWPLYMWPFQKPNYTEIRQYCNHWRNFADIDDSWKSIKSILDWTSFNQERIVDVAGPGGWNDPDMLVIGNFGLSWNQQVTQMALWAIMAAPLFMSNDLRHISPQAKALLQDKDVIAINQDPLGKQGYQLRQGDNFEVWERPLSGLAWAVAMINRQEIGGPRSYTIAVASLGKGVACNPACFITQLLPVKRKLGFYEWTSRLRSHINPTGTVLLQLENTMQMSLKDLL

>NP_001880.2

MATGQKLMRAVRVFEFGGPEVLKLRSDIAVPIPKDHQVLIKVHACGVNPVETYIRSGTYSRKPLLPYTPGSDVAGVIEAVGDNASAFKKGDRVFTSSTISGGYAEYALAADHTVYKLPEKLDFKQGAAIGIPYFTAYRALIHSACVKAGESVLVHGASGGVGLAACQIARAYGLKILGTAGTEEGQKIVLQNGAHEVFNHREVNYIDKIKKYVGEKGIDIIIEMLANVNLSKDLSLLSHGGRVIVVGSRGTIEINPRDTMAKESSIIGVTLFSSTKEEFQQYAAALQAGMEIGWLKPVIGSQYPLEKVAEAHENIIHGSGATGKMILLL

1243 NP_002120.1 NP_008830.2

>NP_002120.1

MGKGDPNKPRGKMSSYAFFVQTCREEHKKKHPDSSVNFAEFSKKCSERWKTMSAKEKSKFEDMAKSDKARYDREMKNYVPPKGDKKGKKKDPNAPKRPPSAFFLFCSEHRPKIKSEHPGLSIGDTAKKLGEMWSEQSAKDKQPYEQKAAKLKEKYEKDIAAYRAKGKSEAGKKGPGRPTGSKKKNEPEDEEEEEEEEDEDEEEEDEDEE

>NP_008830.2

MAALSGVRWLTRALVSAGNPGAWRGLSTSAAAHAASRSQAEDVRVEGSFPVTMLPGDGVGPELMHAVKEVFKAAAVPVEFQEHHLSEVQNMASEEKLEQVLSSMKENKVAIIGKIHTPMEYKGELASYDMRLRRKLDLFANVVHVKSLPGYMTRHNNLDLVIIREQTEGEYSSLEHESARGVIECLKIVTRAKSQRIAKFAFDYATKKGRGKVTAVHKANIMKLGDGLFLQCCEEVAELYPKIKFETMIIDNCCMQLVQNPYQFDVLVMPNLYGNIIDNLAAGLVGGAGVVPGESYSAEYAVFETGARHPFAQAVGRNIANPTAMLLSASNMLRHLNLEYHSSMIADAVKKVIKVGKVRTRDMGGYSTTTDFIKSVIGHLQTKGS

1244 NP_003838.1 NP_003621.1

>NP_003838.1

MDAFTRFTNQTQGRDRLFRATQYTCMLLRYLLEPKAGKEKVVMKLKKLESSVSTGRKWFRLGNVVHAIQATEQSIHATDLVPRLCLTLANLNRVIYFICDTILWVRSVGLTSGINKEKWRTRAAHHYYYSLLLSLVRDLYEISLQMKRVTCDRAKKEKSASQDPLWFSVAEEETEWLQSFLLLLFRSLKQHPPLLLDTVKNLCDILNPLDQLGIYKSNPGIIGLGGLVSSIAGMITVAYPQMKLKTR

>NP_003621.1

MLRSVWNFLKRHKKKCIFLGTVLGGVYILGKYGQKKIREIQEREAAEYIAQARRQYHFESNQRTCNMTVLSMLPTLREALMQQLNSESLTALLKNRPSNKLEIWEDLKIISFTRSTVAVYSTCMLVVLLRVQLNIIGGYIYLDNAAVGKNGTTILAPPDVQQQYLSSIQHLLGDGLTELITVIKQAVQKVLGSVSLKHSLSLLDLEQKLKEIRNLVEQHKSSSWINKDGSKPLLCHYMMPDEETPLAVQACGLSPRDITTIKLLNETRDMLESPDFSTVLNTCLNRGFSRLLDNMAEFFRPTEQDLQHGNSMNSLSSVSLPLAKIIPIVNGQIHSVCSETPSHFVQDLLTMEQVKDFAANVYEAFSTPQQLEK

1245 NP_057412.1 P40939

>NP_057412.1

MALLGKRCDVPTNGCGPDRWNSAFTRKDEIITSLVSALDSMCSALSKLNAEVACVAVHDESAFVVGTEKGRMFLNARKELQSDFLRFCRGPPWKDPEAEHPKKVQRGEGGGRSLPRSSLEHGSDVYLLRKMVEEVFDVLYSEALGRASVVPLPYERLLREPGLLAVQGLPEGLAFRRPAEYDPKALMAILEHSHRIRFKLKRPLEDGGRDSKALVELNGVSLIPKGSRDCGLHGQAPKVPPQDLPPTATSSSMASFLYSTALPNHAIRELKQEAPSCPLAPSDLGLSRPMPEPKATGAQDFSDCCGQKPTGPGGPLIQNVHASKRILFSIVHDKSEKWDAFIKETEDINTLRECVQILFNSRYAEALGLDHMVPVPYRKIACDPEAVEIVGIPDKIPFKRPCTYGVPKLKRILEERHSIHFIIKRMFDERIFTGNKFTKDTTKLEPASPPEDTSAEVSRATVLDLAGNARSDKGSMSEDCGPGTSGELGGLRPIKIEPEDLDIIQVTVPDPSPTSEEMTDSMPGHLPSEDSGYGMEMLTDKGLSEDARPEERPVEDSHGDVIRPLRKQVELLFNTRYAKAIGISEPVKVPYSKFLMHPEELFVVGLPEGISLRRPNCFGIAKLRKILEASNSIQFVIKRPELLTEGVKEPIMDSQGTASSLGFSPPALPPERDSGDPLVDESLKRQGFQENYDARLSRIDIANTLREQVQDLFNKKYGEALGIKYPVQVPYKRIKSNPGSVIIEGLPPGIPFRKPCTFGSQNLERILAVADKIKFTVTRPFQGLIPKPDEDDANRLGEKVILREQVKELFNEKYGEALGLNRPVLVPYKLIRDSPDAVEVTGLPDDIPFRNPNTYDIHRLEKILKAREHVRMVIINQLQPFAEICNDAKVPAKDSSIPKRKRKRVSEGNSVSSSSSSSSSSSSNPDSVASANQISLVQWPMYMVDYAGLNVQLPGPLNY

>P40939

MVACRAIGILSRFSAFRILRSRGYICRNFTGSSALLTRTHINYGVKGDVAVVRINSPNSKVNTLSKELHSEFSEVMNEIWASDQIRSAVLISSKPGCFIAGADINMLAACKTLQEVTQLSQEAQRIVEKLEKSTKPIVAAINGSCLGGGLEVAISCQYRIATKDRKTVLGTPEVLLGALPGAGGTQRLPKMVGVPAALDMMLTGRSIRADRAKKMGLVDQLVEPLGPGLKPPEERTIEYLEEVAITFAKGLADKKISPKRDKGLVEKLTAYAMTIPFVRQQVYKKVEEKVRKQTKGLYPAPLKIIDVVKTGIEQGSDAGYLCESQKFGELVMTKESKALMGLYHGQVLCKKNKFGAPQKDVKHLAILGAGLMGAGIAQVSVDKGLKTILKDATLTALDRGQQQVFKGLNDKVKKKALTSFERDSIFSNLTGQLDYQGFEKADMVIEAVFEDLSLKHRVLKEVEAVIPDHCIFASNTSALPISEIAAVSKRPEKVIGMHYFSPVDKMQLLEIITTEKTSKDTSASAVAVGLKQGKVIIVVKDGPGFYTTRCLAPMMSEVIRILQEGVDPKKLDSLTTSFGFPVGAATLVDEVGVDVAKHVAEDLGKVFGERFGGGNPELLTQMVSKGFLGRKSGKGFYIYQEGVKRKDLNSDMDSILASLKLPPKSEVSSDEDIQFRLVTRFVNEAVMCLQEGILATPAEGDIGAVFGLGFPPCLGGPFRFVDLYGAQKIVDRLKKYEAAYGKQFTPCQLLADHANSPNKKFYQ

1246 Q15029 NP_742024.1

>Q15029

MDTDLYDEFGNYIGPELDSDEDDDELGRETKDLDEMDDDDDDDDVGDHDDDHPGMEVVLHEDKKYYPTAEEVYGPEVETIVQEEDTQPLTEPIIKPVKTKKFTLMEQTLPVTVYEMDFLADLMDNSELIRNVTLCGHLHHGKTCFVDCLIEQTHPEIRKRYDQDLCYTDILFTEQERGVGIKSTPVTVVLPDTKGKSYLFNIMDTPGHVNFSDEVTAGLRISDGVVLFIDAAEGVMLNTERLIKHAVQERLAVTVCINKIDRLILELKLPPTDAYYKLRHIVDEVNGLISMYSTDENLILSPLLGNVCFSSSQYSICFTLGSFAKIYADTFGDINYQEFAKRLWGDIYFNPKTRKFTKKAPTSSSQRSFVEFILEPLYKILAQVVGDVDTSLPRTLDELGIHLTKEELKLNIRPLLRLVCKKFFGEFTGFVDMCVQHIPSPKVGAKPKIEHTYTGGVDSDLGEAMSDCDPDGPLMCHTTKMYSTDDGVQFHAFGRVLSGTIHAGQPVKVLGENYTLEDEEDSQICTVGRLWISVARYHIEVNRVPAGNWVLIEGVDQPIVKTATITEPRGNEEAQIFRPLKFNTTSVIKIAVEPVNPSELPKMLDGLRKVNKSYPSLTTKVEESGEHVILGTGELYLDCVMHDLRKMYSEIDIKVADPVVTFCETVVETSSLKCFAETPNKKNKITMIAEPLEKGLAEDIENEVVQITWNRKKLGEFFQTKYDWDLLAARSIWAFGPDATGPNILVDDTLPSEVDKALLGSVKDSIVQGFQWGTREGPLCDELIRNVKFKILDAVVAQEPLHRGGGQIIPTARRVVYSAFLMATPRLMEPYYFVEVQAPADCVSAVYTVLARRRGHVTQDAPIPGSPLYTIKAFIPAIDSFGFETDLRTHTQGQAFSLSVFHHWQIVPGDPLDKSIVIRPLEPQPAPHLAREFMIKTRRRKGLSEDVSISKFFDDPMLLELAKQDVVLNYPM

>NP_742024.1

MDTSDLFASCRKGDVGRVRYLLEQRDVEVNVRDKWDSTPLYYACLCGHEELVLYLLANGARCEANTFDGERCLYGALSDPIRRALRDYKQVTASCRRRDYYDDFLQRLLEQGIHSDVVFVVHGKPFRVHRCVLGARSAYFANMLDTKWKGKSVVVLRHPLINPVAFGALLQYLYTGRLDIGVEHVSDCERLAKQCQLWDLLSDLEAKCEKVSEFVASKPGTCVKVLTIEPPPADPRLREDMALLADCALPPELRGDLWELPFPCPDGFNSCPDICFRVAGCSFLCHKAFFCGRSDYFRALLDDHFRESEEPATSGGPPAVTLHGISPDVFTHVLYYMYSDHTELSPEAAYDVLSVADMYLLPGLKRLCGRSLAQMLDEDTVVGVWRVAKLFRLARLEDQCTEYMAKVIEKLVEREDFVEAVKEEAAAVAARQETDSIPLVDDIRFHVASTVQTYSAIEEAQQRLRALEDLLVSIGLDC

1247 NP_065700.1 NP_002890.1

>NP_065700.1

MELSAVGERVFAAEALLKRRIRKGRMEYLVKWKGWSQKYSTWEPEENILDARLLAAFEEREREMELYGPKKRGPKPKTFLLKAQAKAKAKTYEFRSDSARGIRIPYPGRSPQDLASTSRAREGLRNMGLSPPASSTSTSSTCRAEAPRDRDRDRDRDRERDRERERERERERERERERERGTSRVDDKPSSPGDSSKKRGPKPRKELPDPSQRPLGEPSAGLGEYLKGRKLDDTPSGAGKFPAGHSVIQLARRQDSDLVQCGVTSPSSAEATGKLAVDTFPARVIKHRAAFLEAKGQGALDPNGTRVRHGSGPPSSGGGLYRDMGAQGGRPSLIARIPVARILGDPEEESWSPSLTNLEKVVVTDVTSNFLTVTIKESNTDQGFFKEKR

>NP_002890.1

MPVDFTGYWKMLVNENFEEYLRALDVNVALRKIANLLKPDKEIVQDGDHMIIRTLSTFRNYIMDFQVGKEFEEDLTGIDDRKCMTTVSWDGDKLQCVQKGEKEGRGWTQWIEGDELHLEMRVEGVVCKQVFKKVQ

1248 P15586 P22532

>P15586

MRLLPLAPGRLRRGSPRHLPSCSPALLLLVLGGCLGVFGVAAGTRRPNVVLLLTDDQDEVLGGMTPLKKTKALIGEMGMTFSSAYVPSALCCPSRASILTGKYPHNHHVVNNTLEGNCSSKSWQKIQEPNTFPAILRSMCGYQTFFAGKYLNEYGAPDAGGLEHVPLGWSYWYALEKNSKYYNYTLSINGKARKHGENYSVDYLTDVLANVSLDFLDYKSNFEPFFMMIATPAPHSPWTAAPQYQKAFQNVFAPRNKNFNIHGTNKHWLIRQAKTPMTNSSIQFLDNAFRKRWQTLLSVDDLVEKLVKRLEFTGELNNTYIFYTSDNGYHTGQFSLPIDKRQLYEFDIKVPLLVRGPGIKPNQTSKMLVANIDLGPTILDIAGYDLNKTQMDGMSLLPILRGASNLTWRSDVLVEYQGEGRNVTDPTCPSLSPGVSQCFPDCVCEDAYNNTYACVRTMSALWNLQYCEFDDQEVFVEVYNLTADPDQITNIAKTIDPELLGKMNYRLMMLQSCSGPTCRTPGVFDPGYRFDPRLMFSNRGSVRTRRFSKHLL

>P22532

MSYQQQQCKQPCQPPPVCPTPKCPEPCPPPKCPEPCPSPKCPQPCPPQQCQQKYPPVTPSPPCQPKCPPKSK

1249 P41235 Q9Y4B6

>P41235

MRLSKTLVDMDMADYSAALDPAYTTLEFENVQVLTMGNDTSPSEGTNLNAPNSLGVSALCAICGDRATGKHYGASSCDGCKGFFRRSVRKNHMYSCRFSRQCVVDKDKRNQCRYCRLKKCFRAGMKKEAVQNERDRISTRRSSYEDSSLPSINALLQAEVLSRQITSPVSGINGDIRAKKIASIADVCESMKEQLLVLVEWAKYIPAFCELPLDDQVALLRAHAGEHLLLGATKRSMVFKDVLLLGNDYIVPRHCPELAEMSRVSIRILDELVLPFQELQIDDNEYAYLKAIIFFDPDAKGLSDPGKIKRLRSQVQVSLEDYINDRQYDSRGRFGELLLLLPTLQSITWQMIEQIQFIKLFGMAKIDNLLQEMLLGGSPSDAPHAHHPLHPHLMQEHMGTNVIVANTMPTHLSNGQMCEWPRPRGQAATPETPQPSPPGGSGSEPYKLLPGAVATIVKPLSAIPQPTITKQEVI

>Q9Y4B6

MTTVVVHVDSKAELTTLLEQWEKEHGSGQDMVPILTRMSQLIEKETEEYRKGDPDPFDDRHPGRADPECMLGHLLRILFKNDDFMNALVNAYVMTSREPPLNTAACRLLLDIMPGLETAVVFQEKEGIVENLFKWAREADQPLRTYSTGLLGGAMENQDIAANYRDENSQLVAIVLRRLRELQLQEVALRQENKRPSPRKLSSEPLLPLDEEAVDMDYGDMAVDVVDGDQEEASGDMEISFHLDSGHKTSSRVNSTTKPEDGGLKKNKSAKQGDRENFRKAKQKLGFSSSDPDRMFVELSNSSWSEMSPWVIGTNYTLYPMTPAIEQRLILQYLTPLGEYQELLPIFMQLGSRELMMFYIDLKQTNDVLLTFEALKHLASLLLHNKFATEFVAHGGVQKLLEIPRPSMAATGVSMCLYYLSYNQDAMERVCMHPHNVLSDVVNYTLWLMECSHASGCCHATMFFSICFSFRAVLELFDRYDGLRRLVNLISTLEILNLEDQGALLSDDEIFASRQTGKHTCMALRKYFEAHLAIKLEQVKQSLQRTEGGILVHPQPPYKACSYTHEQIVEMMEFLIEYGPAQLYWEPAEVFLKLSCVQLLLQLISIACNWKTYYARNDTVRFALDVLAILTVVPKIQLQLAESVDVLDEAGSTVSTVGISIILGVAEGEFFIHDAEIQKSALQIIINCVCGPDNRISSIGKFISGTPRRKLPQNPKSSEHTLAKMWNVVQSNNGIKVLLSLLSIKMPITDADQIRALACKALVGLSRSSTVRQIISKLPLFSSCQIQQLMKEPVLQDKRSDHVKFCKYAAELIERVSGKPLLIGTDVSLARLQKADVVAQSRISFPEKELLLLIRNHLISKGLGETATVLTKEADLPMTAASHSSAFTPVTAAASPVSLPRTPRIANGIATRLGSHAAVGASAPSAPTAHPQPRPPQGPLALPGPSYAGNSPLIGRISFIRERPSPCNGRKIRVLRQKSDHGAYSQSPAIKKQLDRHLPSPPTLDSIITEYLREQHARCKNPVATCPPFSLFTPHQCPEPKQRRQAPINFTSRLNRRASFPKYGGVDGGCFDRHLIFSRFRPISVFREANEDESGFTCCAFSARERFLMLGTCTGQLKLYNVFSGQEEASYNCHNSAITHLEPSRDGSLLLTSATWSQPLSALWGMKSVFDMKHSFTEDHYVEFSKHSQDRVIGTKGDIAHIYDIQTGNKLLTLFNPDLANNYKRNCATFNPTDDLVLNDGVLWDVRSAQAIHKFDKFNMNISGVFHPNGLEVIINTEIWDLRTFHLLHTVPALDQCRVVFNHTGTVMYGAMLQADDEDDLMEERMKSPFGSSFRTFNATDYKPIATIDVKRNIFDLCTDTKDCYLAVIENQGSMDALNMDTVCRLYEVGRQRLAEDEDEEEDQEEEEQEEEDDDEDDDDTDDLDELDTDQLLEAELEEDDNNENAGEDGDNDFSPSDEELANLLEEGEDGEDEDSDADEEVELILGDTDSSDNSDLEDDIILSLNE

1250 NP_778225.1 NP_006180.1

>NP_778225.1

MPDPSKSAPAPKKGSKKAVTKAQKKDGKKRKRGRKESYSIYVYKVLKQVHPDTGISSKAMGIMNSFVNDIFERIASEASRLAHYNKRSTITSREVQTAVRLLLPGELAKHAVSEGTKAVTKYTSSK

>NP_006180.1

MAEDRPQQPQLDMPLVLDQGLTRQMRLRVESLKQRGEKRQDGEKLLQPAESVYRLNFTQQQRLQFERWNVVLDKPGKVTITGTSQNWTPDLTNLMTRQLLDPTAIFWRKEDSDAIDWNEADALEFGERLSDLAKIRKVMYFLVTFGEGVEPANLKASVVFNQL

1251 Q8IZL9 Q9GZZ1

>Q8IZL9

MDQYCILGRIGEGAHGIVFKAKHVETGEIVALKKVALRRLEDGFPNQALREIKALQEMEDNQYVVQLKAVFPHGGGFVLAFEFMLSDLAEVVRHAQRPLAQAQVKSYLQMLLKGVAFCHANNIVHRDLKPANLLISASGQLKIADFGLARVFSPDGSRLYTHQVATRWYRAPELLYGARQYDQGVDLWSVGCIMGELLNGSPLFPGKNDIEQLCYVLRILGTPNPQVWPELTELPDYNKISFKEQVPMPLEEVLPDVSPQALDLLGQFLLYPPHQRIAASKALLHQYFFTAPLPAHPSELPIPQRLGGPAPKAHPGPPHIHDFHVDRPLEESLLNPELIRPFILEG

>Q9GZZ1

MKGSRIELGDVTPHNIKQLKRLNQVIFPVSYNDKFYKDVLEVGELAKLAYFNDIAVGAVCCRVDHSQNQKRLYIMTLGCLAPYRRLGIGTKMLNHVLNICEKDGTFDNIYLHVQISNESAIDFYRKFGFEIIETKKNYYKRIEPADAHVLQKNLKVPSGQNADVQKTDN

1252 Q96DT0 NP_000662.3

>Q96DT0

MSQPSGGRAPGTRIYSWSCPTVMSPGEKLDPIPDSFILQPPVFHPVVPYVTTIFGGLHAGKMVMLQGVVPLDAHRFQVDFQCGCSLCPRPDIAFHFNPRFHTTKPHVICNTLHGGRWQREARWPHLALRRGSSFLILFLFGNEEVKVSVNGQHFLHFRYRLPLSHVDTLGIFGDILVEAVGFLNINPFVEGSREYPAGHPFLLMSPRLEVPCSHALPQGLSPGQVIIVRGLVLQEPKHFTVSLRDQAAHAPVTLRASFADRTLAWISRWGQKKLISAPFLFYPQRFFEVLLLFQEGGLKLALNGQGLGATSMNQQALEQLRELRISGSVQLYCVHS

>NP_000662.3

MANEVIKCKAAVAWEAGKPLSIEEIEVAPPKAHEVRIKIIATAVCHTDAYTLSGADPEGCFPVILGHEGAGIVESVGEGVTKLKAGDTVIPLYIPQCGECKFCLNPKTNLCQKIRVTQGKGLMPDGTSRFTCKGKTILHYMGTSTFSEYTVVADISVAKIDPLAPLDKVCLLGCGISTGYGAAVNTAKLEPGSVCAVFGLGGVGLAVIMGCKVAGASRIIGVDINKDKFARAKEFGATECINPQDFSKPIQEVLIEMTDGGVDYSFECIGNVKVMRAALEACHKGWGVSVVVGVAASGEEIATRPFQLVTGRTWKGTAFGGWKSVESVPKLVSEYMSKKIKVDEFVTHNLSFDEINKAFELMHSGKSIRTVVKI

1253 A6NDE4 NP_004127.1

>A6NDE4

MVEADHPGKLFIGGLNRETNEKMLKAVFGKHGPISEVLLIKDRTSKSRGFAFITFENPADAKNAAKDMNGKSLHGKAIKVEQAKKPSFQSGGRRRPPASSRNRSPSGSLRSARGSRGGTRGWLPSQEGHLDDGGYTPDLKMSYSRGLIPVKRGPSSRSGGPPPKKSAPSAVARSNSWMGSQGPMSQRRENYGVPPRRATISSWRNDRMSTRHDGYATNDGNHPSCQETRDYAPPSRGYAYRDNGHSNRDEHSSRGYRNHRSSRETRDYAPPSRGHAYRDYGHSRRDESYSRGYRNRRSSRETREYAPPSRGHGYRDYGHSRRHESYSRGYRNHPSSRETRDYAPPHRDYAYRDYGHSSWDEHSSRGYSYHDGYGEALGRDHSEHLSGSSYRDALQRYGTSHGAPPARGPRMSYGGSTCHAYSNTRDRYGRSWESYSSCGDFHYCDREHVCRKDQRNPPSLGRVLPDPREAYGSSSYVASIVDGGESRSEKGDSSRY

>NP_004127.1

MDAPKAGYAFEYLIETLNDSSHKKFFDVSKLGTKYDVLPYSIRVLLEAAVRNCDGFLMKKEDVMNILDWKTKQSNVEVPFFPARVLLQDFTGIPAMVDFAAMREAVKTLGGDPEKVHPACPTDLTVDHSLQIDFSKCAIQNAPNPGGGDLQKAGKLSPLKVQPKKLPCRGQTTCRGSCDSGELGRNSGTFSSQIENTPILCPFHLQPVPEPETVLKNQEVEFGRNRERLQFFKWSSRVFKNVAVIPPGTGMAHQINLEYLSRVVFEEKDLLFPDSVVGTDSHITMVNGLGILGWGVGGIETEAVMLGLPVSLTLPEVVGCELTGSSNPFVTSIDVVLGITKHLRQVGVAGKFVEFFGSGVSQLSIVDRTTIANMCPEYGAILSFFPVDNVTLKHLEHTGFSKAKLESMETYLKAVKLFRNDQNSSGEPEYSQVIQINLNSIVPSVSGPKRPQDRVAVTDMKSDFQACLNEKVGFKGFQIAAEKQKDIVSIHYEGSEYKLSHGSVVIAAVISCTNNCNPSVMLAAGLLAKKAVEAGLRVKPYIRTSLSPGSGMVTHYLSSSGVLPYLSKLGFEIVGYGCSTCVGNTAPLSDAVLNAVKQGDLVTCGILSGNKNFEGRLCDCVRANYLASPPLVVAYAIAGTVNIDFQTEPLGTDPTGKNIYLHDIWPSREEVHRVEEEHVILSMFKALKDKIEMGNKRWNSLEAPDSVLFPWDLKSTYIRCPSFFDKLTKEPIALQAIENAHVLLYLGDSVTTDHISPAGSIARNSAAAKYLTNRGLTPREFNSYGARRGNDAVMTRGTFANIKLFNKFIGKPAPKTIHFPSGQTLDVFEAAELYQKEGIPLIILAGKKYGSGNSRDWAAKGPYLLGVKAVLAESYEKIHKDHLIGIGIAPLQFLPGENADSLGLSGRETFSLTFPEELSPGITLNIQTSTGKVFSVIASFEDDVEITLYKHGGLLNFVARKFS

1254 NP_001443.1 Q93015

>NP_001443.1

MTTEGGPPPAPLRRACSPVPGALQAALMSPPPAAAAAAAAAPETTSSSSSSSSASCASSSSSSNSASAPSAACKSAGGGGAGAGSGGAKKASSGLRRPEKPPYSYIALIVMAIQSSPSKRLTLSEIYQFLQARFPFFRGAYQGWKNSVRHNLSLNECFIKLPKGLGRPGKGHYWTIDPASEFMFEEGSFRRRPRGFRRKCQALKPMYHRVVSGLGFGASLLPQGFDFQAPPSAPLGCHSQGGYGGLDMMPAGYDAGAGAPSHAHPHHHHHHHVPHMSPNPGSTYMASCPVPAGPGGVGAAGGGGGGDYGPDSSSSPVPSSPAMASAIECHSPYTSPAAHWSSPGASPYLKQPPALTPSSNPAASAGLHSSMSSYSLEQSYLHQNAREDLSVGLPRYQHHSTPVCDRKDFVLNFNGISSFHPSASGSYYHHHHQSVCQDIKPCVM

>Q93015

MELILSTSPAELTLDPACQPKLPLDSTCQPEMTFNPGPTELTLDPEHQPEETPAPSLAELTLEPVHRRPELLDACADLINDQWPRSRTSRLHSLGQSSDAFPLCLMLLSPHPTLEAAPVVVGHARLSRVLNQPQSLLVETVVVARALRGRGFGRRLMEGLEVFARARGFRKLHLTTHDQVHFYTHLGYQLGEPVQGLVFTSRRLPATLLNAFPTAPSPRPPRKAPNLTAQAAPRGPKGPPLPPPPPLPECLTISPPVPSGPPSKSLLETQYQNVRGRPIFWMEKDI

1255 NP_001349.2 NP_006237.1

>NP_001349.2

MSKRHRLDLGEDYPSGKKRAGTDGKDRDRDRDREDRSKDRDRERDRGDREREREKEKEKELRASTNAMLISAGLPPLKASHSAHSTHSAHSTHSTHSAHSTHAGHAGHTSLPQCINPFTNLPHTPRYYDILKKRLQLPVWEYKDRFTDILVRHQSFVLVGETGSGKTTQIPQWCVEYMRSLPGPKRGVACTQPRRVAAMSVAQRVADEMDVMLGQEVGYSIRFEDCSSAKTILKYMTDGMLLREAMNDPLLERYGVIILDEAHERTLATDILMGVLKEVVRQRSDLKVIVMSATLDAGKFQIYFDNCPLLTIPGRTHPVEIFYTPEPERDYLEAAIRTVIQIHMCEEEEGDLLLFLTGQEEIDEACKRIKREVDDLGPEVGDIKIIPLYSTLPPQQQQRIFEPPPPKKQNGAIGRKVVVSTNIAETSLTIDGVVFVIDPGFAKQKVYNPRIRVESLLVTAISKASAQQRAGRAGRTRPGKCFRLYTEKAYKTEMQDNTYPEILRSNLGSVVLQLKKLGIDDLVHFDFMDPPAPETLMRALELLNYLAALNDDGDLTELGSMMAEFPLDPQLAKMVIASCDYNCSNEVLSITAMLSVPQCFVRPTEAKKAADEAKMRFAHIDGDHLTLLNVYHAFKQNHESVQWCYDNFINYRSLMSADNVRQQLSRIMDRFNLPRRSTDFTSRDYYINIRKALVTGYFMQVAHLERTGHYLTVKDNQVVQLHPSTVLDHKPEWVLYNEFVLTTKNYIRTCTDIKPEWLVKIAPQYYDMSNFPQCEAKRQLDRIIAKLQSKEYSQY

>NP_006237.1

MSSAPTTPPSVDKVDGFSRKSVRKARQKRSQSSSQFRSQGKPIELTPLPLLKDVPSSEQPELFLKKLQQCCVIFDFMDTLSDLKMKEYKRSTLNELVDYITISRGCLTEQTYPEVVRMVSCNIFRTLPPSDSNEFDPEEDEPTLEASWPHLQLVYEFFIRFLESQEFQPSIAKKYIDQKFVLQLLELFDSEDPRERDYLKTVLHRIYGKFLGLRAFIRKQINNIFLRFVYETEHFNGVAELLEILGSIINGFALPLKAEHKQFLVKVLIPLHTVRSLSLFHAQLAYCIVQFLEKDPSLTEPVIRGLMKFWPKTCSQKEVMFLGELEEILDVIEPSQFVKIQEPLFKQIAKCVSSPHFQVAERALYYWNNEYIMSLIEENSNVILPIMFSSLYRISKEHWNPAIVALVYNVLKAFMEMNSTMFDELTATYKSDRQREKKKEKEREELWKKLEDLELKRGLRRDGIIPT

1256 Q9H582 NP_112198.1

>Q9H582

MRSFLQQDVNKTKSRLNVLNGLANNMDDLKINTDITGAKEELLDDNNFISDKESGVHKPKDCQTSFQKNNTLTLPEELSKDKSENALSGGQSSLFIHAGAPTVSSENFILPKGAAVNGPVSHSSLTKTSNMNKGSVSLTTGQPVDQPTTESCSTLKVAADLQLSTPQKASQHQVLFLLSDVAHAKNPTHSNKKLPTSASVGCDIQNSVGSNIKSDGTLINQVEVGEDGEDLLVKDDCVNTVTGISSGTDGFRSENDTNWDPQKEFIQFLMTNEETVDKAPPHSKIGLEKKRKRKMDVSKITRYTEDCFSDSNCVPNKSKMQEVDFLEQNEELQAVDSQKYALSKVKPESTDEDLESVDAFQHLIYNPDKCGEESSPVHTSTFLSNTLKKKCEESDSESPATFSTEEPSFYPCTKCNVNFREKKHLHRHMMYHLDGNSHFRHLNVPRPYACRECGRTFRDRNSLLKHMIIHQERRQKLMEEIRELKELQDEGRSARLQCPQCVFGTNCPKTFVQHAKTHEKDKRYYCCEECNFMAVTENELECHRGIAHGAVVKCPMVTSDIAQRKTQKKTFMKDSVVGSSKKSATYICKMCPFTTSAKSVLKKHTEYLHSSSCVDSFGSPLGLDKRKNDILEEPVDSDSTKTLTKQQSTTFPKNSALKQDVKRTFGSTSQSSSFSKIHKRPHRIQKARKSIAQSGVNMCNQNSSPHKNVTIKSSVDQKPKYFHQAAKEKSNAKANSHYLYRHKYENYRMIKKSGESYPVHFKKEEASSLNSLHLFSSSSNSHNNFISDPHKPDAKRPESFKDHRRVAVKRVIKESKKESSVGGEDLDSYPDFLHKMTVVVLQKLNSAEKKDSYETEDESSWDNVELGDYTTQAIEDETYSDINQEHVNLFPLFKSKVEGQEPGENATLSYDQNDGFYFEYYEDTGSNNFLHEIHDPQHLETADASLSKHSSVFHWTDLSLEKKSCPYCPATFETGVGLSNHVRGHLHRAGLSYEARHVVSPEQIATSDKMQHFKRTGTGTPVKRVRKAIEKSETTSEHTCQLCGGWFDTKIGLSNHVRGHLKRLGKTKWDAHKSPICVLNEMMQNEEKYEKILKALNSRRIIPRPFVAQKLASSDDFISQNVIPLEAYRNGLKTEALSVSASEEEGLNFLNEYDETKPELPSGKKNQSLTLIELLKNKRMGEERNSAISPQKIHNQTARKRFVQKCVLPLNEDSPLMYQPQKMDLTMHSALDCKQKKSRSRSGSKKKMLTLPHGADEVYILRCRFCGLVFRGPLSVQEDWIKHLQRHIVNANLPRTGAGMVEVTSLLKKPASITETSFSLLMAEAAS

>NP_112198.1

MLKNKGHSSKKDNLAVNAVALQDHILHDLQLRNLSVADHSKTQVQKKENKSLKRDTKAIIDTGLKKTTQCPKLEDSEKEYVLDPKPPPLTLAQKLGLIGPPPPPLSSDEWEKVKQRSLLQGDSVQPCPICKEEFELRPQVLLSCSHVFHKACLQAFEKFTNKKTCPLCRKNQYQTRVIHDGARLFRIKCVTRIQAYWRGCVVRKWYRNLRKTVPPTDAKLRKKFFEKKFTEISHRILCSYNTNIEELFAEIDQCLAINRSVLQQLEEKCGHEITEEEWEKIQVQALRRETHECSICLAPLSAAGGQRVGAGRRSREMALLSCSHVFHHACLLALEEFSVGDRPPFHACPLCRSCYQKKILEC

1257 NP_116027.2 NP_071933.2

>NP_116027.2

MEVAPEQPRWMAHPAVLNAQHPDSHHPGLAHNYMEPAQLLPPDEVDVFFNHLDSQGNPYYANPAHARARVSYSPAHARLTGGQMCRPHLLHSPGLPWLDGGKAALSAAAAHHHNPWTVSPFSKTPLHPSAAGGPGGPLSVYPGAGGGSGGGSGSSVASLTPTAAHSGSHLFGFPPTPPKEVSPDPSTTGAASPASSSAGGSAARGEDKDGVKYQVSLTESMKMESGSPLRPGLATMGTQPATHHPIPTYPSYVPAAAHDYSSGLFHPGGFLGGPASSFTPKQRSKARSCSEGRECVNCGATATPLWRRDGTGHYLCNACGLYHKMNGQNRPLIKPKRRLSAARRAGTCCANCQTTTTTLWRRNANGDPVCNACGLYYKLHNVNRPLTMKKEGIQTRNRKMSNKSKKSKKGAECFEELSKCMQEKSSPFSAAALAGHMAPVGHLPPFSHSGHILPTPTPIHPSSSLSFGHPHPSSMVTAMG

>NP_071933.2

MQNVINTVKGKALEVAEYLTPVLKESKFKETGVITPEEFVAAGDHLVHHCPTWQWATGEELKVKAYLPTGKQFLVTKNVPCYKRCKQMEYSDELEAIIEEDDGDGGWVDTYHNTGITGITEAVKEITLENKDNIRLQDCSALCEEEEDEDEGEAADMEEYEESGLLETDEATLDTRKIVEACKAKTDAGGEDAILQTRTYDLYITYDKYYQTPRLWLFGYDEQRQPLTVEHMYEDISQDHVKKTVTIENHPHLPPPPMCSVHPCRHAEVMKKIIETVAEGGGELGVHMYLLIFLKFVQAVIPTIEYDYTRHFTM

1258 Q5H9F3 P82663

>Q5H9F3

MISTAPLYSGVHNWTSSDRIRMCGINEERRAPLSDEESTTGDCQHFGSQEFCVSSSFSKVELTAVGSGSNARGADPDGSATEKLGHKSEDKPDDPQPKMDYAGNVAEAEGFLVPLSSPGDGLKLPASDSAEASNSRADCSWTPLNTQMSKQVDCSPAGVKALDSRQGVGEKNTFILATLGTGVPVEGTLPLVTTNFSPLPAPICPPAPGSASVPHSVPDAFQVPLSVPAPVPHSGLVPVQVATSVPAPSPPLAPVPALAPAPPSVPTLISDSNPLSVSASVLVPVPASAPPSGPVPLSAPAPAPLSVPVSAPPLALIQAPVPPSAPTLVLAPVPTPVLAPMPASTPPAAPAPPSVPMPTPTPSSGPPSTPTLIPAFAPTPVPAPTPAPIFTPAPTPMPAATPAAIPTSAPIPASFSLSRVCFPAAQAPAMQKVPLSFQPGTVLTPSQPLVYIPPPSCGQPLSVATLPTTLGVSSTLTLPVLPSYLQDRCLPGVLASPELRSYPYAFSVARPLTSDSKLVSLEVNRLPCTSPSGSTTTQPAPDGVPGPLADTSLVTASAKVLPTPQPLLPAPSGSSAPPHPAKMPSGTEQQTEGTSVTFSPLKSPPQLEREMASPPECSEMPLDLSSKSNRQKLPLPNQRKTPPMPVLTPVHTSSKALLSTVLSRSQRTTQAAGGNVTSCLGSTSSPFVIFPEIVRNGDPSTWVKNSTALISTIPGTYVGVANPVPASLLLNKDPNLGLNRDPRHLPKQEPISIIDQGEPKGTGATCGKKGSQAGAEGQPSTVKRYTPARIAPGLPGCQTKELSLWKPTGPANIYPRCSVNGKPTSTQVLPVGWSPYHQASLLSIGISSAGQLTPSQGAPIRPTSVVSEFSGVPSLSSSEAVHGLPEGQPRPGGSFVPEQDPVTKNKTCRIAAKPYEEQVNPVLLTLSPQTGTLALSVQPSGGDIRMNQGPEESESHLCSDSTPKMEGPQGACGLKLAGDTKPKNQVLATYMSHELVLATPQNLPKMPELPLLPHDSHPKELILDVVPSSRRGSSTERPQLGSQVDLGRVKMEKVDGDVVFNLATCFRADGLPVAPQRGQAEVRAKAGQARVKQESVGVFACKNKWQPDDVTESLPPKKMKCGKEKDSEEQQLQPQAKAVVRSSHRPKCRKLPSDPQESTKKSPRGASDSGKEHNGVRGKHKHRKPTKPESQSPGKRADSHEEGSLEKKAKSSFRDFIPVVLSTRTRSQSGSICSSFAGMADSDMGSQEVFPTEEEEEVTPTPAKRRKVRKTQRDTQYRSHHAQDKSLLSQGRRHLWRAREMPWRTEAARQMWDTNEEEEEEEEEGLLKRKKRRRQKSRKYQTGEYLTEQEDEQRRKGRADLKARKQKTSSSQSLEHRLRNRNLLLPNKVQGISDSPNGFLPNNLEEPACLENSEKPSGKRKCKTKHMATVSEEAKDVVLYCLQKDSEDVNHRDNAGYTALHEACSRGWTDILNILLEHGANVNCSAQDGTRPVHDAVVNDNLETIWLLLSYGADPTLATYSGQTAMKLASSDTMKRFLSDHLSDLQGRAEGDPGVSWDFYSSSVLEEKDGFACDLLHNPPGSSDQEGDDPMEEDDFMFELSDKPLLPCYNLQVSVSRGPCNWFLFSDVLKRLKLSSRIFQARFPHFEITTMPKAEFYRQVASSQLLTPAERPGGLDDRSPPGSSETVELVRYEPDLLRLLGSEVEFQSCNS

>P82663

MPMKGRFPIRRTLQYLSQGNVVFKDSVKVMTVNYNTHGELGEGARKFVFFNIPQIQYKNPWVQIMMFKNMTPSPFLRFYLDSGEQVLVDVETKSNKEIMEHIRKILGKNEETLREEEEEKKQLSHPANFGPRKYCLRECICEVEGQVPCPSLVPLPKEMRGKYKAALKADAQD

1259 Q9UL17 NP_055683.3

>Q9UL17

MGIVEPGCGDMLTGTEPMPGSDEGRAPGADPQHRYFYPEPGAQDADERRGGGSLGSPYPGGALVPAPPSRFLGAYAYPPRPQAAGFPGAGESFPPPADAEGYQPGEGYAAPDPRAGLYPGPREDYALPAGLEVSGKLRVALNNHLLWSKFNQHQTEMIITKQGRRMFPFLSFTVAGLEPTSHYRMFVDVVLVDQHHWRYQSGKWVQCGKAEGSMPGNRLYVHPDSPNTGAHWMRQEVSFGKLKLTNNKGASNNVTQMIVLQSLHKYQPRLHIVEVNDGEPEAACNASNTHIFTFQETQFIAVTAYQNAEITQLKIDNNPFAKGFRENFESMYTSVDTSIPSPPGPNCQFLGGDHYSPLLPNQYPVPSRFYPDLPGQAKDVVPQAYWLGAPRDHSYEAEFRAVSMKPAFLPSAPGPTMSYYRGQEVLAPGAGWPVAPQYPPKMGPASWFRPMRTLPMEPGPGGSEGRGPEDQGPPLVWTEIAPIRPESSDSGLGEGDSKRRRVSPYPSSGDSSSPAGAPSPFDKEAEGQFYNYFPN

>NP_055683.3

MPLSSPNAAATASDMDKNSGSNSSSASSGSSKGQQPPRSASAGPAGESKPKSDGKNSSGSKRYNRKRELSYPKNESFNNQSRRSSSQKSKTFNKMPPQRGGGSSKLFSSSFNGGRRDEVAEAQRAEFSPAQFSGPKKINLNHLLNFTFEPRGQTGHFEGSGHGSWGKRNKWGHKPFNKELFLQANCQFVVSEDQDYTAHFADPDTLVNWDFVEQVRICSHEVPSCPICLYPPTAAKITRCGHIFCWACILHYLSLSEKTWSKCPICYSSVHKKDLKSVVATESHQYVVGDTITMQLMKREKGVLVALPKSKWMNVDHPIHLGDEQHSQYSKLLLASKEQVLHRVVLEEKVALEQQLAEEKHTPESCFIEAAIQELKTREEALSGLAGSRREVTGVVAALEQLVLMAPLAKESVFQPRKGVLEYLSAFDEETTEVCSLDTPSRPLALPLVEEEEAVSEPEPEGLPEACDDLELADDNLKEGTICTESSQQEPITKSGFTRLSSSPCYYFYQAEDGQHMFLHPVNVRCLVREYGSLERSPEKISATVVEIAGYSMSEDVRQRHRYLSHLPLTCEFSICELALQPPVVSKETLEMFSDDIEKRKRQRQKKAREERRRERRIEIEENKKQGKYPEVHIPLENLQQFPAFNSYTCSSDSALGPTSTEGHGALSISPLSRSPGSHADFLLTPLSPTASQGSPSFCVGSLEEDSPFPSFAQMLRVGKAKADVWPKTAPKKDENSLVPPAPVDSDGESDNSDRVPVPSFQNSFSQAIEAAFMKLDTPATSDPLSEEKGGKKRKKQKQKLLFSTSVVHTK

1260 NP_665906.1 NP_005116.1

>NP_665906.1

MSESSSKSSQPLASKQEKDGTEKRGRGRPRKQPPVSPGTALVGSQKEPSEVPTPKRPRGRPKGSKNKGAAKTRKTTTTPGRKPRGRPKKLEKEEEEGISQESSEEEQ

>NP_005116.1

MASDSGNQGTLCTLEFAVQMTCQSCVDAVRKSLQGVAGVQDVEVHLEDQMVLVHTTLPSQEVQALLEGTGRQAVLKGMGSGQLQNLGAAVAILGGPGTVQGVVRFLQLTPERCLIEGTIDGLEPGLHGLHVHQYGDLTNNCNSCGNHFNPDGASHGGPQDSDRHRGDLGNVRADADGRAIFRMEDEQLKVWDVIGRSLIIDEGEDDLGRGGHPLSKITGNSGERLACGIIARSAGLFQNPKQICSCDGLTIWEERGRPIAGKGRKESAQPPAHL

1261 NP_009153.3 NP_004125.3

>NP_009153.3

MASRRKSTTPCMVLASEQDPDLELISDLDEGPPVLTPVENTRAESISSDEEVHESVDSDNQQNKKVEGGYECKYCTFQTPDLNMFTFHVDSEHPNVVLNSSYVCVECNFLTKRYDALSEHNLKYHPGEENFKLTMVKRNNQTIFEQTINDLTFDGSFVKEENAEQAESTEVSSSGISISKTPIMKMMKNKVENKRIAVHHNSVEDVPEEKENEIKPDREEIVENPSSSASESNTSTSIVNRIHPSTASTVVTPAAVLPGLAQVITAVSAQQNSNLIPKVLIPVNSIPTYNAALDNNPLLLNTYNKFPYPTMSEITVLSAQAKYTEEQIKIWFSAQRLKHGVSWTPEEVEEARRKQFNGTVHTVPQTITVIPTHISTGSNGLPSILQTCQIVGQPGLVLTQVAGTNTLPVTAPIALTVAGVPSQNNIQKSQVPAAQPTAETKPATAAVPTSQSVKHETALVNPDSFGIRAKKTKEQLAELKVSYLKNQFPHDSEIIRLMKITGLTKGEIKKWFSDTRYNQRNSKSNQCLHLNNDSSTTIIIDSSDETTESPTVGTAQPKQSWNPFPDFTPQKFKEKTAEQLRVLQASFLNSSVLTDEELNRLRAQTKLTRREIDAWFTEKKKSKALKEEKMEIDESNAGSSKEEAGETSPADESGAPKSGSTGKICKKTPEQLHMLKSAFVRTQWPSPEEYDKLAKESGLARTDIVSWFGDTRYAWKNGNLKWYYYYQSANSSSMNGLSSLRKRGRGRPKGRGRGRPRGRPRGSKRINNWDRGPSLIKFKTGTAILKDYYLKHKFLNEQDLDELVNKSHMGYEQVREWFAERQRRSELGIELFEENEEEDEVIDDQEEDEEETDDSDTWEPPRHVKRKLSKSDD

>NP_004125.3

MISASRAAAARLVGAAASRGPTAARHQDSWNGLSHEAFRLVSRRDYASEAIKGAVVGIDLGTTNSCVAVMEGKQAKVLENAEGARTTPSVVAFTADGERLVGMPAKRQAVTNPNNTFYATKRLIGRRYDDPEVQKDIKNVPFKIVRASNGDAWVEAHGKLYSPSQIGAFVLMKMKETAENYLGHTAKNAVITVPAYFNDSQRQATKDAGQISGLNVLRVINEPTAAALAYGLDKSEDKVIAVYDLGGGTFDISILEIQKGVFEVKSTNGDTFLGGEDFDQALLRHIVKEFKRETGVDLTKDNMALQRVREAAEKAKCELSSSVQTDINLPYLTMDSSGPKHLNMKLTRAQFEGIVTDLIRRTIAPCQKAMQDAEVSKSDIGEVILVGGMTRMPKVQQTVQDLFGRAPSKAVNPDEAVAIGAAIQGGVLAGDVTDVLLLDVTPLSLGIETLGGVFTKLINRNTTIPTKKSQVFSTAADGQTQVEIKVCQGEREMAGDNKLLGQFTLIGIPPAPRGVPQIEVTFDIDANGIVHVSAKDKGTGREQQIVIQSSGGLSKDDIENMVKNAEKYAEEDRRKKERVEAVNMAEGIIHDTETKMEEFKDQLPADECNKLKEEISKMRELLARKDSETGENIRQAASSLQQASLKLFEMAYKKMASEREGSGSSGTGEQKEDQKEEKQ

1262 NP_005021.2 Q38SD2

>NP_005021.2

MSAAVTAGKLARAPADPGKAGVPGVAAPGAPAAAPPAKEIPEVLVDPRSRRRYVRGRFLGKGGFAKCFEISDADTKEVFAGKIVPKSLLLKPHQREKMSMEISIHRSLAHQHVVGFHGFFEDNDFVFVVLELCRRRSLLELHKRRKALTEPEARYYLRQIVLGCQYLHRNRVIHRDLKLGNLFLNEDLEVKIGDFGLATKVEYDGERKKTLCGTPNYIAPEVLSKKGHSFEVDVWSIGCIMYTLLVGKPPFETSCLKETYLRIKKNEYSIPKHINPVAASLIQKMLQTDPTARPTINELLNDEFFTSGYIPARLPITCLTIPPRFSIAPSSLDPSNRKPLTVLNKGLENPLPERPREKEEPVVRETGEVVDCHLSDMLQQLHSVNASKPSERGLVRQEEAEDPACIPIFWVSKWVDYSDKYGLGYQLCDNSVGVLFNDSTRLILYNDGDSLQYIERDGTESYLTVSSHPNSLMKKITLLKYFRNYMSEHLLKAGANITPREGDELARLPYLRTWFRTRSAIILHLSNGSVQINFFQDHTKLILCPLMAAVTYIDEKRDFRTYRLSLLEEYGCCKELASRLRYARTMVDKLLSSRSASNRLKAS

>Q38SD2

MSQRPPSMYWCVGPEESAVCPERAMETLNGAGDTGGKPSTRGGDPAARSRRTEGIRAAYRRGDRGGARDLLEEACDQCASQLEKGQLLSIPAAYGDLEMVRYLLSKRLVELPTEPTDDNPAVVAAYFGHTAVVQNTLPTEPTDDNPAVVAAYFGHTAVVQELLESLPGPCSPQRLLNWMLALACQRGHLGVVKLLVLTHGADPESYAVRKNEFPVIVRLPLYAAIKSGNEDIAIFLLRHGAYFCSYILLDSPDPSKHLLRKYFIEASPLPSSYPGKTALRVKWSHLRLPWVDLDWLIDISCQITELDLSANCLATLPSVIPWGLINLRKLNLSDNHLGELPGVQSSDEIICSRLLEIDISSNKLSHLPPGFLHLSKLQKLTASKNCLEKLFEEENATNWIGLRKLQELDISDNKLTELPALFLHSFKSLNSLNVSRNNLKVFPDPWACPLKCCKASRNALECLPDKMAVFWKNHLKDVDFSENALKEVPLGLFQLDALMFLRLQGNQLAALPPQEKWTCRQLKTLDLSRNQLGKNEDGLKTKRIAFFTTRGRQRSGTEAASVLEFPAFLSESLEVLCLNDNHLDTVPPSVCLLKSLSELYLGNNPGLRELPPELGQLGNLWQLDTEDLTISNVPAEIQKEGPKAMLSYLRAQLRKAEKCKLMKMIIVGPPRQGKSTLLEILQTGRAPQVVHGEATIRTTKWELQRPAGSRAKVESVEFNVWDIGGPASMATVNQCFFTDKALYVVVWNLALGEEAVANLQFWLLNIEAKAPNAVVLVVGTHLDLIEAKFRVERIATLRAYVLALCRSPSGSRATGFPDITFKHLHEISCKSLEGQEGLRQLIFHVTCSMKDVGSTIGCQRLAGRLIPRSYLSLQEAVLAEQQRRSRDDDVQYLTDRQLEQLVEQTPDNDIKDYEDLQSAISFLIETGTLLHFPDTSHGLRNLYFLDPIWLSECLQRIFNIKGSRSVAKNGVIRAEDLRMLLVGTGFTQQTEEQYFQFLAKFEIALPVANDSYLLPHLLPSKPGLDTHGMRHPTANTIQRVFKMSFVPVGFWQRFIARMLISLAEMDLQLFENKKNTKSRNRKVTIYSFTGNQRNRCSTFRVKRNQTIYWQEGLLVTFDGGYLSVESSDVNWKKKKSGGMKIVCQSEVRDFSAMAFITDHVNSLIDQWFPALTATESDGTPLMEQYVPCPVCETAWAQHTDPSEKSEDVQYFDMEDCVLTAIERDFISCPRHPDLPVPLQELVPELFMTDFPARLFLENSKLEHSEDEGSVLGQGGSGTVIYRARYQGQPVAVKRFHIKKFKNFANVPADTMLRHLRATDAMKNFSEFRQEASMLHALQHPCIVALIGISIHPLCFALELAPLSSLNTVLSENARDSSFIPLGHMLTQKIAYQIASGLAYLHKKNIIFCDLKSDNILVWSLDVKEHINIKLSDYGISRQSFHEGALGVEGTPGYQAPEIRPRIVYDEKVDMFSYGMVLYELLSGQRPALGHHQLQIAKKLSKGIRPVLGQPEEVQFRRLQALMMECWDTKPEKRPLALSVVSQMKDPTFATFMYELCCGKQTAFFSSQGQEYTVVFWDGKEESRNYTVVNTEKGLMEVQRMCCPGMKVSCQLQVQRSLWTATEDQKIYIYTLKGMCPLNTPQQALDTPAVVTCFLAVPVIKKNSYLVLAGLADGLVAVFPVVRGTPKDSCSYLCSHTANRSKFSIADEDARQNPYPVKAMEVVNSGSEVWYSNGPGLLVIDCASLEICRRLEPYMAPSMVTSVVCSSEGRGEEVVWCLDDKANSLVMYHSTTYQLCARYFCGVPSPLRDMFPVRPLDTEPPAASHTANPKVPEGDSIADVSIMYSEELGTQILIHQESLTDYCSMSSYSSSPPRQAARSPSSLPSSPASSSSVPFSTDCEDSDMLHTPGAASDRSEHDLTPMDGETFSQHLQAVKILAVRDLIWVPRRGGDVIVIGLEKDSGAQRGRVIAVLKARELTPHGVLVDAAVVAKDTVVCTFENENTEWCLAVWRGWGAREFDIFYQSYEELGRLEACTRKRR

1263 NP_004621.2 P57723

>NP_004621.2

MATGANATPLDFPSKKRKRSRWNQDTMEQKTVIPGMPTVIPPGLTREQERAYIVQLQIEDLTRKLRTGDLGIPPNPEDRSPSPEPIYNSEGKRLNTREFRTRKKLEEERHNLITEMVALNPDFKPPADYKPPATRVSDKVMIPQDEYPEINFVGLLIGPRGNTLKNIEKECNAKIMIRGKGSVKEGKVGRKDGQMLPGEDEPLHALVTANTMENVKKAVEQIRNILKQGIETPEDQNDLRKMQLRELARLNGTLREDDNRILRPWQSSETRSITNTTVCTKCGGAGHIASDCKFQRPGDPQSAQDKARMDKEYLSLMAELGEAPVPASVGSTSGPATTPLASAPRPAAPANNPPPPSLMSTTQSRPPWMNSGPSESRPYHGMHGGGPGGPGGGPHSFPHPLPSLTGGHGGHPMQHNPNGPPPPWMQPPPPPMNQGPHPPGHHGPPPMDQYLGSTPVGSGVYRLHQGKGMMPPPPMGMMPPPPPPPSGQPPPPPSGPLPPWQQQQQQPPPPPPPSSSMASSTPLPWQQNTTTTTTSAGTGSIPPWQQQQAAAAASPGAPQMQGNPTMVPLPPGVQPPLPPGAPPPPPPPPPGSAGMMYAPPPPPPPPMDPSNFVTMMGMGVAGMPPFGMPPAPPPPPPQN

>P57723

MSGSDGGLEEEPELSITLTLRMLMHGKEVGSIIGKKGETVKRIREQSSARITISEGSCPERITTITGSTAAVFHAVSMIAFKLDEDLCAAPANGGNVSRPPVTLRLVIPASQCGSLIGKAGTKIKEIRETTGAQVQVAGDLLPNSTERAVTVSGVPDAIILCVRQICAVILESPPKGATIPYHPSLSLGTVLLSANQGFSVQGQYGAVTPAEVTKLQQLSSHAVPFATPSVVPGLDPGTQTSSQEFLVPNDLIGCVIGRQGSKISEIRQMSGAHIKIGNQAEGAGERHVTITGSPVSIALAQYLITACLETAKSTSGGTPSSAPADLPAPFSPPLTALPTAPPGLLGTPYAISLSNFIGLKPMPFLALPPASPGPPPGLAAYTAKMAAANGSKKAERQKFSPY

1264 NP_056975.1 NP_055433.2

>NP_056975.1

MSAAIAALAASYGSGSGSESDSDSESSRCPLPAADSLMHLTKSPSSKPSLAVAVDSAPEVAVKEDLETGVHLDPAVKEVQYNPTYETMFAPEFGPENPFRTQQMAAPRNMLSGYAEPAHINDFMFEQQRRTFATYGYALDPSLDNHQVSAKYIGSVEEAEKNQGLTVFETGQKKTEKRKKFKENDASNIDGFLGPWAKYVDEKDVAKPSEEEQKELDEITAKRQKKGKQEEEKPGEEKTILHVKEMYDYQGRSYLHIPQDVGVNLRSTMPPEKCYLPKKQIHVWSGHTKGVSAVRLFPLSGHLLLSCSMDCKIKLWEVYGERRCLRTFIGHSKAVRDICFNTAGTQFLSAAYDRYLKLWDTETGQCISRFTNRKVPYCVKFNPDEDKQNLFVAGMSDKKIVQWDIRSGEIVQEYDRHLGAVNTIVFVDENRRFVSTSDDKSLRVWEWDIPVDFKYIAEPSMHSMPAVTLSPNGKWLACQSMDNQILIFGAQNRFRLNKKKIFKGHMVAGYACQVDFSPDMSYVISGDGNGKLNIWDWKTTKLYSRFKAHDKVCIGAVWHPHETSKVITCGWDGLIKLWD

>NP_055433.2

MNWRFVELLYFLFIWGRISVQPSHQEPAGTDQHVSKEFDWLISDRGPFHHSRSYLSFVERHRQGFTTRYKIYREFARWKVRNTAIERRDLVRHPVPLMPEFQRSIRLLGRRPTTQQFIDTIIKKYGTHLLISATLGGEEALTMYMDKSRLDRKSGNATQSVEALHQLASSYFVDRDGTMRRLHEIQISTGAIKVTETRTGPLGCNSYDNLDSVSSVLLQSTESKLHLQGLQIIFPQYLQEKFVQSALSYIMCNGEGEYLCQNSQCRCQCAEEFPQCNCPITDIQIMEYTLANMAKSWAEAYKDLENSDEFKSFMKRLPSNHFLTIGSIHQHWGNDWDLQNRYKLLQSATEAQRQKIQRTARKLFGLSVRCRHNPNHQLPRERTIQQWLARVQSLLYCNENGFWGTFLESQRSCVCHGSTTLCQRPIPCVIGGNNSCAMCSLANISLCGSCNKGYKLYRGRCEPQNVDSERSEQFISFETDLDFQDLELKYLLQKMDSRLYVHTTFISNEIRLDTFFDPRWRKRMSLTLKSNKNRMDFIHMVIGMSMRICQMRNSSLDPMFFVYVNPFSGSHSEGWNMPFGEFGYPRWEKIRLQNSQCYNWTLLLGNRWKTFFETVHIYLRSRTRLPTLLRNETGQGPVDLSDPSKRQFYIKISDVQVFGYSLRFNADLLRSAVQQVNQSYTQGGQFYSSSSVMLLLLDIRDRINRLAPPVAPGKPQLDLFSCMLKHRLKLTNSEIIRVNHALDLYNTEILKQSDQMTAKLC

1265 P0C1S8 Q2M3C7

>P0C1S8

MDDKDIDKELRQKLNFSYCEETEIEGQKKVEESREASSQTPEKGEVQDSEAKGTPPWTPLSNVHELDTSSEKDKESPDQILRTPVSHPLKCPETPAQPDSRSKLLPSDSPSTPKTMLSRLVISPTGKLPSRGPKHLKLTPAPLKDEMTSLALVNINPFTPESYKKLFLQSGGKRKIRGDLEEAGPEEGKGGLPAKRCVLRETNMASRYEKEFLEVEKIGVGEFGTVYKCIKRLDGCVYAIKRSMKTFTELSNENSALHEVYAHAVLGHHPHVVRYYSSWAEDDHMIIQNEYCNGGSLQAAISENTKSGNHFEEPKLKDILLQISLGLNYIHNSSMVHLDIKPSNIFICHKMQSESSGVIEEVENEADWFLSANVMYKIGDLGHATSINKPKVEEGDSRFLANEILQEDYRHLPKADIFALGLTIAVAAGAESLPTNGAAWHHIRKGNFPDVPQELSESFSSLLKNMIQPDAEQRPSAAALARNTVLRPSLGKTEELQQQLNLEKFKTATLERELREAQQAQSPQGYTHHGDTGVSGTHTGSRSTKRLVGGKSARSSSFTSGEREPLH

>Q2M3C7

MDGNSLLSVPSNLESSRMYDVLEPQQGRGCGSSGSGPGNSITACKKVLRSNSLLESTDYWLQNQRMPCQIGFVEDKSENCASVCFVNLDVNKDECSTEHLQQKLVNVSPDLPKLISSMNVQQPKENEIVVLSGLASGNLQADFEVSQCPWLPDICLVQCARGNRPNSTNCIIFEINKFLIGLELVQERQLHLETNILKLEDDTNCSLSSIEEDFLTASEHLEEESEVDESRNDYENINVSANVLESKQLKGATQVEWNCNKEKWLYALEDKYINKYPTPLIKTERSPENLTKNTALQSLDPSAKPSQWKREAVGNGRQATHYYHSEAFKGQMEKSQALYIPKDAYFSMMDKDVPSACAVAEQRSNLNPGDHEDTRNALPPRQDGEVTTGKYATNLAESVLQDAFIRLSQSQSTLPQESAVSVSVGSSLLPSCYSTKDTVVSRSWNELPKIVVVQSPDGSDAAPQPGISSWPEMEVSVETSSILSGENSSRQPQSALEVALACAATVIGTISSPQATERLKMEQVVSNFPPGSSGALQTQAPQGLKEPSINEYSFPSALCGMTQVASAVAVCGLGEREEVTCSVAPSGSLPPAAEASEAMPPLCGLASMELGKEAIAKGLLKEAALVLTRPNTYSSIGDFLDSMNRRIMETASKSQTLCSENVVRNELAHTLSNVILRHSIDEVHHKNMIIDPNDNRHSSEILDTLMESTNQLLLDVICFTFKKMSHIVRLGECPAVLSKETIRRRETEPSCQPSDPGASQAWTKATESSSSSPLSNSHNTSLVINNLVDGMYSKQDKGGVRPGLFKNPTLQSQLSRSHRVPDSSTATTSSKEIYLKGIAGEDTKSPHHSENECRASSEGQRSPTVSQSRSGSQEAEESIHPNTQEKYNCATSRINEVQVNLSLLGDDLLLPAQSTLQTKHPDIYCITDFAEELADTVVSMATEIAAICLDNSSGKQPWFCAWKRGSEFLMTPNVPCRSLKRKKESQGSGTAVRKHKPPRLSEIKRKTDEHPELKEKLMNRVVDESMNLEDVPDSVNLFANEVAAKIMNLTEFSMVDGMWQAQGYPRNRLLSGDRWSRLKASSCESIPEEDSEARAYVNSLGLMSTLSQPVSRASSVSKQSSCESITDEFSRFMVNQMENEGRGFELLLDYYAGKNASSILNSAMQQACRKSDHLSVRPSCPSKQSSTESITEEFYRYMLRDIERDSRESASSRRSSQDWTAGLLSPSLRSPVCHRQSSMPDSRSPCSRLTVNVPIKANSLDGFAQNCPQDFLSVQPVSSASSSGLCKSDSCLYRRGGTDHITNMLIHETWASSIEALMRKNKIIVDDAEEADTEPVSGGSPSQAEKCANRLAASRMCSGPTLLVQESLDCPRKDSVTECKQPPVSSLSKTASLTNHSPLDSKKETSSCQDPVPINHKRRSLCSREVPLIQIETDQREACAGEPEPFLSKSSLLEEAEGHSNDKNIPDVVRGGDTAVSACQIHSDSLDTRDVPEAEASTEARAPDEAPNPPSSSEESTGSWTQLANEEDNPDDTSSFLQLSERSMSNGNSSATSSLGIMDLDIYQESMPSSPMINELVEEKKILKGQSESTEAPASGPPTGTASPQRSLLVINFDLEPECPDAELRATLQWIAASELGIPTIYFKKSQENRIEKFLDVVQLVHRKSWKVGDIFHAVVQYCKMHEEQKDGRLSLFDWLLELG

1266 NP_002156.2 Q8WVN8

>NP_002156.2

MKVASGSTATAAAGPSCALKAGKTASGAGEVVRCLSEQSVAISRCAGGAGARLPALLDEQQVNVLLYDMNGCYSRLKELVPTLPQNRKVSKVEILQHVIDYIRDLQLELNSESEVGTPGGRGLPVRAPLSTLNGEISALTAEAACVPADDRILCR

>Q8WVN8

MSVSGLKAELKFLASIFDKNHERFRIVSWKLDELHCQFLVPQQGSPHSLPPPLTLHCNITESYPSSSPIWFVDSEDPNLTSVLERLEDTKNNNLLRQQLKWLICELCSLYNLPKHLDVEMLDQPLPTGQNGTTEEVTSEEEEEEEEMAEDIEDLDHYEMKEEEPISGKKSEDEGIEKENLAILEKIRKTQRQDHLNGAVSGSVQASDRLMKELRDIYRSQSYKTGIYSVELINDSLYDWHVKLQKVDPDSPLHSDLQILKEKEGIEYILLNFSFKDNFPFDPPFVRVVLPVLSGGYVLGGGALCMELLTKQGWSSAYSIESVIMQINATLVKGKARVQFGANKNQYNLARAQQSYNSIVQIHEKNGWYTPPKEDG

1267 NP_004233.1 O75170

>NP_004233.1

MPKRKSPENTEGKDGSKVTKQEPTRRSARLSAKPAPPKPEPKPRKTSAKKEPGAKISRGAKGKKEEKQEAGKEGTAPSENGETKAEEAQKTESVDNEGE

>O75170

MFWKFDLNTTSHVDKLLDKEHVTLQELMDEDDILQECKAQNQKLLDFLCRQQCMEELVSLITQDPPLDMEEKVRFKYPNTACELLTCDVPQISDRLGGDESLLSLLYDFLDHEPPLNPLLASFFSKTIGNLIARKTEQVITFLKKKDKFISLVLKHIGTSALMDLLLRLVSCVEPAGLRQDVLHWLNEEKVIQRLVELIHPSQDEDRQSNASQTLCDIVRLGRDQGSQLQEALEPDPLLTALESQDCVEQLLKNMFDGDRTESCLVSGTQVLLTLLETRRVGTEGLVDSFSQGLERSYAVSSSVLHGIEPRLKDFHQLLLNPPKKKAILTTIGVLEEPLGNARLHGARLMAALLHTNTPSINQELCRLNTMDLLLDLFFKYTWNNFLHFQVELCIAAILSHAAREERTEASGSESRVEPPHENGNRSLETPQPAASLPDNTMVTHLFQKCCLVQRILEAWEANDHTQAAGGMRRGNMGHLTRIANAVVQNLERGPVQTHISEVIRGLPADCRGRWESFVEETLTETNRRNTVDLVSTHHLHSSSEDEDIEGAFPNELSLQQAFSDYQIQQMTANFVDQFGFNDEEFADQDDNINAPFDRIAEINFNIDADEDSPSAALFEACCSDRIQPFDDDEDEDIWEDSDTRCAARVMARPRFGAPHASESCSKNGPERGGQDGKASLEAHRDAPGAGAPPAPGKKEAPPVEGDSEGAMWTAVFDEPANSTPTAPGVVRDVGSSVWAAGTSAPEEKGWAKFTDFQPFCCSESGPRCSSPVDTECSHAEGSRSQGPEKASQASYFAVSPASPCAWNVCVTRKAPLLASDSSSSGGSHSEDGDQKAASAMDAVSRGPGREAPPLPTVARTEEAVGRVGCADSRLLSPACPAPKEVTAAPAVAVPPEATVAITTALSKAGPAIPTPAVSSALAVAVPLGPIMAVTAAPAMVATLGTVTKDGKTDAPPEGAALNGPV

1268 NP_115705.2 BAB16421.1

>NP_115705.2

MATAMYLEHYLDSIENLPCELQRNFQLMRELDQRTEDKKAEIDILAAEYISTVKTLSPDQRVERLQKIQNAYSKCKEYSDDKVQLAMQTYEMVDKHIRRLDADLARFEADLKDKMEGSDFESSGGRGLKKGRGQKEKRGSRGRGRRTSEEDTPKKKKHKGGSEFTDTILSVHPSDVLDMPVDPNEPTYCLCHQVSYGEMIGCDNPDCPIEWFHFACVDLTTKPKGKWFCPRCVQEKRKKK

>BAB16421.1

MGSSSEASFRSAQASCSGARRQGLGRGDQNLSVMPPNGRAQTHTPGWVSDPLVLGAQVHGGCRGIEALSVSSGSWSSATVWILTGLGLGLSRPFLPGATVLRDRPLGSAFELSYDQKKAPLRLQ

1269 NP_004292.1 NP_001753.1

>NP_004292.1

MSGGVYGGDEVGALVFDIGSYTVRAGYAGEDCPKVDFPTAIGMVVERDDGSTLMEIDGDKGKQGGPTYYIDTNALRVPRENMEAISPLKNGMVEDWDSFQAILDHTYKMHVKSEASLHPVLMSEAPWNTRAKREKLTELMFEHYNIPAFFLCKTAVLTAFANGRSTGLILDSGATHTTAIPVHDGYVLQQGIVKSPLAGDFITMQCRELFQEMNIELVPPYMIASKEAVREGSPANWKRKEKLPQVTRSWHNYMCNCVIQDFQASVLQVSDSTYDEQVAAQMPTVHYEFPNGYNCDFGAERLKIPEGLFDPSNVKGLSGNTMLGVSHVVTTSVGMCDIDIRPGLYGSVIVAGGNTLIQSFTDRLNRELSQKTPPSMRLKLIANNTTVERRFSSWIGGSILASLGTFQQMWISKQEYEEGGKQCVERKCP

>NP_001753.1

MAAVKTLNPKAEVARAQAALAVNISAARGLQDVLRTNLGPKGTMKMLVSGAGDIKLTKDGNVLLHEMQIQHPTASLIAKVATAQDDITGDGTTSNVLIIGELLKQADLYISEGLHPRIITEGFEAAKEKALQFLEEVKVSREMDRETLIDVARTSLRTKVHAELADVLTEAVVDSILAIKKQDEPIDLFMIEIMEMKHKSETDTSLIRGLVLDHGARHPDMKKRVEDAYILTCNVSLEYEKTEVNSGFFYKSAEEREKLVKAERKFIEDRVKKIIELKRKVCGDSDKGFVVINQKGIDPFSLDALSKEGIVALRRAKRRNMERLTLACGGVALNSFDDLSPDCLGHAGLVYEYTLGEEKFTFIEKCNNPRSVTLLIKGPNKHTLTQIKDAVRDGLRAVKNAIDDGCVVPGAGAVEVAMAEALIKHKPSVKGRAQLGVQAFADALLIIPKVLAQNSGFDLQETLVKIQAEHSESGQLVGVDLNTGEPMVAAEVGVWDNYCVKKQLLHSCTVIATNILLVDEIMRAGMSSLKG

1270 NP_004517.2 NP_056442.2

>NP_004517.2

MAESSESFTMASSPAQRRRGNDPLTSSPGRSSRRTDALTSSPGRDLPPFEDESEGLLGTEGPLEEEEDGEELIGDGMERDYRAIPELDAYEAEGLALDDEDVEELTASQREAAERAMRQRDREAGRGLGRMRRGLLYDSDEEDEERPARKRRQVERATEDGEEDEEMIESIENLEDLKGHSVREWVSMAGPRLEIHHRFKNFLRTHVDSHGHNVFKERISDMCKENRESLVVNYEDLAAREHVLAYFLPEAPAELLQIFDEAALEVVLAMYPKYDRITNHIHVRISHLPLVEELRSLRQLHLNQLIRTSGVVTSCTGVLPQLSMVKYNCNKCNFVLGPFCQSQNQEVKPGSCPECQSAGPFEVNMEETIYQNYQRIRIQESPGKVAAGRLPRSKDAILLADLVDSCKPGDEIELTGIYHNNYDGSLNTANGFPVFATVILANHVAKKDNKVAVGELTDEDVKMITSLSKDQQIGEKIFASIAPSIYGHEDIKRGLALALFGGEPKNPGGKHKVRGDINVLLCGDPGTAKSQFLKYIEKVSSRAIFTTGQGASAVGLTAYVQRHPVSREWTLEAGALVLADRGVCLIDEFDKMNDQDRTSIHEAMEQQSISISKAGIVTSLQARCTVIAAANPIGGRYDPSLTFSENVDLTEPIISRFDILCVVRDTVDPVQDEMLARFVVGSHVRHHPSNKEEEGLANGSAAEPAMPNTYGVEPLPQEVLKKYIIYAKERVHPKLNQMDQDKVAKMYSDLRKESMATGSIPITVRHIESMIRMAEAHARIHLRDYVIEDDVNMAIRVMLESFIDTQKFSVMRSMRKTFARYLSFRRDNNELLLFILKQLVAEQVTYQRNRFGAQQDTIEVPEKDLVDKARQINIHNLSAFYDSELFRMNKFSHDLKRKMILQQF

>NP_056442.2

MDALKSAGRALIRSPSLAKQSWGGGGRHRKLPENWTDTRETLLEGMLFSLKYLGMTLVEQPKGEELSAAAIKRIVATAKASGKKLQKVTLKVSPRGIILTDNLTNQLIENVSIYRISYCTADKMHDKVFAYIAQSQHNQSLECHAFLCTKRKMAQAVTLTVAQAFKVAFEFWQVSKEEKEKRDKASQEGGDVLGARQDCTPSLKSLVATGNLLDLEETAKAPLSTVSANTTNMDEVPRPQALSGSSVVWELDDGLDEAFSRLAQSRTNPQVLDTGLTAQDMHYAQCLSPVDWDKPDSSGTEQDDLFSF

1271 NP_003066.2 O75879

>NP_003066.2

MAVRKKDGGPNVKYYEAADTVTQFDNVRLWLGKNYKKYIQAEPPTNKSLSSLVVQLLQFQEEVFGKHVSNAPLTKLPIKCFLDFKAGGSLCHILAAAYKFKSDQGWRRYDFQNPSRMDRNVEMFMTIEKSLVQNNCLSRPNIFLCPEIEPKLLGKLKDIIKRHQGTVTEDKNNASHVVYPVPGNLEEEEWVRPVMKRDKQVLLHWGYYPDSYDTWIPASEIEASVEDAPTPEKPRKVHAKWILDTDTFNEWMNEEDYEVNDDKNPVSRRKKISAKTLTDEVNSPDSDRRDKKGGNYKKRKRSPSPSPTPEAKKKNAKKGPSTPYTKSKRGHREEEQEDLTKDMDEPSPVPNVEEVTLPKTVNTKKDSESAPVKGGTMTDLDEQEDESMETTGKDEDENSTGNKGEQTKNPDLHEDNVTEQTHHIIIPSYAAWFDYNSVHAIERRALPEFFNGKNKSKTPEIYLAYRNFMIDTYRLNPQEYLTSTACRRNLAGDVCAIMRVHAFLEQWGLINYQVDAESRPTPMGPPPTSHFHVLADTPSGLVPLQPKTPQQTSASQQMLNFPDKGKEKPTDMQNFGLRTDMYTKKNVPSKSKAAASATREWTEQETLLLLEALEMYKDDWNKVSEHVGSRTQDECILHFLRLPIEDPYLEDSEASLGPLAYQPIPFSQSGNPVMSTVAFLASVVDPRVASAAAKSALEEFSKMKEEVPTALVEAHVRKVEEAAKVTGKADPAFGLESSGIAGTTSDEPERIEESGNDEARVEGQATDEKKEPKEPREGGGAIEEEAKEKTSEAPKKDEEKGKEGDSEKESEKSDGDPIVDPEKEKEPKEGQEEVLKEVVESEGERKTKVERDIGEGNLSTAAAAALAAAAVKAKHLAAVEERKIKSLVALLVETQMKKLEIKLRHFEELETIMDREREALEYQRQQLLADRQAFHMEQLKYAEMRARQQHFQQMHQQQQQPPPALPPGSQPIPPTGAAGPPAVHGLAVAPASVVPAPAGSGAPPGSLGPSEQIGQAGSTAGPQQQQPAGAPQPGAVPPGVPPPGPHGPSPFPNQQTPPSMMPGAVPGSGHPGVAGNAPLGLPFGMPPPPPPPAPSIIPFGSLADSISINLPAPPNLHGHHHHLPFAPGTLPPPNLPVSMANPLHPNLPATTTMPSSLPLGPGLGSAAAQSPAIVAAVQGNLLPSASPLPDPGTPLPPDPTAPSPGTVTPVPPPQ

>O75879

MAAPMLRWGCRGRRWAFARVDGGSCHRRGAPTGSTSNQIRGESSVAQQPLHTAQKTRKGEHKWAAVVGLEIHAQISSNSKLFSGSQVRFSAPPNSLVSFFDASLPGTLPVLNRRCVEAAVMTGLALNCHINKKSLFDRKHYFYADLPAGYQITQQRLPIAVNGSLIYGVCAGKKQSQVIPKTVRIKQIQLEQDSGKSLHDNLRSQTLIDLNRAGVGLLEVVLEPDMSCGEEAATAVRELQLILQALGTSQANMAEGQLRVDANISVHHPGEPLGVRTEVKNLNSIRFLAKAIDYEIQRQINELENGGEILNETRSFHHKLGCTMSMRDKEGKQDYRFMPEPNLPPLVLYDATSLPAGADPQQVINIDQIRETLPELPSVTREKLVQQYGMLLEHSFTLLNEVGLLEFFQNVIKETRAEPKKVTSWVLNTFLGYLKQQNLAVSESPVTPSALAELLDLLDSRTISSSAAKQVFEELWKREGKTPGQIVSEKQLELMQDQGALEQLCHSVMEAHPQVVMDVKNRNPRAINKLIGLVRKATQSRADPVMIKEILEKKLSL

1272 Q16560 Q99551

>Q16560

MNDWMPIAKEYDPLKAGSIDGTDEDPHDRAVWRAMLARYVPNKGVIGDPLLTLFVARLNLQTKEDKLKEVFSRYGDIRRLRLVRDLVTGFSKGYAFIEYKEERAVIKAYRDADGLVIDQHEIFVDYELERTLKGWIPRRLGGGLGGKKESGQLRFGGRDRPFRKPINLPVVKNDLYREGKRERRERSRSRERHWDSRTRDRDHDRGREKRWQEREPTRVWPDNDWERERDFRDDRIKGREKKERGK

>Q99551

MQSLSLGQTSISKGLNYLTIMAPGNLWHMRNNFLFGSRCWMTRFSAENIFKSVSFRLFGVKCHNTDSEPLKNEDLLKNLLTMGVDIDMARKRQPGVFHRMITNEQDLKMFLLSKGASKEVIASIISRYPRAITRTPENLSKRWDLWRKIVTSDLEIVNILERSPESFFRSNNNLNLENNIKFLYSVGLTRKCLCRLLTNAPRTFSNSLDLNKQMVEFLQAAGLSLGHNDPADFVRKIIFKNPFILIQSTKRVKANIEFLRSTFNLNSEELLVLICGPGAEILDLSNDYARRSYANIKEKLFSLGCTEEEVQKFVLSYPDVIFLAEKKFNDKIDCLMEENISISQIIENPRVLDSSISTLKSRIKELVNAGCNLSTLNITLLSWSKKRYEAKLKKLSRFA

1273 NP_002219.1 Q58A45

>NP_002219.1

MTAKMETTFYDDALNASFLPSESGPYGYSNPKILKQSMTLNLADPVGSLKPHLRAKNSDLLTSPDVGLLKLASPELERLIIQSSNGHITTTPTPTQFLCPKNVTDEQEGFAEGFVRALAELHSQNTLPSVTSAAQPVNGAGMVAPAVASVAGGSGSGGFSASLHSEPPVYANLSNFNPGALSSGGGAPSYGAAGLAFPAQPQQQQQPPHHLPQQMPVQHPRLQALKEEPQTVPEMPGETPPLSPIDMESQERIKAERKRMRNRIAASKCRKRKLERIARLEEKVKTLKAQNSELASTANMLREQVAQLKQKVMNHVNSGCQLMLTQQLQTF

>Q58A45

MDGGALTDTSLTDSYFSTSFIGVNGFGSPVETKYPLMQRMTNSSSSPSLLNDSAKPYSAHDPLTSPASSLFNDFGALNISQRRKPRKYRLGMLEERLVPMGSKARKAKNPIGCLADRCKSGVPINMVWWNRVTENNLQTPNPTASEFIPKGGSTSRLSNVSQSNMSAFSQVFSHPSMGSPATAGLAPGMSLSAGSSPLHSPKITPHTSPAPRRRSHTPNPASYMVPSSASTSVNNPVSQTPSSGQVIQKETVGGTTYFYTDTTPAPLTGMVFPNYHIYPPTAPHVAYMQPKANAPSFFMADELRQELINRHLITMAQIDQADMPAVPTEVDSYHSLFPLEPLPPPNRIQKSSNFGYITSCYKAVNSKDDLPYCLRRIHGFRLVNTKCMVLVDMWKKIQHSNIVTLREVFTTKAFAEPSLVFAYDFHAGGETMMSRHFNDPNADAYFTKRKWGQHEGPLPRQHAGLLPESLIWAYIVQLSSALRTIHTAGLACRVMDPTKILITGKTRLRVNCVGVFDVLTFDNSQNNNPLALMAQYQQADLISLGKVVLALACNSLAGIQRENLQKAMELVTINYSSDLKNLILYLLTDQNRMRSVNDIMPMIGARFYTQLDAAQMRNDVIEEDLAKEVQNGRLFRLLAKLGTINERPEFQKDPTWSETGDRYLLKLFRDHLFHQVTEAGAPWIDLSHIISCLNKLDAGVPEKISLISRDEKSVLVVTYSDLKRCFENTFQELIAAANGQL

1274 O43474 Q13084

>O43474

MRQPPGESDMAVSDALLPSFSTFASGPAGREKTLRQAGAPNNRWREELSHMKRLPPVLPGRPYDLAAATVATDLESGGAGAACGGSNLAPLPRRETEEFNDLLDLDFILSNSLTHPPESVAATVSSSASASSSSSPSSSGPASAPSTCSFTYPIRAGNDPGVAPGGTGGGLLYGRESAPPPTAPFNLADINDVSPSGGFVAELLRPELDPVYIPPQQPQPPGGGLMGKFVLKASLSAPGSEYGSPSVISVSKGSPDGSHPVVVAPYNGGPPRTCPKIKQEAVSSCTHLGAGPPLSNGHRPAAHDFPLGRQLPSRTTPTLGLEEVLSSRDCHPALPLPPGFHPHPGPNYPSFLPDQMQPQVPPLHYQGQSRGFVARAGEPCVCWPHFGTHGMMLTPPSSPLELMPPGSCMPEEPKPKRGRRSWPRKRTATHTCDYAGCGKTYTKSSHLKAHLRTHTGEKPYHCDWDGCGWKFARSDELTRHYRKHTGHRPFQCQKCDRAFSRSDHLALHMKRHF

>Q13084

MAFTASQRWEEGQARQIGFRFRWALNPERLAMPLHKYPVWLWKRLQLREGICSRLPGHYLRSLEEERTPTPVHYRPHGAKFKINPKNGQRERVEDVPIPIYFPPESQRGLWGGEGWILGQIYANNDKLSKRLKKVWKPQLFEREFYSEILDKKFTVTVTMRTLDLIDEAYGLDFYILKTPKEDLCSKFGMDLKRGMLLRLARQDPQLHPEDPERRAAIYDKYKEFAIPEEEAEWVGLTLEEAIEKQRLLEEKDPVPLFKIYVAELIQQLQQQALSEPAVVQKRASGQ

1275 P17036 NP_079017.1

>P17036

METQADLVSQEPQALLDSALPSKVPAFSDKDSLGDEMLAAALLKAKSQELVTFEDVAVYFIRKEWKRLEPAQRDLYRDVMLENYGNVFSLDRETRTENDQEISEDTRSHGVLLGRFQKDISQGLKFKEAYEREVSLKRPLGNSPGERLNRKMPDFGQVTVEEKLTPRGERSEKYNDFGNSFTVNSNLISHQRLPVGDRPHKCDECSKSFNRTSDLIQHQRIHTGEKPYECNECGKAFSQSSHLIQHQRIHTGEKPYECSDCGKTFSCSSALILHRRIHTGEKPYECNECGKTFSWSSTLTHHQRIHTGEKPYACNECGKAFSRSSTLIHHQRIHTGEKPYECNECGKAFSQSSHLYQHQRIHTGEKPYECMECGGKFTYSSGLIQHQRIHTGENPYECSECGKAFRYSSALVRHQRIHTGEKPLNGIGMSKSSLRVTTELNIREST

>NP_079017.1

MEEAEELLLEGKKALQLAREPRLGLDLGWNPSGEGCTQGLKDVPPEPTRDILALKSLPRGLALGPSLAKEQRLGVWCVGDPLQPGLLWGPLEEESASKEKGEGVKPRQEENLSLGPWGDVCACEQSSGWTSLVQRGRLESEGNVAPVRISERLHLQVYQLVLPGSELLLWPQPSSEGPSLTQPGLDKEAAVAVVTEVESAVQQEVASPGEDAAEPCIDPGSQSPSGIQAENMVSPGLKFPTQDRISKDSQPLGPLLQDGDVDEECPAQAQMPPELQSNSATQQDPDGSGASFSSSARGTQPHGYLAKKLHSPSDQCPPRAKTPEPGAQQSGFPTLSRSPPGPAGSSPKQGRRYRCGECGKAFLQLCHLKKHAFVHTGHKPFLCTECGKSYSSEESFKAHMLGHRGVRPFPCPQCDKAYGTQRDLKEHQVVHSGARPFACDQCGKAFARRPSLRLHRKTHQVPAAPAPCPCPVCGRPLANQGSLRNHMRLHTGEKPFLCPHCGRAFRQRGNLRGHLRLHTGERPYRCPHCADAFPQLPELRRHLISHTGEAHLCPVCGKALRDPHTLRAHERLHSGERPFPCPQCGRAYTLATKLRRHLKSHLEDKPYRCPTCGMGYTLPQSLRRHQLSHRPEAPCSPPSVPSAASEPTVVLLQAEPQLLDTHREEEVSPARDVVEVTISESQEKCFVVPEEPDAAPSLVLIHKDMGLGAWAEVVEVEMGT

1276 NP_006089.1 NP_006248.1

>NP_006089.1

MTEQMTLRGTLKGHNGWVTQIATTPQFPDMILSASRDKTIIMWKLTRDETNYGIPQRALRGHSHFVSDVVISSDGQFALSGSWDGTLRLWDLTTGTTTRRFVGHTKDVLSVAFSSDNRQIVSGSRDKTIKLWNTLGVCKYTVQDESHSEWVSCVRFSPNSSNPIIVSCGWDKLVKVWNLANCKLKTNHIGHTGYLNTVTVSPDGSLCASGGKDGQAMLWDLNEGKHLYTLDGGDIINALCFSPNRYWLCAATGPSIKIWDLEGKIIVDELKQEVISTSSKAEPPQCTSLAWSADGQTLFAGYTDNLVRVWQVTIGTR

>NP_006248.1

MSPFLRIGLSNFDCGSCQSCQGEAVNPYCAVLVKEYVESENGQMYIQKKPTMYPPWDSTFDAHINKGRVMQIIVKGKNVDLISETTVELYSLAERCRKNNGKTEIWLELKPQGRMLMNARYFLEMSDTKDMNEFETEGFFALHQRRGAIKQAKVHHVKCHEFTATFFPQPTFCSVCHEFVWGLNKQGYQCRQCNAAIHKKCIDKVIAKCTGSAINSRETMFHKERFKIDMPHRFKVYNYKSPTFCEHCGTLLWGLARQGLKCDACGMNVHHRCQTKVANLCGINQKLMAEALAMIESTQQARCLRDTEQIFREGPVEIGLPCSIKNEARPPCLPTPGKREPQGISWESPLDEVDKMCHLPEPELNKERPSLQIKLKIEDFILHKMLGKGSFGKVFLAEFKKTNQFFAIKALKKDVVLMDDDVECTMVEKRVLSLAWEHPFLTHMFCTFQTKENLFFVMEYLNGGDLMYHIQSCHKFDLSRATFYAAEIILGLQFLHSKGIVYRDLKLDNILLDKDGHIKIADFGMCKENMLGDAKTNTFCGTPDYIAPEILLGQKYNHSVDWWSFGVLLYEMLIGQSPFHGQDEEELFHSIRMDNPFYPRWLEKEAKDLLVKLFVREPEKRLGVRGDIRQHPLFREINWEELERKEIDPPFRPKVKSPFDCSNFDKEFLNEKPRLSFADRALINSMDQNMFRNFSFMNPGMERLIS

1277 Q12946 NP_055433.2

>Q12946

MSSAPEKQQPPHGGGGGGGGGGGAAMDPASSGPSKAKKTNAGIRRPEKPPYSYIALIVMAIQSSPTKRLTLSEIYQFLQSRFPFFRGSYQGWKNSVRHNLSLNECFIKLPKGLGRPGKGHYWTIDPASEFMFEEGSFRRRPRGFRRKCQALKPMYSMMNGLGFNHLPDTYGFQGSAGGLSCPPNSLALEGGLGMMNGHLPGNVDGMALPSHSVPHLPSNGGHSYMGGCGGAAAGEYPHHDSSVPASPLLPTGAGGVMEPHAVYSGSAAAWPPSASAALNSGASYIKQQPLSPCNPAANPLSGSLSTHSLEQPYLHQNSHNAPAELQGIPRYHSQSPSMCDRKEFVFSFNAMASSSMHSAGGGSYYHQQVTYQDIKPCVM

>NP_055433.2

MNWRFVELLYFLFIWGRISVQPSHQEPAGTDQHVSKEFDWLISDRGPFHHSRSYLSFVERHRQGFTTRYKIYREFARWKVRNTAIERRDLVRHPVPLMPEFQRSIRLLGRRPTTQQFIDTIIKKYGTHLLISATLGGEEALTMYMDKSRLDRKSGNATQSVEALHQLASSYFVDRDGTMRRLHEIQISTGAIKVTETRTGPLGCNSYDNLDSVSSVLLQSTESKLHLQGLQIIFPQYLQEKFVQSALSYIMCNGEGEYLCQNSQCRCQCAEEFPQCNCPITDIQIMEYTLANMAKSWAEAYKDLENSDEFKSFMKRLPSNHFLTIGSIHQHWGNDWDLQNRYKLLQSATEAQRQKIQRTARKLFGLSVRCRHNPNHQLPRERTIQQWLARVQSLLYCNENGFWGTFLESQRSCVCHGSTTLCQRPIPCVIGGNNSCAMCSLANISLCGSCNKGYKLYRGRCEPQNVDSERSEQFISFETDLDFQDLELKYLLQKMDSRLYVHTTFISNEIRLDTFFDPRWRKRMSLTLKSNKNRMDFIHMVIGMSMRICQMRNSSLDPMFFVYVNPFSGSHSEGWNMPFGEFGYPRWEKIRLQNSQCYNWTLLLGNRWKTFFETVHIYLRSRTRLPTLLRNETGQGPVDLSDPSKRQFYIKISDVQVFGYSLRFNADLLRSAVQQVNQSYTQGGQFYSSSSVMLLLLDIRDRINRLAPPVAPGKPQLDLFSCMLKHRLKLTNSEIIRVNHALDLYNTEILKQSDQMTAKLC

1278 Q9H3D4 Q9BUE6

>Q9H3D4

MNFETSRCATLQYCPDPYIQRFVETPAHFSWKESYYRSTMSQSTQTNEFLSPEVFQHIWDFLEQPICSVQPIDLNFVDEPSEDGATNKIEISMDCIRMQDSDLSDPMWPQYTNLGLLNSMDQQIQNGSSSTSPYNTDHAQNSVTAPSPYAQPSSTFDALSPSPAIPSNTDYPGPHSFDVSFQQSSTAKSATWTYSTELKKLYCQIAKTCPIQIKVMTPPPQGAVIRAMPVYKKAEHVTEVVKRCPNHELSREFNEGQIAPPSHLIRVEGNSHAQYVEDPITGRQSVLVPYEPPQVGTEFTTVLYNFMCNSSCVGGMNRRPILIIVTLETRDGQVLGRRCFEARICACPGRDRKADEDSIRKQQVSDSTKNGDGTKRPFRQNTHGIQMTSIKKRRSPDDELLYLPVRGRETYEMLLKIKESLELMQYLPQHTIETYRQQQQQQHQHLLQKQTSIQSPSSYGNSSPPLNKMNSMNKLPSVSQLINPQQRNALTPTTIPDGMGANIPMMGTHMPMAGDMNGLSPTQALPPPLSMPSTSHCTPPPPYPTDCSIVSFLARLGCSSCLDYFTTQGLTTIYQIEHYSMDDLASLKIPEQFRHAIWKGILDHRQLHEFSSPSHLLRTPSSASTVSVGSSETRGERVIDAVRFTLRQTISFPPRDEWNDFNFDMDARRNKQQRIKEEGE

>Q9BUE6

MSASLVRATVRAVSKRKLQPTRAALTLTPSAVNKIKQLLKDKPEHVGVKVGVRTRGCNGLSYTLEYTKTKGDSDEEVIQDGVRVFIEKKAQLTLLGTEMDYVEDKLSSEFVFNNPNIKGTCGCGESFNI

1279 NP_116062.2 O15131

>NP_116062.2

MASPQGGQIAIAMRLRNQLQSVYKMDPLRNEEEVRVKIKDLNEHIVCCLCAGYFVDATTITECLHTFCKSCIVKYLQTSKYCPMCNIKIHETQPLLNLKLDRVMQDIVYKLVPGLQDSEEKRIREFYQSRGLDRVTQPTGEEPALSNLGLPFSSFDHSKAHYYRYDEQLNLCLERLSSGKDKNKSVLQNKYVRCSVRAEVRHLRRVLCHRLMLNPQHVQLLFDNEVLPDHMTMKQIWLSRWFGKPSPLLLQYSVKEKRR

>O15131

MASPGKDNYRMKSYKNKALNPQEMRRRREEEGIQLRKQKREEQLFKRRNVYLPRNDESMLESPIQDPDISSTVPIPEEEVVTTDMVQMIFSNNADQQLTATQKFRKLLSKEPNPPIDQVIQKPGVVQRFVKFLERNENCTLQFEAAWALTNIASGTFLHTKVVIETGAVPIFIKLLNSEHEDVQEQAVWALGNIAGDNAECRDFVLNCEILPPLLELLTNSNRLTTTRNAVWALSNLCRGKNPPPNFSKVSPCLNVLSRLLFSSDPDVLADVCWALSYLSDGPNDKIQAVIDSGVCRRLVELLMHNDYKVVSPALRAVGNIVTGDDIQTQVILNCSALPCLLHLLSSPKESIRKEACWTVSNITAGNRAQIQAVIDANIFPVLIEILQKAEFRTRKEAAWAITNATSGGTPEQIRYLVALGCIKPLCDLLTVMDSKIVQVALNGLENILRLGEQESKQNGIGINPYCALIEEAYGLDKIEFLQSHENQEIYQKAFDLIEHYFGVEEDDPSIVPQVDENQQQFIFQQQEAPMDGFQL

1280 Q5H9F3 NP_001167.2

>Q5H9F3

MISTAPLYSGVHNWTSSDRIRMCGINEERRAPLSDEESTTGDCQHFGSQEFCVSSSFSKVELTAVGSGSNARGADPDGSATEKLGHKSEDKPDDPQPKMDYAGNVAEAEGFLVPLSSPGDGLKLPASDSAEASNSRADCSWTPLNTQMSKQVDCSPAGVKALDSRQGVGEKNTFILATLGTGVPVEGTLPLVTTNFSPLPAPICPPAPGSASVPHSVPDAFQVPLSVPAPVPHSGLVPVQVATSVPAPSPPLAPVPALAPAPPSVPTLISDSNPLSVSASVLVPVPASAPPSGPVPLSAPAPAPLSVPVSAPPLALIQAPVPPSAPTLVLAPVPTPVLAPMPASTPPAAPAPPSVPMPTPTPSSGPPSTPTLIPAFAPTPVPAPTPAPIFTPAPTPMPAATPAAIPTSAPIPASFSLSRVCFPAAQAPAMQKVPLSFQPGTVLTPSQPLVYIPPPSCGQPLSVATLPTTLGVSSTLTLPVLPSYLQDRCLPGVLASPELRSYPYAFSVARPLTSDSKLVSLEVNRLPCTSPSGSTTTQPAPDGVPGPLADTSLVTASAKVLPTPQPLLPAPSGSSAPPHPAKMPSGTEQQTEGTSVTFSPLKSPPQLEREMASPPECSEMPLDLSSKSNRQKLPLPNQRKTPPMPVLTPVHTSSKALLSTVLSRSQRTTQAAGGNVTSCLGSTSSPFVIFPEIVRNGDPSTWVKNSTALISTIPGTYVGVANPVPASLLLNKDPNLGLNRDPRHLPKQEPISIIDQGEPKGTGATCGKKGSQAGAEGQPSTVKRYTPARIAPGLPGCQTKELSLWKPTGPANIYPRCSVNGKPTSTQVLPVGWSPYHQASLLSIGISSAGQLTPSQGAPIRPTSVVSEFSGVPSLSSSEAVHGLPEGQPRPGGSFVPEQDPVTKNKTCRIAAKPYEEQVNPVLLTLSPQTGTLALSVQPSGGDIRMNQGPEESESHLCSDSTPKMEGPQGACGLKLAGDTKPKNQVLATYMSHELVLATPQNLPKMPELPLLPHDSHPKELILDVVPSSRRGSSTERPQLGSQVDLGRVKMEKVDGDVVFNLATCFRADGLPVAPQRGQAEVRAKAGQARVKQESVGVFACKNKWQPDDVTESLPPKKMKCGKEKDSEEQQLQPQAKAVVRSSHRPKCRKLPSDPQESTKKSPRGASDSGKEHNGVRGKHKHRKPTKPESQSPGKRADSHEEGSLEKKAKSSFRDFIPVVLSTRTRSQSGSICSSFAGMADSDMGSQEVFPTEEEEEVTPTPAKRRKVRKTQRDTQYRSHHAQDKSLLSQGRRHLWRAREMPWRTEAARQMWDTNEEEEEEEEEGLLKRKKRRRQKSRKYQTGEYLTEQEDEQRRKGRADLKARKQKTSSSQSLEHRLRNRNLLLPNKVQGISDSPNGFLPNNLEEPACLENSEKPSGKRKCKTKHMATVSEEAKDVVLYCLQKDSEDVNHRDNAGYTALHEACSRGWTDILNILLEHGANVNCSAQDGTRPVHDAVVNDNLETIWLLLSYGADPTLATYSGQTAMKLASSDTMKRFLSDHLSDLQGRAEGDPGVSWDFYSSSVLEEKDGFACDLLHNPPGSSDQEGDDPMEEDDFMFELSDKPLLPCYNLQVSVSRGPCNWFLFSDVLKRLKLSSRIFQARFPHFEITTMPKAEFYRQVASSQLLTPAERPGGLDDRSPPGSSETVELVRYEPDLLRLLGSEVEFQSCNS

>NP_001167.2

MLGLDACELGAQLLELLRLALCARVLLADKEGGPPAVDEVLDEAVPEYRAPGRKSLLEIRQLDPDDRSLAKYKRVLLGPLPPAVDPSLPNVQVTRLTLLSEQAPGPVVMDLTGDLAVLKDQVFVLKEGVDYRVKISFKVHREIVSGLKCLHHTYRRGLRVDKTVYMVGSYGPSAQEYEFVTPVEEAPRGALVRGPYLVVSLFTDDDRTHHLSWEWGLCICQDWKD

1281 NP_001781.2 NP_004299.1

>NP_001781.2

MSTELFSSTREEGSSGSGPSFRSNQRKMLNLLLERDTSFTVCPDVPRTPVGKFLGDSANLSILSGGTPKRCLDLSNLSSGEITATQLTTSADLDETGHLDSSGLQEVHLAGMNHDQHLMKCSPAQLLCSTPNGLDRGHRKRDAMCSSSANKENDNGNLVDSEMKYLGSPITTVPKLDKNPNLGEDQAEEISDELMEFSLKDQEAKVSRSGLYRSPSMPENLNRPRLKQVEKFKDNTIPDKVKKKYFSGQGKLRKGLCLKKTVSLCDITITQMLEEDSNQGHLIGDFSKVCALPTVSGKHQDLKYVNPETVAALLSGKFQGLIEKFYVIDCRYPYEYLGGHIQGALNLYSQEELFNFFLKKPIVPLDTQKRIIIVFHCEFSSERGPRMCRCLREEDRSLNQYPALYYPELYILKGGYRDFFPEYMELCEPQSYCPMHHQDHKTELLRCRSQSKVQEGERQLREQIALLVKDMSP

>NP_004299.1

MDPLSELQDDLTLDDTSEALNQLKLASIDEKNWPSDEMPDFPKSDDSKSSSPELVTHLKWDDPYYDIARHQIVEVAGDDKYGRKIIVFSACRMPPSHQLDHSKLLGYLKHTLDQYVESDYTLLYLHHGLTSDNKPSLSWLRDAYREFDRKYKKNIKALYIVHPTMFIKTLLILFKPLISFKFGQKIFYVNYLSELSEHVKLEQLGIPRQVLKYDDFLKSTQKSPATAPKPMPPRPPLPNQQFGVSLQHLQEKNPEQEPIPIVLRETVAYLQAHALTTEGIFRRSANTQVVREVQQKYNMGLPVDFDQYNELHLPAVILKTFLRELPEPLLTFDLYPHVVGFLNIDESQRVPATLQVLQTLPEENYQVLRFLTAFLVQISAHSDQNKMTNTNLAVVFGPNLLWAKDAAITLKAINPINTFTKFLLDHQGELFPSPDPSGL

1282 O15156 P35558

>O15156

MGSPEDDLIGIPFPDHSSELLSCLNEQRQLGHLCDLTIRTQGLEYRTHRAVLAACSHYFKKLFTEGGGGAVMGAGGSGTATGGAGAGVCELDFVGPEALGALLEFAYTATLTTSSANMPAVLQAARLLEIPCVIAACMEILQGSGLEAPSPDEDDCERARQYLEAFATATASGVPNGEDSPPQVPLPPPPPPPPRPVARRSRKPRKAFLQTKGARANHLVPEVPTVPAHPLTYEEEEVAGRVGSSGGSGPGDSYSPPTGTASPPEGPQSYEPYEGEEEEEELVYPPAYGLAQGGGPPLSPEELGSDEDAIDPDLMAYLSSLHQDNLAPGLDSQDKLVRKRRSQMPQECPVCHKIIHGAGKLPRHMRTHTGEKPFACEVCGVRFTRNDKLKIHMRKHTGERPYSCPHCPARFLHSYDLKNHMHLHTGDRPYECHLCHKAFAKEDHLQRHLKGQNCLEVRTRRRRKDDAPPHYPPPSTAAASPAGLDLSNGHLDTFRLSLARFWEQSAPTGPPVSTPGPPDDDEEEGAPTTPQAEGAMESS

>P35558

MPPQLQNGLNLSAKVVQGSLDSLPQAVREFLENNAELCQPDHIHICDGSEEENGRLLGQMEEEGILRRLKKYDNCWLALTDPRDVARIESKTVIVTQEQRDTVPIPKTGLSQLGRWMSEEDFEKAFNARFPGCMKGRTMYVIPFSMGPLGSPLSKIGIELTDSPYVVASMRIMTRMGTPVLEAVGDGEFVKCLHSVGCPLPLQKPLVNNWPCNPELTLIAHLPDRREIISFGSGYGGNSLLGKKCFALRMASRLAKEEGWLAEHMLILGITNPEGEKKYLAAAFPSACGKTNLAMMNPSLPGWKVECVGDDIAWMKFDAQGHLRAINPENGFFGVAPGTSVKTNPNAIKTIQKNTIFTNVAETSDGGVYWEGIDEPLASGVTITSWKNKEWSSEDGEPCAHPNSRFCTPASQCPIIDAAWESPEGVPIEGIIFGGRRPAGVPLVYEALSWQHGVFVGAAMRSEATAAAEHKGKIIMHDPFAMRPFFGYNFGKYLAHWLSMAQHPAAKLPKIFHVNWFRKDKEGKFLWPGFGENSRVLEWMFNRIDGKASTKLTPIGYIPKEDALNLKGLGHINMMELFSISKEFWEKEVEDIEKYLEDQVNADLPCEIEREILALKQRISQM

1283 P10243 Q9BRJ2

>P10243

MAKRSRSEDEDDDLQYADHDYEVPQQKGLKKLWNRVKWTRDEDDKLKKLVEQHGTDDWTLIASHLQNRSDFQCQHRWQKVLNPELIKGPWTKEEDQRVIELVQKYGPKRWSLIAKHLKGRIGKQCRERWHNHLNPEVKKSSWTEEEDRIIYEAHKRLGNRWAEIAKLLPGRTDNSIKNHWNSTMRRKVEQEGYLQDGIKSERSSSKLQHKPCAAMDHMQTQNQFYIPVQIPGYQYVSPEGNCIEHVQPTSAFIQQPFIDEDPDKEKKIKELEMLLMSAENEVRRKRIPSQPGSFSSWSGSFLMDDNMSNTLNSLDEHTSEFYSMDENQPVSAQQNSPTKFLAVEANAVLSSLQTIPEFAETLELIESDPVAWSDVTSFDISDAAASPIKSTPVKLMRIQHNEGAMECQFNVSLVLEGKKNTCNGGNSEAVPLTSPNIAKFSTPPAILRKKRKMRVGHSPGSELRDGSLNDGGNMALKHTPLKTLPFSPSQFFNTCPGNEQLNIENPSFTSTPICGQKALITTPLHKETTPKDQKENVGFRTPTIRRSILGTTPRTPTPFKNALAAQEKKYGPLKIVSQPLAFLEEDIREVLKEETGTDLFLKEEDEPAYKSCKQENTASGKKVRKSLVLDNWEKEESGTQLLTEDISDMQSENRFTTSLLMIPLLEIHDNRCNLIPEKQDINSTNKTYTLTKKKPNPNTSKVVKLEKNLQSNCEWETVVYGKTEDQLIMTEQARRYLSTYTATSSTSRALIL

>Q9BRJ2

MAAPIPQGFSCLSRFLGWWFRQPVLVTQSAAIVPVRTKKRFTPPIYQPKFKTEKEFMQHARKAGLVIPPEKSDRSIHLACTAGIFDAYVPPEGDARISSLSKEGLIERTERMKKTMASQVSIRRIKDYDANFKIKDFPEKAKDIFIEAHLCLNNSDHDRLHTLVTEHCFPDMTWDIKYKTVRWSFVESLEPSHVVQVRCSSMMNQGNVYGQITVRMHTRQTLAIYDRFGRLMYGQEDVPKDVLEYVVFEKQLTNPYGSWRMHTKIVPPWAPPKQPILKTVMIPGPQLKPEEEYEEAQGEAQKPQLA

1284 NP_001229.1 Q86XT4

>NP_001229.1

MPRERRERDAKERDTMKEDGGAEFSARSRKRKANVTVFLQDPDEEMAKIDRTARDQCGSQPWDNNAVCADPCSLIPTPDKEDDDRVYPNSTCKPRIIAPSRGSPLPVLSWANREEVWKIMLNKEKTYLRDQHFLEQHPLLQPKMRAILLDWLMEVCEVYKLHRETFYLAQDFFDRYMATQENVVKTLLQLIGISSLFIAAKLEEIYPPKLHQFAYVTDGACSGDEILTMELMIMKALKWRLSPLTIVSWLNVYMQVAYLNDLHEVLLPQYPQQIFIQIAELLDLCVLDVDCLEFPYGILAASALYHFSSSELMQKVSGYQWCDIENCVKWMVPFAMVIRETGSSKLKHFRGVADEDAHNIQTHRDSLDLLDKARAKKAMLSEQNRASPLPSGLLTPPQSGKKQSSGPEMA

>Q86XT4

MAWQVSLLELEDWLQCPICLEVFKEPLMLQCGHSYCKGCLVSLSCHLDAELRCPVCRQAVDGSSSLPNVSLARVIEALRLPGDPEPKVCVHHRNPLSLFCEKDQELICGLCGLLGSHQHHPVTPVSTVYSRMKEELAALISELKQEQKKVDELIAKLVNNRTRIVNESDVFSWVIRREFQELHHLVDEEKARCLEGIGGHTRGLVASLDMQLEQAQGTRERLAQAECVLEQFGNEDHHKFIRKFHSMASRAEMPQARPLEGAFSPISFKPGLHQADIKLTVWKRLFRKVLPAPEPLKLDPATAHPLLELSKGNTVVQCGLLAQRRASQPERFDYSTCVLASRGFSCGRHYWEVVVGSKSDWRLGVIKGTASRKGKLNRSPEHGVWLIGLKEGRVYEAFACPRVPLPVAGHPHRIGLYLHYEQGELTFFDADRPDDLRPLYTFQADFQGKLYPILDTCWHERGSNSLPMVLPPPSGPGPLSPEQPTKL

1285 NP_037479.1 NP_001024.1

>NP_037479.1

MSKSGFGSYGSISAADGASGGSDQLCERDATPAIKTQRPKVRIQDVVPCNVNQLLSSTVFDPVFKVRGIIVSQVSIVGVIRGAEKASNHICYKIDDMTAKPIEARQWFGREKVKQVTPLSVGVYVKVFGILKCPTGTKSLEVLKIHVLEDMNEFTVHILETVNAHMMLDKARRDTTVESVPVSPSEVNDAGDNDESHRNFIQDEVLRLIHECPHQEGKSIHELRAQLCDLSVKAIKEAIDYLTVEGHIYPTVDREHFKSAD

>NP_001024.1

MHVIKRDGRQERVMFDKITSRIQKLCYGLNMDFVDPAQITMKVIQGLYSGVTTVELDTLAAETAATLTTKHPDYAILAARIAVSNLHKETKKVFSDVMEDLYNYINPHNGKHSPMVAKSTLDIVLANKDRLNSAIIYDRDFSYNYFGFKTLERSYLLKINGKVAERPQHMLMRVSVGIHKEDIDAAIETYNLLSERWFTHASPTLFNAGTNRPQLSSCFLLSMKDDSIEGIYDTLKQCALISKSAGGIGVAVSCIRATGSYIAGTNGNSNGLVPMLRVYNNTARYVDQGGNKRPGAFAIYLEPWHLDIFEFLDLKKNTGKEEQRARDLFFALWIPDLFMKRVETNQDWSLMCPNECPGLDEVWGEEFEKLYASYEKQGRVRKVVKAQQLWYAIIESQTETGTPYMLYKDSCNRKSNQQNLGTIKCSNLCTEIVEYTSKDEVAVCNLASLALNMYVTSEHTYDFKKLAEVTKVVVRNLNKIIDINYYPVPEACLSNKRHRPIGIGVQGLADAFILMRYPFESAEAQLLNKQIFETIYYGALEASCDLAKEQGPYETYEGSPVSKGILQYDMWNVTPTDLWDWKVLKEKIAKYGIRNSLLIAPMPTASTAQILGNNESIEPYTSNIYTRRVLSGEFQIVNPHLLKDLTERGLWHEEMKNQIIACNGSIQSIPEIPDDLKQLYKTVWEISQKTVLKMAAERGAFIDQSQSLNIHIAEPNYGKLTSMHFYGWKQGLKTGMYYLRTRPAANPIQFTLNKEKLKDKEKVSKEEEEKERNTAAMVCSLENRDECLMCGS

1286 NP_008924.1 NP_003795.2

>NP_008924.1

MAAGTAAALAFLSQESRTRAGGVGGLRVPAPVTMDSFFFGCELSGHTRSFTFKVEEEDDAEHVLALTMLCLTEGAKDECNVVEVVARNHDHQEIAVPVANLKLSCQPMLSLDDFQLQPPVTFRLKSGSGPVRITGRHQIVTMSNDVSEEESEEEEEDSDEEEVELCPILPAKKQGGRP

>NP_003795.2

MQPDMSLNVIKMKSSDFLESAELDSGGFGKVSLCFHRTQGLMIMKTVYKGPNCIEHNEALLEEAKMMNRLRHSRVVKLLGVIIEEGKYSLVMEYMEKGNLMHVLKAEMSTPLSVKGRIILEIIEGMCYLHGKGVIHKDLKPENILVDNDFHIKIADLGLASFKMWSKLNNEEHNELREVDGTAKKNGGTLYYMAPEHLNDVNAKPTEKSDVYSFAVVLWAIFANKEPYENAICEQQLIMCIKSGNRPDVDDITEYCPREIISLMKLCWEANPEARPTFPGIEEKFRPFYLSQLEESVEEDVKSLKKEYSNENAVVKRMQSLQLDCVAVPSSRSNSATEQPGSLHSSQGLGMGPVEESWFAPSLEHPQEENEPSLQSKLQDEANYHLYGSRMDRQTKQQPRQNVAYNREEERRRRVSHDPFAQQRPYENFQNTEGKGTAYSSAASHGNAVHQPSGLTSQPQVLYQNNGLYSSHGFGTRPLDPGTAGPRVWYRPIPSHMPSLHNIPVPETNYLGNTPTMPFSSLPPTDESIKYTIYNSTGIQIGAYNYMEIGGTSSSLLDSTNTNFKEEPAAKYQAIFDNTTSLTDKHLDPIRENLGKHWKNCARKLGFTQSQIDEIDHDYERDGLKEKVYQMLQKWVMREGIKGATVGKLAQALHQCSRIDLLSSLIYVSQN

1287 Q92925 NP_061833.1

>Q92925

MSPGNRMPMARLAGGTPCWLPIWCSSSASTWHPTHHDGSIPKTPACAPAQPPMPAQRRGLKRRKMADKVLPQRIRELVPESQAYMDLLAFERKLDQTIARKRMEIQEAIKKPLTQKRKLRIYISNTFSPSKAEGDSAGTAGTPGGTPAGDKVASWELRVEGKLLDDPSKQKRKFSSFFKSLVIELDKELYGPDGHLVEWYWMPTTQETDGFQVKRPGDLNVKCTLLLMLDHQPPQYKLDPRLARLLGVHTQTRAAIMQALWLYIKHNQLQDGHEREYINCNRYFRQIFSCGRLRFSEIPMKLAGLLQHPDPIVINHVISVDPNDQKKTACYDIDVEVDDPLKAQMSNFLASTTNQQEIASLDVKIHETIESINQLKTQRDFMLSFSTDPQDFIQEWLRSQRRDLKIITDVIGNPEEERRAAFYHQPWAQEAVGRHIFAKVQQRRQELEQVLGIRLT

>NP_061833.1

MVDSVYRTRSLGVAAEGLPDQYADGEAARVWQLYIGDTRSRTAEYKAWLLGLLRQHGCQRVLDVACGTGVDSIMLVEEGFSVTSVDASDKMLKYALKERWNRRHEPAFDKWVIEEANWMTLDKDVPQSAEGGFDAVICLGNSFAHLPDCKGDQSEHRLALKNIASMVRAGGLLVIDHRNYDHILSTGCAPPGKNIYYKSDLTKDVTTSVLIVNNKAHMVTLDYTVQVPGAGQDGSPGLSKFRLSYYPHCLASFTELLQAAFGGKCQHSVLGDFKPYKPGQTYIPCYFIHVLKRTD

1288 O75526 P82675

>O75526

MVEADRPGKLFIGGLNLETDEKALEAEFGKYGRIVEVLLMKDRETNKSRGFAFVTFESPADAKAAARDMNGKSLDGKAIKVAQATKPAFESSRRGPPPPRSRGRPRFLRGTRGGGGGPRRSPSRGGPDDDGGYTADFDLRPSRAPMPMKRGPPPRRVGPPPKRAAPSGPARSSGGGMRGRALAVRGRDGYSGPPRREPLPPRRDPYLGPRDEGYSSRDGYSSRDYREPRGFAPSPGEYTHRDYGHSSVRDDCPLRGYSDRDGYGGRDRDYGDHLSRGSHREPFESYGELRGAAPGRGTPPSYGGGGRYEEYRGYSPDAYSGGRDSYSSSYGRSDRYSRGRHRVGRPDRGLSLSMERGCPPQRDSYSRSGCRVPRGGGRLGGRLERGGGRSRY

>P82675

MATAVRAVGCLPVLCSGTAGHLLGRQCSLNTLPAASILAWKSVLGNGHLSSLGTRDTHPYASLSRALQTQCCISSPSHLMSQQYRPYSFFTKLTADELWKGALAETGAGAKKGRGKRTKKKKRKDLNRGQIIGEGRYGFLWPGLNVPLMKNGAVQTIAQRSKEEQEKVEADMIQQREEWDRKKKMKVKRERGWSGNSWGGISLGPPDPGPCGETYEDFDTRILEVRNVFTMTAKEGRKKSIRVLVAVGNGKGAAGFSIGKATDRMDAFRKAKNRAVHHLHYIERYEDHTIFHDISLRFKRTHIKMKKQPKGYGLRCHRAIITICRLIGIKDMYAKVSGSINMLSLTQGLFRGLSRQETHQQLADKKGLHVVEIREECGPLPIVVASPRGPLRKDPEPEDEVPDVKLDWEDVKTAQGMKRSVWSNLKRAAT

1289 Q96LA8 Q9H4A3

>Q96LA8

MSQPKKRKLESGGGGEGGEGTEEEDGAEREAALERPRRTKRERDQLYYECYSDVSVHEEMIADRVRTDAYRLGILRNWAALRGKTVLDVGAGTGILSIFCAQAGARRVYAVEASAIWQQAREVVRFNGLEDRVHVLPGPVETVELPEQVDAIVSEWMGYGLLHESMLSSVLHARTKWLKEGGLLLPASAELFIAPISDQMLEWRLGFWSQVKQHYGVDMSCLEGFATRCLMGHSEIVVQGLSGEDVLARPQRFAQLELSRAGLEQELEAGVGGRFRCSCYGSAPMHGFAIWFQVTFPGGESEKPLVLSTSPFHPATHWKQALLYLNEPVQVEQDTDVSGEITLLPSRDNPRRLRVLLRYKVGDQEEKTKDFAMED

>Q9H4A3

MSGGAAEKQSSTPGSLFLSPPAPAPKNGSSSDSSVGEKLGAAAADAVTGRTEEYRRRRHTMDKDSRGAAATTTTTEHRFFRRSVICDSNATALELPGLPLSLPQPSIPAAVPQSAPPEPHREETVTATATSQVAQQPPAAAAPGEQAVAGPAPSTVPSSTSKDRPVSQPSLVGSKEEPPPARSGSGGGSAKEPQEERSQQQDDIEELETKAVGMSNDGRFLKFDIEIGRGSFKTVYKGLDTETTVEVAWCELQDRKLTKSERQRFKEEAEMLKGLQHPNIVRFYDSWESTVKGKKCIVLVTELMTSGTLKTYLKRFKVMKIKVLRSWCRQILKGLQFLHTRTPPIIHRDLKCDNIFITGPTGSVKIGDLGLATLKRASFAKSVIGTPEFMAPEMYEEKYDESVDVYAFGMCMLEMATSEYPYSECQNAAQIYRRVTSGVKPASFDKVAIPEVKEIIEGCIRQNKDERYSIKDLLNHAFFQEETGVRVELAEEDDGEKIAIKLWLRIEDIKKLKGKYKDNEAIEFSFDLERDVPEDVAQEMVESGYVCEGDHKTMAKAIKDRVSLIKRKREQRQLVREEQEKKKQEESSLKQQVEQSSASQTGIKQLPSASTGIPTASTTSASVSTQVEPEEPEADQHQQLQYQQPSISVLSDGTVDSGQGSSVFTESRVSSQQTVSYGSQHEQAHSTGTVPGHIPSTVQAQSQPHGVYPPSSVAQGQSQGQPSSSSLTGVSSSQPIQHPQQQQGIQQTAPPQQTVQYSLSQTSTSSEATTAQPVSQPQAPQVLPQVSAGKQLPVSQPVPTIQGEPQIPVATQPSVVPVHSGAHFLPVGQPLPTPLLPQYPVSQIPISTPHVSTAQTGFSSLPITMAAGITQPLLTLASSATTAAIPGVSTVVPSQLPTLLQPVTQLPSQVHPQLLQPAVQSMGIPANLGQAAEVPLSSGDVLYQGFPPRLPPQYPGDSNIAPSSNVASVCIHSTVLSPPMPTEVLATPGYFPTVVQPYVESNLLVPMGGVGGQVQVSQPGGSLAQAPTTSSQQAVLESTQGVSQVAPAEPVAVAQPQATQPTTLASSVDSAHSDVASGMSDGNENVPSSSGRHEGRTTKRHYRKSVRSRSRHEKTSRPKLRILNVSNKGDRVVECQLETHNRKMVTFKFDLDGDNPEEIATIMVNNDFILAIERESFVDQVREIIEKADEMLSEDVSVEPEGDQGLESLQGKDDYGFSGSQKLEGEFKQPIPASSMPQQIGIPTSSLTQVVHSAGRRFIVSPVPESRLRESKVFPSEITDTVAASTAQSPGMNLSHSASSLSLQQAFSELRRAQMTEGPNTAPPNFSHTGPTFPVVPPFLSSIAGVPTTAAATAPVPATSSPPNDISTSVIQSEVTVPTEEGIAGVATSTGVVTSGGLPIPPVSESPVLSSVVSSITIPAVVSISTTSPSLQVPTSTSEIVVSSTALYPSVTVSATSASAGGSTATPGPKPPAVVSQQAAGSTTVGATLTSVSTTTSFPSTASQLSIQLSSSTSTPTLAETVVVSAHSLDKTSHSSTTGLAFSLSAPSSSSSPGAGVSSYISQPGGLHPLVIPSVIASTPILPQAAGPTSTPLLPQVPSIPPLVQPVANVPAVQQTLIHSQPQPALLPNQPHTHCPEVDSDTQPKAPGIDDIKTLEEKLRSLFSEHSSSGAQHASVSLETSLVIESTVTPGIPTTAVAPSKLLTSTTSTCLPPTNLPLGTVALPVTPVVTPGQVSTPVSTTTSGVKPGTAPSKPPLTKAPVLPVGTELPAGTLPSEQLPPFPGPSLTQSQQPLEDLDAQLRRTLSPEIITVTSAVGPVSMAAPTAITEAGTQPQKGVSQVKEGPVLATSSGAGVFKMGRFQVSVAADGAQKEGKNKSEDAKSVHFESSTSESSVLSSSSPESTLVKPEPNGITIPGISSDVPESAHKTTASEAKSDTGQPTKVGRFQVTTTANKVGRFSVSKTEDKITDTKKEGPVASPPFMDLEQAVLPAVIPKKEKPELSEPSHLNGPSSDPEAAFLSRDVDDGSGSPHSPHQLSSKSLPSQNLSQSLSNSFNSSYMSSDNESDIEDEDLKLELRRLRDKHLKEIQDLQSRQKHEIESLYTKLGKVPPAVIIPPAAPLSGRRRRPTKSKGSKSSRSSSLGNKSPQLSGNLSGQSAASVLHPQQTLHPPGNIPESGQNQLLQPLKPSPSSDNLYSAFTSDGAISVPSLSAPGQGTSSTNTVGATVNSQAAQAQPPAMTSSRKGTFTDDLHKLVDNWARDAMNLSGRRGSKGHMNYEGPGMARKFSAPGQLCISMTSNLGGSAPISAASATSLGHFTKSMCPPQQYGFPATPFGAQWSGTGGPAPQPLGQFQPVGTASLQNFNISNLQKSISNPPGSNLRTT

1290 NP_000297.1 NP_002564.1

>NP_000297.1

MSCQAFTSADTFIPLNSDASATLPLIMHHSAAECLPVSNHATNVMSTATGLHYSVPSCHYGNQPSTYGVMAGSLTPCLYKFPDHTLSHGFPPIHQPLLAEDPTAADFKQELRRKSKLVEEPIDMDSPEIRELEKFANEFKVRRIKLGYTQTNVGEALAAVHGSEFSQTTICRFENLQLSFKNACKLKAILSKWLEEAEQVGALYNEKVGANERKRKRRTTISIAAKDALERHFGEQNKPSSQEIMRMAEELNLEKEVVRVWFCNRRQREKRVKTSLNQSLFSISKEHLECR

>NP_002564.1

MSGEENPASKPTPVQDVQGDGRWMSLHHRFVADSKDKEPEVVFIGDSLVQLMHQCEIWRELFSPLHALNFGIGGDGTQHVLWRLENGELEHIRPKIVVVWVGTNNHGHTAEQVTGGIKAIVQLVNERQPQARVVVLGLLPRGQHPNPLREKNRQVNELVRAALAGHPRAHFLDADPGFVHSDGTISHHDMYDYLHLSRLGYTPVCRALHSLLLRLLAQDQGQGAPLLEPAP

1291 NP_001317.1 NP_057564.3

>NP_001317.1

MSGDGATEQAAEYVPEKVKKAEKKLEENPYDLDAWSILIREAQNQPIDKARKTYERLVAQFPSSGRFWKLYIEAEIKAKNYDKVEKLFQRCLMKVLHIDLWKCYLSYVRETKGKLPSYKEKMAQAYDFALDKIGMEIMSYQIWVDYINFLKGVEAVGSYAENQRITAVRRVYQRGCVNPMINIEQLWRDYNKYEEGINIHLAKKMIEDRSRDYMNARRVAKEYETVMKGLDRNAPSVPPQNTPQEAQQVDMWKKYIQWEKSNPLRTEDQTLITKRVMFAYEQCLLVLGHHPDIWYEAAQYLEQSSKLLAEKGDMNNAKLFSDEAANIYERAISTLLKKNMLLYFAYADYEESRMKYEKVHSIYNRLLAIEDIDPTLVYIQYMKFARRAEGIKSGRMIFKKAREDTRTRHHVYVTAALMEYYCSKDKSVAFKIFELGLKKYGDIPEYVLAYIDYLSHLNEDNNTRVLFERVLTSGSLPPEKSGEIWARFLAFESNIGDLASILKVEKRRFTAFKEEYEGKETALLVDRYKFMDLYPCSASELKALGYKDVSRAKLAAIIPDPVVAPSIVPVLKDEVDRKPEYPKPDTQQMIPFQPRHLAPPGLHPVPGGVFPVPPAAVVLMKLLPPPICFQGPFVQVDELMEIFRRCKIPNTVEEAVRIITGGAPELAVEGNGPVESNAVLTKAVKRPNEDSDEDEEKGAVVPPVHDIYRARQQKRIR

>NP_057564.3

MKDPSRSSTSPSIINEDVIINGHSHEDDNPFAEYMWMENEEEFNRQIEEELWEEEFIERCFQEMLEEEEEHEWFIPARDLPQTMDQIQDQFNDLVISDGSSLEDLVVKSNLNPNAKEFVPGVKYGNI

1292 Q6ECI4 O15305

>Q6ECI4

MKSQEEVEVAGIKLCKAMSLGSVTFTDVAIDFSQDEWEWLNLAQRSLYKKVMLENYRNLVSVGLCISKPDVISLLEQEKDPWVIKGGMNRGLCPDLECVWVTKSLSLNQDIYEEKLPPAIIMERLKSYDLECSTLGKNWKCEDLFERELVNQKTHFRQETITHIDTLIEKRDHSNKSGTVFHLNTLSYIKQIFPMEERIFNFHTDKKSLKTHSVVKKHKQDRGEKKLLKCNDCEKIFSKISTLTLHQRIHTGEKPYECIECGKAFSQSAHLAQHQRIHTGEKPFECTECGKAFSQNAHLVQHQRVHTGEKPYQCKQCNKAFSQLAHLAQHQRVHTGEKPYECIECGKAFSDCSSLAHHRRIHTGKRPYECIDCGKAFRQNASLIRHRRYYHTGEKPFDCIDCGKAFTDHIGLIQHKRIHTGERPYKCNVCGKAFSHGSSLTVHQRIHTGEKPYECNICEKAFSHRGSLTLHQRVHTGEKPYECKECGKAFRQSTHLAHHQRIHTGEKPYECKECSKTFSQNAHLAQHQKIHTGEKPYECKECGKAFSQIAHLVQHQRVHTGEKPYECIECGKAFSDGSYLVQHQRLHSGKRPYECLECGKAFRQRASLICHQRCHTGEKPYECNVCGKAFSHRKSLTLHQRIHTGEKPYECKECSKAFSQVAHLTLHKRIHTGERPYECKECGKAFRQSVHLAHHQRIHTGESSVILSSALPYHQVL

>O15305

MAAPGPALCLFDVDGTLTAPRQKITKEMDDFLQKLRQKIKIGVVGGSDFEKVQEQLGNDVVEKYDYVFPENGLVAYKDGKLLCRQNIQSHLGEALIQDLINYCLSYIAKIKLPKKRGTFIEFRNGMLNVSPIGRSCSQEERIEFYELDKKENIRQKFVADLRKEFAGKGLTFSIGGQISFDVFPDGWDKRYCLRHVENDGYKTIYFFGDKTMPGGNDHEIFTDPRTMGYSVTAPEDTRRICELLFS

1293 NP_694964.3 Q06124

>NP_694964.3

MSKLPADSSVPQTGAANGDRDVPQAEVGRGRREPAPAQPEEAGEGAMAAARGGPVPAAREGRMAAARAAPAAAARGAPVAAAALARAAAAGRESPAAAAAREARMAEVARLLGEPVDEEGPEGRPRSRHGNGGLAALPYLRLRHPLSVLGINYQQFLRHYLENYPIAPGRIQELEERRRRFVEACRAREAAFDAEYQRNPHRVDLDILTFTIALTASEVINPLIEELGCDKFINRE

>Q06124

MTSRRWFHPNITGVEAENLLLTRGVDGSFLARPSKSNPGDFTLSVRRNGAVTHIKIQNTGDYYDLYGGEKFATLAELVQYYMEHHGQLKEKNGDVIELKYPLNCADPTSERWFHGHLSGKEAEKLLTEKGKHGSFLVRESQSHPGDFVLSVRTGDDKGESNDGKSKVTHVMIRCQELKYDVGGGERFDSLTDLVEHYKKNPMVETLGTVLQLKQPLNTTRINAAEIESRVRELSKLAETTDKVKQGFWEEFETLQQQECKLLYSRKEGQRQENKNKNRYKNILPFDHTRVVLHDGDPNEPVSDYINANIIMPEFETKCNNSKPKKSYIATQGCLQNTVNDFWRMVFQENSRVIVMTTKEVERGKSKCVKYWPDEYALKEYGVMRVRNVKESAAHDYTLRELKLSKVGQALLQGNTERTVWQYHFRTWPDHGVPSDPGGVLDFLEEVHHKQESIMDAGPVVVHCSAGIGRTGTFIVIDILIDIIREKGVDCDIDVPKTIQMVRSQRSGMVQTEAQYRFIYMAVQHYIETLQRRIEEEQKSKRKGHEYTNIKYSLADQTSGDQSPLPPCTPTPPCAEMREDSARVYENVGLMQQQKSFR

1294 P0C665 NP_000282.1

>P0C665

MDRRRMALRPGSRRPTAFFFHSRWLVPNLLAFFLGLSGAGPIHLPMPWPNGRRHRVLDPHTQLSTHEAPGRWKPVAPRRMKACPQVLLEW

>NP_000282.1

MSLSNKLTLDKLDVKGKRVVMRVDFNVPMKNNQITNNQRIKAAVPSIKFCLDNGAKSVVLMSHLGRPDGVPMPDKYSLEPVAVELKSLLGKDVLFLKDCVGPEVEKACANPAAGSVILLENLRFHVEEEGKGKDASGNKVKAEPAKIEAFRASLSKLGDVYVNDAFGTAHRAHSSMVGVNLPQKAGGFLMKKELNYFAKALESPERPFLAILGGAKVADKIQLINNMLDKVNEMIIGGGMAFTFLKVLNNMEIGTSLFDEEGAKIVKDLMSKAEKNGVKITLPVDFVTADKFDENAKTGQATVASGIPAGWMGLDCGPESSKKYAEAVTRAKQIVWNGPVGVFEWEAFARGTKALMDEVVKATSRGCITIIGGGDTATCCAKWNTEDKVSHVSTGGGASLELLEGKVLPGVDALSNI

1295 NP_005316.1 NP_005554.1

>NP_005316.1

MSETVPPAPAASAAPEKPLAGKKAKKPAKAAAASKKKPAGPSVSELIVQAASSSKERGGVSLAALKKALAAAGYDVEKNNSRIKLGIKSLVSKGTLVQTKGTGASGSFKLNKKASSVETKPGASKVATKTKATGASKKLKKATGASKKSVKTPKKAKKPAATRKSSKNPKKPKTVKPKKVAKSPAKAKAVKPKAAKARVTKPKTAKPKKAAPKKK

>NP_005554.1

MASSDIQVKELEKRASGQAFELILSPRSKESVPEFPLSPPKKKDLSLEEIQKKLEAAEERRKSHEAEVLKQLAEKREHEKEVLQKAIEENNNFSKMAEEKLTHKMEANKENREAQMAAKLERLREKDKHIEEVRKNKESKDPADETEAD

1296 NP_071910.3 P82921

>NP_071910.3

MHTPPALPRRFQGGGRVRTPGSHRQGKDNLERDPSGGCVPDFLPQAQDSNHFIMESLFCESSGDSSLEKEFLGAPVGPSVSTPNSQHSSPSRSLSANSIKVEMYSDEESSRLLGPDERLLEKDDSVIVEDSLSEPLGYCDGSGPEPHSPGGIRLPNGKLKCDVCGMVCIGPNVLMVHKRSHTGERPFHCNQCGASFTQKGNLLRHIKLHSGEKPFKCPFCNYACRRRDALTGHLRTHSVSSPTVGKPYKCNYCGRSYKQQSTLEEHKERCHNYLQSLSTEAQALAGQPGDEIRDLEMVPDSMLHSSSERPTFIDRLANSLTKRKRSTPQKFVGEKQMRFSLSDLPYDVNSGGYEKDVELVAHHSLEPGFGSSLAFVGAEHLRPLRLPPTNCISELTPVISSVYTQMQPLPGRLELPGSREAGEGPEDLADGGPLLYRPRGPLTDPGASPSNGCQDSTDTESNHEDRVAGVVSLPQGPPPQPPPTIVVGRHSPAYAKEDPKPQEGLLRGTPGPSKEVLRVVGESGEPVKAFKCEHCRILFLDHVMFTIHMGCHGFRDPFECNICGYHSQDRYEFSSHIVRGEHKVG

>P82921

MAKHLKFIARTVMVQEGNVESAYRTLNRILTMDGLIEDIKHRRYYEKPCRRRQRESYERCRRIYNMEMARKINFLMRKNRADPWQGC

1297 NP_000240.1 NP_001893.2

>NP_000240.1

MSFVAGVIRRLDETVVNRIAAGEVIQRPANAIKEMIENCLDAKSTSIQVIVKEGGLKLIQIQDNGTGIRKEDLDIVCERFTTSKLQSFEDLASISTYGFRGEALASISHVAHVTITTKTADGKCAYRASYSDGKLKAPPKPCAGNQGTQITVEDLFYNIATRRKALKNPSEEYGKILEVVGRYSVHNAGISFSVKKQGETVADVRTLPNASTVDNIRSIFGNAVSRELIEIGCEDKTLAFKMNGYISNANYSVKKCIFLLFINHRLVESTSLRKAIETVYAAYLPKNTHPFLYLSLEISPQNVDVNVHPTKHEVHFLHEESILERVQQHIESKLLGSNSSRMYFTQTLLPGLAGPSGEMVKSTTSLTSSSTSGSSDKVYAHQMVRTDSREQKLDAFLQPLSKPLSSQPQAIVTEDKTDISSGRARQQDEEMLELPAPAEVAAKNQSLEGDTTKGTSEMSEKRGPTSSNPRKRHREDSDVEMVEDDSRKEMTAACTPRRRIINLTSVLSLQEEINEQGHEVLREMLHNHSFVGCVNPQWALAQHQTKLYLLNTTKLSEELFYQILIYDFANFGVLRLSEPAPLFDLAMLALDSPESGWTEEDGPKEGLAEYIVEFLKKKAEMLADYFSLEIDEEGNLIGLPLLIDNYVPPLEGLPIFILRLATEVNWDEEKECFESLSKECAMFYSIRKQYISEESTLSGQQSEVPGSIPNSWKWTVEHIVYKALRSHILPPKHFTEDGNILQLANLPDLYKVFERC

>NP_001893.2

MQEKDASSQGFLPHFQHFATQAIHVGQDPEQWTSRAVVPPISLSTTFKQGAPGQHSGFEYSRSGNPTRNCLEKAVAALDGAKYCLAFASGLAATVTITHLLKAGDQIICMDDVYGGTNRYFRQVASEFGLKISFVDCSKIKLLEAAITPETKLVWIETPTNPTQKVIDIEGCAHIVHKHGDIILVVDNTFMSPYFQRPLALGADISMYSATKYMNGHSDVVMGLVSVNCESLHNRLRFLQNSLGAVPSPIDCYLCNRGLKTLHVRMEKHFKNGMAVAQFLESNPWVEKVIYPGLPSHPQHELVKRQCTGCTGMVTFYIKGTLQHAEIFLKNLKLFTLAESLGGFESLAELPAIMTHASVLKNDRDVLGISDTLIRLSVGLEDEEDLLEDLDQALKAAHPPSGSHS

1298 NP_055126.1 NP_003741.1

>NP_055126.1

MPQAFLLGSIHEPAGALMEPQPCPGSLAESFLEEELRLNAELSQLQFSEPVGIIYNPVEYAWEPHRNYVTRYCQGPKEVLFLGMNPGPFGMAQTGVPFGEVSMVRDWLGIVGPVLTPPQEHPKRPVLGLECPQSEVSGARFWGFFRNLCGQPEVFFHHCFVHNLCPLLFLAPSGRNLTPAELPAKQREQLLGICDAALCRQVQLLGVRLVVGVGRLAEQRARRALAGLMPEVQVEGLLHPSPRNPQANKGWEAVAKERLNELGLLPLLLK

>NP_003741.1

MPAYFQRPENALKRANEFLEVGKKQPALDVLYDVMKSKKHRTWQKIHEPIMLKYLELCVDLRKSHLAKEGLYQYKNICQQVNIKSLEDVVRAYLKMAEEKTEAAKEESQQMVLDIEDLDNIQTPESVLLSAVSGEDTQDRTDRLLLTPWVKFLWESYRQCLDLLRNNSRVERLYHDIAQQAFKFCLQYTRKAEFRKLCDNLRMHLSQIQRHHNQSTAINLNNPESQSMHLETRLVQLDSAISMELWQEAFKAVEDIHGLFSLSKKPPKPQLMANYYNKVSTVFWKSGNALFHASTLHRLYHLSREMRKNLTQDEMQRMSTRVLLATLSIPITPERTDIARLLDMDGIIVEKQRRLATLLGLQAPPTRIGLINDMVRFNVLQYVVPEVKDLYNWLEVEFNPLKLCERVTKVLNWVREQPEKEPELQQYVPQLQNNTILRLLQQVSQIYQSIEFSRLTSLVPFVDAFQLERAIVDAARHCDLQVRIDHTSRTLSFGSDLNYATREDAPIGPHLQSMPSEQIRNQLTAMSSVLAKALEVIKPAHILQEKEEQHQLAVTAYLKNSRKEHQRILARRQTIEERKERLESLNIQREKEELEQREAELQKVRKAEEERLRQEAKEREKERILQEHEQIKKKTVRERLEQIKKTELGAKAFKDIDIEDLEELDPDFIMAKQVEQLEKEKKELQERLKNQEKKIDYFERAKRLEEIPLIKSAYEEQRIKDMDLWEQQEEERITTMQLEREKALEHKNRMSRMLEDRDLFVMRLKAARQSVYEEKLKQFEERLAEERHNRLEERKRQRKEERRITYYREKEEEEQRRAEEQMLKEREERERAERAKREEELREYQERVKKLEEVERKKRQRELEIEERERRREEERRLGDSSLSRKDSRWGDRDSEGTWRKGPEADSEWRRGPPEKEWRRGEGRDEDRSHRRDEERPRRLGDDEDREPSLRPDDDRVPRRGMDDDRGPRRGPEEDRFSRRGADDDRPSWRNTDDDRPPRRIADEDRGNWRHADDDRPPRRGLDEDRGSWRTADEDRGPRRGMDDDRGPRRGGADDERSSWRNADDDRGPRRGLDDDRGPRRGMDDDRGPRRGMDDDRGPRRGMDDDRGPRRGLDDDRGPWRNADDDRIPRRGAEDDRGPWRNMDDDRLSRRADDDRFPRRGDDSRPGPWRPLVKPGGWREKEKAREESWGPPRESRPSEEREWDREKERDRDNQDREENDKDPERERDRERDVDREDRFRRPRDEGGWRRGPAEESSSWRDSSRRDDRDRDDRRRERDDRRDLRERRDLRDDRDRRGPPLRSEREEVSSWRRADDRKDDRVEERDPPRRVPPPALSRDRERDRDREREGEKEKASWRAEKDRESLRRTKNETDEDGWTTVRR

1299 Q6EKJ0 NP_003493.1

>Q6EKJ0

MAQVAVSTLPVEEESSSETRMVVTFLVSALESMCKELAKSKAEVACIAVYETDVFVVGTERGCAFVNARTDFQKDFAKYCVAEGLCEVKPPCPVNGMQVHSGETEILRKAVEDYFCFCYGKALGTTVMVPVPYEKMLRDQSAVVVQGLPEGVAFQHPENYDLATLKWILENKAGISFIINRPFLGPESQLGGPGMVTDAERSIVSPSESCGPINVKTEPMEDSGISLKAEAVSVKKESEDPNYYQYNMQGSHPSSTSNEVIEMELPMEDSTPLVPSEEPNEDPEAEVKIEGNTNSSSVTNSAAGVEDLNIVQVTVPDNEKERLSSIEKIKQLREQVNDLFSRKFGEAIGVDFPVKVPYRKITFNPGCVVIDGMPPGVVFKAPGYLEISSMRRILEAAEFIKFTVIRPLPGLELSNVGKRKIDQEGRVFQEKWERAYFFVEVQNIPTCLICKQSMSVSKEYNLRRHYQTNHSKHYDQYTERMRDEKLHELKKGLRKYLLGSSDTECPEQKQVFANPSPTQKSPVQPVEDLAGNLWEKLREKIRSFVAYSIAIDEITDINNTTQLAIFIRGVDENFDVSEELLDTVPMTGTKSGNEIFLRVEKSLKKFCINWSRLVSVASTGTPAMVDANNGLVTKLKSRVATFCKGAELKSICCIIHPESLCAQKLKMDHVMDVVVKSVNWICSRGLNHSEFTTLLYELDSQYGSLLYYTEIKWLSRGLVLKRFFESLEEIDSFMSSRGKPLPQLSSIDWIRDLAFLVDMTMHLNALNISLQGHSQIVTQMYDLIRAFLAKLCLWETHLTRNNLAHFPTLKLVSRNESDGLNYIPKIAELKTEFQKRLSDFKLYESELTLFSSPFSTKIDSVHEELQMEVIDLQCNTVLKTKYDKVGIPEFYKYLWGSYPKYKHHCAKILSMFGSTYICEQLFSIMKLSKTKYCSQLKDSQWDSVLHIAT

>NP_003493.1

MNIQEQGFPLDLGASFTEDAPRPPVPGEEGELVSTDPRPASYSFCSGKGVGIKGETSTATPRRSDLDLGYEPEGSASPTPPYLKWAESLHSLLDDQDGISLFRTFLKQEGCADLLDFWFACTGFRKLEPCDSNEEKRLKLARAIYRKYILDNNGIVSRQTKPATKSFIKGCIMKQLIDPAMFDQAQTEIQATMEENTYPSFLKSDIYLEYTRTGSESPKVCSDQSSGSGTGKGISGYLPTLNEDEEWKCDQDMDEDDGRDAAPPGRLPQKLLLETAAPRVSSSRRYSEGREFRYGSWREPVNPYYVNAGYALAPATSANDSEQQSLSSDADTLSLTDSSVDGIPPYRIRKQHRREMQESVQVNGRVPLPHIPRTYRVPKEVRVEPQKFAEELIHRLEAVQRTREAEEKLEERLKRVRMEEEGEDGDPSSGPPGPCHKLPPAPAWHHFPPRCVDMGCAGLRDAHEENPESILDEHVQRVLRTPGRQSPGPGHRSPDSGHVAKMPVALGGAASGHGKHVPKSGAKLDAAGLHHHRHVHHHVHHSTARPKEQVEAEATRRAQSSFAWGLEPHSHGARSRGYSESVGAAPNASDGLAHSGKVGVACKRNAKKAESGKSASTEVPGASEDAEKNQKIMQWIIEGEKEISRHRRTGHGSSGTRKPQPHENSRPLSLEHPWAGPQLRTSVQPSHLFIQDPTMPPHPAPNPLTQLEEARRRLEEEEKRASRAPSKQRYVQEVMRRGRACVRPACAPVLHVVPAVSDMELSETETRSQRKVGGGSAQPCDSIVVAYYFCGEPIPYRTLVRGRAVTLGQFKELLTKKGSYRYYFKKVSDEFDCGVVFEEVREDEAVLPVFEEKIIGKVEKVD

1300 NP_001665.1 NP_001484.1

>NP_001665.1

MMLQHPGQVSASEVSASAIVPCLSPPGSLVFEDFANLTPFVKEELRFAIQNKHLCHRMSSALESVTVSDRPLGVSITKAEVAPEEDERKKRRRERNKIAAAKCRNKKKEKTECLQKESEKLESVNAELKAQIEELKNEKQHLIYMLNLHRPTCIVRAQNGRTPEDERNLFIQQIKEGTLQS

>NP_001484.1

MDEEYDVIVLGTGLTECILSGIMSVNGKKVLHMDRNPYYGGESSSITPLEELYKRFQLLEGPPESMGRGRDWNVDLIPKFLMANGQLVKMLLYTEVTRYLDFKVVEGSFVYKGGKIYKVPSTETEALASNLMGMFEKRRFRKFLVFVANFDENDPKTFEGVDPQTTSMRDVYRKFDLGQDVIDFTGHALALYRTDDYLDQPCLETVNRIKLYSESLARYGKSPYLYPLYGLGELPQGFARLSAIYGGTYMLNKPVDDIIMENGKVVGVKSEGEVARCKQLICDPSYIPDRVRKAGQVIRIICILSHPIKNTNDANSCQIIIPQNQVNRKSDIYVCMISYAHNVAAQGKYIAIASTTVETTDPEKEVEPALELLEPIDQKFVAISDLYEPIDDGCESQVFCSCSYDATTHFETTCNDIKDIYKRMAGTAFDFENMKRKQNDVFGEAEQ

1301 NP_003081.2 O60783

>NP_003081.2

MVKLTAELIEQAAQYTNAVRDRELDLRGYKIPVIENLGATLDQFDAIDFSDNEIRKLDGFPLLRRLKTLLVNNNRICRIGEGLDQALPCLTELILTNNSLVELGDLDPLASLKSLTYLSILRNPVTNKKHYRLYVIYKVPQVRVLDFQKVKLKERQEAEKMFKGKRGAQLAKDIARRSKTFNPGAGLPTDKKKGGPSPGDVEAIKNAIANASTLAEVERLKGLLQSGQIPGRERRSGPTDDGEEEMEEDTVTNGS

>O60783

MAAFMLGSLLRTFKQMVPSSASGQVRSHYVDWRMWRDVKRRKMAYEYADERLRINSLRKNTILPKILQDVADEEIAALPRDSCPVRIRNRCVMTSRPRGVKRRWRLSRIVFRHLADHGQLSGIQRATW

1302 Q7Z7J5 NP_000058.1

>Q7Z7J5

MSDANLDSSKKNFLEGEVDDEESVILTLVPVKDDANMEQMEPSVSSTSDVKLEKPKKYNPGHLLQTNEQFTAPQKARCKIPALPLPTILPPINKVCRDTLRDWCQQLGLSTNGKKIEVYLRLHRHAYPEQRQDMPEMSQETRLQRCSRKRKAVTKRARLQRSYEMNERAEETNTVEVITSAPGAMLASWARIAARAVQPKALNSCSIPVSVEAFLMQASGVRWCVVHGRLLSADTKGWVRLQFHAGQAWVPTTHRRMISLFLLPACIFPSPGIEDNMLCPDCAKRNKKMMKRLMTVEK

>NP_000058.1

MSHHWGYGKHNGPEHWHKDFPIAKGERQSPVDIDTHTAKYDPSLKPLSVSYDQATSLRILNNGHAFNVEFDDSQDKAVLKGGPLDGTYRLIQFHFHWGSLDGQGSEHTVDKKKYAAELHLVHWNTKYGDFGKAVQQPDGLAVLGIFLKVGSAKPGLQKVVDVLDSIKTKGKSADFTNFDPRGLLPESLDYWTYPGSLTTPPLLECVTWIVLKEPISVSSEQVLKFRKLNFNGEGEPEELMVDNWRPAQPLKNRQIKASFK

1303 Q9NWU2 NP_000058.1

>Q9NWU2

MSYAEKPDEITKDEWMEKLNNLHVQRADMNRLIMNYLVTEGFKEAAEKFRMESGIEPSVDLETLDERIKIREMILKGQIQEAIALINSLHPELLDTNRYLYFHLQQQHLIELIRQRETEAALEFAQTQLAEQGEESRECLTEMERTLALLAFDSPEESPFGDLLHTMQRQKVWSEVNQAVLDYENRESTPKLAKLLKLLLWAQNELDQKKVKYPKMTDLSKGVIEEPK

>NP_000058.1

MSHHWGYGKHNGPEHWHKDFPIAKGERQSPVDIDTHTAKYDPSLKPLSVSYDQATSLRILNNGHAFNVEFDDSQDKAVLKGGPLDGTYRLIQFHFHWGSLDGQGSEHTVDKKKYAAELHLVHWNTKYGDFGKAVQQPDGLAVLGIFLKVGSAKPGLQKVVDVLDSIKTKGKSADFTNFDPRGLLPESLDYWTYPGSLTTPPLLECVTWIVLKEPISVSSEQVLKFRKLNFNGEGEPEELMVDNWRPAQPLKNRQIKASFK

1304 NP_002543.2 NP_004138.1

>NP_002543.2

MSSRKSKSNSLIHTECLSQVQRILRERFCRQSPHSNLFGVQVQYKHLSELLKRTALHGESNSVLIIGPRGSGKTMLINHALKELMEIEEVSENVLQVHLNGLLQINDKIALKEITRQLNLENVVGDKVFGSFAENLSFLLEALKKGDRTSSCPVIFILDEFDLFAHHKNQTLLYNLFDISQSAQTPIAVIGLTCRLDILELLEKRVKSRFSHRQIHLMNSFGFPQYVKIFKEQLSLPAEFPDKVFAEKWNENVQYLSEDRSVQEVLQKHFNISKNLRSLHMLLMLALNRVTASHPFMTAVDLMEASQLCSMDSKANIVHGLSVLEICLIIAMKHLNDIYEEEPFNFQMVYNEFQKFVQRKAHSVYNFEKPVVMKAFEHLQQLELIKPMERTSGNSQREYQLMKLLLDNTQIMNALQKYPNCPTDVRQWATSSLSWL

>NP_004138.1

MSSTLAKIAEIEAEMARTQKNKATAHHLGLLKARLAKLRRELITPKGGGGGGPGEGFDVAKTGDARIGFVGFPSVGKSTLLSNLAGVYSEVAAYEFTTLTTVPGVIRYKGAKIQLLDLPGIIEGAKDGKGRGRQVIAVARTCNLILIVLDVLKPLGHKKIIENELEGFGIRLNSKPPNIGFKKKDKGGINLTATCPQSELDAETVKSILAEYKIHNADVTLRSDATADDLIDVVEGNRVYIPCIYVLNKIDQISIEELDIIYKVPHCVPISAHHRWNFDDLLEKIWDYLKLVRIYTKPKGQLPDYTSPVVLPYSRTTVEDFCMKIHKNLIKEFKYALVWGLSVKHNPQKVGKDHTLEDEDVIQIVKK

1305 NP_000957.1 Q9UGC7

>NP_000957.1

MATNKERLFAAGALGPGSGYPGAGFPFAFPGALRGSPPFEMLSPSFRGLGQPDLPKEMASLSVETQSTSSEEMVPSSPSPPPPPRVYKPCFVCNDKSSGYHYGVSSCEGCKGFFRRSIQKNMVYTCHRDKNCIINKVTRNRCQYCRLQKCFEVGMSKEAVRNDRNKKKKEVKEEGSPDSYELSPQLEELITKVSKAHQETFPSLCQLGKYTTNSSADHRVQLDLGLWDKFSELATKCIIKIVEFAKRLPGFTGLSIADQITLLKAACLDILMLRICTRYTPEQDTMTFSDGLTLNRTQMHNAGFGPLTDLVFAFAGQLLPLEMDDTETGLLSAICLICGDRMDLEEPEKVDKLQEPLLEALRLYARRRRPSQPYMFPRMLMKITDLRGISTKGAERAITLKMEIPGPMPPLIREMLENPEMFEDDSSQPGPHPNASSEDEVPGGQGKGGLKSPA

>Q9UGC7

MRSRVLWGAARWLWPRRAVGPARRPLSSGSPPLEELFTRGGPLRTFLERQAGSEAHLKVRRPELLAVIKLLNEKERELRETEHLLHDENEDLRKLAENEITLCQKEITQLKHQIILLLVPSEETDENDLILEVTAGVGGQEAMLFTSEIFDMYQQYAAFKRWHFETLEYFPSELGGLRHASASIGGSEAYRHMKFEGGVHRVQRVPKTEKQGRVHTSTMTVAILPQPTEINLVINPKDLRIDTKRASGAGGQHVNTTDSAVRIVHLPTGVVSECQQERSQLKNKELAMTKLRAKLYSMHLEEEINKRQNARKIQIGSKGRSEKIRTYNFPQNRVTDHRINKTLHDLETFMQGDYLLDELVQSLKEYADYESLVEIISQKV

1306 P14316 NP_004560.1

>P14316

MPVERMRMRPWLEEQINSNTIPGLKWLNKEKKIFQIPWMHAARHGWDVEKDAPLFRNWAIHTGKHQPGVDKPDPKTWKANFRCAMNSLPDIEEVKDKSIKKGNNAFRVYRMLPLSERPSKKGKKPKTEKEDKVKHIKQEPVESSLGLSNGVSDLSPEYAVLTSTIKNEVDSTVNIIVVGQSHLDSNIENQEIVTNPPDICQVVEVTTESDEQPVSMSELYPLQISPVSSYAESETTDSVPSDEESAEGRPHWRKRNIEGKQYLSNMGTRGSYLLPGMASFVTSNKPDLQVTIKEESNPVPYNSSWPPFQDLPLSSSMTPASSSSRPDRETRASVIKKTSDITQARVKSC

>NP_004560.1

MEDERSFSDICGGRLALQRRYYSPSCREFCLSCPRLSLRSLTAVTCTVWLAAYGLFTLCENSMILSAAIFITLLGLLGYLHFVKIDQETLLIIDSLGIQMTSSYASGKESTTFIEMGKVKDIVINEAIYMQKVIYYLCILLKDPVEPHGISQVVPVFQSAKPRLDCLIEVYRSCQEILAHQKATSTSP

1307 Q9Y4A5 NP_056449.1

>Q9Y4A5

MAFVATQGATVVDQTTLMKKYLQFVAALTDVNTPDETKLKMMQEVSENFENVTSSPQYSTFLEHIIPRFLTFLQDGEVQFLQEKPAQQLRKLVLEIIHRIPTNEHLRPHTKNVLSVMFRFLETENEENVLICLRIIIELHKQFRPPITQEIHHFLDFVKQIYKELPKVVNRYFENPQVIPENTVPPPEMVGMITTIAVKVNPEREDSETRTHSIIPRGSLSLKVLAELPIIVVLMYQLYKLNIHNVVAEFVPLIMNTIAIQVSAQARQHKLYNKELYADFIAAQIKTLSFLAYIIRIYQELVTKYSQQMVKGMLQLLSNCPAETAHLRKELLIAAKHILTTELRNQFIPCMDKLFDESILIGSGYTARETLRPLAYSTLADLVHHVRQHLPLSDLSLAVQLFAKNIDDESLPSSIQTMSCKLLLNLVDCIRSKSEQESGNGRDVLMRMLEVFVLKFHTIARYQLSAIFKKCKPQSELGAVEAALPGVPTAPAAPGPAPSPAPVPAPPPPPPPPPPATPVTPAPVPPFEKQGEKDKEDKQTFQVTDCRSLVKTLVCGVKTITWGITSCKAPGEAQFIPNKQLQPKETQIYIKLVKYAMQALDIYQVQIAGNGQTYIRVANCQTVRMKEEKEVLEHFAGVFTMMNPLTFKEIFQTTVPYMVERISKNYALQIVANSFLANPTTSALFATILVEYLLDRLPEMGSNVELSNLYLKLFKLVFGSVSLFAAENEQMLKPHLHKIVNSSMELAQTAKEPYNYFLLLRALFRSIGGGSHDLLYQEFLPLLPNLLQGLNMLQSGLHKQHMKDLFVELCLTVPVRLSSLLPYLPMLMDPLVSALNGSQTLVSQGLRTLELCVDNLQPDFLYDHIQPVRAELMQALWRTLRNPADSISHVAYRVLGKFGGSNRKMLKESQKLHYVVTEVQGPSITVEFSDCKASLQLPMEKAIETALDCLKSANTEPYYRRQAWEVIKCFLVAMMSLEDNKHALYQLLAHPNFTEKTIPNVIISHRYKAQDTPARKTFEQALTGAFMSAVIKDLRPSALPFVASLIRHYTMVAVAQQCGPFLLPCYQVGSQPSTAMFHSEENGSKGMDPLVLIDAIAICMAYEEKELCKIGEVALAVIFDVASIILGSKERACQLPLFSYIVERLCACCYEQAWYAKLGGVVSIKFLMERLPLTWVLQNQQTFLKALLFVMMDLTGEVSNGAVAMAKTTLEQLLMRCATPLKDEERAEEIVAAQEKSFHHVTHDLVREVTSPNSTVRKQAMHSLQVLAQVTGKSVTVIMEPHKEVLQDMVPPKKHLLRHQPANAQIGLMEGNTFCTTLQPRLFTMDLNVVEHKVFYTELLNLCEAEDSALTKLPCYKSLPSLVPLRIAALNALAACNYLPQSREKIIAALFKALNSTNSELQEAGEACMRKFLEGATIEVDQIHTHMRPLLMMLGDYRSLTLNVVNRLTSVTRLFPNSFNDKFCDQMMQHLRKWMEVVVITHKGGQRSDGNESISECGRCPLSPFCQFEEMKICSAIINLFHLIPAAPQTLVKPLLEVVMKTERAMLIEAGSPFREPLIKFLTRHPSQTVELFMMEATLNDPQWSRMFMSFLKHKDARPLRDVLAANPNRFITLLLPGGAQTAVRPGSPSTSTMRLDLQFQAIKIISIIVKNDDSWLASQHSLVSQLRRVWVSENFQERHRKENMAATNWKEPKLLAYCLLNYCKRNYGDIELLFQLLRAFTGRFLCNMTFLKEYMEEEIPKNYSIAQKRALFFRFVDFNDPNFGDELKAKVLQHILNPAFLYSFEKGEGEQLLGPPNPEGDNPESITSVFITKVLDPEKQADMLDSLRIYLLQYATLLVEHAPHHIHDNNKNRNSKLRRLMTFAWPCLLSKACVDPACKYSGHLLLAHIIAKFAIHKKIVLQVFHSLLKAHAMEARAIVRQAMAILTPAVPARMEDGHQMLTHWTRKIIVEEGHTVPQLVHILHLIVQHFKVYYPVRHHLVQHMVSAMQRLGFTPSVTIEQRRLAVDLSEVVIKWELQRIKDQQPDSDMDPNSSGEGVNSVSSSIKRGLSVDSAQEVKRFRTATGAISAVFGRSQSLPGADSLLAKPIDKQHTDTVVNFLIRVACQVNDNTNTAGSPGEVLSRRCVNLLKTALRPDMWPKSELKLQWFDKLLMTVEQPNQVNYGNICTGLEVLSFLLTVLQSPAILSSFKPLQRGIAACMTCGNTKVLRAVHSLLSRLMSIFPTEPSTSSVASKYEELECLYAAVGKVIYEGLTNYEKATNANPSQLFGTLMILKSACSNNPSYIDRLISVFMRSLQKMVREHLNPQAASGSTEATSGTSELVMLSLELVKTRLAVMSMEMRKNFIQAILTSLIEKSPDAKILRAVVKIVEEWVKNNSPMAANQTPTLREKSILLVKMMTYIEKRFPEDLELNAQFLDLVNYVYRDETLSGSELTAKLEPAFLSGLRCAQPLIRAKFFEVFDNSMKRRVYERLLYVTCSQNWEAMGNHFWIKQCIELLLAVCEKSTPIGTSCQGAMLPSITNVINLADSHDRAAFAMVTHVKQEPRERENSESKEEDVEIDIELAPGDQTSTPKTKELSEKDIGNQLHMLTNRHDKFLDTLREVKTGALLSAFVQLCHISTTLAEKTWVQLFPRLWKILSDRQQHALAGEISPFLCSGSHQVQRDCQPSALNCFVEAMSQCVPPIPIRPCVLKYLGKTHNLWFRSTLMLEHQAFEKGLSLQIKPKQTTEFYEQESITPPQQEILDSLAELYSLLQEEDMWAGLWQKRCKYSETATAIAYEQHGFFEQAQESYEKAMDKAKKEHERSNASPAIFPEYQLWEDHWIRCSKELNQWEALTEYGQSKGHINPYLVLECAWRVSNWTAMKEALVQVEVSCPKEMAWKVNMYRGYLAICHPEEQQLSFIERLVEMASSLAIREWRRLPHVVSHVHTPLLQAAQQIIELQEAAQINAGLQPTNLGRNNSLHDMKTVVKTWRNRLPIVSDDLSHWSSIFMWRQHHYQGKPTWSGMHSSSIVTAYENSSQHDPSSNNAMLGVHASASAIIQYGKIARKQGLVNVALDILSRIHTIPTVPIVDCFQKIRQQVKCYLQLAGVMGKNECMQGLEVIESTNLKYFTKEMTAEFYALKGMFLAQINKSEEANKAFSAAVQMHDVLVKAWAMWGDYLENIFVKERQLHLGVSAITCYLHACRHQNESKSRKYLAKVLWLLSFDDDKNTLADAVDKYCIGVPPIQWLAWIPQLLTCLVGSEGKLLLNLISQVGRVYPQAVYFPIRTLYLTLKIEQRERYKSDPGPIRATAPMWRCSRIMHMQRELHPTLLSSLEGIVDQMVWFRENWHEEVLRQLQQGLAKCYSVAFEKSGAVSDAKITPHTLNFVKKLVSTFGVGLENVSNVSTMFSSAASESLARRAQATAQDPVFQKLKGQFTTDFDFSVPGSMKLHNLISKLKKWIKILEAKTKQLPKFFLIEEKCRFLSNFSAQTAEVEIPGEFLMPKPTHYYIKIARFMPRVEIVQKHNTAARRLYIRGHNGKIYPYLVMNDACLTESRREERVLQLLRLLNPCLEKRKETTKRHLFFTVPRVVAVSPQMRLVEDNPSSLSLVEIYKQRCAKKGIEHDNPISRYYDRLATVQARGTQASHQVLRDILKEVQSNMVPRSMLKEWALHTFPNATDYWTFRKMFTIQLALIGFAEFVLHLNRLNPEMLQIAQDTGKLNVAYFRFDINDATGDLDANRPVPFRLTPNISEFLTTIGVSGPLTASMIAVARCFAQPNFKVDGILKTVLRDEIIAWHKKTQEDTSSPLSAAGQPENMDSQQLVSLVQKAVTAIMTRLHNLAQFEGGESKVNTLVAAANSLDNLCRMDPAWHPWL

>NP_056449.1

MANVPWAEVCEKFQAALALSRVELHKNPEKEPYKSKYSARALLEEVKALLGPAPEDEDERPEAEDGPGAGDHALGLPAEVVEPEGPVAQRAVRLAVIEFHLGVNHIDTEELSAGEEHLVKCLRLLRRYRLSHDCISLCIQAQNNLGILWSEREEIETAQAYLESSEALYNQYMKEVGSPPLDPTERFLPEEEKLTEQERSKRFEKVYTHNLYYLAQVYQHLEMFEKAAHYCHSTLKRQLEHNAYHPIEWAINAATLSQFYINKLCFMEARHCLSAANVIFGQTGKISATEDTPEAEGEVPELYHQRKGEIARCWIKYCLTLMQNAQLSMQDNIGELDLDKQSELRALRKKELDEEESIRKKAVQFGTGELCDAISAVEEKVSYLRPLDFEEARELFLLGQHYVFEAKEFFQIDGYVTDHIEVVQDHSALFKVLAFFETDMERRCKMHKRRIAMLEPLTVDLNPQYYLLVNRQIQFEIAHAYYDMMDLKVAIADRLRDPDSHIVKKINNLNKSALKYYQLFLDSLRDPNKVFPEHIGEDVLRPAMLAKFRVARLYGKIITADPKKELENLATSLEHYKFIVDYCEKHPEAAQEIEVELELSKEMVSLLPTKMERFRTKMALT

1308 P57053 Q58A44

>P57053

MPEPAKSAPAPKKGSKKAVTKAQKKDGRKRKRSRKESYSVYVYKVLKQVHPDTGISSKAMGIMNSFVNDIFERIAGEASRLPHYNKRSTITSREIQTAVRLLLPGELAKHAVSEGTKAVTKYTSAK

>Q58A44

MWILSNLMGTSEEGNLLSTVSPTVKALFGKTRVSPIFPFSPRSPFQPLIPRTPGSPWGPVGPASPLGPGFPIGPMGPGKPVGPKGPMLPLGPSGPVGPTSPLFPFCP

1309 NP_004805.2 NP_005179.2

>NP_004805.2

MIEQQKRKGPELPLVPVKRQRHELLLGAGSGPGAGQQQATPGALLQAGPPRCSSLQAPIMLLSGHEGEVYCCKFHPNGSTLASAGFDRLILLWNVYGDCDNYATLKGHSGAVMELHYNTDGSMLFSASTDKTVAVWDSETGERVKRLKGHTSFVNSCYPARRGPQLVCTGSDDGTVKLWDIRKKAAIQTFQNTYQVLAVTFNDTSDQIISGGIDNDIKVWDLRQNKLTYTMRGHADSVTGLSLSSEGSYLLSNAMDNTVRVWDVRPFAPKERCVKIFQGNVHNFEKNLLRCSWSPDGSKIAAGSADRFVYVWDTTSRRILYKLPGHAGSINEVAFHPDEPIIISASSDKRLYMGEIQ

>NP_005179.2

MAGNVKKSSGAGGGSGSGGSGSGGLIGLMKDAFQPHHHHHHHLSPHPPGTVDKKMVEKCWKLMDKVVRLCQNPKLALKNSPPYILDLLPDTYQHLRTILSRYEGKMETLGENEYFRVFMENLMKKTKQTISLFKEGKERMYEENSQPRRNLTKLSLIFSHMLAELKGIFPSGLFQGDTFRITKADAAEFWRKAFGEKTIVPWKSFRQALHEVHPISSGLEAMALKSTIDLTCNDYISVFEFDIFTRLFQPWSSLLRNWNSLAVTHPGYMAFLTYDEVKARLQKFIHKPGSYIFRLSCTRLGQWAIGYVTADGNILQTIPHNKPLFQALIDGFREGFYLFPDGRNQNPDLTGLCEPTPQDHIKVTQEQYELYCEMGSTFQLCKICAENDKDVKIEPCGHLMCTSCLTSWQESEGQGCPFCRCEIKGTEPIVVDPFDPRGSGSLLRQGAEGAPSPNYDDDDDERADDTLFMMKELAGAKVERPPSPFSMAPQASLPPVPPRLDLLPQRVCVPSSASALGTASKAASGSLHKDKPLPVPPTLRDLPPPPPPDRPYSVGAESRPQRRPLPCTPGDCPSRDKLPPVPSSRLGDSWLPRPIPKVPVSAPSSSDPWTGRELTNRHSLPFSLPSQMEPRPDVPRLGSTFSLDTSMSMNSSPLVGPECDHPKIKPSSSANAIYSLAARPLPVPKLPPGEQCEGEEDTEYMTPSSRPLRPLDTSQSSRACDCDQQIDSCTYEAMYNIQSQAPSITESSTFGEGNLAAAHANTGPEESENEDDGYDVPKPPVPAVLARRTLSDISNASSSFGWLSLDGDPTTNVTEGSQVPERPPKPFPRRINSERKAGSCQQGSGPAASAATASPQLSSEIENLMSQGYSYQDIQKALVIAQNNIEMAKNILREFVSISSPAHVAT

1310 NP_112235.2 Q68J44

>NP_112235.2

MVPGSEGPARAGSVVADVVFVIEGTANLGPYFEGLRKHYLLPAIEYFNGGPPAETDFGGDYGGTQYSLVVFNTVDCAPESYVQCHAPTSSAYEFVTWLDGIKFMGGGGESCSLIAEGLSTALQLFDDFKKMREQIGQTHRVCLLICNSPPYLLPAVESTTYSGCTTENLVQQIGERGIHFSIVSPRKLPALRLLFEKAAPPALLEPLQPPTDVSQDPRHMVLVRGLVLPVGGGSAPGPLQSKQPVPLPPAAPSGATLSAAPQQPLPPVPPQYQVPGNLSAAQVAAQNAVEAAKNQKAGLGPRFSPITPLQQAAPGVGPPFSQAPAPQLPPGPPGAPKPPPASQPSLVSTVAPGSGLAPTAQPGAPSMAGTVAPGGVSGPSPAQLGAPALGGQQSVSNKLLAWSGVLEWQEKPKPASVDANTKLTRSLPCQVYVNHGENLKTEQWPQKLIMQLIPQQLLTTLGPLFRNSRMVQFHFTNKDLESLKGLYRIMGNGFAGCVHFPHTAPCEVRVLMLLYSSKKKIFMGLIPYDQSGFVNGIRQVITNHKQVQQQKLEQQQRGMGGQQAPPGLGPILEDQARPSQNLLQLRPPQPQPQGTVGASGATGQPQPQGTAQPPPGAPQGPPGAASGPPPPGPILRPQNPGANPQLRSLLLNPPPPQTGVPPPQASLHHLQPPGAPALLPPPHQGLGQPQLGPPLLHPPPAQSWPAQLPPRAPLPGQMLLSGGPRGPVPQPGLQPSVMEDDILMDLI

>Q68J44

MTSGEVKTSLKNAYSSAKRLSPKMEEEGEEEDYCTPGAFELERLFWKGSPQYTHVNEVWPKLYIGDEATALDRYRLQKAGFTHVLNAAHGRWNVDTGPDYYRDMDIQYHGVEADDLPTFDLSVFFYPAAAFIDRALSDDHSKILVHCVMGRSRSATLVLAYLMIHKDMTLVDAIQQVAKNRCVLPNRGFLKQLRELDKQLVQQRRRSQRQDGEEEDGREL

1311 NP_079058.1 NP_004125.3

>NP_079058.1

MEELEQGLLMQPWAWLQLAENSLLAKVFITKQGYALLVSDLQQVWHEQVDTSVVSQRAKELNKRLTAPPAAFLCHLDNLLRPLLKDAAHPSEATFSCDCVADALILRVRSELSGLPFYWNFHCMLASPSLVSQHLIRPLMGMSLALQCQVRELATLLHMKDLEIQDYQESGATLIRDRLKTEPFEENSFLEQFMIEKLPEACSIGDGKPFVMNLQDLYMAVTTQEVQVGQKHQGAGDPHTSNSASLQGIDSQCVNQPEQLVSSAPTLSAPEKESTGTSGPLQRPQLSKVKRKKPRGLFS

>NP_004125.3

MISASRAAAARLVGAAASRGPTAARHQDSWNGLSHEAFRLVSRRDYASEAIKGAVVGIDLGTTNSCVAVMEGKQAKVLENAEGARTTPSVVAFTADGERLVGMPAKRQAVTNPNNTFYATKRLIGRRYDDPEVQKDIKNVPFKIVRASNGDAWVEAHGKLYSPSQIGAFVLMKMKETAENYLGHTAKNAVITVPAYFNDSQRQATKDAGQISGLNVLRVINEPTAAALAYGLDKSEDKVIAVYDLGGGTFDISILEIQKGVFEVKSTNGDTFLGGEDFDQALLRHIVKEFKRETGVDLTKDNMALQRVREAAEKAKCELSSSVQTDINLPYLTMDSSGPKHLNMKLTRAQFEGIVTDLIRRTIAPCQKAMQDAEVSKSDIGEVILVGGMTRMPKVQQTVQDLFGRAPSKAVNPDEAVAIGAAIQGGVLAGDVTDVLLLDVTPLSLGIETLGGVFTKLINRNTTIPTKKSQVFSTAADGQTQVEIKVCQGEREMAGDNKLLGQFTLIGIPPAPRGVPQIEVTFDIDANGIVHVSAKDKGTGREQQIVIQSSGGLSKDDIENMVKNAEKYAEEDRRKKERVEAVNMAEGIIHDTETKMEEFKDQLPADECNKLKEEISKMRELLARKDSETGENIRQAASSLQQASLKLFEMAYKKMASEREGSGSSGTGEQKEDQKEEKQ

1312 CAA58827.1 NP_004369.1

>CAA58827.1

MADEEEDPTFEEENEEIGGGAEGGQGKRKRLFSKELRCMMYGFGDDQNPYTESVDILEDLVIEFITEMTHKAMSIGRQGRVQVEDIVFLIRKDPRKFARVKDLLTMNEELKRARKAFDEANYGS

>NP_004369.1

MPNFAGTWKMRSSENFDELLKALGVNAMLRKVAVAAASKPHVEIRQDGDQFYIKTSTTVRTTEINFKVGEGFEEETVDGRKCRSLATWENENKIHCTQTLLEGDGPKTYWTRELANDELILTFGADDVVCTRIYVRE

1313 P31249 NP_004369.1

>P31249

MLFEQGQQALELPECTMQKAAYYENPGLFGGYGYSKTTDTYGYSTPHQPYPPPAAASSLDTDYPGSACSIQSSAPLRAPAHKGAELNGSCMRPGTGNSQGGGGGSQPPGLNSEQQPPQPPPPPPTLPPSSPTNPGGGVPAKKPKGGPNASSSSATISKQIFPWMKESRQNSKQKNSCATAGESCEDKSPPGPASKRVRTAYTSAQLVELEKEFHFNRYLCRPRRVEMANLLNLTERQIKIWFQNRRMKYKKDQKAKGILHSPASQSPERSPPLGGAAGHVAYSGQLPPVPGLAYDAPSPPAFAKSQPNMYGLAAYTAPLSSCLPQQKRYAAPEFEPHPMASNGGGFASANLQGSPVYVGGNFVESMAPASGPVFNLGHLSHPSSASVDYSCAAQIPGNHHHGPCDPHPTYTDLSAHHSSQGRLPEAPKLTHL

>NP_004369.1

MPNFAGTWKMRSSENFDELLKALGVNAMLRKVAVAAASKPHVEIRQDGDQFYIKTSTTVRTTEINFKVGEGFEEETVDGRKCRSLATWENENKIHCTQTLLEGDGPKTYWTRELANDELILTFGADDVVCTRIYVRE

1314 P15848 NP_001005.1

>P15848

MGPRGAASLPRGPGPRRLLLPVVLPLLLLLLLAPPGSGAGASRPPHLVFLLADDLGWNDVGFHGSRIRTPHLDALAAGGVLLDNYYTQPLCTPSRSQLLTGRYQIRTGLQHQIIWPCQPSCVPLDEKLLPQLLKEAGYTTHMVGKWHLGMYRKECLPTRRGFDTYFGYLLGSEDYYSHERCTLIDALNVTRCALDFRDGEEVATGYKNMYSTNIFTKRAIALITNHPPEKPLFLYLALQSVHEPLQVPEEYLKPYDFIQDKNRHHYAGMVSLMDEAVGNVTAALKSSGLWNNTVFIFSTDNGGQTLAGGNNWPLRGRKWSLWEGGVRGVGFVASPLLKQKGVKNRELIHISDWLPTLVKLARGHTNGTKPLDGFDVWKTISEGSPSPRIELLHNIDPNFVDSSPCPRNSMAPAKDDSSLPEYSAFNTSVHAAIRHGNWKLLTGYPGCGYWFPPPSQYNVSEIPSSDPPTKTLWLFDIDRDPEERHDLSREYPHIVTKLLSRLQFYHKHSVPVYFPAQDPRCDPKATGVWGPWM

>NP_001005.1

MLMPKKNRIAIYELLFKEGVMVAKKDVHMPKHPELADKNVPNLHVMKAMQSLKSRGYVKEQFAWRHFYWYLTNEGIQYLRDYLHLPPEIVPATLRRSRPETGRPRPKGLEGERPARLTRGEADRDTYRRSAVPPGADKKAEAGAGSATEFQFRGGFGRGRGQPPQ

1315 Q00444 NP_550438.1

>Q00444

MSSYVANSFYKQSPNIPAYNMQTCGNYGSASEVQASRYCYGGLDLSITFPPPAPSNSLHGVDMAANPRAHPDRPACSAAAAPGHAPGRDEAAPLNPGMYSQKAARPALEERAKSSGEIKEEQAQTGQPAGLSQPPAPPQIYPWMTKLHMSHETDGKRSRTSYTRYQTLELEKEFHFNRYLTRRRRIEIANNLCLNERQIKIWFQNRRMKWKKDSKMKSKEAL

>NP_550438.1

MAAGRLFLSRLRAPFSSMAKSPLEGVSSSRGLHAGRGPRRLSIEGNIAVGKSTFVKLLTKTYPEWHVATEPVATWQNIQAAGTQKACTAQSLGNLLDMMYREPARWSYTFQTFSFLSRLKVQLEPFPEKLLQARKPVQIFERSVYSDRYIFAKNLFENGSLSDIEWHIYQDWHSFLLWEFASRITLHGFIYLQASPQVCLKRLYQRAREEEKGIELAYLEQLHGQHEAWLIHKTTKLHFEALMNIPVLVLDVNDDFSEEVTKQEDLMREVNTFVKNL

1316 Q9NV88 Q96P71

>Q9NV88

MKLYCLSGHPTLPCNVLKFKSTTIMLDCGLDMTSTLNFLPLPLVQSPRLSNLPGWSLKDGNAFLDKELKECSGHVFVDSVPEFCLPETELIDLSTVDVILISNYHCMMALPYITEHTGFTGTVYATEPTVQIGRLLMEELVNFIERVPKAQSASLWKNKDIQRLLPSPLKDAVEVSTWRRCYTMQEVNSALSKIQLVGYSQKIELFGAVQVTPLSSGYALGSSNWIIQSHYEKVSYVSGSSLLTTHPQPMDQASLKNSDVLVLTGLTQIPTANPDGMVGEFCSNLALTVRNGGNVLVPCYPSGVIYDLLECLYQYIDSAGLSSVPLYFISPVANSSLEFSQIFAEWLCHNKQSKVYLPEPPFPHAELIQTNKLKHYPSIHGDFSNDFRQPCVVFTGHPSLRFGDVVHFMELWGKSSLNTVIFTEPDFSYLEALAPYQPLAMKCIYCPIDTRLNFIQVSKLLKEVQPLHVVCPEQYTQPPPAQSHRMDLMIDCQPPAMSYRRAEVLALPFKRRYEKIEIMPELADSLVPMEIKPGISLATVSAVLHTKDNKHLLQPPPRPAQPTSGKKRKRVSDDVPDCKVLKPLLSGSIPVEQFVQTLEKHGFSDIKVEDTAKGHIVLLQEAETLIQIEEDSTHIICDNDEMLRVRLRDLVLKFLQKF

>Q96P71

MACAGLLTVCLLRPPAPQPQPQTPRHPQLAPDPGPAGHTLFQDVFRRADKNDDGKLSFEEFQNYFADGVLSLGELQELFSGIDGHLTDNLETEKLCDYFSEHLGVYRPVLAALESLNRAVLAAMDATKLEYERASKVDQFVTRFLLRETVSQLQALQSSLEGASDTLEAQAHGWRSDAESVEAQSRLCGSRRAGRRALRSVSRSSTWSPGSSDTGRSSEAEMQWRLQVNRLQELIDQLECKVRAVGPGPHKGGPSWYPPEPGPCWRPGPHSVPSQAPRLEPLREEDLAKGPDLHILMAQRQVQVAEEGLQDFHRALRCYVDFTGAQSHCLHVSAQKMLDGASFTLYEFWQDEASWRRHQQSPGSKAFQRILIDHLRAPDTLTTVFFPASWWIMNNN

1317 NP_006521.1 Q9BRV8

>NP_006521.1

MFKRMAEFGPDSGGRVKGVTIVKPIVYGNVARYFGKKREEDGHTHQWTVYVKPYRNEDMSAYVKKIQFKLHESYGNPLRVVTKPPYEITETGWGEFEIIIKIFFIDPNERPVTLYHLLKLFQSDTNAMLGKKTVVSEFYDEMIFQDPTAMMQQLLTTSRQLTLGAYKHETEFAELEVKTREKLEAAKKKTSFEIAELKERLKASRETINCLKNEIRKLEEDDQAKDI

>Q9BRV8

MSCTIEKILTDAKTLLERLREHDAAAESLVDQSAALHRRVAAMREAGTALPDQYQEDASDMKDMSKYKPHILLSQENTQIRDLQQENRELWISLEEHQDALELIMSKYRKQMLQLMVAKKAVDAEPVLKAHQSHSAEIESQIDRICEMGEVMRKAVQVDDDQFCKIQEKLAQLELENKELRELLSISSESLQARKENSMDTASQAIK

1318 NP_665906.1 NP_000841.1

>NP_665906.1

MSESSSKSSQPLASKQEKDGTEKRGRGRPRKQPPVSPGTALVGSQKEPSEVPTPKRPRGRPKGSKNKGAAKTRKTTTTPGRKPRGRPKKLEKEEEEGISQESSEEEQ

>NP_000841.1

MSMTLGYWDIRGLAHAIRLLLEYTDSSYEEKKYTMGDAPDYDRSQWLNEKFKLGLDFPNLPYLIDGAHKITQSNAILCYIARKHNLCGETEEEKIRVDILENQAMDVSNQLARVCYSPDFEKLKPEYLEELPTMMQHFSQFLGKRPWFVGDKITFVDFLAYDVLDLHRIFEPNCLDAFPNLKDFISRFEGLEKISAYMKSSRFLPKPLYTRVAVWGNK

1319 NP_057662.3 NP_000377.1

>NP_057662.3

MATAAAAAAVMAPPGCPGSCPNFAVVCSFLERYGPLLDLPELPFPELERVLQAPPPDVGNGEVPKELVELHLKLMRKIGKSVTADRWEKYLIKICQEFNSTWAWEMEKKGYLEMSVECKLALLKYLCECQFDDNLKFKNIINEEDADTMRLQPIGRDKDGLMYWYQLDQDHNVRMYIEEQDDQDGSSWKCIVRNRNELAETLALLKAQIDPVLLKNSSQQDNSSRESPSLEDEETKKEEETPKQEEQKESEKMKSEEQPMDLENRSTANVLEETTVKKEKEDEKELVKLPVIVKLEKPLPENEEKKIIKEESDSFKENVKPIKVEVKECRADPKDTKSSMEKPVAQEPERIEFGGNIKSSHEITEKSTEETEKLKNDQQAKIPLKKREIKLSDDFDSPVKGPLCKSVTPTKEFLKDEIKQEEETCKRISTITALGHEGKQLVNGEVSDERVAPNFKTEPIETKFYETKEESYSPSKDRNIITEGNGTESLNSVITSMKTGELEKETAPLRKDADSSISVLEIHSQKAQIEEPDPPEMETSLDSSEMAKDLSSKTALSSTESCTMKGEEKSPKTKKDKRPPILECLEKLEKSKKTFLDKDAQRLSPIPEEVPKSTLESEKPGSPEAAETSPPSNIIDHCEKLASEKEVVECQSTSTVGGQSVKKVDLETLKEDSEFTKVEMDNLDNAQTSGIEEPSETKGSMQKSKFKYKLVPEEETTASENTEITSERQKEGIKLTIRISSRKKKPDSPPKVLEPENKQEKTEKEEEKTNVGRTLRRSPRISRPTAKVAEIRDQKADKKRGEGEDEVEEESTALQKTDKKEILKKSEKDTNSKVSKVKPKGKVRWTGSRTRGRWKYSSNDESEGSGSEKSSAASEEEEEKESEEAILADDDEPCKKCGLPNHPELILLCDSCDSGYHTACLRPPLMIIPDGEWFCPPCQHKLLCEKLEEQLQDLDVALKKKERAERRKERLVYVGISIENIIPPQEPDFSEDQEEKKKDSKKSKANLLERRSTRTRKCISYRFDEFDEAIDEAIEDDIKEADGGGVGRGKDISTITGHRGKDISTILDEERKENKRPQRAAAARRKKRRRLNDLDSDSNLDEEESEDEFKISDGSQDEFVVSDENPDESEEDPPSNDDSDTDFCSRRLRRHPSRPMRQSRRLRRKTPKKKYSDDDEEEESEENSRDSESDFSDDFSDDFVETRRRRSRRNQKRQINYKEDSESDGSQKSLRRGKEIRRVHKRRLSSSESEESYLSKNSEDDELAKESKRSVRKRGRSTDEYSEADEEEEEEEGKPSRKRLHRIETDEEESCDNAHGDANQPARDSQPRVLPSEQESTKKPYRIESDEEEDFENVGKVGSPLDYSLVDLPSTNGQSPGKAIENLIGKPTEKSQTPKDNSTASASLASNGTSGGQEAGAPEEEEDELLRVTDLVDYVCNSEQL

>NP_000377.1

MSSSGLNSEKVAALIQKLNSDPQFVLAQNVGTTHDLLDICLKRATVQRAQHVFQHAVPQEGKPITNQKSSGRCWIFSCLNVMRLPFMKKLNIEEFEFSQSYLFFWDKVERCYFFLSAFVDTAQRKEPEDGRLVQFLLMNPANDGGQWDMLVNIVEKYGVIPKKCFPESYTTEATRRMNDILNHKMREFCIRLRNLVHSGATKGEISATQDVMMEEIFRVVCICLGNPPETFTWEYRDKDKNYQKIGPITPLEFYREHVKPLFNMEDKICLVNDPRPQHKYNKLYTVEYLSNMVGGRKTLYNNQPIDFLKKMVAASIKDGEAVWFGCDVGKHFNSKLGLSDMNLYDHELVFGVSLKNMNKAERLTFGESLMTHAMTFTAVSEKDDQDGAFTKWRVENSWGEDHGHKGYLCMTDEWFSEYVYEVVVDRKHVPEEVLAVLEQEPIILPAWDPMGALAE

1320 Q8NDX5 P11586

>Q8NDX5

MDTEPNPGTSSVSTTTSSTTTTTITTSSSRMQQPQISVYSGSDRHAVQVIQQALHRPPSSAAQYLQQMYAAQQQHLMLHTAALQQQHLSSSQLQSLAAVQASLSSGRPSTSPTGSVTQQSSMSQTSINLSTSPTPAQLISRSQASSSTSGSITQQTMLLGSTSPTLTASQAQMYLRAQMLIFTPATTVAAVQSDIPVVSSSSSSSCQSAATQVQNLTLRSQKLGVLSSSQNGPPKSTSQTQSLTICHNKTTVTSSKISQRDPSPESNKKGESPSLESRSTAVTRTSSIHQLIAPASYSPIQPHSLIKHQQIPLHSPPSKVSHHQLILQQQQQQIQPITLQNSTQDPPPSQHCIPLQNHGLPPAPSNAQSQHCSPIQSHPSPLTVSPNQSQSAQQSVVVSPPPPHSPSQSPTIIIHPQALIQPHPLVSSALQPGPNLQQSTANQVQATAQLNLPSHLPLPASPVVHIGPVQQSALVSPGQQIVSPSHQQYSSLQSSPIPIASPPQMSTSPPAQIPPLPLQSMQSLQVQPEILSQGQVLVQNALVSEEELPAAEALVQLPFQTLPPPQTVAVNLQVQPPAPVDPPVVYQVEDVCEEEMPEESDECVRMDRTPPPPTLSPAAITVGRGEDLTSEHPLLEQVELPAVASVSASVIKSPSDPSHVSVPPPPLLLPAATTRSNSTSMHSSIPSIENKPPQAIVKPQILTHVIEGFVIQEGLEPFPVSRSSLLIEQPVKKRPLLDNQVINSVCVQPELQNNTKHADNSSDTEMEDMIAEETLEEMDSELLKCEFCGKMGYANEFLRSKRFCTMSCAKRYNVSCSKKFALSRWNRKPDNQSLGHRGRRPSGPDGAAREHILRQLPITYPSAEEDLASHEDSVPSAMTTRLRRQSERERERELRDVRIRKMPENSDLLPVAQTEPSIWTVDDVWAFIHSLPGCQDIADEFRAQEIDGQALLLLKEDHLMSAMNIKLGPALKICARINSLKES

>P11586

MAPAEILNGKEISAQIRARLKNQVTQLKEQVPGFTPRLAILQVGNRDDSNLYINVKLKAAEEIGIKATHIKLPRTTTESEVMKYITSLNEDSTVHGFLVQLPLDSENSINTEEVINAIAPEKDVDGLTSINAGRLARGDLNDCFIPCTPKGCLELIKETGVPIAGRHAVVVGRSKIVGAPMHDLLLWNNATVTTCHSKTAHLDEEVNKGDILVVATGQPEMVKGEWIKPGAIVIDCGINYVPDDKKPNGRKVVGDVAYDEAKERASFITPVPGGVGPMTVAMLMQSTVESAKRFLEKFKPGKWMIQYNNLNLKTPVPSDIDISRSCKPKPIGKLAREIGLLSEEVELYGETKAKVLLSALERLKHRPDGKYVVVTGITPTPLGEGKSTTTIGLVQALGAHLYQNVFACVRQPSQGPTFGIKGGAAGGGYSQVIPMEEFNLHLTGDIHAITAANNLVAAAIDARIFHELTQTDKALFNRLVPSVNGVRRFSDIQIRRLKRLGIEKTDPTTLTDEEINRFARLDIDPETITWQRVLDTNDRFLRKITIGQAPTEKGHTRTAQFDISVASEIMAVLALTTSLEDMRERLGKMVVASSKKGEPVSAEDLGVSGALTVLMKDAIKPNLMQTLEGTPVFVHAGPFANIAHGNSSIIADRIALKLVGPEGFVVTEAGFGADIGMEKFFNIKCRYSGLCPHVVVLVATVRALKMHGGGPTVTAGLPLPKAYIQENLELVEKGFSNLKKQIENARMFGIPVVVAVNAFKTDTESELDLISRLSREHGAFDAVKCTHWAEGGKGALALAQAVQRAAQAPSSFQLLYDLKLPVEDKIRIIAQKIYGADDIELLPEAQHKAEVYTKQGFGNLPICMAKTHLSLSHNPEQKGVPTGFILPIRDIRASVGAGFLYPLVGTMSTMPGLPTRPCFYDIDLDPETEQVNGLF

1321 NP_291028.3 NP_037399.2

>NP_291028.3

MAAARATTPADGEEPAPEAEALAAARERSSRFLSGLELVKQGAEARVFRGRFQGRAAVIKHRFPKGYRHPALEARLGRRRTVQEARALLRCRRAGISAPVVFFVDYASNCLYMEEIEGSVTVRDYIQSTMETEKTPQGLSNLAKTIGQVLARMHDEDLIHGDLTTSNMLLKPPLEQLNIVLIDFGLSFISALPEDKGVDLYVLEKAFLSTHPNTETVFEAFLKSYSTSSKKARPVLKKLDEVRLRGRKRSMVG

>NP_037399.2

MRSMKALQKALSRAGSHCGRGGWGHPSRSPLLGGGVRHHLSEAAAQGRETPHSHQPQHQDHDSSESGMLSRLGDLLFYTIAEGQERIPIHKFTTALKATGLQTSDPRLRDCMSEMHRVVQESSSGGLLDRDLFRKCVSSNIVLLTQAFRKKFVIPDFEEFTGHVDRIFEDVKELTGGKVAAYIPQLAKSNPDLWGVSLCTVDGQRHSVGHTKIPFCLQSCVKPLTYAISISTLGTDYVHKFVGKEPSGLRYNKLSLNEEGIPHNPMVNAGAIVVSSLIKMDCNKAEKFDFVLQYLNKMAGNEYMGFSNATFQSEKETGDRNYAIGYYLKEKKCFPKGVDMMAALDLYFQLCSVEVTCESGSVMAATLANGGICPITGESVLSAEAVRNTLSLMHSCGMYDFSGQFAFHVGLPAKSAVSGAILLVVPNVMGMMCLSPPLDKLGNSHRGTSFCQKLVSLFNFHNYDNLRHCARKLDPRREGAEIRNKTVVNLLFAAYSGDVSALRRFALSAMDMEQKDYDSRTALHVAAAEGHIEVVKFLIEACKVNPFAKDRWGNIPLDDAVQFNHLEVVKLLQDYQDSYTLSETQAEAAAEALSKENLESMV

1322 P54803 Q93088

>P54803

MAEWLLSASWQRRAKAMTAAAGSAGRAAVPLLLCALLAPGGAYVLDDSDGLGREFDGIGAVSGGGATSRLLVNYPEPYRSQILDYLFKPNFGASLHILKVEIGGDGQTTDGTEPSHMHYALDENYFRGYEWWLMKEAKKRNPNITLIGLPWSFPGWLGKGFDWPYVNLQLTAYYVVTWIVGAKRYHDLDIDYIGIWNERSYNANYIKILRKMLNYQGLQRVKIIASDNLWESISASMLLDAELFKVVDVIGAHYPGTHSAKDAKLTGKKLWSSEDFSTLNSDMGAGCWGRILNQNYINGYMTSTIAWNLVASYYEQLPYGRCGLMTAQEPWSGHYVVESPVWVSAHTTQFTQPGWYYLKTVGHLEKGGSYVALTDGLGNLTIIIETMSHKHSKCIRPFLPYFNVSQQFATFVLKGSFSEIPELQVWYTKLGKTSERFLFKQLDSLWLLDSDGSFTLSLHEDELFTLTTLTTGRKGSYPLPPKSQPFPSTYKDDFNVDYPFFSEAPNFADQTGVFEYFTNIEDPGEHHFTLRQVLNQRPITWAADASNTISIIGDYNWTNLTIKCDVYIETPDTGGVFIAGRVNKGGILIRSARGIFFWIFANGSYRVTGDLAGWIIYALGRVEVTAKKWYTLTLTIKGHFASGMLNDKSLWTDIPVNFPKNGWAAIGTHSFEFAQFDNFLVEATR

>Q93088

MPPVGGKKAKKGILERLNAGEIVIGDGGFVFALEKRGYVKAGPWTPEAAVEHPEAVRQLHREFLRAGSNVMQTFTFYASEDKLENRGNYVLEKISGQEVNEAACDIARQVADEGDALVAGGVSQTPSYLSCKSETEVKKVFLQQLEVFMKKNVDFLIAEYFEHVEEAVWAVETLIASGKPVAATMCIGPEGDLHGVPPGECAVRLVKAGASIIGVNCHFDPTISLKTVKLMKEGLEAARLKAHLMSQPLAYHTPDCNKQGFIDLPEFPFGLEPRVATRWDIQKYAREAYNLGVRYIGGCCGFEPYHIRAIAEELAPERGFLPPASEKHGSWGSGLDMHTKPWVRARARKEYWENLRIASGRPYNPSMSKPDGWGVTKGTAELMQQKEATTEQQLKELFEKQKFKSQ

1323 P41229 NP_056449.1

>P41229

MEPGSDDFLPPPECPVFEPSWAEFRDPLGYIAKIRPIAEKSGICKIRPPADWQPPFAVEVDNFRFTPRIQRLNELEAQTRVKLNYLDQIAKFWEIQGSSLKIPNVERRILDLYSLSKIVVEEGGYEAICKDRRWARVAQRLNYPPGKNIGSLLRSHYERIVYPYEMYQSGANLVQCNTRPFDNEEKDKEYKPHSIPLRQSVQPSKFNSYGRRAKRLQPDPEPTEEDIEKNPELKKLQIYGAGPKMMGLGLMAKDKTLRKKDKEGPECPPTVVVKEELGGDVKVESTSPKTFLESKEELSHSPEPCTKMTMRLRRNHSNAQFIESYVCRMCSRGDEDDKLLLCDGCDDNYHIFCLLPPLPEIPKGVWRCPKCVMAECKRPPEAFGFEQATREYTLQSFGEMADSFKADYFNMPVHMVPTELVEKEFWRLVNSIEEDVTVEYGADIHSKEFGSGFPVSDSKRHLTPEEEEYATSGWNLNVMPVLEQSVLCHINADISGMKVPWLYVGMVFSAFCWHIEDHWSYSINYLHWGEPKTWYGVPSLAAEHLEEVMKKLTPELFDSQPDLLHQLVTLMNPNTLMSHGVPVVRTNQCAGEFVITFPRAYHSGFNQGYNFAEAVNFCTADWLPAGRQCIEHYRRLRRYCVFSHEELICKMAACPEKLDLNLAAAVHKEMFIMVQEERRLRKALLEKGITEAEREAFELLPDDERQCIKCKTTCFLSALACYDCPDGLVCLSHINDLCKCSSSRQYLRYRYTLDELPAMLHKLKVRAESFDTWANKVRVALEVEDGRKRSLEELRALESEARERRFPNSELLQQLKNCLSEAEACVSRALGLVSGQEAGPHRVAGLQMTLTELRAFLDQMNNLPCAMHQIGDVKGVLEQVEAYQAEAREALASLPSSPGLLQSLLERGRQLGVEVPEAQQLQRQVEQARWLDEVKRTLAPSARRGTLAVMRGLLVAGASVAPSPAVDKAQAELQELLTIAERWEEKAHLCLEARQKHPPATLEAIIREAENIPVHLPNIQALKEALAKARAWIADVDEIQNGDHYPCLDDLEGLVAVGRDLPVGLEELRQLELQVLTAHSWREKASKTFLKKNSCYTLLEVLCPCADAGSDSTKRSRWMEKELGLYKSDTELLGLSAQDLRDPGSVIVAFKEGEQKEKEGILQLRRTNSAKPSPLASSSTASSTTSICVCGQVLAGAGALQCDLCQDWFHGRCVSVPRLLSSPRPNPTSSPLLAWWEWDTKFLCPLCMRSRRPRLETILALLVALQRLPVRLPEGEALQCLTERAISWQGRARQALASEDVTALLGRLAELRQRLQAEPRPEEPPNYPAAPASDPLREGSGKDMPKVQGLLENGDSVTSPEKVAPEEGSGKRDLELLSSLLPQLTGPVLELPEATRAPLEELMMEGDLLEVTLDENHSIWQLLQAGQPPDLERIRTLLELEKAERHGSRARGRALERRRRRKVDRGGEGDDPAREELEPKRVRSSGPEAEEVQEEEELEEETGGEGPPAPIPTTGSPSTQENQNGLEPAEGTTSGPSAPFSTLTPRLHLPCPQQPPQQQL

>NP_056449.1

MANVPWAEVCEKFQAALALSRVELHKNPEKEPYKSKYSARALLEEVKALLGPAPEDEDERPEAEDGPGAGDHALGLPAEVVEPEGPVAQRAVRLAVIEFHLGVNHIDTEELSAGEEHLVKCLRLLRRYRLSHDCISLCIQAQNNLGILWSEREEIETAQAYLESSEALYNQYMKEVGSPPLDPTERFLPEEEKLTEQERSKRFEKVYTHNLYYLAQVYQHLEMFEKAAHYCHSTLKRQLEHNAYHPIEWAINAATLSQFYINKLCFMEARHCLSAANVIFGQTGKISATEDTPEAEGEVPELYHQRKGEIARCWIKYCLTLMQNAQLSMQDNIGELDLDKQSELRALRKKELDEEESIRKKAVQFGTGELCDAISAVEEKVSYLRPLDFEEARELFLLGQHYVFEAKEFFQIDGYVTDHIEVVQDHSALFKVLAFFETDMERRCKMHKRRIAMLEPLTVDLNPQYYLLVNRQIQFEIAHAYYDMMDLKVAIADRLRDPDSHIVKKINNLNKSALKYYQLFLDSLRDPNKVFPEHIGEDVLRPAMLAKFRVARLYGKIITADPKKELENLATSLEHYKFIVDYCEKHPEAAQEIEVELELSKEMVSLLPTKMERFRTKMALT

1324 NP_001291.3 Q9H4K7

>NP_001291.3

MDVLPMCSIFQELQIVHETGYFSALPSLEEYWQQTCLELERYLQSEPCYVSASEIKFDSQEDLWTKIILAREKKEESELKISSSPPEDTLISPSFCYNLETNSLNSDVSSESSDSSEELSPTAKFTSDPIGEVLVSSGKLSSSVTSTPPSSPELSREPSQLWGCVPGELPSPGKVRSGTSGKPGDKGNGDASPDGRRRVHRCHFNGCRKVYTKSSHLKAHQRTHTGEKPYRCSWEGCEWRFARSDELTRHFRKHTGAKPFKCSHCDRCFSRSDHLALHMKRHL

>Q9H4K7

MAPARCFSARLRTVFQGVGHWALSTWAGLKPSRLLPQRASPRLLSVGRADLAKHQELPGKKLLSEKKLKRYFVDYRRVLVCGGNGGAGASCFHSEPRKEFGGPDGGDGGNGGHVILRVDQQVKSLSSVLSRYQGFSGEDGGSKNCFGRSGAVLYIRVPVGTLVKEGGRVVADLSCVGDEYIAALGGAGGKGNRFFLANNNRAPVTCTPGQPGQQRVLHLELKTVAHAGMVGFPNAGKSSLLRAISNARPAVASYPFTTLKPHVGIVHYEGHLQIAVADIPGIIRGAHQNRGLGSAFLRHIERCRFLLFVVDLSQPEPWTQVDDLKYELEMYEKGLSARPHAIVANKIDLPEAQANLSQLRDHLGQEVIVLSALTGENLEQLLLHLKVLYDAYAEAELGQGRQPLRW

1325 NP_004166.1 NP_004754.1

>NP_004166.1

MSIGVPIKVLHEAEGHIVTCETNTGEVYRGKLIEAEDNMNCQMSNITVTYRDGRVAQLEQVYIRGSKIRFLILPDMLKNAPMLKSMKNKNQGSGAGRGKAAILKAQVAARGRGRGMGRGNIFQKRR

>NP_004754.1

MFRKGKKRHSSSSSQSSEISTKSKSVDSSLGGLSRSSTVASLDTDSTKSSGQSNNNSDTCAEFRIKYVGAIEKLKLSEGKGLEGPLDLINYIDVAQQDGKLPFVPPEEEFIMGVSKYGIKVSTSDQYDVLHRHALYLIIRMVCYDDGLGAGKSLLALKTTDASNEEYSLWVYQCNSLEQAQAICKVLSTAFDSVLTSEKP

1326 NP_006157.1 NP_005978.2

>NP_006157.1

MTMDGDSSTTDASQLGISADYIGGSHYVIQPHDDTEDSMNDHEDTNGSKESFREQDIYLPIANVARIMKNAIPQTGKIAKDAKECVQECVSEFISFITSEASERCHQEKRKTINGEDILFAMSTLGFDSYVEPLKLYLQKFREAMKGEKGIGGAVTATDGLSEELTEEAFTNQLPAGLITTDGQQQNVMVYTTSYQQISGVQQIQFS

>NP_005978.2

MNSQQQKQPCTPPPQPQQQQVKQPCQPPPQEPCIPKTKEPCHPKVPEPCHPKVPEPCQPKVPEPCQPKVPEPCPSTVTPAPAQQKTKQK

1327 NP_057953.1 Q9Y6M4

>NP_057953.1

MDLEKNYPTPRTSRTGHGGVNQLGGVFVNGRPLPDVVRQRIVELAHQGVRPCDISRQLRVSHGCVSKILGRYYETGSIKPGVIGGSKPKVATPKVVEKIAEYKRQNPTMFAWEIRDRLLAERVCDNDTVPSVSSINRIIRTKVQQPPNQPVPASSHSIVSTGSVTQVSSVSTDSAGSSYSISGILGITSPSADTNKRKRDEGIQESPVPNGHSLPGRDFLRKQMRGDLFTQQQLEVLDRVFERQHYSDIFTTTEPIKPEQTTEYSAMASLAGGLDDMKANLASPTPADIGSSVPGPQSYPIVTGRDLASTTLPGYPPHVPPAGQGSYSAPTLTGMVPGSEFSGSPYSHPQYSSYNDSWRFPNPGLLGSPYYYSAAARGAAPPAAATAYDRH

>Q9Y6M4

MENKKKDKDKSDDRMARPSGRSGHNTRGTGSSSSGVLMVGPNFRVGKKIGCGNFGELRLGKNLYTNEYVAIKLEPMKSRAPQLHLEYRFYKQLGSGDGIPQVYYFGPCGKYNAMVLELLGPSLEDLFDLCDRTFSLKTVLMIAIQLISRMEYVHSKNLIYRDVKPENFLIGRPGNKTQQVIHIIDFGLAKEYIDPETKKHIPYREHKSLTGTARYMSINTHLGKEQSRRDDLEALGHMFMYFLRGSLPWQGLKADTLKERYQKIGDTKRATPIEVLCENFPEMATYLRYVRRLDFFEKPDYDYLRKLFTDLFDRKGYMFDYEYDWIGKQLPTPVGAVQQDPALSSNREAHQHRDKMQQSKNQSADHRAAWDSQQANPHHLRAHLAADRHGGSVQVVSSTNGELNTDDPTAGRSNAPITAPTEVEVMDETKCCCFFKRRKRKTIQRHK

1328 P50539 NP_057356.1

>P50539

MERVKMINVQRLLEAAEFLERRERECEHGYASSFPSMPSPRLQHSKPPRRLSRAQKHSSGSSNTSTANRSTHNELEKNRRAHLRLCLERLKVLIPLGPDCTRHTTLGLLNKAKAHIKKLEEAERKSQHQLENLEREQRFLKWRLEQLQGPQEMERIRMDSIGSTISSDRSDSEREEIEVDVESTEFSHGEVDNISTTSISDIDDHSSLPSIGSDEGYSSASVKLSFTS

>NP_057356.1

MQLEIKVALNFIISYLYNKLPRRRADLFGEELERLLKKKYEGHWYPEKPLKGSGFRCVHIGEMVDPVVELAAKRSGLAVEDVRANVPEELSVWIDPFEVSYQIGEKGAVKVLYLDDSEGCGAPELDKEIKSSFNPDAQVFVPIGSQDSSLSNSPSPSFGQSPSPTFIPRSAQPITFTTASFAATKFGSTKMKKGGGAASGGGVASSGAGGQQPPQQPRMARSPTNSLLKHKSLSLSMHSLNFITANPAPQSQLSPNAKEFVYNGGGSPSLFFDAADGQGSGTPGPFGGSGAGTCNSSSFDMAQVFGGGANSLFLEKTPFVEGLSYNLNTMQYPSQQFQPVVLAN

1329 Q6UB99 Q9H2J4

>Q6UB99

MPKGGCPKAPQQEELPLSSDMVEKQTGKKDKDKVSLTKTPKLERGDGGKEVRERASKRKLPFTAGANGEQKDSDTEKQGPERKRIKKEPVTRKAGLLFGMGLSGIRAGYPLSERQQVALLMQMTAEESANSPVDTTPKHPSQSTVCQKGTPNSASKTKDKVNKRNERGETRLHRAAIRGDARRIKELISEGADVNVKDFAGWTALHEACNRGYYDVAKQLLAAGAEVNTKGLDDDTPLHDAANNGHYKVVKLLLRYGGNPQQSNRKGETPLKVANSPTMVNLLLGKGTYTSSEESSTESSEEEDAPSFAPSSSVDGNNTDSEFEKGLKHKAKNPEPQKATAPVKDEYEFDEDDEQDRVPPVDDKHLLKKDYRKETKSNSFISIPKMEVKSYTKNNTIAPKKASHRILSDTSDEEDASVTVGTGEKLRLSAHTILPGSKTREPSNAKQQKEKNKVKKKRKKETKGREVRFGKRSDKFCSSESESESSESGEDDRDSLGSSGCLKGSPLVLKDPSLFSSLSASSTSSHGSSAAQKQNPSHTDQHTKHWRTDNWKTISSPAWSEVSSLSDSTRTRLTSESDYSSEGSSVESLKPVRKRQEHRKRASLSEKKSPFLSSAEGAVPKLDKEGKVVKKHKTKHKHKNKEKGQCSISQELKLKSFTYEYEDSKQKSDKAILLENDLSTENKLKVLKHDRDHFKKEEKLSKMKLEEKEWLFKDEKSLKRIKDTNKDISRSFREEKDRSNKAEKERSLKEKSPKEEKLRLYKEERKKKSKDRPSKLEKKNDLKEDKISKEKEKIFKEDKEKLKKEKVYREDSAFDEYCNKNQFLENEDTKFSLSDDQRDRWFSDLSDSSFDFKGEDSWDSPVTDYRDMKSDSVAKLILETVKEDSKERRRDSRAREKRDYREPFFRKKDRDYLDKNSEKRKEQTEKHKSVPGYLSEKDKKRRESAEAGRDRKDALESCKERRDGRAKPEEVHREELKECGCESGFKDKSDGDFGKGLEPWERHHPAREKEKKDGPDKERKEKTKPERYKEKSSDKDKSEKSILEKCQKDKEFDKCFKEKKDTKEKHKDTHGKDKERKASLDQGKEKKEKAFPGIISEDFSEKKDDKKGKEKSWYIADIFTDESEDDRDSCMGSGFKMGEASDLPRTDGLQEKEEGREAYASDRHRKSSDKQHPERQKDKEPRDRRKDRGAADAGRDKKEKVFEKHKEKKDKESTEKYKDRKDRASVDSTQDKKNKQKLPEKAEKKHAAEDKAKSKHKEKSDKEHSKERKSSRSADAEKSLLEKLEEEALHEYREDSNDKISEVSSDSFTDRGQEPGLTAFLEVSFTEPPGDDKPRESACLPEKLKEKERHRHSSSSSKKSHDRERAKKEKAEKKEKGEDYKEGGSRKDSGQYEKDFLEADAYGVSYNMKADIEDELDKTIELFSTEKKDKNDSEREPSKKIEKELKPYGSSAINILKEKKKREKHREKWRDEKERHRDRHADGLLRHHRDELLRHHRDEQKPATRDKDSPPRVLKDKSRDEGPRLGDAKLKEKFKDGAEKEKGDPVKMSNGNDKVAPSKDPGKKDARPREKLLGDGDLMMTSFERMLSQKDLEIEERHKRHKERMKQMEKLRHRSGDPKLKEKAKPADDGRKKGLDIPAKKPPGLDPPFKDKKLKESTPIPPAAENKLHPASGADSKDWLAGPHMKEVLPASPRPDQSRPTGVPTPTSVLSCPSYEEVMHTPRTPSCSADDYADLVFDCADSQHSTPVPTAPTSACSPSFFDRFSVASSGLSENASQAPARPLSTNLYRSVSVDIRRTPEEEFSVGDKLFRQQSVPAASSYDSPMPPSMEDRAPLPPVPAEKFACLSPGYYSPDYGLPSPKVDALHCPPAAVVTVTPSPEGVFSSLQAKPSPSPRAELLVPSLEGALPPDLDTSEDQQATAAIIPPEPSYLEPLDEGPFSAVITEEPVEWAHPSEQALASSLIGGTSENPVSWPVGSDLLLKSPQRFPESPKRFCPADPLHSAAPGPFSASEAPYPAPPASPAPYALPVAEPGLEDVKDGVDAVPAAISTSEAAPYAPPSGLESFFSNCKSLPEAPLDVAPEPACVAAVAQVEALGPLENSFLDGSRGLSHLGQVEPVPWADAFAGPEDDLDLGPFSLPELPLQTKDAADGEAEPVEESLAPPEEMPPGAPGVINGGDVSTVVAEEPPALPPDQASTRLPAELEPEPSGEPKLDVALEAAVEAETVPEERARGDPDSSVEPAPVPPEQRPLGSGDQGAEAEGPPAASLCAPDGPAPNTVAQAQAADGAGPEDDTEASRAAAPAEGPPGGIQPEAAEPKPTAEAPKAPRVEEIPQRMTRNRAQMLANQSKQGPPPSEKECAPTPAPVTRAKARGSEDDDAQAQHPRKRRFQRSTQQLQQQLNTSTQQTREVIQQTLAAIVDAIKLDAIEPYHSDRANPYFEYLQIRKKIEEKRKILCCITPQAPQCYAEYVTYTGSYLLDGKPLSKLHIPVIAPPPSLAEPLKELFRQQEAVRGKLRLQHSIEREKLIVSCEQEILRVHCRAARTIANQAVPFSACTMLLDSEVYNMPLESQGDENKSVRDRFNARQFISWLQDVDDKYDRMKTCLLMRQQHEAAALNAVQRMEWQLKVQELDPAGHKSLCVNEVPSFYVPMVDVNDDFVLLPA

>Q9H2J4

MQDPNADTEWNDILRKKGILPPKESLKELEEEAEEEQRILQQSVVKTYEDMTLEELEDHEDEFNEEDERAIEMYRRRRLAEWKATKLKNKFGEVLEISGKDYVQEVTKAGEGLWVILHLYKQGIPLCALINQHLSGLARKFPDVKFIKAISTTCIPNYPDRNLPTIFVYLEGDIKAQFIGPLVFGGMNLTRDELEWKLSESGAIMTDLEENPKKPIEDVLLSSVRRSVLMKRDSDSEGD

1330 NP_003787.2 P35236

>NP_003787.2

MEAPTVETPPDPSPPSAPAPALVPLRAPDVARLREEQEKVVTNCQERIQHWKKVDNDYNALRERLSTLPDKLSYNIMVPFGPFAFMPGKLVHTNEVTVLLGDNWFAKCSAKQAVGLVEHRKEHVRKTIDDLKKVMKNFESRVEFTEDLQKMSDAAGDIVDIREEIKCDFEFKAKHRIAHKPHSKPKTSDIFEADIANDVKSKDLLADKELWARLEELERQEELLGELDSKPDTVIANGEDTTSSEEEKEDRNTNVNAMHQVTDSHTPCHKDVASSEPFSGQVNSQLNCSVNGSSSYHSDDDDDDDDDDDDDNIDDDDGDNDHEALGVGDNSIPTIYFSHTVEPKRVRINTGKNTTLKFSEKKEEAKRKRKNSTGSGHSAQELPTIRTPADIYRAFVDVVNGEYVPRKSILKSRSRENSVCSDTSESSAAEFDDRRGVLRSISCEEATCSDTSESILEEEPQENQKKLLPLSVTPEAFSGTVIEKEFVSPSLTPPPAIAHPALPTIPERKEVLLEASEETGKRVSKFKAARLQQKD

>P35236

MVQAHGGRSRAQPLTLSLGAAMTQPPPEKTPAKKHVRLQERRGSNVALMLDVRSLGAVEPICSVNTPREVTLHFLRTAGHPLTRWALQRQPPSPKQLEEEFLKIPSNFVSPEDLDIPGHASKDRYKTILPNPQSRVCLGRAQSQEDGDYINANYIRGYDGKEKVYIATQGPMPNTVSDFWEMVWQEEVSLIVMLTQLREGKEKCVHYWPTEEETYGPFQIRIQDMKECPEYTVRQLTIQYQEERRSVKHILFSAWPDHQTPESAGPLLRLVAEVEESPETAAHPGPIVVHCSAGIGRTGCFIATRIGCQQLKARGEVDILGIVCQLRLDRGGMIQTAEQYQFLHHTLALYAGQLPEEPSP

1331 NP_006512.2 Q86XT4

>NP_006512.2

MSHAAEPARDGVEASAEGPRAVFVLLEERRPADSAQLLSLNSLLPESGIVADIELENVLDPDSFYELKSQPLPLRSSLPISLQATPATPATLSASSSAGGSRTPAMSSSSSSRVLLRQQLMRAQAQEQERRERREQAAAAPFPSPAPASPAISVVGVSAGGHTLSRPPPAQVPREVLKVQTHLENPTRYHLQQARRQQVKQYLSTTLGPKLASQALTPPPGPASAQPLPAPEAAHTTGPTGSAPNSPMALLTIGSSSEKEIDDVIDEIISLESSYNDEMLSYLPGGTTGLQLPSTLPVSGNLLDVYSSQGVATPAITVSNSCPAELPNIKREISETEAKALLKERQKKDNHNLIERRRRFNINDRIKELGTLIPKSSDPEMRWNKGTILKASVDYIRKLQKEQQRSKDLESRQRSLEQANRSLQLRIQELELQAQIHGLPVPPTPGLLSLATTSASDSLKPEQLDIEEEGRPGAATFHVGGGPAQNAPHQQPPAPPSDALLDLHFPSDHLGDLGDPFHLGLEDILMEEEEGVVGGLSGGALSPLRAASDPLLSSVSPAVSKASSRRSSFSMEEES

>Q86XT4

MAWQVSLLELEDWLQCPICLEVFKEPLMLQCGHSYCKGCLVSLSCHLDAELRCPVCRQAVDGSSSLPNVSLARVIEALRLPGDPEPKVCVHHRNPLSLFCEKDQELICGLCGLLGSHQHHPVTPVSTVYSRMKEELAALISELKQEQKKVDELIAKLVNNRTRIVNESDVFSWVIRREFQELHHLVDEEKARCLEGIGGHTRGLVASLDMQLEQAQGTRERLAQAECVLEQFGNEDHHKFIRKFHSMASRAEMPQARPLEGAFSPISFKPGLHQADIKLTVWKRLFRKVLPAPEPLKLDPATAHPLLELSKGNTVVQCGLLAQRRASQPERFDYSTCVLASRGFSCGRHYWEVVVGSKSDWRLGVIKGTASRKGKLNRSPEHGVWLIGLKEGRVYEAFACPRVPLPVAGHPHRIGLYLHYEQGELTFFDADRPDDLRPLYTFQADFQGKLYPILDTCWHERGSNSLPMVLPPPSGPGPLSPEQPTKL

1332 Q53T94 NP_066953.1

>Q53T94

MDLEEAEEFKERCTQCAAVSWGLTDEGKYYCTSCHNVTERYQEVTNTDLIPNTQIKALNRGLKKKNNTEKGWDWYVCEGFQYILYQQAEALKNLGVGPELKNDVLHNFWKRYLQKSKQAYCKNPVYTTGRKPTVLEDNLSHSDWASEPELLSDVSCPPFLESGAESQSDIHTRKPFPVSKASQSETSVCSGSLDGVEYSQRKEKGIVKMTMPQTLAFCYLSLLWQREAITLSDLLRFVEEDHIPYINAFQHFPEQMKLYGRDRGIFGIESWPDYEDIYKKTVEVGTFLDLPRFPDITEDCYLHPNILCMKYLMEVNLPDEMHSLTCHVVKMTGMGEVDFLTFDPIAKMAKTVKYDVQAVAIIVVVLKLLFLLDDSFEWSLSNLAEKHNEKNKKDKPWFDFRKWYQIMKKAFDEKKQKWEEARAKYLWKSEKPLYYSFVDKPVAYKKREMVVNLQKQFSTLVESTATAGKKSPSSFQFNWTEEDTDRTCFHGHSLQGVLKEKGQSLLTKNSLYWLSTQKFCRCYCTHVTTYEESNYSLSYQFILNLFSFLLRIKTSLLHEEVSLVEKKLFEKKYSVKRKKSRSKKVRRH

>NP_066953.1

MVNPTVFFDIAVDGEPLGRVSFELFADKVPKTAENFRALSTGEKGFGYKGSCFHRIIPGFMCQGGDFTRHNGTGGKSIYGEKFEDENFILKHTGPGILSMANAGPNTNGSQFFICTAKTEWLDGKHVVFGKVKEGMNIVEAMERFGSRNGKTSKKITIADCGQLE

1333 Q7RTV3 Q9BRV8

>Q7RTV3

MIRGFEAPMAENPPPPPPPVIFCHDSPKRVLVSVIRTTPIKPTCGGGGEPEPPPPLIPTSPGFSDFMVYPWRWGENAHNVTLSPGAAGAAASAALPAAAAAEHSGLRGRGAPPPAASASAAASGGEDEEEASSPDSGHLKDGIRRGRPRADTVRDLINEGEHSSSRIRCNICNRVFPREKSLQAHKRTHTGERPYLCDYPDCGKAFVQSGQLKTHQRLHTGEKPFVCSENGCLSRFTHANRHCPKHPYARLKREEPTDTLSKHQAADNKAAAEWLARYWEMREQRTPTLKGKLVQKADQEQQDPLEYLQSDEEDDEKRGAQRRLQEQRERLHGALALIELANLTGAPLRQ

>Q9BRV8

MSCTIEKILTDAKTLLERLREHDAAAESLVDQSAALHRRVAAMREAGTALPDQYQEDASDMKDMSKYKPHILLSQENTQIRDLQQENRELWISLEEHQDALELIMSKYRKQMLQLMVAKKAVDAEPVLKAHQSHSAEIESQIDRICEMGEVMRKAVQVDDDQFCKIQEKLAQLELENKELRELLSISSESLQARKENSMDTASQAIK

1334 NP_005988.1 Q8N573

>NP_005988.1

MSLAGGRAPRKTAGNRLSGLLEAEEEDEFYQTTYGGFTEESGDDEYQGDQSDTEDEVDSDFDIDEGDEPSSDGEAEEPRRKRRVVTKAYKEPLKSLRPRKVNTPAGSSQKAREEKALLPLELQDDGSDSRKSMRQSTAEHTRQTFLRVQERQGQSRRRKGPHCERPLTQEELLREAKITEELNLRSLETYERLEADKKKQVHKKRKCPGPIITYHSVTVPLVGEPGPKEENVDIEGLDPAPSVSALTPHAGTGPVNPPARCSRTFITFSDDATFEEWFPQGRPPKVPVREVCPVTHRPALYRDPVTDIPYATARAFKIIREAYKKYITAHGLPPTASALGPGPPPPEPLPGSGPRALRQKIVIK

>Q8N573

MSFQKPKGTIEYTVESRDSLNSIALKFDTTPNELVQLNKLFSRAVVTGQVLYVPDPEYVSSVESSPSLSPVSPLSPTSSEAEFDKTTNPDVHPTEATPSSTFTGIRPARVVSSTSEEEEAFTEKFLKINCKYITSGKGTVSGVLLVTPNNIMFDPHKNDPLVQENGCEEYGIMCPMEEVMSAAMYKEILDSKIKESLPIDIDQLSGRDFCHSKKMTGSNTEEIDSRIRDAGNDSASTAPRSTEESLSEDVFTESELSPIREELVSSDELRQDKSSGASSESVQTVNQAEVESLTVKSESTGTPGHLRSDTEHSTNEVGTLCHKTDLNNLEMAIKEDQIADNFQGISGPKEDSTSIKGNSDQDSFLHENSLHQEESQKENMPCGETAEFKQKQSVNKGKQGKEQNQDSQTEAEELRKLWKTHTMQQTKQQRENIQQVSQKEAKHKITSADGHIESSALLKEKQRHRLHKFLCLRVGKPMRKTFVSQASATMQQYAQRDKKHEYWFAVPQERTDHLYAFFIQWSPEIYAEDTGEYTREPGFIVVKKIEESETIEDSSNQAAAREWEITTREDINSKQVATVKADLESESFRPNLSDPSELLLPDQIEKLTKHLPPRTIGYPWTLVYGTGKHGTSLKTLYRTMTGLDTPVLMVIKDSDGQVFGALASEPLKVSDGFYGTGETFVFTFCPEFEVFKWTGDNMFFIKGDMDSLAFGGGGGEFALWLDGDLYHGRSHSCKTFGNRTLSKKEDFFIQDIEIWAFE

1335 NP_001370.1 P46459

>NP_001370.1

MPARTAPARVPTLAVPAISLPDDVRRRLKDLERDSLTEKECVKEKLNLLHEFLQTEIKNQLCDLETKLRKEELSEEGYLAKVKSLLNKDLSLENGAHAYNREVNGRLENGNQARSEARRVGMADANSPPKPLSKPRTPRRSKSDGEAKPEPSPSPRITRKSTRQTTITSHFAKGPAKRKPQEESERAKSDESIKEEDKDQDEKRRRVTSRERVARPLPAEEPERAKSGTRTEKEEERDEKEEKRLRSQTKEPTPKQKLKEEPDREARAGVQADEDEDGDEKDEKKHRSQPKDLAAKRRPEEKEPEKVNPQISDEKDEDEKEEKRRKTTPKEPTEKKMARAKTVMNSKTHPPKCIQCGQYLDDPDLKYGQHPPDAVDEPQMLTNEKLSIFDANESGFESYEALPQHKLTCFSVYCKHGHLCPIDTGLIEKNIELFFSGSAKPIYDDDPSLEGGVNGKNLGPINEWWITGFDGGEKALIGFSTSFAEYILMDPSPEYAPIFGLMQEKIYISKIVVEFLQSNSDSTYEDLINKIETTVPPSGLNLNRFTEDSLLRHAQFVVEQVESYDEAGDSDEQPIFLTPCMRDLIKLAGVTLGQRRAQARRQTIRHSTREKDRGPTKATTTKLVYQIFDTFFAEQIEKDDREDKENAFKRRRCGVCEVCQQPECGKCKACKDMVKFGGSGRSKQACQERRCPNMAMKEADDDEEVDDNIPEMPSPKKMHQGKKKKQNKNRISWVGEAVKTDGKKSYYKKVCIDAETLEVGDCVSVIPDDSSKPLYLARVTALWEDSSNGQMFHAHWFCAGTDTVLGATSDPLELFLVDECEDMQLSYIHSKVKVIYKAPSENWAMEGGMDPESLLEGDDGKTYFYQLWYDQDYARFESPPKTQPTEDNKFKFCVSCARLAEMRQKEIPRVLEQLEDLDSRVLYYSATKNGILYRVGDGVYLPPEAFTFNIKLSSPVKRPRKEPVDEDLYPEHYRKYSDYIKGSNLDAPEPYRIGRIKEIFCPKKSNGRPNETDIKIRVNKFYRPENTHKSTPASYHADINLLYWSDEEAVVDFKAVQGRCTVEYGEDLPECVQVYSMGGPNRFYFLEAYNAKSKSFEDPPNHARSPGNKGKGKGKGKGKPKSQACEPSEPEIEIKLPKLRTLDVFSGCGGLSEGFHQAGISDTLWAIEMWDPAAQAFRLNNPGSTVFTEDCNILLKLVMAGETTNSRGQRLPQKGDVEMLCGGPPCQGFSGMNRFNSRTYSKFKNSLVVSFLSYCDYYRPRFFLLENVRNFVSFKRSMVLKLTLRCLVRMGYQCTFGVLQAGQYGVAQTRRRAIILAAAPGEKLPLFPEPLHVFAPRACQLSVVVDDKKFVSNITRLSSGPFRTITVRDTMSDLPEVRNGASALEISYNGEPQSWFQRQLRGAQYQPILRDHICKDMSALVAARMRHIPLAPGSDWRDLPNIEVRLSDGTMARKLRYTHHDRKNGRSSSGALRGVCSCVEAGKACDPAARQFNTLIPWCLPHTGNRHNHWAGLYGRLEWDGFFSTTVTNPEPMGKQGRVLHPEQHRVVSVRECARSQGFPDTYRLFGNILDKHRQVGNAVPPPLAKAIGLEIKLCMLAKARESASAKIKEEEAAKD

>P46459

MAGRSMQAARCPTDELSLTNCAVVNEKDFQSGQHVIVRTSPNHRYTFTLKTHPSVVPGSIAFSLPQRKWAGLSIGQEIEVSLYTFDKAKQCIGTMTIEIDFLQKKSIDSNPYDTDKMAAEFIQQFNNQAFSVGQQLVFSFNEKLFGLLVKDIEAMDPSILKGEPATGKRQKIEVGLVVGNSQVAFEKAENSSLNLIGKAKTKENRQSIINPDWNFEKMGIGGLDKEFSDIFRRAFASRVFPPEIVEQMGCKHVKGILLYGPPGCGKTLLARQIGKMLNAREPKVVNGPEILNKYVGESEANIRKLFADAEEEQRRLGANSGLHIIIFDEIDAICKQRGSMAGSTGVHDTVVNQLLSKIDGVEQLNNILVIGMTNRPDLIDEALLRPGRLEVKMEIGLPDEKGRLQILHIHTARMRGHQLLSADVDIKELAVETKNFSGAELEGLVRAAQSTAMNRHIKASTKVEVDMEKAESLQVTRGDFLASLENDIKPAFGTNQEDYASYIMNGIIKWGDPVTRVLDDGELLVQQTKNSDRTPLVSVLLEGPPHSGKTALAAKIAEESNFPFIKICSPDKMIGFSETAKCQAMKKIFDDAYKSQLSCVVVDDIERLLDYVPIGPRFSNLVLQALLVLLKKAPPQGRKLLIIGTTSRKDVLQEMEMLNAFSTTIHVPNIATGEQLLEALELLGNFKDKERTTIAQQVKGKKVWIGIKKLLMLIEMSLQMDPEYRVRKFLALLREEGASPLDFD

1336 Q96S96 Q969P6

>Q96S96

MGWTMRLVTAALLLGLMMVVTGDEDENSPCAHEALLDEDTLFCQGLEVFYPELGNIGCKVVPDCNNYRQKITSWMEPIVKFPGAVDGATYILVMVDPDAPSRAEPRQRFWRHWLVTDIKGADLKKGKIQGQELSAYQAPSPPAHSGFHRYQFFVYLQEGKVISLLPKENKTRGSWKMDRFLNRFHLGEPEASTQFMTQNYQDSPTLQAPRERASEPKHKNQAEIAAC

>Q969P6

MRVVRLLRLRAALTLLGEVPRRPASRGVPGSRRTQKGSGARWEKEKHEDGVKWRQLEHKGPYFAPPYEPLPDGVRFFYEGRPVRLSVAAEEVATFYGRMLDHEYTTKEVFRKNFFNDWRKEMAVEEREVIKSLDKCDFTEIHRYFVDKAAARKVLSREEKQKLKEEAEKLQQEFGYCILDGHQEKIGNFKIEPPGLFRGRGDHPKMGMLKRRITPEDVVINCSRDSKIPEPPAGHQWKEVRSDNTVTWLAAWTESVQNSIKYIMLNPCSKLKGETAWQKFETARRLRGFVDEIRSQYRADWKSREMKTRQRAVALYFIDKLALRAGNEKEDGEAADTVGCCSLRVEHVQLHPEADGCQHVVEFDFLGKDCIRYYNRVPVEKPVYKNLQLFMENKDPRDDLFDRLTTTSLNKHLQELMDGLTAKVFRTYNASITLQEQLRALTRAEDSIAAKILSYNRANRVVAILCNHQRATPSTFEKSMQNLQTKIQAKKEQVAEARAELRRARAEHKAQGDGKSRSVLEKKRRLLEKLQEQLAQLSVQATDKEENKQVALGTSKLNYLDPRISIAWCKRFRVPVEKIYSKTQRERFAWALAMAGEDFEF

1337 Q9BXL5 Q8IUQ0

>Q9BXL5

MDLGKDQSHLKHHQTPDPHQEENHSPEVIGTWSLRNRELLRKRKAEVHEKETSQWLFGEQKKRKQQRTGKGNRRGRKRQQNTELKVEPQPQIEKEIVEKALAPIEKKTEPPGSITKVFPSVASPQKVVPEEHFSEICQESNIYQENFSEYQEIAVQNHSSETCQHVSEPEDLSPKMYQEISVLQDNSSKICQDMKEPEDNSPNTCQVISVIQDHPFKMYQDMAKREDLAPKMCQEAAVPKILPCPTSEDTADLAGCSLQAYPKPDVPKGYILDTDQNPAEPEEYNETDQGIAETEGLFPKIQEIAEPKDLSTKTHQESAEPKYLPHKTCNEIIVPKAPSHKTIQETPHSEDYSIEINQETPGSEKYSPETYQEIPGLEEYSPEIYQETSQLEEYSPEIYQETPGPEDLSTETYKNKDVPKECFPEPHQETGGPQGQDPKAHQEDAKDAYTFPQEMKEKPKEEPGIPAILNESHPENDVYSYVLF

>Q8IUQ0

MGPVSLLPKYQKLNTWNGDLAKMTHLQAGLSPETIEKARLELNENPDVLHQDIQQVRDMIITRPDIGFLRTDDAFILRFLRARKFHQADAFRLLAQYFQYRQLNLDMFKNFKADDPGIKRALIDGFPGVLENRDHYGRKILLLFAANWDQSRNSFTDILRAILLSLEVLIEDPELQINGFILIIDWSNFSFKQASKLTPSILKLAIEGLQDSFPARFGGVHFVNQPWYIHALYTLIKPFLKDKTRKRIFLHGNNLNSLHQLIHPEFLPSEFGGTLPPYDMGTWARTLLGPDYSDENDYTHTSYNAMHVKHTSSNLERECSPKLMKRSQSVVEAGTLKHEEKGENENTQPLLALD

1338 Q8NFD5 NP_001344.2

>Q8NFD5

MAHNAGAAAAAGTHSAKSGGSEAALKEGGSAAALSSSSSSSAAAAAASSSSSSGPGSAMETGLLPNHKLKTVGEAPAAPPHQQHHHHHHAHHHHHHAHHLHHHHALQQQLNQFQQQQQQQQQQQQQQQQQQHPISNNNSLGGAGGGAPQPGPDMEQPQHGGAKDSAAGGQADPPGPPLLSKPGDEDDAPPKMGEPAGGRYEHPGLGALGTQQPPVAVPGGGGGPAAVPEFNNYYGSAAPASGGPGGRAGPCFDQHGGQQSPGMGMMHSASAAAAGAPGSMDPLQNSHEGYPNSQCNHYPGYSRPGAGGGGGGGGGGGGGSGGGGGGGGAGAGGAGAGAVAAAAAAAAAAAGGGGGGGYGGSSAGYGVLSSPRQQGGGMMMGPGGGGAASLSKAAAGSAAGGFQRFAGQNQHPSGATPTLNQLLTSPSPMMRSYGGSYPEYSSPSAPPPPPSQPQSQAAAAGAAAGGQQAAAGMGLGKDMGAQYAAASPAWAAAQQRSHPAMSPGTPGPTMGRSQGSPMDPMVMKRPQLYGMGSNPHSQPQQSSPYPGGSYGPPGPQRYPIGIQGRTPGAMAGMQYPQQQMPPQYGQQGVSGYCQQGQQPYYSQQPQPPHLPPQAQYLPSQSQQRYQPQQDMSQEGYGTRSQPPLAPGKPNHEDLNLIQQERPSSLPDLSGSIDDLPTGTEATLSSAVSASGSTSSQGDQSNPAQSPFSPHASPHLSSIPGGPSPSPVGSPVGSNQSRSGPISPASIPGSQMPPQPPGSQSESSSHPALSQSPMPQERGFMAGTQRNPQMAQYGPQQTGPSMSPHPSPGGQMHAGISSFQQSNSSGTYGPQMSQYGPQGNYSRPPAYSGVPSASYSGPGPGMGISANNQMHGQGPSQPCGAVPLGRMPSAGMQNRPFPGNMSSMTPSSPGMSQQGGPGMGPPMPTVNRKAQEAAAAVMQAAANSAQSRQGSFPGMNQSGLMASSSPYSQPMNNSSSLMNTQAPPYSMAPAMVNSSAASVGLADMMSPGESKLPLPLKADGKEEGTPQPESKSKKSSSSTTTGEKITKVYELGNEPERKLWVDRYLTFMEERGSPVSSLPAVGKKPLDLFRLYVCVKEIGGLAQVNKNKKWRELATNLNVGTSSSAASSLKKQYIQYLFAFECKIERGEEPPPEVFSTGDTKKQPKLQPPSPANSGSLQGPQTPQSTGSNSMAEVPGDLKPPTPASTPHGQMTPMQGGRSSTISVHDPFSDVSDSSFPKRNSMTPNAPYQQGMSMPDVMGRMPYEPNKDPFGGMRKVPGSSEPFMTQGQMPNSSMQDMYNQSPSGAMSNLGMGQRQQFPYGASYDRRHEPYGQQYPGQGPPSGQPPYGGHQPGLYPQQPNYKRHMDGMYGPPAKRHEGDMYNMQYSSQQQEMYNQYGGSYSGPDRRPIQGQYPYPYSRERMQGPGQIQTHGIPPQMMGGPLQSSSSEGPQQNMWAARNDMPYPYQNRQGPGGPTQAPPYPGMNRTDDMMVPDQRINHESQWPSHVSQRQPYMSSSASMQPITRPPQPSYQTPPSLPNHISRAPSPASFQRSLENRMSPSKSPFLPSMKMQKVMPTVPTSQVTGPPPQPPPIRREITFPPGSVEASQPVLKQRRKITSKDIVTPEAWRVMMSLKSGLLAESTWALDTINILLYDDSTVATFNLSQLSGFLELLVEYFRKCLIDIFGILMEYEVGDPSQKALDHNAARKDDSQSLADDSGKEEEDAECIDDDEEDEEDEEEDSEKTESDEKSSIALTAPDAAADPKEKPKQASKFDKLPIKIVKKNNLFVVDRSDKLGRVQEFNSGLLHWQLGGGDTTEHIQTHFESKMEIPPRRRPPPPLSSAGRKKEQEGKGDSEEQQEKSIIATIDDVLSARPGALPEDANPGPQTESSKFPFGIQQAKSHRNIKLLEDEPRSRDETPLCTIAHWQDSLAKRCICVSNIVRSLSFVPGNDAEMSKHPGLVLILGKLILLHHEHPERKRAPQTYEKEEDEDKGVACSKDEWWWDCLEVLRDNTLVTLANISGQLDLSAYTESICLPILDGLLHWMVCPSAEAQDPFPTVGPNSVLSPQRLVLETLCKLSIQDNNVDLILATPPFSRQEKFYATLVRYVGDRKNPVCREMSMALLSNLAQGDALAARAIAVQKGSIGNLISFLEDGVTMAQYQQSQHNLMHMQPPPLEPPSVDMMCRAAKALLAMARVDENRSEFLLHEGRLLDISISAVLNSLVASVICDVLFQIGQL

>NP_001344.2

MDSKYQCVKLNDGHFMPVLGFGTYAPAEVPKSKALEATKLAIEAGFRHIDSAHLYNNEEQVGLAIRSKIADGSVKREDIFYTSKLWCNSHRPELVRPALERSLKNLQLDYVDLYLIHFPVSVKPGEEVIPKDENGKILFDTVDLCATWEAVEKCKDAGLAKSIGVSNFNRRQLEMILNKPGLKYKPVCNQVECHPYFNQRKLLDFCKSKDIVLVAYSALGSHREEPWVDPNSPVLLEDPVLCALAKKHKRTPALIALRYQLQRGVVVLAKSYNEQRIRQNVQVFEFQLTSEEMKAIDGLNRNVRYLTLDIFAGPPNYPFSDEY

1339 Q3ZCT1 O75897

>Q3ZCT1

MIGMLESLQHESDLLQHDQIHTGEKPYECNECRKTFSLKQNLVEHKKMHTGEKSHECTECGKVCSRVSSLTLHLRSHTGKKAYKCNKCGKAFSQKENFLSHQKHHTGEKPYECEKVSIQMPTIIRHQKNHTGTKPYACKECGKAFNGKAYLTEHEKIHTGEKPFECNQCGRAFSQKQYLIKHQNIHTGKKPFKCSECGKAFSQKENLIIHQRIHTGEKPYECKGCGKAFIQKSSLIRHQRSHTGEKPYTCKECGKAFSGKSNLTEHEKIHIGEKPYKCNECGTIFRQKQYLIKHHNIHTGEKPYECNKCGKAFSRITSLIVHVRIHTGDKPYECKVCGKAFCQSSSLTVHMRSHTGEKPYGCNECGKAFSQFSTLALHMRIHTGEKPYQCSECGKAFSQKSHHIRHQRIHTH

>O75897

MALHDMEDFTFDGTKRLSVNYVKGILQPTDTCDIWDKIWNFQAKPDDLLISTYPKAGTTWTQEIVELIQNEGDVEKSKRAPTHQRFPFLEMKIPSLGSGLEQAHAMPSPRILKTHLPFHLLPPSLLEKNCKIIYVARNPKDNMVSYYHFQRMNKALPAPGTWEEYFETFLAGKVCWGSWHEHVKGWWEAKDKHRILYLFYEDMKKNPKHEIQKLAEFIGKKLDDKVLDKIVHYTSFDVMKQNPMANYSSIPAEIMDHSISPFMRKGAVGDWKKHFTVAQNERFDEDYKKKMTDTRLTFHFQF

1340 Q08999 NP_001024.1

>Q08999

MPSGGDQSPPPPPPPPAAAASDEEEEDDGEAEDAAPPAESPTPQIQQRFDELCSRLNMDEAARAEAWDSYRSMSESYTLEGNDLHWLACALYVACRKSVPTVSKGTVEGNYVSLTRILKCSEQSLIEFFNKMKKWEDMANLPPHFRERTERLERNFTVSAVIFKKYEPIFQDIFKYPQEEQPRQQRGRKQRRQPCTVSEIFHFCWVLFIYAKGNFPMISDDLVNSYHLLLCALDLVYGNALQCSNRKELVNPNFKGLSEDFHAKDSKPSSDPPCIIEKLCSLHDGLVLEAKGIKEHFWKPYIRKLYEKKLLKGKEENLTGFLEPGNFGESFKAINKAYEEYVLSVGNLDERIFLGEDAEEEIGTLSRCLNAGSGTETAERVQMKNILQQHFDKSKALRISTPLTGVRYIKENSPCVTPVSTATHSLSRLHTMLTGLRNAPSEKLEQILRTCSRDPTQAIANRLKEMFEIYSQHFQPDEDFSNCAKEIASKHFRFAEMLYYKVLESVIEQEQKRLGDMDLSGILEQDAFHRSLLACCLEVVTFSYKPPGNFPFITEIFDVPLYHFYKVIEVFIRAEDGLCREVVKHLNQIEEQILDHLAWKPESPLWEKIRDNENRVPTCEEVMPPQNLERADEICIAGSPLTPRRVTEVRADTGGLGRSITSPTTLYDRYSSPPASTTRRRLFVENDSPSDGGTPGRMPPQPLVNAVPVQNVSGETVSVTPVPGQTLVTMATATVTANNGQTVTIPVQGIANENGGITFFPVQVNVGGQAQAVTGSIQPLSAQALAGSLSSQQVTGTTLQVPGQVAIQQISPGGQQQKQGQSVTSSSNRPRKTSSLSLFFRKVYHLAAVRLRDLCAKLDISDELRKKIWTCFEFSIIQCPELMMDRHLDQLLMCAIYVMAKVTKEDKSFQNIMRCYRTQPQARSQVYRSVLIKGKRKRRNSGSSDSRSHQNSPTELNKDRTSRDSSPVMRSSSTLPVPQPSSAPPTPTRLTGANSDMEEEERGDLIQFYNNIYIKQIKTFAMKYSQANMDAPPLSPYPFVRTGSPRRIQLSQNHPVYISPHKNETMLSPREKIFYYFSNSPSKRLREINSMIRTGETPTKKRGILLEDGSESPAKRICPENHSALLRRLQDVANDRGSH

>NP_001024.1

MHVIKRDGRQERVMFDKITSRIQKLCYGLNMDFVDPAQITMKVIQGLYSGVTTVELDTLAAETAATLTTKHPDYAILAARIAVSNLHKETKKVFSDVMEDLYNYINPHNGKHSPMVAKSTLDIVLANKDRLNSAIIYDRDFSYNYFGFKTLERSYLLKINGKVAERPQHMLMRVSVGIHKEDIDAAIETYNLLSERWFTHASPTLFNAGTNRPQLSSCFLLSMKDDSIEGIYDTLKQCALISKSAGGIGVAVSCIRATGSYIAGTNGNSNGLVPMLRVYNNTARYVDQGGNKRPGAFAIYLEPWHLDIFEFLDLKKNTGKEEQRARDLFFALWIPDLFMKRVETNQDWSLMCPNECPGLDEVWGEEFEKLYASYEKQGRVRKVVKAQQLWYAIIESQTETGTPYMLYKDSCNRKSNQQNLGTIKCSNLCTEIVEYTSKDEVAVCNLASLALNMYVTSEHTYDFKKLAEVTKVVVRNLNKIIDINYYPVPEACLSNKRHRPIGIGVQGLADAFILMRYPFESAEAQLLNKQIFETIYYGALEASCDLAKEQGPYETYEGSPVSKGILQYDMWNVTPTDLWDWKVLKEKIAKYGIRNSLLIAPMPTASTAQILGNNESIEPYTSNIYTRRVLSGEFQIVNPHLLKDLTERGLWHEEMKNQIIACNGSIQSIPEIPDDLKQLYKTVWEISQKTVLKMAAERGAFIDQSQSLNIHIAEPNYGKLTSMHFYGWKQGLKTGMYYLRTRPAANPIQFTLNKEKLKDKEKVSKEEEEKERNTAAMVCSLENRDECLMCGS

1341 NP_002752.1 NP_064530.1

>NP_002752.1

MARYRCCRSQSRSRYYRQRQRSRRRRRRSCQTRRRAMRCCRPRYRPRCRRH

>NP_064530.1

MDFNMKKLASDAGIFFTRAVQFTEEKFGQAEKTELDAHFENLLARADSTKNWTEKILRQTEVLLQPNPSARVEEFLYEKLDRKVPSRVTNGELLAQYMADAASELGPTTPYGKTLIKVAEAEKQLGAAERDFIHTASISFLTPLRNFLEGDWKTISKERRLLQNRRLDLDACKARLKKAKAAEAKATTVPDFQETRPRNYILSASASALWNDEVDKAEQELRVAQTEFDRQAEVTRLLLEGISSTHVNHLRCLHEFVKSQTTYYAQCYRHMLDLQKQLGRFPGTFVGTTEPASPPLSSTSPTTAAATMPVVPSVASLAPPGEASLCLEEVAPPASGTRKARVLYDYEAADSSELALLADELITVYSLPGMDPDWLIGERGNKKGKVPVTYLELLS

1342 Q14938 Q9BR61

>Q14938

MYSPYCLTQDEFHPFIEALLPHVRAFSYTWFNLQARKRKYFKKHEKRMSKDEERAVKDELLGEKPEIKQKWASRLLAKLRKDIRPEFREDFVLTITGKKPPCCVLSNPDQKGKIRRIDCLRQADKVWRLDLVMVILFKGIPLESTDGERLYKSPQCSNPGLCVQPHHIGVTIKELDLYLAYFVHTPESGQSDSSNQQGDADIKPLPNGHLSFQDCFVTSGVWNVTELVRVSQTPVATASGPNFSLADLESPSYYNINQVTLGRRSITSPPSTSTTKRPKSIDDSEMESPVDDVFYPGTGRSPAAGSSQSSGWPNDVDAGPASLKKSGKLDFCSALSSQGSSPRMAFTHHPLPVLAGVRPGSPRATASALHFPSTSIIQQSSPYFTHPTIRYHHHHGQDSLKEFVQFVCSDGSGQATGQPNGSGQGKVPGSFLLPPPPPVARPVPLPMPDSKSTSTAPDGAALTPPSPSFATTGASSANRFVSIGPRDGNFLNIPQQSQSWFL

>Q9BR61

MASSFLPAGAITGDSGGELSSGDDSGEVEFPHSPEIEETSCLAELFEKAAAHLQGLIQVASREQLLYLYARYKQVKVGNCNTPKPSFFDFEGKQKWEAWKALGDSSPSQAMQEYIAVVKKLDPGWNPQIPEKKGKEANTGFGGPVISSLYHEETIREEDKNIFDYCRENNIDHITKAIKSKNVDVNVKDEEGRALLHWACDRGHKELVTVLLQHRADINCQDNEGQTALHYASACEFLDIVELLLQSGADPTLRDQDGCLPEEVTGCKTVSLVLQRHTTGKA

1343 Q9P0K8 NP_006535.1

>Q9P0K8

MASDLESSLTSIDWLPQLTLRATIEKLGSASQAGPPGSSRKCSPGSPTDPNATLSKDEAAVHQDGKPRYSYATLITYAINSSPAKKMTLSEIYRWICDNFPYYKNAGIGWKNSIRHNLSLNKCFRKVPRPRDDPGKGSYWTIDTCPDISRKRRHPPDDDLSQDSPEQEASKSPRGGVAGSGEASLPPEGNPQMSLQSPTSIASYSQGTGSVDGGAVAAGASGRESAEGPPPLYNTNHDFKFSYSEINFQDLSWSFRNLYKSMLEKSSSSSQHGFSSLLGDIPPSNNYYMYQQQQPPPPQQQQQQQQPPQPPPQQSQPQQQQAPAQGPSAVGGAPPLHTPSTDGCTPPGGKQAGAEGYGPPPVMAMHPPPLQHGGYHPHQHHPHSHPAQQPPPPQPQAQGQAPINNTGFAFPSDWCSNIDSLKESFKMVNRLNWSSIEQSQFSELMESLRQAEQKNWTLDQHHIANLCDSLNHFLTQTGHVPPQGGTHRPPAPARIADSCALTSGKQESAMSQVNSYGHPQAPHLYPGPSPMYPIPTQDSAGYNRPAHHMVPRPSVPPPGANEEIPDDFDWDLIT

>NP_006535.1

MATTAELFEEPFVADEYIERLVWRTPGGGSRGGPEAFDPKRLLEEFVNHIQELQIMDERIQRKVEKLEQQCQKEAKEFAKKVQELQKSNQVAFQHFQELDEHISYVATKVCHLGDQLEGVNTPRQRAVEAQKLMKYFNEFLDGELKSDVFTNSEKIKEAADIIQKLHLIAQELPFDRFSEVKSKIASKYHDLECQLIQEFTSAQRRGEISRMREVAAVLLHFKGYSHCVDVYIKQCQEGAYLRNDIFEDAGILCQRVNKQVGDIFSNPETVLAKLIQNVFEIKLQSFVKEQLEECRKSDAEQYLKNLYDLYTRTTNLSSKLMEFNLGTDKQTFLSKLIKSIFISYLENYIEVETGYLKSRSAMILQRYYDSKNHQKRSIGTGGIQDLKERIRQRTNLPLGPSIDTHGETFLSQEVVVNLLQETKQAFERCHRLSDPSDLPRNAFRIFTILVEFLCIEHIDYALETGLAGIPSSDSRNANLYFLDVVQQANTIFHLFDKQFNDHLMPLISSSPKLSECLQKKKEIIEQMEMKLDTGIDRTLNCMIGQMKHILAAEQKKTDFKPEDENNVLIQYTNACVKVCAYVRKQVEKIKNSMDGKNVDTVLMELGVRFHRLIYEHLQQYSYSCMGGMLAICDVAEYRKCAKDFKIPMVLHLFDTLHALCNLLVVAPDNLKQVCSGEQLANLDKNILHSFVQLRADYRSARLARHFS

1344 NP_000225.1 Q9UBR1

>NP_000225.1

MQRSIMSFFHPKKEGKAKKPEKEASNSSRETEPPPKAALKEWNGVVSESDSPVKRPGRKAARVLGSEGEEEDEALSPAKGQKPALDCSQVSPPRPATSPENNASLSDTSPMDSSPSGIPKRRTARKQLPKRTIQEVLEEQSEDEDREAKRKKEEEEEETPKESLTEAEVATEKEGEDGDQPTTPPKPLKTSKAETPTESVSEPEVATKQELQEEEEQTKPPRRAPKTLSSFFTPRKPAVKKEVKEEEPGAPGKEGAAEGPLDPSGYNPAKNNYHPVEDACWKPGQKVPYLAVARTFEKIEEVSARLRMVETLSNLLRSVVALSPPDLLPVLYLSLNHLGPPQQGLELGVGDGVLLKAVAQATGRQLESVRAEAAEKGDVGLVAENSRSTQRLMLPPPPLTASGVFSKFRDIARLTGSASTAKKIDIIKGLFVACRHSEARFIARSLSGRLRLGLAEQSVLAALSQAVSLTPPGQEFPPAMVDAGKGKTAEARKTWLEEQGMILKQTFCEVPDLDRIIPVLLEHGLERLPEHCKLSPGIPLKPMLAHPTRGISEVLKRFEEAAFTCEYKYDGQRAQIHALEGGEVKIFSRNQEDNTGKYPDIISRIPKIKLPSVTSFILDTEAVAWDREKKQIQPFQVLTTRKRKEVDASEIQVQVCLYAFDLIYLNGESLVREPLSRRRQLLRENFVETEGEFVFATSLDTKDIEQIAEFLEQSVKDSCEGLMVKTLDVDATYEIAKRSHNWLKLKKDYLDGVGDTLDLVVIGAYLGRGKRAGRYGGFLLASYDEDSEELQAICKLGTGFSDEELEEHHQSLKALVLPSPRPYVRIDGAVIPDHWLDPSAVWEVKCADLSLSPIYPAARGLVDSDKGISLRFPRFIRVREDKQPEQATTSAQVACLYRKQSQIQNQQGEDSGSDPEDTY

>Q9UBR1

MAGAEWKSLEECLEKHLPLPDLQEVKRVLYGKELRKLDLPREAFEAASREDFELQGYAFEAAEEQLRRPRIVHVGLVQNRIPLPANAPVAEQVSALHRRIKAIVEVAAMCGVNIICFQEAWTMPFAFCTREKLPWTEFAESAEDGPTTRFCQKLAKNHDMVVVSPILERDSEHGDVLWNTAVVISNSGAVLGKTRKNHIPRVGDFNESTYYMEGNLGHPVFQTQFGRIAVNICYGRHHPLNWLMYSINGAEIIFNPSATIGALSESLWPIEARNAAIANHCFTCAINRVGTEHFPNEFTSGDGKKAHQDFGYFYGSSYVAAPDSSRTPGLSRSRDGLLVAKLDLNLCQQVNDVWNFKMTGRYEMYARELAEAVKSNYSPTIVKE

1345 NP_848511.1 Q9BYC8

>NP_848511.1

MERARPEPPPQPRPLRPAPPPLPVEGTSFWAAAMEPPPSSPTLSAAASATLASSCGEAVASGLQPAVRRLLQVKPEQVLLLPQPQAQNEEAAASSAQARLLQFRPDLRLLQPPTASDGATSRPELHPVQPLALHVKAKKQKLGPSLDQSVGPRGAVETGPRASRVVKLEGPGPALGYFRGDEKGKLEAEEVMRDSMQGGAGKSPAAIREGVIKTEEPERLLEDCRLGAEPASNGLVHGSAEVILAPTSGAFGPHQQDLRIPLTLHTVPPGARIQFQGAPPSELIRLTKVPLTPVPTKMQSLLEPSVKIETKDVPLTVLPSDAGIPDTPFSKDRNGHVKRPMNAFMVWARIHRPALAKANPAANNAEISVQLGLEWNKLSEEQKKPYYDEAQKIKEKHREEFPGWVYQPRPGKRKRFPLSVSNVFSGTTQNIISTNPTTVYPYRSPTYSVVIPSLQNPITHPVGETSPAIQLPTPAVQSPSPVTLFQPSVSSAAQVAVQDPSLPVYPALPPQRFTGPSQTDTHQLHSEATHTVKQPTPVSLESANRISSSASTAHARFATSTIQPPREYSSVSPCPRSAPIPQASPIPHPHVYQPPPLGHPATLFGTPPRFSFHHPYFLPGPHYFPSSTCPYSRPPFGYGNFPSSMPECLSYYEDRYPKHEGIFSTLNRDYSFRDYSSECTHSENSRSCENMNGTSYYNSHSHSGEENLNPVPQLDIGTLENVFTAPTSTPSSIQQVNVTDSDEEEEEKVLRDL

>Q9BYC8

MALAMLVLVVSPWSAARGVLRNYWERLLRKLPQSRPGFPSPPWGPALAVQGPAMFTEPANDTSGSKENSSLLDSIFWMAAPKNRRTIEVNRCRRRNPQKLIKVKNNIDVCPECGHLKQKHVLCAYCYEKVCKETAEIRRQIGKQEGGPFKAPTIETVVLYTGETPSEQDQGKRIIERDRKRPSWFTQN

1346 Q13523 NP_002100.2

>Q13523

MAAAETQSLREQPEMEDANSEKSINEENGEVSEDQSQNKHSRHKKKKHKHRSKHKKHKHSSEEDKDKKHKHKHKHKKHKRKEVIDASDKEGMSPAKRTKLDDLALLEDLEKQRALIKAELDNELMEGKVQSGMGLILQGYESGSEEEGEIHEKARNGNRSSTRSSSTKGKLELVDNKITTKKRSKSRSKERTRHRSDKKKSKGGIEIVKEKTTRSKSKERKKSKSPSKRSKSQDQARKSKSPTLRRRSQEKIGKARSPTDDKVKIEDKSKSKDRKKSPIINESRSRDRGKKSRSPVDLRGKSKDRRSRSKERKSKRSETDKEKKPIKSPSKDASSGKENRSPSRRPGRSPKRRSLSPKPRDKSRRSRSPLLNDRRSKQSKSPSRTLSPGRRAKSRSLERKRREPERRRLSSPRTRPRDDILSRRERSKDASPINRWSPTRRRSRSPIRRRSRSPLRRSRSPRRRSRSPRRRDRGRRSRSRLRRRSRSRGGRRRRSRSKVKEDKFKGSLSEGMKVEQESSSDDNLEDFDVEEEDEEALIEQRRIQRQAIVQKYKYLAEDSNMSVPSEPSSPQSSTRTRSPSPDDILERVAADVKEYERENVDTFEASVKAKHNLMTVEQNNGSSQKKLLAPDMFTESDDMFAAYFDSARLRAAGIGKDFKENPNLRDNWTDAEGYYRVNIGEVLDKRYNVYGYTGQGVFSNVVRARDNARANQEVAVKIIRNNELMQKTGLKELEFLKKLNDADPDDKFHCLRLFRHFYHKQHLCLVFEPLSMNLREVLKKYGKDVGLHIKAVRSYSQQLFLALKLLKRCNILHADIKPDNILVNESKTILKLCDFGSASHVADNDITPYLVSRFYRAPEIIIGKSYDYGIDMWSVGCTLYELYTGKILFPGKTNNHMLKLAMDLKGKMPNKMIRKGVFKDQHFDQNLNFMYIEVDKVTEREKVTVMSTINPTKDLLADLIGCQRLPEDQRKKVHQLKDLLDQILMLDPAKRISINQALQHAFIQEKI

>NP_002100.2

MAERAALEELVKLQGERVRGLKQQKASAELIEEEVAKLLKLKAQLGPDESKQKFVLKTPKGTRDYSPRQMAVREKVFDVIIRCFKRHGAEVIDTPVFELKETLMGKYGEDSKLIYDLKDQGGELLSLRYDLTVPFARYLAMNKLTNIKRYHIAKVYRRDNPAMTRGRYREFYQCDFDIAGNFDPMIPDAECLKIMCEILSSLQIGDFLVKVNDRRILDGMFAICGVSDSKFRTICSSVDKLDKVSWEEVKNEMVGEKGLAPEVADRIGDYVQQHGGVSLVEQLLQDPKLSQNKQALEGLGDLKLLFEYLTLFGIDDKISFDLSLARGLDYYTGVIYEAVLLQTPAQAGEEPLGVGSVAAGGRYDGLVGMFDPKGRKVPCVGLSIGVERIFSIVEQRLEALEEKIRTTETQVLVASAQKKLLEERLKLVSELWDAGIKAELLYKKNPKLLNQLQYCEEAGIPLVAIIGEQELKDGVIKLRSVTSREEVDVRREDLVEEIKRRTGQPLCIC

1347 NP_039269.2 Q16698

>NP_039269.2

MSLAFKIFFPQTLRALSRKELCLFRKHHWRDVRQFSQWSETDLLHGHPLFLRRKPVLSFQGSHLRSRATYLVFLPGLHVGLCSGPCEMAEQRFCVDYAKRGTAGCKKCKEKIVKGVCRIGKVVPNPFSESGGDMKEWYHIKCMFEKLERARATTKKIEDLTELEGWEELEDNEKEQITQHIADLSSKAAGTPKKKAVVQAKLTTTGQVTSPVKGASFVTSTNPRKFSGFSAKPNNSGEAPSSPTPKRSLSSSKCDPRHKDCLLREFRKLCAMVADNPSYNTKTQIIQDFLRKGSAGDGFHGDVYLTVKLLLPGVIKTVYNLNDKQIVKLFSRIFNCNPDDMARDLEQGDVSETIRVFFEQSKSFPPAAKSLLTIQEVDEFLLRLSKLTKEDEQQQALQDIASRCTANDLKCIIRLIKHDLKMNSGAKHVLDALDPNAYEAFKASRNLQDVVERVLHNAQEVEKEPGQRRALSVQASLMTPVQPMLAEACKSVEYAMKKCPNGMFSEIKYDGERVQVHKNGDHFSYFSRSLKPVLPHKVAHFKDYIPQAFPGGHSMILDSEVLLIDNKTGKPLPFGTLGVHKKAAFQDANVCLFVFDCIYFNDVSLMDRPLCERRKFLHDNMVEIPNRIMFSEMKRVTKALDLADMITRVIQEGLEGLVLKDVKGTYEPGKRHWLKVKKDYLNEGAMADTADLVVLGAFYGQGSKGGMMSIFLMGCYDPGSQKWCTVTKCAGGHDDATLARLQNELDMVKISKDPSKIPSWLKVNKIYYPDFIVPDPKKAAVWEITGAEFSKSEAHTADGISIRFPRCTRIRDDKDWKSATNLPQLKELYQLSKEKADFTVVAGDEGSSTTGGSSEENKGPSGSAVSRKAPSKPSASTKKAEGKLSNSNSKDGNMQTAKPSAMKVGEKLATKSSPVKVGEKRKAADETLCQTKVLLDIFTGVRLYLPPSTPDFSRLRRYFVAFDGDLVQEFDMTSATHVLGSRDKNPAAQQVSPEWIWACIRKRRLVAPC

>Q16698

MKLPARVFFTLGSRLPCGLAPRRFFSYGTKILYQNTEALQSKFFSPLQKAMLPPNSFQGKVAFITGGGTGLGKGMTTLLSSLGAQCVIASRKMDVLKATAEQISSQTGNKVHAIQCDVRDPDMVQNTVSELIKVAGHPNIVINNAAGNFISPTERLSPNAWKTITDIVLNGTAFVTLEIGKQLIKAQKGAAFLSITTIYAETGSGFVVPSASAKAGVEAMSKSLAAEWGKYGMRFNVIQPGPIKTKGAFSRLDPTGTFEKEMIGRIPCGRLGTVEELANLAAFLCSDYASWINGAVIKFDGGEEVLISGEFNDLRKVTKEQWDTIEELIRKTKGS

1348 P39880 P11586

>P39880

MLCVRGARLKRELDATATVLANRQDESEQSRKRLIEQSREFKKNTPEDLRKQVAPLLKSFQGEIDALSKRSKEAEAAFLNVYKRLIDVPDPVPALDLGQQLQLKVQRLHDIETENQKLRETLEEYNKEFAEVKNQEVTIKALKEKIREYEQTLKNQAETIALEKEQKLQNDFAEKERKLQETQMSTTSKLEEAEHKVQSLQTALEKTRTELFDLKTKYDEETTAKADEIEMIMTDLERANQRAEVAQREAETLREQLSSANHSLQLASQIQKAPDVEQAIEVLTRSSLEVELAAKEREIAQLVEDVQRLQASLTKLRENSASQISQLEQQLSAKNSTLKQLEEKLKGQADYEEVKKELNILKSMEFAPSEGAGTQDAAKPLEVLLLEKNRSLQSENAALRISNSDLSGSARRKGKDQPESRRPGSLPAPPPSQLPRNPGEQASNTNGTHQFSPAGLSQDFFSSSLASPSLPLASTGKFALNSLLQRQLMQSFYSKAMQEAGSTSMIFSTGPYSTNSISSQSPLQQSPDVNGMAPSPSQSESAGSVSEGEEMDTAEIARQVKEQLIKHNIGQRIFGHYVLGLSQGSVSEILARPKPWNKLTVRGKEPFHKMKQFLSDEQNILALRSIQGRQRENPGQSLNRLFQEVPKRRNGSEGNITTRIRASETGSDEAIKSILEQAKRELQVQKTAEPAQPSSASGSGNSDDAIRSILQQARREMEAQQAALDPALKQAPLSQSDITILTPKLLSTSPMPTVSSYPPLAISLKKPSAAPEAGASALPNPPALKKEAQDAPGLDPQGAADCAQGVLRQVKNEVGRSGAWKDHWWSAVQPERRNAASSEEAKAEETGGGKEKGSGGSGGGSQPRAERSQLQGPSSSEYWKEWPSAESPYSQSSELSLTGASRSETPQNSPLPSSPIVPMSKPTKPSVPPLTPEQYEVYMYQEVDTIELTRQVKEKLAKNGICQRIFGEKVLGLSQGSVSDMLSRPKPWSKLTQKGREPFIRMQLWLNGELGQGVLPVQGQQQGPVLHSVTSLQDPLQQGCVSSESTPKTSASCSPAPESPMSSSESVKSLTELVQQPCPPIEASKDSKPPEPSDPPASDSQPTTPLPLSGHSALSIQELVAMSPELDTYGITKRVKEVLTDNNLGQRLFGETILGLTQGSVSDLLARPKPWHKLSLKGREPFVRMQLWLNDPNNVEKLMDMKRMEKKAYMKRRHSSVSDSQPCEPPSVGTEYSQGASPQPQHQLKKPRVVLAPEEKEALKRAYQQKPYPSPKTIEDLATQLNLKTSTVINWFHNYRSRIRRELFIEEIQAGSQGQAGASDSPSARSGRAAPSSEGDSCDGVEATEGPGSADTEEPKSQGEAEREEVPRPAEQTEPPPSGTPGPDDARDDDHEGGPVEGPGPLPSPASATATAAPAAPEDAATSAAAAPGEGPAAPSSAPPPSNSSSSSAPRRPSSLQSLFGLPEAAGARDSRDNPLRKKKAANLNSIIHRLEKAASREEPIEWEF

>P11586

MAPAEILNGKEISAQIRARLKNQVTQLKEQVPGFTPRLAILQVGNRDDSNLYINVKLKAAEEIGIKATHIKLPRTTTESEVMKYITSLNEDSTVHGFLVQLPLDSENSINTEEVINAIAPEKDVDGLTSINAGRLARGDLNDCFIPCTPKGCLELIKETGVPIAGRHAVVVGRSKIVGAPMHDLLLWNNATVTTCHSKTAHLDEEVNKGDILVVATGQPEMVKGEWIKPGAIVIDCGINYVPDDKKPNGRKVVGDVAYDEAKERASFITPVPGGVGPMTVAMLMQSTVESAKRFLEKFKPGKWMIQYNNLNLKTPVPSDIDISRSCKPKPIGKLAREIGLLSEEVELYGETKAKVLLSALERLKHRPDGKYVVVTGITPTPLGEGKSTTTIGLVQALGAHLYQNVFACVRQPSQGPTFGIKGGAAGGGYSQVIPMEEFNLHLTGDIHAITAANNLVAAAIDARIFHELTQTDKALFNRLVPSVNGVRRFSDIQIRRLKRLGIEKTDPTTLTDEEINRFARLDIDPETITWQRVLDTNDRFLRKITIGQAPTEKGHTRTAQFDISVASEIMAVLALTTSLEDMRERLGKMVVASSKKGEPVSAEDLGVSGALTVLMKDAIKPNLMQTLEGTPVFVHAGPFANIAHGNSSIIADRIALKLVGPEGFVVTEAGFGADIGMEKFFNIKCRYSGLCPHVVVLVATVRALKMHGGGPTVTAGLPLPKAYIQENLELVEKGFSNLKKQIENARMFGIPVVVAVNAFKTDTESELDLISRLSREHGAFDAVKCTHWAEGGKGALALAQAVQRAAQAPSSFQLLYDLKLPVEDKIRIIAQKIYGADDIELLPEAQHKAEVYTKQGFGNLPICMAKTHLSLSHNPEQKGVPTGFILPIRDIRASVGAGFLYPLVGTMSTMPGLPTRPCFYDIDLDPETEQVNGLF

1349 NP_056076.1 P0C7P0

>NP_056076.1

MVALRGLGSGLQPWCPLDLRLEWVDTVWELDFTETEPLDPSIEAEIIETGLAAFTKLYESLLPFATGEHGSMESIWTFFIENNVSHSTLVALFYHFVQIVHKKNVSVQYREYGLHAAGLYFLLLEVPGSVANQVFHPVMFDKCIQTLKKSWPQESNLNRKRKKEQPKSSQANPGRHRKRGKPPRREDIEMDEIIEEQEDENICFSARDLSQIRNAIFHLLKNFLRLLPKFSLKEKPQCVQNCIEVFVSLTNFEPVLHECHVTQARALNQAKYIPELAYYGLYLLCSPIHGEGDKVISCVFHQMLSVILMLEVGEGSHRAPLAVTSQVINCRNQAVQFISALVDELKESIFPVVRILLQHICAKVVDKSEYRTFAAQSLVQLLSKLPCGEYAMFIAWLYKYSRSSKIPHRVFTLDVVLALLELPEREVDNTLSLEHQKFLKHKFLVQEIMFDRCLDKAPTVRSKALSSFAHCLELTVTSASESILELLINSPTFSVIESHPGTLLRNSSAFSYQRQTSNRSEPSGEINIDSSGETVGSGERCVMAMLRRRIRDEKTNVRKSALQVLVSILKHCDVSGMKEDLWILQDQCRDPAVSVRKQALQSLTELLMAQPRCVQIQKAWLRGVVPVVMDCESTVQEKALEFLDQLLLQNIRHHSHFHSGDDSQVLAWALLTLLTTESQELSRYLNKAFHIWSKKEKFSPTFINNVISHTGTEHSAPAWMLLSKIAGSSPRLDYSRIIQSWEKISSQQNPNSNTLGHILCVIGHIAKHLPKSTRDKVTDAVKCKLNGFQWSLEVISSAVDALQRLCRASAETPAEEQELLTQVCGDVLSTCEHRLSNIVLKENGTGNMDEDLLVKYIFTLGDIAQLCPARVEKRIFLLIQSVLASSADADHSPSSQGSSEAPASQPPPQVRGSVMPSVIRAHAIITLGKLCLQHEDLAKKSIPALVRELEVCEDVAVRNNVIIVMCDLCIRYTIMVDKYIPNISMCLKDSDPFIRKQTLILLTNLLQEEFVKWKGSLFFRFVSTLIDSHPDIASFGEFCLAHLLLKRNPVMFFQHFIECIFHFNNYEKHEKYNKFPQSEREKRLFSLKGKSNKERRMKIYKFLLEHFTDEQRFNITSKICLSILACFADGILPLDLDASELLSDTFEVLSSKEIKLLAMRSKPDKDLLMEEDDMALANVVMQEAQKKLISQVQKRNFIENIIPIIISLKTVLEKNKIPALRELMHYLREVMQDYRDELKDFFAVDKQLASELEYDMKKYQEQLVQEQELAKHADVAGTAGGAEVAPVAQVALCLETVPVPAGQENPAMSPAVSQPCTPRASAGHVAVSSPTPETGPLQRLLPKARPMSLSTIAILNSVKKAVESKSRHRSRSLGVLPFTLNSGSPEKTCSQVSSYSLEQESNGEIEHVTKRAISTPEKSISDVTFGAGVSYIGTPRTPSSAKEKIEGRSQGNDILCLSLPDKPPPQPQQWNVRSPARNKDTPACSRRSLRKTPLKTAN

>P0C7P0

MRGAGAILRPAARGARDLNPRRDISSWLAQWFPRTPARSVVALKTPIKVELVAGKTYRWCVCGRSKKQPFCDGSHFFQRTGLSPLKFKAQETRMVALCTCKATQRPPYCDGTHRSERVQKAEVGSPL

1350 NP_000390.2 NP_005557.1

>NP_000390.2

MMTAKAVDKIPVTLSGFVHQLSDNIYPVEDLAATSVTIFPNAELGGPFDQMNGVAGDGMINIDMTGEKRSLDLPYPSSFAPVSAPRNQTFTYMGKFSIDPQYPGASCYPEGIINIVSAGILQGVTSPASTTASSSVTSASPNPLATGPLGVCTMSQTQPDLDHLYSPPPPPPPYSGCAGDLYQDPSAFLSAATTSTSSSLAYPPPPSYPSPKPATDPGLFPMIPDYPGFFPSQCQRDLHGTAGPDRKPFPCPLDTLRVPPPLTPLSTIRNFTLGGPSAGVTGPGASGGSEGPRLPGSSSAAAAAAAAAAYNPHHLPLRPILRPRKYPNRPSKTPVHERPYPCPAEGCDRRFSRSDELTRHIRIHTGHKPFQCRICMRNFSRSDHLTTHIRTHTGEKPFACDYCGRKFARSDERKRHTKIHLRQKERKSSAPSASVPAPSTASCSGGVQPGGTLCSSNSSSLGGGPLAPCSSRTRTP

>NP_005557.1

MATLKDQLIYNLLKEEQTPQNKITVVGVGAVGMACAISILMKDLADELALVDVIEDKLKGEMMDLQHGSLFLRTPKIVSGKDYNVTANSKLVIITAGARQQEGESRLNLVQRNVNIFKFIIPNVVKYSPNCKLLIVSNPVDILTYVAWKISGFPKNRVIGSGCNLDSARFRYLMGERLGVHPLSCHGWVLGEHGDSSVPVWSGMNVAGVSLKTLHPDLGTDKDKEQWKEVHKQVVESAYEVIKLKGYTSWAIGLSVADLAESIMKNLRRVHPVSTMIKGLYGIKDDVFLSVPCILGQNGISDLVKVTLTSEEEARLKKSADTLWGIQKELQF

1351 NP_004217.1 NP_037399.2

>NP_004217.1

MSRRRFDCRSISGLLTTTPQIPIKMENFNNFYILTSKELGRGKFAVVRQCISKSTGQEYAAKFLKKRRRGQDCRAEILHEIAVLELAKSCPRVINLHEVYENTSEIILILEYAAGGEIFSLCLPELAEMVSENDVIRLIKQILEGVYYLHQNNIVHLDLKPQNILLSSIYPLGDIKIVDFGMSRKIGHACELREIMGTPEYLAPEILNYDPITTATDMWNIGIIAYMLLTHTSPFVGEDNQETYLNISQVNVDYSEETFSSVSQLATDFIQSLLVKNPEKRPTAEICLSHSWLQQWDFENLFHPEETSSSSQTQDHSVRSSEDKTSKSSCNGTCGDREDKENIPEDSSMVSKRFRFDDSLPNPHELVSDLLC

>NP_037399.2

MRSMKALQKALSRAGSHCGRGGWGHPSRSPLLGGGVRHHLSEAAAQGRETPHSHQPQHQDHDSSESGMLSRLGDLLFYTIAEGQERIPIHKFTTALKATGLQTSDPRLRDCMSEMHRVVQESSSGGLLDRDLFRKCVSSNIVLLTQAFRKKFVIPDFEEFTGHVDRIFEDVKELTGGKVAAYIPQLAKSNPDLWGVSLCTVDGQRHSVGHTKIPFCLQSCVKPLTYAISISTLGTDYVHKFVGKEPSGLRYNKLSLNEEGIPHNPMVNAGAIVVSSLIKMDCNKAEKFDFVLQYLNKMAGNEYMGFSNATFQSEKETGDRNYAIGYYLKEKKCFPKGVDMMAALDLYFQLCSVEVTCESGSVMAATLANGGICPITGESVLSAEAVRNTLSLMHSCGMYDFSGQFAFHVGLPAKSAVSGAILLVVPNVMGMMCLSPPLDKLGNSHRGTSFCQKLVSLFNFHNYDNLRHCARKLDPRREGAEIRNKTVVNLLFAAYSGDVSALRRFALSAMDMEQKDYDSRTALHVAAAEGHIEVVKFLIEACKVNPFAKDRWGNIPLDDAVQFNHLEVVKLLQDYQDSYTLSETQAEAAAEALSKENLESMV

1352 P57053 NP_002826.2

>P57053

MPEPAKSAPAPKKGSKKAVTKAQKKDGRKRKRSRKESYSVYVYKVLKQVHPDTGISSKAMGIMNSFVNDIFERIAGEASRLPHYNKRSTITSREIQTAVRLLLPGELAKHAVSEGTKAVTKYTSAK

>NP_002826.2

MEQVEILRKFIQRVQAMKSPDHNGEDNFARDFMRLRRLSTKYRTEKIYPTATGEKEENVKKNRYKDILPFDHSRVKLTLKTPSQDSDYINANFIKGVYGPKAYVATQGPLANTVIDFWRMIWEYNVVIIVMACREFEMGRKKCERYWPLYGEDPITFAPFKISCEDEQARTDYFIRTLLLEFQNESRRLYQFHYVNWPDHDVPSSFDSILDMISLMRKYQEHEDVPICIHCSAGCGRTGAICAIDYTWNLLKAGKIPEEFNVFNLIQEMRTQRHSAVQTKEQYELVHRAIAQLFEKQLQLYEIHGAQKIADGVNEINTENMISSIEPEKQDSPPPKPPRTRSCLVEGDAKEEILQPPEPHPVPPILTPSPPSAFPTVTTVWQDNDRYHPKPVLHMVSSEQHSADLNRNYSKSTELPGKNESTIEQIDKKLERNLSFEIKKVPLQEGPKSFDGNTLLNRGHAIKIKSASPCIADKISKPQELSSDLNVGDTSQNSCVDCSVTQSNKVSVTPPEESQNSDTPPRPDRLPLDEKGHVTWSFHGPENAIPIPDLSEGNSSDINYQTRKTVSLTPSPTTQVETPDLVDHDNTSPLFRTPLSFTNPLHSDDSDSDERNSDGAVTQNKTNISTASATVSAATSTESISTRKVLPMSIARHNIAGTTHSGAEKDVDVSEDSPPPLPERTPESFVLASEHNTPVRSEWSELQSQERSEQKKSEGLITSENEKCDHPAGGIHYEMCIECPPTFSDKREQISENPTEATDIGFGNRCGKPKGPRDPPSEWT

1353 O15178 NP_005411.1

>O15178

MSSPGTESAGKSLQYRVDHLLSAVENELQAGSEKGDPTERELRVGLEESELWLRFKELTNEMIVTKNGRRMFPVLKVNVSGLDPNAMYSFLLDFVAADNHRWKYVNGEWVPGGKPEPQAPSCVYIHPDSPNFGAHWMKAPVSFSKVKLTNKLNGGGQIMLNSLHKYEPRIHIVRVGGPQRMITSHCFPETQFIAVTAYQNEEITALKIKYNPFAKAFLDAKERSDHKEMMEEPGDSQQPGYSQWGWLLPGTSTLCPPANPHPQFGGALSLPSTHSCDRYPTLRSHRSSPYPSPYAHRNNSPTYSDNSPACLSMLQSHDNWSSLGMPAHPSMLPVSHNASPPTSSSQYPSLWSVSNGAVTPGSQAAAVSNGLGAQFFRGSPAHYTPLTHPVSAPSSSGSPLYEGAAAATDIVDSQYDAAAQGRLIASWTPVSPPSM

>NP_005411.1

MNSELDYYEKFEEVHGILMYKDFVKYWDNVEAFQARPDDLVIATYPKSGTTWVSEIVYMIYKEGDVEKCKEDVIFNRIPFLECRKENLMNGVKQLDEMNSPRIVKTHLPPELLPASFWEKDCKIIYLCRNAKDVAVSFYYFFLMVAGHPNPGSFPEFVEKFMQGQVPYGSWYKHVKSWWEKGKSPRVLFLFYEDLKEDIRKEVIKLIHFLERKPSEELVDRIIHHTSFQEMKNNPSTNYTTLPDEIMNQKLSPFMRKGITGDWKNHFTVALNEKFDKHYEQQMKESTLKFRTEI

1354 NP_055028.2 Q58A44

>NP_055028.2

MSSSGTLSNYYVDSLIGHEGDEVFAARFGPPGPGAQGRPAGVADGPAATAAEFASCSFAPRSAVFSASWSAVPSQPPAAAAMSGLYHPYVPPPPLAASASEPGRYVRSWMEPLPGFPGGAGGGGGGGGGGPGRGPSPGPSGPANGRHYGIKPETRAAPAPATAASTTSSSSTSLSSSSKRTECSVARESQGSSGPEFSCNSFLQEKAAAATGGTGPGAGIGAATGTGGSSEPSACSDHPIPGCSLKEEEKQHSQPQQQQLDPNNPAANWIHARSTRKKRCPYTKYQTLELEKEFLFNMYLTRDRRYEVARILNLTERQVKIWFQNRRMKMKKMSKEKCPKGD

>Q58A44

MWILSNLMGTSEEGNLLSTVSPTVKALFGKTRVSPIFPFSPRSPFQPLIPRTPGSPWGPVGPASPLGPGFPIGPMGPGKPVGPKGPMLPLGPSGPVGPTSPLFPFCP

1355 NP_008823.1 NP_000934.1

>NP_008823.1

MKGDTRHLNGEEDAGGREDSILVNGACSDQSSDSPPILEAIRTPEIRGRRSSSRLSKREVSSLLSYTQDLTGDGDGEDGDGSDTPVMPKLFRETRTRSESPAVRTRNNNSVSSRERHRPSPRSTRGRQGRNHVDESPVEFPATRSLRRRATASAGTPWPSPPSSYLTIDLTDDTEDTHGTPQSSSTPYARLAQDSQQGGMESPQVEADSGDGDSSEYQDGKEFGIGDLVWGKIKGFSWWPAMVVSWKATSKRQAMSGMRWVQWFGDGKFSEVSADKLVALGLFSQHFNLATFNKLVSYRKAMYHALEKARVRAGKTFPSSPGDSLEDQLKPMLEWAHGGFKPTGIEGLKPNNTQPVVNKSKVRRAGSRKLESRKYENKTRRRTADDSATSDYCPAPKRLKTNCYNNGKDRGDEDQSREQMASDVANNKSSLEDGCLSCGRKNPVSFHPLFEGGLCQTCRDRFLELFYMYDDDGYQSYCTVCCEGRELLLCSNTSCCRCFCVECLEVLVGTGTAAEAKLQEPWSCYMCLPQRCHGVLRRRKDWNVRLQAFFTSDTGLEYEAPKLYPAIPAARRRPIRVLSLFDGIATGYLVLKELGIKVGKYVASEVCEESIAVGTVKHEGNIKYVNDVRNITKKNIEEWGPFDLVIGGSPCNDLSNVNPARKGLYEGTGRLFFEFYHLLNYSRPKEGDDRPFFWMFENVVAMKVGDKRDISRFLECNPVMIDAIKVSAAHRARYFWGNLPGMNRPVIASKNDKLELQDCLEYNRIAKLKKVQTITTKSNSIKQGKNQLFPVVMNGKEDVLWCTELERIFGFPVHYTDVSNMGRGARQKLLGRSWSVPVIRHLFAPLKDYFACE

>NP_000934.1

MGPGPRLLLPLVLCVGLGALVFSSGAEGFRKRGPSVTAKVFFDVRIGDKDVGRIVIGLFGKVVPKTVENFVALATGEKGYGYKGSKFHRVIKDFMIQGGDITTGDGTGGVSIYGETFPDENFKLKHYGIGWVSMANAGPDTNGSQFFITLTKPTWLDGKHVVFGKVIDGMTVVHSIELQATDGHDRPLTNCSIINSGKIDVKTPFVVEIADW

1356 NP_006185.1 Q13084

>NP_006185.1

MEPAFGEVNQLGGVFVNGRPLPNAIRLRIVELAQLGIRPCDISRQLRVSHGCVSKILARYNETGSILPGAIGGSKPRVTTPTVVKHIRTYKQRDPGIFAWEIRDRLLADGVCDKYNVPSVSSISRILRNKIGNLAQQGHYDSYKQHQPTPQPALPYNHIYSYPSPITAAAAKVPTPPGVPAIPGSVAMPRTWPSSHSVTDILGIRSITDQVSDSSPYHSPKVEEWSSLGRNNFPAAAPHAVNGLEKGALEQEAKYGQAPNGLPAVGSFVSASSMAPYPTPAQVSPYMTYSAAPSGYVAGHGWQHAGGTSLSPHNCDIPASLAFKGMQAAREGSHSVTASAL

>Q13084

MAFTASQRWEEGQARQIGFRFRWALNPERLAMPLHKYPVWLWKRLQLREGICSRLPGHYLRSLEEERTPTPVHYRPHGAKFKINPKNGQRERVEDVPIPIYFPPESQRGLWGGEGWILGQIYANNDKLSKRLKKVWKPQLFEREFYSEILDKKFTVTVTMRTLDLIDEAYGLDFYILKTPKEDLCSKFGMDLKRGMLLRLARQDPQLHPEDPERRAAIYDKYKEFAIPEEEAEWVGLTLEEAIEKQRLLEEKDPVPLFKIYVAELIQQLQQQALSEPAVVQKRASGQ

1357 NP_003784.2 Q9BXL7

>NP_003784.2

MAPWLQLLSLLGLLPGAVAAPAQPRAASFQAWGPPSPELLAPTRFALEMFNRGRAAGTRAVLGLVRGRVRRAGQGSLYSLEATLEEPPCNDPMVCRLPVSKKTLLCSFQVLDELGRHVLLRKDCGPVDTKVPGAGEPKSAFTQGSAMISSLSQNHPDNRNETFSSVISLLNEDPLSQDLPVKMASIFKNFVITYNRTYESKEEARWRLSVFVNNMVRAQKIQALDRGTAQYGVTKFSDLTEEEFRTIYLNTLLRKEPGNKMKQAKSVGDLAPPEWDWRSKGAVTKVKDQGMCGSCWAFSVTGNVEGQWFLNQGTLLSLSEQELLDCDKMDKACMGGLPSNAYSAIKNLGGLETEDDYSYQGHMQSCNFSAEKAKVYINDSVELSQNEQKLAAWLAKRGPISVAINAFGMQFYRHGISRPLRPLCSPWLIDHAVLLVGYGNRSDVPFWAIKNSWGTDWGEKGYYYLHRGSGACGVNTMASSAVVD

>Q9BXL7

MPGGGPEMDDYMETLKDEEDALWENVECNRHMLSRYINPAKLTPYLRQCKVIDEQDEDEVLNAPMLPSKINRAGRLLDILHTKGQRGYVVFLESLEFYYPELYKLVTGKEPTRRFSTIVVEEGHEGLTHFLMNEVIKLQQQMKAKDLQRCELLARLRQLEDEKKQMTLTRVELLTFQERYYKMKEERDSYNDELVKVKDDNYNLAMRYAQLSEEKNMAVMRSRDLQLEIDQLKHRLNKMEEECKLERNQSLKLKNDIENRPKKEQVLELERENEMLKTKNQELQSIIQAGKRSLPDSDKAILDILEHDRKEALEDRQELVNRIYNLQEEARQAEELRDKYLEEKEDLELKCSTLGKDCEMYKHRMNTVMLQLEEVERERDQAFHSRDEAQTQYSQCLIEKDKYRKQIRELEEKNDEMRIEMVRREACIVNLESKLRRLSKDSNNLDQSLPRNLPVTIISQDFGDASPRTNGQEADDSSTSEESPEDSKYFLPYHPPQRRMNLKGIQLQRAKSPISLKRTSDFQAKGHEEEGTDASPSSCGSLPITNSFTKMQPPRSRSSIMSITAEPPGNDSIVRRYKEDAPHRSTVEEDNDSGGFDALDLDDDSHERYSFGPSSIHSSSSSHQSEGLDAYDLEQVNLMFRKFSLERPFRPSVTSVGHVRGPGPSVQHTTLNGDSLTSQLTLLGGNARGSFVHSVKPGSLAEKAGLREGHQLLLLEGCIRGERQSVPLDTCTKEEAHWTIQRCSGPVTLHYKVNHEGYRKLVKDMEDGLITSGDSFYIRLNLNISSQLDACTMSLKCDDVVHVRDTMYQDRHEWLCARVDPFTDHDLDMGTIPSYSRAQQLLLVKLQRLMHRGSREEVDGTHHTLRALRNTLQPEEALSTSDPRVSPRLSRASFLFGQLLQFVSRSENKYKRMNSNERVRIISGSPLGSLARSSLDATKLLTEKQEELDPESELGKNLSLIPYSLVRAFYCERRRPVLFTPTVLAKTLVQRLLNSGGAMEFTICKSDIVTRDEFLRRQKTETIIYSREKNPNAFECIAPANIEAVAAKNKHCLLEAGIGCTRDLIKSNIYPIVLFIRVCEKNIKRFRKLLPRPETEEEFLRVCRLKEKELEALPCLYATVEPDMWGSVEELLRVVKDKIGEEQRKTIWVDEDQL

1358 NP_002583.1 NP_057461.1

>NP_002583.1

MFEARLVQGSILKKVLEALKDLINEACWDISSSGVNLQSMDSSHVSLVQLTLRSEGFDTYRCDRNLAMGVNLTSMSKILKCAGNEDIITLRAEDNADTLALVFEAPNQEKVSDYEMKLMDLDVEQLGIPEQEYSCVVKMPSGEFARICRDLSHIGDAVVISCAKDGVKFSASGELGNGNIKLSQTSNVDKEEEAVTIEMNEPVQLTFALRYLNFFTKATPLSSTVTLSMSADVPLVVEYKIADMGHLKYYLAPKIEDEEGS

>NP_057461.1

MSEEFEANTMDSLVDMPFATVDIQDDCGITDEPQINLKRSQENEWVKSDQVKKRKKKRKDYQPNYFLSIPITNKEIIKGIKILQNAIIQQDERLAKAMVSDGSFHITLLVMQLLNEDEVNIGIDALLELKPFIEELLQGKHLTLPFQGIGTFGNQVGFVKLAEGDHVNSLLEIAETANRTFQEKGILVGESRSFKPHLTFMKLSKSPWLRKNGVKKIDPDLYEKFISHRFGEEILYRIDLCSMLKKKQSNGYYHCESSIVIGEKNGGEPDDAELVRLSKRLVENAVLKAVQQYLEETQNKNKPGEGSSVKTEAADQNGNDNENNRK

1359 Q96LA8 NP_434700.2

>Q96LA8

MSQPKKRKLESGGGGEGGEGTEEEDGAEREAALERPRRTKRERDQLYYECYSDVSVHEEMIADRVRTDAYRLGILRNWAALRGKTVLDVGAGTGILSIFCAQAGARRVYAVEASAIWQQAREVVRFNGLEDRVHVLPGPVETVELPEQVDAIVSEWMGYGLLHESMLSSVLHARTKWLKEGGLLLPASAELFIAPISDQMLEWRLGFWSQVKQHYGVDMSCLEGFATRCLMGHSEIVVQGLSGEDVLARPQRFAQLELSRAGLEQELEAGVGGRFRCSCYGSAPMHGFAIWFQVTFPGGESEKPLVLSTSPFHPATHWKQALLYLNEPVQVEQDTDVSGEITLLPSRDNPRRLRVLLRYKVGDQEEKTKDFAMED

>NP_434700.2

MSDYENDDECWSVLEGFRVTLTSVIDPSRITPYLRQCKVLNPDDEEQVLSDPNLVIRKRKVGVLLDILQRTGHKGYVAFLESLELYYPQLYKKVTGKEPARVFSMIIDASGESGLTQLLMTEVMKLQKKVQDLTALLSSKDDFIKELRVKDSLLRKHQERVQRLKEECEAGSRELKRCKEENYDLAMRLAHQSEEKGAALMRNRDLQLEIDQLKHSLMKAEDDCKVERKHTLKLRHAMEQRPSQELLWELQQEKALLQARVQELEASVQEGKLDRSSPYIQVLEEDWRQALRDHQEQANTIFSLRKDLRQGEARRLRCMEEKEMFELQCLALRKDSKMYKDRIEAILLQMEEVAIERDQAIATREELHAQHARGLQEKDALRKQVRELGEKADELQLQVFQCEAQLLAVEGRLRRQQLETLVLSSDLEDGSPRRSQELSLPQDLEDTQLSDKGCLAGGGSPKQPFAALHQEQVLRNPHDAGLSSGEPPEKERRRLKESFENYRRKRALRKMQKGWRQGEEDRENTTGSDNTDTEGS

1360 NP_061134.1 Q99707

>NP_061134.1

MSQGLPAAGSVLQRSVAAPGNQPQPQPQQQSPEDDDRKVRRREKNRVAAQRSRKKQTQKADKLHEEYESLEQENTMLRREIGKLTEELKHLTEALKEHEKMCPLLLCPMNFVPVPPRPDPVAGCLPR

>Q99707

MSPALQDLSQPEGLKKTLRDEINAILQKRIMVLDGGMGTMIQREKLNEEHFRGQEFKDHARPLKGNNDILSITQPDVIYQIHKEYLLAGADIIETNTFSSTSIAQADYGLEHLAYRMNMCSAGVARKAAEEVTLQTGIKRFVAGALGPTNKTLSVSPSVERPDYRNITFDELVEAYQEQAKGLLDGGVDILLIETIFDTANAKAALFALQNLFEEKYAPRPIFISGTIVDKSGRTLSGQTGEGFVISVSHGEPLCIGLNCALGAAEMRPFIEIIGKCTTAYVLCYPNAGLPNTFGDYDETPSMMAKHLKDFAMDGLVNIVGGCCGSTPDHIREIAEAVKNCKPRVPPATAFEGHMLLSGLEPFRIGPYTNFVNIGERCNVAGSRKFAKLIMAGNYEEALCVAKVQVEMGAQVLDVNMDDGMLDGPSAMTRFCNLIASEPDIAKVPLCIDSSNFAVIEAGLKCCQGKCIVNSISLKEGEDDFLEKARKIKKYGAAMVVMAFDEEGQATETDTKIRVCTRAYHLLVKKLGFNPNDIIFDPNILTIGTGMEEHNLYAINFIHATKVIKETLPGARISGGLSNLSFSFRGMEAIREAMHGVFLYHAIKSGMDMGIVNAGNLPVYDDIHKELLQLCEDLIWNKDPEATEKLLRYAQTQGTGGKKVIQTDEWRNGPVEERLEYALVKGIEKHIIEDTEEARLNQKKYPRPLNIIEGPLMNGMKIVGDLFGAGKMFLPQVIKSARVMKKAVGHLIPFMEKEREETRVLNGTVEEEDPYQGTIVLATVKGDVHDIGKNIVGVVLGCNNFRVIDLGVMTPCDKILKAALDHKADIIGLSGLITPSLDEMIFVAKEMERLAIRIPLLIGGATTSKTHTAVKIAPRYSAPVIHVLDASKSVVVCSQLLDENLKDEYFEEIMEEYEDIRQDHYESLKERRYLPLSQARKSGFQMDWLSEPHPVKPTFIGTQVFEDYDLQKLVDYIDWKPFFDVWQLRGKYPNRGFPKIFNDKTVGGEARKVYDDAHNMLNTLISQKKLRARGVVGFWPAQSIQDDIHLYAEAAVPQAAEPIATFYGLRQQAEKDSASTEPYYCLSDFIAPLHSGIRDYLGLFAVACFGVEELSKAYEDDGDDYSSIMVKALGDRLAEAFAEELHERVRRELWAYCGSEQLDVADLRRLRYKGIRPAPGYPSQPDHTEKLTMWRLADIEQSTGIRLTESLAMAPASAVSGLYFSNLKSKYFAVGKISKDQVEDYALRKNISVAEVEKWLGPILGYDTD

1361 NP_003398.1 NP_003115.1

>NP_003398.1

MDLTAIYESLLSLSPDVPVPSDHGGTESSPGWGSSGPWSLSPSDSSPSGVTSRLPGRSTSLVEGRSCGWVPPPPGFAPLAPRLGPELSPSPTSPTATSTTPSRYKTELCRTFSESGRCRYGAKCQFAHGLGELRQANRHPKYKTELCHKFYLQGRCPYGSRCHFIHNPSEDLAAPGHPPVLRQSISFSGLPSGRRTSPPPPGLAGPSLSSSSFSPSSSPPPPGDLPLSPSAFSAAPGTPLARRDPTPVCCPSCRRATPISVWGPLGGLVRTPSVQSLGSDPDEYASSGSSLGGSDSPVFEAGVFAPPQPVAAPRRLPIFNRISVSE

>NP_003115.1

MEGGLGRAVCLLTGASRGFGRTLAPLLASLLSPGSVLVLSARNDEALRQLEAELGAERSGLRVVRVPADLGAEAGLQQLLGALRELPRPKGLQRLLLINNAGSLGDVSKGFVDLSDSTQVNNYWALNLTSMLCLTSSVLKAFPDSPGLNRTVVNISSLCALQPFKGWALYCAGKAARDMLFQVLALEEPNVRVLNYAPGPLDTDMQQLARETSVDPDMRKGLQELKAKGKLVDCKVSAQKLLSLLEKDEFKSGAHVDFYDK

1362 NP_005634.1 P0C7P0

>NP_005634.1

MDDAHESPSDKGGETGESDETAAVPGDPGATDTDGIPEETDGDADVDLKEAAAEEGELESQDVSDLTTVEREDSSLLNPAAKKLKIDTKEKKEKKQKVDEDEIQKMQILVSSFSEEQLNRYEMYRRSAFPKAAIKRLIQSITGTSVSQNVVIAMSGISKVFVGEVVEEALDVCEKWGEMPPLQPKHMREAVRRLKSKGQIPNSKHKKIIFF

>P0C7P0

MRGAGAILRPAARGARDLNPRRDISSWLAQWFPRTPARSVVALKTPIKVELVAGKTYRWCVCGRSKKQPFCDGSHFFQRTGLSPLKFKAQETRMVALCTCKATQRPPYCDGTHRSERVQKAEVGSPL

1363 NP_570722.2 NP_055683.3

>NP_570722.2

MPLQVSDYSWQQTKTAVFLSLPLKGVCVRDTDVFCTENYLKVNFPPFLFEAFLYAPIDDESSKAKIGNDTIVFTLYKKEAAMWETLSVTGVDKEMMQRIREKSILQAQERAKEATEAKAAAKREDQKYALSVMMKIEEEERKKIEDMKENERIKATKALEAWKEYQRKAEEQKKIQREEKLCQKEKQIKEERKKIKYKSLTRNLASRNLAPKGRNSENIFTEKLKEDSIPAPRSVGSIKINFTPRVFPTALRESQVAEEEEWLHKQAEARRAMNTDIAELCDLKEEEKNPEWLKDKGNKLFATENYLAAINAYNLAIRLNNKMPLLYLNRAACHLKLKNLHKAIEDSSKALELLMPPVTDNANARMKAHVRRGTAFCQLELYVEGLQDYEAALKIDPSNKIVQIDAEKIRNVIQGTELKS

>NP_055683.3

MPLSSPNAAATASDMDKNSGSNSSSASSGSSKGQQPPRSASAGPAGESKPKSDGKNSSGSKRYNRKRELSYPKNESFNNQSRRSSSQKSKTFNKMPPQRGGGSSKLFSSSFNGGRRDEVAEAQRAEFSPAQFSGPKKINLNHLLNFTFEPRGQTGHFEGSGHGSWGKRNKWGHKPFNKELFLQANCQFVVSEDQDYTAHFADPDTLVNWDFVEQVRICSHEVPSCPICLYPPTAAKITRCGHIFCWACILHYLSLSEKTWSKCPICYSSVHKKDLKSVVATESHQYVVGDTITMQLMKREKGVLVALPKSKWMNVDHPIHLGDEQHSQYSKLLLASKEQVLHRVVLEEKVALEQQLAEEKHTPESCFIEAAIQELKTREEALSGLAGSRREVTGVVAALEQLVLMAPLAKESVFQPRKGVLEYLSAFDEETTEVCSLDTPSRPLALPLVEEEEAVSEPEPEGLPEACDDLELADDNLKEGTICTESSQQEPITKSGFTRLSSSPCYYFYQAEDGQHMFLHPVNVRCLVREYGSLERSPEKISATVVEIAGYSMSEDVRQRHRYLSHLPLTCEFSICELALQPPVVSKETLEMFSDDIEKRKRQRQKKAREERRRERRIEIEENKKQGKYPEVHIPLENLQQFPAFNSYTCSSDSALGPTSTEGHGALSISPLSRSPGSHADFLLTPLSPTASQGSPSFCVGSLEEDSPFPSFAQMLRVGKAKADVWPKTAPKKDENSLVPPAPVDSDGESDNSDRVPVPSFQNSFSQAIEAAFMKLDTPATSDPLSEEKGGKKRKKQKQKLLFSTSVVHTK

1364 NP_002544.1 Q9NVV4

>NP_002544.1

MPHLENVVLCRESQVSILQSLFGERHHFSFPSIFIYGHTASGKTYVTQTLLKTLELPHVFVNCVECFTLRLLLEQILNKLNHLSSSEDGCSTEITCETFNDFVRLFKQVTTAENLKDQTVYIVLDKAEYLRDMEANLLPGFLRLQELADRNVTVLFLSEIVWEKFRPNTGCFEPFVLYFPDYSIGNLQKILSHDHPPEYSADFYAAYINILLGVFYTVCRDLKELRHLAVLNFPKYCEPVVKGEASERDTRKLWRNIEPHLKKAMQTVYLREISSSQWEKLQKDDTDPGQLKGLSAHTHVELPYYSKFILIAAYLASYNPARTDKRFFLKHHGKIKKTNFLKKHEKTSNHLLGPKPFPLDRLLAILYSIVDSRVAPTANIFSQITSLVTLQLLTLVGHDDQLDGPKYKCTVSLDFIRAIARTVNFDIIKYLYDFL

>Q9NVV4

MAVPGVGLLTRLNLCARRRTRVQRPIVRLLSCPGTVAKDLRRDEQPSGSVETGFEDKIPKRRFSEMQNERREQAQRTVLIHCPEKISENKFLKYLSQFGPINNHFFYESFGLYAVVEFCQKESIGSLQNGTHTPSTAMETAIPFRSRFFNLKLKNQTSERSRVRSSNQLPRSNKQLFELLCYAESIDDQLNTLLKEFQLTEENTKLRYLTCSLIEDMAAAYFPDCIVRPFGSSVNTFGKLGCDLDMFLDLDETRNLSAHKISGNFLMEFQVKNVPSERIATQKILSVLGECLDHFGPGCVGVQKILNARCPLVRFSHQASGFQCDLTTNNRIALTSSELLYIYGALDSRVRALVFSVRCWARAHSLTSSIPGAWITNFSLTMMVIFFLQRRSPPILPTLDSLKTLADAEDKCVIEGNNCTFVRDLSRIKPSQNTETLELLLKEFFEYFGNFAFDKNSINIRQGREQNKPDSSPLYIQNPFETSLNISKNVSQSQLQKFVDLARESAWILQQEDTDRPSISSNRPWGLVSLLLPSAPNRKSFTKKKSNKFAIETVKNLLESLKGNRTENFTKTSGKRTISTQT

1365 NP_002422.1 NP_000036.2

>NP_002422.1

MDDQGCPRCKTTKYRNPSLKLMVNVCGHTLCESCVDLLFVRGAGNCPECGTPLRKSNFRVQLFEDPTVDKEVEIRKKVLKIYNKREEDFPSLREYNDFLEEVEEIVFNLTNNVDLDNTKKKMEIYQKENKDVIQKNKLKLTREQEELEEALEVERQENEQRRLFIQKEEQLQQILKRKNKQAFLDELESSDLPVALLLAQHKDRSTQLEMQLEKPKPVKPVTFSTGIKMGQHISLAPIHKLEEALYEYQPLQIETYGPHVPELEMLGRLGYLNHVRAASPQDLAGGYTSSLACHRALQDAFSGLFWQPS

>NP_000036.2

MSAKSRTIGIIGAPFSKGQPRGGVEEGPTVLRKAGLLEKLKEQECDVKDYGDLPFADIPNDSPFQIVKNPRSVGKASEQLAGKVAEVKKNGRISLVLGGDHSLAIGSISGHARVHPDLGVIWVDAHTDINTPLTTTSGNLHGQPVSFLLKELKGKIPDVPGFSWVTPCISAKDIVYIGLRDVDPGEHYILKTLGIKYFSMTEVDRLGIGKVMEETLSYLLGRKKRPIHLSFDVDGLDPSFTPATGTPVVGGLTYREGLYITEEIYKTGLLSGLDIMEVNPSLGKTPEEVTRTVNTAVAITLACFGLAREGNHKPIDYLNPPK

1366 O00115 Q9NYK5

>O00115

MIPLLLAALLCVPAGALTCYGDSGQPVDWFVVYKLPALRGSGEAAQRGLQYKYLDESSGGWRDGRALINSPEGAVGRSLQPLYRSNTSQLAFLLYNDQPPQPSKAQDSSMRGHTKGVLLLDHDGGFWLVHSVPNFPPPASSAAYSWPHSACTYGQTLLCVSFPFAQFSKMGKQLTYTYPWVYNYQLEGIFAQEFPDLENVVKGHHVSQEPWNSSITLTSQAGAVFQSFAKFSKFGDDLYSGWLAAALGTNLQVQFWHKTVGILPSNCSDIWQVLNVNQIAFPGPAGPSFNSTEDHSKWCVSPKGPWTCVGDMNRNQGEEQRGGGTLCAQLPALWKAFQPLVKNYQPCNGMARKPSRAYKI

>Q9NYK5

MEALAMGSRALRLWLVAPGGGIKWRFIATSPASQLSPTELTEMRNDLFNKEKARQLSLTPRTEKIEVKHVGKTDPGTVFVMNKNISTPYSCAMHLSEWYCRKSILALVDGQPWDMYKPLTKSCEIKFLTFKDCDPGEVNKAYWRSCAMMMGCVIERAFKDEYMVNLVRAPEVPVISGAFCYDVVLDSKLDEWMPTKENLRSFTKDAHALIYKDLPFETLEVEAKVALEIFQHSKYKVDFIEEKASQNPERIVKLHRIGDFIDVSEGPLIPRTSICFQYEVSAVHNLQPTQPSLIRRFQGVSLPVHLRAHFTIWDKLLERSRKMVTEDQSKATEECTST

1367 NP_006038.2 NP_004591.2

>NP_006038.2

MAVVIRLQGLPIVAGTMDIRHFFSGLTIPDGGVHIVGGELGEAFIVFATDEDARLGMMRTGGTIKGSKVTLLLSSKTEMQNMIELSRRRFETANLDIPPANASRSGPPPSSGMSSRVNLPTTVSNFNNPSPSVVTATTSVHESNKNIQTFSTASVGTAPPNMGASFGSPTFSSTVPSTASPMNTVPPPPIPPIPAMPSLPPMPSIPPIPVPPPVPTLPPVPPVPPIPPVPSVPPMTPLPPMSGMPPLNPPPVAPLPAGMNGSGAPMNLNNNLNPMFLGPLNPVNPIQMNSQSSVKPLPINPDDLYVSVHGMPFSAMENDVRDFFHGLRVDAVHLLKDHVGRNNGNGLVKFLSPQDTFEALKRNRMLMIQRYVEVSPATERQWVAAGGHITFKQNMGPSGQTHPPPQTLPRSKSPSGQKRSRSRSPHEAGFCVYLKGLPFEAENKHVIDFFKKLDIVEDSIYIAYGPNGKATGEGFVEFRNEADYKAALCRHKQYMGNRFIQVHPITKKGMLEKIDMIRKRLQNFSYDQREMILNPEGDVNSAKVCAHITNIPFSITKMDVLQFLEGIPVDENAVHVLVDNNGQGLGQALVQFKNEDDARKSERLHRKKLNGREAFVHVVTLEDMREIEKNPPAQGKKGLKMPVPGNPAVPGMPNAGLPGVGLPSAGLPGAGLPSTGLPGSAITSAGLPGAGMPSAGIPSAGGEEHAFLTVGSKEANNGPPFNFPGNFGGSNAFGPPIPPPGLGGGAFGDARPGMPSVGNSGLPGLGLDVPGFGGGPNNLSGPSGFGGGPQNFGNGPGSLGGPPGFGSGPPGLGSAPGHLGGPPAFGPGPGPGPGPGPIHIGGPPGFASSSGKPGPTVIKVQNMPFTVSIDEILDFFYGYQVIPGSVCLKYNEKGMPTGEAMVAFESRDEATAAVIDLNDRPIGSRKVKLVLG

>NP_004591.2

MEESVNQMQPLNEKQIANSQDGYVWQVTDMNRLHRFLCFGSEGGTYYIKEQKLGLENAEALIRLIEDGRGCEVIQEIKSFSQEGRTTKQEPMLFALAICSQCSDISTKQAAFKAVSEVCRIPTHLFTFIQFKKDLKESMKCGMWGRALRKAIADWYNEKGGMALALAVTKYKQRNGWSHKDLLRLSHLKPSSEGLAIVTKYITKGWKEVHELYKEKALSVETEKLLKYLEAVEKVKRTRDELEVIHLIEEHRLVREHLLTNHLKSKEVWKALLQEMPLTALLRNLGKMTANSVLEPGNSEVSLVCEKLCNEKLLKKARIHPFHILIALETYKTGHGLRGKLKWRPDEEILKALDAAFYKTFKTVEPTGKRFLLAVDVSASMNQRVLGSILNASTVAAAMCMVVTRTEKDSYVVAFSDEMVPCPVTTDMTLQQVLMAMSQIPAGGTDCSLPMIWAQKTNTPADVFIVFTDNETFAGGVHPAIALREYRKKMDIPAKLIVCGMTSNGFTIADPDDRGMLDMCGFDTGALDVIRNFTLDMI

1368 Q86V15 Q9P278

>Q86V15

MDLGTAEGTRCTDPPAGKPAMAPKRKGGLKLNAICAKLSRQVVVEKRADAGSHTEGSPSQPRDQERSGPESGAARAPRSEEDKRRAVIEKWVNGEYSEEPAPTPVLGRIAREGLELPPEGVYMVQPQGCSDEEDHAEEPSKDGGALEEKDSDGAASKEDSGPSTRQASGEASSLRDYAASTMTEFLGMFGYDDQNTRDELARKISFEKLHAGSTPEAATSSMLPTSEDTLSKRARFSKYEEYIRKLKAGEQLSWPAPSTKTEERVGKEVVGTLPGLRLPSSTAHLETKATILPLPSHSSVQMQNLVARASKYDFFIQKLKTGENLRPQNGSTYKKPSKYDLENVKYLHLFKPGEGSPDMGGAIAFKTGKVGRPSKYDVRGIQKPGPAKVPPTPSLAPAPLASVPSAPSAPGPGPEPPASLSFNTPEYLKSTFSKTDSITTGTVSTVKNGLPTDKPAVTEDVNIYQKYIARFSGSQHCGHIHCAYQYREHYHCLDPECNYQRFTSKQDVIRHYNMHKKRDNSLQHGFMRFSPLDDCSVYYHGCHLNGKSTHYHCMQVGCNKVYTSTSDVMTHENFHKKNTQLINDGFQRFRATEDCGTADCQFYGQKTTHFHCRRPGCTFTFKNKCDIEKHKSYHIKDDAYAKDGFKKFYKYEECKYEGCVYSKATNHFHCIRAGCGFTFTSTSQMTSHKRKHERRHIRSSGALGLPPSLLGAKDTEHEESSNDDLVDFSALSSKNSSLSASPTSQQSSASLAAATAATEAGPSATKPPNSKISGLLPQGLPGSIPLALALSNSGLPTPTPYFPILAGRGSTSLPVGTPSLLGAVSSGSAASATPDTPTLVASGAGDSAPVAAASVPAPPASIMERISASKGLISPMMARLAAAALKPSATFDPGSGQQVTPARFPPAQVKPEPGESTGAPGPHEASQDRSLDLTVKEPSNESNGHAVPANSSLLSSLMNKMSQGNPGLGSLLNIKAEAEGSPAAEPSPFLGKAVKALVQEKLAEPWKVYLRRFGTKDFCDGQCDFLHKAHFHCVVEECGALFSTLDGAIKHANFHFRTEGGAAKGNTEAAFPASAAETKPPMAPSSPPVPPVTTATVSSLEGPAPSPASVPSTPTLLAWKQLASTIPQMPQIPASVPHLPASPLATTSLENAKPQVKPGFLQFQENDPCLATDCKYANKFHFHCLFGNCKYVCKTSGKAESHCLDHINPNNNLVNVRDQFAYYSLQCLCPNQHCEFRMRGHYHCLRTGCYFVTNITTKLPWHIKKHEKAERRAANGFKYFTKREECGRLGCKYNQVSSHFHCIREGCQFSFLLKHQMTSHARKHMRRMLGKNFDRVPPSQGPPGLMDAETDECMDYTGCSPGAMSSESSTMDRSCSSTPVGNESTAAGNTISMPTASGAKKRFWIIEDMSPFGKRRKTASSRKMLDEGMMLEGFRRFDLYEDCKDAACQFSLKVTHYHCTRENCGYKFCGRTHMYKHAQHHDRVDNLVLDDFKRFKASLSRHFADCPFSGTSTHFHCLRCRFRCTDSTKVTAHRKHHGKQDVISAAGFRQFSSSADCAVPDCKYKLKCSHFHCTFPGCRHTVVGMSQMDSHKRKHEKQERGEPAAEGPAPGPPISLDGSLSLGAEPGSLLFLQSAAAGLGLALGDAGDPGPPDAAAPGPREGAAAAAAAAGESSQEDEEEELELPEEEAEDDEDEDDDEDDDDEDDDEDDDDEDLRTDSEESLPEAAAEAAGAGARTPALAALAALGAPGPAPTAASSP

>Q9P278

MAPTLLQKLFNKRGSSGSSAAASAQGRAPKEGPAFSWSCSEFDLNEIRLIVYQDCDRRGRQVLFDSKAVQKIEEVTAQKTEDVPIKISAKCCQGSSSVSSSSSSSISSHSSSGGSSHHAKEQLPKYQYTRPASDVNMLGEMMFGSVAMSYKGSTLKIHYIRSPPQLMISKVFSARMGSFCGSTNNLQDSFEYINQDPNLGKLNTNQNSLGPCRTGSNLAHSTPVDMPSRGQNEDRDSGIARSASLSSLLITPFPSPSSSTSSSSSYQRRWLRSQTTSLENGIIPRRSTDETFSLAEETCSSNPAMVRRKKIAISIIFSLCEKEEAQRNFQDFFFSHFPLFESHMNRLKSAIEKAMISCRKIAESSLRVQFYVSRLMEALGEFRGTIWNLYSVPRIAEPVWLTMMSGTLEKNQLCQRFLKEFTLLIEQINKNQFFAALLTAVLTYHLAWVPTVMPVDHPPIKAFSEKRTSQSVNMLAKTHPYNPLWAQLGDLYGAIGSPVRLTRTVVVGKQKDLVQRILYVLTYFLRCSELQENQLTWSGNHGEGDQVLNGSKIITALEKGEVEESEYVVITVRNEPALVPPILPPTAAERHNPWPTGFPECPEGTDSRDLGLKPDKEANRRPEQGSEACSAGCLGPASDASWKPQNAFCGDEKNKEAPQDGSSRLPSCEVLGAGMKMDQQAVCELLKVEMPTRLPDRSVAWPCPDRHLREKPSLEKVTFQIGSFASPESDFESRMKKMEERVKACGPSLEASEAADVAQDPQVSRSPFKPGFQENVCCPQNRLSEGDEGESDKGFAEDRGSRNDMAADIAGQLSHAADLGTASHGAGGTGGRRLEATRGLYVKAAEGPVLEPVAPRCVQRGPGLVAGANIPCGDDNKKANFRTEGDIPRNESSDSALGDSDDEACASAMLDLGHGGDRTGGSLEVELPLPRSQSISTQNVRNFGRSLLAGYCPTYMPDLVLHGTGSDEKLKQCLVADLVHTVHHPVLDEPIAEAVCIIADTDKWSVQVATSQRKVTDNMKLGQDVLVSSQVSSLLQSILQLYKLHLPADFCIMHLEDRLQEMYLKSKMLSEYLRGHTRVHVKELGVVLGIESNDLPLLTAIASTHSPYVAQILL

1369 NP_006224.1 NP_002684.1

>NP_006224.1

MEPDGTYEPGFVGIRFCQECNNMLYPKEDKENRILLYACRNCDYQQEADNSCIYVNKITHEVDELTQIIADVSQDPTLPRTEDHPCQKCGHKEAVFFQSHSARAEDAMRLYYVCTAPHCGHRWTE

>NP_002684.1

MSRLLWRKVAGATVGPGPVPAPGRWVSSSVPASDPSDGQRRRQQQQQQQQQQQQQPQQPQVLSSEGGQLRHNPLDIQMLSRGLHEQIFGQGGEMPGEAAVRRSVEHLQKHGLWGQPAVPLPDVELRLPPLYGDNLDQHFRLLAQKQSLPYLEAANLLLQAQLPPKPPAWAWAEGWTRYGPEGEAVPVAIPEERALVFDVEVCLAEGTCPTLAVAISPSAWYSWCSQRLVEERYSWTSQLSPADLIPLEVPTGASSPTQRDWQEQLVVGHNVSFDRAHIREQYLIQGSRMRFLDTMSMHMAISGLSSFQRSLWIAAKQGKHKVQPPTKQGQKSQRKARRGPAISSWDWLDISSVNSLAEVHRLYVGGPPLEKEPRELFVKGTMKDIRENFQDLMQYCAQDVWATHEVFQQQLPLFLERCPHPVTLAGMLEMGVSYLPVNQNWERYLAEAQGTYEELQREMKKSLMDLANDACQLLSGERYKEDPWLWDLEWDLQEFKQKKAKKVKKEPATASKLPIEGAGAPGDPMDQEDLGPCSEEEEFQQDVMARACLQKLKGTTELLPKRPQHLPGHPGWYRKLCPRLDDPAWTPGPSLLSLQMRVTPKLMALTWDGFPLHYSERHGWGYLVPGRRDNLAKLPTGTTLESAGVVCPYRAIESLYRKHCLEQGKQQLMPQEAGLAEEFLLTDNSAIWQTVEELDYLEVEAEAKMENLRAAVPGQPLALTARGGPKDTQPSYHHGNGPYNDVDIPGCWFFKLPHKDGNSCNVGSPFAKDFLPKMEDGTLQAGPGGASGPRALEINKMISFWRNAHKRISSQMVVWLPRSALPRAVIRHPDYDEEGLYGAILPQVVTAGTITRRAVEPTWLTASNARPDRVGSELKAMVQAPPGYTLVGADVDSQELWIAAVLGDAHFAGMHGCTAFGWMTLQGRKSRGTDLHSKTATTVGISREHAKIFNYGRIYGAGQPFAERLLMQFNHRLTQQEAAEKAQQMYAATKGLRWYRLSDEGEWLVRELNLPVDRTEGGWISLQDLRKVQRETARKSQWKKWEVVAERAWKGGTESEMFNKLESIATSDIPRTPVLGCCISRALEPSAVQEEFMTSRVNWVVQSSAVDYLHLMLVAMKWLFEEFAIDGRFCISIHDEVRYLVREEDRYRAALALQITNLLTRCMFAYKLGLNDLPQSVAFFSAVDIDRCLRKEVTMDCKTPSNPTGMERRYGIPQGEALDIYQIIELTKGSLEKRSQPGP

1370 Q99081 NP_001024.1

>Q99081

MNPQQQRMAAIGTDKELSDLLDFSAMFSPPVNSGKTRPTTLGSSQFSGSGIDERGGTTSWGTSGQPSPSYDSSRGFTDSPHYSDHLNDSRLGAHEGLSPTPFMNSNLMGKTSERGSFSLYSRDTGLPGCQSSLLRQDLGLGSPAQLSSSGKPGTAYYSFSATSSRRRPLHDSAALDPLQAKKVRKVPPGLPSSVYAPSPNSDDFNRESPSYPSPKPPTSMFASTFFMQDGTHNSSDLWSSSNGMSQPGFGGILGTSTSHMSQSSSYGNLHSHDRLSYPPHSVSPTDINTSLPPMSSFHRGSTSSSPYVAASHTPPINGSDSILGTRGNAAGSSQTGDALGKALASIYSPDHTSSSFPSNPSTPVGSPSPLTGTSQWPRPGGQAPSSPSYENSLHSLQSRMEDRLDRLDDAIHVLRNHAVGPSTSLPAGHSDIHSLLGPSHNAPIGSLNSNYGGSSLVASSRSASMVGTHREDSVSLNGNHSVLSSTVTTSSTDLNHKTQENYRGGLQSQSGTVVTTEIKTENKEKDENLHEPPSSDDMKSDDESSQKDIKVSSRGRTSSTNEDEDLNPEQKIEREKERRMANNARERLRVRDINEAFKELGRMCQLHLKSEKPQTKLLILHQAVAVILSLEQQVRERNLNPKAACLKRREEEKVSAVSAEPPTTLPGTHPGLSETTNPMGHM

>NP_001024.1

MHVIKRDGRQERVMFDKITSRIQKLCYGLNMDFVDPAQITMKVIQGLYSGVTTVELDTLAAETAATLTTKHPDYAILAARIAVSNLHKETKKVFSDVMEDLYNYINPHNGKHSPMVAKSTLDIVLANKDRLNSAIIYDRDFSYNYFGFKTLERSYLLKINGKVAERPQHMLMRVSVGIHKEDIDAAIETYNLLSERWFTHASPTLFNAGTNRPQLSSCFLLSMKDDSIEGIYDTLKQCALISKSAGGIGVAVSCIRATGSYIAGTNGNSNGLVPMLRVYNNTARYVDQGGNKRPGAFAIYLEPWHLDIFEFLDLKKNTGKEEQRARDLFFALWIPDLFMKRVETNQDWSLMCPNECPGLDEVWGEEFEKLYASYEKQGRVRKVVKAQQLWYAIIESQTETGTPYMLYKDSCNRKSNQQNLGTIKCSNLCTEIVEYTSKDEVAVCNLASLALNMYVTSEHTYDFKKLAEVTKVVVRNLNKIIDINYYPVPEACLSNKRHRPIGIGVQGLADAFILMRYPFESAEAQLLNKQIFETIYYGALEASCDLAKEQGPYETYEGSPVSKGILQYDMWNVTPTDLWDWKVLKEKIAKYGIRNSLLIAPMPTASTAQILGNNESIEPYTSNIYTRRVLSGEFQIVNPHLLKDLTERGLWHEEMKNQIIACNGSIQSIPEIPDDLKQLYKTVWEISQKTVLKMAAERGAFIDQSQSLNIHIAEPNYGKLTSMHFYGWKQGLKTGMYYLRTRPAANPIQFTLNKEKLKDKEKVSKEEEEKERNTAAMVCSLENRDECLMCGS

1371 NP_003649.2 Q16643

>NP_003649.2

MHCHAELRLSSPGQLKAARRRYKTFMIDEILSKETCDYFEKLSLYSVCPSLVVRPKPLHSCTGSPSLRAYPLLSVITRQPTVISHLVPATPGIAQALSCHQVTEAVSAEAPGGEALASSESETEQPTPRQKKPRRSRTIFTELQLMGLEKKFQKQKYLSTPDRLDLAQSLGLTQLQVKTWYQNRRMKWKKMVLKGGQEAPTKPKGRPKKNSIPTSEEIEAEEKMNSQAQGQEQLEPSQGQEELCEAQEPKARDVPLEMAEPPDPPQELPIPSSEPPPLS

>Q16643

MAGVSFSGHRLELLAAYEEVIREESAADWALYTYEDGSDDLKLAASGEGGLQELSGHFENQKVMYGFCSVKDSQAALPKYVLINWVGEDVPDARKCACASHVAKVAEFFQGVDVIVNASSVEDIDAGAIGQRLSNGLARLSSPVLHRLRLREDENAEPVGTTYQKTDAAVEMKRINREQFWEQAKKEEELRKEEERKKALDERLRFEQERMEQERQEQEERERRYREREQQIEEHRRKQQTLEAEEAKRRLKEQSIFGDHRDEEEETHMKKSESEVEEAAAIIAQRPDNPREFFKQQERVASASAGSCDVPSPFNHRPGSHLDSHRRMAPTPIPTRSPSDSSTASTPVAEQIERALDEVTSSQPPPLPPPPPPAQETQEPSPILDSEETRAAAPQAWAGPMEEPPQAQAPPRGPGSPAEDLMFMESAEQAVLAAPVEPATADATEIHDAADTIETDTATADTTVANNVPPAATSLIDLWPGNGEGASTLQGEPRAPTPPSGTEVTLAEVPLLDEVAPEPLLPAGEGCATLLNFDELPEPPATFCDPEEVEGESLAAPQTPTLPSALEELEQEQEPEPHLLTNGETTQKEGTQASEGYFSQSQEEEFAQSEELCAKAPPPVFYNKPPEIDITCWDADPVPEEEEGFEGGD

1372 NP_443082.2 Q96RP9

>NP_443082.2

MAASVRQARSLLGVAATLAPGSRGYRARPPPRRRPGPRWPDPEDLLTPRWQLGPRYAAKQFARYGAASGVVPGSLWPSPEQLRELEAEEREWYPSLATMQESLRVKQLAEEQKRREREQHIAECMAKMPQMIVNWQQQQRENWEKAQADKERRARLQAEAQELLGYQVDPRSARFQELLQDLEKKERKRLKEEKQKRKKEARAAALAAAVAQDPAASGAPSS

>Q96RP9

MRLLGAAAVAALGRGRAPASLGWQRKQVNWKACRWSSSGVIPNEKIRNIGISAHIDSGKTTLTERVLYYTGRIAKMHEVKGKDGVGAVMDSMELERQRGITIQSAATYTMWKDVNINIIDTPGHVDFTIEVERALRVLDGAVLVLCAVGGVQCQTMTVNRQMKRYNVPFLTFINKLDRMGSNPARALQQMRSKLNHNAAFMQIPMGLEGNFKGIVDLIEERAIYFDGDFGQIVRYGEIPAELRAAATDHRQELIECVANSDEQLGEMFLEEKIPSISDLKLAIRRATLKRSFTPVFLGSALKNKGVQPLLDAVLEYLPNPSEVQNYAILNKEDDSKEKTKILMNSSRDNSHPFVGLAFKLEVGRFGQLTYVRSYQGELKKGDTIYNTRTRKKVRLQRLARMHADMMEDVEEVYAGDICALFGIDCASGDTFTDKANSGLSMESIHVPDPVISIAMKPSNKNDLEKFSKGIGRFTREDPTFKVYFDTENKETVISGMGELHLEIYAQRLEREYGCPCITGKPKVAFRETITAPVPFDFTHKKQSGGAGQYGKVIGVLEPLDPEDYTKLEFSDETFGSNIPKQFVPAVEKGFLDACEKGPLSGHKLSGLRFVLQDGAHHMVDSNEISFIRAGEGALKQALANATLCILEPIMAVEVVAPNEFQGQVIAGINRRHGVITGQDGVEDYFTLYADVPLNDMFGYSTELRSCTEGKGEYTMEYSRYQPCLPSTQEDVINKYLEATGQLPVKKGKAKN

1373 NP_004796.1 NP_005470.2

>NP_004796.1

MAAGGSDPRAGDVEEDASQLIFPKEFETAETLLNSEVHMLLEHRKQQNESAEDEQELSEVFMKTLNYTARFSRFKNRETIASVRSLLLQKKLHKFELACLANLCPETAEESKALIPSLEGRFEDEELQQILDDIQTKRSFQY

>NP_005470.2

MPCRREEEEEAGEEAEGEEEEEDSFLLLQQSVALGSSGEVDRLVAQIGETLQLDAAQHSPASPCGPPGAPLRAPGPLAAAVPADKARSPAVPLLLPPALAETVGPAPPGVLRCALGDRGRVRGRAAPYCVAELATGPSALSPLPPQADLDGPPGAGKQGIPQPLSGPCRRGWLRGAAASRRLQQRRGSQPETRTGDDDPHRLLQQLVLSGNLIKEAVRRLHSRRLQLRAKLPQRPLLGPLSAPVHEPPSPRSPRAACSDPGASGRAQLRTGDGVLVPGS

1374 P31267 NP_060767.2

>P31267

MSSYFVNPTFPGSLPSGQDSFLGQLPLYQAGYDALRPFPASYGASSLPDKTYTSPCFYQQSNSVLACNRASYEYGASCFYSDKDLSGASPSGSGKQRGPGDYLHFSPEQQYKPDSSSGQGKALHDEGADRKYTSPVYPWMQRMNSCAGAVYGSHGRRGRQTYTRYQTLELEKEFHFNRYLTRRRRIEIANALCLTERQIKIWFQNRRMKWKKENKLINSTQPSGEDSEAKAGE

>NP_060767.2

MAAAALGSSSGSASPAVAELCQNTPETFLEASKLLLTYADNILRNPNDEKYRSIRIGNTAFSTRLLPVRGAVECLFEMGFEEGETHLIFPKKASVEQLQKIRDLIAIERSSRLDGSNKSHKVKSSQQPAASTQLPTTPSSNPSGLNQHTRNRQGQSSDPPSASTVAADSAILEVLQSNIQHVLVYENPALQEKALACIPVQELKRKSQEKLSRARKLDKGINISDEDFLLLELLHWFKEEFFHWVNNVLCSKCGGQTRSRDRSLLPSDDELKWGAKEVEDHYCDACQFSNRFPRYNNPEKLLETRCGRCGEWANCFTLCCRAVGFEARYVWDYTDHVWTEVYSPSQQRWLHCDACEDVCDKPLLYEIGWGKKLSYVIAFSKDEVVDVTWRYSCKHEEVIARRTKVKEALLRDTINGLNKQRQLFLSENRRKELLQRIIVELVEFISPKTPKPGELGGRISGSVAWRVARGEMGLQRKETLFIPCENEKISKQLHLCYNIVKDRYVRVSNNNQTISGWENGVWKMESIFRKVETDWHMVYLARKEGSSFAYISWKFECGSVGLKVDSISIRTSSQTFQTGTVEWKLRSDTAQVELTGDNSLHSYADFSGATEVILEAELSRGDGDVAWQHTQLFRQSLNDHEENCLEIIIKFSDL

1375 NP_056023.3 NP_001025.1

>NP_056023.3

MPKSGFTKPIQSENSDSDSNMVEKPYGRKSKDKIASYSKTPKIERSDVSKEMKEKSSMKRKLPFTISPSRNEERDSDTDSDPGHTSENWGERLISSYRTYSEKEGPEKKKTKKEAGNKKSTPVSILFGYPLSERKQMALLMQMTARDNSPDSTPNHPSQTTPAQKKTPSSSSRQKDKVNKRNERGETPLHMAAIRGDVKQVKELISLGANVNVKDFAGWTPLHEACNVGYYDVAKILIAAGADVNTQGLDDDTPLHDSASSGHRDIVKLLLRHGGNPFQANKHGERPVDVAETEELELLLKREVPLSDDDESYTDSEEAQSVNPSSVDENIDSETEKDSLICESKQILPSKTPLPSALDEYEFKDDDDEEINKMIDDRHILRKEQRKENEPEAEKTHLFAKQEKAFYPKSFKSKKQKPSRVLYSSTESSDEEALQNKKISTSCSVIPETSNSDMQTKKEYVVSGEHKQKGKVKRKLKNQNKNKENQELKQEKEGKENTRITNLTVNTGLDCSEKTREEGNFRKSFSPKDDTSLHLFHISTGKSPKHSCGLSEKQSTPLKQEHTKTCLSPGSSEMSLQPDLVRYDNTESEFLPESSSVKSCKHKEKSKHQKDFHLEFGEKSNAKIKDEDHSPTFENSDCTLKKMDKEGKTLKKHKLKHKEREKEKHKKEIEGEKEKYKTKDSAKELQRSVEFDREFWKENFFKSDETEDLFLNMEHESLTLEKKSKLEKNIKDDKSTKEKHVSKERNFKEERDKIKKESEKSFREEKIKDLKEERENIPTDKDSEFTSLGMSAIEESIGLHLVEKEIDIEKQEKHIKESKEKPEKRSQIKEKDIEKMERKTFEKEKKIKHEHKSEKDKLDLSECVDKIKEKDKLYSHHTEKCHKEGEKSKNTAAIKKTDDREKSREKMDRKHDKEKPEKERHLAESKEKHLMEKKNKQSDNSEYSKSEKGKNKEKDRELDKKEKSRDKESINITNSKHIQEEKKSSIVDGNKAQHEKPLSLKEKTKDEPLKTPDGKEKDKKDKDIDRYKERDKHKDKIQINSLLKLKSEADKPKPKSSPASKDTRPKEKRLVNDDLMQTSFERMLSLKDLEIEQWHKKHKEKIKQKEKERLRNRNCLELKIKDKEKTKHTPTESKNKELTRSKSSEVTDAYTKEKQPKDAVSNRSQSVDTKNVMTLGKSSFVSDNSLNRSPRSENEKPGLSSRSVSMISVASSEDSCHTTVTTPRPPVEYDSDFMLESSESQMSFSQSPFLSIAKSPALHERELDSLADLPERIKPPYANRLSTSHLRSSSVEDVKLIISEGRPTIEVRRCSMPSVICEHTKQFQTISEESNQGSLLTVPGDTSPSPKPEVFSNVPERDLSNVSNIHSSFATSPTGASNSKYVSADRNLIKNTAPVNTVMDSPVHLEPSSQVGVIQNKSWEMPVDRLETLSTRDFICPNSNIPDQESSLQSFCNSENKVLKENADFLSLRQTELPGNSCAQDPASFMPPQQPCSFPSQSLSDAESISKHMSLSYVANQEPGILQQKNAVQIISSALDTDNESTKDTENTFVLGDVQKTDAFVPVYSDSTIQEASPNFEKAYTLPVLPSEKDFNGSDASTQLNTHYAFSKLTYKSSSGHEVENSTTDTQVISHEKENKLESLVLTHLSRCDSDLCEMNAGMPKGNLNEQDPKHCPESEKCLLSIEDEESQQSILSSLENHSQQSTQPEMHKYGQLVKVELEENAEDDKTENQIPQRMTRNKANTMANQSKQILASCTLLSEKDSESSSPRGRIRLTEDDDPQIHHPRKRKVSRVPQPVQVSPSLLQAKEKTQQSLAAIVDSLKLDEIQPYSSERANPYFEYLHIRKKIEEKRKLLCSVIPQAPQYYDEYVTFNGSYLLDGNPLSKICIPTITPPPSLSDPLKELFRQQEVVRMKLRLQHSIEREKLIVSNEQEVLRVHYRAARTLANQTLPFSACTVLLDAEVYNVPLDSQSDDSKTSVRDRFNARQFMSWLQDVDDKFDKLKTCLLMRQQHEAAALNAVQRLEWQLKLQELDPATYKSISIYEIQEFYVPLVDVNDDFELTPI

>NP_001025.1

MLSLRVPLAPITDPQQLQLSPLKGLSLVDKENTPPALSGTRVLASKTARRIFQEPTEPKTKAAAPGVEDEPLLRENPRRFVIFPIEYHDIWQMYKKAEASFWTAEEVDLSKDIQHWESLKPEERYFISHVLAFFAASDGIVNENLVERFSQEVQITEARCFYGFQIAMENIHSEMYSLLIDTYIKDPKEREFLFNAIETMPCVKKKADWALRWIGDKEATYGERVVAFAAVEGIFFSGSFASIFWLKKRGLMPGLTFSNELISRDEGLHCDFACLMFKHLVHKPSEERVREIIINAVRIEQEFLTEALPVKLIGMNCTLMKQYIEFVADRLMLELGFSKVFRVENPFDFMENISLEGKTNFFEKRVGEYQRMGVMSSPTENSFTLDADF

1376 NP_003074.1 NP_001748.1

>NP_003074.1

MKPPPRRRAAPARYLGEVTGPATWSAREKRQLVRLLQARQGQPEPDATELARELRGRSEAEIRVFLQQLKGRVAREAIQKVHPGGLQGPRRREAQPPAPIEVWTDLAEKITGPLEEALAVAFSQVLTIAATEPVTLLHSKPPKPTQARGKPLLLSAPGGQEDPAPEIPSSAPAAPSSAPRTPDPAPEKPSESSAGPSTEEDFAVDFEKIYKYLSSVSRSGRSPELSAAESAVVLDLLMSLPEELPLLPCTALVEHMTETYLRLTAPQPIPAGGSLGPAAEGDGAGSKAPEETPPATEKAEHSELKSPWQAAGICPLNPFLVPLELLGRAATPAR

>NP_001748.1

MSSGIHVALVTGGNKGIGLAIVRDLCRLFSGDVVLTARDVTRGQAAVQQLQAEGLSPRFHQLDIDDLQSIRALRDFLRKEYGGLDVLVNNAGIAFKVADPTPFHIQAEVTMKTNFFGTRDVCTELLPLIKPQGRVVNVSSIMSVRALKSCSPELQQKFRSETITEEELVGLMNKFVEDTKKGVHQKEGWPSSAYGVTKIGVTVLSRIHARKLSEQRKGDKILLNACCPGWVRTDMAGPKATKSPEEGAETPVYLALLPPDAEGPHGQFVSEKRVEQW

1377 NP_005925.2 NP_060217.1

>NP_005925.2

MDNQCTVQVRLELGHRAQLRKKPTTEGFTHDWMVFVRGPEQCDIQHFVEKVVFWLHDSFPKPRRVCKEPPYKVEESGYAGFIMPIEVHFKNKEEPRKVCFTYDLFLNLEGNPPVNHLRCEKLTFNNPTTEFRYKLLRAGGVMVMPEGADTVSRPSPDYPMLPTIPLSAFSDPKKTKPSHGSKDANKESSKTSKPHKVTKEHRERPRKDSESKSSSKELEREQAKSSKDTSRKLGEGRLPKEEKAPPPKAAFKEPKMALKETKLESTSPKGGPPPPPPPPPRASSKRPATADSPKPSAKKQKKSSSKGSRSAPGTSPRTSSSSSFSDKKPAKDKSSTRGEKVKAESEPREAKKALEVEESNSEDEASFKSESAQSSPSNSSSSSDSSSDSDFEPSQNHSQGPLRSMVEDLQSEESDEDDSSSGEEAAGKTNPGRDSRLSFSDSESDNSADSSLPSREPPPPQKPPPPNSKVSGRRSPESCSKPEKILKKGTYDKAYTDELVELHRRLMALRERNVLQQIVNLIEETGHFNVTNTTFDFDLFSLDETTVRKLQSCLEAVAT

>NP_060217.1

MLTDSGGGGTSFEEDLDSVAPRSAPAGASEPPPPGGVGLGIRTVRLFGEAGPASGVGSSGGGGSGSGTGGGDAALDFKLAAAVLRTGGGGGASGSDEDEVSEVESFILDQEDLDNPVLKTTSEIFLSSTAEGADLRTVDPETQARLEALLEAAGIGKLSTADGKAFADPEVLRRLTSSVSCALDEAAAALTRMKAENSHNAGQVDTRSLAEACSDGDVNAVRKLLDEGRSVNEHTEEGESLLCLACSAGYYELAQVLLAMHANVEDRGNKGDITPLMAASSGGYLDIVKLLLLHDADVNSQSATGNTALTYACAGGFVDIVKVLLNEGANIEDHNENGHTPLMEAASAGHVEVARVLLDHGAGINTHSNEFKESALTLACYKGHLDMVRFLLEAGADQEHKTDEMHTALMEACMDGHVEVARLLLDSGAQVNMPADSFESPLTLAACGGHVELAALLIERGANLEEVNDEGYTPLMEAAREGHEEMVALLLAQGANINAQTEETQETALTLACCGGFSEVADFLIKAGADIELGCSTPLMEASQEGHLELVKYLLASGANVHATTATGDTALTYACENGHTDVADVLLQAGADLEHESEGGRTPLMKAARAGHLCTVQFLISKGANVNRATANNDHTVVSLACAGGHLAVVELLLAHGADPTHRLKDGSTMLIEAAKGGHTNVVSYLLDYPNNVLSVPTTDVSQLPPPSQDQSQVPRVPTHTLAMVVPPQEPDRTSQENSPALLGVQKGTSKQKSSSLQVADQDLLPSFHPYQPLECIVEETEGKLNELGQRISAIEKAQLKSLELIQGEPLNKDKIEELKKNREEQVQKKKKILKELQKVERQLQMKTQQQFTKEYLETKGQKDTVSLHQQCSHRGVFPEGEGDGSLPEDHFSELPQVDTILFKDNDVDDEQQSPPSAEQIDFVPVQPLSSPQCNFSSDLGSNGTNSLELQKVSGNQQIVGQPQIAITGHDQGLLVQEPDGLMVATPAQTLTDTLDDLIAAVSTRVPTGSNSSSQTTECLTPESCSQTTSNVASQSMPPVYPSVDIDAHTESNHDTALTLACAGGHEELVSVLIARDAKIEHRDKKGFTPLILAATAGHVGVVEILLDKGGDIEAQSERTKDTPLSLACSGGRQEVVDLLLARGANKEHRNVSDYTPLSLAASGGYVNIIKILLNAGAEINSRTGSKLGISPLMLAAMNGHVPAVKLLLDMGSDINAQIETNRNTALTLACFQGRAEVVSLLLDRKANVEHRAKTGLTPLMEAASGGYAEVGRVLLDKGADVNAPPVPSSRDTALTIAADKGHYKFCELLIHRGAHIDVRNKKGNTPLWLASNGGHFDVVQLLVQAGADVDAADNRKITPLMSAFRKGHVKVVQYLVKEVNQFPSDIECMRYIATITDKELLKKCHQCVETIVKAKDQQAAEANKNASILLKELDLEKSREESRKQALAAKREKRKEKRKKKKEEQKRKQEEDEENKPKENSELPEDEDEEENDEDVEQEVPIEPPSATTTTTIGISATSATFTNVFGKKRANVVTTPSTNRKNKKNKTKETPPTAHLILPEQHMSLAQQKADKNKINGEPRGGGAGGNSDSDNLDSTDCNSESSSGGKSQELNFVMDVNSSKYPSLLLHSQEEKTSTATSKTQTRLEGEVTPNSLSTSYKTVSLPLSSPNIKLNLTSPKRGQKREEGWKEVVRRSKKLSVPASVVSRIMGRGGCNITAIQDVTGAHIDVDKQKDKNGERMITIRGGTESTRYAVQLINALIQDPAKELEDLIPKNHIRTPASTKSIHANFSSGVGTTAASSKNAFPLGAPTLVTSQATTLSTFQPANKLNKNVPTNVRSSFPVSLPLAYPHPHFALLAAQTMQQIRHPRLPMAQFGGTFSPSPNTWGPFPVRPVNPGNTNSSPKHNNTSRLPNQNGTVLPSESAGLATASCPITVSSVVAASQQLCVTNTRTPSSVRKQLFACVPKTSPPATVISSVTSTCSSLPSVSSAPITSGQAPTTFLPASTSQAQLSSQKMESFSAVPPTKEKVSTQDQPMANLCTPSSTANSCSSSASNTPGAPETHPSSSPTPTSSNTQEEAQPSSVSDLSPMSMPFASNSEPAPLTLTSPRMVAADNQDTSNLPQLAVPAPRVSHRMQPRGSFYSMVPNATIHQDPQSIFVTNPVTLTPPQGPPAAVQLSSAVNIMNGSQMHINPANKSLPPTFGPATLFNHFSSLFDSSQVPANQGWGDGPLSSRVATDASFTVQSAFLGNSVLGHLENMHPDNSKAPGFRPPSQRVSTSPVGLPSIDPSGSSPSSSSAPLASFSGIPGTRVFLQGPAPVGTPSFNRQHFSPHPWTSASNSSTSAPPTLGQPKGVSASQDRKIPPPIGTERLARIRQGGSVAQAPAGTSFVAPVGHSGIWSFGVNAVSEGLSGWSQSVMGNHPMHQQLSDPSTFSQHQPMERDDSGMVAPSNIFHQPMASGFVDFSKGLPISMYGGTIIPSHPQLADVPGGPLFNGLHNPDPAWNPMIKVIQNSTECTDAQQIWPGTWAPHIGNMHLKYVN

1378 Q6NYC1 NP_005908.1

>Q6NYC1

MNHKSKKRIREAKRSARPELKDSLDWTRHNYYESFSLSPAAVADNVERADALQLSVEEFVERYERPYKPVVLLNAQEGWSAQEKWTLERLKRKYRNQKFKCGEDNDGYSVKMKMKYYIEYMESTRDDSPLYIFDSSYGEHPKRRKLLEDYKVPKFFTDDLFQYAGEKRRPPYRWFVMGPPRSGTGIHIDPLGTSAWNALVQGHKRWCLFPTSTPRELIKVTRDEGGNQQDEAITWFNVIYPRTQLPTWPPEFKPLEILQKPGETVFVPGGWWHVVLNLDTTIAITQNFASSTNFPVVWHKTVRGRPKLSRKWYRILKQEHPELAVLADSVDLQESTGIASDSSSDSSSSSSSSSSDSDSECESGSEGDGTVHRRKKRRTCSMVGNGDTTSQDDCVSKERSSSR

>NP_005908.1

MSEPIRVLVTGAAGQIAYSLLYSIGNGSVFGKDQPIILVLLDITPMMGVLDGVLMELQDCALPLLKDVIATDKEDVAFKDLDVAILVGSMPRREGMERKDLLKANVKIFKSQGAALDKYAKKSVKVIVVGNPANTNCLTASKSAPSIPKENFSCLTRLDHNRAKAQIALKLGVTANDVKNVIIWGNHSSTQYPDVNHAKVKLQGKEVGVYEALKDDSWLKGEFVTTVQQRGAAVIKARKLSSAMSAAKAICDHVRDIWFGTPEGEFVSMGVISDGNSYGVPDDLLYSFPVVIKNKTWKFVEGLPINDFSREKMDLTAKELTEEKESAFEFLSSA

1379 NP_060624.1 NP_005195.2

>NP_060624.1

MAKVSVLNVAVLENPSPFHSPFRFEISFECSEALADDLEWKIIYVGSAESEEFDQILDSVLVGPVPAGRHMFVFQADAPNPSLIPETDAVGVTVVLITCTYHGQEFIRVGYYVNNEYLNPELRENPPMKPDFSQLQRNILASNPRVTRFHINWDNNMDRLEAIETQDPSLGCGLPLNCTPIKGLGLPGCIPGLLPENSMDCI

>NP_005195.2

MEYMSTGSDNKEEIDLLIKHLNVSDVIDIMENLYASEEPAVYEPSLMTMCQDSNQNDERSKSLLLSGQEVPWLSSVRYGTVEDLLAFANHISNTAKHFYGQRPQESGILLNMVITPQNGRYQIDSDVLLIPWKLTYRNIGSDFIPRGAFGKVYLAQDIKTKKRMACKLIPVDQFKPSDVEIQACFRHENIAELYGAVLWGETVHLFMEAGEGGSVLEKLESCGPMREFEIIWVTKHVLKGLDFLHSKKVIHHDIKPSNIVFMSTKAVLVDFGLSVQMTEDVYFPKDLRGTEIYMSPEVILCRGHSTKADIYSLGATLIHMQTGTPPWVKRYPRSAYPSYLYIIHKQAPPLEDIADDCSPGMRELIEASLERNPNHRPRAADLLKHEALNPPREDQPRCQSLDSALLERKRLLSRKELELPENIADSSCTGSTEESEMLKRQRSLYIDLGALAGYFNLVRGPPTLEYG

1380 NP_004705.1 NP_001705.2

>NP_004705.1

MAVPPGHGPFSGFPGPQEHTQVLPDVRLLPRRLPLAFRDATSAPLRKLSVDLIKTYKHINEVYYAKKKRRAQQAPPQDSSNKKEKKVLNHGYDDDNHDYIVRSGERWLERYEIDSLIGKGSFGQVVKAYDHQTQELVAIKIIKNKKAFLNQAQIELRLLELMNQHDTEMKYYIVHLKRHFMFRNHLCLVFELLSYNLYDLLRNTHFRGVSLNLTRKLAQQLCTALLFLATPELSIIHCDLKPENILLCNPKRSAIKIVDFGSSCQLGQRIYQYIQSRFYRSPEVLLGTPYDLAIDMWSLGCILVEMHTGEPLFSGSNEVDQMNRIVEVLGIPPAAMLDQAPKARKYFERLPGGGWTLRRTKELRKDYQGPGTRRLQEVLGVQTGGPGGRRAGEPGHSPADYLRFQDLVLRMLEYEPAARISPLGALQHGFFRRTADEATNTGPAGSSASTSPAPLDTCPSSSTASSISSSGGSSGSSSDNRTYRYSNRYCGGPGPPITDCEMNSPQVPPSQPLRPWAGGDVPHKTHQAPASASSLPGTGAQLPPQPRYLGRPPSPTSPPPPELMDVSLVGGPADCSPPHPAPAPQHPAASALRTRMTGGRPPLPPPDDPATLGPHLGLRGVPQSTAASS

>NP_001705.2

MAAEEVLQTVDHYKTEIERLTKELTETTHEKIQAAEYGLVVLEEKLTLKQQYDELEAEYDSLKQELEQLKEAFGQSFSIHRKVAEDGETREETLLQESASKEAYYLGKILEMQNELKQSRAVVTNVQAENERLTAVVQDLKENNEMVELQRIRMKDEIREYKFREARLLQDYTELEEENITLQKLVSTLKQNQVEYEGLKHEIKRFEEETVLLNSQLEDAIRLKEIAEHQLEEALETLKNEREQKNNLRKELSQYISLNDNHISISVDGLKFAEDGSEPNNDDKMNGHIHGPLVKLNGDYRTPTLRKGESLNPVSDLFSELNISEIQKLKQQLMQVEREKAILLANLQESQTQLEHTKGALTEQHERVHRLTEHVNAMRGLQSSKELKAELDGEKGRDSGEEAHDYEVDINGLEILECKYRVAVTEVIDLKAEIKALKEKYNKSVENYTDEKAKYESKIQMYDEQVTSLEKTTKESGEKMAHMEKELQKMTSIANENHSTLNTAQDELVTFSEELAQLYHHVCLCNNETPNRVMLDYYRQSRVTRSGSLKGPDDPRGLLSPRLARRGVSSPVETRTSSEPVAKESTEASKEPSPTKTPTISPVITAPPSSPVLDTSDIRKEPMNIYNLNAIIRDQIKHLQKAVDRSLQLSRQRAAARELAPMIDKDKEALMEEILKLKSLLSTKREQIATLRAVLKANKQTAEVALANLKNKYENEKAMVTETMTKLRNELKALKEDAATFSSLRAMFATRCDEYVTQLDEMQRQLAAAEDEKKTLNTLLRMAIQQKLALTQRLEDLEFDHEQSRRSKGKLGKSKIGSPKVSGEASVTVPTIDTYLLHSQGPQTPNIRVSSGTQRKRQFSPSLCDQSRPRTSGASYLQNLLRVPPDPTSTESFLLKGPPSMSEFIQGHRLSKEKRLTVAPPDCQQPAASVPPQCSQLAGRQDCPTVSPDTALPEEQPHSSSQCAPLHCLSKPPHP

1381 NP_002470.2 Q9Y2R5

>NP_002470.2

MELYETSPYFYQEPRFYDGENYLPVHLQGFEPPGYERTELTLSPEAPGPLEDKGLGTPEHCPGQCLPWACKVCKRKSVSVDRRRAATLREKRRLKKVNEAFEALKRSTLLNPNQRLPKVEILRSAIQYIERLQALLSSLNQEERDLRYRGGGGPQPGVPSECSSHSASCSPEWGSALEFSANPGDHLLTADPTDAHNLHSLTSIVDSITVEDVSVAFPDETMPN

>Q9Y2R5

MSVVRSSVHARWIVGKVIGTKMQKTAKVRVTRLVLDPYLLKYFNKRKTYFAHDALQQCTVGDIVLLRALPVPRAKHVKHELAEIVFKVGKVIDPVTGKPCAGTTYLESPLSSETTQLSKNLEELNISSAQ

1382 NP_001978.1 NP_002631.3

>NP_001978.1

MSETPAQCSIKQERISYTPPESPVPSYASSTPLHVPVPRALRMEEDSIRLPAHLRLQPIYWSRDDVAQWLKWAENEFSLRPIDSNTFEMNGKALLLLTKEDFRYRSPHSGDVLYELLQHILKQRKPRILFSPFFHPGNSIHTQPEVILHQNHEEDNCVQRTPRPSVDNVHHNPPTIELLHRSRSPITTNHRPSPDPEQRPLRSPLDNMIRRLSPAERAQGPRPHQENNHQESYPLSVSPMENNHCPASSESHPKPSSPRQESTRVIQLMPSPIMHPLILNPRHSVDFKQSRLSEDGLHREGKPINLSHREDLAYMNHIMVSVSPPEEHAMPIGRIADCRLLWDYVYQLLSDSRYENFIRWEDKESKIFRIVDPNGLARLWGNHKNRTNMTYEKMSRALRHYYKLNIIRKEPGQRLLFRFMKTPDEIMSGRTDRLEHLESQELDEQIYQEDEC

>NP_002631.3

MDDLCEANGTFAISLFKILGEEDNSRNVFFSPMSISSALAMVFMGAKGSTAAQMSQALCLYKDGDIHRGFQSLLSEVNRTGTQYLLRTANRLFGEKTCDFLPDFKEYCQKFYQAELEELSFAEDTEECRKHINDWVAEKTEGKISEVLDAGTVDPLTKLVLVNAIYFKGKWNEQFDRKYTRGMLFKTNEEKKTVQMMFKEAKFKMGYADEVHTQVLELPYVEEELSMVILLPDDNTDLAVVEKALTYEKFKAWTNSEKLTKSKVQVFLPRLKLEESYDLEPFLRRLGMIDAFDEAKADFSGMSTEKNVPLSKVAHKCFVEVNEEGTEAAAATAVVRNSRCSRMEPRFCADHPFLFFIRHHKTNCILFCGRFSSP

1383 NP_002583.1 NP_001227.1

>NP_002583.1

MFEARLVQGSILKKVLEALKDLINEACWDISSSGVNLQSMDSSHVSLVQLTLRSEGFDTYRCDRNLAMGVNLTSMSKILKCAGNEDIITLRAEDNADTLALVFEAPNQEKVSDYEMKLMDLDVEQLGIPEQEYSCVVKMPSGEFARICRDLSHIGDAVVISCAKDGVKFSASGELGNGNIKLSQTSNVDKEEEAVTIEMNEPVQLTFALRYLNFFTKATPLSSTVTLSMSADVPLVVEYKIADMGHLKYYLAPKIEDEEGS

>NP_001227.1

MSSCSRVALVTGANRGIGLAIARELCRQFSGDVVLTARDVARGQAAVQQLQAEGLSPRFHQLDIDDLQSIRALRDFLRKEYGGLNVLVNNAAVAFKSDDPMPFDIKAEMTLKTNFFATRNMCNELLPIMKPHGRVVNISSLQCLRAFENCSEDLQERFHSETLTEGDLVDLMKKFVEDTKNEVHEREGWPNSPYGVSKLGVTVLSRILARRLDEKRKADRILVNACCPGPVKTDMDGKDSIRTVEEGAETPVYLALLPPDATEPQGQLVHDKVVQNW

1384 NP_005635.1 NP_002940.2

>NP_005635.1

MNQFGPSALINLSNFSSIKPEPASTPPQGSMANSTAVVKIPGTPGAGGRLSPENNQVLTKKKLQDLVREVDPNEQLDEDVEEMLLQIADDFIESVVTAACQLARHRKSSTLEVKDVQLHLERQWNMWIPGFGSEEIRPYKKACTTEAHKQRMALIRKTTKK

>NP_002940.2

MLPAAARPLWGPCLGLRAAAFRLARRQVPCVCAVRHMRSSGHQRCEALAGAPLDNAPKEYPPKIQQLVQDIASLTLLEISDLNELLKKTLKIQDVGLVPMGGVMSGAVPAAAAQEAVEEDIPIAKERTHFTVRLTEAKPVDKVKLIKEIKNYIQGINLVQAKKLVESLPQEIKANVAKAEAEKIKAALEAVGGTVVLE

1385 P28347 Q7RTR2

>P28347

MEPSSWSGSESPAENMERMSDSADKPIDNDAEGVWSPDIEQSFQEALAIYPPCGRRKIILSDEGKMYGRNELIARYIKLRTGKTRTRKQVSSHIQVLARRKSRDFHSKLKDQTAKDKALQHMAAMSSAQIVSATAIHNKLGLPGIPRPTFPGAPGFWPGMIQTGQPGSSQDVKPFVQQAYPIQPAVTAPIPGFEPASAPAPSVPAWQGRSIGTTKLRLVEFSAFLEQQRDPDSYNKHLFVHIGHANHSYSDPLLESVDIRQIYDKFPEKKGGLKELFGKGPQNAFFLVKFWADLNCNIQDDAGAFYGVTSQYESSENMTVTCSTKVCSFGKQVVEKVETEYARFENGRFVYRINRSPMCEYMINFIHKLKHLPEKYMMNSVLENFTILLVVTNRDTQETLLCMACVFEVSNSEHGAQHHIYRLVKD

>Q7RTR2

MRKQEVRTGREAGQGHGTGSPAEQVKALMDLLAGKGSQGSQAPQALDRTPDAPLGPCSNDSRIQRHRKALLSKVGGGPELGGPWHRLASLLLVEGLTDLQLREHDFTQVEATRGGGHPARTVALDRLFLPLSRVSVPPRVSITIGVAGMGKTTLVRHFVRLWAHGQVGKDFSLVLPLTFRDLNTHEKLCADRLICSVFPHVGEPSLAVAVPARALLILDGLDECRTPLDFSNTVACTDPKKEIPVDHLITNIIRGNLFPEVSIWITSRPSASGQIPGGLVDRMTEIRGFNEEEIKVCLEQMFPEDQALLGWMLSQVQADRALYLMCTVPAFCRLTGMALGHLWRSRTGPQDAELWPPRTLCELYSWYFRMALSGEGQEKGKASPRIEQVAHGGRKMVGTLGRLAFHGLLKKKYVFYEQDMKAFGVDLALLQGAPCSCFLQREETLASSVAYCFTHLSLQEFVAAAYYYGASRRAIFDLFTESGVSWPRLGFLTHFRSAAQRAMQAEDGRLDVFLRFLSGLLSPRVNALLAGSLLAQGEHQAYRTQVAELLQGCLRPDAAVCARAINVLHCLHELQHTELARSVEEAMESGALARLTGPAHRAALAYLLQVSDACAQEANLSLSLSQGVLQSLLPQLLYCRKLRLDTNQFQDPVMELLGSVLSGKDCRIQKISLAENQISNKGAKALARSLLVNRSLTSLDLRGNSIGPQGAKALADALKINRTLTSLSLQGNTVRDDGARSMAEALASNRTLSMLHLQKNSIGPMGAQRMADALKQNRSLKELMFSSNSIGDGGAKALAEALKVNQGLESLDLQSNSISDAGVAALMGALCTNQTLLSLSLRENSISPEGAQAIAHALCANSTLKNLDLTANLLHDQGARAIAVAVRENRTLTSLHLQWNFIQAGAAQALGQALQLNRSLTSLDLQENAIGDDGACAVARALKVNTALTALYLQVASIGASGAQVLGEALAVNRTLEILDLRGNAIGVAGAKALANALKVNSSLRRLNLQENSLGMDGAICIATALSGNHRLQHINLQGNHIGDSGARMISEAIKTNAPTCTVEM

1386 P35453 NP_054765.1

>P35453

MSRAGSWDMDGLRADGGGAGGAPASSSSSSVAAAAASGQCRGFLSAPVFAGTHSGRAAAAAAAAAAAAAAASGFAYPGTSERTGSSSSSSSSAVVAARPEAPPAKECPAPTPAAAAAAPPSAPALGYGYHFGNGYYSCRMSHGVGLQQNALKSSPHASLGGFPVEKYMDVSGLASSSVPANEVPARAKEVSFYQGYTSPYQHVPGYIDMVSTFGSGEPRHEAYISMEGYQSWTLANGWNSQVYCTKDQPQGSHFWKSSFPGDVALNQPDMCVYRRGRKKRVPYTKLQLKELENEYAINKFINKDKRRRISAATNLSERQVTIWFQNRRVKDKKIVSKLKDTVS

>NP_054765.1

MAASVLNTVLRRLPMLSLFRGSHRVQVPLQTLCTKAPSEEDSLSSVPISPYKDEPWKYLESEEYQERYGSRPVWADYRRNHKGGVPPQRTRKTCIRRNKVVGNPCPICRDHKLHVDFRNVKLLEQFVCAHTGIIFYAPYTGVCVKQHKRLTQAIQKARDHGLLIYHIPQVEPRDLDFSTSHGAVSATPPAPTLVSGDPWYPWYNWKQPPERELSRLRRLYQGHLQEESGPPPESMPKMPPRTPAEASSTGQTGPQSAL

1387 NP_001252.1 NP_005116.1

>NP_001252.1

MAKQYDSVECPFCDEVSKYEKLAKIGQGTFGEVFKARHRKTGQKVALKKVLMENEKEGFPITALREIKILQLLKHENVVNLIEICRTKASPYNRCKGSIYLVFDFCEHDLAGLLSNVLVKFTLSEIKRVMQMLLNGLYYIHRNKILHRDMKAANVLITRDGVLKLADFGLARAFSLAKNSQPNRYTNRVVTLWYRPPELLLGERDYGPPIDLWGAGCIMAEMWTRSPIMQGNTEQHQLALISQLCGSITPEVWPNVDNYELYEKLELVKGQKRKVKDRLKAYVRDPYALDLIDKLLVLDPAQRIDSDDALNHDFFWSDPMPSDLKGMLSTHLTSMFEYLAPPRRKGSQITQQSTNQSRNPATTNQTEFERVF

>NP_005116.1

MASDSGNQGTLCTLEFAVQMTCQSCVDAVRKSLQGVAGVQDVEVHLEDQMVLVHTTLPSQEVQALLEGTGRQAVLKGMGSGQLQNLGAAVAILGGPGTVQGVVRFLQLTPERCLIEGTIDGLEPGLHGLHVHQYGDLTNNCNSCGNHFNPDGASHGGPQDSDRHRGDLGNVRADADGRAIFRMEDEQLKVWDVIGRSLIIDEGEDDLGRGGHPLSKITGNSGERLACGIIARSAGLFQNPKQICSCDGLTIWEERGRPIAGKGRKESAQPPAHL

1388 NP_002135.2 Q9UBR1

>NP_002135.2

MDYNRMNSFLEYPLCNRGPSAYSAHSAPTSFPPSSAQAVDSYASEGRYGGGLSSPAFQQNSGYPAQQPPSTLGVPFPSSAPSGYAPAACSPSYGPSQYYPLGQSEGDGGYFHPSSYGAQLGGLSDGYGAGGAGPGPYPPQHPPYGNEQTASFAPAYADLLSEDKETPCPSEPNTPTARTFDWMKVKRNPPKTAKVSEPGLGSPSGLRTNFTTRQLTELEKEFHFNKYLSRARRVEIAATLELNETQVKIWFQNRRMKQKKREREEGRVPPAPPGCPKEAAGDASDQSTCTSPEASPSSVTS

>Q9UBR1

MAGAEWKSLEECLEKHLPLPDLQEVKRVLYGKELRKLDLPREAFEAASREDFELQGYAFEAAEEQLRRPRIVHVGLVQNRIPLPANAPVAEQVSALHRRIKAIVEVAAMCGVNIICFQEAWTMPFAFCTREKLPWTEFAESAEDGPTTRFCQKLAKNHDMVVVSPILERDSEHGDVLWNTAVVISNSGAVLGKTRKNHIPRVGDFNESTYYMEGNLGHPVFQTQFGRIAVNICYGRHHPLNWLMYSINGAEIIFNPSATIGALSESLWPIEARNAAIANHCFTCAINRVGTEHFPNEFTSGDGKKAHQDFGYFYGSSYVAAPDSSRTPGLSRSRDGLLVAKLDLNLCQQVNDVWNFKMTGRYEMYARELAEAVKSNYSPTIVKE

1389 Q13105 P51970

>Q13105

MDFPQHSQHVLEQLNQQRQLGLLCDCTFVVDGVHFKAHKAVLAACSEYFKMLFVDQKDVVHLDISNAAGLGQVLEFMYTAKLSLSPENVDDVLAVATFLQMQDIITACHALKSLAEPATSPGGNAEALATEGGDKRAKEEKVATSTLSRLEQAGRSTPIGPSRDLKEERGGQAQSAASGAEQTEKADAPREPPPVELKPDPTSGMAAAEAEAALSESSEQEMEVEPARKGEEEQKEQEEQEEEGAGPAEVKEEGSQLENGEAPEENENEESAGTDSGQELGSEARGLRSGTYGDRTESKAYGSVIHKCEDCGKEFTHTGNFKRHIRIHTGEKPFSCRECSKAFSDPAACKAHEKTHSPLKPYGCEECGKSYRLISLLNLHKKRHSGEARYRCEDCGKLFTTSGNLKRHQLVHSGEKPYQCDYCGRSFSDPTSKMRHLETHDTDKEHKCPHCDKKFNQVGNLKAHLKIHIADGPLKCRECGKQFTTSGNLKRHLRIHSGEKPYVCIHCQRQFADPGALQRHVRIHTGEKPCQCVMCGKAFTQASSLIAHVRQHTGEKPYVCERCGKRFVQSSQLANHIRHHDNIRPHKCSVCSKAFVNVGDLSKHIIIHTGEKPYLCDKCGRGFNRVDNLRSHVKTVHQGKAGIKILEPEEGSEVSVVTVDDMVTLATEALAATAVTQLTVVPVGAAVTADETEVLKAEISKAVKQVQEEDPNTHILYACDSCGDKFLDANSLAQHVRIHTAQALVMFQTDADFYQQYGPGGTWPAGQVLQAGELVFRPRDGAEGQPALAETSPTAPECPPPAE

>P51970

MPGIVELPTLEELKVDEVKISSAVLKAAAHHYGAQCDKPNKEFMLCRWEEKDPRRCLEEGKLVNKCALDFFRQIKRHCAEPFTEYWTCIDYTGQQLFRHCRKQQAKFDECVLDKLGWVRPDLGELSKVTKVKTDRPLPENPYHSRPRPDPSPEIEGDLQPATHGSRFYFWTK

1390 AAH09210.1 NP_003215.1

>AAH09210.1

MAASRWARKAVVLLCASDLLLLLLLLPPPGSCAAEGSPGTPDESTPPPRKKKKDIRDYNDADMARLLEQWEKDDDIEEGDLPEHKRPSAPVDFSKIDPSKPESILKMTKKGKTLMMFVTVSGSPTEKETEEITSLWQGSLFNANYDVQRFIVGSDRAIFMLRDGSYAWEIKDFLVGQDRCADVTLEGQVYPGKGGGSKEKNKTKQDKGKKKKEGDLKSRSSKEENRAGNKREDL

>NP_003215.1

MYTLLSGLYKYMFQKDEYCILILGLDNAGKTTFLEQSKTRFNKNYKGMSLSKITTTVGLNIGTVDVGKARLMFWDLGGQEELQSLWDKYYAECHGVIYVIDSTDEERLAESKQAFEKVVTSEALCGVPVLVLANKQDVETCLSIPDIKTAFSDCTSKIGRRDCLTQACSALTGKGVREGIEWMVKCVVRNVHRPPRQRDIT

1391 NP_059990.2 Q9UDY6

>NP_059990.2

MEQVAEGARVTAVPVSAADSTEELAEVEEGVGVVGEDNDAAARGAEAFGDSEEDGEDVFEVEKILDMKTEGGKVLYKVRWKGYTSDDDTWEPEIHLEDCKEVLLEFRKKIAENKAKAVRKDIQRLSLNNDIFEANSDSDQQSETKEDTSPKKKKKKLRQREEKSPDDLKKKKAKAGKLKDKSKPDLESSLESLVFDLRTKKRISEAKEELKESKKPKKDEVKETKELKKVKKGEIRDLKTKTREDPKENRKTKKEKFVESQVESESSVLNDSPFPEDDSEGLHSDSREEKQNTKSARERAGQDMGLEHGFEKPLDSAMSAEEDTDVRGRRKKKTPRKAEDTRENRKLENKNAFLEKKTVPKKQRNQDRSKSAAELEKLMPVSAQTPKGRRLSGEERGLWSTDSAEEDKETKRNESKEKYQKRHDSDKEEKGRKEPKGLKTLKEIRNAFDLFKLTPEEKNDVSENNRKREEIPLDFKTIDDHKTKENKQSLKERRNTRDETDTWAYIAAEGDQEVLDSVCQADENSDGRQQILSLGMDLQLEWMKLEDFQKHLDGKDENFAATDAIPSNVLRDAVKNGDYITVKVALNSNEEYNLDQEDSSGMTLVMLAAAGGQDDLLRLLITKGAKVNGRQKNGTTALIHAAEKNFLTTVAILLEAGAFVNVQQSNGETALMKACKRGNSDIVRLVIECGADCNILSKHQNSALHFAKQSNNVLVYDLLKNHLETLSRVAEETIKDYFEARLALLEPVFPIACHRLCEGPDFSTDFNYKPPQNIPEGSGILLFIFHANFLGKEVIARLCGPCSVQAVVLNDKFQLPVFLDSHFVYSFSPVAGPNKLFIRLTEAPSAKVKLLIGAYRVQLQ

>Q9UDY6

MASAASVTSLADEVNCPICQGTLREPVTIDCGHNFCRACLTRYCEIPGPDLEESPTCPLCKEPFRPGSFRPNWQLANVVENIERLQLVSTLGLGEEDVCQEHGEKIYFFCEDDEMQLCVVCREAGEHATHTMRFLEDAAAPYREQIHKCLKCLRKEREEIQEIQSRENKRMQVLLTQVSTKRQQVISEFAHLRKFLEEQQSILLAQLESQDGDILRQRDEFDLLVAGEICRFSALIEELEEKNERPARELLTDIRSTLIRCETRKCRKPVAVSPELGQRIRDFPQQALPLQREMKMFLEKLCFELDYEPAHISLDPQTSHPKLLLSEDHQRAQFSYKWQNSPDNPQRFDRATCVLAHTGITGGRHTWVVSIDLAHGGSCTVGVVSEDVQRKGELRLRPEEGVWAVRLAWGFVSALGSFPTRLTLKEQPRQVRVSLDYEVGWVTFTNAVTREPIYTFTASFTRKVIPFFGLWGRGSSFSLSS

1392 NP_003008.1 P21399

>NP_003008.1

MHRDSCPLDCKVYVGNLGNNGNKTELERAFGYYGPLRSVWVARNPPGFAFVEFEDPRDAADAVRELDGRTLCGCRVRVELSNGEKRSRNRGPPPSWGRRPRDDYRRRSPPPRRRSPRRRSFSRSRSRSLSRDRRRERSLSRERNHKPSRSFSRSRSRSRSNERK

>P21399

MSNPFAHLAEPLDPVQPGKKFFNLNKLEDSRYGRLPFSIRVLLEAAIRNCDEFLVKKQDIENILHWNVTQHKNIEVPFKPARVILQDFTGVPAVVDFAAMRDAVKKLGGDPEKINPVCPADLVIDHSIQVDFNRRADSLQKNQDLEFERNRERFEFLKWGSQAFHNMRIIPPGSGIIHQVNLEYLARVVFDQDGYYYPDSLVGTDSHTTMIDGLGILGWGVGGIEAEAVMLGQPISMVLPQVIGYRLMGKPHPLVTSTDIVLTITKHLRQVGVVGKFVEFFGPGVAQLSIADRATIANMCPEYGATAAFFPVDEVSITYLVQTGRDEEKLKYIKKYLQAVGMFRDFNDPSQDPDFTQVVELDLKTVVPCCSGPKRPQDKVAVSDMKKDFESCLGAKQGFKGFQVAPEHHNDHKTFIYDNTEFTLAHGSVVIAAITSCTNTSNPSVMLGAGLLAKKAVDAGLNVMPYIKTSLSPGSGVVTYYLQESGVMPYLSQLGFDVVGYGCMTCIGNSGPLPEPVVEAITQGDLVAVGVLSGNRNFEGRVHPNTRANYLASPPLVIAYAIAGTIRIDFEKEPLGVNAKGQQVFLKDIWPTRDEIQAVERQYVIPGMFKEVYQKIETVNESWNALATPSDKLFFWNSKSTYIKSPPFFENLTLDLQPPKSIVDAYVLLNLGDSVTTDHISPAGNIARNSPAARYLTNRGLTPREFNSYGSRRGNDAVMARGTFANIRLLNRFLNKQAPQTIHLPSGEILDVFDAAERYQQAGLPLIVLAGKEYGAGSSRDWAAKGPFLLGIKAVLAESYERIHRSNLVGMGVIPLEYLPGENADALGLTGQERYTIIIPENLKPQMKVQVKLDTGKTFQAVMRFDTDVELTYFLNGGILNYMIRKMAK

1393 NP_036418.1 NP_005407.1

>NP_036418.1

MSSRKQGSQPRGQQSAEEENFKKPTRSNMQRSKMRGASSGKKTAGPQQKNLEPALPGRWGGRSAENPPSGSVRKTRKNKQKTPGNGDGGSTSEAPQPPRKKRARADPTVESEEAFKNRMEVKVKIPEELKPWLVEDWDLVTRQKQLFQLPAKKNVDAILEEYANCKKSQGNVDNKEYAVNEVVAGIKEYFNVMLGTQLLYKFERPQYAEILLAHPDAPMSQVYGAPHLLRLFVRIGAMLAYTPLDEKSLALLLGYLHDFLKYLAKNSASLFTASDYKVASAEYHRKAL

>NP_005407.1

MSSYQQKQTFTPPPQLQQQQVKQPSQPPPQEIFVPTTKEPCHSKVPQPGNTKIPEPGCTKVPEPGCTKVPEPGCTKVPEPGCTKVPEPGCTKVPEPGCTKVPEPGYTKVPEPGSIKVPDQGFIKFPEPGAIKVPEQGYTKVPVPGYTKLPEPCPSTVTPGPAQQKTKQK

1394 A0AVK6 Q99469

>A0AVK6

MENEKENLFCEPHKRGLMKTPLKESTTANIVLAEIQPDFGPLTTPTKPKEGSQGEPWTPTANLKMLISAVSPEIRNRDQKRGLFDNRSGLPEAKDCIHEHLSGDEFEKSQPSRKEKSLGLLCHKFLARYPNYPNPAVNNDICLDEVAEELNVERRRIYDIVNVLESLHMVSRLAKNRYTWHGRHNLNKTLGTLKSIGEENKYAEQIMMIKKKEYEQEFDFIKSYSIEDHIIKSNTGPNGHPDMCFVELPGVEFRAASVNSRKDKSLRVMSQKFVMLFLVSTPQIVSLEVAAKILIGEDHVEDLDKSKFKTKIRRLYDIANVLSSLDLIKKVHVTEERGRKPAFKWTGPEISPNTSGSSPVIHFTPSDLEVRRSSKENCAKNLFSTRGKPNFTRHPSLIKLVKSIESDRRKINSAPSSPIKTNKAESSQNSAPFPSKMAQLAAICKMQLEEQSSESRQKVKVQLARSGPCKPVAPLDPPVNAEMELTAPSLIQPLGMVPLIPSPLSSAVPLILPQAPSGPSYAIYLQPTQAHQSVTPPQGLSPTVCTTHSSKATGSKDSTDATTEKAANDTSKASASTRPGSLLPAPERQGAKSRTREPAGERGSKRASMLEDSGSKKKFKEDLKGLENVSATLFPSGYLIPLTQCSSLGAESILSGKENSSALSPNHRIYSSPIAGVIPVTSSELTAVNFPSFHVTPLKLMVSPTSVAAVPVGNSPALASSHPVPIQNPSSAIVNFTLQHLGLISPNVQLSASPGSGIVPVSPRIESVNVAPENAGTQQGRATNYDSPVPGQSQPNGQSVAVTGAQQPVPVTPKGSQLVAESFFRTPGGPTKPTSSSCMDFEGANKTSLGTLFVPQRKLEVSTEDVH

>Q99469

MIPPSSPREDGVDGLPKEAVGAEQPPSPASTSSQESKLQKLKRSLSFKTKSLRSKSADNFFQRTNSEDMKLQAHMVAEISPSSSPLPAPGSLTSTPARAGLHPGGKAHAFQEYIFKKPTFCDVCNHMIVGTNAKHGLRCKACKMSIHHKCTDGLAPQRCMGKLPKGFRRYYSSPLLIHEQFGCIKEVMPIACGNKVDPVYETLRFGTSLAQRTKKGSSGSGSDSPHRTSTSDLVEVPEEANGPGGGYDLRKRSNSVFTYPENGTDDFRDPAKNINHQGSLSKDPLQMNTYVALYKFVPQENEDLEMRPGDIITLLEDSNEDWWKGKIQDRIGFFPANFVQRLQQNEKIFRCVRTFIGCKEQGQITLKENQICVSSEEEQDGFIRVLSGKKKGLIPLDVLENI

1395 NP_116294.1 O75879

>NP_116294.1

MSLYDDLGVETSDSKTEGWSKNFKLLQSQLQVKKAALTQAKSQRTKQSTVLAPVIDLKRGGSSDDRQIVDTPPHVAAGLKDPVPSGFSAGEVLIPLADEYDPMFPNDYEKVVKRQREERQRQRELERQKEIEEREKRRKDRHEASGFARRPDPDSDEDEDYERERRKRSMGGAAIAPPTSLVEKDKELPRDFPYEEDSRPRSQSSKAAIPPPVYEEQDRPRSPTGPSNSFLANMGGTVAHKIMQKYGFREGQGLGKHEQGLSTALSVEKTSKRGGKIIVGDATEKDASKKSDSNPLTEILKCPTKVVLLRNMVGAGEVDEDLEVETKEECEKYGKVGKCVIFEIPGAPDDEAVRIFLEFERVESAIKAVVDLNGRYFGGRVVKACFYNLDKFRVLDLAEQV

>O75879

MAAPMLRWGCRGRRWAFARVDGGSCHRRGAPTGSTSNQIRGESSVAQQPLHTAQKTRKGEHKWAAVVGLEIHAQISSNSKLFSGSQVRFSAPPNSLVSFFDASLPGTLPVLNRRCVEAAVMTGLALNCHINKKSLFDRKHYFYADLPAGYQITQQRLPIAVNGSLIYGVCAGKKQSQVIPKTVRIKQIQLEQDSGKSLHDNLRSQTLIDLNRAGVGLLEVVLEPDMSCGEEAATAVRELQLILQALGTSQANMAEGQLRVDANISVHHPGEPLGVRTEVKNLNSIRFLAKAIDYEIQRQINELENGGEILNETRSFHHKLGCTMSMRDKEGKQDYRFMPEPNLPPLVLYDATSLPAGADPQQVINIDQIRETLPELPSVTREKLVQQYGMLLEHSFTLLNEVGLLEFFQNVIKETRAEPKKVTSWVLNTFLGYLKQQNLAVSESPVTPSALAELLDLLDSRTISSSAAKQVFEELWKREGKTPGQIVSEKQLELMQDQGALEQLCHSVMEAHPQVVMDVKNRNPRAINKLIGLVRKATQSRADPVMIKEILEKKLSL

1396 NP_002688.2 NP_005779.1

>NP_002688.2

MNNPSETSKPSMESGDGNTGTQTNGLDFQKQPVPVGGAISTAQAQAFLGHLHQVQLAGTSLQAAAQSLNVQSKSNEESGDSQQPSQPSQQPSVQAAIPQTQLMLAGGQITGLTLTPAQQQLLLQQAQAQAQLLAAAVQQHSASQQHSAAGATISASAATPMTQIPLSQPIQIAQDLQQLQQLQQQNLNLQQFVLVHPTTNLQPAQFIISQTPQGQQGLLQAQNLLTQLPQQSQANLLQSQPSITLTSQPATPTRTIAATPIQTLPQSQSTPKRIDTPSLEEPSDLEELEQFAKTFKQRRIKLGFTQGDVGLAMGKLYGNDFSQTTISRFEALNLSFKNMCKLKPLLEKWLNDAENLSSDSSLSSPSALNSPGIEGLSRRRKKRTSIETNIRVALEKSFLENQKPTSEEITMIADQLNMEKEVIRVWFCNRRQKEKRINPPSSGGTSSSPIKAIFPSPTSLVATTPSLVTSSAATTLTVSPVLPLTSAAVTNLSVTGTSDTTSNNTATVISTAPPASSAVTSPSLSPSPSASASTSEASSASETSTTQTTSTPLSSPLGTSQVMVTASGLQTAAAAALQGAAQLPANASLAAMAAAAGLNPSLMAPSQFAAGGALLSLNPGTLSGALSPALMSNSTLATIQALASGGSLPITSLDATGNLVFANAGGAPNIVTAPLFLNPQNLSLLTSNPVSLVSAAAASAGNSAPVASLHATSTSAESIQNSLFTVASASGAASTTTTASKAQ

>NP_005779.1

MCSLASGATGGRGAVENEEDLPELSDSGDEAAWEDEDDADLPHGKQQTPCLFCNRLFTSAEETFSHCKSEHQFNIDSMVHKHGLEFYGYIKLINFIRLKNPTVEYMNSIYNPVPWEKEEYLKPVLEDDLLLQFDVEDLYEPVSVPFSYPNGLSENTSVVEKLKHMEARALSAEAALARAREDLQKMKQFAQDFVMHTDVRTCSSSTSVIADLQEDEDGVYFSSYGHYGIHEEMLKDKIRTESYRDFIYQNPHIFKDKVVLDVGCGTGILSMFAAKAGAKKVLGVDQSEILYQAMDIIRLNKLEDTITLIKGKIEEVHLPVEKVDVIISEWMGYFLLFESMLDSVLYAKNKYLAKGGSVYPDICTISLVAVSDVNKHADRIAFWDDVYGFKMSCMKKAVIPEAVVEVLDPKTLISEPCGIKHIDCHTTSISDLEFSSDFTLKITRTSMCTAIAGYFDIYFEKNCHNRVVFSTGPQSTKTHWKQTVFLLEKPFSVKAGEALKGKVTVHKNKKDPRSLTVTLTLNNSTQTYGLQ

1397 Q9NV88 NP_066961.2

>Q9NV88

MKLYCLSGHPTLPCNVLKFKSTTIMLDCGLDMTSTLNFLPLPLVQSPRLSNLPGWSLKDGNAFLDKELKECSGHVFVDSVPEFCLPETELIDLSTVDVILISNYHCMMALPYITEHTGFTGTVYATEPTVQIGRLLMEELVNFIERVPKAQSASLWKNKDIQRLLPSPLKDAVEVSTWRRCYTMQEVNSALSKIQLVGYSQKIELFGAVQVTPLSSGYALGSSNWIIQSHYEKVSYVSGSSLLTTHPQPMDQASLKNSDVLVLTGLTQIPTANPDGMVGEFCSNLALTVRNGGNVLVPCYPSGVIYDLLECLYQYIDSAGLSSVPLYFISPVANSSLEFSQIFAEWLCHNKQSKVYLPEPPFPHAELIQTNKLKHYPSIHGDFSNDFRQPCVVFTGHPSLRFGDVVHFMELWGKSSLNTVIFTEPDFSYLEALAPYQPLAMKCIYCPIDTRLNFIQVSKLLKEVQPLHVVCPEQYTQPPPAQSHRMDLMIDCQPPAMSYRRAEVLALPFKRRYEKIEIMPELADSLVPMEIKPGISLATVSAVLHTKDNKHLLQPPPRPAQPTSGKKRKRVSDDVPDCKVLKPLLSGSIPVEQFVQTLEKHGFSDIKVEDTAKGHIVLLQEAETLIQIEEDSTHIICDNDEMLRVRLRDLVLKFLQKF

>NP_066961.2

MAAASVTPPGSLELLQPGFSKTLLGTKLEAKYLCSACRNVLRRPFQAQCGHRYCSFCLASILSSGPQNCAACVHEGIYEEGISILESSSAFPDNAARREVESLPAVCPSDGCTWKGTLKEYESCHEGRCPLMLTECPACKGLVRLGEKERHLEHECPERSLSCRHCRAPCCGADVKAHHEVCPKFPLTCDGCGKKKIPREKFQDHVKTCGKCRVPCRFHAIGCLETVEGEKQQEHEVQWLREHLAMLLSSVLEAKPLLGDQSHAGSELLQRCESLEKKTATFENIVCVLNREVERVAMTAEACSRQHRLDQDKIEALSSKVQQLERSIGLKDLAMADLEQKVLEMEASTYDGVFIWKISDFARKRQEAVAGRIPAIFSPAFYTSRYGYKMCLRIYLNGDGTGRGTHLSLFFVVMKGPNDALLRWPFNQKVTLMLLDQNNREHVIDAFRPDVTSSSFQRPVNDMNIASGCPLFCPVSKMEAKNSYVRDDAIFIKAIVDLTGL

1398 NP_006531.1 P43897

>NP_006531.1

MSGMGENTSDPSRAETRKRKECPDQLGPSPKRNTEKRNREQENKYIEELAELIFANFNDIDNFNFKPDKCAILKETVKQIRQIKEQEKAAAANIDEVQKSDVSSTGQGVIDKDALGPMMLEALDGFFFVVNLEGNVVFVSENVTQYLRYNQEELMNKSVYSILHVGDHTEFVKNLLPKSIVNGGSWSGEPPRRNSHTFNCRMLVKPLPDSEEEGHDNQEAHQKYETMQCFAVSQPKSIKEEGEDLQSCLICVARRVPMKERPVLPSSESFTTRQDLQGKITSLDTSTMRAAMKPGWEDLVRRCIQKFHAQHEGESVSYAKRHHHEVLRQGLAFSQIYRFSLSDGTLVAAQTKSKLIRSQTTNEPQLVISLHMLHREQNVCVMNPDLTGQTMGKPLNPISSNSPAHQALCSGNPGQDMTLSSNINFPINGPKEQMGMPMGRFGGSGGMNHVSGMQATTPQGSNYALKMNSPSQSSPGMNPGQPTSMLSPRHRMSPGVAGSPRIPPSQFSPAGSLHSPVGVCSSTGNSHSYTNSSLNALQALSEGHGVSLGSSLASPDLKMGNLQNSPVNMNPPPLSKMGSLDSKDCFGLYGEPSEGTTGQAESSCHPGEQKETNDPNLPPAVSSERADGQSRLHDSKGQTKLLQLLTTKSDQMEPSPLASSLSDTNKDSTGSLPGSGSTHGTSLKEKHKILHRLLQDSSSPVDLAKLTAEATGKDLSQESSSTAPGSEVTIKQEPVSPKKKENALLRYLLDKDDTKDIGLPEITPKLERLDSKTDPASNTKLIAMKTEKEEMSFEPGDQPGSELDNLEEILDDLQNSQLPQLFPDTRPGAPAGSVDKQAIINDLMQLTAENSPVTPVGAQKTALRISQSTFNNPRPGQLGRLLPNQNLPLDITLQSPTGAGPFPPIRNSSPYSVIPQPGMMGNQGMIGNQGNLGNSSTGMIGNSASRPTMPSGEWAPQSSAVRVTCAATTSAMNRPVQGGMIRNPAASIPMRPSSQPGQRQTLQSQVMNIGPSELEMNMGGPQYSQQQAPPNQTAPWPESILPIDQASFASQNRQPFGSSPDDLLCPHPAAESPSDEGALLDQLYLALRNFDGLEEIDRALGIPELVSQSQAVDPEQFSSQDSNIMLEQKAPVFPQQYASQAQMAQGSYSPMQDPNFHTMGQRPSYATLRMQPRPGLRPTGLVQNQPNQLRLQLQHRLQAQQNRQPLMNQISNVSNVNLTLRPGVPTQAPINAQMLAQRQREILNQHLRQRQMHQQQQVQQRTLMMRGQGLNMTPSMVAPSGMPATMSNPRIPQANAQQFPFPPNYGISQQPDPGFTGATTPQSPLMSPRMAHTQSPMMQQSQANPAYQAPSDINGWAQGNMGGNSMFSQQSPPHFGQQANTSMYSNNMNINVSMATNTGGMSSMNQMTGQISMTSVTSVPTSGLSSMGPEQVNDPALRGGNLFPNQLPGMDMIKQEGDTTRKYC

>P43897

MSLLRSLRVFLVARTGSYPAGSLLRQSPQPRHTFYAGPRLSASASSKELLMKLRRKTGYSFVNCKKALETCGGDLKQAEIWLHKEAQKEGWSKAAKLQGRKTKEGLIGLLQEGNTTVLVEVNCETDFVSRNLKFQLLVQQVALGTMMHCQTLKDQPSAYSKGFLNSSELSGLPAGPDREGSLKDQLALAIGKLGENMILKRAAWVKVPSGFYVGSYVHGAMQSPSLHKLVLGKYGALVICETSEQKTNLEDVGRRLGQHVVGMAPLSVGSLDDEPGGEAETKMLSQPYLLDPSITLGQYVQPQGVSVVDFVRFECGEGEEAAETE

1399 Q9UBR2 P49411

>Q9UBR2

MARRGPGWRPLLLLVLLAGAAQGGLYFRRGQTCYRPLRGDGLAPLGRSTYPRPHEYLSPADLPKSWDWRNVDGVNYASITRNQHIPQYCGSCWAHASTSAMADRINIKRKGAWPSTLLSVQNVIDCGNAGSCEGGNDLSVWDYAHQHGIPDETCNNYQAKDQECDKFNQCGTCNEFKECHAIRNYTLWRVGDYGSLSGREKMMAEIYANGPISCGIMATERLANYTGGIYAEYQDTTYINHVVSVAGWGISDGTEYWIVRNSWGEPWGERGWLRIVTSTYKDGKGARYNLAIEEHCTFGDPIV

>P49411

MAAATLLRATPHFSGLAAGRTFLLQGLLRLLKAPALPLLCRGLAVEAKKTYVRDKPHVNVGTIGHVDHGKTTLTAAITKILAEGGGAKFKKYEEIDNAPEERARGITINAAHVEYSTAARHYAHTDCPGHADYVKNMITGTAPLDGCILVVAANDGPMPQTREHLLLARQIGVEHVVVYVNKADAVQDSEMVELVELEIRELLTEFGYKGEETPVIVGSALCALEGRDPELGLKSVQKLLDAVDTYIPVPARDLEKPFLLPVEAVYSVPGRGTVVTGTLERGILKKGDECELLGHSKNIRTVVTGIEMFHKSLERAEAGDNLGALVRGLKREDLRRGLVMVKPGSIKPHQKVEAQVYILSKEEGGRHKPFVSHFMPVMFSLTWDMACRIILPPEKELAMPGEDLKFNLILRQPMILEKGQRFTLRDGNRTIGTGLVTNTLAMTEEEKNIKWG

1400 NP_690856.1 P14598

>NP_690856.1

MWIQVRTIDGSKTCTIEDVSRKATIEELRERVWALFDVRPECQRLFYRGKQLENGYTLFDYDVGLNDIIQLLVRPDPDHLPGTSTQIEAKPCSNSPPKVKKAPRVGPSNQPSTSARARLIDPGFGIYKVNELVDARDVGLGAWFEAHIHSVTRASDGQSRGKTPLKNGSSCKRTNGNIKHKSKENTNKLDSVPSTSNSDCVAADEDVIYHIQYDEYPESGTLEMNVKDLRPRARTILKWNELNVGDVVMVNYNVESPGQRGFWFDAEITTLKTISRTKKELRVKIFLGGSEGTLNDCKIISVDEIFKIERPGAHPLSFADGKFLRRNDPECDLCGGDPEKKCHSCSCRVCGGKHEPNMQLLCDECNVAYHIYCLNPPLDKVPEEEYWYCPSCKTDSSEVVKAGERLKMSKKKAKMPSASTESRRDWGRGMACVGRTRECTIVPSNHYGPIPGIPVGSTWRFRVQVSEAGVHRPHVGGIHGRSNDGAYSLVLAGGFADEVDRGDEFTYTGSGGKNLAGNKRIGAPSADQTLTNMNRALALNCDAPLDDKIGAESRNWRAGKPVRVIRSFKGRKISKYAPEEGNRYDGIYKVVKYWPEISSSHGFLVWRYLLRRDDVEPAPWTSEGIERSRRLCLRLQYPAGYPSDKEGKKPKGQSKKQPSGTTKRPISDDDCPSASKVYKASDSAEAIEAFQLTPQQQHLIREDCQNQKLWDEVLSHLVEGPNFLKKLEQSFMCVCCQELVYQPVTTECFHNVCKDCLQRSFKAQVFSCPACRHDLGQNYIMIPNEILQTLLDLFFPGYSKGR

>P14598

MGDTFIRHIALLGFEKRFVPSQHYVYMFLVKWQDLSEKVVYRRFTEIYEFHKTLKEMFPIEAGAINPENRIIPHLPAPKWFDGQRAAENRQGTLTEYCSTLMSLPTKISRCPHLLDFFKVRPDDLKLPTDNQTKKPETYLMPKDGKSTATDITGPIILQTYRAIADYEKTSGSEMALSTGDVVEVVEKSESGWWFCQMKAKRGWIPASFLEPLDSPDETEDPEPNYAGEPYVAIKAYTAVEGDEVSLLEGEAVEVIHKLLDGWWVIRKDDVTGYFPSMYLQKSGQDVSQAQRQIKRGAPPRRSSIRNAHSIHQRSRKRLSQDAYRRNSVRFLQQRRRQARPGPQSPGSPLEEERQTQRSKPQPAVPPRPSADLILNRCSESTKRKLASAV

1401 NP_003508.1 P82912

>NP_003508.1

MSGRGKQGGKARAKAKSRSSRAGLQFPVGRVHRLLRKGNYAERVGAGAPVYMAAVLEYLTAEILELAGNAARDNKKTRIIPRHLQLAIRNDEELNKLLGKVTIAQGGVLPNIQAVLLPKKTESHKAKSK

>P82912

MQAVRNAGSRFLRSWTWPQTAGRVVARTPAGTICTGARQLQDAAAKQKVEQNAAPSHTKFSIYPPIPGEESSLRWAGKKFEEIPIAHIKASHNNTQIQVVSASNEPLAFASCGTEGFRNAKKGTGIAAQTAGIAAAARAKQKGVIHIRVVVKGLGPGRLSAMHGLIMGGLEVISITDNTPIPHNGCRPRKARKL

1402 Q16385 P25398

>Q16385

MNGDDAFARRPTVGAQIPEKIQKAFDDIAKYFSKEEWEKMKASEKIFYVYMKRKYEAMTKLGFKATLPPFMCNKRAEDFQGNDLDNDPNRGNQVERPQMTFGRLQGISPKIMPKKPAEEGNDSEEVPEASGPQNDGKELCPPGKPTTSEKIHERSGPKRGEHAWTHRLRERKQLVIYEEISDPEEDDE

>P25398

MAEEGIAAGGVMDVNTALQEVLKTALIHDGLARGIREAAKALDKRQAHLCVLASNCDEPMYVKLVEALCAEHQINLIKVDDNKKLGEWVGLCKIDREGKPRKVVGCSCVVVKDYGKESQAKDVIEEYFKCKK

1403 NP_073207.1 P51116

>NP_073207.1

MTSSYGHVLERQPALGGRLDSPGNLDTLQAKKNFSVSHLLDLEEAGDMVAAQADENVGEAGRSLLESPGLTSGSDTPQQDNDQLNSEEKKKRKQRRNRTTFNSSQLQALERVFERTHYPDAFVREDLARRVNLTEARVQVWFQNRRAKFRRNERAMLANKNASLLKSYSGDVTAVEQPIVPRPAPRPTDYLSWGTASPYSAMATYSATCANNSPAQGINMANSIANLRLKAKEYSLQRNQVPTVN

>P51116

MGGLASGGDVEPGLPVEVRGSNGAFYKGFVKDVHEDSVTIFFENNWQSERQIPFGDVRLPPPADYNKEITEGDEVEVYSRANEQEPCGWWLARVRMMKGDFYVIEYAACDATYNEIVTLERLRPVNPNPLATKGSFFKVTMAVPEDLREACSNENVHKEFKKALGANCIFLNITNSELFILSTTEAPVKRASLLGDMHFRSLRTKLLLMSRNEEATKHLETSKQLAAAFQEEFTVREDLMGLAIGTHGANIQQARKVPGVTAIELGEETCTFRIYGETPEACRQARSYLEFSEDSVQVPRNLVGKVIGKNGKVIQEIVDKSGVVRVRVEGDNDKKNPREEGMVPFIFVGTRENISNAQALLEYHLSYLQEVEQLRLERLQIDEQLRQIGLGFRPPGSGRGSGGSDKAGYSTDESSSSSLHATRTYGGSYGGRGRGRRTGGPAYGPSSDVSTASETESEKREEPNRAGPGDRDPPTRGEESRRRPTGGRGRGPPPAPRPTSRYNSSSISSVLKDPDSNPYSLLDTSEPEPPVDSEPGEPPPASARRRRSRRRRTDEDRTVMDGGLESDGPNMTENGLEDESRPQRRNRSRRRRNRGNRTDGSISGDRQPVTVADYISRAESQSRQRPPLERTKPSEDSLSGQKGDSVSKLPKGPSENGELSAPLELGSMVNGVS

1404 NP_073149.1 Q9Y6G3

>NP_073149.1

MSSYFVNPLFSKYKAGESLEPAYYDCRFPQSVGRSHALVYGPGGSAPGFQHASHHVQDFFHHGTSGISNSGYQQNPCSLSCHGDASKFYGYEALPRQSLYGAQQEASVVQYPDCKSSANTNSSEGQGHLNQNSSPSLMFPWMRPHAPGRRSGRQTYSRYQTLELEKEFLFNPYLTRKRRIEVSHALGLTERQVKIWFQNRRMKWKKENNKDKLPGARDEEKVEEEGNEEEEKEEEEKEENKD

>Q9Y6G3

MAVAAVKWVMSKRTILKHLFPVQNGALYCVCHKSTYSPLPDDYNCNVELALTSDGRTIVCYHPSVDIPYEHTKPIPRPDPVHNNEETHDQVLKTRLEEKVEHLEEGPMIEQLSKMFFTTKHRWYPHGRYHRCRKNLNPPKDR

1405 Q8IZU0 P48728

>Q8IZU0

MAAWGKKHAGKDPVRDECEERNRFTETREEDVTDEHGEREPFAETDEHTGANTKKPEDTAEDLTAKRKRMKMDKTCSKTKNKSKHALRKKQLKRQKRDYIHSLKLLNVLEEYITDEQKEEEEEEGEEEELIRIFQEQQKKWQQYRSVRRERLKEMKLLRDQFVKALEDFEDLCDRVFSDEDSELDN

>P48728

MQRAVSVVARLGFRLQAFPPALCRPLSCAQEVLRRTPLYDFHLAHGGKMVAFAGWSLPVQYRDSHTDSHLHTRQHCSLFDVSHMLQTKILGSDRVKLMESLVVGDIAELRPNQGTLSLFTNEAGGILDDLIVTNTSEGHLYVVSNAGCWEKDLALMQDKVRELQNQGRDVGLEVLDNALLALQGPTAAQVLQAGVADDLRKLPFMTSAVMEVFGVSGCRVTRCGYTGEDGVEISVPVAGAVHLATAILKNPEVKLAGLAARDSLRLEAGLCLYGNDIDEHTTPVEGSLSWTLGKRRRAAMDFPGAKVIVPQLKGRVQRRRVGLMCEGAPMRAHSPILNMEGTKIGTVTSGCPSPSLKKNVAMGYVPCEYSRPGTMLLVEVRRKQQMAVVSKMPFVPTNYYTLK

1406 Q9BWW7 P50226

>Q9BWW7

MPRSFLVKKVKLDAFSSADLESAYGRARSDLGAPLHDKGYLSDYVGPSSVYDGDAEAALLKGPSPEPMYAAAVRGELGPAAAGSAPPPTPRPELATAAGGYINGDAAVSEGYAADAFFITDGRSRRKASNAGSAAAPSTASAAAPDGDAGGGGGAGGRSLGSGPGGRGGTRAGAGTEARAGPGAAGAGGRHACGECGKTYATSSNLSRHKQTHRSLDSQLARRCPTCGKVYVSMPAMAMHLLTHDLRHKCGVCGKAFSRPWLLQGHMRSHTGEKPFGCAHCGKAFADRSNLRAHMQTHSAFKHFQCKRCKKSFALKSYLNKHYESACFKGGAGGPAAPAPPQLSPVQA

>P50226

MELIQDTSRPPLEYVKGVPLIKYFAEALGPLQSFQARPDDLLISTYPKSGTTWVSQILDMIYQGGDLEKCHRAPIFMRVPFLEFKVPGIPSGMETLKNTPAPRLLKTHLPLALLPQTLLDQKVKVVYVARNAKDVAVSYYHFYHMAKVYPHPGTWESFLEKFMAGEVSYGSWYQHVQEWWELSRTHPVLYLFYEDMKENPKREIQKILEFVGRSLPEETVDLMVEHTSFKEMKKTPMTNYTTVRREFMDHSISPFMRKGMAGDWKTTFTVAQNERFDADYAEKMAGCSLSFRSEL

1407 NP_001955.1 NP_006825.1

>NP_001955.1

MAAAKAEMQLMSPLQISDPFGSFPHSPTMDNYPKLEEMMLLSNGAPQFLGAAGAPEGSGSNSSSSSSGGGGGGGGGSNSSSSSSTFNPQADTGEQPYEHLTAESFPDISLNNEKVLVETSYPSQTTRLPPITYTGRFSLEPAPNSGNTLWPEPLFSLVSGLVSMTNPPASSSSAPSPAASSASASQSPPLSCAVPSNDSSPIYSAAPTFPTPNTDIFPEPQSQAFPGSAGTALQYPPPAYPAAKGGFQVPMIPDYLFPQQQGDLGLGTPDQKPFQGLESRTQQPSLTPLSTIKAFATQSGSQDLKALNTSYQSQLIKPSRMRKYPNRPSKTPPHERPYACPVESCDRRFSRSDELTRHIRIHTGQKPFQCRICMRNFSRSDHLTTHIRTHTGEKPFACDICGRKFARSDERKRHTKIHLRQKDKKADKSVVASSATSSLSSYPSPVATSYPSPVTTSYPSPATTSYPSPVPTSFSSPGSSTYPSPVHSGFPSPSVATTYSSVPPAFPAQVSSFPSSAVTNSFSASTGLSDMTATFSPRTIEIC

>NP_006825.1

MAGGGAGDPGLGAAAAPAPETREHLFKVLVIGELGVGKTSIIKRYVHQLFSQHYRATIGVDFALKVLNWDSRTLVRLQLWDIAGQERFGNMTRVYYKEAVGAFVVFDISRSSTFEAVLKWKSDLDSKVHLPNGSPIPAVLLANKCDQNKDSSQSPSQVDQFCKEHGFAGWFETSAKDNINIEEAARFLVEKILVNHQSFPNEENDVDKIKLDQETLRAENKSQCC

1408 NP_006222.2 Q7Z7A3

>NP_006222.2

MSLRSGGRRRADPGADGEASRDDGATSSVSALKRLERSQWTDKMDLRFGFERLKEPGEKTGWLINMHPTEILDEDKRLGSAVDYYFIQDDGSRFKVALPYKPYFYIATRKGCEREVSSFLSKKFQGKIAKVETVPKEDLDLPNHLVGLKRNYIRLSFHTVEDLVKVRKEISPAVKKNREQDHASDAYTALLSSVLQRGGVITDEEETSKKIADQLDNIVDMREYDVPYHIRLSIDLKIHVAHWYNVRYRGNAFPVEITRRDDLVERPDPVVLAFDIETTKLPLKFPDAETDQIMMISYMIDGQGYLITNREIVSEDIEDFEFTPKPEYEGPFCVFNEPDEAHLIQRWFEHVQETKPTIMVTYNGDFFDWPFVEARAAVHGLSMQQEIGFQKDSQGEYKAPQCIHMDCLRWVKRDSYLPVGSHNLKAAAKAKLGYDPVELDPEDMCRMATEQPQTLATYSVSDAVATYYLYMKYVHPFIFALCTIIPMEPDEVLRKGSGTLCEALLMVQAFHANIIFPNKQEQEFNKLTDDGHVLDSETYVGGHVEALESGVFRSDIPCRFRMNPAAFDFLLQRVEKTLRHALEEEEKVPVEQVTNFEEVCDEIKSKLASLKDVPSRIECPLIYHLDVGAMYPNIILTNRLQPSAMVDEATCAACDFNKPGANCQRKMAWQWRGEFMPASRSEYHRIQHQLESEKFPPLFPEGPARAFHELSREEQAKYEKRRLADYCRKAYKKIHITKVEERLTTICQRENSFYVDTVRAFRDRRYEFKGLHKVWKKKLSAAVEVGDAAEVKRCKNMEVLYDSLQLAHKCILNSFYGYVMRKGARWYSMEMAGIVCFTGANIITQARELIEQIGRPLELDTDGIWCVLPNSFPENFVFKTTNVKKPKVTISYPGAMLNIMVKEGFTNDQYQELAEPSSLTYVTRSENSIFFEVDGPYLAMILPASKEEGKKLKKRYAVFNEDGSLAELKGFEVKRRGELQLIKIFQSSVFEAFLKGSTLEEVYGSVAKVADYWLDVLYSKAANMPDSELFELISENRSMSRKLEDYGEQKSTSISTAKRLAEFLGDQMVKDAGLSCRYIISRKPEGSPVTERAIPLAIFQAEPTVRKHFLRKWLKSSSLQDFDIRAILDWDYYIERLGSAIQKIITIPAALQQVKNPVPRVKHPDWLHKKLLEKNDVYKQKKISELFTLEGRRQVTMAEASEDSPRPSAPDMEDFGLVKLPHPAAPVTVKRKRVLWESQEESQDLTPTVPWQEILGQPPALGTSQEEWLVWLRFHKKKWQLQARQRLARRKRQRLESAEGVLRPGAIRDGPATGLGSFLRRTARSILDLPWQIVQISETSQAGLFRLWALVGSDLHCIRLSIPRVFYVNQRVAKAEEGASYRKVNRVLPRSNMVYNLYEYSVPEDMYQEHINEINAELSAPDIEGVYETQVPLLFRALVHLGCVCVVNKQLVRHLSGWEAETFALEHLEMRSLAQFSYLEPGSIRHIYLYHHAQAHKALFGIFIPSQRRASVFVLDTVRSNQMPSLGALYSAEHGLLLEKVGPELLPPPKHTFEVRAETDLKTICRAIQRFLLAYKEERRGPTLIAVQSSWELKRLASEIPVLEEFPLVPICVADKINYGVLDWQRHGARRMIRHYLNLDTCLSQAFEMSRYFHIPIGNLPEDISTFGSDLFFARHLQRHNHLLWLSPTARPDLGGKEADDNCLVMEFDDQATVEINSSGCYSTVCVELDLQNLAVNTILQSHHVNDMEGADSMGISFDVIQQASLEDMITGGQAASAPASYDETALCSNTFRILKSMVVGWVKEITQYHNIYADNQVMHFYRWLRSPSSLLHDPALHRTLHNMMKKLFLQLIAEFKRLGSSVIYANFNRIILCTKKRRVEDAIAYVEYITSSIHSKETFHSLTISFSRCWEFLLWMDPSNYGGIKGKVSSRIHCGLQDSQKAGGAEDEQENEDDEEERDGEEEEEAEESNVEDLLENNWNILQFLPQAASCQNYFLMIVSAYIVAVYHCMKDGLRRSAPGSTPVRRRGASQLSQEAEGAVGALPGMITFSQDYVANELTQSFFTITQKIQKKVTGSRNSTELSEMFPVLPGSHLLLNNPALEFIKYVCKVLSLDTNITNQVNKLNRDLLRLVDVGEFSEEAQFRDPCRSYVLPEVICRSCNFCRDLDLCKDSSFSEDGAVLPQWLCSNCQAPYDSSAIEMTLVEVLQKKLMAFTLQDLVCLKCRGVKETSMPVYCSCAGDFALTIHTQVFMEQIGIFRNIAQHYGMSYLLETLEWLLQKNPQLGH

>Q7Z7A3

MPAPPCASCHAARAALRRPLSGQALCGACFCAAFEAEVLHTVLAGRLLPPGAVVAVGASGGKDSTVLAHVLRALAPRLGISLQLVAVDEGIGGYRDAALAAVRRQAARWELPLTVVAYEDLFGGWTMDAVARSTAGSGRSRSCCTFCGVLRRRALEEGARRVGATHIVTGHNADDMAETVLMNFLRGDAGRLARGGGLGSPGEGGALPRCRPLQFASQKEVVLYAHFRRLDYFSEECVYAPEAFRGHARDLLKRLEAARPSAVLDLVHSAERLALAPAARPPRPGACSRCGALASRALCQACALLDGLNRGRPRLAIGKGRRGLDEEATPGTPGDPARPPASKAVPTF

1409 AAG28389.1 P07864

>AAG28389.1

MSTEGGFGGTSSSDAQQSLQSFWPRVMEEIRNLTVKDFRVQELPLARIKKIMKLDEDVKMISAEAPVLFAKAAQIFITELTLRAWIHTEDNKRRTLQRNDIAMAITKFDQFDFLIDIVPRDELKPPKRQEEVRQSVTPAEPVQYYFTLAQQPTAVQVQGQQQGQQTTSSTTTIQPGQIIIAQPQQGQTTPVTMQVGEGQQVQIVQAQPQGQAQQAQSGTGQTMQVMQQIITNTGEIQQIPVQLNAGQLQYIRLAQPVSGTQVVQGQIQTLATNAQQGQRNASQGKPRRCLKETLQITQTEVQQGQQQFSQFTDGQRNSVQQARVSELTGEAEPREVKATGNSTPCTSSLPTTHPPSHRAGASCVCCSQPQQSSTSPPPSDALQWVVVEVSGTPNQLETHRELHAPLPGMTSLSPLHPSQQLYQIQQVTMPAGQDLAQPMFIQSANQPSDGQAPQVTGD

>P07864

MSTVKEQLIEKLIEDDENSQCKITIVGTGAVGMACAISILLKDLADELALVDVALDKLKGEMMDLQHGSLFFSTSKITSGKDYSVSANSRIVIVTAGARQQEGETRLALVQRNVAIMKSIIPAIVHYSPDCKILVVSNPVDILTYIVWKISGLPVTRVIGSGCNLDSARFRYLIGEKLGVHPTSCHGWIIGEHGDSSVPLWSGVNVAGVALKTLDPKLGTDSDKEHWKNIHKQVIQSAYEIIKLKGYTSWAIGLSVMDLVGSILKNLRRVHPVSTMVKGLYGIKEELFLSIPCVLGRNGVSDVVKINLNSEEEALFKKSAETLWNIQKDLIF

1410 NP_005632.1 NP_055973.2

>NP_005632.1

MAEEKKLKLSNTVLPSESMKVVAESMGIAQIQEETCQLLTDEVSYRIKEIAQDALKFMHMGKRQKLTTSDIDYALKLKNVEPLYGFHAQEFIPFRFASGGGRELYFYEEKEVDLSDIINTPLPRVPLDVCLKAHWLSIEGCQPAIPENPPPAPKEQQKAEATEPLKSAKPGQEEDGPLKGKGQGATTADGKGKEKKAPPLLEGAPLRLKPRSIHELSVEQQLYYKEITEACVGSCEAKRAEALQSIATDPGLYQMLPRFSTFISEGVRVNVVQNNLALLIYLMRMVKALMDNPTLYLEKYVHELIPAVMTCIVSRQLCLRPDVDNHWALRDFAARLVAQICKHFSTTTNNIQSRITKTFTKSWVDEKTPWTTRYGSIAGLAELGHDVIKTLILPRLQQEGERIRSVLDGPVLSNIDRIGADHVQSLLLKHCAPVLAKLRPPPDNQDAYRAEFGSLGPLLCSQVVKARAQAALQAQQVNRTTLTITQPRPTLTLSQAPQPGPRTPGLLKVPGSIALPVQTLVSARAAAPPQPSPPPTKFIVMSSSSSAPSTQQVLSLSTSAPGSGSTTTSPVTTTVPSVQPIVKLVSTATTAPPSTAPSGPGSVQKYIVVSLPPTGEGKGGPTSHPSPVPPPASSPSPLSGSALCGGKQEAGDSPPPAPGTPKANGSQPNSGSPQPAP

>NP_055973.2

MAHTTKVNGSASGKAGDILSGDQDKEQKDPYFVETPYGYQLDLDFLKYVDDIQKGNTIKRLNIQKRRKPSVPCPEPRTTSGQQGIWTSTESLSSSNSDDNKQCPNFLIARSQVTSTPISKPPPPLETSLPFLTIPENRQLPPPSPQLPKHNLHVTKTLMETRRRLEQERATMQMTPGEFRRPRLASFGGMGTTSSLPSFVGSGNHNPAKHQLQNGYQGNGDYGSYAPAAPTTSSMGSSIRHSPLSSGISTPVTNVSPMHLQHIREQMAIALKRLKELEEQVRTIPVLQVKISVLQEEKRQLVSQLKNQRAASQINVCGVRKRSYSAGNASQLEQLSRARRSGGELYIDYEEEEMETVEQSTQRIKEFRQLTADMQALEQKIQDSSCEASSELRENGECRSVAVGAEENMNDIVVYHRGSRSCKDAAVGTLVEMRNCGVSVTEAMLGVMTEADKEIELQQQTIESLKEKIYRLEVQLRETTHDREMTKLKQELQAAGSRKKVDKATMAQPLVFSKVVEAVVQTRDQMVGSHMDLVDTCVGTSVETNSVGISCQPECKNKVVGPELPMNWWIVKERVEMHDRCAGRSVEMCDKSVSVEVSVCETGSNTEESVNDLTLLKTNLNLKEVRSIGCGDCSVDVTVCSPKECASRGVNTEAVSQVEAAVMAVPRTADQDTSTDLEQVHQFTNTETATLIESCTNTCLSTLDKQTSTQTVETRTVAVGEGRVKDINSSTKTRSIGVGTLLSGHSGFDRPSAVKTKESGVGQININDNYLVGLKMRTIACGPPQLTVGLTASRRSVGVGDDPVGESLENPQPQAPLGMMTGLDHYIERIQKLLAEQQTLLAENYSELAEAFGEPHSQMGSLNSQLISTLSSINSVMKSASTEELRNPDFQKTSLGKITGNYLGYTCKCGGLQSGSPLSSQTSQPEQEVGTSEGKPISSLDAFPTQEGTLSPVNLTDDQIAAGLYACTNNESTLKSIMKKKDGNKDSNGAKKNLQFVGINGGYETTSSDDSSSDESSSSESDDECDVIEYPLEEEEEEEDEDTRGMAEGHHAVNIEGLKSARVEDEMQVQECEPEKVEIRERYELSEKMLSACNLLKNTINDPKALTSKDMRFCLNTLQHEWFRVSSQKSAIPAMVGDYIAAFEAISPDVLRYVINLADGNGNTALHYSVSHSNFEIVKLLLDADVCNVDHQNKAGYTPIMLAALAAVEAEKDMRIVEELFGCGDVNAKASQAGQTALMLAVSHGRIDMVKGLLACGADVNIQDDEGSTALMCASEHGHVEIVKLLLAQPGCNGHLEDNDGSTALSIALEAGHKDIAVLLYAHVNFAKAQSPGTPRLGRKTSPGPTHRGSFD

1411 Q96T88 P51580

>Q96T88

MWIQVRTMDGRQTHTVDSLSRLTKVEELRRKIQELFHVEPGLQRLFYRGKQMEDGHTLFDYEVRLNDTIQLLVRQSLVLPHSTKERDSELSDTDSGCCLGQSESDKSSTHGEAAAETDSRPADEDMWDETELGLYKVNEYVDARDTNMGAWFEAQVVRVTRKAPSRDEPCSSTSRPALEEDVIYHVKYDDYPENGVVQMNSRDVRARARTIIKWQDLEVGQVVMLNYNPDNPKERGFWYDAEISRKRETRTARELYANVVLGDDSLNDCRIIFVDEVFKIERPGEGSPMVDNPMRRKSGPSCKHCKDDVNRLCRVCACHLCGGRQDPDKQLMCDECDMAFHIYCLDPPLSSVPSEDEWYCPECRNDASEVVLAGERLRESKKKAKMASATSSSQRDWGKGMACVGRTKECTIVPSNHYGPIPGIPVGTMWRFRVQVSESGVHRPHVAGIHGRSNDGAYSLVLAGGYEDDVDHGNFFTYTGSGGRDLSGNKRTAEQSCDQKLTNTNRALALNCFAPINDQEGAEAKDWRSGKPVRVVRNVKGGKNSKYAPAEGNRYDGIYKVVKYWPEKGKSGFLVWRYLLRRDDDEPGPWTKEGKDRIKKLGLTMQYPEGYLEALANREREKENSKREEEEQQEGGFASPRTGKGKWKRKSAGGGPSRAGSPRRTSKKTKVEPYSLTAQQSSLIREDKSNAKLWNEVLASLKDRPASGSPFQLFLSKVEETFQCICCQELVFRPITTVCQHNVCKDCLDRSFRAQVFSCPACRYDLGRSYAMQVNQPLQTVLNQLFPGYGNGR

>P51580

MDGTRTSLDIEEYSDTEVQKNQVLTLEEWQDKWVNGKTAFHQEQGHQLLKKHLDTFLKGKSGLRVFFPLCGKAVEMKWFADRGHSVVGVEISELGIQEFFTEQNLSYSEEPITEIPGTKVFKSSSGNISLYCCSIFDLPRTNIGKFDMIWDRGALVAINPGDRKCYADTMFSLLGKKFQYLLCVLSYDPTKHPGPPFYVPHAEIERLFGKICNIRCLEKVDAFEERHKSWGIDCLFEKLYLLTEK

1412 NP_001092755.1 NP_002826.2

>NP_001092755.1

MSDAAVDTSSEITTKDLKEKKEVVEEAENGRDAPANGNAENEENGEQEADNEVDEEEEEGGEEEEEEEEGDGEEEDGDEDEEAESATGKRAAEDDEDDDVDTKKQKTDEDD

>NP_002826.2

MEQVEILRKFIQRVQAMKSPDHNGEDNFARDFMRLRRLSTKYRTEKIYPTATGEKEENVKKNRYKDILPFDHSRVKLTLKTPSQDSDYINANFIKGVYGPKAYVATQGPLANTVIDFWRMIWEYNVVIIVMACREFEMGRKKCERYWPLYGEDPITFAPFKISCEDEQARTDYFIRTLLLEFQNESRRLYQFHYVNWPDHDVPSSFDSILDMISLMRKYQEHEDVPICIHCSAGCGRTGAICAIDYTWNLLKAGKIPEEFNVFNLIQEMRTQRHSAVQTKEQYELVHRAIAQLFEKQLQLYEIHGAQKIADGVNEINTENMISSIEPEKQDSPPPKPPRTRSCLVEGDAKEEILQPPEPHPVPPILTPSPPSAFPTVTTVWQDNDRYHPKPVLHMVSSEQHSADLNRNYSKSTELPGKNESTIEQIDKKLERNLSFEIKKVPLQEGPKSFDGNTLLNRGHAIKIKSASPCIADKISKPQELSSDLNVGDTSQNSCVDCSVTQSNKVSVTPPEESQNSDTPPRPDRLPLDEKGHVTWSFHGPENAIPIPDLSEGNSSDINYQTRKTVSLTPSPTTQVETPDLVDHDNTSPLFRTPLSFTNPLHSDDSDSDERNSDGAVTQNKTNISTASATVSAATSTESISTRKVLPMSIARHNIAGTTHSGAEKDVDVSEDSPPPLPERTPESFVLASEHNTPVRSEWSELQSQERSEQKKSEGLITSENEKCDHPAGGIHYEMCIECPPTFSDKREQISENPTEATDIGFGNRCGKPKGPRDPPSEWT

1413 Q14188 NP_056053.1

>Q14188

MTAKNVGLTSTNAEVRGFIDQNLSPTKGNISFVAFPVSNTNSPTKILPKTLGPINVNVGPQMIISTPQRLTSSGSVLIGSPYTPAPAMVTQTHIAEATGWVPGDRKRARKFIDSDFSESKRSKKGDKNGKGLRHFSMKVCEKVQRKGTTSYNEVADELVSEFTNSNNHLAADSAYDQKNIRRRVYDALNVLMAMNIISKEKKEIKWIGLPTNSAQECQNLEIEKQRRIERIKQKRAQLQELLLQQIAFKNLVQRNRQNEQQNQGPPALNSTIQLPFIIINTSRKTVIDCSISSDKFEYLFNFDNTFEIHDDIEVLKRMGMSFGLESGKCSLEDLKLAKSLVPKALEGYITDISTGPSWLNQGLLLNSTQSVSNLDLTTGATLPQSSVNQGLCLDAEVALATGQFLAPNSHQSSSAASHCSESRGETPCSFNDEDEEDDEEDSSSPE

>NP_056053.1

MPRPELPLPEGWEEARDFDGKVYYIDHTNRTTSWIDPRDRYTKPLTFADCISDELPLGWEEAYDPQVGDYFIDHNTKTTQIEDPRVQWRREQEHMLKDYLVVAQEALSAQKEIYQVKQQRLELAQQEYQQLHAVWEHKLGSQVSLVSGSSSSSKYDPEILKAEIATAKSRVNKLKREMVHLQHELQFKERGFQTLKKIDKKMSDAQGSYKLDEAQAVLRETKAIKKAITCGEKEKQDLIKSLAMLKDGFRTDRGSHSDLWSSSSSLESSSFPLPKQYLDVSSQTDISGSFGINSNNQLAEKVRLRLRYEEAKRRIANLKIQLAKLDSEAWPGVLDSERDRLILINEKEELLKEMRFISPRKWTQGEVEQLEMARKRLEKDLQAARDTQSKALTERLKLNSKRNQLVRELEEATRQVATLHSQLKSLSSSMQSLSSGSSPGSLTSSRGSLVASSLDSSTSASFTDLYYDPFEQLDSELQSKVEFLLLEGATGFRPSGCITTIHEDEVAKTQKAEGGGRLQALRSLSGTPKSMTSLSPRSSLSSPSPPCSPLMADPLLAGDAFLNSLEFEDPELSATLCELSLGNSAQERYRLEEPGTEGKQLGQAVNTAQGCGLKVACVSAAVSDESVAGDSGVYEASVQRLGASEAAAFDSDESEAVGATRIQIALKYDEKNKQFAILIIQLSNLSALLQQQDQKVNIRVAVLPCSESTTCLFRTRPLDASDTLVFNEVFWVSMSYPALHQKTLRVDVCTTDRSHLEECLGGAQISLAEVCRSGERSTRWYNLLSYKYLKKQSRELKPVGVMAPASGPASTDAVSALLEQTAVELEKRQEGRSSTQTLEDSWRYEETSENEAVAEEEEEEVEEEEGEEDVFTEKASPDMDGYPALKVDKETNTETPAPSPTVVRPKDRRVGTPSQGPFLRGSTIIRSKTFSPGPQSQYVCRLNRSDSDSSTLSKKPPFVRNSLERRSVRMKRPSSVKSLRSERLIRTSLDLELDLQATRTWHSQLTQEISVLKELKEQLEQAKSHGEKELPQWLREDERFRLLLRMLEKRQMDRAEHKGELQTDKMMRAAAKDVHRLRGQSCKEPPEVQSFREKMAFFTRPRMNIPALSADDV

1414 Q9HCU8 Q9H2W6

>Q9HCU8

MGRKRLITDSYPVVKRREGPAGHSKGELAPELGEEPQPRDEEEAELELLRQFDLAWQYGPCTGITRLQRWCRAKQMGLEPPPEVWQVLKTHPGDPRFQCSLWHLYPL

>Q9H2W6

MAAPVRRTLLGVAGGWRRFERLWAGSLSSRSLALAAAPSSNGSPWRLLGALCLQRPPVVSKPLTPLQEEMASLLQQIEIERSLYSDHELRALDENQRLAKKKADLHDEEDEQDILLAQDLEDMWEQKFLQFKLGARITEADEKNDRTSLNRKLDRNLVLLVREKFGDQDVWILPQAEWQPGETLRGTAERTLATLSENNMEAKFLGNAPCGHYTFKFPQAMRTESNLGAKVFFFKALLLTGDFSQAGNKGHHVWVTKDELGDYLKPKYLAQVRRFVSDL

1415 NP_620147.1 NP_065842.1

>NP_620147.1

MADMEDLFGSDADSEAERKDSDSGSDSDSDQENAASGSNASGSESDQDERGDSGQPSNKELFGDDSEDEGASHHSGSDNHSERSDNRSEASERSDHEDNDPSDVDQHSGSEAPNDDEDEGHRSDGGSHHSEAEGSEKAHSDDEKWGREDKSDQSDDEKIQNSDDEERAQGSDEDKLQNSDDDEKMQNTDDEERPQLSDDERQQLSEEEKANSDDERPVASDNDDEKQNSDDEEQPQLSDEEKMQNSDDERPQASDEEHRHSDDEEEQDHKSESARGSDSEDEVLRMKRKNAIASDSEADSDTEVPKDNSGTMDLFGGADDISSGSDGEDKPPTPGQPVDENGLPQDQQEEEPIPETRIEVEIPKVNTDLGNDLYFVKLPNFLSVEPRPFDPQYYEDEFEDEEMLDEEGRTRLKLKVENTIRWRIRRDEEGNEIKESNARIVKWSDGSMSLHLGNEVFDVYKAPLQGDHNHLFIRQGTGLQGQAVFKTKLTFRPHSTDSATHRKMTLSLADRCSKTQKIRILPMAGRDPECQRTEMIKKEEERLRASIRRESQQRRMREKQHQRGLSASYLEPDRYDEEEEGEESISLAAIKNRYKGGIREERARIYSSDSDEGSEEDKAQRLLKAKKLTSDEEGEPSGKRKAEDDDKANKKHKKYVISDEEEEDDD

>NP_065842.1

MPSTNRAGSLKDPEIAELFFKEDPEKLFTDLREIGHGSFGAVYFARDVRTNEVVAIKKMSYSGKQSTEKWQDIIKEVKFLQRIKHPNSIEYKGCYLREHTAWLVMEYCLGSASDLLEVHKKPLQEVEIAAITHGALQGLAYLHSHTMIHRDIKAGNILLTEPGQVKLADFGSASMASPANSFVGTPYWMAPEVILAMDEGQYDGKVDVWSLGITCIELAERKPPLFNMNAMSALYHIAQNESPTLQSNEWSDYFRNFVDSCLQKIPQDRPTSEELLKHIFVLRERPETVLIDLIQRTKDAVRELDNLQYRKMKKLLFQEAHNGPAVEAQEEEEEQDHGVGRTGTVNSVGSNQSIPSMSISASSQSSSVNSLPDVSDDKSELDMMEGDHTVMSNSSVIHLKPEEENYREEGDPRTRASDPQSPPQVSRHKSHYRNREHFATIRTASLVTRQMQEHEQDSELREQMSGYKRMRRQHQKQLMTLENKLKAEMDEHRLRLDKDLETQRNNFAAEMEKLIKKHQAAMEKEAKVMSNEEKKFQQHIQAQQKKELNSFLESQKREYKLRKEQLKEELNENQSTPKKEKQEWLSKQKENIQHFQAEEEANLLRRQRQYLELECRRFKRRMLLGRHNLEQDLVREELNKRQTQKDLEHAMLLRQHESMQELEFRHLNTIQKMRCELIRLQHQTELTNQLEYNKRRERELRRKHVMEVRQQPKSLKSKELQIKKQFQDTCKIQTRQYKALRNHLLETTPKSEHKAVLKRLKEEQTRKLAILAEQYDHSINEMLSTQALRLDEAQEAECQVLKMQLQQELELLNAYQSKIKMQAEAQHDRELRELEQRVSLRRALLEQKIEEEMLALQNERTERIRSLLERQAREIEAFDSESMRLGFSNMVLSNLSPEAFSHSYPGASGWSHNPTGGPGPHWGHPMGGPPQAWGHPMQGGPQPWGHPSGPMQGVPRGSSMGVRNSPQALRRTASGGRTEQGMSRSTSVTSQISNGSHMSYT

1416 O60231 Q9H2W6

>O60231

MATPAGLERWVQDELHSVLGLSERHVAQFLIGTAQRCTSAEEFVQRLRDTDTLDLSGPARDFALRLWNKVPRKAVVEKPARAAEREARALLEKNRSYRLLEDSEESSEETVSRAGSSLQKKRKKRKHLRKKREEEEEEEASEKGKKKTGGSKQQTEKPESEDEWERTERERLQDLEERDAFAERVRQRDKDRTRNVLERSDKKAYEEAQKRLKMAEEDRKAMVPELRKKSRREYLAKREREKLEDLEAELADEEFLFGDVELSRHERQELKYKRRVRDLAREYRAAGEQEKLEATNRYHMPKETRGQPARAVDLVEEESGAPGEEQRRWEEARLGAASLKFGARDAASQEPKYQLVLEEEETIEFVRATQLQGDEEPSAPPTSTQAQQKESIQAVRRSLPVFPFREELLAAIANHQVLIIEGETGSGKTTQIPQYLFEEGYTNKGMKIACTQPRRVAAMSVAARVAREMGVKLGNEVGYSIRFEDCTSERTVLRYMTDGMLLREFLSEPDLASYSVVMVDEAHERTLHTDILFGLIKDVARFRPELKVLVASATMDTARFSTFFDDAPVFRIPGRRFPVDIFYTKAPEADYLEACVVSVLQIHVTQPPGDILVFLTGQEEIEAACEMLQDRCRRLGSKIRELLVLPIYANLPSDMQARIFQPTPPGARKVVVATNIAETSLTIEGIIYVLDPGFCKQKSYNPRTGMESLTVTPCSKASANQRAGRAGRVAAGKCFRLYTAWAYQHELEETTVPEIQRTSLGNVVLLLKSLGIHDLMHFDFLDPPPYETLLLALEQLYALGALNHLGELTTSGRKMAELPVDPMLSKMILASEKYSCSEEILTVAAMLSVNNSIFYRPKDKVVHADNARVNFFLPGGDHLVLLNVYTQWAESGYSSQWCYENFVQFRSMRRARDVREQLEGLLERVEVGLSSCQGDYIRVRKAITAGYFYHTARLTRSGYRTVKQQQTVFIHPNSSLFEQQPRWLLYHELVLTTKEFMRQVLEIESSWLLEVAPHYYKAKELEDPHAKKMPKKIGKTREELG

>Q9H2W6

MAAPVRRTLLGVAGGWRRFERLWAGSLSSRSLALAAAPSSNGSPWRLLGALCLQRPPVVSKPLTPLQEEMASLLQQIEIERSLYSDHELRALDENQRLAKKKADLHDEEDEQDILLAQDLEDMWEQKFLQFKLGARITEADEKNDRTSLNRKLDRNLVLLVREKFGDQDVWILPQAEWQPGETLRGTAERTLATLSENNMEAKFLGNAPCGHYTFKFPQAMRTESNLGAKVFFFKALLLTGDFSQAGNKGHHVWVTKDELGDYLKPKYLAQVRRFVSDL

1417 NP_001537.1 NP_056000.1

>NP_001537.1

MKAVSPVRPSGRKAPSGCGGGELALRCLAEHGHSLGGSAAAAAAAAAARCKAAEAAADEPALCLQCDMNDCYSRLRRLVPTIPPNKKVSKVEILQHVIDYILDLQLALETHPALLRQPPPPAPPHHPAGTCPAAPPRTPLTALNTDPAGAVNKQGDSILCR

>NP_056000.1

MTLLITGDSIVSAEAVWDHVTMANRELAFKAGDVIKVLDASNKDWWWGQIDDEEGWFPASFVRLWVNQEDEVEEGPSDVQNGHLDPNSDCLCLGRPLQNRDQMRANVINEIMSTERHYIKHLKDICEGYLKQCRKRRDMFSDEQLKVIFGNIEDIYRFQMGFVRDLEKQYNNDDPHLSEIGPCFLEHQDGFWIYSEYCNNHLDACMELSKLMKDSRYQHFFEACRLLQQMIDIAIDGFLLTPVQKICKYPLQLAELLKYTAQDHSDYRYVAAALAVMRNVTQQINERKRRLENIDKIAQWQASVLDWEGEDILDRSSELIYTGEMAWIYQPYGRNQQRVFFLFDHQMVLCKKDLIRRDILYYKGRIDMDKYEVVDIEDGRDDDFNVSMKNAFKLHNKETEEIHLFFAKKLEEKIRWLRAFREERKMVQEDEKIGFEISENQKRQAAMTVRKVPKQKGVNSARSVPPSYPPPQDPLNHGQYLVPDGIAQSQVFEFTEPKRSQSPFWQNFSRLTPFKK

1418 NP_849194.1 O75251

>NP_849194.1

MSAEASGPAAAAAPSLEAPKPSGLEPGPAAYGLKPLTPNSKYVKLNVGGSLHYTTLRTLTGQDTMLKAMFSGRVEVLTDAGGWVLIDRSGRHFGTILNYLRDGSVPLPESTRELGELLGEARYYLVQGLIEDCQLALQQKRETLSPLCLIPMVTSPREEQQLLASTSKPVVKLLHNRSNNKYSYTSTSDDNLLKNIELFDKLALRFHGRLLFLKDVLGDEICCWSFYGQGRKIAEVCCTSIVYATEKKQTKVEFPEARIFEETLNILIYETPRGPDPALLEATGGAAGAGGAGRGEDEENREHRVRRIHVRRHITHDERPHGQQIVFKD

>O75251

MAVLSAPGLRGFRILGLRSSVGPAVQARGVHQSVATDGPSSTQPALPKARAVAPKPSSRGEYVVAKLDDLVNWARRSSLWPMTFGLACCAVEMMHMAAPRYDMDRFGVVFRASPRQSDVMIVAGTLTNKMAPALRKVYDQMPEPRYVVSMGSCANGGGYYHYSYSVVRGCDRIVPVDIYIPGCPPTAEALLYGILQLQRKIKRERRLQIWYRR

1419 NP_004588.1 NP_061821.1

>NP_004588.1

MSLLNKPKSEMTPEELQKREEEEFNTGPLSVLTQSVKNNTQVLINCRNNKKLLGRVKAFDRHCNMVLENVKEMWTEVPKSGKGKKKSKPVNKDRYISKMFLRGDSVIVVLRNPLIAGK

>NP_061821.1

MSIAGVAAQEIRVPLKTGFLHNGRAMGNMRKTYWSSRSEFKNNFLNIDPITMAYSLNSSAQERLIPLGHASKSAPMNGHCFAENGPSQKSSLPPLLIPPSENLGPHEEDQVVCGFKKLTVNGVCASTPPLTPIKNSPSLFPCAPLCERGSRPLPPLPISEALSLDDTDCEVEFLTSSDTDFLLEDSTLSDFKYDVPGRRSFRGCGQINYAYFDTPAVSAADLSYVSDQNGGVPDPNPPPPQTHRRLRRSHSGPAGSFNKPAIRISNCCIHRASPNSDEDKPEVPPRVPIPPRPVKPDYRRWSAEVTSSTYSDEDRPPKVPPREPLSPSNSRTPSPKSLPSYLNGVMPPTQSFAPDPKYVSSKALQRQNSEGSASKVPCILPIIENGKKVSSTHYYLLPERPPYLDKYEKFFREAEETNGGAQIQPLPADCGISSATEKPDSKTKMDLGGHVKRKHLSYVVSP

1420 NP_057219.1 NP_005470.2

>NP_057219.1

MQEGELAISPISPVAAMPPLGTHVQARCEAQINLLGEGGICKLPGRLRIQPALWSREDVLHWLRWAEQEYSLPCTAEHGFEMNGRALCILTKDDFRHRAPSSGDVLYELLQYIKTQRRALVCGPFFGGIFRLKTPTQHSPVPPEEVTGPSQMDTRRGHLLQPPDPGLTSNFGHLDDPGLARWTPGKEESLNLCHCAELGCRTQGVCSFPAMPQAPIDGRIADCRLLWDYVYQLLLDTRYEPYIKWEDKDAKIFRVVDPNGLARLWGNHKNRVNMTYEKMSRALRHYYKLNIIKKEPGQKLLFRFLKTPGKMVQDKHSHLEPLESQEQDRIEFKDKRPEISP

>NP_005470.2

MPCRREEEEEAGEEAEGEEEEEDSFLLLQQSVALGSSGEVDRLVAQIGETLQLDAAQHSPASPCGPPGAPLRAPGPLAAAVPADKARSPAVPLLLPPALAETVGPAPPGVLRCALGDRGRVRGRAAPYCVAELATGPSALSPLPPQADLDGPPGAGKQGIPQPLSGPCRRGWLRGAAASRRLQQRRGSQPETRTGDDDPHRLLQQLVLSGNLIKEAVRRLHSRRLQLRAKLPQRPLLGPLSAPVHEPPSPRSPRAACSDPGASGRAQLRTGDGVLVPGS

1421 NP_054790.2 NP_004332.2

>NP_054790.2

MVLDDLPNLEDIYTSLCSSTMEDSEMDFDSGLEDDDTKSDSILEDSTIFVAFKGNIDDKDFKWKLDAILKNVPNLLHMESSKLKVQKVEPWNSVRVTFNIPREAAERLRILAQSNNQQLRDLGILSVQIEGEGAINLALAQNRSQDVRMNGPMGAGNSVRMEAGFPMASGPGIIRMNNPATVMIPPGGNVSSSMMAPGPNPELQPRTPRPASQSDAMDPLLSGLHIQQQSHPSGSLAPPHHPMQPVSVNRQMNPANFPQLQQQQQQQQQQQQQQQQQQQQQQQQQLQARPPQQHQQQQPQGIRPQFTAPTQVPVPPGWNQLPSGALQPPPAQGSLGTMTANQGWKKAPLPGPMQQQLQARPSLATVQTPSHPPPPYPFGSQQASQAHTNFPQMSNPGQFTAPQMKSLQGGPSRVPTPLQQPHLTNKSPASSPSSFQQGSPASSPTVNQTQQQMGPRPPQNNPLPQGFQQPVSSPGRNPMVQQGNVPPNFMVMQQQPPNQGPQSLHPGLGGMPKRLPPGFSAGQANPNFMQGQVPSTTATTPGNSGAPQLQANQNVQHAGGQGAGPPQNQMQVSHGPPNMMQPSLMGIHGNMNNQQAGTSGVPQVNLSNMQGQPQQGPPSQLMGMHQQIVPSQGQMVQQQGTLNPQNPMILSRAQLMPQGQMMVNPPSQNLGPSPQRMTPPKQMLSQQGPQMMAPHNQMMGPQGQVLLQQNPMIEQIMTNQMQGNKQQFNTQNQSNVMPGPAQIMRGPTPNMQGNMVQFTGQMSGQMLPQQGPVNNSPSQVMGIQGQVLRPPGPSPHMAQQHGDPATTANNDVSLSQMMPDVSIQQTNMVPPHVQAMQGNSASGNHFSGHGMSFNAPFSGAPNGNQMSCGQNPGFPVNKDVTLTSPLLVNLLQSDISAGHFGVNNKQNNTNANKPKKKKPPRKKKNSQQDLNTPDTRPAGLEEADQPPLPGEQGINLDNSGPKLPEFSNRPPGYPSQPVEQRPLQQMPPQLMQHVAPPPQPPQQQPQPQLPQQQQPPPPSQPQSQQQQQQQQQMMMMLMMQQDPKSVRLPVSQNVHPPRGPLNPDSQRMPMQQSGSVPVMVSLQGPASVPPSPDKQRMPMPVNTPLGSNSRKMVYQESPQNPSSSPLAEMASLPEASGSEAPSVPGGPNNMPSHVVLPQNQLMMTGPKPGPSPLSATQGATPQQPPVNSLPSSHGHHFPNVAAPTQTSRPKTPNRASPRPYYPQTPNNRPPSTEPSEISLSPERLNASIAGLFPPQINIPLPPRPNLNRGFDQQGLNPTTLKAIGQAPSNLTMNPSNFATPQTHKLDSVVVNSGKQSNSGATKRASPSNSRRSSPGSSRKTTPSPGRQNSKAPKLTLASQTNAALLQNVELPRNVLVSPTPLANPPVPGSFPNNSGLNPQNSTVSVAAVGGVVEDNKESLNVPQDSDCQNSQSRKEQVNIELKAVPAQEVKMVVPEDQSKKDGQPSDPNKLPSVEENKNLVSPAMREAPTSLSQLLDNSGAPNVTIKPPGLTDLEVTPPVVSGEDLKKASVIPTLQDLSSSKEPSNSLNLPHSNELCSSLVHPELSEVSSNVAPSIPPVMSRPVSSSSISTPLPPNQITVFVTSNPITTSANTSAALPTHLQSALMSTVVTMPNAGSKVMVSEGQSAAQSNARPQFITPVFINSSSIIQVMKGSQPSTIPAAPLTTNSGLMPPSVAVVGPLHIPQNIKFSSAPVPPNALSSSPAPNIQTGRPLVLSSRATPVQLPSPPCTSSPVVPSHPPVQQVKELNPDEASPQVNTSADQNTLPSSQSTTMVSPLLTNSPGSSGNRRSPVSSSKGKGKVDKIGQILLTKACKKVTGSLEKGEEQYGADGETEGQGLDTTAPGLMGTEQLSTELDSKTPTPPAPTLLKMTSSPVGPGTASAGPSLPGGALPTSVRSIVTTLVPSELISAVPTTKSNHGGIASESLAGGLVEEKVGSHPELLPSIAPSQNLVSKETSTTALQASVARPELEVNAAIVSGQSSEPKEIVEKSKIPGRRNSRTEEPTVASESVENGHRKRSSRPASASSSTKDITSAVQSKRRKSK

>NP_004332.2

MAALVLEDGSVLRGQPFGAAVSTAGEVVFQTGMVGYPEALTDPSYKAQILVLTYPLIGNYGIPPDEMDEFGLCKWFESSGIHVAALVVGECCPTPSHWSATRTLHEWLQQHGIPGLQGVDTRELTKKLREQGSLLGKLVQNGTEPSSLPFLDPNARPLVPEVSIKTPRVFNTGGAPRILALDCGLKYNQIRCLCQRGAEVTVVPWDHALDSQEYEGLFLSNGPGDPASYPSVVSTLSRVLSEPNPRPVFGICLGHQLLALAIGAKTYKMRYGNRGHNQPCLLVGSGRCFLTSQNHGFAVETDSLPADWAPLFTNANDGSNEGIVHNSLPFFSVQFHPEHQAGPSDMELLFDIFLETVKEATAGNPGGQTVRERLTERLCPPGIPTPGSGLPPPRKVLILGSGGLSIGQAGEFDYSGSQAIKALKEENIQTLLINPNIATVQTSQGLADKVYFLPITPHYVTQVIRNERPDGVLLTFGGQTALNCGVELTKAGVLARYGVRVLGTPVETIELTEDRRAFAARMAEIGEHVAPSEAANSLEQAQAAAERLGYPVLVRAAFALGGLGSGFASNREELSALVAPAFAHTSQVLVDKSLKGWKEIEYEVVRDAYGNCVTVCNMENLDPLGIHTGESIVVAPSQTLNDREYQLLRQTAIKVTQHLGIVGECNVQYALNPESEQYYIIEVNARLSRSSALASKATGYPLAYVAAKLALGIPLPELRNSVTGGTAAFEPSVDYCVVKIPRWDLSKFLRVSTKIGSCMKSVGEVMGIGRSFEEAFQKALRMVDENCVGFDHTVKPVSDMELETPTDKRIFVVAAALWAGYSVDRLYELTRIDRWFLHRMKRIIAHAQLLEQHRGQPLPPDLLQQAKCLGFSDKQIALAVLSTELAVRKLRQELGICPAVKQIDTVAAEWPAQTNYLYLTYWGTTHDLTFRTPHVLVLGSGVYRIGSSVEFDWCAVGCIQQLRKMGYKTIMVNYNPETVSTDYDMCDRLYFDEISFEVVMDIYELENPEGVILSMGGQLPNNMAMALHRQQCRVLGTSPEAIDSAENRFKFSRLLDTIGISQPQWRELSDLESARQFCQTVGYPCVVRPSYVLSGAAMNVAYTDGDLERFLSSAAAVSKEHPVVISKFIQEAKEIDVDAVASDGVVAAIAISEHVENAGVHSGDATLVTPPQDITAKTLERIKAIVHAVGQELQVTGPFNLQLIAKDDQLKVIECNVRVSRSFPFVSKTLGVDLVALATRVIMGEEVEPVGLMTGSGVVGVKVPQFSFSRLAGADVVLGVEMTSTGEVAGFGESRCEAYLKAMLSTGFKIPKKNILLTIGSYKNKSELLPTVRLLESLGYSLYASLGTADFYTEHGVKVTAVDWHFEEAVDGECPPQRSILEQLAEKNFELVINLSMRGAGGRRLSSFVTKGYRTRRLAADFSVPLIIDIKCTKLFVEALGQIGPAPPLKVHVDCMTSQKLVRLPGLIDVHVHLREPGGTHKEDFASGTAAALAGGITMVCAMPNTRPPIIDAPALALAQKLAEAGARCDFALFLGASSENAGTLGTVAGSAAGLKLYLNETFSELRLDSVVQWMEHFETWPSHLPIVAHAEQQTVAAVLMVAQLTQRSVHICHVARKEEILLIKAAKARGLPVTCEVAPHHLFLSHDDLERLGPGKGEVRPELGSRQDVEALWENMAVIDCFASDHAPHTLEEKCGSRPPPGFPGLETMLPLLLTAVSEGRLSLDDLLQRLHHNPRRIFHLPPQEDTYVEVDLEHEWTIPSHMPFSKAHWTPFEGQKVKGTVRRVVLRGEVAYIDGQVLVPPGYGQDVRKWPQGAVPQLPPSAPATSEMTTTPERPRRGIPGLPDGRFHLPPRIHRASDPGLPAEEPKEKSSRKVAEPELMGTPDGTCYPPPPVPRQASPQNLGTPGLLHPQTSPLLHSLVGQHILSVQQFTKDQMSHLFNVAHTLRMMVQKERSLDILKGKVMASMFYEVSTRTSSSFAAAMARLGGAVLSFSEATSSVQKGESLADSVQTMSCYADVVVLRHPQPGAVELAAKHCRRPVINAGDGVGEHPTQALLDIFTIREELGTVNGMTITMVGDLKHGRTVHSLACLLTQYRVSLRYVAPPSLRMPPTVRAFVASRGTKQEEFESIEEALPDTDVLYMTRIQKERFGSTQEYEACFGQFILTPHIMTRAKKKMVVMHPMPRVNEISVEVDSDPRAAYFRQAENGMYIRMALLATVLGRF

1422 NP_892017.2 NP_071761.1

>NP_892017.2

MAAAVAVAAASRRQSCYLCDLPRMPWAMIWDFTEPVCRGCVNYEGADRVEFVIETARQLKRAHGCFPEGRSPPGAAASAAAKPPPLSAKDILLQQQQQLGHGGPEAAPRAPQALERYPLAAAAERPPRLGSDFGSSRPAASLAQPPTPQPPPVNGILVPNGFSKLEEPPELNRQSPNPRRGHAVPPTLVPLMNGSATPLPTALGLGGRAAASLAAVSGTAAASLGSAQPTDLGAHKRPASVSSSAAVEHEQREAAAKEKQPPPPAHRGPADSLSTAAGAAELSAEGAGKSRGSGEQDWVNRPKTVRDTLLALHQHGHSGPFESKFKKEPALTAGRLLGFEANGANGSKAVARTARKRKPSPEPEGEVGPPKINGEAQPWLSTSTEGLKIPMTPTSSFVSPPPPTASPHSNRTTPPEAAQNGQSPMAALILVADNAGGSHASKDANQVHSTTRRNSNSPPSPSSMNQRRLGPREVGGQGAGNTGGLEPVHPASLPDSSLATSAPLCCTLCHERLEDTHFVQCPSVPSHKFCFPCSRQSIKQQGASGEVYCPSGEKCPLVGSNVPWAFMQGEIATILAGDVKVKKERDS

>NP_071761.1

MWIPVVGLPRRLRLSALAGAGRFCILGSEAATRKHLPARNHCGLSDSSPQLWPEPDFRNPPRKASKASLDFKRYVTDRRLAETLAQIYLGKPSRPPHLLLECNPGPGILTQALLEAGAKVVALESDKTFIPHLESLGKNLDGKLRVIHCDFFKLDPRSGGVIKPPAMSSRGLFKNLGIEAVPWTADIPLKVVGMFPSRGEKRALWKLAYDLYSCTSIYKFGRIEVNMFIGEKEFQKLMADPGNPDLYHVLSVIWQLACEIKVLHMEPWSSFDIYTRKGPLENPKRRELLDQLQQKLYLIQMIPRQNLFTKNLTPMNYNIFFHLLKHCFGRRSATVIDHLRSLTPLDARDILMQIGKQEDEKVVNMHPQDFKTLFETIERSKDCAYKWLYDETLEDR

1423 O60341 Q9BSE5

>O60341

MLSGKKAAAAAAAAAAAATGTEAGPGTAGGSENGSEVAAQPAGLSGPAEVGPGAVGERTPRKKEPPRASPPGGLAEPPGSAGPQAGPTVVPGSATPMETGIAETPEGRRTSRRKRAKVEYREMDESLANLSEDEYYSEEERNAKAEKEKKLPPPPPQAPPEEENESEPEEPSGVEGAAFQSRLPHDRMTSQEAACFPDIISGPQQTQKVFLFIRNRTLQLWLDNPKIQLTFEATLQQLEAPYNSDTVLVHRVHSYLERHGLINFGIYKRIKPLPTKKTGKVIIIGSGVSGLAAARQLQSFGMDVTLLEARDRVGGRVATFRKGNYVADLGAMVVTGLGGNPMAVVSKQVNMELAKIKQKCPLYEANGQAVPKEKDEMVEQEFNRLLEATSYLSHQLDFNVLNNKPVSLGQALEVVIQLQEKHVKDEQIEHWKKIVKTQEELKELLNKMVNLKEKIKELHQQYKEASEVKPPRDITAEFLVKSKHRDLTALCKEYDELAETQGKLEEKLQELEANPPSDVYLSSRDRQILDWHFANLEFANATPLSTLSLKHWDQDDDFEFTGSHLTVRNGYSCVPVALAEGLDIKLNTAVRQVRYTASGCEVIAVNTRSTSQTFIYKCDAVLCTLPLGVLKQQPPAVQFVPPLPEWKTSAVQRMGFGNLNKVVLCFDRVFWDPSVNLFGHVGSTTASRGELFLFWNLYKAPILLALVAGEAAGIMENISDDVIVGRCLAILKGIFGSSAVPQPKETVVSRWRADPWARGSYSYVAAGSSGNDYDLMAQPITPGPSIPGAPQPIPRLFFAGEHTIRNYPATVHGALLSGLREAGRIADQFLGAMYTLPRQATPGVPAQQSPSM

>Q9BSE5

MLRLLASGCARGPGPGVGARPAAGLFHPGRRQSRQASDAPRNQPPSPEFVARPVGVCSMMRLPVQTSPEGLDAAFIGVPLDTGTSNRPGARFGPRRIREESVMLGTVNPSTGALPFQSLMVADLGDVNVNLYNLQDSCRRIQEAYEKIVAAGCIPLTLGGDHTITYPILQAMAKKHGPVGLLHVDAHTDTTDKALGEKLYHGAPFRRCVDEGLLDCKRVVQIGIRGSSTTLDPYRYNRSQGFRVVLAEDCWMKSLVPLMGEVRQQMGGKPIYISFDIDALDPAYAPGTGTPEIAGLTPSQALEIIRGCQGLNVMGCDLVEVSPPYDLSGNTALLAANLLFEMLCALPKVTTV

1424 P10275 Q9P0M9

>P10275

MEVQLGLGRVYPRPPSKTYRGAFQNLFQSVREVIQNPGPRHPEAASAAPPGASLLLLQQQQQQQQQQQQQQQQQQQQQETSPRQQQQQQGEDGSPQAHRRGPTGYLVLDEEQQPSQPQSALECHPERGCVPEPGAAVAASKGLPQQLPAPPDEDDSAAPSTLSLLGPTFPGLSSCSADLKDILSEASTMQLLQQQQQEAVSEGSSSGRAREASGAPTSSKDNYLGGTSTISDNAKELCKAVSVSMGLGVEALEHLSPGEQLRGDCMYAPLLGVPPAVRPTPCAPLAECKGSLLDDSAGKSTEDTAEYSPFKGGYTKGLEGESLGCSGSAAAGSSGTLELPSTLSLYKSGALDEAAAYQSRDYYNFPLALAGPPPPPPPPHPHARIKLENPLDYGSAWAAAAAQCRYGDLASLHGAGAAGPGSGSPSAAASSSWHTLFTAEEGQLYGPCGGGGGGGGGGGGGGGGGGGGGGGGEAGAVAPYGYTRPPQGLAGQESDFTAPDVWYPGGMVSRVPYPSPTCVKSEMGPWMDSYSGPYGDMRLETARDHVLPIDYYFPPQKTCLICGDEASGCHYGALTCGSCKVFFKRAAEGKQKYLCASRNDCTIDKFRRKNCPSCRLRKCYEAGMTLGARKLKKLGNLKLQEEGEASSTTSPTEETTQKLTVSHIEGYECQPIFLNVLEAIEPGVVCAGHDNNQPDSFAALLSSLNELGERQLVHVVKWAKALPGFRNLHVDDQMAVIQYSWMGLMVFAMGWRSFTNVNSRMLYFAPDLVFNEYRMHKSRMYSQCVRMRHLSQEFGWLQITPQEFLCMKALLLFSIIPVDGLKNQKFFDELRMNYIKELDRIIACKRKNPTSCSRRFYQLTKLLDSVQPIARELHQFTFDLLIKSHMVSVDFPEMMAEIISVQVPKILSGKVKPIYFHTQ

>Q9P0M9

MASVVLALRTRTAVTSLLSPTPATALAVRYASKKSGGSSKNLGGKSSGRRQGIKKMEGHYVHAGNIIATQRHFRWHPGAHVGVGKNKCLYALEEGIVRYTKEVYVPHPRNTEAVDLITRLPKGAVLYKTFVHVVPAKPEGTFKLVAML

1425 P50548 NP_036361.1

>P50548

MKTPADTGFAFPDWAYKPESSPGSRQIQLWHFILELLRKEEYQGVIAWQGDYGEFVIKDPDEVARLWGVRKCKPQMNYDKLSRALRYYYNKRILHKTKGKRFTYKFNFNKLVLVNYPFIDVGLAGGAVPQSAPPVPSGGSHFRFPPSTPSEVLSPTEDPRSPPACSSSSSSLFSAVVARRLGRGSVSDCSDGTSELEEPLGEDPRARPPGPPDLGAFRGPPLARLPHDPGVFRVYPRPRGGPEPLSPFPVSPLAGPGSLLPPQLSPALPMTPTHLAYTPSPTLSPMYPSGGGGPSGSGGGSHFSFSPEDMKRYLQAHTQSVYNYHLSPRAFLHYPGLVVPQPQRPDKCPLPPMAPETPPVPSSASSSSSSSSSPFKFKLQPPPLGRRQRAAGEKAVAGADKSGGSAGGLAEGAGALAPPPPPPQIKVEPISEGESEEVEVTDISDEDEEDGEVFKTPRAPPAPPKPEPGEAPGASQCMPLKLRFKRRWSEDCRLEGGGGPAGGFEDEGEDKKVRGEGPGEAGGPLTPRRVSSDLQHATAQLSLEHRDS

>NP_036361.1

MSTSWSDRLQNAADMPANMDKHALKKYRREAYHRVFVNRSLAMEKIKCFGFDMDYTLAVYKSPEYESLGFELTVERLVSIGYPQELLSFAYDSTFPTRGLVFDTLYGNLLKVDAYGNLLVCAHGFNFIRGPETREQYPNKFIQRDDTERFYILNTLFNLPETYLLACLVDFFTNCPRYTSCETGFKDGDLFMSYRSMFQDVRDAVDWVHYKGSLKEKTVENLEKYVVKDGKLPLLLSRMKEVGKVFLATNSDYKYTDKIMTYLFDFPHGPKPGSSHRPWQSYFDLILVDARKPLFFGEGTVLRQVDTKTGKLKIGTYTGPLQHGIVYSGGSSDTICDLLGAKGKDILYIGDHIFGDILKSKKRQGWRTFLVIPELAQELHVWTDKSSLFEELQSLDIFLAELYKHLDSSSNERPDISSIQRRIKKVTHDMDMCYGMMGSLFRSGSRQTLFASQVMRYADLYAASFINLLYYPFSYLFRAAHVLMPHESTVEHTHVDINEMESPLATRNRTSVDFKDTDYKRHQLTRSISEIKPPNLFPLAPQEITHCHDEDDDEEEEEEEE

1426 P01106 NP_064530.1

>P01106

MPLNVSFTNRNYDLDYDSVQPYFYCDEEENFYQQQQQSELQPPAPSEDIWKKFELLPTPPLSPSRRSGLCSPSYVAVTPFSLRGDNDGGGGSFSTADQLEMVTELLGGDMVNQSFICDPDDETFIKNIIIQDCMWSGFSAAAKLVSEKLASYQAARKDSGSPNPARGHSVCSTSSLYLQDLSAAASECIDPSVVFPYPLNDSSSPKSCASQDSSAFSPSSDSLLSSTESSPQGSPEPLVLHEETPPTTSSDSEEEQEDEEEIDVVSVEKRQAPGKRSESGSPSAGGHSKPPHSPLVLKRCHVSTHQHNYAAPPSTRKDYPAAKRVKLDSVRVLRQISNNRKCTSPRSSDTEENVKRRTHNVLERQRRNELKRSFFALRDQIPELENNEKAPKVVILKKATAYILSVQAEEQKLISEEDLLRKRREQLKHKLEQLRNSCA

>NP_064530.1

MDFNMKKLASDAGIFFTRAVQFTEEKFGQAEKTELDAHFENLLARADSTKNWTEKILRQTEVLLQPNPSARVEEFLYEKLDRKVPSRVTNGELLAQYMADAASELGPTTPYGKTLIKVAEAEKQLGAAERDFIHTASISFLTPLRNFLEGDWKTISKERRLLQNRRLDLDACKARLKKAKAAEAKATTVPDFQETRPRNYILSASASALWNDEVDKAEQELRVAQTEFDRQAEVTRLLLEGISSTHVNHLRCLHEFVKSQTTYYAQCYRHMLDLQKQLGRFPGTFVGTTEPASPPLSSTSPTTAAATMPVVPSVASLAPPGEASLCLEEVAPPASGTRKARVLYDYEAADSSELALLADELITVYSLPGMDPDWLIGERGNKKGKVPVTYLELLS

1427 NP_065090.1 BAC04299.1

>NP_065090.1

MADGKAGDEKPEKSQRAGAAGGPEEEAEKPVKTKTVSSSNGGESSSRSAEKRSAEEEAADLPTKPTKISKFGFAIGSQTTKKASAISIKLGSSKPKETVPTLAPKTLSVAAAFNEDEDSEPEEMPPEAKMRMKNIGRDTPTSAGPNSFNKGKHGFSDNQKLWERNIKSHLGNVHDQDN

>BAC04299.1

MEVLRPQLIRIDGRNYRKNPVQEQTYQHEEDEEDFYQGSMECADEPCDAYEVEQTPQGFRSTLRAPSLLYNLIHLNTSNDCGFQKITLDCQNIYTWKSRHIVGKRGDTRKKIEMETKTSISIPKPGQDGEIVITGQHRNGVISARTRIDVLLDTFRRKQPFTHFLAFFLNEVEVQEGFLRFQEEVLAKCSMDHGVDSSIFQNPKKLHLTIGMLVLLSEEEIQQTCEMLQQCKEEFINDISGGKPLEVEMAGIEYMNDDPGMVDVLYAKVHMKDGSNRLQELVDRVLERFQASGLIVKEWNSVKLHATVMNTLFRKDPNAEGRYNLYTAEGKYIFKERESFDGRNILKSFALLPRLEYNDAISAHCNLCLPGSSDSPASASQVAGITGVSDAYSQSLPGKS

1428 NP_003446.2 P29144

>NP_003446.2

MATAVEPEDQDLWEEEGILMVKLEDDFTCRPESVLQRDDPVLETSHQNFRRFRYQEAASPREALIRLRELCHQWLRPERRTKEQILELLVLEQFLTVLPGELQSWVRGQRPESGEEAVTLVEGLQKQPRRPRRWVTVHVHGQEVLSEETVHLGVEPESPNELQDPVQSSTPEQSPEETTQSPDLGAPAEQRPHQEEELQTLQESEVPVPEDPDLPAERSSGDSEMVALLTALSQGLVTFKDVAVCFSQDQWSDLDPTQKEFYGEYVLEEDCGIVVSLSFPIPRPDEISQVREEEPWVPDIQEPQETQEPEILSFTYTGDRSKDEEECLEQEDLSLEDIHRPVLGEPEIHQTPDWEIVFEDNPGRLNERRFGTNISQVNSFVNLRETTPVHPLLGRHHDCSVCGKSFTCNSHLVRHLRTHTGEKPYKCMECGKSYTRSSHLARHQKVHKMNAPYKYPLNRKNLEETSPVTQAERTPSVEKPYRCDDCGKHFRWTSDLVRHQRTHTGEKPFFCTICGKSFSQKSVLTTHQRIHLGGKPYLCGECGEDFSEHRRYLAHRKTHAAEELYLCSECGRCFTHSAAFAKHLRGHASVRPCRCNECGKSFSRRDHLVRHQRTHTGEKPFTCPTCGKSFSRGYHLIRHQRTHSEKTS

>P29144

MATAATEEPFPFHGLLPKKETGAASFLCRYPEYDGRGVLIAVLDTGVDPGAPGMQVTTDGKPKIVDIIDTTGSGDVNTATEVEPKDGEIVGLSGRVLKIPASWTNPSGKYHIGIKNGYDFYPKALKERIQKERKEKIWDPVHRVALAEACRKQEEFDVANNGSSQANKLIKEELQSQVELLNSFEKKYSDPGPVYDCLVWHDGEVWRACIDSNEDGDLSKSTVLRNYKEAQEYGSFGTAEMLNYSVNIYDDGNLLSIVTSGGAHGTHVASIAAGHFPEEPERNGVAPGAQILSIKIGDTRLSTMETGTGLIRAMIEVINHKCDLVNYSYGEATHWPNSGRICEVINEAVWKHNIIYVSSAGNNGPCLSTVGCPGGTTSSVIGVGAYVSPDMMVAEYSLREKLPANQYTWSSRGPSADGALGVSISAPGGAIASVPNWTLRGTQLMNGTSMSSPNACGGIALILSGLKANNIDYTVHSVRRALENTAVKADNIEVFAQGHGIIQVDKAYDYLVQNTSFANKLGFTVTVGNNRGIYLRDPVQVAAPSDHGVGIEPVFPENTENSEKISLQLHLALTSNSSWVQCPSHLELMNQCRHINIRVDPRGLREGLHYTEVCGYDIASPNAGPLFRVPITAVIAAKVNESSHYDLAFTDVHFKPGQIRRHFIEVPEGATWAEVTVCSCSSEVSAKFVLHAVQLVKQRAYRSHEFYKFCSLPEKGTLTEAFPVLGGKAIEFCIARWWASLSDVNIDYTISFHGIVCTAPQLNIHASEGINRFDVQSSLKYEDLAPCITLKNWVQTLRPVSAKTKPLGSRDVLPNNRQLYEMVLTYNFHQPKSGEVTPSCPLLCELLYESEFDSQLWIIFDQNKRQMGSGDAYPHQYSLKLEKGDYTIRLQIRHEQISDLERLKDLPFIVSHRLSNTLSLDIHENHSFALLGKKKSSNLTLPPKYNQPFFVTSLPDDKIPKGAGPGCYLAGSLTLSKTELGKKADVIPVHYYLIPPPTKTKNGSKDKEKDSEKEKDLKEEFTEALRDLKIQWMTKLDSSDIYNELKETYPNYLPLYVARLHQLDAEKERMKRLNEIVDAANAVISHIDQTALAVYIAMKTDPRPDAATIKNDMDKQKSTLVDALCRKGCALADHLLHTQAQDGAISTDAEGKEEEGESPLDSLAETFWETTKWTDLFDNKVLTFAYKHALVNKMYGRGLKFATKLVEEKPTKENWKNCIQLMKLLGWTHCASFTENWLPIMYPPDYCVF

1429 P08651 NP_000894.1

>P08651

MYSSPLCLTQDEFHPFIEALLPHVRAFAYTWFNLQARKRKYFKKHEKRMSKDEERAVKDELLGEKPEVKQKWASRLLAKLRKDIRPECREDFVLSITGKKAPGCVLSNPDQKGKMRRIDCLRQADKVWRLDLVMVILFKGIPLESTDGERLVKAAQCGHPVLCVQPHHIGVAVKELDLYLAYFVRERDAEQSGSPRTGMGSDQEDSKPITLDTTDFQESFVTSGVFSVTELIQVSRTPVVTGTGPNFSLGELQGHLAYDLNPASTGLRRTLPSTSSSGSKRHKSGSMEEDVDTSPGGDYYTSPSSPTSSSRNWTEDMEGGISSPVKKTEMDKSPFNSPSPQDSPRLSSFTQHHRPVIAVHSGIARSPHPSSALHFPTTSILPQTASTYFPHTAIRYPPHLNPQDPLKDLVSLACDPASQQPGPLNGSGQLKMPSHCLSAQMLAPPPPGLPRLALPPATKPATTSEGGATSPTSPSYSPPDTSPANRSFVGLGPRDPAGIYQAQSWYLG

>NP_000894.1

MVGRRALIVLAHSERTSFNYAMKEAAAAALKKKGWEVVESDLYAMNFNPIISRKDITGKLKDPANFQYPAESVLAYKEGHLSPDIVAEQKKLEAADLVIFQFPLQWFGVPAILKGWFERVFIGEFAYTYAAMYDKGPFRSKKAVLSITTGGSGSMYSLQGIHGDMNVILWPIQSGILHFCGFQVLEPQLTYSIGHTPADARIQILEGWKKRLENIWDETPLYFAPSSLFDLNFQAGFLMKKEVQDEEKNKKFGLSVGHHLGKSIPTDNQIKARK

1430 NP_003400.2 NP_068707.1

>NP_003400.2

MEFFISMSETIKYNDDDHKTLFLKTLNEQRLEGEFCDIAIVVEDVKFRAHRCVLAACSTYFKKLFKKLEVDSSSVIEIDFLRSDIFEEVLNYMYTAKISVKKEDVNLMMSSGQILGIRFLDKLCSQKRDVSSPDENNGQSKSKYCLKINRPIGDAADTQDDDVEEIGDQDDSPSDDTVEGTPPSQEDGKSPTTTLRVQEAILKELGSEEVRKVNCYGQEVESMETPESKDLGSQTPQALTFNDGMSEVKDEQTPGWTTAASDMKFEYLLYGHHREQIACQACGKTFSDEGRLRKHEKLHTADRPFVCEMCTKGFTTQAHLKEHLKIHTGYKPYSCEVCGKSFIRAPDLKKHERVHSNERPFACHMCDKAFKHKSHLKDHERRHRGEKPFVCGSCTKAFAKASDLKRHENNMHSERKQVTPSAIQSETEQLQAAAMAAEAEQQLETIACS

>NP_068707.1

MIEESGNKRKTMAEKRQLFIEMRAQNFDVIRLSTYRTACKLRFVQKRCNLHLVDIWNMIEAFRDNGLNTLDHTTEISVSRLETVISSIYYQLNKRLPSTHQISVEQSISLLLNFMIAAYDSEGRGKLTVFSVKAMLATMCGGKMLDKLRYVFSQMSDSNGLMIFSKFDQFLKEVLKLPTAVFEGPSFGYTEHSVRTCFPQQRKIMLNMFLDTMMADPPPQCLVWLPLMHRLAHVENVFHPVECSYCRCESMMGFRYRCQQCHNYQLCQNCFWRGHAGGPHSNQHQMKEHSSWKSPAKKLSHAISKSLGCVPTREPPHPVFPEQPEKPLDLAHIVPPRPLTNMNDTMVSHMSSGVPTPTKRLQYSQDIPSHLADEHALIASYVARLQHCARVLDSPSRLDEEHRLIARYAARLAAEAGNVTRPPTDLSFNFDANKQQRQLIAELENKNREILQEIQRLRLEHEQASQPTPEKAQQNPTLLAELRLLRQRKDELEQRMSALQESRRELMVQLEELMKLLKEEEQKQAAQATGSPHTSPTHGGGRPMPMPVRSTSAGSTPTHCPQDSLSGVGGDVQEAFAQGTRRNLRNDLLVAADSITNTMSSLVKELHSAEEGAEEEEEKMQNGKDRG

1431 P16402 P50225

>P16402

MSETAPLAPTIPAPAEKTPVKKKAKKAGATAGKRKASGPPVSELITKAVAASKERSGVSLAALKKALAAAGYDVEKNNSRIKLGLKSLVSKGTLVQTKGTGASGSFKLNKKAASGEGKPKAKKAGAAKPRKPAGAAKKPKKVAGAATPKKSIKKTPKKVKKPATAAGTKKVAKSAKKVKTPQPKKAAKSPAKAKAPKPKAAKPKSGKPKVTKAKKAAPKKK

>P50225

MELIQDTSRPPLEYVKGVPLIKYFAEALGPLQSFQARPDDLLISTYPKSGTTWVSQILDMIYQGGDLEKCHRAPIFMRVPFLEFKAPGIPSGMETLKDTPAPRLLKTHLPLALLPQTLLDQKVKVVYVARNAKDVAVSYYHFYHMAKVHPEPGTWDSFLEKFMVGEVSYGSWYQHVQEWWELSRTHPVLYLFYEDMKENPKREIQKILEFVGRSLPEETVDFMVQHTSFKEMKKNPMTNYTTVPQEFMDHSISPFMRKGMAGDWKTTFTVAQNERFDADYAEKMAGCSLSFRSEL

1432 O00115 AAH07654.1

>O00115

MIPLLLAALLCVPAGALTCYGDSGQPVDWFVVYKLPALRGSGEAAQRGLQYKYLDESSGGWRDGRALINSPEGAVGRSLQPLYRSNTSQLAFLLYNDQPPQPSKAQDSSMRGHTKGVLLLDHDGGFWLVHSVPNFPPPASSAAYSWPHSACTYGQTLLCVSFPFAQFSKMGKQLTYTYPWVYNYQLEGIFAQEFPDLENVVKGHHVSQEPWNSSITLTSQAGAVFQSFAKFSKFGDDLYSGWLAAALGTNLQVQFWHKTVGILPSNCSDIWQVLNVNQIAFPGPAGPSFNSTEDHSKWCVSPKGPWTCVGDMNRNQGEEQRGGGTLCAQLPALWKAFQPLVKNYQPCNGMARKPSRAYKI

>AAH07654.1

MLSSCVRPVPTTVRFVDSLICNSSRSFMDLKALLSSLNDFASLSFAESWDNVGLLVEPSPPHTVNTLFLTNDLTEEVMEEVLQKKADLILSYHPPIFRPMKRITWNTWKERLVIRALENRVGIYSPHTAYDAAPQGVNNWLAKGLGACTSRPIHPSKAPNYPTEGNHRVEFNVNYTQDLDKVMSAVKGIDGVSVTSFSARTGNEEQTRINLNCTQKALMQVVDFLSRNKQLYQKTEILSLEKPLLLHTGMGRLCTLDESVSLATMIDRIKRHLKLSHIRLALGVGRTLESQVKVVALCAGSGSSVLQGVEADLYLTGEMSHHDTLDAASQGINVILCEHSNTERGFLSDLRDMLDSHLENKINIILSETDRDPLQVV

1433 NP_005186.2 NP_006592.3

>NP_005186.2

MSAALFSLDGPARGAPWPAEPAPFYEPGRAGKPGRGAEPGALGEPGAAAPAMYDDESAIDFSAYIDSMAAVPTLELCHDELFADLFNSNHKAGGAGPLELLPGGPARPLGPGPAAPRLLKREPDWGDGDAPGSLLPAQVAACAQTVVSLAAAGQPTPPTSPEPPRSSPRQTPAPGPAREKSAGKRGPDRGSPEYRQRRERNNIAVRKSRDKAKRRNQEMQQKLVELSAENEKLHQRVEQLTRDLAGLRQFFKQLPSPPFLPAAGTADCR

>NP_006592.3

MQPASAKWYDRRDYVFIEFCVEDSKDVNVNFEKSKLTFSCLGGSDNFKHLNEIDLFHCIDPNDSKHKRTDRSILCCLRKGESGQSWPRLTKERAKLNWLSVDFNNWKDWEDDSDEDMSNFDRFSEMMNNMGGDEDVDLPEVDGADDDSQDSDDEKMPDLE

1434 O60356 Q9NR19

>O60356

MATFPPATSAPQQPPGPEDEDSSLDESDLYSLAHSYLGGGGRKGRTKREAAANTNRPSPGGHERKLVTKLQNSERKKRGARR

>Q9NR19

MGLPEERVRSGSGSRGQEEAGAGGRARSWSPPPEVSRSAHVPSLQRYRELHRRSVEEPREFWGDIAKEFYWKTPCPGPFLRYNFDVTKGKIFIEWMKGATTNICYNVLDRNVHEKKLGDKVAFYWEGNEPGETTQITYHQLLVQVCQFSNVLRKQGIQKGDRVAIYMPMIPELVVAMLACARIGALHSIVFAGFSSESLCERILDSSCSLLITTDAFYRGEKLVNLKELADEALQKCQEKGFPVRCCIVVKHLGRAELGMGDSTSQSPPIKRSCPDVQISWNQGIDLWWHELMQEAGDECEPEWCDAEDPLFILYTSGSTGKPKGVVHTVGGYMLYVATTFKYVFDFHAEDVFWCTADIGWITGHSYVTYGPLANGATSVLFEGIPTYPDVNRLWSIVDKYKVTKFYTAPTAIRLLMKFGDEPVTKHSRASLQVLGTVGEPINPEAWLWYHRVVGAQRCPIVDTFWQTETGGHMLTPLPGATPMKPGSATFPFFGVAPAILNESGEELEGEAEGYLVFKQPWPGIMRTVYGNHERFETTYFKKFPGYYVTGDGCQRDQDGYYWITGRIDDMLNVSGHLLSTAEVESALVEHEAVAEAAVVGHPHPVKGECLYCFVTLCDGHTFSPKLTEELKKQIREKIGPIATPDYIQNAPGLPKTRSGKIMRRVLRKIAQNDHDLGDMSTVADPSVISHLFSHRCLTIQ

1435 NP_001028733.1 Q9BZQ8

>NP_001028733.1

MWEKMETKTIVYDLDTSGGLMEQIQALLAPPKTDEAEKRSRKPEKEPRRSGRATNHDSCDSCKEGGDLLCCDHCPAAFHLQCCNPPLSEEMLPPGEWMCHRCTVRRKKREQKKELGHVNGLVDKSGKRTTSPSSDTDLLDRSASKTELKAIAHARILERRASRPGTPTSSASTETPTSEQNDVDEDIIDVDEEPVAAEPDYVQPQLRRPFELLIAAAMERNPTQFQLPNELTCTTALPGSSKRRRKEETTGKNVKKTQHELDHNGLVPLPVKVCFTCNRSCRVAPLIQCDYCPLLFHMDCLEPPLTAMPLGRWMCPNHIEHVVLNQKNMTLSNRCQVFDRFQDTVSQHVVKVDFLNRIHKKHPPNRRVLQSVKRRSLKVPDAIKSQYQFPPPLIAPAAIRDGELICNGIPEESQMHLLNSEHLATQAEQQEWLCSVVALQCSILKHLSAKQMPSHWDSEQTEKADIKPVIVTDSSVTTSLQTADKTPTPSHYPLSCPSGISTQNSLSCSPPHQSPALEDIGCSSCAEKSKKTPCGTANGPVNTEVKANGPHLYSSPTDSTDPRRLPGANTPLPGLSHRQGWPRPLTPPAAGGLQNHTVGIIVKTENATGPSSCPQRSLVPVPSLPPSIPSSCASIENTSTLQRKTVQSQIGPPLTDSRPLGSPPNATRVLTPPQAAGDGILATTANQRFSSPAPSSDGKVSPGTLSIGSALTVPSFPANSTAMVDLTNSLRAFMDVNGEIEINMLDEKLIKFLALQRIHQLFPSRVQPSPGSVGTHQLASGGHHIEVQRKEVQARAVFYPLLGLGGAVNMCYRTLYIGTGADMDVCLTNYGHCNYVSGKHACIFYDENTKHYELLNYSEHGTTVDNVLYSCDFSEKTPPTPPSSIVAKVQSVIRRRRHQKQDEEPSEEAAMMSSQAQGPQRRPCNCKASSSSLIGGSGAGWEGTALLHHGSYIKLGCLQFVFSITEFATKQPKGDASLLQDGVLAEKLSLKPHQGPVLRSNSVP

>Q9BZQ8

MGGSASSQLDEGKCAYIRGKTEAAIKNFSPYYSRQYSVAFCNHVRTEVEQQRDLTSQFLKTKPPLAPGTILYEAELSQFSEDIKKWKERYVVVKNDYAVESYENKEAYQRGAAPKCRILPAGGKVLTSEDEYNLLSDRHFPDPLASSEKENTQPFVVLPKEFPVYLWQPFFRHGYFCFHEAADQKRFSALLSDCVRHLNHDYMKQMTFEAQAFLEAVQFFRQEKGHYGSWEMITGDEIQILSNLVMEELLPTLQTDLLPKMKGKKNDRKRTWLGLLEEAYTLVQHQVSEGLSALKEECRALTKGLEGTIRSDMDQIVNSKNYLIGKIKAMVAQPAEKSCLESVQPFLASILEELMGPVSSGFSEVRVLFEKEVNEVSQNFQTTKDSVQLKEHLDRLMNLPLHSVKMEPCYTKVNLLHERLQDLKSRFRFPHIDLVVQRTQNYMQELMENAVFTFEQLLSPHLQGEASKTAVAIEKVKLRVLKQYDYDSSTIRKKIFQEALVQITLPTVQKALASTCKPELQKYEQFIFADHTNMIHVENVYEEILHQILLDETLKVIKEAAILKKHNLFEDNMALPSESVSSLTDLKPPTGSNQASPARRASAILPGVLGSETLSNEVFQESEEEKQPEVPSSLAKGESLSLPGPSPPPDGTEQVIISRVDDPVVNPVATEDTAGLPGTCSSELEFGGTLEDEEPAQEEPEPITASGSLKALRKLLTASVEVPVDSAPVMEEDTNGESHVPQENEEEEEKEPSQAAAIHPDNCEESEVSEREAQPPCPEAHGEELGGFPEVGSPASPPASGGLTEEPLGPMEGELPGEACTLTAHEGRGGKCTEEGDASQQEGCTLGSDPICLSESQVSEEQEEMGGQSSAAQATASVNAEEIKVARIHECQWVVEDAPNPDVLLSHKDDVKEGEGGQESFPELPSEE

1436 NP_005180.1 NP_004835.2

>NP_005180.1

MEELSSVGEQVFAAECILSKRLRKGKLEYLVKWRGWSSKHNSWEPEENILDPRLLLAFQKKEHEKEVQNRKRGKRPRGRPRKLTAMSSCSRRSKLKEPDAPSKSKSSSSSSSSTSSSSSSDEEDDSDLDAKRGPRGRETHPVPQKKAQILVAKPELKDPIRKKRGRKPLPPEQKATRRPVSLAKVLKTARKDLGAPASKLPPPLSAPVAGLAALKAHAKEACGGPSAMATPENLASLMKGMASSPGRGGISWQSSIVHYMNRMTQSQAQAASRLALKAQATNKCGLGLDLKVRTQKGELGMSPPGSKIPKAPSGGAVEQKVGNTGGPPHTHGASRVPAGCPGPQPAPTQELSLQVLDLQSVKNGMPGVGLLARHATATKGVPATNPAPGKGTGSGLIGASGATMPTDTSKSEKLASRAVAPPTPASKRDCVKGSATPSGQESRTAPGEARKAATLPEMSAGEESSSSDSDPDSASPPSTGQNPSVSVQTSQDWKPTRSLIEHVFVTDVTANLITVTVKESPTSVGFFNLRHY

>NP_004835.2

MDAALKRSRSEEPAEILPPARDEEEEEEEGMEQGLEEEEEVDPRIQGELEKLNQSTDDINRRETELEDARQKFRSVLVEATVKLDELVKKIGKAVEDSKPYWEARRVARQAQLEAQKATQDFQRATEVLRAAKETISLAEQRLLEDDKRQFDSAWQEMLNHATQRVMEAEQTKTRSELVHKETAARYNAAMGRMRQLEKKLKRAINKSKPYFELKAKYYVQLEQLKKTVDDLQAKLTLAKGEYKMALKNLEMISDEIHERRRSSAMGPRGCGVGAEGSSTSVEDLPGSKPEPDAISVASEAFEDDSCSNFVSEDDSETQSVSSFSSGPTSPSEMPDQFPAVVRPGSLDLPSPVSLSEFGMMFPVLGPRSECSGASSPECEVERGDRAEGAENKTSDKANNNRGLSSSSGSGGSSKSQSSTSPEGQALENRMKQLSLQCSKGRDGIIADIKMVQIG

1437 NP_002898.2 Q9HBG6

>NP_002898.2

MASVSALTEELDSITSELHAVEIQIQELTERQQELIQKKKVLTKKIKQCLEDSDAGASNEYDSSPAAWNKEDFPWSGKVKDILQNVFKLEKFRPLQLETINVTMAGKEVFLVMPTGGGKSLCYQLPALCSDGFTLVICPLISLMEDQLMVLKQLGISATMLNASSSKEHVKWVHAEMVNKNSELKLIYVTPEKIAKSKMFMSRLEKAYEARRFTRIAVDEVHCCSQWGHDFRPDYKALGILKRQFPNASLIGLTATATNHVLTDAQKILCIEKCFTFTASFNRPNLYYEVRQKPSNTEDFIEDIVKLINGRYKGQSGIIYCFSQKDSEQVTVSLQNLGIHAGAYHANLEPEDKTTVHRKWSANEIQVVVATVAFGMGIDKPDVRFVIHHSMSKSMENYYQESGRAGRDDMKADCILYYGFGDIFRISSMVVMENVGQQKLYEMVSYCQNISKCRRVLMAQHFDEVWNSEACNKMCDNCCKDSAFERKNITEYCRDLIKILKQAEELNEKLTPLKLIDSWMGKGAAKLRVAGVVAPTLPREDLEKIIAHFLIQQYLKEDYSFTAYATISYLKIGPKANLLNNEAHAITMQVTKSTQNSFRAESSQTCHSEQGDKKMEEKNSGNFQKKAANMLQQSGSKNTGAKKRKIDDA

>Q9HBG6

MRAVLTWRDKAEHCINDIAFKPDGTQLILAAGSRLLVYDTSDGTLLQPLKGHKDTVYCVAYAKDGKRFASGSADKSVIIWTSKLEGILKYTHNDAIQCVSYNPITHQLASCSSSDFGLWSPEQKSVSKHKSSSKIICCSWTNDGQYLALGMFNGIISIRNKNGEEKVKIERPGGSLSPIWSICWNPSSRWESFWMNRENEDAEDVIVNRYIQEIPSTLKSAVYSSQGSEAEEEEPEEEDDSPRDDNLEERNDILAVADWGQKVSFYQLSGKQIGKDRALNFDPCCISYFTKGEYILLGGSDKQVSLFTKDGVRLGTVGEQNSWVWTCQAKPDSNYVVVGCQDGTISFYQLIFSTVHGLYKDRYAYRDSMTDVIVQHLITEQKVRIKCKELVKKIAIYRNRLAIQLPEKILIYELYSEDLSDMHYRVKEKIIKKFECNLLVVCANHIILCQEKRLQCLSFSGVKEREWQMESLIRYIKVIGGPPGREGLLVGLKNGQILKIFVDNLFAIVLLKQATAVRCLDMSASRKKLAVVDENDTCLVYDIDTKELLFQEPNANSVAWNTQCEDMLCFSGGGYLNIKASTFPVHRQKLQGFVVGYNGSKIFCLHVFSISAVEVPQSAPMYQYLDRKLFKEAYQIACLGVTDTDWRELAMEALEGLDFETAKKAFIRVQDLRYLELISSIEERKKRGETNNDLFLADVFSYQGKFHEAAKLYKRSGHENLALEMYTDLCMFEYAKDFLGSGDPKETKMLITKQADWARNIKEPKAAVEMYISAGEHVKAIEICGDHGWVDMLIDIARKLDKAEREPLLLCATYLKKLDSPGYAAETYLKMGDLKSLVQLHVETQRWDEAFALGEKHPEFKDDIYMPYAQWLAENDRFEEAQKAFHKAGRQREAVQVLEQLTNNAVAESRFNDAAYYYWMLSMQCLDIAQDPAQKDTMLGKFYHFQRLAELYHGYHAIHRHTEDPFSVHRPETLFNISRFLLHSLPKDTPSGISKVKILFTLAKQSKALGAYRLARHAYDKLRGLYIPARFQKSIELGTLTIRAKPFHDSEELVPLCYRCSTNNPLLNNLGNVCINCRQPFIFSASSYDVLHLVEFYLEEGITDEEAISLIDLEVLRPKRDDRQLEIANNSSQILRLVETKDSIGDEDPFTAKLSFEQGGSEFVPVVVSRLVLRSMSRRDVLIKRWPPPLRWQYFRSLLPDASITMCPSCFQMFHSEDYELLVLQHGCCPYCRRCKDDPGP

1438 Q9UEE5 P48729

>Q9UEE5

MIPLEKPGSGGSSPGATSGSGRAGRGLSGPCRPPPPPQARGLLTEIRAVVRTEPFQDGYSLCPGRELGRGKFAVVRKCIKKDSGKEFAAKFMRKRRKGQDCRMEIIHEIAVLELAQDNPWVINLHEVYETASEMILVLEYAAGGEIFDQCVADREEAFKEKDVQRLMRQILEGVHFLHTRDVVHLDLKPQNILLTSESPLGDIKIVDFGLSRILKNSEELREIMGTPEYVAPEILSYDPISMATDMWSIGVLTYVMLTGISPFLGNDKQETFLNISQMNLSYSEEEFDVLSESAVDFIRTLLVKKPEDRATAEECLKHPWLTQSSIQEPSFRMEKALEEANALQEGHSVPEINSDTDKSETEESIVTEELIVVTSYTLGQCRQSEKEKMEQKAISKRFKFEEPLLQEIPGEFIY

>P48729

MASSSGSKAEFIVGGKYKLVRKIGSGSFGDIYLAINITNGEEVAVKLESQKARHPQLLYESKLYKILQGGVGIPHIRWYGQEKDYNVLVMDLLGPSLEDLFNFCSRRFTMKTVLMLADQMISRIEYVHTKNFIHRDIKPDNFLMGIGRHCNKLFLIDFGLAKKYRDNRTRQHIPYREDKNLTGTARYASINAHLGIEQSRRDDMESLGYVLMYFNRTSLPWQGLKAATKKQKYEKISEKKMSTPVEVLCKGFPAEFAMYLNYCRGLRFEEAPDYMYLRQLFRILFRTLNHQYDYTFDWTMLKQKAAQQAASSSGQGQQAQTPTGKQTDKTKSNMKGF

1439 NP_036558.3 NP_110438.1

>NP_036558.3

MFLYNLTLQRATGISFAIHGNFSGTKQQEIVVSRGKILELLRPDPNTGKVHTLLTVEVFGVIRSLMAFRLTGGTKDYIVVGSDSGRIVILEYQPSKNMFEKIHQETFGKSGCRRIVPGQFLAVDPKGRAVMISAIEKQKLVYILNRDAAARLTISSPLEAHKANTLVYHVVGVDVGFENPMFACLEMDYEEADNDPTGEAAANTQQTLTFYELDLGLNHVVRKYSEPLEEHGNFLITVPGGSDGPSGVLICSENYITYKNFGDQPDIRCPIPRRRNDLDDPERGMIFVCSATHKTKSMFFFLAQTEQGDIFKITLETDEDMVTEIRLKYFDTVPVAAAMCVLKTGFLFVASEFGNHYLYQIAHLGDDDEEPEFSSAMPLEEGDTFFFQPRPLKNLVLVDELDSLSPILFCQIADLANEDTPQLYVACGRGPRSSLRVLRHGLEVSEMAVSELPGNPNAVWTVRRHIEDEFDAYIIVSFVNATLVLSIGETVEEVTDSGFLGTTPTLSCSLLGDDALVQVYPDGIRHIRADKRVNEWKTPGKKTIVKCAVNQRQVVIALTGGELVYFEMDPSGQLNEYTERKEMSADVVCMSLANVPPGEQRSRFLAVGLVDNTVRIISLDPSDCLQPLSMQALPAQPESLCIVEMGGTEKQDELGERGSIGFLYLNIGLQNGVLLRTVLDPVTGDLSDTRTRYLGSRPVKLFRVRMQGQEAVLAMSSRSWLSYSYQSRFHLTPLSYETLEFASGFASEQCPEGIVAISTNTLRILALEKLGAVFNQVAFPLQYTPRKFVIHPESNNLIIIETDHNAYTEATKAQRKQQMAEEMVEAAGEDERELAAEMAAAFLNENLPESIFGAPKAGNGQWASVIRVMNPIQGNTLDLVQLEQNEAAFSVAVCRFSNTGEDWYVLVGVAKDLILNPRSVAGGFVYTYKLVNNGEKLEFLHKTPVEEVPAAIAPFQGRVLIGVGKLLRVYDLGKKKLLRKCENKHIANYISGIQTIGHRVIVSDVQESFIWVRYKRNENQLIIFADDTYPRWVTTASLLDYDTVAGADKFGNICVVRLPPNTNDEVDEDPTGNKALWDRGLLNGASQKAEVIMNYHVGETVLSLQKTTLIPGGSESLVYTTLSGGIGILVPFTSHEDHDFFQHVEMHLRSEHPPLCGRDHLSFRSYYFPVKNVIDGDLCEQFNSMEPNKQKNVSEELDRTPPEVSKKLEDIRTRYAF

>NP_110438.1

MLRALSRLGAGTPCRPRAPLVLPARGRKTRHDPLAKSKIERVNMPPAVDPAEFFVLMERYQHYRQTVRALRMEFVSEVQRKVHEARAGVLAERKALKDAAEHRELMAWNQAENRRLHELRIARLRQEEREQEQRQALEQARKAEEVQAWAQRKEREVLQLQEEVKNFITRENLEARVEAALDSRKNYNWAITREGLVVRPQRRDS

1440 NP_036363.2 NP_002684.1

>NP_036363.2

MNQNTTEPVAATETLAEVPEHVLRGLPEEVRLFPSAVDKTRIGVWATKPILKGKKFGPFVGDKKKRSQVKNNVYMWEVYYPNLGWMCIDATDPEKGNWLRYVNWACSGEEQNLFPLEINRAIYYKTLKPIAPGEELLVWYNGEDNPEIAAAIEEERASARSKRSSPKSRKGKKKSQENKNKGNKIQDIQLKTSEPDFTSANMRDSAEGPKEDEEKPSASALEQPATLQEVASQEVPPELATPAPAWEPQPEPDERLEAAACEVNDLGEEEEEEEEEDEEEEEDDDDDELEDEGEEEASMPNENSVKEPEIRCDEKPEDLLEEPKTTSEETLEDCSEVTPAMQIPRTKEEANGDVFETFMFPCQHCERKFTTKQGLERHMHIHISTVNHAFKCKYCGKAFGTQINRRRHERRHEAGLKRKPSQTLQPSEDLADGKASGENVASKDDSSPPSLGPDCLIMNSEKASQDTINSSVVEENGEVKELHPCKYCKKVFGTHTNMRRHQRRVHERHLIPKGVRRKGGLEEPQPPAEQAQATQNVYVPSTEPEEEGEADDVYIMDISSNISENLNYYIDGKIQTNNNTSNCDVIEMESASADLYGINCLLTPVTVEITQNIKTTQVPVTEDLPKEPLGSTNSEAKKRRTASPPALPKIKAETDSDPMVPSCSLSLPLSISTTEAVSFHKEKSVYLSSKLKQLLQTQDKLTPAGISATEIAKLGPVCVSAPASMLPVTSSRFKRRTSSPPSSPQHSPALRDFGKPSDGKAAWTDAGLTSKKSKLESHSDSPAWSLSGRDERETVSPPCFDEYKMSKEWTASSAFSSVCNQQPLDLSSGVKQKAEGTGKTPVQWESVLDLSVHKKHCSDSEGKEFKESHSVQPTCSAVKKRKPTTCMLQKVLLNEYNGIDLPVENPADGTRSPSPCKSLEAQPDPDLGPGSGFPAPTVESTPDVCPSSPALQTPSLSSGQLPPLLIPTDPSSPPPCPPVLTVATPPPPLLPTVPLPAPSSSASPHPCPSPLSNATAQSPLPILSPTVSPSPSPIPPVEPLMSAASPGPPTLSSSSSSSSSSSSFSSSSSSSSPSPPPLSAISSVVSSGDNLEASLPMISFKQEELENEGLKPREEPQSAAEQDVVVQETFNKNFVCNVCESPFLSIKDLTKHLSIHAEEWPFKCEFCVQLFKDKTDLSEHRFLLHGVGNIFVCSVCKKEFAFLCNLQQHQRDLHPDKVCTHHEFESGTLRPQNFTDPSKAHVEHMQSLPEDPLETSKEEEELNDSSEELYTTIKIMASGIKTKDPDVRLGLNQHYPSFKPPPFQYHHRNPMGIGVTATNFTTHNIPQTFTTAIRCTKCGKGVDNMPELHKHILACASASDKKRYTPKKNPVPLKQTVQPKNGVVVLDNSGKNAFRRMGQPKRLNFSVELSKMSSNKLKLNALKKKNQLVQKAILQKNKSAKQKADLKNACESSSHICPYCNREFTYIGSLNKHAAFSCPKKPLSPPKKKVSHSSKKGGHSSPASSDKNSNSNHRRRTADAEIKMQSMQTPLGKTRARSSGPTQVPLPSSSFRSKQNVKFAASVKSKKPSSSSLRNSSPIRMAKITHVEGKKPKAVAKNHSAQLSSKTSRSLHVRVQKSKAVLQSKSTLASKKRTDRFNIKSRERSGGPVTRSLQLAAAADLSENKREDGSAKQELKDFSYSLRLASRCSPPAAPYITRQYRKVKAPAAAQFQGPFFKE

>NP_002684.1

MSRLLWRKVAGATVGPGPVPAPGRWVSSSVPASDPSDGQRRRQQQQQQQQQQQQQPQQPQVLSSEGGQLRHNPLDIQMLSRGLHEQIFGQGGEMPGEAAVRRSVEHLQKHGLWGQPAVPLPDVELRLPPLYGDNLDQHFRLLAQKQSLPYLEAANLLLQAQLPPKPPAWAWAEGWTRYGPEGEAVPVAIPEERALVFDVEVCLAEGTCPTLAVAISPSAWYSWCSQRLVEERYSWTSQLSPADLIPLEVPTGASSPTQRDWQEQLVVGHNVSFDRAHIREQYLIQGSRMRFLDTMSMHMAISGLSSFQRSLWIAAKQGKHKVQPPTKQGQKSQRKARRGPAISSWDWLDISSVNSLAEVHRLYVGGPPLEKEPRELFVKGTMKDIRENFQDLMQYCAQDVWATHEVFQQQLPLFLERCPHPVTLAGMLEMGVSYLPVNQNWERYLAEAQGTYEELQREMKKSLMDLANDACQLLSGERYKEDPWLWDLEWDLQEFKQKKAKKVKKEPATASKLPIEGAGAPGDPMDQEDLGPCSEEEEFQQDVMARACLQKLKGTTELLPKRPQHLPGHPGWYRKLCPRLDDPAWTPGPSLLSLQMRVTPKLMALTWDGFPLHYSERHGWGYLVPGRRDNLAKLPTGTTLESAGVVCPYRAIESLYRKHCLEQGKQQLMPQEAGLAEEFLLTDNSAIWQTVEELDYLEVEAEAKMENLRAAVPGQPLALTARGGPKDTQPSYHHGNGPYNDVDIPGCWFFKLPHKDGNSCNVGSPFAKDFLPKMEDGTLQAGPGGASGPRALEINKMISFWRNAHKRISSQMVVWLPRSALPRAVIRHPDYDEEGLYGAILPQVVTAGTITRRAVEPTWLTASNARPDRVGSELKAMVQAPPGYTLVGADVDSQELWIAAVLGDAHFAGMHGCTAFGWMTLQGRKSRGTDLHSKTATTVGISREHAKIFNYGRIYGAGQPFAERLLMQFNHRLTQQEAAEKAQQMYAATKGLRWYRLSDEGEWLVRELNLPVDRTEGGWISLQDLRKVQRETARKSQWKKWEVVAERAWKGGTESEMFNKLESIATSDIPRTPVLGCCISRALEPSAVQEEFMTSRVNWVVQSSAVDYLHLMLVAMKWLFEEFAIDGRFCISIHDEVRYLVREEDRYRAALALQITNLLTRCMFAYKLGLNDLPQSVAFFSAVDIDRCLRKEVTMDCKTPSNPTGMERRYGIPQGEALDIYQIIELTKGSLEKRSQPGP

1441 NP_037406.1 P50226

>NP_037406.1

MDPRGILKAFPKRQKIHADASSKVLAKIPRREEGEEAEEWLSSLRAHVVRTGIGRARAELFEKQIVQHGGQLCPAQGPGVTHIVVDEGMDYERALRLLRLPQLPPGAQLVKSAWLSLCLQERRLVDVAGFSIFIPSRYLDHPQPSKAEQDASIPPGTHEALLQTALSPPPPPTRPVSPPQKAKEAPNTQAQPISDDEASDGEETQVSAADLEALISGHYPTSLEGDCEPSPAPAVLDKWVCAQPSSQKATNHNLHITEKLEVLAKAYSVQGDKWRALGYAKAINALKSFHKPVTSYQEACSIPGIGKRMAEKIIEILESGHLRKLDHISESVPVLELFSNIWGAGTKTAQMWYQQGFRSLEDIRSQASLTTQQAIGLKHYSDFLERMPREEATEIEQTVQKAAQAFNSGLLCVACGSYRRGKATCGDVDVLITHPDGRSHRGIFSRLLDSLRQEGFLTDDLVSQEENGQQQKYLGVCRLPGPGRRHRRLDIIVVPYSEFACALLYFTGSAHFNRSMRALAKTKGMSLSEHALSTAVVRNTHGCKVGPGRVLPTPTEKDVFRLLGLPYREPAERDW

>P50226

MELIQDTSRPPLEYVKGVPLIKYFAEALGPLQSFQARPDDLLISTYPKSGTTWVSQILDMIYQGGDLEKCHRAPIFMRVPFLEFKVPGIPSGMETLKNTPAPRLLKTHLPLALLPQTLLDQKVKVVYVARNAKDVAVSYYHFYHMAKVYPHPGTWESFLEKFMAGEVSYGSWYQHVQEWWELSRTHPVLYLFYEDMKENPKREIQKILEFVGRSLPEETVDLMVEHTSFKEMKKTPMTNYTTVRREFMDHSISPFMRKGMAGDWKTTFTVAQNERFDADYAEKMAGCSLSFRSEL

1442 Q8N5A5 NP_055165.2

>Q8N5A5

MDEESLESALQTYRAQLQQVELALGAGLDSSEQADLRQLQGDLKELIELTEASLVSVRKSSLLAALDEERPGRQEDAEYQAFREAITEAVEAPAAARGSGSETVPKAEAGPESAAGGQEEEEGEDEEELSGTKVSAPYYSSWGTLEYHNAMVVGTEEAEDGSAGVRVLYLYPTHKSLKPCPFFLEGKCRFKENCRFSHGQVVSLDELRPFQDPDLSSLQAGSACLAKHQDGLWHAARITDVDNGYYTVKFDSLLLREAVVEGDGILPPLRTEATESDSDSDGTGDSSYARVVGSDAVDSAQSSALCPSLAVVGSDAVDSGTCSSAFAGWEVHTRGIGSRLLTKMGYEFGKGLGRHAEGRVEPIHAVVLPRGKSLDQCVETLQKQTRVGKAGTNKPPRCRGRGARPGGRPAPRNVFDFLNEKLQGQAPGALEAGAAPAGRRSKDMYHASKSAKRALSLRLFQTEEKIERTQRDIRSIQEALARNAGRHSVASAQLQEKLAGAQRQLGQLRAQEAGLQQEQRKADTHKKMTEF

>NP_055165.2

MHSEAEESKEVATDVFNSKNLAVQAQKKILGKMVSKSIATTLIDDTSSEVLDELYRVTREYTQNKKEAEKIIKNLIKTVIKLAILYRNNQFNQDELALMEKFKKKVHQLAMTVVSFHQVDYTFDRNVLSRLLNECREMLHQIIQRHLTAKSHGRVNNVFDHFSDCEFLAALYNPFGNFKPHLQKLCDGINKMLDEENI

1443 Q9Y6W3 Q06124

>Q9Y6W3

MDATALERDAVQFARLAVQRDHEGRYSEAVFYYKEAAQALIYAEMAGSSLENIQEKITEYLERVQALHSAVQSKSADPLKSKHQLDLERAHFLVTQAFDEDEKENVEDAIELYTEAVDLCLKTSYETADKVLQNKLKQLARQALDRAEALSEPLTKPVGKISSTSVKPKPPPVRAHFPLGANPFLERPQSFISPQSCDAQGQRYTAEEIEVLRTTSKINGIEYVPFMNVDLRERFAYPMPFCDRWGKLPLSPKQKTTFSKWVRPEDLTNNPTMIYTVSSFSIKQTIVSDCSFVASLAISAAYERRFNKKLITGIIYPQNKDGEPEYNPCGKYMVKLHLNGVPRKVIIDDQLPVDHKGELLCSYSNNKSELWVSLIEKAYMKVMGGYDFPGSNSNIDLHALTGWIPERIAMHSDSQTFSKDNSFRMLYQRFHKGDVLITASTGMMTEAEGEKWGLVPTHAYAVLDIREFKGLRFIQLKNPWSHLRWKGRYSENDVKNWTPELQKYLNFDPRTAQKIDNGIFWISWDDLCQYYDVIYLSWNPGLFKESTCIHSTWDAKQGPVKDAYSLANNPQYKLEVQCPQGGAAVWVLLSRHITDKDDFANNREFITMVVYKTDGKKVYYPADPPPYIDGIRINSPHYLTKIKLTTPGTHTFTLVVSQYEKQNTIHYTVRVYSACSFTFSKIPSPYTLSKRINGKWSGQSAGGCGNFQETHKNNPIYQFHIEKTGPLLIELRGPRQYSVGFEVVTVSTLGDPGPHGFLRKSSGDYRCGFCYLELENIPSGIFNIIPSTFLPKQEGPFFLDFNSIIPIKITQLQ

>Q06124

MTSRRWFHPNITGVEAENLLLTRGVDGSFLARPSKSNPGDFTLSVRRNGAVTHIKIQNTGDYYDLYGGEKFATLAELVQYYMEHHGQLKEKNGDVIELKYPLNCADPTSERWFHGHLSGKEAEKLLTEKGKHGSFLVRESQSHPGDFVLSVRTGDDKGESNDGKSKVTHVMIRCQELKYDVGGGERFDSLTDLVEHYKKNPMVETLGTVLQLKQPLNTTRINAAEIESRVRELSKLAETTDKVKQGFWEEFETLQQQECKLLYSRKEGQRQENKNKNRYKNILPFDHTRVVLHDGDPNEPVSDYINANIIMPEFETKCNNSKPKKSYIATQGCLQNTVNDFWRMVFQENSRVIVMTTKEVERGKSKCVKYWPDEYALKEYGVMRVRNVKESAAHDYTLRELKLSKVGQALLQGNTERTVWQYHFRTWPDHGVPSDPGGVLDFLEEVHHKQESIMDAGPVVVHCSAGIGRTGTFIVIDILIDIIREKGVDCDIDVPKTIQMVRSQRSGMVQTEAQYRFIYMAVQHYIETLQRRIEEEQKSKRKGHEYTNIKYSLADQTSGDQSPLPPCTPTPPCAEMREDSARVYENVGLMQQQKSFR

1444 NP_002303.2 NP_002717.3

>NP_002303.2

MAASQTSQTVASHVPFADLCSTLERIQKSKGRAEKIRHFREFLDSWRKFHDALHKNHKDVTDSFYPAMRLILPQLERERMAYGIKETMLAKLYIELLNLPRDGKDALKLLNYRTPTGTHGDAGDFAMIAYFVLKPRCLQKGSLTIQQVNDLLDSIASNNSAKRKDLIKKSLLQLITQSSALEQKWLIRMIIKDLKLGVSQQTIFSVFHNDAAELHNVTTDLEKVCRQLHDPSVGLSDISITLFSAFKPMLAAIADIEHIEKDMKHQSFYIETKLDGERMQMHKDGDVYKYFSRNGYNYTDQFGASPTEGSLTPFIHNAFKADIQICILDGEMMAYNPNTQTFMQKGTKFDIKRMVEDSDLQTCYCVFDVLMVNNKKLGHETLRKRYEILSSIFTPIPGRIEIVQKTQAHTKNEVIDALNEAIDKREEGIMVKQPLSIYKPDKRGEGWLKIKPEYVSGLMDELDILIVGGYWGKGSRGGMMSHFLCAVAEKPPPGEKPSVFHTLSRVGSGCTMKELYDLGLKLAKYWKPFHRKAPPSSILCGTEKPEVYIEPCNSVIVQIKAAEIVPSDMYKTGCTLRFPRIEKIRDDKEWHECMTLDDLEQLRGKASGKLASKHLYIGGDDEPQEKKRKAAPKMKKVIGIIEHLKAPNLTNVNKISNIFEDVEFCVMSGTDSQPKPDLENRIAEFGGYIVQNPGPDTYCVIAGSENIRVKNIILSNKHDVVKPAWLLECFKTKSFVPWQPRFMIHMCPSTKEHFAREYDCYGDSYFIDTDLNQLKEVFSGIKNSNEQTPEEMASLIADLEYRYSWDCSPLSMFRRHTVYLDSYAVINDLSTKNEGTRLAIKALELRFHGAKVVSCLAEGVSHVIIGEDHSRVADFKAFRRTFKRKFKILKESWVTDSIDKCELQEENQYLI

>NP_002717.3

MLSLQYPDVYRDETAVQDYHGHKICDPYAWLEDPDSEQTKAFVEAQNKITVPFLEQCPIRGLYKERMTELYDYPKYSCHFKKGKRYFYFYNTGLQNQRVLYVQDSLEGEARVFLDPNILSDDGTVALRGYAFSEDGEYFAYGLSASGSDWVTIKFMKVDGAKELPDVLERVKFSCMAWTHDGKGMFYNSYPQQDGKSDGTETSTNLHQKLYYHVLGTDQSEDILCAEFPDEPKWMGGAELSDDGRYVLLSIREGCDPVNRLWYCDLQQESSGIAGILKWVKLIDNFEGEYDYVTNEGTVFTFKTNRQSPNYRVINIDFRDPEESKWKVLVPEHEKDVLEWIACVRSNFLVLCYLHDVKNILQLHDLTTGALLKTFPLDVGSIVGYSGQKKDTEIFYQFTSFLSPGIIYHCDLTKEELEPRVFREVTVKGIDASDYQTVQIFYPSKDGTKIPMFIVHKKGIKLDGSHPAFLYGYGGFNISITPNYSVSRLIFVRHMGGILAVANIRGGGEYGETWHKGGILANKQNCFDDFQCAAEYLIKEGYTSPKRLTINGGSNGGLLVAACANQRPDLFGCVIAQVGVMDMLKFHKYTIGHAWTTDYGCSDSKQHFEWLVKYSPLHNVKLPEADDIQYPSMLLLTADHDDRVVPLHSLKFIATLQYIVGRSRKQSNPLLIHVDTKAGHGAGKPTAKVIEEVSDMFAFIARCLNVDWIP

1445 NP_073598.1 P51970

>NP_073598.1

MAASQCLCCSKFLFQRQNLACFLTNPHCGSLVNADGHGEVWTDWNNMSKFFQYGWRCTTNENTYSNRTLMGNWNQERYDLRNIVQPKPLPSQFGHYFETTYDTSYNNKMPLSTHRFKREPHWFPGHQPELDPPRYKCTEKSTYMNSYSKP

>P51970

MPGIVELPTLEELKVDEVKISSAVLKAAAHHYGAQCDKPNKEFMLCRWEEKDPRRCLEEGKLVNKCALDFFRQIKRHCAEPFTEYWTCIDYTGQQLFRHCRKQQAKFDECVLDKLGWVRPDLGELSKVTKVKTDRPLPENPYHSRPRPDPSPEIEGDLQPATHGSRFYFWTK

1446 P28715 NP_803187.1

>P28715

MGVQGLWKLLECSGRQVSPEALEGKILAVDISIWLNQALKGVRDRHGNSIENPHLLTLFHRLCKLLFFRIRPIFVFDGDAPLLKKQTLVKRRQRKDLASSDSRKTTEKLLKTFLKRQAIKTAFRSKRDEALPSLTQVRRENDLYVLPPLQEEEKHSSEEEDEKEWQERMNQKQALQEEFFHNPQAIDIESEDFSSLPPEVKHEILTDMKEFTKRRRTLFEAMPEESDDFSQYQLKGLLKKNYLNQHIEHVQKEMNQQHSGHIRRQYEDEGGFLKEVESRRVVSEDTSHYILIKGIQAKTVAEVDSESLPSSSKMHGMSFDVKSSPCEKLKTEKEPDATPPSPRTLLAMQAALLGSSSEEELESENRRQARGRNAPAAVDEGSISPRTLSAIKRALDDDEDVKVCAGDDVQTGGPGAEEMRINSSTENSDEGLKVRDGKGIPFTATLASSSVNSAEEHVASTNEGREPTDSVPKEQMSLVHVGTEAFPISDESMIKDRKDRLPLESAVVRHSDAPGLPNGRELTPASPTCTNSVSKNETHAEVLEQQNELCPYESKFDSSLLSSDDETKCKPNSASEVIGPVSLQETSSIVSVPSEAVDNVENVVSFNAKEHENFLETIQEQQTTESAGQDLISIPKAVEPMEIDSEESESDGSFIEVQSVISDEELQAEFPETSKPPSEQGEEELVGTREGEAPAESESLLRDNSERDDVDGEPQEAEKDAEDSLHEWQDINLEELETLESNLLAQQNSLKAQKQQQERIAATVTGQMFLESQELLRLFGIPYIQAPMEAEAQCAILDLTDQTSGTITDDSDIWLFGARHVYRNFFNKNKFVEYYQYVDFHNQLGLDRNKLINLAYLLGSDYTEGIPTVGCVTAMEILNEFPGHGLEPLLKFSEWWHEAQKNPKIRPNPHDTKVKKKLRTLQLTPGFPNPAVAEAYLKPVVDDSKGSFLWGKPDLDKIREFCQRYFGWNRTKTDESLFPVLKQLDAQQTQLRIDSFFRLAQQEKEDAKRIKSQRLNRAVTCMLRKEKEAAASEIEAVSVAMEKEFELLDKAKGKTQKRGITNTLEESSSLKRKRLSDSKGKNTCGGFLGETCLSESSDGSSSEDAESSSLMNVQRRTAAKEPKTSASDSQNSVKEAPVKNGGATTSSSSDSDDDGGKEKMVLVTARSVFGKKRRKLRRARGRKRKT

>NP_803187.1

MKSPALQPLSMAGLQLMTPASSPMGPFFGLPWQQEAIHDNIYTPRKYQVELLEAALDHNTIVCLNTGSGKTFIAVLLTKELSYQIRGDFSRNGKRTVFLVNSANQVAQQVSAVRTHSDLKVGEYSNLEVNASWTKERWNQEFTKHQVLIMTCYVALNVLKNGYLSLSDINLLVFDECHLAILDHPYREIMKLCENCPSCPRILGLTASILNGKCDPEELEEKIQKLEKILKSNAETATDLVVLDRYTSQPCEIVVDCGPFTDRSGLYERLLMELEEALNFINDCNISVHSKERDSTLISKQILSDCRAVLVVLGPWCADKVAGMMVRELQKYIKHEQEELHRKFLLFTDTFLRKIHALCEEHFSPASLDLKFVTPKVIKLLEILRKYKPYERQQFESVEWYNNRNQDNYVSWSDSEDDDEDEEIEEKEKPETNFPSPFTNILCGIIFVERRYTAVVLNRLIKEAGKQDPELAYISSNFITGHGIGKNQPRNKQMEAEFRKQEEVLRKFRAHETNLLIATSIVEEGVDIPKCNLVVRFDLPTEYRSYVQSKGRARAPISNYIMLADTDKIKSFEEDLKTYKAIEKILRNKCSKSVDTGETDIDPVMDDDDVFPPYVLRPDDGGPRVTINTAIGHINRYCARLPSDPFTHLAPKCRTRELPDGTFYSTLYLPINSPLRASIVGPPMSCVRLAERVVALICCEKLHKIGELDDHLMPVGKETVKYEEELDLHDEEETSVPGRPGSTKRRQCYPKAIPECLRDSYPRPDQPCYLYVIGMVLTTPLPDELNFRRRKLYPPEDTTRCFGILTAKPIPQIPHFPVYTRSGEVTISIELKKSGFMLSLQMLELITRLHQYIFSHILRLEKPALEFKPTDADSAYCVLPLNVVNDSSTLDIDFKFMEDIEKSEARIGIPSTKYTKETPFVFKLEDYQDAVIIPRYRNFDQPHRFYVADVYTDLTPLSKFPSPEYETFAEYYKTKYNLDLTNLNQPLLDVDHTSSRLNLLTPRHLNQKGKALPLSSAEKRKAKWESLQNKQILVPELCAIHPIPASLWRKAVCLPSILYRLHCLLTAEELRAQTASDAGVGVRSLPADFRYPNLDFGWKKSIDSKSFISISNSSSAENDNYCKHSTIVPENAAHQGANRTSSLENHDQMSVNCRTLLSESPGKLHVEVSADLTAINGLSYNQNLANGSYDLANRDFCQGNQLNYYKQEIPVQPTTSYSIQNLYSYENQPQPSDECTLLSNKYLDGNANKSTSDGSPVMAVMPGTTDTIQVLKGRMDSEQSPSIGYSSRTLGPNPGLILQALTLSNASDGFNLERLEMLGDSFLKHAITTYLFCTYPDAHEGRLSYMRSKKVSNCNLYRLGKKKGLPSRMVVSIFDPPVNWLPPGYVVNQDKSNTDKWEKDEMTKDCMLANGKLDEDYEEEDEEEESLMWRAPKEEADYEDDFLEYDQEHIRFIDNMLMGSGAFVKKISLSPFSTTDSAYEWKMPKKSSLGSMPFSSDFEDFDYSSWDAMCYLDPSKAVEEDDFVVGFWNPSEENCGVDTGKQSISYDLHTEQCIADKSIADCVEALLGCYLTSCGERAAQLFLCSLGLKVLPVIKRTDREKALCPTRENFNSQQKNLSVSCAAASVASSRSSVLKDSEYGCLKIPPRCMFDHPDADKTLNHLISGFENFEKKINYRFKNKAYLLQAFTHASYHYNTITDCYQRLEFLGDAILDYLITKHLYEDPRQHSPGVLTDLRSALVNNTIFASLAVKYDYHKYFKAVSPELFHVIDDFVQFQLEKNEMQGMDSELRRSEEDEEKEEDIEVPKAMGDIFESLAGAIYMDSGMSLETVWQVYYPMMRPLIEKFSANVPRSPVRELLEMEPETAKFSPAERTYDGKVRVTVEVVGKGKFKGVGRSYRIAKSAAARRALRSLKANQPQVPNS

1447 NP_001343.2 NP_004126.1

>NP_001343.2

MARPVSDRTPAPLLLGGPAGTPPGGGALLGLRSLLQGTSKPKEPASCLLKEKERKAALPAATTPGPGLETAGPADAPAGAVVGGGSPRGRPGPVPAPGLLAPLLWERTLPFGDVEYVDLDAFLLEHGLPPSPPPPGGPSPEPSPARTPAPSPGPGSCGSASPRSSPGHAPARAALGTASGHRAGLTSRDTPSPVDPDTVEVLMTFEPDPADLALSSIPGHETFDPRRHRFSEEELKPQPIMKKARKIQVPEEQKDEKYWSRRYKNNEAAKRSRDARRLKENQISVRAAFLEKENALLRQEVVAVRQELSHYRAVLSRYQAQHGAL

>NP_004126.1

MALKVATVAGSAAKAVLGPALLCRPWEVLGAHEVPSRNIFSEQTIPPSAKYGGRHTVTMIPGDGIGPELMLHVKSVFRHACVPVDFEEVHVSSNADEEDIRNAIMAIRRNRVALKGNIETNHNLPPSHKSRNNILRTSLDLYANVIHCKSLPGVVTRHKDIDILIVRENTEGEYSSLEHESVAGVVESLKIITKAKSLRIAEYAFKLAQESGRKKVTAVHKANIMKLGDGLFLQCCREVAARYPQITFENMIVDNTTMQLVSRPQQFDVMVMPNLYGNIVNNVCAGLVGGPGLVAGANYGHVYAVFETATRNTGKSIANKNIANPTATLLASCMMLDHLKLHSYATSIRKAVLASMDNENMHTPDIGGQGTTSEAIQDVIRHIRVINGRAVEA

1448 Q9NWU2 NP_055199.1

>Q9NWU2

MSYAEKPDEITKDEWMEKLNNLHVQRADMNRLIMNYLVTEGFKEAAEKFRMESGIEPSVDLETLDERIKIREMILKGQIQEAIALINSLHPELLDTNRYLYFHLQQQHLIELIRQRETEAALEFAQTQLAEQGEESRECLTEMERTLALLAFDSPEESPFGDLLHTMQRQKVWSEVNQAVLDYENRESTPKLAKLLKLLLWAQNELDQKKVKYPKMTDLSKGVIEEPK

>NP_055199.1

MLWSGCRRFGARLGCLPGGLRVLVQTGHRSLTSCIDPSMGLNEEQKEFQKVAFDFAAREMAPNMAEWDQKELFPVDVMRKAAQLGFGGVYIQTDVGGSGLSRLDTSVIFEALATGCTSTTAYISIHNMCAWMIDSFGNEEQRHKFCPPLCTMEKFASYCLTEPGSGSDAASLLTSAKKQGDHYILNGSKAFISGAGESDIYVVMCRTGGPGPKGISCIVVEKGTPGLSFGKKEKKVGWNSQPTRAVIFEDCAVPVANRIGSEGQGFLIAVRGLNGGRINIASCSLGAAHASVILTRDHLNVRKQFGEPLASNQYLQFTLADMATRLVAARLMVRNAAVALQEERKDAVALCSMAKLFATDECFAICNQALQMHGGYGYLKDYAVQQYVRDSRVHQILEGSNEVMRILISRSLLQE

1449 Q9UKN8 NP_005411.1

>Q9UKN8

MNTADQARVGPADDGPAPSGEEEGEGGGEAGGKEPAADAAPGPSAAFRLMVTRREPAVKLQYAVSGLEPLAWSEDHRVSVSTARSIAVLELICDVHNPGQDLVIHRTSVPAPLNSCLLKVGSKTEVAECKEKFAASKDPTVSQTFMLDRVFNPEGKALPPMRGFKYTSWSPMGCDANGRCLLAALTMDNRLTIQANLNRLQWVQLVDLTEIYGERLYETSYRLSKNEAPEGNLGDFAEFQRRHSMQTPVRMEWSGICTTQQVKHNNECRDVGSVLLAVLFENGNIAVWQFQLPFVGKESISSCNTIESGITSPSVLFWWEYEHNNRKMSGLIVGSAFGPIKILPVNLKAVKGYFTLRQPVILWKEMDQLPVHSIKCVPLYHPYQKCSCSLVVAARGSYVFWCLLLISKAGLNVHNSHVTGLHSLPIVSMTADKQNGTVYTCSSDGKVRQLIPIFTDVALKFEHQLIKLSDVFGSVRTHGIAVSPCGAYLAIITTEGMINGLHPVNKNYQVQFVTLKTFEEAAAQLLESSVQNLFKQVDLIDLVRWKILKDKHIPQFLQEALEKKIESSGVTYFWRFKLFLLRILYQSMQKTPSEALWKPTHEDSKILLVDSPGMGNADDEQQEEGTSSKQVVKQGLQERSKEGDVEEPTDDSLPTTGDAGGREPMEEKLLEIQGKIEAVEMHLTREHMKRVLGEVYLHTWITENTSIPTRGLCNFLMSDEEYDDRTARVLIGHISKKMNKQTFPEHCSLCKEILPFTDRKQAVCSNGHIWLRCFLTYQSCQSLIYRRCLLHDSIARHPAPEDPDWIKRLLQSPCPFCDSPVF

>NP_005411.1

MNSELDYYEKFEEVHGILMYKDFVKYWDNVEAFQARPDDLVIATYPKSGTTWVSEIVYMIYKEGDVEKCKEDVIFNRIPFLECRKENLMNGVKQLDEMNSPRIVKTHLPPELLPASFWEKDCKIIYLCRNAKDVAVSFYYFFLMVAGHPNPGSFPEFVEKFMQGQVPYGSWYKHVKSWWEKGKSPRVLFLFYEDLKEDIRKEVIKLIHFLERKPSEELVDRIIHHTSFQEMKNNPSTNYTTLPDEIMNQKLSPFMRKGITGDWKNHFTVALNEKFDKHYEQQMKESTLKFRTEI

1450 NP_904357.1 Q8WXI4

>NP_904357.1

MPFLGQDWRSPGWSWIKTEDGWKRCESCSQKLERENNRCNISHSIILNSEDGEIFNNEEHEYASKKRKKDHFRNDTNTQSFYREKWIYVHKESTKERHGYCTLGEAFNRLDFSSAIQDIRRFNYVVKLLQLIAKSQLTSLSGVAQKNYFNILDKIVQKVLDDHHNPRLIKDLLQDLSSTLCILIRGVGKSVLVGNINIWICRLETILAWQQQLQDLQMTKQVNNGLTLSDLPLHMLNNILYRFSDGWDIITLGQVTPTLYMLSEDRQLWKKLCQYHFAEKQFCRHLILSEKGHIEWKLMYFALQKHYPAKEQYGDTLHFCRHCSILFWKDYHLALLFKDSGHPCTAADPDSCFTPVSPQHFIDLFKF

>Q8WXI4

MIQNVGNHLRRGLASVFSNRTSRKSALRAGNDSAMADGEGYRNPTEVQMSQLVLPCHTNQRGELSVGQLLKWIDTTACLSAERHAGCPCVTASMDDIYFEHTISVGQVVNIKAKVNRAFNSSMEVGIQVASEDLCSEKQWNVCKALATFVARREITKVKLKQITPRTEEEKMEHSVAAERRRMRLVYADTIKDLLANCAIQGDLESRDCSRMVPAEKTRVESVELVLPPHANHQGNTFGGQIMAWMENVATIAASRLCRAHPTLKAIEMFHFRGPSQVGDRLVLKAIVNNAFKHSMEVGVCVEAYRQEAETHRRHINSAFMTFVVLDADDQPQLLPWIRPQPGDGERRYREASARKKIRLDRKYIVSCKQTEVPLSVPWDPSNQVYLSYNNVSSLKMLVAKDNWVLSSEISQVRLYTLEDDKFLSFHMEMVVHVDAAQAFLLLSDLRQRPEWDKHYRSVELVQQVDEDDAIYHVTSPALGGHTKPQDFVILASRRKPCDNGDPYVIALRSVTLPTHRETPEYRRGETLCSGFCLWREGDQLTKCCWVRVSLTELVSASGFYSWGLESRSKGRRSDGWNGKLAGGHLSTLKAIPVAKINSRFGYLQDT

1451 NP_002350.1 NP_036232.1

>NP_002350.1

MTTPNKGNKALKVKREPGENGTSLTDEELVTMSVRELNQHLRGLSKEEIVQLKQRRRTLKNRGYAASCRVKRVTQKEELEKQKAELQQEVEKLASENASMKLELDALRSKYEALQTFARTVARSPVAPARGPLAAGLGPLVPGKVAATSVITIVKSKTDARS

>NP_036232.1

MQVAMNGKARKEAVQTAAKELLKFVNRSPSPFHAVAECRNRLLQAGFSELKETEKWNIKPESKYFMTRNSSTIIAFAVGGQYVPGNGFSLIGAHTDSPCLRVKRRSRRSQVGFQQVGVETYGGGIWSTWFDRDLTLAGRVIVKCPTSGRLEQQLVHVERPILRIPHLAIHLQRNINENFGPNTEMHLVPILATAIQEELEKGTPEPGPLNAVDERHHSVLMSLLCAHLGLSPKDIVEMELCLADTQPAVLGGAYDEFIFAPRLDNLHSCFCALQALIDSCAGPGSLATEPHVRMVTLYDNEEVGSESAQGAQSLLTELVLRRISASCQHPTAFEEAIPKSFMISADMAHAVHPNYLDKHEENHRPLFHKGPVIKVNSKQRYASNAVSEALIREVANKVKVPLQDLMVRNDTPCGTTIGPILASRLGLRVLDLGSPQLAMHSIREMACTTGVLQTLTLFKGFFELFPSLSHNLLVD

1452 O95766 NP_001025.1

>O95766

MAAAAAGAGSGPWAAQEKQFPPALLSFFIYNPRFGPREGQEENKILFYHPNEVEKNEKIRNVGLCEAIVQFTRTFSPSKPAKSLHTQKNRQFFNEPEENFWMVMVVRNPIIEKQSKDGKPVIEYQEEELLDKVYSSVLRQCYSMYKLFNGTFLKAMEDGGVKLLKERLEKFFHRYLQTLHLQSCDLLDIFGGISFFPLDKMTYLKIQSFINRMEESLNIVKYTAFLYNDQLIWSGLEQDDMRILYKYLTTSLFPRHIEPELAGRDSPIRAEMPGNLQHYGRFLTGPLNLNDPDAKCRFPKIFVNTDDTYEELHLIVYKAMSAAVCFMIDASVHPTLDFCRRLDSIVGPQLTVLASDICEQFNINKRMSGSEKEPQFKFIYFNHMNLAEKSTVHMRKTPSVSLTSVHPDLMKILGDINSDFTRVDEDEEIIVKAMSDYWVVGKKSDRRELYVILNQKNANLIEVNEEVKKLCATQFNNIFFLD

>NP_001025.1

MLSLRVPLAPITDPQQLQLSPLKGLSLVDKENTPPALSGTRVLASKTARRIFQEPTEPKTKAAAPGVEDEPLLRENPRRFVIFPIEYHDIWQMYKKAEASFWTAEEVDLSKDIQHWESLKPEERYFISHVLAFFAASDGIVNENLVERFSQEVQITEARCFYGFQIAMENIHSEMYSLLIDTYIKDPKEREFLFNAIETMPCVKKKADWALRWIGDKEATYGERVVAFAAVEGIFFSGSFASIFWLKKRGLMPGLTFSNELISRDEGLHCDFACLMFKHLVHKPSEERVREIIINAVRIEQEFLTEALPVKLIGMNCTLMKQYIEFVADRLMLELGFSKVFRVENPFDFMENISLEGKTNFFEKRVGEYQRMGVMSSPTENSFTLDADF

1453 NP_004951.1 NP_071445.1

>NP_004951.1

MASNDYTQQATQSYGAYPTQPGQGYSQQSSQPYGQQSYSGYSQSTDTSGYGQSSYSSYGQSQNTGYGTQSTPQGYGSTGGYGSSQSSQSSYGQQSSYPGYGQQPAPSSTSGSYGSSSQSSSYGQPQSGSYSQQPSYGGQQQSYGQQQSYNPPQGYGQQNQYNSSSGGGGGGGGGGNYGQDQSSMSSGGGSGGGYGNQDQSGGGGSGGYGQQDRGGRGRGGSGGGGGGGGGGYNRSSGGYEPRGRGGGRGGRGGMGGSDRGGFNKFGGPRDQGSRHDSEQDNSDNNTIFVQGLGENVTIESVADYFKQIGIIKTNKKTGQPMINLYTDRETGKLKGEATVSFDDPPSAKAAIDWFDGKEFSGNPIKVSFATRRADFNRGGGNGRGGRGRGGPMGRGGYGGGGSGGGGRGGFPSGGGGGGGQQRAGDWKCPNPTCENMNFSWRNECNQCKAPKPDGPGGGPGGSHMGGNYGDDRRGGRGGYDRGGYRGRGGDRGGFRGGRGGGDRGGFGPGKMDSRGEHRQDRRERPY

>NP_071445.1

MGEEGGSASHDEEERASVLLGHSPGCEMCSQEAFQAQRSQLVELLVSGSLEGFESVLDWLLSWEVLSWEDYEGFHLLGQPLSHLARRLLDTVWNKGTWACQKLIAAAQEAQADSQSPKLHGCWDPHSLHPARDLQSHRPAIVRRLHSHVENMLDLAWERGFVSQYECDEIRLPIFTPSQRARRLLDLATVKANGLAAFLLQHVQELPVPLALPLEAATCKKYMAKLRTTVSAQSRFLSTYDGAETLCLEDIYTENVLEVWADVGMAGPPQKSPATLGLEELFSTPGHLNDDADTVLVVGEAGSGKSTLLQRLHLLWAAGQDFQEFLFVFPFSCRQLQCMAKPLSVRTLLFEHCCWPDVGQEDIFQLLLDHPDRVLLTFDGFDEFKFRFTDRERHCSPTDPTSVQTLLFNLLQGNLLKNARKVVTSRPAAVSAFLRKYIRTEFNLKGFSEQGIELYLRKRHHEPGVADRLIRLLQETSALHGLCHLPVFSWMVSKCHQELLLQEGGSPKTTTDMYLLILQHFLLHATPPDSASQGLGPSLLRGRLPTLLHLGRLALWGLGMCCYVFSAQQLQAAQVSPDDISLGFLVRAKGVVPGSTAPLEFLHITFQCFFAAFYLALSADVPPALLRHLFNCGRPGNSPMARLLPTMCIQASEGKDSSVAALLQKAEPHNLQITAAFLAGLLSREHWGLLAECQTSEKALLRRQACARWCLARSLRKHFHSIPPAAPGEAKSVHAMPGFIWLIRSLYEMQEERLARKAARGLNVGHLKLTFCSVGPTECAALAFVLQHLRRPVALQLDYNSVGDIGVEQLLPCLGVCKALYLRDNNISDRGICKLIECALHCEQLQKLALFNNKLTDGCAHSMAKLLACRQNFLALRLGNNYITAAGAQVLAEGLRGNTSLQFLGFWGNRVGDEGAQALAEALGDHQSLRWLSLVGNNIGSVGAQALALMLAKNVMLEELCLEENHLQDEGVCSLAEGLKKNSSLKILKLSNNCITYLGAEALLQALERNDTILEVWLRGNTFSLEEVDKLGCRDTRLLL

1454 NP_006158.2 Q8TE04

>NP_006158.2

MLRVPEPRPGEAKAEGAAPPTPSKPLTSFLIQDILRDGAQRQGGRTSSQRQRDPEPEPEPEPEGGRSRAGAQNDQLSTGPRAAPEEAETLAETEPERHLGSYLLDSENTSGALPRLPQTPKQPQKRSRAAFSHTQVIELERKFSHQKYLSAPERAHLAKNLKLTETQVKIWFQNRRYKTKRKQLSSELGDLEKHSSLPALKEEAFSRASLVSVYNSYPYYPYLYCVGSWSPAFW

>Q8TE04

MLKLVGGGGGQDWACSVAGTSLGGEEAAFEVARPGDQGKAGGGSPGWGCAGIPDSAPGAGVLQAGAVGPARGGQGAEEVGESAGGGEERRVRHPQAPALRLLNRKPQGGSGEIKTPENDLQRGRLSRGPRTAPPAPGMGDRSGQQERSVPHSPGAPVGTSAAAVNGLLHNGFHPPPVQPPHVCSRGPVGGSDAAPQRLPLLPELQPQPLLPQHDSPAKKCRLRRRMDSGRKNRPPFPWFGMDIGGTLVKLVYFEPKDITAEEEQEEVENLKSIRKYLTSNTAYGKTGIRDVHLELKNLTMCGRKGNLHFIRFPSCAMHRFIQMGSEKNFSSLHTTLCATGGGAFKFEEDFRMIADLQLHKLDELDCLIQGLLYVDSVGFNGKPECYYFENPTNPELCQKKPYCLDNPYPMLLVNMGSGVSILAVYSKDNYKRVTGTSLGGGTFLGLCCLLTGCETFEEALEMAAKGDSTNVDKLVKDIYGGDYERFGLQGSAVASSFGNMMSKEKRDSISKEDLARATLVTITNNIGSIARMCALNENIDRVVFVGNFLRINMVSMKLLAYAMDFWSKGQLKALFLEHEGYFGAVGALLELFKMTDDK

1455 NP_002004.1 NP_002878.2

>NP_002004.1

MAAAVPQRAWTVEQLRSEQLPKKDIIKFLQEHGSDSFLAEHKLLGNIKNVAKTANKDHLVTAYNHLFETKRFKGTESISKVSEQVKNVKLNEDKPKETKSEETLDEGPPKYTKSVLKKGDKTNFPKKGDVVHCWYTGTLQDGTVFDTNIQTSAKKKKNAKPLSFKVGVGKVIRGWDEALLTMSKGEKARLEIEPEWAYGKKGQPDAKIPPNAKLTFEVELVDID

>NP_002878.2

MDVLVSECSARLLQQEEEIKSLTAEIDRLKNCGCLGASPNLEQLQEENLKLKYRLNILRKSLQAERNKPTKNMINIISRLQEVFGHAIKAAYPDLENPPLLVTPSQQAKFGDYQCNSAMGISQMLKTKEQKVNPREIAENITKHLPDNECIEKVEIAGPGFINVHLRKDFVSEQLTSLLVNGVQLPALGENKKVIVDFSSPNIAKEMHVGHLRSTIIGESISRLFEFAGYDVLRLNHVGDWGTQFGMLIAHLQDKFPDYLTVSPPIGDLQVFYKESKKRFDTEEEFKKRAYQCVVLLQGKNPDITKAWKLICDVSRQELNKIYDALDVSLIERGESFYQDRMNDIVKEFEDRGFVQVDDGRKIVFVPGCSIPLTIVKSDGGYTYDTSDLAAIKQRLFEEKADMIIYVVDNGQSVHFQTIFAAAQMIGWYDPKVTRVFHAGFGVVLGEDKKKFKTRSGETVRLMDLLGEGLKRSMDKLKEKERDKVLTAEELNAAQTSVAYGCIKYADLSHNRLNDYIFSFDKMLDDRGNTAAYLLYAFTRIRSIARLANIDEEMLQKAARETKILLDHEKEWKLGRCILRFPEILQKILDDLFLHTLCDYIYELATAFTEFYDSCYCVEKDRQTGKILKVNMWRMLLCEAVAAVMAKGFDILGIKPVQRM

1456 NP_002690.3 NP_057740.2

>NP_002690.3

MATTAQYLPRGPGGGAGGTGPLMHPDAAAAAAAAAAAERLHAGAAYREVQKLMHHEWLGAGAGHPVGLAHPQWLPTGGGGGGDWAGGPHLEHGKAGGGGTGRADDGGGGGGFHARLVHQGAAHAGAAWAQGSTAHHLGPAMSPSPGASGGHQPQPLGLYAQAAYPGGGGGGLAGMLAAGGGGAGPGLHHALHEDGHEAQLEPSPPPHLGAHGHAHGHAHAGGLHAAAAHLHPGAGGGGSSVGEHSDEDAPSSDDLEQFAKQFKQRRIKLGFTQADVGLALGTLYGNVFSQTTICRFEALQLSFKNMCKLKPLLNKWLEETDSSSGSPTNLDKIAAQGRKRKKRTSIEVGVKGALESHFLKCPKPSAHEITGLADSLQLEKEVVRVWFCNRRQKEKRMTPAAGAGHPPMDDVYAPGELGPGGGGASPPSAPPPPPPAALHHHHHHTLPGSVQ

>NP_057740.2

MEESDSEKTTEKENLGPRMDPPLGEPEGSLGWVLPNTAMKKKVLLMGKSGSGKTSMRSIIFANYIARDTRRLGATILDRIHSLQINSSLSTYSLVDSVGNTKTFDVEHSHVRFLGNLVLNLWDCGGQDTFMENYFTSQRDNIFRNVEVLIYVFDVESRELEKDMHYYQSCLEAILQNSPDAKIFCLVHKMDLVQEDQRDLIFKEREEDLRRLSRPLECSCFRTSIWDETLYKAWSSIVYQLIPNVQQLEMNLRNFAEIIEADEVLLFERATFLVISHYQCKEQRDAHRFEKISNIIKQFKLSCSKLAASFQSMEVRNSNFAAFIDIFTSNTYVMVVMSDPSIPSAATLINIRNARKHFEKLERVDGPKQCLLMR

1457 AAA93070.1 NP_008878.3

>AAA93070.1

MSGCRVFIGRLNPAAREKDVERFFKGYGRIRDIDLKRGFGFVEFEDPRDADDAVYELDGKELCSERVTIEHARARSRGGRGRGRYSDRFSSRRPRNDRRNAPPVRTENRLIVENLSSRVSWQDLKDFMRQAGEVTFADAHRPKLNEGVVEFASYGDLKNAIEKLSGKEINGRKIKLIEGSKRHSRSRSRSRSRTRSSSRSRSRSRSRSRKSYSRSRSRSRSRSRSKSRSVSRSPVPEKSQKRGSSSRSKSPASVDRQRSRSRSRSRSVDSGN

>NP_008878.3

MASGGSGGVSVPALWSEVNRYGQNGDFTRALKTVNKILQINKDDVTALHCKVVCLIQNGSFKEALNVINTHTKVLANNSLSFEKAYCEYRLNRIENALKTIESANQQTDKLKELYGQVLYRLERYDECLAVYRDLVRNSQDDYDEERKTNLSAVVAAQSNWEKVVPENLGLQEGTHELCYNTACALIGQGQLNQAMKILQKAEDLCRRSLSEDTDGTEEDPQAELAIIHGQMAYILQLQGRTEEALQLYNQIIKLKPTDVGLLAVIANNIITINKDQNVFDSKKKVKLTNAEGVEFKLSKKQLQAIEFNKALLAMYTNQAEQCRKISASLQSQSPEHLLPVLIQAAQLCREKQHTKAIELLQEFSDQHPENAAEIKLTMAQLKISQGNISKACLILRSIEELKHKPGMVSALVTMYSHEEDIDSAIEVFTQAIQWYQNHQPKSPAHLSLIREAANFKLKYGRKKEAISDLQQLWKQNPKDIHTLAQLISAYSLVDPEKAKALSKHLPSSDSMSLKVDVEALENSAGATYIRKKGGKVTGDSQPKEQGQGDLKKKKKKKKGKLPKNYDPKVTPDPERWLPMRERSYYRGRKKGKKKDQIGKGTQGATAGASSELDASKTVSSPPTSPRPGSAATVSASTSNIIPPRHQKPAGAPATKKKQQQKKKKGGKGGW

1458 Q9Y5B0 NP_002475.2

>Q9Y5B0

MEVPAAGRVPAEGAPTAAVAEVRCPGPAPLRLLEWRVAAGAAVRIGSVLAVFEAAASAQSAGASQSRVASGGCVRPARPERRLRSERAGVVRELCAQPGQVVAPGAVLVRLEGCSHPVVMKGLCAECGQDLTQLQSKNGKQQVPLSTATVSMVHSVPELMVSSEQAEQLGREDQQRLHRNRKLVLMVDLDQTLIHTTEQHCQQMSNKGIFHFQLGRGEPMLHTRLRPHCKDFLEKIAKLYELHVFTFGSRLYAHTIAGFLDPEKKLFSHRILSRDECIDPFSKTGNLRNLFPCGDSMVCIIDDREDVWKFAPNLITVKKYVYFQGTGDMNAPPGSRESQTRKKVNHSRGTEVSEPSPPVRDPEGVTQAPGVEPSNGLEKPARELNGSEAATPRDSPRPGKPDERDIWPPAQAPTSSQELAGAPEPQGSCAQGGRVAPGQRPAQGATGTDLDFDLSSDSESSSESEGTKSSSSASDGESEGKRGRQKPKAAPEGAGALAQGSSLEPGRPAAPSLPGEAEPGAHAPDKEPELGGQEEGERDGLCGLGNGCADRKEAETESQNSELSGVTAGESLDQSMEEEEEEDTDEDDHLIYLEEILVRVHTDYYAKYDRYLNKEIEEAPDIRKIVPELKSKVLADVAIIFSGLHPTNFPIEKTREHYHATALGAKILTRLVLSPDAPDRATHLIAARAGTEKVLQAQECGHLHVVNPDWLWSCLERWDKVEEQLFPLRDDHTKAQRENSPAAFPDREGVPPTALFHPMPVLPKAQPGPEVRIYDSNTGKLIRTGARGPPAPSSSLPIRQEPSSFRAVPPPQPQMFGEELPDAQDGEQPGPSRRKRQPSMSETMPLYTLCKEDLESMDKEVDDILGEGSDDSDSEKRRPEEQEEEPQPRKPGTRRGADARAPASSERSAAGGRGPRGHKRKLNEEDAASESSRESSNEDEGSSSEADEMAKALEAELNDLM

>NP_002475.2

MEEVPHDCPGADSAQAGRGASCQGCPNQRLCASGAGATPDTAIEEIKEKMKTVKHKILVLSGKGGVGKSTFSAHLAHGLAEDENTQIALLDIDICGPSIPKIMGLEGEQVHQSGSGWSPVYVEDNLGVMSVGFLLSSPDDAVIWRGPKKNGMIKQFLRDVDWGEVDYLIVDTPPGTSDEHLSVVRYLATAHIDGAVIITTPQEVSLQDVRKEINFCRKVKLPIIGVVENMSGFICPKCKKESQIFPPTTGGAELMCQDLEVPLLGRVPLDPLIGKNCDKGQSFFIDAPDSPATLAYRSIIQRIQEFCNLHQSKEENLISS

1459 P13349 NP_742024.1

>P13349

MDVMDGCQFSPSEYFYDGSCIPSPEGEFGDEFVPRVAAFGAHKAELQGSDEDEHVRAPTGHHQAGHCLMWACKACKRKSTTMDRRKAATMRERRRLKKVNQAFETLKRCTTTNPNQRLPKVEILRNAIRYIESLQELLREQVENYYSLPGQSCSEPTSPTSNCSDGMPECNSPVWSRKSSTFDSIYCPDVSNVYATDKNSLSSLDCLSNIVDRITSSEQPGLPLQDLASLSPVASTDSQPATPGASSSRLIYHVL

>NP_742024.1

MDTSDLFASCRKGDVGRVRYLLEQRDVEVNVRDKWDSTPLYYACLCGHEELVLYLLANGARCEANTFDGERCLYGALSDPIRRALRDYKQVTASCRRRDYYDDFLQRLLEQGIHSDVVFVVHGKPFRVHRCVLGARSAYFANMLDTKWKGKSVVVLRHPLINPVAFGALLQYLYTGRLDIGVEHVSDCERLAKQCQLWDLLSDLEAKCEKVSEFVASKPGTCVKVLTIEPPPADPRLREDMALLADCALPPELRGDLWELPFPCPDGFNSCPDICFRVAGCSFLCHKAFFCGRSDYFRALLDDHFRESEEPATSGGPPAVTLHGISPDVFTHVLYYMYSDHTELSPEAAYDVLSVADMYLLPGLKRLCGRSLAQMLDEDTVVGVWRVAKLFRLARLEDQCTEYMAKVIEKLVEREDFVEAVKEEAAAVAARQETDSIPLVDDIRFHVASTVQTYSAIEEAQQRLRALEDLLVSIGLDC

1460 NP_057662.3 Q06278

>NP_057662.3

MATAAAAAAVMAPPGCPGSCPNFAVVCSFLERYGPLLDLPELPFPELERVLQAPPPDVGNGEVPKELVELHLKLMRKIGKSVTADRWEKYLIKICQEFNSTWAWEMEKKGYLEMSVECKLALLKYLCECQFDDNLKFKNIINEEDADTMRLQPIGRDKDGLMYWYQLDQDHNVRMYIEEQDDQDGSSWKCIVRNRNELAETLALLKAQIDPVLLKNSSQQDNSSRESPSLEDEETKKEEETPKQEEQKESEKMKSEEQPMDLENRSTANVLEETTVKKEKEDEKELVKLPVIVKLEKPLPENEEKKIIKEESDSFKENVKPIKVEVKECRADPKDTKSSMEKPVAQEPERIEFGGNIKSSHEITEKSTEETEKLKNDQQAKIPLKKREIKLSDDFDSPVKGPLCKSVTPTKEFLKDEIKQEEETCKRISTITALGHEGKQLVNGEVSDERVAPNFKTEPIETKFYETKEESYSPSKDRNIITEGNGTESLNSVITSMKTGELEKETAPLRKDADSSISVLEIHSQKAQIEEPDPPEMETSLDSSEMAKDLSSKTALSSTESCTMKGEEKSPKTKKDKRPPILECLEKLEKSKKTFLDKDAQRLSPIPEEVPKSTLESEKPGSPEAAETSPPSNIIDHCEKLASEKEVVECQSTSTVGGQSVKKVDLETLKEDSEFTKVEMDNLDNAQTSGIEEPSETKGSMQKSKFKYKLVPEEETTASENTEITSERQKEGIKLTIRISSRKKKPDSPPKVLEPENKQEKTEKEEEKTNVGRTLRRSPRISRPTAKVAEIRDQKADKKRGEGEDEVEEESTALQKTDKKEILKKSEKDTNSKVSKVKPKGKVRWTGSRTRGRWKYSSNDESEGSGSEKSSAASEEEEEKESEEAILADDDEPCKKCGLPNHPELILLCDSCDSGYHTACLRPPLMIIPDGEWFCPPCQHKLLCEKLEEQLQDLDVALKKKERAERRKERLVYVGISIENIIPPQEPDFSEDQEEKKKDSKKSKANLLERRSTRTRKCISYRFDEFDEAIDEAIEDDIKEADGGGVGRGKDISTITGHRGKDISTILDEERKENKRPQRAAAARRKKRRRLNDLDSDSNLDEEESEDEFKISDGSQDEFVVSDENPDESEEDPPSNDDSDTDFCSRRLRRHPSRPMRQSRRLRRKTPKKKYSDDDEEEESEENSRDSESDFSDDFSDDFVETRRRRSRRNQKRQINYKEDSESDGSQKSLRRGKEIRRVHKRRLSSSESEESYLSKNSEDDELAKESKRSVRKRGRSTDEYSEADEEEEEEEGKPSRKRLHRIETDEEESCDNAHGDANQPARDSQPRVLPSEQESTKKPYRIESDEEEDFENVGKVGSPLDYSLVDLPSTNGQSPGKAIENLIGKPTEKSQTPKDNSTASASLASNGTSGGQEAGAPEEEEDELLRVTDLVDYVCNSEQL

>Q06278

MDRASELLFYVNGRKVIEKNVDPETMLLPYLRKKLRLTGTKYGCGGGGCGACTVMISRYNPITKRIRHHPANACLIPICSLYGAAVTTVEGIGSTHTRIHPVQERIAKCHGTQCGFCTPGMVMSIYTLLRNHPEPTLDQLTDALGGNLCRCTGYRPIIDACKTFCKTSGCCQSKENGVCCLDQGINGLPEFEEGSKTSPKLFAEEEFLPLDPTQELIFPPELMIMAEKQSQRTRVFGSERMMWFSPVTLKELLEFKFKYPQAPVIMGNTSVGPEVKFKGVFHPVIISPDRIEELSVVNHAYNGLTLGAGLSLAQVKDILADVVQKLPEEKTQMYHALLKHLGTLAGSQIRNMASLGGHIISRHPDSDLNPILAVGNCTLNLLSKEGKRQIPLNEQFLSKCPNADLKPQEILVSVNIPYSRKWEFVSAFRQAQRQENALAIVNSGMRVFFGEGDGIIRELCISYGGVGPATICAKNSCQKLIGRHWNEQMLDIACRLILNEVSLLGSAPGGKVEFKRTLIISFLFKFYLEVSQILKKMDPVHYPSLADKYESALEDLHSKHHCSTLKYQNIGPKQHPEDPIGHPIMHLSGVKHATGEAIYCDDMPLVDQELFLTFVTSSRAHAKIVSIDLSEALSMPGVVDIMTAEHLSDVNSFCFFTEAEKFLATDKVFCVGQLVCAVLADSEVQAKRAAKRVKIVYQDLEPLILTIEESIQHNSSFKPERKLEYGNVDEAFKVVDQILEGEIHMGGQEHFYMETQSMLVVPKGEDQEMDVYVSTQFPKYIQDIVASTLKLPANKVMCHVRRVGGAFGGKVLKTGIIAAVTAFAANKHGRAVRCVLERGEDMLITGGRHPYLGKYKAGFMNDGRILALDMEHYSNAGASLDESLFVIEMGLLKMDNAYKFPNLRCRGWACRTNLPSNTAFRGFGFPQAALITESCITEVAAKCGLSPEKVRIINMYKEIDQTPYKQEINAKNLIQCWRECMAMSSYSLRKVAVEKFNAENYWKKKGLAMVPLKFPVGLGSRAAGQAAALVHIYLDGSVLVTHGGIEMGQGVHTKMIQVVSRELRMPMSNVHLRGTSTETVPNANISGGSVVADLNGLAVKDACQTLLKRLEPIISKNPKGTWKDWAQTAFDESINLSAVGYFRGYESDMNWEKGEGQPFEYFVYGAACSEVEIDCLTGDHKNIRTDIVMDVGCSINPAIDIGQIEGAFIQGMGLYTIEELNYSPQGILHTRGPDQYKIPAICDMPTELHIALLPPSQNSNTLYSSKGLGESGVFLGCSVFFAIHDAVSAARQERGLHGPLTLNSPLTPEKIRMACEDKFTKMIPRDEPGSYVPWNVPI

1461 NP_003007.2 NP_006262.1

>NP_003007.2

MSYGRPPPDVEGMTSLKVDNLTYRTSPDTLRRVFEKYGRVGDVYIPRDRYTKESRGFAFVRFHDKRDAEDAMDAMDGAVLDGRELRVQMARYGRPPDSHHSRRGPPPRRYGGGGYGRRSRSPRRRRRSRSRSRSRSRSRSRSRYSRSKSRSRTRSRSRSTSKSRSARRSKSKSSSVSRSRSRSRSRSRSRSPPPVSKRESKSRSRSKSPPKSPEEEGAVSS

>NP_006262.1

MGSELETAMETLINVFHAHSGKEGDKYKLSKKELKELLQTELSGFLDAQKDVDAVDKVMKELDENGDGEVDFQEYVVLVAALTVACNNFFWENS

1462 NP_055392.1 NP_005908.1

>NP_055392.1

MRRKGRCHRGSAARHPSSPCSVKHSPTRETLTYAQAQRMVEIEIEGRLHRISIFDPLEIILEDDLTAQEMSECNSNKENSERPPVCLRTKRHKNNRVKKKNEALPSAHGTPASASALPEPKVRIVEYSPPSAPRRPPVYYKFIEKSAEELDNEVEYDMDEEDYAWLEIVNEKRKGDCVPAVSQSMFEFLMDRFEKESHCENQKQGEQQSLIDEDAVCCICMDGECQNSNVILFCDMCNLAVHQECYGVPYIPEGQWLCRHCLQSRARPADCVLCPNKGGAFKKTDDDRWGHVVCALWIPEVGFANTVFIEPIDGVRNIPPARWKLTCYLCKQKGVGACIQCHKANCYTAFHVTCAQKAGLYMKMEPVKELTGGGTTFSVRKTAYCDVHTPPGCTRRPLNIYGDVEMKNGVCRKESSVKTVRSTSKVRKKAKKAKKALAEPCAVLPTVCAPYIPPQRLNRIANQVAIQRKKQFVERAHSYWLLKRLSRNGAPLLRRLQSSLQSQRSSQQRENDEEMKAAKEKLKYWQRLRHDLERARLLIELLRKREKLKREQVKVEQVAMELRLTPLTVLLRSVLDQLQDKDPARIFAQPVSLKEVPDYLDHIKHPMDFATMRKRLEAQGYKNLHEFEEDFDLIIDNCMKYNARDTVFYRAAVRLRDQGGVVLRQARREVDSIGLEEASGMHLPERPAAAPRRPFSWEDVDRLLDPANRAHLGLEEQLRELLDMLDLTCAMKSSGSRSKRAKLLKKEIALLRNKLSQQHSQPLPTGPGLEGFEEDGAALGPEAGEEVLPRLETLLQPRKRSRSTCGDSEVEEESPGKRLDAGLTNGFGGARSEQEPGGGLGRKATPRRRCASESSISSSNSPLCDSSFNAPKCGRGKPALVRRHTLEDRSELISCIENGNYAKAARIAAEVGQSSMWISTDAAASVLEPLKVVWAKCSGYPSYPALIIDPKMPRVPGHHNGVTIPAPPLDVLKIGEHMQTKSDEKLFLVLFFDNKRSWQWLPKSKMVPLGIDETIDKLKMMEGRNSSIRKAVRIAFDRAMNHLSRVHGEPTSDLSDID

>NP_005908.1

MSEPIRVLVTGAAGQIAYSLLYSIGNGSVFGKDQPIILVLLDITPMMGVLDGVLMELQDCALPLLKDVIATDKEDVAFKDLDVAILVGSMPRREGMERKDLLKANVKIFKSQGAALDKYAKKSVKVIVVGNPANTNCLTASKSAPSIPKENFSCLTRLDHNRAKAQIALKLGVTANDVKNVIIWGNHSSTQYPDVNHAKVKLQGKEVGVYEALKDDSWLKGEFVTTVQQRGAAVIKARKLSSAMSAAKAICDHVRDIWFGTPEGEFVSMGVISDGNSYGVPDDLLYSFPVVIKNKTWKFVEGLPINDFSREKMDLTAKELTEEKESAFEFLSSA

1463 P0C665 P20591

>P0C665

MDRRRMALRPGSRRPTAFFFHSRWLVPNLLAFFLGLSGAGPIHLPMPWPNGRRHRVLDPHTQLSTHEAPGRWKPVAPRRMKACPQVLLEW

>P20591

MVVSEVDIAKADPAAASHPLLLNGDATVAQKNPGSVAENNLCSQYEEKVRPCIDLIDSLRALGVEQDLALPAIAVIGDQSSGKSSVLEALSGVALPRGSGIVTRCPLVLKLKKLVNEDKWRGKVSYQDYEIEISDASEVEKEINKAQNAIAGEGMGISHELITLEISSRDVPDLTLIDLPGITRVAVGNQPADIGYKIKTLIKKYIQRQETISLVVVPSNVDIATTEALSMAQEVDPEGDRTIGILTKPDLVDKGTEDKVVDVVRNLVFHLKKGYMIVKCRGQQEIQDQLSLSEALQREKIFFENHPYFRDLLEEGKATVPCLAEKLTSELITHICKSLPLLENQIKETHQRITEELQKYGVDIPEDENEKMFFLIDKVNAFNQDITALMQGEETVGEEDIRLFTRLRHEFHKWSTIIENNFQEGHKILSRKIQKFENQYRGRELPGFVNYRTFETIVKQQIKALEEPAVDMLHTVTDMVRLAFTDVSIKNFEEFFNLHRTAKSKIEDIRAEQEREGEKLIRLHFQMEQIVYCQDQVYRGALQKVREKELEEEKKKKSWDFGAFQSSSATDSSMEEIFQHLMAYHQEASKRISSHIPLIIQFFMLQTYGQQLQKAMLQLLQDKDTYSWLLKERSDTSDKRKFLKERLARLTQARRRLAQFPG

1464 NP_064706.1 P17706

>NP_064706.1

MSIETLLEAARFLEWQAQQQQRAREEQERLRLEQEREQEQKKANSLARLAHTLPVEEPRMEAPPLPLSPPAPPPAPPPPLATPAPLTVIPIPVVTNSPQPLPPPPPLPAAAQPLPLAPRQPALVGAPGLSIKEPAPLPSRPQVPTPAPLLPDSKATIPPNGSPKPLQPLPTPVLTIAPHPGVQPQLAPQQPPPPTLGTLKLAPAEEVKSSEQKKRPGGIGTREVHNKLEKNRRAHLKECFETLKRNIPNVDDKKTSNLSVLRTALRYIQSLKRKEKEYEHEMERLAREKIATQQRLAELKHELSQWMDVLEIDRVLRQTGQPEDDQASTSTASEGEDNIDEDMEEDRAGLGPPKLSHRPQPELLKSTLPPPSTTPAPLPPHPHPHPHSVALPPAHLPVQQQQPQQKTPLPAPPPPPAAPAQTLVPAPAHLVATAGGGSTVIAHTATTHASVIQTVNHVLQGPGGKHIAHIAPSAPSPAVQLAPATPPIGHITVHPATLNHVAHLGSQLPLYPQPVAVSHIAHTLSHQQVNGTAGLGPPATVMAKPAVGAQVVHHPQLVGQTVLNPVTMVTMPSFPVSTLKLA

>P17706

MPTTIEREFEELDTQRRWQPLYLEIRNESHDYPHRVAKFPENRNRNRYRDVSPYDHSRVKLQNAENDYINASLVDIEEAQRSYILTQGPLPNTCCHFWLMVWQQKTKAVVMLNRIVEKESVKCAQYWPTDDQEMLFKETGFSVKLLSEDVKSYYTVHLLQLENINSGETRTISHFHYTTWPDFGVPESPASFLNFLFKVRESGSLNPDHGPAVIHCSAGIGRSGTFSLVDTCLVLMEKGDDINIKQVLLNMRKYRMGLIQTPDQLRFSYMAIIEGAKCIKGDSSIQKRWKELSKEDLSPAFDHSPNKIMTEKYNGNRIGLEEEKLTGDRCTGLSSKMQDTMEENSESALRKRIREDRKATTAQKVQQMKQRLNENERKRKRWLYWQPILTKMGFMSVILVGAFVGWTLFFQQNAL

1465 NP_002135.2 O94788

>NP_002135.2

MDYNRMNSFLEYPLCNRGPSAYSAHSAPTSFPPSSAQAVDSYASEGRYGGGLSSPAFQQNSGYPAQQPPSTLGVPFPSSAPSGYAPAACSPSYGPSQYYPLGQSEGDGGYFHPSSYGAQLGGLSDGYGAGGAGPGPYPPQHPPYGNEQTASFAPAYADLLSEDKETPCPSEPNTPTARTFDWMKVKRNPPKTAKVSEPGLGSPSGLRTNFTTRQLTELEKEFHFNKYLSRARRVEIAATLELNETQVKIWFQNRRMKQKKREREEGRVPPAPPGCPKEAAGDASDQSTCTSPEASPSSVTS

>O94788

MTSSKIEMPGEVKADPAALMASLHLLPSPTPNLEIKYTKIFINNEWQNSESGRVFPVYNPATGEQVCEVQEADKADIDKAVQAARLAFSLGSVWRRMDASERGRLLDKLADLVERDRAVLATMESLNGGKPFLQAFYVDLQGVIKTFRYYAGWADKIHGMTIPVDGDYFTFTRHEPIGVCGQIIPWNFPLLMFAWKIAPALCCGNTVVIKPAEQTPLSALYMGALIKEAGFPPGVINILPGYGPTAGAAIASHIGIDKIAFTGSTEVGKLIQEAAGRSNLKRVTLELGGKSPNIIFADADLDYAVEQAHQGVFFNQGQCCTAGSRIFVEESIYEEFVRRSVERAKRRVVGSPFDPTTEQGPQIDKKQYNKILELIQSGVAEGAKLECGGKGLGRKGFFIEPTVFSNVTDDMRIAKEEIFGPVQEILRFKTMDEVIERANNSDFGLVAAVFTNDINKALTVSSAMQAGTVWINCYNALNAQSPFGGFKMSGNGREMGEFGLREYSEVKTVTVKIPQKNS

1466 NP_060589.1 NP_004324.2

>NP_060589.1

MANEEDDPVVQEIDVYLAKSLAEKLYLFQYPVRPASMTYDDIPHLSAKIKPKQQKVELEMAIDTLNPNYCRSKGEQIALNVDGACADETSTYSSKLMDKQTFCSSQTTSNTSRYAAALYRQGELHLTPLHGILQLRPSFSYLDKADAKHREREAANEAGDSSQDEAEDDVKQITVRFSRPESEQARQRRVQSYEFLQKKHAEEPWVHLHYYGLRDSRSEHERQYLLCPGSSGVENTELVKSPSEYLMMLMPPSQEEEKDKPVAPSNVLSMAQLRTLPLADQIKILMKNVKVMPFANLMSLLGPSIDSVAVLRGIQKVAMLVQGNWVVKSDILYPKDSSSPHSGVPAEVLCRGRDFVMWKFTQSRWVVRKEVATVTKLCAEDVKDFLEHMAVVRINKGWEFILPYDGEFIKKHPDVVQRQHMLWTGIQAKLEKVYNLVKETMPKKPDAQSGPAGLVCGDQRIQVAKTKAQQNHALLERELQRRKEQLRVPAVPPGVRIKEEPVSEEGEEDEEQEAEEEPMDTSPSGLHSKLANGLPLGRAAGTDSFNGHPPQGCASTPVARELKAFVEATFQRQFVLTLSELKRLFNLHLASLPPGHTLFSGISDRMLQDTVLAAGCKQILVPFPPQTAASPDEQKVFALWESGDMSDQHRQVLLEIFSKNYRVRRNMIQSRLTQECGEDLSKQEVDKVLKDCCVSYGGMWYLKGTVQS

>NP_004324.2

MAALSGGGGGGAEPGQALFNGDMEPEAGAGAGAAASSAADPAIPEEVWNIKQMIKLTQEHIEALLDKFGGEHNPPSIYLEAYEEYTSKLDALQQREQQLLESLGNGTDFSVSSSASMDTVTSSSSSSLSVLPSSLSVFQNPTDVARSNPKSPQKPIVRVFLPNKQRTVVPARCGVTVRDSLKKALMMRGLIPECCAVYRIQDGEKKPIGWDTDISWLTGEELHVEVLENVPLTTHNFVRKTFFTLAFCDFCRKLLFQGFRCQTCGYKFHQRCSTEVPLMCVNYDQLDLLFVSKFFEHHPIPQEEASLAETALTSGSSPSAPASDSIGPQILTSPSPSKSIPIPQPFRPADEDHRNQFGQRDRSSSAPNVHINTIEPVNIDDLIRDQGFRGDGGSTTGLSATPPASLPGSLTNVKALQKSPGPQRERKSSSSSEDRNRMKTLGRRDSSDDWEIPDGQITVGQRIGSGSFGTVYKGKWHGDVAVKMLNVTAPTPQQLQAFKNEVGVLRKTRHVNILLFMGYSTKPQLAIVTQWCEGSSLYHHLHIIETKFEMIKLIDIARQTAQGMDYLHAKSIIHRDLKSNNIFLHEDLTVKIGDFGLATVKSRWSGSHQFEQLSGSILWMAPEVIRMQDKNPYSFQSDVYAFGIVLYELMTGQLPYSNINNRDQIIFMVGRGYLSPDLSKVRSNCPKAMKRLMAECLKKKRDERPLFPQILASIELLARSLPKIHRSASEPSLNRAGFQTEDFSLYACASPKTPIQAGGYGAFPVH

1467 O00472 Q93008

>O00472

MAAGGTGGLREEQRYGLSCGRLGQDNITVLHVKLTETAIRALETYQSHKNLIPFRPSIQFQGLHGLVKIPKNDPLNEVHNFNFYLSNVGKDNPQGSFDCIQQTFSSSGASQLNCLGFIQDKITVCATNDSYQMTRERMTQAEEESRNRSTKVIKPGGPYVGKRVQIRKAPQAVSDTVPERKRSTPMNPANTIRKTHSSSTISQRPYRDRVIHLLALKAYKKPELLARLQKDGVNQKDKNSLGAILQQVANLNSKDLSYTLKDYVFKELQRDWPGYSEIDRRSLESVLSRKLNPSQNAAGTSRSESPVCSSRDAVSSPQKRLLDSEFIDPLMNKKARISHLTNRVPPTLNGHLNPTSEKSAAGLPLPPAAAAIPTPPPLPSTYLPISHPPQIVNSNSNSPSTPEGRGTQDLPVDSFSQNDSIYEDQQDKYTSRTSLETLPPGSVLLKCPKPMEENHSMSHKKSKKKSKKHKEKDQIKKHDIETIEEKEEDLKREEEIAKLNNSSPNSSGGVKEDCTASMEPSAIELPDYLIKYIAIVSYEQRQNYKDDFNAEYDEYRALHARMETVARRFIKLDAQRKRLSPGSKEYQNVHEEVLQEYQKIKQSSPNYHEEKYRCEYLHNKLAHIKRLIGEFDQQQAESWS

>Q93008

MTATTRGSPVGGNDNQGQAPDGQSQPPLQQNQTSSPDSSNENSPATPPDEQGQGDAPPQLEDEEPAFPHTDLAKLDDMINRPRWVVPVLPKGELEVLLEAAIDLSKKGLDVKSEACQRFFRDGLTISFTKILTDEAVSGWKFEIHVSLVELCVAKLSQDWFPLLELLAMALNPHCKFHIYNGTRPCESVSSSVQLPEDELFARSPDPRSPKGWLVDLLNKFGTLNGFQILHDRFINGSALNVQIIAALIKPFGQCYEFLTLHTVKKYFLPIIEMVPQFLENLTDEELKKEAKNEAKNDALSMIIKSLKNLASRVPGQEETVKNLEIFRLKMILRLLQISSFNGKMNALNEVNKVISSVSYYTHRHGNPEEEEWLTAERMAEWIQQNNILSIVLRDSLHQPQYVEKLEKILRFVIKEKALTLQDLDNIWAAQAGKHEAIVKNVHDLLAKLAWDFSPEQLDHLFDCFKASWTNASKKQREKLLELIRRLAEDDKDGVMAHKVLNLLWNLAHSDDVPVDIMDLALSAHIKILDYSCSQDRDTQKIQWIDRFIEELRTNDKWVIPALKQIREICSLFGEAPQNLSQTQRSPHVFYRHDLINQLQHNHALVTLVAENLATYMESMRLYARDHEDYDPQTVRLGSRYSHVQEVQERLNFLRFLLKDGQLWLCAPQAKQIWKCLAENAVYLCDREACFKWYSKLMGDEPDLDPDINKDFFESNVLQLDPSLLTENGMKCFERFFKAVNCREGKLVAKRRAYMMDDLELIGLDYLWRVVIQSNDDIASRAIDLLKEIYTNLGPRLQVNQVVIHEDFIQSCFDRLKASYDTLCVLDGDKDSVNCARQEAVRMVRVLTVLREYINECDSDYHEERTILPMSRAFRGKHLSFVVRFPNQGRQVDDLEVWSHTNDTIGSVRRCILNRIKANVAHTKIELFVGGELIDPADDRKLIGQLNLKDKSLITAKLTQISSNMPSSPDSSSDSSTGSPGNHGNHYSDGPNPEVESCLPGVIMSLHPRYISFLWQVADLGSSLNMPPLRDGARVLMKLMPPDSTTIEKLRAICLDHAKLGESSLSPSLDSLFFGPSASQVLYLTEVVYALLMPAGAPLADDSSDFQFHFLKSGGLPLVLSMLTRNNFLPNADMETRRGAYLNALKIAKLLLTAIGYGHVRAVAEACQPGVEGVNPMTQINQVTHDQAVVLQSALQSIPNPSSECMLRNVSVRLAQQISDEASRYMPDICVIRAIQKIIWASGCGSLQLVFSPNEEITKIYEKTNAGNEPDLEDEQVCCEALEVMTLCFALIPTALDALSKEKAWQTFIIDLLLHCHSKTVRQVAQEQFFLMCTRCCMGHRPLLFFITLLFTVLGSTARERAKHSGDYFTLLRHLLNYAYNSNINVPNAEVLLNNEIDWLKRIRDDVKRTGETGIEETILEGHLGVTKELLAFQTSEKKFHIGCEKGGANLIKELIDDFIFPASNVYLQYMRNGELPAEQAIPVCGSPPTINAGFELLVALAVGCVRNLKQIVDSLTEMYYIGTAITTCEALTEWEYLPPVGPRPPKGFVGLKNAGATCYMNSVIQQLYMIPSIRNGILAIEGTGSDVDDDMSGDEKQDNESNVDPRDDVFGYPQQFEDKPALSKTEDRKEYNIGVLRHLQVIFGHLAASRLQYYVPRGFWKQFRLWGEPVNLREQHDALEFFNSLVDSLDEALKALGHPAMLSKVLGGSFADQKICQGCPHRYECEESFTTLNVDIRNHQNLLDSLEQYVKGDLLEGANAYHCEKCNKKVDTVKRLLIKKLPPVLAIQLKRFDYDWERECAIKFNDYFEFPRELDMEPYTVAGVAKLEGDNVNPESQLIQQSEQSESETAGSTKYRLVGVLVHSGQASGGHYYSYIIQRNGGDGERNRWYKFDDGDVTECKMDDDEEMKNQCFGGEYMGEVFDHMMKRMSYRRQKRWWNAYILFYERMDTIDQDDELIRYISELAITTRPHQIIMPSAIERSVRKQNVQFMHNRMQYSMEYFQFMKKLLTCNGVYLNPPPGQDHLLPEAEEITMISIQLAARFLFTTGFHTKKVVRGSASDWYDALCILLRHSKNVRFWFAHNVLFNVSNRFSEYLLECPSAEVRGAFAKLIVFIAHFSLQDGPCPSPFASPGPSSQAYDNLSLSDHLLRAVLNLLRREVSEHGRHLQQYFNLFVMYANLGVAEKTQLLKLSVPATFMLVSLDEGPGPPIKYQYAELGKLYSVVSQLIRCCNVSSRMQSSINGNPPLPNPFGDPNLSQPIMPIQQNVADILFVRTSYVKKIIEDCSNSEETVKLLRFCCWENPQFSSTVLSELLWQVAYSYTYELRPYLDLLLQILLIEDSWQTHRIHNALKGIPDDRDGLFDTIQRSKNHYQKRAYQCIKCMVALFSNCPVAYQILQGNGDLKRKWTWAVEWLGDELERRPYTGNPQYTYNNWSPPVQSNETSNGYFLERSHSARMTLAKACELCPEEEPDDQDAPDEHESPPPEDAPLYPHSPGSQYQQNNHVHGQPYTGPAAHHMNNPQRTGQRAQENYEGSEEVSPPQTKDQ

1468 NP_002575.1 NP_008996.1

>NP_002575.1

MAALPGTVPRMMRPAPGQNYPRTGFPLEVSTPLGQGRVNQLGGVFINGRPLPNHIRHKIVEMAHHGIRPCVISRQLRVSHGCVSKILCRYQETGSIRPGAIGGSKPRQVATPDVEKKIEEYKRENPGMFSWEIRDRLLKDGHCDRSTVPSGLVSSISRVLRIKFGKKEEEDEADKKEDDGEKKAKHSIDGILGDKGNRLDEGSDVESEPDLPLKRKQRRSRTTFTAEQLEELEKAFERTHYPDIYTREELAQRTKLTEARVQVWFSNRRARWRKQAGANQLAAFNHLLPGGFPPTGMPTLPPYQLPDSTYPTTTISQDGGSTVHRPQPLPPSTMHQGGLAAAAAAADTSSAYGARHSFSSYSDSFMNPAAPSNHMNPVSNGLSPQVMSILGNPSAVPPQPQADFSISPLHGGLDSATSISASCSQRADSIKPGDSLPTSQAYCPPTYSTTGYSVDPVAGYQYGQYGQSECLVPWASPVPIPSPTPRASCLFMESYKVVSGWGMSISQMEKLKSSQMEQFT

>NP_008996.1

MVDYSVWDHIEVSDDEDETHPNIDTASLFRWRHQARVERMEQFQKEKEELDRGCRECKRKVAECQRKLKELEVAEGGKAELERLQAEAQQLRKEERSWEQKLEEMRKKEKSMPWNVDTLSKDGFSKSMVNTKPEKTEEDSEEVREQKHKTFVEKYEKQIKHFGMLRRWDDSQKYLSDNVHLVCEETANYLVIWCIDLEVEEKCALMEQVAHQTIVMQFILELAKSLKVDPRACFRQFFTKIKTADRQYMEGFNDELEAFKERVRGRAKLRIEKAMKEYEEEERKKRLGPGGLDPVEVYESLPEELQKCFDVKDVQMLQDAISKMDPTDAKYHMQRCIDSGLWVPNSKASEAKEGEEAGPGDPLLEAVPKTGDEKDVSV

1469 NP_003101.3 Q9BY32

>NP_003101.3

MSDPQTSMAATAAVSPSDYLQPAASTTQDSQPSPLALLAATCSKIGPPAVEAAVTPPAPPQPTPRKLVPIKPAPLPLSPGKNSFGILSSKGNILQIQGSQLSASYPGGQLVFAIQNPTMINKGTRSNANIQYQAVPQIQASNSQTIQVQPNLTNQIQIIPGTNQAIITPSPSSHKPVPIKPAPIQKSSTTTTPVQSGANVVKLTGGGGNVTLTLPVNNLVNASDTGAPTQLLTESPPTPLSKTNKKARKKSLPASQPPVAVAEQVETVLIETTADNIIQAGNNLLIVQSPGGGQPAVVQQVQVVPPKAEQQQVVQIPQQALRVVQAASATLPTVPQKPSQNFQIQAAEPTPTQVYIRTPSGEVQTVLVQDSPPATAAATSNTTCSSPASRAPHLSGTSKKHSAAILRKERPLPKIAPAGSIISLNAAQLAAAAQAMQTININGVQVQGVPVTITNTGGQQQLTVQNVSGNNLTISGLSPTQIQLQMEQALAGETQPGEKRRRMACTCPNCKDGEKRSGEQGKKKHVCHIPDCGKTFRKTSLLRAHVRLHTGERPFVCNWFFCGKRFTRSDELQRHARTHTGDKRFECAQCQKRFMRSDHLTKHYKTHLVTKNL

>Q9BY32

MAASLVGKKIVFVTGNAKKLEEVVQILGDKFPCTLVAQKIDLPEYQGEPDEISIQKCQEAVRQVQGPVLVEDTCLCFNALGGLPGPYIKWFLEKLKPEGLHQLLAGFEDKSAYALCTFALSTGDPSQPVRLFRGRTSGRIVAPRGCQDFGWDPCFQPDGYEQTYAEMPKAEKNAVSHRFRALLELQEYFGSLAA

1470 NP_001942.2 NP_000934.1

>NP_001942.2

MAAAEPASSGQQAPAGQGQGQRPPPQPPQAQAPQPPPPPQLGGAGGGSSRHEKSLGLLTTKFVSLLQEAKDGVLDLKAAADTLAVRQKRRIYDITNVLEGIDLIEKKSKNSIQWKGVGAGCNTKEVIDRLRYLKAEIEDLELKERELDQQKLWLQQSIKNVMDDSINNRFSYVTHEDICNCFNGDTLLAIQAPSGTQLEVPIPEMGQNGQKKYQINLKSHSGPIHVLLINKESSSSKPVVFPVPPPDDLTQPSSQSLTPVTPQKSSMATQNLPEQHVSERSQALQQTSATDISSAGSISGDIIDELMSSDVFPLLRLSPTPADDYNFNLDDNEGVCDLFDVQILNY

>NP_000934.1

MGPGPRLLLPLVLCVGLGALVFSSGAEGFRKRGPSVTAKVFFDVRIGDKDVGRIVIGLFGKVVPKTVENFVALATGEKGYGYKGSKFHRVIKDFMIQGGDITTGDGTGGVSIYGETFPDENFKLKHYGIGWVSMANAGPDTNGSQFFITLTKPTWLDGKHVVFGKVIDGMTVVHSIELQATDGHDRPLTNCSIINSGKIDVKTPFVVEIADW

1471 Q13523 Q9NP31

>Q13523

MAAAETQSLREQPEMEDANSEKSINEENGEVSEDQSQNKHSRHKKKKHKHRSKHKKHKHSSEEDKDKKHKHKHKHKKHKRKEVIDASDKEGMSPAKRTKLDDLALLEDLEKQRALIKAELDNELMEGKVQSGMGLILQGYESGSEEEGEIHEKARNGNRSSTRSSSTKGKLELVDNKITTKKRSKSRSKERTRHRSDKKKSKGGIEIVKEKTTRSKSKERKKSKSPSKRSKSQDQARKSKSPTLRRRSQEKIGKARSPTDDKVKIEDKSKSKDRKKSPIINESRSRDRGKKSRSPVDLRGKSKDRRSRSKERKSKRSETDKEKKPIKSPSKDASSGKENRSPSRRPGRSPKRRSLSPKPRDKSRRSRSPLLNDRRSKQSKSPSRTLSPGRRAKSRSLERKRREPERRRLSSPRTRPRDDILSRRERSKDASPINRWSPTRRRSRSPIRRRSRSPLRRSRSPRRRSRSPRRRDRGRRSRSRLRRRSRSRGGRRRRSRSKVKEDKFKGSLSEGMKVEQESSSDDNLEDFDVEEEDEEALIEQRRIQRQAIVQKYKYLAEDSNMSVPSEPSSPQSSTRTRSPSPDDILERVAADVKEYERENVDTFEASVKAKHNLMTVEQNNGSSQKKLLAPDMFTESDDMFAAYFDSARLRAAGIGKDFKENPNLRDNWTDAEGYYRVNIGEVLDKRYNVYGYTGQGVFSNVVRARDNARANQEVAVKIIRNNELMQKTGLKELEFLKKLNDADPDDKFHCLRLFRHFYHKQHLCLVFEPLSMNLREVLKKYGKDVGLHIKAVRSYSQQLFLALKLLKRCNILHADIKPDNILVNESKTILKLCDFGSASHVADNDITPYLVSRFYRAPEIIIGKSYDYGIDMWSVGCTLYELYTGKILFPGKTNNHMLKLAMDLKGKMPNKMIRKGVFKDQHFDQNLNFMYIEVDKVTEREKVTVMSTINPTKDLLADLIGCQRLPEDQRKKVHQLKDLLDQILMLDPAKRISINQALQHAFIQEKI

>Q9NP31

MEFPLAQICPQGSHEAPIPTFSTFQITDMTRRSCQNLGYTAASPQAPEAASNTGNAERAEEVPGEGSLFLQAETRAWFQKTQAHWLLQHGAAPAWFHGFITRREAERLLEPKPQGCYLVRFSESAVTFVLTYRSRTCCRHFLLAQLRDGRHVVLGEDSAHARLQDLLLHYTAHPLSPYGETLTEPLARQTPEPAGLSLRTEESNFGSKSQDPNPQYSPIIKQGQAPVPMQKEGAGEKEPSQLLRPKPPIPAKPQLPPEVYTIPVPRHRPAPRPKPSNPIYNEPDEPIAFYAMGRGSPGEAPSNIYVEVEDEGLPATLGHPVLRKSWSRPVPGGQNTGGSQLHSENSVIGQGPPLPHQPPPAWRHTLPHNLSRQVLQDRGQAWLPLGPPQ

1472 O75132 NP_003671.1

>O75132

MENNLKTCPKEDGDFVSDKIKFKIEEEDDDGIPPDSLERMDFKSEQEDMKQTDSGGERAGLGGTGCSCKPPGKYLSAESEDDYGALFSQYSSTLYDVAMEAVTQSLLSSRNMSSRKKSPAWKHFFISPRDSTKAICMYCVKEFSRGKNEKDLSTSCLMRHVRRAHPTVLIQENGSVSAVSSFPSPSLLLPPQPADAGDLSTILSPIKLVQKVASKIPSPDRITEESVSVVSSEEISSDMSVSEKCGREEALVGSSPHLPALHYDEPAENLAEKSLPLPKSTSGSRRRSAVWKHFYLSPLDNSKAVCIHCMNEFSRGKNGKDLGTSCLIRHMWRAHRAIVLQENGGTGIPPLYSTPPTLLPSLLPPEGELSSVSSSPVKPVRESPSASSSPDRLTEDLQSHLNPGDGLMEDVAAFSSSDDVGEASASSPEKQQADGLSPRLFESGAIFQQNKKVMKRLKSEVWHHFSLAPMDSLKAECRYCGCAISRGKKGDVGTSCLMRHLYRRHPEVVGSQKGFLGASLANSPYATLASAESSSSKLTDLPTVVTKNNQVMFPVNSKKTSKLWNHFSICSADSTKVVCLHCGRTISRGKKPTNLGTSCLLRHLQRFHSNVLKTEVSETARPSSPDTRVPRGTELSGASSFDDTNEKFYDSHPVAKKITSLIAEMIALDLQPYSFVDNVGFNRLLEYLKPQYSLPAPSYFSRTAIPGMYDNVKQIIMSHLKEAESGVIHFTSGIWMSNQTREYLTLTAHWVSFESPARPRCDDHHCSALLDVSQVDCDYSGNSIQKQLECWWEAWVTSTGLQVGITVTDNASIGKTLNEGEHSSVQCFSHTVNLIVSEAIKSQRMVQNLLSLARKICERVHRSPKAKEKLAELQREYALPQHHLIQDVPSKWSTSFHMLERLIEQKRAINEMSVECNFRELISCDQWEVMQSVCRALKPFEAASREMSTQMSTLSQVIPMVHILNRKVEMLFEETMGIDTMLRSLKEAMVSRLSATLHDPRYVFATLLDPRYKASLFTEEEAEQYKQDLIRELELMNSTSEDVAASHRCDAGSPSKDSAAEENLWSLVAKVKKKDPREKLPEAMVLAYLEEEVLEHSCDPLTYWNLKKASWPGLSALAVRFLGCPPSIVPSEKLFNTPTENGSLGQSRLMMEHFEKLIFLKVNLPLIYFQY

>NP_003671.1

MGDAPSPEEKLHLITRNLQEVLGEEKLKEILKERELKIYWGTATTGKPHVAYFVPMSKIADFLKAGCEVTILFADLHAYLDNMKAPWELLELRVSYYENVIKAMLESIGVPLEKLKFIKGTDYQLSKEYTLDVYRLSSVVTQHDSKKAGAEVVKQVEHPLLSGLLYPGLQALDEEYLKVDAQFGGIDQRKIFTFAEKYLPALGYSKRVHLMNPMVPGLTGSKMSSSEEESKIDLLDRKEDVKKKLKKAFCEPGNVENNGVLSFIKHVLFPLKSEFVILRDEKWGGNKTYTAYVDLEKDFAAEVVHPGDLKNSVEVALNKLLDPIREKFNTPALKKLASAAYPDPSKQKPMAKGPAKNSEPEEVIPSRLDIRVGKIITVEKHPDADSLYVEKIDVGEAEPRTVVSGLVQFVPKEELQDRLVVVLCNLKPQKMRGVESQGMLLCASIEGINRQVEPLDPPAGSAPGEHVFVKGYEKGQPDEELKPKKKVFEKLQADFKISEECIAQWKQTNFMTKLGSISCKSLKGGNIS

1473 Q9Y4A5 Q9BXL7

>Q9Y4A5

MAFVATQGATVVDQTTLMKKYLQFVAALTDVNTPDETKLKMMQEVSENFENVTSSPQYSTFLEHIIPRFLTFLQDGEVQFLQEKPAQQLRKLVLEIIHRIPTNEHLRPHTKNVLSVMFRFLETENEENVLICLRIIIELHKQFRPPITQEIHHFLDFVKQIYKELPKVVNRYFENPQVIPENTVPPPEMVGMITTIAVKVNPEREDSETRTHSIIPRGSLSLKVLAELPIIVVLMYQLYKLNIHNVVAEFVPLIMNTIAIQVSAQARQHKLYNKELYADFIAAQIKTLSFLAYIIRIYQELVTKYSQQMVKGMLQLLSNCPAETAHLRKELLIAAKHILTTELRNQFIPCMDKLFDESILIGSGYTARETLRPLAYSTLADLVHHVRQHLPLSDLSLAVQLFAKNIDDESLPSSIQTMSCKLLLNLVDCIRSKSEQESGNGRDVLMRMLEVFVLKFHTIARYQLSAIFKKCKPQSELGAVEAALPGVPTAPAAPGPAPSPAPVPAPPPPPPPPPPATPVTPAPVPPFEKQGEKDKEDKQTFQVTDCRSLVKTLVCGVKTITWGITSCKAPGEAQFIPNKQLQPKETQIYIKLVKYAMQALDIYQVQIAGNGQTYIRVANCQTVRMKEEKEVLEHFAGVFTMMNPLTFKEIFQTTVPYMVERISKNYALQIVANSFLANPTTSALFATILVEYLLDRLPEMGSNVELSNLYLKLFKLVFGSVSLFAAENEQMLKPHLHKIVNSSMELAQTAKEPYNYFLLLRALFRSIGGGSHDLLYQEFLPLLPNLLQGLNMLQSGLHKQHMKDLFVELCLTVPVRLSSLLPYLPMLMDPLVSALNGSQTLVSQGLRTLELCVDNLQPDFLYDHIQPVRAELMQALWRTLRNPADSISHVAYRVLGKFGGSNRKMLKESQKLHYVVTEVQGPSITVEFSDCKASLQLPMEKAIETALDCLKSANTEPYYRRQAWEVIKCFLVAMMSLEDNKHALYQLLAHPNFTEKTIPNVIISHRYKAQDTPARKTFEQALTGAFMSAVIKDLRPSALPFVASLIRHYTMVAVAQQCGPFLLPCYQVGSQPSTAMFHSEENGSKGMDPLVLIDAIAICMAYEEKELCKIGEVALAVIFDVASIILGSKERACQLPLFSYIVERLCACCYEQAWYAKLGGVVSIKFLMERLPLTWVLQNQQTFLKALLFVMMDLTGEVSNGAVAMAKTTLEQLLMRCATPLKDEERAEEIVAAQEKSFHHVTHDLVREVTSPNSTVRKQAMHSLQVLAQVTGKSVTVIMEPHKEVLQDMVPPKKHLLRHQPANAQIGLMEGNTFCTTLQPRLFTMDLNVVEHKVFYTELLNLCEAEDSALTKLPCYKSLPSLVPLRIAALNALAACNYLPQSREKIIAALFKALNSTNSELQEAGEACMRKFLEGATIEVDQIHTHMRPLLMMLGDYRSLTLNVVNRLTSVTRLFPNSFNDKFCDQMMQHLRKWMEVVVITHKGGQRSDGNESISECGRCPLSPFCQFEEMKICSAIINLFHLIPAAPQTLVKPLLEVVMKTERAMLIEAGSPFREPLIKFLTRHPSQTVELFMMEATLNDPQWSRMFMSFLKHKDARPLRDVLAANPNRFITLLLPGGAQTAVRPGSPSTSTMRLDLQFQAIKIISIIVKNDDSWLASQHSLVSQLRRVWVSENFQERHRKENMAATNWKEPKLLAYCLLNYCKRNYGDIELLFQLLRAFTGRFLCNMTFLKEYMEEEIPKNYSIAQKRALFFRFVDFNDPNFGDELKAKVLQHILNPAFLYSFEKGEGEQLLGPPNPEGDNPESITSVFITKVLDPEKQADMLDSLRIYLLQYATLLVEHAPHHIHDNNKNRNSKLRRLMTFAWPCLLSKACVDPACKYSGHLLLAHIIAKFAIHKKIVLQVFHSLLKAHAMEARAIVRQAMAILTPAVPARMEDGHQMLTHWTRKIIVEEGHTVPQLVHILHLIVQHFKVYYPVRHHLVQHMVSAMQRLGFTPSVTIEQRRLAVDLSEVVIKWELQRIKDQQPDSDMDPNSSGEGVNSVSSSIKRGLSVDSAQEVKRFRTATGAISAVFGRSQSLPGADSLLAKPIDKQHTDTVVNFLIRVACQVNDNTNTAGSPGEVLSRRCVNLLKTALRPDMWPKSELKLQWFDKLLMTVEQPNQVNYGNICTGLEVLSFLLTVLQSPAILSSFKPLQRGIAACMTCGNTKVLRAVHSLLSRLMSIFPTEPSTSSVASKYEELECLYAAVGKVIYEGLTNYEKATNANPSQLFGTLMILKSACSNNPSYIDRLISVFMRSLQKMVREHLNPQAASGSTEATSGTSELVMLSLELVKTRLAVMSMEMRKNFIQAILTSLIEKSPDAKILRAVVKIVEEWVKNNSPMAANQTPTLREKSILLVKMMTYIEKRFPEDLELNAQFLDLVNYVYRDETLSGSELTAKLEPAFLSGLRCAQPLIRAKFFEVFDNSMKRRVYERLLYVTCSQNWEAMGNHFWIKQCIELLLAVCEKSTPIGTSCQGAMLPSITNVINLADSHDRAAFAMVTHVKQEPRERENSESKEEDVEIDIELAPGDQTSTPKTKELSEKDIGNQLHMLTNRHDKFLDTLREVKTGALLSAFVQLCHISTTLAEKTWVQLFPRLWKILSDRQQHALAGEISPFLCSGSHQVQRDCQPSALNCFVEAMSQCVPPIPIRPCVLKYLGKTHNLWFRSTLMLEHQAFEKGLSLQIKPKQTTEFYEQESITPPQQEILDSLAELYSLLQEEDMWAGLWQKRCKYSETATAIAYEQHGFFEQAQESYEKAMDKAKKEHERSNASPAIFPEYQLWEDHWIRCSKELNQWEALTEYGQSKGHINPYLVLECAWRVSNWTAMKEALVQVEVSCPKEMAWKVNMYRGYLAICHPEEQQLSFIERLVEMASSLAIREWRRLPHVVSHVHTPLLQAAQQIIELQEAAQINAGLQPTNLGRNNSLHDMKTVVKTWRNRLPIVSDDLSHWSSIFMWRQHHYQGKPTWSGMHSSSIVTAYENSSQHDPSSNNAMLGVHASASAIIQYGKIARKQGLVNVALDILSRIHTIPTVPIVDCFQKIRQQVKCYLQLAGVMGKNECMQGLEVIESTNLKYFTKEMTAEFYALKGMFLAQINKSEEANKAFSAAVQMHDVLVKAWAMWGDYLENIFVKERQLHLGVSAITCYLHACRHQNESKSRKYLAKVLWLLSFDDDKNTLADAVDKYCIGVPPIQWLAWIPQLLTCLVGSEGKLLLNLISQVGRVYPQAVYFPIRTLYLTLKIEQRERYKSDPGPIRATAPMWRCSRIMHMQRELHPTLLSSLEGIVDQMVWFRENWHEEVLRQLQQGLAKCYSVAFEKSGAVSDAKITPHTLNFVKKLVSTFGVGLENVSNVSTMFSSAASESLARRAQATAQDPVFQKLKGQFTTDFDFSVPGSMKLHNLISKLKKWIKILEAKTKQLPKFFLIEEKCRFLSNFSAQTAEVEIPGEFLMPKPTHYYIKIARFMPRVEIVQKHNTAARRLYIRGHNGKIYPYLVMNDACLTESRREERVLQLLRLLNPCLEKRKETTKRHLFFTVPRVVAVSPQMRLVEDNPSSLSLVEIYKQRCAKKGIEHDNPISRYYDRLATVQARGTQASHQVLRDILKEVQSNMVPRSMLKEWALHTFPNATDYWTFRKMFTIQLALIGFAEFVLHLNRLNPEMLQIAQDTGKLNVAYFRFDINDATGDLDANRPVPFRLTPNISEFLTTIGVSGPLTASMIAVARCFAQPNFKVDGILKTVLRDEIIAWHKKTQEDTSSPLSAAGQPENMDSQQLVSLVQKAVTAIMTRLHNLAQFEGGESKVNTLVAAANSLDNLCRMDPAWHPWL

>Q9BXL7

MPGGGPEMDDYMETLKDEEDALWENVECNRHMLSRYINPAKLTPYLRQCKVIDEQDEDEVLNAPMLPSKINRAGRLLDILHTKGQRGYVVFLESLEFYYPELYKLVTGKEPTRRFSTIVVEEGHEGLTHFLMNEVIKLQQQMKAKDLQRCELLARLRQLEDEKKQMTLTRVELLTFQERYYKMKEERDSYNDELVKVKDDNYNLAMRYAQLSEEKNMAVMRSRDLQLEIDQLKHRLNKMEEECKLERNQSLKLKNDIENRPKKEQVLELERENEMLKTKNQELQSIIQAGKRSLPDSDKAILDILEHDRKEALEDRQELVNRIYNLQEEARQAEELRDKYLEEKEDLELKCSTLGKDCEMYKHRMNTVMLQLEEVERERDQAFHSRDEAQTQYSQCLIEKDKYRKQIRELEEKNDEMRIEMVRREACIVNLESKLRRLSKDSNNLDQSLPRNLPVTIISQDFGDASPRTNGQEADDSSTSEESPEDSKYFLPYHPPQRRMNLKGIQLQRAKSPISLKRTSDFQAKGHEEEGTDASPSSCGSLPITNSFTKMQPPRSRSSIMSITAEPPGNDSIVRRYKEDAPHRSTVEEDNDSGGFDALDLDDDSHERYSFGPSSIHSSSSSHQSEGLDAYDLEQVNLMFRKFSLERPFRPSVTSVGHVRGPGPSVQHTTLNGDSLTSQLTLLGGNARGSFVHSVKPGSLAEKAGLREGHQLLLLEGCIRGERQSVPLDTCTKEEAHWTIQRCSGPVTLHYKVNHEGYRKLVKDMEDGLITSGDSFYIRLNLNISSQLDACTMSLKCDDVVHVRDTMYQDRHEWLCARVDPFTDHDLDMGTIPSYSRAQQLLLVKLQRLMHRGSREEVDGTHHTLRALRNTLQPEEALSTSDPRVSPRLSRASFLFGQLLQFVSRSENKYKRMNSNERVRIISGSPLGSLARSSLDATKLLTEKQEELDPESELGKNLSLIPYSLVRAFYCERRRPVLFTPTVLAKTLVQRLLNSGGAMEFTICKSDIVTRDEFLRRQKTETIIYSREKNPNAFECIAPANIEAVAAKNKHCLLEAGIGCTRDLIKSNIYPIVLFIRVCEKNIKRFRKLLPRPETEEEFLRVCRLKEKELEALPCLYATVEPDMWGSVEELLRVVKDKIGEEQRKTIWVDEDQL

1474 Q9BXL5 Q9P0M9

>Q9BXL5

MDLGKDQSHLKHHQTPDPHQEENHSPEVIGTWSLRNRELLRKRKAEVHEKETSQWLFGEQKKRKQQRTGKGNRRGRKRQQNTELKVEPQPQIEKEIVEKALAPIEKKTEPPGSITKVFPSVASPQKVVPEEHFSEICQESNIYQENFSEYQEIAVQNHSSETCQHVSEPEDLSPKMYQEISVLQDNSSKICQDMKEPEDNSPNTCQVISVIQDHPFKMYQDMAKREDLAPKMCQEAAVPKILPCPTSEDTADLAGCSLQAYPKPDVPKGYILDTDQNPAEPEEYNETDQGIAETEGLFPKIQEIAEPKDLSTKTHQESAEPKYLPHKTCNEIIVPKAPSHKTIQETPHSEDYSIEINQETPGSEKYSPETYQEIPGLEEYSPEIYQETSQLEEYSPEIYQETPGPEDLSTETYKNKDVPKECFPEPHQETGGPQGQDPKAHQEDAKDAYTFPQEMKEKPKEEPGIPAILNESHPENDVYSYVLF

>Q9P0M9

MASVVLALRTRTAVTSLLSPTPATALAVRYASKKSGGSSKNLGGKSSGRRQGIKKMEGHYVHAGNIIATQRHFRWHPGAHVGVGKNKCLYALEEGIVRYTKEVYVPHPRNTEAVDLITRLPKGAVLYKTFVHVVPAKPEGTFKLVAML

1475 NP_005924.2 NP_055605.2

>NP_005924.2

MAHSCRWRFPARPGTTGGGGGGGRRGLGGAPRQRVPALLLPPGPPVGGGGPGAPPSPPAVAAAAAAAGSSGAGVPGGAAAASAASSSSASSSSSSSSSASSGPALLRVGPGFDAALQVSAAIGTNLRRFRAVFGESGGGGGSGEDEQFLGFGSDEEVRVRSPTRSPSVKTSPRKPRGRPRSGSDRNSAILSDPSVFSPLNKSETKSGDKIKKKDSKSIEKKRGRPPTFPGVKIKITHGKDISELPKGNKEDSLKKIKRTPSATFQQATKIKKLRAGKLSPLKSKFKTGKLQIGRKGVQIVRRRGRPPSTERIKTPSGLLINSELEKPQKVRKDKEGTPPLTKEDKTVVRQSPRRIKPVRIIPSSKRTDATIAKQLLQRAKKGAQKKIEKEAAQLQGRKVKTQVKNIRQFIMPVVSAISSRIIKTPRRFIEDEDYDPPIKIARLESTPNSRFSAPSCGSSEKSSAASQHSSQMSSDSSRSSSPSVDTSTDSQASEEIQVLPEERSDTPEVHPPLPISQSPENESNDRRSRRYSVSERSFGSRTTKKLSTLQSAPQQQTSSSPPPPLLTPPPPLQPASSISDHTPWLMPPTIPLASPFLPASTAPMQGKRKSILREPTFRWTSLKHSRSEPQYFSSAKYAKEGLIRKPIFDNFRPPPLTPEDVGFASGFSASGTAASARLFSPLHSGTRFDMHKRSPLLRAPRFTPSEAHSRIFESVTLPSNRTSAGTSSSGVSNRKRKRKVFSPIRSEPRSPSHSMRTRSGRLSSSELSPLTPPSSVSSSLSISVSPLATSALNPTFTFPSHSLTQSGESAEKNQRPRKQTSAPAEPFSSSSPTPLFPWFTPGSQTERGRNKDKAPEELSKDRDADKSVEKDKSRERDREREKENKRESRKEKRKKGSEIQSSSALYPVGRVSKEKVVGEDVATSSSAKKATGRKKSSSHDSGTDITSVTLGDTTAVKTKILIKKGRGNLEKTNLDLGPTAPSLEKEKTLCLSTPSSSTVKHSTSSIGSMLAQADKLPMTDKRVASLLKKAKAQLCKIEKSKSLKQTDQPKAQGQESDSSETSVRGPRIKHVCRRAAVALGRKRAVFPDDMPTLSALPWEEREKILSSMGNDDKSSIAGSEDAEPLAPPIKPIKPVTRNKAPQEPPVKKGRRSRRCGQCPGCQVPEDCGVCTNCLDKPKFGGRNIKKQCCKMRKCQNLQWMPSKAYLQKQAKAVKKKEKKSKTSEKKDSKESSVVKNVVDSSQKPTPSAREDPAPKKSSSEPPPRKPVEEKSEEGNVSAPGPESKQATTPASRKSSKQVSQPALVIPPQPPTTGPPRKEVPKTTPSEPKKKQPPPPESGPEQSKQKKVAPRPSIPVKQKPKEKEKPPPVNKQENAGTLNILSTLSNGNSSKQKIPADGVHRIRVDFKEDCEAENVWEMGGLGILTSVPITPRVVCFLCASSGHVEFVYCQVCCEPFHKFCLEENERPLEDQLENWCCRRCKFCHVCGRQHQATKQLLECNKCRNSYHPECLGPNYPTKPTKKKKVWICTKCVRCKSCGSTTPGKGWDAQWSHDFSLCHDCAKLFAKGNFCPLCDKCYDDDDYESKMMQCGKCDRWVHSKCENLSDEMYEILSNLPESVAYTCVNCTERHPAEWRLALEKELQISLKQVLTALLNSRTTSHLLRYRQAAKPPDLNPETEESIPSRSSPEGPDPPVLTEVSKQDDQQPLDLEGVKRKMDQGNYTSVLEFSDDIVKIIQAAINSDGGQPEIKKANSMVKSFFIRQMERVFPWFSVKKSRFWEPNKVSSNSGMLPNAVLPPSLDHNYAQWQEREENSHTEQPPLMKKIIPAPKPKGPGEPDSPTPLHPPTPPILSTDRSREDSPELNPPPGIEDNRQCALCLTYGDDSANDAGRLLYIGQNEWTHVNCALWSAEVFEDDDGSLKNVHMAVIRGKQLRCEFCQKPGATVGCCLTSCTSNYHFMCSRAKNCVFLDDKKVYCQRHRDLIKGEVVPENGFEVFRRVFVDFEGISLRRKFLNGLEPENIHMMIGSMTIDCLGILNDLSDCEDKLFPIGYQCSRVYWSTTDARKRCVYTCKIVECRPPVVEPDINSTVEHDENRTIAHSPTSFTESSSKESQNTAEIISPPSPDRPPHSQTSGSCYYHVISKVPRIRTPSYSPTQRSPGCRPLPSAGSPTPTTHEIVTVGDPLLSSGLRSIGSRRHSTSSLSPQRSKLRIMSPMRTGNTYSRNNVSSVSTTGTATDLESSAKVVDHVLGPLNSSTSLGQNTSTSSNLQRTVVTVGNKNSHLDGSSSSEMKQSSASDLVSKSSSLKGEKTKVLSSKSSEGSAHNVAYPGIPKLAPQVHNTTSRELNVSKIGSFAEPSSVSFSSKEALSFPHLHLRGQRNDRDQHTDSTQSANSSPDEDTEVKTLKLSGMSNRSSIINEHMGSSSRDRRQKGKKSCKETFKEKHSSKSFLEPGQVTTGEEGNLKPEFMDEVLTPEYMGQRPCNNVSSDKIGDKGLSMPGVPKAPPMQVEGSAKELQAPRKRTVKVTLTPLKMENESQSKNALKESSPASPLQIESTSPTEPISASENPGDGPVAQPSPNNTSCQDSQSNNYQNLPVQDRNLMLPDGPKPQEDGSFKRRYPRRSARARSNMFFGLTPLYGVRSYGEEDIPFYSSSTGKKRGKRSAEGQVDGADDLSTSDEDDLYYYNFTRTVISSGGEERLASHNLFREEEQCDLPKISQLDGVDDGTESDTSVTATTRKSSQIPKRNGKENGTENLKIDRPEDAGEKEHVTKSSVGHKNEPKMDNCHSVSRVKTQGQDSLEAQLSSLESSRRVHTSTPSDKNLLDTYNTELLKSDSDNNNSDDCGNILPSDIMDFVLKNTPSMQALGESPESSSSELLNLGEGLGLDSNREKDMGLFEVFSQQLPTTEPVDSSVSSSISAEEQFELPLELPSDLSVLTTRSPTVPSQNPSRLAVISDSGEKRVTITEKSVASSESDPALLSPGVDPTPEGHMTPDHFIQGHMDADHISSPPCGSVEQGHGNNQDLTRNSSTPGLQVPVSPTVPIQNQKYVPNSTDSPGPSQISNAAVQTTPPHLKPATEKLIVVNQNMQPLYVLQTLPNGVTQKIQLTSSVSSTPSVMETNTSVLGPMGGGLTLTTGLNPSLPTSQSLFPSASKGLLPMSHHQHLHSFPAATQSSFPPNISNPPSGLLIGVQPPPDPQLLVSESSQRTDLSTTVATPSSGLKKRPISRLQTRKNKKLAPSSTPSNIAPSDVVSNMTLINFTPSQLPNHPSLLDLGSLNTSSHRTVPNIIKRSKSSIMYFEPAPLLPQSVGGTAATAAGTSTISQDTSHLTSGSVSGLASSSSVLNVVSMQTTTTPTSSASVPGHVTLTNPRLLGTPDIGSISNLLIKASQQSLGIQDQPVALPPSSGMFPQLGTSQTPSTAAITAASSICVLPSTQTTGITAASPSGEADEHYQLQHVNQLLASKTGIHSSQRDLDSASGPQVSNFTQTVDAPNSMGLEQNKALSSAVQASPTSPGGSPSSPSSGQRSASPSVPGPTKPKPKTKRFQLPLDKGNGKKHKVSHLRTSSSEAHIPDQETTSLTSGTGTPGAEAEQQDTASVEQSSQKECGQPAGQVAVLPEVQVTQNPANEQESAEPKTVEEEESNFSSPLMLWLQQEQKRKESITEKKPKKGLVFEISSDDGFQICAESIEDAWKSLTDKVQEARSNARLKQLSFAGVNGLRMLGILHDAVVFLIEQLSGAKHCRNYKFRFHKPEEANEPPLNPHGSARAEVHLRKSAFDMFNFLASKHRQPPEYNPNDEEEEEVQLKSARRATSMDLPMPMRFRHLKKTSKEAVGVYRSPIHGRGLFCKRNIDAGEMVIEYAGNVIRSIQTDKREKYYDSKGIGCYMFRIDDSEVVDATMHGNAARFINHSCEPNCYSRVINIDGQKHIVIFAMRKIYRGEELTYDYKFPIEDASNKLPCNCGAKKCRKFLN

>NP_055605.2

MSKKGRNKGEKPEALIVALQAANEDLRTKLTDIQIELHQEKSKVSKLEREKTQEAKRIRELEQRKHTVLVTELKAKLHEEKMKELQAVRENLIKQHEQEMSRTVKVRDGEIQRLKSALCALRDGSSDKVRTALTIEAREEARKLFDTERLKLLQEIADLKTAKKQVDEALSNMIQADKIKAGDLRSEHQSHQEAISKIKWESERDIRRLMDEIKAKDRIIFSLEKELETQTGYVQKLQLQKEALDEQLFLVKEAECNMSSPKREIPGRAGDGSEHCSSPDLRRNQKRIAELNATIRKLEDRNTLLGDERNELLKRVRETEKQCKPLLERNKCLAKRNDELMVSLQRMEEKLKAVTKENSEMREKITSHPPLKKLKSLNDLDQANEEQETEFLKLQVIEQQNIIDELTRDREKLIRRRKHRRSSKPIKRPVLDPFIGYDEDSMDSETSSMASFRTDRTPATPDDDLDESLAAEESELRFRQLTKEYQALQRAYALLQEQTGGIIDAEREAKAQEQLQAEVLRYKAKIEDLEATLAQKGQDSHWVEDKQLFIKRNQELLEKIEKQEAENHRLQQELQDARDQNELLEFRNLELEERERRSPPFNLQIHPFSDGVSALQIYCMKEGVKDVNIPDLIKQLDILGDNGNLRNEEQVAIIQASTVLSLAEKWIQQIEGAEAALHQKMMELESDMEQFCKIKGYLEEELDYRKQALDQAYMRIQELEATLYNALQQETVIKFGELLSEKQQEELRTAVEKLRRQMLRKSREYDCQILQERMELLQQAHQRIRDLEDKTDIQKRQIKDLEEKSNRKHG

1476 NP_006684.1 NP_002717.3

>NP_006684.1

MQEIIASVDHIKFDLEIAVEQQLGAQPLPFPGMDKSGAAVCEFFLKAACGKGGMCPFRHISGEKTVVCKHWLRGLCKKGDQCEFLHEYDMTKMPECYFYSKFGECSNKECPFLHIDPESKIKDCPWYDRGFCKHGPLCRHRHTRRVICVNYLVGFCPEGPSCKFMHPRFELPMGTTEQPPLPQQTQPPAKQSNNPPLQRSSSLIQLTSQNSSPNQQRTPQVIGVMQSQNSSAGNRGPRPLEQVTCYKCGEKGHYANRCTKGHLAFLSGQ

>NP_002717.3

MLSLQYPDVYRDETAVQDYHGHKICDPYAWLEDPDSEQTKAFVEAQNKITVPFLEQCPIRGLYKERMTELYDYPKYSCHFKKGKRYFYFYNTGLQNQRVLYVQDSLEGEARVFLDPNILSDDGTVALRGYAFSEDGEYFAYGLSASGSDWVTIKFMKVDGAKELPDVLERVKFSCMAWTHDGKGMFYNSYPQQDGKSDGTETSTNLHQKLYYHVLGTDQSEDILCAEFPDEPKWMGGAELSDDGRYVLLSIREGCDPVNRLWYCDLQQESSGIAGILKWVKLIDNFEGEYDYVTNEGTVFTFKTNRQSPNYRVINIDFRDPEESKWKVLVPEHEKDVLEWIACVRSNFLVLCYLHDVKNILQLHDLTTGALLKTFPLDVGSIVGYSGQKKDTEIFYQFTSFLSPGIIYHCDLTKEELEPRVFREVTVKGIDASDYQTVQIFYPSKDGTKIPMFIVHKKGIKLDGSHPAFLYGYGGFNISITPNYSVSRLIFVRHMGGILAVANIRGGGEYGETWHKGGILANKQNCFDDFQCAAEYLIKEGYTSPKRLTINGGSNGGLLVAACANQRPDLFGCVIAQVGVMDMLKFHKYTIGHAWTTDYGCSDSKQHFEWLVKYSPLHNVKLPEADDIQYPSMLLLTADHDDRVVPLHSLKFIATLQYIVGRSRKQSNPLLIHVDTKAGHGAGKPTAKVIEEVSDMFAFIARCLNVDWIP

1477 NP_056943.1 O14561

>NP_056943.1

MANSANTNTVPKLYRSVIEDVINDVRDIFLDDGVDEQVLMELKTLWENKLMQSRAVDGFHSEEQQLLLQVQQQHQPQQQQHHHHHHHQQAQPQQTVPQQAQTQQVLIPASQQATAPQVIVPDSKLIQHMNASNMSAAATAATLALPAGVTPVQQILTNSGQLLQVVRAANGAQYIFQPQQSVVLQQQVIPQMQPGGVQAPVIQQVLAPLPGGISPQTGVIIQPQQILFTGNKTQVIPTTVAAPTPAQAQITATGQQQPQAQPAQTQAPLVLQVDGTGDTSSEEDEDEEEDYDDDEEEDKEKDGAEDGQVEEEPLNSEDDVSDEEGQELFDTENVVVCQYDKIHRSKNKWKFHLKDGIMNLNGRDYIFSKAIGDAEW

>O14561

MASRVLSAYVSRLPAAFAPLPRVRMLAVARPLSTALCSAGTQTRLGTLQPALVLAQVPGRVTQLCRQYSDMPPLTLEGIQDRVLYVLKLYDKIDPEKLSVNSHFMKDLGLDSLDQVEIIMAMEDEFGFEIPDIDAEKLMCPQEIVDYIADKKDVYE

1478 NP_003404.1 Q8IVS2

>NP_003404.1

MTMLLDGGPQFPGLGVGSFGAPRHHEMPNREPAGMGLNPFGDSTHAAAAAAAAAAFKLSPAAAHDLSSGQSSAFTPQGSGYANALGHHHHHHHHHHHTSQVPSYGGAASAAFNSTREFLFRQRSSGLSEAASGGGQHGLFAGSASSLHAPAGIPEPPSYLLFPGLHEQGAGHPSPTGHVDNNQVHLGLRGELFGRADPYRPVASPRTDPYAAGAQFPNYSPMNMNMGVNVAAHHGPGAFFRYMRQPIKQELSCKWIDEAQLSRPKKSCDRTFSTMHELVTHVTMEHVGGPEQNNHVCYWEECPREGKSFKAKYKLVNHIRVHTGEKPFPCPFPGCGKIFARSENLKIHKRTHTGEKPFKCEFEGCDRRFANSSDRKKHMHVHTSDKPYICKVCDKSYTHPSSLRKHMKVHESQGSDSSPAASSGYESSTPPAIASANSKDTTKTPSAVQTSTSHNPGLPPNFNEWYV

>Q8IVS2

MSVRVARVAWVRGLGASYRRGASSFPVPPPGAQGVAELLRDATGAEEEAPWAATERRMPGQCSVLLFPGQGSQVVGMGRGLLNYPRVRELYAAARRVLGYDLLELSLHGPQETLDRTVHCQPAIFVASLAAVEKLHHLQPSVIENCVAAAGFSVGEFAALVFAGAMEFAEGLYAVKIRAEAMQEASEAVPSGMLSVLGQPQSKFNFACLEAREHCKSLGIENPVCEVSNYLFPDCRVISGHQEALRFLQKNSSKFHFRRTRMLPVSGAFHTRLMEPAVEPLTQALKAVDIKKPLVSVYSNVHAHRYRHPGHIHKLLAQQLVSPVKWEQTMHAIYERKKGRGFPQTFEVGPGRQLGAILKSCNMQAWKSYSAVDVLQTLEHVDLDPQEPPR

1479 Q15723 Q7Z7A3

>Q15723

MTSAVVDSGGTILELSSNGVENQEESEKVSEYPAVIVEPVPSARLEQGYAAQVLVYDDETYMMQDVAEEQEVETENVETVEASVHSSNAHCTDKTIEAAEALLHMESPTCLRDSRSPVEVFVPPCVSTPEFIHAAMRPDVITETVVEVSTEESEPMDTSPIPTSPDSHEPMKKKKVGRKPKTQQSPISNGSPELGIKKKPREGKGNTTYLWEFLLDLLQDKNTCPRYIKWTQREKGIFKLVDSKAVSKLWGKHKNKPDMNYETMGRALRYYYQRGILAKVEGQRLVYQFKDMPKNIVVIDDDKSETCNEDLAGTTDEKSLERVSLSAESLLKAASSVRSGKNSSPINCSRAEKGVARVVNITSPGHDASSRSPTTTASVSATAAPRTVRVAMQVPVVMTSLGQKISTVAVQSVNAGAPLITSTSPTTATSPKVVIQTIPTVMPASTENGDKITMQPAKIITIPATQLAQCQLQTKSNLTGSGSINIVGTPLAVRALTPVSIAHGTPVMRLSMPTQQASGQTPPRVISAVIKGPEVKSEAVAKKQEHDVKTLQLVEEKPADGNKTVTHVVVVSAPSAIALPVTMKTEGLVTCEK

>Q7Z7A3

MPAPPCASCHAARAALRRPLSGQALCGACFCAAFEAEVLHTVLAGRLLPPGAVVAVGASGGKDSTVLAHVLRALAPRLGISLQLVAVDEGIGGYRDAALAAVRRQAARWELPLTVVAYEDLFGGWTMDAVARSTAGSGRSRSCCTFCGVLRRRALEEGARRVGATHIVTGHNADDMAETVLMNFLRGDAGRLARGGGLGSPGEGGALPRCRPLQFASQKEVVLYAHFRRLDYFSEECVYAPEAFRGHARDLLKRLEAARPSAVLDLVHSAERLALAPAARPPRPGACSRCGALASRALCQACALLDGLNRGRPRLAIGKGRRGLDEEATPGTPGDPARPPASKAVPTF

1480 Q13105 NP_060383.2

>Q13105

MDFPQHSQHVLEQLNQQRQLGLLCDCTFVVDGVHFKAHKAVLAACSEYFKMLFVDQKDVVHLDISNAAGLGQVLEFMYTAKLSLSPENVDDVLAVATFLQMQDIITACHALKSLAEPATSPGGNAEALATEGGDKRAKEEKVATSTLSRLEQAGRSTPIGPSRDLKEERGGQAQSAASGAEQTEKADAPREPPPVELKPDPTSGMAAAEAEAALSESSEQEMEVEPARKGEEEQKEQEEQEEEGAGPAEVKEEGSQLENGEAPEENENEESAGTDSGQELGSEARGLRSGTYGDRTESKAYGSVIHKCEDCGKEFTHTGNFKRHIRIHTGEKPFSCRECSKAFSDPAACKAHEKTHSPLKPYGCEECGKSYRLISLLNLHKKRHSGEARYRCEDCGKLFTTSGNLKRHQLVHSGEKPYQCDYCGRSFSDPTSKMRHLETHDTDKEHKCPHCDKKFNQVGNLKAHLKIHIADGPLKCRECGKQFTTSGNLKRHLRIHSGEKPYVCIHCQRQFADPGALQRHVRIHTGEKPCQCVMCGKAFTQASSLIAHVRQHTGEKPYVCERCGKRFVQSSQLANHIRHHDNIRPHKCSVCSKAFVNVGDLSKHIIIHTGEKPYLCDKCGRGFNRVDNLRSHVKTVHQGKAGIKILEPEEGSEVSVVTVDDMVTLATEALAATAVTQLTVVPVGAAVTADETEVLKAEISKAVKQVQEEDPNTHILYACDSCGDKFLDANSLAQHVRIHTAQALVMFQTDADFYQQYGPGGTWPAGQVLQAGELVFRPRDGAEGQPALAETSPTAPECPPPAE

>NP_060383.2

MEQPWPPPGPWSLPRAEGEAEEESDFDVFPSSPRCPQLPGGGAQMYSHGIELACQKQKEFVKSSVACKWNLAEAQQKLGSLALHNSESLDQEHAKAQTAVSELRQREEEWRQKEEALVQREKMCLWSTDAISKDVFNKSFINQDKRKDTEDEDKSESFMQKYEQKIRHFGMLSRWDDSQRFLSDHPYLVCEETAKYLILWCFHLEAEKKGALMEQIAHQAVVMQFIMEMAKNCNVDPRGCFRLFFQKAKAEEEGYFEAFKNELEAFKSRVRLYSQSQSFQPMTVQNHVPHSGVGSIGLLESLPQNPDYLQYSISTALCSLNSVVHKEDDEPKMMDTV

1481 NP_079273.2 NP_076425.1

>NP_079273.2

MASPDRSKRKILKAKKTMPLSCRKQVEMLNKSRNVEALKTAIGSNVPSGNQSFSPSVITRTTEITKCSPSENGASSLDSNKNSISEKSKVFSQNCIKPVEEIVHSETKLEQVVCSYQKPSRTTESPSRVFTEEAKDSLNTSENDSEHQTNVTRSLFEHEGACSLKSSCCPPSVLSGVVQMPESTVTSTVGDKKTDQMVFHLETNSNSESHDKRQSDNILCSEDSGFVPVEKTPNLVNSVTSNNCADDILKTDECSRTSISNCESADSTWQSSLDTNNNSHYQKKRMFSENEENVKRMKTSEQINENICVSLERQTAFLEQVRHLIQQEIYSINYELFDKKLKELNQRIGKTECRNKHEGIADKLLAKIAKLQRRIKTVLLFQRNCLKPNMLSSNGASKVANSEAMILDKNLESVNSPIEKSSVNYEPSNPSEKGSKKINLSSDQNKSVSESNNDDVMLISVESPNLTTPITSNPTDTRKITSGNSSNSPNAEVMAVQKKLDSIIDLTKEGLSNCNTESPVSPLESHSKAASNSKETTPLAQNAVQVPESFEHLPPLPEPPAPLPELVDKTRDTLPPQKPELKVKRVFRPNGIALTWNITKINPKCAPVESYHLFLCHENSNNKLIWKKIGEIKALPLPMACTLSQFLASNRYYFTVQSKDIFGRYGPFCDIKSIPGFSENLT

>NP_076425.1

MARKKVRPRLIAELARRVRALREQLNRPRDSQLYAVDYETLTRPFSGRRLPVRAWADVRRESRLLQLLGRLPLFGLGRLVTRKSWLWQHDEPCYWRLTRVRPDYTAQNLDHGKAWGILTFKGKTESEAREIEHVMYHDWRLVPKHEEEAFTAFTPAPEDSLASVPYPPLLRAMIIAERQKNGDTSTEEPMLNVQRIRMEPWDYPAKQEDKGRAKGTPV

1482 NP_068556.1 NP_872578.1

>NP_068556.1

MLGAVKMEGHEPSDWSSYYAEPEGYSSVSNMNAGLGMNGMNTYMSMSAAAMGSGSGNMSAGSMNMSSYVGAGMSPSLAGMSPGAGAMAGMGGSAGAAGVAGMGPHLSPSLSPLGGQAAGAMGGLAPYANMNSMSPMYGQAGLSRARDPKTYRRSYTHAKPPYSYISLITMAIQQSPNKMLTLSEIYQWIMDLFPFYRQNQQRWQNSIRHSLSFNDCFLKVPRSPDKPGKGSFWTLHPDSGNMFENGCYLRRQKRFKCEKQLALKEAAGAAGSGKKAAAGAQASQAQLGEAAGPASETPAGTESPHSSASPCQEHKRGGLGELKGTPAAALSPPEPAPSPGQQQQAAAHLLGPPHHPGLPPEAHLKPEHHYAFNHPFSINNLMSSEQQHHHSHHHHQPHKMDLKAYEQVMHYPGYGSPMPGSLAMGPVTNKTGLDASPLAADTSYYQGVYSRPIMNSS

>NP_872578.1

MAAPCVSYGGAVSYRLLLWGRGSLARKQGLWKTAAPELQTNVRSQILRLRHTAFVIPKKNVPTSKRETYTEDFIKKQIEEFNIGKRHLANMMGEDPETFTQEDIDRAIAYLFPSGLFEKRARPVMKHPEQIFPRQRAIQWGEDGRPFHYLFYTGKQSYYSLMHDVYGMLLNLEKHQSHLQAKSLLPEKTVTRDVIGSRWLIKEELEEMLVEKLSDLDYMQFIRLLEKLLTSQCGAAEEEFVQRFRRSVTLESKKQLIEPVQYDEQGMAFSKSEGKRKTAKAEAIVYKHGSGRIKVNGIDYQLYFPITQDREQLMFPFHFVDRLGKHDVTCTVSGGGRSAQAGAIRLAMAKALCSFVTEDEVEWMRQAGLLTTDPRVRERKKPGQEGARRKFTWKKR

1483 NP_006692.1 NP_001379.1

>NP_006692.1

MSYMLPHLHNGWQVDQAILSEEDRVVVIRFGHDWDPTCMKMDEVLYSIAEKVKNFAVIYLVDITEVPDFNKMYELYDPCTVMFFFRNKHIMIDLGTGNNNKINWAMEDKQEMVDIIETVYRGARKGRGLVVSPKDYSTKYRY

>NP_001379.1

MGILEKISEIEKEIARTQKNKATEYHLGLLKAKLAKYRAQLLEPSKSASSKGEGFDVMKSGDARVALIGFPSVGKSTFLSLMTSTASEAASYEFTTLTCIPGVIEYKGANIQLLDLPGIIEGAAQGKGRGRQVIAVARTADVIIMMLDATKGEVQRSLLEKELESVGIRLNKHKPNIYFKPKKGGGISFNSTVTLTQCSEKLVQLILHEYKIFNAEVLFREDCSPDEFIDVIVGNRVYMPCLYVYNKIDQISMEEVDRLARKPNSVVISCGMKLNLDYLLEMLWEYLALTCIYTKKRGQRPDFTDAIILRKGASVEHVCHRIHRSLASQFKYALVWGTSTKYSPQRVGLTHTMEHEDVIQIVKK

1484 Q96JN0 NP_056177.3

>Q96JN0

MQRMIQQFAAEYTSKNSSTQDPSQPNSTKNQSLPKASPVTTSPTAATTQNPVLSKLLMADQDSPLDLTVRKSQSEPSEQDGVLDLSTKKSPCAGSTSLSHSPGCSSTQGNGRPGRPSQYRPDGLRSGDGVPPRSLQDGTREGFGHSTSLKVPLARSLQISEELLSRNQLSTAASLGPSGLQNHGQHLILSREASWAKPHYEFNLSRMKFRGNGALSNISDLPFLAENSAFPKMALQAKQDGKKDVSHSSPVDLKIPQVRGMDLSWESRTGDQYSYSSLVMGSQTESALSKKLRAILPKQSRKSMLDAGPDSWGSDAEQSTSGQPYPTSDQEGDPGSKQPRKKRGRYRQYNSEILEEAISVVMSGKMSVSKAQSIYGIPHSTLEYKVKERLGTLKNPPKKKMKLMRSEGPDVSVKIELDPQGEAAQSANESKNE

>NP_056177.3

MTPSEGARAGTGRELEMLDSLLALGGLVLLRDSVEWEGRSLLKALVKKSALCGEQVHILGCEVSEEEFREGFDSDINNRLVYHDFFRDPLNWSKTEEAFPGGPLGALRAMCKRTDPVPVTIALDSLSWLLLRLPCTTLCQVLHAVSHQDSCPGDSSSVGKVSVLGLLHEELHGPGPVGALSSLAQTEVTLGGTMGQASAHILCRRPRQRPTDQTQWFSILPDFSLDLQEGPSVESQPYSDPHIPPVDPTTHLTFNLHLSKKEREARDSLILPFQFSSEKQQALLRPRPGQATSHIFYEPDAYDDLDQEDPDDDLDI

1485 Q9H3D4 Q9ULW8

>Q9H3D4

MNFETSRCATLQYCPDPYIQRFVETPAHFSWKESYYRSTMSQSTQTNEFLSPEVFQHIWDFLEQPICSVQPIDLNFVDEPSEDGATNKIEISMDCIRMQDSDLSDPMWPQYTNLGLLNSMDQQIQNGSSSTSPYNTDHAQNSVTAPSPYAQPSSTFDALSPSPAIPSNTDYPGPHSFDVSFQQSSTAKSATWTYSTELKKLYCQIAKTCPIQIKVMTPPPQGAVIRAMPVYKKAEHVTEVVKRCPNHELSREFNEGQIAPPSHLIRVEGNSHAQYVEDPITGRQSVLVPYEPPQVGTEFTTVLYNFMCNSSCVGGMNRRPILIIVTLETRDGQVLGRRCFEARICACPGRDRKADEDSIRKQQVSDSTKNGDGTKRPFRQNTHGIQMTSIKKRRSPDDELLYLPVRGRETYEMLLKIKESLELMQYLPQHTIETYRQQQQQQHQHLLQKQTSIQSPSSYGNSSPPLNKMNSMNKLPSVSQLINPQQRNALTPTTIPDGMGANIPMMGTHMPMAGDMNGLSPTQALPPPLSMPSTSHCTPPPPYPTDCSIVSFLARLGCSSCLDYFTTQGLTTIYQIEHYSMDDLASLKIPEQFRHAIWKGILDHRQLHEFSSPSHLLRTPSSASTVSVGSSETRGERVIDAVRFTLRQTISFPPRDEWNDFNFDMDARRNKQQRIKEEGE

>Q9ULW8

MSLQRIVRVSLEHPTSAVCVAGVETLVDIYGSVPEGTEMFEVYGTPGVDIYISPNMERGRERADTRRWRFDATLEIIVVMNSPSNDLNDSHVQISYHSSHEPLPLAYAVLYLTCVDISLDCDLNCEGRQDRNFVDKRQWVWGPSGYGGILLVNCDRDDPSCDVQDNCDQHVHCLQDLEDMSVMVLRTQGPAALFDDHKLVLHTSSYDAKRAQVFHICGPEDVCEAYRHVLGQDKVSYEVPRLHGDEERFFVEGLSFPDAGFTGLISFHVTLLDDSNEDFSASPIFTDTVVFRVAPWIMTPSTLPPLEVYVCRVRNNTCFVDAVAELARKAGCKLTICPQAENRNDRWIQDEMELGYVQAPHKTLPVVFDSPRNGELQDFPYKRILGPDFGYVTREPRDRSVSGLDSFGNLEVSPPVVANGKEYPLGRILIGGNLPGSSGRRVTQVVRDFLHAQKVQPPVELFVDWLAVGHVDEFLSFVPAPDGKGFRMLLASPGACFKLFQEKQKCGHGRALLFQGVVDDEQVKTISINQVLSNKDLINYNKFVQSCIDWNREVLKRELGLAECDIIDIPQLFKTERKKATAFFPDLVNMLVLGKHLGIPKPFGPIINGCCCLEEKVRSLLEPLGLHCTFIDDFTPYHMLHGEVHCGTNVCRKPFSFKWWNMVP

1486 NP_057272.1 Q9NX20

>NP_057272.1

MSGGVYGGDEVGALVFDIGSFSVRAGYAGEDCPKADFPTTVGLLAAEEGGGLELEGDKEKKGKIFHIDTNALHVPRDGAEVMSPLKNGMIEDWECFRAILDHTYSKHVKSEPNLHPVLMSEAPWNTRAKREKLTELMFEQYNIPAFFLCKTAVLTAFANGRSTGLVLDSGATHTTAIPVHDGYVLQQGIVKSPLAGDFISMQCRELFQEMAIDIIPPYMIAAKEPVREGAPPNWKKKEKLPQVSKSWHNYMCNEVIQDFQASVLQVSDSPYDEQVAAQMPTVHYEMPNGYNTDYGAERLRIPEGLFDPSNVKGLSGNTMLGVGHVVTTSIGMCDIDIRPGLYGSVIVTGGNTLLQGFTDRLNRELSQKTPPSMRLKLIASNSTMERKFSPWIGGSILASLGTFQQMWISKQEYEEGGKQCVERKCP

>Q9NX20

MWRLLARASAPLLRVPLSDSWALLPASAGVKTLLPVPSFEDVSIPEKPKLRFIERAPLVPKVRREPKNLSDIRGPSTEATEFTEGNFAILALGGGYLHWGHFEMMRLTINRSMDPKNMFAIWRVPAPFKPITRKSVGHRMGGGKGAIDHYVTPVKAGRLVVEMGGRCEFEEVQGFLDQVAHKLPFAAKAVSRGTLEKMRKDQEERERNNQNPWTFERIATANMLGIRKVLSPYDLTHKGKYWGKFYMPKRV

1487 Q9BXL5 Q9Y2Q9

>Q9BXL5

MDLGKDQSHLKHHQTPDPHQEENHSPEVIGTWSLRNRELLRKRKAEVHEKETSQWLFGEQKKRKQQRTGKGNRRGRKRQQNTELKVEPQPQIEKEIVEKALAPIEKKTEPPGSITKVFPSVASPQKVVPEEHFSEICQESNIYQENFSEYQEIAVQNHSSETCQHVSEPEDLSPKMYQEISVLQDNSSKICQDMKEPEDNSPNTCQVISVIQDHPFKMYQDMAKREDLAPKMCQEAAVPKILPCPTSEDTADLAGCSLQAYPKPDVPKGYILDTDQNPAEPEEYNETDQGIAETEGLFPKIQEIAEPKDLSTKTHQESAEPKYLPHKTCNEIIVPKAPSHKTIQETPHSEDYSIEINQETPGSEKYSPETYQEIPGLEEYSPEIYQETSQLEEYSPEIYQETPGPEDLSTETYKNKDVPKECFPEPHQETGGPQGQDPKAHQEDAKDAYTFPQEMKEKPKEEPGIPAILNESHPENDVYSYVLF

>Q9Y2Q9

MAALCRTRAVAAESHFLRVFLFFRPFRGVGTESGSESGSSNAKEPKTRAGGFASALERHSELLQKVEPLQKGSPKNVESFASMLRHSPLTQMGPAKDKLVIGRIFHIVENDLYIDFGGKFHCVCRRPEVDGEKYQKGTRVRLRLLDLELTSRFLGATTDTTVLEANAVLLGIQESKDSRSKEEHHEK

1488 NP_001197.1 NP_006262.1

>NP_001197.1

MSAAAYMDFVAAQCLVSISNRAAVPEHGVAPDAERLRLPEREVTKEHGDPGDTWKDYCTLVTIAKSLLDLNKYRPIQTPSVCSDSLESPDEDMGSDSDVTTESGSSPSHSPEERQDPGSAPSPLSLLHPGVAAKGKHASEKRHKCPYSGCGKVYGKSSHLKAHYRVHTGERPFPCTWPDCLKKFSRSDELTRHYRTHTGEKQFRCPLCEKRFMRSDHLTKHARRHTEFHPSMIKRSKKALANAL

>NP_006262.1

MGSELETAMETLINVFHAHSGKEGDKYKLSKKELKELLQTELSGFLDAQKDVDAVDKVMKELDENGDGEVDFQEYVVLVAALTVACNNFFWENS

1489 NP_778225.1 NP_064530.1

>NP_778225.1

MPDPSKSAPAPKKGSKKAVTKAQKKDGKKRKRGRKESYSIYVYKVLKQVHPDTGISSKAMGIMNSFVNDIFERIASEASRLAHYNKRSTITSREVQTAVRLLLPGELAKHAVSEGTKAVTKYTSSK

>NP_064530.1

MDFNMKKLASDAGIFFTRAVQFTEEKFGQAEKTELDAHFENLLARADSTKNWTEKILRQTEVLLQPNPSARVEEFLYEKLDRKVPSRVTNGELLAQYMADAASELGPTTPYGKTLIKVAEAEKQLGAAERDFIHTASISFLTPLRNFLEGDWKTISKERRLLQNRRLDLDACKARLKKAKAAEAKATTVPDFQETRPRNYILSASASALWNDEVDKAEQELRVAQTEFDRQAEVTRLLLEGISSTHVNHLRCLHEFVKSQTTYYAQCYRHMLDLQKQLGRFPGTFVGTTEPASPPLSSTSPTTAAATMPVVPSVASLAPPGEASLCLEEVAPPASGTRKARVLYDYEAADSSELALLADELITVYSLPGMDPDWLIGERGNKKGKVPVTYLELLS

1490 NP_005641.1 Q9BXL7

>NP_005641.1

MQSFREQSSYHGNQQSYPQEVHGSSRLEEFSPRQAQMFQNFGGTGGSSGSSGSGSGGGRRGAAAAAAAMASETSGHQGYQGFRKEAGDFYYMAGNKDPVTTGTPQPPQRRPSGPVQSYGPPQGSSFGNQYGSEGHVGQFQAQHSGLGGVSHYQQDYTGPFSPGSAQYQQQASSQQQQQQVQQLRQQLYQSHQPLPQATGQPASSSSHLQPMQRPSTLPSSAAGYQLRVGQFGQHYQSSASSSSSSSFPSPQRFSQSGQSYDGSYNVNAGSQYEGHNVGSNAQAYGTQSNYSYQPQSMKNFEQAKIPQGTQQGQQQQQPQQQQHPSQHVMQYTNAATKLPLQSQVGQYNQPEVPVRSPMQFHQNFSPISNPSPAASVVQSPSCSSTPSPLMQTGENLQCGQGSVPMGSRNRILQLMPQLSPTPSMMPSPNSHAAGFKGFGLEGVPEKRLTDPGLSSLSALSTQVANLPNTVQHMLLSDALTPQKKTSKRPSSSKKADSCTNSEGSSQPEEQLKSPMAESLDGGCSSSSEDQGERVRQLSGQSTSSDTTYKGGASEKAGSSPAQGAQNEPPRLNASPAAREEATSPGAKDMPLSSDGNPKVNEKTVGVIVSREAMTGRVEKPGGQDKGSQEDDPAATQRPPSNGGAKETSHASLPQPEPPGGGGSKGNKNGDNNSNHNGEGNGQSGHSAAGPGFTSRTEPSKSPGSLRYSYKDSFGSAVPRNVSGFPQYPTGQEKGDFTGHGERKGRNEKFPSLLQEVLQGYHHHPDRRYSRSTQEHQGMAGSLEGTTRPNVLVSQTNELASRGLLNKSIGSLLENPHWGPWERKSSSTAPEMKQINLTDYPIPRKFEIEPQSSAHEPGGSLSERRSVICDISPLRQIVRDPGAHSLGHMSADTRIGRNDRLNPTLSQSVILPGGLVSMETKLKSQSGQIKEEDFEQSKSQASFNNKKSGDHCHPPSIKHESYRGNASPGAATHDSLSDYGPQDSRPTPMRRVPGRVGGREGMRGRSPSQYHDFAEKLKMSPGRSRGPGGDPHHMNPHMTFSERANRSSLHTPFSPNSETLASAYHANTRAHAYGDPNAGLNSQLHYKRQMYQQQPEEYKDWSSGSAQGVIAAAQHRQEGPRKSPRQQQFLDRVRSPLKNDKDGMMYGPPVGTYHDPSAQEAGRCLMSSDGLPNKGMELKHGSQKLQESCWDLSRQTSPAKSSGPPGMSSQKRYGPPHETDGHGLAEATQSSKPGSVMLRLPGQEDHSSQNPLIMRRRVRSFISPIPSKRQSQDVKNSSTEDKGRLLHSSKEGADKAFNSYAHLSHSQDIKSIPKRDSSKDLPSPDSRNCPAVTLTSPAKTKILPPRKGRGLKLEAIVQKITSPNIRRSASSNSAEAGGDTVTLDDILSLKSGPPEGGSVAVQDADIEKRKGEVASDLVSPANQELHVEKPLPRSSEEWRGSVDDKVKTETHAETVTAGKEPPGAMTSTTSQKPGSNQGRPDGSLGGTAPLIFPDSKNVPPVGILAPEANPKAEEKENDTVTISPKQEGFPPKGYFPSGKKKGRPIGSVNKQKKQQQPPPPPPQPPQIPEGSADGEPKPKKQRQRRERRKPGAQPRKRKTKQAVPIVEPQEPEIKLKYATQPLDKTDAKNKSFYPYIHVVNKCELGAVCTIINAEEEEQTKLVRGRKGQRSLTPPPSSTESKALPASSFMLQGPVVTESSVMGHLVCCLCGKWASYRNMGDLFGPFYPQDYAATLPKNPPPKRATEMQSKVKVRHKSASNGSKTDTEEEEEQQQQQKEQRSLAAHPRFKRRHRSEDCGGGPRSLSRGLPCKKAATEGSSEKTVLDSKPSVPTTSEGGPELELQIPELPLDSNEFWVHEGCILWANGIYLVCGRLYGLQEALEIAREMKCSHCQEAGATLGCYNKGCSFRYHYPCAIDADCLLHEENFSVRCPKHKPPLPCPLPPLQNKTAKGSLSTEQSERG

>Q9BXL7

MPGGGPEMDDYMETLKDEEDALWENVECNRHMLSRYINPAKLTPYLRQCKVIDEQDEDEVLNAPMLPSKINRAGRLLDILHTKGQRGYVVFLESLEFYYPELYKLVTGKEPTRRFSTIVVEEGHEGLTHFLMNEVIKLQQQMKAKDLQRCELLARLRQLEDEKKQMTLTRVELLTFQERYYKMKEERDSYNDELVKVKDDNYNLAMRYAQLSEEKNMAVMRSRDLQLEIDQLKHRLNKMEEECKLERNQSLKLKNDIENRPKKEQVLELERENEMLKTKNQELQSIIQAGKRSLPDSDKAILDILEHDRKEALEDRQELVNRIYNLQEEARQAEELRDKYLEEKEDLELKCSTLGKDCEMYKHRMNTVMLQLEEVERERDQAFHSRDEAQTQYSQCLIEKDKYRKQIRELEEKNDEMRIEMVRREACIVNLESKLRRLSKDSNNLDQSLPRNLPVTIISQDFGDASPRTNGQEADDSSTSEESPEDSKYFLPYHPPQRRMNLKGIQLQRAKSPISLKRTSDFQAKGHEEEGTDASPSSCGSLPITNSFTKMQPPRSRSSIMSITAEPPGNDSIVRRYKEDAPHRSTVEEDNDSGGFDALDLDDDSHERYSFGPSSIHSSSSSHQSEGLDAYDLEQVNLMFRKFSLERPFRPSVTSVGHVRGPGPSVQHTTLNGDSLTSQLTLLGGNARGSFVHSVKPGSLAEKAGLREGHQLLLLEGCIRGERQSVPLDTCTKEEAHWTIQRCSGPVTLHYKVNHEGYRKLVKDMEDGLITSGDSFYIRLNLNISSQLDACTMSLKCDDVVHVRDTMYQDRHEWLCARVDPFTDHDLDMGTIPSYSRAQQLLLVKLQRLMHRGSREEVDGTHHTLRALRNTLQPEEALSTSDPRVSPRLSRASFLFGQLLQFVSRSENKYKRMNSNERVRIISGSPLGSLARSSLDATKLLTEKQEELDPESELGKNLSLIPYSLVRAFYCERRRPVLFTPTVLAKTLVQRLLNSGGAMEFTICKSDIVTRDEFLRRQKTETIIYSREKNPNAFECIAPANIEAVAAKNKHCLLEAGIGCTRDLIKSNIYPIVLFIRVCEKNIKRFRKLLPRPETEEEFLRVCRLKEKELEALPCLYATVEPDMWGSVEELLRVVKDKIGEEQRKTIWVDEDQL

1491 P60008 Q01064

>P60008

MLHASTIWHLRSTPPRRKQWGHCDPHRILVASEVTTEITSPTPAPRAQVCGGQPWVTVLDPLSGHTGREAERHFATVSISAVELKYCHGWRPAGQRVPSKTATGQRTCAKPCQKPSTSKVILRAVADKGTCKYVSLATLKKAVSTTGYDMARNAYHFKRVLKGLVDKGSAGSFTLGKKQASKSKLKVKRQRQQRWRSGQRPFGQHRSLLGSKQGHKRLIKGVRRVAKCHCN

>Q01064

MELSPRSPPEMLEESDCPSPLELKSAPSKKMWIKLRSLLRYMVKQLENGEINIEELKKNLEYTASLLEAVYIDETRQILDTEDELQELRSDAVPSEVRDWLASTFTQQARAKGRRAEEKPKFRSIVHAVQAGIFVERMFRRTYTSVGPTYSTAVLNCLKNLDLWCFDVFSLNQAADDHALRTIVFELLTRHNLISRFKIPTVFLMSFLDALETGYGKYKNPYHNQIHAADVTQTVHCFLLRTGMVHCLSEIELLAIIFAAAIHDYEHTGTTNSFHIQTKSECAIVYNDRSVLENHHISSVFRLMQDDEMNIFINLTKDEFVELRALVIEMVLATDMSCHFQQVKTMKTALQQLERIDKPKALSLLLHAADISHPTKQWLVHSRWTKALMEEFFRQGDKEAELGLPFSPLCDRTSTLVAQSQIGFIDFIVEPTFSVLTDVAEKSVQPLADEDSKSKNQPSFQWRQPSLDVEVGDPNPDVVSFRSTWVKRIQENKQKWKERAASGITNQMSIDELSPCEEEAPPSPAEDEHNQNGNLD

1492 P34059 NP_003354.2

>P34059

MAAVVAATRWWQLLLVLSAAGMGASGAPQPPNILLLLMDDMGWGDLGVYGEPSRETPNLDRMAAEGLLFPNFYSANPLCSPSRAALLTGRLPIRNGFYTTNAHARNAYTPQEIVGGIPDSEQLLPELLKKAGYVSKIVGKWHLGHRPQFHPLKHGFDEWFGSPNCHFGPYDNKARPNIPVYRDWEMVGRYYEEFPINLKTGEANLTQIYLQEALDFIKRQARHHPFFLYWAVDATHAPVYASKPFLGTSQRGRYGDAVREIDDSIGKILELLQDLHVADNTFVFFTSDNGAALISAPEQGGSNGPFLCGKQTTFEGGMREPALAWWPGHVTAGQVSHQLGSIMDLFTTSLALAGLTPPSDRAIDGLNLLPTLLQGRLMDRPIFYYRGDTLMAATLGQHKAHFWTWTNSWENFRQGIDFCPGQNVSGVTTHNLEDHTKLPLIFHLGRDPGERFPLSFASAEYQEALSRITSVVQQHQEALVPAQPQLNVCNWAVMNWAPPGCEKLGKCLTPPESIPKKCLWSH

>NP_003354.2

MAEGGGCRERPDAETQKSELGPLMRTTLQRGAQWYLIDSRWFKQWKKYVGFDSWDMYNVGEHNLFPGPIDNSGLFSDPESQTLKEHLIDELDYVLVPTEAWNKLLNWYGCVEGQQPIVRKVVEHGLFVKHCKVEVYLLELKLCENSDPTNVLSCHFSKADTIATIEKEMRKLFNIPAERETRLWNKYMSNTYEQLSKLDNTVQDAGLYQGQVLVIEPQNEDGTWPRQTLQSKSSTAPSRNFTTSPKSSASPYSSVSASLIANGDSTSTCGMHSSGVSRGGSGFSASYNCQEPPSSHIQPGLCGLGNLGNTCFMNSALQCLSNTAPLTDYFLKDEYEAEINRDNPLGMKGEIAEAYAELIKQMWSGRDAHVAPRMFKTQVGRFAPQFSGYQQQDSQELLAFLLDGLHEDLNRVKKKPYLELKDANGRPDAVVAKEAWENHRLRNDSVIVDTFHGLFKSTLVCPECAKVSVTFDPFCYLTLPLPLKKDRVMEVFLVPADPHCRPTQYRVTVPLMGAVSDLCEALSRLSGIAAENMVVADVYNHRFHKIFQMDEGLNHIMPRDDIFVYEVCSTSVDGSECVTLPVYFRERKSRPSSTSSASALYGQPLLLSVPKHKLTLESLYQAVCDRISRYVKQPLPDEFGSSPLEPGACNGSRNSCEGEDEEEMEHQEEGKEQLSETEGSGEDEPGNDPSETTQKKIKGQPCPKRLFTFSLVNSYGTADINSLAADGKLLKLNSRSTLAMDWDSETRRLYYDEQESEAYEKHVSMLQPQKKKKTTVALRDCIELFTTMETLGEHDPWYCPNCKKHQQATKKFDLWSLPKILVVHLKRFSYNRYWRDKLDTVVEFPIRGLNMSEFVCNLSARPYVYDLIAVSNHYGAMGVGHYTAYAKNKLNGKWYYFDDSNVSLASEDQIVTKAAYVLFYQRRDDEFYKTPSLSSSGSSDGGTRPSSSQQGFGDDEACSMDTN

1493 NP_002157.2 Q9Y3D5

>NP_002157.2

MKAFSPVRSVRKNSLSDHSLGISRSKTPVDDPMSLLYNMNDCYSKLKELVPSIPQNKKVSKMEILQHVIDYILDLQIALDSHPTIVSLHHQRPGQNQASRTPLTTLNTDISILSLQASEFPSELMSNDSKALCG

>Q9Y3D5

MAAVVAVCGGLGRKKLTHLVTAAVSLTHPGTHTVLWRRGCSQQVSSNEDLPISMENPYKEPLKKCILCGKHVDYKNVQLLSQFVSPFTGCIYGRHITGLCGKKQKEITKAIKRAQIMGFMPVTYKDPAYLKDPKVCNIRYRE

1494 NP_002683.2 NP_000847.2

>NP_002683.2

MAPERLRSRALSAFKLRGLLLRGEAIKYLTEALQSISELELEDKLEKIINAVEKQPLSSNMIERSVVEAAVQECSQSVDETIEHVFNIIGAFDIPRFVYNSERKKFLPLLMTNHPAPNLFGTPRDKAEMFRERYTILHQRTHRHELFTPPVIGSHPDESGSKFQLKTIETLLGSTTKIGDAIVLGMITQLKEGKFFLEDPTGTVQLDLSKAQFHSGLYTEACFVLAEGWFEDQVFHVNAFGFPPTEPSSTTRAYYGNINFFGGPSNTSVKTSAKLKQLEEENKDAMFVFLSDVWLDQVEVLEKLRIMFAGYSPAPPTCFILCGNFSSAPYGKNQVQALKDSLKTLADIICEYPDIHQSSRFVFVPGPEDPGFGSILPRPPLAESITNEFRQRVPFSVFTTNPCRIQYCTQEITVFREDLVNKMCRNCVRFPSSNLAIPNHFVKTILSQGHLTPLPLYVCPVYWAYDYALRVYPVPDLLVIADKYDPFTTTNTECLCINPGSFPRSGFSFKVFYPSNKTVEDSKLQGF

>NP_000847.2

MFCTKLKDLKITGECPFSLLAPGQVPNESSEEAAGSSESCKATVPICQDIPEKNIQESLPQRKTSRSRVYLHTLAESICKLIFPEFERLNVALQRTLAKHKIKESRKSLEREDFEKTIAEQAVAAGVPVEVIKESLGEEVFKICYEEDENILGVVGGTLKDFLNSFSTLLKQSSHCQEAGKRGRLEDASILCLDKEDDFLHVYYFFPKRTTSLILPGIIKAAAHVLYETEVEVSLMPPCFHNDCSEFVNQPYLLYSVHMKSTKPSLSPSKPQSSLVIPTSLFCKTFPFHFMFDKDMTILQFGNGIRRLMNRRDFQGKPNFEEYFEILTPKINQTFSGIMTMLNMQFVVRVRRWDNSVKKSSRVMDLKGQMIYIVESSAILFLGSPCVDRLEDFTGRGLYLSDIPIHNALRDVVLIGEQARAQDGLKKRLGKLKATLEQAHQALEEEKKKTVDLLCSIFPCEVAQQLWQGQVVQAKKFSNVTMLFSDIVGFTAICSQCSPLQVITMLNALYTRFDQQCGELDVYKVETIGDAYCVAGGLHKESDTHAVQIALMALKMMELSDEVMSPHGEPIKMRIGLHSGSVFAGVVGVKMPRYCLFGNNVTLANKFESCSVPRKINVSPTTYRLLKDCPGFVFTPRSREELPPNFPSEIPGICHFLDAYQQGTNSKPCFQKKDVEDGNANFLGKASGID

1495 Q03060 NP_065845.1

>Q03060

MSKCARKKYIKTNPRQMTMETVESQHDGSITASLTESKSAHVQTQTGQNSIPALAQVSVAGSGTRRGSPAVTLVQLPSGQTIHVQGVIQTPQPWVIQSSEIHTVQVAAIAETDESAESEGVIDSHKRREILSRRPSYRKILNELSSDVPGVPKIEEERSEEEGTPPSIATMAVPTSIYQTSTGQYIAIAQGGTIQISNPGSDGVQGLQALTMTNSGAPPPGATIVQYAAQSADGTQQFFVPGSQVVVQDEETELAPSHMAAATGDMPTYQIRAPTAALPQGVVMAASPGSLHSPQQLAEEATRKRELRLMKNREAAKECRRRKKEYVKCLESRVAVLEVQNKKLIEELETLKDICSPKTDY

>NP_065845.1

MTTKRKIIGRLVPCRCFRGEEEIISVLDYSHCSLQQVPKEVFNFERTLEELYLDANQIEELPKQLFNCQALRKLSIPDNDLSNLPTTIASLVNLKELDISKNGVQEFPENIKCCKCLTIIEASVNPISKLPDGFTQLLNLTQLYLNDAFLEFLPANFGRLVKLRILELRENHLKTLPKSMHKLAQLERLDLGNNEFGELPEVLDQIQNLRELWMDNNALQVLPGSIGKLKMLVYLDMSKNRIETVDMDISGCEALEDLLLSSNMLQQLPDSIGLLKKLTTLKVDDNQLTMLPNTIGNLSLLEEFDCSCNELESLPSTIGYLHSLRTLAVDENFLPELPREIGSCKNVTVMSLRSNKLEFLPEEIGQMQKLRVLNLSDNRLKNLPFSFTKLKELAALWLSDNQSKALIPLQTEAHPETKQRVLTNYMFPQQPRGDEDFQSDSDSFNPTLWEEQRQQRMTVAFEFEDKKEDDENAGKVKDLSCQAPWERGQRGITLQPARLSGDCCTPWARCDQQIQDMPVPQNDPQLAWGCISGLQQERSMCTPLPVAAQSTTLPSLSGRQVEINLKRYPTPYPEDLKNMVKSVQNLVGKPSHGVRVENSNPTANTEQTVKEKYEHKWPVAPKEITVEDSFVHPANEMRIGELHPSLAETPLYPPKLVLLGKDKKESTDESEVDKTHCLNNSVSSGTYSDYSPSQASSGSSNTRVKVGSLQTTAKDAVHNSLWGNRIAPSFPQPLDSKPLLSQREAVPPGNIPQRPDRLPMSDTFTDNWTDGSHYDNTGFVAEETTAENANSNPLLSSKSRSTSSHGRRPLIRQDRIVGVPLELEQSTHRHTPETEVPPSNPWQNWTRTPSPFEDRTAFPSKLETTPTTSPLPERKEHIKESTEIPSPFSPGVPWEYHDSNPNRSLSNVFSQIHCRPESSKGVISISKSTERLSPLMKDIKSNKFKKSQSIDEIDIGTYKVYNIPLENYASGSDHLGSHERPDKMLGPEHGMSSMSRSQSVPMLDDEMLTYGSSKGPQQQKASMTKKVYQFDQSFNPQGSVEVKAEKRIPPPFQHNPEYVQQASKNIAKDLISPRAYRGYPPMEQMFSFSQPSVNEDAVVNAQFASQGARAGFLRRADSLVSATEMAMFRRVNEPHELPPTDRYGRPPYRGGLDRQSSVTVTESQFLKRNGRYEDEHPSYQEVKAQAGSFPVKNLTQRRPLSARSYSTESYGASQTRPVSARPTMAALLEKIPSDYNLGNYGDKPSDNSDLKTRPTPVKGEESCGKMPADWRQQLLRHIEARRLDRNAAYKHNTVNLGMLPYGGISAMHAGRSMTLNLQTKSKFDHQELPLQKTPSQQSNILDNGQEDVSPSGQWNPYPLGRRDVPPDTITKKAGSHIQTLMGSQSLQHRSREQQPYEGNINKVTIQQFQSPLPIQIPSSQATRGPQPGRCLIQTKGQRSMDGYPEQFCVRIEKNPGLGFSISGGISGQGNPFKPSDKGIFVTRVQPDGPASNLLQPGDKILQANGHSFVHMEHEKAVLLLKSFQNTVDLVIQRELTV

1496 NP_055064.1 O43504

>NP_055064.1

METRPTALMSSTVAAAAPAAGAASRKESPGRWGLGEDPTGVSPSLQCRVCGDSSSGKHYGIYACNGCSGFFKRSVRRRLIYRCQVGAGMCPVDKAHRNQCQACRLKKCLQAGMNQDAVQNERQPRSTAQVHLDSMESNTESRPESLVAPPAPAGRSPRGPTPMSAARALGHHFMASLITAETCAKLEPEDADENIDVTSNDPEFPSSPYSSSSPCGLDSIHETSARLLFMAVKWAKNLPVFSSLPFRDQVILLEEAWSELFLLGAIQWSLPLDSCPLLAPPEASAAGGAQGRLTLASMETRVLQETISRFRALAVDPTEFACMKALVLFKPETRGLKDPEHVEALQDQSQVMLSQHSKAHHPSQPVRFGKLLLLLPSLRFITAERIELLFFRKTIGNTPMEKLLCDMFKN

>O43504

MEATLEQHLEDTMKNPSIVGVLCTDSQGLNLGCRGTLSDEHAGVISVLAQQAAKLTSDPTDIPVVCLESDNGNIMIQKHDGITVAVHKMAS

1497 NP_073622.2 NP_001503.1

>NP_073622.2

MCKMAIIPDWLRSHPHTRKFTHSRPHSSPCRVYSRNGSPNKFRSSSTTAVANPTLSSLDVKRILFQKITDRGDELQKAFQLLDTGQNLTVSKSELRRIITDFLMPLTREQFQDVLAQIPLSTSGTVPYLAFLSRFGGIDLYINGIKRGGGNEMNCCRTLRELEIQVGEKVFKNIKTVMKAFELIDVNKTGLVRPQELRRVLETFCMKLRDEEYEKFSKHYNIHKDTAVDYNVFLKNLSINNDLNLRYCMGNQEVSLENQQAKNSKKERLLGSASSEDIWRNYSLDEIERNFCLQLSKSYEKVEKALSAGDPCKGGYVSFNYLKIVLDTFVYQIPRRIFIQLMKRFGLKATTKINWKQFLTSFHEPQGLQVSSKGPLTKRNSINSRNESHKENIITKLFRHTEDHSASLKKALLIINTKPDGPITREEFRYILNCMAVKLSDSEFKELMQMLDPGDTGVVNTSMFIDLIEENCRMRKTSPCTDAKTPFLLAWDSVEEIVHDTITRNLQAFYNMLRSYDLGDTGRIGRNNFKKIMHVFCPFLTNAHFIKLCSKIQDIGSGRILYKKLLACIGIDGPPTVSPVLVPKDQLLSEHLQKDEQQQPDLSERTKLTEDKTTLTKKMTTEEVIEKFKKCIQQQDPAFKKRFLDFSKEPNGKINVHDFKKVLEDTGMPMDDDQYALLTTKIGFEKEGMSYLDFAAGFEDPPMRGPETTPPQPPTPSKSYVNSHFITAEECLKLFPRRLKESFRDPYSAFFKTDADRDGIINMHDLHRLLLHLLLNLKDDEFERFLGLLGLRLSVTLNFREFQNLCEKRPWRTDEAPQRLIRPKQKVADSELACEQAHQYLVTKAKNRWSDLSKNFLETDNEGNGILRRRDIKNALYGFDIPLTPREFEKLWARYDTEGKGHITYQEFLQKLGINYSPAVHRPCAEDYFNFMGHFTKPQQLQEEMKELQQSTEKAVAARDKLMDRHQDISKAFTKTDQSKTNYISICKMQEVLEECGCSLTEGELTHLLNSWGVSRHDNAINYLDFLRAVENSKSTGAQPKEKEESMPINFATLNPQEAVRKIQEVVESSQLALSTAFSALDKEDTGFVKATEFGQVLKDFCYKLTDNQYHYFLRKLRIHLTPYINWKYFLQNFSCFLEETADEWAEKMPKGPPPTSPKATADRDILARLHKAVTSHYHAITQEFENFDTMKTNTISREEFRAICNRRVQILTDEQFDRLWNEMPVNAKGRLKYPDFLSRFSSETAATPMATGDSAVAQRGSSVPDVSEGTRSALSLPTQELRPGSKSQSHPCTPASTTVIPGTPPLQNCDPIESRLRKRIQGCWRQLLKECKEKDVARQGDINASDFLALVEKFNLDISKEECQQLIIKYDLKSNGKFAYCDFIQSCVLLLKAKESSLMHRMKIQNAHKMKEAGAETPSFYSALLRIQPKIVHCWRPMRRTFKSYDEAGTGLLSVADFRTVLRQYSINLSEEEFFHILEYYDKTLSSKISYNDFLRAFLQ

>NP_001503.1

MAARPKLHYPNGRGRMESVRWVLAAAGVEFDEEFLETKEQLYKLQDGNHLLFQQVPMVEIDGMKLVQTRSILHYIADKHNLFGKNLKERTLIDMYVEGTLDLLELLIMHPFLKPDDQQKEVVNMAQKAIIRYFPVFEKILRGHGQSFLVGNQLSLADVILLQTILALEEKIPNILSAFPFLQEYTVKLSNIPTIKRFLEPGSKKKPPPDEIYVRTVYNIFRP

1498 NP_003507.1 Q14894

>NP_003507.1

MSGRGKQGGKARAKAKSRSSRAGLQFPVGRVHRLLRKGNYAERVGAGAPVYMAAVLEYLTAEILELAGNAARDNKKTRIIPRHLQLAIRNDEELNKLLGKVTIAQGGVLPNIQAVLLPKKTESHHKAKGK

>Q14894

MSRVPAFLSAAEVEEHLRSSSLLIPPLETALANFSSGPEGGVMQPVRTVVPVTKHRGYLGVMPAYSAAEDALTTKLVTFYEDRGITSVVPSHQATVLLFEPSNGTLLAVMDGNVITAKRTAAVSAIATKFLKPPSSEVLCILGAGVQAYSHYEIFTEQFSFKEVRIWNRTKENAEKFADTVQGEVRVCSSVQEAVAGADVIITVTLATEPILFGEWVKPGAHINAVGASRPDWRELDDELMKEAVLYVDSQEAALKESGDVLLSGAEIFAELGEVIKGVKPAHCEKTTVFKSLGMAVEDTVAAKLIYDSWSSGK

1499 Q9NQ33 NP_055165.2

>Q9NQ33

MDNRGNSSLPDKLPIFPDSARLPLTRSFYLEPMVTFHVHPEAPVSSPYSEELPRLPFPSDSLILGNYSEPCPFSFPMPYPNYRGCEYSYGPAFTRKRNERERQRVKCVNEGYAQLRHHLPEEYLEKRLSKVETLRAAIKYINYLQSLLYPDKAETKNNPGKVSSMIATTSHHADPMFRIV

>NP_055165.2

MHSEAEESKEVATDVFNSKNLAVQAQKKILGKMVSKSIATTLIDDTSSEVLDELYRVTREYTQNKKEAEKIIKNLIKTVIKLAILYRNNQFNQDELALMEKFKKKVHQLAMTVVSFHQVDYTFDRNVLSRLLNECREMLHQIIQRHLTAKSHGRVNNVFDHFSDCEFLAALYNPFGNFKPHLQKLCDGINKMLDEENI

1500 NP_004796.1 NP_002563.1

>NP_004796.1

MAAGGSDPRAGDVEEDASQLIFPKEFETAETLLNSEVHMLLEHRKQQNESAEDEQELSEVFMKTLNYTARFSRFKNRETIASVRSLLLQKKLHKFELACLANLCPETAEESKALIPSLEGRFEDEELQQILDDIQTKRSFQY

>NP_002563.1

MSQGDSNPAAIPHAAEDIQGDDRWMSQHNRFVLDCKDKEPDVLFVGDSMVQLMQQYEIWRELFSPLHALNFGIGGDTTRHVLWRLKNGELENIKPKVIVVWVGTNNHENTAEEVAGGIEAIVQLINTRQPQAKIIVLGLLPRGEKPNPLRQKNAKVNQLLKVSLPKLANVQLLDTDGGFVHSDGAISCHDMFDFLHLTGGGYAKICKPLHELIMQLLEETPEEKQTTIA

1501 NP_006185.1 NP_071403.1

>NP_006185.1

MEPAFGEVNQLGGVFVNGRPLPNAIRLRIVELAQLGIRPCDISRQLRVSHGCVSKILARYNETGSILPGAIGGSKPRVTTPTVVKHIRTYKQRDPGIFAWEIRDRLLADGVCDKYNVPSVSSISRILRNKIGNLAQQGHYDSYKQHQPTPQPALPYNHIYSYPSPITAAAAKVPTPPGVPAIPGSVAMPRTWPSSHSVTDILGIRSITDQVSDSSPYHSPKVEEWSSLGRNNFPAAAPHAVNGLEKGALEQEAKYGQAPNGLPAVGSFVSASSMAPYPTPAQVSPYMTYSAAPSGYVAGHGWQHAGGTSLSPHNCDIPASLAFKGMQAAREGSHSVTASAL

>NP_071403.1

MAALRLLASVLGRGVPAGGSGLALSQGCARCFATSPRLRAKFYADPVEMVKDISDGATVMIGGFGLCGIPENLIAALLRTRVKDLQVVSSNVGVEDFGLGLLLAARQVRRIVCSYVGENTLCESQYLAGELELELTPQGTLAERIRAGGAGVPAFYTPTGYGTLVQEGGAPIRYTPDGHLALMSQPREVREFNGDHFLLERAIRADFALVKGWKADRAGNVVFRRSARNFNVPMCKAADVTAVEVEEIVEVGAFPPEDIHVPNIYVDRVIKGQKYEKRIERLTILKEEDGDAGKEEDARTRIIRRAALEFEDGMYANLGIGIPLLASNFISPSMTVHLHSENGILGLGPFPTEDEVDADLINAGKQTVTVLPGGCFFASDDSFAMIRGGHIQLTMLGAMQVSKYGDLANWMIPGKKVKGMGGAMDLVSSQKTRVVVTMQHCTKDNTPKIMEKCTMPLTGKRCVDRIITEKAVFDVHRKKELTLRELWEGLTVDDIKKSTGCAFAVSPNLRPMQQVAP

1502 NP_001528.1 Q8TE04

>NP_001528.1

MAETDPKTVQDLTSVVQTLLQQMQDKFQTMSDQIIGRIDDMSSRIDDLEKNIADLMTQAGVEELESENKIPATQKS

>Q8TE04

MLKLVGGGGGQDWACSVAGTSLGGEEAAFEVARPGDQGKAGGGSPGWGCAGIPDSAPGAGVLQAGAVGPARGGQGAEEVGESAGGGEERRVRHPQAPALRLLNRKPQGGSGEIKTPENDLQRGRLSRGPRTAPPAPGMGDRSGQQERSVPHSPGAPVGTSAAAVNGLLHNGFHPPPVQPPHVCSRGPVGGSDAAPQRLPLLPELQPQPLLPQHDSPAKKCRLRRRMDSGRKNRPPFPWFGMDIGGTLVKLVYFEPKDITAEEEQEEVENLKSIRKYLTSNTAYGKTGIRDVHLELKNLTMCGRKGNLHFIRFPSCAMHRFIQMGSEKNFSSLHTTLCATGGGAFKFEEDFRMIADLQLHKLDELDCLIQGLLYVDSVGFNGKPECYYFENPTNPELCQKKPYCLDNPYPMLLVNMGSGVSILAVYSKDNYKRVTGTSLGGGTFLGLCCLLTGCETFEEALEMAAKGDSTNVDKLVKDIYGGDYERFGLQGSAVASSFGNMMSKEKRDSISKEDLARATLVTITNNIGSIARMCALNENIDRVVFVGNFLRINMVSMKLLAYAMDFWSKGQLKALFLEHEGYFGAVGALLELFKMTDDK

1503 Q9H6T0 Q9BZE1

>Q9H6T0

MTPPPPPPPPPGPDPAADPAADPCPWPGSLVVLFGATAGALGRDLGSDETDLILLVWQVVEPRSRQVGTLHKSLVRAEAAALSTQCREASGLSADSLARAEPLDKVLQQFSQLVNGDVALLGGGPYMLCTDGQQLLRQVLHPEASRKNLVLPDMFFSFYDLRREFHMQHPSTCPARDLTVATMAQGLGLETDATEDDFGVWEVKTMVAVILHLLKEPSSQLFSKPEVIKQKYETGPCSDSTVPCPYSSKADVVDSETVVRARGLPWQSSDQDVARFFKGLNVARGGVALCLNAQGRRNGEALIRFVDSEQRDLALQRHKHHMGVRYIEVYKATGEEFVKIAGGTSLEVARFLSREDQVILRLRGLPFSAGPTDVLGFLGPECPVTGGTEGLLFVRHPDGRPTGDAFALFACEELAQAALRRHKGMLGKRYIELFRSTAAEVQQVLNRYASGPLLPTLTAPLLPIPFPLAPGTGRDCVRLRGLPYTATIEDILSFLGEAAADIRPHGVHMVLNQQGRPSGDAFIQMTSAERALAAAQRCHKKVMKERYVEVVPCSTEEMSRVLMGGTLGRSGMSPPPCKLPCLSPPTYTTFQATPTLIPTETAALYPSSALLPAARVPAAPTPVAYYPGPATQLYLNYTAYYPSPPVSPTTVGYLTTPTAALASAPTSVLSQSGALVRMQGVPYTAGMKDLLSVFQAYQLPADDYTSLMPVGDPPRTVLQAPKEWVCL

>Q9BZE1

MALASGPARRALAGSGQLGLGGFGAPRRGAYEWGVRSTRKSEPPPLDRVYEIPGLEPITFAGKMHFVPWLARPIFPPWDRGYKDPRFYRSPPLHEHPLYKDQACYIFHHRCRLLEGVKQALWLTKTKLIEGLPEKVLSLVDDPRNHIENQDECVLNVISHARLWQTTEEIPKRETYCPVIVDNLIQLCKSQILKHPSLARRICVQNSTFSATWNRESLLLQVRGSGGARLSTKDPLPTIASREEIEATKNHVLETFYPISPIIDLHECNIYDVKNDTGFQEGYPYPYPHTLYLLDKANLRPHRLQPDQLRAKMILFAFGSALAQARLLYGNDAKVLEQPVVVQSVGTDGRVFHFLVFQLNTTDLDCNEGVKNLAWVDSDQLLYQHFWCLPVIKKRVVVEPVGPVGFKPETFRKFLALYLHGAA

1504 Q5TB30 Q12931

>Q5TB30

MESQGVPPGPYRATKLWNEVTTSFRAGMPLRKHRQHFKKYGNCFTAGEAVDWLYDLLRNNSNFGPEVTRQQTIQLLRKFLKNHVIEDIKGRWGSENVDDNNQLFRFPATSPLKTLPRRYPELRKNNIENFSKDKDSIFKLRNLSRRTPKRHGLHLSQENGEKIKHEIINEDQENAIDNRELSQEDVEEVWRYVILIYLQTILGVPSLEEVINPKQVIPQYIMYNMANTSKRGVVILQNKSDDLPHWVLSAMKCLANWPRSNDMNNPTYVGFERDVFRTIADYFLDLPEPLLTFEYYELFVNILVVCGYITVSDRSSGIHKIQDDPQSSKFLHLNNLNSFKSTECLLLSLLHREKNKEESDSTERLQISNPGFQERCAKKMQLVNLRNRRVSANDIMGGSCHNLIGLSNMHDLSSNSKPRCCSLEGIVDVPGNSSKEASSVFHQSFPNIEGQNNKLFLESKPKQEFLLNLHSEENIQKPFSAGFKRTSTLTVQDQEELCNGKCKSKQLCRSQSLLLRSSTRRNSYINTPVAEIIMKPNVGQGSTSVQTAMESELGESSATINKRLCKSTIELSENSLLPASSMLTGTQSLLQPHLERVAIDALQLCCLLLPPPNRRKLQLLMRMISRMSQNVDMPKLHDAMGTRSLMIHTFSRCVLCCAEEVDLDELLAGRLVSFLMDHHQEILQVPSYLQTAVEKHLDYLKKGHVSIVSLKILCNSQLQVANSSQNSLIKKGDFISSHKQPLKVRNVDRIWDSNSYMTLSIPPSLISVLILCLTMAPSHDEECG

>Q12931

MARELRALLLWGRRLRPLLRAPALAAVPGGKPILCPRRTTAQLGPRRNPAWSLQAGRLFSTQTAEDKEEPLHSIISSTESVQGSTSKHEFQAETKKLLDIVARSLYSEKEVFIRELISNASDALEKLRHKLVSDGQALPEMEIHLQTNAEKGTITIQDTGIGMTQEELVSNLGTIARSGSKAFLDALQNQAEASSKIIGQFGVGFYSAFMVADRVEVYSRSAAPGSLGYQWLSDGSGVFEIAEASGVRTGTKIIIHLKSDCKEFSSEARVRDVVTKYSNFVSFPLYLNGRRMNTLQAIWMMDPKDVREWQHEEFYRYVAQAHDKPRYTLHYKTDAPLNIRSIFYVPDMKPSMFDVSRELGSSVALYSRKVLIQTKATDILPKWLRFIRGVVDSEDIPLNLSRELLQESALIRKLRDVLQQRLIKFFIDQSKKDAEKYAKFFEDYGLFMREGIVTATEQEVKEDIAKLLRYESSALPSGQLTSLSEYASRMRAGTRNIYYLCAPNRHLAEHSPYYEAMKKKDTEVLFCFEQFDELTLLHLREFDKKKLISVETDIVVDHYKEEKFEDRSPAAECLSEKETEELMAWMRNVLGSRVTNVKVTLRLDTHPAMVTVLEMGAARHFLRMQQLAKTQEERAQLLQPTLEINPRHALIKKLNQLRASEPGLAQLLVDQIYENAMIAAGLVDDPRAMVGRLNELLVKALERH

1505 NP_057157.1 NP_003671.1

>NP_057157.1

MARPRPREYKAGDLVFAKMKGYPHWPARIDELPEGAVKPPANKYPIFFFGTHETAFLGPKDLFPYKEYKDKFGKSNKRKGFNEGLWEIENNPGVKFTGYQAIQQQSSSETEGEGGNTADASSEEEGDRVEEDGKGKRKNEKAGSKRKKSYTSKKSSKQSRKSPGDEDDKDCKEEENKSSSEGGDAGNDTRNTTSDLQKTSEGT

>NP_003671.1

MGDAPSPEEKLHLITRNLQEVLGEEKLKEILKERELKIYWGTATTGKPHVAYFVPMSKIADFLKAGCEVTILFADLHAYLDNMKAPWELLELRVSYYENVIKAMLESIGVPLEKLKFIKGTDYQLSKEYTLDVYRLSSVVTQHDSKKAGAEVVKQVEHPLLSGLLYPGLQALDEEYLKVDAQFGGIDQRKIFTFAEKYLPALGYSKRVHLMNPMVPGLTGSKMSSSEEESKIDLLDRKEDVKKKLKKAFCEPGNVENNGVLSFIKHVLFPLKSEFVILRDEKWGGNKTYTAYVDLEKDFAAEVVHPGDLKNSVEVALNKLLDPIREKFNTPALKKLASAAYPDPSKQKPMAKGPAKNSEPEEVIPSRLDIRVGKIITVEKHPDADSLYVEKIDVGEAEPRTVVSGLVQFVPKEELQDRLVVVLCNLKPQKMRGVESQGMLLCASIEGINRQVEPLDPPAGSAPGEHVFVKGYEKGQPDEELKPKKKVFEKLQADFKISEECIAQWKQTNFMTKLGSISCKSLKGGNIS

1506 NP_054885.1 Q8N142

>NP_054885.1

MAASSSGEKEKERLGGGLGVAGGNSTRERLLSALEDLEVLSRELIEMLAISRNQKLLQAGEENQVLELLIHRDGEFQELMKLALNQGKIHHEMQVLEKEVEKRDSDIQQLQKQLKEAEQILATAVYQAKEKLKSIEKARKGAISSEEIIKYAHRISASNAVCAPLTWVPGDPRRPYPTDLEMRSGLLGQMNNPSTNGVNGHLPGDALAAGRLPDVLAPQYPWQSNDMSMNMLPPNHSSDFLLEPPGHNKENEDDVEIMSTDSSSSSSESD

>Q8N142

MSGTRASNDRPPGAGGVKRGRLQQEAAATGSRVTVVLGAQWGDEGKGKVVDLLATDADIISRCQGGNNAGHTVVVDGKEYDFHLLPSGIINTKAVSFIGNGVVIHLPGLFEEAEKNEKKGLKDWEKRLIISDRAHLVFDFHQAVDGLQEVQRQAQEGKNIGTTKKGIGPTYSSKAARTGLRICDLLSDFDEFSSRFKNLAHQHQSMFPTLEIDIEGQLKRLKGFAERIRPMVRDGVYFMYEALHGPPKKILVEGANAALLDIDFGTYPFVTSSNCTVGGVCTGLGIPPQNIGDVYGVVKAYTTRVGIGAFPTEQINEIGGLLQTRGHEWGVTTGRKRRCGWLDLMILRYAHMVNGFTALALTKLDILDVLGEVKVGVSYKLNGKRIPYFPANQEMLQKVEVEYETLPGWKADTTGARRWEDLPPQAQNYIRFVENHVGVAVKWVGVGKSRESMIQLF

1507 Q06330 P82664

>Q06330

MDHTEGLPAEEPPAHAPSPGKFGERPPPKRLTREAMRNYLKERGDQTVLILHAKVAQKSYGNEKRFFCPPPCVYLMGSGWKKKKEQMERDGCSEQESQPCAFIGIGNSDQEMQQLNLEGKNYCTAKTLYISDSDKRKHFMLSVKMFYGNSDDIGVFLSKRIKVISKPSKKKQSLKNADLCIASGTKVALFNRLRSQTVSTRYLHVEGGNFHASSQQWGAFFIHLLDDDESEGEEFTVRDGYIHYGQTVKLVCSVTGMALPRLIIRKVDKQTALLDADDPVSQLHKCAFYLKDTERMYLCLSQERIIQFQATPCPKEPNKEMINDGASWTIISTDKAEYTFYEGMGPVLAPVTPVPVVESLQLNGGGDVAMLELTGQNFTPNLRVWFGDVEAETMYRCGESMLCVVPDISAFREGWRWVRQPVQVPVTLVRNDGIIYSTSLTFTYTPEPGPRPHCSAAGAILRANSSQVPPNESNTNSEGSYTNASTNSTSVTSSTATVVS

>P82664

MAARTAFGAVCRRLWQGLGNFSVNTSKGNTAKNGGLLLSTNMKWVQFSNLHVDVPKDLTKPVVTISDEPDILYKRLSVLVKGHDKAVLDSYEYFAVLAAKELGISIKVHEPPRKIERFTLLQSVHIYKKHRVQYEMRTLYRCLELEHLTGSTADVYLEYIQRNLPEGVAMEVTKTQLEQLPEHIKEPIWETLSEEKEESKS

1508 NP_003176.2 NP_005026.3

>NP_003176.2

MAAGSDLLDEVFFNSEVDEKVVSDLVGSLESQLAASAAHHHHLAPRTPEVRAAAAGALGNHVVSGSPAGAAGAGPAAPAEGAPGAAPEPPPAGRARPGGGGPQRPGPPSPRRPLVPAGPAPPAAKLRPPPEGSAGSCAPVPAAAAVAAGPEPAPAGPAKPAGPAALAARAGPGPGPGPGPGPGPGPGKPAGPGAAQTLNGSAALLNSHHAAAPAVSLVNNGPAALLPLPKPAAPGTVIQTPPFVGAAAPPAPAAPSPPAAPAPAAPAAAPPPPPPAPATLARPPGHPAGPPTAAPAVPPPAAAQNGGSAGAAPAPAPAAGGPAGVSGQPGPGAAAAAPAPGVKAESPKRVVQAAPPAAQTLAASGPASTAASMVIGPTMQGALPSPAAVPPPAPGTPTGLPKGAAGAVTQSLSRTPTATTSGIRATLTPTVLAPRLPQPPQNPTNIQNFQLPPGMVLVRSENGQLLMIPQQALAQMQAQAHAQPQTTMAPRPATPTSAPPVQISTVQAPGTPIIARQVTPTTIIKQVSQAQTTVQPSATLQRSPGVQPQLVLGGAAQTASLGTATAVQTGTPQRTVPGATTTSSAATETMENVKKCKNFLSTLIKLASSGKQSTETAANVKELVQNLLDGKIEAEDFTSRLYRELNSSPQPYLVPFLKRSLPALRQLTPDSAAFIQQSQQQPPPPTSQATTALTAVVLSSSVQRTAGKTAATVTSALQPPVLSLTQPTQVGVGKQGQPTPLVIQQPPKPGALIRPPQVTLTQTPMVALRQPHNRIMLTTPQQIQLNPLQPVPVVKPAVLPGTKALSAVSAQAAAAQKNKLKEPGGGSFRDDDDINDVASMAGVNLSEESARILATNSELVGTLTRSCKDETFLLQAPLQRRILEIGKKHGITELHPDVVSYVSHATQQRLQNLVEKISETAQQKNFSYKDDDRYEQASDVRAQLKFFEQLDQIEKQRKDEQEREILMRAAKSRSRQEDPEQLRLKQKAKEMQQQELAQMRQRDANLTALAAIGPRKKRKVDCPGPGSGAEGSGPGSVVPGSSGVGTPRQFTRQRITRVNLRDLIFCLENERETSHSLLLYKAFLK

>NP_005026.3

MSALCWGRGAAGLKRALRPCGRPGLPGKEGTAGGVCGPRRSSSASPQEQDQDRRKDWGHVELLEVLQARVRQLQAESVSEVVVNRVDVARLPECGSGDGSLQPPRKVQMGAKDATPVPCGRWAKILEKDKRTQQMRMQRLKAKLQMPFQSGEFKALTRRLQVEPRLLSKQMAGCLEDCTRQAPESPWEEQLARLLQEAPGKLSLDVEQAPSGQHSQAQLSGQQQRLLAFFKCCLLTDQLPLAHHLLVVHHGQRQKRKLLTLDMYNAVMLGWARQGAFKELVYVLFMVKDAGLTPDLLSYAAALQCMGRQDQDAGTIERCLEQMSQEGLKLQALFTAVLLSEEDRATVLKAVHKVKPTFSLPPQLPPPVNTSKLLRDVYAKDGRVSYPKLHLPLKTLQCLFEKQLHMELASRVCVVSVEKPTLPSKEVKHARKTLKTLRDQWEKALCRALRETKNRLEREVYEGRFSLYPFLCLLDEREVVRMLLQVLQALPAQGESFTTLARELSARTFSRHVVQRQRVSGQVQALQNHYRKYLCLLASDAEVPEPCLPRQYWEELGAPEALREQPWPLPVQMELGKLLAEMLVQATQMPCSLDKPHRSSRLVPVLYHVYSFRNVQQIGILKPHPAYVQLLEKAAEPTLTFEAVDVPMLCPPLPWTSPHSGAFLLSPTKLMRTVEGATQHQELLETCPPTALHGALDALTQLGNCAWRVNGRVLDLVLQLFQAKGCPQLGVPAPPSEAPQPPEAHLPHSAAPARKAELRRELAHCQKVAREMHSLRAEALYRLSLAQHLRDRVFWLPHNMDFRGRTYPCPPHFNHLGSDVARALLEFAQGRPLGPHGLDWLKIHLVNLTGLKKREPLRKRLAFAEEVMDDILDSADQPLTGRKWWMGAEEPWQTLACCMEVANAVRASDPAAYVSHLPVHQDGSCNGLQHYAALGRDSVGAASVNLEPSDVPQDVYSGVAAQVEVFRRQDAQRGMRVAQVLEGFITRKVVKQTVMTVVYGVTRYGGRLQIEKRLRELSDFPQEFVWEASHYLVRQVFKSLQEMFSGTRAIQHWLTESARLISHMGSVVEWVTPLGVPVIQPYRLDSKVKQIGGGIQSITYTHNGDISRKPNTRKQKNGFPPNFIHSLDSSHMMLTALHCYRKGLTFVSVHDCYWTHAADVSVMNQVCREQFVRLHSEPILQDLSRFLVKRFCSEPQKILEASQLKETLQAVPKPGAFDLEQVKRSTYFFS

1509 P11308 NP_006215.1

>P11308

MIQTVPDPAAHIKEALSVVSEDQSLFECAYGTPHLAKTEMTASSSSDYGQTSKMSPRVPQQDWLSQPPARVTIKMECNPSQVNGSRNSPDECSVAKGGKMVGSPDTVGMNYGSYMEEKHMPPPNMTTNERRVIVPADPTLWSTDHVRQWLEWAVKEYGLPDVNILLFQNIDGKELCKMTKDDFQRLTPSYNADILLSHLHYLRETPLPHLTSDDVDKALQNSPRLMHARNTGGAAFIFPNTSVYPEATQRITTRPDLPYEPPRRSAWTGHGHPTPQSKAAQPSPSTVPKTEDQRPQLDPYQILGPTSSRLANPGSGQIQLWQFLLELLSDSSNSSCITWEGTNGEFKMTDPDEVARRWGERKSKPNMNYDKLSRALRYYYDKNIMTKVHGKRYAYKFDFHGIAQALQPHPPESSLYKYPSDLPYMGSYHAHPQKMNFVAPHPPALPVTSSSFFAAPNPYWNSPTGGIYPNTRLPTSHMPSHLGTYY

>NP_006215.1

MVLLKEYRVILPVSVDEYQVGQLYSVAEASKNETGGGEGVEVLVNEPYEKDGEKGQYTHKIYHLQSKVPTFVRMLAPEGALNIHEKAWNAYPYCRTVITNEYMKEDFLIKIETWHKPDLGTQENVHKLEPEAWKHVEAVYIDIADRSQVLSKDYKAEEDPAKFKSIKTGRGPLGPNWKQELVNQKDCPYMCAYKLVTVKFKWWGLQNKVENFIHKQERRLFTNFHRQLFCWLDKWVDLTMDDIRRMEEETKRQLDEMRQKDPVKGMTADD

1510 NP_005997.2 NP_653288.1

>NP_005997.2

MDLTKMGMIQLQNPSHPTGLLCKANQMRLAGTLCDVVIMVDSQEFHAHRTVLACTSKMFEILFHRNSQHYTLDFLSPKTFQQILEYAYTATLQAKAEDLDDLLYAAEILEIEYLEEQCLKMLETIQASDDNDTEATMADGGAEEEEDRKARYLKNIFISKHSSEESGYASVAGQSLPGPMVDQSPSVSTSFGLSAMSPTKAAVDSLMTIGQSLLQGTLQPPAGPEEPTLAGGGRHPGVAEVKTEMMQVDEVPSQDSPGAAESSISGGMGDKVEERGKEGPGTPTRSSVITSARELHYGREESAEQVPPPAEAGQAPTGRPEHPAPPPEKHLGIYSVLPNHKADAVLSMPSSVTSGLHVQPALAVSMDFSTYGGLLPQGFIQRELFSKLGELAVGMKSESRTIGEQCSVCGVELPDNEAVEQHRKLHSGMKTYGCELCGKRFLDSLRLRMHLLAHSAGAKAFVCDQCGAQFSKEDALETHRQTHTGTDMAVFCLLCGKRFQAQSALQQHMEVHAGVRSYICSECNRTFPSHTALKRHLRSHTGDHPYECEFCGSCFRDESTLKSHKRIHTGEKPYECNGCGKKFSLKHQLETHYRVHTGEKPFECKLCHQRSRDYSAMIKHLRTHNGASPYQCTICTEYCPSLSSMQKHMKGHKPEEIPPDWRIEKTYLYLCYV

>NP_653288.1

MLRTAGRDGLCRLSTYLEELEAVELKKFKLYLGTATELGEGKIPWGSMEKAGPLEMAQLLITHFGPEEAWRLALSTFERINRKDLWERGQREDLVRDTPPGGPSSLGNQSTCLLEVSLVTPRKDPQETYRDYVRRKFRLMEDRNARLGECVNLSHRYTRLLLVKEHSNPMQVQQQLLDTGRGHARTVGHQASPIKIETLFEPDEERPEPPRTVVMQGAAGIGKSMLAHKVMLDWADGKLFQGRFDYLFYINCREMNQSATECSMQDLIFSCWPEPSAPLQELIRVPERLLFIIDGFDELKPSFHDPQGPWCLCWEEKRPTELLLNSLIRKKLLPELSLLITTRPTALEKLHRLLEHPRHVEILGFSEAERKEYFYKYFHNAEQAGQVFNYVRDNEPLFTMCFVPLVCWVVCTCLQQQLEGGGLLRQTSRTTTAVYMLYLLSLMQPKPGAPRLQPPPNQRGLCSLAADGLWNQKILFEEQDLRKHGLDGEDVSAFLNMNIFQKDINCERYYSFIHLSFQEFFAAMYYILDEGEGGAGPDQDVTRLLTEYAFSERSFLALTSRFLFGLLNEETRSHLEKSLCWKVSPHIKMDLLQWIQSKAQSDGSTLQQGSLEFFSCLYEIQEEEFIQQALSHFQVIVVSNIASKMEHMVSSFCLKRCRSAQVLHLYGATYSADGEDRARCSAGAHTLLVQLPERTVLLDAYSEHLAAALCTNPNLIELSLYRNALGSRGVKLLCQGLRHPNCKLQNLRLKRCRISSSACEDLSAALIANKNLTRMDLSGNGVGFPGMMLLCEGLRHPQCRLQMIQLRKCQLESGACQEMASVLGTNPHLVELDLTGNALEDLGLRLLCQGLRHPVCRLRTLWLKICRLTAAACDELASTLSVNQSLRELDLSLNELGDLGVLLLCEGLRHPTCKLQTLRLGICRLGSAACEGLSVVLQANHNLRELDLSFNDLGDWGLWLLAEGLQHPACRLQKLWLDSCGLTAKACENLYFTLGINQTLTDLYLTNNALGDTGVRLLCKRLSHPGCKLRVLWLFGMDLNKMTHSRLAALRVTKPYLDIGC

1511 NP_006181.1 NP_777283.1

>NP_006181.1

MSKPELKEDKMLEVHFVGDDDVLNHILDREGGAKLKKERAQLLVNPKKIIKKPEYDLEEDDQEVLKDQNYVEIMGRDVQESLKNGSATGGGNKVYSFQNRKHSEKMAKLASELAKTPQKSVSFSLKNDPEITINVPQSSKGHSASDKVQPKNNDKSEFLSTAPRSLRKRLIVPRSHSDSESEYSASNSEDDEGVAQEHEEDTNAVIFSQKIQAQNRVVSAPVGKETPSKRMKRDKTSDLVEEYFEAHSSSKVLTSDRTLQKLKRAKLDQQTLRNLLSKVSPSFSAELKQLNQQYEKLFHKWMLQLHLGFNIVLYGLGSKRDLLERFRTTMLQDSIHVVINGFFPGISVKSVLNSITEEVLDHMGTFRSILDQLDWIVNKFKEDSSLELFLLIHNLDSQMLRGEKSQQIIGQLSSLHNIYLIASIDHLNAPLMWDHAKQSLFNWLWYETTTYSPYTEETSYENSLLVKQSGSLPLSSLTHVLRSLTPNARGIFRLLIKYQLDNQDNPSYIGLSFQDFYQQCREAFLVNSDLTLRAQLTEFRDHKLIRTKKGTDGVEYLLIPVDNGTLTDFLEKEEEEA

>NP_777283.1

MNTNDAKEYLARREIPQLFESLLNGLMCSKPEDPVEYLESCLQKVKELGGCDKVKWDTFVSQEKKTLPPLNGGQSRRSFLRNVMPENSNFPYRRYDRLPPIHQFSIESDTDLSETAELIEEYEVFDPTRPRPKIILVIGGPGSGKGTQSLKIAERYGFQYISVGELLRKKIHSTSSNRKWSLIAKIITTGELAPQETTITEIKQKLMQIPDEEGIVIDGFPRDVAQALSFEDQICTPDLVVFLACANQRLKERLLKRAEQQGRPDDNVKATQRRLMNFKQNAAPLVKYFQEKGLIMTFDADRDEDEVFYDISMAVDNKLFPNKEAAAGSSDLDPSMILDTGEIIDTGSDYEDQGDDQLNVFGEDTMGGFMEDLRKCKIIFIIGGPGSGKGTQCEKLVEKYGFTHLSTGELLREELASESERSKLIRDIMERGDLVPSGIVLELLKEAMVASLGDTRGFLIDGYPREVKQGEEFGRRIGDPQLVICMDCSADTMTNRLLQRSRSSLPVDDTTKTIAKRLEAYYRASIPVIAYYETKTQLHKINAEGTPEDVFLQLCTAIDSIF

1512 Q01851 Q8N427

>Q01851

MMSMNSKQPHFAMHPTLPEHKYPSLHSSSEAIRRACLPTPPLQSNLFASLDETLLARAEALAAVDIAVSQGKSHPFKPDATYHTMNSVPCTSTSTVPLAHHHHHHHHHQALEPGDLLDHISSPSLALMAGAGGAGAAAGGGGAHDGPGGGGGPGGGGGPGGGGPGGGGGGGPGGGGGGPGGGLLGGSAHPHPHMHSLGHLSHPAAAAAMNMPSGLPHPGLVAAAAHHGAAAAAAAAAAGQVAAASAAAAVVGAAGLASICDSDTDPRELEAFAERFKQRRIKLGVTQADVGSALANLKIPGVGSLSQSTICRFESLTLSHNNMIALKPILQAWLEEAEGAQREKMNKPELFNGGEKKRKRTSIAAPEKRSLEAYFAVQPRPSSEKIAAIAEKLDLKKNVVRVWFCNQRQKQKRMKFSATY

>Q8N427

MASKKREVQLQTVINNQSLWDEMLQNKGLTVIDVYQAWCGPCRAMQPLFRKLKNELNEDEILHFAVAEADNIVTLQPFRDKCEPVFLFSVNGKIIEKIQGANAPLVNKKVINLIDEERKIAAGEMARPQYPEIPLVDSDSEVSEESPCESVQELYSIAIIKPDAVISKKVLEIKRKITKAGFIIEAEHKTVLTEEQVVNFYSRIADQCDFEEFVSFMTSGLSYILVVSQGSKHNPPSEETEPQTDTEPNERSEDQPEVEAQVTPGMMKNKQDSLQEYLERQHLAQLCDIEEDAANVAKFMDAFFPDFKKMKSMKLEKTLALLRPNLFHERKDDVLRIIKDEDFKILEQRQVVLSEKEAQALCKEYENEDYFNKLIENMTSGPSLALVLLRDNGLQYWKQLLGPRTVEEAIEYFPESLCAQFAMDSLPVNQLYGSDSLETAEREIQHFFPLQSTLGLIKPHATSEQREQILKIVKEAGFDLTQVKKMFLTPEQIEKIYPKVTGKDFYKDLLEMLSVGPSMVMILTKWNAVAEWRRLMGPTDPEEAKLLSPDSIRAQFGISKLKNIVHGASNAYEAKEVVNRLFEDPEEN

1513 NP_003186.1 NP_000377.1

>NP_003186.1

MMGKEEEIARIARRLDKMVTKKSAEGAMDLLRELKAMPITLHLLQSTRVGMSVNALRKQSSDEEVIALAKSLIKSWKKLLDASDAKARERGRGMPLPTSSRDASEAPDPSRKRPELPRAPSTPRITTFPPVPVTCDAVRNKCREMLTAALQTDHDHVAIGADCERLSAQIEECIFRDVGNTDMKYKNRVRSRISNLKDAKNPDLRRNVLCGAITPQQIAVMTSEEMASDELKEIRKAMTKEAIREHQMARTGGTQTDLFTCGKCRKKNCTYTQVQTRSSDEPMTTFVVCNECGNRWKFC

>NP_000377.1

MSSSGLNSEKVAALIQKLNSDPQFVLAQNVGTTHDLLDICLKRATVQRAQHVFQHAVPQEGKPITNQKSSGRCWIFSCLNVMRLPFMKKLNIEEFEFSQSYLFFWDKVERCYFFLSAFVDTAQRKEPEDGRLVQFLLMNPANDGGQWDMLVNIVEKYGVIPKKCFPESYTTEATRRMNDILNHKMREFCIRLRNLVHSGATKGEISATQDVMMEEIFRVVCICLGNPPETFTWEYRDKDKNYQKIGPITPLEFYREHVKPLFNMEDKICLVNDPRPQHKYNKLYTVEYLSNMVGGRKTLYNNQPIDFLKKMVAASIKDGEAVWFGCDVGKHFNSKLGLSDMNLYDHELVFGVSLKNMNKAERLTFGESLMTHAMTFTAVSEKDDQDGAFTKWRVENSWGEDHGHKGYLCMTDEWFSEYVYEVVVDRKHVPEEVLAVLEQEPIILPAWDPMGALAE

1514 NP_003963.1 NP_006651.2

>NP_003963.1

MAVSRLDRLFILLDTGTTPVTRKAAAQQLGEVVKLHPHELNNLLSKVLIYLRSANWDTRIAAGQAVEAIVKNVPEWNPVPRTRQEPTSESSMEDSPTTERLNFDRFDICRLLQHGASLLGSAGAEFEVQDEKSGEVDPKERIARQRKLLQKKLGLNMGEAIGMSTEELFNDEDLDYTPTSASFVNKQPTLQAAELIDSEFRAGMSNRQKNKAKRMAKLFAKQRSRDAVETNEKSNDSTDGEPEEKRRKIANVVINQSANDSKVLIDNIPDSSSLIEETNEWPLESFCEELCNDLFNPSWEVRHGAGTGLREILKAHGKSGGKMGDSTLEEMIQQHQEWLEDLVIRLLCVFALDRFGDFVSDEVVAPVRETCAQTLGVVLKHMNETGVHKTVDVLLKLLTQEQWEVRHGGLLGIKYALAVRQDVINTLLPKVLTRIIEGLQDLDDDVRAVAAASLVPVVESLVYLQTQKVPFIINTLWDALLELDDLTASTNSIMTLLSSLLTYPQVQQCSIQQSLTVLVPRVWPFLHHTISSVRRAALETLFTLLSTQDQNSSSWLIPILPDMLRHIFQFCVLESSQEILDLIHKVWMELLSKASVQYVVAAACPWMGAWLCLMMQPSHLPIDLNMLLEVKARAKEKTGGKVRQGQSQNKEVLQEYIAGADTIMEDPATRDFVVMRARMMAAKLLGALCCCICDPGVNVVTQEIKPAESLGQLLLFHLNSKSALQRISVALVICEWAALQKECKAVTLAVQPRLLDILSEHLYYDEIAVPFTRMQNECKQLISSLADVHIEVGNRVNNNVLTIDQASDLVTTVFNEATSSFDLNPQVLQQLDSKRQQVQMTVTETNQEWQVLQLRVHTFAACAVVSLQQLPEKLNPIIKPLMETIKKEENTLVQNYAAQCIAKLLQQCTTRTPCPNSKIIKNLCSSLCVDPYLTPCVTCPVPTQSGQENSKGSTSEKDGMHHTVTKHRGIITLYRHQKAAFAITSRRGPTPKAVKAQIADLPAGSSGNILVELDEAQKPYLVQRRGAEFALTTIVKHFGGEMAVKLPHLWDAMVGPLRNTIDINNFDGKSLLDKGDSPAQELVNSLQVFETAAASMDSELHPLLVQHLPHLYMCLQYPSTAVRHMAARCVGVMSKIATMETMNIFLEKVLPWLGAIDDSVKQEGAIEALACVMEQLDVGIVPYIVLLVVPVLGRMSDQTDSVRFMATQCFATLIRLMPLEAGIPDPPNMSAELIQLKAKERHFLEQLLDGKKLENYKIPVPINAELRKYQQDGVNWLAFLNKYKLHGILCDDMGLGKTLQSICILAGDHCHRAQEYARSKLAECMPLPSLVVCPPTLTGHWVDEVGKFCSREYLNPLHYTGPPTERIRLQHQVKRHNLIVASYDVVRNDIDFFRNIKFNYCILDEGHVIKNGKTKLSKAVKQLTANYRIILSGTPIQNNVLELWSLFDFLMPGFLGTERQFAARYGKPILASRDARSSSREQEAGVLAMDALHRQVLPFLLRRMKEDVLQDLPPKIIQDYYCTLSPLQVQLYEDFAKSRAKCDVDETVSSATLSEETEKPKLKATGHVFQALQYLRKLCNHPALVLTPQHPEFKTTAEKLAVQNSSLHDIQHAPKLSALKQLLLDCGLGNGSTSESGTESVVAQHRILIFCQLKSMLDIVEHDLLKPHLPSVTYLRLDGSIPPGQRHSIVSRFNNDPSIDVLLLTTHVGGLGLNLTGADTVVFVEHDWNPMRDLQAMDRAHRIGQKRVVNVYRLITRGTLEEKIMGLQKFKMNIANTVISQENSSLQSMGTDQLLDLFTLDKDGKAEKADTSTSGKASMKSILENLSDLWDQEQYDSEYSLENFMHSLK

>NP_006651.2

MPSCGACTCGAAAVRLITSSLASAQRGISGGRIHMSVLGRLGTFETQILQRAPLRSFTETPAYFASKDGISKDGSGDGNKKSASEGSSKKSGSGNSGKGGNQLRCPKCGDLCTHVETFVSSTRFVKCEKCHHFFVVLSEADSKKSIIKEPESAAEAVKLAFQQKPPPPPKKIYNYLDKYVVGQSFAKKVLSVAVYNHYKRIYNNIPANLRQQAEVEKQTSLTPRELEIRRREDEYRFTKLLQIAGISPHGNALGASMQQQVNQQIPQEKRGGEVLDSSHDDIKLEKSNILLLGPTGSGKTLLAQTLAKCLDVPFAICDCTTLTQAGYVGEDIESVIAKLLQDANYNVEKAQQGIVFLDEVDKIGSVPGIHQLRDVGGEGVQQGLLKLLEGTIVNVPEKNSRKLRGETVQVDTTNILFVASGAFNGLDRIISRRKNEKYLGFGTPSNLGKGRRAAAAADLANRSGESNTHQDIEEKDRLLRHVEARDLIEFGMIPEFVGRLPVVVPLHSLDEKTLVQILTEPRNAVIPQYQALFSMDKCELNVTEDALKAIARLALERKTGARGLRSIMEKLLLEPMFEVPNSDIVCVEVDKEVVEGKKEPGYIRAPTKESSEEEYDSGVEEEGWPRQADAANS

1515 NP_000779.1 NP_478144.1

>NP_000779.1

MATPPKRSCPSFSASSEGTRIKKISIEGNIAAGKSTFVNILKQLCEDWEVVPEPVARWCNVQSTQDEFEELTMSQKNGGNVLQMMYEKPERWSFTFQTYACLSRIRAQLASLNGKLKDAEKPVLFFERSVYSDRYIFASNLYESECMNETEWTIYQDWHDWMNNQFGQSLELDGIIYLQATPETCLHRIYLRGRNEEQGIPLEYLEKLHYKHESWLLHRTLKTNFDYLQEVPILTLDVNEDFKDKYESLVEKVKEFLSTL

>NP_478144.1

MHPPPPAAAMDFSQNSLFGYMEDLQELTIIERPVRRSLKTPEEIERLTVDEDLSDIERAVYLLSAGQDVQGTSVIANLPFLMRQNPTETLRRVLPKVREALHVAGVEMQLTAAMSFLTILQDESVSIHAYTHSFLQVILLHLEHRDTGVSNAWLETLLSVIEVLPKETLRHEILNPLVSKAQLSQTVQSRLVSCKILGKLTNKFDAHTIKREILPLVKSLCQDVEYEVRSCMCRQLENIAQGIGTELTKSVVLPELIELSRDEGSSVRLAAFETLVNLLDIFDTDDRSQTILPLVKSFCEKSFKADESILISLSFHLGKLCHGLYGIFTPDQHLRFLEFYKKLCTLGLQQENGHNENQIPPQILEQEKKYISVRKNCAYNFPAMIVFVDPKNFHMELYSTFFCLCHDPEVPVRYTIAICFYEVSKLLNSGVYLIHKELITLLQDESLEVLDALIDHLPEILELMSTGGESSVQENKLSSLPDLIPALTAAEQRAAASLKWRTHEKLLQKYACLPHVISSDQIYYRFLQRMFTIMMTNNVLPVQKAASRTLCIFLRYNRKQEQRHEVIQKLIEQLGQGKSYWNRLRFLDTCEFIIEIFSKSFFCKYFFLPAIELTHDPVANVRMKLCYLLPKVKSTLKIPADKHLLQQLEMCVRKLLCQEKDKDVLAIVKRTVLELDRMEMSMDAFQKKFYEKDLLDQEKEREELLLLEMEQLEKEKQQNDGRPMSDKMFEKKRRDTKTPTQSLPKNIPISVPGPSSVTPSTSKEIKKSKLIRSQSFNNQAFHAKYGNLEKCASKSSTTGYTTSVSGLGKTSVLSLADDSFRTRNASSVPSSFSPNTPLPSTSRGTGNSVDPKSSGSKDTQPRKATLKSRKSNP

1516 Q96A08 Q6XE24

>Q96A08

MPEVSSKGATISKKGFKKAVVKTQKKEGKKRKRTRKESYSIYIYKVLKQVHPDTGISSKAMSIMNSFVTDIFERIASEASRLAHYSKRSTISSREIQTAVRLLLPGELAKHAVSEGTKAVTKYTSSK

>Q6XE24

MGKRLDQPQMYPQYTYYYPHYLQTKQSYAPAPHPMAPPSPSTNSSSNNSSNNSSGEQLSKTNLYIRGLPPGTTDQDLIKLCQPYGKIVSTKAILDKNTNQCKGYGFVDFDSPAAAQKAVASLKANGVQAQMAKQQEQDPTNLYISNLPISMDEQELENMLKPFGHVISTRILRDANGVSRGVGFARMESTEKCEVVIQHFNGKYLKTPPGIPAPSEPLLCKFADGGQKKRQNQSKYTQNGRPWPREGEAGMALTYDPTAAIQNGFYSSPYSIATNRMIPQTSITPFIAASPVSTYQVQSTSWMPHPPYVMQPTGAVITPTMDHPMSMQPANMMGPLTQQMNHLSLGTTGTIQSQDRIMILHQLLCQYMTAAAPMQGTYIPQYTPVPPTAVSIEGVVADTSPQTVAPSSQDTSGQQQQIAVDTSNEHAPAYSYQQSKP

1517 Q5QNW6 Q12849

>Q5QNW6

MPDPAKSAPAPKKGSKKAVTKVQKKDGKKRKRSRKESYSVYVYKVLKQVHPDTGISSKAMGIMNSFVNDIFERIAGEASRLAHYNKRSTITSREIQTAVRLLLPGELAKHAVSEGTKAVTKYTSSK

>Q12849

MAGTRWVLGALLRGCGCNCSSCRRTGAACLPFYSAAGSIPSGVSGRRRLLLLLGAAAAAASQTRGLQTGPVPPGRLAGPPAVATSAAAAAAASYSALRASLLPQSLAAAAAVPTRSYSQESKTTYLEDLPPPPEYELAPSKLEEEVDDVFLIRAQGLPWSCTMEDVLNFFSDCRIRNGENGIHFLLNRDGKRRGDALIEMESEQDVQKALEKHRMYMGQRYVEVYEINNEDVDALMKSLQVKSSPVVNDGVVRLRGLPYSCNEKDIVDFFAGLNIVDITFVMDYRGRRKTGEAYVQFEEPEMANQALLKHREEIGNRYIEIFPSRRNEVRTHVGSYKGKKIASFPTAKYITEPEMVFEEHEVNEDIQPMTAFESEKEIELPKEVPEKLPEAADFGTTSSLHFVHMRGLPFQANAQDIINFFAPLKPVRITMEYSSSGKATGEADVHFETHEDAVAAMLKDRSHVHHRYIELFLNSCPKGK

1518 Q9Y3Z3 Q16719

>Q9Y3Z3

MQRADSEQPSKRPRCDDSPRTPSNTPSAEADWSPGLELHPDYKTWGPEQVCSFLRRGGFEEPVLLKNIRENEITGALLPCLDESRFENLGVSSLGERKKLLSYIQRLVQIHVDTMKVINDPIHGHIELHPLLVRIIDTPQFQRLRYIKQLGGGYYVFPGASHNRFEHSLGVGYLAGCLVHALGEKQPELQISERDVLCVQIAGLCHDLGHGPFSHMFDGRFIPLARPEVKWTHEQGSVMMFEHLINSNGIKPVMEQYGLIPEEDICFIKEQIVGPLESPVEDSLWPYKGRPENKSFLYEIVSNKRNGIDVDKWDYFARDCHHLGIQNNFDYKRFIKFARVCEVDNELRICARDKEVGNLYDMFHTRNSLHRRAYQHKVGNIIDTMITDAFLKADDYIEITGAGGKKYRISTAIDDMEAYTKLTDNIFLEILYSTDPKLKDAREILKQIEYRNLFKYVGETQPTGQIKIKREDYESLPKEVASAKPKVLLDVKLKAEDFIVDVINMDYGMQEKNPIDHVSFYCKTAPNRAIRITKNQVSQLLPEKFAEQLIRVYCKKVDRKSLYAARQYFVQWCADRNFTKPQDGDVIAPLITPQKKEWNDSTSVQNPTRLREASKSRVQLFKDDPM

>Q16719

MEPSSLELPADTVQRIAAELKCHPTDERVALHLDEEDKLRHFRECFYIPKIQDLPPVDLSLVNKDENAIYFLGNSLGLQPKMVKTYLEEELDKWAKIAAYGHEVGKRPWITGDESIVGLMKDIVGANEKEIALMNALTVNLHLLMLSFFKPTPKRYKILLEAKAFPSDHYAIESQLQLHGLNIEESMRMIKPREGEETLRIEDILEVIEKEGDSIAVILFSGVHFYTGQHFNIPAITKAGQAKGCYVGFDLAHAVGNVELYLHDWGVDFACWCSYKYLNAGAGGIAGAFIHEKHAHTIKPALVGWFGHELSTRFKMDNKLQLIPGVCGFRISNPPILLVCSLHASLEIFKQATMKALRKKSVLLTGYLEYLIKHNYGKDKAATKKPVVNIITPSHVEERGCQLTITFSVPNKDVFQELEKRGVVCDKRNPNGIRVAPVPLYNSFHDVYKFTNLLTSILDSAETKN

1519 Q9NS39 O75170

>Q9NS39

MASVLGSGRGSGGLSSQLKCKSKRRRRRRSKRKDKVSILSTFLAPFKHLSPGITNTEDDDTLSTSSAEVKENRNVGNLAARPPPSGDRARGGAPGAKRKRPLEEGNGGHLCKLQLVWKKLSWSVAPKNALVQLHELRPGLQYRTVSQTGPVHAPVFAVAVEVNGLTFEGTGPTKKKAKMRAAELALRSFVQFPNACQAHLAMGGGPGPGTDFTSDQADFPDTLFQEFEPPAPRPGLAGGRPGDAALLSAAYGRRRLLCRALDLVGPTPATPAAPGERNPVVLLNRLRAGLRYVCLAEPAERRARSFVMAVSVDGRTFEGSGRSKKLARGQAAQAALQELFDIQMPGHAPGRARRTPMPQEFADSISQLVTQKFREVTTDLTPMHARHKALAGIVMTKGLDARQAQVVALSSGTKCISGEHLSDQGLVVNDCHAEVVARRAFLHFLYTQLELHLSKRREDSERSIFVRLKEGGYRLRENILFHLYVSTSPCGDARLHSPYEITTDLHSSKHLVRKFRGHLRTKIESGEGTVPVRGPSAVQTWDGVLLGEQLITMSCTDKIARWNVLGLQGALLSHFVEPVYLQSIVVGSLHHTGHLARVMSHRMEGVGQLPASYRHNRPLLSGVSDAEARQPGKSPPFSMNWVVGSADLEIINATTGRRSCGGPSRLCKHVLSARWARLYGRLSTRTPSPGDTPSMYCEAKLGAHTYQSVKQQLFKAFQKAGLGTWVRKPPEQQQFLLTL

>O75170

MFWKFDLNTTSHVDKLLDKEHVTLQELMDEDDILQECKAQNQKLLDFLCRQQCMEELVSLITQDPPLDMEEKVRFKYPNTACELLTCDVPQISDRLGGDESLLSLLYDFLDHEPPLNPLLASFFSKTIGNLIARKTEQVITFLKKKDKFISLVLKHIGTSALMDLLLRLVSCVEPAGLRQDVLHWLNEEKVIQRLVELIHPSQDEDRQSNASQTLCDIVRLGRDQGSQLQEALEPDPLLTALESQDCVEQLLKNMFDGDRTESCLVSGTQVLLTLLETRRVGTEGLVDSFSQGLERSYAVSSSVLHGIEPRLKDFHQLLLNPPKKKAILTTIGVLEEPLGNARLHGARLMAALLHTNTPSINQELCRLNTMDLLLDLFFKYTWNNFLHFQVELCIAAILSHAAREERTEASGSESRVEPPHENGNRSLETPQPAASLPDNTMVTHLFQKCCLVQRILEAWEANDHTQAAGGMRRGNMGHLTRIANAVVQNLERGPVQTHISEVIRGLPADCRGRWESFVEETLTETNRRNTVDLVSTHHLHSSSEDEDIEGAFPNELSLQQAFSDYQIQQMTANFVDQFGFNDEEFADQDDNINAPFDRIAEINFNIDADEDSPSAALFEACCSDRIQPFDDDEDEDIWEDSDTRCAARVMARPRFGAPHASESCSKNGPERGGQDGKASLEAHRDAPGAGAPPAPGKKEAPPVEGDSEGAMWTAVFDEPANSTPTAPGVVRDVGSSVWAAGTSAPEEKGWAKFTDFQPFCCSESGPRCSSPVDTECSHAEGSRSQGPEKASQASYFAVSPASPCAWNVCVTRKAPLLASDSSSSGGSHSEDGDQKAASAMDAVSRGPGREAPPLPTVARTEEAVGRVGCADSRLLSPACPAPKEVTAAPAVAVPPEATVAITTALSKAGPAIPTPAVSSALAVAVPLGPIMAVTAAPAMVATLGTVTKDGKTDAPPEGAALNGPV

1520 NP_904357.1 P51649

>NP_904357.1

MPFLGQDWRSPGWSWIKTEDGWKRCESCSQKLERENNRCNISHSIILNSEDGEIFNNEEHEYASKKRKKDHFRNDTNTQSFYREKWIYVHKESTKERHGYCTLGEAFNRLDFSSAIQDIRRFNYVVKLLQLIAKSQLTSLSGVAQKNYFNILDKIVQKVLDDHHNPRLIKDLLQDLSSTLCILIRGVGKSVLVGNINIWICRLETILAWQQQLQDLQMTKQVNNGLTLSDLPLHMLNNILYRFSDGWDIITLGQVTPTLYMLSEDRQLWKKLCQYHFAEKQFCRHLILSEKGHIEWKLMYFALQKHYPAKEQYGDTLHFCRHCSILFWKDYHLALLFKDSGHPCTAADPDSCFTPVSPQHFIDLFKF

>P51649

MATCIWLRSCGARRLGSTFPGCRLRPRAGGLVPASGPAPGPAQLRCYAGRLAGLSAALLRTDSFVGGRWLPAAATFPVQDPASGAALGMVADCGVREARAAVRAAYEAFCRWREVSAKERSSLLRKWYNLMIQNKDDLARIITAESGKPLKEAHGEILYSAFFLEWFSEEARRVYGDIIHTPAKDRRALVLKQPIGVAAVITPWNFPSAMITRKVGAALAAGCTVVVKPAEDTPFSALALAELASQAGIPSGVYNVIPCSRKNAKEVGEAICTDPLVSKISFTGSTTTGKILLHHAANSVKRVSMELGGLAPFIVFDSANVDQAVAGAMASKFRNTGQTCVCSNQFLVQRGIHDAFVKAFAEAMKKNLRVGNGFEEGTTQGPLINEKAVEKVEKQVNDAVSKGATVVTGGKRHQLGKNFFEPTLLCNVTQDMLCTHEETFGPLAPVIKFDTEEEAIAIANAADVGLAGYFYSQDPAQIWRVAEQLEVGMVGVNEGLISSVECPFGGVKQSGLGREGSKYGIDEYLELKYVCYGGL

1521 NP_000312.2 Q96G61

>NP_000312.2

MPPKTPRKTAATAAAAAAEPPAPPPPPPPEEDPEQDSGPEDLPLVRLEFEETEEPDFTALCQKLKIPDHVRERAWLTWEKVSSVDGVLGGYIQKKKELWGICIFIAAVDLDEMSFTFTELQKNIEISVHKFFNLLKEIDTSTKVDNAMSRLLKKYDVLFALFSKLERTCELIYLTQPSSSISTEINSALVLKVSWITFLLAKGEVLQMEDDLVISFQLMLCVLDYFIKLSPPMLLKEPYKTAVIPINGSPRTPRRGQNRSARIAKQLENDTRIIEVLCKEHECNIDEVKNVYFKNFIPFMNSLGLVTSNGLPEVENLSKRYEEIYLKNKDLDARLFLDHDKTLQTDSIDSFETQRTPRKSNLDEEVNVIPPHTPVRTVMNTIQQLMMILNSASDQPSENLISYFNNCTVNPKESILKRVKDIGYIFKEKFAKAVGQGCVEIGSQRYKLGVRLYYRVMESMLKSEEERLSIQNFSKLLNDNIFHMSLLACALEVVMATYSRSTSQNLDSGTDLSFPWILNVLNLKAFDFYKVIESFIKAEGNLTREMIKHLERCEHRIMESLAWLSDSPLFDLIKQSKDREGPTDHLESACPLNLPLQNNHTAADMYLSPVRSPKKKGSTTRVNSTANAETQATSAFQTQKPLKSTSLSLFYKKVYRLAYLRLNTLCERLLSEHPELEHIIWTLFQHTLQNEYELMRDRHLDQIMMCSMYGICKVKNIDLKFKIIVTAYKDLPHAVQETFKRVLIKEEEYDSIIVFYNSVFMQRLKTNILQYASTRPPTLSPIPHIPRSPYKFPSSPLRIPGGNIYISPLKSPYKISEGLPTPTKMTPRSRILVSIGESFGTSEKFQKINQMVCNSDRVLKRSAEGSNPPKPLKKLRFDIEGSDEADGSKHLPGESKFQQKLAEMTSTRTRMQKQKMNDSMDTSNKEEK

>Q96G61

MKCKPNQTRTYDPEGFKKRAACLCFRSEREDEVLLVSSSRYPDRWIVPGGGMEPEEEPGGAAVREVYEEAGVKGKLGRLLGVFEQNQDRKHRTYVYVLTVTELLEDWEDSVSIGRKREWFKVEDAIKVLQCHKPVHAEYLEKLKLGGSPTNGNSMAPSSPDSDP

1522 Q12946 NP_001005.1

>Q12946

MSSAPEKQQPPHGGGGGGGGGGGAAMDPASSGPSKAKKTNAGIRRPEKPPYSYIALIVMAIQSSPTKRLTLSEIYQFLQSRFPFFRGSYQGWKNSVRHNLSLNECFIKLPKGLGRPGKGHYWTIDPASEFMFEEGSFRRRPRGFRRKCQALKPMYSMMNGLGFNHLPDTYGFQGSAGGLSCPPNSLALEGGLGMMNGHLPGNVDGMALPSHSVPHLPSNGGHSYMGGCGGAAAGEYPHHDSSVPASPLLPTGAGGVMEPHAVYSGSAAAWPPSASAALNSGASYIKQQPLSPCNPAANPLSGSLSTHSLEQPYLHQNSHNAPAELQGIPRYHSQSPSMCDRKEFVFSFNAMASSSMHSAGGGSYYHQQVTYQDIKPCVM

>NP_001005.1

MLMPKKNRIAIYELLFKEGVMVAKKDVHMPKHPELADKNVPNLHVMKAMQSLKSRGYVKEQFAWRHFYWYLTNEGIQYLRDYLHLPPEIVPATLRRSRPETGRPRPKGLEGERPARLTRGEADRDTYRRSAVPPGADKKAEAGAGSATEFQFRGGFGRGRGQPPQ

1523 NP_112573.1 NP_003192.1

>NP_112573.1

MPQLGGGGGGGGGGSGGGGGSSAGAAGGGDDLGANDELIPFQDEGGEEQEPSSDSASAQRDLDEVKSSLVNESENQSSSSDSEAERRPQPVRDTFQKPRDYFAEVRRPQDSAFFKGPPYPGYPFLMIPDLSSPYLSNGPLSPGGARTYLQMKWPLLDVPSSATVKDTRSPSPAHLSNKVPVVQHPHHMHPLTPLITYSNDHFSPGSPPTHLSPEIDPKTGIPRPPHPSELSPYYPLSPGAVGQIPHPLGWLVPQQGQPMYSLPPGGFRHPYPALAMNASMSSLVSSRFSPHMVAPAHPGLPTSGIPHPAIVSPIVKQEPAPPSLSPAVSVKSPVTVKKEEEKKPHVKKPLNAFMLYMKEMRAKVVAECTLKESAAINQILGRKWHNLSREEQAKYYELARKERQLHSQLYPTWSARDNYGKKKKRKREKQLSQTQSQQQVQEAEGALASKSKKPCVQYLPPEKPCDSPASSHGSMLDSPATPSAALASPAAPAATHSEQAQPLSLTTKPETRAQLALHSAAFLSAKAAASSSGQMGSQPPLLSRPLPLGSMPTALLASPPSFPATLHAHQALPVLQAQPLSLVTKSAH

>NP_003192.1

MAFLRSMWGVLSALGRSGAELCTGCGSRLRSPFSFVYLPRWFSSVLASCPKKPVSSYLRFSKEQLPIFKAQNPDAKTTELIRRIAQRWRELPDSKKKIYQDAYRAEWQVYKEEISRFKEQLTPSQIMSLEKEIMDKHLKRKAMTKKKELTLLGKPKRPRSAYNVYVAERFQEAKGDSPQEKLKTVKENWKNLSDSEKELYIQHAKEDETRYHNEMKSWEEQMIEVGRKDLLRRTIKKQRKYGAEEC

1524 P54803 NP_071761.1

>P54803

MAEWLLSASWQRRAKAMTAAAGSAGRAAVPLLLCALLAPGGAYVLDDSDGLGREFDGIGAVSGGGATSRLLVNYPEPYRSQILDYLFKPNFGASLHILKVEIGGDGQTTDGTEPSHMHYALDENYFRGYEWWLMKEAKKRNPNITLIGLPWSFPGWLGKGFDWPYVNLQLTAYYVVTWIVGAKRYHDLDIDYIGIWNERSYNANYIKILRKMLNYQGLQRVKIIASDNLWESISASMLLDAELFKVVDVIGAHYPGTHSAKDAKLTGKKLWSSEDFSTLNSDMGAGCWGRILNQNYINGYMTSTIAWNLVASYYEQLPYGRCGLMTAQEPWSGHYVVESPVWVSAHTTQFTQPGWYYLKTVGHLEKGGSYVALTDGLGNLTIIIETMSHKHSKCIRPFLPYFNVSQQFATFVLKGSFSEIPELQVWYTKLGKTSERFLFKQLDSLWLLDSDGSFTLSLHEDELFTLTTLTTGRKGSYPLPPKSQPFPSTYKDDFNVDYPFFSEAPNFADQTGVFEYFTNIEDPGEHHFTLRQVLNQRPITWAADASNTISIIGDYNWTNLTIKCDVYIETPDTGGVFIAGRVNKGGILIRSARGIFFWIFANGSYRVTGDLAGWIIYALGRVEVTAKKWYTLTLTIKGHFASGMLNDKSLWTDIPVNFPKNGWAAIGTHSFEFAQFDNFLVEATR

>NP_071761.1

MWIPVVGLPRRLRLSALAGAGRFCILGSEAATRKHLPARNHCGLSDSSPQLWPEPDFRNPPRKASKASLDFKRYVTDRRLAETLAQIYLGKPSRPPHLLLECNPGPGILTQALLEAGAKVVALESDKTFIPHLESLGKNLDGKLRVIHCDFFKLDPRSGGVIKPPAMSSRGLFKNLGIEAVPWTADIPLKVVGMFPSRGEKRALWKLAYDLYSCTSIYKFGRIEVNMFIGEKEFQKLMADPGNPDLYHVLSVIWQLACEIKVLHMEPWSSFDIYTRKGPLENPKRRELLDQLQQKLYLIQMIPRQNLFTKNLTPMNYNIFFHLLKHCFGRRSATVIDHLRSLTPLDARDILMQIGKQEDEKVVNMHPQDFKTLFETIERSKDCAYKWLYDETLEDR

1525 NP_002157.2 Q08495

>NP_002157.2

MKAFSPVRSVRKNSLSDHSLGISRSKTPVDDPMSLLYNMNDCYSKLKELVPSIPQNKKVSKMEILQHVIDYILDLQIALDSHPTIVSLHHQRPGQNQASRTPLTTLNTDISILSLQASEFPSELMSNDSKALCG

>Q08495

MERLQKQPLTSPGSVSPSRDSSVPGSPSSIVAKMDNQVLGYKDLAAIPKDKAILDIERPDLMIYEPHFTYSLLEHVELPRSRERSLSPKSTSPPPSPEVWADSRSPGIISQASAPRTTGTPRTSLPHFHHPETSRPDSNIYKKPPIYKQRESVGGSPQTKHLIEDLIIESSKFPAAQPPDPNQPAKIETDYWPCPPSLAVVETEWRKRKASRRGAEEEEEEEDDDSGEEMKALRERQREELSKVTSNLGKMILKEEMEKSLPIRRKTRSLPDRTPFHTSLHQGTSKSSSLPAYGRTTLSRLQSTEFSPSGSETGSPGLQNGEGQRGRMDRGNSLPCVLEQKIYPYEMLVVTNKGRTKLPPGVDRMRLERHLSAEDFSRVFAMSPEEFGKLALWKRNELKKKASLF

1526 NP_006070.2 Q9NRJ4

>NP_006070.2

MADHMMAMNHGRFPDGTNGLHHHPAHRMGMGQFPSPHHHQQQQPQHAFNALMGEHIHYGAGNMNATSGIRHAMGPGTVNGGHPPSALAPAARFNNSQFMGPPVASQGGSLPASMQLQKLNNQYFNHHPYPHNHYMPDLHPAAGHQMNGTNQHFRDCNPKHSGGSSTPGGSGGSSTPGGSGSSSGGGAGSSNSGGGSGSGNMPASVAHVPAAMLPPNVIDTDFIDEEVLMSLVIEMGLDRIKELPELWLGQNEFDFMTDFVCKQQPSRVSC

>Q9NRJ4

MYAAVEHGPVLCSDSNILCLSWKGRVPKSEKEKPVCRRRYYEEGWLATGNGRGVVGVTFTSSHCRRDRSTPQRINFNLRGHNSEVVLVRWNEPYQKLATCDADGGIFVWIQYEGRWSVELVNDRGAQVSDFTWSHDGTQALISYRDGFVLVGSVSGQRHWSSEINLESQITCGIWTPDDQQVLFGTADGQVIVMDCHGRMLAHVLLHESDGVLGMSWNYPIFLVEDSSESDTDSDDYAPPQDGPAAYPIPVQNIKPLLTVSFTSGDISLMNNYDDLSPTVIRSGLKEVVAQWCTQGDLLAVAGMERQTQLGELPNGPLLKSAMVKFYNVRGEHIFTLDTLVQRPIISICWGHRDSRLLMASGPALYVVRVEHRVSSLQLLCQQAIASTLREDKDVSKLTLPPRLCSYLSTAFIPTIKPPIPDPNNMRDFVSYPSAGNERLHCTMKRTEDDPEVGGPCYTLYLEYLGGLVPILKGRRISKLRPEFVIMDPRTDSKPDEIYGNSLISTVIDSCNCSDSSDIELSDDWAAKKSPKISRASKSPKLPRISIEARKSPKLPRAAQELSRSPRLPLRKPSVGSPSLTRREFPFEDITQHNYLAQVTSNIWGTKFKIVGLAAFLPTNLGAVIYKTSLLHLQPRQMTIYLPEVRKISMDYINLPVFNPNVFSEDEDDLPVTGASGVPENSPPCTVNIPIAPIHSSAQAMSPTQSIGLVQSLLANQNVQLDVLTNQTTAVGTAEHAGDSATQYPVSNRYSNPGQVIFGSVEMGRIIQNPPPLSLPPPPQGPMQLSTVGHGDRDHEHLQKSAKALRPTPQLAAEGDAVVFSAPQEVQVTKINPPPPYPGTIPAAPTTAAPPPPLPPPQPPVDVCLKKGDFSLYPTSVHYQTPLGYERITTFDSSGNVEEVCRPRTRMLCSQNTYTLPGPGSSATLRLTATEKKVPQPCSSATLNRLTVPRYSIPTGDPPPYPEIASQLAQGRGAAQRSDNSLIHATLRRNNREATLKMAQLADSPRAPLQPLAKSKGGPGGVVTQLPARPPPALYTCSQCSGTGPSSQPGASLAHTASASPLASQSSYSLLSPPDSARDRTDYVNSAFTEDEALSQHCQLEKPLRHPPLPEAAVTLKRPPPYQWDPMLGEDVWVPQERTAQTSGPNPLKLSSLMLSQGQHLDVSRLPFISPKSPASPTATFQTGYGMGVPYPGSYNNPPLPGVQAPCSPKDALSPTQFAQQEPAVVLQPLYPPSLSYCTLPPMYPGSSTCSSLQLPPVALHPWSSYSACPPMQNPQGTLPPKPHLVVEKPLVSPPPADLQSHLGTEVMVETADNFQEVLSLTESPVPQRTEKFGKKNRKRLDSRAEEGSVQAITEGKVKKEARTLSDFNSLISSPHLGREKKKVKSQKDQLKSKKLNKTNEFQDSSESEPELFISGDELMNQSQGSRKGWKSKRSPRAAGELEEAKCRRASEKEDGRLGSQGFVYVMANKQPLWNEATQVYQLDFGGRVTQESAKNFQIELEGRQVMQFGRIDGSAYILDFQYPFSAVQAFAVALANVTQRLK

1527 Q13461 Q8N573

>Q13461

MAGRSDMDPPAAFSGFPALPAVAPSGPPPSPLAGAEPGREPEEAAAGRGEAAPTPAPGPGRRRRRPLQRGKPPYSYIALIAMALAHAPGRRLTLAAIYRFITERFAFYRDSPRKWQNSIRHNLTLNDCFVKVPREPGNPGKGNYWTLDPAAADMFDNGSFLRRRKRFKRAELPAHAAAAPGPPLPFPYAPYAPAPGPALLVPPPSAGPGPSPPARLFSVDSLVNLQPELAGLGAPEPPCCAAPDAAAAAFPPCAAAASPPLYSQVPDRLVLPATRPGPGPLPAEPLLALAGPAAALGPLSPGEAYLRQPGFASGLERYL

>Q8N573

MSFQKPKGTIEYTVESRDSLNSIALKFDTTPNELVQLNKLFSRAVVTGQVLYVPDPEYVSSVESSPSLSPVSPLSPTSSEAEFDKTTNPDVHPTEATPSSTFTGIRPARVVSSTSEEEEAFTEKFLKINCKYITSGKGTVSGVLLVTPNNIMFDPHKNDPLVQENGCEEYGIMCPMEEVMSAAMYKEILDSKIKESLPIDIDQLSGRDFCHSKKMTGSNTEEIDSRIRDAGNDSASTAPRSTEESLSEDVFTESELSPIREELVSSDELRQDKSSGASSESVQTVNQAEVESLTVKSESTGTPGHLRSDTEHSTNEVGTLCHKTDLNNLEMAIKEDQIADNFQGISGPKEDSTSIKGNSDQDSFLHENSLHQEESQKENMPCGETAEFKQKQSVNKGKQGKEQNQDSQTEAEELRKLWKTHTMQQTKQQRENIQQVSQKEAKHKITSADGHIESSALLKEKQRHRLHKFLCLRVGKPMRKTFVSQASATMQQYAQRDKKHEYWFAVPQERTDHLYAFFIQWSPEIYAEDTGEYTREPGFIVVKKIEESETIEDSSNQAAAREWEITTREDINSKQVATVKADLESESFRPNLSDPSELLLPDQIEKLTKHLPPRTIGYPWTLVYGTGKHGTSLKTLYRTMTGLDTPVLMVIKDSDGQVFGALASEPLKVSDGFYGTGETFVFTFCPEFEVFKWTGDNMFFIKGDMDSLAFGGGGGEFALWLDGDLYHGRSHSCKTFGNRTLSKKEDFFIQDIEIWAFE

1528 NP_001291.3 NP_002475.2

>NP_001291.3

MDVLPMCSIFQELQIVHETGYFSALPSLEEYWQQTCLELERYLQSEPCYVSASEIKFDSQEDLWTKIILAREKKEESELKISSSPPEDTLISPSFCYNLETNSLNSDVSSESSDSSEELSPTAKFTSDPIGEVLVSSGKLSSSVTSTPPSSPELSREPSQLWGCVPGELPSPGKVRSGTSGKPGDKGNGDASPDGRRRVHRCHFNGCRKVYTKSSHLKAHQRTHTGEKPYRCSWEGCEWRFARSDELTRHFRKHTGAKPFKCSHCDRCFSRSDHLALHMKRHL

>NP_002475.2

MEEVPHDCPGADSAQAGRGASCQGCPNQRLCASGAGATPDTAIEEIKEKMKTVKHKILVLSGKGGVGKSTFSAHLAHGLAEDENTQIALLDIDICGPSIPKIMGLEGEQVHQSGSGWSPVYVEDNLGVMSVGFLLSSPDDAVIWRGPKKNGMIKQFLRDVDWGEVDYLIVDTPPGTSDEHLSVVRYLATAHIDGAVIITTPQEVSLQDVRKEINFCRKVKLPIIGVVENMSGFICPKCKKESQIFPPTTGGAELMCQDLEVPLLGRVPLDPLIGKNCDKGQSFFIDAPDSPATLAYRSIIQRIQEFCNLHQSKEENLISS

1529 Q6ZRS2 NP_000174.1

>Q6ZRS2

MQSSPSPAHPQLPVLQTQMVSDGMTGSNPVSPASSSSPASSGAGGISPQHIAQDSSLDGPPGPPDGATVPLEGFSLSQAADLANKGPKWEKSHAEIAEQAKHEAEIETRIAELRKEGFWSLKRLPKVPEPPRPKGHWDYLCEEMQWLSADFAQERRWKRGVARKVVRMVIRHHEEQRQKEERARREEQAKLRRIASTMAKDVRQFWSNVEKVVQFKQQSRLEEKRKKALDLHLDFIVGQTEKYSDLLSQSLNQPLTSSKAGSSPCLGSSSAASSPPPPASRLDDEDGDFQPQEDEEEDDEETIEVEEQQEGNDAEAQRREIELLRREGELPLEELLRSLPPQLLEGPSSPSQTPSSHDSDTRDGPEEGAEEEPPQVLEIKPPPSAVTQRNKQPWHPDEDDEEFTANEEEAEDEEDTIAAEEQLEGEVDHAMELSELAREGELSMEELLQQYAGAYAPGSGSSEDEDEDEVDANSSDCEPEGPVEAEEPPQEDSSSQSDSVEDRSEDEEDEHSEEEETSGSSASEESESEESEDAQSQSQADEEEEDDDFGVEYLLARDEEQSEADAGSGPPTPGPTTLGPKKEITDIAAAAESLQPKGYTLATTQVKTPIPLLLRGQLREYQHIGLDWLVTMYEKKLNGILADEMGLGKTIQTISLLAHLACEKGNWGPHLIIVPTSVMLNWEMELKRWCPSFKILTYYGAQKERKLKRQGWTKPNAFHVCITSYKLVLQDHQAFRRKNWRYLILDEAQNIKNFKSQRWQSLLNFNSQRRLLLTGTPLQNSLMELWSLMHFLMPHVFQSHREFKEWFSNPLTGMIEGSQEYNEGLVKRLHKVLRPFLLRRVKVDVEKQMPKKYEHVIRCRLSKRQRCLYDDFMAQTTTKETLATGHFMSVINILMQLRKVCNHPNLFDPRPVTSPFITPGICFSTASLVLRATDVHPLQRIDMGRFDLIGLEGRVSRYEADTFLPRHRLSRRVLLEVATAPDPPPRPKPVKMKVNRMLQPVPKQEGRTVVVVNNPRAPLGPVPVRPPPGPELSAQPTPGPVPQVLPASLMVSASPAGPPLIPASRPPGPVLLPPLQPNSGSLPQVLPSPLGVLSGTSRPPTPTLSLKPTPPAPVRLSPAPPPGSSSLLKPLTVPPGYTFPPAAATTASTTTATATTTAVPAPTPAPQRLILSPDMQARLPSGEVVSIGQLASLAQRPVANAGGSKPLTFQIQGNKLTLTGAQVRQLAVGQPRPLQRNVVHLVSAGGQHHLISQPAHVALIQAVAPTPGPTPVSVLPSSTPSTTPAPTGLSLPLAANQVPPTMVNNTGVVKIVVRQAPRDGLTPVPPLAPAPRPPSSGLPAVLNPRPTLTPGRLPTPTLGTARAPMPTPTLVRPLLKLVHSPSPEVSASAPGAAPLTISSPLHVPSSLPGPASSPMPIPNSSPLASPVSSTVSVPLSSSLPISVPTTLPAPASAPLTIPISAPLTVSASGPALLTSVTPPLAPVVPAAPGPPSLAPSGASPSASALTLGLATAPSLSSSQTPGHPLLLAPTSSHVPGLNSTVAPACSPVLVPASALASPFPSAPNPAPAQASLLAPASSASQALATPLAPMAAPQTAILAPSPAPPLAPLPVLAPSPGAAPVLASSQTPVPVMAPSSTPGTSLASASPVPAPTPVLAPSSTQTMLPAPVPSPLPSPASTQTLALAPALAPTLGGSSPSQTLSLGTGNPQGPFPTQTLSLTPASSLVPTPAQTLSLAPGPPLGPTQTLSLAPAPPLAPASPVGPAPAHTLTLAPASSSASLLAPASVQTLTLSPAPVPTLGPAAAQTLALAPASTQSPASQASSLVVSASGAAPLPVTMVSRLPVSKDEPDTLTLRSGPPSPPSTATSFGGPRPRRQPPPPPRSPFYLDSLEEKRKRQRSERLERIFQLSEAHGALAPVYGTEVLDFCTLPQPVASPIGPRSPGPSHPTFWTYTEAAHRAVLFPQQRLDQLSEIIERFIFVMPPVEAPPPSLHACHPPPWLAPRQAAFQEQLASELWPRARPLHRIVCNMRTQFPDLRLIQYDCGKLQTLAVLLRQLKAEGHRVLIFTQMTRMLDVLEQFLTYHGHLYLRLDGSTRVEQRQALMERFNADKRIFCFILSTRSGGVGVNLTGADTVVFYDSDWNPTMDAQAQDRCHRIGQTRDVHIYRLISERTVEENILKKANQKRMLGDMAIEGGNFTTAYFKQQTIRELFDMPLEEPSSSSVPSAPEEEEETVASKQTHILEQALCRAEDEEDIRAATQAKAEQVAELAEFNENDGFPAGEGEEAGRPGAEDEEMSRAEQEIAALVEQLTPIERYAMKFLEASLEEVSREELKQAEEQVEAARKDLDQAKEEVFRLPQEEEEGPGAGDESSCGTGGGTHRRSKKAKAPERPGTRVSERLRGARAETQGANHTPVISAHQTRSTTTPPRCSPARERVPRPAPRPRPTPASAPAAIPALVPVPVSAPVPISAPNPITILPVHILPSPPPPSQIPPCSSPACTPPPACTPPPAHTPPPAQTCLVTPSSPLLLGPPSVPISASVTNLPLGLRPEAELCAQALASPESLELASVASSETSSLSLVPPKDLLPVAVEILPVSEKNLSLTPSAPSLTLEAGSIPNGQEQEAPDSAEGTTLTVLPEGEELPLCVSESNGLELPPSAASDEPLQEPLEADRTSEELTEAKTPTSSPEKPQELVTAEVAAPSTSSSATSSPEGPSPARPPRRRTSADVEIRGQGTGRPGQPPGPKVLRKLPGRLVTVVEEKELVRRRRQQRGAASTLVPGVSETSASPGSPSVRSMSGPESSPPIGGPCEAAPSSSLPTPPQQPFIARRHIELGVTGGGSPENGDGALLAITPPAVKRRRGRPPKKNRSPADAGRGVDEAPSSTLKGKTNGADPVPGPETLIVADPVLEPQLIPGPQPLGPQPVHRPNPLLSPVEKRRRGRPPKARDLPIPGTISSAGDGNSESRTQPPPHPSPLTPLPPLLVCPTATVANTVTTVTISTSPPKRKRGRPPKNPPSPRPSQLPVLDRDSTSVLESCGLGRRRQPQGQGESEGSSSDEDGSRPLTRLARLRLEAEGMRGRKSGGSMVVAVIQDDLDLADSGPGGLELTPPVVSLTPKLRSTRLRPGSLVPPLETEKLPRKRAGAPVGGSPGLAKRGRLQPPSPLGPEGSVEESEAEASGEEEEGDGTPRRRPGPRRLVGTTNQGDQRILRSSAPPSLAGPAVSHRGRKAKT

>NP_000174.1

MTILTYPFKNLPTASKWALRFSIRPLSCSSQLRAAPAVQTKTKKTLAKPNIRNVVVVDGVRTPFLLSGTSYKDLMPHDLARAALTGLLHRTSVPKEVVDYIIFGTVIQEVKTSNVAREAALGAGFSDKTPAHTVTMACISANQAMTTGVGLIASGQCDVIVAGGVELMSDVPIRHSRKMRKLMLDLNKAKSMGQRLSLISKFRFNFLAPELPAVSEFSTSETMGHSADRLAAAFAVSRLEQDEYALRSHSLAKKAQDEGLLSDVVPFKVPGKDTVTKDNGIRPSSLEQMAKLKPAFIKPYGTVTAANSSFLTDGASAMLIMAEEKALAMGYKPKAYLRDFMYVSQDPKDQLLLGPTYATPKVLEKAGLTMNDIDAFEFHEAFSGQILANFKAMDSDWFAENYMGRKTKVGLPPLEKFNNWGGSLSLGHPFGATGCRLVMAAANRLRKEGGQYGLVAACAAGGQGHAMIVEAYPK

1530 P10253 NP_113608.1

>P10253

MGVRHPPCSHRLLAVCALVSLATAALLGHILLHDFLLVPRELSGSSPVLEETHPAHQQGASRPGPRDAQAHPGRPRAVPTQCDVPPNSRFDCAPDKAITQEQCEARGCCYIPAKQGLQGAQMGQPWCFFPPSYPSYKLENLSSSEMGYTATLTRTTPTFFPKDILTLRLDVMMETENRLHFTIKDPANRRYEVPLETPHVHSRAPSPLYSVEFSEEPFGVIVHRQLDGRVLLNTTVAPLFFADQFLQLSTSLPSQYITGLAEHLSPLMLSTSWTRITLWNRDLAPTPGANLYGSHPFYLALEDGGSAHGVFLLNSNAMDVVLQPSPALSWRSTGGILDVYIFLGPEPKSVVQQYLDVVGYPFMPPYWGLGFHLCRWGYSSTAITRQVVENMTRAHFPLDVQWNDLDYMDSRRDFTFNKDGFRDFPAMVQELHQGGRRYMMIVDPAISSSGPAGSYRPYDEGLRRGVFITNETGQPLIGKVWPGSTAFPDFTNPTALAWWEDMVAEFHDQVPFDGMWIDMNEPSNFIRGSEDGCPNNELENPPYVPGVVGGTLQAATICASSHQFLSTHYNLHNLYGLTEAIASHRALVKARGTRPFVISRSTFAGHGRYAGHWTGDVWSSWEQLASSVPEILQFNLLGVPLVGADVCGFLGNTSEELCVRWTQLGAFYPFMRNHNSLLSLPQEPYSFSEPAQQAMRKALTLRYALLPHLYTLFHQAHVAGETVARPLFLEFPKDSSTWTVDHQLLWGEALLITPVLQAGKAEVTGYFPLGTWYDLQTVPIEALGSLPPPPAAPREPAIHSEGQWVTLPAPLDTINVHLRAGYIIPLQGPGLTTTESRQQPMALAVALTKGGEARGELFWDDGESLEVLERGAYTQVIFLARNNTIVNELVRVTSEGAGLQLQKVTVLGVATAPQQVLSNGVPVSNFTYSPDTKVLDICVSLLMGEQFLVSWC

>NP_113608.1

MAAPVVTAPGRALLRAGAGRLLRGGVQELLRPRHEGNAPDLACNFSLSQNRGTVIVERWWKVPLAGEGRKPRLHRRHRVYKLVEDTKHRPKENLELILTQSVENVGVRGDLVSVKKSLGRNRLLPQGLAVYASPENKKLFEEEKLLRQEGKLEKIQTKAGEATVKFLKSCRLEVGMKNNVKWELNPEIVARHFFKNLGVVVAPHTLKLPEEPITRWGEYWCEVTVNGLDTVRVPMSVVNFEKPKTKRYKYWLAQQAAKAMAPTSPQI

1531 NP_002373.3 NP_055595.2

>NP_002373.3

MSDNDDIEVESDEEQPRFQSAADKRAHHNALERKRRDHIKDSFHSLRDSVPSLQGEKASRAQILDKATEYIQYMRRKNHTHQQDIDDLKRQNALLEQQVRALEKARSSAQLQTNYPSSDNSLYTNAKGSTISAFDGGSDSSSESEPEEPQSRKKLRMEAS

>NP_055595.2

MVGELRYREFRVPLGPGLHAYPDELIRQRVGHDGHPEYQIRWLILRRGDEGDGGSGQVDCKAEHILLWMSKDEIYANCHKMLGEDGQVIGPSQESAGEVGALDKSVLEEMETDVKSLIQRALRQLEECVGTIPPAPLLHTVHVLSAYASIEPLTGVFKDPRVLDLLMHMLSSPDYQIRWSAGRMIQALSSHDAGTRTQILLSLSQQEAIEKHLDFDSRCALLALFAQATLSEHPMSFEGIQLPQVPGRVLFSLVKRYLHVTSLLDQLNDSAAEPGAQNTSAPEELSGERGQLELEFSMAMGTLISELVQAMRWDQASDRPRSSARSPGSIFQPQLADVSPGLPAAQAQPSFRRSRRFRPRSEFASGNTYALYVRDTLQPGMRVRMLDDYEEISAGDEGEFRQSNNGVPPVQVFWESTGRTYWVHWHMLEILGFEEDIEDMVEADEYQGAVASRVLGRALPAWRWRPMTELYAVPYVLPEDEDTEECEHLTLAEWWELLFFIKKLDGPDHQEVLQILQENLDGEILDDEILAELAVPIELAQDLLLTLPQRLNDSALRDLINCHVYKKYGPEALAGNQAYPSLLEAQEDVLLLDAQAQAKDSEDAAKVEAKEPPSQSPNTPLQRLVEGYGPAGKILLDLEQALSSEGTQENKVKPLLLQLQRQPQPFLALMQSLDTPETNRTLHLTVLRILKQLVDFPEALLLPWHEAVDACMACLRSPNTDREVLQELIFFLHRLTSVSRDYAVVLNQLGARDAISKALEKHLGKLELAQELRDMVFKCEKHAHLYRKLITNILGGCIQMVLGQIEDHRRTHQPINIPFFDVFLRYLCQGSSVEVKEDKCWEKVEVSSNPHRASKLTDHNPKTYWESNGSAGSHYITLHMRRGILIRQLTLLVASEDSSYMPARVVVCGGDSTSSLHTELNSVNVMPSASRVILLENLTRFWPIIQIRIKRCQQGGIDTRIRGLEILGPKPTFWPVFREQLCRHTRLFYMVRAQAWSQDMAEDRRSLLHLSSRLNGALRQEQNFADRFLPDDEAAQALGKTCWEALVSPVVQNITSPDEDGISPLGWLLDQYLECQEAVFNPQSRGPAFFSRVRRLTHLLVHVEPCEAPPPVVATPRPKGRNRSHDWSSLATRGLPSSIMRNLTRCWRAVVEKQVNNFLTSSWRDDDFVPRYCEHFNILQNSSSELFGPRAAFLLALQNGCAGALLKLPFLKAAHVSEQFARHIDQQIQGSRIGGAQEMERLAQLQQCLQAVLIFSGLEIATTFEHYYQHYMADRLLGVVSSWLEGAVLEQIGPCFPNRLPQQMLQSLSTSKELQRQFHVYQLQQLDQELLKLEDTEKKIQVGLGASGKEHKSEKEEEAGAAAVVDVAEGEEEEEENEDLYYEGAMPEVSVLVLSRHSWPVASICHTLNPRTCLPSYLRGTLNRYSNFYNKSQSHPALERGSQRRLQWTWLGWAELQFGNQTLHVSTVQMWLLLYLNDLKAVSVESLLAFSGLSADMLNQAIGPLTSSRGPLDLHEQKDIPGGVLKIRDGSKEPRSRWDIVRLIPPQTYLQAEGEDGQNLEKRRNLLNCLIVRILKAHGDEGLHIDQLVCLVLEAWQKGPCPPRGLVSSLGKGSACSSTDVLSCILHLLGKGTLRRHDDRPQVLSYAVPVTVMEPHTESLNPGSSGPNPPLTFHTLQIRSRGVPYASCTATQSFSTFR

1532 NP_612565.1 Q96M27

>NP_612565.1

MKSARAKTPRKPTVKKGSQTNLKDPVGVYCRVRPLGFPDQECCIEVINNTTVQLHTPEGYRLNRNGDYKETQYSFKQVFGTHTTQKELFDVVANPLVNDLIHGKNGLLFTYGVTGSGKTHTMTGSPGEGGLLPRCLDMIFNSIGSFQAKRYVFKSNDRNSMDIQCEVDALLERQKREAMPNPKTSSSKRQVDPEFADMITVQEFCKAEEVDEDSVYGVFVSYIEIYNNYIYDLLEEVPFDPIKPKPPQSKLLREDKNHNMYVAGCTEVEVKSTEEAFEVFWRGQKKRRIANTHLNRESSRSHSVFNIKLVQAPLDADGDNVLQEKEQITISQLSLVDLAGSERTNRTRAEGNRLREAGNINQSLMTLRTCMDVLRENQMYGTNKMVPYRDSKLTHLFKNYFDGEGKVRMIVCVNPKAEDYEENLQVMRFAEVTQEVEVARPVDKAICGLTPGRRYRNQPRGPVGNEPLVTDVVLQSFPPLPSCEILDINDEQTLPRLIEALEKRHNLRQMMIDEFNKQSNAFKALLQEFDNAVLSKENHMQGKLNEKEKMISGQKLEIERLEKKNKTLEYKIEILEKTTTIYEEDKRNLQQELETQNQKLQRQFSDKRRLEARLQGMVTETTMKWEKECERRVAAKQLEMQNKLWVKDEKLKQLKAIVTEPKTEKPERPSRERDREKVTQRSVSPSPVPLSSNYIAQISNGQQLMSQPQLHRRSNSCSSISVASCISEWEQKIPTYNTPLKVTSIARRRQQEPGQSKTCIVSDRRRGMYWTEGREVVPTFRNEIEIEEDHCGRLLFQPDQNAPPIRLRHRRSRSAGDRWVDHKPASNMQTETVMQPHVPHAITVSVANEKALAKCEKYMLTHQELASDGEIETKLIKGDIYKTRGGGQSVQFTDIETLKQESPNGSRKRRSSTVAPAQPDGAESEWTDVETRCSVAVEMRAGSQLGPGYQHHAQPKRKKP

>Q96M27

MMEESGIETTPPGTPPPNPAGLAATAMSSTPVPLAATSSFSSPNVSSMESFPPLAYSTPQPPLPPVRPSAPLPFVPPPAVPSVPPLVTSMPPPVSPSTAAAFGNPPVSHFPPSTSAPNTLLPAPPSGPPISGFSVGSTYDITRGHAGRAPQTPLMPSFSAPSGTGLLPTPITQQASLTSLAQGTGTTSAITFPEEQEDPRITRGQDEASAGGIWGFIKGVAGNPMVKSVLDKTKHSVESMITTLDPGMAPYIKSGGELDIVVTSNKEVKVAAVRDAFQEVFGLAVVVGEAGQSNIAPQPVGYAAGLKGAQERIDSLRRTGVIHEKQTAVSVENFIAELLPDKWFDIGCLVVEDPVHGIHLETFTQATPVPLEFVQQAQSLTPQDYNLRWSGLLVTVGEVLEKSLLNVSRTDWHMAFTGMSRRQMIYSAARAIAGMYKQRLPPRTV

1533 NP_000371.1 Q99757

>NP_000371.1

MAAADGALPEAAALEQPAELPASVRASIERKRQRALMLRQARLAARPYSATAAAATGGMANVKAAPKIIDTGGGFILEEEEEEEQKIGKVVHQPGPVMEFDYVICEECGKEFMDSYLMNHFDLPTCDNCRDADDKHKLITKTEAKQEYLLKDCDLEKREPPLKFIVKKNPHHSQWGDMKLYLKLQIVKRSLEVWGSQEALEEAKEVRQENREKMKQKKFDKKVKELRRAVRSSVWKRETIVHQHEYGPEENLEDDMYRKTCTMCGHELTYEKM

>Q99757

MAQRLLLRRFLASVISRKPSQGQWPPLTSRALQTPQCSPGGLTVTPNPARTIYTTRISLTTFNIQDGPDFQDRVVNSETPVVVDFHAQWCGPCKILGPRLEKMVAKQHGKVVMAKVDIDDHTDLAIEYEVSAVPTVLAMKNGDVVDKFVGIKDEDQLEAFLKKLIG

1534 NP_071345.2 NP_002055.1

>NP_071345.2

MMQHASPAPALTMMATQNVPPPPYQDSPQMTATAQPPSKAQAVHISAPSAAASTPVPSAPIDPQAQLEADKRAVYRHPLFPLLTLLFEKCEQATQGSECITSASFDVDIENFVHQQEQEHKPFFSDDPELDNLMVKAIQVLRIHLLELEKVNELCKDFCNRYITCLKTKMHSDNLLRNDLGGPYSPNQPSINLHSQDLLQNSPNSMSGVSNNPQGIVVPASALQQGNIAMTTVNSQVVSGGALYQPVTMVTSQGQVVTQAIPQGAIQIQNTQVNLDLTSLLDNEDKKSKNKRGVLPKHATNIMRSWLFQHLMHPYPTEDEKRQIAAQTNLTLLQVNNWFINARRRILQPMLDASNPDPAPKAKKIKSQHRPTQRFWPNSIAAGVLQQQGGAPGTNPDGSINLDNLQSLSSDSATMAMQQAMMAAHDDSLDGTEEEDEDEMEEEEEEELEEEVDELQTTNVSDLGLEHSDSLE

>NP_002055.1

MAQEFVNCKIQPGKVVVFIKPTCPYCRRAQEILSQLPIKQGLLEFVDITATNHTNEIQDYLQQLTGARTVPRVFIGKDCIGGCSDLVSLQQSGELLTRLKQIGALQ

1535 NP_002583.1 NP_071761.1

>NP_002583.1

MFEARLVQGSILKKVLEALKDLINEACWDISSSGVNLQSMDSSHVSLVQLTLRSEGFDTYRCDRNLAMGVNLTSMSKILKCAGNEDIITLRAEDNADTLALVFEAPNQEKVSDYEMKLMDLDVEQLGIPEQEYSCVVKMPSGEFARICRDLSHIGDAVVISCAKDGVKFSASGELGNGNIKLSQTSNVDKEEEAVTIEMNEPVQLTFALRYLNFFTKATPLSSTVTLSMSADVPLVVEYKIADMGHLKYYLAPKIEDEEGS

>NP_071761.1

MWIPVVGLPRRLRLSALAGAGRFCILGSEAATRKHLPARNHCGLSDSSPQLWPEPDFRNPPRKASKASLDFKRYVTDRRLAETLAQIYLGKPSRPPHLLLECNPGPGILTQALLEAGAKVVALESDKTFIPHLESLGKNLDGKLRVIHCDFFKLDPRSGGVIKPPAMSSRGLFKNLGIEAVPWTADIPLKVVGMFPSRGEKRALWKLAYDLYSCTSIYKFGRIEVNMFIGEKEFQKLMADPGNPDLYHVLSVIWQLACEIKVLHMEPWSSFDIYTRKGPLENPKRRELLDQLQQKLYLIQMIPRQNLFTKNLTPMNYNIFFHLLKHCFGRRSATVIDHLRSLTPLDARDILMQIGKQEDEKVVNMHPQDFKTLFETIERSKDCAYKWLYDETLEDR

1536 Q9UJC5 P22532

>Q9UJC5

MVIRVFIASSSGFVAIKKKQQDVVRFLEANKIEFEEVDITMSEEQRQWMYKNVPPEKKPTQGNPLPPQIFNGDRYCGDYDSFFESKESNTVFSFLGLKPRLASKAEP

>P22532

MSYQQQQCKQPCQPPPVCPTPKCPEPCPPPKCPEPCPSPKCPQPCPPQQCQQKYPPVTPSPPCQPKCPPKSK

1537 NP_291028.3 NP_008996.1

>NP_291028.3

MAAARATTPADGEEPAPEAEALAAARERSSRFLSGLELVKQGAEARVFRGRFQGRAAVIKHRFPKGYRHPALEARLGRRRTVQEARALLRCRRAGISAPVVFFVDYASNCLYMEEIEGSVTVRDYIQSTMETEKTPQGLSNLAKTIGQVLARMHDEDLIHGDLTTSNMLLKPPLEQLNIVLIDFGLSFISALPEDKGVDLYVLEKAFLSTHPNTETVFEAFLKSYSTSSKKARPVLKKLDEVRLRGRKRSMVG

>NP_008996.1

MVDYSVWDHIEVSDDEDETHPNIDTASLFRWRHQARVERMEQFQKEKEELDRGCRECKRKVAECQRKLKELEVAEGGKAELERLQAEAQQLRKEERSWEQKLEEMRKKEKSMPWNVDTLSKDGFSKSMVNTKPEKTEEDSEEVREQKHKTFVEKYEKQIKHFGMLRRWDDSQKYLSDNVHLVCEETANYLVIWCIDLEVEEKCALMEQVAHQTIVMQFILELAKSLKVDPRACFRQFFTKIKTADRQYMEGFNDELEAFKERVRGRAKLRIEKAMKEYEEEERKKRLGPGGLDPVEVYESLPEELQKCFDVKDVQMLQDAISKMDPTDAKYHMQRCIDSGLWVPNSKASEAKEGEEAGPGDPLLEAVPKTGDEKDVSV

1538 P50219 P51970

>P50219

MEKSKNFRIDALLAVDPPRAASAQSAPLALVTSLAAAASGTGGGGGGGGASGGTSGSCSPASSEPPAAPADRLRAESPSPPRLLAAHCALLPKPGFLGAGGGGGGTGGGHGGPHHHAHPGAAAAAAAAAAAAAAGGLALGLHPGGAQGGAGLPAQAALYGHPVYGYSAAAAAAALAGQHPALSYSYPQVQGAHPAHPADPIKLGAGTFQLDQWLRASTAGMILPKMPDFNSQAQSNLLGKCRRPRTAFTSQQLLELEHQFKLNKYLSRPKRFEVATSLMLTETQVKIWFQNRRMKWKRSKKAKEQAAQEAEKQKGGGGGAGKGGAEEPGAEELLGPPAPGDKGSGRRLRDLRDSDPEEDEDEDDEDHFPYSNGASVHAASSDCSSEDDSPPPRPSHQPAPQ

>P51970

MPGIVELPTLEELKVDEVKISSAVLKAAAHHYGAQCDKPNKEFMLCRWEEKDPRRCLEEGKLVNKCALDFFRQIKRHCAEPFTEYWTCIDYTGQQLFRHCRKQQAKFDECVLDKLGWVRPDLGELSKVTKVKTDRPLPENPYHSRPRPDPSPEIEGDLQPATHGSRFYFWTK

1539 NP_006312.1 Q2M3C7

>NP_006312.1

MSVDMNSQGSDSNEEDYDPNCEEEEEEEEDDPGDIEDYYVGVASDVEQQGADAFDPEEYQFTCLTYKESEGALNEHMTSLASVLKVSHSVAKLILVNFHWQVSEILDRYKSNSAQLLVEARVQPNPSKHVPTSHPPHHCAVCMQFVRKENLLSLACQHQFCRSCWEQHCSVLVKDGVGVGVSCMAQDCPLRTPEDFVFPLLPNEELREKYRRYLFRDYVESHYQLQLCPGADCPMVIRVQEPRARRVQCNRCNEVFCFKCRQMYHAPTDCATIRKWLTKCADDSETANYISAHTKDCPKCNICIEKNGGCNHMQCSKCKHDFCWMCLGDWKTHGSEYYECSRYKENPDIVNQSQQAQAREALKKYLFYFERWENHNKSLQLEAQTYQRIHEKIQERVMNNLGTWIDWQYLQNAAKLLAKCRYTLQYTYPYAYYMESGPRKKLFEYQQAQLEAEIENLSWKVERADSYDRGDLENQMHIAEQRRRTLLKDFHDT

>Q2M3C7

MDGNSLLSVPSNLESSRMYDVLEPQQGRGCGSSGSGPGNSITACKKVLRSNSLLESTDYWLQNQRMPCQIGFVEDKSENCASVCFVNLDVNKDECSTEHLQQKLVNVSPDLPKLISSMNVQQPKENEIVVLSGLASGNLQADFEVSQCPWLPDICLVQCARGNRPNSTNCIIFEINKFLIGLELVQERQLHLETNILKLEDDTNCSLSSIEEDFLTASEHLEEESEVDESRNDYENINVSANVLESKQLKGATQVEWNCNKEKWLYALEDKYINKYPTPLIKTERSPENLTKNTALQSLDPSAKPSQWKREAVGNGRQATHYYHSEAFKGQMEKSQALYIPKDAYFSMMDKDVPSACAVAEQRSNLNPGDHEDTRNALPPRQDGEVTTGKYATNLAESVLQDAFIRLSQSQSTLPQESAVSVSVGSSLLPSCYSTKDTVVSRSWNELPKIVVVQSPDGSDAAPQPGISSWPEMEVSVETSSILSGENSSRQPQSALEVALACAATVIGTISSPQATERLKMEQVVSNFPPGSSGALQTQAPQGLKEPSINEYSFPSALCGMTQVASAVAVCGLGEREEVTCSVAPSGSLPPAAEASEAMPPLCGLASMELGKEAIAKGLLKEAALVLTRPNTYSSIGDFLDSMNRRIMETASKSQTLCSENVVRNELAHTLSNVILRHSIDEVHHKNMIIDPNDNRHSSEILDTLMESTNQLLLDVICFTFKKMSHIVRLGECPAVLSKETIRRRETEPSCQPSDPGASQAWTKATESSSSSPLSNSHNTSLVINNLVDGMYSKQDKGGVRPGLFKNPTLQSQLSRSHRVPDSSTATTSSKEIYLKGIAGEDTKSPHHSENECRASSEGQRSPTVSQSRSGSQEAEESIHPNTQEKYNCATSRINEVQVNLSLLGDDLLLPAQSTLQTKHPDIYCITDFAEELADTVVSMATEIAAICLDNSSGKQPWFCAWKRGSEFLMTPNVPCRSLKRKKESQGSGTAVRKHKPPRLSEIKRKTDEHPELKEKLMNRVVDESMNLEDVPDSVNLFANEVAAKIMNLTEFSMVDGMWQAQGYPRNRLLSGDRWSRLKASSCESIPEEDSEARAYVNSLGLMSTLSQPVSRASSVSKQSSCESITDEFSRFMVNQMENEGRGFELLLDYYAGKNASSILNSAMQQACRKSDHLSVRPSCPSKQSSTESITEEFYRYMLRDIERDSRESASSRRSSQDWTAGLLSPSLRSPVCHRQSSMPDSRSPCSRLTVNVPIKANSLDGFAQNCPQDFLSVQPVSSASSSGLCKSDSCLYRRGGTDHITNMLIHETWASSIEALMRKNKIIVDDAEEADTEPVSGGSPSQAEKCANRLAASRMCSGPTLLVQESLDCPRKDSVTECKQPPVSSLSKTASLTNHSPLDSKKETSSCQDPVPINHKRRSLCSREVPLIQIETDQREACAGEPEPFLSKSSLLEEAEGHSNDKNIPDVVRGGDTAVSACQIHSDSLDTRDVPEAEASTEARAPDEAPNPPSSSEESTGSWTQLANEEDNPDDTSSFLQLSERSMSNGNSSATSSLGIMDLDIYQESMPSSPMINELVEEKKILKGQSESTEAPASGPPTGTASPQRSLLVINFDLEPECPDAELRATLQWIAASELGIPTIYFKKSQENRIEKFLDVVQLVHRKSWKVGDIFHAVVQYCKMHEEQKDGRLSLFDWLLELG

1540 Q9UBR2 Q9BZE1

>Q9UBR2

MARRGPGWRPLLLLVLLAGAAQGGLYFRRGQTCYRPLRGDGLAPLGRSTYPRPHEYLSPADLPKSWDWRNVDGVNYASITRNQHIPQYCGSCWAHASTSAMADRINIKRKGAWPSTLLSVQNVIDCGNAGSCEGGNDLSVWDYAHQHGIPDETCNNYQAKDQECDKFNQCGTCNEFKECHAIRNYTLWRVGDYGSLSGREKMMAEIYANGPISCGIMATERLANYTGGIYAEYQDTTYINHVVSVAGWGISDGTEYWIVRNSWGEPWGERGWLRIVTSTYKDGKGARYNLAIEEHCTFGDPIV

>Q9BZE1

MALASGPARRALAGSGQLGLGGFGAPRRGAYEWGVRSTRKSEPPPLDRVYEIPGLEPITFAGKMHFVPWLARPIFPPWDRGYKDPRFYRSPPLHEHPLYKDQACYIFHHRCRLLEGVKQALWLTKTKLIEGLPEKVLSLVDDPRNHIENQDECVLNVISHARLWQTTEEIPKRETYCPVIVDNLIQLCKSQILKHPSLARRICVQNSTFSATWNRESLLLQVRGSGGARLSTKDPLPTIASREEIEATKNHVLETFYPISPIIDLHECNIYDVKNDTGFQEGYPYPYPHTLYLLDKANLRPHRLQPDQLRAKMILFAFGSALAQARLLYGNDAKVLEQPVVVQSVGTDGRVFHFLVFQLNTTDLDCNEGVKNLAWVDSDQLLYQHFWCLPVIKKRVVVEPVGPVGFKPETFRKFLALYLHGAA

1541 NP_055909.2 P07203

>NP_055909.2

MVSGPLALRWCAWAGRGDMGPDMELPSHSKQLLLQLNQQRTKGFLCDVIIMVENSIFRAHKNVLAASSIYFKSLVLHDNLINLDTDMVSSTVFQQILDFIYTGKLLPSDQPAEPNFSTLLTAASYLQLPELAALCRRKLKRAGKPFGSGRAGSTGMGRPPRSQRLSTASVIQARYQGLVDGRKGAHAPQELPQAKGSDDELFLGGSNQDSVQGLGRAVCPAGGEAGLGGCSSSTNGSSGGCEQELGLDLSKKSPPLPPATPGPHLTPDDAAQLSDSQHGSPPAASAPPVANSASYSELGGTPDEPMDLEGAEDNHLSLLEAPGGQPRKSLRHSTRKKEWGKKEPVAGSPFERREAGPKGPCPGEEGEGVGDRVPNGILASGAGPSGPYGEPPYPCKEEEENGKDASEDSAQSGSEGGSGHASAHYMYRQEGYETVSYGDNLYVCIPCAKGFPSSEQLNAHVETHTEEELFIKEEGAYETGSGGAEEEAEDLSAPSAAYTAEPRPFKCSVCEKTYKDPATLRQHEKTHWLTRPFPCNICGKMFTQRGTMTRHMRSHLGLKPFACDECGMRFTRQYRLTEHMRVHSGEKPYECQLCGGKFTQQRNLISHLRMHTSPS

>P07203

MCAARLAAAAAQSVYAFSARPLAGGEPVSLGSLRGKVLLIENVASLUGTTVRDYTQMNELQRRLGPRGLVVLGFPCNQFGHQENAKNEEILNSLKYVRPGGGFEPNFMLFEKCEVNGAGAHPLFAFLREALPAPSDDATALMTDPKLITWSPVCRNDVAWNFEKFLVGPDGVPLRRYSRRFQTIDIEPDIEALLSQGPSCA

1542 NP_076921.1 Q12849

>NP_076921.1

MSSYFVNSLFSKYKTGESLRPNYYDCGFAQDLGGRPTVVYGPSSGGSFQHPSQIQEFYHGPSSLSTAPYQQNPCAVACHGDPGNFYGYDPLQRQSLFGAQDPDLVQYADCKLAAASGLGEEAEGSEQSPSPTQLFPWMRPQAAAGRRRGRQTYSRYQTLELEKEFLFNPYLTRKRRIEVSHALGLTERQVKIWFQNRRMKWKKENNKDKFPSSKCEQEELEKQKLERAPEAADEGDAQKGDKK

>Q12849

MAGTRWVLGALLRGCGCNCSSCRRTGAACLPFYSAAGSIPSGVSGRRRLLLLLGAAAAAASQTRGLQTGPVPPGRLAGPPAVATSAAAAAAASYSALRASLLPQSLAAAAAVPTRSYSQESKTTYLEDLPPPPEYELAPSKLEEEVDDVFLIRAQGLPWSCTMEDVLNFFSDCRIRNGENGIHFLLNRDGKRRGDALIEMESEQDVQKALEKHRMYMGQRYVEVYEINNEDVDALMKSLQVKSSPVVNDGVVRLRGLPYSCNEKDIVDFFAGLNIVDITFVMDYRGRRKTGEAYVQFEEPEMANQALLKHREEIGNRYIEIFPSRRNEVRTHVGSYKGKKIASFPTAKYITEPEMVFEEHEVNEDIQPMTAFESEKEIELPKEVPEKLPEAADFGTTSSLHFVHMRGLPFQANAQDIINFFAPLKPVRITMEYSSSGKATGEADVHFETHEDAVAAMLKDRSHVHHRYIELFLNSCPKGK

1543 NP_002909.3 NP_001340.2

>NP_002909.3

MATQAYTELQAAPPPSQPPQAPPQAQPQPPPPPPPAAPQPPQPPTAAATPQPQYVTELQSPQPQAQPPGGQKQYVTELPAVPAPSQPTGAPTPSPAPQQYIVVTVSEGAMRASETVSEASPGSTASQTGVPTQVVQQVQGTQQRLLVQTSVQAKPGHVSPLQLTNIQVPQQALPTQRLVVQSAAPGSKGGQVSLTVHGTQQVHSPPEQSPVQANSSSSKTAGAPTGTVPQQLQVHGVQQSVPVTQERSVVQATPQAPKPGPVQPLTVQGLQPVHVAQEVQQLQQVPVPHVYSSQVQYVEGGDASYTASAIRSSTYSYPETPLYTQTASTSYYEAAGTATQVSTPATSQAVASSGSMPMYVSGSQVVASSASTGAGASNSSGGGGSGGGGGGGGGGGGGGSGSTGGGGSGAGTYVIQGGYMLGSASQSYSHTTRASPATVQWLLDNYETAEGVSLPRSTLYCHYLLHCQEQKLEPVNAASFGKLIRSVFMGLRTRRLGTRGNSKYHYYGLRIKASSPLLRLMEDQQHMAMRGQPFSQKQRLKPIQKMEGMTNGVAVGQQPSTGLSDISAQVQQYQQFLDASRSLPDFTELDLQGKVLPEGVGPGDIKAFQVLYREHCEAIVDVMVNLQFTLVETLWKTFWRYNLSQPSEAPPLAVHDEAEKRLPKAILVLLSKFEPVLQWTKHCDNVLYQGLVEILIPDVLRPIPSALTQAIRNFAKSLESWLTHAMVNIPEEMLRVKVAAAGAFAQTLRRYTSLNHLAQAARAVLQNTAQINQMLSDLNRVDFANVQEQASWVCRCEDRVVQRLEQDFKVTLQQQNSLEQWAAWLDGVVSQVLKPYQGSAGFPKAAKLFLLKWSFYSSMVIRDLTLRSAASFGSFHLIRLLYDEYMYYLIEHRVAQAKGETPIAVMGEFANLATSLNPLDPDKDEEEEEEEESEDELPQDISLAAGGESPALGPETLEPPAKLARTDARGLFVQALPSS

>NP_001340.2

MPSASASRKSQEKPREIMDAAEDYAKERYGISSMIQSQEKPDRVLVRVRDLTIQKADEVVWVRARVHTSRAKGKQCFLVLRQQQFNVQALVAVGDHASKQMVKFAANINKESIVDVEGVVRKVNQKIGSCTQQDVELHVQKIYVISLAEPRLPLQLDDAVRPEAEGEEEGRATVNQDTRLDNRVIDLRTSTSQAVFRLQSGICHLFRETLINKGFVEIQTPKIISAASEGGANVFTVSYFKNNAYLAQSPQLYKQMCICADFEKVFSIGPVFRAEDSNTHRHLTEFVGLDIEMAFNYHYHEVMEEIADTMVQIFKGLQERFQTEIQTVNKQFPCEPFKFLEPTLRLEYCEALAMLREAGVEMGDEDDLSTPNEKLLGHLVKEKYDTDFYILDKYPLAVRPFYTMPDPRNPKQSNSYDMFMRGEEILSGAQRIHDPQLLTERALHHGIDLEKIKAYIDSFRFGAPPHAGGGIGLERVTMLFLGLHNVRQTSMFPRDPKRLTP

1544 NP_004575.1 NP_116119.2

>NP_004575.1

MKCLVTGGNVKVLGKAVHSLSRIGDELYLEPLEDGLSLRTVNSSRSAYACFLFAPLFFQQYQAATPGQDLLRCKILMKSFLSVFRSLAMLEKTVEKCCISLNGRSSRLVVQLHCKFGVRKTHNLSFQDCESLQAVFDPASCPHMLRAPARVLGEAVLPFSPALAEVTLGIGRGRRVILRSYHEEEADSTAKAMVTEMCLGEEDFQQLQAQEGVAITFCLKEFRGLLSFAESANLNLSIHFDAPGRPAIFTIKDSLLDGHFVLATLSDTDSHSQDLGSPERHQPVPQLQAHSTPHPDDFANDDIDSYMIAMETTIGNEGSRVLPSISLSPGPQPPKSPGPHSEEEDEAEPSTVPGTPPPKKFRSLFFGSILAPVRSPQGPSPVLAEDSEGEG

>NP_116119.2

MEFLKTCVLRRNACTAVCFWRSKVVQKPSVRRISTTSPRSTVMPAWVIDKYGKNEVLRFTQNMMMPIIHYPNEVIVKVHAASVNPIDVNMRSGYGATALNMKRDPLHVKIKGEEFPLTLGRDVSGVVMECGLDVKYFKPGDEVWAAVPPWKQGTLSEFVVVSGNEVSHKPKSLTHTQAASLPYVALTAWSAINKVGGLNDKNCTGKRVLILGASGGVGTFAIQVMKAWDAHVTAVCSQDASELVRKLGADDVIDYKSGSVEEQLKSLKPFDFILDNVGGSTETWAPDFLKKWSGATYVTLVTPFLLNMDRLGIADGMLQTGVTVGSKALKHFWKGVHYRWAFFMASGPCLDDIAELVDAGKIRPVIEQTFPFSKVPEAFLKVERGHARGKTVINVV

1545 Q9BSH3 NP_002291.1

>Q9BSH3

MSRVLVPCHVKGSVALQVGDVRTSQGRPGVLVIDVTFPSVAPFELQEITFKNYYTAFLSIRVRQYTSAHTPAKWVTCLRDYCLMPDPHSEEGAQEYVSLFKHQMLCDMARISELRLILRQPSPLWLSFTVEELQIYQQGPKSPSVTFPKWLSHPVPCEQPALLREGLPDPSRVSSEVQQMWALTEMIRASHTSARIGRFDVDGCYDLNLLSYT

>NP_002291.1

MATLKEKLIAPVAEEEATVPNNKITVVGVGQVGMACAISILGKSLADELALVDVLEDKLKGEMMDLQHGSLFLQTPKIVADKDYSVTANSKIVVVTAGVRQQEGESRLNLVQRNVNVFKFIIPQIVKYSPDCIIIVVSNPVDILTYVTWKLSGLPKHRVIGSGCNLDSARFRYLMAEKLGIHPSSCHGWILGEHGDSSVAVWSGVNVAGVSLQELNPEMGTDNDSENWKEVHKMVVESAYEVIKLKGYTNWAIGLSVADLIESMLKNLSRIHPVSTMVKGMYGIENEVFLSLPCILNARGLTSVINQKLKDDEVAQLKKSADTLWDIQKDLKDL

1546 NP_005241.1 Q8WUN7

>NP_005241.1

MSHLFDPRLPALAASPMLYLYGPERPGLPLAFAPAAALAASGRAETPQKPPYSYIALIAMAIQDAPEQRVTLNGIYQFIMDRFPFYHDNRQGWQNSIRHNLSLNDCFVKVPREKGRPGKGSYWTLDPRCLDMFENGNYRRRKRKPKPGPGAPEAKRPRAETHQRSAEAQPEAGSGAGGSGPAISRLQAAPAGPSPLLDGPSPPAPLHWPGTASPNEDAGDAAQGAAAVAVGQAARTGDGPGSPLRPASRSSPKSSDKSKSFSIDSILAGKQGQKPPSGDELLGGAKPGPGGRLGASLLAASSSLRPPFNASLMLDPHVQGGFYQLGIPFLSYFPLQVPDTVLHFQ

>Q8WUN7

MGGCVGAQHDSSGSLNENSEGTGVALGRNQPLKKEKPKWKSDYPMTDGQLRSKRDEFWDTAPAFEGRKEIWDALKAAAHAFESNDHELAQAIIDGANITLPHGALTECYDELGNRYQLPVYCLAPPINMIEEKSDIETLDIPEPPPNSGYECQLRLRLSTGKDLKLVVRSTDTVFHMKRRLHAAEGVEPGSQRWFFSGRPLTDKMKFEELKIPKDYVVQVIVSQPVQNPTPVEN

1547 NP_006707.1 Q14914

>NP_006707.1

MNSGAMRIHSKGHFQGGIQVKNEKNRPSLKSLKTDNRPEKSKCKPLWGKVFYLDLPSVTISEKLQKDIKDLGGRVEEFLSKDISYLISNKKEAKFAQTLGRISPVPSPESAYTAETTSPHPSHDGSSFKSPDTVCLSRGKLLVEKAIKDHDFIPSNSILSNALSWGVKILHIDDIRYYIEQKKKELYLLKKSSTSVRDGGKRVGSGAQKTRTGRLKKPFVKVEDMSQLYRPFYLQLTNMPFINYSIQKPCSPFDVDKPSSMQKQTQVKLRIQTDGDKYGGTSIQLQLKEKKKKGYCECCLQKYEDLETHLLSEQHRNFAQSNQYQVVDDIVSKLVFDFVEYEKDTPKKKRIKYSVGSLSPVSASVLKKTEQKEKVELQHISQKDCQEDDTTVKEQNFLYKETQETEKKLLFISEPIPHPSNELRGLNEKMSNKCSMLSTAEDDIRQNFTQLPLHKNKQECILDISEHTLSENDLEELRVDHYKCNIQASVHVSDFSTDNSGSQPKQKSDTVLFPAKDLKEKDLHSIFTHDSGLITINSSQEHLTVQAKAPFHTPPEEPNECDFKNMDSLPSGKIHRKVKIILGRNRKENLEPNAEFDKRTEFITQEENRICSSPVQSLLDLFQTSEEKSEFLGFTSYTEKSGICNVLDIWEEENSDNLLTAFFSSPSTSTFTGF

>Q14914

MVRTKTWTLKKHFVGYPTNSDFELKTAELPPLKNGEVLLEALFLTVDPYMRVAAKRLKEGDTMMGQQVAKVVESKNVALPKGTIVLASPGWTTHSISDGKDLEKLLTEWPDTIPLSLALGTVGMPGLTAYFGLLEICGVKGGETVMVNAAAGAVGSVVGQIAKLKGCKVVGAVGSDEKVAYLQKLGFDVVFNYKTVESLEETLKKASPDGYDCYFDNVGGEFSNTVIGQMKKFGRIAICGAISTYNRTGPLPPGPPPEIVIYQELRMEAFVVYRWQGDARQKALKDLLKWVLEGKIQYKEYIIEGFENMPAAFMGMLKGDNLGKTIVKA

1548 NP_076921.1 NP_004172.2

>NP_076921.1

MSSYFVNSLFSKYKTGESLRPNYYDCGFAQDLGGRPTVVYGPSSGGSFQHPSQIQEFYHGPSSLSTAPYQQNPCAVACHGDPGNFYGYDPLQRQSLFGAQDPDLVQYADCKLAAASGLGEEAEGSEQSPSPTQLFPWMRPQAAAGRRRGRQTYSRYQTLELEKEFLFNPYLTRKRRIEVSHALGLTERQVKIWFQNRRMKWKKENNKDKFPSSKCEQEELEKQKLERAPEAADEGDAQKGDKK

>NP_004172.2

MQLKPMEINPEMLNKVLSRLGVAGQWRFVDVLGLEEESLGSVPAPACALLLLFPLTAQHENFRKKQIEELKGQEVSPKVYFMKQTIGNSCGTIGLIHAVANNQDKLGFEDGSVLKQFLSETEKMSPEDRAKCFEKNEAIQAAHDAVAQEGQCRVDDKVNFHFILFNNVDGHLYELDGRMPFPVNHGASSEDTLLKDAAKVCREFTEREQGEVRFSAVALCKAA

1549 Q9UKI9 NP_005527.1

>Q9UKI9

MVNLESMHTDIKMSGDVADSTDARSTLSQVEPGNDRNGLDFNRQIKTEDLSDSLQQTLSHRPCHLSQGPAMMSGNQMSGLNASPCQDMASLHPLQQLVLVPGHLQSVSQFLLSQTQPGQQGLQPNLLPFPQQQSGLLLPQTGPGLASQAFGRPGLPGSSLEPHLEASQHLPVPKHLPSSGGADEPSDLEELEKFAKTFKQRRIKLGFTQGDVGLAMGKLYGNDFSQTTISRFEALNLSFKNMCKLKPLLEKWLNDAESSPSDPSVSTPSSYPSLSEVFGRKRKKRTSIETNIRLTLEKRFQDNPKPSSEEISMIAEQLSMEKEVVRVWFCNRRQKEKRINCPVATPIKPPVYNSRLVSPSGSLGPLSVPPVHSTMPGTVTSSCSPGNNSRPSSPGSGLHASSPTASQNNSKAAVNSASSFNSSGSWYRWNHSTYLH

>NP_005527.1

MADPWQECMDYAVTLARQAGEVVCEAIKNEMNVMLKSSPVDLVTATDQKVEKMLISSIKEKYPSHSFIGEESVAAGEKSILTDNPTWIIDPIDGTTNFVHRFPFVAVSIGFAVNKKIEFGVVYSCVEGKMYTARKGKGAFCNGQKLQVSQQEDITKSLLVTELGSSRTPETVRMVLSNMEKLFCIPVHGIRSVGTAAVNMCLVATGGADAYYEMGIHCWDVAGAGIIVTEAGGVLMDVTGGPFDLMSRRVIAANNRILAERIAKEIQVIPLQRDDED

1550 Q8TBZ0 Q9Y4K4

>Q8TBZ0

MSPEKQHREEDEVDSVLLSASKILNSSEGVKESGCSDTEYGCIAESENQIQPQSALKVLQQQLESFQALRMQTLQNVSMVQSEISEILNKSIIEVENPQFSSEKNLVFGTRIEKDLPTENQEENLSMEKSHHFEDSKTLHSVEEKLSGDSVNSLPQSVNVPSQIHSEDTLTLRTSTDNLSSNIIIHPSENSDILKNYNNFYRFLPTAPPNVMSQADTVILDKSKITVPFLKHGFCENLDDICHSIKQMKEELQKSHDGEVALTNELQTLQTDPDVHRNGKYDMSPIHQDKMNFIKEENLDGNLNEDIKSKRISELEALVKKLLPFRETVSKFHVHFCRKCKKLSKSEMHRGKKNEKNNKEIPITGKNITDLKFHSRVPRYTLSFLDQTKHEMKDKERQPFLVKQGSIISENEKTSKVNSVTEQCVAKIQYLQNYLKESVQIQKKVMELESENLNLKSKMKPLIFTTQSLIQKVETYEKQLKNLVEEKSTIQSKLSKTEEYSKECLKEFKKIISKYNVLQGQNKTLEEKNIQLSLEKQQMMEALDQLKSKEHKTQSDMAIVNNENNRMSIEMEAMKTNILLIQDEKEMLEKKTHQLLKEKSSLGNELKESQLEIIQLKEKERLAKTEQETLLQIIETVKDEKLNLETTLQESTAARQIMEREIENIQTYQSTAEENFLQEIKNAKSEASIYKNSLSEIGKECEMLSKMVMETKTDNQILKEELKKHSQENIKFENSISRLTEDKILLENYVRSIENERDTLEFEMRHLQREYLSLSDKICNQHNDPSKTTYISRREKFHFDNYTHEDTSSPQSRPLASDLKGYFKVKDRTLKHH

>Q9Y4K4

MEAPLRPAADILRRNPQQDYELVQRVGSGTYGDVYKARNVHTGELAAVKIIKLEPGDDFSLIQQEIFMVKECKHCNIVAYFGSYLSREKLWICMEYCGGGSLQDIYHVTGPLSELQIAYVCRETLQGLAYLHTKGKMHRDIKGANILLTDHGDVKLADFGVAAKITATIAKRKSFIGTPYWMAPEVAAVEKNGGYNQLCDIWAVGITAIELGELQPPMFDLHPMRALFLMSKSNFQPPKLKDKTKWSSTFHNFVKIALTKNPKKRPTAERLLTHTFVAQPGLSRALAVELLDKVNNPDNHAHYTEADDDDFEPHAIIRHTIRSTNRNARAERTASEINFDKLQFEPPLRKETEARDEMGLSSDPNFMLQWNPFVDGANTGKSTSKRAIPPPLPPKPRISSYPEDNFPDEEKASTIKHCPDSESRAPQILRRQSSPSCGPVAETSSIGNGDGISKLMSENTEGSAQAPQLPRKNDKRDFPKPAINGLPPTPKVLMGACFSKVFDGCPLKINCATSWIHPDTKDQYIIFGTEDGIYTLNLNELHEATMEQLFPRKCTWLYVINNTLMSLSEGKTFQLYSHNLIALFEHAKKPGLAAHIQTHRFPDRILPRKFALTTKIPDTKGCHKCCIVRNPYTGHKYLCGALQSGIVLLQWYEPMQKFMLIKHFDFPLPSPLNVFEMLVIPEQEYPMVCVAISKGTESNQVVQFETINLNSASSWFTEIGAGSQQLDSIHVTQLERDTVLVCLDKFVKIVNLQGKLKSSKKLASELSFDFRIESVVCLQDSVLAFWKHGMQGKSFKSDEVTQEISDETRVFRLLGSDRVVVLESRPTENPTAHSNLYILAGHENSY

1551 NP_005595.2 P51580

>NP_005595.2

MATAASNHYSLLTSSASIVHAEPPGGMQQGAGGYREAQSLVQGDYGALQSNGHPLSHAHQWITALSHGGGGGGGGGGGGGGGGGGGGGDGSPWSTSPLGQPDIKPSVVVQQGGRGDELHGPGALQQQHQQQQQQQQQQQQQQQQQQQQQRPPHLVHHAANHHPGPGAWRSAAAAAHLPPSMGASNGGLLYSQPSFTVNGMLGAGGQPAGLHHHGLRDAHDEPHHADHHPHPHSHPHQQPPPPPPPQGPPGHPGAHHDPHSDEDTPTSDDLEQFAKQFKQRRIKLGFTQADVGLALGTLYGNVFSQTTICRFEALQLSFKNMCKLKPLLNKWLEEADSSSGSPTSIDKIAAQGRKRKKRTSIEVSVKGALESHFLKCPKPSAQEITSLADSLQLEKEVVRVWFCNRRQKEKRMTPPGGTLPGAEDVYGGSRDTPPHHGVQTPVQ

>P51580

MDGTRTSLDIEEYSDTEVQKNQVLTLEEWQDKWVNGKTAFHQEQGHQLLKKHLDTFLKGKSGLRVFFPLCGKAVEMKWFADRGHSVVGVEISELGIQEFFTEQNLSYSEEPITEIPGTKVFKSSSGNISLYCCSIFDLPRTNIGKFDMIWDRGALVAINPGDRKCYADTMFSLLGKKFQYLLCVLSYDPTKHPGPPFYVPHAEIERLFGKICNIRCLEKVDAFEERHKSWGIDCLFEKLYLLTEK

1552 Q99581 Q8N7A1

>Q99581

MRQSGASQPLLINMYLPDPVGDGLFKDGKNPSWGPLSPAVQKGSGQIQLWQFLLELLADRANAGCIAWEGGHGEFKLTDPDEVARRWGERKSKPNMNYDKLSRALRYYYDKNIMSKVHGKRYAYRFDFQGLAQACQPPPAHAHAAAAAAAAAAAAQDGALYKLPAGLAPLPFPGLSKLNLMAASAGVAPAGFSYWPGPGPAATAAAATAALYPSPSLQPPPGPFGAVAAASHLGGHYH

>Q8N7A1

MADSQLFCVAEERSGHCAVVDGNFLYVWGGYVSIEDNEVYLPNDEIWTYDIDSGLWRMHLMEGELPASMSGSCGACINGKLYIFGGYDDKGYSNRLYFVNLRTRDETYIWEKITDFEGQPPTPRDKLSCWVYKDRLIYFGGYGCRRHSELQDCFDVHDASWEEQIFWGWHNDVHIFDTKTQTWFQPEIKGGVPPQPRAAHTCAVLGNKGYIFGGRVLQTRMNDLHYLNLDTWTWSGRITINGESPKHRSWHTLTPIADDKLFLCGGLSADNIPLSDGWIHNVTTNCWKQLTHLPKTRPRLWHTACLGKENEIMVFGGSKDDLLALDTGHCNDLLIFQTQPYSLLRSCLDCIGKNSIMLESQISLLPPKLLQQVLKKITFWAAANHREEQRVQKEETENKYQWISSN

1553 NP_060589.1 NP_055904.1

>NP_060589.1

MANEEDDPVVQEIDVYLAKSLAEKLYLFQYPVRPASMTYDDIPHLSAKIKPKQQKVELEMAIDTLNPNYCRSKGEQIALNVDGACADETSTYSSKLMDKQTFCSSQTTSNTSRYAAALYRQGELHLTPLHGILQLRPSFSYLDKADAKHREREAANEAGDSSQDEAEDDVKQITVRFSRPESEQARQRRVQSYEFLQKKHAEEPWVHLHYYGLRDSRSEHERQYLLCPGSSGVENTELVKSPSEYLMMLMPPSQEEEKDKPVAPSNVLSMAQLRTLPLADQIKILMKNVKVMPFANLMSLLGPSIDSVAVLRGIQKVAMLVQGNWVVKSDILYPKDSSSPHSGVPAEVLCRGRDFVMWKFTQSRWVVRKEVATVTKLCAEDVKDFLEHMAVVRINKGWEFILPYDGEFIKKHPDVVQRQHMLWTGIQAKLEKVYNLVKETMPKKPDAQSGPAGLVCGDQRIQVAKTKAQQNHALLERELQRRKEQLRVPAVPPGVRIKEEPVSEEGEEDEEQEAEEEPMDTSPSGLHSKLANGLPLGRAAGTDSFNGHPPQGCASTPVARELKAFVEATFQRQFVLTLSELKRLFNLHLASLPPGHTLFSGISDRMLQDTVLAAGCKQILVPFPPQTAASPDEQKVFALWESGDMSDQHRQVLLEIFSKNYRVRRNMIQSRLTQECGEDLSKQEVDKVLKDCCVSYGGMWYLKGTVQS

>NP_055904.1

MVGERHAGDLMVPLGPRLQAYPEELIRQRPGHDGHPEYLIRWSVLKCGEVGKVGVEEGKAEHILMWLSAPEVYANCPGLLGERALSKGLQHEPAGVSGSFPRDPGGLDEVAMGEMEADVQALVRRAARQLAESGTPSLTAAVLHTIHVLSAYASIGPLTGVFRETGALDLLMHMLCNPEPQIRRSAGKMLQALAAHDAGSRAHVLLSLSQQDGIEQHMDFDSRYTLLELFAETTSSEEHCMAFEGIHLPQIPGKLLFSLVKRYLCVTSLLDQLNSSPELGAGDQSSPCATREKSRGQRELEFSMAVGNLISELVRSMGWARNLSEQGMSPPRPTRSIFQPYISGPSLLLPTIVTTPRRQGWVFRQRSEFSSRSGYGEYVQQTLQPGMRVRMLDDYEEISAGDEGEFRQSNNGIPPVQVFWQSTGRTYWVHWHMLEILGPEEATEDKASAAVEKGAGATVLGTAFPSWDWNPMDGLYPLPYLQPEPQKNERVGYLTQAEWWELLFFIKKLDLCEQQPIFQNLWKNLDETLGEKALGEISVSVEMAESLLQVLSSRFEGSTLNDLLNSQIYTKYGLLSNEPSSSSTSRNHSCTPDPEEESKSEASFSEEETESLKAKAEAPKTEAEPTKTRTETPMAQSDSQLFNQLLVTEGMTLPTEMKEAASEMARALRGPGPRSSLDQHVAAVVATVQISSLDTNLQLSGLSALSQAVEEVTERDHPLVRPDRSLREKLVKMLVELLTNQVGEKMVVVQALRLLYLLMTKHEWRPLFAREGGIYAVLVCMQEYKTSVLVQQAGLAALKMLAVASSSEIPTFVTGRDSIHSLFDAQMTREIFASIDSATRPGSESLLLTVPAAVILMLNTEGCSSAARNGLLLLNLLLCNHHTLGDQIITQELRDTLFRHSGIAPRTEPMPTTRTILMMLLNRYSEPPGSPERAALETPIIQGQDGSPELLIRSLVGGPSAELLLDLERVLCREGSPGGAVRPLLKRLQQETQPFLLLLRTLDAPGPNKTLLLSVLRVITRLLDFPEAMVLPWHEVLEPCLNCLSGPSSDSEIVQELTCFLHRLASMHKDYAVVLCCLGAKEILSKVLDKHSAQLLLGCELRDLVTECEKYAQLYSNLTSSILAGCIQMVLGQIEDHRRTHQPINIPFFDVFLRHLCQGSSVEVKEDKCWEKVEVSSNPHRASKLTDHNPKTYWESNGSTGSHYITLHMHRGVLVRQLTLLVASEDSSYMPARVVVFGGDSTSCIGTELNTVNVMPSASRVILLENLNRFWPIIQIRIKRCQQGGIDTRVRGVEVLGPKPTFWPLFREQLCRRTCLFYTIRAQAWSRDIAEDHRRLLQLCPRLNRVLRHEQNFADRFLPDDEAAQALGKTCWEALVSPLVQNITSPDAEGVSALGWLLDQYLEQRETSRNPLSRAASFASRVRRLCHLLVHVEPPPGPSPEPSTRPFSKNSKGRDRSPAPSPVLPSSSLRNITQCWLSVVQEQVSRFLAAAWRAPDFVPRYCKLYEHLQRAGSELFGPRAAFMLALRSGFSGALLQQSFLTAAHMSEQFARYIDQQIQGGLIGGAPGVEMLGQLQRHLEPIMVLSGLELATTFEHFYQHYMADRLLSFGSSWLEGAVLEQIGLCFPNRLPQLMLQSLSTSEELQRQFHLFQLQRLDKLFLEQEDEEEKRLEEEEEEEEEEEAEKELFIEDPSPAISILVLSPRCWPVSPLCYLYHPRKCLPTEFCDALDRFSSFYSQSQNHPVLDMGPHRRLQWTWLGRAELQFGKQILHVSTVQMWLLLKFNQTEEVSVETLLKDSDLSPELLLQALVPLTSGNGPLTLHEGQDFPHGGVLRLHEPGPQRSGEALWLIPPQAYLNVEKDEGRTLEQKRNLLSCLLVRILKAHGEKGLHIDQLVCLVLEAWQKGPNPPGTLGHTVAGGVACTSTDVLSCILHLLGQGYVKRRDDRPQILMYAAPEPMGPCRGQADVPFCGSQSETSKPSPEAVATLASLQLPAGRTMSPQEVEGLMKQTVRQVQETLNLEPDVAQHLLAHSHWGAEQLLQSYSEDPEPLLLAAGLCVHQAQAVPVRPDHCPVCVSPLGCDDDLPSLCCMHYCCKSCWNEYLTTRIEQNLVLNCTCPIADCPAQPTGAFIRAIVSSPEVISKYEKALLRGYVESCSNLTWCTNPQGCDRILCRQGLGCGTTCSKCGWASCFNCSFPEAHYPASCGHMSQWVDDGGYYDGMSVEAQSKHLAKLISKRCPSCQAPIEKNEGCLHMTCAKCNHGFCWRCLKSWKPNHKDYYNCSAMVSKAARQEKRFQDYNERCTFHHQAREFAVNLRNRVSAIHEVPPPRSFTFLNDACQGLEQARKVLAYACVYSFYSQDAEYMDVVEQQTENLELHTNALQILLEETLLRCRDLASSLRLLRADCLSTGMELLRRIQERLLAILQHSAQDFRVGLQSPSVEAWEAKGPNMPGSQPQASSGPEAEEEEEDDEDDVPEWQQDEFDEELDNDSFSYDESENLDQETFFFGDEEEDEDEAYD

1554 NP_001036111.1 NP_001937.2

>NP_001036111.1

MALVFQFGQPVRAQPLPGLCHGKLIRTNACDVCNSTDLPEVEIISLLEEQLPHYKLRADTIYGYDHDDWLHTPLISPDANIDLTTEQIEETLKYFLLCAERVGQMTKTYNDIDAVTRLLEEKERDLELAARIGQSLLKKNKTLTERNELLEEQVEHIREEVSQLRHELSMKDELLQFYTSAAEESEPESVCSTPLKRNESSSSVQNYFHLDSLQKKLKDLEEENVVLRSEASQLKTETITYEEKEQQLVNDCVKELRDANVQIASISEELAKKTEDAARQQEEITHLLSQIVDLQKKAKACAVENEELVQHLGAAKDAQRQLTAELRELEDKYAECMEMLHEAQEELKNLRNKTMPNTTSRRYHSLGLFPMDSLAAEIEGTMRKELQLEEAESPDITHQKRVFETVRNINQVVKQRSLTPSPMNIPGSNQSSAMNSLLSSCVSTPRSSFYGSDIGNVVLDNKTNSIILETEAADLGNDERSKKPGTPGTPGSHDLETALRRLSLRRENYLSERRFFEEEQERKLQELAEKGELRSGSLTPTESIMSLGTHSRFSEFTGFSGMSFSSRSYLPEKLQIVKPLEGSATLHHWQQLAQPHLGGILDPRPGVVTKGFRTLDVDLDEVYCLNDFEEDDTGDHISLPRLATSTPVQHPETSAHHPGKCMSQTNSTFTFTTCRILHPSDELTRVTPSLNSAPTPACGSTSHLKSTPVATPCTPRRLSLAESFTNTRESTTTMSTSLGLVWLLKERGISAAVYDPQSWDRAGRGSLLHSYTPKMAVIPSTPPNSPMQTPTSSPPSFEFKCTSPPYDNFLASKPASSILREVREKNVRSSESQTDVSVSNLNLVDKVRRFGVAKVVNSGRAHVPTLTEEQGPLLCGPPGPAPALVPRGLVPEGLPLRCPTVTSAIGGLQLNSGIRRNRSFPTMVGSSMQMKAPVTLTSGILMGAKLSKQTSLR

>NP_001937.2

MIDTLRPVPFASEMAISKTVAWLNEQLELGNERLLLMDCRPQELYESSHIESAINVAIPGIMLRRLQKGNLPVRALFTRGEDRDRFTRRCGTDTVVLYDESSSDWNENTGGESVLGLLLKKLKDEGCRAFYLEGGFSKFQAEFSLHCETNLDGSCSSSSPPLPVLGLGGLRISSDSSSDIESDLDRDPNSATDSDGSPLSNSQPSFPVEILPFLYLGCAKDSTNLDVLEEFGIKYILNVTPNLPNLFENAGEFKYKQIPISDHWSQNLSQFFPEAISFIDEARGKNCGVLVHCLAGISRSVTVTVAYLMQKLNLSMNDAYDIVKMKKSNISPNFNFMGQLLDFERTLGLSSPCDNRVPAQQLYFTTPSNQNVYQVDSLQST

1555 NP_001252.1 Q92665

>NP_001252.1

MAKQYDSVECPFCDEVSKYEKLAKIGQGTFGEVFKARHRKTGQKVALKKVLMENEKEGFPITALREIKILQLLKHENVVNLIEICRTKASPYNRCKGSIYLVFDFCEHDLAGLLSNVLVKFTLSEIKRVMQMLLNGLYYIHRNKILHRDMKAANVLITRDGVLKLADFGLARAFSLAKNSQPNRYTNRVVTLWYRPPELLLGERDYGPPIDLWGAGCIMAEMWTRSPIMQGNTEQHQLALISQLCGSITPEVWPNVDNYELYEKLELVKGQKRKVKDRLKAYVRDPYALDLIDKLLVLDPAQRIDSDDALNHDFFWSDPMPSDLKGMLSTHLTSMFEYLAPPRRKGSQITQQSTNQSRNPATTNQTEFERVF

>Q92665

MFPRVSTFLPLRPLSRHPLSSGSPETSAAAIMLLTVRHGTVRYRSSALLARTKNNIQRYFGTNSVICSKKDKQSVRTEETSKETSESQDSEKENTKKDLLGIIKGMKVELSTVNVRTTKPPKRRPLKSLEATLGRLRRATEYAPKKRIEPLSPELVAAASAVADSLPFDKQTTKSELLSQLQQHEEESRAQRDAKRPKISFSNIISDMKVARSATARVRSRPELRIQFDEGYDNYPGQEKTDDLKKRKNIFTGKRLNIFDMMAVTKEAPETDTSPSLWDVEFAKQLATVNEQPLQNGFEELIQWTKEGKLWEFPINNEAGFDDDGSEFHEHIFLEKHLESFPKQGPIRHFMELVTCGLSKNPYLSVKQKVEHIEWFRNYFNEKKDILKESNIQFN

1556 NP_002135.2 Q9H2W6

>NP_002135.2

MDYNRMNSFLEYPLCNRGPSAYSAHSAPTSFPPSSAQAVDSYASEGRYGGGLSSPAFQQNSGYPAQQPPSTLGVPFPSSAPSGYAPAACSPSYGPSQYYPLGQSEGDGGYFHPSSYGAQLGGLSDGYGAGGAGPGPYPPQHPPYGNEQTASFAPAYADLLSEDKETPCPSEPNTPTARTFDWMKVKRNPPKTAKVSEPGLGSPSGLRTNFTTRQLTELEKEFHFNKYLSRARRVEIAATLELNETQVKIWFQNRRMKQKKREREEGRVPPAPPGCPKEAAGDASDQSTCTSPEASPSSVTS

>Q9H2W6

MAAPVRRTLLGVAGGWRRFERLWAGSLSSRSLALAAAPSSNGSPWRLLGALCLQRPPVVSKPLTPLQEEMASLLQQIEIERSLYSDHELRALDENQRLAKKKADLHDEEDEQDILLAQDLEDMWEQKFLQFKLGARITEADEKNDRTSLNRKLDRNLVLLVREKFGDQDVWILPQAEWQPGETLRGTAERTLATLSENNMEAKFLGNAPCGHYTFKFPQAMRTESNLGAKVFFFKALLLTGDFSQAGNKGHHVWVTKDELGDYLKPKYLAQVRRFVSDL

1557 NP_060274.3 NP_064632.2

>NP_060274.3

MKEPRIFPRERPTPWTRAPLPPRGRLDGSLGPQGGPVLNTGHPLGVNSDPFLMAAGSLGGNLTPFPRNPSPFPASSGSLASNPAPFPAGARDPSMASFPRGMNPTGTGAVSFPRPGGLLGPGPGPGPTLNPRTGALPGPGPLSNPRLGGLPGPGPMSNPRAGGLLGAGPDPRGGGPMGPGSGPNLRAGVLLTSGNGPPNPRPVGLGPGPNPNLRSGFLGTNPAPRSGVFPGPGLGPNPRPSGLGPGPNLDARAGGLLGTGSGLNLRMAGPQGLDLAPILRAAGLLGANSASFSQASGNMGTSPSSMARVPGPMGPNSGPSSRGIGLPGPNPSPMSRAPGPIGPNSAHFSRPVGPMGVNANPFPRGAGSSAFSQSSGTLASNPATFQRSAGLQGSNPTIFPRASGPLGPNPANFPRATGLQGPSPTTFPRSTGPLGPGQVTFPRPAAGHLGPSPAGPVGINPAPFTRPTGTLGLNPASFPRMNGPAGKSFVPFPRVGSLPGTNPAAFPRPGGPMAAMYPNGMLPP

>NP_064632.2

MAAILGDTIMVAKGLVKLTQAAVETHLQHLGIGGELIMAARALQSTAVEQIGMFLGKVQGQDKHEEYFAENFGGPEGEFHFSVPHAAGASTDFSSASAPDQSAPPSLGHAHSEGPAPAYVASGPFREAGFPGQASSPLGRANGRLFANPRDSFSAMGFQRRFFHQDQSPVGGLTAEDIEKARQAKARPENKQHKQTLSEHARERKVPVTRIGRLANFGGLAVGLGFGALAEVAKKSLRSEDPSGKKAVLGSSPFLSEANAERIVRTLCKVRGAALKLGQMLSIQDDAFINPHLAKIFERVRQSADFMPLKQMMKTLNNDLGPNWRDKLEYFEERPFAAASIGQVHLARMKGGREVAMKIQYPGVAQSINSDVNNLMAVLNMSNMLPEGLFPEHLIDVLRRELALECDYQREAACARKFRDLLKGHPFFYVPEIVDELCSPHVLTTELVSGFPLDQAEGLSQEIRNEICYNILVLCLRELFEFHFMQTDPNWSNFFYDPQQHKVALLDFGATREYDRSFTDLYIQIIRAAADRDRETVRAKSIEMKFLTGYEVKVMEDAHLDAILILGEAFASDEPFDFGTQSTTEKIHNLIPVMLRHRLVPPPEETYSLHRKMGGSFLICSKLKARFPCKAMFEEAYSNYCKRQAQQ

1558 Q86Y25 Q8WVN8

>Q86Y25

MAVDLLSAQEPVTFRDVAVFFSQDEWLHLDSAQRALYREVMLENYSSLVSLGIPFSMPKLIHQLQQGEDPCMVEREVPSDTRLGFKTWLETEALPHRQDIFIEETSQGMVKKESIKDGHWDINFEEAVEFESEIEEEQEKKPLRQMIDSHEKTISEDGNHTSLELGKSLFTNTALVTQQSVPIERIPNMYYTFGKDFKQNFDLMKCFQIYPGGKPHICNECGKSFKQNLHLIEHQRIHTGEKPYKCNECEKTFSHRSSLLSHQRIHTGEKPYKCNECEKAFSNSSTLIKHLRVHTGEKPYRCRECGKAFSQCSTLTVHQRIHTGEKLYKCGECEKAFNCRAKLHRHQRIHTGEKPYKCSECGKGYSQFTSLAEHQRFHTGEQLYTCLECGRTFTRIVTLIEHQRIHTGQKPYQCNECEKAFNQYSSFNEHRKIHTGEKLYTCEECGKAFGCKSNLYRHQRIHTGEKPYQCNQCGKAFSQYSFLTEHERIHTGEKLYKCMECGKAYSYRSNLCRHKKVHTKEKLYKWKEYGKPFICSSSLTQYQRFFKGDKAYEV

>Q8WVN8

MSVSGLKAELKFLASIFDKNHERFRIVSWKLDELHCQFLVPQQGSPHSLPPPLTLHCNITESYPSSSPIWFVDSEDPNLTSVLERLEDTKNNNLLRQQLKWLICELCSLYNLPKHLDVEMLDQPLPTGQNGTTEEVTSEEEEEEEEMAEDIEDLDHYEMKEEEPISGKKSEDEGIEKENLAILEKIRKTQRQDHLNGAVSGSVQASDRLMKELRDIYRSQSYKTGIYSVELINDSLYDWHVKLQKVDPDSPLHSDLQILKEKEGIEYILLNFSFKDNFPFDPPFVRVVLPVLSGGYVLGGGALCMELLTKQGWSSAYSIESVIMQINATLVKGKARVQFGANKNQYNLARAQQSYNSIVQIHEKNGWYTPPKEDG

1559 NP_076922.1 NP_008878.3

>NP_076922.1

MSISGTLSSYYVDSIISHESEDAPPAKFPSGQYASSRQPGHAEHLEFPSCSFQPKAPVFGASWAPLSPHASGSLPSVYHPYIQPQGVPPAESRYLRTWLEPAPRGEAAPGQGQAAVKAEPLLGAPGELLKQGTPEYSLETSAGREAVLSNQRPGYGDNKICEGSEDKERPDQTNPSANWLHARSSRKKRCPYTKYQTLELEKEFLFNMYLTRDRRHEVARLLNLSERQVKIWFQNRRMKMKKMNKEQGKE

>NP_008878.3

MASGGSGGVSVPALWSEVNRYGQNGDFTRALKTVNKILQINKDDVTALHCKVVCLIQNGSFKEALNVINTHTKVLANNSLSFEKAYCEYRLNRIENALKTIESANQQTDKLKELYGQVLYRLERYDECLAVYRDLVRNSQDDYDEERKTNLSAVVAAQSNWEKVVPENLGLQEGTHELCYNTACALIGQGQLNQAMKILQKAEDLCRRSLSEDTDGTEEDPQAELAIIHGQMAYILQLQGRTEEALQLYNQIIKLKPTDVGLLAVIANNIITINKDQNVFDSKKKVKLTNAEGVEFKLSKKQLQAIEFNKALLAMYTNQAEQCRKISASLQSQSPEHLLPVLIQAAQLCREKQHTKAIELLQEFSDQHPENAAEIKLTMAQLKISQGNISKACLILRSIEELKHKPGMVSALVTMYSHEEDIDSAIEVFTQAIQWYQNHQPKSPAHLSLIREAANFKLKYGRKKEAISDLQQLWKQNPKDIHTLAQLISAYSLVDPEKAKALSKHLPSSDSMSLKVDVEALENSAGATYIRKKGGKVTGDSQPKEQGQGDLKKKKKKKKGKLPKNYDPKVTPDPERWLPMRERSYYRGRKKGKKKDQIGKGTQGATAGASSELDASKTVSSPPTSPRPGSAATVSASTSNIIPPRHQKPAGAPATKKKQQQKKKKGGKGGW

1560 NP_490595.1 NP_001079.1

>NP_490595.1

MASGRGASSRWFFTREQLENTPSRRCGVEADKELSCRQQAANLIQEMGQRLNVSQLTINTAIVYMHRFYMHHSFTKFNKNIISSTALFLAAKVEEQARKLEHVIKVAHACLHPLEPLLDTKCDAYLQQTQELVILETIMLQTLGFEITIEHPHTDVVKCTQLVRASKDLAQTSYFMATNSLHLTTFCLQYKPTVIACVCIHLACKWSNWEIPVSTDGKHWWEYVDPTVTLELLDELTHEFLQILEKTPNRLKKIRNWRANQAARKPKVDGQVSETPLLGSSLVQNSILVDSVTGVPTNPSFQKPSTSAFPAPVPLNSGNISVQDSHTSDNLSMLATGMPSTSYGLSSHQEWPQHQDSARTEQLYSQKQETSLSGSQYNINFQQGPSISLHSGLHHRPDKISDHSSVKQEYTHKAGSSKHHGPISTTPGIIPQKMSLDKYREKRKLETLDLDVRDHYIAAQVEQQHKQGQSQAASSSSVTSPIKMKIPIANTEKYMADKKEKSGSLKLRIPIPPTDKSASKEELKMKIKVSSSERHSSSDEGSGKSKHSSPHISRDHKEKHKEHPSSRHHTSSHKHSHSHSGSSSGGSKHSADGIPPTVLRSPVGLSSDGISSSSSSSRKRLHVNDASHNHHSKMSKSSKSSGSSSSSSSSVKQYISSHNSVFNHPLPPPPPVTYQVGYGHLSTLVKLDKKPVETNGPDANHEYSTSSQHMDYKDTFDMLDSLLSAQGMNM

>NP_001079.1

MSTQSTHPLKPEAPRLPPGIPESPSCQRRHTLPASEFRCLTPEDAVSAFEIEREAFISVLGVCPLYLDEIRHFLTLCPELSLGWFEEGCLVAFIIGSLWDKERLMQESLTLHRSGGHIAHLHVLAVHRAFRQQGRGPILLWRYLHHLGSQPAVRRAALMCEDALVPFYERFSFHAVGPCAITVGSLTFMELHCSLRGHPFLRRNSGC

1561 NP_001343.2 O75343

>NP_001343.2

MARPVSDRTPAPLLLGGPAGTPPGGGALLGLRSLLQGTSKPKEPASCLLKEKERKAALPAATTPGPGLETAGPADAPAGAVVGGGSPRGRPGPVPAPGLLAPLLWERTLPFGDVEYVDLDAFLLEHGLPPSPPPPGGPSPEPSPARTPAPSPGPGSCGSASPRSSPGHAPARAALGTASGHRAGLTSRDTPSPVDPDTVEVLMTFEPDPADLALSSIPGHETFDPRRHRFSEEELKPQPIMKKARKIQVPEEQKDEKYWSRRYKNNEAAKRSRDARRLKENQISVRAAFLEKENALLRQEVVAVRQELSHYRAVLSRYQAQHGAL

>O75343

MSGYDRMLRTLGGNLMEFIENLDALHSYLALSYQEMNAPSFRVERGADGKMFLHYYSDRSGLCHIVPGIIEAVAKDFFDIDVIMDILDMNEEVERTGKKEHVVFLIVQKAHRKMRKTKPKRLQDSQGMERDQEALQAAFLKMKEKYLNVSACPVKKSHWDVVRSIVMFGKGHLMNTFEPIYPERLWIEEKTFCNAFPFHIVFDESLQVKQARVNIQKYVPGLQTQNIQLDEYFSIIHPQVTFNIFSIRRFINSQFVLKTRREMMPVAWQSRTTLKLQGQMIWMESMWCMVYLCSPKLRSLQELEELNMHLSDIAPNDTTRDLILLNQQRLAEIELSNQLERKKEELQVLSKHLAIEKKKTETLLYAMLPKHVANQLREGKKVAAGEFKSCTILFSDVVTFTNICTACEPIQIVNVLNSMYSKFDRLTSVHAVYKVETIGDAYMVVGGVPVPIGNHAQRVANFALGMRISAKEVTNPVTGEPIQLRVGIHTGPVLADVVGDKMPRYCLFGDTVNTASRMESHGLPNKVHLSPTAYRALKNQGFKIIERGEIEVKGKGRMTTYFLIQNLNATEDEIMGRSKTPVDHKGSTQKASLPTTKLQGSVQPSCPEHSSLASWLL

1562 NP_003358.1 Q9H4A3

>NP_003358.1

MDMLDPGLDPAASATAAAAASHDKGPEAEEGVELQEGGDGPGAEEQTAVAITSVQQAAFGDHNIQYQFRTETNGGQVTYRVVQVTDGQLDGQGDTAGAVSVVSTAAFAGGQQAVTQVGVDGAAQRPGPAAASVPPGPAAPFPLAVIQNPFSNGGSPAAEAVSGEARFAYFPASSVGDTTAVSVQTTDQSLQAGGQFYVMMTPQDVLQTGTQRTIAPRTHPYSPKIDGTRTPRDERRRAQHNEVERRRRDKINNWIVQLSKIIPDCNADNSKTGASKGGILSKACDYIRELRQTNQRMQETFKEAERLQMDNELLRQQIEELKNENALLRAQLQQHNLEMVGEGTRQ

>Q9H4A3

MSGGAAEKQSSTPGSLFLSPPAPAPKNGSSSDSSVGEKLGAAAADAVTGRTEEYRRRRHTMDKDSRGAAATTTTTEHRFFRRSVICDSNATALELPGLPLSLPQPSIPAAVPQSAPPEPHREETVTATATSQVAQQPPAAAAPGEQAVAGPAPSTVPSSTSKDRPVSQPSLVGSKEEPPPARSGSGGGSAKEPQEERSQQQDDIEELETKAVGMSNDGRFLKFDIEIGRGSFKTVYKGLDTETTVEVAWCELQDRKLTKSERQRFKEEAEMLKGLQHPNIVRFYDSWESTVKGKKCIVLVTELMTSGTLKTYLKRFKVMKIKVLRSWCRQILKGLQFLHTRTPPIIHRDLKCDNIFITGPTGSVKIGDLGLATLKRASFAKSVIGTPEFMAPEMYEEKYDESVDVYAFGMCMLEMATSEYPYSECQNAAQIYRRVTSGVKPASFDKVAIPEVKEIIEGCIRQNKDERYSIKDLLNHAFFQEETGVRVELAEEDDGEKIAIKLWLRIEDIKKLKGKYKDNEAIEFSFDLERDVPEDVAQEMVESGYVCEGDHKTMAKAIKDRVSLIKRKREQRQLVREEQEKKKQEESSLKQQVEQSSASQTGIKQLPSASTGIPTASTTSASVSTQVEPEEPEADQHQQLQYQQPSISVLSDGTVDSGQGSSVFTESRVSSQQTVSYGSQHEQAHSTGTVPGHIPSTVQAQSQPHGVYPPSSVAQGQSQGQPSSSSLTGVSSSQPIQHPQQQQGIQQTAPPQQTVQYSLSQTSTSSEATTAQPVSQPQAPQVLPQVSAGKQLPVSQPVPTIQGEPQIPVATQPSVVPVHSGAHFLPVGQPLPTPLLPQYPVSQIPISTPHVSTAQTGFSSLPITMAAGITQPLLTLASSATTAAIPGVSTVVPSQLPTLLQPVTQLPSQVHPQLLQPAVQSMGIPANLGQAAEVPLSSGDVLYQGFPPRLPPQYPGDSNIAPSSNVASVCIHSTVLSPPMPTEVLATPGYFPTVVQPYVESNLLVPMGGVGGQVQVSQPGGSLAQAPTTSSQQAVLESTQGVSQVAPAEPVAVAQPQATQPTTLASSVDSAHSDVASGMSDGNENVPSSSGRHEGRTTKRHYRKSVRSRSRHEKTSRPKLRILNVSNKGDRVVECQLETHNRKMVTFKFDLDGDNPEEIATIMVNNDFILAIERESFVDQVREIIEKADEMLSEDVSVEPEGDQGLESLQGKDDYGFSGSQKLEGEFKQPIPASSMPQQIGIPTSSLTQVVHSAGRRFIVSPVPESRLRESKVFPSEITDTVAASTAQSPGMNLSHSASSLSLQQAFSELRRAQMTEGPNTAPPNFSHTGPTFPVVPPFLSSIAGVPTTAAATAPVPATSSPPNDISTSVIQSEVTVPTEEGIAGVATSTGVVTSGGLPIPPVSESPVLSSVVSSITIPAVVSISTTSPSLQVPTSTSEIVVSSTALYPSVTVSATSASAGGSTATPGPKPPAVVSQQAAGSTTVGATLTSVSTTTSFPSTASQLSIQLSSSTSTPTLAETVVVSAHSLDKTSHSSTTGLAFSLSAPSSSSSPGAGVSSYISQPGGLHPLVIPSVIASTPILPQAAGPTSTPLLPQVPSIPPLVQPVANVPAVQQTLIHSQPQPALLPNQPHTHCPEVDSDTQPKAPGIDDIKTLEEKLRSLFSEHSSSGAQHASVSLETSLVIESTVTPGIPTTAVAPSKLLTSTTSTCLPPTNLPLGTVALPVTPVVTPGQVSTPVSTTTSGVKPGTAPSKPPLTKAPVLPVGTELPAGTLPSEQLPPFPGPSLTQSQQPLEDLDAQLRRTLSPEIITVTSAVGPVSMAAPTAITEAGTQPQKGVSQVKEGPVLATSSGAGVFKMGRFQVSVAADGAQKEGKNKSEDAKSVHFESSTSESSVLSSSSPESTLVKPEPNGITIPGISSDVPESAHKTTASEAKSDTGQPTKVGRFQVTTTANKVGRFSVSKTEDKITDTKKEGPVASPPFMDLEQAVLPAVIPKKEKPELSEPSHLNGPSSDPEAAFLSRDVDDGSGSPHSPHQLSSKSLPSQNLSQSLSNSFNSSYMSSDNESDIEDEDLKLELRRLRDKHLKEIQDLQSRQKHEIESLYTKLGKVPPAVIIPPAAPLSGRRRRPTKSKGSKSSRSSSLGNKSPQLSGNLSGQSAASVLHPQQTLHPPGNIPESGQNQLLQPLKPSPSSDNLYSAFTSDGAISVPSLSAPGQGTSSTNTVGATVNSQAAQAQPPAMTSSRKGTFTDDLHKLVDNWARDAMNLSGRRGSKGHMNYEGPGMARKFSAPGQLCISMTSNLGGSAPISAASATSLGHFTKSMCPPQQYGFPATPFGAQWSGTGGPAPQPLGQFQPVGTASLQNFNISNLQKSISNPPGSNLRTT

1563 Q9P0K8 Q9H6S3

>Q9P0K8

MASDLESSLTSIDWLPQLTLRATIEKLGSASQAGPPGSSRKCSPGSPTDPNATLSKDEAAVHQDGKPRYSYATLITYAINSSPAKKMTLSEIYRWICDNFPYYKNAGIGWKNSIRHNLSLNKCFRKVPRPRDDPGKGSYWTIDTCPDISRKRRHPPDDDLSQDSPEQEASKSPRGGVAGSGEASLPPEGNPQMSLQSPTSIASYSQGTGSVDGGAVAAGASGRESAEGPPPLYNTNHDFKFSYSEINFQDLSWSFRNLYKSMLEKSSSSSQHGFSSLLGDIPPSNNYYMYQQQQPPPPQQQQQQQQPPQPPPQQSQPQQQQAPAQGPSAVGGAPPLHTPSTDGCTPPGGKQAGAEGYGPPPVMAMHPPPLQHGGYHPHQHHPHSHPAQQPPPPQPQAQGQAPINNTGFAFPSDWCSNIDSLKESFKMVNRLNWSSIEQSQFSELMESLRQAEQKNWTLDQHHIANLCDSLNHFLTQTGHVPPQGGTHRPPAPARIADSCALTSGKQESAMSQVNSYGHPQAPHLYPGPSPMYPIPTQDSAGYNRPAHHMVPRPSVPPPGANEEIPDDFDWDLIT

>Q9H6S3

MSQSGAVSCCPGATNGSLGRSDGVAKMSPKDLFEQRKKYSNSNVIMHETSQYHVQHLATFIMDKSEAITSVDDAIRKLVQLSSKEKIWTQEMLLQVNDQSLRLLDIESQEELEDFPLPTVQRSQTVLNQLRYPSVLLLVCQDSEQSKPDVHFFHCDEVEAELVHEDIESALADCRLGKKMRPQTLKGHQEKIRQRQSILPPPQGPAPIPFQHRGGDSPEAKNRVGPQVPLSEPGFRRRESQEEPRAVLAQKIEKETQILNCALDDIEWFVARLQKAAEAFKQLNQRKKGKKKGKKAPAEGVLTLRARPPSEGEFIDCFQKIKLAINLLAKLQKHIQNPSAAELVHFLFGPLDLIVNTCSGPDIARSVSCPLLSRDAVDFLRGHLVPKEMSLWESLGESWMRPRSEWPREPQVPLYVPKFHSGWEPPVDVLQEAPWEVEGLASAPIEEVSPVSRQSIRNSQKHSPTSEPTPPGDALPPVSSPHTHRGYQPTPAMAKYVKILYDFTARNANELSVLKDEVLEVLEDGRQWWKLRSRSGQAGYVPCNILGEARPEDAGAPFEQAGQKYWGPASPTHKLPPSFPGNKDELMQHMDEVNDELIRKISNIRAQPQRHFRVERSQPVSQPLTYESGPDEVRAWLEAKAFSPRIVENLGILTGPQLFSLNKEELKKVCGEEGVRVYSQLTMQKAFLEKQQSGSELEELMNKFHSMNQRRGEDS

1564 NP_005850.1 NP_001386.1

>NP_005850.1

MADRDSGSEQGGAALGSGGSLGHPGSGSGSGGGGGGGGGGGGSGGGGGGAPGGLQHETQELASKRVDIQNKRFYLDVKQNAKGRFLKIAEVGAGGNKSRLTLSMSVAVEFRDYLGDFIEHYAQLGPSQPPDLAQAQDEPRRALKSEFLVRENRKYYMDLKENQRGRFLRIRQTVNRGPGLGSTQGQTIALPAQGLIEFRDALAKLIDDYGVEEEPAELPEGTSLTVDNKRFFFDVGSNKYGVFMRVSEVKPTYRNSITVPYKVWAKFGHTFCKYSEEMKKIQEKQREKRAACEQLHQQQQQQQEETAAATLLLQGEEEGEED

>NP_001386.1

MEGLGRSCLWLRRELSPPRPRLLLLDCRSRELYESARIGGALSVALPALLLRRLRRGSLSVRALLPGPPLQPPPPAPVLLYDQGGGRRRRGEAEAEAEEWEAESVLGTLLQKLREEGYLAYYLQGGFSRFQAECPHLCETSLAGRAGSSMAPVPGPVPVVGLGSLCLGSDCSDAESEADRDSMSCGLDSEGATPPPVGLRASFPVQILPNLYLGSARDSANLESLAKLGIRYILNVTPNLPNFFEKNGDFHYKQIPISDHWSQNLSRFFPEAIEFIDEALSQNCGVLVHCLAGVSRSVTVTVAYLMQKLHLSLNDAYDLVKRKKSNISPNFNFMGQLLDFERSLRLEERHSQEQGSGGQASAASNPPSFFTTPTSDGAFELAPT

1565 NP_005739.2 NP_115865.1

>NP_005739.2

MGDKKSPTRPKRQPKPSSDEGYWDCSVCTFRNSAEAFKCMMCDVRKGTSTRKPRPVSQLVAQQVTQQFVPPTQSKKEKKDKVEKEKSEKETTSKKNSHKKTRPRLKNVDRSSAQHLEVTVGDLTVIITDFKEKTKSPPASSAASADQHSQSGSSSDNTERGMSRSSSPRGEASSLNGESH

>NP_115865.1

MPRYELALILKAMQRPETAATLKRTIEALMDRGAIVRDLENLGERALPYRISAHSQQHNRGGYFLVDFYAPTAAVESMVEHLSRDIDVIRGNIVKHPLTQELKECEGIVPVPLAEKLYSTKKRKK

1566 Q8NFU5 Q9BU02

>Q8NFU5

MATEPPSPLRVEAPGPPEMRTSPAIESTPEGTPQPAGGRLRFLNGCVPLSHQVAGHMYGKDKVGILQHPDGTVLKQLQPPPRGPRELEFYNMVYAADCFDGVLLELRKYLPKYYGIWSPPTAPNDLYLKLEDVTHKFNKPCIMDVKIGQKSYDPFASSEKIQQQVSKYPLMEEIGFLVLGMRVYHVHSDSYETENQHYGRSLTKETIKDGVSRFFHNGYCLRKDAVAASIQKIEKILQWFENQKQLNFYASSLLFVYEGSSQPTTTKLNDRTLAEKFLSKGQLSDTEVLEYNNNFHVLSSTANGKIESSVGKSLSKMYARHRKIYTKKHHSQTSLKVENLEQDNGWKSMSQEHLNGNVLSQLEKVFYHLPTGCQEIAEVEVRMIDFAHVFPSNTIDEGYVYGLKHLISVLRSILDN

>Q9BU02

MAQGLIEVERKFLPGPGTEERLQELGGTLEYRVTFRDTYYDTPELSLMQADHWLRRREDSGWELKCPGAAGVLGPHTEYKELTAEPTIVAQLCKVLRADGLGAGDVAAVLGPLGLQEVASFVTKRSAWKLVLLGADEEEPQLRVDLDTADFGYAVGEVEALVHEEAEVPTALEKIHRLSSMLGVPAQETAPAKLIVYLQRFRPQDYQRLLEVNSSRERPQETEDPDHCLG

1567 Q96A08 Q7L0Y3

>Q96A08

MPEVSSKGATISKKGFKKAVVKTQKKEGKKRKRTRKESYSIYIYKVLKQVHPDTGISSKAMSIMNSFVTDIFERIASEASRLAHYSKRSTISSREIQTAVRLLLPGELAKHAVSEGTKAVTKYTSSK

>Q7L0Y3

MAAFLKMSVSVNFFRPFTRFLVPFTLHRKRNNLTILQRYMSSKIPAVTYPKNESTPPSEELELDKWKTTMKSSVQEECVSTISSSKDEDPLAATREFIEMWRLLGREVPEHITEEELKTLMECVSNTAKKKYLKYLYTKEKVKKARQIKKEMKAAAREEAKNIKLLETTEEDKQKNFLFLRLWDRNMDIAMGWKGAQAMQFGQPLVFDMAYENYMKRKELQNTVSQLLESEGWNRRNVDPFHIYFCNLKIDGALHRELVKRYQEKWDKLLLTSTEKSHVDLFPKDSIIYLTADSPNVMTTFRHDKVYVIGSFVDKSMQPGTSLAKAKRLNLATECLPLDKYLQWEIGNKNLTLDQMIRILLCLKNNGNWQEALQFVPKRKHTGFLEISQHSQEFINRLKKAKT

1568 NP_002688.2 NP_006576.2

>NP_002688.2

MNNPSETSKPSMESGDGNTGTQTNGLDFQKQPVPVGGAISTAQAQAFLGHLHQVQLAGTSLQAAAQSLNVQSKSNEESGDSQQPSQPSQQPSVQAAIPQTQLMLAGGQITGLTLTPAQQQLLLQQAQAQAQLLAAAVQQHSASQQHSAAGATISASAATPMTQIPLSQPIQIAQDLQQLQQLQQQNLNLQQFVLVHPTTNLQPAQFIISQTPQGQQGLLQAQNLLTQLPQQSQANLLQSQPSITLTSQPATPTRTIAATPIQTLPQSQSTPKRIDTPSLEEPSDLEELEQFAKTFKQRRIKLGFTQGDVGLAMGKLYGNDFSQTTISRFEALNLSFKNMCKLKPLLEKWLNDAENLSSDSSLSSPSALNSPGIEGLSRRRKKRTSIETNIRVALEKSFLENQKPTSEEITMIADQLNMEKEVIRVWFCNRRQKEKRINPPSSGGTSSSPIKAIFPSPTSLVATTPSLVTSSAATTLTVSPVLPLTSAAVTNLSVTGTSDTTSNNTATVISTAPPASSAVTSPSLSPSPSASASTSEASSASETSTTQTTSTPLSSPLGTSQVMVTASGLQTAAAAALQGAAQLPANASLAAMAAAAGLNPSLMAPSQFAAGGALLSLNPGTLSGALSPALMSNSTLATIQALASGGSLPITSLDATGNLVFANAGGAPNIVTAPLFLNPQNLSLLTSNPVSLVSAAAASAGNSAPVASLHATSTSAESIQNSLFTVASASGAASTTTTASKAQ

>NP_006576.2

MALHVPKAPGFAQMLKEGAKHFSGLEEAVYRNIQACKELAQTTRTAYGPNGMNKMVINHLEKLFVTNDAATILRELEVQHPAAKMIVMASHMQEQEVGDGTNFVLVFAGALLELAEELLRIGLSVSEVIEGYEIACRKAHEILPNLVCCSAKNLRDIDEVSSLLRTSIMSKQYGNEVFLAKLIAQACVSIFPDSGHFNVDNIRVCKILGSGISSSSVLHGMVFKKETEGDVTSVKDAKIAVYSCPFDGMITETKGTVLIKTAEELMNFSKGEENLMDAQVKAIADTGANVVVTGGKVADMALHYANKYNIMLVRLNSKWDLRRLCKTVGATALPRLTPPVLEEMGHCDSVYLSEVGDTQVVVFKHEKEDGAISTIVLRGSTDNLMDDIERAVDDGVNTFKVLTRDKRLVPGGGATEIELAKQITSYGETCPGLEQYAIKKFAEAFEAIPRALAENSGVKANEVISKLYAVHQEGNKNVGLDIEAEVPAVKDMLEAGILDTYLGKYWAIKLATNAAVTVLRVDQIIMAKPAGGPKPPSGKKDWDDDQND

1569 NP_068809.1 NP_001503.1

>NP_068809.1

MSDNEDNFDGDDFDDVEEDEGLDDLENAEEEGQENVEILPSGERPQANQKRITTPYMTKYERARVLGTRALQIAMCAPVMVELEGETDPLLIAMKELKARKIPIIIRRYLPDGSYEDWGVDELIITD

>NP_001503.1

MAARPKLHYPNGRGRMESVRWVLAAAGVEFDEEFLETKEQLYKLQDGNHLLFQQVPMVEIDGMKLVQTRSILHYIADKHNLFGKNLKERTLIDMYVEGTLDLLELLIMHPFLKPDDQQKEVVNMAQKAIIRYFPVFEKILRGHGQSFLVGNQLSLADVILLQTILALEEKIPNILSAFPFLQEYTVKLSNIPTIKRFLEPGSKKKPPPDEIYVRTVYNIFRP

1570 NP_003914.1 Q9H0U6

>NP_003914.1

MGPCSGSRLGPPEAESPSQPPKRRKKRYLRHDKPPYTYLAMIALVIQAAPSRRLKLAQIIRQVQAVFPFFREDYEGWKDSIRHNLSSNRCFRKVPKDPAKPQAKGNFWAVDVSLIPAEALRLQNTALCRRWQNGGARGAFAKDLGPYVLHGRPYRPPSPPPPPSEGFSIKSLLGGSGEGAPWPGLAPQSSPVPAGTGNSGEEAVPTPPLPSSERPLWPLCPLPGPTRVEGETVQGGAIGPSTLSPEPRAWPLHLLQGTAVPGGRSSGGHRASLWGQLPTSYLPIYTPNVVMPLAPPPTSCPQCPSTSPAYWGVAPETRGPPGLLCDLDALFQGVPPNKSIYDVWVSHPRDLAAPGPGWLLSWCSL

>Q9H0U6

MALRSRFWGLFSVCRNPGCRFAALSTSSEPAAKPEVDPVENEAVAPEFTNRNPRNLELLSVARKERGWRTVFPSREFWHRLRVIRTQHHVEALVEHQNGKVVVSASTREWAIKKHLYSTRNVVACESIGRVLAQRCLEAGINFMVYQPTPWEAASDSMKRLQSAMTEGGVVLREPQRIYE

1571 NP_612565.1 NP_075066.1

>NP_612565.1

MKSARAKTPRKPTVKKGSQTNLKDPVGVYCRVRPLGFPDQECCIEVINNTTVQLHTPEGYRLNRNGDYKETQYSFKQVFGTHTTQKELFDVVANPLVNDLIHGKNGLLFTYGVTGSGKTHTMTGSPGEGGLLPRCLDMIFNSIGSFQAKRYVFKSNDRNSMDIQCEVDALLERQKREAMPNPKTSSSKRQVDPEFADMITVQEFCKAEEVDEDSVYGVFVSYIEIYNNYIYDLLEEVPFDPIKPKPPQSKLLREDKNHNMYVAGCTEVEVKSTEEAFEVFWRGQKKRRIANTHLNRESSRSHSVFNIKLVQAPLDADGDNVLQEKEQITISQLSLVDLAGSERTNRTRAEGNRLREAGNINQSLMTLRTCMDVLRENQMYGTNKMVPYRDSKLTHLFKNYFDGEGKVRMIVCVNPKAEDYEENLQVMRFAEVTQEVEVARPVDKAICGLTPGRRYRNQPRGPVGNEPLVTDVVLQSFPPLPSCEILDINDEQTLPRLIEALEKRHNLRQMMIDEFNKQSNAFKALLQEFDNAVLSKENHMQGKLNEKEKMISGQKLEIERLEKKNKTLEYKIEILEKTTTIYEEDKRNLQQELETQNQKLQRQFSDKRRLEARLQGMVTETTMKWEKECERRVAAKQLEMQNKLWVKDEKLKQLKAIVTEPKTEKPERPSRERDREKVTQRSVSPSPVPLSSNYIAQISNGQQLMSQPQLHRRSNSCSSISVASCISEWEQKIPTYNTPLKVTSIARRRQQEPGQSKTCIVSDRRRGMYWTEGREVVPTFRNEIEIEEDHCGRLLFQPDQNAPPIRLRHRRSRSAGDRWVDHKPASNMQTETVMQPHVPHAITVSVANEKALAKCEKYMLTHQELASDGEIETKLIKGDIYKTRGGGQSVQFTDIETLKQESPNGSRKRRSSTVAPAQPDGAESEWTDVETRCSVAVEMRAGSQLGPGYQHHAQPKRKKP

>NP_075066.1

MASGLVRLLQQGHRCLLAPVAPKLVPPVRGVKKGFRAAFRFQKELERQRLLRCPPPPVRRSEKPNWDYHAEIQAFGHRLQENFSLDLLKTAFVNSCYIKSEEAKRQQLGIEKEAVLLNLKSNQELSEQGTSFSQTCLTQFLEDEYPDMPTEGIKNLVDFLTGEEVVCHVARNLAVEQLTLSEEFPVPPAVLQQTFFAVIGALLQSSGPERTALFIRDFLITQMTGKELFEMWKIINPMGLLVEELKKRNVSAPESRLTRQSGGTTALPLYFVGLYCDKKLIAEGPGETVLVAEEEAARVALRKLYGFTENRRPWNYSKPKETLRAEKSITAS

1572 P37275 Q01064

>P37275

MADGPRCKRRKQANPRRNNVTNYNTVVETNSDSDDEDKLHIVEEESVTDAADCEGVPEDDLPTDQTVLPGRSSEREGNAKNCWEDDRKEGQEILGPEAQADEAGCTVKDDECESDAENEQNHDPNVEEFLQQQDTAVIFPEAPEEDQRQGTPEASGHDENGTPDAFSQLLTCPYCDRGYKRFTSLKEHIKYRHEKNEDNFSCSLCSYTFAYRTQLERHMTSHKSGRDQRHVTQSGCNRKFKCTECGKAFKYKHHLKEHLRIHSGEKPYECPNCKKRFSHSGSYSSHISSKKCISLIPVNGRPRTGLKTSQCSSPSLSASPGSPTRPQIRQKIENKPLQEQLSVNQIKTEPVDYEFKPIVVASGINCSTPLQNGVFTGGGPLQATSSPQGMVQAVVLPTVGLVSPISINLSDIQNVLKVAVDGNVIRQVLENNQANLASKEQETINASPIQQGGHSVISAISLPLVDQDGTTKIIINYSLEQPSQLQVVPQNLKKENPVATNSCKSEKLPEDLTVKSEKDKSFEGGVNDSTCLLCDDCPGDINALPELKHYDLKQPTQPPPLPAAEAEKPESSVSSATGDGNLSPSQPPLKNLLSLLKAYYALNAQPSAEELSKIADSVNLPLDVVKKWFEKMQAGQISVQSSEPSSPEPGKVNIPAKNNDQPQSANANEPQDSTVNLQSPLKMTNSPVLPVGSTTNGSRSSTPSPSPLNLSSSRNTQGYLYTAEGAQEEPQVEPLDLSLPKQQGELLERSTITSVYQNSVYSVQEEPLNLSCAKKEPQKDSCVTDSEPVVNVIPPSANPINIAIPTVTAQLPTIVAIADQNSVPCLRALAANKQTILIPQVAYTYSTTVSPAVQEPPLKVIQPNGNQDERQDTSSEGVSNVEDQNDSDSTPPKKKMRKTENGMYACDLCDKIFQKSSSLLRHKYEHTGKRPHECGICKKAFKHKHHLIEHMRLHSGEKPYQCDKCGKRFSHSGSYSQHMNHRYSYCKREAEERDSTEQEEAGPEILSNEHVGARASPSQGDSDERESLTREEDEDSEKEEEEEDKEMEELQEEKECEKPQGDEEEEEEEEEVEEEEVEEAENEGEEAKTEGLMKDDRAESQASSLGQKVGESSEQVSEEKTNEA

>Q01064

MELSPRSPPEMLEESDCPSPLELKSAPSKKMWIKLRSLLRYMVKQLENGEINIEELKKNLEYTASLLEAVYIDETRQILDTEDELQELRSDAVPSEVRDWLASTFTQQARAKGRRAEEKPKFRSIVHAVQAGIFVERMFRRTYTSVGPTYSTAVLNCLKNLDLWCFDVFSLNQAADDHALRTIVFELLTRHNLISRFKIPTVFLMSFLDALETGYGKYKNPYHNQIHAADVTQTVHCFLLRTGMVHCLSEIELLAIIFAAAIHDYEHTGTTNSFHIQTKSECAIVYNDRSVLENHHISSVFRLMQDDEMNIFINLTKDEFVELRALVIEMVLATDMSCHFQQVKTMKTALQQLERIDKPKALSLLLHAADISHPTKQWLVHSRWTKALMEEFFRQGDKEAELGLPFSPLCDRTSTLVAQSQIGFIDFIVEPTFSVLTDVAEKSVQPLADEDSKSKNQPSFQWRQPSLDVEVGDPNPDVVSFRSTWVKRIQENKQKWKERAASGITNQMSIDELSPCEEEAPPSPAEDEHNQNGNLD

1573 NP_066986.1 NP_055683.3

>NP_066986.1

MNTLQGPVSFKDVAVDFTQEEWQQLDPDEKITYRDVMLENYSHLVSVGYDTTKPNVIIKLEQGEEPWIMGGEFPCQHSPEAWRVDDLIERIQENEDKHSRQAACINSKTLTEEKENTFSQIYMETSLVPSSIIAHNCVSCGKNLESISQLISSDGSYARTKPDECNECGKTYHGEKMCEFNQNGDTYSHNEENILQKISILEKPFEYNECMEALDNEAVFIAHKRAYIGEKPYEWNDSGPDFIQMSNFNAYQRSQMEMKPFECSECGKSFCKKSKFIIHQRAHTGEKPYECNVCGKSFSQKGTLTVHRRSHLEEKPYKCNECGKTFCQKLHLTQHLRTHSGEKPYECSECGKTFCQKTHLTLHQRNHSGERPYPCNECGKSFSRKSALSDHQRTHTGEKLYKCNECGKSYYRKSTLITHQRTHTGEKPYQCSECGKFFSRVSYLTIHYRSHLEEKPYECNECGKTFNLNSAFIRHRKVHTEEKSHECSECGKFSQLYLTDHHTAHLEEKPYECNECGKTFLVNSAFDGHQPLPKGEKSYECNVCGKLFNELSYYTEHYRSHSEEKPYGCSECGKTFSHNSSLFRHQRVHTGEKPYECYECGKFFSQKSYLTIHHRIHSGEKPYECSKCGKVFSRMSNLTVHYRSHSGEKPYECNECGKVFSQKSYLTVHYRTHSGEKPYECNECGKKFHHRSAFNSHQRIHRRGNMNVLDVENL

>NP_055683.3

MPLSSPNAAATASDMDKNSGSNSSSASSGSSKGQQPPRSASAGPAGESKPKSDGKNSSGSKRYNRKRELSYPKNESFNNQSRRSSSQKSKTFNKMPPQRGGGSSKLFSSSFNGGRRDEVAEAQRAEFSPAQFSGPKKINLNHLLNFTFEPRGQTGHFEGSGHGSWGKRNKWGHKPFNKELFLQANCQFVVSEDQDYTAHFADPDTLVNWDFVEQVRICSHEVPSCPICLYPPTAAKITRCGHIFCWACILHYLSLSEKTWSKCPICYSSVHKKDLKSVVATESHQYVVGDTITMQLMKREKGVLVALPKSKWMNVDHPIHLGDEQHSQYSKLLLASKEQVLHRVVLEEKVALEQQLAEEKHTPESCFIEAAIQELKTREEALSGLAGSRREVTGVVAALEQLVLMAPLAKESVFQPRKGVLEYLSAFDEETTEVCSLDTPSRPLALPLVEEEEAVSEPEPEGLPEACDDLELADDNLKEGTICTESSQQEPITKSGFTRLSSSPCYYFYQAEDGQHMFLHPVNVRCLVREYGSLERSPEKISATVVEIAGYSMSEDVRQRHRYLSHLPLTCEFSICELALQPPVVSKETLEMFSDDIEKRKRQRQKKAREERRRERRIEIEENKKQGKYPEVHIPLENLQQFPAFNSYTCSSDSALGPTSTEGHGALSISPLSRSPGSHADFLLTPLSPTASQGSPSFCVGSLEEDSPFPSFAQMLRVGKAKADVWPKTAPKKDENSLVPPAPVDSDGESDNSDRVPVPSFQNSFSQAIEAAFMKLDTPATSDPLSEEKGGKKRKKQKQKLLFSTSVVHTK

1574 NP_001197.1 NP_004127.1

>NP_001197.1

MSAAAYMDFVAAQCLVSISNRAAVPEHGVAPDAERLRLPEREVTKEHGDPGDTWKDYCTLVTIAKSLLDLNKYRPIQTPSVCSDSLESPDEDMGSDSDVTTESGSSPSHSPEERQDPGSAPSPLSLLHPGVAAKGKHASEKRHKCPYSGCGKVYGKSSHLKAHYRVHTGERPFPCTWPDCLKKFSRSDELTRHYRTHTGEKQFRCPLCEKRFMRSDHLTKHARRHTEFHPSMIKRSKKALANAL

>NP_004127.1

MDAPKAGYAFEYLIETLNDSSHKKFFDVSKLGTKYDVLPYSIRVLLEAAVRNCDGFLMKKEDVMNILDWKTKQSNVEVPFFPARVLLQDFTGIPAMVDFAAMREAVKTLGGDPEKVHPACPTDLTVDHSLQIDFSKCAIQNAPNPGGGDLQKAGKLSPLKVQPKKLPCRGQTTCRGSCDSGELGRNSGTFSSQIENTPILCPFHLQPVPEPETVLKNQEVEFGRNRERLQFFKWSSRVFKNVAVIPPGTGMAHQINLEYLSRVVFEEKDLLFPDSVVGTDSHITMVNGLGILGWGVGGIETEAVMLGLPVSLTLPEVVGCELTGSSNPFVTSIDVVLGITKHLRQVGVAGKFVEFFGSGVSQLSIVDRTTIANMCPEYGAILSFFPVDNVTLKHLEHTGFSKAKLESMETYLKAVKLFRNDQNSSGEPEYSQVIQINLNSIVPSVSGPKRPQDRVAVTDMKSDFQACLNEKVGFKGFQIAAEKQKDIVSIHYEGSEYKLSHGSVVIAAVISCTNNCNPSVMLAAGLLAKKAVEAGLRVKPYIRTSLSPGSGMVTHYLSSSGVLPYLSKLGFEIVGYGCSTCVGNTAPLSDAVLNAVKQGDLVTCGILSGNKNFEGRLCDCVRANYLASPPLVVAYAIAGTVNIDFQTEPLGTDPTGKNIYLHDIWPSREEVHRVEEEHVILSMFKALKDKIEMGNKRWNSLEAPDSVLFPWDLKSTYIRCPSFFDKLTKEPIALQAIENAHVLLYLGDSVTTDHISPAGSIARNSAAAKYLTNRGLTPREFNSYGARRGNDAVMTRGTFANIKLFNKFIGKPAPKTIHFPSGQTLDVFEAAELYQKEGIPLIILAGKKYGSGNSRDWAAKGPYLLGVKAVLAESYEKIHKDHLIGIGIAPLQFLPGENADSLGLSGRETFSLTFPEELSPGITLNIQTSTGKVFSVIASFEDDVEITLYKHGGLLNFVARKFS

1575 O00462 NP_758958.3

>O00462

MRLHLLLLLALCGAGTTAAELSYSLRGNWSICNGNGSLELPGAVPGCVHSALFQQGLIQDSYYRFNDLNYRWVSLDNWTYSKEFKIPFEISKWQKVNLILEGVDTVSKILFNEVTIGETDNMFNRYSFDITNVVRDVNSIELRFQSAVLYAAQQSKAHTRYQVPPDCPPLVQKGECHVNFVRKEQCSFSWDWGPSFPTQGIWKDVRIEAYNICHLNYFTFSPIYDKSAQEWNLEIESTFDVVSSKPVGGQVIVAIPKLQTQQTYSIELQPGKRIVELFVNISKNITVETWWPHGHGNQTGYNMTVLFELDGGLNIEKSAKVYFRTVELIEEPIKGSPGLSFYFKINGFPIFLKGSNWIPADSFQDRVTSELLRLLLQSVVDANMNTLRVWGGGIYEQDEFYELCDELGIMVWQDFMFACALYPTDQGFLDSVTAEVAYQIKRLKSHPSIIIWSGNNENEEALMMNWYHISFTDRPIYIKDYVTLYVKNIRELVLAGDKSRPFITSSPTNGAETVAEAWVSQNPNSNYFGDVHFYDYISDCWNWKVFPKARFASEYGYQSWPSFSTLEKVSSTEDWSFNSKFSLHRQHHEGGNKQMLYQAGLHFKLPQSTDPLRTFKDTIYLTQVMQAQCVKTETEFYRRSRSEIVDQQGHTMGALYWQLNDIWQAPSWASLEYGGKWKMLHYFAQNFFAPLLPVGFENENTFYIYGVSDLHSDYSMTLSVRVHTWSSLEPVCSRVTERFVMKGGEAVCLYEEPVSELLRRCGNCTRESCVVSFYLSADHELLSPTNYHFLSSPKEAVGLCKAQITAIISQQGDIFVFDLETSAVAPFVWLDVGSIPGRFSDNGFLMTEKTRTILFYPWEPTSKNELEQSFHVTSLTDIY

>NP_758958.3

MLEKLMGADSLQLFRSRYTLGKIYFIGFQRSILLSKSENSLNSIAKETEEGRETVTRKEGWKRRHEDGYLEMAQRHLQRSLCPWVSYLPQPYAELEEVSSHVGKVFMARNYEFLAYEASKDRRQPLERMWTCNYNQQKDQSCNHKEITSTKAE

1576 P02545 Q9H4K7

>P02545

METPSQRRATRSGAQASSTPLSPTRITRLQEKEDLQELNDRLAVYIDRVRSLETENAGLRLRITESEEVVSREVSGIKAAYEAELGDARKTLDSVAKERARLQLELSKVREEFKELKARNTKKEGDLIAAQARLKDLEALLNSKEAALSTALSEKRTLEGELHDLRGQVAKLEAALGEAKKQLQDEMLRRVDAENRLQTMKEELDFQKNIYSEELRETKRRHETRLVEIDNGKQREFESRLADALQELRAQHEDQVEQYKKELEKTYSAKLDNARQSAERNSNLVGAAHEELQQSRIRIDSLSAQLSQLQKQLAAKEAKLRDLEDSLARERDTSRRLLAEKEREMAEMRARMQQQLDEYQELLDIKLALDMEIHAYRKLLEGEEERLRLSPSPTSQRSRGRASSHSSQTQGGGSVTKKRKLESTESRSSFSQHARTSGRVAVEEVDEEGKFVRLRNKSNEDQSMGNWQIKRQNGDDPLLTYRFPPKFTLKAGQVVTIWAAGAGATHSPPTDLVWKAQNTWGCGNSLRTALINSTGEEVAMRKLVRSVTVVEDDEDEDGDDLLHHHHGSHCSSSGDPAEYNLRSRTVLCGTCGQPADKASASGSGAQVGGPISSGSSASSVTVTRSYRSVGGSGGGSFGDNLVTRSYLLGNSSPRTQSPQNCSIM

>Q9H4K7

MAPARCFSARLRTVFQGVGHWALSTWAGLKPSRLLPQRASPRLLSVGRADLAKHQELPGKKLLSEKKLKRYFVDYRRVLVCGGNGGAGASCFHSEPRKEFGGPDGGDGGNGGHVILRVDQQVKSLSSVLSRYQGFSGEDGGSKNCFGRSGAVLYIRVPVGTLVKEGGRVVADLSCVGDEYIAALGGAGGKGNRFFLANNNRAPVTCTPGQPGQQRVLHLELKTVAHAGMVGFPNAGKSSLLRAISNARPAVASYPFTTLKPHVGIVHYEGHLQIAVADIPGIIRGAHQNRGLGSAFLRHIERCRFLLFVVDLSQPEPWTQVDDLKYELEMYEKGLSARPHAIVANKIDLPEAQANLSQLRDHLGQEVIVLSALTGENLEQLLLHLKVLYDAYAEAELGQGRQPLRW

1577 P09067 NP_055300.1

>P09067

MSSYFVNSFSGRYPNGPDYQLLNYGSGSSLSGSYRDPAAMHTGSYGYNYNGMDLSVNRSSASSSHFGAVGESSRAFPAPAQEPRFRQAASSCSLSSPESLPCTNGDSHGAKPSASSPSDQATSASSSANFTEIDEASASSEPEEAASQLSSPSLARAQPEPMATSTAAPEGQTPQIFPWMRKLHISHDMTGPDGKRARTAYTRYQTLELEKEFHFNRYLTRRRRIEIAHALCLSERQIKIWFQNRRMKWKKDNKLKSMSLATAGSAFQP

>NP_055300.1

MPNYKLTYFNMRGRAEIIRYIFAYLDIQYEDHRIEQADWPEIKSTLPFGKIPILEVDGLTLHQSLAIARYLTKNTDLAGNTEMEQCHVDAIVDTLDDFMSCFPWAEKKQDVKEQMFNELLTYNAPHLMQDLDTYLGGREWLIGNSVTWADFYWEICSTTLLVFKPDLLDNHPRLVTLRKKVQAIPAVANWIKRRPQTKL

1578 NP_004955.2 NP_006083.1

>NP_004955.2

MAQTQGTRRKVCYYYDGDVGNYYYGQGHPMKPHRIRMTHNLLLNYGLYRKMEIYRPHKANAEEMTKYHSDDYIKFLRSIRPDNMSEYSKQMQRFNVGEDCPVFDGLFEFCQLSTGGSVASAVKLNKQQTDIAVNWAGGLHHAKKSEASGFCYVNDIVLAILELLKYHQRVLYIDIDIHHGDGVEEAFYTTDRVMTVSFHKYGEYFPGTGDLRDIGAGKGKYYAVNYPLRDGIDDESYEAIFKPVMSKVMEMFQPSAVVLQCGSDSLSGDRLGCFNLTIKGHAKCVEFVKSFNLPMLMLGGGGYTIRNVARCWTYETAVALDTEIPNELPYNDYFEYFGPDFKLHISPSNMTNQNTNEYLEKIKQRLFENLRMLPHAPGVQMQAIPEDAIPEESGDEDEDDPDKRISICSSDKRIACEEEFSDSEEEGEGGRKNSSNFKKAKRVKTEDEKEKDPEEKKEVTEEEKTKEEKPEAKGVKEEVKLA

>NP_006083.1

MEEQGHSEMEIIPSESHPHIQLLKSNRELLVTHIRNTQCLVDNLLKNDYFSAEDAEIVCACPTQPDKVRKILDLVQSKGEEVSEFFLYLLQQLADAYVDLRPWLLEIGFSPSLLTQSKVVVNTDPVSRYTQQLRHHLGRDSKFVLCYAQKEELLLEEIYMDTIMELVGFSNESLGSLNSLACLLDHTTGILNEQGETIFILGDAGVGKSMLLQRLQSLWATGRLDAGVKFFFHFRCRMFSCFKESDRLCLQDLLFKHYCYPERDPEEVFAFLLRFPHVALFTFDGLDELHSDLDLSRVPDSSCPWEPAHPLVLLANLLSGKLLKGASKLLTARTGIEVPRQFLRKKVLLRGFSPSHLRAYARRMFPERALQDRLLSQLEANPNLCSLCSVPLFCWIIFRCFQHFRAAFEGSPQLPDCTMTLTDVFLLVTEVHLNRMQPSSLVQRNTRSPVETLHAGRDTLCSLGQVAHRGMEKSLFVFTQEEVQASGLQERDMQLGFLRALPELGPGGDQQSYEFFHLTLQAFFTAFFLVLDDRVGTQELLRFFQEWMPPAGAATTSCYPPFLPFQCLQGSGPAREDLFKNKDHFQFTNLFLCGLLSKAKQKLLRHLVPAAALRRKRKALWAHLFSSLRGYLKSLPRVQVESFNQVQAMPTFIWMLRCIYETQSQKVGQLAARGICANYLKLTYCNACSADCSALSFVLHHFPKRLALDLDNNNLNDYGVRELQPCFSRLTVLRLSVNQITDGGVKVLSEELTKYKIVTYLGLYNNQITDVGARYVTKILDECKGLTHLKLGKNKITSEGGKYLALAVKNSKSISEVGMWGNQVGDEGAKAFAEALRNHPSLTTLSLASNGISTEGGKSLARALQQNTSLEILWLTQNELNDEVAESLAEMLKVNQTLKHLWLIQNQITAKGTAQLADALQSNTGITEICLNGNLIKPEEAKVYEDEKRIICF

1579 NP_150093.1 Q58A44

>NP_150093.1

MADGDSGSERGGGGGPCGFQPASRGGGEQETQELASKRLDIQNKRFYLDVKQNAKGRFLKIAEVGAGGSKSRLTLSMAVAAEFRDSLGDFIEHYAQLGPSSPEQLAAGAEEGGGPRRALKSEFLVRENRKYYLDLKENQRGRFLRIRQTVNRGGGGFGAGPGPGGLQSGQTIALPAQGLIEFRDALAKLIDDYGGEDDELAGGPGGGAGGPGGGLYGELPEGTSITVDSKRFFFDVGCNKYGVFLRVSEVKPSYRNAITVPFKAWGKFGGAFCRYADEMKEIQERQRDKLYERRGGGSGGGEESEGEEVDED

>Q58A44

MWILSNLMGTSEEGNLLSTVSPTVKALFGKTRVSPIFPFSPRSPFQPLIPRTPGSPWGPVGPASPLGPGFPIGPMGPGKPVGPKGPMLPLGPSGPVGPTSPLFPFCP

1580 P34059 Q9UBW5

>P34059

MAAVVAATRWWQLLLVLSAAGMGASGAPQPPNILLLLMDDMGWGDLGVYGEPSRETPNLDRMAAEGLLFPNFYSANPLCSPSRAALLTGRLPIRNGFYTTNAHARNAYTPQEIVGGIPDSEQLLPELLKKAGYVSKIVGKWHLGHRPQFHPLKHGFDEWFGSPNCHFGPYDNKARPNIPVYRDWEMVGRYYEEFPINLKTGEANLTQIYLQEALDFIKRQARHHPFFLYWAVDATHAPVYASKPFLGTSQRGRYGDAVREIDDSIGKILELLQDLHVADNTFVFFTSDNGAALISAPEQGGSNGPFLCGKQTTFEGGMREPALAWWPGHVTAGQVSHQLGSIMDLFTTSLALAGLTPPSDRAIDGLNLLPTLLQGRLMDRPIFYYRGDTLMAATLGQHKAHFWTWTNSWENFRQGIDFCPGQNVSGVTTHNLEDHTKLPLIFHLGRDPGERFPLSFASAEYQEALSRITSVVQQHQEALVPAQPQLNVCNWAVMNWAPPGCEKLGKCLTPPESIPKKCLWSH

>Q9UBW5

MAEGKAGGAAGLFAKQVQKKFSRAQEKVLQKLGKAVETKDERFEQSASNFYQQQAEGHKLYKDLKNFLSAVKVMHESSKRVSETLQEIYSSEWDGHEELKAIVWNNDLLWEDYEEKLADQAVRTMEIYVAQFSEIKERIAKRGRKLVDYDSARHHLEAVQNAKKKDEAKTAKAEEEFNKAQTVFEDLNQELLEELPILYNSRIGCYVTIFQNISNLRDVFYREMSKLNHNLYEVMSKLEKQHSNKVFVVKGLSSSSRRSLVISPPVRTATVSSPLTSPTSPSTLSLKSESESVSATEDLAPDAAQGEDNSEIKELLEEEEIEKEGSEASSSEEDEPLPACNGPAQAQPSPTTERAKSQEEVLPSSTTPSPGGALSPSGQPSSSATEVVLRTRTASEGSEQPKKRASIQRTSAPPSRPPPPRATASPRPSSGNIPSSPTASGGGSPTSPRASLGTGTASPRTSLEVSPNPEPPEKPVRTPEAKENENIHNQNPEELCTSPTLMTSQVASEPGEAKKMEDKEKDNKLISANSSEGQDQLQVSMVPENNNLTAPEPQEEVSTSENPQL

1581 NP_003063.2 Q9UGC7

>NP_003063.2

MSTPDPPLGGTPRPGPSPGPGPSPGAMLGPSPGPSPGSAHSMMGPSPGPPSAGHPIPTQGPGGYPQDNMHQMHKPMESMHEKGMSDDPRYNQMKGMGMRSGGHAGMGPPPSPMDQHSQGYPSPLGGSEHASSPVPASGPSSGPQMSSGPGGAPLDGADPQALGQQNRGPTPFNQNQLHQLRAQIMAYKMLARGQPLPDHLQMAVQGKRPMPGMQQQMPTLPPPSVSATGPGPGPGPGPGPGPGPAPPNYSRPHGMGGPNMPPPGPSGVPPGMPGQPPGGPPKPWPEGPMANAAAPTSTPQKLIPPQPTGRPSPAPPAVPPAASPVMPPQTQSPGQPAQPAPMVPLHQKQSRITPIQKPRGLDPVEILQEREYRLQARIAHRIQELENLPGSLAGDLRTKATIELKALRLLNFQRQLRQEVVVCMRRDTALETALNAKAYKRSKRQSLREARITEKLEKQQKIEQERKRRQKHQEYLNSILQHAKDFKEYHRSVTGKIQKLTKAVATYHANTEREQKKENERIEKERMRRLMAEDEEGYRKLIDQKKDKRLAYLLQQTDEYVANLTELVRQHKAAQVAKEKKKKKKKKKAENAEGQTPAIGPDGEPLDETSQMSDLPVKVIHVESGKILTGTDAPKAGQLEAWLEMNPGYEVAPRSDSEESGSEEEEEEEEEEQPQAAQPPTLPVEEKKKIPDPDSDDVSEVDARHIIENAKQDVDDEYGVSQALARGLQSYYAVAHAVTERVDKQSALMVNGVLKQYQIKGLEWLVSLYNNNLNGILADEMGLGKTIQTIALITYLMEHKRINGPFLIIVPLSTLSNWAYEFDKWAPSVVKVSYKGSPAARRAFVPQLRSGKFNVLLTTYEYIIKDKHILAKIRWKYMIVDEGHRMKNHHCKLTQVLNTHYVAPRRLLLTGTPLQNKLPELWALLNFLLPTIFKSCSTFEQWFNAPFAMTGEKVDLNEEETILIIRRLHKVLRPFLLRRLKKEVEAQLPEKVEYVIKCDMSALQRVLYRHMQAKGVLLTDGSEKDKKGKGGTKTLMNTIMQLRKICNHPYMFQHIEESFSEHLGFTGGIVQGLDLYRASGKFELLDRILPKLRATNHKVLLFCQMTSLMTIMEDYFAYRGFKYLRLDGTTKAEDRGMLLKTFNEPGSEYFIFLLSTRAGGLGLNLQSADTVIIFDSDWNPHQDLQAQDRAHRIGQQNEVRVLRLCTVNSVEEKILAAAKYKLNVDQKVIQAGMFDQKSSSHERRAFLQAILEHEEQDESRHCSTGSGSASFAHTAPPPAGVNPDLEEPPLKEEDEVPDDETVNQMIARHEEEFDLFMRMDLDRRREEARNPKRKPRLMEEDELPSWIIKDDAEVERLTCEEEEEKMFGRGSRHRKEVDYSDSLTEKQWLKAIEEGTLEEIEEEVRQKKSSRKRKRDSDAGSSTPTTSTRSRDKDDESKKQKKRGRPPAEKLSPNPPNLTKKMKKIVDAVIKYKDSSSGRQLSEVFIQLPSRKELPEYYELIRKPVDFKKIKERIRNHKYRSLNDLEKDVMLLCQNAQTFNLEGSLIYEDSIVLQSVFTSVRQKIEKEDDSEGEESEEEEEGEEEGSESESRSVKVKIKLGRKEKAQDRLKGGRRRPSRGSRAKPVVSDDDSEEEQEEDRSGSGSEED

>Q9UGC7

MRSRVLWGAARWLWPRRAVGPARRPLSSGSPPLEELFTRGGPLRTFLERQAGSEAHLKVRRPELLAVIKLLNEKERELRETEHLLHDENEDLRKLAENEITLCQKEITQLKHQIILLLVPSEETDENDLILEVTAGVGGQEAMLFTSEIFDMYQQYAAFKRWHFETLEYFPSELGGLRHASASIGGSEAYRHMKFEGGVHRVQRVPKTEKQGRVHTSTMTVAILPQPTEINLVINPKDLRIDTKRASGAGGQHVNTTDSAVRIVHLPTGVVSECQQERSQLKNKELAMTKLRAKLYSMHLEEEINKRQNARKIQIGSKGRSEKIRTYNFPQNRVTDHRINKTLHDLETFMQGDYLLDELVQSLKEYADYESLVEIISQKV

1582 Q15306 NP_002070.1

>Q15306

MNLEGGGRGGEFGMSAVSCGNGKLRQWLIDQIDSGKYPGLVWENEEKSIFRIPWKHAGKQDYNREEDAALFKAWALFKGKFREGIDKPDPPTWKTRLRCALNKSNDFEELVERSQLDISDPYKVYRIVPEGAKKGAKQLTLEDPQMSMSHPYTMTTPYPSLPAQQVHNYMMPPLDRSWRDYVPDQPHPEIPYQCPMTFGPRGHHWQGPACENGCQVTGTFYACAPPESQAPGVPTEPSIRSAEALAFSDCRLHICLYYREILVKELTTSSPEGCRISHGHTYDASNLDQVLFPYPEDNGQRKNIEKLLSHLERGVVLWMAPDGLYAKRLCQSRIYWDGPLALCNDRPNKLERDQTCKLFDTQQFLSELQAFAHHGRSLPRFQVTLCFGEEFPDPQRQRKLITAHVEPLLARQLYYFAQQNSGHFLRGYDLPEHISNPEDYHRSIRHSSIQE

>NP_002070.1

MAPPSVFAEVPQAQPVLVFKLTADFREDPDPRKVNLGVGAYRTDDCHPWVLPVVKKVEQKIANDNSLNHEYLPILGLAEFRSCASRLALGDDSPALKEKRVGGVQSLGGTGALRIGADFLARWYNGTNNKNTPVYVSSPTWENHNAVFSAAGFKDIRSYRYWDAEKRGLDLQGFLNDLENAPEFSIVVLHACAHNPTGIDPTPEQWKQIASVMKHRFLFPFFDSAYQGFASGNLERDAWAIRYFVSEGFEFFCAQSFSKNFGLYNERVGNLTVVGKEPESILQVLSQMEKIVRITWSNPPAQGARIVASTLSNPELFEEWTGNVKTMADRILTMRSELRARLEALKTPGTWNHITDQIGMFSFTGLNPKQVEYLVNEKHIYLLPSGRINVSGLTTKNLDYVATSIHEAVTKIQ

1583 NP_037393.1 NP_000895.1

>NP_037393.1

MAWDMCNQDSESVWSDIECAALVGEDQPLCPDLPELDLSELDVNDLDTDSFLGGLKWCSDQSEIISNQYNNEPSNIFEKIDEENEANLLAVLTETLDSLPVDEDGLPSFDALTDGDVTTDNEASPSSMPDGTPPPQEAEEPSLLKKLLLAPANTQLSYNECSGLSTQNHANHNHRIRTNPAIVKTENSWSNKAKSICQQQKPQRRPCSELLKYLTTNDDPPHTKPTENRNSSRDKCTSKKKSHTQSQSQHLQAKPTTLSLPLTPESPNDPKGSPFENKTIERTLSVELSGTAGLTPPTTPPHKANQDNPFRASPKLKSSCKTVVPPPSKKPRYSESSGTQGNNSTKKGPEQSELYAQLSKSSVLTGGHEERKTKRPSLRLFGDHDYCQSINSKTEILINISQELQDSRQLENKDVSSDWQGQICSSTDSDQCYLRETLEASKQVSPCSTRKQLQDQEIRAELNKHFGHPSQAVFDDEADKTGELRDSDFSNEQFSKLPMFINSGLAMDGLFDDSEDESDKLSYPWDGTQSYSLFNVSPSCSSFNSPCRDSVSPPKSLFSQRPQRMRSRSRSFSRHRSCSRSPYSRSRSRSPGSRSSSRSCYYYESSHYRHRTHRNSPLYVRSRSRSPYSRRPRYDSYEEYQHERLKREEYRREYEKRESERAKQRERQRQKAIEERRVIYVGKIRPDTTRTELRDRFEVFGEIEECTVNLRDDGDSYGFITYRYTCDAFAALENGYTLRRSNETDFELYFCGRKQFFKSNYADLDSNSDDFDPASTKSKYDSLDFDSLLKEAQRSLRR

>NP_000895.1

MAGKKVLIVYAHQEPKSFNGSLKNVAVDELSRQGCTVTVSDLYAMNFEPRATDKDITGTLSNPEVFNYGVETHEAYKQRSLASDITDEQKKVREADLVIFQFPLYWFSVPAILKGWMDRVLCQGFAFDIPGFYDSGLLQGKLALLSVTTGGTAEMYTKTGVNGDSRYFLWPLQHGTLHFCGFKVLAPQISFAPEIASEEERKGMVAAWSQRLQTIWKEEPIPCTAHWHFGQ

1584 NP_003251.2 Q93015

>NP_003251.2

MYPQGRHPTPLQSGQPFKFSILEICDRIKEEFQFLQAQYHSLKLECEKLASEKTEMQRHYVMYYEMSYGLNIEMHKQAEIVKRLSGICAQIIPFLTQEHQQQVLQAVERAKQVTVGELNSLIGQQLQPLSHHAPPVPLTPRPAGLVGGSATGLLALSGALAAQAQLAAAVKEDRAGVEAEGSRVERAPSRSASPSPPESLVEEERPSGPGGGGKQRADEKEPSGPYESDEDKSDYNLVVDEDQPSEPPSPATTPCGKVPICIPARRDLVDSPASLASSLGSPLPRAKELILNDLPASTPASKSCDSSPPQDASTPGPSSASHLCQLAAKPAPSTDSVALRSPLTLSSPFTTSFSLGSHSTLNGDLSVPSSYVSLHLSPQVSSSVVYGRSPVMAFESHPHLRGSSVSSSLPSIPGGKPAYSFHVSADGQMQPVPFPSDALVGAGIPRHARQLHTLAHGEVVCAVTISGSTQHVYTGGKGCVKVWDVGQPGAKTPVAQLDCLNRDNYIRSCKLLPDGRSLIVGGEASTLSIWDLAAPTPRIKAELTSSAPACYALAVSPDAKVCFSCCSDGNIVVWDLQNQTMVRQFQGHTDGASCIDISDYGTRLWTGGLDNTVRCWDLREGRQLQQHDFSSQIFSLGHCPNQDWLAVGMESSNVEILHVRKPEKYQLHLHESCVLSLKFASCGRWFVSTGKDNLLNAWRTPYGASIFQSKESSSVLSCDISRNNKYIVTGSGDKKATVYEVVY

>Q93015

MELILSTSPAELTLDPACQPKLPLDSTCQPEMTFNPGPTELTLDPEHQPEETPAPSLAELTLEPVHRRPELLDACADLINDQWPRSRTSRLHSLGQSSDAFPLCLMLLSPHPTLEAAPVVVGHARLSRVLNQPQSLLVETVVVARALRGRGFGRRLMEGLEVFARARGFRKLHLTTHDQVHFYTHLGYQLGEPVQGLVFTSRRLPATLLNAFPTAPSPRPPRKAPNLTAQAAPRGPKGPPLPPPPPLPECLTISPPVPSGPPSKSLLETQYQNVRGRPIFWMEKDI

1585 NP_008882.2 NP_036205.1

>NP_008882.2

MAALAEEQTEVAVKLEPEGPPTLLPPQAGDGAGEGSGGTTNNGPNGGGGNVAASSSTGGDGGTPKPTVAVSAAAPAGAAPVPAAAPDAGAPHDRQTLLAVLQFLRQSKLREAEEALRREAGLLEEAVAGSGAPGEVDSAGAEVTSALLSRVTASAPGPAAPDPPGTGASGATVVSGSASGPAAPGKVGSVAVEDQPDVSAVLSAYNQQGDPTMYEEYYSGLKHFIECSLDCHRAELSQLFYPLFVHMYLELVYNQHENEAKSFFEKFHGDQECYYQDDLRVLSSLTKKEHMKGNETMLDFRTSKFVLRISRDSYQLLKRHLQEKQNNQIWNIVQEHLYIDIFDGMPRSKQQIDAMVGSLAGEAKREANKSKVFFGLLKEPEIEVPLDDEDEEGENEEGKPKKKKPKKDSIGSKSKKQDPNAPPQNRIPLPELKDSDKLDKIMNMKETTKRVRLGPDCLPSICFYTFLNAYQGLTAVDVTDDSSLIAGGFADSTVRVWSVTPKKLRSVKQASDLSLIDKESDDVLERIMDEKTASELKILYGHSGPVYGASFSPDRNYLLSSSEDGTVRLWSLQTFTCLVGYKGHNYPVWDTQFSPYGYYFVSGGHDRVARLWATDHYQPLRIFAGHLADVNCTRFHPNSNYVATGSADRTVRLWDVLNGNCVRIFTGHKGPIHSLTFSPNGRFLATGATDGRVLLWDIGHGLMVGELKGHTDTVCSLRFSRDGEILASGSMDNTVRLWDAIKAFEDLETDDFTTATGHINLPENSQELLLGTYMTKSTPVVHLHFTRRNLVLAAGAYSPQ

>NP_036205.1

MASMGTLAFDEYGRPFLIIKDQDRKSRLMGLEALKSHIMAAKAVANTMRTSLGPNGLDKMMVDKDGDVTVTNDGATILSMMDVDHQIAKLMVELSKSQDDEIGDGTTGVVVLAGALLEEAEQLLDRGIHPIRIADGYEQAARVAIEHLDKISDSVLVDIKDTEPLIQTAKTTLGSKVVNSCHRQMAEIAVNAVLTVADMERRDVDFELIKVEGKVGGRLEDTKLIKGVIVDKDFSHPQMPKKVEDAKIAILTCPFEPPKPKTKHKLDVTSVEDYKALQKYEKEKFEEMIQQIKETGANLAICQWGFDDEANHLLLQNNLPAVRWVGGPEIELIAIATGGRIVPRFSELTAEKLGFAGLVQEISFGTTKDKMLVIEQCKNSRAVTIFIRGGNKMIIEEAKRSLHDALCVIRNLIRDNRVVYGGGAAEISCALAVSQEADKCPTLEQYAMRAFADALEVIPMALSENSGMNPIQTMTEVRARQVKEMNPALGIDCLHKGTNDMKQQHVIETLIGKKQQISLATQMVRMILKIDDIRKPGESEE

1586 NP_976225.1 Q9UGC7

>NP_976225.1

MSTKNFRVSDGDWICPDKKCGNVNFARRTSCNRCGREKTTEAKMMKAGGTEIGKTLAEKSRGLFSANDWQCKTCSNVNWARRSECNMCNTPKYAKLEERTGYGGGFNERENVEYIEREESDGEYDEFGRKKKKYRGKAVGPASILKEVEDKESEGEEEDEDEDLSKYKLDEDEDEDDADLSKYNLDASEEEDSNKKKSNRRSRSKSRSSHSRSSSRSSSPSSSRSRSRSRSRSSSSSQSRSRSSSRERSRSRGSKSRSSSRSHRGSSSPRKRSYSSSSSSPERNRKRSRSRSSSSGDRKKRRTRSRSPERRHRSSSGSSHSGSRSSSKKK

>Q9UGC7

MRSRVLWGAARWLWPRRAVGPARRPLSSGSPPLEELFTRGGPLRTFLERQAGSEAHLKVRRPELLAVIKLLNEKERELRETEHLLHDENEDLRKLAENEITLCQKEITQLKHQIILLLVPSEETDENDLILEVTAGVGGQEAMLFTSEIFDMYQQYAAFKRWHFETLEYFPSELGGLRHASASIGGSEAYRHMKFEGGVHRVQRVPKTEKQGRVHTSTMTVAILPQPTEINLVINPKDLRIDTKRASGAGGQHVNTTDSAVRIVHLPTGVVSECQQERSQLKNKELAMTKLRAKLYSMHLEEEINKRQNARKIQIGSKGRSEKIRTYNFPQNRVTDHRINKTLHDLETFMQGDYLLDELVQSLKEYADYESLVEIISQKV

1587 NP_056975.1 NP_000340.2

>NP_056975.1

MSAAIAALAASYGSGSGSESDSDSESSRCPLPAADSLMHLTKSPSSKPSLAVAVDSAPEVAVKEDLETGVHLDPAVKEVQYNPTYETMFAPEFGPENPFRTQQMAAPRNMLSGYAEPAHINDFMFEQQRRTFATYGYALDPSLDNHQVSAKYIGSVEEAEKNQGLTVFETGQKKTEKRKKFKENDASNIDGFLGPWAKYVDEKDVAKPSEEEQKELDEITAKRQKKGKQEEEKPGEEKTILHVKEMYDYQGRSYLHIPQDVGVNLRSTMPPEKCYLPKKQIHVWSGHTKGVSAVRLFPLSGHLLLSCSMDCKIKLWEVYGERRCLRTFIGHSKAVRDICFNTAGTQFLSAAYDRYLKLWDTETGQCISRFTNRKVPYCVKFNPDEDKQNLFVAGMSDKKIVQWDIRSGEIVQEYDRHLGAVNTIVFVDENRRFVSTSDDKSLRVWEWDIPVDFKYIAEPSMHSMPAVTLSPNGKWLACQSMDNQILIFGAQNRFRLNKKKIFKGHMVAGYACQVDFSPDMSYVISGDGNGKLNIWDWKTTKLYSRFKAHDKVCIGAVWHPHETSKVITCGWDGLIKLWD

>NP_000340.2

MLLATFKLCAGSSYRHMRNMKGLRQQAVMAISQELNRRALGGPTPSTWINQVRRRSSLLGSRLEETLYSDQELAYLQQGEEAMQKALGILSNQEGWKKESQQDNGDKVMSKVVPDVGKVFRLEVVVDQPMERLYEELVERMEAMGEWNPNVKEIKVLQKIGKDTFITHELAAEAAGNLVGPRDFVSVRCAKRRGSTCVLAGMATDFGNMPEQKGVIRAEHGPTCMVLHPLAGSPSKTKLTWLLSIDLKGWLPKSIINQVLSQTQVDFANHLRKRLESHPASEARC

1588 Q6ECI4 P53004

>Q6ECI4

MKSQEEVEVAGIKLCKAMSLGSVTFTDVAIDFSQDEWEWLNLAQRSLYKKVMLENYRNLVSVGLCISKPDVISLLEQEKDPWVIKGGMNRGLCPDLECVWVTKSLSLNQDIYEEKLPPAIIMERLKSYDLECSTLGKNWKCEDLFERELVNQKTHFRQETITHIDTLIEKRDHSNKSGTVFHLNTLSYIKQIFPMEERIFNFHTDKKSLKTHSVVKKHKQDRGEKKLLKCNDCEKIFSKISTLTLHQRIHTGEKPYECIECGKAFSQSAHLAQHQRIHTGEKPFECTECGKAFSQNAHLVQHQRVHTGEKPYQCKQCNKAFSQLAHLAQHQRVHTGEKPYECIECGKAFSDCSSLAHHRRIHTGKRPYECIDCGKAFRQNASLIRHRRYYHTGEKPFDCIDCGKAFTDHIGLIQHKRIHTGERPYKCNVCGKAFSHGSSLTVHQRIHTGEKPYECNICEKAFSHRGSLTLHQRVHTGEKPYECKECGKAFRQSTHLAHHQRIHTGEKPYECKECSKTFSQNAHLAQHQKIHTGEKPYECKECGKAFSQIAHLVQHQRVHTGEKPYECIECGKAFSDGSYLVQHQRLHSGKRPYECLECGKAFRQRASLICHQRCHTGEKPYECNVCGKAFSHRKSLTLHQRIHTGEKPYECKECSKAFSQVAHLTLHKRIHTGERPYECKECGKAFRQSVHLAHHQRIHTGESSVILSSALPYHQVL

>P53004

MNAEPERKFGVVVVGVGRAGSVRMRDLRNPHPSSAFLNLIGFVSRRELGSIDGVQQISLEDALSSQEVEVAYICSESSSHEDYIRQFLNAGKHVLVEYPMTLSLAAAQELWELAEQKGKVLHEEHVELLMEEFAFLKKEVVGKDLLKGSLLFTAGPLEEERFGFPAFSGISRLTWLVSLFGELSLVSATLEERKEDQYMKMTVCLETEKKSPLSWIEEKGPGLKRNRYLSFHFKSGSLENVPNVGVNKNIFLKDQNIFVQKLLGQFSEKELAAEKKRILHCLGLAEEIQKYCCSRK

1589 NP_001315.1 NP_004484.1

>NP_001315.1

MYRTKVGLKDRQQLYKLIISQLLYDGYISIANGLINEIKPQSVCAPSEQLLHLIKLGMENDDTAVQYAIGRSDTVAPGTGIDLEFDADVQTMSPEASEYETCYVTSHKGPCRVATYSRDGQLIATGSADASIKILDTERMLAKSAMPIEVMMNETAQQNMENHPVIRTLYDHVDEVTCLAFHPTEQILASGSRDYTLKLFDYSKPSAKRAFKYIQEAEMLRSISFHPSGDFILVGTQHPTLRLYDINTFQCFVSCNPQDQHTDAICSVNYNSSANMYVTGSKDGCIKLWDGVSNRCITTFEKAHDGAEVCSAIFSKNSKYILSSGKDSVAKLWEISTGRTLVRYTGAGLSGRQVHRTQAVFNHTEDYVLLPDERTISLCCWDSRTAERRNLLSLGHNNIVRCIVHSPTNPGFMTCSDDFRARFWYRRSTTD

>NP_004484.1

MAAACRSVKGLVAVITGGASGLGLATAERLVGQGASAVLLDLPNSGGEAQAKKLGNNCVFAPADVTSEKDVQTALALAKGKFGRVDVAVNCAGIAVASKTYNLKKGQTHTLEDFQRVLDVNLMGTFNVIRLVAGEMGQNEPDQGGQRGVIINTASVAAFEGQVGQAAYSASKGGIVGMTLPIARDLAPIGIRVMTIAPGLFGTPLLTSLPEKVCNFLASQVPFPSRLGDPAEYAHLVQAIIENPFLNGEVIRLDGAIRMQP

1590 Q15170 NP_061821.1

>Q15170

MDKPRKENEEEPQSRPRPMRRGLRWSTLPKSSPPRSSLRRSSPRRRSSFLRSSCLSSCLRCSSRRTPSAGLSRKDLFEGRPPMEQPPCGVGKHKLEEGSFKERLARSRPQFRGDIHGRNLSNEEMIQAADELEEMKRVRNKLMIMHWKAKRSRPYPI

>NP_061821.1

MSIAGVAAQEIRVPLKTGFLHNGRAMGNMRKTYWSSRSEFKNNFLNIDPITMAYSLNSSAQERLIPLGHASKSAPMNGHCFAENGPSQKSSLPPLLIPPSENLGPHEEDQVVCGFKKLTVNGVCASTPPLTPIKNSPSLFPCAPLCERGSRPLPPLPISEALSLDDTDCEVEFLTSSDTDFLLEDSTLSDFKYDVPGRRSFRGCGQINYAYFDTPAVSAADLSYVSDQNGGVPDPNPPPPQTHRRLRRSHSGPAGSFNKPAIRISNCCIHRASPNSDEDKPEVPPRVPIPPRPVKPDYRRWSAEVTSSTYSDEDRPPKVPPREPLSPSNSRTPSPKSLPSYLNGVMPPTQSFAPDPKYVSSKALQRQNSEGSASKVPCILPIIENGKKVSSTHYYLLPERPPYLDKYEKFFREAEETNGGAQIQPLPADCGISSATEKPDSKTKMDLGGHVKRKHLSYVVSP

1591 NP_000440.1 Q8WVM0

>NP_000440.1

MAEDEPDAKSPKTGGRAPPGGAEAGEPTTLLQRLRGTISKAVQNKVEGILQDVQKFSDNDKLYLYLQLPSGPTTGDKSSEPSTLSNEEYMYAYRWIRNHLEEHTDTCLPKQSVYDAYRKYCESLACCRPLSTANFGKIIREIFPDIKARRLGGRGQSKYCYSGIRRKTLVSMPPLPGLDLKGSESPEMGPEVTPAPRDELVEAACALTCDWAERILKRSFSSIVEVARFLLQQHLISARSAHAHVLKAMGLAEEDEHAPRERSSKPKNGLENPEGGAHKKPERLAQPPKDLEARTGAGPLARGERKKSVVESSAPGANNLQVNALVARLPLLLPRAPRSLIPPIPVSPPILAPRLSSGALKVATLPLSSRAGAPPAAVPIINMILPTVPALPGPGPGPGRAPPGGLTQPRGTENREVGIGGDQGPHDKGVKRTAEVPVSEASGQAPPAKAAKQDIEDTASDAKRKRGRPRKKSGGSGERNSTPLKSAAAMESAQSSRLPWETWGSGGEGNSAGGAERPGPMGEAEKGAVLAQGQGDGTVSKGGRGPGSQHTKEAEDKIPLVPSKVSVIKGSRSQKEAFPLAKGEVDTAPQGNKDLKEHVLQSSLSQEHKDPKATPP

>Q8WVM0

MAASGKLSTCRLPPLPTIREIIKLLRLQAAKQLSQNFLLDLRLTDKIVRKAGNLTNAYVYEVGPGPGGITRSILNADVAELLVVEKDTRFIPGLQMLSDAAPGKLRIVHGDVLTFKVEKAFSESLKRPWEDDPPNVHIIGNLPFSVSTPLIIKWLENISCRDGPFVYGRTQMTLTFQKEVAERLAANTGSKQRSRLSVMAQYLCNVRHIFTIPGQAFVPKPEVDVGVVHFTPLIQPKIEQPFKLVEKVVQNVFQFRRKYCHRGLRMLFPEAQRLESTGRLLELADIDPTLRPRQLSISHFKSLCDVYRKMCDEDPQLFAYNFREELKRRKSKNEEKEEDDAENYRL

1592 NP_003874.2 NP_057330.2

>NP_003874.2

MAKTVAYFYDPDVGNFHYGAGHPMKPHRLALTHSLVLHYGLYKKMIVFKPYQASQHDMCRFHSEDYIDFLQRVSPTNMQGFTKSLNAFNVGDDCPVFPGLFEFCSRYTGASLQGATQLNNKICDIAINWAGGLHHAKKFEASGFCYVNDIVIGILELLKYHPRVLYIDIDIHHGDGVQEAFYLTDRVMTVSFHKYGNYFFPGTGDMYEVGAESGRYYCLNVPLRDGIDDQSYKHLFQPVINQVVDFYQPTCIVLQCGADSLGCDRLGCFNLSIRGHGECVEYVKSFNIPLLVLGGGGYTVRNVARCWTYETSLLVEEAISEELPYSEYFEYFAPDFTLHPDVSTRIENQNSRQYLDQIRQTIFENLKMLNHAPSVQIHDVPADLLTYDRTDEADAEERGPEENYSRPEAPNEFYDGDHDNDKESDVEI

>NP_057330.2

MATGTRYAGKVVVVTGGGRGIGAGIVRAFVNSGARVVICDKDESGGRALEQELPGAVFILCDVTQEDDVKTLVSETIRRFGRLDCVVNNAGHHPPPQRPEETSAQGFRQLLELNLLGTYTLTKLALPYLRKSQGNVINISSLVGAIGQAQAVPYVATKGAVTAMTKALALDESPYGVRVNCISPGNIWTPLWEELAALMPDPRATIREGMLAQPLGRMGQPAEVGAAAVFLASEANFCTGIELLVTGGAELGYGCKASRSTPVDAPDIPS

1593 NP_006692.1 NP_071906.1

>NP_006692.1

MSYMLPHLHNGWQVDQAILSEEDRVVVIRFGHDWDPTCMKMDEVLYSIAEKVKNFAVIYLVDITEVPDFNKMYELYDPCTVMFFFRNKHIMIDLGTGNNNKINWAMEDKQEMVDIIETVYRGARKGRGLVVSPKDYSTKYRY

>NP_071906.1

MDALVEDDICILNHEKAHKRDTVTPVSIYSGDESVASHFALVTAYEDIKKRLKDSEKENSLLKKRIRFLEEKLIARFEEETSSVGREQVNKAYHAYREVCIDRDNLKSKLDKMNKDNSESLKVLNEQLQSKEVELLQLRTEVETQQVMRNLNPPSSNWEVEKLSCDLKIHGLEQELELMRKECSDLKIELQKAKQTDPYQEDNLKSRDLQKLSISSDNMQHAYWELKREMSNLHLVTQVQAELLRKLKTSTAIKKACAPVGCSEDLGRDSTKLHLMNFTATYTRHPPLLPNGKALCHTTSSPLPGDVKVLSEKAILQSWTDNERSIPNDGTCFQEHSSYGRNSLEDNSWVFPSPPKSSETAFGETKTKTLPLPNLPPLHYLDQHNQNCLYKN

1594 NP_071394.2 NP_055433.2

>NP_071394.2

MTGEVGSEVHLEINDPNVISQEEADSPSDSGQGSYETIGPLSEGDSDEEIFVSKKLKNRKVLQDSDSETEDTNASPEKTTYDSAEEENKENLYAGKNTKIKRIYKTVADSDESYMEKSLYQENLEAQVKPCLELSLQSGNSTDFTTDRKSSKKHIHDKEGTAGKAKVKSKRRLEKEERKMEKIRQLKKKETKNQEDDVEQPFNDSGCLLVDKDLFETGLEDENNSPLEDEESLESIRAAVKNKVKKHKKKEPSLESGVHSFEEGSELSKGTTRKERKAARLSKEALKQLHSETQRLIRESALNLPYHMPENKTIHDFFKRKPRPTCHGNAMALLKSSKYQSSHHKEIIDTANTTEMNSDHHSKGSEQTTGAENEVETNALPVVSKETQIITGSDESCRKDLVKNEELEIQEKQKQSDIRPSPGDSSVLQQESNFLGNNHSEECQVGGLVAFEPHALEGEGPQNPEETDEKVEEPEQQNKSSAVGPPEKVRRFTLDRLKQLGVDVSIKPRLGADEDSFVILEPETNRELEALKQRFWKHANPAAKPRAGQTVNVNVIVKDMGTDGKEELKADVVPVTLAPKKLDGASHTKPGEKLQVLKAKLQEAMKLRRFEERQKRQALFKLDNEDGFEEEEEEEEEMTDESEEDGEEKVEKEEKEEELEEEEEKEEEEEEEGNQETAEFLLSSEEIETKDEKEMDKENNDGSSEIGKAVGFLSVPKSLSSDSTLLLFKDSSSKMGYFPTEEKSETDENSGKQPSKLDEDDSCSLLTKESSHNSSFELIGSTIPSYQPCNRQTGRGTSFFPTAGGFRSPSPGLFRASLVSSASKSSGKLSEPSLPIEDSQDLYNASPEPKTLFLGAGDFQFCLEDDTQSQLLDADGFLNVRNHRNQYQALKPRLPLASMDENAMDANMDELLDLCTGKFTSQAEKHLPRKSDKKENMEELLNLCSGKFTSQDASTPASSELNKQEKESSMGDPMEEALALCSGSFPTDKEEEDEEEEFGDFRLVSNDNEFDSDEDEHSDSGNDLALEDHEDDDEEELLKRSEKLKRQMRLRKYLEDEAEVSGSDVGSEDEYDGEEIDEYEEDVIDEVLPSDEELQSQIKKIHMKTMLDDDKRQLRLYQERYLADGDLHSDGPGRMRKFRWKNIDDASQMDLFHRDSDDDQTEEQLDESEARWRKERIEREQWLRDMAQQGKITAEEEEEIGEDSQFMILAKKVTAKALQKNASRPMVIQESKSLLRNPFEAIRPGSAQQVKTGSLLNQPKAVLQKLAALSDHNPSAPRNSRNFVFHTLSPVKAEAAKESSKSQVKKRGPSFMTSPSPKHLKTDDSTSGLTRSIFKYLES

>NP_055433.2

MNWRFVELLYFLFIWGRISVQPSHQEPAGTDQHVSKEFDWLISDRGPFHHSRSYLSFVERHRQGFTTRYKIYREFARWKVRNTAIERRDLVRHPVPLMPEFQRSIRLLGRRPTTQQFIDTIIKKYGTHLLISATLGGEEALTMYMDKSRLDRKSGNATQSVEALHQLASSYFVDRDGTMRRLHEIQISTGAIKVTETRTGPLGCNSYDNLDSVSSVLLQSTESKLHLQGLQIIFPQYLQEKFVQSALSYIMCNGEGEYLCQNSQCRCQCAEEFPQCNCPITDIQIMEYTLANMAKSWAEAYKDLENSDEFKSFMKRLPSNHFLTIGSIHQHWGNDWDLQNRYKLLQSATEAQRQKIQRTARKLFGLSVRCRHNPNHQLPRERTIQQWLARVQSLLYCNENGFWGTFLESQRSCVCHGSTTLCQRPIPCVIGGNNSCAMCSLANISLCGSCNKGYKLYRGRCEPQNVDSERSEQFISFETDLDFQDLELKYLLQKMDSRLYVHTTFISNEIRLDTFFDPRWRKRMSLTLKSNKNRMDFIHMVIGMSMRICQMRNSSLDPMFFVYVNPFSGSHSEGWNMPFGEFGYPRWEKIRLQNSQCYNWTLLLGNRWKTFFETVHIYLRSRTRLPTLLRNETGQGPVDLSDPSKRQFYIKISDVQVFGYSLRFNADLLRSAVQQVNQSYTQGGQFYSSSSVMLLLLDIRDRINRLAPPVAPGKPQLDLFSCMLKHRLKLTNSEIIRVNHALDLYNTEILKQSDQMTAKLC

1595 Q15306 O95749

>Q15306

MNLEGGGRGGEFGMSAVSCGNGKLRQWLIDQIDSGKYPGLVWENEEKSIFRIPWKHAGKQDYNREEDAALFKAWALFKGKFREGIDKPDPPTWKTRLRCALNKSNDFEELVERSQLDISDPYKVYRIVPEGAKKGAKQLTLEDPQMSMSHPYTMTTPYPSLPAQQVHNYMMPPLDRSWRDYVPDQPHPEIPYQCPMTFGPRGHHWQGPACENGCQVTGTFYACAPPESQAPGVPTEPSIRSAEALAFSDCRLHICLYYREILVKELTTSSPEGCRISHGHTYDASNLDQVLFPYPEDNGQRKNIEKLLSHLERGVVLWMAPDGLYAKRLCQSRIYWDGPLALCNDRPNKLERDQTCKLFDTQQFLSELQAFAHHGRSLPRFQVTLCFGEEFPDPQRQRKLITAHVEPLLARQLYYFAQQNSGHFLRGYDLPEHISNPEDYHRSIRHSSIQE

>O95749

MEKTQETVQRILLEPYKYLLQLPGKQVRTKLSQAFNHWLKVPEDKLQIIIEVTEMLHNASLLIDDIEDNSKLRRGFPVAHSIYGIPSVINSANYVYFLGLEKVLTLDHPDAVKLFTRQLLELHQGQGLDIYWRDNYTCPTEEEYKAMVLQKTGGLFGLAVGLMQLFSDYKEDLKPLLNTLGLFFQIRDDYANLHSKEYSENKSFCEDLTEGKFSFPTIHAIWSRPESTQVQNILRQRTENIDIKKYCVHYLEDVGSFEYTRNTLKELEAKAYKQIDARGGNPELVALVKHLSKMFKEENE

1596 NP_005135.2 NP_056449.1

>NP_005135.2

MESTPSFLKGTPTWEKTAPENGIVRQEPGSPPRDGLHHGPLCLGEPAPFWRGVLSTPDSWLPPGFPQGPKDMLPLVEGEGPQNGERKVNWLGSKEGLRWKEAMLTHPLAFCGPACPPRCGPLMPEHSGGHLKSDPVAFRPWHCPFLLETKILERAPFWVPTCLPPYLVSGLPPEHPCDWPLTPHPWVYSGGQPKVPSAFSLGSKGFYYKDPSIPRLAKEPLAAAEPGLFGLNSGGHLQRAGEAERPSLHQRDGEMGAGRQQNPCPLFLGQPDTVPWTSWPACPPGLVHTLGNVWAGPGDGNLGYQLGPPATPRCPSPEPPVTQRGCCSSYPPTKGGGLGPCGKCQEGLEGGASGASEPSEEVNKASGPRACPPSHHTKLKKTWLTRHSEQFECPRGCPEVEERPVARLRALKRAGSPEVQGAMGSPAPKRPPDPFPGTAEQGAGGWQEVRDTSIGNKDVDSGQHDEQKGPQDGQASLQDPGLQDIPCLALPAKLAQCQSCAQAAGEGGGHACHSQQVRRSPLGGELQQEEDTATNSSSEEGPGSGPDSRLSTGLAKHLLSGLGDRLCRLLRREREALAWAQREGQGPAVTEDSPGIPRCCSRCHHGLFNTHWRCPRCSHRLCVACGRVAGTGRAREKAGFQEQSAEECTQEAGHAACSLMLTQFVSSQALAELSTAMHQVWVKFDIRGHCPCQADARVWAPGDAGQQKESTQKTPPTPQPSCNGDTHRTKSIKEETPDSAETPAEDRAGRGPLPCPSLCELLASTAVKLCLGHERIHMAFAPVTPALPSDDRITNILDSIIAQVVERKIQEKALGPGLRAGPGLRKGLGLPLSPVRPRLPPPGALLWLQEPQPCPRRGFHLFQEHWRQGQPVLVSGIQRTLQGNLWGTEALGALGGQVQALSPLGPPQPSSLGSTTFWEGFSWPELRPKSDEGSVLLLHRALGDEDTSRVENLAASLPLPEYCALHGKLNLASYLPPGLALRPLEPQLWAAYGVSPHRGHLGTKNLCVEVADLVSILVHADTPLPAWHRAQKDFLSGLDGEGLWSPGSQVSTVWHVFRAQDAQRIRRFLQMVCPAGAGALEPGAPGSCYLDAGLRRRLREEWGVSCWTLLQAPGEAVLVPAGAPHQVQGLVSTVSVTQHFLSPETSALSAQLCHQGPSLPPDCHLLYAQMDWAVFQAVKVAVGTLQEAK

>NP_056449.1

MANVPWAEVCEKFQAALALSRVELHKNPEKEPYKSKYSARALLEEVKALLGPAPEDEDERPEAEDGPGAGDHALGLPAEVVEPEGPVAQRAVRLAVIEFHLGVNHIDTEELSAGEEHLVKCLRLLRRYRLSHDCISLCIQAQNNLGILWSEREEIETAQAYLESSEALYNQYMKEVGSPPLDPTERFLPEEEKLTEQERSKRFEKVYTHNLYYLAQVYQHLEMFEKAAHYCHSTLKRQLEHNAYHPIEWAINAATLSQFYINKLCFMEARHCLSAANVIFGQTGKISATEDTPEAEGEVPELYHQRKGEIARCWIKYCLTLMQNAQLSMQDNIGELDLDKQSELRALRKKELDEEESIRKKAVQFGTGELCDAISAVEEKVSYLRPLDFEEARELFLLGQHYVFEAKEFFQIDGYVTDHIEVVQDHSALFKVLAFFETDMERRCKMHKRRIAMLEPLTVDLNPQYYLLVNRQIQFEIAHAYYDMMDLKVAIADRLRDPDSHIVKKINNLNKSALKYYQLFLDSLRDPNKVFPEHIGEDVLRPAMLAKFRVARLYGKIITADPKKELENLATSLEHYKFIVDYCEKHPEAAQEIEVELELSKEMVSLLPTKMERFRTKMALT

1597 P10071 NP_002291.1

>P10071

MEAQSHSSTTTEKKKVENSIVKCSTRTDVSEKAVASSTTSNEDESPGQTYHRERRNAITMQPQNVQGLSKVSEEPSTSSDERASLIKKEIHGSLPHVAEPSVPYRGTVFAMDPRNGYMEPHYHPPHLFPAFHPPVPIDARHHEGRYHYDPSPIPPLHMTSALSSSPTYPDLPFIRISPHRNPAAASESPFSPPHPYINPYMDYIRSLHSSPSLSMISATRGLSPTDAPHAGVSPAEYYHQMALLTGQRSPYADIIPSAATAGTGAIHMEYLHAMDSTRFSSPRLSARPSRKRTLSISPLSDHSFDLQTMIRTSPNSLVTILNNSRSSSSASGSYGHLSASAISPALSFTYSSAPVSLHMHQQILSRQQSLGSAFGHSPPLIHPAPTFPTQRPIPGIPTVLNPVQVSSGPSESSQNKPTSESAVSSTGDPMHNKRSKIKPDEDLPSPGARGQQEQPEGTTLVKEEGDKDESKQEPEVIYETNCHWEGCAREFDTQEQLVHHINNDHIHGEKKEFVCRWLDCSREQKPFKAQYMLVVHMRRHTGEKPHKCTFEGCTKAYSRLENLKTHLRSHTGEKPYVCEHEGCNKAFSNASDRAKHQNRTHSNEKPYVCKIPGCTKRYTDPSSLRKHVKTVHGPEAHVTKKQRGDIHPRPPPPRDSGSHSQSRSPGRPTQGALGEQQDLSNTTSKREECLQVKTVKAEKPMTSQPSPGGQSSCSSQQSPISNYSNSGLELPLTDGGSIGDLSAIDETPIMDSTISTATTALALQARRNPAGTKWMEHVKLERLKQVNGMFPRLNPILPPKAPAVSPLIGNGTQSNNTCSLGGPMTLLPGRSDLSGVDVTMLNMLNRRDSSASTISSAYLSSRRSSGISPCFSSRRSSEASQAEGRPQNVSVADSYDPISTDASRRSSEASQSDGLPSLLSLTPAQQYRLKAKYAAATGGPPPTPLPNMERMSLKTRLALLGDALEPGVALPPVHAPRRCSDGGAHGYGRRHLQPHDALGHGVRRASDPVRTGSEGLALPRVPRFSSLSSCNPPAMATSAEKRSLVLQNYTRPEGGQSRNFHSSPCPPSITENVTLESLTMDADANLNDEDFLPDDVVQYLNSQNQAGYEQHFPSALPDDSKVPHGPGDFDAPGLPDSHAGQQFHALEQPCPEGSKTDLPIQWNEVSSGSADLSSSKLKCGPRPAVPQTRAFGFCNGMVVHPQNPLRSGPAGGYQTLGENSNPYGGPEHLMLHNSPGSGTSGNAFHEQPCKAPQYGNCLNRQPVAPGALDGACGAGIQASKLKSTPMQGSGGQLNFGLPVAPNESAGSMVNGMQNQDPVGQGYLAHQLLGDSMQHPGAGRPGQQMLGQISATSHINIYQGPESCLPGAHGMGSQPSSLAVVRGYQPCASFGGSRRQAMPRDSLALQSGQLSDTSQTCRVNGIKMEMKGQPHPLCSNLQNYSGQFYDQTVGFSQQDTKAGSFSISDASCLLQGTSAKNSELLSPGANQVTSTVDSLDSHDLEGVQIDFDAIIDDGDHSSLMSGALSPSIIQNLSHSSSRLTTPRASLPFPALSMSTTNMAIGDMSSLLTSLAEESKFLAVMQ

>NP_002291.1

MATLKEKLIAPVAEEEATVPNNKITVVGVGQVGMACAISILGKSLADELALVDVLEDKLKGEMMDLQHGSLFLQTPKIVADKDYSVTANSKIVVVTAGVRQQEGESRLNLVQRNVNVFKFIIPQIVKYSPDCIIIVVSNPVDILTYVTWKLSGLPKHRVIGSGCNLDSARFRYLMAEKLGIHPSSCHGWILGEHGDSSVAVWSGVNVAGVSLQELNPEMGTDNDSENWKEVHKMVVESAYEVIKLKGYTNWAIGLSVADLIESMLKNLSRIHPVSTMVKGMYGIENEVFLSLPCILNARGLTSVINQKLKDDEVAQLKKSADTLWDIQKDLKDL

1598 NP_000225.1 NP_006819.2

>NP_000225.1

MQRSIMSFFHPKKEGKAKKPEKEASNSSRETEPPPKAALKEWNGVVSESDSPVKRPGRKAARVLGSEGEEEDEALSPAKGQKPALDCSQVSPPRPATSPENNASLSDTSPMDSSPSGIPKRRTARKQLPKRTIQEVLEEQSEDEDREAKRKKEEEEEETPKESLTEAEVATEKEGEDGDQPTTPPKPLKTSKAETPTESVSEPEVATKQELQEEEEQTKPPRRAPKTLSSFFTPRKPAVKKEVKEEEPGAPGKEGAAEGPLDPSGYNPAKNNYHPVEDACWKPGQKVPYLAVARTFEKIEEVSARLRMVETLSNLLRSVVALSPPDLLPVLYLSLNHLGPPQQGLELGVGDGVLLKAVAQATGRQLESVRAEAAEKGDVGLVAENSRSTQRLMLPPPPLTASGVFSKFRDIARLTGSASTAKKIDIIKGLFVACRHSEARFIARSLSGRLRLGLAEQSVLAALSQAVSLTPPGQEFPPAMVDAGKGKTAEARKTWLEEQGMILKQTFCEVPDLDRIIPVLLEHGLERLPEHCKLSPGIPLKPMLAHPTRGISEVLKRFEEAAFTCEYKYDGQRAQIHALEGGEVKIFSRNQEDNTGKYPDIISRIPKIKLPSVTSFILDTEAVAWDREKKQIQPFQVLTTRKRKEVDASEIQVQVCLYAFDLIYLNGESLVREPLSRRRQLLRENFVETEGEFVFATSLDTKDIEQIAEFLEQSVKDSCEGLMVKTLDVDATYEIAKRSHNWLKLKKDYLDGVGDTLDLVVIGAYLGRGKRAGRYGGFLLASYDEDSEELQAICKLGTGFSDEELEEHHQSLKALVLPSPRPYVRIDGAVIPDHWLDPSAVWEVKCADLSLSPIYPAARGLVDSDKGISLRFPRFIRVREDKQPEQATTSAQVACLYRKQSQIQNQQGEDSGSDPEDTY

>NP_006819.2

MALPRLTGALRSFSNVTKQDNYNEEVADLKIKRSKLHEQVLDLGLTWKKIIKFLNEKLEKSKMQSINEDLKDILHAAKQIVGTDNGREAIESGAAFLFMTFHLKDSVGHKETKAIKQMFGPFPSSSATAACNATNRIISHFSQDDLTALVQMTEKEHGDRVFFGKNLAFSFDMHDLDHFDELPINGETQKTISLDYKKFLNEHLQEACTPELKPVEKTNGSFLWCEVEKYLNSTLKEMTEVPRVEDLCCTLYDMLASIKSGDELQDELFELLGPEGLELIEKLLQNRITIVDRFLNSSNDHRFQALQDNCKKILGENAKPNYGCQVTIQSEQEKQLMKQYRREEKRIARREKKAGEDLEVSEGLMCFDPKELRIQREQALLNARSVPILSRQRDADVEKIHYPHVYDSQAEAMKTSAFIAGAKMILPEGIQRENNKLYEEVRIPYSEPMPLSFEEKPVYIQDLDEIGQLAFKGMKRLNRIQSIVFETAYNTNENMLICAPTGAGKTNIAMLTVLHEIRQHFQQGVIKKNEFKIVYVAPMKALAAEMTDYFSRRLEPLGIIVKELTGDMQLSKSEILRTQMLVTTPEKWDVVTRKSVGDVALSQIVRLLILDEVHLLHEDRGPVLESIVARTLRQVESTQSMIRILGLSATLPNYLDVATFLHVNPYIGLFFFDGRFRPVPLGQTFLGIKCANKMQQLNNMDEVCYENVLKQVKAGHQVMVFVHARNATVRTAMSLIERAKNCGHIPFFFPTQGHDYVLAEKQVQRSRNKQVRELFPDGFSIHHAGMLRQDRNLVENLFSNGHIKVLVCTATLAWGVNLPAHAVIIKGTQIYAAKRGSFVDLGILDVMQIFGRAGRPQFDKFGEGIIITTHDKLSHYLTLLTQRNPIESQFLESLADNLNAEIALGTVTNVEEAVKWISYTYLYVRMRANPLAYGISHKAYQIDPTLRKHREQLVIEVGRKLDKAQMIRFEERTGYFSSTDLGRTASHYYIKYNTIETFNELFDAHKTEGDIFAIVSKAEEFDQIKVREEEIEELDTLLSNFCELSTPGGVENSYGKINILLQTYISRGEMDSFSLISDSAYVAQNAARIVRALFEIALRKRWPTMTYRLLNLSKVIDKRLWGWASPLRQFSILPPHILTRLEEKKLTVDKLKDMRKDEIGHILHHVNIGLKVKQCVHQIPSVMMEASIQPITRTVLRVTLSIYADFTWNDQVHGTVGEPWWIWVEDPTNDHIYHSEYFLALKKQVISKEAQLLVFTIPIFEPLPSQYYIRAVSDRWLGAEAVCIINFQHLILPERHPPHTELLDLQPLPITALGCKAYEALYNFSHFNPVQTQIFHTLYHTDCNVLLGAPTGSGKTVAAELAIFRVFNKYPTSKAVYIAPLKALVRERMDDWKVRIEEKLGKKVIELTGDVTPDMKSIAKADLIVTTPEKWDGVSRSWQNRNYVQQVTILIIDEIHLLGEERGPVLEVIVSRTNFISSHTEKPVRIVGLSTALANARDLADWLNIKQMGLFNFRPSVRPVPLEVHIQGFPGQHYCPRMASMNKPAFQAIRSHSPAKPVLIFVSSRRQTRLTALELIAFLATEEDPKQWLNMDEREMENIIATVRDSNLKLTLAFGIGMHHAGLHERDRKTVEELFVNCKVQVLIATSTLAWGVNFPAHLVIIKGTEYYDGKTRRYVDFPITDVLQMMGRAGRPQFDDQGKAVILVHDIKKDFYKKFLYEPFPVESSLLGVLSDHLNAEIAGGTITSKQDALDYITWTYFFRRLIMNPSYYNLGDVSHDSVNKFLSHLIEKSLIELELSYCIEIGEDNRSIEPLTYGRIASYYYLKHQTVKMFKDRLKPECSTEELLSILSDAEEYTDLPVRHNEDHMNSELAKCLPIESNPHSFDSPHTKAHLLLQAHLSRAMLPCPDYDTDTKTVLDQALRVCQAMLDVAANQGWLVTVLNITNLIQMVIQGRWLKDSSLLTLPNIENHHLHLFKKWKPIMKGPHARGRTSIESLPELIHACGGKDHVFSSMVESELHAAKTKQAWNFLSHLPVINVGISVKGSWDDLVEGHNELSVSTLTADKRDDNKWIKLHADQEYVLQVSLQRVHFGFHKGKPESCAVTPRFPKSKDEGWFLILGEVDKRELIALKRVGYIRNHHVASLSFYTPEIPGRYIYTLYFMSDCYLGLDQQYDIYLNVTQASLSAQVNTKVSDSLTDLALK

1599 NP_001007227.1 Q9NP74

>NP_001007227.1

MSRVPSPPPPAEMSSGPVAESWCYTQIKVVKFSYMWTINNFSFCREEMGEVIKSSTFSSGANDKLKWCLRVNPKGLDEESKDYLSLYLLLVSCPKSEVRAKFKFSILNAKGEETKAMESQRAYRFVQGKDWGFKKFIRRDFLLDEANGLLPDDKLTLFCEVSVVQDSVNISGQNTMNMVKVPECRLADELGGLWENSRFTDCCLCVAGQEFQAHKAILAARSPVFSAMFEHEMEESKKNRVEINDVEPEVFKEMMCFIYTGKAPNLDKMADDLLAAADKYALERLKVMCEDALCSNLSVENAAEILILADLHSADQLKTQAVDFINYHASDVLETSGWKSMVVSHPHLVAEAYRSLASAQCPFLGPPRKRLKQS

>Q9NP74

MEEAELVKGRLQAITDKRKIQEEISQKRLKIEEDKLKHQHLKKKALREKWLLDGISSGKEQEEMKKQNQQDQHQIQVLEQSILRLEKEIQDLEKAELQISTKEEAILKKLKSIERTTEDIIRSVKVEREERAEESIEDIYANIPDLPKSYIPSRLRKEINEEKEDDEQNRKALYAMEIKVEKDLKTGESTVLSSIPLPSDDFKGTGIKVYDDGQKSVYAVSSNHSAAYNGTDGLAPVEVEELLRQASERNSKSPTEYHEPVYANPFYRPTTPQRETVTPGPNFQERIKIKTNGLGIGVNESIHNMGNGLSEERGNNFNHISPIPPVPHPRSVIQQAEEKLHTPQKRLMTPWEESNVMQDKDAPSPKPRLSPRETIFGKSEHQNSSPTCQEDEEDVRYNIVHSLPPDINDTEPVTMIFMGYQQAEDSEEDKKFLTGYDGIIHAELVVIDDEEEEDEGEAEKPSYHPIAPHSQVYQPAKPTPLPRKRSEASPHENTNHKSPHKNSISLKEQEESLGSPVHHSPFDAQTTGDGTEDPSLTALRMRMAKLGKKVI

1600 NP_006181.1 NP_659491.4

>NP_006181.1

MSKPELKEDKMLEVHFVGDDDVLNHILDREGGAKLKKERAQLLVNPKKIIKKPEYDLEEDDQEVLKDQNYVEIMGRDVQESLKNGSATGGGNKVYSFQNRKHSEKMAKLASELAKTPQKSVSFSLKNDPEITINVPQSSKGHSASDKVQPKNNDKSEFLSTAPRSLRKRLIVPRSHSDSESEYSASNSEDDEGVAQEHEEDTNAVIFSQKIQAQNRVVSAPVGKETPSKRMKRDKTSDLVEEYFEAHSSSKVLTSDRTLQKLKRAKLDQQTLRNLLSKVSPSFSAELKQLNQQYEKLFHKWMLQLHLGFNIVLYGLGSKRDLLERFRTTMLQDSIHVVINGFFPGISVKSVLNSITEEVLDHMGTFRSILDQLDWIVNKFKEDSSLELFLLIHNLDSQMLRGEKSQQIIGQLSSLHNIYLIASIDHLNAPLMWDHAKQSLFNWLWYETTTYSPYTEETSYENSLLVKQSGSLPLSSLTHVLRSLTPNARGIFRLLIKYQLDNQDNPSYIGLSFQDFYQQCREAFLVNSDLTLRAQLTEFRDHKLIRTKKGTDGVEYLLIPVDNGTLTDFLEKEEEEA

>NP_659491.4

MDNKISPEAQVAELELDAVIGFNGHVPTGLKCHPDQEHMIYPLGCTVLIQAINTKEQNFLQGHGNNVSCLAISRSGEYIASGQVTFMGFKADIILWDYKNRELLARLSLHKGKIEALAFSPNDLYLVSLGGPDDGSVVVWSIAKRDAICGSPAAGLNVGNATNVIFSRCRDEMFMTAGNGTIRVWELDLPNRKIWPTECQTGQLKRIVMSIGVDDDDSFFYLGTTTGDILKMNPRTKLLTDVGPAKDKFSLGVSAIRCLKMGGLLVGSGAGLLVFCKSPGYKPIKKIQLQGGITSITLRGEGHQFLVGTEESHIYRVSFTDFKETLIATCHFDAVEDIVFPFGTAELFATCAKKDIRVWHTSSNRELLRITVPNMTCHGIDFMRDGKSIISAWNDGKIRAFAPETGRLMYVINNAHRIGVTAIATTSDCKRVISGGGEGEVRVWQIGCQTQKLEEALKEHKSSVSCIRVKRNNEECVTASTDGTCIIWDLVRLRRNQMILANTLFQCVCYHPEEFQIITSGTDRKIAYWEVFDGTVIRELEGSLSGSINGMDITQEGVHFVTGGNDHLVKVWDYNEGEVTHVGVGHSGNITRIRISPGNQYIVSVSADGAILRWKYPYTS

1601 Q96FI4 NP_001344.2

>Q96FI4

MPEGPELHLASQFVNEACRALVFGGCVEKSSVSRNPEVPFESSAYRISASARGKELRLILSPLPGAQPQQEPLALVFRFGMSGSFQLVPREELPRHAHLRFYTAPPGPRLALCFVDIRRFGRWDLGGKWQPGRGPCVLQEYQQFRENVLRNLADKAFDRPICEALLDQRFFNGIGNYLRAEILYRLKIPPFEKARSVLEALQQHRPSPELTLSQKIRTKLQNPDLLELCHSVPKEVVQLGGKGYGSESGEEDFAAFRAWLRCYGMPGMSSLQDRHGRTIWFQGDPGPLAPKGRKSRKKKSKATQLSPEDRVEDALPPSKAPSRTRRAKRDLPKRTATQRPEGTSLQQDPEAPTVPKKGRRKGRQAASGHCRPRKVKADIPSLEPEGTSAS

>NP_001344.2

MDSKYQCVKLNDGHFMPVLGFGTYAPAEVPKSKALEATKLAIEAGFRHIDSAHLYNNEEQVGLAIRSKIADGSVKREDIFYTSKLWCNSHRPELVRPALERSLKNLQLDYVDLYLIHFPVSVKPGEEVIPKDENGKILFDTVDLCATWEAVEKCKDAGLAKSIGVSNFNRRQLEMILNKPGLKYKPVCNQVECHPYFNQRKLLDFCKSKDIVLVAYSALGSHREEPWVDPNSPVLLEDPVLCALAKKHKRTPALIALRYQLQRGVVVLAKSYNEQRIRQNVQVFEFQLTSEEMKAIDGLNRNVRYLTLDIFAGPPNYPFSDEY

1602 NP_036418.1 NP_077006.1

>NP_036418.1

MSSRKQGSQPRGQQSAEEENFKKPTRSNMQRSKMRGASSGKKTAGPQQKNLEPALPGRWGGRSAENPPSGSVRKTRKNKQKTPGNGDGGSTSEAPQPPRKKRARADPTVESEEAFKNRMEVKVKIPEELKPWLVEDWDLVTRQKQLFQLPAKKNVDAILEEYANCKKSQGNVDNKEYAVNEVVAGIKEYFNVMLGTQLLYKFERPQYAEILLAHPDAPMSQVYGAPHLLRLFVRIGAMLAYTPLDEKSLALLLGYLHDFLKYLAKNSASLFTASDYKVASAEYHRKAL

>NP_077006.1

MGKKLDLSKLTDEEAQHVLEVVQRDFDLRRKEEERLEALKGKIKKESSKRELLSDTAHLNETHCARCLQPYQLLVNSKRQCLECGLFTCKSCGRVHPEEQGWICDPCHLARVVKIGSLEWYYEHVKARFKRFGSAKVIRSLHGRLQGGAGPELISEERSGDSDQTDEDGEPGSEAQAQAQPFGSKKKRLLSVHDFDFEGDSDDSTQPQGHSLHLSSVPEARDSPQSLTDESCSEKAAPHKAEGLEEADTGASGCHSHPEEQPTSISPSRHGALAELCPPGGSHRMALGTAAALGSNVIRNEQLPLQYLADVDTSDEESIRAHVMASHHSKRRGRASSESQIFELNKHISAVECLLTYLENTVVPPLAKGLGAGVRTEADVEEEALRRKLEELTSNVSDQETSSEEEEAKDEKAEPNRDKSVGPLPQADPEVGTAAHQTNRQEKSPQDPGDPVQYNRTTDEELSELEDRVAVTASEVQQAESEVSDIESRIAALRAAGLTVKPSGKPRRKSNLPIFLPRVAGKLGKRPEDPNADPSSEAKAMAVPYLLRRKFSNSLKSQGKDDDSFDRKSVYRGSLTQRNPNARKGMASHTFAKPVVAHQS

1603 NP_006730.2 NP_758958.3

>NP_006730.2

MSGFDDPGIFYSDSFGGDAQADEGQARKSQLQRRFKEFLRQYRVGTDRTGFTFKYRDELKRHYNLGEYWIEVEMEDLASFDEDLADYLYKQPAEHLQLLEEAAKEVADEVTRPRPSGEEVLQDIQVMLKSDASPSSIRSLKSDMMSHLVKIPGIIIAASAVRAKATRISIQCRSCRNTLTNIAMRPGLEGYALPRKCNTDQAGRPKCPLDPYFIMPDKCKCVDFQTLKLQELPDAVPHGEMPRHMQLYCDRYLCDKVVPGNRVTIMGIYSIKKFGLTTSRGRDRVGVGIRSSYIRVLGIQVDTDGSGRSFAGAVSPQEEEEFRRLAALPNVYEVISKSIAPSIFGGTDMKKAIACLLFGGSRKRLPDGLTRRGDINLLMLGDPGTAKSQLLKFVEKCSPIGVYTSGKGSSAAGLTASVMRDPSSRNFIMEGGAMVLADGGVVCIDEFDKMREDDRVAIHEAMEQQTISIAKAGITTTLNSRCSVLAAANSVFGRWDETKGEDNIDFMPTILSRFDMIFIVKDEHNEERDVMLAKHVITLHVSALTQTQAVEGEIDLAKLKKFIAYCRVKCGPRLSAEAAEKLKNRYIIMRSGARQHERDSDRRSSIPITVRQLEAIVRIAEALSKMKLQPFATEADVEEALRLFQVSTLDAALSGTLSGVEGFTSQEDQEMLSRIEKQLKRRFAIGSQVSEHSIIKDFTKQKYPEHAIHKVLQLMLRRGEIQHRMQRKVLYRLK

>NP_758958.3

MLEKLMGADSLQLFRSRYTLGKIYFIGFQRSILLSKSENSLNSIAKETEEGRETVTRKEGWKRRHEDGYLEMAQRHLQRSLCPWVSYLPQPYAELEEVSSHVGKVFMARNYEFLAYEASKDRRQPLERMWTCNYNQQKDQSCNHKEITSTKAE

1604 NP_003790.1 NP_000476.1

>NP_003790.1

MANSAKAEEYEKMSLEQAKASVNSETESSFNINENTTASGTGLSEKTSVCRQVDIARKRKEFEDDLVKESSSCGKDTPSKKRKLDPEIVPEEKDCGDAEGNSKKRKRETEDVPKDKSSTGDGTQNKRKIALEDVPEKQKNLEEGHSSTVAAHYNELQEVGLEKRSQSRIFYLRNFNNWMKSVLIGEFLEKVRQKKKRDITVLDLGCGKGGDLLKWKKGRINKLVCTDIADVSVKQCQQRYEDMKNRRDSEYIFSAEFITADSSKELLIDKFRDPQMCFDICSCQFVCHYSFESYEQADMMLRNACERLSPGGYFIGTTPNSFELIRRLEASETESFGNEIYTVKFQKKGDYPLFGCKYDFNLEGVVDVPEFLVYFPLLNEMAKKYNMKLVYKKTFLEFYEEKIKNNENKMLLKRMQALEPYPANESSKLVSEKVDDYEHAAKYMKNSQVRLPLGTLSKSEWEATSIYLVFAFEKQQ

>NP_000476.1

MADSELQLVEQRIRSFPDFPTPGVVFRDISPVLKDPASFRAAIGLLARHLKATHGGRIDYIAGLDSRGFLFGPSLAQELGLGCVLIRKRGKLPGPTLWASYSLEYGKAELEIQKDALEPGQRVVVVDDLLATGGTMNAACELLGRLQAEVLECVSLVELTSLKGREKLAPVPFFSLLQYE

1605 P14316 Q8WVM0

>P14316

MPVERMRMRPWLEEQINSNTIPGLKWLNKEKKIFQIPWMHAARHGWDVEKDAPLFRNWAIHTGKHQPGVDKPDPKTWKANFRCAMNSLPDIEEVKDKSIKKGNNAFRVYRMLPLSERPSKKGKKPKTEKEDKVKHIKQEPVESSLGLSNGVSDLSPEYAVLTSTIKNEVDSTVNIIVVGQSHLDSNIENQEIVTNPPDICQVVEVTTESDEQPVSMSELYPLQISPVSSYAESETTDSVPSDEESAEGRPHWRKRNIEGKQYLSNMGTRGSYLLPGMASFVTSNKPDLQVTIKEESNPVPYNSSWPPFQDLPLSSSMTPASSSSRPDRETRASVIKKTSDITQARVKSC

>Q8WVM0

MAASGKLSTCRLPPLPTIREIIKLLRLQAAKQLSQNFLLDLRLTDKIVRKAGNLTNAYVYEVGPGPGGITRSILNADVAELLVVEKDTRFIPGLQMLSDAAPGKLRIVHGDVLTFKVEKAFSESLKRPWEDDPPNVHIIGNLPFSVSTPLIIKWLENISCRDGPFVYGRTQMTLTFQKEVAERLAANTGSKQRSRLSVMAQYLCNVRHIFTIPGQAFVPKPEVDVGVVHFTPLIQPKIEQPFKLVEKVVQNVFQFRRKYCHRGLRMLFPEAQRLESTGRLLELADIDPTLRPRQLSISHFKSLCDVYRKMCDEDPQLFAYNFREELKRRKSKNEEKEEDDAENYRL

1606 NP_004796.1 Q9ULW8

>NP_004796.1

MAAGGSDPRAGDVEEDASQLIFPKEFETAETLLNSEVHMLLEHRKQQNESAEDEQELSEVFMKTLNYTARFSRFKNRETIASVRSLLLQKKLHKFELACLANLCPETAEESKALIPSLEGRFEDEELQQILDDIQTKRSFQY

>Q9ULW8

MSLQRIVRVSLEHPTSAVCVAGVETLVDIYGSVPEGTEMFEVYGTPGVDIYISPNMERGRERADTRRWRFDATLEIIVVMNSPSNDLNDSHVQISYHSSHEPLPLAYAVLYLTCVDISLDCDLNCEGRQDRNFVDKRQWVWGPSGYGGILLVNCDRDDPSCDVQDNCDQHVHCLQDLEDMSVMVLRTQGPAALFDDHKLVLHTSSYDAKRAQVFHICGPEDVCEAYRHVLGQDKVSYEVPRLHGDEERFFVEGLSFPDAGFTGLISFHVTLLDDSNEDFSASPIFTDTVVFRVAPWIMTPSTLPPLEVYVCRVRNNTCFVDAVAELARKAGCKLTICPQAENRNDRWIQDEMELGYVQAPHKTLPVVFDSPRNGELQDFPYKRILGPDFGYVTREPRDRSVSGLDSFGNLEVSPPVVANGKEYPLGRILIGGNLPGSSGRRVTQVVRDFLHAQKVQPPVELFVDWLAVGHVDEFLSFVPAPDGKGFRMLLASPGACFKLFQEKQKCGHGRALLFQGVVDDEQVKTISINQVLSNKDLINYNKFVQSCIDWNREVLKRELGLAECDIIDIPQLFKTERKKATAFFPDLVNMLVLGKHLGIPKPFGPIINGCCCLEEKVRSLLEPLGLHCTFIDDFTPYHMLHGEVHCGTNVCRKPFSFKWWNMVP

1607 NP_001795.2 NP_005554.1

>NP_001795.2

MYVGYVLDKDSPVYPGPARPASLGLGPQAYGPPAPPPAPPQYPDFSSYSHVEPAPAPPTAWGAPFPAPKDDWAAAYGPGPAAPAASPASLAFGPPPDFSPVPAPPGPGPGLLAQPLGGPGTPSSPGAQRPTPYEWMRRSVAAGGGGGSGKTRTKDKYRVVYTDHQRLELEKEFHYSRYITIRRKSELAANLGLTERQVKIWFQNRRAKERKVNKKKQQQQQPPQPPMAHDITATPAGPSLGGLCPSNTSLLATSSPMPVKEEFLP

>NP_005554.1

MASSDIQVKELEKRASGQAFELILSPRSKESVPEFPLSPPKKKDLSLEEIQKKLEAAEERRKSHEAEVLKQLAEKREHEKEVLQKAIEENNNFSKMAEEKLTHKMEANKENREAQMAAKLERLREKDKHIEEVRKNKESKDPADETEAD

1608 NP_000928.1 NP_001619.1

>NP_000928.1

MHGGGPPSGDSACPLRTIKRVQFGVLSPDELKRMSVTEGGIKYPETTEGGRPKLGGLMDPRQGVIERTGRCQTCAGNMTECPGHFGHIELAKPVFHVGFLVKTMKVLRCVCFFCSKLLVDSNNPKIKDILAKSKGQPKKRLTHVYDLCKGKNICEGGEEMDNKFGVEQPEGDEDLTKEKGHGGCGRYQPRIRRSGLELYAEWKHVNEDSQEKKILLSPERVHEIFKRISDEECFVLGMEPRYARPEWMIVTVLPVPPLSVRPAVVMQGSARNQDDLTHKLADIVKINNQLRRNEQNGAAAHVIAEDVKLLQFHVATMVDNELPGLPRAMQKSGRPLKSLKQRLKGKEGRVRGNLMGKRVDFSARTVITPDPNLSIDQVGVPRSIAANMTFAEIVTPFNIDRLQELVRRGNSQYPGAKYIIRDNGDRIDLRFHPKPSDLHLQTGYKVERHMCDGDIVIFNRQPTLHKMSMMGHRVRILPWSTFRLNLSVTTPYNADFDGDEMNLHLPQSLETRAEIQELAMVPRMIVTPQSNRPVMGIVQDTLTAVRKFTKRDVFLERGEVMNLLMFLSTWDGKVPQPAILKPRPLWTGKQIFSLIIPGHINCIRTHSTHPDDEDSGPYKHISPGDTKVVVENGELIMGILCKKSLGTSAGSLVHISYLEMGHDITRLFYSNIQTVINNWLLIEGHTIGIGDSIADSKTYQDIQNTIKKAKQDVIEVIEKAHNNELEPTPGNTLRQTFENQVNRILNDARDKTGSSAQKSLSEYNNFKSMVVSGAKGSKINISQVIAVVGQQNVEGKRIPFGFKHRTLPHFIKDDYGPESRGFVENSYLAGLTPTEFFFHAMGGREGLIDTAVKTAETGYIQRRLIKSMESVMVKYDATVRNSINQVVQLRYGEDGLAGESVEFQNLATLKPSNKAFEKKFRFDYTNERALRRTLQEDLVKDVLSNAHIQNELEREFERMREDREVLRVIFPTGDSKVVLPCNLLRMIWNAQKIFHINPRLPSDLHPIKVVEGVKELSKKLVIVNGDDPLSRQAQENATLLFNIHLRSTLCSRRMAEEFRLSGEAFDWLLGEIESKFNQAIAHPGEMVGALAAQSLGEPATQMTLNTFHYAGVSAKNVTLGVPRLKELINISKKPKTPSLTVFLLGQSARDAERAKDILCRLEHTTLRKVTANTAIYYDPNPQSTVVAEDQEWVNVYYEMPDFDVARISPWLLRVELDRKHMTDRKLTMEQIAEKINAGFGDDLNCIFNDDNAEKLVLRIRIMNSDENKMQEEEEVVDKMDDDVFLRCIESNMLTDMTLQGIEQISKVYMHLPQTDNKKKIIITEDGEFKALQEWILETDGVSLMRVLSEKDVDPVRTTSNDIVEIFTVLGIEAVRKALERELYHVISFDGSYVNYRHLALLCDTMTCRGHLMAITRHGVNRQDTGPLMKCSFEETVDVLMEAAAHGESDPMKGVSENIMLGQLAPAGTGCFDLLLDAEKCKYGMEIPTNIPGLGAAGPTGMFFGSAPSPMGGISPAMTPWNQGATPAYGAWSPSVGSGMTPGAAGFSPSAASDASGFSPGYSPAWSPTPGSPGSPGPSSPYIPSPGGAMSPSYSPTSPAYEPRSPGGYTPQSPSYSPTSPSYSPTSPSYSPTSPNYSPTSPSYSPTSPSYSPTSPSYSPTSPSYSPTSPSYSPTSPSYSPTSPSYSPTSPSYSPTSPSYSPTSPSYSPTSPSYSPTSPSYSPTSPSYSPTSPSYSPTSPSYSPTSPNYSPTSPNYTPTSPSYSPTSPSYSPTSPNYTPTSPNYSPTSPSYSPTSPSYSPTSPSYSPSSPRYTPQSPTYTPSSPSYSPSSPSYSPTSPKYTPTSPSYSPSSPEYTPTSPKYSPTSPKYSPTSPKYSPTSPTYSPTTPKYSPTSPTYSPTSPVYTPTSPKYSPTSPTYSPTSPKYSPTSPTYSPTSPKGSTYSPTSPGYSPTSPTYSLTSPAISPDDSDEEN

>NP_001619.1

MASRLLLNNGAKMPILGLGTWKSPPGQVTEAVKVAIDVGYRHIDCAHVYQNENEVGVAIQEKLREQVVKREELFIVSKLWCTYHEKGLVKGACQKTLSDLKLDYLDLYLIHWPTGFKPGKEFFPLDESGNVVPSDTNILDTWAAMEELVDEGLVKAIGISNFNHLQVEMILNKPGLKYKPAVNQIECHPYLTQEKLIQYCQSKGIVVTAYSPLGSPDRPWAKPEDPSLLEDPRIKAIAAKHNKTTAQVLIRFPMQRNLVVIPKSVTPERIAENFKVFDFELSSQDMTTLLSYNRNWRVCALLSCTSHKDYPFHEEF

1609 NP_002189.1 NP_036205.1

>NP_002189.1

MPITRMRMRPWLEMQINSNQIPGLIWINKEEMIFQIPWKHAAKHGWDINKDACLFRSWAIHTGRYKAGEKEPDPKTWKANFRCAMNSLPDIEEVKDQSRNKGSSAVRVYRMLPPLTKNQRKERKSKSSRDAKSKAKRKSCGDSSPDTFSDGLSSSTLPDDHSSYTVPGYMQDLEVEQALTPALSPCAVSSTLPDWHIPVEVVPDSTSDLYNFQVSPMPSTSEATTDEDEEGKLPEDIMKLLEQSEWQPTNVDGKGYLLNEPGVQPTSVYGDFSCKEEPEIDSPGGDIGLSLQRVFTDLKNMDATWLDSLLTPVRLPSIQAIPCAP

>NP_036205.1

MASMGTLAFDEYGRPFLIIKDQDRKSRLMGLEALKSHIMAAKAVANTMRTSLGPNGLDKMMVDKDGDVTVTNDGATILSMMDVDHQIAKLMVELSKSQDDEIGDGTTGVVVLAGALLEEAEQLLDRGIHPIRIADGYEQAARVAIEHLDKISDSVLVDIKDTEPLIQTAKTTLGSKVVNSCHRQMAEIAVNAVLTVADMERRDVDFELIKVEGKVGGRLEDTKLIKGVIVDKDFSHPQMPKKVEDAKIAILTCPFEPPKPKTKHKLDVTSVEDYKALQKYEKEKFEEMIQQIKETGANLAICQWGFDDEANHLLLQNNLPAVRWVGGPEIELIAIATGGRIVPRFSELTAEKLGFAGLVQEISFGTTKDKMLVIEQCKNSRAVTIFIRGGNKMIIEEAKRSLHDALCVIRNLIRDNRVVYGGGAAEISCALAVSQEADKCPTLEQYAMRAFADALEVIPMALSENSGMNPIQTMTEVRARQVKEMNPALGIDCLHKGTNDMKQQHVIETLIGKKQQISLATQMVRMILKIDDIRKPGESEE

1610 NP_055909.2 NP_036232.1

>NP_055909.2

MVSGPLALRWCAWAGRGDMGPDMELPSHSKQLLLQLNQQRTKGFLCDVIIMVENSIFRAHKNVLAASSIYFKSLVLHDNLINLDTDMVSSTVFQQILDFIYTGKLLPSDQPAEPNFSTLLTAASYLQLPELAALCRRKLKRAGKPFGSGRAGSTGMGRPPRSQRLSTASVIQARYQGLVDGRKGAHAPQELPQAKGSDDELFLGGSNQDSVQGLGRAVCPAGGEAGLGGCSSSTNGSSGGCEQELGLDLSKKSPPLPPATPGPHLTPDDAAQLSDSQHGSPPAASAPPVANSASYSELGGTPDEPMDLEGAEDNHLSLLEAPGGQPRKSLRHSTRKKEWGKKEPVAGSPFERREAGPKGPCPGEEGEGVGDRVPNGILASGAGPSGPYGEPPYPCKEEEENGKDASEDSAQSGSEGGSGHASAHYMYRQEGYETVSYGDNLYVCIPCAKGFPSSEQLNAHVETHTEEELFIKEEGAYETGSGGAEEEAEDLSAPSAAYTAEPRPFKCSVCEKTYKDPATLRQHEKTHWLTRPFPCNICGKMFTQRGTMTRHMRSHLGLKPFACDECGMRFTRQYRLTEHMRVHSGEKPYECQLCGGKFTQQRNLISHLRMHTSPS

>NP_036232.1

MQVAMNGKARKEAVQTAAKELLKFVNRSPSPFHAVAECRNRLLQAGFSELKETEKWNIKPESKYFMTRNSSTIIAFAVGGQYVPGNGFSLIGAHTDSPCLRVKRRSRRSQVGFQQVGVETYGGGIWSTWFDRDLTLAGRVIVKCPTSGRLEQQLVHVERPILRIPHLAIHLQRNINENFGPNTEMHLVPILATAIQEELEKGTPEPGPLNAVDERHHSVLMSLLCAHLGLSPKDIVEMELCLADTQPAVLGGAYDEFIFAPRLDNLHSCFCALQALIDSCAGPGSLATEPHVRMVTLYDNEEVGSESAQGAQSLLTELVLRRISASCQHPTAFEEAIPKSFMISADMAHAVHPNYLDKHEENHRPLFHKGPVIKVNSKQRYASNAVSEALIREVANKVKVPLQDLMVRNDTPCGTTIGPILASRLGLRVLDLGSPQLAMHSIREMACTTGVLQTLTLFKGFFELFPSLSHNLLVD

1611 NP_002156.2 Q99487

>NP_002156.2

MKVASGSTATAAAGPSCALKAGKTASGAGEVVRCLSEQSVAISRCAGGAGARLPALLDEQQVNVLLYDMNGCYSRLKELVPTLPQNRKVSKVEILQHVIDYIRDLQLELNSESEVGTPGGRGLPVRAPLSTLNGEISALTAEAACVPADDRILCR

>Q99487

MGVNQSVGFPPVTGPHLVGCGDVMEGQNLQGSFFRLFYPCQKAEETMEQPLWIPRYEYCTGLAEYLQFNKRCGGLLFNLAVGSCRLPVSWNGPFKTKDSGYPLIIFSHGLGAFRTLYSAFCMELASRGFVVAVPEHRDRSAATTYFCKQAPEENQPTNESLQEEWIPFRRVEEGEKEFHVRNPQVHQRVSECLRVLKILQEVTAGQTVFNILPGGLDLMTLKGNIDMSRVAVMGHSFGGATAILALAKETQFRCAVALDAWMFPLERDFYPKARGPVFFINTEKFQTMESVNLMKKICAQHEQSRIITVLGSVHRSQTDFAFVTGNLIGKFFSTETRGSLDPYEGQEVMVRAMLAFLQKHLDLKEDYNQWNNLIEGIGPSLTPGAPHHLSSL

1612 NP_004588.1 NP_000404.1

>NP_004588.1

MSLLNKPKSEMTPEELQKREEEEFNTGPLSVLTQSVKNNTQVLINCRNNKKLLGRVKAFDRHCNMVLENVKEMWTEVPKSGKGKKKSKPVNKDRYISKMFLRGDSVIVVLRNPLIAGK

>NP_000404.1

MARTVVLITGCSSGIGLHLAVRLASDPSQSFKVYATLRDLKTQGRLWEAARALACPPGSLETLQLDVRDSKSVAAARERVTEGRVDVLVCNAGLGLLGPLEALGEDAVASVLDVNVVGTVRMLQAFLPDMKRRGSGRVLVTGSVGGLMGLPFNDVYCASKFALEGLCESLAVLLLPFGVHLSLIECGPVHTAFMEKVLGSPEEVLDRTDIHTFHRFYQYLAHSKQVFREAAQNPEEVAEVFLTALRAPKPTLRYFTTERFLPLLRMRLDDPSGSNYVTAMHREVFGDVPAKAEAGAEAGGGAGPGAEDEAGRSAVGDPELGDPPAAPQ

1613 NP_001721.2 Q92526

>NP_001721.2

MATRVLSMSARLGPVPQPPAPQDEPVFAQLKPVLGAANPARDAALFPGEELKHAHHRPQAQPAPAQAPQPAQPPATGPRLPPEDLVQTRCEMEKYLTPQLPPVPIIPEHKKYRRDSASVVDQFFTDTEGLPYSINMNVFLPDITHLRTGLYKSQRPCVTHIKTEPVAIFSHQSETTAPPPAPTQALPEFTSIFSSHQTAAPEVNNIFIKQELPTPDLHLSVPTQQGHLYQLLNTPDLDMPSSTNQTAAMDTLNVSMSAAMAGLNTHTSAVPQTAVKQFQGMPPCTYTMPSQFLPQQATYFPPSPPSSEPGSPDRQAEMLQNLTPPPSYAATIASKLAIHNPNLPTTLPVNSQNIQPVRYNRRSNPDLEKRRIHYCDYPGCTKVYTKSSHLKAHLRTHTGEKPYKCTWEGCDWRFARSDELTRHYRKHTGAKPFQCGVCNRSFSRSDHLALHMKRHQN

>Q92526

MAAIKAVNSKAEVARAQAALAVNICAARGLQDVLRTNLGPKGTMKMLASGAGDIKLTKDGNVLLDEMQIQHPTASLIAKVATAQDDVTGDGTTSNVLIIGELLKQADLYISEGLHPRIIAEGFEAAKIKALEVLEEVKVTKEMKRKILLDVARTSLQTKVHAELADVLTEVVVDSVLAVRRPGYPIDLFMVEIMEMKHKLGTDTKLIQGLVLDHGARHPDMKKRVEDAFILICNVSLEYEKTEVNSGFFYKTAEEKEKLVKAERKFIEDRVQKIIDLKDKVCAQSNKGFVVINQKGIDPFSLDSLAKHGIVALRRAKRRNMERLSLACGGMAVNSFEDLTVDCLGHAGLVYEYTLGEEKFTFIEECVNPCSVTLLVKGPNKHTLTQVKDAIRDGLRAIKNAIEDGCMVPGAGAIEVAMAEALVTYKNSIKGRARLGVQAFADALLIIPKVLAQNAGYDPQETLVKVQAEHVESKQLVGVDLNTGEPMVAADAGVWDNYCVKKQLLHSCTVIATNILLVDEIMRAGMSSLK

1614 NP_003085.1 NP_009197.1

>NP_003085.1

MAYRGQGQKVQKVMVQPINLIFRYLQNRSRIQVWLYEQVNMRIEGCIIGFDEYMNLVLDDAEEIHSKTKSRKQLGRIMLKGDNITLLQSVSN

>NP_009197.1

MAASAAAAELQASGGPRHPVCLLVLGMAGSGKTTFVQRLTGHLHAQGTPPYVINLDPAVHEVPFPANIDIRDTVKYKEVMKQYGLGPNGGIVTSLNLFATRFDQVMKFIEKAQNMSKYVLIDTPGQIEVFTWSASGTIITEALASSFPTVVIYVMDTSRSTNPVTFMSNMLYACSILYKTKLPFIVVMNKTDIIDHSFAVEWMQDFEAFQDALNQETTYVSNLTRSMSLVLDEFYSSLRVVGVSAVLGTGLDELFVQVTSAAEEYEREYRPEYERLKKSLANAESQQQREQLERLRKDMGSVALDAGTAKDSLSPVLHPSDLILTRGTLDEEDEEADSDTDDIDHRVTEESHEEPAFQNFMQESMAQYWKRNNK

1615 A6NDE4 NP_075066.1

>A6NDE4

MVEADHPGKLFIGGLNRETNEKMLKAVFGKHGPISEVLLIKDRTSKSRGFAFITFENPADAKNAAKDMNGKSLHGKAIKVEQAKKPSFQSGGRRRPPASSRNRSPSGSLRSARGSRGGTRGWLPSQEGHLDDGGYTPDLKMSYSRGLIPVKRGPSSRSGGPPPKKSAPSAVARSNSWMGSQGPMSQRRENYGVPPRRATISSWRNDRMSTRHDGYATNDGNHPSCQETRDYAPPSRGYAYRDNGHSNRDEHSSRGYRNHRSSRETRDYAPPSRGHAYRDYGHSRRDESYSRGYRNRRSSRETREYAPPSRGHGYRDYGHSRRHESYSRGYRNHPSSRETRDYAPPHRDYAYRDYGHSSWDEHSSRGYSYHDGYGEALGRDHSEHLSGSSYRDALQRYGTSHGAPPARGPRMSYGGSTCHAYSNTRDRYGRSWESYSSCGDFHYCDREHVCRKDQRNPPSLGRVLPDPREAYGSSSYVASIVDGGESRSEKGDSSRY

>NP_075066.1

MASGLVRLLQQGHRCLLAPVAPKLVPPVRGVKKGFRAAFRFQKELERQRLLRCPPPPVRRSEKPNWDYHAEIQAFGHRLQENFSLDLLKTAFVNSCYIKSEEAKRQQLGIEKEAVLLNLKSNQELSEQGTSFSQTCLTQFLEDEYPDMPTEGIKNLVDFLTGEEVVCHVARNLAVEQLTLSEEFPVPPAVLQQTFFAVIGALLQSSGPERTALFIRDFLITQMTGKELFEMWKIINPMGLLVEELKKRNVSAPESRLTRQSGGTTALPLYFVGLYCDKKLIAEGPGETVLVAEEEAARVALRKLYGFTENRRPWNYSKPKETLRAEKSITAS

1616 NP_006730.2 P35237

>NP_006730.2

MSGFDDPGIFYSDSFGGDAQADEGQARKSQLQRRFKEFLRQYRVGTDRTGFTFKYRDELKRHYNLGEYWIEVEMEDLASFDEDLADYLYKQPAEHLQLLEEAAKEVADEVTRPRPSGEEVLQDIQVMLKSDASPSSIRSLKSDMMSHLVKIPGIIIAASAVRAKATRISIQCRSCRNTLTNIAMRPGLEGYALPRKCNTDQAGRPKCPLDPYFIMPDKCKCVDFQTLKLQELPDAVPHGEMPRHMQLYCDRYLCDKVVPGNRVTIMGIYSIKKFGLTTSRGRDRVGVGIRSSYIRVLGIQVDTDGSGRSFAGAVSPQEEEEFRRLAALPNVYEVISKSIAPSIFGGTDMKKAIACLLFGGSRKRLPDGLTRRGDINLLMLGDPGTAKSQLLKFVEKCSPIGVYTSGKGSSAAGLTASVMRDPSSRNFIMEGGAMVLADGGVVCIDEFDKMREDDRVAIHEAMEQQTISIAKAGITTTLNSRCSVLAAANSVFGRWDETKGEDNIDFMPTILSRFDMIFIVKDEHNEERDVMLAKHVITLHVSALTQTQAVEGEIDLAKLKKFIAYCRVKCGPRLSAEAAEKLKNRYIIMRSGARQHERDSDRRSSIPITVRQLEAIVRIAEALSKMKLQPFATEADVEEALRLFQVSTLDAALSGTLSGVEGFTSQEDQEMLSRIEKQLKRRFAIGSQVSEHSIIKDFTKQKYPEHAIHKVLQLMLRRGEIQHRMQRKVLYRLK

>P35237

MDVLAEANGTFALNLLKTLGKDNSKNVFFSPMSMSCALAMVYMGAKGNTAAQMAQILSFNKSGGGGDIHQGFQSLLTEVNKTGTQYLLRMANRLFGEKSCDFLSSFRDSCQKFYQAEMEELDFISAVEKSRKHINTWVAEKTEGKIAELLSPGSVDPLTRLVLVNAVYFRGNWDEQFDKENTEERLFKVSKNEEKPVQMMFKQSTFKKTYIGEIFTQILVLPYVGKELNMIIMLPDETTDLRTVEKELTYEKFVEWTRLDMMDEEEVEVSLPRFKLEESYDMESVLRNLGMTDAFELGKADFSGMSQTDLSLSKVVHKSFVEVNEEGTEAAAATAAIMMMRCARFVPRFCADHPFLFFIQHSKTNGILFCGRFSSP

1617 P22304 NP_036429.2

>P22304

MPPPRTGRGLLWLGLVLSSVCVALGSETQANSTTDALNVLLIIVDDLRPSLGCYGDKLVRSPNIDQLASHSLLFQNAFAQQAVCAPSRVSFLTGRRPDTTRLYDFNSYWRVHAGNFSTIPQYFKENGYVTMSVGKVFHPGISSNHTDDSPYSWSFPPYHPSSEKYENTKTCRGPDGELHANLLCPVDVLDVPEGTLPDKQSTEQAIQLLEKMKTSASPFFLAVGYHKPHIPFRYPKEFQKLYPLENITLAPDPEVPDGLPPVAYNPWMDIRQREDVQALNISVPYGPIPVDFQRKIRQSYFASVSYLDTQVGRLLSALDDLQLANSTIIAFTSDHGWALGEHGEWAKYSNFDVATHVPLIFYVPGRTASLPEAGEKLFPYLDPFDSASQLMEPGRQSMDLVELVSLFPTLAGLAGLQVPPRCPVPSFHVELCREGKNLLKHFRFRDLEEDPYLPGNPRELIAYSQYPRPSDIPQWNSDKPSLKDIKIMGYSIRTIDYRYTVWVGFNPDEFLANFSDIHAGELYFVDSDPLQDHNMYNDSQGGDLFQLLMP

>NP_036429.2

MVMEKPSPLLVGREFVRQYYTLLNKAPEYLHRFYGRNSSYVHGGVDASGKPQEAVYGQNDIHHKVLSLNFSECHTKIRHVDAHATLSDGVVVQVMGLLSNSGQPERKFMQTFVLAPEGSVPNKFYVHNDMFRYEDEVFGDSEPELDEESEDEVEEEQEERQPSPEPVQENANSGYYEAHPVTNGIEEPLEESSHEPEPEPESETKTEELKPQVEEKNLEELEEKSTTPPPAEPVSLPQEPPKAFSWASVTSKNLPPSGTVSSSGIPPHVKAPVSQPRVEAKPEVQSQPPRVREQRPRERPGFPPRGPRPGRGDMEQNDSDNRRIIRYPDSHQLFVGNLPHDIDENELKEFFMSFGNVVELRINTKGVGGKLPNFGFVVFDDSEPVQRILIAKPIMFRGEVRLNVEEKKTRAARERETRGGGDDRRDIRRNDRGPGGPRGIVGGGMMRDRDGRGPPPRGGMAQKLGSGRGTGQMEGRFTGQRR

1618 Q58WW2 Q9Y4B6

>Q58WW2

MSRGGSYPHLLWDVRKRSLGLEDPSRLRSRYLGRREFIQRLKLEATLNVHDGCVNTICWNDTGEYILSGSDDTKLVISNPYSRKVLTTIRSGHRANIFSAKFLPCTNDKQIVSCSGDGVIFYTNVEQDAETNRQCQFTCHYGTTYEIMTVPNDPYTFLSCGEDGTVRWFDTRIKTSCTKEDCKDDILINCRRAATSVAICPPIPYYLAVGCSDSSVRIYDRRMLGTRATGNYAGRGTTGMVARFIPSHLNNKSCRVTSLCYSEDGQEILVSYSSDYIYLFDPKDDTARELKTPSAEERREELRQPPVKRLRLRGDWSDTGPRARPESERERDGEQSPNVSLMQRMSDMLSRWFEEASEVAQSNRGRGRSRPRGGTSQSDISTLPTVPSSPDLEVSETAMEVDTPAEQFLQPSTSSTMSAQAHSTSSPTESPHSTPLLSSPDSEQRQSVEASGHHTHHQSDNNNEKLSPKPGTGEPVLSLHYSTEGTTTSTIKLNFTDEWSSIASSSRGIGSHCKSEGQEESFVPQSSVQPPEGDSETKAPEESSEDVTKYQEGVSAENPVENHINITQSDKFTAKPLDSNSGERNDLNLDRSCGVPEESASSEKAKEPETSDQTSTESATNENNTNPEPQFQTEATGPSAHEETSTRDSALQDTDDSDDDPVLIPGARYRAGPGDRRSAVARIQEFFRRRKERKEMEELDTLNIRRPLVKMVYKGHRNSRTMIKEANFWGANFVMSGSDCGHIFIWDRHTAEHLMLLEADNHVVNCLQPHPFDPILASSGIDYDIKIWSPLEESRIFNRKLADEVITRNELMLEETRNTITVPASFMLRMLASLNHIRADRLEGDRSEGSGQENENEDEE

>Q9Y4B6

MTTVVVHVDSKAELTTLLEQWEKEHGSGQDMVPILTRMSQLIEKETEEYRKGDPDPFDDRHPGRADPECMLGHLLRILFKNDDFMNALVNAYVMTSREPPLNTAACRLLLDIMPGLETAVVFQEKEGIVENLFKWAREADQPLRTYSTGLLGGAMENQDIAANYRDENSQLVAIVLRRLRELQLQEVALRQENKRPSPRKLSSEPLLPLDEEAVDMDYGDMAVDVVDGDQEEASGDMEISFHLDSGHKTSSRVNSTTKPEDGGLKKNKSAKQGDRENFRKAKQKLGFSSSDPDRMFVELSNSSWSEMSPWVIGTNYTLYPMTPAIEQRLILQYLTPLGEYQELLPIFMQLGSRELMMFYIDLKQTNDVLLTFEALKHLASLLLHNKFATEFVAHGGVQKLLEIPRPSMAATGVSMCLYYLSYNQDAMERVCMHPHNVLSDVVNYTLWLMECSHASGCCHATMFFSICFSFRAVLELFDRYDGLRRLVNLISTLEILNLEDQGALLSDDEIFASRQTGKHTCMALRKYFEAHLAIKLEQVKQSLQRTEGGILVHPQPPYKACSYTHEQIVEMMEFLIEYGPAQLYWEPAEVFLKLSCVQLLLQLISIACNWKTYYARNDTVRFALDVLAILTVVPKIQLQLAESVDVLDEAGSTVSTVGISIILGVAEGEFFIHDAEIQKSALQIIINCVCGPDNRISSIGKFISGTPRRKLPQNPKSSEHTLAKMWNVVQSNNGIKVLLSLLSIKMPITDADQIRALACKALVGLSRSSTVRQIISKLPLFSSCQIQQLMKEPVLQDKRSDHVKFCKYAAELIERVSGKPLLIGTDVSLARLQKADVVAQSRISFPEKELLLLIRNHLISKGLGETATVLTKEADLPMTAASHSSAFTPVTAAASPVSLPRTPRIANGIATRLGSHAAVGASAPSAPTAHPQPRPPQGPLALPGPSYAGNSPLIGRISFIRERPSPCNGRKIRVLRQKSDHGAYSQSPAIKKQLDRHLPSPPTLDSIITEYLREQHARCKNPVATCPPFSLFTPHQCPEPKQRRQAPINFTSRLNRRASFPKYGGVDGGCFDRHLIFSRFRPISVFREANEDESGFTCCAFSARERFLMLGTCTGQLKLYNVFSGQEEASYNCHNSAITHLEPSRDGSLLLTSATWSQPLSALWGMKSVFDMKHSFTEDHYVEFSKHSQDRVIGTKGDIAHIYDIQTGNKLLTLFNPDLANNYKRNCATFNPTDDLVLNDGVLWDVRSAQAIHKFDKFNMNISGVFHPNGLEVIINTEIWDLRTFHLLHTVPALDQCRVVFNHTGTVMYGAMLQADDEDDLMEERMKSPFGSSFRTFNATDYKPIATIDVKRNIFDLCTDTKDCYLAVIENQGSMDALNMDTVCRLYEVGRQRLAEDEDEEEDQEEEEQEEEDDDEDDDDTDDLDELDTDQLLEAELEEDDNNENAGEDGDNDFSPSDEELANLLEEGEDGEDEDSDADEEVELILGDTDSSDNSDLEDDIILSLNE

1619 NP_000367.1 Q14249

>NP_000367.1

MEAMAASTSLPDPGDFDRNVPRICGVCGDRATGFHFNAMTCEGCKGFFRRSMKRKALFTCPFNGDCRITKDNRRHCQACRLKRCVDIGMMKEFILTDEEVQRKREMILKRKEEEALKDSLRPKLSEEQQRIIAILLDAHHKTYDPTYSDFCQFRPPVRVNDGGGSHPSRPNSRHTPSFSGDSSSSCSDHCITSSDMMDSSSFSNLDLSEEDSDDPSVTLELSQLSMLPHLADLVSYSIQKVIGFAKMIPGFRDLTSEDQIVLLKSSAIEVIMLRSNESFTMDDMSWTCGNQDYKYRVSDVTKAGHSLELIEPLIKFQVGLKKLNLHEEEHVLLMAICIVSPDRPGVQDAALIEAIQDRLSNTLQTYIRCRHPPPGSHLLYAKMIQKLADLRSLNEEHSKQYRCLSFQPECSMKLTPLVLEVFGNEIS

>Q14249

MRALRAGLTLALGAGLGAVVEGWRRRREDARAAPGLLGRLPVLPVAAAAELPPVPGGPRGPGELAKYGLPGLAQLKSRESYVLCYDPRTRGALWVVEQLRPERLRGDGDRRECDFREDDSVHAYHRATNADYRGSGFDRGHLAAAANHRWSQKAMDDTFYLSNVAPQVPHLNQNAWNNLEKYSRSLTRSYQNVYVCTGPLFLPRTEADGKSYVKYQVIGKNHVAVPTHFFKVLILEAAGGQIELRTYVMPNAPVDEAIPLERFLVPIESIERASGLLFVPNILARAGSLKAITAGSK

1620 NP_000225.1 NP_002717.3

>NP_000225.1

MQRSIMSFFHPKKEGKAKKPEKEASNSSRETEPPPKAALKEWNGVVSESDSPVKRPGRKAARVLGSEGEEEDEALSPAKGQKPALDCSQVSPPRPATSPENNASLSDTSPMDSSPSGIPKRRTARKQLPKRTIQEVLEEQSEDEDREAKRKKEEEEEETPKESLTEAEVATEKEGEDGDQPTTPPKPLKTSKAETPTESVSEPEVATKQELQEEEEQTKPPRRAPKTLSSFFTPRKPAVKKEVKEEEPGAPGKEGAAEGPLDPSGYNPAKNNYHPVEDACWKPGQKVPYLAVARTFEKIEEVSARLRMVETLSNLLRSVVALSPPDLLPVLYLSLNHLGPPQQGLELGVGDGVLLKAVAQATGRQLESVRAEAAEKGDVGLVAENSRSTQRLMLPPPPLTASGVFSKFRDIARLTGSASTAKKIDIIKGLFVACRHSEARFIARSLSGRLRLGLAEQSVLAALSQAVSLTPPGQEFPPAMVDAGKGKTAEARKTWLEEQGMILKQTFCEVPDLDRIIPVLLEHGLERLPEHCKLSPGIPLKPMLAHPTRGISEVLKRFEEAAFTCEYKYDGQRAQIHALEGGEVKIFSRNQEDNTGKYPDIISRIPKIKLPSVTSFILDTEAVAWDREKKQIQPFQVLTTRKRKEVDASEIQVQVCLYAFDLIYLNGESLVREPLSRRRQLLRENFVETEGEFVFATSLDTKDIEQIAEFLEQSVKDSCEGLMVKTLDVDATYEIAKRSHNWLKLKKDYLDGVGDTLDLVVIGAYLGRGKRAGRYGGFLLASYDEDSEELQAICKLGTGFSDEELEEHHQSLKALVLPSPRPYVRIDGAVIPDHWLDPSAVWEVKCADLSLSPIYPAARGLVDSDKGISLRFPRFIRVREDKQPEQATTSAQVACLYRKQSQIQNQQGEDSGSDPEDTY

>NP_002717.3

MLSLQYPDVYRDETAVQDYHGHKICDPYAWLEDPDSEQTKAFVEAQNKITVPFLEQCPIRGLYKERMTELYDYPKYSCHFKKGKRYFYFYNTGLQNQRVLYVQDSLEGEARVFLDPNILSDDGTVALRGYAFSEDGEYFAYGLSASGSDWVTIKFMKVDGAKELPDVLERVKFSCMAWTHDGKGMFYNSYPQQDGKSDGTETSTNLHQKLYYHVLGTDQSEDILCAEFPDEPKWMGGAELSDDGRYVLLSIREGCDPVNRLWYCDLQQESSGIAGILKWVKLIDNFEGEYDYVTNEGTVFTFKTNRQSPNYRVINIDFRDPEESKWKVLVPEHEKDVLEWIACVRSNFLVLCYLHDVKNILQLHDLTTGALLKTFPLDVGSIVGYSGQKKDTEIFYQFTSFLSPGIIYHCDLTKEELEPRVFREVTVKGIDASDYQTVQIFYPSKDGTKIPMFIVHKKGIKLDGSHPAFLYGYGGFNISITPNYSVSRLIFVRHMGGILAVANIRGGGEYGETWHKGGILANKQNCFDDFQCAAEYLIKEGYTSPKRLTINGGSNGGLLVAACANQRPDLFGCVIAQVGVMDMLKFHKYTIGHAWTTDYGCSDSKQHFEWLVKYSPLHNVKLPEADDIQYPSMLLLTADHDDRVVPLHSLKFIATLQYIVGRSRKQSNPLLIHVDTKAGHGAGKPTAKVIEEVSDMFAFIARCLNVDWIP

1621 NP_001197.1 Q7Z7A3

>NP_001197.1

MSAAAYMDFVAAQCLVSISNRAAVPEHGVAPDAERLRLPEREVTKEHGDPGDTWKDYCTLVTIAKSLLDLNKYRPIQTPSVCSDSLESPDEDMGSDSDVTTESGSSPSHSPEERQDPGSAPSPLSLLHPGVAAKGKHASEKRHKCPYSGCGKVYGKSSHLKAHYRVHTGERPFPCTWPDCLKKFSRSDELTRHYRTHTGEKQFRCPLCEKRFMRSDHLTKHARRHTEFHPSMIKRSKKALANAL

>Q7Z7A3

MPAPPCASCHAARAALRRPLSGQALCGACFCAAFEAEVLHTVLAGRLLPPGAVVAVGASGGKDSTVLAHVLRALAPRLGISLQLVAVDEGIGGYRDAALAAVRRQAARWELPLTVVAYEDLFGGWTMDAVARSTAGSGRSRSCCTFCGVLRRRALEEGARRVGATHIVTGHNADDMAETVLMNFLRGDAGRLARGGGLGSPGEGGALPRCRPLQFASQKEVVLYAHFRRLDYFSEECVYAPEAFRGHARDLLKRLEAARPSAVLDLVHSAERLALAPAARPPRPGACSRCGALASRALCQACALLDGLNRGRPRLAIGKGRRGLDEEATPGTPGDPARPPASKAVPTF

1622 NP_877433.2 NP_000934.1

>NP_877433.2

MRHNQMCCETPPTVTVYVKSGSNRSHQPKKPITLKRPICKDNWQAFEKNTHNNNKSKRPKGPCLVIQRQDMTAFFKLFDDDLIQDFLWMDCCCKIADKYLLAMTFVYFKRAKFTISEHTRINFFIALYLANTVEEDEEETKYEIFPWALGKNWRKLFPNFLKLRDQLWDRIDYRAIVSRRCCEEVMAIAPTHYIWQRERSVHHSGAVRNYNRDEVQLPRGPSATPVDCSLCGKKRRYVRLGLSSSSSLSSHTAGVTEKHSQDSYNSLSMDIIGDPSQAYTGSEVVNDHQSNKGKKTNFLKKDKSMEWFTGSEE

>NP_000934.1

MGPGPRLLLPLVLCVGLGALVFSSGAEGFRKRGPSVTAKVFFDVRIGDKDVGRIVIGLFGKVVPKTVENFVALATGEKGYGYKGSKFHRVIKDFMIQGGDITTGDGTGGVSIYGETFPDENFKLKHYGIGWVSMANAGPDTNGSQFFITLTKPTWLDGKHVVFGKVIDGMTVVHSIELQATDGHDRPLTNCSIINSGKIDVKTPFVVEIADW

1623 NP_002544.1 Q93088

>NP_002544.1

MPHLENVVLCRESQVSILQSLFGERHHFSFPSIFIYGHTASGKTYVTQTLLKTLELPHVFVNCVECFTLRLLLEQILNKLNHLSSSEDGCSTEITCETFNDFVRLFKQVTTAENLKDQTVYIVLDKAEYLRDMEANLLPGFLRLQELADRNVTVLFLSEIVWEKFRPNTGCFEPFVLYFPDYSIGNLQKILSHDHPPEYSADFYAAYINILLGVFYTVCRDLKELRHLAVLNFPKYCEPVVKGEASERDTRKLWRNIEPHLKKAMQTVYLREISSSQWEKLQKDDTDPGQLKGLSAHTHVELPYYSKFILIAAYLASYNPARTDKRFFLKHHGKIKKTNFLKKHEKTSNHLLGPKPFPLDRLLAILYSIVDSRVAPTANIFSQITSLVTLQLLTLVGHDDQLDGPKYKCTVSLDFIRAIARTVNFDIIKYLYDFL

>Q93088

MPPVGGKKAKKGILERLNAGEIVIGDGGFVFALEKRGYVKAGPWTPEAAVEHPEAVRQLHREFLRAGSNVMQTFTFYASEDKLENRGNYVLEKISGQEVNEAACDIARQVADEGDALVAGGVSQTPSYLSCKSETEVKKVFLQQLEVFMKKNVDFLIAEYFEHVEEAVWAVETLIASGKPVAATMCIGPEGDLHGVPPGECAVRLVKAGASIIGVNCHFDPTISLKTVKLMKEGLEAARLKAHLMSQPLAYHTPDCNKQGFIDLPEFPFGLEPRVATRWDIQKYAREAYNLGVRYIGGCCGFEPYHIRAIAEELAPERGFLPPASEKHGSWGSGLDMHTKPWVRARARKEYWENLRIASGRPYNPSMSKPDGWGVTKGTAELMQQKEATTEQQLKELFEKQKFKSQ

1624 NP_055331.1 P36915

>NP_055331.1

MADKRKLQGEIDRCLKKVSEGVEQFEDIWQKLHNAANANQKEKYEADLKKEIKKLQRLRDQIKTWVASNEIKDKRQLIDNRKLIETQMERFKVVERETKTKAYSKEGLGLAQKVDPAQKEKEEVGQWLTNTIDTLNMQVDQFESEVESLSVQTRKKKGDKDKQDRIEGLKRHIEKHRYHVRMLETILRMLDNDSILVDAIRKIKDDVEYYVDSSQDPDFEENEFLYDDLDLEDIPQALVATSPPSHSHMEDEIFNQSSSTPTSTTSSSPIPPSPANCTTENSEDDKKRGRSTDSEVSQSPAKNGSKPVHSNQHPQSPAVPPTYPSGPPPAASALSTTPGNNGVPAPAAPPSALGPKASPAPSHNSGTPAPYAQAVAPPAPSGPSTTQPRPPSVQPSGGGGGGSGGGGSSSSSNSSAGGGAGKQNGATSYSSVVADSPAEVALSSSGGNNASSQALGPPSGPHNPPPSTSKEPSAAAPTGAGGVAPGSGNNSGGPSLLVPLPVNPPSSPTPSFSDAKAAGALLNGPPQFSTAPEIKAPEPLSSLKSMAERAAISSGIEDPVPTLHLTERDIILSSTSAPPASAQPPLQLSEVNIPLSLGVCPLGPVPLTKEQLYQQAMEEAAWHHMPHPSDSERIRQYLPRNPCPTPPYHHQMPPPHSDTVEFYQRLSTETLFFIFYYLEGTKAQYLAAKALKKQSWRFHTKYMMWFQRHEEPKTITDEFEQGTYIYFDYEKWGQRKKEGFTFEYRYLEDRDLQ

>P36915

MPRKKPFSVKQKKKQLQDKRERKRGLQDGLRSSSNSRSGSRERREEQTDTSDGESVTHHIRRLNQQPSQGLGPRGYDPNRYRLHFERDSREEVERRKRAAREQVLQPVSAELLELDIREVYQPGSVLDFPRRPPWSYEMSKEQLMSQEERSFQDYLGKIHGAYSSEKLSYFEHNLETWRQLWRVLEMSDIVLLITDIRHPVVNFPPALYEYVTGELGLALVLVLNKVDLAPPALVVAWKHYFHQHYPQLHVVLFTSFPRDPRTPQDPSSVLKKSRRRGRGWTRALGPEQLLRACEAITVGKVDLSSWREKIARDVAGATWGNGSGEEEEEEDGPAVLVEQQTDSAMEPTGPTQERYKDGVVTIGCVGFPNVGKSSLINGLVGRKVVSVSRTPGHTRYFQTYFLTPSVKLCDCPGLIFPSLLPRQLQVLAGIYPIAQIQEPYTAVGYLASRIPVQALLHLRHPEAEDPSAEHPWCAWDICEAWAEKRGYKTAKAARNDVYRAANSLLRLAVDGRLSLCFHPPGYSEQKGTWESHPETTELVVLQGRVGPAGDEEEEEEEELSSSCEEEGEEDRDADEEGEGDEETPTSAPGSSLAGRNPYALLGEDEC

1625 NP_003007.2 Q8TE68

>NP_003007.2

MSYGRPPPDVEGMTSLKVDNLTYRTSPDTLRRVFEKYGRVGDVYIPRDRYTKESRGFAFVRFHDKRDAEDAMDAMDGAVLDGRELRVQMARYGRPPDSHHSRRGPPPRRYGGGGYGRRSRSPRRRRRSRSRSRSRSRSRSRSRYSRSKSRSRTRSRSRSTSKSRSARRSKSKSSSVSRSRSRSRSRSRSRSPPPVSKRESKSRSRSKSPPKSPEEEGAVSS

>Q8TE68

MSTTTGPEAAPKPSAKSIYEQRKRYSTVVMADVSQYPVNHLVTFCLGEDDGVHTVEDASRKLAVMDSQGRVWAQEMLLRVSPDHVTLLDPASKEELESYPLGAIVRCDAVMPPGRSRSLLLLVCQEPERAQPDVHFFQGLRLGAELIREDIQGALHNYRSGRGERRAAALRATQEELQRDRSPAAETPPLQRRPSVRAVISTVERGAGRGRPQAKPIPEAEEAQRPEPVGTSSNADSASPDLGPRGPDLAVLQAEREVDILNHVFDDVESFVSRLQKSAEAARVLEHRERGRRSRRRAAGEGLLTLRAKPPSEAEYTDVLQKIKYAFSLLARLRGNIADPSSPELLHFLFGPLQMIVNTSGGPEFASSVRRPHLTSDAVALLRDNVTPRENELWTSLGDSWTRPGLELSPEEGPPYRPEFFSGWEPPVTDPQSRAWEDPVEKQLQHERRRRQQSAPQVAVNGHRDLEPESEPQLESETAGKWVLCNYDFQARNSSELSVKQRDVLEVLDDSRKWWKVRDPAGQEGYVPYNILTPYPGPRLHHSQSPARSLNSTPPPPPAPAPAPPPALARPRWDRPRWDSCDSLNGLDPSEKEKFSQMLIVNEELQARLAQGRSGPSRAVPGPRAPEPQLSPGSDASEVRAWLQAKGFSSGTVDALGVLTGAQLFSLQREELRAVSPEEGARVYSQVTVQRSLLEDKEKVSELEAVMEKQKKKVEGEVEMEVI

1626 NP_001942.2 NP_003121.1

>NP_001942.2

MAAAEPASSGQQAPAGQGQGQRPPPQPPQAQAPQPPPPPQLGGAGGGSSRHEKSLGLLTTKFVSLLQEAKDGVLDLKAAADTLAVRQKRRIYDITNVLEGIDLIEKKSKNSIQWKGVGAGCNTKEVIDRLRYLKAEIEDLELKERELDQQKLWLQQSIKNVMDDSINNRFSYVTHEDICNCFNGDTLLAIQAPSGTQLEVPIPEMGQNGQKKYQINLKSHSGPIHVLLINKESSSSKPVVFPVPPPDDLTQPSSQSLTPVTPQKSSMATQNLPEQHVSERSQALQQTSATDISSAGSISGDIIDELMSSDVFPLLRLSPTPADDYNFNLDDNEGVCDLFDVQILNY

>NP_003121.1

MAYPGHPGAGGGYYPGGYGGAPGGPAFPGQTQDPLYGYFAAVAGQDGQIDADELQRCLTQSGIAGGYKPFNLETCRLMVSMLDRDMSGTMGFNEFKELWAVLNGWRQHFISFDTDRSGTVDPQELQKALTTMGFRLSPQAVNSIAKRYSTNGKITFDDYIACCVKLRALTDSFRRRDTAQQGVVNFPYDDFIQCVMSV

1627 NP_001231.2 P56277

>NP_001231.2

MEGERKNNNKRWYFTREQLENSPSRRFGVDPDKELSYRQQAANLLQDMGQRLNVSQLTINTAIVYMHRFYMIQSFTQFPGNSVAPAALFLAAKVEEQPKKLEHVIKVAHTCLHPQESLPDTRSEAYLQQVQDLVILESIILQTLGFELTIDHPHTHVVKCTQLVRASKDLAQTSYFMATNSLHLTTFSLQYTPPVVACVCIHLACKWSNWEIPVSTDGKHWWEYVDATVTLELLDELTHEFLQILEKTPNRLKRIWNWRACEAAKKTKADDRGTDEKTSEQTILNMISQSSSDTTIAGLMSMSTSTTSAVPSLPVSEESSSNLTSVEMLPGKRWLSSQPSFKLEPTQGHRTSENLALTGVDHSLPQDGSNAFISQKQNSKSVPSAKVSLKEYRAKHAEELAAQKRQLENMEANVKSQYAYAAQNLLSHHDSHSSVILKMPIEGSENPERPFLEKADKTALKMRIPVAGGDKAASSKPEEIKMRIKVHAAADKHNSVEDSVTKSREHKEKHKTHPSNHHHHHNHHSHKHSHSQLPVGTGNKRPGDPKHSSQTSNLAHKTYSLSSSFSSSSSTRKRGPSEETGGAVFDHPAKIAKSTKSSSLNFSFPSLPTMGQMPGHSSDTSGLSFSQPSCKTRVPHSKLDKGPTGANGHNTTQTIDYQDTVNMLHSLLSAQGVQPTQPTAFEFVRPYSDYLNPRSGGISSRSGNTDKPRPPPLPSEPPPPLPPLPK

>P56277

MPQKDPCQKQACEIQKCLQANSYMESKCQAVIQELRKCCAQYPKGRSVVCSGFEKEEEENLTRKSASK

1628 NP_620147.1 NP_115927.1

>NP_620147.1

MADMEDLFGSDADSEAERKDSDSGSDSDSDQENAASGSNASGSESDQDERGDSGQPSNKELFGDDSEDEGASHHSGSDNHSERSDNRSEASERSDHEDNDPSDVDQHSGSEAPNDDEDEGHRSDGGSHHSEAEGSEKAHSDDEKWGREDKSDQSDDEKIQNSDDEERAQGSDEDKLQNSDDDEKMQNTDDEERPQLSDDERQQLSEEEKANSDDERPVASDNDDEKQNSDDEEQPQLSDEEKMQNSDDERPQASDEEHRHSDDEEEQDHKSESARGSDSEDEVLRMKRKNAIASDSEADSDTEVPKDNSGTMDLFGGADDISSGSDGEDKPPTPGQPVDENGLPQDQQEEEPIPETRIEVEIPKVNTDLGNDLYFVKLPNFLSVEPRPFDPQYYEDEFEDEEMLDEEGRTRLKLKVENTIRWRIRRDEEGNEIKESNARIVKWSDGSMSLHLGNEVFDVYKAPLQGDHNHLFIRQGTGLQGQAVFKTKLTFRPHSTDSATHRKMTLSLADRCSKTQKIRILPMAGRDPECQRTEMIKKEEERLRASIRRESQQRRMREKQHQRGLSASYLEPDRYDEEEEGEESISLAAIKNRYKGGIREERARIYSSDSDEGSEEDKAQRLLKAKKLTSDEEGEPSGKRKAEDDDKANKKHKKYVISDEEEEDDD

>NP_115927.1

MQCLAAALKDETNMSGGGEQADILPANYVVKDRWKVLKKIGGGGFGEIYEAMDLLTRENVALKVESAQQPKQVLKMEVAVLKKLQGKDHVCRFIGCGRNEKFNYVVMQLQGRNLADLRRSQPRGTFTLSTTLRLGKQILESIEAIHSVGFLHRDIKPSNFAMGRLPSTYRKCYMLDFGLARQYTNTTGDVRPPRNVAGFRGTVRYASVNAHKNREMGRHDDLWSLFYMLVEFAVGQLPWRKIKDKEQVGMIKEKYEHRMLLKHMPSEFHLFLDHIASLDYFTKPDYQLIMSVFENSMKERGIAENEAFDWEKAGTDALLSTSTSTPPQQNTRQTAAMFGVVNVTPVPGDLLRENTEDVLQGEHLSDQENAPPILPGRPSEGLGPSPHLVPHPGGPEAEVWEETDVNRNKLRINIGKSPCVEEEQSRGMGVPSSPVRAPPDSPTTPVRSLRYRRVNSPESERLSTADGRVELPERRSRMDLPGSPSRQACSSQPAQMLSVDTGHADRQASGRMDVSASVEQEALSNAFRSVPLAEEEDFDSKEWVIIDKETELKDFPPGAEPSTSGTTDEEPEELRPLPEEGEERRRLGAEPTVRPRGRSMQALAEEDLQHLPPQPLPPQLSQGDGRSETSQPPTPGSPSHSPLHSGPRPRRRESDPTGPQRQVFSVAPPFEVNGLPRAVPLSLPYQDFKRDLSDYRERARLLNRVRRVGFSHMLLTTPQVPLAPVQPQANGKEEEEEEEEDEEEEEEDEEEEEEEEEEEEEEEEEEEEEEEAAAAVALGEVLGPRSGSSSEGSERSTDRSQEGAPSTLLADDQKESRGRASMADGDLEPEEGSKTLVLVSPGDMKKSPVTAELAPDPDLGTLAALTPQHERPQPTGSQLDVSEPGTLSSVLKSEPKPPGPGAGLGAGTVTTGVGGVAVTSSPFTKVERTFVHIAEKTHLNVMSSGGQALRSEEFSAGGELGLELASDGGAVEEGARAPLENGLALSGLNGAEIEGSALSGAPRETPSEMATNSLPNGPALADGPAPVSPLEPSPEKVATISPRRHAMPGSRPRSRIPVLLSEEDTGSEPSGSLSAKERWSKRARPQQDLARLVMEKRQGRLLLRLASGASSSSSEEQRRASETLSGTGSEEDTPASEPAAALPRKSGRAAATRSRIPRPIGLRMPMPVAAQQPASRSHGAAPALDTAITSRLQLQTPPGSATAADLRPKQPPGRGLGPGRAQAGARPPAPRSPRLPASTSAARNASASPRSQSLSRRESPSPSHQARPGVPPPRGVPPARAQPDGTPSPGGSKKGPRGKLQAQRATTKGRAGGAEGRAGAR

1629 NP_006221.1 NP_071445.1

>NP_006221.1

MFSEQAAQRAHTLLSPPSANNATFARVPVATYTNSSQPFRLGERSFSRQYAHIYATRLIQMRPFLENRAQQHWGSGVGVKKLCELQPEEKCCVVGTLFKAMPLQPSILREVSEEHNLLPQPPRSKYIHPDDELVLEDELQRIKLKGTIDVSKLVTGTVLAVFGSVRDDGKFLVEDYCFADLAPQKPAPPLDTDRFVLLVSGLGLGGGGGESLLGTQLLVDVVTGQLGDEGEQCSAAHVSRVILAGNLLSHSTQSRDSINKAKYLTKKTQAASVEAVKMLDEILLQLSASVPVDVMPGEFDPTNYTLPQQPLHPCMFPLATAYSTLQLVTNPYQATIDGVRFLGTSGQNVSDIFRYSSMEDHLEILEWTLRVRHISPTAPDTLGCYPFYKTDPFIFPECPHVYFCGNTPSFGSKIIRGPEDQTVLLVTVPDFSATQTACLVNLRSLACQPISFSGFGAEDDDLGGLGLGP

>NP_071445.1

MGEEGGSASHDEEERASVLLGHSPGCEMCSQEAFQAQRSQLVELLVSGSLEGFESVLDWLLSWEVLSWEDYEGFHLLGQPLSHLARRLLDTVWNKGTWACQKLIAAAQEAQADSQSPKLHGCWDPHSLHPARDLQSHRPAIVRRLHSHVENMLDLAWERGFVSQYECDEIRLPIFTPSQRARRLLDLATVKANGLAAFLLQHVQELPVPLALPLEAATCKKYMAKLRTTVSAQSRFLSTYDGAETLCLEDIYTENVLEVWADVGMAGPPQKSPATLGLEELFSTPGHLNDDADTVLVVGEAGSGKSTLLQRLHLLWAAGQDFQEFLFVFPFSCRQLQCMAKPLSVRTLLFEHCCWPDVGQEDIFQLLLDHPDRVLLTFDGFDEFKFRFTDRERHCSPTDPTSVQTLLFNLLQGNLLKNARKVVTSRPAAVSAFLRKYIRTEFNLKGFSEQGIELYLRKRHHEPGVADRLIRLLQETSALHGLCHLPVFSWMVSKCHQELLLQEGGSPKTTTDMYLLILQHFLLHATPPDSASQGLGPSLLRGRLPTLLHLGRLALWGLGMCCYVFSAQQLQAAQVSPDDISLGFLVRAKGVVPGSTAPLEFLHITFQCFFAAFYLALSADVPPALLRHLFNCGRPGNSPMARLLPTMCIQASEGKDSSVAALLQKAEPHNLQITAAFLAGLLSREHWGLLAECQTSEKALLRRQACARWCLARSLRKHFHSIPPAAPGEAKSVHAMPGFIWLIRSLYEMQEERLARKAARGLNVGHLKLTFCSVGPTECAALAFVLQHLRRPVALQLDYNSVGDIGVEQLLPCLGVCKALYLRDNNISDRGICKLIECALHCEQLQKLALFNNKLTDGCAHSMAKLLACRQNFLALRLGNNYITAAGAQVLAEGLRGNTSLQFLGFWGNRVGDEGAQALAEALGDHQSLRWLSLVGNNIGSVGAQALALMLAKNVMLEELCLEENHLQDEGVCSLAEGLKKNSSLKILKLSNNCITYLGAEALLQALERNDTILEVWLRGNTFSLEEVDKLGCRDTRLLL

1630 NP_005185.2 NP_036361.1

>NP_005185.2

MQRLVAWDPACLPLPPPPPAFKSMEVANFYYEADCLAAAYGGKAAPAAPPAARPGPRPPAGELGSIGDHERAIDFSPYLEPLGAPQAPAPATATDTFEAAPPAPAPAPASSGQHHDFLSDLFSDDYGGKNCKKPAEYGYVSLGRLGAAKGALHPGCFAPLHPPPPPPPPPAELKAEPGFEPADCKRKEEAGAPGGGAGMAAGFPYALRAYLGYQAVPSGSSGSLSTSSSSSPPGTPSPADAKAPPTACYAGAAPAPSQVKSKAKKTVDKHSDEYKIRRERNNIAVRKSRDKAKMRNLETQHKVLELTAENERLQKKVEQLSRELSTLRNLFKQLPEPLLASSGHC

>NP_036361.1

MSTSWSDRLQNAADMPANMDKHALKKYRREAYHRVFVNRSLAMEKIKCFGFDMDYTLAVYKSPEYESLGFELTVERLVSIGYPQELLSFAYDSTFPTRGLVFDTLYGNLLKVDAYGNLLVCAHGFNFIRGPETREQYPNKFIQRDDTERFYILNTLFNLPETYLLACLVDFFTNCPRYTSCETGFKDGDLFMSYRSMFQDVRDAVDWVHYKGSLKEKTVENLEKYVVKDGKLPLLLSRMKEVGKVFLATNSDYKYTDKIMTYLFDFPHGPKPGSSHRPWQSYFDLILVDARKPLFFGEGTVLRQVDTKTGKLKIGTYTGPLQHGIVYSGGSSDTICDLLGAKGKDILYIGDHIFGDILKSKKRQGWRTFLVIPELAQELHVWTDKSSLFEELQSLDIFLAELYKHLDSSSNERPDISSIQRRIKKVTHDMDMCYGMMGSLFRSGSRQTLFASQVMRYADLYAASFINLLYYPFSYLFRAAHVLMPHESTVEHTHVDINEMESPLATRNRTSVDFKDTDYKRHQLTRSISEIKPPNLFPLAPQEITHCHDEDDDEEEEEEEE

1631 Q15022 NP_004332.2

>Q15022

MAPQKHGGGGGGGSGPSAGSGGGGFGGSAAVAAATASGGKSGGGSCGGGGSYSASSSSSAAAAAGAAVLPVKKPKMEHVQADHELFLQAFEKPTQIYRFLRTRNLIAPIFLHRTLTYMSHRNSRTNIKRKTFKVDDMLSKVEKMKGEQESHSLSAHLQLTFTGFFHKNDKPSPNSENEQNSVTLEVLLVKVCHKKRKDVSCPIRQVPTGKKQVPLNPDLNQTKPGNFPSLAVSSNEFEPSNSHMVKSYSLLFRVTRPGRREFNGMINGETNENIDVNEELPARRKRNREDGEKTFVAQMTVFDKNRRLQLLDGEYEVAMQEMEECPISKKRATWETILDGKRLPPFETFSQGPTLQFTLRWTGETNDKSTAPIAKPLATRNSESLHQENKPGSVKPTQTIAVKESLTTDLQTRKEKDTPNENRQKLRIFYQFLYNNNTRQQTEARDDLHCPWCTLNCRKLYSLLKHLKLCHSRFIFNYVYHPKGARIDVSINECYDGSYAGNPQDIHRQPGFAFSRNGPVKRTPITHILVCRPKRTKASMSEFLESEDGEVEQQRTYSSGHNRLYFHSDTCLPLRPQEMEVDSEDEKDPEWLREKTITQIEEFSDVNEGEKEVMKLWNLHVMKHGFIADNQMNHACMLFVENYGQKIIKKNLCRNFMLHLVSMHDFNLISIMSIDKAVTKLREMQQKLEKGESASPANEEITEEQNGTANGFSEINSKEKALETDSVSGVSKQSKKQKL

>NP_004332.2

MAALVLEDGSVLRGQPFGAAVSTAGEVVFQTGMVGYPEALTDPSYKAQILVLTYPLIGNYGIPPDEMDEFGLCKWFESSGIHVAALVVGECCPTPSHWSATRTLHEWLQQHGIPGLQGVDTRELTKKLREQGSLLGKLVQNGTEPSSLPFLDPNARPLVPEVSIKTPRVFNTGGAPRILALDCGLKYNQIRCLCQRGAEVTVVPWDHALDSQEYEGLFLSNGPGDPASYPSVVSTLSRVLSEPNPRPVFGICLGHQLLALAIGAKTYKMRYGNRGHNQPCLLVGSGRCFLTSQNHGFAVETDSLPADWAPLFTNANDGSNEGIVHNSLPFFSVQFHPEHQAGPSDMELLFDIFLETVKEATAGNPGGQTVRERLTERLCPPGIPTPGSGLPPPRKVLILGSGGLSIGQAGEFDYSGSQAIKALKEENIQTLLINPNIATVQTSQGLADKVYFLPITPHYVTQVIRNERPDGVLLTFGGQTALNCGVELTKAGVLARYGVRVLGTPVETIELTEDRRAFAARMAEIGEHVAPSEAANSLEQAQAAAERLGYPVLVRAAFALGGLGSGFASNREELSALVAPAFAHTSQVLVDKSLKGWKEIEYEVVRDAYGNCVTVCNMENLDPLGIHTGESIVVAPSQTLNDREYQLLRQTAIKVTQHLGIVGECNVQYALNPESEQYYIIEVNARLSRSSALASKATGYPLAYVAAKLALGIPLPELRNSVTGGTAAFEPSVDYCVVKIPRWDLSKFLRVSTKIGSCMKSVGEVMGIGRSFEEAFQKALRMVDENCVGFDHTVKPVSDMELETPTDKRIFVVAAALWAGYSVDRLYELTRIDRWFLHRMKRIIAHAQLLEQHRGQPLPPDLLQQAKCLGFSDKQIALAVLSTELAVRKLRQELGICPAVKQIDTVAAEWPAQTNYLYLTYWGTTHDLTFRTPHVLVLGSGVYRIGSSVEFDWCAVGCIQQLRKMGYKTIMVNYNPETVSTDYDMCDRLYFDEISFEVVMDIYELENPEGVILSMGGQLPNNMAMALHRQQCRVLGTSPEAIDSAENRFKFSRLLDTIGISQPQWRELSDLESARQFCQTVGYPCVVRPSYVLSGAAMNVAYTDGDLERFLSSAAAVSKEHPVVISKFIQEAKEIDVDAVASDGVVAAIAISEHVENAGVHSGDATLVTPPQDITAKTLERIKAIVHAVGQELQVTGPFNLQLIAKDDQLKVIECNVRVSRSFPFVSKTLGVDLVALATRVIMGEEVEPVGLMTGSGVVGVKVPQFSFSRLAGADVVLGVEMTSTGEVAGFGESRCEAYLKAMLSTGFKIPKKNILLTIGSYKNKSELLPTVRLLESLGYSLYASLGTADFYTEHGVKVTAVDWHFEEAVDGECPPQRSILEQLAEKNFELVINLSMRGAGGRRLSSFVTKGYRTRRLAADFSVPLIIDIKCTKLFVEALGQIGPAPPLKVHVDCMTSQKLVRLPGLIDVHVHLREPGGTHKEDFASGTAAALAGGITMVCAMPNTRPPIIDAPALALAQKLAEAGARCDFALFLGASSENAGTLGTVAGSAAGLKLYLNETFSELRLDSVVQWMEHFETWPSHLPIVAHAEQQTVAAVLMVAQLTQRSVHICHVARKEEILLIKAAKARGLPVTCEVAPHHLFLSHDDLERLGPGKGEVRPELGSRQDVEALWENMAVIDCFASDHAPHTLEEKCGSRPPPGFPGLETMLPLLLTAVSEGRLSLDDLLQRLHHNPRRIFHLPPQEDTYVEVDLEHEWTIPSHMPFSKAHWTPFEGQKVKGTVRRVVLRGEVAYIDGQVLVPPGYGQDVRKWPQGAVPQLPPSAPATSEMTTTPERPRRGIPGLPDGRFHLPPRIHRASDPGLPAEEPKEKSSRKVAEPELMGTPDGTCYPPPPVPRQASPQNLGTPGLLHPQTSPLLHSLVGQHILSVQQFTKDQMSHLFNVAHTLRMMVQKERSLDILKGKVMASMFYEVSTRTSSSFAAAMARLGGAVLSFSEATSSVQKGESLADSVQTMSCYADVVVLRHPQPGAVELAAKHCRRPVINAGDGVGEHPTQALLDIFTIREELGTVNGMTITMVGDLKHGRTVHSLACLLTQYRVSLRYVAPPSLRMPPTVRAFVASRGTKQEEFESIEEALPDTDVLYMTRIQKERFGSTQEYEACFGQFILTPHIMTRAKKKMVVMHPMPRVNEISVEVDSDPRAAYFRQAENGMYIRMALLATVLGRF

1632 NP_003502.1 NP_001815.2

>NP_003502.1

MSGRGKQGGKARAKAKTRSSRAGLQFPVGRVHRLLRKGNYAERVGAGAPVYLAAVLEYLTAEILELAGNAARDNKKTRIIPRHLQLAIRNDEELNKLLGKVTIAQGGVLPNIQAVLLPKKTESHHKAKGK

>NP_001815.2

MPFGNTHNKFKLNYKPEEEYPDLSKHNNHMAKVLTLELYKKLRDKETPSGFTVDDVIQTGVDNPGHPFIMTVGCVAGDEESYEVFKELFDPIISDRHGGYKPTDKHKTDLNHENLKGGDDLDPNYVLSSRVRTGRSIKGYTLPPHCSRGERRAVEKLSVEALNSLTGEFKGKYYPLKSMTEKEQQQLIDDHFLFDKPVSPLLLASGMARDWPDARGIWHNDNKSFLVWVNEEDHLRVISMEKGGNMKEVFRRFCVGLQKIEEIFKKAGHPFMWNQHLGYVLTCPSNLGTGLRGGVHVKLAHLSKHPKFEEILTRLRLQKRGTGGVDTAAVGSVFDVSNADRLGSSEVEQVQLVVDGVKLMVEMEKKLEKGQSIDDMIPAQK

1633 Q9H2W2 NP_077726.1

>Q9H2W2

MATAESRALQFAEGAAFPAYRAPHAGGALLPPPSPAAALLPAPPAGPGPATFAGFLGRDPGPAPPPPASLGSPAPPKGAAAPSASQRRKRTSFSAEQLQLLELVFRRTRYPDIHLRERLAALTLLPESRIQVWFQNRRAKSRRQSGKSFQPLARPEIILNHCAPGTETKCLKPQLPLEVDVNCLPEPNGVGGGISDSSSQGQNFETCSPLSEDIGSKLDSWEEHIFSAFGNF

>NP_077726.1

MGDEDWEAEINPHMSSYVPIFEKDRYSGENGDNFNRTPASSSEMDDGPSRRDHFMKSGFASGRNFGNRDAGECNKRDNTSTMGGFGVGKSFGNRGFSNSRFEDGDSSGFWRESSNDCEDNPTRNRGFSKRGGYRDGNNSEASGPYRRGGRGSFRGCRGGFGLGSPNNDLDPDECMQRTGGLFGSRRPVLSGTGNGDTSQSRSGSGSERGGYKGLNEEVITGSGKNSWKSEAEGGESSDTQGPKVTYIPPPPPEDEDSIFAHYQTGINFDKYDTILVEVSGHDAPPAILTFEEANLCQTLNNNIAKAGYTKLTPVQKYSIPIILAGRDLMACAQTGSGKTAAFLLPILAHMMHDGITASRFKELQEPECIIVAPTRELVNQIYLEARKFSFGTCVRAVVIYGGTQLGHSIRQIVQGCNILCATPGRLMDIIGKEKIGLKQIKYLVLDEADRMLDMGFGPEMKKLISCPGMPSKEQRQTLMFSATFPEEIQRLAAEFLKSNYLFVAVGQVGGACRDVQQTVLQVGQFSKREKLVEILRNIGDERTMVFVETKKKADFIATFLCQEKISTTSIHGDREQREREQALGDFRFGKCPVLVATSVAARGLDIENVQHVINFDLPSTIDEYVHRIGRTGRCGNTGRAISFFDLESDNHLAQPLVKVLTDAQQDVPAWLEEIAFSTYIPGFSGSTRGNVFASVDTRKGKSTLNTAGFSSSQAPNPVDDESWD

1634 NP_002193.2 NP_066565.1

>NP_002193.2

MGDMGDPPKKKRLISLCVGCGNQIHDQYILRVSPDLEWHAACLKCAECNQYLDESCTCFVRDGKTYCKRDYIRLYGIKCAKCSIGFSKNDFVMRARSKVYHIECFRCVACSRQLIPGDEFALREDGLFCRADHDVVERASLGAGDPLSPLHPARPLQMAAEPISARQPALRPHVHKQPEKTTRVRTVLNEKQLHTLRTCYAANPRPDALMKEQLVEMTGLSPRVIRVWFQNKRCKDKKRSIMMKQLQQQQPNDKTNIQGMTGTPMVAASPERHDGGLQANPVEVQSYQPPWKVLSDFALQSDIDQPAFQQLVNFSEGGPGSNSTGSEVASMSSQLPDTPNSMVASPIEA

>NP_066565.1

MADESETAVKPPAPPLPQMMEGNGNGHEHCSDCENEEDNSYNRGGLSPANDTGAKKKKKKQKKKKEKGSETDSAQDQPVKMNSLPAERIQEIQKAIELFSVGQGPAKTMEEASKRSYQFWDTQPVPKLGEVVNTHGPVEPDKDNIRQEPYTLPQGFTWDALDLGDRGVLKELYTLLNENYVEDDDNMFRFDYSPEFLLWALRPPGWLPQWHCGVRVVSSRKLVGFISAIPANIHIYDTEKKMVEINFLCVHKKLRSKRVAPVLIREITRRVHLEGIFQAVYTAGVVLPKPVGTCRYWHRSLNPRKLIEVKFSHLSRNMTMQRTMKLYRLPETPKTAGLRPMETKDIPVVHQLLTRYLKQFHLTPVMSQEEVEHWFYPQENIIDTFVVENANGEVTDFLSFYTLPSTIMNHPTHKSLKAAYSFYNVHTQTPLLDLMSDALVLAKMKGFDVFNALDLMENKTFLEKLKFGIGDGNLQYYLYNWKCPSMGAEKVGLVLQ

1635 NP_002351.1 Q9BRJ2

>NP_002351.1

MTTNPKPNKALKVKKEAGENAPVLSDDELVSMSVRELNQHLRGLTKEEVTRLKQRRRTLKNRGYAASCRIKRVTQKEELERQRVELQQEVEKLARENSSMRLELDALRSKYEALQTFARTVARGPVAPSKVATTSVITIVKSTELSSTSVPFSAAS

>Q9BRJ2

MAAPIPQGFSCLSRFLGWWFRQPVLVTQSAAIVPVRTKKRFTPPIYQPKFKTEKEFMQHARKAGLVIPPEKSDRSIHLACTAGIFDAYVPPEGDARISSLSKEGLIERTERMKKTMASQVSIRRIKDYDANFKIKDFPEKAKDIFIEAHLCLNNSDHDRLHTLVTEHCFPDMTWDIKYKTVRWSFVESLEPSHVVQVRCSSMMNQGNVYGQITVRMHTRQTLAIYDRFGRLMYGQEDVPKDVLEYVVFEKQLTNPYGSWRMHTKIVPPWAPPKQPILKTVMIPGPQLKPEEEYEEAQGEAQKPQLA

1636 NP_003063.2 P49589

>NP_003063.2

MSTPDPPLGGTPRPGPSPGPGPSPGAMLGPSPGPSPGSAHSMMGPSPGPPSAGHPIPTQGPGGYPQDNMHQMHKPMESMHEKGMSDDPRYNQMKGMGMRSGGHAGMGPPPSPMDQHSQGYPSPLGGSEHASSPVPASGPSSGPQMSSGPGGAPLDGADPQALGQQNRGPTPFNQNQLHQLRAQIMAYKMLARGQPLPDHLQMAVQGKRPMPGMQQQMPTLPPPSVSATGPGPGPGPGPGPGPGPAPPNYSRPHGMGGPNMPPPGPSGVPPGMPGQPPGGPPKPWPEGPMANAAAPTSTPQKLIPPQPTGRPSPAPPAVPPAASPVMPPQTQSPGQPAQPAPMVPLHQKQSRITPIQKPRGLDPVEILQEREYRLQARIAHRIQELENLPGSLAGDLRTKATIELKALRLLNFQRQLRQEVVVCMRRDTALETALNAKAYKRSKRQSLREARITEKLEKQQKIEQERKRRQKHQEYLNSILQHAKDFKEYHRSVTGKIQKLTKAVATYHANTEREQKKENERIEKERMRRLMAEDEEGYRKLIDQKKDKRLAYLLQQTDEYVANLTELVRQHKAAQVAKEKKKKKKKKKAENAEGQTPAIGPDGEPLDETSQMSDLPVKVIHVESGKILTGTDAPKAGQLEAWLEMNPGYEVAPRSDSEESGSEEEEEEEEEEQPQAAQPPTLPVEEKKKIPDPDSDDVSEVDARHIIENAKQDVDDEYGVSQALARGLQSYYAVAHAVTERVDKQSALMVNGVLKQYQIKGLEWLVSLYNNNLNGILADEMGLGKTIQTIALITYLMEHKRINGPFLIIVPLSTLSNWAYEFDKWAPSVVKVSYKGSPAARRAFVPQLRSGKFNVLLTTYEYIIKDKHILAKIRWKYMIVDEGHRMKNHHCKLTQVLNTHYVAPRRLLLTGTPLQNKLPELWALLNFLLPTIFKSCSTFEQWFNAPFAMTGEKVDLNEEETILIIRRLHKVLRPFLLRRLKKEVEAQLPEKVEYVIKCDMSALQRVLYRHMQAKGVLLTDGSEKDKKGKGGTKTLMNTIMQLRKICNHPYMFQHIEESFSEHLGFTGGIVQGLDLYRASGKFELLDRILPKLRATNHKVLLFCQMTSLMTIMEDYFAYRGFKYLRLDGTTKAEDRGMLLKTFNEPGSEYFIFLLSTRAGGLGLNLQSADTVIIFDSDWNPHQDLQAQDRAHRIGQQNEVRVLRLCTVNSVEEKILAAAKYKLNVDQKVIQAGMFDQKSSSHERRAFLQAILEHEEQDESRHCSTGSGSASFAHTAPPPAGVNPDLEEPPLKEEDEVPDDETVNQMIARHEEEFDLFMRMDLDRRREEARNPKRKPRLMEEDELPSWIIKDDAEVERLTCEEEEEKMFGRGSRHRKEVDYSDSLTEKQWLKAIEEGTLEEIEEEVRQKKSSRKRKRDSDAGSSTPTTSTRSRDKDDESKKQKKRGRPPAEKLSPNPPNLTKKMKKIVDAVIKYKDSSSGRQLSEVFIQLPSRKELPEYYELIRKPVDFKKIKERIRNHKYRSLNDLEKDVMLLCQNAQTFNLEGSLIYEDSIVLQSVFTSVRQKIEKEDDSEGEESEEEEEGEEEGSESESRSVKVKIKLGRKEKAQDRLKGGRRRPSRGSRAKPVVSDDDSEEEQEEDRSGSGSEED

>P49589

MADSSGQQGKGRRVQPQWSPPAGTQPCRLHLYNSLTRNKEVFIPQDGKKVTWYCCGPTVYDASHMGHARSYISFDILRRVLKDYFKFDVFYCMNITDIDDKIIKRARQNHLFEQYREKRPEAAQLLEDVQAALKPFSVKLNETTDPDKKQMLERIQHAVQLATEPLEKAVQSRLTGEEVNSCVEVLLEEAKDLLSDWLDSTLGCDVTDNSIFSKLPKFWEGDFHRDMEALNVLPPDVLTRVSEYVPEIVNFVQKIVDNGYGYVSNGSVYFDTAKFASSEKHSYGKLVPEAVGDQKALQEGEGDLSISADRLSEKRSPNDFALWKASKPGEPSWPCPWGKGRPGWHIECSAMAGTLLGASMDIHGGGFDLRFPHHDNELAQSEAYFENDCWVRYFLHTGHLTIAGCKMSKSLKNFITIKDALKKHSARQLRLAFLMHSWKDTLDYSSNTMESALQYEKFLNEFFLNVKDILRAPVDITGQFEKWGEEEAELNKNFYDKKTAIHKALCDNVDTRTVMEEMRALVSQCNLYMAARKAVRKRPNQALLENIALYLTHMLKIFGAVEEDSSLGFPVGGPGTSLSLEATVMPYLQVLSEFREGVRKIAREQKVPEILQLSDALRDNILPELGVRFEDHEGLPTVVKLVDRNTLLKEREEKRRVEEEKRKKKEEAARRKQEQEAAKLAKMKIPPSEMFLSETDKYSKFDENGLPTHDMEGKELSKGQAKKLKKLFEAQEKLYKEYLQMAQNGSFQ

1637 NP_036377.1 NP_071445.1

>NP_036377.1

MALTSFLPAPTQLSQDQLEAEEKARSQRSRQTSLVSSRREPPPYGYRKGWIPRLLEDFGDGGAFPEIHVAQYPLDMGRKKKMSNALAIQVDSEGKIKYDAIARQGQSKDKVIYSKYTDLVPKEVMNADDPDLQRPDEEAIKEITEKTRVALEKSVSQKVAAAMPVRAADKLAPAQYIRYTPSQQGVAFNSGAKQRVIRMVEMQKDPMEPPRFKINKKIPRGPPSPPAPVMHSPSRKMTVKEQQEWKIPPCISNWKNAKGYTIPLDKRLAADGRGLQTVHINENFAKLAEALYIADRKAREAVEMRAQVERKMAQKEKEKHEEKLREMAQKARERRAGIKTHVEKEDGEARERDEIRHDRRKERQHDRNLSRAAPDKRSKLQRNENRDISEVIALGVPNPRTSNEVQYDQRLFNQSKGMDSGFAGGEDEIYNVYDQAWRGGKDMAQSIYRPSKNLDKDMYGDDLEARIKTNRFVPDKEFSGSDRRQRGREGPVQFEEDPFGLDKFLEEAKQHGGSKRPSDSSRPKEHEHEGKKRRKE

>NP_071445.1

MGEEGGSASHDEEERASVLLGHSPGCEMCSQEAFQAQRSQLVELLVSGSLEGFESVLDWLLSWEVLSWEDYEGFHLLGQPLSHLARRLLDTVWNKGTWACQKLIAAAQEAQADSQSPKLHGCWDPHSLHPARDLQSHRPAIVRRLHSHVENMLDLAWERGFVSQYECDEIRLPIFTPSQRARRLLDLATVKANGLAAFLLQHVQELPVPLALPLEAATCKKYMAKLRTTVSAQSRFLSTYDGAETLCLEDIYTENVLEVWADVGMAGPPQKSPATLGLEELFSTPGHLNDDADTVLVVGEAGSGKSTLLQRLHLLWAAGQDFQEFLFVFPFSCRQLQCMAKPLSVRTLLFEHCCWPDVGQEDIFQLLLDHPDRVLLTFDGFDEFKFRFTDRERHCSPTDPTSVQTLLFNLLQGNLLKNARKVVTSRPAAVSAFLRKYIRTEFNLKGFSEQGIELYLRKRHHEPGVADRLIRLLQETSALHGLCHLPVFSWMVSKCHQELLLQEGGSPKTTTDMYLLILQHFLLHATPPDSASQGLGPSLLRGRLPTLLHLGRLALWGLGMCCYVFSAQQLQAAQVSPDDISLGFLVRAKGVVPGSTAPLEFLHITFQCFFAAFYLALSADVPPALLRHLFNCGRPGNSPMARLLPTMCIQASEGKDSSVAALLQKAEPHNLQITAAFLAGLLSREHWGLLAECQTSEKALLRRQACARWCLARSLRKHFHSIPPAAPGEAKSVHAMPGFIWLIRSLYEMQEERLARKAARGLNVGHLKLTFCSVGPTECAALAFVLQHLRRPVALQLDYNSVGDIGVEQLLPCLGVCKALYLRDNNISDRGICKLIECALHCEQLQKLALFNNKLTDGCAHSMAKLLACRQNFLALRLGNNYITAAGAQVLAEGLRGNTSLQFLGFWGNRVGDEGAQALAEALGDHQSLRWLSLVGNNIGSVGAQALALMLAKNVMLEELCLEENHLQDEGVCSLAEGLKKNSSLKILKLSNNCITYLGAEALLQALERNDTILEVWLRGNTFSLEEVDKLGCRDTRLLL

1638 NP_056464.1 P82914

>NP_056464.1

MASVQASRRQWCYLCDLPKMPWAMVWDFSEAVCRGCVNFEGADRIELLIDAARQLKRSHVLPEGRSPGPPALKHPATKDLAAAAAQGPQLPPPQAQPQPSGTGGGVSGQDRYDRATSSGRLPLPSPALEYTLGSRLANGLGREEAVAEGARRALLGSMPGLMPPGLLAAAVSGLGSRGLTLAPGLSPARPLFGSDFEKEKQQRNADCLAELNEAMRGRAEEWHGRPKAVREQLLALSACAPFNVRFKKDHGLVGRVFAFDATARPPGYEFELKLFTEYPCGSGNVYAGVLAVARQMFHDALREPGKALASSGFKYLEYERRHGSGEWRQLGELLTDGVRSFREPAPAEALPQQYPEPAPAALCGPPPRAPSRNLAPTPRRRKASPEPEGEAAGKMTTEEQQQRHWVAPGGPYSAETPGVPSPIAALKNVAEALGHSPKDPGGGGGPVRAGGASPAASSTAQPPTQHRLVARNGEAEVSPTAGAEAVSGGGSGTGATPGAPLCCTLCRERLEDTHFVQCPSVPGHKFCFPCSREFIKAQGPAGEVYCPSGDKCPLVGSSVPWAFMQGEIATILAGDIKVKKERDP

>P82914

MLRVAWRTLSLIRTRAVTQVLVPGLPGGGSAKFPFNQWGLQPRSLLLQAARGYVVRKPAQSRLDDDPPPSTLLKDYQNVPGIEKVDDVVKRLLSLEMANKKEMLKIKQEQFMKKIVANPEDTRSLEARIIALSVKIRSYEEHLEKHRKDKAHKRYLLMSIDQRKKMLKNLRNTNYDVFEKICWGLGIEYTFPPLYYRRAHRRFVTKKALCIRVFQETQKLKKRRRALKAAAAAQKQAKRRNPDSPAKAIPKTLKDSQ

1639 NP_003087.1 NP_001937.2

>NP_003087.1

MSKAHPPELKKFMDKKLSLKLNGGRHVQGILRGFDPFMNLVIDECVEMATSGQQNNIGMVVIRGNSIIMLEALERV

>NP_001937.2

MIDTLRPVPFASEMAISKTVAWLNEQLELGNERLLLMDCRPQELYESSHIESAINVAIPGIMLRRLQKGNLPVRALFTRGEDRDRFTRRCGTDTVVLYDESSSDWNENTGGESVLGLLLKKLKDEGCRAFYLEGGFSKFQAEFSLHCETNLDGSCSSSSPPLPVLGLGGLRISSDSSSDIESDLDRDPNSATDSDGSPLSNSQPSFPVEILPFLYLGCAKDSTNLDVLEEFGIKYILNVTPNLPNLFENAGEFKYKQIPISDHWSQNLSQFFPEAISFIDEARGKNCGVLVHCLAGISRSVTVTVAYLMQKLNLSMNDAYDIVKMKKSNISPNFNFMGQLLDFERTLGLSSPCDNRVPAQQLYFTTPSNQNVYQVDSLQST

1640 NP_997001.1 Q6XUX3

>NP_997001.1

MVNVLKGVLIECDPAMKQFLLYLDESNALGKKFIIQDIDDTHVFVIAELVNVLQERVGELMDQNAFSLTQK

>Q6XUX3

MEGDGVPWGSEPVSGPGPGGGGMIRELCRGFGRYRRYLGRLRQNLRETQKFFRDIKCSHNHTCLSSLTGGGGAERGPAGDVAETGLQAGQLSCISFPPKEEKYLQQIVDCLPCILILGQDCNVKCQLLNLLLGVQVLPTTKLGSEESCKLRRLRFTYGTQTRVSLALPGQYELVHTLVAHQGNWETIPEEDLEVQENNEDAAHVLAELEVTMHHALLQEVDVVVAPCQGLRPTVDVLGDLVNDFLPVITYALHKDELSERDEQELQEIRKYFSFPVFFFKVPKLGSEIIDSSTRRMESERSPLYRQLIDLGYLSSSHWNCGAPGQDTKAQSMLVEQSEKLRHLSTFSHQVLQTRLVDAAKALNLVHCHCLDIFINQAFDMQRDLQITPKRLEYTRKKENELYESLMNIANRKQEEMKDMIVETLNTMKEELLDDATNMEFKDVIVPENGEPVGTREIKCCIRQIQELIISRLNQAVANKLISSVDYLRESFVGTLERCLQSLEKSQDVSVHITSNYLKQILNAAYHVEVTFHSGSSVTRMLWEQIKQIIQRITWVSPPAITLEWKRKVAQEAIESLSASKLAKSICSQFRTRLNSSHEAFAASLRQLEAGHSGRLEKTEDLWLRVRKDHAPRLARLSLESRSLQDVLLHRKPKLGQELGRGQYGVVYLCDNWGGHFPCALKSVVPPDEKHWNDLALEFHYMRSLPKHERLVDLHGSVIDYNYGGGSSIAVLLIMERLHRDLYTGLKAGLTLETRLQIALDVVEGIRFLHSQGLVHRDIKLKNVLLDKQNRAKITDLGFCKPEAMMSGSIVGTPIHMAPELFTGKYDNSVDVYAFGILFWYICSGSVKLPEAFERCASKDHLWNNVRRGARPERLPVFDEECWQLMEACWDGDPLKRPLLGIVQPMLQGIMNRLCKSNSEQPNRGLDDST

1641 Q12857 Q9NWF9

>Q12857

MYSPLCLTQDEFHPFIEALLPHVRAFAYTWFNLQARKRKYFKKHEKRMSKEEERAVKDELLSEKPEVKQKWASRLLAKLRKDIRPEYREDFVLTVTGKKPPCCVLSNPDQKGKMRRIDCLRQADKVWRLDLVMVILFKGIPLESTDGERLVKSPQCSNPGLCVQPHHIGVSVKELDLYLAYFVHAADSSQSESPSQPSDADIKDQPENGHLGFQDSFVTSGVFSVTELVRVSQTPIAAGTGPNFSLSDLESSSYYSMSPGAMRRSLPSTSSTSSTKRLKSVEDEMDSPGEEPFYTGQGRSPGSGSQSSGWHEVEPGMPSPTTLKKSEKSGFSSPSPSQTSSLGTAFTQHHRPVITGPRASPHATPSTLHFPTSPIIQQPGPYFSHPAIRYHPQETLKEFVQLVCPDAGQQAGQVGFLNPNGSSQGKVHNPFLPTPMLPPPPPPPMARPVPLPVPDTKPPTTSTEGGAASPTSPTYSTPSTSPANRFVSVGPRDPSFVNIPQQTQSWYLG

>Q9NWF9

MEEGNNNEEVIHLNNFHCHRGQEWINLRDGPITISDSSDEERIPMLVTPAPQQHEEEDLDDDVILTEDDSEDDYGEFLDLGPPGISEFTKPSGQTEREPKPGPSHNQAANDIVNPRSEQKVIILEEGSLLYTESDPLETQNQSSEDSETELLSNLGESAALADDQAIEEDCWLDHPYFQSLNQQPREITNQVVPQERQPEAELGRLLFQHEFPGPAFPRPEPQQGGISGPSSPQPAHPLGEFEDQQLASDDEEPGPAFPMQESQEPNLENIWGQEAAEVDQELVELLVKETEARFPDVANGFIEEIIHFKNYYDLNVLCNFLLENPDYPKREDRIIINPSSSLLASQDETKLPKIDFFDYSKLTPLDQRCFIQAADLLMADFKVLSSQDIKWALHELKGHYAITRKALSDAIKKWQELSPETSGKRKKRKQMNQYSYIDFKFEQGDIKIEKRMFFLENKRRHCRSYDRRALLPAVQQEQEFYEQKIKEMAEHEDFLLALQMNEEQYQKDGQLIECRCCYGEFPFEELTQCADAHLFCKECLIRYAQEAVFGSGKLELSCMEGSCTCSFPTSELEKVLPQTILYKYYERKAEEEVAAAYADELVRCPSCSFPALLDSDVKRFSCPNPHCRKETCRKCQGLWKEHNGLTCEELAEKDDIKYRTSIEEKMTAARIRKCHKCGTGLIKSEGCNRMSCRCGAQMCYLCRVSINGYDHFCQHPRSPGAPCQECSRCSLWTDPTEDDEKLIEEIQKEAEEEQKRKNGENTFKRIGPPLEKPVEKVQRVEALPRPVPQNLPQPQMPPYAFAHPPFPLPPVRPVFNNFPLNMGPIPAPYVPPLPNVRVNYDFGPIHMPLEHNLPMHFGPQPRHRF

1642 NP_001528.1 Q01581

>NP_001528.1

MAETDPKTVQDLTSVVQTLLQQMQDKFQTMSDQIIGRIDDMSSRIDDLEKNIADLMTQAGVEELESENKIPATQKS

>Q01581

MPGSLPLNAEACWPKDVGIVALEIYFPSQYVDQAELEKYDGVDAGKYTIGLGQAKMGFCTDREDINSLCMTVVQNLMERNNLSYDCIGRLEVGTETIIDKSKSVKTNLMQLFEESGNTDIEGIDTTNACYGGTAAVFNAVNWIESSSWDGRYALVVAGDIAVYATGNARPTGGVGAVALLIGPNAPLIFERGLRGTHMQHAYDFYKPDMLSEYPIVDGKLSIQCYLSALDRCYSVYCKKIHAQWQKEGNDKDFTLNDFGFMIFHSPYCKLVQKSLARMLLNDFLNDQNRDKNSIYSGLEAFGDVKLEDTYFDRDVEKAFMKASSELFSQKTKASLLVSNQNGNMYTSSVYGSLASVLAQYSPQQLAGKRIGVFSYGSGLAATLYSLKVTQDATPGSALDKITASLCDLKSRLDSRTGVAPDVFAENMKLREDTHHLVNYIPQGSIDSLFEGTWYLVRVDEKHRRTYARRPTPNDDTLDEGVGLVHSNIATEHIPSPAKKVPRLPATAAEPEAAVISNGEH

1643 NP_003204.2 O15305

>NP_003204.2

MEGTAGTITSNEWSSPTSPEGSTASGGSQALDKPIDNDAEGVWSPDIEQSFQEALAIYPPCGRRKIILSDEGKMYGRNELIARYIKLRTGKTRTRKQVSSHIQVLARRKAREIQAKLKDQAAKDKALQSMAAMSSAQIISATAFHSSMALARGPGRPAVSGFWQGALPGQAGTSHDVKPFSQQTYAVQPPLPLPGFESPAGPAPSPSAPPAPPWQGRSVASSKLWMLEFSAFLEQQQDPDTYNKHLFVHIGQSSPSYSDPYLEAVDIRQIYDKFPEKKGGLKDLFERGPSNAFFLVKFWADLNTNIEDEGSSFYGVSSQYESPENMIITCSTKVCSFGKQVVEKVETEYARYENGHYSYRIHRSPLCEYMINFIHKLKHLPEKYMMNSVLENFTILQVVTNRDTQETLLCIAYVFEVSASEHGAQHHIYRLVKE

>O15305

MAAPGPALCLFDVDGTLTAPRQKITKEMDDFLQKLRQKIKIGVVGGSDFEKVQEQLGNDVVEKYDYVFPENGLVAYKDGKLLCRQNIQSHLGEALIQDLINYCLSYIAKIKLPKKRGTFIEFRNGMLNVSPIGRSCSQEERIEFYELDKKENIRQKFVADLRKEFAGKGLTFSIGGQISFDVFPDGWDKRYCLRHVENDGYKTIYFFGDKTMPGGNDHEIFTDPRTMGYSVTAPEDTRRICELLFS

1644 NP_904357.1 NP_434700.2

>NP_904357.1

MPFLGQDWRSPGWSWIKTEDGWKRCESCSQKLERENNRCNISHSIILNSEDGEIFNNEEHEYASKKRKKDHFRNDTNTQSFYREKWIYVHKESTKERHGYCTLGEAFNRLDFSSAIQDIRRFNYVVKLLQLIAKSQLTSLSGVAQKNYFNILDKIVQKVLDDHHNPRLIKDLLQDLSSTLCILIRGVGKSVLVGNINIWICRLETILAWQQQLQDLQMTKQVNNGLTLSDLPLHMLNNILYRFSDGWDIITLGQVTPTLYMLSEDRQLWKKLCQYHFAEKQFCRHLILSEKGHIEWKLMYFALQKHYPAKEQYGDTLHFCRHCSILFWKDYHLALLFKDSGHPCTAADPDSCFTPVSPQHFIDLFKF

>NP_434700.2

MSDYENDDECWSVLEGFRVTLTSVIDPSRITPYLRQCKVLNPDDEEQVLSDPNLVIRKRKVGVLLDILQRTGHKGYVAFLESLELYYPQLYKKVTGKEPARVFSMIIDASGESGLTQLLMTEVMKLQKKVQDLTALLSSKDDFIKELRVKDSLLRKHQERVQRLKEECEAGSRELKRCKEENYDLAMRLAHQSEEKGAALMRNRDLQLEIDQLKHSLMKAEDDCKVERKHTLKLRHAMEQRPSQELLWELQQEKALLQARVQELEASVQEGKLDRSSPYIQVLEEDWRQALRDHQEQANTIFSLRKDLRQGEARRLRCMEEKEMFELQCLALRKDSKMYKDRIEAILLQMEEVAIERDQAIATREELHAQHARGLQEKDALRKQVRELGEKADELQLQVFQCEAQLLAVEGRLRRQQLETLVLSSDLEDGSPRRSQELSLPQDLEDTQLSDKGCLAGGGSPKQPFAALHQEQVLRNPHDAGLSSGEPPEKERRRLKESFENYRRKRALRKMQKGWRQGEEDRENTTGSDNTDTEGS

1645 NP_002189.1 NP_075066.1

>NP_002189.1

MPITRMRMRPWLEMQINSNQIPGLIWINKEEMIFQIPWKHAAKHGWDINKDACLFRSWAIHTGRYKAGEKEPDPKTWKANFRCAMNSLPDIEEVKDQSRNKGSSAVRVYRMLPPLTKNQRKERKSKSSRDAKSKAKRKSCGDSSPDTFSDGLSSSTLPDDHSSYTVPGYMQDLEVEQALTPALSPCAVSSTLPDWHIPVEVVPDSTSDLYNFQVSPMPSTSEATTDEDEEGKLPEDIMKLLEQSEWQPTNVDGKGYLLNEPGVQPTSVYGDFSCKEEPEIDSPGGDIGLSLQRVFTDLKNMDATWLDSLLTPVRLPSIQAIPCAP

>NP_075066.1

MASGLVRLLQQGHRCLLAPVAPKLVPPVRGVKKGFRAAFRFQKELERQRLLRCPPPPVRRSEKPNWDYHAEIQAFGHRLQENFSLDLLKTAFVNSCYIKSEEAKRQQLGIEKEAVLLNLKSNQELSEQGTSFSQTCLTQFLEDEYPDMPTEGIKNLVDFLTGEEVVCHVARNLAVEQLTLSEEFPVPPAVLQQTFFAVIGALLQSSGPERTALFIRDFLITQMTGKELFEMWKIINPMGLLVEELKKRNVSAPESRLTRQSGGTTALPLYFVGLYCDKKLIAEGPGETVLVAEEEAARVALRKLYGFTENRRPWNYSKPKETLRAEKSITAS

1646 Q13510 NP_006750.3

>Q13510

MPGRSCVALVLLAAAVSCAVAQHAPPWTEDCRKSTYPPSGPTYRGAVPWYTINLDLPPYKRWHELMLDKAPVLKVIVNSLKNMINTFVPSGKIMQVVDEKLPGLLGNFPGPFEEEMKGIAAVTDIPLGEIISFNIFYELFTICTSIVAEDKKGHLIHGRNMDFGVFLGWNINNDTWVITEQLKPLTVNLDFQRNNKTVFKASSFAGYVGMLTGFKPGLFSLTLNERFSINGGYLGILEWILGKKDVMWIGFLTRTVLENSTSYEEAKNLLTKTKILAPAYFILGGNQSGEGCVITRDRKESLDVYELDAKQGRWYVVQTNYDRWKHPFFLDDRRTPAKMCLNRTSQENISFETMYDVLSTKPVLNKLTVYTTLIDVTKGQFETYLRDCPDPCIGW

>NP_006750.3

MSRFVQDLSKAMSQDGASQFQEVIRQELELSVKKELEKILTTASSHEFEHTKKDLDGFRKLFHRFLQEKGPSVDWGKIQRPPEDSIQPYEKIKARGLPDNISSVLNKLVVVKLNGGLGTSMGCKGPKSLIGVRNENTFLDLTVQQIEHLNKTYNTDVPLVLMNSFNTDEDTKKILQKYNHCRVKIYTFNQSRYPRINKESLLPVAKDVSYSGENTEAWYPPGHGDIYASFYNSGLLDTFIGEGKEYIFVSNIDNLGATVDLYILNHLMNPPNGKRCEFVMEVTNKTRADVKGGTLTQYEGKLRLVEIAQVPKAHVDEFKSVSKFKIFNTNNLWISLAAVKRLQEQNAIDMEIIVNAKTLDGGLNVIQLETAVGAAIKSFENSLGINVPRSRFLPVKTTSDLLLVMSNLYSLNAGSLTMSEKREFPTVPLVKLGSSFTKVQDYLRRFESIPDMLELDHLTVSGDVTFGKNVSLKGTVIIIANHGDRIDIPPGAVLENKIVSGNLRILDH

1647 NP_055412.2 NP_004865.1

>NP_055412.2

MVVTRSARAKASIQAASAESSGQKSFAANGIQAHPESSTGSDARTTAESQTTGKQSLIPRTPKARKRKSRTTGSLPKGTEPSTDGETSEAESNYSVSEHHDTILRVTRRRQILIACSPVSSVRKKPKVTPTKESYTEEIVSEAESHVSGISRIVLPTEKTTGARRSKAKSLTDPSQESHTEAISDAETSSSDISFSGIATRRTRSMQRKLKAQTEKKDSKIVPGNEKQIVGTPVNSEDSDTRQTSHLQARSLSEINKPNFYNNDFDDDFSHRSSENILTVHEQANVESLKETKQNCKDLDEDANGITDEGKEINEKSSQLKNLSELQDTSLQQLVSQRHSTPQNKNAVSVHSNLNSEAVMKSLTQTFATVEVGRWNNNKKSPIKASDLTKFGDCGGSDDEEESTVISVSEDMNSEGNVDFECDTKLYTSAPNTSQGKDNSVLLVLSSDESQQSENSENEEDTLCFVENSGQRESLSGDTGSLSCDNALFVIDTTPGMSADKNFYLEEEDKASEVAIEEEKEEEEDEKSEEDSSDHDENEDEFSDEEDFLNSTKAKLLKLTSSSIDPGLSIKQLGGLYINFNADKLQSNKRTLTQIKEKKKNELLQKAVITPDFEKNHCVPPYSESKYQLQKKRRKERQKTAGDGWFGMKAPEMTNELKNDLKALKMRASMDPKRFYKKNDRDGFPKYFQIGTIVDNPADFYHSRIPKKQRKRTIVEELLADSEFRRYNRRKYSEIMAEKAANAAGKKFRKKKKFRN

>NP_004865.1

MSALRRSGYGPSDGPSYGRYYGPGGGDVPVHPPPPLYPLRPEPPQPPISWRVRGGGPAETTWLGEGGGGDGYYPSGGAWPEPGRAGGSHQEQPPYPSYNSNYWNSTARSRAPYPSTYPVRPELQGQSLNSYTNGAYGPTYPPGPGANTASYSGAYYAPGYTQTSYSTEVPSTYRSSGNSPTPVSRWIYPQQDCQTEAPPLRGQVPGYPPSQNPGMTLPHYPYGDGNRSVPQSGPTVRPQEDAWASPGAYGMGGRYPWPSSAPSAPPGNLYMTESTSPWPSSGSPQSPPSPPVQQPKDSSYPYSQSDQSMNRHNFPCSVHQYESSGTVNNDDSDLLDSQVQYSAEPQLYGNATSDHPNNQDQSSSLPEECVPSDESTPPSIKKIIHVLEKVQYLEQEVEEFVGKKTDKAYWLLEEMLTKELLELDSVETGGQDSVRQARKEAVCKIQAILEKLEKKGL

1648 NP_000866.1 NP_001146.2

>NP_000866.1

MKSGSGGGSPTSLWGLLFLSAALSLWPTSGEICGPGIDIRNDYQQLKRLENCTVIEGYLHILLISKAEDYRSYRFPKLTVITEYLLLFRVAGLESLGDLFPNLTVIRGWKLFYNYALVIFEMTNLKDIGLYNLRNITRGAIRIEKNADLCYLSTVDWSLILDAVSNNYIVGNKPPKECGDLCPGTMEEKPMCEKTTINNEYNYRCWTTNRCQKMCPSTCGKRACTENNECCHPECLGSCSAPDNDTACVACRHYYYAGVCVPACPPNTYRFEGWRCVDRDFCANILSAESSDSEGFVIHDGECMQECPSGFIRNGSQSMYCIPCEGPCPKVCEEEKKTKTIDSVTSAQMLQGCTIFKGNLLINIRRGNNIASELENFMGLIEVVTGYVKIRHSHALVSLSFLKNLRLILGEEQLEGNYSFYVLDNQNLQQLWDWDHRNLTIKAGKMYFAFNPKLCVSEIYRMEEVTGTKGRQSKGDINTRNNGERASCESDVLHFTSTTTSKNRIIITWHRYRPPDYRDLISFTVYYKEAPFKNVTEYDGQDACGSNSWNMVDVDLPPNKDVEPGILLHGLKPWTQYAVYVKAVTLTMVENDHIRGAKSEILYIRTNASVPSIPLDVLSASNSSSQLIVKWNPPSLPNGNLSYYIVRWQRQPQDGYLYRHNYCSKDKIPIRKYADGTIDIEEVTENPKTEVCGGEKGPCCACPKTEAEKQAEKEEAEYRKVFENFLHNSIFVPRPERKRRDVMQVANTTMSSRSRNTTAADTYNITDPEELETEYPFFESRVDNKERTVISNLRPFTLYRIDIHSCNHEAEKLGCSASNFVFARTMPAEGADDIPGPVTWEPRPENSIFLKWPEPENPNGLILMYEIKYGSQVEDQRECVSRQEYRKYGGAKLNRLNPGNYTARIQATSLSGNGSWTDPVFFYVQAKTGYENFIHLIIALPVAVLLIVGGLVIMLYVFHRKRNNSRLGNGVLYASVNPEYFSAADVYVPDEWEVAREKITMSRELGQGSFGMVYEGVAKGVVKDEPETRVAIKTVNEAASMRERIEFLNEASVMKEFNCHHVVRLLGVVSQGQPTLVIMELMTRGDLKSYLRSLRPEMENNPVLAPPSLSKMIQMAGEIADGMAYLNANKFVHRDLAARNCMVAEDFTVKIGDFGMTRDIYETDYYRKGGKGLLPVRWMSPESLKDGVFTTYSDVWSFGVVLWEIATLAEQPYQGLSNEQVLRFVMEGGLLDKPDNCPDMLFELMRMCWQYNPKMRPSFLEIISSIKEEMEPGFREVSFYYSEENKLPEPEELDLEPENMESVPLDPSASSSSLPLPDRHSGHKAENGPGPGVLVLRASFDERQPYAHMNGGRKNERALPLPQSSTC

>NP_001146.2

MAKPAQGAKYRGSIHDFPGFDPNQDAEALYTAMKGFGSDKEAILDIITSRSNRQRQEVCQSYKSLYGKDLIADLKYELTGKFERLIVGLMRPPAYCDAKEIKDAISGIGTDEKCLIEILASRTNEQMHQLVAAYKDAYERDLEADIIGDTSGHFQKMLVVLLQGTREEDDVVSEDLVQQDVQDLYEAGELKWGTDEAQFIYILGNRSKQHLRLVFDEYLKTTGKPIEASIRGELSGDFEKLMLAVVKCIRSTPEYFAERLFKAMKGLGTRDNTLIRIMVSRSELDMLDIREIFRTKYEKSLYSMIKNDTSGEYKKTLLKLSGGDDDAAGQFFPEAAQVAYQMWELSAVARVELKGTVRPANDFNPDADAKALRKAMKGLGTDEDTIIDIITHRSNVQRQQIRQTFKSHFGRDLMTDLKSEISGDLARLILGLMMPPAHYDAKQLKKAMEGAGTDEKALIEILATRTNAEIRAINEAYKEDYHKSLEDALSSDTSGHFRRILISLATGHREEGGENLDQAREDAQVAAEILEIADTPSGDKTSLETRFMTILCTRSYPHLRRVFQEFIKMTNYDVEHTIKKEMSGDVRDAFVAIVQSVKNKPLFFADKLYKSMKGAGTDEKTLTRIMVSRSEIDLLNIRREFIEKYDKSLHQAIEGDTSGDFLKALLALCGGED

1649 Q15170 NP_001893.2

>Q15170

MDKPRKENEEEPQSRPRPMRRGLRWSTLPKSSPPRSSLRRSSPRRRSSFLRSSCLSSCLRCSSRRTPSAGLSRKDLFEGRPPMEQPPCGVGKHKLEEGSFKERLARSRPQFRGDIHGRNLSNEEMIQAADELEEMKRVRNKLMIMHWKAKRSRPYPI

>NP_001893.2

MQEKDASSQGFLPHFQHFATQAIHVGQDPEQWTSRAVVPPISLSTTFKQGAPGQHSGFEYSRSGNPTRNCLEKAVAALDGAKYCLAFASGLAATVTITHLLKAGDQIICMDDVYGGTNRYFRQVASEFGLKISFVDCSKIKLLEAAITPETKLVWIETPTNPTQKVIDIEGCAHIVHKHGDIILVVDNTFMSPYFQRPLALGADISMYSATKYMNGHSDVVMGLVSVNCESLHNRLRFLQNSLGAVPSPIDCYLCNRGLKTLHVRMEKHFKNGMAVAQFLESNPWVEKVIYPGLPSHPQHELVKRQCTGCTGMVTFYIKGTLQHAEIFLKNLKLFTLAESLGGFESLAELPAIMTHASVLKNDRDVLGISDTLIRLSVGLEDEEDLLEDLDQALKAAHPPSGSHS

1650 Q13642 NP_001441.4

>Q13642

MAEKFDCHYCRDPLQGKKYVQKDGHHCCLKCFDKFCANTCVECRKPIGADSKEVHYKNRFWHDTCFRCAKCLHPLANETFVAKDNKILCNKCTTREDSPKCKGCFKAIVAGDQNVEYKGTVWHKDCFTCSNCKQVIGTGSFFPKGEDFYCVTCHETKFAKHCVKCNKAITSGGITYQDQPWHADCFVCVTCSKKLAGQRFTAVEDQYYCVDCYKNFVAKKCAGCKNPITGKRTVSRVSHPVSKARKPPVCHGKRLPLTLFPSANLRGRHPGGERTCPSWVVVLYRKNRSLAAPRGPGLVKAPVWWPMKDNPGTTTASTAKNAP

>NP_001441.4

MTERFDCHHCNESLFGKKYILREESPYCVVCFETLFANTCEECGKPIGCDCKDLSYKDRHWHEACFHCSQCRNSLVDKPFAAKEDQLLCTDCYSNEYSSKCQECKKTIMPGTRKMEYKGSSWHETCFICHRCQQPIGTKSFIPKDNQNFCVPCYEKQHAMQCVQCKKPITTGGVTYREQPWHKECFVCTACRKQLSGQRFTARDDFAYCLNCFCDLYAKKCAGCTNPISGLGGTKYISFEERQWHNDCFNCKKCSLSLVGRGFLTERDDILCPDCGKDI

1651 NP_877433.2 NP_002684.1

>NP_877433.2

MRHNQMCCETPPTVTVYVKSGSNRSHQPKKPITLKRPICKDNWQAFEKNTHNNNKSKRPKGPCLVIQRQDMTAFFKLFDDDLIQDFLWMDCCCKIADKYLLAMTFVYFKRAKFTISEHTRINFFIALYLANTVEEDEEETKYEIFPWALGKNWRKLFPNFLKLRDQLWDRIDYRAIVSRRCCEEVMAIAPTHYIWQRERSVHHSGAVRNYNRDEVQLPRGPSATPVDCSLCGKKRRYVRLGLSSSSSLSSHTAGVTEKHSQDSYNSLSMDIIGDPSQAYTGSEVVNDHQSNKGKKTNFLKKDKSMEWFTGSEE

>NP_002684.1

MSRLLWRKVAGATVGPGPVPAPGRWVSSSVPASDPSDGQRRRQQQQQQQQQQQQQPQQPQVLSSEGGQLRHNPLDIQMLSRGLHEQIFGQGGEMPGEAAVRRSVEHLQKHGLWGQPAVPLPDVELRLPPLYGDNLDQHFRLLAQKQSLPYLEAANLLLQAQLPPKPPAWAWAEGWTRYGPEGEAVPVAIPEERALVFDVEVCLAEGTCPTLAVAISPSAWYSWCSQRLVEERYSWTSQLSPADLIPLEVPTGASSPTQRDWQEQLVVGHNVSFDRAHIREQYLIQGSRMRFLDTMSMHMAISGLSSFQRSLWIAAKQGKHKVQPPTKQGQKSQRKARRGPAISSWDWLDISSVNSLAEVHRLYVGGPPLEKEPRELFVKGTMKDIRENFQDLMQYCAQDVWATHEVFQQQLPLFLERCPHPVTLAGMLEMGVSYLPVNQNWERYLAEAQGTYEELQREMKKSLMDLANDACQLLSGERYKEDPWLWDLEWDLQEFKQKKAKKVKKEPATASKLPIEGAGAPGDPMDQEDLGPCSEEEEFQQDVMARACLQKLKGTTELLPKRPQHLPGHPGWYRKLCPRLDDPAWTPGPSLLSLQMRVTPKLMALTWDGFPLHYSERHGWGYLVPGRRDNLAKLPTGTTLESAGVVCPYRAIESLYRKHCLEQGKQQLMPQEAGLAEEFLLTDNSAIWQTVEELDYLEVEAEAKMENLRAAVPGQPLALTARGGPKDTQPSYHHGNGPYNDVDIPGCWFFKLPHKDGNSCNVGSPFAKDFLPKMEDGTLQAGPGGASGPRALEINKMISFWRNAHKRISSQMVVWLPRSALPRAVIRHPDYDEEGLYGAILPQVVTAGTITRRAVEPTWLTASNARPDRVGSELKAMVQAPPGYTLVGADVDSQELWIAAVLGDAHFAGMHGCTAFGWMTLQGRKSRGTDLHSKTATTVGISREHAKIFNYGRIYGAGQPFAERLLMQFNHRLTQQEAAEKAQQMYAATKGLRWYRLSDEGEWLVRELNLPVDRTEGGWISLQDLRKVQRETARKSQWKKWEVVAERAWKGGTESEMFNKLESIATSDIPRTPVLGCCISRALEPSAVQEEFMTSRVNWVVQSSAVDYLHLMLVAMKWLFEEFAIDGRFCISIHDEVRYLVREEDRYRAALALQITNLLTRCMFAYKLGLNDLPQSVAFFSAVDIDRCLRKEVTMDCKTPSNPTGMERRYGIPQGEALDIYQIIELTKGSLEKRSQPGP

1652 NP_003494.1 NP_653288.1

>NP_003494.1

MEASLGIQMDEPMAFSPQRDRFQAEGSLKKNEQNFKLAGVKKDIEKLYEAVPQLSNVFKIEDKIGEGTFSSVYLATAQLQVGPEEKIALKHLIPTSHPIRIAAELQCLTVAGGQDNVMGVKYCFRKNDHVVIAMPYLEHESFLDILNSLSFQEVREYMLNLFKALKRIHQFGIVHRDVKPSNFLYNRRLKKYALVDFGLAQGTHDTKIELLKFVQSEAQQERCSQNKSHIITGNKIPLSGPVPKELDQQSTTKASVKRPYTNAQIQIKQGKDGKEGSVGLSVQRSVFGERNFNIHSSISHESPAVKLMKQSKTVDVLSRKLATKKKAISTKVMNSAVMRKTASSCPASLTCDCYATDKVCSICLSRRQQVAPRAGTPGFRAPEVLTKCPNQTTAIDMWSAGVIFLSLLSGRYPFYKASDDLTALAQIMTIRGSRETIQAAKTFGKSILCSKEVPAQDLRKLCERLRGMDSSTPKLTSDIQGHASHQPAISEKTDHKASCLVQTPPGQYSGNSFKKGDSNSCEHCFDEYNTNLEGWNEVPDEAYDLLDKLLDLNPASRITAEEALLHPFFKDMSL

>NP_653288.1

MLRTAGRDGLCRLSTYLEELEAVELKKFKLYLGTATELGEGKIPWGSMEKAGPLEMAQLLITHFGPEEAWRLALSTFERINRKDLWERGQREDLVRDTPPGGPSSLGNQSTCLLEVSLVTPRKDPQETYRDYVRRKFRLMEDRNARLGECVNLSHRYTRLLLVKEHSNPMQVQQQLLDTGRGHARTVGHQASPIKIETLFEPDEERPEPPRTVVMQGAAGIGKSMLAHKVMLDWADGKLFQGRFDYLFYINCREMNQSATECSMQDLIFSCWPEPSAPLQELIRVPERLLFIIDGFDELKPSFHDPQGPWCLCWEEKRPTELLLNSLIRKKLLPELSLLITTRPTALEKLHRLLEHPRHVEILGFSEAERKEYFYKYFHNAEQAGQVFNYVRDNEPLFTMCFVPLVCWVVCTCLQQQLEGGGLLRQTSRTTTAVYMLYLLSLMQPKPGAPRLQPPPNQRGLCSLAADGLWNQKILFEEQDLRKHGLDGEDVSAFLNMNIFQKDINCERYYSFIHLSFQEFFAAMYYILDEGEGGAGPDQDVTRLLTEYAFSERSFLALTSRFLFGLLNEETRSHLEKSLCWKVSPHIKMDLLQWIQSKAQSDGSTLQQGSLEFFSCLYEIQEEEFIQQALSHFQVIVVSNIASKMEHMVSSFCLKRCRSAQVLHLYGATYSADGEDRARCSAGAHTLLVQLPERTVLLDAYSEHLAAALCTNPNLIELSLYRNALGSRGVKLLCQGLRHPNCKLQNLRLKRCRISSSACEDLSAALIANKNLTRMDLSGNGVGFPGMMLLCEGLRHPQCRLQMIQLRKCQLESGACQEMASVLGTNPHLVELDLTGNALEDLGLRLLCQGLRHPVCRLRTLWLKICRLTAAACDELASTLSVNQSLRELDLSLNELGDLGVLLLCEGLRHPTCKLQTLRLGICRLGSAACEGLSVVLQANHNLRELDLSFNDLGDWGLWLLAEGLQHPACRLQKLWLDSCGLTAKACENLYFTLGINQTLTDLYLTNNALGDTGVRLLCKRLSHPGCKLRVLWLFGMDLNKMTHSRLAALRVTKPYLDIGC

1653 NP_079485.1 NP_004369.1

>NP_079485.1

MSKLSFRARALDASKPLPVFRCEDLPDLHEYASINRAVPQMPTGMEKEEESEHHLQRAISAQQVYGEKRDNMVIPVPEAESNIAYYESIYPGEFKMPKQLIHIQPFSLDAEQPDYDLDSEDEVFVNKLKKKMDICPLQFEEMIDRLEKGSGQQPVSLQEAKLLLKEDDELIREVYEYWIKKRKNCRGPSLIPSVKQEKRDGSSTNDPYVAFRRRTEKMQTRKNRKNDEASYEKMLKLRRDLSRAVTILEMIKRREKSKRELLHLTLEIMEKRYNLGDYNGEIMSEVMAQRQPMKPTYAIPIIPITNSSQFKHQEAMDVKEFKVNKQDKADLIRPKRKYEKKPKVLPSSAAATPQQTSPAALPVFNAKDLNQYDFPSSDEEPLSQVLSGSSEAEEDNDPDGPFAFRRKAGCQYYAPHLDQTGNWPWTSPKDGGLGDVRYRYCLTTLTVPQRCIGFARRRVGRGGRVLLDRAHSDYDSVFHHLDLEMLSSPQHSPVNQFANTSETNTSDKSFSKDLSQILVNIKSCRWRHFRPRTPSLHDSDNDELSCRKLYRSINRTGTAQPGTQTCSTSTQSKSSSGSAHFAFTAEQYQQHQQQLALMQKQQLAQIQQQQANSNSSTNTSQNLASNQQKSGFRLNIQGLERTLQGFVSKTLDSASAQFAASALVTSEQLMGFKMKDDVVLGIGVNGVLPASGVYKGLHLSSTTPTALVHTSPSTAGSALLQPSNITQTSSSHSALSHQVTAANSATTQVLIGNNIRLTVPSSVATVNSIAPINARHIPRTLSAVPSSALKLAAAANCQVSKVPSSSSVDSVPRENHESEKPALNNIADNTVAMEVT

>NP_004369.1

MPNFAGTWKMRSSENFDELLKALGVNAMLRKVAVAAASKPHVEIRQDGDQFYIKTSTTVRTTEINFKVGEGFEEETVDGRKCRSLATWENENKIHCTQTLLEGDGPKTYWTRELANDELILTFGADDVVCTRIYVRE

1654 NP_008816.3 P11245

>NP_008816.3

MEGCDSPVVSGKDNGCGIPQHQQWTELNSTHLPDKPSSMEQSTGESHGPLDSLRAPFNERLAESTASAGPPSEPASKEVTCNECSASFASLQTYMEHHCPSARPPPPLREESASDTGEEGDEESDVENLAGEIVYQPDGSAYIVESLSQLTQGGGACGSGSGSGPLPSLFLNSLPGAGGKQGDPSCAAPVYPQIINTFHIASSFGKWFEGPDQAFPNTSALAGLSPVLHSFRVFDVRHKSNKDYLNSDGSAKSSCVSKDVPNNVDLSKFDGFVLYGKRKPILMCFLCKLSFGYVRSFVTHAVHDHRMTLSEDERKILSNKNISAIIQGIGKDKEPLVSFLEPKNKNFQHPLVSTANLIGPGHSFYGKFSGIRMEGEEALPAGSAAGPEQPQAGLLTPSTLLNLGGLTSSVLKTPITSVPLGPLASSPTKSSEGKDSGAAEGEKQEVGDGDCFSEKVEPAEEEAEEEEEEEEAEEEEEEEEEEEEEEEDEGCKGLFPSELDEELEDRPHEEPGAAAGSSSKKDLALSNQSISNSPLMPNVLQTLSRGTASTSSNSASSFVVFDGANRRNRLSFNSEGVRANVAEGGRRLDFADESANKDNATAPEPNESTEGDDGGFVPHHQHAGSLCELGVGECPSGSGVECPKCDTVLGSSRSLGGHMTMMHSRNSCKTLKCPKCNWHYKYQQTLEAHMKEKHPEPGGSCVYCKSGQPHPRLARGESYTCGYKPFRCEVCNYSTTTKGNLSIHMQSDKHLNNMQNLQNGGGEQVFSHTAGAAAAAVAAAAAAANISSSCGAPSPTKPKTKPTWRCEVCDYETNVARNLRIHMTSEKHMHNMMLLQQNMTQIQHNRHLGLGSLPSPAEAELYQYYLAQNMNLPNLKMDSAASDAQFMMSGFQLDPAGPMAAMTPALVGGEIPLDMRLGGGQLVSEELMNLGESFIQTNDPSLKLFQCAVCNKFTTDNLDMLGLHMNVERSLSEDEWKAVMGDSYQCKLCRYNTQLKANFQLHCKTDKHVQKYQLVAHIKEGGKANEWRLKCVAIGNPVHLKCNACDYYTNSLEKLRLHTVNSRHEASLKLYKHLQQHESGVEGESCYYHCVLCNYSTKAKLNLIQHVRSMKHQRSESLRKLQRLQKGLPEEDEDLGQIFTIRRCPSTDPEEAIEDVEGPSETAADPEELAKDQEGGASSSQAEKELTDSPATSKRISFPGSSESPLSSKRPKTAEEIKPEQMYQCPYCKYSNADVNRLRVHAMTQHSVQPMLRCPLCQDMLNNKIHLQLHLTHLHSVAPDCVEKLIMTVTTPEMVMPSSMFLPAAVPDRDGNSNLEEAGKQPETSEDLGKNILPSASTEQSGDLKPSPADPGSVREDSGFICWKKGCNQVFKTSAALQTHFNEVHAKRPQLPVSDRHVYKYRCNQCSLAFKTIEKLQLHSQYHVIRAATMCCLCQRSFRTFQALKKHLETSHLELSEADIQQLYGGLLANGDLLAMGDPTLAEDHTIIVEEDKEEESDLEDKQSPTGSDSGSVQEDSGSEPKRALPFRKGPNFTMEKFLDPSRPYKCTVCKESFTQKNILLVHYNSVSHLHKLKRALQESATGQPEPTSSPDNKPFKCNTCNVAYSQSSTLEIHMRSVLHQTKARAAKLEAASGSSNGTGNSSSISLSSSTPSPVSTSGSNTFTTSNPSSAGIAPSSNLLSQVPTESVGMPPLGNPIGANIASPSEPKEANRKKLADMIASRQQQQQQQQQQQQQQQQQQQAQTLAQAQAQVQAHLQQELQQQAALIQSQLFNPTLLPHFPMTTETLLQLQQQQHLLFPFYIPSAEFQLNPEVSLPVTSGALTLTGTGPGLLEDLKAQVQVPQQSHQQILPQQQQNQLSIAQSHSALLQPSQHPEKKNKLVIKEKEKESQRERDSAEGGEGNTGPKETLPDALKAKEKKELAPGGGSEPSMLPPRIASDARGNATKALLENFGFELVIQYNENKQKVQKKNGKTDQGENLEKLECDSCGKLFSNILILKSHQEHVHQNYFPFKQLERFAKQYRDHYDKLYPLRPQTPEPPPPPPPPPPPPLPAAPPQPASTPAIPASAPPITSPTIAPAQPSVPLTQLSMPMELPIFSPLMMQTMPLQTLPAQLPPQLGPVEPLPADLAQLYQHQLNPTLLQQQNKRPRTRITDDQLRVLRQYFDINNSPSEEQIKEMADKSGLPQKVIKHWFRNTLFKERQRNKDSPYNFSNPPITSLEELKIDSRPPSPEPPKQEYWGSKRSSRTRFTDYQLRVLQDFFDANAYPKDDEFEQLSNLLNLPTRVIVVWFQNARQKARKNYENQGEGKDGERRELTNDRYIRTSNLNYQCKKCSLVFQRIFDLIKHQKKLCYKDEDEEGQDDSQNEDSMDAMEILTPTSSSCSTPMPSQAYSAPAPSANNTASSAFLQLTAEAEELATFNSKTEAGDEKPKLAEAPSAQPNQTQEKQGQPKPELQQQEQPEQKTNTPQQKLPQLVSLPSLPQPPPQAPPPQCPLPQSSPSPSQLSHLPLKPLHTSTPQQLANLPPQLIPYQCDQCKLAFPSFEHWQEHQQLHFLSAQNQFIHPQFLDRSLDMPFMLFDPSNPLLASQLLSGAIPQIPASSATSPSTPTSTMNTLKRKLEEKASASPGENDSGTGGEEPQRDKRLRTTITPEQLEILYQKYLLDSNPTRKMLDHIAHEVGLKKRVVQVWFQNTRARERKGQFRAVGPAQAHRRCPFCRALFKAKTALEAHIRSRHWHEAKRAGYNLTLSAMLLDCDGGLQMKGDIFDGTSFSHLPPSSSDGQGVPLSPVSKTMELSPRTLLSPSSIKVEGIEDFESPSMSSVNLNFDQTKLDNDDCSSVNTAITDTTTGDEGNADNDSATGIATETKSSSAPNEGLTKAAMMAMSEYEDRLSSGLVSPAPSFYSKEYDNEGTVDYSETSSLADPCSPSPGASGSAGKSGDSGDRPGQKRFRTQMTNLQLKVLKSCFNDYRTPTMLECEVLGNDIGLPKRVVQVWFQNARAKEKKSKLSMAKHFGINQTSYEGPKTECTLCGIKYSARLSVRDHIFSQQHISKVKDTIGSQLDKEKEYFDPATVRQLMAQQELDRIKKANEVLGLAAQQQGMFDNTPLQALNLPTAYPALQGIPPVLLPGLNSPSLPGFTPSNTALTSPKPNLMGLPSTTVPSPGLPTSGLPNKPSSASLSSPTPAQATMAMGPQQPPQQQQQQQQPQVQQPPPPPAAQPPPTPQLPLQQQQQRKDKDSEKVKEKEKAHKGKGEPLPVPKKEKGEAPTATAATISAPLPTMEYAVDPAQLQALQAALTSDPTALLTSQFLPYFVPGFSPYYAPQIPGALQSGYLQPMYGMEGLFPYSPALSQALMGLSPGSLLQQYQQYQQSLQEAIQQQQQRQLQQQQQQKVQQQQPKASQTPVPPGAPSPDKDPAKESPKPEEQKNTPREVSPLLPKLPEEPEAESKSADSLYDPFIVPKVQYKLVCRKCQAGFSDEEAARSHLKSLCFFGQSVVNLQEMVLHVPTGGGGGGSGGGGGGGGGGGGGGSYHCLACESALCGEEALSQHLESALHKHRTITRAARNAKEHPSLLPHSACFPDPSTASTSQSAAHSNDSPPPPSAAAPSSASPHASRKSWPQVVSRASAAKPPSFPPLSSSSTVTSSSCSTSGVQPSMPTDDYSEESDTDLSQKSDGPASPVEGPKDPSCPKDSGLTSVGTDTFRL

>P11245

MDIEAYFERIGYKNSRNKLDLETLTDILEHQIRAVPFENLNMHCGQAMELGLEAIFDHIVRRNRGGWCLQVNQLLYWALTTIGFQTTMLGGYFYIPPVNKYSTGMVHLLLQVTIDGRNYIVDAGSGSSSQMWQPLELISGKDQPQVPCIFCLTEERGIWYLDQIRREQYITNKEFLNSHLLPKKKHQKIYLFTLEPRTIEDFESMNTYLQTSPTSSFITTSFCSLQTPEGVYCLVGFILTYRKFNYKDNTDLVEFKTLTEEEVEEVLKNIFKISLGRNLVPKPGDGSLTI

1655 Q06416 NP_001393.1

>Q06416

MAGHLASDFAFSPPPGGGGDGPWGAEPGWVDPLTWLSFQGPPGGPGIGPGVGPGSEVWGIPPCPPPYELCGGMAYCGPQVGVGLVPQGGLETSQPESEAGVGVESNSNGASPEPCTVPPGAVKLEKEKLEQNPEKSQDIKALQKELEQFAKLLKQKRITLGYTQADVGLILGVLFGKVFSQKTICRFEALQLSFKNICKLRPLLQKWVEEADNNENLQEICKAETLMQARKRKRTSIENRVRGNLENLFLQCPKPTLQISHIAQQLGLEKDVVRVWFCNRRQKGKRSSSDYAQREDFEAAGSPFSGGPVSFPPAPGPHFGTPGYGSPHFTALYSSVPFPEGEVFPPVSVITLGSPMHSN

>NP_001393.1

MGKEKTHINIVVIGHVDSGKSTTTGHLIYKCGGIDKRTIEKFEKEAAEMGKGSFKYAWVLDKLKAERERGITIDISLWKFETSKYYVTIIDAPGHRDFIKNMITGTSQADCAVLIVAAGVGEFEAGISKNGQTREHALLAYTLGVKQLIVGVNKMDSTEPPYSQKRYEEIVKEVSTYIKKIGYNPDTVAFVPISGWNGDNMLEPSANMPWFKGWKVTRKDGNASGTTLLEALDCILPPTRPTDKPLRLPLQDVYKIGGIGTVPVGRVETGVLKPGMVVTFAPVNVTTEVKSVEMHHEALSEALPGDNVGFNVKNVSVKDVRRGNVAGDSKNDPPMEAAGFTAQVIILNHPGQISAGYAPVLDCHTAHIACKFAELKEKIDRRSGKKLEDGPKFLKSGDAAIVDMVPGKPMCVESFSDYPPLGRFAVRDMRQTVAVGVIKAVDKKAAGAGKVTKSAQKAQKAK

1656 NP_000391.1 NP_055165.2

>NP_000391.1

MKLNVDGLLVYFPYDYIYPEQFSYMRELKRTLDAKGHGVLEMPSGTGKTVSLLALIMAYQRAYPLEVTKLIYCSRTVPEIEKVIEELRKLLNFYEKQEGEKLPFLGLALSSRKNLCIHPEVTPLRFGKDVDGKCHSLTASYVRAQYQHDTSLPHCRFYEEFDAHGREVPLPAGIYNLDDLKALGRRQGWCPYFLARYSILHANVVVYSYHYLLDPKIADLVSKELARKAVVVFDEAHNIDNVCIDSMSVNLTRRTLDRCQGNLETLQKTVLRIKETDEQRLRDEYRRLVEGLREASAARETDAHLANPVLPDEVLQEAVPGSIRTAEHFLGFLRRLLEYVKWRLRVQHVVQESPPAFLSGLAQRVCIQRKPLRFCAERLRSLLHTLEITDLADFSPLTLLANFATLVSTYAKGFTIIIEPFDDRTPTIANPILHFSCMDASLAIKPVFERFQSVIITSGTLSPLDIYPKILDFHPVTMATFTMTLARVCLCPMIIGRGNDQVAISSKFETREDIAVIRNYGNLLLEMSAVVPDGIVAFFTSYQYMESTVASWYEQGILENIQRNKLLFIETQDGAETSVALEKYQEACENGRGAILLSVARGKVSEGIDFVHHYGRAVIMFGVPYVYTQSRILKARLEYLRDQFQIRENDFLTFDAMRHAAQCVGRAIRGKTDYGLMVFADKRFARGDKRGKLPRWIQEHLTDANLNLTVDEGVQVAKYFLRQMAQPFHREDQLGLSLLSLEQLESEETLKRIEQIAQQL

>NP_055165.2

MHSEAEESKEVATDVFNSKNLAVQAQKKILGKMVSKSIATTLIDDTSSEVLDELYRVTREYTQNKKEAEKIIKNLIKTVIKLAILYRNNQFNQDELALMEKFKKKVHQLAMTVVSFHQVDYTFDRNVLSRLLNECREMLHQIIQRHLTAKSHGRVNNVFDHFSDCEFLAALYNPFGNFKPHLQKLCDGINKMLDEENI

1657 NP_061134.1 NP_065723.1

>NP_061134.1

MSQGLPAAGSVLQRSVAAPGNQPQPQPQQQSPEDDDRKVRRREKNRVAAQRSRKKQTQKADKLHEEYESLEQENTMLRREIGKLTEELKHLTEALKEHEKMCPLLLCPMNFVPVPPRPDPVAGCLPR

>NP_065723.1

MGQCRSANAEDAQEFSDVERAIETLIKNFHQYSVEGGKETLTPSELRDLVTQQLPHLMPSNCGLEEKIANLGSCNDSKLEFRSFWELIGEAAKSVKLERPVRGH

1658 NP_003661.1 NP_002961.1

>NP_003661.1

MERIPSAQPPPACLPKAPGLEHGDLPGMYPAHMYQVYKSRRGIKRSEDSKETYKLPHRLIEKKRRDRINECIAQLKDLLPEHLKLTTLGHLEKAVVLELTLKHVKALTNLIDQQQQKIIALQSGLQAGELSGRNVETGQEMFCSGFQTCAREVLQYLAKHENTRDLKSSQLVTHLHRVVSELLQGGTSRKPSDPAPKVMDFKEKPSSPAKGSEGPGKNCVPVIQRTFAHSSGEQSGSDTDTDSGYGGESEKGDLRSEQPCFKSDHGRRFTMGERIGAIKQESEEPPTKKNRMQLSDDEGHFTSSDLISSPFLGPHPHQPPFCLPFYLIPPSATAYLPMLEKCWYPTSVPVLYPGLNASAAALSSFMNPDKISAPLLMPQRLPSPLPAHPSVDSSVLLQALKPIPPLNLETKD

>NP_002961.1

MAKFVIRPATAADCSDILRLIKELAKYEYMEEQVILTEKDLLEDGFGEHPFYHCLVAEVPKEHWTPEGHSIVGFAMYYFTYDPWIGKLLYLEDFFVMSDYRGFGIGSEILKNLSQVAMRCRCSSMHFLVAEWNEPSINFYKRRGASDLSSEEGWRLFKIDKEYLLKMATEE

1659 Q5VVJ2 NP_005172.1

>Q5VVJ2

MAAEEADVDIEGDVVAAAGAQPGSGENTASVLQKDHYLDSSWRTENGLIPWTLDNTISEENRAVIEKMLLEEEYYLSKKSQPEKVWLDQKEDDKKYMKSLQKTAKIMVHSPTKPASYSVKWTIEEKELFEQGLAKFGRRWTKISKLIGSRTVLQVKSYARQYFKNKVKCGLDKETPNQKTGHNLQVKNEDKGTKAWTPSCLRGRADPNLNAVKIEKLSDDEEVDITDEVDELSSQTPQKNSSSDLLLDFPNSKMHETNQGEFITSDSQEALFSKSSRGCLQNEKQDETLSSSEITLWTEKQSNGDKKSIELNDQKFNELIKNCNKHDGRGIIVDARQLPSPEPCEIQKNLNDNEMLFHSCQMVEESHEEEELKPPEQEIEIDRNIIQEEEKQAIPEFFEGRQAKTPERYLKIRNYILDQWEICKPKYLNKTSVRPGLKNCGDVNCIGRIHTYLELIGAINFGCEQAVYNRPQTVDKVRIRDRKDAVEAYQLAQRLQSMRTRRRRVRDPWGNWCDAKDLEGQTFEHLSAEELAKRREEEKGRPVKSLKVPRPTKSSFDPFQLIPCNFFSEEKQEPFQVKVASEALLIMDLHAHVSMAEVIGLLGGRYSEVDKVVEVCAAEPCNSLSTGLQCEMDPVSQTQASETLAVRGFSVIGWYHSHPAFDPNPSLRDIDTQAKYQSYFSRGGAKFIGMIVSPYNRNNPLPYSQITCLVISEEISPDGSYRLPYKFEVQQMLEEPQWGLVFEKTRWIIEKYRLSHSSVPMDKIFRRDSDLTCLQKLLECMRKTLSKVTNCFMAEEFLTEIENLFLSNYKSNQENGVTEENCTKELLM

>NP_005172.1

MAKEWGYASHNGPDHWHELFPNAKGENQSPVELHTKDIRHDPSLQPWSVSYDGGSAKTILNNGKTCRVVFDDTYDRSMLRGGPLPGPYRLRQFHLHWGSSDDHGSEHTVDGVKYAAELHLVHWNPKYNTFKEALKQRDGIAVIGIFLKIGHENGEFQIFLDALDKIKTKGKEAPFTKFDPSCLFPACRDYWTYQGSFTTPPCEECIVWLLLKEPMTVSSDQMAKLRSLLSSAENEPPVPLVSNWRPPQPINNRVVRASFK

1660 Q5M775 Q9NWF9

>Q5M775

MRSAAKPWNPAIRAGGHGPDRVRPLPAASSGMKSSKSSTSLAFESRLSRLKRASSEDTLNKPGSTAASGVVRLKKTATAGAISELTESRLRSGTGAFTTTKRTGIPAPREFSVTVSRERSVPRGPSNPRKSVSSPTSSNTPTPTKHLRTPSTKPKQENEGGEKAALESQVRELLAEAKAKDSEINRLRSELKKYKEKRTLNAEGTDALGPNVDGTSVSPGDTEPMIRALEEKNKNFQKELSDLEEENRVLKEKLIYLEHSPNSEGAASHTGDSSCPTSITQESSFGSPTGNQMSSDIDEYKKNIHGNALRTSGSSSSDVTKASLSPDASDFEHITAETPSRPLSSTSNPFKSSKCSTAGSSPNSVSELSLASLTEKIQKMEENHHSTAEELQATLQELSDQQQMVQELTAENEKLVDEKTILETSFHQHRERAEQLSQENEKLMNLLQERVKNEEPTTQEGKIIELEQKCTGILEQGRFEREKLLNIQQQLTCSLRKVEEENQGALEMIKRLKEENEKLNEFLELERHNNNMMAKTLEECRVTLEGLKMENGSLKSHLQGEKQKATEASAVEQTAESCEVQEMLKVARAEKDLLELSCNELRQELLKANGEIKHVSSLLAKVEKDYSYLKEICDHQAEQLSRTSLKLQEKASESDAEIKDMKETIFELEDQVEQHRAVKLHNNQLISELESSVIKLEEQKSDLERQLKTLTKQMKEETEEWRRFQADLQTAVVVANDIKCEAQQELRTVKRKLLEEEEKNARLQKELGDVQGHGRVVTSRAAPPPVDEEPESSEVDAAGRWPGVCVSRTSPTPPESATTVKSLIKSFDLGRPGGAGQNISVHKTPRSPLSGIPVRTAPAAAVSPMQRHSTYSSVRPASRGVTQRLDLPDLPLSDILKGRTETLKPDPHLRKSPSLESLSRPPSLGFGDTRLLSASTRAWKPQSKLSVERKDPLAALAREYGGSKRNALLKWCQKKTQGYANIDITNFSSSWSDGLAFCALLHTYLPAHIPYQELNSQEKKRNLLLAFEAAESVGIKPSLELSEMLYTDRPDWQSVMQYVAQIYKYFET

>Q9NWF9

MEEGNNNEEVIHLNNFHCHRGQEWINLRDGPITISDSSDEERIPMLVTPAPQQHEEEDLDDDVILTEDDSEDDYGEFLDLGPPGISEFTKPSGQTEREPKPGPSHNQAANDIVNPRSEQKVIILEEGSLLYTESDPLETQNQSSEDSETELLSNLGESAALADDQAIEEDCWLDHPYFQSLNQQPREITNQVVPQERQPEAELGRLLFQHEFPGPAFPRPEPQQGGISGPSSPQPAHPLGEFEDQQLASDDEEPGPAFPMQESQEPNLENIWGQEAAEVDQELVELLVKETEARFPDVANGFIEEIIHFKNYYDLNVLCNFLLENPDYPKREDRIIINPSSSLLASQDETKLPKIDFFDYSKLTPLDQRCFIQAADLLMADFKVLSSQDIKWALHELKGHYAITRKALSDAIKKWQELSPETSGKRKKRKQMNQYSYIDFKFEQGDIKIEKRMFFLENKRRHCRSYDRRALLPAVQQEQEFYEQKIKEMAEHEDFLLALQMNEEQYQKDGQLIECRCCYGEFPFEELTQCADAHLFCKECLIRYAQEAVFGSGKLELSCMEGSCTCSFPTSELEKVLPQTILYKYYERKAEEEVAAAYADELVRCPSCSFPALLDSDVKRFSCPNPHCRKETCRKCQGLWKEHNGLTCEELAEKDDIKYRTSIEEKMTAARIRKCHKCGTGLIKSEGCNRMSCRCGAQMCYLCRVSINGYDHFCQHPRSPGAPCQECSRCSLWTDPTEDDEKLIEEIQKEAEEEQKRKNGENTFKRIGPPLEKPVEKVQRVEALPRPVPQNLPQPQMPPYAFAHPPFPLPPVRPVFNNFPLNMGPIPAPYVPPLPNVRVNYDFGPIHMPLEHNLPMHFGPQPRHRF

1661 O75526 Q08495

>O75526

MVEADRPGKLFIGGLNLETDEKALEAEFGKYGRIVEVLLMKDRETNKSRGFAFVTFESPADAKAAARDMNGKSLDGKAIKVAQATKPAFESSRRGPPPPRSRGRPRFLRGTRGGGGGPRRSPSRGGPDDDGGYTADFDLRPSRAPMPMKRGPPPRRVGPPPKRAAPSGPARSSGGGMRGRALAVRGRDGYSGPPRREPLPPRRDPYLGPRDEGYSSRDGYSSRDYREPRGFAPSPGEYTHRDYGHSSVRDDCPLRGYSDRDGYGGRDRDYGDHLSRGSHREPFESYGELRGAAPGRGTPPSYGGGGRYEEYRGYSPDAYSGGRDSYSSSYGRSDRYSRGRHRVGRPDRGLSLSMERGCPPQRDSYSRSGCRVPRGGGRLGGRLERGGGRSRY

>Q08495

MERLQKQPLTSPGSVSPSRDSSVPGSPSSIVAKMDNQVLGYKDLAAIPKDKAILDIERPDLMIYEPHFTYSLLEHVELPRSRERSLSPKSTSPPPSPEVWADSRSPGIISQASAPRTTGTPRTSLPHFHHPETSRPDSNIYKKPPIYKQRESVGGSPQTKHLIEDLIIESSKFPAAQPPDPNQPAKIETDYWPCPPSLAVVETEWRKRKASRRGAEEEEEEEDDDSGEEMKALRERQREELSKVTSNLGKMILKEEMEKSLPIRRKTRSLPDRTPFHTSLHQGTSKSSSLPAYGRTTLSRLQSTEFSPSGSETGSPGLQNGEGQRGRMDRGNSLPCVLEQKIYPYEMLVVTNKGRTKLPPGVDRMRLERHLSAEDFSRVFAMSPEEFGKLALWKRNELKKKASLF

1662 NP_006749.1 Q9UDX5

>NP_006749.1

MAEYLASIFGTEKDKVNCSFYFKIGACRHGDRCSRLHNKPTFSQTIALLNIYRNPQNSSQSADGLRCAVSDVEMQEHYDEFFEEVFTEMEEKYGEVEEMNVCDNLGDHLVGNVYVKFRREEDAEKAVIDLNNRWFNGQPIHAELSPVTDFREACCRQYEMGECTRGGFCNFMHLKPISRELRRELYGRRRKKHRSRSRSRERRSRSRDRGRGGGGGGGGGGGGRERDRRRSRDRERSGRF

>Q9UDX5

MSEPQPRGAERDLYRDTWVRYLGYANEVGEAFRSLVPAAVVWLSYGVASSYVLADAIDKGKKAGEVPSPEAGRSARVTVAVVDTFVWQALASVAIPGFTINRVCAASLYVLGTATRWPLAVRKWTTTALGLLTIPIIIHPIDRSVDFLLDSSLRKLYPTVGKPSSS

1663 NP_067015.2 Q9NX20

>NP_067015.2

MNDFDECGQSAASMYLPGCAYYVAPSDFASKPSFLSQPSSCQMTFPYSSNLAPHVQPVREVAFRDYGLERAKWPYRGGGGGGSAGGGSSGGGPGGGGGGAGGYAPYYAAAAAAAAAAAAAEEAAMQRELLPPAGRRPDVLFKAPEPVCAAPGPPHGPAGAASNFYSAVGRNGILPQGFDQFYEAAPGPPFAGPQPPPPPAPPQPEGAADKGDPRTGAGGGGGSPCTKATPGSEPKGAAEGSGGDGEGPPGEAGAEKSSSAVAPQRSRKKRCPYTKYQIRELEREFFFNVYINKEKRLQLSRMLNLTDRQVKIWFQNRRMKEKKLNRDRLQYFTGNPLF

>Q9NX20

MWRLLARASAPLLRVPLSDSWALLPASAGVKTLLPVPSFEDVSIPEKPKLRFIERAPLVPKVRREPKNLSDIRGPSTEATEFTEGNFAILALGGGYLHWGHFEMMRLTINRSMDPKNMFAIWRVPAPFKPITRKSVGHRMGGGKGAIDHYVTPVKAGRLVVEMGGRCEFEEVQGFLDQVAHKLPFAAKAVSRGTLEKMRKDQEERERNNQNPWTFERIATANMLGIRKVLSPYDLTHKGKYWGKFYMPKRV

1664 NP_000607.1 P01857

>NP_000607.1

MNRGVPFRHLLLVLQLALLPAATQGKKVVLGKKGDTVELTCTASQKKSIQFHWKNSNQIKILGNQGSFLTKGPSKLNDRADSRRSLWDQGNFPLIIKNLKIEDSDTYICEVEDQKEEVQLLVFGLTANSDTHLLQGQSLTLTLESPPGSSPSVQCRSPRGKNIQGGKTLSVSQLELQDSGTWTCTVLQNQKKVEFKIDIVVLAFQKASSIVYKKEGEQVEFSFPLAFTVEKLTGSGELWWQAERASSSKSWITFDLKNKEVSVKRVTQDPKLQMGKKLPLHLTLPQALPQYAGSGNLTLALEAKTGKLHQEVNLVVMRATQLQKNLTCEVWGPTSPKLMLSLKLENKEAKVSKREKAVWVLNPEAGMWQCLLSDSGQVLLESNIKVLPTWSTPVQPMALIVLGGVAGLLLFIGLGIFFCVRCRHRRRQAERMSQIKRLLSEKKTCQCPHRFQKTCSPI

>P01857

ASTKGPSVFPLAPSSKSTSGGTAALGCLVKDYFPEPVTVSWNSGALTSGVHTFPAVLQSSGLYSLSSVVTVPSSSLGTQTYICNVNHKPSNTKVDKKVEPKSCDKTHTCPPCPAPELLGGPSVFLFPPKPKDTLMISRTPEVTCVVVDVSHEDPEVKFNWYVDGVEVHNAKTKPREEQYNSTYRVVSVLTVLHQDWLNGKEYKCKVSNKALPAPIEKTISKAKGQPREPQVYTLPPSRDELTKNQVSLTCLVKGFYPSDIAVEWESNGQPENNYKTTPPVLDSDGSFFLYSKLTVDKSRWQQGNVFSCSVMHEALHNHYTQKSLSLSPGK

1665 P01106 NP_071369.4

>P01106

MPLNVSFTNRNYDLDYDSVQPYFYCDEEENFYQQQQQSELQPPAPSEDIWKKFELLPTPPLSPSRRSGLCSPSYVAVTPFSLRGDNDGGGGSFSTADQLEMVTELLGGDMVNQSFICDPDDETFIKNIIIQDCMWSGFSAAAKLVSEKLASYQAARKDSGSPNPARGHSVCSTSSLYLQDLSAAASECIDPSVVFPYPLNDSSSPKSCASQDSSAFSPSSDSLLSSTESSPQGSPEPLVLHEETPPTTSSDSEEEQEDEEEIDVVSVEKRQAPGKRSESGSPSAGGHSKPPHSPLVLKRCHVSTHQHNYAAPPSTRKDYPAAKRVKLDSVRVLRQISNNRKCTSPRSSDTEENVKRRTHNVLERQRRNELKRSFFALRDQIPELENNEKAPKVVILKKATAYILSVQAEEQKLISEEDLLRKRREQLKHKLEQLRNSCA

>NP_071369.4

MPPPSDIVKVAIEWPGANAQLLEIDQKRPLASIIKEVCDGWSLPNPEYYTLRYADGPQLYITEQTRSDIKNGTILQLAISPSRAARQLMERTQSSNMETRLDAMKELAKLSADVTFATEFINMDGIIVLTRLVESGTKLLSHYSEMLAFTLTAFLELMDHGIVSWDMVSITFIKQIAGYVSQPMVDVSILQRSLAILESMVLNSQSLYQKIAEEITVGQLISHLQVSNQEIQTYAIALINALFLKAPEDKRQDMANAFAQKHLRSIILNHVIRGNRPIKTEMAHQLYVLQVLTFNLLEERMMTKMDPNDQAQRDIIFELRRIAFDAESDPSNAPGSGTEKRKAMYTKDYKMLGFTNHINPAMDFTQTPPGMLALDNMLYLAKVHQDTYIRIVLENSSREDKHECPFGRSAIELTKMLCEILQVGELPNEGRNDYHPMFFTHDRAFEELFGICIQLLNKTWKEMRATAEDFNKVMQVVREQITRALPSKPNSLDQFKSKLRSLSYSEILRLRQSERMSQDDFQSPPIVELREKIQPEILELIKQQRLNRLCEGSSFRKIGNRRRQERFWYCRLALNHKVLHYGDLDDNPQGEVTFESLQEKIPVADIKAIVTGKDCPHMKEKSALKQNKEVLELAFSILYDPDETLNFIAPNKYEYCIWIDGLSALLGKDMSSELTKSDLDTLLSMEMKLRLLDLENIQIPEAPPPIPKEPSSYDFVYHYG

1666 NP_001003801.1 Q6UB28

>NP_001003801.1

MAADEVAGGARKATKSKLFEFLVHGVRPGMPSGARMPHQGAPMGPPGSPYMGSPAVRPGLAPAGMEPARKRAAPPPGQSQAQSQGQPVPTAPARSRSAKRRKMADKILPQRIRELVPESQAYMDLLAFERKLDQTIMRKRVDIQEALKRPMKQKRKLRLYISNTFNPAKPDAEDSDGSIASWELRVEGKLLDDPSKQKRKFSSFFKSLVIELDKDLYGPDNHLVEWHRTPTTQETDGFQVKRPGDLSVRCTLLLMLDYQPPQFKLDPRLARLLGLHTQSRSAIVQALWQYVKTNRLQDSHDKEYINGDKYFQQIFDCPRLKFSEIPQRLTALLLPPDPIVINHVISVDPSDQKKTACYDIDVEVEEPLKGQMSSFLLSTANQQEISALDSKIHETIESINQLKIQRDFMLSFSRDPKGYVQDLLRSQSRDLKVMTDVAGNPEEERRAEFYHQPWSQEAVSRYFYCKIQQRRQELEQSLVVRNT

>Q6UB28

MAAPSGVHLLVRRGSHRIFSSPLNHIYLHKQSSSQQRRNFFFRRQRDISHSIVLPAAVSSAHPVPKHIKKPDYVTTGIVPDWGDSIEVKNEDQIQGLHQACQLARHVLLLAGKSLKVDMTTEEIDALVHREIISHNAYPSPLGYGGFPKSVCTSVNNVLCHGIPDSRPLQDGDIINIDVTVYYNGYHGDTSETFLVGNVDECGKKLVEVARRCRDEAIAACRAGAPFSVIGNTISHITHQNGFQVCPHFVGHGIGSYFHGHPEIWHHANDSDLPMEEGMAFTIEPIITEGSPEFKVLEDAWTVVSLDNQRSAQFEHTVLITSRGAQILTKLPHEA

1667 NP_002884.1 NP_001158.2

>NP_002884.1

MASKEMFEDTVEERVINEEYKIWKKNTPFLYDLVMTHALQWPSLTVQWLPEVTKPEGKDYALHWLVLGTHTSDEQNHLVVARVHIPNDDAQFDASHCDSDKGEFGGFGSVTGKIECEIKINHEGEVNRARYMPQNPHIIATKTPSSDVLVFDYTKHPAKPDPSGECNPDLRLRGHQKEGYGLSWNSNLSGHLLSASDDHTVCLWDINAGPKEGKIVDAKAIFTGHSAVVEDVAWHLLHESLFGSVADDQKLMIWDTRSNTTSKPSHLVDAHTAEVNCLSFNPYSEFILATGSADKTVALWDLRNLKLKLHTFESHKDEIFQVHWSPHNETILASSGTDRRLNVWDLSKIGEEQSAEDAEDGPPELLFIHGGHTAKISDFSWNPNEPWVICSVSEDNIMQIWQMAENIYNDEESDVTTSELEGQGS

>NP_001158.2

MTFNSFEGSKTCVPADINKEEEFVEEFNRLKTFANFPSGSPVSASTLARAGFLYTGEGDTVRCFSCHAAVDRWQYGDSAVGRHRKVSPNCRFINGFYLENSATQSTNSGIQNGQYKVENYLGSRDHFALDRPSETHADYLLRTGQVVDISDTIYPRNPAMYSEEARLKSFQNWPDYAHLTPRELASAGLYYTGIGDQVQCFCCGGKLKNWEPCDRAWSEHRRHFPNCFFVLGRNLNIRSESDAVSSDRNFPNSTNLPRNPSMADYEARIFTFGTWIYSVNKEQLARAGFYALGEGDKVKCFHCGGGLTDWKPSEDPWEQHAKWYPGCKYLLEQKGQEYINNIHLTHSLEECLVRTTEKTPSLTRRIDDTIFQNPMVQEAIRMGFSFKDIKKIMEEKIQISGSNYKSLEVLVADLVNAQKDSMQDESSQTSLQKEISTEEQLRRLQEEKLCKICMDRNIAIVFVPCGHLVTCKQCAEAVDKCPMCYTVITFKQKIFMS

1668 NP_000449.1 Q06124

>NP_000449.1

MVSKLTSLQQELLSALLSSGVTKEVLVQALEELLPSPNFGVKLETLPLSPGSGAEPDTKPVFHTLTNGHAKGRLSGDEGSEDGDDYDTPPILKELQALNTEEAAEQRAEVDRMLSEDPWRAAKMIKGYMQQHNIPQREVVDVTGLNQSHLSQHLNKGTPMKTQKRAALYTWYVRKQREILRQFNQTVQSSGNMTDKSSQDQLLFLFPEFSQQSHGPGQSDDACSEPTNKKMRRNRFKWGPASQQILYQAYDRQKNPSKEEREALVEECNRAECLQRGVSPSKAHGLGSNLVTEVRVYNWFANRRKEEAFRQKLAMDAYSSNQTHSLNPLLSHGSPHHQPSSSPPNKLSGVRYSQQGNNEITSSSTISHHGNSAMVTSQSVLQQVSPASLDPGHNLLSPDGKMISVSGGGLPPVSTLTNIHSLSHHNPQQSQNLIMTPLSGVMAIAQSLNTSQAQSVPVINSVAGSLAALQPVQFSQQLHSPHQQPLMQQSPGSHMAQQPFMAAVTQLQNSHMYAHKQEPPQYSHTSRFPSAMVVTDTSSISTLTNMSSSKQCPLQAW

>Q06124

MTSRRWFHPNITGVEAENLLLTRGVDGSFLARPSKSNPGDFTLSVRRNGAVTHIKIQNTGDYYDLYGGEKFATLAELVQYYMEHHGQLKEKNGDVIELKYPLNCADPTSERWFHGHLSGKEAEKLLTEKGKHGSFLVRESQSHPGDFVLSVRTGDDKGESNDGKSKVTHVMIRCQELKYDVGGGERFDSLTDLVEHYKKNPMVETLGTVLQLKQPLNTTRINAAEIESRVRELSKLAETTDKVKQGFWEEFETLQQQECKLLYSRKEGQRQENKNKNRYKNILPFDHTRVVLHDGDPNEPVSDYINANIIMPEFETKCNNSKPKKSYIATQGCLQNTVNDFWRMVFQENSRVIVMTTKEVERGKSKCVKYWPDEYALKEYGVMRVRNVKESAAHDYTLRELKLSKVGQALLQGNTERTVWQYHFRTWPDHGVPSDPGGVLDFLEEVHHKQESIMDAGPVVVHCSAGIGRTGTFIVIDILIDIIREKGVDCDIDVPKTIQMVRSQRSGMVQTEAQYRFIYMAVQHYIETLQRRIEEEQKSKRKGHEYTNIKYSLADQTSGDQSPLPPCTPTPPCAEMREDSARVYENVGLMQQQKSFR

1669 NP_055436.2 Q12849

>NP_055436.2

MVMSSYMVNSKYVDPKFPPCEEYLQGGYLGEQGADYYGGGAQGADFQPPGLYPRPDFGEQPFGGSGPGPGSALPARGHGQEPGGPGGHYAAPGEPCPAPPAPPPAPLPGARAYSQSDPKQPPSGTALKQPAVVYPWMKKVHVNSVNPNYTGGEPKRSRTAYTRQQVLELEKEFHFNRYLTRRRRIEIAHTLCLSERQIKIWFQNRRMKWKKDHKLPNTKGRSSSSSSSSSCSSSVAPSQHLQPMAKDHHTDLTTL

>Q12849

MAGTRWVLGALLRGCGCNCSSCRRTGAACLPFYSAAGSIPSGVSGRRRLLLLLGAAAAAASQTRGLQTGPVPPGRLAGPPAVATSAAAAAAASYSALRASLLPQSLAAAAAVPTRSYSQESKTTYLEDLPPPPEYELAPSKLEEEVDDVFLIRAQGLPWSCTMEDVLNFFSDCRIRNGENGIHFLLNRDGKRRGDALIEMESEQDVQKALEKHRMYMGQRYVEVYEINNEDVDALMKSLQVKSSPVVNDGVVRLRGLPYSCNEKDIVDFFAGLNIVDITFVMDYRGRRKTGEAYVQFEEPEMANQALLKHREEIGNRYIEIFPSRRNEVRTHVGSYKGKKIASFPTAKYITEPEMVFEEHEVNEDIQPMTAFESEKEIELPKEVPEKLPEAADFGTTSSLHFVHMRGLPFQANAQDIINFFAPLKPVRITMEYSSSGKATGEADVHFETHEDAVAAMLKDRSHVHHRYIELFLNSCPKGK

1670 NP_004588.1 NP_659451.1

>NP_004588.1

MSLLNKPKSEMTPEELQKREEEEFNTGPLSVLTQSVKNNTQVLINCRNNKKLLGRVKAFDRHCNMVLENVKEMWTEVPKSGKGKKKSKPVNKDRYISKMFLRGDSVIVVLRNPLIAGK

>NP_659451.1

MEELLPDGQIWANMDPEERMLAAATAFTHICAGQGEGDVRREAQSIQYDPYSKASVAPGKRPALPVQLQYPHVESNVPSETVSEASQRLRKPVMKRKVLRRKPDGEVLVTDESIISESESGTENDQDLWDLRQRLMNVQFQEDKESSFDVSQKFNLPHEYQGISQDQLICSLQREGMGSPAYEQDLIVASRPKSFILPKLDQLSRNRGKTDRVARYFEYKRDWDSIRLPGEDHRKELRWGVREQMLCRAEPQSKPQHIYVPNNYLVPTEKKRSALRWGVRCDLANGVIPRKLPFPLSPS

1671 NP_055586.1 NP_001344.2

>NP_055586.1

MSGPGNKRAAGDGGSGPPEKKLSREEKTTTTLIEPIRLGGISSTEEMDLKVLQFKNKKLAERLEQRQACEDELRERIEKLEKRQATDDATLLIVNRYWAQLDETVEALLRCHESQGELSSAPEAPGTQEGPTCDGTPLPEPGTSELRDPLLMQLRPPLSEPALAFVVALGASSSEEVELELQGRMEFSKAAVSRVVEASDRLQRRVEELCQRVYSRGDSEPLSEAAQAHTRELGRENRRLQDLATQLQEKHHRISLEYSELQDKVTSAETKVLEMETTVEDLQWDIEKLRKREQKLNKHLAEALEQLNSGYYVSGSSSGFQGGQITLSMQKFEMLNAELEENQELANSRMAELEKLQAELQGAVRTNERLKVALRSLPEEVVRETGEYRMLQAQFSLLYNESLQVKTQLDEARGLLLATKNSHLRHIEHMESDELGLQKKLRTEVIQLEDTLAQVRKEYEMLRIEFEQNLAANEQAGPINREMRHLISSLQNHNHQLKGDAQRYKRKLREVQAEIGKLRAQASGSAHSTPNLGHPEDSGVSAPAPGKEEGGPGPVSTPDNRKEMAPVPGTTTTTTSVKKEELVPSEEDFQGITPGAQGPSSRGREPEARPKRELREREGPSLGPPPVASALSRADREKAKVEETKRKESELLKGLRAELKKAQESQKEMKLLLDMYKSAPKEQRDKVQLMAAERKAKAEVDELRSRIRELEERDRRESKKIADEDALRRIRQAEEQIEHLQRKLGATKQEEEALLSEMDVTGQAFEDMQEQNGRLLQQLREKDDANFKLMSERIKANQIHKLLREEKDELGEQVLGLKSQVDAQLLTVQKLEEKERALQGSLGGVEKELTLRSQALELNKRKAVEAAQLAEDLKVQLEHVQTRLREIQPCLAESRAAREKESFNLKRAQEDISRLRRKLEKQRKVEVYADADEILQEEIKEYKARLTCPCCNTRKKDAVLTKCFHVFCFECVRGRYEARQRKCPKCNAAFGAHDFHRIYIS

>NP_001344.2

MDSKYQCVKLNDGHFMPVLGFGTYAPAEVPKSKALEATKLAIEAGFRHIDSAHLYNNEEQVGLAIRSKIADGSVKREDIFYTSKLWCNSHRPELVRPALERSLKNLQLDYVDLYLIHFPVSVKPGEEVIPKDENGKILFDTVDLCATWEAVEKCKDAGLAKSIGVSNFNRRQLEMILNKPGLKYKPVCNQVECHPYFNQRKLLDFCKSKDIVLVAYSALGSHREEPWVDPNSPVLLEDPVLCALAKKHKRTPALIALRYQLQRGVVVLAKSYNEQRIRQNVQVFEFQLTSEEMKAIDGLNRNVRYLTLDIFAGPPNYPFSDEY

1672 P35453 NP_006432.1

>P35453

MSRAGSWDMDGLRADGGGAGGAPASSSSSSVAAAAASGQCRGFLSAPVFAGTHSGRAAAAAAAAAAAAAAASGFAYPGTSERTGSSSSSSSSAVVAARPEAPPAKECPAPTPAAAAAAPPSAPALGYGYHFGNGYYSCRMSHGVGLQQNALKSSPHASLGGFPVEKYMDVSGLASSSVPANEVPARAKEVSFYQGYTSPYQHVPGYIDMVSTFGSGEPRHEAYISMEGYQSWTLANGWNSQVYCTKDQPQGSHFWKSSFPGDVALNQPDMCVYRRGRKKRVPYTKLQLKELENEYAINKFINKDKRRRISAATNLSERQVTIWFQNRRVKDKKIVSKLKDTVS

>NP_006432.1

MAAAAVSSAKRSLRGELKQRLRAMSAEERLRQSRVLSQKVIAHSEYQKSKRISIFLSMQDEIETEEIIKDIFQRGKICFIPRYRFQSNHMDMVRIESPEEISLLPKTSWNIPQPGEGDVREEALSTGGLDLIFMPGLGFDKHGNRLGRGKGYYDAYLKRCLQHQEVKPYTLALAFKEQICLQVPVNENDMKVDEVLYEDSSTA

1673 Q8IXH6 Q9P0J6

>Q8IXH6

MFQRLSSLFFSTPSPPEDPDCPRAFVSEEDEVDGWLIIDLPDSYAAPPSPGAAPAPAGRPPPAPSLMDESWFVTPPACFTAEGPGLGPARLQSSPLEDLLIEHPSMSVYVTGSTIVLEPGSPSPLPDAALPDGDLSEGELTPARREPRAARHAAPLPARAALLEKAGQVRRLQRARQRAERHALSAKAVQRQNRARESRPRRSKNQSSFIYQPCQRQFNY

>Q9P0J6

MANLFIRKMVNPLLYLSRHTVKPRALSTFLFGSIRGAAPVAVEPGAAVRSLLSPGLLPHLLPALGFKNKTVLKKRCKDCYLVKRRGRWYVYCKTHPRHKQRQM

1674 NP_006029.1 NP_036232.1

>NP_006029.1

MGKPSSMDTKFKDDLFRKYVQFHESKVDTTTSRQRPGSDECLRVAASTLLSLHKVDPFYRFRLIQFYEVVESSLRSLSSSSLRALHGAFSMLETVGINLFLYPWKKEFRSIKTYTGPFVYYVKSTLLEEDIRAILSCMGYTPELGTAYKLRELVETLQVKMVSFELFLAKVECEQMLEIHSQVKDKGYSELDIVSERKSSAEDVRGCSDALRRRAEGREHLTASMSRVALQKSASERAAKDYYKPRVTKPSRSVDAYDSYWESRKPPLKASLSLRKEPVATDVGDDLKDEIIRPSPSLLTMASSPHGSPDVLPPASPSNGPALLRGTYFSTQDDVDLYTDSEPRATYRRQDALRPDVWLLRNDAHSLYHKRSPPAKESALSKCQSCGLSCSSSLCQRCDSLLTCPPASKPSAFPSKASTHDSLAHGASLREKYPGQTQGLDRLPHLHSKSKPSTTPTSRCGFCNRPGATNTCTQCSKVSCDACLSAYHYDPCYKKSELHKFMPNNQLNYKSTQLSHLVYR

>NP_036232.1

MQVAMNGKARKEAVQTAAKELLKFVNRSPSPFHAVAECRNRLLQAGFSELKETEKWNIKPESKYFMTRNSSTIIAFAVGGQYVPGNGFSLIGAHTDSPCLRVKRRSRRSQVGFQQVGVETYGGGIWSTWFDRDLTLAGRVIVKCPTSGRLEQQLVHVERPILRIPHLAIHLQRNINENFGPNTEMHLVPILATAIQEELEKGTPEPGPLNAVDERHHSVLMSLLCAHLGLSPKDIVEMELCLADTQPAVLGGAYDEFIFAPRLDNLHSCFCALQALIDSCAGPGSLATEPHVRMVTLYDNEEVGSESAQGAQSLLTELVLRRISASCQHPTAFEEAIPKSFMISADMAHAVHPNYLDKHEENHRPLFHKGPVIKVNSKQRYASNAVSEALIREVANKVKVPLQDLMVRNDTPCGTTIGPILASRLGLRVLDLGSPQLAMHSIREMACTTGVLQTLTLFKGFFELFPSLSHNLLVD

1675 O75030 NP_006397.1

>O75030

MQSESGIVPDFEVGEEFHEEPKTYYELKSQPLKSSSSAEHPGASKPPISSSSMTSRILLRQQLMREQMQEQERREQQQKLQAAQFMQQRVPVSQTPAINVSVPTTLPSATQVPMEVLKVQTHLENPTKYHIQQAQRQQVKQYLSTTLANKHANQVLSLPCPNQPGDHVMPPVPGSSAPNSPMAMLTLNSNCEKEGFYKFEEQNRAESECPGMNTHSRASCMQMDDVIDDIISLESSYNEEILGLMDPALQMANTLPVSGNLIDLYGNQGLPPPGLTISNSCPANLPNIKRELTACIFPTESEARALAKERQKKDNHNLIERRRRFNINDRIKELGTLIPKSNDPDMRWNKGTILKASVDYIRKLQREQQRAKELENRQKKLEHANRHLLLRIQELEMQARAHGLSLIPSTGLCSPDLVNRIIKQEPVLENCSQDLLQHHADLTCTTTLDLTDGTITFNNNLGTGTEANQAYSVPTKMGSKLEDILMDDTLSPVGVTDPLLSSVSPGASKTSSRRSSMSMEETEHTC

>NP_006397.1

MEALPLLAATTPDHGRHRRLLLLPLLLFLLPAGAVQGWETEERPRTREEECHFYAGGQVYPGEASRVSVADHSLHLSKAKISKPAPYWEGTAVIDGEFKELKLTDYRGKYLVFFFYPLDFTFVCPTEIIAFGDRLEEFRSINTEVVACSVDSQFTHLAWINTPRRQGGLGPIRIPLLSDLTHQISKDYGVYLEDSGHTLRGLFIIDDKGILRQITLNDLPVGRSVDETLRLVQAFQYTDKHGEVCPAGWKPGSETIIPDPAGKLKYFDKLN

1676 NP_001929.1 O14772

>NP_001929.1

MASSSGNDDDLTIPRAAINKMIKETLPNVRVANDARELVVNCCTEFIHLISSEANEICNKSEKKTISPEHVIQALESLGFGSYISEVKEVLQECKTVALKRRKASSRLENLGIPEEELLRQQQELFAKARQQQAELAQQEWLQMQQAAQQAQLAAASASASNQAGSSQDEEDDDDI

>O14772

MAAARDPPEVSLREATQRKLRRFSELRGKLVARGEFWDIVAITAADEKQELAYNQQLSEKLKRKELPLGVQYHVFVDPAGAKIGNGGSTLCALQCLEKLYGDKWNSFTILLIHSGGYSQRLPNASALGKIFTALPLGNPIYQMLELKLAMYIDFPLNMNPGILVTCADDIELYSIGEFEFIRFDKPGFTALAHPSSLTIGTTHGVFVLDPFDDLKHRDLEYRSCHRFLHKPSIEKMYQFNAVCRPGNFCQQDFAGGDIADLKLDSDYVYTDSLFYMDHKSAKMLLAFYEKIGTLSCEIDAYGDFLQALGPGATVEYTRNTSNVIKEESELVEMRQRIFHLLKGTSLNVVVLNNSKFYHIGTTEEYLFYFTSDNSLKSELGLQSITFSIFPDIPECSGKTSCIIQSILDSRCSVAPGSVVEYSRLGPDVSVGENCIISGSYILTKAALPAHSFVCSLSLKMNRCLKYATMAFGVQDNLKKSVKTLSDIKLLQFFGVCFLSCLDVWNLKVTEELFSGNKTCLSLWTARIFPVCSSLSDSVITSLKMLNAVKNKSAFSLNSYKLLSIEEMLIYKDVEDMITYREQIFLEISLKSSLM

1677 NP_003654.3 P82921

>NP_003654.3

MERFVVTAPPARNRSKTALYVTPLDRVTEFGGELHEDGGKLFCTSCNVVLNHVRKSAISDHLKSKTHTKRKAEFEEQNVRKKQRPLTASLQCNSTAQTEKVSVIQDFVKMCLEANIPLEKADHPAVRAFLSRHVKNGGSIPKSDQLRRAYLPDGYENENQLLNSQDC

>P82921

MAKHLKFIARTVMVQEGNVESAYRTLNRILTMDGLIEDIKHRRYYEKPCRRRQRESYERCRRIYNMEMARKINFLMRKNRADPWQGC

1678 NP_056986.2 NP_001268.2

>NP_056986.2

MTSIHFVVHPLPGTEDQLNDRLREVSEKLNKYNLNSHPPLNVLEQATIKQCVVGPNHAAFLLEDGRVCRIGFSVQPDRLELGKPDNNDGSKLNSNSGAGRTSRPGRTSDSPWFLSGSETLGRLAGNTLGSRWSSGVGGSGGGSSGRSSAGARDSRRQTRVIRTGRDRGSGLLGSQPQPVIPASVIPEELISQAQVVLQGKSRSVIIRELQRTNLDVNLAVNNLLSRDDEDGDDGDDTASESYLPGEDLMSLLDADIHSAHPSVIIDADAMFSEDISYFGYPSFRRSSLSRLGSSRVLLLPLERDSELLRERESVLRLRERRWLDGASFDNERGSTSKEGEPNLDKKNTPVQSPVSLGEDLQWWPDKDGTKFICIGALYSELLAVSSKGELYQWKWSESEPYRNAQNPSLHHPRATFLGLTNEKIVLLSANSIRATVATENNKVATWVDETLSSVASKLEHTAQTYSELQGERIVSLHCCALYTCAQLENSLYWWGVVPFSQRKKMLEKARAKNKKPKSSAGISSMPNITVGTQVCLRNNPLYHAGAVAFSISAGIPKVGVLMESVWNMNDSCRFQLRSPESLKNMEKASKTTEAKPESKQEPVKTEMGPPPSPASTCSDASSIASSASMPYKRRRSTPAPKEEEKVNEEQWSLREVVFVEDVKNVPVGKVLKVDGAYVAVKFPGTSSNTNCQNSSGPDADPSSLLQDCRLLRIDELQVVKTGGTPKVPDCFQRTPKKLCIPEKTEILAVNVDSKGVHAVLKTGNWVRYCIFDLATGKAEQENNFPTSSIAFLGQNERNVAIFTAGQESPIILRDGNGTIYPMAKDCMGGIRDPDWLDLPPISSLGMGVHSLINLPANSTIKKKAAVIIMAVEKQTLMQHILRCDYEACRQYLMNLEQAVVLEQNLQMLQTFISHRCDGNRNILHACVSVCFPTSNKETKEEEEAERSERNTFAERLSAVEAIANAISVVSSNGPGNRAGSSSSRSLRLREMMRRSLRAAGLGRHEAGASSSDHQDPVSPPIAPPSWVPDPPAMDPDGDIDFILAPAVGSLTTAATGTGQGPSTSTIPGPSTEPSVVESKDRKANAHFILKLLCDSVVLQPYLRELLSAKDARGMTPFMSAVSGRAYPAAITILETAQKIAKAEISSSEKEEDVFMGMVCPSGTNPDDSPLYVLCCNDTCSFTWTGAEHINQDIFECRTCGLLESLCCCTECARVCHKGHDCKLKRTSPTAYCDCWEKCKCKTLIAGQKSARLDLLYRLLTATNLVTLPNSRGEHLLLFLVQTVARQTVEHCQYRPPRIREDRNRKTASPEDSDMPDHDLEPPRFAQLALERVLQDWNALKSMIMFGSQENKDPLSASSRIGHLLPEEQVYLNQQSGTIRLDCFTHCLIVKCTADILLLDTLLGTLVKELQNKYTPGRREEAIAVTMRFLRSVARVFVILSVEMASSKKKNNFIPQPIGKCKRVFQALLPYAVEELCNVAESLIVPVRMGIARPTAPFTLASTSIDAMQGSEELFSVEPLPPRPSSDQSSSSSQSQSSYIIRNPQQRRISQSQPVRGRDEEQDDIVSADVEEVEVVEGVAGEEDHHDEQEEHGEENAEAEGQHDEHDEDGSDMELDLLAAAETESDSESNHSNQDNASGRRSVVTAATAGSEAGASSVPAFFSEDDSQSNDSSDSDSSSSQSDDIEQETFMLDEPLERTTNSSHANGAAQAPRSMQWAVRNTQHQRAASTAPSSTSTPAASSAGLIYIDPSNLRRSGTISTSAAAAAAALEASNASSYLTSASSLARAYSIVIRQISDLMGLIPKYNHLVYSQIPAAVKLTYQDAVNLQNYVEEKLIPTWNWMVSIMDSTEAQLRYGSALASAGDPGHPNHPLHASQNSARRERMTAREEASLRTLEGRRRATLLSARQGMMSARGDFLNYALSLMRSHNDEHSDVLPVLDVCSLKHVAYVFQALIYWIKAMNQQTTLDTPQLERKRTRELLELGIDNEDSEHENDDDTNQSATLNDKDDDSLPAETGQNHPFFRRSDSMTFLGCIPPNPFEVPLAEAIPLADQPHLLQPNARKEDLFGRPSQGLYSSSASSGKCLMEVTVDRNCLEVLPTKMSYAANLKNVMNMQNRQKKEGEEQPVLPEETESSKPGPSAHDLAAQLKSSLLAEIGLTESEGPPLTSFRPQCSFMGMVISHDMLLGRWRLSLELFGRVFMEDVGAEPGSILTELGGFEVKESKFRREMEKLRNQQSRDLSLEVDRDRDLLIQQTMRQLNNHFGRRCATTPMAVHRVKVTFKDEPGEGSGVARSFYTAIAQAFLSNEKLPNLECIQNANKGTHTSLMQRLRNRGERDREREREREMRRSSGLRAGSRRDRDRDFRRQLSIDTRPFRPASEGNPSDDPEPLPAHRQALGERLYPRVQAMQPAFASKITGMLLELSPAQLLLLLASEDSLRARVDEAMELIIAHGRENGADSILDLGLVDSSEKVQQENRKRHGSSRSVVDMDLDDTDDGDDNAPLFYQPGKRGFYTPRPGKNTEARLNCFRNIGRILGLCLLQNELCPITLNRHVIKVLLGRKVNWHDFAFFDPVMYESLRQLILASQSSDADAVFSAMDLAFAIDLCKEEGGGQVELIPNGVNIPVTPQNVYEYVRKYAEHRMLVVAEQPLHAMRKGLLDVLPKNSLEDLTAEDFRLLVNGCGEVNVQMLISFTSFNDESGENAEKLLQFKRWFWSIVEKMSMTERQDLVYFWTSSPSLPASEEGFQPMPSITIRPPDDQHLPTANTCISRLYVPLYSSKQILKQKLLLAIKTKNFGFV

>NP_001268.2

MKTKFCTGGEAEPSPLGLLLSCGSGSAAPAPGVGQQRDAASDLESKQLGGQQPPLALPPPPPLPLPLPLPQPPPPQPPADEQPEPRTRRRAYLWCKEFLPGAWRGLREDEFHISVIRGGLSNMLFQCSLPDTTATLGDEPRKVLLRLYGAILQMRSCNKEGSEQAQKENEFQGAEAMVLESVMFAILAERSLGPKLYGIFPQGRLEQFIPSRRLDTEELSLPDISAEIAEKMATFHGMKMPFNKEPKWLFGTMEKYLKEVLRIKFTEESRIKKLHKLLSYNLPLELENLRSLLESTPSPVVFCHNDCQEGNILLLEGRENSEKQKLMLIDFEYSSYNYRGFDIGNHFCEWMYDYSYEKYPFFRANIRKYPTKKQQLHFISSYLPAFQNDFENLSTEEKSIIKEEMLLEVNRFALASHFLWGLWSIVQAKISSIEFGYMDYAQARFDAYFHQKRKLGV

1679 Q15029 Q3I5F7

>Q15029

MDTDLYDEFGNYIGPELDSDEDDDELGRETKDLDEMDDDDDDDDVGDHDDDHPGMEVVLHEDKKYYPTAEEVYGPEVETIVQEEDTQPLTEPIIKPVKTKKFTLMEQTLPVTVYEMDFLADLMDNSELIRNVTLCGHLHHGKTCFVDCLIEQTHPEIRKRYDQDLCYTDILFTEQERGVGIKSTPVTVVLPDTKGKSYLFNIMDTPGHVNFSDEVTAGLRISDGVVLFIDAAEGVMLNTERLIKHAVQERLAVTVCINKIDRLILELKLPPTDAYYKLRHIVDEVNGLISMYSTDENLILSPLLGNVCFSSSQYSICFTLGSFAKIYADTFGDINYQEFAKRLWGDIYFNPKTRKFTKKAPTSSSQRSFVEFILEPLYKILAQVVGDVDTSLPRTLDELGIHLTKEELKLNIRPLLRLVCKKFFGEFTGFVDMCVQHIPSPKVGAKPKIEHTYTGGVDSDLGEAMSDCDPDGPLMCHTTKMYSTDDGVQFHAFGRVLSGTIHAGQPVKVLGENYTLEDEEDSQICTVGRLWISVARYHIEVNRVPAGNWVLIEGVDQPIVKTATITEPRGNEEAQIFRPLKFNTTSVIKIAVEPVNPSELPKMLDGLRKVNKSYPSLTTKVEESGEHVILGTGELYLDCVMHDLRKMYSEIDIKVADPVVTFCETVVETSSLKCFAETPNKKNKITMIAEPLEKGLAEDIENEVVQITWNRKKLGEFFQTKYDWDLLAARSIWAFGPDATGPNILVDDTLPSEVDKALLGSVKDSIVQGFQWGTREGPLCDELIRNVKFKILDAVVAQEPLHRGGGQIIPTARRVVYSAFLMATPRLMEPYYFVEVQAPADCVSAVYTVLARRRGHVTQDAPIPGSPLYTIKAFIPAIDSFGFETDLRTHTQGQAFSLSVFHHWQIVPGDPLDKSIVIRPLEPQPAPHLAREFMIKTRRRKGLSEDVSISKFFDDPMLLELAKQDVVLNYPM

>Q3I5F7

MLQHPKVKGPSIALLGFSKGGDLCLSMASFLKGITATVLINACVANTVAPLHYKDMIIPKLVDDLGKVKITKSGFLTFMDTWSNPLEEHNHQSLVPLEKAQVPFLFIVGMDDQSWKSEFYAQIASERLQAHGKERPQIICYPETGHCIDPPYFPPSRASVHAVLGEAIFYGGEPKAHSKAQVDAWQQIQTFFHKHLNGKKSVKHSKI

1680 NP_055596.3 NP_001001936.1

>NP_055596.3

MKLYVFLVNTGTTLTFDTELTVQTVADLKHAIQSKYKIAIQHQVLVVNGGECMAADRRVCTYSAGTDTNPIFLFNKEMILCDRPPAIPKTTFSTENDMEIKVEESLMMPAVFHTVASRTQLALEMYEVAKKLCSFCEGLVHDEHLQHQGWAAIMANLEDCSNSYQKLLFKFESIYSNYLQSIEDIKLKLTHLGTAVSVMAKIPLLECLTRHSYRECLGRLDSLPEHEDSEKAEMKRSTELVLSPDMPRTTNESLLTSFPKSVEHVSPDTADAESGKEIRESCQSTVHQQDETTIDTKDGDLPFFNVSLLDWINVQDRPNDVESLVRKCFDSMSRLDPRIIRPFIAECRQTIAKLDNQNMKAIKGLEDRLYALDQMIASCGRLVNEQKELAQGFLANQKRAENLKDASVLPDLCLSHANQLMIMLQNHRKLLDIKQKCTTAKQELANNLHVRLKWCCFVMLHADQDGEKLQALLRLVIELLERVKIVEALSTVPQMYCLAVVEVVRRKMFIKHYREWAGALVKDGKRLYEAEKSKRESFGKLFRKSFLRNRLFRGLDSWPPSFCTQKPRKFDCELPDISLKDLQFLQSFCPSEVQPFLRVPLLCDFEPLHQHVLALHNLVKAAQSLDEMSQTITDLLSEQKASVSQTSPQSASSPRMESTAGITTTTSPRTPPPLTVQDPLCPAVCPLEELSPDSIDAHTFDFETIPHPNIEQTIHQVSLDLDSLAESPESDFMSAVNEFVIEENLSSPNPISDPQSPEMMVESLYSSVINAIDSRRMQDTNVCGKEDFGDHTSLNVQLERCRVVAQDSHFSIQTIKEDLCHFRTFVQKEQCDFSNSLKCTAVEIRNIIEKVKCSLEITLKEKHQKELLSLKNEYEGKLDGLIKETEENENKIKKLKGELVCLEEVLQNKDNEFALVKHEKEAVICLQNEKDQKLLEMENIMHSQNCEIKELKQSREIVLEDLKKLHVENDEKLQLLRAELQSLEQSHLKELEDTLQVRHIQEFEKVMTDHRVSLEELKKENQQIINQIQESHAEIIQEKEKQLQELKLKVSDLSDTRCKLEVELALKEAETDEIKILLEESRAQQKETLKSLLEQETENLRTEISKLNQKIQDNNENYQVGLAELRTLMTIEKDQCISELISRHEEESNILKAELNKVTSLHNQAFEIEKNLKEQIIELQSKLDSELSALERQKDEKITQQEEKYEAIIQNLEKDRQKLVSSQEQDREQLIQKLNCEKDEAIQTALKEFKLEREVVEKELLEKVKHLENQIAKSPAIDSTRGDSSSLVAELQEKLQEEKAKFLEQLEEQEKRKNEEMQNVRTSLIAEQQTNFNTVLTREKMRKENIINDLSDKLKSTMQQQERDKDLIESLSEDRARLLEEKKKLEEEVSKLRSSSFVPSPYVATAPELYGACAPELPGESDRSAVETADEGRVDSAMETSMMSVQENIHMLSEEKQRIMLLERTLQLKEEENKRLNQRLMSQSMSSVSSRHSEKIAIRDFQVGDLVLIILDERHDNYVLFTVSPTLYFLHSESLPALDLKPGEGASGASRRPWVLGKVMEKEYCQAKKAQNRFKVPLGTKFYRVKAVSWNKKV

>NP_001001936.1

MERYKALEQLLTELDDFLKILDQENLSSTALVKKSCLAELLRLYTKSSSSDEEYIYMNKVTINKQQNAESQGKAPEEQGLLPNGEPSQHSSAPQKSLPDLPPPKMIPERKQLAIPKTESPEGYYEEAEPYDTSLNEDGEAVSSSYESYDEEDGSKGKSAPYQWPSPEAGIELMRDARICAFLWRKKWLGQWAKQLCVIKDNRLLCYKSSKDHSPQLDVNLLGSSVIHKEKQVRKKEHKLKITPMNADVIVLGLQSKDQAEQWLRVIQEVSGLPSEGASEGNQYTPDAQRFNCQKPDIAEKYLSASEYGSSVDGHPEVPETKDVKKKCSAGLKLSNLMNLGRKKSTSLEPVERSLETSSYLNVLVNSQWKSRWCSVRDNHLHFYQDRNRSKVAQQPLSLVGCEVVPDPSPDHLYSFRILHKGEELAKLEAKSSEEMGHWLGLLLSESGSKTDPEEFTYDYVDADRVSCIVSAAKNSLLLMQRKFSEPNTYIDGLPSQDRQEELYDDVDLSELTAAVEPTEEATPVADDPNERESDRVYLDLTPVKSFLHGPSSAQAQASSPTLSCLDNATEALPADSGPGPTPDEPCIKCPENLGEQQLESLEPEDPSLRITTVKIQTEQQRISFPPSCPDAVVATPPGASPPVKDRLRVTSAEIKLGKNRTEAEVKRYTEEKERLEKKKEEIRGHLAQLRKEKRELKETLLKCTDKEVLASLEQKLKEIDEECRGEESRRVDLELSIMEVKDNLKKAEAGPVTLGTTVDTTHLENVSPRPKAVTPASAPDCTPVNSATTLKNRPLSVVVTGKGTVLQKAKEWEKKGAS

1681 Q92925 P53677

>Q92925

MSPGNRMPMARLAGGTPCWLPIWCSSSASTWHPTHHDGSIPKTPACAPAQPPMPAQRRGLKRRKMADKVLPQRIRELVPESQAYMDLLAFERKLDQTIARKRMEIQEAIKKPLTQKRKLRIYISNTFSPSKAEGDSAGTAGTPGGTPAGDKVASWELRVEGKLLDDPSKQKRKFSSFFKSLVIELDKELYGPDGHLVEWYWMPTTQETDGFQVKRPGDLNVKCTLLLMLDHQPPQYKLDPRLARLLGVHTQTRAAIMQALWLYIKHNQLQDGHEREYINCNRYFRQIFSCGRLRFSEIPMKLAGLLQHPDPIVINHVISVDPNDQKKTACYDIDVEVDDPLKAQMSNFLASTTNQQEIASLDVKIHETIESINQLKTQRDFMLSFSTDPQDFIQEWLRSQRRDLKIITDVIGNPEEERRAAFYHQPWAQEAVGRHIFAKVQQRRQELEQVLGIRLT

>P53677

MIHSLFLINSSGDIFLEKHWKSVVSRSVCDYFFEAQERATEAENVPPVIPTPHHYLLSVYRHKIFFVAVIQTEVPPLFVIEFLHRVVDTFQDYFGVCSEPVIKDNVVVVYEVLEEMLDNGFPLATESNILKELIKPPTILRTVVNTITGSTNVGDQLPTGQLSVVPWRRTGVKYTNNEAYFDVIEEIDAIIDKSGSTITAEIQGVIDACVKLTGMPDLTLSFMNPRLLDDVSFHPCVRFKRWESERILSFIPPDGNFRLLSYHVSAQNLVAIPVYVKHNISFRDSSSLGRFEITVGPKQTMGKTIEGVTVTSQMPKGVLNMSLTPSQGTHTFDPVTKMLSWDVGKINPQKLPSLKGTMSLQAGASKPDENPTINLQFKIQQLAISGLKVNRLDMYGEKYKPFKGIKYMTKAGKFQVRT

1682 NP_005230.1 NP_001167.2

>NP_005230.1

MNDFGIKNMDQVAPVANSYRGTLKRQPAFDTFDGSLFAVFPSLNEEQTLQEVPTGLDSISHDSANCELPLLTPCSKAVMSQALKATFSGFKKEQRRLGIPKNPWLWSEQQVCQWLLWATNEFSLVNVNLQRFGMNGQMLCNLGKERFLELAPDFVGDILWEHLEQMIKENQEKTEDQYEENSHLTSVPHWINSNTLGFGTEQAPYGMQTQNYPKGGLLDSMCPASTPSVLSSEQEFQMFPKSRLSSVSVTYCSVSQDFPGSNLNLLTNNSGTPKDHDSPENGADSFESSDSLLQSWNSQSSLLDVQRVPSFESFEDDCSQSLCLNKPTMSFKDYIQERSDPVEQGKPVIPAAVLAGFTGSGPIQLWQFLLELLSDKSCQSFISWTGDGWEFKLADPDEVARRWGKRKNKPKMNYEKLSRGLRYYYDKNIIHKTSGKRYVYRFVCDLQNLLGFTPEELHAILGVQPDTED

>NP_001167.2

MLGLDACELGAQLLELLRLALCARVLLADKEGGPPAVDEVLDEAVPEYRAPGRKSLLEIRQLDPDDRSLAKYKRVLLGPLPPAVDPSLPNVQVTRLTLLSEQAPGPVVMDLTGDLAVLKDQVFVLKEGVDYRVKISFKVHREIVSGLKCLHHTYRRGLRVDKTVYMVGSYGPSAQEYEFVTPVEEAPRGALVRGPYLVVSLFTDDDRTHHLSWEWGLCICQDWKD

1683 NP_003191.1 P29144

>NP_003191.1

MNQPQRMAPVGTDKELSDLLDFSMMFPLPVTNGKGRPASLAGAQFGGSGLEDRPSSGSWGSGDQSSSSFDPSRTFSEGTHFTESHSSLSSSTFLGPGLGGKSGERGAYASFGRDAGVGGLTQAGFLSGELALNSPGPLSPSGMKGTSQYYPSYSGSSRRRAADGSLDTQPKKVRKVPPGLPSSVYPPSSGEDYGRDATAYPSAKTPSSTYPAPFYVADGSLHPSAELWSPPGQAGFGPMLGGGSSPLPLPPGSGPVGSSGSSSTFGGLHQHERMGYQLHGAEVNGGLPSASSFSSAPGATYGGVSSHTPPVSGADSLLGSRGTTAGSSGDALGKALASIYSPDHSSNNFSSSPSTPVGSPQGLAGTSQWPRAGAPGALSPSYDGGLHGLQSKIEDHLDEAIHVLRSHAVGTAGDMHTLLPGHGALASGFTGPMSLGGRHAGLVGGSHPEDGLAGSTSLMHNHAALPSQPGTLPDLSRPPDSYSGLGRAGATAAASEIKREEKEDEENTSAADHSEEEKKELKAPRARTSPDEDEDDLLPPEQKAEREKERRVANNARERLRVRDINEAFKELGRMCQLHLNSEKPQTKLLILHQAVSVILNLEQQVRERNLNPKAACLKRREEEKVSGVVGDPQMVLSAPHPGLSEAHNPAGHM

>P29144

MATAATEEPFPFHGLLPKKETGAASFLCRYPEYDGRGVLIAVLDTGVDPGAPGMQVTTDGKPKIVDIIDTTGSGDVNTATEVEPKDGEIVGLSGRVLKIPASWTNPSGKYHIGIKNGYDFYPKALKERIQKERKEKIWDPVHRVALAEACRKQEEFDVANNGSSQANKLIKEELQSQVELLNSFEKKYSDPGPVYDCLVWHDGEVWRACIDSNEDGDLSKSTVLRNYKEAQEYGSFGTAEMLNYSVNIYDDGNLLSIVTSGGAHGTHVASIAAGHFPEEPERNGVAPGAQILSIKIGDTRLSTMETGTGLIRAMIEVINHKCDLVNYSYGEATHWPNSGRICEVINEAVWKHNIIYVSSAGNNGPCLSTVGCPGGTTSSVIGVGAYVSPDMMVAEYSLREKLPANQYTWSSRGPSADGALGVSISAPGGAIASVPNWTLRGTQLMNGTSMSSPNACGGIALILSGLKANNIDYTVHSVRRALENTAVKADNIEVFAQGHGIIQVDKAYDYLVQNTSFANKLGFTVTVGNNRGIYLRDPVQVAAPSDHGVGIEPVFPENTENSEKISLQLHLALTSNSSWVQCPSHLELMNQCRHINIRVDPRGLREGLHYTEVCGYDIASPNAGPLFRVPITAVIAAKVNESSHYDLAFTDVHFKPGQIRRHFIEVPEGATWAEVTVCSCSSEVSAKFVLHAVQLVKQRAYRSHEFYKFCSLPEKGTLTEAFPVLGGKAIEFCIARWWASLSDVNIDYTISFHGIVCTAPQLNIHASEGINRFDVQSSLKYEDLAPCITLKNWVQTLRPVSAKTKPLGSRDVLPNNRQLYEMVLTYNFHQPKSGEVTPSCPLLCELLYESEFDSQLWIIFDQNKRQMGSGDAYPHQYSLKLEKGDYTIRLQIRHEQISDLERLKDLPFIVSHRLSNTLSLDIHENHSFALLGKKKSSNLTLPPKYNQPFFVTSLPDDKIPKGAGPGCYLAGSLTLSKTELGKKADVIPVHYYLIPPPTKTKNGSKDKEKDSEKEKDLKEEFTEALRDLKIQWMTKLDSSDIYNELKETYPNYLPLYVARLHQLDAEKERMKRLNEIVDAANAVISHIDQTALAVYIAMKTDPRPDAATIKNDMDKQKSTLVDALCRKGCALADHLLHTQAQDGAISTDAEGKEEEGESPLDSLAETFWETTKWTDLFDNKVLTFAYKHALVNKMYGRGLKFATKLVEEKPTKENWKNCIQLMKLLGWTHCASFTENWLPIMYPPDYCVF

1684 Q9Y5Q8 O00142

>Q9Y5Q8

MAAEAADLGLGAAVPVELRRERRMVCVEYPGVVRDVAKMLPTLGGEEGVSRIYADPTKRLELYFRPKDPYCHPVCANRFSTSSLLLRIRKRTRRQKGVLGTEAHSEVTFDMEILGIISTIYKFQGMSDFQYLAVHTEAGGKHTSMYDKVLMLRPEKEAFFHQELPLYIPPPIFSRLDAPVDYFYRPETQHREGYNNPPISGENLIGLSRARRPHNAIFVNFEDEEVPKQPLEAAAQTWRRVCTNPVDRKVEEELRKLFDIRPIWSRNAVKANISVHPDKLKVLLPFIAYYMITGPWRSLWIRFGYDPRKNPDAKIYQVLDFRIRCGMKHGYAPSDLPVKAKRSTYNYSLPITVKKTSSQLVTMHDLKQGLGPSGTSGARKPASSKYKLKDSVYIFREGALPPYRQMFYQLCDLNVEELQKIIHRNDGAENSCTERDGWCLPKTSDELRDTMSLMIRQTIRSKRPALFSSSAKADGGKEQLTYESGEDEEDEEEEEEEEEDFKPSDGSENEMETEILDYV

>O00142

MLLWPLRGWAARALRCFGPGSRGSPASGPGPRRVQRRAWPPDKEQEKEKKSVICVEGNIASGKTTCLEFFSNATDVEVLTEPVSKWRNVRGHNPLGLMYHDASRWGLTLQTYVQLTMLDRHTRPQVSSVRLMERSIHSARYIFVENLYRSGKMPEVDYVVLSEWFDWILRNMDVSVDLIVYLRTNPETCYQRLKKRCREEEKVIPLEYLEAIHHLHEEWLIKGSLFPMAAPVLVIEADHHMERMLELFEQNRDRILTPENRKHCP

1685 Q9BWT1 NP_005116.1

>Q9BWT1

MDARRVPQKDLRVKKNLKKFRYVKLISMETSSSSDDSCDSFASDNFANTRLQSVREGCRTRSQCRHSGPLRVAMKFPARSTRGATNKKAESRQPSENSVTDSNSDSEDESGMNFLEKRALNIKQNKAMLAKLMSELESFPGSFRGRHPLPGSDSQSRRPRRRTFPGVASRRNPERRARPLTRSRSRILGSLDALPMEEEEEEDKYMLVRKRKTVDGYMNEDDLPRSRRSRSSVTLPHIIRPVEEITEEELENVCSNSREKIYNRSLGSTCHQCRQKTIDTKTNCRNPDCWGVRGQFCGPCLRNRYGEEVRDALLDPNWHCPPCRGICNCSFCRQRDGRCATGVLVYLAKYHGFGNVHAYLKSLKQEFEMQA

>NP_005116.1

MASDSGNQGTLCTLEFAVQMTCQSCVDAVRKSLQGVAGVQDVEVHLEDQMVLVHTTLPSQEVQALLEGTGRQAVLKGMGSGQLQNLGAAVAILGGPGTVQGVVRFLQLTPERCLIEGTIDGLEPGLHGLHVHQYGDLTNNCNSCGNHFNPDGASHGGPQDSDRHRGDLGNVRADADGRAIFRMEDEQLKVWDVIGRSLIIDEGEDDLGRGGHPLSKITGNSGERLACGIIARSAGLFQNPKQICSCDGLTIWEERGRPIAGKGRKESAQPPAHL

1686 NP_055597.1 NP_056981.2

>NP_055597.1

MSRVRDAGCVAAGIVIGAGAWYCVYKYTRGRDQTKKRMAKPKNRAVAGTGARARAGLRAGFTIDLGSGFSPPTPVRAEAEDRAQDEASALDTVGAEAVAPAASSAEAQSGAGSQAQEADGAGVGPKAESVVGAAMASAIAPPPGVTEALGAAEAPAMAGAPKVAEAPREAETSRAAVPPGTVVPTEAAAPTEVTEGPGVAAPTKVAEAPGVASPTEAAEAPVPATPTGAAAPTGAAESPGTSGSPRTAVVPGTSAAKKATPGAHTGAIPKATSATGAVPKGGGKGVTRSRNGGKGKGKKSKVEVDELGMGFRPGDGAAAAAAASANGGQAFLAEVPDSEEGESGWTDTESDSDSEPETQRRGRGRRPVAMQKRPFPYEIDEILGVRDLRKVLALLQKSDDPFIQQVALLTLSNNANYSCNQETIRKLGGLPIIANMINKTDPHIKEKALMAMNNLSENYENQGRLQVYMNKVMDDIMASNLNSAVQVVGLKFLTNMTITNDYQHLLVNSIANFFRLLSQGGGKIKVEILKILSNFAENPDMLKKLLSTQVPASFSSLYNSYVESEILINALTLFEIIYDNLRAEVFNYREFNKGSLFYLCTTSGVCVKKIRALANHHDLLVKVKVIKLVNKF

>NP_056981.2

MAAELVEAKNMVMSFRVSDLQMLLGFVGRSKSGLKHELVTRALQLVQFDCSPELFKKIKELYETRYAKKNSEPAPQPHRPLDPLTMHSTYDRAGAVPRTPLAGPNIDYPVLYGKYLNGLGRLPAKTLKPEVRLVKLPFFNMLDELLKPTELVPQNNEKLQESPCIFALTPRQVELIRNSRELQPGVKAVQVVLRICYSDTSCPQEDQYPPNIAVKVNHSYCSVPGYYPSNKPGVEPKRPCRPINLTHLMYLSSATNRITVTWGNYGKSYSVALYLVRQLTSSELLQRLKTIGVKHPELCKALVKEKLRLDPDSEIATTGVRVSLICPLVKMRLSVPCRAETCAHLQCFDAVFYLQMNEKKPTWMCPVCDKPAPYDQLIIDGLLSKILSECEDADEIEYLVDGSWCPIRAEKERSCSPQGAILVLGPSDANGLLPAPSVNGSGALGSTGGGGPVGSMENGKPGADVVDLTLDSSSSSEDEEEEEEEEEDEDEEGPRPKRRCPFQKGLVPAC

1687 NP_477097.1 P35237

>NP_477097.1

MSRRSSRLQAKQQPQPSQTESPQEAQIIQAKKRKTTQDVKKRREEVTKKHQYEIRNCWPPVLSGGISPCIIIETPHKEIGTSDFSRFTNYRFKNLFINPSPLPDLSWGCSKEVWLNMLKKESRYVHDKHFEVLHSDLEPQMRSILLDWLLEVCEVYTLHRETFYLAQDFFDRFMLTQKDINKNMLQLIGITSLFIASKLEEIYAPKLQEFAYVTDGACSEEDILRMELIILKALKWELCPVTIISWLNLFLQVDALKDAPKVLLPQYSQETFIQIAQLLDLCILAIDSLEFQYRILTAAALCHFTSIEVVKKASGLEWDSISECVDWMVPFVNVVKSTSPVKLKTFKKIPMEDRHNIQTHTNYLAMLEEVNYINTFRKGGQLSPVCNGGIMTPPKSTEKPPGKH

>P35237

MDVLAEANGTFALNLLKTLGKDNSKNVFFSPMSMSCALAMVYMGAKGNTAAQMAQILSFNKSGGGGDIHQGFQSLLTEVNKTGTQYLLRMANRLFGEKSCDFLSSFRDSCQKFYQAEMEELDFISAVEKSRKHINTWVAEKTEGKIAELLSPGSVDPLTRLVLVNAVYFRGNWDEQFDKENTEERLFKVSKNEEKPVQMMFKQSTFKKTYIGEIFTQILVLPYVGKELNMIIMLPDETTDLRTVEKELTYEKFVEWTRLDMMDEEEVEVSLPRFKLEESYDMESVLRNLGMTDAFELGKADFSGMSQTDLSLSKVVHKSFVEVNEEGTEAAAATAAIMMMRCARFVPRFCADHPFLFFIQHSKTNGILFCGRFSSP

1688 NP_003484.1 NP_112176.1

>NP_003484.1

MARTKQTARKSTGGKAPRKQLATKVARKSAPATGGVKKPHRYRPGTVALREIRRYQKSTELLIRKLPFQRLMREIAQDFKTDLRFQSSAVMALQEACESYLVGLFEDTNLCVIHAKRVTIMPKDIQLARRIRGERA

>NP_112176.1

MAAPLSVEVEFGGGAELLFDGIKKHRVTLPGQEEPWDIRNLLIWIKKNLLKERPELFIQGDSVRPGILVLINDADWELLGELDYQLQDQDSVLFISTLHGG

1689 NP_003241.2 NP_056273.2

>NP_003241.2

MEQKPSKVECGSDPEENSARSPDGKRKRKNGQCSLKTSMSGYIPSYLDKDEQCVVCGDKATGYHYRCITCEGCKGFFRRTIQKNLHPTYSCKYDSCCVIDKITRNQCQLCRFKKCIAVGMAMDLVLDDSKRVAKRKLIEQNRERRRKEEMIRSLQQRPEPTPEEWDLIHIATEAHRSTNAQGSHWKQRRKFLPDDIGQSPIVSMPDGDKVDLEAFSEFTKIITPAITRVVDFAKKLPMFSELPCEDQIILLKGCCMEIMSLRAAVRYDPESDTLTLSGEMAVKREQLKNGGLGVVSDAIFELGKSLSAFNLDDTEVALLQAVLLMSTDRSGLLCVDKIEKSQEAYLLAFEHYVNHRKHNIPHFWPKLLMKEREVQSSILYKGAAAEGRPGGSLGVHPEGQQLLGMHVVQGPQVRQLEQQLGEAGSLQGPVLQHQSPKSPQQRLLELLHRSGILHARAVCGEDDSSEADSPSSSEEEPEVCEDLAGNAASP

>NP_056273.2

MEFAELIKTPRVDNVVLHRPFYPAVEGTLCLTGHHLILSSRQDNTEELWLLHSNIDAIDKRFVGSLGTIIIKCKDFRIIQLDIPGMEECLNIASSIEALSTLDSITLMYPFFYRPMFEVIEDGWHSFLPEQEFELYSSATSEWRLSYVNKEFAVCPSYPPIVTVPKSIDDEALRKVATFRHGGRFPVLSYYHKKNGMVIMRSGQPLTGTNGRRCKEDEKLINATLRAGKRGYIIDTRSLNVAQQTRAKGGGFEQEAHYPQWRRIHKSIERYHILQESLIKLVEACNDQTHNMDRWLSKLEASNWLTHIKEILTTACLAAQCIDREGASILIHGTEGTDSTLQVTSLAQIILEPRSRTIRGFEALIEREWLQAGHPFQQRCAQSAYCNTKQKWEAPVFLLFLDCVWQILRQFPCSFEFNENFLIMLFEHAYASQFGTFLGNNESERCKLKLQQKTMSLWSWVNQPSELSKFTNPLFEANNLVIWPSVAPQSLPLWEGIFLRWNRSSKYLDEAYEEMVNIIEYNKELQAKVNILRRQLAELETEDGMQESP

1690 NP_001665.1 NP_037392.1

>NP_001665.1

MMLQHPGQVSASEVSASAIVPCLSPPGSLVFEDFANLTPFVKEELRFAIQNKHLCHRMSSALESVTVSDRPLGVSITKAEVAPEEDERKKRRRERNKIAAAKCRNKKKEKTECLQKESEKLESVNAELKAQIEELKNEKQHLIYMLNLHRPTCIVRAQNGRTPEDERNLFIQQIKEGTLQS

>NP_037392.1

MAGKKNVLSSLAVYAEDSEPESDGEAGIEAVGSAAEEKGGLVSDAYGEDDFSRLGGDEDGYEEEEDENSRQSEDDDSETEKPEADDPKDNTEAEKRDPQELVASFSERVRNMSPDEIKIPPEPPGRCSNHLQDKIQKLYERKIKEGMDMNYIIQRKKEFRNPSIYEKLIQFCAIDELGTNYPKDMFDPHGWSEDSYYEALAKAQKIEMDKLEKAKKERTKIEFVTGTKKGTTTNATSTTTTTASTAVADAQKRKSKWDSAIPVTTIAQPTILTTTATLPAVVTVTTSASGSKTTVISAVGTIVKKAKQ

1691 Q96AV8 NP_001880.2

>Q96AV8

MEVNCLTLKDLISPRQPRLDFAVEDGENAQKENIFVDRSRMAPKTPIKNEPIDLSKQKKFTPERNPITPVKLVDRQQAEPWTPTANLKMLISAASPDIRDREKKKGLFRPIENKDDAFTDSLQLDVVGDSAVDEFEKQRPSRKQKSLGLLCQKFLARYPSYPLSTEKTTISLDEVAVSLGVERRRIYDIVNVLESLHLVSRVAKNQYGWHGRHSLPKTLRNLQRLGEEQKYEEQMAYLQQKELDLIDYKFGERKKDGDPDSQEQQLLDFSEPDCPSSSANSRKDKSLRIMSQKFVMLFLVSKTKIVTLDVAAKILIEESQDAPDHSKFKTKVRRLYDIANVLTSLALIKKVHVTEERGRKPAFKWIGPVDFSSSDEELVDVSASVLPELKRETYGQIQVCAKQKLARHGSFNTVQASERIQRKVNSEPSSPYREEQGSGGYSLEIGSLAAVYRQKIEDNSQGKAFASKRVVPPSSSLDPVAPFPVLSVDPEYCVNPLAHPVFSVAQTDLQAFSMQNGLNGQVDVSLASAASAVESLKPALLAGQPLVYVPSASLFMLYGSLQEGPASGSGSERDDRSSEAPATVELSSAPSAQKRLCEERKPQEEDEPATKRQSREYEDGPLSLVMPKKPSDSTDLASPKTMGNRASIPLKDIHVNGQLPAAEEISGKATANSLVSSEWGNPSRNTDVEKPSKENESTKEPSLLQYLCVQSPAGLNGFNVLLSGSQTPPTVGPSSGQLPSFSVPCMVLPSPPLGPFPVLYSPAMPGPVSSTLGALPNTGPVNFSLPGLGSIAQLLVGPTAVVNPKSSTLPSADPQLQSQPSLNLSPVMSRSHSVVQQPESPVYVGHPVSVVKLQQSPVPVTPKSIQRTHRETFFKTPGSLGDPVLKRRERNQSRNTSSAQRRLEIPSGGAD

>NP_001880.2

MATGQKLMRAVRVFEFGGPEVLKLRSDIAVPIPKDHQVLIKVHACGVNPVETYIRSGTYSRKPLLPYTPGSDVAGVIEAVGDNASAFKKGDRVFTSSTISGGYAEYALAADHTVYKLPEKLDFKQGAAIGIPYFTAYRALIHSACVKAGESVLVHGASGGVGLAACQIARAYGLKILGTAGTEEGQKIVLQNGAHEVFNHREVNYIDKIKKYVGEKGIDIIIEMLANVNLSKDLSLLSHGGRVIVVGSRGTIEINPRDTMAKESSIIGVTLFSSTKEEFQQYAAALQAGMEIGWLKPVIGSQYPLEKVAEAHENIIHGSGATGKMILLL

1692 NP_005513.1 NP_742024.1

>NP_005513.1

MDNARMNSFLEYPILSSGDSGTCSARAYPSDHRITTFQSCAVSANSCGGDDRFLVGRGVQIGSPHHHHHHHHHHPQPATYQTSGNLGVSYSHSSCGPSYGSQNFSAPYSPYALNQEADVSGGYPQCAPAVYSGNLSSPMVQHHHHHQGYAGGAVGSPQYIHHSYGQEHQSLALATYNNSLSPLHASHQEACRSPASETSSPAQTFDWMKVKRNPPKTGKVGEYGYLGQPNAVRTNFTTKQLTELEKEFHFNKYLTRARRVEIAASLQLNETQVKIWFQNRRMKQKKREKEGLLPISPATPPGNDEKAEESSEKSSSSPCVPSPGSSTSDTLTTSH

>NP_742024.1

MDTSDLFASCRKGDVGRVRYLLEQRDVEVNVRDKWDSTPLYYACLCGHEELVLYLLANGARCEANTFDGERCLYGALSDPIRRALRDYKQVTASCRRRDYYDDFLQRLLEQGIHSDVVFVVHGKPFRVHRCVLGARSAYFANMLDTKWKGKSVVVLRHPLINPVAFGALLQYLYTGRLDIGVEHVSDCERLAKQCQLWDLLSDLEAKCEKVSEFVASKPGTCVKVLTIEPPPADPRLREDMALLADCALPPELRGDLWELPFPCPDGFNSCPDICFRVAGCSFLCHKAFFCGRSDYFRALLDDHFRESEEPATSGGPPAVTLHGISPDVFTHVLYYMYSDHTELSPEAAYDVLSVADMYLLPGLKRLCGRSLAQMLDEDTVVGVWRVAKLFRLARLEDQCTEYMAKVIEKLVEREDFVEAVKEEAAAVAARQETDSIPLVDDIRFHVASTVQTYSAIEEAQQRLRALEDLLVSIGLDC

1693 NP_003529.1 NP_055595.2

>NP_003529.1

MSGRGKGGKGLGKGGAKRHRKVLRDNIQGITKPAIRRLARRGGVKRISGLIYEETRGVLKVFLENVIRDAVTYTEHAKRKTVTAMDVVYALKRQGRTLYGFGG

>NP_055595.2

MVGELRYREFRVPLGPGLHAYPDELIRQRVGHDGHPEYQIRWLILRRGDEGDGGSGQVDCKAEHILLWMSKDEIYANCHKMLGEDGQVIGPSQESAGEVGALDKSVLEEMETDVKSLIQRALRQLEECVGTIPPAPLLHTVHVLSAYASIEPLTGVFKDPRVLDLLMHMLSSPDYQIRWSAGRMIQALSSHDAGTRTQILLSLSQQEAIEKHLDFDSRCALLALFAQATLSEHPMSFEGIQLPQVPGRVLFSLVKRYLHVTSLLDQLNDSAAEPGAQNTSAPEELSGERGQLELEFSMAMGTLISELVQAMRWDQASDRPRSSARSPGSIFQPQLADVSPGLPAAQAQPSFRRSRRFRPRSEFASGNTYALYVRDTLQPGMRVRMLDDYEEISAGDEGEFRQSNNGVPPVQVFWESTGRTYWVHWHMLEILGFEEDIEDMVEADEYQGAVASRVLGRALPAWRWRPMTELYAVPYVLPEDEDTEECEHLTLAEWWELLFFIKKLDGPDHQEVLQILQENLDGEILDDEILAELAVPIELAQDLLLTLPQRLNDSALRDLINCHVYKKYGPEALAGNQAYPSLLEAQEDVLLLDAQAQAKDSEDAAKVEAKEPPSQSPNTPLQRLVEGYGPAGKILLDLEQALSSEGTQENKVKPLLLQLQRQPQPFLALMQSLDTPETNRTLHLTVLRILKQLVDFPEALLLPWHEAVDACMACLRSPNTDREVLQELIFFLHRLTSVSRDYAVVLNQLGARDAISKALEKHLGKLELAQELRDMVFKCEKHAHLYRKLITNILGGCIQMVLGQIEDHRRTHQPINIPFFDVFLRYLCQGSSVEVKEDKCWEKVEVSSNPHRASKLTDHNPKTYWESNGSAGSHYITLHMRRGILIRQLTLLVASEDSSYMPARVVVCGGDSTSSLHTELNSVNVMPSASRVILLENLTRFWPIIQIRIKRCQQGGIDTRIRGLEILGPKPTFWPVFREQLCRHTRLFYMVRAQAWSQDMAEDRRSLLHLSSRLNGALRQEQNFADRFLPDDEAAQALGKTCWEALVSPVVQNITSPDEDGISPLGWLLDQYLECQEAVFNPQSRGPAFFSRVRRLTHLLVHVEPCEAPPPVVATPRPKGRNRSHDWSSLATRGLPSSIMRNLTRCWRAVVEKQVNNFLTSSWRDDDFVPRYCEHFNILQNSSSELFGPRAAFLLALQNGCAGALLKLPFLKAAHVSEQFARHIDQQIQGSRIGGAQEMERLAQLQQCLQAVLIFSGLEIATTFEHYYQHYMADRLLGVVSSWLEGAVLEQIGPCFPNRLPQQMLQSLSTSKELQRQFHVYQLQQLDQELLKLEDTEKKIQVGLGASGKEHKSEKEEEAGAAAVVDVAEGEEEEEENEDLYYEGAMPEVSVLVLSRHSWPVASICHTLNPRTCLPSYLRGTLNRYSNFYNKSQSHPALERGSQRRLQWTWLGWAELQFGNQTLHVSTVQMWLLLYLNDLKAVSVESLLAFSGLSADMLNQAIGPLTSSRGPLDLHEQKDIPGGVLKIRDGSKEPRSRWDIVRLIPPQTYLQAEGEDGQNLEKRRNLLNCLIVRILKAHGDEGLHIDQLVCLVLEAWQKGPCPPRGLVSSLGKGSACSSTDVLSCILHLLGKGTLRRHDDRPQVLSYAVPVTVMEPHTESLNPGSSGPNPPLTFHTLQIRSRGVPYASCTATQSFSTFR

1694 NP_055367.2 NP_001025.1

>NP_055367.2

MTQEYDNKRPVLVLQNEALYPQRRSYTSEDEAWKSFLENPLTAATKAMMSINGDEDSAAALGLLYDYYKVPRERRSSTAKPEVEHPEPDHSKRNSIPIVTEQPLISAGENRVQVLKNVPFNIVLPHGNQLGIDKRGHLTAPDTTVTVSIATMPTHSIKTETQPHGFAVGIPPAVYHPEPTERVVVFDRNLNTDQFSSGAQAPNAQRRTPDSTFSETFKEGVQEVFFPSDLSLRMPGMNSEDYVFDSVSGNNFEYTLEASKSLRQKPGDSTMTYLNKGQFYPITLKEVSSSEGIHHPISKVRSVIMVVFAEDKSREDQLRHWKYWHSRQHTAKQRCIDIADYKESFNTISNIEEIAYNAISFTWDINDEAKVFISVNCLSTDFSSQKGVKGLPLNIQVDTYSYNNRSNKPVHRAYCQIKVFCDKGAERKIRDEERKQSKRKVSDVKVPLLPSHKRMDITVFKPFIDLDTQPVLFIPDVHFANLQRGTHVLPIASEELEGEGSVLKRGPYGTEDDFAVPPSTKLARIEEPKRVLLYVRKESEEVFDALMLKTPSLKGLMEAISDKYDVPHDKIGKIFKKCKKGILVNMDDNIVKHYSNEDTFQLQIEEAGGSYKLTLTEI

>NP_001025.1

MLSLRVPLAPITDPQQLQLSPLKGLSLVDKENTPPALSGTRVLASKTARRIFQEPTEPKTKAAAPGVEDEPLLRENPRRFVIFPIEYHDIWQMYKKAEASFWTAEEVDLSKDIQHWESLKPEERYFISHVLAFFAASDGIVNENLVERFSQEVQITEARCFYGFQIAMENIHSEMYSLLIDTYIKDPKEREFLFNAIETMPCVKKKADWALRWIGDKEATYGERVVAFAAVEGIFFSGSFASIFWLKKRGLMPGLTFSNELISRDEGLHCDFACLMFKHLVHKPSEERVREIIINAVRIEQEFLTEALPVKLIGMNCTLMKQYIEFVADRLMLELGFSKVFRVENPFDFMENISLEGKTNFFEKRVGEYQRMGVMSSPTENSFTLDADF

1695 NP_059105.2 NP_005078.2

>NP_059105.2

MTCPRNVTPNSYAEPLAAPGGGERYSRSAGMYMQSGSDFNCGVMRGCGLAPSLSKRDEGSSPSLALNTYPSYLSQLDSWGDPKAAYRLEQPVGRPLSSCSYPPSVKEENVCCMYSAEKRAKSGPEAALYSHPLPESCLGEHEVPVPSYYRASPSYSALDKTPHCSGANDFEAPFEQRASLNPRAEHLESPQLGGKVSFPETPKSDSQTPSPNEIKTEQSLAGPKGSPSESEKERAKAADSSPDTSDNEAKEEIKAENTTGNWLTAKSGRKKRCPYTKHQTLELEKEFLFNMYLTRERRLEISKTINLTDRQVKIWFQNRRMKLKKMNRENRIRELTSNFNFT

>NP_005078.2

MAELTVEVRGSNGAFYKGFIKDVHEDSLTVVFENNWQPERQVPFNEVRLPPPPDIKKEISEGDEVEVYSRANDQEPCGWWLAKVRMMKGEFYVIEYAACDATYNEIVTFERLRPVNQNKTVKKNTFFKCTVDVPEDLREACANENAHKDFKKAVGACRIFYHPETTQLMILSASEATVKRVNILSDMHLRSIRTKLMLMSRNEEATKHLECTKQLAAAFHEEFVVREDLMGLAIGTHGSNIQQARKVPGVTAIELDEDTGTFRIYGESADAVKKARGFLEFVEDFIQVPRNLVGKVIGKNGKVIQEIVDKSGVVRVRIEGDNENKLPREDGMVPFVFVGTKESIGNVQVLLEYHIAYLKEVEQLRMERLQIDEQLRQIGSRSYSGRGRGRRGPNYTSGYGTNSELSNPSETESERKDELSDWSLAGEDDRDSRHQRDSRRRPGGRGRSVSGGRGRGGPRGGKSSISSVLKDPDSNPYSLLDNTESDQTADTDASESHHSTNRRRRSRRRRTDEDAVLMDGMTESDTASVNENGLVTVADYISRAESQSRQRNLPRETLAKNKKEMAKDVIEEHGPSEKAINGPTSASGDDISKLQRTPGEEKINTLKEENTQEAAVLNGVS

1696 NP_001964.2 NP_057330.2

>NP_001964.2

MDSAITLWQFLLQLLQKPQNKHMICWTSNDGQFKLLQAEEVARLWGIRKNKPNMNYDKLSRALRYYYVKNIIKKVNGQKFVYKFVSYPEILNMDPMTVGRIEGDCESLNFSEVSSSSKDVENGGKDKPPQPGAKTSSRNDYIHSGLYSSFTLNSLNSSNVKLFKLIKTENPAEKLAEKKSPQEPTPSVIKFVTTPSKKPPVEPVAATISIGPSISPSSEETIQALETLVSPKLPSLEAPTSASNVMTAFATTPPISSIPPLQEPPRTPSPPLSSHPDIDTDIDSVASQPMELPENLSLEPKDQDSVLLEKDKVNNSSRSKKPKGLELAPTLVITSSDPSPLGILSPSLPTASLTPAFFSQTPIILTPSPLLSSIHFWSTLSPVAPLSPARLQGANTLFQFPSVLNSHGPFTLSGLDGPSTPGPFSPDLQKT

>NP_057330.2

MATGTRYAGKVVVVTGGGRGIGAGIVRAFVNSGARVVICDKDESGGRALEQELPGAVFILCDVTQEDDVKTLVSETIRRFGRLDCVVNNAGHHPPPQRPEETSAQGFRQLLELNLLGTYTLTKLALPYLRKSQGNVINISSLVGAIGQAQAVPYVATKGAVTAMTKALALDESPYGVRVNCISPGNIWTPLWEELAALMPDPRATIREGMLAQPLGRMGQPAEVGAAAVFLASEANFCTGIELLVTGGAELGYGCKASRSTPVDAPDIPS

1697 P02545 Q13085

>P02545

METPSQRRATRSGAQASSTPLSPTRITRLQEKEDLQELNDRLAVYIDRVRSLETENAGLRLRITESEEVVSREVSGIKAAYEAELGDARKTLDSVAKERARLQLELSKVREEFKELKARNTKKEGDLIAAQARLKDLEALLNSKEAALSTALSEKRTLEGELHDLRGQVAKLEAALGEAKKQLQDEMLRRVDAENRLQTMKEELDFQKNIYSEELRETKRRHETRLVEIDNGKQREFESRLADALQELRAQHEDQVEQYKKELEKTYSAKLDNARQSAERNSNLVGAAHEELQQSRIRIDSLSAQLSQLQKQLAAKEAKLRDLEDSLARERDTSRRLLAEKEREMAEMRARMQQQLDEYQELLDIKLALDMEIHAYRKLLEGEEERLRLSPSPTSQRSRGRASSHSSQTQGGGSVTKKRKLESTESRSSFSQHARTSGRVAVEEVDEEGKFVRLRNKSNEDQSMGNWQIKRQNGDDPLLTYRFPPKFTLKAGQVVTIWAAGAGATHSPPTDLVWKAQNTWGCGNSLRTALINSTGEEVAMRKLVRSVTVVEDDEDEDGDDLLHHHHGSHCSSSGDPAEYNLRSRTVLCGTCGQPADKASASGSGAQVGGPISSGSSASSVTVTRSYRSVGGSGGGSFGDNLVTRSYLLGNSSPRTQSPQNCSIM

>Q13085

MDEPSPLAQPLELNQHSRFIIGSVSEDNSEDEISNLVKLDLLEEKEGSLSPASVGSDTLSDLGISSLQDGLALHIRSSMSGLHLVKQGRDRKKIDSQRDFTVASPAEFVTRFGGNKVIEKVLIANNGIAAVKCMRSIRRWSYEMFRNERAIRFVVMVTPEDLKANAEYIKMADHYVPVPGGPNNNNYANVELILDIAKRIPVQAVWAGWGHASENPKLPELLLKNGIAFMGPPSQAMWALGDKIASSIVAQTAGIPTLPWSGSGLRVDWQENDFSKRILNVPQELYEKGYVKDVDDGLQAAEEVGYPVMIKASEGGGGKGIRKVNNADDFPNLFRQVQAEVPGSPIFVMRLAKQSRHLEVQILADQYGNAISLFGRDCSVQRRHQKIIEEAPATIATPAVFEHMEQCAVKLAKMVGYVSAGTVEYLYSQDGSFYFLELNPRLQVEHPCTEMVADVNLPAAQLQIAMGIPLYRIKDIRMMYGVSPWGDSPIDFEDSAHVPCPRGHVIAARITSENPDEGFKPSSGTVQELNFRSNKNVWGYFSVAAAGGLHEFADSQFGHCFSWGENREEAISNMVVALKELSIRGDFRTTVEYLIKLLETESFQMNRIDTGWLDRLIAEKVQAERPDTMLGVVCGALHVADVSLRNSVSNFLHSLERGQVLPAHTLLNTVDVELIYEGVKYVLKVTRQSPNSYVVIMNGSCVEVDVHRLSDGGLLLSYDGSSYTTYMKEEVDRYRITIGNKTCVFEKENDPSVMRSPSAGKLIQYIVEDGGHVFAGQCYAEIEVMKMVMTLTAVESGCIHYVKRPGAALDPGCVLAKMQLDNPSKVQQAELHTGSLPRIQSTALRGEKLHRVFHYVLDNLVNVMNGYCLPDPFFSSKVKDWVERLMKTLRDPSLPLLELQDIMTSVSGRIPPNVEKSIKKEMAQYASNITSVLCQFPSQQIANILDSHAATLNRKSEREVFFMNTQSIVQLVQRYRSGIRGHMKAVVMDLLRQYLRVETQFQNGHYDKCVFALREENKSDMNTVLNYIFSHAQVTKKNLLVTMLIDQLCGRDPTLTDELLNILTELTQLSKTTNAKVALRARQVLIASHLPSYELRHNQVESIFLSAIDMYGHQFCIENLQKLILSETSIFDVLPNFFYHSNQVVRMAALEVYVRRAYIAYELNSVQHRQLKDNTCVVEFQFMLPTSHPNRGNIPTLNRMSFSSNLNHYGMTHVASVSDVLLDNSFTPPCQRMGGMVSFRTFEDFVRIFDEVMGCFSDSPPQSPTFPEAGHTSLYDEDKVPRDEPIHILNVAIKTDCDIEDDRLAAMFREFTQQNKATLVDHGIRRLTFLVAQKDFRKQVNYEVDRRFHREFPKFFTFRARDKFEEDRIYRHLEPALAFQLELNRMRNFDLTAIPCANHKMHLYLGAAKVEVGTEVTDYRFFVRAIIRHSDLVTKEASFEYLQNEGERLLLEAMDELEVAFNNTNVRTDCNHIFLNFVPTVIMDPSKIEESVRSMVMRYGSRLWKLRVLQAELKINIRLTPTGKAIPIRLFLTNESGYYLDISLYKEVTDSRTAQIMFQAYGDKQGPLHGMLINTPYVTKDLLQSKRFQAQSLGTTYIYDIPEMFRQSLIKLWESMSTQAFLPSPPLPSDMLTYTELVLDDQGQLVHMNRLPGGNEIGMVAWKMTFKSPEYPEGRDIIVIGNDITYRIGSFGPQEDLLFLRASELARAEGIPRIYVSANSGARIGLAEEIRHMFHVAWVDPEDPYKGYRYLYLTPQDYKRVSALNSVHCEHVEDEGESRYKITDIIGKEEGIGPENLRGSGMIAGESSLAYNEIITISLVTCRAIGIGAYLVRLGQRTIQVENSHLILTGAGALNKVLGREVYTSNNQLGGIQIMHNNGVTHCTVCDDFEGVFTVLHWLSYMPKSVHSSVPLLNSKDPIDRIIEFVPTKTPYDPRWMLAGRPHPTQKGQWLSGFFDYGSFSEIMQPWAQTVVVGRARLGGIPVGVVAVETRTVELSIPADPANLDSEAKIIQQAGQVWFPDSAFKTYQAIKDFNREGLPLMVFANWRGFSGGMKDMYDQVLKFGAYIVDGLRECCQPVLVYIPPQAELRGGSWVVIDSSINPRHMEMYADRESRGSVLEPEGTVEIKFRRKDLVKTMRRVDPVYIHLAERLGTPELSTAERKELENKLKEREEFLIPIYHQVAVQFADLHDTPGRMQEKGVISDILDWKTSRTFFYWRLRRLLLEDLVKKKIHNANPELTDGQIQAMLRRWFVEVEGTVKAYVWDNNKDLAEWLEKQLTEEDGVHSVIEENIKCISRDYVLKQIRSLVQANPEVAMDSIIHMTQHISPTQRAEVIRILSTMDSPST

1698 Q6ISB3 NP_203526.1

>Q6ISB3

MSQESDNNKRLVALVPMPSDPPFNTRRAYTSEDEAWKSYLENPLTAATKAMMSINGDEDSAAALGLLYDYYKVPRDKRLLSVSKASDSQEDQEKRNCLGTSEAQSNLSGGENRVQVLKTVPVNLSLNQDHLENSKREQYSISFPESSAIIPVSGITVVKAEDFTPVFMAPPVHYPRGDGEEQRVVIFEQTQYDVPSLATHSAYLKDDQRSTPDSTYSESFKDAATEKFRSASVGAEEYMYDQTSSGTFQYTLEATKSLRQKQGEGPMTYLNKGQFYAITLSETGDNKCFRHPISKVRSVVMVVFSEDKNRDEQLKYWKYWHSRQHTAKQRVLDIADYKESFNTIGNIEEIAYNAVSFTWDVNEEAKIFITVNCLSTDFSSQKGVKGLPLMIQIDTYSYNNRSNKPIHRAYCQIKVFCDKGAERKIRDEERKQNRKKGKGQASQTQCNSSSDGKLAAIPLQKKSDITYFKTMPDLHSQPVLFIPDVHFANLQRTGQVYYNTDDEREGGSVLVKRMFRPMEEEFGPVPSKQMKEEGTKRVLLYVRKETDDVFDALMLKSPTVKGLMEAISEKYGLPVEKIAKLYKKSKKGILVNMDDNIIEHYSNEDTFILNMESMVEGFKVTLMEI

>NP_203526.1

MSWSGLLHGLNTSLTCGPALVPRLWATCSMATLNQMHRLGPPKRPPRKLGPTEGRPQLKGVVLCTFTRKPKKPNSANRKCCRVRLSTGREAVCFIPGEGHTLQEHQIVLVEGGRTQDLPGVKLTVVRGKYDCGHVQKK

1699 P10275 O75394

>P10275

MEVQLGLGRVYPRPPSKTYRGAFQNLFQSVREVIQNPGPRHPEAASAAPPGASLLLLQQQQQQQQQQQQQQQQQQQQQETSPRQQQQQQGEDGSPQAHRRGPTGYLVLDEEQQPSQPQSALECHPERGCVPEPGAAVAASKGLPQQLPAPPDEDDSAAPSTLSLLGPTFPGLSSCSADLKDILSEASTMQLLQQQQQEAVSEGSSSGRAREASGAPTSSKDNYLGGTSTISDNAKELCKAVSVSMGLGVEALEHLSPGEQLRGDCMYAPLLGVPPAVRPTPCAPLAECKGSLLDDSAGKSTEDTAEYSPFKGGYTKGLEGESLGCSGSAAAGSSGTLELPSTLSLYKSGALDEAAAYQSRDYYNFPLALAGPPPPPPPPHPHARIKLENPLDYGSAWAAAAAQCRYGDLASLHGAGAAGPGSGSPSAAASSSWHTLFTAEEGQLYGPCGGGGGGGGGGGGGGGGGGGGGGGGEAGAVAPYGYTRPPQGLAGQESDFTAPDVWYPGGMVSRVPYPSPTCVKSEMGPWMDSYSGPYGDMRLETARDHVLPIDYYFPPQKTCLICGDEASGCHYGALTCGSCKVFFKRAAEGKQKYLCASRNDCTIDKFRRKNCPSCRLRKCYEAGMTLGARKLKKLGNLKLQEEGEASSTTSPTEETTQKLTVSHIEGYECQPIFLNVLEAIEPGVVCAGHDNNQPDSFAALLSSLNELGERQLVHVVKWAKALPGFRNLHVDDQMAVIQYSWMGLMVFAMGWRSFTNVNSRMLYFAPDLVFNEYRMHKSRMYSQCVRMRHLSQEFGWLQITPQEFLCMKALLLFSIIPVDGLKNQKFFDELRMNYIKELDRIIACKRKNPTSCSRRFYQLTKLLDSVQPIARELHQFTFDLLIKSHMVSVDFPEMMAEIISVQVPKILSGKVKPIYFHTQ

>O75394

MFLSAVFFAKSKSKNILVRMVSEAGTGFCFNTKRNRLREKLTLLHYDPVVKQRVLFVEKKKIRSL

1700 NP_060624.1 Q9Y3D5

>NP_060624.1

MAKVSVLNVAVLENPSPFHSPFRFEISFECSEALADDLEWKIIYVGSAESEEFDQILDSVLVGPVPAGRHMFVFQADAPNPSLIPETDAVGVTVVLITCTYHGQEFIRVGYYVNNEYLNPELRENPPMKPDFSQLQRNILASNPRVTRFHINWDNNMDRLEAIETQDPSLGCGLPLNCTPIKGLGLPGCIPGLLPENSMDCI

>Q9Y3D5

MAAVVAVCGGLGRKKLTHLVTAAVSLTHPGTHTVLWRRGCSQQVSSNEDLPISMENPYKEPLKKCILCGKHVDYKNVQLLSQFVSPFTGCIYGRHITGLCGKKQKEITKAIKRAQIMGFMPVTYKDPAYLKDPKVCNIRYRE

1701 NP_003916.1 Q9BYN0

>NP_003916.1

MGTTGLESLSLGDRGAAPTVTSSERLVPDPPNDLRKEDVAMELERVGEDEEQMMIKRSSECNPLLQEPIASAQFGATAGTECRKSVPCGWERVVKQRLFGKTAGRFDVYFISPQGLKFRSKSSLANYLHKNGETSLKPEDFDFTVLSKRGIKSRYKDCSMAALTSHLQNQSNNSNWNLRTRSKCKKDVFMPPSSSSELQESRGLSNFTSTHLLLKEDEGVDDVNFRKVRKPKGKVTILKGIPIKKTKKGCRKSCSGFVQSDSKRESVCNKADAESEPVAQKSQLDRTVCISDAGACGETLSVTSEENSLVKKKERSLSSGSNFCSEQKTSGIINKFCSAKDSEHNEKYEDTFLESEEIGTKVEVVERKEHLHTDILKRGSEMDNNCSPTRKDFTGEKIFQEDTIPRTQIERRKTSLYFSSKYNKEALSPPRRKAFKKWTPPRSPFNLVQETLFHDPWKLLIATIFLNRTSGKMAIPVLWKFLEKYPSAEVARTADWRDVSELLKPLGLYDLRAKTIVKFSDEYLTKQWKYPIELHGIGKYGNDSYRIFCVNEWKQVHPEDHKLNKYHDWLWENHEKLSLS

>Q9BYN0

MGLRAGGTLGRAGAGRGAPEGPGPSGGAQGGSIHSGRIAAVHNVPLSVLIRPLPSVLDPAKVQSLVDTIREDPDSVPPIDVLWIKGAQGGDYFYSFGGCHRYAAYQQLQRETIPAKLVQSTLSDLRVYLGASTPDLQ

1702 Q969F1 NP_002264.1

>Q969F1

MAAAADERSPEDGEDEEEEEQLVLVELSGIIDSDFLSKCENKCKVLGIDTERPILQVDSCVFAGEYEDTLGTCVIFEENVEHADTEGNNKTVLKYKCHTMKKLSMTRTLLTEKKEGEENIGGVEWLQIKDNDFSYRPNMICNFLHENEDEEVVASAPDKSLELEEEEIQMNDSSNLSCEQEKPMHLEIEDSGPLIDIPSETEGSVFMETQMLP

>NP_002264.1

MSIRVTQKSYKVSTSGPRAFSSRSYTSGPGSRISSSSFSRVGSSNFRGGLGGGYGGASGMGGITAVTVNQSLLSPLVLEVDPNIQAVRTQEKEQIKTLNNKFASFIDKVRFLEQQNKMLETKWSLLQQQKTARSNMDNMFESYINNLRRQLETLGQEKLKLEAELGNMQGLVEDFKNKYEDEINKRTEMENEFVLIKKDVDEAYMNKVELESRLEGLTDEINFLRQLYEEEIRELQSQISDTSVVLSMDNSRSLDMDSIIAEVKAQYEDIANRSRAEAESMYQIKYEELQSLAGKHGDDLRRTKTEISEMNRNISRLQAEIEGLKGQRASLEAAIADAEQRGELAIKDANAKLSELEAALQRAKQDMARQLREYQELMNVKLALDIEIATYRKLLEGEESRLESGMQNMSIHTKTTSGYAGGLSSAYGGLTSPGLSYSLGSSFGSGAGSSSFSRTSSSRAVVVKKIETRDGKLVSESSDVLPK

1703 P04066 NP_000476.1

>P04066

MRAPGMRSRPAGPALLLLLLFLGAAESVRRAQPPRRYTPDWPSLDSRPLPAWFDEAKFGVFIHWGVFSVPAWGSEWFWWHWQGEGRPQYQRFMRDNYPPGFSYADFGPQFTARFFHPEEWADLFQAAGAKYVVLTTKHHEGFTNWPSPVSWNWNSKDVGPHRDLVGELGTALRKRNIRYGLYHSLLEWFHPLYLLDKKNGFKTQHFVSAKTMPELYDLVNSYKPDLIWSDGEWECPDTYWNSTNFLSWLYNDSPVKDEVVVNDRWGQNCSCHHGGYYNCEDKFKPQSLPDHKWEMCTSIDKFSWGYRRDMALSDVTEESEIISELVQTVSLGGNYLLNIGPTKDGLIVPIFQERLLAVGKWLSINGEAIYASKPWRVQWEKNTTSVWYTSKGSAVYAIFLHWPENGVLNLESPITTSTTKITMLGIQGDLKWSTDPDKGLFISLPQLPPSAVPAEFAWTIKLTGVK

>NP_000476.1

MADSELQLVEQRIRSFPDFPTPGVVFRDISPVLKDPASFRAAIGLLARHLKATHGGRIDYIAGLDSRGFLFGPSLAQELGLGCVLIRKRGKLPGPTLWASYSLEYGKAELEIQKDALEPGQRVVVVDDLLATGGTMNAACELLGRLQAEVLECVSLVELTSLKGREKLAPVPFFSLLQYE

1704 NP_068556.1 Q9NX20

>NP_068556.1

MLGAVKMEGHEPSDWSSYYAEPEGYSSVSNMNAGLGMNGMNTYMSMSAAAMGSGSGNMSAGSMNMSSYVGAGMSPSLAGMSPGAGAMAGMGGSAGAAGVAGMGPHLSPSLSPLGGQAAGAMGGLAPYANMNSMSPMYGQAGLSRARDPKTYRRSYTHAKPPYSYISLITMAIQQSPNKMLTLSEIYQWIMDLFPFYRQNQQRWQNSIRHSLSFNDCFLKVPRSPDKPGKGSFWTLHPDSGNMFENGCYLRRQKRFKCEKQLALKEAAGAAGSGKKAAAGAQASQAQLGEAAGPASETPAGTESPHSSASPCQEHKRGGLGELKGTPAAALSPPEPAPSPGQQQQAAAHLLGPPHHPGLPPEAHLKPEHHYAFNHPFSINNLMSSEQQHHHSHHHHQPHKMDLKAYEQVMHYPGYGSPMPGSLAMGPVTNKTGLDASPLAADTSYYQGVYSRPIMNSS

>Q9NX20

MWRLLARASAPLLRVPLSDSWALLPASAGVKTLLPVPSFEDVSIPEKPKLRFIERAPLVPKVRREPKNLSDIRGPSTEATEFTEGNFAILALGGGYLHWGHFEMMRLTINRSMDPKNMFAIWRVPAPFKPITRKSVGHRMGGGKGAIDHYVTPVKAGRLVVEMGGRCEFEEVQGFLDQVAHKLPFAAKAVSRGTLEKMRKDQEERERNNQNPWTFERIATANMLGIRKVLSPYDLTHKGKYWGKFYMPKRV

1705 NP_254280.1 AAH07654.1

>NP_254280.1

MSGRGKQGGKARAKAKSRSSRAGLQFPVGRVHRLLRKGNYSERVGAGAPVYLAAVLEYLTAEILELAGNAARDNKKTRIIPRHLQLAIRNDEELNKLLGRVTIAQGGVLPNIQAVLLPKKTESHHKAKGK

>AAH07654.1

MLSSCVRPVPTTVRFVDSLICNSSRSFMDLKALLSSLNDFASLSFAESWDNVGLLVEPSPPHTVNTLFLTNDLTEEVMEEVLQKKADLILSYHPPIFRPMKRITWNTWKERLVIRALENRVGIYSPHTAYDAAPQGVNNWLAKGLGACTSRPIHPSKAPNYPTEGNHRVEFNVNYTQDLDKVMSAVKGIDGVSVTSFSARTGNEEQTRINLNCTQKALMQVVDFLSRNKQLYQKTEILSLEKPLLLHTGMGRLCTLDESVSLATMIDRIKRHLKLSHIRLALGVGRTLESQVKVVALCAGSGSSVLQGVEADLYLTGEMSHHDTLDAASQGINVILCEHSNTERGFLSDLRDMLDSHLENKINIILSETDRDPLQVV

1706 NP_002519.1 Q92526

>NP_002519.1

MCSPQESGMTALSARMLTRSRSLGPGAGPRGCREEPGPLRRREAAAEARKSHSPVKRPRKAQRLRVAYEGSDSEKGEGAEPLKVPVWEPQDWQQQLVNIRAMRNKKDAPVDHLGTEHCYDSSAPPKVRRYQVLLSLMLSSQTKDQVTAGAMQRLRARGLTVDSILQTDDATLGKLIYPVGFWRSKVKYIKQTSAILQQHYGGDIPASVAELVALPGVGPKMAHLAMAVAWGTVSGIAVDTHVHRIANRLRWTKKATKSPEETRAALEEWLPRELWHEINGLLVGFGQQTCLPVHPRCHACLNQALCPAAQGL

>Q92526

MAAIKAVNSKAEVARAQAALAVNICAARGLQDVLRTNLGPKGTMKMLASGAGDIKLTKDGNVLLDEMQIQHPTASLIAKVATAQDDVTGDGTTSNVLIIGELLKQADLYISEGLHPRIIAEGFEAAKIKALEVLEEVKVTKEMKRKILLDVARTSLQTKVHAELADVLTEVVVDSVLAVRRPGYPIDLFMVEIMEMKHKLGTDTKLIQGLVLDHGARHPDMKKRVEDAFILICNVSLEYEKTEVNSGFFYKTAEEKEKLVKAERKFIEDRVQKIIDLKDKVCAQSNKGFVVINQKGIDPFSLDSLAKHGIVALRRAKRRNMERLSLACGGMAVNSFEDLTVDCLGHAGLVYEYTLGEEKFTFIEECVNPCSVTLLVKGPNKHTLTQVKDAIRDGLRAIKNAIEDGCMVPGAGAIEVAMAEALVTYKNSIKGRARLGVQAFADALLIIPKVLAQNAGYDPQETLVKVQAEHVESKQLVGVDLNTGEPMVAADAGVWDNYCVKKQLLHSCTVIATNILLVDEIMRAGMSSLK

1707 NP_006224.1 NP_055199.1

>NP_006224.1

MEPDGTYEPGFVGIRFCQECNNMLYPKEDKENRILLYACRNCDYQQEADNSCIYVNKITHEVDELTQIIADVSQDPTLPRTEDHPCQKCGHKEAVFFQSHSARAEDAMRLYYVCTAPHCGHRWTE

>NP_055199.1

MLWSGCRRFGARLGCLPGGLRVLVQTGHRSLTSCIDPSMGLNEEQKEFQKVAFDFAAREMAPNMAEWDQKELFPVDVMRKAAQLGFGGVYIQTDVGGSGLSRLDTSVIFEALATGCTSTTAYISIHNMCAWMIDSFGNEEQRHKFCPPLCTMEKFASYCLTEPGSGSDAASLLTSAKKQGDHYILNGSKAFISGAGESDIYVVMCRTGGPGPKGISCIVVEKGTPGLSFGKKEKKVGWNSQPTRAVIFEDCAVPVANRIGSEGQGFLIAVRGLNGGRINIASCSLGAAHASVILTRDHLNVRKQFGEPLASNQYLQFTLADMATRLVAARLMVRNAAVALQEERKDAVALCSMAKLFATDECFAICNQALQMHGGYGYLKDYAVQQYVRDSRVHQILEGSNEVMRILISRSLLQE

1708 NP_149073.1 P40261

>NP_149073.1

MATETVELHKLKLAELKQECLARGLETKGIKQDLIHRLQAYLEEHAEEEANEEDVLGDETEEEETKPIELPVKEEEPPEKTVDVAAEKKVVKITSEIPQTERMQKRAERFNVPVSLESKKAARAARFGISSVPTKGLSSDNKPMVNLDKLKERAQRFGLNVSSISRKSEDDEKLKKRKERFGIVTSSAGTGTTEDTEAKKRKRAERFGIA

>P40261

MESGFTSKDTYLSHFNPRDYLEKYYKFGSRHSAESQILKHLLKNLFKIFCLDGVKGDLLIDIGSGPTIYQLLSACESFKEIVVTDYSDQNLQELEKWLKKEPEAFDWSPVVTYVCDLEGNRVKGPEKEEKLRQAVKQVLKCDVTQSQPLGAVPLPPADCVLSTLCLDAACPDLPTYCRALRNLGSLLKPGGFLVIMDALKSSYYMIGEQKFSSLPLGREAVEAAVKEAGYTIEWFEVISQSYSSTMANNEGLFSLVARKLSRPL

1709 Q16560 NP_005026.3

>Q16560

MNDWMPIAKEYDPLKAGSIDGTDEDPHDRAVWRAMLARYVPNKGVIGDPLLTLFVARLNLQTKEDKLKEVFSRYGDIRRLRLVRDLVTGFSKGYAFIEYKEERAVIKAYRDADGLVIDQHEIFVDYELERTLKGWIPRRLGGGLGGKKESGQLRFGGRDRPFRKPINLPVVKNDLYREGKRERRERSRSRERHWDSRTRDRDHDRGREKRWQEREPTRVWPDNDWERERDFRDDRIKGREKKERGK

>NP_005026.3

MSALCWGRGAAGLKRALRPCGRPGLPGKEGTAGGVCGPRRSSSASPQEQDQDRRKDWGHVELLEVLQARVRQLQAESVSEVVVNRVDVARLPECGSGDGSLQPPRKVQMGAKDATPVPCGRWAKILEKDKRTQQMRMQRLKAKLQMPFQSGEFKALTRRLQVEPRLLSKQMAGCLEDCTRQAPESPWEEQLARLLQEAPGKLSLDVEQAPSGQHSQAQLSGQQQRLLAFFKCCLLTDQLPLAHHLLVVHHGQRQKRKLLTLDMYNAVMLGWARQGAFKELVYVLFMVKDAGLTPDLLSYAAALQCMGRQDQDAGTIERCLEQMSQEGLKLQALFTAVLLSEEDRATVLKAVHKVKPTFSLPPQLPPPVNTSKLLRDVYAKDGRVSYPKLHLPLKTLQCLFEKQLHMELASRVCVVSVEKPTLPSKEVKHARKTLKTLRDQWEKALCRALRETKNRLEREVYEGRFSLYPFLCLLDEREVVRMLLQVLQALPAQGESFTTLARELSARTFSRHVVQRQRVSGQVQALQNHYRKYLCLLASDAEVPEPCLPRQYWEELGAPEALREQPWPLPVQMELGKLLAEMLVQATQMPCSLDKPHRSSRLVPVLYHVYSFRNVQQIGILKPHPAYVQLLEKAAEPTLTFEAVDVPMLCPPLPWTSPHSGAFLLSPTKLMRTVEGATQHQELLETCPPTALHGALDALTQLGNCAWRVNGRVLDLVLQLFQAKGCPQLGVPAPPSEAPQPPEAHLPHSAAPARKAELRRELAHCQKVAREMHSLRAEALYRLSLAQHLRDRVFWLPHNMDFRGRTYPCPPHFNHLGSDVARALLEFAQGRPLGPHGLDWLKIHLVNLTGLKKREPLRKRLAFAEEVMDDILDSADQPLTGRKWWMGAEEPWQTLACCMEVANAVRASDPAAYVSHLPVHQDGSCNGLQHYAALGRDSVGAASVNLEPSDVPQDVYSGVAAQVEVFRRQDAQRGMRVAQVLEGFITRKVVKQTVMTVVYGVTRYGGRLQIEKRLRELSDFPQEFVWEASHYLVRQVFKSLQEMFSGTRAIQHWLTESARLISHMGSVVEWVTPLGVPVIQPYRLDSKVKQIGGGIQSITYTHNGDISRKPNTRKQKNGFPPNFIHSLDSSHMMLTALHCYRKGLTFVSVHDCYWTHAADVSVMNQVCREQFVRLHSEPILQDLSRFLVKRFCSEPQKILEASQLKETLQAVPKPGAFDLEQVKRSTYFFS

1710 NP_002220.1 Q8TF09

>NP_002220.1

MCTKMEQPFYHDDSYTATGYGRAPGGLSLHDYKLLKPSLAVNLADPYRSLKAPGARGPGPEGGGGGSYFSGQGSDTGASLKLASSELERLIVPNSNGVITTTPTPPGQYFYPRGGGSGGGAGGAGGGVTEEQEGFADGFVKALDDLHKMNHVTPPNVSLGATGGPPAGPGGVYAGPEPPPVYTNLSSYSPASASSGGAGAAVGTGSSYPTTTISYLPHAPPFAGGHPAQLGLGRGASTFKEEPQTVPEARSRDATPPVSPINMEDQERIKVERKRLRNRLAATKCRKRKLERIARLEDKVKTLKAENAGLSSTAGLLREQVAQLKQKVMTHVSNGCQLLLGVKGHAF

>Q8TF09

MAEVEETLKRIQSHKGVIGTMVVNAEGIPIRTTLDNSTTVQYAGLLHHLTMKAKSTVRDIDPQNDLTFLRIRSKKHEIMVAPDKEYLLIVIQNPCE

1711 Q99879 P22528

>Q99879

MPEPVKSAPVPKKGSKKAINKAQKKDGKKRKRSRKESYSVYVYKVLKQVHPDTGISSKAMGIMNSFVNDIFERIAGEASRLAHYNKRSTITSREIQTAVRLLLPGELAKHAVSEGTKAVTKYTSSK

>P22528

MSSQQQKQPCTPPPQLQQQQVKQPCQPPPQEPCIPKTKEPCHPKVPEPCHPKVPEPCQPKVPEPCHPKVPEPCPSIVTPAPAQQKTKQK

1712 P36915 Q68DY1

>P36915

MPRKKPFSVKQKKKQLQDKRERKRGLQDGLRSSSNSRSGSRERREEQTDTSDGESVTHHIRRLNQQPSQGLGPRGYDPNRYRLHFERDSREEVERRKRAAREQVLQPVSAELLELDIREVYQPGSVLDFPRRPPWSYEMSKEQLMSQEERSFQDYLGKIHGAYSSEKLSYFEHNLETWRQLWRVLEMSDIVLLITDIRHPVVNFPPALYEYVTGELGLALVLVLNKVDLAPPALVVAWKHYFHQHYPQLHVVLFTSFPRDPRTPQDPSSVLKKSRRRGRGWTRALGPEQLLRACEAITVGKVDLSSWREKIARDVAGATWGNGSGEEEEEEDGPAVLVEQQTDSAMEPTGPTQERYKDGVVTIGCVGFPNVGKSSLINGLVGRKVVSVSRTPGHTRYFQTYFLTPSVKLCDCPGLIFPSLLPRQLQVLAGIYPIAQIQEPYTAVGYLASRIPVQALLHLRHPEAEDPSAEHPWCAWDICEAWAEKRGYKTAKAARNDVYRAANSLLRLAVDGRLSLCFHPPGYSEQKGTWESHPETTELVVLQGRVGPAGDEEEEEEEELSSSCEEEGEEDRDADEEGEGDEETPTSAPGSSLAGRNPYALLGEDEC

>Q68DY1

MGPLQFRDVAIEFSLEEWHCLDTAQRNLYRNVMLENYSNLVFLGITVSKPDLITCLEQGRKPLTMKRNEMIAKPSVMCSHFAQDLWPEQSMKDSFQKVVLRRYEKCEHDNLQLKKGCISVDECKVHKEGYNELNQCLTTTPRKICQCDKYVKVLHQFPNSNGQKRGHTGKKPFKYIECGKAFKQFSTLTTHKKIHTGGKPYKCEECGKAFNHSCSLTRHKKIHTGEKPYKCEECGKAFKHSSTLTTHKRNHTGEKPYKCDKCGKAFMSSSTLSKHEIIHTEKKPYKCEECGKAFNRSSTLTTHKIIHTGEKPYKCEECDKAFKYSYTLTTHKRIHTEDKPYKCEECGKAFKYSSTLTTHKRIHTGEKPYKCEECGKAFKRSSDLTTHKIIHTGEKPYKCEECGKAFKYSSNLTTHKKIHTGERPYKCEECGKAFNQSSILTTHRRIHTGEKFYKCEECGKAFKCSSNLTTHKKIHTGERPYKCEECGKAFNQSSILTTHERIILERNSTNVKNVAKPSSGPHTLLHIR

1713 NP_115696.2 NP_005179.2

>NP_115696.2

MAGRPHPYDGNSSDPENWDRKLHSRPRKLYKHSSTSSRIAKGGVDHTKMSLHGASGGHERSRDRRRSSDRSRDSSHERTESQLTPCIRNVTSPTRQHHVEREKDHSSSRPSSPRPQKASPNGSISSAGNSSRNSSQSSSDGSCKTAGEMVFVYENAKEGARNIRTSERVTLIVDNTRFVVDPSIFTAQPNTMLGRMFGSGREHNFTRPNEKGEYEVAEGIGSTVFRAILDYYKTGIIRCPDGISIPELREACDYLCISFEYSTIKCRDLSALMHELSNDGARRQFEFYLEEMILPLMVASAQSGERECHIVVLTDDDVVDWDEEYPPQMGEEYSQIIYSTKLYRFFKYIENRDVAKSVLKERGLKKIRLGIEGYPTYKEKVKKRPGGRPEVIYNYVQRPFIRMSWEKEEGKSRHVDFQCVKSKSITNLAAAAADIPQDQLVVMHPTPQVDELDILPIHPPSGNSDLDPDAQNPML

>NP_005179.2

MAGNVKKSSGAGGGSGSGGSGSGGLIGLMKDAFQPHHHHHHHLSPHPPGTVDKKMVEKCWKLMDKVVRLCQNPKLALKNSPPYILDLLPDTYQHLRTILSRYEGKMETLGENEYFRVFMENLMKKTKQTISLFKEGKERMYEENSQPRRNLTKLSLIFSHMLAELKGIFPSGLFQGDTFRITKADAAEFWRKAFGEKTIVPWKSFRQALHEVHPISSGLEAMALKSTIDLTCNDYISVFEFDIFTRLFQPWSSLLRNWNSLAVTHPGYMAFLTYDEVKARLQKFIHKPGSYIFRLSCTRLGQWAIGYVTADGNILQTIPHNKPLFQALIDGFREGFYLFPDGRNQNPDLTGLCEPTPQDHIKVTQEQYELYCEMGSTFQLCKICAENDKDVKIEPCGHLMCTSCLTSWQESEGQGCPFCRCEIKGTEPIVVDPFDPRGSGSLLRQGAEGAPSPNYDDDDDERADDTLFMMKELAGAKVERPPSPFSMAPQASLPPVPPRLDLLPQRVCVPSSASALGTASKAASGSLHKDKPLPVPPTLRDLPPPPPPDRPYSVGAESRPQRRPLPCTPGDCPSRDKLPPVPSSRLGDSWLPRPIPKVPVSAPSSSDPWTGRELTNRHSLPFSLPSQMEPRPDVPRLGSTFSLDTSMSMNSSPLVGPECDHPKIKPSSSANAIYSLAARPLPVPKLPPGEQCEGEEDTEYMTPSSRPLRPLDTSQSSRACDCDQQIDSCTYEAMYNIQSQAPSITESSTFGEGNLAAAHANTGPEESENEDDGYDVPKPPVPAVLARRTLSDISNASSSFGWLSLDGDPTTNVTEGSQVPERPPKPFPRRINSERKAGSCQQGSGPAASAATASPQLSSEIENLMSQGYSYQDIQKALVIAQNNIEMAKNILREFVSISSPAHVAT

1714 Q16385 Q9NY74

>Q16385

MNGDDAFARRPTVGAQIPEKIQKAFDDIAKYFSKEEWEKMKASEKIFYVYMKRKYEAMTKLGFKATLPPFMCNKRAEDFQGNDLDNDPNRGNQVERPQMTFGRLQGISPKIMPKKPAEEGNDSEEVPEASGPQNDGKELCPPGKPTTSEKIHERSGPKRGEHAWTHRLRERKQLVIYEEISDPEEDDE

>Q9NY74

MSRRRKHDDSPSPKKTPHKTVAAEECGSVVEPGRRRLRSARGSWPCGAREGPPGPVRQREQPPTAALCSKSNPEERYETPKRALKMDSLSSSFSSPNDPDGQNDIFWDQNSPLTKQLGKGRKKQIYTTDSDEISHIVNRIAPQDEKPTTNSMLDMWIGETAIPCTPSVAKGKSRAKISCTKLKTQSQEEELMKLAKQFDKNMEELDVIQEQNKRNYDFTQMISETEILSNYKDNIQMWSLHNIVPEIDNATKKPIKGNTKISVANNQNSSQKPFDQIAEAAFNAIFDGSTQKCSGQLSQELPEAFWSTSNTTFVKTNALKEEKIITNETLVIEKLSNKTPRSLSSQVDTPIMTKSCVTSCTKEPETSNKYIDAFTTSDFEDDWENLLGSEPFAMQNIDMPELFPSKTAHVTDQKEICTFNSKTVKNTSRANTSPDARLGDSKVLQDLSSKTYDRELIDAEYRFSPNSNKSNKLSTGNKMKFENSSNKIVIQDEIQNCIVTSNLTKIKEDILTNSTEASERKSALNTRYSNEQKNKCILNQSIKAPVNTDLFGSANLGSKTSVSNPNQTSASKVGSFFDDWNDPSFANEIIKACHQLDNTWEADDVDDDLLYQACDDIERLTQQQDIRKDSKTSESICEINNNSEHGAKLTQQQDIRKDSKTSESICEINNNSEHGAKNMFAISKQGSNLVQSKHLNPGSISVQTSLTNSSQIDKPMKMEKGEMYGNSPRFLGATNLTMYSKISNCQINNLHVSYTNTDVPIQVNSSKLVLPGSSSLNVTSDHMNTEITTYKKKLSTNQPCHKTVTDEAQSNLNTTVGFSKFTFTRMKNSQILSQFNQNCITGSMSDTKITQGVEKKKGVNPLLEEAVGQQSLVKLSESLKQSSKEEEEKNRKCSPEEIQRKRQEALVRRMAKARASSVNAAPTSFL

1715 NP_996670.1 P30838

>NP_996670.1

MAPKQDPKPKFQEGERVLCFHGPLLYEAKCVKVAIKDKQVKYFIHYSGWNKKSAVRPRRSEKSLKTHEDIVALFPVPEGAPSVHHPLLTSSWDEWVPESRVLKYVDTNLQKQRELQKANQEQYAEGKMRGAAPGKKTSGLQQKNVEVKTKKNKQKTPGNGDGGSTSETPQPPRKKRARVDPTVENEETFMNRVEVKVKIPEELKPWLVDDWDLITRQKQLFYLPAKKNVDSILEDYANYKKSRGNTDNKEYAVNEVVAGIKEYFNVMLGTQLLYKFERPQYAEILADHPDAPMSQVYGAPHLLRLFVRIGAMLAYTPLDEKSLALLLNYLHDFLKYLAKNSATLFSASDYEVAPPEYHRKAV

>P30838

MSKISEAVKRARAAFSSGRTRPLQFRIQQLEALQRLIQEQEQELVGALAADLHKNEWNAYYEEVVYVLEEIEYMIQKLPEWAADEPVEKTPQTQQDELYIHSEPLGVVLVIGTWNYPFNLTIQPMVGAIAAGNAVVLKPSELSENMASLLATIIPQYLDKDLYPVINGGVPETTELLKERFDHILYTGSTGVGKIIMTAAAKHLTPVTLELGGKSPCYVDKNCDLDVACRRIAWGKFMNSGQTCVAPDYILCDPSIQNQIVEKLKKSLKEFYGEDAKKSRDYGRIISARHFQRVMGLIEGQKVAYGGTGDAATRYIAPTILTDVDPQSPVMQEEIFGPVLPIVCVRSLEEAIQFINQREKPLALYMFSSNDKVIKKMIAETSSGGVAANDVIVHITLHSLPFGGVGNSGMGSYHGKKSFETFSHRRSCLVRPLMNDEGLKVRYPPSPAKMTQH

1716 NP_005595.2 NP_001753.1

>NP_005595.2

MATAASNHYSLLTSSASIVHAEPPGGMQQGAGGYREAQSLVQGDYGALQSNGHPLSHAHQWITALSHGGGGGGGGGGGGGGGGGGGGGDGSPWSTSPLGQPDIKPSVVVQQGGRGDELHGPGALQQQHQQQQQQQQQQQQQQQQQQQQQRPPHLVHHAANHHPGPGAWRSAAAAAHLPPSMGASNGGLLYSQPSFTVNGMLGAGGQPAGLHHHGLRDAHDEPHHADHHPHPHSHPHQQPPPPPPPQGPPGHPGAHHDPHSDEDTPTSDDLEQFAKQFKQRRIKLGFTQADVGLALGTLYGNVFSQTTICRFEALQLSFKNMCKLKPLLNKWLEEADSSSGSPTSIDKIAAQGRKRKKRTSIEVSVKGALESHFLKCPKPSAQEITSLADSLQLEKEVVRVWFCNRRQKEKRMTPPGGTLPGAEDVYGGSRDTPPHHGVQTPVQ

>NP_001753.1

MAAVKTLNPKAEVARAQAALAVNISAARGLQDVLRTNLGPKGTMKMLVSGAGDIKLTKDGNVLLHEMQIQHPTASLIAKVATAQDDITGDGTTSNVLIIGELLKQADLYISEGLHPRIITEGFEAAKEKALQFLEEVKVSREMDRETLIDVARTSLRTKVHAELADVLTEAVVDSILAIKKQDEPIDLFMIEIMEMKHKSETDTSLIRGLVLDHGARHPDMKKRVEDAYILTCNVSLEYEKTEVNSGFFYKSAEEREKLVKAERKFIEDRVKKIIELKRKVCGDSDKGFVVINQKGIDPFSLDALSKEGIVALRRAKRRNMERLTLACGGVALNSFDDLSPDCLGHAGLVYEYTLGEEKFTFIEKCNNPRSVTLLIKGPNKHTLTQIKDAVRDGLRAVKNAIDDGCVVPGAGAVEVAMAEALIKHKPSVKGRAQLGVQAFADALLIIPKVLAQNSGFDLQETLVKIQAEHSESGQLVGVDLNTGEPMVAAEVGVWDNYCVKKQLLHSCTVIATNILLVDEIMRAGMSSLKG

1717 NP_612211.1 Q9BZQ8

>NP_612211.1

MFVLVEMVDTVRIPPWQFERKLNDSIAEELNKKLANKVVYNVGLCICLFDITKLEDAYVFPGDGASHTKVHFRCVVFHPFLDEILIGKIKGCSPEGVHVSLGFFDDILIPPESLQQPAKFDEAEQVWVWEYETEEGAHDLYMDTGEEIRFRVVDESFVDTSPTGPSSADATTSSEELPKKEAPYTLVGSISEPGLGLLSWWTSN

>Q9BZQ8

MGGSASSQLDEGKCAYIRGKTEAAIKNFSPYYSRQYSVAFCNHVRTEVEQQRDLTSQFLKTKPPLAPGTILYEAELSQFSEDIKKWKERYVVVKNDYAVESYENKEAYQRGAAPKCRILPAGGKVLTSEDEYNLLSDRHFPDPLASSEKENTQPFVVLPKEFPVYLWQPFFRHGYFCFHEAADQKRFSALLSDCVRHLNHDYMKQMTFEAQAFLEAVQFFRQEKGHYGSWEMITGDEIQILSNLVMEELLPTLQTDLLPKMKGKKNDRKRTWLGLLEEAYTLVQHQVSEGLSALKEECRALTKGLEGTIRSDMDQIVNSKNYLIGKIKAMVAQPAEKSCLESVQPFLASILEELMGPVSSGFSEVRVLFEKEVNEVSQNFQTTKDSVQLKEHLDRLMNLPLHSVKMEPCYTKVNLLHERLQDLKSRFRFPHIDLVVQRTQNYMQELMENAVFTFEQLLSPHLQGEASKTAVAIEKVKLRVLKQYDYDSSTIRKKIFQEALVQITLPTVQKALASTCKPELQKYEQFIFADHTNMIHVENVYEEILHQILLDETLKVIKEAAILKKHNLFEDNMALPSESVSSLTDLKPPTGSNQASPARRASAILPGVLGSETLSNEVFQESEEEKQPEVPSSLAKGESLSLPGPSPPPDGTEQVIISRVDDPVVNPVATEDTAGLPGTCSSELEFGGTLEDEEPAQEEPEPITASGSLKALRKLLTASVEVPVDSAPVMEEDTNGESHVPQENEEEEEKEPSQAAAIHPDNCEESEVSEREAQPPCPEAHGEELGGFPEVGSPASPPASGGLTEEPLGPMEGELPGEACTLTAHEGRGGKCTEEGDASQQEGCTLGSDPICLSESQVSEEQEEMGGQSSAAQATASVNAEEIKVARIHECQWVVEDAPNPDVLLSHKDDVKEGEGGQESFPELPSEE

1718 O00115 NP_004138.1

>O00115

MIPLLLAALLCVPAGALTCYGDSGQPVDWFVVYKLPALRGSGEAAQRGLQYKYLDESSGGWRDGRALINSPEGAVGRSLQPLYRSNTSQLAFLLYNDQPPQPSKAQDSSMRGHTKGVLLLDHDGGFWLVHSVPNFPPPASSAAYSWPHSACTYGQTLLCVSFPFAQFSKMGKQLTYTYPWVYNYQLEGIFAQEFPDLENVVKGHHVSQEPWNSSITLTSQAGAVFQSFAKFSKFGDDLYSGWLAAALGTNLQVQFWHKTVGILPSNCSDIWQVLNVNQIAFPGPAGPSFNSTEDHSKWCVSPKGPWTCVGDMNRNQGEEQRGGGTLCAQLPALWKAFQPLVKNYQPCNGMARKPSRAYKI

>NP_004138.1

MSSTLAKIAEIEAEMARTQKNKATAHHLGLLKARLAKLRRELITPKGGGGGGPGEGFDVAKTGDARIGFVGFPSVGKSTLLSNLAGVYSEVAAYEFTTLTTVPGVIRYKGAKIQLLDLPGIIEGAKDGKGRGRQVIAVARTCNLILIVLDVLKPLGHKKIIENELEGFGIRLNSKPPNIGFKKKDKGGINLTATCPQSELDAETVKSILAEYKIHNADVTLRSDATADDLIDVVEGNRVYIPCIYVLNKIDQISIEELDIIYKVPHCVPISAHHRWNFDDLLEKIWDYLKLVRIYTKPKGQLPDYTSPVVLPYSRTTVEDFCMKIHKNLIKEFKYALVWGLSVKHNPQKVGKDHTLEDEDVIQIVKK

1719 NP_005404.1 NP_003076.1

>NP_005404.1

MVFRSPLDLYSSHFLLPNFADSHHRSILLASSGGGNGAGGGGGAGGGSGGGNGAGGGGAGGAGGGGGGGSRAPPEELSMFQLPTLNFSPEQVASVCETLEETGDIERLGRFLWSLPVAPGACEAINKHESILRARAVVAFHTGNFRDLYHILENHKFTKESHGKLQAMWLEAHYQEAEKLRGRPLGPVDKYRVRKKFPLPRTIWDGEQKTHCFKERTRSLLREWYLQDPYPNPSKKRELAQATGLTPTQVGNWFKNRRQRDRAAAAKNRLQHQAIGPSGMRSLAEPGCPTHGSAESPSTAASPTTSVSSLTERADTGTSILSVTSSDSECDV

>NP_003076.1

MDVFMKGLSMAKEGVVAAAEKTKQGVTEAAEKTKEGVLYVGSKTREGVVQGVASVAEKTKEQASHLGGAVFSGAGNIAAATGLVKREEFPTDLKPEEVAQEAAEEPLIEPLMEPEGESYEDPPQEEYQEYEPEA

1720 Q9GZZ0 Q8WXC3

>Q9GZZ0

MSSYLEYVSCSSSGGVGGDVLSLAPKFCRSDARPVALQPAFPLGNGDGAFVSCLPLAAARPSPSPPAAPARPSVPPPAAPQYAQCTLEGAYEPGAAPAAAAGGADYGFLGSGPAYDFPGVLGRAADDGGSHVHYATSAVFSGGGSFLLSGQVDYAAFGEPGPFPACLKASADGHPGAFQTASPAPGTYPKSVSPASGLPAAFSTFEWMKVKRNASKKGKLAEYGAASPSSAIRTNFSTKQLTELEKEFHFNKYLTRARRIEIANCLHLNDTQVKIWFQNRRMKQKKREREGLLATAIPVAPLQLPLSGTTPTKFIKNPGSPSQSQEPS

>Q8WXC3

MGTKREAILKVLENLTPEELKKFKMKLGTVPLREGFERIPRGALGQLDIVDLTDKLVASYYEDYAAELVVAVLRDMRMLEEAARLQRAA

1721 NP_003519.1 NP_659491.4

>NP_003519.1

MPEPAKSAPAPKKGSKKAVTKAQKKDGKKRKRSRKESYSIYVYKVLKQVHPDTGISSKAMGIMNSFVNDIFERIAGEASRLAHYNKRSTITSREIQTAVRLLLPGELAKHAVSEGTKAVTKYTSSK

>NP_659491.4

MDNKISPEAQVAELELDAVIGFNGHVPTGLKCHPDQEHMIYPLGCTVLIQAINTKEQNFLQGHGNNVSCLAISRSGEYIASGQVTFMGFKADIILWDYKNRELLARLSLHKGKIEALAFSPNDLYLVSLGGPDDGSVVVWSIAKRDAICGSPAAGLNVGNATNVIFSRCRDEMFMTAGNGTIRVWELDLPNRKIWPTECQTGQLKRIVMSIGVDDDDSFFYLGTTTGDILKMNPRTKLLTDVGPAKDKFSLGVSAIRCLKMGGLLVGSGAGLLVFCKSPGYKPIKKIQLQGGITSITLRGEGHQFLVGTEESHIYRVSFTDFKETLIATCHFDAVEDIVFPFGTAELFATCAKKDIRVWHTSSNRELLRITVPNMTCHGIDFMRDGKSIISAWNDGKIRAFAPETGRLMYVINNAHRIGVTAIATTSDCKRVISGGGEGEVRVWQIGCQTQKLEEALKEHKSSVSCIRVKRNNEECVTASTDGTCIIWDLVRLRRNQMILANTLFQCVCYHPEEFQIITSGTDRKIAYWEVFDGTVIRELEGSLSGSINGMDITQEGVHFVTGGNDHLVKVWDYNEGEVTHVGVGHSGNITRIRISPGNQYIVSVSADGAILRWKYPYTS

1722 P53999 Q8N573

>P53999

MPKSKELVSSSSSGSDSDSEVDKKLKRKKQVAPEKPVKKQKTGETSRALSSSKQSSSSRDDNMFQIGKMRYVSVRDFKGKVLIDIREYWMDPEGEMKPGRKGISLNPEQWSQLKEQISDIDDAVRKL

>Q8N573

MSFQKPKGTIEYTVESRDSLNSIALKFDTTPNELVQLNKLFSRAVVTGQVLYVPDPEYVSSVESSPSLSPVSPLSPTSSEAEFDKTTNPDVHPTEATPSSTFTGIRPARVVSSTSEEEEAFTEKFLKINCKYITSGKGTVSGVLLVTPNNIMFDPHKNDPLVQENGCEEYGIMCPMEEVMSAAMYKEILDSKIKESLPIDIDQLSGRDFCHSKKMTGSNTEEIDSRIRDAGNDSASTAPRSTEESLSEDVFTESELSPIREELVSSDELRQDKSSGASSESVQTVNQAEVESLTVKSESTGTPGHLRSDTEHSTNEVGTLCHKTDLNNLEMAIKEDQIADNFQGISGPKEDSTSIKGNSDQDSFLHENSLHQEESQKENMPCGETAEFKQKQSVNKGKQGKEQNQDSQTEAEELRKLWKTHTMQQTKQQRENIQQVSQKEAKHKITSADGHIESSALLKEKQRHRLHKFLCLRVGKPMRKTFVSQASATMQQYAQRDKKHEYWFAVPQERTDHLYAFFIQWSPEIYAEDTGEYTREPGFIVVKKIEESETIEDSSNQAAAREWEITTREDINSKQVATVKADLESESFRPNLSDPSELLLPDQIEKLTKHLPPRTIGYPWTLVYGTGKHGTSLKTLYRTMTGLDTPVLMVIKDSDGQVFGALASEPLKVSDGFYGTGETFVFTFCPEFEVFKWTGDNMFFIKGDMDSLAFGGGGGEFALWLDGDLYHGRSHSCKTFGNRTLSKKEDFFIQDIEIWAFE

1723 NP_003403.2 NP_002444.2

>NP_003403.2

MLLDAGPQYPAIGVTTFGASRHHSAGDVAERDVGLGINPFADGMGAFKLNPSSHELASAGQTAFTSQAPGYAAAAALGHHHHPGHVGSYSSAAFNSTRDFLFRNRGFGDAAAAASAQHSLFAASAGGFGGPHGHTDAAGHLLFPGLHEQAAGHASPNVVNGQMRLGFSGDMYPRPEQYGQVTSPRSEHYAAPQLHGYGPMNVNMAAHHGAGAFFRYMRQPIKQELICKWIEPEQLANPKKSCNKTFSTMHELVTHVTVEHVGGPEQSNHICFWEECPREGKPFKAKYKLVNHIRVHTGEKPFPCPFPGCGKVFARSENLKIHKRTHTGEKPFKCEFEGCDRRFANSSDRKKHMHVHTSDKPYLCKMCDKSYTHPSSLRKHMKVHESSSQGSQPSPAASSGYESSTPPTIVSPSTDNPTTSSLSPSSSAVHHTAGHSALSSNFNEWYV

>NP_002444.2

MNQKLLKLENLLRFHTIYRQLHSLCQRRALRQWRHGFSSAYPVWTAQLCAWPWPTDVLTGAALSQYRLLVTKKEEGPWKSQLSSTKSKKVVEVWIGMTIEELARAMEKNTDYVYEALLNTDIDIDSLEADSHLDEVWIKEVITKAGMKLKWSKLKQDKVRKNKDAVRRPQADPALLTPRSPVVTIMGHVDHGKTTLLDKFRKTQVAAVETGGITQHIGAFLVSLPSGEKITFLDTPGHAAFSAMRARGAQVTDIVVLVVAADDGVMKQTVESIQHAKDAQVPIILAVNKCDKAEADPEKVKKELLAYDVVCEDYGGDVQAVPVSALTGDNLMALAEATVALAEMLELKADPNGPVEGTVIESFTDKGRGLVTTAIIQRGTLRKGSVLVAGKCWAKVRLMFDENGKTIDEAYPSMPVGITGWRDLPSAGEEILEVESEPRAREVVDWRKYEQEQEKGQEDLKIIEEKRKEHKEAHQKAREKYGHLLWKKRSILRFLERKEQIPLKPKEKRERDSNVLSVIIKGDVDGSVEAILNIIDTYDASHECELELVHFGVGDVSANDVNLAETFDGVIYGFNVNAGNVIQQSAAKKGVKIKLHKIIYRLVEDLQEELSSRLPCAVEEHPVGEASILATFSVTEGKKKVPVAGCRVQKGQLEKQKKFKLTRNGHVIWKGSLTSLKHHKDDISIVKTGMDCGLSLDEDNMEFQVGDRIVCYEEKQIQAKTSWDPGF

1724 NP_005135.2 NP_001938.1

>NP_005135.2

MESTPSFLKGTPTWEKTAPENGIVRQEPGSPPRDGLHHGPLCLGEPAPFWRGVLSTPDSWLPPGFPQGPKDMLPLVEGEGPQNGERKVNWLGSKEGLRWKEAMLTHPLAFCGPACPPRCGPLMPEHSGGHLKSDPVAFRPWHCPFLLETKILERAPFWVPTCLPPYLVSGLPPEHPCDWPLTPHPWVYSGGQPKVPSAFSLGSKGFYYKDPSIPRLAKEPLAAAEPGLFGLNSGGHLQRAGEAERPSLHQRDGEMGAGRQQNPCPLFLGQPDTVPWTSWPACPPGLVHTLGNVWAGPGDGNLGYQLGPPATPRCPSPEPPVTQRGCCSSYPPTKGGGLGPCGKCQEGLEGGASGASEPSEEVNKASGPRACPPSHHTKLKKTWLTRHSEQFECPRGCPEVEERPVARLRALKRAGSPEVQGAMGSPAPKRPPDPFPGTAEQGAGGWQEVRDTSIGNKDVDSGQHDEQKGPQDGQASLQDPGLQDIPCLALPAKLAQCQSCAQAAGEGGGHACHSQQVRRSPLGGELQQEEDTATNSSSEEGPGSGPDSRLSTGLAKHLLSGLGDRLCRLLRREREALAWAQREGQGPAVTEDSPGIPRCCSRCHHGLFNTHWRCPRCSHRLCVACGRVAGTGRAREKAGFQEQSAEECTQEAGHAACSLMLTQFVSSQALAELSTAMHQVWVKFDIRGHCPCQADARVWAPGDAGQQKESTQKTPPTPQPSCNGDTHRTKSIKEETPDSAETPAEDRAGRGPLPCPSLCELLASTAVKLCLGHERIHMAFAPVTPALPSDDRITNILDSIIAQVVERKIQEKALGPGLRAGPGLRKGLGLPLSPVRPRLPPPGALLWLQEPQPCPRRGFHLFQEHWRQGQPVLVSGIQRTLQGNLWGTEALGALGGQVQALSPLGPPQPSSLGSTTFWEGFSWPELRPKSDEGSVLLLHRALGDEDTSRVENLAASLPLPEYCALHGKLNLASYLPPGLALRPLEPQLWAAYGVSPHRGHLGTKNLCVEVADLVSILVHADTPLPAWHRAQKDFLSGLDGEGLWSPGSQVSTVWHVFRAQDAQRIRRFLQMVCPAGAGALEPGAPGSCYLDAGLRRRLREEWGVSCWTLLQAPGEAVLVPAGAPHQVQGLVSTVSVTQHFLSPETSALSAQLCHQGPSLPPDCHLLYAQMDWAVFQAVKVAVGTLQEAK

>NP_001938.1

MPCKSAEWLQEELEARGGASLLLLDCRPHELFESSHIETAINLAIPGLMLRRLRKGNLPIRSIIPNHADKERFATRCKAATVLLYDEATAEWQPEPGAPASVLGLLLQKLRDDGCQAYYLQGGFNKFQTEYSEHCETNVDSSSSPSSSPPTSVLGLGGLRISSDCSDGESDRELPSSATESDGSPVPSSQPAFPVQILPYLYLGCAKDSTNLDVLGKYGIKYILNVTPNLPNAFEHGGEFTYKQIPISDHWSQNLSQFFPEAISFIDEARSKKCGVLVHCLAGISRSVTVTVAYLMQKMNLSLNDAYDFVKRKKSNISPNFNFMGQLLDFERTLGLSSPCDNHASSEQLYFSTPTNHNLFPLNTLEST

1725 O94953 NP_056238.2

>O94953

MGSEDHGAQNPSCKIMTFRPTMEEFKDFNKYVAYIESQGAHRAGLAKIIPPKEWKPRQTYDDIDDVVIPAPIQQVVTGQSGLFTQYNIQKKAMTVGEYRRLANSEKYCTPRHQDFDDLERKYWKNLTFVSPIYGADISGSLYDDDVAQWNIGSLRTILDMVERECGTIIEGVNTPYLYFGMWKTTFAWHTEDMDLYSINYLHFGEPKSWYAIPPEHGKRLERLAIGFFPGSSQGCDAFLRHKMTLISPIILKKYGIPFSRITQEAGEFMITFPYGYHAGFNHGFNCAESTNFATLRWIDYGKVATQCTCRKDMVKISMDVFVRILQPERYELWKQGKDLTVLDHTRPTALTSPELSSWSASRASLKAKLLRRSHRKRSQPKKPKPEDPKFPGEGTAGAALLEEAGGSVKEEAGPEVDPEEEEEEPQPLPHGREAEGAEEDGRGKLRPTKAKSERKKKSFGLLPPQLPPPPAHFPSEEALWLPSPLEPPVLGPGPAAMEESPLPAPLNVVPPEVPSEELEAKPRPIIPMLYVVPRPGKAAFNQEHVSCQQAFEHFAQKGPTWKEPVSPMELTGPEDGAASSGAGRMETKARAGEGQAPSTFSKLKMEIKKSRRHPLGRPPTRSPLSVVKQEASSDEEASPFSGEEDVSDPDALRPLLSLQWKNRAASFQAERKFNAAAARTEPYCAICTLFYPYCQALQTEKEAPIASLGKGCPATLPSKSRQKTRPLIPEMCFTSGGENTEPLPANSYIGDDGTSPLIACGKCCLQVHASCYGIRPELVNEGWTCSRCAAHAWTAECCLCNLRGGALQMTTDRRWIHVICAIAVPEARFLNVIERHPVDISAIPEQRWKLKCVYCRKRMKKVSGACIQCSYEHCSTSFHVTCAHAAGVLMEPDDWPYVVSITCLKHKSGGHAVQLLRAVSLGQVVITKNRNGLYYRCRVIGAASQTCYEVNFDDGSYSDNLYPESITSRDCVQLGPPSEGELVELRWTDGNLYKAKFISSVTSHIYQVEFEDGSQLTVKRGDIFTLEEELPKRVRSRLSLSTGAPQEPAFSGEEAKAAKRPRVGTPLATEDSGRSQDYVAFVESLLQVQGRPGAPF

>NP_056238.2

MVFPAKRFCLVPSMEGVRWAFSCGTWLPSRAEWLLAVRSIQPEEKERIGQFVFARDAKAAMAGRLMIRKLVAEKLNIPWNHIRLQRTAKGKPVLAKDSSNPYPNFNFNISHQGDYAVLAAEPELQVGIDIMKTSFPGRGSIPEFFHIMKRKFTNKEWETIRSFKDEWTQLDMFYRNWALKESFIKAIGVGLGFELQRLEFDLSPLNLDIGQVYKETRLFLDGEEEKEWAFEESKIDEHHFVAVALRKPDGSRHQDVPSQDDSKPTQRQFTILNFNDLMSSAVPMTPEDPSFWDCFCFTEEIPIRNGTKS

1726 NP_004836.2 Q6UB35

>NP_004836.2

MPVVTTDAESETGIPKSLSNEPPSETMEEIEHTCPQPRLTLTAPAPFADETNCQCQAPHEKLTIAQARLGTPADRPVRVYADGIFDLFHSGHARALMQAKTLFPNSYLLVGVCSDDLTHKFKGFTVMNEAERYEALRHCRYVDEVIRDAPWTLTPEFLEKHKIDFVAHDDIPYSSAGSDDVYKHIKEAGMFVPTQRTEGISTSDIITRIVRDYDVYARRNLQRGYTAKELNVSFINEKRYRFQNQVDKMKEKVKNVEERSKEFVNRVEEKSHDLIQKWEEKSREFIGNFLELFGPDGAWKQMFQERSSRMLQALSPKQSPVSSPTRSRSPSRSPSPTFSWLPLKTSPPSSPKAASASISSMSEGDEDEK

>Q6UB35

MGTRLPLVLRQLRRPPQPPGPPRRLRVPCRASSGGGGGGGGGREGLLGQRRPQDGQARSSCSPGGRTPAARDSIVREVIQNSKEVLSLLQEKNPAFKPVLAIIQAGDDNLMQEINQNLAEEAGLNITHICLPPDSSEAEIIDEILKINEDTRVHGLALQISENLFSNKVLNALKPEKDVDGVTDINLGKLVRGDAHECFVSPVAKAVIELLEKSGVNLDGKKILVVGAHGSLEAALQCLFQRKGSMTMSIQWKTRQLQSKLHEADIVVLGSPKPEEIPLTWIQPGTTVLNCSHDFLSGKVGCGSPRIHFGGLIEEDDVILLAAALRIQNMVSSGRRWLREQQHRRWRLHCLKLQPLSPVPSDIEISRGQTPKAVDVLAKEIGLLADEIEIYGKSKAKVRLSVLERLKDQADGKYVLVAGITPTPLGEGKSTVTIGLVQALTAHLNVNSFACLRQPSQGPTFGVKGGAAGGGYAQVIPMEEFNLHLTGDIHAITAANNLLAAAIDTRILHENTQTDKALYNRLVPLVNGVREFSEIQLARLKKLGINKTDPSTLTEEEVSKFARLDIDPSTITWQRVLDTNDRFLRKITIGQGNTEKGHYRQAQFDIAVASEIMAVLALTDSLADMKARLGRMVVASDKSGQPVTADDLGVTGALTVLMKDAIKPNLMQTLEGTPVFVHAGPFANIAHGNSSVLADKIALKLVGEEGFVVTEAGFGADIGMEKFFNIKCRASGLVPNVVVLVATVRALKMHGGGPSVTAGVPLKKEYTEENIQLVADGCCNLQKQIQITQLFGVPVVVALNVFKTDTRAEIDLVCELAKRAGAFDAVPCYHWSVGGKGSVDLARAVREAASKRSRFQFLYDVQVPIVDKIRTIAQAVYGAKDIELSPEAQAKIDRYTQQGFGNLPICMAKTHLSLSHQPDKKGVPRDFILPISDVRASIGAGFIYPLVGTMSTMPGLPTRPCFYDIDLDTETEQVKGLF

1727 Q9H6T0 P59044

>Q9H6T0

MTPPPPPPPPPGPDPAADPAADPCPWPGSLVVLFGATAGALGRDLGSDETDLILLVWQVVEPRSRQVGTLHKSLVRAEAAALSTQCREASGLSADSLARAEPLDKVLQQFSQLVNGDVALLGGGPYMLCTDGQQLLRQVLHPEASRKNLVLPDMFFSFYDLRREFHMQHPSTCPARDLTVATMAQGLGLETDATEDDFGVWEVKTMVAVILHLLKEPSSQLFSKPEVIKQKYETGPCSDSTVPCPYSSKADVVDSETVVRARGLPWQSSDQDVARFFKGLNVARGGVALCLNAQGRRNGEALIRFVDSEQRDLALQRHKHHMGVRYIEVYKATGEEFVKIAGGTSLEVARFLSREDQVILRLRGLPFSAGPTDVLGFLGPECPVTGGTEGLLFVRHPDGRPTGDAFALFACEELAQAALRRHKGMLGKRYIELFRSTAAEVQQVLNRYASGPLLPTLTAPLLPIPFPLAPGTGRDCVRLRGLPYTATIEDILSFLGEAAADIRPHGVHMVLNQQGRPSGDAFIQMTSAERALAAAQRCHKKVMKERYVEVVPCSTEEMSRVLMGGTLGRSGMSPPPCKLPCLSPPTYTTFQATPTLIPTETAALYPSSALLPAARVPAAPTPVAYYPGPATQLYLNYTAYYPSPPVSPTTVGYLTTPTAALASAPTSVLSQSGALVRMQGVPYTAGMKDLLSVFQAYQLPADDYTSLMPVGDPPRTVLQAPKEWVCL

>P59044

MDQPEAPCSSTGPRLAVARELLLAALEELSQEQLKRFRHKLRDVGPDGRSIPWGRLERADAVDLAEQLAQFYGPEPALEVARKTLKRADARDVAAQLQERRLQRLGLGSGTLLSVSEYKKKYREHVLQLHARVKERNARSVKITKRFTKLLIAPESAAPEEAMGPAEEPEPGRARRSDTHTFNRLFRRDEEGRRPLTVVLQGPAGIGKTMAAKKILYDWAAGKLYQGQVDFAFFMPCGELLERPGTRSLADLILDQCPDRGAPVPQMLAQPQRLLFILDGADELPALGGPEAAPCTDPFEAASGARVLGGLLSKALLPTALLLVTTRAAAPGRLQGRLCSPQCAEVRGFSDKDKKKYFYKYFRDERRAERAYRFVKENETLFALCFVPFVCWIVCTVLRQQLELGRDLSRTSKTTTSVYLLFITSVLSSAPVADGPRLQGDLRNLCRLAREGVLGRRAQFAEKELEQLELRGSKVQTLFLSKKELPGVLETEVTYQFIDQSFQEFLAALSYLLEDGGVPRTAAGGVGTLLRGDAQPHSHLVLTTRFLFGLLSAERMRDIERHFGCMVSERVKQEALRWVQGQGQGCPGVAPEVTEGAKGLEDTEEPEEEEEGEEPNYPLELLYCLYETQEDAFVRQALCRFPELALQRVRFCRMDVAVLSYCVRCCPAGQALRLISCRLVAAQEKKKKSLGKRLQASLGGGSSSQGTTKQLPASLLHPLFQAMTDPLCHLSSLTLSHCKLPDAVCRDLSEALRAAPALTELGLLHNRLSEAGLRMLSEGLAWPQCRVQTVRVQLPDPQRGLQYLVGMLRQSPALTTLDLSGCQLPAPMVTYLCAVLQHQGCGLQTLSLASVELSEQSLQELQAVKRAKPDLVITHPALDGHPQPPKELISTF

1728 Q9NV88 NP_002940.2

>Q9NV88

MKLYCLSGHPTLPCNVLKFKSTTIMLDCGLDMTSTLNFLPLPLVQSPRLSNLPGWSLKDGNAFLDKELKECSGHVFVDSVPEFCLPETELIDLSTVDVILISNYHCMMALPYITEHTGFTGTVYATEPTVQIGRLLMEELVNFIERVPKAQSASLWKNKDIQRLLPSPLKDAVEVSTWRRCYTMQEVNSALSKIQLVGYSQKIELFGAVQVTPLSSGYALGSSNWIIQSHYEKVSYVSGSSLLTTHPQPMDQASLKNSDVLVLTGLTQIPTANPDGMVGEFCSNLALTVRNGGNVLVPCYPSGVIYDLLECLYQYIDSAGLSSVPLYFISPVANSSLEFSQIFAEWLCHNKQSKVYLPEPPFPHAELIQTNKLKHYPSIHGDFSNDFRQPCVVFTGHPSLRFGDVVHFMELWGKSSLNTVIFTEPDFSYLEALAPYQPLAMKCIYCPIDTRLNFIQVSKLLKEVQPLHVVCPEQYTQPPPAQSHRMDLMIDCQPPAMSYRRAEVLALPFKRRYEKIEIMPELADSLVPMEIKPGISLATVSAVLHTKDNKHLLQPPPRPAQPTSGKKRKRVSDDVPDCKVLKPLLSGSIPVEQFVQTLEKHGFSDIKVEDTAKGHIVLLQEAETLIQIEEDSTHIICDNDEMLRVRLRDLVLKFLQKF

>NP_002940.2

MLPAAARPLWGPCLGLRAAAFRLARRQVPCVCAVRHMRSSGHQRCEALAGAPLDNAPKEYPPKIQQLVQDIASLTLLEISDLNELLKKTLKIQDVGLVPMGGVMSGAVPAAAAQEAVEEDIPIAKERTHFTVRLTEAKPVDKVKLIKEIKNYIQGINLVQAKKLVESLPQEIKANVAKAEAEKIKAALEAVGGTVVLE

1729 NP_003075.1 NP_000958.1

>NP_003075.1

MAEGSRGGPTCSGVGGRQDPVSGSGGCNFPEYELPELNTRAFHVGAFGELWRGRLRGAGDLSLREPPASALPGSQAADSDREDAAVARDLDCSLEAAAELRAVCGLDKLKCLEDGEDPEVIPENTDLVTLGVRKRFLEHREETITIDRACRQETFVYEMESHAIGKKPENSADMIEEGELILSVNILYPVIFHKHKEHKPYQTMLVLGSQKLTQLRDSIRCVSDLQIGGEFSNTPDQAPEHISKDLYKSAFFYFEGTFYNDKRYPECRDLSRTIIEWSESHDRGYGKFQTARMEDFTFNDLCIKLGFPYLYCHQGDCEHVIVITDIRLVHHDDCLDRTLYPLLIKKHWLWTRKCFVCKMYTARWVTNNDSFAPEDPCFFCDVCFRMLHYDSEGNKLGEFLAYPYVDPGTFN

>NP_000958.1

MSHRKFSAPRHGSLGFLPRKRSSRHRGKVKSFPKDDPSKPVHLTAFLGYKAGMTHIVREVDRPGSKVNKKEVVEAVTIVETPPMVVVGIVGYVETPRGLRTFKTVFAEHISDECKRRFYKNWHKSKKKAFTKYCKKWQDEDGKKQLEKDFSSMKKYCQVIRVIAHTQMRLLPLRQKKAHLMEIQVNGGTVAEKLDWARERLEQQVPVNQVFGQDEMIDVIGVTKGKGYKGVTSRWHTKKLPRKTHRGLRKVACIGAWHPARVAFSVARAGQKGYHHRTEINKKIYKIGQGYLIKDGKLIKNNASTDYDLSDKSINPLGGFVHYGEVTNDFVMLKGCVVGTKKRVLTLRKSLLVQTKRRALEKIDLKFIDTTSKFGHGRFQTMEEKKAFMGPLKKDRIAKEEGA

1730 NP_149020.2 O15540

>NP_149020.2

MLLPLPPQSSKPVPKKSQSSKIVPSHHDPSEKTGENCQTKISPSSLQESPSSLQGALKKRSAFEDLTNASQCQPVQPKKEANKEFVKVVSKKINRNTHALGLAKKNKRNLKWHKLEVTPVVASTTVVPNIMEKPLILDISTTSKTPNTEEASLFRKPLVLKEEPTIEDETLINKSLSLKKCSNHEEVSLLEKLQPLQEESDSDDAFVIEPMTFKKTHKTEEAAITKKTLSLKKKMCASQRKQSCQEESLAVQDVNMEEDSFFMESMSFKKKPKTEESIPTHKLSSLKKKCTIYGKICHFRKPPVLQTTICGAMSSIKKPTTEKETLFQELSVLQEKHTTEHEMSILKKSLALQKTNFKEDSLVKESLAFKKKPSTEEAIMMPVILKEQCMTEGKRSRLKPLVLQEITSGEKSLIMKPLSIKEKPSTEKESFSQEPSALQKKHTTQEEVSILKEPSSLLKSPTEESPFDEALAFTKKCTIEEAPPTKKPLILKRKHATQGTMSHLKKPLILQTTSGEKSLIKEPLPFKEEKVSLKKKCTTQEMMSICPELLDFQDMIGEDKNSFFMEPMSFRKNPTTEETVLTKTSLSLQEKKITQGKMSHLKKPLVLQKITSEEESFYKKLLPFKMKSTTEEKFLSQEPSALKEKHTTLQEVSLSKESLAIQEKATTEEEFSQELFSLHVKHTNKSGSLFQEALVLQEKTDAEEDSLKNLLALQEKSTMEEESLINKLLALKEELSAEAATNIQTQLSLKKKSTSHGKVFFLKKQLALNETINEEEFLNKQPLALEGYPSIAEGETLFKKLLAMQEEPSIEKEAVLKEPTIDTEAHFKEPLALQEEPSTEKEAVLKEPSVDTEAHFKETLALQEKPSIEQEALFKRHSALWEKPSTEKETIFKESLDLQEKPSIKKETLLKKPLALKMSTINEAVLFEDMIALNEKPTTGKELSFKEPLALQESPTYKEDTFLKTLLVPQVGTSPNVSSTAPESITSKSSIATMTSVGKSGTINEAFLFEDMITLNEKPTTGKELSFKEPLALQESPTCKEDTFLETFLIPQIGTSPYVFSTTPESITEKSSIATMTSVGKSRTTTESSACESASDKPVSPQAKGTPKEITPREDIDEDSSDPSFNPMYAKEIFSYMKEREEQFILTDYMNRQIEITSDMRAILVDWLVEVQVSFEMTHETLYLAVKLVDLYLMKAVCKKDKLQLLGATAFMIAAKFEEHNSPRVDDFVYICDDNYQRSEVLSMEINILNVLKCDINIPIAYHFLRRYARCIHTNMKTLTLSRYICEMTLQEYHYVQEKASKLAAASLLLALYMKKLGYWVPFLEHYSGYSISELHPLVRQLNKLLTFSSYDSLKAVYYKYSHPVFFEVAKIPALDMLKLEEILNCDCEAQGLVL

>O15540

MVEAFCATWKLTNSQNFDEYMKALGVGFATRQVGNVTKPTVIISQEGDKVVIRTLSTFKNTEISFQLGEEFDETTADDRNCKSVVSLDGDKLVHIQKWDGKETNFVREIKDGKMVMTLTFGDVVAVRHYEKA

1731 NP_003060.2 NP_076425.1

>NP_003060.2

MEQDTAAVAATVAAADATATIVVIEDEQPGPSTSQEEGAAAAATEATAATEKGEKKKEKNVSSFQLKLAAKAPKSEKEMDPEYEEKMKADRAKRFEFLLKQTELFAHFIQPSAQKSPTSPLNMKLGRPRIKKDEKQSLISAGDYRHRRTEQEEDEELLSESRKTSNVCIRFEVSPSYVKGGPLRDYQIRGLNWLISLYENGVNGILADEMGLGKTLQTIALLGYLKHYRNIPGPHMVLVPKSTLHNWMNEFKRWVPSLRVICFVGDKDARAAFIRDEMMPGEWDVCVTSYEMVIKEKSVFKKFHWRYLVIDEAHRIKNEKSKLSEIVREFKSTNRLLLTGTPLQNNLHELWALLNFLLPDVFNSADDFDSWFDTKNCLGDQKLVERLHAVLKPFLLRRIKTDVEKSLPPKKEIKIYLGLSKMQREWYTKILMKDIDVLNSSGKMDKMRLLNILMQLRKCCNHPYLFDGAEPGPPYTTDEHIVSNSGKMVVLDKLLAKLKEQGSRVLIFSQMTRLLDILEDYCMWRGYEYCRLDGQTPHEEREDKFLEVEFLGQREAIEAFNAPNSSKFIFMLSTRAGGLGINLASADVVILYDSDWNPQVDLQAMDRAHRIGQKKPVRVFRLITDNTVEERIVERAEIKLRLDSIVIQQGRLIDQQSNKLAKEEMLQMIRHGATHVFASKESELTDEDITTILERGEKKTAEMNERLQKMGESSLRNFRMDIEQSLYKFEGEDYREKQKLGMVEWIEPPKRERKANYAVDAYFREALRVSEPKIPKAPRPPKQPNVQDFQFFPPRLFELLEKEILYYRKTIGYKVPRNPDIPNPALAQREEQKKIDGAEPLTPEETEEKEKLLTQGFTNWTKRDFNQFIKANEKYGRDDIDNIAREVEGKSPEEVMEYSAVFWERCNELQDIEKIMAQIERGEARIQRRISIKKALDAKIARYKAPFHQLRIQYGTSKGKNYTEEEDRFLICMLHKMGFDRENVYEELRQCVRNAPQFRFDWFIKSRTAMEFQRRCNTLISLIEKENMEIEERERAEKKKRATKTPMVKFSAFS

>NP_076425.1

MARKKVRPRLIAELARRVRALREQLNRPRDSQLYAVDYETLTRPFSGRRLPVRAWADVRRESRLLQLLGRLPLFGLGRLVTRKSWLWQHDEPCYWRLTRVRPDYTAQNLDHGKAWGILTFKGKTESEAREIEHVMYHDWRLVPKHEEEAFTAFTPAPEDSLASVPYPPLLRAMIIAERQKNGDTSTEEPMLNVQRIRMEPWDYPAKQEDKGRAKGTPV

1732 NP_057662.3 Q93088

>NP_057662.3

MATAAAAAAVMAPPGCPGSCPNFAVVCSFLERYGPLLDLPELPFPELERVLQAPPPDVGNGEVPKELVELHLKLMRKIGKSVTADRWEKYLIKICQEFNSTWAWEMEKKGYLEMSVECKLALLKYLCECQFDDNLKFKNIINEEDADTMRLQPIGRDKDGLMYWYQLDQDHNVRMYIEEQDDQDGSSWKCIVRNRNELAETLALLKAQIDPVLLKNSSQQDNSSRESPSLEDEETKKEEETPKQEEQKESEKMKSEEQPMDLENRSTANVLEETTVKKEKEDEKELVKLPVIVKLEKPLPENEEKKIIKEESDSFKENVKPIKVEVKECRADPKDTKSSMEKPVAQEPERIEFGGNIKSSHEITEKSTEETEKLKNDQQAKIPLKKREIKLSDDFDSPVKGPLCKSVTPTKEFLKDEIKQEEETCKRISTITALGHEGKQLVNGEVSDERVAPNFKTEPIETKFYETKEESYSPSKDRNIITEGNGTESLNSVITSMKTGELEKETAPLRKDADSSISVLEIHSQKAQIEEPDPPEMETSLDSSEMAKDLSSKTALSSTESCTMKGEEKSPKTKKDKRPPILECLEKLEKSKKTFLDKDAQRLSPIPEEVPKSTLESEKPGSPEAAETSPPSNIIDHCEKLASEKEVVECQSTSTVGGQSVKKVDLETLKEDSEFTKVEMDNLDNAQTSGIEEPSETKGSMQKSKFKYKLVPEEETTASENTEITSERQKEGIKLTIRISSRKKKPDSPPKVLEPENKQEKTEKEEEKTNVGRTLRRSPRISRPTAKVAEIRDQKADKKRGEGEDEVEEESTALQKTDKKEILKKSEKDTNSKVSKVKPKGKVRWTGSRTRGRWKYSSNDESEGSGSEKSSAASEEEEEKESEEAILADDDEPCKKCGLPNHPELILLCDSCDSGYHTACLRPPLMIIPDGEWFCPPCQHKLLCEKLEEQLQDLDVALKKKERAERRKERLVYVGISIENIIPPQEPDFSEDQEEKKKDSKKSKANLLERRSTRTRKCISYRFDEFDEAIDEAIEDDIKEADGGGVGRGKDISTITGHRGKDISTILDEERKENKRPQRAAAARRKKRRRLNDLDSDSNLDEEESEDEFKISDGSQDEFVVSDENPDESEEDPPSNDDSDTDFCSRRLRRHPSRPMRQSRRLRRKTPKKKYSDDDEEEESEENSRDSESDFSDDFSDDFVETRRRRSRRNQKRQINYKEDSESDGSQKSLRRGKEIRRVHKRRLSSSESEESYLSKNSEDDELAKESKRSVRKRGRSTDEYSEADEEEEEEEGKPSRKRLHRIETDEEESCDNAHGDANQPARDSQPRVLPSEQESTKKPYRIESDEEEDFENVGKVGSPLDYSLVDLPSTNGQSPGKAIENLIGKPTEKSQTPKDNSTASASLASNGTSGGQEAGAPEEEEDELLRVTDLVDYVCNSEQL

>Q93088

MPPVGGKKAKKGILERLNAGEIVIGDGGFVFALEKRGYVKAGPWTPEAAVEHPEAVRQLHREFLRAGSNVMQTFTFYASEDKLENRGNYVLEKISGQEVNEAACDIARQVADEGDALVAGGVSQTPSYLSCKSETEVKKVFLQQLEVFMKKNVDFLIAEYFEHVEEAVWAVETLIASGKPVAATMCIGPEGDLHGVPPGECAVRLVKAGASIIGVNCHFDPTISLKTVKLMKEGLEAARLKAHLMSQPLAYHTPDCNKQGFIDLPEFPFGLEPRVATRWDIQKYAREAYNLGVRYIGGCCGFEPYHIRAIAEELAPERGFLPPASEKHGSWGSGLDMHTKPWVRARARKEYWENLRIASGRPYNPSMSKPDGWGVTKGTAELMQQKEATTEQQLKELFEKQKFKSQ

1733 Q8TF76 P09001

>Q8TF76

MAASLPGPGSRLFRTYGAADGRRQRRPGREAAQWFPPQDRRRFFNSSGSSDASIGDPSQSDDPDDPDDPDFPGSPVRRRRRCPGGRVPKDRPSLTVTPKRWKLRARPSLTVTPRRLGLRARPPQKCSTPCGPLRLPPFPSRDSGRLSPDLSVCGQPRDGDELGISASLFSSLASPCPGSPTPRDSVISIGTSACLVAASAVPSDLHLPEVSLDRASLPCSQEEATGGAKDTRMVHQTRASLRSVLFGLMNSGTPEDSEFRADGKNMRESCCKRKLVVGNGPEGPGLSSTGKRRATGQDSCQERGLQEAVRREHQEASVPKGRIVPRGTDRLERTRSSRKSKHQEATETSLLHSHRFKKGQKLGKDSFPTQDLTPLQNACFWTKTRASFSFHKKKIVTDVSEVCSIYTTATSLSGSLLSECSNRPVMNRTSGAPSSWHSSSMYLLSPLNTLSISNKKASDAEKVYGECSQKGPVPFSHCLPTEKLQRCEKIGEGVFGEVFQTIADHTPVAIKIIAIEGPDLVNGSHQKTFEEILPEIIISKELSLLSGEVCNRTEGFIGLNSVHCVQGSYPPLLLKAWDHYNSTKGSANDRPDFFKDDQLFIVLEFEFGGIDLEQMRTKLSSLATAKSILHQLTASLAVAEASLRFEHRDLHWGNVLLKKTSLKKLHYTLNGKSSTIPSCGLQVSIIDYTLSRLERDGIVVFCDVSMDEDLFTGDGDYQFDIYRLMKKENNNRWGEYHPYSNVLWLHYLTDKMLKQMTFKTKCNTPAMKQIKRKIQEFHRTMLNFSSATDLLCQHSLFK

>P09001

MPGWRLLTQVGAQVLGRLGDGLGAALGPGNRTHIWLFVRGLHGKSGTWWDEHLSEENVPFIKQLVSDEDKAQLASKLCPLKDEPWPIHPWEPGSFRVGLIALKLGMMPLWTKDGQKHVVTLLQVQDCHVLKYTSKENCNGKMATLSVGGKTVSRFRKATSILEFYRELGLPPKQTVKIFNITDNAAIKPGTPLYAAHFRPGQYVDVTAKTIGKGFQGVMKRWGFKGQPATHGQTKTHRRPGAVATGDIGRVWPGTKMPGKMGNIYRTEYGLKVWRINTKHNIIYVNGSVPGHKNCLVKVKDSKLPAYKDLGKNLPFPTYFPDGDEEELPEDLYDENVCQPGAPSITFA

1734 NP_055138.2 Q9BRJ2

>NP_055138.2

MERVNDASCGPSGCYTYQVSRHSTEMLHNLNQQRKNGGRFCDVLLRVGDESFPAHRAVLAACSEYFESVFSAQLGDGGAADGGPADVGGATAAPGGGAGGSRELEMHTISSKVFGDILDFAYTSRIVVRLESFPELMTAAKFLLMRSVIEICQEVIKQSNVQILVPPARADIMLFRPPGTSDLGFPLDMTNGAALAANSNGIAGSMQPEEEAARAAGAAIAGQASLPVLPGVDRLPMVAGPLSPQLLTSPFPSVASSAPPLTGKRGRGRPRKANLLDSMFGSPGGLREAGILPCGLCGKVFTDANRLRQHEAQHGVTSLQLGYIDLPPPRLGENGLPISEDPDGPRKRSRTRKQVACEICGKIFRDVYHLNRHKLSHSGEKPYSCPVCGLRFKRKDRMSYHVRSHDGSVGKPYICQSCGKGFSRPDHLNGHIKQVHTSERPHKCQTCNASFATRDRLRSHLACHEDKVPCQVCGKYLRAAYMADHLKKHSEGPSNFCSICNRGFSSASYLKVHVKTHHGVPLPQVSRHQEPILNGGAAFHCARTYGNKEGQKCSHQDPIESSDSYGDLSDASDLKTPEKQSANGSFSCDMAVPKNKMESDGEKKYPCPECGSFFRSKSYLNKHIQKVHVRALGGPLGDLGPALGSPFSPQQNMSLLESFGFQIVQSAFASSLVDPEVDQQPMGPEGK

>Q9BRJ2

MAAPIPQGFSCLSRFLGWWFRQPVLVTQSAAIVPVRTKKRFTPPIYQPKFKTEKEFMQHARKAGLVIPPEKSDRSIHLACTAGIFDAYVPPEGDARISSLSKEGLIERTERMKKTMASQVSIRRIKDYDANFKIKDFPEKAKDIFIEAHLCLNNSDHDRLHTLVTEHCFPDMTWDIKYKTVRWSFVESLEPSHVVQVRCSSMMNQGNVYGQITVRMHTRQTLAIYDRFGRLMYGQEDVPKDVLEYVVFEKQLTNPYGSWRMHTKIVPPWAPPKQPILKTVMIPGPQLKPEEEYEEAQGEAQKPQLA

1735 NP_057953.1 Q96HA7

>NP_057953.1

MDLEKNYPTPRTSRTGHGGVNQLGGVFVNGRPLPDVVRQRIVELAHQGVRPCDISRQLRVSHGCVSKILGRYYETGSIKPGVIGGSKPKVATPKVVEKIAEYKRQNPTMFAWEIRDRLLAERVCDNDTVPSVSSINRIIRTKVQQPPNQPVPASSHSIVSTGSVTQVSSVSTDSAGSSYSISGILGITSPSADTNKRKRDEGIQESPVPNGHSLPGRDFLRKQMRGDLFTQQQLEVLDRVFERQHYSDIFTTTEPIKPEQTTEYSAMASLAGGLDDMKANLASPTPADIGSSVPGPQSYPIVTGRDLASTTLPGYPPHVPPAGQGSYSAPTLTGMVPGSEFSGSPYSHPQYSSYNDSWRFPNPGLLGSPYYYSAAARGAAPPAAATAYDRH

>Q96HA7

MSLERELRQLSKAKAKAQRAGQRREEAALCHQLGELLAGHGRYAEALEQHWQELQLRERADDPLGCAVAHRKIGERLAEMEDYPAALQHQHQYLELAHSLRNHTELQRAWATIGRTHLDIYDHCQSRDALLQAQAAFEKSLAIVDEELEGTLAQGELNEMRTRLYLNLGLTFESLQQTALCNDYFRKSIFLAEQNHLYEDLFRARYNLGTIHWRAGQHSQAMRCLEGARECAHTMRKRFMESECCVVIAQVLQDLGDFLAAKRALKKAYRLGSQKPVQRAAICQNLQHVLAVVRLQQQLEEAEGRDPQGAMVICEQLGDLFSKAGDFPRAAEAYQKQLRFAELLDRPGAERAIIHVSLATTLGDMKDHHGAVRHYEEELRLRSGNVLEEAKTWLNIALSREEAGDAYELLAPCFQKALSCAQQAQRPQLQRQVLQHLHTVQLRLQPQEAPETETRLRELSVAEDEDEEEEAEEAAATAESEALEAGEVELSEGEDDTDGLTPQLEEDEELQGHLGRRKGSKWNRRNDMGETLLHRACIEGQLRRVQDLVRQGHPLNPRDYCGWTPLHEACNYGHLEIVRFLLDHGAAVDDPGGQGCEGITPLHDALNCGHFEVAELLLERGASVTLRTRKGLSPLETLQQWVKLYRRDLDLETRQKARAMEMLLQAAASGQDPHSSQAFHTPSSLLFDPETSPPLSPCPEPPSNSTRLPEASQAHVRVSPGQAAPAMARPRRSRHGPASSSSSSEGEDSAGPARPSQKRPRCSATAQRVAAWTPGPASNREAATASTSRAAYQAAIRGVGSAQSRLGPGPPRGHSKALAPQAALIPEEECLAGDWLELDMPLTRSRRPRPRGTGDNRRPSSTSGSDSEESRPRARAKQVRLTCMQSCSAPVNAGPSSLASEPPGSPSTPRVSEPSGDSSAAGQPLGPAPPPPIRVRVQVQDHLFLIPVPHSSDTHSVAWLAEQAAQRYYQTCGLLPRLTLRKEGALLAPQDLIPDVLQSNDEVLAEVTSWDLPPLTDRYRRACQSLGQGEHQQVLQAVELQGLGLSFSACSLALDQAQLTPLLRALKLHTALRELRLAGNRLGDKCVAELVAALGTMPSLALLDLSSNHLGPEGLRQLAMGLPGQATLQSLEELDLSMNPLGDGCGQSLASLLHACPLLSTLRLQACGFGPSFFLSHQTALGSAFQDAEHLKTLSLSYNALGAPALARTLQSLPAGTLLHLELSSVAAGKGDSDLMEPVFRYLAKEGCALAHLTLSANHLGDKAVRDLCRCLSLCPSLISLDLSANPEISCASLEELLSTLQKRPQGLSFLGLSGCAVQGPLGLGLWDKIAAQLRELQLCSRRLCAEDRDALRQLQPSRPGPGECTLDHGSKLFFRRL

1736 NP_000779.1 NP_006535.1

>NP_000779.1

MATPPKRSCPSFSASSEGTRIKKISIEGNIAAGKSTFVNILKQLCEDWEVVPEPVARWCNVQSTQDEFEELTMSQKNGGNVLQMMYEKPERWSFTFQTYACLSRIRAQLASLNGKLKDAEKPVLFFERSVYSDRYIFASNLYESECMNETEWTIYQDWHDWMNNQFGQSLELDGIIYLQATPETCLHRIYLRGRNEEQGIPLEYLEKLHYKHESWLLHRTLKTNFDYLQEVPILTLDVNEDFKDKYESLVEKVKEFLSTL

>NP_006535.1

MATTAELFEEPFVADEYIERLVWRTPGGGSRGGPEAFDPKRLLEEFVNHIQELQIMDERIQRKVEKLEQQCQKEAKEFAKKVQELQKSNQVAFQHFQELDEHISYVATKVCHLGDQLEGVNTPRQRAVEAQKLMKYFNEFLDGELKSDVFTNSEKIKEAADIIQKLHLIAQELPFDRFSEVKSKIASKYHDLECQLIQEFTSAQRRGEISRMREVAAVLLHFKGYSHCVDVYIKQCQEGAYLRNDIFEDAGILCQRVNKQVGDIFSNPETVLAKLIQNVFEIKLQSFVKEQLEECRKSDAEQYLKNLYDLYTRTTNLSSKLMEFNLGTDKQTFLSKLIKSIFISYLENYIEVETGYLKSRSAMILQRYYDSKNHQKRSIGTGGIQDLKERIRQRTNLPLGPSIDTHGETFLSQEVVVNLLQETKQAFERCHRLSDPSDLPRNAFRIFTILVEFLCIEHIDYALETGLAGIPSSDSRNANLYFLDVVQQANTIFHLFDKQFNDHLMPLISSSPKLSECLQKKKEIIEQMEMKLDTGIDRTLNCMIGQMKHILAAEQKKTDFKPEDENNVLIQYTNACVKVCAYVRKQVEKIKNSMDGKNVDTVLMELGVRFHRLIYEHLQQYSYSCMGGMLAICDVAEYRKCAKDFKIPMVLHLFDTLHALCNLLVVAPDNLKQVCSGEQLANLDKNILHSFVQLRADYRSARLARHFS

1737 NP_003160.2 Q9BU02

>NP_003160.2

MSDSEDSNFSEEEDSERSSDGEEAEVDEERRSAAGSEKEEEPEDEEEEEEEEEYDEEEEEEDDDRPPKKPRHGGFILDEADVDDEYEDEDQWEDGAEDILEKEEIEASNIDNVVLDEDRSGARRLQNLWRDQREEELGEYYMKKYAKSSVGETVYGGSDELSDDITQQQLLPGVKDPNLWTVKCKIGEERATAISLMRKFIAYQFTDTPLQIKSVVAPEHVKGYIYVEAYKQTHVKQAIEGVGNLRLGYWNQQMVPIKEMTDVLKVVKEVANLKPKSWVRLKRGIYKDDIAQVDYVEPSQNTISLKMIPRIDYDRIKARMSLKDWFAKRKKFKRPPQRLFDAEKIRSLGGDVASDGDFLIFEGNRYSRKGFLFKSFAMSAVITEGVKPTLSELEKFEDQPEGIDLEVVTESTGKEREHNFQPGDNVEVCEGELINLQGKILSVDGNKITIMPKHEDLKDMLEFPAQELRKYFKMGDHVKVIAGRFEGDTGLIVRVEENFVILFSDLTMHELKVLPRDLQLCSETASGVDVGGQHEWGELVQLDPQTVGVIVRLERETFQVLNMYGKVVTVRHQAVTRKKDNRFAVALDSEQNNIHVKDIVKVIDGPHSGREGEIRHLFRSFAFLHCKKLVENGGMFVCKTRHLVLAGGSKPRDVTNFTVGGFAPMSPRISSPMHPSAGGQRGGFGSPGGGSGGMSRGRGRRDNELIGQTVRISQGPYKGYIGVVKDATESTARVELHSTCQTISVDRQRLTTVGSRRPGGMTSTYGRTPMYGSQTPMYGSGSRTPMYGSQTPLQDGSRTPHYGSQTPLHDGSRTPAQSGAWDPNNPNTPSRAEEEYEYAFDDEPTPSPQAYGGTPNPQTPGYPDPSSPQVNPQYNPQTPGTPAMYNTDQFSPYAAPSPQGSYQPSPSPQSYHQVAPSPAGYQNTHSPASYHPTPSPMAYQASPSPSPVGYSPMTPGAPSPGGYNPHTPGSGIEQNSSDWVTTDIQVKVRDTYLDTQVVGQTGVIRSVTGGMCSVYLKDSEKVVSISSEHLEPITPTKNNKVKVILGEDREATGVLLSIDGEDGIVRMDLDEQLKILNLRFLGKLLEA

>Q9BU02

MAQGLIEVERKFLPGPGTEERLQELGGTLEYRVTFRDTYYDTPELSLMQADHWLRRREDSGWELKCPGAAGVLGPHTEYKELTAEPTIVAQLCKVLRADGLGAGDVAAVLGPLGLQEVASFVTKRSAWKLVLLGADEEEPQLRVDLDTADFGYAVGEVEALVHEEAEVPTALEKIHRLSSMLGVPAQETAPAKLIVYLQRFRPQDYQRLLEVNSSRERPQETEDPDHCLG

1738 Q9UBR2 Q9UPP2

>Q9UBR2

MARRGPGWRPLLLLVLLAGAAQGGLYFRRGQTCYRPLRGDGLAPLGRSTYPRPHEYLSPADLPKSWDWRNVDGVNYASITRNQHIPQYCGSCWAHASTSAMADRINIKRKGAWPSTLLSVQNVIDCGNAGSCEGGNDLSVWDYAHQHGIPDETCNNYQAKDQECDKFNQCGTCNEFKECHAIRNYTLWRVGDYGSLSGREKMMAEIYANGPISCGIMATERLANYTGGIYAEYQDTTYINHVVSVAGWGISDGTEYWIVRNSWGEPWGERGWLRIVTSTYKDGKGARYNLAIEEHCTFGDPIV

>Q9UPP2

MESLLENPVRAVLYLKELTAIVQNQQSLIHTQRERIDELERRLDELSAENRSLWEHQQLLQAQPPPGLVPPSSAPLPAAPATAPAAAARAQEPLQDQGQRSAAAPHPAPDRPPRQHHGQLLEQPQRGPGSRAHTPQSPHKHLGTQGAVTDKEKERPPSCCAAAGALLQHKSPSALGKGVLSRRPENETVLHQFCCPAADACSDLASQSDGSCTQAGGGMEDSVVAAAAVAAGRPSAHAPKAQAQELQEEEERPGAGAASPRAGPQHKASPGRQQPALATALCPHAPAASDYELSLDLKNKQIEMLEHKYGGHLVSRRAACTIQTAFRQYQLSKNFEKIRNSLLESRLPRRISLRKVRSPTAESLAAEKALMEGYGLVGLPLVRSPSLPPTFAGTLTELEDSFTEQVQSLAKSIDDALSTWSLKTMCSLRESGAYQLHQALQAAAGPPGLEAEGRAPESAGPGPGDDAAETPGLPPAHSGTLMMAFRDVTVQIANQNISVSSSTALSVANCLGAQTVQAPAEPAAGKAEQGETSGREAPEAPAVGREDASAEDSCAEAAASGAADGATAPKTEEEEEEEETAEVGRGAEAEAGDLEQLSSSSTSTKSAKSGSEASASASKDALQAMILSLPRYHCENPASCKSPTLSTDTLRKRLYRIGLNLFNINPDKGIQFLISRGFIPDTPIGVAHFLLQRKGLSRQMIGEFLGNSKKQFNRDVLDCVVDEMDFSSMELDEALRKFQAHIRVQGEAQKVERLIEAFSQRYCMCNPEVVQQFHNPDTIFILAFAIILLNTDMYSPNIKPDRKMMLEDFIRNLRGVDDGADIPRELVVGIYERIQQKELKSNEDHVTYVTKVEKSIVGMKTVLSVPHRRLVCCSRLFEVTDVNKLQKQAAHQREVFLFNDLLVILKLCPKKKSSSTYTFCKSVGLLGMQFQLFENEYYSHGITLVTPLSGSEKKQVLHFCALGSDEMQKFVEDLKESIAEVTELEQIRIEWELEKQQGTKTLSFKPCGAQGDPQSKQGSPTAKREAALRERPAESTVEVSIHNRLQTSQHNSGLGAERGAPVPPPDLQPSPPRQQTPPLPPPPPTPPGTLVQCQQIVKVIVLDKPCLARMEPLLSQALSCYTSSSSDSCGSTPLGGPGSPVKVTHQPPLPPPPPPYNHPHQFCPPGSLLHGHRYSSGSRSLV

1739 NP_002136.1 Q6XE24

>NP_002136.1

MNFEFEREIGFINSQPSLAECLTSFPAVLETFQTSSIKESTLIPPPPPFEQTFPSLQPGASTLQRPRSQKRAEDGPALPPPPPPPLPAAPPAPEFPWMKEKKSAKKPSQSATSPSPAASAVPASGVGSPADGLGLPEAGGGGARRLRTAYTNTQLLELEKEFHFNKYLCRPRRVEIAALLDLTERQVKVWFQNRRMKHKRQTQHREPPDGEPACPGALEDICDPAEEPAASPGGPSASRAAWEACCHPPEVVPGALSADPRPLAVRLEGAGASSPGCALRGAGGLEPGPLPEDVFSGRQDSPFLPDLNFFAADSCLQLSGGLSPSLQGSLDSPVPFSEEELDFFTSTLCAIDLQFP

>Q6XE24

MGKRLDQPQMYPQYTYYYPHYLQTKQSYAPAPHPMAPPSPSTNSSSNNSSNNSSGEQLSKTNLYIRGLPPGTTDQDLIKLCQPYGKIVSTKAILDKNTNQCKGYGFVDFDSPAAAQKAVASLKANGVQAQMAKQQEQDPTNLYISNLPISMDEQELENMLKPFGHVISTRILRDANGVSRGVGFARMESTEKCEVVIQHFNGKYLKTPPGIPAPSEPLLCKFADGGQKKRQNQSKYTQNGRPWPREGEAGMALTYDPTAAIQNGFYSSPYSIATNRMIPQTSITPFIAASPVSTYQVQSTSWMPHPPYVMQPTGAVITPTMDHPMSMQPANMMGPLTQQMNHLSLGTTGTIQSQDRIMILHQLLCQYMTAAAPMQGTYIPQYTPVPPTAVSIEGVVADTSPQTVAPSSQDTSGQQQQIAVDTSNEHAPAYSYQQSKP

1740 NP_000312.2 NP_000605.1

>NP_000312.2

MPPKTPRKTAATAAAAAAEPPAPPPPPPPEEDPEQDSGPEDLPLVRLEFEETEEPDFTALCQKLKIPDHVRERAWLTWEKVSSVDGVLGGYIQKKKELWGICIFIAAVDLDEMSFTFTELQKNIEISVHKFFNLLKEIDTSTKVDNAMSRLLKKYDVLFALFSKLERTCELIYLTQPSSSISTEINSALVLKVSWITFLLAKGEVLQMEDDLVISFQLMLCVLDYFIKLSPPMLLKEPYKTAVIPINGSPRTPRRGQNRSARIAKQLENDTRIIEVLCKEHECNIDEVKNVYFKNFIPFMNSLGLVTSNGLPEVENLSKRYEEIYLKNKDLDARLFLDHDKTLQTDSIDSFETQRTPRKSNLDEEVNVIPPHTPVRTVMNTIQQLMMILNSASDQPSENLISYFNNCTVNPKESILKRVKDIGYIFKEKFAKAVGQGCVEIGSQRYKLGVRLYYRVMESMLKSEEERLSIQNFSKLLNDNIFHMSLLACALEVVMATYSRSTSQNLDSGTDLSFPWILNVLNLKAFDFYKVIESFIKAEGNLTREMIKHLERCEHRIMESLAWLSDSPLFDLIKQSKDREGPTDHLESACPLNLPLQNNHTAADMYLSPVRSPKKKGSTTRVNSTANAETQATSAFQTQKPLKSTSLSLFYKKVYRLAYLRLNTLCERLLSEHPELEHIIWTLFQHTLQNEYELMRDRHLDQIMMCSMYGICKVKNIDLKFKIIVTAYKDLPHAVQETFKRVLIKEEEYDSIIVFYNSVFMQRLKTNILQYASTRPPTLSPIPHIPRSPYKFPSSPLRIPGGNIYISPLKSPYKISEGLPTPTKMTPRSRILVSIGESFGTSEKFQKINQMVCNSDRVLKRSAEGSNPPKPLKKLRFDIEGSDEADGSKHLPGESKFQQKLAEMTSTRTRMQKQKMNDSMDTSNKEEK

>NP_000605.1

MAFTEHSPLTPHRRDLCSRSIWLARKIRSDLTALTESYVKHQGLNKNINLDSADGMPVASTDQWSELTEAERLQENLQAYRTFHVLLARLLEDQQVHFTPTEGDFHQAIHTLLLQVAAFAYQIEELMILLEYKIPRNEADGMPINVGDGGLFEKKLWGLKVLQELSQWTVRSIHDLRFISSHQTGIPARGSHYIANNKKM

1741 Q8IUM7 NP_071390.1

>Q8IUM7

MYRSTKGASKARRDQINAEIRNLKELLPLAEADKVRLSYLHIMSLACIYTRKGVFFAGGTPLAGPTGLLSAQELEDIVAALPGFLLVFTAEGKLLYLSESVSEHLGHSMVDLVAQGDSIYDIIDPADHLTVRQQLTLPSALDTDRLFRCRFNTSKSLRRQSAGNKLVLIRGRFHAHPPGAYWAGNPVFTAFCAPLEPRPRPGPGPGPGPASLFLAMFQSRHAKDLALLDISESVLIYLGFERSELLCKSWYGLLHPEDLAHASAQHYRLLAESGDIQAEMVVRLQAKTGGWAWIYCLLYSEGPEGPITANNYPISDMEAWSLRQQLNSEDTQAAYVLGTPTMLPSFPENILSQEECSSTNPLFTAALGAPRSTSFPSAPELSVVSASEELPRPSKELDFSYLTFPSGPEPSLQAELSKDLVCTPPYTPHQPGGCAFLFSLHEPFQTHLPTPSSTLQEQLTPSTATFSDQLTPSSATFPDPLTSPLQGQLTETSVRSYEDQLTPCTSTFPDQLLPSTATFPEPLGSPAHEQLTPPSTAFQAHLDSPSQTFPEQLSPNPTKTYFAQEGCSFLYEKLPPSPSSPGNGDCTLLALAQLRGPLSVDVPLVPEGLLTPEASPVKQSFFHYSEKEQNEIDRLIQQISQLAQGMDRPFSAEAGTGGLEPLGGLEPLDSNLSLSGAGPPVLSLDLKPWKCQELDFLADPDNMFLEETPVEDIFMDLSTPDPSEEWGSGDPEAEGPGGAPSPCNNLSPEDHSFLEDLATYETAFETGVSAFPYDGFTDELHQLQSQVQDSFHEDGSGGEPTF

>NP_071390.1

MEAERPQEEEDGEQGPPQDEEGWPPPNSTTRPWRSAPPSPPPPGTRHTALGPRSASLLSLQTELLLDLVAEAQSRRLEEQRATFYTPQNPSSLAPAPLRPLEDREQLYSTILSHQCQRMEAQRSEPPLPPGGQELLELLLRVQGGGRMEEQRSRPPTHTC

1742 Q9BPZ2 NP_008996.1

>Q9BPZ2

MKTPNAQEAEGQQTRAAAGRATGSANMTKKKVSQKKQRGRPSSQPRRNIVGCRISHGWKEGDEPITQWKGTVLDQVPINPSLYLVKYDGIDCVYGLELHRDERVLSLKILSDRVASSHISDANLANTIIGKAVEHMFEGEHGSKDEWRGMVLAQAPIMKAWFYITYEKDPVLYMYQLLDDYKEGDLRIMPESSESPPTEREPGGVVDGLIGKHVEYTKEDGSKRIGMVIHQVEAKPSVYFIKFDDDFHIYVYDLVKKS

>NP_008996.1

MVDYSVWDHIEVSDDEDETHPNIDTASLFRWRHQARVERMEQFQKEKEELDRGCRECKRKVAECQRKLKELEVAEGGKAELERLQAEAQQLRKEERSWEQKLEEMRKKEKSMPWNVDTLSKDGFSKSMVNTKPEKTEEDSEEVREQKHKTFVEKYEKQIKHFGMLRRWDDSQKYLSDNVHLVCEETANYLVIWCIDLEVEEKCALMEQVAHQTIVMQFILELAKSLKVDPRACFRQFFTKIKTADRQYMEGFNDELEAFKERVRGRAKLRIEKAMKEYEEEERKKRLGPGGLDPVEVYESLPEELQKCFDVKDVQMLQDAISKMDPTDAKYHMQRCIDSGLWVPNSKASEAKEGEEAGPGDPLLEAVPKTGDEKDVSV

1743 Q9Y675 NP_001631.3

>Q9Y675

MERARDRLHLRRTTEQHVPEVEVQVKRRRTASLSNQECQLYPRRSQQQQVPVVDFQAELRQAFLAETPRGG

>NP_001631.3

MERQVLLSEPEEAAALYRGLSRQPALSAACLGPEVTTQYGGQYRTVHTEWTQRDLERMENIRFCRQYLVFHDGDSVVFAGPAGNSVETRGELLSRESPSGTMKAVLRKAGGTGPGEEKQFLEVWEKNRKLKSFNLSALEKHGPVYEDDCFGCLSWSHSETHLLYVAEKKRPKAESFFQTKALDVSASDDEIARLKKPDQAIKGDQFVFYEDWGENMVSKSIPVLCVLDVESGNISVLEGVPENVSPGQAFWAPGDAGVVFVGWWHEPFRLGIRFCTNRRSALYYVDLIGGKCELLSDDSLAVSSPRLSPDQCRIVYLQYPSLIPHHQCSQLCLYDWYTKVTSVVVDVVPRQLGENFSGIYCSLLPLGCWSADSQRVVFDSAQRSRQDLFAVDTQVGTVTSLTAGGSGGSWKLLTIDQDLMVAQFSTPSLPPTLKVGFLPSAGKEQSVLWVSLEEAEPIPDIHWGIRVLQPPPEQENVQYAGLDFEAILLQPGSPPDKTQVPMVVMPHGGPHSSFVTAWMLFPAMLCKMGFAVLLVNYRGSTGFGQDSILSLPGNVGHQDVKDVQFAVEQVLQEEHFDASHVALMGGSHGGFISCHLIGQYPETYRACVARNPVINIASMLGSTDIPDWCVVEAGFPFSSDCLPDLSVWAEMLDKSPIRYIPQVKTPLLLMLGQEDRRVPFKQGMEYYRALKTRNVPVRLLLYPKSTHALSEVEVESDSFMNAVLWLRTHLGS

1744 NP_002348.1 P49589

>NP_002348.1

MAAAVRMNIQMLLEAADYLERREREAEHGYASMLPYNNKDRDALKRRNKSKKNNSSSRSTHNEMEKNRRAHLRLCLEKLKGLVPLGPESSRHTTLSLLTKAKLHIKKLEDCDRKAVHQIDQLQREQRHLKRQLEKLGIERIRMDSIGSTVSSERSDSDREEIDVDVESTDYLTGDLDWSSSSVSDSDERGSMQSLGSDEGYSSTSIKRIKLQDSHKACLGL

>P49589

MADSSGQQGKGRRVQPQWSPPAGTQPCRLHLYNSLTRNKEVFIPQDGKKVTWYCCGPTVYDASHMGHARSYISFDILRRVLKDYFKFDVFYCMNITDIDDKIIKRARQNHLFEQYREKRPEAAQLLEDVQAALKPFSVKLNETTDPDKKQMLERIQHAVQLATEPLEKAVQSRLTGEEVNSCVEVLLEEAKDLLSDWLDSTLGCDVTDNSIFSKLPKFWEGDFHRDMEALNVLPPDVLTRVSEYVPEIVNFVQKIVDNGYGYVSNGSVYFDTAKFASSEKHSYGKLVPEAVGDQKALQEGEGDLSISADRLSEKRSPNDFALWKASKPGEPSWPCPWGKGRPGWHIECSAMAGTLLGASMDIHGGGFDLRFPHHDNELAQSEAYFENDCWVRYFLHTGHLTIAGCKMSKSLKNFITIKDALKKHSARQLRLAFLMHSWKDTLDYSSNTMESALQYEKFLNEFFLNVKDILRAPVDITGQFEKWGEEEAELNKNFYDKKTAIHKALCDNVDTRTVMEEMRALVSQCNLYMAARKAVRKRPNQALLENIALYLTHMLKIFGAVEEDSSLGFPVGGPGTSLSLEATVMPYLQVLSEFREGVRKIAREQKVPEILQLSDALRDNILPELGVRFEDHEGLPTVVKLVDRNTLLKEREEKRRVEEEKRKKKEEAARRKQEQEAAKLAKMKIPPSEMFLSETDKYSKFDENGLPTHDMEGKELSKGQAKKLKKLFEAQEKLYKEYLQMAQNGSFQ

1745 NP_060624.1 NP_056238.2

>NP_060624.1

MAKVSVLNVAVLENPSPFHSPFRFEISFECSEALADDLEWKIIYVGSAESEEFDQILDSVLVGPVPAGRHMFVFQADAPNPSLIPETDAVGVTVVLITCTYHGQEFIRVGYYVNNEYLNPELRENPPMKPDFSQLQRNILASNPRVTRFHINWDNNMDRLEAIETQDPSLGCGLPLNCTPIKGLGLPGCIPGLLPENSMDCI

>NP_056238.2

MVFPAKRFCLVPSMEGVRWAFSCGTWLPSRAEWLLAVRSIQPEEKERIGQFVFARDAKAAMAGRLMIRKLVAEKLNIPWNHIRLQRTAKGKPVLAKDSSNPYPNFNFNISHQGDYAVLAAEPELQVGIDIMKTSFPGRGSIPEFFHIMKRKFTNKEWETIRSFKDEWTQLDMFYRNWALKESFIKAIGVGLGFELQRLEFDLSPLNLDIGQVYKETRLFLDGEEEKEWAFEESKIDEHHFVAVALRKPDGSRHQDVPSQDDSKPTQRQFTILNFNDLMSSAVPMTPEDPSFWDCFCFTEEIPIRNGTKS

1746 NP_001370.1 NP_004799.1

>NP_001370.1

MPARTAPARVPTLAVPAISLPDDVRRRLKDLERDSLTEKECVKEKLNLLHEFLQTEIKNQLCDLETKLRKEELSEEGYLAKVKSLLNKDLSLENGAHAYNREVNGRLENGNQARSEARRVGMADANSPPKPLSKPRTPRRSKSDGEAKPEPSPSPRITRKSTRQTTITSHFAKGPAKRKPQEESERAKSDESIKEEDKDQDEKRRRVTSRERVARPLPAEEPERAKSGTRTEKEEERDEKEEKRLRSQTKEPTPKQKLKEEPDREARAGVQADEDEDGDEKDEKKHRSQPKDLAAKRRPEEKEPEKVNPQISDEKDEDEKEEKRRKTTPKEPTEKKMARAKTVMNSKTHPPKCIQCGQYLDDPDLKYGQHPPDAVDEPQMLTNEKLSIFDANESGFESYEALPQHKLTCFSVYCKHGHLCPIDTGLIEKNIELFFSGSAKPIYDDDPSLEGGVNGKNLGPINEWWITGFDGGEKALIGFSTSFAEYILMDPSPEYAPIFGLMQEKIYISKIVVEFLQSNSDSTYEDLINKIETTVPPSGLNLNRFTEDSLLRHAQFVVEQVESYDEAGDSDEQPIFLTPCMRDLIKLAGVTLGQRRAQARRQTIRHSTREKDRGPTKATTTKLVYQIFDTFFAEQIEKDDREDKENAFKRRRCGVCEVCQQPECGKCKACKDMVKFGGSGRSKQACQERRCPNMAMKEADDDEEVDDNIPEMPSPKKMHQGKKKKQNKNRISWVGEAVKTDGKKSYYKKVCIDAETLEVGDCVSVIPDDSSKPLYLARVTALWEDSSNGQMFHAHWFCAGTDTVLGATSDPLELFLVDECEDMQLSYIHSKVKVIYKAPSENWAMEGGMDPESLLEGDDGKTYFYQLWYDQDYARFESPPKTQPTEDNKFKFCVSCARLAEMRQKEIPRVLEQLEDLDSRVLYYSATKNGILYRVGDGVYLPPEAFTFNIKLSSPVKRPRKEPVDEDLYPEHYRKYSDYIKGSNLDAPEPYRIGRIKEIFCPKKSNGRPNETDIKIRVNKFYRPENTHKSTPASYHADINLLYWSDEEAVVDFKAVQGRCTVEYGEDLPECVQVYSMGGPNRFYFLEAYNAKSKSFEDPPNHARSPGNKGKGKGKGKGKPKSQACEPSEPEIEIKLPKLRTLDVFSGCGGLSEGFHQAGISDTLWAIEMWDPAAQAFRLNNPGSTVFTEDCNILLKLVMAGETTNSRGQRLPQKGDVEMLCGGPPCQGFSGMNRFNSRTYSKFKNSLVVSFLSYCDYYRPRFFLLENVRNFVSFKRSMVLKLTLRCLVRMGYQCTFGVLQAGQYGVAQTRRRAIILAAAPGEKLPLFPEPLHVFAPRACQLSVVVDDKKFVSNITRLSSGPFRTITVRDTMSDLPEVRNGASALEISYNGEPQSWFQRQLRGAQYQPILRDHICKDMSALVAARMRHIPLAPGSDWRDLPNIEVRLSDGTMARKLRYTHHDRKNGRSSSGALRGVCSCVEAGKACDPAARQFNTLIPWCLPHTGNRHNHWAGLYGRLEWDGFFSTTVTNPEPMGKQGRVLHPEQHRVVSVRECARSQGFPDTYRLFGNILDKHRQVGNAVPPPLAKAIGLEIKLCMLAKARESASAKIKEEEAAKD

>NP_004799.1

MAEDSESAASQQSLELDDQDTCGIDGDNEEETEHAKGSPGGYLGAKKKKKKQKRKKEKPNSGGTKSDSASDSQEIKIQQPSKNPSVPMQKLQDIQRAMELLSACQGPARNIDEAAKHRYQFWDTQPVPKLDEVITSHGAIEPDKDNVRQEPYSLPQGFMWDTLDLSDAEVLKELYTLLNENYVEDDDNMFRFDYSPEFLLWALRPPGWLLQWHCGVRVSSNKKLVGFISAIPANIRIYDSVKKMVEINFLCVHKKLRSKRVAPVLIREITRRVNLEGIFQAVYTAGVVLPKPIATCRYWHRSLNPRKLVEVKFSHLSRNMTLQRTMKLYRLPDVTKTSGLRPMEPKDIKSVRELINTYLKQFHLAPVMDEEEVAHWFLPREHIIDTFVVESPNGKLTDFLSFYTLPSTVMHHPAHKSLKAAYSFYNIHTETPLLDLMSDALILAKSKGFDVFNALDLMENKTFLEKLKFGIGDGNLQYYLYNWRCPGTDSEKVGLVLQ

1747 Q02962 Q8NFG4

>Q02962

MDMHCKADPFSAMHPGHGGVNQLGGVFVNGRPLPDVVRQRIVELAHQGVRPCDISRQLRVSHGCVSKILGRYYETGSIKPGVIGGSKPKVATPKVVDKIAEYKRQNPTMFAWEIRDRLLAEGICDNDTVPSVSSINRIIRTKVQQPFHPTPDGAGTGVTAPGHTIVPSTASPPVSSASNDPVGSYSINGILGIPRSNGEKRKRDEVEVYTDPAHIRGGGGLHLVWTLRDVSEGSVPNGDSQSGVDSLRKHLRADTFTQQQLEALDRVFERPSYPDVFQASEHIKSEQGNEYSLPALTPGLDEVKSSLSASTNPELGSNVSGTQTYPVVTGRDMASTTLPGYPPHVPPTGQGSYPTSTLAGMVPGSEFSGNPYSHPQYTAYNEAWRFSNPALLSSPYYYSAAPRGSAPAAAAAAYDRH

>Q8NFG4

MNAIVALCHFCELHGPRTLFCTEVLHAPLPQGDGNEDSPGQGEQAEEEEGGIQMNSRMRAHSPAEGASVESSSPGPKKSDMCEGCRSLAAGHPGYISHDKETSIKYVSHQHPSHPQLFSIVRQACVRSLSCEVCPGREGPIFFGDEQHGFVFSHTFFIKDSLARGFQRWYSIITIMMDRIYLINSWPFLLGKVRGIIDELQGKALKVFEAEQFGCPQRAQRMNTAFTPFLHQRNGNAARSLTSLTSDDNLWACLHTSFAWLLKACGSRLTEKLLEGAPTEDTLVQMEKLADLEEESESWDNSEAEEEEKAPVLPESTEGRELTQGPAESSSLSGCGSWQPRKLPVFKSLRHMRQVLGAPSFRMLAWHVLMGNQVIWKSRDVDLVQSAFEVLRTMLPVGCVRIIPYSSQYEEAYRCNFLGLSPHVQIPPHVLSSEFAVIVEVHAAARSTLHPVGCEDDQSLSKYEFVVTSGSPVAADRVGPTILNKIEAALTNQNLSVDVVDQCLVCLKEEWMNKVKVLFKFTKVDSRPKEDTQKLLSILGASEEDNVKLLKFWMTGLSKTYKSHLMSTVRSPTASESRN

1748 A6H8Y1 Q9H2J4

>A6H8Y1

MFRRARLSVKPNVRPGVGARGSTASNPQRGRESPRPPEPATDSASKPAEPTDVPTVDFGGAEPQEKAPRSSTEKTGGDNDVEESSRSSSTVSQRRKRISSTSSLVKSSVSVPSESHPLSTINQEAPQPTATSTKEKQPCSDRYRIYKAQKLREMLKEELRKEKKQWKNKYAINESQRPPDRSKMTMRDFIYYLPDNNPMTSSLEQEKKTEKPSTPVQTREQEGKSTPNAEDNEMEEETDDGPLLVPRVKVAEDGSIILDEESLTVEVLRTKGPCVVEENDPIFERGSTTTYSSFRKNYYSKPWSNKETDMFFLAISMVGTDFSMIGQLFPHRARIEIKNKFKREEKTNGWRIDKAFQEKRPFDFDFFAHLLQKVLAEEEKRKQKSVKNHSLKEKKSTKPRKNVKVKKVACEGVNNDPDESMSSRISDTERSQKDAQTVEEESLTLSREDAEQVALEVDLNQKKRRRKKQDGANELGVNNLLENATVQAGPSKGEKHKNKCQAIRPELKEGECSKEQMLSCTQNIDGIVGFASTEKVEKRTDPILSLSNQQDATSVATESSESSTSDLPSFEVGIRALCEVNNAEGSCIEERNVDLKNNSLEIDQTENVKPMLRGRFQRPKPNLSRAGKKSVLSQGKTESESKNSHSKTSVEKNHVEKDKMNTLDILRMETTERENPEAETVSVLGEKNCLQEGSQLKALRPVQVRGRLQKPKPNAGKAAERKEILISQEEIGANVEKNENESCADRDTPQHMEDQSCKDFEEEDVILQPEKNDSFQNVQPDEPKVLNECLSVQENNKANKLNQVPILRTRFQKPKPNIGRGTGRREISSKEEVLEKILVSGEMAAALRETVRLDTSPKEMVPAEINTKEMQSDLKETGRRAISPREKILDVIDDTIEMETGLKAMGREICLREKTPEVIDATEEIDKDLEEAGRREISPQKNGPEEVKPLGEVETDLKATGNESSPREKTPEVTDATEEIDKNLEETGRRKISPRENGPEEVKPVDEMETDLNATGRESSPREKTPEVIDATEEIDLEETEREVSPQENGLEEVKPLGEMETDLKATGRDSFPRGKTPEVIDAIEEIEIDLEETEREISPQENGLEEVKPLGEMQTDLKATGREISPREKTPEVIDATEEIDKDLEETGRREISPEENGPEEVKPVDEMETDLKTTGREGSSREKTREVIDAAEVIETDLEETEREISPQENGPEEVKPVGKMETDLKEIREEISQREKVLAEFSAIREKEIDLKETGKRDIPMMEKVSGKMAVVEEMEADLKETGKENFRERGSEEICVTEEKVAELKQTGKTDISPRENELEETSTSRQTDTHLMQSGSNDFSAMPSLDIQNISSEVLSMMHTPVEEKRNSEKEVSSHFSHFKISSQTHESDKTEVQGIQSPDVPEQFSDINLSKSLPQEQKPLEIKPAPFVRSRFKRPKPNLARAALKRETTESEKYIYEKKSETKKMETIVMQENNEQTDTLPSQHDEASLMISREKDTLGHRNEEAVILPCTQTERNLSPSNSCEPKEESQSAPVQKNDSVVSVGTNNVNTFQQEMKESVIQTARQVRGRLQRPRPNIRKTGQRQIVDKGEAKGIIKEGRTILPKDETEKKVLTVSNSQIETEIEVPSSAVPEHRMYENQSQVVLVENLHVNKTNETIRHENKPYVPSSAQMTRRKFQKAKPNLGRAHSKKEEPVLEKVTTDQSKEGKPEDHLLQKGASNTQLLLKEKAELLTSLEVSARKDCVGSKESALAKIDAELEEVGPSRRVGEETVGDNSPSSVVEEQYLNKLTSCPQPLNETSYSKIALDGKTTISSTSEYERNRGERRSHKKFKPNVTRGRGSKRVRGKTSKKEPRASKAMLVTLRASQEEDDDADDFESDYEEESYHLAPEEVNKAPVFVPVGLRSPEPVSAQIEETMEELEITVNVPDVGCIAVVEHELPNTDVTTEEMKQEENLSVPFEMTTSEHIQDEPGTNDGSTEAAITLLTMGDLVLQSEISSEQGDVGVCILPHVHSKDKSHIPSSLDNVNHKIVHECQELSSPVITTSPASFEENKIVLEEQSSREEISLMEKVKENATPTRNTISKVTSNLRIRSRLAKPKPNLEKTLGTNRLDDYQEVSSLCVTKGAEMETQRETEKNASKATELENKNLGPVTTAENKDQSKLACVHGIKGTSISSEVNLTERNENQEESSQEVHMLSVAPVASSETGPCTLGLDRGLGENSVEEPQIKDSKGDSVLTLPVPEYTPTSIPEVQQENIINPQDLTVNLVANVPQDGEDEQAFILTLVEIPANAVEEFTDATAQFMPNPLLPAPILVKSVNTEERGDMSICLPATSVGQDAMGLSISGRDNSKKPPDNLDLVSRKRFQCRLDKNDHIPPAKKRSLTLRDDCQEYTTEVHSKELTNVFEETGESHKGQDIFLTSGSTLTTPEPQRQQVEAAFQSRGSRSPDACMDKNVPQLPQDEMIVSDKEERTDAAPKSQQMDSRTSSSKASLSRPGRRPLGFLSLICSKNSLESDEPMQVHSKKRLKPLIPGLRKKLKRSNPFNESQEKNRESSDLLPSPSVITTQSENISSSATQVSCDQPLLKEGYKSAQKRAPQGEATTVSEYFFNDIFIEVDETE

>Q9H2J4

MQDPNADTEWNDILRKKGILPPKESLKELEEEAEEEQRILQQSVVKTYEDMTLEELEDHEDEFNEEDERAIEMYRRRRLAEWKATKLKNKFGEVLEISGKDYVQEVTKAGEGLWVILHLYKQGIPLCALINQHLSGLARKFPDVKFIKAISTTCIPNYPDRNLPTIFVYLEGDIKAQFIGPLVFGGMNLTRDELEWKLSESGAIMTDLEENPKKPIEDVLLSSVRRSVLMKRDSDSEGD

1749 P20823 Q9NWU1

>P20823

MVSKLSQLQTELLAALLESGLSKEALIQALGEPGPYLLAGEGPLDKGESCGGGRGELAELPNGLGETRGSEDETDDDGEDFTPPILKELENLSPEEAAHQKAVVETLLQEDPWRVAKMVKSYLQQHNIPQREVVDTTGLNQSHLSQHLNKGTPMKTQKRAALYTWYVRKQREVAQQFTHAGQGGLIEEPTGDELPTKKGRRNRFKWGPASQQILFQAYERQKNPSKEERETLVEECNRAECIQRGVSPSQAQGLGSNLVTEVRVYNWFANRRKEEAFRHKLAMDTYSGPPPGPGPGPALPAHSSPGLPPPALSPSKVHGVRYGQPATSETAEVPSSSGGPLVTVSTPLHQVSPTGLEPSHSLLSTEAKLVSAAGGPLPPVSTLTALHSLEQTSPGLNQQPQNLIMASLPGVMTIGPGEPASLGPTFTNTGASTLVIGLASTQAQSVPVINSMGSSLTTLQPVQFSQPLHPSYQQPLMPPVQSHVTQSPFMATMAQLQSPHALYSHKPEVAQYTHTGLLPQTMLITDTTNLSALASLTPTKQVFTSDTEASSESGLHTPASQATTLHVPSQDPAGIQHLQPAHRLSASPTVSSSSLVLYQSSDSSNGQSHLLPSNHSVIETFISTQMASSSQ

>Q9NWU1

MSNCLQNFLKITSTRLLCSRLCQQLRSKRKFFGTVPISRLHRRVVITGIGLVTPLGVGTHLVWDRLIGGESGIVSLVGEEYKSIPCSVAAYVPRGSDEGQFNEQNFVSKSDIKSMSSPTIMAIGAAELAMKDSGWHPQSEADQVATGVAIGMGMIPLEVVSETALNFQTKGYNKVSPFFVPKILVNMAAGQVSIRYKLKGPNHAVSTACTTGAHAVGDSFRFIAHGDADVMVAGGTDSCISPLSLAGFSRARALSTNSDPKLACRPFHPKRDGFVMGEGAAVLVLEEYEHAVQRRARIYAEVLGYGLSGDAGHITAPDPEGEGALRCMAAALKDAGVQPEEISYINAHATSTPLGDAAENKAIKHLFKDHAYALAVSSTKGATGHLLGAAGAVEAAFTTLACYYQKLPPTLNLDCSEPEFDLNYVPLKAQEWKTEKRFIGLTNSFGFGGTNATLCIAGL

1750 NP_003502.1 NP_001007554.1

>NP_003502.1

MSGRGKQGGKARAKAKTRSSRAGLQFPVGRVHRLLRKGNYAERVGAGAPVYLAAVLEYLTAEILELAGNAARDNKKTRIIPRHLQLAIRNDEELNKLLGKVTIAQGGVLPNIQAVLLPKKTESHHKAKGK

>NP_001007554.1

MSFDPNLLHNNGHNGYPNGTSAALRETGVIEKLLTSYGFIQCSERQARLFFHCSQYNGNLQDLKVGDDVEFEVSSDRRTGKPIAVKLVKIKQEILPEERMNGQVVCAVPHNLESKSPAAPGQSPTGSVCYERNGEVFYLTYTPEDVEGNVQLETGDKINFVIDNNKHTGAVSARNIMLLKKKQARCQGVVCAMKEAFGFIERGDVVKEIFFHYSEFKGDLETLQPGDDVEFTIKDRNGKEVATDVRLLPQGTVIFEDISIEHFEGTVTKVIPKVPSKNQNDPLPGRIKVDFVIPKELPFGDKDTKSKVTLLEGDHVRFNISTDRRDKLERATNIEVLSNTFQFTNEAREMGVIAAMRDGFGFIKCVDRDVRMFFHFSEILDGNQLHIADEVEFTVVPDMLSAQRNHAIRIKKLPKGTVSFHSHSDHRFLGTVEKEATFSNPKTTSPNKGKEKEAEDGIIAYDDCGVKLTIAFQAKDVEGSTSPQIGDKVEFSISDKQRPGQQVATCVRLLGRNSNSKRLLGYVATLKDNFGFIETANHDKEIFFHYSEFSGDVDSLELGDMVEYSLSKGKGNKVSAEKVNKTHSVNGITEEADPTIYSGKVIRPLRSVDPTQTEYQGMIEIVEEGDMKGEVYPFGIVGMANKGDCLQKGESVKFQLCVLGQNAQTMAYNITPLRRATVECVKDQFGFINYEVGDSKKLFFHVKEVQDGIELQAGDEVEFSVILNQRTGKCSACNVWRVCEGPKAVAAPRPDRLVNRLKNITLDDASAPRLMVLRQPRGPDNSMGFGAERKIRQAGVID

1751 NP_002948.1 Q9GZZ1

>NP_002948.1

MDTKHFLPLDFSTQVNSSLTSPTGRGSMAAPSLHPSLGPGIGSPGQLHSPISTLSSPINGMGPPFSVISSPMGPHSMSVPTTPTLGFSTGSPQLSSPMNPVSSSEDIKPPLGLNGVLKVPAHPSGNMASFTKHICAICGDRSSGKHYGVYSCEGCKGFFKRTVRKDLTYTCRDNKDCLIDKRQRNRCQYCRYQKCLAMGMKREAVQEERQRGKDRNENEVESTSSANEDMPVERILEAELAVEPKTETYVEANMGLNPSSPNDPVTNICQAADKQLFTLVEWAKRIPHFSELPLDDQVILLRAGWNELLIASFSHRSIAVKDGILLATGLHVHRNSAHSAGVGAIFDRVLTELVSKMRDMQMDKTELGCLRAIVLFNPDSKGLSNPAEVEALREKVYASLEAYCKHKYPEQPGRFAKLLLRLPALRSIGLKCLEHLFFFKLIGDTPIDTFLMEMLEAPHQMT

>Q9GZZ1

MKGSRIELGDVTPHNIKQLKRLNQVIFPVSYNDKFYKDVLEVGELAKLAYFNDIAVGAVCCRVDHSQNQKRLYIMTLGCLAPYRRLGIGTKMLNHVLNICEKDGTFDNIYLHVQISNESAIDFYRKFGFEIIETKKNYYKRIEPADAHVLQKNLKVPSGQNADVQKTDN

1752 NP_075555.1 O75648

>NP_075555.1

MMASYPEPEDAAGALLAPETGRTVKEPEGPPPSPGKGGGGGGGTAPEKPDPAQKPPYSYVALIAMAIRESAEKRLTLSGIYQYIIAKFPFYEKNKKGWQNSIRHNLSLNECFIKVPREGGGERKGNYWTLDPACEDMFEKGNYRRRRRMKRPFRPPPAHFQPGKGLFGAGGAAGGCGVAGAGADGYGYLAPPKYLQSGFLNNSWPLPQPPSPMPYASCQMAAAAAAAAAAAAAAGPGSPGAAAVVKGLAGPAASYGPYTRVQSMALPPGVVNSYNGLGGPPAAPPPPPHPHPHPHAHHLHAAAAPPPAPPHHGAAAPPPGQLSPASPATAAPPAPAPTSAPGLQFACARQPELAMMHCSYWDHDSKTGALHSRLDL

>O75648

MQALRHVVCALSGGVDSAVAALLLRRRGYQVTGVFMKNWDSLDEHGVCTADKDCEDAYRVCQILDIPFHQVSYVKEYWNDVFSDFLNEYEKGRTPNPDIVCNKHIKFSCFFHYAVDNLGADAIATGHYARTSLEDEEVFEQKHVKKPEGLFRNRFEVRNAVKLLQAADSFKDQTFFLSQVSQDALRRTIFPLGGLTKEFVKKIAAENRLHHVLQKKESMGMCFIGKRNFEHFLLQYLQPRPGHFISIEDNKVLGTHKGWFLYTLGQRANIGGLREPWYVVEKDSVKGDVFVAPRTDHPALYRDLLRTSRVHWIAEEPPAALVRDKMMECHFRFRHQMALVPCVLTLNQDGTVWVTAVQAVRALATGQFAVFYKGDECLGSGKILRLGPSAYTLQKGQRRAGMATESPSDSPEDGPGLSPLL

1753 Q9UDW3 NP_071445.1

>Q9UDW3

MGKRYFCDYCDRSFQDNLHNRKKHLNGLQHLKAKKVWYDMFRDAAAILLDEQNKRPCRKFLLTGQCDFGSNCRFSHMSERDLQELSIQVEEERRAREWLLDAPELPEGHLEDWLEKRAKRLSSAPSSRAEPIRTTVFQYPVGWPPVQELPPSLRAPPPGGWPLQPRVQWG

>NP_071445.1

MGEEGGSASHDEEERASVLLGHSPGCEMCSQEAFQAQRSQLVELLVSGSLEGFESVLDWLLSWEVLSWEDYEGFHLLGQPLSHLARRLLDTVWNKGTWACQKLIAAAQEAQADSQSPKLHGCWDPHSLHPARDLQSHRPAIVRRLHSHVENMLDLAWERGFVSQYECDEIRLPIFTPSQRARRLLDLATVKANGLAAFLLQHVQELPVPLALPLEAATCKKYMAKLRTTVSAQSRFLSTYDGAETLCLEDIYTENVLEVWADVGMAGPPQKSPATLGLEELFSTPGHLNDDADTVLVVGEAGSGKSTLLQRLHLLWAAGQDFQEFLFVFPFSCRQLQCMAKPLSVRTLLFEHCCWPDVGQEDIFQLLLDHPDRVLLTFDGFDEFKFRFTDRERHCSPTDPTSVQTLLFNLLQGNLLKNARKVVTSRPAAVSAFLRKYIRTEFNLKGFSEQGIELYLRKRHHEPGVADRLIRLLQETSALHGLCHLPVFSWMVSKCHQELLLQEGGSPKTTTDMYLLILQHFLLHATPPDSASQGLGPSLLRGRLPTLLHLGRLALWGLGMCCYVFSAQQLQAAQVSPDDISLGFLVRAKGVVPGSTAPLEFLHITFQCFFAAFYLALSADVPPALLRHLFNCGRPGNSPMARLLPTMCIQASEGKDSSVAALLQKAEPHNLQITAAFLAGLLSREHWGLLAECQTSEKALLRRQACARWCLARSLRKHFHSIPPAAPGEAKSVHAMPGFIWLIRSLYEMQEERLARKAARGLNVGHLKLTFCSVGPTECAALAFVLQHLRRPVALQLDYNSVGDIGVEQLLPCLGVCKALYLRDNNISDRGICKLIECALHCEQLQKLALFNNKLTDGCAHSMAKLLACRQNFLALRLGNNYITAAGAQVLAEGLRGNTSLQFLGFWGNRVGDEGAQALAEALGDHQSLRWLSLVGNNIGSVGAQALALMLAKNVMLEELCLEENHLQDEGVCSLAEGLKKNSSLKILKLSNNCITYLGAEALLQALERNDTILEVWLRGNTFSLEEVDKLGCRDTRLLL

1754 Q8N9N5 NP_071347.2

>Q8N9N5

MMSEHDLADVVQIAVEDLSPDHPVVLENHVVTDEDEPALKRQRLEINCQDPSIKTICLRLDSIEAKLQALEATCKSLEEKLDLVTNKQHSPIQVPMVAGSPLGATQTCNKVRCVVPQTTVILNNDRQNAIVAKMEDPLSNRAPDSLENVISNAVPGRRQNTIVVKVPGQEDSHHEDGESGSEASDSVSSCGQAGSQSIGSNVTLITLNSEEDYPNGTWLGDENNPEMRVRCAIIPSDMLHISTNCRTAEKMALTLLDYLFHREVQAVSNLSGQGKHGKKQLDPLTIYGIRCHLFYKFGITESDWYRIKQSIDSKCRTAWRRKQRGQSLAVKSFSRRTPNSSSYCPSESMMSTPPPASELPQPQPQPQALHYALANAQQVQIHQIGEDGQVQVGHLHIAQVPQGEQVQITQDSEGNLQIHHVGQDGQLLEATRIPCLLAPSVFKASSGQVLQGAQLIAVASSDPAAAGVDGSPLQGSDIQVQYVQLAPVSDHTAGAQTAEALQPTLQPEMQLEHGAIQIQ

>NP_071347.2

MASKGAGMSFSRKSYRLTSDAEKSRVTGIVQEKLLNDYLNRIFSSSEHAPPAATSRKPLNFQNLPEHLDQLLQVDNEEEESQGQVEGRLGPSTVVLDHTGGFEGLLLVDDDLLGVIGHSNFGTIRSTTCVYKGKWLYEVLISSQGLMQIGWCTISCRFNQEEGVGDTHNSYAYDGNRVRKWNVTTTNYGKAWAAGDIVSCLIDLDDGTLSFCLNGVSLGTAFENLSRGLGMAYFPAISLSFKESVAFNFGSRPLRYPVAGYRPLQDPPSADLVRAQRLLGCFRAVLSVELDPVEGRLLDKESSKWRLRGQPTVLLTLAHIFHHFAPLLRKVYLVEAVLMSFLLGIVEKGTPTQAQSVVHQVLDLLWLFMEDYEVQDCLKQLMMSLLRLYRFSPIVPDLGLQIHYLRLTIAILRHEKSRKFLLSNVLFDVLRSVVFFYIKSPLRVEEAGLQELIPTTWWPHCSSREGKESTEMKEETAEERLRRRAYERGCQRLRKRIEVVEELQVQILKLLLDNKDDNGGEASRYIFLTKFRKFLQENASGRGNMPMLCPPEYMVCFLHRLISALRYYWDEYKASNPHASFSEEAYIPPQVFYNGKVDYFDLQRLGGLLSHLRKTLKDDLASKANIVIDPLELQSTAMDDLDEDEEPAPAMAQRPMQALAVGGPLPLPRPGWLSSPTLGRANRFLSTAAVSLMTPRRPLSTSEKVKVRTLSVEQRTREDIEGSHWNEGLLLGRPPEEPEQPLTENSLLEVLDGAVMMYNLSVHQQLGKMVGVSDDVNEYAMALRDTEDKLRRCPKRRKDILAELTKSQKVFSEKLDHLSRRLAWVHATVYSQEKMLDIYWLLRVCLRTIEHGDRTGSLFAFMPEFYLSVAINSYSALKNYFGPVHSMEELPGYEETLTRLAAILAKHFADARIVGTDIRDSLMQALASYVCYPHSLRAVERIPEEQRIAMVRNLLAPYEQRPWAQTNWILVRLWRGCGFGYRYTRLPHLLKTKLEDANLPSLQKPCPSTLLQQHMADLLQQGPDVAPSFLNSVLNQLNWAFSEFIGMIQEIQQAAERLERNFVDSRQLKVCATCFDLSVSLLRVLEMTITLVPEIFLDWTRPTSEMLLRRLAQLLNQVLNRVTAERNLFDRVVTLRLPGLESVDHYPILVAVTGILVQLLVRGPASEREQATSVLLADPCFQLRSICYLLGQPEPPAPGTALPAPDRKRFSLQSYADYISADELAQVEQMLAHLTSASAQAAAASLPTSEEDLCPICYAHPISAVFQPCGHKSCKACINQHLMNNKDCFFCKTTIVSVEDWEKGANTSTTSSAA

1755 NP_006615.1 Q9NVS2

>NP_006615.1

MSRVHGMHPKETTRQLSLAVKDGLIVETLTVGCKGSKAGIEQEGYWLPGDEIDWETENHDWYCFECHLPGEVLICDLCFRVYHSKCLSDEFRLRDSSSPWQCPVCRSIKKKNTNKQEMGTYLRFIVSRMKERAIDLNKKGKDNKHPMYRRLVHSAVDVPTIQEKVNEGKYRSYEEFKADAQLLLHNTVIFYGADSEQADIARMLYKDTCHELDELQLCKNCFYLSNARPDNWFCYPCIPNHELVWAKMKGFGFWPAKVMQKEDNQVDVRFFGHHHQRAWIPSENIQDITVNIHRLHVKRSMGWKKACDELELHQRFLREGRFWKSKNEDRGEEEAESSISSTSNEQLKVTQEPRAKKGRRNQSVEPKKEEPEPETEAVSSSQEIPTMPQPIEKVSVSTQTKKLSASSPRMLHRSTQTTNDGVCQSMCHDKYTKIFNDFKDRMKSDHKRETERVVREALEKLRSEMEEEKRQAVNKAVANMQGEMDRKCKQVKEKCKEEFVEEIKKLATQHKQLISQTKKKQWCYNCEEEAMYHCCWNTSYCSIKCQQEHWHAEHKRTCRRKR

>Q9NVS2

MAALKALVSGCGRLLRGLLAGPAATSWSRLPARGFREVVETQEGKTTIIEGRITATPKESPNPPNPSGQCPICRWNLKHKYNYDDVLLLSQFIRPHGGMLPRKITGLCQEEHRKIEECVKMAHRAGLLPNHRPRLPEGVVPKSKPQLNRYLTRWAPGSVKPIYKKGPRWNRVRMPVGSPLLRDNVCYSRTPWKLYH

1756 NP_002031.2 NP_060322.1

>NP_002031.2

MTKREAEELIEIEIDGTEKAECTEESIVEQTYAPAECVSQAIDINEPIGNLKKLLEPRLQCSLDAHEICLQDIQLDPERSLFDQGVKTDGTVQLSVQVISYQGIEPKLNILEIVKPADTVEVVIDPDAHHAESEAHLVEEAQVITLDGTKHITTISDETSEQVTRWAAALEGYRKEQERLGIPYDPIQWSTDQVLHWVVWVMKEFSMTDIDLTTLNISGRELCSLNQEDFFQRVPRGEILWSHLELLRKYVLASQEQQMNEIVTIDQPVQIIPASVQSATPTTIKVINSSAKAAKVQRAPRISGEDRSSPGNRTGNNGQIQLWQFLLELLTDKDARDCISWVGDEGEFKLNQPELVAQKWGQRKNKPTMNYEKLSRALRYYYDGDMICKVQGKRFVYKFVCDLKTLIGYSAAELNRLVTECEQKKLAKMQLHGIAQPVTAVALATASLQTEKDN

>NP_060322.1

MVSSAQMGFNLQALLEQLSQDELSKFKYLITTFSLAHELQKIPHKEVDKADGKQLVEILTTHCDSYWVEMASLQVFEKMHRMDLSERAKDEVREAALKSFNKRKPLSLGITRKERPPLDVDEMLERFKTEAQAFTETKGNVICLGKEVFKGKKPDKDNRCRYILKTKFREMWKSWPGDSKEVQVMAERYKMLIPFSNPRVLPGPFSYTVVLYGPAGLGKTTLAQKLMLDWAEDNLIHKFKYAFYLSCRELSRLGPCSFAELVFRDWPELQDDIPHILAQARKILFVIDGFDELGAAPGALIEDICGDWEKKKPVPVLLGSLLNRVMLPKAALLVTTRPRALRDLRILAEEPIYIRVEGFLEEDRRAYFLRHFGDEDQAMRAFELMRSNAALFQLGSAPAVCWIVCTTLKLQMEKGEDPVPTCLTRTGLFLRFLCSRFPQGAQLRGALRTLSLLAAQGLWAQTSVLHREDLERLGVQESDLRLFLDGDILRQDRVSKGCYSFIHLSFQQFLTALFYTLEKEEEEDRDGHTWDIGDVQKLLSGVERLRNPDLIQAGYYSFGLANEKRAKELEATFGCRMSPDIKQELLRCDISCKGGHSTVTDLQELLGCLYESQEEELVKEVMAQFKEISLHLNAVDVVPSSFCVKHCRNLQKMSLQVIKENLPENVTASESDAEVERSQDDQHMLPFWTDLCSIFGSNKDLMGLAINDSFLSASLVRILCEQIASDTCHLQRVVFKNISPADAHRNLCLALRGHKTVTYLTLQGNDQDDMFPALCEVLRHPECNLRYLGLVSCSATTQQWADLSLALEVNQSLTCVNLSDNELLDEGAKLLYTTLRHPKCFLQRLSLENCHLTEANCKDLAAVLVVSRELTHLCLAKNPIGNTGVKFLCEGLRYPECKLQTLVLWNCDITSDGCCDLTKLLQEKSSLLCLDLGLNHIGVKGMKFLCEALRKPLCNLRCLWLWGCSIPPFSCEDLCSALSCNQSLVTLDLGQNPLGSSGVKMLFETLTCSSGTLRTLRLKIDDFNDELNKLLEEIEEKNPQLIIDTEKHHPWAERPSSHDFMI

1757 NP_001508.1 NP_064632.2

>NP_001508.1

MESTPSRGLNRVHLQCRNLQEFLGGLSPGVLDRLYGHPATCLAVFRELPSLAKNWVMRMLFLEQPLPQAAVALWVKKEFSKAQEESTGLLSGLRIWHTQLLPGGLQGLILNPIFRQNLRIALLGGGKAWSDDTSQLGPDKHARDVPSLDKYAEERWEVVLHFMVGSPSAAVSQDLAQLLSQAGLMKSTEPGEPPCITSAGFQFLLLDTPAQLWYFMLQYLQTAQSRGMDLVEILSFLFQLSFSTLGKDYSVEGMSDSLLNFLQHLREFGLVFQRKRKSRRYYPTRLAINLSSGVSGAGGTVHQPGFIVVETNYRLYAYTESELQIALIALFSEMLYRFPNMVVAQVTRESVQQAIASGITAQQIIHFLRTRAHPVMLKQTPVLPPTITDQIRLWELERDRLRFTEGVLYNQFLSQVDFELLLAHARELGVLVFENSAKRLMVVTPAGHSDVKRFWKRQKHSS

>NP_064632.2

MAAILGDTIMVAKGLVKLTQAAVETHLQHLGIGGELIMAARALQSTAVEQIGMFLGKVQGQDKHEEYFAENFGGPEGEFHFSVPHAAGASTDFSSASAPDQSAPPSLGHAHSEGPAPAYVASGPFREAGFPGQASSPLGRANGRLFANPRDSFSAMGFQRRFFHQDQSPVGGLTAEDIEKARQAKARPENKQHKQTLSEHARERKVPVTRIGRLANFGGLAVGLGFGALAEVAKKSLRSEDPSGKKAVLGSSPFLSEANAERIVRTLCKVRGAALKLGQMLSIQDDAFINPHLAKIFERVRQSADFMPLKQMMKTLNNDLGPNWRDKLEYFEERPFAAASIGQVHLARMKGGREVAMKIQYPGVAQSINSDVNNLMAVLNMSNMLPEGLFPEHLIDVLRRELALECDYQREAACARKFRDLLKGHPFFYVPEIVDELCSPHVLTTELVSGFPLDQAEGLSQEIRNEICYNILVLCLRELFEFHFMQTDPNWSNFFYDPQQHKVALLDFGATREYDRSFTDLYIQIIRAAADRDRETVRAKSIEMKFLTGYEVKVMEDAHLDAILILGEAFASDEPFDFGTQSTTEKIHNLIPVMLRHRLVPPPEETYSLHRKMGGSFLICSKLKARFPCKAMFEEAYSNYCKRQAQQ

1758 O76039 Q49AM1

>O76039

MKIPNIGNVMNKFEILGVVGEGAYGVVLKCRHKETHEIVAIKKFKDSEENEEVKETTLRELKMLRTLKQENIVELKEAFRRRGKLYLVFEYVEKNMLELLEEMPNGVPPEKVKSYIYQLIKAIHWCHKNDIVHRDIKPENLLISHNDVLKLCDFGFARNLSEGNNANYTEYVATRWYRSPELLLGAPYGKSVDMWSVGCILGELSDGQPLFPGESEIDQLFTIQKVLGPLPSEQMKLFYSNPRFHGLRFPAVNHPQSLERRYLGILNSVLLDLMKNLLKLDPADRYLTEQCLNHPTFQTQRLLDRSPSRSAKRKPYHVESSTLSNRNQAGKSTALQSHHRSNSKDIQNLSVGLPRADEGLPANESFLNGNLAGASLSPLHTKTYQASSQPGSTSKDLTNNNIPHLLSPKEAKSKTEFDFNIDPKPSEGPGTKYLKSNSRSQQNRHSFMESSQSKAGTLQPNEKQSRHSYIDTIPQSSRSPSYRTKAKSHGALSDSKSVSNLSEARAQIAEPSTSRYFPSSCLDLNSPTSPTPTRHSDTRTLLSPSGRNNRNEGTLDSRRTTTRHSKTMEELKLPEHMDSSHSHSLSAPHESFSYGLGYTSPFSSQQRPHRHSMYVTRDKVRAKGLDGSLSIGQGMAARANSLQLLSPQPGEQLPPEMTVARSSVKETSREGTSSFHTRQKSEGGVYHDPHSDDGTAPKENRHLYNDPVPRRVGSFYRVPSPRPDNSFHENNVSTRVSSLPSESSSGTNHSKRQPAFDPWKSPENISHSEQLKEKEKQGFFRSMKKKKKKSQTVPNSDSPDLLTLQKSIHSASTPSSRPKEWRPEKISDLQTQSQPLKSLRKLLHLSSASNHPASSDPRFQPLTAQQTKNSFSEIRIHPLSQASGGSSNIRQEPAPKGRPALQLPDGGCDGRRQRHHSGPQDRRFMLRTTEQQGEYFCCGDPKKPHTPCVPNRALHRPISSPAPYPVLQVRGTSMCPTLQVRGTDAFSCPTQQSGFSFFVRHVMREALIHRAQVNQAALLTYHENAALTGK

>Q49AM1

MLWKLLLRSQSCRLCSFRKMRSPPKYRPFLACFTYTTDKQSSKENTRTVEKLYKCSVDIRKIRRLKGWVLLEDETYVEEIANILQELGADETAVASILERCPEAIVCSPTAVNTQRKLWQLVCKNEEELIKLIEQFPESFFTIKDQENQKLNVQFFQELGLKNVVISRLLTAAPNVFHNPVEKNKQMVRILQESYLDVGGSEANMKVWLLKLLSQNPFILLNSPTAIKETLEFLQEQGFTSFEILQLLSKLKGFLFQLCPRSIQNSISFSKNAFKCTDHDLKQLVLKCPALLYYSVPVLEERMQGLLREGISIAQIRETPMVLELTPQIVQYRIRKLNSSGYRIKDGHLANLNGSKKEFEANFGKIQAKKVRPLFNPVAPLNVEE

1759 Q8IXQ6 NP_005407.1

>Q8IXQ6

MDFSMVAGAAAYNEKSGRITSLSLLFQKVFAQIFPQWRKGNTEECLPYKCSETGALGENYSWQIPINHNDFKILKNNERQLCEVLQNKFGCISTLVSPVQEGNSKSLQVFRKMLTPRIELSVWKDDLTTHAVDAVVNAANEDLLHGGGLALALVKAGGFEIQEESKQFVARYGKVSAGEIAVTGAGRLPCKQIIHAVGPRWMEWDKQGCTGKLQRAIVSILNYVIYKNTHIKTVAIPALSSGIFQFPLNLCTKTIVETIRVSLQGKPMMSNLKEIHLVSNEDPTVAAFKAASEFILGKSELGQETTPSFNAMVVNNLTLQIVQGHIEWQTADVIVNSVNPHDITVGPVAKSILQQAGVEMKSEFLATKAKQFQRSQLVLVTKGFNLFCKYIYHVLWHSEFPKPQILKHAMKECLEKCIEQNITSISFPALGTGNMEIKKETAAEILFDEVLTFAKDHVKHQLTVKFVIFPTDLEIYKAFSSEMAKRSKMLSLNNYSVPQSTREEKRENGLEARSPAINLMGFNVEEMYEAHAWIQRILSLQNHHIIENNHILYLGRKEHDILSQLQKTSSVSITEIISPGRTELEIEGARADLIEVVMNIEDMLCKVQEEMARKKERGLWRSLGQWTIQQQKTQDEMKENIIFLKCPVPPTQELLDQKKQFEKCGLQVLKVEKIDNEVLMAAFQRKKKMMEEKLHRQPVSHRLFQQVPYQFCNVVCRVGFQRMYSTPCDPKYGAGIYFTKNLKNLAEKAKKISAADKLIYVFEAEVLTGFFCQGHPLNIVPPPLSPGAIDGHDSVVDNVSSPETFVIFSGMQAIPQYLWTCTQEYVQSQDYSSGPMRPFAQHPWRGFASGSPVD

>NP_005407.1

MSSYQQKQTFTPPPQLQQQQVKQPSQPPPQEIFVPTTKEPCHSKVPQPGNTKIPEPGCTKVPEPGCTKVPEPGCTKVPEPGCTKVPEPGCTKVPEPGCTKVPEPGYTKVPEPGSIKVPDQGFIKFPEPGAIKVPEQGYTKVPVPGYTKLPEPCPSTVTPGPAQQKTKQK

1760 NP_003963.1 NP_056000.1

>NP_003963.1

MAVSRLDRLFILLDTGTTPVTRKAAAQQLGEVVKLHPHELNNLLSKVLIYLRSANWDTRIAAGQAVEAIVKNVPEWNPVPRTRQEPTSESSMEDSPTTERLNFDRFDICRLLQHGASLLGSAGAEFEVQDEKSGEVDPKERIARQRKLLQKKLGLNMGEAIGMSTEELFNDEDLDYTPTSASFVNKQPTLQAAELIDSEFRAGMSNRQKNKAKRMAKLFAKQRSRDAVETNEKSNDSTDGEPEEKRRKIANVVINQSANDSKVLIDNIPDSSSLIEETNEWPLESFCEELCNDLFNPSWEVRHGAGTGLREILKAHGKSGGKMGDSTLEEMIQQHQEWLEDLVIRLLCVFALDRFGDFVSDEVVAPVRETCAQTLGVVLKHMNETGVHKTVDVLLKLLTQEQWEVRHGGLLGIKYALAVRQDVINTLLPKVLTRIIEGLQDLDDDVRAVAAASLVPVVESLVYLQTQKVPFIINTLWDALLELDDLTASTNSIMTLLSSLLTYPQVQQCSIQQSLTVLVPRVWPFLHHTISSVRRAALETLFTLLSTQDQNSSSWLIPILPDMLRHIFQFCVLESSQEILDLIHKVWMELLSKASVQYVVAAACPWMGAWLCLMMQPSHLPIDLNMLLEVKARAKEKTGGKVRQGQSQNKEVLQEYIAGADTIMEDPATRDFVVMRARMMAAKLLGALCCCICDPGVNVVTQEIKPAESLGQLLLFHLNSKSALQRISVALVICEWAALQKECKAVTLAVQPRLLDILSEHLYYDEIAVPFTRMQNECKQLISSLADVHIEVGNRVNNNVLTIDQASDLVTTVFNEATSSFDLNPQVLQQLDSKRQQVQMTVTETNQEWQVLQLRVHTFAACAVVSLQQLPEKLNPIIKPLMETIKKEENTLVQNYAAQCIAKLLQQCTTRTPCPNSKIIKNLCSSLCVDPYLTPCVTCPVPTQSGQENSKGSTSEKDGMHHTVTKHRGIITLYRHQKAAFAITSRRGPTPKAVKAQIADLPAGSSGNILVELDEAQKPYLVQRRGAEFALTTIVKHFGGEMAVKLPHLWDAMVGPLRNTIDINNFDGKSLLDKGDSPAQELVNSLQVFETAAASMDSELHPLLVQHLPHLYMCLQYPSTAVRHMAARCVGVMSKIATMETMNIFLEKVLPWLGAIDDSVKQEGAIEALACVMEQLDVGIVPYIVLLVVPVLGRMSDQTDSVRFMATQCFATLIRLMPLEAGIPDPPNMSAELIQLKAKERHFLEQLLDGKKLENYKIPVPINAELRKYQQDGVNWLAFLNKYKLHGILCDDMGLGKTLQSICILAGDHCHRAQEYARSKLAECMPLPSLVVCPPTLTGHWVDEVGKFCSREYLNPLHYTGPPTERIRLQHQVKRHNLIVASYDVVRNDIDFFRNIKFNYCILDEGHVIKNGKTKLSKAVKQLTANYRIILSGTPIQNNVLELWSLFDFLMPGFLGTERQFAARYGKPILASRDARSSSREQEAGVLAMDALHRQVLPFLLRRMKEDVLQDLPPKIIQDYYCTLSPLQVQLYEDFAKSRAKCDVDETVSSATLSEETEKPKLKATGHVFQALQYLRKLCNHPALVLTPQHPEFKTTAEKLAVQNSSLHDIQHAPKLSALKQLLLDCGLGNGSTSESGTESVVAQHRILIFCQLKSMLDIVEHDLLKPHLPSVTYLRLDGSIPPGQRHSIVSRFNNDPSIDVLLLTTHVGGLGLNLTGADTVVFVEHDWNPMRDLQAMDRAHRIGQKRVVNVYRLITRGTLEEKIMGLQKFKMNIANTVISQENSSLQSMGTDQLLDLFTLDKDGKAEKADTSTSGKASMKSILENLSDLWDQEQYDSEYSLENFMHSLK

>NP_056000.1

MTLLITGDSIVSAEAVWDHVTMANRELAFKAGDVIKVLDASNKDWWWGQIDDEEGWFPASFVRLWVNQEDEVEEGPSDVQNGHLDPNSDCLCLGRPLQNRDQMRANVINEIMSTERHYIKHLKDICEGYLKQCRKRRDMFSDEQLKVIFGNIEDIYRFQMGFVRDLEKQYNNDDPHLSEIGPCFLEHQDGFWIYSEYCNNHLDACMELSKLMKDSRYQHFFEACRLLQQMIDIAIDGFLLTPVQKICKYPLQLAELLKYTAQDHSDYRYVAAALAVMRNVTQQINERKRRLENIDKIAQWQASVLDWEGEDILDRSSELIYTGEMAWIYQPYGRNQQRVFFLFDHQMVLCKKDLIRRDILYYKGRIDMDKYEVVDIEDGRDDDFNVSMKNAFKLHNKETEEIHLFFAKKLEEKIRWLRAFREERKMVQEDEKIGFEISENQKRQAAMTVRKVPKQKGVNSARSVPPSYPPPQDPLNHGQYLVPDGIAQSQVFEFTEPKRSQSPFWQNFSRLTPFKK

1761 O60264 P04183

>O60264

MSSAAEPPPPPPPESAPSKPAASIASGGSNSSNKGGPEGVAAQAVASAASAGPADAEMEEIFDDASPGKQKEIQEPDPTYEEKMQTDRANRFEYLLKQTELFAHFIQPAAQKTPTSPLKMKPGRPRIKKDEKQNLLSVGDYRHRRTEQEEDEELLTESSKATNVCTRFEDSPSYVKWGKLRDYQVRGLNWLISLYENGINGILADEMGLGKTLQTISLLGYMKHYRNIPGPHMVLVPKSTLHNWMSEFKRWVPTLRSVCLIGDKEQRAAFVRDVLLPGEWDVCVTSYEMLIKEKSVFKKFNWRYLVIDEAHRIKNEKSKLSEIVREFKTTNRLLLTGTPLQNNLHELWSLLNFLLPDVFNSADDFDSWFDTNNCLGDQKLVERLHMVLRPFLLRRIKADVEKSLPPKKEVKIYVGLSKMQREWYTRILMKDIDILNSAGKMDKMRLLNILMQLRKCCNHPYLFDGAEPGPPYTTDMHLVTNSGKMVVLDKLLPKLKEQGSRVLIFSQMTRVLDILEDYCMWRNYEYCRLDGQTPHDERQDSINAYNEPNSTKFVFMLSTRAGGLGINLATADVVILYDSDWNPQVDLQAMDRAHRIGQTKTVRVFRFITDNTVEERIVERAEMKLRLDSIVIQQGRLVDQNLNKIGKDEMLQMIRHGATHVFASKESEITDEDIDGILERGAKKTAEMNEKLSKMGESSLRNFTMDTESSVYNFEGEDYREKQKIAFTEWIEPPKRERKANYAVDAYFREALRVSEPKAPKAPRPPKQPNVQDFQFFPPRLFELLEKEILFYRKTIGYKVPRNPELPNAAQAQKEEQLKIDEAESLNDEELEEKEKLLTQGFTNWNKRDFNQFIKANEKWGRDDIENIAREVEGKTPEEVIEYSAVFWERCNELQDIEKIMAQIERGEARIQRRISIKKALDTKIGRYKAPFHQLRISYGTNKGKNYTEEEDRFLICMLHKLGFDKENVYDELRQCIRNSPQFRFDWFLKSRTAMELQRRCNTLITLIERENMELEEKEKAEKKKRGPKPSTQKRKMDGAPDGRGRKKKLKL

>P04183

MSCINLPTVLPGSPSKTRGQIQVILGPMFSGKSTELMRRVRRFQIAQYKCLVIKYAKDTRYSSSFCTHDRNTMEALPACLLRDVAQEALGVAVIGIDEGQFFPDIVEFCEAMANAGKTVIVAALDGTFQRKPFGAILNLVPLAESVVKLTAVCMECFREAAYTKRLGTEKEVEVIGGADKYHSVCRLCYFKKASGQPAGPDNKENCPVPGKPGEAVAARKLFAPQQILQCSPAN

1762 NP_057415.1 NP_004050.3

>NP_057415.1

MNEYPKKRKRKTLHPSRYSDSSGISRIADGFNGIFSDHCYSVCSMRQPDLKYFDNKDDDSDTETSNDLPKFADGIKARNRNQNYLVPSPVLRILDHTAFSTEKSADIVICDEECDSPESVNQQTQEESPIEVHTAEDVPIAVEVHAISEDYDIETENNSSESLQDQTDEEPPAKLCKILDKSQALNVTAQQKWPLLRANSSGLYKCELCEFNSKYFSDLKQHMILKHKRTDSNVCRVCKESFSTNMLLIEHAKLHEEDPYICKYCDYKTVIFENLSQHIADTHFSDHLYWCEQCDVQFSSSSELYLHFQEHSCDEQYLCQFCEHETNDPEDLHSHVVNEHACKLIELSDKYNNGEHGQYSLLSKITFDKCKNFFVCQVCGFRSRLHTNVNRHVAIEHTKIFPHVCDDCGKGFSSMLEYCKHLNSHLSEGIYLCQYCEYSTGQIEDLKIHLDFKHSADLPHKCSDCLMRFGNERELISHLPVHETT

>NP_004050.3

MAKQLQARRLDGIDYNPWVEFVKLASEHDVVNLGQGFPDFPPPDFAVEAFQHAVSGDFMLNQYTKTFGYPPLTKILASFFGELLGQEIDPLRNVLVTVGGYGALFTAFQALVDEGDEVIIIEPFFDCYEPMTMMAGGRPVFVSLKPGPIQNGELGSSSNWQLDPMELAGKFTSRTKALVLNTPNNPLGKVFSREELELVASLCQQHDVVCITDEVYQWMVYDGHQHISIASLPGMWERTLTIGSAGKTFSATGWKVGWVLGPDHIMKHLRTVHQNSVFHCPTQSQAAVAESFEREQLLFRQPSSYFVQFPQAMQRCRDHMIRSLQSVGLKPIIPQGSYFLITDISDFKRKMPDLPGAVDEPYDRRFVKWMIKNKGLVAIPVSIFYSVPHQKHFDHYIRFCFVKDEATLQAMDEKLRKWKVEL

1763 NP_003063.2 NP_036205.1

>NP_003063.2

MSTPDPPLGGTPRPGPSPGPGPSPGAMLGPSPGPSPGSAHSMMGPSPGPPSAGHPIPTQGPGGYPQDNMHQMHKPMESMHEKGMSDDPRYNQMKGMGMRSGGHAGMGPPPSPMDQHSQGYPSPLGGSEHASSPVPASGPSSGPQMSSGPGGAPLDGADPQALGQQNRGPTPFNQNQLHQLRAQIMAYKMLARGQPLPDHLQMAVQGKRPMPGMQQQMPTLPPPSVSATGPGPGPGPGPGPGPGPAPPNYSRPHGMGGPNMPPPGPSGVPPGMPGQPPGGPPKPWPEGPMANAAAPTSTPQKLIPPQPTGRPSPAPPAVPPAASPVMPPQTQSPGQPAQPAPMVPLHQKQSRITPIQKPRGLDPVEILQEREYRLQARIAHRIQELENLPGSLAGDLRTKATIELKALRLLNFQRQLRQEVVVCMRRDTALETALNAKAYKRSKRQSLREARITEKLEKQQKIEQERKRRQKHQEYLNSILQHAKDFKEYHRSVTGKIQKLTKAVATYHANTEREQKKENERIEKERMRRLMAEDEEGYRKLIDQKKDKRLAYLLQQTDEYVANLTELVRQHKAAQVAKEKKKKKKKKKAENAEGQTPAIGPDGEPLDETSQMSDLPVKVIHVESGKILTGTDAPKAGQLEAWLEMNPGYEVAPRSDSEESGSEEEEEEEEEEQPQAAQPPTLPVEEKKKIPDPDSDDVSEVDARHIIENAKQDVDDEYGVSQALARGLQSYYAVAHAVTERVDKQSALMVNGVLKQYQIKGLEWLVSLYNNNLNGILADEMGLGKTIQTIALITYLMEHKRINGPFLIIVPLSTLSNWAYEFDKWAPSVVKVSYKGSPAARRAFVPQLRSGKFNVLLTTYEYIIKDKHILAKIRWKYMIVDEGHRMKNHHCKLTQVLNTHYVAPRRLLLTGTPLQNKLPELWALLNFLLPTIFKSCSTFEQWFNAPFAMTGEKVDLNEEETILIIRRLHKVLRPFLLRRLKKEVEAQLPEKVEYVIKCDMSALQRVLYRHMQAKGVLLTDGSEKDKKGKGGTKTLMNTIMQLRKICNHPYMFQHIEESFSEHLGFTGGIVQGLDLYRASGKFELLDRILPKLRATNHKVLLFCQMTSLMTIMEDYFAYRGFKYLRLDGTTKAEDRGMLLKTFNEPGSEYFIFLLSTRAGGLGLNLQSADTVIIFDSDWNPHQDLQAQDRAHRIGQQNEVRVLRLCTVNSVEEKILAAAKYKLNVDQKVIQAGMFDQKSSSHERRAFLQAILEHEEQDESRHCSTGSGSASFAHTAPPPAGVNPDLEEPPLKEEDEVPDDETVNQMIARHEEEFDLFMRMDLDRRREEARNPKRKPRLMEEDELPSWIIKDDAEVERLTCEEEEEKMFGRGSRHRKEVDYSDSLTEKQWLKAIEEGTLEEIEEEVRQKKSSRKRKRDSDAGSSTPTTSTRSRDKDDESKKQKKRGRPPAEKLSPNPPNLTKKMKKIVDAVIKYKDSSSGRQLSEVFIQLPSRKELPEYYELIRKPVDFKKIKERIRNHKYRSLNDLEKDVMLLCQNAQTFNLEGSLIYEDSIVLQSVFTSVRQKIEKEDDSEGEESEEEEEGEEEGSESESRSVKVKIKLGRKEKAQDRLKGGRRRPSRGSRAKPVVSDDDSEEEQEEDRSGSGSEED

>NP_036205.1

MASMGTLAFDEYGRPFLIIKDQDRKSRLMGLEALKSHIMAAKAVANTMRTSLGPNGLDKMMVDKDGDVTVTNDGATILSMMDVDHQIAKLMVELSKSQDDEIGDGTTGVVVLAGALLEEAEQLLDRGIHPIRIADGYEQAARVAIEHLDKISDSVLVDIKDTEPLIQTAKTTLGSKVVNSCHRQMAEIAVNAVLTVADMERRDVDFELIKVEGKVGGRLEDTKLIKGVIVDKDFSHPQMPKKVEDAKIAILTCPFEPPKPKTKHKLDVTSVEDYKALQKYEKEKFEEMIQQIKETGANLAICQWGFDDEANHLLLQNNLPAVRWVGGPEIELIAIATGGRIVPRFSELTAEKLGFAGLVQEISFGTTKDKMLVIEQCKNSRAVTIFIRGGNKMIIEEAKRSLHDALCVIRNLIRDNRVVYGGGAAEISCALAVSQEADKCPTLEQYAMRAFADALEVIPMALSENSGMNPIQTMTEVRARQVKEMNPALGIDCLHKGTNDMKQQHVIETLIGKKQQISLATQMVRMILKIDDIRKPGESEE

1764 NP_001505.1 NP_005979.1

>NP_001505.1

MASTSRLDALPRVTCPNHPDAILVEDYRAGDMICPECGLVVGDRVIDVGSEWRTFSNDKATKDPSRVGDSQNPLLSDGDLSTMIGKGTGAASFDEFGNSKYQNRRTMSSSDRAMMNAFKEITTMADRINLPRNIVDRTNNLFKQVYEQKSLKGRANDAIASACLYIACRQEGVPRTFKEICAVSRISKKEIGRCFKLILKALETSVDLITTGDFMSRFCSNLCLPKQVQMAATHIARKAVELDLVPGRSPISVAAAAIYMASQASAEKRTQKEIGDIAGVADVTIRQSYRLIYPRAPDLFPTDFKFDTPVDKLPQL

>NP_005979.1

MSYQQQQCKQPCQPPPVCPTPKCPEPCPPPKCPEPCPPPKCPQPCPPQQCQQKYPPVTPSPPCQSKYPPKSK

1765 NP_003503.1 NP_000658.1

>NP_003503.1

MSGRGKQGGKARAKAKSRSSRAGLQFPVGRVHRLLRKGNYAERVGAGAPVYLAAVLEYLTAEILELAGNAARDNKKTRIIPRHLQLAIRNDEELNKLLGRVTIAQGGVLPNIQAVLLPKKTESHHKAKGK

>NP_000658.1

MSTAGKVIKCKAAVLWELKKPFSIEEVEVAPPKAHEVRIKMVAVGICGTDDHVVSGTMVTPLPVILGHEAAGIVESVGEGVTTVKPGDKVIPLAIPQCGKCRICKNPESNYCLKNDVSNPQGTLQDGTSRFTCRRKPIHHFLGISTFSQYTVVDENAVAKIDAASPLEKVCLIGCGFSTGYGSAVNVAKVTPGSTCAVFGLGGVGLSAIMGCKAAGAARIIAVDINKDKFAKAKELGATECINPQDYKKPIQEVLKEMTDGGVDFSFEVIGRLDTMMASLLCCHEACGTSVIVGVPPDSQNLSMNPMLLLTGRTWKGAILGGFKSKECVPKLVADFMAKKFSLDALITHVLPFEKINEGFDLLHSGKSIRTILMF

1766 P17861 Q8NEL9

>P17861

MVVVAAAPNPADGTPKVLLLSGQPASAAGAPAGQALPLMVPAQRGASPEAASGGLPQARKRQRLTHLSPEEKALRRKLKNRVAAQTARDRKKARMSELEQQVVDLEEENQKLLLENQLLREKTHGLVVENQELRQRLGMDALVAEEEAEAKGNEVRPVAGSAESAALRLRAPLQQVQAQLSPLQNISPWILAVLTLQIQSLISCWAFWTTWTQSCSSNALPQSLPAWRSSQRSTQKDPVPYQPPFLCQWGRHQPSWKPLMN

>Q8NEL9

MNYPGRGSPRSPEHNGRGGGGGAWELGSDARPAFGGGVCCFEHLPGGDPDDGDVPLALLRGEPGLHLAPGTDDHNHHLALDPCLSDENYDFSSAESGSSLRYYSEGESGGGGSSLSLHPPQQPPLVPTNSGGGGATGGSPGERKRTRLGGPAARHRYEVVTELGPEEVRWFYKEDKKTWKPFIGYDSLRIELAFRTLLQTTGARPQGGDRDGDHVCSPTGPASSSGEDDDEDRACGFCQSTTGHEPEMVELVNIEPVCVRGGLYEVDVTQGECYPVYWNQADKIPVMRGQWFIDGTWQPLEEEESNLIEQEHLNCFRGQQMQENFDIEVSKSIDGKDAVHSFKLSRNHVDWHSVDEVYLYSDATTSKIARTVTQKLGFSKASSSGTRLHRGYVEEATLEDKPSQTTHIVFVVHGIGQKMDQGRIIKNTAMMREAARKIEERHFSNHATHVEFLPVEWRSKLTLDGDTVDSITPDKVRGLRDMLNSSAMDIMYYTSPLYRDELVKGLQQELNRLYSLFCSRNPDFEEKGGKVSIVSHSLGCVITYDIMTGWNPVRLYEQLLQKEEELPDERWMSYEERHLLDELYITKRRLKEIEERLHGLKASSMTQTPALKFKVENFFCMGSPLAVFLALRGIRPGNTGSQDHILPREICNRLLNIFHPTDPVAYRLEPLILKHYSNISPVQIHWYNTSNPLPYEHMKPSFLNPAKEPTSVSENEGISTIPSPVTSPVLSRRHYGESITNIGKASILGAASIGKGLGGMLFSRFGRSSTTQSSETSKDSMEDEKKPVASPSATTVGTQTLPHSSSGFLDSAYFRLQESFFNLPQLLFPENVMQNKDNALVELDHRIDFELREGLVESRYWSAVTSHTAYWSSLDVALFLLTFMYKHEHDDDAKPNLDPI

1767 NP_001871.2 NP_003795.2

>NP_001871.2

MKFKLHVNSARQYKDLWNMSDDKPFLCTAPGCGQRFTNEDHLAVHKHKHEMTLKFGPARNDSVIVADQTPTPTRFLKNCEEVGLFNELASPFENEFKKASEDDIKKMPLDLSPLATPIIRSKIEEPSVVETTHQDSPLPHPESTTSDEKEVPLAQTAQPTSAIVRPASLQVPNVLLTSSDSSVIIQQAVPSPTSSTVITQAPSSNRPIVPVPGPFPLLLHLPNGQTMPVAIPASITSSNVHVPAAVPLVRPVTMVPSVPGIPGPSSPQPVQSEAKMRLKAALTQQHPPVTNGDTVKGHGSGLVRTQSEESRPQSLQQPATSTTETPASPAHTTPQTQSTSGRRRRAANEDPDEKRRKFLERNRAAASRCRQKRKVWVQSLEKKAEDLSSLNGQLQSEVTLLRNEVAQLKQLLLAHKDCPVTAMQKKSGYHTADKDDSSEDISVPSSPHTEAIQHSSVSTSNGVSSTSKAEAVATSVLTQMADQSTEPALSQIVMAPSSQSQPSGS

>NP_003795.2

MQPDMSLNVIKMKSSDFLESAELDSGGFGKVSLCFHRTQGLMIMKTVYKGPNCIEHNEALLEEAKMMNRLRHSRVVKLLGVIIEEGKYSLVMEYMEKGNLMHVLKAEMSTPLSVKGRIILEIIEGMCYLHGKGVIHKDLKPENILVDNDFHIKIADLGLASFKMWSKLNNEEHNELREVDGTAKKNGGTLYYMAPEHLNDVNAKPTEKSDVYSFAVVLWAIFANKEPYENAICEQQLIMCIKSGNRPDVDDITEYCPREIISLMKLCWEANPEARPTFPGIEEKFRPFYLSQLEESVEEDVKSLKKEYSNENAVVKRMQSLQLDCVAVPSSRSNSATEQPGSLHSSQGLGMGPVEESWFAPSLEHPQEENEPSLQSKLQDEANYHLYGSRMDRQTKQQPRQNVAYNREEERRRRVSHDPFAQQRPYENFQNTEGKGTAYSSAASHGNAVHQPSGLTSQPQVLYQNNGLYSSHGFGTRPLDPGTAGPRVWYRPIPSHMPSLHNIPVPETNYLGNTPTMPFSSLPPTDESIKYTIYNSTGIQIGAYNYMEIGGTSSSLLDSTNTNFKEEPAAKYQAIFDNTTSLTDKHLDPIRENLGKHWKNCARKLGFTQSQIDEIDHDYERDGLKEKVYQMLQKWVMREGIKGATVGKLAQALHQCSRIDLLSSLIYVSQN

1768 NP_036354.1 NP_000404.1

>NP_036354.1

MTPLVSRLSRLWAIMRKPRAAVGSGHRKQAASQEGRQKHAKNNSQAKPSACDGMIAECPGAPAGLARQPEEVVLQASVSSYHLFRDVAEVTAFRGSLLSWYDQEKRDLPWRRRAEDEMDLDRRAYAVWVSEVMLQQTQVATVINYYTGWMQKWPTLQDLASASLEEVNQLWAGLGYYSRGRRLQEGARKVVEELGGHMPRTAETLQQLLPGVGRYTAGAIASIAFGQATGVVDGNVARVLCRVRAIGADPSSTLVSQQLWGLAQQLVDPARPGDFNQAAMELGATVCTPQRPLCSQCPVESLCRARQRVEQEQLLASGSLSGSPDVEECAPNTGQCHLCLPPSEPWDQTLGVVNFPRKASRKPPREESSATCVLEQPGALGAQILLVQRPNSGLLAGLWEFPSVTWEPSEQLQRKALLQELQRWAGPLPATHLRHLGEVVHTFSHIKLTYQVYGLALEGQTPVTTVPPGARWLTQEEFHTAAVSTAMKKVFRVYQGQQPGTCMGSKRSQVSSPCSRKKPRMGQQVLDNFFRSHISTDAHSLNSAAQ

>NP_000404.1

MARTVVLITGCSSGIGLHLAVRLASDPSQSFKVYATLRDLKTQGRLWEAARALACPPGSLETLQLDVRDSKSVAAARERVTEGRVDVLVCNAGLGLLGPLEALGEDAVASVLDVNVVGTVRMLQAFLPDMKRRGSGRVLVTGSVGGLMGLPFNDVYCASKFALEGLCESLAVLLLPFGVHLSLIECGPVHTAFMEKVLGSPEEVLDRTDIHTFHRFYQYLAHSKQVFREAAQNPEEVAEVFLTALRAPKPTLRYFTTERFLPLLRMRLDDPSGSNYVTAMHREVFGDVPAKAEAGAEAGGGAGPGAEDEAGRSAVGDPELGDPPAAPQ

1769 NP_006158.2 Q6UB28

>NP_006158.2

MLRVPEPRPGEAKAEGAAPPTPSKPLTSFLIQDILRDGAQRQGGRTSSQRQRDPEPEPEPEPEGGRSRAGAQNDQLSTGPRAAPEEAETLAETEPERHLGSYLLDSENTSGALPRLPQTPKQPQKRSRAAFSHTQVIELERKFSHQKYLSAPERAHLAKNLKLTETQVKIWFQNRRYKTKRKQLSSELGDLEKHSSLPALKEEAFSRASLVSVYNSYPYYPYLYCVGSWSPAFW

>Q6UB28

MAAPSGVHLLVRRGSHRIFSSPLNHIYLHKQSSSQQRRNFFFRRQRDISHSIVLPAAVSSAHPVPKHIKKPDYVTTGIVPDWGDSIEVKNEDQIQGLHQACQLARHVLLLAGKSLKVDMTTEEIDALVHREIISHNAYPSPLGYGGFPKSVCTSVNNVLCHGIPDSRPLQDGDIINIDVTVYYNGYHGDTSETFLVGNVDECGKKLVEVARRCRDEAIAACRAGAPFSVIGNTISHITHQNGFQVCPHFVGHGIGSYFHGHPEIWHHANDSDLPMEEGMAFTIEPIITEGSPEFKVLEDAWTVVSLDNQRSAQFEHTVLITSRGAQILTKLPHEA

1770 Q15306 NP_003493.1

>Q15306

MNLEGGGRGGEFGMSAVSCGNGKLRQWLIDQIDSGKYPGLVWENEEKSIFRIPWKHAGKQDYNREEDAALFKAWALFKGKFREGIDKPDPPTWKTRLRCALNKSNDFEELVERSQLDISDPYKVYRIVPEGAKKGAKQLTLEDPQMSMSHPYTMTTPYPSLPAQQVHNYMMPPLDRSWRDYVPDQPHPEIPYQCPMTFGPRGHHWQGPACENGCQVTGTFYACAPPESQAPGVPTEPSIRSAEALAFSDCRLHICLYYREILVKELTTSSPEGCRISHGHTYDASNLDQVLFPYPEDNGQRKNIEKLLSHLERGVVLWMAPDGLYAKRLCQSRIYWDGPLALCNDRPNKLERDQTCKLFDTQQFLSELQAFAHHGRSLPRFQVTLCFGEEFPDPQRQRKLITAHVEPLLARQLYYFAQQNSGHFLRGYDLPEHISNPEDYHRSIRHSSIQE

>NP_003493.1

MNIQEQGFPLDLGASFTEDAPRPPVPGEEGELVSTDPRPASYSFCSGKGVGIKGETSTATPRRSDLDLGYEPEGSASPTPPYLKWAESLHSLLDDQDGISLFRTFLKQEGCADLLDFWFACTGFRKLEPCDSNEEKRLKLARAIYRKYILDNNGIVSRQTKPATKSFIKGCIMKQLIDPAMFDQAQTEIQATMEENTYPSFLKSDIYLEYTRTGSESPKVCSDQSSGSGTGKGISGYLPTLNEDEEWKCDQDMDEDDGRDAAPPGRLPQKLLLETAAPRVSSSRRYSEGREFRYGSWREPVNPYYVNAGYALAPATSANDSEQQSLSSDADTLSLTDSSVDGIPPYRIRKQHRREMQESVQVNGRVPLPHIPRTYRVPKEVRVEPQKFAEELIHRLEAVQRTREAEEKLEERLKRVRMEEEGEDGDPSSGPPGPCHKLPPAPAWHHFPPRCVDMGCAGLRDAHEENPESILDEHVQRVLRTPGRQSPGPGHRSPDSGHVAKMPVALGGAASGHGKHVPKSGAKLDAAGLHHHRHVHHHVHHSTARPKEQVEAEATRRAQSSFAWGLEPHSHGARSRGYSESVGAAPNASDGLAHSGKVGVACKRNAKKAESGKSASTEVPGASEDAEKNQKIMQWIIEGEKEISRHRRTGHGSSGTRKPQPHENSRPLSLEHPWAGPQLRTSVQPSHLFIQDPTMPPHPAPNPLTQLEEARRRLEEEEKRASRAPSKQRYVQEVMRRGRACVRPACAPVLHVVPAVSDMELSETETRSQRKVGGGSAQPCDSIVVAYYFCGEPIPYRTLVRGRAVTLGQFKELLTKKGSYRYYFKKVSDEFDCGVVFEEVREDEAVLPVFEEKIIGKVEKVD

1771 NP_073624.2 NP_004171.2

>NP_073624.2

MENSEKTEVVLLACGSFNPITNMHLRLFELAKDYMNGTGRYTVVKGIISPVGDAYKKKGLIPAYHRVIMAELATKNSKWVEVDTWESLQKEWKETLKVLRHHQEKLEASDCDHQQNSPTLERPGRKRKWTETQDSSQKKSLEPKTKAVPKVKLLCGADLLESFAVPNLWKSEDITQIVANYGLICVTRAGNDAQKFIYESDVLWKHRSNIHVVNEWIANDISSTKIRRALRRGQSIRYLVPDLVQEYIEKHNLYSSESEDRNAGVILAPLQRNTAEAKT

>NP_004171.2

MDKNIGEQLNKAYEAFRQACMDRDSAVKELQQKTENYEQRIREQQEQLSLQQTIIDKLKSQLLLVNSTQDNNYGCVPLLEDSETRKNNLTLDQPQDKVISGIAREKLPKVRRQEVSSPRKETSARSLGSPLLHERGNIEKTFWDLKEEFHKICMLAKAQKDHLSKLNIPDTATETQCSVPIQCTDKTDKQEALFKPQAKDDINRGAPSITSVTPRGLCRDEEDTSFESLSKFNVKFPPMDNDSTFLHSTPERPGILSPATSEAVCQEKFNMEFRDNPGNFVKTEETLFEIQGIDPIASAIQNLKTTDKTKPSNLVNTCIRTTLDRAACLPPGDHNALYVNSFPLLDPSDAPFPSLDSPGKAIRGPQQPIWKPFPNQDSDSVVLSGTDSELHIPRVCEFCQAVFPPSITSRGDFLRHLNSHFNGET

1772 Q14839 Q9Y4K4

>Q14839

MASGLGSPSPCSAGSEEEDMDALLNNSLPPPHPENEEDPEEDLSETETPKLKKKKKPKKPRDPKIPKSKRQKKERMLLCRQLGDSSGEGPEFVEEEEEVALRSDSEGSDYTPGKKKKKKLGPKKEKKSKSKRKEEEEEDDDDDDSKEPKSSAQLLEDWGMEDIDHVFSEEDYRTLTNYKAFSQFVRPLIAAKNPKIAVSKMMMVLGAKWREFSTNNPFKGSSGASVAAAAAAAVAVVESMVTATEVAPPPPPVEVPIRKAKTKEGKGPNARRKPKGSPRVPDAKKPKPKKVAPLKIKLGGFGSKRKRSSSEDDDLDVESDFDDASINSYSVSDGSTSRSSRSRKKLRTTKKKKKGEEEVTAVDGYETDHQDYCEVCQQGGEIILCDTCPRAYHMVCLDPDMEKAPEGKWSCPHCEKEGIQWEAKEDNSEGEEILEEVGGDLEEEDDHHMEFCRVCKDGGELLCCDTCPSSYHIHCLNPPLPEIPNGEWLCPRCTCPALKGKVQKILIWKWGQPPSPTPVPRPPDADPNTPSPKPLEGRPERQFFVKWQGMSYWHCSWVSELQLELHCQVMFRNYQRKNDMDEPPSGDFGGDEEKSRKRKNKDPKFAEMEERFYRYGIKPEWMMIHRILNHSVDKKGHVHYLIKWRDLPYDQASWESEDVEIQDYDLFKQSYWNHRELMRGEEGRPGKKLKKVKLRKLERPPETPTVDPTVKYERQPEYLDATGGTLHPYQMEGLNWLRFSWAQGTDTILADEMGLGKTVQTAVFLYSLYKEGHSKGPFLVSAPLSTIINWEREFEMWAPDMYVVTYVGDKDSRAIIRENEFSFEDNAIRGGKKASRMKKEASVKFHVLLTSYELITIDMAILGSIDWACLIVDEAHRLKNNQSKFFRVLNGYSLQHKLLLTGTPLQNNLEELFHLLNFLTPERFHNLEGFLEEFADIAKEDQIKKLHDMLGPHMLRRLKADVFKNMPSKTELIVRVELSPMQKKYYKYILTRNFEALNARGGGNQVSLLNVVMDLKKCCNHPYLFPVAAMEAPKMPNGMYDGSALIRASGKLLLLQKMLKNLKEGGHRVLIFSQMTKMLDLLEDFLEHEGYKYERIDGGITGNMRQEAIDRFNAPGAQQFCFLLSTRAGGLGINLATADTVIIYDSDWNPHNDIQAFSRAHRIGQNKKVMIYRFVTRASVEERITQVAKKKMMLTHLVVRPGLGSKTGSMSKQELDDILKFGTEELFKDEATDGGGDNKEGEDSSVIHYDDKAIERLLDRNQDETEDTELQGMNEYLSSFKVAQYVVREEEMGEEEEVEREIIKQEESVDPDYWEKLLRHHYEQQQEDLARNLGKGKRIRKQVNYNDGSQEDRDWQDDQSDNQSDYSVASEEGDEDFDERSEAPRRPSRKGLRNDKDKPLPPLLARVGGNIEVLGFNARQRKAFLNAIMRYGMPPQDAFTTQWLVRDLRGKSEKEFKAYVSLFMRHLCEPGADGAETFADGVPREGLSRQHVLTRIGVMSLIRKKVQEFEHVNGRWSMPELAEVEENKKMSQPGSPSPKTPTPSTPGDTQPNTPAPVPPAEDGIKIEENSLKEEESIEGEKEVKSTAPETAIECTQAPAPASEDEKVVVEPPEGEEKVEKAEVKERTEEPMETEPKGAADVEKVEEKSAIDLTPIVVEDKEEKKEEEEKKEVMLQNGETPKDLNDEKQKKNIKQRFMFNIADGGFTELHSLWQNEERAATVTKKTYEIWHRRHDYWLLAGIINHGYARWQDIQNDPRYAILNEPFKGEMNRGNFLEIKNKFLARRFKLLEQALVIEEQLRRAAYLNMSEDPSHPSMALNTRFAEVECLAESHQHLSKESMAGNKPANAVLHKVLKQLEELLSDMKADVTRLPATIARIPPVAVRLQMSERNILSRLANRAPEPTPQQVAQQQ

>Q9Y4K4

MEAPLRPAADILRRNPQQDYELVQRVGSGTYGDVYKARNVHTGELAAVKIIKLEPGDDFSLIQQEIFMVKECKHCNIVAYFGSYLSREKLWICMEYCGGGSLQDIYHVTGPLSELQIAYVCRETLQGLAYLHTKGKMHRDIKGANILLTDHGDVKLADFGVAAKITATIAKRKSFIGTPYWMAPEVAAVEKNGGYNQLCDIWAVGITAIELGELQPPMFDLHPMRALFLMSKSNFQPPKLKDKTKWSSTFHNFVKIALTKNPKKRPTAERLLTHTFVAQPGLSRALAVELLDKVNNPDNHAHYTEADDDDFEPHAIIRHTIRSTNRNARAERTASEINFDKLQFEPPLRKETEARDEMGLSSDPNFMLQWNPFVDGANTGKSTSKRAIPPPLPPKPRISSYPEDNFPDEEKASTIKHCPDSESRAPQILRRQSSPSCGPVAETSSIGNGDGISKLMSENTEGSAQAPQLPRKNDKRDFPKPAINGLPPTPKVLMGACFSKVFDGCPLKINCATSWIHPDTKDQYIIFGTEDGIYTLNLNELHEATMEQLFPRKCTWLYVINNTLMSLSEGKTFQLYSHNLIALFEHAKKPGLAAHIQTHRFPDRILPRKFALTTKIPDTKGCHKCCIVRNPYTGHKYLCGALQSGIVLLQWYEPMQKFMLIKHFDFPLPSPLNVFEMLVIPEQEYPMVCVAISKGTESNQVVQFETINLNSASSWFTEIGAGSQQLDSIHVTQLERDTVLVCLDKFVKIVNLQGKLKSSKKLASELSFDFRIESVVCLQDSVLAFWKHGMQGKSFKSDEVTQEISDETRVFRLLGSDRVVVLESRPTENPTAHSNLYILAGHENSY

1773 NP_001460.1 NP_005495.2

>NP_001460.1

MSGWESYYKTEGDEEAEEEQEENLEASGDYKYSGRDSLIFLVDASKAMFESQSEDELTPFDMSIQCIQSVYISKIISSDRDLLAVVFYGTEKDKNSVNFKNIYVLQELDNPGAKRILELDQFKGQQGQKRFQDMMGHGSDYSLSEVLWVCANLFSDVQFKMSHKRIMLFTNEDNPHGNDSAKASRARTKAGDLRDTGIFLDLMHLKKPGGFDISLFYRDIISIAEDEDLRVHFEESSKLEDLLRKVRAKETRKRALSRLKLKLNKDIVISVGIYNLVQKALKPPPIKLYRETNEPVKTKTRTFNTSTGGLLLPSDTKRSQIYGSRQIILEKEETEELKRFDDPGLMLMGFKPLVLLKKHHYLRPSLFVYPEESLVIGSSTLFSALLIKCLEKEVAALCRYTPRRNIPPYFVALVPQEEELDDQKIQVTPPGFQLVFLPFADDKRKMPFTEKIMATPEQVGKMKAIVEKLRFTYRSDSFENPVLQQHFRNLEALALDLMEPEQAVDLTLPKVEAMNKRLGSLVDEFKELVYPPDYNPEGKVTKRKHDNEGSGSKRPKVEYSEEELKTHISKGTLGKFTVPMLKEACRAYGLKSGLKKQELLEALTKHFQD

>NP_005495.2

MKDCSNGCSAECTGEGGSKEVVGTFKAKDLIVTPATILKEKPDPNNLVFGTVFTDHMLTVEWSSEFGWEKPHIKPLQNLSLHPGSSALHYAVELFEGLKAFRGVDNKIRLFQPNLNMDRMYRSAVRATLPVFDKEELLECIQQLVKLDQEWVPYSTSASLYIRPTFIGTEPSLGVKKPTKALLFVLLSPVGPYFSSGTFNPVSLWANPKYVRAWKGGTGDCKMGGNYGSSLFAQCEAVDNGCQQVLWLYGEDHQITEVGTMNLFLYWINEDGEEELATPPLDGIILPGVTRRCILDLAHQWGEFKVSERYLTMDDLTTALEGNRVREMFGSGTACVVCPVSDILYKGETIHIPTMENGPKLASRILSKLTDIQYGREESDWTIVLS

1774 NP_056023.3 NP_003945.2

>NP_056023.3

MPKSGFTKPIQSENSDSDSNMVEKPYGRKSKDKIASYSKTPKIERSDVSKEMKEKSSMKRKLPFTISPSRNEERDSDTDSDPGHTSENWGERLISSYRTYSEKEGPEKKKTKKEAGNKKSTPVSILFGYPLSERKQMALLMQMTARDNSPDSTPNHPSQTTPAQKKTPSSSSRQKDKVNKRNERGETPLHMAAIRGDVKQVKELISLGANVNVKDFAGWTPLHEACNVGYYDVAKILIAAGADVNTQGLDDDTPLHDSASSGHRDIVKLLLRHGGNPFQANKHGERPVDVAETEELELLLKREVPLSDDDESYTDSEEAQSVNPSSVDENIDSETEKDSLICESKQILPSKTPLPSALDEYEFKDDDDEEINKMIDDRHILRKEQRKENEPEAEKTHLFAKQEKAFYPKSFKSKKQKPSRVLYSSTESSDEEALQNKKISTSCSVIPETSNSDMQTKKEYVVSGEHKQKGKVKRKLKNQNKNKENQELKQEKEGKENTRITNLTVNTGLDCSEKTREEGNFRKSFSPKDDTSLHLFHISTGKSPKHSCGLSEKQSTPLKQEHTKTCLSPGSSEMSLQPDLVRYDNTESEFLPESSSVKSCKHKEKSKHQKDFHLEFGEKSNAKIKDEDHSPTFENSDCTLKKMDKEGKTLKKHKLKHKEREKEKHKKEIEGEKEKYKTKDSAKELQRSVEFDREFWKENFFKSDETEDLFLNMEHESLTLEKKSKLEKNIKDDKSTKEKHVSKERNFKEERDKIKKESEKSFREEKIKDLKEERENIPTDKDSEFTSLGMSAIEESIGLHLVEKEIDIEKQEKHIKESKEKPEKRSQIKEKDIEKMERKTFEKEKKIKHEHKSEKDKLDLSECVDKIKEKDKLYSHHTEKCHKEGEKSKNTAAIKKTDDREKSREKMDRKHDKEKPEKERHLAESKEKHLMEKKNKQSDNSEYSKSEKGKNKEKDRELDKKEKSRDKESINITNSKHIQEEKKSSIVDGNKAQHEKPLSLKEKTKDEPLKTPDGKEKDKKDKDIDRYKERDKHKDKIQINSLLKLKSEADKPKPKSSPASKDTRPKEKRLVNDDLMQTSFERMLSLKDLEIEQWHKKHKEKIKQKEKERLRNRNCLELKIKDKEKTKHTPTESKNKELTRSKSSEVTDAYTKEKQPKDAVSNRSQSVDTKNVMTLGKSSFVSDNSLNRSPRSENEKPGLSSRSVSMISVASSEDSCHTTVTTPRPPVEYDSDFMLESSESQMSFSQSPFLSIAKSPALHERELDSLADLPERIKPPYANRLSTSHLRSSSVEDVKLIISEGRPTIEVRRCSMPSVICEHTKQFQTISEESNQGSLLTVPGDTSPSPKPEVFSNVPERDLSNVSNIHSSFATSPTGASNSKYVSADRNLIKNTAPVNTVMDSPVHLEPSSQVGVIQNKSWEMPVDRLETLSTRDFICPNSNIPDQESSLQSFCNSENKVLKENADFLSLRQTELPGNSCAQDPASFMPPQQPCSFPSQSLSDAESISKHMSLSYVANQEPGILQQKNAVQIISSALDTDNESTKDTENTFVLGDVQKTDAFVPVYSDSTIQEASPNFEKAYTLPVLPSEKDFNGSDASTQLNTHYAFSKLTYKSSSGHEVENSTTDTQVISHEKENKLESLVLTHLSRCDSDLCEMNAGMPKGNLNEQDPKHCPESEKCLLSIEDEESQQSILSSLENHSQQSTQPEMHKYGQLVKVELEENAEDDKTENQIPQRMTRNKANTMANQSKQILASCTLLSEKDSESSSPRGRIRLTEDDDPQIHHPRKRKVSRVPQPVQVSPSLLQAKEKTQQSLAAIVDSLKLDEIQPYSSERANPYFEYLHIRKKIEEKRKLLCSVIPQAPQYYDEYVTFNGSYLLDGNPLSKICIPTITPPPSLSDPLKELFRQQEVVRMKLRLQHSIEREKLIVSNEQEVLRVHYRAARTLANQTLPFSACTVLLDAEVYNVPLDSQSDDSKTSVRDRFNARQFMSWLQDVDDKFDKLKTCLLMRQQHEAAALNAVQRLEWQLKLQELDPATYKSISIYEIQEFYVPLVDVNDDFELTPI

>NP_003945.2

MAVMEMACPGAPGSAVGQQKELPKAKEKTPPLGKKQSSVYKLEAVEKSPVFCGKWEILNDVITKGTAKEGSEAGPAAISIIAQAECENSQEFSPTFSERIFIAGSKQYSQSESLDQIPNNVAHATEGKMARVCWKGKRRSKARKKRKKKSSKSLAHAGVALAKPLPRTPEQESCTIPVQEDESPLGAPYVRNTPQFTKPLKEPGLGQLCFKQLGEGLRPALPRSELHKLISPLQCLNHVWKLHHPQDGGPLPLPTHPFPYSRLPHPFPFHPLQPWKPHPLESFLGKLACVDSQKPLPDPHLSKLACVDSPKPLPGPHLEPSCLSRGAHEKFSVEEYLVHALQGSVSSGQAHSLTSLAKTWAARGSRSREPSPKTEDNEGVLLTEKLKPVDYEYREEVHWATHQLRLGRGSFGEVHRMEDKQTGFQCAVKKVRLEVFRAEELMACAGLTSPRIVPLYGAVREGPWVNIFMELLEGGSLGQLVKEQGCLPEDRALYYLGQALEGLEYLHSRRILHGDVKADNVLLSSDGSHAALCDFGHAVCLQPDGLGKSLLTGDYIPGTETHMAPEVVLGRSCDAKVDVWSSCCMMLHMLNGCHPWTQFFRGPLCLKIASEPPPVREIPPSCAPLTAQAIQEGLRKEPIHRVSAAELGGKVNRALQQVGGLKSPWRGEYKEPRHPPPNQANYHQTLHAQPRELSPRAPGPRPAEETTGRAPKLQPPLPPEPPEPNKSPPLTLSKEESGMWEPLPLSSLEPAPARNPSSPERKATVPEQELQQLEIELFLNSLSQPFSLEEQEQILSCLSIDSLSLSDDSEKNPSKASQSSRDTLSSGVHSWSSQAEARSSSWNMVLARGRPTDTPSYFNGVKVQIQSLNGEHLHIREFHRVKVGDIATGISSQIPAAAFSLVTKDGQPVRYDMEVPDSGIDLQCTLAPDGSFAWSWRVKHGQLENRP

1775 Q9BTC8 Q9P278

>Q9BTC8

MAANMYRVGDYVYFENSSSNPYLIRRIEELNKTASGNVEAKVVCFYRRRDISNTLIMLADKHAKEIEEESETTVEADLTDKQKHQLKHRELFLSRQYESLPATHIRGKCSVALLNETESVLSYLDKEDTFFYSLVYDPSLKTLLADKGEIRVGPRYQADIPEMLLEGESDEREQSKLEVKVWDPNSPLTDRQIDQFLVVARAVGTFARALDCSSSVRQPSLHMSAAAASRDITLFHAMDTLYRHSYDLSSAISVLVPLGGPVLCRDEMEEWSASEASLFEEALEKYGKDFNDIRQDFLPWKSLTSIIEYYYMWKTTDRYVQQKRLKAAEAESKLKQVYIPTYSKPNPNQISTSNGKPGAVNGAVGTTFQPQNPLLGRACESCYATQSHQWYSWGPPNMQCRLCAICWLYWKKYGGLKMPTQSEEEKLSPSPTTEDPRVRSHVSRQAMQGMPVRNTGSPKSAVKTRQAFFLHTTYFTKFARQVCKNTLRLRQAARRPFVAINYAAIRAEYADRHAELSGSPLKSKSTRKPLACIIGYLEIHPAKKPNVIRSTPSLQTPTTKRMLTTPNHTSLSILGKRNYSHHNGLDELTCCVSD

>Q9P278

MAPTLLQKLFNKRGSSGSSAAASAQGRAPKEGPAFSWSCSEFDLNEIRLIVYQDCDRRGRQVLFDSKAVQKIEEVTAQKTEDVPIKISAKCCQGSSSVSSSSSSSISSHSSSGGSSHHAKEQLPKYQYTRPASDVNMLGEMMFGSVAMSYKGSTLKIHYIRSPPQLMISKVFSARMGSFCGSTNNLQDSFEYINQDPNLGKLNTNQNSLGPCRTGSNLAHSTPVDMPSRGQNEDRDSGIARSASLSSLLITPFPSPSSSTSSSSSYQRRWLRSQTTSLENGIIPRRSTDETFSLAEETCSSNPAMVRRKKIAISIIFSLCEKEEAQRNFQDFFFSHFPLFESHMNRLKSAIEKAMISCRKIAESSLRVQFYVSRLMEALGEFRGTIWNLYSVPRIAEPVWLTMMSGTLEKNQLCQRFLKEFTLLIEQINKNQFFAALLTAVLTYHLAWVPTVMPVDHPPIKAFSEKRTSQSVNMLAKTHPYNPLWAQLGDLYGAIGSPVRLTRTVVVGKQKDLVQRILYVLTYFLRCSELQENQLTWSGNHGEGDQVLNGSKIITALEKGEVEESEYVVITVRNEPALVPPILPPTAAERHNPWPTGFPECPEGTDSRDLGLKPDKEANRRPEQGSEACSAGCLGPASDASWKPQNAFCGDEKNKEAPQDGSSRLPSCEVLGAGMKMDQQAVCELLKVEMPTRLPDRSVAWPCPDRHLREKPSLEKVTFQIGSFASPESDFESRMKKMEERVKACGPSLEASEAADVAQDPQVSRSPFKPGFQENVCCPQNRLSEGDEGESDKGFAEDRGSRNDMAADIAGQLSHAADLGTASHGAGGTGGRRLEATRGLYVKAAEGPVLEPVAPRCVQRGPGLVAGANIPCGDDNKKANFRTEGDIPRNESSDSALGDSDDEACASAMLDLGHGGDRTGGSLEVELPLPRSQSISTQNVRNFGRSLLAGYCPTYMPDLVLHGTGSDEKLKQCLVADLVHTVHHPVLDEPIAEAVCIIADTDKWSVQVATSQRKVTDNMKLGQDVLVSSQVSSLLQSILQLYKLHLPADFCIMHLEDRLQEMYLKSKMLSEYLRGHTRVHVKELGVVLGIESNDLPLLTAIASTHSPYVAQILL

1776 Q9BWT3 Q9UGK3

>Q9BWT3

MKEMSANTVLDSQRQQKHYGITSPISLASPKEIDHIYTQKLIDAMKPFGVFEDEEELNHRLVVLGKLNNLVKEWISDVSESKNLPPSVVATVGGKIFTFGSYRLGVHTKGADIDALCVAPRHVERSDFFQSFFEKLKHQDGIRNLRAVEDAFVPVIKFEFDGIEIDLVFARLAIQTISDNLDLRDDSRLRSLDIRCIRSLNGCRVTDEILHLVPNKETFRLTLRAVKLWAKRRGIYSNMLGFLGGVSWAMLVARTCQLYPNAAASTLVHKFFLVFSKWEWPNPVLLKQPEESNLNLPVWDPRVNPSDRYHLMPIITPAYPQQNSTYNVSTSTRTVMVEEFKQGLAVTDEILQGKSDWSKLLEPPNFFQKYRHYIVLTASASTEENHLEWVGLVESKIRVLVGNLERNEFITLAHVNPQSFPGNKEHHKDNNYVSMWFLGIIFRRVENAESVNIDLTYDIQSFTDTVYRQANNINMLKEGMKIEATHVKKKQLHHYLPAEILQKKKKQSLSDVNRSSGGLQSKRLSLDSSCLDSSRDTDNGTPFNSPASKSDSPSVGETERNSAEPAAVIVEKPLSVPPAQGLSIPVIGAKVDSTVKTVSPPTVCTIPTVVGRNVIPRITTPHNPAQGQPHLNGMSNITKTVTPKRSHSPSIDGTPKRLKDVEKFIRLESTFKDPRTAEERKRKSVDAIGGESMPIPTIDTSRKKRLPSKELPDSSSPVPANNIRVIKNSIRLTLNR

>Q9UGK3

MASALRPPRVPKPKGVLPSHYYESFLEKKGPCDRDYKKFWAGLQGLTIYFYNSNRDFQHVEKLNLGAFEKLTDEIPWGSSRDPGTHFSLILRDQEIKFKVETLECREMWKGFILTVVELRVPTDLTLLPGHLYMMSEVLAKEEARRALETPSCFLKVSRLEAQLLLERYPECGNLLLRPSGDGADGVSVTTRQMHNGTHVVRHYKVKREGPKYVIDVEQPFSCTSLDAVVNYFVSHTKKALVPFLLDEDYEKVLGYVEADKENGENVWVAPSAPGPGPAPCTGGPKPLSPASSQDKLPPLPPLPNQEENYVTPIGDGPAVDYENQDVASSSWPVILKPKKLPKPPAKLPKPPVGPKPEPKVFNGGLGRKLPVSSAQPLFPTAGLADMTAELQKKLEKRRALEH

1777 Q9UEE5 NP_000845.1

>Q9UEE5

MIPLEKPGSGGSSPGATSGSGRAGRGLSGPCRPPPPPQARGLLTEIRAVVRTEPFQDGYSLCPGRELGRGKFAVVRKCIKKDSGKEFAAKFMRKRRKGQDCRMEIIHEIAVLELAQDNPWVINLHEVYETASEMILVLEYAAGGEIFDQCVADREEAFKEKDVQRLMRQILEGVHFLHTRDVVHLDLKPQNILLTSESPLGDIKIVDFGLSRILKNSEELREIMGTPEYVAPEILSYDPISMATDMWSIGVLTYVMLTGISPFLGNDKQETFLNISQMNLSYSEEEFDVLSESAVDFIRTLLVKKPEDRATAEECLKHPWLTQSSIQEPSFRMEKALEEANALQEGHSVPEINSDTDKSETEESIVTEELIVVTSYTLGQCRQSEKEKMEQKAISKRFKFEEPLLQEIPGEFIY

>NP_000845.1

MGLELFLDLVSQPSRAVYIFAKKNGIPLELRTVDLVKGQHKSKEFLQINSLGKLPTLKDGDFILTESSAILIYLSCKYQTPDHWYPSDLQARARVHEYLGWHADCIRGTFGIPLWVQVLGPLIGVQVPEEKVERNRTAMDQALQWLEDKFLGDRPFLAGQQVTLADLMALEELMQPVALGYELFEGRPRLAAWRGRVEAFLGAELCQEAHSIILSILEQAAKKTLPTPSPEAYQAMLLRIARIP

1778 NP_003503.1 NP_005411.1

>NP_003503.1

MSGRGKQGGKARAKAKSRSSRAGLQFPVGRVHRLLRKGNYAERVGAGAPVYLAAVLEYLTAEILELAGNAARDNKKTRIIPRHLQLAIRNDEELNKLLGRVTIAQGGVLPNIQAVLLPKKTESHHKAKGK

>NP_005411.1

MNSELDYYEKFEEVHGILMYKDFVKYWDNVEAFQARPDDLVIATYPKSGTTWVSEIVYMIYKEGDVEKCKEDVIFNRIPFLECRKENLMNGVKQLDEMNSPRIVKTHLPPELLPASFWEKDCKIIYLCRNAKDVAVSFYYFFLMVAGHPNPGSFPEFVEKFMQGQVPYGSWYKHVKSWWEKGKSPRVLFLFYEDLKEDIRKEVIKLIHFLERKPSEELVDRIIHHTSFQEMKNNPSTNYTTLPDEIMNQKLSPFMRKGITGDWKNHFTVALNEKFDKHYEQQMKESTLKFRTEI

1779 NP_005490.1 NP_003115.1

>NP_005490.1

MALSRGLPRELAEAVAGGRVLVVGAGGIGCELLKNLVLTGFSHIDLIDLDTIDVSNLNRQFLFQKKHVGRSKAQVAKESVLQFYPKANIVAYHDSIMNPDYNVEFFRQFILVMNALDNRAARNHVNRMCLAADVPLIESGTAGYLGQVTTIKKGVTECYECHPKPTQRTFPGCTIRNTPSEPIHCIVWAKYLFNQLFGEEDADQEVSPDRADPEAAWEPTEAEARARASNEDGDIKRISTKEWAKSTGYDPVKLFTKLFKDDIRYLLTMDKLWRKRKPPVPLDWAEVQSQGEETNASDQQNEPQLGLKDQQVLDVKSYARLFSKSIETLRVHLAEKGDGAELIWDKDDPSAMDFVTSAANLRMHIFSMNMKSRFDIKSMAGNIIPAIATTNAVIAGLIVLEGLKILSGKIDQCRTIFLNKQPNPRKKLLVPCALDPPNPNCYVCASKPEVTVRLNVHKVTVLTLQDKIVKEKFAMVAPDVQIEDGKGTILISSEEGETEANNHKKLSEFGIRNGSRLQADDFLQDYTLLINILHSEDLGKDVEFEVVGDAPEKVGPKQAEDAAKSITNGSDDGAQPSTSTAQEQDDVLIVDSDEEDSSNNADVSEEERSRKRKLDEKENLSAKRSRIEQKEELDDVIALD

>NP_003115.1

MEGGLGRAVCLLTGASRGFGRTLAPLLASLLSPGSVLVLSARNDEALRQLEAELGAERSGLRVVRVPADLGAEAGLQQLLGALRELPRPKGLQRLLLINNAGSLGDVSKGFVDLSDSTQVNNYWALNLTSMLCLTSSVLKAFPDSPGLNRTVVNISSLCALQPFKGWALYCAGKAARDMLFQVLALEEPNVRVLNYAPGPLDTDMQQLARETSVDPDMRKGLQELKAKGKLVDCKVSAQKLLSLLEKDEFKSGAHVDFYDK

1780 NP_004070.3 Q58A44

>NP_004070.3

MKRLVCVLLVCSSAVAQLHKDPTLDHHWHLWKKTYGKQYKEKNEEAVRRLIWEKNLKFVMLHNLEHSMGMHSYDLGMNHLGDMTSEEVMSLMSSLRVPSQWQRNITYKSNPNRILPDSVDWREKGCVTEVKYQGSCGACWAFSAVGALEAQLKLKTGKLVSLSAQNLVDCSTEKYGNKGCNGGFMTTAFQYIIDNKGIDSDASYPYKAMDQKCQYDSKYRAATCSKYTELPYGREDVLKEAVANKGPVSVGVDARHPSFFLYRSGVYYEPSCTQNVNHGVLVVGYGDLNGKEYWLVKNSWGHNFGEEGYIRMARNKGNHCGIASFPSYPEI

>Q58A44

MWILSNLMGTSEEGNLLSTVSPTVKALFGKTRVSPIFPFSPRSPFQPLIPRTPGSPWGPVGPASPLGPGFPIGPMGPGKPVGPKGPMLPLGPSGPVGPTSPLFPFCP

1781 NP_006512.2 NP_004865.1

>NP_006512.2

MSHAAEPARDGVEASAEGPRAVFVLLEERRPADSAQLLSLNSLLPESGIVADIELENVLDPDSFYELKSQPLPLRSSLPISLQATPATPATLSASSSAGGSRTPAMSSSSSSRVLLRQQLMRAQAQEQERRERREQAAAAPFPSPAPASPAISVVGVSAGGHTLSRPPPAQVPREVLKVQTHLENPTRYHLQQARRQQVKQYLSTTLGPKLASQALTPPPGPASAQPLPAPEAAHTTGPTGSAPNSPMALLTIGSSSEKEIDDVIDEIISLESSYNDEMLSYLPGGTTGLQLPSTLPVSGNLLDVYSSQGVATPAITVSNSCPAELPNIKREISETEAKALLKERQKKDNHNLIERRRRFNINDRIKELGTLIPKSSDPEMRWNKGTILKASVDYIRKLQKEQQRSKDLESRQRSLEQANRSLQLRIQELELQAQIHGLPVPPTPGLLSLATTSASDSLKPEQLDIEEEGRPGAATFHVGGGPAQNAPHQQPPAPPSDALLDLHFPSDHLGDLGDPFHLGLEDILMEEEEGVVGGLSGGALSPLRAASDPLLSSVSPAVSKASSRRSSFSMEEES

>NP_004865.1

MSALRRSGYGPSDGPSYGRYYGPGGGDVPVHPPPPLYPLRPEPPQPPISWRVRGGGPAETTWLGEGGGGDGYYPSGGAWPEPGRAGGSHQEQPPYPSYNSNYWNSTARSRAPYPSTYPVRPELQGQSLNSYTNGAYGPTYPPGPGANTASYSGAYYAPGYTQTSYSTEVPSTYRSSGNSPTPVSRWIYPQQDCQTEAPPLRGQVPGYPPSQNPGMTLPHYPYGDGNRSVPQSGPTVRPQEDAWASPGAYGMGGRYPWPSSAPSAPPGNLYMTESTSPWPSSGSPQSPPSPPVQQPKDSSYPYSQSDQSMNRHNFPCSVHQYESSGTVNNDDSDLLDSQVQYSAEPQLYGNATSDHPNNQDQSSSLPEECVPSDESTPPSIKKIIHVLEKVQYLEQEVEEFVGKKTDKAYWLLEEMLTKELLELDSVETGGQDSVRQARKEAVCKIQAILEKLEKKGL

1782 NP_055136.1 Q06278

>NP_055136.1

MGSELIGRLAPRLGLAEPDMLRKAEEYLRLSRVKCVGLSARTTETSSAVMCLDLAASWMKCPLDRAYLIKLSGLNKETYQSCLKSFECLLGLNSNIGIRDLAVQFSCIEAVNMASKILKSYESSLPQTQQVDLDLSRPLFTSAALLSACKILKLKVDKNKMVATSGVKKAIFDRLCKQLEKIGQQVDREPGDVATPPRKRKKIVVEAPAKEMEKVEEMPHKPQKDEDLTQDYEEWKRKILENAASAQKATAE

>Q06278

MDRASELLFYVNGRKVIEKNVDPETMLLPYLRKKLRLTGTKYGCGGGGCGACTVMISRYNPITKRIRHHPANACLIPICSLYGAAVTTVEGIGSTHTRIHPVQERIAKCHGTQCGFCTPGMVMSIYTLLRNHPEPTLDQLTDALGGNLCRCTGYRPIIDACKTFCKTSGCCQSKENGVCCLDQGINGLPEFEEGSKTSPKLFAEEEFLPLDPTQELIFPPELMIMAEKQSQRTRVFGSERMMWFSPVTLKELLEFKFKYPQAPVIMGNTSVGPEVKFKGVFHPVIISPDRIEELSVVNHAYNGLTLGAGLSLAQVKDILADVVQKLPEEKTQMYHALLKHLGTLAGSQIRNMASLGGHIISRHPDSDLNPILAVGNCTLNLLSKEGKRQIPLNEQFLSKCPNADLKPQEILVSVNIPYSRKWEFVSAFRQAQRQENALAIVNSGMRVFFGEGDGIIRELCISYGGVGPATICAKNSCQKLIGRHWNEQMLDIACRLILNEVSLLGSAPGGKVEFKRTLIISFLFKFYLEVSQILKKMDPVHYPSLADKYESALEDLHSKHHCSTLKYQNIGPKQHPEDPIGHPIMHLSGVKHATGEAIYCDDMPLVDQELFLTFVTSSRAHAKIVSIDLSEALSMPGVVDIMTAEHLSDVNSFCFFTEAEKFLATDKVFCVGQLVCAVLADSEVQAKRAAKRVKIVYQDLEPLILTIEESIQHNSSFKPERKLEYGNVDEAFKVVDQILEGEIHMGGQEHFYMETQSMLVVPKGEDQEMDVYVSTQFPKYIQDIVASTLKLPANKVMCHVRRVGGAFGGKVLKTGIIAAVTAFAANKHGRAVRCVLERGEDMLITGGRHPYLGKYKAGFMNDGRILALDMEHYSNAGASLDESLFVIEMGLLKMDNAYKFPNLRCRGWACRTNLPSNTAFRGFGFPQAALITESCITEVAAKCGLSPEKVRIINMYKEIDQTPYKQEINAKNLIQCWRECMAMSSYSLRKVAVEKFNAENYWKKKGLAMVPLKFPVGLGSRAAGQAAALVHIYLDGSVLVTHGGIEMGQGVHTKMIQVVSRELRMPMSNVHLRGTSTETVPNANISGGSVVADLNGLAVKDACQTLLKRLEPIISKNPKGTWKDWAQTAFDESINLSAVGYFRGYESDMNWEKGEGQPFEYFVYGAACSEVEIDCLTGDHKNIRTDIVMDVGCSINPAIDIGQIEGAFIQGMGLYTIEELNYSPQGILHTRGPDQYKIPAICDMPTELHIALLPPSQNSNTLYSSKGLGESGVFLGCSVFFAIHDAVSAARQERGLHGPLTLNSPLTPEKIRMACEDKFTKMIPRDEPGSYVPWNVPI

1783 Q9UKI9 NP_005403.2

>Q9UKI9

MVNLESMHTDIKMSGDVADSTDARSTLSQVEPGNDRNGLDFNRQIKTEDLSDSLQQTLSHRPCHLSQGPAMMSGNQMSGLNASPCQDMASLHPLQQLVLVPGHLQSVSQFLLSQTQPGQQGLQPNLLPFPQQQSGLLLPQTGPGLASQAFGRPGLPGSSLEPHLEASQHLPVPKHLPSSGGADEPSDLEELEKFAKTFKQRRIKLGFTQGDVGLAMGKLYGNDFSQTTISRFEALNLSFKNMCKLKPLLEKWLNDAESSPSDPSVSTPSSYPSLSEVFGRKRKKRTSIETNIRLTLEKRFQDNPKPSSEEISMIAEQLSMEKEVVRVWFCNRRQKEKRINCPVATPIKPPVYNSRLVSPSGSLGPLSVPPVHSTMPGTVTSSCSPGNNSRPSSPGSGLHASSPTASQNNSKAAVNSASSFNSSGSWYRWNHSTYLH

>NP_005403.2

MLYFSLFWAARPLQRCGQLVRMAIRAQHSNAAQTQTGEANRGWTGQESLSDSDPEMWELLQREKDRQCRGLELIASENFCSRAALEALGSCLNNKYSEGYPGKRYYGGAEVVDEIELLCQRRALEAFDLDPAQWGVNVQPYSGSPANLAVYTALLQPHDRIMGLDLPDGGHLTHGYMSDVKRISATSIFFESMPYKLNPKTGLIDYNQLALTARLFRPRLIIAGTSAYARLIDYARMREVCDEVKAHLLADMAHISGLVAAKVIPSPFKHADIVTTTTHKTLRGARSGLIFYRKGVKAVDPKTGREIPYTFEDRINFAVFPSLQGGPHNHAIAAVAVALKQACTPMFREYSLQVLKNARAMADALLERGYSLVSGGTDNHLVLVDLRPKGLDGARAERVLELVSITANKNTCPGDRSAITPGGLRLGAPALTSRQFREDDFRRVVDFIDEGVNIGLEVKSKTAKLQDFKSFLLKDSETSQRLANLRQRVEQFARAFPMPGFDEH

1784 NP_003211.1 Q8N183

>NP_003211.1

MLWKLTDNIKYEDCEDRHDGTSNGTARLPQLGTVGQSPYTSAPPLSHTPNADFQPPYFPPPYQPIYPQSQDPYSHVNDPYSLNPLHAQPQPQHPGWPGQRQSQESGLLHTHRGLPHQLSGLDPRRDYRRHEDLLHGPHALSSGLGDLSIHSLPHAIEEVPHVEDPGINIPDQTVIKKGPVSLSKSNSNAVSAIPINKDNLFGGVVNPNEVFCSVPGRLSLLSSTSKYKVTVAEVQRRLSPPECLNASLLGGVLRRAKSKNGGRSLREKLDKIGLNLPAGRRKAANVTLLTSLVEGEAVHLARDFGYVCETEFPAKAVAEFLNRQHSDPNEQVTRKNMLLATKQICKEFTDLLAQDRSPLGNSRPNPILEPGIQSCLTHFNLISHGFGSPAVCAAVTALQNYLTEALKAMDKMYLSNNPNSHTDNNAKSSDKEEKHRK

>Q8N183

MGWSQDLFRALWRSLSREVKEHVGTDQFGNKYYYIPQYKNWRGQTIREKRIVEAANKKEVDYEAGDIPTEWEAWIRRTRKTPPTMEEILKNEKHREEIKIKSQDFYEKEKLLSKETSEELLPPPVQTQIKGHASAPYFGKEEPSVAPSSTGKTFQPGSWMPRDGKSHNQ

1785 NP_076920.1 Q9UPP2

>NP_076920.1

MAMSSFLINSNYVDPKFPPCEEYSQSDYLPSDHSPGYYAGGQRRESSFQPEAGFGRRAACTVQRYAACRDPGPPPPPPPPPPPPPPPGLSPRAPAPPPAGALLPEPGQRCEAVSSSPPPPPCAQNPLHPSPSHSACKEPVVYPWMRKVHVSTVNPNYAGGEPKRSRTAYTRQQVLELEKEFHYNRYLTRRRRVEIAHALCLSERQIKIWFQNRRMKWKKDHKLPNTKIRSGGAAGSAGGPPGRPNGGPRAL

>Q9UPP2

MESLLENPVRAVLYLKELTAIVQNQQSLIHTQRERIDELERRLDELSAENRSLWEHQQLLQAQPPPGLVPPSSAPLPAAPATAPAAAARAQEPLQDQGQRSAAAPHPAPDRPPRQHHGQLLEQPQRGPGSRAHTPQSPHKHLGTQGAVTDKEKERPPSCCAAAGALLQHKSPSALGKGVLSRRPENETVLHQFCCPAADACSDLASQSDGSCTQAGGGMEDSVVAAAAVAAGRPSAHAPKAQAQELQEEEERPGAGAASPRAGPQHKASPGRQQPALATALCPHAPAASDYELSLDLKNKQIEMLEHKYGGHLVSRRAACTIQTAFRQYQLSKNFEKIRNSLLESRLPRRISLRKVRSPTAESLAAEKALMEGYGLVGLPLVRSPSLPPTFAGTLTELEDSFTEQVQSLAKSIDDALSTWSLKTMCSLRESGAYQLHQALQAAAGPPGLEAEGRAPESAGPGPGDDAAETPGLPPAHSGTLMMAFRDVTVQIANQNISVSSSTALSVANCLGAQTVQAPAEPAAGKAEQGETSGREAPEAPAVGREDASAEDSCAEAAASGAADGATAPKTEEEEEEEETAEVGRGAEAEAGDLEQLSSSSTSTKSAKSGSEASASASKDALQAMILSLPRYHCENPASCKSPTLSTDTLRKRLYRIGLNLFNINPDKGIQFLISRGFIPDTPIGVAHFLLQRKGLSRQMIGEFLGNSKKQFNRDVLDCVVDEMDFSSMELDEALRKFQAHIRVQGEAQKVERLIEAFSQRYCMCNPEVVQQFHNPDTIFILAFAIILLNTDMYSPNIKPDRKMMLEDFIRNLRGVDDGADIPRELVVGIYERIQQKELKSNEDHVTYVTKVEKSIVGMKTVLSVPHRRLVCCSRLFEVTDVNKLQKQAAHQREVFLFNDLLVILKLCPKKKSSSTYTFCKSVGLLGMQFQLFENEYYSHGITLVTPLSGSEKKQVLHFCALGSDEMQKFVEDLKESIAEVTELEQIRIEWELEKQQGTKTLSFKPCGAQGDPQSKQGSPTAKREAALRERPAESTVEVSIHNRLQTSQHNSGLGAERGAPVPPPDLQPSPPRQQTPPLPPPPPTPPGTLVQCQQIVKVIVLDKPCLARMEPLLSQALSCYTSSSSDSCGSTPLGGPGSPVKVTHQPPLPPPPPPYNHPHQFCPPGSLLHGHRYSSGSRSLV

1786 Q86Y25 NP_065078.1

>Q86Y25

MAVDLLSAQEPVTFRDVAVFFSQDEWLHLDSAQRALYREVMLENYSSLVSLGIPFSMPKLIHQLQQGEDPCMVEREVPSDTRLGFKTWLETEALPHRQDIFIEETSQGMVKKESIKDGHWDINFEEAVEFESEIEEEQEKKPLRQMIDSHEKTISEDGNHTSLELGKSLFTNTALVTQQSVPIERIPNMYYTFGKDFKQNFDLMKCFQIYPGGKPHICNECGKSFKQNLHLIEHQRIHTGEKPYKCNECEKTFSHRSSLLSHQRIHTGEKPYKCNECEKAFSNSSTLIKHLRVHTGEKPYRCRECGKAFSQCSTLTVHQRIHTGEKLYKCGECEKAFNCRAKLHRHQRIHTGEKPYKCSECGKGYSQFTSLAEHQRFHTGEQLYTCLECGRTFTRIVTLIEHQRIHTGQKPYQCNECEKAFNQYSSFNEHRKIHTGEKLYTCEECGKAFGCKSNLYRHQRIHTGEKPYQCNQCGKAFSQYSFLTEHERIHTGEKLYKCMECGKAYSYRSNLCRHKKVHTKEKLYKWKEYGKPFICSSSLTQYQRFFKGDKAYEV

>NP_065078.1

MGKGCKVVVCGLLSVGKTAILEQLLYGNHTIGMEDCETMEDVYMASVETDRGVKEQLHLYDTRGLQEGVELPKHYFSFADGFVLVYSVNNLESFQRVELLKKEIDKFKDKKEVAIVVLGNKIDLSEQRQVDAEVAQQWAKSEKVRLWEVTVTDRKTLIEPFTLLASKLSQPQSKSSFPLPGRKNKGNSNSEN

1787 NP_001417.3 NP_056053.1

>NP_001417.3

MEEQQPEPKSQRDSALGAAAAATPGGLSLSLSPGASGSSGSGSDGDSVPVSPQPAPPSPPAAPCLPPLAHHPHLPPHPPPPPPQHLAAPAHQPQPAAQLHRTTNFFIDNILRPDFGCKKEQPPPQLLVAAAARGGAGGGGRVERDRGQTAAGRDPVHPLGTRAPGAASLLCAPDANCGPPDGSQPAAAGAGASKAGNPAAAAAAAAAAVAAAAAAAAAKPSDTGGGGSGGGAGSPGAQGTKYPEHGNPAILLMGSANGGPVVKTDSQQPLVWPAWVYCTRYSDRPSSGPRTRKLKKKKNEKEDKRPRTAFTAEQLQRLKAEFQANRYITEQRRQTLAQELSLNESQIKIWFQNKRAKIKKATGIKNGLALHLMAQGLYNHSTTTVQDKDESE

>NP_056053.1

MPRPELPLPEGWEEARDFDGKVYYIDHTNRTTSWIDPRDRYTKPLTFADCISDELPLGWEEAYDPQVGDYFIDHNTKTTQIEDPRVQWRREQEHMLKDYLVVAQEALSAQKEIYQVKQQRLELAQQEYQQLHAVWEHKLGSQVSLVSGSSSSSKYDPEILKAEIATAKSRVNKLKREMVHLQHELQFKERGFQTLKKIDKKMSDAQGSYKLDEAQAVLRETKAIKKAITCGEKEKQDLIKSLAMLKDGFRTDRGSHSDLWSSSSSLESSSFPLPKQYLDVSSQTDISGSFGINSNNQLAEKVRLRLRYEEAKRRIANLKIQLAKLDSEAWPGVLDSERDRLILINEKEELLKEMRFISPRKWTQGEVEQLEMARKRLEKDLQAARDTQSKALTERLKLNSKRNQLVRELEEATRQVATLHSQLKSLSSSMQSLSSGSSPGSLTSSRGSLVASSLDSSTSASFTDLYYDPFEQLDSELQSKVEFLLLEGATGFRPSGCITTIHEDEVAKTQKAEGGGRLQALRSLSGTPKSMTSLSPRSSLSSPSPPCSPLMADPLLAGDAFLNSLEFEDPELSATLCELSLGNSAQERYRLEEPGTEGKQLGQAVNTAQGCGLKVACVSAAVSDESVAGDSGVYEASVQRLGASEAAAFDSDESEAVGATRIQIALKYDEKNKQFAILIIQLSNLSALLQQQDQKVNIRVAVLPCSESTTCLFRTRPLDASDTLVFNEVFWVSMSYPALHQKTLRVDVCTTDRSHLEECLGGAQISLAEVCRSGERSTRWYNLLSYKYLKKQSRELKPVGVMAPASGPASTDAVSALLEQTAVELEKRQEGRSSTQTLEDSWRYEETSENEAVAEEEEEEVEEEEGEEDVFTEKASPDMDGYPALKVDKETNTETPAPSPTVVRPKDRRVGTPSQGPFLRGSTIIRSKTFSPGPQSQYVCRLNRSDSDSSTLSKKPPFVRNSLERRSVRMKRPSSVKSLRSERLIRTSLDLELDLQATRTWHSQLTQEISVLKELKEQLEQAKSHGEKELPQWLREDERFRLLLRMLEKRQMDRAEHKGELQTDKMMRAAAKDVHRLRGQSCKEPPEVQSFREKMAFFTRPRMNIPALSADDV

1788 NP_006707.1 NP_000365.3

>NP_006707.1

MNSGAMRIHSKGHFQGGIQVKNEKNRPSLKSLKTDNRPEKSKCKPLWGKVFYLDLPSVTISEKLQKDIKDLGGRVEEFLSKDISYLISNKKEAKFAQTLGRISPVPSPESAYTAETTSPHPSHDGSSFKSPDTVCLSRGKLLVEKAIKDHDFIPSNSILSNALSWGVKILHIDDIRYYIEQKKKELYLLKKSSTSVRDGGKRVGSGAQKTRTGRLKKPFVKVEDMSQLYRPFYLQLTNMPFINYSIQKPCSPFDVDKPSSMQKQTQVKLRIQTDGDKYGGTSIQLQLKEKKKKGYCECCLQKYEDLETHLLSEQHRNFAQSNQYQVVDDIVSKLVFDFVEYEKDTPKKKRIKYSVGSLSPVSASVLKKTEQKEKVELQHISQKDCQEDDTTVKEQNFLYKETQETEKKLLFISEPIPHPSNELRGLNEKMSNKCSMLSTAEDDIRQNFTQLPLHKNKQECILDISEHTLSENDLEELRVDHYKCNIQASVHVSDFSTDNSGSQPKQKSDTVLFPAKDLKEKDLHSIFTHDSGLITINSSQEHLTVQAKAPFHTPPEEPNECDFKNMDSLPSGKIHRKVKIILGRNRKENLEPNAEFDKRTEFITQEENRICSSPVQSLLDLFQTSEEKSEFLGFTSYTEKSGICNVLDIWEEENSDNLLTAFFSSPSTSTFTGF

>NP_000365.3

MEANGLGPQGFPELKNDTFLRAAWGEETDYTPVWCMRQAGRYLPEFRETRAAQDFFSTCRSPEACCELTLQPLRRFPLDAAIIFSDILVVPQALGMEVTMVPGKGPSFPEPLREEQDLERLRDPEVVASELGYVFQAITLTRQRLAGRVPLIGFAGAPWTLMTYMVEGGGSSTMAQAKRWLYQRPQASHQLLRILTDALVPYLVGQVVAGAQALQLFESHAGHLGPQLFNKFALPYIRDVAKQVKARLREAGLAPVPMIIFAKDGHFALEELAQAGYEVVGLDWTVAPKKARECVGKTVTLQGNLDPCALYASEEEIGQLVKQMLDDFGPHRYIANLGHGLYPDMDPEHVGAFVDAVHKHSRLLRQN

1789 NP_066403.2 Q16540

>NP_066403.2

MARTKQTARKSTGGKAPRKQLATKAARKSAPATGGVKKPHRYRPGTVALREIRRYQKSTELLIRKLPFQRLVREIAQDFKTDLRFQSSAVMALQEASEAYLVGLFEDTNLCAIHAKRVTIMPKDIQLARRIRGERA

>Q16540

MARNVVYPLYRLGGPQLRVFRTNFFIQLVRPGVAQPEDTVQFRIPMEMTRVDLRNYLEGIYNVPVAAVRTRVQHGSNKRRDHRNVRIKKPDYKVAYVQLAHGQTFTFPDLFPEKDESPEGSAADDLYSMLEEERQQRQSSDPRRGGVPSWFGL

1790 NP_003933.1 Q8TE04

>NP_003933.1

MGDEDDDESCAVELRITEANLTGHEEKVSVENFELLKVLGTGAYGKVFLVRKAGGHDAGKLYAMKVLRKAALVQRAKTQEHTRTERSVLELVRQAPFLVTLHYAFQTDAKLHLILDYVSGGEMFTHLYQRQYFKEAEVRVYGGEIVLALEHLHKLGIIYRDLKLENVLLDSEGHIVLTDFGLSKEFLTEEKERTFSFCGTIEYMAPEIIRSKTGHGKAVDWWSLGILLFELLTGASPFTLEGERNTQAEVSRRILKCSPPFPPRIGPVAQDLLQRLLCKDPKKRLGAGPQGAQEVRNHPFFQGLDWVALAARKIPAPFRPQIRSELDVGNFAEEFTRLEPVYSPPGSPPPGDPRIFQGYSFVAPSILFDHNNAVMTDGLEAPGAGDRPGRAAVARSAMMQDSPFFQQYELDLREPALGQGSFSVCRRCRQRQSGQEFAVKILSRRLEANTQREVAALRLCQSHPNVVNLHEVHHDQLHTYLVLELLRGGELLEHIRKKRHFSESEASQILRSLVSAVSFMHEEAGVVHRDLKPENILYADDTPGAPVKIIDFGFARLRPQSPGVPMQTPCFTLQYAAPELLAQQGYDESCDLWSLGVILYMMLSGQVPFQGASGQGGQSQAAEIMCKIREGRFSLDGEAWQGVSEEAKELVRGLLTVDPAKRLKLEGLRGSSWLQDGSARSSPPLRTPDVLESSGPAVRSGLNATFMAFNRGKREGFFLKSVENAPLAKRRKQKLRSATASRRGSPAPANPGRAPVASKGAPRRANGPLPPS

>Q8TE04

MLKLVGGGGGQDWACSVAGTSLGGEEAAFEVARPGDQGKAGGGSPGWGCAGIPDSAPGAGVLQAGAVGPARGGQGAEEVGESAGGGEERRVRHPQAPALRLLNRKPQGGSGEIKTPENDLQRGRLSRGPRTAPPAPGMGDRSGQQERSVPHSPGAPVGTSAAAVNGLLHNGFHPPPVQPPHVCSRGPVGGSDAAPQRLPLLPELQPQPLLPQHDSPAKKCRLRRRMDSGRKNRPPFPWFGMDIGGTLVKLVYFEPKDITAEEEQEEVENLKSIRKYLTSNTAYGKTGIRDVHLELKNLTMCGRKGNLHFIRFPSCAMHRFIQMGSEKNFSSLHTTLCATGGGAFKFEEDFRMIADLQLHKLDELDCLIQGLLYVDSVGFNGKPECYYFENPTNPELCQKKPYCLDNPYPMLLVNMGSGVSILAVYSKDNYKRVTGTSLGGGTFLGLCCLLTGCETFEEALEMAAKGDSTNVDKLVKDIYGGDYERFGLQGSAVASSFGNMMSKEKRDSISKEDLARATLVTITNNIGSIARMCALNENIDRVVFVGNFLRINMVSMKLLAYAMDFWSKGQLKALFLEHEGYFGAVGALLELFKMTDDK

1791 Q9GZZ0 NP_996895.1

>Q9GZZ0

MSSYLEYVSCSSSGGVGGDVLSLAPKFCRSDARPVALQPAFPLGNGDGAFVSCLPLAAARPSPSPPAAPARPSVPPPAAPQYAQCTLEGAYEPGAAPAAAAGGADYGFLGSGPAYDFPGVLGRAADDGGSHVHYATSAVFSGGGSFLLSGQVDYAAFGEPGPFPACLKASADGHPGAFQTASPAPGTYPKSVSPASGLPAAFSTFEWMKVKRNASKKGKLAEYGAASPSSAIRTNFSTKQLTELEKEFHFNKYLTRARRIEIANCLHLNDTQVKIWFQNRRMKQKKREREGLLATAIPVAPLQLPLSGTTPTKFIKNPGSPSQSQEPS

>NP_996895.1

MIHSLFLINCSGDIFLEKHWKSVVSQSVCDYFFEAQEKAADVENVPPVISTPHHYLISIYRDKLFFVSVIQTEVPPLFVIEFLHRVADTFQDYFGECSEAAIKDNVVIVYELLEEMLDNGFPLATESNILKELIKPPTILRSVVNSITGSSNVGDTLPTGQLSNIPWRRAGVKYTNNEAYFDVVEEIDAIIDKSGSTVFAEIQGVIDACIKLSGMPDLSLSFMNPRLLDDVSFHPCIRFKRWESERVLSFIPPDGNFRLISYRVSSQNLVAIPVYVKHSISFKENSSCGRFDITIGPKQNMGKTIEGITVTVHMPKVVLNMNLTPTQGSYTFDPVTKVLTWDVGKITPQKLPSLKGLVNLQSGAPKPEENPSLNIQFKIQQLAISGLKVNRLDMYGEKYKPFKGVKYVTKAGKFQVRT

1792 NP_006212.1 NP_005026.3

>NP_006212.1

MADEEKLPPGWEKRMSRSSGRVYYFNHITNASQWERPSGNSSSGGKNGQGEPARVRCSHLLVKHSQSRRPSSWRQEKITRTKEEALELINGYIQKIKSGEEDFESLASQFSDCSSAKARGDLGAFSRGQMQKPFEDASFALRTGEMSGPVFTDSGIHIILRTE

>NP_005026.3

MSALCWGRGAAGLKRALRPCGRPGLPGKEGTAGGVCGPRRSSSASPQEQDQDRRKDWGHVELLEVLQARVRQLQAESVSEVVVNRVDVARLPECGSGDGSLQPPRKVQMGAKDATPVPCGRWAKILEKDKRTQQMRMQRLKAKLQMPFQSGEFKALTRRLQVEPRLLSKQMAGCLEDCTRQAPESPWEEQLARLLQEAPGKLSLDVEQAPSGQHSQAQLSGQQQRLLAFFKCCLLTDQLPLAHHLLVVHHGQRQKRKLLTLDMYNAVMLGWARQGAFKELVYVLFMVKDAGLTPDLLSYAAALQCMGRQDQDAGTIERCLEQMSQEGLKLQALFTAVLLSEEDRATVLKAVHKVKPTFSLPPQLPPPVNTSKLLRDVYAKDGRVSYPKLHLPLKTLQCLFEKQLHMELASRVCVVSVEKPTLPSKEVKHARKTLKTLRDQWEKALCRALRETKNRLEREVYEGRFSLYPFLCLLDEREVVRMLLQVLQALPAQGESFTTLARELSARTFSRHVVQRQRVSGQVQALQNHYRKYLCLLASDAEVPEPCLPRQYWEELGAPEALREQPWPLPVQMELGKLLAEMLVQATQMPCSLDKPHRSSRLVPVLYHVYSFRNVQQIGILKPHPAYVQLLEKAAEPTLTFEAVDVPMLCPPLPWTSPHSGAFLLSPTKLMRTVEGATQHQELLETCPPTALHGALDALTQLGNCAWRVNGRVLDLVLQLFQAKGCPQLGVPAPPSEAPQPPEAHLPHSAAPARKAELRRELAHCQKVAREMHSLRAEALYRLSLAQHLRDRVFWLPHNMDFRGRTYPCPPHFNHLGSDVARALLEFAQGRPLGPHGLDWLKIHLVNLTGLKKREPLRKRLAFAEEVMDDILDSADQPLTGRKWWMGAEEPWQTLACCMEVANAVRASDPAAYVSHLPVHQDGSCNGLQHYAALGRDSVGAASVNLEPSDVPQDVYSGVAAQVEVFRRQDAQRGMRVAQVLEGFITRKVVKQTVMTVVYGVTRYGGRLQIEKRLRELSDFPQEFVWEASHYLVRQVFKSLQEMFSGTRAIQHWLTESARLISHMGSVVEWVTPLGVPVIQPYRLDSKVKQIGGGIQSITYTHNGDISRKPNTRKQKNGFPPNFIHSLDSSHMMLTALHCYRKGLTFVSVHDCYWTHAADVSVMNQVCREQFVRLHSEPILQDLSRFLVKRFCSEPQKILEASQLKETLQAVPKPGAFDLEQVKRSTYFFS

1793 NP_003511.1 P82664

>NP_003511.1

MPEPSKSAPAPKKGSKKAVTKAQKKDGKKRKRSRKESYSVYVYKVLKQVHPDTGISSKAMGIMNSFVNDIFERIAGEASRLAHYNKRSTITSREIQTAVRLLLPGELAKHAVSEGTKAVTKYTSSK

>P82664

MAARTAFGAVCRRLWQGLGNFSVNTSKGNTAKNGGLLLSTNMKWVQFSNLHVDVPKDLTKPVVTISDEPDILYKRLSVLVKGHDKAVLDSYEYFAVLAAKELGISIKVHEPPRKIERFTLLQSVHIYKKHRVQYEMRTLYRCLELEHLTGSTADVYLEYIQRNLPEGVAMEVTKTQLEQLPEHIKEPIWETLSEEKEESKS

1794 O43316 Q96T66

>O43316

MHQDGISSMNQLGGLFVNGRPLPLDTRQQIVRLAVSGMRPCDISRILKVSNGCVSKILGRYYRTGVLEPKGIGGSKPRLATPPVVARIAQLKGECPALFAWEIQRQLCAEGLCTQDKTPSVSSINRVLRALQEDQGLPCTRLRSPAVLAPAVLTPHSGSETPRGTHPGTGHRNRTIFSPSQAEALEKEFQRGQYPDSVARGKLATATSLPEDTVRVWFSNRRAKWRRQEKLKWEMQLPGASQGLTVPRVAPGIISAQQSPGSVPTAALPALEPLGPSCYQLCWATAPERCLSDTPPKACLKPCWDCGSFLLPVIAPSCVDVAWPCLDASLAHHLIGGAGKATPTHFSHWP

>Q96T66

MKSRIPVVLLACGSFNPITNMHLRMFEVARDHLHQTGMYQVIQGIISPVNDTYGKKDLAASHHRVAMARLALQTSDWIRVDPWESEQAQWMETVKVLRHHHSKLLRSPPQMEGPDHGKALFSTPAAVPELKLLCGADVLKTFQTPNLWKDAHIQEIVEKFGLVCVGRVGHDPKGYIAESPILRMHQHNIHLAKEPVQNEISATYIRRALGQGQSVKYLIPDAVITYIKDHGLYTKGSTWKGKSTQSTEGKTS

1795 NP_037500.2 Q8WVM0

>NP_037500.2

MVGGLKRKHSDLEEEEERWEWSPAGLQSYQQALLRISLDKVQRSLGPRAPSLRRHVLIHNTLQQLQAALRLAPAPALPPEPLFLGEEDFSLSATIGSILRELDTSMDGTEPPQNPVTPLGLQNEVPPQPDPVFLEALSSRYLGDSGLDDFFLDIDTSAVEKEPARAPPEPPHNLFCAPGSWEWNELDHIMEIILGS

>Q8WVM0

MAASGKLSTCRLPPLPTIREIIKLLRLQAAKQLSQNFLLDLRLTDKIVRKAGNLTNAYVYEVGPGPGGITRSILNADVAELLVVEKDTRFIPGLQMLSDAAPGKLRIVHGDVLTFKVEKAFSESLKRPWEDDPPNVHIIGNLPFSVSTPLIIKWLENISCRDGPFVYGRTQMTLTFQKEVAERLAANTGSKQRSRLSVMAQYLCNVRHIFTIPGQAFVPKPEVDVGVVHFTPLIQPKIEQPFKLVEKVVQNVFQFRRKYCHRGLRMLFPEAQRLESTGRLLELADIDPTLRPRQLSISHFKSLCDVYRKMCDEDPQLFAYNFREELKRRKSKNEEKEEDDAENYRL

1796 NP_003911.1 Q16822

>NP_003911.1

MDLHMMNCELLATCSALGYLEGDTYHKEPDCLESVKDLIRYLRHEDETRDVRQQLGAAQILQSDLLPILTQHHQDKPLFDAVIRLMVNLTQPALLCFGNLPKEPSFRHHFLQVLTYLQAYKEAFASEKAFGVLSETLYELLQLGWEERQEEDNLLIERILLLVRNILHVPADLDQEKKIDDDASAHDQLLWAIHLSGLDDLLLFLASSSAEEQWSLHVLEIVSLMFRDQNPEQLAGVGQGRLAQERSADFAELEVLRQREMAEKKTRALQRGNRHSRFGGSYIVQGLKSIGERDLIFHKGLHNLRNYSSDLGKQPKKVPKRRQAARELSIQRRSALNVRLFLRDFCSEFLENCYNRLMGSVKDHLLREKAQQHDETYYMWALAFFMAFNRAASFRPGLVSETLSVRTFHFIEQNLTNYYEMMLTDRKEAASWARRMHLALKAYQELLATVNEMDLSPDEAVRESSRIIKNNIFYVMEYRELFLALFRKFDERCQPRSFLRDLVETTHLFLKMLERFCRSRGNLVVQNKQKKRRKKKKKVLDQAIVSGNVPSSPEEVEAVWPALAEQLQCCAQNSELSMDSVVPFDAASEVPVEEQRAEAMVRIQDCLLAGQAPQALTLLRSAREVWPEGDVFGSQDISPEEEIQLLKQILSAPLPRQQGPEERGAEEEEEEEEEEEEELQVVQVSEKEFNFLDYLKRFACSTVVRAYVLLLRSYQQNSAHTNHCIVKMLHRLAHDLKMEALLFQLSVFCLFNRLLSDPAAGAYKELVTFAKYILGKFFALAAVNQKAFVELLFWKNTAVVREMTEGYGSLDDRSSSRRAPTWSPEEEAHLQELYLANKDVEGQDVVEAILAHLNTVPRTRKQIIHHLVQMGLADSVKDFQRKGTHIVLWTGDQELELQRLFEEFRDSDDVLGHIMKNITAKRSRARIVDKLLALGLVAERRELYKKRQKKLASSILPNGAESLKDFCQEDLEEEENLPEEDSEEEEEGGSEAEQVQGSLVLSNENLGQSLHQEGFSIPLLWLQNCLIRAADDREEDGCSQAVPLVPLTEENEEAMENEQFQQLLRKLGVRPPASGQETFWRIPAKLSPTQLRRAAASLSQPEEEQKLQPELQPKVPGEQGSDEEHCKEHRAQALRALLLAHKKKAGLASPEEEDAVGKEPLKAAPKKRQLLDSDEEQEEDEGRNRAPELGAPGIQKKKRYQIEDDEDD

>Q16822
[truncated: 38,873,780 more chars]
